# First characterization of PIWI-interacting RNA clusters in a cichlid fish with a B chromosome

**DOI:** 10.1186/s12915-022-01403-2

**Published:** 2022-09-21

**Authors:** Jordana Inácio Nascimento Oliveira, Adauto Lima Cardoso, Ivan Rodrigo Wolf, Rogério Antônio de Oliveira, Cesar Martins

**Affiliations:** 1 Department of Structural and Functional Biology, Institute of Bioscience at Botucatu, São Paulo State University (UNESP), Botucatu, SP 18618-689 Brazil; 2 Department of Biostatistics, Plant Biology, Parasitology and Zoology, Institute of Bioscience at Botucatu, São Paulo State University (UNESP), Botucatu, SP Brazil

**Keywords:** Arms race, African cichlid fish, Genome evolution, piRNAs, Small noncoding RNAs, Supernumerary chromosome, Transposable elements

## Abstract

**Background:**

B chromosomes are extra elements found in several eukaryote species. Usually, they do not express a phenotype in the host. However, advances in bioinformatics over the last decades have allowed us to describe several genes and molecular functions related to B chromosomes. These advances enable investigations of the relationship between the B chromosome and the host to understand how this element has been preserved in genomes. However, considering that transposable elements (TEs) are highly abundant in this supernumerary chromosome, there is a lack of knowledge concerning the dynamics of TE control in B-carrying cells. Thus, the present study characterized PIWI-interacting RNA (piRNA) clusters and pathways responsible for silencing the mobilization of TEs in gonads of the cichlid fish *Astatotilapia latifasciata* carrying the B chromosome.

**Results:**

Through small RNA-seq and genome assembly, we predicted and annotated piRNA clusters in the *A. latifasciata* genome for the first time. We observed that these clusters had biased expression related to sex and the presence of the B chromosome. Furthermore, three piRNA clusters, named *curupira*, were identified in the B chromosome. Two of them were expressed exclusively in gonads of samples with the B chromosome. The composition of these *curupira* sequences was derived from LTR, LINE, and DNA elements, representing old and recent transposition events in the *A. latifasciata* genome and the B chromosome. The presence of the B chromosome also affected the expression of piRNA pathway genes. The mitochondrial cardiolipin hydrolase-like (*pld6*) gene is present in the B chromosome, as previously reported, and an increase in its expression was detected in gonads with the B chromosome.

**Conclusions:**

Due to the high abundance of TEs in the B chromosome, it was possible to investigate the origin of piRNA from these jumping genes. We hypothesize that the B chromosome has evolved its own genomic guardians to prevent uncontrolled TE mobilization. Furthermore, we also detected an expression bias in the presence of the B chromosome over *A. latifasciata* piRNA clusters and pathway genes.

**Supplementary Information:**

The online version contains supplementary material available at 10.1186/s12915-022-01403-2.

## Background

B chromosomes are extra elements found in several species, including fungi, plants, and animals [1]. They were first described as supernumerary chromosomes in an insect species more than a century ago [2]. The name “B” distinguishes this extra element from the regular chromosome complement (the “A” complement) [3]. Current estimates show that B chromosomes are present in approximately 15% of karyotyped eukaryotic species [1].

In general, intraspecific origins of B chromosomes arise from a protochromosome derived from an abnormal event during meiosis, such as chromosome breaks during nondisjunction [4]. These proto-B chromosomes are then invaded by A complement and/or organellar (mitochondrion and chloroplast) sequences, which increase the diversity of their genomic content [5]. For this reason, B chromosomes are often called “mosaic elements” and have accumulated several types of sequences, such as satellite DNA [6], transposable elements (TEs) [7], pseudogenes [8], retrogenes [9], protein-coding genes [10–12], long noncoding RNAs [13], and small noncoding RNAs [14, 15].

The B chromosome is generally recognized as an inert element without genetic activity based on its heterochromatic characteristics [16]. However, recent advances in molecular biology and bioinformatics have made it possible to identify the expression of B chromosome sequences [17], also known as “B genes.” The B chromosome of a fungal species, for example, carries a gene that confers antibiotic resistance against a host compound, making the individuals more infectious [18]. Additionally, in rye, RNA slicer activity for an Argonaute-like B chromosome copy has been observed in vitro [10], and some B chromosome peptides have been identified by mass spectrometry [19]. Furthermore, several studies have shown that B chromosomes can affect the expression of A complement sequences [14, 20] or affect various biological processes in the cell [21].

Repetitive DNA is abundant in B chromosomes, especially in the large numbers of TEs [22]. The impact of transposition on the host genome due to the presence of the B chromosome is not well understood. Although TEs are important for genome evolution, mobilization must be controlled to maintain genome integrity to avoid deleterious mutations [23].

PIWI-interacting RNAs (piRNAs) are small noncoding sequences that are responsible for targeting TEs and promoting their silencing through the piRNA pathway in animals [24]. The piRNAs originate from degenerated TE regions, the TE “junkyards” [25], forming piRNA clusters that are first transcribed as long RNAs and subsequently processed in the cytoplasm via a Dicer-independent pathway [26, 27]. In the cytoplasm, piRNA cluster transcripts are processed into small RNAs (piRNAs) in two ways: via primary piRNAs and the ping-pong cycle. In primary or phased piRNA processing, the endonuclease mitochondrial cardiolipin hydrolase (PLD6), a *Zucchini* homolog in *Drosophila*, cleaves piRNA cluster transcripts into mature piRNAs, allowing these sequences to bind PIWI proteins and form an RNA-inducing silencing complex and halting the mobilization of target TEs. Furthermore, PIWI proteins are able to process piRNA cluster transcripts into piRNA sequences through the ping-pong cycle, which increases the variability of piRNAs [24]. Fish species carry two PIWI proteins encoded by *piwi-like* genes (*piwil1* and *piwil2*) [28–30]. In addition to the cytoplasm silencing pathway, the PIWI-piRNA complex can be directed to the nucleus to silence TE transcription, attracting methylation machinery to the TE chromosome region [31, 32]. For this reason, piRNAs are commonly called “genome guardians,” as they prevent the uncontrolled mobilization of TEs and thus help maintain genome integrity [33, 34].

How the B chromosome and its excess TEs affect piRNAs or vice versa has not yet been investigated. Few studies have examined the relationship between the B chromosome and small RNAs, and most of them have been related to microRNAs [14, 15, 20]. Thus, this study was designed to investigate the relationship of piRNAs and B chromosomes by examining the African cichlid fish species *Astatotilapia latifasciata* as a model organism. Furthermore, this is the first characterization of piRNA clusters in a cichlid species.

The B chromosome of *A. latifasciata* is a metacentric chromosome similar in size to the first pair of A complement [35]. Heteromorphic sex chromosomes have not been identified in this species, and the B chromosome can be found in one-third of the population. Previous studies have already characterized the B chromosome and the *A. latifasciata* genome through molecular and large-scale approaches, such as annotation of the coding genes [36], TE content [22], epigenetic DNA modifications [37, 38], genome assembly [39], proteome and transcriptome [40], lncRNAs [13], and miRNAs [20]. Among the coding genes, *pld6*, which is important for primary piRNA generation, is present in the B chromosome of *A. latifasciata* [41].

Through RNA-seq and genomic data, the present study reports how the accumulation of TEs through B chromosome evolution could be followed by piRNA pathway expression. We suggest that the B chromosome accumulates TEs that are controlled by its own piRNAs. This coevolution generates a cycle that allows B chromosome maintenance, perpetuation, and accumulation in cells and individuals.

## Results

### Characterization of piRNA clusters in *A. latifasciata*

**Fig. 1.**
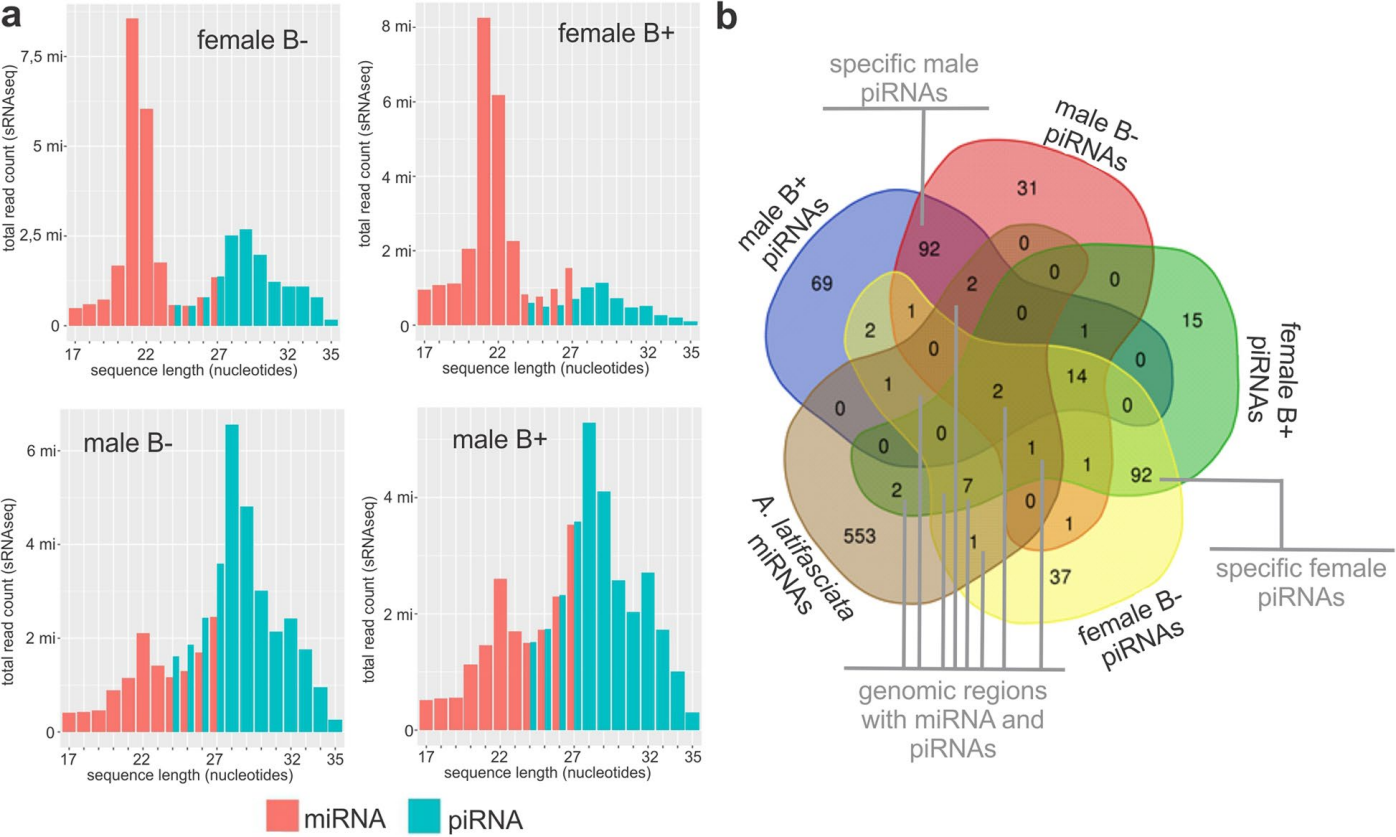
Data on miRNAs and piRNAs in *A. latifasciata*. **a** Histogram of filtered datasets for each miRNA and piRNA analysis. **b** Genomic miRNA and piRNA Venn diagram depicting all the *A. latifasciata* small RNA genomic annotations. B−, samples without the B chromosome; B+, samples with the B chromosome

The sRNA-seq libraries of gonads from FB− (female without the B chromosome), FB+ (female with the B chromosome), MB− (male without the B chromosome), and MB+ (male with the B chromosome) *A. latifasciata* were filtered by size to characterize piRNAs and compare them with miRNAs from *A. latifasciata* [20], highlighting the two different length distributions in each type of small RNA dataset (Fig. 1a). MiRNAs showed a higher abundance of 21-22 nucleotide sequences, while putative piRNAs showed more instances of 28-29 nucleotide sequences, the most common length for these two classes [27]. Additionally, miRNAs showed more expression than piRNAs in females, while the opposite was observed in males. Nonetheless, since protection using periodate oxidation was not performed to protect only piRNAs, this dataset might have contained small degraded RNAs. We reduced this bias through piRNA prediction to detect patterns of these molecules for reliable characterization, as presented below.

By prediction of piRNA clusters, which consists of finding patterns among sRNA-seq data aligned to a reference genome (see the “Methods” section), we found 372 piRNA clusters in *A. latifasciata*. The shortest piRNA cluster had 1006 base pairs (bp), while the longest piRNA cluster had 19,333 bp. The numbers of transcribed piRNA clusters in each group were 160 in FB−, 135 in FB+, 146 in MB−, and 184 in MB+ (Fig. 1b and Additional file 1). Some clusters were specifically expressed in determinate groups (either female/male, or groups with the presence/absence of the B chromosome). This suggests that the B chromosome and sex influence piRNA cluster expression, as discussed below.

The clusters of each group were combined to standardize the nomenclature for piRNA clusters of *A. latifasciata* (for more information about the clusters, such as name, genome localization, size, and expression, see Additional file 1).

To verify the reliability of the prediction, we compared the miRNA and piRNA distributions since it is known that these molecules are present in different genomic regions. While piRNAs are located in repetitive regions, miRNAs are close to euchromatin in gene-rich regions, and we expected to find piRNA clusters that were not superposed on miRNAs. The contigs that carried the expressed piRNAs predicted in each sample (FB−, FB+, MB−, MB+) and the annotated miRNA genes [20] were included in a Venn diagram, showing the number of contigs that carried either piRNAs or miRNAs. The *A. latifasciata* genome carried 941 contigs according to sRNA annotation (piRNA or miRNA); of these, 569 contigs carried miRNA genes, while 372 carried piRNA clusters (Fig. 1b). Only 16 contigs of the *A. latifasciata* genome carried both miRNA and piRNA genes. Among them, 13 contigs contained pre-miRNA sequences (miRNA genes that transcribe the hairpin structure) located inside piRNA clusters (Fig. 1b and Table 1).

**Table 1:** Colocalization of miRNA and piRNA sequences in the *A. latifasciata* genome. *S*, strand; *T:S*; transcription and strand

Contig	miRNA			piRNA			
	miRNA start: end	S	miRNA ID	piRNA cluster start: end	T:S	piRNA cluster	Gonad expression
**miRNAs and piRNAs not superposed**							
NODE_413592	23358: 23437	+	novel_27198	5015: 11188	Mono:−	161	MB−, MB+
NODE_659880	5542: 5604	+	novel_40237	1: 5034	Mono:−	297	FB+ FB−
NODE_530411	42958: 43045	+	novel_33801	46001: 52942	Mono:+	230	FB+ FB−
**miRNAs and piRNAs superposed**							
NODE_540432	60460: 60520	−	miR-17b-1	58002: 63335	Mono:+	235	FB−, FB+, MB−, FB+
	61062: 61118	+	novel-34307				
	61235: 61302	+	novel-34310				
	61365: 61424	+	novel-34311				
	61471: 61530	+	novel-34313				
NODE_374673	36480: 36543	+	miR-146b	35407: 36661	Mono:+	146	FB−, FB+, MB−
NODE_347176	36121: 36181	+	miR-21-1	35371: 38453	Mono:−	134	FB−, FB+
NODE_34239	37421: 37486	+	miR-30a-1	36149: 39395	Mono:+	133	FB−, MB+
	37756: 37816	+	miR-30b				
NODE_158712	303: 365	+	miR-10c	304: 3733	Mono:+	33	FB+
NODE_566122	1109: 1162	+	novel_35680	2: 5452		248	FB+
NODE_749189	27328: 27349	+	miR-19a-5p	25083: 30798	Mono:+	330	FB−, FB+, MB−, FB+
	27674: 27696	+	miR-19b-5p				
NODE_319315	10039: 10120	+	novel_21996	7: 10614	Mono:+	127	MB−, MB+
NODE_6163	99: 153	+	novel_675	10: 4241	Bi:+/ −	270	FB+ FB−
NODE_27951	2471: 2536	+	novel_2628	6: 3836	Mono:+	107	FB+ FB−
NODE_960524	12443: 12504	+	mir-21a	12344: 14456	Mono:−	368	FB+ FB−
NODE_581740	1963: 2049	−	novel_36349	1: 4010	Mono:−	253	FB+ FB−
NODE_81238	848: 916	−	novel_6616	9: 2856	Mono:−	344	FB−

We conclude that sRNA-seq followed by different predictions makes it possible to distinguish piRNAs and miRNAs in the *A. latifasciata* genome.

**Fig. 2.**
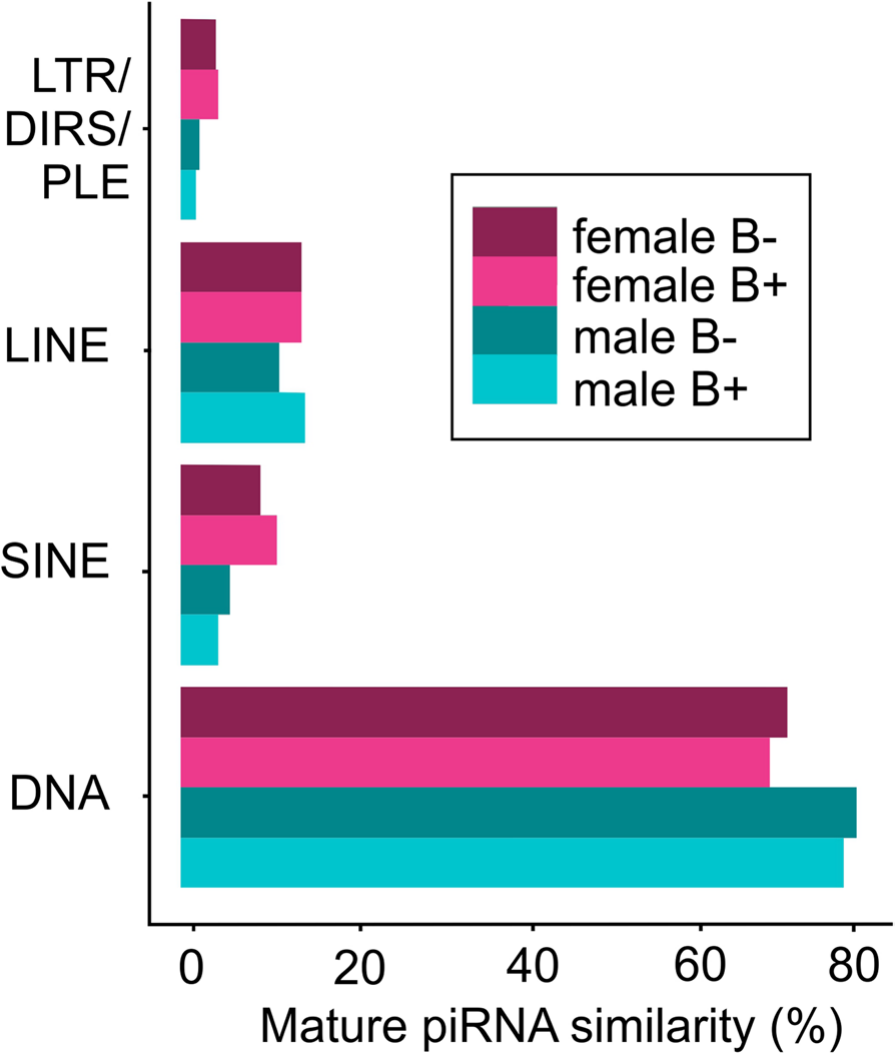
Similarity of piRNAs with TEs. Each group (FB−, FB+, MB−, MB+) was analyzed separately. The total (100%) corresponds to the number of piRNA molecules in a certain group. The percentages indicate the relative number of sequences that present TE similarity in each TE subclass

Based on the prediction of mature piRNAs in the clusters, we analyzed their similarity with TEs, identifying the types of TEs that could be silenced by the piRNAs (Fig. 2). Most of the mature piRNAs in all groups (FB−, FB+, MB−, and MB+) exhibited similarity to DNA transposons, followed by LINE elements. SINEs and LTRs were less well represented in the piRNA sequences. This finding indicates that each type of piRNA is more likely to silence certain TE families (discussed in more detail below).

### The *A. latifasciata* B chromosome carries piRNA clusters

Taking into account the similarity to the B chromosome and the entire standard genome, it is not possible to recover the assembled B chromosome based only on Illumina data [39]. An alternative strategy to find B chromosome copies is through the coverage ratio approach [36]. This method consists of comparing the genomic read alignment of sequenced B− and B+ samples to the reference genome (*A. latifasciata* assembly). Via this comparison, it is possible to detect genomic regions with higher coverage in B+ sequencing (B-blocks) [39]. Based on the annotated piRNA clusters on the *A. latifasciata* reference genome described in the previous section, we applied the coverage ratio method to find piRNAs in the B chromosome by comparing the B− and B+ unassembled reads to *A. latifasciata* contigs with piRNA cluster annotation. When a contig that carried a piRNA cluster presented more read coverage from B+ samples, we concluded that the sequence was copied in the B chromosome (see the “Methods” section).

**Fig. 3.**
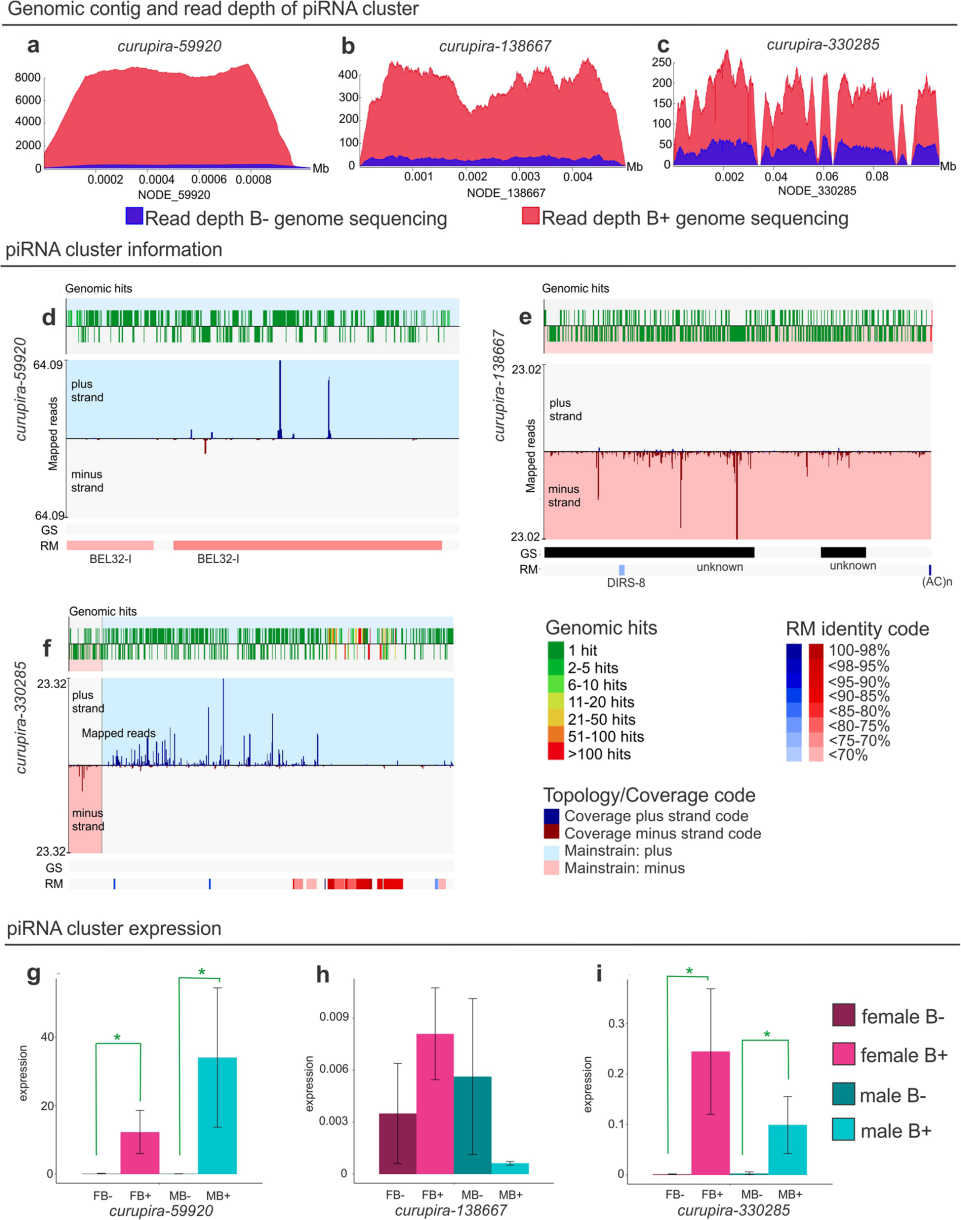
Description of piRNA clusters in the B chromosome (named *curupira*). **a**–**c** Depth coverage graphs of piRNA clusters comparing B− (blue) and B+ (red) genomic reads. **d**–**f** piRNA cluster characterization. The genomic hits on the top indicate how many times each small RNA was aligned over the genome. Green hits indicate exclusive matches to that genome region. The graphs show the piRNA sequences from the small RNA-seq dataset aligned to piRNA clusters (mapped reads); blue and red regions indicate plus and minus strands, respectively. GS, gene set from genome annotation; RM, RepeatMasker identity. **g**–**i** piRNA cluster expression in gonads. The *Y* axis shows the expression based on the ∆∆Cq method, and the *X* axis shows the samples. Green asterisks represent significant differential expression (*p* < 0.0001)

Three piRNA clusters were located in contigs with higher genomic read coverage in B+ sequencing (Fig. 3a–c), suggesting that these piRNA clusters were present in the B chromosome and were enriched in TEs (Fig. 3d–f). All the clusters were composed mostly of piRNAs with few alignment hits (genomic hits, green graph on top), which indicates fewer biases in small RNA-seq alignment anywhere in the genome (Fig. 3d–f). Additionally, RT–qPCR of precursor transcripts (the piRNA cluster) containing regions without TE similarity to avoid background interference showed that two piRNA clusters had significant differential expression in the B+ samples (Fig. 3g–i). These three piRNA clusters found in the B chromosome were named *curupira* (*curu*), followed by the contig number derived from the cluster: *curupira-59920* (*curu-59920*), *curupira-138667* (*curu-138667*), and *curupira-330285* (*curu-330285*). Curupira is a famous character in Brazilian folklore who protects forests against hunters; he is the guardian of the forest. The *curupira* clusters are the guardians of the genome from the B chromosome.

The first cluster, *curupira-59920* (cluster 263 in Additional file 1), was 1027 bp long and was predicted to be in the contig NODE_59920. This contig had approximately 300× coverage when considering the B− genomic reads, while the B+ genomic read count showed approximately 9000× coverage (Fig. 3a). The piRNA cluster corresponded to the entire contig of the reference genome and showed similarity to BEL/PAO retroelements (Fig. 3d). Regarding piRNA transcription characteristics, this cluster was monodirectional, wherein the processed piRNAs were derived from the plus strand. This cluster was expressed only in B+ samples, including both males and females (Fig. 3g).

*Curupira-138667* (cluster 19, Additional file 1) was 4986 bp in size, which is almost the complete reference contig size. The B− genome reads provided approximately 60× coverage, and the B+ genome reads provided approximately 400× coverage (Fig. 3b). Additionally, this contig showed similarity to a small portion of the retroelement DIRS (Fig. 3e). This cluster was monodirectional, with expression on the minus strand. There was no significant difference in expression between the groups (Fig. 3h).

**Fig. 4.**
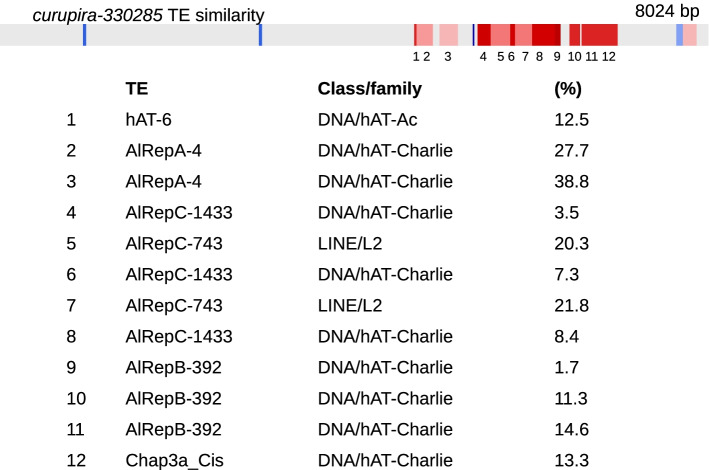
RepeatMasker similarity of cluster 131 (*curupira*-330285). Details for the TEs and other repeats of the *curupira*-330285 cluster are shown at the top of the figure. TE matches are colored blue for the plus strand and red for the minus strand. The list describes each TE match, including the percentage of similarity between the piRNA cluster and the TE fragment match

The *curupira-330285* cluster (cluster 131, Additional file 1) was 8024 bp long and corresponded to 80% of the contig size. Regarding coverage, the B− genome reads provided 60× coverage, and the B+ genome reads provided 200× coverage (Fig. 3c). Both contigs and clusters were enriched in several TEs and other simple repeats (Fig. 3f). The elements *hAT* and LINE were represented in this cluster, and the details are described in Fig. 4. Furthermore, this cluster showed bidirectionality; in other words, transcription occurred on both strands. Expression was also observed in B+ samples, including both males and females (Fig. 3i).

The coverage ratio is a qualitative indicator, and it is not possible to determine how many copies of piRNA clusters are present on the B chromosome. The very large difference in coverage might be due to other TEs, which are also present in the piRNA clusters. The evidence that the piRNA cluster located in the B chromosome is the piRNAs from sRNA came from B+ samples and was validated with cluster expression analysis.

Table 2 shows a summary of the three piRNA clusters found in the B chromosome. Finally, of the three piRNA clusters, two were expressed in only the B+ samples (Table 2). This suggests that the paralog sequences in the *A. latifasciata* genome reference (NODE_59920 and NODE_330285) are neither piRNA clusters nor expressed in autosomes.

**Table 2:** Description of piRNA clusters in the B chromosome

piRNA cluster name	piRNA cluster ID	Size (bp)	Reference genome localization	TE enrichment	T:S	RT–PCR of gonads
*curupira-59920*	263	1027	NODE_59920	Retroelement (LTR/PAO)	Mono:+	B+ samples
*curupira-138667*	19	4986	NODE_138667	Retroelement (LTR/DIRS)	Mono:+	Upregulated in B+ females
*curupira-330285*	131	8024	NODE_330285	Transposon and retrotransposon (DNA/hAT, LINE/L2)	Di:+/−	B+ samples

### Transposable elements and piRNA clusters in the *A. latifasciata* genome

The *curupira* clusters are composed of TEs that have already been identified in the B chromosome [22]. Therefore, we ran RepeatMasker to analyze the landscape of specific TE families by comparing the B− and B+ genome assemblies.

**Fig. 5.**
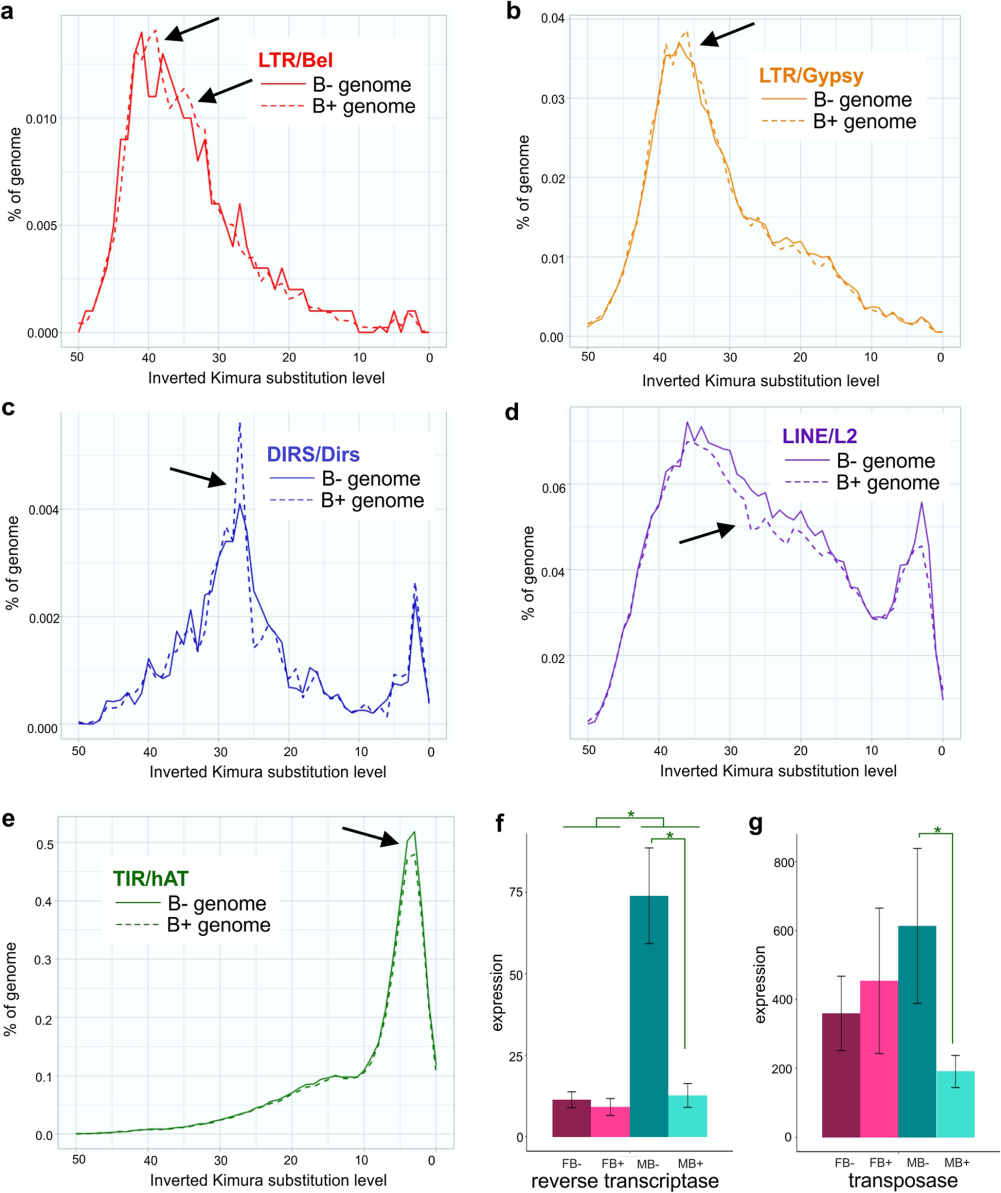
Transposable elements in *A. latifasciata*. **a**–**e** Kimura landscapes for nucleotide substitution rates. Black arrows highlight pointed divergence between B− and B+ assemblies, indicating putative B+ chromosome TE diversification. **f**, **g** RVT and DDE expression in gonads. The *Y* axis shows expression based on the ∆∆Cq method, and the *X* axis indicates the samples (FB−, FB+, MB−, MB+). The green asterisk represents significant differential expression. FB−, female without B; FB+, female with B; MB−, male without B; MB+, male with B

Landscape analysis revealed differences between B− and B+ TE insertion events in duplicated sequences. Extracting the Kimura values from the landscape of specific TE families from B− and B+ allowed us to infer the time at which TEs from B chromosomes could have emerged and differentiated from the A TE sequences (Fig. 5a–e). The BEL/PAO, L2, and *hAT* families are present in the B chromosome and make up the piRNA clusters in the B chromosome. The Gypsy family is not present in *curupira* clusters but is present in the B chromosome [22]. These duplicated families in the B chromosome carried B-specific events in the Kimura landscape (Fig. 5a–e). We hypothesize that these degeneration events could have contributed to the origin of the *curupira* cluster.

The representative major classes of TEs composing the three *curupira* sequences were LTR retrotransposons (PAO and DIRS), non-LTR retrotransposons (LINE/L2), and the *hAT* DNA transposon (Table 2). Compared to all the selected TEs, the LTR/PAO present in *curupira-59920* was representative of an older process of genome invasion. Although the LTR/DIRS sequences were acquired during newer waves of invasion, one was older, and the other was as recent as the new *hAT* element dispersion. The LINE/L2 element was indicative of constant invasion without initiation of genomic degeneration (Fig. 5d).

Comparisons of the B− and B+ TE levels revealed different copy numbers among the assemblies, and these differences suggest a *curupira-59920* origin. This piRNA cluster was not expressed in B− samples, indicating that the original copy was simply a TE region. However, in the B+ genome, this process enabled the piRNA cluster to originate in the B chromosome.

### Presence of the B chromosome and transposition domain expression

To determine whether the presence of the B chromosome affects the expression of transposition domains, we performed reverse transcriptase (RVT) and transposase (DDE) RT–qPCR (Fig. 5f–g). The sequences annotated in the *A. latifasciata* transcriptome that corresponded to RVT and DDE (regardless of the TE family) were aligned to obtain the consensus sequences, which were used to design RT–qPCR primers for common regions of each gene (Additional file 2). The RVT domain was highly expressed in males compared to females, while the presence of the B chromosome was correlated with reduced expression of RVT in testes (*p* value < 0.003) (Fig. 5f). In turn, for DDE, no significant differences were observed between the sexes. In contrast, the presence of the B chromosome was correlated with reduced expression in the testes (*p* value <0.04) (Fig. 5g).

### piRNA pathway in *A. latifasciata* and the B chromosome

**Fig. 6.**
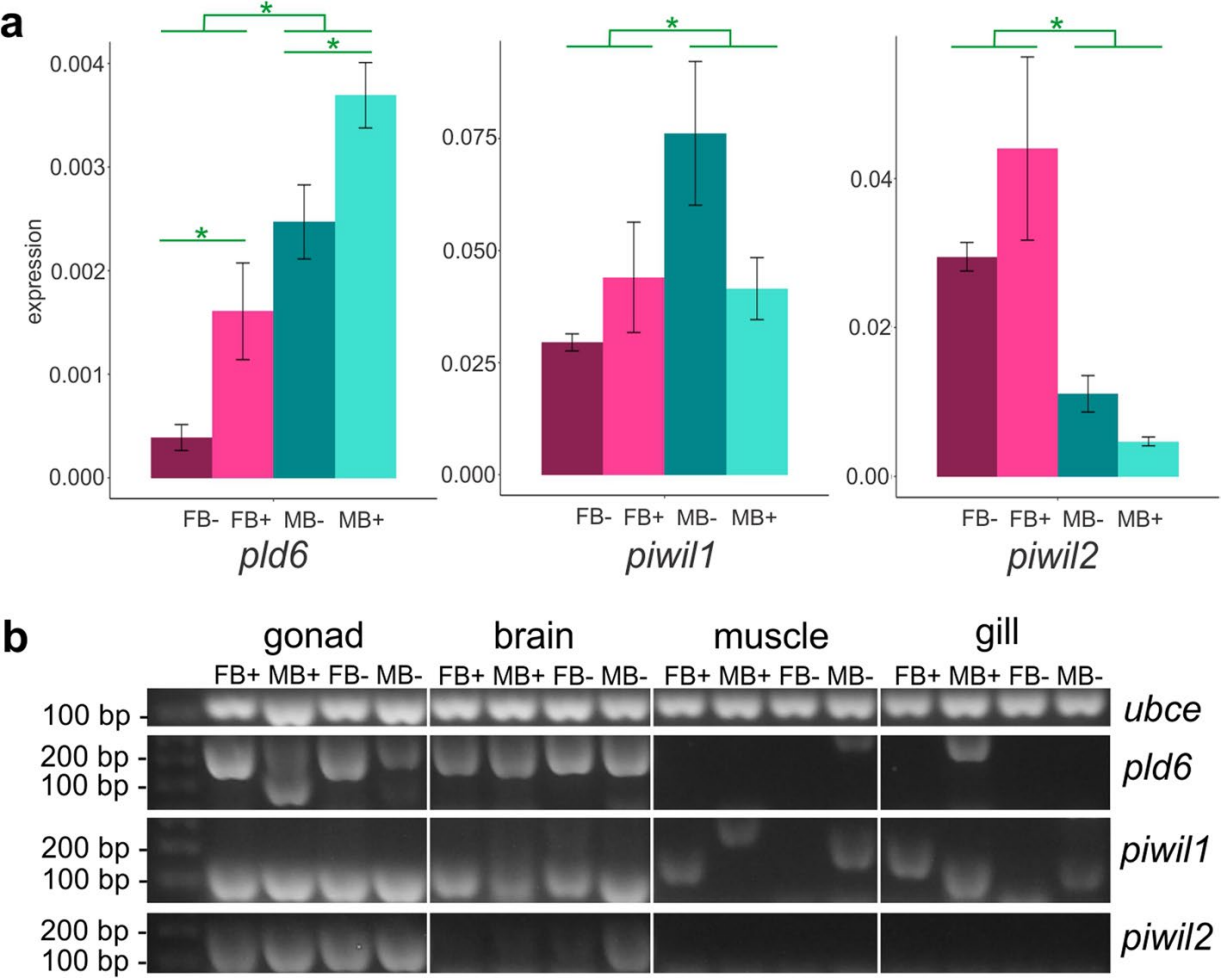
Expression of piRNA pathway genes in *A. latifasciata*. **a***pld6*, *piwil1*, and *piwil2* gene expression in gonads. The *Y* axis shows the expression based on the ∆∆Cq method, and the *X* axis shows the samples. Green asterisks represent significant differential expression between the groups (*p* <0.001). **b** PCR of piRNA pathway genes expressed in the gonad, brain, muscle, and gills

We evaluated the expression levels of genes related to the piRNA pathway (*pld6*, *piwil1*, and *piwi2*) in the testes and ovaries of B− and B+ samples (Fig. 6a). Among these genes, the *pld6* gene has been previously reported to be present in the B chromosome using Illumina sequencing [36].

The expression levels of *pld6* in both male and female B samples were significantly higher (*p* = 0.0017), indicating that the *pld6* B-copy could be active and contribute to increased expression in B samples (Fig. 6a). There was no significant difference in the expression levels of *piwil1* and *piwil2* in the presence of the B chromosome (*p* <0.09 and *p* < 0.08, respectively). Conversely, there was a difference in expression among males and females for the three analyzed genes (*p* <0.0001). While *pld6* and *piwil1* were highly expressed in males, *piwil2* was more expressed in females.

We confirmed that these piRNA pathway genes were expressed in the gonad and found evidence for the transcription of these genes in somatic samples, such as brain, muscle, and gill samples (Fig. 6b). The *pld6* gene was also expressed in the brain in both males and females, with or without the B chromosome. Expression in muscle and gills was observed only in B− and B+ males, respectively. The expression of *piwil1* was detected in the brain and gills in all samples, and the expression of *piwil1* in muscle was not detected in B− females. It was possible to observe some variations in the detected amplicons in muscle and gills, suggesting the existence of differences in mRNA processing in these tissues. We also detected *piwil2* expression in the brain, which was clear in the B− male and background samples, suggesting truly low expression in other samples. Additionally, there was no evidence of *piwil2* expression in the muscle and gills. These data suggested that sex and the presence of the B chromosome could affect the expression of piRNA pathway genes in somatic tissues.

**Fig. 7.**
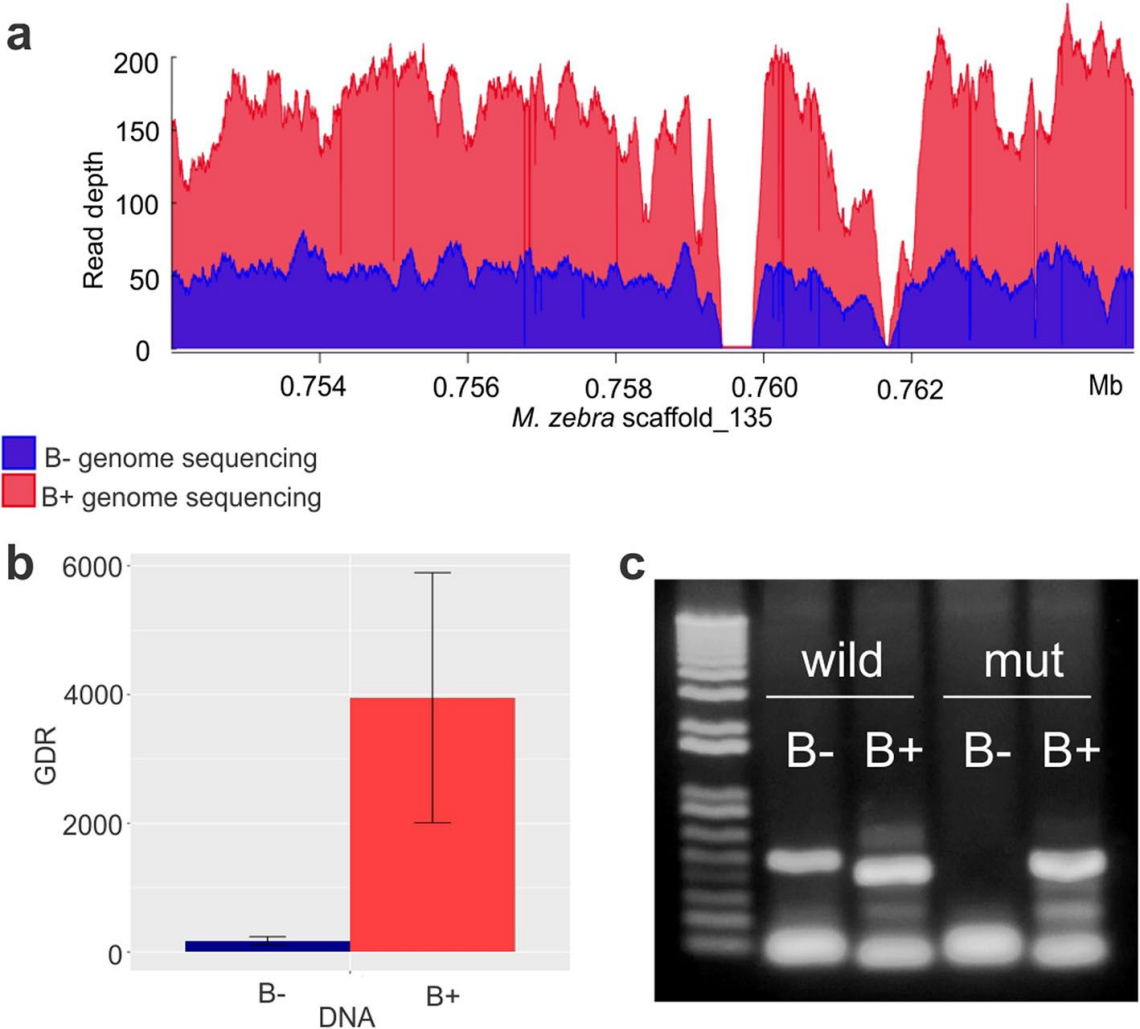
*pld6* B-copy characterization. **a** Coverage chart of the B− (blue) and B+ (red) reads aligned against *M. zebra pld6*. **b ***pld6* gene dosage ratio (GDR) from B− (blue) and B+ (red) DNA samples (*p* <0.0001). The *Y* axis shows the GDR based on the ∆∆Cq method. **c** B-specific copy amplification. Wild is a nonmutated primer, and mut is a primer with a B-specific sequence that was found in the B+ genome sequenced reads. FB−, female without B; FB+, female with B; MB−, male without B; MB+, male with B

In addition to analyzing *pld6* expression, we conducted genome investigations to detect the B chromosome copy. Due to fragmentation of the draft genome, it was not possible to recover the complete *pld6* sequence from the *A. latifasciata* assembly; thus, the *Metriaclima zebra* assembly was used as a reference genome. The *pld6* gene was present in scaffold_135 of the *M. zebra* genome, and the *A. latifasciata* B− and B+ sequencing read alignments in this region revealed a difference in coverage in the B+ data compared with the B− data. The reads of the B+ genome had approximately 200× coverage, while those of the B− genome had approximately 60× coverage (Fig. 7a). Gene dose ratio (GDR) analyses indicated the occurrence of more copies in the B+ samples, confirming the existence of copies of this gene in the B chromosome (Fig. 7b). It was possible to observe several polymorphisms in the *A. latifasciata* reads compared to the *M. zebra* reads. In addition to the difference in coverage, several B-specific mutations located in the *pld6* introns were identified, indicating that only the B+ reads carried that SNP (Additional file 3). One of them was used to construct a primer to amplify the B-specific copy of *pld6* (Fig. 7c and Additional file 3).

We performed a standalone blastx in *A. latifasciata* to assemble the transcriptome (available in [40]) to find the *M. zebra pld6* sequence (XM_004567832). This search identified two transcripts corresponding to *A. latifasciata pld6.* When we compared the nonassembled B− and B+ mRNA-seq results, it was not possible to distinguish whether one of the transcripts was specific to the B+ samples. The presence of B-specific mutations located in introns could explain the absence of such mutations among mRNA transcripts. These sequences were used to construct a phylogenetic tree based on the CDS, including other species, as described below.

**Fig. 8.**
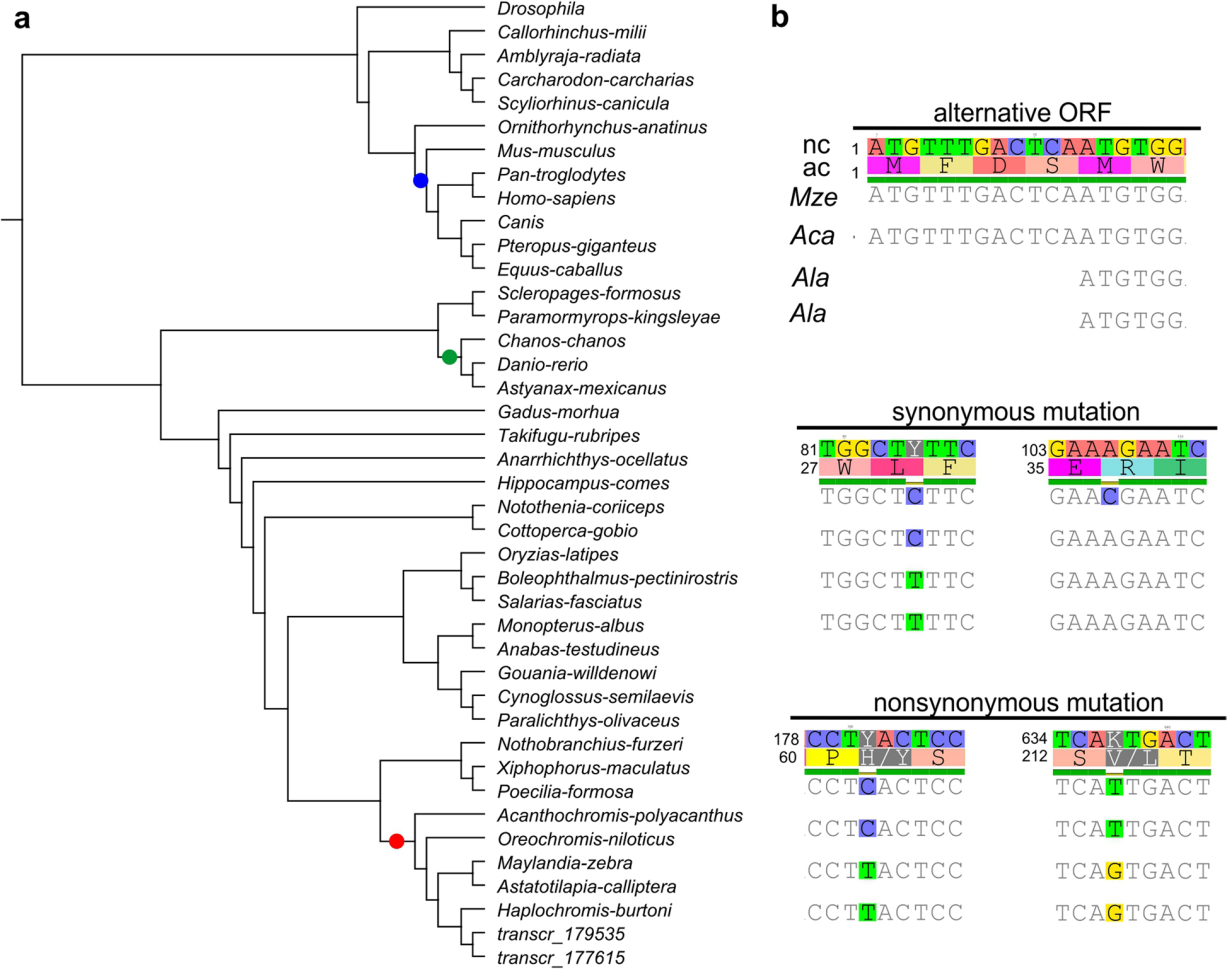
*A. latifasciata pld6* transcript comparison. **a ***Pld6* phylogenetic tree; transcr_179535 and transcr_177615 are *A. latifasciata* sequences from its transcriptome. The colored dots represent the nodes under diversifying selection. **b** Nucleotides (nc) and amino acids (ac) of *M. zebra* (*Mze*), *Astatotilapia caliptera* (*Aca*), and *A. latifasciata* (*Ala*) *pld6* CDS alignment. An initial alternative frame is detected in the *A. latifasciata* transcripts. Two detected synonymous mutations are shared among the *Astatotilapia* genus. Finally, two nonsynonymous mutations represent changes in the amino acid sequences in *A. latifasciata* PLD6

Some studies have revealed the rapid evolution of piRNA pathway genes in teleosts [28, 30]. On the basis of this evidence, we investigated the *pld6*-selective pressure in different ways. We manually identified the CDS regions of *pld6* sequences from 41 species: *Drosophila melanogaster*, seven mammals, four Chondrichthyes, 27 teleosts, and two *A. latifasciata* sequences recovered from the transcriptome. These sequences were used to construct a phylogenetic tree and perform evolutionary analysis. The *A. latifasciata pld6* sequences are well located in the cichlid clade (Fig. 8a). The BUSTED test provided evidence (*p* <0.03) of gene-wide episodic diversifying selection in the selected test branches in the phylogeny. This means that at least one tested branch had undergone diversifying selection. For this reason, we performed the aBSREL test, which identified those branches that were under diversifying selection. After correction, three nodes in the tree were determined to be under selection, including mammals, Cypriformes, and cichlids (Fig. 8a).

To search for sites under selection, the FUBAR and FEL tests revealed 2 sites that were subjected to diversifying positive selection. In contrast, 121 sites were under negative selection (*p* <0.1) (Additional file 4).

We also compared alignments of the *A. latifasciata pld6* sequence to *M. zebra* and *Astatotilapia caliptera*. The mismatches in the alignment represent (1) the specificity of the *Astatotilapia* genus, where *Astatotilapia* species and *M. zebra* showed differences, and (2) *A. latifasciata*-specific nucleotides, where this species differs from *M. zebra* and *A. caliptera* (Fig. 8b).

## Discussion

Based on sRNA-seq, we identified the genomic loci of piRNA clusters expressed in the gonads of *A. latifasciata*, which is a cichlid fish. There are no available data on the piRNA sequences of any African cichlid, and the species most closely related to *A. latifasciata* with piRNA annotation deposited in the piRBase is *Danio rerio* [42–44]. We also tested the *M. zebra* genome as a reference to predict the piRNA clusters using *A. latifasciata* sRNA-seq with no success (data not shown). Probability, because in contrast to miRNAs, piRNA sequences are poorly conserved and are considered species-specific [27, 45]. miRNAs and piRNAs also differ in length and genomic distribution [27]. piRNAs are longer than miRNAs and are frequently found in heterochromatic regions [46], while miRNAs are more common in intergenic regions [47]. Based on this knowledge, our characterization was efficient in differentiating the two small RNA classes from the same sRNA-seq data, and most miRNAs were not superposed on or colocalized to piRNAs (Fig. 1).

In addition to piRNA cluster annotation, we identified miRNA genes that were colocalized with piRNA sequences. For piRNA and miRNA predictions, we used the same sRNA-seq libraries, which is why the analysis considered several specific characteristics of each molecule. In the case of miRNA, the steps adopted were as described in [20], and in addition to the putative miRNAs from the sRNA-seq alignment to the reference genome, the secondary structures of the miRNA genes (hairpins) were considered. Here, as detailed in the results, the putative piRNAs from sRNA-seq were filtered based on several piRNA characteristics done in cluster prediction. Consequently, the few colocalized sequences raised some questions. We questioned whether the piRNA cluster evolved after the miRNA or whether the miRNA evolved from the piRNA cluster. As a third possibility, piRNA clusters carry small RNA sequences, potentially with miRNA secondary structures, thus serving as miRNAs from piRNA clusters. PiRNA sequences were first described a few years ago [48], and with ongoing assessments of the piRNA pathway, new related proteins, pathways, and functions have been identified. Recently, new functions have been presented for piRNA targeting, and a weak pairing similar to miRNA:mRNA interactions has been detected [49]. Thus, we suggest that the *A. latifasciata* piRNA clusters carry miRNA-like sequences, and these sequences together facilitate TE silencing (Fig. 1).

Furthermore, in comparisons of the miRNAs and piRNAs of *A. latifasciata*, the miRNAs were more highly expressed than the piRNAs in the female gonad, while the expression of piRNAs was higher in the testis (Fig. 1a). There are two explanations for these findings, which concern the different activities of the piRNA pathway and TEs in the ovary and testes [26, 28], both of which will be further discussed. These phenomena are also reflected in the expression of the clusters shown in Fig. 1b. Differences are observed between samples; in addition to sex bias [26, 50, 51], we suggest a B chromosome bias for piRNA cluster activity.

Upon analyzing the similarity of piRNAs with TEs, we found that the most mature sequences coincided with DNA transposons (Fig. 2). This information is consistent with the TE landscape of *A. latifasciata*, which is mostly composed of DNA transposons, mainly the *hAT* family, members of which are younger and functionally active elements [22]. This finding indicates that more piRNAs are derived from highly expressed elements originating from either piRNA cluster processing or TE silencing in the ping-pong cycle [52, 53]. However, we detected fewer piRNAs matching LTR elements, which are the oldest and least highly expressed in the *A. latifasciata* genome [22]. Therefore, we did not identify any significant sex or B chromosome bias under this aspect.

In addition to the B chromosome bias for piRNA activity identified in the gonads, three piRNA clusters, called *curupira-59920*, *curupira-138667*, and *curupira-330285*, were identified in this extra chromosome (Fig. 3). The piRNA description in the B chromosome was determined only in the wasp B chromosome by identifying the putative piRNA length range using sRNA-seq [15]. Here, we performed the first detailed characterization of piRNA clusters encoded by the B chromosome.

Additionally, our findings revealed a connection among the *A. latifasciata* B chromosome TEs. The three *curupira* clusters were enriched in B chromosome TEs that were identified using in situ techniques [22]. Furthermore, the BEL/PAO family was present in the B chromosome, but its expression was not detected [22]. This family is representative of the *curupira-59920* cluster, which was exclusively expressed in B chromosome samples. Therefore, we hypothesize that *curupira-59920* is a B piRNA cluster exclusively and is able to control LTR elements, such as the BEL/PAO family members (Fig. 3).

The *hAT* and other transposons represent the most recent invasions in the *A. latifasciata* genome [22], and this type of TE-derived piRNA could be more active in silencing young transposons. Conversely, as shown in Fig. 5a, LTR elements are the oldest TEs in the *A. latifasciata* genome [22]. Consistent with this finding, LINE elements have exhibited mobilization over time, starting with LTRs and continuing to date (Fig. 5d, [22]). In contrast, TEs that spread in the second burst followed by the third newest wave were composed of *hAT* elements [22]. Thus, the piRNAs and TEs constantly evolved together [23, 54, 55]. We found piRNA clusters in the B chromosome composed of both old and new insertions (Figs. 3 and 5).

These results reinforce our hypothesis that old and new TE insertions could have originated piRNA clusters [54] in the B chromosome. Thus, we hypothesize that *curupira-59920* represents the first piRNA cluster to arise in the B chromosome due to early LTR/PAO invasion followed by sudden degeneration (Fig. 5c). Moreover, *curupira-330285* could represent a younger piRNA in the B chromosome, as there is evidence for newer *hAT* and constant LINE insertions. Additionally, we hypothesize that the original paralog copy in the *A. latifasciata* genome is merely a TE region, while in the B chromosome, these copied regions evolved in piRNA clusters. Therefore, we observed the expression of *curupira-59920* and *curupira-330285* only in the B+ samples (Fig. 2).

In addition to the impact of the B chromosome, we reported sex-related effects on TE expression (Fig. 5f, g). Considering the differences between ovaries and testes, it was possible to detect differences in TE expression in each tissue and in the meiosis phase [56]. This observation indicates why the RVT and DDE domains have different expression levels between the sexes (Fig. 5f, g [22]).

TE *loci* are a “trap” to generate new piRNAs and promote their silencing [23, 57]. Due to heterochromatic and TE enrichment, nonrecombined chromosomes evolve piRNA clusters that carry biased expression [50, 51, 58]. We hypothesize that the B chromosome is a harbor for the evolution of new piRNAs and orchestrates its own TE silencing to guarantee the maintenance of Bs in the host genome [23, 31]. Another idea that could support the origin of new piRNAs in the B chromosome is that proposed by Kofler [57]. Using *Drosophila* as a model and following dozens of simulations, Kofler found a minimum piRNA size (proportion of piRNA clusters) in the genome that was needed to control TE mobilization and avoid extinction. In this way, the origin of piRNA clusters in the B chromosome could help to maintain host fitness and prevent the elimination of the population carrying the B chromosome and its extra TEs.

We also investigated the influence of the presence of the B chromosome on piRNA pathway genes. We confirmed the presence of *pld6* in the B chromosome and verified that the gene was more highly expressed in gonads carrying the B chromosome (Figs. 6 and 7). The coding region of the *pld6* B chromosome copy was complete [36], and based on this evidence and our results, we conclude that the *pld6* B chromosome copy contributes to the piRNA pathway. This evidence suggests why the *pld6* B-copy had no significant mutations in its CDS region. The rapid adaptive evolution observed in piRNAs of teleost genes and the high variability of TEs found in these genomes [28–30] could prevent degeneration of the *pld6* B-copy [8] due to selective pressure on this gene (Fig. 8).

In contrast, we did not observe any influence of the presence of the B chromosome on *piwil1* and *piwil2* gonad expression. TE insertion does not affect the expression of genes involved in piRNA biogenesis [59], which could explain why we were unable to identify a clear expression pattern between the *piwil1* and *piwil2* genes when comparing samples with or without the B chromosome. In teleosts, *piwil1* and *piwil2* are usually more highly expressed in the testes [26, 28, 29]; here, we observed this pattern for only the *piwil1* gene, while *piwil2* was upregulated in females compared with males (Fig. 7a).

However, we detected the expression of these genes in other tissues (Fig. 7b). In Nile tilapia, the expression of the *piwil1* and *piwil2* genes has been detected in muscle [29]; here, only *piwil1* was expressed in the brain, muscle, and gills. In contrast, this pattern is not observed in teleosts, as no expression was detected in the soma tissue of *Cyprinus carpio* and *Scophthalmus maximus* [60, 61]. The faster evolution of the piRNA pathway genes in the cichlid appears to be orchestrated by the diversity of TEs compared to the other groups, contributing to adaptive processes in the TE silencing pathway (Fig. 8, [30, 62]). We also observed the expression of piRNA pathway genes in tissues other than gonads of cichlid species, suggesting that these genes could have functions other than TE silencing; however, further investigation is required [29, 30].

## Conclusions

Although the B chromosome is currently recognized as an inert element enriched in TEs, the *A. latifasciata* B genome has accumulated new TE insertions involved in a piRNA arms-race pathway. The existence of piRNA clusters in the B chromosome could contribute to the ratio of genome size versus piRNA size to prevent the elimination of the B chromosome in the species. Furthermore, the *pld6* gene copied with 100% integrity in the B chromosome provides additional evidence for the impact of the B chromosome on the piRNA pathway. These data provide several molecular evolutionary lines of evidence for novel features that ensure B chromosome survival in the host genome. The B chromosome carries its own guardians.

## Methods

### Sample collection and small RNA sequencing

DNA and RNA samples were obtained from the *A. latifasciata* fish population maintained at the aquarium facility at the Integrative Genomics Laboratory of São Paulo State University, Botucatu (SP), Brazil (Protocol no. 769–2015). All the fish were genotyped for the presence/absence of B chromosomes using the marker for B chromosomes [63] in extracted caudal fin DNA [64] and were maintained in different aquariums until use. These materials were also used for quantitative PCR (qPCR), as described below. The fish were euthanized by immersion in 1% eugenol for 3 min. Total gonadal RNA was extracted from three females and three males with B chromosomes (B+) and without B chromosomes (B−), totaling 12 samples, following the manufacturer’s protocol for TRIzol^TM^. *A. latifasciata* is a continuous breeder (data not shown), and the same has been reported for its sister species *A. burtoni*, which means that collection of gonads did not interfere with the reproductive period [65]. These samples were shipped for large-scale small RNA sequencing by Illumina HiSeq (Sequencing Service at LC Sciences - Houston, TX, USA). This sequencing is a single-end, small-fragment method to identify ncRNA.

### Library filtering and piRNA characterization

The raw data from sRNA-seq were filtered using the FastX Toolkit [66], trimming the sequences, removing the adapters, and selecting reads ranging from 24 to 35 nucleotides in length to obtain mature piRNA transcripts [67] (Additional file 5, Table S01). This small RNA dataset is available at the NCBI database, accession number PRJNA675585 [20, 68].

The filtered and selected reads of each library (female without B, FB−; female with B, FB+; male without B, MB−; and male with B, MB+) were collapsed using the script TBr2_collapse.pl [67] and aligned against the *A. latifasciata* reference genome (40) with RNAmapper.pl [69]. These steps are mandatory for piRNA prediction. The *A. latifasciata* genome was assembled using Illumina reads and is available at Bioproject accession PRJNA369442 [39, 70]; it has been visualized in [71].

*A. latifasciata* piRNA cluster prediction was performed using proTRAC [67] based on the following aspects: (1) the *A. latifasciata* genome [39], (2) small RNA-seq mapping of the genome (from the RNAmapper.pl step), and (3) RepeatMasker annotation [67]. Based on these data, proTRAC filtered the putative clusters by considering some characteristics: minimum fraction of sRNA hits with 1T(U) or 10A (0.75), minimum fraction of sRNAs with typical piRNA length (0.75 with 24–35 nt), and minimum size of piRNA cluster (1000 bp). All these filtered characteristics showed confidence in the predicted piRNA cluster [67]. Finally, piRNA predictions were returned as output information for the length of piRNA clusters, the expression of piRNAs in the piRNA clusters, and the piRNA activity (mono- or bidirectional). The predicted piRNA clusters are shown in Additional file 1.

To analyze miRNA and piRNA distributions over the *A. latifasciata* genome, the genomic localization (*A. latifasciata* contigs) of piRNAs (this study) and miRNAs [20] was compared in a Venn diagram [72].

### Identification of piRNA clusters on the B chromosome

The genomic location of the predicted piRNA clusters was proposed to validate the presence of piRNAs in the B chromosome through the “coverage ratio” [36]. This strategy consists of aligning the B− and B+ genomic reads against the reference genome (the genomic contigs with piRNA clusters from the *A. latifasciata* assembly). Due to the duplicated sequence composition of the B chromosome, higher coverage of B+ genomic reads (at least twice) than B− genomic reads was expected in alignments. Read alignments were performed using the default parameters in Bowtie [73] for the *A. latifasciata* assembled genome and its B− and B+ unassembled filtered genomic read samples (available at Bioproject accession no. PRJN369442 [39] and visualized in [71]). The contigs with a B+/B− coverage ratio >2 were selected as putative sequences belonging to the B chromosome. To visualize the depth of coverage, the nonassembled reads (B− and B+) from the selected contigs containing piRNA clusters that presented higher coverage in the B+ alignments were extracted and subjected to bedcov analysis using Bedtools v2.29.2 [74]. The depth of coverage of the selected B contigs was visualized using the R package Sushi [75].

### Validation of the *pld6* genomic copy in the B chromosome

The mitochondrial cardiolipin hydrolase-like (*pld6*) gene was detected in the *M. zebra* genome [76], and this genomic location was used to check for the presence of *pld6* in *A. latifasciata* based on an alignment of B− and B+ samples against the *M. zebra* genome available in SaciBase [71]. The presence of B+ was detected by “coverage ratio analysis” as previously described.

To validate the *pld6* duplication in the B chromosome, we determined the gene dose ratio (GDR) through qPCR using B− and B+ DNA samples as follows: 95 °C for 20 s; 34 cycles of 95 °C for 3 s, and 60 °C for 30 s; melting curve generation at 95 °C for 15 s, 60 °C for 1 min, and 95 °C for 15 s. qPCR was carried out using SYBR Green qPCR Master Mix (High ROX) Ampliqon (HY-K0521), and the results were analyzed by the ΔΔCq [77] based on the hypoxanthine phosphoribosyl transferase 1 (*hprt1*) single-copy gene as a reference, calculated by Q-Gene software [78].

The B-specific mutations in the *pld6* copy were investigated by manually checking the polymorphisms present in only the B+ genomic reads aligned to the *M. zebra* genome (Additional file 3). The polymorphisms present in all the read samples (B− and B+) were considered *A. latifasciata* SNPs and were not considered in this analysis. The SNP described in Additional file 3 was chosen to construct primers for the B-specific mutation as described below. Conventional PCR was performed using primers for the *pld6* gene and the *pld6* B-specific copy amplified from genomic DNA of B− and B+ genotyped individuals. Conventional genomic PCR was performed using recombinant T*aq* DNA polymerase (Invitrogen-10342-053) as follows: 94 °C for 5 min; 35 cycles of 94 °C for 1 min, 50 to 60 °C for 45 s, and 72 °C for 10 min; and 72 °C for 10 min. The results were verified by agarose gel electrophoresis (1%). The primers used are described in Additional file 5.

### Molecular evolution analysis

To construct a phylogenetic tree for the molecular evolution analysis, the *pld6* mRNA sequences of 40 vertebrate species and a fly species were downloaded from the NCBI database. The sequences covered the following groups: an insect species, 7 mammalian species, 4 Chondrichthyes species, 27 teleost species (including 5 cichlids), and the two *pld6* sequences from the *A. latifasciata* transcriptome. The *A. latifasciata pld6* sequences were obtained through a standalone Blast 2.2.31 [79] of the *M. zebra* mRNA against the assembled *A. latifasciata* transcriptome [40]. *D. melanogaster* was included due to the good description of piRNA biogenesis, which is a good parameter to compare with the available data. We also included Chondrichthyes because there has been no study on *pld6* in this group. We included all available cichlid sequences due to our interest in *A. latifasciata pld6* characterization. The accession IDs of all sequences and the *A. latifasciata* sequence are described in Additional file 6 and Additional file 7.

The CDS region of each *pld6* gene was extracted using Geneious 7.1.3 [80]. The CDSs were submitted to a codon alignment, also in Geneious, and used to construct a neighbor-joining tree based on the Tamura-Nei genetic distance model. The *pld6* codon alignment and the tree were used to perform several tests for molecular evolution analysis by hypothesis testing using the HyPhy v2.5 program [81]. Based on the BUSTED method, it was possible to determine whether the *pld6* gene had experienced episodic selection events [82]. From these results, the aBSREL method was employed to determine which branch in the tree was under positive selection [83]. In addition to the previous branch site models, we also employed FEL and FUBAR to determine which sites in the CDS could be under pervasive selection, either positive or purifying selection [84, 85].

### Reverse transcriptase (RVT) and transposase (DDE) consensus domain alignment

The identification of DDE (transposase) and RVT (reverse transcriptase) domains in the *A. latifasciata* transcriptome [40] was performed using the standalone version of the Hidden Markov model, HMMER 3.3 [86], with the PFAM 34.0 protein database [87]. Sequences corresponding to the DDE and RVT domains (*E*-value <0.01 and acc >0.8) were selected to perform nucleotide alignments in Geneious 7.1.3 [80]. Based on these alignments, for each DDE and RVT domain, a consensus sequence was generated and used for primer design in Primer3Plus [88] to perform RT–PCR. The primers and the domain alignments are shown in Additional files 2 and 5, respectively.

### Transposon element landscape by family

Repetitive sequences of B− and B+ *A. latifasciata* assemblies were identified using RepeatMasker 4.1.0 [89]. A custom library based on RepeatModeler [90] and merged with Repbase Update 20181026 [91] was used to run RepeatMasker with -s, -l, and -a parameters.

The repeat landscape and Kimura divergence values were generated using calcdivergencefromalign.pl and repeatlandscape.pl, both RepeatMasker pipelines that use repetitive annotation as input. TEs that are present in *curupira* clusters and validated in the B chromosome [22] were selected to generate a comparative Kimura chart of B− and B+ assemblies.

### Functional validation (RT–PCR and RT–qPCR)

Total RNA from the gonads, brain, gills, and muscle of four males and females genotyped as B− and B+ was extracted using TRIzol (see the “Sample collection and small RNA sequencing”), resulting in 16 samples for each tissue, followed by cDNA synthesis using an Applied Biosystems™ High-Capacity cDNA Reverse Transcription Kit (10400745). RT–qPCR was performed using SYBR Green qPCR Master Mix (High ROX) Ampliqon (HY-K0521) with the following cycles: 95 °C for 20 s; 34 cycles of 95 °C for 3 s and 60° for 30 s; melting curve generation at 95 °C for 15 s, 60 °C for 1 min, and 95 °C for 15 s. The expression based on the ΔΔCq method was analyzed using the ubiquitin-conjugating enzyme (*ubce*) as a reference through Q-Gene software [78]. Statistical analyses were performed using generalized linear models considering the gamma distribution, due to its flexibility for continuous variables, among the asymmetric distributions. The presence or absence of the B chromosome was coded as a binary variable, for example, 1 or 0, respectively. Likewise, sex was categorized as 1 or 0 for males or females, respectively. The models adjusted for gene expression allowed us to assess whether the effects of the B chromosome and sex were statistically significant (*p* <0.05). All statistical analyses were performed using the statistical software SAS (Statistical Analysis Systems, version 9.3; SAS Institute Inc., Cary, North Carolina, USA). The primers are listed in Additional file 5.

## Supplementary Information

**Additional file 1. **Zipped folder with fasta and interactive html piRNA cluster information for the *A. latifasciata* genome. The nomenclature is as follows: number-pirna-cluster_sex_B-presence (f, female; m, male; 0b, without B chromosome; 1b, with B chromosome).

**Additional file 2.** PDF file containing reverse transcriptase and transposase alignment information.

**Additional file 3. **PDF file containing the *pld6* B-mutation alignment.

**Additional file 4.** Spreadsheet of FEL and FUBAR selection site analysis.

**Additional file 5. **PDF file containing **Table S01** Small RNA sequencing **Table S02** Primer sequences.

**Additional file 6. **PDF file containing **Table S03***Pld6* NCBI ID accession numbers.

**Additional file 7. **Fasta file containing *A. latifasciata pld6* sequences from the transcriptome.

## Data Availability

The *A. latifasciata* assembly is available under Bioproject accession PRJNA369442 [39, 70] and can be visualized at SACI base [71]. The *A. latifasciata* piRNA sequences are shown in the additional files. The small RNA-seq libraries were deposited in the NCBI database (Bioproject PRJNA675585 [68]).

## Abbreviations

B−: Samples without B chromosomes
B+: Samples with B chromosomes
GDR: Gene dose ratio
miRNA: MicroRNA
PCR: Polymerase chain reaction
piRNA: PIWI-interacting RNA
qPCR: Quantitative real-time PCR
RT–qPCR: Reverse transcription-qPCR
sRNA-seq: Small RNA sequencing
TE: Transposable element
