# Supplementary material for: First characterization of PIWI-interacting RNA clusters in a cichlid fish with a B chromosome
Source: BMC Biol. 2022 Sep 21;20:204. doi: 10.1186/s12915-022-01403-2 (PMC9490952; doi:10.1186/s12915-022-01403-2)
Supplement: Supplementary file 1 — Additional file 1. Zipped folder with fasta and interactive html piRNA cluster information for the A. latifasciata genome. The nomenclature is as follows: number-pirna-cluster_sex_B-presence (f, female; m, male; 0b, without B chromosome; 1b, with B chromosome). [file 12915_2022_1403_MOESM1_ESM.zip › 100_f0b.html]

piRNA cluster 100\_f0b 56


Predicted piRNA cluster no. 100\_f0b
  

Show proTRAC run info
Hide proTRAC run info

/\  
                \_\_\_\_\_\_\_\_\_\_\_\_\_\_\_\_\_\_\_\_\_\_\_/\\_\_\_ /  \\_\_\_\_\_\_\_  
               I                      /  \  /    \      I  
               I     pro             /    \/      \     I  
               I        TRAC        /               \   I  
               I   \_\_\_\_\_\_\_\_\_\_\_\_\_\_\_\_/\_\_\_\_\_\_\_\_\_\_\_\_\_\_\_\_\_\\_ I  
               I   \              /                     I  
               I    \            /                      I  
               I     \  /\      /       V.2.4.2         I  
               I      \/  \    /                        I  
               I\_\_\_\_\_\_\_\_\_\_\_\  /\_\_\_\_\_\_\_\_\_\_\_\_\_\_\_\_\_\_\_\_\_\_\_\_\_I  
                            \/  
  
  
================================= proTRAC ====================================  
VERSION: .......... 2.4.2  
LAST MODIFIED: .... 11. May 2018  
  
Please cite:  
Rosenkranz D, Zischler H. proTRAC - a software for probabilistic piRNA cluster  
detection, visualization and analysis. 2012. BMC Bioinformatics 13:5.  
  
  
Contact:  
David Rosenkranz  
Institute of Organismic and Molecular Evolutionary Biology  
Dept. Anthropology, small RNA group  
Johannes Gutenberg University Mainz  
email: rosenkranz@uni-mainz.de  
  
You can find the latest proTRAC version at:  
http://sourceforge.net/projects/protrac/files  
http://www.smallRNAgroup-mainz.de/software  
==============================================================================  
  
PARAMETERS:  
Map file: ...............piwi-femeas-0B.fa-collapse.map  
Genome file: ............../../../0B\_ala\_genome.fa  
RepeatMasker annotation: Alatifasciata-all0B-maryan-v2.fa\_corrected.out  
GeneSet:................./guest-storage/Data/annotation/Alatifasciata\_all0B\_maryan-v2\_out2017.gff  
  
Significant (p<=0.01) hit density will be calculated based  
on observed hit distribution.  
  
Sliding window size: ........................................ 5000 bp  
Sliding window increament: .................................. 1000 bp  
Normalize each hit by number of genomic hits: ............... yes  
Normalize each hit by number of sequence reads: ............. yes  
Normalize values (-> per million mapped reads): ............. yes  
Min. fraction of hits with 1T(U) or 10A: .................... 0.75  
Alternatively: Min. fraction of hits with 1T(U) and 10A: .... 0.5  
Min. fraction of hits with typical piRNA length: ............ 0.75  
Typical piRNA length: ....................................... 24-32 nt  
Min. size of a piRNA cluster: ............................... 1000 bp.  
Min. number of hits (absolute): ............................. 0  
Min. number of hits (normalized): ........................... 0  
Min. fraction of hits on the mainstrand: .................... 0.75  
Top fraction of mapped sequences (in terms of read counts): . 1%  
Top fraction accounts for max. n% of sequence reads: ........ 90%  
Min. fraction of hits on each arm of a bidirectional cluster: 0.05  
Output html file for each cluster: .......................... yes  
Output a summary table: ..................................... yes  
Output a FASTA file for each cluster (piRNA sequences): ..... yes  
Output a FASTA file comprising cluster sequences: ........... yes  
Output a GTF file for predicted piRNA clusters: ..............yes  
Search DNA motifs in clusters: .............................. yes  
Output flanking sequences: +/- .............................. 0 bp  
Output ~.pTi file: .......................................... no  
==============================================================================  
  
  
Genome size (without gaps): ............ 758543724 bp  
Gaps (N/X/-): .......................... 417479 bp  
Mapped reads: .......................... 13052187  
Non-identical sequences: ............... 3338911  
Genomic hits: .......................... 28737726  
Significant densitiy of mapped reads: .. 470.083249848448 reads/kb

Show proTRAC cluster info
Hide proTRAC cluster info

|  |  |
| --- | --- |
| Location | NODE\_260454\_length\_5643\_cov\_34.903244 |
| Coordinates | 3-5765 |
| Size [bp] | 5763 |
| Sequence hit loci | 1312 |
| Mapped reads (normalized) | 3327.9 |
| Mapped reads (normalized) per kb | 577.5 |
| Normalized reads with 1T (1U) | 82.1% |
| Normalized reads with 10A | 36.8% |
| Normalized reads with length 24-32 nt | 98.1% |
| Normalized reads on the main strand(s) | 90.5% |
| Predicted directionality | mono:minus |

100%

0%

1T (1U)  
reads

10A reads

24-32 nt  
reads

reads on mainstrand

**Either the amount of reads with 1T (1U) OR 10A has to exceed 75% (set with option: -1Tor10A)  
Alternatively the amount of reads with 1T (1U) AND 10A has to exceed 50% (set with option: -1Tand10A)  
Minimum amount of reads with preferred size is 75% (set with option: -pisize)  
Minimum amount of reads on the main strand(s) is 75% (set with option: -clstrand)**

Show read coverage
Hide read coverage

WHAT DO I SEE HERE?  
This chart shows the location of mapped sequence reads within a predicted piRNA cluster. The color refers to the number of genomic hits produced by the sequence read in question. A dark red bar indicates that this sequence read produces many other hits elsewhere in the genome. Many adjacent red or yellow bars can indicate the presence of a multi-copy element such as transposons or rRNA genes. A dark green bar indicates that this sequence read maps uniquely to this locus.

1 hit

2-5 hits

6-10 hits

11-20 hits

21-50 hits

51-100 hits

> 100 hits

NODE\_260454\_length\_5643\_cov\_34.903244

3

5765

Gene Set

RepeatMasker

Mapped  
Reads

23.21

plus strand

minus strand

23.21

Region: NODE\_260454\_length\_5643\_cov\_34.903244 8017-8. Max. coverage (+): 0. Max coverage (-): 0

Region: NODE\_260454\_length\_5643\_cov\_34.903244 9-20. Max. coverage (+): 0. Max coverage (-): 0

Region: NODE\_260454\_length\_5643\_cov\_34.903244 21-31. Max. coverage (+): 0. Max coverage (-): 0

Region: NODE\_260454\_length\_5643\_cov\_34.903244 32-43. Max. coverage (+): 0. Max coverage (-): 0

Region: NODE\_260454\_length\_5643\_cov\_34.903244 44-54. Max. coverage (+): 0.15. Max coverage (-): 0

Region: NODE\_260454\_length\_5643\_cov\_34.903244 55-66. Max. coverage (+): 0. Max coverage (-): 0

Region: NODE\_260454\_length\_5643\_cov\_34.903244 67-77. Max. coverage (+): 0. Max coverage (-): 0.15

Region: NODE\_260454\_length\_5643\_cov\_34.903244 78-89. Max. coverage (+): 1.3. Max coverage (-): 0

Region: NODE\_260454\_length\_5643\_cov\_34.903244 90-100. Max. coverage (+): 0. Max coverage (-): 0

Region: NODE\_260454\_length\_5643\_cov\_34.903244 101-112. Max. coverage (+): 0. Max coverage (-): 0.04

Region: NODE\_260454\_length\_5643\_cov\_34.903244 113-124. Max. coverage (+): 0. Max coverage (-): 0

Region: NODE\_260454\_length\_5643\_cov\_34.903244 125-135. Max. coverage (+): 0.08. Max coverage (-): 0

Region: NODE\_260454\_length\_5643\_cov\_34.903244 136-147. Max. coverage (+): 0. Max coverage (-): 0

Region: NODE\_260454\_length\_5643\_cov\_34.903244 148-158. Max. coverage (+): 0. Max coverage (-): 0

Region: NODE\_260454\_length\_5643\_cov\_34.903244 159-170. Max. coverage (+): 0. Max coverage (-): 0

Region: NODE\_260454\_length\_5643\_cov\_34.903244 171-181. Max. coverage (+): 0.05. Max coverage (-): 0

Region: NODE\_260454\_length\_5643\_cov\_34.903244 182-193. Max. coverage (+): 0.02. Max coverage (-): 0.94

Region: NODE\_260454\_length\_5643\_cov\_34.903244 194-204. Max. coverage (+): 0.02. Max coverage (-): 0.94

Region: NODE\_260454\_length\_5643\_cov\_34.903244 205-216. Max. coverage (+): 0. Max coverage (-): 0

Region: NODE\_260454\_length\_5643\_cov\_34.903244 217-227. Max. coverage (+): 0. Max coverage (-): 0

Region: NODE\_260454\_length\_5643\_cov\_34.903244 228-239. Max. coverage (+): 0. Max coverage (-): 0

Region: NODE\_260454\_length\_5643\_cov\_34.903244 240-250. Max. coverage (+): 0. Max coverage (-): 0

Region: NODE\_260454\_length\_5643\_cov\_34.903244 251-262. Max. coverage (+): 0.08. Max coverage (-): 0.03

Region: NODE\_260454\_length\_5643\_cov\_34.903244 263-273. Max. coverage (+): 0.03. Max coverage (-): 0.01

Region: NODE\_260454\_length\_5643\_cov\_34.903244 274-285. Max. coverage (+): 0.08. Max coverage (-): 0

Region: NODE\_260454\_length\_5643\_cov\_34.903244 286-296. Max. coverage (+): 0. Max coverage (-): 0

Region: NODE\_260454\_length\_5643\_cov\_34.903244 297-308. Max. coverage (+): 0.07. Max coverage (-): 0

Region: NODE\_260454\_length\_5643\_cov\_34.903244 309-319. Max. coverage (+): 0.05. Max coverage (-): 0

Region: NODE\_260454\_length\_5643\_cov\_34.903244 320-331. Max. coverage (+): 0. Max coverage (-): 0

Region: NODE\_260454\_length\_5643\_cov\_34.903244 332-343. Max. coverage (+): 0. Max coverage (-): 0.31

Region: NODE\_260454\_length\_5643\_cov\_34.903244 344-354. Max. coverage (+): 0. Max coverage (-): 0

Region: NODE\_260454\_length\_5643\_cov\_34.903244 355-366. Max. coverage (+): 0.23. Max coverage (-): 0

Region: NODE\_260454\_length\_5643\_cov\_34.903244 367-377. Max. coverage (+): 0.08. Max coverage (-): 0

Region: NODE\_260454\_length\_5643\_cov\_34.903244 378-389. Max. coverage (+): 0.01. Max coverage (-): 0

Region: NODE\_260454\_length\_5643\_cov\_34.903244 390-400. Max. coverage (+): 0. Max coverage (-): 0.02

Region: NODE\_260454\_length\_5643\_cov\_34.903244 401-412. Max. coverage (+): 0. Max coverage (-): 0.02

Region: NODE\_260454\_length\_5643\_cov\_34.903244 413-423. Max. coverage (+): 0.12. Max coverage (-): 0

Region: NODE\_260454\_length\_5643\_cov\_34.903244 424-435. Max. coverage (+): 0. Max coverage (-): 0

Region: NODE\_260454\_length\_5643\_cov\_34.903244 436-446. Max. coverage (+): 0. Max coverage (-): 0

Region: NODE\_260454\_length\_5643\_cov\_34.903244 447-458. Max. coverage (+): 0. Max coverage (-): 0

Region: NODE\_260454\_length\_5643\_cov\_34.903244 459-469. Max. coverage (+): 0. Max coverage (-): 0

Region: NODE\_260454\_length\_5643\_cov\_34.903244 470-481. Max. coverage (+): 0. Max coverage (-): 0

Region: NODE\_260454\_length\_5643\_cov\_34.903244 482-492. Max. coverage (+): 0. Max coverage (-): 0

Region: NODE\_260454\_length\_5643\_cov\_34.903244 493-504. Max. coverage (+): 0. Max coverage (-): 0

Region: NODE\_260454\_length\_5643\_cov\_34.903244 505-515. Max. coverage (+): 0.08. Max coverage (-): 0

Region: NODE\_260454\_length\_5643\_cov\_34.903244 516-527. Max. coverage (+): 0. Max coverage (-): 0

Region: NODE\_260454\_length\_5643\_cov\_34.903244 528-538. Max. coverage (+): 0. Max coverage (-): 0

Region: NODE\_260454\_length\_5643\_cov\_34.903244 539-550. Max. coverage (+): 0. Max coverage (-): 0

Region: NODE\_260454\_length\_5643\_cov\_34.903244 551-562. Max. coverage (+): 0. Max coverage (-): 0

Region: NODE\_260454\_length\_5643\_cov\_34.903244 563-573. Max. coverage (+): 0. Max coverage (-): 0

Region: NODE\_260454\_length\_5643\_cov\_34.903244 574-585. Max. coverage (+): 0. Max coverage (-): 0

Region: NODE\_260454\_length\_5643\_cov\_34.903244 586-596. Max. coverage (+): 0. Max coverage (-): 0

Region: NODE\_260454\_length\_5643\_cov\_34.903244 597-608. Max. coverage (+): 0. Max coverage (-): 0

Region: NODE\_260454\_length\_5643\_cov\_34.903244 609-619. Max. coverage (+): 0.02. Max coverage (-): 0

Region: NODE\_260454\_length\_5643\_cov\_34.903244 620-631. Max. coverage (+): 0. Max coverage (-): 0

Region: NODE\_260454\_length\_5643\_cov\_34.903244 632-642. Max. coverage (+): 0. Max coverage (-): 0

Region: NODE\_260454\_length\_5643\_cov\_34.903244 643-654. Max. coverage (+): 0. Max coverage (-): 0

Region: NODE\_260454\_length\_5643\_cov\_34.903244 655-665. Max. coverage (+): 0. Max coverage (-): 0

Region: NODE\_260454\_length\_5643\_cov\_34.903244 666-677. Max. coverage (+): 0. Max coverage (-): 0.38

Region: NODE\_260454\_length\_5643\_cov\_34.903244 678-688. Max. coverage (+): 0. Max coverage (-): 0.31

Region: NODE\_260454\_length\_5643\_cov\_34.903244 689-700. Max. coverage (+): 0. Max coverage (-): 0

Region: NODE\_260454\_length\_5643\_cov\_34.903244 701-711. Max. coverage (+): 0. Max coverage (-): 0

Region: NODE\_260454\_length\_5643\_cov\_34.903244 712-723. Max. coverage (+): 0. Max coverage (-): 0

Region: NODE\_260454\_length\_5643\_cov\_34.903244 724-734. Max. coverage (+): 0. Max coverage (-): 0

Region: NODE\_260454\_length\_5643\_cov\_34.903244 735-746. Max. coverage (+): 0. Max coverage (-): 0

Region: NODE\_260454\_length\_5643\_cov\_34.903244 747-757. Max. coverage (+): 0. Max coverage (-): 0

Region: NODE\_260454\_length\_5643\_cov\_34.903244 758-769. Max. coverage (+): 0. Max coverage (-): 0

Region: NODE\_260454\_length\_5643\_cov\_34.903244 770-781. Max. coverage (+): 0. Max coverage (-): 0

Region: NODE\_260454\_length\_5643\_cov\_34.903244 782-792. Max. coverage (+): 0. Max coverage (-): 0

Region: NODE\_260454\_length\_5643\_cov\_34.903244 793-804. Max. coverage (+): 0. Max coverage (-): 0

Region: NODE\_260454\_length\_5643\_cov\_34.903244 805-815. Max. coverage (+): 0. Max coverage (-): 0

Region: NODE\_260454\_length\_5643\_cov\_34.903244 816-827. Max. coverage (+): 0. Max coverage (-): 0

Region: NODE\_260454\_length\_5643\_cov\_34.903244 828-838. Max. coverage (+): 0. Max coverage (-): 0

Region: NODE\_260454\_length\_5643\_cov\_34.903244 839-850. Max. coverage (+): 0. Max coverage (-): 0

Region: NODE\_260454\_length\_5643\_cov\_34.903244 851-861. Max. coverage (+): 0. Max coverage (-): 0

Region: NODE\_260454\_length\_5643\_cov\_34.903244 862-873. Max. coverage (+): 0. Max coverage (-): 0

Region: NODE\_260454\_length\_5643\_cov\_34.903244 874-884. Max. coverage (+): 0. Max coverage (-): 0

Region: NODE\_260454\_length\_5643\_cov\_34.903244 885-896. Max. coverage (+): 0. Max coverage (-): 0

Region: NODE\_260454\_length\_5643\_cov\_34.903244 897-907. Max. coverage (+): 0. Max coverage (-): 0

Region: NODE\_260454\_length\_5643\_cov\_34.903244 908-919. Max. coverage (+): 0. Max coverage (-): 0

Region: NODE\_260454\_length\_5643\_cov\_34.903244 920-930. Max. coverage (+): 0. Max coverage (-): 0

Region: NODE\_260454\_length\_5643\_cov\_34.903244 931-942. Max. coverage (+): 0. Max coverage (-): 0

Region: NODE\_260454\_length\_5643\_cov\_34.903244 943-953. Max. coverage (+): 0. Max coverage (-): 0

Region: NODE\_260454\_length\_5643\_cov\_34.903244 954-965. Max. coverage (+): 0. Max coverage (-): 0

Region: NODE\_260454\_length\_5643\_cov\_34.903244 966-976. Max. coverage (+): 0. Max coverage (-): 0

Region: NODE\_260454\_length\_5643\_cov\_34.903244 977-988. Max. coverage (+): 0. Max coverage (-): 0

Region: NODE\_260454\_length\_5643\_cov\_34.903244 989-999. Max. coverage (+): 0. Max coverage (-): 0

Region: NODE\_260454\_length\_5643\_cov\_34.903244 1000-1011. Max. coverage (+): 0. Max coverage (-): 0

Region: NODE\_260454\_length\_5643\_cov\_34.903244 1012-1023. Max. coverage (+): 0. Max coverage (-): 0

Region: NODE\_260454\_length\_5643\_cov\_34.903244 1024-1034. Max. coverage (+): 0. Max coverage (-): 0

Region: NODE\_260454\_length\_5643\_cov\_34.903244 1035-1046. Max. coverage (+): 0. Max coverage (-): 0

Region: NODE\_260454\_length\_5643\_cov\_34.903244 1047-1057. Max. coverage (+): 0. Max coverage (-): 0

Region: NODE\_260454\_length\_5643\_cov\_34.903244 1058-1069. Max. coverage (+): 0. Max coverage (-): 0

Region: NODE\_260454\_length\_5643\_cov\_34.903244 1070-1080. Max. coverage (+): 0. Max coverage (-): 0

Region: NODE\_260454\_length\_5643\_cov\_34.903244 1081-1092. Max. coverage (+): 0. Max coverage (-): 0

Region: NODE\_260454\_length\_5643\_cov\_34.903244 1093-1103. Max. coverage (+): 0. Max coverage (-): 0

Region: NODE\_260454\_length\_5643\_cov\_34.903244 1104-1115. Max. coverage (+): 0. Max coverage (-): 0

Region: NODE\_260454\_length\_5643\_cov\_34.903244 1116-1126. Max. coverage (+): 0. Max coverage (-): 0

Region: NODE\_260454\_length\_5643\_cov\_34.903244 1127-1138. Max. coverage (+): 0. Max coverage (-): 0

Region: NODE\_260454\_length\_5643\_cov\_34.903244 1139-1149. Max. coverage (+): 0. Max coverage (-): 0

Region: NODE\_260454\_length\_5643\_cov\_34.903244 1150-1161. Max. coverage (+): 0. Max coverage (-): 0

Region: NODE\_260454\_length\_5643\_cov\_34.903244 1162-1172. Max. coverage (+): 0. Max coverage (-): 0

Region: NODE\_260454\_length\_5643\_cov\_34.903244 1173-1184. Max. coverage (+): 0. Max coverage (-): 0

Region: NODE\_260454\_length\_5643\_cov\_34.903244 1185-1195. Max. coverage (+): 0. Max coverage (-): 0

Region: NODE\_260454\_length\_5643\_cov\_34.903244 1196-1207. Max. coverage (+): 0. Max coverage (-): 0

Region: NODE\_260454\_length\_5643\_cov\_34.903244 1208-1218. Max. coverage (+): 0. Max coverage (-): 0

Region: NODE\_260454\_length\_5643\_cov\_34.903244 1219-1230. Max. coverage (+): 0.08. Max coverage (-): 0

Region: NODE\_260454\_length\_5643\_cov\_34.903244 1231-1242. Max. coverage (+): 0.08. Max coverage (-): 0

Region: NODE\_260454\_length\_5643\_cov\_34.903244 1243-1253. Max. coverage (+): 0. Max coverage (-): 0

Region: NODE\_260454\_length\_5643\_cov\_34.903244 1254-1265. Max. coverage (+): 0. Max coverage (-): 0

Region: NODE\_260454\_length\_5643\_cov\_34.903244 1266-1276. Max. coverage (+): 0. Max coverage (-): 0

Region: NODE\_260454\_length\_5643\_cov\_34.903244 1277-1288. Max. coverage (+): 0. Max coverage (-): 0

Region: NODE\_260454\_length\_5643\_cov\_34.903244 1289-1299. Max. coverage (+): 0. Max coverage (-): 0

Region: NODE\_260454\_length\_5643\_cov\_34.903244 1300-1311. Max. coverage (+): 0. Max coverage (-): 0.04

Region: NODE\_260454\_length\_5643\_cov\_34.903244 1312-1322. Max. coverage (+): 0. Max coverage (-): 0.02

Region: NODE\_260454\_length\_5643\_cov\_34.903244 1323-1334. Max. coverage (+): 0. Max coverage (-): 0

Region: NODE\_260454\_length\_5643\_cov\_34.903244 1335-1345. Max. coverage (+): 0. Max coverage (-): 0

Region: NODE\_260454\_length\_5643\_cov\_34.903244 1346-1357. Max. coverage (+): 0. Max coverage (-): 0.08

Region: NODE\_260454\_length\_5643\_cov\_34.903244 1358-1368. Max. coverage (+): 0. Max coverage (-): 0

Region: NODE\_260454\_length\_5643\_cov\_34.903244 1369-1380. Max. coverage (+): 0. Max coverage (-): 0

Region: NODE\_260454\_length\_5643\_cov\_34.903244 1381-1391. Max. coverage (+): 0. Max coverage (-): 0

Region: NODE\_260454\_length\_5643\_cov\_34.903244 1392-1403. Max. coverage (+): 0.08. Max coverage (-): 0

Region: NODE\_260454\_length\_5643\_cov\_34.903244 1404-1414. Max. coverage (+): 0. Max coverage (-): 0

Region: NODE\_260454\_length\_5643\_cov\_34.903244 1415-1426. Max. coverage (+): 0. Max coverage (-): 0

Region: NODE\_260454\_length\_5643\_cov\_34.903244 1427-1437. Max. coverage (+): 0. Max coverage (-): 0

Region: NODE\_260454\_length\_5643\_cov\_34.903244 1438-1449. Max. coverage (+): 0. Max coverage (-): 0

Region: NODE\_260454\_length\_5643\_cov\_34.903244 1450-1461. Max. coverage (+): 0. Max coverage (-): 0

Region: NODE\_260454\_length\_5643\_cov\_34.903244 1462-1472. Max. coverage (+): 0. Max coverage (-): 0

Region: NODE\_260454\_length\_5643\_cov\_34.903244 1473-1484. Max. coverage (+): 0. Max coverage (-): 0

Region: NODE\_260454\_length\_5643\_cov\_34.903244 1485-1495. Max. coverage (+): 0. Max coverage (-): 0

Region: NODE\_260454\_length\_5643\_cov\_34.903244 1496-1507. Max. coverage (+): 0. Max coverage (-): 0

Region: NODE\_260454\_length\_5643\_cov\_34.903244 1508-1518. Max. coverage (+): 0. Max coverage (-): 0

Region: NODE\_260454\_length\_5643\_cov\_34.903244 1519-1530. Max. coverage (+): 0. Max coverage (-): 0

Region: NODE\_260454\_length\_5643\_cov\_34.903244 1531-1541. Max. coverage (+): 0. Max coverage (-): 0

Region: NODE\_260454\_length\_5643\_cov\_34.903244 1542-1553. Max. coverage (+): 0. Max coverage (-): 0

Region: NODE\_260454\_length\_5643\_cov\_34.903244 1554-1564. Max. coverage (+): 0. Max coverage (-): 0

Region: NODE\_260454\_length\_5643\_cov\_34.903244 1565-1576. Max. coverage (+): 0. Max coverage (-): 0

Region: NODE\_260454\_length\_5643\_cov\_34.903244 1577-1587. Max. coverage (+): 0. Max coverage (-): 0

Region: NODE\_260454\_length\_5643\_cov\_34.903244 1588-1599. Max. coverage (+): 0. Max coverage (-): 0

Region: NODE\_260454\_length\_5643\_cov\_34.903244 1600-1610. Max. coverage (+): 0. Max coverage (-): 0

Region: NODE\_260454\_length\_5643\_cov\_34.903244 1611-1622. Max. coverage (+): 0. Max coverage (-): 0

Region: NODE\_260454\_length\_5643\_cov\_34.903244 1623-1633. Max. coverage (+): 0. Max coverage (-): 0

Region: NODE\_260454\_length\_5643\_cov\_34.903244 1634-1645. Max. coverage (+): 0. Max coverage (-): 0

Region: NODE\_260454\_length\_5643\_cov\_34.903244 1646-1656. Max. coverage (+): 0. Max coverage (-): 0

Region: NODE\_260454\_length\_5643\_cov\_34.903244 1657-1668. Max. coverage (+): 0. Max coverage (-): 0

Region: NODE\_260454\_length\_5643\_cov\_34.903244 1669-1680. Max. coverage (+): 0. Max coverage (-): 0

Region: NODE\_260454\_length\_5643\_cov\_34.903244 1681-1691. Max. coverage (+): 0. Max coverage (-): 0

Region: NODE\_260454\_length\_5643\_cov\_34.903244 1692-1703. Max. coverage (+): 0. Max coverage (-): 0

Region: NODE\_260454\_length\_5643\_cov\_34.903244 1704-1714. Max. coverage (+): 0. Max coverage (-): 0

Region: NODE\_260454\_length\_5643\_cov\_34.903244 1715-1726. Max. coverage (+): 0. Max coverage (-): 0

Region: NODE\_260454\_length\_5643\_cov\_34.903244 1727-1737. Max. coverage (+): 0. Max coverage (-): 0

Region: NODE\_260454\_length\_5643\_cov\_34.903244 1738-1749. Max. coverage (+): 0. Max coverage (-): 0

Region: NODE\_260454\_length\_5643\_cov\_34.903244 1750-1760. Max. coverage (+): 0. Max coverage (-): 0

Region: NODE\_260454\_length\_5643\_cov\_34.903244 1761-1772. Max. coverage (+): 0. Max coverage (-): 0

Region: NODE\_260454\_length\_5643\_cov\_34.903244 1773-1783. Max. coverage (+): 0. Max coverage (-): 0

Region: NODE\_260454\_length\_5643\_cov\_34.903244 1784-1795. Max. coverage (+): 0.01. Max coverage (-): 0

Region: NODE\_260454\_length\_5643\_cov\_34.903244 1796-1806. Max. coverage (+): 0.01. Max coverage (-): 0

Region: NODE\_260454\_length\_5643\_cov\_34.903244 1807-1818. Max. coverage (+): 0. Max coverage (-): 0

Region: NODE\_260454\_length\_5643\_cov\_34.903244 1819-1829. Max. coverage (+): 0.01. Max coverage (-): 0

Region: NODE\_260454\_length\_5643\_cov\_34.903244 1830-1841. Max. coverage (+): 0. Max coverage (-): 0

Region: NODE\_260454\_length\_5643\_cov\_34.903244 1842-1852. Max. coverage (+): 0. Max coverage (-): 0

Region: NODE\_260454\_length\_5643\_cov\_34.903244 1853-1864. Max. coverage (+): 0. Max coverage (-): 0

Region: NODE\_260454\_length\_5643\_cov\_34.903244 1865-1875. Max. coverage (+): 0. Max coverage (-): 0

Region: NODE\_260454\_length\_5643\_cov\_34.903244 1876-1887. Max. coverage (+): 0. Max coverage (-): 0

Region: NODE\_260454\_length\_5643\_cov\_34.903244 1888-1899. Max. coverage (+): 0. Max coverage (-): 0

Region: NODE\_260454\_length\_5643\_cov\_34.903244 1900-1910. Max. coverage (+): 0. Max coverage (-): 0

Region: NODE\_260454\_length\_5643\_cov\_34.903244 1911-1922. Max. coverage (+): 0. Max coverage (-): 0

Region: NODE\_260454\_length\_5643\_cov\_34.903244 1923-1933. Max. coverage (+): 0.01. Max coverage (-): 0

Region: NODE\_260454\_length\_5643\_cov\_34.903244 1934-1945. Max. coverage (+): 0. Max coverage (-): 0.01

Region: NODE\_260454\_length\_5643\_cov\_34.903244 1946-1956. Max. coverage (+): 0. Max coverage (-): 0.01

Region: NODE\_260454\_length\_5643\_cov\_34.903244 1957-1968. Max. coverage (+): 0. Max coverage (-): 0

Region: NODE\_260454\_length\_5643\_cov\_34.903244 1969-1979. Max. coverage (+): 0. Max coverage (-): 0

Region: NODE\_260454\_length\_5643\_cov\_34.903244 1980-1991. Max. coverage (+): 0. Max coverage (-): 0

Region: NODE\_260454\_length\_5643\_cov\_34.903244 1992-2002. Max. coverage (+): 0. Max coverage (-): 0

Region: NODE\_260454\_length\_5643\_cov\_34.903244 2003-2014. Max. coverage (+): 0.01. Max coverage (-): 0

Region: NODE\_260454\_length\_5643\_cov\_34.903244 2015-2025. Max. coverage (+): 0. Max coverage (-): 0

Region: NODE\_260454\_length\_5643\_cov\_34.903244 2026-2037. Max. coverage (+): 0. Max coverage (-): 0

Region: NODE\_260454\_length\_5643\_cov\_34.903244 2038-2048. Max. coverage (+): 0. Max coverage (-): 0

Region: NODE\_260454\_length\_5643\_cov\_34.903244 2049-2060. Max. coverage (+): 0. Max coverage (-): 0

Region: NODE\_260454\_length\_5643\_cov\_34.903244 2061-2071. Max. coverage (+): 0. Max coverage (-): 0

Region: NODE\_260454\_length\_5643\_cov\_34.903244 2072-2083. Max. coverage (+): 0. Max coverage (-): 0

Region: NODE\_260454\_length\_5643\_cov\_34.903244 2084-2094. Max. coverage (+): 0. Max coverage (-): 0

Region: NODE\_260454\_length\_5643\_cov\_34.903244 2095-2106. Max. coverage (+): 0. Max coverage (-): 0

Region: NODE\_260454\_length\_5643\_cov\_34.903244 2107-2118. Max. coverage (+): 0. Max coverage (-): 0

Region: NODE\_260454\_length\_5643\_cov\_34.903244 2119-2129. Max. coverage (+): 0. Max coverage (-): 0

Region: NODE\_260454\_length\_5643\_cov\_34.903244 2130-2141. Max. coverage (+): 0. Max coverage (-): 0

Region: NODE\_260454\_length\_5643\_cov\_34.903244 2142-2152. Max. coverage (+): 0. Max coverage (-): 0

Region: NODE\_260454\_length\_5643\_cov\_34.903244 2153-2164. Max. coverage (+): 0. Max coverage (-): 0

Region: NODE\_260454\_length\_5643\_cov\_34.903244 2165-2175. Max. coverage (+): 0. Max coverage (-): 0

Region: NODE\_260454\_length\_5643\_cov\_34.903244 2176-2187. Max. coverage (+): 0. Max coverage (-): 0

Region: NODE\_260454\_length\_5643\_cov\_34.903244 2188-2198. Max. coverage (+): 0. Max coverage (-): 0

Region: NODE\_260454\_length\_5643\_cov\_34.903244 2199-2210. Max. coverage (+): 0. Max coverage (-): 0

Region: NODE\_260454\_length\_5643\_cov\_34.903244 2211-2221. Max. coverage (+): 0. Max coverage (-): 0

Region: NODE\_260454\_length\_5643\_cov\_34.903244 2222-2233. Max. coverage (+): 0. Max coverage (-): 0

Region: NODE\_260454\_length\_5643\_cov\_34.903244 2234-2244. Max. coverage (+): 0. Max coverage (-): 0

Region: NODE\_260454\_length\_5643\_cov\_34.903244 2245-2256. Max. coverage (+): 0. Max coverage (-): 0

Region: NODE\_260454\_length\_5643\_cov\_34.903244 2257-2267. Max. coverage (+): 0. Max coverage (-): 0

Region: NODE\_260454\_length\_5643\_cov\_34.903244 2268-2279. Max. coverage (+): 0. Max coverage (-): 0

Region: NODE\_260454\_length\_5643\_cov\_34.903244 2280-2290. Max. coverage (+): 0. Max coverage (-): 0

Region: NODE\_260454\_length\_5643\_cov\_34.903244 2291-2302. Max. coverage (+): 0.11. Max coverage (-): 0

Region: NODE\_260454\_length\_5643\_cov\_34.903244 2303-2313. Max. coverage (+): 0. Max coverage (-): 0.15

Region: NODE\_260454\_length\_5643\_cov\_34.903244 2314-2325. Max. coverage (+): 0. Max coverage (-): 0.15

Region: NODE\_260454\_length\_5643\_cov\_34.903244 2326-2337. Max. coverage (+): 0. Max coverage (-): 0.08

Region: NODE\_260454\_length\_5643\_cov\_34.903244 2338-2348. Max. coverage (+): 0. Max coverage (-): 0.15

Region: NODE\_260454\_length\_5643\_cov\_34.903244 2349-2360. Max. coverage (+): 0. Max coverage (-): 0

Region: NODE\_260454\_length\_5643\_cov\_34.903244 2361-2371. Max. coverage (+): 0.08. Max coverage (-): 0.08

Region: NODE\_260454\_length\_5643\_cov\_34.903244 2372-2383. Max. coverage (+): 0.08. Max coverage (-): 0

Region: NODE\_260454\_length\_5643\_cov\_34.903244 2384-2394. Max. coverage (+): 0. Max coverage (-): 0.08

Region: NODE\_260454\_length\_5643\_cov\_34.903244 2395-2406. Max. coverage (+): 0. Max coverage (-): 0.08

Region: NODE\_260454\_length\_5643\_cov\_34.903244 2407-2417. Max. coverage (+): 0. Max coverage (-): 0

Region: NODE\_260454\_length\_5643\_cov\_34.903244 2418-2429. Max. coverage (+): 0. Max coverage (-): 0.08

Region: NODE\_260454\_length\_5643\_cov\_34.903244 2430-2440. Max. coverage (+): 0. Max coverage (-): 0

Region: NODE\_260454\_length\_5643\_cov\_34.903244 2441-2452. Max. coverage (+): 0. Max coverage (-): 0.08

Region: NODE\_260454\_length\_5643\_cov\_34.903244 2453-2463. Max. coverage (+): 0.08. Max coverage (-): 0

Region: NODE\_260454\_length\_5643\_cov\_34.903244 2464-2475. Max. coverage (+): 0. Max coverage (-): 0.15

Region: NODE\_260454\_length\_5643\_cov\_34.903244 2476-2486. Max. coverage (+): 0. Max coverage (-): 0.08

Region: NODE\_260454\_length\_5643\_cov\_34.903244 2487-2498. Max. coverage (+): 0. Max coverage (-): 0

Region: NODE\_260454\_length\_5643\_cov\_34.903244 2499-2509. Max. coverage (+): 0. Max coverage (-): 0

Region: NODE\_260454\_length\_5643\_cov\_34.903244 2510-2521. Max. coverage (+): 0. Max coverage (-): 0.08

Region: NODE\_260454\_length\_5643\_cov\_34.903244 2522-2532. Max. coverage (+): 0. Max coverage (-): 0.31

Region: NODE\_260454\_length\_5643\_cov\_34.903244 2533-2544. Max. coverage (+): 0. Max coverage (-): 0

Region: NODE\_260454\_length\_5643\_cov\_34.903244 2545-2556. Max. coverage (+): 0. Max coverage (-): 0

Region: NODE\_260454\_length\_5643\_cov\_34.903244 2557-2567. Max. coverage (+): 0. Max coverage (-): 0.61

Region: NODE\_260454\_length\_5643\_cov\_34.903244 2568-2579. Max. coverage (+): 0. Max coverage (-): 0.31

Region: NODE\_260454\_length\_5643\_cov\_34.903244 2580-2590. Max. coverage (+): 0.08. Max coverage (-): 0.15

Region: NODE\_260454\_length\_5643\_cov\_34.903244 2591-2602. Max. coverage (+): 0. Max coverage (-): 0.23

Region: NODE\_260454\_length\_5643\_cov\_34.903244 2603-2613. Max. coverage (+): 0. Max coverage (-): 0

Region: NODE\_260454\_length\_5643\_cov\_34.903244 2614-2625. Max. coverage (+): 0. Max coverage (-): 0

Region: NODE\_260454\_length\_5643\_cov\_34.903244 2626-2636. Max. coverage (+): 0. Max coverage (-): 0

Region: NODE\_260454\_length\_5643\_cov\_34.903244 2637-2648. Max. coverage (+): 0. Max coverage (-): 1

Region: NODE\_260454\_length\_5643\_cov\_34.903244 2649-2659. Max. coverage (+): 0. Max coverage (-): 0.23

Region: NODE\_260454\_length\_5643\_cov\_34.903244 2660-2671. Max. coverage (+): 0. Max coverage (-): 0.61

Region: NODE\_260454\_length\_5643\_cov\_34.903244 2672-2682. Max. coverage (+): 0.08. Max coverage (-): 0

Region: NODE\_260454\_length\_5643\_cov\_34.903244 2683-2694. Max. coverage (+): 0.08. Max coverage (-): 0

Region: NODE\_260454\_length\_5643\_cov\_34.903244 2695-2705. Max. coverage (+): 0. Max coverage (-): 0

Region: NODE\_260454\_length\_5643\_cov\_34.903244 2706-2717. Max. coverage (+): 0. Max coverage (-): 0.15

Region: NODE\_260454\_length\_5643\_cov\_34.903244 2718-2728. Max. coverage (+): 0. Max coverage (-): 4.37

Region: NODE\_260454\_length\_5643\_cov\_34.903244 2729-2740. Max. coverage (+): 0. Max coverage (-): 0.38

Region: NODE\_260454\_length\_5643\_cov\_34.903244 2741-2751. Max. coverage (+): 0. Max coverage (-): 0.61

Region: NODE\_260454\_length\_5643\_cov\_34.903244 2752-2763. Max. coverage (+): 0. Max coverage (-): 0.69

Region: NODE\_260454\_length\_5643\_cov\_34.903244 2764-2775. Max. coverage (+): 0. Max coverage (-): 1

Region: NODE\_260454\_length\_5643\_cov\_34.903244 2776-2786. Max. coverage (+): 0.15. Max coverage (-): 0

Region: NODE\_260454\_length\_5643\_cov\_34.903244 2787-2798. Max. coverage (+): 0. Max coverage (-): 0.38

Region: NODE\_260454\_length\_5643\_cov\_34.903244 2799-2809. Max. coverage (+): 0. Max coverage (-): 1.46

Region: NODE\_260454\_length\_5643\_cov\_34.903244 2810-2821. Max. coverage (+): 0.31. Max coverage (-): 20.15

Region: NODE\_260454\_length\_5643\_cov\_34.903244 2822-2832. Max. coverage (+): 0.15. Max coverage (-): 1

Region: NODE\_260454\_length\_5643\_cov\_34.903244 2833-2844. Max. coverage (+): 0.15. Max coverage (-): 0.08

Region: NODE\_260454\_length\_5643\_cov\_34.903244 2845-2855. Max. coverage (+): 0. Max coverage (-): 0.31

Region: NODE\_260454\_length\_5643\_cov\_34.903244 2856-2867. Max. coverage (+): 0.84. Max coverage (-): 2.22

Region: NODE\_260454\_length\_5643\_cov\_34.903244 2868-2878. Max. coverage (+): 0.23. Max coverage (-): 0

Region: NODE\_260454\_length\_5643\_cov\_34.903244 2879-2890. Max. coverage (+): 0.08. Max coverage (-): 0.23

Region: NODE\_260454\_length\_5643\_cov\_34.903244 2891-2901. Max. coverage (+): 0.08. Max coverage (-): 1.07

Region: NODE\_260454\_length\_5643\_cov\_34.903244 2902-2913. Max. coverage (+): 0. Max coverage (-): 0.08

Region: NODE\_260454\_length\_5643\_cov\_34.903244 2914-2924. Max. coverage (+): 0. Max coverage (-): 0.08

Region: NODE\_260454\_length\_5643\_cov\_34.903244 2925-2936. Max. coverage (+): 0. Max coverage (-): 0.08

Region: NODE\_260454\_length\_5643\_cov\_34.903244 2937-2947. Max. coverage (+): 0.04. Max coverage (-): 2.76

Region: NODE\_260454\_length\_5643\_cov\_34.903244 2948-2959. Max. coverage (+): 0.77. Max coverage (-): 0.15

Region: NODE\_260454\_length\_5643\_cov\_34.903244 2960-2970. Max. coverage (+): 0.31. Max coverage (-): 0.08

Region: NODE\_260454\_length\_5643\_cov\_34.903244 2971-2982. Max. coverage (+): 0. Max coverage (-): 0.08

Region: NODE\_260454\_length\_5643\_cov\_34.903244 2983-2993. Max. coverage (+): 0. Max coverage (-): 0

Region: NODE\_260454\_length\_5643\_cov\_34.903244 2994-3005. Max. coverage (+): 0. Max coverage (-): 0

Region: NODE\_260454\_length\_5643\_cov\_34.903244 3006-3017. Max. coverage (+): 0. Max coverage (-): 6.36

Region: NODE\_260454\_length\_5643\_cov\_34.903244 3018-3028. Max. coverage (+): 0.08. Max coverage (-): 0

Region: NODE\_260454\_length\_5643\_cov\_34.903244 3029-3040. Max. coverage (+): 0.08. Max coverage (-): 0

Region: NODE\_260454\_length\_5643\_cov\_34.903244 3041-3051. Max. coverage (+): 0. Max coverage (-): 13.02

Region: NODE\_260454\_length\_5643\_cov\_34.903244 3052-3063. Max. coverage (+): 0. Max coverage (-): 15.94

Region: NODE\_260454\_length\_5643\_cov\_34.903244 3064-3074. Max. coverage (+): 0. Max coverage (-): 0

Region: NODE\_260454\_length\_5643\_cov\_34.903244 3075-3086. Max. coverage (+): 0. Max coverage (-): 0.08

Region: NODE\_260454\_length\_5643\_cov\_34.903244 3087-3097. Max. coverage (+): 0. Max coverage (-): 0

Region: NODE\_260454\_length\_5643\_cov\_34.903244 3098-3109. Max. coverage (+): 0.08. Max coverage (-): 0

Region: NODE\_260454\_length\_5643\_cov\_34.903244 3110-3120. Max. coverage (+): 0. Max coverage (-): 0.15

Region: NODE\_260454\_length\_5643\_cov\_34.903244 3121-3132. Max. coverage (+): 0.08. Max coverage (-): 0

Region: NODE\_260454\_length\_5643\_cov\_34.903244 3133-3143. Max. coverage (+): 0.15. Max coverage (-): 1.3

Region: NODE\_260454\_length\_5643\_cov\_34.903244 3144-3155. Max. coverage (+): 0. Max coverage (-): 1.3

Region: NODE\_260454\_length\_5643\_cov\_34.903244 3156-3166. Max. coverage (+): 0.15. Max coverage (-): 0.31

Region: NODE\_260454\_length\_5643\_cov\_34.903244 3167-3178. Max. coverage (+): 0.15. Max coverage (-): 0.15

Region: NODE\_260454\_length\_5643\_cov\_34.903244 3179-3189. Max. coverage (+): 0. Max coverage (-): 0

Region: NODE\_260454\_length\_5643\_cov\_34.903244 3190-3201. Max. coverage (+): 0. Max coverage (-): 0.38

Region: NODE\_260454\_length\_5643\_cov\_34.903244 3202-3212. Max. coverage (+): 0. Max coverage (-): 0.23

Region: NODE\_260454\_length\_5643\_cov\_34.903244 3213-3224. Max. coverage (+): 0. Max coverage (-): 0.08

Region: NODE\_260454\_length\_5643\_cov\_34.903244 3225-3236. Max. coverage (+): 0. Max coverage (-): 2.15

Region: NODE\_260454\_length\_5643\_cov\_34.903244 3237-3247. Max. coverage (+): 0.54. Max coverage (-): 0.23

Region: NODE\_260454\_length\_5643\_cov\_34.903244 3248-3259. Max. coverage (+): 3.98. Max coverage (-): 0.15

Region: NODE\_260454\_length\_5643\_cov\_34.903244 3260-3270. Max. coverage (+): 0. Max coverage (-): 4.98

Region: NODE\_260454\_length\_5643\_cov\_34.903244 3271-3282. Max. coverage (+): 1.69. Max coverage (-): 0

Region: NODE\_260454\_length\_5643\_cov\_34.903244 3283-3293. Max. coverage (+): 0. Max coverage (-): 0.69

Region: NODE\_260454\_length\_5643\_cov\_34.903244 3294-3305. Max. coverage (+): 0. Max coverage (-): 1.53

Region: NODE\_260454\_length\_5643\_cov\_34.903244 3306-3316. Max. coverage (+): 0. Max coverage (-): 0.15

Region: NODE\_260454\_length\_5643\_cov\_34.903244 3317-3328. Max. coverage (+): 0. Max coverage (-): 0.08

Region: NODE\_260454\_length\_5643\_cov\_34.903244 3329-3339. Max. coverage (+): 0. Max coverage (-): 0.61

Region: NODE\_260454\_length\_5643\_cov\_34.903244 3340-3351. Max. coverage (+): 0. Max coverage (-): 0.19

Region: NODE\_260454\_length\_5643\_cov\_34.903244 3352-3362. Max. coverage (+): 0. Max coverage (-): 0

Region: NODE\_260454\_length\_5643\_cov\_34.903244 3363-3374. Max. coverage (+): 0. Max coverage (-): 0.23

Region: NODE\_260454\_length\_5643\_cov\_34.903244 3375-3385. Max. coverage (+): 0. Max coverage (-): 0.08

Region: NODE\_260454\_length\_5643\_cov\_34.903244 3386-3397. Max. coverage (+): 0. Max coverage (-): 0

Region: NODE\_260454\_length\_5643\_cov\_34.903244 3398-3408. Max. coverage (+): 0. Max coverage (-): 0.15

Region: NODE\_260454\_length\_5643\_cov\_34.903244 3409-3420. Max. coverage (+): 0. Max coverage (-): 0.08

Region: NODE\_260454\_length\_5643\_cov\_34.903244 3421-3431. Max. coverage (+): 0. Max coverage (-): 0

Region: NODE\_260454\_length\_5643\_cov\_34.903244 3432-3443. Max. coverage (+): 0. Max coverage (-): 0.08

Region: NODE\_260454\_length\_5643\_cov\_34.903244 3444-3455. Max. coverage (+): 0. Max coverage (-): 0.08

Region: NODE\_260454\_length\_5643\_cov\_34.903244 3456-3466. Max. coverage (+): 0. Max coverage (-): 0

Region: NODE\_260454\_length\_5643\_cov\_34.903244 3467-3478. Max. coverage (+): 0. Max coverage (-): 0.61

Region: NODE\_260454\_length\_5643\_cov\_34.903244 3479-3489. Max. coverage (+): 0. Max coverage (-): 1.38

Region: NODE\_260454\_length\_5643\_cov\_34.903244 3490-3501. Max. coverage (+): 3.06. Max coverage (-): 0

Region: NODE\_260454\_length\_5643\_cov\_34.903244 3502-3512. Max. coverage (+): 0.08. Max coverage (-): 0.38

Region: NODE\_260454\_length\_5643\_cov\_34.903244 3513-3524. Max. coverage (+): 0.08. Max coverage (-): 0.38

Region: NODE\_260454\_length\_5643\_cov\_34.903244 3525-3535. Max. coverage (+): 0. Max coverage (-): 0.84

Region: NODE\_260454\_length\_5643\_cov\_34.903244 3536-3547. Max. coverage (+): 0. Max coverage (-): 0.04

Region: NODE\_260454\_length\_5643\_cov\_34.903244 3548-3558. Max. coverage (+): 0. Max coverage (-): 0.04

Region: NODE\_260454\_length\_5643\_cov\_34.903244 3559-3570. Max. coverage (+): 0. Max coverage (-): 0.23

Region: NODE\_260454\_length\_5643\_cov\_34.903244 3571-3581. Max. coverage (+): 0. Max coverage (-): 0.15

Region: NODE\_260454\_length\_5643\_cov\_34.903244 3582-3593. Max. coverage (+): 0. Max coverage (-): 0.08

Region: NODE\_260454\_length\_5643\_cov\_34.903244 3594-3604. Max. coverage (+): 0. Max coverage (-): 3.22

Region: NODE\_260454\_length\_5643\_cov\_34.903244 3605-3616. Max. coverage (+): 0.08. Max coverage (-): 2.91

Region: NODE\_260454\_length\_5643\_cov\_34.903244 3617-3627. Max. coverage (+): 0.08. Max coverage (-): 0.23

Region: NODE\_260454\_length\_5643\_cov\_34.903244 3628-3639. Max. coverage (+): 0.08. Max coverage (-): 0

Region: NODE\_260454\_length\_5643\_cov\_34.903244 3640-3650. Max. coverage (+): 0.08. Max coverage (-): 0

Region: NODE\_260454\_length\_5643\_cov\_34.903244 3651-3662. Max. coverage (+): 0. Max coverage (-): 0.08

Region: NODE\_260454\_length\_5643\_cov\_34.903244 3663-3674. Max. coverage (+): 0.08. Max coverage (-): 0.15

Region: NODE\_260454\_length\_5643\_cov\_34.903244 3675-3685. Max. coverage (+): 0.19. Max coverage (-): 0

Region: NODE\_260454\_length\_5643\_cov\_34.903244 3686-3697. Max. coverage (+): 0.04. Max coverage (-): 0.08

Region: NODE\_260454\_length\_5643\_cov\_34.903244 3698-3708. Max. coverage (+): 0. Max coverage (-): 0.08

Region: NODE\_260454\_length\_5643\_cov\_34.903244 3709-3720. Max. coverage (+): 0.38. Max coverage (-): 0.15

Region: NODE\_260454\_length\_5643\_cov\_34.903244 3721-3731. Max. coverage (+): 0. Max coverage (-): 0.61

Region: NODE\_260454\_length\_5643\_cov\_34.903244 3732-3743. Max. coverage (+): 0. Max coverage (-): 0.15

Region: NODE\_260454\_length\_5643\_cov\_34.903244 3744-3754. Max. coverage (+): 0. Max coverage (-): 0.08

Region: NODE\_260454\_length\_5643\_cov\_34.903244 3755-3766. Max. coverage (+): 0.04. Max coverage (-): 0

Region: NODE\_260454\_length\_5643\_cov\_34.903244 3767-3777. Max. coverage (+): 0.04. Max coverage (-): 0

Region: NODE\_260454\_length\_5643\_cov\_34.903244 3778-3789. Max. coverage (+): 0. Max coverage (-): 1.23

Region: NODE\_260454\_length\_5643\_cov\_34.903244 3790-3800. Max. coverage (+): 0. Max coverage (-): 8.81

Region: NODE\_260454\_length\_5643\_cov\_34.903244 3801-3812. Max. coverage (+): 0.27. Max coverage (-): 0.38

Region: NODE\_260454\_length\_5643\_cov\_34.903244 3813-3823. Max. coverage (+): 0. Max coverage (-): 0

Region: NODE\_260454\_length\_5643\_cov\_34.903244 3824-3835. Max. coverage (+): 0. Max coverage (-): 0

Region: NODE\_260454\_length\_5643\_cov\_34.903244 3836-3846. Max. coverage (+): 0. Max coverage (-): 0

Region: NODE\_260454\_length\_5643\_cov\_34.903244 3847-3858. Max. coverage (+): 0. Max coverage (-): 0

Region: NODE\_260454\_length\_5643\_cov\_34.903244 3859-3869. Max. coverage (+): 0. Max coverage (-): 0.15

Region: NODE\_260454\_length\_5643\_cov\_34.903244 3870-3881. Max. coverage (+): 0. Max coverage (-): 0

Region: NODE\_260454\_length\_5643\_cov\_34.903244 3882-3893. Max. coverage (+): 0.08. Max coverage (-): 0

Region: NODE\_260454\_length\_5643\_cov\_34.903244 3894-3904. Max. coverage (+): 0. Max coverage (-): 2.6

Region: NODE\_260454\_length\_5643\_cov\_34.903244 3905-3916. Max. coverage (+): 0. Max coverage (-): 1.61

Region: NODE\_260454\_length\_5643\_cov\_34.903244 3917-3927. Max. coverage (+): 0. Max coverage (-): 0

Region: NODE\_260454\_length\_5643\_cov\_34.903244 3928-3939. Max. coverage (+): 0. Max coverage (-): 0

Region: NODE\_260454\_length\_5643\_cov\_34.903244 3940-3950. Max. coverage (+): 0. Max coverage (-): 5.13

Region: NODE\_260454\_length\_5643\_cov\_34.903244 3951-3962. Max. coverage (+): 0. Max coverage (-): 0.61

Region: NODE\_260454\_length\_5643\_cov\_34.903244 3963-3973. Max. coverage (+): 0. Max coverage (-): 0.08

Region: NODE\_260454\_length\_5643\_cov\_34.903244 3974-3985. Max. coverage (+): 0. Max coverage (-): 0.23

Region: NODE\_260454\_length\_5643\_cov\_34.903244 3986-3996. Max. coverage (+): 0. Max coverage (-): 0.46

Region: NODE\_260454\_length\_5643\_cov\_34.903244 3997-4008. Max. coverage (+): 0. Max coverage (-): 1.15

Region: NODE\_260454\_length\_5643\_cov\_34.903244 4009-4019. Max. coverage (+): 0. Max coverage (-): 1.15

Region: NODE\_260454\_length\_5643\_cov\_34.903244 4020-4031. Max. coverage (+): 0.61. Max coverage (-): 0

Region: NODE\_260454\_length\_5643\_cov\_34.903244 4032-4042. Max. coverage (+): 0.54. Max coverage (-): 0.23

Region: NODE\_260454\_length\_5643\_cov\_34.903244 4043-4054. Max. coverage (+): 0. Max coverage (-): 0.23

Region: NODE\_260454\_length\_5643\_cov\_34.903244 4055-4065. Max. coverage (+): 0. Max coverage (-): 0.15

Region: NODE\_260454\_length\_5643\_cov\_34.903244 4066-4077. Max. coverage (+): 0. Max coverage (-): 0.08

Region: NODE\_260454\_length\_5643\_cov\_34.903244 4078-4088. Max. coverage (+): 0. Max coverage (-): 0.69

Region: NODE\_260454\_length\_5643\_cov\_34.903244 4089-4100. Max. coverage (+): 0.31. Max coverage (-): 0

Region: NODE\_260454\_length\_5643\_cov\_34.903244 4101-4112. Max. coverage (+): 0. Max coverage (-): 0.38

Region: NODE\_260454\_length\_5643\_cov\_34.903244 4113-4123. Max. coverage (+): 0. Max coverage (-): 0.31

Region: NODE\_260454\_length\_5643\_cov\_34.903244 4124-4135. Max. coverage (+): 0.15. Max coverage (-): 0.04

Region: NODE\_260454\_length\_5643\_cov\_34.903244 4136-4146. Max. coverage (+): 0.08. Max coverage (-): 0.08

Region: NODE\_260454\_length\_5643\_cov\_34.903244 4147-4158. Max. coverage (+): 0.08. Max coverage (-): 0.15

Region: NODE\_260454\_length\_5643\_cov\_34.903244 4159-4169. Max. coverage (+): 0. Max coverage (-): 0.15

Region: NODE\_260454\_length\_5643\_cov\_34.903244 4170-4181. Max. coverage (+): 0.04. Max coverage (-): 0

Region: NODE\_260454\_length\_5643\_cov\_34.903244 4182-4192. Max. coverage (+): 0. Max coverage (-): 0.38

Region: NODE\_260454\_length\_5643\_cov\_34.903244 4193-4204. Max. coverage (+): 0. Max coverage (-): 0

Region: NODE\_260454\_length\_5643\_cov\_34.903244 4205-4215. Max. coverage (+): 0. Max coverage (-): 0

Region: NODE\_260454\_length\_5643\_cov\_34.903244 4216-4227. Max. coverage (+): 0. Max coverage (-): 0

Region: NODE\_260454\_length\_5643\_cov\_34.903244 4228-4238. Max. coverage (+): 0. Max coverage (-): 0

Region: NODE\_260454\_length\_5643\_cov\_34.903244 4239-4250. Max. coverage (+): 0. Max coverage (-): 0

Region: NODE\_260454\_length\_5643\_cov\_34.903244 4251-4261. Max. coverage (+): 0. Max coverage (-): 0.08

Region: NODE\_260454\_length\_5643\_cov\_34.903244 4262-4273. Max. coverage (+): 0.08. Max coverage (-): 0.08

Region: NODE\_260454\_length\_5643\_cov\_34.903244 4274-4284. Max. coverage (+): 0.15. Max coverage (-): 0

Region: NODE\_260454\_length\_5643\_cov\_34.903244 4285-4296. Max. coverage (+): 0. Max coverage (-): 0

Region: NODE\_260454\_length\_5643\_cov\_34.903244 4297-4307. Max. coverage (+): 0. Max coverage (-): 1.53

Region: NODE\_260454\_length\_5643\_cov\_34.903244 4308-4319. Max. coverage (+): 0. Max coverage (-): 0.08

Region: NODE\_260454\_length\_5643\_cov\_34.903244 4320-4331. Max. coverage (+): 0.08. Max coverage (-): 0.15

Region: NODE\_260454\_length\_5643\_cov\_34.903244 4332-4342. Max. coverage (+): 0. Max coverage (-): 0.69

Region: NODE\_260454\_length\_5643\_cov\_34.903244 4343-4354. Max. coverage (+): 1. Max coverage (-): 0

Region: NODE\_260454\_length\_5643\_cov\_34.903244 4355-4365. Max. coverage (+): 0. Max coverage (-): 0.31

Region: NODE\_260454\_length\_5643\_cov\_34.903244 4366-4377. Max. coverage (+): 0.08. Max coverage (-): 0.38

Region: NODE\_260454\_length\_5643\_cov\_34.903244 4378-4388. Max. coverage (+): 0.08. Max coverage (-): 0.77

Region: NODE\_260454\_length\_5643\_cov\_34.903244 4389-4400. Max. coverage (+): 0. Max coverage (-): 0.84

Region: NODE\_260454\_length\_5643\_cov\_34.903244 4401-4411. Max. coverage (+): 0.08. Max coverage (-): 0

Region: NODE\_260454\_length\_5643\_cov\_34.903244 4412-4423. Max. coverage (+): 0. Max coverage (-): 0.31

Region: NODE\_260454\_length\_5643\_cov\_34.903244 4424-4434. Max. coverage (+): 0. Max coverage (-): 0.23

Region: NODE\_260454\_length\_5643\_cov\_34.903244 4435-4446. Max. coverage (+): 0. Max coverage (-): 0.15

Region: NODE\_260454\_length\_5643\_cov\_34.903244 4447-4457. Max. coverage (+): 0. Max coverage (-): 3.06

Region: NODE\_260454\_length\_5643\_cov\_34.903244 4458-4469. Max. coverage (+): 0. Max coverage (-): 0.08

Region: NODE\_260454\_length\_5643\_cov\_34.903244 4470-4480. Max. coverage (+): 0. Max coverage (-): 0

Region: NODE\_260454\_length\_5643\_cov\_34.903244 4481-4492. Max. coverage (+): 0. Max coverage (-): 0.15

Region: NODE\_260454\_length\_5643\_cov\_34.903244 4493-4503. Max. coverage (+): 0.08. Max coverage (-): 0.23

Region: NODE\_260454\_length\_5643\_cov\_34.903244 4504-4515. Max. coverage (+): 0. Max coverage (-): 0.23

Region: NODE\_260454\_length\_5643\_cov\_34.903244 4516-4526. Max. coverage (+): 0. Max coverage (-): 0

Region: NODE\_260454\_length\_5643\_cov\_34.903244 4527-4538. Max. coverage (+): 0. Max coverage (-): 0.38

Region: NODE\_260454\_length\_5643\_cov\_34.903244 4539-4550. Max. coverage (+): 0.08. Max coverage (-): 0.31

Region: NODE\_260454\_length\_5643\_cov\_34.903244 4551-4561. Max. coverage (+): 0.08. Max coverage (-): 0.15

Region: NODE\_260454\_length\_5643\_cov\_34.903244 4562-4573. Max. coverage (+): 0.08. Max coverage (-): 4.37

Region: NODE\_260454\_length\_5643\_cov\_34.903244 4574-4584. Max. coverage (+): 0.15. Max coverage (-): 0

Region: NODE\_260454\_length\_5643\_cov\_34.903244 4585-4596. Max. coverage (+): 0. Max coverage (-): 0.15

Region: NODE\_260454\_length\_5643\_cov\_34.903244 4597-4607. Max. coverage (+): 0. Max coverage (-): 0.15

Region: NODE\_260454\_length\_5643\_cov\_34.903244 4608-4619. Max. coverage (+): 0. Max coverage (-): 0.38

Region: NODE\_260454\_length\_5643\_cov\_34.903244 4620-4630. Max. coverage (+): 0. Max coverage (-): 3.37

Region: NODE\_260454\_length\_5643\_cov\_34.903244 4631-4642. Max. coverage (+): 0. Max coverage (-): 0

Region: NODE\_260454\_length\_5643\_cov\_34.903244 4643-4653. Max. coverage (+): 0. Max coverage (-): 0

Region: NODE\_260454\_length\_5643\_cov\_34.903244 4654-4665. Max. coverage (+): 0. Max coverage (-): 10.04

Region: NODE\_260454\_length\_5643\_cov\_34.903244 4666-4676. Max. coverage (+): 0.38. Max coverage (-): 2.15

Region: NODE\_260454\_length\_5643\_cov\_34.903244 4677-4688. Max. coverage (+): 0.69. Max coverage (-): 2.91

Region: NODE\_260454\_length\_5643\_cov\_34.903244 4689-4699. Max. coverage (+): 0. Max coverage (-): 0.61

Region: NODE\_260454\_length\_5643\_cov\_34.903244 4700-4711. Max. coverage (+): 0.08. Max coverage (-): 0

Region: NODE\_260454\_length\_5643\_cov\_34.903244 4712-4722. Max. coverage (+): 0.08. Max coverage (-): 0

Region: NODE\_260454\_length\_5643\_cov\_34.903244 4723-4734. Max. coverage (+): 0.08. Max coverage (-): 0.69

Region: NODE\_260454\_length\_5643\_cov\_34.903244 4735-4745. Max. coverage (+): 0.08. Max coverage (-): 0.08

Region: NODE\_260454\_length\_5643\_cov\_34.903244 4746-4757. Max. coverage (+): 0. Max coverage (-): 0

Region: NODE\_260454\_length\_5643\_cov\_34.903244 4758-4769. Max. coverage (+): 0. Max coverage (-): 0.08

Region: NODE\_260454\_length\_5643\_cov\_34.903244 4770-4780. Max. coverage (+): 0. Max coverage (-): 0.69

Region: NODE\_260454\_length\_5643\_cov\_34.903244 4781-4792. Max. coverage (+): 0. Max coverage (-): 0

Region: NODE\_260454\_length\_5643\_cov\_34.903244 4793-4803. Max. coverage (+): 0.08. Max coverage (-): 0

Region: NODE\_260454\_length\_5643\_cov\_34.903244 4804-4815. Max. coverage (+): 0. Max coverage (-): 2.07

Region: NODE\_260454\_length\_5643\_cov\_34.903244 4816-4826. Max. coverage (+): 0. Max coverage (-): 0.46

Region: NODE\_260454\_length\_5643\_cov\_34.903244 4827-4838. Max. coverage (+): 0. Max coverage (-): 0.08

Region: NODE\_260454\_length\_5643\_cov\_34.903244 4839-4849. Max. coverage (+): 0. Max coverage (-): 0.38

Region: NODE\_260454\_length\_5643\_cov\_34.903244 4850-4861. Max. coverage (+): 0.08. Max coverage (-): 0

Region: NODE\_260454\_length\_5643\_cov\_34.903244 4862-4872. Max. coverage (+): 0. Max coverage (-): 0

Region: NODE\_260454\_length\_5643\_cov\_34.903244 4873-4884. Max. coverage (+): 0. Max coverage (-): 0.38

Region: NODE\_260454\_length\_5643\_cov\_34.903244 4885-4895. Max. coverage (+): 0. Max coverage (-): 3.37

Region: NODE\_260454\_length\_5643\_cov\_34.903244 4896-4907. Max. coverage (+): 0. Max coverage (-): 0.08

Region: NODE\_260454\_length\_5643\_cov\_34.903244 4908-4918. Max. coverage (+): 0. Max coverage (-): 0.69

Region: NODE\_260454\_length\_5643\_cov\_34.903244 4919-4930. Max. coverage (+): 0. Max coverage (-): 0.69

Region: NODE\_260454\_length\_5643\_cov\_34.903244 4931-4941. Max. coverage (+): 0. Max coverage (-): 0

Region: NODE\_260454\_length\_5643\_cov\_34.903244 4942-4953. Max. coverage (+): 0. Max coverage (-): 0

Region: NODE\_260454\_length\_5643\_cov\_34.903244 4954-4964. Max. coverage (+): 0. Max coverage (-): 0.08

Region: NODE\_260454\_length\_5643\_cov\_34.903244 4965-4976. Max. coverage (+): 0. Max coverage (-): 0

Region: NODE\_260454\_length\_5643\_cov\_34.903244 4977-4987. Max. coverage (+): 0. Max coverage (-): 0.08

Region: NODE\_260454\_length\_5643\_cov\_34.903244 4988-4999. Max. coverage (+): 0. Max coverage (-): 0

Region: NODE\_260454\_length\_5643\_cov\_34.903244 5000-5011. Max. coverage (+): 0. Max coverage (-): 0.08

Region: NODE\_260454\_length\_5643\_cov\_34.903244 5012-5022. Max. coverage (+): 0. Max coverage (-): 0.08

Region: NODE\_260454\_length\_5643\_cov\_34.903244 5023-5034. Max. coverage (+): 0. Max coverage (-): 0.08

Region: NODE\_260454\_length\_5643\_cov\_34.903244 5035-5045. Max. coverage (+): 0. Max coverage (-): 0.92

Region: NODE\_260454\_length\_5643\_cov\_34.903244 5046-5057. Max. coverage (+): 0. Max coverage (-): 1.15

Region: NODE\_260454\_length\_5643\_cov\_34.903244 5058-5068. Max. coverage (+): 0. Max coverage (-): 0.08

Region: NODE\_260454\_length\_5643\_cov\_34.903244 5069-5080. Max. coverage (+): 0. Max coverage (-): 0.08

Region: NODE\_260454\_length\_5643\_cov\_34.903244 5081-5091. Max. coverage (+): 0. Max coverage (-): 0.08

Region: NODE\_260454\_length\_5643\_cov\_34.903244 5092-5103. Max. coverage (+): 0. Max coverage (-): 0

Region: NODE\_260454\_length\_5643\_cov\_34.903244 5104-5114. Max. coverage (+): 0. Max coverage (-): 1.38

Region: NODE\_260454\_length\_5643\_cov\_34.903244 5115-5126. Max. coverage (+): 0.23. Max coverage (-): 23.21

Region: NODE\_260454\_length\_5643\_cov\_34.903244 5127-5137. Max. coverage (+): 0.04. Max coverage (-): 1.23

Region: NODE\_260454\_length\_5643\_cov\_34.903244 5138-5149. Max. coverage (+): 0. Max coverage (-): 0

Region: NODE\_260454\_length\_5643\_cov\_34.903244 5150-5160. Max. coverage (+): 0. Max coverage (-): 0

Region: NODE\_260454\_length\_5643\_cov\_34.903244 5161-5172. Max. coverage (+): 0. Max coverage (-): 0

Region: NODE\_260454\_length\_5643\_cov\_34.903244 5173-5183. Max. coverage (+): 0. Max coverage (-): 1.69

Region: NODE\_260454\_length\_5643\_cov\_34.903244 5184-5195. Max. coverage (+): 0. Max coverage (-): 1.76

Region: NODE\_260454\_length\_5643\_cov\_34.903244 5196-5206. Max. coverage (+): 0. Max coverage (-): 0.69

Region: NODE\_260454\_length\_5643\_cov\_34.903244 5207-5218. Max. coverage (+): 0. Max coverage (-): 0

Region: NODE\_260454\_length\_5643\_cov\_34.903244 5219-5230. Max. coverage (+): 0. Max coverage (-): 0

Region: NODE\_260454\_length\_5643\_cov\_34.903244 5231-5241. Max. coverage (+): 0.15. Max coverage (-): 0.08

Region: NODE\_260454\_length\_5643\_cov\_34.903244 5242-5253. Max. coverage (+): 0. Max coverage (-): 0.08

Region: NODE\_260454\_length\_5643\_cov\_34.903244 5254-5264. Max. coverage (+): 0. Max coverage (-): 0.38

Region: NODE\_260454\_length\_5643\_cov\_34.903244 5265-5276. Max. coverage (+): 0. Max coverage (-): 0.08

Region: NODE\_260454\_length\_5643\_cov\_34.903244 5277-5287. Max. coverage (+): 0. Max coverage (-): 0.15

Region: NODE\_260454\_length\_5643\_cov\_34.903244 5288-5299. Max. coverage (+): 0. Max coverage (-): 0.73

Region: NODE\_260454\_length\_5643\_cov\_34.903244 5300-5310. Max. coverage (+): 0. Max coverage (-): 0.11

Region: NODE\_260454\_length\_5643\_cov\_34.903244 5311-5322. Max. coverage (+): 0.08. Max coverage (-): 0.54

Region: NODE\_260454\_length\_5643\_cov\_34.903244 5323-5333. Max. coverage (+): 0. Max coverage (-): 0

Region: NODE\_260454\_length\_5643\_cov\_34.903244 5334-5345. Max. coverage (+): 0.61. Max coverage (-): 0.15

Region: NODE\_260454\_length\_5643\_cov\_34.903244 5346-5356. Max. coverage (+): 0.08. Max coverage (-): 0.61

Region: NODE\_260454\_length\_5643\_cov\_34.903244 5357-5368. Max. coverage (+): 0. Max coverage (-): 0

Region: NODE\_260454\_length\_5643\_cov\_34.903244 5369-5379. Max. coverage (+): 0. Max coverage (-): 0

Region: NODE\_260454\_length\_5643\_cov\_34.903244 5380-5391. Max. coverage (+): 0. Max coverage (-): 0

Region: NODE\_260454\_length\_5643\_cov\_34.903244 5392-5402. Max. coverage (+): 0. Max coverage (-): 0.08

Region: NODE\_260454\_length\_5643\_cov\_34.903244 5403-5414. Max. coverage (+): 0. Max coverage (-): 0.15

Region: NODE\_260454\_length\_5643\_cov\_34.903244 5415-5425. Max. coverage (+): 0. Max coverage (-): 21.15

Region: NODE\_260454\_length\_5643\_cov\_34.903244 5426-5437. Max. coverage (+): 0. Max coverage (-): 1.95

Region: NODE\_260454\_length\_5643\_cov\_34.903244 5438-5449. Max. coverage (+): 0.08. Max coverage (-): 0.08

Region: NODE\_260454\_length\_5643\_cov\_34.903244 5450-5460. Max. coverage (+): 0. Max coverage (-): 0.61

Region: NODE\_260454\_length\_5643\_cov\_34.903244 5461-5472. Max. coverage (+): 0. Max coverage (-): 1.07

Region: NODE\_260454\_length\_5643\_cov\_34.903244 5473-5483. Max. coverage (+): 0. Max coverage (-): 1.3

Region: NODE\_260454\_length\_5643\_cov\_34.903244 5484-5495. Max. coverage (+): 0. Max coverage (-): 0.46

Region: NODE\_260454\_length\_5643\_cov\_34.903244 5496-5506. Max. coverage (+): 0. Max coverage (-): 0.08

Region: NODE\_260454\_length\_5643\_cov\_34.903244 5507-5518. Max. coverage (+): 0. Max coverage (-): 1.92

Region: NODE\_260454\_length\_5643\_cov\_34.903244 5519-5529. Max. coverage (+): 0. Max coverage (-): 0

Region: NODE\_260454\_length\_5643\_cov\_34.903244 5530-5541. Max. coverage (+): 0.15. Max coverage (-): 0.08

Region: NODE\_260454\_length\_5643\_cov\_34.903244 5542-5552. Max. coverage (+): 0. Max coverage (-): 0.23

Region: NODE\_260454\_length\_5643\_cov\_34.903244 5553-5564. Max. coverage (+): 0. Max coverage (-): 0

Region: NODE\_260454\_length\_5643\_cov\_34.903244 5565-5575. Max. coverage (+): 0. Max coverage (-): 0

Region: NODE\_260454\_length\_5643\_cov\_34.903244 5576-5587. Max. coverage (+): 0. Max coverage (-): 0

Region: NODE\_260454\_length\_5643\_cov\_34.903244 5588-5598. Max. coverage (+): 0. Max coverage (-): 0

Region: NODE\_260454\_length\_5643\_cov\_34.903244 5599-5610. Max. coverage (+): 0. Max coverage (-): 0

Region: NODE\_260454\_length\_5643\_cov\_34.903244 5611-5621. Max. coverage (+): 0. Max coverage (-): 0

Region: NODE\_260454\_length\_5643\_cov\_34.903244 5622-5633. Max. coverage (+): 0. Max coverage (-): 0

Region: NODE\_260454\_length\_5643\_cov\_34.903244 5634-5644. Max. coverage (+): 0. Max coverage (-): 0

Region: NODE\_260454\_length\_5643\_cov\_34.903244 5645-5656. Max. coverage (+): 0. Max coverage (-): 0

Region: NODE\_260454\_length\_5643\_cov\_34.903244 5657-5668. Max. coverage (+): 0.15. Max coverage (-): 0.15

Region: NODE\_260454\_length\_5643\_cov\_34.903244 5669-5679. Max. coverage (+): 0.46. Max coverage (-): 0.31

Region: NODE\_260454\_length\_5643\_cov\_34.903244 5680-5691. Max. coverage (+): 0.08. Max coverage (-): 0.38

Region: NODE\_260454\_length\_5643\_cov\_34.903244 5692-5702. Max. coverage (+): 0. Max coverage (-): 1.15

Region: NODE\_260454\_length\_5643\_cov\_34.903244 5703-5714. Max. coverage (+): 0.09. Max coverage (-): 0.31

Region: NODE\_260454\_length\_5643\_cov\_34.903244 5715-5725. Max. coverage (+): 0. Max coverage (-): 0

Region: NODE\_260454\_length\_5643\_cov\_34.903244 5726-5737. Max. coverage (+): 0.04. Max coverage (-): 0.01

Region: NODE\_260454\_length\_5643\_cov\_34.903244 5738-5748. Max. coverage (+): 0.03. Max coverage (-): 0.01

Region: NODE\_260454\_length\_5643\_cov\_34.903244 5749-5760. Max. coverage (+): 0. Max coverage (-): 0

Region: NODE\_260454\_length\_5643\_cov\_34.903244 5761-. Max. coverage (+): 0. Max coverage (-): 0

RepeatMasker Color Code

**+**

100-98% Identity

<98-95% Identity

<95-90% Identity

<90-85% Identity

<85-80% Identity

<80-75% Identity

<75-70% Identity

<70% Identity

**-**

Gene Set Color Code

**+**

Gene

Pseudogene

Other

**-**

Topology/Coverage Color Code

Coverage Plus Strand

Coverage Minus Strand

Mainstrand: Plus

Mainstrand: Minus

Complementary Strand

Flanking Region  
(if option -flank >0)

Gene Set Annotation  
  
RepeatMasker Annotation  

**1. RTE-1\_GA**: 1-63 (+), Divergence to consensus: 8.1%  
**2. AlRepD-4371**: 61-416 (+), Divergence to consensus: 12.4%  
**3. AlRepB-60**: 417-460 (+), Divergence to consensus: 4.5%  
**4. AlRepB-60**: 460-489 (+), Divergence to consensus: 6.7%  
**5. AlRepD-1578**: 496-1253 (+), Divergence to consensus: 21%  
**6. AlRepB-727**: 1314-1568 (-), Divergence to consensus: 23%  
**7. AlRepB-127**: 1533-1584 (+), Divergence to consensus: 15.7%  
**8. AlRepB-127**: 1572-1971 (+), Divergence to consensus: 13.4%  
**9. AlRepB-727**: 1853-2091 (-), Divergence to consensus: 4.6%  
**10. AlRepB-727**: 2163-2266 (-), Divergence to consensus: 3.9%  
**11. AlRepB-727**: 2267-2318 (+), Divergence to consensus: 3.6%  
**12. AlRepE-1478**: 2463-2568 (+), Divergence to consensus: 15.5%  
**13. EnSpm-17\_DR**: 3635-3768 (+), Divergence to consensus: 33.9%  
**14. (TTTG)n**: 3830-3882 (+), Divergence to consensus: 23.9%  
**15. EnSpm-17\_DR**: 3897-3970 (+), Divergence to consensus: 24.3%  
**16. EnSpm-5\_DR**: 4127-4253 (+), Divergence to consensus: 34.2%  
**17. EnSpm-2\_DR**: 4318-4446 (+), Divergence to consensus: 34.1%  
**18. EnSpm-17\_DR**: 4662-4860 (+), Divergence to consensus: 40.8%  
**19. EnSpm-17\_DR**: 5298-5559 (+), Divergence to consensus: 29.8%  
**20. (A)n**: 5609-5632 (+), Divergence to consensus: 8.9%

  
Transcription Factor Binding Sites  

**RHOXF1** (Sequence: GGATTA (-): 60)  
**RHOXF1** (Sequence: AGCTCA (-): 80)  
**RHOXF1** (Sequence: AGATCA (-): 1223)  
**RHOXF1** (Sequence: AGATCA (-): 1385)  
**RHOXF1** (Sequence: AGCTTA (-): 1416)  
**RHOXF1** (Sequence: AGATTA (-): 1459)  
**RHOXF1** (Sequence: AGCTTA (-): 2227)  
**RHOXF1** (Sequence: GGATCA (-): 2410)  
**RHOXF1** (Sequence: AGATCA (-): 3142)  
**RHOXF1** (Sequence: AGCTCA (-): 3466)  
**RHOXF1** (Sequence: TAATCC (+): 364)  
**RHOXF1** (Sequence: TGAGCT (+): 482)  
**RHOXF1** (Sequence: TGAGCC (+): 1719)  
**RHOXF1** (Sequence: TGAGCT (+): 2807)  
**RHOXF1** (Sequence: TGATCC (+): 3271)  
**RHOXF1** (Sequence: TGAGCT (+): 3464)  
**RHOXF1** (Sequence: TGATCC (+): 3912)  
**RHOXF1** (Sequence: TAAGCC (+): 4913)  
**RHOXF1** (Sequence: TAATCC (+): 5044)  
**Lhx8** (Sequence: TTAATTAA (-): 1529)  
**Gata4** (Sequence: GTTATCT (+): 1631)  
**Gata4** (Sequence: GTTATCT (+): 3762)  
**SOX9** (Sequence: AACAATGG (-): 1055)  
**SOX9** (Sequence: AACAATAA (-): 1614)  
**Sox5** (Sequence: ATTGTT (+): 987)  
**Sox5** (Sequence: ATTGTT (+): 1358)  
**Sox5** (Sequence: ATTGTT (+): 1929)  
**Sox5** (Sequence: ATTGTT (+): 2354)  
**Sox5** (Sequence: ATTGTT (+): 5680)  
**SOX9** (Sequence: CCATTGTT (+): 1927)  
**FOXO1** (Sequence: AAAAACAGC (-): 4792)  
**Nobox** (Sequence: TAATTACT (+): 2459)  
**Nobox** (Sequence: TAATTACC (+): 4950)  
**POU2F1** (Sequence: ATTAGAATA (-): 254)  
**Rhox11** (Sequence: TGCTGTTAT (+): 3020)  
**Rhox11** (Sequence: TTAACAGCA (-): 3372)  
**Rhox11** (Sequence: AAAACAGCA (-): 4793)  
**Gata4** (Sequence: AGATAAC (-): 4272)  
**Sox5** (Sequence: AACAAT (-): 166)  
**Sox5** (Sequence: AACAAT (-): 1055)  
**Sox5** (Sequence: AACAAT (-): 1614)  
**Sox5** (Sequence: AACAAT (-): 3610)  
**POU2F1** (Sequence: TATTCAAAT (+): 5239)
