# Supplementary material for: First characterization of PIWI-interacting RNA clusters in a cichlid fish with a B chromosome
Source: BMC Biol. 2022 Sep 21;20:204. doi: 10.1186/s12915-022-01403-2 (PMC9490952; doi:10.1186/s12915-022-01403-2)
Supplement: Supplementary file 1 — Additional file 1. Zipped folder with fasta and interactive html piRNA cluster information for the A. latifasciata genome. The nomenclature is as follows: number-pirna-cluster_sex_B-presence (f, female; m, male; 0b, without B chromosome; 1b, with B chromosome). [file 12915_2022_1403_MOESM1_ESM.zip › 100_f1b.html]

piRNA cluster 100\_f1b 52


Predicted piRNA cluster no. 100\_f1b
  

Show proTRAC run info
Hide proTRAC run info

/\  
                \_\_\_\_\_\_\_\_\_\_\_\_\_\_\_\_\_\_\_\_\_\_\_/\\_\_\_ /  \\_\_\_\_\_\_\_  
               I                      /  \  /    \      I  
               I     pro             /    \/      \     I  
               I        TRAC        /               \   I  
               I   \_\_\_\_\_\_\_\_\_\_\_\_\_\_\_\_/\_\_\_\_\_\_\_\_\_\_\_\_\_\_\_\_\_\\_ I  
               I   \              /                     I  
               I    \            /                      I  
               I     \  /\      /       V.2.4.2         I  
               I      \/  \    /                        I  
               I\_\_\_\_\_\_\_\_\_\_\_\  /\_\_\_\_\_\_\_\_\_\_\_\_\_\_\_\_\_\_\_\_\_\_\_\_\_I  
                            \/  
  
  
================================= proTRAC ====================================  
VERSION: .......... 2.4.2  
LAST MODIFIED: .... 11. May 2018  
  
Please cite:  
Rosenkranz D, Zischler H. proTRAC - a software for probabilistic piRNA cluster  
detection, visualization and analysis. 2012. BMC Bioinformatics 13:5.  
  
  
Contact:  
David Rosenkranz  
Institute of Organismic and Molecular Evolutionary Biology  
Dept. Anthropology, small RNA group  
Johannes Gutenberg University Mainz  
email: rosenkranz@uni-mainz.de  
  
You can find the latest proTRAC version at:  
http://sourceforge.net/projects/protrac/files  
http://www.smallRNAgroup-mainz.de/software  
==============================================================================  
  
PARAMETERS:  
Map file: ...............piwi-femeas-1B.fa-collapse.map  
Genome file: ............../../../0B\_ala\_genome.fa  
RepeatMasker annotation: Alatifasciata-all0B-maryan-v2.fa\_corrected.out  
GeneSet:................./guest-storage/Data/annotation/Alatifasciata\_all0B\_maryan-v2\_out2017.gff  
  
Significant (p<=0.01) hit density will be calculated based  
on observed hit distribution.  
  
Sliding window size: ........................................ 5000 bp  
Sliding window increament: .................................. 1000 bp  
Normalize each hit by number of genomic hits: ............... yes  
Normalize each hit by number of sequence reads: ............. yes  
Normalize values (-> per million mapped reads): ............. yes  
Min. fraction of hits with 1T(U) or 10A: .................... 0.75  
Alternatively: Min. fraction of hits with 1T(U) and 10A: .... 0.5  
Min. fraction of hits with typical piRNA length: ............ 0.75  
Typical piRNA length: ....................................... 24-32 nt  
Min. size of a piRNA cluster: ............................... 1000 bp.  
Min. number of hits (absolute): ............................. 0  
Min. number of hits (normalized): ........................... 0  
Min. fraction of hits on the mainstrand: .................... 0.75  
Top fraction of mapped sequences (in terms of read counts): . 1%  
Top fraction accounts for max. n% of sequence reads: ........ 90%  
Min. fraction of hits on each arm of a bidirectional cluster: 0.05  
Output html file for each cluster: .......................... yes  
Output a summary table: ..................................... yes  
Output a FASTA file for each cluster (piRNA sequences): ..... yes  
Output a FASTA file comprising cluster sequences: ........... yes  
Output a GTF file for predicted piRNA clusters: ..............yes  
Search DNA motifs in clusters: .............................. yes  
Output flanking sequences: +/- .............................. 0 bp  
Output ~.pTi file: .......................................... no  
==============================================================================  
  
  
Genome size (without gaps): ............ 758543724 bp  
Gaps (N/X/-): .......................... 417479 bp  
Mapped reads: .......................... 10641844  
Non-identical sequences: ............... 2832837  
Genomic hits: .......................... 26056853  
Significant densitiy of mapped reads: .. 368.713530323068 reads/kb

Show proTRAC cluster info
Hide proTRAC cluster info

|  |  |
| --- | --- |
| Location | NODE\_260454\_length\_5643\_cov\_34.903244 |
| Coordinates | 5-5765 |
| Size [bp] | 5761 |
| Sequence hit loci | 1165 |
| Mapped reads (normalized) | 2557.7 |
| Mapped reads (normalized) per kb | 444 |
| Normalized reads with 1T (1U) | 82.6% |
| Normalized reads with 10A | 35.3% |
| Normalized reads with length 24-32 nt | 99.2% |
| Normalized reads on the main strand(s) | 91.6% |
| Predicted directionality | mono:minus |

100%

0%

1T (1U)  
reads

10A reads

24-32 nt  
reads

reads on mainstrand

**Either the amount of reads with 1T (1U) OR 10A has to exceed 75% (set with option: -1Tor10A)  
Alternatively the amount of reads with 1T (1U) AND 10A has to exceed 50% (set with option: -1Tand10A)  
Minimum amount of reads with preferred size is 75% (set with option: -pisize)  
Minimum amount of reads on the main strand(s) is 75% (set with option: -clstrand)**

Show read coverage
Hide read coverage

WHAT DO I SEE HERE?  
This chart shows the location of mapped sequence reads within a predicted piRNA cluster. The color refers to the number of genomic hits produced by the sequence read in question. A dark red bar indicates that this sequence read produces many other hits elsewhere in the genome. Many adjacent red or yellow bars can indicate the presence of a multi-copy element such as transposons or rRNA genes. A dark green bar indicates that this sequence read maps uniquely to this locus.

1 hit

2-5 hits

6-10 hits

11-20 hits

21-50 hits

51-100 hits

> 100 hits

NODE\_260454\_length\_5643\_cov\_34.903244

5

5765

Gene Set

RepeatMasker

Mapped  
Reads

23.4

plus strand

minus strand

23.4

Region: NODE\_260454\_length\_5643\_cov\_34.903244 46002-10. Max. coverage (+): 0. Max coverage (-): 0

Region: NODE\_260454\_length\_5643\_cov\_34.903244 11-22. Max. coverage (+): 0. Max coverage (-): 0

Region: NODE\_260454\_length\_5643\_cov\_34.903244 23-33. Max. coverage (+): 0.01. Max coverage (-): 0

Region: NODE\_260454\_length\_5643\_cov\_34.903244 34-45. Max. coverage (+): 0.01. Max coverage (-): 0

Region: NODE\_260454\_length\_5643\_cov\_34.903244 46-56. Max. coverage (+): 0. Max coverage (-): 0

Region: NODE\_260454\_length\_5643\_cov\_34.903244 57-68. Max. coverage (+): 0. Max coverage (-): 0

Region: NODE\_260454\_length\_5643\_cov\_34.903244 69-79. Max. coverage (+): 0. Max coverage (-): 0.28

Region: NODE\_260454\_length\_5643\_cov\_34.903244 80-91. Max. coverage (+): 1.88. Max coverage (-): 0

Region: NODE\_260454\_length\_5643\_cov\_34.903244 92-102. Max. coverage (+): 0. Max coverage (-): 0

Region: NODE\_260454\_length\_5643\_cov\_34.903244 103-114. Max. coverage (+): 0.05. Max coverage (-): 0

Region: NODE\_260454\_length\_5643\_cov\_34.903244 115-125. Max. coverage (+): 0. Max coverage (-): 0

Region: NODE\_260454\_length\_5643\_cov\_34.903244 126-137. Max. coverage (+): 0. Max coverage (-): 0

Region: NODE\_260454\_length\_5643\_cov\_34.903244 138-149. Max. coverage (+): 0. Max coverage (-): 0

Region: NODE\_260454\_length\_5643\_cov\_34.903244 150-160. Max. coverage (+): 0. Max coverage (-): 0

Region: NODE\_260454\_length\_5643\_cov\_34.903244 161-172. Max. coverage (+): 0. Max coverage (-): 0.02

Region: NODE\_260454\_length\_5643\_cov\_34.903244 173-183. Max. coverage (+): 0.13. Max coverage (-): 0.02

Region: NODE\_260454\_length\_5643\_cov\_34.903244 184-195. Max. coverage (+): 0. Max coverage (-): 0.72

Region: NODE\_260454\_length\_5643\_cov\_34.903244 196-206. Max. coverage (+): 0. Max coverage (-): 0.02

Region: NODE\_260454\_length\_5643\_cov\_34.903244 207-218. Max. coverage (+): 0. Max coverage (-): 0

Region: NODE\_260454\_length\_5643\_cov\_34.903244 219-229. Max. coverage (+): 0. Max coverage (-): 0

Region: NODE\_260454\_length\_5643\_cov\_34.903244 230-241. Max. coverage (+): 0. Max coverage (-): 0

Region: NODE\_260454\_length\_5643\_cov\_34.903244 242-252. Max. coverage (+): 0. Max coverage (-): 0

Region: NODE\_260454\_length\_5643\_cov\_34.903244 253-264. Max. coverage (+): 0.09. Max coverage (-): 0.02

Region: NODE\_260454\_length\_5643\_cov\_34.903244 265-275. Max. coverage (+): 0.05. Max coverage (-): 0

Region: NODE\_260454\_length\_5643\_cov\_34.903244 276-287. Max. coverage (+): 0.05. Max coverage (-): 0

Region: NODE\_260454\_length\_5643\_cov\_34.903244 288-298. Max. coverage (+): 0. Max coverage (-): 0

Region: NODE\_260454\_length\_5643\_cov\_34.903244 299-310. Max. coverage (+): 0.22. Max coverage (-): 0

Region: NODE\_260454\_length\_5643\_cov\_34.903244 311-321. Max. coverage (+): 0.09. Max coverage (-): 0

Region: NODE\_260454\_length\_5643\_cov\_34.903244 322-333. Max. coverage (+): 0. Max coverage (-): 0

Region: NODE\_260454\_length\_5643\_cov\_34.903244 334-344. Max. coverage (+): 0. Max coverage (-): 0.09

Region: NODE\_260454\_length\_5643\_cov\_34.903244 345-356. Max. coverage (+): 0. Max coverage (-): 0

Region: NODE\_260454\_length\_5643\_cov\_34.903244 357-367. Max. coverage (+): 0.19. Max coverage (-): 0

Region: NODE\_260454\_length\_5643\_cov\_34.903244 368-379. Max. coverage (+): 0.01. Max coverage (-): 0

Region: NODE\_260454\_length\_5643\_cov\_34.903244 380-390. Max. coverage (+): 0. Max coverage (-): 0

Region: NODE\_260454\_length\_5643\_cov\_34.903244 391-402. Max. coverage (+): 0. Max coverage (-): 0.04

Region: NODE\_260454\_length\_5643\_cov\_34.903244 403-414. Max. coverage (+): 0.11. Max coverage (-): 0

Region: NODE\_260454\_length\_5643\_cov\_34.903244 415-425. Max. coverage (+): 0.13. Max coverage (-): 0

Region: NODE\_260454\_length\_5643\_cov\_34.903244 426-437. Max. coverage (+): 0. Max coverage (-): 0

Region: NODE\_260454\_length\_5643\_cov\_34.903244 438-448. Max. coverage (+): 0. Max coverage (-): 0

Region: NODE\_260454\_length\_5643\_cov\_34.903244 449-460. Max. coverage (+): 0. Max coverage (-): 0

Region: NODE\_260454\_length\_5643\_cov\_34.903244 461-471. Max. coverage (+): 0. Max coverage (-): 0

Region: NODE\_260454\_length\_5643\_cov\_34.903244 472-483. Max. coverage (+): 0. Max coverage (-): 0

Region: NODE\_260454\_length\_5643\_cov\_34.903244 484-494. Max. coverage (+): 0. Max coverage (-): 0

Region: NODE\_260454\_length\_5643\_cov\_34.903244 495-506. Max. coverage (+): 0. Max coverage (-): 0

Region: NODE\_260454\_length\_5643\_cov\_34.903244 507-517. Max. coverage (+): 0. Max coverage (-): 0

Region: NODE\_260454\_length\_5643\_cov\_34.903244 518-529. Max. coverage (+): 0. Max coverage (-): 0

Region: NODE\_260454\_length\_5643\_cov\_34.903244 530-540. Max. coverage (+): 0. Max coverage (-): 0

Region: NODE\_260454\_length\_5643\_cov\_34.903244 541-552. Max. coverage (+): 0. Max coverage (-): 0

Region: NODE\_260454\_length\_5643\_cov\_34.903244 553-563. Max. coverage (+): 0. Max coverage (-): 0

Region: NODE\_260454\_length\_5643\_cov\_34.903244 564-575. Max. coverage (+): 0. Max coverage (-): 0

Region: NODE\_260454\_length\_5643\_cov\_34.903244 576-586. Max. coverage (+): 0. Max coverage (-): 0

Region: NODE\_260454\_length\_5643\_cov\_34.903244 587-598. Max. coverage (+): 0. Max coverage (-): 0

Region: NODE\_260454\_length\_5643\_cov\_34.903244 599-609. Max. coverage (+): 0. Max coverage (-): 0

Region: NODE\_260454\_length\_5643\_cov\_34.903244 610-621. Max. coverage (+): 0.01. Max coverage (-): 0

Region: NODE\_260454\_length\_5643\_cov\_34.903244 622-632. Max. coverage (+): 0. Max coverage (-): 0

Region: NODE\_260454\_length\_5643\_cov\_34.903244 633-644. Max. coverage (+): 0. Max coverage (-): 0

Region: NODE\_260454\_length\_5643\_cov\_34.903244 645-655. Max. coverage (+): 0. Max coverage (-): 0

Region: NODE\_260454\_length\_5643\_cov\_34.903244 656-667. Max. coverage (+): 0. Max coverage (-): 0

Region: NODE\_260454\_length\_5643\_cov\_34.903244 668-679. Max. coverage (+): 0. Max coverage (-): 0.28

Region: NODE\_260454\_length\_5643\_cov\_34.903244 680-690. Max. coverage (+): 0.09. Max coverage (-): 0

Region: NODE\_260454\_length\_5643\_cov\_34.903244 691-702. Max. coverage (+): 0. Max coverage (-): 0

Region: NODE\_260454\_length\_5643\_cov\_34.903244 703-713. Max. coverage (+): 0. Max coverage (-): 0

Region: NODE\_260454\_length\_5643\_cov\_34.903244 714-725. Max. coverage (+): 0. Max coverage (-): 0

Region: NODE\_260454\_length\_5643\_cov\_34.903244 726-736. Max. coverage (+): 0. Max coverage (-): 0

Region: NODE\_260454\_length\_5643\_cov\_34.903244 737-748. Max. coverage (+): 0. Max coverage (-): 0

Region: NODE\_260454\_length\_5643\_cov\_34.903244 749-759. Max. coverage (+): 0. Max coverage (-): 0

Region: NODE\_260454\_length\_5643\_cov\_34.903244 760-771. Max. coverage (+): 0. Max coverage (-): 0

Region: NODE\_260454\_length\_5643\_cov\_34.903244 772-782. Max. coverage (+): 0. Max coverage (-): 0

Region: NODE\_260454\_length\_5643\_cov\_34.903244 783-794. Max. coverage (+): 0. Max coverage (-): 0

Region: NODE\_260454\_length\_5643\_cov\_34.903244 795-805. Max. coverage (+): 0. Max coverage (-): 0

Region: NODE\_260454\_length\_5643\_cov\_34.903244 806-817. Max. coverage (+): 0. Max coverage (-): 0

Region: NODE\_260454\_length\_5643\_cov\_34.903244 818-828. Max. coverage (+): 0. Max coverage (-): 0

Region: NODE\_260454\_length\_5643\_cov\_34.903244 829-840. Max. coverage (+): 0. Max coverage (-): 0

Region: NODE\_260454\_length\_5643\_cov\_34.903244 841-851. Max. coverage (+): 0. Max coverage (-): 0

Region: NODE\_260454\_length\_5643\_cov\_34.903244 852-863. Max. coverage (+): 0. Max coverage (-): 0

Region: NODE\_260454\_length\_5643\_cov\_34.903244 864-874. Max. coverage (+): 0. Max coverage (-): 0

Region: NODE\_260454\_length\_5643\_cov\_34.903244 875-886. Max. coverage (+): 0. Max coverage (-): 0

Region: NODE\_260454\_length\_5643\_cov\_34.903244 887-897. Max. coverage (+): 0. Max coverage (-): 0

Region: NODE\_260454\_length\_5643\_cov\_34.903244 898-909. Max. coverage (+): 0. Max coverage (-): 0

Region: NODE\_260454\_length\_5643\_cov\_34.903244 910-920. Max. coverage (+): 0.09. Max coverage (-): 0

Region: NODE\_260454\_length\_5643\_cov\_34.903244 921-932. Max. coverage (+): 0. Max coverage (-): 0

Region: NODE\_260454\_length\_5643\_cov\_34.903244 933-944. Max. coverage (+): 0. Max coverage (-): 0

Region: NODE\_260454\_length\_5643\_cov\_34.903244 945-955. Max. coverage (+): 0. Max coverage (-): 0

Region: NODE\_260454\_length\_5643\_cov\_34.903244 956-967. Max. coverage (+): 0. Max coverage (-): 0

Region: NODE\_260454\_length\_5643\_cov\_34.903244 968-978. Max. coverage (+): 0. Max coverage (-): 0

Region: NODE\_260454\_length\_5643\_cov\_34.903244 979-990. Max. coverage (+): 0. Max coverage (-): 0

Region: NODE\_260454\_length\_5643\_cov\_34.903244 991-1001. Max. coverage (+): 0. Max coverage (-): 0

Region: NODE\_260454\_length\_5643\_cov\_34.903244 1002-1013. Max. coverage (+): 0. Max coverage (-): 0

Region: NODE\_260454\_length\_5643\_cov\_34.903244 1014-1024. Max. coverage (+): 0. Max coverage (-): 0

Region: NODE\_260454\_length\_5643\_cov\_34.903244 1025-1036. Max. coverage (+): 0. Max coverage (-): 0

Region: NODE\_260454\_length\_5643\_cov\_34.903244 1037-1047. Max. coverage (+): 0. Max coverage (-): 0

Region: NODE\_260454\_length\_5643\_cov\_34.903244 1048-1059. Max. coverage (+): 0. Max coverage (-): 0

Region: NODE\_260454\_length\_5643\_cov\_34.903244 1060-1070. Max. coverage (+): 0. Max coverage (-): 0

Region: NODE\_260454\_length\_5643\_cov\_34.903244 1071-1082. Max. coverage (+): 0. Max coverage (-): 0

Region: NODE\_260454\_length\_5643\_cov\_34.903244 1083-1093. Max. coverage (+): 0. Max coverage (-): 0

Region: NODE\_260454\_length\_5643\_cov\_34.903244 1094-1105. Max. coverage (+): 0. Max coverage (-): 0

Region: NODE\_260454\_length\_5643\_cov\_34.903244 1106-1116. Max. coverage (+): 0. Max coverage (-): 0

Region: NODE\_260454\_length\_5643\_cov\_34.903244 1117-1128. Max. coverage (+): 0. Max coverage (-): 0

Region: NODE\_260454\_length\_5643\_cov\_34.903244 1129-1139. Max. coverage (+): 0. Max coverage (-): 0

Region: NODE\_260454\_length\_5643\_cov\_34.903244 1140-1151. Max. coverage (+): 0. Max coverage (-): 0

Region: NODE\_260454\_length\_5643\_cov\_34.903244 1152-1162. Max. coverage (+): 0. Max coverage (-): 0

Region: NODE\_260454\_length\_5643\_cov\_34.903244 1163-1174. Max. coverage (+): 0. Max coverage (-): 0

Region: NODE\_260454\_length\_5643\_cov\_34.903244 1175-1186. Max. coverage (+): 0. Max coverage (-): 0

Region: NODE\_260454\_length\_5643\_cov\_34.903244 1187-1197. Max. coverage (+): 0. Max coverage (-): 0

Region: NODE\_260454\_length\_5643\_cov\_34.903244 1198-1209. Max. coverage (+): 0. Max coverage (-): 0

Region: NODE\_260454\_length\_5643\_cov\_34.903244 1210-1220. Max. coverage (+): 0. Max coverage (-): 0

Region: NODE\_260454\_length\_5643\_cov\_34.903244 1221-1232. Max. coverage (+): 0. Max coverage (-): 0

Region: NODE\_260454\_length\_5643\_cov\_34.903244 1233-1243. Max. coverage (+): 0. Max coverage (-): 0

Region: NODE\_260454\_length\_5643\_cov\_34.903244 1244-1255. Max. coverage (+): 0. Max coverage (-): 0

Region: NODE\_260454\_length\_5643\_cov\_34.903244 1256-1266. Max. coverage (+): 0. Max coverage (-): 0

Region: NODE\_260454\_length\_5643\_cov\_34.903244 1267-1278. Max. coverage (+): 0. Max coverage (-): 0

Region: NODE\_260454\_length\_5643\_cov\_34.903244 1279-1289. Max. coverage (+): 0. Max coverage (-): 0

Region: NODE\_260454\_length\_5643\_cov\_34.903244 1290-1301. Max. coverage (+): 0. Max coverage (-): 0

Region: NODE\_260454\_length\_5643\_cov\_34.903244 1302-1312. Max. coverage (+): 0. Max coverage (-): 0

Region: NODE\_260454\_length\_5643\_cov\_34.903244 1313-1324. Max. coverage (+): 0. Max coverage (-): 0.02

Region: NODE\_260454\_length\_5643\_cov\_34.903244 1325-1335. Max. coverage (+): 0. Max coverage (-): 0

Region: NODE\_260454\_length\_5643\_cov\_34.903244 1336-1347. Max. coverage (+): 0. Max coverage (-): 0

Region: NODE\_260454\_length\_5643\_cov\_34.903244 1348-1358. Max. coverage (+): 0. Max coverage (-): 0

Region: NODE\_260454\_length\_5643\_cov\_34.903244 1359-1370. Max. coverage (+): 0. Max coverage (-): 0

Region: NODE\_260454\_length\_5643\_cov\_34.903244 1371-1381. Max. coverage (+): 0. Max coverage (-): 0

Region: NODE\_260454\_length\_5643\_cov\_34.903244 1382-1393. Max. coverage (+): 0. Max coverage (-): 0

Region: NODE\_260454\_length\_5643\_cov\_34.903244 1394-1404. Max. coverage (+): 0. Max coverage (-): 0

Region: NODE\_260454\_length\_5643\_cov\_34.903244 1405-1416. Max. coverage (+): 0. Max coverage (-): 0

Region: NODE\_260454\_length\_5643\_cov\_34.903244 1417-1427. Max. coverage (+): 0. Max coverage (-): 0

Region: NODE\_260454\_length\_5643\_cov\_34.903244 1428-1439. Max. coverage (+): 0. Max coverage (-): 0

Region: NODE\_260454\_length\_5643\_cov\_34.903244 1440-1451. Max. coverage (+): 0. Max coverage (-): 0

Region: NODE\_260454\_length\_5643\_cov\_34.903244 1452-1462. Max. coverage (+): 0. Max coverage (-): 0

Region: NODE\_260454\_length\_5643\_cov\_34.903244 1463-1474. Max. coverage (+): 0. Max coverage (-): 0

Region: NODE\_260454\_length\_5643\_cov\_34.903244 1475-1485. Max. coverage (+): 0. Max coverage (-): 0

Region: NODE\_260454\_length\_5643\_cov\_34.903244 1486-1497. Max. coverage (+): 0. Max coverage (-): 0

Region: NODE\_260454\_length\_5643\_cov\_34.903244 1498-1508. Max. coverage (+): 0. Max coverage (-): 0

Region: NODE\_260454\_length\_5643\_cov\_34.903244 1509-1520. Max. coverage (+): 0. Max coverage (-): 0

Region: NODE\_260454\_length\_5643\_cov\_34.903244 1521-1531. Max. coverage (+): 0. Max coverage (-): 0

Region: NODE\_260454\_length\_5643\_cov\_34.903244 1532-1543. Max. coverage (+): 0. Max coverage (-): 0

Region: NODE\_260454\_length\_5643\_cov\_34.903244 1544-1554. Max. coverage (+): 0. Max coverage (-): 0

Region: NODE\_260454\_length\_5643\_cov\_34.903244 1555-1566. Max. coverage (+): 0. Max coverage (-): 0.09

Region: NODE\_260454\_length\_5643\_cov\_34.903244 1567-1577. Max. coverage (+): 0. Max coverage (-): 0

Region: NODE\_260454\_length\_5643\_cov\_34.903244 1578-1589. Max. coverage (+): 0. Max coverage (-): 0

Region: NODE\_260454\_length\_5643\_cov\_34.903244 1590-1600. Max. coverage (+): 0. Max coverage (-): 0

Region: NODE\_260454\_length\_5643\_cov\_34.903244 1601-1612. Max. coverage (+): 0. Max coverage (-): 0

Region: NODE\_260454\_length\_5643\_cov\_34.903244 1613-1623. Max. coverage (+): 0. Max coverage (-): 0

Region: NODE\_260454\_length\_5643\_cov\_34.903244 1624-1635. Max. coverage (+): 0. Max coverage (-): 0

Region: NODE\_260454\_length\_5643\_cov\_34.903244 1636-1646. Max. coverage (+): 0. Max coverage (-): 0

Region: NODE\_260454\_length\_5643\_cov\_34.903244 1647-1658. Max. coverage (+): 0. Max coverage (-): 0

Region: NODE\_260454\_length\_5643\_cov\_34.903244 1659-1669. Max. coverage (+): 0. Max coverage (-): 0

Region: NODE\_260454\_length\_5643\_cov\_34.903244 1670-1681. Max. coverage (+): 0. Max coverage (-): 0

Region: NODE\_260454\_length\_5643\_cov\_34.903244 1682-1692. Max. coverage (+): 0. Max coverage (-): 0

Region: NODE\_260454\_length\_5643\_cov\_34.903244 1693-1704. Max. coverage (+): 0. Max coverage (-): 0

Region: NODE\_260454\_length\_5643\_cov\_34.903244 1705-1716. Max. coverage (+): 0. Max coverage (-): 0

Region: NODE\_260454\_length\_5643\_cov\_34.903244 1717-1727. Max. coverage (+): 0. Max coverage (-): 0

Region: NODE\_260454\_length\_5643\_cov\_34.903244 1728-1739. Max. coverage (+): 0. Max coverage (-): 0

Region: NODE\_260454\_length\_5643\_cov\_34.903244 1740-1750. Max. coverage (+): 0. Max coverage (-): 0

Region: NODE\_260454\_length\_5643\_cov\_34.903244 1751-1762. Max. coverage (+): 0.01. Max coverage (-): 0

Region: NODE\_260454\_length\_5643\_cov\_34.903244 1763-1773. Max. coverage (+): 0. Max coverage (-): 0

Region: NODE\_260454\_length\_5643\_cov\_34.903244 1774-1785. Max. coverage (+): 0. Max coverage (-): 0

Region: NODE\_260454\_length\_5643\_cov\_34.903244 1786-1796. Max. coverage (+): 0.01. Max coverage (-): 0

Region: NODE\_260454\_length\_5643\_cov\_34.903244 1797-1808. Max. coverage (+): 0.01. Max coverage (-): 0

Region: NODE\_260454\_length\_5643\_cov\_34.903244 1809-1819. Max. coverage (+): 0. Max coverage (-): 0

Region: NODE\_260454\_length\_5643\_cov\_34.903244 1820-1831. Max. coverage (+): 0.01. Max coverage (-): 0

Region: NODE\_260454\_length\_5643\_cov\_34.903244 1832-1842. Max. coverage (+): 0. Max coverage (-): 0

Region: NODE\_260454\_length\_5643\_cov\_34.903244 1843-1854. Max. coverage (+): 0. Max coverage (-): 0

Region: NODE\_260454\_length\_5643\_cov\_34.903244 1855-1865. Max. coverage (+): 0. Max coverage (-): 0

Region: NODE\_260454\_length\_5643\_cov\_34.903244 1866-1877. Max. coverage (+): 0. Max coverage (-): 0

Region: NODE\_260454\_length\_5643\_cov\_34.903244 1878-1888. Max. coverage (+): 0. Max coverage (-): 0

Region: NODE\_260454\_length\_5643\_cov\_34.903244 1889-1900. Max. coverage (+): 0. Max coverage (-): 0

Region: NODE\_260454\_length\_5643\_cov\_34.903244 1901-1911. Max. coverage (+): 0. Max coverage (-): 0

Region: NODE\_260454\_length\_5643\_cov\_34.903244 1912-1923. Max. coverage (+): 0. Max coverage (-): 0.01

Region: NODE\_260454\_length\_5643\_cov\_34.903244 1924-1934. Max. coverage (+): 0.03. Max coverage (-): 0

Region: NODE\_260454\_length\_5643\_cov\_34.903244 1935-1946. Max. coverage (+): 0. Max coverage (-): 0.02

Region: NODE\_260454\_length\_5643\_cov\_34.903244 1947-1957. Max. coverage (+): 0. Max coverage (-): 0.01

Region: NODE\_260454\_length\_5643\_cov\_34.903244 1958-1969. Max. coverage (+): 0.04. Max coverage (-): 0

Region: NODE\_260454\_length\_5643\_cov\_34.903244 1970-1981. Max. coverage (+): 0. Max coverage (-): 0

Region: NODE\_260454\_length\_5643\_cov\_34.903244 1982-1992. Max. coverage (+): 0. Max coverage (-): 0

Region: NODE\_260454\_length\_5643\_cov\_34.903244 1993-2004. Max. coverage (+): 0. Max coverage (-): 0

Region: NODE\_260454\_length\_5643\_cov\_34.903244 2005-2015. Max. coverage (+): 0. Max coverage (-): 0

Region: NODE\_260454\_length\_5643\_cov\_34.903244 2016-2027. Max. coverage (+): 0. Max coverage (-): 0

Region: NODE\_260454\_length\_5643\_cov\_34.903244 2028-2038. Max. coverage (+): 0. Max coverage (-): 0.01

Region: NODE\_260454\_length\_5643\_cov\_34.903244 2039-2050. Max. coverage (+): 0. Max coverage (-): 0

Region: NODE\_260454\_length\_5643\_cov\_34.903244 2051-2061. Max. coverage (+): 0.01. Max coverage (-): 0

Region: NODE\_260454\_length\_5643\_cov\_34.903244 2062-2073. Max. coverage (+): 0. Max coverage (-): 0

Region: NODE\_260454\_length\_5643\_cov\_34.903244 2074-2084. Max. coverage (+): 0. Max coverage (-): 0

Region: NODE\_260454\_length\_5643\_cov\_34.903244 2085-2096. Max. coverage (+): 0. Max coverage (-): 0

Region: NODE\_260454\_length\_5643\_cov\_34.903244 2097-2107. Max. coverage (+): 0. Max coverage (-): 0

Region: NODE\_260454\_length\_5643\_cov\_34.903244 2108-2119. Max. coverage (+): 0. Max coverage (-): 0

Region: NODE\_260454\_length\_5643\_cov\_34.903244 2120-2130. Max. coverage (+): 0. Max coverage (-): 0

Region: NODE\_260454\_length\_5643\_cov\_34.903244 2131-2142. Max. coverage (+): 0. Max coverage (-): 0

Region: NODE\_260454\_length\_5643\_cov\_34.903244 2143-2153. Max. coverage (+): 0. Max coverage (-): 0

Region: NODE\_260454\_length\_5643\_cov\_34.903244 2154-2165. Max. coverage (+): 0. Max coverage (-): 0

Region: NODE\_260454\_length\_5643\_cov\_34.903244 2166-2176. Max. coverage (+): 0. Max coverage (-): 0

Region: NODE\_260454\_length\_5643\_cov\_34.903244 2177-2188. Max. coverage (+): 0.01. Max coverage (-): 0

Region: NODE\_260454\_length\_5643\_cov\_34.903244 2189-2199. Max. coverage (+): 0. Max coverage (-): 0

Region: NODE\_260454\_length\_5643\_cov\_34.903244 2200-2211. Max. coverage (+): 0. Max coverage (-): 0

Region: NODE\_260454\_length\_5643\_cov\_34.903244 2212-2222. Max. coverage (+): 0.03. Max coverage (-): 0

Region: NODE\_260454\_length\_5643\_cov\_34.903244 2223-2234. Max. coverage (+): 0. Max coverage (-): 0

Region: NODE\_260454\_length\_5643\_cov\_34.903244 2235-2246. Max. coverage (+): 0. Max coverage (-): 0

Region: NODE\_260454\_length\_5643\_cov\_34.903244 2247-2257. Max. coverage (+): 0. Max coverage (-): 0

Region: NODE\_260454\_length\_5643\_cov\_34.903244 2258-2269. Max. coverage (+): 0. Max coverage (-): 0

Region: NODE\_260454\_length\_5643\_cov\_34.903244 2270-2280. Max. coverage (+): 0. Max coverage (-): 0

Region: NODE\_260454\_length\_5643\_cov\_34.903244 2281-2292. Max. coverage (+): 0. Max coverage (-): 0

Region: NODE\_260454\_length\_5643\_cov\_34.903244 2293-2303. Max. coverage (+): 0.3. Max coverage (-): 0.09

Region: NODE\_260454\_length\_5643\_cov\_34.903244 2304-2315. Max. coverage (+): 0. Max coverage (-): 0

Region: NODE\_260454\_length\_5643\_cov\_34.903244 2316-2326. Max. coverage (+): 0. Max coverage (-): 0

Region: NODE\_260454\_length\_5643\_cov\_34.903244 2327-2338. Max. coverage (+): 0.19. Max coverage (-): 0.09

Region: NODE\_260454\_length\_5643\_cov\_34.903244 2339-2349. Max. coverage (+): 0. Max coverage (-): 0.09

Region: NODE\_260454\_length\_5643\_cov\_34.903244 2350-2361. Max. coverage (+): 0. Max coverage (-): 0.09

Region: NODE\_260454\_length\_5643\_cov\_34.903244 2362-2372. Max. coverage (+): 0. Max coverage (-): 0.09

Region: NODE\_260454\_length\_5643\_cov\_34.903244 2373-2384. Max. coverage (+): 0. Max coverage (-): 0.09

Region: NODE\_260454\_length\_5643\_cov\_34.903244 2385-2395. Max. coverage (+): 0. Max coverage (-): 0

Region: NODE\_260454\_length\_5643\_cov\_34.903244 2396-2407. Max. coverage (+): 0. Max coverage (-): 0.09

Region: NODE\_260454\_length\_5643\_cov\_34.903244 2408-2418. Max. coverage (+): 0. Max coverage (-): 0

Region: NODE\_260454\_length\_5643\_cov\_34.903244 2419-2430. Max. coverage (+): 0. Max coverage (-): 0

Region: NODE\_260454\_length\_5643\_cov\_34.903244 2431-2441. Max. coverage (+): 0. Max coverage (-): 0

Region: NODE\_260454\_length\_5643\_cov\_34.903244 2442-2453. Max. coverage (+): 0. Max coverage (-): 0

Region: NODE\_260454\_length\_5643\_cov\_34.903244 2454-2464. Max. coverage (+): 0. Max coverage (-): 0

Region: NODE\_260454\_length\_5643\_cov\_34.903244 2465-2476. Max. coverage (+): 0. Max coverage (-): 0.19

Region: NODE\_260454\_length\_5643\_cov\_34.903244 2477-2487. Max. coverage (+): 0. Max coverage (-): 0.09

Region: NODE\_260454\_length\_5643\_cov\_34.903244 2488-2499. Max. coverage (+): 0. Max coverage (-): 0.09

Region: NODE\_260454\_length\_5643\_cov\_34.903244 2500-2511. Max. coverage (+): 0.09. Max coverage (-): 0

Region: NODE\_260454\_length\_5643\_cov\_34.903244 2512-2522. Max. coverage (+): 0. Max coverage (-): 0.56

Region: NODE\_260454\_length\_5643\_cov\_34.903244 2523-2534. Max. coverage (+): 0. Max coverage (-): 0.28

Region: NODE\_260454\_length\_5643\_cov\_34.903244 2535-2545. Max. coverage (+): 0. Max coverage (-): 0.09

Region: NODE\_260454\_length\_5643\_cov\_34.903244 2546-2557. Max. coverage (+): 0. Max coverage (-): 0

Region: NODE\_260454\_length\_5643\_cov\_34.903244 2558-2568. Max. coverage (+): 0. Max coverage (-): 0.38

Region: NODE\_260454\_length\_5643\_cov\_34.903244 2569-2580. Max. coverage (+): 0. Max coverage (-): 0.09

Region: NODE\_260454\_length\_5643\_cov\_34.903244 2581-2591. Max. coverage (+): 0. Max coverage (-): 0.09

Region: NODE\_260454\_length\_5643\_cov\_34.903244 2592-2603. Max. coverage (+): 0. Max coverage (-): 0.09

Region: NODE\_260454\_length\_5643\_cov\_34.903244 2604-2614. Max. coverage (+): 0. Max coverage (-): 0

Region: NODE\_260454\_length\_5643\_cov\_34.903244 2615-2626. Max. coverage (+): 0. Max coverage (-): 0

Region: NODE\_260454\_length\_5643\_cov\_34.903244 2627-2637. Max. coverage (+): 0. Max coverage (-): 0

Region: NODE\_260454\_length\_5643\_cov\_34.903244 2638-2649. Max. coverage (+): 0. Max coverage (-): 0.85

Region: NODE\_260454\_length\_5643\_cov\_34.903244 2650-2660. Max. coverage (+): 0. Max coverage (-): 0

Region: NODE\_260454\_length\_5643\_cov\_34.903244 2661-2672. Max. coverage (+): 0. Max coverage (-): 0.09

Region: NODE\_260454\_length\_5643\_cov\_34.903244 2673-2683. Max. coverage (+): 0.09. Max coverage (-): 0

Region: NODE\_260454\_length\_5643\_cov\_34.903244 2684-2695. Max. coverage (+): 0.09. Max coverage (-): 0.09

Region: NODE\_260454\_length\_5643\_cov\_34.903244 2696-2706. Max. coverage (+): 0. Max coverage (-): 0.09

Region: NODE\_260454\_length\_5643\_cov\_34.903244 2707-2718. Max. coverage (+): 0. Max coverage (-): 0.56

Region: NODE\_260454\_length\_5643\_cov\_34.903244 2719-2729. Max. coverage (+): 0. Max coverage (-): 3.1

Region: NODE\_260454\_length\_5643\_cov\_34.903244 2730-2741. Max. coverage (+): 0. Max coverage (-): 0.28

Region: NODE\_260454\_length\_5643\_cov\_34.903244 2742-2752. Max. coverage (+): 0.09. Max coverage (-): 1.32

Region: NODE\_260454\_length\_5643\_cov\_34.903244 2753-2764. Max. coverage (+): 0. Max coverage (-): 0.85

Region: NODE\_260454\_length\_5643\_cov\_34.903244 2765-2776. Max. coverage (+): 0. Max coverage (-): 0.85

Region: NODE\_260454\_length\_5643\_cov\_34.903244 2777-2787. Max. coverage (+): 0. Max coverage (-): 0

Region: NODE\_260454\_length\_5643\_cov\_34.903244 2788-2799. Max. coverage (+): 0. Max coverage (-): 0.94

Region: NODE\_260454\_length\_5643\_cov\_34.903244 2800-2810. Max. coverage (+): 0.19. Max coverage (-): 5.54

Region: NODE\_260454\_length\_5643\_cov\_34.903244 2811-2822. Max. coverage (+): 0.09. Max coverage (-): 7.8

Region: NODE\_260454\_length\_5643\_cov\_34.903244 2823-2833. Max. coverage (+): 0.09. Max coverage (-): 1.13

Region: NODE\_260454\_length\_5643\_cov\_34.903244 2834-2845. Max. coverage (+): 0.09. Max coverage (-): 0

Region: NODE\_260454\_length\_5643\_cov\_34.903244 2846-2856. Max. coverage (+): 0. Max coverage (-): 0.47

Region: NODE\_260454\_length\_5643\_cov\_34.903244 2857-2868. Max. coverage (+): 0.28. Max coverage (-): 2.44

Region: NODE\_260454\_length\_5643\_cov\_34.903244 2869-2879. Max. coverage (+): 0.09. Max coverage (-): 0

Region: NODE\_260454\_length\_5643\_cov\_34.903244 2880-2891. Max. coverage (+): 0. Max coverage (-): 0.94

Region: NODE\_260454\_length\_5643\_cov\_34.903244 2892-2902. Max. coverage (+): 0. Max coverage (-): 1.41

Region: NODE\_260454\_length\_5643\_cov\_34.903244 2903-2914. Max. coverage (+): 0. Max coverage (-): 0

Region: NODE\_260454\_length\_5643\_cov\_34.903244 2915-2925. Max. coverage (+): 0.28. Max coverage (-): 0.09

Region: NODE\_260454\_length\_5643\_cov\_34.903244 2926-2937. Max. coverage (+): 0.28. Max coverage (-): 0.28

Region: NODE\_260454\_length\_5643\_cov\_34.903244 2938-2948. Max. coverage (+): 0. Max coverage (-): 3.24

Region: NODE\_260454\_length\_5643\_cov\_34.903244 2949-2960. Max. coverage (+): 0.47. Max coverage (-): 0.19

Region: NODE\_260454\_length\_5643\_cov\_34.903244 2961-2971. Max. coverage (+): 0.09. Max coverage (-): 0

Region: NODE\_260454\_length\_5643\_cov\_34.903244 2972-2983. Max. coverage (+): 0. Max coverage (-): 0.09

Region: NODE\_260454\_length\_5643\_cov\_34.903244 2984-2994. Max. coverage (+): 0. Max coverage (-): 0.09

Region: NODE\_260454\_length\_5643\_cov\_34.903244 2995-3006. Max. coverage (+): 0. Max coverage (-): 0.09

Region: NODE\_260454\_length\_5643\_cov\_34.903244 3007-3018. Max. coverage (+): 0. Max coverage (-): 6.81

Region: NODE\_260454\_length\_5643\_cov\_34.903244 3019-3029. Max. coverage (+): 0.19. Max coverage (-): 0

Region: NODE\_260454\_length\_5643\_cov\_34.903244 3030-3041. Max. coverage (+): 0.19. Max coverage (-): 0

Region: NODE\_260454\_length\_5643\_cov\_34.903244 3042-3052. Max. coverage (+): 0. Max coverage (-): 16.54

Region: NODE\_260454\_length\_5643\_cov\_34.903244 3053-3064. Max. coverage (+): 0. Max coverage (-): 17.1

Region: NODE\_260454\_length\_5643\_cov\_34.903244 3065-3075. Max. coverage (+): 0. Max coverage (-): 0

Region: NODE\_260454\_length\_5643\_cov\_34.903244 3076-3087. Max. coverage (+): 0. Max coverage (-): 0

Region: NODE\_260454\_length\_5643\_cov\_34.903244 3088-3098. Max. coverage (+): 0. Max coverage (-): 0

Region: NODE\_260454\_length\_5643\_cov\_34.903244 3099-3110. Max. coverage (+): 0. Max coverage (-): 0.09

Region: NODE\_260454\_length\_5643\_cov\_34.903244 3111-3121. Max. coverage (+): 0. Max coverage (-): 0.09

Region: NODE\_260454\_length\_5643\_cov\_34.903244 3122-3133. Max. coverage (+): 0.09. Max coverage (-): 0

Region: NODE\_260454\_length\_5643\_cov\_34.903244 3134-3144. Max. coverage (+): 0.19. Max coverage (-): 0.94

Region: NODE\_260454\_length\_5643\_cov\_34.903244 3145-3156. Max. coverage (+): 0. Max coverage (-): 1.03

Region: NODE\_260454\_length\_5643\_cov\_34.903244 3157-3167. Max. coverage (+): 0. Max coverage (-): 0.38

Region: NODE\_260454\_length\_5643\_cov\_34.903244 3168-3179. Max. coverage (+): 0. Max coverage (-): 0.28

Region: NODE\_260454\_length\_5643\_cov\_34.903244 3180-3190. Max. coverage (+): 0. Max coverage (-): 0.19

Region: NODE\_260454\_length\_5643\_cov\_34.903244 3191-3202. Max. coverage (+): 0. Max coverage (-): 0.28

Region: NODE\_260454\_length\_5643\_cov\_34.903244 3203-3213. Max. coverage (+): 0.19. Max coverage (-): 0.56

Region: NODE\_260454\_length\_5643\_cov\_34.903244 3214-3225. Max. coverage (+): 0.19. Max coverage (-): 0.28

Region: NODE\_260454\_length\_5643\_cov\_34.903244 3226-3236. Max. coverage (+): 0. Max coverage (-): 6.48

Region: NODE\_260454\_length\_5643\_cov\_34.903244 3237-3248. Max. coverage (+): 3.19. Max coverage (-): 0.94

Region: NODE\_260454\_length\_5643\_cov\_34.903244 3249-3259. Max. coverage (+): 3.19. Max coverage (-): 0.09

Region: NODE\_260454\_length\_5643\_cov\_34.903244 3260-3271. Max. coverage (+): 0. Max coverage (-): 4.89

Region: NODE\_260454\_length\_5643\_cov\_34.903244 3272-3283. Max. coverage (+): 0.66. Max coverage (-): 0.09

Region: NODE\_260454\_length\_5643\_cov\_34.903244 3284-3294. Max. coverage (+): 0. Max coverage (-): 0.28

Region: NODE\_260454\_length\_5643\_cov\_34.903244 3295-3306. Max. coverage (+): 0. Max coverage (-): 1.6

Region: NODE\_260454\_length\_5643\_cov\_34.903244 3307-3317. Max. coverage (+): 0. Max coverage (-): 0.09

Region: NODE\_260454\_length\_5643\_cov\_34.903244 3318-3329. Max. coverage (+): 0. Max coverage (-): 0.28

Region: NODE\_260454\_length\_5643\_cov\_34.903244 3330-3340. Max. coverage (+): 0. Max coverage (-): 0.42

Region: NODE\_260454\_length\_5643\_cov\_34.903244 3341-3352. Max. coverage (+): 0. Max coverage (-): 0.09

Region: NODE\_260454\_length\_5643\_cov\_34.903244 3353-3363. Max. coverage (+): 0. Max coverage (-): 0.09

Region: NODE\_260454\_length\_5643\_cov\_34.903244 3364-3375. Max. coverage (+): 0. Max coverage (-): 0.19

Region: NODE\_260454\_length\_5643\_cov\_34.903244 3376-3386. Max. coverage (+): 0. Max coverage (-): 0

Region: NODE\_260454\_length\_5643\_cov\_34.903244 3387-3398. Max. coverage (+): 0. Max coverage (-): 0

Region: NODE\_260454\_length\_5643\_cov\_34.903244 3399-3409. Max. coverage (+): 0. Max coverage (-): 0.09

Region: NODE\_260454\_length\_5643\_cov\_34.903244 3410-3421. Max. coverage (+): 0. Max coverage (-): 0

Region: NODE\_260454\_length\_5643\_cov\_34.903244 3422-3432. Max. coverage (+): 0. Max coverage (-): 0

Region: NODE\_260454\_length\_5643\_cov\_34.903244 3433-3444. Max. coverage (+): 0. Max coverage (-): 0.09

Region: NODE\_260454\_length\_5643\_cov\_34.903244 3445-3455. Max. coverage (+): 0. Max coverage (-): 0.28

Region: NODE\_260454\_length\_5643\_cov\_34.903244 3456-3467. Max. coverage (+): 0. Max coverage (-): 0

Region: NODE\_260454\_length\_5643\_cov\_34.903244 3468-3478. Max. coverage (+): 0. Max coverage (-): 0.66

Region: NODE\_260454\_length\_5643\_cov\_34.903244 3479-3490. Max. coverage (+): 0. Max coverage (-): 1.6

Region: NODE\_260454\_length\_5643\_cov\_34.903244 3491-3501. Max. coverage (+): 2.73. Max coverage (-): 0

Region: NODE\_260454\_length\_5643\_cov\_34.903244 3502-3513. Max. coverage (+): 0. Max coverage (-): 0.85

Region: NODE\_260454\_length\_5643\_cov\_34.903244 3514-3524. Max. coverage (+): 0. Max coverage (-): 0.28

Region: NODE\_260454\_length\_5643\_cov\_34.903244 3525-3536. Max. coverage (+): 0. Max coverage (-): 1.6

Region: NODE\_260454\_length\_5643\_cov\_34.903244 3537-3548. Max. coverage (+): 0. Max coverage (-): 0

Region: NODE\_260454\_length\_5643\_cov\_34.903244 3549-3559. Max. coverage (+): 0. Max coverage (-): 0.09

Region: NODE\_260454\_length\_5643\_cov\_34.903244 3560-3571. Max. coverage (+): 0. Max coverage (-): 0.19

Region: NODE\_260454\_length\_5643\_cov\_34.903244 3572-3582. Max. coverage (+): 0. Max coverage (-): 0.28

Region: NODE\_260454\_length\_5643\_cov\_34.903244 3583-3594. Max. coverage (+): 0. Max coverage (-): 0.19

Region: NODE\_260454\_length\_5643\_cov\_34.903244 3595-3605. Max. coverage (+): 0. Max coverage (-): 3.1

Region: NODE\_260454\_length\_5643\_cov\_34.903244 3606-3617. Max. coverage (+): 0.09. Max coverage (-): 0.28

Region: NODE\_260454\_length\_5643\_cov\_34.903244 3618-3628. Max. coverage (+): 0.09. Max coverage (-): 0.19

Region: NODE\_260454\_length\_5643\_cov\_34.903244 3629-3640. Max. coverage (+): 0. Max coverage (-): 0.09

Region: NODE\_260454\_length\_5643\_cov\_34.903244 3641-3651. Max. coverage (+): 0. Max coverage (-): 0

Region: NODE\_260454\_length\_5643\_cov\_34.903244 3652-3663. Max. coverage (+): 0.09. Max coverage (-): 0.47

Region: NODE\_260454\_length\_5643\_cov\_34.903244 3664-3674. Max. coverage (+): 0. Max coverage (-): 0.38

Region: NODE\_260454\_length\_5643\_cov\_34.903244 3675-3686. Max. coverage (+): 0.09. Max coverage (-): 0

Region: NODE\_260454\_length\_5643\_cov\_34.903244 3687-3697. Max. coverage (+): 0. Max coverage (-): 0

Region: NODE\_260454\_length\_5643\_cov\_34.903244 3698-3709. Max. coverage (+): 0. Max coverage (-): 0.05

Region: NODE\_260454\_length\_5643\_cov\_34.903244 3710-3720. Max. coverage (+): 0.47. Max coverage (-): 0.09

Region: NODE\_260454\_length\_5643\_cov\_34.903244 3721-3732. Max. coverage (+): 0. Max coverage (-): 0.38

Region: NODE\_260454\_length\_5643\_cov\_34.903244 3733-3743. Max. coverage (+): 0. Max coverage (-): 0.28

Region: NODE\_260454\_length\_5643\_cov\_34.903244 3744-3755. Max. coverage (+): 0. Max coverage (-): 0.38

Region: NODE\_260454\_length\_5643\_cov\_34.903244 3756-3766. Max. coverage (+): 0.09. Max coverage (-): 0.05

Region: NODE\_260454\_length\_5643\_cov\_34.903244 3767-3778. Max. coverage (+): 0.05. Max coverage (-): 0

Region: NODE\_260454\_length\_5643\_cov\_34.903244 3779-3789. Max. coverage (+): 0.09. Max coverage (-): 1.03

Region: NODE\_260454\_length\_5643\_cov\_34.903244 3790-3801. Max. coverage (+): 0.09. Max coverage (-): 9.3

Region: NODE\_260454\_length\_5643\_cov\_34.903244 3802-3813. Max. coverage (+): 0. Max coverage (-): 0

Region: NODE\_260454\_length\_5643\_cov\_34.903244 3814-3824. Max. coverage (+): 0. Max coverage (-): 0

Region: NODE\_260454\_length\_5643\_cov\_34.903244 3825-3836. Max. coverage (+): 0. Max coverage (-): 0

Region: NODE\_260454\_length\_5643\_cov\_34.903244 3837-3847. Max. coverage (+): 0. Max coverage (-): 0

Region: NODE\_260454\_length\_5643\_cov\_34.903244 3848-3859. Max. coverage (+): 0. Max coverage (-): 0

Region: NODE\_260454\_length\_5643\_cov\_34.903244 3860-3870. Max. coverage (+): 0. Max coverage (-): 0.09

Region: NODE\_260454\_length\_5643\_cov\_34.903244 3871-3882. Max. coverage (+): 0. Max coverage (-): 0.09

Region: NODE\_260454\_length\_5643\_cov\_34.903244 3883-3893. Max. coverage (+): 0.09. Max coverage (-): 0

Region: NODE\_260454\_length\_5643\_cov\_34.903244 3894-3905. Max. coverage (+): 0. Max coverage (-): 1.79

Region: NODE\_260454\_length\_5643\_cov\_34.903244 3906-3916. Max. coverage (+): 0. Max coverage (-): 0.38

Region: NODE\_260454\_length\_5643\_cov\_34.903244 3917-3928. Max. coverage (+): 0. Max coverage (-): 0.09

Region: NODE\_260454\_length\_5643\_cov\_34.903244 3929-3939. Max. coverage (+): 0. Max coverage (-): 0

Region: NODE\_260454\_length\_5643\_cov\_34.903244 3940-3951. Max. coverage (+): 0. Max coverage (-): 7.05

Region: NODE\_260454\_length\_5643\_cov\_34.903244 3952-3962. Max. coverage (+): 0. Max coverage (-): 0.09

Region: NODE\_260454\_length\_5643\_cov\_34.903244 3963-3974. Max. coverage (+): 0. Max coverage (-): 0.23

Region: NODE\_260454\_length\_5643\_cov\_34.903244 3975-3985. Max. coverage (+): 0. Max coverage (-): 0.28

Region: NODE\_260454\_length\_5643\_cov\_34.903244 3986-3997. Max. coverage (+): 0. Max coverage (-): 0.28

Region: NODE\_260454\_length\_5643\_cov\_34.903244 3998-4008. Max. coverage (+): 0. Max coverage (-): 1.69

Region: NODE\_260454\_length\_5643\_cov\_34.903244 4009-4020. Max. coverage (+): 0. Max coverage (-): 2.96

Region: NODE\_260454\_length\_5643\_cov\_34.903244 4021-4031. Max. coverage (+): 0.75. Max coverage (-): 0

Region: NODE\_260454\_length\_5643\_cov\_34.903244 4032-4043. Max. coverage (+): 0.56. Max coverage (-): 0.09

Region: NODE\_260454\_length\_5643\_cov\_34.903244 4044-4054. Max. coverage (+): 0. Max coverage (-): 0.28

Region: NODE\_260454\_length\_5643\_cov\_34.903244 4055-4066. Max. coverage (+): 0. Max coverage (-): 0.19

Region: NODE\_260454\_length\_5643\_cov\_34.903244 4067-4078. Max. coverage (+): 0. Max coverage (-): 0.19

Region: NODE\_260454\_length\_5643\_cov\_34.903244 4079-4089. Max. coverage (+): 0.09. Max coverage (-): 0.66

Region: NODE\_260454\_length\_5643\_cov\_34.903244 4090-4101. Max. coverage (+): 0.28. Max coverage (-): 0

Region: NODE\_260454\_length\_5643\_cov\_34.903244 4102-4112. Max. coverage (+): 0.09. Max coverage (-): 0.47

Region: NODE\_260454\_length\_5643\_cov\_34.903244 4113-4124. Max. coverage (+): 0. Max coverage (-): 0.09

Region: NODE\_260454\_length\_5643\_cov\_34.903244 4125-4135. Max. coverage (+): 0.28. Max coverage (-): 0.09

Region: NODE\_260454\_length\_5643\_cov\_34.903244 4136-4147. Max. coverage (+): 0. Max coverage (-): 0.09

Region: NODE\_260454\_length\_5643\_cov\_34.903244 4148-4158. Max. coverage (+): 0. Max coverage (-): 0

Region: NODE\_260454\_length\_5643\_cov\_34.903244 4159-4170. Max. coverage (+): 0. Max coverage (-): 0.38

Region: NODE\_260454\_length\_5643\_cov\_34.903244 4171-4181. Max. coverage (+): 0. Max coverage (-): 0

Region: NODE\_260454\_length\_5643\_cov\_34.903244 4182-4193. Max. coverage (+): 0. Max coverage (-): 0.38

Region: NODE\_260454\_length\_5643\_cov\_34.903244 4194-4204. Max. coverage (+): 0. Max coverage (-): 0.09

Region: NODE\_260454\_length\_5643\_cov\_34.903244 4205-4216. Max. coverage (+): 0. Max coverage (-): 0

Region: NODE\_260454\_length\_5643\_cov\_34.903244 4217-4227. Max. coverage (+): 0. Max coverage (-): 0.09

Region: NODE\_260454\_length\_5643\_cov\_34.903244 4228-4239. Max. coverage (+): 0. Max coverage (-): 0.09

Region: NODE\_260454\_length\_5643\_cov\_34.903244 4240-4250. Max. coverage (+): 0. Max coverage (-): 0.09

Region: NODE\_260454\_length\_5643\_cov\_34.903244 4251-4262. Max. coverage (+): 0.09. Max coverage (-): 0.09

Region: NODE\_260454\_length\_5643\_cov\_34.903244 4263-4273. Max. coverage (+): 0.28. Max coverage (-): 0.28

Region: NODE\_260454\_length\_5643\_cov\_34.903244 4274-4285. Max. coverage (+): 0.09. Max coverage (-): 0.19

Region: NODE\_260454\_length\_5643\_cov\_34.903244 4286-4296. Max. coverage (+): 0.09. Max coverage (-): 0

Region: NODE\_260454\_length\_5643\_cov\_34.903244 4297-4308. Max. coverage (+): 0. Max coverage (-): 1.6

Region: NODE\_260454\_length\_5643\_cov\_34.903244 4309-4319. Max. coverage (+): 0. Max coverage (-): 0.09

Region: NODE\_260454\_length\_5643\_cov\_34.903244 4320-4331. Max. coverage (+): 0.09. Max coverage (-): 0.19

Region: NODE\_260454\_length\_5643\_cov\_34.903244 4332-4343. Max. coverage (+): 0. Max coverage (-): 1.03

Region: NODE\_260454\_length\_5643\_cov\_34.903244 4344-4354. Max. coverage (+): 0.47. Max coverage (-): 0

Region: NODE\_260454\_length\_5643\_cov\_34.903244 4355-4366. Max. coverage (+): 0. Max coverage (-): 0.94

Region: NODE\_260454\_length\_5643\_cov\_34.903244 4367-4377. Max. coverage (+): 0. Max coverage (-): 1.13

Region: NODE\_260454\_length\_5643\_cov\_34.903244 4378-4389. Max. coverage (+): 0. Max coverage (-): 0.94

Region: NODE\_260454\_length\_5643\_cov\_34.903244 4390-4400. Max. coverage (+): 0. Max coverage (-): 1.13

Region: NODE\_260454\_length\_5643\_cov\_34.903244 4401-4412. Max. coverage (+): 0. Max coverage (-): 0

Region: NODE\_260454\_length\_5643\_cov\_34.903244 4413-4423. Max. coverage (+): 0. Max coverage (-): 0.66

Region: NODE\_260454\_length\_5643\_cov\_34.903244 4424-4435. Max. coverage (+): 0. Max coverage (-): 0.47

Region: NODE\_260454\_length\_5643\_cov\_34.903244 4436-4446. Max. coverage (+): 0. Max coverage (-): 0.19

Region: NODE\_260454\_length\_5643\_cov\_34.903244 4447-4458. Max. coverage (+): 0. Max coverage (-): 3.1

Region: NODE\_260454\_length\_5643\_cov\_34.903244 4459-4469. Max. coverage (+): 0.09. Max coverage (-): 0.09

Region: NODE\_260454\_length\_5643\_cov\_34.903244 4470-4481. Max. coverage (+): 0.09. Max coverage (-): 0

Region: NODE\_260454\_length\_5643\_cov\_34.903244 4482-4492. Max. coverage (+): 0.09. Max coverage (-): 0.38

Region: NODE\_260454\_length\_5643\_cov\_34.903244 4493-4504. Max. coverage (+): 0. Max coverage (-): 0.38

Region: NODE\_260454\_length\_5643\_cov\_34.903244 4505-4515. Max. coverage (+): 0.09. Max coverage (-): 0.38

Region: NODE\_260454\_length\_5643\_cov\_34.903244 4516-4527. Max. coverage (+): 0.09. Max coverage (-): 0.09

Region: NODE\_260454\_length\_5643\_cov\_34.903244 4528-4538. Max. coverage (+): 0. Max coverage (-): 0.28

Region: NODE\_260454\_length\_5643\_cov\_34.903244 4539-4550. Max. coverage (+): 0. Max coverage (-): 0.19

Region: NODE\_260454\_length\_5643\_cov\_34.903244 4551-4561. Max. coverage (+): 0. Max coverage (-): 0.19

Region: NODE\_260454\_length\_5643\_cov\_34.903244 4562-4573. Max. coverage (+): 0. Max coverage (-): 6.11

Region: NODE\_260454\_length\_5643\_cov\_34.903244 4574-4584. Max. coverage (+): 0. Max coverage (-): 0.09

Region: NODE\_260454\_length\_5643\_cov\_34.903244 4585-4596. Max. coverage (+): 0. Max coverage (-): 0.28

Region: NODE\_260454\_length\_5643\_cov\_34.903244 4597-4608. Max. coverage (+): 0. Max coverage (-): 0.28

Region: NODE\_260454\_length\_5643\_cov\_34.903244 4609-4619. Max. coverage (+): 0.09. Max coverage (-): 0

Region: NODE\_260454\_length\_5643\_cov\_34.903244 4620-4631. Max. coverage (+): 0. Max coverage (-): 3.66

Region: NODE\_260454\_length\_5643\_cov\_34.903244 4632-4642. Max. coverage (+): 0. Max coverage (-): 0

Region: NODE\_260454\_length\_5643\_cov\_34.903244 4643-4654. Max. coverage (+): 0. Max coverage (-): 0.09

Region: NODE\_260454\_length\_5643\_cov\_34.903244 4655-4665. Max. coverage (+): 0. Max coverage (-): 6.2

Region: NODE\_260454\_length\_5643\_cov\_34.903244 4666-4677. Max. coverage (+): 0.09. Max coverage (-): 1.69

Region: NODE\_260454\_length\_5643\_cov\_34.903244 4678-4688. Max. coverage (+): 0.09. Max coverage (-): 2.44

Region: NODE\_260454\_length\_5643\_cov\_34.903244 4689-4700. Max. coverage (+): 0. Max coverage (-): 0.94

Region: NODE\_260454\_length\_5643\_cov\_34.903244 4701-4711. Max. coverage (+): 0.19. Max coverage (-): 0

Region: NODE\_260454\_length\_5643\_cov\_34.903244 4712-4723. Max. coverage (+): 0.19. Max coverage (-): 0

Region: NODE\_260454\_length\_5643\_cov\_34.903244 4724-4734. Max. coverage (+): 0.09. Max coverage (-): 0.38

Region: NODE\_260454\_length\_5643\_cov\_34.903244 4735-4746. Max. coverage (+): 0.05. Max coverage (-): 0.05

Region: NODE\_260454\_length\_5643\_cov\_34.903244 4747-4757. Max. coverage (+): 0.05. Max coverage (-): 0

Region: NODE\_260454\_length\_5643\_cov\_34.903244 4758-4769. Max. coverage (+): 0. Max coverage (-): 0.09

Region: NODE\_260454\_length\_5643\_cov\_34.903244 4770-4780. Max. coverage (+): 0. Max coverage (-): 0.47

Region: NODE\_260454\_length\_5643\_cov\_34.903244 4781-4792. Max. coverage (+): 0. Max coverage (-): 0.09

Region: NODE\_260454\_length\_5643\_cov\_34.903244 4793-4803. Max. coverage (+): 0. Max coverage (-): 0

Region: NODE\_260454\_length\_5643\_cov\_34.903244 4804-4815. Max. coverage (+): 0. Max coverage (-): 0.85

Region: NODE\_260454\_length\_5643\_cov\_34.903244 4816-4826. Max. coverage (+): 0. Max coverage (-): 0.56

Region: NODE\_260454\_length\_5643\_cov\_34.903244 4827-4838. Max. coverage (+): 0. Max coverage (-): 0

Region: NODE\_260454\_length\_5643\_cov\_34.903244 4839-4850. Max. coverage (+): 0. Max coverage (-): 0.38

Region: NODE\_260454\_length\_5643\_cov\_34.903244 4851-4861. Max. coverage (+): 0. Max coverage (-): 0.09

Region: NODE\_260454\_length\_5643\_cov\_34.903244 4862-4873. Max. coverage (+): 0. Max coverage (-): 0

Region: NODE\_260454\_length\_5643\_cov\_34.903244 4874-4884. Max. coverage (+): 0. Max coverage (-): 0.28

Region: NODE\_260454\_length\_5643\_cov\_34.903244 4885-4896. Max. coverage (+): 0.09. Max coverage (-): 3.01

Region: NODE\_260454\_length\_5643\_cov\_34.903244 4897-4907. Max. coverage (+): 0. Max coverage (-): 0.38

Region: NODE\_260454\_length\_5643\_cov\_34.903244 4908-4919. Max. coverage (+): 0. Max coverage (-): 0.94

Region: NODE\_260454\_length\_5643\_cov\_34.903244 4920-4930. Max. coverage (+): 0. Max coverage (-): 0.85

Region: NODE\_260454\_length\_5643\_cov\_34.903244 4931-4942. Max. coverage (+): 0. Max coverage (-): 0

Region: NODE\_260454\_length\_5643\_cov\_34.903244 4943-4953. Max. coverage (+): 0. Max coverage (-): 0.09

Region: NODE\_260454\_length\_5643\_cov\_34.903244 4954-4965. Max. coverage (+): 0. Max coverage (-): 0.09

Region: NODE\_260454\_length\_5643\_cov\_34.903244 4966-4976. Max. coverage (+): 0. Max coverage (-): 0

Region: NODE\_260454\_length\_5643\_cov\_34.903244 4977-4988. Max. coverage (+): 0. Max coverage (-): 0

Region: NODE\_260454\_length\_5643\_cov\_34.903244 4989-4999. Max. coverage (+): 0. Max coverage (-): 0.09

Region: NODE\_260454\_length\_5643\_cov\_34.903244 5000-5011. Max. coverage (+): 0.09. Max coverage (-): 0.09

Region: NODE\_260454\_length\_5643\_cov\_34.903244 5012-5022. Max. coverage (+): 0. Max coverage (-): 0

Region: NODE\_260454\_length\_5643\_cov\_34.903244 5023-5034. Max. coverage (+): 0. Max coverage (-): 0.09

Region: NODE\_260454\_length\_5643\_cov\_34.903244 5035-5045. Max. coverage (+): 0. Max coverage (-): 0.19

Region: NODE\_260454\_length\_5643\_cov\_34.903244 5046-5057. Max. coverage (+): 0. Max coverage (-): 0.38

Region: NODE\_260454\_length\_5643\_cov\_34.903244 5058-5068. Max. coverage (+): 0. Max coverage (-): 0

Region: NODE\_260454\_length\_5643\_cov\_34.903244 5069-5080. Max. coverage (+): 0.09. Max coverage (-): 0.09

Region: NODE\_260454\_length\_5643\_cov\_34.903244 5081-5091. Max. coverage (+): 0. Max coverage (-): 0.09

Region: NODE\_260454\_length\_5643\_cov\_34.903244 5092-5103. Max. coverage (+): 0.09. Max coverage (-): 0

Region: NODE\_260454\_length\_5643\_cov\_34.903244 5104-5115. Max. coverage (+): 0.09. Max coverage (-): 4.51

Region: NODE\_260454\_length\_5643\_cov\_34.903244 5116-5126. Max. coverage (+): 0. Max coverage (-): 23.4

Region: NODE\_260454\_length\_5643\_cov\_34.903244 5127-5138. Max. coverage (+): 0. Max coverage (-): 0.61

Region: NODE\_260454\_length\_5643\_cov\_34.903244 5139-5149. Max. coverage (+): 0.09. Max coverage (-): 0

Region: NODE\_260454\_length\_5643\_cov\_34.903244 5150-5161. Max. coverage (+): 0.05. Max coverage (-): 0.05

Region: NODE\_260454\_length\_5643\_cov\_34.903244 5162-5172. Max. coverage (+): 0. Max coverage (-): 0.09

Region: NODE\_260454\_length\_5643\_cov\_34.903244 5173-5184. Max. coverage (+): 0. Max coverage (-): 2.07

Region: NODE\_260454\_length\_5643\_cov\_34.903244 5185-5195. Max. coverage (+): 0. Max coverage (-): 2.07

Region: NODE\_260454\_length\_5643\_cov\_34.903244 5196-5207. Max. coverage (+): 0. Max coverage (-): 0.28

Region: NODE\_260454\_length\_5643\_cov\_34.903244 5208-5218. Max. coverage (+): 0. Max coverage (-): 0

Region: NODE\_260454\_length\_5643\_cov\_34.903244 5219-5230. Max. coverage (+): 0. Max coverage (-): 0

Region: NODE\_260454\_length\_5643\_cov\_34.903244 5231-5241. Max. coverage (+): 0.09. Max coverage (-): 0

Region: NODE\_260454\_length\_5643\_cov\_34.903244 5242-5253. Max. coverage (+): 0. Max coverage (-): 0.28

Region: NODE\_260454\_length\_5643\_cov\_34.903244 5254-5264. Max. coverage (+): 0. Max coverage (-): 0.56

Region: NODE\_260454\_length\_5643\_cov\_34.903244 5265-5276. Max. coverage (+): 0. Max coverage (-): 0.28

Region: NODE\_260454\_length\_5643\_cov\_34.903244 5277-5287. Max. coverage (+): 0. Max coverage (-): 0

Region: NODE\_260454\_length\_5643\_cov\_34.903244 5288-5299. Max. coverage (+): 0. Max coverage (-): 1.22

Region: NODE\_260454\_length\_5643\_cov\_34.903244 5300-5310. Max. coverage (+): 0. Max coverage (-): 0.05

Region: NODE\_260454\_length\_5643\_cov\_34.903244 5311-5322. Max. coverage (+): 0. Max coverage (-): 0.66

Region: NODE\_260454\_length\_5643\_cov\_34.903244 5323-5333. Max. coverage (+): 0. Max coverage (-): 0.09

Region: NODE\_260454\_length\_5643\_cov\_34.903244 5334-5345. Max. coverage (+): 0.66. Max coverage (-): 0

Region: NODE\_260454\_length\_5643\_cov\_34.903244 5346-5356. Max. coverage (+): 0. Max coverage (-): 0.94

Region: NODE\_260454\_length\_5643\_cov\_34.903244 5357-5368. Max. coverage (+): 0. Max coverage (-): 0

Region: NODE\_260454\_length\_5643\_cov\_34.903244 5369-5380. Max. coverage (+): 0. Max coverage (-): 0

Region: NODE\_260454\_length\_5643\_cov\_34.903244 5381-5391. Max. coverage (+): 0. Max coverage (-): 0

Region: NODE\_260454\_length\_5643\_cov\_34.903244 5392-5403. Max. coverage (+): 0. Max coverage (-): 0

Region: NODE\_260454\_length\_5643\_cov\_34.903244 5404-5414. Max. coverage (+): 0. Max coverage (-): 0.19

Region: NODE\_260454\_length\_5643\_cov\_34.903244 5415-5426. Max. coverage (+): 0. Max coverage (-): 9.02

Region: NODE\_260454\_length\_5643\_cov\_34.903244 5427-5437. Max. coverage (+): 0. Max coverage (-): 0.7

Region: NODE\_260454\_length\_5643\_cov\_34.903244 5438-5449. Max. coverage (+): 0. Max coverage (-): 0.14

Region: NODE\_260454\_length\_5643\_cov\_34.903244 5450-5460. Max. coverage (+): 0. Max coverage (-): 0.75

Region: NODE\_260454\_length\_5643\_cov\_34.903244 5461-5472. Max. coverage (+): 0. Max coverage (-): 0.75

Region: NODE\_260454\_length\_5643\_cov\_34.903244 5473-5483. Max. coverage (+): 0. Max coverage (-): 1.03

Region: NODE\_260454\_length\_5643\_cov\_34.903244 5484-5495. Max. coverage (+): 0. Max coverage (-): 0.38

Region: NODE\_260454\_length\_5643\_cov\_34.903244 5496-5506. Max. coverage (+): 0. Max coverage (-): 0

Region: NODE\_260454\_length\_5643\_cov\_34.903244 5507-5518. Max. coverage (+): 0. Max coverage (-): 3.48

Region: NODE\_260454\_length\_5643\_cov\_34.903244 5519-5529. Max. coverage (+): 0. Max coverage (-): 0.09

Region: NODE\_260454\_length\_5643\_cov\_34.903244 5530-5541. Max. coverage (+): 0.28. Max coverage (-): 0.09

Region: NODE\_260454\_length\_5643\_cov\_34.903244 5542-5552. Max. coverage (+): 0. Max coverage (-): 0.19

Region: NODE\_260454\_length\_5643\_cov\_34.903244 5553-5564. Max. coverage (+): 0. Max coverage (-): 0.09

Region: NODE\_260454\_length\_5643\_cov\_34.903244 5565-5575. Max. coverage (+): 0. Max coverage (-): 0

Region: NODE\_260454\_length\_5643\_cov\_34.903244 5576-5587. Max. coverage (+): 0. Max coverage (-): 0

Region: NODE\_260454\_length\_5643\_cov\_34.903244 5588-5598. Max. coverage (+): 0. Max coverage (-): 0

Region: NODE\_260454\_length\_5643\_cov\_34.903244 5599-5610. Max. coverage (+): 0. Max coverage (-): 0

Region: NODE\_260454\_length\_5643\_cov\_34.903244 5611-5621. Max. coverage (+): 0. Max coverage (-): 0

Region: NODE\_260454\_length\_5643\_cov\_34.903244 5622-5633. Max. coverage (+): 0. Max coverage (-): 0

Region: NODE\_260454\_length\_5643\_cov\_34.903244 5634-5645. Max. coverage (+): 0. Max coverage (-): 0

Region: NODE\_260454\_length\_5643\_cov\_34.903244 5646-5656. Max. coverage (+): 0. Max coverage (-): 0

Region: NODE\_260454\_length\_5643\_cov\_34.903244 5657-5668. Max. coverage (+): 0.09. Max coverage (-): 0

Region: NODE\_260454\_length\_5643\_cov\_34.903244 5669-5679. Max. coverage (+): 0.19. Max coverage (-): 0.19

Region: NODE\_260454\_length\_5643\_cov\_34.903244 5680-5691. Max. coverage (+): 0. Max coverage (-): 0.19

Region: NODE\_260454\_length\_5643\_cov\_34.903244 5692-5702. Max. coverage (+): 0.09. Max coverage (-): 1.69

Region: NODE\_260454\_length\_5643\_cov\_34.903244 5703-5714. Max. coverage (+): 0.19. Max coverage (-): 0.09

Region: NODE\_260454\_length\_5643\_cov\_34.903244 5715-5725. Max. coverage (+): 0.01. Max coverage (-): 0

Region: NODE\_260454\_length\_5643\_cov\_34.903244 5726-5737. Max. coverage (+): 0.04. Max coverage (-): 0

Region: NODE\_260454\_length\_5643\_cov\_34.903244 5738-5748. Max. coverage (+): 0.02. Max coverage (-): 0

Region: NODE\_260454\_length\_5643\_cov\_34.903244 5749-5760. Max. coverage (+): 0. Max coverage (-): 0

Region: NODE\_260454\_length\_5643\_cov\_34.903244 5761-. Max. coverage (+): 0. Max coverage (-): 0

RepeatMasker Color Code

**+**

100-98% Identity

<98-95% Identity

<95-90% Identity

<90-85% Identity

<85-80% Identity

<80-75% Identity

<75-70% Identity

<70% Identity

**-**

Gene Set Color Code

**+**

Gene

Pseudogene

Other

**-**

Topology/Coverage Color Code

Coverage Plus Strand

Coverage Minus Strand

Mainstrand: Plus

Mainstrand: Minus

Complementary Strand

Flanking Region  
(if option -flank >0)

Gene Set Annotation  
  
RepeatMasker Annotation  

**1. RTE-1\_GA**: 1-63 (+), Divergence to consensus: 8.1%  
**2. AlRepD-4371**: 61-416 (+), Divergence to consensus: 12.4%  
**3. AlRepB-60**: 417-460 (+), Divergence to consensus: 4.5%  
**4. AlRepB-60**: 460-489 (+), Divergence to consensus: 6.7%  
**5. AlRepD-1578**: 496-1253 (+), Divergence to consensus: 21%  
**6. AlRepB-727**: 1314-1568 (-), Divergence to consensus: 23%  
**7. AlRepB-127**: 1533-1584 (+), Divergence to consensus: 15.7%  
**8. AlRepB-127**: 1572-1971 (+), Divergence to consensus: 13.4%  
**9. AlRepB-727**: 1853-2091 (-), Divergence to consensus: 4.6%  
**10. AlRepB-727**: 2163-2266 (-), Divergence to consensus: 3.9%  
**11. AlRepB-727**: 2267-2318 (+), Divergence to consensus: 3.6%  
**12. AlRepE-1478**: 2463-2568 (+), Divergence to consensus: 15.5%  
**13. EnSpm-17\_DR**: 3635-3768 (+), Divergence to consensus: 33.9%  
**14. (TTTG)n**: 3830-3882 (+), Divergence to consensus: 23.9%  
**15. EnSpm-17\_DR**: 3897-3970 (+), Divergence to consensus: 24.3%  
**16. EnSpm-5\_DR**: 4127-4253 (+), Divergence to consensus: 34.2%  
**17. EnSpm-2\_DR**: 4318-4446 (+), Divergence to consensus: 34.1%  
**18. EnSpm-17\_DR**: 4662-4860 (+), Divergence to consensus: 40.8%  
**19. EnSpm-17\_DR**: 5298-5559 (+), Divergence to consensus: 29.8%  
**20. (A)n**: 5609-5632 (+), Divergence to consensus: 8.9%

  
Transcription Factor Binding Sites  

**RHOXF1** (Sequence: GGATTA (-): 60)  
**RHOXF1** (Sequence: AGCTCA (-): 80)  
**RHOXF1** (Sequence: AGATCA (-): 1223)  
**RHOXF1** (Sequence: AGATCA (-): 1385)  
**RHOXF1** (Sequence: AGCTTA (-): 1416)  
**RHOXF1** (Sequence: AGATTA (-): 1459)  
**RHOXF1** (Sequence: AGCTTA (-): 2227)  
**RHOXF1** (Sequence: GGATCA (-): 2410)  
**RHOXF1** (Sequence: AGATCA (-): 3142)  
**RHOXF1** (Sequence: AGCTCA (-): 3466)  
**RHOXF1** (Sequence: TAATCC (+): 364)  
**RHOXF1** (Sequence: TGAGCT (+): 482)  
**RHOXF1** (Sequence: TGAGCC (+): 1719)  
**RHOXF1** (Sequence: TGAGCT (+): 2807)  
**RHOXF1** (Sequence: TGATCC (+): 3271)  
**RHOXF1** (Sequence: TGAGCT (+): 3464)  
**RHOXF1** (Sequence: TGATCC (+): 3912)  
**RHOXF1** (Sequence: TAAGCC (+): 4913)  
**RHOXF1** (Sequence: TAATCC (+): 5044)  
**Lhx8** (Sequence: TTAATTAA (-): 1529)  
**Gata4** (Sequence: GTTATCT (+): 1631)  
**Gata4** (Sequence: GTTATCT (+): 3762)  
**SOX9** (Sequence: AACAATGG (-): 1055)  
**SOX9** (Sequence: AACAATAA (-): 1614)  
**Sox5** (Sequence: ATTGTT (+): 987)  
**Sox5** (Sequence: ATTGTT (+): 1358)  
**Sox5** (Sequence: ATTGTT (+): 1929)  
**Sox5** (Sequence: ATTGTT (+): 2354)  
**Sox5** (Sequence: ATTGTT (+): 5680)  
**SOX9** (Sequence: CCATTGTT (+): 1927)  
**FOXO1** (Sequence: AAAAACAGC (-): 4792)  
**Nobox** (Sequence: TAATTACT (+): 2459)  
**Nobox** (Sequence: TAATTACC (+): 4950)  
**POU2F1** (Sequence: ATTAGAATA (-): 254)  
**Rhox11** (Sequence: TGCTGTTAT (+): 3020)  
**Rhox11** (Sequence: TTAACAGCA (-): 3372)  
**Rhox11** (Sequence: AAAACAGCA (-): 4793)  
**Gata4** (Sequence: AGATAAC (-): 4272)  
**Sox5** (Sequence: AACAAT (-): 166)  
**Sox5** (Sequence: AACAAT (-): 1055)  
**Sox5** (Sequence: AACAAT (-): 1614)  
**Sox5** (Sequence: AACAAT (-): 3610)  
**POU2F1** (Sequence: TATTCAAAT (+): 5239)
