# Supplementary material for: First characterization of PIWI-interacting RNA clusters in a cichlid fish with a B chromosome
Source: BMC Biol. 2022 Sep 21;20:204. doi: 10.1186/s12915-022-01403-2 (PMC9490952; doi:10.1186/s12915-022-01403-2)
Supplement: Supplementary file 1 — Additional file 1. Zipped folder with fasta and interactive html piRNA cluster information for the A. latifasciata genome. The nomenclature is as follows: number-pirna-cluster_sex_B-presence (f, female; m, male; 0b, without B chromosome; 1b, with B chromosome). [file 12915_2022_1403_MOESM1_ESM.zip › 101_m0b.html]

piRNA cluster 101\_m0b 50


Predicted piRNA cluster no. 101\_m0b
  

Show proTRAC run info
Hide proTRAC run info

/\  
                \_\_\_\_\_\_\_\_\_\_\_\_\_\_\_\_\_\_\_\_\_\_\_/\\_\_\_ /  \\_\_\_\_\_\_\_  
               I                      /  \  /    \      I  
               I     pro             /    \/      \     I  
               I        TRAC        /               \   I  
               I   \_\_\_\_\_\_\_\_\_\_\_\_\_\_\_\_/\_\_\_\_\_\_\_\_\_\_\_\_\_\_\_\_\_\\_ I  
               I   \              /                     I  
               I    \            /                      I  
               I     \  /\      /       V.2.4.2         I  
               I      \/  \    /                        I  
               I\_\_\_\_\_\_\_\_\_\_\_\  /\_\_\_\_\_\_\_\_\_\_\_\_\_\_\_\_\_\_\_\_\_\_\_\_\_I  
                            \/  
  
  
================================= proTRAC ====================================  
VERSION: .......... 2.4.2  
LAST MODIFIED: .... 11. May 2018  
  
Please cite:  
Rosenkranz D, Zischler H. proTRAC - a software for probabilistic piRNA cluster  
detection, visualization and analysis. 2012. BMC Bioinformatics 13:5.  
  
  
Contact:  
David Rosenkranz  
Institute of Organismic and Molecular Evolutionary Biology  
Dept. Anthropology, small RNA group  
Johannes Gutenberg University Mainz  
email: rosenkranz@uni-mainz.de  
  
You can find the latest proTRAC version at:  
http://sourceforge.net/projects/protrac/files  
http://www.smallRNAgroup-mainz.de/software  
==============================================================================  
  
PARAMETERS:  
Map file: ...............piwi-machos-0B.fa-collapse.map  
Genome file: ............../../../0B\_ala\_genome.fa  
RepeatMasker annotation: Alatifasciata-all0B-maryan-v2.fa\_corrected.out  
GeneSet:................./guest-storage/Data/annotation/Alatifasciata\_all0B\_maryan-v2\_out2017.gff  
  
Significant (p<=0.01) hit density will be calculated based  
on observed hit distribution.  
  
Sliding window size: ........................................ 5000 bp  
Sliding window increament: .................................. 1000 bp  
Normalize each hit by number of genomic hits: ............... yes  
Normalize each hit by number of sequence reads: ............. yes  
Normalize values (-> per million mapped reads): ............. yes  
Min. fraction of hits with 1T(U) or 10A: .................... 0.75  
Alternatively: Min. fraction of hits with 1T(U) and 10A: .... 0.5  
Min. fraction of hits with typical piRNA length: ............ 0.75  
Typical piRNA length: ....................................... 24-32 nt  
Min. size of a piRNA cluster: ............................... 1000 bp.  
Min. number of hits (absolute): ............................. 0  
Min. number of hits (normalized): ........................... 0  
Min. fraction of hits on the mainstrand: .................... 0.75  
Top fraction of mapped sequences (in terms of read counts): . 1%  
Top fraction accounts for max. n% of sequence reads: ........ 90%  
Min. fraction of hits on each arm of a bidirectional cluster: 0.05  
Output html file for each cluster: .......................... yes  
Output a summary table: ..................................... yes  
Output a FASTA file for each cluster (piRNA sequences): ..... yes  
Output a FASTA file comprising cluster sequences: ........... yes  
Output a GTF file for predicted piRNA clusters: ..............yes  
Search DNA motifs in clusters: .............................. yes  
Output flanking sequences: +/- .............................. 0 bp  
Output ~.pTi file: .......................................... no  
==============================================================================  
  
  
Genome size (without gaps): ............ 758543724 bp  
Gaps (N/X/-): .......................... 417479 bp  
Mapped reads: .......................... 24765598  
Non-identical sequences: ............... 6158275  
Genomic hits: .......................... 53103584  
Significant densitiy of mapped reads: .. 763.098963422187 reads/kb

Show proTRAC cluster info
Hide proTRAC cluster info

|  |  |
| --- | --- |
| Location | NODE\_261184\_length\_10127\_cov\_26.726177 |
| Coordinates | 1-10230 |
| Size [bp] | 10230 |
| Sequence hit loci | 6095 |
| Mapped reads (normalized) | 23571.2 |
| Mapped reads (normalized) per kb | 2304.1 |
| Normalized reads with 1T (1U) | 84.2% |
| Normalized reads with 10A | 39.8% |
| Normalized reads with length 24-32 nt | 99.3% |
| Normalized reads on the main strand(s) | 93.7% |
| Predicted directionality | mono:plus |

100%

0%

1T (1U)  
reads

10A reads

24-32 nt  
reads

reads on mainstrand

**Either the amount of reads with 1T (1U) OR 10A has to exceed 75% (set with option: -1Tor10A)  
Alternatively the amount of reads with 1T (1U) AND 10A has to exceed 50% (set with option: -1Tand10A)  
Minimum amount of reads with preferred size is 75% (set with option: -pisize)  
Minimum amount of reads on the main strand(s) is 75% (set with option: -clstrand)**

Show read coverage
Hide read coverage

WHAT DO I SEE HERE?  
This chart shows the location of mapped sequence reads within a predicted piRNA cluster. The color refers to the number of genomic hits produced by the sequence read in question. A dark red bar indicates that this sequence read produces many other hits elsewhere in the genome. Many adjacent red or yellow bars can indicate the presence of a multi-copy element such as transposons or rRNA genes. A dark green bar indicates that this sequence read maps uniquely to this locus.

1 hit

2-5 hits

6-10 hits

11-20 hits

21-50 hits

51-100 hits

> 100 hits

NODE\_261184\_length\_10127\_cov\_26.726177

1

10230

Gene Set

RepeatMasker

Mapped  
Reads

85.93

plus strand

minus strand

85.93

Region: NODE\_261184\_length\_10127\_cov\_26.726177 7390-11. Max. coverage (+): 0.01. Max coverage (-): 0

Region: NODE\_261184\_length\_10127\_cov\_26.726177 12-31. Max. coverage (+): 0. Max coverage (-): 0.01

Region: NODE\_261184\_length\_10127\_cov\_26.726177 32-52. Max. coverage (+): 5.32. Max coverage (-): 0.01

Region: NODE\_261184\_length\_10127\_cov\_26.726177 53-72. Max. coverage (+): 0.01. Max coverage (-): 0.01

Region: NODE\_261184\_length\_10127\_cov\_26.726177 73-93. Max. coverage (+): 0.01. Max coverage (-): 0

Region: NODE\_261184\_length\_10127\_cov\_26.726177 94-113. Max. coverage (+): 0. Max coverage (-): 0

Region: NODE\_261184\_length\_10127\_cov\_26.726177 114-133. Max. coverage (+): 0.04. Max coverage (-): 0

Region: NODE\_261184\_length\_10127\_cov\_26.726177 134-154. Max. coverage (+): 0. Max coverage (-): 0

Region: NODE\_261184\_length\_10127\_cov\_26.726177 155-174. Max. coverage (+): 0. Max coverage (-): 0

Region: NODE\_261184\_length\_10127\_cov\_26.726177 175-195. Max. coverage (+): 0. Max coverage (-): 0

Region: NODE\_261184\_length\_10127\_cov\_26.726177 196-215. Max. coverage (+): 0. Max coverage (-): 0

Region: NODE\_261184\_length\_10127\_cov\_26.726177 216-236. Max. coverage (+): 0. Max coverage (-): 0.04

Region: NODE\_261184\_length\_10127\_cov\_26.726177 237-256. Max. coverage (+): 0. Max coverage (-): 0

Region: NODE\_261184\_length\_10127\_cov\_26.726177 257-277. Max. coverage (+): 0. Max coverage (-): 0

Region: NODE\_261184\_length\_10127\_cov\_26.726177 278-297. Max. coverage (+): 0. Max coverage (-): 0.32

Region: NODE\_261184\_length\_10127\_cov\_26.726177 298-318. Max. coverage (+): 0. Max coverage (-): 0

Region: NODE\_261184\_length\_10127\_cov\_26.726177 319-338. Max. coverage (+): 0. Max coverage (-): 0

Region: NODE\_261184\_length\_10127\_cov\_26.726177 339-359. Max. coverage (+): 0.02. Max coverage (-): 0

Region: NODE\_261184\_length\_10127\_cov\_26.726177 360-379. Max. coverage (+): 0. Max coverage (-): 0

Region: NODE\_261184\_length\_10127\_cov\_26.726177 380-399. Max. coverage (+): 0. Max coverage (-): 0

Region: NODE\_261184\_length\_10127\_cov\_26.726177 400-420. Max. coverage (+): 0.19. Max coverage (-): 0.04

Region: NODE\_261184\_length\_10127\_cov\_26.726177 421-440. Max. coverage (+): 0.16. Max coverage (-): 0.04

Region: NODE\_261184\_length\_10127\_cov\_26.726177 441-461. Max. coverage (+): 0. Max coverage (-): 0.85

Region: NODE\_261184\_length\_10127\_cov\_26.726177 462-481. Max. coverage (+): 0.4. Max coverage (-): 0.01

Region: NODE\_261184\_length\_10127\_cov\_26.726177 482-502. Max. coverage (+): 0.08. Max coverage (-): 0.08

Region: NODE\_261184\_length\_10127\_cov\_26.726177 503-522. Max. coverage (+): 0. Max coverage (-): 0

Region: NODE\_261184\_length\_10127\_cov\_26.726177 523-543. Max. coverage (+): 0.02. Max coverage (-): 0.03

Region: NODE\_261184\_length\_10127\_cov\_26.726177 544-563. Max. coverage (+): 0.02. Max coverage (-): 0

Region: NODE\_261184\_length\_10127\_cov\_26.726177 564-584. Max. coverage (+): 0. Max coverage (-): 0.08

Region: NODE\_261184\_length\_10127\_cov\_26.726177 585-604. Max. coverage (+): 0. Max coverage (-): 0.01

Region: NODE\_261184\_length\_10127\_cov\_26.726177 605-625. Max. coverage (+): 0.05. Max coverage (-): 0.01

Region: NODE\_261184\_length\_10127\_cov\_26.726177 626-645. Max. coverage (+): 0.01. Max coverage (-): 0.04

Region: NODE\_261184\_length\_10127\_cov\_26.726177 646-665. Max. coverage (+): 0. Max coverage (-): 0.01

Region: NODE\_261184\_length\_10127\_cov\_26.726177 666-686. Max. coverage (+): 0. Max coverage (-): 0.04

Region: NODE\_261184\_length\_10127\_cov\_26.726177 687-706. Max. coverage (+): 0. Max coverage (-): 0

Region: NODE\_261184\_length\_10127\_cov\_26.726177 707-727. Max. coverage (+): 0.04. Max coverage (-): 0.02

Region: NODE\_261184\_length\_10127\_cov\_26.726177 728-747. Max. coverage (+): 0. Max coverage (-): 0

Region: NODE\_261184\_length\_10127\_cov\_26.726177 748-768. Max. coverage (+): 0. Max coverage (-): 0

Region: NODE\_261184\_length\_10127\_cov\_26.726177 769-788. Max. coverage (+): 0. Max coverage (-): 0.02

Region: NODE\_261184\_length\_10127\_cov\_26.726177 789-809. Max. coverage (+): 0.12. Max coverage (-): 0.28

Region: NODE\_261184\_length\_10127\_cov\_26.726177 810-829. Max. coverage (+): 0.04. Max coverage (-): 0.28

Region: NODE\_261184\_length\_10127\_cov\_26.726177 830-850. Max. coverage (+): 0.04. Max coverage (-): 0.27

Region: NODE\_261184\_length\_10127\_cov\_26.726177 851-870. Max. coverage (+): 0.08. Max coverage (-): 0.12

Region: NODE\_261184\_length\_10127\_cov\_26.726177 871-891. Max. coverage (+): 0.09. Max coverage (-): 0.09

Region: NODE\_261184\_length\_10127\_cov\_26.726177 892-911. Max. coverage (+): 0. Max coverage (-): 0.02

Region: NODE\_261184\_length\_10127\_cov\_26.726177 912-931. Max. coverage (+): 0.02. Max coverage (-): 0.02

Region: NODE\_261184\_length\_10127\_cov\_26.726177 932-952. Max. coverage (+): 0.01. Max coverage (-): 0

Region: NODE\_261184\_length\_10127\_cov\_26.726177 953-972. Max. coverage (+): 0.04. Max coverage (-): 0

Region: NODE\_261184\_length\_10127\_cov\_26.726177 973-993. Max. coverage (+): 0. Max coverage (-): 0.02

Region: NODE\_261184\_length\_10127\_cov\_26.726177 994-1013. Max. coverage (+): 0.2. Max coverage (-): 0

Region: NODE\_261184\_length\_10127\_cov\_26.726177 1014-1034. Max. coverage (+): 0.12. Max coverage (-): 0.08

Region: NODE\_261184\_length\_10127\_cov\_26.726177 1035-1054. Max. coverage (+): 0.05. Max coverage (-): 0.04

Region: NODE\_261184\_length\_10127\_cov\_26.726177 1055-1075. Max. coverage (+): 0. Max coverage (-): 0.02

Region: NODE\_261184\_length\_10127\_cov\_26.726177 1076-1095. Max. coverage (+): 0.06. Max coverage (-): 0

Region: NODE\_261184\_length\_10127\_cov\_26.726177 1096-1116. Max. coverage (+): 0.24. Max coverage (-): 0.04

Region: NODE\_261184\_length\_10127\_cov\_26.726177 1117-1136. Max. coverage (+): 0.32. Max coverage (-): 0.04

Region: NODE\_261184\_length\_10127\_cov\_26.726177 1137-1156. Max. coverage (+): 0.04. Max coverage (-): 0

Region: NODE\_261184\_length\_10127\_cov\_26.726177 1157-1177. Max. coverage (+): 0.01. Max coverage (-): 0.03

Region: NODE\_261184\_length\_10127\_cov\_26.726177 1178-1197. Max. coverage (+): 0.04. Max coverage (-): 0

Region: NODE\_261184\_length\_10127\_cov\_26.726177 1198-1218. Max. coverage (+): 0.2. Max coverage (-): 0.02

Region: NODE\_261184\_length\_10127\_cov\_26.726177 1219-1238. Max. coverage (+): 0.04. Max coverage (-): 0

Region: NODE\_261184\_length\_10127\_cov\_26.726177 1239-1259. Max. coverage (+): 0.03. Max coverage (-): 0.02

Region: NODE\_261184\_length\_10127\_cov\_26.726177 1260-1279. Max. coverage (+): 0.2. Max coverage (-): 0.04

Region: NODE\_261184\_length\_10127\_cov\_26.726177 1280-1300. Max. coverage (+): 0.04. Max coverage (-): 0.04

Region: NODE\_261184\_length\_10127\_cov\_26.726177 1301-1320. Max. coverage (+): 0.01. Max coverage (-): 0.02

Region: NODE\_261184\_length\_10127\_cov\_26.726177 1321-1341. Max. coverage (+): 0.08. Max coverage (-): 0.04

Region: NODE\_261184\_length\_10127\_cov\_26.726177 1342-1361. Max. coverage (+): 0.32. Max coverage (-): 0.08

Region: NODE\_261184\_length\_10127\_cov\_26.726177 1362-1382. Max. coverage (+): 0.24. Max coverage (-): 0.04

Region: NODE\_261184\_length\_10127\_cov\_26.726177 1383-1402. Max. coverage (+): 0.24. Max coverage (-): 0.2

Region: NODE\_261184\_length\_10127\_cov\_26.726177 1403-1422. Max. coverage (+): 2.46. Max coverage (-): 0.08

Region: NODE\_261184\_length\_10127\_cov\_26.726177 1423-1443. Max. coverage (+): 1.13. Max coverage (-): 0.04

Region: NODE\_261184\_length\_10127\_cov\_26.726177 1444-1463. Max. coverage (+): 0.04. Max coverage (-): 0

Region: NODE\_261184\_length\_10127\_cov\_26.726177 1464-1484. Max. coverage (+): 0.08. Max coverage (-): 0

Region: NODE\_261184\_length\_10127\_cov\_26.726177 1485-1504. Max. coverage (+): 0. Max coverage (-): 0.12

Region: NODE\_261184\_length\_10127\_cov\_26.726177 1505-1525. Max. coverage (+): 0.12. Max coverage (-): 0.04

Region: NODE\_261184\_length\_10127\_cov\_26.726177 1526-1545. Max. coverage (+): 0.16. Max coverage (-): 0.08

Region: NODE\_261184\_length\_10127\_cov\_26.726177 1546-1566. Max. coverage (+): 1.74. Max coverage (-): 0.24

Region: NODE\_261184\_length\_10127\_cov\_26.726177 1567-1586. Max. coverage (+): 0.12. Max coverage (-): 0.28

Region: NODE\_261184\_length\_10127\_cov\_26.726177 1587-1607. Max. coverage (+): 1.21. Max coverage (-): 0.08

Region: NODE\_261184\_length\_10127\_cov\_26.726177 1608-1627. Max. coverage (+): 0.77. Max coverage (-): 0.44

Region: NODE\_261184\_length\_10127\_cov\_26.726177 1628-1648. Max. coverage (+): 0.77. Max coverage (-): 0.12

Region: NODE\_261184\_length\_10127\_cov\_26.726177 1649-1668. Max. coverage (+): 3.35. Max coverage (-): 0.36

Region: NODE\_261184\_length\_10127\_cov\_26.726177 1669-1688. Max. coverage (+): 3.35. Max coverage (-): 0.61

Region: NODE\_261184\_length\_10127\_cov\_26.726177 1689-1709. Max. coverage (+): 0.6. Max coverage (-): 0.57

Region: NODE\_261184\_length\_10127\_cov\_26.726177 1710-1729. Max. coverage (+): 0.06. Max coverage (-): 0.06

Region: NODE\_261184\_length\_10127\_cov\_26.726177 1730-1750. Max. coverage (+): 3.07. Max coverage (-): 0.04

Region: NODE\_261184\_length\_10127\_cov\_26.726177 1751-1770. Max. coverage (+): 0.86. Max coverage (-): 0.04

Region: NODE\_261184\_length\_10127\_cov\_26.726177 1771-1791. Max. coverage (+): 0.12. Max coverage (-): 4.85

Region: NODE\_261184\_length\_10127\_cov\_26.726177 1792-1811. Max. coverage (+): 0.2. Max coverage (-): 0

Region: NODE\_261184\_length\_10127\_cov\_26.726177 1812-1832. Max. coverage (+): 0.08. Max coverage (-): 0.24

Region: NODE\_261184\_length\_10127\_cov\_26.726177 1833-1852. Max. coverage (+): 1.33. Max coverage (-): 0

Region: NODE\_261184\_length\_10127\_cov\_26.726177 1853-1873. Max. coverage (+): 0.23. Max coverage (-): 0.05

Region: NODE\_261184\_length\_10127\_cov\_26.726177 1874-1893. Max. coverage (+): 0.73. Max coverage (-): 0.05

Region: NODE\_261184\_length\_10127\_cov\_26.726177 1894-1914. Max. coverage (+): 0.89. Max coverage (-): 0.12

Region: NODE\_261184\_length\_10127\_cov\_26.726177 1915-1934. Max. coverage (+): 0.52. Max coverage (-): 0.97

Region: NODE\_261184\_length\_10127\_cov\_26.726177 1935-1954. Max. coverage (+): 0.12. Max coverage (-): 0.2

Region: NODE\_261184\_length\_10127\_cov\_26.726177 1955-1975. Max. coverage (+): 2.22. Max coverage (-): 0.2

Region: NODE\_261184\_length\_10127\_cov\_26.726177 1976-1995. Max. coverage (+): 11.1. Max coverage (-): 0

Region: NODE\_261184\_length\_10127\_cov\_26.726177 1996-2016. Max. coverage (+): 0.73. Max coverage (-): 0

Region: NODE\_261184\_length\_10127\_cov\_26.726177 2017-2036. Max. coverage (+): 0.04. Max coverage (-): 0

Region: NODE\_261184\_length\_10127\_cov\_26.726177 2037-2057. Max. coverage (+): 0. Max coverage (-): 0.04

Region: NODE\_261184\_length\_10127\_cov\_26.726177 2058-2077. Max. coverage (+): 0. Max coverage (-): 0

Region: NODE\_261184\_length\_10127\_cov\_26.726177 2078-2098. Max. coverage (+): 0. Max coverage (-): 0

Region: NODE\_261184\_length\_10127\_cov\_26.726177 2099-2118. Max. coverage (+): 0.08. Max coverage (-): 0.04

Region: NODE\_261184\_length\_10127\_cov\_26.726177 2119-2139. Max. coverage (+): 0.69. Max coverage (-): 0

Region: NODE\_261184\_length\_10127\_cov\_26.726177 2140-2159. Max. coverage (+): 0.24. Max coverage (-): 0.24

Region: NODE\_261184\_length\_10127\_cov\_26.726177 2160-2179. Max. coverage (+): 0.36. Max coverage (-): 0.04

Region: NODE\_261184\_length\_10127\_cov\_26.726177 2180-2200. Max. coverage (+): 0. Max coverage (-): 0

Region: NODE\_261184\_length\_10127\_cov\_26.726177 2201-2220. Max. coverage (+): 0.04. Max coverage (-): 0

Region: NODE\_261184\_length\_10127\_cov\_26.726177 2221-2241. Max. coverage (+): 0.04. Max coverage (-): 0

Region: NODE\_261184\_length\_10127\_cov\_26.726177 2242-2261. Max. coverage (+): 7.27. Max coverage (-): 0.04

Region: NODE\_261184\_length\_10127\_cov\_26.726177 2262-2282. Max. coverage (+): 0.24. Max coverage (-): 0

Region: NODE\_261184\_length\_10127\_cov\_26.726177 2283-2302. Max. coverage (+): 9.61. Max coverage (-): 0.04

Region: NODE\_261184\_length\_10127\_cov\_26.726177 2303-2323. Max. coverage (+): 80.68. Max coverage (-): 0.16

Region: NODE\_261184\_length\_10127\_cov\_26.726177 2324-2343. Max. coverage (+): 0.2. Max coverage (-): 0.24

Region: NODE\_261184\_length\_10127\_cov\_26.726177 2344-2364. Max. coverage (+): 0.89. Max coverage (-): 0.16

Region: NODE\_261184\_length\_10127\_cov\_26.726177 2365-2384. Max. coverage (+): 2.22. Max coverage (-): 0.24

Region: NODE\_261184\_length\_10127\_cov\_26.726177 2385-2405. Max. coverage (+): 2.22. Max coverage (-): 0.04

Region: NODE\_261184\_length\_10127\_cov\_26.726177 2406-2425. Max. coverage (+): 0.16. Max coverage (-): 0

Region: NODE\_261184\_length\_10127\_cov\_26.726177 2426-2445. Max. coverage (+): 0.12. Max coverage (-): 1.94

Region: NODE\_261184\_length\_10127\_cov\_26.726177 2446-2466. Max. coverage (+): 29.76. Max coverage (-): 0.04

Region: NODE\_261184\_length\_10127\_cov\_26.726177 2467-2486. Max. coverage (+): 0.28. Max coverage (-): 0

Region: NODE\_261184\_length\_10127\_cov\_26.726177 2487-2507. Max. coverage (+): 0.16. Max coverage (-): 0

Region: NODE\_261184\_length\_10127\_cov\_26.726177 2508-2527. Max. coverage (+): 2.58. Max coverage (-): 1.29

Region: NODE\_261184\_length\_10127\_cov\_26.726177 2528-2548. Max. coverage (+): 2.71. Max coverage (-): 0.12

Region: NODE\_261184\_length\_10127\_cov\_26.726177 2549-2568. Max. coverage (+): 18.9. Max coverage (-): 0.04

Region: NODE\_261184\_length\_10127\_cov\_26.726177 2569-2589. Max. coverage (+): 3.03. Max coverage (-): 0.04

Region: NODE\_261184\_length\_10127\_cov\_26.726177 2590-2609. Max. coverage (+): 1.32. Max coverage (-): 0

Region: NODE\_261184\_length\_10127\_cov\_26.726177 2610-2630. Max. coverage (+): 3.41. Max coverage (-): 0.03

Region: NODE\_261184\_length\_10127\_cov\_26.726177 2631-2650. Max. coverage (+): 0.93. Max coverage (-): 1.09

Region: NODE\_261184\_length\_10127\_cov\_26.726177 2651-2671. Max. coverage (+): 0.04. Max coverage (-): 0

Region: NODE\_261184\_length\_10127\_cov\_26.726177 2672-2691. Max. coverage (+): 0.4. Max coverage (-): 0.09

Region: NODE\_261184\_length\_10127\_cov\_26.726177 2692-2711. Max. coverage (+): 0.97. Max coverage (-): 0

Region: NODE\_261184\_length\_10127\_cov\_26.726177 2712-2732. Max. coverage (+): 0.02. Max coverage (-): 0.04

Region: NODE\_261184\_length\_10127\_cov\_26.726177 2733-2752. Max. coverage (+): 1.36. Max coverage (-): 0.04

Region: NODE\_261184\_length\_10127\_cov\_26.726177 2753-2773. Max. coverage (+): 0.93. Max coverage (-): 0

Region: NODE\_261184\_length\_10127\_cov\_26.726177 2774-2793. Max. coverage (+): 0.04. Max coverage (-): 0.08

Region: NODE\_261184\_length\_10127\_cov\_26.726177 2794-2814. Max. coverage (+): 0. Max coverage (-): 0.08

Region: NODE\_261184\_length\_10127\_cov\_26.726177 2815-2834. Max. coverage (+): 0.08. Max coverage (-): 0.57

Region: NODE\_261184\_length\_10127\_cov\_26.726177 2835-2855. Max. coverage (+): 6.54. Max coverage (-): 0

Region: NODE\_261184\_length\_10127\_cov\_26.726177 2856-2875. Max. coverage (+): 0.48. Max coverage (-): 0.04

Region: NODE\_261184\_length\_10127\_cov\_26.726177 2876-2896. Max. coverage (+): 10.9. Max coverage (-): 0.04

Region: NODE\_261184\_length\_10127\_cov\_26.726177 2897-2916. Max. coverage (+): 0.61. Max coverage (-): 0

Region: NODE\_261184\_length\_10127\_cov\_26.726177 2917-2937. Max. coverage (+): 0.97. Max coverage (-): 1.62

Region: NODE\_261184\_length\_10127\_cov\_26.726177 2938-2957. Max. coverage (+): 13.08. Max coverage (-): 0.2

Region: NODE\_261184\_length\_10127\_cov\_26.726177 2958-2977. Max. coverage (+): 0.28. Max coverage (-): 0.12

Region: NODE\_261184\_length\_10127\_cov\_26.726177 2978-2998. Max. coverage (+): 1.53. Max coverage (-): 0.2

Region: NODE\_261184\_length\_10127\_cov\_26.726177 2999-3018. Max. coverage (+): 0.15. Max coverage (-): 0.19

Region: NODE\_261184\_length\_10127\_cov\_26.726177 3019-3039. Max. coverage (+): 3.59. Max coverage (-): 0.12

Region: NODE\_261184\_length\_10127\_cov\_26.726177 3040-3059. Max. coverage (+): 0.24. Max coverage (-): 0.04

Region: NODE\_261184\_length\_10127\_cov\_26.726177 3060-3080. Max. coverage (+): 0.22. Max coverage (-): 0

Region: NODE\_261184\_length\_10127\_cov\_26.726177 3081-3100. Max. coverage (+): 0.12. Max coverage (-): 0.04

Region: NODE\_261184\_length\_10127\_cov\_26.726177 3101-3121. Max. coverage (+): 0.57. Max coverage (-): 0

Region: NODE\_261184\_length\_10127\_cov\_26.726177 3122-3141. Max. coverage (+): 0.44. Max coverage (-): 0.06

Region: NODE\_261184\_length\_10127\_cov\_26.726177 3142-3162. Max. coverage (+): 0.44. Max coverage (-): 0.04

Region: NODE\_261184\_length\_10127\_cov\_26.726177 3163-3182. Max. coverage (+): 2.75. Max coverage (-): 0.08

Region: NODE\_261184\_length\_10127\_cov\_26.726177 3183-3202. Max. coverage (+): 0.08. Max coverage (-): 0

Region: NODE\_261184\_length\_10127\_cov\_26.726177 3203-3223. Max. coverage (+): 0. Max coverage (-): 0

Region: NODE\_261184\_length\_10127\_cov\_26.726177 3224-3243. Max. coverage (+): 0.36. Max coverage (-): 0

Region: NODE\_261184\_length\_10127\_cov\_26.726177 3244-3264. Max. coverage (+): 0.44. Max coverage (-): 0

Region: NODE\_261184\_length\_10127\_cov\_26.726177 3265-3284. Max. coverage (+): 0.36. Max coverage (-): 0

Region: NODE\_261184\_length\_10127\_cov\_26.726177 3285-3305. Max. coverage (+): 0.12. Max coverage (-): 0

Region: NODE\_261184\_length\_10127\_cov\_26.726177 3306-3325. Max. coverage (+): 0.04. Max coverage (-): 0.04

Region: NODE\_261184\_length\_10127\_cov\_26.726177 3326-3346. Max. coverage (+): 0.12. Max coverage (-): 0

Region: NODE\_261184\_length\_10127\_cov\_26.726177 3347-3366. Max. coverage (+): 0.12. Max coverage (-): 0

Region: NODE\_261184\_length\_10127\_cov\_26.726177 3367-3387. Max. coverage (+): 0.38. Max coverage (-): 0.01

Region: NODE\_261184\_length\_10127\_cov\_26.726177 3388-3407. Max. coverage (+): 0.36. Max coverage (-): 0

Region: NODE\_261184\_length\_10127\_cov\_26.726177 3408-3428. Max. coverage (+): 1.53. Max coverage (-): 0.2

Region: NODE\_261184\_length\_10127\_cov\_26.726177 3429-3448. Max. coverage (+): 1.86. Max coverage (-): 0.16

Region: NODE\_261184\_length\_10127\_cov\_26.726177 3449-3468. Max. coverage (+): 17.48. Max coverage (-): 3.03

Region: NODE\_261184\_length\_10127\_cov\_26.726177 3469-3489. Max. coverage (+): 15.91. Max coverage (-): 0.04

Region: NODE\_261184\_length\_10127\_cov\_26.726177 3490-3509. Max. coverage (+): 1.86. Max coverage (-): 0.04

Region: NODE\_261184\_length\_10127\_cov\_26.726177 3510-3530. Max. coverage (+): 0. Max coverage (-): 0.08

Region: NODE\_261184\_length\_10127\_cov\_26.726177 3531-3550. Max. coverage (+): 0.02. Max coverage (-): 0.04

Region: NODE\_261184\_length\_10127\_cov\_26.726177 3551-3571. Max. coverage (+): 0.03. Max coverage (-): 0

Region: NODE\_261184\_length\_10127\_cov\_26.726177 3572-3591. Max. coverage (+): 0.01. Max coverage (-): 0.08

Region: NODE\_261184\_length\_10127\_cov\_26.726177 3592-3612. Max. coverage (+): 0.67. Max coverage (-): 0

Region: NODE\_261184\_length\_10127\_cov\_26.726177 3613-3632. Max. coverage (+): 0. Max coverage (-): 0

Region: NODE\_261184\_length\_10127\_cov\_26.726177 3633-3653. Max. coverage (+): 0.11. Max coverage (-): 0

Region: NODE\_261184\_length\_10127\_cov\_26.726177 3654-3673. Max. coverage (+): 0. Max coverage (-): 0

Region: NODE\_261184\_length\_10127\_cov\_26.726177 3674-3694. Max. coverage (+): 0.08. Max coverage (-): 0

Region: NODE\_261184\_length\_10127\_cov\_26.726177 3695-3714. Max. coverage (+): 0. Max coverage (-): 0.2

Region: NODE\_261184\_length\_10127\_cov\_26.726177 3715-3734. Max. coverage (+): 0. Max coverage (-): 0

Region: NODE\_261184\_length\_10127\_cov\_26.726177 3735-3755. Max. coverage (+): 0. Max coverage (-): 0

Region: NODE\_261184\_length\_10127\_cov\_26.726177 3756-3775. Max. coverage (+): 0.52. Max coverage (-): 0

Region: NODE\_261184\_length\_10127\_cov\_26.726177 3776-3796. Max. coverage (+): 0.04. Max coverage (-): 0

Region: NODE\_261184\_length\_10127\_cov\_26.726177 3797-3816. Max. coverage (+): 0.12. Max coverage (-): 0

Region: NODE\_261184\_length\_10127\_cov\_26.726177 3817-3837. Max. coverage (+): 0. Max coverage (-): 0.02

Region: NODE\_261184\_length\_10127\_cov\_26.726177 3838-3857. Max. coverage (+): 0.09. Max coverage (-): 0.01

Region: NODE\_261184\_length\_10127\_cov\_26.726177 3858-3878. Max. coverage (+): 0.16. Max coverage (-): 0.02

Region: NODE\_261184\_length\_10127\_cov\_26.726177 3879-3898. Max. coverage (+): 3.43. Max coverage (-): 0.04

Region: NODE\_261184\_length\_10127\_cov\_26.726177 3899-3919. Max. coverage (+): 0.12. Max coverage (-): 0.04

Region: NODE\_261184\_length\_10127\_cov\_26.726177 3920-3939. Max. coverage (+): 0.04. Max coverage (-): 0.2

Region: NODE\_261184\_length\_10127\_cov\_26.726177 3940-3960. Max. coverage (+): 0.2. Max coverage (-): 0

Region: NODE\_261184\_length\_10127\_cov\_26.726177 3961-3980. Max. coverage (+): 0. Max coverage (-): 0.04

Region: NODE\_261184\_length\_10127\_cov\_26.726177 3981-4000. Max. coverage (+): 0.17. Max coverage (-): 0.24

Region: NODE\_261184\_length\_10127\_cov\_26.726177 4001-4021. Max. coverage (+): 0.12. Max coverage (-): 0.08

Region: NODE\_261184\_length\_10127\_cov\_26.726177 4022-4041. Max. coverage (+): 0.73. Max coverage (-): 0.04

Region: NODE\_261184\_length\_10127\_cov\_26.726177 4042-4062. Max. coverage (+): 0.28. Max coverage (-): 0

Region: NODE\_261184\_length\_10127\_cov\_26.726177 4063-4082. Max. coverage (+): 0.24. Max coverage (-): 0

Region: NODE\_261184\_length\_10127\_cov\_26.726177 4083-4103. Max. coverage (+): 1.98. Max coverage (-): 0

Region: NODE\_261184\_length\_10127\_cov\_26.726177 4104-4123. Max. coverage (+): 2.12. Max coverage (-): 0.02

Region: NODE\_261184\_length\_10127\_cov\_26.726177 4124-4144. Max. coverage (+): 0.04. Max coverage (-): 0.04

Region: NODE\_261184\_length\_10127\_cov\_26.726177 4145-4164. Max. coverage (+): 0. Max coverage (-): 0.04

Region: NODE\_261184\_length\_10127\_cov\_26.726177 4165-4185. Max. coverage (+): 0.2. Max coverage (-): 0

Region: NODE\_261184\_length\_10127\_cov\_26.726177 4186-4205. Max. coverage (+): 0.24. Max coverage (-): 0.16

Region: NODE\_261184\_length\_10127\_cov\_26.726177 4206-4225. Max. coverage (+): 0.85. Max coverage (-): 0.04

Region: NODE\_261184\_length\_10127\_cov\_26.726177 4226-4246. Max. coverage (+): 0.04. Max coverage (-): 0

Region: NODE\_261184\_length\_10127\_cov\_26.726177 4247-4266. Max. coverage (+): 0.12. Max coverage (-): 0.08

Region: NODE\_261184\_length\_10127\_cov\_26.726177 4267-4287. Max. coverage (+): 2.46. Max coverage (-): 0.08

Region: NODE\_261184\_length\_10127\_cov\_26.726177 4288-4307. Max. coverage (+): 0.57. Max coverage (-): 0.04

Region: NODE\_261184\_length\_10127\_cov\_26.726177 4308-4328. Max. coverage (+): 0.4. Max coverage (-): 0

Region: NODE\_261184\_length\_10127\_cov\_26.726177 4329-4348. Max. coverage (+): 0.08. Max coverage (-): 0.44

Region: NODE\_261184\_length\_10127\_cov\_26.726177 4349-4369. Max. coverage (+): 0.2. Max coverage (-): 0.16

Region: NODE\_261184\_length\_10127\_cov\_26.726177 4370-4389. Max. coverage (+): 0.44. Max coverage (-): 0.16

Region: NODE\_261184\_length\_10127\_cov\_26.726177 4390-4410. Max. coverage (+): 0.08. Max coverage (-): 0.04

Region: NODE\_261184\_length\_10127\_cov\_26.726177 4411-4430. Max. coverage (+): 3.51. Max coverage (-): 0.04

Region: NODE\_261184\_length\_10127\_cov\_26.726177 4431-4451. Max. coverage (+): 0.65. Max coverage (-): 0

Region: NODE\_261184\_length\_10127\_cov\_26.726177 4452-4471. Max. coverage (+): 0.08. Max coverage (-): 0

Region: NODE\_261184\_length\_10127\_cov\_26.726177 4472-4491. Max. coverage (+): 0.89. Max coverage (-): 0

Region: NODE\_261184\_length\_10127\_cov\_26.726177 4492-4512. Max. coverage (+): 0.28. Max coverage (-): 0

Region: NODE\_261184\_length\_10127\_cov\_26.726177 4513-4532. Max. coverage (+): 0.81. Max coverage (-): 0.16

Region: NODE\_261184\_length\_10127\_cov\_26.726177 4533-4553. Max. coverage (+): 1.66. Max coverage (-): 0.04

Region: NODE\_261184\_length\_10127\_cov\_26.726177 4554-4573. Max. coverage (+): 0.52. Max coverage (-): 0.12

Region: NODE\_261184\_length\_10127\_cov\_26.726177 4574-4594. Max. coverage (+): 0.2. Max coverage (-): 0.2

Region: NODE\_261184\_length\_10127\_cov\_26.726177 4595-4614. Max. coverage (+): 0.32. Max coverage (-): 0.04

Region: NODE\_261184\_length\_10127\_cov\_26.726177 4615-4635. Max. coverage (+): 0.32. Max coverage (-): 0.12

Region: NODE\_261184\_length\_10127\_cov\_26.726177 4636-4655. Max. coverage (+): 5.77. Max coverage (-): 0

Region: NODE\_261184\_length\_10127\_cov\_26.726177 4656-4676. Max. coverage (+): 0.36. Max coverage (-): 0.2

Region: NODE\_261184\_length\_10127\_cov\_26.726177 4677-4696. Max. coverage (+): 3.88. Max coverage (-): 0

Region: NODE\_261184\_length\_10127\_cov\_26.726177 4697-4717. Max. coverage (+): 0.77. Max coverage (-): 0.04

Region: NODE\_261184\_length\_10127\_cov\_26.726177 4718-4737. Max. coverage (+): 0.77. Max coverage (-): 0.73

Region: NODE\_261184\_length\_10127\_cov\_26.726177 4738-4757. Max. coverage (+): 3.92. Max coverage (-): 0

Region: NODE\_261184\_length\_10127\_cov\_26.726177 4758-4778. Max. coverage (+): 0.32. Max coverage (-): 0.04

Region: NODE\_261184\_length\_10127\_cov\_26.726177 4779-4798. Max. coverage (+): 1.66. Max coverage (-): 0

Region: NODE\_261184\_length\_10127\_cov\_26.726177 4799-4819. Max. coverage (+): 2.22. Max coverage (-): 0.04

Region: NODE\_261184\_length\_10127\_cov\_26.726177 4820-4839. Max. coverage (+): 0.48. Max coverage (-): 0.04

Region: NODE\_261184\_length\_10127\_cov\_26.726177 4840-4860. Max. coverage (+): 33.64. Max coverage (-): 0.2

Region: NODE\_261184\_length\_10127\_cov\_26.726177 4861-4880. Max. coverage (+): 0.08. Max coverage (-): 0

Region: NODE\_261184\_length\_10127\_cov\_26.726177 4881-4901. Max. coverage (+): 0.08. Max coverage (-): 0.16

Region: NODE\_261184\_length\_10127\_cov\_26.726177 4902-4921. Max. coverage (+): 0.12. Max coverage (-): 0

Region: NODE\_261184\_length\_10127\_cov\_26.726177 4922-4942. Max. coverage (+): 0.08. Max coverage (-): 0.04

Region: NODE\_261184\_length\_10127\_cov\_26.726177 4943-4962. Max. coverage (+): 0.04. Max coverage (-): 0

Region: NODE\_261184\_length\_10127\_cov\_26.726177 4963-4983. Max. coverage (+): 0.32. Max coverage (-): 0.2

Region: NODE\_261184\_length\_10127\_cov\_26.726177 4984-5003. Max. coverage (+): 0.24. Max coverage (-): 0.08

Region: NODE\_261184\_length\_10127\_cov\_26.726177 5004-5023. Max. coverage (+): 0.24. Max coverage (-): 0

Region: NODE\_261184\_length\_10127\_cov\_26.726177 5024-5044. Max. coverage (+): 3.47. Max coverage (-): 0.08

Region: NODE\_261184\_length\_10127\_cov\_26.726177 5045-5064. Max. coverage (+): 2.95. Max coverage (-): 0

Region: NODE\_261184\_length\_10127\_cov\_26.726177 5065-5085. Max. coverage (+): 5.21. Max coverage (-): 0.04

Region: NODE\_261184\_length\_10127\_cov\_26.726177 5086-5105. Max. coverage (+): 6.22. Max coverage (-): 0.52

Region: NODE\_261184\_length\_10127\_cov\_26.726177 5106-5126. Max. coverage (+): 2.22. Max coverage (-): 0.73

Region: NODE\_261184\_length\_10127\_cov\_26.726177 5127-5146. Max. coverage (+): 2.66. Max coverage (-): 0.32

Region: NODE\_261184\_length\_10127\_cov\_26.726177 5147-5167. Max. coverage (+): 0.93. Max coverage (-): 0.12

Region: NODE\_261184\_length\_10127\_cov\_26.726177 5168-5187. Max. coverage (+): 7.79. Max coverage (-): 0.08

Region: NODE\_261184\_length\_10127\_cov\_26.726177 5188-5208. Max. coverage (+): 1.45. Max coverage (-): 0.04

Region: NODE\_261184\_length\_10127\_cov\_26.726177 5209-5228. Max. coverage (+): 9.37. Max coverage (-): 0.12

Region: NODE\_261184\_length\_10127\_cov\_26.726177 5229-5248. Max. coverage (+): 0.28. Max coverage (-): 0

Region: NODE\_261184\_length\_10127\_cov\_26.726177 5249-5269. Max. coverage (+): 1.53. Max coverage (-): 0.04

Region: NODE\_261184\_length\_10127\_cov\_26.726177 5270-5289. Max. coverage (+): 3.67. Max coverage (-): 0.04

Region: NODE\_261184\_length\_10127\_cov\_26.726177 5290-5310. Max. coverage (+): 1.62. Max coverage (-): 0

Region: NODE\_261184\_length\_10127\_cov\_26.726177 5311-5330. Max. coverage (+): 0. Max coverage (-): 0

Region: NODE\_261184\_length\_10127\_cov\_26.726177 5331-5351. Max. coverage (+): 39.41. Max coverage (-): 0

Region: NODE\_261184\_length\_10127\_cov\_26.726177 5352-5371. Max. coverage (+): 0.08. Max coverage (-): 0.08

Region: NODE\_261184\_length\_10127\_cov\_26.726177 5372-5392. Max. coverage (+): 10.05. Max coverage (-): 0.24

Region: NODE\_261184\_length\_10127\_cov\_26.726177 5393-5412. Max. coverage (+): 0.57. Max coverage (-): 0

Region: NODE\_261184\_length\_10127\_cov\_26.726177 5413-5433. Max. coverage (+): 0.36. Max coverage (-): 0.08

Region: NODE\_261184\_length\_10127\_cov\_26.726177 5434-5453. Max. coverage (+): 0.28. Max coverage (-): 0.08

Region: NODE\_261184\_length\_10127\_cov\_26.726177 5454-5474. Max. coverage (+): 3.39. Max coverage (-): 0

Region: NODE\_261184\_length\_10127\_cov\_26.726177 5475-5494. Max. coverage (+): 1.01. Max coverage (-): 0.08

Region: NODE\_261184\_length\_10127\_cov\_26.726177 5495-5514. Max. coverage (+): 0.48. Max coverage (-): 0.08

Region: NODE\_261184\_length\_10127\_cov\_26.726177 5515-5535. Max. coverage (+): 3.23. Max coverage (-): 0

Region: NODE\_261184\_length\_10127\_cov\_26.726177 5536-5555. Max. coverage (+): 9.65. Max coverage (-): 0.16

Region: NODE\_261184\_length\_10127\_cov\_26.726177 5556-5576. Max. coverage (+): 7.43. Max coverage (-): 4.2

Region: NODE\_261184\_length\_10127\_cov\_26.726177 5577-5596. Max. coverage (+): 1.37. Max coverage (-): 0.04

Region: NODE\_261184\_length\_10127\_cov\_26.726177 5597-5617. Max. coverage (+): 1.21. Max coverage (-): 0.16

Region: NODE\_261184\_length\_10127\_cov\_26.726177 5618-5637. Max. coverage (+): 1.09. Max coverage (-): 0

Region: NODE\_261184\_length\_10127\_cov\_26.726177 5638-5658. Max. coverage (+): 0.2. Max coverage (-): 0.04

Region: NODE\_261184\_length\_10127\_cov\_26.726177 5659-5678. Max. coverage (+): 5.05. Max coverage (-): 0.32

Region: NODE\_261184\_length\_10127\_cov\_26.726177 5679-5699. Max. coverage (+): 5.05. Max coverage (-): 0.08

Region: NODE\_261184\_length\_10127\_cov\_26.726177 5700-5719. Max. coverage (+): 2.58. Max coverage (-): 0.12

Region: NODE\_261184\_length\_10127\_cov\_26.726177 5720-5740. Max. coverage (+): 0.16. Max coverage (-): 0

Region: NODE\_261184\_length\_10127\_cov\_26.726177 5741-5760. Max. coverage (+): 1.78. Max coverage (-): 0.04

Region: NODE\_261184\_length\_10127\_cov\_26.726177 5761-5780. Max. coverage (+): 2.42. Max coverage (-): 0.04

Region: NODE\_261184\_length\_10127\_cov\_26.726177 5781-5801. Max. coverage (+): 13.04. Max coverage (-): 0.04

Region: NODE\_261184\_length\_10127\_cov\_26.726177 5802-5821. Max. coverage (+): 0.57. Max coverage (-): 0.04

Region: NODE\_261184\_length\_10127\_cov\_26.726177 5822-5842. Max. coverage (+): 1.41. Max coverage (-): 0.28

Region: NODE\_261184\_length\_10127\_cov\_26.726177 5843-5862. Max. coverage (+): 85.93. Max coverage (-): 0

Region: NODE\_261184\_length\_10127\_cov\_26.726177 5863-5883. Max. coverage (+): 0.12. Max coverage (-): 0.04

Region: NODE\_261184\_length\_10127\_cov\_26.726177 5884-5903. Max. coverage (+): 6.3. Max coverage (-): 0.08

Region: NODE\_261184\_length\_10127\_cov\_26.726177 5904-5924. Max. coverage (+): 1.05. Max coverage (-): 0.04

Region: NODE\_261184\_length\_10127\_cov\_26.726177 5925-5944. Max. coverage (+): 1.62. Max coverage (-): 0.24

Region: NODE\_261184\_length\_10127\_cov\_26.726177 5945-5965. Max. coverage (+): 1.01. Max coverage (-): 0

Region: NODE\_261184\_length\_10127\_cov\_26.726177 5966-5985. Max. coverage (+): 3.67. Max coverage (-): 0.08

Region: NODE\_261184\_length\_10127\_cov\_26.726177 5986-6006. Max. coverage (+): 7.03. Max coverage (-): 0.28

Region: NODE\_261184\_length\_10127\_cov\_26.726177 6007-6026. Max. coverage (+): 0.77. Max coverage (-): 0.97

Region: NODE\_261184\_length\_10127\_cov\_26.726177 6027-6046. Max. coverage (+): 2.02. Max coverage (-): 0.04

Region: NODE\_261184\_length\_10127\_cov\_26.726177 6047-6067. Max. coverage (+): 0.04. Max coverage (-): 0.12

Region: NODE\_261184\_length\_10127\_cov\_26.726177 6068-6087. Max. coverage (+): 0.04. Max coverage (-): 0

Region: NODE\_261184\_length\_10127\_cov\_26.726177 6088-6108. Max. coverage (+): 0.08. Max coverage (-): 0.44

Region: NODE\_261184\_length\_10127\_cov\_26.726177 6109-6128. Max. coverage (+): 25.32. Max coverage (-): 0.44

Region: NODE\_261184\_length\_10127\_cov\_26.726177 6129-6149. Max. coverage (+): 0.04. Max coverage (-): 0.04

Region: NODE\_261184\_length\_10127\_cov\_26.726177 6150-6169. Max. coverage (+): 1.41. Max coverage (-): 0

Region: NODE\_261184\_length\_10127\_cov\_26.726177 6170-6190. Max. coverage (+): 0.4. Max coverage (-): 0.08

Region: NODE\_261184\_length\_10127\_cov\_26.726177 6191-6210. Max. coverage (+): 3.27. Max coverage (-): 0

Region: NODE\_261184\_length\_10127\_cov\_26.726177 6211-6231. Max. coverage (+): 0.2. Max coverage (-): 0.04

Region: NODE\_261184\_length\_10127\_cov\_26.726177 6232-6251. Max. coverage (+): 0.2. Max coverage (-): 0

Region: NODE\_261184\_length\_10127\_cov\_26.726177 6252-6271. Max. coverage (+): 0.16. Max coverage (-): 0

Region: NODE\_261184\_length\_10127\_cov\_26.726177 6272-6292. Max. coverage (+): 0.2. Max coverage (-): 0

Region: NODE\_261184\_length\_10127\_cov\_26.726177 6293-6312. Max. coverage (+): 0.77. Max coverage (-): 0.08

Region: NODE\_261184\_length\_10127\_cov\_26.726177 6313-6333. Max. coverage (+): 0.4. Max coverage (-): 0.16

Region: NODE\_261184\_length\_10127\_cov\_26.726177 6334-6353. Max. coverage (+): 0.12. Max coverage (-): 0.04

Region: NODE\_261184\_length\_10127\_cov\_26.726177 6354-6374. Max. coverage (+): 0. Max coverage (-): 0

Region: NODE\_261184\_length\_10127\_cov\_26.726177 6375-6394. Max. coverage (+): 0. Max coverage (-): 0

Region: NODE\_261184\_length\_10127\_cov\_26.726177 6395-6415. Max. coverage (+): 0.73. Max coverage (-): 0

Region: NODE\_261184\_length\_10127\_cov\_26.726177 6416-6435. Max. coverage (+): 9.65. Max coverage (-): 0.04

Region: NODE\_261184\_length\_10127\_cov\_26.726177 6436-6456. Max. coverage (+): 2.14. Max coverage (-): 0.04

Region: NODE\_261184\_length\_10127\_cov\_26.726177 6457-6476. Max. coverage (+): 0.85. Max coverage (-): 0.08

Region: NODE\_261184\_length\_10127\_cov\_26.726177 6477-6497. Max. coverage (+): 0.52. Max coverage (-): 0.04

Region: NODE\_261184\_length\_10127\_cov\_26.726177 6498-6517. Max. coverage (+): 0.77. Max coverage (-): 0.04

Region: NODE\_261184\_length\_10127\_cov\_26.726177 6518-6537. Max. coverage (+): 0.04. Max coverage (-): 0

Region: NODE\_261184\_length\_10127\_cov\_26.726177 6538-6558. Max. coverage (+): 0.52. Max coverage (-): 0.04

Region: NODE\_261184\_length\_10127\_cov\_26.726177 6559-6578. Max. coverage (+): 1.86. Max coverage (-): 0.08

Region: NODE\_261184\_length\_10127\_cov\_26.726177 6579-6599. Max. coverage (+): 1.53. Max coverage (-): 0

Region: NODE\_261184\_length\_10127\_cov\_26.726177 6600-6619. Max. coverage (+): 0.44. Max coverage (-): 0.08

Region: NODE\_261184\_length\_10127\_cov\_26.726177 6620-6640. Max. coverage (+): 0.97. Max coverage (-): 0.04

Region: NODE\_261184\_length\_10127\_cov\_26.726177 6641-6660. Max. coverage (+): 0.2. Max coverage (-): 0.97

Region: NODE\_261184\_length\_10127\_cov\_26.726177 6661-6681. Max. coverage (+): 0.32. Max coverage (-): 0.93

Region: NODE\_261184\_length\_10127\_cov\_26.726177 6682-6701. Max. coverage (+): 0.97. Max coverage (-): 0.24

Region: NODE\_261184\_length\_10127\_cov\_26.726177 6702-6722. Max. coverage (+): 0.2. Max coverage (-): 0.24

Region: NODE\_261184\_length\_10127\_cov\_26.726177 6723-6742. Max. coverage (+): 7.07. Max coverage (-): 0.28

Region: NODE\_261184\_length\_10127\_cov\_26.726177 6743-6763. Max. coverage (+): 3.63. Max coverage (-): 0.32

Region: NODE\_261184\_length\_10127\_cov\_26.726177 6764-6783. Max. coverage (+): 0.04. Max coverage (-): 0

Region: NODE\_261184\_length\_10127\_cov\_26.726177 6784-6803. Max. coverage (+): 0.36. Max coverage (-): 0

Region: NODE\_261184\_length\_10127\_cov\_26.726177 6804-6824. Max. coverage (+): 0.04. Max coverage (-): 0.04

Region: NODE\_261184\_length\_10127\_cov\_26.726177 6825-6844. Max. coverage (+): 0.12. Max coverage (-): 0

Region: NODE\_261184\_length\_10127\_cov\_26.726177 6845-6865. Max. coverage (+): 0.08. Max coverage (-): 0.04

Region: NODE\_261184\_length\_10127\_cov\_26.726177 6866-6885. Max. coverage (+): 2.42. Max coverage (-): 0.2

Region: NODE\_261184\_length\_10127\_cov\_26.726177 6886-6906. Max. coverage (+): 1.86. Max coverage (-): 0.01

Region: NODE\_261184\_length\_10127\_cov\_26.726177 6907-6926. Max. coverage (+): 1.17. Max coverage (-): 0.01

Region: NODE\_261184\_length\_10127\_cov\_26.726177 6927-6947. Max. coverage (+): 0.16. Max coverage (-): 0

Region: NODE\_261184\_length\_10127\_cov\_26.726177 6948-6967. Max. coverage (+): 0.03. Max coverage (-): 0

Region: NODE\_261184\_length\_10127\_cov\_26.726177 6968-6988. Max. coverage (+): 0.12. Max coverage (-): 0

Region: NODE\_261184\_length\_10127\_cov\_26.726177 6989-7008. Max. coverage (+): 0.12. Max coverage (-): 0.08

Region: NODE\_261184\_length\_10127\_cov\_26.726177 7009-7029. Max. coverage (+): 0.87. Max coverage (-): 0.08

Region: NODE\_261184\_length\_10127\_cov\_26.726177 7030-7049. Max. coverage (+): 0.08. Max coverage (-): 0

Region: NODE\_261184\_length\_10127\_cov\_26.726177 7050-7069. Max. coverage (+): 0.12. Max coverage (-): 0.05

Region: NODE\_261184\_length\_10127\_cov\_26.726177 7070-7090. Max. coverage (+): 0.4. Max coverage (-): 0

Region: NODE\_261184\_length\_10127\_cov\_26.726177 7091-7110. Max. coverage (+): 0.04. Max coverage (-): 0

Region: NODE\_261184\_length\_10127\_cov\_26.726177 7111-7131. Max. coverage (+): 0.44. Max coverage (-): 0.04

Region: NODE\_261184\_length\_10127\_cov\_26.726177 7132-7151. Max. coverage (+): 0.2. Max coverage (-): 0.05

Region: NODE\_261184\_length\_10127\_cov\_26.726177 7152-7172. Max. coverage (+): 0.08. Max coverage (-): 0.04

Region: NODE\_261184\_length\_10127\_cov\_26.726177 7173-7192. Max. coverage (+): 0. Max coverage (-): 0

Region: NODE\_261184\_length\_10127\_cov\_26.726177 7193-7213. Max. coverage (+): 0.04. Max coverage (-): 0

Region: NODE\_261184\_length\_10127\_cov\_26.726177 7214-7233. Max. coverage (+): 0.01. Max coverage (-): 0.24

Region: NODE\_261184\_length\_10127\_cov\_26.726177 7234-7254. Max. coverage (+): 0.25. Max coverage (-): 0

Region: NODE\_261184\_length\_10127\_cov\_26.726177 7255-7274. Max. coverage (+): 0.4. Max coverage (-): 0

Region: NODE\_261184\_length\_10127\_cov\_26.726177 7275-7294. Max. coverage (+): 0.04. Max coverage (-): 0

Region: NODE\_261184\_length\_10127\_cov\_26.726177 7295-7315. Max. coverage (+): 0.29. Max coverage (-): 0

Region: NODE\_261184\_length\_10127\_cov\_26.726177 7316-7335. Max. coverage (+): 0.04. Max coverage (-): 0

Region: NODE\_261184\_length\_10127\_cov\_26.726177 7336-7356. Max. coverage (+): 0.36. Max coverage (-): 0.08

Region: NODE\_261184\_length\_10127\_cov\_26.726177 7357-7376. Max. coverage (+): 0.08. Max coverage (-): 0.08

Region: NODE\_261184\_length\_10127\_cov\_26.726177 7377-7397. Max. coverage (+): 0.08. Max coverage (-): 0

Region: NODE\_261184\_length\_10127\_cov\_26.726177 7398-7417. Max. coverage (+): 0.08. Max coverage (-): 0.04

Region: NODE\_261184\_length\_10127\_cov\_26.726177 7418-7438. Max. coverage (+): 0.16. Max coverage (-): 0

Region: NODE\_261184\_length\_10127\_cov\_26.726177 7439-7458. Max. coverage (+): 0. Max coverage (-): 0.04

Region: NODE\_261184\_length\_10127\_cov\_26.726177 7459-7479. Max. coverage (+): 0.04. Max coverage (-): 0.01

Region: NODE\_261184\_length\_10127\_cov\_26.726177 7480-7499. Max. coverage (+): 0.05. Max coverage (-): 0.01

Region: NODE\_261184\_length\_10127\_cov\_26.726177 7500-7520. Max. coverage (+): 0.01. Max coverage (-): 0

Region: NODE\_261184\_length\_10127\_cov\_26.726177 7521-7540. Max. coverage (+): 0.03. Max coverage (-): 0.02

Region: NODE\_261184\_length\_10127\_cov\_26.726177 7541-7560. Max. coverage (+): 0.01. Max coverage (-): 0

Region: NODE\_261184\_length\_10127\_cov\_26.726177 7561-7581. Max. coverage (+): 0.04. Max coverage (-): 0

Region: NODE\_261184\_length\_10127\_cov\_26.726177 7582-7601. Max. coverage (+): 0.04. Max coverage (-): 0

Region: NODE\_261184\_length\_10127\_cov\_26.726177 7602-7622. Max. coverage (+): 0.26. Max coverage (-): 0

Region: NODE\_261184\_length\_10127\_cov\_26.726177 7623-7642. Max. coverage (+): 0.04. Max coverage (-): 0

Region: NODE\_261184\_length\_10127\_cov\_26.726177 7643-7663. Max. coverage (+): 0.08. Max coverage (-): 0.08

Region: NODE\_261184\_length\_10127\_cov\_26.726177 7664-7683. Max. coverage (+): 0.12. Max coverage (-): 0

Region: NODE\_261184\_length\_10127\_cov\_26.726177 7684-7704. Max. coverage (+): 0. Max coverage (-): 0

Region: NODE\_261184\_length\_10127\_cov\_26.726177 7705-7724. Max. coverage (+): 0.04. Max coverage (-): 0.03

Region: NODE\_261184\_length\_10127\_cov\_26.726177 7725-7745. Max. coverage (+): 1.76. Max coverage (-): 0

Region: NODE\_261184\_length\_10127\_cov\_26.726177 7746-7765. Max. coverage (+): 0.04. Max coverage (-): 0

Region: NODE\_261184\_length\_10127\_cov\_26.726177 7766-7786. Max. coverage (+): 0.4. Max coverage (-): 0

Region: NODE\_261184\_length\_10127\_cov\_26.726177 7787-7806. Max. coverage (+): 0.45. Max coverage (-): 0.04

Region: NODE\_261184\_length\_10127\_cov\_26.726177 7807-7826. Max. coverage (+): 0.89. Max coverage (-): 0.02

Region: NODE\_261184\_length\_10127\_cov\_26.726177 7827-7847. Max. coverage (+): 0.05. Max coverage (-): 0.01

Region: NODE\_261184\_length\_10127\_cov\_26.726177 7848-7867. Max. coverage (+): 0.55. Max coverage (-): 0.01

Region: NODE\_261184\_length\_10127\_cov\_26.726177 7868-7888. Max. coverage (+): 0.16. Max coverage (-): 0

Region: NODE\_261184\_length\_10127\_cov\_26.726177 7889-7908. Max. coverage (+): 0.08. Max coverage (-): 0

Region: NODE\_261184\_length\_10127\_cov\_26.726177 7909-7929. Max. coverage (+): 0. Max coverage (-): 0

Region: NODE\_261184\_length\_10127\_cov\_26.726177 7930-7949. Max. coverage (+): 0.89. Max coverage (-): 0

Region: NODE\_261184\_length\_10127\_cov\_26.726177 7950-7970. Max. coverage (+): 0.44. Max coverage (-): 0

Region: NODE\_261184\_length\_10127\_cov\_26.726177 7971-7990. Max. coverage (+): 0.69. Max coverage (-): 0

Region: NODE\_261184\_length\_10127\_cov\_26.726177 7991-8011. Max. coverage (+): 0.05. Max coverage (-): 0

Region: NODE\_261184\_length\_10127\_cov\_26.726177 8012-8031. Max. coverage (+): 0.09. Max coverage (-): 0

Region: NODE\_261184\_length\_10127\_cov\_26.726177 8032-8052. Max. coverage (+): 0.04. Max coverage (-): 0

Region: NODE\_261184\_length\_10127\_cov\_26.726177 8053-8072. Max. coverage (+): 0.08. Max coverage (-): 0

Region: NODE\_261184\_length\_10127\_cov\_26.726177 8073-8092. Max. coverage (+): 0.4. Max coverage (-): 0

Region: NODE\_261184\_length\_10127\_cov\_26.726177 8093-8113. Max. coverage (+): 0.2. Max coverage (-): 0

Region: NODE\_261184\_length\_10127\_cov\_26.726177 8114-8133. Max. coverage (+): 0.4. Max coverage (-): 0

Region: NODE\_261184\_length\_10127\_cov\_26.726177 8134-8154. Max. coverage (+): 0. Max coverage (-): 0.04

Region: NODE\_261184\_length\_10127\_cov\_26.726177 8155-8174. Max. coverage (+): 0. Max coverage (-): 0.04

Region: NODE\_261184\_length\_10127\_cov\_26.726177 8175-8195. Max. coverage (+): 0.04. Max coverage (-): 0

Region: NODE\_261184\_length\_10127\_cov\_26.726177 8196-8215. Max. coverage (+): 0.04. Max coverage (-): 0

Region: NODE\_261184\_length\_10127\_cov\_26.726177 8216-8236. Max. coverage (+): 0.24. Max coverage (-): 0

Region: NODE\_261184\_length\_10127\_cov\_26.726177 8237-8256. Max. coverage (+): 0. Max coverage (-): 0.04

Region: NODE\_261184\_length\_10127\_cov\_26.726177 8257-8277. Max. coverage (+): 1.62. Max coverage (-): 0.04

Region: NODE\_261184\_length\_10127\_cov\_26.726177 8278-8297. Max. coverage (+): 1.25. Max coverage (-): 0.16

Region: NODE\_261184\_length\_10127\_cov\_26.726177 8298-8317. Max. coverage (+): 0.16. Max coverage (-): 0.04

Region: NODE\_261184\_length\_10127\_cov\_26.726177 8318-8338. Max. coverage (+): 0.65. Max coverage (-): 0

Region: NODE\_261184\_length\_10127\_cov\_26.726177 8339-8358. Max. coverage (+): 0.57. Max coverage (-): 0.01

Region: NODE\_261184\_length\_10127\_cov\_26.726177 8359-8379. Max. coverage (+): 1.45. Max coverage (-): 0.2

Region: NODE\_261184\_length\_10127\_cov\_26.726177 8380-8399. Max. coverage (+): 0.77. Max coverage (-): 0.04

Region: NODE\_261184\_length\_10127\_cov\_26.726177 8400-8420. Max. coverage (+): 0.93. Max coverage (-): 0

Region: NODE\_261184\_length\_10127\_cov\_26.726177 8421-8440. Max. coverage (+): 0.12. Max coverage (-): 0

Region: NODE\_261184\_length\_10127\_cov\_26.726177 8441-8461. Max. coverage (+): 0.32. Max coverage (-): 0

Region: NODE\_261184\_length\_10127\_cov\_26.726177 8462-8481. Max. coverage (+): 0.04. Max coverage (-): 0

Region: NODE\_261184\_length\_10127\_cov\_26.726177 8482-8502. Max. coverage (+): 0.59. Max coverage (-): 0

Region: NODE\_261184\_length\_10127\_cov\_26.726177 8503-8522. Max. coverage (+): 0.04. Max coverage (-): 0.05

Region: NODE\_261184\_length\_10127\_cov\_26.726177 8523-8543. Max. coverage (+): 0.36. Max coverage (-): 0.05

Region: NODE\_261184\_length\_10127\_cov\_26.726177 8544-8563. Max. coverage (+): 0.24. Max coverage (-): 0.07

Region: NODE\_261184\_length\_10127\_cov\_26.726177 8564-8583. Max. coverage (+): 0.04. Max coverage (-): 0.02

Region: NODE\_261184\_length\_10127\_cov\_26.726177 8584-8604. Max. coverage (+): 0.12. Max coverage (-): 0.16

Region: NODE\_261184\_length\_10127\_cov\_26.726177 8605-8624. Max. coverage (+): 0.48. Max coverage (-): 0.04

Region: NODE\_261184\_length\_10127\_cov\_26.726177 8625-8645. Max. coverage (+): 0.4. Max coverage (-): 0.12

Region: NODE\_261184\_length\_10127\_cov\_26.726177 8646-8665. Max. coverage (+): 1.35. Max coverage (-): 0.08

Region: NODE\_261184\_length\_10127\_cov\_26.726177 8666-8686. Max. coverage (+): 2.02. Max coverage (-): 0.08

Region: NODE\_261184\_length\_10127\_cov\_26.726177 8687-8706. Max. coverage (+): 0.28. Max coverage (-): 0.04

Region: NODE\_261184\_length\_10127\_cov\_26.726177 8707-8727. Max. coverage (+): 0.32. Max coverage (-): 0.08

Region: NODE\_261184\_length\_10127\_cov\_26.726177 8728-8747. Max. coverage (+): 8.82. Max coverage (-): 0

Region: NODE\_261184\_length\_10127\_cov\_26.726177 8748-8768. Max. coverage (+): 0.09. Max coverage (-): 0.09

Region: NODE\_261184\_length\_10127\_cov\_26.726177 8769-8788. Max. coverage (+): 0.37. Max coverage (-): 0.01

Region: NODE\_261184\_length\_10127\_cov\_26.726177 8789-8809. Max. coverage (+): 0.02. Max coverage (-): 0

Region: NODE\_261184\_length\_10127\_cov\_26.726177 8810-8829. Max. coverage (+): 0.97. Max coverage (-): 0.04

Region: NODE\_261184\_length\_10127\_cov\_26.726177 8830-8849. Max. coverage (+): 0.33. Max coverage (-): 0.08

Region: NODE\_261184\_length\_10127\_cov\_26.726177 8850-8870. Max. coverage (+): 0.42. Max coverage (-): 0.1

Region: NODE\_261184\_length\_10127\_cov\_26.726177 8871-8890. Max. coverage (+): 0.61. Max coverage (-): 0.04

Region: NODE\_261184\_length\_10127\_cov\_26.726177 8891-8911. Max. coverage (+): 0.04. Max coverage (-): 0.04

Region: NODE\_261184\_length\_10127\_cov\_26.726177 8912-8931. Max. coverage (+): 0.16. Max coverage (-): 0.01

Region: NODE\_261184\_length\_10127\_cov\_26.726177 8932-8952. Max. coverage (+): 3.23. Max coverage (-): 0

Region: NODE\_261184\_length\_10127\_cov\_26.726177 8953-8972. Max. coverage (+): 0.22. Max coverage (-): 0

Region: NODE\_261184\_length\_10127\_cov\_26.726177 8973-8993. Max. coverage (+): 0.19. Max coverage (-): 0.01

Region: NODE\_261184\_length\_10127\_cov\_26.726177 8994-9013. Max. coverage (+): 0.52. Max coverage (-): 0.1

Region: NODE\_261184\_length\_10127\_cov\_26.726177 9014-9034. Max. coverage (+): 0.52. Max coverage (-): 0

Region: NODE\_261184\_length\_10127\_cov\_26.726177 9035-9054. Max. coverage (+): 0.36. Max coverage (-): 0.04

Region: NODE\_261184\_length\_10127\_cov\_26.726177 9055-9075. Max. coverage (+): 0.32. Max coverage (-): 0.08

Region: NODE\_261184\_length\_10127\_cov\_26.726177 9076-9095. Max. coverage (+): 0.04. Max coverage (-): 0.26

Region: NODE\_261184\_length\_10127\_cov\_26.726177 9096-9115. Max. coverage (+): 0.04. Max coverage (-): 0.01

Region: NODE\_261184\_length\_10127\_cov\_26.726177 9116-9136. Max. coverage (+): 0.03. Max coverage (-): 0

Region: NODE\_261184\_length\_10127\_cov\_26.726177 9137-9156. Max. coverage (+): 0. Max coverage (-): 0.01

Region: NODE\_261184\_length\_10127\_cov\_26.726177 9157-9177. Max. coverage (+): 0. Max coverage (-): 0

Region: NODE\_261184\_length\_10127\_cov\_26.726177 9178-9197. Max. coverage (+): 0. Max coverage (-): 0

Region: NODE\_261184\_length\_10127\_cov\_26.726177 9198-9218. Max. coverage (+): 0. Max coverage (-): 0

Region: NODE\_261184\_length\_10127\_cov\_26.726177 9219-9238. Max. coverage (+): 0. Max coverage (-): 0

Region: NODE\_261184\_length\_10127\_cov\_26.726177 9239-9259. Max. coverage (+): 0. Max coverage (-): 0

Region: NODE\_261184\_length\_10127\_cov\_26.726177 9260-9279. Max. coverage (+): 0. Max coverage (-): 0

Region: NODE\_261184\_length\_10127\_cov\_26.726177 9280-9300. Max. coverage (+): 0. Max coverage (-): 0

Region: NODE\_261184\_length\_10127\_cov\_26.726177 9301-9320. Max. coverage (+): 0.64. Max coverage (-): 0

Region: NODE\_261184\_length\_10127\_cov\_26.726177 9321-9340. Max. coverage (+): 0.68. Max coverage (-): 0

Region: NODE\_261184\_length\_10127\_cov\_26.726177 9341-9361. Max. coverage (+): 0.11. Max coverage (-): 0.01

Region: NODE\_261184\_length\_10127\_cov\_26.726177 9362-9381. Max. coverage (+): 0.02. Max coverage (-): 0

Region: NODE\_261184\_length\_10127\_cov\_26.726177 9382-9402. Max. coverage (+): 3.67. Max coverage (-): 0

Region: NODE\_261184\_length\_10127\_cov\_26.726177 9403-9422. Max. coverage (+): 0.4. Max coverage (-): 0

Region: NODE\_261184\_length\_10127\_cov\_26.726177 9423-9443. Max. coverage (+): 0.32. Max coverage (-): 0.07

Region: NODE\_261184\_length\_10127\_cov\_26.726177 9444-9463. Max. coverage (+): 0.02. Max coverage (-): 0.01

Region: NODE\_261184\_length\_10127\_cov\_26.726177 9464-9484. Max. coverage (+): 0.16. Max coverage (-): 0

Region: NODE\_261184\_length\_10127\_cov\_26.726177 9485-9504. Max. coverage (+): 0.02. Max coverage (-): 1.09

Region: NODE\_261184\_length\_10127\_cov\_26.726177 9505-9525. Max. coverage (+): 4.17. Max coverage (-): 1.22

Region: NODE\_261184\_length\_10127\_cov\_26.726177 9526-9545. Max. coverage (+): 0.04. Max coverage (-): 0

Region: NODE\_261184\_length\_10127\_cov\_26.726177 9546-9566. Max. coverage (+): 0.06. Max coverage (-): 0

Region: NODE\_261184\_length\_10127\_cov\_26.726177 9567-9586. Max. coverage (+): 0.35. Max coverage (-): 0.01

Region: NODE\_261184\_length\_10127\_cov\_26.726177 9587-9606. Max. coverage (+): 0.12. Max coverage (-): 0.01

Region: NODE\_261184\_length\_10127\_cov\_26.726177 9607-9627. Max. coverage (+): 0.05. Max coverage (-): 0.01

Region: NODE\_261184\_length\_10127\_cov\_26.726177 9628-9647. Max. coverage (+): 0.01. Max coverage (-): 0

Region: NODE\_261184\_length\_10127\_cov\_26.726177 9648-9668. Max. coverage (+): 0.24. Max coverage (-): 0.04

Region: NODE\_261184\_length\_10127\_cov\_26.726177 9669-9688. Max. coverage (+): 0.26. Max coverage (-): 0.1

Region: NODE\_261184\_length\_10127\_cov\_26.726177 9689-9709. Max. coverage (+): 0.36. Max coverage (-): 0.24

Region: NODE\_261184\_length\_10127\_cov\_26.726177 9710-9729. Max. coverage (+): 0.08. Max coverage (-): 0

Region: NODE\_261184\_length\_10127\_cov\_26.726177 9730-9750. Max. coverage (+): 0.77. Max coverage (-): 0

Region: NODE\_261184\_length\_10127\_cov\_26.726177 9751-9770. Max. coverage (+): 0.07. Max coverage (-): 0.04

Region: NODE\_261184\_length\_10127\_cov\_26.726177 9771-9791. Max. coverage (+): 0.4. Max coverage (-): 0.04

Region: NODE\_261184\_length\_10127\_cov\_26.726177 9792-9811. Max. coverage (+): 2.99. Max coverage (-): 0

Region: NODE\_261184\_length\_10127\_cov\_26.726177 9812-9832. Max. coverage (+): 0.06. Max coverage (-): 0

Region: NODE\_261184\_length\_10127\_cov\_26.726177 9833-9852. Max. coverage (+): 0. Max coverage (-): 0.12

Region: NODE\_261184\_length\_10127\_cov\_26.726177 9853-9872. Max. coverage (+): 0.77. Max coverage (-): 0

Region: NODE\_261184\_length\_10127\_cov\_26.726177 9873-9893. Max. coverage (+): 0.12. Max coverage (-): 0

Region: NODE\_261184\_length\_10127\_cov\_26.726177 9894-9913. Max. coverage (+): 0.08. Max coverage (-): 0.08

Region: NODE\_261184\_length\_10127\_cov\_26.726177 9914-9934. Max. coverage (+): 0.24. Max coverage (-): 0

Region: NODE\_261184\_length\_10127\_cov\_26.726177 9935-9954. Max. coverage (+): 0.2. Max coverage (-): 0.04

Region: NODE\_261184\_length\_10127\_cov\_26.726177 9955-9975. Max. coverage (+): 0.08. Max coverage (-): 0.04

Region: NODE\_261184\_length\_10127\_cov\_26.726177 9976-9995. Max. coverage (+): 0. Max coverage (-): 0.02

Region: NODE\_261184\_length\_10127\_cov\_26.726177 9996-10016. Max. coverage (+): 0.24. Max coverage (-): 0

Region: NODE\_261184\_length\_10127\_cov\_26.726177 10017-10036. Max. coverage (+): 0.89. Max coverage (-): 0

Region: NODE\_261184\_length\_10127\_cov\_26.726177 10037-10057. Max. coverage (+): 0.97. Max coverage (-): 0

Region: NODE\_261184\_length\_10127\_cov\_26.726177 10058-10077. Max. coverage (+): 0.04. Max coverage (-): 0.08

Region: NODE\_261184\_length\_10127\_cov\_26.726177 10078-10098. Max. coverage (+): 0.93. Max coverage (-): 0.28

Region: NODE\_261184\_length\_10127\_cov\_26.726177 10099-10118. Max. coverage (+): 0.93. Max coverage (-): 0

Region: NODE\_261184\_length\_10127\_cov\_26.726177 10119-10138. Max. coverage (+): 0.04. Max coverage (-): 0

Region: NODE\_261184\_length\_10127\_cov\_26.726177 10139-10159. Max. coverage (+): 0.16. Max coverage (-): 0

Region: NODE\_261184\_length\_10127\_cov\_26.726177 10160-10179. Max. coverage (+): 0.03. Max coverage (-): 0

Region: NODE\_261184\_length\_10127\_cov\_26.726177 10180-10200. Max. coverage (+): 0. Max coverage (-): 0

Region: NODE\_261184\_length\_10127\_cov\_26.726177 10201-10220. Max. coverage (+): 0. Max coverage (-): 0.04

Region: NODE\_261184\_length\_10127\_cov\_26.726177 10221-. Max. coverage (+): 0. Max coverage (-): 0

RepeatMasker Color Code

**+**

100-98% Identity

<98-95% Identity

<95-90% Identity

<90-85% Identity

<85-80% Identity

<80-75% Identity

<75-70% Identity

<70% Identity

**-**

Gene Set Color Code

**+**

Gene

Pseudogene

Other

**-**

Topology/Coverage Color Code

Coverage Plus Strand

Coverage Minus Strand

Mainstrand: Plus

Mainstrand: Minus

Complementary Strand

Flanking Region  
(if option -flank >0)

Gene Set Annotation  
  
RepeatMasker Annotation  

**1. DNAX-24\_DR**: 1-63 (-), Divergence to consensus: 22.2%  
**2. AlRepB-250**: 13-66 (-), Divergence to consensus: 3.7%  
**3. AlRepB-65**: 67-185 (+), Divergence to consensus: 20.3%  
**4. SINE\_AFC**: 185-506 (-), Divergence to consensus: 15.9%  
**5. Merlin-1\_DR**: 432-568 (-), Divergence to consensus: 39.1%  
**6. AlRepB-65**: 517-578 (+), Divergence to consensus: 12.9%  
**7. AlRepD-3818**: 536-643 (-), Divergence to consensus: 31.7%  
**8. AlRepB-65**: 628-1292 (+), Divergence to consensus: 8.8%  
**9. AlRepD-382**: 1351-1657 (+), Divergence to consensus: 34.6%  
**10. (TATTTT)n**: 2013-2091 (+), Divergence to consensus: 31.9%  
**11. A-rich**: 2206-2254 (+), Divergence to consensus: 27.3%  
**12. (TCCTC)n**: 2405-2438 (+), Divergence to consensus: 15.7%  
**13. AlRepB-127**: 3479-3899 (+), Divergence to consensus: 7.8%  
**14. AlRepE-1026**: 3904-4064 (-), Divergence to consensus: 31.7%  
**15. AlRepE-174**: 4156-4990 (-), Divergence to consensus: 44.5%  
**16. CryptonV-1\_DR**: 4992-5894 (-), Divergence to consensus: 43.1%  
**17. (TTTTA)n**: 6073-6098 (+), Divergence to consensus: 12.1%  
**18. AlRepC-1574**: 6900-6947 (-), Divergence to consensus: 21%  
**19. AlRepD-1790**: 6936-7179 (+), Divergence to consensus: 18.5%  
**20. AlRepC-146**: 7219-7765 (-), Divergence to consensus: 21.9%  
**21. AlRepA-118**: 7802-7894 (+), Divergence to consensus: 24.8%  
**22. AlRepC-146**: 8072-8252 (-), Divergence to consensus: 25.1%  
**23. Furousha1**: 8494-8625 (-), Divergence to consensus: 37.2%  
**24. Furousha1**: 8704-9138 (-), Divergence to consensus: 35.4%  
**25. Furousha2**: 9300-9744 (-), Divergence to consensus: 36%  
**26. AlRepC-2154**: 10115-10217 (-), Divergence to consensus: 27.6%

  
Transcription Factor Binding Sites  

**RFX4\_2** (Sequence: CCTGGATAC (+): 2725)  
**RHOXF1** (Sequence: AGATCA (-): 645)  
**RHOXF1** (Sequence: AGCTCA (-): 2479)  
**RHOXF1** (Sequence: AGATTA (-): 3637)  
**RHOXF1** (Sequence: AGCTCA (-): 4263)  
**RHOXF1** (Sequence: AGCTCA (-): 4335)  
**RHOXF1** (Sequence: AGCTCA (-): 4410)  
**RHOXF1** (Sequence: AGCTCA (-): 4650)  
**RHOXF1** (Sequence: AGCTCA (-): 4674)  
**RHOXF1** (Sequence: AGCTTA (-): 5424)  
**RHOXF1** (Sequence: GGCTCA (-): 5525)  
**RHOXF1** (Sequence: GGATCA (-): 5722)  
**RHOXF1** (Sequence: AGCTTA (-): 5830)  
**RHOXF1** (Sequence: AGCTTA (-): 6189)  
**RHOXF1** (Sequence: AGATTA (-): 6204)  
**RHOXF1** (Sequence: GGCTCA (-): 6327)  
**RHOXF1** (Sequence: GGATTA (-): 6591)  
**RHOXF1** (Sequence: AGCTCA (-): 6806)  
**RHOXF1** (Sequence: AGATTA (-): 7689)  
**RHOXF1** (Sequence: GGATCA (-): 8581)  
**RHOXF1** (Sequence: AGCTTA (-): 9042)  
**RHOXF1** (Sequence: GGCTTA (-): 9812)  
**RHOXF1** (Sequence: GGATCA (-): 9890)  
**RHOXF1** (Sequence: TGAGCC (+): 492)  
**RHOXF1** (Sequence: TAAGCT (+): 697)  
**RHOXF1** (Sequence: TAATCC (+): 827)  
**RHOXF1** (Sequence: TGATCT (+): 1888)  
**RHOXF1** (Sequence: TGATCT (+): 1939)  
**RHOXF1** (Sequence: TAATCC (+): 1989)  
**RHOXF1** (Sequence: TGATCT (+): 2527)  
**RHOXF1** (Sequence: TAATCC (+): 3004)  
**RHOXF1** (Sequence: TAAGCC (+): 3993)  
**RHOXF1** (Sequence: TGATCC (+): 5491)  
**RHOXF1** (Sequence: TGATCT (+): 5769)  
**RHOXF1** (Sequence: TAATCC (+): 6623)  
**RHOXF1** (Sequence: TAAGCC (+): 6819)  
**RHOXF1** (Sequence: TAAGCT (+): 7087)  
**RHOXF1** (Sequence: TAAGCT (+): 7218)  
**RHOXF1** (Sequence: TGATCT (+): 8977)  
**RHOXF1** (Sequence: TGATCT (+): 9069)  
**RHOXF1** (Sequence: TGATCC (+): 9766)  
**Gata4** (Sequence: CTTATCT (+): 2658)  
**POU5F1** (Sequence: TTTGCAT (-): 541)  
**POU5F1** (Sequence: TTTGCAT (-): 1696)  
**POU5F1** (Sequence: TTTGCAT (-): 7463)  
**RFX4\_2** (Sequence: GTATCCATG (-): 1312)  
**SOX9** (Sequence: AACAATAG (-): 6414)  
**SOX9** (Sequence: AACAATGG (-): 9674)  
**FOXO3\_mmu** (Sequence: TGTTTACA (-): 517)  
**FOXO3\_mmu** (Sequence: TGTTTTGA (-): 4077)  
**FOXO3\_mmu** (Sequence: TGTTTTCA (-): 5124)  
**Sox5** (Sequence: ATTGTT (+): 79)  
**Sox5** (Sequence: ATTGTT (+): 1503)  
**Sox5** (Sequence: ATTGTT (+): 3318)  
**Sox5** (Sequence: ATTGTT (+): 4085)  
**Sox5** (Sequence: ATTGTT (+): 6275)  
**Sox5** (Sequence: ATTGTT (+): 7412)  
**Sox5** (Sequence: ATTGTT (+): 8614)  
**FIGLA** (Sequence: TCCAGCTGGT (-): 3490)  
**FIGLA** (Sequence: TCCACCTGGT (-): 8940)  
**SOX9** (Sequence: TCATTGTT (+): 8612)  
**FOXO1** (Sequence: AAAAACAGC (-): 139)  
**FOXO1** (Sequence: AAAAACAAC (-): 2150)  
**FOXO1** (Sequence: AAAAACAAC (-): 2777)  
**FOXO1** (Sequence: AAAAACAAG (-): 6547)  
**FOXO3\_hsa** (Sequence: ATGTTTAC (-): 516)  
**FOXP1** (Sequence: TGTTTAC (-): 517)  
**FOXP1** (Sequence: TGTTTAC (-): 5589)  
**POU2F1** (Sequence: ATTTGAATA (-): 2342)  
**POU2F1** (Sequence: ATTTGAATA (-): 9789)  
**Rhox11** (Sequence: TGCTGTTTT (+): 4074)  
**Rhox11** (Sequence: TGGTGTTTT (+): 6946)  
**Rhox11** (Sequence: AAAACAGCA (-): 140)  
**Sox5** (Sequence: AACAAT (-): 749)  
**Sox5** (Sequence: AACAAT (-): 912)  
**Sox5** (Sequence: AACAAT (-): 2333)  
**Sox5** (Sequence: AACAAT (-): 6309)  
**Sox5** (Sequence: AACAAT (-): 6414)  
**Sox5** (Sequence: AACAAT (-): 6500)  
**Sox5** (Sequence: AACAAT (-): 9674)  
**POU2F1** (Sequence: TATTCAAAT (+): 2238)  
**POU2F1** (Sequence: TATGCAAAT (+): 3343)  
**POU2F1** (Sequence: TATTCAAAT (+): 8191)  
**POU5F1** (Sequence: ATGCAAA (+): 3344)
