# Supplementary material for: First characterization of PIWI-interacting RNA clusters in a cichlid fish with a B chromosome
Source: BMC Biol. 2022 Sep 21;20:204. doi: 10.1186/s12915-022-01403-2 (PMC9490952; doi:10.1186/s12915-022-01403-2)
Supplement: Supplementary file 1 — Additional file 1. Zipped folder with fasta and interactive html piRNA cluster information for the A. latifasciata genome. The nomenclature is as follows: number-pirna-cluster_sex_B-presence (f, female; m, male; 0b, without B chromosome; 1b, with B chromosome). [file 12915_2022_1403_MOESM1_ESM.zip › 101_m1b.html]

piRNA cluster 101\_m1b 62


Predicted piRNA cluster no. 101\_m1b
  

Show proTRAC run info
Hide proTRAC run info

/\  
                \_\_\_\_\_\_\_\_\_\_\_\_\_\_\_\_\_\_\_\_\_\_\_/\\_\_\_ /  \\_\_\_\_\_\_\_  
               I                      /  \  /    \      I  
               I     pro             /    \/      \     I  
               I        TRAC        /               \   I  
               I   \_\_\_\_\_\_\_\_\_\_\_\_\_\_\_\_/\_\_\_\_\_\_\_\_\_\_\_\_\_\_\_\_\_\\_ I  
               I   \              /                     I  
               I    \            /                      I  
               I     \  /\      /       V.2.4.2         I  
               I      \/  \    /                        I  
               I\_\_\_\_\_\_\_\_\_\_\_\  /\_\_\_\_\_\_\_\_\_\_\_\_\_\_\_\_\_\_\_\_\_\_\_\_\_I  
                            \/  
  
  
================================= proTRAC ====================================  
VERSION: .......... 2.4.2  
LAST MODIFIED: .... 11. May 2018  
  
Please cite:  
Rosenkranz D, Zischler H. proTRAC - a software for probabilistic piRNA cluster  
detection, visualization and analysis. 2012. BMC Bioinformatics 13:5.  
  
  
Contact:  
David Rosenkranz  
Institute of Organismic and Molecular Evolutionary Biology  
Dept. Anthropology, small RNA group  
Johannes Gutenberg University Mainz  
email: rosenkranz@uni-mainz.de  
  
You can find the latest proTRAC version at:  
http://sourceforge.net/projects/protrac/files  
http://www.smallRNAgroup-mainz.de/software  
==============================================================================  
  
PARAMETERS:  
Map file: ...............piwi-machos-1B.fa-collapse.map  
Genome file: ............../../../0B\_ala\_genome.fa  
RepeatMasker annotation: Alatifasciata-all0B-maryan-v2.fa\_corrected.out  
GeneSet:................./guest-storage/Data/annotation/Alatifasciata\_all0B\_maryan-v2\_out2017.gff  
  
Significant (p<=0.01) hit density will be calculated based  
on observed hit distribution.  
  
Sliding window size: ........................................ 5000 bp  
Sliding window increament: .................................. 1000 bp  
Normalize each hit by number of genomic hits: ............... yes  
Normalize each hit by number of sequence reads: ............. yes  
Normalize values (-> per million mapped reads): ............. yes  
Min. fraction of hits with 1T(U) or 10A: .................... 0.75  
Alternatively: Min. fraction of hits with 1T(U) and 10A: .... 0.5  
Min. fraction of hits with typical piRNA length: ............ 0.75  
Typical piRNA length: ....................................... 24-32 nt  
Min. size of a piRNA cluster: ............................... 1000 bp.  
Min. number of hits (absolute): ............................. 0  
Min. number of hits (normalized): ........................... 0  
Min. fraction of hits on the mainstrand: .................... 0.75  
Top fraction of mapped sequences (in terms of read counts): . 1%  
Top fraction accounts for max. n% of sequence reads: ........ 90%  
Min. fraction of hits on each arm of a bidirectional cluster: 0.05  
Output html file for each cluster: .......................... yes  
Output a summary table: ..................................... yes  
Output a FASTA file for each cluster (piRNA sequences): ..... yes  
Output a FASTA file comprising cluster sequences: ........... yes  
Output a GTF file for predicted piRNA clusters: ..............yes  
Search DNA motifs in clusters: .............................. yes  
Output flanking sequences: +/- .............................. 0 bp  
Output ~.pTi file: .......................................... no  
==============================================================================  
  
  
Genome size (without gaps): ............ 758543724 bp  
Gaps (N/X/-): .......................... 417479 bp  
Mapped reads: .......................... 26973943  
Non-identical sequences: ............... 6209225  
Genomic hits: .......................... 48438990  
Significant densitiy of mapped reads: .. 821.144211136946 reads/kb

Show proTRAC cluster info
Hide proTRAC cluster info

|  |  |
| --- | --- |
| Location | NODE\_261184\_length\_10127\_cov\_26.726177 |
| Coordinates | 1-10248 |
| Size [bp] | 10248 |
| Sequence hit loci | 7721 |
| Mapped reads (normalized) | 39850.7 |
| Mapped reads (normalized) per kb | 3888.6 |
| Normalized reads with 1T (1U) | 87.7% |
| Normalized reads with 10A | 34.9% |
| Normalized reads with length 24-32 nt | 99% |
| Normalized reads on the main strand(s) | 94.8% |
| Predicted directionality | mono:plus |

100%

0%

1T (1U)  
reads

10A reads

24-32 nt  
reads

reads on mainstrand

**Either the amount of reads with 1T (1U) OR 10A has to exceed 75% (set with option: -1Tor10A)  
Alternatively the amount of reads with 1T (1U) AND 10A has to exceed 50% (set with option: -1Tand10A)  
Minimum amount of reads with preferred size is 75% (set with option: -pisize)  
Minimum amount of reads on the main strand(s) is 75% (set with option: -clstrand)**

Show read coverage
Hide read coverage

WHAT DO I SEE HERE?  
This chart shows the location of mapped sequence reads within a predicted piRNA cluster. The color refers to the number of genomic hits produced by the sequence read in question. A dark red bar indicates that this sequence read produces many other hits elsewhere in the genome. Many adjacent red or yellow bars can indicate the presence of a multi-copy element such as transposons or rRNA genes. A dark green bar indicates that this sequence read maps uniquely to this locus.

1 hit

2-5 hits

6-10 hits

11-20 hits

21-50 hits

51-100 hits

> 100 hits

NODE\_261184\_length\_10127\_cov\_26.726177

1

10248

Gene Set

RepeatMasker

Mapped  
Reads

205.98

plus strand

minus strand

205.98

Region: NODE\_261184\_length\_10127\_cov\_26.726177 7390-11. Max. coverage (+): 0.01. Max coverage (-): 0

Region: NODE\_261184\_length\_10127\_cov\_26.726177 12-31. Max. coverage (+): 0.01. Max coverage (-): 0

Region: NODE\_261184\_length\_10127\_cov\_26.726177 32-52. Max. coverage (+): 1.72. Max coverage (-): 0

Region: NODE\_261184\_length\_10127\_cov\_26.726177 53-72. Max. coverage (+): 0.01. Max coverage (-): 0.04

Region: NODE\_261184\_length\_10127\_cov\_26.726177 73-93. Max. coverage (+): 0.06. Max coverage (-): 0.04

Region: NODE\_261184\_length\_10127\_cov\_26.726177 94-113. Max. coverage (+): 0.04. Max coverage (-): 0

Region: NODE\_261184\_length\_10127\_cov\_26.726177 114-134. Max. coverage (+): 0.07. Max coverage (-): 0

Region: NODE\_261184\_length\_10127\_cov\_26.726177 135-154. Max. coverage (+): 0. Max coverage (-): 0

Region: NODE\_261184\_length\_10127\_cov\_26.726177 155-175. Max. coverage (+): 0. Max coverage (-): 0

Region: NODE\_261184\_length\_10127\_cov\_26.726177 176-195. Max. coverage (+): 0. Max coverage (-): 0

Region: NODE\_261184\_length\_10127\_cov\_26.726177 196-216. Max. coverage (+): 0. Max coverage (-): 0

Region: NODE\_261184\_length\_10127\_cov\_26.726177 217-236. Max. coverage (+): 0. Max coverage (-): 0

Region: NODE\_261184\_length\_10127\_cov\_26.726177 237-257. Max. coverage (+): 0. Max coverage (-): 0

Region: NODE\_261184\_length\_10127\_cov\_26.726177 258-277. Max. coverage (+): 0. Max coverage (-): 0

Region: NODE\_261184\_length\_10127\_cov\_26.726177 278-298. Max. coverage (+): 0. Max coverage (-): 0.06

Region: NODE\_261184\_length\_10127\_cov\_26.726177 299-318. Max. coverage (+): 0. Max coverage (-): 0

Region: NODE\_261184\_length\_10127\_cov\_26.726177 319-339. Max. coverage (+): 0. Max coverage (-): 0

Region: NODE\_261184\_length\_10127\_cov\_26.726177 340-359. Max. coverage (+): 0.01. Max coverage (-): 0

Region: NODE\_261184\_length\_10127\_cov\_26.726177 360-380. Max. coverage (+): 0. Max coverage (-): 0

Region: NODE\_261184\_length\_10127\_cov\_26.726177 381-400. Max. coverage (+): 0.01. Max coverage (-): 0

Region: NODE\_261184\_length\_10127\_cov\_26.726177 401-421. Max. coverage (+): 0.24. Max coverage (-): 0

Region: NODE\_261184\_length\_10127\_cov\_26.726177 422-441. Max. coverage (+): 0.44. Max coverage (-): 0.11

Region: NODE\_261184\_length\_10127\_cov\_26.726177 442-462. Max. coverage (+): 0. Max coverage (-): 0.64

Region: NODE\_261184\_length\_10127\_cov\_26.726177 463-482. Max. coverage (+): 0.27. Max coverage (-): 0

Region: NODE\_261184\_length\_10127\_cov\_26.726177 483-503. Max. coverage (+): 0.04. Max coverage (-): 0.04

Region: NODE\_261184\_length\_10127\_cov\_26.726177 504-523. Max. coverage (+): 0.04. Max coverage (-): 0

Region: NODE\_261184\_length\_10127\_cov\_26.726177 524-544. Max. coverage (+): 0.02. Max coverage (-): 0.05

Region: NODE\_261184\_length\_10127\_cov\_26.726177 545-564. Max. coverage (+): 0.02. Max coverage (-): 0

Region: NODE\_261184\_length\_10127\_cov\_26.726177 565-585. Max. coverage (+): 0.04. Max coverage (-): 0

Region: NODE\_261184\_length\_10127\_cov\_26.726177 586-605. Max. coverage (+): 0. Max coverage (-): 0.01

Region: NODE\_261184\_length\_10127\_cov\_26.726177 606-626. Max. coverage (+): 0.06. Max coverage (-): 0.01

Region: NODE\_261184\_length\_10127\_cov\_26.726177 627-646. Max. coverage (+): 0.01. Max coverage (-): 0.03

Region: NODE\_261184\_length\_10127\_cov\_26.726177 647-667. Max. coverage (+): 0. Max coverage (-): 0.04

Region: NODE\_261184\_length\_10127\_cov\_26.726177 668-687. Max. coverage (+): 0. Max coverage (-): 0

Region: NODE\_261184\_length\_10127\_cov\_26.726177 688-708. Max. coverage (+): 0. Max coverage (-): 0

Region: NODE\_261184\_length\_10127\_cov\_26.726177 709-728. Max. coverage (+): 0. Max coverage (-): 0.02

Region: NODE\_261184\_length\_10127\_cov\_26.726177 729-749. Max. coverage (+): 0.04. Max coverage (-): 0

Region: NODE\_261184\_length\_10127\_cov\_26.726177 750-769. Max. coverage (+): 0.04. Max coverage (-): 0

Region: NODE\_261184\_length\_10127\_cov\_26.726177 770-790. Max. coverage (+): 0. Max coverage (-): 0.01

Region: NODE\_261184\_length\_10127\_cov\_26.726177 791-810. Max. coverage (+): 0.04. Max coverage (-): 0.3

Region: NODE\_261184\_length\_10127\_cov\_26.726177 811-831. Max. coverage (+): 0. Max coverage (-): 0.26

Region: NODE\_261184\_length\_10127\_cov\_26.726177 832-851. Max. coverage (+): 0. Max coverage (-): 0.22

Region: NODE\_261184\_length\_10127\_cov\_26.726177 852-872. Max. coverage (+): 0. Max coverage (-): 0.04

Region: NODE\_261184\_length\_10127\_cov\_26.726177 873-892. Max. coverage (+): 0.03. Max coverage (-): 0.05

Region: NODE\_261184\_length\_10127\_cov\_26.726177 893-913. Max. coverage (+): 0. Max coverage (-): 0.02

Region: NODE\_261184\_length\_10127\_cov\_26.726177 914-933. Max. coverage (+): 0.02. Max coverage (-): 0

Region: NODE\_261184\_length\_10127\_cov\_26.726177 934-954. Max. coverage (+): 0.11. Max coverage (-): 0.01

Region: NODE\_261184\_length\_10127\_cov\_26.726177 955-974. Max. coverage (+): 0.11. Max coverage (-): 0

Region: NODE\_261184\_length\_10127\_cov\_26.726177 975-995. Max. coverage (+): 0.03. Max coverage (-): 0.01

Region: NODE\_261184\_length\_10127\_cov\_26.726177 996-1015. Max. coverage (+): 0.04. Max coverage (-): 0.07

Region: NODE\_261184\_length\_10127\_cov\_26.726177 1016-1036. Max. coverage (+): 0.04. Max coverage (-): 0.11

Region: NODE\_261184\_length\_10127\_cov\_26.726177 1037-1056. Max. coverage (+): 0.09. Max coverage (-): 0.04

Region: NODE\_261184\_length\_10127\_cov\_26.726177 1057-1077. Max. coverage (+): 0. Max coverage (-): 0.01

Region: NODE\_261184\_length\_10127\_cov\_26.726177 1078-1097. Max. coverage (+): 0.04. Max coverage (-): 0

Region: NODE\_261184\_length\_10127\_cov\_26.726177 1098-1118. Max. coverage (+): 0.22. Max coverage (-): 0.04

Region: NODE\_261184\_length\_10127\_cov\_26.726177 1119-1138. Max. coverage (+): 0.15. Max coverage (-): 0

Region: NODE\_261184\_length\_10127\_cov\_26.726177 1139-1159. Max. coverage (+): 0. Max coverage (-): 0

Region: NODE\_261184\_length\_10127\_cov\_26.726177 1160-1179. Max. coverage (+): 0.04. Max coverage (-): 0

Region: NODE\_261184\_length\_10127\_cov\_26.726177 1180-1200. Max. coverage (+): 0.07. Max coverage (-): 0.04

Region: NODE\_261184\_length\_10127\_cov\_26.726177 1201-1220. Max. coverage (+): 0.07. Max coverage (-): 0.07

Region: NODE\_261184\_length\_10127\_cov\_26.726177 1221-1241. Max. coverage (+): 0.09. Max coverage (-): 0.02

Region: NODE\_261184\_length\_10127\_cov\_26.726177 1242-1261. Max. coverage (+): 0.04. Max coverage (-): 0.02

Region: NODE\_261184\_length\_10127\_cov\_26.726177 1262-1281. Max. coverage (+): 0.15. Max coverage (-): 0.07

Region: NODE\_261184\_length\_10127\_cov\_26.726177 1282-1302. Max. coverage (+): 0.07. Max coverage (-): 0.07

Region: NODE\_261184\_length\_10127\_cov\_26.726177 1303-1322. Max. coverage (+): 0.04. Max coverage (-): 0

Region: NODE\_261184\_length\_10127\_cov\_26.726177 1323-1343. Max. coverage (+): 0.11. Max coverage (-): 0.04

Region: NODE\_261184\_length\_10127\_cov\_26.726177 1344-1363. Max. coverage (+): 0.19. Max coverage (-): 0.07

Region: NODE\_261184\_length\_10127\_cov\_26.726177 1364-1384. Max. coverage (+): 0.33. Max coverage (-): 0

Region: NODE\_261184\_length\_10127\_cov\_26.726177 1385-1404. Max. coverage (+): 0.11. Max coverage (-): 0.15

Region: NODE\_261184\_length\_10127\_cov\_26.726177 1405-1425. Max. coverage (+): 1.68. Max coverage (-): 0.15

Region: NODE\_261184\_length\_10127\_cov\_26.726177 1426-1445. Max. coverage (+): 0.19. Max coverage (-): 0.04

Region: NODE\_261184\_length\_10127\_cov\_26.726177 1446-1466. Max. coverage (+): 0. Max coverage (-): 0

Region: NODE\_261184\_length\_10127\_cov\_26.726177 1467-1486. Max. coverage (+): 0.04. Max coverage (-): 0.04

Region: NODE\_261184\_length\_10127\_cov\_26.726177 1487-1507. Max. coverage (+): 0.04. Max coverage (-): 0.07

Region: NODE\_261184\_length\_10127\_cov\_26.726177 1508-1527. Max. coverage (+): 0.15. Max coverage (-): 0

Region: NODE\_261184\_length\_10127\_cov\_26.726177 1528-1548. Max. coverage (+): 0.11. Max coverage (-): 0.04

Region: NODE\_261184\_length\_10127\_cov\_26.726177 1549-1568. Max. coverage (+): 1.37. Max coverage (-): 0.26

Region: NODE\_261184\_length\_10127\_cov\_26.726177 1569-1589. Max. coverage (+): 0.3. Max coverage (-): 0.37

Region: NODE\_261184\_length\_10127\_cov\_26.726177 1590-1609. Max. coverage (+): 0.56. Max coverage (-): 0.04

Region: NODE\_261184\_length\_10127\_cov\_26.726177 1610-1630. Max. coverage (+): 0.78. Max coverage (-): 0.15

Region: NODE\_261184\_length\_10127\_cov\_26.726177 1631-1650. Max. coverage (+): 1.82. Max coverage (-): 0.11

Region: NODE\_261184\_length\_10127\_cov\_26.726177 1651-1671. Max. coverage (+): 9.16. Max coverage (-): 0.22

Region: NODE\_261184\_length\_10127\_cov\_26.726177 1672-1691. Max. coverage (+): 0.78. Max coverage (-): 0.26

Region: NODE\_261184\_length\_10127\_cov\_26.726177 1692-1712. Max. coverage (+): 0.82. Max coverage (-): 0.04

Region: NODE\_261184\_length\_10127\_cov\_26.726177 1713-1732. Max. coverage (+): 0.11. Max coverage (-): 0.07

Region: NODE\_261184\_length\_10127\_cov\_26.726177 1733-1753. Max. coverage (+): 0.89. Max coverage (-): 0.04

Region: NODE\_261184\_length\_10127\_cov\_26.726177 1754-1773. Max. coverage (+): 0.67. Max coverage (-): 0.04

Region: NODE\_261184\_length\_10127\_cov\_26.726177 1774-1794. Max. coverage (+): 0.3. Max coverage (-): 4.75

Region: NODE\_261184\_length\_10127\_cov\_26.726177 1795-1814. Max. coverage (+): 0.08. Max coverage (-): 0.04

Region: NODE\_261184\_length\_10127\_cov\_26.726177 1815-1835. Max. coverage (+): 0.59. Max coverage (-): 0.19

Region: NODE\_261184\_length\_10127\_cov\_26.726177 1836-1855. Max. coverage (+): 1.01. Max coverage (-): 0.04

Region: NODE\_261184\_length\_10127\_cov\_26.726177 1856-1876. Max. coverage (+): 0.25. Max coverage (-): 0.09

Region: NODE\_261184\_length\_10127\_cov\_26.726177 1877-1896. Max. coverage (+): 0.48. Max coverage (-): 0.02

Region: NODE\_261184\_length\_10127\_cov\_26.726177 1897-1917. Max. coverage (+): 0.26. Max coverage (-): 0.96

Region: NODE\_261184\_length\_10127\_cov\_26.726177 1918-1937. Max. coverage (+): 0.63. Max coverage (-): 0.93

Region: NODE\_261184\_length\_10127\_cov\_26.726177 1938-1958. Max. coverage (+): 0.26. Max coverage (-): 0.22

Region: NODE\_261184\_length\_10127\_cov\_26.726177 1959-1978. Max. coverage (+): 2.6. Max coverage (-): 0.26

Region: NODE\_261184\_length\_10127\_cov\_26.726177 1979-1999. Max. coverage (+): 7.27. Max coverage (-): 0

Region: NODE\_261184\_length\_10127\_cov\_26.726177 2000-2019. Max. coverage (+): 0.04. Max coverage (-): 0

Region: NODE\_261184\_length\_10127\_cov\_26.726177 2020-2040. Max. coverage (+): 0.04. Max coverage (-): 0.04

Region: NODE\_261184\_length\_10127\_cov\_26.726177 2041-2060. Max. coverage (+): 0. Max coverage (-): 0.04

Region: NODE\_261184\_length\_10127\_cov\_26.726177 2061-2081. Max. coverage (+): 0. Max coverage (-): 0

Region: NODE\_261184\_length\_10127\_cov\_26.726177 2082-2101. Max. coverage (+): 0.07. Max coverage (-): 0

Region: NODE\_261184\_length\_10127\_cov\_26.726177 2102-2122. Max. coverage (+): 0.19. Max coverage (-): 0.07

Region: NODE\_261184\_length\_10127\_cov\_26.726177 2123-2142. Max. coverage (+): 0.89. Max coverage (-): 0

Region: NODE\_261184\_length\_10127\_cov\_26.726177 2143-2163. Max. coverage (+): 0.56. Max coverage (-): 0.15

Region: NODE\_261184\_length\_10127\_cov\_26.726177 2164-2183. Max. coverage (+): 0.26. Max coverage (-): 0

Region: NODE\_261184\_length\_10127\_cov\_26.726177 2184-2204. Max. coverage (+): 0.01. Max coverage (-): 0

Region: NODE\_261184\_length\_10127\_cov\_26.726177 2205-2224. Max. coverage (+): 0.04. Max coverage (-): 0

Region: NODE\_261184\_length\_10127\_cov\_26.726177 2225-2245. Max. coverage (+): 0.07. Max coverage (-): 0

Region: NODE\_261184\_length\_10127\_cov\_26.726177 2246-2265. Max. coverage (+): 12.83. Max coverage (-): 0.07

Region: NODE\_261184\_length\_10127\_cov\_26.726177 2266-2286. Max. coverage (+): 4.37. Max coverage (-): 0.15

Region: NODE\_261184\_length\_10127\_cov\_26.726177 2287-2306. Max. coverage (+): 54.02. Max coverage (-): 0.11

Region: NODE\_261184\_length\_10127\_cov\_26.726177 2307-2327. Max. coverage (+): 29.51. Max coverage (-): 0.07

Region: NODE\_261184\_length\_10127\_cov\_26.726177 2328-2347. Max. coverage (+): 0.11. Max coverage (-): 0.04

Region: NODE\_261184\_length\_10127\_cov\_26.726177 2348-2368. Max. coverage (+): 2.08. Max coverage (-): 0.44

Region: NODE\_261184\_length\_10127\_cov\_26.726177 2369-2388. Max. coverage (+): 2.15. Max coverage (-): 0.3

Region: NODE\_261184\_length\_10127\_cov\_26.726177 2389-2409. Max. coverage (+): 0.21. Max coverage (-): 0.04

Region: NODE\_261184\_length\_10127\_cov\_26.726177 2410-2429. Max. coverage (+): 0.25. Max coverage (-): 0

Region: NODE\_261184\_length\_10127\_cov\_26.726177 2430-2450. Max. coverage (+): 95.39. Max coverage (-): 2.67

Region: NODE\_261184\_length\_10127\_cov\_26.726177 2451-2470. Max. coverage (+): 94.09. Max coverage (-): 0

Region: NODE\_261184\_length\_10127\_cov\_26.726177 2471-2491. Max. coverage (+): 0.11. Max coverage (-): 0.11

Region: NODE\_261184\_length\_10127\_cov\_26.726177 2492-2511. Max. coverage (+): 0.15. Max coverage (-): 1.74

Region: NODE\_261184\_length\_10127\_cov\_26.726177 2512-2532. Max. coverage (+): 3.23. Max coverage (-): 2.6

Region: NODE\_261184\_length\_10127\_cov\_26.726177 2533-2552. Max. coverage (+): 0.07. Max coverage (-): 0.07

Region: NODE\_261184\_length\_10127\_cov\_26.726177 2553-2573. Max. coverage (+): 28.43. Max coverage (-): 0.04

Region: NODE\_261184\_length\_10127\_cov\_26.726177 2574-2593. Max. coverage (+): 3.11. Max coverage (-): 0.06

Region: NODE\_261184\_length\_10127\_cov\_26.726177 2594-2614. Max. coverage (+): 0.89. Max coverage (-): 0

Region: NODE\_261184\_length\_10127\_cov\_26.726177 2615-2634. Max. coverage (+): 3.35. Max coverage (-): 0.04

Region: NODE\_261184\_length\_10127\_cov\_26.726177 2635-2655. Max. coverage (+): 2. Max coverage (-): 1.56

Region: NODE\_261184\_length\_10127\_cov\_26.726177 2656-2675. Max. coverage (+): 0.11. Max coverage (-): 0.04

Region: NODE\_261184\_length\_10127\_cov\_26.726177 2676-2696. Max. coverage (+): 0.19. Max coverage (-): 0

Region: NODE\_261184\_length\_10127\_cov\_26.726177 2697-2716. Max. coverage (+): 1.22. Max coverage (-): 0.03

Region: NODE\_261184\_length\_10127\_cov\_26.726177 2717-2737. Max. coverage (+): 0.07. Max coverage (-): 0.04

Region: NODE\_261184\_length\_10127\_cov\_26.726177 2738-2757. Max. coverage (+): 2.62. Max coverage (-): 0.01

Region: NODE\_261184\_length\_10127\_cov\_26.726177 2758-2778. Max. coverage (+): 0.41. Max coverage (-): 0.15

Region: NODE\_261184\_length\_10127\_cov\_26.726177 2779-2798. Max. coverage (+): 0.19. Max coverage (-): 0.04

Region: NODE\_261184\_length\_10127\_cov\_26.726177 2799-2819. Max. coverage (+): 0. Max coverage (-): 0

Region: NODE\_261184\_length\_10127\_cov\_26.726177 2820-2839. Max. coverage (+): 0.15. Max coverage (-): 0.82

Region: NODE\_261184\_length\_10127\_cov\_26.726177 2840-2860. Max. coverage (+): 7.12. Max coverage (-): 0.02

Region: NODE\_261184\_length\_10127\_cov\_26.726177 2861-2880. Max. coverage (+): 10.97. Max coverage (-): 0.06

Region: NODE\_261184\_length\_10127\_cov\_26.726177 2881-2901. Max. coverage (+): 4. Max coverage (-): 0

Region: NODE\_261184\_length\_10127\_cov\_26.726177 2902-2921. Max. coverage (+): 0.67. Max coverage (-): 0.04

Region: NODE\_261184\_length\_10127\_cov\_26.726177 2922-2942. Max. coverage (+): 11.27. Max coverage (-): 2.6

Region: NODE\_261184\_length\_10127\_cov\_26.726177 2943-2962. Max. coverage (+): 4.3. Max coverage (-): 0

Region: NODE\_261184\_length\_10127\_cov\_26.726177 2963-2983. Max. coverage (+): 1.17. Max coverage (-): 0.17

Region: NODE\_261184\_length\_10127\_cov\_26.726177 2984-3003. Max. coverage (+): 0.83. Max coverage (-): 0.01

Region: NODE\_261184\_length\_10127\_cov\_26.726177 3004-3024. Max. coverage (+): 0.73. Max coverage (-): 0.24

Region: NODE\_261184\_length\_10127\_cov\_26.726177 3025-3044. Max. coverage (+): 6.59. Max coverage (-): 0

Region: NODE\_261184\_length\_10127\_cov\_26.726177 3045-3065. Max. coverage (+): 0.04. Max coverage (-): 0

Region: NODE\_261184\_length\_10127\_cov\_26.726177 3066-3085. Max. coverage (+): 0.34. Max coverage (-): 0

Region: NODE\_261184\_length\_10127\_cov\_26.726177 3086-3106. Max. coverage (+): 0.22. Max coverage (-): 0.09

Region: NODE\_261184\_length\_10127\_cov\_26.726177 3107-3126. Max. coverage (+): 1.33. Max coverage (-): 0.07

Region: NODE\_261184\_length\_10127\_cov\_26.726177 3127-3147. Max. coverage (+): 0.48. Max coverage (-): 0.09

Region: NODE\_261184\_length\_10127\_cov\_26.726177 3148-3167. Max. coverage (+): 0.67. Max coverage (-): 0.04

Region: NODE\_261184\_length\_10127\_cov\_26.726177 3168-3188. Max. coverage (+): 0.52. Max coverage (-): 0.15

Region: NODE\_261184\_length\_10127\_cov\_26.726177 3189-3208. Max. coverage (+): 0.11. Max coverage (-): 0.04

Region: NODE\_261184\_length\_10127\_cov\_26.726177 3209-3229. Max. coverage (+): 0.15. Max coverage (-): 0.07

Region: NODE\_261184\_length\_10127\_cov\_26.726177 3230-3249. Max. coverage (+): 0.67. Max coverage (-): 0.04

Region: NODE\_261184\_length\_10127\_cov\_26.726177 3250-3270. Max. coverage (+): 0.19. Max coverage (-): 0.04

Region: NODE\_261184\_length\_10127\_cov\_26.726177 3271-3290. Max. coverage (+): 0.22. Max coverage (-): 0.04

Region: NODE\_261184\_length\_10127\_cov\_26.726177 3291-3311. Max. coverage (+): 0.26. Max coverage (-): 0.04

Region: NODE\_261184\_length\_10127\_cov\_26.726177 3312-3331. Max. coverage (+): 0.22. Max coverage (-): 0.19

Region: NODE\_261184\_length\_10127\_cov\_26.726177 3332-3352. Max. coverage (+): 0.11. Max coverage (-): 0

Region: NODE\_261184\_length\_10127\_cov\_26.726177 3353-3372. Max. coverage (+): 0.07. Max coverage (-): 0.01

Region: NODE\_261184\_length\_10127\_cov\_26.726177 3373-3393. Max. coverage (+): 0.51. Max coverage (-): 0

Region: NODE\_261184\_length\_10127\_cov\_26.726177 3394-3413. Max. coverage (+): 0.04. Max coverage (-): 0.15

Region: NODE\_261184\_length\_10127\_cov\_26.726177 3414-3434. Max. coverage (+): 1.41. Max coverage (-): 0.26

Region: NODE\_261184\_length\_10127\_cov\_26.726177 3435-3454. Max. coverage (+): 2.34. Max coverage (-): 1.59

Region: NODE\_261184\_length\_10127\_cov\_26.726177 3455-3475. Max. coverage (+): 12.42. Max coverage (-): 1

Region: NODE\_261184\_length\_10127\_cov\_26.726177 3476-3495. Max. coverage (+): 0.15. Max coverage (-): 0

Region: NODE\_261184\_length\_10127\_cov\_26.726177 3496-3516. Max. coverage (+): 1.78. Max coverage (-): 0.07

Region: NODE\_261184\_length\_10127\_cov\_26.726177 3517-3536. Max. coverage (+): 0.04. Max coverage (-): 0

Region: NODE\_261184\_length\_10127\_cov\_26.726177 3537-3557. Max. coverage (+): 0.01. Max coverage (-): 0.01

Region: NODE\_261184\_length\_10127\_cov\_26.726177 3558-3577. Max. coverage (+): 0.05. Max coverage (-): 0.03

Region: NODE\_261184\_length\_10127\_cov\_26.726177 3578-3598. Max. coverage (+): 0.92. Max coverage (-): 0.03

Region: NODE\_261184\_length\_10127\_cov\_26.726177 3599-3618. Max. coverage (+): 0.87. Max coverage (-): 0

Region: NODE\_261184\_length\_10127\_cov\_26.726177 3619-3639. Max. coverage (+): 0. Max coverage (-): 0

Region: NODE\_261184\_length\_10127\_cov\_26.726177 3640-3659. Max. coverage (+): 0.09. Max coverage (-): 0

Region: NODE\_261184\_length\_10127\_cov\_26.726177 3660-3680. Max. coverage (+): 0.19. Max coverage (-): 0

Region: NODE\_261184\_length\_10127\_cov\_26.726177 3681-3700. Max. coverage (+): 0.19. Max coverage (-): 0.26

Region: NODE\_261184\_length\_10127\_cov\_26.726177 3701-3721. Max. coverage (+): 0. Max coverage (-): 0.11

Region: NODE\_261184\_length\_10127\_cov\_26.726177 3722-3741. Max. coverage (+): 0. Max coverage (-): 0

Region: NODE\_261184\_length\_10127\_cov\_26.726177 3742-3762. Max. coverage (+): 0.04. Max coverage (-): 0.04

Region: NODE\_261184\_length\_10127\_cov\_26.726177 3763-3782. Max. coverage (+): 0.53. Max coverage (-): 0

Region: NODE\_261184\_length\_10127\_cov\_26.726177 3783-3803. Max. coverage (+): 0.07. Max coverage (-): 0.11

Region: NODE\_261184\_length\_10127\_cov\_26.726177 3804-3823. Max. coverage (+): 0.11. Max coverage (-): 0

Region: NODE\_261184\_length\_10127\_cov\_26.726177 3824-3843. Max. coverage (+): 0.04. Max coverage (-): 0

Region: NODE\_261184\_length\_10127\_cov\_26.726177 3844-3864. Max. coverage (+): 0.2. Max coverage (-): 0.01

Region: NODE\_261184\_length\_10127\_cov\_26.726177 3865-3884. Max. coverage (+): 3.37. Max coverage (-): 0.04

Region: NODE\_261184\_length\_10127\_cov\_26.726177 3885-3905. Max. coverage (+): 0.19. Max coverage (-): 0.04

Region: NODE\_261184\_length\_10127\_cov\_26.726177 3906-3925. Max. coverage (+): 0.07. Max coverage (-): 0.07

Region: NODE\_261184\_length\_10127\_cov\_26.726177 3926-3946. Max. coverage (+): 0.11. Max coverage (-): 0.15

Region: NODE\_261184\_length\_10127\_cov\_26.726177 3947-3966. Max. coverage (+): 0.04. Max coverage (-): 0.04

Region: NODE\_261184\_length\_10127\_cov\_26.726177 3967-3987. Max. coverage (+): 0.04. Max coverage (-): 0.15

Region: NODE\_261184\_length\_10127\_cov\_26.726177 3988-4007. Max. coverage (+): 0.22. Max coverage (-): 0.19

Region: NODE\_261184\_length\_10127\_cov\_26.726177 4008-4028. Max. coverage (+): 0. Max coverage (-): 0.07

Region: NODE\_261184\_length\_10127\_cov\_26.726177 4029-4048. Max. coverage (+): 0.67. Max coverage (-): 0.02

Region: NODE\_261184\_length\_10127\_cov\_26.726177 4049-4069. Max. coverage (+): 0.11. Max coverage (-): 0

Region: NODE\_261184\_length\_10127\_cov\_26.726177 4070-4089. Max. coverage (+): 0.07. Max coverage (-): 0

Region: NODE\_261184\_length\_10127\_cov\_26.726177 4090-4110. Max. coverage (+): 0.96. Max coverage (-): 0.04

Region: NODE\_261184\_length\_10127\_cov\_26.726177 4111-4130. Max. coverage (+): 0.04. Max coverage (-): 0.04

Region: NODE\_261184\_length\_10127\_cov\_26.726177 4131-4151. Max. coverage (+): 0. Max coverage (-): 0.04

Region: NODE\_261184\_length\_10127\_cov\_26.726177 4152-4171. Max. coverage (+): 0.37. Max coverage (-): 0.04

Region: NODE\_261184\_length\_10127\_cov\_26.726177 4172-4192. Max. coverage (+): 0.19. Max coverage (-): 0

Region: NODE\_261184\_length\_10127\_cov\_26.726177 4193-4212. Max. coverage (+): 0.07. Max coverage (-): 0.15

Region: NODE\_261184\_length\_10127\_cov\_26.726177 4213-4233. Max. coverage (+): 1.04. Max coverage (-): 0

Region: NODE\_261184\_length\_10127\_cov\_26.726177 4234-4253. Max. coverage (+): 0.11. Max coverage (-): 0.04

Region: NODE\_261184\_length\_10127\_cov\_26.726177 4254-4274. Max. coverage (+): 0.44. Max coverage (-): 0.04

Region: NODE\_261184\_length\_10127\_cov\_26.726177 4275-4294. Max. coverage (+): 2.11. Max coverage (-): 0.04

Region: NODE\_261184\_length\_10127\_cov\_26.726177 4295-4315. Max. coverage (+): 0.22. Max coverage (-): 0.11

Region: NODE\_261184\_length\_10127\_cov\_26.726177 4316-4335. Max. coverage (+): 0.48. Max coverage (-): 0.3

Region: NODE\_261184\_length\_10127\_cov\_26.726177 4336-4356. Max. coverage (+): 0.11. Max coverage (-): 0.15

Region: NODE\_261184\_length\_10127\_cov\_26.726177 4357-4376. Max. coverage (+): 0.11. Max coverage (-): 0.15

Region: NODE\_261184\_length\_10127\_cov\_26.726177 4377-4397. Max. coverage (+): 1.08. Max coverage (-): 0.04

Region: NODE\_261184\_length\_10127\_cov\_26.726177 4398-4417. Max. coverage (+): 0.07. Max coverage (-): 0.07

Region: NODE\_261184\_length\_10127\_cov\_26.726177 4418-4438. Max. coverage (+): 5.64. Max coverage (-): 0.07

Region: NODE\_261184\_length\_10127\_cov\_26.726177 4439-4458. Max. coverage (+): 0.15. Max coverage (-): 0

Region: NODE\_261184\_length\_10127\_cov\_26.726177 4459-4479. Max. coverage (+): 1.63. Max coverage (-): 0.07

Region: NODE\_261184\_length\_10127\_cov\_26.726177 4480-4499. Max. coverage (+): 1.63. Max coverage (-): 0.15

Region: NODE\_261184\_length\_10127\_cov\_26.726177 4500-4520. Max. coverage (+): 0.63. Max coverage (-): 0.07

Region: NODE\_261184\_length\_10127\_cov\_26.726177 4521-4540. Max. coverage (+): 2.67. Max coverage (-): 0.19

Region: NODE\_261184\_length\_10127\_cov\_26.726177 4541-4561. Max. coverage (+): 3.3. Max coverage (-): 0.19

Region: NODE\_261184\_length\_10127\_cov\_26.726177 4562-4581. Max. coverage (+): 0.78. Max coverage (-): 0.19

Region: NODE\_261184\_length\_10127\_cov\_26.726177 4582-4602. Max. coverage (+): 0.33. Max coverage (-): 0.33

Region: NODE\_261184\_length\_10127\_cov\_26.726177 4603-4622. Max. coverage (+): 0.19. Max coverage (-): 0.11

Region: NODE\_261184\_length\_10127\_cov\_26.726177 4623-4643. Max. coverage (+): 9.23. Max coverage (-): 0.15

Region: NODE\_261184\_length\_10127\_cov\_26.726177 4644-4663. Max. coverage (+): 3.19. Max coverage (-): 0.04

Region: NODE\_261184\_length\_10127\_cov\_26.726177 4664-4684. Max. coverage (+): 2.22. Max coverage (-): 0.22

Region: NODE\_261184\_length\_10127\_cov\_26.726177 4685-4704. Max. coverage (+): 5.97. Max coverage (-): 0.04

Region: NODE\_261184\_length\_10127\_cov\_26.726177 4705-4725. Max. coverage (+): 2.04. Max coverage (-): 0.04

Region: NODE\_261184\_length\_10127\_cov\_26.726177 4726-4745. Max. coverage (+): 5.34. Max coverage (-): 1.37

Region: NODE\_261184\_length\_10127\_cov\_26.726177 4746-4766. Max. coverage (+): 5.26. Max coverage (-): 0.04

Region: NODE\_261184\_length\_10127\_cov\_26.726177 4767-4786. Max. coverage (+): 1.04. Max coverage (-): 0

Region: NODE\_261184\_length\_10127\_cov\_26.726177 4787-4807. Max. coverage (+): 4.3. Max coverage (-): 0.11

Region: NODE\_261184\_length\_10127\_cov\_26.726177 4808-4827. Max. coverage (+): 0.19. Max coverage (-): 0.11

Region: NODE\_261184\_length\_10127\_cov\_26.726177 4828-4848. Max. coverage (+): 66.62. Max coverage (-): 0.22

Region: NODE\_261184\_length\_10127\_cov\_26.726177 4849-4868. Max. coverage (+): 0.15. Max coverage (-): 0.04

Region: NODE\_261184\_length\_10127\_cov\_26.726177 4869-4889. Max. coverage (+): 0.15. Max coverage (-): 0.04

Region: NODE\_261184\_length\_10127\_cov\_26.726177 4890-4909. Max. coverage (+): 0.11. Max coverage (-): 0.19

Region: NODE\_261184\_length\_10127\_cov\_26.726177 4910-4930. Max. coverage (+): 0.26. Max coverage (-): 0

Region: NODE\_261184\_length\_10127\_cov\_26.726177 4931-4950. Max. coverage (+): 0.04. Max coverage (-): 0.04

Region: NODE\_261184\_length\_10127\_cov\_26.726177 4951-4971. Max. coverage (+): 0.15. Max coverage (-): 0

Region: NODE\_261184\_length\_10127\_cov\_26.726177 4972-4991. Max. coverage (+): 0.19. Max coverage (-): 0

Region: NODE\_261184\_length\_10127\_cov\_26.726177 4992-5012. Max. coverage (+): 0.56. Max coverage (-): 0

Region: NODE\_261184\_length\_10127\_cov\_26.726177 5013-5032. Max. coverage (+): 2.41. Max coverage (-): 0.15

Region: NODE\_261184\_length\_10127\_cov\_26.726177 5033-5053. Max. coverage (+): 7.9. Max coverage (-): 0.19

Region: NODE\_261184\_length\_10127\_cov\_26.726177 5054-5073. Max. coverage (+): 0.15. Max coverage (-): 0

Region: NODE\_261184\_length\_10127\_cov\_26.726177 5074-5094. Max. coverage (+): 10.64. Max coverage (-): 0.93

Region: NODE\_261184\_length\_10127\_cov\_26.726177 5095-5114. Max. coverage (+): 3.15. Max coverage (-): 1.71

Region: NODE\_261184\_length\_10127\_cov\_26.726177 5115-5135. Max. coverage (+): 4.34. Max coverage (-): 1.08

Region: NODE\_261184\_length\_10127\_cov\_26.726177 5136-5155. Max. coverage (+): 2.78. Max coverage (-): 0.22

Region: NODE\_261184\_length\_10127\_cov\_26.726177 5156-5176. Max. coverage (+): 4.86. Max coverage (-): 0.11

Region: NODE\_261184\_length\_10127\_cov\_26.726177 5177-5196. Max. coverage (+): 1.26. Max coverage (-): 0.52

Region: NODE\_261184\_length\_10127\_cov\_26.726177 5197-5217. Max. coverage (+): 14.35. Max coverage (-): 0.11

Region: NODE\_261184\_length\_10127\_cov\_26.726177 5218-5237. Max. coverage (+): 0.52. Max coverage (-): 0

Region: NODE\_261184\_length\_10127\_cov\_26.726177 5238-5258. Max. coverage (+): 1.41. Max coverage (-): 0.11

Region: NODE\_261184\_length\_10127\_cov\_26.726177 5259-5278. Max. coverage (+): 7.08. Max coverage (-): 0.07

Region: NODE\_261184\_length\_10127\_cov\_26.726177 5279-5299. Max. coverage (+): 2.11. Max coverage (-): 0

Region: NODE\_261184\_length\_10127\_cov\_26.726177 5300-5319. Max. coverage (+): 2.15. Max coverage (-): 0.07

Region: NODE\_261184\_length\_10127\_cov\_26.726177 5320-5340. Max. coverage (+): 0.78. Max coverage (-): 0.07

Region: NODE\_261184\_length\_10127\_cov\_26.726177 5341-5360. Max. coverage (+): 91.87. Max coverage (-): 0.04

Region: NODE\_261184\_length\_10127\_cov\_26.726177 5361-5381. Max. coverage (+): 4.63. Max coverage (-): 0.56

Region: NODE\_261184\_length\_10127\_cov\_26.726177 5382-5401. Max. coverage (+): 4.93. Max coverage (-): 0.04

Region: NODE\_261184\_length\_10127\_cov\_26.726177 5402-5422. Max. coverage (+): 0.59. Max coverage (-): 0.07

Region: NODE\_261184\_length\_10127\_cov\_26.726177 5423-5442. Max. coverage (+): 0.41. Max coverage (-): 0.11

Region: NODE\_261184\_length\_10127\_cov\_26.726177 5443-5463. Max. coverage (+): 0.22. Max coverage (-): 0.04

Region: NODE\_261184\_length\_10127\_cov\_26.726177 5464-5483. Max. coverage (+): 3.15. Max coverage (-): 0

Region: NODE\_261184\_length\_10127\_cov\_26.726177 5484-5504. Max. coverage (+): 3.11. Max coverage (-): 0.07

Region: NODE\_261184\_length\_10127\_cov\_26.726177 5505-5524. Max. coverage (+): 3. Max coverage (-): 0

Region: NODE\_261184\_length\_10127\_cov\_26.726177 5525-5545. Max. coverage (+): 0.7. Max coverage (-): 0

Region: NODE\_261184\_length\_10127\_cov\_26.726177 5546-5565. Max. coverage (+): 13.42. Max coverage (-): 0.26

Region: NODE\_261184\_length\_10127\_cov\_26.726177 5566-5586. Max. coverage (+): 13.31. Max coverage (-): 0.22

Region: NODE\_261184\_length\_10127\_cov\_26.726177 5587-5606. Max. coverage (+): 3.71. Max coverage (-): 0.11

Region: NODE\_261184\_length\_10127\_cov\_26.726177 5607-5627. Max. coverage (+): 1.48. Max coverage (-): 0.22

Region: NODE\_261184\_length\_10127\_cov\_26.726177 5628-5647. Max. coverage (+): 2.37. Max coverage (-): 0.04

Region: NODE\_261184\_length\_10127\_cov\_26.726177 5648-5668. Max. coverage (+): 1.78. Max coverage (-): 0.26

Region: NODE\_261184\_length\_10127\_cov\_26.726177 5669-5688. Max. coverage (+): 7.75. Max coverage (-): 0.15

Region: NODE\_261184\_length\_10127\_cov\_26.726177 5689-5709. Max. coverage (+): 0.48. Max coverage (-): 0.19

Region: NODE\_261184\_length\_10127\_cov\_26.726177 5710-5729. Max. coverage (+): 3.52. Max coverage (-): 0

Region: NODE\_261184\_length\_10127\_cov\_26.726177 5730-5750. Max. coverage (+): 0.44. Max coverage (-): 0

Region: NODE\_261184\_length\_10127\_cov\_26.726177 5751-5770. Max. coverage (+): 2.89. Max coverage (-): 0.15

Region: NODE\_261184\_length\_10127\_cov\_26.726177 5771-5791. Max. coverage (+): 23.73. Max coverage (-): 0.04

Region: NODE\_261184\_length\_10127\_cov\_26.726177 5792-5811. Max. coverage (+): 1.19. Max coverage (-): 0.15

Region: NODE\_261184\_length\_10127\_cov\_26.726177 5812-5832. Max. coverage (+): 0.48. Max coverage (-): 0.15

Region: NODE\_261184\_length\_10127\_cov\_26.726177 5833-5852. Max. coverage (+): 46.16. Max coverage (-): 0.67

Region: NODE\_261184\_length\_10127\_cov\_26.726177 5853-5873. Max. coverage (+): 205.98. Max coverage (-): 0.04

Region: NODE\_261184\_length\_10127\_cov\_26.726177 5874-5893. Max. coverage (+): 1.08. Max coverage (-): 0.07

Region: NODE\_261184\_length\_10127\_cov\_26.726177 5894-5914. Max. coverage (+): 15.2. Max coverage (-): 0.3

Region: NODE\_261184\_length\_10127\_cov\_26.726177 5915-5934. Max. coverage (+): 1.63. Max coverage (-): 0.07

Region: NODE\_261184\_length\_10127\_cov\_26.726177 5935-5955. Max. coverage (+): 3.45. Max coverage (-): 0.19

Region: NODE\_261184\_length\_10127\_cov\_26.726177 5956-5975. Max. coverage (+): 2.93. Max coverage (-): 0

Region: NODE\_261184\_length\_10127\_cov\_26.726177 5976-5996. Max. coverage (+): 7.97. Max coverage (-): 0.44

Region: NODE\_261184\_length\_10127\_cov\_26.726177 5997-6016. Max. coverage (+): 9.6. Max coverage (-): 1.78

Region: NODE\_261184\_length\_10127\_cov\_26.726177 6017-6037. Max. coverage (+): 1.78. Max coverage (-): 0.15

Region: NODE\_261184\_length\_10127\_cov\_26.726177 6038-6057. Max. coverage (+): 2.89. Max coverage (-): 0.07

Region: NODE\_261184\_length\_10127\_cov\_26.726177 6058-6078. Max. coverage (+): 0.11. Max coverage (-): 0.07

Region: NODE\_261184\_length\_10127\_cov\_26.726177 6079-6098. Max. coverage (+): 0.33. Max coverage (-): 0.19

Region: NODE\_261184\_length\_10127\_cov\_26.726177 6099-6119. Max. coverage (+): 34.29. Max coverage (-): 0.33

Region: NODE\_261184\_length\_10127\_cov\_26.726177 6120-6139. Max. coverage (+): 1.15. Max coverage (-): 0.04

Region: NODE\_261184\_length\_10127\_cov\_26.726177 6140-6160. Max. coverage (+): 6.19. Max coverage (-): 0.15

Region: NODE\_261184\_length\_10127\_cov\_26.726177 6161-6180. Max. coverage (+): 2.56. Max coverage (-): 0.19

Region: NODE\_261184\_length\_10127\_cov\_26.726177 6181-6201. Max. coverage (+): 5.75. Max coverage (-): 0.19

Region: NODE\_261184\_length\_10127\_cov\_26.726177 6202-6221. Max. coverage (+): 0.15. Max coverage (-): 0.04

Region: NODE\_261184\_length\_10127\_cov\_26.726177 6222-6242. Max. coverage (+): 0.67. Max coverage (-): 0.15

Region: NODE\_261184\_length\_10127\_cov\_26.726177 6243-6262. Max. coverage (+): 0.07. Max coverage (-): 0

Region: NODE\_261184\_length\_10127\_cov\_26.726177 6263-6283. Max. coverage (+): 0.15. Max coverage (-): 0.07

Region: NODE\_261184\_length\_10127\_cov\_26.726177 6284-6303. Max. coverage (+): 0.48. Max coverage (-): 0.04

Region: NODE\_261184\_length\_10127\_cov\_26.726177 6304-6324. Max. coverage (+): 1.37. Max coverage (-): 1.15

Region: NODE\_261184\_length\_10127\_cov\_26.726177 6325-6344. Max. coverage (+): 0.04. Max coverage (-): 0.74

Region: NODE\_261184\_length\_10127\_cov\_26.726177 6345-6365. Max. coverage (+): 0.22. Max coverage (-): 0

Region: NODE\_261184\_length\_10127\_cov\_26.726177 6366-6385. Max. coverage (+): 0.33. Max coverage (-): 0.04

Region: NODE\_261184\_length\_10127\_cov\_26.726177 6386-6405. Max. coverage (+): 2.19. Max coverage (-): 0.04

Region: NODE\_261184\_length\_10127\_cov\_26.726177 6406-6426. Max. coverage (+): 30.55. Max coverage (-): 0

Region: NODE\_261184\_length\_10127\_cov\_26.726177 6427-6446. Max. coverage (+): 2.82. Max coverage (-): 0.15

Region: NODE\_261184\_length\_10127\_cov\_26.726177 6447-6467. Max. coverage (+): 5.04. Max coverage (-): 0.07

Region: NODE\_261184\_length\_10127\_cov\_26.726177 6468-6487. Max. coverage (+): 1.74. Max coverage (-): 0.07

Region: NODE\_261184\_length\_10127\_cov\_26.726177 6488-6508. Max. coverage (+): 1.96. Max coverage (-): 0.26

Region: NODE\_261184\_length\_10127\_cov\_26.726177 6509-6528. Max. coverage (+): 1.37. Max coverage (-): 0

Region: NODE\_261184\_length\_10127\_cov\_26.726177 6529-6549. Max. coverage (+): 0.11. Max coverage (-): 0

Region: NODE\_261184\_length\_10127\_cov\_26.726177 6550-6569. Max. coverage (+): 0.93. Max coverage (-): 0.11

Region: NODE\_261184\_length\_10127\_cov\_26.726177 6570-6590. Max. coverage (+): 2.63. Max coverage (-): 0.37

Region: NODE\_261184\_length\_10127\_cov\_26.726177 6591-6610. Max. coverage (+): 0.15. Max coverage (-): 0

Region: NODE\_261184\_length\_10127\_cov\_26.726177 6611-6631. Max. coverage (+): 1.22. Max coverage (-): 0.11

Region: NODE\_261184\_length\_10127\_cov\_26.726177 6632-6651. Max. coverage (+): 2.26. Max coverage (-): 0

Region: NODE\_261184\_length\_10127\_cov\_26.726177 6652-6672. Max. coverage (+): 0.33. Max coverage (-): 1.85

Region: NODE\_261184\_length\_10127\_cov\_26.726177 6673-6692. Max. coverage (+): 1.22. Max coverage (-): 0.44

Region: NODE\_261184\_length\_10127\_cov\_26.726177 6693-6713. Max. coverage (+): 1.37. Max coverage (-): 0.89

Region: NODE\_261184\_length\_10127\_cov\_26.726177 6714-6733. Max. coverage (+): 5.04. Max coverage (-): 0.07

Region: NODE\_261184\_length\_10127\_cov\_26.726177 6734-6754. Max. coverage (+): 0.93. Max coverage (-): 1.26

Region: NODE\_261184\_length\_10127\_cov\_26.726177 6755-6774. Max. coverage (+): 0.19. Max coverage (-): 0

Region: NODE\_261184\_length\_10127\_cov\_26.726177 6775-6795. Max. coverage (+): 0.85. Max coverage (-): 0.07

Region: NODE\_261184\_length\_10127\_cov\_26.726177 6796-6815. Max. coverage (+): 0.33. Max coverage (-): 0.04

Region: NODE\_261184\_length\_10127\_cov\_26.726177 6816-6836. Max. coverage (+): 0.07. Max coverage (-): 0

Region: NODE\_261184\_length\_10127\_cov\_26.726177 6837-6856. Max. coverage (+): 0.04. Max coverage (-): 0

Region: NODE\_261184\_length\_10127\_cov\_26.726177 6857-6877. Max. coverage (+): 0.41. Max coverage (-): 0.11

Region: NODE\_261184\_length\_10127\_cov\_26.726177 6878-6897. Max. coverage (+): 2.82. Max coverage (-): 0

Region: NODE\_261184\_length\_10127\_cov\_26.726177 6898-6918. Max. coverage (+): 0.59. Max coverage (-): 0.04

Region: NODE\_261184\_length\_10127\_cov\_26.726177 6919-6938. Max. coverage (+): 1.22. Max coverage (-): 0.01

Region: NODE\_261184\_length\_10127\_cov\_26.726177 6939-6959. Max. coverage (+): 0.01. Max coverage (-): 0

Region: NODE\_261184\_length\_10127\_cov\_26.726177 6960-6979. Max. coverage (+): 0.04. Max coverage (-): 0

Region: NODE\_261184\_length\_10127\_cov\_26.726177 6980-7000. Max. coverage (+): 0.26. Max coverage (-): 0.04

Region: NODE\_261184\_length\_10127\_cov\_26.726177 7001-7020. Max. coverage (+): 1.26. Max coverage (-): 0.01

Region: NODE\_261184\_length\_10127\_cov\_26.726177 7021-7041. Max. coverage (+): 0.14. Max coverage (-): 0.13

Region: NODE\_261184\_length\_10127\_cov\_26.726177 7042-7061. Max. coverage (+): 0.14. Max coverage (-): 0.07

Region: NODE\_261184\_length\_10127\_cov\_26.726177 7062-7082. Max. coverage (+): 0.11. Max coverage (-): 0.04

Region: NODE\_261184\_length\_10127\_cov\_26.726177 7083-7102. Max. coverage (+): 0.07. Max coverage (-): 0

Region: NODE\_261184\_length\_10127\_cov\_26.726177 7103-7123. Max. coverage (+): 0.48. Max coverage (-): 0.04

Region: NODE\_261184\_length\_10127\_cov\_26.726177 7124-7143. Max. coverage (+): 0.52. Max coverage (-): 0.11

Region: NODE\_261184\_length\_10127\_cov\_26.726177 7144-7164. Max. coverage (+): 0.15. Max coverage (-): 0

Region: NODE\_261184\_length\_10127\_cov\_26.726177 7165-7184. Max. coverage (+): 0. Max coverage (-): 0

Region: NODE\_261184\_length\_10127\_cov\_26.726177 7185-7205. Max. coverage (+): 0. Max coverage (-): 0.07

Region: NODE\_261184\_length\_10127\_cov\_26.726177 7206-7225. Max. coverage (+): 0.04. Max coverage (-): 0.08

Region: NODE\_261184\_length\_10127\_cov\_26.726177 7226-7246. Max. coverage (+): 1.98. Max coverage (-): 0.55

Region: NODE\_261184\_length\_10127\_cov\_26.726177 7247-7266. Max. coverage (+): 0.7. Max coverage (-): 0

Region: NODE\_261184\_length\_10127\_cov\_26.726177 7267-7287. Max. coverage (+): 0.15. Max coverage (-): 0

Region: NODE\_261184\_length\_10127\_cov\_26.726177 7288-7307. Max. coverage (+): 0.11. Max coverage (-): 0

Region: NODE\_261184\_length\_10127\_cov\_26.726177 7308-7328. Max. coverage (+): 0.3. Max coverage (-): 0

Region: NODE\_261184\_length\_10127\_cov\_26.726177 7329-7348. Max. coverage (+): 0.07. Max coverage (-): 0

Region: NODE\_261184\_length\_10127\_cov\_26.726177 7349-7369. Max. coverage (+): 0.63. Max coverage (-): 0.07

Region: NODE\_261184\_length\_10127\_cov\_26.726177 7370-7389. Max. coverage (+): 0.11. Max coverage (-): 0

Region: NODE\_261184\_length\_10127\_cov\_26.726177 7390-7410. Max. coverage (+): 0.11. Max coverage (-): 0.07

Region: NODE\_261184\_length\_10127\_cov\_26.726177 7411-7430. Max. coverage (+): 0.07. Max coverage (-): 0.04

Region: NODE\_261184\_length\_10127\_cov\_26.726177 7431-7451. Max. coverage (+): 0.07. Max coverage (-): 0

Region: NODE\_261184\_length\_10127\_cov\_26.726177 7452-7471. Max. coverage (+): 0.07. Max coverage (-): 0

Region: NODE\_261184\_length\_10127\_cov\_26.726177 7472-7492. Max. coverage (+): 0.01. Max coverage (-): 0

Region: NODE\_261184\_length\_10127\_cov\_26.726177 7493-7512. Max. coverage (+): 0.05. Max coverage (-): 0

Region: NODE\_261184\_length\_10127\_cov\_26.726177 7513-7533. Max. coverage (+): 0.02. Max coverage (-): 0.02

Region: NODE\_261184\_length\_10127\_cov\_26.726177 7534-7553. Max. coverage (+): 0.02. Max coverage (-): 0.01

Region: NODE\_261184\_length\_10127\_cov\_26.726177 7554-7574. Max. coverage (+): 0. Max coverage (-): 0

Region: NODE\_261184\_length\_10127\_cov\_26.726177 7575-7594. Max. coverage (+): 0.11. Max coverage (-): 0.04

Region: NODE\_261184\_length\_10127\_cov\_26.726177 7595-7615. Max. coverage (+): 0.44. Max coverage (-): 0

Region: NODE\_261184\_length\_10127\_cov\_26.726177 7616-7635. Max. coverage (+): 0.04. Max coverage (-): 0.04

Region: NODE\_261184\_length\_10127\_cov\_26.726177 7636-7656. Max. coverage (+): 0.19. Max coverage (-): 0.04

Region: NODE\_261184\_length\_10127\_cov\_26.726177 7657-7676. Max. coverage (+): 0.07. Max coverage (-): 0.07

Region: NODE\_261184\_length\_10127\_cov\_26.726177 7677-7697. Max. coverage (+): 0. Max coverage (-): 0

Region: NODE\_261184\_length\_10127\_cov\_26.726177 7698-7717. Max. coverage (+): 0.02. Max coverage (-): 0

Region: NODE\_261184\_length\_10127\_cov\_26.726177 7718-7738. Max. coverage (+): 2.39. Max coverage (-): 0.02

Region: NODE\_261184\_length\_10127\_cov\_26.726177 7739-7758. Max. coverage (+): 0.07. Max coverage (-): 0

Region: NODE\_261184\_length\_10127\_cov\_26.726177 7759-7779. Max. coverage (+): 0.04. Max coverage (-): 0.04

Region: NODE\_261184\_length\_10127\_cov\_26.726177 7780-7799. Max. coverage (+): 0.12. Max coverage (-): 0

Region: NODE\_261184\_length\_10127\_cov\_26.726177 7800-7820. Max. coverage (+): 0.39. Max coverage (-): 0.11

Region: NODE\_261184\_length\_10127\_cov\_26.726177 7821-7840. Max. coverage (+): 0.03. Max coverage (-): 0.01

Region: NODE\_261184\_length\_10127\_cov\_26.726177 7841-7861. Max. coverage (+): 0.67. Max coverage (-): 0

Region: NODE\_261184\_length\_10127\_cov\_26.726177 7862-7881. Max. coverage (+): 0.66. Max coverage (-): 0

Region: NODE\_261184\_length\_10127\_cov\_26.726177 7882-7902. Max. coverage (+): 0.15. Max coverage (-): 0

Region: NODE\_261184\_length\_10127\_cov\_26.726177 7903-7922. Max. coverage (+): 0.11. Max coverage (-): 0.04

Region: NODE\_261184\_length\_10127\_cov\_26.726177 7923-7943. Max. coverage (+): 1.22. Max coverage (-): 0.04

Region: NODE\_261184\_length\_10127\_cov\_26.726177 7944-7963. Max. coverage (+): 2.37. Max coverage (-): 0.07

Region: NODE\_261184\_length\_10127\_cov\_26.726177 7964-7984. Max. coverage (+): 5.56. Max coverage (-): 0.04

Region: NODE\_261184\_length\_10127\_cov\_26.726177 7985-8004. Max. coverage (+): 0.05. Max coverage (-): 0.01

Region: NODE\_261184\_length\_10127\_cov\_26.726177 8005-8025. Max. coverage (+): 1.12. Max coverage (-): 0.01

Region: NODE\_261184\_length\_10127\_cov\_26.726177 8026-8045. Max. coverage (+): 0.3. Max coverage (-): 0

Region: NODE\_261184\_length\_10127\_cov\_26.726177 8046-8066. Max. coverage (+): 0.19. Max coverage (-): 0.04

Region: NODE\_261184\_length\_10127\_cov\_26.726177 8067-8086. Max. coverage (+): 1.3. Max coverage (-): 0

Region: NODE\_261184\_length\_10127\_cov\_26.726177 8087-8107. Max. coverage (+): 0.67. Max coverage (-): 0

Region: NODE\_261184\_length\_10127\_cov\_26.726177 8108-8127. Max. coverage (+): 1.33. Max coverage (-): 0

Region: NODE\_261184\_length\_10127\_cov\_26.726177 8128-8148. Max. coverage (+): 0. Max coverage (-): 0

Region: NODE\_261184\_length\_10127\_cov\_26.726177 8149-8168. Max. coverage (+): 0. Max coverage (-): 0.11

Region: NODE\_261184\_length\_10127\_cov\_26.726177 8169-8189. Max. coverage (+): 0.13. Max coverage (-): 0

Region: NODE\_261184\_length\_10127\_cov\_26.726177 8190-8209. Max. coverage (+): 0. Max coverage (-): 0

Region: NODE\_261184\_length\_10127\_cov\_26.726177 8210-8230. Max. coverage (+): 1.89. Max coverage (-): 0

Region: NODE\_261184\_length\_10127\_cov\_26.726177 8231-8250. Max. coverage (+): 0.04. Max coverage (-): 0

Region: NODE\_261184\_length\_10127\_cov\_26.726177 8251-8271. Max. coverage (+): 0.22. Max coverage (-): 0

Region: NODE\_261184\_length\_10127\_cov\_26.726177 8272-8291. Max. coverage (+): 8.64. Max coverage (-): 0.59

Region: NODE\_261184\_length\_10127\_cov\_26.726177 8292-8312. Max. coverage (+): 1.59. Max coverage (-): 0.63

Region: NODE\_261184\_length\_10127\_cov\_26.726177 8313-8332. Max. coverage (+): 0.48. Max coverage (-): 0.04

Region: NODE\_261184\_length\_10127\_cov\_26.726177 8333-8353. Max. coverage (+): 0.93. Max coverage (-): 0.09

Region: NODE\_261184\_length\_10127\_cov\_26.726177 8354-8373. Max. coverage (+): 3.52. Max coverage (-): 0.11

Region: NODE\_261184\_length\_10127\_cov\_26.726177 8374-8394. Max. coverage (+): 2.3. Max coverage (-): 0.04

Region: NODE\_261184\_length\_10127\_cov\_26.726177 8395-8414. Max. coverage (+): 2.89. Max coverage (-): 0.04

Region: NODE\_261184\_length\_10127\_cov\_26.726177 8415-8435. Max. coverage (+): 0.59. Max coverage (-): 0.07

Region: NODE\_261184\_length\_10127\_cov\_26.726177 8436-8455. Max. coverage (+): 0.59. Max coverage (-): 0.04

Region: NODE\_261184\_length\_10127\_cov\_26.726177 8456-8476. Max. coverage (+): 0.56. Max coverage (-): 0

Region: NODE\_261184\_length\_10127\_cov\_26.726177 8477-8496. Max. coverage (+): 1.48. Max coverage (-): 0.01

Region: NODE\_261184\_length\_10127\_cov\_26.726177 8497-8517. Max. coverage (+): 0.07. Max coverage (-): 0.02

Region: NODE\_261184\_length\_10127\_cov\_26.726177 8518-8537. Max. coverage (+): 0.7. Max coverage (-): 0.07

Region: NODE\_261184\_length\_10127\_cov\_26.726177 8538-8558. Max. coverage (+): 0.7. Max coverage (-): 0.01

Region: NODE\_261184\_length\_10127\_cov\_26.726177 8559-8578. Max. coverage (+): 0.11. Max coverage (-): 0.06

Region: NODE\_261184\_length\_10127\_cov\_26.726177 8579-8599. Max. coverage (+): 0.11. Max coverage (-): 0.22

Region: NODE\_261184\_length\_10127\_cov\_26.726177 8600-8619. Max. coverage (+): 0.37. Max coverage (-): 0.22

Region: NODE\_261184\_length\_10127\_cov\_26.726177 8620-8640. Max. coverage (+): 1.04. Max coverage (-): 0.02

Region: NODE\_261184\_length\_10127\_cov\_26.726177 8641-8660. Max. coverage (+): 1.91. Max coverage (-): 0

Region: NODE\_261184\_length\_10127\_cov\_26.726177 8661-8681. Max. coverage (+): 0.96. Max coverage (-): 0.07

Region: NODE\_261184\_length\_10127\_cov\_26.726177 8682-8701. Max. coverage (+): 1.3. Max coverage (-): 0

Region: NODE\_261184\_length\_10127\_cov\_26.726177 8702-8722. Max. coverage (+): 0.52. Max coverage (-): 0.04

Region: NODE\_261184\_length\_10127\_cov\_26.726177 8723-8742. Max. coverage (+): 14.49. Max coverage (-): 0.04

Region: NODE\_261184\_length\_10127\_cov\_26.726177 8743-8763. Max. coverage (+): 0.5. Max coverage (-): 0.19

Region: NODE\_261184\_length\_10127\_cov\_26.726177 8764-8783. Max. coverage (+): 0.82. Max coverage (-): 0.05

Region: NODE\_261184\_length\_10127\_cov\_26.726177 8784-8804. Max. coverage (+): 0.08. Max coverage (-): 0

Region: NODE\_261184\_length\_10127\_cov\_26.726177 8805-8824. Max. coverage (+): 0.52. Max coverage (-): 0

Region: NODE\_261184\_length\_10127\_cov\_26.726177 8825-8845. Max. coverage (+): 0.33. Max coverage (-): 0

Region: NODE\_261184\_length\_10127\_cov\_26.726177 8846-8865. Max. coverage (+): 0.03. Max coverage (-): 0.25

Region: NODE\_261184\_length\_10127\_cov\_26.726177 8866-8886. Max. coverage (+): 0.79. Max coverage (-): 0.01

Region: NODE\_261184\_length\_10127\_cov\_26.726177 8887-8906. Max. coverage (+): 0.03. Max coverage (-): 0.05

Region: NODE\_261184\_length\_10127\_cov\_26.726177 8907-8927. Max. coverage (+): 0.06. Max coverage (-): 0

Region: NODE\_261184\_length\_10127\_cov\_26.726177 8928-8947. Max. coverage (+): 6.82. Max coverage (-): 0

Region: NODE\_261184\_length\_10127\_cov\_26.726177 8948-8967. Max. coverage (+): 0. Max coverage (-): 0.01

Region: NODE\_261184\_length\_10127\_cov\_26.726177 8968-8988. Max. coverage (+): 0.36. Max coverage (-): 0

Region: NODE\_261184\_length\_10127\_cov\_26.726177 8989-9008. Max. coverage (+): 0.01. Max coverage (-): 0.23

Region: NODE\_261184\_length\_10127\_cov\_26.726177 9009-9029. Max. coverage (+): 0.85. Max coverage (-): 0

Region: NODE\_261184\_length\_10127\_cov\_26.726177 9030-9049. Max. coverage (+): 0.37. Max coverage (-): 0

Region: NODE\_261184\_length\_10127\_cov\_26.726177 9050-9070. Max. coverage (+): 0.22. Max coverage (-): 0.04

Region: NODE\_261184\_length\_10127\_cov\_26.726177 9071-9090. Max. coverage (+): 0.11. Max coverage (-): 0.22

Region: NODE\_261184\_length\_10127\_cov\_26.726177 9091-9111. Max. coverage (+): 0.02. Max coverage (-): 0.01

Region: NODE\_261184\_length\_10127\_cov\_26.726177 9112-9131. Max. coverage (+): 0.03. Max coverage (-): 0.01

Region: NODE\_261184\_length\_10127\_cov\_26.726177 9132-9152. Max. coverage (+): 0.06. Max coverage (-): 0

Region: NODE\_261184\_length\_10127\_cov\_26.726177 9153-9172. Max. coverage (+): 0. Max coverage (-): 0

Region: NODE\_261184\_length\_10127\_cov\_26.726177 9173-9193. Max. coverage (+): 0. Max coverage (-): 0

Region: NODE\_261184\_length\_10127\_cov\_26.726177 9194-9213. Max. coverage (+): 0. Max coverage (-): 0

Region: NODE\_261184\_length\_10127\_cov\_26.726177 9214-9234. Max. coverage (+): 0. Max coverage (-): 0

Region: NODE\_261184\_length\_10127\_cov\_26.726177 9235-9254. Max. coverage (+): 0. Max coverage (-): 0

Region: NODE\_261184\_length\_10127\_cov\_26.726177 9255-9275. Max. coverage (+): 0. Max coverage (-): 0

Region: NODE\_261184\_length\_10127\_cov\_26.726177 9276-9295. Max. coverage (+): 0. Max coverage (-): 0

Region: NODE\_261184\_length\_10127\_cov\_26.726177 9296-9316. Max. coverage (+): 0.07. Max coverage (-): 0

Region: NODE\_261184\_length\_10127\_cov\_26.726177 9317-9336. Max. coverage (+): 2.23. Max coverage (-): 0.01

Region: NODE\_261184\_length\_10127\_cov\_26.726177 9337-9357. Max. coverage (+): 0.1. Max coverage (-): 0

Region: NODE\_261184\_length\_10127\_cov\_26.726177 9358-9377. Max. coverage (+): 0.06. Max coverage (-): 0

Region: NODE\_261184\_length\_10127\_cov\_26.726177 9378-9398. Max. coverage (+): 2.15. Max coverage (-): 0

Region: NODE\_261184\_length\_10127\_cov\_26.726177 9399-9418. Max. coverage (+): 0.22. Max coverage (-): 0.04

Region: NODE\_261184\_length\_10127\_cov\_26.726177 9419-9439. Max. coverage (+): 0.22. Max coverage (-): 0.06

Region: NODE\_261184\_length\_10127\_cov\_26.726177 9440-9459. Max. coverage (+): 0.04. Max coverage (-): 0.07

Region: NODE\_261184\_length\_10127\_cov\_26.726177 9460-9480. Max. coverage (+): 0.19. Max coverage (-): 0.04

Region: NODE\_261184\_length\_10127\_cov\_26.726177 9481-9500. Max. coverage (+): 0.19. Max coverage (-): 0.78

Region: NODE\_261184\_length\_10127\_cov\_26.726177 9501-9521. Max. coverage (+): 4.13. Max coverage (-): 1.25

Region: NODE\_261184\_length\_10127\_cov\_26.726177 9522-9541. Max. coverage (+): 1.84. Max coverage (-): 0.01

Region: NODE\_261184\_length\_10127\_cov\_26.726177 9542-9562. Max. coverage (+): 0.04. Max coverage (-): 0

Region: NODE\_261184\_length\_10127\_cov\_26.726177 9563-9582. Max. coverage (+): 0.12. Max coverage (-): 0

Region: NODE\_261184\_length\_10127\_cov\_26.726177 9583-9603. Max. coverage (+): 0.14. Max coverage (-): 0.02

Region: NODE\_261184\_length\_10127\_cov\_26.726177 9604-9623. Max. coverage (+): 0.06. Max coverage (-): 0.01

Region: NODE\_261184\_length\_10127\_cov\_26.726177 9624-9644. Max. coverage (+): 0.02. Max coverage (-): 0.01

Region: NODE\_261184\_length\_10127\_cov\_26.726177 9645-9664. Max. coverage (+): 0.04. Max coverage (-): 0

Region: NODE\_261184\_length\_10127\_cov\_26.726177 9665-9685. Max. coverage (+): 0.04. Max coverage (-): 0.02

Region: NODE\_261184\_length\_10127\_cov\_26.726177 9686-9705. Max. coverage (+): 0.63. Max coverage (-): 0.19

Region: NODE\_261184\_length\_10127\_cov\_26.726177 9706-9726. Max. coverage (+): 0.04. Max coverage (-): 0

Region: NODE\_261184\_length\_10127\_cov\_26.726177 9727-9746. Max. coverage (+): 0.89. Max coverage (-): 0

Region: NODE\_261184\_length\_10127\_cov\_26.726177 9747-9767. Max. coverage (+): 0.19. Max coverage (-): 0.07

Region: NODE\_261184\_length\_10127\_cov\_26.726177 9768-9787. Max. coverage (+): 0.56. Max coverage (-): 0

Region: NODE\_261184\_length\_10127\_cov\_26.726177 9788-9808. Max. coverage (+): 5.19. Max coverage (-): 0

Region: NODE\_261184\_length\_10127\_cov\_26.726177 9809-9828. Max. coverage (+): 0.19. Max coverage (-): 0

Region: NODE\_261184\_length\_10127\_cov\_26.726177 9829-9849. Max. coverage (+): 0. Max coverage (-): 0.52

Region: NODE\_261184\_length\_10127\_cov\_26.726177 9850-9869. Max. coverage (+): 1.48. Max coverage (-): 0.37

Region: NODE\_261184\_length\_10127\_cov\_26.726177 9870-9890. Max. coverage (+): 0.17. Max coverage (-): 0

Region: NODE\_261184\_length\_10127\_cov\_26.726177 9891-9910. Max. coverage (+): 0. Max coverage (-): 0

Region: NODE\_261184\_length\_10127\_cov\_26.726177 9911-9931. Max. coverage (+): 0.48. Max coverage (-): 0

Region: NODE\_261184\_length\_10127\_cov\_26.726177 9932-9951. Max. coverage (+): 0.85. Max coverage (-): 0

Region: NODE\_261184\_length\_10127\_cov\_26.726177 9952-9972. Max. coverage (+): 0.22. Max coverage (-): 0.04

Region: NODE\_261184\_length\_10127\_cov\_26.726177 9973-9992. Max. coverage (+): 0. Max coverage (-): 0.04

Region: NODE\_261184\_length\_10127\_cov\_26.726177 9993-10013. Max. coverage (+): 0.43. Max coverage (-): 0

Region: NODE\_261184\_length\_10127\_cov\_26.726177 10014-10033. Max. coverage (+): 0.82. Max coverage (-): 0.02

Region: NODE\_261184\_length\_10127\_cov\_26.726177 10034-10054. Max. coverage (+): 3. Max coverage (-): 0.07

Region: NODE\_261184\_length\_10127\_cov\_26.726177 10055-10074. Max. coverage (+): 0.19. Max coverage (-): 0.04

Region: NODE\_261184\_length\_10127\_cov\_26.726177 10075-10095. Max. coverage (+): 2.48. Max coverage (-): 0.65

Region: NODE\_261184\_length\_10127\_cov\_26.726177 10096-10115. Max. coverage (+): 2.48. Max coverage (-): 0

Region: NODE\_261184\_length\_10127\_cov\_26.726177 10116-10136. Max. coverage (+): 0.04. Max coverage (-): 0

Region: NODE\_261184\_length\_10127\_cov\_26.726177 10137-10156. Max. coverage (+): 0.07. Max coverage (-): 0

Region: NODE\_261184\_length\_10127\_cov\_26.726177 10157-10177. Max. coverage (+): 0.04. Max coverage (-): 0

Region: NODE\_261184\_length\_10127\_cov\_26.726177 10178-10197. Max. coverage (+): 0. Max coverage (-): 0

Region: NODE\_261184\_length\_10127\_cov\_26.726177 10198-10218. Max. coverage (+): 0.04. Max coverage (-): 0.04

Region: NODE\_261184\_length\_10127\_cov\_26.726177 10219-10238. Max. coverage (+): 0.04. Max coverage (-): 0

Region: NODE\_261184\_length\_10127\_cov\_26.726177 10239-. Max. coverage (+): 0. Max coverage (-): 0

RepeatMasker Color Code

**+**

100-98% Identity

<98-95% Identity

<95-90% Identity

<90-85% Identity

<85-80% Identity

<80-75% Identity

<75-70% Identity

<70% Identity

**-**

Gene Set Color Code

**+**

Gene

Pseudogene

Other

**-**

Topology/Coverage Color Code

Coverage Plus Strand

Coverage Minus Strand

Mainstrand: Plus

Mainstrand: Minus

Complementary Strand

Flanking Region  
(if option -flank >0)

Gene Set Annotation  
  
RepeatMasker Annotation  

**1. DNAX-24\_DR**: 1-63 (-), Divergence to consensus: 22.2%  
**2. AlRepB-250**: 13-66 (-), Divergence to consensus: 3.7%  
**3. AlRepB-65**: 67-185 (+), Divergence to consensus: 20.3%  
**4. SINE\_AFC**: 185-506 (-), Divergence to consensus: 15.9%  
**5. Merlin-1\_DR**: 432-568 (-), Divergence to consensus: 39.1%  
**6. AlRepB-65**: 517-578 (+), Divergence to consensus: 12.9%  
**7. AlRepD-3818**: 536-643 (-), Divergence to consensus: 31.7%  
**8. AlRepB-65**: 628-1292 (+), Divergence to consensus: 8.8%  
**9. AlRepD-382**: 1351-1657 (+), Divergence to consensus: 34.6%  
**10. (TATTTT)n**: 2013-2091 (+), Divergence to consensus: 31.9%  
**11. A-rich**: 2206-2254 (+), Divergence to consensus: 27.3%  
**12. (TCCTC)n**: 2405-2438 (+), Divergence to consensus: 15.7%  
**13. AlRepB-127**: 3479-3899 (+), Divergence to consensus: 7.8%  
**14. AlRepE-1026**: 3904-4064 (-), Divergence to consensus: 31.7%  
**15. AlRepE-174**: 4156-4990 (-), Divergence to consensus: 44.5%  
**16. CryptonV-1\_DR**: 4992-5894 (-), Divergence to consensus: 43.1%  
**17. (TTTTA)n**: 6073-6098 (+), Divergence to consensus: 12.1%  
**18. AlRepC-1574**: 6900-6947 (-), Divergence to consensus: 21%  
**19. AlRepD-1790**: 6936-7179 (+), Divergence to consensus: 18.5%  
**20. AlRepC-146**: 7219-7765 (-), Divergence to consensus: 21.9%  
**21. AlRepA-118**: 7802-7894 (+), Divergence to consensus: 24.8%  
**22. AlRepC-146**: 8072-8252 (-), Divergence to consensus: 25.1%  
**23. Furousha1**: 8494-8625 (-), Divergence to consensus: 37.2%  
**24. Furousha1**: 8704-9138 (-), Divergence to consensus: 35.4%  
**25. Furousha2**: 9300-9744 (-), Divergence to consensus: 36%  
**26. AlRepC-2154**: 10115-10217 (-), Divergence to consensus: 27.6%

  
Transcription Factor Binding Sites  

**RFX4\_2** (Sequence: CCTGGATAC (+): 2725)  
**RHOXF1** (Sequence: AGATCA (-): 645)  
**RHOXF1** (Sequence: AGCTCA (-): 2479)  
**RHOXF1** (Sequence: AGATTA (-): 3637)  
**RHOXF1** (Sequence: AGCTCA (-): 4263)  
**RHOXF1** (Sequence: AGCTCA (-): 4335)  
**RHOXF1** (Sequence: AGCTCA (-): 4410)  
**RHOXF1** (Sequence: AGCTCA (-): 4650)  
**RHOXF1** (Sequence: AGCTCA (-): 4674)  
**RHOXF1** (Sequence: AGCTTA (-): 5424)  
**RHOXF1** (Sequence: GGCTCA (-): 5525)  
**RHOXF1** (Sequence: GGATCA (-): 5722)  
**RHOXF1** (Sequence: AGCTTA (-): 5830)  
**RHOXF1** (Sequence: AGCTTA (-): 6189)  
**RHOXF1** (Sequence: AGATTA (-): 6204)  
**RHOXF1** (Sequence: GGCTCA (-): 6327)  
**RHOXF1** (Sequence: GGATTA (-): 6591)  
**RHOXF1** (Sequence: AGCTCA (-): 6806)  
**RHOXF1** (Sequence: AGATTA (-): 7689)  
**RHOXF1** (Sequence: GGATCA (-): 8581)  
**RHOXF1** (Sequence: AGCTTA (-): 9042)  
**RHOXF1** (Sequence: GGCTTA (-): 9812)  
**RHOXF1** (Sequence: GGATCA (-): 9890)  
**RHOXF1** (Sequence: TGAGCC (+): 492)  
**RHOXF1** (Sequence: TAAGCT (+): 697)  
**RHOXF1** (Sequence: TAATCC (+): 827)  
**RHOXF1** (Sequence: TGATCT (+): 1888)  
**RHOXF1** (Sequence: TGATCT (+): 1939)  
**RHOXF1** (Sequence: TAATCC (+): 1989)  
**RHOXF1** (Sequence: TGATCT (+): 2527)  
**RHOXF1** (Sequence: TAATCC (+): 3004)  
**RHOXF1** (Sequence: TAAGCC (+): 3993)  
**RHOXF1** (Sequence: TGATCC (+): 5491)  
**RHOXF1** (Sequence: TGATCT (+): 5769)  
**RHOXF1** (Sequence: TAATCC (+): 6623)  
**RHOXF1** (Sequence: TAAGCC (+): 6819)  
**RHOXF1** (Sequence: TAAGCT (+): 7087)  
**RHOXF1** (Sequence: TAAGCT (+): 7218)  
**RHOXF1** (Sequence: TGATCT (+): 8977)  
**RHOXF1** (Sequence: TGATCT (+): 9069)  
**RHOXF1** (Sequence: TGATCC (+): 9766)  
**Gata4** (Sequence: CTTATCT (+): 2658)  
**POU5F1** (Sequence: TTTGCAT (-): 541)  
**POU5F1** (Sequence: TTTGCAT (-): 1696)  
**POU5F1** (Sequence: TTTGCAT (-): 7463)  
**RFX4\_2** (Sequence: GTATCCATG (-): 1312)  
**SOX9** (Sequence: AACAATAG (-): 6414)  
**SOX9** (Sequence: AACAATGG (-): 9674)  
**FOXO3\_mmu** (Sequence: TGTTTACA (-): 517)  
**FOXO3\_mmu** (Sequence: TGTTTTGA (-): 4077)  
**FOXO3\_mmu** (Sequence: TGTTTTCA (-): 5124)  
**Sox5** (Sequence: ATTGTT (+): 79)  
**Sox5** (Sequence: ATTGTT (+): 1503)  
**Sox5** (Sequence: ATTGTT (+): 3318)  
**Sox5** (Sequence: ATTGTT (+): 4085)  
**Sox5** (Sequence: ATTGTT (+): 6275)  
**Sox5** (Sequence: ATTGTT (+): 7412)  
**Sox5** (Sequence: ATTGTT (+): 8614)  
**FIGLA** (Sequence: TCCAGCTGGT (-): 3490)  
**FIGLA** (Sequence: TCCACCTGGT (-): 8940)  
**SOX9** (Sequence: TCATTGTT (+): 8612)  
**FOXO1** (Sequence: AAAAACAGC (-): 139)  
**FOXO1** (Sequence: AAAAACAAC (-): 2150)  
**FOXO1** (Sequence: AAAAACAAC (-): 2777)  
**FOXO1** (Sequence: AAAAACAAG (-): 6547)  
**FOXO3\_hsa** (Sequence: ATGTTTAC (-): 516)  
**FOXP1** (Sequence: TGTTTAC (-): 517)  
**FOXP1** (Sequence: TGTTTAC (-): 5589)  
**POU2F1** (Sequence: ATTTGAATA (-): 2342)  
**POU2F1** (Sequence: ATTTGAATA (-): 9789)  
**Rhox11** (Sequence: TGCTGTTTT (+): 4074)  
**Rhox11** (Sequence: TGGTGTTTT (+): 6946)  
**Rhox11** (Sequence: AAAACAGCA (-): 140)  
**Sox5** (Sequence: AACAAT (-): 749)  
**Sox5** (Sequence: AACAAT (-): 912)  
**Sox5** (Sequence: AACAAT (-): 2333)  
**Sox5** (Sequence: AACAAT (-): 6309)  
**Sox5** (Sequence: AACAAT (-): 6414)  
**Sox5** (Sequence: AACAAT (-): 6500)  
**Sox5** (Sequence: AACAAT (-): 9674)  
**POU2F1** (Sequence: TATTCAAAT (+): 2238)  
**POU2F1** (Sequence: TATGCAAAT (+): 3343)  
**POU2F1** (Sequence: TATTCAAAT (+): 8191)  
**POU5F1** (Sequence: ATGCAAA (+): 3344)
