# Supplementary material for: First characterization of PIWI-interacting RNA clusters in a cichlid fish with a B chromosome
Source: BMC Biol. 2022 Sep 21;20:204. doi: 10.1186/s12915-022-01403-2 (PMC9490952; doi:10.1186/s12915-022-01403-2)
Supplement: Supplementary file 1 — Additional file 1. Zipped folder with fasta and interactive html piRNA cluster information for the A. latifasciata genome. The nomenclature is as follows: number-pirna-cluster_sex_B-presence (f, female; m, male; 0b, without B chromosome; 1b, with B chromosome). [file 12915_2022_1403_MOESM1_ESM.zip › 102_f0b.html]

piRNA cluster 102\_f0b 57


Predicted piRNA cluster no. 102\_f0b
  

Show proTRAC run info
Hide proTRAC run info

/\  
                \_\_\_\_\_\_\_\_\_\_\_\_\_\_\_\_\_\_\_\_\_\_\_/\\_\_\_ /  \\_\_\_\_\_\_\_  
               I                      /  \  /    \      I  
               I     pro             /    \/      \     I  
               I        TRAC        /               \   I  
               I   \_\_\_\_\_\_\_\_\_\_\_\_\_\_\_\_/\_\_\_\_\_\_\_\_\_\_\_\_\_\_\_\_\_\\_ I  
               I   \              /                     I  
               I    \            /                      I  
               I     \  /\      /       V.2.4.2         I  
               I      \/  \    /                        I  
               I\_\_\_\_\_\_\_\_\_\_\_\  /\_\_\_\_\_\_\_\_\_\_\_\_\_\_\_\_\_\_\_\_\_\_\_\_\_I  
                            \/  
  
  
================================= proTRAC ====================================  
VERSION: .......... 2.4.2  
LAST MODIFIED: .... 11. May 2018  
  
Please cite:  
Rosenkranz D, Zischler H. proTRAC - a software for probabilistic piRNA cluster  
detection, visualization and analysis. 2012. BMC Bioinformatics 13:5.  
  
  
Contact:  
David Rosenkranz  
Institute of Organismic and Molecular Evolutionary Biology  
Dept. Anthropology, small RNA group  
Johannes Gutenberg University Mainz  
email: rosenkranz@uni-mainz.de  
  
You can find the latest proTRAC version at:  
http://sourceforge.net/projects/protrac/files  
http://www.smallRNAgroup-mainz.de/software  
==============================================================================  
  
PARAMETERS:  
Map file: ...............piwi-femeas-0B.fa-collapse.map  
Genome file: ............../../../0B\_ala\_genome.fa  
RepeatMasker annotation: Alatifasciata-all0B-maryan-v2.fa\_corrected.out  
GeneSet:................./guest-storage/Data/annotation/Alatifasciata\_all0B\_maryan-v2\_out2017.gff  
  
Significant (p<=0.01) hit density will be calculated based  
on observed hit distribution.  
  
Sliding window size: ........................................ 5000 bp  
Sliding window increament: .................................. 1000 bp  
Normalize each hit by number of genomic hits: ............... yes  
Normalize each hit by number of sequence reads: ............. yes  
Normalize values (-> per million mapped reads): ............. yes  
Min. fraction of hits with 1T(U) or 10A: .................... 0.75  
Alternatively: Min. fraction of hits with 1T(U) and 10A: .... 0.5  
Min. fraction of hits with typical piRNA length: ............ 0.75  
Typical piRNA length: ....................................... 24-32 nt  
Min. size of a piRNA cluster: ............................... 1000 bp.  
Min. number of hits (absolute): ............................. 0  
Min. number of hits (normalized): ........................... 0  
Min. fraction of hits on the mainstrand: .................... 0.75  
Top fraction of mapped sequences (in terms of read counts): . 1%  
Top fraction accounts for max. n% of sequence reads: ........ 90%  
Min. fraction of hits on each arm of a bidirectional cluster: 0.05  
Output html file for each cluster: .......................... yes  
Output a summary table: ..................................... yes  
Output a FASTA file for each cluster (piRNA sequences): ..... yes  
Output a FASTA file comprising cluster sequences: ........... yes  
Output a GTF file for predicted piRNA clusters: ..............yes  
Search DNA motifs in clusters: .............................. yes  
Output flanking sequences: +/- .............................. 0 bp  
Output ~.pTi file: .......................................... no  
==============================================================================  
  
  
Genome size (without gaps): ............ 758543724 bp  
Gaps (N/X/-): .......................... 417479 bp  
Mapped reads: .......................... 13052187  
Non-identical sequences: ............... 3338911  
Genomic hits: .......................... 28737726  
Significant densitiy of mapped reads: .. 470.083249848448 reads/kb

Show proTRAC cluster info
Hide proTRAC cluster info

|  |  |
| --- | --- |
| Location | NODE\_268840\_length\_12752\_cov\_27.436010 |
| Coordinates | 1-8027 |
| Size [bp] | 8027 |
| Sequence hit loci | 1769 |
| Mapped reads (normalized) | 5968.6 |
| Mapped reads (normalized) per kb | 743.6 |
| Normalized reads with 1T (1U) | 85.4% |
| Normalized reads with 10A | 32% |
| Normalized reads with length 24-32 nt | 98.6% |
| Normalized reads on the main strand(s) | 98.2% |
| Predicted directionality | mono:minus |

100%

0%

1T (1U)  
reads

10A reads

24-32 nt  
reads

reads on mainstrand

**Either the amount of reads with 1T (1U) OR 10A has to exceed 75% (set with option: -1Tor10A)  
Alternatively the amount of reads with 1T (1U) AND 10A has to exceed 50% (set with option: -1Tand10A)  
Minimum amount of reads with preferred size is 75% (set with option: -pisize)  
Minimum amount of reads on the main strand(s) is 75% (set with option: -clstrand)**

Show read coverage
Hide read coverage

WHAT DO I SEE HERE?  
This chart shows the location of mapped sequence reads within a predicted piRNA cluster. The color refers to the number of genomic hits produced by the sequence read in question. A dark red bar indicates that this sequence read produces many other hits elsewhere in the genome. Many adjacent red or yellow bars can indicate the presence of a multi-copy element such as transposons or rRNA genes. A dark green bar indicates that this sequence read maps uniquely to this locus.

1 hit

2-5 hits

6-10 hits

11-20 hits

21-50 hits

51-100 hits

> 100 hits

NODE\_268840\_length\_12752\_cov\_27.436010

1

8027

Gene Set

RepeatMasker

Mapped  
Reads

55.24

plus strand

minus strand

55.24

Region: NODE\_268840\_length\_12752\_cov\_27.436010 5762-9. Max. coverage (+): 0. Max coverage (-): 0.08

Region: NODE\_268840\_length\_12752\_cov\_27.436010 10-25. Max. coverage (+): 0. Max coverage (-): 0.05

Region: NODE\_268840\_length\_12752\_cov\_27.436010 26-41. Max. coverage (+): 0.01. Max coverage (-): 0.08

Region: NODE\_268840\_length\_12752\_cov\_27.436010 42-57. Max. coverage (+): 0. Max coverage (-): 11.03

Region: NODE\_268840\_length\_12752\_cov\_27.436010 58-73. Max. coverage (+): 0.04. Max coverage (-): 0

Region: NODE\_268840\_length\_12752\_cov\_27.436010 74-89. Max. coverage (+): 0.08. Max coverage (-): 0

Region: NODE\_268840\_length\_12752\_cov\_27.436010 90-105. Max. coverage (+): 0. Max coverage (-): 0.08

Region: NODE\_268840\_length\_12752\_cov\_27.436010 106-121. Max. coverage (+): 0. Max coverage (-): 0

Region: NODE\_268840\_length\_12752\_cov\_27.436010 122-137. Max. coverage (+): 0. Max coverage (-): 0

Region: NODE\_268840\_length\_12752\_cov\_27.436010 138-153. Max. coverage (+): 0. Max coverage (-): 0

Region: NODE\_268840\_length\_12752\_cov\_27.436010 154-169. Max. coverage (+): 0. Max coverage (-): 0.08

Region: NODE\_268840\_length\_12752\_cov\_27.436010 170-185. Max. coverage (+): 0. Max coverage (-): 0.08

Region: NODE\_268840\_length\_12752\_cov\_27.436010 186-201. Max. coverage (+): 0. Max coverage (-): 0

Region: NODE\_268840\_length\_12752\_cov\_27.436010 202-217. Max. coverage (+): 0. Max coverage (-): 0

Region: NODE\_268840\_length\_12752\_cov\_27.436010 218-233. Max. coverage (+): 0. Max coverage (-): 0

Region: NODE\_268840\_length\_12752\_cov\_27.436010 234-249. Max. coverage (+): 0. Max coverage (-): 0

Region: NODE\_268840\_length\_12752\_cov\_27.436010 250-265. Max. coverage (+): 0. Max coverage (-): 0

Region: NODE\_268840\_length\_12752\_cov\_27.436010 266-281. Max. coverage (+): 0. Max coverage (-): 0.08

Region: NODE\_268840\_length\_12752\_cov\_27.436010 282-297. Max. coverage (+): 0. Max coverage (-): 0.23

Region: NODE\_268840\_length\_12752\_cov\_27.436010 298-314. Max. coverage (+): 0. Max coverage (-): 0.23

Region: NODE\_268840\_length\_12752\_cov\_27.436010 315-330. Max. coverage (+): 0. Max coverage (-): 0.08

Region: NODE\_268840\_length\_12752\_cov\_27.436010 331-346. Max. coverage (+): 0.08. Max coverage (-): 0.08

Region: NODE\_268840\_length\_12752\_cov\_27.436010 347-362. Max. coverage (+): 0. Max coverage (-): 0.08

Region: NODE\_268840\_length\_12752\_cov\_27.436010 363-378. Max. coverage (+): 0. Max coverage (-): 0

Region: NODE\_268840\_length\_12752\_cov\_27.436010 379-394. Max. coverage (+): 0. Max coverage (-): 0

Region: NODE\_268840\_length\_12752\_cov\_27.436010 395-410. Max. coverage (+): 0. Max coverage (-): 0.38

Region: NODE\_268840\_length\_12752\_cov\_27.436010 411-426. Max. coverage (+): 0.08. Max coverage (-): 0.08

Region: NODE\_268840\_length\_12752\_cov\_27.436010 427-442. Max. coverage (+): 0. Max coverage (-): 0

Region: NODE\_268840\_length\_12752\_cov\_27.436010 443-458. Max. coverage (+): 0. Max coverage (-): 0.08

Region: NODE\_268840\_length\_12752\_cov\_27.436010 459-474. Max. coverage (+): 0. Max coverage (-): 0

Region: NODE\_268840\_length\_12752\_cov\_27.436010 475-490. Max. coverage (+): 0. Max coverage (-): 0.23

Region: NODE\_268840\_length\_12752\_cov\_27.436010 491-506. Max. coverage (+): 0. Max coverage (-): 0

Region: NODE\_268840\_length\_12752\_cov\_27.436010 507-522. Max. coverage (+): 0. Max coverage (-): 0

Region: NODE\_268840\_length\_12752\_cov\_27.436010 523-538. Max. coverage (+): 0. Max coverage (-): 1.53

Region: NODE\_268840\_length\_12752\_cov\_27.436010 539-554. Max. coverage (+): 0. Max coverage (-): 2.53

Region: NODE\_268840\_length\_12752\_cov\_27.436010 555-570. Max. coverage (+): 0.08. Max coverage (-): 2.07

Region: NODE\_268840\_length\_12752\_cov\_27.436010 571-586. Max. coverage (+): 0. Max coverage (-): 0.69

Region: NODE\_268840\_length\_12752\_cov\_27.436010 587-603. Max. coverage (+): 0.15. Max coverage (-): 0.15

Region: NODE\_268840\_length\_12752\_cov\_27.436010 604-619. Max. coverage (+): 0. Max coverage (-): 0.77

Region: NODE\_268840\_length\_12752\_cov\_27.436010 620-635. Max. coverage (+): 0.23. Max coverage (-): 0.15

Region: NODE\_268840\_length\_12752\_cov\_27.436010 636-651. Max. coverage (+): 0.15. Max coverage (-): 2.91

Region: NODE\_268840\_length\_12752\_cov\_27.436010 652-667. Max. coverage (+): 0.15. Max coverage (-): 0.15

Region: NODE\_268840\_length\_12752\_cov\_27.436010 668-683. Max. coverage (+): 0. Max coverage (-): 0.38

Region: NODE\_268840\_length\_12752\_cov\_27.436010 684-699. Max. coverage (+): 0. Max coverage (-): 0

Region: NODE\_268840\_length\_12752\_cov\_27.436010 700-715. Max. coverage (+): 0. Max coverage (-): 0.61

Region: NODE\_268840\_length\_12752\_cov\_27.436010 716-731. Max. coverage (+): 0.08. Max coverage (-): 0

Region: NODE\_268840\_length\_12752\_cov\_27.436010 732-747. Max. coverage (+): 0. Max coverage (-): 0

Region: NODE\_268840\_length\_12752\_cov\_27.436010 748-763. Max. coverage (+): 0. Max coverage (-): 0.15

Region: NODE\_268840\_length\_12752\_cov\_27.436010 764-779. Max. coverage (+): 0. Max coverage (-): 0.15

Region: NODE\_268840\_length\_12752\_cov\_27.436010 780-795. Max. coverage (+): 0. Max coverage (-): 0

Region: NODE\_268840\_length\_12752\_cov\_27.436010 796-811. Max. coverage (+): 0. Max coverage (-): 0.08

Region: NODE\_268840\_length\_12752\_cov\_27.436010 812-827. Max. coverage (+): 0. Max coverage (-): 1.07

Region: NODE\_268840\_length\_12752\_cov\_27.436010 828-843. Max. coverage (+): 0. Max coverage (-): 0

Region: NODE\_268840\_length\_12752\_cov\_27.436010 844-859. Max. coverage (+): 0. Max coverage (-): 0

Region: NODE\_268840\_length\_12752\_cov\_27.436010 860-875. Max. coverage (+): 0. Max coverage (-): 0.08

Region: NODE\_268840\_length\_12752\_cov\_27.436010 876-891. Max. coverage (+): 0. Max coverage (-): 0

Region: NODE\_268840\_length\_12752\_cov\_27.436010 892-908. Max. coverage (+): 0. Max coverage (-): 0

Region: NODE\_268840\_length\_12752\_cov\_27.436010 909-924. Max. coverage (+): 0. Max coverage (-): 0

Region: NODE\_268840\_length\_12752\_cov\_27.436010 925-940. Max. coverage (+): 0. Max coverage (-): 0.15

Region: NODE\_268840\_length\_12752\_cov\_27.436010 941-956. Max. coverage (+): 0.08. Max coverage (-): 0.31

Region: NODE\_268840\_length\_12752\_cov\_27.436010 957-972. Max. coverage (+): 0. Max coverage (-): 0

Region: NODE\_268840\_length\_12752\_cov\_27.436010 973-988. Max. coverage (+): 0. Max coverage (-): 0

Region: NODE\_268840\_length\_12752\_cov\_27.436010 989-1004. Max. coverage (+): 0. Max coverage (-): 2.6

Region: NODE\_268840\_length\_12752\_cov\_27.436010 1005-1020. Max. coverage (+): 0. Max coverage (-): 0

Region: NODE\_268840\_length\_12752\_cov\_27.436010 1021-1036. Max. coverage (+): 0. Max coverage (-): 0.23

Region: NODE\_268840\_length\_12752\_cov\_27.436010 1037-1052. Max. coverage (+): 0. Max coverage (-): 0.61

Region: NODE\_268840\_length\_12752\_cov\_27.436010 1053-1068. Max. coverage (+): 0.08. Max coverage (-): 0.08

Region: NODE\_268840\_length\_12752\_cov\_27.436010 1069-1084. Max. coverage (+): 0. Max coverage (-): 0.23

Region: NODE\_268840\_length\_12752\_cov\_27.436010 1085-1100. Max. coverage (+): 0. Max coverage (-): 0.15

Region: NODE\_268840\_length\_12752\_cov\_27.436010 1101-1116. Max. coverage (+): 0. Max coverage (-): 3.52

Region: NODE\_268840\_length\_12752\_cov\_27.436010 1117-1132. Max. coverage (+): 0.46. Max coverage (-): 0.54

Region: NODE\_268840\_length\_12752\_cov\_27.436010 1133-1148. Max. coverage (+): 0. Max coverage (-): 1

Region: NODE\_268840\_length\_12752\_cov\_27.436010 1149-1164. Max. coverage (+): 0. Max coverage (-): 0.69

Region: NODE\_268840\_length\_12752\_cov\_27.436010 1165-1180. Max. coverage (+): 0. Max coverage (-): 1.53

Region: NODE\_268840\_length\_12752\_cov\_27.436010 1181-1197. Max. coverage (+): 0. Max coverage (-): 2.53

Region: NODE\_268840\_length\_12752\_cov\_27.436010 1198-1213. Max. coverage (+): 0. Max coverage (-): 0

Region: NODE\_268840\_length\_12752\_cov\_27.436010 1214-1229. Max. coverage (+): 0. Max coverage (-): 0

Region: NODE\_268840\_length\_12752\_cov\_27.436010 1230-1245. Max. coverage (+): 0. Max coverage (-): 0.08

Region: NODE\_268840\_length\_12752\_cov\_27.436010 1246-1261. Max. coverage (+): 0. Max coverage (-): 0.69

Region: NODE\_268840\_length\_12752\_cov\_27.436010 1262-1277. Max. coverage (+): 0. Max coverage (-): 0.69

Region: NODE\_268840\_length\_12752\_cov\_27.436010 1278-1293. Max. coverage (+): 0. Max coverage (-): 0

Region: NODE\_268840\_length\_12752\_cov\_27.436010 1294-1309. Max. coverage (+): 0. Max coverage (-): 0

Region: NODE\_268840\_length\_12752\_cov\_27.436010 1310-1325. Max. coverage (+): 0. Max coverage (-): 0.46

Region: NODE\_268840\_length\_12752\_cov\_27.436010 1326-1341. Max. coverage (+): 0. Max coverage (-): 0.46

Region: NODE\_268840\_length\_12752\_cov\_27.436010 1342-1357. Max. coverage (+): 0.08. Max coverage (-): 0.15

Region: NODE\_268840\_length\_12752\_cov\_27.436010 1358-1373. Max. coverage (+): 0. Max coverage (-): 0.84

Region: NODE\_268840\_length\_12752\_cov\_27.436010 1374-1389. Max. coverage (+): 0. Max coverage (-): 0.54

Region: NODE\_268840\_length\_12752\_cov\_27.436010 1390-1405. Max. coverage (+): 0. Max coverage (-): 1.99

Region: NODE\_268840\_length\_12752\_cov\_27.436010 1406-1421. Max. coverage (+): 0.15. Max coverage (-): 6.36

Region: NODE\_268840\_length\_12752\_cov\_27.436010 1422-1437. Max. coverage (+): 0. Max coverage (-): 0.46

Region: NODE\_268840\_length\_12752\_cov\_27.436010 1438-1453. Max. coverage (+): 0.08. Max coverage (-): 1.15

Region: NODE\_268840\_length\_12752\_cov\_27.436010 1454-1469. Max. coverage (+): 0.08. Max coverage (-): 0

Region: NODE\_268840\_length\_12752\_cov\_27.436010 1470-1485. Max. coverage (+): 0. Max coverage (-): 0.92

Region: NODE\_268840\_length\_12752\_cov\_27.436010 1486-1502. Max. coverage (+): 0. Max coverage (-): 0

Region: NODE\_268840\_length\_12752\_cov\_27.436010 1503-1518. Max. coverage (+): 0. Max coverage (-): 0.23

Region: NODE\_268840\_length\_12752\_cov\_27.436010 1519-1534. Max. coverage (+): 0. Max coverage (-): 0.61

Region: NODE\_268840\_length\_12752\_cov\_27.436010 1535-1550. Max. coverage (+): 0. Max coverage (-): 0.08

Region: NODE\_268840\_length\_12752\_cov\_27.436010 1551-1566. Max. coverage (+): 0. Max coverage (-): 0.15

Region: NODE\_268840\_length\_12752\_cov\_27.436010 1567-1582. Max. coverage (+): 0. Max coverage (-): 0.15

Region: NODE\_268840\_length\_12752\_cov\_27.436010 1583-1598. Max. coverage (+): 0. Max coverage (-): 0

Region: NODE\_268840\_length\_12752\_cov\_27.436010 1599-1614. Max. coverage (+): 0. Max coverage (-): 0

Region: NODE\_268840\_length\_12752\_cov\_27.436010 1615-1630. Max. coverage (+): 0. Max coverage (-): 0.31

Region: NODE\_268840\_length\_12752\_cov\_27.436010 1631-1646. Max. coverage (+): 0. Max coverage (-): 0.46

Region: NODE\_268840\_length\_12752\_cov\_27.436010 1647-1662. Max. coverage (+): 0. Max coverage (-): 0

Region: NODE\_268840\_length\_12752\_cov\_27.436010 1663-1678. Max. coverage (+): 0. Max coverage (-): 0.08

Region: NODE\_268840\_length\_12752\_cov\_27.436010 1679-1694. Max. coverage (+): 0. Max coverage (-): 0.08

Region: NODE\_268840\_length\_12752\_cov\_27.436010 1695-1710. Max. coverage (+): 0. Max coverage (-): 0.15

Region: NODE\_268840\_length\_12752\_cov\_27.436010 1711-1726. Max. coverage (+): 0. Max coverage (-): 0.77

Region: NODE\_268840\_length\_12752\_cov\_27.436010 1727-1742. Max. coverage (+): 0. Max coverage (-): 0.08

Region: NODE\_268840\_length\_12752\_cov\_27.436010 1743-1758. Max. coverage (+): 0. Max coverage (-): 1.99

Region: NODE\_268840\_length\_12752\_cov\_27.436010 1759-1774. Max. coverage (+): 0. Max coverage (-): 1.99

Region: NODE\_268840\_length\_12752\_cov\_27.436010 1775-1791. Max. coverage (+): 0. Max coverage (-): 0.05

Region: NODE\_268840\_length\_12752\_cov\_27.436010 1792-1807. Max. coverage (+): 0. Max coverage (-): 0.69

Region: NODE\_268840\_length\_12752\_cov\_27.436010 1808-1823. Max. coverage (+): 0.08. Max coverage (-): 0.31

Region: NODE\_268840\_length\_12752\_cov\_27.436010 1824-1839. Max. coverage (+): 0.08. Max coverage (-): 0.46

Region: NODE\_268840\_length\_12752\_cov\_27.436010 1840-1855. Max. coverage (+): 0. Max coverage (-): 0.15

Region: NODE\_268840\_length\_12752\_cov\_27.436010 1856-1871. Max. coverage (+): 0.31. Max coverage (-): 0.31

Region: NODE\_268840\_length\_12752\_cov\_27.436010 1872-1887. Max. coverage (+): 0. Max coverage (-): 0.23

Region: NODE\_268840\_length\_12752\_cov\_27.436010 1888-1903. Max. coverage (+): 0. Max coverage (-): 0.15

Region: NODE\_268840\_length\_12752\_cov\_27.436010 1904-1919. Max. coverage (+): 0. Max coverage (-): 0.08

Region: NODE\_268840\_length\_12752\_cov\_27.436010 1920-1935. Max. coverage (+): 0. Max coverage (-): 0.54

Region: NODE\_268840\_length\_12752\_cov\_27.436010 1936-1951. Max. coverage (+): 0. Max coverage (-): 0.38

Region: NODE\_268840\_length\_12752\_cov\_27.436010 1952-1967. Max. coverage (+): 0. Max coverage (-): 0.08

Region: NODE\_268840\_length\_12752\_cov\_27.436010 1968-1983. Max. coverage (+): 0. Max coverage (-): 0

Region: NODE\_268840\_length\_12752\_cov\_27.436010 1984-1999. Max. coverage (+): 0. Max coverage (-): 0.23

Region: NODE\_268840\_length\_12752\_cov\_27.436010 2000-2015. Max. coverage (+): 0. Max coverage (-): 0.23

Region: NODE\_268840\_length\_12752\_cov\_27.436010 2016-2031. Max. coverage (+): 0.08. Max coverage (-): 0.23

Region: NODE\_268840\_length\_12752\_cov\_27.436010 2032-2047. Max. coverage (+): 0. Max coverage (-): 0.08

Region: NODE\_268840\_length\_12752\_cov\_27.436010 2048-2063. Max. coverage (+): 0. Max coverage (-): 0.08

Region: NODE\_268840\_length\_12752\_cov\_27.436010 2064-2079. Max. coverage (+): 0. Max coverage (-): 0.08

Region: NODE\_268840\_length\_12752\_cov\_27.436010 2080-2096. Max. coverage (+): 0. Max coverage (-): 0

Region: NODE\_268840\_length\_12752\_cov\_27.436010 2097-2112. Max. coverage (+): 0. Max coverage (-): 0.46

Region: NODE\_268840\_length\_12752\_cov\_27.436010 2113-2128. Max. coverage (+): 0. Max coverage (-): 0.15

Region: NODE\_268840\_length\_12752\_cov\_27.436010 2129-2144. Max. coverage (+): 0. Max coverage (-): 0

Region: NODE\_268840\_length\_12752\_cov\_27.436010 2145-2160. Max. coverage (+): 0. Max coverage (-): 0

Region: NODE\_268840\_length\_12752\_cov\_27.436010 2161-2176. Max. coverage (+): 0. Max coverage (-): 0

Region: NODE\_268840\_length\_12752\_cov\_27.436010 2177-2192. Max. coverage (+): 0. Max coverage (-): 0.08

Region: NODE\_268840\_length\_12752\_cov\_27.436010 2193-2208. Max. coverage (+): 0. Max coverage (-): 0.08

Region: NODE\_268840\_length\_12752\_cov\_27.436010 2209-2224. Max. coverage (+): 0. Max coverage (-): 0

Region: NODE\_268840\_length\_12752\_cov\_27.436010 2225-2240. Max. coverage (+): 0. Max coverage (-): 0

Region: NODE\_268840\_length\_12752\_cov\_27.436010 2241-2256. Max. coverage (+): 0. Max coverage (-): 0

Region: NODE\_268840\_length\_12752\_cov\_27.436010 2257-2272. Max. coverage (+): 0. Max coverage (-): 0.15

Region: NODE\_268840\_length\_12752\_cov\_27.436010 2273-2288. Max. coverage (+): 0. Max coverage (-): 0.54

Region: NODE\_268840\_length\_12752\_cov\_27.436010 2289-2304. Max. coverage (+): 0. Max coverage (-): 0.15

Region: NODE\_268840\_length\_12752\_cov\_27.436010 2305-2320. Max. coverage (+): 0. Max coverage (-): 0.13

Region: NODE\_268840\_length\_12752\_cov\_27.436010 2321-2336. Max. coverage (+): 0. Max coverage (-): 0

Region: NODE\_268840\_length\_12752\_cov\_27.436010 2337-2352. Max. coverage (+): 0. Max coverage (-): 0

Region: NODE\_268840\_length\_12752\_cov\_27.436010 2353-2368. Max. coverage (+): 0. Max coverage (-): 0.05

Region: NODE\_268840\_length\_12752\_cov\_27.436010 2369-2385. Max. coverage (+): 0. Max coverage (-): 0

Region: NODE\_268840\_length\_12752\_cov\_27.436010 2386-2401. Max. coverage (+): 0.03. Max coverage (-): 0

Region: NODE\_268840\_length\_12752\_cov\_27.436010 2402-2417. Max. coverage (+): 0.03. Max coverage (-): 0

Region: NODE\_268840\_length\_12752\_cov\_27.436010 2418-2433. Max. coverage (+): 0. Max coverage (-): 0

Region: NODE\_268840\_length\_12752\_cov\_27.436010 2434-2449. Max. coverage (+): 0. Max coverage (-): 0.08

Region: NODE\_268840\_length\_12752\_cov\_27.436010 2450-2465. Max. coverage (+): 0. Max coverage (-): 0

Region: NODE\_268840\_length\_12752\_cov\_27.436010 2466-2481. Max. coverage (+): 0. Max coverage (-): 0

Region: NODE\_268840\_length\_12752\_cov\_27.436010 2482-2497. Max. coverage (+): 0. Max coverage (-): 0.03

Region: NODE\_268840\_length\_12752\_cov\_27.436010 2498-2513. Max. coverage (+): 0. Max coverage (-): 0.18

Region: NODE\_268840\_length\_12752\_cov\_27.436010 2514-2529. Max. coverage (+): 0. Max coverage (-): 0.05

Region: NODE\_268840\_length\_12752\_cov\_27.436010 2530-2545. Max. coverage (+): 0. Max coverage (-): 0

Region: NODE\_268840\_length\_12752\_cov\_27.436010 2546-2561. Max. coverage (+): 0. Max coverage (-): 4.37

Region: NODE\_268840\_length\_12752\_cov\_27.436010 2562-2577. Max. coverage (+): 0. Max coverage (-): 0.11

Region: NODE\_268840\_length\_12752\_cov\_27.436010 2578-2593. Max. coverage (+): 0. Max coverage (-): 0.1

Region: NODE\_268840\_length\_12752\_cov\_27.436010 2594-2609. Max. coverage (+): 0.04. Max coverage (-): 7.36

Region: NODE\_268840\_length\_12752\_cov\_27.436010 2610-2625. Max. coverage (+): 0.08. Max coverage (-): 1.38

Region: NODE\_268840\_length\_12752\_cov\_27.436010 2626-2641. Max. coverage (+): 0. Max coverage (-): 6.67

Region: NODE\_268840\_length\_12752\_cov\_27.436010 2642-2657. Max. coverage (+): 0. Max coverage (-): 0.77

Region: NODE\_268840\_length\_12752\_cov\_27.436010 2658-2673. Max. coverage (+): 0. Max coverage (-): 2.22

Region: NODE\_268840\_length\_12752\_cov\_27.436010 2674-2690. Max. coverage (+): 0. Max coverage (-): 2.22

Region: NODE\_268840\_length\_12752\_cov\_27.436010 2691-2706. Max. coverage (+): 0.04. Max coverage (-): 0.73

Region: NODE\_268840\_length\_12752\_cov\_27.436010 2707-2722. Max. coverage (+): 0. Max coverage (-): 0

Region: NODE\_268840\_length\_12752\_cov\_27.436010 2723-2738. Max. coverage (+): 0. Max coverage (-): 0.08

Region: NODE\_268840\_length\_12752\_cov\_27.436010 2739-2754. Max. coverage (+): 0. Max coverage (-): 0.04

Region: NODE\_268840\_length\_12752\_cov\_27.436010 2755-2770. Max. coverage (+): 0. Max coverage (-): 0.11

Region: NODE\_268840\_length\_12752\_cov\_27.436010 2771-2786. Max. coverage (+): 0. Max coverage (-): 0.46

Region: NODE\_268840\_length\_12752\_cov\_27.436010 2787-2802. Max. coverage (+): 0.08. Max coverage (-): 0.31

Region: NODE\_268840\_length\_12752\_cov\_27.436010 2803-2818. Max. coverage (+): 0. Max coverage (-): 0.54

Region: NODE\_268840\_length\_12752\_cov\_27.436010 2819-2834. Max. coverage (+): 0. Max coverage (-): 0.54

Region: NODE\_268840\_length\_12752\_cov\_27.436010 2835-2850. Max. coverage (+): 0. Max coverage (-): 0.5

Region: NODE\_268840\_length\_12752\_cov\_27.436010 2851-2866. Max. coverage (+): 0.08. Max coverage (-): 0.08

Region: NODE\_268840\_length\_12752\_cov\_27.436010 2867-2882. Max. coverage (+): 0. Max coverage (-): 3.52

Region: NODE\_268840\_length\_12752\_cov\_27.436010 2883-2898. Max. coverage (+): 0. Max coverage (-): 0.08

Region: NODE\_268840\_length\_12752\_cov\_27.436010 2899-2914. Max. coverage (+): 0. Max coverage (-): 0.08

Region: NODE\_268840\_length\_12752\_cov\_27.436010 2915-2930. Max. coverage (+): 0. Max coverage (-): 0.08

Region: NODE\_268840\_length\_12752\_cov\_27.436010 2931-2946. Max. coverage (+): 0. Max coverage (-): 0.08

Region: NODE\_268840\_length\_12752\_cov\_27.436010 2947-2962. Max. coverage (+): 0. Max coverage (-): 2.3

Region: NODE\_268840\_length\_12752\_cov\_27.436010 2963-2979. Max. coverage (+): 0.04. Max coverage (-): 0.23

Region: NODE\_268840\_length\_12752\_cov\_27.436010 2980-2995. Max. coverage (+): 0. Max coverage (-): 1.4

Region: NODE\_268840\_length\_12752\_cov\_27.436010 2996-3011. Max. coverage (+): 0.15. Max coverage (-): 0.41

Region: NODE\_268840\_length\_12752\_cov\_27.436010 3012-3027. Max. coverage (+): 0.15. Max coverage (-): 0.38

Region: NODE\_268840\_length\_12752\_cov\_27.436010 3028-3043. Max. coverage (+): 0. Max coverage (-): 0.38

Region: NODE\_268840\_length\_12752\_cov\_27.436010 3044-3059. Max. coverage (+): 0. Max coverage (-): 0.73

Region: NODE\_268840\_length\_12752\_cov\_27.436010 3060-3075. Max. coverage (+): 0. Max coverage (-): 0.92

Region: NODE\_268840\_length\_12752\_cov\_27.436010 3076-3091. Max. coverage (+): 0. Max coverage (-): 0.23

Region: NODE\_268840\_length\_12752\_cov\_27.436010 3092-3107. Max. coverage (+): 0. Max coverage (-): 0.11

Region: NODE\_268840\_length\_12752\_cov\_27.436010 3108-3123. Max. coverage (+): 0. Max coverage (-): 0

Region: NODE\_268840\_length\_12752\_cov\_27.436010 3124-3139. Max. coverage (+): 0. Max coverage (-): 0.02

Region: NODE\_268840\_length\_12752\_cov\_27.436010 3140-3155. Max. coverage (+): 0. Max coverage (-): 0

Region: NODE\_268840\_length\_12752\_cov\_27.436010 3156-3171. Max. coverage (+): 0. Max coverage (-): 0.08

Region: NODE\_268840\_length\_12752\_cov\_27.436010 3172-3187. Max. coverage (+): 0. Max coverage (-): 0.08

Region: NODE\_268840\_length\_12752\_cov\_27.436010 3188-3203. Max. coverage (+): 0. Max coverage (-): 0.08

Region: NODE\_268840\_length\_12752\_cov\_27.436010 3204-3219. Max. coverage (+): 0. Max coverage (-): 0.08

Region: NODE\_268840\_length\_12752\_cov\_27.436010 3220-3235. Max. coverage (+): 0. Max coverage (-): 10.65

Region: NODE\_268840\_length\_12752\_cov\_27.436010 3236-3251. Max. coverage (+): 0. Max coverage (-): 10.99

Region: NODE\_268840\_length\_12752\_cov\_27.436010 3252-3267. Max. coverage (+): 0.31. Max coverage (-): 0.08

Region: NODE\_268840\_length\_12752\_cov\_27.436010 3268-3284. Max. coverage (+): 0.08. Max coverage (-): 3.68

Region: NODE\_268840\_length\_12752\_cov\_27.436010 3285-3300. Max. coverage (+): 0. Max coverage (-): 0.54

Region: NODE\_268840\_length\_12752\_cov\_27.436010 3301-3316. Max. coverage (+): 0. Max coverage (-): 5.36

Region: NODE\_268840\_length\_12752\_cov\_27.436010 3317-3332. Max. coverage (+): 0.08. Max coverage (-): 0.34

Region: NODE\_268840\_length\_12752\_cov\_27.436010 3333-3348. Max. coverage (+): 0.08. Max coverage (-): 0.04

Region: NODE\_268840\_length\_12752\_cov\_27.436010 3349-3364. Max. coverage (+): 0. Max coverage (-): 1.47

Region: NODE\_268840\_length\_12752\_cov\_27.436010 3365-3380. Max. coverage (+): 0. Max coverage (-): 2.26

Region: NODE\_268840\_length\_12752\_cov\_27.436010 3381-3396. Max. coverage (+): 0.15. Max coverage (-): 6.05

Region: NODE\_268840\_length\_12752\_cov\_27.436010 3397-3412. Max. coverage (+): 0. Max coverage (-): 13.18

Region: NODE\_268840\_length\_12752\_cov\_27.436010 3413-3428. Max. coverage (+): 0. Max coverage (-): 1.61

Region: NODE\_268840\_length\_12752\_cov\_27.436010 3429-3444. Max. coverage (+): 0. Max coverage (-): 0.23

Region: NODE\_268840\_length\_12752\_cov\_27.436010 3445-3460. Max. coverage (+): 0. Max coverage (-): 0.19

Region: NODE\_268840\_length\_12752\_cov\_27.436010 3461-3476. Max. coverage (+): 0. Max coverage (-): 3.33

Region: NODE\_268840\_length\_12752\_cov\_27.436010 3477-3492. Max. coverage (+): 0. Max coverage (-): 0.04

Region: NODE\_268840\_length\_12752\_cov\_27.436010 3493-3508. Max. coverage (+): 0. Max coverage (-): 0.08

Region: NODE\_268840\_length\_12752\_cov\_27.436010 3509-3524. Max. coverage (+): 0. Max coverage (-): 0.08

Region: NODE\_268840\_length\_12752\_cov\_27.436010 3525-3540. Max. coverage (+): 0. Max coverage (-): 25.47

Region: NODE\_268840\_length\_12752\_cov\_27.436010 3541-3556. Max. coverage (+): 0.34. Max coverage (-): 1.13

Region: NODE\_268840\_length\_12752\_cov\_27.436010 3557-3573. Max. coverage (+): 0.05. Max coverage (-): 8.58

Region: NODE\_268840\_length\_12752\_cov\_27.436010 3574-3589. Max. coverage (+): 0. Max coverage (-): 16.26

Region: NODE\_268840\_length\_12752\_cov\_27.436010 3590-3605. Max. coverage (+): 0.34. Max coverage (-): 0

Region: NODE\_268840\_length\_12752\_cov\_27.436010 3606-3621. Max. coverage (+): 0. Max coverage (-): 17.47

Region: NODE\_268840\_length\_12752\_cov\_27.436010 3622-3637. Max. coverage (+): 0.33. Max coverage (-): 3.72

Region: NODE\_268840\_length\_12752\_cov\_27.436010 3638-3653. Max. coverage (+): 0. Max coverage (-): 0.15

Region: NODE\_268840\_length\_12752\_cov\_27.436010 3654-3669. Max. coverage (+): 0. Max coverage (-): 6.44

Region: NODE\_268840\_length\_12752\_cov\_27.436010 3670-3685. Max. coverage (+): 0. Max coverage (-): 0.05

Region: NODE\_268840\_length\_12752\_cov\_27.436010 3686-3701. Max. coverage (+): 0. Max coverage (-): 0.03

Region: NODE\_268840\_length\_12752\_cov\_27.436010 3702-3717. Max. coverage (+): 0. Max coverage (-): 0

Region: NODE\_268840\_length\_12752\_cov\_27.436010 3718-3733. Max. coverage (+): 0. Max coverage (-): 1.74

Region: NODE\_268840\_length\_12752\_cov\_27.436010 3734-3749. Max. coverage (+): 0. Max coverage (-): 0.13

Region: NODE\_268840\_length\_12752\_cov\_27.436010 3750-3765. Max. coverage (+): 0. Max coverage (-): 0

Region: NODE\_268840\_length\_12752\_cov\_27.436010 3766-3781. Max. coverage (+): 0. Max coverage (-): 0.31

Region: NODE\_268840\_length\_12752\_cov\_27.436010 3782-3797. Max. coverage (+): 0. Max coverage (-): 0.1

Region: NODE\_268840\_length\_12752\_cov\_27.436010 3798-3813. Max. coverage (+): 0.08. Max coverage (-): 0.03

Region: NODE\_268840\_length\_12752\_cov\_27.436010 3814-3829. Max. coverage (+): 0. Max coverage (-): 0.41

Region: NODE\_268840\_length\_12752\_cov\_27.436010 3830-3845. Max. coverage (+): 0.08. Max coverage (-): 0.05

Region: NODE\_268840\_length\_12752\_cov\_27.436010 3846-3861. Max. coverage (+): 0. Max coverage (-): 0.08

Region: NODE\_268840\_length\_12752\_cov\_27.436010 3862-3878. Max. coverage (+): 0. Max coverage (-): 6.17

Region: NODE\_268840\_length\_12752\_cov\_27.436010 3879-3894. Max. coverage (+): 0. Max coverage (-): 0

Region: NODE\_268840\_length\_12752\_cov\_27.436010 3895-3910. Max. coverage (+): 0. Max coverage (-): 0.42

Region: NODE\_268840\_length\_12752\_cov\_27.436010 3911-3926. Max. coverage (+): 0. Max coverage (-): 0.54

Region: NODE\_268840\_length\_12752\_cov\_27.436010 3927-3942. Max. coverage (+): 0. Max coverage (-): 0.61

Region: NODE\_268840\_length\_12752\_cov\_27.436010 3943-3958. Max. coverage (+): 0. Max coverage (-): 0.08

Region: NODE\_268840\_length\_12752\_cov\_27.436010 3959-3974. Max. coverage (+): 0. Max coverage (-): 0.11

Region: NODE\_268840\_length\_12752\_cov\_27.436010 3975-3990. Max. coverage (+): 0. Max coverage (-): 0.77

Region: NODE\_268840\_length\_12752\_cov\_27.436010 3991-4006. Max. coverage (+): 0. Max coverage (-): 1

Region: NODE\_268840\_length\_12752\_cov\_27.436010 4007-4022. Max. coverage (+): 0. Max coverage (-): 0.11

Region: NODE\_268840\_length\_12752\_cov\_27.436010 4023-4038. Max. coverage (+): 0. Max coverage (-): 0.04

Region: NODE\_268840\_length\_12752\_cov\_27.436010 4039-4054. Max. coverage (+): 0. Max coverage (-): 0.1

Region: NODE\_268840\_length\_12752\_cov\_27.436010 4055-4070. Max. coverage (+): 0. Max coverage (-): 0

Region: NODE\_268840\_length\_12752\_cov\_27.436010 4071-4086. Max. coverage (+): 0. Max coverage (-): 0.05

Region: NODE\_268840\_length\_12752\_cov\_27.436010 4087-4102. Max. coverage (+): 0. Max coverage (-): 0.03

Region: NODE\_268840\_length\_12752\_cov\_27.436010 4103-4118. Max. coverage (+): 0.04. Max coverage (-): 0.77

Region: NODE\_268840\_length\_12752\_cov\_27.436010 4119-4134. Max. coverage (+): 0.04. Max coverage (-): 0.31

Region: NODE\_268840\_length\_12752\_cov\_27.436010 4135-4150. Max. coverage (+): 0.04. Max coverage (-): 0.03

Region: NODE\_268840\_length\_12752\_cov\_27.436010 4151-4167. Max. coverage (+): 0. Max coverage (-): 0.38

Region: NODE\_268840\_length\_12752\_cov\_27.436010 4168-4183. Max. coverage (+): 0. Max coverage (-): 0.11

Region: NODE\_268840\_length\_12752\_cov\_27.436010 4184-4199. Max. coverage (+): 0. Max coverage (-): 0.19

Region: NODE\_268840\_length\_12752\_cov\_27.436010 4200-4215. Max. coverage (+): 0. Max coverage (-): 0.23

Region: NODE\_268840\_length\_12752\_cov\_27.436010 4216-4231. Max. coverage (+): 0. Max coverage (-): 0.2

Region: NODE\_268840\_length\_12752\_cov\_27.436010 4232-4247. Max. coverage (+): 0. Max coverage (-): 0.14

Region: NODE\_268840\_length\_12752\_cov\_27.436010 4248-4263. Max. coverage (+): 0. Max coverage (-): 0.06

Region: NODE\_268840\_length\_12752\_cov\_27.436010 4264-4279. Max. coverage (+): 0.08. Max coverage (-): 0

Region: NODE\_268840\_length\_12752\_cov\_27.436010 4280-4295. Max. coverage (+): 0. Max coverage (-): 0.23

Region: NODE\_268840\_length\_12752\_cov\_27.436010 4296-4311. Max. coverage (+): 0. Max coverage (-): 0

Region: NODE\_268840\_length\_12752\_cov\_27.436010 4312-4327. Max. coverage (+): 0. Max coverage (-): 0

Region: NODE\_268840\_length\_12752\_cov\_27.436010 4328-4343. Max. coverage (+): 0. Max coverage (-): 0.69

Region: NODE\_268840\_length\_12752\_cov\_27.436010 4344-4359. Max. coverage (+): 0. Max coverage (-): 0.15

Region: NODE\_268840\_length\_12752\_cov\_27.436010 4360-4375. Max. coverage (+): 0.08. Max coverage (-): 0.08

Region: NODE\_268840\_length\_12752\_cov\_27.436010 4376-4391. Max. coverage (+): 0. Max coverage (-): 4.14

Region: NODE\_268840\_length\_12752\_cov\_27.436010 4392-4407. Max. coverage (+): 0. Max coverage (-): 1.23

Region: NODE\_268840\_length\_12752\_cov\_27.436010 4408-4423. Max. coverage (+): 0.23. Max coverage (-): 0

Region: NODE\_268840\_length\_12752\_cov\_27.436010 4424-4439. Max. coverage (+): 0. Max coverage (-): 0.15

Region: NODE\_268840\_length\_12752\_cov\_27.436010 4440-4455. Max. coverage (+): 0.23. Max coverage (-): 0

Region: NODE\_268840\_length\_12752\_cov\_27.436010 4456-4472. Max. coverage (+): 0. Max coverage (-): 0.08

Region: NODE\_268840\_length\_12752\_cov\_27.436010 4473-4488. Max. coverage (+): 0. Max coverage (-): 0.15

Region: NODE\_268840\_length\_12752\_cov\_27.436010 4489-4504. Max. coverage (+): 0. Max coverage (-): 0.38

Region: NODE\_268840\_length\_12752\_cov\_27.436010 4505-4520. Max. coverage (+): 0.08. Max coverage (-): 0.08

Region: NODE\_268840\_length\_12752\_cov\_27.436010 4521-4536. Max. coverage (+): 0.08. Max coverage (-): 0.31

Region: NODE\_268840\_length\_12752\_cov\_27.436010 4537-4552. Max. coverage (+): 0. Max coverage (-): 0.61

Region: NODE\_268840\_length\_12752\_cov\_27.436010 4553-4568. Max. coverage (+): 0. Max coverage (-): 1.3

Region: NODE\_268840\_length\_12752\_cov\_27.436010 4569-4584. Max. coverage (+): 0. Max coverage (-): 0.08

Region: NODE\_268840\_length\_12752\_cov\_27.436010 4585-4600. Max. coverage (+): 0. Max coverage (-): 0.15

Region: NODE\_268840\_length\_12752\_cov\_27.436010 4601-4616. Max. coverage (+): 0. Max coverage (-): 0.11

Region: NODE\_268840\_length\_12752\_cov\_27.436010 4617-4632. Max. coverage (+): 0. Max coverage (-): 0.15

Region: NODE\_268840\_length\_12752\_cov\_27.436010 4633-4648. Max. coverage (+): 0. Max coverage (-): 16.55

Region: NODE\_268840\_length\_12752\_cov\_27.436010 4649-4664. Max. coverage (+): 0. Max coverage (-): 0.31

Region: NODE\_268840\_length\_12752\_cov\_27.436010 4665-4680. Max. coverage (+): 0.08. Max coverage (-): 0.15

Region: NODE\_268840\_length\_12752\_cov\_27.436010 4681-4696. Max. coverage (+): 0. Max coverage (-): 0.61

Region: NODE\_268840\_length\_12752\_cov\_27.436010 4697-4712. Max. coverage (+): 0. Max coverage (-): 0.23

Region: NODE\_268840\_length\_12752\_cov\_27.436010 4713-4728. Max. coverage (+): 0. Max coverage (-): 0.08

Region: NODE\_268840\_length\_12752\_cov\_27.436010 4729-4744. Max. coverage (+): 0.08. Max coverage (-): 0.31

Region: NODE\_268840\_length\_12752\_cov\_27.436010 4745-4761. Max. coverage (+): 0. Max coverage (-): 0.46

Region: NODE\_268840\_length\_12752\_cov\_27.436010 4762-4777. Max. coverage (+): 0. Max coverage (-): 5.98

Region: NODE\_268840\_length\_12752\_cov\_27.436010 4778-4793. Max. coverage (+): 0.08. Max coverage (-): 7.2

Region: NODE\_268840\_length\_12752\_cov\_27.436010 4794-4809. Max. coverage (+): 0. Max coverage (-): 4.1

Region: NODE\_268840\_length\_12752\_cov\_27.436010 4810-4825. Max. coverage (+): 0. Max coverage (-): 1.07

Region: NODE\_268840\_length\_12752\_cov\_27.436010 4826-4841. Max. coverage (+): 0. Max coverage (-): 2.3

Region: NODE\_268840\_length\_12752\_cov\_27.436010 4842-4857. Max. coverage (+): 0. Max coverage (-): 0.38

Region: NODE\_268840\_length\_12752\_cov\_27.436010 4858-4873. Max. coverage (+): 0. Max coverage (-): 2.99

Region: NODE\_268840\_length\_12752\_cov\_27.436010 4874-4889. Max. coverage (+): 0. Max coverage (-): 0.23

Region: NODE\_268840\_length\_12752\_cov\_27.436010 4890-4905. Max. coverage (+): 0. Max coverage (-): 54.47

Region: NODE\_268840\_length\_12752\_cov\_27.436010 4906-4921. Max. coverage (+): 0. Max coverage (-): 55.24

Region: NODE\_268840\_length\_12752\_cov\_27.436010 4922-4937. Max. coverage (+): 0.15. Max coverage (-): 0.15

Region: NODE\_268840\_length\_12752\_cov\_27.436010 4938-4953. Max. coverage (+): 0. Max coverage (-): 0.84

Region: NODE\_268840\_length\_12752\_cov\_27.436010 4954-4969. Max. coverage (+): 0. Max coverage (-): 2.07

Region: NODE\_268840\_length\_12752\_cov\_27.436010 4970-4985. Max. coverage (+): 0.08. Max coverage (-): 0.77

Region: NODE\_268840\_length\_12752\_cov\_27.436010 4986-5001. Max. coverage (+): 0.08. Max coverage (-): 0.38

Region: NODE\_268840\_length\_12752\_cov\_27.436010 5002-5017. Max. coverage (+): 0. Max coverage (-): 0.54

Region: NODE\_268840\_length\_12752\_cov\_27.436010 5018-5033. Max. coverage (+): 0.08. Max coverage (-): 0

Region: NODE\_268840\_length\_12752\_cov\_27.436010 5034-5049. Max. coverage (+): 0. Max coverage (-): 0.69

Region: NODE\_268840\_length\_12752\_cov\_27.436010 5050-5066. Max. coverage (+): 0. Max coverage (-): 0.15

Region: NODE\_268840\_length\_12752\_cov\_27.436010 5067-5082. Max. coverage (+): 0. Max coverage (-): 1.23

Region: NODE\_268840\_length\_12752\_cov\_27.436010 5083-5098. Max. coverage (+): 0. Max coverage (-): 0.84

Region: NODE\_268840\_length\_12752\_cov\_27.436010 5099-5114. Max. coverage (+): 0. Max coverage (-): 0.15

Region: NODE\_268840\_length\_12752\_cov\_27.436010 5115-5130. Max. coverage (+): 0. Max coverage (-): 0

Region: NODE\_268840\_length\_12752\_cov\_27.436010 5131-5146. Max. coverage (+): 0. Max coverage (-): 0

Region: NODE\_268840\_length\_12752\_cov\_27.436010 5147-5162. Max. coverage (+): 0. Max coverage (-): 0.08

Region: NODE\_268840\_length\_12752\_cov\_27.436010 5163-5178. Max. coverage (+): 0. Max coverage (-): 0.15

Region: NODE\_268840\_length\_12752\_cov\_27.436010 5179-5194. Max. coverage (+): 0. Max coverage (-): 1.61

Region: NODE\_268840\_length\_12752\_cov\_27.436010 5195-5210. Max. coverage (+): 0. Max coverage (-): 0.15

Region: NODE\_268840\_length\_12752\_cov\_27.436010 5211-5226. Max. coverage (+): 0. Max coverage (-): 0.15

Region: NODE\_268840\_length\_12752\_cov\_27.436010 5227-5242. Max. coverage (+): 0. Max coverage (-): 0.31

Region: NODE\_268840\_length\_12752\_cov\_27.436010 5243-5258. Max. coverage (+): 0. Max coverage (-): 0.15

Region: NODE\_268840\_length\_12752\_cov\_27.436010 5259-5274. Max. coverage (+): 0. Max coverage (-): 0.23

Region: NODE\_268840\_length\_12752\_cov\_27.436010 5275-5290. Max. coverage (+): 0. Max coverage (-): 0.23

Region: NODE\_268840\_length\_12752\_cov\_27.436010 5291-5306. Max. coverage (+): 0. Max coverage (-): 0.15

Region: NODE\_268840\_length\_12752\_cov\_27.436010 5307-5322. Max. coverage (+): 0. Max coverage (-): 0

Region: NODE\_268840\_length\_12752\_cov\_27.436010 5323-5338. Max. coverage (+): 0. Max coverage (-): 0.08

Region: NODE\_268840\_length\_12752\_cov\_27.436010 5339-5355. Max. coverage (+): 0. Max coverage (-): 0

Region: NODE\_268840\_length\_12752\_cov\_27.436010 5356-5371. Max. coverage (+): 0. Max coverage (-): 0.23

Region: NODE\_268840\_length\_12752\_cov\_27.436010 5372-5387. Max. coverage (+): 0. Max coverage (-): 1.3

Region: NODE\_268840\_length\_12752\_cov\_27.436010 5388-5403. Max. coverage (+): 0. Max coverage (-): 0

Region: NODE\_268840\_length\_12752\_cov\_27.436010 5404-5419. Max. coverage (+): 0. Max coverage (-): 0.54

Region: NODE\_268840\_length\_12752\_cov\_27.436010 5420-5435. Max. coverage (+): 0. Max coverage (-): 0.31

Region: NODE\_268840\_length\_12752\_cov\_27.436010 5436-5451. Max. coverage (+): 0. Max coverage (-): 0

Region: NODE\_268840\_length\_12752\_cov\_27.436010 5452-5467. Max. coverage (+): 0. Max coverage (-): 0.15

Region: NODE\_268840\_length\_12752\_cov\_27.436010 5468-5483. Max. coverage (+): 0.08. Max coverage (-): 0.15

Region: NODE\_268840\_length\_12752\_cov\_27.436010 5484-5499. Max. coverage (+): 0.08. Max coverage (-): 0.23

Region: NODE\_268840\_length\_12752\_cov\_27.436010 5500-5515. Max. coverage (+): 0.08. Max coverage (-): 0.15

Region: NODE\_268840\_length\_12752\_cov\_27.436010 5516-5531. Max. coverage (+): 0.08. Max coverage (-): 0.15

Region: NODE\_268840\_length\_12752\_cov\_27.436010 5532-5547. Max. coverage (+): 0. Max coverage (-): 0.15

Region: NODE\_268840\_length\_12752\_cov\_27.436010 5548-5563. Max. coverage (+): 0. Max coverage (-): 0.23

Region: NODE\_268840\_length\_12752\_cov\_27.436010 5564-5579. Max. coverage (+): 0. Max coverage (-): 0.38

Region: NODE\_268840\_length\_12752\_cov\_27.436010 5580-5595. Max. coverage (+): 0. Max coverage (-): 0.46

Region: NODE\_268840\_length\_12752\_cov\_27.436010 5596-5611. Max. coverage (+): 0. Max coverage (-): 0.38

Region: NODE\_268840\_length\_12752\_cov\_27.436010 5612-5627. Max. coverage (+): 0. Max coverage (-): 0

Region: NODE\_268840\_length\_12752\_cov\_27.436010 5628-5643. Max. coverage (+): 0. Max coverage (-): 0

Region: NODE\_268840\_length\_12752\_cov\_27.436010 5644-5660. Max. coverage (+): 0. Max coverage (-): 0.15

Region: NODE\_268840\_length\_12752\_cov\_27.436010 5661-5676. Max. coverage (+): 0. Max coverage (-): 0.08

Region: NODE\_268840\_length\_12752\_cov\_27.436010 5677-5692. Max. coverage (+): 0. Max coverage (-): 0

Region: NODE\_268840\_length\_12752\_cov\_27.436010 5693-5708. Max. coverage (+): 0. Max coverage (-): 0.08

Region: NODE\_268840\_length\_12752\_cov\_27.436010 5709-5724. Max. coverage (+): 0. Max coverage (-): 0

Region: NODE\_268840\_length\_12752\_cov\_27.436010 5725-5740. Max. coverage (+): 0. Max coverage (-): 0.08

Region: NODE\_268840\_length\_12752\_cov\_27.436010 5741-5756. Max. coverage (+): 0. Max coverage (-): 0.08

Region: NODE\_268840\_length\_12752\_cov\_27.436010 5757-5772. Max. coverage (+): 0.08. Max coverage (-): 0.23

Region: NODE\_268840\_length\_12752\_cov\_27.436010 5773-5788. Max. coverage (+): 0. Max coverage (-): 1.37

Region: NODE\_268840\_length\_12752\_cov\_27.436010 5789-5804. Max. coverage (+): 0. Max coverage (-): 1.34

Region: NODE\_268840\_length\_12752\_cov\_27.436010 5805-5820. Max. coverage (+): 0.01. Max coverage (-): 0.07

Region: NODE\_268840\_length\_12752\_cov\_27.436010 5821-5836. Max. coverage (+): 0. Max coverage (-): 0.23

Region: NODE\_268840\_length\_12752\_cov\_27.436010 5837-5852. Max. coverage (+): 0. Max coverage (-): 0.19

Region: NODE\_268840\_length\_12752\_cov\_27.436010 5853-5868. Max. coverage (+): 0.15. Max coverage (-): 0.04

Region: NODE\_268840\_length\_12752\_cov\_27.436010 5869-5884. Max. coverage (+): 0. Max coverage (-): 0

Region: NODE\_268840\_length\_12752\_cov\_27.436010 5885-5900. Max. coverage (+): 0. Max coverage (-): 0

Region: NODE\_268840\_length\_12752\_cov\_27.436010 5901-5916. Max. coverage (+): 0. Max coverage (-): 0

Region: NODE\_268840\_length\_12752\_cov\_27.436010 5917-5932. Max. coverage (+): 0. Max coverage (-): 0

Region: NODE\_268840\_length\_12752\_cov\_27.436010 5933-5949. Max. coverage (+): 0. Max coverage (-): 0

Region: NODE\_268840\_length\_12752\_cov\_27.436010 5950-5965. Max. coverage (+): 0. Max coverage (-): 0

Region: NODE\_268840\_length\_12752\_cov\_27.436010 5966-5981. Max. coverage (+): 0. Max coverage (-): 0

Region: NODE\_268840\_length\_12752\_cov\_27.436010 5982-5997. Max. coverage (+): 0. Max coverage (-): 0

Region: NODE\_268840\_length\_12752\_cov\_27.436010 5998-6013. Max. coverage (+): 0. Max coverage (-): 0

Region: NODE\_268840\_length\_12752\_cov\_27.436010 6014-6029. Max. coverage (+): 0. Max coverage (-): 0

Region: NODE\_268840\_length\_12752\_cov\_27.436010 6030-6045. Max. coverage (+): 0. Max coverage (-): 0

Region: NODE\_268840\_length\_12752\_cov\_27.436010 6046-6061. Max. coverage (+): 0. Max coverage (-): 0

Region: NODE\_268840\_length\_12752\_cov\_27.436010 6062-6077. Max. coverage (+): 0. Max coverage (-): 0.08

Region: NODE\_268840\_length\_12752\_cov\_27.436010 6078-6093. Max. coverage (+): 0. Max coverage (-): 0

Region: NODE\_268840\_length\_12752\_cov\_27.436010 6094-6109. Max. coverage (+): 0. Max coverage (-): 0

Region: NODE\_268840\_length\_12752\_cov\_27.436010 6110-6125. Max. coverage (+): 0. Max coverage (-): 0

Region: NODE\_268840\_length\_12752\_cov\_27.436010 6126-6141. Max. coverage (+): 0. Max coverage (-): 0

Region: NODE\_268840\_length\_12752\_cov\_27.436010 6142-6157. Max. coverage (+): 0. Max coverage (-): 0

Region: NODE\_268840\_length\_12752\_cov\_27.436010 6158-6173. Max. coverage (+): 0. Max coverage (-): 0

Region: NODE\_268840\_length\_12752\_cov\_27.436010 6174-6189. Max. coverage (+): 0. Max coverage (-): 0

Region: NODE\_268840\_length\_12752\_cov\_27.436010 6190-6205. Max. coverage (+): 0. Max coverage (-): 0

Region: NODE\_268840\_length\_12752\_cov\_27.436010 6206-6221. Max. coverage (+): 0. Max coverage (-): 0

Region: NODE\_268840\_length\_12752\_cov\_27.436010 6222-6237. Max. coverage (+): 0. Max coverage (-): 0

Region: NODE\_268840\_length\_12752\_cov\_27.436010 6238-6254. Max. coverage (+): 0. Max coverage (-): 0

Region: NODE\_268840\_length\_12752\_cov\_27.436010 6255-6270. Max. coverage (+): 0. Max coverage (-): 0

Region: NODE\_268840\_length\_12752\_cov\_27.436010 6271-6286. Max. coverage (+): 0. Max coverage (-): 0

Region: NODE\_268840\_length\_12752\_cov\_27.436010 6287-6302. Max. coverage (+): 0. Max coverage (-): 0

Region: NODE\_268840\_length\_12752\_cov\_27.436010 6303-6318. Max. coverage (+): 0. Max coverage (-): 0

Region: NODE\_268840\_length\_12752\_cov\_27.436010 6319-6334. Max. coverage (+): 0. Max coverage (-): 0

Region: NODE\_268840\_length\_12752\_cov\_27.436010 6335-6350. Max. coverage (+): 0. Max coverage (-): 0

Region: NODE\_268840\_length\_12752\_cov\_27.436010 6351-6366. Max. coverage (+): 0. Max coverage (-): 0

Region: NODE\_268840\_length\_12752\_cov\_27.436010 6367-6382. Max. coverage (+): 0. Max coverage (-): 0

Region: NODE\_268840\_length\_12752\_cov\_27.436010 6383-6398. Max. coverage (+): 0. Max coverage (-): 0

Region: NODE\_268840\_length\_12752\_cov\_27.436010 6399-6414. Max. coverage (+): 0. Max coverage (-): 0

Region: NODE\_268840\_length\_12752\_cov\_27.436010 6415-6430. Max. coverage (+): 0. Max coverage (-): 0

Region: NODE\_268840\_length\_12752\_cov\_27.436010 6431-6446. Max. coverage (+): 0. Max coverage (-): 0

Region: NODE\_268840\_length\_12752\_cov\_27.436010 6447-6462. Max. coverage (+): 0. Max coverage (-): 0

Region: NODE\_268840\_length\_12752\_cov\_27.436010 6463-6478. Max. coverage (+): 0. Max coverage (-): 0

Region: NODE\_268840\_length\_12752\_cov\_27.436010 6479-6494. Max. coverage (+): 0. Max coverage (-): 0.03

Region: NODE\_268840\_length\_12752\_cov\_27.436010 6495-6510. Max. coverage (+): 0. Max coverage (-): 1.37

Region: NODE\_268840\_length\_12752\_cov\_27.436010 6511-6526. Max. coverage (+): 0. Max coverage (-): 0

Region: NODE\_268840\_length\_12752\_cov\_27.436010 6527-6543. Max. coverage (+): 0. Max coverage (-): 0

Region: NODE\_268840\_length\_12752\_cov\_27.436010 6544-6559. Max. coverage (+): 0. Max coverage (-): 0

Region: NODE\_268840\_length\_12752\_cov\_27.436010 6560-6575. Max. coverage (+): 0. Max coverage (-): 0

Region: NODE\_268840\_length\_12752\_cov\_27.436010 6576-6591. Max. coverage (+): 0. Max coverage (-): 0

Region: NODE\_268840\_length\_12752\_cov\_27.436010 6592-6607. Max. coverage (+): 0. Max coverage (-): 0

Region: NODE\_268840\_length\_12752\_cov\_27.436010 6608-6623. Max. coverage (+): 0. Max coverage (-): 0

Region: NODE\_268840\_length\_12752\_cov\_27.436010 6624-6639. Max. coverage (+): 0. Max coverage (-): 0

Region: NODE\_268840\_length\_12752\_cov\_27.436010 6640-6655. Max. coverage (+): 0. Max coverage (-): 0

Region: NODE\_268840\_length\_12752\_cov\_27.436010 6656-6671. Max. coverage (+): 0. Max coverage (-): 0.08

Region: NODE\_268840\_length\_12752\_cov\_27.436010 6672-6687. Max. coverage (+): 0. Max coverage (-): 0

Region: NODE\_268840\_length\_12752\_cov\_27.436010 6688-6703. Max. coverage (+): 0. Max coverage (-): 0

Region: NODE\_268840\_length\_12752\_cov\_27.436010 6704-6719. Max. coverage (+): 0. Max coverage (-): 0.04

Region: NODE\_268840\_length\_12752\_cov\_27.436010 6720-6735. Max. coverage (+): 0. Max coverage (-): 0

Region: NODE\_268840\_length\_12752\_cov\_27.436010 6736-6751. Max. coverage (+): 0. Max coverage (-): 0.08

Region: NODE\_268840\_length\_12752\_cov\_27.436010 6752-6767. Max. coverage (+): 0. Max coverage (-): 0.04

Region: NODE\_268840\_length\_12752\_cov\_27.436010 6768-6783. Max. coverage (+): 0. Max coverage (-): 0.14

Region: NODE\_268840\_length\_12752\_cov\_27.436010 6784-6799. Max. coverage (+): 0. Max coverage (-): 2.89

Region: NODE\_268840\_length\_12752\_cov\_27.436010 6800-6815. Max. coverage (+): 0.15. Max coverage (-): 0.08

Region: NODE\_268840\_length\_12752\_cov\_27.436010 6816-6831. Max. coverage (+): 0. Max coverage (-): 0.38

Region: NODE\_268840\_length\_12752\_cov\_27.436010 6832-6848. Max. coverage (+): 0. Max coverage (-): 0.2

Region: NODE\_268840\_length\_12752\_cov\_27.436010 6849-6864. Max. coverage (+): 0. Max coverage (-): 0.39

Region: NODE\_268840\_length\_12752\_cov\_27.436010 6865-6880. Max. coverage (+): 0. Max coverage (-): 0.39

Region: NODE\_268840\_length\_12752\_cov\_27.436010 6881-6896. Max. coverage (+): 0. Max coverage (-): 0.23

Region: NODE\_268840\_length\_12752\_cov\_27.436010 6897-6912. Max. coverage (+): 0. Max coverage (-): 0.38

Region: NODE\_268840\_length\_12752\_cov\_27.436010 6913-6928. Max. coverage (+): 0. Max coverage (-): 0.08

Region: NODE\_268840\_length\_12752\_cov\_27.436010 6929-6944. Max. coverage (+): 0.23. Max coverage (-): 0.15

Region: NODE\_268840\_length\_12752\_cov\_27.436010 6945-6960. Max. coverage (+): 0. Max coverage (-): 0

Region: NODE\_268840\_length\_12752\_cov\_27.436010 6961-6976. Max. coverage (+): 0. Max coverage (-): 0

Region: NODE\_268840\_length\_12752\_cov\_27.436010 6977-6992. Max. coverage (+): 0. Max coverage (-): 0

Region: NODE\_268840\_length\_12752\_cov\_27.436010 6993-7008. Max. coverage (+): 0. Max coverage (-): 0.04

Region: NODE\_268840\_length\_12752\_cov\_27.436010 7009-7024. Max. coverage (+): 0.23. Max coverage (-): 0.04

Region: NODE\_268840\_length\_12752\_cov\_27.436010 7025-7040. Max. coverage (+): 0.38. Max coverage (-): 0

Region: NODE\_268840\_length\_12752\_cov\_27.436010 7041-7056. Max. coverage (+): 0. Max coverage (-): 0

Region: NODE\_268840\_length\_12752\_cov\_27.436010 7057-7072. Max. coverage (+): 0. Max coverage (-): 0.61

Region: NODE\_268840\_length\_12752\_cov\_27.436010 7073-7088. Max. coverage (+): 0. Max coverage (-): 0.09

Region: NODE\_268840\_length\_12752\_cov\_27.436010 7089-7104. Max. coverage (+): 0. Max coverage (-): 0

Region: NODE\_268840\_length\_12752\_cov\_27.436010 7105-7120. Max. coverage (+): 0. Max coverage (-): 0.61

Region: NODE\_268840\_length\_12752\_cov\_27.436010 7121-7137. Max. coverage (+): 0.08. Max coverage (-): 0.54

Region: NODE\_268840\_length\_12752\_cov\_27.436010 7138-7153. Max. coverage (+): 0. Max coverage (-): 0.54

Region: NODE\_268840\_length\_12752\_cov\_27.436010 7154-7169. Max. coverage (+): 0. Max coverage (-): 0

Region: NODE\_268840\_length\_12752\_cov\_27.436010 7170-7185. Max. coverage (+): 0. Max coverage (-): 0.15

Region: NODE\_268840\_length\_12752\_cov\_27.436010 7186-7201. Max. coverage (+): 0. Max coverage (-): 0.15

Region: NODE\_268840\_length\_12752\_cov\_27.436010 7202-7217. Max. coverage (+): 0. Max coverage (-): 0.04

Region: NODE\_268840\_length\_12752\_cov\_27.436010 7218-7233. Max. coverage (+): 0. Max coverage (-): 0.38

Region: NODE\_268840\_length\_12752\_cov\_27.436010 7234-7249. Max. coverage (+): 0. Max coverage (-): 0.31

Region: NODE\_268840\_length\_12752\_cov\_27.436010 7250-7265. Max. coverage (+): 0. Max coverage (-): 0

Region: NODE\_268840\_length\_12752\_cov\_27.436010 7266-7281. Max. coverage (+): 0. Max coverage (-): 0

Region: NODE\_268840\_length\_12752\_cov\_27.436010 7282-7297. Max. coverage (+): 0. Max coverage (-): 0

Region: NODE\_268840\_length\_12752\_cov\_27.436010 7298-7313. Max. coverage (+): 0. Max coverage (-): 0.08

Region: NODE\_268840\_length\_12752\_cov\_27.436010 7314-7329. Max. coverage (+): 0. Max coverage (-): 0

Region: NODE\_268840\_length\_12752\_cov\_27.436010 7330-7345. Max. coverage (+): 0. Max coverage (-): 0

Region: NODE\_268840\_length\_12752\_cov\_27.436010 7346-7361. Max. coverage (+): 0. Max coverage (-): 0

Region: NODE\_268840\_length\_12752\_cov\_27.436010 7362-7377. Max. coverage (+): 0. Max coverage (-): 0

Region: NODE\_268840\_length\_12752\_cov\_27.436010 7378-7393. Max. coverage (+): 0. Max coverage (-): 0

Region: NODE\_268840\_length\_12752\_cov\_27.436010 7394-7409. Max. coverage (+): 0. Max coverage (-): 0

Region: NODE\_268840\_length\_12752\_cov\_27.436010 7410-7425. Max. coverage (+): 0.04. Max coverage (-): 0

Region: NODE\_268840\_length\_12752\_cov\_27.436010 7426-7442. Max. coverage (+): 0. Max coverage (-): 0

Region: NODE\_268840\_length\_12752\_cov\_27.436010 7443-7458. Max. coverage (+): 0. Max coverage (-): 0

Region: NODE\_268840\_length\_12752\_cov\_27.436010 7459-7474. Max. coverage (+): 0. Max coverage (-): 0

Region: NODE\_268840\_length\_12752\_cov\_27.436010 7475-7490. Max. coverage (+): 0. Max coverage (-): 0.02

Region: NODE\_268840\_length\_12752\_cov\_27.436010 7491-7506. Max. coverage (+): 0. Max coverage (-): 0.15

Region: NODE\_268840\_length\_12752\_cov\_27.436010 7507-7522. Max. coverage (+): 0. Max coverage (-): 0.15

Region: NODE\_268840\_length\_12752\_cov\_27.436010 7523-7538. Max. coverage (+): 0. Max coverage (-): 0.08

Region: NODE\_268840\_length\_12752\_cov\_27.436010 7539-7554. Max. coverage (+): 0. Max coverage (-): 3.06

Region: NODE\_268840\_length\_12752\_cov\_27.436010 7555-7570. Max. coverage (+): 0. Max coverage (-): 0.15

Region: NODE\_268840\_length\_12752\_cov\_27.436010 7571-7586. Max. coverage (+): 0. Max coverage (-): 0

Region: NODE\_268840\_length\_12752\_cov\_27.436010 7587-7602. Max. coverage (+): 0. Max coverage (-): 0

Region: NODE\_268840\_length\_12752\_cov\_27.436010 7603-7618. Max. coverage (+): 0. Max coverage (-): 0

Region: NODE\_268840\_length\_12752\_cov\_27.436010 7619-7634. Max. coverage (+): 0. Max coverage (-): 0

Region: NODE\_268840\_length\_12752\_cov\_27.436010 7635-7650. Max. coverage (+): 0. Max coverage (-): 0.08

Region: NODE\_268840\_length\_12752\_cov\_27.436010 7651-7666. Max. coverage (+): 0. Max coverage (-): 0

Region: NODE\_268840\_length\_12752\_cov\_27.436010 7667-7682. Max. coverage (+): 0. Max coverage (-): 0.08

Region: NODE\_268840\_length\_12752\_cov\_27.436010 7683-7698. Max. coverage (+): 0. Max coverage (-): 0.08

Region: NODE\_268840\_length\_12752\_cov\_27.436010 7699-7714. Max. coverage (+): 0. Max coverage (-): 0

Region: NODE\_268840\_length\_12752\_cov\_27.436010 7715-7731. Max. coverage (+): 0. Max coverage (-): 0.08

Region: NODE\_268840\_length\_12752\_cov\_27.436010 7732-7747. Max. coverage (+): 0. Max coverage (-): 0

Region: NODE\_268840\_length\_12752\_cov\_27.436010 7748-7763. Max. coverage (+): 0. Max coverage (-): 0

Region: NODE\_268840\_length\_12752\_cov\_27.436010 7764-7779. Max. coverage (+): 0. Max coverage (-): 0.08

Region: NODE\_268840\_length\_12752\_cov\_27.436010 7780-7795. Max. coverage (+): 0. Max coverage (-): 0.15

Region: NODE\_268840\_length\_12752\_cov\_27.436010 7796-7811. Max. coverage (+): 0. Max coverage (-): 0.88

Region: NODE\_268840\_length\_12752\_cov\_27.436010 7812-7827. Max. coverage (+): 0.15. Max coverage (-): 0.08

Region: NODE\_268840\_length\_12752\_cov\_27.436010 7828-7843. Max. coverage (+): 0. Max coverage (-): 0.31

Region: NODE\_268840\_length\_12752\_cov\_27.436010 7844-7859. Max. coverage (+): 0. Max coverage (-): 1.03

Region: NODE\_268840\_length\_12752\_cov\_27.436010 7860-7875. Max. coverage (+): 0. Max coverage (-): 0.01

Region: NODE\_268840\_length\_12752\_cov\_27.436010 7876-7891. Max. coverage (+): 0. Max coverage (-): 0

Region: NODE\_268840\_length\_12752\_cov\_27.436010 7892-7907. Max. coverage (+): 0. Max coverage (-): 0.08

Region: NODE\_268840\_length\_12752\_cov\_27.436010 7908-7923. Max. coverage (+): 0. Max coverage (-): 0

Region: NODE\_268840\_length\_12752\_cov\_27.436010 7924-7939. Max. coverage (+): 0. Max coverage (-): 0

Region: NODE\_268840\_length\_12752\_cov\_27.436010 7940-7955. Max. coverage (+): 0. Max coverage (-): 0.08

Region: NODE\_268840\_length\_12752\_cov\_27.436010 7956-7971. Max. coverage (+): 0. Max coverage (-): 0

Region: NODE\_268840\_length\_12752\_cov\_27.436010 7972-7987. Max. coverage (+): 0. Max coverage (-): 0

Region: NODE\_268840\_length\_12752\_cov\_27.436010 7988-8003. Max. coverage (+): 0.15. Max coverage (-): 0.08

Region: NODE\_268840\_length\_12752\_cov\_27.436010 8004-8019. Max. coverage (+): 0. Max coverage (-): 0.08

Region: NODE\_268840\_length\_12752\_cov\_27.436010 8020-. Max. coverage (+): 0. Max coverage (-): 0

RepeatMasker Color Code

**+**

100-98% Identity

<98-95% Identity

<95-90% Identity

<90-85% Identity

<85-80% Identity

<80-75% Identity

<75-70% Identity

<70% Identity

**-**

Gene Set Color Code

**+**

Gene

Pseudogene

Other

**-**

Topology/Coverage Color Code

Coverage Plus Strand

Coverage Minus Strand

Mainstrand: Plus

Mainstrand: Minus

Complementary Strand

Flanking Region  
(if option -flank >0)

Gene Set Annotation  
  
RepeatMasker Annotation  

**1. AlRepD-8475**: 8-92 (+), Divergence to consensus: 22.4%  
**2. (AGCT)n**: 806-829 (+), Divergence to consensus: 8.8%  
**3. TE-X-5\_DR**: 1342-1422 (+), Divergence to consensus: 28.4%  
**4. TE-X-4\_DR**: 1388-1530 (-), Divergence to consensus: 36.3%  
**5. (TGTCTG)n**: 4313-4350 (+), Divergence to consensus: 19.6%  
**6. (AC)n**: 4611-4630 (+), Divergence to consensus: 0%  
**7. AlRepB-392**: 5557-5737 (+), Divergence to consensus: 12.2%  
**8. AlRepD-1024**: 5786-5892 (+), Divergence to consensus: 10.3%  
**9. AlRepC-1280**: 5891-5963 (+), Divergence to consensus: 5.5%  
**10. SINE2-1\_AFC**: 6272-6337 (-), Divergence to consensus: 17.9%  
**11. Harbinger-2N1\_DR**: 6338-6482 (+), Divergence to consensus: 16.2%  
**12. AlRepD-1024**: 6496-6891 (+), Divergence to consensus: 14.8%  
**13. AlRepB-392**: 6892-6924 (+), Divergence to consensus: 12.1%  
**14. AlRepB-392**: 7067-7441 (+), Divergence to consensus: 17.1%  
**15. AlRepC-1433**: 7444-7893 (+), Divergence to consensus: 24.8%

  
Transcription Factor Binding Sites  

**RHOXF1** (Sequence: GGATCA (-): 667)  
**RHOXF1** (Sequence: AGCTTA (-): 1165)  
**RHOXF1** (Sequence: AGATCA (-): 1632)  
**RHOXF1** (Sequence: GGATTA (-): 2360)  
**RHOXF1** (Sequence: AGATTA (-): 2383)  
**RHOXF1** (Sequence: AGATTA (-): 2467)  
**RHOXF1** (Sequence: GGCTCA (-): 2604)  
**RHOXF1** (Sequence: AGATCA (-): 2987)  
**RHOXF1** (Sequence: GGATCA (-): 3415)  
**RHOXF1** (Sequence: AGCTCA (-): 3849)  
**RHOXF1** (Sequence: AGCTTA (-): 4442)  
**RHOXF1** (Sequence: GGATCA (-): 4515)  
**RHOXF1** (Sequence: AGATCA (-): 4578)  
**RHOXF1** (Sequence: AGCTCA (-): 5042)  
**RHOXF1** (Sequence: GGCTTA (-): 5058)  
**RHOXF1** (Sequence: AGATTA (-): 5348)  
**RHOXF1** (Sequence: AGATTA (-): 5672)  
**RHOXF1** (Sequence: AGATCA (-): 5675)  
**RHOXF1** (Sequence: AGCTTA (-): 5853)  
**RHOXF1** (Sequence: AGCTCA (-): 6204)  
**RHOXF1** (Sequence: AGCTTA (-): 6562)  
**RHOXF1** (Sequence: AGCTTA (-): 7098)  
**RHOXF1** (Sequence: GGATCA (-): 7549)  
**RHOXF1** (Sequence: TAAGCT (+): 65)  
**RHOXF1** (Sequence: TAAGCT (+): 187)  
**RHOXF1** (Sequence: TAATCT (+): 2836)  
**RHOXF1** (Sequence: TGAGCT (+): 3819)  
**RHOXF1** (Sequence: TGAGCT (+): 4393)  
**RHOXF1** (Sequence: TGAGCT (+): 4440)  
**RHOXF1** (Sequence: TGATCT (+): 5681)  
**RHOXF1** (Sequence: TAATCC (+): 6214)  
**RHOXF1** (Sequence: TGAGCT (+): 7892)  
**Lhx8** (Sequence: CTAATTAG (-): 91)  
**Gata4** (Sequence: CTTATCT (+): 1167)  
**Gata4** (Sequence: CTTATCT (+): 3185)  
**Gata4** (Sequence: CTTATCT (+): 3994)  
**POU5F1** (Sequence: TTTGCAT (-): 865)  
**POU5F1** (Sequence: TTTGCAT (-): 7013)  
**POU5F1** (Sequence: TTTGCAT (-): 7214)  
**RFX4\_2** (Sequence: GTATCCATG (-): 4038)  
**RFX4\_1** (Sequence: GTTGCTAGG (-): 2426)  
**SOX9** (Sequence: AACAATAG (-): 5065)  
**FOXO1** (Sequence: GTTGTTTAT (+): 3663)  
**FOXO3\_mmu** (Sequence: TGTTTTCC (-): 268)  
**FOXO3\_mmu** (Sequence: TGTTTAGC (-): 276)  
**FOXO3\_mmu** (Sequence: TGTTTTCA (-): 5172)  
**Sox5** (Sequence: ATTGTT (+): 111)  
**Sox5** (Sequence: ATTGTT (+): 904)  
**Sox5** (Sequence: ATTGTT (+): 1002)  
**Sox5** (Sequence: ATTGTT (+): 1859)  
**Sox5** (Sequence: ATTGTT (+): 1952)  
**Sox5** (Sequence: ATTGTT (+): 2210)  
**Sox5** (Sequence: ATTGTT (+): 5858)  
**Sox5** (Sequence: ATTGTT (+): 5917)  
**Sox5** (Sequence: ATTGTT (+): 6316)  
**Sox5** (Sequence: ATTGTT (+): 6567)  
**SOX9** (Sequence: TTATTGTT (+): 5856)  
**SOX9** (Sequence: TTATTGTT (+): 6565)  
**Nobox** (Sequence: ACTAATTA (-): 90)  
**Rhox11** (Sequence: TGGTGTATT (+): 1106)  
**Gata4** (Sequence: AGATAAG (-): 6673)  
**Sox5** (Sequence: AACAAT (-): 770)  
**Sox5** (Sequence: AACAAT (-): 5065)  
**POU2F1** (Sequence: TATTTTAAT (+): 894)  
**POU5F1** (Sequence: ATGCAAA (+): 2540)  
**Mybl1\_1** (Sequence: AACCGTTA (+): 5507)
