# Supplementary material for: First characterization of PIWI-interacting RNA clusters in a cichlid fish with a B chromosome
Source: BMC Biol. 2022 Sep 21;20:204. doi: 10.1186/s12915-022-01403-2 (PMC9490952; doi:10.1186/s12915-022-01403-2)
Supplement: Supplementary file 1 — Additional file 1. Zipped folder with fasta and interactive html piRNA cluster information for the A. latifasciata genome. The nomenclature is as follows: number-pirna-cluster_sex_B-presence (f, female; m, male; 0b, without B chromosome; 1b, with B chromosome). [file 12915_2022_1403_MOESM1_ESM.zip › 102_f1b.html]

piRNA cluster 102\_f1b 53


Predicted piRNA cluster no. 102\_f1b
  

Show proTRAC run info
Hide proTRAC run info

/\  
                \_\_\_\_\_\_\_\_\_\_\_\_\_\_\_\_\_\_\_\_\_\_\_/\\_\_\_ /  \\_\_\_\_\_\_\_  
               I                      /  \  /    \      I  
               I     pro             /    \/      \     I  
               I        TRAC        /               \   I  
               I   \_\_\_\_\_\_\_\_\_\_\_\_\_\_\_\_/\_\_\_\_\_\_\_\_\_\_\_\_\_\_\_\_\_\\_ I  
               I   \              /                     I  
               I    \            /                      I  
               I     \  /\      /       V.2.4.2         I  
               I      \/  \    /                        I  
               I\_\_\_\_\_\_\_\_\_\_\_\  /\_\_\_\_\_\_\_\_\_\_\_\_\_\_\_\_\_\_\_\_\_\_\_\_\_I  
                            \/  
  
  
================================= proTRAC ====================================  
VERSION: .......... 2.4.2  
LAST MODIFIED: .... 11. May 2018  
  
Please cite:  
Rosenkranz D, Zischler H. proTRAC - a software for probabilistic piRNA cluster  
detection, visualization and analysis. 2012. BMC Bioinformatics 13:5.  
  
  
Contact:  
David Rosenkranz  
Institute of Organismic and Molecular Evolutionary Biology  
Dept. Anthropology, small RNA group  
Johannes Gutenberg University Mainz  
email: rosenkranz@uni-mainz.de  
  
You can find the latest proTRAC version at:  
http://sourceforge.net/projects/protrac/files  
http://www.smallRNAgroup-mainz.de/software  
==============================================================================  
  
PARAMETERS:  
Map file: ...............piwi-femeas-1B.fa-collapse.map  
Genome file: ............../../../0B\_ala\_genome.fa  
RepeatMasker annotation: Alatifasciata-all0B-maryan-v2.fa\_corrected.out  
GeneSet:................./guest-storage/Data/annotation/Alatifasciata\_all0B\_maryan-v2\_out2017.gff  
  
Significant (p<=0.01) hit density will be calculated based  
on observed hit distribution.  
  
Sliding window size: ........................................ 5000 bp  
Sliding window increament: .................................. 1000 bp  
Normalize each hit by number of genomic hits: ............... yes  
Normalize each hit by number of sequence reads: ............. yes  
Normalize values (-> per million mapped reads): ............. yes  
Min. fraction of hits with 1T(U) or 10A: .................... 0.75  
Alternatively: Min. fraction of hits with 1T(U) and 10A: .... 0.5  
Min. fraction of hits with typical piRNA length: ............ 0.75  
Typical piRNA length: ....................................... 24-32 nt  
Min. size of a piRNA cluster: ............................... 1000 bp.  
Min. number of hits (absolute): ............................. 0  
Min. number of hits (normalized): ........................... 0  
Min. fraction of hits on the mainstrand: .................... 0.75  
Top fraction of mapped sequences (in terms of read counts): . 1%  
Top fraction accounts for max. n% of sequence reads: ........ 90%  
Min. fraction of hits on each arm of a bidirectional cluster: 0.05  
Output html file for each cluster: .......................... yes  
Output a summary table: ..................................... yes  
Output a FASTA file for each cluster (piRNA sequences): ..... yes  
Output a FASTA file comprising cluster sequences: ........... yes  
Output a GTF file for predicted piRNA clusters: ..............yes  
Search DNA motifs in clusters: .............................. yes  
Output flanking sequences: +/- .............................. 0 bp  
Output ~.pTi file: .......................................... no  
==============================================================================  
  
  
Genome size (without gaps): ............ 758543724 bp  
Gaps (N/X/-): .......................... 417479 bp  
Mapped reads: .......................... 10641844  
Non-identical sequences: ............... 2832837  
Genomic hits: .......................... 26056853  
Significant densitiy of mapped reads: .. 368.713530323068 reads/kb

Show proTRAC cluster info
Hide proTRAC cluster info

|  |  |
| --- | --- |
| Location | NODE\_268840\_length\_12752\_cov\_27.436010 |
| Coordinates | 2-8810 |
| Size [bp] | 8809 |
| Sequence hit loci | 1651 |
| Mapped reads (normalized) | 5363 |
| Mapped reads (normalized) per kb | 608.8 |
| Normalized reads with 1T (1U) | 84.4% |
| Normalized reads with 10A | 30.1% |
| Normalized reads with length 24-32 nt | 98.9% |
| Normalized reads on the main strand(s) | 98.3% |
| Predicted directionality | mono:minus |

100%

0%

1T (1U)  
reads

10A reads

24-32 nt  
reads

reads on mainstrand

**Either the amount of reads with 1T (1U) OR 10A has to exceed 75% (set with option: -1Tor10A)  
Alternatively the amount of reads with 1T (1U) AND 10A has to exceed 50% (set with option: -1Tand10A)  
Minimum amount of reads with preferred size is 75% (set with option: -pisize)  
Minimum amount of reads on the main strand(s) is 75% (set with option: -clstrand)**

Show read coverage
Hide read coverage

WHAT DO I SEE HERE?  
This chart shows the location of mapped sequence reads within a predicted piRNA cluster. The color refers to the number of genomic hits produced by the sequence read in question. A dark red bar indicates that this sequence read produces many other hits elsewhere in the genome. Many adjacent red or yellow bars can indicate the presence of a multi-copy element such as transposons or rRNA genes. A dark green bar indicates that this sequence read maps uniquely to this locus.

1 hit

2-5 hits

6-10 hits

11-20 hits

21-50 hits

51-100 hits

> 100 hits

NODE\_268840\_length\_12752\_cov\_27.436010

2

8810

Gene Set

RepeatMasker

Mapped  
Reads

62.02

plus strand

minus strand

62.02

Region: NODE\_268840\_length\_12752\_cov\_27.436010 5762-10. Max. coverage (+): 0. Max coverage (-): 0.03

Region: NODE\_268840\_length\_12752\_cov\_27.436010 11-28. Max. coverage (+): 0.03. Max coverage (-): 0.03

Region: NODE\_268840\_length\_12752\_cov\_27.436010 29-46. Max. coverage (+): 0. Max coverage (-): 0.09

Region: NODE\_268840\_length\_12752\_cov\_27.436010 47-63. Max. coverage (+): 0. Max coverage (-): 6.86

Region: NODE\_268840\_length\_12752\_cov\_27.436010 64-81. Max. coverage (+): 0.09. Max coverage (-): 0.09

Region: NODE\_268840\_length\_12752\_cov\_27.436010 82-98. Max. coverage (+): 0. Max coverage (-): 0

Region: NODE\_268840\_length\_12752\_cov\_27.436010 99-116. Max. coverage (+): 0. Max coverage (-): 0.09

Region: NODE\_268840\_length\_12752\_cov\_27.436010 117-134. Max. coverage (+): 0. Max coverage (-): 0

Region: NODE\_268840\_length\_12752\_cov\_27.436010 135-151. Max. coverage (+): 0. Max coverage (-): 0.09

Region: NODE\_268840\_length\_12752\_cov\_27.436010 152-169. Max. coverage (+): 0. Max coverage (-): 0.19

Region: NODE\_268840\_length\_12752\_cov\_27.436010 170-186. Max. coverage (+): 0. Max coverage (-): 0.09

Region: NODE\_268840\_length\_12752\_cov\_27.436010 187-204. Max. coverage (+): 0. Max coverage (-): 0

Region: NODE\_268840\_length\_12752\_cov\_27.436010 205-222. Max. coverage (+): 0. Max coverage (-): 0.09

Region: NODE\_268840\_length\_12752\_cov\_27.436010 223-239. Max. coverage (+): 0. Max coverage (-): 0.09

Region: NODE\_268840\_length\_12752\_cov\_27.436010 240-257. Max. coverage (+): 0. Max coverage (-): 0

Region: NODE\_268840\_length\_12752\_cov\_27.436010 258-275. Max. coverage (+): 0. Max coverage (-): 0

Region: NODE\_268840\_length\_12752\_cov\_27.436010 276-292. Max. coverage (+): 0. Max coverage (-): 0

Region: NODE\_268840\_length\_12752\_cov\_27.436010 293-310. Max. coverage (+): 0. Max coverage (-): 0.38

Region: NODE\_268840\_length\_12752\_cov\_27.436010 311-327. Max. coverage (+): 0. Max coverage (-): 0.56

Region: NODE\_268840\_length\_12752\_cov\_27.436010 328-345. Max. coverage (+): 0. Max coverage (-): 0.09

Region: NODE\_268840\_length\_12752\_cov\_27.436010 346-363. Max. coverage (+): 0. Max coverage (-): 0.09

Region: NODE\_268840\_length\_12752\_cov\_27.436010 364-380. Max. coverage (+): 0. Max coverage (-): 0

Region: NODE\_268840\_length\_12752\_cov\_27.436010 381-398. Max. coverage (+): 0. Max coverage (-): 0

Region: NODE\_268840\_length\_12752\_cov\_27.436010 399-416. Max. coverage (+): 0. Max coverage (-): 0.56

Region: NODE\_268840\_length\_12752\_cov\_27.436010 417-433. Max. coverage (+): 0.09. Max coverage (-): 0

Region: NODE\_268840\_length\_12752\_cov\_27.436010 434-451. Max. coverage (+): 0. Max coverage (-): 0.09

Region: NODE\_268840\_length\_12752\_cov\_27.436010 452-468. Max. coverage (+): 0. Max coverage (-): 0

Region: NODE\_268840\_length\_12752\_cov\_27.436010 469-486. Max. coverage (+): 0. Max coverage (-): 0.09

Region: NODE\_268840\_length\_12752\_cov\_27.436010 487-504. Max. coverage (+): 0. Max coverage (-): 0.09

Region: NODE\_268840\_length\_12752\_cov\_27.436010 505-521. Max. coverage (+): 0. Max coverage (-): 0

Region: NODE\_268840\_length\_12752\_cov\_27.436010 522-539. Max. coverage (+): 0. Max coverage (-): 1.6

Region: NODE\_268840\_length\_12752\_cov\_27.436010 540-556. Max. coverage (+): 0.19. Max coverage (-): 1.69

Region: NODE\_268840\_length\_12752\_cov\_27.436010 557-574. Max. coverage (+): 0.19. Max coverage (-): 1.32

Region: NODE\_268840\_length\_12752\_cov\_27.436010 575-592. Max. coverage (+): 0.09. Max coverage (-): 0.28

Region: NODE\_268840\_length\_12752\_cov\_27.436010 593-609. Max. coverage (+): 0. Max coverage (-): 0.09

Region: NODE\_268840\_length\_12752\_cov\_27.436010 610-627. Max. coverage (+): 0. Max coverage (-): 1.5

Region: NODE\_268840\_length\_12752\_cov\_27.436010 628-645. Max. coverage (+): 0.09. Max coverage (-): 2.54

Region: NODE\_268840\_length\_12752\_cov\_27.436010 646-662. Max. coverage (+): 0. Max coverage (-): 3.38

Region: NODE\_268840\_length\_12752\_cov\_27.436010 663-680. Max. coverage (+): 0.09. Max coverage (-): 0.19

Region: NODE\_268840\_length\_12752\_cov\_27.436010 681-697. Max. coverage (+): 0. Max coverage (-): 0.09

Region: NODE\_268840\_length\_12752\_cov\_27.436010 698-715. Max. coverage (+): 0.09. Max coverage (-): 1.6

Region: NODE\_268840\_length\_12752\_cov\_27.436010 716-733. Max. coverage (+): 0.09. Max coverage (-): 0.28

Region: NODE\_268840\_length\_12752\_cov\_27.436010 734-750. Max. coverage (+): 0. Max coverage (-): 0

Region: NODE\_268840\_length\_12752\_cov\_27.436010 751-768. Max. coverage (+): 0. Max coverage (-): 0.28

Region: NODE\_268840\_length\_12752\_cov\_27.436010 769-786. Max. coverage (+): 0. Max coverage (-): 0.09

Region: NODE\_268840\_length\_12752\_cov\_27.436010 787-803. Max. coverage (+): 0. Max coverage (-): 0.09

Region: NODE\_268840\_length\_12752\_cov\_27.436010 804-821. Max. coverage (+): 0. Max coverage (-): 0.75

Region: NODE\_268840\_length\_12752\_cov\_27.436010 822-838. Max. coverage (+): 0. Max coverage (-): 1.13

Region: NODE\_268840\_length\_12752\_cov\_27.436010 839-856. Max. coverage (+): 0. Max coverage (-): 0.09

Region: NODE\_268840\_length\_12752\_cov\_27.436010 857-874. Max. coverage (+): 0. Max coverage (-): 0.09

Region: NODE\_268840\_length\_12752\_cov\_27.436010 875-891. Max. coverage (+): 0. Max coverage (-): 0

Region: NODE\_268840\_length\_12752\_cov\_27.436010 892-909. Max. coverage (+): 0. Max coverage (-): 0

Region: NODE\_268840\_length\_12752\_cov\_27.436010 910-926. Max. coverage (+): 0. Max coverage (-): 0

Region: NODE\_268840\_length\_12752\_cov\_27.436010 927-944. Max. coverage (+): 0. Max coverage (-): 0.19

Region: NODE\_268840\_length\_12752\_cov\_27.436010 945-962. Max. coverage (+): 0. Max coverage (-): 0.19

Region: NODE\_268840\_length\_12752\_cov\_27.436010 963-979. Max. coverage (+): 0. Max coverage (-): 0.09

Region: NODE\_268840\_length\_12752\_cov\_27.436010 980-997. Max. coverage (+): 0. Max coverage (-): 3.01

Region: NODE\_268840\_length\_12752\_cov\_27.436010 998-1015. Max. coverage (+): 0. Max coverage (-): 0.09

Region: NODE\_268840\_length\_12752\_cov\_27.436010 1016-1032. Max. coverage (+): 0. Max coverage (-): 0

Region: NODE\_268840\_length\_12752\_cov\_27.436010 1033-1050. Max. coverage (+): 0. Max coverage (-): 1.03

Region: NODE\_268840\_length\_12752\_cov\_27.436010 1051-1067. Max. coverage (+): 0.09. Max coverage (-): 0.09

Region: NODE\_268840\_length\_12752\_cov\_27.436010 1068-1085. Max. coverage (+): 0. Max coverage (-): 0

Region: NODE\_268840\_length\_12752\_cov\_27.436010 1086-1103. Max. coverage (+): 0.09. Max coverage (-): 4.32

Region: NODE\_268840\_length\_12752\_cov\_27.436010 1104-1120. Max. coverage (+): 0.19. Max coverage (-): 4.23

Region: NODE\_268840\_length\_12752\_cov\_27.436010 1121-1138. Max. coverage (+): 0.19. Max coverage (-): 1.03

Region: NODE\_268840\_length\_12752\_cov\_27.436010 1139-1155. Max. coverage (+): 0. Max coverage (-): 1.22

Region: NODE\_268840\_length\_12752\_cov\_27.436010 1156-1173. Max. coverage (+): 0. Max coverage (-): 0.28

Region: NODE\_268840\_length\_12752\_cov\_27.436010 1174-1191. Max. coverage (+): 0. Max coverage (-): 3.76

Region: NODE\_268840\_length\_12752\_cov\_27.436010 1192-1208. Max. coverage (+): 0. Max coverage (-): 0.09

Region: NODE\_268840\_length\_12752\_cov\_27.436010 1209-1226. Max. coverage (+): 0. Max coverage (-): 0.09

Region: NODE\_268840\_length\_12752\_cov\_27.436010 1227-1244. Max. coverage (+): 0. Max coverage (-): 0.19

Region: NODE\_268840\_length\_12752\_cov\_27.436010 1245-1261. Max. coverage (+): 0. Max coverage (-): 0.47

Region: NODE\_268840\_length\_12752\_cov\_27.436010 1262-1279. Max. coverage (+): 0. Max coverage (-): 1.69

Region: NODE\_268840\_length\_12752\_cov\_27.436010 1280-1296. Max. coverage (+): 0. Max coverage (-): 0

Region: NODE\_268840\_length\_12752\_cov\_27.436010 1297-1314. Max. coverage (+): 0. Max coverage (-): 0

Region: NODE\_268840\_length\_12752\_cov\_27.436010 1315-1332. Max. coverage (+): 0. Max coverage (-): 0.47

Region: NODE\_268840\_length\_12752\_cov\_27.436010 1333-1349. Max. coverage (+): 0. Max coverage (-): 0.66

Region: NODE\_268840\_length\_12752\_cov\_27.436010 1350-1367. Max. coverage (+): 0.28. Max coverage (-): 1.13

Region: NODE\_268840\_length\_12752\_cov\_27.436010 1368-1385. Max. coverage (+): 0. Max coverage (-): 0.47

Region: NODE\_268840\_length\_12752\_cov\_27.436010 1386-1402. Max. coverage (+): 0.09. Max coverage (-): 3.66

Region: NODE\_268840\_length\_12752\_cov\_27.436010 1403-1420. Max. coverage (+): 0. Max coverage (-): 6.77

Region: NODE\_268840\_length\_12752\_cov\_27.436010 1421-1437. Max. coverage (+): 0. Max coverage (-): 1.88

Region: NODE\_268840\_length\_12752\_cov\_27.436010 1438-1455. Max. coverage (+): 0. Max coverage (-): 0.94

Region: NODE\_268840\_length\_12752\_cov\_27.436010 1456-1473. Max. coverage (+): 0.09. Max coverage (-): 1.69

Region: NODE\_268840\_length\_12752\_cov\_27.436010 1474-1490. Max. coverage (+): 0.09. Max coverage (-): 1.6

Region: NODE\_268840\_length\_12752\_cov\_27.436010 1491-1508. Max. coverage (+): 0. Max coverage (-): 0.56

Region: NODE\_268840\_length\_12752\_cov\_27.436010 1509-1525. Max. coverage (+): 0. Max coverage (-): 0.28

Region: NODE\_268840\_length\_12752\_cov\_27.436010 1526-1543. Max. coverage (+): 0. Max coverage (-): 0.56

Region: NODE\_268840\_length\_12752\_cov\_27.436010 1544-1561. Max. coverage (+): 0. Max coverage (-): 0.47

Region: NODE\_268840\_length\_12752\_cov\_27.436010 1562-1578. Max. coverage (+): 0. Max coverage (-): 0.38

Region: NODE\_268840\_length\_12752\_cov\_27.436010 1579-1596. Max. coverage (+): 0. Max coverage (-): 0.09

Region: NODE\_268840\_length\_12752\_cov\_27.436010 1597-1614. Max. coverage (+): 0.09. Max coverage (-): 0

Region: NODE\_268840\_length\_12752\_cov\_27.436010 1615-1631. Max. coverage (+): 0.09. Max coverage (-): 0.28

Region: NODE\_268840\_length\_12752\_cov\_27.436010 1632-1649. Max. coverage (+): 0. Max coverage (-): 0.47

Region: NODE\_268840\_length\_12752\_cov\_27.436010 1650-1666. Max. coverage (+): 0. Max coverage (-): 0

Region: NODE\_268840\_length\_12752\_cov\_27.436010 1667-1684. Max. coverage (+): 0. Max coverage (-): 0.09

Region: NODE\_268840\_length\_12752\_cov\_27.436010 1685-1702. Max. coverage (+): 0. Max coverage (-): 0.09

Region: NODE\_268840\_length\_12752\_cov\_27.436010 1703-1719. Max. coverage (+): 0. Max coverage (-): 0.38

Region: NODE\_268840\_length\_12752\_cov\_27.436010 1720-1737. Max. coverage (+): 0. Max coverage (-): 0.66

Region: NODE\_268840\_length\_12752\_cov\_27.436010 1738-1754. Max. coverage (+): 0.09. Max coverage (-): 0

Region: NODE\_268840\_length\_12752\_cov\_27.436010 1755-1772. Max. coverage (+): 0. Max coverage (-): 0.19

Region: NODE\_268840\_length\_12752\_cov\_27.436010 1773-1790. Max. coverage (+): 0. Max coverage (-): 0.28

Region: NODE\_268840\_length\_12752\_cov\_27.436010 1791-1807. Max. coverage (+): 0. Max coverage (-): 0.85

Region: NODE\_268840\_length\_12752\_cov\_27.436010 1808-1825. Max. coverage (+): 0. Max coverage (-): 0.09

Region: NODE\_268840\_length\_12752\_cov\_27.436010 1826-1843. Max. coverage (+): 0. Max coverage (-): 0.47

Region: NODE\_268840\_length\_12752\_cov\_27.436010 1844-1860. Max. coverage (+): 0. Max coverage (-): 0.19

Region: NODE\_268840\_length\_12752\_cov\_27.436010 1861-1878. Max. coverage (+): 0. Max coverage (-): 0.28

Region: NODE\_268840\_length\_12752\_cov\_27.436010 1879-1895. Max. coverage (+): 0. Max coverage (-): 0.09

Region: NODE\_268840\_length\_12752\_cov\_27.436010 1896-1913. Max. coverage (+): 0. Max coverage (-): 0.09

Region: NODE\_268840\_length\_12752\_cov\_27.436010 1914-1931. Max. coverage (+): 0. Max coverage (-): 0.12

Region: NODE\_268840\_length\_12752\_cov\_27.436010 1932-1948. Max. coverage (+): 0. Max coverage (-): 0.31

Region: NODE\_268840\_length\_12752\_cov\_27.436010 1949-1966. Max. coverage (+): 0. Max coverage (-): 0.09

Region: NODE\_268840\_length\_12752\_cov\_27.436010 1967-1984. Max. coverage (+): 0. Max coverage (-): 0.09

Region: NODE\_268840\_length\_12752\_cov\_27.436010 1985-2001. Max. coverage (+): 0. Max coverage (-): 0

Region: NODE\_268840\_length\_12752\_cov\_27.436010 2002-2019. Max. coverage (+): 0. Max coverage (-): 0.28

Region: NODE\_268840\_length\_12752\_cov\_27.436010 2020-2036. Max. coverage (+): 0. Max coverage (-): 0.19

Region: NODE\_268840\_length\_12752\_cov\_27.436010 2037-2054. Max. coverage (+): 0. Max coverage (-): 0.09

Region: NODE\_268840\_length\_12752\_cov\_27.436010 2055-2072. Max. coverage (+): 0. Max coverage (-): 0.19

Region: NODE\_268840\_length\_12752\_cov\_27.436010 2073-2089. Max. coverage (+): 0. Max coverage (-): 0

Region: NODE\_268840\_length\_12752\_cov\_27.436010 2090-2107. Max. coverage (+): 0. Max coverage (-): 0.66

Region: NODE\_268840\_length\_12752\_cov\_27.436010 2108-2124. Max. coverage (+): 0. Max coverage (-): 0.66

Region: NODE\_268840\_length\_12752\_cov\_27.436010 2125-2142. Max. coverage (+): 0.09. Max coverage (-): 0

Region: NODE\_268840\_length\_12752\_cov\_27.436010 2143-2160. Max. coverage (+): 0. Max coverage (-): 0

Region: NODE\_268840\_length\_12752\_cov\_27.436010 2161-2177. Max. coverage (+): 0. Max coverage (-): 0.09

Region: NODE\_268840\_length\_12752\_cov\_27.436010 2178-2195. Max. coverage (+): 0. Max coverage (-): 0.09

Region: NODE\_268840\_length\_12752\_cov\_27.436010 2196-2213. Max. coverage (+): 0. Max coverage (-): 0.19

Region: NODE\_268840\_length\_12752\_cov\_27.436010 2214-2230. Max. coverage (+): 0. Max coverage (-): 0.09

Region: NODE\_268840\_length\_12752\_cov\_27.436010 2231-2248. Max. coverage (+): 0. Max coverage (-): 0.09

Region: NODE\_268840\_length\_12752\_cov\_27.436010 2249-2265. Max. coverage (+): 0. Max coverage (-): 0

Region: NODE\_268840\_length\_12752\_cov\_27.436010 2266-2283. Max. coverage (+): 0. Max coverage (-): 0.85

Region: NODE\_268840\_length\_12752\_cov\_27.436010 2284-2301. Max. coverage (+): 0. Max coverage (-): 0.09

Region: NODE\_268840\_length\_12752\_cov\_27.436010 2302-2318. Max. coverage (+): 0. Max coverage (-): 0.03

Region: NODE\_268840\_length\_12752\_cov\_27.436010 2319-2336. Max. coverage (+): 0. Max coverage (-): 0

Region: NODE\_268840\_length\_12752\_cov\_27.436010 2337-2354. Max. coverage (+): 0. Max coverage (-): 0

Region: NODE\_268840\_length\_12752\_cov\_27.436010 2355-2371. Max. coverage (+): 0. Max coverage (-): 0.13

Region: NODE\_268840\_length\_12752\_cov\_27.436010 2372-2389. Max. coverage (+): 0. Max coverage (-): 0

Region: NODE\_268840\_length\_12752\_cov\_27.436010 2390-2406. Max. coverage (+): 0. Max coverage (-): 0

Region: NODE\_268840\_length\_12752\_cov\_27.436010 2407-2424. Max. coverage (+): 0. Max coverage (-): 0

Region: NODE\_268840\_length\_12752\_cov\_27.436010 2425-2442. Max. coverage (+): 0. Max coverage (-): 0.03

Region: NODE\_268840\_length\_12752\_cov\_27.436010 2443-2459. Max. coverage (+): 0. Max coverage (-): 0.06

Region: NODE\_268840\_length\_12752\_cov\_27.436010 2460-2477. Max. coverage (+): 0. Max coverage (-): 0

Region: NODE\_268840\_length\_12752\_cov\_27.436010 2478-2494. Max. coverage (+): 0. Max coverage (-): 0

Region: NODE\_268840\_length\_12752\_cov\_27.436010 2495-2512. Max. coverage (+): 0. Max coverage (-): 0.38

Region: NODE\_268840\_length\_12752\_cov\_27.436010 2513-2530. Max. coverage (+): 0. Max coverage (-): 0.03

Region: NODE\_268840\_length\_12752\_cov\_27.436010 2531-2547. Max. coverage (+): 0. Max coverage (-): 0

Region: NODE\_268840\_length\_12752\_cov\_27.436010 2548-2565. Max. coverage (+): 0. Max coverage (-): 4.7

Region: NODE\_268840\_length\_12752\_cov\_27.436010 2566-2583. Max. coverage (+): 0. Max coverage (-): 0.23

Region: NODE\_268840\_length\_12752\_cov\_27.436010 2584-2600. Max. coverage (+): 0. Max coverage (-): 1.03

Region: NODE\_268840\_length\_12752\_cov\_27.436010 2601-2618. Max. coverage (+): 0.05. Max coverage (-): 10.43

Region: NODE\_268840\_length\_12752\_cov\_27.436010 2619-2635. Max. coverage (+): 0.19. Max coverage (-): 5.07

Region: NODE\_268840\_length\_12752\_cov\_27.436010 2636-2653. Max. coverage (+): 0.05. Max coverage (-): 1.46

Region: NODE\_268840\_length\_12752\_cov\_27.436010 2654-2671. Max. coverage (+): 0.09. Max coverage (-): 0.28

Region: NODE\_268840\_length\_12752\_cov\_27.436010 2672-2688. Max. coverage (+): 0. Max coverage (-): 1.88

Region: NODE\_268840\_length\_12752\_cov\_27.436010 2689-2706. Max. coverage (+): 0.05. Max coverage (-): 0.8

Region: NODE\_268840\_length\_12752\_cov\_27.436010 2707-2723. Max. coverage (+): 0. Max coverage (-): 0.09

Region: NODE\_268840\_length\_12752\_cov\_27.436010 2724-2741. Max. coverage (+): 0. Max coverage (-): 0.09

Region: NODE\_268840\_length\_12752\_cov\_27.436010 2742-2759. Max. coverage (+): 0. Max coverage (-): 0

Region: NODE\_268840\_length\_12752\_cov\_27.436010 2760-2776. Max. coverage (+): 0. Max coverage (-): 0.14

Region: NODE\_268840\_length\_12752\_cov\_27.436010 2777-2794. Max. coverage (+): 0. Max coverage (-): 0.09

Region: NODE\_268840\_length\_12752\_cov\_27.436010 2795-2812. Max. coverage (+): 0. Max coverage (-): 0.19

Region: NODE\_268840\_length\_12752\_cov\_27.436010 2813-2829. Max. coverage (+): 0. Max coverage (-): 0.47

Region: NODE\_268840\_length\_12752\_cov\_27.436010 2830-2847. Max. coverage (+): 0. Max coverage (-): 0.09

Region: NODE\_268840\_length\_12752\_cov\_27.436010 2848-2864. Max. coverage (+): 0.09. Max coverage (-): 0.09

Region: NODE\_268840\_length\_12752\_cov\_27.436010 2865-2882. Max. coverage (+): 0. Max coverage (-): 2.35

Region: NODE\_268840\_length\_12752\_cov\_27.436010 2883-2900. Max. coverage (+): 0. Max coverage (-): 0.05

Region: NODE\_268840\_length\_12752\_cov\_27.436010 2901-2917. Max. coverage (+): 0. Max coverage (-): 0.09

Region: NODE\_268840\_length\_12752\_cov\_27.436010 2918-2935. Max. coverage (+): 0.09. Max coverage (-): 0.09

Region: NODE\_268840\_length\_12752\_cov\_27.436010 2936-2953. Max. coverage (+): 0. Max coverage (-): 3.29

Region: NODE\_268840\_length\_12752\_cov\_27.436010 2954-2970. Max. coverage (+): 0.05. Max coverage (-): 2.54

Region: NODE\_268840\_length\_12752\_cov\_27.436010 2971-2988. Max. coverage (+): 0.05. Max coverage (-): 0.5

Region: NODE\_268840\_length\_12752\_cov\_27.436010 2989-3005. Max. coverage (+): 0. Max coverage (-): 1.38

Region: NODE\_268840\_length\_12752\_cov\_27.436010 3006-3023. Max. coverage (+): 0. Max coverage (-): 0.56

Region: NODE\_268840\_length\_12752\_cov\_27.436010 3024-3041. Max. coverage (+): 0.19. Max coverage (-): 0.28

Region: NODE\_268840\_length\_12752\_cov\_27.436010 3042-3058. Max. coverage (+): 0. Max coverage (-): 0.42

Region: NODE\_268840\_length\_12752\_cov\_27.436010 3059-3076. Max. coverage (+): 0.05. Max coverage (-): 0.61

Region: NODE\_268840\_length\_12752\_cov\_27.436010 3077-3093. Max. coverage (+): 0. Max coverage (-): 0.05

Region: NODE\_268840\_length\_12752\_cov\_27.436010 3094-3111. Max. coverage (+): 0. Max coverage (-): 0.07

Region: NODE\_268840\_length\_12752\_cov\_27.436010 3112-3129. Max. coverage (+): 0. Max coverage (-): 0.05

Region: NODE\_268840\_length\_12752\_cov\_27.436010 3130-3146. Max. coverage (+): 0. Max coverage (-): 0.02

Region: NODE\_268840\_length\_12752\_cov\_27.436010 3147-3164. Max. coverage (+): 0. Max coverage (-): 0.09

Region: NODE\_268840\_length\_12752\_cov\_27.436010 3165-3182. Max. coverage (+): 0. Max coverage (-): 0.19

Region: NODE\_268840\_length\_12752\_cov\_27.436010 3183-3199. Max. coverage (+): 0. Max coverage (-): 0.09

Region: NODE\_268840\_length\_12752\_cov\_27.436010 3200-3217. Max. coverage (+): 0. Max coverage (-): 0

Region: NODE\_268840\_length\_12752\_cov\_27.436010 3218-3234. Max. coverage (+): 0. Max coverage (-): 4.46

Region: NODE\_268840\_length\_12752\_cov\_27.436010 3235-3252. Max. coverage (+): 0. Max coverage (-): 4.75

Region: NODE\_268840\_length\_12752\_cov\_27.436010 3253-3270. Max. coverage (+): 0.19. Max coverage (-): 0.19

Region: NODE\_268840\_length\_12752\_cov\_27.436010 3271-3287. Max. coverage (+): 0. Max coverage (-): 3.57

Region: NODE\_268840\_length\_12752\_cov\_27.436010 3288-3305. Max. coverage (+): 0.19. Max coverage (-): 0.38

Region: NODE\_268840\_length\_12752\_cov\_27.436010 3306-3322. Max. coverage (+): 0. Max coverage (-): 5.17

Region: NODE\_268840\_length\_12752\_cov\_27.436010 3323-3340. Max. coverage (+): 0.09. Max coverage (-): 0.09

Region: NODE\_268840\_length\_12752\_cov\_27.436010 3341-3358. Max. coverage (+): 0.05. Max coverage (-): 2.02

Region: NODE\_268840\_length\_12752\_cov\_27.436010 3359-3375. Max. coverage (+): 0.05. Max coverage (-): 2.77

Region: NODE\_268840\_length\_12752\_cov\_27.436010 3376-3393. Max. coverage (+): 0.09. Max coverage (-): 10.24

Region: NODE\_268840\_length\_12752\_cov\_27.436010 3394-3411. Max. coverage (+): 0.09. Max coverage (-): 29.69

Region: NODE\_268840\_length\_12752\_cov\_27.436010 3412-3428. Max. coverage (+): 0.09. Max coverage (-): 1.5

Region: NODE\_268840\_length\_12752\_cov\_27.436010 3429-3446. Max. coverage (+): 0. Max coverage (-): 0.19

Region: NODE\_268840\_length\_12752\_cov\_27.436010 3447-3463. Max. coverage (+): 0. Max coverage (-): 3.81

Region: NODE\_268840\_length\_12752\_cov\_27.436010 3464-3481. Max. coverage (+): 0. Max coverage (-): 4.51

Region: NODE\_268840\_length\_12752\_cov\_27.436010 3482-3499. Max. coverage (+): 0. Max coverage (-): 0

Region: NODE\_268840\_length\_12752\_cov\_27.436010 3500-3516. Max. coverage (+): 0. Max coverage (-): 0

Region: NODE\_268840\_length\_12752\_cov\_27.436010 3517-3534. Max. coverage (+): 0. Max coverage (-): 26.03

Region: NODE\_268840\_length\_12752\_cov\_27.436010 3535-3552. Max. coverage (+): 0.43. Max coverage (-): 0.75

Region: NODE\_268840\_length\_12752\_cov\_27.436010 3553-3569. Max. coverage (+): 0.06. Max coverage (-): 0.13

Region: NODE\_268840\_length\_12752\_cov\_27.436010 3570-3587. Max. coverage (+): 0. Max coverage (-): 15.74

Region: NODE\_268840\_length\_12752\_cov\_27.436010 3588-3604. Max. coverage (+): 0.42. Max coverage (-): 0.05

Region: NODE\_268840\_length\_12752\_cov\_27.436010 3605-3622. Max. coverage (+): 0. Max coverage (-): 18.04

Region: NODE\_268840\_length\_12752\_cov\_27.436010 3623-3640. Max. coverage (+): 0.22. Max coverage (-): 4.09

Region: NODE\_268840\_length\_12752\_cov\_27.436010 3641-3657. Max. coverage (+): 0. Max coverage (-): 0.28

Region: NODE\_268840\_length\_12752\_cov\_27.436010 3658-3675. Max. coverage (+): 0. Max coverage (-): 11.37

Region: NODE\_268840\_length\_12752\_cov\_27.436010 3676-3692. Max. coverage (+): 0.03. Max coverage (-): 0.06

Region: NODE\_268840\_length\_12752\_cov\_27.436010 3693-3710. Max. coverage (+): 0. Max coverage (-): 0

Region: NODE\_268840\_length\_12752\_cov\_27.436010 3711-3728. Max. coverage (+): 0. Max coverage (-): 0.66

Region: NODE\_268840\_length\_12752\_cov\_27.436010 3729-3745. Max. coverage (+): 0. Max coverage (-): 0.09

Region: NODE\_268840\_length\_12752\_cov\_27.436010 3746-3763. Max. coverage (+): 0. Max coverage (-): 0

Region: NODE\_268840\_length\_12752\_cov\_27.436010 3764-3781. Max. coverage (+): 0. Max coverage (-): 0.14

Region: NODE\_268840\_length\_12752\_cov\_27.436010 3782-3798. Max. coverage (+): 0.03. Max coverage (-): 0.06

Region: NODE\_268840\_length\_12752\_cov\_27.436010 3799-3816. Max. coverage (+): 0. Max coverage (-): 0.03

Region: NODE\_268840\_length\_12752\_cov\_27.436010 3817-3833. Max. coverage (+): 0. Max coverage (-): 0.19

Region: NODE\_268840\_length\_12752\_cov\_27.436010 3834-3851. Max. coverage (+): 0. Max coverage (-): 0.05

Region: NODE\_268840\_length\_12752\_cov\_27.436010 3852-3869. Max. coverage (+): 0. Max coverage (-): 3.52

Region: NODE\_268840\_length\_12752\_cov\_27.436010 3870-3886. Max. coverage (+): 0. Max coverage (-): 0

Region: NODE\_268840\_length\_12752\_cov\_27.436010 3887-3904. Max. coverage (+): 0. Max coverage (-): 0.56

Region: NODE\_268840\_length\_12752\_cov\_27.436010 3905-3922. Max. coverage (+): 0. Max coverage (-): 0.14

Region: NODE\_268840\_length\_12752\_cov\_27.436010 3923-3939. Max. coverage (+): 0. Max coverage (-): 0.66

Region: NODE\_268840\_length\_12752\_cov\_27.436010 3940-3957. Max. coverage (+): 0. Max coverage (-): 0.09

Region: NODE\_268840\_length\_12752\_cov\_27.436010 3958-3974. Max. coverage (+): 0. Max coverage (-): 0.33

Region: NODE\_268840\_length\_12752\_cov\_27.436010 3975-3992. Max. coverage (+): 0. Max coverage (-): 0.47

Region: NODE\_268840\_length\_12752\_cov\_27.436010 3993-4010. Max. coverage (+): 0. Max coverage (-): 0.38

Region: NODE\_268840\_length\_12752\_cov\_27.436010 4011-4027. Max. coverage (+): 0. Max coverage (-): 0.09

Region: NODE\_268840\_length\_12752\_cov\_27.436010 4028-4045. Max. coverage (+): 0. Max coverage (-): 0.06

Region: NODE\_268840\_length\_12752\_cov\_27.436010 4046-4062. Max. coverage (+): 0. Max coverage (-): 0.07

Region: NODE\_268840\_length\_12752\_cov\_27.436010 4063-4080. Max. coverage (+): 0. Max coverage (-): 0.03

Region: NODE\_268840\_length\_12752\_cov\_27.436010 4081-4098. Max. coverage (+): 0. Max coverage (-): 0

Region: NODE\_268840\_length\_12752\_cov\_27.436010 4099-4115. Max. coverage (+): 0. Max coverage (-): 0.23

Region: NODE\_268840\_length\_12752\_cov\_27.436010 4116-4133. Max. coverage (+): 0. Max coverage (-): 0.38

Region: NODE\_268840\_length\_12752\_cov\_27.436010 4134-4151. Max. coverage (+): 0.05. Max coverage (-): 0.05

Region: NODE\_268840\_length\_12752\_cov\_27.436010 4152-4168. Max. coverage (+): 0. Max coverage (-): 0.33

Region: NODE\_268840\_length\_12752\_cov\_27.436010 4169-4186. Max. coverage (+): 0. Max coverage (-): 0.19

Region: NODE\_268840\_length\_12752\_cov\_27.436010 4187-4203. Max. coverage (+): 0. Max coverage (-): 0.09

Region: NODE\_268840\_length\_12752\_cov\_27.436010 4204-4221. Max. coverage (+): 0.02. Max coverage (-): 0.19

Region: NODE\_268840\_length\_12752\_cov\_27.436010 4222-4239. Max. coverage (+): 0.02. Max coverage (-): 0

Region: NODE\_268840\_length\_12752\_cov\_27.436010 4240-4256. Max. coverage (+): 0. Max coverage (-): 0.04

Region: NODE\_268840\_length\_12752\_cov\_27.436010 4257-4274. Max. coverage (+): 0. Max coverage (-): 0

Region: NODE\_268840\_length\_12752\_cov\_27.436010 4275-4291. Max. coverage (+): 0. Max coverage (-): 0.28

Region: NODE\_268840\_length\_12752\_cov\_27.436010 4292-4309. Max. coverage (+): 0. Max coverage (-): 0.09

Region: NODE\_268840\_length\_12752\_cov\_27.436010 4310-4327. Max. coverage (+): 0. Max coverage (-): 0.19

Region: NODE\_268840\_length\_12752\_cov\_27.436010 4328-4344. Max. coverage (+): 0. Max coverage (-): 0.19

Region: NODE\_268840\_length\_12752\_cov\_27.436010 4345-4362. Max. coverage (+): 0. Max coverage (-): 0.09

Region: NODE\_268840\_length\_12752\_cov\_27.436010 4363-4380. Max. coverage (+): 0. Max coverage (-): 1.88

Region: NODE\_268840\_length\_12752\_cov\_27.436010 4381-4397. Max. coverage (+): 0.09. Max coverage (-): 1.6

Region: NODE\_268840\_length\_12752\_cov\_27.436010 4398-4415. Max. coverage (+): 0.09. Max coverage (-): 0.09

Region: NODE\_268840\_length\_12752\_cov\_27.436010 4416-4432. Max. coverage (+): 0. Max coverage (-): 0.09

Region: NODE\_268840\_length\_12752\_cov\_27.436010 4433-4450. Max. coverage (+): 0.09. Max coverage (-): 0.09

Region: NODE\_268840\_length\_12752\_cov\_27.436010 4451-4468. Max. coverage (+): 0.09. Max coverage (-): 0.09

Region: NODE\_268840\_length\_12752\_cov\_27.436010 4469-4485. Max. coverage (+): 0. Max coverage (-): 0.28

Region: NODE\_268840\_length\_12752\_cov\_27.436010 4486-4503. Max. coverage (+): 0. Max coverage (-): 0.09

Region: NODE\_268840\_length\_12752\_cov\_27.436010 4504-4521. Max. coverage (+): 0. Max coverage (-): 0

Region: NODE\_268840\_length\_12752\_cov\_27.436010 4522-4538. Max. coverage (+): 0. Max coverage (-): 0.56

Region: NODE\_268840\_length\_12752\_cov\_27.436010 4539-4556. Max. coverage (+): 0. Max coverage (-): 0.75

Region: NODE\_268840\_length\_12752\_cov\_27.436010 4557-4573. Max. coverage (+): 0. Max coverage (-): 0.75

Region: NODE\_268840\_length\_12752\_cov\_27.436010 4574-4591. Max. coverage (+): 0. Max coverage (-): 0.19

Region: NODE\_268840\_length\_12752\_cov\_27.436010 4592-4609. Max. coverage (+): 0. Max coverage (-): 0.19

Region: NODE\_268840\_length\_12752\_cov\_27.436010 4610-4626. Max. coverage (+): 0. Max coverage (-): 0.19

Region: NODE\_268840\_length\_12752\_cov\_27.436010 4627-4644. Max. coverage (+): 0.09. Max coverage (-): 18.04

Region: NODE\_268840\_length\_12752\_cov\_27.436010 4645-4661. Max. coverage (+): 0.09. Max coverage (-): 1.6

Region: NODE\_268840\_length\_12752\_cov\_27.436010 4662-4679. Max. coverage (+): 0. Max coverage (-): 0.28

Region: NODE\_268840\_length\_12752\_cov\_27.436010 4680-4697. Max. coverage (+): 0. Max coverage (-): 0.66

Region: NODE\_268840\_length\_12752\_cov\_27.436010 4698-4714. Max. coverage (+): 0. Max coverage (-): 0.09

Region: NODE\_268840\_length\_12752\_cov\_27.436010 4715-4732. Max. coverage (+): 0. Max coverage (-): 0

Region: NODE\_268840\_length\_12752\_cov\_27.436010 4733-4750. Max. coverage (+): 0. Max coverage (-): 0.19

Region: NODE\_268840\_length\_12752\_cov\_27.436010 4751-4767. Max. coverage (+): 0. Max coverage (-): 5.83

Region: NODE\_268840\_length\_12752\_cov\_27.436010 4768-4785. Max. coverage (+): 0.19. Max coverage (-): 0.19

Region: NODE\_268840\_length\_12752\_cov\_27.436010 4786-4802. Max. coverage (+): 0. Max coverage (-): 9.96

Region: NODE\_268840\_length\_12752\_cov\_27.436010 4803-4820. Max. coverage (+): 0. Max coverage (-): 0.56

Region: NODE\_268840\_length\_12752\_cov\_27.436010 4821-4838. Max. coverage (+): 0.66. Max coverage (-): 3.19

Region: NODE\_268840\_length\_12752\_cov\_27.436010 4839-4855. Max. coverage (+): 0.38. Max coverage (-): 0.75

Region: NODE\_268840\_length\_12752\_cov\_27.436010 4856-4873. Max. coverage (+): 0. Max coverage (-): 4.23

Region: NODE\_268840\_length\_12752\_cov\_27.436010 4874-4890. Max. coverage (+): 0. Max coverage (-): 0.28

Region: NODE\_268840\_length\_12752\_cov\_27.436010 4891-4908. Max. coverage (+): 0. Max coverage (-): 62.02

Region: NODE\_268840\_length\_12752\_cov\_27.436010 4909-4926. Max. coverage (+): 0.09. Max coverage (-): 20.3

Region: NODE\_268840\_length\_12752\_cov\_27.436010 4927-4943. Max. coverage (+): 0.09. Max coverage (-): 0.19

Region: NODE\_268840\_length\_12752\_cov\_27.436010 4944-4961. Max. coverage (+): 0. Max coverage (-): 1.32

Region: NODE\_268840\_length\_12752\_cov\_27.436010 4962-4979. Max. coverage (+): 0. Max coverage (-): 1.5

Region: NODE\_268840\_length\_12752\_cov\_27.436010 4980-4996. Max. coverage (+): 0. Max coverage (-): 0.38

Region: NODE\_268840\_length\_12752\_cov\_27.436010 4997-5014. Max. coverage (+): 0. Max coverage (-): 0.56

Region: NODE\_268840\_length\_12752\_cov\_27.436010 5015-5031. Max. coverage (+): 0. Max coverage (-): 0.19

Region: NODE\_268840\_length\_12752\_cov\_27.436010 5032-5049. Max. coverage (+): 0.09. Max coverage (-): 0.75

Region: NODE\_268840\_length\_12752\_cov\_27.436010 5050-5067. Max. coverage (+): 0. Max coverage (-): 0.09

Region: NODE\_268840\_length\_12752\_cov\_27.436010 5068-5084. Max. coverage (+): 0. Max coverage (-): 2.16

Region: NODE\_268840\_length\_12752\_cov\_27.436010 5085-5102. Max. coverage (+): 0. Max coverage (-): 0.28

Region: NODE\_268840\_length\_12752\_cov\_27.436010 5103-5120. Max. coverage (+): 0. Max coverage (-): 0

Region: NODE\_268840\_length\_12752\_cov\_27.436010 5121-5137. Max. coverage (+): 0. Max coverage (-): 0

Region: NODE\_268840\_length\_12752\_cov\_27.436010 5138-5155. Max. coverage (+): 0. Max coverage (-): 0

Region: NODE\_268840\_length\_12752\_cov\_27.436010 5156-5172. Max. coverage (+): 0. Max coverage (-): 0

Region: NODE\_268840\_length\_12752\_cov\_27.436010 5173-5190. Max. coverage (+): 0. Max coverage (-): 0.38

Region: NODE\_268840\_length\_12752\_cov\_27.436010 5191-5208. Max. coverage (+): 0.09. Max coverage (-): 0.09

Region: NODE\_268840\_length\_12752\_cov\_27.436010 5209-5225. Max. coverage (+): 0. Max coverage (-): 0

Region: NODE\_268840\_length\_12752\_cov\_27.436010 5226-5243. Max. coverage (+): 0. Max coverage (-): 0.38

Region: NODE\_268840\_length\_12752\_cov\_27.436010 5244-5260. Max. coverage (+): 0. Max coverage (-): 0.19

Region: NODE\_268840\_length\_12752\_cov\_27.436010 5261-5278. Max. coverage (+): 0. Max coverage (-): 0.09

Region: NODE\_268840\_length\_12752\_cov\_27.436010 5279-5296. Max. coverage (+): 0. Max coverage (-): 0.19

Region: NODE\_268840\_length\_12752\_cov\_27.436010 5297-5313. Max. coverage (+): 0. Max coverage (-): 0

Region: NODE\_268840\_length\_12752\_cov\_27.436010 5314-5331. Max. coverage (+): 0. Max coverage (-): 0.09

Region: NODE\_268840\_length\_12752\_cov\_27.436010 5332-5349. Max. coverage (+): 0. Max coverage (-): 0

Region: NODE\_268840\_length\_12752\_cov\_27.436010 5350-5366. Max. coverage (+): 0. Max coverage (-): 0

Region: NODE\_268840\_length\_12752\_cov\_27.436010 5367-5384. Max. coverage (+): 0. Max coverage (-): 0.47

Region: NODE\_268840\_length\_12752\_cov\_27.436010 5385-5401. Max. coverage (+): 0. Max coverage (-): 0

Region: NODE\_268840\_length\_12752\_cov\_27.436010 5402-5419. Max. coverage (+): 0. Max coverage (-): 0.28

Region: NODE\_268840\_length\_12752\_cov\_27.436010 5420-5437. Max. coverage (+): 0. Max coverage (-): 0.38

Region: NODE\_268840\_length\_12752\_cov\_27.436010 5438-5454. Max. coverage (+): 0. Max coverage (-): 0

Region: NODE\_268840\_length\_12752\_cov\_27.436010 5455-5472. Max. coverage (+): 0. Max coverage (-): 0.09

Region: NODE\_268840\_length\_12752\_cov\_27.436010 5473-5490. Max. coverage (+): 0.09. Max coverage (-): 0

Region: NODE\_268840\_length\_12752\_cov\_27.436010 5491-5507. Max. coverage (+): 0. Max coverage (-): 0.19

Region: NODE\_268840\_length\_12752\_cov\_27.436010 5508-5525. Max. coverage (+): 0. Max coverage (-): 0.09

Region: NODE\_268840\_length\_12752\_cov\_27.436010 5526-5542. Max. coverage (+): 0. Max coverage (-): 0

Region: NODE\_268840\_length\_12752\_cov\_27.436010 5543-5560. Max. coverage (+): 0. Max coverage (-): 0.19

Region: NODE\_268840\_length\_12752\_cov\_27.436010 5561-5578. Max. coverage (+): 0. Max coverage (-): 0.56

Region: NODE\_268840\_length\_12752\_cov\_27.436010 5579-5595. Max. coverage (+): 0. Max coverage (-): 0.38

Region: NODE\_268840\_length\_12752\_cov\_27.436010 5596-5613. Max. coverage (+): 0. Max coverage (-): 0.38

Region: NODE\_268840\_length\_12752\_cov\_27.436010 5614-5630. Max. coverage (+): 0. Max coverage (-): 0

Region: NODE\_268840\_length\_12752\_cov\_27.436010 5631-5648. Max. coverage (+): 0. Max coverage (-): 0

Region: NODE\_268840\_length\_12752\_cov\_27.436010 5649-5666. Max. coverage (+): 0. Max coverage (-): 0

Region: NODE\_268840\_length\_12752\_cov\_27.436010 5667-5683. Max. coverage (+): 0. Max coverage (-): 0

Region: NODE\_268840\_length\_12752\_cov\_27.436010 5684-5701. Max. coverage (+): 0. Max coverage (-): 0

Region: NODE\_268840\_length\_12752\_cov\_27.436010 5702-5719. Max. coverage (+): 0. Max coverage (-): 0

Region: NODE\_268840\_length\_12752\_cov\_27.436010 5720-5736. Max. coverage (+): 0. Max coverage (-): 0

Region: NODE\_268840\_length\_12752\_cov\_27.436010 5737-5754. Max. coverage (+): 0. Max coverage (-): 0

Region: NODE\_268840\_length\_12752\_cov\_27.436010 5755-5771. Max. coverage (+): 0. Max coverage (-): 0.09

Region: NODE\_268840\_length\_12752\_cov\_27.436010 5772-5789. Max. coverage (+): 0. Max coverage (-): 0.6

Region: NODE\_268840\_length\_12752\_cov\_27.436010 5790-5807. Max. coverage (+): 0. Max coverage (-): 0.06

Region: NODE\_268840\_length\_12752\_cov\_27.436010 5808-5824. Max. coverage (+): 0. Max coverage (-): 0.02

Region: NODE\_268840\_length\_12752\_cov\_27.436010 5825-5842. Max. coverage (+): 0. Max coverage (-): 0.38

Region: NODE\_268840\_length\_12752\_cov\_27.436010 5843-5859. Max. coverage (+): 0. Max coverage (-): 0.41

Region: NODE\_268840\_length\_12752\_cov\_27.436010 5860-5877. Max. coverage (+): 0. Max coverage (-): 0.02

Region: NODE\_268840\_length\_12752\_cov\_27.436010 5878-5895. Max. coverage (+): 0. Max coverage (-): 0

Region: NODE\_268840\_length\_12752\_cov\_27.436010 5896-5912. Max. coverage (+): 0. Max coverage (-): 0

Region: NODE\_268840\_length\_12752\_cov\_27.436010 5913-5930. Max. coverage (+): 0. Max coverage (-): 0

Region: NODE\_268840\_length\_12752\_cov\_27.436010 5931-5948. Max. coverage (+): 0. Max coverage (-): 0

Region: NODE\_268840\_length\_12752\_cov\_27.436010 5949-5965. Max. coverage (+): 0. Max coverage (-): 0

Region: NODE\_268840\_length\_12752\_cov\_27.436010 5966-5983. Max. coverage (+): 0. Max coverage (-): 0

Region: NODE\_268840\_length\_12752\_cov\_27.436010 5984-6000. Max. coverage (+): 0. Max coverage (-): 0

Region: NODE\_268840\_length\_12752\_cov\_27.436010 6001-6018. Max. coverage (+): 0. Max coverage (-): 0

Region: NODE\_268840\_length\_12752\_cov\_27.436010 6019-6036. Max. coverage (+): 0. Max coverage (-): 0

Region: NODE\_268840\_length\_12752\_cov\_27.436010 6037-6053. Max. coverage (+): 0. Max coverage (-): 0

Region: NODE\_268840\_length\_12752\_cov\_27.436010 6054-6071. Max. coverage (+): 0. Max coverage (-): 0

Region: NODE\_268840\_length\_12752\_cov\_27.436010 6072-6089. Max. coverage (+): 0. Max coverage (-): 0

Region: NODE\_268840\_length\_12752\_cov\_27.436010 6090-6106. Max. coverage (+): 0. Max coverage (-): 0

Region: NODE\_268840\_length\_12752\_cov\_27.436010 6107-6124. Max. coverage (+): 0. Max coverage (-): 0

Region: NODE\_268840\_length\_12752\_cov\_27.436010 6125-6141. Max. coverage (+): 0. Max coverage (-): 0

Region: NODE\_268840\_length\_12752\_cov\_27.436010 6142-6159. Max. coverage (+): 0. Max coverage (-): 0

Region: NODE\_268840\_length\_12752\_cov\_27.436010 6160-6177. Max. coverage (+): 0. Max coverage (-): 0

Region: NODE\_268840\_length\_12752\_cov\_27.436010 6178-6194. Max. coverage (+): 0. Max coverage (-): 0

Region: NODE\_268840\_length\_12752\_cov\_27.436010 6195-6212. Max. coverage (+): 0. Max coverage (-): 0

Region: NODE\_268840\_length\_12752\_cov\_27.436010 6213-6229. Max. coverage (+): 0. Max coverage (-): 0

Region: NODE\_268840\_length\_12752\_cov\_27.436010 6230-6247. Max. coverage (+): 0. Max coverage (-): 0

Region: NODE\_268840\_length\_12752\_cov\_27.436010 6248-6265. Max. coverage (+): 0. Max coverage (-): 0

Region: NODE\_268840\_length\_12752\_cov\_27.436010 6266-6282. Max. coverage (+): 0. Max coverage (-): 0

Region: NODE\_268840\_length\_12752\_cov\_27.436010 6283-6300. Max. coverage (+): 0. Max coverage (-): 0

Region: NODE\_268840\_length\_12752\_cov\_27.436010 6301-6318. Max. coverage (+): 0. Max coverage (-): 0

Region: NODE\_268840\_length\_12752\_cov\_27.436010 6319-6335. Max. coverage (+): 0. Max coverage (-): 0

Region: NODE\_268840\_length\_12752\_cov\_27.436010 6336-6353. Max. coverage (+): 0. Max coverage (-): 0

Region: NODE\_268840\_length\_12752\_cov\_27.436010 6354-6370. Max. coverage (+): 0. Max coverage (-): 0

Region: NODE\_268840\_length\_12752\_cov\_27.436010 6371-6388. Max. coverage (+): 0. Max coverage (-): 0

Region: NODE\_268840\_length\_12752\_cov\_27.436010 6389-6406. Max. coverage (+): 0. Max coverage (-): 0

Region: NODE\_268840\_length\_12752\_cov\_27.436010 6407-6423. Max. coverage (+): 0. Max coverage (-): 0

Region: NODE\_268840\_length\_12752\_cov\_27.436010 6424-6441. Max. coverage (+): 0. Max coverage (-): 0

Region: NODE\_268840\_length\_12752\_cov\_27.436010 6442-6458. Max. coverage (+): 0. Max coverage (-): 0

Region: NODE\_268840\_length\_12752\_cov\_27.436010 6459-6476. Max. coverage (+): 0. Max coverage (-): 0

Region: NODE\_268840\_length\_12752\_cov\_27.436010 6477-6494. Max. coverage (+): 0. Max coverage (-): 0

Region: NODE\_268840\_length\_12752\_cov\_27.436010 6495-6511. Max. coverage (+): 0. Max coverage (-): 0.6

Region: NODE\_268840\_length\_12752\_cov\_27.436010 6512-6529. Max. coverage (+): 0. Max coverage (-): 0

Region: NODE\_268840\_length\_12752\_cov\_27.436010 6530-6547. Max. coverage (+): 0. Max coverage (-): 0

Region: NODE\_268840\_length\_12752\_cov\_27.436010 6548-6564. Max. coverage (+): 0. Max coverage (-): 0

Region: NODE\_268840\_length\_12752\_cov\_27.436010 6565-6582. Max. coverage (+): 0. Max coverage (-): 0

Region: NODE\_268840\_length\_12752\_cov\_27.436010 6583-6599. Max. coverage (+): 0. Max coverage (-): 0

Region: NODE\_268840\_length\_12752\_cov\_27.436010 6600-6617. Max. coverage (+): 0. Max coverage (-): 0

Region: NODE\_268840\_length\_12752\_cov\_27.436010 6618-6635. Max. coverage (+): 0. Max coverage (-): 0

Region: NODE\_268840\_length\_12752\_cov\_27.436010 6636-6652. Max. coverage (+): 0. Max coverage (-): 0

Region: NODE\_268840\_length\_12752\_cov\_27.436010 6653-6670. Max. coverage (+): 0. Max coverage (-): 0.19

Region: NODE\_268840\_length\_12752\_cov\_27.436010 6671-6688. Max. coverage (+): 0. Max coverage (-): 0.19

Region: NODE\_268840\_length\_12752\_cov\_27.436010 6689-6705. Max. coverage (+): 0. Max coverage (-): 0

Region: NODE\_268840\_length\_12752\_cov\_27.436010 6706-6723. Max. coverage (+): 0. Max coverage (-): 0

Region: NODE\_268840\_length\_12752\_cov\_27.436010 6724-6740. Max. coverage (+): 0. Max coverage (-): 0.19

Region: NODE\_268840\_length\_12752\_cov\_27.436010 6741-6758. Max. coverage (+): 0. Max coverage (-): 0.09

Region: NODE\_268840\_length\_12752\_cov\_27.436010 6759-6776. Max. coverage (+): 0.03. Max coverage (-): 0.05

Region: NODE\_268840\_length\_12752\_cov\_27.436010 6777-6793. Max. coverage (+): 0.03. Max coverage (-): 1.55

Region: NODE\_268840\_length\_12752\_cov\_27.436010 6794-6811. Max. coverage (+): 0.19. Max coverage (-): 0.02

Region: NODE\_268840\_length\_12752\_cov\_27.436010 6812-6828. Max. coverage (+): 0.01. Max coverage (-): 0.09

Region: NODE\_268840\_length\_12752\_cov\_27.436010 6829-6846. Max. coverage (+): 0.01. Max coverage (-): 0.22

Region: NODE\_268840\_length\_12752\_cov\_27.436010 6847-6864. Max. coverage (+): 0. Max coverage (-): 0.23

Region: NODE\_268840\_length\_12752\_cov\_27.436010 6865-6881. Max. coverage (+): 0.09. Max coverage (-): 0.23

Region: NODE\_268840\_length\_12752\_cov\_27.436010 6882-6899. Max. coverage (+): 0. Max coverage (-): 0.09

Region: NODE\_268840\_length\_12752\_cov\_27.436010 6900-6917. Max. coverage (+): 0. Max coverage (-): 0.66

Region: NODE\_268840\_length\_12752\_cov\_27.436010 6918-6934. Max. coverage (+): 0. Max coverage (-): 0.38

Region: NODE\_268840\_length\_12752\_cov\_27.436010 6935-6952. Max. coverage (+): 0. Max coverage (-): 0

Region: NODE\_268840\_length\_12752\_cov\_27.436010 6953-6969. Max. coverage (+): 0. Max coverage (-): 0

Region: NODE\_268840\_length\_12752\_cov\_27.436010 6970-6987. Max. coverage (+): 0. Max coverage (-): 0

Region: NODE\_268840\_length\_12752\_cov\_27.436010 6988-7005. Max. coverage (+): 0. Max coverage (-): 0

Region: NODE\_268840\_length\_12752\_cov\_27.436010 7006-7022. Max. coverage (+): 0.16. Max coverage (-): 0

Region: NODE\_268840\_length\_12752\_cov\_27.436010 7023-7040. Max. coverage (+): 0.25. Max coverage (-): 0.09

Region: NODE\_268840\_length\_12752\_cov\_27.436010 7041-7058. Max. coverage (+): 0. Max coverage (-): 0

Region: NODE\_268840\_length\_12752\_cov\_27.436010 7059-7075. Max. coverage (+): 0. Max coverage (-): 1.22

Region: NODE\_268840\_length\_12752\_cov\_27.436010 7076-7093. Max. coverage (+): 0. Max coverage (-): 0.12

Region: NODE\_268840\_length\_12752\_cov\_27.436010 7094-7110. Max. coverage (+): 0.09. Max coverage (-): 0.75

Region: NODE\_268840\_length\_12752\_cov\_27.436010 7111-7128. Max. coverage (+): 0. Max coverage (-): 0

Region: NODE\_268840\_length\_12752\_cov\_27.436010 7129-7146. Max. coverage (+): 0. Max coverage (-): 0.66

Region: NODE\_268840\_length\_12752\_cov\_27.436010 7147-7163. Max. coverage (+): 0.05. Max coverage (-): 0.09

Region: NODE\_268840\_length\_12752\_cov\_27.436010 7164-7181. Max. coverage (+): 0.09. Max coverage (-): 0.28

Region: NODE\_268840\_length\_12752\_cov\_27.436010 7182-7198. Max. coverage (+): 0. Max coverage (-): 0

Region: NODE\_268840\_length\_12752\_cov\_27.436010 7199-7216. Max. coverage (+): 0. Max coverage (-): 0

Region: NODE\_268840\_length\_12752\_cov\_27.436010 7217-7234. Max. coverage (+): 0. Max coverage (-): 0.66

Region: NODE\_268840\_length\_12752\_cov\_27.436010 7235-7251. Max. coverage (+): 0. Max coverage (-): 0.66

Region: NODE\_268840\_length\_12752\_cov\_27.436010 7252-7269. Max. coverage (+): 0. Max coverage (-): 0

Region: NODE\_268840\_length\_12752\_cov\_27.436010 7270-7287. Max. coverage (+): 0. Max coverage (-): 0.05

Region: NODE\_268840\_length\_12752\_cov\_27.436010 7288-7304. Max. coverage (+): 0. Max coverage (-): 0

Region: NODE\_268840\_length\_12752\_cov\_27.436010 7305-7322. Max. coverage (+): 0. Max coverage (-): 0.09

Region: NODE\_268840\_length\_12752\_cov\_27.436010 7323-7339. Max. coverage (+): 0. Max coverage (-): 0

Region: NODE\_268840\_length\_12752\_cov\_27.436010 7340-7357. Max. coverage (+): 0. Max coverage (-): 0

Region: NODE\_268840\_length\_12752\_cov\_27.436010 7358-7375. Max. coverage (+): 0. Max coverage (-): 0

Region: NODE\_268840\_length\_12752\_cov\_27.436010 7376-7392. Max. coverage (+): 0. Max coverage (-): 0

Region: NODE\_268840\_length\_12752\_cov\_27.436010 7393-7410. Max. coverage (+): 0. Max coverage (-): 0.02

Region: NODE\_268840\_length\_12752\_cov\_27.436010 7411-7427. Max. coverage (+): 0. Max coverage (-): 0

Region: NODE\_268840\_length\_12752\_cov\_27.436010 7428-7445. Max. coverage (+): 0. Max coverage (-): 0

Region: NODE\_268840\_length\_12752\_cov\_27.436010 7446-7463. Max. coverage (+): 0. Max coverage (-): 0

Region: NODE\_268840\_length\_12752\_cov\_27.436010 7464-7480. Max. coverage (+): 0. Max coverage (-): 0

Region: NODE\_268840\_length\_12752\_cov\_27.436010 7481-7498. Max. coverage (+): 0. Max coverage (-): 0.19

Region: NODE\_268840\_length\_12752\_cov\_27.436010 7499-7516. Max. coverage (+): 0. Max coverage (-): 0.09

Region: NODE\_268840\_length\_12752\_cov\_27.436010 7517-7533. Max. coverage (+): 0. Max coverage (-): 0

Region: NODE\_268840\_length\_12752\_cov\_27.436010 7534-7551. Max. coverage (+): 0.19. Max coverage (-): 0.85

Region: NODE\_268840\_length\_12752\_cov\_27.436010 7552-7568. Max. coverage (+): 0. Max coverage (-): 0.09

Region: NODE\_268840\_length\_12752\_cov\_27.436010 7569-7586. Max. coverage (+): 0. Max coverage (-): 0.09

Region: NODE\_268840\_length\_12752\_cov\_27.436010 7587-7604. Max. coverage (+): 0. Max coverage (-): 0

Region: NODE\_268840\_length\_12752\_cov\_27.436010 7605-7621. Max. coverage (+): 0. Max coverage (-): 0

Region: NODE\_268840\_length\_12752\_cov\_27.436010 7622-7639. Max. coverage (+): 0. Max coverage (-): 0

Region: NODE\_268840\_length\_12752\_cov\_27.436010 7640-7657. Max. coverage (+): 0. Max coverage (-): 0.09

Region: NODE\_268840\_length\_12752\_cov\_27.436010 7658-7674. Max. coverage (+): 0. Max coverage (-): 0

Region: NODE\_268840\_length\_12752\_cov\_27.436010 7675-7692. Max. coverage (+): 0. Max coverage (-): 0.19

Region: NODE\_268840\_length\_12752\_cov\_27.436010 7693-7709. Max. coverage (+): 0. Max coverage (-): 0

Region: NODE\_268840\_length\_12752\_cov\_27.436010 7710-7727. Max. coverage (+): 0. Max coverage (-): 0

Region: NODE\_268840\_length\_12752\_cov\_27.436010 7728-7745. Max. coverage (+): 0. Max coverage (-): 0

Region: NODE\_268840\_length\_12752\_cov\_27.436010 7746-7762. Max. coverage (+): 0. Max coverage (-): 0.09

Region: NODE\_268840\_length\_12752\_cov\_27.436010 7763-7780. Max. coverage (+): 0. Max coverage (-): 0.19

Region: NODE\_268840\_length\_12752\_cov\_27.436010 7781-7797. Max. coverage (+): 0. Max coverage (-): 0.09

Region: NODE\_268840\_length\_12752\_cov\_27.436010 7798-7815. Max. coverage (+): 0. Max coverage (-): 0.56

Region: NODE\_268840\_length\_12752\_cov\_27.436010 7816-7833. Max. coverage (+): 0. Max coverage (-): 0.85

Region: NODE\_268840\_length\_12752\_cov\_27.436010 7834-7850. Max. coverage (+): 0. Max coverage (-): 0.28

Region: NODE\_268840\_length\_12752\_cov\_27.436010 7851-7868. Max. coverage (+): 0. Max coverage (-): 0.94

Region: NODE\_268840\_length\_12752\_cov\_27.436010 7869-7886. Max. coverage (+): 0. Max coverage (-): 0.09

Region: NODE\_268840\_length\_12752\_cov\_27.436010 7887-7903. Max. coverage (+): 0. Max coverage (-): 0

Region: NODE\_268840\_length\_12752\_cov\_27.436010 7904-7921. Max. coverage (+): 0. Max coverage (-): 0

Region: NODE\_268840\_length\_12752\_cov\_27.436010 7922-7938. Max. coverage (+): 0. Max coverage (-): 0

Region: NODE\_268840\_length\_12752\_cov\_27.436010 7939-7956. Max. coverage (+): 0. Max coverage (-): 0

Region: NODE\_268840\_length\_12752\_cov\_27.436010 7957-7974. Max. coverage (+): 0. Max coverage (-): 0

Region: NODE\_268840\_length\_12752\_cov\_27.436010 7975-7991. Max. coverage (+): 0. Max coverage (-): 0

Region: NODE\_268840\_length\_12752\_cov\_27.436010 7992-8009. Max. coverage (+): 0. Max coverage (-): 0

Region: NODE\_268840\_length\_12752\_cov\_27.436010 8010-8026. Max. coverage (+): 0. Max coverage (-): 0.09

Region: NODE\_268840\_length\_12752\_cov\_27.436010 8027-8044. Max. coverage (+): 0. Max coverage (-): 0.09

Region: NODE\_268840\_length\_12752\_cov\_27.436010 8045-8062. Max. coverage (+): 0. Max coverage (-): 0

Region: NODE\_268840\_length\_12752\_cov\_27.436010 8063-8079. Max. coverage (+): 0. Max coverage (-): 0.09

Region: NODE\_268840\_length\_12752\_cov\_27.436010 8080-8097. Max. coverage (+): 0. Max coverage (-): 0.09

Region: NODE\_268840\_length\_12752\_cov\_27.436010 8098-8115. Max. coverage (+): 0. Max coverage (-): 0

Region: NODE\_268840\_length\_12752\_cov\_27.436010 8116-8132. Max. coverage (+): 0. Max coverage (-): 0

Region: NODE\_268840\_length\_12752\_cov\_27.436010 8133-8150. Max. coverage (+): 0.09. Max coverage (-): 1.22

Region: NODE\_268840\_length\_12752\_cov\_27.436010 8151-8167. Max. coverage (+): 0. Max coverage (-): 1.03

Region: NODE\_268840\_length\_12752\_cov\_27.436010 8168-8185. Max. coverage (+): 0. Max coverage (-): 0

Region: NODE\_268840\_length\_12752\_cov\_27.436010 8186-8203. Max. coverage (+): 0. Max coverage (-): 0

Region: NODE\_268840\_length\_12752\_cov\_27.436010 8204-8220. Max. coverage (+): 0. Max coverage (-): 0.09

Region: NODE\_268840\_length\_12752\_cov\_27.436010 8221-8238. Max. coverage (+): 0. Max coverage (-): 0.09

Region: NODE\_268840\_length\_12752\_cov\_27.436010 8239-8256. Max. coverage (+): 0. Max coverage (-): 0.19

Region: NODE\_268840\_length\_12752\_cov\_27.436010 8257-8273. Max. coverage (+): 0. Max coverage (-): 0

Region: NODE\_268840\_length\_12752\_cov\_27.436010 8274-8291. Max. coverage (+): 0. Max coverage (-): 0.09

Region: NODE\_268840\_length\_12752\_cov\_27.436010 8292-8308. Max. coverage (+): 0. Max coverage (-): 0.09

Region: NODE\_268840\_length\_12752\_cov\_27.436010 8309-8326. Max. coverage (+): 0. Max coverage (-): 0.38

Region: NODE\_268840\_length\_12752\_cov\_27.436010 8327-8344. Max. coverage (+): 0. Max coverage (-): 0.28

Region: NODE\_268840\_length\_12752\_cov\_27.436010 8345-8361. Max. coverage (+): 0. Max coverage (-): 0.19

Region: NODE\_268840\_length\_12752\_cov\_27.436010 8362-8379. Max. coverage (+): 0. Max coverage (-): 0.19

Region: NODE\_268840\_length\_12752\_cov\_27.436010 8380-8396. Max. coverage (+): 0. Max coverage (-): 0.09

Region: NODE\_268840\_length\_12752\_cov\_27.436010 8397-8414. Max. coverage (+): 0. Max coverage (-): 0

Region: NODE\_268840\_length\_12752\_cov\_27.436010 8415-8432. Max. coverage (+): 0. Max coverage (-): 0.09

Region: NODE\_268840\_length\_12752\_cov\_27.436010 8433-8449. Max. coverage (+): 0. Max coverage (-): 0

Region: NODE\_268840\_length\_12752\_cov\_27.436010 8450-8467. Max. coverage (+): 0. Max coverage (-): 0.09

Region: NODE\_268840\_length\_12752\_cov\_27.436010 8468-8485. Max. coverage (+): 0. Max coverage (-): 0.09

Region: NODE\_268840\_length\_12752\_cov\_27.436010 8486-8502. Max. coverage (+): 0. Max coverage (-): 0.28

Region: NODE\_268840\_length\_12752\_cov\_27.436010 8503-8520. Max. coverage (+): 0. Max coverage (-): 0

Region: NODE\_268840\_length\_12752\_cov\_27.436010 8521-8537. Max. coverage (+): 0. Max coverage (-): 0

Region: NODE\_268840\_length\_12752\_cov\_27.436010 8538-8555. Max. coverage (+): 0. Max coverage (-): 0

Region: NODE\_268840\_length\_12752\_cov\_27.436010 8556-8573. Max. coverage (+): 0. Max coverage (-): 0

Region: NODE\_268840\_length\_12752\_cov\_27.436010 8574-8590. Max. coverage (+): 0. Max coverage (-): 0

Region: NODE\_268840\_length\_12752\_cov\_27.436010 8591-8608. Max. coverage (+): 0. Max coverage (-): 0.09

Region: NODE\_268840\_length\_12752\_cov\_27.436010 8609-8626. Max. coverage (+): 0. Max coverage (-): 0.09

Region: NODE\_268840\_length\_12752\_cov\_27.436010 8627-8643. Max. coverage (+): 0. Max coverage (-): 0

Region: NODE\_268840\_length\_12752\_cov\_27.436010 8644-8661. Max. coverage (+): 0. Max coverage (-): 0.38

Region: NODE\_268840\_length\_12752\_cov\_27.436010 8662-8678. Max. coverage (+): 0. Max coverage (-): 0.28

Region: NODE\_268840\_length\_12752\_cov\_27.436010 8679-8696. Max. coverage (+): 0. Max coverage (-): 0.09

Region: NODE\_268840\_length\_12752\_cov\_27.436010 8697-8714. Max. coverage (+): 0. Max coverage (-): 1.79

Region: NODE\_268840\_length\_12752\_cov\_27.436010 8715-8731. Max. coverage (+): 0. Max coverage (-): 0

Region: NODE\_268840\_length\_12752\_cov\_27.436010 8732-8749. Max. coverage (+): 0. Max coverage (-): 0.56

Region: NODE\_268840\_length\_12752\_cov\_27.436010 8750-8766. Max. coverage (+): 0.09. Max coverage (-): 0.28

Region: NODE\_268840\_length\_12752\_cov\_27.436010 8767-8784. Max. coverage (+): 0. Max coverage (-): 0.19

Region: NODE\_268840\_length\_12752\_cov\_27.436010 8785-8802. Max. coverage (+): 0. Max coverage (-): 0.75

Region: NODE\_268840\_length\_12752\_cov\_27.436010 8803-. Max. coverage (+): 0. Max coverage (-): 0

RepeatMasker Color Code

**+**

100-98% Identity

<98-95% Identity

<95-90% Identity

<90-85% Identity

<85-80% Identity

<80-75% Identity

<75-70% Identity

<70% Identity

**-**

Gene Set Color Code

**+**

Gene

Pseudogene

Other

**-**

Topology/Coverage Color Code

Coverage Plus Strand

Coverage Minus Strand

Mainstrand: Plus

Mainstrand: Minus

Complementary Strand

Flanking Region  
(if option -flank >0)

Gene Set Annotation  
  
RepeatMasker Annotation  

**1. AlRepD-8475**: 8-92 (+), Divergence to consensus: 22.4%  
**2. (AGCT)n**: 806-829 (+), Divergence to consensus: 8.8%  
**3. TE-X-5\_DR**: 1342-1422 (+), Divergence to consensus: 28.4%  
**4. TE-X-4\_DR**: 1388-1530 (-), Divergence to consensus: 36.3%  
**5. (TGTCTG)n**: 4313-4350 (+), Divergence to consensus: 19.6%  
**6. (AC)n**: 4611-4630 (+), Divergence to consensus: 0%  
**7. AlRepB-392**: 5557-5737 (+), Divergence to consensus: 12.2%  
**8. AlRepD-1024**: 5786-5892 (+), Divergence to consensus: 10.3%  
**9. AlRepC-1280**: 5891-5963 (+), Divergence to consensus: 5.5%  
**10. SINE2-1\_AFC**: 6272-6337 (-), Divergence to consensus: 17.9%  
**11. Harbinger-2N1\_DR**: 6338-6482 (+), Divergence to consensus: 16.2%  
**12. AlRepD-1024**: 6496-6891 (+), Divergence to consensus: 14.8%  
**13. AlRepB-392**: 6892-6924 (+), Divergence to consensus: 12.1%  
**14. AlRepB-392**: 7067-7441 (+), Divergence to consensus: 17.1%  
**15. AlRepC-1433**: 7444-7893 (+), Divergence to consensus: 24.8%  
**16. AlRepC-1574**: 8262-8401 (+), Divergence to consensus: 33.2%  
**17. AlRepC-1574**: 8507-8664 (+), Divergence to consensus: 21.8%  
**18. AlRepD-209**: 8777-8839 (-), Divergence to consensus: 15.8%

  
Transcription Factor Binding Sites  

**RHOXF1** (Sequence: GGATCA (-): 667)  
**RHOXF1** (Sequence: AGCTTA (-): 1165)  
**RHOXF1** (Sequence: AGATCA (-): 1632)  
**RHOXF1** (Sequence: GGATTA (-): 2360)  
**RHOXF1** (Sequence: AGATTA (-): 2383)  
**RHOXF1** (Sequence: AGATTA (-): 2467)  
**RHOXF1** (Sequence: GGCTCA (-): 2604)  
**RHOXF1** (Sequence: AGATCA (-): 2987)  
**RHOXF1** (Sequence: GGATCA (-): 3415)  
**RHOXF1** (Sequence: AGCTCA (-): 3849)  
**RHOXF1** (Sequence: AGCTTA (-): 4442)  
**RHOXF1** (Sequence: GGATCA (-): 4515)  
**RHOXF1** (Sequence: AGATCA (-): 4578)  
**RHOXF1** (Sequence: AGCTCA (-): 5042)  
**RHOXF1** (Sequence: GGCTTA (-): 5058)  
**RHOXF1** (Sequence: AGATTA (-): 5348)  
**RHOXF1** (Sequence: AGATTA (-): 5672)  
**RHOXF1** (Sequence: AGATCA (-): 5675)  
**RHOXF1** (Sequence: AGCTTA (-): 5853)  
**RHOXF1** (Sequence: AGCTCA (-): 6204)  
**RHOXF1** (Sequence: AGCTTA (-): 6562)  
**RHOXF1** (Sequence: AGCTTA (-): 7098)  
**RHOXF1** (Sequence: GGATCA (-): 7549)  
**RHOXF1** (Sequence: AGATCA (-): 8058)  
**RHOXF1** (Sequence: TAAGCT (+): 65)  
**RHOXF1** (Sequence: TAAGCT (+): 187)  
**RHOXF1** (Sequence: TAATCT (+): 2836)  
**RHOXF1** (Sequence: TGAGCT (+): 3819)  
**RHOXF1** (Sequence: TGAGCT (+): 4393)  
**RHOXF1** (Sequence: TGAGCT (+): 4440)  
**RHOXF1** (Sequence: TGATCT (+): 5681)  
**RHOXF1** (Sequence: TAATCC (+): 6214)  
**RHOXF1** (Sequence: TGAGCT (+): 7892)  
**RHOXF1** (Sequence: TAATCC (+): 8788)  
**Lhx8** (Sequence: CTAATTAG (-): 91)  
**Gata4** (Sequence: CTTATCT (+): 1167)  
**Gata4** (Sequence: CTTATCT (+): 3185)  
**Gata4** (Sequence: CTTATCT (+): 3994)  
**POU5F1** (Sequence: TTTGCAT (-): 865)  
**POU5F1** (Sequence: TTTGCAT (-): 7013)  
**POU5F1** (Sequence: TTTGCAT (-): 7214)  
**POU5F1** (Sequence: TTTGCAT (-): 8076)  
**RFX4\_2** (Sequence: GTATCCATG (-): 4038)  
**RFX4\_2** (Sequence: GTATCCAGG (-): 8310)  
**RFX4\_1** (Sequence: GTTGCTAGG (-): 2426)  
**SOX9** (Sequence: AACAATAG (-): 5065)  
**FOXO1** (Sequence: GTTGTTTAT (+): 3663)  
**FOXO3\_mmu** (Sequence: TGTTTTCC (-): 268)  
**FOXO3\_mmu** (Sequence: TGTTTAGC (-): 276)  
**FOXO3\_mmu** (Sequence: TGTTTTCA (-): 5172)  
**FOXO3\_mmu** (Sequence: TGTTTTGC (-): 8073)  
**Sox5** (Sequence: ATTGTT (+): 111)  
**Sox5** (Sequence: ATTGTT (+): 904)  
**Sox5** (Sequence: ATTGTT (+): 1002)  
**Sox5** (Sequence: ATTGTT (+): 1859)  
**Sox5** (Sequence: ATTGTT (+): 1952)  
**Sox5** (Sequence: ATTGTT (+): 2210)  
**Sox5** (Sequence: ATTGTT (+): 5858)  
**Sox5** (Sequence: ATTGTT (+): 5917)  
**Sox5** (Sequence: ATTGTT (+): 6316)  
**Sox5** (Sequence: ATTGTT (+): 6567)  
**Sox5** (Sequence: ATTGTT (+): 8747)  
**SOX9** (Sequence: TTATTGTT (+): 5856)  
**SOX9** (Sequence: TTATTGTT (+): 6565)  
**FOXO3\_mmu** (Sequence: GCAAAACA (+): 8028)  
**FOXO3\_mmu** (Sequence: GGAAAACA (+): 8116)  
**FOXO3\_mmu** (Sequence: GGAAAACA (+): 8294)  
**FOXO3\_mmu** (Sequence: TCTAAACA (+): 8443)  
**Nobox** (Sequence: ACTAATTA (-): 90)  
**Nobox** (Sequence: ACCAATTA (-): 8727)  
**FOXO1** (Sequence: GAAAACAAC (-): 8295)  
**Nobox** (Sequence: TAATTGCT (+): 8549)  
**Rhox11** (Sequence: TGGTGTATT (+): 1106)  
**Gata4** (Sequence: AGATAAG (-): 6673)  
**Sox5** (Sequence: AACAAT (-): 770)  
**Sox5** (Sequence: AACAAT (-): 5065)  
**POU2F1** (Sequence: TATTTTAAT (+): 894)  
**POU5F1** (Sequence: ATGCAAA (+): 2540)  
**Mybl1\_1** (Sequence: AACCGTTA (+): 5507)
