# Supplementary material for: First characterization of PIWI-interacting RNA clusters in a cichlid fish with a B chromosome
Source: BMC Biol. 2022 Sep 21;20:204. doi: 10.1186/s12915-022-01403-2 (PMC9490952; doi:10.1186/s12915-022-01403-2)
Supplement: Supplementary file 1 — Additional file 1. Zipped folder with fasta and interactive html piRNA cluster information for the A. latifasciata genome. The nomenclature is as follows: number-pirna-cluster_sex_B-presence (f, female; m, male; 0b, without B chromosome; 1b, with B chromosome). [file 12915_2022_1403_MOESM1_ESM.zip › 102_m0b.html]

piRNA cluster 102\_m0b 51


Predicted piRNA cluster no. 102\_m0b
  

Show proTRAC run info
Hide proTRAC run info

/\  
                \_\_\_\_\_\_\_\_\_\_\_\_\_\_\_\_\_\_\_\_\_\_\_/\\_\_\_ /  \\_\_\_\_\_\_\_  
               I                      /  \  /    \      I  
               I     pro             /    \/      \     I  
               I        TRAC        /               \   I  
               I   \_\_\_\_\_\_\_\_\_\_\_\_\_\_\_\_/\_\_\_\_\_\_\_\_\_\_\_\_\_\_\_\_\_\\_ I  
               I   \              /                     I  
               I    \            /                      I  
               I     \  /\      /       V.2.4.2         I  
               I      \/  \    /                        I  
               I\_\_\_\_\_\_\_\_\_\_\_\  /\_\_\_\_\_\_\_\_\_\_\_\_\_\_\_\_\_\_\_\_\_\_\_\_\_I  
                            \/  
  
  
================================= proTRAC ====================================  
VERSION: .......... 2.4.2  
LAST MODIFIED: .... 11. May 2018  
  
Please cite:  
Rosenkranz D, Zischler H. proTRAC - a software for probabilistic piRNA cluster  
detection, visualization and analysis. 2012. BMC Bioinformatics 13:5.  
  
  
Contact:  
David Rosenkranz  
Institute of Organismic and Molecular Evolutionary Biology  
Dept. Anthropology, small RNA group  
Johannes Gutenberg University Mainz  
email: rosenkranz@uni-mainz.de  
  
You can find the latest proTRAC version at:  
http://sourceforge.net/projects/protrac/files  
http://www.smallRNAgroup-mainz.de/software  
==============================================================================  
  
PARAMETERS:  
Map file: ...............piwi-machos-0B.fa-collapse.map  
Genome file: ............../../../0B\_ala\_genome.fa  
RepeatMasker annotation: Alatifasciata-all0B-maryan-v2.fa\_corrected.out  
GeneSet:................./guest-storage/Data/annotation/Alatifasciata\_all0B\_maryan-v2\_out2017.gff  
  
Significant (p<=0.01) hit density will be calculated based  
on observed hit distribution.  
  
Sliding window size: ........................................ 5000 bp  
Sliding window increament: .................................. 1000 bp  
Normalize each hit by number of genomic hits: ............... yes  
Normalize each hit by number of sequence reads: ............. yes  
Normalize values (-> per million mapped reads): ............. yes  
Min. fraction of hits with 1T(U) or 10A: .................... 0.75  
Alternatively: Min. fraction of hits with 1T(U) and 10A: .... 0.5  
Min. fraction of hits with typical piRNA length: ............ 0.75  
Typical piRNA length: ....................................... 24-32 nt  
Min. size of a piRNA cluster: ............................... 1000 bp.  
Min. number of hits (absolute): ............................. 0  
Min. number of hits (normalized): ........................... 0  
Min. fraction of hits on the mainstrand: .................... 0.75  
Top fraction of mapped sequences (in terms of read counts): . 1%  
Top fraction accounts for max. n% of sequence reads: ........ 90%  
Min. fraction of hits on each arm of a bidirectional cluster: 0.05  
Output html file for each cluster: .......................... yes  
Output a summary table: ..................................... yes  
Output a FASTA file for each cluster (piRNA sequences): ..... yes  
Output a FASTA file comprising cluster sequences: ........... yes  
Output a GTF file for predicted piRNA clusters: ..............yes  
Search DNA motifs in clusters: .............................. yes  
Output flanking sequences: +/- .............................. 0 bp  
Output ~.pTi file: .......................................... no  
==============================================================================  
  
  
Genome size (without gaps): ............ 758543724 bp  
Gaps (N/X/-): .......................... 417479 bp  
Mapped reads: .......................... 24765598  
Non-identical sequences: ............... 6158275  
Genomic hits: .......................... 53103584  
Significant densitiy of mapped reads: .. 763.098963422187 reads/kb

Show proTRAC cluster info
Hide proTRAC cluster info

|  |  |
| --- | --- |
| Location | NODE\_268840\_length\_12752\_cov\_27.436010 |
| Coordinates | 1-12873 |
| Size [bp] | 12873 |
| Sequence hit loci | 9929 |
| Mapped reads (normalized) | 32332 |
| Mapped reads (normalized) per kb | 2511.6 |
| Normalized reads with 1T (1U) | 76.3% |
| Normalized reads with 10A | 29.5% |
| Normalized reads with length 24-32 nt | 99% |
| Normalized reads on the main strand(s) | 88.7% |
| Predicted directionality | mono:minus |

100%

0%

1T (1U)  
reads

10A reads

24-32 nt  
reads

reads on mainstrand

**Either the amount of reads with 1T (1U) OR 10A has to exceed 75% (set with option: -1Tor10A)  
Alternatively the amount of reads with 1T (1U) AND 10A has to exceed 50% (set with option: -1Tand10A)  
Minimum amount of reads with preferred size is 75% (set with option: -pisize)  
Minimum amount of reads on the main strand(s) is 75% (set with option: -clstrand)**

Show read coverage
Hide read coverage

WHAT DO I SEE HERE?  
This chart shows the location of mapped sequence reads within a predicted piRNA cluster. The color refers to the number of genomic hits produced by the sequence read in question. A dark red bar indicates that this sequence read produces many other hits elsewhere in the genome. Many adjacent red or yellow bars can indicate the presence of a multi-copy element such as transposons or rRNA genes. A dark green bar indicates that this sequence read maps uniquely to this locus.

1 hit

2-5 hits

6-10 hits

11-20 hits

21-50 hits

51-100 hits

> 100 hits

NODE\_268840\_length\_12752\_cov\_27.436010

1

12873

Gene Set

RepeatMasker

Mapped  
Reads

123.98

plus strand

minus strand

123.98

Region: NODE\_268840\_length\_12752\_cov\_27.436010 10222-13. Max. coverage (+): 0. Max coverage (-): 0.04

Region: NODE\_268840\_length\_12752\_cov\_27.436010 14-39. Max. coverage (+): 0.02. Max coverage (-): 0.04

Region: NODE\_268840\_length\_12752\_cov\_27.436010 40-65. Max. coverage (+): 0.12. Max coverage (-): 0.44

Region: NODE\_268840\_length\_12752\_cov\_27.436010 66-91. Max. coverage (+): 1.23. Max coverage (-): 0.05

Region: NODE\_268840\_length\_12752\_cov\_27.436010 92-116. Max. coverage (+): 0.04. Max coverage (-): 0.12

Region: NODE\_268840\_length\_12752\_cov\_27.436010 117-142. Max. coverage (+): 0.2. Max coverage (-): 0.16

Region: NODE\_268840\_length\_12752\_cov\_27.436010 143-168. Max. coverage (+): 0. Max coverage (-): 0.32

Region: NODE\_268840\_length\_12752\_cov\_27.436010 169-194. Max. coverage (+): 0.04. Max coverage (-): 0.32

Region: NODE\_268840\_length\_12752\_cov\_27.436010 195-219. Max. coverage (+): 0.08. Max coverage (-): 0.16

Region: NODE\_268840\_length\_12752\_cov\_27.436010 220-245. Max. coverage (+): 0.04. Max coverage (-): 0.65

Region: NODE\_268840\_length\_12752\_cov\_27.436010 246-271. Max. coverage (+): 0.08. Max coverage (-): 0.32

Region: NODE\_268840\_length\_12752\_cov\_27.436010 272-297. Max. coverage (+): 0.04. Max coverage (-): 0.32

Region: NODE\_268840\_length\_12752\_cov\_27.436010 298-322. Max. coverage (+): 0.04. Max coverage (-): 1.25

Region: NODE\_268840\_length\_12752\_cov\_27.436010 323-348. Max. coverage (+): 0.12. Max coverage (-): 1.33

Region: NODE\_268840\_length\_12752\_cov\_27.436010 349-374. Max. coverage (+): 0. Max coverage (-): 0.32

Region: NODE\_268840\_length\_12752\_cov\_27.436010 375-400. Max. coverage (+): 0. Max coverage (-): 0.04

Region: NODE\_268840\_length\_12752\_cov\_27.436010 401-425. Max. coverage (+): 0.65. Max coverage (-): 3.43

Region: NODE\_268840\_length\_12752\_cov\_27.436010 426-451. Max. coverage (+): 0.04. Max coverage (-): 0.4

Region: NODE\_268840\_length\_12752\_cov\_27.436010 452-477. Max. coverage (+): 0. Max coverage (-): 0.08

Region: NODE\_268840\_length\_12752\_cov\_27.436010 478-503. Max. coverage (+): 0.12. Max coverage (-): 0.32

Region: NODE\_268840\_length\_12752\_cov\_27.436010 504-528. Max. coverage (+): 0. Max coverage (-): 0.24

Region: NODE\_268840\_length\_12752\_cov\_27.436010 529-554. Max. coverage (+): 0.04. Max coverage (-): 2.18

Region: NODE\_268840\_length\_12752\_cov\_27.436010 555-580. Max. coverage (+): 1.62. Max coverage (-): 1.13

Region: NODE\_268840\_length\_12752\_cov\_27.436010 581-606. Max. coverage (+): 0.89. Max coverage (-): 0.2

Region: NODE\_268840\_length\_12752\_cov\_27.436010 607-631. Max. coverage (+): 0.97. Max coverage (-): 1.13

Region: NODE\_268840\_length\_12752\_cov\_27.436010 632-657. Max. coverage (+): 0.93. Max coverage (-): 4.24

Region: NODE\_268840\_length\_12752\_cov\_27.436010 658-683. Max. coverage (+): 1.62. Max coverage (-): 1.53

Region: NODE\_268840\_length\_12752\_cov\_27.436010 684-709. Max. coverage (+): 0. Max coverage (-): 0.04

Region: NODE\_268840\_length\_12752\_cov\_27.436010 710-734. Max. coverage (+): 0.24. Max coverage (-): 4.48

Region: NODE\_268840\_length\_12752\_cov\_27.436010 735-760. Max. coverage (+): 0. Max coverage (-): 0.16

Region: NODE\_268840\_length\_12752\_cov\_27.436010 761-786. Max. coverage (+): 0. Max coverage (-): 0.24

Region: NODE\_268840\_length\_12752\_cov\_27.436010 787-811. Max. coverage (+): 0.04. Max coverage (-): 0.32

Region: NODE\_268840\_length\_12752\_cov\_27.436010 812-837. Max. coverage (+): 0.04. Max coverage (-): 3.07

Region: NODE\_268840\_length\_12752\_cov\_27.436010 838-863. Max. coverage (+): 0.04. Max coverage (-): 0.08

Region: NODE\_268840\_length\_12752\_cov\_27.436010 864-889. Max. coverage (+): 0.04. Max coverage (-): 0.52

Region: NODE\_268840\_length\_12752\_cov\_27.436010 890-914. Max. coverage (+): 0. Max coverage (-): 0.04

Region: NODE\_268840\_length\_12752\_cov\_27.436010 915-940. Max. coverage (+): 0.08. Max coverage (-): 0.08

Region: NODE\_268840\_length\_12752\_cov\_27.436010 941-966. Max. coverage (+): 0.12. Max coverage (-): 0.57

Region: NODE\_268840\_length\_12752\_cov\_27.436010 967-992. Max. coverage (+): 0.12. Max coverage (-): 19.91

Region: NODE\_268840\_length\_12752\_cov\_27.436010 993-1017. Max. coverage (+): 0.08. Max coverage (-): 19.06

Region: NODE\_268840\_length\_12752\_cov\_27.436010 1018-1043. Max. coverage (+): 0.08. Max coverage (-): 4.64

Region: NODE\_268840\_length\_12752\_cov\_27.436010 1044-1069. Max. coverage (+): 0.08. Max coverage (-): 0.16

Region: NODE\_268840\_length\_12752\_cov\_27.436010 1070-1095. Max. coverage (+): 0.52. Max coverage (-): 0.57

Region: NODE\_268840\_length\_12752\_cov\_27.436010 1096-1120. Max. coverage (+): 2.22. Max coverage (-): 2.5

Region: NODE\_268840\_length\_12752\_cov\_27.436010 1121-1146. Max. coverage (+): 2.22. Max coverage (-): 4.32

Region: NODE\_268840\_length\_12752\_cov\_27.436010 1147-1172. Max. coverage (+): 0.28. Max coverage (-): 0.52

Region: NODE\_268840\_length\_12752\_cov\_27.436010 1173-1198. Max. coverage (+): 0.32. Max coverage (-): 2.83

Region: NODE\_268840\_length\_12752\_cov\_27.436010 1199-1223. Max. coverage (+): 0.08. Max coverage (-): 0.12

Region: NODE\_268840\_length\_12752\_cov\_27.436010 1224-1249. Max. coverage (+): 0.2. Max coverage (-): 0.61

Region: NODE\_268840\_length\_12752\_cov\_27.436010 1250-1275. Max. coverage (+): 0.04. Max coverage (-): 1.66

Region: NODE\_268840\_length\_12752\_cov\_27.436010 1276-1301. Max. coverage (+): 0.08. Max coverage (-): 0.2

Region: NODE\_268840\_length\_12752\_cov\_27.436010 1302-1326. Max. coverage (+): 0.08. Max coverage (-): 2.06

Region: NODE\_268840\_length\_12752\_cov\_27.436010 1327-1352. Max. coverage (+): 0.08. Max coverage (-): 0.28

Region: NODE\_268840\_length\_12752\_cov\_27.436010 1353-1378. Max. coverage (+): 1.25. Max coverage (-): 3.63

Region: NODE\_268840\_length\_12752\_cov\_27.436010 1379-1404. Max. coverage (+): 0.69. Max coverage (-): 2.75

Region: NODE\_268840\_length\_12752\_cov\_27.436010 1405-1429. Max. coverage (+): 1.09. Max coverage (-): 6.62

Region: NODE\_268840\_length\_12752\_cov\_27.436010 1430-1455. Max. coverage (+): 0.24. Max coverage (-): 1.33

Region: NODE\_268840\_length\_12752\_cov\_27.436010 1456-1481. Max. coverage (+): 0.24. Max coverage (-): 1.17

Region: NODE\_268840\_length\_12752\_cov\_27.436010 1482-1507. Max. coverage (+): 0.69. Max coverage (-): 1.49

Region: NODE\_268840\_length\_12752\_cov\_27.436010 1508-1532. Max. coverage (+): 0. Max coverage (-): 5.17

Region: NODE\_268840\_length\_12752\_cov\_27.436010 1533-1558. Max. coverage (+): 0.08. Max coverage (-): 4.48

Region: NODE\_268840\_length\_12752\_cov\_27.436010 1559-1584. Max. coverage (+): 0.16. Max coverage (-): 0.65

Region: NODE\_268840\_length\_12752\_cov\_27.436010 1585-1610. Max. coverage (+): 0.97. Max coverage (-): 0.16

Region: NODE\_268840\_length\_12752\_cov\_27.436010 1611-1635. Max. coverage (+): 0.04. Max coverage (-): 1.21

Region: NODE\_268840\_length\_12752\_cov\_27.436010 1636-1661. Max. coverage (+): 0.04. Max coverage (-): 2.75

Region: NODE\_268840\_length\_12752\_cov\_27.436010 1662-1687. Max. coverage (+): 0. Max coverage (-): 1.78

Region: NODE\_268840\_length\_12752\_cov\_27.436010 1688-1713. Max. coverage (+): 0.12. Max coverage (-): 0.73

Region: NODE\_268840\_length\_12752\_cov\_27.436010 1714-1738. Max. coverage (+): 0.04. Max coverage (-): 5.49

Region: NODE\_268840\_length\_12752\_cov\_27.436010 1739-1764. Max. coverage (+): 0.2. Max coverage (-): 3.67

Region: NODE\_268840\_length\_12752\_cov\_27.436010 1765-1790. Max. coverage (+): 0.04. Max coverage (-): 0.81

Region: NODE\_268840\_length\_12752\_cov\_27.436010 1791-1816. Max. coverage (+): 0.24. Max coverage (-): 4.16

Region: NODE\_268840\_length\_12752\_cov\_27.436010 1817-1841. Max. coverage (+): 0.32. Max coverage (-): 1.17

Region: NODE\_268840\_length\_12752\_cov\_27.436010 1842-1867. Max. coverage (+): 1.21. Max coverage (-): 1.74

Region: NODE\_268840\_length\_12752\_cov\_27.436010 1868-1893. Max. coverage (+): 1.33. Max coverage (-): 1.25

Region: NODE\_268840\_length\_12752\_cov\_27.436010 1894-1919. Max. coverage (+): 0.16. Max coverage (-): 0.32

Region: NODE\_268840\_length\_12752\_cov\_27.436010 1920-1944. Max. coverage (+): 0.01. Max coverage (-): 4.74

Region: NODE\_268840\_length\_12752\_cov\_27.436010 1945-1970. Max. coverage (+): 0.04. Max coverage (-): 0.4

Region: NODE\_268840\_length\_12752\_cov\_27.436010 1971-1996. Max. coverage (+): 0.2. Max coverage (-): 0.89

Region: NODE\_268840\_length\_12752\_cov\_27.436010 1997-2022. Max. coverage (+): 0.12. Max coverage (-): 1.9

Region: NODE\_268840\_length\_12752\_cov\_27.436010 2023-2047. Max. coverage (+): 0.08. Max coverage (-): 0.36

Region: NODE\_268840\_length\_12752\_cov\_27.436010 2048-2073. Max. coverage (+): 0.04. Max coverage (-): 0.36

Region: NODE\_268840\_length\_12752\_cov\_27.436010 2074-2099. Max. coverage (+): 0.08. Max coverage (-): 0.73

Region: NODE\_268840\_length\_12752\_cov\_27.436010 2100-2125. Max. coverage (+): 0.24. Max coverage (-): 2.46

Region: NODE\_268840\_length\_12752\_cov\_27.436010 2126-2150. Max. coverage (+): 0.93. Max coverage (-): 0.04

Region: NODE\_268840\_length\_12752\_cov\_27.436010 2151-2176. Max. coverage (+): 0. Max coverage (-): 0.93

Region: NODE\_268840\_length\_12752\_cov\_27.436010 2177-2202. Max. coverage (+): 0.08. Max coverage (-): 0.36

Region: NODE\_268840\_length\_12752\_cov\_27.436010 2203-2228. Max. coverage (+): 0. Max coverage (-): 0.73

Region: NODE\_268840\_length\_12752\_cov\_27.436010 2229-2253. Max. coverage (+): 0.04. Max coverage (-): 0.16

Region: NODE\_268840\_length\_12752\_cov\_27.436010 2254-2279. Max. coverage (+): 0.08. Max coverage (-): 2.34

Region: NODE\_268840\_length\_12752\_cov\_27.436010 2280-2305. Max. coverage (+): 0.08. Max coverage (-): 2.18

Region: NODE\_268840\_length\_12752\_cov\_27.436010 2306-2331. Max. coverage (+): 0.01. Max coverage (-): 0.23

Region: NODE\_268840\_length\_12752\_cov\_27.436010 2332-2356. Max. coverage (+): 0.03. Max coverage (-): 0.12

Region: NODE\_268840\_length\_12752\_cov\_27.436010 2357-2382. Max. coverage (+): 0.07. Max coverage (-): 0.51

Region: NODE\_268840\_length\_12752\_cov\_27.436010 2383-2408. Max. coverage (+): 0.07. Max coverage (-): 0.23

Region: NODE\_268840\_length\_12752\_cov\_27.436010 2409-2433. Max. coverage (+): 0.01. Max coverage (-): 0.19

Region: NODE\_268840\_length\_12752\_cov\_27.436010 2434-2459. Max. coverage (+): 0.05. Max coverage (-): 0.38

Region: NODE\_268840\_length\_12752\_cov\_27.436010 2460-2485. Max. coverage (+): 0.05. Max coverage (-): 0.09

Region: NODE\_268840\_length\_12752\_cov\_27.436010 2486-2511. Max. coverage (+): 0.01. Max coverage (-): 2.71

Region: NODE\_268840\_length\_12752\_cov\_27.436010 2512-2536. Max. coverage (+): 0.05. Max coverage (-): 0.16

Region: NODE\_268840\_length\_12752\_cov\_27.436010 2537-2562. Max. coverage (+): 0. Max coverage (-): 64.65

Region: NODE\_268840\_length\_12752\_cov\_27.436010 2563-2588. Max. coverage (+): 0.09. Max coverage (-): 2.18

Region: NODE\_268840\_length\_12752\_cov\_27.436010 2589-2614. Max. coverage (+): 0.28. Max coverage (-): 75.14

Region: NODE\_268840\_length\_12752\_cov\_27.436010 2615-2639. Max. coverage (+): 5.61. Max coverage (-): 2.42

Region: NODE\_268840\_length\_12752\_cov\_27.436010 2640-2665. Max. coverage (+): 0.57. Max coverage (-): 6.6

Region: NODE\_268840\_length\_12752\_cov\_27.436010 2666-2691. Max. coverage (+): 0.59. Max coverage (-): 1.94

Region: NODE\_268840\_length\_12752\_cov\_27.436010 2692-2717. Max. coverage (+): 0.5. Max coverage (-): 0.2

Region: NODE\_268840\_length\_12752\_cov\_27.436010 2718-2742. Max. coverage (+): 0.04. Max coverage (-): 0.28

Region: NODE\_268840\_length\_12752\_cov\_27.436010 2743-2768. Max. coverage (+): 0.02. Max coverage (-): 0.55

Region: NODE\_268840\_length\_12752\_cov\_27.436010 2769-2794. Max. coverage (+): 0.36. Max coverage (-): 1.86

Region: NODE\_268840\_length\_12752\_cov\_27.436010 2795-2820. Max. coverage (+): 0.16. Max coverage (-): 3.19

Region: NODE\_268840\_length\_12752\_cov\_27.436010 2821-2845. Max. coverage (+): 0.04. Max coverage (-): 2.02

Region: NODE\_268840\_length\_12752\_cov\_27.436010 2846-2871. Max. coverage (+): 0.4. Max coverage (-): 4.12

Region: NODE\_268840\_length\_12752\_cov\_27.436010 2872-2897. Max. coverage (+): 0.02. Max coverage (-): 5.09

Region: NODE\_268840\_length\_12752\_cov\_27.436010 2898-2923. Max. coverage (+): 0. Max coverage (-): 0.24

Region: NODE\_268840\_length\_12752\_cov\_27.436010 2924-2948. Max. coverage (+): 0.32. Max coverage (-): 0.12

Region: NODE\_268840\_length\_12752\_cov\_27.436010 2949-2974. Max. coverage (+): 0.48. Max coverage (-): 0.81

Region: NODE\_268840\_length\_12752\_cov\_27.436010 2975-3000. Max. coverage (+): 0.28. Max coverage (-): 1.49

Region: NODE\_268840\_length\_12752\_cov\_27.436010 3001-3026. Max. coverage (+): 0.16. Max coverage (-): 0.69

Region: NODE\_268840\_length\_12752\_cov\_27.436010 3027-3051. Max. coverage (+): 0.08. Max coverage (-): 3.92

Region: NODE\_268840\_length\_12752\_cov\_27.436010 3052-3077. Max. coverage (+): 0.06. Max coverage (-): 1.03

Region: NODE\_268840\_length\_12752\_cov\_27.436010 3078-3103. Max. coverage (+): 0.16. Max coverage (-): 0.25

Region: NODE\_268840\_length\_12752\_cov\_27.436010 3104-3129. Max. coverage (+): 0.09. Max coverage (-): 0.27

Region: NODE\_268840\_length\_12752\_cov\_27.436010 3130-3154. Max. coverage (+): 0.03. Max coverage (-): 0.16

Region: NODE\_268840\_length\_12752\_cov\_27.436010 3155-3180. Max. coverage (+): 0. Max coverage (-): 0.79

Region: NODE\_268840\_length\_12752\_cov\_27.436010 3181-3206. Max. coverage (+): 0. Max coverage (-): 0.24

Region: NODE\_268840\_length\_12752\_cov\_27.436010 3207-3232. Max. coverage (+): 0.02. Max coverage (-): 0.26

Region: NODE\_268840\_length\_12752\_cov\_27.436010 3233-3257. Max. coverage (+): 1.94. Max coverage (-): 15.77

Region: NODE\_268840\_length\_12752\_cov\_27.436010 3258-3283. Max. coverage (+): 1.98. Max coverage (-): 2.62

Region: NODE\_268840\_length\_12752\_cov\_27.436010 3284-3309. Max. coverage (+): 0.75. Max coverage (-): 2.75

Region: NODE\_268840\_length\_12752\_cov\_27.436010 3310-3335. Max. coverage (+): 1.39. Max coverage (-): 1.64

Region: NODE\_268840\_length\_12752\_cov\_27.436010 3336-3360. Max. coverage (+): 0.26. Max coverage (-): 0.7

Region: NODE\_268840\_length\_12752\_cov\_27.436010 3361-3386. Max. coverage (+): 0.06. Max coverage (-): 13.43

Region: NODE\_268840\_length\_12752\_cov\_27.436010 3387-3412. Max. coverage (+): 1.45. Max coverage (-): 4.64

Region: NODE\_268840\_length\_12752\_cov\_27.436010 3413-3438. Max. coverage (+): 0. Max coverage (-): 2.3

Region: NODE\_268840\_length\_12752\_cov\_27.436010 3439-3463. Max. coverage (+): 0.08. Max coverage (-): 4.12

Region: NODE\_268840\_length\_12752\_cov\_27.436010 3464-3489. Max. coverage (+): 0.02. Max coverage (-): 5.57

Region: NODE\_268840\_length\_12752\_cov\_27.436010 3490-3515. Max. coverage (+): 0. Max coverage (-): 0

Region: NODE\_268840\_length\_12752\_cov\_27.436010 3516-3541. Max. coverage (+): 0.04. Max coverage (-): 1.21

Region: NODE\_268840\_length\_12752\_cov\_27.436010 3542-3566. Max. coverage (+): 0.09. Max coverage (-): 0.36

Region: NODE\_268840\_length\_12752\_cov\_27.436010 3567-3592. Max. coverage (+): 0.04. Max coverage (-): 1.19

Region: NODE\_268840\_length\_12752\_cov\_27.436010 3593-3618. Max. coverage (+): 0.1. Max coverage (-): 1.17

Region: NODE\_268840\_length\_12752\_cov\_27.436010 3619-3644. Max. coverage (+): 0.08. Max coverage (-): 1.03

Region: NODE\_268840\_length\_12752\_cov\_27.436010 3645-3669. Max. coverage (+): 0. Max coverage (-): 0.91

Region: NODE\_268840\_length\_12752\_cov\_27.436010 3670-3695. Max. coverage (+): 0.01. Max coverage (-): 0.15

Region: NODE\_268840\_length\_12752\_cov\_27.436010 3696-3721. Max. coverage (+): 0. Max coverage (-): 0.05

Region: NODE\_268840\_length\_12752\_cov\_27.436010 3722-3747. Max. coverage (+): 0.02. Max coverage (-): 1.87

Region: NODE\_268840\_length\_12752\_cov\_27.436010 3748-3772. Max. coverage (+): 0.02. Max coverage (-): 0.36

Region: NODE\_268840\_length\_12752\_cov\_27.436010 3773-3798. Max. coverage (+): 0.16. Max coverage (-): 0.12

Region: NODE\_268840\_length\_12752\_cov\_27.436010 3799-3824. Max. coverage (+): 0.03. Max coverage (-): 0.82

Region: NODE\_268840\_length\_12752\_cov\_27.436010 3825-3850. Max. coverage (+): 0.84. Max coverage (-): 0.2

Region: NODE\_268840\_length\_12752\_cov\_27.436010 3851-3875. Max. coverage (+): 0.15. Max coverage (-): 6.14

Region: NODE\_268840\_length\_12752\_cov\_27.436010 3876-3901. Max. coverage (+): 0.04. Max coverage (-): 2.42

Region: NODE\_268840\_length\_12752\_cov\_27.436010 3902-3927. Max. coverage (+): 0.1. Max coverage (-): 0.67

Region: NODE\_268840\_length\_12752\_cov\_27.436010 3928-3953. Max. coverage (+): 0.04. Max coverage (-): 4.12

Region: NODE\_268840\_length\_12752\_cov\_27.436010 3954-3978. Max. coverage (+): 0.06. Max coverage (-): 1.05

Region: NODE\_268840\_length\_12752\_cov\_27.436010 3979-4004. Max. coverage (+): 0.08. Max coverage (-): 0.86

Region: NODE\_268840\_length\_12752\_cov\_27.436010 4005-4030. Max. coverage (+): 0.03. Max coverage (-): 0.52

Region: NODE\_268840\_length\_12752\_cov\_27.436010 4031-4055. Max. coverage (+): 0.05. Max coverage (-): 0.47

Region: NODE\_268840\_length\_12752\_cov\_27.436010 4056-4081. Max. coverage (+): 0.04. Max coverage (-): 0.4

Region: NODE\_268840\_length\_12752\_cov\_27.436010 4082-4107. Max. coverage (+): 0.13. Max coverage (-): 0.92

Region: NODE\_268840\_length\_12752\_cov\_27.436010 4108-4133. Max. coverage (+): 0.4. Max coverage (-): 3.57

Region: NODE\_268840\_length\_12752\_cov\_27.436010 4134-4158. Max. coverage (+): 0.09. Max coverage (-): 0.73

Region: NODE\_268840\_length\_12752\_cov\_27.436010 4159-4184. Max. coverage (+): 0.32. Max coverage (-): 1.57

Region: NODE\_268840\_length\_12752\_cov\_27.436010 4185-4210. Max. coverage (+): 0.02. Max coverage (-): 1.23

Region: NODE\_268840\_length\_12752\_cov\_27.436010 4211-4236. Max. coverage (+): 0.08. Max coverage (-): 1.17

Region: NODE\_268840\_length\_12752\_cov\_27.436010 4237-4261. Max. coverage (+): 0.02. Max coverage (-): 0.21

Region: NODE\_268840\_length\_12752\_cov\_27.436010 4262-4287. Max. coverage (+): 0.2. Max coverage (-): 0.81

Region: NODE\_268840\_length\_12752\_cov\_27.436010 4288-4313. Max. coverage (+): 0.04. Max coverage (-): 0.4

Region: NODE\_268840\_length\_12752\_cov\_27.436010 4314-4339. Max. coverage (+): 0.12. Max coverage (-): 2.02

Region: NODE\_268840\_length\_12752\_cov\_27.436010 4340-4364. Max. coverage (+): 1.37. Max coverage (-): 3.92

Region: NODE\_268840\_length\_12752\_cov\_27.436010 4365-4390. Max. coverage (+): 0.93. Max coverage (-): 33.76

Region: NODE\_268840\_length\_12752\_cov\_27.436010 4391-4416. Max. coverage (+): 0.57. Max coverage (-): 11.06

Region: NODE\_268840\_length\_12752\_cov\_27.436010 4417-4442. Max. coverage (+): 0.32. Max coverage (-): 1.78

Region: NODE\_268840\_length\_12752\_cov\_27.436010 4443-4467. Max. coverage (+): 0.16. Max coverage (-): 1.45

Region: NODE\_268840\_length\_12752\_cov\_27.436010 4468-4493. Max. coverage (+): 0.52. Max coverage (-): 1.53

Region: NODE\_268840\_length\_12752\_cov\_27.436010 4494-4519. Max. coverage (+): 0.36. Max coverage (-): 2.3

Region: NODE\_268840\_length\_12752\_cov\_27.436010 4520-4545. Max. coverage (+): 0.36. Max coverage (-): 3.96

Region: NODE\_268840\_length\_12752\_cov\_27.436010 4546-4570. Max. coverage (+): 0.16. Max coverage (-): 4.93

Region: NODE\_268840\_length\_12752\_cov\_27.436010 4571-4596. Max. coverage (+): 0.69. Max coverage (-): 0.36

Region: NODE\_268840\_length\_12752\_cov\_27.436010 4597-4622. Max. coverage (+): 0.04. Max coverage (-): 0.4

Region: NODE\_268840\_length\_12752\_cov\_27.436010 4623-4648. Max. coverage (+): 0.44. Max coverage (-): 2.95

Region: NODE\_268840\_length\_12752\_cov\_27.436010 4649-4673. Max. coverage (+): 0.44. Max coverage (-): 2.66

Region: NODE\_268840\_length\_12752\_cov\_27.436010 4674-4699. Max. coverage (+): 0. Max coverage (-): 4.76

Region: NODE\_268840\_length\_12752\_cov\_27.436010 4700-4725. Max. coverage (+): 1.05. Max coverage (-): 0.24

Region: NODE\_268840\_length\_12752\_cov\_27.436010 4726-4751. Max. coverage (+): 1.33. Max coverage (-): 1.49

Region: NODE\_268840\_length\_12752\_cov\_27.436010 4752-4776. Max. coverage (+): 0.12. Max coverage (-): 1.25

Region: NODE\_268840\_length\_12752\_cov\_27.436010 4777-4802. Max. coverage (+): 0.93. Max coverage (-): 1.47

Region: NODE\_268840\_length\_12752\_cov\_27.436010 4803-4828. Max. coverage (+): 0.2. Max coverage (-): 0.89

Region: NODE\_268840\_length\_12752\_cov\_27.436010 4829-4854. Max. coverage (+): 0.44. Max coverage (-): 2.99

Region: NODE\_268840\_length\_12752\_cov\_27.436010 4855-4879. Max. coverage (+): 0.16. Max coverage (-): 1.41

Region: NODE\_268840\_length\_12752\_cov\_27.436010 4880-4905. Max. coverage (+): 0.36. Max coverage (-): 8.92

Region: NODE\_268840\_length\_12752\_cov\_27.436010 4906-4931. Max. coverage (+): 1.13. Max coverage (-): 10.5

Region: NODE\_268840\_length\_12752\_cov\_27.436010 4932-4957. Max. coverage (+): 0.12. Max coverage (-): 1.74

Region: NODE\_268840\_length\_12752\_cov\_27.436010 4958-4982. Max. coverage (+): 0.24. Max coverage (-): 12.07

Region: NODE\_268840\_length\_12752\_cov\_27.436010 4983-5008. Max. coverage (+): 0.36. Max coverage (-): 0.61

Region: NODE\_268840\_length\_12752\_cov\_27.436010 5009-5034. Max. coverage (+): 0.73. Max coverage (-): 1.09

Region: NODE\_268840\_length\_12752\_cov\_27.436010 5035-5060. Max. coverage (+): 0.04. Max coverage (-): 0.81

Region: NODE\_268840\_length\_12752\_cov\_27.436010 5061-5085. Max. coverage (+): 0.08. Max coverage (-): 1.33

Region: NODE\_268840\_length\_12752\_cov\_27.436010 5086-5111. Max. coverage (+): 0.04. Max coverage (-): 0.61

Region: NODE\_268840\_length\_12752\_cov\_27.436010 5112-5137. Max. coverage (+): 0. Max coverage (-): 0.12

Region: NODE\_268840\_length\_12752\_cov\_27.436010 5138-5163. Max. coverage (+): 0.04. Max coverage (-): 0.12

Region: NODE\_268840\_length\_12752\_cov\_27.436010 5164-5188. Max. coverage (+): 0.16. Max coverage (-): 4.76

Region: NODE\_268840\_length\_12752\_cov\_27.436010 5189-5214. Max. coverage (+): 1.29. Max coverage (-): 0.65

Region: NODE\_268840\_length\_12752\_cov\_27.436010 5215-5240. Max. coverage (+): 0.16. Max coverage (-): 1.37

Region: NODE\_268840\_length\_12752\_cov\_27.436010 5241-5266. Max. coverage (+): 0.08. Max coverage (-): 2.75

Region: NODE\_268840\_length\_12752\_cov\_27.436010 5267-5291. Max. coverage (+): 0.04. Max coverage (-): 1.01

Region: NODE\_268840\_length\_12752\_cov\_27.436010 5292-5317. Max. coverage (+): 0.04. Max coverage (-): 0.32

Region: NODE\_268840\_length\_12752\_cov\_27.436010 5318-5343. Max. coverage (+): 0.16. Max coverage (-): 0.32

Region: NODE\_268840\_length\_12752\_cov\_27.436010 5344-5369. Max. coverage (+): 0.04. Max coverage (-): 0.2

Region: NODE\_268840\_length\_12752\_cov\_27.436010 5370-5394. Max. coverage (+): 0.04. Max coverage (-): 6.34

Region: NODE\_268840\_length\_12752\_cov\_27.436010 5395-5420. Max. coverage (+): 0. Max coverage (-): 3.86

Region: NODE\_268840\_length\_12752\_cov\_27.436010 5421-5446. Max. coverage (+): 0.08. Max coverage (-): 3.07

Region: NODE\_268840\_length\_12752\_cov\_27.436010 5447-5472. Max. coverage (+): 0.08. Max coverage (-): 1.25

Region: NODE\_268840\_length\_12752\_cov\_27.436010 5473-5497. Max. coverage (+): 2.06. Max coverage (-): 3.39

Region: NODE\_268840\_length\_12752\_cov\_27.436010 5498-5523. Max. coverage (+): 1.01. Max coverage (-): 1.98

Region: NODE\_268840\_length\_12752\_cov\_27.436010 5524-5549. Max. coverage (+): 0.48. Max coverage (-): 0.89

Region: NODE\_268840\_length\_12752\_cov\_27.436010 5550-5575. Max. coverage (+): 0.73. Max coverage (-): 1.98

Region: NODE\_268840\_length\_12752\_cov\_27.436010 5576-5600. Max. coverage (+): 0. Max coverage (-): 0.24

Region: NODE\_268840\_length\_12752\_cov\_27.436010 5601-5626. Max. coverage (+): 0.08. Max coverage (-): 0

Region: NODE\_268840\_length\_12752\_cov\_27.436010 5627-5652. Max. coverage (+): 0. Max coverage (-): 0.36

Region: NODE\_268840\_length\_12752\_cov\_27.436010 5653-5677. Max. coverage (+): 0. Max coverage (-): 0.44

Region: NODE\_268840\_length\_12752\_cov\_27.436010 5678-5703. Max. coverage (+): 0. Max coverage (-): 0.34

Region: NODE\_268840\_length\_12752\_cov\_27.436010 5704-5729. Max. coverage (+): 0.04. Max coverage (-): 0.36

Region: NODE\_268840\_length\_12752\_cov\_27.436010 5730-5755. Max. coverage (+): 0. Max coverage (-): 0.2

Region: NODE\_268840\_length\_12752\_cov\_27.436010 5756-5780. Max. coverage (+): 0.04. Max coverage (-): 0.52

Region: NODE\_268840\_length\_12752\_cov\_27.436010 5781-5806. Max. coverage (+): 0.01. Max coverage (-): 0.46

Region: NODE\_268840\_length\_12752\_cov\_27.436010 5807-5832. Max. coverage (+): 0. Max coverage (-): 0.42

Region: NODE\_268840\_length\_12752\_cov\_27.436010 5833-5858. Max. coverage (+): 0.02. Max coverage (-): 0.24

Region: NODE\_268840\_length\_12752\_cov\_27.436010 5859-5883. Max. coverage (+): 0.07. Max coverage (-): 0.09

Region: NODE\_268840\_length\_12752\_cov\_27.436010 5884-5909. Max. coverage (+): 0. Max coverage (-): 0

Region: NODE\_268840\_length\_12752\_cov\_27.436010 5910-5935. Max. coverage (+): 0.01. Max coverage (-): 0.05

Region: NODE\_268840\_length\_12752\_cov\_27.436010 5936-5961. Max. coverage (+): 0. Max coverage (-): 0.01

Region: NODE\_268840\_length\_12752\_cov\_27.436010 5962-5986. Max. coverage (+): 0. Max coverage (-): 0

Region: NODE\_268840\_length\_12752\_cov\_27.436010 5987-6012. Max. coverage (+): 0. Max coverage (-): 0

Region: NODE\_268840\_length\_12752\_cov\_27.436010 6013-6038. Max. coverage (+): 0. Max coverage (-): 0

Region: NODE\_268840\_length\_12752\_cov\_27.436010 6039-6064. Max. coverage (+): 0. Max coverage (-): 0

Region: NODE\_268840\_length\_12752\_cov\_27.436010 6065-6089. Max. coverage (+): 0. Max coverage (-): 0

Region: NODE\_268840\_length\_12752\_cov\_27.436010 6090-6115. Max. coverage (+): 0. Max coverage (-): 0

Region: NODE\_268840\_length\_12752\_cov\_27.436010 6116-6141. Max. coverage (+): 0. Max coverage (-): 0

Region: NODE\_268840\_length\_12752\_cov\_27.436010 6142-6167. Max. coverage (+): 0. Max coverage (-): 0

Region: NODE\_268840\_length\_12752\_cov\_27.436010 6168-6192. Max. coverage (+): 0. Max coverage (-): 0

Region: NODE\_268840\_length\_12752\_cov\_27.436010 6193-6218. Max. coverage (+): 0. Max coverage (-): 0

Region: NODE\_268840\_length\_12752\_cov\_27.436010 6219-6244. Max. coverage (+): 0. Max coverage (-): 0

Region: NODE\_268840\_length\_12752\_cov\_27.436010 6245-6270. Max. coverage (+): 0. Max coverage (-): 0.04

Region: NODE\_268840\_length\_12752\_cov\_27.436010 6271-6295. Max. coverage (+): 0. Max coverage (-): 0

Region: NODE\_268840\_length\_12752\_cov\_27.436010 6296-6321. Max. coverage (+): 0. Max coverage (-): 0

Region: NODE\_268840\_length\_12752\_cov\_27.436010 6322-6347. Max. coverage (+): 0. Max coverage (-): 0

Region: NODE\_268840\_length\_12752\_cov\_27.436010 6348-6373. Max. coverage (+): 0. Max coverage (-): 0

Region: NODE\_268840\_length\_12752\_cov\_27.436010 6374-6398. Max. coverage (+): 0. Max coverage (-): 0

Region: NODE\_268840\_length\_12752\_cov\_27.436010 6399-6424. Max. coverage (+): 0. Max coverage (-): 0

Region: NODE\_268840\_length\_12752\_cov\_27.436010 6425-6450. Max. coverage (+): 0. Max coverage (-): 0

Region: NODE\_268840\_length\_12752\_cov\_27.436010 6451-6476. Max. coverage (+): 0. Max coverage (-): 0

Region: NODE\_268840\_length\_12752\_cov\_27.436010 6477-6501. Max. coverage (+): 0.01. Max coverage (-): 0.46

Region: NODE\_268840\_length\_12752\_cov\_27.436010 6502-6527. Max. coverage (+): 0.01. Max coverage (-): 0.01

Region: NODE\_268840\_length\_12752\_cov\_27.436010 6528-6553. Max. coverage (+): 0. Max coverage (-): 0

Region: NODE\_268840\_length\_12752\_cov\_27.436010 6554-6579. Max. coverage (+): 0. Max coverage (-): 0

Region: NODE\_268840\_length\_12752\_cov\_27.436010 6580-6604. Max. coverage (+): 0. Max coverage (-): 0

Region: NODE\_268840\_length\_12752\_cov\_27.436010 6605-6630. Max. coverage (+): 0. Max coverage (-): 0

Region: NODE\_268840\_length\_12752\_cov\_27.436010 6631-6656. Max. coverage (+): 0.02. Max coverage (-): 0.04

Region: NODE\_268840\_length\_12752\_cov\_27.436010 6657-6682. Max. coverage (+): 0.28. Max coverage (-): 0.65

Region: NODE\_268840\_length\_12752\_cov\_27.436010 6683-6707. Max. coverage (+): 0.12. Max coverage (-): 0.2

Region: NODE\_268840\_length\_12752\_cov\_27.436010 6708-6733. Max. coverage (+): 0.1. Max coverage (-): 0.2

Region: NODE\_268840\_length\_12752\_cov\_27.436010 6734-6759. Max. coverage (+): 0.04. Max coverage (-): 0

Region: NODE\_268840\_length\_12752\_cov\_27.436010 6760-6785. Max. coverage (+): 0.08. Max coverage (-): 0.14

Region: NODE\_268840\_length\_12752\_cov\_27.436010 6786-6810. Max. coverage (+): 0.24. Max coverage (-): 0.69

Region: NODE\_268840\_length\_12752\_cov\_27.436010 6811-6836. Max. coverage (+): 0.04. Max coverage (-): 0.16

Region: NODE\_268840\_length\_12752\_cov\_27.436010 6837-6862. Max. coverage (+): 0. Max coverage (-): 0.16

Region: NODE\_268840\_length\_12752\_cov\_27.436010 6863-6888. Max. coverage (+): 0.12. Max coverage (-): 1.19

Region: NODE\_268840\_length\_12752\_cov\_27.436010 6889-6913. Max. coverage (+): 0.12. Max coverage (-): 0.61

Region: NODE\_268840\_length\_12752\_cov\_27.436010 6914-6939. Max. coverage (+): 2.14. Max coverage (-): 0.52

Region: NODE\_268840\_length\_12752\_cov\_27.436010 6940-6965. Max. coverage (+): 0.08. Max coverage (-): 0.04

Region: NODE\_268840\_length\_12752\_cov\_27.436010 6966-6991. Max. coverage (+): 0.16. Max coverage (-): 0.12

Region: NODE\_268840\_length\_12752\_cov\_27.436010 6992-7016. Max. coverage (+): 0.09. Max coverage (-): 0.08

Region: NODE\_268840\_length\_12752\_cov\_27.436010 7017-7042. Max. coverage (+): 0.4. Max coverage (-): 0.16

Region: NODE\_268840\_length\_12752\_cov\_27.436010 7043-7068. Max. coverage (+): 0.12. Max coverage (-): 0.28

Region: NODE\_268840\_length\_12752\_cov\_27.436010 7069-7094. Max. coverage (+): 0.08. Max coverage (-): 0.4

Region: NODE\_268840\_length\_12752\_cov\_27.436010 7095-7119. Max. coverage (+): 0.12. Max coverage (-): 0.73

Region: NODE\_268840\_length\_12752\_cov\_27.436010 7120-7145. Max. coverage (+): 0.24. Max coverage (-): 0.28

Region: NODE\_268840\_length\_12752\_cov\_27.436010 7146-7171. Max. coverage (+): 0.04. Max coverage (-): 0.22

Region: NODE\_268840\_length\_12752\_cov\_27.436010 7172-7197. Max. coverage (+): 0. Max coverage (-): 0.24

Region: NODE\_268840\_length\_12752\_cov\_27.436010 7198-7222. Max. coverage (+): 0.02. Max coverage (-): 0.32

Region: NODE\_268840\_length\_12752\_cov\_27.436010 7223-7248. Max. coverage (+): 0.12. Max coverage (-): 0.4

Region: NODE\_268840\_length\_12752\_cov\_27.436010 7249-7274. Max. coverage (+): 0.04. Max coverage (-): 0.04

Region: NODE\_268840\_length\_12752\_cov\_27.436010 7275-7299. Max. coverage (+): 0.02. Max coverage (-): 0.1

Region: NODE\_268840\_length\_12752\_cov\_27.436010 7300-7325. Max. coverage (+): 0.08. Max coverage (-): 0.08

Region: NODE\_268840\_length\_12752\_cov\_27.436010 7326-7351. Max. coverage (+): 0.08. Max coverage (-): 0.12

Region: NODE\_268840\_length\_12752\_cov\_27.436010 7352-7377. Max. coverage (+): 0.04. Max coverage (-): 0.2

Region: NODE\_268840\_length\_12752\_cov\_27.436010 7378-7402. Max. coverage (+): 0. Max coverage (-): 0.01

Region: NODE\_268840\_length\_12752\_cov\_27.436010 7403-7428. Max. coverage (+): 0.02. Max coverage (-): 0.12

Region: NODE\_268840\_length\_12752\_cov\_27.436010 7429-7454. Max. coverage (+): 0. Max coverage (-): 0.08

Region: NODE\_268840\_length\_12752\_cov\_27.436010 7455-7480. Max. coverage (+): 0.04. Max coverage (-): 0.03

Region: NODE\_268840\_length\_12752\_cov\_27.436010 7481-7505. Max. coverage (+): 0.44. Max coverage (-): 2.35

Region: NODE\_268840\_length\_12752\_cov\_27.436010 7506-7531. Max. coverage (+): 0.28. Max coverage (-): 0.57

Region: NODE\_268840\_length\_12752\_cov\_27.436010 7532-7557. Max. coverage (+): 0.08. Max coverage (-): 7.91

Region: NODE\_268840\_length\_12752\_cov\_27.436010 7558-7583. Max. coverage (+): 0. Max coverage (-): 0.61

Region: NODE\_268840\_length\_12752\_cov\_27.436010 7584-7608. Max. coverage (+): 0. Max coverage (-): 0.89

Region: NODE\_268840\_length\_12752\_cov\_27.436010 7609-7634. Max. coverage (+): 0.04. Max coverage (-): 0

Region: NODE\_268840\_length\_12752\_cov\_27.436010 7635-7660. Max. coverage (+): 0. Max coverage (-): 0.44

Region: NODE\_268840\_length\_12752\_cov\_27.436010 7661-7686. Max. coverage (+): 0. Max coverage (-): 0.36

Region: NODE\_268840\_length\_12752\_cov\_27.436010 7687-7711. Max. coverage (+): 0.1. Max coverage (-): 0.16

Region: NODE\_268840\_length\_12752\_cov\_27.436010 7712-7737. Max. coverage (+): 0. Max coverage (-): 0.04

Region: NODE\_268840\_length\_12752\_cov\_27.436010 7738-7763. Max. coverage (+): 0.08. Max coverage (-): 0.32

Region: NODE\_268840\_length\_12752\_cov\_27.436010 7764-7789. Max. coverage (+): 0.04. Max coverage (-): 1.13

Region: NODE\_268840\_length\_12752\_cov\_27.436010 7790-7814. Max. coverage (+): 0.32. Max coverage (-): 5.83

Region: NODE\_268840\_length\_12752\_cov\_27.436010 7815-7840. Max. coverage (+): 0.52. Max coverage (-): 7.31

Region: NODE\_268840\_length\_12752\_cov\_27.436010 7841-7866. Max. coverage (+): 0.57. Max coverage (-): 3.33

Region: NODE\_268840\_length\_12752\_cov\_27.436010 7867-7892. Max. coverage (+): 0.14. Max coverage (-): 0.48

Region: NODE\_268840\_length\_12752\_cov\_27.436010 7893-7917. Max. coverage (+): 1.01. Max coverage (-): 2.02

Region: NODE\_268840\_length\_12752\_cov\_27.436010 7918-7943. Max. coverage (+): 0.57. Max coverage (-): 0.52

Region: NODE\_268840\_length\_12752\_cov\_27.436010 7944-7969. Max. coverage (+): 0.04. Max coverage (-): 0.36

Region: NODE\_268840\_length\_12752\_cov\_27.436010 7970-7995. Max. coverage (+): 0.08. Max coverage (-): 0.16

Region: NODE\_268840\_length\_12752\_cov\_27.436010 7996-8020. Max. coverage (+): 0.57. Max coverage (-): 0.73

Region: NODE\_268840\_length\_12752\_cov\_27.436010 8021-8046. Max. coverage (+): 0.08. Max coverage (-): 0.48

Region: NODE\_268840\_length\_12752\_cov\_27.436010 8047-8072. Max. coverage (+): 0.16. Max coverage (-): 0.2

Region: NODE\_268840\_length\_12752\_cov\_27.436010 8073-8098. Max. coverage (+): 0. Max coverage (-): 0.93

Region: NODE\_268840\_length\_12752\_cov\_27.436010 8099-8123. Max. coverage (+): 0. Max coverage (-): 1.01

Region: NODE\_268840\_length\_12752\_cov\_27.436010 8124-8149. Max. coverage (+): 0.65. Max coverage (-): 18.49

Region: NODE\_268840\_length\_12752\_cov\_27.436010 8150-8175. Max. coverage (+): 0.04. Max coverage (-): 12.11

Region: NODE\_268840\_length\_12752\_cov\_27.436010 8176-8201. Max. coverage (+): 0. Max coverage (-): 2.38

Region: NODE\_268840\_length\_12752\_cov\_27.436010 8202-8226. Max. coverage (+): 0.32. Max coverage (-): 0.52

Region: NODE\_268840\_length\_12752\_cov\_27.436010 8227-8252. Max. coverage (+): 0.2. Max coverage (-): 1.98

Region: NODE\_268840\_length\_12752\_cov\_27.436010 8253-8278. Max. coverage (+): 0.52. Max coverage (-): 0.77

Region: NODE\_268840\_length\_12752\_cov\_27.436010 8279-8304. Max. coverage (+): 0.2. Max coverage (-): 1.33

Region: NODE\_268840\_length\_12752\_cov\_27.436010 8305-8329. Max. coverage (+): 0.2. Max coverage (-): 5.25

Region: NODE\_268840\_length\_12752\_cov\_27.436010 8330-8355. Max. coverage (+): 0.04. Max coverage (-): 0.28

Region: NODE\_268840\_length\_12752\_cov\_27.436010 8356-8381. Max. coverage (+): 0.2. Max coverage (-): 1.29

Region: NODE\_268840\_length\_12752\_cov\_27.436010 8382-8407. Max. coverage (+): 0.12. Max coverage (-): 0.52

Region: NODE\_268840\_length\_12752\_cov\_27.436010 8408-8432. Max. coverage (+): 0.04. Max coverage (-): 0.65

Region: NODE\_268840\_length\_12752\_cov\_27.436010 8433-8458. Max. coverage (+): 0.4. Max coverage (-): 0.28

Region: NODE\_268840\_length\_12752\_cov\_27.436010 8459-8484. Max. coverage (+): 0.2. Max coverage (-): 0.32

Region: NODE\_268840\_length\_12752\_cov\_27.436010 8485-8510. Max. coverage (+): 0.12. Max coverage (-): 1.25

Region: NODE\_268840\_length\_12752\_cov\_27.436010 8511-8535. Max. coverage (+): 0.16. Max coverage (-): 2.3

Region: NODE\_268840\_length\_12752\_cov\_27.436010 8536-8561. Max. coverage (+): 0. Max coverage (-): 0.16

Region: NODE\_268840\_length\_12752\_cov\_27.436010 8562-8587. Max. coverage (+): 0.16. Max coverage (-): 0.2

Region: NODE\_268840\_length\_12752\_cov\_27.436010 8588-8613. Max. coverage (+): 0.12. Max coverage (-): 2.32

Region: NODE\_268840\_length\_12752\_cov\_27.436010 8614-8638. Max. coverage (+): 0. Max coverage (-): 0.12

Region: NODE\_268840\_length\_12752\_cov\_27.436010 8639-8664. Max. coverage (+): 0. Max coverage (-): 0.85

Region: NODE\_268840\_length\_12752\_cov\_27.436010 8665-8690. Max. coverage (+): 0. Max coverage (-): 0.24

Region: NODE\_268840\_length\_12752\_cov\_27.436010 8691-8716. Max. coverage (+): 0.04. Max coverage (-): 0.24

Region: NODE\_268840\_length\_12752\_cov\_27.436010 8717-8741. Max. coverage (+): 0. Max coverage (-): 0.04

Region: NODE\_268840\_length\_12752\_cov\_27.436010 8742-8767. Max. coverage (+): 0.08. Max coverage (-): 0.2

Region: NODE\_268840\_length\_12752\_cov\_27.436010 8768-8793. Max. coverage (+): 0.04. Max coverage (-): 0.57

Region: NODE\_268840\_length\_12752\_cov\_27.436010 8794-8819. Max. coverage (+): 0. Max coverage (-): 0

Region: NODE\_268840\_length\_12752\_cov\_27.436010 8820-8844. Max. coverage (+): 0. Max coverage (-): 0

Region: NODE\_268840\_length\_12752\_cov\_27.436010 8845-8870. Max. coverage (+): 0. Max coverage (-): 0

Region: NODE\_268840\_length\_12752\_cov\_27.436010 8871-8896. Max. coverage (+): 0. Max coverage (-): 0

Region: NODE\_268840\_length\_12752\_cov\_27.436010 8897-8921. Max. coverage (+): 0. Max coverage (-): 0

Region: NODE\_268840\_length\_12752\_cov\_27.436010 8922-8947. Max. coverage (+): 0. Max coverage (-): 0

Region: NODE\_268840\_length\_12752\_cov\_27.436010 8948-8973. Max. coverage (+): 0. Max coverage (-): 0

Region: NODE\_268840\_length\_12752\_cov\_27.436010 8974-8999. Max. coverage (+): 0. Max coverage (-): 0

Region: NODE\_268840\_length\_12752\_cov\_27.436010 9000-9024. Max. coverage (+): 0. Max coverage (-): 0

Region: NODE\_268840\_length\_12752\_cov\_27.436010 9025-9050. Max. coverage (+): 0. Max coverage (-): 0

Region: NODE\_268840\_length\_12752\_cov\_27.436010 9051-9076. Max. coverage (+): 0. Max coverage (-): 0.08

Region: NODE\_268840\_length\_12752\_cov\_27.436010 9077-9102. Max. coverage (+): 0. Max coverage (-): 1.05

Region: NODE\_268840\_length\_12752\_cov\_27.436010 9103-9127. Max. coverage (+): 0.12. Max coverage (-): 0.08

Region: NODE\_268840\_length\_12752\_cov\_27.436010 9128-9153. Max. coverage (+): 0. Max coverage (-): 0.08

Region: NODE\_268840\_length\_12752\_cov\_27.436010 9154-9179. Max. coverage (+): 0.08. Max coverage (-): 2.14

Region: NODE\_268840\_length\_12752\_cov\_27.436010 9180-9205. Max. coverage (+): 0. Max coverage (-): 1.05

Region: NODE\_268840\_length\_12752\_cov\_27.436010 9206-9230. Max. coverage (+): 0.04. Max coverage (-): 0.61

Region: NODE\_268840\_length\_12752\_cov\_27.436010 9231-9256. Max. coverage (+): 0. Max coverage (-): 0.08

Region: NODE\_268840\_length\_12752\_cov\_27.436010 9257-9282. Max. coverage (+): 0. Max coverage (-): 0.04

Region: NODE\_268840\_length\_12752\_cov\_27.436010 9283-9308. Max. coverage (+): 0.08. Max coverage (-): 0.2

Region: NODE\_268840\_length\_12752\_cov\_27.436010 9309-9333. Max. coverage (+): 0.04. Max coverage (-): 0.04

Region: NODE\_268840\_length\_12752\_cov\_27.436010 9334-9359. Max. coverage (+): 0.32. Max coverage (-): 0.24

Region: NODE\_268840\_length\_12752\_cov\_27.436010 9360-9385. Max. coverage (+): 0.24. Max coverage (-): 0.31

Region: NODE\_268840\_length\_12752\_cov\_27.436010 9386-9411. Max. coverage (+): 0.04. Max coverage (-): 0.16

Region: NODE\_268840\_length\_12752\_cov\_27.436010 9412-9436. Max. coverage (+): 0.01. Max coverage (-): 0

Region: NODE\_268840\_length\_12752\_cov\_27.436010 9437-9462. Max. coverage (+): 0.01. Max coverage (-): 0.02

Region: NODE\_268840\_length\_12752\_cov\_27.436010 9463-9488. Max. coverage (+): 0.04. Max coverage (-): 0

Region: NODE\_268840\_length\_12752\_cov\_27.436010 9489-9514. Max. coverage (+): 0.05. Max coverage (-): 0.03

Region: NODE\_268840\_length\_12752\_cov\_27.436010 9515-9539. Max. coverage (+): 0.06. Max coverage (-): 0.01

Region: NODE\_268840\_length\_12752\_cov\_27.436010 9540-9565. Max. coverage (+): 0. Max coverage (-): 0

Region: NODE\_268840\_length\_12752\_cov\_27.436010 9566-9591. Max. coverage (+): 0. Max coverage (-): 0

Region: NODE\_268840\_length\_12752\_cov\_27.436010 9592-9617. Max. coverage (+): 0.3. Max coverage (-): 0.08

Region: NODE\_268840\_length\_12752\_cov\_27.436010 9618-9642. Max. coverage (+): 0.01. Max coverage (-): 0.03

Region: NODE\_268840\_length\_12752\_cov\_27.436010 9643-9668. Max. coverage (+): 0.04. Max coverage (-): 0.11

Region: NODE\_268840\_length\_12752\_cov\_27.436010 9669-9694. Max. coverage (+): 0.04. Max coverage (-): 0.08

Region: NODE\_268840\_length\_12752\_cov\_27.436010 9695-9720. Max. coverage (+): 0.04. Max coverage (-): 0.48

Region: NODE\_268840\_length\_12752\_cov\_27.436010 9721-9745. Max. coverage (+): 0.04. Max coverage (-): 0.44

Region: NODE\_268840\_length\_12752\_cov\_27.436010 9746-9771. Max. coverage (+): 0. Max coverage (-): 0.16

Region: NODE\_268840\_length\_12752\_cov\_27.436010 9772-9797. Max. coverage (+): 0. Max coverage (-): 0.32

Region: NODE\_268840\_length\_12752\_cov\_27.436010 9798-9823. Max. coverage (+): 0.04. Max coverage (-): 1.09

Region: NODE\_268840\_length\_12752\_cov\_27.436010 9824-9848. Max. coverage (+): 0.2. Max coverage (-): 0.44

Region: NODE\_268840\_length\_12752\_cov\_27.436010 9849-9874. Max. coverage (+): 0.12. Max coverage (-): 0.04

Region: NODE\_268840\_length\_12752\_cov\_27.436010 9875-9900. Max. coverage (+): 0.04. Max coverage (-): 0.04

Region: NODE\_268840\_length\_12752\_cov\_27.436010 9901-9926. Max. coverage (+): 0. Max coverage (-): 0

Region: NODE\_268840\_length\_12752\_cov\_27.436010 9927-9951. Max. coverage (+): 0. Max coverage (-): 0.12

Region: NODE\_268840\_length\_12752\_cov\_27.436010 9952-9977. Max. coverage (+): 0.04. Max coverage (-): 0

Region: NODE\_268840\_length\_12752\_cov\_27.436010 9978-10003. Max. coverage (+): 0.04. Max coverage (-): 0

Region: NODE\_268840\_length\_12752\_cov\_27.436010 10004-10029. Max. coverage (+): 0.12. Max coverage (-): 0

Region: NODE\_268840\_length\_12752\_cov\_27.436010 10030-10054. Max. coverage (+): 0.08. Max coverage (-): 0.02

Region: NODE\_268840\_length\_12752\_cov\_27.436010 10055-10080. Max. coverage (+): 0.08. Max coverage (-): 0

Region: NODE\_268840\_length\_12752\_cov\_27.436010 10081-10106. Max. coverage (+): 0.3. Max coverage (-): 0.16

Region: NODE\_268840\_length\_12752\_cov\_27.436010 10107-10132. Max. coverage (+): 0.08. Max coverage (-): 0.08

Region: NODE\_268840\_length\_12752\_cov\_27.436010 10133-10157. Max. coverage (+): 0.08. Max coverage (-): 0.17

Region: NODE\_268840\_length\_12752\_cov\_27.436010 10158-10183. Max. coverage (+): 0.16. Max coverage (-): 0.08

Region: NODE\_268840\_length\_12752\_cov\_27.436010 10184-10209. Max. coverage (+): 0.08. Max coverage (-): 0.04

Region: NODE\_268840\_length\_12752\_cov\_27.436010 10210-10235. Max. coverage (+): 0.4. Max coverage (-): 0.24

Region: NODE\_268840\_length\_12752\_cov\_27.436010 10236-10260. Max. coverage (+): 0.08. Max coverage (-): 0.16

Region: NODE\_268840\_length\_12752\_cov\_27.436010 10261-10286. Max. coverage (+): 0.04. Max coverage (-): 0.89

Region: NODE\_268840\_length\_12752\_cov\_27.436010 10287-10312. Max. coverage (+): 0. Max coverage (-): 0

Region: NODE\_268840\_length\_12752\_cov\_27.436010 10313-10338. Max. coverage (+): 0.08. Max coverage (-): 0.12

Region: NODE\_268840\_length\_12752\_cov\_27.436010 10339-10363. Max. coverage (+): 0.04. Max coverage (-): 0.12

Region: NODE\_268840\_length\_12752\_cov\_27.436010 10364-10389. Max. coverage (+): 1.05. Max coverage (-): 0.32

Region: NODE\_268840\_length\_12752\_cov\_27.436010 10390-10415. Max. coverage (+): 0.32. Max coverage (-): 0.08

Region: NODE\_268840\_length\_12752\_cov\_27.436010 10416-10441. Max. coverage (+): 0.04. Max coverage (-): 0.08

Region: NODE\_268840\_length\_12752\_cov\_27.436010 10442-10466. Max. coverage (+): 0.16. Max coverage (-): 0

Region: NODE\_268840\_length\_12752\_cov\_27.436010 10467-10492. Max. coverage (+): 0. Max coverage (-): 0

Region: NODE\_268840\_length\_12752\_cov\_27.436010 10493-10518. Max. coverage (+): 0. Max coverage (-): 0

Region: NODE\_268840\_length\_12752\_cov\_27.436010 10519-10543. Max. coverage (+): 0. Max coverage (-): 0.04

Region: NODE\_268840\_length\_12752\_cov\_27.436010 10544-10569. Max. coverage (+): 0. Max coverage (-): 0

Region: NODE\_268840\_length\_12752\_cov\_27.436010 10570-10595. Max. coverage (+): 0.12. Max coverage (-): 0

Region: NODE\_268840\_length\_12752\_cov\_27.436010 10596-10621. Max. coverage (+): 0.04. Max coverage (-): 0

Region: NODE\_268840\_length\_12752\_cov\_27.436010 10622-10646. Max. coverage (+): 0. Max coverage (-): 0

Region: NODE\_268840\_length\_12752\_cov\_27.436010 10647-10672. Max. coverage (+): 0.08. Max coverage (-): 0

Region: NODE\_268840\_length\_12752\_cov\_27.436010 10673-10698. Max. coverage (+): 0. Max coverage (-): 0

Region: NODE\_268840\_length\_12752\_cov\_27.436010 10699-10724. Max. coverage (+): 0. Max coverage (-): 2.47

Region: NODE\_268840\_length\_12752\_cov\_27.436010 10725-10749. Max. coverage (+): 0.02. Max coverage (-): 0.74

Region: NODE\_268840\_length\_12752\_cov\_27.436010 10750-10775. Max. coverage (+): 0.14. Max coverage (-): 0.53

Region: NODE\_268840\_length\_12752\_cov\_27.436010 10776-10801. Max. coverage (+): 0.14. Max coverage (-): 0.61

Region: NODE\_268840\_length\_12752\_cov\_27.436010 10802-10827. Max. coverage (+): 0.08. Max coverage (-): 0.09

Region: NODE\_268840\_length\_12752\_cov\_27.436010 10828-10852. Max. coverage (+): 0. Max coverage (-): 0.16

Region: NODE\_268840\_length\_12752\_cov\_27.436010 10853-10878. Max. coverage (+): 0. Max coverage (-): 1.02

Region: NODE\_268840\_length\_12752\_cov\_27.436010 10879-10904. Max. coverage (+): 0. Max coverage (-): 0.08

Region: NODE\_268840\_length\_12752\_cov\_27.436010 10905-10930. Max. coverage (+): 0.04. Max coverage (-): 0.04

Region: NODE\_268840\_length\_12752\_cov\_27.436010 10931-10955. Max. coverage (+): 0. Max coverage (-): 0.32

Region: NODE\_268840\_length\_12752\_cov\_27.436010 10956-10981. Max. coverage (+): 0.08. Max coverage (-): 0.09

Region: NODE\_268840\_length\_12752\_cov\_27.436010 10982-11007. Max. coverage (+): 0.08. Max coverage (-): 0.04

Region: NODE\_268840\_length\_12752\_cov\_27.436010 11008-11033. Max. coverage (+): 0.09. Max coverage (-): 0.04

Region: NODE\_268840\_length\_12752\_cov\_27.436010 11034-11058. Max. coverage (+): 0.69. Max coverage (-): 0.16

Region: NODE\_268840\_length\_12752\_cov\_27.436010 11059-11084. Max. coverage (+): 0.12. Max coverage (-): 0.52

Region: NODE\_268840\_length\_12752\_cov\_27.436010 11085-11110. Max. coverage (+): 0.08. Max coverage (-): 0.48

Region: NODE\_268840\_length\_12752\_cov\_27.436010 11111-11136. Max. coverage (+): 0. Max coverage (-): 0.48

Region: NODE\_268840\_length\_12752\_cov\_27.436010 11137-11161. Max. coverage (+): 0. Max coverage (-): 0.04

Region: NODE\_268840\_length\_12752\_cov\_27.436010 11162-11187. Max. coverage (+): 0.02. Max coverage (-): 0

Region: NODE\_268840\_length\_12752\_cov\_27.436010 11188-11213. Max. coverage (+): 0.16. Max coverage (-): 1.25

Region: NODE\_268840\_length\_12752\_cov\_27.436010 11214-11239. Max. coverage (+): 0.08. Max coverage (-): 0.44

Region: NODE\_268840\_length\_12752\_cov\_27.436010 11240-11264. Max. coverage (+): 0.85. Max coverage (-): 0.29

Region: NODE\_268840\_length\_12752\_cov\_27.436010 11265-11290. Max. coverage (+): 0.03. Max coverage (-): 0.12

Region: NODE\_268840\_length\_12752\_cov\_27.436010 11291-11316. Max. coverage (+): 0.04. Max coverage (-): 0.65

Region: NODE\_268840\_length\_12752\_cov\_27.436010 11317-11342. Max. coverage (+): 0.04. Max coverage (-): 2.91

Region: NODE\_268840\_length\_12752\_cov\_27.436010 11343-11367. Max. coverage (+): 3.43. Max coverage (-): 1.74

Region: NODE\_268840\_length\_12752\_cov\_27.436010 11368-11393. Max. coverage (+): 1.7. Max coverage (-): 0.85

Region: NODE\_268840\_length\_12752\_cov\_27.436010 11394-11419. Max. coverage (+): 0.12. Max coverage (-): 6.3

Region: NODE\_268840\_length\_12752\_cov\_27.436010 11420-11445. Max. coverage (+): 0.16. Max coverage (-): 0.98

Region: NODE\_268840\_length\_12752\_cov\_27.436010 11446-11470. Max. coverage (+): 0.28. Max coverage (-): 0.49

Region: NODE\_268840\_length\_12752\_cov\_27.436010 11471-11496. Max. coverage (+): 1.21. Max coverage (-): 0.57

Region: NODE\_268840\_length\_12752\_cov\_27.436010 11497-11522. Max. coverage (+): 0.04. Max coverage (-): 3.51

Region: NODE\_268840\_length\_12752\_cov\_27.436010 11523-11548. Max. coverage (+): 0.32. Max coverage (-): 5.49

Region: NODE\_268840\_length\_12752\_cov\_27.436010 11549-11573. Max. coverage (+): 3.47. Max coverage (-): 3.01

Region: NODE\_268840\_length\_12752\_cov\_27.436010 11574-11599. Max. coverage (+): 0.08. Max coverage (-): 123.98

Region: NODE\_268840\_length\_12752\_cov\_27.436010 11600-11625. Max. coverage (+): 0.42. Max coverage (-): 116.35

Region: NODE\_268840\_length\_12752\_cov\_27.436010 11626-11651. Max. coverage (+): 0.22. Max coverage (-): 0.18

Region: NODE\_268840\_length\_12752\_cov\_27.436010 11652-11676. Max. coverage (+): 2.69. Max coverage (-): 0.77

Region: NODE\_268840\_length\_12752\_cov\_27.436010 11677-11702. Max. coverage (+): 1.62. Max coverage (-): 1.53

Region: NODE\_268840\_length\_12752\_cov\_27.436010 11703-11728. Max. coverage (+): 1.45. Max coverage (-): 4.18

Region: NODE\_268840\_length\_12752\_cov\_27.436010 11729-11754. Max. coverage (+): 3.23. Max coverage (-): 2.14

Region: NODE\_268840\_length\_12752\_cov\_27.436010 11755-11779. Max. coverage (+): 0.12. Max coverage (-): 10.3

Region: NODE\_268840\_length\_12752\_cov\_27.436010 11780-11805. Max. coverage (+): 0.44. Max coverage (-): 0.32

Region: NODE\_268840\_length\_12752\_cov\_27.436010 11806-11831. Max. coverage (+): 1.01. Max coverage (-): 2.26

Region: NODE\_268840\_length\_12752\_cov\_27.436010 11832-11857. Max. coverage (+): 0.07. Max coverage (-): 4.64

Region: NODE\_268840\_length\_12752\_cov\_27.436010 11858-11882. Max. coverage (+): 0.24. Max coverage (-): 4.89

Region: NODE\_268840\_length\_12752\_cov\_27.436010 11883-11908. Max. coverage (+): 1.94. Max coverage (-): 3.89

Region: NODE\_268840\_length\_12752\_cov\_27.436010 11909-11934. Max. coverage (+): 1.94. Max coverage (-): 1.37

Region: NODE\_268840\_length\_12752\_cov\_27.436010 11935-11960. Max. coverage (+): 0.57. Max coverage (-): 5.53

Region: NODE\_268840\_length\_12752\_cov\_27.436010 11961-11985. Max. coverage (+): 0.08. Max coverage (-): 0.61

Region: NODE\_268840\_length\_12752\_cov\_27.436010 11986-12011. Max. coverage (+): 0.04. Max coverage (-): 0.04

Region: NODE\_268840\_length\_12752\_cov\_27.436010 12012-12037. Max. coverage (+): 0. Max coverage (-): 1.86

Region: NODE\_268840\_length\_12752\_cov\_27.436010 12038-12063. Max. coverage (+): 0.89. Max coverage (-): 0.2

Region: NODE\_268840\_length\_12752\_cov\_27.436010 12064-12088. Max. coverage (+): 0.52. Max coverage (-): 0.24

Region: NODE\_268840\_length\_12752\_cov\_27.436010 12089-12114. Max. coverage (+): 0.32. Max coverage (-): 0.02

Region: NODE\_268840\_length\_12752\_cov\_27.436010 12115-12140. Max. coverage (+): 0.14. Max coverage (-): 0.52

Region: NODE\_268840\_length\_12752\_cov\_27.436010 12141-12165. Max. coverage (+): 0.04. Max coverage (-): 0.04

Region: NODE\_268840\_length\_12752\_cov\_27.436010 12166-12191. Max. coverage (+): 0.08. Max coverage (-): 0.16

Region: NODE\_268840\_length\_12752\_cov\_27.436010 12192-12217. Max. coverage (+): 0.02. Max coverage (-): 0

Region: NODE\_268840\_length\_12752\_cov\_27.436010 12218-12243. Max. coverage (+): 0. Max coverage (-): 0

Region: NODE\_268840\_length\_12752\_cov\_27.436010 12244-12268. Max. coverage (+): 0. Max coverage (-): 0

Region: NODE\_268840\_length\_12752\_cov\_27.436010 12269-12294. Max. coverage (+): 0.85. Max coverage (-): 3.51

Region: NODE\_268840\_length\_12752\_cov\_27.436010 12295-12320. Max. coverage (+): 1.7. Max coverage (-): 0.24

Region: NODE\_268840\_length\_12752\_cov\_27.436010 12321-12346. Max. coverage (+): 0.05. Max coverage (-): 0.31

Region: NODE\_268840\_length\_12752\_cov\_27.436010 12347-12371. Max. coverage (+): 0.01. Max coverage (-): 0

Region: NODE\_268840\_length\_12752\_cov\_27.436010 12372-12397. Max. coverage (+): 0.01. Max coverage (-): 0.02

Region: NODE\_268840\_length\_12752\_cov\_27.436010 12398-12423. Max. coverage (+): 0.26. Max coverage (-): 0.02

Region: NODE\_268840\_length\_12752\_cov\_27.436010 12424-12449. Max. coverage (+): 0.26. Max coverage (-): 0.01

Region: NODE\_268840\_length\_12752\_cov\_27.436010 12450-12474. Max. coverage (+): 0.06. Max coverage (-): 0.01

Region: NODE\_268840\_length\_12752\_cov\_27.436010 12475-12500. Max. coverage (+): 0. Max coverage (-): 0.01

Region: NODE\_268840\_length\_12752\_cov\_27.436010 12501-12526. Max. coverage (+): 0.01. Max coverage (-): 0

Region: NODE\_268840\_length\_12752\_cov\_27.436010 12527-12552. Max. coverage (+): 0.01. Max coverage (-): 0.02

Region: NODE\_268840\_length\_12752\_cov\_27.436010 12553-12577. Max. coverage (+): 0.02. Max coverage (-): 0

Region: NODE\_268840\_length\_12752\_cov\_27.436010 12578-12603. Max. coverage (+): 0. Max coverage (-): 0.01

Region: NODE\_268840\_length\_12752\_cov\_27.436010 12604-12629. Max. coverage (+): 0. Max coverage (-): 0.04

Region: NODE\_268840\_length\_12752\_cov\_27.436010 12630-12655. Max. coverage (+): 0.02. Max coverage (-): 0

Region: NODE\_268840\_length\_12752\_cov\_27.436010 12656-12680. Max. coverage (+): 0. Max coverage (-): 0

Region: NODE\_268840\_length\_12752\_cov\_27.436010 12681-12706. Max. coverage (+): 0. Max coverage (-): 0

Region: NODE\_268840\_length\_12752\_cov\_27.436010 12707-12732. Max. coverage (+): 0. Max coverage (-): 0.01

Region: NODE\_268840\_length\_12752\_cov\_27.436010 12733-12758. Max. coverage (+): 0.01. Max coverage (-): 0

Region: NODE\_268840\_length\_12752\_cov\_27.436010 12759-12783. Max. coverage (+): 0. Max coverage (-): 0

Region: NODE\_268840\_length\_12752\_cov\_27.436010 12784-12809. Max. coverage (+): 0. Max coverage (-): 0.93

Region: NODE\_268840\_length\_12752\_cov\_27.436010 12810-12835. Max. coverage (+): 0.03. Max coverage (-): 0.01

Region: NODE\_268840\_length\_12752\_cov\_27.436010 12836-12861. Max. coverage (+): 0.03. Max coverage (-): 0

Region: NODE\_268840\_length\_12752\_cov\_27.436010 12862-. Max. coverage (+): 0. Max coverage (-): 0

RepeatMasker Color Code

**+**

100-98% Identity

<98-95% Identity

<95-90% Identity

<90-85% Identity

<85-80% Identity

<80-75% Identity

<75-70% Identity

<70% Identity

**-**

Gene Set Color Code

**+**

Gene

Pseudogene

Other

**-**

Topology/Coverage Color Code

Coverage Plus Strand

Coverage Minus Strand

Mainstrand: Plus

Mainstrand: Minus

Complementary Strand

Flanking Region  
(if option -flank >0)

Gene Set Annotation  
  
RepeatMasker Annotation  

**1. AlRepD-8475**: 8-92 (+), Divergence to consensus: 22.4%  
**2. (AGCT)n**: 806-829 (+), Divergence to consensus: 8.8%  
**3. TE-X-5\_DR**: 1342-1422 (+), Divergence to consensus: 28.4%  
**4. TE-X-4\_DR**: 1388-1530 (-), Divergence to consensus: 36.3%  
**5. (TGTCTG)n**: 4313-4350 (+), Divergence to consensus: 19.6%  
**6. (AC)n**: 4611-4630 (+), Divergence to consensus: 0%  
**7. AlRepB-392**: 5557-5737 (+), Divergence to consensus: 12.2%  
**8. AlRepD-1024**: 5786-5892 (+), Divergence to consensus: 10.3%  
**9. AlRepC-1280**: 5891-5963 (+), Divergence to consensus: 5.5%  
**10. SINE2-1\_AFC**: 6272-6337 (-), Divergence to consensus: 17.9%  
**11. Harbinger-2N1\_DR**: 6338-6482 (+), Divergence to consensus: 16.2%  
**12. AlRepD-1024**: 6496-6891 (+), Divergence to consensus: 14.8%  
**13. AlRepB-392**: 6892-6924 (+), Divergence to consensus: 12.1%  
**14. AlRepB-392**: 7067-7441 (+), Divergence to consensus: 17.1%  
**15. AlRepC-1433**: 7444-7893 (+), Divergence to consensus: 24.8%  
**16. AlRepC-1574**: 8262-8401 (+), Divergence to consensus: 33.2%  
**17. AlRepC-1574**: 8507-8664 (+), Divergence to consensus: 21.8%  
**18. AlRepD-209**: 8777-8839 (-), Divergence to consensus: 15.8%  
**19. AlRepD-209**: 9054-9215 (-), Divergence to consensus: 22.1%  
**20. AlRepB-128**: 9306-9383 (-), Divergence to consensus: 26.7%  
**21. AlRepD-4636**: 9381-9425 (+), Divergence to consensus: 15.5%  
**22. AlRepB-438**: 9414-9720 (-), Divergence to consensus: 5.2%  
**23. AlRepD-4636**: 9722-9944 (+), Divergence to consensus: 22.4%  
**24. AlRepB-250**: 9989-10119 (-), Divergence to consensus: 26.4%  
**25. AlRepB-128**: 10120-10210 (-), Divergence to consensus: 20.5%  
**26. AlRepD-880**: 10365-10513 (-), Divergence to consensus: 28.9%  
**27. L1-1\_AFC**: 10709-10854 (-), Divergence to consensus: 12%  
**28. AlRepB-923**: 10853-11083 (+), Divergence to consensus: 16.2%  
**29. AlRepB-185**: 11084-11283 (+), Divergence to consensus: 19.7%  
**30. Harbinger-3\_BF**: 11856-11991 (+), Divergence to consensus: 33.1%  
**31. hAT-N21\_DR**: 12314-12326 (-), Divergence to consensus: 19.2%  
**32. AlRepC-436**: 12327-12410 (-), Divergence to consensus: 1.2%  
**33. AlRepB-234**: 12398-12796 (+), Divergence to consensus: 6.6%  
**34. AlRepB-234**: 12796-12876 (+), Divergence to consensus: 16.1%

  
Transcription Factor Binding Sites  

**RHOXF1** (Sequence: GGATCA (-): 667)  
**RHOXF1** (Sequence: AGCTTA (-): 1165)  
**RHOXF1** (Sequence: AGATCA (-): 1632)  
**RHOXF1** (Sequence: GGATTA (-): 2360)  
**RHOXF1** (Sequence: AGATTA (-): 2383)  
**RHOXF1** (Sequence: AGATTA (-): 2467)  
**RHOXF1** (Sequence: GGCTCA (-): 2604)  
**RHOXF1** (Sequence: AGATCA (-): 2987)  
**RHOXF1** (Sequence: GGATCA (-): 3415)  
**RHOXF1** (Sequence: AGCTCA (-): 3849)  
**RHOXF1** (Sequence: AGCTTA (-): 4442)  
**RHOXF1** (Sequence: GGATCA (-): 4515)  
**RHOXF1** (Sequence: AGATCA (-): 4578)  
**RHOXF1** (Sequence: AGCTCA (-): 5042)  
**RHOXF1** (Sequence: GGCTTA (-): 5058)  
**RHOXF1** (Sequence: AGATTA (-): 5348)  
**RHOXF1** (Sequence: AGATTA (-): 5672)  
**RHOXF1** (Sequence: AGATCA (-): 5675)  
**RHOXF1** (Sequence: AGCTTA (-): 5853)  
**RHOXF1** (Sequence: AGCTCA (-): 6204)  
**RHOXF1** (Sequence: AGCTTA (-): 6562)  
**RHOXF1** (Sequence: AGCTTA (-): 7098)  
**RHOXF1** (Sequence: GGATCA (-): 7549)  
**RHOXF1** (Sequence: AGATCA (-): 8058)  
**RHOXF1** (Sequence: GGATTA (-): 10134)  
**RHOXF1** (Sequence: AGATTA (-): 11054)  
**RHOXF1** (Sequence: GGCTCA (-): 12032)  
**RHOXF1** (Sequence: AGATCA (-): 12379)  
**RHOXF1** (Sequence: AGCTTA (-): 12445)  
**RHOXF1** (Sequence: TAAGCT (+): 65)  
**RHOXF1** (Sequence: TAAGCT (+): 187)  
**RHOXF1** (Sequence: TAATCT (+): 2836)  
**RHOXF1** (Sequence: TGAGCT (+): 3819)  
**RHOXF1** (Sequence: TGAGCT (+): 4393)  
**RHOXF1** (Sequence: TGAGCT (+): 4440)  
**RHOXF1** (Sequence: TGATCT (+): 5681)  
**RHOXF1** (Sequence: TAATCC (+): 6214)  
**RHOXF1** (Sequence: TGAGCT (+): 7892)  
**RHOXF1** (Sequence: TAATCC (+): 8788)  
**RHOXF1** (Sequence: TGAGCT (+): 9125)  
**RHOXF1** (Sequence: TGATCT (+): 9192)  
**RHOXF1** (Sequence: TAAGCT (+): 9195)  
**RHOXF1** (Sequence: TAATCT (+): 9372)  
**RHOXF1** (Sequence: TGAGCC (+): 9642)  
**RHOXF1** (Sequence: TAAGCT (+): 11029)  
**RHOXF1** (Sequence: TGATCC (+): 12273)  
**Lhx8** (Sequence: CTAATTAG (-): 91)  
**Gata4** (Sequence: CTTATCT (+): 1167)  
**Gata4** (Sequence: CTTATCT (+): 3185)  
**Gata4** (Sequence: CTTATCT (+): 3994)  
**POU5F1** (Sequence: TTTGCAT (-): 865)  
**POU5F1** (Sequence: TTTGCAT (-): 7013)  
**POU5F1** (Sequence: TTTGCAT (-): 7214)  
**POU5F1** (Sequence: TTTGCAT (-): 8076)  
**POU5F1** (Sequence: TTTGCAT (-): 9999)  
**POU5F1** (Sequence: TTTGCAT (-): 10826)  
**RFX4\_2** (Sequence: GTATCCATG (-): 4038)  
**RFX4\_2** (Sequence: GTATCCAGG (-): 8310)  
**RFX4\_1** (Sequence: GTTGCTAGG (-): 2426)  
**SOX9** (Sequence: AACAATAG (-): 5065)  
**SOX9** (Sequence: AACAATAA (-): 9591)  
**SOX9** (Sequence: AACAATGG (-): 11423)  
**FOXO1** (Sequence: GTTGTTTAT (+): 3663)  
**FOXO1** (Sequence: GTTGTTTAT (+): 10870)  
**FOXO1** (Sequence: GTTGTTTTC (+): 12124)  
**FOXO3\_mmu** (Sequence: TGTTTTCC (-): 268)  
**FOXO3\_mmu** (Sequence: TGTTTAGC (-): 276)  
**FOXO3\_mmu** (Sequence: TGTTTTCA (-): 5172)  
**FOXO3\_mmu** (Sequence: TGTTTTGC (-): 8073)  
**FOXO3\_mmu** (Sequence: TGTTTTCC (-): 12126)  
**FOXO3\_mmu** (Sequence: TGTTTACA (-): 12627)  
**Sox5** (Sequence: ATTGTT (+): 111)  
**Sox5** (Sequence: ATTGTT (+): 904)  
**Sox5** (Sequence: ATTGTT (+): 1002)  
**Sox5** (Sequence: ATTGTT (+): 1859)  
**Sox5** (Sequence: ATTGTT (+): 1952)  
**Sox5** (Sequence: ATTGTT (+): 2210)  
**Sox5** (Sequence: ATTGTT (+): 5858)  
**Sox5** (Sequence: ATTGTT (+): 5917)  
**Sox5** (Sequence: ATTGTT (+): 6316)  
**Sox5** (Sequence: ATTGTT (+): 6567)  
**Sox5** (Sequence: ATTGTT (+): 8747)  
**Sox5** (Sequence: ATTGTT (+): 10734)  
**Sox5** (Sequence: ATTGTT (+): 10835)  
**Sox5** (Sequence: ATTGTT (+): 12222)  
**FIGLA** (Sequence: AACAGGTGGA (-): 10154)  
**SOX9** (Sequence: TTATTGTT (+): 5856)  
**SOX9** (Sequence: TTATTGTT (+): 6565)  
**SOX9** (Sequence: TTATTGTT (+): 10833)  
**FOXO3\_mmu** (Sequence: GCAAAACA (+): 8028)  
**FOXO3\_mmu** (Sequence: GGAAAACA (+): 8116)  
**FOXO3\_mmu** (Sequence: GGAAAACA (+): 8294)  
**FOXO3\_mmu** (Sequence: TCTAAACA (+): 8443)  
**Nobox** (Sequence: ACTAATTA (-): 90)  
**Nobox** (Sequence: ACCAATTA (-): 8727)  
**Nobox** (Sequence: AGTAATTA (-): 12562)  
**FOXO1** (Sequence: GAAAACAAC (-): 8295)  
**FOXO1** (Sequence: ATAAACAGG (-): 10123)  
**FOXO3\_hsa** (Sequence: TTGTTTAC (-): 12626)  
**FOXP1** (Sequence: TGTTTAC (-): 11278)  
**FOXP1** (Sequence: TGTTTAC (-): 12627)  
**Nobox** (Sequence: TAATTGCT (+): 8549)  
**Nobox** (Sequence: TAATTAGT (+): 10250)  
**Rhox11** (Sequence: TGGTGTATT (+): 1106)  
**Gata4** (Sequence: AGATAAG (-): 6673)  
**Sox5** (Sequence: AACAAT (-): 770)  
**Sox5** (Sequence: AACAAT (-): 5065)  
**Sox5** (Sequence: AACAAT (-): 9591)  
**Sox5** (Sequence: AACAAT (-): 10387)  
**Sox5** (Sequence: AACAAT (-): 11423)  
**POU2F1** (Sequence: TATTTTAAT (+): 894)  
**POU2F1** (Sequence: TATTTTAAT (+): 12531)  
**POU5F1** (Sequence: ATGCAAA (+): 2540)  
**Mybl1\_1** (Sequence: AACCGTTA (+): 5507)
