# Supplementary material for: First characterization of PIWI-interacting RNA clusters in a cichlid fish with a B chromosome
Source: BMC Biol. 2022 Sep 21;20:204. doi: 10.1186/s12915-022-01403-2 (PMC9490952; doi:10.1186/s12915-022-01403-2)
Supplement: Supplementary file 1 — Additional file 1. Zipped folder with fasta and interactive html piRNA cluster information for the A. latifasciata genome. The nomenclature is as follows: number-pirna-cluster_sex_B-presence (f, female; m, male; 0b, without B chromosome; 1b, with B chromosome). [file 12915_2022_1403_MOESM1_ESM.zip › 102_m1b.html]

piRNA cluster 102\_m1b 63


Predicted piRNA cluster no. 102\_m1b
  

Show proTRAC run info
Hide proTRAC run info

/\  
                \_\_\_\_\_\_\_\_\_\_\_\_\_\_\_\_\_\_\_\_\_\_\_/\\_\_\_ /  \\_\_\_\_\_\_\_  
               I                      /  \  /    \      I  
               I     pro             /    \/      \     I  
               I        TRAC        /               \   I  
               I   \_\_\_\_\_\_\_\_\_\_\_\_\_\_\_\_/\_\_\_\_\_\_\_\_\_\_\_\_\_\_\_\_\_\\_ I  
               I   \              /                     I  
               I    \            /                      I  
               I     \  /\      /       V.2.4.2         I  
               I      \/  \    /                        I  
               I\_\_\_\_\_\_\_\_\_\_\_\  /\_\_\_\_\_\_\_\_\_\_\_\_\_\_\_\_\_\_\_\_\_\_\_\_\_I  
                            \/  
  
  
================================= proTRAC ====================================  
VERSION: .......... 2.4.2  
LAST MODIFIED: .... 11. May 2018  
  
Please cite:  
Rosenkranz D, Zischler H. proTRAC - a software for probabilistic piRNA cluster  
detection, visualization and analysis. 2012. BMC Bioinformatics 13:5.  
  
  
Contact:  
David Rosenkranz  
Institute of Organismic and Molecular Evolutionary Biology  
Dept. Anthropology, small RNA group  
Johannes Gutenberg University Mainz  
email: rosenkranz@uni-mainz.de  
  
You can find the latest proTRAC version at:  
http://sourceforge.net/projects/protrac/files  
http://www.smallRNAgroup-mainz.de/software  
==============================================================================  
  
PARAMETERS:  
Map file: ...............piwi-machos-1B.fa-collapse.map  
Genome file: ............../../../0B\_ala\_genome.fa  
RepeatMasker annotation: Alatifasciata-all0B-maryan-v2.fa\_corrected.out  
GeneSet:................./guest-storage/Data/annotation/Alatifasciata\_all0B\_maryan-v2\_out2017.gff  
  
Significant (p<=0.01) hit density will be calculated based  
on observed hit distribution.  
  
Sliding window size: ........................................ 5000 bp  
Sliding window increament: .................................. 1000 bp  
Normalize each hit by number of genomic hits: ............... yes  
Normalize each hit by number of sequence reads: ............. yes  
Normalize values (-> per million mapped reads): ............. yes  
Min. fraction of hits with 1T(U) or 10A: .................... 0.75  
Alternatively: Min. fraction of hits with 1T(U) and 10A: .... 0.5  
Min. fraction of hits with typical piRNA length: ............ 0.75  
Typical piRNA length: ....................................... 24-32 nt  
Min. size of a piRNA cluster: ............................... 1000 bp.  
Min. number of hits (absolute): ............................. 0  
Min. number of hits (normalized): ........................... 0  
Min. fraction of hits on the mainstrand: .................... 0.75  
Top fraction of mapped sequences (in terms of read counts): . 1%  
Top fraction accounts for max. n% of sequence reads: ........ 90%  
Min. fraction of hits on each arm of a bidirectional cluster: 0.05  
Output html file for each cluster: .......................... yes  
Output a summary table: ..................................... yes  
Output a FASTA file for each cluster (piRNA sequences): ..... yes  
Output a FASTA file comprising cluster sequences: ........... yes  
Output a GTF file for predicted piRNA clusters: ..............yes  
Search DNA motifs in clusters: .............................. yes  
Output flanking sequences: +/- .............................. 0 bp  
Output ~.pTi file: .......................................... no  
==============================================================================  
  
  
Genome size (without gaps): ............ 758543724 bp  
Gaps (N/X/-): .......................... 417479 bp  
Mapped reads: .......................... 26973943  
Non-identical sequences: ............... 6209225  
Genomic hits: .......................... 48438990  
Significant densitiy of mapped reads: .. 821.144211136946 reads/kb

Show proTRAC cluster info
Hide proTRAC cluster info

|  |  |
| --- | --- |
| Location | NODE\_268840\_length\_12752\_cov\_27.436010 |
| Coordinates | 6-12873 |
| Size [bp] | 12868 |
| Sequence hit loci | 10489 |
| Mapped reads (normalized) | 37564.9 |
| Mapped reads (normalized) per kb | 2919.3 |
| Normalized reads with 1T (1U) | 78% |
| Normalized reads with 10A | 28.6% |
| Normalized reads with length 24-32 nt | 98.9% |
| Normalized reads on the main strand(s) | 92.1% |
| Predicted directionality | mono:minus |

100%

0%

1T (1U)  
reads

10A reads

24-32 nt  
reads

reads on mainstrand

**Either the amount of reads with 1T (1U) OR 10A has to exceed 75% (set with option: -1Tor10A)  
Alternatively the amount of reads with 1T (1U) AND 10A has to exceed 50% (set with option: -1Tand10A)  
Minimum amount of reads with preferred size is 75% (set with option: -pisize)  
Minimum amount of reads on the main strand(s) is 75% (set with option: -clstrand)**

Show read coverage
Hide read coverage

WHAT DO I SEE HERE?  
This chart shows the location of mapped sequence reads within a predicted piRNA cluster. The color refers to the number of genomic hits produced by the sequence read in question. A dark red bar indicates that this sequence read produces many other hits elsewhere in the genome. Many adjacent red or yellow bars can indicate the presence of a multi-copy element such as transposons or rRNA genes. A dark green bar indicates that this sequence read maps uniquely to this locus.

1 hit

2-5 hits

6-10 hits

11-20 hits

21-50 hits

51-100 hits

> 100 hits

NODE\_268840\_length\_12752\_cov\_27.436010

6

12873

Gene Set

RepeatMasker

Mapped  
Reads

112.2

plus strand

minus strand

112.2

Region: NODE\_268840\_length\_12752\_cov\_27.436010 10240-18. Max. coverage (+): 0. Max coverage (-): 0.05

Region: NODE\_268840\_length\_12752\_cov\_27.436010 19-44. Max. coverage (+): 0.19. Max coverage (-): 0.11

Region: NODE\_268840\_length\_12752\_cov\_27.436010 45-70. Max. coverage (+): 0.04. Max coverage (-): 0.78

Region: NODE\_268840\_length\_12752\_cov\_27.436010 71-96. Max. coverage (+): 0.61. Max coverage (-): 0.11

Region: NODE\_268840\_length\_12752\_cov\_27.436010 97-121. Max. coverage (+): 0. Max coverage (-): 0.15

Region: NODE\_268840\_length\_12752\_cov\_27.436010 122-147. Max. coverage (+): 0.04. Max coverage (-): 0.07

Region: NODE\_268840\_length\_12752\_cov\_27.436010 148-173. Max. coverage (+): 0. Max coverage (-): 0.19

Region: NODE\_268840\_length\_12752\_cov\_27.436010 174-199. Max. coverage (+): 0.04. Max coverage (-): 0.52

Region: NODE\_268840\_length\_12752\_cov\_27.436010 200-224. Max. coverage (+): 0. Max coverage (-): 0.56

Region: NODE\_268840\_length\_12752\_cov\_27.436010 225-250. Max. coverage (+): 0.04. Max coverage (-): 0.74

Region: NODE\_268840\_length\_12752\_cov\_27.436010 251-276. Max. coverage (+): 0.04. Max coverage (-): 0.15

Region: NODE\_268840\_length\_12752\_cov\_27.436010 277-301. Max. coverage (+): 0. Max coverage (-): 0.26

Region: NODE\_268840\_length\_12752\_cov\_27.436010 302-327. Max. coverage (+): 0. Max coverage (-): 1.08

Region: NODE\_268840\_length\_12752\_cov\_27.436010 328-353. Max. coverage (+): 0.15. Max coverage (-): 0.96

Region: NODE\_268840\_length\_12752\_cov\_27.436010 354-379. Max. coverage (+): 0.04. Max coverage (-): 0.07

Region: NODE\_268840\_length\_12752\_cov\_27.436010 380-404. Max. coverage (+): 0.04. Max coverage (-): 0.19

Region: NODE\_268840\_length\_12752\_cov\_27.436010 405-430. Max. coverage (+): 0.56. Max coverage (-): 3.15

Region: NODE\_268840\_length\_12752\_cov\_27.436010 431-456. Max. coverage (+): 0.04. Max coverage (-): 0.37

Region: NODE\_268840\_length\_12752\_cov\_27.436010 457-482. Max. coverage (+): 0. Max coverage (-): 0.3

Region: NODE\_268840\_length\_12752\_cov\_27.436010 483-507. Max. coverage (+): 0.11. Max coverage (-): 0.48

Region: NODE\_268840\_length\_12752\_cov\_27.436010 508-533. Max. coverage (+): 0. Max coverage (-): 3.45

Region: NODE\_268840\_length\_12752\_cov\_27.436010 534-559. Max. coverage (+): 1.26. Max coverage (-): 1.67

Region: NODE\_268840\_length\_12752\_cov\_27.436010 560-585. Max. coverage (+): 0.04. Max coverage (-): 1.15

Region: NODE\_268840\_length\_12752\_cov\_27.436010 586-610. Max. coverage (+): 0.63. Max coverage (-): 0.41

Region: NODE\_268840\_length\_12752\_cov\_27.436010 611-636. Max. coverage (+): 0.63. Max coverage (-): 1.89

Region: NODE\_268840\_length\_12752\_cov\_27.436010 637-662. Max. coverage (+): 0.26. Max coverage (-): 4.56

Region: NODE\_268840\_length\_12752\_cov\_27.436010 663-688. Max. coverage (+): 1.37. Max coverage (-): 1.82

Region: NODE\_268840\_length\_12752\_cov\_27.436010 689-713. Max. coverage (+): 0.04. Max coverage (-): 16.13

Region: NODE\_268840\_length\_12752\_cov\_27.436010 714-739. Max. coverage (+): 0.11. Max coverage (-): 16.35

Region: NODE\_268840\_length\_12752\_cov\_27.436010 740-765. Max. coverage (+): 0. Max coverage (-): 0.33

Region: NODE\_268840\_length\_12752\_cov\_27.436010 766-790. Max. coverage (+): 0.04. Max coverage (-): 0.37

Region: NODE\_268840\_length\_12752\_cov\_27.436010 791-816. Max. coverage (+): 0.04. Max coverage (-): 1.33

Region: NODE\_268840\_length\_12752\_cov\_27.436010 817-842. Max. coverage (+): 0. Max coverage (-): 3.04

Region: NODE\_268840\_length\_12752\_cov\_27.436010 843-868. Max. coverage (+): 0. Max coverage (-): 0.67

Region: NODE\_268840\_length\_12752\_cov\_27.436010 869-893. Max. coverage (+): 0.07. Max coverage (-): 1.26

Region: NODE\_268840\_length\_12752\_cov\_27.436010 894-919. Max. coverage (+): 0. Max coverage (-): 0.22

Region: NODE\_268840\_length\_12752\_cov\_27.436010 920-945. Max. coverage (+): 0.3. Max coverage (-): 0.22

Region: NODE\_268840\_length\_12752\_cov\_27.436010 946-971. Max. coverage (+): 0.15. Max coverage (-): 0.67

Region: NODE\_268840\_length\_12752\_cov\_27.436010 972-996. Max. coverage (+): 0. Max coverage (-): 26.73

Region: NODE\_268840\_length\_12752\_cov\_27.436010 997-1022. Max. coverage (+): 0.22. Max coverage (-): 0.22

Region: NODE\_268840\_length\_12752\_cov\_27.436010 1023-1048. Max. coverage (+): 0.04. Max coverage (-): 5

Region: NODE\_268840\_length\_12752\_cov\_27.436010 1049-1074. Max. coverage (+): 0.56. Max coverage (-): 0.41

Region: NODE\_268840\_length\_12752\_cov\_27.436010 1075-1099. Max. coverage (+): 0.56. Max coverage (-): 0.41

Region: NODE\_268840\_length\_12752\_cov\_27.436010 1100-1125. Max. coverage (+): 1.11. Max coverage (-): 4.19

Region: NODE\_268840\_length\_12752\_cov\_27.436010 1126-1151. Max. coverage (+): 0.04. Max coverage (-): 5.12

Region: NODE\_268840\_length\_12752\_cov\_27.436010 1152-1176. Max. coverage (+): 0.07. Max coverage (-): 2.45

Region: NODE\_268840\_length\_12752\_cov\_27.436010 1177-1202. Max. coverage (+): 0.3. Max coverage (-): 2.45

Region: NODE\_268840\_length\_12752\_cov\_27.436010 1203-1228. Max. coverage (+): 0. Max coverage (-): 0.07

Region: NODE\_268840\_length\_12752\_cov\_27.436010 1229-1254. Max. coverage (+): 0.11. Max coverage (-): 2.15

Region: NODE\_268840\_length\_12752\_cov\_27.436010 1255-1279. Max. coverage (+): 0.07. Max coverage (-): 2.45

Region: NODE\_268840\_length\_12752\_cov\_27.436010 1280-1305. Max. coverage (+): 0. Max coverage (-): 0.3

Region: NODE\_268840\_length\_12752\_cov\_27.436010 1306-1331. Max. coverage (+): 0.19. Max coverage (-): 3

Region: NODE\_268840\_length\_12752\_cov\_27.436010 1332-1357. Max. coverage (+): 0.26. Max coverage (-): 0.15

Region: NODE\_268840\_length\_12752\_cov\_27.436010 1358-1382. Max. coverage (+): 0.33. Max coverage (-): 2.52

Region: NODE\_268840\_length\_12752\_cov\_27.436010 1383-1408. Max. coverage (+): 0.22. Max coverage (-): 4.75

Region: NODE\_268840\_length\_12752\_cov\_27.436010 1409-1434. Max. coverage (+): 0.56. Max coverage (-): 6.38

Region: NODE\_268840\_length\_12752\_cov\_27.436010 1435-1460. Max. coverage (+): 0.04. Max coverage (-): 0.96

Region: NODE\_268840\_length\_12752\_cov\_27.436010 1461-1485. Max. coverage (+): 0.15. Max coverage (-): 1.19

Region: NODE\_268840\_length\_12752\_cov\_27.436010 1486-1511. Max. coverage (+): 0.44. Max coverage (-): 0.89

Region: NODE\_268840\_length\_12752\_cov\_27.436010 1512-1537. Max. coverage (+): 0.15. Max coverage (-): 5.45

Region: NODE\_268840\_length\_12752\_cov\_27.436010 1538-1563. Max. coverage (+): 0.04. Max coverage (-): 1.56

Region: NODE\_268840\_length\_12752\_cov\_27.436010 1564-1588. Max. coverage (+): 0.22. Max coverage (-): 0.3

Region: NODE\_268840\_length\_12752\_cov\_27.436010 1589-1614. Max. coverage (+): 0.26. Max coverage (-): 0.26

Region: NODE\_268840\_length\_12752\_cov\_27.436010 1615-1640. Max. coverage (+): 0.07. Max coverage (-): 4.08

Region: NODE\_268840\_length\_12752\_cov\_27.436010 1641-1665. Max. coverage (+): 0.15. Max coverage (-): 0.44

Region: NODE\_268840\_length\_12752\_cov\_27.436010 1666-1691. Max. coverage (+): 0.07. Max coverage (-): 1

Region: NODE\_268840\_length\_12752\_cov\_27.436010 1692-1717. Max. coverage (+): 0.26. Max coverage (-): 4.34

Region: NODE\_268840\_length\_12752\_cov\_27.436010 1718-1743. Max. coverage (+): 0.11. Max coverage (-): 9.97

Region: NODE\_268840\_length\_12752\_cov\_27.436010 1744-1768. Max. coverage (+): 0.04. Max coverage (-): 6.86

Region: NODE\_268840\_length\_12752\_cov\_27.436010 1769-1794. Max. coverage (+): 0.19. Max coverage (-): 2.6

Region: NODE\_268840\_length\_12752\_cov\_27.436010 1795-1820. Max. coverage (+): 0.19. Max coverage (-): 3.08

Region: NODE\_268840\_length\_12752\_cov\_27.436010 1821-1846. Max. coverage (+): 0.52. Max coverage (-): 1.52

Region: NODE\_268840\_length\_12752\_cov\_27.436010 1847-1871. Max. coverage (+): 1. Max coverage (-): 2.26

Region: NODE\_268840\_length\_12752\_cov\_27.436010 1872-1897. Max. coverage (+): 0.07. Max coverage (-): 0.7

Region: NODE\_268840\_length\_12752\_cov\_27.436010 1898-1923. Max. coverage (+): 0.22. Max coverage (-): 0.44

Region: NODE\_268840\_length\_12752\_cov\_27.436010 1924-1949. Max. coverage (+): 0.04. Max coverage (-): 3.3

Region: NODE\_268840\_length\_12752\_cov\_27.436010 1950-1974. Max. coverage (+): 0.04. Max coverage (-): 0.63

Region: NODE\_268840\_length\_12752\_cov\_27.436010 1975-2000. Max. coverage (+): 0.48. Max coverage (-): 2.15

Region: NODE\_268840\_length\_12752\_cov\_27.436010 2001-2026. Max. coverage (+): 0.07. Max coverage (-): 1.82

Region: NODE\_268840\_length\_12752\_cov\_27.436010 2027-2052. Max. coverage (+): 0.07. Max coverage (-): 0.37

Region: NODE\_268840\_length\_12752\_cov\_27.436010 2053-2077. Max. coverage (+): 0. Max coverage (-): 0.59

Region: NODE\_268840\_length\_12752\_cov\_27.436010 2078-2103. Max. coverage (+): 0.04. Max coverage (-): 0.82

Region: NODE\_268840\_length\_12752\_cov\_27.436010 2104-2129. Max. coverage (+): 0.44. Max coverage (-): 2.85

Region: NODE\_268840\_length\_12752\_cov\_27.436010 2130-2154. Max. coverage (+): 0. Max coverage (-): 0.07

Region: NODE\_268840\_length\_12752\_cov\_27.436010 2155-2180. Max. coverage (+): 0. Max coverage (-): 1.15

Region: NODE\_268840\_length\_12752\_cov\_27.436010 2181-2206. Max. coverage (+): 0.07. Max coverage (-): 0.78

Region: NODE\_268840\_length\_12752\_cov\_27.436010 2207-2232. Max. coverage (+): 0.04. Max coverage (-): 1.63

Region: NODE\_268840\_length\_12752\_cov\_27.436010 2233-2257. Max. coverage (+): 0.04. Max coverage (-): 0.22

Region: NODE\_268840\_length\_12752\_cov\_27.436010 2258-2283. Max. coverage (+): 0.04. Max coverage (-): 4.56

Region: NODE\_268840\_length\_12752\_cov\_27.436010 2284-2309. Max. coverage (+): 0.04. Max coverage (-): 0.82

Region: NODE\_268840\_length\_12752\_cov\_27.436010 2310-2335. Max. coverage (+): 0.02. Max coverage (-): 0.84

Region: NODE\_268840\_length\_12752\_cov\_27.436010 2336-2360. Max. coverage (+): 0.07. Max coverage (-): 0.31

Region: NODE\_268840\_length\_12752\_cov\_27.436010 2361-2386. Max. coverage (+): 0.04. Max coverage (-): 0.59

Region: NODE\_268840\_length\_12752\_cov\_27.436010 2387-2412. Max. coverage (+): 0.01. Max coverage (-): 0.21

Region: NODE\_268840\_length\_12752\_cov\_27.436010 2413-2438. Max. coverage (+): 0. Max coverage (-): 0.51

Region: NODE\_268840\_length\_12752\_cov\_27.436010 2439-2463. Max. coverage (+): 0.06. Max coverage (-): 0.61

Region: NODE\_268840\_length\_12752\_cov\_27.436010 2464-2489. Max. coverage (+): 0.05. Max coverage (-): 0.01

Region: NODE\_268840\_length\_12752\_cov\_27.436010 2490-2515. Max. coverage (+): 0.22. Max coverage (-): 2.62

Region: NODE\_268840\_length\_12752\_cov\_27.436010 2516-2540. Max. coverage (+): 0.04. Max coverage (-): 0.17

Region: NODE\_268840\_length\_12752\_cov\_27.436010 2541-2566. Max. coverage (+): 0.04. Max coverage (-): 112.2

Region: NODE\_268840\_length\_12752\_cov\_27.436010 2567-2592. Max. coverage (+): 0.2. Max coverage (-): 2.56

Region: NODE\_268840\_length\_12752\_cov\_27.436010 2593-2618. Max. coverage (+): 0.39. Max coverage (-): 73.81

Region: NODE\_268840\_length\_12752\_cov\_27.436010 2619-2643. Max. coverage (+): 2.19. Max coverage (-): 7.27

Region: NODE\_268840\_length\_12752\_cov\_27.436010 2644-2669. Max. coverage (+): 0.19. Max coverage (-): 7.14

Region: NODE\_268840\_length\_12752\_cov\_27.436010 2670-2695. Max. coverage (+): 0.24. Max coverage (-): 2.6

Region: NODE\_268840\_length\_12752\_cov\_27.436010 2696-2721. Max. coverage (+): 0.3. Max coverage (-): 0.22

Region: NODE\_268840\_length\_12752\_cov\_27.436010 2722-2746. Max. coverage (+): 0.07. Max coverage (-): 0.33

Region: NODE\_268840\_length\_12752\_cov\_27.436010 2747-2772. Max. coverage (+): 0.07. Max coverage (-): 0.78

Region: NODE\_268840\_length\_12752\_cov\_27.436010 2773-2798. Max. coverage (+): 0.32. Max coverage (-): 2.15

Region: NODE\_268840\_length\_12752\_cov\_27.436010 2799-2824. Max. coverage (+): 0.26. Max coverage (-): 4.52

Region: NODE\_268840\_length\_12752\_cov\_27.436010 2825-2849. Max. coverage (+): 0.04. Max coverage (-): 4.12

Region: NODE\_268840\_length\_12752\_cov\_27.436010 2850-2875. Max. coverage (+): 0.37. Max coverage (-): 6.43

Region: NODE\_268840\_length\_12752\_cov\_27.436010 2876-2901. Max. coverage (+): 0.04. Max coverage (-): 2.6

Region: NODE\_268840\_length\_12752\_cov\_27.436010 2902-2927. Max. coverage (+): 0. Max coverage (-): 0.48

Region: NODE\_268840\_length\_12752\_cov\_27.436010 2928-2952. Max. coverage (+): 0.59. Max coverage (-): 0.48

Region: NODE\_268840\_length\_12752\_cov\_27.436010 2953-2978. Max. coverage (+): 0.3. Max coverage (-): 2.02

Region: NODE\_268840\_length\_12752\_cov\_27.436010 2979-3004. Max. coverage (+): 0.09. Max coverage (-): 1.52

Region: NODE\_268840\_length\_12752\_cov\_27.436010 3005-3029. Max. coverage (+): 0.33. Max coverage (-): 0.78

Region: NODE\_268840\_length\_12752\_cov\_27.436010 3030-3055. Max. coverage (+): 0.04. Max coverage (-): 3.41

Region: NODE\_268840\_length\_12752\_cov\_27.436010 3056-3081. Max. coverage (+): 0.04. Max coverage (-): 1.02

Region: NODE\_268840\_length\_12752\_cov\_27.436010 3082-3107. Max. coverage (+): 0.1. Max coverage (-): 0.56

Region: NODE\_268840\_length\_12752\_cov\_27.436010 3108-3132. Max. coverage (+): 0.11. Max coverage (-): 0.06

Region: NODE\_268840\_length\_12752\_cov\_27.436010 3133-3158. Max. coverage (+): 0.04. Max coverage (-): 0.11

Region: NODE\_268840\_length\_12752\_cov\_27.436010 3159-3184. Max. coverage (+): 0.04. Max coverage (-): 0.69

Region: NODE\_268840\_length\_12752\_cov\_27.436010 3185-3210. Max. coverage (+): 0.04. Max coverage (-): 0.44

Region: NODE\_268840\_length\_12752\_cov\_27.436010 3211-3235. Max. coverage (+): 0. Max coverage (-): 7.4

Region: NODE\_268840\_length\_12752\_cov\_27.436010 3236-3261. Max. coverage (+): 0.74. Max coverage (-): 18.72

Region: NODE\_268840\_length\_12752\_cov\_27.436010 3262-3287. Max. coverage (+): 1.74. Max coverage (-): 2.37

Region: NODE\_268840\_length\_12752\_cov\_27.436010 3288-3313. Max. coverage (+): 0.52. Max coverage (-): 1.35

Region: NODE\_268840\_length\_12752\_cov\_27.436010 3314-3338. Max. coverage (+): 1.15. Max coverage (-): 0.3

Region: NODE\_268840\_length\_12752\_cov\_27.436010 3339-3364. Max. coverage (+): 0.5. Max coverage (-): 0.57

Region: NODE\_268840\_length\_12752\_cov\_27.436010 3365-3390. Max. coverage (+): 0.04. Max coverage (-): 13.83

Region: NODE\_268840\_length\_12752\_cov\_27.436010 3391-3416. Max. coverage (+): 1.78. Max coverage (-): 4.45

Region: NODE\_268840\_length\_12752\_cov\_27.436010 3417-3441. Max. coverage (+): 0.04. Max coverage (-): 0.56

Region: NODE\_268840\_length\_12752\_cov\_27.436010 3442-3467. Max. coverage (+): 0. Max coverage (-): 5.97

Region: NODE\_268840\_length\_12752\_cov\_27.436010 3468-3493. Max. coverage (+): 0. Max coverage (-): 0.2

Region: NODE\_268840\_length\_12752\_cov\_27.436010 3494-3518. Max. coverage (+): 0. Max coverage (-): 0

Region: NODE\_268840\_length\_12752\_cov\_27.436010 3519-3544. Max. coverage (+): 0.02. Max coverage (-): 2.35

Region: NODE\_268840\_length\_12752\_cov\_27.436010 3545-3570. Max. coverage (+): 0.14. Max coverage (-): 0.39

Region: NODE\_268840\_length\_12752\_cov\_27.436010 3571-3596. Max. coverage (+): 0.02. Max coverage (-): 1.91

Region: NODE\_268840\_length\_12752\_cov\_27.436010 3597-3621. Max. coverage (+): 0.04. Max coverage (-): 1.45

Region: NODE\_268840\_length\_12752\_cov\_27.436010 3622-3647. Max. coverage (+): 0.14. Max coverage (-): 0.63

Region: NODE\_268840\_length\_12752\_cov\_27.436010 3648-3673. Max. coverage (+): 0. Max coverage (-): 0.83

Region: NODE\_268840\_length\_12752\_cov\_27.436010 3674-3699. Max. coverage (+): 0.02. Max coverage (-): 0.1

Region: NODE\_268840\_length\_12752\_cov\_27.436010 3700-3724. Max. coverage (+): 0. Max coverage (-): 2.6

Region: NODE\_268840\_length\_12752\_cov\_27.436010 3725-3750. Max. coverage (+): 0.02. Max coverage (-): 2.61

Region: NODE\_268840\_length\_12752\_cov\_27.436010 3751-3776. Max. coverage (+): 0.02. Max coverage (-): 0.41

Region: NODE\_268840\_length\_12752\_cov\_27.436010 3777-3802. Max. coverage (+): 0.17. Max coverage (-): 0.11

Region: NODE\_268840\_length\_12752\_cov\_27.436010 3803-3827. Max. coverage (+): 0.09. Max coverage (-): 0.89

Region: NODE\_268840\_length\_12752\_cov\_27.436010 3828-3853. Max. coverage (+): 0.83. Max coverage (-): 0.23

Region: NODE\_268840\_length\_12752\_cov\_27.436010 3854-3879. Max. coverage (+): 0.07. Max coverage (-): 9.14

Region: NODE\_268840\_length\_12752\_cov\_27.436010 3880-3905. Max. coverage (+): 0.06. Max coverage (-): 2.21

Region: NODE\_268840\_length\_12752\_cov\_27.436010 3906-3930. Max. coverage (+): 0.06. Max coverage (-): 1.58

Region: NODE\_268840\_length\_12752\_cov\_27.436010 3931-3956. Max. coverage (+): 0.11. Max coverage (-): 2.97

Region: NODE\_268840\_length\_12752\_cov\_27.436010 3957-3982. Max. coverage (+): 0.09. Max coverage (-): 1.19

Region: NODE\_268840\_length\_12752\_cov\_27.436010 3983-4007. Max. coverage (+): 0.04. Max coverage (-): 1.71

Region: NODE\_268840\_length\_12752\_cov\_27.436010 4008-4033. Max. coverage (+): 0.01. Max coverage (-): 0.82

Region: NODE\_268840\_length\_12752\_cov\_27.436010 4034-4059. Max. coverage (+): 0.02. Max coverage (-): 0.52

Region: NODE\_268840\_length\_12752\_cov\_27.436010 4060-4085. Max. coverage (+): 0.06. Max coverage (-): 0.61

Region: NODE\_268840\_length\_12752\_cov\_27.436010 4086-4110. Max. coverage (+): 0.06. Max coverage (-): 0.74

Region: NODE\_268840\_length\_12752\_cov\_27.436010 4111-4136. Max. coverage (+): 0.17. Max coverage (-): 4.23

Region: NODE\_268840\_length\_12752\_cov\_27.436010 4137-4162. Max. coverage (+): 0.14. Max coverage (-): 4.99

Region: NODE\_268840\_length\_12752\_cov\_27.436010 4163-4188. Max. coverage (+): 0.16. Max coverage (-): 1.54

Region: NODE\_268840\_length\_12752\_cov\_27.436010 4189-4213. Max. coverage (+): 0. Max coverage (-): 1.04

Region: NODE\_268840\_length\_12752\_cov\_27.436010 4214-4239. Max. coverage (+): 0.07. Max coverage (-): 1.61

Region: NODE\_268840\_length\_12752\_cov\_27.436010 4240-4265. Max. coverage (+): 0.2. Max coverage (-): 0.69

Region: NODE\_268840\_length\_12752\_cov\_27.436010 4266-4291. Max. coverage (+): 0.23. Max coverage (-): 1.52

Region: NODE\_268840\_length\_12752\_cov\_27.436010 4292-4316. Max. coverage (+): 0.07. Max coverage (-): 0.26

Region: NODE\_268840\_length\_12752\_cov\_27.436010 4317-4342. Max. coverage (+): 0.11. Max coverage (-): 3.67

Region: NODE\_268840\_length\_12752\_cov\_27.436010 4343-4368. Max. coverage (+): 1. Max coverage (-): 2.89

Region: NODE\_268840\_length\_12752\_cov\_27.436010 4369-4393. Max. coverage (+): 0.7. Max coverage (-): 68.66

Region: NODE\_268840\_length\_12752\_cov\_27.436010 4394-4419. Max. coverage (+): 0.48. Max coverage (-): 12.86

Region: NODE\_268840\_length\_12752\_cov\_27.436010 4420-4445. Max. coverage (+): 0.19. Max coverage (-): 3.04

Region: NODE\_268840\_length\_12752\_cov\_27.436010 4446-4471. Max. coverage (+): 0.04. Max coverage (-): 1.59

Region: NODE\_268840\_length\_12752\_cov\_27.436010 4472-4496. Max. coverage (+): 0.33. Max coverage (-): 2.41

Region: NODE\_268840\_length\_12752\_cov\_27.436010 4497-4522. Max. coverage (+): 0.15. Max coverage (-): 3.08

Region: NODE\_268840\_length\_12752\_cov\_27.436010 4523-4548. Max. coverage (+): 0.04. Max coverage (-): 3.63

Region: NODE\_268840\_length\_12752\_cov\_27.436010 4549-4574. Max. coverage (+): 0.04. Max coverage (-): 5.64

Region: NODE\_268840\_length\_12752\_cov\_27.436010 4575-4599. Max. coverage (+): 0.22. Max coverage (-): 0.26

Region: NODE\_268840\_length\_12752\_cov\_27.436010 4600-4625. Max. coverage (+): 0.04. Max coverage (-): 0.26

Region: NODE\_268840\_length\_12752\_cov\_27.436010 4626-4651. Max. coverage (+): 0.22. Max coverage (-): 2.63

Region: NODE\_268840\_length\_12752\_cov\_27.436010 4652-4677. Max. coverage (+): 0.04. Max coverage (-): 2.3

Region: NODE\_268840\_length\_12752\_cov\_27.436010 4678-4702. Max. coverage (+): 1.08. Max coverage (-): 4.34

Region: NODE\_268840\_length\_12752\_cov\_27.436010 4703-4728. Max. coverage (+): 1.08. Max coverage (-): 0.22

Region: NODE\_268840\_length\_12752\_cov\_27.436010 4729-4754. Max. coverage (+): 1.85. Max coverage (-): 1.45

Region: NODE\_268840\_length\_12752\_cov\_27.436010 4755-4780. Max. coverage (+): 0.04. Max coverage (-): 2.08

Region: NODE\_268840\_length\_12752\_cov\_27.436010 4781-4805. Max. coverage (+): 1.26. Max coverage (-): 1.33

Region: NODE\_268840\_length\_12752\_cov\_27.436010 4806-4831. Max. coverage (+): 0.15. Max coverage (-): 2.45

Region: NODE\_268840\_length\_12752\_cov\_27.436010 4832-4857. Max. coverage (+): 1.15. Max coverage (-): 2.78

Region: NODE\_268840\_length\_12752\_cov\_27.436010 4858-4882. Max. coverage (+): 0.07. Max coverage (-): 1.82

Region: NODE\_268840\_length\_12752\_cov\_27.436010 4883-4908. Max. coverage (+): 0.19. Max coverage (-): 16.02

Region: NODE\_268840\_length\_12752\_cov\_27.436010 4909-4934. Max. coverage (+): 0.37. Max coverage (-): 5.71

Region: NODE\_268840\_length\_12752\_cov\_27.436010 4935-4960. Max. coverage (+): 0.3. Max coverage (-): 1.08

Region: NODE\_268840\_length\_12752\_cov\_27.436010 4961-4985. Max. coverage (+): 0.22. Max coverage (-): 13.46

Region: NODE\_268840\_length\_12752\_cov\_27.436010 4986-5011. Max. coverage (+): 0.22. Max coverage (-): 1.41

Region: NODE\_268840\_length\_12752\_cov\_27.436010 5012-5037. Max. coverage (+): 0.33. Max coverage (-): 1.22

Region: NODE\_268840\_length\_12752\_cov\_27.436010 5038-5063. Max. coverage (+): 0.04. Max coverage (-): 0.85

Region: NODE\_268840\_length\_12752\_cov\_27.436010 5064-5088. Max. coverage (+): 0.04. Max coverage (-): 1.08

Region: NODE\_268840\_length\_12752\_cov\_27.436010 5089-5114. Max. coverage (+): 0. Max coverage (-): 0.47

Region: NODE\_268840\_length\_12752\_cov\_27.436010 5115-5140. Max. coverage (+): 0. Max coverage (-): 0.11

Region: NODE\_268840\_length\_12752\_cov\_27.436010 5141-5166. Max. coverage (+): 0.07. Max coverage (-): 0.07

Region: NODE\_268840\_length\_12752\_cov\_27.436010 5167-5191. Max. coverage (+): 0.15. Max coverage (-): 4.41

Region: NODE\_268840\_length\_12752\_cov\_27.436010 5192-5217. Max. coverage (+): 1.26. Max coverage (-): 0.59

Region: NODE\_268840\_length\_12752\_cov\_27.436010 5218-5243. Max. coverage (+): 0.07. Max coverage (-): 1.3

Region: NODE\_268840\_length\_12752\_cov\_27.436010 5244-5269. Max. coverage (+): 0. Max coverage (-): 1.59

Region: NODE\_268840\_length\_12752\_cov\_27.436010 5270-5294. Max. coverage (+): 0.15. Max coverage (-): 0.7

Region: NODE\_268840\_length\_12752\_cov\_27.436010 5295-5320. Max. coverage (+): 0.11. Max coverage (-): 1

Region: NODE\_268840\_length\_12752\_cov\_27.436010 5321-5346. Max. coverage (+): 0.15. Max coverage (-): 0.19

Region: NODE\_268840\_length\_12752\_cov\_27.436010 5347-5371. Max. coverage (+): 0. Max coverage (-): 0.59

Region: NODE\_268840\_length\_12752\_cov\_27.436010 5372-5397. Max. coverage (+): 0.07. Max coverage (-): 6.19

Region: NODE\_268840\_length\_12752\_cov\_27.436010 5398-5423. Max. coverage (+): 0.04. Max coverage (-): 6.36

Region: NODE\_268840\_length\_12752\_cov\_27.436010 5424-5449. Max. coverage (+): 0.15. Max coverage (-): 1.67

Region: NODE\_268840\_length\_12752\_cov\_27.436010 5450-5474. Max. coverage (+): 0.07. Max coverage (-): 0.82

Region: NODE\_268840\_length\_12752\_cov\_27.436010 5475-5500. Max. coverage (+): 1.45. Max coverage (-): 3.34

Region: NODE\_268840\_length\_12752\_cov\_27.436010 5501-5526. Max. coverage (+): 1. Max coverage (-): 0.52

Region: NODE\_268840\_length\_12752\_cov\_27.436010 5527-5552. Max. coverage (+): 0.37. Max coverage (-): 1.15

Region: NODE\_268840\_length\_12752\_cov\_27.436010 5553-5577. Max. coverage (+): 0.56. Max coverage (-): 1.3

Region: NODE\_268840\_length\_12752\_cov\_27.436010 5578-5603. Max. coverage (+): 0. Max coverage (-): 0.3

Region: NODE\_268840\_length\_12752\_cov\_27.436010 5604-5629. Max. coverage (+): 0.04. Max coverage (-): 0.07

Region: NODE\_268840\_length\_12752\_cov\_27.436010 5630-5655. Max. coverage (+): 0. Max coverage (-): 0.56

Region: NODE\_268840\_length\_12752\_cov\_27.436010 5656-5680. Max. coverage (+): 0.07. Max coverage (-): 0.19

Region: NODE\_268840\_length\_12752\_cov\_27.436010 5681-5706. Max. coverage (+): 0. Max coverage (-): 0.45

Region: NODE\_268840\_length\_12752\_cov\_27.436010 5707-5732. Max. coverage (+): 0. Max coverage (-): 0.12

Region: NODE\_268840\_length\_12752\_cov\_27.436010 5733-5757. Max. coverage (+): 0. Max coverage (-): 0.22

Region: NODE\_268840\_length\_12752\_cov\_27.436010 5758-5783. Max. coverage (+): 0.11. Max coverage (-): 0.56

Region: NODE\_268840\_length\_12752\_cov\_27.436010 5784-5809. Max. coverage (+): 0. Max coverage (-): 0.72

Region: NODE\_268840\_length\_12752\_cov\_27.436010 5810-5835. Max. coverage (+): 0. Max coverage (-): 0.35

Region: NODE\_268840\_length\_12752\_cov\_27.436010 5836-5860. Max. coverage (+): 0.04. Max coverage (-): 0.03

Region: NODE\_268840\_length\_12752\_cov\_27.436010 5861-5886. Max. coverage (+): 0.05. Max coverage (-): 0.02

Region: NODE\_268840\_length\_12752\_cov\_27.436010 5887-5912. Max. coverage (+): 0. Max coverage (-): 0

Region: NODE\_268840\_length\_12752\_cov\_27.436010 5913-5938. Max. coverage (+): 0. Max coverage (-): 0.04

Region: NODE\_268840\_length\_12752\_cov\_27.436010 5939-5963. Max. coverage (+): 0. Max coverage (-): 0

Region: NODE\_268840\_length\_12752\_cov\_27.436010 5964-5989. Max. coverage (+): 0. Max coverage (-): 0

Region: NODE\_268840\_length\_12752\_cov\_27.436010 5990-6015. Max. coverage (+): 0. Max coverage (-): 0

Region: NODE\_268840\_length\_12752\_cov\_27.436010 6016-6041. Max. coverage (+): 0. Max coverage (-): 0

Region: NODE\_268840\_length\_12752\_cov\_27.436010 6042-6066. Max. coverage (+): 0. Max coverage (-): 0

Region: NODE\_268840\_length\_12752\_cov\_27.436010 6067-6092. Max. coverage (+): 0. Max coverage (-): 0

Region: NODE\_268840\_length\_12752\_cov\_27.436010 6093-6118. Max. coverage (+): 0. Max coverage (-): 0

Region: NODE\_268840\_length\_12752\_cov\_27.436010 6119-6144. Max. coverage (+): 0. Max coverage (-): 0

Region: NODE\_268840\_length\_12752\_cov\_27.436010 6145-6169. Max. coverage (+): 0. Max coverage (-): 0

Region: NODE\_268840\_length\_12752\_cov\_27.436010 6170-6195. Max. coverage (+): 0. Max coverage (-): 0

Region: NODE\_268840\_length\_12752\_cov\_27.436010 6196-6221. Max. coverage (+): 0. Max coverage (-): 0

Region: NODE\_268840\_length\_12752\_cov\_27.436010 6222-6246. Max. coverage (+): 0. Max coverage (-): 0

Region: NODE\_268840\_length\_12752\_cov\_27.436010 6247-6272. Max. coverage (+): 0. Max coverage (-): 0.04

Region: NODE\_268840\_length\_12752\_cov\_27.436010 6273-6298. Max. coverage (+): 0. Max coverage (-): 0

Region: NODE\_268840\_length\_12752\_cov\_27.436010 6299-6324. Max. coverage (+): 0. Max coverage (-): 0

Region: NODE\_268840\_length\_12752\_cov\_27.436010 6325-6349. Max. coverage (+): 0. Max coverage (-): 0

Region: NODE\_268840\_length\_12752\_cov\_27.436010 6350-6375. Max. coverage (+): 0. Max coverage (-): 0

Region: NODE\_268840\_length\_12752\_cov\_27.436010 6376-6401. Max. coverage (+): 0. Max coverage (-): 0.01

Region: NODE\_268840\_length\_12752\_cov\_27.436010 6402-6427. Max. coverage (+): 0. Max coverage (-): 0

Region: NODE\_268840\_length\_12752\_cov\_27.436010 6428-6452. Max. coverage (+): 0. Max coverage (-): 0

Region: NODE\_268840\_length\_12752\_cov\_27.436010 6453-6478. Max. coverage (+): 0. Max coverage (-): 0

Region: NODE\_268840\_length\_12752\_cov\_27.436010 6479-6504. Max. coverage (+): 0. Max coverage (-): 0.72

Region: NODE\_268840\_length\_12752\_cov\_27.436010 6505-6530. Max. coverage (+): 0. Max coverage (-): 0.02

Region: NODE\_268840\_length\_12752\_cov\_27.436010 6531-6555. Max. coverage (+): 0. Max coverage (-): 0

Region: NODE\_268840\_length\_12752\_cov\_27.436010 6556-6581. Max. coverage (+): 0.01. Max coverage (-): 0.02

Region: NODE\_268840\_length\_12752\_cov\_27.436010 6582-6607. Max. coverage (+): 0. Max coverage (-): 0

Region: NODE\_268840\_length\_12752\_cov\_27.436010 6608-6633. Max. coverage (+): 0. Max coverage (-): 0

Region: NODE\_268840\_length\_12752\_cov\_27.436010 6634-6658. Max. coverage (+): 0.02. Max coverage (-): 0.22

Region: NODE\_268840\_length\_12752\_cov\_27.436010 6659-6684. Max. coverage (+): 0.33. Max coverage (-): 0.67

Region: NODE\_268840\_length\_12752\_cov\_27.436010 6685-6710. Max. coverage (+): 0.04. Max coverage (-): 0.09

Region: NODE\_268840\_length\_12752\_cov\_27.436010 6711-6735. Max. coverage (+): 0.07. Max coverage (-): 0.32

Region: NODE\_268840\_length\_12752\_cov\_27.436010 6736-6761. Max. coverage (+): 0.04. Max coverage (-): 0.04

Region: NODE\_268840\_length\_12752\_cov\_27.436010 6762-6787. Max. coverage (+): 0.09. Max coverage (-): 0.67

Region: NODE\_268840\_length\_12752\_cov\_27.436010 6788-6813. Max. coverage (+): 0.41. Max coverage (-): 0.95

Region: NODE\_268840\_length\_12752\_cov\_27.436010 6814-6838. Max. coverage (+): 0.07. Max coverage (-): 0.15

Region: NODE\_268840\_length\_12752\_cov\_27.436010 6839-6864. Max. coverage (+): 0.02. Max coverage (-): 1.38

Region: NODE\_268840\_length\_12752\_cov\_27.436010 6865-6890. Max. coverage (+): 0.11. Max coverage (-): 1.38

Region: NODE\_268840\_length\_12752\_cov\_27.436010 6891-6916. Max. coverage (+): 0.11. Max coverage (-): 0.56

Region: NODE\_268840\_length\_12752\_cov\_27.436010 6917-6941. Max. coverage (+): 1.33. Max coverage (-): 0.48

Region: NODE\_268840\_length\_12752\_cov\_27.436010 6942-6967. Max. coverage (+): 0. Max coverage (-): 0

Region: NODE\_268840\_length\_12752\_cov\_27.436010 6968-6993. Max. coverage (+): 0.33. Max coverage (-): 0.22

Region: NODE\_268840\_length\_12752\_cov\_27.436010 6994-7019. Max. coverage (+): 0.11. Max coverage (-): 0.04

Region: NODE\_268840\_length\_12752\_cov\_27.436010 7020-7044. Max. coverage (+): 0.82. Max coverage (-): 0.41

Region: NODE\_268840\_length\_12752\_cov\_27.436010 7045-7070. Max. coverage (+): 0.11. Max coverage (-): 0.44

Region: NODE\_268840\_length\_12752\_cov\_27.436010 7071-7096. Max. coverage (+): 0.04. Max coverage (-): 0.67

Region: NODE\_268840\_length\_12752\_cov\_27.436010 7097-7122. Max. coverage (+): 0.04. Max coverage (-): 0.37

Region: NODE\_268840\_length\_12752\_cov\_27.436010 7123-7147. Max. coverage (+): 0.26. Max coverage (-): 0.22

Region: NODE\_268840\_length\_12752\_cov\_27.436010 7148-7173. Max. coverage (+): 0.07. Max coverage (-): 0.26

Region: NODE\_268840\_length\_12752\_cov\_27.436010 7174-7199. Max. coverage (+): 0. Max coverage (-): 0.11

Region: NODE\_268840\_length\_12752\_cov\_27.436010 7200-7224. Max. coverage (+): 0. Max coverage (-): 0.96

Region: NODE\_268840\_length\_12752\_cov\_27.436010 7225-7250. Max. coverage (+): 0.04. Max coverage (-): 0.63

Region: NODE\_268840\_length\_12752\_cov\_27.436010 7251-7276. Max. coverage (+): 0.07. Max coverage (-): 0.07

Region: NODE\_268840\_length\_12752\_cov\_27.436010 7277-7302. Max. coverage (+): 0.04. Max coverage (-): 0.07

Region: NODE\_268840\_length\_12752\_cov\_27.436010 7303-7327. Max. coverage (+): 0.07. Max coverage (-): 0.11

Region: NODE\_268840\_length\_12752\_cov\_27.436010 7328-7353. Max. coverage (+): 0.07. Max coverage (-): 0.15

Region: NODE\_268840\_length\_12752\_cov\_27.436010 7354-7379. Max. coverage (+): 0.07. Max coverage (-): 0.15

Region: NODE\_268840\_length\_12752\_cov\_27.436010 7380-7405. Max. coverage (+): 0. Max coverage (-): 0.04

Region: NODE\_268840\_length\_12752\_cov\_27.436010 7406-7430. Max. coverage (+): 0.06. Max coverage (-): 0.11

Region: NODE\_268840\_length\_12752\_cov\_27.436010 7431-7456. Max. coverage (+): 0. Max coverage (-): 0.04

Region: NODE\_268840\_length\_12752\_cov\_27.436010 7457-7482. Max. coverage (+): 0.06. Max coverage (-): 0.03

Region: NODE\_268840\_length\_12752\_cov\_27.436010 7483-7508. Max. coverage (+): 0.22. Max coverage (-): 3.18

Region: NODE\_268840\_length\_12752\_cov\_27.436010 7509-7533. Max. coverage (+): 0.07. Max coverage (-): 0.59

Region: NODE\_268840\_length\_12752\_cov\_27.436010 7534-7559. Max. coverage (+): 0.11. Max coverage (-): 9.9

Region: NODE\_268840\_length\_12752\_cov\_27.436010 7560-7585. Max. coverage (+): 0. Max coverage (-): 0.41

Region: NODE\_268840\_length\_12752\_cov\_27.436010 7586-7610. Max. coverage (+): 0. Max coverage (-): 1.22

Region: NODE\_268840\_length\_12752\_cov\_27.436010 7611-7636. Max. coverage (+): 0. Max coverage (-): 0.07

Region: NODE\_268840\_length\_12752\_cov\_27.436010 7637-7662. Max. coverage (+): 0. Max coverage (-): 0.33

Region: NODE\_268840\_length\_12752\_cov\_27.436010 7663-7688. Max. coverage (+): 0. Max coverage (-): 0.48

Region: NODE\_268840\_length\_12752\_cov\_27.436010 7689-7713. Max. coverage (+): 0.04. Max coverage (-): 0.07

Region: NODE\_268840\_length\_12752\_cov\_27.436010 7714-7739. Max. coverage (+): 0. Max coverage (-): 0.04

Region: NODE\_268840\_length\_12752\_cov\_27.436010 7740-7765. Max. coverage (+): 0.07. Max coverage (-): 0.22

Region: NODE\_268840\_length\_12752\_cov\_27.436010 7766-7791. Max. coverage (+): 0.04. Max coverage (-): 1.48

Region: NODE\_268840\_length\_12752\_cov\_27.436010 7792-7816. Max. coverage (+): 0.19. Max coverage (-): 8.28

Region: NODE\_268840\_length\_12752\_cov\_27.436010 7817-7842. Max. coverage (+): 0.7. Max coverage (-): 8.01

Region: NODE\_268840\_length\_12752\_cov\_27.436010 7843-7868. Max. coverage (+): 0.48. Max coverage (-): 3.91

Region: NODE\_268840\_length\_12752\_cov\_27.436010 7869-7894. Max. coverage (+): 0.22. Max coverage (-): 0.44

Region: NODE\_268840\_length\_12752\_cov\_27.436010 7895-7919. Max. coverage (+): 0.37. Max coverage (-): 0.93

Region: NODE\_268840\_length\_12752\_cov\_27.436010 7920-7945. Max. coverage (+): 0.37. Max coverage (-): 0.74

Region: NODE\_268840\_length\_12752\_cov\_27.436010 7946-7971. Max. coverage (+): 0.04. Max coverage (-): 0.22

Region: NODE\_268840\_length\_12752\_cov\_27.436010 7972-7997. Max. coverage (+): 0.07. Max coverage (-): 0.19

Region: NODE\_268840\_length\_12752\_cov\_27.436010 7998-8022. Max. coverage (+): 0.59. Max coverage (-): 0.3

Region: NODE\_268840\_length\_12752\_cov\_27.436010 8023-8048. Max. coverage (+): 0.11. Max coverage (-): 0.7

Region: NODE\_268840\_length\_12752\_cov\_27.436010 8049-8074. Max. coverage (+): 0.19. Max coverage (-): 0.07

Region: NODE\_268840\_length\_12752\_cov\_27.436010 8075-8099. Max. coverage (+): 0.04. Max coverage (-): 1.04

Region: NODE\_268840\_length\_12752\_cov\_27.436010 8100-8125. Max. coverage (+): 0. Max coverage (-): 0.59

Region: NODE\_268840\_length\_12752\_cov\_27.436010 8126-8151. Max. coverage (+): 0.44. Max coverage (-): 19.61

Region: NODE\_268840\_length\_12752\_cov\_27.436010 8152-8177. Max. coverage (+): 0.07. Max coverage (-): 1.56

Region: NODE\_268840\_length\_12752\_cov\_27.436010 8178-8202. Max. coverage (+): 0.04. Max coverage (-): 1.59

Region: NODE\_268840\_length\_12752\_cov\_27.436010 8203-8228. Max. coverage (+): 0.26. Max coverage (-): 0.56

Region: NODE\_268840\_length\_12752\_cov\_27.436010 8229-8254. Max. coverage (+): 0.3. Max coverage (-): 2.04

Region: NODE\_268840\_length\_12752\_cov\_27.436010 8255-8280. Max. coverage (+): 0.33. Max coverage (-): 1.85

Region: NODE\_268840\_length\_12752\_cov\_27.436010 8281-8305. Max. coverage (+): 0.19. Max coverage (-): 1.45

Region: NODE\_268840\_length\_12752\_cov\_27.436010 8306-8331. Max. coverage (+): 0.11. Max coverage (-): 4.23

Region: NODE\_268840\_length\_12752\_cov\_27.436010 8332-8357. Max. coverage (+): 0. Max coverage (-): 0.11

Region: NODE\_268840\_length\_12752\_cov\_27.436010 8358-8383. Max. coverage (+): 0.41. Max coverage (-): 0.7

Region: NODE\_268840\_length\_12752\_cov\_27.436010 8384-8408. Max. coverage (+): 0.07. Max coverage (-): 0.61

Region: NODE\_268840\_length\_12752\_cov\_27.436010 8409-8434. Max. coverage (+): 0.04. Max coverage (-): 0.56

Region: NODE\_268840\_length\_12752\_cov\_27.436010 8435-8460. Max. coverage (+): 0.3. Max coverage (-): 0.33

Region: NODE\_268840\_length\_12752\_cov\_27.436010 8461-8486. Max. coverage (+): 0.22. Max coverage (-): 0.41

Region: NODE\_268840\_length\_12752\_cov\_27.436010 8487-8511. Max. coverage (+): 0.15. Max coverage (-): 1.08

Region: NODE\_268840\_length\_12752\_cov\_27.436010 8512-8537. Max. coverage (+): 0.19. Max coverage (-): 2.6

Region: NODE\_268840\_length\_12752\_cov\_27.436010 8538-8563. Max. coverage (+): 0.04. Max coverage (-): 0.22

Region: NODE\_268840\_length\_12752\_cov\_27.436010 8564-8588. Max. coverage (+): 0.26. Max coverage (-): 0.3

Region: NODE\_268840\_length\_12752\_cov\_27.436010 8589-8614. Max. coverage (+): 0.11. Max coverage (-): 2.37

Region: NODE\_268840\_length\_12752\_cov\_27.436010 8615-8640. Max. coverage (+): 0. Max coverage (-): 0.15

Region: NODE\_268840\_length\_12752\_cov\_27.436010 8641-8666. Max. coverage (+): 0.04. Max coverage (-): 0.67

Region: NODE\_268840\_length\_12752\_cov\_27.436010 8667-8691. Max. coverage (+): 0.04. Max coverage (-): 0.19

Region: NODE\_268840\_length\_12752\_cov\_27.436010 8692-8717. Max. coverage (+): 0.04. Max coverage (-): 0.22

Region: NODE\_268840\_length\_12752\_cov\_27.436010 8718-8743. Max. coverage (+): 0.07. Max coverage (-): 0.04

Region: NODE\_268840\_length\_12752\_cov\_27.436010 8744-8769. Max. coverage (+): 0.19. Max coverage (-): 0.11

Region: NODE\_268840\_length\_12752\_cov\_27.436010 8770-8794. Max. coverage (+): 0. Max coverage (-): 1.41

Region: NODE\_268840\_length\_12752\_cov\_27.436010 8795-8820. Max. coverage (+): 0. Max coverage (-): 0

Region: NODE\_268840\_length\_12752\_cov\_27.436010 8821-8846. Max. coverage (+): 0. Max coverage (-): 0

Region: NODE\_268840\_length\_12752\_cov\_27.436010 8847-8872. Max. coverage (+): 0. Max coverage (-): 0

Region: NODE\_268840\_length\_12752\_cov\_27.436010 8873-8897. Max. coverage (+): 0. Max coverage (-): 0

Region: NODE\_268840\_length\_12752\_cov\_27.436010 8898-8923. Max. coverage (+): 0. Max coverage (-): 0

Region: NODE\_268840\_length\_12752\_cov\_27.436010 8924-8949. Max. coverage (+): 0. Max coverage (-): 0

Region: NODE\_268840\_length\_12752\_cov\_27.436010 8950-8974. Max. coverage (+): 0. Max coverage (-): 0

Region: NODE\_268840\_length\_12752\_cov\_27.436010 8975-9000. Max. coverage (+): 0. Max coverage (-): 0

Region: NODE\_268840\_length\_12752\_cov\_27.436010 9001-9026. Max. coverage (+): 0. Max coverage (-): 0

Region: NODE\_268840\_length\_12752\_cov\_27.436010 9027-9052. Max. coverage (+): 0. Max coverage (-): 0

Region: NODE\_268840\_length\_12752\_cov\_27.436010 9053-9077. Max. coverage (+): 0. Max coverage (-): 0

Region: NODE\_268840\_length\_12752\_cov\_27.436010 9078-9103. Max. coverage (+): 0. Max coverage (-): 0.74

Region: NODE\_268840\_length\_12752\_cov\_27.436010 9104-9129. Max. coverage (+): 0.05. Max coverage (-): 0.15

Region: NODE\_268840\_length\_12752\_cov\_27.436010 9130-9155. Max. coverage (+): 0.04. Max coverage (-): 0.26

Region: NODE\_268840\_length\_12752\_cov\_27.436010 9156-9180. Max. coverage (+): 0. Max coverage (-): 0.74

Region: NODE\_268840\_length\_12752\_cov\_27.436010 9181-9206. Max. coverage (+): 0.04. Max coverage (-): 1.3

Region: NODE\_268840\_length\_12752\_cov\_27.436010 9207-9232. Max. coverage (+): 0.04. Max coverage (-): 0.3

Region: NODE\_268840\_length\_12752\_cov\_27.436010 9233-9258. Max. coverage (+): 0.11. Max coverage (-): 0

Region: NODE\_268840\_length\_12752\_cov\_27.436010 9259-9283. Max. coverage (+): 0. Max coverage (-): 0

Region: NODE\_268840\_length\_12752\_cov\_27.436010 9284-9309. Max. coverage (+): 0.04. Max coverage (-): 0.3

Region: NODE\_268840\_length\_12752\_cov\_27.436010 9310-9335. Max. coverage (+): 0.04. Max coverage (-): 0.04

Region: NODE\_268840\_length\_12752\_cov\_27.436010 9336-9361. Max. coverage (+): 0.3. Max coverage (-): 0.26

Region: NODE\_268840\_length\_12752\_cov\_27.436010 9362-9386. Max. coverage (+): 0.22. Max coverage (-): 0.3

Region: NODE\_268840\_length\_12752\_cov\_27.436010 9387-9412. Max. coverage (+): 0. Max coverage (-): 0.15

Region: NODE\_268840\_length\_12752\_cov\_27.436010 9413-9438. Max. coverage (+): 0.01. Max coverage (-): 0

Region: NODE\_268840\_length\_12752\_cov\_27.436010 9439-9463. Max. coverage (+): 0.01. Max coverage (-): 0.01

Region: NODE\_268840\_length\_12752\_cov\_27.436010 9464-9489. Max. coverage (+): 0.01. Max coverage (-): 0.01

Region: NODE\_268840\_length\_12752\_cov\_27.436010 9490-9515. Max. coverage (+): 0.11. Max coverage (-): 0.02

Region: NODE\_268840\_length\_12752\_cov\_27.436010 9516-9541. Max. coverage (+): 0.02. Max coverage (-): 0

Region: NODE\_268840\_length\_12752\_cov\_27.436010 9542-9566. Max. coverage (+): 0. Max coverage (-): 0

Region: NODE\_268840\_length\_12752\_cov\_27.436010 9567-9592. Max. coverage (+): 0. Max coverage (-): 0

Region: NODE\_268840\_length\_12752\_cov\_27.436010 9593-9618. Max. coverage (+): 0.21. Max coverage (-): 0.01

Region: NODE\_268840\_length\_12752\_cov\_27.436010 9619-9644. Max. coverage (+): 0. Max coverage (-): 0

Region: NODE\_268840\_length\_12752\_cov\_27.436010 9645-9669. Max. coverage (+): 0.03. Max coverage (-): 0.05

Region: NODE\_268840\_length\_12752\_cov\_27.436010 9670-9695. Max. coverage (+): 0. Max coverage (-): 0.22

Region: NODE\_268840\_length\_12752\_cov\_27.436010 9696-9721. Max. coverage (+): 0.04. Max coverage (-): 0.67

Region: NODE\_268840\_length\_12752\_cov\_27.436010 9722-9747. Max. coverage (+): 0.11. Max coverage (-): 0.22

Region: NODE\_268840\_length\_12752\_cov\_27.436010 9748-9772. Max. coverage (+): 0. Max coverage (-): 0.04

Region: NODE\_268840\_length\_12752\_cov\_27.436010 9773-9798. Max. coverage (+): 0. Max coverage (-): 0.3

Region: NODE\_268840\_length\_12752\_cov\_27.436010 9799-9824. Max. coverage (+): 0.19. Max coverage (-): 0.89

Region: NODE\_268840\_length\_12752\_cov\_27.436010 9825-9850. Max. coverage (+): 0.19. Max coverage (-): 0.56

Region: NODE\_268840\_length\_12752\_cov\_27.436010 9851-9875. Max. coverage (+): 0.11. Max coverage (-): 0.04

Region: NODE\_268840\_length\_12752\_cov\_27.436010 9876-9901. Max. coverage (+): 0.04. Max coverage (-): 0.02

Region: NODE\_268840\_length\_12752\_cov\_27.436010 9902-9927. Max. coverage (+): 0. Max coverage (-): 0.04

Region: NODE\_268840\_length\_12752\_cov\_27.436010 9928-9952. Max. coverage (+): 0.04. Max coverage (-): 0.04

Region: NODE\_268840\_length\_12752\_cov\_27.436010 9953-9978. Max. coverage (+): 0. Max coverage (-): 0.04

Region: NODE\_268840\_length\_12752\_cov\_27.436010 9979-10004. Max. coverage (+): 0.04. Max coverage (-): 0

Region: NODE\_268840\_length\_12752\_cov\_27.436010 10005-10030. Max. coverage (+): 0.04. Max coverage (-): 0

Region: NODE\_268840\_length\_12752\_cov\_27.436010 10031-10055. Max. coverage (+): 0.04. Max coverage (-): 0

Region: NODE\_268840\_length\_12752\_cov\_27.436010 10056-10081. Max. coverage (+): 0.04. Max coverage (-): 0.11

Region: NODE\_268840\_length\_12752\_cov\_27.436010 10082-10107. Max. coverage (+): 0.17. Max coverage (-): 0.11

Region: NODE\_268840\_length\_12752\_cov\_27.436010 10108-10133. Max. coverage (+): 0.07. Max coverage (-): 0.11

Region: NODE\_268840\_length\_12752\_cov\_27.436010 10134-10158. Max. coverage (+): 0.04. Max coverage (-): 0.13

Region: NODE\_268840\_length\_12752\_cov\_27.436010 10159-10184. Max. coverage (+): 0.07. Max coverage (-): 0.05

Region: NODE\_268840\_length\_12752\_cov\_27.436010 10185-10210. Max. coverage (+): 0.04. Max coverage (-): 0.07

Region: NODE\_268840\_length\_12752\_cov\_27.436010 10211-10236. Max. coverage (+): 0.15. Max coverage (-): 0.15

Region: NODE\_268840\_length\_12752\_cov\_27.436010 10237-10261. Max. coverage (+): 0.04. Max coverage (-): 0.11

Region: NODE\_268840\_length\_12752\_cov\_27.436010 10262-10287. Max. coverage (+): 0. Max coverage (-): 0.26

Region: NODE\_268840\_length\_12752\_cov\_27.436010 10288-10313. Max. coverage (+): 0.04. Max coverage (-): 0

Region: NODE\_268840\_length\_12752\_cov\_27.436010 10314-10339. Max. coverage (+): 0.07. Max coverage (-): 0.19

Region: NODE\_268840\_length\_12752\_cov\_27.436010 10340-10364. Max. coverage (+): 0.04. Max coverage (-): 0.15

Region: NODE\_268840\_length\_12752\_cov\_27.436010 10365-10390. Max. coverage (+): 0.96. Max coverage (-): 0.07

Region: NODE\_268840\_length\_12752\_cov\_27.436010 10391-10416. Max. coverage (+): 0.11. Max coverage (-): 0

Region: NODE\_268840\_length\_12752\_cov\_27.436010 10417-10441. Max. coverage (+): 0.04. Max coverage (-): 0.02

Region: NODE\_268840\_length\_12752\_cov\_27.436010 10442-10467. Max. coverage (+): 0.07. Max coverage (-): 0

Region: NODE\_268840\_length\_12752\_cov\_27.436010 10468-10493. Max. coverage (+): 0.04. Max coverage (-): 0

Region: NODE\_268840\_length\_12752\_cov\_27.436010 10494-10519. Max. coverage (+): 0.04. Max coverage (-): 0

Region: NODE\_268840\_length\_12752\_cov\_27.436010 10520-10544. Max. coverage (+): 0. Max coverage (-): 0.07

Region: NODE\_268840\_length\_12752\_cov\_27.436010 10545-10570. Max. coverage (+): 0. Max coverage (-): 0.04

Region: NODE\_268840\_length\_12752\_cov\_27.436010 10571-10596. Max. coverage (+): 0.04. Max coverage (-): 0

Region: NODE\_268840\_length\_12752\_cov\_27.436010 10597-10622. Max. coverage (+): 0. Max coverage (-): 0

Region: NODE\_268840\_length\_12752\_cov\_27.436010 10623-10647. Max. coverage (+): 0. Max coverage (-): 0.07

Region: NODE\_268840\_length\_12752\_cov\_27.436010 10648-10673. Max. coverage (+): 0.04. Max coverage (-): 0

Region: NODE\_268840\_length\_12752\_cov\_27.436010 10674-10699. Max. coverage (+): 0.04. Max coverage (-): 0

Region: NODE\_268840\_length\_12752\_cov\_27.436010 10700-10725. Max. coverage (+): 0.04. Max coverage (-): 2.22

Region: NODE\_268840\_length\_12752\_cov\_27.436010 10726-10750. Max. coverage (+): 0.02. Max coverage (-): 0.52

Region: NODE\_268840\_length\_12752\_cov\_27.436010 10751-10776. Max. coverage (+): 0.19. Max coverage (-): 0.28

Region: NODE\_268840\_length\_12752\_cov\_27.436010 10777-10802. Max. coverage (+): 0.04. Max coverage (-): 0.37

Region: NODE\_268840\_length\_12752\_cov\_27.436010 10803-10827. Max. coverage (+): 0.04. Max coverage (-): 0.04

Region: NODE\_268840\_length\_12752\_cov\_27.436010 10828-10853. Max. coverage (+): 0.04. Max coverage (-): 0.22

Region: NODE\_268840\_length\_12752\_cov\_27.436010 10854-10879. Max. coverage (+): 0. Max coverage (-): 0.69

Region: NODE\_268840\_length\_12752\_cov\_27.436010 10880-10905. Max. coverage (+): 0.04. Max coverage (-): 0.07

Region: NODE\_268840\_length\_12752\_cov\_27.436010 10906-10930. Max. coverage (+): 0.04. Max coverage (-): 0.07

Region: NODE\_268840\_length\_12752\_cov\_27.436010 10931-10956. Max. coverage (+): 0. Max coverage (-): 0.04

Region: NODE\_268840\_length\_12752\_cov\_27.436010 10957-10982. Max. coverage (+): 0. Max coverage (-): 0.04

Region: NODE\_268840\_length\_12752\_cov\_27.436010 10983-11008. Max. coverage (+): 0.04. Max coverage (-): 0.07

Region: NODE\_268840\_length\_12752\_cov\_27.436010 11009-11033. Max. coverage (+): 0. Max coverage (-): 0.07

Region: NODE\_268840\_length\_12752\_cov\_27.436010 11034-11059. Max. coverage (+): 0.26. Max coverage (-): 0.07

Region: NODE\_268840\_length\_12752\_cov\_27.436010 11060-11085. Max. coverage (+): 0.11. Max coverage (-): 0.41

Region: NODE\_268840\_length\_12752\_cov\_27.436010 11086-11111. Max. coverage (+): 0.04. Max coverage (-): 0.22

Region: NODE\_268840\_length\_12752\_cov\_27.436010 11112-11136. Max. coverage (+): 0.11. Max coverage (-): 0.19

Region: NODE\_268840\_length\_12752\_cov\_27.436010 11137-11162. Max. coverage (+): 0. Max coverage (-): 0.11

Region: NODE\_268840\_length\_12752\_cov\_27.436010 11163-11188. Max. coverage (+): 0. Max coverage (-): 0

Region: NODE\_268840\_length\_12752\_cov\_27.436010 11189-11214. Max. coverage (+): 0.07. Max coverage (-): 0.67

Region: NODE\_268840\_length\_12752\_cov\_27.436010 11215-11239. Max. coverage (+): 0. Max coverage (-): 0.38

Region: NODE\_268840\_length\_12752\_cov\_27.436010 11240-11265. Max. coverage (+): 0.37. Max coverage (-): 0.16

Region: NODE\_268840\_length\_12752\_cov\_27.436010 11266-11291. Max. coverage (+): 0.04. Max coverage (-): 0.22

Region: NODE\_268840\_length\_12752\_cov\_27.436010 11292-11316. Max. coverage (+): 0.02. Max coverage (-): 0.82

Region: NODE\_268840\_length\_12752\_cov\_27.436010 11317-11342. Max. coverage (+): 0. Max coverage (-): 2.74

Region: NODE\_268840\_length\_12752\_cov\_27.436010 11343-11368. Max. coverage (+): 1.3. Max coverage (-): 1.3

Region: NODE\_268840\_length\_12752\_cov\_27.436010 11369-11394. Max. coverage (+): 0.56. Max coverage (-): 0.26

Region: NODE\_268840\_length\_12752\_cov\_27.436010 11395-11419. Max. coverage (+): 0.37. Max coverage (-): 5.52

Region: NODE\_268840\_length\_12752\_cov\_27.436010 11420-11445. Max. coverage (+): 0.22. Max coverage (-): 0.59

Region: NODE\_268840\_length\_12752\_cov\_27.436010 11446-11471. Max. coverage (+): 0.04. Max coverage (-): 0.24

Region: NODE\_268840\_length\_12752\_cov\_27.436010 11472-11497. Max. coverage (+): 0.78. Max coverage (-): 0.26

Region: NODE\_268840\_length\_12752\_cov\_27.436010 11498-11522. Max. coverage (+): 0.04. Max coverage (-): 4.71

Region: NODE\_268840\_length\_12752\_cov\_27.436010 11523-11548. Max. coverage (+): 0.15. Max coverage (-): 6.86

Region: NODE\_268840\_length\_12752\_cov\_27.436010 11549-11574. Max. coverage (+): 2.19. Max coverage (-): 2.08

Region: NODE\_268840\_length\_12752\_cov\_27.436010 11575-11600. Max. coverage (+): 0.26. Max coverage (-): 38.76

Region: NODE\_268840\_length\_12752\_cov\_27.436010 11601-11625. Max. coverage (+): 0.95. Max coverage (-): 33.61

Region: NODE\_268840\_length\_12752\_cov\_27.436010 11626-11651. Max. coverage (+): 0.15. Max coverage (-): 0.2

Region: NODE\_268840\_length\_12752\_cov\_27.436010 11652-11677. Max. coverage (+): 1.09. Max coverage (-): 0.82

Region: NODE\_268840\_length\_12752\_cov\_27.436010 11678-11703. Max. coverage (+): 1.08. Max coverage (-): 1.15

Region: NODE\_268840\_length\_12752\_cov\_27.436010 11704-11728. Max. coverage (+): 0.89. Max coverage (-): 2.84

Region: NODE\_268840\_length\_12752\_cov\_27.436010 11729-11754. Max. coverage (+): 1.85. Max coverage (-): 2.45

Region: NODE\_268840\_length\_12752\_cov\_27.436010 11755-11780. Max. coverage (+): 0.11. Max coverage (-): 9.79

Region: NODE\_268840\_length\_12752\_cov\_27.436010 11781-11805. Max. coverage (+): 0.15. Max coverage (-): 0.26

Region: NODE\_268840\_length\_12752\_cov\_27.436010 11806-11831. Max. coverage (+): 0.31. Max coverage (-): 1.67

Region: NODE\_268840\_length\_12752\_cov\_27.436010 11832-11857. Max. coverage (+): 0. Max coverage (-): 2.52

Region: NODE\_268840\_length\_12752\_cov\_27.436010 11858-11883. Max. coverage (+): 0.04. Max coverage (-): 2.67

Region: NODE\_268840\_length\_12752\_cov\_27.436010 11884-11908. Max. coverage (+): 0.89. Max coverage (-): 2

Region: NODE\_268840\_length\_12752\_cov\_27.436010 11909-11934. Max. coverage (+): 0.96. Max coverage (-): 0.89

Region: NODE\_268840\_length\_12752\_cov\_27.436010 11935-11960. Max. coverage (+): 0.48. Max coverage (-): 3.97

Region: NODE\_268840\_length\_12752\_cov\_27.436010 11961-11986. Max. coverage (+): 0.04. Max coverage (-): 0.26

Region: NODE\_268840\_length\_12752\_cov\_27.436010 11987-12011. Max. coverage (+): 0.04. Max coverage (-): 0.11

Region: NODE\_268840\_length\_12752\_cov\_27.436010 12012-12037. Max. coverage (+): 0.04. Max coverage (-): 1.08

Region: NODE\_268840\_length\_12752\_cov\_27.436010 12038-12063. Max. coverage (+): 0.41. Max coverage (-): 0.11

Region: NODE\_268840\_length\_12752\_cov\_27.436010 12064-12089. Max. coverage (+): 0.48. Max coverage (-): 0.13

Region: NODE\_268840\_length\_12752\_cov\_27.436010 12090-12114. Max. coverage (+): 0.3. Max coverage (-): 0.04

Region: NODE\_268840\_length\_12752\_cov\_27.436010 12115-12140. Max. coverage (+): 0.22. Max coverage (-): 0.19

Region: NODE\_268840\_length\_12752\_cov\_27.436010 12141-12166. Max. coverage (+): 0.07. Max coverage (-): 0

Region: NODE\_268840\_length\_12752\_cov\_27.436010 12167-12191. Max. coverage (+): 0.26. Max coverage (-): 0.07

Region: NODE\_268840\_length\_12752\_cov\_27.436010 12192-12217. Max. coverage (+): 0.04. Max coverage (-): 0.04

Region: NODE\_268840\_length\_12752\_cov\_27.436010 12218-12243. Max. coverage (+): 0. Max coverage (-): 0

Region: NODE\_268840\_length\_12752\_cov\_27.436010 12244-12269. Max. coverage (+): 0. Max coverage (-): 0

Region: NODE\_268840\_length\_12752\_cov\_27.436010 12270-12294. Max. coverage (+): 0.59. Max coverage (-): 1.3

Region: NODE\_268840\_length\_12752\_cov\_27.436010 12295-12320. Max. coverage (+): 1.19. Max coverage (-): 0.22

Region: NODE\_268840\_length\_12752\_cov\_27.436010 12321-12346. Max. coverage (+): 0.05. Max coverage (-): 0.19

Region: NODE\_268840\_length\_12752\_cov\_27.436010 12347-12372. Max. coverage (+): 0. Max coverage (-): 0

Region: NODE\_268840\_length\_12752\_cov\_27.436010 12373-12397. Max. coverage (+): 0.02. Max coverage (-): 0.01

Region: NODE\_268840\_length\_12752\_cov\_27.436010 12398-12423. Max. coverage (+): 0.24. Max coverage (-): 0.01

Region: NODE\_268840\_length\_12752\_cov\_27.436010 12424-12449. Max. coverage (+): 0.24. Max coverage (-): 0

Region: NODE\_268840\_length\_12752\_cov\_27.436010 12450-12475. Max. coverage (+): 0.09. Max coverage (-): 0.02

Region: NODE\_268840\_length\_12752\_cov\_27.436010 12476-12500. Max. coverage (+): 0. Max coverage (-): 0.02

Region: NODE\_268840\_length\_12752\_cov\_27.436010 12501-12526. Max. coverage (+): 0.01. Max coverage (-): 0

Region: NODE\_268840\_length\_12752\_cov\_27.436010 12527-12552. Max. coverage (+): 0.01. Max coverage (-): 0.01

Region: NODE\_268840\_length\_12752\_cov\_27.436010 12553-12578. Max. coverage (+): 0.01. Max coverage (-): 0

Region: NODE\_268840\_length\_12752\_cov\_27.436010 12579-12603. Max. coverage (+): 0. Max coverage (-): 0.02

Region: NODE\_268840\_length\_12752\_cov\_27.436010 12604-12629. Max. coverage (+): 0. Max coverage (-): 0.01

Region: NODE\_268840\_length\_12752\_cov\_27.436010 12630-12655. Max. coverage (+): 0. Max coverage (-): 0

Region: NODE\_268840\_length\_12752\_cov\_27.436010 12656-12680. Max. coverage (+): 0. Max coverage (-): 0

Region: NODE\_268840\_length\_12752\_cov\_27.436010 12681-12706. Max. coverage (+): 0. Max coverage (-): 0

Region: NODE\_268840\_length\_12752\_cov\_27.436010 12707-12732. Max. coverage (+): 0. Max coverage (-): 0

Region: NODE\_268840\_length\_12752\_cov\_27.436010 12733-12758. Max. coverage (+): 0. Max coverage (-): 0

Region: NODE\_268840\_length\_12752\_cov\_27.436010 12759-12783. Max. coverage (+): 0. Max coverage (-): 0

Region: NODE\_268840\_length\_12752\_cov\_27.436010 12784-12809. Max. coverage (+): 0. Max coverage (-): 0.96

Region: NODE\_268840\_length\_12752\_cov\_27.436010 12810-12835. Max. coverage (+): 0.05. Max coverage (-): 0.01

Region: NODE\_268840\_length\_12752\_cov\_27.436010 12836-12861. Max. coverage (+): 0.02. Max coverage (-): 0

Region: NODE\_268840\_length\_12752\_cov\_27.436010 12862-. Max. coverage (+): 0. Max coverage (-): 0

RepeatMasker Color Code

**+**

100-98% Identity

<98-95% Identity

<95-90% Identity

<90-85% Identity

<85-80% Identity

<80-75% Identity

<75-70% Identity

<70% Identity

**-**

Gene Set Color Code

**+**

Gene

Pseudogene

Other

**-**

Topology/Coverage Color Code

Coverage Plus Strand

Coverage Minus Strand

Mainstrand: Plus

Mainstrand: Minus

Complementary Strand

Flanking Region  
(if option -flank >0)

Gene Set Annotation  
  
RepeatMasker Annotation  

**1. AlRepD-8475**: 8-92 (+), Divergence to consensus: 22.4%  
**2. (AGCT)n**: 806-829 (+), Divergence to consensus: 8.8%  
**3. TE-X-5\_DR**: 1342-1422 (+), Divergence to consensus: 28.4%  
**4. TE-X-4\_DR**: 1388-1530 (-), Divergence to consensus: 36.3%  
**5. (TGTCTG)n**: 4313-4350 (+), Divergence to consensus: 19.6%  
**6. (AC)n**: 4611-4630 (+), Divergence to consensus: 0%  
**7. AlRepB-392**: 5557-5737 (+), Divergence to consensus: 12.2%  
**8. AlRepD-1024**: 5786-5892 (+), Divergence to consensus: 10.3%  
**9. AlRepC-1280**: 5891-5963 (+), Divergence to consensus: 5.5%  
**10. SINE2-1\_AFC**: 6272-6337 (-), Divergence to consensus: 17.9%  
**11. Harbinger-2N1\_DR**: 6338-6482 (+), Divergence to consensus: 16.2%  
**12. AlRepD-1024**: 6496-6891 (+), Divergence to consensus: 14.8%  
**13. AlRepB-392**: 6892-6924 (+), Divergence to consensus: 12.1%  
**14. AlRepB-392**: 7067-7441 (+), Divergence to consensus: 17.1%  
**15. AlRepC-1433**: 7444-7893 (+), Divergence to consensus: 24.8%  
**16. AlRepC-1574**: 8262-8401 (+), Divergence to consensus: 33.2%  
**17. AlRepC-1574**: 8507-8664 (+), Divergence to consensus: 21.8%  
**18. AlRepD-209**: 8777-8839 (-), Divergence to consensus: 15.8%  
**19. AlRepD-209**: 9054-9215 (-), Divergence to consensus: 22.1%  
**20. AlRepB-128**: 9306-9383 (-), Divergence to consensus: 26.7%  
**21. AlRepD-4636**: 9381-9425 (+), Divergence to consensus: 15.5%  
**22. AlRepB-438**: 9414-9720 (-), Divergence to consensus: 5.2%  
**23. AlRepD-4636**: 9722-9944 (+), Divergence to consensus: 22.4%  
**24. AlRepB-250**: 9989-10119 (-), Divergence to consensus: 26.4%  
**25. AlRepB-128**: 10120-10210 (-), Divergence to consensus: 20.5%  
**26. AlRepD-880**: 10365-10513 (-), Divergence to consensus: 28.9%  
**27. L1-1\_AFC**: 10709-10854 (-), Divergence to consensus: 12%  
**28. AlRepB-923**: 10853-11083 (+), Divergence to consensus: 16.2%  
**29. AlRepB-185**: 11084-11283 (+), Divergence to consensus: 19.7%  
**30. Harbinger-3\_BF**: 11856-11991 (+), Divergence to consensus: 33.1%  
**31. hAT-N21\_DR**: 12314-12326 (-), Divergence to consensus: 19.2%  
**32. AlRepC-436**: 12327-12410 (-), Divergence to consensus: 1.2%  
**33. AlRepB-234**: 12398-12796 (+), Divergence to consensus: 6.6%  
**34. AlRepB-234**: 12796-12876 (+), Divergence to consensus: 16.1%

  
Transcription Factor Binding Sites  

**RHOXF1** (Sequence: GGATCA (-): 667)  
**RHOXF1** (Sequence: AGCTTA (-): 1165)  
**RHOXF1** (Sequence: AGATCA (-): 1632)  
**RHOXF1** (Sequence: GGATTA (-): 2360)  
**RHOXF1** (Sequence: AGATTA (-): 2383)  
**RHOXF1** (Sequence: AGATTA (-): 2467)  
**RHOXF1** (Sequence: GGCTCA (-): 2604)  
**RHOXF1** (Sequence: AGATCA (-): 2987)  
**RHOXF1** (Sequence: GGATCA (-): 3415)  
**RHOXF1** (Sequence: AGCTCA (-): 3849)  
**RHOXF1** (Sequence: AGCTTA (-): 4442)  
**RHOXF1** (Sequence: GGATCA (-): 4515)  
**RHOXF1** (Sequence: AGATCA (-): 4578)  
**RHOXF1** (Sequence: AGCTCA (-): 5042)  
**RHOXF1** (Sequence: GGCTTA (-): 5058)  
**RHOXF1** (Sequence: AGATTA (-): 5348)  
**RHOXF1** (Sequence: AGATTA (-): 5672)  
**RHOXF1** (Sequence: AGATCA (-): 5675)  
**RHOXF1** (Sequence: AGCTTA (-): 5853)  
**RHOXF1** (Sequence: AGCTCA (-): 6204)  
**RHOXF1** (Sequence: AGCTTA (-): 6562)  
**RHOXF1** (Sequence: AGCTTA (-): 7098)  
**RHOXF1** (Sequence: GGATCA (-): 7549)  
**RHOXF1** (Sequence: AGATCA (-): 8058)  
**RHOXF1** (Sequence: GGATTA (-): 10134)  
**RHOXF1** (Sequence: AGATTA (-): 11054)  
**RHOXF1** (Sequence: GGCTCA (-): 12032)  
**RHOXF1** (Sequence: AGATCA (-): 12379)  
**RHOXF1** (Sequence: AGCTTA (-): 12445)  
**RHOXF1** (Sequence: TAAGCT (+): 65)  
**RHOXF1** (Sequence: TAAGCT (+): 187)  
**RHOXF1** (Sequence: TAATCT (+): 2836)  
**RHOXF1** (Sequence: TGAGCT (+): 3819)  
**RHOXF1** (Sequence: TGAGCT (+): 4393)  
**RHOXF1** (Sequence: TGAGCT (+): 4440)  
**RHOXF1** (Sequence: TGATCT (+): 5681)  
**RHOXF1** (Sequence: TAATCC (+): 6214)  
**RHOXF1** (Sequence: TGAGCT (+): 7892)  
**RHOXF1** (Sequence: TAATCC (+): 8788)  
**RHOXF1** (Sequence: TGAGCT (+): 9125)  
**RHOXF1** (Sequence: TGATCT (+): 9192)  
**RHOXF1** (Sequence: TAAGCT (+): 9195)  
**RHOXF1** (Sequence: TAATCT (+): 9372)  
**RHOXF1** (Sequence: TGAGCC (+): 9642)  
**RHOXF1** (Sequence: TAAGCT (+): 11029)  
**RHOXF1** (Sequence: TGATCC (+): 12273)  
**Lhx8** (Sequence: CTAATTAG (-): 91)  
**Gata4** (Sequence: CTTATCT (+): 1167)  
**Gata4** (Sequence: CTTATCT (+): 3185)  
**Gata4** (Sequence: CTTATCT (+): 3994)  
**POU5F1** (Sequence: TTTGCAT (-): 865)  
**POU5F1** (Sequence: TTTGCAT (-): 7013)  
**POU5F1** (Sequence: TTTGCAT (-): 7214)  
**POU5F1** (Sequence: TTTGCAT (-): 8076)  
**POU5F1** (Sequence: TTTGCAT (-): 9999)  
**POU5F1** (Sequence: TTTGCAT (-): 10826)  
**RFX4\_2** (Sequence: GTATCCATG (-): 4038)  
**RFX4\_2** (Sequence: GTATCCAGG (-): 8310)  
**RFX4\_1** (Sequence: GTTGCTAGG (-): 2426)  
**SOX9** (Sequence: AACAATAG (-): 5065)  
**SOX9** (Sequence: AACAATAA (-): 9591)  
**SOX9** (Sequence: AACAATGG (-): 11423)  
**FOXO1** (Sequence: GTTGTTTAT (+): 3663)  
**FOXO1** (Sequence: GTTGTTTAT (+): 10870)  
**FOXO1** (Sequence: GTTGTTTTC (+): 12124)  
**FOXO3\_mmu** (Sequence: TGTTTTCC (-): 268)  
**FOXO3\_mmu** (Sequence: TGTTTAGC (-): 276)  
**FOXO3\_mmu** (Sequence: TGTTTTCA (-): 5172)  
**FOXO3\_mmu** (Sequence: TGTTTTGC (-): 8073)  
**FOXO3\_mmu** (Sequence: TGTTTTCC (-): 12126)  
**FOXO3\_mmu** (Sequence: TGTTTACA (-): 12627)  
**Sox5** (Sequence: ATTGTT (+): 111)  
**Sox5** (Sequence: ATTGTT (+): 904)  
**Sox5** (Sequence: ATTGTT (+): 1002)  
**Sox5** (Sequence: ATTGTT (+): 1859)  
**Sox5** (Sequence: ATTGTT (+): 1952)  
**Sox5** (Sequence: ATTGTT (+): 2210)  
**Sox5** (Sequence: ATTGTT (+): 5858)  
**Sox5** (Sequence: ATTGTT (+): 5917)  
**Sox5** (Sequence: ATTGTT (+): 6316)  
**Sox5** (Sequence: ATTGTT (+): 6567)  
**Sox5** (Sequence: ATTGTT (+): 8747)  
**Sox5** (Sequence: ATTGTT (+): 10734)  
**Sox5** (Sequence: ATTGTT (+): 10835)  
**Sox5** (Sequence: ATTGTT (+): 12222)  
**FIGLA** (Sequence: AACAGGTGGA (-): 10154)  
**SOX9** (Sequence: TTATTGTT (+): 5856)  
**SOX9** (Sequence: TTATTGTT (+): 6565)  
**SOX9** (Sequence: TTATTGTT (+): 10833)  
**FOXO3\_mmu** (Sequence: GCAAAACA (+): 8028)  
**FOXO3\_mmu** (Sequence: GGAAAACA (+): 8116)  
**FOXO3\_mmu** (Sequence: GGAAAACA (+): 8294)  
**FOXO3\_mmu** (Sequence: TCTAAACA (+): 8443)  
**Nobox** (Sequence: ACTAATTA (-): 90)  
**Nobox** (Sequence: ACCAATTA (-): 8727)  
**Nobox** (Sequence: AGTAATTA (-): 12562)  
**FOXO1** (Sequence: GAAAACAAC (-): 8295)  
**FOXO1** (Sequence: ATAAACAGG (-): 10123)  
**FOXO3\_hsa** (Sequence: TTGTTTAC (-): 12626)  
**FOXP1** (Sequence: TGTTTAC (-): 11278)  
**FOXP1** (Sequence: TGTTTAC (-): 12627)  
**Nobox** (Sequence: TAATTGCT (+): 8549)  
**Nobox** (Sequence: TAATTAGT (+): 10250)  
**Rhox11** (Sequence: TGGTGTATT (+): 1106)  
**Gata4** (Sequence: AGATAAG (-): 6673)  
**Sox5** (Sequence: AACAAT (-): 770)  
**Sox5** (Sequence: AACAAT (-): 5065)  
**Sox5** (Sequence: AACAAT (-): 9591)  
**Sox5** (Sequence: AACAAT (-): 10387)  
**Sox5** (Sequence: AACAAT (-): 11423)  
**POU2F1** (Sequence: TATTTTAAT (+): 894)  
**POU2F1** (Sequence: TATTTTAAT (+): 12531)  
**POU5F1** (Sequence: ATGCAAA (+): 2540)  
**Mybl1\_1** (Sequence: AACCGTTA (+): 5507)
