# Supplementary material for: First characterization of PIWI-interacting RNA clusters in a cichlid fish with a B chromosome
Source: BMC Biol. 2022 Sep 21;20:204. doi: 10.1186/s12915-022-01403-2 (PMC9490952; doi:10.1186/s12915-022-01403-2)
Supplement: Supplementary file 1 — Additional file 1. Zipped folder with fasta and interactive html piRNA cluster information for the A. latifasciata genome. The nomenclature is as follows: number-pirna-cluster_sex_B-presence (f, female; m, male; 0b, without B chromosome; 1b, with B chromosome). [file 12915_2022_1403_MOESM1_ESM.zip › 103_m1b.html]

piRNA cluster 103\_m1b 10


Predicted piRNA cluster no. 103\_m1b
  

Show proTRAC run info
Hide proTRAC run info

/\  
                \_\_\_\_\_\_\_\_\_\_\_\_\_\_\_\_\_\_\_\_\_\_\_/\\_\_\_ /  \\_\_\_\_\_\_\_  
               I                      /  \  /    \      I  
               I     pro             /    \/      \     I  
               I        TRAC        /               \   I  
               I   \_\_\_\_\_\_\_\_\_\_\_\_\_\_\_\_/\_\_\_\_\_\_\_\_\_\_\_\_\_\_\_\_\_\\_ I  
               I   \              /                     I  
               I    \            /                      I  
               I     \  /\      /       V.2.4.2         I  
               I      \/  \    /                        I  
               I\_\_\_\_\_\_\_\_\_\_\_\  /\_\_\_\_\_\_\_\_\_\_\_\_\_\_\_\_\_\_\_\_\_\_\_\_\_I  
                            \/  
  
  
================================= proTRAC ====================================  
VERSION: .......... 2.4.2  
LAST MODIFIED: .... 11. May 2018  
  
Please cite:  
Rosenkranz D, Zischler H. proTRAC - a software for probabilistic piRNA cluster  
detection, visualization and analysis. 2012. BMC Bioinformatics 13:5.  
  
  
Contact:  
David Rosenkranz  
Institute of Organismic and Molecular Evolutionary Biology  
Dept. Anthropology, small RNA group  
Johannes Gutenberg University Mainz  
email: rosenkranz@uni-mainz.de  
  
You can find the latest proTRAC version at:  
http://sourceforge.net/projects/protrac/files  
http://www.smallRNAgroup-mainz.de/software  
==============================================================================  
  
PARAMETERS:  
Map file: ...............piwi-machos-1B.fa-collapse.map  
Genome file: ............../../../0B\_ala\_genome.fa  
RepeatMasker annotation: Alatifasciata-all0B-maryan-v2.fa\_corrected.out  
GeneSet:................./guest-storage/Data/annotation/Alatifasciata\_all0B\_maryan-v2\_out2017.gff  
  
Significant (p<=0.01) hit density will be calculated based  
on observed hit distribution.  
  
Sliding window size: ........................................ 5000 bp  
Sliding window increament: .................................. 1000 bp  
Normalize each hit by number of genomic hits: ............... yes  
Normalize each hit by number of sequence reads: ............. yes  
Normalize values (-> per million mapped reads): ............. yes  
Min. fraction of hits with 1T(U) or 10A: .................... 0.75  
Alternatively: Min. fraction of hits with 1T(U) and 10A: .... 0.5  
Min. fraction of hits with typical piRNA length: ............ 0.75  
Typical piRNA length: ....................................... 24-32 nt  
Min. size of a piRNA cluster: ............................... 1000 bp.  
Min. number of hits (absolute): ............................. 0  
Min. number of hits (normalized): ........................... 0  
Min. fraction of hits on the mainstrand: .................... 0.75  
Top fraction of mapped sequences (in terms of read counts): . 1%  
Top fraction accounts for max. n% of sequence reads: ........ 90%  
Min. fraction of hits on each arm of a bidirectional cluster: 0.05  
Output html file for each cluster: .......................... yes  
Output a summary table: ..................................... yes  
Output a FASTA file for each cluster (piRNA sequences): ..... yes  
Output a FASTA file comprising cluster sequences: ........... yes  
Output a GTF file for predicted piRNA clusters: ..............yes  
Search DNA motifs in clusters: .............................. yes  
Output flanking sequences: +/- .............................. 0 bp  
Output ~.pTi file: .......................................... no  
==============================================================================  
  
  
Genome size (without gaps): ............ 758543724 bp  
Gaps (N/X/-): .......................... 417479 bp  
Mapped reads: .......................... 26973943  
Non-identical sequences: ............... 6209225  
Genomic hits: .......................... 48438990  
Significant densitiy of mapped reads: .. 821.144211136946 reads/kb

Show proTRAC cluster info
Hide proTRAC cluster info

|  |  |
| --- | --- |
| Location | NODE\_26968\_length\_1640\_cov\_67.407928 |
| Coordinates | 8-1700 |
| Size [bp] | 1693 |
| Sequence hit loci | 1543 |
| Mapped reads (normalized) | 4518.3 |
| Mapped reads (normalized) per kb | 2668.8 |
| Normalized reads with 1T (1U) | 68.2% |
| Normalized reads with 10A | 53.1% |
| Normalized reads with length 24-32 nt | 98.8% |
| Normalized reads on the main strand(s) | 83.7% |
| Predicted directionality | mono:minus |

100%

0%

1T (1U)  
reads

10A reads

24-32 nt  
reads

reads on mainstrand

**Either the amount of reads with 1T (1U) OR 10A has to exceed 75% (set with option: -1Tor10A)  
Alternatively the amount of reads with 1T (1U) AND 10A has to exceed 50% (set with option: -1Tand10A)  
Minimum amount of reads with preferred size is 75% (set with option: -pisize)  
Minimum amount of reads on the main strand(s) is 75% (set with option: -clstrand)**

Show read coverage
Hide read coverage

WHAT DO I SEE HERE?  
This chart shows the location of mapped sequence reads within a predicted piRNA cluster. The color refers to the number of genomic hits produced by the sequence read in question. A dark red bar indicates that this sequence read produces many other hits elsewhere in the genome. Many adjacent red or yellow bars can indicate the presence of a multi-copy element such as transposons or rRNA genes. A dark green bar indicates that this sequence read maps uniquely to this locus.

1 hit

2-5 hits

6-10 hits

11-20 hits

21-50 hits

51-100 hits

> 100 hits

NODE\_26968\_length\_1640\_cov\_67.407928

8

1700

Gene Set

RepeatMasker

Mapped  
Reads

27.21

plus strand

minus strand

27.21

Region: NODE\_26968\_length\_1640\_cov\_67.407928 1756-9. Max. coverage (+): 0. Max coverage (-): 0.05

Region: NODE\_26968\_length\_1640\_cov\_67.407928 10-13. Max. coverage (+): 0.05. Max coverage (-): 0.23

Region: NODE\_26968\_length\_1640\_cov\_67.407928 14-16. Max. coverage (+): 0.04. Max coverage (-): 0.09

Region: NODE\_26968\_length\_1640\_cov\_67.407928 17-19. Max. coverage (+): 0.01. Max coverage (-): 0.25

Region: NODE\_26968\_length\_1640\_cov\_67.407928 20-23. Max. coverage (+): 0. Max coverage (-): 0.1

Region: NODE\_26968\_length\_1640\_cov\_67.407928 24-26. Max. coverage (+): 0. Max coverage (-): 0

Region: NODE\_26968\_length\_1640\_cov\_67.407928 27-30. Max. coverage (+): 0.01. Max coverage (-): 0.09

Region: NODE\_26968\_length\_1640\_cov\_67.407928 31-33. Max. coverage (+): 0.4. Max coverage (-): 0.07

Region: NODE\_26968\_length\_1640\_cov\_67.407928 34-36. Max. coverage (+): 0.28. Max coverage (-): 0.05

Region: NODE\_26968\_length\_1640\_cov\_67.407928 37-40. Max. coverage (+): 0.02. Max coverage (-): 0.05

Region: NODE\_26968\_length\_1640\_cov\_67.407928 41-43. Max. coverage (+): 0. Max coverage (-): 0.01

Region: NODE\_26968\_length\_1640\_cov\_67.407928 44-46. Max. coverage (+): 0. Max coverage (-): 0.02

Region: NODE\_26968\_length\_1640\_cov\_67.407928 47-50. Max. coverage (+): 0.01. Max coverage (-): 0.02

Region: NODE\_26968\_length\_1640\_cov\_67.407928 51-53. Max. coverage (+): 0. Max coverage (-): 0.01

Region: NODE\_26968\_length\_1640\_cov\_67.407928 54-57. Max. coverage (+): 0. Max coverage (-): 0.01

Region: NODE\_26968\_length\_1640\_cov\_67.407928 58-60. Max. coverage (+): 0.4. Max coverage (-): 0.49

Region: NODE\_26968\_length\_1640\_cov\_67.407928 61-63. Max. coverage (+): 0.4. Max coverage (-): 0.04

Region: NODE\_26968\_length\_1640\_cov\_67.407928 64-67. Max. coverage (+): 0.07. Max coverage (-): 0.04

Region: NODE\_26968\_length\_1640\_cov\_67.407928 68-70. Max. coverage (+): 0.04. Max coverage (-): 0.04

Region: NODE\_26968\_length\_1640\_cov\_67.407928 71-74. Max. coverage (+): 0.06. Max coverage (-): 0.04

Region: NODE\_26968\_length\_1640\_cov\_67.407928 75-77. Max. coverage (+): 0. Max coverage (-): 0

Region: NODE\_26968\_length\_1640\_cov\_67.407928 78-80. Max. coverage (+): 0.04. Max coverage (-): 0.04

Region: NODE\_26968\_length\_1640\_cov\_67.407928 81-84. Max. coverage (+): 0. Max coverage (-): 0.11

Region: NODE\_26968\_length\_1640\_cov\_67.407928 85-87. Max. coverage (+): 0.19. Max coverage (-): 0.19

Region: NODE\_26968\_length\_1640\_cov\_67.407928 88-90. Max. coverage (+): 0.19. Max coverage (-): 0

Region: NODE\_26968\_length\_1640\_cov\_67.407928 91-94. Max. coverage (+): 0.01. Max coverage (-): 0

Region: NODE\_26968\_length\_1640\_cov\_67.407928 95-97. Max. coverage (+): 0. Max coverage (-): 0

Region: NODE\_26968\_length\_1640\_cov\_67.407928 98-101. Max. coverage (+): 0. Max coverage (-): 0

Region: NODE\_26968\_length\_1640\_cov\_67.407928 102-104. Max. coverage (+): 0. Max coverage (-): 0

Region: NODE\_26968\_length\_1640\_cov\_67.407928 105-107. Max. coverage (+): 0. Max coverage (-): 0

Region: NODE\_26968\_length\_1640\_cov\_67.407928 108-111. Max. coverage (+): 0. Max coverage (-): 0

Region: NODE\_26968\_length\_1640\_cov\_67.407928 112-114. Max. coverage (+): 0. Max coverage (-): 0.11

Region: NODE\_26968\_length\_1640\_cov\_67.407928 115-118. Max. coverage (+): 0. Max coverage (-): 0.07

Region: NODE\_26968\_length\_1640\_cov\_67.407928 119-121. Max. coverage (+): 0. Max coverage (-): 0.04

Region: NODE\_26968\_length\_1640\_cov\_67.407928 122-124. Max. coverage (+): 0.04. Max coverage (-): 0.04

Region: NODE\_26968\_length\_1640\_cov\_67.407928 125-128. Max. coverage (+): 0.04. Max coverage (-): 0

Region: NODE\_26968\_length\_1640\_cov\_67.407928 129-131. Max. coverage (+): 0. Max coverage (-): 0

Region: NODE\_26968\_length\_1640\_cov\_67.407928 132-134. Max. coverage (+): 0. Max coverage (-): 0.07

Region: NODE\_26968\_length\_1640\_cov\_67.407928 135-138. Max. coverage (+): 0.04. Max coverage (-): 0.11

Region: NODE\_26968\_length\_1640\_cov\_67.407928 139-141. Max. coverage (+): 0. Max coverage (-): 0.04

Region: NODE\_26968\_length\_1640\_cov\_67.407928 142-145. Max. coverage (+): 0.07. Max coverage (-): 0.11

Region: NODE\_26968\_length\_1640\_cov\_67.407928 146-148. Max. coverage (+): 0. Max coverage (-): 0.11

Region: NODE\_26968\_length\_1640\_cov\_67.407928 149-151. Max. coverage (+): 0. Max coverage (-): 0

Region: NODE\_26968\_length\_1640\_cov\_67.407928 152-155. Max. coverage (+): 0. Max coverage (-): 0.15

Region: NODE\_26968\_length\_1640\_cov\_67.407928 156-158. Max. coverage (+): 0. Max coverage (-): 0.44

Region: NODE\_26968\_length\_1640\_cov\_67.407928 159-162. Max. coverage (+): 0. Max coverage (-): 1.15

Region: NODE\_26968\_length\_1640\_cov\_67.407928 163-165. Max. coverage (+): 0.04. Max coverage (-): 0.11

Region: NODE\_26968\_length\_1640\_cov\_67.407928 166-168. Max. coverage (+): 0.04. Max coverage (-): 0.11

Region: NODE\_26968\_length\_1640\_cov\_67.407928 169-172. Max. coverage (+): 0. Max coverage (-): 0

Region: NODE\_26968\_length\_1640\_cov\_67.407928 173-175. Max. coverage (+): 0.04. Max coverage (-): 0

Region: NODE\_26968\_length\_1640\_cov\_67.407928 176-178. Max. coverage (+): 0.04. Max coverage (-): 0.04

Region: NODE\_26968\_length\_1640\_cov\_67.407928 179-182. Max. coverage (+): 0.04. Max coverage (-): 0.11

Region: NODE\_26968\_length\_1640\_cov\_67.407928 183-185. Max. coverage (+): 0.04. Max coverage (-): 2

Region: NODE\_26968\_length\_1640\_cov\_67.407928 186-189. Max. coverage (+): 0.04. Max coverage (-): 2.37

Region: NODE\_26968\_length\_1640\_cov\_67.407928 190-192. Max. coverage (+): 0. Max coverage (-): 0.19

Region: NODE\_26968\_length\_1640\_cov\_67.407928 193-195. Max. coverage (+): 0.04. Max coverage (-): 0.11

Region: NODE\_26968\_length\_1640\_cov\_67.407928 196-199. Max. coverage (+): 0. Max coverage (-): 0.04

Region: NODE\_26968\_length\_1640\_cov\_67.407928 200-202. Max. coverage (+): 0.22. Max coverage (-): 0.07

Region: NODE\_26968\_length\_1640\_cov\_67.407928 203-206. Max. coverage (+): 2.97. Max coverage (-): 0

Region: NODE\_26968\_length\_1640\_cov\_67.407928 207-209. Max. coverage (+): 0.11. Max coverage (-): 0.04

Region: NODE\_26968\_length\_1640\_cov\_67.407928 210-212. Max. coverage (+): 0.07. Max coverage (-): 0.04

Region: NODE\_26968\_length\_1640\_cov\_67.407928 213-216. Max. coverage (+): 0.33. Max coverage (-): 0.04

Region: NODE\_26968\_length\_1640\_cov\_67.407928 217-219. Max. coverage (+): 0.11. Max coverage (-): 0.04

Region: NODE\_26968\_length\_1640\_cov\_67.407928 220-223. Max. coverage (+): 0.33. Max coverage (-): 0

Region: NODE\_26968\_length\_1640\_cov\_67.407928 224-226. Max. coverage (+): 0.04. Max coverage (-): 0.07

Region: NODE\_26968\_length\_1640\_cov\_67.407928 227-229. Max. coverage (+): 0. Max coverage (-): 1.11

Region: NODE\_26968\_length\_1640\_cov\_67.407928 230-233. Max. coverage (+): 0.02. Max coverage (-): 2

Region: NODE\_26968\_length\_1640\_cov\_67.407928 234-236. Max. coverage (+): 0.02. Max coverage (-): 2.71

Region: NODE\_26968\_length\_1640\_cov\_67.407928 237-239. Max. coverage (+): 0.02. Max coverage (-): 0.7

Region: NODE\_26968\_length\_1640\_cov\_67.407928 240-243. Max. coverage (+): 0.04. Max coverage (-): 0.06

Region: NODE\_26968\_length\_1640\_cov\_67.407928 244-246. Max. coverage (+): 0.02. Max coverage (-): 0.04

Region: NODE\_26968\_length\_1640\_cov\_67.407928 247-250. Max. coverage (+): 0.04. Max coverage (-): 0.04

Region: NODE\_26968\_length\_1640\_cov\_67.407928 251-253. Max. coverage (+): 0.3. Max coverage (-): 0.46

Region: NODE\_26968\_length\_1640\_cov\_67.407928 254-256. Max. coverage (+): 0. Max coverage (-): 0.2

Region: NODE\_26968\_length\_1640\_cov\_67.407928 257-260. Max. coverage (+): 0. Max coverage (-): 0.17

Region: NODE\_26968\_length\_1640\_cov\_67.407928 261-263. Max. coverage (+): 0. Max coverage (-): 0.19

Region: NODE\_26968\_length\_1640\_cov\_67.407928 264-267. Max. coverage (+): 0. Max coverage (-): 0.07

Region: NODE\_26968\_length\_1640\_cov\_67.407928 268-270. Max. coverage (+): 0.01. Max coverage (-): 0

Region: NODE\_26968\_length\_1640\_cov\_67.407928 271-273. Max. coverage (+): 0.01. Max coverage (-): 0

Region: NODE\_26968\_length\_1640\_cov\_67.407928 274-277. Max. coverage (+): 0. Max coverage (-): 0

Region: NODE\_26968\_length\_1640\_cov\_67.407928 278-280. Max. coverage (+): 0. Max coverage (-): 0

Region: NODE\_26968\_length\_1640\_cov\_67.407928 281-283. Max. coverage (+): 0. Max coverage (-): 0.56

Region: NODE\_26968\_length\_1640\_cov\_67.407928 284-287. Max. coverage (+): 0. Max coverage (-): 0.37

Region: NODE\_26968\_length\_1640\_cov\_67.407928 288-290. Max. coverage (+): 0. Max coverage (-): 0

Region: NODE\_26968\_length\_1640\_cov\_67.407928 291-294. Max. coverage (+): 0. Max coverage (-): 0

Region: NODE\_26968\_length\_1640\_cov\_67.407928 295-297. Max. coverage (+): 0. Max coverage (-): 0

Region: NODE\_26968\_length\_1640\_cov\_67.407928 298-300. Max. coverage (+): 0. Max coverage (-): 0

Region: NODE\_26968\_length\_1640\_cov\_67.407928 301-304. Max. coverage (+): 0.07. Max coverage (-): 0

Region: NODE\_26968\_length\_1640\_cov\_67.407928 305-307. Max. coverage (+): 0. Max coverage (-): 0

Region: NODE\_26968\_length\_1640\_cov\_67.407928 308-311. Max. coverage (+): 0.04. Max coverage (-): 0

Region: NODE\_26968\_length\_1640\_cov\_67.407928 312-314. Max. coverage (+): 0. Max coverage (-): 0.11

Region: NODE\_26968\_length\_1640\_cov\_67.407928 315-317. Max. coverage (+): 0. Max coverage (-): 0.15

Region: NODE\_26968\_length\_1640\_cov\_67.407928 318-321. Max. coverage (+): 0. Max coverage (-): 0.04

Region: NODE\_26968\_length\_1640\_cov\_67.407928 322-324. Max. coverage (+): 0. Max coverage (-): 0.04

Region: NODE\_26968\_length\_1640\_cov\_67.407928 325-327. Max. coverage (+): 0. Max coverage (-): 0

Region: NODE\_26968\_length\_1640\_cov\_67.407928 328-331. Max. coverage (+): 0.04. Max coverage (-): 0.07

Region: NODE\_26968\_length\_1640\_cov\_67.407928 332-334. Max. coverage (+): 0. Max coverage (-): 0.04

Region: NODE\_26968\_length\_1640\_cov\_67.407928 335-338. Max. coverage (+): 0. Max coverage (-): 0.04

Region: NODE\_26968\_length\_1640\_cov\_67.407928 339-341. Max. coverage (+): 0. Max coverage (-): 0

Region: NODE\_26968\_length\_1640\_cov\_67.407928 342-344. Max. coverage (+): 0. Max coverage (-): 0

Region: NODE\_26968\_length\_1640\_cov\_67.407928 345-348. Max. coverage (+): 0. Max coverage (-): 0.04

Region: NODE\_26968\_length\_1640\_cov\_67.407928 349-351. Max. coverage (+): 0. Max coverage (-): 0.15

Region: NODE\_26968\_length\_1640\_cov\_67.407928 352-355. Max. coverage (+): 0.04. Max coverage (-): 8.9

Region: NODE\_26968\_length\_1640\_cov\_67.407928 356-358. Max. coverage (+): 0.04. Max coverage (-): 0.15

Region: NODE\_26968\_length\_1640\_cov\_67.407928 359-361. Max. coverage (+): 0.11. Max coverage (-): 0.14

Region: NODE\_26968\_length\_1640\_cov\_67.407928 362-365. Max. coverage (+): 0.04. Max coverage (-): 0.15

Region: NODE\_26968\_length\_1640\_cov\_67.407928 366-368. Max. coverage (+): 0. Max coverage (-): 0

Region: NODE\_26968\_length\_1640\_cov\_67.407928 369-371. Max. coverage (+): 0.04. Max coverage (-): 0.04

Region: NODE\_26968\_length\_1640\_cov\_67.407928 372-375. Max. coverage (+): 0.15. Max coverage (-): 0.04

Region: NODE\_26968\_length\_1640\_cov\_67.407928 376-378. Max. coverage (+): 0.04. Max coverage (-): 0.04

Region: NODE\_26968\_length\_1640\_cov\_67.407928 379-382. Max. coverage (+): 0.04. Max coverage (-): 0.04

Region: NODE\_26968\_length\_1640\_cov\_67.407928 383-385. Max. coverage (+): 0. Max coverage (-): 0.04

Region: NODE\_26968\_length\_1640\_cov\_67.407928 386-388. Max. coverage (+): 0. Max coverage (-): 0

Region: NODE\_26968\_length\_1640\_cov\_67.407928 389-392. Max. coverage (+): 0.04. Max coverage (-): 0.04

Region: NODE\_26968\_length\_1640\_cov\_67.407928 393-395. Max. coverage (+): 0.11. Max coverage (-): 0.19

Region: NODE\_26968\_length\_1640\_cov\_67.407928 396-399. Max. coverage (+): 0.07. Max coverage (-): 0.04

Region: NODE\_26968\_length\_1640\_cov\_67.407928 400-402. Max. coverage (+): 0.04. Max coverage (-): 0

Region: NODE\_26968\_length\_1640\_cov\_67.407928 403-405. Max. coverage (+): 0. Max coverage (-): 0

Region: NODE\_26968\_length\_1640\_cov\_67.407928 406-409. Max. coverage (+): 0.11. Max coverage (-): 0.15

Region: NODE\_26968\_length\_1640\_cov\_67.407928 410-412. Max. coverage (+): 0.11. Max coverage (-): 0.15

Region: NODE\_26968\_length\_1640\_cov\_67.407928 413-416. Max. coverage (+): 0.19. Max coverage (-): 0.11

Region: NODE\_26968\_length\_1640\_cov\_67.407928 417-419. Max. coverage (+): 0.07. Max coverage (-): 0.07

Region: NODE\_26968\_length\_1640\_cov\_67.407928 420-422. Max. coverage (+): 0. Max coverage (-): 0.07

Region: NODE\_26968\_length\_1640\_cov\_67.407928 423-426. Max. coverage (+): 0. Max coverage (-): 0.74

Region: NODE\_26968\_length\_1640\_cov\_67.407928 427-429. Max. coverage (+): 0. Max coverage (-): 0.85

Region: NODE\_26968\_length\_1640\_cov\_67.407928 430-432. Max. coverage (+): 0.07. Max coverage (-): 0.41

Region: NODE\_26968\_length\_1640\_cov\_67.407928 433-436. Max. coverage (+): 0.04. Max coverage (-): 1

Region: NODE\_26968\_length\_1640\_cov\_67.407928 437-439. Max. coverage (+): 0.04. Max coverage (-): 2.19

Region: NODE\_26968\_length\_1640\_cov\_67.407928 440-443. Max. coverage (+): 0.04. Max coverage (-): 2.45

Region: NODE\_26968\_length\_1640\_cov\_67.407928 444-446. Max. coverage (+): 0.19. Max coverage (-): 0.37

Region: NODE\_26968\_length\_1640\_cov\_67.407928 447-449. Max. coverage (+): 0.15. Max coverage (-): 0.11

Region: NODE\_26968\_length\_1640\_cov\_67.407928 450-453. Max. coverage (+): 0.04. Max coverage (-): 0.11

Region: NODE\_26968\_length\_1640\_cov\_67.407928 454-456. Max. coverage (+): 0. Max coverage (-): 0.41

Region: NODE\_26968\_length\_1640\_cov\_67.407928 457-460. Max. coverage (+): 0.67. Max coverage (-): 0.15

Region: NODE\_26968\_length\_1640\_cov\_67.407928 461-463. Max. coverage (+): 0.78. Max coverage (-): 0

Region: NODE\_26968\_length\_1640\_cov\_67.407928 464-466. Max. coverage (+): 0.52. Max coverage (-): 0

Region: NODE\_26968\_length\_1640\_cov\_67.407928 467-470. Max. coverage (+): 0. Max coverage (-): 0.15

Region: NODE\_26968\_length\_1640\_cov\_67.407928 471-473. Max. coverage (+): 0.04. Max coverage (-): 0.44

Region: NODE\_26968\_length\_1640\_cov\_67.407928 474-476. Max. coverage (+): 0. Max coverage (-): 0.33

Region: NODE\_26968\_length\_1640\_cov\_67.407928 477-480. Max. coverage (+): 0. Max coverage (-): 0.33

Region: NODE\_26968\_length\_1640\_cov\_67.407928 481-483. Max. coverage (+): 0. Max coverage (-): 0.07

Region: NODE\_26968\_length\_1640\_cov\_67.407928 484-487. Max. coverage (+): 0. Max coverage (-): 0.11

Region: NODE\_26968\_length\_1640\_cov\_67.407928 488-490. Max. coverage (+): 0.07. Max coverage (-): 0.26

Region: NODE\_26968\_length\_1640\_cov\_67.407928 491-493. Max. coverage (+): 1.2. Max coverage (-): 0.41

Region: NODE\_26968\_length\_1640\_cov\_67.407928 494-497. Max. coverage (+): 1.33. Max coverage (-): 0.02

Region: NODE\_26968\_length\_1640\_cov\_67.407928 498-500. Max. coverage (+): 0.18. Max coverage (-): 0.01

Region: NODE\_26968\_length\_1640\_cov\_67.407928 501-504. Max. coverage (+): 0.07. Max coverage (-): 0.23

Region: NODE\_26968\_length\_1640\_cov\_67.407928 505-507. Max. coverage (+): 0.01. Max coverage (-): 0.44

Region: NODE\_26968\_length\_1640\_cov\_67.407928 508-510. Max. coverage (+): 0.07. Max coverage (-): 0.44

Region: NODE\_26968\_length\_1640\_cov\_67.407928 511-514. Max. coverage (+): 0. Max coverage (-): 0.52

Region: NODE\_26968\_length\_1640\_cov\_67.407928 515-517. Max. coverage (+): 0. Max coverage (-): 0.52

Region: NODE\_26968\_length\_1640\_cov\_67.407928 518-520. Max. coverage (+): 0. Max coverage (-): 0.85

Region: NODE\_26968\_length\_1640\_cov\_67.407928 521-524. Max. coverage (+): 0.11. Max coverage (-): 1.26

Region: NODE\_26968\_length\_1640\_cov\_67.407928 525-527. Max. coverage (+): 0.19. Max coverage (-): 0.26

Region: NODE\_26968\_length\_1640\_cov\_67.407928 528-531. Max. coverage (+): 0. Max coverage (-): 0.82

Region: NODE\_26968\_length\_1640\_cov\_67.407928 532-534. Max. coverage (+): 0.04. Max coverage (-): 0.63

Region: NODE\_26968\_length\_1640\_cov\_67.407928 535-537. Max. coverage (+): 0.04. Max coverage (-): 0.59

Region: NODE\_26968\_length\_1640\_cov\_67.407928 538-541. Max. coverage (+): 0.04. Max coverage (-): 0.04

Region: NODE\_26968\_length\_1640\_cov\_67.407928 542-544. Max. coverage (+): 0.19. Max coverage (-): 0.04

Region: NODE\_26968\_length\_1640\_cov\_67.407928 545-548. Max. coverage (+): 0. Max coverage (-): 0.04

Region: NODE\_26968\_length\_1640\_cov\_67.407928 549-551. Max. coverage (+): 0. Max coverage (-): 0.04

Region: NODE\_26968\_length\_1640\_cov\_67.407928 552-554. Max. coverage (+): 0. Max coverage (-): 0.48

Region: NODE\_26968\_length\_1640\_cov\_67.407928 555-558. Max. coverage (+): 0. Max coverage (-): 27.21

Region: NODE\_26968\_length\_1640\_cov\_67.407928 559-561. Max. coverage (+): 0. Max coverage (-): 25.32

Region: NODE\_26968\_length\_1640\_cov\_67.407928 562-564. Max. coverage (+): 0.04. Max coverage (-): 3.6

Region: NODE\_26968\_length\_1640\_cov\_67.407928 565-568. Max. coverage (+): 0.04. Max coverage (-): 0.93

Region: NODE\_26968\_length\_1640\_cov\_67.407928 569-571. Max. coverage (+): 0. Max coverage (-): 2.52

Region: NODE\_26968\_length\_1640\_cov\_67.407928 572-575. Max. coverage (+): 0. Max coverage (-): 1.59

Region: NODE\_26968\_length\_1640\_cov\_67.407928 576-578. Max. coverage (+): 0.26. Max coverage (-): 0.3

Region: NODE\_26968\_length\_1640\_cov\_67.407928 579-581. Max. coverage (+): 0.26. Max coverage (-): 0.3

Region: NODE\_26968\_length\_1640\_cov\_67.407928 582-585. Max. coverage (+): 0.19. Max coverage (-): 0.11

Region: NODE\_26968\_length\_1640\_cov\_67.407928 586-588. Max. coverage (+): 0.52. Max coverage (-): 0.11

Region: NODE\_26968\_length\_1640\_cov\_67.407928 589-592. Max. coverage (+): 0.7. Max coverage (-): 0.11

Region: NODE\_26968\_length\_1640\_cov\_67.407928 593-595. Max. coverage (+): 0.07. Max coverage (-): 0.04

Region: NODE\_26968\_length\_1640\_cov\_67.407928 596-598. Max. coverage (+): 0.11. Max coverage (-): 0.04

Region: NODE\_26968\_length\_1640\_cov\_67.407928 599-602. Max. coverage (+): 0.04. Max coverage (-): 0

Region: NODE\_26968\_length\_1640\_cov\_67.407928 603-605. Max. coverage (+): 0.04. Max coverage (-): 0

Region: NODE\_26968\_length\_1640\_cov\_67.407928 606-609. Max. coverage (+): 0.19. Max coverage (-): 0

Region: NODE\_26968\_length\_1640\_cov\_67.407928 610-612. Max. coverage (+): 0.11. Max coverage (-): 0.11

Region: NODE\_26968\_length\_1640\_cov\_67.407928 613-615. Max. coverage (+): 0.15. Max coverage (-): 1.59

Region: NODE\_26968\_length\_1640\_cov\_67.407928 616-619. Max. coverage (+): 0.19. Max coverage (-): 0.15

Region: NODE\_26968\_length\_1640\_cov\_67.407928 620-622. Max. coverage (+): 0.04. Max coverage (-): 0.22

Region: NODE\_26968\_length\_1640\_cov\_67.407928 623-625. Max. coverage (+): 0. Max coverage (-): 0.07

Region: NODE\_26968\_length\_1640\_cov\_67.407928 626-629. Max. coverage (+): 0.11. Max coverage (-): 0.07

Region: NODE\_26968\_length\_1640\_cov\_67.407928 630-632. Max. coverage (+): 0.26. Max coverage (-): 0

Region: NODE\_26968\_length\_1640\_cov\_67.407928 633-636. Max. coverage (+): 0.69. Max coverage (-): 0

Region: NODE\_26968\_length\_1640\_cov\_67.407928 637-639. Max. coverage (+): 0.06. Max coverage (-): 0

Region: NODE\_26968\_length\_1640\_cov\_67.407928 640-642. Max. coverage (+): 0.02. Max coverage (-): 0.15

Region: NODE\_26968\_length\_1640\_cov\_67.407928 643-646. Max. coverage (+): 0. Max coverage (-): 1.33

Region: NODE\_26968\_length\_1640\_cov\_67.407928 647-649. Max. coverage (+): 0. Max coverage (-): 16.68

Region: NODE\_26968\_length\_1640\_cov\_67.407928 650-653. Max. coverage (+): 0. Max coverage (-): 2.11

Region: NODE\_26968\_length\_1640\_cov\_67.407928 654-656. Max. coverage (+): 0.07. Max coverage (-): 0.41

Region: NODE\_26968\_length\_1640\_cov\_67.407928 657-659. Max. coverage (+): 0.11. Max coverage (-): 0.52

Region: NODE\_26968\_length\_1640\_cov\_67.407928 660-663. Max. coverage (+): 0. Max coverage (-): 0.89

Region: NODE\_26968\_length\_1640\_cov\_67.407928 664-666. Max. coverage (+): 0. Max coverage (-): 0.04

Region: NODE\_26968\_length\_1640\_cov\_67.407928 667-669. Max. coverage (+): 0. Max coverage (-): 0.37

Region: NODE\_26968\_length\_1640\_cov\_67.407928 670-673. Max. coverage (+): 0. Max coverage (-): 0.37

Region: NODE\_26968\_length\_1640\_cov\_67.407928 674-676. Max. coverage (+): 0.04. Max coverage (-): 0.26

Region: NODE\_26968\_length\_1640\_cov\_67.407928 677-680. Max. coverage (+): 0.04. Max coverage (-): 0.37

Region: NODE\_26968\_length\_1640\_cov\_67.407928 681-683. Max. coverage (+): 0. Max coverage (-): 0.26

Region: NODE\_26968\_length\_1640\_cov\_67.407928 684-686. Max. coverage (+): 0. Max coverage (-): 3.48

Region: NODE\_26968\_length\_1640\_cov\_67.407928 687-690. Max. coverage (+): 0. Max coverage (-): 0

Region: NODE\_26968\_length\_1640\_cov\_67.407928 691-693. Max. coverage (+): 0. Max coverage (-): 0

Region: NODE\_26968\_length\_1640\_cov\_67.407928 694-697. Max. coverage (+): 0.11. Max coverage (-): 0.11

Region: NODE\_26968\_length\_1640\_cov\_67.407928 698-700. Max. coverage (+): 0.04. Max coverage (-): 0.11

Region: NODE\_26968\_length\_1640\_cov\_67.407928 701-703. Max. coverage (+): 0.05. Max coverage (-): 0.01

Region: NODE\_26968\_length\_1640\_cov\_67.407928 704-707. Max. coverage (+): 0.06. Max coverage (-): 0.05

Region: NODE\_26968\_length\_1640\_cov\_67.407928 708-710. Max. coverage (+): 0.01. Max coverage (-): 0.19

Region: NODE\_26968\_length\_1640\_cov\_67.407928 711-713. Max. coverage (+): 0. Max coverage (-): 0.15

Region: NODE\_26968\_length\_1640\_cov\_67.407928 714-717. Max. coverage (+): 0. Max coverage (-): 0.26

Region: NODE\_26968\_length\_1640\_cov\_67.407928 718-720. Max. coverage (+): 0. Max coverage (-): 0.41

Region: NODE\_26968\_length\_1640\_cov\_67.407928 721-724. Max. coverage (+): 0.04. Max coverage (-): 0.07

Region: NODE\_26968\_length\_1640\_cov\_67.407928 725-727. Max. coverage (+): 0. Max coverage (-): 0.07

Region: NODE\_26968\_length\_1640\_cov\_67.407928 728-730. Max. coverage (+): 0.04. Max coverage (-): 0.04

Region: NODE\_26968\_length\_1640\_cov\_67.407928 731-734. Max. coverage (+): 0.04. Max coverage (-): 0.04

Region: NODE\_26968\_length\_1640\_cov\_67.407928 735-737. Max. coverage (+): 0. Max coverage (-): 0.44

Region: NODE\_26968\_length\_1640\_cov\_67.407928 738-741. Max. coverage (+): 0. Max coverage (-): 2.82

Region: NODE\_26968\_length\_1640\_cov\_67.407928 742-744. Max. coverage (+): 0. Max coverage (-): 1.3

Region: NODE\_26968\_length\_1640\_cov\_67.407928 745-747. Max. coverage (+): 0.15. Max coverage (-): 0.63

Region: NODE\_26968\_length\_1640\_cov\_67.407928 748-751. Max. coverage (+): 0.15. Max coverage (-): 1.52

Region: NODE\_26968\_length\_1640\_cov\_67.407928 752-754. Max. coverage (+): 0.11. Max coverage (-): 1.52

Region: NODE\_26968\_length\_1640\_cov\_67.407928 755-757. Max. coverage (+): 0.41. Max coverage (-): 0.15

Region: NODE\_26968\_length\_1640\_cov\_67.407928 758-761. Max. coverage (+): 0.11. Max coverage (-): 0.07

Region: NODE\_26968\_length\_1640\_cov\_67.407928 762-764. Max. coverage (+): 0. Max coverage (-): 0

Region: NODE\_26968\_length\_1640\_cov\_67.407928 765-768. Max. coverage (+): 0.11. Max coverage (-): 2.19

Region: NODE\_26968\_length\_1640\_cov\_67.407928 769-771. Max. coverage (+): 0.15. Max coverage (-): 2.19

Region: NODE\_26968\_length\_1640\_cov\_67.407928 772-774. Max. coverage (+): 0.11. Max coverage (-): 0.37

Region: NODE\_26968\_length\_1640\_cov\_67.407928 775-778. Max. coverage (+): 0.07. Max coverage (-): 0.48

Region: NODE\_26968\_length\_1640\_cov\_67.407928 779-781. Max. coverage (+): 0.07. Max coverage (-): 0.44

Region: NODE\_26968\_length\_1640\_cov\_67.407928 782-785. Max. coverage (+): 0. Max coverage (-): 0.33

Region: NODE\_26968\_length\_1640\_cov\_67.407928 786-788. Max. coverage (+): 0.04. Max coverage (-): 0.04

Region: NODE\_26968\_length\_1640\_cov\_67.407928 789-791. Max. coverage (+): 0.63. Max coverage (-): 0.15

Region: NODE\_26968\_length\_1640\_cov\_67.407928 792-795. Max. coverage (+): 0.63. Max coverage (-): 0.26

Region: NODE\_26968\_length\_1640\_cov\_67.407928 796-798. Max. coverage (+): 0.19. Max coverage (-): 0

Region: NODE\_26968\_length\_1640\_cov\_67.407928 799-802. Max. coverage (+): 0.04. Max coverage (-): 0

Region: NODE\_26968\_length\_1640\_cov\_67.407928 803-805. Max. coverage (+): 0. Max coverage (-): 0.07

Region: NODE\_26968\_length\_1640\_cov\_67.407928 806-808. Max. coverage (+): 0.04. Max coverage (-): 0.07

Region: NODE\_26968\_length\_1640\_cov\_67.407928 809-812. Max. coverage (+): 0. Max coverage (-): 0

Region: NODE\_26968\_length\_1640\_cov\_67.407928 813-815. Max. coverage (+): 0.15. Max coverage (-): 0

Region: NODE\_26968\_length\_1640\_cov\_67.407928 816-818. Max. coverage (+): 0.15. Max coverage (-): 0.59

Region: NODE\_26968\_length\_1640\_cov\_67.407928 819-822. Max. coverage (+): 0.04. Max coverage (-): 0.19

Region: NODE\_26968\_length\_1640\_cov\_67.407928 823-825. Max. coverage (+): 0.07. Max coverage (-): 0.15

Region: NODE\_26968\_length\_1640\_cov\_67.407928 826-829. Max. coverage (+): 0.26. Max coverage (-): 0.15

Region: NODE\_26968\_length\_1640\_cov\_67.407928 830-832. Max. coverage (+): 0. Max coverage (-): 0.11

Region: NODE\_26968\_length\_1640\_cov\_67.407928 833-835. Max. coverage (+): 0. Max coverage (-): 0

Region: NODE\_26968\_length\_1640\_cov\_67.407928 836-839. Max. coverage (+): 0. Max coverage (-): 0.04

Region: NODE\_26968\_length\_1640\_cov\_67.407928 840-842. Max. coverage (+): 0. Max coverage (-): 0.04

Region: NODE\_26968\_length\_1640\_cov\_67.407928 843-846. Max. coverage (+): 0. Max coverage (-): 0

Region: NODE\_26968\_length\_1640\_cov\_67.407928 847-849. Max. coverage (+): 0. Max coverage (-): 0

Region: NODE\_26968\_length\_1640\_cov\_67.407928 850-852. Max. coverage (+): 0. Max coverage (-): 0.04

Region: NODE\_26968\_length\_1640\_cov\_67.407928 853-856. Max. coverage (+): 0. Max coverage (-): 0.07

Region: NODE\_26968\_length\_1640\_cov\_67.407928 857-859. Max. coverage (+): 0. Max coverage (-): 0.04

Region: NODE\_26968\_length\_1640\_cov\_67.407928 860-862. Max. coverage (+): 0.3. Max coverage (-): 0

Region: NODE\_26968\_length\_1640\_cov\_67.407928 863-866. Max. coverage (+): 0.3. Max coverage (-): 0

Region: NODE\_26968\_length\_1640\_cov\_67.407928 867-869. Max. coverage (+): 0. Max coverage (-): 0

Region: NODE\_26968\_length\_1640\_cov\_67.407928 870-873. Max. coverage (+): 0. Max coverage (-): 0.07

Region: NODE\_26968\_length\_1640\_cov\_67.407928 874-876. Max. coverage (+): 0. Max coverage (-): 0.67

Region: NODE\_26968\_length\_1640\_cov\_67.407928 877-879. Max. coverage (+): 0. Max coverage (-): 0.59

Region: NODE\_26968\_length\_1640\_cov\_67.407928 880-883. Max. coverage (+): 0. Max coverage (-): 0.11

Region: NODE\_26968\_length\_1640\_cov\_67.407928 884-886. Max. coverage (+): 0. Max coverage (-): 0

Region: NODE\_26968\_length\_1640\_cov\_67.407928 887-890. Max. coverage (+): 0. Max coverage (-): 0

Region: NODE\_26968\_length\_1640\_cov\_67.407928 891-893. Max. coverage (+): 0. Max coverage (-): 0.04

Region: NODE\_26968\_length\_1640\_cov\_67.407928 894-896. Max. coverage (+): 0. Max coverage (-): 0

Region: NODE\_26968\_length\_1640\_cov\_67.407928 897-900. Max. coverage (+): 0. Max coverage (-): 3.45

Region: NODE\_26968\_length\_1640\_cov\_67.407928 901-903. Max. coverage (+): 0.11. Max coverage (-): 0.19

Region: NODE\_26968\_length\_1640\_cov\_67.407928 904-906. Max. coverage (+): 0.04. Max coverage (-): 0.26

Region: NODE\_26968\_length\_1640\_cov\_67.407928 907-910. Max. coverage (+): 0.07. Max coverage (-): 0.04

Region: NODE\_26968\_length\_1640\_cov\_67.407928 911-913. Max. coverage (+): 0.04. Max coverage (-): 0.3

Region: NODE\_26968\_length\_1640\_cov\_67.407928 914-917. Max. coverage (+): 0. Max coverage (-): 0.22

Region: NODE\_26968\_length\_1640\_cov\_67.407928 918-920. Max. coverage (+): 0. Max coverage (-): 0.3

Region: NODE\_26968\_length\_1640\_cov\_67.407928 921-923. Max. coverage (+): 0.22. Max coverage (-): 0.04

Region: NODE\_26968\_length\_1640\_cov\_67.407928 924-927. Max. coverage (+): 0.22. Max coverage (-): 0

Region: NODE\_26968\_length\_1640\_cov\_67.407928 928-930. Max. coverage (+): 0. Max coverage (-): 0

Region: NODE\_26968\_length\_1640\_cov\_67.407928 931-934. Max. coverage (+): 0. Max coverage (-): 0.63

Region: NODE\_26968\_length\_1640\_cov\_67.407928 935-937. Max. coverage (+): 0. Max coverage (-): 0.19

Region: NODE\_26968\_length\_1640\_cov\_67.407928 938-940. Max. coverage (+): 0. Max coverage (-): 0.67

Region: NODE\_26968\_length\_1640\_cov\_67.407928 941-944. Max. coverage (+): 0.04. Max coverage (-): 0.74

Region: NODE\_26968\_length\_1640\_cov\_67.407928 945-947. Max. coverage (+): 0.07. Max coverage (-): 0.07

Region: NODE\_26968\_length\_1640\_cov\_67.407928 948-951. Max. coverage (+): 0.07. Max coverage (-): 0.04

Region: NODE\_26968\_length\_1640\_cov\_67.407928 952-954. Max. coverage (+): 0.22. Max coverage (-): 0.04

Region: NODE\_26968\_length\_1640\_cov\_67.407928 955-957. Max. coverage (+): 0.22. Max coverage (-): 0

Region: NODE\_26968\_length\_1640\_cov\_67.407928 958-961. Max. coverage (+): 0.04. Max coverage (-): 0.04

Region: NODE\_26968\_length\_1640\_cov\_67.407928 962-964. Max. coverage (+): 0.04. Max coverage (-): 0

Region: NODE\_26968\_length\_1640\_cov\_67.407928 965-967. Max. coverage (+): 0. Max coverage (-): 0

Region: NODE\_26968\_length\_1640\_cov\_67.407928 968-971. Max. coverage (+): 0. Max coverage (-): 0.15

Region: NODE\_26968\_length\_1640\_cov\_67.407928 972-974. Max. coverage (+): 0. Max coverage (-): 0.04

Region: NODE\_26968\_length\_1640\_cov\_67.407928 975-978. Max. coverage (+): 0. Max coverage (-): 0.04

Region: NODE\_26968\_length\_1640\_cov\_67.407928 979-981. Max. coverage (+): 0. Max coverage (-): 0

Region: NODE\_26968\_length\_1640\_cov\_67.407928 982-984. Max. coverage (+): 0. Max coverage (-): 0.04

Region: NODE\_26968\_length\_1640\_cov\_67.407928 985-988. Max. coverage (+): 0. Max coverage (-): 0.04

Region: NODE\_26968\_length\_1640\_cov\_67.407928 989-991. Max. coverage (+): 0. Max coverage (-): 0

Region: NODE\_26968\_length\_1640\_cov\_67.407928 992-995. Max. coverage (+): 0. Max coverage (-): 0

Region: NODE\_26968\_length\_1640\_cov\_67.407928 996-998. Max. coverage (+): 0. Max coverage (-): 0

Region: NODE\_26968\_length\_1640\_cov\_67.407928 999-1001. Max. coverage (+): 0. Max coverage (-): 0.04

Region: NODE\_26968\_length\_1640\_cov\_67.407928 1002-1005. Max. coverage (+): 0. Max coverage (-): 0.04

Region: NODE\_26968\_length\_1640\_cov\_67.407928 1006-1008. Max. coverage (+): 0. Max coverage (-): 0

Region: NODE\_26968\_length\_1640\_cov\_67.407928 1009-1011. Max. coverage (+): 0.19. Max coverage (-): 0.04

Region: NODE\_26968\_length\_1640\_cov\_67.407928 1012-1015. Max. coverage (+): 0.19. Max coverage (-): 0.04

Region: NODE\_26968\_length\_1640\_cov\_67.407928 1016-1018. Max. coverage (+): 0. Max coverage (-): 0

Region: NODE\_26968\_length\_1640\_cov\_67.407928 1019-1022. Max. coverage (+): 0. Max coverage (-): 0

Region: NODE\_26968\_length\_1640\_cov\_67.407928 1023-1025. Max. coverage (+): 0. Max coverage (-): 0.04

Region: NODE\_26968\_length\_1640\_cov\_67.407928 1026-1028. Max. coverage (+): 0. Max coverage (-): 0.07

Region: NODE\_26968\_length\_1640\_cov\_67.407928 1029-1032. Max. coverage (+): 0. Max coverage (-): 0.07

Region: NODE\_26968\_length\_1640\_cov\_67.407928 1033-1035. Max. coverage (+): 0.04. Max coverage (-): 0.07

Region: NODE\_26968\_length\_1640\_cov\_67.407928 1036-1039. Max. coverage (+): 0.11. Max coverage (-): 0.15

Region: NODE\_26968\_length\_1640\_cov\_67.407928 1040-1042. Max. coverage (+): 0.11. Max coverage (-): 0.07

Region: NODE\_26968\_length\_1640\_cov\_67.407928 1043-1045. Max. coverage (+): 0.07. Max coverage (-): 0.15

Region: NODE\_26968\_length\_1640\_cov\_67.407928 1046-1049. Max. coverage (+): 0.04. Max coverage (-): 0.63

Region: NODE\_26968\_length\_1640\_cov\_67.407928 1050-1052. Max. coverage (+): 0.11. Max coverage (-): 1.37

Region: NODE\_26968\_length\_1640\_cov\_67.407928 1053-1055. Max. coverage (+): 0.37. Max coverage (-): 1.26

Region: NODE\_26968\_length\_1640\_cov\_67.407928 1056-1059. Max. coverage (+): 0.33. Max coverage (-): 0.11

Region: NODE\_26968\_length\_1640\_cov\_67.407928 1060-1062. Max. coverage (+): 0.26. Max coverage (-): 0.07

Region: NODE\_26968\_length\_1640\_cov\_67.407928 1063-1066. Max. coverage (+): 0. Max coverage (-): 0.41

Region: NODE\_26968\_length\_1640\_cov\_67.407928 1067-1069. Max. coverage (+): 0. Max coverage (-): 0.07

Region: NODE\_26968\_length\_1640\_cov\_67.407928 1070-1072. Max. coverage (+): 0. Max coverage (-): 0.26

Region: NODE\_26968\_length\_1640\_cov\_67.407928 1073-1076. Max. coverage (+): 0. Max coverage (-): 0.22

Region: NODE\_26968\_length\_1640\_cov\_67.407928 1077-1079. Max. coverage (+): 0. Max coverage (-): 0

Region: NODE\_26968\_length\_1640\_cov\_67.407928 1080-1083. Max. coverage (+): 0. Max coverage (-): 0.04

Region: NODE\_26968\_length\_1640\_cov\_67.407928 1084-1086. Max. coverage (+): 0. Max coverage (-): 0.04

Region: NODE\_26968\_length\_1640\_cov\_67.407928 1087-1089. Max. coverage (+): 0. Max coverage (-): 0

Region: NODE\_26968\_length\_1640\_cov\_67.407928 1090-1093. Max. coverage (+): 0. Max coverage (-): 0

Region: NODE\_26968\_length\_1640\_cov\_67.407928 1094-1096. Max. coverage (+): 0. Max coverage (-): 0

Region: NODE\_26968\_length\_1640\_cov\_67.407928 1097-1099. Max. coverage (+): 0. Max coverage (-): 0

Region: NODE\_26968\_length\_1640\_cov\_67.407928 1100-1103. Max. coverage (+): 0. Max coverage (-): 0

Region: NODE\_26968\_length\_1640\_cov\_67.407928 1104-1106. Max. coverage (+): 0. Max coverage (-): 0.04

Region: NODE\_26968\_length\_1640\_cov\_67.407928 1107-1110. Max. coverage (+): 0. Max coverage (-): 0.07

Region: NODE\_26968\_length\_1640\_cov\_67.407928 1111-1113. Max. coverage (+): 0. Max coverage (-): 0.04

Region: NODE\_26968\_length\_1640\_cov\_67.407928 1114-1116. Max. coverage (+): 0. Max coverage (-): 0.04

Region: NODE\_26968\_length\_1640\_cov\_67.407928 1117-1120. Max. coverage (+): 0. Max coverage (-): 0.11

Region: NODE\_26968\_length\_1640\_cov\_67.407928 1121-1123. Max. coverage (+): 0. Max coverage (-): 0.19

Region: NODE\_26968\_length\_1640\_cov\_67.407928 1124-1127. Max. coverage (+): 0. Max coverage (-): 0.11

Region: NODE\_26968\_length\_1640\_cov\_67.407928 1128-1130. Max. coverage (+): 0. Max coverage (-): 0.04

Region: NODE\_26968\_length\_1640\_cov\_67.407928 1131-1133. Max. coverage (+): 0. Max coverage (-): 0

Region: NODE\_26968\_length\_1640\_cov\_67.407928 1134-1137. Max. coverage (+): 0. Max coverage (-): 0.19

Region: NODE\_26968\_length\_1640\_cov\_67.407928 1138-1140. Max. coverage (+): 0.04. Max coverage (-): 0.04

Region: NODE\_26968\_length\_1640\_cov\_67.407928 1141-1144. Max. coverage (+): 0.04. Max coverage (-): 0

Region: NODE\_26968\_length\_1640\_cov\_67.407928 1145-1147. Max. coverage (+): 0. Max coverage (-): 0

Region: NODE\_26968\_length\_1640\_cov\_67.407928 1148-1150. Max. coverage (+): 0. Max coverage (-): 0

Region: NODE\_26968\_length\_1640\_cov\_67.407928 1151-1154. Max. coverage (+): 0. Max coverage (-): 0

Region: NODE\_26968\_length\_1640\_cov\_67.407928 1155-1157. Max. coverage (+): 0. Max coverage (-): 0

Region: NODE\_26968\_length\_1640\_cov\_67.407928 1158-1160. Max. coverage (+): 0. Max coverage (-): 0

Region: NODE\_26968\_length\_1640\_cov\_67.407928 1161-1164. Max. coverage (+): 0. Max coverage (-): 0.04

Region: NODE\_26968\_length\_1640\_cov\_67.407928 1165-1167. Max. coverage (+): 0. Max coverage (-): 0.19

Region: NODE\_26968\_length\_1640\_cov\_67.407928 1168-1171. Max. coverage (+): 0.04. Max coverage (-): 1.15

Region: NODE\_26968\_length\_1640\_cov\_67.407928 1172-1174. Max. coverage (+): 0.04. Max coverage (-): 0.59

Region: NODE\_26968\_length\_1640\_cov\_67.407928 1175-1177. Max. coverage (+): 0. Max coverage (-): 1.22

Region: NODE\_26968\_length\_1640\_cov\_67.407928 1178-1181. Max. coverage (+): 0.04. Max coverage (-): 1.04

Region: NODE\_26968\_length\_1640\_cov\_67.407928 1182-1184. Max. coverage (+): 0.19. Max coverage (-): 0.44

Region: NODE\_26968\_length\_1640\_cov\_67.407928 1185-1188. Max. coverage (+): 0.19. Max coverage (-): 0.19

Region: NODE\_26968\_length\_1640\_cov\_67.407928 1189-1191. Max. coverage (+): 0.04. Max coverage (-): 0

Region: NODE\_26968\_length\_1640\_cov\_67.407928 1192-1194. Max. coverage (+): 0.19. Max coverage (-): 0.04

Region: NODE\_26968\_length\_1640\_cov\_67.407928 1195-1198. Max. coverage (+): 0.15. Max coverage (-): 0.04

Region: NODE\_26968\_length\_1640\_cov\_67.407928 1199-1201. Max. coverage (+): 0. Max coverage (-): 0

Region: NODE\_26968\_length\_1640\_cov\_67.407928 1202-1204. Max. coverage (+): 0. Max coverage (-): 0.04

Region: NODE\_26968\_length\_1640\_cov\_67.407928 1205-1208. Max. coverage (+): 0. Max coverage (-): 0.07

Region: NODE\_26968\_length\_1640\_cov\_67.407928 1209-1211. Max. coverage (+): 0.07. Max coverage (-): 0.04

Region: NODE\_26968\_length\_1640\_cov\_67.407928 1212-1215. Max. coverage (+): 0.07. Max coverage (-): 0.04

Region: NODE\_26968\_length\_1640\_cov\_67.407928 1216-1218. Max. coverage (+): 0. Max coverage (-): 0.04

Region: NODE\_26968\_length\_1640\_cov\_67.407928 1219-1221. Max. coverage (+): 0. Max coverage (-): 0

Region: NODE\_26968\_length\_1640\_cov\_67.407928 1222-1225. Max. coverage (+): 0. Max coverage (-): 0.41

Region: NODE\_26968\_length\_1640\_cov\_67.407928 1226-1228. Max. coverage (+): 0. Max coverage (-): 0.56

Region: NODE\_26968\_length\_1640\_cov\_67.407928 1229-1232. Max. coverage (+): 0. Max coverage (-): 0.19

Region: NODE\_26968\_length\_1640\_cov\_67.407928 1233-1235. Max. coverage (+): 0. Max coverage (-): 0

Region: NODE\_26968\_length\_1640\_cov\_67.407928 1236-1238. Max. coverage (+): 0. Max coverage (-): 0

Region: NODE\_26968\_length\_1640\_cov\_67.407928 1239-1242. Max. coverage (+): 0. Max coverage (-): 0.04

Region: NODE\_26968\_length\_1640\_cov\_67.407928 1243-1245. Max. coverage (+): 0. Max coverage (-): 0.3

Region: NODE\_26968\_length\_1640\_cov\_67.407928 1246-1248. Max. coverage (+): 0. Max coverage (-): 0.3

Region: NODE\_26968\_length\_1640\_cov\_67.407928 1249-1252. Max. coverage (+): 0.04. Max coverage (-): 0.15

Region: NODE\_26968\_length\_1640\_cov\_67.407928 1253-1255. Max. coverage (+): 0.04. Max coverage (-): 0

Region: NODE\_26968\_length\_1640\_cov\_67.407928 1256-1259. Max. coverage (+): 0. Max coverage (-): 0

Region: NODE\_26968\_length\_1640\_cov\_67.407928 1260-1262. Max. coverage (+): 0. Max coverage (-): 0

Region: NODE\_26968\_length\_1640\_cov\_67.407928 1263-1265. Max. coverage (+): 0. Max coverage (-): 0

Region: NODE\_26968\_length\_1640\_cov\_67.407928 1266-1269. Max. coverage (+): 0. Max coverage (-): 0.04

Region: NODE\_26968\_length\_1640\_cov\_67.407928 1270-1272. Max. coverage (+): 0. Max coverage (-): 0.04

Region: NODE\_26968\_length\_1640\_cov\_67.407928 1273-1276. Max. coverage (+): 0.02. Max coverage (-): 0.15

Region: NODE\_26968\_length\_1640\_cov\_67.407928 1277-1279. Max. coverage (+): 0.07. Max coverage (-): 0.07

Region: NODE\_26968\_length\_1640\_cov\_67.407928 1280-1282. Max. coverage (+): 0.07. Max coverage (-): 0.04

Region: NODE\_26968\_length\_1640\_cov\_67.407928 1283-1286. Max. coverage (+): 0.04. Max coverage (-): 0

Region: NODE\_26968\_length\_1640\_cov\_67.407928 1287-1289. Max. coverage (+): 0. Max coverage (-): 0

Region: NODE\_26968\_length\_1640\_cov\_67.407928 1290-1292. Max. coverage (+): 0.19. Max coverage (-): 0

Region: NODE\_26968\_length\_1640\_cov\_67.407928 1293-1296. Max. coverage (+): 0.33. Max coverage (-): 0

Region: NODE\_26968\_length\_1640\_cov\_67.407928 1297-1299. Max. coverage (+): 0.15. Max coverage (-): 0.15

Region: NODE\_26968\_length\_1640\_cov\_67.407928 1300-1303. Max. coverage (+): 0.19. Max coverage (-): 0.74

Region: NODE\_26968\_length\_1640\_cov\_67.407928 1304-1306. Max. coverage (+): 0.11. Max coverage (-): 0.63

Region: NODE\_26968\_length\_1640\_cov\_67.407928 1307-1309. Max. coverage (+): 0.04. Max coverage (-): 0.15

Region: NODE\_26968\_length\_1640\_cov\_67.407928 1310-1313. Max. coverage (+): 0.15. Max coverage (-): 0.11

Region: NODE\_26968\_length\_1640\_cov\_67.407928 1314-1316. Max. coverage (+): 0.44. Max coverage (-): 0.04

Region: NODE\_26968\_length\_1640\_cov\_67.407928 1317-1320. Max. coverage (+): 0.41. Max coverage (-): 0.04

Region: NODE\_26968\_length\_1640\_cov\_67.407928 1321-1323. Max. coverage (+): 0.33. Max coverage (-): 0

Region: NODE\_26968\_length\_1640\_cov\_67.407928 1324-1326. Max. coverage (+): 0.15. Max coverage (-): 0.07

Region: NODE\_26968\_length\_1640\_cov\_67.407928 1327-1330. Max. coverage (+): 0. Max coverage (-): 0.07

Region: NODE\_26968\_length\_1640\_cov\_67.407928 1331-1333. Max. coverage (+): 0. Max coverage (-): 0.19

Region: NODE\_26968\_length\_1640\_cov\_67.407928 1334-1337. Max. coverage (+): 0.04. Max coverage (-): 0.3

Region: NODE\_26968\_length\_1640\_cov\_67.407928 1338-1340. Max. coverage (+): 0.07. Max coverage (-): 0.19

Region: NODE\_26968\_length\_1640\_cov\_67.407928 1341-1343. Max. coverage (+): 0.04. Max coverage (-): 0

Region: NODE\_26968\_length\_1640\_cov\_67.407928 1344-1347. Max. coverage (+): 0. Max coverage (-): 0.04

Region: NODE\_26968\_length\_1640\_cov\_67.407928 1348-1350. Max. coverage (+): 0. Max coverage (-): 0.04

Region: NODE\_26968\_length\_1640\_cov\_67.407928 1351-1353. Max. coverage (+): 0.04. Max coverage (-): 0

Region: NODE\_26968\_length\_1640\_cov\_67.407928 1354-1357. Max. coverage (+): 0.11. Max coverage (-): 0.07

Region: NODE\_26968\_length\_1640\_cov\_67.407928 1358-1360. Max. coverage (+): 0.15. Max coverage (-): 0.04

Region: NODE\_26968\_length\_1640\_cov\_67.407928 1361-1364. Max. coverage (+): 0.04. Max coverage (-): 0.59

Region: NODE\_26968\_length\_1640\_cov\_67.407928 1365-1367. Max. coverage (+): 0. Max coverage (-): 0.63

Region: NODE\_26968\_length\_1640\_cov\_67.407928 1368-1370. Max. coverage (+): 0. Max coverage (-): 0.04

Region: NODE\_26968\_length\_1640\_cov\_67.407928 1371-1374. Max. coverage (+): 0. Max coverage (-): 0.15

Region: NODE\_26968\_length\_1640\_cov\_67.407928 1375-1377. Max. coverage (+): 0.02. Max coverage (-): 0.11

Region: NODE\_26968\_length\_1640\_cov\_67.407928 1378-1381. Max. coverage (+): 0.02. Max coverage (-): 0.52

Region: NODE\_26968\_length\_1640\_cov\_67.407928 1382-1384. Max. coverage (+): 0.07. Max coverage (-): 0.47

Region: NODE\_26968\_length\_1640\_cov\_67.407928 1385-1387. Max. coverage (+): 0.16. Max coverage (-): 0.17

Region: NODE\_26968\_length\_1640\_cov\_67.407928 1388-1391. Max. coverage (+): 2.69. Max coverage (-): 0.01

Region: NODE\_26968\_length\_1640\_cov\_67.407928 1392-1394. Max. coverage (+): 2.64. Max coverage (-): 0.02

Region: NODE\_26968\_length\_1640\_cov\_67.407928 1395-1397. Max. coverage (+): 0.3. Max coverage (-): 0.02

Region: NODE\_26968\_length\_1640\_cov\_67.407928 1398-1401. Max. coverage (+): 0.26. Max coverage (-): 0

Region: NODE\_26968\_length\_1640\_cov\_67.407928 1402-1404. Max. coverage (+): 0.02. Max coverage (-): 0

Region: NODE\_26968\_length\_1640\_cov\_67.407928 1405-1408. Max. coverage (+): 0. Max coverage (-): 0

Region: NODE\_26968\_length\_1640\_cov\_67.407928 1409-1411. Max. coverage (+): 0. Max coverage (-): 0

Region: NODE\_26968\_length\_1640\_cov\_67.407928 1412-1414. Max. coverage (+): 0. Max coverage (-): 0

Region: NODE\_26968\_length\_1640\_cov\_67.407928 1415-1418. Max. coverage (+): 0. Max coverage (-): 0

Region: NODE\_26968\_length\_1640\_cov\_67.407928 1419-1421. Max. coverage (+): 0. Max coverage (-): 0.11

Region: NODE\_26968\_length\_1640\_cov\_67.407928 1422-1425. Max. coverage (+): 0. Max coverage (-): 0.11

Region: NODE\_26968\_length\_1640\_cov\_67.407928 1426-1428. Max. coverage (+): 0. Max coverage (-): 0

Region: NODE\_26968\_length\_1640\_cov\_67.407928 1429-1431. Max. coverage (+): 0. Max coverage (-): 0

Region: NODE\_26968\_length\_1640\_cov\_67.407928 1432-1435. Max. coverage (+): 0. Max coverage (-): 0

Region: NODE\_26968\_length\_1640\_cov\_67.407928 1436-1438. Max. coverage (+): 0. Max coverage (-): 0

Region: NODE\_26968\_length\_1640\_cov\_67.407928 1439-1441. Max. coverage (+): 0. Max coverage (-): 0

Region: NODE\_26968\_length\_1640\_cov\_67.407928 1442-1445. Max. coverage (+): 0. Max coverage (-): 0.11

Region: NODE\_26968\_length\_1640\_cov\_67.407928 1446-1448. Max. coverage (+): 0. Max coverage (-): 0.11

Region: NODE\_26968\_length\_1640\_cov\_67.407928 1449-1452. Max. coverage (+): 0. Max coverage (-): 0.02

Region: NODE\_26968\_length\_1640\_cov\_67.407928 1453-1455. Max. coverage (+): 0. Max coverage (-): 0.02

Region: NODE\_26968\_length\_1640\_cov\_67.407928 1456-1458. Max. coverage (+): 0. Max coverage (-): 0.32

Region: NODE\_26968\_length\_1640\_cov\_67.407928 1459-1462. Max. coverage (+): 0. Max coverage (-): 0.3

Region: NODE\_26968\_length\_1640\_cov\_67.407928 1463-1465. Max. coverage (+): 0. Max coverage (-): 0.05

Region: NODE\_26968\_length\_1640\_cov\_67.407928 1466-1469. Max. coverage (+): 0.04. Max coverage (-): 0.24

Region: NODE\_26968\_length\_1640\_cov\_67.407928 1470-1472. Max. coverage (+): 0.04. Max coverage (-): 0

Region: NODE\_26968\_length\_1640\_cov\_67.407928 1473-1475. Max. coverage (+): 0. Max coverage (-): 0

Region: NODE\_26968\_length\_1640\_cov\_67.407928 1476-1479. Max. coverage (+): 0. Max coverage (-): 0.07

Region: NODE\_26968\_length\_1640\_cov\_67.407928 1480-1482. Max. coverage (+): 0. Max coverage (-): 0.07

Region: NODE\_26968\_length\_1640\_cov\_67.407928 1483-1485. Max. coverage (+): 0.01. Max coverage (-): 0.09

Region: NODE\_26968\_length\_1640\_cov\_67.407928 1486-1489. Max. coverage (+): 0.04. Max coverage (-): 0.08

Region: NODE\_26968\_length\_1640\_cov\_67.407928 1490-1492. Max. coverage (+): 0.07. Max coverage (-): 0

Region: NODE\_26968\_length\_1640\_cov\_67.407928 1493-1496. Max. coverage (+): 0.07. Max coverage (-): 0

Region: NODE\_26968\_length\_1640\_cov\_67.407928 1497-1499. Max. coverage (+): 0.07. Max coverage (-): 0

Region: NODE\_26968\_length\_1640\_cov\_67.407928 1500-1502. Max. coverage (+): 0. Max coverage (-): 0

Region: NODE\_26968\_length\_1640\_cov\_67.407928 1503-1506. Max. coverage (+): 0. Max coverage (-): 0

Region: NODE\_26968\_length\_1640\_cov\_67.407928 1507-1509. Max. coverage (+): 0. Max coverage (-): 0

Region: NODE\_26968\_length\_1640\_cov\_67.407928 1510-1513. Max. coverage (+): 0. Max coverage (-): 0

Region: NODE\_26968\_length\_1640\_cov\_67.407928 1514-1516. Max. coverage (+): 0. Max coverage (-): 0.04

Region: NODE\_26968\_length\_1640\_cov\_67.407928 1517-1519. Max. coverage (+): 0. Max coverage (-): 0.52

Region: NODE\_26968\_length\_1640\_cov\_67.407928 1520-1523. Max. coverage (+): 0. Max coverage (-): 0.48

Region: NODE\_26968\_length\_1640\_cov\_67.407928 1524-1526. Max. coverage (+): 0. Max coverage (-): 0.07

Region: NODE\_26968\_length\_1640\_cov\_67.407928 1527-1530. Max. coverage (+): 0. Max coverage (-): 0.07

Region: NODE\_26968\_length\_1640\_cov\_67.407928 1531-1533. Max. coverage (+): 0. Max coverage (-): 0

Region: NODE\_26968\_length\_1640\_cov\_67.407928 1534-1536. Max. coverage (+): 0.07. Max coverage (-): 0

Region: NODE\_26968\_length\_1640\_cov\_67.407928 1537-1540. Max. coverage (+): 0.44. Max coverage (-): 0

Region: NODE\_26968\_length\_1640\_cov\_67.407928 1541-1543. Max. coverage (+): 0.26. Max coverage (-): 0

Region: NODE\_26968\_length\_1640\_cov\_67.407928 1544-1546. Max. coverage (+): 0.07. Max coverage (-): 0

Region: NODE\_26968\_length\_1640\_cov\_67.407928 1547-1550. Max. coverage (+): 0. Max coverage (-): 0

Region: NODE\_26968\_length\_1640\_cov\_67.407928 1551-1553. Max. coverage (+): 0. Max coverage (-): 0

Region: NODE\_26968\_length\_1640\_cov\_67.407928 1554-1557. Max. coverage (+): 0. Max coverage (-): 0

Region: NODE\_26968\_length\_1640\_cov\_67.407928 1558-1560. Max. coverage (+): 0. Max coverage (-): 0

Region: NODE\_26968\_length\_1640\_cov\_67.407928 1561-1563. Max. coverage (+): 0. Max coverage (-): 0

Region: NODE\_26968\_length\_1640\_cov\_67.407928 1564-1567. Max. coverage (+): 0. Max coverage (-): 0

Region: NODE\_26968\_length\_1640\_cov\_67.407928 1568-1570. Max. coverage (+): 0. Max coverage (-): 0

Region: NODE\_26968\_length\_1640\_cov\_67.407928 1571-1574. Max. coverage (+): 0. Max coverage (-): 0.11

Region: NODE\_26968\_length\_1640\_cov\_67.407928 1575-1577. Max. coverage (+): 0. Max coverage (-): 0.85

Region: NODE\_26968\_length\_1640\_cov\_67.407928 1578-1580. Max. coverage (+): 0. Max coverage (-): 0.89

Region: NODE\_26968\_length\_1640\_cov\_67.407928 1581-1584. Max. coverage (+): 0. Max coverage (-): 0.26

Region: NODE\_26968\_length\_1640\_cov\_67.407928 1585-1587. Max. coverage (+): 0. Max coverage (-): 0.19

Region: NODE\_26968\_length\_1640\_cov\_67.407928 1588-1590. Max. coverage (+): 0. Max coverage (-): 0.11

Region: NODE\_26968\_length\_1640\_cov\_67.407928 1591-1594. Max. coverage (+): 0. Max coverage (-): 0

Region: NODE\_26968\_length\_1640\_cov\_67.407928 1595-1597. Max. coverage (+): 0. Max coverage (-): 0.04

Region: NODE\_26968\_length\_1640\_cov\_67.407928 1598-1601. Max. coverage (+): 0.02. Max coverage (-): 0.06

Region: NODE\_26968\_length\_1640\_cov\_67.407928 1602-1604. Max. coverage (+): 0. Max coverage (-): 0

Region: NODE\_26968\_length\_1640\_cov\_67.407928 1605-1607. Max. coverage (+): 0.04. Max coverage (-): 0.14

Region: NODE\_26968\_length\_1640\_cov\_67.407928 1608-1611. Max. coverage (+): 0.34. Max coverage (-): 0.14

Region: NODE\_26968\_length\_1640\_cov\_67.407928 1612-1614. Max. coverage (+): 0.24. Max coverage (-): 0.03

Region: NODE\_26968\_length\_1640\_cov\_67.407928 1615-1618. Max. coverage (+): 0.01. Max coverage (-): 0.02

Region: NODE\_26968\_length\_1640\_cov\_67.407928 1619-1621. Max. coverage (+): 0.04. Max coverage (-): 0.07

Region: NODE\_26968\_length\_1640\_cov\_67.407928 1622-1624. Max. coverage (+): 0.04. Max coverage (-): 0.59

Region: NODE\_26968\_length\_1640\_cov\_67.407928 1625-1628. Max. coverage (+): 0. Max coverage (-): 3.04

Region: NODE\_26968\_length\_1640\_cov\_67.407928 1629-1631. Max. coverage (+): 0. Max coverage (-): 3.93

Region: NODE\_26968\_length\_1640\_cov\_67.407928 1632-1634. Max. coverage (+): 0.04. Max coverage (-): 1.89

Region: NODE\_26968\_length\_1640\_cov\_67.407928 1635-1638. Max. coverage (+): 0.07. Max coverage (-): 0.26

Region: NODE\_26968\_length\_1640\_cov\_67.407928 1639-1641. Max. coverage (+): 0.1. Max coverage (-): 0.04

Region: NODE\_26968\_length\_1640\_cov\_67.407928 1642-1645. Max. coverage (+): 0.02. Max coverage (-): 0

Region: NODE\_26968\_length\_1640\_cov\_67.407928 1646-1648. Max. coverage (+): 0.01. Max coverage (-): 0

Region: NODE\_26968\_length\_1640\_cov\_67.407928 1649-1651. Max. coverage (+): 0.01. Max coverage (-): 0

Region: NODE\_26968\_length\_1640\_cov\_67.407928 1652-1655. Max. coverage (+): 0.01. Max coverage (-): 0

Region: NODE\_26968\_length\_1640\_cov\_67.407928 1656-1658. Max. coverage (+): 0. Max coverage (-): 0

Region: NODE\_26968\_length\_1640\_cov\_67.407928 1659-1662. Max. coverage (+): 0. Max coverage (-): 0

Region: NODE\_26968\_length\_1640\_cov\_67.407928 1663-1665. Max. coverage (+): 0. Max coverage (-): 0

Region: NODE\_26968\_length\_1640\_cov\_67.407928 1666-1668. Max. coverage (+): 0. Max coverage (-): 0

Region: NODE\_26968\_length\_1640\_cov\_67.407928 1669-1672. Max. coverage (+): 0.01. Max coverage (-): 0.01

Region: NODE\_26968\_length\_1640\_cov\_67.407928 1673-1675. Max. coverage (+): 0.02. Max coverage (-): 0.01

Region: NODE\_26968\_length\_1640\_cov\_67.407928 1676-1678. Max. coverage (+): 0.01. Max coverage (-): 0

Region: NODE\_26968\_length\_1640\_cov\_67.407928 1679-1682. Max. coverage (+): 0. Max coverage (-): 0

Region: NODE\_26968\_length\_1640\_cov\_67.407928 1683-1685. Max. coverage (+): 0. Max coverage (-): 0

Region: NODE\_26968\_length\_1640\_cov\_67.407928 1686-1689. Max. coverage (+): 0. Max coverage (-): 0

Region: NODE\_26968\_length\_1640\_cov\_67.407928 1690-1692. Max. coverage (+): 0. Max coverage (-): 0

Region: NODE\_26968\_length\_1640\_cov\_67.407928 1693-1695. Max. coverage (+): 0. Max coverage (-): 0

Region: NODE\_26968\_length\_1640\_cov\_67.407928 1696-1699. Max. coverage (+): 0. Max coverage (-): 0

Region: NODE\_26968\_length\_1640\_cov\_67.407928 1700-. Max. coverage (+): 0. Max coverage (-): 0

RepeatMasker Color Code

**+**

100-98% Identity

<98-95% Identity

<95-90% Identity

<90-85% Identity

<85-80% Identity

<80-75% Identity

<75-70% Identity

<70% Identity

**-**

Gene Set Color Code

**+**

Gene

Pseudogene

Other

**-**

Topology/Coverage Color Code

Coverage Plus Strand

Coverage Minus Strand

Mainstrand: Plus

Mainstrand: Minus

Complementary Strand

Flanking Region  
(if option -flank >0)

Gene Set Annotation  
  
RepeatMasker Annotation  

**1. AlRepD-288**: 27-159 (+), Divergence to consensus: 18.2%  
**2. AlRepD-2481**: 175-219 (-), Divergence to consensus: 13.3%  
**3. AlRepA-118**: 262-304 (+), Divergence to consensus: 18.6%  
**4. TC1\_TF**: 309-540 (-), Divergence to consensus: 26.3%  
**5. TC1\_TF**: 530-856 (-), Divergence to consensus: 29.1%  
**6. AlRepB-161**: 906-1012 (+), Divergence to consensus: 17.8%  
**7. AlRepB-161**: 1029-1209 (+), Divergence to consensus: 28.8%  
**8. AlRepB-103**: 1218-1251 (+), Divergence to consensus: 11.8%  
**9. AlRepC-1745**: 1314-1522 (+), Divergence to consensus: 27.4%  
**10. AlRepC-1745**: 1604-1704 (+), Divergence to consensus: 18%

  
Transcription Factor Binding Sites  

**RHOXF1** (Sequence: AGCTTA (-): 104)  
**RHOXF1** (Sequence: AGCTCA (-): 810)  
**RHOXF1** (Sequence: AGCTCA (-): 1227)  
**RHOXF1** (Sequence: AGCTTA (-): 1466)  
**RHOXF1** (Sequence: TGAGCC (+): 261)  
**RHOXF1** (Sequence: TGAGCC (+): 645)  
**RHOXF1** (Sequence: TAAGCT (+): 808)  
**RHOXF1** (Sequence: TGAGCT (+): 948)  
**RHOXF1** (Sequence: TGAGCC (+): 1097)  
**RHOXF1** (Sequence: TGAGCC (+): 1204)  
**RHOXF1** (Sequence: TGAGCC (+): 1377)  
**Lhx8** (Sequence: TTAATTAA (-): 785)  
**Gata4** (Sequence: CTTATCT (+): 106)  
**SOX9** (Sequence: AACAATAA (-): 1403)  
**FOXO3\_mmu** (Sequence: TGTTTACA (-): 275)  
**FOXO3\_mmu** (Sequence: TGTTTACA (-): 298)  
**FIGLA** (Sequence: TCCAGCTGGT (-): 33)  
**FOXP1** (Sequence: TGTTTAC (-): 275)  
**FOXP1** (Sequence: TGTTTAC (-): 298)  
**Rhox11** (Sequence: TGGTGTTTT (+): 90)  
**Rhox11** (Sequence: ATAACACCG (-): 1592)  
**Gata4** (Sequence: AGATAAC (-): 139)  
**Sox5** (Sequence: AACAAT (-): 1403)
