# Supplementary material for: First characterization of PIWI-interacting RNA clusters in a cichlid fish with a B chromosome
Source: BMC Biol. 2022 Sep 21;20:204. doi: 10.1186/s12915-022-01403-2 (PMC9490952; doi:10.1186/s12915-022-01403-2)
Supplement: Supplementary file 1 — Additional file 1. Zipped folder with fasta and interactive html piRNA cluster information for the A. latifasciata genome. The nomenclature is as follows: number-pirna-cluster_sex_B-presence (f, female; m, male; 0b, without B chromosome; 1b, with B chromosome). [file 12915_2022_1403_MOESM1_ESM.zip › 104_m1b.html]

piRNA cluster 104\_m1b 64


Predicted piRNA cluster no. 104\_m1b
  

Show proTRAC run info
Hide proTRAC run info

/\  
                \_\_\_\_\_\_\_\_\_\_\_\_\_\_\_\_\_\_\_\_\_\_\_/\\_\_\_ /  \\_\_\_\_\_\_\_  
               I                      /  \  /    \      I  
               I     pro             /    \/      \     I  
               I        TRAC        /               \   I  
               I   \_\_\_\_\_\_\_\_\_\_\_\_\_\_\_\_/\_\_\_\_\_\_\_\_\_\_\_\_\_\_\_\_\_\\_ I  
               I   \              /                     I  
               I    \            /                      I  
               I     \  /\      /       V.2.4.2         I  
               I      \/  \    /                        I  
               I\_\_\_\_\_\_\_\_\_\_\_\  /\_\_\_\_\_\_\_\_\_\_\_\_\_\_\_\_\_\_\_\_\_\_\_\_\_I  
                            \/  
  
  
================================= proTRAC ====================================  
VERSION: .......... 2.4.2  
LAST MODIFIED: .... 11. May 2018  
  
Please cite:  
Rosenkranz D, Zischler H. proTRAC - a software for probabilistic piRNA cluster  
detection, visualization and analysis. 2012. BMC Bioinformatics 13:5.  
  
  
Contact:  
David Rosenkranz  
Institute of Organismic and Molecular Evolutionary Biology  
Dept. Anthropology, small RNA group  
Johannes Gutenberg University Mainz  
email: rosenkranz@uni-mainz.de  
  
You can find the latest proTRAC version at:  
http://sourceforge.net/projects/protrac/files  
http://www.smallRNAgroup-mainz.de/software  
==============================================================================  
  
PARAMETERS:  
Map file: ...............piwi-machos-1B.fa-collapse.map  
Genome file: ............../../../0B\_ala\_genome.fa  
RepeatMasker annotation: Alatifasciata-all0B-maryan-v2.fa\_corrected.out  
GeneSet:................./guest-storage/Data/annotation/Alatifasciata\_all0B\_maryan-v2\_out2017.gff  
  
Significant (p<=0.01) hit density will be calculated based  
on observed hit distribution.  
  
Sliding window size: ........................................ 5000 bp  
Sliding window increament: .................................. 1000 bp  
Normalize each hit by number of genomic hits: ............... yes  
Normalize each hit by number of sequence reads: ............. yes  
Normalize values (-> per million mapped reads): ............. yes  
Min. fraction of hits with 1T(U) or 10A: .................... 0.75  
Alternatively: Min. fraction of hits with 1T(U) and 10A: .... 0.5  
Min. fraction of hits with typical piRNA length: ............ 0.75  
Typical piRNA length: ....................................... 24-32 nt  
Min. size of a piRNA cluster: ............................... 1000 bp.  
Min. number of hits (absolute): ............................. 0  
Min. number of hits (normalized): ........................... 0  
Min. fraction of hits on the mainstrand: .................... 0.75  
Top fraction of mapped sequences (in terms of read counts): . 1%  
Top fraction accounts for max. n% of sequence reads: ........ 90%  
Min. fraction of hits on each arm of a bidirectional cluster: 0.05  
Output html file for each cluster: .......................... yes  
Output a summary table: ..................................... yes  
Output a FASTA file for each cluster (piRNA sequences): ..... yes  
Output a FASTA file comprising cluster sequences: ........... yes  
Output a GTF file for predicted piRNA clusters: ..............yes  
Search DNA motifs in clusters: .............................. yes  
Output flanking sequences: +/- .............................. 0 bp  
Output ~.pTi file: .......................................... no  
==============================================================================  
  
  
Genome size (without gaps): ............ 758543724 bp  
Gaps (N/X/-): .......................... 417479 bp  
Mapped reads: .......................... 26973943  
Non-identical sequences: ............... 6209225  
Genomic hits: .......................... 48438990  
Significant densitiy of mapped reads: .. 821.144211136946 reads/kb

Show proTRAC cluster info
Hide proTRAC cluster info

|  |  |
| --- | --- |
| Location | NODE\_271279\_length\_1703\_cov\_19.421610 |
| Coordinates | 1-1767 |
| Size [bp] | 1767 |
| Sequence hit loci | 1491 |
| Mapped reads (normalized) | 5192.4 |
| Mapped reads (normalized) per kb | 2938.5 |
| Normalized reads with 1T (1U) | 85.8% |
| Normalized reads with 10A | 18% |
| Normalized reads with length 24-32 nt | 97.5% |
| Normalized reads on the main strand(s) | 96.8% |
| Predicted directionality | mono:minus |

100%

0%

1T (1U)  
reads

10A reads

24-32 nt  
reads

reads on mainstrand

**Either the amount of reads with 1T (1U) OR 10A has to exceed 75% (set with option: -1Tor10A)  
Alternatively the amount of reads with 1T (1U) AND 10A has to exceed 50% (set with option: -1Tand10A)  
Minimum amount of reads with preferred size is 75% (set with option: -pisize)  
Minimum amount of reads on the main strand(s) is 75% (set with option: -clstrand)**

Show read coverage
Hide read coverage

WHAT DO I SEE HERE?  
This chart shows the location of mapped sequence reads within a predicted piRNA cluster. The color refers to the number of genomic hits produced by the sequence read in question. A dark red bar indicates that this sequence read produces many other hits elsewhere in the genome. Many adjacent red or yellow bars can indicate the presence of a multi-copy element such as transposons or rRNA genes. A dark green bar indicates that this sequence read maps uniquely to this locus.

1 hit

2-5 hits

6-10 hits

11-20 hits

21-50 hits

51-100 hits

> 100 hits

NODE\_271279\_length\_1703\_cov\_19.421610

1

1767

Gene Set

RepeatMasker

Mapped  
Reads

26.82

plus strand

minus strand

26.82

Region: NODE\_271279\_length\_1703\_cov\_19.421610 12863-2. Max. coverage (+): 0. Max coverage (-): 0

Region: NODE\_271279\_length\_1703\_cov\_19.421610 3-6. Max. coverage (+): 0. Max coverage (-): 0

Region: NODE\_271279\_length\_1703\_cov\_19.421610 7-9. Max. coverage (+): 0. Max coverage (-): 0

Region: NODE\_271279\_length\_1703\_cov\_19.421610 10-13. Max. coverage (+): 0. Max coverage (-): 0

Region: NODE\_271279\_length\_1703\_cov\_19.421610 14-16. Max. coverage (+): 0. Max coverage (-): 0

Region: NODE\_271279\_length\_1703\_cov\_19.421610 17-20. Max. coverage (+): 0. Max coverage (-): 0

Region: NODE\_271279\_length\_1703\_cov\_19.421610 21-23. Max. coverage (+): 0. Max coverage (-): 0

Region: NODE\_271279\_length\_1703\_cov\_19.421610 24-27. Max. coverage (+): 0. Max coverage (-): 0

Region: NODE\_271279\_length\_1703\_cov\_19.421610 28-31. Max. coverage (+): 0. Max coverage (-): 0

Region: NODE\_271279\_length\_1703\_cov\_19.421610 32-34. Max. coverage (+): 0. Max coverage (-): 0

Region: NODE\_271279\_length\_1703\_cov\_19.421610 35-38. Max. coverage (+): 0. Max coverage (-): 0

Region: NODE\_271279\_length\_1703\_cov\_19.421610 39-41. Max. coverage (+): 0. Max coverage (-): 0

Region: NODE\_271279\_length\_1703\_cov\_19.421610 42-45. Max. coverage (+): 0. Max coverage (-): 0

Region: NODE\_271279\_length\_1703\_cov\_19.421610 46-48. Max. coverage (+): 0. Max coverage (-): 0

Region: NODE\_271279\_length\_1703\_cov\_19.421610 49-52. Max. coverage (+): 0. Max coverage (-): 0

Region: NODE\_271279\_length\_1703\_cov\_19.421610 53-55. Max. coverage (+): 0. Max coverage (-): 0

Region: NODE\_271279\_length\_1703\_cov\_19.421610 56-59. Max. coverage (+): 0. Max coverage (-): 0

Region: NODE\_271279\_length\_1703\_cov\_19.421610 60-62. Max. coverage (+): 0. Max coverage (-): 0

Region: NODE\_271279\_length\_1703\_cov\_19.421610 63-66. Max. coverage (+): 0. Max coverage (-): 0

Region: NODE\_271279\_length\_1703\_cov\_19.421610 67-69. Max. coverage (+): 0. Max coverage (-): 0

Region: NODE\_271279\_length\_1703\_cov\_19.421610 70-73. Max. coverage (+): 0. Max coverage (-): 0

Region: NODE\_271279\_length\_1703\_cov\_19.421610 74-76. Max. coverage (+): 0. Max coverage (-): 0

Region: NODE\_271279\_length\_1703\_cov\_19.421610 77-80. Max. coverage (+): 0. Max coverage (-): 0

Region: NODE\_271279\_length\_1703\_cov\_19.421610 81-84. Max. coverage (+): 0. Max coverage (-): 0

Region: NODE\_271279\_length\_1703\_cov\_19.421610 85-87. Max. coverage (+): 0. Max coverage (-): 0

Region: NODE\_271279\_length\_1703\_cov\_19.421610 88-91. Max. coverage (+): 0. Max coverage (-): 0

Region: NODE\_271279\_length\_1703\_cov\_19.421610 92-94. Max. coverage (+): 0. Max coverage (-): 0

Region: NODE\_271279\_length\_1703\_cov\_19.421610 95-98. Max. coverage (+): 0. Max coverage (-): 0

Region: NODE\_271279\_length\_1703\_cov\_19.421610 99-101. Max. coverage (+): 0. Max coverage (-): 0

Region: NODE\_271279\_length\_1703\_cov\_19.421610 102-105. Max. coverage (+): 0. Max coverage (-): 0

Region: NODE\_271279\_length\_1703\_cov\_19.421610 106-108. Max. coverage (+): 0. Max coverage (-): 0

Region: NODE\_271279\_length\_1703\_cov\_19.421610 109-112. Max. coverage (+): 0. Max coverage (-): 0

Region: NODE\_271279\_length\_1703\_cov\_19.421610 113-115. Max. coverage (+): 0. Max coverage (-): 0

Region: NODE\_271279\_length\_1703\_cov\_19.421610 116-119. Max. coverage (+): 0. Max coverage (-): 0

Region: NODE\_271279\_length\_1703\_cov\_19.421610 120-122. Max. coverage (+): 0. Max coverage (-): 0

Region: NODE\_271279\_length\_1703\_cov\_19.421610 123-126. Max. coverage (+): 0. Max coverage (-): 0

Region: NODE\_271279\_length\_1703\_cov\_19.421610 127-129. Max. coverage (+): 0. Max coverage (-): 0

Region: NODE\_271279\_length\_1703\_cov\_19.421610 130-133. Max. coverage (+): 0. Max coverage (-): 0

Region: NODE\_271279\_length\_1703\_cov\_19.421610 134-137. Max. coverage (+): 0. Max coverage (-): 0.01

Region: NODE\_271279\_length\_1703\_cov\_19.421610 138-140. Max. coverage (+): 0. Max coverage (-): 0.01

Region: NODE\_271279\_length\_1703\_cov\_19.421610 141-144. Max. coverage (+): 0. Max coverage (-): 0

Region: NODE\_271279\_length\_1703\_cov\_19.421610 145-147. Max. coverage (+): 0. Max coverage (-): 0

Region: NODE\_271279\_length\_1703\_cov\_19.421610 148-151. Max. coverage (+): 0. Max coverage (-): 0

Region: NODE\_271279\_length\_1703\_cov\_19.421610 152-154. Max. coverage (+): 0. Max coverage (-): 0

Region: NODE\_271279\_length\_1703\_cov\_19.421610 155-158. Max. coverage (+): 0. Max coverage (-): 0

Region: NODE\_271279\_length\_1703\_cov\_19.421610 159-161. Max. coverage (+): 0. Max coverage (-): 0

Region: NODE\_271279\_length\_1703\_cov\_19.421610 162-165. Max. coverage (+): 0. Max coverage (-): 0

Region: NODE\_271279\_length\_1703\_cov\_19.421610 166-168. Max. coverage (+): 0. Max coverage (-): 0

Region: NODE\_271279\_length\_1703\_cov\_19.421610 169-172. Max. coverage (+): 0. Max coverage (-): 0

Region: NODE\_271279\_length\_1703\_cov\_19.421610 173-175. Max. coverage (+): 0. Max coverage (-): 0

Region: NODE\_271279\_length\_1703\_cov\_19.421610 176-179. Max. coverage (+): 0. Max coverage (-): 0

Region: NODE\_271279\_length\_1703\_cov\_19.421610 180-183. Max. coverage (+): 0. Max coverage (-): 0

Region: NODE\_271279\_length\_1703\_cov\_19.421610 184-186. Max. coverage (+): 0. Max coverage (-): 0

Region: NODE\_271279\_length\_1703\_cov\_19.421610 187-190. Max. coverage (+): 0. Max coverage (-): 0

Region: NODE\_271279\_length\_1703\_cov\_19.421610 191-193. Max. coverage (+): 0. Max coverage (-): 0

Region: NODE\_271279\_length\_1703\_cov\_19.421610 194-197. Max. coverage (+): 0. Max coverage (-): 0

Region: NODE\_271279\_length\_1703\_cov\_19.421610 198-200. Max. coverage (+): 0. Max coverage (-): 0

Region: NODE\_271279\_length\_1703\_cov\_19.421610 201-204. Max. coverage (+): 0. Max coverage (-): 0

Region: NODE\_271279\_length\_1703\_cov\_19.421610 205-207. Max. coverage (+): 0. Max coverage (-): 0

Region: NODE\_271279\_length\_1703\_cov\_19.421610 208-211. Max. coverage (+): 0. Max coverage (-): 0

Region: NODE\_271279\_length\_1703\_cov\_19.421610 212-214. Max. coverage (+): 0. Max coverage (-): 0

Region: NODE\_271279\_length\_1703\_cov\_19.421610 215-218. Max. coverage (+): 0.01. Max coverage (-): 0.18

Region: NODE\_271279\_length\_1703\_cov\_19.421610 219-221. Max. coverage (+): 0.02. Max coverage (-): 1.35

Region: NODE\_271279\_length\_1703\_cov\_19.421610 222-225. Max. coverage (+): 0.01. Max coverage (-): 14.75

Region: NODE\_271279\_length\_1703\_cov\_19.421610 226-228. Max. coverage (+): 0.01. Max coverage (-): 14.49

Region: NODE\_271279\_length\_1703\_cov\_19.421610 229-232. Max. coverage (+): 0. Max coverage (-): 6.48

Region: NODE\_271279\_length\_1703\_cov\_19.421610 233-236. Max. coverage (+): 0. Max coverage (-): 0.38

Region: NODE\_271279\_length\_1703\_cov\_19.421610 237-239. Max. coverage (+): 0. Max coverage (-): 0.27

Region: NODE\_271279\_length\_1703\_cov\_19.421610 240-243. Max. coverage (+): 0.02. Max coverage (-): 0.11

Region: NODE\_271279\_length\_1703\_cov\_19.421610 244-246. Max. coverage (+): 0.16. Max coverage (-): 0.29

Region: NODE\_271279\_length\_1703\_cov\_19.421610 247-250. Max. coverage (+): 0.18. Max coverage (-): 0.31

Region: NODE\_271279\_length\_1703\_cov\_19.421610 251-253. Max. coverage (+): 0.01. Max coverage (-): 0.13

Region: NODE\_271279\_length\_1703\_cov\_19.421610 254-257. Max. coverage (+): 0.03. Max coverage (-): 0.4

Region: NODE\_271279\_length\_1703\_cov\_19.421610 258-260. Max. coverage (+): 0.02. Max coverage (-): 0.87

Region: NODE\_271279\_length\_1703\_cov\_19.421610 261-264. Max. coverage (+): 0.03. Max coverage (-): 1.32

Region: NODE\_271279\_length\_1703\_cov\_19.421610 265-267. Max. coverage (+): 0.01. Max coverage (-): 1.53

Region: NODE\_271279\_length\_1703\_cov\_19.421610 268-271. Max. coverage (+): 0.11. Max coverage (-): 13.69

Region: NODE\_271279\_length\_1703\_cov\_19.421610 272-274. Max. coverage (+): 0.08. Max coverage (-): 13.87

Region: NODE\_271279\_length\_1703\_cov\_19.421610 275-278. Max. coverage (+): 0.01. Max coverage (-): 0.04

Region: NODE\_271279\_length\_1703\_cov\_19.421610 279-281. Max. coverage (+): 0.01. Max coverage (-): 0.04

Region: NODE\_271279\_length\_1703\_cov\_19.421610 282-285. Max. coverage (+): 0.01. Max coverage (-): 0.07

Region: NODE\_271279\_length\_1703\_cov\_19.421610 286-289. Max. coverage (+): 0.12. Max coverage (-): 0.11

Region: NODE\_271279\_length\_1703\_cov\_19.421610 290-292. Max. coverage (+): 0.13. Max coverage (-): 0.07

Region: NODE\_271279\_length\_1703\_cov\_19.421610 293-296. Max. coverage (+): 0.01. Max coverage (-): 0.04

Region: NODE\_271279\_length\_1703\_cov\_19.421610 297-299. Max. coverage (+): 0. Max coverage (-): 0

Region: NODE\_271279\_length\_1703\_cov\_19.421610 300-303. Max. coverage (+): 0. Max coverage (-): 0

Region: NODE\_271279\_length\_1703\_cov\_19.421610 304-306. Max. coverage (+): 0. Max coverage (-): 0.01

Region: NODE\_271279\_length\_1703\_cov\_19.421610 307-310. Max. coverage (+): 0. Max coverage (-): 1.56

Region: NODE\_271279\_length\_1703\_cov\_19.421610 311-313. Max. coverage (+): 0. Max coverage (-): 1.58

Region: NODE\_271279\_length\_1703\_cov\_19.421610 314-317. Max. coverage (+): 0. Max coverage (-): 0.47

Region: NODE\_271279\_length\_1703\_cov\_19.421610 318-320. Max. coverage (+): 0. Max coverage (-): 1.14

Region: NODE\_271279\_length\_1703\_cov\_19.421610 321-324. Max. coverage (+): 0.01. Max coverage (-): 1.89

Region: NODE\_271279\_length\_1703\_cov\_19.421610 325-327. Max. coverage (+): 0.1. Max coverage (-): 2.28

Region: NODE\_271279\_length\_1703\_cov\_19.421610 328-331. Max. coverage (+): 0.72. Max coverage (-): 2.95

Region: NODE\_271279\_length\_1703\_cov\_19.421610 332-334. Max. coverage (+): 0.74. Max coverage (-): 0.52

Region: NODE\_271279\_length\_1703\_cov\_19.421610 335-338. Max. coverage (+): 0.04. Max coverage (-): 2.97

Region: NODE\_271279\_length\_1703\_cov\_19.421610 339-342. Max. coverage (+): 0.11. Max coverage (-): 0.19

Region: NODE\_271279\_length\_1703\_cov\_19.421610 343-345. Max. coverage (+): 0.2. Max coverage (-): 0.43

Region: NODE\_271279\_length\_1703\_cov\_19.421610 346-349. Max. coverage (+): 0.17. Max coverage (-): 0.99

Region: NODE\_271279\_length\_1703\_cov\_19.421610 350-352. Max. coverage (+): 0.03. Max coverage (-): 0.17

Region: NODE\_271279\_length\_1703\_cov\_19.421610 353-356. Max. coverage (+): 0.03. Max coverage (-): 0.15

Region: NODE\_271279\_length\_1703\_cov\_19.421610 357-359. Max. coverage (+): 0. Max coverage (-): 0

Region: NODE\_271279\_length\_1703\_cov\_19.421610 360-363. Max. coverage (+): 0. Max coverage (-): 0.09

Region: NODE\_271279\_length\_1703\_cov\_19.421610 364-366. Max. coverage (+): 0. Max coverage (-): 0.28

Region: NODE\_271279\_length\_1703\_cov\_19.421610 367-370. Max. coverage (+): 0. Max coverage (-): 0.12

Region: NODE\_271279\_length\_1703\_cov\_19.421610 371-373. Max. coverage (+): 0. Max coverage (-): 0.04

Region: NODE\_271279\_length\_1703\_cov\_19.421610 374-377. Max. coverage (+): 0. Max coverage (-): 0.23

Region: NODE\_271279\_length\_1703\_cov\_19.421610 378-380. Max. coverage (+): 0.02. Max coverage (-): 0.21

Region: NODE\_271279\_length\_1703\_cov\_19.421610 381-384. Max. coverage (+): 0.02. Max coverage (-): 0.08

Region: NODE\_271279\_length\_1703\_cov\_19.421610 385-387. Max. coverage (+): 0. Max coverage (-): 14.12

Region: NODE\_271279\_length\_1703\_cov\_19.421610 388-391. Max. coverage (+): 0.01. Max coverage (-): 14.01

Region: NODE\_271279\_length\_1703\_cov\_19.421610 392-395. Max. coverage (+): 0.04. Max coverage (-): 1.32

Region: NODE\_271279\_length\_1703\_cov\_19.421610 396-398. Max. coverage (+): 0.01. Max coverage (-): 2.52

Region: NODE\_271279\_length\_1703\_cov\_19.421610 399-402. Max. coverage (+): 0.01. Max coverage (-): 2.52

Region: NODE\_271279\_length\_1703\_cov\_19.421610 403-405. Max. coverage (+): 0.1. Max coverage (-): 0.22

Region: NODE\_271279\_length\_1703\_cov\_19.421610 406-409. Max. coverage (+): 0.15. Max coverage (-): 0.1

Region: NODE\_271279\_length\_1703\_cov\_19.421610 410-412. Max. coverage (+): 0. Max coverage (-): 0.02

Region: NODE\_271279\_length\_1703\_cov\_19.421610 413-416. Max. coverage (+): 0.02. Max coverage (-): 0.23

Region: NODE\_271279\_length\_1703\_cov\_19.421610 417-419. Max. coverage (+): 0.06. Max coverage (-): 0.26

Region: NODE\_271279\_length\_1703\_cov\_19.421610 420-423. Max. coverage (+): 0.01. Max coverage (-): 0.14

Region: NODE\_271279\_length\_1703\_cov\_19.421610 424-426. Max. coverage (+): 0. Max coverage (-): 0.07

Region: NODE\_271279\_length\_1703\_cov\_19.421610 427-430. Max. coverage (+): 0. Max coverage (-): 0.07

Region: NODE\_271279\_length\_1703\_cov\_19.421610 431-433. Max. coverage (+): 0. Max coverage (-): 0

Region: NODE\_271279\_length\_1703\_cov\_19.421610 434-437. Max. coverage (+): 0. Max coverage (-): 0.04

Region: NODE\_271279\_length\_1703\_cov\_19.421610 438-440. Max. coverage (+): 0. Max coverage (-): 0

Region: NODE\_271279\_length\_1703\_cov\_19.421610 441-444. Max. coverage (+): 0. Max coverage (-): 0

Region: NODE\_271279\_length\_1703\_cov\_19.421610 445-448. Max. coverage (+): 0. Max coverage (-): 0.19

Region: NODE\_271279\_length\_1703\_cov\_19.421610 449-451. Max. coverage (+): 0. Max coverage (-): 0.21

Region: NODE\_271279\_length\_1703\_cov\_19.421610 452-455. Max. coverage (+): 0.04. Max coverage (-): 0.2

Region: NODE\_271279\_length\_1703\_cov\_19.421610 456-458. Max. coverage (+): 0. Max coverage (-): 0.2

Region: NODE\_271279\_length\_1703\_cov\_19.421610 459-462. Max. coverage (+): 0. Max coverage (-): 0.2

Region: NODE\_271279\_length\_1703\_cov\_19.421610 463-465. Max. coverage (+): 0. Max coverage (-): 0.04

Region: NODE\_271279\_length\_1703\_cov\_19.421610 466-469. Max. coverage (+): 0. Max coverage (-): 0.02

Region: NODE\_271279\_length\_1703\_cov\_19.421610 470-472. Max. coverage (+): 0.01. Max coverage (-): 0

Region: NODE\_271279\_length\_1703\_cov\_19.421610 473-476. Max. coverage (+): 0.01. Max coverage (-): 0.01

Region: NODE\_271279\_length\_1703\_cov\_19.421610 477-479. Max. coverage (+): 0. Max coverage (-): 0.04

Region: NODE\_271279\_length\_1703\_cov\_19.421610 480-483. Max. coverage (+): 0. Max coverage (-): 0.02

Region: NODE\_271279\_length\_1703\_cov\_19.421610 484-486. Max. coverage (+): 0. Max coverage (-): 0

Region: NODE\_271279\_length\_1703\_cov\_19.421610 487-490. Max. coverage (+): 0. Max coverage (-): 1.58

Region: NODE\_271279\_length\_1703\_cov\_19.421610 491-493. Max. coverage (+): 0. Max coverage (-): 0.12

Region: NODE\_271279\_length\_1703\_cov\_19.421610 494-497. Max. coverage (+): 0.23. Max coverage (-): 0.35

Region: NODE\_271279\_length\_1703\_cov\_19.421610 498-501. Max. coverage (+): 0.36. Max coverage (-): 0.93

Region: NODE\_271279\_length\_1703\_cov\_19.421610 502-504. Max. coverage (+): 0.14. Max coverage (-): 0.91

Region: NODE\_271279\_length\_1703\_cov\_19.421610 505-508. Max. coverage (+): 0.08. Max coverage (-): 0.07

Region: NODE\_271279\_length\_1703\_cov\_19.421610 509-511. Max. coverage (+): 0.04. Max coverage (-): 0.09

Region: NODE\_271279\_length\_1703\_cov\_19.421610 512-515. Max. coverage (+): 0. Max coverage (-): 0.67

Region: NODE\_271279\_length\_1703\_cov\_19.421610 516-518. Max. coverage (+): 0. Max coverage (-): 0.5

Region: NODE\_271279\_length\_1703\_cov\_19.421610 519-522. Max. coverage (+): 0. Max coverage (-): 0.04

Region: NODE\_271279\_length\_1703\_cov\_19.421610 523-525. Max. coverage (+): 0.01. Max coverage (-): 0.54

Region: NODE\_271279\_length\_1703\_cov\_19.421610 526-529. Max. coverage (+): 0.02. Max coverage (-): 14.89

Region: NODE\_271279\_length\_1703\_cov\_19.421610 530-532. Max. coverage (+): 0.05. Max coverage (-): 15.03

Region: NODE\_271279\_length\_1703\_cov\_19.421610 533-536. Max. coverage (+): 0.05. Max coverage (-): 2.63

Region: NODE\_271279\_length\_1703\_cov\_19.421610 537-539. Max. coverage (+): 0. Max coverage (-): 0.04

Region: NODE\_271279\_length\_1703\_cov\_19.421610 540-543. Max. coverage (+): 0. Max coverage (-): 0.12

Region: NODE\_271279\_length\_1703\_cov\_19.421610 544-547. Max. coverage (+): 0.01. Max coverage (-): 0.16

Region: NODE\_271279\_length\_1703\_cov\_19.421610 548-550. Max. coverage (+): 0.01. Max coverage (-): 0.04

Region: NODE\_271279\_length\_1703\_cov\_19.421610 551-554. Max. coverage (+): 0.01. Max coverage (-): 0

Region: NODE\_271279\_length\_1703\_cov\_19.421610 555-557. Max. coverage (+): 0. Max coverage (-): 0.04

Region: NODE\_271279\_length\_1703\_cov\_19.421610 558-561. Max. coverage (+): 0. Max coverage (-): 0.04

Region: NODE\_271279\_length\_1703\_cov\_19.421610 562-564. Max. coverage (+): 0. Max coverage (-): 0.03

Region: NODE\_271279\_length\_1703\_cov\_19.421610 565-568. Max. coverage (+): 0. Max coverage (-): 0.06

Region: NODE\_271279\_length\_1703\_cov\_19.421610 569-571. Max. coverage (+): 0. Max coverage (-): 0.85

Region: NODE\_271279\_length\_1703\_cov\_19.421610 572-575. Max. coverage (+): 0. Max coverage (-): 18.48

Region: NODE\_271279\_length\_1703\_cov\_19.421610 576-578. Max. coverage (+): 0. Max coverage (-): 25.82

Region: NODE\_271279\_length\_1703\_cov\_19.421610 579-582. Max. coverage (+): 0. Max coverage (-): 0.5

Region: NODE\_271279\_length\_1703\_cov\_19.421610 583-585. Max. coverage (+): 0. Max coverage (-): 0.13

Region: NODE\_271279\_length\_1703\_cov\_19.421610 586-589. Max. coverage (+): 0. Max coverage (-): 0.35

Region: NODE\_271279\_length\_1703\_cov\_19.421610 590-592. Max. coverage (+): 0.33. Max coverage (-): 0.35

Region: NODE\_271279\_length\_1703\_cov\_19.421610 593-596. Max. coverage (+): 0.35. Max coverage (-): 0.41

Region: NODE\_271279\_length\_1703\_cov\_19.421610 597-600. Max. coverage (+): 0.02. Max coverage (-): 2.17

Region: NODE\_271279\_length\_1703\_cov\_19.421610 601-603. Max. coverage (+): 0. Max coverage (-): 26.82

Region: NODE\_271279\_length\_1703\_cov\_19.421610 604-607. Max. coverage (+): 0.02. Max coverage (-): 26.67

Region: NODE\_271279\_length\_1703\_cov\_19.421610 608-610. Max. coverage (+): 0.02. Max coverage (-): 4.45

Region: NODE\_271279\_length\_1703\_cov\_19.421610 611-614. Max. coverage (+): 0. Max coverage (-): 0.82

Region: NODE\_271279\_length\_1703\_cov\_19.421610 615-617. Max. coverage (+): 0. Max coverage (-): 6.06

Region: NODE\_271279\_length\_1703\_cov\_19.421610 618-621. Max. coverage (+): 0. Max coverage (-): 8.58

Region: NODE\_271279\_length\_1703\_cov\_19.421610 622-624. Max. coverage (+): 0. Max coverage (-): 0.09

Region: NODE\_271279\_length\_1703\_cov\_19.421610 625-628. Max. coverage (+): 0.37. Max coverage (-): 1.61

Region: NODE\_271279\_length\_1703\_cov\_19.421610 629-631. Max. coverage (+): 0.04. Max coverage (-): 1.61

Region: NODE\_271279\_length\_1703\_cov\_19.421610 632-635. Max. coverage (+): 0. Max coverage (-): 0.07

Region: NODE\_271279\_length\_1703\_cov\_19.421610 636-638. Max. coverage (+): 0. Max coverage (-): 0.12

Region: NODE\_271279\_length\_1703\_cov\_19.421610 639-642. Max. coverage (+): 0.07. Max coverage (-): 0.12

Region: NODE\_271279\_length\_1703\_cov\_19.421610 643-645. Max. coverage (+): 0.01. Max coverage (-): 0.11

Region: NODE\_271279\_length\_1703\_cov\_19.421610 646-649. Max. coverage (+): 0.1. Max coverage (-): 0.11

Region: NODE\_271279\_length\_1703\_cov\_19.421610 650-653. Max. coverage (+): 0.1. Max coverage (-): 0.04

Region: NODE\_271279\_length\_1703\_cov\_19.421610 654-656. Max. coverage (+): 0. Max coverage (-): 0.02

Region: NODE\_271279\_length\_1703\_cov\_19.421610 657-660. Max. coverage (+): 0.01. Max coverage (-): 0.01

Region: NODE\_271279\_length\_1703\_cov\_19.421610 661-663. Max. coverage (+): 0. Max coverage (-): 0

Region: NODE\_271279\_length\_1703\_cov\_19.421610 664-667. Max. coverage (+): 0. Max coverage (-): 0

Region: NODE\_271279\_length\_1703\_cov\_19.421610 668-670. Max. coverage (+): 0. Max coverage (-): 0

Region: NODE\_271279\_length\_1703\_cov\_19.421610 671-674. Max. coverage (+): 0. Max coverage (-): 0

Region: NODE\_271279\_length\_1703\_cov\_19.421610 675-677. Max. coverage (+): 0. Max coverage (-): 0

Region: NODE\_271279\_length\_1703\_cov\_19.421610 678-681. Max. coverage (+): 0. Max coverage (-): 0

Region: NODE\_271279\_length\_1703\_cov\_19.421610 682-684. Max. coverage (+): 0. Max coverage (-): 0

Region: NODE\_271279\_length\_1703\_cov\_19.421610 685-688. Max. coverage (+): 0. Max coverage (-): 0

Region: NODE\_271279\_length\_1703\_cov\_19.421610 689-691. Max. coverage (+): 0. Max coverage (-): 0

Region: NODE\_271279\_length\_1703\_cov\_19.421610 692-695. Max. coverage (+): 0. Max coverage (-): 0

Region: NODE\_271279\_length\_1703\_cov\_19.421610 696-698. Max. coverage (+): 0. Max coverage (-): 0

Region: NODE\_271279\_length\_1703\_cov\_19.421610 699-702. Max. coverage (+): 0. Max coverage (-): 0.02

Region: NODE\_271279\_length\_1703\_cov\_19.421610 703-706. Max. coverage (+): 0. Max coverage (-): 0.01

Region: NODE\_271279\_length\_1703\_cov\_19.421610 707-709. Max. coverage (+): 0. Max coverage (-): 0.02

Region: NODE\_271279\_length\_1703\_cov\_19.421610 710-713. Max. coverage (+): 0. Max coverage (-): 0

Region: NODE\_271279\_length\_1703\_cov\_19.421610 714-716. Max. coverage (+): 0. Max coverage (-): 0

Region: NODE\_271279\_length\_1703\_cov\_19.421610 717-720. Max. coverage (+): 0. Max coverage (-): 0.04

Region: NODE\_271279\_length\_1703\_cov\_19.421610 721-723. Max. coverage (+): 0. Max coverage (-): 0.04

Region: NODE\_271279\_length\_1703\_cov\_19.421610 724-727. Max. coverage (+): 0. Max coverage (-): 0

Region: NODE\_271279\_length\_1703\_cov\_19.421610 728-730. Max. coverage (+): 0. Max coverage (-): 0.04

Region: NODE\_271279\_length\_1703\_cov\_19.421610 731-734. Max. coverage (+): 0. Max coverage (-): 0

Region: NODE\_271279\_length\_1703\_cov\_19.421610 735-737. Max. coverage (+): 0. Max coverage (-): 0

Region: NODE\_271279\_length\_1703\_cov\_19.421610 738-741. Max. coverage (+): 0. Max coverage (-): 0.01

Region: NODE\_271279\_length\_1703\_cov\_19.421610 742-744. Max. coverage (+): 0.12. Max coverage (-): 0.06

Region: NODE\_271279\_length\_1703\_cov\_19.421610 745-748. Max. coverage (+): 0. Max coverage (-): 0.25

Region: NODE\_271279\_length\_1703\_cov\_19.421610 749-751. Max. coverage (+): 0.04. Max coverage (-): 0.06

Region: NODE\_271279\_length\_1703\_cov\_19.421610 752-755. Max. coverage (+): 0.02. Max coverage (-): 0.3

Region: NODE\_271279\_length\_1703\_cov\_19.421610 756-759. Max. coverage (+): 0. Max coverage (-): 0.09

Region: NODE\_271279\_length\_1703\_cov\_19.421610 760-762. Max. coverage (+): 0. Max coverage (-): 0

Region: NODE\_271279\_length\_1703\_cov\_19.421610 763-766. Max. coverage (+): 0. Max coverage (-): 0

Region: NODE\_271279\_length\_1703\_cov\_19.421610 767-769. Max. coverage (+): 0. Max coverage (-): 0

Region: NODE\_271279\_length\_1703\_cov\_19.421610 770-773. Max. coverage (+): 0. Max coverage (-): 0

Region: NODE\_271279\_length\_1703\_cov\_19.421610 774-776. Max. coverage (+): 0. Max coverage (-): 0

Region: NODE\_271279\_length\_1703\_cov\_19.421610 777-780. Max. coverage (+): 0. Max coverage (-): 0

Region: NODE\_271279\_length\_1703\_cov\_19.421610 781-783. Max. coverage (+): 0. Max coverage (-): 0

Region: NODE\_271279\_length\_1703\_cov\_19.421610 784-787. Max. coverage (+): 0. Max coverage (-): 0

Region: NODE\_271279\_length\_1703\_cov\_19.421610 788-790. Max. coverage (+): 0. Max coverage (-): 0

Region: NODE\_271279\_length\_1703\_cov\_19.421610 791-794. Max. coverage (+): 0. Max coverage (-): 0

Region: NODE\_271279\_length\_1703\_cov\_19.421610 795-797. Max. coverage (+): 0. Max coverage (-): 0

Region: NODE\_271279\_length\_1703\_cov\_19.421610 798-801. Max. coverage (+): 0. Max coverage (-): 0

Region: NODE\_271279\_length\_1703\_cov\_19.421610 802-804. Max. coverage (+): 0. Max coverage (-): 0

Region: NODE\_271279\_length\_1703\_cov\_19.421610 805-808. Max. coverage (+): 0. Max coverage (-): 0

Region: NODE\_271279\_length\_1703\_cov\_19.421610 809-812. Max. coverage (+): 0. Max coverage (-): 0

Region: NODE\_271279\_length\_1703\_cov\_19.421610 813-815. Max. coverage (+): 0. Max coverage (-): 0

Region: NODE\_271279\_length\_1703\_cov\_19.421610 816-819. Max. coverage (+): 0. Max coverage (-): 0

Region: NODE\_271279\_length\_1703\_cov\_19.421610 820-822. Max. coverage (+): 0. Max coverage (-): 0

Region: NODE\_271279\_length\_1703\_cov\_19.421610 823-826. Max. coverage (+): 0. Max coverage (-): 0

Region: NODE\_271279\_length\_1703\_cov\_19.421610 827-829. Max. coverage (+): 0. Max coverage (-): 0

Region: NODE\_271279\_length\_1703\_cov\_19.421610 830-833. Max. coverage (+): 0. Max coverage (-): 0

Region: NODE\_271279\_length\_1703\_cov\_19.421610 834-836. Max. coverage (+): 0. Max coverage (-): 0

Region: NODE\_271279\_length\_1703\_cov\_19.421610 837-840. Max. coverage (+): 0. Max coverage (-): 0

Region: NODE\_271279\_length\_1703\_cov\_19.421610 841-843. Max. coverage (+): 0. Max coverage (-): 0

Region: NODE\_271279\_length\_1703\_cov\_19.421610 844-847. Max. coverage (+): 0. Max coverage (-): 0

Region: NODE\_271279\_length\_1703\_cov\_19.421610 848-850. Max. coverage (+): 0. Max coverage (-): 0

Region: NODE\_271279\_length\_1703\_cov\_19.421610 851-854. Max. coverage (+): 0. Max coverage (-): 0

Region: NODE\_271279\_length\_1703\_cov\_19.421610 855-857. Max. coverage (+): 0. Max coverage (-): 0

Region: NODE\_271279\_length\_1703\_cov\_19.421610 858-861. Max. coverage (+): 0. Max coverage (-): 0

Region: NODE\_271279\_length\_1703\_cov\_19.421610 862-865. Max. coverage (+): 0. Max coverage (-): 0

Region: NODE\_271279\_length\_1703\_cov\_19.421610 866-868. Max. coverage (+): 0. Max coverage (-): 0

Region: NODE\_271279\_length\_1703\_cov\_19.421610 869-872. Max. coverage (+): 0. Max coverage (-): 0

Region: NODE\_271279\_length\_1703\_cov\_19.421610 873-875. Max. coverage (+): 0. Max coverage (-): 0

Region: NODE\_271279\_length\_1703\_cov\_19.421610 876-879. Max. coverage (+): 0. Max coverage (-): 0

Region: NODE\_271279\_length\_1703\_cov\_19.421610 880-882. Max. coverage (+): 0. Max coverage (-): 0

Region: NODE\_271279\_length\_1703\_cov\_19.421610 883-886. Max. coverage (+): 0. Max coverage (-): 0

Region: NODE\_271279\_length\_1703\_cov\_19.421610 887-889. Max. coverage (+): 0. Max coverage (-): 0

Region: NODE\_271279\_length\_1703\_cov\_19.421610 890-893. Max. coverage (+): 0. Max coverage (-): 0

Region: NODE\_271279\_length\_1703\_cov\_19.421610 894-896. Max. coverage (+): 0. Max coverage (-): 0

Region: NODE\_271279\_length\_1703\_cov\_19.421610 897-900. Max. coverage (+): 0. Max coverage (-): 0

Region: NODE\_271279\_length\_1703\_cov\_19.421610 901-903. Max. coverage (+): 0. Max coverage (-): 0

Region: NODE\_271279\_length\_1703\_cov\_19.421610 904-907. Max. coverage (+): 0. Max coverage (-): 0

Region: NODE\_271279\_length\_1703\_cov\_19.421610 908-911. Max. coverage (+): 0. Max coverage (-): 0

Region: NODE\_271279\_length\_1703\_cov\_19.421610 912-914. Max. coverage (+): 0. Max coverage (-): 0.04

Region: NODE\_271279\_length\_1703\_cov\_19.421610 915-918. Max. coverage (+): 0. Max coverage (-): 0.04

Region: NODE\_271279\_length\_1703\_cov\_19.421610 919-921. Max. coverage (+): 0. Max coverage (-): 0

Region: NODE\_271279\_length\_1703\_cov\_19.421610 922-925. Max. coverage (+): 0. Max coverage (-): 0

Region: NODE\_271279\_length\_1703\_cov\_19.421610 926-928. Max. coverage (+): 0. Max coverage (-): 0

Region: NODE\_271279\_length\_1703\_cov\_19.421610 929-932. Max. coverage (+): 0. Max coverage (-): 0

Region: NODE\_271279\_length\_1703\_cov\_19.421610 933-935. Max. coverage (+): 0. Max coverage (-): 0

Region: NODE\_271279\_length\_1703\_cov\_19.421610 936-939. Max. coverage (+): 0. Max coverage (-): 0

Region: NODE\_271279\_length\_1703\_cov\_19.421610 940-942. Max. coverage (+): 0. Max coverage (-): 0

Region: NODE\_271279\_length\_1703\_cov\_19.421610 943-946. Max. coverage (+): 0. Max coverage (-): 0

Region: NODE\_271279\_length\_1703\_cov\_19.421610 947-949. Max. coverage (+): 0. Max coverage (-): 0

Region: NODE\_271279\_length\_1703\_cov\_19.421610 950-953. Max. coverage (+): 0. Max coverage (-): 0

Region: NODE\_271279\_length\_1703\_cov\_19.421610 954-956. Max. coverage (+): 0. Max coverage (-): 0

Region: NODE\_271279\_length\_1703\_cov\_19.421610 957-960. Max. coverage (+): 0. Max coverage (-): 0

Region: NODE\_271279\_length\_1703\_cov\_19.421610 961-964. Max. coverage (+): 0. Max coverage (-): 0

Region: NODE\_271279\_length\_1703\_cov\_19.421610 965-967. Max. coverage (+): 0. Max coverage (-): 0

Region: NODE\_271279\_length\_1703\_cov\_19.421610 968-971. Max. coverage (+): 0. Max coverage (-): 0

Region: NODE\_271279\_length\_1703\_cov\_19.421610 972-974. Max. coverage (+): 0. Max coverage (-): 0

Region: NODE\_271279\_length\_1703\_cov\_19.421610 975-978. Max. coverage (+): 0. Max coverage (-): 0

Region: NODE\_271279\_length\_1703\_cov\_19.421610 979-981. Max. coverage (+): 0. Max coverage (-): 0

Region: NODE\_271279\_length\_1703\_cov\_19.421610 982-985. Max. coverage (+): 0. Max coverage (-): 0

Region: NODE\_271279\_length\_1703\_cov\_19.421610 986-988. Max. coverage (+): 0. Max coverage (-): 0

Region: NODE\_271279\_length\_1703\_cov\_19.421610 989-992. Max. coverage (+): 0. Max coverage (-): 0

Region: NODE\_271279\_length\_1703\_cov\_19.421610 993-995. Max. coverage (+): 0. Max coverage (-): 0

Region: NODE\_271279\_length\_1703\_cov\_19.421610 996-999. Max. coverage (+): 0. Max coverage (-): 0

Region: NODE\_271279\_length\_1703\_cov\_19.421610 1000-1002. Max. coverage (+): 0. Max coverage (-): 0

Region: NODE\_271279\_length\_1703\_cov\_19.421610 1003-1006. Max. coverage (+): 0. Max coverage (-): 0

Region: NODE\_271279\_length\_1703\_cov\_19.421610 1007-1009. Max. coverage (+): 0. Max coverage (-): 0

Region: NODE\_271279\_length\_1703\_cov\_19.421610 1010-1013. Max. coverage (+): 0. Max coverage (-): 0

Region: NODE\_271279\_length\_1703\_cov\_19.421610 1014-1017. Max. coverage (+): 0. Max coverage (-): 0

Region: NODE\_271279\_length\_1703\_cov\_19.421610 1018-1020. Max. coverage (+): 0. Max coverage (-): 0

Region: NODE\_271279\_length\_1703\_cov\_19.421610 1021-1024. Max. coverage (+): 0. Max coverage (-): 0

Region: NODE\_271279\_length\_1703\_cov\_19.421610 1025-1027. Max. coverage (+): 0. Max coverage (-): 0

Region: NODE\_271279\_length\_1703\_cov\_19.421610 1028-1031. Max. coverage (+): 0. Max coverage (-): 0

Region: NODE\_271279\_length\_1703\_cov\_19.421610 1032-1034. Max. coverage (+): 0. Max coverage (-): 0

Region: NODE\_271279\_length\_1703\_cov\_19.421610 1035-1038. Max. coverage (+): 0. Max coverage (-): 0

Region: NODE\_271279\_length\_1703\_cov\_19.421610 1039-1041. Max. coverage (+): 0. Max coverage (-): 0

Region: NODE\_271279\_length\_1703\_cov\_19.421610 1042-1045. Max. coverage (+): 0. Max coverage (-): 0

Region: NODE\_271279\_length\_1703\_cov\_19.421610 1046-1048. Max. coverage (+): 0. Max coverage (-): 0

Region: NODE\_271279\_length\_1703\_cov\_19.421610 1049-1052. Max. coverage (+): 0. Max coverage (-): 0

Region: NODE\_271279\_length\_1703\_cov\_19.421610 1053-1055. Max. coverage (+): 0. Max coverage (-): 0

Region: NODE\_271279\_length\_1703\_cov\_19.421610 1056-1059. Max. coverage (+): 0. Max coverage (-): 0

Region: NODE\_271279\_length\_1703\_cov\_19.421610 1060-1062. Max. coverage (+): 0. Max coverage (-): 0

Region: NODE\_271279\_length\_1703\_cov\_19.421610 1063-1066. Max. coverage (+): 0. Max coverage (-): 0

Region: NODE\_271279\_length\_1703\_cov\_19.421610 1067-1070. Max. coverage (+): 0. Max coverage (-): 0

Region: NODE\_271279\_length\_1703\_cov\_19.421610 1071-1073. Max. coverage (+): 0. Max coverage (-): 0

Region: NODE\_271279\_length\_1703\_cov\_19.421610 1074-1077. Max. coverage (+): 0.02. Max coverage (-): 0

Region: NODE\_271279\_length\_1703\_cov\_19.421610 1078-1080. Max. coverage (+): 0.02. Max coverage (-): 0

Region: NODE\_271279\_length\_1703\_cov\_19.421610 1081-1084. Max. coverage (+): 0. Max coverage (-): 0

Region: NODE\_271279\_length\_1703\_cov\_19.421610 1085-1087. Max. coverage (+): 0. Max coverage (-): 0.02

Region: NODE\_271279\_length\_1703\_cov\_19.421610 1088-1091. Max. coverage (+): 0.02. Max coverage (-): 0.02

Region: NODE\_271279\_length\_1703\_cov\_19.421610 1092-1094. Max. coverage (+): 0.02. Max coverage (-): 0

Region: NODE\_271279\_length\_1703\_cov\_19.421610 1095-1098. Max. coverage (+): 0. Max coverage (-): 0

Region: NODE\_271279\_length\_1703\_cov\_19.421610 1099-1101. Max. coverage (+): 0. Max coverage (-): 0

Region: NODE\_271279\_length\_1703\_cov\_19.421610 1102-1105. Max. coverage (+): 0. Max coverage (-): 0

Region: NODE\_271279\_length\_1703\_cov\_19.421610 1106-1108. Max. coverage (+): 0. Max coverage (-): 0

Region: NODE\_271279\_length\_1703\_cov\_19.421610 1109-1112. Max. coverage (+): 0. Max coverage (-): 0

Region: NODE\_271279\_length\_1703\_cov\_19.421610 1113-1115. Max. coverage (+): 0. Max coverage (-): 0

Region: NODE\_271279\_length\_1703\_cov\_19.421610 1116-1119. Max. coverage (+): 0. Max coverage (-): 0

Region: NODE\_271279\_length\_1703\_cov\_19.421610 1120-1123. Max. coverage (+): 0. Max coverage (-): 0

Region: NODE\_271279\_length\_1703\_cov\_19.421610 1124-1126. Max. coverage (+): 0. Max coverage (-): 0

Region: NODE\_271279\_length\_1703\_cov\_19.421610 1127-1130. Max. coverage (+): 0. Max coverage (-): 0

Region: NODE\_271279\_length\_1703\_cov\_19.421610 1131-1133. Max. coverage (+): 0. Max coverage (-): 0

Region: NODE\_271279\_length\_1703\_cov\_19.421610 1134-1137. Max. coverage (+): 0. Max coverage (-): 0

Region: NODE\_271279\_length\_1703\_cov\_19.421610 1138-1140. Max. coverage (+): 0. Max coverage (-): 0

Region: NODE\_271279\_length\_1703\_cov\_19.421610 1141-1144. Max. coverage (+): 0. Max coverage (-): 0

Region: NODE\_271279\_length\_1703\_cov\_19.421610 1145-1147. Max. coverage (+): 0. Max coverage (-): 0

Region: NODE\_271279\_length\_1703\_cov\_19.421610 1148-1151. Max. coverage (+): 0. Max coverage (-): 0

Region: NODE\_271279\_length\_1703\_cov\_19.421610 1152-1154. Max. coverage (+): 0. Max coverage (-): 0

Region: NODE\_271279\_length\_1703\_cov\_19.421610 1155-1158. Max. coverage (+): 0.01. Max coverage (-): 0.02

Region: NODE\_271279\_length\_1703\_cov\_19.421610 1159-1161. Max. coverage (+): 0.01. Max coverage (-): 0.42

Region: NODE\_271279\_length\_1703\_cov\_19.421610 1162-1165. Max. coverage (+): 0. Max coverage (-): 0.41

Region: NODE\_271279\_length\_1703\_cov\_19.421610 1166-1168. Max. coverage (+): 0. Max coverage (-): 0

Region: NODE\_271279\_length\_1703\_cov\_19.421610 1169-1172. Max. coverage (+): 0.01. Max coverage (-): 0

Region: NODE\_271279\_length\_1703\_cov\_19.421610 1173-1176. Max. coverage (+): 0.01. Max coverage (-): 0

Region: NODE\_271279\_length\_1703\_cov\_19.421610 1177-1179. Max. coverage (+): 0.07. Max coverage (-): 0

Region: NODE\_271279\_length\_1703\_cov\_19.421610 1180-1183. Max. coverage (+): 0.07. Max coverage (-): 0

Region: NODE\_271279\_length\_1703\_cov\_19.421610 1184-1186. Max. coverage (+): 0. Max coverage (-): 0

Region: NODE\_271279\_length\_1703\_cov\_19.421610 1187-1190. Max. coverage (+): 0.01. Max coverage (-): 0

Region: NODE\_271279\_length\_1703\_cov\_19.421610 1191-1193. Max. coverage (+): 0. Max coverage (-): 0

Region: NODE\_271279\_length\_1703\_cov\_19.421610 1194-1197. Max. coverage (+): 0. Max coverage (-): 0

Region: NODE\_271279\_length\_1703\_cov\_19.421610 1198-1200. Max. coverage (+): 0. Max coverage (-): 0.02

Region: NODE\_271279\_length\_1703\_cov\_19.421610 1201-1204. Max. coverage (+): 0. Max coverage (-): 0.02

Region: NODE\_271279\_length\_1703\_cov\_19.421610 1205-1207. Max. coverage (+): 0. Max coverage (-): 0

Region: NODE\_271279\_length\_1703\_cov\_19.421610 1208-1211. Max. coverage (+): 0. Max coverage (-): 0.02

Region: NODE\_271279\_length\_1703\_cov\_19.421610 1212-1214. Max. coverage (+): 0. Max coverage (-): 0

Region: NODE\_271279\_length\_1703\_cov\_19.421610 1215-1218. Max. coverage (+): 0. Max coverage (-): 0

Region: NODE\_271279\_length\_1703\_cov\_19.421610 1219-1221. Max. coverage (+): 0. Max coverage (-): 0

Region: NODE\_271279\_length\_1703\_cov\_19.421610 1222-1225. Max. coverage (+): 0. Max coverage (-): 0

Region: NODE\_271279\_length\_1703\_cov\_19.421610 1226-1229. Max. coverage (+): 0. Max coverage (-): 0.04

Region: NODE\_271279\_length\_1703\_cov\_19.421610 1230-1232. Max. coverage (+): 0. Max coverage (-): 0.06

Region: NODE\_271279\_length\_1703\_cov\_19.421610 1233-1236. Max. coverage (+): 0. Max coverage (-): 0.04

Region: NODE\_271279\_length\_1703\_cov\_19.421610 1237-1239. Max. coverage (+): 0. Max coverage (-): 0.05

Region: NODE\_271279\_length\_1703\_cov\_19.421610 1240-1243. Max. coverage (+): 0. Max coverage (-): 0.05

Region: NODE\_271279\_length\_1703\_cov\_19.421610 1244-1246. Max. coverage (+): 0. Max coverage (-): 0

Region: NODE\_271279\_length\_1703\_cov\_19.421610 1247-1250. Max. coverage (+): 0. Max coverage (-): 0

Region: NODE\_271279\_length\_1703\_cov\_19.421610 1251-1253. Max. coverage (+): 0. Max coverage (-): 0

Region: NODE\_271279\_length\_1703\_cov\_19.421610 1254-1257. Max. coverage (+): 0. Max coverage (-): 0

Region: NODE\_271279\_length\_1703\_cov\_19.421610 1258-1260. Max. coverage (+): 0. Max coverage (-): 0

Region: NODE\_271279\_length\_1703\_cov\_19.421610 1261-1264. Max. coverage (+): 0. Max coverage (-): 0

Region: NODE\_271279\_length\_1703\_cov\_19.421610 1265-1267. Max. coverage (+): 0.05. Max coverage (-): 0

Region: NODE\_271279\_length\_1703\_cov\_19.421610 1268-1271. Max. coverage (+): 0.05. Max coverage (-): 0

Region: NODE\_271279\_length\_1703\_cov\_19.421610 1272-1275. Max. coverage (+): 0. Max coverage (-): 0

Region: NODE\_271279\_length\_1703\_cov\_19.421610 1276-1278. Max. coverage (+): 0. Max coverage (-): 0

Region: NODE\_271279\_length\_1703\_cov\_19.421610 1279-1282. Max. coverage (+): 0. Max coverage (-): 0

Region: NODE\_271279\_length\_1703\_cov\_19.421610 1283-1285. Max. coverage (+): 0. Max coverage (-): 0

Region: NODE\_271279\_length\_1703\_cov\_19.421610 1286-1289. Max. coverage (+): 0. Max coverage (-): 0

Region: NODE\_271279\_length\_1703\_cov\_19.421610 1290-1292. Max. coverage (+): 0. Max coverage (-): 0

Region: NODE\_271279\_length\_1703\_cov\_19.421610 1293-1296. Max. coverage (+): 0.01. Max coverage (-): 0.01

Region: NODE\_271279\_length\_1703\_cov\_19.421610 1297-1299. Max. coverage (+): 0.01. Max coverage (-): 0.1

Region: NODE\_271279\_length\_1703\_cov\_19.421610 1300-1303. Max. coverage (+): 0.01. Max coverage (-): 0.14

Region: NODE\_271279\_length\_1703\_cov\_19.421610 1304-1306. Max. coverage (+): 0.03. Max coverage (-): 0.01

Region: NODE\_271279\_length\_1703\_cov\_19.421610 1307-1310. Max. coverage (+): 0.03. Max coverage (-): 0

Region: NODE\_271279\_length\_1703\_cov\_19.421610 1311-1313. Max. coverage (+): 0.01. Max coverage (-): 0.02

Region: NODE\_271279\_length\_1703\_cov\_19.421610 1314-1317. Max. coverage (+): 0.08. Max coverage (-): 0.02

Region: NODE\_271279\_length\_1703\_cov\_19.421610 1318-1320. Max. coverage (+): 0.08. Max coverage (-): 0.03

Region: NODE\_271279\_length\_1703\_cov\_19.421610 1321-1324. Max. coverage (+): 0. Max coverage (-): 0.05

Region: NODE\_271279\_length\_1703\_cov\_19.421610 1325-1328. Max. coverage (+): 0. Max coverage (-): 0.49

Region: NODE\_271279\_length\_1703\_cov\_19.421610 1329-1331. Max. coverage (+): 0. Max coverage (-): 0.47

Region: NODE\_271279\_length\_1703\_cov\_19.421610 1332-1335. Max. coverage (+): 0. Max coverage (-): 0.01

Region: NODE\_271279\_length\_1703\_cov\_19.421610 1336-1338. Max. coverage (+): 0.01. Max coverage (-): 0.01

Region: NODE\_271279\_length\_1703\_cov\_19.421610 1339-1342. Max. coverage (+): 0.01. Max coverage (-): 0.01

Region: NODE\_271279\_length\_1703\_cov\_19.421610 1343-1345. Max. coverage (+): 0.01. Max coverage (-): 0

Region: NODE\_271279\_length\_1703\_cov\_19.421610 1346-1349. Max. coverage (+): 0.02. Max coverage (-): 0

Region: NODE\_271279\_length\_1703\_cov\_19.421610 1350-1352. Max. coverage (+): 0.01. Max coverage (-): 0

Region: NODE\_271279\_length\_1703\_cov\_19.421610 1353-1356. Max. coverage (+): 0. Max coverage (-): 0

Region: NODE\_271279\_length\_1703\_cov\_19.421610 1357-1359. Max. coverage (+): 0. Max coverage (-): 0.02

Region: NODE\_271279\_length\_1703\_cov\_19.421610 1360-1363. Max. coverage (+): 0.02. Max coverage (-): 0.04

Region: NODE\_271279\_length\_1703\_cov\_19.421610 1364-1366. Max. coverage (+): 0.02. Max coverage (-): 0.04

Region: NODE\_271279\_length\_1703\_cov\_19.421610 1367-1370. Max. coverage (+): 0. Max coverage (-): 0.01

Region: NODE\_271279\_length\_1703\_cov\_19.421610 1371-1373. Max. coverage (+): 0.04. Max coverage (-): 0.01

Region: NODE\_271279\_length\_1703\_cov\_19.421610 1374-1377. Max. coverage (+): 0.83. Max coverage (-): 0

Region: NODE\_271279\_length\_1703\_cov\_19.421610 1378-1381. Max. coverage (+): 0.83. Max coverage (-): 0

Region: NODE\_271279\_length\_1703\_cov\_19.421610 1382-1384. Max. coverage (+): 0.06. Max coverage (-): 0

Region: NODE\_271279\_length\_1703\_cov\_19.421610 1385-1388. Max. coverage (+): 0.07. Max coverage (-): 0.06

Region: NODE\_271279\_length\_1703\_cov\_19.421610 1389-1391. Max. coverage (+): 0.11. Max coverage (-): 0.19

Region: NODE\_271279\_length\_1703\_cov\_19.421610 1392-1395. Max. coverage (+): 0.09. Max coverage (-): 0.3

Region: NODE\_271279\_length\_1703\_cov\_19.421610 1396-1398. Max. coverage (+): 0. Max coverage (-): 0.06

Region: NODE\_271279\_length\_1703\_cov\_19.421610 1399-1402. Max. coverage (+): 0.02. Max coverage (-): 0.06

Region: NODE\_271279\_length\_1703\_cov\_19.421610 1403-1405. Max. coverage (+): 0.02. Max coverage (-): 0

Region: NODE\_271279\_length\_1703\_cov\_19.421610 1406-1409. Max. coverage (+): 0. Max coverage (-): 0

Region: NODE\_271279\_length\_1703\_cov\_19.421610 1410-1412. Max. coverage (+): 0. Max coverage (-): 0

Region: NODE\_271279\_length\_1703\_cov\_19.421610 1413-1416. Max. coverage (+): 0.02. Max coverage (-): 0.06

Region: NODE\_271279\_length\_1703\_cov\_19.421610 1417-1419. Max. coverage (+): 0.02. Max coverage (-): 0.04

Region: NODE\_271279\_length\_1703\_cov\_19.421610 1420-1423. Max. coverage (+): 0. Max coverage (-): 0.04

Region: NODE\_271279\_length\_1703\_cov\_19.421610 1424-1426. Max. coverage (+): 0. Max coverage (-): 0.04

Region: NODE\_271279\_length\_1703\_cov\_19.421610 1427-1430. Max. coverage (+): 0.02. Max coverage (-): 0.02

Region: NODE\_271279\_length\_1703\_cov\_19.421610 1431-1434. Max. coverage (+): 0.02. Max coverage (-): 0.02

Region: NODE\_271279\_length\_1703\_cov\_19.421610 1435-1437. Max. coverage (+): 0.07. Max coverage (-): 0.02

Region: NODE\_271279\_length\_1703\_cov\_19.421610 1438-1441. Max. coverage (+): 0.07. Max coverage (-): 0.02

Region: NODE\_271279\_length\_1703\_cov\_19.421610 1442-1444. Max. coverage (+): 0. Max coverage (-): 0

Region: NODE\_271279\_length\_1703\_cov\_19.421610 1445-1448. Max. coverage (+): 0.02. Max coverage (-): 0.02

Region: NODE\_271279\_length\_1703\_cov\_19.421610 1449-1451. Max. coverage (+): 0.07. Max coverage (-): 0.02

Region: NODE\_271279\_length\_1703\_cov\_19.421610 1452-1455. Max. coverage (+): 0.07. Max coverage (-): 0.06

Region: NODE\_271279\_length\_1703\_cov\_19.421610 1456-1458. Max. coverage (+): 0.02. Max coverage (-): 0.02

Region: NODE\_271279\_length\_1703\_cov\_19.421610 1459-1462. Max. coverage (+): 0.02. Max coverage (-): 0.02

Region: NODE\_271279\_length\_1703\_cov\_19.421610 1463-1465. Max. coverage (+): 0.02. Max coverage (-): 0.02

Region: NODE\_271279\_length\_1703\_cov\_19.421610 1466-1469. Max. coverage (+): 0. Max coverage (-): 0

Region: NODE\_271279\_length\_1703\_cov\_19.421610 1470-1472. Max. coverage (+): 0.09. Max coverage (-): 0

Region: NODE\_271279\_length\_1703\_cov\_19.421610 1473-1476. Max. coverage (+): 0.09. Max coverage (-): 0

Region: NODE\_271279\_length\_1703\_cov\_19.421610 1477-1479. Max. coverage (+): 0.04. Max coverage (-): 0

Region: NODE\_271279\_length\_1703\_cov\_19.421610 1480-1483. Max. coverage (+): 0.09. Max coverage (-): 0.02

Region: NODE\_271279\_length\_1703\_cov\_19.421610 1484-1487. Max. coverage (+): 0.04. Max coverage (-): 0.01

Region: NODE\_271279\_length\_1703\_cov\_19.421610 1488-1490. Max. coverage (+): 0. Max coverage (-): 0

Region: NODE\_271279\_length\_1703\_cov\_19.421610 1491-1494. Max. coverage (+): 0. Max coverage (-): 0

Region: NODE\_271279\_length\_1703\_cov\_19.421610 1495-1497. Max. coverage (+): 0. Max coverage (-): 0

Region: NODE\_271279\_length\_1703\_cov\_19.421610 1498-1501. Max. coverage (+): 0. Max coverage (-): 0

Region: NODE\_271279\_length\_1703\_cov\_19.421610 1502-1504. Max. coverage (+): 0. Max coverage (-): 0

Region: NODE\_271279\_length\_1703\_cov\_19.421610 1505-1508. Max. coverage (+): 0. Max coverage (-): 0.04

Region: NODE\_271279\_length\_1703\_cov\_19.421610 1509-1511. Max. coverage (+): 0. Max coverage (-): 0

Region: NODE\_271279\_length\_1703\_cov\_19.421610 1512-1515. Max. coverage (+): 0. Max coverage (-): 0

Region: NODE\_271279\_length\_1703\_cov\_19.421610 1516-1518. Max. coverage (+): 0. Max coverage (-): 0

Region: NODE\_271279\_length\_1703\_cov\_19.421610 1519-1522. Max. coverage (+): 0. Max coverage (-): 0

Region: NODE\_271279\_length\_1703\_cov\_19.421610 1523-1525. Max. coverage (+): 0. Max coverage (-): 0

Region: NODE\_271279\_length\_1703\_cov\_19.421610 1526-1529. Max. coverage (+): 0. Max coverage (-): 0

Region: NODE\_271279\_length\_1703\_cov\_19.421610 1530-1532. Max. coverage (+): 0. Max coverage (-): 0

Region: NODE\_271279\_length\_1703\_cov\_19.421610 1533-1536. Max. coverage (+): 0. Max coverage (-): 0

Region: NODE\_271279\_length\_1703\_cov\_19.421610 1537-1540. Max. coverage (+): 0. Max coverage (-): 0

Region: NODE\_271279\_length\_1703\_cov\_19.421610 1541-1543. Max. coverage (+): 0. Max coverage (-): 0

Region: NODE\_271279\_length\_1703\_cov\_19.421610 1544-1547. Max. coverage (+): 0. Max coverage (-): 0

Region: NODE\_271279\_length\_1703\_cov\_19.421610 1548-1550. Max. coverage (+): 0. Max coverage (-): 0

Region: NODE\_271279\_length\_1703\_cov\_19.421610 1551-1554. Max. coverage (+): 0. Max coverage (-): 0.04

Region: NODE\_271279\_length\_1703\_cov\_19.421610 1555-1557. Max. coverage (+): 0. Max coverage (-): 0.11

Region: NODE\_271279\_length\_1703\_cov\_19.421610 1558-1561. Max. coverage (+): 0. Max coverage (-): 0.11

Region: NODE\_271279\_length\_1703\_cov\_19.421610 1562-1564. Max. coverage (+): 0. Max coverage (-): 0.09

Region: NODE\_271279\_length\_1703\_cov\_19.421610 1565-1568. Max. coverage (+): 0. Max coverage (-): 0.02

Region: NODE\_271279\_length\_1703\_cov\_19.421610 1569-1571. Max. coverage (+): 0. Max coverage (-): 0.02

Region: NODE\_271279\_length\_1703\_cov\_19.421610 1572-1575. Max. coverage (+): 0. Max coverage (-): 0

Region: NODE\_271279\_length\_1703\_cov\_19.421610 1576-1578. Max. coverage (+): 0. Max coverage (-): 0

Region: NODE\_271279\_length\_1703\_cov\_19.421610 1579-1582. Max. coverage (+): 0. Max coverage (-): 0.02

Region: NODE\_271279\_length\_1703\_cov\_19.421610 1583-1585. Max. coverage (+): 0. Max coverage (-): 0.02

Region: NODE\_271279\_length\_1703\_cov\_19.421610 1586-1589. Max. coverage (+): 0. Max coverage (-): 0

Region: NODE\_271279\_length\_1703\_cov\_19.421610 1590-1593. Max. coverage (+): 0. Max coverage (-): 0

Region: NODE\_271279\_length\_1703\_cov\_19.421610 1594-1596. Max. coverage (+): 0. Max coverage (-): 0

Region: NODE\_271279\_length\_1703\_cov\_19.421610 1597-1600. Max. coverage (+): 0. Max coverage (-): 0

Region: NODE\_271279\_length\_1703\_cov\_19.421610 1601-1603. Max. coverage (+): 0. Max coverage (-): 0

Region: NODE\_271279\_length\_1703\_cov\_19.421610 1604-1607. Max. coverage (+): 0. Max coverage (-): 0

Region: NODE\_271279\_length\_1703\_cov\_19.421610 1608-1610. Max. coverage (+): 0. Max coverage (-): 0

Region: NODE\_271279\_length\_1703\_cov\_19.421610 1611-1614. Max. coverage (+): 0. Max coverage (-): 0

Region: NODE\_271279\_length\_1703\_cov\_19.421610 1615-1617. Max. coverage (+): 0. Max coverage (-): 0

Region: NODE\_271279\_length\_1703\_cov\_19.421610 1618-1621. Max. coverage (+): 0. Max coverage (-): 0

Region: NODE\_271279\_length\_1703\_cov\_19.421610 1622-1624. Max. coverage (+): 0. Max coverage (-): 0

Region: NODE\_271279\_length\_1703\_cov\_19.421610 1625-1628. Max. coverage (+): 0. Max coverage (-): 0

Region: NODE\_271279\_length\_1703\_cov\_19.421610 1629-1631. Max. coverage (+): 0. Max coverage (-): 0

Region: NODE\_271279\_length\_1703\_cov\_19.421610 1632-1635. Max. coverage (+): 0. Max coverage (-): 0

Region: NODE\_271279\_length\_1703\_cov\_19.421610 1636-1639. Max. coverage (+): 0. Max coverage (-): 0

Region: NODE\_271279\_length\_1703\_cov\_19.421610 1640-1642. Max. coverage (+): 0. Max coverage (-): 0

Region: NODE\_271279\_length\_1703\_cov\_19.421610 1643-1646. Max. coverage (+): 0. Max coverage (-): 0

Region: NODE\_271279\_length\_1703\_cov\_19.421610 1647-1649. Max. coverage (+): 0. Max coverage (-): 0

Region: NODE\_271279\_length\_1703\_cov\_19.421610 1650-1653. Max. coverage (+): 0. Max coverage (-): 0

Region: NODE\_271279\_length\_1703\_cov\_19.421610 1654-1656. Max. coverage (+): 0. Max coverage (-): 0

Region: NODE\_271279\_length\_1703\_cov\_19.421610 1657-1660. Max. coverage (+): 0. Max coverage (-): 0

Region: NODE\_271279\_length\_1703\_cov\_19.421610 1661-1663. Max. coverage (+): 0. Max coverage (-): 0

Region: NODE\_271279\_length\_1703\_cov\_19.421610 1664-1667. Max. coverage (+): 0. Max coverage (-): 0

Region: NODE\_271279\_length\_1703\_cov\_19.421610 1668-1670. Max. coverage (+): 0. Max coverage (-): 0

Region: NODE\_271279\_length\_1703\_cov\_19.421610 1671-1674. Max. coverage (+): 0. Max coverage (-): 0

Region: NODE\_271279\_length\_1703\_cov\_19.421610 1675-1677. Max. coverage (+): 0. Max coverage (-): 0

Region: NODE\_271279\_length\_1703\_cov\_19.421610 1678-1681. Max. coverage (+): 0. Max coverage (-): 0

Region: NODE\_271279\_length\_1703\_cov\_19.421610 1682-1684. Max. coverage (+): 0. Max coverage (-): 0

Region: NODE\_271279\_length\_1703\_cov\_19.421610 1685-1688. Max. coverage (+): 0. Max coverage (-): 0

Region: NODE\_271279\_length\_1703\_cov\_19.421610 1689-1692. Max. coverage (+): 0. Max coverage (-): 0

Region: NODE\_271279\_length\_1703\_cov\_19.421610 1693-1695. Max. coverage (+): 0. Max coverage (-): 0

Region: NODE\_271279\_length\_1703\_cov\_19.421610 1696-1699. Max. coverage (+): 0. Max coverage (-): 0

Region: NODE\_271279\_length\_1703\_cov\_19.421610 1700-1702. Max. coverage (+): 0. Max coverage (-): 0

Region: NODE\_271279\_length\_1703\_cov\_19.421610 1703-1706. Max. coverage (+): 0. Max coverage (-): 0

Region: NODE\_271279\_length\_1703\_cov\_19.421610 1707-1709. Max. coverage (+): 0. Max coverage (-): 0

Region: NODE\_271279\_length\_1703\_cov\_19.421610 1710-1713. Max. coverage (+): 0. Max coverage (-): 0

Region: NODE\_271279\_length\_1703\_cov\_19.421610 1714-1716. Max. coverage (+): 0. Max coverage (-): 0

Region: NODE\_271279\_length\_1703\_cov\_19.421610 1717-1720. Max. coverage (+): 0. Max coverage (-): 0

Region: NODE\_271279\_length\_1703\_cov\_19.421610 1721-1723. Max. coverage (+): 0. Max coverage (-): 0

Region: NODE\_271279\_length\_1703\_cov\_19.421610 1724-1727. Max. coverage (+): 0. Max coverage (-): 0

Region: NODE\_271279\_length\_1703\_cov\_19.421610 1728-1730. Max. coverage (+): 0. Max coverage (-): 0

Region: NODE\_271279\_length\_1703\_cov\_19.421610 1731-1734. Max. coverage (+): 0. Max coverage (-): 0

Region: NODE\_271279\_length\_1703\_cov\_19.421610 1735-1737. Max. coverage (+): 0. Max coverage (-): 0

Region: NODE\_271279\_length\_1703\_cov\_19.421610 1738-1741. Max. coverage (+): 0.01. Max coverage (-): 0

Region: NODE\_271279\_length\_1703\_cov\_19.421610 1742-1745. Max. coverage (+): 0.01. Max coverage (-): 0

Region: NODE\_271279\_length\_1703\_cov\_19.421610 1746-1748. Max. coverage (+): 0. Max coverage (-): 0

Region: NODE\_271279\_length\_1703\_cov\_19.421610 1749-1752. Max. coverage (+): 0. Max coverage (-): 0

Region: NODE\_271279\_length\_1703\_cov\_19.421610 1753-1755. Max. coverage (+): 0. Max coverage (-): 0

Region: NODE\_271279\_length\_1703\_cov\_19.421610 1756-1759. Max. coverage (+): 0. Max coverage (-): 0

Region: NODE\_271279\_length\_1703\_cov\_19.421610 1760-1762. Max. coverage (+): 0. Max coverage (-): 0

Region: NODE\_271279\_length\_1703\_cov\_19.421610 1763-1766. Max. coverage (+): 0. Max coverage (-): 0

Region: NODE\_271279\_length\_1703\_cov\_19.421610 1767-. Max. coverage (+): 0. Max coverage (-): 0

RepeatMasker Color Code

**+**

100-98% Identity

<98-95% Identity

<95-90% Identity

<90-85% Identity

<85-80% Identity

<80-75% Identity

<75-70% Identity

<70% Identity

**-**

Gene Set Color Code

**+**

Gene

Pseudogene

Other

**-**

Topology/Coverage Color Code

Coverage Plus Strand

Coverage Minus Strand

Mainstrand: Plus

Mainstrand: Minus

Complementary Strand

Flanking Region  
(if option -flank >0)

Gene Set Annotation  
  
RepeatMasker Annotation  

**1. AlRepC-690**: 1-229 (+), Divergence to consensus: 13.8%  
**2. AlRepB-420**: 203-473 (+), Divergence to consensus: 37.6%  
**3. ISL2EU-7\_CGi**: 611-671 (-), Divergence to consensus: 21.7%

  
Transcription Factor Binding Sites  

**RHOXF1** (Sequence: TGAGCT (+): 307)  
**RHOXF1** (Sequence: TAAGCT (+): 318)  
**RHOXF1** (Sequence: TGAGCC (+): 1138)  
**FOXO3\_mmu** (Sequence: TGTTTAGA (-): 149)  
**Sox5** (Sequence: ATTGTT (+): 657)  
**Sox5** (Sequence: ATTGTT (+): 1445)  
**Sox5** (Sequence: ATTGTT (+): 1613)  
**FIGLA** (Sequence: TACAGCTGGA (-): 1338)  
**SOX9** (Sequence: TTATTGTT (+): 1443)  
**SOX9** (Sequence: TTATTGTT (+): 1611)  
**Nobox** (Sequence: AGCAATTA (-): 187)  
**Gata4** (Sequence: AGATAAG (-): 88)
