# Supplementary material for: First characterization of PIWI-interacting RNA clusters in a cichlid fish with a B chromosome
Source: BMC Biol. 2022 Sep 21;20:204. doi: 10.1186/s12915-022-01403-2 (PMC9490952; doi:10.1186/s12915-022-01403-2)
Supplement: Supplementary file 1 — Additional file 1. Zipped folder with fasta and interactive html piRNA cluster information for the A. latifasciata genome. The nomenclature is as follows: number-pirna-cluster_sex_B-presence (f, female; m, male; 0b, without B chromosome; 1b, with B chromosome). [file 12915_2022_1403_MOESM1_ESM.zip › 105_m1b.html]

piRNA cluster 105\_m1b 65


Predicted piRNA cluster no. 105\_m1b
  

Show proTRAC run info
Hide proTRAC run info

/\  
                \_\_\_\_\_\_\_\_\_\_\_\_\_\_\_\_\_\_\_\_\_\_\_/\\_\_\_ /  \\_\_\_\_\_\_\_  
               I                      /  \  /    \      I  
               I     pro             /    \/      \     I  
               I        TRAC        /               \   I  
               I   \_\_\_\_\_\_\_\_\_\_\_\_\_\_\_\_/\_\_\_\_\_\_\_\_\_\_\_\_\_\_\_\_\_\\_ I  
               I   \              /                     I  
               I    \            /                      I  
               I     \  /\      /       V.2.4.2         I  
               I      \/  \    /                        I  
               I\_\_\_\_\_\_\_\_\_\_\_\  /\_\_\_\_\_\_\_\_\_\_\_\_\_\_\_\_\_\_\_\_\_\_\_\_\_I  
                            \/  
  
  
================================= proTRAC ====================================  
VERSION: .......... 2.4.2  
LAST MODIFIED: .... 11. May 2018  
  
Please cite:  
Rosenkranz D, Zischler H. proTRAC - a software for probabilistic piRNA cluster  
detection, visualization and analysis. 2012. BMC Bioinformatics 13:5.  
  
  
Contact:  
David Rosenkranz  
Institute of Organismic and Molecular Evolutionary Biology  
Dept. Anthropology, small RNA group  
Johannes Gutenberg University Mainz  
email: rosenkranz@uni-mainz.de  
  
You can find the latest proTRAC version at:  
http://sourceforge.net/projects/protrac/files  
http://www.smallRNAgroup-mainz.de/software  
==============================================================================  
  
PARAMETERS:  
Map file: ...............piwi-machos-1B.fa-collapse.map  
Genome file: ............../../../0B\_ala\_genome.fa  
RepeatMasker annotation: Alatifasciata-all0B-maryan-v2.fa\_corrected.out  
GeneSet:................./guest-storage/Data/annotation/Alatifasciata\_all0B\_maryan-v2\_out2017.gff  
  
Significant (p<=0.01) hit density will be calculated based  
on observed hit distribution.  
  
Sliding window size: ........................................ 5000 bp  
Sliding window increament: .................................. 1000 bp  
Normalize each hit by number of genomic hits: ............... yes  
Normalize each hit by number of sequence reads: ............. yes  
Normalize values (-> per million mapped reads): ............. yes  
Min. fraction of hits with 1T(U) or 10A: .................... 0.75  
Alternatively: Min. fraction of hits with 1T(U) and 10A: .... 0.5  
Min. fraction of hits with typical piRNA length: ............ 0.75  
Typical piRNA length: ....................................... 24-32 nt  
Min. size of a piRNA cluster: ............................... 1000 bp.  
Min. number of hits (absolute): ............................. 0  
Min. number of hits (normalized): ........................... 0  
Min. fraction of hits on the mainstrand: .................... 0.75  
Top fraction of mapped sequences (in terms of read counts): . 1%  
Top fraction accounts for max. n% of sequence reads: ........ 90%  
Min. fraction of hits on each arm of a bidirectional cluster: 0.05  
Output html file for each cluster: .......................... yes  
Output a summary table: ..................................... yes  
Output a FASTA file for each cluster (piRNA sequences): ..... yes  
Output a FASTA file comprising cluster sequences: ........... yes  
Output a GTF file for predicted piRNA clusters: ..............yes  
Search DNA motifs in clusters: .............................. yes  
Output flanking sequences: +/- .............................. 0 bp  
Output ~.pTi file: .......................................... no  
==============================================================================  
  
  
Genome size (without gaps): ............ 758543724 bp  
Gaps (N/X/-): .......................... 417479 bp  
Mapped reads: .......................... 26973943  
Non-identical sequences: ............... 6209225  
Genomic hits: .......................... 48438990  
Significant densitiy of mapped reads: .. 821.144211136946 reads/kb

Show proTRAC cluster info
Hide proTRAC cluster info

|  |  |
| --- | --- |
| Location | NODE\_273527\_length\_1611\_cov\_28.567970 |
| Coordinates | 1-1675 |
| Size [bp] | 1675 |
| Sequence hit loci | 2532 |
| Mapped reads (normalized) | 9804.7 |
| Mapped reads (normalized) per kb | 5853.6 |
| Normalized reads with 1T (1U) | 79.9% |
| Normalized reads with 10A | 56.9% |
| Normalized reads with length 24-32 nt | 98.9% |
| Normalized reads on the main strand(s) | 91.9% |
| Predicted directionality | bi:minus-plus (split between 236 and 236) |

100%

0%

1T (1U)  
reads

10A reads

24-32 nt  
reads

reads on mainstrand

**Either the amount of reads with 1T (1U) OR 10A has to exceed 75% (set with option: -1Tor10A)  
Alternatively the amount of reads with 1T (1U) AND 10A has to exceed 50% (set with option: -1Tand10A)  
Minimum amount of reads with preferred size is 75% (set with option: -pisize)  
Minimum amount of reads on the main strand(s) is 75% (set with option: -clstrand)**

Show read coverage
Hide read coverage

WHAT DO I SEE HERE?  
This chart shows the location of mapped sequence reads within a predicted piRNA cluster. The color refers to the number of genomic hits produced by the sequence read in question. A dark red bar indicates that this sequence read produces many other hits elsewhere in the genome. Many adjacent red or yellow bars can indicate the presence of a multi-copy element such as transposons or rRNA genes. A dark green bar indicates that this sequence read maps uniquely to this locus.

1 hit

2-5 hits

6-10 hits

11-20 hits

21-50 hits

51-100 hits

> 100 hits

NODE\_273527\_length\_1611\_cov\_28.567970

1

1675

Gene Set

RepeatMasker

Mapped  
Reads

60.28

plus strand

minus strand

60.28

Region: NODE\_273527\_length\_1611\_cov\_28.567970 1768-2. Max. coverage (+): 0. Max coverage (-): 0.06

Region: NODE\_273527\_length\_1611\_cov\_28.567970 3-6. Max. coverage (+): 0.01. Max coverage (-): 0.01

Region: NODE\_273527\_length\_1611\_cov\_28.567970 7-9. Max. coverage (+): 0.01. Max coverage (-): 0.05

Region: NODE\_273527\_length\_1611\_cov\_28.567970 10-12. Max. coverage (+): 0.97. Max coverage (-): 0.1

Region: NODE\_273527\_length\_1611\_cov\_28.567970 13-16. Max. coverage (+): 0.25. Max coverage (-): 0.15

Region: NODE\_273527\_length\_1611\_cov\_28.567970 17-19. Max. coverage (+): 0. Max coverage (-): 0.35

Region: NODE\_273527\_length\_1611\_cov\_28.567970 20-22. Max. coverage (+): 0. Max coverage (-): 0.17

Region: NODE\_273527\_length\_1611\_cov\_28.567970 23-26. Max. coverage (+): 0.02. Max coverage (-): 0.11

Region: NODE\_273527\_length\_1611\_cov\_28.567970 27-29. Max. coverage (+): 0.02. Max coverage (-): 2.54

Region: NODE\_273527\_length\_1611\_cov\_28.567970 30-32. Max. coverage (+): 0.26. Max coverage (-): 0.09

Region: NODE\_273527\_length\_1611\_cov\_28.567970 33-36. Max. coverage (+): 0.91. Max coverage (-): 0.02

Region: NODE\_273527\_length\_1611\_cov\_28.567970 37-39. Max. coverage (+): 0.11. Max coverage (-): 0

Region: NODE\_273527\_length\_1611\_cov\_28.567970 40-42. Max. coverage (+): 0.07. Max coverage (-): 0

Region: NODE\_273527\_length\_1611\_cov\_28.567970 43-46. Max. coverage (+): 0.26. Max coverage (-): 0.04

Region: NODE\_273527\_length\_1611\_cov\_28.567970 47-49. Max. coverage (+): 0.11. Max coverage (-): 0

Region: NODE\_273527\_length\_1611\_cov\_28.567970 50-52. Max. coverage (+): 0.15. Max coverage (-): 0.04

Region: NODE\_273527\_length\_1611\_cov\_28.567970 53-56. Max. coverage (+): 0.19. Max coverage (-): 0.33

Region: NODE\_273527\_length\_1611\_cov\_28.567970 57-59. Max. coverage (+): 0.37. Max coverage (-): 0

Region: NODE\_273527\_length\_1611\_cov\_28.567970 60-62. Max. coverage (+): 0.26. Max coverage (-): 0.15

Region: NODE\_273527\_length\_1611\_cov\_28.567970 63-66. Max. coverage (+): 0.07. Max coverage (-): 0.04

Region: NODE\_273527\_length\_1611\_cov\_28.567970 67-69. Max. coverage (+): 0.22. Max coverage (-): 0.07

Region: NODE\_273527\_length\_1611\_cov\_28.567970 70-73. Max. coverage (+): 0.22. Max coverage (-): 0.41

Region: NODE\_273527\_length\_1611\_cov\_28.567970 74-76. Max. coverage (+): 0. Max coverage (-): 0.19

Region: NODE\_273527\_length\_1611\_cov\_28.567970 77-79. Max. coverage (+): 0. Max coverage (-): 0

Region: NODE\_273527\_length\_1611\_cov\_28.567970 80-83. Max. coverage (+): 0. Max coverage (-): 0

Region: NODE\_273527\_length\_1611\_cov\_28.567970 84-86. Max. coverage (+): 0.04. Max coverage (-): 0

Region: NODE\_273527\_length\_1611\_cov\_28.567970 87-89. Max. coverage (+): 0.04. Max coverage (-): 0.07

Region: NODE\_273527\_length\_1611\_cov\_28.567970 90-93. Max. coverage (+): 0.04. Max coverage (-): 0.04

Region: NODE\_273527\_length\_1611\_cov\_28.567970 94-96. Max. coverage (+): 0. Max coverage (-): 0

Region: NODE\_273527\_length\_1611\_cov\_28.567970 97-99. Max. coverage (+): 0. Max coverage (-): 0.04

Region: NODE\_273527\_length\_1611\_cov\_28.567970 100-103. Max. coverage (+): 0. Max coverage (-): 0

Region: NODE\_273527\_length\_1611\_cov\_28.567970 104-106. Max. coverage (+): 0. Max coverage (-): 0.11

Region: NODE\_273527\_length\_1611\_cov\_28.567970 107-109. Max. coverage (+): 0. Max coverage (-): 0.11

Region: NODE\_273527\_length\_1611\_cov\_28.567970 110-113. Max. coverage (+): 0. Max coverage (-): 0.02

Region: NODE\_273527\_length\_1611\_cov\_28.567970 114-116. Max. coverage (+): 0. Max coverage (-): 0

Region: NODE\_273527\_length\_1611\_cov\_28.567970 117-119. Max. coverage (+): 0. Max coverage (-): 0.02

Region: NODE\_273527\_length\_1611\_cov\_28.567970 120-123. Max. coverage (+): 0. Max coverage (-): 0.22

Region: NODE\_273527\_length\_1611\_cov\_28.567970 124-126. Max. coverage (+): 0. Max coverage (-): 0.28

Region: NODE\_273527\_length\_1611\_cov\_28.567970 127-129. Max. coverage (+): 0.05. Max coverage (-): 0.15

Region: NODE\_273527\_length\_1611\_cov\_28.567970 130-133. Max. coverage (+): 0.05. Max coverage (-): 0.02

Region: NODE\_273527\_length\_1611\_cov\_28.567970 134-136. Max. coverage (+): 0.03. Max coverage (-): 0.04

Region: NODE\_273527\_length\_1611\_cov\_28.567970 137-140. Max. coverage (+): 0.02. Max coverage (-): 0

Region: NODE\_273527\_length\_1611\_cov\_28.567970 141-143. Max. coverage (+): 0.02. Max coverage (-): 0

Region: NODE\_273527\_length\_1611\_cov\_28.567970 144-146. Max. coverage (+): 0. Max coverage (-): 0

Region: NODE\_273527\_length\_1611\_cov\_28.567970 147-150. Max. coverage (+): 0.04. Max coverage (-): 0.11

Region: NODE\_273527\_length\_1611\_cov\_28.567970 151-153. Max. coverage (+): 0. Max coverage (-): 0.22

Region: NODE\_273527\_length\_1611\_cov\_28.567970 154-156. Max. coverage (+): 0. Max coverage (-): 0.19

Region: NODE\_273527\_length\_1611\_cov\_28.567970 157-160. Max. coverage (+): 0. Max coverage (-): 0.15

Region: NODE\_273527\_length\_1611\_cov\_28.567970 161-163. Max. coverage (+): 0.04. Max coverage (-): 0

Region: NODE\_273527\_length\_1611\_cov\_28.567970 164-166. Max. coverage (+): 0.22. Max coverage (-): 0.07

Region: NODE\_273527\_length\_1611\_cov\_28.567970 167-170. Max. coverage (+): 0.3. Max coverage (-): 0

Region: NODE\_273527\_length\_1611\_cov\_28.567970 171-173. Max. coverage (+): 0.1. Max coverage (-): 0

Region: NODE\_273527\_length\_1611\_cov\_28.567970 174-176. Max. coverage (+): 0. Max coverage (-): 0.01

Region: NODE\_273527\_length\_1611\_cov\_28.567970 177-180. Max. coverage (+): 0.02. Max coverage (-): 8.44

Region: NODE\_273527\_length\_1611\_cov\_28.567970 181-183. Max. coverage (+): 0.02. Max coverage (-): 15.99

Region: NODE\_273527\_length\_1611\_cov\_28.567970 184-186. Max. coverage (+): 0. Max coverage (-): 0.3

Region: NODE\_273527\_length\_1611\_cov\_28.567970 187-190. Max. coverage (+): 0.17. Max coverage (-): 0.48

Region: NODE\_273527\_length\_1611\_cov\_28.567970 191-193. Max. coverage (+): 0.13. Max coverage (-): 1.44

Region: NODE\_273527\_length\_1611\_cov\_28.567970 194-196. Max. coverage (+): 0.05. Max coverage (-): 0.17

Region: NODE\_273527\_length\_1611\_cov\_28.567970 197-200. Max. coverage (+): 0.03. Max coverage (-): 0.11

Region: NODE\_273527\_length\_1611\_cov\_28.567970 201-203. Max. coverage (+): 0.02. Max coverage (-): 0.09

Region: NODE\_273527\_length\_1611\_cov\_28.567970 204-207. Max. coverage (+): 0.03. Max coverage (-): 0.03

Region: NODE\_273527\_length\_1611\_cov\_28.567970 208-210. Max. coverage (+): 0.03. Max coverage (-): 0.04

Region: NODE\_273527\_length\_1611\_cov\_28.567970 211-213. Max. coverage (+): 0. Max coverage (-): 0.04

Region: NODE\_273527\_length\_1611\_cov\_28.567970 214-217. Max. coverage (+): 0.01. Max coverage (-): 0.51

Region: NODE\_273527\_length\_1611\_cov\_28.567970 218-220. Max. coverage (+): 0.02. Max coverage (-): 0.53

Region: NODE\_273527\_length\_1611\_cov\_28.567970 221-223. Max. coverage (+): 0.02. Max coverage (-): 0.89

Region: NODE\_273527\_length\_1611\_cov\_28.567970 224-227. Max. coverage (+): 0. Max coverage (-): 0.95

Region: NODE\_273527\_length\_1611\_cov\_28.567970 228-230. Max. coverage (+): 0. Max coverage (-): 0.84

Region: NODE\_273527\_length\_1611\_cov\_28.567970 231-233. Max. coverage (+): 0. Max coverage (-): 0.49

Region: NODE\_273527\_length\_1611\_cov\_28.567970 234-237. Max. coverage (+): 0.04. Max coverage (-): 0.09

Region: NODE\_273527\_length\_1611\_cov\_28.567970 238-240. Max. coverage (+): 0.04. Max coverage (-): 0.04

Region: NODE\_273527\_length\_1611\_cov\_28.567970 241-243. Max. coverage (+): 0.02. Max coverage (-): 0.04

Region: NODE\_273527\_length\_1611\_cov\_28.567970 244-247. Max. coverage (+): 0.15. Max coverage (-): 0.01

Region: NODE\_273527\_length\_1611\_cov\_28.567970 248-250. Max. coverage (+): 0.02. Max coverage (-): 0

Region: NODE\_273527\_length\_1611\_cov\_28.567970 251-253. Max. coverage (+): 0. Max coverage (-): 0

Region: NODE\_273527\_length\_1611\_cov\_28.567970 254-257. Max. coverage (+): 0. Max coverage (-): 0

Region: NODE\_273527\_length\_1611\_cov\_28.567970 258-260. Max. coverage (+): 0. Max coverage (-): 0

Region: NODE\_273527\_length\_1611\_cov\_28.567970 261-263. Max. coverage (+): 0. Max coverage (-): 0

Region: NODE\_273527\_length\_1611\_cov\_28.567970 264-267. Max. coverage (+): 0.01. Max coverage (-): 0.01

Region: NODE\_273527\_length\_1611\_cov\_28.567970 268-270. Max. coverage (+): 0.01. Max coverage (-): 0

Region: NODE\_273527\_length\_1611\_cov\_28.567970 271-274. Max. coverage (+): 0. Max coverage (-): 0

Region: NODE\_273527\_length\_1611\_cov\_28.567970 275-277. Max. coverage (+): 0. Max coverage (-): 0.01

Region: NODE\_273527\_length\_1611\_cov\_28.567970 278-280. Max. coverage (+): 0.09. Max coverage (-): 0.01

Region: NODE\_273527\_length\_1611\_cov\_28.567970 281-284. Max. coverage (+): 0.51. Max coverage (-): 0.04

Region: NODE\_273527\_length\_1611\_cov\_28.567970 285-287. Max. coverage (+): 0.33. Max coverage (-): 0.04

Region: NODE\_273527\_length\_1611\_cov\_28.567970 288-290. Max. coverage (+): 0.11. Max coverage (-): 0

Region: NODE\_273527\_length\_1611\_cov\_28.567970 291-294. Max. coverage (+): 0.96. Max coverage (-): 0.11

Region: NODE\_273527\_length\_1611\_cov\_28.567970 295-297. Max. coverage (+): 0.85. Max coverage (-): 0.11

Region: NODE\_273527\_length\_1611\_cov\_28.567970 298-300. Max. coverage (+): 0.01. Max coverage (-): 0.04

Region: NODE\_273527\_length\_1611\_cov\_28.567970 301-304. Max. coverage (+): 0.02. Max coverage (-): 0.01

Region: NODE\_273527\_length\_1611\_cov\_28.567970 305-307. Max. coverage (+): 0.05. Max coverage (-): 0.02

Region: NODE\_273527\_length\_1611\_cov\_28.567970 308-310. Max. coverage (+): 0.01. Max coverage (-): 0.03

Region: NODE\_273527\_length\_1611\_cov\_28.567970 311-314. Max. coverage (+): 0.04. Max coverage (-): 0.02

Region: NODE\_273527\_length\_1611\_cov\_28.567970 315-317. Max. coverage (+): 0.04. Max coverage (-): 0

Region: NODE\_273527\_length\_1611\_cov\_28.567970 318-320. Max. coverage (+): 0.07. Max coverage (-): 0.01

Region: NODE\_273527\_length\_1611\_cov\_28.567970 321-324. Max. coverage (+): 0.62. Max coverage (-): 0.01

Region: NODE\_273527\_length\_1611\_cov\_28.567970 325-327. Max. coverage (+): 0.63. Max coverage (-): 0

Region: NODE\_273527\_length\_1611\_cov\_28.567970 328-330. Max. coverage (+): 0.13. Max coverage (-): 0

Region: NODE\_273527\_length\_1611\_cov\_28.567970 331-334. Max. coverage (+): 0.17. Max coverage (-): 0.02

Region: NODE\_273527\_length\_1611\_cov\_28.567970 335-337. Max. coverage (+): 0.22. Max coverage (-): 0.02

Region: NODE\_273527\_length\_1611\_cov\_28.567970 338-341. Max. coverage (+): 1.52. Max coverage (-): 0.19

Region: NODE\_273527\_length\_1611\_cov\_28.567970 342-344. Max. coverage (+): 0.54. Max coverage (-): 0.28

Region: NODE\_273527\_length\_1611\_cov\_28.567970 345-347. Max. coverage (+): 0.15. Max coverage (-): 0.38

Region: NODE\_273527\_length\_1611\_cov\_28.567970 348-351. Max. coverage (+): 0.14. Max coverage (-): 0.4

Region: NODE\_273527\_length\_1611\_cov\_28.567970 352-354. Max. coverage (+): 0.36. Max coverage (-): 0.1

Region: NODE\_273527\_length\_1611\_cov\_28.567970 355-357. Max. coverage (+): 0.2. Max coverage (-): 0.04

Region: NODE\_273527\_length\_1611\_cov\_28.567970 358-361. Max. coverage (+): 0.53. Max coverage (-): 0.01

Region: NODE\_273527\_length\_1611\_cov\_28.567970 362-364. Max. coverage (+): 0.01. Max coverage (-): 0.02

Region: NODE\_273527\_length\_1611\_cov\_28.567970 365-367. Max. coverage (+): 0.39. Max coverage (-): 0.02

Region: NODE\_273527\_length\_1611\_cov\_28.567970 368-371. Max. coverage (+): 0.38. Max coverage (-): 0.04

Region: NODE\_273527\_length\_1611\_cov\_28.567970 372-374. Max. coverage (+): 0.07. Max coverage (-): 0.04

Region: NODE\_273527\_length\_1611\_cov\_28.567970 375-377. Max. coverage (+): 0.15. Max coverage (-): 0.04

Region: NODE\_273527\_length\_1611\_cov\_28.567970 378-381. Max. coverage (+): 0.32. Max coverage (-): 0.01

Region: NODE\_273527\_length\_1611\_cov\_28.567970 382-384. Max. coverage (+): 0.1. Max coverage (-): 0.01

Region: NODE\_273527\_length\_1611\_cov\_28.567970 385-387. Max. coverage (+): 0. Max coverage (-): 0.04

Region: NODE\_273527\_length\_1611\_cov\_28.567970 388-391. Max. coverage (+): 0.03. Max coverage (-): 0.04

Region: NODE\_273527\_length\_1611\_cov\_28.567970 392-394. Max. coverage (+): 0.04. Max coverage (-): 0

Region: NODE\_273527\_length\_1611\_cov\_28.567970 395-397. Max. coverage (+): 0.06. Max coverage (-): 0.26

Region: NODE\_273527\_length\_1611\_cov\_28.567970 398-401. Max. coverage (+): 0.33. Max coverage (-): 0.3

Region: NODE\_273527\_length\_1611\_cov\_28.567970 402-404. Max. coverage (+): 0.44. Max coverage (-): 0

Region: NODE\_273527\_length\_1611\_cov\_28.567970 405-408. Max. coverage (+): 0.52. Max coverage (-): 0

Region: NODE\_273527\_length\_1611\_cov\_28.567970 409-411. Max. coverage (+): 0.04. Max coverage (-): 0

Region: NODE\_273527\_length\_1611\_cov\_28.567970 412-414. Max. coverage (+): 0.07. Max coverage (-): 0

Region: NODE\_273527\_length\_1611\_cov\_28.567970 415-418. Max. coverage (+): 0.19. Max coverage (-): 0

Region: NODE\_273527\_length\_1611\_cov\_28.567970 419-421. Max. coverage (+): 0.11. Max coverage (-): 0

Region: NODE\_273527\_length\_1611\_cov\_28.567970 422-424. Max. coverage (+): 0.22. Max coverage (-): 0.02

Region: NODE\_273527\_length\_1611\_cov\_28.567970 425-428. Max. coverage (+): 0.13. Max coverage (-): 0.02

Region: NODE\_273527\_length\_1611\_cov\_28.567970 429-431. Max. coverage (+): 0. Max coverage (-): 0.03

Region: NODE\_273527\_length\_1611\_cov\_28.567970 432-434. Max. coverage (+): 0.01. Max coverage (-): 0.03

Region: NODE\_273527\_length\_1611\_cov\_28.567970 435-438. Max. coverage (+): 0.01. Max coverage (-): 0.04

Region: NODE\_273527\_length\_1611\_cov\_28.567970 439-441. Max. coverage (+): 0.01. Max coverage (-): 0

Region: NODE\_273527\_length\_1611\_cov\_28.567970 442-444. Max. coverage (+): 0. Max coverage (-): 0.01

Region: NODE\_273527\_length\_1611\_cov\_28.567970 445-448. Max. coverage (+): 0.03. Max coverage (-): 0.04

Region: NODE\_273527\_length\_1611\_cov\_28.567970 449-451. Max. coverage (+): 0.04. Max coverage (-): 0.04

Region: NODE\_273527\_length\_1611\_cov\_28.567970 452-454. Max. coverage (+): 0. Max coverage (-): 0.01

Region: NODE\_273527\_length\_1611\_cov\_28.567970 455-458. Max. coverage (+): 0. Max coverage (-): 0

Region: NODE\_273527\_length\_1611\_cov\_28.567970 459-461. Max. coverage (+): 0. Max coverage (-): 0

Region: NODE\_273527\_length\_1611\_cov\_28.567970 462-464. Max. coverage (+): 0. Max coverage (-): 0.04

Region: NODE\_273527\_length\_1611\_cov\_28.567970 465-468. Max. coverage (+): 0. Max coverage (-): 0

Region: NODE\_273527\_length\_1611\_cov\_28.567970 469-471. Max. coverage (+): 0.52. Max coverage (-): 0

Region: NODE\_273527\_length\_1611\_cov\_28.567970 472-475. Max. coverage (+): 9.23. Max coverage (-): 0

Region: NODE\_273527\_length\_1611\_cov\_28.567970 476-478. Max. coverage (+): 22.5. Max coverage (-): 0

Region: NODE\_273527\_length\_1611\_cov\_28.567970 479-481. Max. coverage (+): 2.95. Max coverage (-): 0

Region: NODE\_273527\_length\_1611\_cov\_28.567970 482-485. Max. coverage (+): 0.06. Max coverage (-): 0.02

Region: NODE\_273527\_length\_1611\_cov\_28.567970 486-488. Max. coverage (+): 0. Max coverage (-): 0.02

Region: NODE\_273527\_length\_1611\_cov\_28.567970 489-491. Max. coverage (+): 0.07. Max coverage (-): 0.19

Region: NODE\_273527\_length\_1611\_cov\_28.567970 492-495. Max. coverage (+): 0.01. Max coverage (-): 0.58

Region: NODE\_273527\_length\_1611\_cov\_28.567970 496-498. Max. coverage (+): 0.01. Max coverage (-): 0.28

Region: NODE\_273527\_length\_1611\_cov\_28.567970 499-501. Max. coverage (+): 0.01. Max coverage (-): 0.01

Region: NODE\_273527\_length\_1611\_cov\_28.567970 502-505. Max. coverage (+): 0.02. Max coverage (-): 0.01

Region: NODE\_273527\_length\_1611\_cov\_28.567970 506-508. Max. coverage (+): 1.17. Max coverage (-): 0

Region: NODE\_273527\_length\_1611\_cov\_28.567970 509-511. Max. coverage (+): 1.84. Max coverage (-): 0

Region: NODE\_273527\_length\_1611\_cov\_28.567970 512-515. Max. coverage (+): 1.38. Max coverage (-): 0.01

Region: NODE\_273527\_length\_1611\_cov\_28.567970 516-518. Max. coverage (+): 0.11. Max coverage (-): 0.1

Region: NODE\_273527\_length\_1611\_cov\_28.567970 519-521. Max. coverage (+): 0.04. Max coverage (-): 0.11

Region: NODE\_273527\_length\_1611\_cov\_28.567970 522-525. Max. coverage (+): 0. Max coverage (-): 0.01

Region: NODE\_273527\_length\_1611\_cov\_28.567970 526-528. Max. coverage (+): 0.01. Max coverage (-): 0.03

Region: NODE\_273527\_length\_1611\_cov\_28.567970 529-531. Max. coverage (+): 0.01. Max coverage (-): 0.03

Region: NODE\_273527\_length\_1611\_cov\_28.567970 532-535. Max. coverage (+): 0.06. Max coverage (-): 0

Region: NODE\_273527\_length\_1611\_cov\_28.567970 536-538. Max. coverage (+): 0.07. Max coverage (-): 0

Region: NODE\_273527\_length\_1611\_cov\_28.567970 539-542. Max. coverage (+): 0.04. Max coverage (-): 0.07

Region: NODE\_273527\_length\_1611\_cov\_28.567970 543-545. Max. coverage (+): 0. Max coverage (-): 0.07

Region: NODE\_273527\_length\_1611\_cov\_28.567970 546-548. Max. coverage (+): 0. Max coverage (-): 0

Region: NODE\_273527\_length\_1611\_cov\_28.567970 549-552. Max. coverage (+): 0. Max coverage (-): 0

Region: NODE\_273527\_length\_1611\_cov\_28.567970 553-555. Max. coverage (+): 0. Max coverage (-): 0

Region: NODE\_273527\_length\_1611\_cov\_28.567970 556-558. Max. coverage (+): 0. Max coverage (-): 0

Region: NODE\_273527\_length\_1611\_cov\_28.567970 559-562. Max. coverage (+): 0. Max coverage (-): 0

Region: NODE\_273527\_length\_1611\_cov\_28.567970 563-565. Max. coverage (+): 0. Max coverage (-): 0.02

Region: NODE\_273527\_length\_1611\_cov\_28.567970 566-568. Max. coverage (+): 0.04. Max coverage (-): 0.04

Region: NODE\_273527\_length\_1611\_cov\_28.567970 569-572. Max. coverage (+): 0.04. Max coverage (-): 0.01

Region: NODE\_273527\_length\_1611\_cov\_28.567970 573-575. Max. coverage (+): 0.01. Max coverage (-): 0

Region: NODE\_273527\_length\_1611\_cov\_28.567970 576-578. Max. coverage (+): 0. Max coverage (-): 0

Region: NODE\_273527\_length\_1611\_cov\_28.567970 579-582. Max. coverage (+): 0. Max coverage (-): 0

Region: NODE\_273527\_length\_1611\_cov\_28.567970 583-585. Max. coverage (+): 0. Max coverage (-): 0

Region: NODE\_273527\_length\_1611\_cov\_28.567970 586-588. Max. coverage (+): 0. Max coverage (-): 0

Region: NODE\_273527\_length\_1611\_cov\_28.567970 589-592. Max. coverage (+): 0.29. Max coverage (-): 0

Region: NODE\_273527\_length\_1611\_cov\_28.567970 593-595. Max. coverage (+): 0.02. Max coverage (-): 0

Region: NODE\_273527\_length\_1611\_cov\_28.567970 596-598. Max. coverage (+): 0. Max coverage (-): 0

Region: NODE\_273527\_length\_1611\_cov\_28.567970 599-602. Max. coverage (+): 0. Max coverage (-): 0

Region: NODE\_273527\_length\_1611\_cov\_28.567970 603-605. Max. coverage (+): 0. Max coverage (-): 0.03

Region: NODE\_273527\_length\_1611\_cov\_28.567970 606-609. Max. coverage (+): 0. Max coverage (-): 0.06

Region: NODE\_273527\_length\_1611\_cov\_28.567970 610-612. Max. coverage (+): 0.01. Max coverage (-): 0.07

Region: NODE\_273527\_length\_1611\_cov\_28.567970 613-615. Max. coverage (+): 0. Max coverage (-): 0.05

Region: NODE\_273527\_length\_1611\_cov\_28.567970 616-619. Max. coverage (+): 0. Max coverage (-): 0.06

Region: NODE\_273527\_length\_1611\_cov\_28.567970 620-622. Max. coverage (+): 0.07. Max coverage (-): 0

Region: NODE\_273527\_length\_1611\_cov\_28.567970 623-625. Max. coverage (+): 0.07. Max coverage (-): 0

Region: NODE\_273527\_length\_1611\_cov\_28.567970 626-629. Max. coverage (+): 0.15. Max coverage (-): 0

Region: NODE\_273527\_length\_1611\_cov\_28.567970 630-632. Max. coverage (+): 0.22. Max coverage (-): 0

Region: NODE\_273527\_length\_1611\_cov\_28.567970 633-635. Max. coverage (+): 0.15. Max coverage (-): 0

Region: NODE\_273527\_length\_1611\_cov\_28.567970 636-639. Max. coverage (+): 0.07. Max coverage (-): 0

Region: NODE\_273527\_length\_1611\_cov\_28.567970 640-642. Max. coverage (+): 0. Max coverage (-): 0

Region: NODE\_273527\_length\_1611\_cov\_28.567970 643-645. Max. coverage (+): 0.04. Max coverage (-): 0.07

Region: NODE\_273527\_length\_1611\_cov\_28.567970 646-649. Max. coverage (+): 0.07. Max coverage (-): 0.33

Region: NODE\_273527\_length\_1611\_cov\_28.567970 650-652. Max. coverage (+): 0.07. Max coverage (-): 0.04

Region: NODE\_273527\_length\_1611\_cov\_28.567970 653-655. Max. coverage (+): 0. Max coverage (-): 0

Region: NODE\_273527\_length\_1611\_cov\_28.567970 656-659. Max. coverage (+): 0.04. Max coverage (-): 0

Region: NODE\_273527\_length\_1611\_cov\_28.567970 660-662. Max. coverage (+): 3.56. Max coverage (-): 0

Region: NODE\_273527\_length\_1611\_cov\_28.567970 663-665. Max. coverage (+): 3.53. Max coverage (-): 0

Region: NODE\_273527\_length\_1611\_cov\_28.567970 666-669. Max. coverage (+): 0.07. Max coverage (-): 0

Region: NODE\_273527\_length\_1611\_cov\_28.567970 670-672. Max. coverage (+): 0.07. Max coverage (-): 0

Region: NODE\_273527\_length\_1611\_cov\_28.567970 673-676. Max. coverage (+): 0.11. Max coverage (-): 0

Region: NODE\_273527\_length\_1611\_cov\_28.567970 677-679. Max. coverage (+): 0. Max coverage (-): 0

Region: NODE\_273527\_length\_1611\_cov\_28.567970 680-682. Max. coverage (+): 0. Max coverage (-): 0.04

Region: NODE\_273527\_length\_1611\_cov\_28.567970 683-686. Max. coverage (+): 0. Max coverage (-): 0.04

Region: NODE\_273527\_length\_1611\_cov\_28.567970 687-689. Max. coverage (+): 0. Max coverage (-): 0.07

Region: NODE\_273527\_length\_1611\_cov\_28.567970 690-692. Max. coverage (+): 0. Max coverage (-): 0

Region: NODE\_273527\_length\_1611\_cov\_28.567970 693-696. Max. coverage (+): 0.3. Max coverage (-): 0

Region: NODE\_273527\_length\_1611\_cov\_28.567970 697-699. Max. coverage (+): 2.22. Max coverage (-): 0

Region: NODE\_273527\_length\_1611\_cov\_28.567970 700-702. Max. coverage (+): 1.52. Max coverage (-): 0

Region: NODE\_273527\_length\_1611\_cov\_28.567970 703-706. Max. coverage (+): 3.45. Max coverage (-): 0.11

Region: NODE\_273527\_length\_1611\_cov\_28.567970 707-709. Max. coverage (+): 0.37. Max coverage (-): 0.15

Region: NODE\_273527\_length\_1611\_cov\_28.567970 710-712. Max. coverage (+): 0.15. Max coverage (-): 0.04

Region: NODE\_273527\_length\_1611\_cov\_28.567970 713-716. Max. coverage (+): 0.15. Max coverage (-): 0.04

Region: NODE\_273527\_length\_1611\_cov\_28.567970 717-719. Max. coverage (+): 0.33. Max coverage (-): 0

Region: NODE\_273527\_length\_1611\_cov\_28.567970 720-722. Max. coverage (+): 0.07. Max coverage (-): 0.78

Region: NODE\_273527\_length\_1611\_cov\_28.567970 723-726. Max. coverage (+): 0.07. Max coverage (-): 0.74

Region: NODE\_273527\_length\_1611\_cov\_28.567970 727-729. Max. coverage (+): 0.04. Max coverage (-): 0.56

Region: NODE\_273527\_length\_1611\_cov\_28.567970 730-732. Max. coverage (+): 4.37. Max coverage (-): 0.48

Region: NODE\_273527\_length\_1611\_cov\_28.567970 733-736. Max. coverage (+): 3.63. Max coverage (-): 0.37

Region: NODE\_273527\_length\_1611\_cov\_28.567970 737-739. Max. coverage (+): 2. Max coverage (-): 0.04

Region: NODE\_273527\_length\_1611\_cov\_28.567970 740-743. Max. coverage (+): 1.33. Max coverage (-): 0.11

Region: NODE\_273527\_length\_1611\_cov\_28.567970 744-746. Max. coverage (+): 0.93. Max coverage (-): 0.3

Region: NODE\_273527\_length\_1611\_cov\_28.567970 747-749. Max. coverage (+): 0.78. Max coverage (-): 0.33

Region: NODE\_273527\_length\_1611\_cov\_28.567970 750-753. Max. coverage (+): 0.78. Max coverage (-): 0.15

Region: NODE\_273527\_length\_1611\_cov\_28.567970 754-756. Max. coverage (+): 0.26. Max coverage (-): 0.11

Region: NODE\_273527\_length\_1611\_cov\_28.567970 757-759. Max. coverage (+): 0.7. Max coverage (-): 0.11

Region: NODE\_273527\_length\_1611\_cov\_28.567970 760-763. Max. coverage (+): 0.44. Max coverage (-): 0.04

Region: NODE\_273527\_length\_1611\_cov\_28.567970 764-766. Max. coverage (+): 0.11. Max coverage (-): 0

Region: NODE\_273527\_length\_1611\_cov\_28.567970 767-769. Max. coverage (+): 0.07. Max coverage (-): 0

Region: NODE\_273527\_length\_1611\_cov\_28.567970 770-773. Max. coverage (+): 0.04. Max coverage (-): 0.04

Region: NODE\_273527\_length\_1611\_cov\_28.567970 774-776. Max. coverage (+): 0.15. Max coverage (-): 0.04

Region: NODE\_273527\_length\_1611\_cov\_28.567970 777-779. Max. coverage (+): 0.19. Max coverage (-): 0

Region: NODE\_273527\_length\_1611\_cov\_28.567970 780-783. Max. coverage (+): 0.04. Max coverage (-): 0

Region: NODE\_273527\_length\_1611\_cov\_28.567970 784-786. Max. coverage (+): 0. Max coverage (-): 0

Region: NODE\_273527\_length\_1611\_cov\_28.567970 787-789. Max. coverage (+): 0. Max coverage (-): 0

Region: NODE\_273527\_length\_1611\_cov\_28.567970 790-793. Max. coverage (+): 0. Max coverage (-): 0.52

Region: NODE\_273527\_length\_1611\_cov\_28.567970 794-796. Max. coverage (+): 0. Max coverage (-): 0.48

Region: NODE\_273527\_length\_1611\_cov\_28.567970 797-799. Max. coverage (+): 0. Max coverage (-): 0.7

Region: NODE\_273527\_length\_1611\_cov\_28.567970 800-803. Max. coverage (+): 0.41. Max coverage (-): 0.11

Region: NODE\_273527\_length\_1611\_cov\_28.567970 804-806. Max. coverage (+): 0.3. Max coverage (-): 0.11

Region: NODE\_273527\_length\_1611\_cov\_28.567970 807-810. Max. coverage (+): 0.44. Max coverage (-): 0.04

Region: NODE\_273527\_length\_1611\_cov\_28.567970 811-813. Max. coverage (+): 2.82. Max coverage (-): 0

Region: NODE\_273527\_length\_1611\_cov\_28.567970 814-816. Max. coverage (+): 3.6. Max coverage (-): 0

Region: NODE\_273527\_length\_1611\_cov\_28.567970 817-820. Max. coverage (+): 0.7. Max coverage (-): 0

Region: NODE\_273527\_length\_1611\_cov\_28.567970 821-823. Max. coverage (+): 0.22. Max coverage (-): 0

Region: NODE\_273527\_length\_1611\_cov\_28.567970 824-826. Max. coverage (+): 0.22. Max coverage (-): 0.04

Region: NODE\_273527\_length\_1611\_cov\_28.567970 827-830. Max. coverage (+): 0.07. Max coverage (-): 0.04

Region: NODE\_273527\_length\_1611\_cov\_28.567970 831-833. Max. coverage (+): 0.07. Max coverage (-): 0

Region: NODE\_273527\_length\_1611\_cov\_28.567970 834-836. Max. coverage (+): 0. Max coverage (-): 0

Region: NODE\_273527\_length\_1611\_cov\_28.567970 837-840. Max. coverage (+): 0. Max coverage (-): 0

Region: NODE\_273527\_length\_1611\_cov\_28.567970 841-843. Max. coverage (+): 0.04. Max coverage (-): 0

Region: NODE\_273527\_length\_1611\_cov\_28.567970 844-846. Max. coverage (+): 0.07. Max coverage (-): 0.07

Region: NODE\_273527\_length\_1611\_cov\_28.567970 847-850. Max. coverage (+): 0.11. Max coverage (-): 0

Region: NODE\_273527\_length\_1611\_cov\_28.567970 851-853. Max. coverage (+): 0.22. Max coverage (-): 0.04

Region: NODE\_273527\_length\_1611\_cov\_28.567970 854-856. Max. coverage (+): 0. Max coverage (-): 3

Region: NODE\_273527\_length\_1611\_cov\_28.567970 857-860. Max. coverage (+): 0. Max coverage (-): 2.97

Region: NODE\_273527\_length\_1611\_cov\_28.567970 861-863. Max. coverage (+): 0. Max coverage (-): 0

Region: NODE\_273527\_length\_1611\_cov\_28.567970 864-866. Max. coverage (+): 0. Max coverage (-): 0

Region: NODE\_273527\_length\_1611\_cov\_28.567970 867-870. Max. coverage (+): 0. Max coverage (-): 0

Region: NODE\_273527\_length\_1611\_cov\_28.567970 871-873. Max. coverage (+): 0.33. Max coverage (-): 0

Region: NODE\_273527\_length\_1611\_cov\_28.567970 874-877. Max. coverage (+): 0.37. Max coverage (-): 0.04

Region: NODE\_273527\_length\_1611\_cov\_28.567970 878-880. Max. coverage (+): 0.3. Max coverage (-): 0.04

Region: NODE\_273527\_length\_1611\_cov\_28.567970 881-883. Max. coverage (+): 0.07. Max coverage (-): 0

Region: NODE\_273527\_length\_1611\_cov\_28.567970 884-887. Max. coverage (+): 1.04. Max coverage (-): 0

Region: NODE\_273527\_length\_1611\_cov\_28.567970 888-890. Max. coverage (+): 1. Max coverage (-): 0

Region: NODE\_273527\_length\_1611\_cov\_28.567970 891-893. Max. coverage (+): 0.04. Max coverage (-): 0

Region: NODE\_273527\_length\_1611\_cov\_28.567970 894-897. Max. coverage (+): 0. Max coverage (-): 0

Region: NODE\_273527\_length\_1611\_cov\_28.567970 898-900. Max. coverage (+): 0. Max coverage (-): 0

Region: NODE\_273527\_length\_1611\_cov\_28.567970 901-903. Max. coverage (+): 0. Max coverage (-): 0

Region: NODE\_273527\_length\_1611\_cov\_28.567970 904-907. Max. coverage (+): 0. Max coverage (-): 0

Region: NODE\_273527\_length\_1611\_cov\_28.567970 908-910. Max. coverage (+): 0. Max coverage (-): 0

Region: NODE\_273527\_length\_1611\_cov\_28.567970 911-913. Max. coverage (+): 0. Max coverage (-): 1.52

Region: NODE\_273527\_length\_1611\_cov\_28.567970 914-917. Max. coverage (+): 0.04. Max coverage (-): 1.56

Region: NODE\_273527\_length\_1611\_cov\_28.567970 918-920. Max. coverage (+): 0.04. Max coverage (-): 0.22

Region: NODE\_273527\_length\_1611\_cov\_28.567970 921-923. Max. coverage (+): 0.07. Max coverage (-): 0.22

Region: NODE\_273527\_length\_1611\_cov\_28.567970 924-927. Max. coverage (+): 0.89. Max coverage (-): 0.04

Region: NODE\_273527\_length\_1611\_cov\_28.567970 928-930. Max. coverage (+): 1.93. Max coverage (-): 0.07

Region: NODE\_273527\_length\_1611\_cov\_28.567970 931-933. Max. coverage (+): 3.23. Max coverage (-): 0.15

Region: NODE\_273527\_length\_1611\_cov\_28.567970 934-937. Max. coverage (+): 0.93. Max coverage (-): 0.15

Region: NODE\_273527\_length\_1611\_cov\_28.567970 938-940. Max. coverage (+): 1.22. Max coverage (-): 0

Region: NODE\_273527\_length\_1611\_cov\_28.567970 941-944. Max. coverage (+): 0.56. Max coverage (-): 0

Region: NODE\_273527\_length\_1611\_cov\_28.567970 945-947. Max. coverage (+): 0.93. Max coverage (-): 0

Region: NODE\_273527\_length\_1611\_cov\_28.567970 948-950. Max. coverage (+): 0.22. Max coverage (-): 0

Region: NODE\_273527\_length\_1611\_cov\_28.567970 951-954. Max. coverage (+): 0.07. Max coverage (-): 0

Region: NODE\_273527\_length\_1611\_cov\_28.567970 955-957. Max. coverage (+): 0.04. Max coverage (-): 0

Region: NODE\_273527\_length\_1611\_cov\_28.567970 958-960. Max. coverage (+): 1.56. Max coverage (-): 0

Region: NODE\_273527\_length\_1611\_cov\_28.567970 961-964. Max. coverage (+): 1.78. Max coverage (-): 0

Region: NODE\_273527\_length\_1611\_cov\_28.567970 965-967. Max. coverage (+): 0.26. Max coverage (-): 0

Region: NODE\_273527\_length\_1611\_cov\_28.567970 968-970. Max. coverage (+): 0.2. Max coverage (-): 0

Region: NODE\_273527\_length\_1611\_cov\_28.567970 971-974. Max. coverage (+): 0. Max coverage (-): 0

Region: NODE\_273527\_length\_1611\_cov\_28.567970 975-977. Max. coverage (+): 0. Max coverage (-): 0

Region: NODE\_273527\_length\_1611\_cov\_28.567970 978-980. Max. coverage (+): 0. Max coverage (-): 0

Region: NODE\_273527\_length\_1611\_cov\_28.567970 981-984. Max. coverage (+): 0.48. Max coverage (-): 0

Region: NODE\_273527\_length\_1611\_cov\_28.567970 985-987. Max. coverage (+): 0.48. Max coverage (-): 0.04

Region: NODE\_273527\_length\_1611\_cov\_28.567970 988-990. Max. coverage (+): 0.26. Max coverage (-): 0.04

Region: NODE\_273527\_length\_1611\_cov\_28.567970 991-994. Max. coverage (+): 0.04. Max coverage (-): 0

Region: NODE\_273527\_length\_1611\_cov\_28.567970 995-997. Max. coverage (+): 0. Max coverage (-): 0

Region: NODE\_273527\_length\_1611\_cov\_28.567970 998-1000. Max. coverage (+): 0. Max coverage (-): 0

Region: NODE\_273527\_length\_1611\_cov\_28.567970 1001-1004. Max. coverage (+): 0. Max coverage (-): 0

Region: NODE\_273527\_length\_1611\_cov\_28.567970 1005-1007. Max. coverage (+): 0. Max coverage (-): 0

Region: NODE\_273527\_length\_1611\_cov\_28.567970 1008-1011. Max. coverage (+): 0.04. Max coverage (-): 0

Region: NODE\_273527\_length\_1611\_cov\_28.567970 1012-1014. Max. coverage (+): 0.04. Max coverage (-): 0

Region: NODE\_273527\_length\_1611\_cov\_28.567970 1015-1017. Max. coverage (+): 0.22. Max coverage (-): 0

Region: NODE\_273527\_length\_1611\_cov\_28.567970 1018-1021. Max. coverage (+): 0.22. Max coverage (-): 0.7

Region: NODE\_273527\_length\_1611\_cov\_28.567970 1022-1024. Max. coverage (+): 0.33. Max coverage (-): 0.11

Region: NODE\_273527\_length\_1611\_cov\_28.567970 1025-1027. Max. coverage (+): 0.22. Max coverage (-): 0.07

Region: NODE\_273527\_length\_1611\_cov\_28.567970 1028-1031. Max. coverage (+): 0.22. Max coverage (-): 0.07

Region: NODE\_273527\_length\_1611\_cov\_28.567970 1032-1034. Max. coverage (+): 0.3. Max coverage (-): 0.07

Region: NODE\_273527\_length\_1611\_cov\_28.567970 1035-1037. Max. coverage (+): 0.07. Max coverage (-): 0.11

Region: NODE\_273527\_length\_1611\_cov\_28.567970 1038-1041. Max. coverage (+): 0.07. Max coverage (-): 0.15

Region: NODE\_273527\_length\_1611\_cov\_28.567970 1042-1044. Max. coverage (+): 0.15. Max coverage (-): 0.15

Region: NODE\_273527\_length\_1611\_cov\_28.567970 1045-1047. Max. coverage (+): 1.45. Max coverage (-): 0.15

Region: NODE\_273527\_length\_1611\_cov\_28.567970 1048-1051. Max. coverage (+): 2.67. Max coverage (-): 0

Region: NODE\_273527\_length\_1611\_cov\_28.567970 1052-1054. Max. coverage (+): 4.56. Max coverage (-): 0

Region: NODE\_273527\_length\_1611\_cov\_28.567970 1055-1057. Max. coverage (+): 4.08. Max coverage (-): 0.19

Region: NODE\_273527\_length\_1611\_cov\_28.567970 1058-1061. Max. coverage (+): 0.11. Max coverage (-): 0.19

Region: NODE\_273527\_length\_1611\_cov\_28.567970 1062-1064. Max. coverage (+): 0.22. Max coverage (-): 0.04

Region: NODE\_273527\_length\_1611\_cov\_28.567970 1065-1067. Max. coverage (+): 0.11. Max coverage (-): 0.02

Region: NODE\_273527\_length\_1611\_cov\_28.567970 1068-1071. Max. coverage (+): 0.01. Max coverage (-): 0.05

Region: NODE\_273527\_length\_1611\_cov\_28.567970 1072-1074. Max. coverage (+): 0.01. Max coverage (-): 0.08

Region: NODE\_273527\_length\_1611\_cov\_28.567970 1075-1078. Max. coverage (+): 0.06. Max coverage (-): 0.05

Region: NODE\_273527\_length\_1611\_cov\_28.567970 1079-1081. Max. coverage (+): 0.06. Max coverage (-): 0

Region: NODE\_273527\_length\_1611\_cov\_28.567970 1082-1084. Max. coverage (+): 0.02. Max coverage (-): 0

Region: NODE\_273527\_length\_1611\_cov\_28.567970 1085-1088. Max. coverage (+): 0.5. Max coverage (-): 0.02

Region: NODE\_273527\_length\_1611\_cov\_28.567970 1089-1091. Max. coverage (+): 0.11. Max coverage (-): 0.02

Region: NODE\_273527\_length\_1611\_cov\_28.567970 1092-1094. Max. coverage (+): 0.15. Max coverage (-): 0.02

Region: NODE\_273527\_length\_1611\_cov\_28.567970 1095-1098. Max. coverage (+): 1. Max coverage (-): 0

Region: NODE\_273527\_length\_1611\_cov\_28.567970 1099-1101. Max. coverage (+): 0.56. Max coverage (-): 0

Region: NODE\_273527\_length\_1611\_cov\_28.567970 1102-1104. Max. coverage (+): 1.37. Max coverage (-): 0.04

Region: NODE\_273527\_length\_1611\_cov\_28.567970 1105-1108. Max. coverage (+): 0.89. Max coverage (-): 0.04

Region: NODE\_273527\_length\_1611\_cov\_28.567970 1109-1111. Max. coverage (+): 0.48. Max coverage (-): 0.11

Region: NODE\_273527\_length\_1611\_cov\_28.567970 1112-1114. Max. coverage (+): 0.11. Max coverage (-): 0.19

Region: NODE\_273527\_length\_1611\_cov\_28.567970 1115-1118. Max. coverage (+): 0.89. Max coverage (-): 1.26

Region: NODE\_273527\_length\_1611\_cov\_28.567970 1119-1121. Max. coverage (+): 0.85. Max coverage (-): 1.33

Region: NODE\_273527\_length\_1611\_cov\_28.567970 1122-1124. Max. coverage (+): 0.11. Max coverage (-): 0.33

Region: NODE\_273527\_length\_1611\_cov\_28.567970 1125-1128. Max. coverage (+): 0.07. Max coverage (-): 0.3

Region: NODE\_273527\_length\_1611\_cov\_28.567970 1129-1131. Max. coverage (+): 2. Max coverage (-): 0.19

Region: NODE\_273527\_length\_1611\_cov\_28.567970 1132-1134. Max. coverage (+): 1.93. Max coverage (-): 0.04

Region: NODE\_273527\_length\_1611\_cov\_28.567970 1135-1138. Max. coverage (+): 17.13. Max coverage (-): 0

Region: NODE\_273527\_length\_1611\_cov\_28.567970 1139-1141. Max. coverage (+): 17.57. Max coverage (-): 0.11

Region: NODE\_273527\_length\_1611\_cov\_28.567970 1142-1145. Max. coverage (+): 1.89. Max coverage (-): 0.22

Region: NODE\_273527\_length\_1611\_cov\_28.567970 1146-1148. Max. coverage (+): 7.16. Max coverage (-): 0.22

Region: NODE\_273527\_length\_1611\_cov\_28.567970 1149-1151. Max. coverage (+): 7.75. Max coverage (-): 0.07

Region: NODE\_273527\_length\_1611\_cov\_28.567970 1152-1155. Max. coverage (+): 1.74. Max coverage (-): 0.09

Region: NODE\_273527\_length\_1611\_cov\_28.567970 1156-1158. Max. coverage (+): 0.39. Max coverage (-): 0.07

Region: NODE\_273527\_length\_1611\_cov\_28.567970 1159-1161. Max. coverage (+): 4. Max coverage (-): 0.04

Region: NODE\_273527\_length\_1611\_cov\_28.567970 1162-1165. Max. coverage (+): 9.19. Max coverage (-): 0.07

Region: NODE\_273527\_length\_1611\_cov\_28.567970 1166-1168. Max. coverage (+): 13.74. Max coverage (-): 0.07

Region: NODE\_273527\_length\_1611\_cov\_28.567970 1169-1171. Max. coverage (+): 6.47. Max coverage (-): 0.06

Region: NODE\_273527\_length\_1611\_cov\_28.567970 1172-1175. Max. coverage (+): 0.02. Max coverage (-): 0.06

Region: NODE\_273527\_length\_1611\_cov\_28.567970 1176-1178. Max. coverage (+): 0. Max coverage (-): 0.13

Region: NODE\_273527\_length\_1611\_cov\_28.567970 1179-1181. Max. coverage (+): 0.11. Max coverage (-): 0.15

Region: NODE\_273527\_length\_1611\_cov\_28.567970 1182-1185. Max. coverage (+): 0.15. Max coverage (-): 0.06

Region: NODE\_273527\_length\_1611\_cov\_28.567970 1186-1188. Max. coverage (+): 0.35. Max coverage (-): 0.04

Region: NODE\_273527\_length\_1611\_cov\_28.567970 1189-1191. Max. coverage (+): 1.13. Max coverage (-): 0

Region: NODE\_273527\_length\_1611\_cov\_28.567970 1192-1195. Max. coverage (+): 1.08. Max coverage (-): 0.22

Region: NODE\_273527\_length\_1611\_cov\_28.567970 1196-1198. Max. coverage (+): 0.11. Max coverage (-): 0.22

Region: NODE\_273527\_length\_1611\_cov\_28.567970 1199-1201. Max. coverage (+): 0.24. Max coverage (-): 0.02

Region: NODE\_273527\_length\_1611\_cov\_28.567970 1202-1205. Max. coverage (+): 0.24. Max coverage (-): 0.06

Region: NODE\_273527\_length\_1611\_cov\_28.567970 1206-1208. Max. coverage (+): 0.04. Max coverage (-): 0.07

Region: NODE\_273527\_length\_1611\_cov\_28.567970 1209-1212. Max. coverage (+): 0.3. Max coverage (-): 0.07

Region: NODE\_273527\_length\_1611\_cov\_28.567970 1213-1215. Max. coverage (+): 0.11. Max coverage (-): 0

Region: NODE\_273527\_length\_1611\_cov\_28.567970 1216-1218. Max. coverage (+): 0.07. Max coverage (-): 0.11

Region: NODE\_273527\_length\_1611\_cov\_28.567970 1219-1222. Max. coverage (+): 0.07. Max coverage (-): 0.11

Region: NODE\_273527\_length\_1611\_cov\_28.567970 1223-1225. Max. coverage (+): 0.07. Max coverage (-): 0

Region: NODE\_273527\_length\_1611\_cov\_28.567970 1226-1228. Max. coverage (+): 0.04. Max coverage (-): 0

Region: NODE\_273527\_length\_1611\_cov\_28.567970 1229-1232. Max. coverage (+): 0.04. Max coverage (-): 0

Region: NODE\_273527\_length\_1611\_cov\_28.567970 1233-1235. Max. coverage (+): 0.15. Max coverage (-): 0

Region: NODE\_273527\_length\_1611\_cov\_28.567970 1236-1238. Max. coverage (+): 0.13. Max coverage (-): 0

Region: NODE\_273527\_length\_1611\_cov\_28.567970 1239-1242. Max. coverage (+): 0.72. Max coverage (-): 0.09

Region: NODE\_273527\_length\_1611\_cov\_28.567970 1243-1245. Max. coverage (+): 0.87. Max coverage (-): 0.07

Region: NODE\_273527\_length\_1611\_cov\_28.567970 1246-1248. Max. coverage (+): 0.33. Max coverage (-): 0.04

Region: NODE\_273527\_length\_1611\_cov\_28.567970 1249-1252. Max. coverage (+): 0.26. Max coverage (-): 0.04

Region: NODE\_273527\_length\_1611\_cov\_28.567970 1253-1255. Max. coverage (+): 0.82. Max coverage (-): 0.11

Region: NODE\_273527\_length\_1611\_cov\_28.567970 1256-1258. Max. coverage (+): 0.85. Max coverage (-): 0.11

Region: NODE\_273527\_length\_1611\_cov\_28.567970 1259-1262. Max. coverage (+): 12.79. Max coverage (-): 0.11

Region: NODE\_273527\_length\_1611\_cov\_28.567970 1263-1265. Max. coverage (+): 60.28. Max coverage (-): 0.04

Region: NODE\_273527\_length\_1611\_cov\_28.567970 1266-1268. Max. coverage (+): 51.79. Max coverage (-): 0.07

Region: NODE\_273527\_length\_1611\_cov\_28.567970 1269-1272. Max. coverage (+): 3.19. Max coverage (-): 0.07

Region: NODE\_273527\_length\_1611\_cov\_28.567970 1273-1275. Max. coverage (+): 0.09. Max coverage (-): 0.02

Region: NODE\_273527\_length\_1611\_cov\_28.567970 1276-1279. Max. coverage (+): 0.2. Max coverage (-): 0.02

Region: NODE\_273527\_length\_1611\_cov\_28.567970 1280-1282. Max. coverage (+): 0.17. Max coverage (-): 0.02

Region: NODE\_273527\_length\_1611\_cov\_28.567970 1283-1285. Max. coverage (+): 0.17. Max coverage (-): 0

Region: NODE\_273527\_length\_1611\_cov\_28.567970 1286-1289. Max. coverage (+): 0.52. Max coverage (-): 0

Region: NODE\_273527\_length\_1611\_cov\_28.567970 1290-1292. Max. coverage (+): 0. Max coverage (-): 0

Region: NODE\_273527\_length\_1611\_cov\_28.567970 1293-1295. Max. coverage (+): 0.04. Max coverage (-): 0

Region: NODE\_273527\_length\_1611\_cov\_28.567970 1296-1299. Max. coverage (+): 1.15. Max coverage (-): 0

Region: NODE\_273527\_length\_1611\_cov\_28.567970 1300-1302. Max. coverage (+): 1.2. Max coverage (-): 0

Region: NODE\_273527\_length\_1611\_cov\_28.567970 1303-1305. Max. coverage (+): 0.57. Max coverage (-): 0

Region: NODE\_273527\_length\_1611\_cov\_28.567970 1306-1309. Max. coverage (+): 0.04. Max coverage (-): 0

Region: NODE\_273527\_length\_1611\_cov\_28.567970 1310-1312. Max. coverage (+): 0. Max coverage (-): 0

Region: NODE\_273527\_length\_1611\_cov\_28.567970 1313-1315. Max. coverage (+): 0. Max coverage (-): 0.33

Region: NODE\_273527\_length\_1611\_cov\_28.567970 1316-1319. Max. coverage (+): 0. Max coverage (-): 0.33

Region: NODE\_273527\_length\_1611\_cov\_28.567970 1320-1322. Max. coverage (+): 0.04. Max coverage (-): 0.48

Region: NODE\_273527\_length\_1611\_cov\_28.567970 1323-1325. Max. coverage (+): 1.56. Max coverage (-): 0.37

Region: NODE\_273527\_length\_1611\_cov\_28.567970 1326-1329. Max. coverage (+): 1.78. Max coverage (-): 0.48

Region: NODE\_273527\_length\_1611\_cov\_28.567970 1330-1332. Max. coverage (+): 0.11. Max coverage (-): 0.04

Region: NODE\_273527\_length\_1611\_cov\_28.567970 1333-1335. Max. coverage (+): 2.04. Max coverage (-): 0.04

Region: NODE\_273527\_length\_1611\_cov\_28.567970 1336-1339. Max. coverage (+): 2.11. Max coverage (-): 0.06

Region: NODE\_273527\_length\_1611\_cov\_28.567970 1340-1342. Max. coverage (+): 0. Max coverage (-): 0.07

Region: NODE\_273527\_length\_1611\_cov\_28.567970 1343-1346. Max. coverage (+): 0. Max coverage (-): 0.02

Region: NODE\_273527\_length\_1611\_cov\_28.567970 1347-1349. Max. coverage (+): 0.02. Max coverage (-): 0

Region: NODE\_273527\_length\_1611\_cov\_28.567970 1350-1352. Max. coverage (+): 0.02. Max coverage (-): 0

Region: NODE\_273527\_length\_1611\_cov\_28.567970 1353-1356. Max. coverage (+): 0.11. Max coverage (-): 0

Region: NODE\_273527\_length\_1611\_cov\_28.567970 1357-1359. Max. coverage (+): 0.54. Max coverage (-): 0

Region: NODE\_273527\_length\_1611\_cov\_28.567970 1360-1362. Max. coverage (+): 1.37. Max coverage (-): 0

Region: NODE\_273527\_length\_1611\_cov\_28.567970 1363-1366. Max. coverage (+): 3.67. Max coverage (-): 0

Region: NODE\_273527\_length\_1611\_cov\_28.567970 1367-1369. Max. coverage (+): 3. Max coverage (-): 0

Region: NODE\_273527\_length\_1611\_cov\_28.567970 1370-1372. Max. coverage (+): 1.08. Max coverage (-): 0

Region: NODE\_273527\_length\_1611\_cov\_28.567970 1373-1376. Max. coverage (+): 0.93. Max coverage (-): 0.02

Region: NODE\_273527\_length\_1611\_cov\_28.567970 1377-1379. Max. coverage (+): 0.28. Max coverage (-): 0.02

Region: NODE\_273527\_length\_1611\_cov\_28.567970 1380-1382. Max. coverage (+): 0.07. Max coverage (-): 0

Region: NODE\_273527\_length\_1611\_cov\_28.567970 1383-1386. Max. coverage (+): 0.03. Max coverage (-): 0

Region: NODE\_273527\_length\_1611\_cov\_28.567970 1387-1389. Max. coverage (+): 0.05. Max coverage (-): 0.02

Region: NODE\_273527\_length\_1611\_cov\_28.567970 1390-1392. Max. coverage (+): 0.37. Max coverage (-): 0.04

Region: NODE\_273527\_length\_1611\_cov\_28.567970 1393-1396. Max. coverage (+): 15.29. Max coverage (-): 0.02

Region: NODE\_273527\_length\_1611\_cov\_28.567970 1397-1399. Max. coverage (+): 28.25. Max coverage (-): 0

Region: NODE\_273527\_length\_1611\_cov\_28.567970 1400-1402. Max. coverage (+): 16.48. Max coverage (-): 0

Region: NODE\_273527\_length\_1611\_cov\_28.567970 1403-1406. Max. coverage (+): 0.02. Max coverage (-): 0

Region: NODE\_273527\_length\_1611\_cov\_28.567970 1407-1409. Max. coverage (+): 0.39. Max coverage (-): 0

Region: NODE\_273527\_length\_1611\_cov\_28.567970 1410-1413. Max. coverage (+): 0.46. Max coverage (-): 0.04

Region: NODE\_273527\_length\_1611\_cov\_28.567970 1414-1416. Max. coverage (+): 0.09. Max coverage (-): 0.04

Region: NODE\_273527\_length\_1611\_cov\_28.567970 1417-1419. Max. coverage (+): 0. Max coverage (-): 0

Region: NODE\_273527\_length\_1611\_cov\_28.567970 1420-1423. Max. coverage (+): 0.04. Max coverage (-): 0.04

Region: NODE\_273527\_length\_1611\_cov\_28.567970 1424-1426. Max. coverage (+): 0.04. Max coverage (-): 0.04

Region: NODE\_273527\_length\_1611\_cov\_28.567970 1427-1429. Max. coverage (+): 1.06. Max coverage (-): 0

Region: NODE\_273527\_length\_1611\_cov\_28.567970 1430-1433. Max. coverage (+): 1.08. Max coverage (-): 0

Region: NODE\_273527\_length\_1611\_cov\_28.567970 1434-1436. Max. coverage (+): 0.11. Max coverage (-): 0

Region: NODE\_273527\_length\_1611\_cov\_28.567970 1437-1439. Max. coverage (+): 0.02. Max coverage (-): 0

Region: NODE\_273527\_length\_1611\_cov\_28.567970 1440-1443. Max. coverage (+): 0.07. Max coverage (-): 0

Region: NODE\_273527\_length\_1611\_cov\_28.567970 1444-1446. Max. coverage (+): 0.07. Max coverage (-): 0

Region: NODE\_273527\_length\_1611\_cov\_28.567970 1447-1449. Max. coverage (+): 0.11. Max coverage (-): 0

Region: NODE\_273527\_length\_1611\_cov\_28.567970 1450-1453. Max. coverage (+): 0.3. Max coverage (-): 0

Region: NODE\_273527\_length\_1611\_cov\_28.567970 1454-1456. Max. coverage (+): 0.22. Max coverage (-): 0

Region: NODE\_273527\_length\_1611\_cov\_28.567970 1457-1459. Max. coverage (+): 0.02. Max coverage (-): 0.07

Region: NODE\_273527\_length\_1611\_cov\_28.567970 1460-1463. Max. coverage (+): 0.04. Max coverage (-): 0.07

Region: NODE\_273527\_length\_1611\_cov\_28.567970 1464-1466. Max. coverage (+): 0.07. Max coverage (-): 0

Region: NODE\_273527\_length\_1611\_cov\_28.567970 1467-1469. Max. coverage (+): 0.06. Max coverage (-): 0.02

Region: NODE\_273527\_length\_1611\_cov\_28.567970 1470-1473. Max. coverage (+): 0.17. Max coverage (-): 0.02

Region: NODE\_273527\_length\_1611\_cov\_28.567970 1474-1476. Max. coverage (+): 0.02. Max coverage (-): 0

Region: NODE\_273527\_length\_1611\_cov\_28.567970 1477-1480. Max. coverage (+): 0. Max coverage (-): 0.07

Region: NODE\_273527\_length\_1611\_cov\_28.567970 1481-1483. Max. coverage (+): 0.06. Max coverage (-): 0.2

Region: NODE\_273527\_length\_1611\_cov\_28.567970 1484-1486. Max. coverage (+): 0.09. Max coverage (-): 0.17

Region: NODE\_273527\_length\_1611\_cov\_28.567970 1487-1490. Max. coverage (+): 0.2. Max coverage (-): 0.11

Region: NODE\_273527\_length\_1611\_cov\_28.567970 1491-1493. Max. coverage (+): 0.32. Max coverage (-): 0

Region: NODE\_273527\_length\_1611\_cov\_28.567970 1494-1496. Max. coverage (+): 6.28. Max coverage (-): 0

Region: NODE\_273527\_length\_1611\_cov\_28.567970 1497-1500. Max. coverage (+): 6.3. Max coverage (-): 0.02

Region: NODE\_273527\_length\_1611\_cov\_28.567970 1501-1503. Max. coverage (+): 1.37. Max coverage (-): 0.02

Region: NODE\_273527\_length\_1611\_cov\_28.567970 1504-1506. Max. coverage (+): 0.19. Max coverage (-): 0.02

Region: NODE\_273527\_length\_1611\_cov\_28.567970 1507-1510. Max. coverage (+): 0.09. Max coverage (-): 0.02

Region: NODE\_273527\_length\_1611\_cov\_28.567970 1511-1513. Max. coverage (+): 0.02. Max coverage (-): 0

Region: NODE\_273527\_length\_1611\_cov\_28.567970 1514-1516. Max. coverage (+): 0.02. Max coverage (-): 0

Region: NODE\_273527\_length\_1611\_cov\_28.567970 1517-1520. Max. coverage (+): 0.02. Max coverage (-): 0

Region: NODE\_273527\_length\_1611\_cov\_28.567970 1521-1523. Max. coverage (+): 0. Max coverage (-): 0

Region: NODE\_273527\_length\_1611\_cov\_28.567970 1524-1526. Max. coverage (+): 0. Max coverage (-): 0

Region: NODE\_273527\_length\_1611\_cov\_28.567970 1527-1530. Max. coverage (+): 0.02. Max coverage (-): 0

Region: NODE\_273527\_length\_1611\_cov\_28.567970 1531-1533. Max. coverage (+): 0. Max coverage (-): 0

Region: NODE\_273527\_length\_1611\_cov\_28.567970 1534-1536. Max. coverage (+): 0. Max coverage (-): 0

Region: NODE\_273527\_length\_1611\_cov\_28.567970 1537-1540. Max. coverage (+): 0. Max coverage (-): 0

Region: NODE\_273527\_length\_1611\_cov\_28.567970 1541-1543. Max. coverage (+): 0. Max coverage (-): 0

Region: NODE\_273527\_length\_1611\_cov\_28.567970 1544-1547. Max. coverage (+): 0. Max coverage (-): 0

Region: NODE\_273527\_length\_1611\_cov\_28.567970 1548-1550. Max. coverage (+): 0. Max coverage (-): 0

Region: NODE\_273527\_length\_1611\_cov\_28.567970 1551-1553. Max. coverage (+): 0. Max coverage (-): 0.15

Region: NODE\_273527\_length\_1611\_cov\_28.567970 1554-1557. Max. coverage (+): 0.04. Max coverage (-): 0.15

Region: NODE\_273527\_length\_1611\_cov\_28.567970 1558-1560. Max. coverage (+): 1.67. Max coverage (-): 0.04

Region: NODE\_273527\_length\_1611\_cov\_28.567970 1561-1563. Max. coverage (+): 6.82. Max coverage (-): 0.04

Region: NODE\_273527\_length\_1611\_cov\_28.567970 1564-1567. Max. coverage (+): 6.08. Max coverage (-): 0

Region: NODE\_273527\_length\_1611\_cov\_28.567970 1568-1570. Max. coverage (+): 2.26. Max coverage (-): 0.11

Region: NODE\_273527\_length\_1611\_cov\_28.567970 1571-1573. Max. coverage (+): 1.96. Max coverage (-): 0.24

Region: NODE\_273527\_length\_1611\_cov\_28.567970 1574-1577. Max. coverage (+): 1.28. Max coverage (-): 0.26

Region: NODE\_273527\_length\_1611\_cov\_28.567970 1578-1580. Max. coverage (+): 1.24. Max coverage (-): 0.15

Region: NODE\_273527\_length\_1611\_cov\_28.567970 1581-1583. Max. coverage (+): 0.15. Max coverage (-): 0.19

Region: NODE\_273527\_length\_1611\_cov\_28.567970 1584-1587. Max. coverage (+): 0.15. Max coverage (-): 0.37

Region: NODE\_273527\_length\_1611\_cov\_28.567970 1588-1590. Max. coverage (+): 0. Max coverage (-): 0.32

Region: NODE\_273527\_length\_1611\_cov\_28.567970 1591-1593. Max. coverage (+): 0. Max coverage (-): 0.22

Region: NODE\_273527\_length\_1611\_cov\_28.567970 1594-1597. Max. coverage (+): 0.48. Max coverage (-): 0.04

Region: NODE\_273527\_length\_1611\_cov\_28.567970 1598-1600. Max. coverage (+): 2.19. Max coverage (-): 0

Region: NODE\_273527\_length\_1611\_cov\_28.567970 1601-1603. Max. coverage (+): 3.56. Max coverage (-): 0

Region: NODE\_273527\_length\_1611\_cov\_28.567970 1604-1607. Max. coverage (+): 10.64. Max coverage (-): 0.04

Region: NODE\_273527\_length\_1611\_cov\_28.567970 1608-1610. Max. coverage (+): 10.6. Max coverage (-): 0.3

Region: NODE\_273527\_length\_1611\_cov\_28.567970 1611-1614. Max. coverage (+): 0.56. Max coverage (-): 0.32

Region: NODE\_273527\_length\_1611\_cov\_28.567970 1615-1617. Max. coverage (+): 0. Max coverage (-): 0.22

Region: NODE\_273527\_length\_1611\_cov\_28.567970 1618-1620. Max. coverage (+): 0. Max coverage (-): 0.46

Region: NODE\_273527\_length\_1611\_cov\_28.567970 1621-1624. Max. coverage (+): 0.02. Max coverage (-): 0.33

Region: NODE\_273527\_length\_1611\_cov\_28.567970 1625-1627. Max. coverage (+): 0.06. Max coverage (-): 0.04

Region: NODE\_273527\_length\_1611\_cov\_28.567970 1628-1630. Max. coverage (+): 0.11. Max coverage (-): 0.06

Region: NODE\_273527\_length\_1611\_cov\_28.567970 1631-1634. Max. coverage (+): 0.61. Max coverage (-): 0.06

Region: NODE\_273527\_length\_1611\_cov\_28.567970 1635-1637. Max. coverage (+): 0.7. Max coverage (-): 0.04

Region: NODE\_273527\_length\_1611\_cov\_28.567970 1638-1640. Max. coverage (+): 0.35. Max coverage (-): 0.04

Region: NODE\_273527\_length\_1611\_cov\_28.567970 1641-1644. Max. coverage (+): 0.35. Max coverage (-): 0.11

Region: NODE\_273527\_length\_1611\_cov\_28.567970 1645-1647. Max. coverage (+): 0.3. Max coverage (-): 0.06

Region: NODE\_273527\_length\_1611\_cov\_28.567970 1648-1650. Max. coverage (+): 0.32. Max coverage (-): 0

Region: NODE\_273527\_length\_1611\_cov\_28.567970 1651-1654. Max. coverage (+): 0.06. Max coverage (-): 0

Region: NODE\_273527\_length\_1611\_cov\_28.567970 1655-1657. Max. coverage (+): 0. Max coverage (-): 0

Region: NODE\_273527\_length\_1611\_cov\_28.567970 1658-1660. Max. coverage (+): 0. Max coverage (-): 0

Region: NODE\_273527\_length\_1611\_cov\_28.567970 1661-1664. Max. coverage (+): 0. Max coverage (-): 0

Region: NODE\_273527\_length\_1611\_cov\_28.567970 1665-1667. Max. coverage (+): 0. Max coverage (-): 0

Region: NODE\_273527\_length\_1611\_cov\_28.567970 1668-1670. Max. coverage (+): 0. Max coverage (-): 0

Region: NODE\_273527\_length\_1611\_cov\_28.567970 1671-1674. Max. coverage (+): 0. Max coverage (-): 0

Region: NODE\_273527\_length\_1611\_cov\_28.567970 1675-. Max. coverage (+): 0. Max coverage (-): 0

RepeatMasker Color Code

**+**

100-98% Identity

<98-95% Identity

<95-90% Identity

<90-85% Identity

<85-80% Identity

<80-75% Identity

<75-70% Identity

<70% Identity

**-**

Gene Set Color Code

**+**

Gene

Pseudogene

Other

**-**

Topology/Coverage Color Code

Coverage Plus Strand

Coverage Minus Strand

Mainstrand: Plus

Mainstrand: Minus

Complementary Strand

Flanking Region  
(if option -flank >0)

Gene Set Annotation  
  
RepeatMasker Annotation  

**1. AlRepD-82**: 298-850 (+), Divergence to consensus: 15.7%

  
Transcription Factor Binding Sites  

**RHOXF1** (Sequence: AGATTA (-): 252)  
**RHOXF1** (Sequence: AGCTCA (-): 1171)  
**RHOXF1** (Sequence: AGATTA (-): 1342)  
**RHOXF1** (Sequence: AGCTTA (-): 1455)  
**RHOXF1** (Sequence: AGATCA (-): 1614)  
**RHOXF1** (Sequence: TAATCT (+): 786)  
**Lhx8** (Sequence: TTAATTAA (-): 263)  
**SOX9** (Sequence: AACAATAA (-): 578)  
**FOXO1** (Sequence: CTTGTTTTC (+): 867)  
**FOXO3\_mmu** (Sequence: TCAAAACA (+): 1370)  
**SPZ1** (Sequence: AGGGTTACAG (+): 1057)  
**Nobox** (Sequence: TAATTGCT (+): 366)  
**Sox5** (Sequence: AACAAT (-): 578)  
**POU2F1** (Sequence: TATGTAAAT (+): 1233)  
**POU5F1** (Sequence: ATGCAAA (+): 1111)  
**POU5F1** (Sequence: ATGCAAA (+): 1608)
