# Supplementary material for: First characterization of PIWI-interacting RNA clusters in a cichlid fish with a B chromosome
Source: BMC Biol. 2022 Sep 21;20:204. doi: 10.1186/s12915-022-01403-2 (PMC9490952; doi:10.1186/s12915-022-01403-2)
Supplement: Supplementary file 1 — Additional file 1. Zipped folder with fasta and interactive html piRNA cluster information for the A. latifasciata genome. The nomenclature is as follows: number-pirna-cluster_sex_B-presence (f, female; m, male; 0b, without B chromosome; 1b, with B chromosome). [file 12915_2022_1403_MOESM1_ESM.zip › 106_f0b.html]

piRNA cluster 106\_f0b 10


Predicted piRNA cluster no. 106\_f0b
  

Show proTRAC run info
Hide proTRAC run info

/\  
                \_\_\_\_\_\_\_\_\_\_\_\_\_\_\_\_\_\_\_\_\_\_\_/\\_\_\_ /  \\_\_\_\_\_\_\_  
               I                      /  \  /    \      I  
               I     pro             /    \/      \     I  
               I        TRAC        /               \   I  
               I   \_\_\_\_\_\_\_\_\_\_\_\_\_\_\_\_/\_\_\_\_\_\_\_\_\_\_\_\_\_\_\_\_\_\\_ I  
               I   \              /                     I  
               I    \            /                      I  
               I     \  /\      /       V.2.4.2         I  
               I      \/  \    /                        I  
               I\_\_\_\_\_\_\_\_\_\_\_\  /\_\_\_\_\_\_\_\_\_\_\_\_\_\_\_\_\_\_\_\_\_\_\_\_\_I  
                            \/  
  
  
================================= proTRAC ====================================  
VERSION: .......... 2.4.2  
LAST MODIFIED: .... 11. May 2018  
  
Please cite:  
Rosenkranz D, Zischler H. proTRAC - a software for probabilistic piRNA cluster  
detection, visualization and analysis. 2012. BMC Bioinformatics 13:5.  
  
  
Contact:  
David Rosenkranz  
Institute of Organismic and Molecular Evolutionary Biology  
Dept. Anthropology, small RNA group  
Johannes Gutenberg University Mainz  
email: rosenkranz@uni-mainz.de  
  
You can find the latest proTRAC version at:  
http://sourceforge.net/projects/protrac/files  
http://www.smallRNAgroup-mainz.de/software  
==============================================================================  
  
PARAMETERS:  
Map file: ...............piwi-femeas-0B.fa-collapse.map  
Genome file: ............../../../0B\_ala\_genome.fa  
RepeatMasker annotation: Alatifasciata-all0B-maryan-v2.fa\_corrected.out  
GeneSet:................./guest-storage/Data/annotation/Alatifasciata\_all0B\_maryan-v2\_out2017.gff  
  
Significant (p<=0.01) hit density will be calculated based  
on observed hit distribution.  
  
Sliding window size: ........................................ 5000 bp  
Sliding window increament: .................................. 1000 bp  
Normalize each hit by number of genomic hits: ............... yes  
Normalize each hit by number of sequence reads: ............. yes  
Normalize values (-> per million mapped reads): ............. yes  
Min. fraction of hits with 1T(U) or 10A: .................... 0.75  
Alternatively: Min. fraction of hits with 1T(U) and 10A: .... 0.5  
Min. fraction of hits with typical piRNA length: ............ 0.75  
Typical piRNA length: ....................................... 24-32 nt  
Min. size of a piRNA cluster: ............................... 1000 bp.  
Min. number of hits (absolute): ............................. 0  
Min. number of hits (normalized): ........................... 0  
Min. fraction of hits on the mainstrand: .................... 0.75  
Top fraction of mapped sequences (in terms of read counts): . 1%  
Top fraction accounts for max. n% of sequence reads: ........ 90%  
Min. fraction of hits on each arm of a bidirectional cluster: 0.05  
Output html file for each cluster: .......................... yes  
Output a summary table: ..................................... yes  
Output a FASTA file for each cluster (piRNA sequences): ..... yes  
Output a FASTA file comprising cluster sequences: ........... yes  
Output a GTF file for predicted piRNA clusters: ..............yes  
Search DNA motifs in clusters: .............................. yes  
Output flanking sequences: +/- .............................. 0 bp  
Output ~.pTi file: .......................................... no  
==============================================================================  
  
  
Genome size (without gaps): ............ 758543724 bp  
Gaps (N/X/-): .......................... 417479 bp  
Mapped reads: .......................... 13052187  
Non-identical sequences: ............... 3338911  
Genomic hits: .......................... 28737726  
Significant densitiy of mapped reads: .. 470.083249848448 reads/kb

Show proTRAC cluster info
Hide proTRAC cluster info

|  |  |
| --- | --- |
| Location | NODE\_27459\_length\_24731\_cov\_33.167362 |
| Coordinates | 3475-11926 |
| Size [bp] | 8452 |
| Sequence hit loci | 1452 |
| Mapped reads (normalized) | 3467.2 |
| Mapped reads (normalized) per kb | 410.2 |
| Normalized reads with 1T (1U) | 86.5% |
| Normalized reads with 10A | 28.4% |
| Normalized reads with length 24-32 nt | 99% |
| Normalized reads on the main strand(s) | 91.4% |
| Predicted directionality | mono:plus |

100%

0%

1T (1U)  
reads

10A reads

24-32 nt  
reads

reads on mainstrand

**Either the amount of reads with 1T (1U) OR 10A has to exceed 75% (set with option: -1Tor10A)  
Alternatively the amount of reads with 1T (1U) AND 10A has to exceed 50% (set with option: -1Tand10A)  
Minimum amount of reads with preferred size is 75% (set with option: -pisize)  
Minimum amount of reads on the main strand(s) is 75% (set with option: -clstrand)**

Show read coverage
Hide read coverage

WHAT DO I SEE HERE?  
This chart shows the location of mapped sequence reads within a predicted piRNA cluster. The color refers to the number of genomic hits produced by the sequence read in question. A dark red bar indicates that this sequence read produces many other hits elsewhere in the genome. Many adjacent red or yellow bars can indicate the presence of a multi-copy element such as transposons or rRNA genes. A dark green bar indicates that this sequence read maps uniquely to this locus.

1 hit

2-5 hits

6-10 hits

11-20 hits

21-50 hits

51-100 hits

> 100 hits

NODE\_27459\_length\_24731\_cov\_33.167362

3475

11926

Gene Set

RepeatMasker

Mapped  
Reads

71.56

plus strand

minus strand

71.56

Region: NODE\_27459\_length\_24731\_cov\_33.167362 1356-3483. Max. coverage (+): 0. Max coverage (-): 0.01

Region: NODE\_27459\_length\_24731\_cov\_33.167362 3484-3500. Max. coverage (+): 0. Max coverage (-): 0

Region: NODE\_27459\_length\_24731\_cov\_33.167362 3501-3517. Max. coverage (+): 0. Max coverage (-): 0

Region: NODE\_27459\_length\_24731\_cov\_33.167362 3518-3534. Max. coverage (+): 0. Max coverage (-): 0

Region: NODE\_27459\_length\_24731\_cov\_33.167362 3535-3551. Max. coverage (+): 0. Max coverage (-): 0

Region: NODE\_27459\_length\_24731\_cov\_33.167362 3552-3567. Max. coverage (+): 0.01. Max coverage (-): 0

Region: NODE\_27459\_length\_24731\_cov\_33.167362 3568-3584. Max. coverage (+): 0.01. Max coverage (-): 0.01

Region: NODE\_27459\_length\_24731\_cov\_33.167362 3585-3601. Max. coverage (+): 0. Max coverage (-): 0

Region: NODE\_27459\_length\_24731\_cov\_33.167362 3602-3618. Max. coverage (+): 0. Max coverage (-): 0

Region: NODE\_27459\_length\_24731\_cov\_33.167362 3619-3635. Max. coverage (+): 0. Max coverage (-): 0

Region: NODE\_27459\_length\_24731\_cov\_33.167362 3636-3652. Max. coverage (+): 0.08. Max coverage (-): 0

Region: NODE\_27459\_length\_24731\_cov\_33.167362 3653-3669. Max. coverage (+): 0. Max coverage (-): 0

Region: NODE\_27459\_length\_24731\_cov\_33.167362 3670-3686. Max. coverage (+): 0. Max coverage (-): 0.01

Region: NODE\_27459\_length\_24731\_cov\_33.167362 3687-3703. Max. coverage (+): 0. Max coverage (-): 0

Region: NODE\_27459\_length\_24731\_cov\_33.167362 3704-3720. Max. coverage (+): 0. Max coverage (-): 0

Region: NODE\_27459\_length\_24731\_cov\_33.167362 3721-3737. Max. coverage (+): 0.08. Max coverage (-): 0

Region: NODE\_27459\_length\_24731\_cov\_33.167362 3738-3753. Max. coverage (+): 0. Max coverage (-): 0

Region: NODE\_27459\_length\_24731\_cov\_33.167362 3754-3770. Max. coverage (+): 0. Max coverage (-): 0

Region: NODE\_27459\_length\_24731\_cov\_33.167362 3771-3787. Max. coverage (+): 0. Max coverage (-): 0

Region: NODE\_27459\_length\_24731\_cov\_33.167362 3788-3804. Max. coverage (+): 0. Max coverage (-): 0

Region: NODE\_27459\_length\_24731\_cov\_33.167362 3805-3821. Max. coverage (+): 0. Max coverage (-): 0

Region: NODE\_27459\_length\_24731\_cov\_33.167362 3822-3838. Max. coverage (+): 0. Max coverage (-): 0

Region: NODE\_27459\_length\_24731\_cov\_33.167362 3839-3855. Max. coverage (+): 0. Max coverage (-): 0

Region: NODE\_27459\_length\_24731\_cov\_33.167362 3856-3872. Max. coverage (+): 0. Max coverage (-): 0.08

Region: NODE\_27459\_length\_24731\_cov\_33.167362 3873-3889. Max. coverage (+): 0. Max coverage (-): 0

Region: NODE\_27459\_length\_24731\_cov\_33.167362 3890-3906. Max. coverage (+): 0. Max coverage (-): 0.01

Region: NODE\_27459\_length\_24731\_cov\_33.167362 3907-3922. Max. coverage (+): 0. Max coverage (-): 0.01

Region: NODE\_27459\_length\_24731\_cov\_33.167362 3923-3939. Max. coverage (+): 0. Max coverage (-): 0

Region: NODE\_27459\_length\_24731\_cov\_33.167362 3940-3956. Max. coverage (+): 0. Max coverage (-): 0

Region: NODE\_27459\_length\_24731\_cov\_33.167362 3957-3973. Max. coverage (+): 0. Max coverage (-): 0

Region: NODE\_27459\_length\_24731\_cov\_33.167362 3974-3990. Max. coverage (+): 0.05. Max coverage (-): 0

Region: NODE\_27459\_length\_24731\_cov\_33.167362 3991-4007. Max. coverage (+): 0. Max coverage (-): 0.03

Region: NODE\_27459\_length\_24731\_cov\_33.167362 4008-4024. Max. coverage (+): 0. Max coverage (-): 0.03

Region: NODE\_27459\_length\_24731\_cov\_33.167362 4025-4041. Max. coverage (+): 0. Max coverage (-): 0

Region: NODE\_27459\_length\_24731\_cov\_33.167362 4042-4058. Max. coverage (+): 0. Max coverage (-): 0

Region: NODE\_27459\_length\_24731\_cov\_33.167362 4059-4075. Max. coverage (+): 0. Max coverage (-): 0

Region: NODE\_27459\_length\_24731\_cov\_33.167362 4076-4091. Max. coverage (+): 0. Max coverage (-): 0

Region: NODE\_27459\_length\_24731\_cov\_33.167362 4092-4108. Max. coverage (+): 0. Max coverage (-): 0

Region: NODE\_27459\_length\_24731\_cov\_33.167362 4109-4125. Max. coverage (+): 0. Max coverage (-): 0

Region: NODE\_27459\_length\_24731\_cov\_33.167362 4126-4142. Max. coverage (+): 0. Max coverage (-): 0

Region: NODE\_27459\_length\_24731\_cov\_33.167362 4143-4159. Max. coverage (+): 0. Max coverage (-): 0

Region: NODE\_27459\_length\_24731\_cov\_33.167362 4160-4176. Max. coverage (+): 0. Max coverage (-): 0

Region: NODE\_27459\_length\_24731\_cov\_33.167362 4177-4193. Max. coverage (+): 0. Max coverage (-): 0

Region: NODE\_27459\_length\_24731\_cov\_33.167362 4194-4210. Max. coverage (+): 0. Max coverage (-): 0

Region: NODE\_27459\_length\_24731\_cov\_33.167362 4211-4227. Max. coverage (+): 0. Max coverage (-): 0

Region: NODE\_27459\_length\_24731\_cov\_33.167362 4228-4244. Max. coverage (+): 0. Max coverage (-): 0

Region: NODE\_27459\_length\_24731\_cov\_33.167362 4245-4261. Max. coverage (+): 0. Max coverage (-): 0

Region: NODE\_27459\_length\_24731\_cov\_33.167362 4262-4277. Max. coverage (+): 0. Max coverage (-): 0

Region: NODE\_27459\_length\_24731\_cov\_33.167362 4278-4294. Max. coverage (+): 0. Max coverage (-): 0

Region: NODE\_27459\_length\_24731\_cov\_33.167362 4295-4311. Max. coverage (+): 0. Max coverage (-): 0

Region: NODE\_27459\_length\_24731\_cov\_33.167362 4312-4328. Max. coverage (+): 0. Max coverage (-): 0

Region: NODE\_27459\_length\_24731\_cov\_33.167362 4329-4345. Max. coverage (+): 0. Max coverage (-): 0

Region: NODE\_27459\_length\_24731\_cov\_33.167362 4346-4362. Max. coverage (+): 0. Max coverage (-): 0

Region: NODE\_27459\_length\_24731\_cov\_33.167362 4363-4379. Max. coverage (+): 0. Max coverage (-): 0

Region: NODE\_27459\_length\_24731\_cov\_33.167362 4380-4396. Max. coverage (+): 0. Max coverage (-): 0

Region: NODE\_27459\_length\_24731\_cov\_33.167362 4397-4413. Max. coverage (+): 0. Max coverage (-): 0

Region: NODE\_27459\_length\_24731\_cov\_33.167362 4414-4430. Max. coverage (+): 0. Max coverage (-): 0

Region: NODE\_27459\_length\_24731\_cov\_33.167362 4431-4446. Max. coverage (+): 0. Max coverage (-): 0

Region: NODE\_27459\_length\_24731\_cov\_33.167362 4447-4463. Max. coverage (+): 0. Max coverage (-): 0

Region: NODE\_27459\_length\_24731\_cov\_33.167362 4464-4480. Max. coverage (+): 0. Max coverage (-): 0

Region: NODE\_27459\_length\_24731\_cov\_33.167362 4481-4497. Max. coverage (+): 0. Max coverage (-): 0

Region: NODE\_27459\_length\_24731\_cov\_33.167362 4498-4514. Max. coverage (+): 0. Max coverage (-): 0

Region: NODE\_27459\_length\_24731\_cov\_33.167362 4515-4531. Max. coverage (+): 0. Max coverage (-): 0

Region: NODE\_27459\_length\_24731\_cov\_33.167362 4532-4548. Max. coverage (+): 0. Max coverage (-): 0

Region: NODE\_27459\_length\_24731\_cov\_33.167362 4549-4565. Max. coverage (+): 0. Max coverage (-): 0

Region: NODE\_27459\_length\_24731\_cov\_33.167362 4566-4582. Max. coverage (+): 0. Max coverage (-): 0

Region: NODE\_27459\_length\_24731\_cov\_33.167362 4583-4599. Max. coverage (+): 0. Max coverage (-): 0

Region: NODE\_27459\_length\_24731\_cov\_33.167362 4600-4616. Max. coverage (+): 0.08. Max coverage (-): 0

Region: NODE\_27459\_length\_24731\_cov\_33.167362 4617-4632. Max. coverage (+): 0.08. Max coverage (-): 0

Region: NODE\_27459\_length\_24731\_cov\_33.167362 4633-4649. Max. coverage (+): 0. Max coverage (-): 0

Region: NODE\_27459\_length\_24731\_cov\_33.167362 4650-4666. Max. coverage (+): 0. Max coverage (-): 0

Region: NODE\_27459\_length\_24731\_cov\_33.167362 4667-4683. Max. coverage (+): 0. Max coverage (-): 0

Region: NODE\_27459\_length\_24731\_cov\_33.167362 4684-4700. Max. coverage (+): 0. Max coverage (-): 0

Region: NODE\_27459\_length\_24731\_cov\_33.167362 4701-4717. Max. coverage (+): 0. Max coverage (-): 0

Region: NODE\_27459\_length\_24731\_cov\_33.167362 4718-4734. Max. coverage (+): 0. Max coverage (-): 0

Region: NODE\_27459\_length\_24731\_cov\_33.167362 4735-4751. Max. coverage (+): 0. Max coverage (-): 0

Region: NODE\_27459\_length\_24731\_cov\_33.167362 4752-4768. Max. coverage (+): 0. Max coverage (-): 0

Region: NODE\_27459\_length\_24731\_cov\_33.167362 4769-4785. Max. coverage (+): 0. Max coverage (-): 0

Region: NODE\_27459\_length\_24731\_cov\_33.167362 4786-4801. Max. coverage (+): 0. Max coverage (-): 0

Region: NODE\_27459\_length\_24731\_cov\_33.167362 4802-4818. Max. coverage (+): 0. Max coverage (-): 0

Region: NODE\_27459\_length\_24731\_cov\_33.167362 4819-4835. Max. coverage (+): 0. Max coverage (-): 0

Region: NODE\_27459\_length\_24731\_cov\_33.167362 4836-4852. Max. coverage (+): 0. Max coverage (-): 0

Region: NODE\_27459\_length\_24731\_cov\_33.167362 4853-4869. Max. coverage (+): 0. Max coverage (-): 0

Region: NODE\_27459\_length\_24731\_cov\_33.167362 4870-4886. Max. coverage (+): 0. Max coverage (-): 0

Region: NODE\_27459\_length\_24731\_cov\_33.167362 4887-4903. Max. coverage (+): 0. Max coverage (-): 0

Region: NODE\_27459\_length\_24731\_cov\_33.167362 4904-4920. Max. coverage (+): 0. Max coverage (-): 0.15

Region: NODE\_27459\_length\_24731\_cov\_33.167362 4921-4937. Max. coverage (+): 0.08. Max coverage (-): 0

Region: NODE\_27459\_length\_24731\_cov\_33.167362 4938-4954. Max. coverage (+): 0. Max coverage (-): 0

Region: NODE\_27459\_length\_24731\_cov\_33.167362 4955-4971. Max. coverage (+): 0.08. Max coverage (-): 0

Region: NODE\_27459\_length\_24731\_cov\_33.167362 4972-4987. Max. coverage (+): 0.08. Max coverage (-): 0

Region: NODE\_27459\_length\_24731\_cov\_33.167362 4988-5004. Max. coverage (+): 0. Max coverage (-): 0

Region: NODE\_27459\_length\_24731\_cov\_33.167362 5005-5021. Max. coverage (+): 0. Max coverage (-): 0

Region: NODE\_27459\_length\_24731\_cov\_33.167362 5022-5038. Max. coverage (+): 0. Max coverage (-): 0

Region: NODE\_27459\_length\_24731\_cov\_33.167362 5039-5055. Max. coverage (+): 0. Max coverage (-): 0

Region: NODE\_27459\_length\_24731\_cov\_33.167362 5056-5072. Max. coverage (+): 0. Max coverage (-): 0

Region: NODE\_27459\_length\_24731\_cov\_33.167362 5073-5089. Max. coverage (+): 0. Max coverage (-): 0

Region: NODE\_27459\_length\_24731\_cov\_33.167362 5090-5106. Max. coverage (+): 0.08. Max coverage (-): 0

Region: NODE\_27459\_length\_24731\_cov\_33.167362 5107-5123. Max. coverage (+): 0. Max coverage (-): 0

Region: NODE\_27459\_length\_24731\_cov\_33.167362 5124-5140. Max. coverage (+): 0. Max coverage (-): 0

Region: NODE\_27459\_length\_24731\_cov\_33.167362 5141-5156. Max. coverage (+): 0. Max coverage (-): 0

Region: NODE\_27459\_length\_24731\_cov\_33.167362 5157-5173. Max. coverage (+): 0. Max coverage (-): 0

Region: NODE\_27459\_length\_24731\_cov\_33.167362 5174-5190. Max. coverage (+): 0. Max coverage (-): 0

Region: NODE\_27459\_length\_24731\_cov\_33.167362 5191-5207. Max. coverage (+): 0. Max coverage (-): 0

Region: NODE\_27459\_length\_24731\_cov\_33.167362 5208-5224. Max. coverage (+): 0. Max coverage (-): 0

Region: NODE\_27459\_length\_24731\_cov\_33.167362 5225-5241. Max. coverage (+): 0. Max coverage (-): 0

Region: NODE\_27459\_length\_24731\_cov\_33.167362 5242-5258. Max. coverage (+): 0. Max coverage (-): 0

Region: NODE\_27459\_length\_24731\_cov\_33.167362 5259-5275. Max. coverage (+): 0. Max coverage (-): 0.08

Region: NODE\_27459\_length\_24731\_cov\_33.167362 5276-5292. Max. coverage (+): 0.08. Max coverage (-): 0.08

Region: NODE\_27459\_length\_24731\_cov\_33.167362 5293-5309. Max. coverage (+): 0.15. Max coverage (-): 0.08

Region: NODE\_27459\_length\_24731\_cov\_33.167362 5310-5325. Max. coverage (+): 0.08. Max coverage (-): 0

Region: NODE\_27459\_length\_24731\_cov\_33.167362 5326-5342. Max. coverage (+): 0.08. Max coverage (-): 0

Region: NODE\_27459\_length\_24731\_cov\_33.167362 5343-5359. Max. coverage (+): 0. Max coverage (-): 0.08

Region: NODE\_27459\_length\_24731\_cov\_33.167362 5360-5376. Max. coverage (+): 0. Max coverage (-): 0.08

Region: NODE\_27459\_length\_24731\_cov\_33.167362 5377-5393. Max. coverage (+): 0.08. Max coverage (-): 0

Region: NODE\_27459\_length\_24731\_cov\_33.167362 5394-5410. Max. coverage (+): 0. Max coverage (-): 0

Region: NODE\_27459\_length\_24731\_cov\_33.167362 5411-5427. Max. coverage (+): 0. Max coverage (-): 0.08

Region: NODE\_27459\_length\_24731\_cov\_33.167362 5428-5444. Max. coverage (+): 0. Max coverage (-): 0

Region: NODE\_27459\_length\_24731\_cov\_33.167362 5445-5461. Max. coverage (+): 0. Max coverage (-): 0

Region: NODE\_27459\_length\_24731\_cov\_33.167362 5462-5478. Max. coverage (+): 0.08. Max coverage (-): 0

Region: NODE\_27459\_length\_24731\_cov\_33.167362 5479-5495. Max. coverage (+): 0.15. Max coverage (-): 0

Region: NODE\_27459\_length\_24731\_cov\_33.167362 5496-5511. Max. coverage (+): 0.08. Max coverage (-): 0.15

Region: NODE\_27459\_length\_24731\_cov\_33.167362 5512-5528. Max. coverage (+): 0.15. Max coverage (-): 0.15

Region: NODE\_27459\_length\_24731\_cov\_33.167362 5529-5545. Max. coverage (+): 0.23. Max coverage (-): 0.08

Region: NODE\_27459\_length\_24731\_cov\_33.167362 5546-5562. Max. coverage (+): 0. Max coverage (-): 0.15

Region: NODE\_27459\_length\_24731\_cov\_33.167362 5563-5579. Max. coverage (+): 0.15. Max coverage (-): 0

Region: NODE\_27459\_length\_24731\_cov\_33.167362 5580-5596. Max. coverage (+): 0.08. Max coverage (-): 0

Region: NODE\_27459\_length\_24731\_cov\_33.167362 5597-5613. Max. coverage (+): 0.08. Max coverage (-): 0

Region: NODE\_27459\_length\_24731\_cov\_33.167362 5614-5630. Max. coverage (+): 0. Max coverage (-): 0

Region: NODE\_27459\_length\_24731\_cov\_33.167362 5631-5647. Max. coverage (+): 0. Max coverage (-): 0.08

Region: NODE\_27459\_length\_24731\_cov\_33.167362 5648-5664. Max. coverage (+): 0.23. Max coverage (-): 0.08

Region: NODE\_27459\_length\_24731\_cov\_33.167362 5665-5680. Max. coverage (+): 0.08. Max coverage (-): 0

Region: NODE\_27459\_length\_24731\_cov\_33.167362 5681-5697. Max. coverage (+): 0.08. Max coverage (-): 0

Region: NODE\_27459\_length\_24731\_cov\_33.167362 5698-5714. Max. coverage (+): 0.08. Max coverage (-): 0

Region: NODE\_27459\_length\_24731\_cov\_33.167362 5715-5731. Max. coverage (+): 0. Max coverage (-): 0

Region: NODE\_27459\_length\_24731\_cov\_33.167362 5732-5748. Max. coverage (+): 0. Max coverage (-): 0

Region: NODE\_27459\_length\_24731\_cov\_33.167362 5749-5765. Max. coverage (+): 0.23. Max coverage (-): 0

Region: NODE\_27459\_length\_24731\_cov\_33.167362 5766-5782. Max. coverage (+): 0. Max coverage (-): 0

Region: NODE\_27459\_length\_24731\_cov\_33.167362 5783-5799. Max. coverage (+): 0. Max coverage (-): 0

Region: NODE\_27459\_length\_24731\_cov\_33.167362 5800-5816. Max. coverage (+): 0. Max coverage (-): 0

Region: NODE\_27459\_length\_24731\_cov\_33.167362 5817-5833. Max. coverage (+): 0. Max coverage (-): 0

Region: NODE\_27459\_length\_24731\_cov\_33.167362 5834-5850. Max. coverage (+): 0. Max coverage (-): 0

Region: NODE\_27459\_length\_24731\_cov\_33.167362 5851-5866. Max. coverage (+): 0. Max coverage (-): 0.08

Region: NODE\_27459\_length\_24731\_cov\_33.167362 5867-5883. Max. coverage (+): 0. Max coverage (-): 0

Region: NODE\_27459\_length\_24731\_cov\_33.167362 5884-5900. Max. coverage (+): 0. Max coverage (-): 0

Region: NODE\_27459\_length\_24731\_cov\_33.167362 5901-5917. Max. coverage (+): 0. Max coverage (-): 0

Region: NODE\_27459\_length\_24731\_cov\_33.167362 5918-5934. Max. coverage (+): 0. Max coverage (-): 0

Region: NODE\_27459\_length\_24731\_cov\_33.167362 5935-5951. Max. coverage (+): 0.08. Max coverage (-): 0.23

Region: NODE\_27459\_length\_24731\_cov\_33.167362 5952-5968. Max. coverage (+): 0.31. Max coverage (-): 0

Region: NODE\_27459\_length\_24731\_cov\_33.167362 5969-5985. Max. coverage (+): 0.15. Max coverage (-): 0

Region: NODE\_27459\_length\_24731\_cov\_33.167362 5986-6002. Max. coverage (+): 0. Max coverage (-): 0

Region: NODE\_27459\_length\_24731\_cov\_33.167362 6003-6019. Max. coverage (+): 0.08. Max coverage (-): 0.08

Region: NODE\_27459\_length\_24731\_cov\_33.167362 6020-6035. Max. coverage (+): 0. Max coverage (-): 0

Region: NODE\_27459\_length\_24731\_cov\_33.167362 6036-6052. Max. coverage (+): 0. Max coverage (-): 0

Region: NODE\_27459\_length\_24731\_cov\_33.167362 6053-6069. Max. coverage (+): 0. Max coverage (-): 0

Region: NODE\_27459\_length\_24731\_cov\_33.167362 6070-6086. Max. coverage (+): 0. Max coverage (-): 0

Region: NODE\_27459\_length\_24731\_cov\_33.167362 6087-6103. Max. coverage (+): 0. Max coverage (-): 0

Region: NODE\_27459\_length\_24731\_cov\_33.167362 6104-6120. Max. coverage (+): 0. Max coverage (-): 0

Region: NODE\_27459\_length\_24731\_cov\_33.167362 6121-6137. Max. coverage (+): 0. Max coverage (-): 0

Region: NODE\_27459\_length\_24731\_cov\_33.167362 6138-6154. Max. coverage (+): 0. Max coverage (-): 0

Region: NODE\_27459\_length\_24731\_cov\_33.167362 6155-6171. Max. coverage (+): 0. Max coverage (-): 0

Region: NODE\_27459\_length\_24731\_cov\_33.167362 6172-6188. Max. coverage (+): 0. Max coverage (-): 0

Region: NODE\_27459\_length\_24731\_cov\_33.167362 6189-6204. Max. coverage (+): 0.01. Max coverage (-): 0

Region: NODE\_27459\_length\_24731\_cov\_33.167362 6205-6221. Max. coverage (+): 0. Max coverage (-): 0.01

Region: NODE\_27459\_length\_24731\_cov\_33.167362 6222-6238. Max. coverage (+): 0.01. Max coverage (-): 0.01

Region: NODE\_27459\_length\_24731\_cov\_33.167362 6239-6255. Max. coverage (+): 0.01. Max coverage (-): 0

Region: NODE\_27459\_length\_24731\_cov\_33.167362 6256-6272. Max. coverage (+): 0.08. Max coverage (-): 0

Region: NODE\_27459\_length\_24731\_cov\_33.167362 6273-6289. Max. coverage (+): 0. Max coverage (-): 0

Region: NODE\_27459\_length\_24731\_cov\_33.167362 6290-6306. Max. coverage (+): 0. Max coverage (-): 1

Region: NODE\_27459\_length\_24731\_cov\_33.167362 6307-6323. Max. coverage (+): 0.23. Max coverage (-): 0.01

Region: NODE\_27459\_length\_24731\_cov\_33.167362 6324-6340. Max. coverage (+): 0. Max coverage (-): 0.38

Region: NODE\_27459\_length\_24731\_cov\_33.167362 6341-6357. Max. coverage (+): 0.01. Max coverage (-): 1.53

Region: NODE\_27459\_length\_24731\_cov\_33.167362 6358-6374. Max. coverage (+): 0.23. Max coverage (-): 0

Region: NODE\_27459\_length\_24731\_cov\_33.167362 6375-6390. Max. coverage (+): 0. Max coverage (-): 0

Region: NODE\_27459\_length\_24731\_cov\_33.167362 6391-6407. Max. coverage (+): 0. Max coverage (-): 0.08

Region: NODE\_27459\_length\_24731\_cov\_33.167362 6408-6424. Max. coverage (+): 0.08. Max coverage (-): 0.01

Region: NODE\_27459\_length\_24731\_cov\_33.167362 6425-6441. Max. coverage (+): 0. Max coverage (-): 0

Region: NODE\_27459\_length\_24731\_cov\_33.167362 6442-6458. Max. coverage (+): 0.02. Max coverage (-): 0

Region: NODE\_27459\_length\_24731\_cov\_33.167362 6459-6475. Max. coverage (+): 0. Max coverage (-): 0

Region: NODE\_27459\_length\_24731\_cov\_33.167362 6476-6492. Max. coverage (+): 0. Max coverage (-): 0

Region: NODE\_27459\_length\_24731\_cov\_33.167362 6493-6509. Max. coverage (+): 0. Max coverage (-): 0

Region: NODE\_27459\_length\_24731\_cov\_33.167362 6510-6526. Max. coverage (+): 0. Max coverage (-): 0

Region: NODE\_27459\_length\_24731\_cov\_33.167362 6527-6543. Max. coverage (+): 0. Max coverage (-): 0

Region: NODE\_27459\_length\_24731\_cov\_33.167362 6544-6559. Max. coverage (+): 0. Max coverage (-): 0

Region: NODE\_27459\_length\_24731\_cov\_33.167362 6560-6576. Max. coverage (+): 0. Max coverage (-): 0

Region: NODE\_27459\_length\_24731\_cov\_33.167362 6577-6593. Max. coverage (+): 0. Max coverage (-): 0

Region: NODE\_27459\_length\_24731\_cov\_33.167362 6594-6610. Max. coverage (+): 0. Max coverage (-): 0

Region: NODE\_27459\_length\_24731\_cov\_33.167362 6611-6627. Max. coverage (+): 0. Max coverage (-): 0

Region: NODE\_27459\_length\_24731\_cov\_33.167362 6628-6644. Max. coverage (+): 0. Max coverage (-): 0.08

Region: NODE\_27459\_length\_24731\_cov\_33.167362 6645-6661. Max. coverage (+): 0.08. Max coverage (-): 0

Region: NODE\_27459\_length\_24731\_cov\_33.167362 6662-6678. Max. coverage (+): 0. Max coverage (-): 0.15

Region: NODE\_27459\_length\_24731\_cov\_33.167362 6679-6695. Max. coverage (+): 0. Max coverage (-): 0.08

Region: NODE\_27459\_length\_24731\_cov\_33.167362 6696-6712. Max. coverage (+): 0.31. Max coverage (-): 0.03

Region: NODE\_27459\_length\_24731\_cov\_33.167362 6713-6729. Max. coverage (+): 0.31. Max coverage (-): 0.15

Region: NODE\_27459\_length\_24731\_cov\_33.167362 6730-6745. Max. coverage (+): 0.08. Max coverage (-): 0.15

Region: NODE\_27459\_length\_24731\_cov\_33.167362 6746-6762. Max. coverage (+): 0.07. Max coverage (-): 0.23

Region: NODE\_27459\_length\_24731\_cov\_33.167362 6763-6779. Max. coverage (+): 1.38. Max coverage (-): 0.2

Region: NODE\_27459\_length\_24731\_cov\_33.167362 6780-6796. Max. coverage (+): 0.08. Max coverage (-): 0.08

Region: NODE\_27459\_length\_24731\_cov\_33.167362 6797-6813. Max. coverage (+): 0.08. Max coverage (-): 0.08

Region: NODE\_27459\_length\_24731\_cov\_33.167362 6814-6830. Max. coverage (+): 0.61. Max coverage (-): 0.84

Region: NODE\_27459\_length\_24731\_cov\_33.167362 6831-6847. Max. coverage (+): 0.92. Max coverage (-): 0.08

Region: NODE\_27459\_length\_24731\_cov\_33.167362 6848-6864. Max. coverage (+): 0.15. Max coverage (-): 0.38

Region: NODE\_27459\_length\_24731\_cov\_33.167362 6865-6881. Max. coverage (+): 0.23. Max coverage (-): 0.08

Region: NODE\_27459\_length\_24731\_cov\_33.167362 6882-6898. Max. coverage (+): 1.92. Max coverage (-): 0

Region: NODE\_27459\_length\_24731\_cov\_33.167362 6899-6914. Max. coverage (+): 0.08. Max coverage (-): 0

Region: NODE\_27459\_length\_24731\_cov\_33.167362 6915-6931. Max. coverage (+): 0.08. Max coverage (-): 0

Region: NODE\_27459\_length\_24731\_cov\_33.167362 6932-6948. Max. coverage (+): 0.15. Max coverage (-): 0

Region: NODE\_27459\_length\_24731\_cov\_33.167362 6949-6965. Max. coverage (+): 3.06. Max coverage (-): 0.31

Region: NODE\_27459\_length\_24731\_cov\_33.167362 6966-6982. Max. coverage (+): 0.23. Max coverage (-): 0.15

Region: NODE\_27459\_length\_24731\_cov\_33.167362 6983-6999. Max. coverage (+): 0.38. Max coverage (-): 0.15

Region: NODE\_27459\_length\_24731\_cov\_33.167362 7000-7016. Max. coverage (+): 0.38. Max coverage (-): 0.08

Region: NODE\_27459\_length\_24731\_cov\_33.167362 7017-7033. Max. coverage (+): 1.53. Max coverage (-): 0.08

Region: NODE\_27459\_length\_24731\_cov\_33.167362 7034-7050. Max. coverage (+): 1.46. Max coverage (-): 0.38

Region: NODE\_27459\_length\_24731\_cov\_33.167362 7051-7067. Max. coverage (+): 1.23. Max coverage (-): 0.08

Region: NODE\_27459\_length\_24731\_cov\_33.167362 7068-7084. Max. coverage (+): 0.23. Max coverage (-): 0

Region: NODE\_27459\_length\_24731\_cov\_33.167362 7085-7100. Max. coverage (+): 1.23. Max coverage (-): 0

Region: NODE\_27459\_length\_24731\_cov\_33.167362 7101-7117. Max. coverage (+): 0.61. Max coverage (-): 0

Region: NODE\_27459\_length\_24731\_cov\_33.167362 7118-7134. Max. coverage (+): 0.61. Max coverage (-): 0.23

Region: NODE\_27459\_length\_24731\_cov\_33.167362 7135-7151. Max. coverage (+): 0.54. Max coverage (-): 0

Region: NODE\_27459\_length\_24731\_cov\_33.167362 7152-7168. Max. coverage (+): 0.08. Max coverage (-): 0

Region: NODE\_27459\_length\_24731\_cov\_33.167362 7169-7185. Max. coverage (+): 0.08. Max coverage (-): 0.69

Region: NODE\_27459\_length\_24731\_cov\_33.167362 7186-7202. Max. coverage (+): 25.13. Max coverage (-): 0.15

Region: NODE\_27459\_length\_24731\_cov\_33.167362 7203-7219. Max. coverage (+): 0. Max coverage (-): 1.53

Region: NODE\_27459\_length\_24731\_cov\_33.167362 7220-7236. Max. coverage (+): 1.07. Max coverage (-): 0.61

Region: NODE\_27459\_length\_24731\_cov\_33.167362 7237-7253. Max. coverage (+): 71.56. Max coverage (-): 0.46

Region: NODE\_27459\_length\_24731\_cov\_33.167362 7254-7269. Max. coverage (+): 4.75. Max coverage (-): 0.15

Region: NODE\_27459\_length\_24731\_cov\_33.167362 7270-7286. Max. coverage (+): 0.38. Max coverage (-): 0.23

Region: NODE\_27459\_length\_24731\_cov\_33.167362 7287-7303. Max. coverage (+): 0.92. Max coverage (-): 0.08

Region: NODE\_27459\_length\_24731\_cov\_33.167362 7304-7320. Max. coverage (+): 7.43. Max coverage (-): 0

Region: NODE\_27459\_length\_24731\_cov\_33.167362 7321-7337. Max. coverage (+): 0.31. Max coverage (-): 0.46

Region: NODE\_27459\_length\_24731\_cov\_33.167362 7338-7354. Max. coverage (+): 1.99. Max coverage (-): 0

Region: NODE\_27459\_length\_24731\_cov\_33.167362 7355-7371. Max. coverage (+): 0.38. Max coverage (-): 0

Region: NODE\_27459\_length\_24731\_cov\_33.167362 7372-7388. Max. coverage (+): 0.15. Max coverage (-): 0.84

Region: NODE\_27459\_length\_24731\_cov\_33.167362 7389-7405. Max. coverage (+): 10.27. Max coverage (-): 0.08

Region: NODE\_27459\_length\_24731\_cov\_33.167362 7406-7422. Max. coverage (+): 0.31. Max coverage (-): 0.08

Region: NODE\_27459\_length\_24731\_cov\_33.167362 7423-7438. Max. coverage (+): 0.23. Max coverage (-): 0.08

Region: NODE\_27459\_length\_24731\_cov\_33.167362 7439-7455. Max. coverage (+): 2.53. Max coverage (-): 0

Region: NODE\_27459\_length\_24731\_cov\_33.167362 7456-7472. Max. coverage (+): 0.08. Max coverage (-): 0

Region: NODE\_27459\_length\_24731\_cov\_33.167362 7473-7489. Max. coverage (+): 0.23. Max coverage (-): 0.54

Region: NODE\_27459\_length\_24731\_cov\_33.167362 7490-7506. Max. coverage (+): 3.98. Max coverage (-): 0.38

Region: NODE\_27459\_length\_24731\_cov\_33.167362 7507-7523. Max. coverage (+): 3.22. Max coverage (-): 0

Region: NODE\_27459\_length\_24731\_cov\_33.167362 7524-7540. Max. coverage (+): 0.38. Max coverage (-): 0.15

Region: NODE\_27459\_length\_24731\_cov\_33.167362 7541-7557. Max. coverage (+): 1.38. Max coverage (-): 0.15

Region: NODE\_27459\_length\_24731\_cov\_33.167362 7558-7574. Max. coverage (+): 1.23. Max coverage (-): 0

Region: NODE\_27459\_length\_24731\_cov\_33.167362 7575-7591. Max. coverage (+): 0.38. Max coverage (-): 0.08

Region: NODE\_27459\_length\_24731\_cov\_33.167362 7592-7608. Max. coverage (+): 4.06. Max coverage (-): 0

Region: NODE\_27459\_length\_24731\_cov\_33.167362 7609-7624. Max. coverage (+): 0.69. Max coverage (-): 0.23

Region: NODE\_27459\_length\_24731\_cov\_33.167362 7625-7641. Max. coverage (+): 2.22. Max coverage (-): 0

Region: NODE\_27459\_length\_24731\_cov\_33.167362 7642-7658. Max. coverage (+): 2.53. Max coverage (-): 0.31

Region: NODE\_27459\_length\_24731\_cov\_33.167362 7659-7675. Max. coverage (+): 0.31. Max coverage (-): 0

Region: NODE\_27459\_length\_24731\_cov\_33.167362 7676-7692. Max. coverage (+): 0.23. Max coverage (-): 0

Region: NODE\_27459\_length\_24731\_cov\_33.167362 7693-7709. Max. coverage (+): 0. Max coverage (-): 0.08

Region: NODE\_27459\_length\_24731\_cov\_33.167362 7710-7726. Max. coverage (+): 1.07. Max coverage (-): 0

Region: NODE\_27459\_length\_24731\_cov\_33.167362 7727-7743. Max. coverage (+): 0.23. Max coverage (-): 0

Region: NODE\_27459\_length\_24731\_cov\_33.167362 7744-7760. Max. coverage (+): 1.3. Max coverage (-): 0

Region: NODE\_27459\_length\_24731\_cov\_33.167362 7761-7777. Max. coverage (+): 0.31. Max coverage (-): 0.08

Region: NODE\_27459\_length\_24731\_cov\_33.167362 7778-7793. Max. coverage (+): 0.08. Max coverage (-): 0

Region: NODE\_27459\_length\_24731\_cov\_33.167362 7794-7810. Max. coverage (+): 0. Max coverage (-): 0.08

Region: NODE\_27459\_length\_24731\_cov\_33.167362 7811-7827. Max. coverage (+): 0.92. Max coverage (-): 0

Region: NODE\_27459\_length\_24731\_cov\_33.167362 7828-7844. Max. coverage (+): 0. Max coverage (-): 0

Region: NODE\_27459\_length\_24731\_cov\_33.167362 7845-7861. Max. coverage (+): 0.61. Max coverage (-): 0.08

Region: NODE\_27459\_length\_24731\_cov\_33.167362 7862-7878. Max. coverage (+): 0. Max coverage (-): 0

Region: NODE\_27459\_length\_24731\_cov\_33.167362 7879-7895. Max. coverage (+): 0.08. Max coverage (-): 0

Region: NODE\_27459\_length\_24731\_cov\_33.167362 7896-7912. Max. coverage (+): 0.84. Max coverage (-): 0

Region: NODE\_27459\_length\_24731\_cov\_33.167362 7913-7929. Max. coverage (+): 0.23. Max coverage (-): 0

Region: NODE\_27459\_length\_24731\_cov\_33.167362 7930-7946. Max. coverage (+): 0.84. Max coverage (-): 0

Region: NODE\_27459\_length\_24731\_cov\_33.167362 7947-7963. Max. coverage (+): 0.69. Max coverage (-): 0

Region: NODE\_27459\_length\_24731\_cov\_33.167362 7964-7979. Max. coverage (+): 0.08. Max coverage (-): 0.54

Region: NODE\_27459\_length\_24731\_cov\_33.167362 7980-7996. Max. coverage (+): 0.15. Max coverage (-): 0

Region: NODE\_27459\_length\_24731\_cov\_33.167362 7997-8013. Max. coverage (+): 0.15. Max coverage (-): 0

Region: NODE\_27459\_length\_24731\_cov\_33.167362 8014-8030. Max. coverage (+): 0.15. Max coverage (-): 0

Region: NODE\_27459\_length\_24731\_cov\_33.167362 8031-8047. Max. coverage (+): 0.15. Max coverage (-): 0.23

Region: NODE\_27459\_length\_24731\_cov\_33.167362 8048-8064. Max. coverage (+): 0.46. Max coverage (-): 0

Region: NODE\_27459\_length\_24731\_cov\_33.167362 8065-8081. Max. coverage (+): 0.08. Max coverage (-): 0.08

Region: NODE\_27459\_length\_24731\_cov\_33.167362 8082-8098. Max. coverage (+): 2.76. Max coverage (-): 0

Region: NODE\_27459\_length\_24731\_cov\_33.167362 8099-8115. Max. coverage (+): 1.23. Max coverage (-): 0

Region: NODE\_27459\_length\_24731\_cov\_33.167362 8116-8132. Max. coverage (+): 0. Max coverage (-): 0.08

Region: NODE\_27459\_length\_24731\_cov\_33.167362 8133-8148. Max. coverage (+): 0.61. Max coverage (-): 0

Region: NODE\_27459\_length\_24731\_cov\_33.167362 8149-8165. Max. coverage (+): 0.08. Max coverage (-): 0

Region: NODE\_27459\_length\_24731\_cov\_33.167362 8166-8182. Max. coverage (+): 0.08. Max coverage (-): 0

Region: NODE\_27459\_length\_24731\_cov\_33.167362 8183-8199. Max. coverage (+): 0.08. Max coverage (-): 0

Region: NODE\_27459\_length\_24731\_cov\_33.167362 8200-8216. Max. coverage (+): 0. Max coverage (-): 0

Region: NODE\_27459\_length\_24731\_cov\_33.167362 8217-8233. Max. coverage (+): 0.08. Max coverage (-): 0

Region: NODE\_27459\_length\_24731\_cov\_33.167362 8234-8250. Max. coverage (+): 0. Max coverage (-): 0

Region: NODE\_27459\_length\_24731\_cov\_33.167362 8251-8267. Max. coverage (+): 0.84. Max coverage (-): 0.08

Region: NODE\_27459\_length\_24731\_cov\_33.167362 8268-8284. Max. coverage (+): 0.61. Max coverage (-): 0

Region: NODE\_27459\_length\_24731\_cov\_33.167362 8285-8301. Max. coverage (+): 0. Max coverage (-): 0.31

Region: NODE\_27459\_length\_24731\_cov\_33.167362 8302-8317. Max. coverage (+): 0. Max coverage (-): 0.31

Region: NODE\_27459\_length\_24731\_cov\_33.167362 8318-8334. Max. coverage (+): 0.08. Max coverage (-): 0

Region: NODE\_27459\_length\_24731\_cov\_33.167362 8335-8351. Max. coverage (+): 0. Max coverage (-): 0

Region: NODE\_27459\_length\_24731\_cov\_33.167362 8352-8368. Max. coverage (+): 0.08. Max coverage (-): 0

Region: NODE\_27459\_length\_24731\_cov\_33.167362 8369-8385. Max. coverage (+): 0.08. Max coverage (-): 0.15

Region: NODE\_27459\_length\_24731\_cov\_33.167362 8386-8402. Max. coverage (+): 0.23. Max coverage (-): 0

Region: NODE\_27459\_length\_24731\_cov\_33.167362 8403-8419. Max. coverage (+): 0. Max coverage (-): 0.08

Region: NODE\_27459\_length\_24731\_cov\_33.167362 8420-8436. Max. coverage (+): 0.08. Max coverage (-): 0

Region: NODE\_27459\_length\_24731\_cov\_33.167362 8437-8453. Max. coverage (+): 0.15. Max coverage (-): 0

Region: NODE\_27459\_length\_24731\_cov\_33.167362 8454-8470. Max. coverage (+): 19.54. Max coverage (-): 0

Region: NODE\_27459\_length\_24731\_cov\_33.167362 8471-8487. Max. coverage (+): 17.7. Max coverage (-): 0.08

Region: NODE\_27459\_length\_24731\_cov\_33.167362 8488-8503. Max. coverage (+): 0.08. Max coverage (-): 0

Region: NODE\_27459\_length\_24731\_cov\_33.167362 8504-8520. Max. coverage (+): 0.08. Max coverage (-): 0

Region: NODE\_27459\_length\_24731\_cov\_33.167362 8521-8537. Max. coverage (+): 0. Max coverage (-): 0

Region: NODE\_27459\_length\_24731\_cov\_33.167362 8538-8554. Max. coverage (+): 0. Max coverage (-): 0

Region: NODE\_27459\_length\_24731\_cov\_33.167362 8555-8571. Max. coverage (+): 0.15. Max coverage (-): 0

Region: NODE\_27459\_length\_24731\_cov\_33.167362 8572-8588. Max. coverage (+): 0.15. Max coverage (-): 0

Region: NODE\_27459\_length\_24731\_cov\_33.167362 8589-8605. Max. coverage (+): 0.15. Max coverage (-): 0

Region: NODE\_27459\_length\_24731\_cov\_33.167362 8606-8622. Max. coverage (+): 0.08. Max coverage (-): 0

Region: NODE\_27459\_length\_24731\_cov\_33.167362 8623-8639. Max. coverage (+): 0.23. Max coverage (-): 0

Region: NODE\_27459\_length\_24731\_cov\_33.167362 8640-8656. Max. coverage (+): 0. Max coverage (-): 0

Region: NODE\_27459\_length\_24731\_cov\_33.167362 8657-8672. Max. coverage (+): 0.84. Max coverage (-): 0

Region: NODE\_27459\_length\_24731\_cov\_33.167362 8673-8689. Max. coverage (+): 0.31. Max coverage (-): 0

Region: NODE\_27459\_length\_24731\_cov\_33.167362 8690-8706. Max. coverage (+): 0.38. Max coverage (-): 0

Region: NODE\_27459\_length\_24731\_cov\_33.167362 8707-8723. Max. coverage (+): 1.23. Max coverage (-): 0

Region: NODE\_27459\_length\_24731\_cov\_33.167362 8724-8740. Max. coverage (+): 2.15. Max coverage (-): 0

Region: NODE\_27459\_length\_24731\_cov\_33.167362 8741-8757. Max. coverage (+): 0.08. Max coverage (-): 0

Region: NODE\_27459\_length\_24731\_cov\_33.167362 8758-8774. Max. coverage (+): 0. Max coverage (-): 0

Region: NODE\_27459\_length\_24731\_cov\_33.167362 8775-8791. Max. coverage (+): 1.15. Max coverage (-): 0

Region: NODE\_27459\_length\_24731\_cov\_33.167362 8792-8808. Max. coverage (+): 0.15. Max coverage (-): 0

Region: NODE\_27459\_length\_24731\_cov\_33.167362 8809-8825. Max. coverage (+): 1.53. Max coverage (-): 0

Region: NODE\_27459\_length\_24731\_cov\_33.167362 8826-8842. Max. coverage (+): 0.23. Max coverage (-): 0

Region: NODE\_27459\_length\_24731\_cov\_33.167362 8843-8858. Max. coverage (+): 0.15. Max coverage (-): 0

Region: NODE\_27459\_length\_24731\_cov\_33.167362 8859-8875. Max. coverage (+): 0.08. Max coverage (-): 0

Region: NODE\_27459\_length\_24731\_cov\_33.167362 8876-8892. Max. coverage (+): 0.15. Max coverage (-): 0.15

Region: NODE\_27459\_length\_24731\_cov\_33.167362 8893-8909. Max. coverage (+): 0.46. Max coverage (-): 0

Region: NODE\_27459\_length\_24731\_cov\_33.167362 8910-8926. Max. coverage (+): 0.08. Max coverage (-): 0

Region: NODE\_27459\_length\_24731\_cov\_33.167362 8927-8943. Max. coverage (+): 1. Max coverage (-): 0

Region: NODE\_27459\_length\_24731\_cov\_33.167362 8944-8960. Max. coverage (+): 0. Max coverage (-): 0

Region: NODE\_27459\_length\_24731\_cov\_33.167362 8961-8977. Max. coverage (+): 0.38. Max coverage (-): 0

Region: NODE\_27459\_length\_24731\_cov\_33.167362 8978-8994. Max. coverage (+): 0.54. Max coverage (-): 0

Region: NODE\_27459\_length\_24731\_cov\_33.167362 8995-9011. Max. coverage (+): 0.31. Max coverage (-): 0.15

Region: NODE\_27459\_length\_24731\_cov\_33.167362 9012-9027. Max. coverage (+): 0.38. Max coverage (-): 0.08

Region: NODE\_27459\_length\_24731\_cov\_33.167362 9028-9044. Max. coverage (+): 0.23. Max coverage (-): 0

Region: NODE\_27459\_length\_24731\_cov\_33.167362 9045-9061. Max. coverage (+): 0.61. Max coverage (-): 0

Region: NODE\_27459\_length\_24731\_cov\_33.167362 9062-9078. Max. coverage (+): 0.31. Max coverage (-): 0

Region: NODE\_27459\_length\_24731\_cov\_33.167362 9079-9095. Max. coverage (+): 0. Max coverage (-): 0

Region: NODE\_27459\_length\_24731\_cov\_33.167362 9096-9112. Max. coverage (+): 0. Max coverage (-): 0.01

Region: NODE\_27459\_length\_24731\_cov\_33.167362 9113-9129. Max. coverage (+): 0. Max coverage (-): 0

Region: NODE\_27459\_length\_24731\_cov\_33.167362 9130-9146. Max. coverage (+): 0. Max coverage (-): 0

Region: NODE\_27459\_length\_24731\_cov\_33.167362 9147-9163. Max. coverage (+): 0. Max coverage (-): 0

Region: NODE\_27459\_length\_24731\_cov\_33.167362 9164-9180. Max. coverage (+): 0.01. Max coverage (-): 0

Region: NODE\_27459\_length\_24731\_cov\_33.167362 9181-9197. Max. coverage (+): 0. Max coverage (-): 0.02

Region: NODE\_27459\_length\_24731\_cov\_33.167362 9198-9213. Max. coverage (+): 0. Max coverage (-): 0

Region: NODE\_27459\_length\_24731\_cov\_33.167362 9214-9230. Max. coverage (+): 0. Max coverage (-): 0

Region: NODE\_27459\_length\_24731\_cov\_33.167362 9231-9247. Max. coverage (+): 0. Max coverage (-): 0

Region: NODE\_27459\_length\_24731\_cov\_33.167362 9248-9264. Max. coverage (+): 0. Max coverage (-): 0

Region: NODE\_27459\_length\_24731\_cov\_33.167362 9265-9281. Max. coverage (+): 0.08. Max coverage (-): 0

Region: NODE\_27459\_length\_24731\_cov\_33.167362 9282-9298. Max. coverage (+): 0. Max coverage (-): 0

Region: NODE\_27459\_length\_24731\_cov\_33.167362 9299-9315. Max. coverage (+): 0. Max coverage (-): 0

Region: NODE\_27459\_length\_24731\_cov\_33.167362 9316-9332. Max. coverage (+): 0. Max coverage (-): 0

Region: NODE\_27459\_length\_24731\_cov\_33.167362 9333-9349. Max. coverage (+): 1.28. Max coverage (-): 0

Region: NODE\_27459\_length\_24731\_cov\_33.167362 9350-9366. Max. coverage (+): 0. Max coverage (-): 0

Region: NODE\_27459\_length\_24731\_cov\_33.167362 9367-9382. Max. coverage (+): 0.01. Max coverage (-): 0

Region: NODE\_27459\_length\_24731\_cov\_33.167362 9383-9399. Max. coverage (+): 0. Max coverage (-): 0

Region: NODE\_27459\_length\_24731\_cov\_33.167362 9400-9416. Max. coverage (+): 0. Max coverage (-): 0

Region: NODE\_27459\_length\_24731\_cov\_33.167362 9417-9433. Max. coverage (+): 0. Max coverage (-): 0

Region: NODE\_27459\_length\_24731\_cov\_33.167362 9434-9450. Max. coverage (+): 0.04. Max coverage (-): 0

Region: NODE\_27459\_length\_24731\_cov\_33.167362 9451-9467. Max. coverage (+): 0. Max coverage (-): 0.23

Region: NODE\_27459\_length\_24731\_cov\_33.167362 9468-9484. Max. coverage (+): 0.03. Max coverage (-): 0.23

Region: NODE\_27459\_length\_24731\_cov\_33.167362 9485-9501. Max. coverage (+): 0.02. Max coverage (-): 0

Region: NODE\_27459\_length\_24731\_cov\_33.167362 9502-9518. Max. coverage (+): 0. Max coverage (-): 0

Region: NODE\_27459\_length\_24731\_cov\_33.167362 9519-9535. Max. coverage (+): 0. Max coverage (-): 0

Region: NODE\_27459\_length\_24731\_cov\_33.167362 9536-9551. Max. coverage (+): 0. Max coverage (-): 0

Region: NODE\_27459\_length\_24731\_cov\_33.167362 9552-9568. Max. coverage (+): 0. Max coverage (-): 0

Region: NODE\_27459\_length\_24731\_cov\_33.167362 9569-9585. Max. coverage (+): 0. Max coverage (-): 0

Region: NODE\_27459\_length\_24731\_cov\_33.167362 9586-9602. Max. coverage (+): 0. Max coverage (-): 0

Region: NODE\_27459\_length\_24731\_cov\_33.167362 9603-9619. Max. coverage (+): 0.01. Max coverage (-): 0

Region: NODE\_27459\_length\_24731\_cov\_33.167362 9620-9636. Max. coverage (+): 0. Max coverage (-): 0

Region: NODE\_27459\_length\_24731\_cov\_33.167362 9637-9653. Max. coverage (+): 0. Max coverage (-): 0

Region: NODE\_27459\_length\_24731\_cov\_33.167362 9654-9670. Max. coverage (+): 0.03. Max coverage (-): 0

Region: NODE\_27459\_length\_24731\_cov\_33.167362 9671-9687. Max. coverage (+): 0. Max coverage (-): 0

Region: NODE\_27459\_length\_24731\_cov\_33.167362 9688-9704. Max. coverage (+): 0.02. Max coverage (-): 0

Region: NODE\_27459\_length\_24731\_cov\_33.167362 9705-9721. Max. coverage (+): 0. Max coverage (-): 0

Region: NODE\_27459\_length\_24731\_cov\_33.167362 9722-9737. Max. coverage (+): 0.31. Max coverage (-): 0

Region: NODE\_27459\_length\_24731\_cov\_33.167362 9738-9754. Max. coverage (+): 0. Max coverage (-): 0

Region: NODE\_27459\_length\_24731\_cov\_33.167362 9755-9771. Max. coverage (+): 0. Max coverage (-): 0

Region: NODE\_27459\_length\_24731\_cov\_33.167362 9772-9788. Max. coverage (+): 0. Max coverage (-): 0.04

Region: NODE\_27459\_length\_24731\_cov\_33.167362 9789-9805. Max. coverage (+): 0. Max coverage (-): 0

Region: NODE\_27459\_length\_24731\_cov\_33.167362 9806-9822. Max. coverage (+): 0.03. Max coverage (-): 0.03

Region: NODE\_27459\_length\_24731\_cov\_33.167362 9823-9839. Max. coverage (+): 0.01. Max coverage (-): 0

Region: NODE\_27459\_length\_24731\_cov\_33.167362 9840-9856. Max. coverage (+): 0. Max coverage (-): 0

Region: NODE\_27459\_length\_24731\_cov\_33.167362 9857-9873. Max. coverage (+): 0.03. Max coverage (-): 0.02

Region: NODE\_27459\_length\_24731\_cov\_33.167362 9874-9890. Max. coverage (+): 0. Max coverage (-): 0.02

Region: NODE\_27459\_length\_24731\_cov\_33.167362 9891-9906. Max. coverage (+): 0. Max coverage (-): 0

Region: NODE\_27459\_length\_24731\_cov\_33.167362 9907-9923. Max. coverage (+): 0. Max coverage (-): 0

Region: NODE\_27459\_length\_24731\_cov\_33.167362 9924-9940. Max. coverage (+): 0.01. Max coverage (-): 0

Region: NODE\_27459\_length\_24731\_cov\_33.167362 9941-9957. Max. coverage (+): 0. Max coverage (-): 0

Region: NODE\_27459\_length\_24731\_cov\_33.167362 9958-9974. Max. coverage (+): 0. Max coverage (-): 0

Region: NODE\_27459\_length\_24731\_cov\_33.167362 9975-9991. Max. coverage (+): 0. Max coverage (-): 0

Region: NODE\_27459\_length\_24731\_cov\_33.167362 9992-10008. Max. coverage (+): 0. Max coverage (-): 0

Region: NODE\_27459\_length\_24731\_cov\_33.167362 10009-10025. Max. coverage (+): 0. Max coverage (-): 0

Region: NODE\_27459\_length\_24731\_cov\_33.167362 10026-10042. Max. coverage (+): 0. Max coverage (-): 0

Region: NODE\_27459\_length\_24731\_cov\_33.167362 10043-10059. Max. coverage (+): 0. Max coverage (-): 0

Region: NODE\_27459\_length\_24731\_cov\_33.167362 10060-10076. Max. coverage (+): 0.08. Max coverage (-): 0

Region: NODE\_27459\_length\_24731\_cov\_33.167362 10077-10092. Max. coverage (+): 0. Max coverage (-): 0

Region: NODE\_27459\_length\_24731\_cov\_33.167362 10093-10109. Max. coverage (+): 0. Max coverage (-): 0

Region: NODE\_27459\_length\_24731\_cov\_33.167362 10110-10126. Max. coverage (+): 0. Max coverage (-): 0

Region: NODE\_27459\_length\_24731\_cov\_33.167362 10127-10143. Max. coverage (+): 0. Max coverage (-): 0

Region: NODE\_27459\_length\_24731\_cov\_33.167362 10144-10160. Max. coverage (+): 0. Max coverage (-): 0

Region: NODE\_27459\_length\_24731\_cov\_33.167362 10161-10177. Max. coverage (+): 0. Max coverage (-): 0.54

Region: NODE\_27459\_length\_24731\_cov\_33.167362 10178-10194. Max. coverage (+): 0.11. Max coverage (-): 0.6

Region: NODE\_27459\_length\_24731\_cov\_33.167362 10195-10211. Max. coverage (+): 0. Max coverage (-): 0

Region: NODE\_27459\_length\_24731\_cov\_33.167362 10212-10228. Max. coverage (+): 0.04. Max coverage (-): 0

Region: NODE\_27459\_length\_24731\_cov\_33.167362 10229-10245. Max. coverage (+): 0.04. Max coverage (-): 0

Region: NODE\_27459\_length\_24731\_cov\_33.167362 10246-10261. Max. coverage (+): 0. Max coverage (-): 0

Region: NODE\_27459\_length\_24731\_cov\_33.167362 10262-10278. Max. coverage (+): 0. Max coverage (-): 0

Region: NODE\_27459\_length\_24731\_cov\_33.167362 10279-10295. Max. coverage (+): 0. Max coverage (-): 0

Region: NODE\_27459\_length\_24731\_cov\_33.167362 10296-10312. Max. coverage (+): 0. Max coverage (-): 0

Region: NODE\_27459\_length\_24731\_cov\_33.167362 10313-10329. Max. coverage (+): 0. Max coverage (-): 0

Region: NODE\_27459\_length\_24731\_cov\_33.167362 10330-10346. Max. coverage (+): 0. Max coverage (-): 0

Region: NODE\_27459\_length\_24731\_cov\_33.167362 10347-10363. Max. coverage (+): 0. Max coverage (-): 0

Region: NODE\_27459\_length\_24731\_cov\_33.167362 10364-10380. Max. coverage (+): 0. Max coverage (-): 0

Region: NODE\_27459\_length\_24731\_cov\_33.167362 10381-10397. Max. coverage (+): 0.31. Max coverage (-): 0

Region: NODE\_27459\_length\_24731\_cov\_33.167362 10398-10414. Max. coverage (+): 0. Max coverage (-): 0

Region: NODE\_27459\_length\_24731\_cov\_33.167362 10415-10430. Max. coverage (+): 0. Max coverage (-): 0

Region: NODE\_27459\_length\_24731\_cov\_33.167362 10431-10447. Max. coverage (+): 0. Max coverage (-): 0

Region: NODE\_27459\_length\_24731\_cov\_33.167362 10448-10464. Max. coverage (+): 0. Max coverage (-): 0

Region: NODE\_27459\_length\_24731\_cov\_33.167362 10465-10481. Max. coverage (+): 0. Max coverage (-): 0

Region: NODE\_27459\_length\_24731\_cov\_33.167362 10482-10498. Max. coverage (+): 0. Max coverage (-): 0

Region: NODE\_27459\_length\_24731\_cov\_33.167362 10499-10515. Max. coverage (+): 0. Max coverage (-): 0

Region: NODE\_27459\_length\_24731\_cov\_33.167362 10516-10532. Max. coverage (+): 0. Max coverage (-): 0

Region: NODE\_27459\_length\_24731\_cov\_33.167362 10533-10549. Max. coverage (+): 0. Max coverage (-): 0

Region: NODE\_27459\_length\_24731\_cov\_33.167362 10550-10566. Max. coverage (+): 0. Max coverage (-): 0

Region: NODE\_27459\_length\_24731\_cov\_33.167362 10567-10583. Max. coverage (+): 0. Max coverage (-): 0

Region: NODE\_27459\_length\_24731\_cov\_33.167362 10584-10600. Max. coverage (+): 0.08. Max coverage (-): 0

Region: NODE\_27459\_length\_24731\_cov\_33.167362 10601-10616. Max. coverage (+): 0.08. Max coverage (-): 0.04

Region: NODE\_27459\_length\_24731\_cov\_33.167362 10617-10633. Max. coverage (+): 0. Max coverage (-): 0

Region: NODE\_27459\_length\_24731\_cov\_33.167362 10634-10650. Max. coverage (+): 0.03. Max coverage (-): 0.03

Region: NODE\_27459\_length\_24731\_cov\_33.167362 10651-10667. Max. coverage (+): 0.01. Max coverage (-): 0

Region: NODE\_27459\_length\_24731\_cov\_33.167362 10668-10684. Max. coverage (+): 0. Max coverage (-): 0

Region: NODE\_27459\_length\_24731\_cov\_33.167362 10685-10701. Max. coverage (+): 0.03. Max coverage (-): 0.02

Region: NODE\_27459\_length\_24731\_cov\_33.167362 10702-10718. Max. coverage (+): 0. Max coverage (-): 0.02

Region: NODE\_27459\_length\_24731\_cov\_33.167362 10719-10735. Max. coverage (+): 0. Max coverage (-): 0

Region: NODE\_27459\_length\_24731\_cov\_33.167362 10736-10752. Max. coverage (+): 0. Max coverage (-): 0

Region: NODE\_27459\_length\_24731\_cov\_33.167362 10753-10769. Max. coverage (+): 0.01. Max coverage (-): 0

Region: NODE\_27459\_length\_24731\_cov\_33.167362 10770-10785. Max. coverage (+): 0.01. Max coverage (-): 0

Region: NODE\_27459\_length\_24731\_cov\_33.167362 10786-10802. Max. coverage (+): 0. Max coverage (-): 0

Region: NODE\_27459\_length\_24731\_cov\_33.167362 10803-10819. Max. coverage (+): 0.01. Max coverage (-): 0

Region: NODE\_27459\_length\_24731\_cov\_33.167362 10820-10836. Max. coverage (+): 0. Max coverage (-): 0

Region: NODE\_27459\_length\_24731\_cov\_33.167362 10837-10853. Max. coverage (+): 0. Max coverage (-): 0

Region: NODE\_27459\_length\_24731\_cov\_33.167362 10854-10870. Max. coverage (+): 0. Max coverage (-): 0

Region: NODE\_27459\_length\_24731\_cov\_33.167362 10871-10887. Max. coverage (+): 0. Max coverage (-): 0

Region: NODE\_27459\_length\_24731\_cov\_33.167362 10888-10904. Max. coverage (+): 0. Max coverage (-): 0

Region: NODE\_27459\_length\_24731\_cov\_33.167362 10905-10921. Max. coverage (+): 0. Max coverage (-): 0.06

Region: NODE\_27459\_length\_24731\_cov\_33.167362 10922-10938. Max. coverage (+): 0. Max coverage (-): 0.6

Region: NODE\_27459\_length\_24731\_cov\_33.167362 10939-10955. Max. coverage (+): 0.11. Max coverage (-): 0

Region: NODE\_27459\_length\_24731\_cov\_33.167362 10956-10971. Max. coverage (+): 0. Max coverage (-): 0

Region: NODE\_27459\_length\_24731\_cov\_33.167362 10972-10988. Max. coverage (+): 0.04. Max coverage (-): 0

Region: NODE\_27459\_length\_24731\_cov\_33.167362 10989-11005. Max. coverage (+): 0. Max coverage (-): 0

Region: NODE\_27459\_length\_24731\_cov\_33.167362 11006-11022. Max. coverage (+): 0. Max coverage (-): 0

Region: NODE\_27459\_length\_24731\_cov\_33.167362 11023-11039. Max. coverage (+): 0. Max coverage (-): 0

Region: NODE\_27459\_length\_24731\_cov\_33.167362 11040-11056. Max. coverage (+): 0. Max coverage (-): 0

Region: NODE\_27459\_length\_24731\_cov\_33.167362 11057-11073. Max. coverage (+): 0. Max coverage (-): 0

Region: NODE\_27459\_length\_24731\_cov\_33.167362 11074-11090. Max. coverage (+): 0. Max coverage (-): 0

Region: NODE\_27459\_length\_24731\_cov\_33.167362 11091-11107. Max. coverage (+): 0. Max coverage (-): 0

Region: NODE\_27459\_length\_24731\_cov\_33.167362 11108-11124. Max. coverage (+): 0. Max coverage (-): 0

Region: NODE\_27459\_length\_24731\_cov\_33.167362 11125-11140. Max. coverage (+): 0. Max coverage (-): 0

Region: NODE\_27459\_length\_24731\_cov\_33.167362 11141-11157. Max. coverage (+): 0. Max coverage (-): 0

Region: NODE\_27459\_length\_24731\_cov\_33.167362 11158-11174. Max. coverage (+): 0. Max coverage (-): 0

Region: NODE\_27459\_length\_24731\_cov\_33.167362 11175-11191. Max. coverage (+): 0.15. Max coverage (-): 0

Region: NODE\_27459\_length\_24731\_cov\_33.167362 11192-11208. Max. coverage (+): 0. Max coverage (-): 0

Region: NODE\_27459\_length\_24731\_cov\_33.167362 11209-11225. Max. coverage (+): 0. Max coverage (-): 0

Region: NODE\_27459\_length\_24731\_cov\_33.167362 11226-11242. Max. coverage (+): 0. Max coverage (-): 0

Region: NODE\_27459\_length\_24731\_cov\_33.167362 11243-11259. Max. coverage (+): 0. Max coverage (-): 0

Region: NODE\_27459\_length\_24731\_cov\_33.167362 11260-11276. Max. coverage (+): 0. Max coverage (-): 0

Region: NODE\_27459\_length\_24731\_cov\_33.167362 11277-11293. Max. coverage (+): 0. Max coverage (-): 0

Region: NODE\_27459\_length\_24731\_cov\_33.167362 11294-11310. Max. coverage (+): 0. Max coverage (-): 0.08

Region: NODE\_27459\_length\_24731\_cov\_33.167362 11311-11326. Max. coverage (+): 0. Max coverage (-): 0

Region: NODE\_27459\_length\_24731\_cov\_33.167362 11327-11343. Max. coverage (+): 0.15. Max coverage (-): 0

Region: NODE\_27459\_length\_24731\_cov\_33.167362 11344-11360. Max. coverage (+): 0. Max coverage (-): 0

Region: NODE\_27459\_length\_24731\_cov\_33.167362 11361-11377. Max. coverage (+): 0. Max coverage (-): 0

Region: NODE\_27459\_length\_24731\_cov\_33.167362 11378-11394. Max. coverage (+): 0. Max coverage (-): 0

Region: NODE\_27459\_length\_24731\_cov\_33.167362 11395-11411. Max. coverage (+): 0. Max coverage (-): 0

Region: NODE\_27459\_length\_24731\_cov\_33.167362 11412-11428. Max. coverage (+): 0. Max coverage (-): 0

Region: NODE\_27459\_length\_24731\_cov\_33.167362 11429-11445. Max. coverage (+): 0. Max coverage (-): 0

Region: NODE\_27459\_length\_24731\_cov\_33.167362 11446-11462. Max. coverage (+): 0. Max coverage (-): 0

Region: NODE\_27459\_length\_24731\_cov\_33.167362 11463-11479. Max. coverage (+): 0. Max coverage (-): 0

Region: NODE\_27459\_length\_24731\_cov\_33.167362 11480-11495. Max. coverage (+): 0. Max coverage (-): 0.08

Region: NODE\_27459\_length\_24731\_cov\_33.167362 11496-11512. Max. coverage (+): 3.68. Max coverage (-): 0

Region: NODE\_27459\_length\_24731\_cov\_33.167362 11513-11529. Max. coverage (+): 3.6. Max coverage (-): 0

Region: NODE\_27459\_length\_24731\_cov\_33.167362 11530-11546. Max. coverage (+): 0. Max coverage (-): 0

Region: NODE\_27459\_length\_24731\_cov\_33.167362 11547-11563. Max. coverage (+): 0.08. Max coverage (-): 0

Region: NODE\_27459\_length\_24731\_cov\_33.167362 11564-11580. Max. coverage (+): 0. Max coverage (-): 0

Region: NODE\_27459\_length\_24731\_cov\_33.167362 11581-11597. Max. coverage (+): 0. Max coverage (-): 0

Region: NODE\_27459\_length\_24731\_cov\_33.167362 11598-11614. Max. coverage (+): 0.08. Max coverage (-): 0

Region: NODE\_27459\_length\_24731\_cov\_33.167362 11615-11631. Max. coverage (+): 0. Max coverage (-): 0

Region: NODE\_27459\_length\_24731\_cov\_33.167362 11632-11648. Max. coverage (+): 0.08. Max coverage (-): 0

Region: NODE\_27459\_length\_24731\_cov\_33.167362 11649-11664. Max. coverage (+): 0.08. Max coverage (-): 0

Region: NODE\_27459\_length\_24731\_cov\_33.167362 11665-11681. Max. coverage (+): 0.08. Max coverage (-): 0

Region: NODE\_27459\_length\_24731\_cov\_33.167362 11682-11698. Max. coverage (+): 0. Max coverage (-): 0

Region: NODE\_27459\_length\_24731\_cov\_33.167362 11699-11715. Max. coverage (+): 0. Max coverage (-): 0

Region: NODE\_27459\_length\_24731\_cov\_33.167362 11716-11732. Max. coverage (+): 0. Max coverage (-): 0

Region: NODE\_27459\_length\_24731\_cov\_33.167362 11733-11749. Max. coverage (+): 0. Max coverage (-): 0

Region: NODE\_27459\_length\_24731\_cov\_33.167362 11750-11766. Max. coverage (+): 0. Max coverage (-): 0

Region: NODE\_27459\_length\_24731\_cov\_33.167362 11767-11783. Max. coverage (+): 0. Max coverage (-): 0

Region: NODE\_27459\_length\_24731\_cov\_33.167362 11784-11800. Max. coverage (+): 0.31. Max coverage (-): 0

Region: NODE\_27459\_length\_24731\_cov\_33.167362 11801-11817. Max. coverage (+): 0. Max coverage (-): 0

Region: NODE\_27459\_length\_24731\_cov\_33.167362 11818-11834. Max. coverage (+): 0. Max coverage (-): 0

Region: NODE\_27459\_length\_24731\_cov\_33.167362 11835-11850. Max. coverage (+): 0.15. Max coverage (-): 0

Region: NODE\_27459\_length\_24731\_cov\_33.167362 11851-11867. Max. coverage (+): 0.15. Max coverage (-): 0

Region: NODE\_27459\_length\_24731\_cov\_33.167362 11868-11884. Max. coverage (+): 0. Max coverage (-): 0.23

Region: NODE\_27459\_length\_24731\_cov\_33.167362 11885-11901. Max. coverage (+): 0.46. Max coverage (-): 0

Region: NODE\_27459\_length\_24731\_cov\_33.167362 11902-11918. Max. coverage (+): 0. Max coverage (-): 0

Region: NODE\_27459\_length\_24731\_cov\_33.167362 11919-. Max. coverage (+): 0. Max coverage (-): 0

RepeatMasker Color Code

**+**

100-98% Identity

<98-95% Identity

<95-90% Identity

<90-85% Identity

<85-80% Identity

<80-75% Identity

<75-70% Identity

<70% Identity

**-**

Gene Set Color Code

**+**

Gene

Pseudogene

Other

**-**

Topology/Coverage Color Code

Coverage Plus Strand

Coverage Minus Strand

Mainstrand: Plus

Mainstrand: Minus

Complementary Strand

Flanking Region  
(if option -flank >0)

Gene Set Annotation  
  
RepeatMasker Annotation  

**1. AlRepB-738**: 3481-4174 (-), Divergence to consensus: 10.8%  
**2. AlRepA-386**: 4441-4582 (-), Divergence to consensus: 31.3%  
**3. Penelope-1\_AFC**: 4913-5068 (-), Divergence to consensus: 25.9%  
**4. Penelope-1\_AFC**: 5221-5361 (-), Divergence to consensus: 32.8%  
**5. AlRepA-297**: 6124-6490 (+), Divergence to consensus: 30.7%  
**6. AlRepA-297**: 6665-6841 (+), Divergence to consensus: 37.5%  
**7. AlRepB-429**: 9110-9231 (+), Divergence to consensus: 0.8%  
**8. AlRepB-738**: 9325-9773 (+), Divergence to consensus: 8.1%  
**9. AlRepB-738**: 9775-9999 (+), Divergence to consensus: 6.7%  
**10. AlRepB-569**: 10073-10165 (-), Divergence to consensus: 31.8%  
**11. Dong2\_FR**: 10173-10266 (-), Divergence to consensus: 25.5%  
**12. (TTA)n**: 10284-10331 (+), Divergence to consensus: 0%  
**13. EnSpm-17\_HM**: 10332-10381 (-), Divergence to consensus: 18.3%  
**14. AlRepB-738**: 10354-10515 (+), Divergence to consensus: 11.6%  
**15. AlRepB-738**: 10551-10827 (+), Divergence to consensus: 6.2%  
**16. Dong2\_FR**: 10923-11016 (-), Divergence to consensus: 25.5%  
**17. (TTA)n**: 11034-11066 (+), Divergence to consensus: 0%  
**18. EnSpm-17\_HM**: 11067-11120 (-), Divergence to consensus: 20.6%  
**19. RTE-2\_AFC**: 11322-11633 (+), Divergence to consensus: 32.1%  
**20. SINE\_TE**: 11768-11963 (-), Divergence to consensus: 23.6%  
**21. DNA7-N2\_DR**: 11905-11982 (+), Divergence to consensus: 34.6%

  
Transcription Factor Binding Sites  

**RHOXF1** (Sequence: AGATTA (-): 3517)  
**RHOXF1** (Sequence: GGATTA (-): 3597)  
**RHOXF1** (Sequence: AGCTTA (-): 3983)  
**RHOXF1** (Sequence: AGATTA (-): 5927)  
**RHOXF1** (Sequence: GGCTTA (-): 8400)  
**RHOXF1** (Sequence: AGATCA (-): 8402)  
**RHOXF1** (Sequence: GGATTA (-): 9456)  
**RHOXF1** (Sequence: TGAGCC (+): 4492)  
**RHOXF1** (Sequence: TAATCC (+): 4497)  
**RHOXF1** (Sequence: TAAGCT (+): 5732)  
**RHOXF1** (Sequence: TAAGCC (+): 6453)  
**RHOXF1** (Sequence: TGAGCC (+): 6846)  
**RHOXF1** (Sequence: TAATCC (+): 8095)  
**RHOXF1** (Sequence: TAAGCC (+): 8531)  
**RHOXF1** (Sequence: TGATCC (+): 8696)  
**RHOXF1** (Sequence: TGATCC (+): 8700)  
**RHOXF1** (Sequence: TGATCT (+): 8860)  
**RHOXF1** (Sequence: TGAGCT (+): 9004)  
**RHOXF1** (Sequence: TAATCC (+): 9873)  
**RHOXF1** (Sequence: TAATCT (+): 9958)  
**RHOXF1** (Sequence: TAATCC (+): 10701)  
**RHOXF1** (Sequence: TAATCT (+): 10786)  
**RHOXF1** (Sequence: TGAGCT (+): 11213)  
**Lhx8** (Sequence: CTAATTAG (-): 7404)  
**Gata4** (Sequence: CTTATCT (+): 5379)  
**POU5F1** (Sequence: TTTGCAT (-): 5682)  
**POU5F1** (Sequence: TTTGCAT (-): 7582)  
**POU5F1** (Sequence: TTTGCAT (-): 7837)  
**SOX9** (Sequence: AACAATAG (-): 7916)  
**SOX9** (Sequence: AACAATGA (-): 7938)  
**SOX9** (Sequence: AACAATGA (-): 8350)  
**FOXO1** (Sequence: CTTGTTTTT (+): 4795)  
**FOXO1** (Sequence: CCTGTTTAT (+): 7110)  
**FOXO1** (Sequence: CCTGTTTTT (+): 7299)  
**FOXO1** (Sequence: GCTGTTTTT (+): 8250)  
**FOXO3\_mmu** (Sequence: TGTTTTCC (-): 9470)  
**Sox5** (Sequence: ATTGTT (+): 3936)  
**Sox5** (Sequence: ATTGTT (+): 6668)  
**Sox5** (Sequence: ATTGTT (+): 7617)  
**Sox5** (Sequence: ATTGTT (+): 7980)  
**Sox5** (Sequence: ATTGTT (+): 10087)  
**Sox5** (Sequence: ATTGTT (+): 10090)  
**SOX9** (Sequence: TTATTGTT (+): 3934)  
**SOX9** (Sequence: TCATTGTT (+): 7615)  
**SOX9** (Sequence: TCATTGTT (+): 7978)  
**SOX9** (Sequence: TTATTGTT (+): 10085)  
**FOXO3\_mmu** (Sequence: GGAAAACA (+): 3947)  
**FOXO3\_mmu** (Sequence: GCAAAACA (+): 8740)  
**FOXO3\_mmu** (Sequence: GGAAAACA (+): 10123)  
**Nobox** (Sequence: AGTAATTA (-): 4233)  
**Nobox** (Sequence: ACCAATTA (-): 4572)  
**Nobox** (Sequence: ACTAATTA (-): 9258)  
**FOXO1** (Sequence: AAAAACAAC (-): 5037)  
**FOXO1** (Sequence: AAAAACAGG (-): 11386)  
**Nobox** (Sequence: TAATTAGT (+): 4235)  
**Nobox** (Sequence: TAATTAGT (+): 7405)  
**Rhox11** (Sequence: TGCTGTAAA (+): 7852)  
**Rhox11** (Sequence: TGCTGTTTT (+): 8249)  
**Rhox11** (Sequence: TGCTGTTAT (+): 8310)  
**Rhox11** (Sequence: CGGTGTTTT (+): 9467)  
**Rhox11** (Sequence: ATTACAGCG (-): 6030)  
**Sox5** (Sequence: AACAAT (-): 5251)  
**Sox5** (Sequence: AACAAT (-): 7916)  
**Sox5** (Sequence: AACAAT (-): 7938)  
**Sox5** (Sequence: AACAAT (-): 8350)  
**POU2F1** (Sequence: TATTTTAAT (+): 5750)  
**POU5F1** (Sequence: ATGCAAA (+): 11561)
