# Supplementary material for: First characterization of PIWI-interacting RNA clusters in a cichlid fish with a B chromosome
Source: BMC Biol. 2022 Sep 21;20:204. doi: 10.1186/s12915-022-01403-2 (PMC9490952; doi:10.1186/s12915-022-01403-2)
Supplement: Supplementary file 1 — Additional file 1. Zipped folder with fasta and interactive html piRNA cluster information for the A. latifasciata genome. The nomenclature is as follows: number-pirna-cluster_sex_B-presence (f, female; m, male; 0b, without B chromosome; 1b, with B chromosome). [file 12915_2022_1403_MOESM1_ESM.zip › 106_f1b.html]

piRNA cluster 106\_f1b 7


Predicted piRNA cluster no. 106\_f1b
  

Show proTRAC run info
Hide proTRAC run info

/\  
                \_\_\_\_\_\_\_\_\_\_\_\_\_\_\_\_\_\_\_\_\_\_\_/\\_\_\_ /  \\_\_\_\_\_\_\_  
               I                      /  \  /    \      I  
               I     pro             /    \/      \     I  
               I        TRAC        /               \   I  
               I   \_\_\_\_\_\_\_\_\_\_\_\_\_\_\_\_/\_\_\_\_\_\_\_\_\_\_\_\_\_\_\_\_\_\\_ I  
               I   \              /                     I  
               I    \            /                      I  
               I     \  /\      /       V.2.4.2         I  
               I      \/  \    /                        I  
               I\_\_\_\_\_\_\_\_\_\_\_\  /\_\_\_\_\_\_\_\_\_\_\_\_\_\_\_\_\_\_\_\_\_\_\_\_\_I  
                            \/  
  
  
================================= proTRAC ====================================  
VERSION: .......... 2.4.2  
LAST MODIFIED: .... 11. May 2018  
  
Please cite:  
Rosenkranz D, Zischler H. proTRAC - a software for probabilistic piRNA cluster  
detection, visualization and analysis. 2012. BMC Bioinformatics 13:5.  
  
  
Contact:  
David Rosenkranz  
Institute of Organismic and Molecular Evolutionary Biology  
Dept. Anthropology, small RNA group  
Johannes Gutenberg University Mainz  
email: rosenkranz@uni-mainz.de  
  
You can find the latest proTRAC version at:  
http://sourceforge.net/projects/protrac/files  
http://www.smallRNAgroup-mainz.de/software  
==============================================================================  
  
PARAMETERS:  
Map file: ...............piwi-femeas-1B.fa-collapse.map  
Genome file: ............../../../0B\_ala\_genome.fa  
RepeatMasker annotation: Alatifasciata-all0B-maryan-v2.fa\_corrected.out  
GeneSet:................./guest-storage/Data/annotation/Alatifasciata\_all0B\_maryan-v2\_out2017.gff  
  
Significant (p<=0.01) hit density will be calculated based  
on observed hit distribution.  
  
Sliding window size: ........................................ 5000 bp  
Sliding window increament: .................................. 1000 bp  
Normalize each hit by number of genomic hits: ............... yes  
Normalize each hit by number of sequence reads: ............. yes  
Normalize values (-> per million mapped reads): ............. yes  
Min. fraction of hits with 1T(U) or 10A: .................... 0.75  
Alternatively: Min. fraction of hits with 1T(U) and 10A: .... 0.5  
Min. fraction of hits with typical piRNA length: ............ 0.75  
Typical piRNA length: ....................................... 24-32 nt  
Min. size of a piRNA cluster: ............................... 1000 bp.  
Min. number of hits (absolute): ............................. 0  
Min. number of hits (normalized): ........................... 0  
Min. fraction of hits on the mainstrand: .................... 0.75  
Top fraction of mapped sequences (in terms of read counts): . 1%  
Top fraction accounts for max. n% of sequence reads: ........ 90%  
Min. fraction of hits on each arm of a bidirectional cluster: 0.05  
Output html file for each cluster: .......................... yes  
Output a summary table: ..................................... yes  
Output a FASTA file for each cluster (piRNA sequences): ..... yes  
Output a FASTA file comprising cluster sequences: ........... yes  
Output a GTF file for predicted piRNA clusters: ..............yes  
Search DNA motifs in clusters: .............................. yes  
Output flanking sequences: +/- .............................. 0 bp  
Output ~.pTi file: .......................................... no  
==============================================================================  
  
  
Genome size (without gaps): ............ 758543724 bp  
Gaps (N/X/-): .......................... 417479 bp  
Mapped reads: .......................... 10641844  
Non-identical sequences: ............... 2832837  
Genomic hits: .......................... 26056853  
Significant densitiy of mapped reads: .. 368.713530323068 reads/kb

Show proTRAC cluster info
Hide proTRAC cluster info

|  |  |
| --- | --- |
| Location | NODE\_27459\_length\_24731\_cov\_33.167362 |
| Coordinates | 3367-11972 |
| Size [bp] | 8606 |
| Sequence hit loci | 1341 |
| Mapped reads (normalized) | 2884.9 |
| Mapped reads (normalized) per kb | 335.2 |
| Normalized reads with 1T (1U) | 87.2% |
| Normalized reads with 10A | 28.6% |
| Normalized reads with length 24-32 nt | 99.3% |
| Normalized reads on the main strand(s) | 92.9% |
| Predicted directionality | mono:plus |

100%

0%

1T (1U)  
reads

10A reads

24-32 nt  
reads

reads on mainstrand

**Either the amount of reads with 1T (1U) OR 10A has to exceed 75% (set with option: -1Tor10A)  
Alternatively the amount of reads with 1T (1U) AND 10A has to exceed 50% (set with option: -1Tand10A)  
Minimum amount of reads with preferred size is 75% (set with option: -pisize)  
Minimum amount of reads on the main strand(s) is 75% (set with option: -clstrand)**

Show read coverage
Hide read coverage

WHAT DO I SEE HERE?  
This chart shows the location of mapped sequence reads within a predicted piRNA cluster. The color refers to the number of genomic hits produced by the sequence read in question. A dark red bar indicates that this sequence read produces many other hits elsewhere in the genome. Many adjacent red or yellow bars can indicate the presence of a multi-copy element such as transposons or rRNA genes. A dark green bar indicates that this sequence read maps uniquely to this locus.

1 hit

2-5 hits

6-10 hits

11-20 hits

21-50 hits

51-100 hits

> 100 hits

NODE\_27459\_length\_24731\_cov\_33.167362

3367

11972

Gene Set

RepeatMasker

Mapped  
Reads

72.17

plus strand

minus strand

72.17

Region: NODE\_27459\_length\_24731\_cov\_33.167362 1334-3375. Max. coverage (+): 0. Max coverage (-): 0.09

Region: NODE\_27459\_length\_24731\_cov\_33.167362 3376-3392. Max. coverage (+): 0. Max coverage (-): 0

Region: NODE\_27459\_length\_24731\_cov\_33.167362 3393-3410. Max. coverage (+): 0. Max coverage (-): 0

Region: NODE\_27459\_length\_24731\_cov\_33.167362 3411-3427. Max. coverage (+): 0. Max coverage (-): 0

Region: NODE\_27459\_length\_24731\_cov\_33.167362 3428-3444. Max. coverage (+): 0. Max coverage (-): 0

Region: NODE\_27459\_length\_24731\_cov\_33.167362 3445-3461. Max. coverage (+): 0. Max coverage (-): 0

Region: NODE\_27459\_length\_24731\_cov\_33.167362 3462-3478. Max. coverage (+): 0. Max coverage (-): 0

Region: NODE\_27459\_length\_24731\_cov\_33.167362 3479-3496. Max. coverage (+): 0. Max coverage (-): 0

Region: NODE\_27459\_length\_24731\_cov\_33.167362 3497-3513. Max. coverage (+): 0. Max coverage (-): 0

Region: NODE\_27459\_length\_24731\_cov\_33.167362 3514-3530. Max. coverage (+): 0. Max coverage (-): 0

Region: NODE\_27459\_length\_24731\_cov\_33.167362 3531-3547. Max. coverage (+): 0. Max coverage (-): 0

Region: NODE\_27459\_length\_24731\_cov\_33.167362 3548-3564. Max. coverage (+): 0. Max coverage (-): 0

Region: NODE\_27459\_length\_24731\_cov\_33.167362 3565-3582. Max. coverage (+): 0.02. Max coverage (-): 0.01

Region: NODE\_27459\_length\_24731\_cov\_33.167362 3583-3599. Max. coverage (+): 0. Max coverage (-): 0

Region: NODE\_27459\_length\_24731\_cov\_33.167362 3600-3616. Max. coverage (+): 0. Max coverage (-): 0

Region: NODE\_27459\_length\_24731\_cov\_33.167362 3617-3633. Max. coverage (+): 0. Max coverage (-): 0

Region: NODE\_27459\_length\_24731\_cov\_33.167362 3634-3650. Max. coverage (+): 0. Max coverage (-): 0

Region: NODE\_27459\_length\_24731\_cov\_33.167362 3651-3668. Max. coverage (+): 0. Max coverage (-): 0

Region: NODE\_27459\_length\_24731\_cov\_33.167362 3669-3685. Max. coverage (+): 0. Max coverage (-): 0

Region: NODE\_27459\_length\_24731\_cov\_33.167362 3686-3702. Max. coverage (+): 0. Max coverage (-): 0

Region: NODE\_27459\_length\_24731\_cov\_33.167362 3703-3719. Max. coverage (+): 0. Max coverage (-): 0

Region: NODE\_27459\_length\_24731\_cov\_33.167362 3720-3737. Max. coverage (+): 0. Max coverage (-): 0

Region: NODE\_27459\_length\_24731\_cov\_33.167362 3738-3754. Max. coverage (+): 0. Max coverage (-): 0

Region: NODE\_27459\_length\_24731\_cov\_33.167362 3755-3771. Max. coverage (+): 0. Max coverage (-): 0.02

Region: NODE\_27459\_length\_24731\_cov\_33.167362 3772-3788. Max. coverage (+): 0. Max coverage (-): 0

Region: NODE\_27459\_length\_24731\_cov\_33.167362 3789-3805. Max. coverage (+): 0. Max coverage (-): 0

Region: NODE\_27459\_length\_24731\_cov\_33.167362 3806-3823. Max. coverage (+): 0. Max coverage (-): 0

Region: NODE\_27459\_length\_24731\_cov\_33.167362 3824-3840. Max. coverage (+): 0. Max coverage (-): 0

Region: NODE\_27459\_length\_24731\_cov\_33.167362 3841-3857. Max. coverage (+): 0. Max coverage (-): 0.19

Region: NODE\_27459\_length\_24731\_cov\_33.167362 3858-3874. Max. coverage (+): 0. Max coverage (-): 0.09

Region: NODE\_27459\_length\_24731\_cov\_33.167362 3875-3891. Max. coverage (+): 0. Max coverage (-): 0

Region: NODE\_27459\_length\_24731\_cov\_33.167362 3892-3909. Max. coverage (+): 0. Max coverage (-): 0

Region: NODE\_27459\_length\_24731\_cov\_33.167362 3910-3926. Max. coverage (+): 0. Max coverage (-): 0

Region: NODE\_27459\_length\_24731\_cov\_33.167362 3927-3943. Max. coverage (+): 0. Max coverage (-): 0

Region: NODE\_27459\_length\_24731\_cov\_33.167362 3944-3960. Max. coverage (+): 0. Max coverage (-): 0

Region: NODE\_27459\_length\_24731\_cov\_33.167362 3961-3978. Max. coverage (+): 0. Max coverage (-): 0

Region: NODE\_27459\_length\_24731\_cov\_33.167362 3979-3995. Max. coverage (+): 0.05. Max coverage (-): 0

Region: NODE\_27459\_length\_24731\_cov\_33.167362 3996-4012. Max. coverage (+): 0. Max coverage (-): 0

Region: NODE\_27459\_length\_24731\_cov\_33.167362 4013-4029. Max. coverage (+): 0. Max coverage (-): 0

Region: NODE\_27459\_length\_24731\_cov\_33.167362 4030-4046. Max. coverage (+): 0. Max coverage (-): 0

Region: NODE\_27459\_length\_24731\_cov\_33.167362 4047-4064. Max. coverage (+): 0. Max coverage (-): 0

Region: NODE\_27459\_length\_24731\_cov\_33.167362 4065-4081. Max. coverage (+): 0. Max coverage (-): 0

Region: NODE\_27459\_length\_24731\_cov\_33.167362 4082-4098. Max. coverage (+): 0. Max coverage (-): 0

Region: NODE\_27459\_length\_24731\_cov\_33.167362 4099-4115. Max. coverage (+): 0. Max coverage (-): 0

Region: NODE\_27459\_length\_24731\_cov\_33.167362 4116-4132. Max. coverage (+): 0. Max coverage (-): 0

Region: NODE\_27459\_length\_24731\_cov\_33.167362 4133-4150. Max. coverage (+): 0.01. Max coverage (-): 0

Region: NODE\_27459\_length\_24731\_cov\_33.167362 4151-4167. Max. coverage (+): 0. Max coverage (-): 0

Region: NODE\_27459\_length\_24731\_cov\_33.167362 4168-4184. Max. coverage (+): 0. Max coverage (-): 0

Region: NODE\_27459\_length\_24731\_cov\_33.167362 4185-4201. Max. coverage (+): 0. Max coverage (-): 0

Region: NODE\_27459\_length\_24731\_cov\_33.167362 4202-4218. Max. coverage (+): 0. Max coverage (-): 0

Region: NODE\_27459\_length\_24731\_cov\_33.167362 4219-4236. Max. coverage (+): 0. Max coverage (-): 0

Region: NODE\_27459\_length\_24731\_cov\_33.167362 4237-4253. Max. coverage (+): 0. Max coverage (-): 0

Region: NODE\_27459\_length\_24731\_cov\_33.167362 4254-4270. Max. coverage (+): 0. Max coverage (-): 0

Region: NODE\_27459\_length\_24731\_cov\_33.167362 4271-4287. Max. coverage (+): 0. Max coverage (-): 0

Region: NODE\_27459\_length\_24731\_cov\_33.167362 4288-4305. Max. coverage (+): 0. Max coverage (-): 0

Region: NODE\_27459\_length\_24731\_cov\_33.167362 4306-4322. Max. coverage (+): 0. Max coverage (-): 0

Region: NODE\_27459\_length\_24731\_cov\_33.167362 4323-4339. Max. coverage (+): 0. Max coverage (-): 0

Region: NODE\_27459\_length\_24731\_cov\_33.167362 4340-4356. Max. coverage (+): 0. Max coverage (-): 0

Region: NODE\_27459\_length\_24731\_cov\_33.167362 4357-4373. Max. coverage (+): 0. Max coverage (-): 0

Region: NODE\_27459\_length\_24731\_cov\_33.167362 4374-4391. Max. coverage (+): 0. Max coverage (-): 0

Region: NODE\_27459\_length\_24731\_cov\_33.167362 4392-4408. Max. coverage (+): 0. Max coverage (-): 0

Region: NODE\_27459\_length\_24731\_cov\_33.167362 4409-4425. Max. coverage (+): 0. Max coverage (-): 0

Region: NODE\_27459\_length\_24731\_cov\_33.167362 4426-4442. Max. coverage (+): 0. Max coverage (-): 0

Region: NODE\_27459\_length\_24731\_cov\_33.167362 4443-4459. Max. coverage (+): 0. Max coverage (-): 0.09

Region: NODE\_27459\_length\_24731\_cov\_33.167362 4460-4477. Max. coverage (+): 0. Max coverage (-): 0

Region: NODE\_27459\_length\_24731\_cov\_33.167362 4478-4494. Max. coverage (+): 0. Max coverage (-): 0

Region: NODE\_27459\_length\_24731\_cov\_33.167362 4495-4511. Max. coverage (+): 0. Max coverage (-): 0

Region: NODE\_27459\_length\_24731\_cov\_33.167362 4512-4528. Max. coverage (+): 0. Max coverage (-): 0

Region: NODE\_27459\_length\_24731\_cov\_33.167362 4529-4546. Max. coverage (+): 0.09. Max coverage (-): 0

Region: NODE\_27459\_length\_24731\_cov\_33.167362 4547-4563. Max. coverage (+): 0.09. Max coverage (-): 0

Region: NODE\_27459\_length\_24731\_cov\_33.167362 4564-4580. Max. coverage (+): 0.09. Max coverage (-): 0

Region: NODE\_27459\_length\_24731\_cov\_33.167362 4581-4597. Max. coverage (+): 0. Max coverage (-): 0

Region: NODE\_27459\_length\_24731\_cov\_33.167362 4598-4614. Max. coverage (+): 0.09. Max coverage (-): 0

Region: NODE\_27459\_length\_24731\_cov\_33.167362 4615-4632. Max. coverage (+): 0.09. Max coverage (-): 0

Region: NODE\_27459\_length\_24731\_cov\_33.167362 4633-4649. Max. coverage (+): 0. Max coverage (-): 0

Region: NODE\_27459\_length\_24731\_cov\_33.167362 4650-4666. Max. coverage (+): 0. Max coverage (-): 0

Region: NODE\_27459\_length\_24731\_cov\_33.167362 4667-4683. Max. coverage (+): 0. Max coverage (-): 0

Region: NODE\_27459\_length\_24731\_cov\_33.167362 4684-4700. Max. coverage (+): 0. Max coverage (-): 0

Region: NODE\_27459\_length\_24731\_cov\_33.167362 4701-4718. Max. coverage (+): 0. Max coverage (-): 0

Region: NODE\_27459\_length\_24731\_cov\_33.167362 4719-4735. Max. coverage (+): 0. Max coverage (-): 0

Region: NODE\_27459\_length\_24731\_cov\_33.167362 4736-4752. Max. coverage (+): 0. Max coverage (-): 0

Region: NODE\_27459\_length\_24731\_cov\_33.167362 4753-4769. Max. coverage (+): 0. Max coverage (-): 0

Region: NODE\_27459\_length\_24731\_cov\_33.167362 4770-4786. Max. coverage (+): 0.09. Max coverage (-): 0.09

Region: NODE\_27459\_length\_24731\_cov\_33.167362 4787-4804. Max. coverage (+): 0.09. Max coverage (-): 0

Region: NODE\_27459\_length\_24731\_cov\_33.167362 4805-4821. Max. coverage (+): 0. Max coverage (-): 0

Region: NODE\_27459\_length\_24731\_cov\_33.167362 4822-4838. Max. coverage (+): 0. Max coverage (-): 0

Region: NODE\_27459\_length\_24731\_cov\_33.167362 4839-4855. Max. coverage (+): 0. Max coverage (-): 0

Region: NODE\_27459\_length\_24731\_cov\_33.167362 4856-4873. Max. coverage (+): 0. Max coverage (-): 0

Region: NODE\_27459\_length\_24731\_cov\_33.167362 4874-4890. Max. coverage (+): 0. Max coverage (-): 0

Region: NODE\_27459\_length\_24731\_cov\_33.167362 4891-4907. Max. coverage (+): 0.09. Max coverage (-): 0

Region: NODE\_27459\_length\_24731\_cov\_33.167362 4908-4924. Max. coverage (+): 0. Max coverage (-): 0.38

Region: NODE\_27459\_length\_24731\_cov\_33.167362 4925-4941. Max. coverage (+): 0.56. Max coverage (-): 0

Region: NODE\_27459\_length\_24731\_cov\_33.167362 4942-4959. Max. coverage (+): 0. Max coverage (-): 0

Region: NODE\_27459\_length\_24731\_cov\_33.167362 4960-4976. Max. coverage (+): 0.09. Max coverage (-): 0.09

Region: NODE\_27459\_length\_24731\_cov\_33.167362 4977-4993. Max. coverage (+): 0. Max coverage (-): 0

Region: NODE\_27459\_length\_24731\_cov\_33.167362 4994-5010. Max. coverage (+): 0.09. Max coverage (-): 0

Region: NODE\_27459\_length\_24731\_cov\_33.167362 5011-5027. Max. coverage (+): 0. Max coverage (-): 0

Region: NODE\_27459\_length\_24731\_cov\_33.167362 5028-5045. Max. coverage (+): 0. Max coverage (-): 0

Region: NODE\_27459\_length\_24731\_cov\_33.167362 5046-5062. Max. coverage (+): 0. Max coverage (-): 0

Region: NODE\_27459\_length\_24731\_cov\_33.167362 5063-5079. Max. coverage (+): 0. Max coverage (-): 0

Region: NODE\_27459\_length\_24731\_cov\_33.167362 5080-5096. Max. coverage (+): 0. Max coverage (-): 0

Region: NODE\_27459\_length\_24731\_cov\_33.167362 5097-5114. Max. coverage (+): 0.66. Max coverage (-): 0

Region: NODE\_27459\_length\_24731\_cov\_33.167362 5115-5131. Max. coverage (+): 0. Max coverage (-): 0

Region: NODE\_27459\_length\_24731\_cov\_33.167362 5132-5148. Max. coverage (+): 0. Max coverage (-): 0

Region: NODE\_27459\_length\_24731\_cov\_33.167362 5149-5165. Max. coverage (+): 0. Max coverage (-): 0

Region: NODE\_27459\_length\_24731\_cov\_33.167362 5166-5182. Max. coverage (+): 0.19. Max coverage (-): 0

Region: NODE\_27459\_length\_24731\_cov\_33.167362 5183-5200. Max. coverage (+): 0.19. Max coverage (-): 0

Region: NODE\_27459\_length\_24731\_cov\_33.167362 5201-5217. Max. coverage (+): 0. Max coverage (-): 0

Region: NODE\_27459\_length\_24731\_cov\_33.167362 5218-5234. Max. coverage (+): 0. Max coverage (-): 0

Region: NODE\_27459\_length\_24731\_cov\_33.167362 5235-5251. Max. coverage (+): 0. Max coverage (-): 0

Region: NODE\_27459\_length\_24731\_cov\_33.167362 5252-5268. Max. coverage (+): 0. Max coverage (-): 0

Region: NODE\_27459\_length\_24731\_cov\_33.167362 5269-5286. Max. coverage (+): 0.19. Max coverage (-): 0.09

Region: NODE\_27459\_length\_24731\_cov\_33.167362 5287-5303. Max. coverage (+): 0.09. Max coverage (-): 0.19

Region: NODE\_27459\_length\_24731\_cov\_33.167362 5304-5320. Max. coverage (+): 0. Max coverage (-): 0

Region: NODE\_27459\_length\_24731\_cov\_33.167362 5321-5337. Max. coverage (+): 0.19. Max coverage (-): 0

Region: NODE\_27459\_length\_24731\_cov\_33.167362 5338-5354. Max. coverage (+): 0. Max coverage (-): 0

Region: NODE\_27459\_length\_24731\_cov\_33.167362 5355-5372. Max. coverage (+): 0. Max coverage (-): 0.19

Region: NODE\_27459\_length\_24731\_cov\_33.167362 5373-5389. Max. coverage (+): 0.28. Max coverage (-): 0

Region: NODE\_27459\_length\_24731\_cov\_33.167362 5390-5406. Max. coverage (+): 0. Max coverage (-): 0

Region: NODE\_27459\_length\_24731\_cov\_33.167362 5407-5423. Max. coverage (+): 0. Max coverage (-): 0

Region: NODE\_27459\_length\_24731\_cov\_33.167362 5424-5441. Max. coverage (+): 0. Max coverage (-): 0

Region: NODE\_27459\_length\_24731\_cov\_33.167362 5442-5458. Max. coverage (+): 0. Max coverage (-): 0

Region: NODE\_27459\_length\_24731\_cov\_33.167362 5459-5475. Max. coverage (+): 0. Max coverage (-): 0

Region: NODE\_27459\_length\_24731\_cov\_33.167362 5476-5492. Max. coverage (+): 0.09. Max coverage (-): 0

Region: NODE\_27459\_length\_24731\_cov\_33.167362 5493-5509. Max. coverage (+): 0.09. Max coverage (-): 0

Region: NODE\_27459\_length\_24731\_cov\_33.167362 5510-5527. Max. coverage (+): 0.56. Max coverage (-): 0.09

Region: NODE\_27459\_length\_24731\_cov\_33.167362 5528-5544. Max. coverage (+): 0.19. Max coverage (-): 0

Region: NODE\_27459\_length\_24731\_cov\_33.167362 5545-5561. Max. coverage (+): 0.09. Max coverage (-): 0.47

Region: NODE\_27459\_length\_24731\_cov\_33.167362 5562-5578. Max. coverage (+): 0.28. Max coverage (-): 0.09

Region: NODE\_27459\_length\_24731\_cov\_33.167362 5579-5595. Max. coverage (+): 0. Max coverage (-): 0

Region: NODE\_27459\_length\_24731\_cov\_33.167362 5596-5613. Max. coverage (+): 0.38. Max coverage (-): 0

Region: NODE\_27459\_length\_24731\_cov\_33.167362 5614-5630. Max. coverage (+): 0.28. Max coverage (-): 0

Region: NODE\_27459\_length\_24731\_cov\_33.167362 5631-5647. Max. coverage (+): 0. Max coverage (-): 0.09

Region: NODE\_27459\_length\_24731\_cov\_33.167362 5648-5664. Max. coverage (+): 0.09. Max coverage (-): 0.09

Region: NODE\_27459\_length\_24731\_cov\_33.167362 5665-5682. Max. coverage (+): 0. Max coverage (-): 0.09

Region: NODE\_27459\_length\_24731\_cov\_33.167362 5683-5699. Max. coverage (+): 0.09. Max coverage (-): 0.19

Region: NODE\_27459\_length\_24731\_cov\_33.167362 5700-5716. Max. coverage (+): 0. Max coverage (-): 0.19

Region: NODE\_27459\_length\_24731\_cov\_33.167362 5717-5733. Max. coverage (+): 0. Max coverage (-): 0

Region: NODE\_27459\_length\_24731\_cov\_33.167362 5734-5750. Max. coverage (+): 0. Max coverage (-): 0

Region: NODE\_27459\_length\_24731\_cov\_33.167362 5751-5768. Max. coverage (+): 0.38. Max coverage (-): 0

Region: NODE\_27459\_length\_24731\_cov\_33.167362 5769-5785. Max. coverage (+): 0. Max coverage (-): 0

Region: NODE\_27459\_length\_24731\_cov\_33.167362 5786-5802. Max. coverage (+): 0.09. Max coverage (-): 0

Region: NODE\_27459\_length\_24731\_cov\_33.167362 5803-5819. Max. coverage (+): 0. Max coverage (-): 0.09

Region: NODE\_27459\_length\_24731\_cov\_33.167362 5820-5836. Max. coverage (+): 0. Max coverage (-): 0

Region: NODE\_27459\_length\_24731\_cov\_33.167362 5837-5854. Max. coverage (+): 0.09. Max coverage (-): 0

Region: NODE\_27459\_length\_24731\_cov\_33.167362 5855-5871. Max. coverage (+): 0.09. Max coverage (-): 0

Region: NODE\_27459\_length\_24731\_cov\_33.167362 5872-5888. Max. coverage (+): 0. Max coverage (-): 0

Region: NODE\_27459\_length\_24731\_cov\_33.167362 5889-5905. Max. coverage (+): 0. Max coverage (-): 0

Region: NODE\_27459\_length\_24731\_cov\_33.167362 5906-5922. Max. coverage (+): 0. Max coverage (-): 0

Region: NODE\_27459\_length\_24731\_cov\_33.167362 5923-5940. Max. coverage (+): 0.28. Max coverage (-): 0

Region: NODE\_27459\_length\_24731\_cov\_33.167362 5941-5957. Max. coverage (+): 0. Max coverage (-): 0.38

Region: NODE\_27459\_length\_24731\_cov\_33.167362 5958-5974. Max. coverage (+): 0.28. Max coverage (-): 0.09

Region: NODE\_27459\_length\_24731\_cov\_33.167362 5975-5991. Max. coverage (+): 0. Max coverage (-): 0.09

Region: NODE\_27459\_length\_24731\_cov\_33.167362 5992-6009. Max. coverage (+): 0. Max coverage (-): 0

Region: NODE\_27459\_length\_24731\_cov\_33.167362 6010-6026. Max. coverage (+): 0. Max coverage (-): 0

Region: NODE\_27459\_length\_24731\_cov\_33.167362 6027-6043. Max. coverage (+): 0.09. Max coverage (-): 0

Region: NODE\_27459\_length\_24731\_cov\_33.167362 6044-6060. Max. coverage (+): 0. Max coverage (-): 0

Region: NODE\_27459\_length\_24731\_cov\_33.167362 6061-6077. Max. coverage (+): 0.19. Max coverage (-): 0

Region: NODE\_27459\_length\_24731\_cov\_33.167362 6078-6095. Max. coverage (+): 0. Max coverage (-): 0

Region: NODE\_27459\_length\_24731\_cov\_33.167362 6096-6112. Max. coverage (+): 0. Max coverage (-): 0

Region: NODE\_27459\_length\_24731\_cov\_33.167362 6113-6129. Max. coverage (+): 0. Max coverage (-): 0

Region: NODE\_27459\_length\_24731\_cov\_33.167362 6130-6146. Max. coverage (+): 0. Max coverage (-): 0

Region: NODE\_27459\_length\_24731\_cov\_33.167362 6147-6163. Max. coverage (+): 0. Max coverage (-): 0

Region: NODE\_27459\_length\_24731\_cov\_33.167362 6164-6181. Max. coverage (+): 0. Max coverage (-): 0

Region: NODE\_27459\_length\_24731\_cov\_33.167362 6182-6198. Max. coverage (+): 0. Max coverage (-): 0

Region: NODE\_27459\_length\_24731\_cov\_33.167362 6199-6215. Max. coverage (+): 0. Max coverage (-): 0

Region: NODE\_27459\_length\_24731\_cov\_33.167362 6216-6232. Max. coverage (+): 0. Max coverage (-): 0

Region: NODE\_27459\_length\_24731\_cov\_33.167362 6233-6250. Max. coverage (+): 0.01. Max coverage (-): 0

Region: NODE\_27459\_length\_24731\_cov\_33.167362 6251-6267. Max. coverage (+): 0.09. Max coverage (-): 0

Region: NODE\_27459\_length\_24731\_cov\_33.167362 6268-6284. Max. coverage (+): 0.09. Max coverage (-): 0.09

Region: NODE\_27459\_length\_24731\_cov\_33.167362 6285-6301. Max. coverage (+): 0.09. Max coverage (-): 1.13

Region: NODE\_27459\_length\_24731\_cov\_33.167362 6302-6318. Max. coverage (+): 0. Max coverage (-): 1.13

Region: NODE\_27459\_length\_24731\_cov\_33.167362 6319-6336. Max. coverage (+): 1.03. Max coverage (-): 0.09

Region: NODE\_27459\_length\_24731\_cov\_33.167362 6337-6353. Max. coverage (+): 0. Max coverage (-): 0.56

Region: NODE\_27459\_length\_24731\_cov\_33.167362 6354-6370. Max. coverage (+): 0. Max coverage (-): 0

Region: NODE\_27459\_length\_24731\_cov\_33.167362 6371-6387. Max. coverage (+): 0. Max coverage (-): 0.05

Region: NODE\_27459\_length\_24731\_cov\_33.167362 6388-6404. Max. coverage (+): 0. Max coverage (-): 0.09

Region: NODE\_27459\_length\_24731\_cov\_33.167362 6405-6422. Max. coverage (+): 0. Max coverage (-): 0.09

Region: NODE\_27459\_length\_24731\_cov\_33.167362 6423-6439. Max. coverage (+): 0. Max coverage (-): 0.02

Region: NODE\_27459\_length\_24731\_cov\_33.167362 6440-6456. Max. coverage (+): 0.04. Max coverage (-): 0

Region: NODE\_27459\_length\_24731\_cov\_33.167362 6457-6473. Max. coverage (+): 0. Max coverage (-): 0.01

Region: NODE\_27459\_length\_24731\_cov\_33.167362 6474-6490. Max. coverage (+): 0. Max coverage (-): 0

Region: NODE\_27459\_length\_24731\_cov\_33.167362 6491-6508. Max. coverage (+): 0. Max coverage (-): 0

Region: NODE\_27459\_length\_24731\_cov\_33.167362 6509-6525. Max. coverage (+): 0. Max coverage (-): 0

Region: NODE\_27459\_length\_24731\_cov\_33.167362 6526-6542. Max. coverage (+): 0. Max coverage (-): 0

Region: NODE\_27459\_length\_24731\_cov\_33.167362 6543-6559. Max. coverage (+): 0. Max coverage (-): 0

Region: NODE\_27459\_length\_24731\_cov\_33.167362 6560-6577. Max. coverage (+): 0. Max coverage (-): 0

Region: NODE\_27459\_length\_24731\_cov\_33.167362 6578-6594. Max. coverage (+): 0. Max coverage (-): 0

Region: NODE\_27459\_length\_24731\_cov\_33.167362 6595-6611. Max. coverage (+): 0. Max coverage (-): 0

Region: NODE\_27459\_length\_24731\_cov\_33.167362 6612-6628. Max. coverage (+): 0. Max coverage (-): 0

Region: NODE\_27459\_length\_24731\_cov\_33.167362 6629-6645. Max. coverage (+): 0. Max coverage (-): 0

Region: NODE\_27459\_length\_24731\_cov\_33.167362 6646-6663. Max. coverage (+): 0.09. Max coverage (-): 0

Region: NODE\_27459\_length\_24731\_cov\_33.167362 6664-6680. Max. coverage (+): 0.09. Max coverage (-): 0.19

Region: NODE\_27459\_length\_24731\_cov\_33.167362 6681-6697. Max. coverage (+): 0.19. Max coverage (-): 0.18

Region: NODE\_27459\_length\_24731\_cov\_33.167362 6698-6714. Max. coverage (+): 0.38. Max coverage (-): 0

Region: NODE\_27459\_length\_24731\_cov\_33.167362 6715-6731. Max. coverage (+): 0. Max coverage (-): 0

Region: NODE\_27459\_length\_24731\_cov\_33.167362 6732-6749. Max. coverage (+): 0.09. Max coverage (-): 0

Region: NODE\_27459\_length\_24731\_cov\_33.167362 6750-6766. Max. coverage (+): 0.09. Max coverage (-): 0.19

Region: NODE\_27459\_length\_24731\_cov\_33.167362 6767-6783. Max. coverage (+): 1.13. Max coverage (-): 0.09

Region: NODE\_27459\_length\_24731\_cov\_33.167362 6784-6800. Max. coverage (+): 0. Max coverage (-): 0.09

Region: NODE\_27459\_length\_24731\_cov\_33.167362 6801-6818. Max. coverage (+): 0.09. Max coverage (-): 0

Region: NODE\_27459\_length\_24731\_cov\_33.167362 6819-6835. Max. coverage (+): 0.66. Max coverage (-): 0.09

Region: NODE\_27459\_length\_24731\_cov\_33.167362 6836-6852. Max. coverage (+): 1.03. Max coverage (-): 0

Region: NODE\_27459\_length\_24731\_cov\_33.167362 6853-6869. Max. coverage (+): 0.38. Max coverage (-): 0.09

Region: NODE\_27459\_length\_24731\_cov\_33.167362 6870-6886. Max. coverage (+): 3.48. Max coverage (-): 0.09

Region: NODE\_27459\_length\_24731\_cov\_33.167362 6887-6904. Max. coverage (+): 2.82. Max coverage (-): 0

Region: NODE\_27459\_length\_24731\_cov\_33.167362 6905-6921. Max. coverage (+): 0.19. Max coverage (-): 0

Region: NODE\_27459\_length\_24731\_cov\_33.167362 6922-6938. Max. coverage (+): 0.09. Max coverage (-): 0.09

Region: NODE\_27459\_length\_24731\_cov\_33.167362 6939-6955. Max. coverage (+): 2.91. Max coverage (-): 0

Region: NODE\_27459\_length\_24731\_cov\_33.167362 6956-6972. Max. coverage (+): 0.09. Max coverage (-): 0

Region: NODE\_27459\_length\_24731\_cov\_33.167362 6973-6990. Max. coverage (+): 0. Max coverage (-): 0.09

Region: NODE\_27459\_length\_24731\_cov\_33.167362 6991-7007. Max. coverage (+): 0.19. Max coverage (-): 0.28

Region: NODE\_27459\_length\_24731\_cov\_33.167362 7008-7024. Max. coverage (+): 0.19. Max coverage (-): 0.38

Region: NODE\_27459\_length\_24731\_cov\_33.167362 7025-7041. Max. coverage (+): 1.32. Max coverage (-): 0.28

Region: NODE\_27459\_length\_24731\_cov\_33.167362 7042-7058. Max. coverage (+): 2.16. Max coverage (-): 0.09

Region: NODE\_27459\_length\_24731\_cov\_33.167362 7059-7076. Max. coverage (+): 0.19. Max coverage (-): 0.09

Region: NODE\_27459\_length\_24731\_cov\_33.167362 7077-7093. Max. coverage (+): 0.85. Max coverage (-): 0

Region: NODE\_27459\_length\_24731\_cov\_33.167362 7094-7110. Max. coverage (+): 0.66. Max coverage (-): 0.19

Region: NODE\_27459\_length\_24731\_cov\_33.167362 7111-7127. Max. coverage (+): 2.16. Max coverage (-): 0

Region: NODE\_27459\_length\_24731\_cov\_33.167362 7128-7145. Max. coverage (+): 1.22. Max coverage (-): 0

Region: NODE\_27459\_length\_24731\_cov\_33.167362 7146-7162. Max. coverage (+): 1.41. Max coverage (-): 0

Region: NODE\_27459\_length\_24731\_cov\_33.167362 7163-7179. Max. coverage (+): 0.38. Max coverage (-): 0.66

Region: NODE\_27459\_length\_24731\_cov\_33.167362 7180-7196. Max. coverage (+): 24.43. Max coverage (-): 0.19

Region: NODE\_27459\_length\_24731\_cov\_33.167362 7197-7213. Max. coverage (+): 0.38. Max coverage (-): 1.32

Region: NODE\_27459\_length\_24731\_cov\_33.167362 7214-7231. Max. coverage (+): 1.32. Max coverage (-): 0.28

Region: NODE\_27459\_length\_24731\_cov\_33.167362 7232-7248. Max. coverage (+): 5.36. Max coverage (-): 0.47

Region: NODE\_27459\_length\_24731\_cov\_33.167362 7249-7265. Max. coverage (+): 72.17. Max coverage (-): 0.19

Region: NODE\_27459\_length\_24731\_cov\_33.167362 7266-7282. Max. coverage (+): 1.32. Max coverage (-): 0.09

Region: NODE\_27459\_length\_24731\_cov\_33.167362 7283-7299. Max. coverage (+): 0.47. Max coverage (-): 0.38

Region: NODE\_27459\_length\_24731\_cov\_33.167362 7300-7317. Max. coverage (+): 5.36. Max coverage (-): 0.09

Region: NODE\_27459\_length\_24731\_cov\_33.167362 7318-7334. Max. coverage (+): 0.38. Max coverage (-): 0.38

Region: NODE\_27459\_length\_24731\_cov\_33.167362 7335-7351. Max. coverage (+): 1.88. Max coverage (-): 0

Region: NODE\_27459\_length\_24731\_cov\_33.167362 7352-7368. Max. coverage (+): 1.41. Max coverage (-): 0

Region: NODE\_27459\_length\_24731\_cov\_33.167362 7369-7386. Max. coverage (+): 0.28. Max coverage (-): 0.09

Region: NODE\_27459\_length\_24731\_cov\_33.167362 7387-7403. Max. coverage (+): 9.68. Max coverage (-): 0

Region: NODE\_27459\_length\_24731\_cov\_33.167362 7404-7420. Max. coverage (+): 0.56. Max coverage (-): 0

Region: NODE\_27459\_length\_24731\_cov\_33.167362 7421-7437. Max. coverage (+): 0.28. Max coverage (-): 0

Region: NODE\_27459\_length\_24731\_cov\_33.167362 7438-7454. Max. coverage (+): 3.57. Max coverage (-): 0

Region: NODE\_27459\_length\_24731\_cov\_33.167362 7455-7472. Max. coverage (+): 0. Max coverage (-): 0

Region: NODE\_27459\_length\_24731\_cov\_33.167362 7473-7489. Max. coverage (+): 0.28. Max coverage (-): 0

Region: NODE\_27459\_length\_24731\_cov\_33.167362 7490-7506. Max. coverage (+): 4.6. Max coverage (-): 0.09

Region: NODE\_27459\_length\_24731\_cov\_33.167362 7507-7523. Max. coverage (+): 3.48. Max coverage (-): 0

Region: NODE\_27459\_length\_24731\_cov\_33.167362 7524-7540. Max. coverage (+): 1.88. Max coverage (-): 0.09

Region: NODE\_27459\_length\_24731\_cov\_33.167362 7541-7558. Max. coverage (+): 1.03. Max coverage (-): 0.09

Region: NODE\_27459\_length\_24731\_cov\_33.167362 7559-7575. Max. coverage (+): 0.19. Max coverage (-): 0

Region: NODE\_27459\_length\_24731\_cov\_33.167362 7576-7592. Max. coverage (+): 0.38. Max coverage (-): 0.09

Region: NODE\_27459\_length\_24731\_cov\_33.167362 7593-7609. Max. coverage (+): 6.77. Max coverage (-): 0

Region: NODE\_27459\_length\_24731\_cov\_33.167362 7610-7626. Max. coverage (+): 0.28. Max coverage (-): 0.09

Region: NODE\_27459\_length\_24731\_cov\_33.167362 7627-7644. Max. coverage (+): 1.41. Max coverage (-): 0

Region: NODE\_27459\_length\_24731\_cov\_33.167362 7645-7661. Max. coverage (+): 0.66. Max coverage (-): 0.09

Region: NODE\_27459\_length\_24731\_cov\_33.167362 7662-7678. Max. coverage (+): 0.56. Max coverage (-): 0

Region: NODE\_27459\_length\_24731\_cov\_33.167362 7679-7695. Max. coverage (+): 0.19. Max coverage (-): 0

Region: NODE\_27459\_length\_24731\_cov\_33.167362 7696-7713. Max. coverage (+): 0.09. Max coverage (-): 0.19

Region: NODE\_27459\_length\_24731\_cov\_33.167362 7714-7730. Max. coverage (+): 0.47. Max coverage (-): 0.09

Region: NODE\_27459\_length\_24731\_cov\_33.167362 7731-7747. Max. coverage (+): 0.56. Max coverage (-): 0

Region: NODE\_27459\_length\_24731\_cov\_33.167362 7748-7764. Max. coverage (+): 1.32. Max coverage (-): 0

Region: NODE\_27459\_length\_24731\_cov\_33.167362 7765-7781. Max. coverage (+): 0. Max coverage (-): 0.09

Region: NODE\_27459\_length\_24731\_cov\_33.167362 7782-7799. Max. coverage (+): 0.19. Max coverage (-): 0

Region: NODE\_27459\_length\_24731\_cov\_33.167362 7800-7816. Max. coverage (+): 0.47. Max coverage (-): 0

Region: NODE\_27459\_length\_24731\_cov\_33.167362 7817-7833. Max. coverage (+): 0.75. Max coverage (-): 0

Region: NODE\_27459\_length\_24731\_cov\_33.167362 7834-7850. Max. coverage (+): 0.28. Max coverage (-): 0

Region: NODE\_27459\_length\_24731\_cov\_33.167362 7851-7867. Max. coverage (+): 0.66. Max coverage (-): 0

Region: NODE\_27459\_length\_24731\_cov\_33.167362 7868-7885. Max. coverage (+): 0.09. Max coverage (-): 0

Region: NODE\_27459\_length\_24731\_cov\_33.167362 7886-7902. Max. coverage (+): 0.09. Max coverage (-): 0

Region: NODE\_27459\_length\_24731\_cov\_33.167362 7903-7919. Max. coverage (+): 0.38. Max coverage (-): 0

Region: NODE\_27459\_length\_24731\_cov\_33.167362 7920-7936. Max. coverage (+): 0.19. Max coverage (-): 0

Region: NODE\_27459\_length\_24731\_cov\_33.167362 7937-7953. Max. coverage (+): 1.22. Max coverage (-): 0

Region: NODE\_27459\_length\_24731\_cov\_33.167362 7954-7971. Max. coverage (+): 0. Max coverage (-): 0.19

Region: NODE\_27459\_length\_24731\_cov\_33.167362 7972-7988. Max. coverage (+): 0. Max coverage (-): 0.09

Region: NODE\_27459\_length\_24731\_cov\_33.167362 7989-8005. Max. coverage (+): 0.09. Max coverage (-): 0.09

Region: NODE\_27459\_length\_24731\_cov\_33.167362 8006-8022. Max. coverage (+): 0.09. Max coverage (-): 0

Region: NODE\_27459\_length\_24731\_cov\_33.167362 8023-8040. Max. coverage (+): 0.19. Max coverage (-): 0.09

Region: NODE\_27459\_length\_24731\_cov\_33.167362 8041-8057. Max. coverage (+): 0.56. Max coverage (-): 0.09

Region: NODE\_27459\_length\_24731\_cov\_33.167362 8058-8074. Max. coverage (+): 0. Max coverage (-): 0

Region: NODE\_27459\_length\_24731\_cov\_33.167362 8075-8091. Max. coverage (+): 0.19. Max coverage (-): 0

Region: NODE\_27459\_length\_24731\_cov\_33.167362 8092-8108. Max. coverage (+): 3.1. Max coverage (-): 0

Region: NODE\_27459\_length\_24731\_cov\_33.167362 8109-8126. Max. coverage (+): 0.28. Max coverage (-): 0

Region: NODE\_27459\_length\_24731\_cov\_33.167362 8127-8143. Max. coverage (+): 0.28. Max coverage (-): 0

Region: NODE\_27459\_length\_24731\_cov\_33.167362 8144-8160. Max. coverage (+): 0.75. Max coverage (-): 0

Region: NODE\_27459\_length\_24731\_cov\_33.167362 8161-8177. Max. coverage (+): 0.09. Max coverage (-): 0

Region: NODE\_27459\_length\_24731\_cov\_33.167362 8178-8194. Max. coverage (+): 0.19. Max coverage (-): 0

Region: NODE\_27459\_length\_24731\_cov\_33.167362 8195-8212. Max. coverage (+): 0. Max coverage (-): 0

Region: NODE\_27459\_length\_24731\_cov\_33.167362 8213-8229. Max. coverage (+): 0. Max coverage (-): 0

Region: NODE\_27459\_length\_24731\_cov\_33.167362 8230-8246. Max. coverage (+): 0. Max coverage (-): 0.28

Region: NODE\_27459\_length\_24731\_cov\_33.167362 8247-8263. Max. coverage (+): 0.19. Max coverage (-): 0.19

Region: NODE\_27459\_length\_24731\_cov\_33.167362 8264-8281. Max. coverage (+): 0.47. Max coverage (-): 0

Region: NODE\_27459\_length\_24731\_cov\_33.167362 8282-8298. Max. coverage (+): 0. Max coverage (-): 0.09

Region: NODE\_27459\_length\_24731\_cov\_33.167362 8299-8315. Max. coverage (+): 0. Max coverage (-): 0.38

Region: NODE\_27459\_length\_24731\_cov\_33.167362 8316-8332. Max. coverage (+): 0.66. Max coverage (-): 0

Region: NODE\_27459\_length\_24731\_cov\_33.167362 8333-8349. Max. coverage (+): 0.19. Max coverage (-): 0.09

Region: NODE\_27459\_length\_24731\_cov\_33.167362 8350-8367. Max. coverage (+): 0.09. Max coverage (-): 0

Region: NODE\_27459\_length\_24731\_cov\_33.167362 8368-8384. Max. coverage (+): 0.19. Max coverage (-): 0.09

Region: NODE\_27459\_length\_24731\_cov\_33.167362 8385-8401. Max. coverage (+): 0.28. Max coverage (-): 0

Region: NODE\_27459\_length\_24731\_cov\_33.167362 8402-8418. Max. coverage (+): 0.09. Max coverage (-): 0

Region: NODE\_27459\_length\_24731\_cov\_33.167362 8419-8435. Max. coverage (+): 0.09. Max coverage (-): 0

Region: NODE\_27459\_length\_24731\_cov\_33.167362 8436-8453. Max. coverage (+): 0.38. Max coverage (-): 0

Region: NODE\_27459\_length\_24731\_cov\_33.167362 8454-8470. Max. coverage (+): 17.67. Max coverage (-): 0

Region: NODE\_27459\_length\_24731\_cov\_33.167362 8471-8487. Max. coverage (+): 17.29. Max coverage (-): 0

Region: NODE\_27459\_length\_24731\_cov\_33.167362 8488-8504. Max. coverage (+): 0.09. Max coverage (-): 0

Region: NODE\_27459\_length\_24731\_cov\_33.167362 8505-8521. Max. coverage (+): 0. Max coverage (-): 0

Region: NODE\_27459\_length\_24731\_cov\_33.167362 8522-8539. Max. coverage (+): 0. Max coverage (-): 0

Region: NODE\_27459\_length\_24731\_cov\_33.167362 8540-8556. Max. coverage (+): 0.19. Max coverage (-): 0

Region: NODE\_27459\_length\_24731\_cov\_33.167362 8557-8573. Max. coverage (+): 0.09. Max coverage (-): 0

Region: NODE\_27459\_length\_24731\_cov\_33.167362 8574-8590. Max. coverage (+): 0.19. Max coverage (-): 0

Region: NODE\_27459\_length\_24731\_cov\_33.167362 8591-8608. Max. coverage (+): 0.19. Max coverage (-): 0.09

Region: NODE\_27459\_length\_24731\_cov\_33.167362 8609-8625. Max. coverage (+): 0.09. Max coverage (-): 0.09

Region: NODE\_27459\_length\_24731\_cov\_33.167362 8626-8642. Max. coverage (+): 0. Max coverage (-): 0.09

Region: NODE\_27459\_length\_24731\_cov\_33.167362 8643-8659. Max. coverage (+): 0.09. Max coverage (-): 0

Region: NODE\_27459\_length\_24731\_cov\_33.167362 8660-8676. Max. coverage (+): 0.47. Max coverage (-): 0

Region: NODE\_27459\_length\_24731\_cov\_33.167362 8677-8694. Max. coverage (+): 0.66. Max coverage (-): 0

Region: NODE\_27459\_length\_24731\_cov\_33.167362 8695-8711. Max. coverage (+): 0.19. Max coverage (-): 0.09

Region: NODE\_27459\_length\_24731\_cov\_33.167362 8712-8728. Max. coverage (+): 2.26. Max coverage (-): 0

Region: NODE\_27459\_length\_24731\_cov\_33.167362 8729-8745. Max. coverage (+): 1.22. Max coverage (-): 0

Region: NODE\_27459\_length\_24731\_cov\_33.167362 8746-8762. Max. coverage (+): 0. Max coverage (-): 0.09

Region: NODE\_27459\_length\_24731\_cov\_33.167362 8763-8780. Max. coverage (+): 0.28. Max coverage (-): 0

Region: NODE\_27459\_length\_24731\_cov\_33.167362 8781-8797. Max. coverage (+): 0.66. Max coverage (-): 0

Region: NODE\_27459\_length\_24731\_cov\_33.167362 8798-8814. Max. coverage (+): 0.94. Max coverage (-): 0

Region: NODE\_27459\_length\_24731\_cov\_33.167362 8815-8831. Max. coverage (+): 0.19. Max coverage (-): 0

Region: NODE\_27459\_length\_24731\_cov\_33.167362 8832-8849. Max. coverage (+): 0.38. Max coverage (-): 0.09

Region: NODE\_27459\_length\_24731\_cov\_33.167362 8850-8866. Max. coverage (+): 0.38. Max coverage (-): 0

Region: NODE\_27459\_length\_24731\_cov\_33.167362 8867-8883. Max. coverage (+): 0.09. Max coverage (-): 0.19

Region: NODE\_27459\_length\_24731\_cov\_33.167362 8884-8900. Max. coverage (+): 0.56. Max coverage (-): 0

Region: NODE\_27459\_length\_24731\_cov\_33.167362 8901-8917. Max. coverage (+): 0. Max coverage (-): 0

Region: NODE\_27459\_length\_24731\_cov\_33.167362 8918-8935. Max. coverage (+): 0.28. Max coverage (-): 0

Region: NODE\_27459\_length\_24731\_cov\_33.167362 8936-8952. Max. coverage (+): 0.66. Max coverage (-): 0

Region: NODE\_27459\_length\_24731\_cov\_33.167362 8953-8969. Max. coverage (+): 0. Max coverage (-): 0

Region: NODE\_27459\_length\_24731\_cov\_33.167362 8970-8986. Max. coverage (+): 0.28. Max coverage (-): 0.09

Region: NODE\_27459\_length\_24731\_cov\_33.167362 8987-9003. Max. coverage (+): 0.28. Max coverage (-): 0.09

Region: NODE\_27459\_length\_24731\_cov\_33.167362 9004-9021. Max. coverage (+): 0.38. Max coverage (-): 0.09

Region: NODE\_27459\_length\_24731\_cov\_33.167362 9022-9038. Max. coverage (+): 0.38. Max coverage (-): 0

Region: NODE\_27459\_length\_24731\_cov\_33.167362 9039-9055. Max. coverage (+): 0.19. Max coverage (-): 0

Region: NODE\_27459\_length\_24731\_cov\_33.167362 9056-9072. Max. coverage (+): 0.56. Max coverage (-): 0

Region: NODE\_27459\_length\_24731\_cov\_33.167362 9073-9089. Max. coverage (+): 0.09. Max coverage (-): 0

Region: NODE\_27459\_length\_24731\_cov\_33.167362 9090-9107. Max. coverage (+): 0. Max coverage (-): 0.01

Region: NODE\_27459\_length\_24731\_cov\_33.167362 9108-9124. Max. coverage (+): 0. Max coverage (-): 0.01

Region: NODE\_27459\_length\_24731\_cov\_33.167362 9125-9141. Max. coverage (+): 0. Max coverage (-): 0

Region: NODE\_27459\_length\_24731\_cov\_33.167362 9142-9158. Max. coverage (+): 0. Max coverage (-): 0

Region: NODE\_27459\_length\_24731\_cov\_33.167362 9159-9176. Max. coverage (+): 0. Max coverage (-): 0

Region: NODE\_27459\_length\_24731\_cov\_33.167362 9177-9193. Max. coverage (+): 0. Max coverage (-): 0

Region: NODE\_27459\_length\_24731\_cov\_33.167362 9194-9210. Max. coverage (+): 0. Max coverage (-): 0.01

Region: NODE\_27459\_length\_24731\_cov\_33.167362 9211-9227. Max. coverage (+): 0. Max coverage (-): 0

Region: NODE\_27459\_length\_24731\_cov\_33.167362 9228-9244. Max. coverage (+): 0. Max coverage (-): 0

Region: NODE\_27459\_length\_24731\_cov\_33.167362 9245-9262. Max. coverage (+): 0. Max coverage (-): 0

Region: NODE\_27459\_length\_24731\_cov\_33.167362 9263-9279. Max. coverage (+): 0.09. Max coverage (-): 0

Region: NODE\_27459\_length\_24731\_cov\_33.167362 9280-9296. Max. coverage (+): 0. Max coverage (-): 0

Region: NODE\_27459\_length\_24731\_cov\_33.167362 9297-9313. Max. coverage (+): 0. Max coverage (-): 0

Region: NODE\_27459\_length\_24731\_cov\_33.167362 9314-9330. Max. coverage (+): 0. Max coverage (-): 0.02

Region: NODE\_27459\_length\_24731\_cov\_33.167362 9331-9348. Max. coverage (+): 2.69. Max coverage (-): 0

Region: NODE\_27459\_length\_24731\_cov\_33.167362 9349-9365. Max. coverage (+): 0. Max coverage (-): 0

Region: NODE\_27459\_length\_24731\_cov\_33.167362 9366-9382. Max. coverage (+): 0. Max coverage (-): 0

Region: NODE\_27459\_length\_24731\_cov\_33.167362 9383-9399. Max. coverage (+): 0. Max coverage (-): 0

Region: NODE\_27459\_length\_24731\_cov\_33.167362 9400-9417. Max. coverage (+): 0. Max coverage (-): 0

Region: NODE\_27459\_length\_24731\_cov\_33.167362 9418-9434. Max. coverage (+): 0. Max coverage (-): 0

Region: NODE\_27459\_length\_24731\_cov\_33.167362 9435-9451. Max. coverage (+): 0. Max coverage (-): 0

Region: NODE\_27459\_length\_24731\_cov\_33.167362 9452-9468. Max. coverage (+): 0. Max coverage (-): 0.28

Region: NODE\_27459\_length\_24731\_cov\_33.167362 9469-9485. Max. coverage (+): 0.01. Max coverage (-): 0.09

Region: NODE\_27459\_length\_24731\_cov\_33.167362 9486-9503. Max. coverage (+): 0. Max coverage (-): 0

Region: NODE\_27459\_length\_24731\_cov\_33.167362 9504-9520. Max. coverage (+): 0. Max coverage (-): 0

Region: NODE\_27459\_length\_24731\_cov\_33.167362 9521-9537. Max. coverage (+): 0. Max coverage (-): 0

Region: NODE\_27459\_length\_24731\_cov\_33.167362 9538-9554. Max. coverage (+): 0. Max coverage (-): 0

Region: NODE\_27459\_length\_24731\_cov\_33.167362 9555-9571. Max. coverage (+): 0. Max coverage (-): 0

Region: NODE\_27459\_length\_24731\_cov\_33.167362 9572-9589. Max. coverage (+): 0. Max coverage (-): 0

Region: NODE\_27459\_length\_24731\_cov\_33.167362 9590-9606. Max. coverage (+): 0.01. Max coverage (-): 0

Region: NODE\_27459\_length\_24731\_cov\_33.167362 9607-9623. Max. coverage (+): 0.02. Max coverage (-): 0

Region: NODE\_27459\_length\_24731\_cov\_33.167362 9624-9640. Max. coverage (+): 0. Max coverage (-): 0

Region: NODE\_27459\_length\_24731\_cov\_33.167362 9641-9657. Max. coverage (+): 0.02. Max coverage (-): 0

Region: NODE\_27459\_length\_24731\_cov\_33.167362 9658-9675. Max. coverage (+): 0.02. Max coverage (-): 0.05

Region: NODE\_27459\_length\_24731\_cov\_33.167362 9676-9692. Max. coverage (+): 0.01. Max coverage (-): 0

Region: NODE\_27459\_length\_24731\_cov\_33.167362 9693-9709. Max. coverage (+): 0. Max coverage (-): 0

Region: NODE\_27459\_length\_24731\_cov\_33.167362 9710-9726. Max. coverage (+): 0. Max coverage (-): 0

Region: NODE\_27459\_length\_24731\_cov\_33.167362 9727-9744. Max. coverage (+): 0.06. Max coverage (-): 0

Region: NODE\_27459\_length\_24731\_cov\_33.167362 9745-9761. Max. coverage (+): 0. Max coverage (-): 0

Region: NODE\_27459\_length\_24731\_cov\_33.167362 9762-9778. Max. coverage (+): 0. Max coverage (-): 0

Region: NODE\_27459\_length\_24731\_cov\_33.167362 9779-9795. Max. coverage (+): 0. Max coverage (-): 0

Region: NODE\_27459\_length\_24731\_cov\_33.167362 9796-9812. Max. coverage (+): 0. Max coverage (-): 0

Region: NODE\_27459\_length\_24731\_cov\_33.167362 9813-9830. Max. coverage (+): 0.01. Max coverage (-): 0

Region: NODE\_27459\_length\_24731\_cov\_33.167362 9831-9847. Max. coverage (+): 0.01. Max coverage (-): 0

Region: NODE\_27459\_length\_24731\_cov\_33.167362 9848-9864. Max. coverage (+): 0.03. Max coverage (-): 0

Region: NODE\_27459\_length\_24731\_cov\_33.167362 9865-9881. Max. coverage (+): 0.03. Max coverage (-): 0.01

Region: NODE\_27459\_length\_24731\_cov\_33.167362 9882-9898. Max. coverage (+): 0. Max coverage (-): 0.02

Region: NODE\_27459\_length\_24731\_cov\_33.167362 9899-9916. Max. coverage (+): 0. Max coverage (-): 0

Region: NODE\_27459\_length\_24731\_cov\_33.167362 9917-9933. Max. coverage (+): 0. Max coverage (-): 0

Region: NODE\_27459\_length\_24731\_cov\_33.167362 9934-9950. Max. coverage (+): 0.01. Max coverage (-): 0

Region: NODE\_27459\_length\_24731\_cov\_33.167362 9951-9967. Max. coverage (+): 0. Max coverage (-): 0

Region: NODE\_27459\_length\_24731\_cov\_33.167362 9968-9985. Max. coverage (+): 0. Max coverage (-): 0

Region: NODE\_27459\_length\_24731\_cov\_33.167362 9986-10002. Max. coverage (+): 0. Max coverage (-): 0

Region: NODE\_27459\_length\_24731\_cov\_33.167362 10003-10019. Max. coverage (+): 0. Max coverage (-): 0

Region: NODE\_27459\_length\_24731\_cov\_33.167362 10020-10036. Max. coverage (+): 0. Max coverage (-): 0

Region: NODE\_27459\_length\_24731\_cov\_33.167362 10037-10053. Max. coverage (+): 0. Max coverage (-): 0

Region: NODE\_27459\_length\_24731\_cov\_33.167362 10054-10071. Max. coverage (+): 0. Max coverage (-): 0

Region: NODE\_27459\_length\_24731\_cov\_33.167362 10072-10088. Max. coverage (+): 0. Max coverage (-): 0

Region: NODE\_27459\_length\_24731\_cov\_33.167362 10089-10105. Max. coverage (+): 0. Max coverage (-): 0

Region: NODE\_27459\_length\_24731\_cov\_33.167362 10106-10122. Max. coverage (+): 0. Max coverage (-): 0

Region: NODE\_27459\_length\_24731\_cov\_33.167362 10123-10139. Max. coverage (+): 0. Max coverage (-): 0

Region: NODE\_27459\_length\_24731\_cov\_33.167362 10140-10157. Max. coverage (+): 0. Max coverage (-): 0

Region: NODE\_27459\_length\_24731\_cov\_33.167362 10158-10174. Max. coverage (+): 0. Max coverage (-): 0.1

Region: NODE\_27459\_length\_24731\_cov\_33.167362 10175-10191. Max. coverage (+): 0.23. Max coverage (-): 0.42

Region: NODE\_27459\_length\_24731\_cov\_33.167362 10192-10208. Max. coverage (+): 0.19. Max coverage (-): 0

Region: NODE\_27459\_length\_24731\_cov\_33.167362 10209-10225. Max. coverage (+): 0. Max coverage (-): 0

Region: NODE\_27459\_length\_24731\_cov\_33.167362 10226-10243. Max. coverage (+): 0.05. Max coverage (-): 0

Region: NODE\_27459\_length\_24731\_cov\_33.167362 10244-10260. Max. coverage (+): 0. Max coverage (-): 0

Region: NODE\_27459\_length\_24731\_cov\_33.167362 10261-10277. Max. coverage (+): 0. Max coverage (-): 0

Region: NODE\_27459\_length\_24731\_cov\_33.167362 10278-10294. Max. coverage (+): 0. Max coverage (-): 0

Region: NODE\_27459\_length\_24731\_cov\_33.167362 10295-10312. Max. coverage (+): 0. Max coverage (-): 0

Region: NODE\_27459\_length\_24731\_cov\_33.167362 10313-10329. Max. coverage (+): 0. Max coverage (-): 0

Region: NODE\_27459\_length\_24731\_cov\_33.167362 10330-10346. Max. coverage (+): 0. Max coverage (-): 0

Region: NODE\_27459\_length\_24731\_cov\_33.167362 10347-10363. Max. coverage (+): 0. Max coverage (-): 0

Region: NODE\_27459\_length\_24731\_cov\_33.167362 10364-10380. Max. coverage (+): 0. Max coverage (-): 0

Region: NODE\_27459\_length\_24731\_cov\_33.167362 10381-10398. Max. coverage (+): 0.06. Max coverage (-): 0

Region: NODE\_27459\_length\_24731\_cov\_33.167362 10399-10415. Max. coverage (+): 0. Max coverage (-): 0

Region: NODE\_27459\_length\_24731\_cov\_33.167362 10416-10432. Max. coverage (+): 0. Max coverage (-): 0

Region: NODE\_27459\_length\_24731\_cov\_33.167362 10433-10449. Max. coverage (+): 0. Max coverage (-): 0

Region: NODE\_27459\_length\_24731\_cov\_33.167362 10450-10466. Max. coverage (+): 0.09. Max coverage (-): 0

Region: NODE\_27459\_length\_24731\_cov\_33.167362 10467-10484. Max. coverage (+): 0. Max coverage (-): 0

Region: NODE\_27459\_length\_24731\_cov\_33.167362 10485-10501. Max. coverage (+): 0. Max coverage (-): 0

Region: NODE\_27459\_length\_24731\_cov\_33.167362 10502-10518. Max. coverage (+): 0. Max coverage (-): 0

Region: NODE\_27459\_length\_24731\_cov\_33.167362 10519-10535. Max. coverage (+): 0. Max coverage (-): 0

Region: NODE\_27459\_length\_24731\_cov\_33.167362 10536-10553. Max. coverage (+): 0. Max coverage (-): 0

Region: NODE\_27459\_length\_24731\_cov\_33.167362 10554-10570. Max. coverage (+): 0.03. Max coverage (-): 0

Region: NODE\_27459\_length\_24731\_cov\_33.167362 10571-10587. Max. coverage (+): 0. Max coverage (-): 0

Region: NODE\_27459\_length\_24731\_cov\_33.167362 10588-10604. Max. coverage (+): 0. Max coverage (-): 0

Region: NODE\_27459\_length\_24731\_cov\_33.167362 10605-10621. Max. coverage (+): 0. Max coverage (-): 0

Region: NODE\_27459\_length\_24731\_cov\_33.167362 10622-10639. Max. coverage (+): 0. Max coverage (-): 0

Region: NODE\_27459\_length\_24731\_cov\_33.167362 10640-10656. Max. coverage (+): 0. Max coverage (-): 0

Region: NODE\_27459\_length\_24731\_cov\_33.167362 10657-10673. Max. coverage (+): 0.01. Max coverage (-): 0

Region: NODE\_27459\_length\_24731\_cov\_33.167362 10674-10690. Max. coverage (+): 0. Max coverage (-): 0

Region: NODE\_27459\_length\_24731\_cov\_33.167362 10691-10707. Max. coverage (+): 0.03. Max coverage (-): 0

Region: NODE\_27459\_length\_24731\_cov\_33.167362 10708-10725. Max. coverage (+): 0. Max coverage (-): 0.02

Region: NODE\_27459\_length\_24731\_cov\_33.167362 10726-10742. Max. coverage (+): 0. Max coverage (-): 0

Region: NODE\_27459\_length\_24731\_cov\_33.167362 10743-10759. Max. coverage (+): 0. Max coverage (-): 0

Region: NODE\_27459\_length\_24731\_cov\_33.167362 10760-10776. Max. coverage (+): 0.01. Max coverage (-): 0

Region: NODE\_27459\_length\_24731\_cov\_33.167362 10777-10793. Max. coverage (+): 0. Max coverage (-): 0

Region: NODE\_27459\_length\_24731\_cov\_33.167362 10794-10811. Max. coverage (+): 0.01. Max coverage (-): 0

Region: NODE\_27459\_length\_24731\_cov\_33.167362 10812-10828. Max. coverage (+): 0. Max coverage (-): 0

Region: NODE\_27459\_length\_24731\_cov\_33.167362 10829-10845. Max. coverage (+): 0. Max coverage (-): 0

Region: NODE\_27459\_length\_24731\_cov\_33.167362 10846-10862. Max. coverage (+): 0. Max coverage (-): 0

Region: NODE\_27459\_length\_24731\_cov\_33.167362 10863-10880. Max. coverage (+): 0. Max coverage (-): 0

Region: NODE\_27459\_length\_24731\_cov\_33.167362 10881-10897. Max. coverage (+): 0. Max coverage (-): 0

Region: NODE\_27459\_length\_24731\_cov\_33.167362 10898-10914. Max. coverage (+): 0. Max coverage (-): 0

Region: NODE\_27459\_length\_24731\_cov\_33.167362 10915-10931. Max. coverage (+): 0. Max coverage (-): 0.42

Region: NODE\_27459\_length\_24731\_cov\_33.167362 10932-10948. Max. coverage (+): 0.23. Max coverage (-): 0.04

Region: NODE\_27459\_length\_24731\_cov\_33.167362 10949-10966. Max. coverage (+): 0. Max coverage (-): 0

Region: NODE\_27459\_length\_24731\_cov\_33.167362 10967-10983. Max. coverage (+): 0.05. Max coverage (-): 0

Region: NODE\_27459\_length\_24731\_cov\_33.167362 10984-11000. Max. coverage (+): 0.05. Max coverage (-): 0

Region: NODE\_27459\_length\_24731\_cov\_33.167362 11001-11017. Max. coverage (+): 0. Max coverage (-): 0

Region: NODE\_27459\_length\_24731\_cov\_33.167362 11018-11034. Max. coverage (+): 0. Max coverage (-): 0

Region: NODE\_27459\_length\_24731\_cov\_33.167362 11035-11052. Max. coverage (+): 0. Max coverage (-): 0

Region: NODE\_27459\_length\_24731\_cov\_33.167362 11053-11069. Max. coverage (+): 0. Max coverage (-): 0

Region: NODE\_27459\_length\_24731\_cov\_33.167362 11070-11086. Max. coverage (+): 0. Max coverage (-): 0

Region: NODE\_27459\_length\_24731\_cov\_33.167362 11087-11103. Max. coverage (+): 0. Max coverage (-): 0

Region: NODE\_27459\_length\_24731\_cov\_33.167362 11104-11121. Max. coverage (+): 0. Max coverage (-): 0

Region: NODE\_27459\_length\_24731\_cov\_33.167362 11122-11138. Max. coverage (+): 0. Max coverage (-): 0

Region: NODE\_27459\_length\_24731\_cov\_33.167362 11139-11155. Max. coverage (+): 0. Max coverage (-): 0

Region: NODE\_27459\_length\_24731\_cov\_33.167362 11156-11172. Max. coverage (+): 0. Max coverage (-): 0

Region: NODE\_27459\_length\_24731\_cov\_33.167362 11173-11189. Max. coverage (+): 0. Max coverage (-): 0

Region: NODE\_27459\_length\_24731\_cov\_33.167362 11190-11207. Max. coverage (+): 0. Max coverage (-): 0

Region: NODE\_27459\_length\_24731\_cov\_33.167362 11208-11224. Max. coverage (+): 0. Max coverage (-): 0

Region: NODE\_27459\_length\_24731\_cov\_33.167362 11225-11241. Max. coverage (+): 0. Max coverage (-): 0

Region: NODE\_27459\_length\_24731\_cov\_33.167362 11242-11258. Max. coverage (+): 0. Max coverage (-): 0

Region: NODE\_27459\_length\_24731\_cov\_33.167362 11259-11275. Max. coverage (+): 0. Max coverage (-): 0

Region: NODE\_27459\_length\_24731\_cov\_33.167362 11276-11293. Max. coverage (+): 0. Max coverage (-): 0.09

Region: NODE\_27459\_length\_24731\_cov\_33.167362 11294-11310. Max. coverage (+): 0. Max coverage (-): 0.09

Region: NODE\_27459\_length\_24731\_cov\_33.167362 11311-11327. Max. coverage (+): 0. Max coverage (-): 0

Region: NODE\_27459\_length\_24731\_cov\_33.167362 11328-11344. Max. coverage (+): 0. Max coverage (-): 0

Region: NODE\_27459\_length\_24731\_cov\_33.167362 11345-11361. Max. coverage (+): 0.09. Max coverage (-): 0

Region: NODE\_27459\_length\_24731\_cov\_33.167362 11362-11379. Max. coverage (+): 0. Max coverage (-): 0

Region: NODE\_27459\_length\_24731\_cov\_33.167362 11380-11396. Max. coverage (+): 0. Max coverage (-): 0

Region: NODE\_27459\_length\_24731\_cov\_33.167362 11397-11413. Max. coverage (+): 0. Max coverage (-): 0

Region: NODE\_27459\_length\_24731\_cov\_33.167362 11414-11430. Max. coverage (+): 0. Max coverage (-): 0

Region: NODE\_27459\_length\_24731\_cov\_33.167362 11431-11448. Max. coverage (+): 0. Max coverage (-): 0

Region: NODE\_27459\_length\_24731\_cov\_33.167362 11449-11465. Max. coverage (+): 0. Max coverage (-): 0

Region: NODE\_27459\_length\_24731\_cov\_33.167362 11466-11482. Max. coverage (+): 0. Max coverage (-): 0

Region: NODE\_27459\_length\_24731\_cov\_33.167362 11483-11499. Max. coverage (+): 0.38. Max coverage (-): 0

Region: NODE\_27459\_length\_24731\_cov\_33.167362 11500-11516. Max. coverage (+): 3.1. Max coverage (-): 0

Region: NODE\_27459\_length\_24731\_cov\_33.167362 11517-11534. Max. coverage (+): 0. Max coverage (-): 0

Region: NODE\_27459\_length\_24731\_cov\_33.167362 11535-11551. Max. coverage (+): 0.09. Max coverage (-): 0

Region: NODE\_27459\_length\_24731\_cov\_33.167362 11552-11568. Max. coverage (+): 0.09. Max coverage (-): 0

Region: NODE\_27459\_length\_24731\_cov\_33.167362 11569-11585. Max. coverage (+): 0. Max coverage (-): 0

Region: NODE\_27459\_length\_24731\_cov\_33.167362 11586-11602. Max. coverage (+): 0.19. Max coverage (-): 0

Region: NODE\_27459\_length\_24731\_cov\_33.167362 11603-11620. Max. coverage (+): 0.66. Max coverage (-): 0

Region: NODE\_27459\_length\_24731\_cov\_33.167362 11621-11637. Max. coverage (+): 0.09. Max coverage (-): 0

Region: NODE\_27459\_length\_24731\_cov\_33.167362 11638-11654. Max. coverage (+): 0. Max coverage (-): 0

Region: NODE\_27459\_length\_24731\_cov\_33.167362 11655-11671. Max. coverage (+): 0.09. Max coverage (-): 0

Region: NODE\_27459\_length\_24731\_cov\_33.167362 11672-11689. Max. coverage (+): 0. Max coverage (-): 0

Region: NODE\_27459\_length\_24731\_cov\_33.167362 11690-11706. Max. coverage (+): 0. Max coverage (-): 0

Region: NODE\_27459\_length\_24731\_cov\_33.167362 11707-11723. Max. coverage (+): 0. Max coverage (-): 0

Region: NODE\_27459\_length\_24731\_cov\_33.167362 11724-11740. Max. coverage (+): 0. Max coverage (-): 0

Region: NODE\_27459\_length\_24731\_cov\_33.167362 11741-11757. Max. coverage (+): 0. Max coverage (-): 0

Region: NODE\_27459\_length\_24731\_cov\_33.167362 11758-11775. Max. coverage (+): 0. Max coverage (-): 0.09

Region: NODE\_27459\_length\_24731\_cov\_33.167362 11776-11792. Max. coverage (+): 0.47. Max coverage (-): 0

Region: NODE\_27459\_length\_24731\_cov\_33.167362 11793-11809. Max. coverage (+): 0.47. Max coverage (-): 0

Region: NODE\_27459\_length\_24731\_cov\_33.167362 11810-11826. Max. coverage (+): 0. Max coverage (-): 0

Region: NODE\_27459\_length\_24731\_cov\_33.167362 11827-11843. Max. coverage (+): 0. Max coverage (-): 0

Region: NODE\_27459\_length\_24731\_cov\_33.167362 11844-11861. Max. coverage (+): 0.09. Max coverage (-): 0

Region: NODE\_27459\_length\_24731\_cov\_33.167362 11862-11878. Max. coverage (+): 0. Max coverage (-): 0

Region: NODE\_27459\_length\_24731\_cov\_33.167362 11879-11895. Max. coverage (+): 0. Max coverage (-): 0

Region: NODE\_27459\_length\_24731\_cov\_33.167362 11896-11912. Max. coverage (+): 0.09. Max coverage (-): 0

Region: NODE\_27459\_length\_24731\_cov\_33.167362 11913-11929. Max. coverage (+): 0. Max coverage (-): 0

Region: NODE\_27459\_length\_24731\_cov\_33.167362 11930-11947. Max. coverage (+): 0. Max coverage (-): 0.09

Region: NODE\_27459\_length\_24731\_cov\_33.167362 11948-11964. Max. coverage (+): 0. Max coverage (-): 0

Region: NODE\_27459\_length\_24731\_cov\_33.167362 11965-. Max. coverage (+): 0. Max coverage (-): 0

RepeatMasker Color Code

**+**

100-98% Identity

<98-95% Identity

<95-90% Identity

<90-85% Identity

<85-80% Identity

<80-75% Identity

<75-70% Identity

<70% Identity

**-**

Gene Set Color Code

**+**

Gene

Pseudogene

Other

**-**

Topology/Coverage Color Code

Coverage Plus Strand

Coverage Minus Strand

Mainstrand: Plus

Mainstrand: Minus

Complementary Strand

Flanking Region  
(if option -flank >0)

Gene Set Annotation  
  
RepeatMasker Annotation  

**1. AlRepB-738**: 3481-4174 (-), Divergence to consensus: 10.8%  
**2. AlRepA-386**: 4441-4582 (-), Divergence to consensus: 31.3%  
**3. Penelope-1\_AFC**: 4913-5068 (-), Divergence to consensus: 25.9%  
**4. Penelope-1\_AFC**: 5221-5361 (-), Divergence to consensus: 32.8%  
**5. AlRepA-297**: 6124-6490 (+), Divergence to consensus: 30.7%  
**6. AlRepA-297**: 6665-6841 (+), Divergence to consensus: 37.5%  
**7. AlRepB-429**: 9110-9231 (+), Divergence to consensus: 0.8%  
**8. AlRepB-738**: 9325-9773 (+), Divergence to consensus: 8.1%  
**9. AlRepB-738**: 9775-9999 (+), Divergence to consensus: 6.7%  
**10. AlRepB-569**: 10073-10165 (-), Divergence to consensus: 31.8%  
**11. Dong2\_FR**: 10173-10266 (-), Divergence to consensus: 25.5%  
**12. (TTA)n**: 10284-10331 (+), Divergence to consensus: 0%  
**13. EnSpm-17\_HM**: 10332-10381 (-), Divergence to consensus: 18.3%  
**14. AlRepB-738**: 10354-10515 (+), Divergence to consensus: 11.6%  
**15. AlRepB-738**: 10551-10827 (+), Divergence to consensus: 6.2%  
**16. Dong2\_FR**: 10923-11016 (-), Divergence to consensus: 25.5%  
**17. (TTA)n**: 11034-11066 (+), Divergence to consensus: 0%  
**18. EnSpm-17\_HM**: 11067-11120 (-), Divergence to consensus: 20.6%  
**19. RTE-2\_AFC**: 11322-11633 (+), Divergence to consensus: 32.1%  
**20. SINE\_TE**: 11768-11963 (-), Divergence to consensus: 23.6%  
**21. DNA7-N2\_DR**: 11905-11982 (+), Divergence to consensus: 34.6%

  
Transcription Factor Binding Sites  

**RHOXF1** (Sequence: AGATTA (-): 3517)  
**RHOXF1** (Sequence: GGATTA (-): 3597)  
**RHOXF1** (Sequence: AGCTTA (-): 3983)  
**RHOXF1** (Sequence: AGATTA (-): 5927)  
**RHOXF1** (Sequence: GGCTTA (-): 8400)  
**RHOXF1** (Sequence: AGATCA (-): 8402)  
**RHOXF1** (Sequence: GGATTA (-): 9456)  
**RHOXF1** (Sequence: TGAGCC (+): 4492)  
**RHOXF1** (Sequence: TAATCC (+): 4497)  
**RHOXF1** (Sequence: TAAGCT (+): 5732)  
**RHOXF1** (Sequence: TAAGCC (+): 6453)  
**RHOXF1** (Sequence: TGAGCC (+): 6846)  
**RHOXF1** (Sequence: TAATCC (+): 8095)  
**RHOXF1** (Sequence: TAAGCC (+): 8531)  
**RHOXF1** (Sequence: TGATCC (+): 8696)  
**RHOXF1** (Sequence: TGATCC (+): 8700)  
**RHOXF1** (Sequence: TGATCT (+): 8860)  
**RHOXF1** (Sequence: TGAGCT (+): 9004)  
**RHOXF1** (Sequence: TAATCC (+): 9873)  
**RHOXF1** (Sequence: TAATCT (+): 9958)  
**RHOXF1** (Sequence: TAATCC (+): 10701)  
**RHOXF1** (Sequence: TAATCT (+): 10786)  
**RHOXF1** (Sequence: TGAGCT (+): 11213)  
**Lhx8** (Sequence: CTAATTAG (-): 7404)  
**Gata4** (Sequence: CTTATCT (+): 5379)  
**POU5F1** (Sequence: TTTGCAT (-): 5682)  
**POU5F1** (Sequence: TTTGCAT (-): 7582)  
**POU5F1** (Sequence: TTTGCAT (-): 7837)  
**SOX9** (Sequence: AACAATAG (-): 7916)  
**SOX9** (Sequence: AACAATGA (-): 7938)  
**SOX9** (Sequence: AACAATGA (-): 8350)  
**FOXO1** (Sequence: CTTGTTTTT (+): 4795)  
**FOXO1** (Sequence: CCTGTTTAT (+): 7110)  
**FOXO1** (Sequence: CCTGTTTTT (+): 7299)  
**FOXO1** (Sequence: GCTGTTTTT (+): 8250)  
**FOXO3\_mmu** (Sequence: TGTTTTCC (-): 9470)  
**Sox5** (Sequence: ATTGTT (+): 3936)  
**Sox5** (Sequence: ATTGTT (+): 6668)  
**Sox5** (Sequence: ATTGTT (+): 7617)  
**Sox5** (Sequence: ATTGTT (+): 7980)  
**Sox5** (Sequence: ATTGTT (+): 10087)  
**Sox5** (Sequence: ATTGTT (+): 10090)  
**SOX9** (Sequence: TTATTGTT (+): 3934)  
**SOX9** (Sequence: TCATTGTT (+): 7615)  
**SOX9** (Sequence: TCATTGTT (+): 7978)  
**SOX9** (Sequence: TTATTGTT (+): 10085)  
**FOXO3\_mmu** (Sequence: GGAAAACA (+): 3947)  
**FOXO3\_mmu** (Sequence: GCAAAACA (+): 8740)  
**FOXO3\_mmu** (Sequence: GGAAAACA (+): 10123)  
**Nobox** (Sequence: AGTAATTA (-): 4233)  
**Nobox** (Sequence: ACCAATTA (-): 4572)  
**Nobox** (Sequence: ACTAATTA (-): 9258)  
**FOXO1** (Sequence: AAAAACAAC (-): 5037)  
**FOXO1** (Sequence: AAAAACAGG (-): 11386)  
**Nobox** (Sequence: TAATTAGT (+): 4235)  
**Nobox** (Sequence: TAATTAGT (+): 7405)  
**Rhox11** (Sequence: TGCTGTAAA (+): 7852)  
**Rhox11** (Sequence: TGCTGTTTT (+): 8249)  
**Rhox11** (Sequence: TGCTGTTAT (+): 8310)  
**Rhox11** (Sequence: CGGTGTTTT (+): 9467)  
**Rhox11** (Sequence: ATTACAGCG (-): 6030)  
**Sox5** (Sequence: AACAAT (-): 5251)  
**Sox5** (Sequence: AACAAT (-): 7916)  
**Sox5** (Sequence: AACAAT (-): 7938)  
**Sox5** (Sequence: AACAAT (-): 8350)  
**Sox5** (Sequence: AACAAT (-): 11958)  
**POU2F1** (Sequence: TATTTTAAT (+): 5750)  
**POU5F1** (Sequence: ATGCAAA (+): 11561)
