# Supplementary material for: First characterization of PIWI-interacting RNA clusters in a cichlid fish with a B chromosome
Source: BMC Biol. 2022 Sep 21;20:204. doi: 10.1186/s12915-022-01403-2 (PMC9490952; doi:10.1186/s12915-022-01403-2)
Supplement: Supplementary file 1 — Additional file 1. Zipped folder with fasta and interactive html piRNA cluster information for the A. latifasciata genome. The nomenclature is as follows: number-pirna-cluster_sex_B-presence (f, female; m, male; 0b, without B chromosome; 1b, with B chromosome). [file 12915_2022_1403_MOESM1_ESM.zip › 107_f0b.html]

piRNA cluster 107\_f0b 11


Predicted piRNA cluster no. 107\_f0b
  

Show proTRAC run info
Hide proTRAC run info

/\  
                \_\_\_\_\_\_\_\_\_\_\_\_\_\_\_\_\_\_\_\_\_\_\_/\\_\_\_ /  \\_\_\_\_\_\_\_  
               I                      /  \  /    \      I  
               I     pro             /    \/      \     I  
               I        TRAC        /               \   I  
               I   \_\_\_\_\_\_\_\_\_\_\_\_\_\_\_\_/\_\_\_\_\_\_\_\_\_\_\_\_\_\_\_\_\_\\_ I  
               I   \              /                     I  
               I    \            /                      I  
               I     \  /\      /       V.2.4.2         I  
               I      \/  \    /                        I  
               I\_\_\_\_\_\_\_\_\_\_\_\  /\_\_\_\_\_\_\_\_\_\_\_\_\_\_\_\_\_\_\_\_\_\_\_\_\_I  
                            \/  
  
  
================================= proTRAC ====================================  
VERSION: .......... 2.4.2  
LAST MODIFIED: .... 11. May 2018  
  
Please cite:  
Rosenkranz D, Zischler H. proTRAC - a software for probabilistic piRNA cluster  
detection, visualization and analysis. 2012. BMC Bioinformatics 13:5.  
  
  
Contact:  
David Rosenkranz  
Institute of Organismic and Molecular Evolutionary Biology  
Dept. Anthropology, small RNA group  
Johannes Gutenberg University Mainz  
email: rosenkranz@uni-mainz.de  
  
You can find the latest proTRAC version at:  
http://sourceforge.net/projects/protrac/files  
http://www.smallRNAgroup-mainz.de/software  
==============================================================================  
  
PARAMETERS:  
Map file: ...............piwi-femeas-0B.fa-collapse.map  
Genome file: ............../../../0B\_ala\_genome.fa  
RepeatMasker annotation: Alatifasciata-all0B-maryan-v2.fa\_corrected.out  
GeneSet:................./guest-storage/Data/annotation/Alatifasciata\_all0B\_maryan-v2\_out2017.gff  
  
Significant (p<=0.01) hit density will be calculated based  
on observed hit distribution.  
  
Sliding window size: ........................................ 5000 bp  
Sliding window increament: .................................. 1000 bp  
Normalize each hit by number of genomic hits: ............... yes  
Normalize each hit by number of sequence reads: ............. yes  
Normalize values (-> per million mapped reads): ............. yes  
Min. fraction of hits with 1T(U) or 10A: .................... 0.75  
Alternatively: Min. fraction of hits with 1T(U) and 10A: .... 0.5  
Min. fraction of hits with typical piRNA length: ............ 0.75  
Typical piRNA length: ....................................... 24-32 nt  
Min. size of a piRNA cluster: ............................... 1000 bp.  
Min. number of hits (absolute): ............................. 0  
Min. number of hits (normalized): ........................... 0  
Min. fraction of hits on the mainstrand: .................... 0.75  
Top fraction of mapped sequences (in terms of read counts): . 1%  
Top fraction accounts for max. n% of sequence reads: ........ 90%  
Min. fraction of hits on each arm of a bidirectional cluster: 0.05  
Output html file for each cluster: .......................... yes  
Output a summary table: ..................................... yes  
Output a FASTA file for each cluster (piRNA sequences): ..... yes  
Output a FASTA file comprising cluster sequences: ........... yes  
Output a GTF file for predicted piRNA clusters: ..............yes  
Search DNA motifs in clusters: .............................. yes  
Output flanking sequences: +/- .............................. 0 bp  
Output ~.pTi file: .......................................... no  
==============================================================================  
  
  
Genome size (without gaps): ............ 758543724 bp  
Gaps (N/X/-): .......................... 417479 bp  
Mapped reads: .......................... 13052187  
Non-identical sequences: ............... 3338911  
Genomic hits: .......................... 28737726  
Significant densitiy of mapped reads: .. 470.083249848448 reads/kb

Show proTRAC cluster info
Hide proTRAC cluster info

|  |  |
| --- | --- |
| Location | NODE\_27951\_length\_3778\_cov\_35.373741 |
| Coordinates | 6-3836 |
| Size [bp] | 3831 |
| Sequence hit loci | 1778 |
| Mapped reads (normalized) | 5926.2 |
| Mapped reads (normalized) per kb | 1546.9 |
| Normalized reads with 1T (1U) | 84.6% |
| Normalized reads with 10A | 45.2% |
| Normalized reads with length 24-32 nt | 98.8% |
| Normalized reads on the main strand(s) | 95.1% |
| Predicted directionality | mono:plus |

100%

0%

1T (1U)  
reads

10A reads

24-32 nt  
reads

reads on mainstrand

**Either the amount of reads with 1T (1U) OR 10A has to exceed 75% (set with option: -1Tor10A)  
Alternatively the amount of reads with 1T (1U) AND 10A has to exceed 50% (set with option: -1Tand10A)  
Minimum amount of reads with preferred size is 75% (set with option: -pisize)  
Minimum amount of reads on the main strand(s) is 75% (set with option: -clstrand)**

Show read coverage
Hide read coverage

WHAT DO I SEE HERE?  
This chart shows the location of mapped sequence reads within a predicted piRNA cluster. The color refers to the number of genomic hits produced by the sequence read in question. A dark red bar indicates that this sequence read produces many other hits elsewhere in the genome. Many adjacent red or yellow bars can indicate the presence of a multi-copy element such as transposons or rRNA genes. A dark green bar indicates that this sequence read maps uniquely to this locus.

1 hit

2-5 hits

6-10 hits

11-20 hits

21-50 hits

51-100 hits

> 100 hits

NODE\_27951\_length\_3778\_cov\_35.373741

6

3836

Gene Set

RepeatMasker

Mapped  
Reads

65.2

plus strand

minus strand

65.2

Region: NODE\_27951\_length\_3778\_cov\_35.373741 11920-9. Max. coverage (+): 0. Max coverage (-): 0

Region: NODE\_27951\_length\_3778\_cov\_35.373741 10-17. Max. coverage (+): 0. Max coverage (-): 0

Region: NODE\_27951\_length\_3778\_cov\_35.373741 18-25. Max. coverage (+): 0. Max coverage (-): 0

Region: NODE\_27951\_length\_3778\_cov\_35.373741 26-32. Max. coverage (+): 0. Max coverage (-): 0

Region: NODE\_27951\_length\_3778\_cov\_35.373741 33-40. Max. coverage (+): 0.08. Max coverage (-): 0

Region: NODE\_27951\_length\_3778\_cov\_35.373741 41-48. Max. coverage (+): 0. Max coverage (-): 0

Region: NODE\_27951\_length\_3778\_cov\_35.373741 49-55. Max. coverage (+): 0.15. Max coverage (-): 0

Region: NODE\_27951\_length\_3778\_cov\_35.373741 56-63. Max. coverage (+): 0.15. Max coverage (-): 0.08

Region: NODE\_27951\_length\_3778\_cov\_35.373741 64-71. Max. coverage (+): 0.08. Max coverage (-): 0

Region: NODE\_27951\_length\_3778\_cov\_35.373741 72-78. Max. coverage (+): 0. Max coverage (-): 0

Region: NODE\_27951\_length\_3778\_cov\_35.373741 79-86. Max. coverage (+): 0. Max coverage (-): 0

Region: NODE\_27951\_length\_3778\_cov\_35.373741 87-94. Max. coverage (+): 0. Max coverage (-): 0

Region: NODE\_27951\_length\_3778\_cov\_35.373741 95-101. Max. coverage (+): 0. Max coverage (-): 0.08

Region: NODE\_27951\_length\_3778\_cov\_35.373741 102-109. Max. coverage (+): 0. Max coverage (-): 0

Region: NODE\_27951\_length\_3778\_cov\_35.373741 110-117. Max. coverage (+): 0.08. Max coverage (-): 0.08

Region: NODE\_27951\_length\_3778\_cov\_35.373741 118-124. Max. coverage (+): 0.08. Max coverage (-): 0

Region: NODE\_27951\_length\_3778\_cov\_35.373741 125-132. Max. coverage (+): 0.15. Max coverage (-): 0

Region: NODE\_27951\_length\_3778\_cov\_35.373741 133-140. Max. coverage (+): 0.08. Max coverage (-): 0

Region: NODE\_27951\_length\_3778\_cov\_35.373741 141-147. Max. coverage (+): 0.15. Max coverage (-): 0

Region: NODE\_27951\_length\_3778\_cov\_35.373741 148-155. Max. coverage (+): 0.08. Max coverage (-): 0

Region: NODE\_27951\_length\_3778\_cov\_35.373741 156-163. Max. coverage (+): 0.08. Max coverage (-): 0

Region: NODE\_27951\_length\_3778\_cov\_35.373741 164-170. Max. coverage (+): 0.15. Max coverage (-): 0

Region: NODE\_27951\_length\_3778\_cov\_35.373741 171-178. Max. coverage (+): 0. Max coverage (-): 0

Region: NODE\_27951\_length\_3778\_cov\_35.373741 179-186. Max. coverage (+): 0. Max coverage (-): 0.08

Region: NODE\_27951\_length\_3778\_cov\_35.373741 187-193. Max. coverage (+): 0.08. Max coverage (-): 0.31

Region: NODE\_27951\_length\_3778\_cov\_35.373741 194-201. Max. coverage (+): 0.08. Max coverage (-): 0.31

Region: NODE\_27951\_length\_3778\_cov\_35.373741 202-209. Max. coverage (+): 0.08. Max coverage (-): 0.15

Region: NODE\_27951\_length\_3778\_cov\_35.373741 210-216. Max. coverage (+): 0. Max coverage (-): 0

Region: NODE\_27951\_length\_3778\_cov\_35.373741 217-224. Max. coverage (+): 0.54. Max coverage (-): 0

Region: NODE\_27951\_length\_3778\_cov\_35.373741 225-232. Max. coverage (+): 0. Max coverage (-): 0

Region: NODE\_27951\_length\_3778\_cov\_35.373741 233-239. Max. coverage (+): 0.08. Max coverage (-): 0

Region: NODE\_27951\_length\_3778\_cov\_35.373741 240-247. Max. coverage (+): 0.08. Max coverage (-): 0

Region: NODE\_27951\_length\_3778\_cov\_35.373741 248-255. Max. coverage (+): 0.15. Max coverage (-): 0

Region: NODE\_27951\_length\_3778\_cov\_35.373741 256-262. Max. coverage (+): 0.08. Max coverage (-): 0.15

Region: NODE\_27951\_length\_3778\_cov\_35.373741 263-270. Max. coverage (+): 0.46. Max coverage (-): 0.15

Region: NODE\_27951\_length\_3778\_cov\_35.373741 271-278. Max. coverage (+): 0.46. Max coverage (-): 0.08

Region: NODE\_27951\_length\_3778\_cov\_35.373741 279-285. Max. coverage (+): 0.08. Max coverage (-): 0.08

Region: NODE\_27951\_length\_3778\_cov\_35.373741 286-293. Max. coverage (+): 0.46. Max coverage (-): 0.15

Region: NODE\_27951\_length\_3778\_cov\_35.373741 294-300. Max. coverage (+): 0.08. Max coverage (-): 0.15

Region: NODE\_27951\_length\_3778\_cov\_35.373741 301-308. Max. coverage (+): 0.31. Max coverage (-): 0

Region: NODE\_27951\_length\_3778\_cov\_35.373741 309-316. Max. coverage (+): 0. Max coverage (-): 0.08

Region: NODE\_27951\_length\_3778\_cov\_35.373741 317-323. Max. coverage (+): 0. Max coverage (-): 0

Region: NODE\_27951\_length\_3778\_cov\_35.373741 324-331. Max. coverage (+): 0. Max coverage (-): 0

Region: NODE\_27951\_length\_3778\_cov\_35.373741 332-339. Max. coverage (+): 0. Max coverage (-): 0

Region: NODE\_27951\_length\_3778\_cov\_35.373741 340-346. Max. coverage (+): 0. Max coverage (-): 0

Region: NODE\_27951\_length\_3778\_cov\_35.373741 347-354. Max. coverage (+): 0. Max coverage (-): 0

Region: NODE\_27951\_length\_3778\_cov\_35.373741 355-362. Max. coverage (+): 0. Max coverage (-): 0

Region: NODE\_27951\_length\_3778\_cov\_35.373741 363-369. Max. coverage (+): 0. Max coverage (-): 0

Region: NODE\_27951\_length\_3778\_cov\_35.373741 370-377. Max. coverage (+): 0. Max coverage (-): 0

Region: NODE\_27951\_length\_3778\_cov\_35.373741 378-385. Max. coverage (+): 0. Max coverage (-): 0.08

Region: NODE\_27951\_length\_3778\_cov\_35.373741 386-392. Max. coverage (+): 0. Max coverage (-): 0

Region: NODE\_27951\_length\_3778\_cov\_35.373741 393-400. Max. coverage (+): 0. Max coverage (-): 0

Region: NODE\_27951\_length\_3778\_cov\_35.373741 401-408. Max. coverage (+): 0. Max coverage (-): 0

Region: NODE\_27951\_length\_3778\_cov\_35.373741 409-415. Max. coverage (+): 0. Max coverage (-): 0

Region: NODE\_27951\_length\_3778\_cov\_35.373741 416-423. Max. coverage (+): 0. Max coverage (-): 0

Region: NODE\_27951\_length\_3778\_cov\_35.373741 424-431. Max. coverage (+): 0. Max coverage (-): 0

Region: NODE\_27951\_length\_3778\_cov\_35.373741 432-438. Max. coverage (+): 0.31. Max coverage (-): 0

Region: NODE\_27951\_length\_3778\_cov\_35.373741 439-446. Max. coverage (+): 0.38. Max coverage (-): 0.08

Region: NODE\_27951\_length\_3778\_cov\_35.373741 447-454. Max. coverage (+): 0. Max coverage (-): 0.15

Region: NODE\_27951\_length\_3778\_cov\_35.373741 455-461. Max. coverage (+): 0. Max coverage (-): 0.08

Region: NODE\_27951\_length\_3778\_cov\_35.373741 462-469. Max. coverage (+): 1.99. Max coverage (-): 0

Region: NODE\_27951\_length\_3778\_cov\_35.373741 470-477. Max. coverage (+): 2.53. Max coverage (-): 0

Region: NODE\_27951\_length\_3778\_cov\_35.373741 478-484. Max. coverage (+): 0.15. Max coverage (-): 0

Region: NODE\_27951\_length\_3778\_cov\_35.373741 485-492. Max. coverage (+): 0. Max coverage (-): 0

Region: NODE\_27951\_length\_3778\_cov\_35.373741 493-500. Max. coverage (+): 0.31. Max coverage (-): 0

Region: NODE\_27951\_length\_3778\_cov\_35.373741 501-507. Max. coverage (+): 19. Max coverage (-): 0

Region: NODE\_27951\_length\_3778\_cov\_35.373741 508-515. Max. coverage (+): 12.26. Max coverage (-): 0

Region: NODE\_27951\_length\_3778\_cov\_35.373741 516-523. Max. coverage (+): 0.08. Max coverage (-): 0

Region: NODE\_27951\_length\_3778\_cov\_35.373741 524-530. Max. coverage (+): 0.08. Max coverage (-): 0

Region: NODE\_27951\_length\_3778\_cov\_35.373741 531-538. Max. coverage (+): 0. Max coverage (-): 0

Region: NODE\_27951\_length\_3778\_cov\_35.373741 539-546. Max. coverage (+): 0.08. Max coverage (-): 0

Region: NODE\_27951\_length\_3778\_cov\_35.373741 547-553. Max. coverage (+): 0.15. Max coverage (-): 0.08

Region: NODE\_27951\_length\_3778\_cov\_35.373741 554-561. Max. coverage (+): 1.46. Max coverage (-): 0

Region: NODE\_27951\_length\_3778\_cov\_35.373741 562-569. Max. coverage (+): 0.46. Max coverage (-): 0

Region: NODE\_27951\_length\_3778\_cov\_35.373741 570-576. Max. coverage (+): 0.08. Max coverage (-): 0.08

Region: NODE\_27951\_length\_3778\_cov\_35.373741 577-584. Max. coverage (+): 0. Max coverage (-): 0

Region: NODE\_27951\_length\_3778\_cov\_35.373741 585-592. Max. coverage (+): 3.29. Max coverage (-): 0

Region: NODE\_27951\_length\_3778\_cov\_35.373741 593-599. Max. coverage (+): 2.76. Max coverage (-): 0.15

Region: NODE\_27951\_length\_3778\_cov\_35.373741 600-607. Max. coverage (+): 0. Max coverage (-): 0

Region: NODE\_27951\_length\_3778\_cov\_35.373741 608-615. Max. coverage (+): 0. Max coverage (-): 0

Region: NODE\_27951\_length\_3778\_cov\_35.373741 616-622. Max. coverage (+): 0.31. Max coverage (-): 0

Region: NODE\_27951\_length\_3778\_cov\_35.373741 623-630. Max. coverage (+): 0.08. Max coverage (-): 0

Region: NODE\_27951\_length\_3778\_cov\_35.373741 631-638. Max. coverage (+): 0. Max coverage (-): 0.08

Region: NODE\_27951\_length\_3778\_cov\_35.373741 639-645. Max. coverage (+): 0.08. Max coverage (-): 0.08

Region: NODE\_27951\_length\_3778\_cov\_35.373741 646-653. Max. coverage (+): 1.84. Max coverage (-): 0.08

Region: NODE\_27951\_length\_3778\_cov\_35.373741 654-661. Max. coverage (+): 16.63. Max coverage (-): 0

Region: NODE\_27951\_length\_3778\_cov\_35.373741 662-668. Max. coverage (+): 1.69. Max coverage (-): 0

Region: NODE\_27951\_length\_3778\_cov\_35.373741 669-676. Max. coverage (+): 0. Max coverage (-): 1.07

Region: NODE\_27951\_length\_3778\_cov\_35.373741 677-684. Max. coverage (+): 2.15. Max coverage (-): 0.54

Region: NODE\_27951\_length\_3778\_cov\_35.373741 685-691. Max. coverage (+): 2.22. Max coverage (-): 0.08

Region: NODE\_27951\_length\_3778\_cov\_35.373741 692-699. Max. coverage (+): 0.46. Max coverage (-): 0

Region: NODE\_27951\_length\_3778\_cov\_35.373741 700-707. Max. coverage (+): 1.15. Max coverage (-): 0.15

Region: NODE\_27951\_length\_3778\_cov\_35.373741 708-714. Max. coverage (+): 0.77. Max coverage (-): 0.08

Region: NODE\_27951\_length\_3778\_cov\_35.373741 715-722. Max. coverage (+): 0. Max coverage (-): 0

Region: NODE\_27951\_length\_3778\_cov\_35.373741 723-730. Max. coverage (+): 0.54. Max coverage (-): 0.08

Region: NODE\_27951\_length\_3778\_cov\_35.373741 731-737. Max. coverage (+): 0.23. Max coverage (-): 0

Region: NODE\_27951\_length\_3778\_cov\_35.373741 738-745. Max. coverage (+): 0.08. Max coverage (-): 0.15

Region: NODE\_27951\_length\_3778\_cov\_35.373741 746-753. Max. coverage (+): 0.08. Max coverage (-): 0

Region: NODE\_27951\_length\_3778\_cov\_35.373741 754-760. Max. coverage (+): 0. Max coverage (-): 0

Region: NODE\_27951\_length\_3778\_cov\_35.373741 761-768. Max. coverage (+): 0. Max coverage (-): 0.08

Region: NODE\_27951\_length\_3778\_cov\_35.373741 769-776. Max. coverage (+): 0. Max coverage (-): 0

Region: NODE\_27951\_length\_3778\_cov\_35.373741 777-783. Max. coverage (+): 0.08. Max coverage (-): 0

Region: NODE\_27951\_length\_3778\_cov\_35.373741 784-791. Max. coverage (+): 0.15. Max coverage (-): 0

Region: NODE\_27951\_length\_3778\_cov\_35.373741 792-799. Max. coverage (+): 3.91. Max coverage (-): 0

Region: NODE\_27951\_length\_3778\_cov\_35.373741 800-806. Max. coverage (+): 0.54. Max coverage (-): 0

Region: NODE\_27951\_length\_3778\_cov\_35.373741 807-814. Max. coverage (+): 0.54. Max coverage (-): 0.46

Region: NODE\_27951\_length\_3778\_cov\_35.373741 815-822. Max. coverage (+): 0.15. Max coverage (-): 0.08

Region: NODE\_27951\_length\_3778\_cov\_35.373741 823-829. Max. coverage (+): 0.84. Max coverage (-): 0

Region: NODE\_27951\_length\_3778\_cov\_35.373741 830-837. Max. coverage (+): 16.32. Max coverage (-): 0

Region: NODE\_27951\_length\_3778\_cov\_35.373741 838-844. Max. coverage (+): 1.38. Max coverage (-): 0

Region: NODE\_27951\_length\_3778\_cov\_35.373741 845-852. Max. coverage (+): 0.08. Max coverage (-): 0

Region: NODE\_27951\_length\_3778\_cov\_35.373741 853-860. Max. coverage (+): 0. Max coverage (-): 0

Region: NODE\_27951\_length\_3778\_cov\_35.373741 861-867. Max. coverage (+): 0.08. Max coverage (-): 0

Region: NODE\_27951\_length\_3778\_cov\_35.373741 868-875. Max. coverage (+): 1.15. Max coverage (-): 0

Region: NODE\_27951\_length\_3778\_cov\_35.373741 876-883. Max. coverage (+): 0. Max coverage (-): 0

Region: NODE\_27951\_length\_3778\_cov\_35.373741 884-890. Max. coverage (+): 0. Max coverage (-): 0.61

Region: NODE\_27951\_length\_3778\_cov\_35.373741 891-898. Max. coverage (+): 0.08. Max coverage (-): 0.08

Region: NODE\_27951\_length\_3778\_cov\_35.373741 899-906. Max. coverage (+): 5.75. Max coverage (-): 0

Region: NODE\_27951\_length\_3778\_cov\_35.373741 907-913. Max. coverage (+): 13.1. Max coverage (-): 0

Region: NODE\_27951\_length\_3778\_cov\_35.373741 914-921. Max. coverage (+): 0.15. Max coverage (-): 0

Region: NODE\_27951\_length\_3778\_cov\_35.373741 922-929. Max. coverage (+): 0. Max coverage (-): 0

Region: NODE\_27951\_length\_3778\_cov\_35.373741 930-936. Max. coverage (+): 0. Max coverage (-): 0.08

Region: NODE\_27951\_length\_3778\_cov\_35.373741 937-944. Max. coverage (+): 0.15. Max coverage (-): 0

Region: NODE\_27951\_length\_3778\_cov\_35.373741 945-952. Max. coverage (+): 20.84. Max coverage (-): 0

Region: NODE\_27951\_length\_3778\_cov\_35.373741 953-959. Max. coverage (+): 20.69. Max coverage (-): 0

Region: NODE\_27951\_length\_3778\_cov\_35.373741 960-967. Max. coverage (+): 0. Max coverage (-): 0.15

Region: NODE\_27951\_length\_3778\_cov\_35.373741 968-975. Max. coverage (+): 0. Max coverage (-): 0

Region: NODE\_27951\_length\_3778\_cov\_35.373741 976-982. Max. coverage (+): 0. Max coverage (-): 0.08

Region: NODE\_27951\_length\_3778\_cov\_35.373741 983-990. Max. coverage (+): 0. Max coverage (-): 0.08

Region: NODE\_27951\_length\_3778\_cov\_35.373741 991-998. Max. coverage (+): 0.31. Max coverage (-): 0

Region: NODE\_27951\_length\_3778\_cov\_35.373741 999-1005. Max. coverage (+): 0. Max coverage (-): 0.31

Region: NODE\_27951\_length\_3778\_cov\_35.373741 1006-1013. Max. coverage (+): 0. Max coverage (-): 0.23

Region: NODE\_27951\_length\_3778\_cov\_35.373741 1014-1021. Max. coverage (+): 0.31. Max coverage (-): 0

Region: NODE\_27951\_length\_3778\_cov\_35.373741 1022-1028. Max. coverage (+): 2.22. Max coverage (-): 0

Region: NODE\_27951\_length\_3778\_cov\_35.373741 1029-1036. Max. coverage (+): 0.31. Max coverage (-): 0

Region: NODE\_27951\_length\_3778\_cov\_35.373741 1037-1044. Max. coverage (+): 0.15. Max coverage (-): 0

Region: NODE\_27951\_length\_3778\_cov\_35.373741 1045-1051. Max. coverage (+): 0.08. Max coverage (-): 0.23

Region: NODE\_27951\_length\_3778\_cov\_35.373741 1052-1059. Max. coverage (+): 0.69. Max coverage (-): 0.31

Region: NODE\_27951\_length\_3778\_cov\_35.373741 1060-1067. Max. coverage (+): 2.91. Max coverage (-): 0.08

Region: NODE\_27951\_length\_3778\_cov\_35.373741 1068-1074. Max. coverage (+): 0.46. Max coverage (-): 0.08

Region: NODE\_27951\_length\_3778\_cov\_35.373741 1075-1082. Max. coverage (+): 0. Max coverage (-): 0

Region: NODE\_27951\_length\_3778\_cov\_35.373741 1083-1090. Max. coverage (+): 0. Max coverage (-): 0

Region: NODE\_27951\_length\_3778\_cov\_35.373741 1091-1097. Max. coverage (+): 0.38. Max coverage (-): 0

Region: NODE\_27951\_length\_3778\_cov\_35.373741 1098-1105. Max. coverage (+): 4.9. Max coverage (-): 0

Region: NODE\_27951\_length\_3778\_cov\_35.373741 1106-1113. Max. coverage (+): 0.61. Max coverage (-): 0

Region: NODE\_27951\_length\_3778\_cov\_35.373741 1114-1120. Max. coverage (+): 0. Max coverage (-): 0

Region: NODE\_27951\_length\_3778\_cov\_35.373741 1121-1128. Max. coverage (+): 0.61. Max coverage (-): 0

Region: NODE\_27951\_length\_3778\_cov\_35.373741 1129-1136. Max. coverage (+): 0.69. Max coverage (-): 0.08

Region: NODE\_27951\_length\_3778\_cov\_35.373741 1137-1143. Max. coverage (+): 1.23. Max coverage (-): 0.23

Region: NODE\_27951\_length\_3778\_cov\_35.373741 1144-1151. Max. coverage (+): 0.31. Max coverage (-): 0.15

Region: NODE\_27951\_length\_3778\_cov\_35.373741 1152-1159. Max. coverage (+): 0.46. Max coverage (-): 0.23

Region: NODE\_27951\_length\_3778\_cov\_35.373741 1160-1166. Max. coverage (+): 0.46. Max coverage (-): 0

Region: NODE\_27951\_length\_3778\_cov\_35.373741 1167-1174. Max. coverage (+): 4.44. Max coverage (-): 0

Region: NODE\_27951\_length\_3778\_cov\_35.373741 1175-1182. Max. coverage (+): 0.15. Max coverage (-): 0

Region: NODE\_27951\_length\_3778\_cov\_35.373741 1183-1189. Max. coverage (+): 1.38. Max coverage (-): 0

Region: NODE\_27951\_length\_3778\_cov\_35.373741 1190-1197. Max. coverage (+): 3.45. Max coverage (-): 0.08

Region: NODE\_27951\_length\_3778\_cov\_35.373741 1198-1205. Max. coverage (+): 0.08. Max coverage (-): 0.08

Region: NODE\_27951\_length\_3778\_cov\_35.373741 1206-1212. Max. coverage (+): 0. Max coverage (-): 0

Region: NODE\_27951\_length\_3778\_cov\_35.373741 1213-1220. Max. coverage (+): 2.38. Max coverage (-): 0

Region: NODE\_27951\_length\_3778\_cov\_35.373741 1221-1228. Max. coverage (+): 2.38. Max coverage (-): 0

Region: NODE\_27951\_length\_3778\_cov\_35.373741 1229-1235. Max. coverage (+): 0.23. Max coverage (-): 0

Region: NODE\_27951\_length\_3778\_cov\_35.373741 1236-1243. Max. coverage (+): 0.38. Max coverage (-): 0

Region: NODE\_27951\_length\_3778\_cov\_35.373741 1244-1251. Max. coverage (+): 0. Max coverage (-): 0

Region: NODE\_27951\_length\_3778\_cov\_35.373741 1252-1258. Max. coverage (+): 0. Max coverage (-): 0

Region: NODE\_27951\_length\_3778\_cov\_35.373741 1259-1266. Max. coverage (+): 0.38. Max coverage (-): 0

Region: NODE\_27951\_length\_3778\_cov\_35.373741 1267-1274. Max. coverage (+): 0.38. Max coverage (-): 0

Region: NODE\_27951\_length\_3778\_cov\_35.373741 1275-1281. Max. coverage (+): 0. Max coverage (-): 0.08

Region: NODE\_27951\_length\_3778\_cov\_35.373741 1282-1289. Max. coverage (+): 0. Max coverage (-): 0.15

Region: NODE\_27951\_length\_3778\_cov\_35.373741 1290-1297. Max. coverage (+): 0. Max coverage (-): 0.08

Region: NODE\_27951\_length\_3778\_cov\_35.373741 1298-1304. Max. coverage (+): 2.76. Max coverage (-): 0

Region: NODE\_27951\_length\_3778\_cov\_35.373741 1305-1312. Max. coverage (+): 0.54. Max coverage (-): 0.08

Region: NODE\_27951\_length\_3778\_cov\_35.373741 1313-1320. Max. coverage (+): 0.23. Max coverage (-): 0.08

Region: NODE\_27951\_length\_3778\_cov\_35.373741 1321-1327. Max. coverage (+): 0. Max coverage (-): 0

Region: NODE\_27951\_length\_3778\_cov\_35.373741 1328-1335. Max. coverage (+): 0.23. Max coverage (-): 0

Region: NODE\_27951\_length\_3778\_cov\_35.373741 1336-1343. Max. coverage (+): 0.31. Max coverage (-): 0.31

Region: NODE\_27951\_length\_3778\_cov\_35.373741 1344-1350. Max. coverage (+): 0. Max coverage (-): 0

Region: NODE\_27951\_length\_3778\_cov\_35.373741 1351-1358. Max. coverage (+): 0.08. Max coverage (-): 0

Region: NODE\_27951\_length\_3778\_cov\_35.373741 1359-1366. Max. coverage (+): 0.46. Max coverage (-): 0

Region: NODE\_27951\_length\_3778\_cov\_35.373741 1367-1373. Max. coverage (+): 0.15. Max coverage (-): 0

Region: NODE\_27951\_length\_3778\_cov\_35.373741 1374-1381. Max. coverage (+): 0. Max coverage (-): 0

Region: NODE\_27951\_length\_3778\_cov\_35.373741 1382-1388. Max. coverage (+): 0. Max coverage (-): 0

Region: NODE\_27951\_length\_3778\_cov\_35.373741 1389-1396. Max. coverage (+): 0.08. Max coverage (-): 0.77

Region: NODE\_27951\_length\_3778\_cov\_35.373741 1397-1404. Max. coverage (+): 0.15. Max coverage (-): 0.77

Region: NODE\_27951\_length\_3778\_cov\_35.373741 1405-1411. Max. coverage (+): 0.15. Max coverage (-): 0

Region: NODE\_27951\_length\_3778\_cov\_35.373741 1412-1419. Max. coverage (+): 24.75. Max coverage (-): 0

Region: NODE\_27951\_length\_3778\_cov\_35.373741 1420-1427. Max. coverage (+): 0.08. Max coverage (-): 0

Region: NODE\_27951\_length\_3778\_cov\_35.373741 1428-1434. Max. coverage (+): 0.31. Max coverage (-): 0.08

Region: NODE\_27951\_length\_3778\_cov\_35.373741 1435-1442. Max. coverage (+): 0.23. Max coverage (-): 0.15

Region: NODE\_27951\_length\_3778\_cov\_35.373741 1443-1450. Max. coverage (+): 0.23. Max coverage (-): 0.23

Region: NODE\_27951\_length\_3778\_cov\_35.373741 1451-1457. Max. coverage (+): 0. Max coverage (-): 0.31

Region: NODE\_27951\_length\_3778\_cov\_35.373741 1458-1465. Max. coverage (+): 0.08. Max coverage (-): 0.38

Region: NODE\_27951\_length\_3778\_cov\_35.373741 1466-1473. Max. coverage (+): 0.08. Max coverage (-): 0

Region: NODE\_27951\_length\_3778\_cov\_35.373741 1474-1480. Max. coverage (+): 0. Max coverage (-): 0

Region: NODE\_27951\_length\_3778\_cov\_35.373741 1481-1488. Max. coverage (+): 0.08. Max coverage (-): 0.23

Region: NODE\_27951\_length\_3778\_cov\_35.373741 1489-1496. Max. coverage (+): 0.23. Max coverage (-): 0.31

Region: NODE\_27951\_length\_3778\_cov\_35.373741 1497-1503. Max. coverage (+): 0.23. Max coverage (-): 0.23

Region: NODE\_27951\_length\_3778\_cov\_35.373741 1504-1511. Max. coverage (+): 0. Max coverage (-): 0

Region: NODE\_27951\_length\_3778\_cov\_35.373741 1512-1519. Max. coverage (+): 0.15. Max coverage (-): 0

Region: NODE\_27951\_length\_3778\_cov\_35.373741 1520-1526. Max. coverage (+): 2.45. Max coverage (-): 0

Region: NODE\_27951\_length\_3778\_cov\_35.373741 1527-1534. Max. coverage (+): 2.53. Max coverage (-): 0.08

Region: NODE\_27951\_length\_3778\_cov\_35.373741 1535-1542. Max. coverage (+): 0.23. Max coverage (-): 0.08

Region: NODE\_27951\_length\_3778\_cov\_35.373741 1543-1549. Max. coverage (+): 0.77. Max coverage (-): 0

Region: NODE\_27951\_length\_3778\_cov\_35.373741 1550-1557. Max. coverage (+): 0.77. Max coverage (-): 0

Region: NODE\_27951\_length\_3778\_cov\_35.373741 1558-1565. Max. coverage (+): 0.08. Max coverage (-): 0

Region: NODE\_27951\_length\_3778\_cov\_35.373741 1566-1572. Max. coverage (+): 0. Max coverage (-): 0

Region: NODE\_27951\_length\_3778\_cov\_35.373741 1573-1580. Max. coverage (+): 0.15. Max coverage (-): 0

Region: NODE\_27951\_length\_3778\_cov\_35.373741 1581-1588. Max. coverage (+): 0.61. Max coverage (-): 0

Region: NODE\_27951\_length\_3778\_cov\_35.373741 1589-1595. Max. coverage (+): 1.53. Max coverage (-): 0

Region: NODE\_27951\_length\_3778\_cov\_35.373741 1596-1603. Max. coverage (+): 0.84. Max coverage (-): 0

Region: NODE\_27951\_length\_3778\_cov\_35.373741 1604-1611. Max. coverage (+): 1.53. Max coverage (-): 0

Region: NODE\_27951\_length\_3778\_cov\_35.373741 1612-1618. Max. coverage (+): 0.08. Max coverage (-): 0

Region: NODE\_27951\_length\_3778\_cov\_35.373741 1619-1626. Max. coverage (+): 0. Max coverage (-): 0.15

Region: NODE\_27951\_length\_3778\_cov\_35.373741 1627-1634. Max. coverage (+): 0.15. Max coverage (-): 0

Region: NODE\_27951\_length\_3778\_cov\_35.373741 1635-1641. Max. coverage (+): 2.15. Max coverage (-): 0

Region: NODE\_27951\_length\_3778\_cov\_35.373741 1642-1649. Max. coverage (+): 1.69. Max coverage (-): 0

Region: NODE\_27951\_length\_3778\_cov\_35.373741 1650-1657. Max. coverage (+): 0.08. Max coverage (-): 0.23

Region: NODE\_27951\_length\_3778\_cov\_35.373741 1658-1664. Max. coverage (+): 0. Max coverage (-): 1.07

Region: NODE\_27951\_length\_3778\_cov\_35.373741 1665-1672. Max. coverage (+): 0.08. Max coverage (-): 2.15

Region: NODE\_27951\_length\_3778\_cov\_35.373741 1673-1680. Max. coverage (+): 0.23. Max coverage (-): 0.08

Region: NODE\_27951\_length\_3778\_cov\_35.373741 1681-1687. Max. coverage (+): 25.36. Max coverage (-): 0

Region: NODE\_27951\_length\_3778\_cov\_35.373741 1688-1695. Max. coverage (+): 65.2. Max coverage (-): 0

Region: NODE\_27951\_length\_3778\_cov\_35.373741 1696-1703. Max. coverage (+): 0. Max coverage (-): 0

Region: NODE\_27951\_length\_3778\_cov\_35.373741 1704-1710. Max. coverage (+): 0. Max coverage (-): 0.08

Region: NODE\_27951\_length\_3778\_cov\_35.373741 1711-1718. Max. coverage (+): 0.08. Max coverage (-): 0.08

Region: NODE\_27951\_length\_3778\_cov\_35.373741 1719-1726. Max. coverage (+): 0.23. Max coverage (-): 0

Region: NODE\_27951\_length\_3778\_cov\_35.373741 1727-1733. Max. coverage (+): 0.38. Max coverage (-): 0

Region: NODE\_27951\_length\_3778\_cov\_35.373741 1734-1741. Max. coverage (+): 0.08. Max coverage (-): 0.08

Region: NODE\_27951\_length\_3778\_cov\_35.373741 1742-1749. Max. coverage (+): 0.03. Max coverage (-): 0.08

Region: NODE\_27951\_length\_3778\_cov\_35.373741 1750-1756. Max. coverage (+): 0.15. Max coverage (-): 0

Region: NODE\_27951\_length\_3778\_cov\_35.373741 1757-1764. Max. coverage (+): 0.03. Max coverage (-): 0.05

Region: NODE\_27951\_length\_3778\_cov\_35.373741 1765-1772. Max. coverage (+): 0. Max coverage (-): 0.03

Region: NODE\_27951\_length\_3778\_cov\_35.373741 1773-1779. Max. coverage (+): 0. Max coverage (-): 0.03

Region: NODE\_27951\_length\_3778\_cov\_35.373741 1780-1787. Max. coverage (+): 0.38. Max coverage (-): 0

Region: NODE\_27951\_length\_3778\_cov\_35.373741 1788-1795. Max. coverage (+): 0.56. Max coverage (-): 0

Region: NODE\_27951\_length\_3778\_cov\_35.373741 1796-1802. Max. coverage (+): 2.32. Max coverage (-): 0

Region: NODE\_27951\_length\_3778\_cov\_35.373741 1803-1810. Max. coverage (+): 0.41. Max coverage (-): 0

Region: NODE\_27951\_length\_3778\_cov\_35.373741 1811-1818. Max. coverage (+): 0.03. Max coverage (-): 0

Region: NODE\_27951\_length\_3778\_cov\_35.373741 1819-1825. Max. coverage (+): 0.05. Max coverage (-): 0

Region: NODE\_27951\_length\_3778\_cov\_35.373741 1826-1833. Max. coverage (+): 1.74. Max coverage (-): 0

Region: NODE\_27951\_length\_3778\_cov\_35.373741 1834-1841. Max. coverage (+): 0.13. Max coverage (-): 0

Region: NODE\_27951\_length\_3778\_cov\_35.373741 1842-1848. Max. coverage (+): 0.03. Max coverage (-): 0

Region: NODE\_27951\_length\_3778\_cov\_35.373741 1849-1856. Max. coverage (+): 0.03. Max coverage (-): 0.05

Region: NODE\_27951\_length\_3778\_cov\_35.373741 1857-1864. Max. coverage (+): 0.77. Max coverage (-): 0.08

Region: NODE\_27951\_length\_3778\_cov\_35.373741 1865-1871. Max. coverage (+): 1.12. Max coverage (-): 0

Region: NODE\_27951\_length\_3778\_cov\_35.373741 1872-1879. Max. coverage (+): 2.35. Max coverage (-): 0.08

Region: NODE\_27951\_length\_3778\_cov\_35.373741 1880-1887. Max. coverage (+): 0. Max coverage (-): 0.03

Region: NODE\_27951\_length\_3778\_cov\_35.373741 1888-1894. Max. coverage (+): 0.03. Max coverage (-): 0

Region: NODE\_27951\_length\_3778\_cov\_35.373741 1895-1902. Max. coverage (+): 0.31. Max coverage (-): 0.03

Region: NODE\_27951\_length\_3778\_cov\_35.373741 1903-1910. Max. coverage (+): 0.18. Max coverage (-): 0

Region: NODE\_27951\_length\_3778\_cov\_35.373741 1911-1917. Max. coverage (+): 0. Max coverage (-): 0

Region: NODE\_27951\_length\_3778\_cov\_35.373741 1918-1925. Max. coverage (+): 0.1. Max coverage (-): 0.03

Region: NODE\_27951\_length\_3778\_cov\_35.373741 1926-1932. Max. coverage (+): 1.66. Max coverage (-): 0.03

Region: NODE\_27951\_length\_3778\_cov\_35.373741 1933-1940. Max. coverage (+): 1.48. Max coverage (-): 0

Region: NODE\_27951\_length\_3778\_cov\_35.373741 1941-1948. Max. coverage (+): 0.59. Max coverage (-): 0

Region: NODE\_27951\_length\_3778\_cov\_35.373741 1949-1955. Max. coverage (+): 0. Max coverage (-): 0.05

Region: NODE\_27951\_length\_3778\_cov\_35.373741 1956-1963. Max. coverage (+): 0. Max coverage (-): 0.03

Region: NODE\_27951\_length\_3778\_cov\_35.373741 1964-1971. Max. coverage (+): 3.78. Max coverage (-): 0

Region: NODE\_27951\_length\_3778\_cov\_35.373741 1972-1978. Max. coverage (+): 4.09. Max coverage (-): 0

Region: NODE\_27951\_length\_3778\_cov\_35.373741 1979-1986. Max. coverage (+): 0.05. Max coverage (-): 0

Region: NODE\_27951\_length\_3778\_cov\_35.373741 1987-1994. Max. coverage (+): 0.05. Max coverage (-): 0.03

Region: NODE\_27951\_length\_3778\_cov\_35.373741 1995-2001. Max. coverage (+): 0.08. Max coverage (-): 0.03

Region: NODE\_27951\_length\_3778\_cov\_35.373741 2002-2009. Max. coverage (+): 0.08. Max coverage (-): 0.05

Region: NODE\_27951\_length\_3778\_cov\_35.373741 2010-2017. Max. coverage (+): 0.49. Max coverage (-): 0

Region: NODE\_27951\_length\_3778\_cov\_35.373741 2018-2024. Max. coverage (+): 0.03. Max coverage (-): 0.03

Region: NODE\_27951\_length\_3778\_cov\_35.373741 2025-2032. Max. coverage (+): 0. Max coverage (-): 0.03

Region: NODE\_27951\_length\_3778\_cov\_35.373741 2033-2040. Max. coverage (+): 0. Max coverage (-): 0

Region: NODE\_27951\_length\_3778\_cov\_35.373741 2041-2047. Max. coverage (+): 0. Max coverage (-): 0

Region: NODE\_27951\_length\_3778\_cov\_35.373741 2048-2055. Max. coverage (+): 0. Max coverage (-): 0.03

Region: NODE\_27951\_length\_3778\_cov\_35.373741 2056-2063. Max. coverage (+): 0. Max coverage (-): 0.03

Region: NODE\_27951\_length\_3778\_cov\_35.373741 2064-2070. Max. coverage (+): 0.66. Max coverage (-): 0

Region: NODE\_27951\_length\_3778\_cov\_35.373741 2071-2078. Max. coverage (+): 0.64. Max coverage (-): 0

Region: NODE\_27951\_length\_3778\_cov\_35.373741 2079-2086. Max. coverage (+): 0. Max coverage (-): 0

Region: NODE\_27951\_length\_3778\_cov\_35.373741 2087-2093. Max. coverage (+): 0.05. Max coverage (-): 0

Region: NODE\_27951\_length\_3778\_cov\_35.373741 2094-2101. Max. coverage (+): 0. Max coverage (-): 0

Region: NODE\_27951\_length\_3778\_cov\_35.373741 2102-2109. Max. coverage (+): 0.46. Max coverage (-): 0

Region: NODE\_27951\_length\_3778\_cov\_35.373741 2110-2116. Max. coverage (+): 0.31. Max coverage (-): 0

Region: NODE\_27951\_length\_3778\_cov\_35.373741 2117-2124. Max. coverage (+): 0.15. Max coverage (-): 0

Region: NODE\_27951\_length\_3778\_cov\_35.373741 2125-2132. Max. coverage (+): 0.08. Max coverage (-): 0

Region: NODE\_27951\_length\_3778\_cov\_35.373741 2133-2139. Max. coverage (+): 2.76. Max coverage (-): 0

Region: NODE\_27951\_length\_3778\_cov\_35.373741 2140-2147. Max. coverage (+): 2.83. Max coverage (-): 0

Region: NODE\_27951\_length\_3778\_cov\_35.373741 2148-2155. Max. coverage (+): 0. Max coverage (-): 0

Region: NODE\_27951\_length\_3778\_cov\_35.373741 2156-2162. Max. coverage (+): 0. Max coverage (-): 0.08

Region: NODE\_27951\_length\_3778\_cov\_35.373741 2163-2170. Max. coverage (+): 0.08. Max coverage (-): 0

Region: NODE\_27951\_length\_3778\_cov\_35.373741 2171-2178. Max. coverage (+): 0. Max coverage (-): 0

Region: NODE\_27951\_length\_3778\_cov\_35.373741 2179-2185. Max. coverage (+): 0. Max coverage (-): 0

Region: NODE\_27951\_length\_3778\_cov\_35.373741 2186-2193. Max. coverage (+): 0. Max coverage (-): 0

Region: NODE\_27951\_length\_3778\_cov\_35.373741 2194-2201. Max. coverage (+): 0. Max coverage (-): 0

Region: NODE\_27951\_length\_3778\_cov\_35.373741 2202-2208. Max. coverage (+): 0.08. Max coverage (-): 0

Region: NODE\_27951\_length\_3778\_cov\_35.373741 2209-2216. Max. coverage (+): 0.13. Max coverage (-): 0

Region: NODE\_27951\_length\_3778\_cov\_35.373741 2217-2224. Max. coverage (+): 0.46. Max coverage (-): 0

Region: NODE\_27951\_length\_3778\_cov\_35.373741 2225-2231. Max. coverage (+): 0.13. Max coverage (-): 0

Region: NODE\_27951\_length\_3778\_cov\_35.373741 2232-2239. Max. coverage (+): 0.2. Max coverage (-): 0.1

Region: NODE\_27951\_length\_3778\_cov\_35.373741 2240-2247. Max. coverage (+): 0.18. Max coverage (-): 0.08

Region: NODE\_27951\_length\_3778\_cov\_35.373741 2248-2254. Max. coverage (+): 0.1. Max coverage (-): 0

Region: NODE\_27951\_length\_3778\_cov\_35.373741 2255-2262. Max. coverage (+): 0.1. Max coverage (-): 0

Region: NODE\_27951\_length\_3778\_cov\_35.373741 2263-2270. Max. coverage (+): 0. Max coverage (-): 0

Region: NODE\_27951\_length\_3778\_cov\_35.373741 2271-2277. Max. coverage (+): 0. Max coverage (-): 0.13

Region: NODE\_27951\_length\_3778\_cov\_35.373741 2278-2285. Max. coverage (+): 0.03. Max coverage (-): 0.15

Region: NODE\_27951\_length\_3778\_cov\_35.373741 2286-2293. Max. coverage (+): 0.03. Max coverage (-): 0

Region: NODE\_27951\_length\_3778\_cov\_35.373741 2294-2300. Max. coverage (+): 0.79. Max coverage (-): 0

Region: NODE\_27951\_length\_3778\_cov\_35.373741 2301-2308. Max. coverage (+): 0.08. Max coverage (-): 0.03

Region: NODE\_27951\_length\_3778\_cov\_35.373741 2309-2316. Max. coverage (+): 0.08. Max coverage (-): 0

Region: NODE\_27951\_length\_3778\_cov\_35.373741 2317-2323. Max. coverage (+): 0.08. Max coverage (-): 0.03

Region: NODE\_27951\_length\_3778\_cov\_35.373741 2324-2331. Max. coverage (+): 0.38. Max coverage (-): 0

Region: NODE\_27951\_length\_3778\_cov\_35.373741 2332-2339. Max. coverage (+): 0.03. Max coverage (-): 0

Region: NODE\_27951\_length\_3778\_cov\_35.373741 2340-2346. Max. coverage (+): 0.15. Max coverage (-): 0

Region: NODE\_27951\_length\_3778\_cov\_35.373741 2347-2354. Max. coverage (+): 0.05. Max coverage (-): 0

Region: NODE\_27951\_length\_3778\_cov\_35.373741 2355-2362. Max. coverage (+): 0.05. Max coverage (-): 0.03

Region: NODE\_27951\_length\_3778\_cov\_35.373741 2363-2369. Max. coverage (+): 0. Max coverage (-): 0.03

Region: NODE\_27951\_length\_3778\_cov\_35.373741 2370-2377. Max. coverage (+): 0.05. Max coverage (-): 0.05

Region: NODE\_27951\_length\_3778\_cov\_35.373741 2378-2385. Max. coverage (+): 0.94. Max coverage (-): 0

Region: NODE\_27951\_length\_3778\_cov\_35.373741 2386-2392. Max. coverage (+): 0.97. Max coverage (-): 0

Region: NODE\_27951\_length\_3778\_cov\_35.373741 2393-2400. Max. coverage (+): 0. Max coverage (-): 0

Region: NODE\_27951\_length\_3778\_cov\_35.373741 2401-2408. Max. coverage (+): 0.03. Max coverage (-): 0

Region: NODE\_27951\_length\_3778\_cov\_35.373741 2409-2415. Max. coverage (+): 0.08. Max coverage (-): 0

Region: NODE\_27951\_length\_3778\_cov\_35.373741 2416-2423. Max. coverage (+): 0.18. Max coverage (-): 0

Region: NODE\_27951\_length\_3778\_cov\_35.373741 2424-2431. Max. coverage (+): 0.2. Max coverage (-): 0

Region: NODE\_27951\_length\_3778\_cov\_35.373741 2432-2438. Max. coverage (+): 0.03. Max coverage (-): 0

Region: NODE\_27951\_length\_3778\_cov\_35.373741 2439-2446. Max. coverage (+): 1.1. Max coverage (-): 0.03

Region: NODE\_27951\_length\_3778\_cov\_35.373741 2447-2454. Max. coverage (+): 0.03. Max coverage (-): 0

Region: NODE\_27951\_length\_3778\_cov\_35.373741 2455-2461. Max. coverage (+): 0. Max coverage (-): 0

Region: NODE\_27951\_length\_3778\_cov\_35.373741 2462-2469. Max. coverage (+): 0. Max coverage (-): 0.15

Region: NODE\_27951\_length\_3778\_cov\_35.373741 2470-2476. Max. coverage (+): 0.23. Max coverage (-): 0.59

Region: NODE\_27951\_length\_3778\_cov\_35.373741 2477-2484. Max. coverage (+): 0.23. Max coverage (-): 0.06

Region: NODE\_27951\_length\_3778\_cov\_35.373741 2485-2492. Max. coverage (+): 0.05. Max coverage (-): 0.06

Region: NODE\_27951\_length\_3778\_cov\_35.373741 2493-2499. Max. coverage (+): 0.15. Max coverage (-): 0.03

Region: NODE\_27951\_length\_3778\_cov\_35.373741 2500-2507. Max. coverage (+): 0.15. Max coverage (-): 0.03

Region: NODE\_27951\_length\_3778\_cov\_35.373741 2508-2515. Max. coverage (+): 0.23. Max coverage (-): 0

Region: NODE\_27951\_length\_3778\_cov\_35.373741 2516-2522. Max. coverage (+): 0.15. Max coverage (-): 0

Region: NODE\_27951\_length\_3778\_cov\_35.373741 2523-2530. Max. coverage (+): 0.15. Max coverage (-): 0.08

Region: NODE\_27951\_length\_3778\_cov\_35.373741 2531-2538. Max. coverage (+): 0.15. Max coverage (-): 0.15

Region: NODE\_27951\_length\_3778\_cov\_35.373741 2539-2545. Max. coverage (+): 0.11. Max coverage (-): 0.15

Region: NODE\_27951\_length\_3778\_cov\_35.373741 2546-2553. Max. coverage (+): 1.15. Max coverage (-): 0.15

Region: NODE\_27951\_length\_3778\_cov\_35.373741 2554-2561. Max. coverage (+): 1.15. Max coverage (-): 0

Region: NODE\_27951\_length\_3778\_cov\_35.373741 2562-2568. Max. coverage (+): 0.69. Max coverage (-): 0

Region: NODE\_27951\_length\_3778\_cov\_35.373741 2569-2576. Max. coverage (+): 0.04. Max coverage (-): 0

Region: NODE\_27951\_length\_3778\_cov\_35.373741 2577-2584. Max. coverage (+): 0. Max coverage (-): 0

Region: NODE\_27951\_length\_3778\_cov\_35.373741 2585-2591. Max. coverage (+): 0.61. Max coverage (-): 0

Region: NODE\_27951\_length\_3778\_cov\_35.373741 2592-2599. Max. coverage (+): 0. Max coverage (-): 0

Region: NODE\_27951\_length\_3778\_cov\_35.373741 2600-2607. Max. coverage (+): 0.08. Max coverage (-): 0

Region: NODE\_27951\_length\_3778\_cov\_35.373741 2608-2614. Max. coverage (+): 0.15. Max coverage (-): 0

Region: NODE\_27951\_length\_3778\_cov\_35.373741 2615-2622. Max. coverage (+): 0. Max coverage (-): 0.08

Region: NODE\_27951\_length\_3778\_cov\_35.373741 2623-2630. Max. coverage (+): 0.08. Max coverage (-): 0

Region: NODE\_27951\_length\_3778\_cov\_35.373741 2631-2637. Max. coverage (+): 0.46. Max coverage (-): 0

Region: NODE\_27951\_length\_3778\_cov\_35.373741 2638-2645. Max. coverage (+): 0.54. Max coverage (-): 0

Region: NODE\_27951\_length\_3778\_cov\_35.373741 2646-2653. Max. coverage (+): 0. Max coverage (-): 0

Region: NODE\_27951\_length\_3778\_cov\_35.373741 2654-2660. Max. coverage (+): 0.08. Max coverage (-): 0

Region: NODE\_27951\_length\_3778\_cov\_35.373741 2661-2668. Max. coverage (+): 0.08. Max coverage (-): 0.04

Region: NODE\_27951\_length\_3778\_cov\_35.373741 2669-2676. Max. coverage (+): 0.27. Max coverage (-): 0

Region: NODE\_27951\_length\_3778\_cov\_35.373741 2677-2683. Max. coverage (+): 0.03. Max coverage (-): 0

Region: NODE\_27951\_length\_3778\_cov\_35.373741 2684-2691. Max. coverage (+): 0.12. Max coverage (-): 0

Region: NODE\_27951\_length\_3778\_cov\_35.373741 2692-2699. Max. coverage (+): 0.46. Max coverage (-): 0

Region: NODE\_27951\_length\_3778\_cov\_35.373741 2700-2706. Max. coverage (+): 0.19. Max coverage (-): 0

Region: NODE\_27951\_length\_3778\_cov\_35.373741 2707-2714. Max. coverage (+): 0.27. Max coverage (-): 0

Region: NODE\_27951\_length\_3778\_cov\_35.373741 2715-2722. Max. coverage (+): 0.11. Max coverage (-): 0

Region: NODE\_27951\_length\_3778\_cov\_35.373741 2723-2729. Max. coverage (+): 0.19. Max coverage (-): 0.04

Region: NODE\_27951\_length\_3778\_cov\_35.373741 2730-2737. Max. coverage (+): 0.23. Max coverage (-): 0.04

Region: NODE\_27951\_length\_3778\_cov\_35.373741 2738-2745. Max. coverage (+): 0.94. Max coverage (-): 0

Region: NODE\_27951\_length\_3778\_cov\_35.373741 2746-2752. Max. coverage (+): 0.04. Max coverage (-): 0

Region: NODE\_27951\_length\_3778\_cov\_35.373741 2753-2760. Max. coverage (+): 0. Max coverage (-): 0.11

Region: NODE\_27951\_length\_3778\_cov\_35.373741 2761-2768. Max. coverage (+): 0.04. Max coverage (-): 0.11

Region: NODE\_27951\_length\_3778\_cov\_35.373741 2769-2775. Max. coverage (+): 0.08. Max coverage (-): 0

Region: NODE\_27951\_length\_3778\_cov\_35.373741 2776-2783. Max. coverage (+): 0.5. Max coverage (-): 0

Region: NODE\_27951\_length\_3778\_cov\_35.373741 2784-2791. Max. coverage (+): 0.08. Max coverage (-): 0

Region: NODE\_27951\_length\_3778\_cov\_35.373741 2792-2798. Max. coverage (+): 0. Max coverage (-): 0

Region: NODE\_27951\_length\_3778\_cov\_35.373741 2799-2806. Max. coverage (+): 0. Max coverage (-): 0

Region: NODE\_27951\_length\_3778\_cov\_35.373741 2807-2814. Max. coverage (+): 0. Max coverage (-): 0

Region: NODE\_27951\_length\_3778\_cov\_35.373741 2815-2821. Max. coverage (+): 0.15. Max coverage (-): 0

Region: NODE\_27951\_length\_3778\_cov\_35.373741 2822-2829. Max. coverage (+): 0.15. Max coverage (-): 0

Region: NODE\_27951\_length\_3778\_cov\_35.373741 2830-2837. Max. coverage (+): 0.04. Max coverage (-): 0

Region: NODE\_27951\_length\_3778\_cov\_35.373741 2838-2844. Max. coverage (+): 0.04. Max coverage (-): 0

Region: NODE\_27951\_length\_3778\_cov\_35.373741 2845-2852. Max. coverage (+): 0.08. Max coverage (-): 0

Region: NODE\_27951\_length\_3778\_cov\_35.373741 2853-2860. Max. coverage (+): 0.46. Max coverage (-): 0.04

Region: NODE\_27951\_length\_3778\_cov\_35.373741 2861-2867. Max. coverage (+): 0.04. Max coverage (-): 0

Region: NODE\_27951\_length\_3778\_cov\_35.373741 2868-2875. Max. coverage (+): 0.15. Max coverage (-): 0

Region: NODE\_27951\_length\_3778\_cov\_35.373741 2876-2883. Max. coverage (+): 0.15. Max coverage (-): 0

Region: NODE\_27951\_length\_3778\_cov\_35.373741 2884-2890. Max. coverage (+): 0.19. Max coverage (-): 0

Region: NODE\_27951\_length\_3778\_cov\_35.373741 2891-2898. Max. coverage (+): 4.14. Max coverage (-): 0.08

Region: NODE\_27951\_length\_3778\_cov\_35.373741 2899-2906. Max. coverage (+): 1.99. Max coverage (-): 0

Region: NODE\_27951\_length\_3778\_cov\_35.373741 2907-2913. Max. coverage (+): 5.52. Max coverage (-): 0

Region: NODE\_27951\_length\_3778\_cov\_35.373741 2914-2921. Max. coverage (+): 5.36. Max coverage (-): 0

Region: NODE\_27951\_length\_3778\_cov\_35.373741 2922-2929. Max. coverage (+): 0.15. Max coverage (-): 0

Region: NODE\_27951\_length\_3778\_cov\_35.373741 2930-2936. Max. coverage (+): 0.23. Max coverage (-): 0

Region: NODE\_27951\_length\_3778\_cov\_35.373741 2937-2944. Max. coverage (+): 3.91. Max coverage (-): 0

Region: NODE\_27951\_length\_3778\_cov\_35.373741 2945-2952. Max. coverage (+): 0.84. Max coverage (-): 0

Region: NODE\_27951\_length\_3778\_cov\_35.373741 2953-2959. Max. coverage (+): 0.04. Max coverage (-): 0

Region: NODE\_27951\_length\_3778\_cov\_35.373741 2960-2967. Max. coverage (+): 0.04. Max coverage (-): 0

Region: NODE\_27951\_length\_3778\_cov\_35.373741 2968-2975. Max. coverage (+): 0.04. Max coverage (-): 0.04

Region: NODE\_27951\_length\_3778\_cov\_35.373741 2976-2982. Max. coverage (+): 0.08. Max coverage (-): 0.04

Region: NODE\_27951\_length\_3778\_cov\_35.373741 2983-2990. Max. coverage (+): 0.27. Max coverage (-): 0

Region: NODE\_27951\_length\_3778\_cov\_35.373741 2991-2998. Max. coverage (+): 0.31. Max coverage (-): 0

Region: NODE\_27951\_length\_3778\_cov\_35.373741 2999-3005. Max. coverage (+): 0. Max coverage (-): 0

Region: NODE\_27951\_length\_3778\_cov\_35.373741 3006-3013. Max. coverage (+): 0. Max coverage (-): 0.57

Region: NODE\_27951\_length\_3778\_cov\_35.373741 3014-3020. Max. coverage (+): 0. Max coverage (-): 0.11

Region: NODE\_27951\_length\_3778\_cov\_35.373741 3021-3028. Max. coverage (+): 0.27. Max coverage (-): 0.04

Region: NODE\_27951\_length\_3778\_cov\_35.373741 3029-3036. Max. coverage (+): 0.5. Max coverage (-): 0

Region: NODE\_27951\_length\_3778\_cov\_35.373741 3037-3043. Max. coverage (+): 0. Max coverage (-): 0

Region: NODE\_27951\_length\_3778\_cov\_35.373741 3044-3051. Max. coverage (+): 0. Max coverage (-): 0

Region: NODE\_27951\_length\_3778\_cov\_35.373741 3052-3059. Max. coverage (+): 0.04. Max coverage (-): 0

Region: NODE\_27951\_length\_3778\_cov\_35.373741 3060-3066. Max. coverage (+): 0.04. Max coverage (-): 0

Region: NODE\_27951\_length\_3778\_cov\_35.373741 3067-3074. Max. coverage (+): 0.34. Max coverage (-): 0

Region: NODE\_27951\_length\_3778\_cov\_35.373741 3075-3082. Max. coverage (+): 0.04. Max coverage (-): 0.08

Region: NODE\_27951\_length\_3778\_cov\_35.373741 3083-3089. Max. coverage (+): 0. Max coverage (-): 0

Region: NODE\_27951\_length\_3778\_cov\_35.373741 3090-3097. Max. coverage (+): 0.11. Max coverage (-): 0

Region: NODE\_27951\_length\_3778\_cov\_35.373741 3098-3105. Max. coverage (+): 0. Max coverage (-): 0

Region: NODE\_27951\_length\_3778\_cov\_35.373741 3106-3112. Max. coverage (+): 0.08. Max coverage (-): 0.04

Region: NODE\_27951\_length\_3778\_cov\_35.373741 3113-3120. Max. coverage (+): 0.08. Max coverage (-): 0

Region: NODE\_27951\_length\_3778\_cov\_35.373741 3121-3128. Max. coverage (+): 9.31. Max coverage (-): 0

Region: NODE\_27951\_length\_3778\_cov\_35.373741 3129-3135. Max. coverage (+): 10.3. Max coverage (-): 0

Region: NODE\_27951\_length\_3778\_cov\_35.373741 3136-3143. Max. coverage (+): 0.46. Max coverage (-): 0

Region: NODE\_27951\_length\_3778\_cov\_35.373741 3144-3151. Max. coverage (+): 0.19. Max coverage (-): 0

Region: NODE\_27951\_length\_3778\_cov\_35.373741 3152-3158. Max. coverage (+): 0.04. Max coverage (-): 0

Region: NODE\_27951\_length\_3778\_cov\_35.373741 3159-3166. Max. coverage (+): 0.5. Max coverage (-): 0

Region: NODE\_27951\_length\_3778\_cov\_35.373741 3167-3174. Max. coverage (+): 0.27. Max coverage (-): 0

Region: NODE\_27951\_length\_3778\_cov\_35.373741 3175-3181. Max. coverage (+): 0.19. Max coverage (-): 0

Region: NODE\_27951\_length\_3778\_cov\_35.373741 3182-3189. Max. coverage (+): 0.08. Max coverage (-): 0

Region: NODE\_27951\_length\_3778\_cov\_35.373741 3190-3197. Max. coverage (+): 0.04. Max coverage (-): 0

Region: NODE\_27951\_length\_3778\_cov\_35.373741 3198-3204. Max. coverage (+): 0.04. Max coverage (-): 0.27

Region: NODE\_27951\_length\_3778\_cov\_35.373741 3205-3212. Max. coverage (+): 2.11. Max coverage (-): 0.27

Region: NODE\_27951\_length\_3778\_cov\_35.373741 3213-3220. Max. coverage (+): 0.34. Max coverage (-): 0

Region: NODE\_27951\_length\_3778\_cov\_35.373741 3221-3227. Max. coverage (+): 0. Max coverage (-): 0

Region: NODE\_27951\_length\_3778\_cov\_35.373741 3228-3235. Max. coverage (+): 0. Max coverage (-): 0

Region: NODE\_27951\_length\_3778\_cov\_35.373741 3236-3243. Max. coverage (+): 0.19. Max coverage (-): 0

Region: NODE\_27951\_length\_3778\_cov\_35.373741 3244-3250. Max. coverage (+): 0. Max coverage (-): 0

Region: NODE\_27951\_length\_3778\_cov\_35.373741 3251-3258. Max. coverage (+): 0. Max coverage (-): 1.69

Region: NODE\_27951\_length\_3778\_cov\_35.373741 3259-3266. Max. coverage (+): 0. Max coverage (-): 0.65

Region: NODE\_27951\_length\_3778\_cov\_35.373741 3267-3273. Max. coverage (+): 7.24. Max coverage (-): 0

Region: NODE\_27951\_length\_3778\_cov\_35.373741 3274-3281. Max. coverage (+): 7.39. Max coverage (-): 0

Region: NODE\_27951\_length\_3778\_cov\_35.373741 3282-3289. Max. coverage (+): 0.04. Max coverage (-): 0.23

Region: NODE\_27951\_length\_3778\_cov\_35.373741 3290-3296. Max. coverage (+): 0. Max coverage (-): 0.23

Region: NODE\_27951\_length\_3778\_cov\_35.373741 3297-3304. Max. coverage (+): 0.08. Max coverage (-): 0.08

Region: NODE\_27951\_length\_3778\_cov\_35.373741 3305-3312. Max. coverage (+): 1.15. Max coverage (-): 0.08

Region: NODE\_27951\_length\_3778\_cov\_35.373741 3313-3319. Max. coverage (+): 0.23. Max coverage (-): 0

Region: NODE\_27951\_length\_3778\_cov\_35.373741 3320-3327. Max. coverage (+): 0.04. Max coverage (-): 0

Region: NODE\_27951\_length\_3778\_cov\_35.373741 3328-3335. Max. coverage (+): 0. Max coverage (-): 0

Region: NODE\_27951\_length\_3778\_cov\_35.373741 3336-3342. Max. coverage (+): 0. Max coverage (-): 0

Region: NODE\_27951\_length\_3778\_cov\_35.373741 3343-3350. Max. coverage (+): 0. Max coverage (-): 0

Region: NODE\_27951\_length\_3778\_cov\_35.373741 3351-3358. Max. coverage (+): 0.34. Max coverage (-): 0

Region: NODE\_27951\_length\_3778\_cov\_35.373741 3359-3365. Max. coverage (+): 0. Max coverage (-): 0

Region: NODE\_27951\_length\_3778\_cov\_35.373741 3366-3373. Max. coverage (+): 0.15. Max coverage (-): 0

Region: NODE\_27951\_length\_3778\_cov\_35.373741 3374-3381. Max. coverage (+): 0. Max coverage (-): 0

Region: NODE\_27951\_length\_3778\_cov\_35.373741 3382-3388. Max. coverage (+): 0.57. Max coverage (-): 0

Region: NODE\_27951\_length\_3778\_cov\_35.373741 3389-3396. Max. coverage (+): 6.36. Max coverage (-): 0

Region: NODE\_27951\_length\_3778\_cov\_35.373741 3397-3404. Max. coverage (+): 0. Max coverage (-): 0

Region: NODE\_27951\_length\_3778\_cov\_35.373741 3405-3411. Max. coverage (+): 0. Max coverage (-): 0

Region: NODE\_27951\_length\_3778\_cov\_35.373741 3412-3419. Max. coverage (+): 0.08. Max coverage (-): 0

Region: NODE\_27951\_length\_3778\_cov\_35.373741 3420-3427. Max. coverage (+): 0.23. Max coverage (-): 0

Region: NODE\_27951\_length\_3778\_cov\_35.373741 3428-3434. Max. coverage (+): 0.04. Max coverage (-): 0.08

Region: NODE\_27951\_length\_3778\_cov\_35.373741 3435-3442. Max. coverage (+): 0.08. Max coverage (-): 0.08

Region: NODE\_27951\_length\_3778\_cov\_35.373741 3443-3450. Max. coverage (+): 0.08. Max coverage (-): 0

Region: NODE\_27951\_length\_3778\_cov\_35.373741 3451-3457. Max. coverage (+): 1.76. Max coverage (-): 0

Region: NODE\_27951\_length\_3778\_cov\_35.373741 3458-3465. Max. coverage (+): 1.76. Max coverage (-): 0

Region: NODE\_27951\_length\_3778\_cov\_35.373741 3466-3473. Max. coverage (+): 0.08. Max coverage (-): 0

Region: NODE\_27951\_length\_3778\_cov\_35.373741 3474-3480. Max. coverage (+): 0.03. Max coverage (-): 0.03

Region: NODE\_27951\_length\_3778\_cov\_35.373741 3481-3488. Max. coverage (+): 0.03. Max coverage (-): 0

Region: NODE\_27951\_length\_3778\_cov\_35.373741 3489-3496. Max. coverage (+): 0.03. Max coverage (-): 0.05

Region: NODE\_27951\_length\_3778\_cov\_35.373741 3497-3503. Max. coverage (+): 0.05. Max coverage (-): 0.03

Region: NODE\_27951\_length\_3778\_cov\_35.373741 3504-3511. Max. coverage (+): 14.76. Max coverage (-): 0

Region: NODE\_27951\_length\_3778\_cov\_35.373741 3512-3519. Max. coverage (+): 1. Max coverage (-): 0

Region: NODE\_27951\_length\_3778\_cov\_35.373741 3520-3526. Max. coverage (+): 0.31. Max coverage (-): 0

Region: NODE\_27951\_length\_3778\_cov\_35.373741 3527-3534. Max. coverage (+): 0.08. Max coverage (-): 0

Region: NODE\_27951\_length\_3778\_cov\_35.373741 3535-3542. Max. coverage (+): 0. Max coverage (-): 0

Region: NODE\_27951\_length\_3778\_cov\_35.373741 3543-3549. Max. coverage (+): 0. Max coverage (-): 0

Region: NODE\_27951\_length\_3778\_cov\_35.373741 3550-3557. Max. coverage (+): 0. Max coverage (-): 0

Region: NODE\_27951\_length\_3778\_cov\_35.373741 3558-3564. Max. coverage (+): 0.05. Max coverage (-): 0

Region: NODE\_27951\_length\_3778\_cov\_35.373741 3565-3572. Max. coverage (+): 0.05. Max coverage (-): 0

Region: NODE\_27951\_length\_3778\_cov\_35.373741 3573-3580. Max. coverage (+): 0. Max coverage (-): 0

Region: NODE\_27951\_length\_3778\_cov\_35.373741 3581-3587. Max. coverage (+): 0. Max coverage (-): 0.03

Region: NODE\_27951\_length\_3778\_cov\_35.373741 3588-3595. Max. coverage (+): 0.03. Max coverage (-): 0.03

Region: NODE\_27951\_length\_3778\_cov\_35.373741 3596-3603. Max. coverage (+): 0.08. Max coverage (-): 0

Region: NODE\_27951\_length\_3778\_cov\_35.373741 3604-3610. Max. coverage (+): 2.96. Max coverage (-): 0

Region: NODE\_27951\_length\_3778\_cov\_35.373741 3611-3618. Max. coverage (+): 0.05. Max coverage (-): 0

Region: NODE\_27951\_length\_3778\_cov\_35.373741 3619-3626. Max. coverage (+): 0. Max coverage (-): 0

Region: NODE\_27951\_length\_3778\_cov\_35.373741 3627-3633. Max. coverage (+): 0.23. Max coverage (-): 0

Region: NODE\_27951\_length\_3778\_cov\_35.373741 3634-3641. Max. coverage (+): 7. Max coverage (-): 0

Region: NODE\_27951\_length\_3778\_cov\_35.373741 3642-3649. Max. coverage (+): 0.2. Max coverage (-): 0

Region: NODE\_27951\_length\_3778\_cov\_35.373741 3650-3656. Max. coverage (+): 0.03. Max coverage (-): 0

Region: NODE\_27951\_length\_3778\_cov\_35.373741 3657-3664. Max. coverage (+): 0. Max coverage (-): 0.1

Region: NODE\_27951\_length\_3778\_cov\_35.373741 3665-3672. Max. coverage (+): 0. Max coverage (-): 0.03

Region: NODE\_27951\_length\_3778\_cov\_35.373741 3673-3679. Max. coverage (+): 0.13. Max coverage (-): 0

Region: NODE\_27951\_length\_3778\_cov\_35.373741 3680-3687. Max. coverage (+): 16.14. Max coverage (-): 0

Region: NODE\_27951\_length\_3778\_cov\_35.373741 3688-3695. Max. coverage (+): 0. Max coverage (-): 0

Region: NODE\_27951\_length\_3778\_cov\_35.373741 3696-3702. Max. coverage (+): 0.08. Max coverage (-): 0

Region: NODE\_27951\_length\_3778\_cov\_35.373741 3703-3710. Max. coverage (+): 1.38. Max coverage (-): 0

Region: NODE\_27951\_length\_3778\_cov\_35.373741 3711-3718. Max. coverage (+): 0.46. Max coverage (-): 0

Region: NODE\_27951\_length\_3778\_cov\_35.373741 3719-3725. Max. coverage (+): 0.31. Max coverage (-): 0

Region: NODE\_27951\_length\_3778\_cov\_35.373741 3726-3733. Max. coverage (+): 0.08. Max coverage (-): 0

Region: NODE\_27951\_length\_3778\_cov\_35.373741 3734-3741. Max. coverage (+): 0. Max coverage (-): 0

Region: NODE\_27951\_length\_3778\_cov\_35.373741 3742-3748. Max. coverage (+): 0. Max coverage (-): 0

Region: NODE\_27951\_length\_3778\_cov\_35.373741 3749-3756. Max. coverage (+): 0. Max coverage (-): 0

Region: NODE\_27951\_length\_3778\_cov\_35.373741 3757-3764. Max. coverage (+): 0.03. Max coverage (-): 0

Region: NODE\_27951\_length\_3778\_cov\_35.373741 3765-3771. Max. coverage (+): 0.08. Max coverage (-): 0

Region: NODE\_27951\_length\_3778\_cov\_35.373741 3772-3779. Max. coverage (+): 0.03. Max coverage (-): 0

Region: NODE\_27951\_length\_3778\_cov\_35.373741 3780-3787. Max. coverage (+): 0. Max coverage (-): 0.03

Region: NODE\_27951\_length\_3778\_cov\_35.373741 3788-3794. Max. coverage (+): 0.31. Max coverage (-): 0

Region: NODE\_27951\_length\_3778\_cov\_35.373741 3795-3802. Max. coverage (+): 0.05. Max coverage (-): 0.03

Region: NODE\_27951\_length\_3778\_cov\_35.373741 3803-3810. Max. coverage (+): 0.23. Max coverage (-): 0.05

Region: NODE\_27951\_length\_3778\_cov\_35.373741 3811-3817. Max. coverage (+): 0.08. Max coverage (-): 0

Region: NODE\_27951\_length\_3778\_cov\_35.373741 3818-3825. Max. coverage (+): 0. Max coverage (-): 0

Region: NODE\_27951\_length\_3778\_cov\_35.373741 3826-3833. Max. coverage (+): 0. Max coverage (-): 0

Region: NODE\_27951\_length\_3778\_cov\_35.373741 3834-. Max. coverage (+): 0. Max coverage (-): 0

RepeatMasker Color Code

**+**

100-98% Identity

<98-95% Identity

<95-90% Identity

<90-85% Identity

<85-80% Identity

<80-75% Identity

<75-70% Identity

<70% Identity

**-**

Gene Set Color Code

**+**

Gene

Pseudogene

Other

**-**

Topology/Coverage Color Code

Coverage Plus Strand

Coverage Minus Strand

Mainstrand: Plus

Mainstrand: Minus

Complementary Strand

Flanking Region  
(if option -flank >0)

Gene Set Annotation  
  
RepeatMasker Annotation  

**1. AlRepB-358**: 1-57 (+), Divergence to consensus: 1.8%  
**2. A-rich**: 349-387 (+), Divergence to consensus: 20.7%  
**3. RTE-2\_AFC**: 2452-2817 (-), Divergence to consensus: 28.7%  
**4. RTE-2\_AFC**: 2791-2886 (-), Divergence to consensus: 24.1%  
**5. A-rich**: 3537-3588 (+), Divergence to consensus: 25.6%

  
Transcription Factor Binding Sites  

**RHOXF1** (Sequence: AGATTA (-): 502)  
**RHOXF1** (Sequence: AGCTTA (-): 578)  
**RHOXF1** (Sequence: AGATTA (-): 1008)  
**RHOXF1** (Sequence: GGCTTA (-): 1700)  
**RHOXF1** (Sequence: GGCTTA (-): 1897)  
**RHOXF1** (Sequence: AGCTCA (-): 1959)  
**RHOXF1** (Sequence: AGCTCA (-): 2026)  
**RHOXF1** (Sequence: GGATCA (-): 2492)  
**RHOXF1** (Sequence: GGCTCA (-): 3118)  
**RHOXF1** (Sequence: AGATTA (-): 3560)  
**RHOXF1** (Sequence: GGCTCA (-): 3731)  
**RHOXF1** (Sequence: TAATCT (+): 341)  
**RHOXF1** (Sequence: TGAGCC (+): 1822)  
**RHOXF1** (Sequence: TGAGCT (+): 2024)  
**RHOXF1** (Sequence: TAAGCT (+): 2442)  
**RHOXF1** (Sequence: TGAGCT (+): 2714)  
**RHOXF1** (Sequence: TAATCT (+): 2742)  
**RHOXF1** (Sequence: TGAGCC (+): 2787)  
**RHOXF1** (Sequence: TGAGCT (+): 2900)  
**RHOXF1** (Sequence: TGAGCT (+): 3317)  
**SOX9** (Sequence: AACAATAA (-): 1250)  
**FOXO3\_mmu** (Sequence: TGTTTTGC (-): 1069)  
**FOXO3\_mmu** (Sequence: TGTTTTCA (-): 3090)  
**FOXO3\_mmu** (Sequence: GGAAAACA (+): 1725)  
**FOXO3\_mmu** (Sequence: TGAAAACA (+): 3574)  
**Nobox** (Sequence: GGTAATTA (-): 664)  
**FOXO1** (Sequence: AAAAACAAC (-): 362)  
**FOXO1** (Sequence: AAAAACAAG (-): 1942)  
**FOXO1** (Sequence: AAAAACAAG (-): 3584)  
**Rhox11** (Sequence: AATACACCA (-): 1424)  
**Gata4** (Sequence: AGATAAG (-): 1220)  
**Sox5** (Sequence: AACAAT (-): 69)  
**Sox5** (Sequence: AACAAT (-): 420)  
**Sox5** (Sequence: AACAAT (-): 1250)  
**Sox5** (Sequence: AACAAT (-): 2629)  
**Sox5** (Sequence: AACAAT (-): 3106)  
**POU5F1** (Sequence: ATGCAAA (+): 1687)
