# Supplementary material for: First characterization of PIWI-interacting RNA clusters in a cichlid fish with a B chromosome
Source: BMC Biol. 2022 Sep 21;20:204. doi: 10.1186/s12915-022-01403-2 (PMC9490952; doi:10.1186/s12915-022-01403-2)
Supplement: Supplementary file 1 — Additional file 1. Zipped folder with fasta and interactive html piRNA cluster information for the A. latifasciata genome. The nomenclature is as follows: number-pirna-cluster_sex_B-presence (f, female; m, male; 0b, without B chromosome; 1b, with B chromosome). [file 12915_2022_1403_MOESM1_ESM.zip › 107_f1b.html]

piRNA cluster 107\_f1b 8


Predicted piRNA cluster no. 107\_f1b
  

Show proTRAC run info
Hide proTRAC run info

/\  
                \_\_\_\_\_\_\_\_\_\_\_\_\_\_\_\_\_\_\_\_\_\_\_/\\_\_\_ /  \\_\_\_\_\_\_\_  
               I                      /  \  /    \      I  
               I     pro             /    \/      \     I  
               I        TRAC        /               \   I  
               I   \_\_\_\_\_\_\_\_\_\_\_\_\_\_\_\_/\_\_\_\_\_\_\_\_\_\_\_\_\_\_\_\_\_\\_ I  
               I   \              /                     I  
               I    \            /                      I  
               I     \  /\      /       V.2.4.2         I  
               I      \/  \    /                        I  
               I\_\_\_\_\_\_\_\_\_\_\_\  /\_\_\_\_\_\_\_\_\_\_\_\_\_\_\_\_\_\_\_\_\_\_\_\_\_I  
                            \/  
  
  
================================= proTRAC ====================================  
VERSION: .......... 2.4.2  
LAST MODIFIED: .... 11. May 2018  
  
Please cite:  
Rosenkranz D, Zischler H. proTRAC - a software for probabilistic piRNA cluster  
detection, visualization and analysis. 2012. BMC Bioinformatics 13:5.  
  
  
Contact:  
David Rosenkranz  
Institute of Organismic and Molecular Evolutionary Biology  
Dept. Anthropology, small RNA group  
Johannes Gutenberg University Mainz  
email: rosenkranz@uni-mainz.de  
  
You can find the latest proTRAC version at:  
http://sourceforge.net/projects/protrac/files  
http://www.smallRNAgroup-mainz.de/software  
==============================================================================  
  
PARAMETERS:  
Map file: ...............piwi-femeas-1B.fa-collapse.map  
Genome file: ............../../../0B\_ala\_genome.fa  
RepeatMasker annotation: Alatifasciata-all0B-maryan-v2.fa\_corrected.out  
GeneSet:................./guest-storage/Data/annotation/Alatifasciata\_all0B\_maryan-v2\_out2017.gff  
  
Significant (p<=0.01) hit density will be calculated based  
on observed hit distribution.  
  
Sliding window size: ........................................ 5000 bp  
Sliding window increament: .................................. 1000 bp  
Normalize each hit by number of genomic hits: ............... yes  
Normalize each hit by number of sequence reads: ............. yes  
Normalize values (-> per million mapped reads): ............. yes  
Min. fraction of hits with 1T(U) or 10A: .................... 0.75  
Alternatively: Min. fraction of hits with 1T(U) and 10A: .... 0.5  
Min. fraction of hits with typical piRNA length: ............ 0.75  
Typical piRNA length: ....................................... 24-32 nt  
Min. size of a piRNA cluster: ............................... 1000 bp.  
Min. number of hits (absolute): ............................. 0  
Min. number of hits (normalized): ........................... 0  
Min. fraction of hits on the mainstrand: .................... 0.75  
Top fraction of mapped sequences (in terms of read counts): . 1%  
Top fraction accounts for max. n% of sequence reads: ........ 90%  
Min. fraction of hits on each arm of a bidirectional cluster: 0.05  
Output html file for each cluster: .......................... yes  
Output a summary table: ..................................... yes  
Output a FASTA file for each cluster (piRNA sequences): ..... yes  
Output a FASTA file comprising cluster sequences: ........... yes  
Output a GTF file for predicted piRNA clusters: ..............yes  
Search DNA motifs in clusters: .............................. yes  
Output flanking sequences: +/- .............................. 0 bp  
Output ~.pTi file: .......................................... no  
==============================================================================  
  
  
Genome size (without gaps): ............ 758543724 bp  
Gaps (N/X/-): .......................... 417479 bp  
Mapped reads: .......................... 10641844  
Non-identical sequences: ............... 2832837  
Genomic hits: .......................... 26056853  
Significant densitiy of mapped reads: .. 368.713530323068 reads/kb

Show proTRAC cluster info
Hide proTRAC cluster info

|  |  |
| --- | --- |
| Location | NODE\_27951\_length\_3778\_cov\_35.373741 |
| Coordinates | 6-3830 |
| Size [bp] | 3825 |
| Sequence hit loci | 1292 |
| Mapped reads (normalized) | 3095.5 |
| Mapped reads (normalized) per kb | 809.3 |
| Normalized reads with 1T (1U) | 84.4% |
| Normalized reads with 10A | 37.6% |
| Normalized reads with length 24-32 nt | 99.5% |
| Normalized reads on the main strand(s) | 94.8% |
| Predicted directionality | mono:plus |

100%

0%

1T (1U)  
reads

10A reads

24-32 nt  
reads

reads on mainstrand

**Either the amount of reads with 1T (1U) OR 10A has to exceed 75% (set with option: -1Tor10A)  
Alternatively the amount of reads with 1T (1U) AND 10A has to exceed 50% (set with option: -1Tand10A)  
Minimum amount of reads with preferred size is 75% (set with option: -pisize)  
Minimum amount of reads on the main strand(s) is 75% (set with option: -clstrand)**

Show read coverage
Hide read coverage

WHAT DO I SEE HERE?  
This chart shows the location of mapped sequence reads within a predicted piRNA cluster. The color refers to the number of genomic hits produced by the sequence read in question. A dark red bar indicates that this sequence read produces many other hits elsewhere in the genome. Many adjacent red or yellow bars can indicate the presence of a multi-copy element such as transposons or rRNA genes. A dark green bar indicates that this sequence read maps uniquely to this locus.

1 hit

2-5 hits

6-10 hits

11-20 hits

21-50 hits

51-100 hits

> 100 hits

NODE\_27951\_length\_3778\_cov\_35.373741

6

3830

Gene Set

RepeatMasker

Mapped  
Reads

25.18

plus strand

minus strand

25.18

Region: NODE\_27951\_length\_3778\_cov\_35.373741 11966-9. Max. coverage (+): 0. Max coverage (-): 0

Region: NODE\_27951\_length\_3778\_cov\_35.373741 10-17. Max. coverage (+): 0. Max coverage (-): 0

Region: NODE\_27951\_length\_3778\_cov\_35.373741 18-25. Max. coverage (+): 0. Max coverage (-): 0

Region: NODE\_27951\_length\_3778\_cov\_35.373741 26-32. Max. coverage (+): 0. Max coverage (-): 0

Region: NODE\_27951\_length\_3778\_cov\_35.373741 33-40. Max. coverage (+): 0.2. Max coverage (-): 0.05

Region: NODE\_27951\_length\_3778\_cov\_35.373741 41-48. Max. coverage (+): 0. Max coverage (-): 0

Region: NODE\_27951\_length\_3778\_cov\_35.373741 49-55. Max. coverage (+): 0.09. Max coverage (-): 0

Region: NODE\_27951\_length\_3778\_cov\_35.373741 56-63. Max. coverage (+): 0.09. Max coverage (-): 0

Region: NODE\_27951\_length\_3778\_cov\_35.373741 64-71. Max. coverage (+): 0. Max coverage (-): 0

Region: NODE\_27951\_length\_3778\_cov\_35.373741 72-78. Max. coverage (+): 0. Max coverage (-): 0

Region: NODE\_27951\_length\_3778\_cov\_35.373741 79-86. Max. coverage (+): 0. Max coverage (-): 0

Region: NODE\_27951\_length\_3778\_cov\_35.373741 87-93. Max. coverage (+): 0. Max coverage (-): 0

Region: NODE\_27951\_length\_3778\_cov\_35.373741 94-101. Max. coverage (+): 0. Max coverage (-): 0.09

Region: NODE\_27951\_length\_3778\_cov\_35.373741 102-109. Max. coverage (+): 0.09. Max coverage (-): 0

Region: NODE\_27951\_length\_3778\_cov\_35.373741 110-116. Max. coverage (+): 0. Max coverage (-): 0

Region: NODE\_27951\_length\_3778\_cov\_35.373741 117-124. Max. coverage (+): 0. Max coverage (-): 0

Region: NODE\_27951\_length\_3778\_cov\_35.373741 125-132. Max. coverage (+): 0. Max coverage (-): 0

Region: NODE\_27951\_length\_3778\_cov\_35.373741 133-139. Max. coverage (+): 0. Max coverage (-): 0

Region: NODE\_27951\_length\_3778\_cov\_35.373741 140-147. Max. coverage (+): 0. Max coverage (-): 0

Region: NODE\_27951\_length\_3778\_cov\_35.373741 148-155. Max. coverage (+): 0.09. Max coverage (-): 0

Region: NODE\_27951\_length\_3778\_cov\_35.373741 156-162. Max. coverage (+): 0.09. Max coverage (-): 0

Region: NODE\_27951\_length\_3778\_cov\_35.373741 163-170. Max. coverage (+): 0. Max coverage (-): 0

Region: NODE\_27951\_length\_3778\_cov\_35.373741 171-178. Max. coverage (+): 0. Max coverage (-): 0

Region: NODE\_27951\_length\_3778\_cov\_35.373741 179-185. Max. coverage (+): 0. Max coverage (-): 0

Region: NODE\_27951\_length\_3778\_cov\_35.373741 186-193. Max. coverage (+): 0.19. Max coverage (-): 0.09

Region: NODE\_27951\_length\_3778\_cov\_35.373741 194-201. Max. coverage (+): 0.09. Max coverage (-): 0.19

Region: NODE\_27951\_length\_3778\_cov\_35.373741 202-208. Max. coverage (+): 0. Max coverage (-): 0.09

Region: NODE\_27951\_length\_3778\_cov\_35.373741 209-216. Max. coverage (+): 0. Max coverage (-): 0

Region: NODE\_27951\_length\_3778\_cov\_35.373741 217-224. Max. coverage (+): 0.38. Max coverage (-): 0

Region: NODE\_27951\_length\_3778\_cov\_35.373741 225-231. Max. coverage (+): 0.19. Max coverage (-): 0

Region: NODE\_27951\_length\_3778\_cov\_35.373741 232-239. Max. coverage (+): 0.19. Max coverage (-): 0

Region: NODE\_27951\_length\_3778\_cov\_35.373741 240-246. Max. coverage (+): 0.19. Max coverage (-): 0

Region: NODE\_27951\_length\_3778\_cov\_35.373741 247-254. Max. coverage (+): 0. Max coverage (-): 0

Region: NODE\_27951\_length\_3778\_cov\_35.373741 255-262. Max. coverage (+): 0.09. Max coverage (-): 0

Region: NODE\_27951\_length\_3778\_cov\_35.373741 263-269. Max. coverage (+): 0.19. Max coverage (-): 0

Region: NODE\_27951\_length\_3778\_cov\_35.373741 270-277. Max. coverage (+): 0. Max coverage (-): 0

Region: NODE\_27951\_length\_3778\_cov\_35.373741 278-285. Max. coverage (+): 0. Max coverage (-): 0

Region: NODE\_27951\_length\_3778\_cov\_35.373741 286-292. Max. coverage (+): 0.19. Max coverage (-): 0.28

Region: NODE\_27951\_length\_3778\_cov\_35.373741 293-300. Max. coverage (+): 0.09. Max coverage (-): 0.28

Region: NODE\_27951\_length\_3778\_cov\_35.373741 301-308. Max. coverage (+): 0.19. Max coverage (-): 0

Region: NODE\_27951\_length\_3778\_cov\_35.373741 309-315. Max. coverage (+): 0.19. Max coverage (-): 0.09

Region: NODE\_27951\_length\_3778\_cov\_35.373741 316-323. Max. coverage (+): 0. Max coverage (-): 0

Region: NODE\_27951\_length\_3778\_cov\_35.373741 324-331. Max. coverage (+): 0. Max coverage (-): 0

Region: NODE\_27951\_length\_3778\_cov\_35.373741 332-338. Max. coverage (+): 0. Max coverage (-): 0

Region: NODE\_27951\_length\_3778\_cov\_35.373741 339-346. Max. coverage (+): 0. Max coverage (-): 0

Region: NODE\_27951\_length\_3778\_cov\_35.373741 347-354. Max. coverage (+): 0. Max coverage (-): 0

Region: NODE\_27951\_length\_3778\_cov\_35.373741 355-361. Max. coverage (+): 0. Max coverage (-): 0

Region: NODE\_27951\_length\_3778\_cov\_35.373741 362-369. Max. coverage (+): 0. Max coverage (-): 0

Region: NODE\_27951\_length\_3778\_cov\_35.373741 370-377. Max. coverage (+): 0. Max coverage (-): 0

Region: NODE\_27951\_length\_3778\_cov\_35.373741 378-384. Max. coverage (+): 0. Max coverage (-): 0

Region: NODE\_27951\_length\_3778\_cov\_35.373741 385-392. Max. coverage (+): 0. Max coverage (-): 0

Region: NODE\_27951\_length\_3778\_cov\_35.373741 393-399. Max. coverage (+): 0. Max coverage (-): 0

Region: NODE\_27951\_length\_3778\_cov\_35.373741 400-407. Max. coverage (+): 0. Max coverage (-): 0

Region: NODE\_27951\_length\_3778\_cov\_35.373741 408-415. Max. coverage (+): 0. Max coverage (-): 0

Region: NODE\_27951\_length\_3778\_cov\_35.373741 416-422. Max. coverage (+): 0. Max coverage (-): 0

Region: NODE\_27951\_length\_3778\_cov\_35.373741 423-430. Max. coverage (+): 0. Max coverage (-): 0

Region: NODE\_27951\_length\_3778\_cov\_35.373741 431-438. Max. coverage (+): 0.56. Max coverage (-): 0

Region: NODE\_27951\_length\_3778\_cov\_35.373741 439-445. Max. coverage (+): 0.09. Max coverage (-): 0

Region: NODE\_27951\_length\_3778\_cov\_35.373741 446-453. Max. coverage (+): 0. Max coverage (-): 0.09

Region: NODE\_27951\_length\_3778\_cov\_35.373741 454-461. Max. coverage (+): 0. Max coverage (-): 0.28

Region: NODE\_27951\_length\_3778\_cov\_35.373741 462-468. Max. coverage (+): 2.16. Max coverage (-): 0

Region: NODE\_27951\_length\_3778\_cov\_35.373741 469-476. Max. coverage (+): 2.35. Max coverage (-): 0

Region: NODE\_27951\_length\_3778\_cov\_35.373741 477-484. Max. coverage (+): 0. Max coverage (-): 0

Region: NODE\_27951\_length\_3778\_cov\_35.373741 485-491. Max. coverage (+): 0. Max coverage (-): 0

Region: NODE\_27951\_length\_3778\_cov\_35.373741 492-499. Max. coverage (+): 0.19. Max coverage (-): 0

Region: NODE\_27951\_length\_3778\_cov\_35.373741 500-507. Max. coverage (+): 8.65. Max coverage (-): 0

Region: NODE\_27951\_length\_3778\_cov\_35.373741 508-514. Max. coverage (+): 5.26. Max coverage (-): 0

Region: NODE\_27951\_length\_3778\_cov\_35.373741 515-522. Max. coverage (+): 0. Max coverage (-): 0

Region: NODE\_27951\_length\_3778\_cov\_35.373741 523-530. Max. coverage (+): 0.09. Max coverage (-): 0

Region: NODE\_27951\_length\_3778\_cov\_35.373741 531-537. Max. coverage (+): 0. Max coverage (-): 0

Region: NODE\_27951\_length\_3778\_cov\_35.373741 538-545. Max. coverage (+): 0.09. Max coverage (-): 0

Region: NODE\_27951\_length\_3778\_cov\_35.373741 546-552. Max. coverage (+): 0.09. Max coverage (-): 0

Region: NODE\_27951\_length\_3778\_cov\_35.373741 553-560. Max. coverage (+): 0.56. Max coverage (-): 0

Region: NODE\_27951\_length\_3778\_cov\_35.373741 561-568. Max. coverage (+): 0.47. Max coverage (-): 0

Region: NODE\_27951\_length\_3778\_cov\_35.373741 569-575. Max. coverage (+): 0.47. Max coverage (-): 0

Region: NODE\_27951\_length\_3778\_cov\_35.373741 576-583. Max. coverage (+): 0.09. Max coverage (-): 0

Region: NODE\_27951\_length\_3778\_cov\_35.373741 584-591. Max. coverage (+): 1.97. Max coverage (-): 0

Region: NODE\_27951\_length\_3778\_cov\_35.373741 592-598. Max. coverage (+): 1.6. Max coverage (-): 0.09

Region: NODE\_27951\_length\_3778\_cov\_35.373741 599-606. Max. coverage (+): 0. Max coverage (-): 0

Region: NODE\_27951\_length\_3778\_cov\_35.373741 607-614. Max. coverage (+): 0. Max coverage (-): 0

Region: NODE\_27951\_length\_3778\_cov\_35.373741 615-621. Max. coverage (+): 0.19. Max coverage (-): 0

Region: NODE\_27951\_length\_3778\_cov\_35.373741 622-629. Max. coverage (+): 0. Max coverage (-): 0

Region: NODE\_27951\_length\_3778\_cov\_35.373741 630-637. Max. coverage (+): 0.09. Max coverage (-): 0

Region: NODE\_27951\_length\_3778\_cov\_35.373741 638-644. Max. coverage (+): 0. Max coverage (-): 0

Region: NODE\_27951\_length\_3778\_cov\_35.373741 645-652. Max. coverage (+): 0.38. Max coverage (-): 0.09

Region: NODE\_27951\_length\_3778\_cov\_35.373741 653-660. Max. coverage (+): 16.54. Max coverage (-): 0

Region: NODE\_27951\_length\_3778\_cov\_35.373741 661-667. Max. coverage (+): 0.19. Max coverage (-): 0.09

Region: NODE\_27951\_length\_3778\_cov\_35.373741 668-675. Max. coverage (+): 0.09. Max coverage (-): 0.66

Region: NODE\_27951\_length\_3778\_cov\_35.373741 676-683. Max. coverage (+): 2.07. Max coverage (-): 0.28

Region: NODE\_27951\_length\_3778\_cov\_35.373741 684-690. Max. coverage (+): 2.44. Max coverage (-): 0

Region: NODE\_27951\_length\_3778\_cov\_35.373741 691-698. Max. coverage (+): 0.38. Max coverage (-): 0.09

Region: NODE\_27951\_length\_3778\_cov\_35.373741 699-705. Max. coverage (+): 0.75. Max coverage (-): 0.19

Region: NODE\_27951\_length\_3778\_cov\_35.373741 706-713. Max. coverage (+): 0.56. Max coverage (-): 0

Region: NODE\_27951\_length\_3778\_cov\_35.373741 714-721. Max. coverage (+): 0. Max coverage (-): 0

Region: NODE\_27951\_length\_3778\_cov\_35.373741 722-728. Max. coverage (+): 1.22. Max coverage (-): 0

Region: NODE\_27951\_length\_3778\_cov\_35.373741 729-736. Max. coverage (+): 0.09. Max coverage (-): 0

Region: NODE\_27951\_length\_3778\_cov\_35.373741 737-744. Max. coverage (+): 0. Max coverage (-): 0.09

Region: NODE\_27951\_length\_3778\_cov\_35.373741 745-751. Max. coverage (+): 0. Max coverage (-): 0.09

Region: NODE\_27951\_length\_3778\_cov\_35.373741 752-759. Max. coverage (+): 0. Max coverage (-): 0

Region: NODE\_27951\_length\_3778\_cov\_35.373741 760-767. Max. coverage (+): 0. Max coverage (-): 0.09

Region: NODE\_27951\_length\_3778\_cov\_35.373741 768-774. Max. coverage (+): 0. Max coverage (-): 0.09

Region: NODE\_27951\_length\_3778\_cov\_35.373741 775-782. Max. coverage (+): 0. Max coverage (-): 0

Region: NODE\_27951\_length\_3778\_cov\_35.373741 783-790. Max. coverage (+): 0.09. Max coverage (-): 0

Region: NODE\_27951\_length\_3778\_cov\_35.373741 791-797. Max. coverage (+): 3.76. Max coverage (-): 0

Region: NODE\_27951\_length\_3778\_cov\_35.373741 798-805. Max. coverage (+): 3.76. Max coverage (-): 0

Region: NODE\_27951\_length\_3778\_cov\_35.373741 806-813. Max. coverage (+): 0.85. Max coverage (-): 0.47

Region: NODE\_27951\_length\_3778\_cov\_35.373741 814-820. Max. coverage (+): 0.47. Max coverage (-): 0.47

Region: NODE\_27951\_length\_3778\_cov\_35.373741 821-828. Max. coverage (+): 0.47. Max coverage (-): 0

Region: NODE\_27951\_length\_3778\_cov\_35.373741 829-836. Max. coverage (+): 6.67. Max coverage (-): 0

Region: NODE\_27951\_length\_3778\_cov\_35.373741 837-843. Max. coverage (+): 1.6. Max coverage (-): 0

Region: NODE\_27951\_length\_3778\_cov\_35.373741 844-851. Max. coverage (+): 0.19. Max coverage (-): 0

Region: NODE\_27951\_length\_3778\_cov\_35.373741 852-858. Max. coverage (+): 0. Max coverage (-): 0

Region: NODE\_27951\_length\_3778\_cov\_35.373741 859-866. Max. coverage (+): 0. Max coverage (-): 0

Region: NODE\_27951\_length\_3778\_cov\_35.373741 867-874. Max. coverage (+): 1.22. Max coverage (-): 0

Region: NODE\_27951\_length\_3778\_cov\_35.373741 875-881. Max. coverage (+): 0. Max coverage (-): 0

Region: NODE\_27951\_length\_3778\_cov\_35.373741 882-889. Max. coverage (+): 0. Max coverage (-): 0.38

Region: NODE\_27951\_length\_3778\_cov\_35.373741 890-897. Max. coverage (+): 0. Max coverage (-): 0.38

Region: NODE\_27951\_length\_3778\_cov\_35.373741 898-904. Max. coverage (+): 0.19. Max coverage (-): 0

Region: NODE\_27951\_length\_3778\_cov\_35.373741 905-912. Max. coverage (+): 14.66. Max coverage (-): 0

Region: NODE\_27951\_length\_3778\_cov\_35.373741 913-920. Max. coverage (+): 0.28. Max coverage (-): 0

Region: NODE\_27951\_length\_3778\_cov\_35.373741 921-927. Max. coverage (+): 0. Max coverage (-): 0

Region: NODE\_27951\_length\_3778\_cov\_35.373741 928-935. Max. coverage (+): 0. Max coverage (-): 0

Region: NODE\_27951\_length\_3778\_cov\_35.373741 936-943. Max. coverage (+): 0. Max coverage (-): 0

Region: NODE\_27951\_length\_3778\_cov\_35.373741 944-950. Max. coverage (+): 0.09. Max coverage (-): 0

Region: NODE\_27951\_length\_3778\_cov\_35.373741 951-958. Max. coverage (+): 16.16. Max coverage (-): 0

Region: NODE\_27951\_length\_3778\_cov\_35.373741 959-966. Max. coverage (+): 0. Max coverage (-): 0

Region: NODE\_27951\_length\_3778\_cov\_35.373741 967-973. Max. coverage (+): 0. Max coverage (-): 0

Region: NODE\_27951\_length\_3778\_cov\_35.373741 974-981. Max. coverage (+): 0. Max coverage (-): 0

Region: NODE\_27951\_length\_3778\_cov\_35.373741 982-989. Max. coverage (+): 0.09. Max coverage (-): 0

Region: NODE\_27951\_length\_3778\_cov\_35.373741 990-996. Max. coverage (+): 0.47. Max coverage (-): 0

Region: NODE\_27951\_length\_3778\_cov\_35.373741 997-1004. Max. coverage (+): 0.19. Max coverage (-): 0.19

Region: NODE\_27951\_length\_3778\_cov\_35.373741 1005-1011. Max. coverage (+): 0.09. Max coverage (-): 0.38

Region: NODE\_27951\_length\_3778\_cov\_35.373741 1012-1019. Max. coverage (+): 0. Max coverage (-): 0

Region: NODE\_27951\_length\_3778\_cov\_35.373741 1020-1027. Max. coverage (+): 2.44. Max coverage (-): 0

Region: NODE\_27951\_length\_3778\_cov\_35.373741 1028-1034. Max. coverage (+): 0.09. Max coverage (-): 0

Region: NODE\_27951\_length\_3778\_cov\_35.373741 1035-1042. Max. coverage (+): 0.09. Max coverage (-): 0.09

Region: NODE\_27951\_length\_3778\_cov\_35.373741 1043-1050. Max. coverage (+): 0.09. Max coverage (-): 0.09

Region: NODE\_27951\_length\_3778\_cov\_35.373741 1051-1057. Max. coverage (+): 0.94. Max coverage (-): 0.28

Region: NODE\_27951\_length\_3778\_cov\_35.373741 1058-1065. Max. coverage (+): 3.85. Max coverage (-): 0.09

Region: NODE\_27951\_length\_3778\_cov\_35.373741 1066-1073. Max. coverage (+): 3.85. Max coverage (-): 0

Region: NODE\_27951\_length\_3778\_cov\_35.373741 1074-1080. Max. coverage (+): 0. Max coverage (-): 0

Region: NODE\_27951\_length\_3778\_cov\_35.373741 1081-1088. Max. coverage (+): 0. Max coverage (-): 0

Region: NODE\_27951\_length\_3778\_cov\_35.373741 1089-1096. Max. coverage (+): 0.19. Max coverage (-): 0.09

Region: NODE\_27951\_length\_3778\_cov\_35.373741 1097-1103. Max. coverage (+): 2.54. Max coverage (-): 0

Region: NODE\_27951\_length\_3778\_cov\_35.373741 1104-1111. Max. coverage (+): 1.22. Max coverage (-): 0

Region: NODE\_27951\_length\_3778\_cov\_35.373741 1112-1119. Max. coverage (+): 0.75. Max coverage (-): 0

Region: NODE\_27951\_length\_3778\_cov\_35.373741 1120-1126. Max. coverage (+): 0.19. Max coverage (-): 0

Region: NODE\_27951\_length\_3778\_cov\_35.373741 1127-1134. Max. coverage (+): 0.28. Max coverage (-): 0

Region: NODE\_27951\_length\_3778\_cov\_35.373741 1135-1142. Max. coverage (+): 0.38. Max coverage (-): 0

Region: NODE\_27951\_length\_3778\_cov\_35.373741 1143-1149. Max. coverage (+): 0.56. Max coverage (-): 0.19

Region: NODE\_27951\_length\_3778\_cov\_35.373741 1150-1157. Max. coverage (+): 0.38. Max coverage (-): 0.19

Region: NODE\_27951\_length\_3778\_cov\_35.373741 1158-1164. Max. coverage (+): 0.38. Max coverage (-): 0

Region: NODE\_27951\_length\_3778\_cov\_35.373741 1165-1172. Max. coverage (+): 1.13. Max coverage (-): 0

Region: NODE\_27951\_length\_3778\_cov\_35.373741 1173-1180. Max. coverage (+): 0. Max coverage (-): 0

Region: NODE\_27951\_length\_3778\_cov\_35.373741 1181-1187. Max. coverage (+): 0. Max coverage (-): 0

Region: NODE\_27951\_length\_3778\_cov\_35.373741 1188-1195. Max. coverage (+): 2.44. Max coverage (-): 0

Region: NODE\_27951\_length\_3778\_cov\_35.373741 1196-1203. Max. coverage (+): 0. Max coverage (-): 0

Region: NODE\_27951\_length\_3778\_cov\_35.373741 1204-1210. Max. coverage (+): 0. Max coverage (-): 0

Region: NODE\_27951\_length\_3778\_cov\_35.373741 1211-1218. Max. coverage (+): 1.22. Max coverage (-): 0

Region: NODE\_27951\_length\_3778\_cov\_35.373741 1219-1226. Max. coverage (+): 1.22. Max coverage (-): 0

Region: NODE\_27951\_length\_3778\_cov\_35.373741 1227-1233. Max. coverage (+): 0.09. Max coverage (-): 0

Region: NODE\_27951\_length\_3778\_cov\_35.373741 1234-1241. Max. coverage (+): 0.28. Max coverage (-): 0

Region: NODE\_27951\_length\_3778\_cov\_35.373741 1242-1249. Max. coverage (+): 0.19. Max coverage (-): 0

Region: NODE\_27951\_length\_3778\_cov\_35.373741 1250-1256. Max. coverage (+): 0. Max coverage (-): 0

Region: NODE\_27951\_length\_3778\_cov\_35.373741 1257-1264. Max. coverage (+): 0.28. Max coverage (-): 0

Region: NODE\_27951\_length\_3778\_cov\_35.373741 1265-1272. Max. coverage (+): 0.38. Max coverage (-): 0

Region: NODE\_27951\_length\_3778\_cov\_35.373741 1273-1279. Max. coverage (+): 0. Max coverage (-): 0

Region: NODE\_27951\_length\_3778\_cov\_35.373741 1280-1287. Max. coverage (+): 0. Max coverage (-): 0

Region: NODE\_27951\_length\_3778\_cov\_35.373741 1288-1295. Max. coverage (+): 0.09. Max coverage (-): 0

Region: NODE\_27951\_length\_3778\_cov\_35.373741 1296-1302. Max. coverage (+): 2.07. Max coverage (-): 0.09

Region: NODE\_27951\_length\_3778\_cov\_35.373741 1303-1310. Max. coverage (+): 2.26. Max coverage (-): 0

Region: NODE\_27951\_length\_3778\_cov\_35.373741 1311-1317. Max. coverage (+): 0. Max coverage (-): 0

Region: NODE\_27951\_length\_3778\_cov\_35.373741 1318-1325. Max. coverage (+): 0. Max coverage (-): 0

Region: NODE\_27951\_length\_3778\_cov\_35.373741 1326-1333. Max. coverage (+): 0. Max coverage (-): 0

Region: NODE\_27951\_length\_3778\_cov\_35.373741 1334-1340. Max. coverage (+): 0.09. Max coverage (-): 0.09

Region: NODE\_27951\_length\_3778\_cov\_35.373741 1341-1348. Max. coverage (+): 0. Max coverage (-): 0.38

Region: NODE\_27951\_length\_3778\_cov\_35.373741 1349-1356. Max. coverage (+): 0. Max coverage (-): 0

Region: NODE\_27951\_length\_3778\_cov\_35.373741 1357-1363. Max. coverage (+): 0.28. Max coverage (-): 0

Region: NODE\_27951\_length\_3778\_cov\_35.373741 1364-1371. Max. coverage (+): 0.85. Max coverage (-): 0

Region: NODE\_27951\_length\_3778\_cov\_35.373741 1372-1379. Max. coverage (+): 0.09. Max coverage (-): 0

Region: NODE\_27951\_length\_3778\_cov\_35.373741 1380-1386. Max. coverage (+): 0. Max coverage (-): 0

Region: NODE\_27951\_length\_3778\_cov\_35.373741 1387-1394. Max. coverage (+): 0.09. Max coverage (-): 0.28

Region: NODE\_27951\_length\_3778\_cov\_35.373741 1395-1402. Max. coverage (+): 0.66. Max coverage (-): 0.38

Region: NODE\_27951\_length\_3778\_cov\_35.373741 1403-1409. Max. coverage (+): 0.47. Max coverage (-): 0

Region: NODE\_27951\_length\_3778\_cov\_35.373741 1410-1417. Max. coverage (+): 11.56. Max coverage (-): 0

Region: NODE\_27951\_length\_3778\_cov\_35.373741 1418-1425. Max. coverage (+): 2.63. Max coverage (-): 0

Region: NODE\_27951\_length\_3778\_cov\_35.373741 1426-1432. Max. coverage (+): 0.19. Max coverage (-): 0.28

Region: NODE\_27951\_length\_3778\_cov\_35.373741 1433-1440. Max. coverage (+): 0.09. Max coverage (-): 0.19

Region: NODE\_27951\_length\_3778\_cov\_35.373741 1441-1448. Max. coverage (+): 0.28. Max coverage (-): 0.09

Region: NODE\_27951\_length\_3778\_cov\_35.373741 1449-1455. Max. coverage (+): 0.38. Max coverage (-): 0

Region: NODE\_27951\_length\_3778\_cov\_35.373741 1456-1463. Max. coverage (+): 0. Max coverage (-): 0.19

Region: NODE\_27951\_length\_3778\_cov\_35.373741 1464-1470. Max. coverage (+): 0. Max coverage (-): 0.19

Region: NODE\_27951\_length\_3778\_cov\_35.373741 1471-1478. Max. coverage (+): 0.28. Max coverage (-): 0.09

Region: NODE\_27951\_length\_3778\_cov\_35.373741 1479-1486. Max. coverage (+): 0.19. Max coverage (-): 0

Region: NODE\_27951\_length\_3778\_cov\_35.373741 1487-1493. Max. coverage (+): 0.09. Max coverage (-): 0.09

Region: NODE\_27951\_length\_3778\_cov\_35.373741 1494-1501. Max. coverage (+): 0.19. Max coverage (-): 0.47

Region: NODE\_27951\_length\_3778\_cov\_35.373741 1502-1509. Max. coverage (+): 0.09. Max coverage (-): 0.38

Region: NODE\_27951\_length\_3778\_cov\_35.373741 1510-1516. Max. coverage (+): 0. Max coverage (-): 0

Region: NODE\_27951\_length\_3778\_cov\_35.373741 1517-1524. Max. coverage (+): 0.85. Max coverage (-): 0

Region: NODE\_27951\_length\_3778\_cov\_35.373741 1525-1532. Max. coverage (+): 1.97. Max coverage (-): 0

Region: NODE\_27951\_length\_3778\_cov\_35.373741 1533-1539. Max. coverage (+): 0.28. Max coverage (-): 0

Region: NODE\_27951\_length\_3778\_cov\_35.373741 1540-1547. Max. coverage (+): 0.28. Max coverage (-): 0

Region: NODE\_27951\_length\_3778\_cov\_35.373741 1548-1555. Max. coverage (+): 0.56. Max coverage (-): 0

Region: NODE\_27951\_length\_3778\_cov\_35.373741 1556-1562. Max. coverage (+): 0. Max coverage (-): 0

Region: NODE\_27951\_length\_3778\_cov\_35.373741 1563-1570. Max. coverage (+): 0. Max coverage (-): 0

Region: NODE\_27951\_length\_3778\_cov\_35.373741 1571-1578. Max. coverage (+): 0. Max coverage (-): 0

Region: NODE\_27951\_length\_3778\_cov\_35.373741 1579-1585. Max. coverage (+): 0.09. Max coverage (-): 0.09

Region: NODE\_27951\_length\_3778\_cov\_35.373741 1586-1593. Max. coverage (+): 0.47. Max coverage (-): 0.09

Region: NODE\_27951\_length\_3778\_cov\_35.373741 1594-1601. Max. coverage (+): 0.66. Max coverage (-): 0

Region: NODE\_27951\_length\_3778\_cov\_35.373741 1602-1608. Max. coverage (+): 0.47. Max coverage (-): 0

Region: NODE\_27951\_length\_3778\_cov\_35.373741 1609-1616. Max. coverage (+): 0.47. Max coverage (-): 0

Region: NODE\_27951\_length\_3778\_cov\_35.373741 1617-1623. Max. coverage (+): 0.09. Max coverage (-): 0

Region: NODE\_27951\_length\_3778\_cov\_35.373741 1624-1631. Max. coverage (+): 0.19. Max coverage (-): 0

Region: NODE\_27951\_length\_3778\_cov\_35.373741 1632-1639. Max. coverage (+): 0.47. Max coverage (-): 0

Region: NODE\_27951\_length\_3778\_cov\_35.373741 1640-1646. Max. coverage (+): 1.32. Max coverage (-): 0

Region: NODE\_27951\_length\_3778\_cov\_35.373741 1647-1654. Max. coverage (+): 0. Max coverage (-): 0.19

Region: NODE\_27951\_length\_3778\_cov\_35.373741 1655-1662. Max. coverage (+): 0. Max coverage (-): 0.19

Region: NODE\_27951\_length\_3778\_cov\_35.373741 1663-1669. Max. coverage (+): 0. Max coverage (-): 0.66

Region: NODE\_27951\_length\_3778\_cov\_35.373741 1670-1677. Max. coverage (+): 0.28. Max coverage (-): 0.56

Region: NODE\_27951\_length\_3778\_cov\_35.373741 1678-1685. Max. coverage (+): 3.1. Max coverage (-): 0

Region: NODE\_27951\_length\_3778\_cov\_35.373741 1686-1692. Max. coverage (+): 25.18. Max coverage (-): 0.09

Region: NODE\_27951\_length\_3778\_cov\_35.373741 1693-1700. Max. coverage (+): 0. Max coverage (-): 0.09

Region: NODE\_27951\_length\_3778\_cov\_35.373741 1701-1708. Max. coverage (+): 0. Max coverage (-): 0

Region: NODE\_27951\_length\_3778\_cov\_35.373741 1709-1715. Max. coverage (+): 0. Max coverage (-): 0.19

Region: NODE\_27951\_length\_3778\_cov\_35.373741 1716-1723. Max. coverage (+): 0.09. Max coverage (-): 0.09

Region: NODE\_27951\_length\_3778\_cov\_35.373741 1724-1731. Max. coverage (+): 0.47. Max coverage (-): 0

Region: NODE\_27951\_length\_3778\_cov\_35.373741 1732-1738. Max. coverage (+): 0.56. Max coverage (-): 0

Region: NODE\_27951\_length\_3778\_cov\_35.373741 1739-1746. Max. coverage (+): 0.19. Max coverage (-): 0

Region: NODE\_27951\_length\_3778\_cov\_35.373741 1747-1754. Max. coverage (+): 0.25. Max coverage (-): 0

Region: NODE\_27951\_length\_3778\_cov\_35.373741 1755-1761. Max. coverage (+): 0. Max coverage (-): 0

Region: NODE\_27951\_length\_3778\_cov\_35.373741 1762-1769. Max. coverage (+): 0.03. Max coverage (-): 0

Region: NODE\_27951\_length\_3778\_cov\_35.373741 1770-1776. Max. coverage (+): 0. Max coverage (-): 0.06

Region: NODE\_27951\_length\_3778\_cov\_35.373741 1777-1784. Max. coverage (+): 0.19. Max coverage (-): 0

Region: NODE\_27951\_length\_3778\_cov\_35.373741 1785-1792. Max. coverage (+): 0.31. Max coverage (-): 0.03

Region: NODE\_27951\_length\_3778\_cov\_35.373741 1793-1799. Max. coverage (+): 1.44. Max coverage (-): 0

Region: NODE\_27951\_length\_3778\_cov\_35.373741 1800-1807. Max. coverage (+): 1. Max coverage (-): 0

Region: NODE\_27951\_length\_3778\_cov\_35.373741 1808-1815. Max. coverage (+): 0. Max coverage (-): 0

Region: NODE\_27951\_length\_3778\_cov\_35.373741 1816-1822. Max. coverage (+): 0. Max coverage (-): 0.06

Region: NODE\_27951\_length\_3778\_cov\_35.373741 1823-1830. Max. coverage (+): 1.91. Max coverage (-): 0.06

Region: NODE\_27951\_length\_3778\_cov\_35.373741 1831-1838. Max. coverage (+): 1.94. Max coverage (-): 0

Region: NODE\_27951\_length\_3778\_cov\_35.373741 1839-1845. Max. coverage (+): 0. Max coverage (-): 0

Region: NODE\_27951\_length\_3778\_cov\_35.373741 1846-1853. Max. coverage (+): 0.03. Max coverage (-): 0.03

Region: NODE\_27951\_length\_3778\_cov\_35.373741 1854-1861. Max. coverage (+): 0.16. Max coverage (-): 0

Region: NODE\_27951\_length\_3778\_cov\_35.373741 1862-1868. Max. coverage (+): 0.81. Max coverage (-): 0

Region: NODE\_27951\_length\_3778\_cov\_35.373741 1869-1876. Max. coverage (+): 1.5. Max coverage (-): 0

Region: NODE\_27951\_length\_3778\_cov\_35.373741 1877-1884. Max. coverage (+): 0. Max coverage (-): 0.03

Region: NODE\_27951\_length\_3778\_cov\_35.373741 1885-1891. Max. coverage (+): 0.03. Max coverage (-): 0

Region: NODE\_27951\_length\_3778\_cov\_35.373741 1892-1899. Max. coverage (+): 0.66. Max coverage (-): 0

Region: NODE\_27951\_length\_3778\_cov\_35.373741 1900-1907. Max. coverage (+): 0.13. Max coverage (-): 0

Region: NODE\_27951\_length\_3778\_cov\_35.373741 1908-1914. Max. coverage (+): 0. Max coverage (-): 0

Region: NODE\_27951\_length\_3778\_cov\_35.373741 1915-1922. Max. coverage (+): 0.03. Max coverage (-): 0

Region: NODE\_27951\_length\_3778\_cov\_35.373741 1923-1929. Max. coverage (+): 0.41. Max coverage (-): 0

Region: NODE\_27951\_length\_3778\_cov\_35.373741 1930-1937. Max. coverage (+): 1.13. Max coverage (-): 0

Region: NODE\_27951\_length\_3778\_cov\_35.373741 1938-1945. Max. coverage (+): 0.41. Max coverage (-): 0

Region: NODE\_27951\_length\_3778\_cov\_35.373741 1946-1952. Max. coverage (+): 0.28. Max coverage (-): 0

Region: NODE\_27951\_length\_3778\_cov\_35.373741 1953-1960. Max. coverage (+): 0. Max coverage (-): 0

Region: NODE\_27951\_length\_3778\_cov\_35.373741 1961-1968. Max. coverage (+): 0.09. Max coverage (-): 0

Region: NODE\_27951\_length\_3778\_cov\_35.373741 1969-1975. Max. coverage (+): 2.22. Max coverage (-): 0

Region: NODE\_27951\_length\_3778\_cov\_35.373741 1976-1983. Max. coverage (+): 0.38. Max coverage (-): 0

Region: NODE\_27951\_length\_3778\_cov\_35.373741 1984-1991. Max. coverage (+): 0.03. Max coverage (-): 0.03

Region: NODE\_27951\_length\_3778\_cov\_35.373741 1992-1998. Max. coverage (+): 0. Max coverage (-): 0

Region: NODE\_27951\_length\_3778\_cov\_35.373741 1999-2006. Max. coverage (+): 0.09. Max coverage (-): 0

Region: NODE\_27951\_length\_3778\_cov\_35.373741 2007-2014. Max. coverage (+): 0.34. Max coverage (-): 0

Region: NODE\_27951\_length\_3778\_cov\_35.373741 2015-2021. Max. coverage (+): 0.19. Max coverage (-): 0.03

Region: NODE\_27951\_length\_3778\_cov\_35.373741 2022-2029. Max. coverage (+): 0.06. Max coverage (-): 0.06

Region: NODE\_27951\_length\_3778\_cov\_35.373741 2030-2037. Max. coverage (+): 0. Max coverage (-): 0.06

Region: NODE\_27951\_length\_3778\_cov\_35.373741 2038-2044. Max. coverage (+): 0. Max coverage (-): 0

Region: NODE\_27951\_length\_3778\_cov\_35.373741 2045-2052. Max. coverage (+): 0. Max coverage (-): 0

Region: NODE\_27951\_length\_3778\_cov\_35.373741 2053-2060. Max. coverage (+): 0. Max coverage (-): 0

Region: NODE\_27951\_length\_3778\_cov\_35.373741 2061-2067. Max. coverage (+): 0.03. Max coverage (-): 0

Region: NODE\_27951\_length\_3778\_cov\_35.373741 2068-2075. Max. coverage (+): 0.44. Max coverage (-): 0

Region: NODE\_27951\_length\_3778\_cov\_35.373741 2076-2082. Max. coverage (+): 0. Max coverage (-): 0

Region: NODE\_27951\_length\_3778\_cov\_35.373741 2083-2090. Max. coverage (+): 0. Max coverage (-): 0

Region: NODE\_27951\_length\_3778\_cov\_35.373741 2091-2098. Max. coverage (+): 0.03. Max coverage (-): 0

Region: NODE\_27951\_length\_3778\_cov\_35.373741 2099-2105. Max. coverage (+): 0.38. Max coverage (-): 0

Region: NODE\_27951\_length\_3778\_cov\_35.373741 2106-2113. Max. coverage (+): 0.38. Max coverage (-): 0

Region: NODE\_27951\_length\_3778\_cov\_35.373741 2114-2121. Max. coverage (+): 0. Max coverage (-): 0

Region: NODE\_27951\_length\_3778\_cov\_35.373741 2122-2128. Max. coverage (+): 0. Max coverage (-): 0

Region: NODE\_27951\_length\_3778\_cov\_35.373741 2129-2136. Max. coverage (+): 0.33. Max coverage (-): 0

Region: NODE\_27951\_length\_3778\_cov\_35.373741 2137-2144. Max. coverage (+): 1.32. Max coverage (-): 0

Region: NODE\_27951\_length\_3778\_cov\_35.373741 2145-2151. Max. coverage (+): 0. Max coverage (-): 0.05

Region: NODE\_27951\_length\_3778\_cov\_35.373741 2152-2159. Max. coverage (+): 0. Max coverage (-): 0

Region: NODE\_27951\_length\_3778\_cov\_35.373741 2160-2167. Max. coverage (+): 0. Max coverage (-): 0.09

Region: NODE\_27951\_length\_3778\_cov\_35.373741 2168-2174. Max. coverage (+): 0. Max coverage (-): 0

Region: NODE\_27951\_length\_3778\_cov\_35.373741 2175-2182. Max. coverage (+): 0. Max coverage (-): 0

Region: NODE\_27951\_length\_3778\_cov\_35.373741 2183-2190. Max. coverage (+): 0. Max coverage (-): 0

Region: NODE\_27951\_length\_3778\_cov\_35.373741 2191-2197. Max. coverage (+): 0.03. Max coverage (-): 0

Region: NODE\_27951\_length\_3778\_cov\_35.373741 2198-2205. Max. coverage (+): 0.13. Max coverage (-): 0

Region: NODE\_27951\_length\_3778\_cov\_35.373741 2206-2213. Max. coverage (+): 0.06. Max coverage (-): 0

Region: NODE\_27951\_length\_3778\_cov\_35.373741 2214-2220. Max. coverage (+): 0.34. Max coverage (-): 0

Region: NODE\_27951\_length\_3778\_cov\_35.373741 2221-2228. Max. coverage (+): 0.34. Max coverage (-): 0

Region: NODE\_27951\_length\_3778\_cov\_35.373741 2229-2235. Max. coverage (+): 0.25. Max coverage (-): 0

Region: NODE\_27951\_length\_3778\_cov\_35.373741 2236-2243. Max. coverage (+): 0.28. Max coverage (-): 0

Region: NODE\_27951\_length\_3778\_cov\_35.373741 2244-2251. Max. coverage (+): 0.25. Max coverage (-): 0

Region: NODE\_27951\_length\_3778\_cov\_35.373741 2252-2258. Max. coverage (+): 0.03. Max coverage (-): 0

Region: NODE\_27951\_length\_3778\_cov\_35.373741 2259-2266. Max. coverage (+): 0.03. Max coverage (-): 0

Region: NODE\_27951\_length\_3778\_cov\_35.373741 2267-2274. Max. coverage (+): 0. Max coverage (-): 0

Region: NODE\_27951\_length\_3778\_cov\_35.373741 2275-2281. Max. coverage (+): 0. Max coverage (-): 0.06

Region: NODE\_27951\_length\_3778\_cov\_35.373741 2282-2289. Max. coverage (+): 0.03. Max coverage (-): 0.03

Region: NODE\_27951\_length\_3778\_cov\_35.373741 2290-2297. Max. coverage (+): 0.25. Max coverage (-): 0

Region: NODE\_27951\_length\_3778\_cov\_35.373741 2298-2304. Max. coverage (+): 0.25. Max coverage (-): 0

Region: NODE\_27951\_length\_3778\_cov\_35.373741 2305-2312. Max. coverage (+): 0.03. Max coverage (-): 0.06

Region: NODE\_27951\_length\_3778\_cov\_35.373741 2313-2320. Max. coverage (+): 0.03. Max coverage (-): 0

Region: NODE\_27951\_length\_3778\_cov\_35.373741 2321-2327. Max. coverage (+): 0.16. Max coverage (-): 0

Region: NODE\_27951\_length\_3778\_cov\_35.373741 2328-2335. Max. coverage (+): 0.09. Max coverage (-): 0.03

Region: NODE\_27951\_length\_3778\_cov\_35.373741 2336-2343. Max. coverage (+): 0.31. Max coverage (-): 0.03

Region: NODE\_27951\_length\_3778\_cov\_35.373741 2344-2350. Max. coverage (+): 0.28. Max coverage (-): 0

Region: NODE\_27951\_length\_3778\_cov\_35.373741 2351-2358. Max. coverage (+): 0.03. Max coverage (-): 0

Region: NODE\_27951\_length\_3778\_cov\_35.373741 2359-2366. Max. coverage (+): 0.03. Max coverage (-): 0

Region: NODE\_27951\_length\_3778\_cov\_35.373741 2367-2373. Max. coverage (+): 0.13. Max coverage (-): 0

Region: NODE\_27951\_length\_3778\_cov\_35.373741 2374-2381. Max. coverage (+): 1.53. Max coverage (-): 0

Region: NODE\_27951\_length\_3778\_cov\_35.373741 2382-2388. Max. coverage (+): 1.57. Max coverage (-): 0

Region: NODE\_27951\_length\_3778\_cov\_35.373741 2389-2396. Max. coverage (+): 0.5. Max coverage (-): 0

Region: NODE\_27951\_length\_3778\_cov\_35.373741 2397-2404. Max. coverage (+): 0. Max coverage (-): 0

Region: NODE\_27951\_length\_3778\_cov\_35.373741 2405-2411. Max. coverage (+): 0. Max coverage (-): 0

Region: NODE\_27951\_length\_3778\_cov\_35.373741 2412-2419. Max. coverage (+): 0.16. Max coverage (-): 0

Region: NODE\_27951\_length\_3778\_cov\_35.373741 2420-2427. Max. coverage (+): 0.16. Max coverage (-): 0.06

Region: NODE\_27951\_length\_3778\_cov\_35.373741 2428-2434. Max. coverage (+): 0.03. Max coverage (-): 0.03

Region: NODE\_27951\_length\_3778\_cov\_35.373741 2435-2442. Max. coverage (+): 1.66. Max coverage (-): 0

Region: NODE\_27951\_length\_3778\_cov\_35.373741 2443-2450. Max. coverage (+): 1.66. Max coverage (-): 0

Region: NODE\_27951\_length\_3778\_cov\_35.373741 2451-2457. Max. coverage (+): 0. Max coverage (-): 0

Region: NODE\_27951\_length\_3778\_cov\_35.373741 2458-2465. Max. coverage (+): 0. Max coverage (-): 0

Region: NODE\_27951\_length\_3778\_cov\_35.373741 2466-2473. Max. coverage (+): 0. Max coverage (-): 0.34

Region: NODE\_27951\_length\_3778\_cov\_35.373741 2474-2480. Max. coverage (+): 0.09. Max coverage (-): 0.34

Region: NODE\_27951\_length\_3778\_cov\_35.373741 2481-2488. Max. coverage (+): 0.05. Max coverage (-): 0

Region: NODE\_27951\_length\_3778\_cov\_35.373741 2489-2496. Max. coverage (+): 0.16. Max coverage (-): 0

Region: NODE\_27951\_length\_3778\_cov\_35.373741 2497-2503. Max. coverage (+): 0.16. Max coverage (-): 0

Region: NODE\_27951\_length\_3778\_cov\_35.373741 2504-2511. Max. coverage (+): 0.09. Max coverage (-): 0

Region: NODE\_27951\_length\_3778\_cov\_35.373741 2512-2519. Max. coverage (+): 0.19. Max coverage (-): 0.09

Region: NODE\_27951\_length\_3778\_cov\_35.373741 2520-2526. Max. coverage (+): 0.09. Max coverage (-): 0

Region: NODE\_27951\_length\_3778\_cov\_35.373741 2527-2534. Max. coverage (+): 0.28. Max coverage (-): 0

Region: NODE\_27951\_length\_3778\_cov\_35.373741 2535-2541. Max. coverage (+): 0.19. Max coverage (-): 0

Region: NODE\_27951\_length\_3778\_cov\_35.373741 2542-2549. Max. coverage (+): 0.09. Max coverage (-): 0

Region: NODE\_27951\_length\_3778\_cov\_35.373741 2550-2557. Max. coverage (+): 0.66. Max coverage (-): 0

Region: NODE\_27951\_length\_3778\_cov\_35.373741 2558-2564. Max. coverage (+): 0.66. Max coverage (-): 0

Region: NODE\_27951\_length\_3778\_cov\_35.373741 2565-2572. Max. coverage (+): 0.28. Max coverage (-): 0

Region: NODE\_27951\_length\_3778\_cov\_35.373741 2573-2580. Max. coverage (+): 0.05. Max coverage (-): 0

Region: NODE\_27951\_length\_3778\_cov\_35.373741 2581-2587. Max. coverage (+): 0.38. Max coverage (-): 0

Region: NODE\_27951\_length\_3778\_cov\_35.373741 2588-2595. Max. coverage (+): 0.38. Max coverage (-): 0

Region: NODE\_27951\_length\_3778\_cov\_35.373741 2596-2603. Max. coverage (+): 0.08. Max coverage (-): 0

Region: NODE\_27951\_length\_3778\_cov\_35.373741 2604-2610. Max. coverage (+): 0.19. Max coverage (-): 0

Region: NODE\_27951\_length\_3778\_cov\_35.373741 2611-2618. Max. coverage (+): 0. Max coverage (-): 0

Region: NODE\_27951\_length\_3778\_cov\_35.373741 2619-2626. Max. coverage (+): 0. Max coverage (-): 0

Region: NODE\_27951\_length\_3778\_cov\_35.373741 2627-2633. Max. coverage (+): 0. Max coverage (-): 0

Region: NODE\_27951\_length\_3778\_cov\_35.373741 2634-2641. Max. coverage (+): 0.33. Max coverage (-): 0

Region: NODE\_27951\_length\_3778\_cov\_35.373741 2642-2649. Max. coverage (+): 0. Max coverage (-): 0

Region: NODE\_27951\_length\_3778\_cov\_35.373741 2650-2656. Max. coverage (+): 0. Max coverage (-): 0

Region: NODE\_27951\_length\_3778\_cov\_35.373741 2657-2664. Max. coverage (+): 0.05. Max coverage (-): 0

Region: NODE\_27951\_length\_3778\_cov\_35.373741 2665-2672. Max. coverage (+): 0.19. Max coverage (-): 0

Region: NODE\_27951\_length\_3778\_cov\_35.373741 2673-2679. Max. coverage (+): 0.09. Max coverage (-): 0

Region: NODE\_27951\_length\_3778\_cov\_35.373741 2680-2687. Max. coverage (+): 0.18. Max coverage (-): 0

Region: NODE\_27951\_length\_3778\_cov\_35.373741 2688-2694. Max. coverage (+): 1.03. Max coverage (-): 0

Region: NODE\_27951\_length\_3778\_cov\_35.373741 2695-2702. Max. coverage (+): 1.08. Max coverage (-): 0

Region: NODE\_27951\_length\_3778\_cov\_35.373741 2703-2710. Max. coverage (+): 0.09. Max coverage (-): 0.05

Region: NODE\_27951\_length\_3778\_cov\_35.373741 2711-2717. Max. coverage (+): 0.05. Max coverage (-): 0

Region: NODE\_27951\_length\_3778\_cov\_35.373741 2718-2725. Max. coverage (+): 0.13. Max coverage (-): 0

Region: NODE\_27951\_length\_3778\_cov\_35.373741 2726-2733. Max. coverage (+): 0.09. Max coverage (-): 0

Region: NODE\_27951\_length\_3778\_cov\_35.373741 2734-2740. Max. coverage (+): 0.23. Max coverage (-): 0

Region: NODE\_27951\_length\_3778\_cov\_35.373741 2741-2748. Max. coverage (+): 0.19. Max coverage (-): 0

Region: NODE\_27951\_length\_3778\_cov\_35.373741 2749-2756. Max. coverage (+): 0.05. Max coverage (-): 0.05

Region: NODE\_27951\_length\_3778\_cov\_35.373741 2757-2763. Max. coverage (+): 0. Max coverage (-): 0

Region: NODE\_27951\_length\_3778\_cov\_35.373741 2764-2771. Max. coverage (+): 0. Max coverage (-): 0

Region: NODE\_27951\_length\_3778\_cov\_35.373741 2772-2779. Max. coverage (+): 0.23. Max coverage (-): 0

Region: NODE\_27951\_length\_3778\_cov\_35.373741 2780-2786. Max. coverage (+): 0.09. Max coverage (-): 0

Region: NODE\_27951\_length\_3778\_cov\_35.373741 2787-2794. Max. coverage (+): 0.05. Max coverage (-): 0

Region: NODE\_27951\_length\_3778\_cov\_35.373741 2795-2802. Max. coverage (+): 0. Max coverage (-): 0

Region: NODE\_27951\_length\_3778\_cov\_35.373741 2803-2809. Max. coverage (+): 0. Max coverage (-): 0

Region: NODE\_27951\_length\_3778\_cov\_35.373741 2810-2817. Max. coverage (+): 0.23. Max coverage (-): 0

Region: NODE\_27951\_length\_3778\_cov\_35.373741 2818-2825. Max. coverage (+): 0.28. Max coverage (-): 0

Region: NODE\_27951\_length\_3778\_cov\_35.373741 2826-2832. Max. coverage (+): 0.14. Max coverage (-): 0

Region: NODE\_27951\_length\_3778\_cov\_35.373741 2833-2840. Max. coverage (+): 0.19. Max coverage (-): 0

Region: NODE\_27951\_length\_3778\_cov\_35.373741 2841-2847. Max. coverage (+): 0.05. Max coverage (-): 0

Region: NODE\_27951\_length\_3778\_cov\_35.373741 2848-2855. Max. coverage (+): 0.28. Max coverage (-): 0

Region: NODE\_27951\_length\_3778\_cov\_35.373741 2856-2863. Max. coverage (+): 0.28. Max coverage (-): 0.05

Region: NODE\_27951\_length\_3778\_cov\_35.373741 2864-2870. Max. coverage (+): 0.05. Max coverage (-): 0

Region: NODE\_27951\_length\_3778\_cov\_35.373741 2871-2878. Max. coverage (+): 0.05. Max coverage (-): 0

Region: NODE\_27951\_length\_3778\_cov\_35.373741 2879-2886. Max. coverage (+): 0.33. Max coverage (-): 0

Region: NODE\_27951\_length\_3778\_cov\_35.373741 2887-2893. Max. coverage (+): 0.19. Max coverage (-): 0

Region: NODE\_27951\_length\_3778\_cov\_35.373741 2894-2901. Max. coverage (+): 1.32. Max coverage (-): 0

Region: NODE\_27951\_length\_3778\_cov\_35.373741 2902-2909. Max. coverage (+): 0.38. Max coverage (-): 0

Region: NODE\_27951\_length\_3778\_cov\_35.373741 2910-2916. Max. coverage (+): 6.58. Max coverage (-): 0

Region: NODE\_27951\_length\_3778\_cov\_35.373741 2917-2924. Max. coverage (+): 0. Max coverage (-): 0

Region: NODE\_27951\_length\_3778\_cov\_35.373741 2925-2932. Max. coverage (+): 0.09. Max coverage (-): 0

Region: NODE\_27951\_length\_3778\_cov\_35.373741 2933-2939. Max. coverage (+): 0.94. Max coverage (-): 0

Region: NODE\_27951\_length\_3778\_cov\_35.373741 2940-2947. Max. coverage (+): 2.49. Max coverage (-): 0

Region: NODE\_27951\_length\_3778\_cov\_35.373741 2948-2955. Max. coverage (+): 0.75. Max coverage (-): 0.05

Region: NODE\_27951\_length\_3778\_cov\_35.373741 2956-2962. Max. coverage (+): 0.05. Max coverage (-): 0

Region: NODE\_27951\_length\_3778\_cov\_35.373741 2963-2970. Max. coverage (+): 0.05. Max coverage (-): 0

Region: NODE\_27951\_length\_3778\_cov\_35.373741 2971-2978. Max. coverage (+): 0.09. Max coverage (-): 0

Region: NODE\_27951\_length\_3778\_cov\_35.373741 2979-2985. Max. coverage (+): 0. Max coverage (-): 0

Region: NODE\_27951\_length\_3778\_cov\_35.373741 2986-2993. Max. coverage (+): 0.33. Max coverage (-): 0

Region: NODE\_27951\_length\_3778\_cov\_35.373741 2994-3000. Max. coverage (+): 0.09. Max coverage (-): 0

Region: NODE\_27951\_length\_3778\_cov\_35.373741 3001-3008. Max. coverage (+): 0.14. Max coverage (-): 0

Region: NODE\_27951\_length\_3778\_cov\_35.373741 3009-3016. Max. coverage (+): 0. Max coverage (-): 0.23

Region: NODE\_27951\_length\_3778\_cov\_35.373741 3017-3023. Max. coverage (+): 0.05. Max coverage (-): 0.14

Region: NODE\_27951\_length\_3778\_cov\_35.373741 3024-3031. Max. coverage (+): 0.05. Max coverage (-): 0

Region: NODE\_27951\_length\_3778\_cov\_35.373741 3032-3039. Max. coverage (+): 0.61. Max coverage (-): 0

Region: NODE\_27951\_length\_3778\_cov\_35.373741 3040-3046. Max. coverage (+): 0.05. Max coverage (-): 0

Region: NODE\_27951\_length\_3778\_cov\_35.373741 3047-3054. Max. coverage (+): 0.09. Max coverage (-): 0.09

Region: NODE\_27951\_length\_3778\_cov\_35.373741 3055-3062. Max. coverage (+): 0.09. Max coverage (-): 0.05

Region: NODE\_27951\_length\_3778\_cov\_35.373741 3063-3069. Max. coverage (+): 0.14. Max coverage (-): 0

Region: NODE\_27951\_length\_3778\_cov\_35.373741 3070-3077. Max. coverage (+): 0.14. Max coverage (-): 0

Region: NODE\_27951\_length\_3778\_cov\_35.373741 3078-3085. Max. coverage (+): 0. Max coverage (-): 0

Region: NODE\_27951\_length\_3778\_cov\_35.373741 3086-3092. Max. coverage (+): 0. Max coverage (-): 0

Region: NODE\_27951\_length\_3778\_cov\_35.373741 3093-3100. Max. coverage (+): 0.19. Max coverage (-): 0

Region: NODE\_27951\_length\_3778\_cov\_35.373741 3101-3108. Max. coverage (+): 0.05. Max coverage (-): 0

Region: NODE\_27951\_length\_3778\_cov\_35.373741 3109-3115. Max. coverage (+): 0.14. Max coverage (-): 0

Region: NODE\_27951\_length\_3778\_cov\_35.373741 3116-3123. Max. coverage (+): 0.09. Max coverage (-): 0

Region: NODE\_27951\_length\_3778\_cov\_35.373741 3124-3131. Max. coverage (+): 5.87. Max coverage (-): 0

Region: NODE\_27951\_length\_3778\_cov\_35.373741 3132-3138. Max. coverage (+): 2.91. Max coverage (-): 0

Region: NODE\_27951\_length\_3778\_cov\_35.373741 3139-3146. Max. coverage (+): 0.23. Max coverage (-): 0.05

Region: NODE\_27951\_length\_3778\_cov\_35.373741 3147-3153. Max. coverage (+): 0.09. Max coverage (-): 0.05

Region: NODE\_27951\_length\_3778\_cov\_35.373741 3154-3161. Max. coverage (+): 0.05. Max coverage (-): 0

Region: NODE\_27951\_length\_3778\_cov\_35.373741 3162-3169. Max. coverage (+): 0.28. Max coverage (-): 0

Region: NODE\_27951\_length\_3778\_cov\_35.373741 3170-3176. Max. coverage (+): 0.33. Max coverage (-): 0

Region: NODE\_27951\_length\_3778\_cov\_35.373741 3177-3184. Max. coverage (+): 0.14. Max coverage (-): 0

Region: NODE\_27951\_length\_3778\_cov\_35.373741 3185-3192. Max. coverage (+): 0.05. Max coverage (-): 0

Region: NODE\_27951\_length\_3778\_cov\_35.373741 3193-3199. Max. coverage (+): 0. Max coverage (-): 0

Region: NODE\_27951\_length\_3778\_cov\_35.373741 3200-3207. Max. coverage (+): 0.8. Max coverage (-): 0.14

Region: NODE\_27951\_length\_3778\_cov\_35.373741 3208-3215. Max. coverage (+): 2.91. Max coverage (-): 0

Region: NODE\_27951\_length\_3778\_cov\_35.373741 3216-3222. Max. coverage (+): 0.09. Max coverage (-): 0

Region: NODE\_27951\_length\_3778\_cov\_35.373741 3223-3230. Max. coverage (+): 0.09. Max coverage (-): 0

Region: NODE\_27951\_length\_3778\_cov\_35.373741 3231-3238. Max. coverage (+): 0.23. Max coverage (-): 0

Region: NODE\_27951\_length\_3778\_cov\_35.373741 3239-3245. Max. coverage (+): 0.52. Max coverage (-): 0

Region: NODE\_27951\_length\_3778\_cov\_35.373741 3246-3253. Max. coverage (+): 0.09. Max coverage (-): 0.09

Region: NODE\_27951\_length\_3778\_cov\_35.373741 3254-3261. Max. coverage (+): 0.05. Max coverage (-): 1.74

Region: NODE\_27951\_length\_3778\_cov\_35.373741 3262-3268. Max. coverage (+): 0.05. Max coverage (-): 0

Region: NODE\_27951\_length\_3778\_cov\_35.373741 3269-3276. Max. coverage (+): 4.7. Max coverage (-): 0

Region: NODE\_27951\_length\_3778\_cov\_35.373741 3277-3284. Max. coverage (+): 0.05. Max coverage (-): 0

Region: NODE\_27951\_length\_3778\_cov\_35.373741 3285-3291. Max. coverage (+): 0.09. Max coverage (-): 0.09

Region: NODE\_27951\_length\_3778\_cov\_35.373741 3292-3299. Max. coverage (+): 0.09. Max coverage (-): 0.28

Region: NODE\_27951\_length\_3778\_cov\_35.373741 3300-3306. Max. coverage (+): 1.13. Max coverage (-): 0.09

Region: NODE\_27951\_length\_3778\_cov\_35.373741 3307-3314. Max. coverage (+): 1.22. Max coverage (-): 0

Region: NODE\_27951\_length\_3778\_cov\_35.373741 3315-3322. Max. coverage (+): 0.14. Max coverage (-): 0

Region: NODE\_27951\_length\_3778\_cov\_35.373741 3323-3329. Max. coverage (+): 0. Max coverage (-): 0

Region: NODE\_27951\_length\_3778\_cov\_35.373741 3330-3337. Max. coverage (+): 0. Max coverage (-): 0

Region: NODE\_27951\_length\_3778\_cov\_35.373741 3338-3345. Max. coverage (+): 0. Max coverage (-): 0

Region: NODE\_27951\_length\_3778\_cov\_35.373741 3346-3352. Max. coverage (+): 0. Max coverage (-): 0

Region: NODE\_27951\_length\_3778\_cov\_35.373741 3353-3360. Max. coverage (+): 0.19. Max coverage (-): 0

Region: NODE\_27951\_length\_3778\_cov\_35.373741 3361-3368. Max. coverage (+): 0.28. Max coverage (-): 0

Region: NODE\_27951\_length\_3778\_cov\_35.373741 3369-3375. Max. coverage (+): 0.23. Max coverage (-): 0

Region: NODE\_27951\_length\_3778\_cov\_35.373741 3376-3383. Max. coverage (+): 0. Max coverage (-): 0

Region: NODE\_27951\_length\_3778\_cov\_35.373741 3384-3391. Max. coverage (+): 0.19. Max coverage (-): 0

Region: NODE\_27951\_length\_3778\_cov\_35.373741 3392-3398. Max. coverage (+): 3.05. Max coverage (-): 0

Region: NODE\_27951\_length\_3778\_cov\_35.373741 3399-3406. Max. coverage (+): 0. Max coverage (-): 0.09

Region: NODE\_27951\_length\_3778\_cov\_35.373741 3407-3414. Max. coverage (+): 0. Max coverage (-): 0

Region: NODE\_27951\_length\_3778\_cov\_35.373741 3415-3421. Max. coverage (+): 0. Max coverage (-): 0

Region: NODE\_27951\_length\_3778\_cov\_35.373741 3422-3429. Max. coverage (+): 0. Max coverage (-): 0

Region: NODE\_27951\_length\_3778\_cov\_35.373741 3430-3437. Max. coverage (+): 0. Max coverage (-): 0.09

Region: NODE\_27951\_length\_3778\_cov\_35.373741 3438-3444. Max. coverage (+): 0.28. Max coverage (-): 0.09

Region: NODE\_27951\_length\_3778\_cov\_35.373741 3445-3452. Max. coverage (+): 0.09. Max coverage (-): 0

Region: NODE\_27951\_length\_3778\_cov\_35.373741 3453-3459. Max. coverage (+): 0.94. Max coverage (-): 0.09

Region: NODE\_27951\_length\_3778\_cov\_35.373741 3460-3467. Max. coverage (+): 1.22. Max coverage (-): 0.09

Region: NODE\_27951\_length\_3778\_cov\_35.373741 3468-3475. Max. coverage (+): 0.19. Max coverage (-): 0

Region: NODE\_27951\_length\_3778\_cov\_35.373741 3476-3482. Max. coverage (+): 0.03. Max coverage (-): 0.06

Region: NODE\_27951\_length\_3778\_cov\_35.373741 3483-3490. Max. coverage (+): 0. Max coverage (-): 0.06

Region: NODE\_27951\_length\_3778\_cov\_35.373741 3491-3498. Max. coverage (+): 0. Max coverage (-): 0.03

Region: NODE\_27951\_length\_3778\_cov\_35.373741 3499-3505. Max. coverage (+): 0.03. Max coverage (-): 0

Region: NODE\_27951\_length\_3778\_cov\_35.373741 3506-3513. Max. coverage (+): 5.26. Max coverage (-): 0

Region: NODE\_27951\_length\_3778\_cov\_35.373741 3514-3521. Max. coverage (+): 1.38. Max coverage (-): 0

Region: NODE\_27951\_length\_3778\_cov\_35.373741 3522-3528. Max. coverage (+): 0.13. Max coverage (-): 0

Region: NODE\_27951\_length\_3778\_cov\_35.373741 3529-3536. Max. coverage (+): 0. Max coverage (-): 0

Region: NODE\_27951\_length\_3778\_cov\_35.373741 3537-3544. Max. coverage (+): 0.03. Max coverage (-): 0

Region: NODE\_27951\_length\_3778\_cov\_35.373741 3545-3551. Max. coverage (+): 0.03. Max coverage (-): 0

Region: NODE\_27951\_length\_3778\_cov\_35.373741 3552-3559. Max. coverage (+): 0. Max coverage (-): 0

Region: NODE\_27951\_length\_3778\_cov\_35.373741 3560-3567. Max. coverage (+): 0. Max coverage (-): 0

Region: NODE\_27951\_length\_3778\_cov\_35.373741 3568-3574. Max. coverage (+): 0. Max coverage (-): 0

Region: NODE\_27951\_length\_3778\_cov\_35.373741 3575-3582. Max. coverage (+): 0. Max coverage (-): 0

Region: NODE\_27951\_length\_3778\_cov\_35.373741 3583-3590. Max. coverage (+): 0.03. Max coverage (-): 0

Region: NODE\_27951\_length\_3778\_cov\_35.373741 3591-3597. Max. coverage (+): 0.03. Max coverage (-): 0

Region: NODE\_27951\_length\_3778\_cov\_35.373741 3598-3605. Max. coverage (+): 0.16. Max coverage (-): 0.03

Region: NODE\_27951\_length\_3778\_cov\_35.373741 3606-3612. Max. coverage (+): 1.82. Max coverage (-): 0.03

Region: NODE\_27951\_length\_3778\_cov\_35.373741 3613-3620. Max. coverage (+): 0.03. Max coverage (-): 0.03

Region: NODE\_27951\_length\_3778\_cov\_35.373741 3621-3628. Max. coverage (+): 0.03. Max coverage (-): 0.03

Region: NODE\_27951\_length\_3778\_cov\_35.373741 3629-3635. Max. coverage (+): 0.28. Max coverage (-): 0

Region: NODE\_27951\_length\_3778\_cov\_35.373741 3636-3643. Max. coverage (+): 5.14. Max coverage (-): 0

Region: NODE\_27951\_length\_3778\_cov\_35.373741 3644-3651. Max. coverage (+): 0.22. Max coverage (-): 0

Region: NODE\_27951\_length\_3778\_cov\_35.373741 3652-3658. Max. coverage (+): 0.03. Max coverage (-): 0.03

Region: NODE\_27951\_length\_3778\_cov\_35.373741 3659-3666. Max. coverage (+): 0. Max coverage (-): 0.06

Region: NODE\_27951\_length\_3778\_cov\_35.373741 3667-3674. Max. coverage (+): 0.03. Max coverage (-): 0

Region: NODE\_27951\_length\_3778\_cov\_35.373741 3675-3681. Max. coverage (+): 9.18. Max coverage (-): 0

Region: NODE\_27951\_length\_3778\_cov\_35.373741 3682-3689. Max. coverage (+): 9.18. Max coverage (-): 0

Region: NODE\_27951\_length\_3778\_cov\_35.373741 3690-3697. Max. coverage (+): 0. Max coverage (-): 0

Region: NODE\_27951\_length\_3778\_cov\_35.373741 3698-3704. Max. coverage (+): 0. Max coverage (-): 0

Region: NODE\_27951\_length\_3778\_cov\_35.373741 3705-3712. Max. coverage (+): 1.32. Max coverage (-): 0

Region: NODE\_27951\_length\_3778\_cov\_35.373741 3713-3720. Max. coverage (+): 0.38. Max coverage (-): 0

Region: NODE\_27951\_length\_3778\_cov\_35.373741 3721-3727. Max. coverage (+): 0.28. Max coverage (-): 0

Region: NODE\_27951\_length\_3778\_cov\_35.373741 3728-3735. Max. coverage (+): 0. Max coverage (-): 0

Region: NODE\_27951\_length\_3778\_cov\_35.373741 3736-3743. Max. coverage (+): 0. Max coverage (-): 0

Region: NODE\_27951\_length\_3778\_cov\_35.373741 3744-3750. Max. coverage (+): 0. Max coverage (-): 0

Region: NODE\_27951\_length\_3778\_cov\_35.373741 3751-3758. Max. coverage (+): 0. Max coverage (-): 0.03

Region: NODE\_27951\_length\_3778\_cov\_35.373741 3759-3765. Max. coverage (+): 0. Max coverage (-): 0.03

Region: NODE\_27951\_length\_3778\_cov\_35.373741 3766-3773. Max. coverage (+): 0.03. Max coverage (-): 0

Region: NODE\_27951\_length\_3778\_cov\_35.373741 3774-3781. Max. coverage (+): 0.03. Max coverage (-): 0

Region: NODE\_27951\_length\_3778\_cov\_35.373741 3782-3788. Max. coverage (+): 0.03. Max coverage (-): 0

Region: NODE\_27951\_length\_3778\_cov\_35.373741 3789-3796. Max. coverage (+): 0.5. Max coverage (-): 0

Region: NODE\_27951\_length\_3778\_cov\_35.373741 3797-3804. Max. coverage (+): 0.03. Max coverage (-): 0.09

Region: NODE\_27951\_length\_3778\_cov\_35.373741 3805-3811. Max. coverage (+): 0.06. Max coverage (-): 0.13

Region: NODE\_27951\_length\_3778\_cov\_35.373741 3812-3819. Max. coverage (+): 0. Max coverage (-): 0

Region: NODE\_27951\_length\_3778\_cov\_35.373741 3820-3827. Max. coverage (+): 0. Max coverage (-): 0

Region: NODE\_27951\_length\_3778\_cov\_35.373741 3828-. Max. coverage (+): 0. Max coverage (-): 0

RepeatMasker Color Code

**+**

100-98% Identity

<98-95% Identity

<95-90% Identity

<90-85% Identity

<85-80% Identity

<80-75% Identity

<75-70% Identity

<70% Identity

**-**

Gene Set Color Code

**+**

Gene

Pseudogene

Other

**-**

Topology/Coverage Color Code

Coverage Plus Strand

Coverage Minus Strand

Mainstrand: Plus

Mainstrand: Minus

Complementary Strand

Flanking Region  
(if option -flank >0)

Gene Set Annotation  
  
RepeatMasker Annotation  

**1. AlRepB-358**: 1-57 (+), Divergence to consensus: 1.8%  
**2. A-rich**: 349-387 (+), Divergence to consensus: 20.7%  
**3. RTE-2\_AFC**: 2452-2817 (-), Divergence to consensus: 28.7%  
**4. RTE-2\_AFC**: 2791-2886 (-), Divergence to consensus: 24.1%  
**5. A-rich**: 3537-3588 (+), Divergence to consensus: 25.6%

  
Transcription Factor Binding Sites  

**RHOXF1** (Sequence: AGATTA (-): 502)  
**RHOXF1** (Sequence: AGCTTA (-): 578)  
**RHOXF1** (Sequence: AGATTA (-): 1008)  
**RHOXF1** (Sequence: GGCTTA (-): 1700)  
**RHOXF1** (Sequence: GGCTTA (-): 1897)  
**RHOXF1** (Sequence: AGCTCA (-): 1959)  
**RHOXF1** (Sequence: AGCTCA (-): 2026)  
**RHOXF1** (Sequence: GGATCA (-): 2492)  
**RHOXF1** (Sequence: GGCTCA (-): 3118)  
**RHOXF1** (Sequence: AGATTA (-): 3560)  
**RHOXF1** (Sequence: GGCTCA (-): 3731)  
**RHOXF1** (Sequence: TAATCT (+): 341)  
**RHOXF1** (Sequence: TGAGCC (+): 1822)  
**RHOXF1** (Sequence: TGAGCT (+): 2024)  
**RHOXF1** (Sequence: TAAGCT (+): 2442)  
**RHOXF1** (Sequence: TGAGCT (+): 2714)  
**RHOXF1** (Sequence: TAATCT (+): 2742)  
**RHOXF1** (Sequence: TGAGCC (+): 2787)  
**RHOXF1** (Sequence: TGAGCT (+): 2900)  
**RHOXF1** (Sequence: TGAGCT (+): 3317)  
**SOX9** (Sequence: AACAATAA (-): 1250)  
**FOXO3\_mmu** (Sequence: TGTTTTGC (-): 1069)  
**FOXO3\_mmu** (Sequence: TGTTTTCA (-): 3090)  
**FOXO3\_mmu** (Sequence: GGAAAACA (+): 1725)  
**FOXO3\_mmu** (Sequence: TGAAAACA (+): 3574)  
**Nobox** (Sequence: GGTAATTA (-): 664)  
**FOXO1** (Sequence: AAAAACAAC (-): 362)  
**FOXO1** (Sequence: AAAAACAAG (-): 1942)  
**FOXO1** (Sequence: AAAAACAAG (-): 3584)  
**Rhox11** (Sequence: AATACACCA (-): 1424)  
**Gata4** (Sequence: AGATAAG (-): 1220)  
**Sox5** (Sequence: AACAAT (-): 69)  
**Sox5** (Sequence: AACAAT (-): 420)  
**Sox5** (Sequence: AACAAT (-): 1250)  
**Sox5** (Sequence: AACAAT (-): 2629)  
**Sox5** (Sequence: AACAAT (-): 3106)  
**POU5F1** (Sequence: ATGCAAA (+): 1687)
