# Supplementary material for: First characterization of PIWI-interacting RNA clusters in a cichlid fish with a B chromosome
Source: BMC Biol. 2022 Sep 21;20:204. doi: 10.1186/s12915-022-01403-2 (PMC9490952; doi:10.1186/s12915-022-01403-2)
Supplement: Supplementary file 1 — Additional file 1. Zipped folder with fasta and interactive html piRNA cluster information for the A. latifasciata genome. The nomenclature is as follows: number-pirna-cluster_sex_B-presence (f, female; m, male; 0b, without B chromosome; 1b, with B chromosome). [file 12915_2022_1403_MOESM1_ESM.zip › 108_f0b.html]

piRNA cluster 108\_f0b 58


Predicted piRNA cluster no. 108\_f0b
  

Show proTRAC run info
Hide proTRAC run info

/\  
                \_\_\_\_\_\_\_\_\_\_\_\_\_\_\_\_\_\_\_\_\_\_\_/\\_\_\_ /  \\_\_\_\_\_\_\_  
               I                      /  \  /    \      I  
               I     pro             /    \/      \     I  
               I        TRAC        /               \   I  
               I   \_\_\_\_\_\_\_\_\_\_\_\_\_\_\_\_/\_\_\_\_\_\_\_\_\_\_\_\_\_\_\_\_\_\\_ I  
               I   \              /                     I  
               I    \            /                      I  
               I     \  /\      /       V.2.4.2         I  
               I      \/  \    /                        I  
               I\_\_\_\_\_\_\_\_\_\_\_\  /\_\_\_\_\_\_\_\_\_\_\_\_\_\_\_\_\_\_\_\_\_\_\_\_\_I  
                            \/  
  
  
================================= proTRAC ====================================  
VERSION: .......... 2.4.2  
LAST MODIFIED: .... 11. May 2018  
  
Please cite:  
Rosenkranz D, Zischler H. proTRAC - a software for probabilistic piRNA cluster  
detection, visualization and analysis. 2012. BMC Bioinformatics 13:5.  
  
  
Contact:  
David Rosenkranz  
Institute of Organismic and Molecular Evolutionary Biology  
Dept. Anthropology, small RNA group  
Johannes Gutenberg University Mainz  
email: rosenkranz@uni-mainz.de  
  
You can find the latest proTRAC version at:  
http://sourceforge.net/projects/protrac/files  
http://www.smallRNAgroup-mainz.de/software  
==============================================================================  
  
PARAMETERS:  
Map file: ...............piwi-femeas-0B.fa-collapse.map  
Genome file: ............../../../0B\_ala\_genome.fa  
RepeatMasker annotation: Alatifasciata-all0B-maryan-v2.fa\_corrected.out  
GeneSet:................./guest-storage/Data/annotation/Alatifasciata\_all0B\_maryan-v2\_out2017.gff  
  
Significant (p<=0.01) hit density will be calculated based  
on observed hit distribution.  
  
Sliding window size: ........................................ 5000 bp  
Sliding window increament: .................................. 1000 bp  
Normalize each hit by number of genomic hits: ............... yes  
Normalize each hit by number of sequence reads: ............. yes  
Normalize values (-> per million mapped reads): ............. yes  
Min. fraction of hits with 1T(U) or 10A: .................... 0.75  
Alternatively: Min. fraction of hits with 1T(U) and 10A: .... 0.5  
Min. fraction of hits with typical piRNA length: ............ 0.75  
Typical piRNA length: ....................................... 24-32 nt  
Min. size of a piRNA cluster: ............................... 1000 bp.  
Min. number of hits (absolute): ............................. 0  
Min. number of hits (normalized): ........................... 0  
Min. fraction of hits on the mainstrand: .................... 0.75  
Top fraction of mapped sequences (in terms of read counts): . 1%  
Top fraction accounts for max. n% of sequence reads: ........ 90%  
Min. fraction of hits on each arm of a bidirectional cluster: 0.05  
Output html file for each cluster: .......................... yes  
Output a summary table: ..................................... yes  
Output a FASTA file for each cluster (piRNA sequences): ..... yes  
Output a FASTA file comprising cluster sequences: ........... yes  
Output a GTF file for predicted piRNA clusters: ..............yes  
Search DNA motifs in clusters: .............................. yes  
Output flanking sequences: +/- .............................. 0 bp  
Output ~.pTi file: .......................................... no  
==============================================================================  
  
  
Genome size (without gaps): ............ 758543724 bp  
Gaps (N/X/-): .......................... 417479 bp  
Mapped reads: .......................... 13052187  
Non-identical sequences: ............... 3338911  
Genomic hits: .......................... 28737726  
Significant densitiy of mapped reads: .. 470.083249848448 reads/kb

Show proTRAC cluster info
Hide proTRAC cluster info

|  |  |
| --- | --- |
| Location | NODE\_282267\_length\_2958\_cov\_19.565924 |
| Coordinates | 1-3024 |
| Size [bp] | 3024 |
| Sequence hit loci | 979 |
| Mapped reads (normalized) | 2524.6 |
| Mapped reads (normalized) per kb | 834.9 |
| Normalized reads with 1T (1U) | 75.2% |
| Normalized reads with 10A | 15.8% |
| Normalized reads with length 24-32 nt | 97.3% |
| Normalized reads on the main strand(s) | 97% |
| Predicted directionality | mono:minus |

100%

0%

1T (1U)  
reads

10A reads

24-32 nt  
reads

reads on mainstrand

**Either the amount of reads with 1T (1U) OR 10A has to exceed 75% (set with option: -1Tor10A)  
Alternatively the amount of reads with 1T (1U) AND 10A has to exceed 50% (set with option: -1Tand10A)  
Minimum amount of reads with preferred size is 75% (set with option: -pisize)  
Minimum amount of reads on the main strand(s) is 75% (set with option: -clstrand)**

Show read coverage
Hide read coverage

WHAT DO I SEE HERE?  
This chart shows the location of mapped sequence reads within a predicted piRNA cluster. The color refers to the number of genomic hits produced by the sequence read in question. A dark red bar indicates that this sequence read produces many other hits elsewhere in the genome. Many adjacent red or yellow bars can indicate the presence of a multi-copy element such as transposons or rRNA genes. A dark green bar indicates that this sequence read maps uniquely to this locus.

1 hit

2-5 hits

6-10 hits

11-20 hits

21-50 hits

51-100 hits

> 100 hits

NODE\_282267\_length\_2958\_cov\_19.565924

1

3024

Gene Set

RepeatMasker

Mapped  
Reads

38.61

plus strand

minus strand

38.61

Region: NODE\_282267\_length\_2958\_cov\_19.565924 8021-4. Max. coverage (+): 0.06. Max coverage (-): 0.02

Region: NODE\_282267\_length\_2958\_cov\_19.565924 5-10. Max. coverage (+): 0.04. Max coverage (-): 0.04

Region: NODE\_282267\_length\_2958\_cov\_19.565924 11-16. Max. coverage (+): 0.02. Max coverage (-): 0.04

Region: NODE\_282267\_length\_2958\_cov\_19.565924 17-22. Max. coverage (+): 0.04. Max coverage (-): 0.02

Region: NODE\_282267\_length\_2958\_cov\_19.565924 23-28. Max. coverage (+): 0. Max coverage (-): 27.31

Region: NODE\_282267\_length\_2958\_cov\_19.565924 29-34. Max. coverage (+): 0.02. Max coverage (-): 1.19

Region: NODE\_282267\_length\_2958\_cov\_19.565924 35-40. Max. coverage (+): 0. Max coverage (-): 0.15

Region: NODE\_282267\_length\_2958\_cov\_19.565924 41-46. Max. coverage (+): 0. Max coverage (-): 0.15

Region: NODE\_282267\_length\_2958\_cov\_19.565924 47-52. Max. coverage (+): 0. Max coverage (-): 0.08

Region: NODE\_282267\_length\_2958\_cov\_19.565924 53-58. Max. coverage (+): 0. Max coverage (-): 0

Region: NODE\_282267\_length\_2958\_cov\_19.565924 59-64. Max. coverage (+): 0. Max coverage (-): 0

Region: NODE\_282267\_length\_2958\_cov\_19.565924 65-70. Max. coverage (+): 0. Max coverage (-): 0.38

Region: NODE\_282267\_length\_2958\_cov\_19.565924 71-76. Max. coverage (+): 0. Max coverage (-): 0.08

Region: NODE\_282267\_length\_2958\_cov\_19.565924 77-82. Max. coverage (+): 0. Max coverage (-): 0

Region: NODE\_282267\_length\_2958\_cov\_19.565924 83-88. Max. coverage (+): 0. Max coverage (-): 0.77

Region: NODE\_282267\_length\_2958\_cov\_19.565924 89-94. Max. coverage (+): 0. Max coverage (-): 0.11

Region: NODE\_282267\_length\_2958\_cov\_19.565924 95-100. Max. coverage (+): 0. Max coverage (-): 2.22

Region: NODE\_282267\_length\_2958\_cov\_19.565924 101-106. Max. coverage (+): 0. Max coverage (-): 0.15

Region: NODE\_282267\_length\_2958\_cov\_19.565924 107-112. Max. coverage (+): 0. Max coverage (-): 0.08

Region: NODE\_282267\_length\_2958\_cov\_19.565924 113-118. Max. coverage (+): 0. Max coverage (-): 0

Region: NODE\_282267\_length\_2958\_cov\_19.565924 119-124. Max. coverage (+): 0. Max coverage (-): 0.46

Region: NODE\_282267\_length\_2958\_cov\_19.565924 125-131. Max. coverage (+): 0. Max coverage (-): 9.35

Region: NODE\_282267\_length\_2958\_cov\_19.565924 132-137. Max. coverage (+): 0. Max coverage (-): 8.43

Region: NODE\_282267\_length\_2958\_cov\_19.565924 138-143. Max. coverage (+): 0. Max coverage (-): 1.57

Region: NODE\_282267\_length\_2958\_cov\_19.565924 144-149. Max. coverage (+): 0. Max coverage (-): 0

Region: NODE\_282267\_length\_2958\_cov\_19.565924 150-155. Max. coverage (+): 0. Max coverage (-): 0.15

Region: NODE\_282267\_length\_2958\_cov\_19.565924 156-161. Max. coverage (+): 0. Max coverage (-): 0.23

Region: NODE\_282267\_length\_2958\_cov\_19.565924 162-167. Max. coverage (+): 0. Max coverage (-): 0.08

Region: NODE\_282267\_length\_2958\_cov\_19.565924 168-173. Max. coverage (+): 0. Max coverage (-): 18.73

Region: NODE\_282267\_length\_2958\_cov\_19.565924 174-179. Max. coverage (+): 0. Max coverage (-): 16.36

Region: NODE\_282267\_length\_2958\_cov\_19.565924 180-185. Max. coverage (+): 0. Max coverage (-): 38.38

Region: NODE\_282267\_length\_2958\_cov\_19.565924 186-191. Max. coverage (+): 0.04. Max coverage (-): 38.61

Region: NODE\_282267\_length\_2958\_cov\_19.565924 192-197. Max. coverage (+): 0.11. Max coverage (-): 0

Region: NODE\_282267\_length\_2958\_cov\_19.565924 198-203. Max. coverage (+): 0. Max coverage (-): 0.08

Region: NODE\_282267\_length\_2958\_cov\_19.565924 204-209. Max. coverage (+): 0. Max coverage (-): 0.23

Region: NODE\_282267\_length\_2958\_cov\_19.565924 210-215. Max. coverage (+): 0. Max coverage (-): 0

Region: NODE\_282267\_length\_2958\_cov\_19.565924 216-221. Max. coverage (+): 0. Max coverage (-): 0

Region: NODE\_282267\_length\_2958\_cov\_19.565924 222-227. Max. coverage (+): 0. Max coverage (-): 0.61

Region: NODE\_282267\_length\_2958\_cov\_19.565924 228-233. Max. coverage (+): 0. Max coverage (-): 0.61

Region: NODE\_282267\_length\_2958\_cov\_19.565924 234-239. Max. coverage (+): 0. Max coverage (-): 0.54

Region: NODE\_282267\_length\_2958\_cov\_19.565924 240-245. Max. coverage (+): 0. Max coverage (-): 0.15

Region: NODE\_282267\_length\_2958\_cov\_19.565924 246-251. Max. coverage (+): 0.15. Max coverage (-): 0.08

Region: NODE\_282267\_length\_2958\_cov\_19.565924 252-258. Max. coverage (+): 0.15. Max coverage (-): 0

Region: NODE\_282267\_length\_2958\_cov\_19.565924 259-264. Max. coverage (+): 0. Max coverage (-): 3.14

Region: NODE\_282267\_length\_2958\_cov\_19.565924 265-270. Max. coverage (+): 0. Max coverage (-): 29.42

Region: NODE\_282267\_length\_2958\_cov\_19.565924 271-276. Max. coverage (+): 0. Max coverage (-): 2.99

Region: NODE\_282267\_length\_2958\_cov\_19.565924 277-282. Max. coverage (+): 0. Max coverage (-): 0.08

Region: NODE\_282267\_length\_2958\_cov\_19.565924 283-288. Max. coverage (+): 0.04. Max coverage (-): 0

Region: NODE\_282267\_length\_2958\_cov\_19.565924 289-294. Max. coverage (+): 0. Max coverage (-): 0

Region: NODE\_282267\_length\_2958\_cov\_19.565924 295-300. Max. coverage (+): 0. Max coverage (-): 0

Region: NODE\_282267\_length\_2958\_cov\_19.565924 301-306. Max. coverage (+): 0. Max coverage (-): 0.15

Region: NODE\_282267\_length\_2958\_cov\_19.565924 307-312. Max. coverage (+): 0. Max coverage (-): 0.15

Region: NODE\_282267\_length\_2958\_cov\_19.565924 313-318. Max. coverage (+): 0. Max coverage (-): 0

Region: NODE\_282267\_length\_2958\_cov\_19.565924 319-324. Max. coverage (+): 0. Max coverage (-): 0.08

Region: NODE\_282267\_length\_2958\_cov\_19.565924 325-330. Max. coverage (+): 0. Max coverage (-): 0.84

Region: NODE\_282267\_length\_2958\_cov\_19.565924 331-336. Max. coverage (+): 0. Max coverage (-): 0

Region: NODE\_282267\_length\_2958\_cov\_19.565924 337-342. Max. coverage (+): 0. Max coverage (-): 0

Region: NODE\_282267\_length\_2958\_cov\_19.565924 343-348. Max. coverage (+): 0. Max coverage (-): 0.15

Region: NODE\_282267\_length\_2958\_cov\_19.565924 349-354. Max. coverage (+): 0. Max coverage (-): 0

Region: NODE\_282267\_length\_2958\_cov\_19.565924 355-360. Max. coverage (+): 0. Max coverage (-): 0

Region: NODE\_282267\_length\_2958\_cov\_19.565924 361-366. Max. coverage (+): 0. Max coverage (-): 1.84

Region: NODE\_282267\_length\_2958\_cov\_19.565924 367-372. Max. coverage (+): 0. Max coverage (-): 1.07

Region: NODE\_282267\_length\_2958\_cov\_19.565924 373-378. Max. coverage (+): 0. Max coverage (-): 0.46

Region: NODE\_282267\_length\_2958\_cov\_19.565924 379-385. Max. coverage (+): 0. Max coverage (-): 0.08

Region: NODE\_282267\_length\_2958\_cov\_19.565924 386-391. Max. coverage (+): 0. Max coverage (-): 0

Region: NODE\_282267\_length\_2958\_cov\_19.565924 392-397. Max. coverage (+): 0. Max coverage (-): 0

Region: NODE\_282267\_length\_2958\_cov\_19.565924 398-403. Max. coverage (+): 0. Max coverage (-): 0

Region: NODE\_282267\_length\_2958\_cov\_19.565924 404-409. Max. coverage (+): 0. Max coverage (-): 0.23

Region: NODE\_282267\_length\_2958\_cov\_19.565924 410-415. Max. coverage (+): 0. Max coverage (-): 0.38

Region: NODE\_282267\_length\_2958\_cov\_19.565924 416-421. Max. coverage (+): 0. Max coverage (-): 0.46

Region: NODE\_282267\_length\_2958\_cov\_19.565924 422-427. Max. coverage (+): 0. Max coverage (-): 0

Region: NODE\_282267\_length\_2958\_cov\_19.565924 428-433. Max. coverage (+): 0. Max coverage (-): 0

Region: NODE\_282267\_length\_2958\_cov\_19.565924 434-439. Max. coverage (+): 0. Max coverage (-): 0

Region: NODE\_282267\_length\_2958\_cov\_19.565924 440-445. Max. coverage (+): 0. Max coverage (-): 0.15

Region: NODE\_282267\_length\_2958\_cov\_19.565924 446-451. Max. coverage (+): 0. Max coverage (-): 0

Region: NODE\_282267\_length\_2958\_cov\_19.565924 452-457. Max. coverage (+): 0. Max coverage (-): 0

Region: NODE\_282267\_length\_2958\_cov\_19.565924 458-463. Max. coverage (+): 0. Max coverage (-): 0.23

Region: NODE\_282267\_length\_2958\_cov\_19.565924 464-469. Max. coverage (+): 0. Max coverage (-): 0.23

Region: NODE\_282267\_length\_2958\_cov\_19.565924 470-475. Max. coverage (+): 0. Max coverage (-): 0

Region: NODE\_282267\_length\_2958\_cov\_19.565924 476-481. Max. coverage (+): 0. Max coverage (-): 0

Region: NODE\_282267\_length\_2958\_cov\_19.565924 482-487. Max. coverage (+): 0. Max coverage (-): 0

Region: NODE\_282267\_length\_2958\_cov\_19.565924 488-493. Max. coverage (+): 0. Max coverage (-): 0

Region: NODE\_282267\_length\_2958\_cov\_19.565924 494-499. Max. coverage (+): 0. Max coverage (-): 0

Region: NODE\_282267\_length\_2958\_cov\_19.565924 500-506. Max. coverage (+): 0. Max coverage (-): 0

Region: NODE\_282267\_length\_2958\_cov\_19.565924 507-512. Max. coverage (+): 0. Max coverage (-): 0

Region: NODE\_282267\_length\_2958\_cov\_19.565924 513-518. Max. coverage (+): 0. Max coverage (-): 0

Region: NODE\_282267\_length\_2958\_cov\_19.565924 519-524. Max. coverage (+): 0. Max coverage (-): 0

Region: NODE\_282267\_length\_2958\_cov\_19.565924 525-530. Max. coverage (+): 0. Max coverage (-): 0

Region: NODE\_282267\_length\_2958\_cov\_19.565924 531-536. Max. coverage (+): 0. Max coverage (-): 0

Region: NODE\_282267\_length\_2958\_cov\_19.565924 537-542. Max. coverage (+): 0. Max coverage (-): 0.23

Region: NODE\_282267\_length\_2958\_cov\_19.565924 543-548. Max. coverage (+): 0. Max coverage (-): 0.31

Region: NODE\_282267\_length\_2958\_cov\_19.565924 549-554. Max. coverage (+): 0. Max coverage (-): 0

Region: NODE\_282267\_length\_2958\_cov\_19.565924 555-560. Max. coverage (+): 0. Max coverage (-): 0

Region: NODE\_282267\_length\_2958\_cov\_19.565924 561-566. Max. coverage (+): 0. Max coverage (-): 0

Region: NODE\_282267\_length\_2958\_cov\_19.565924 567-572. Max. coverage (+): 0. Max coverage (-): 0.08

Region: NODE\_282267\_length\_2958\_cov\_19.565924 573-578. Max. coverage (+): 0. Max coverage (-): 0.08

Region: NODE\_282267\_length\_2958\_cov\_19.565924 579-584. Max. coverage (+): 0. Max coverage (-): 0

Region: NODE\_282267\_length\_2958\_cov\_19.565924 585-590. Max. coverage (+): 0. Max coverage (-): 0.08

Region: NODE\_282267\_length\_2958\_cov\_19.565924 591-596. Max. coverage (+): 0. Max coverage (-): 0.15

Region: NODE\_282267\_length\_2958\_cov\_19.565924 597-602. Max. coverage (+): 0. Max coverage (-): 0

Region: NODE\_282267\_length\_2958\_cov\_19.565924 603-608. Max. coverage (+): 0. Max coverage (-): 0

Region: NODE\_282267\_length\_2958\_cov\_19.565924 609-614. Max. coverage (+): 0. Max coverage (-): 0

Region: NODE\_282267\_length\_2958\_cov\_19.565924 615-620. Max. coverage (+): 0. Max coverage (-): 0

Region: NODE\_282267\_length\_2958\_cov\_19.565924 621-626. Max. coverage (+): 0. Max coverage (-): 0

Region: NODE\_282267\_length\_2958\_cov\_19.565924 627-633. Max. coverage (+): 0. Max coverage (-): 0

Region: NODE\_282267\_length\_2958\_cov\_19.565924 634-639. Max. coverage (+): 0. Max coverage (-): 0

Region: NODE\_282267\_length\_2958\_cov\_19.565924 640-645. Max. coverage (+): 0. Max coverage (-): 0

Region: NODE\_282267\_length\_2958\_cov\_19.565924 646-651. Max. coverage (+): 0. Max coverage (-): 0

Region: NODE\_282267\_length\_2958\_cov\_19.565924 652-657. Max. coverage (+): 0. Max coverage (-): 0

Region: NODE\_282267\_length\_2958\_cov\_19.565924 658-663. Max. coverage (+): 0. Max coverage (-): 0

Region: NODE\_282267\_length\_2958\_cov\_19.565924 664-669. Max. coverage (+): 0. Max coverage (-): 0

Region: NODE\_282267\_length\_2958\_cov\_19.565924 670-675. Max. coverage (+): 0. Max coverage (-): 0

Region: NODE\_282267\_length\_2958\_cov\_19.565924 676-681. Max. coverage (+): 0. Max coverage (-): 0

Region: NODE\_282267\_length\_2958\_cov\_19.565924 682-687. Max. coverage (+): 0. Max coverage (-): 0

Region: NODE\_282267\_length\_2958\_cov\_19.565924 688-693. Max. coverage (+): 0. Max coverage (-): 0

Region: NODE\_282267\_length\_2958\_cov\_19.565924 694-699. Max. coverage (+): 0. Max coverage (-): 0

Region: NODE\_282267\_length\_2958\_cov\_19.565924 700-705. Max. coverage (+): 0. Max coverage (-): 0

Region: NODE\_282267\_length\_2958\_cov\_19.565924 706-711. Max. coverage (+): 0. Max coverage (-): 0

Region: NODE\_282267\_length\_2958\_cov\_19.565924 712-717. Max. coverage (+): 0. Max coverage (-): 0

Region: NODE\_282267\_length\_2958\_cov\_19.565924 718-723. Max. coverage (+): 0. Max coverage (-): 0.08

Region: NODE\_282267\_length\_2958\_cov\_19.565924 724-729. Max. coverage (+): 0. Max coverage (-): 0.38

Region: NODE\_282267\_length\_2958\_cov\_19.565924 730-735. Max. coverage (+): 0. Max coverage (-): 0

Region: NODE\_282267\_length\_2958\_cov\_19.565924 736-741. Max. coverage (+): 0. Max coverage (-): 0

Region: NODE\_282267\_length\_2958\_cov\_19.565924 742-747. Max. coverage (+): 0. Max coverage (-): 0

Region: NODE\_282267\_length\_2958\_cov\_19.565924 748-753. Max. coverage (+): 0. Max coverage (-): 0

Region: NODE\_282267\_length\_2958\_cov\_19.565924 754-760. Max. coverage (+): 0. Max coverage (-): 0

Region: NODE\_282267\_length\_2958\_cov\_19.565924 761-766. Max. coverage (+): 0. Max coverage (-): 0.08

Region: NODE\_282267\_length\_2958\_cov\_19.565924 767-772. Max. coverage (+): 0. Max coverage (-): 0

Region: NODE\_282267\_length\_2958\_cov\_19.565924 773-778. Max. coverage (+): 0. Max coverage (-): 0.15

Region: NODE\_282267\_length\_2958\_cov\_19.565924 779-784. Max. coverage (+): 0.08. Max coverage (-): 0.15

Region: NODE\_282267\_length\_2958\_cov\_19.565924 785-790. Max. coverage (+): 0. Max coverage (-): 0

Region: NODE\_282267\_length\_2958\_cov\_19.565924 791-796. Max. coverage (+): 0. Max coverage (-): 0

Region: NODE\_282267\_length\_2958\_cov\_19.565924 797-802. Max. coverage (+): 0. Max coverage (-): 0

Region: NODE\_282267\_length\_2958\_cov\_19.565924 803-808. Max. coverage (+): 0. Max coverage (-): 0.08

Region: NODE\_282267\_length\_2958\_cov\_19.565924 809-814. Max. coverage (+): 0. Max coverage (-): 0

Region: NODE\_282267\_length\_2958\_cov\_19.565924 815-820. Max. coverage (+): 0. Max coverage (-): 0

Region: NODE\_282267\_length\_2958\_cov\_19.565924 821-826. Max. coverage (+): 0. Max coverage (-): 0

Region: NODE\_282267\_length\_2958\_cov\_19.565924 827-832. Max. coverage (+): 0. Max coverage (-): 0

Region: NODE\_282267\_length\_2958\_cov\_19.565924 833-838. Max. coverage (+): 0. Max coverage (-): 0

Region: NODE\_282267\_length\_2958\_cov\_19.565924 839-844. Max. coverage (+): 0. Max coverage (-): 0

Region: NODE\_282267\_length\_2958\_cov\_19.565924 845-850. Max. coverage (+): 0. Max coverage (-): 0

Region: NODE\_282267\_length\_2958\_cov\_19.565924 851-856. Max. coverage (+): 0. Max coverage (-): 0

Region: NODE\_282267\_length\_2958\_cov\_19.565924 857-862. Max. coverage (+): 0. Max coverage (-): 0

Region: NODE\_282267\_length\_2958\_cov\_19.565924 863-868. Max. coverage (+): 0. Max coverage (-): 0

Region: NODE\_282267\_length\_2958\_cov\_19.565924 869-874. Max. coverage (+): 0. Max coverage (-): 0

Region: NODE\_282267\_length\_2958\_cov\_19.565924 875-880. Max. coverage (+): 0. Max coverage (-): 0

Region: NODE\_282267\_length\_2958\_cov\_19.565924 881-887. Max. coverage (+): 0. Max coverage (-): 0

Region: NODE\_282267\_length\_2958\_cov\_19.565924 888-893. Max. coverage (+): 0.03. Max coverage (-): 0.03

Region: NODE\_282267\_length\_2958\_cov\_19.565924 894-899. Max. coverage (+): 0. Max coverage (-): 0

Region: NODE\_282267\_length\_2958\_cov\_19.565924 900-905. Max. coverage (+): 0. Max coverage (-): 0

Region: NODE\_282267\_length\_2958\_cov\_19.565924 906-911. Max. coverage (+): 0. Max coverage (-): 0

Region: NODE\_282267\_length\_2958\_cov\_19.565924 912-917. Max. coverage (+): 0. Max coverage (-): 0.08

Region: NODE\_282267\_length\_2958\_cov\_19.565924 918-923. Max. coverage (+): 0. Max coverage (-): 0

Region: NODE\_282267\_length\_2958\_cov\_19.565924 924-929. Max. coverage (+): 0. Max coverage (-): 0.01

Region: NODE\_282267\_length\_2958\_cov\_19.565924 930-935. Max. coverage (+): 0. Max coverage (-): 0

Region: NODE\_282267\_length\_2958\_cov\_19.565924 936-941. Max. coverage (+): 0.07. Max coverage (-): 0

Region: NODE\_282267\_length\_2958\_cov\_19.565924 942-947. Max. coverage (+): 0.04. Max coverage (-): 0

Region: NODE\_282267\_length\_2958\_cov\_19.565924 948-953. Max. coverage (+): 0.04. Max coverage (-): 0

Region: NODE\_282267\_length\_2958\_cov\_19.565924 954-959. Max. coverage (+): 0. Max coverage (-): 0.61

Region: NODE\_282267\_length\_2958\_cov\_19.565924 960-965. Max. coverage (+): 0. Max coverage (-): 1.46

Region: NODE\_282267\_length\_2958\_cov\_19.565924 966-971. Max. coverage (+): 0. Max coverage (-): 0.69

Region: NODE\_282267\_length\_2958\_cov\_19.565924 972-977. Max. coverage (+): 0. Max coverage (-): 0

Region: NODE\_282267\_length\_2958\_cov\_19.565924 978-983. Max. coverage (+): 0. Max coverage (-): 0.08

Region: NODE\_282267\_length\_2958\_cov\_19.565924 984-989. Max. coverage (+): 0. Max coverage (-): 0

Region: NODE\_282267\_length\_2958\_cov\_19.565924 990-995. Max. coverage (+): 0. Max coverage (-): 0

Region: NODE\_282267\_length\_2958\_cov\_19.565924 996-1001. Max. coverage (+): 0. Max coverage (-): 0

Region: NODE\_282267\_length\_2958\_cov\_19.565924 1002-1007. Max. coverage (+): 0. Max coverage (-): 0

Region: NODE\_282267\_length\_2958\_cov\_19.565924 1008-1014. Max. coverage (+): 0. Max coverage (-): 0

Region: NODE\_282267\_length\_2958\_cov\_19.565924 1015-1020. Max. coverage (+): 0. Max coverage (-): 0.08

Region: NODE\_282267\_length\_2958\_cov\_19.565924 1021-1026. Max. coverage (+): 0. Max coverage (-): 0

Region: NODE\_282267\_length\_2958\_cov\_19.565924 1027-1032. Max. coverage (+): 0. Max coverage (-): 0

Region: NODE\_282267\_length\_2958\_cov\_19.565924 1033-1038. Max. coverage (+): 0. Max coverage (-): 0.08

Region: NODE\_282267\_length\_2958\_cov\_19.565924 1039-1044. Max. coverage (+): 0. Max coverage (-): 0.08

Region: NODE\_282267\_length\_2958\_cov\_19.565924 1045-1050. Max. coverage (+): 0. Max coverage (-): 0

Region: NODE\_282267\_length\_2958\_cov\_19.565924 1051-1056. Max. coverage (+): 0. Max coverage (-): 0

Region: NODE\_282267\_length\_2958\_cov\_19.565924 1057-1062. Max. coverage (+): 0. Max coverage (-): 0

Region: NODE\_282267\_length\_2958\_cov\_19.565924 1063-1068. Max. coverage (+): 0. Max coverage (-): 0

Region: NODE\_282267\_length\_2958\_cov\_19.565924 1069-1074. Max. coverage (+): 0. Max coverage (-): 0

Region: NODE\_282267\_length\_2958\_cov\_19.565924 1075-1080. Max. coverage (+): 0.08. Max coverage (-): 0

Region: NODE\_282267\_length\_2958\_cov\_19.565924 1081-1086. Max. coverage (+): 0.08. Max coverage (-): 0

Region: NODE\_282267\_length\_2958\_cov\_19.565924 1087-1092. Max. coverage (+): 0. Max coverage (-): 0.04

Region: NODE\_282267\_length\_2958\_cov\_19.565924 1093-1098. Max. coverage (+): 0. Max coverage (-): 0.03

Region: NODE\_282267\_length\_2958\_cov\_19.565924 1099-1104. Max. coverage (+): 0. Max coverage (-): 0.03

Region: NODE\_282267\_length\_2958\_cov\_19.565924 1105-1110. Max. coverage (+): 0. Max coverage (-): 0

Region: NODE\_282267\_length\_2958\_cov\_19.565924 1111-1116. Max. coverage (+): 0.01. Max coverage (-): 0

Region: NODE\_282267\_length\_2958\_cov\_19.565924 1117-1122. Max. coverage (+): 0.01. Max coverage (-): 0

Region: NODE\_282267\_length\_2958\_cov\_19.565924 1123-1128. Max. coverage (+): 0.01. Max coverage (-): 0

Region: NODE\_282267\_length\_2958\_cov\_19.565924 1129-1134. Max. coverage (+): 0. Max coverage (-): 0

Region: NODE\_282267\_length\_2958\_cov\_19.565924 1135-1141. Max. coverage (+): 0. Max coverage (-): 0

Region: NODE\_282267\_length\_2958\_cov\_19.565924 1142-1147. Max. coverage (+): 0. Max coverage (-): 0

Region: NODE\_282267\_length\_2958\_cov\_19.565924 1148-1153. Max. coverage (+): 0. Max coverage (-): 0

Region: NODE\_282267\_length\_2958\_cov\_19.565924 1154-1159. Max. coverage (+): 0. Max coverage (-): 0

Region: NODE\_282267\_length\_2958\_cov\_19.565924 1160-1165. Max. coverage (+): 0. Max coverage (-): 0

Region: NODE\_282267\_length\_2958\_cov\_19.565924 1166-1171. Max. coverage (+): 0. Max coverage (-): 0

Region: NODE\_282267\_length\_2958\_cov\_19.565924 1172-1177. Max. coverage (+): 0. Max coverage (-): 0

Region: NODE\_282267\_length\_2958\_cov\_19.565924 1178-1183. Max. coverage (+): 0. Max coverage (-): 0.13

Region: NODE\_282267\_length\_2958\_cov\_19.565924 1184-1189. Max. coverage (+): 0. Max coverage (-): 0

Region: NODE\_282267\_length\_2958\_cov\_19.565924 1190-1195. Max. coverage (+): 0.03. Max coverage (-): 0

Region: NODE\_282267\_length\_2958\_cov\_19.565924 1196-1201. Max. coverage (+): 0.04. Max coverage (-): 0

Region: NODE\_282267\_length\_2958\_cov\_19.565924 1202-1207. Max. coverage (+): 0. Max coverage (-): 0

Region: NODE\_282267\_length\_2958\_cov\_19.565924 1208-1213. Max. coverage (+): 0. Max coverage (-): 0

Region: NODE\_282267\_length\_2958\_cov\_19.565924 1214-1219. Max. coverage (+): 0. Max coverage (-): 0.15

Region: NODE\_282267\_length\_2958\_cov\_19.565924 1220-1225. Max. coverage (+): 0. Max coverage (-): 0.46

Region: NODE\_282267\_length\_2958\_cov\_19.565924 1226-1231. Max. coverage (+): 0. Max coverage (-): 0.15

Region: NODE\_282267\_length\_2958\_cov\_19.565924 1232-1237. Max. coverage (+): 0. Max coverage (-): 0

Region: NODE\_282267\_length\_2958\_cov\_19.565924 1238-1243. Max. coverage (+): 0.11. Max coverage (-): 0

Region: NODE\_282267\_length\_2958\_cov\_19.565924 1244-1249. Max. coverage (+): 0.16. Max coverage (-): 0

Region: NODE\_282267\_length\_2958\_cov\_19.565924 1250-1255. Max. coverage (+): 0. Max coverage (-): 0

Region: NODE\_282267\_length\_2958\_cov\_19.565924 1256-1262. Max. coverage (+): 0. Max coverage (-): 0

Region: NODE\_282267\_length\_2958\_cov\_19.565924 1263-1268. Max. coverage (+): 0. Max coverage (-): 0

Region: NODE\_282267\_length\_2958\_cov\_19.565924 1269-1274. Max. coverage (+): 0. Max coverage (-): 0

Region: NODE\_282267\_length\_2958\_cov\_19.565924 1275-1280. Max. coverage (+): 0. Max coverage (-): 0

Region: NODE\_282267\_length\_2958\_cov\_19.565924 1281-1286. Max. coverage (+): 0. Max coverage (-): 0

Region: NODE\_282267\_length\_2958\_cov\_19.565924 1287-1292. Max. coverage (+): 0. Max coverage (-): 0

Region: NODE\_282267\_length\_2958\_cov\_19.565924 1293-1298. Max. coverage (+): 0. Max coverage (-): 0

Region: NODE\_282267\_length\_2958\_cov\_19.565924 1299-1304. Max. coverage (+): 0.01. Max coverage (-): 0

Region: NODE\_282267\_length\_2958\_cov\_19.565924 1305-1310. Max. coverage (+): 0.01. Max coverage (-): 0

Region: NODE\_282267\_length\_2958\_cov\_19.565924 1311-1316. Max. coverage (+): 0. Max coverage (-): 0.04

Region: NODE\_282267\_length\_2958\_cov\_19.565924 1317-1322. Max. coverage (+): 0. Max coverage (-): 0.02

Region: NODE\_282267\_length\_2958\_cov\_19.565924 1323-1328. Max. coverage (+): 0.02. Max coverage (-): 0.02

Region: NODE\_282267\_length\_2958\_cov\_19.565924 1329-1334. Max. coverage (+): 0. Max coverage (-): 0

Region: NODE\_282267\_length\_2958\_cov\_19.565924 1335-1340. Max. coverage (+): 0. Max coverage (-): 0.03

Region: NODE\_282267\_length\_2958\_cov\_19.565924 1341-1346. Max. coverage (+): 0.03. Max coverage (-): 0.01

Region: NODE\_282267\_length\_2958\_cov\_19.565924 1347-1352. Max. coverage (+): 0.05. Max coverage (-): 0.03

Region: NODE\_282267\_length\_2958\_cov\_19.565924 1353-1358. Max. coverage (+): 0.11. Max coverage (-): 0.01

Region: NODE\_282267\_length\_2958\_cov\_19.565924 1359-1364. Max. coverage (+): 0.08. Max coverage (-): 0.05

Region: NODE\_282267\_length\_2958\_cov\_19.565924 1365-1370. Max. coverage (+): 0.08. Max coverage (-): 0.07

Region: NODE\_282267\_length\_2958\_cov\_19.565924 1371-1376. Max. coverage (+): 0.02. Max coverage (-): 0.02

Region: NODE\_282267\_length\_2958\_cov\_19.565924 1377-1382. Max. coverage (+): 0.03. Max coverage (-): 0

Region: NODE\_282267\_length\_2958\_cov\_19.565924 1383-1389. Max. coverage (+): 0.03. Max coverage (-): 0

Region: NODE\_282267\_length\_2958\_cov\_19.565924 1390-1395. Max. coverage (+): 0. Max coverage (-): 0

Region: NODE\_282267\_length\_2958\_cov\_19.565924 1396-1401. Max. coverage (+): 0. Max coverage (-): 0

Region: NODE\_282267\_length\_2958\_cov\_19.565924 1402-1407. Max. coverage (+): 0. Max coverage (-): 0

Region: NODE\_282267\_length\_2958\_cov\_19.565924 1408-1413. Max. coverage (+): 0.01. Max coverage (-): 0

Region: NODE\_282267\_length\_2958\_cov\_19.565924 1414-1419. Max. coverage (+): 0.2. Max coverage (-): 0.02

Region: NODE\_282267\_length\_2958\_cov\_19.565924 1420-1425. Max. coverage (+): 0.22. Max coverage (-): 0.09

Region: NODE\_282267\_length\_2958\_cov\_19.565924 1426-1431. Max. coverage (+): 0.05. Max coverage (-): 0.02

Region: NODE\_282267\_length\_2958\_cov\_19.565924 1432-1437. Max. coverage (+): 0.01. Max coverage (-): 0.18

Region: NODE\_282267\_length\_2958\_cov\_19.565924 1438-1443. Max. coverage (+): 0.04. Max coverage (-): 0.48

Region: NODE\_282267\_length\_2958\_cov\_19.565924 1444-1449. Max. coverage (+): 0. Max coverage (-): 0.22

Region: NODE\_282267\_length\_2958\_cov\_19.565924 1450-1455. Max. coverage (+): 0.03. Max coverage (-): 0.04

Region: NODE\_282267\_length\_2958\_cov\_19.565924 1456-1461. Max. coverage (+): 0.01. Max coverage (-): 0.11

Region: NODE\_282267\_length\_2958\_cov\_19.565924 1462-1467. Max. coverage (+): 0. Max coverage (-): 0

Region: NODE\_282267\_length\_2958\_cov\_19.565924 1468-1473. Max. coverage (+): 0. Max coverage (-): 0

Region: NODE\_282267\_length\_2958\_cov\_19.565924 1474-1479. Max. coverage (+): 0. Max coverage (-): 0

Region: NODE\_282267\_length\_2958\_cov\_19.565924 1480-1485. Max. coverage (+): 0. Max coverage (-): 0

Region: NODE\_282267\_length\_2958\_cov\_19.565924 1486-1491. Max. coverage (+): 0. Max coverage (-): 0

Region: NODE\_282267\_length\_2958\_cov\_19.565924 1492-1497. Max. coverage (+): 0. Max coverage (-): 0

Region: NODE\_282267\_length\_2958\_cov\_19.565924 1498-1503. Max. coverage (+): 0. Max coverage (-): 0

Region: NODE\_282267\_length\_2958\_cov\_19.565924 1504-1509. Max. coverage (+): 0. Max coverage (-): 3.06

Region: NODE\_282267\_length\_2958\_cov\_19.565924 1510-1516. Max. coverage (+): 0. Max coverage (-): 4.67

Region: NODE\_282267\_length\_2958\_cov\_19.565924 1517-1522. Max. coverage (+): 0. Max coverage (-): 0

Region: NODE\_282267\_length\_2958\_cov\_19.565924 1523-1528. Max. coverage (+): 0.02. Max coverage (-): 0

Region: NODE\_282267\_length\_2958\_cov\_19.565924 1529-1534. Max. coverage (+): 0.03. Max coverage (-): 0.02

Region: NODE\_282267\_length\_2958\_cov\_19.565924 1535-1540. Max. coverage (+): 0. Max coverage (-): 0

Region: NODE\_282267\_length\_2958\_cov\_19.565924 1541-1546. Max. coverage (+): 0. Max coverage (-): 0

Region: NODE\_282267\_length\_2958\_cov\_19.565924 1547-1552. Max. coverage (+): 0.01. Max coverage (-): 0.04

Region: NODE\_282267\_length\_2958\_cov\_19.565924 1553-1558. Max. coverage (+): 0.01. Max coverage (-): 0.04

Region: NODE\_282267\_length\_2958\_cov\_19.565924 1559-1564. Max. coverage (+): 0. Max coverage (-): 0.03

Region: NODE\_282267\_length\_2958\_cov\_19.565924 1565-1570. Max. coverage (+): 0.41. Max coverage (-): 0.01

Region: NODE\_282267\_length\_2958\_cov\_19.565924 1571-1576. Max. coverage (+): 0.28. Max coverage (-): 0.02

Region: NODE\_282267\_length\_2958\_cov\_19.565924 1577-1582. Max. coverage (+): 0.06. Max coverage (-): 0.02

Region: NODE\_282267\_length\_2958\_cov\_19.565924 1583-1588. Max. coverage (+): 0.02. Max coverage (-): 0

Region: NODE\_282267\_length\_2958\_cov\_19.565924 1589-1594. Max. coverage (+): 0.01. Max coverage (-): 0

Region: NODE\_282267\_length\_2958\_cov\_19.565924 1595-1600. Max. coverage (+): 0.03. Max coverage (-): 0.04

Region: NODE\_282267\_length\_2958\_cov\_19.565924 1601-1606. Max. coverage (+): 0.04. Max coverage (-): 0.03

Region: NODE\_282267\_length\_2958\_cov\_19.565924 1607-1612. Max. coverage (+): 0. Max coverage (-): 0.04

Region: NODE\_282267\_length\_2958\_cov\_19.565924 1613-1618. Max. coverage (+): 0. Max coverage (-): 0.14

Region: NODE\_282267\_length\_2958\_cov\_19.565924 1619-1624. Max. coverage (+): 0. Max coverage (-): 0.01

Region: NODE\_282267\_length\_2958\_cov\_19.565924 1625-1630. Max. coverage (+): 0.02. Max coverage (-): 0

Region: NODE\_282267\_length\_2958\_cov\_19.565924 1631-1636. Max. coverage (+): 0.03. Max coverage (-): 0

Region: NODE\_282267\_length\_2958\_cov\_19.565924 1637-1643. Max. coverage (+): 0.04. Max coverage (-): 0

Region: NODE\_282267\_length\_2958\_cov\_19.565924 1644-1649. Max. coverage (+): 0.03. Max coverage (-): 0

Region: NODE\_282267\_length\_2958\_cov\_19.565924 1650-1655. Max. coverage (+): 0. Max coverage (-): 0.02

Region: NODE\_282267\_length\_2958\_cov\_19.565924 1656-1661. Max. coverage (+): 0.02. Max coverage (-): 0.02

Region: NODE\_282267\_length\_2958\_cov\_19.565924 1662-1667. Max. coverage (+): 0.02. Max coverage (-): 0

Region: NODE\_282267\_length\_2958\_cov\_19.565924 1668-1673. Max. coverage (+): 0.09. Max coverage (-): 0

Region: NODE\_282267\_length\_2958\_cov\_19.565924 1674-1679. Max. coverage (+): 0.05. Max coverage (-): 0

Region: NODE\_282267\_length\_2958\_cov\_19.565924 1680-1685. Max. coverage (+): 0.02. Max coverage (-): 0.04

Region: NODE\_282267\_length\_2958\_cov\_19.565924 1686-1691. Max. coverage (+): 0. Max coverage (-): 0.02

Region: NODE\_282267\_length\_2958\_cov\_19.565924 1692-1697. Max. coverage (+): 0. Max coverage (-): 0

Region: NODE\_282267\_length\_2958\_cov\_19.565924 1698-1703. Max. coverage (+): 0. Max coverage (-): 0

Region: NODE\_282267\_length\_2958\_cov\_19.565924 1704-1709. Max. coverage (+): 0. Max coverage (-): 0

Region: NODE\_282267\_length\_2958\_cov\_19.565924 1710-1715. Max. coverage (+): 0. Max coverage (-): 0

Region: NODE\_282267\_length\_2958\_cov\_19.565924 1716-1721. Max. coverage (+): 0. Max coverage (-): 0

Region: NODE\_282267\_length\_2958\_cov\_19.565924 1722-1727. Max. coverage (+): 0. Max coverage (-): 0.03

Region: NODE\_282267\_length\_2958\_cov\_19.565924 1728-1733. Max. coverage (+): 0. Max coverage (-): 0.04

Region: NODE\_282267\_length\_2958\_cov\_19.565924 1734-1739. Max. coverage (+): 0.01. Max coverage (-): 0.02

Region: NODE\_282267\_length\_2958\_cov\_19.565924 1740-1745. Max. coverage (+): 0.09. Max coverage (-): 0

Region: NODE\_282267\_length\_2958\_cov\_19.565924 1746-1751. Max. coverage (+): 0.03. Max coverage (-): 0

Region: NODE\_282267\_length\_2958\_cov\_19.565924 1752-1757. Max. coverage (+): 0. Max coverage (-): 0

Region: NODE\_282267\_length\_2958\_cov\_19.565924 1758-1763. Max. coverage (+): 0. Max coverage (-): 0

Region: NODE\_282267\_length\_2958\_cov\_19.565924 1764-1770. Max. coverage (+): 0. Max coverage (-): 0

Region: NODE\_282267\_length\_2958\_cov\_19.565924 1771-1776. Max. coverage (+): 0. Max coverage (-): 0

Region: NODE\_282267\_length\_2958\_cov\_19.565924 1777-1782. Max. coverage (+): 0. Max coverage (-): 0

Region: NODE\_282267\_length\_2958\_cov\_19.565924 1783-1788. Max. coverage (+): 0.02. Max coverage (-): 0

Region: NODE\_282267\_length\_2958\_cov\_19.565924 1789-1794. Max. coverage (+): 0.03. Max coverage (-): 0

Region: NODE\_282267\_length\_2958\_cov\_19.565924 1795-1800. Max. coverage (+): 0. Max coverage (-): 0

Region: NODE\_282267\_length\_2958\_cov\_19.565924 1801-1806. Max. coverage (+): 0. Max coverage (-): 0

Region: NODE\_282267\_length\_2958\_cov\_19.565924 1807-1812. Max. coverage (+): 0. Max coverage (-): 0

Region: NODE\_282267\_length\_2958\_cov\_19.565924 1813-1818. Max. coverage (+): 0. Max coverage (-): 0

Region: NODE\_282267\_length\_2958\_cov\_19.565924 1819-1824. Max. coverage (+): 0.06. Max coverage (-): 0

Region: NODE\_282267\_length\_2958\_cov\_19.565924 1825-1830. Max. coverage (+): 0.08. Max coverage (-): 0.2

Region: NODE\_282267\_length\_2958\_cov\_19.565924 1831-1836. Max. coverage (+): 0.01. Max coverage (-): 0.14

Region: NODE\_282267\_length\_2958\_cov\_19.565924 1837-1842. Max. coverage (+): 0.13. Max coverage (-): 0.01

Region: NODE\_282267\_length\_2958\_cov\_19.565924 1843-1848. Max. coverage (+): 0.02. Max coverage (-): 0.01

Region: NODE\_282267\_length\_2958\_cov\_19.565924 1849-1854. Max. coverage (+): 0.02. Max coverage (-): 0

Region: NODE\_282267\_length\_2958\_cov\_19.565924 1855-1860. Max. coverage (+): 0. Max coverage (-): 0

Region: NODE\_282267\_length\_2958\_cov\_19.565924 1861-1866. Max. coverage (+): 0. Max coverage (-): 0

Region: NODE\_282267\_length\_2958\_cov\_19.565924 1867-1872. Max. coverage (+): 0. Max coverage (-): 0.08

Region: NODE\_282267\_length\_2958\_cov\_19.565924 1873-1878. Max. coverage (+): 0. Max coverage (-): 0

Region: NODE\_282267\_length\_2958\_cov\_19.565924 1879-1884. Max. coverage (+): 0.04. Max coverage (-): 0

Region: NODE\_282267\_length\_2958\_cov\_19.565924 1885-1890. Max. coverage (+): 0. Max coverage (-): 0

Region: NODE\_282267\_length\_2958\_cov\_19.565924 1891-1897. Max. coverage (+): 0. Max coverage (-): 0

Region: NODE\_282267\_length\_2958\_cov\_19.565924 1898-1903. Max. coverage (+): 0. Max coverage (-): 0.31

Region: NODE\_282267\_length\_2958\_cov\_19.565924 1904-1909. Max. coverage (+): 0. Max coverage (-): 0.46

Region: NODE\_282267\_length\_2958\_cov\_19.565924 1910-1915. Max. coverage (+): 0. Max coverage (-): 0.01

Region: NODE\_282267\_length\_2958\_cov\_19.565924 1916-1921. Max. coverage (+): 0. Max coverage (-): 0.01

Region: NODE\_282267\_length\_2958\_cov\_19.565924 1922-1927. Max. coverage (+): 0. Max coverage (-): 0.01

Region: NODE\_282267\_length\_2958\_cov\_19.565924 1928-1933. Max. coverage (+): 0. Max coverage (-): 0.01

Region: NODE\_282267\_length\_2958\_cov\_19.565924 1934-1939. Max. coverage (+): 0. Max coverage (-): 0

Region: NODE\_282267\_length\_2958\_cov\_19.565924 1940-1945. Max. coverage (+): 0. Max coverage (-): 0

Region: NODE\_282267\_length\_2958\_cov\_19.565924 1946-1951. Max. coverage (+): 0. Max coverage (-): 0

Region: NODE\_282267\_length\_2958\_cov\_19.565924 1952-1957. Max. coverage (+): 0. Max coverage (-): 0

Region: NODE\_282267\_length\_2958\_cov\_19.565924 1958-1963. Max. coverage (+): 0.01. Max coverage (-): 0.01

Region: NODE\_282267\_length\_2958\_cov\_19.565924 1964-1969. Max. coverage (+): 0.01. Max coverage (-): 0.15

Region: NODE\_282267\_length\_2958\_cov\_19.565924 1970-1975. Max. coverage (+): 0. Max coverage (-): 0.15

Region: NODE\_282267\_length\_2958\_cov\_19.565924 1976-1981. Max. coverage (+): 0. Max coverage (-): 0.08

Region: NODE\_282267\_length\_2958\_cov\_19.565924 1982-1987. Max. coverage (+): 0. Max coverage (-): 0.16

Region: NODE\_282267\_length\_2958\_cov\_19.565924 1988-1993. Max. coverage (+): 0.03. Max coverage (-): 0.16

Region: NODE\_282267\_length\_2958\_cov\_19.565924 1994-1999. Max. coverage (+): 0.03. Max coverage (-): 0.02

Region: NODE\_282267\_length\_2958\_cov\_19.565924 2000-2005. Max. coverage (+): 0. Max coverage (-): 0

Region: NODE\_282267\_length\_2958\_cov\_19.565924 2006-2011. Max. coverage (+): 0. Max coverage (-): 0

Region: NODE\_282267\_length\_2958\_cov\_19.565924 2012-2018. Max. coverage (+): 0. Max coverage (-): 0

Region: NODE\_282267\_length\_2958\_cov\_19.565924 2019-2024. Max. coverage (+): 0. Max coverage (-): 0.03

Region: NODE\_282267\_length\_2958\_cov\_19.565924 2025-2030. Max. coverage (+): 0. Max coverage (-): 0.36

Region: NODE\_282267\_length\_2958\_cov\_19.565924 2031-2036. Max. coverage (+): 0. Max coverage (-): 0.92

Region: NODE\_282267\_length\_2958\_cov\_19.565924 2037-2042. Max. coverage (+): 0. Max coverage (-): 1.02

Region: NODE\_282267\_length\_2958\_cov\_19.565924 2043-2048. Max. coverage (+): 0. Max coverage (-): 0.45

Region: NODE\_282267\_length\_2958\_cov\_19.565924 2049-2054. Max. coverage (+): 0.08. Max coverage (-): 0

Region: NODE\_282267\_length\_2958\_cov\_19.565924 2055-2060. Max. coverage (+): 0.15. Max coverage (-): 0

Region: NODE\_282267\_length\_2958\_cov\_19.565924 2061-2066. Max. coverage (+): 0.05. Max coverage (-): 0.04

Region: NODE\_282267\_length\_2958\_cov\_19.565924 2067-2072. Max. coverage (+): 0. Max coverage (-): 0.16

Region: NODE\_282267\_length\_2958\_cov\_19.565924 2073-2078. Max. coverage (+): 0. Max coverage (-): 0.27

Region: NODE\_282267\_length\_2958\_cov\_19.565924 2079-2084. Max. coverage (+): 0.12. Max coverage (-): 0.05

Region: NODE\_282267\_length\_2958\_cov\_19.565924 2085-2090. Max. coverage (+): 0.11. Max coverage (-): 0.01

Region: NODE\_282267\_length\_2958\_cov\_19.565924 2091-2096. Max. coverage (+): 0.45. Max coverage (-): 0.01

Region: NODE\_282267\_length\_2958\_cov\_19.565924 2097-2102. Max. coverage (+): 0.33. Max coverage (-): 0.01

Region: NODE\_282267\_length\_2958\_cov\_19.565924 2103-2108. Max. coverage (+): 0. Max coverage (-): 0

Region: NODE\_282267\_length\_2958\_cov\_19.565924 2109-2114. Max. coverage (+): 0. Max coverage (-): 0

Region: NODE\_282267\_length\_2958\_cov\_19.565924 2115-2120. Max. coverage (+): 0.01. Max coverage (-): 0

Region: NODE\_282267\_length\_2958\_cov\_19.565924 2121-2126. Max. coverage (+): 0.04. Max coverage (-): 0.01

Region: NODE\_282267\_length\_2958\_cov\_19.565924 2127-2132. Max. coverage (+): 0. Max coverage (-): 0.04

Region: NODE\_282267\_length\_2958\_cov\_19.565924 2133-2138. Max. coverage (+): 0. Max coverage (-): 0.14

Region: NODE\_282267\_length\_2958\_cov\_19.565924 2139-2145. Max. coverage (+): 0. Max coverage (-): 0.16

Region: NODE\_282267\_length\_2958\_cov\_19.565924 2146-2151. Max. coverage (+): 0. Max coverage (-): 0

Region: NODE\_282267\_length\_2958\_cov\_19.565924 2152-2157. Max. coverage (+): 0.04. Max coverage (-): 0

Region: NODE\_282267\_length\_2958\_cov\_19.565924 2158-2163. Max. coverage (+): 0.03. Max coverage (-): 0

Region: NODE\_282267\_length\_2958\_cov\_19.565924 2164-2169. Max. coverage (+): 0. Max coverage (-): 0

Region: NODE\_282267\_length\_2958\_cov\_19.565924 2170-2175. Max. coverage (+): 0.04. Max coverage (-): 0.01

Region: NODE\_282267\_length\_2958\_cov\_19.565924 2176-2181. Max. coverage (+): 0.01. Max coverage (-): 0.09

Region: NODE\_282267\_length\_2958\_cov\_19.565924 2182-2187. Max. coverage (+): 0. Max coverage (-): 3.5

Region: NODE\_282267\_length\_2958\_cov\_19.565924 2188-2193. Max. coverage (+): 0. Max coverage (-): 3.45

Region: NODE\_282267\_length\_2958\_cov\_19.565924 2194-2199. Max. coverage (+): 0.03. Max coverage (-): 0.01

Region: NODE\_282267\_length\_2958\_cov\_19.565924 2200-2205. Max. coverage (+): 0.15. Max coverage (-): 0

Region: NODE\_282267\_length\_2958\_cov\_19.565924 2206-2211. Max. coverage (+): 0. Max coverage (-): 0.02

Region: NODE\_282267\_length\_2958\_cov\_19.565924 2212-2217. Max. coverage (+): 0. Max coverage (-): 0

Region: NODE\_282267\_length\_2958\_cov\_19.565924 2218-2223. Max. coverage (+): 0.02. Max coverage (-): 0

Region: NODE\_282267\_length\_2958\_cov\_19.565924 2224-2229. Max. coverage (+): 0.01. Max coverage (-): 0

Region: NODE\_282267\_length\_2958\_cov\_19.565924 2230-2235. Max. coverage (+): 0.15. Max coverage (-): 0

Region: NODE\_282267\_length\_2958\_cov\_19.565924 2236-2241. Max. coverage (+): 0.15. Max coverage (-): 0

Region: NODE\_282267\_length\_2958\_cov\_19.565924 2242-2247. Max. coverage (+): 0. Max coverage (-): 0

Region: NODE\_282267\_length\_2958\_cov\_19.565924 2248-2253. Max. coverage (+): 0. Max coverage (-): 0.03

Region: NODE\_282267\_length\_2958\_cov\_19.565924 2254-2259. Max. coverage (+): 0. Max coverage (-): 0.03

Region: NODE\_282267\_length\_2958\_cov\_19.565924 2260-2265. Max. coverage (+): 0. Max coverage (-): 0

Region: NODE\_282267\_length\_2958\_cov\_19.565924 2266-2272. Max. coverage (+): 0.01. Max coverage (-): 0

Region: NODE\_282267\_length\_2958\_cov\_19.565924 2273-2278. Max. coverage (+): 0.04. Max coverage (-): 0

Region: NODE\_282267\_length\_2958\_cov\_19.565924 2279-2284. Max. coverage (+): 0. Max coverage (-): 0

Region: NODE\_282267\_length\_2958\_cov\_19.565924 2285-2290. Max. coverage (+): 0. Max coverage (-): 0.35

Region: NODE\_282267\_length\_2958\_cov\_19.565924 2291-2296. Max. coverage (+): 0.02. Max coverage (-): 0.42

Region: NODE\_282267\_length\_2958\_cov\_19.565924 2297-2302. Max. coverage (+): 0.01. Max coverage (-): 0

Region: NODE\_282267\_length\_2958\_cov\_19.565924 2303-2308. Max. coverage (+): 0. Max coverage (-): 0.07

Region: NODE\_282267\_length\_2958\_cov\_19.565924 2309-2314. Max. coverage (+): 0. Max coverage (-): 0

Region: NODE\_282267\_length\_2958\_cov\_19.565924 2315-2320. Max. coverage (+): 0. Max coverage (-): 0

Region: NODE\_282267\_length\_2958\_cov\_19.565924 2321-2326. Max. coverage (+): 0. Max coverage (-): 0

Region: NODE\_282267\_length\_2958\_cov\_19.565924 2327-2332. Max. coverage (+): 0. Max coverage (-): 0

Region: NODE\_282267\_length\_2958\_cov\_19.565924 2333-2338. Max. coverage (+): 0. Max coverage (-): 0

Region: NODE\_282267\_length\_2958\_cov\_19.565924 2339-2344. Max. coverage (+): 0. Max coverage (-): 0.02

Region: NODE\_282267\_length\_2958\_cov\_19.565924 2345-2350. Max. coverage (+): 0. Max coverage (-): 0

Region: NODE\_282267\_length\_2958\_cov\_19.565924 2351-2356. Max. coverage (+): 0. Max coverage (-): 0

Region: NODE\_282267\_length\_2958\_cov\_19.565924 2357-2362. Max. coverage (+): 0.03. Max coverage (-): 0

Region: NODE\_282267\_length\_2958\_cov\_19.565924 2363-2368. Max. coverage (+): 0.01. Max coverage (-): 0.01

Region: NODE\_282267\_length\_2958\_cov\_19.565924 2369-2374. Max. coverage (+): 0. Max coverage (-): 0.24

Region: NODE\_282267\_length\_2958\_cov\_19.565924 2375-2380. Max. coverage (+): 0. Max coverage (-): 0.04

Region: NODE\_282267\_length\_2958\_cov\_19.565924 2381-2386. Max. coverage (+): 0. Max coverage (-): 0

Region: NODE\_282267\_length\_2958\_cov\_19.565924 2387-2392. Max. coverage (+): 0.03. Max coverage (-): 0

Region: NODE\_282267\_length\_2958\_cov\_19.565924 2393-2399. Max. coverage (+): 0.03. Max coverage (-): 0

Region: NODE\_282267\_length\_2958\_cov\_19.565924 2400-2405. Max. coverage (+): 0. Max coverage (-): 0

Region: NODE\_282267\_length\_2958\_cov\_19.565924 2406-2411. Max. coverage (+): 0. Max coverage (-): 0.02

Region: NODE\_282267\_length\_2958\_cov\_19.565924 2412-2417. Max. coverage (+): 0.01. Max coverage (-): 0.02

Region: NODE\_282267\_length\_2958\_cov\_19.565924 2418-2423. Max. coverage (+): 0.01. Max coverage (-): 0.01

Region: NODE\_282267\_length\_2958\_cov\_19.565924 2424-2429. Max. coverage (+): 0. Max coverage (-): 0.01

Region: NODE\_282267\_length\_2958\_cov\_19.565924 2430-2435. Max. coverage (+): 0. Max coverage (-): 0.04

Region: NODE\_282267\_length\_2958\_cov\_19.565924 2436-2441. Max. coverage (+): 0. Max coverage (-): 0.15

Region: NODE\_282267\_length\_2958\_cov\_19.565924 2442-2447. Max. coverage (+): 0. Max coverage (-): 0.03

Region: NODE\_282267\_length\_2958\_cov\_19.565924 2448-2453. Max. coverage (+): 0. Max coverage (-): 0

Region: NODE\_282267\_length\_2958\_cov\_19.565924 2454-2459. Max. coverage (+): 0. Max coverage (-): 0

Region: NODE\_282267\_length\_2958\_cov\_19.565924 2460-2465. Max. coverage (+): 0. Max coverage (-): 0

Region: NODE\_282267\_length\_2958\_cov\_19.565924 2466-2471. Max. coverage (+): 0. Max coverage (-): 0.08

Region: NODE\_282267\_length\_2958\_cov\_19.565924 2472-2477. Max. coverage (+): 0. Max coverage (-): 0.08

Region: NODE\_282267\_length\_2958\_cov\_19.565924 2478-2483. Max. coverage (+): 0.15. Max coverage (-): 0

Region: NODE\_282267\_length\_2958\_cov\_19.565924 2484-2489. Max. coverage (+): 0.12. Max coverage (-): 0.03

Region: NODE\_282267\_length\_2958\_cov\_19.565924 2490-2495. Max. coverage (+): 0.03. Max coverage (-): 0.04

Region: NODE\_282267\_length\_2958\_cov\_19.565924 2496-2501. Max. coverage (+): 0. Max coverage (-): 0.04

Region: NODE\_282267\_length\_2958\_cov\_19.565924 2502-2507. Max. coverage (+): 0. Max coverage (-): 0.92

Region: NODE\_282267\_length\_2958\_cov\_19.565924 2508-2513. Max. coverage (+): 0. Max coverage (-): 0.15

Region: NODE\_282267\_length\_2958\_cov\_19.565924 2514-2519. Max. coverage (+): 0. Max coverage (-): 0.03

Region: NODE\_282267\_length\_2958\_cov\_19.565924 2520-2526. Max. coverage (+): 0.1. Max coverage (-): 0.01

Region: NODE\_282267\_length\_2958\_cov\_19.565924 2527-2532. Max. coverage (+): 0.17. Max coverage (-): 0.01

Region: NODE\_282267\_length\_2958\_cov\_19.565924 2533-2538. Max. coverage (+): 0.03. Max coverage (-): 0

Region: NODE\_282267\_length\_2958\_cov\_19.565924 2539-2544. Max. coverage (+): 0. Max coverage (-): 0.12

Region: NODE\_282267\_length\_2958\_cov\_19.565924 2545-2550. Max. coverage (+): 0. Max coverage (-): 0.14

Region: NODE\_282267\_length\_2958\_cov\_19.565924 2551-2556. Max. coverage (+): 0. Max coverage (-): 0.02

Region: NODE\_282267\_length\_2958\_cov\_19.565924 2557-2562. Max. coverage (+): 0.05. Max coverage (-): 0

Region: NODE\_282267\_length\_2958\_cov\_19.565924 2563-2568. Max. coverage (+): 0.05. Max coverage (-): 0

Region: NODE\_282267\_length\_2958\_cov\_19.565924 2569-2574. Max. coverage (+): 0. Max coverage (-): 0

Region: NODE\_282267\_length\_2958\_cov\_19.565924 2575-2580. Max. coverage (+): 0. Max coverage (-): 0

Region: NODE\_282267\_length\_2958\_cov\_19.565924 2581-2586. Max. coverage (+): 0. Max coverage (-): 0

Region: NODE\_282267\_length\_2958\_cov\_19.565924 2587-2592. Max. coverage (+): 0. Max coverage (-): 0

Region: NODE\_282267\_length\_2958\_cov\_19.565924 2593-2598. Max. coverage (+): 0. Max coverage (-): 0.54

Region: NODE\_282267\_length\_2958\_cov\_19.565924 2599-2604. Max. coverage (+): 0.08. Max coverage (-): 0.46

Region: NODE\_282267\_length\_2958\_cov\_19.565924 2605-2610. Max. coverage (+): 0. Max coverage (-): 0

Region: NODE\_282267\_length\_2958\_cov\_19.565924 2611-2616. Max. coverage (+): 0. Max coverage (-): 0

Region: NODE\_282267\_length\_2958\_cov\_19.565924 2617-2622. Max. coverage (+): 0. Max coverage (-): 0

Region: NODE\_282267\_length\_2958\_cov\_19.565924 2623-2628. Max. coverage (+): 0. Max coverage (-): 0.18

Region: NODE\_282267\_length\_2958\_cov\_19.565924 2629-2634. Max. coverage (+): 0. Max coverage (-): 0.1

Region: NODE\_282267\_length\_2958\_cov\_19.565924 2635-2640. Max. coverage (+): 0. Max coverage (-): 0

Region: NODE\_282267\_length\_2958\_cov\_19.565924 2641-2646. Max. coverage (+): 0. Max coverage (-): 0

Region: NODE\_282267\_length\_2958\_cov\_19.565924 2647-2653. Max. coverage (+): 0. Max coverage (-): 0

Region: NODE\_282267\_length\_2958\_cov\_19.565924 2654-2659. Max. coverage (+): 0. Max coverage (-): 0

Region: NODE\_282267\_length\_2958\_cov\_19.565924 2660-2665. Max. coverage (+): 0. Max coverage (-): 4.22

Region: NODE\_282267\_length\_2958\_cov\_19.565924 2666-2671. Max. coverage (+): 0. Max coverage (-): 4.12

Region: NODE\_282267\_length\_2958\_cov\_19.565924 2672-2677. Max. coverage (+): 0.01. Max coverage (-): 0.07

Region: NODE\_282267\_length\_2958\_cov\_19.565924 2678-2683. Max. coverage (+): 0. Max coverage (-): 0

Region: NODE\_282267\_length\_2958\_cov\_19.565924 2684-2689. Max. coverage (+): 0. Max coverage (-): 0

Region: NODE\_282267\_length\_2958\_cov\_19.565924 2690-2695. Max. coverage (+): 0. Max coverage (-): 0.31

Region: NODE\_282267\_length\_2958\_cov\_19.565924 2696-2701. Max. coverage (+): 0. Max coverage (-): 0.31

Region: NODE\_282267\_length\_2958\_cov\_19.565924 2702-2707. Max. coverage (+): 0. Max coverage (-): 0.04

Region: NODE\_282267\_length\_2958\_cov\_19.565924 2708-2713. Max. coverage (+): 0. Max coverage (-): 0.04

Region: NODE\_282267\_length\_2958\_cov\_19.565924 2714-2719. Max. coverage (+): 0.11. Max coverage (-): 0.04

Region: NODE\_282267\_length\_2958\_cov\_19.565924 2720-2725. Max. coverage (+): 0.09. Max coverage (-): 0

Region: NODE\_282267\_length\_2958\_cov\_19.565924 2726-2731. Max. coverage (+): 0. Max coverage (-): 0.02

Region: NODE\_282267\_length\_2958\_cov\_19.565924 2732-2737. Max. coverage (+): 0. Max coverage (-): 0.02

Region: NODE\_282267\_length\_2958\_cov\_19.565924 2738-2743. Max. coverage (+): 0. Max coverage (-): 0

Region: NODE\_282267\_length\_2958\_cov\_19.565924 2744-2749. Max. coverage (+): 0. Max coverage (-): 0

Region: NODE\_282267\_length\_2958\_cov\_19.565924 2750-2755. Max. coverage (+): 0.01. Max coverage (-): 0

Region: NODE\_282267\_length\_2958\_cov\_19.565924 2756-2761. Max. coverage (+): 0. Max coverage (-): 0.01

Region: NODE\_282267\_length\_2958\_cov\_19.565924 2762-2767. Max. coverage (+): 0. Max coverage (-): 0.15

Region: NODE\_282267\_length\_2958\_cov\_19.565924 2768-2774. Max. coverage (+): 0. Max coverage (-): 0

Region: NODE\_282267\_length\_2958\_cov\_19.565924 2775-2780. Max. coverage (+): 0. Max coverage (-): 0.08

Region: NODE\_282267\_length\_2958\_cov\_19.565924 2781-2786. Max. coverage (+): 0. Max coverage (-): 0.08

Region: NODE\_282267\_length\_2958\_cov\_19.565924 2787-2792. Max. coverage (+): 0. Max coverage (-): 0

Region: NODE\_282267\_length\_2958\_cov\_19.565924 2793-2798. Max. coverage (+): 0. Max coverage (-): 0.15

Region: NODE\_282267\_length\_2958\_cov\_19.565924 2799-2804. Max. coverage (+): 0. Max coverage (-): 0.23

Region: NODE\_282267\_length\_2958\_cov\_19.565924 2805-2810. Max. coverage (+): 0.16. Max coverage (-): 0

Region: NODE\_282267\_length\_2958\_cov\_19.565924 2811-2816. Max. coverage (+): 0.17. Max coverage (-): 0

Region: NODE\_282267\_length\_2958\_cov\_19.565924 2817-2822. Max. coverage (+): 0. Max coverage (-): 0.15

Region: NODE\_282267\_length\_2958\_cov\_19.565924 2823-2828. Max. coverage (+): 0. Max coverage (-): 0.15

Region: NODE\_282267\_length\_2958\_cov\_19.565924 2829-2834. Max. coverage (+): 0. Max coverage (-): 0.41

Region: NODE\_282267\_length\_2958\_cov\_19.565924 2835-2840. Max. coverage (+): 0. Max coverage (-): 0.08

Region: NODE\_282267\_length\_2958\_cov\_19.565924 2841-2846. Max. coverage (+): 0. Max coverage (-): 0

Region: NODE\_282267\_length\_2958\_cov\_19.565924 2847-2852. Max. coverage (+): 0. Max coverage (-): 0

Region: NODE\_282267\_length\_2958\_cov\_19.565924 2853-2858. Max. coverage (+): 0. Max coverage (-): 0

Region: NODE\_282267\_length\_2958\_cov\_19.565924 2859-2864. Max. coverage (+): 0. Max coverage (-): 0

Region: NODE\_282267\_length\_2958\_cov\_19.565924 2865-2870. Max. coverage (+): 0. Max coverage (-): 0

Region: NODE\_282267\_length\_2958\_cov\_19.565924 2871-2876. Max. coverage (+): 0. Max coverage (-): 0

Region: NODE\_282267\_length\_2958\_cov\_19.565924 2877-2882. Max. coverage (+): 0. Max coverage (-): 0

Region: NODE\_282267\_length\_2958\_cov\_19.565924 2883-2888. Max. coverage (+): 0. Max coverage (-): 0

Region: NODE\_282267\_length\_2958\_cov\_19.565924 2889-2894. Max. coverage (+): 0. Max coverage (-): 0

Region: NODE\_282267\_length\_2958\_cov\_19.565924 2895-2901. Max. coverage (+): 0. Max coverage (-): 0

Region: NODE\_282267\_length\_2958\_cov\_19.565924 2902-2907. Max. coverage (+): 0. Max coverage (-): 0.15

Region: NODE\_282267\_length\_2958\_cov\_19.565924 2908-2913. Max. coverage (+): 0. Max coverage (-): 0.15

Region: NODE\_282267\_length\_2958\_cov\_19.565924 2914-2919. Max. coverage (+): 0. Max coverage (-): 0

Region: NODE\_282267\_length\_2958\_cov\_19.565924 2920-2925. Max. coverage (+): 0. Max coverage (-): 0

Region: NODE\_282267\_length\_2958\_cov\_19.565924 2926-2931. Max. coverage (+): 0. Max coverage (-): 0

Region: NODE\_282267\_length\_2958\_cov\_19.565924 2932-2937. Max. coverage (+): 0. Max coverage (-): 0

Region: NODE\_282267\_length\_2958\_cov\_19.565924 2938-2943. Max. coverage (+): 0. Max coverage (-): 0

Region: NODE\_282267\_length\_2958\_cov\_19.565924 2944-2949. Max. coverage (+): 0. Max coverage (-): 0

Region: NODE\_282267\_length\_2958\_cov\_19.565924 2950-2955. Max. coverage (+): 0. Max coverage (-): 0

Region: NODE\_282267\_length\_2958\_cov\_19.565924 2956-2961. Max. coverage (+): 0. Max coverage (-): 0

Region: NODE\_282267\_length\_2958\_cov\_19.565924 2962-2967. Max. coverage (+): 0. Max coverage (-): 0

Region: NODE\_282267\_length\_2958\_cov\_19.565924 2968-2973. Max. coverage (+): 0. Max coverage (-): 0

Region: NODE\_282267\_length\_2958\_cov\_19.565924 2974-2979. Max. coverage (+): 0. Max coverage (-): 0

Region: NODE\_282267\_length\_2958\_cov\_19.565924 2980-2985. Max. coverage (+): 0. Max coverage (-): 0

Region: NODE\_282267\_length\_2958\_cov\_19.565924 2986-2991. Max. coverage (+): 0. Max coverage (-): 0

Region: NODE\_282267\_length\_2958\_cov\_19.565924 2992-2997. Max. coverage (+): 0. Max coverage (-): 0

Region: NODE\_282267\_length\_2958\_cov\_19.565924 2998-3003. Max. coverage (+): 0. Max coverage (-): 0

Region: NODE\_282267\_length\_2958\_cov\_19.565924 3004-3009. Max. coverage (+): 0. Max coverage (-): 0

Region: NODE\_282267\_length\_2958\_cov\_19.565924 3010-3015. Max. coverage (+): 0. Max coverage (-): 0

Region: NODE\_282267\_length\_2958\_cov\_19.565924 3016-3021. Max. coverage (+): 0. Max coverage (-): 0

Region: NODE\_282267\_length\_2958\_cov\_19.565924 3022-. Max. coverage (+): 0. Max coverage (-): 0

RepeatMasker Color Code

**+**

100-98% Identity

<98-95% Identity

<95-90% Identity

<90-85% Identity

<85-80% Identity

<80-75% Identity

<75-70% Identity

<70% Identity

**-**

Gene Set Color Code

**+**

Gene

Pseudogene

Other

**-**

Topology/Coverage Color Code

Coverage Plus Strand

Coverage Minus Strand

Mainstrand: Plus

Mainstrand: Minus

Complementary Strand

Flanking Region  
(if option -flank >0)

Gene Set Annotation  
  
RepeatMasker Annotation  

**1. (TC)n**: 669-709 (+), Divergence to consensus: 12.2%  
**2. AlRepE-3106**: 868-2997 (-), Divergence to consensus: 5.7%  
**3. (AC)n**: 2998-3022 (+), Divergence to consensus: 0%

  
Transcription Factor Binding Sites  

**RFX4\_2** (Sequence: CATAGATAC (+): 282)  
**RHOXF1** (Sequence: GGCTCA (-): 1011)  
**RHOXF1** (Sequence: AGCTTA (-): 1250)  
**RHOXF1** (Sequence: AGCTTA (-): 1484)  
**RHOXF1** (Sequence: GGATCA (-): 1644)  
**RHOXF1** (Sequence: GGATTA (-): 2470)  
**RHOXF1** (Sequence: AGCTCA (-): 2491)  
**RHOXF1** (Sequence: TAAGCC (+): 646)  
**RHOXF1** (Sequence: TAATCT (+): 896)  
**RHOXF1** (Sequence: TGAGCT (+): 1248)  
**RHOXF1** (Sequence: TAAGCT (+): 2183)  
**RHOXF1** (Sequence: TGAGCC (+): 2301)  
**RHOXF1** (Sequence: TAATCT (+): 2744)  
**RHOXF1** (Sequence: TAATCT (+): 2771)  
**Lhx8** (Sequence: CTAATTAA (-): 708)  
**POU5F1** (Sequence: TTTGCAT (-): 2527)  
**SOX9** (Sequence: AACAATGG (-): 1130)  
**Sox5** (Sequence: ATTGTT (+): 554)  
**FIGLA** (Sequence: ACCAGGTGGA (-): 2463)  
**FOXO3\_mmu** (Sequence: GGAAAACA (+): 111)  
**FOXO3\_mmu** (Sequence: TCAAAACA (+): 2901)  
**Nobox** (Sequence: TAATTGGT (+): 2877)  
**POU2F1** (Sequence: ATTAAAATA (-): 711)  
**Rhox11** (Sequence: TTAACAGCA (-): 2077)  
**Sox5** (Sequence: AACAAT (-): 115)  
**Sox5** (Sequence: AACAAT (-): 1130)  
**Sox5** (Sequence: AACAAT (-): 1570)
