# Supplementary material for: First characterization of PIWI-interacting RNA clusters in a cichlid fish with a B chromosome
Source: BMC Biol. 2022 Sep 21;20:204. doi: 10.1186/s12915-022-01403-2 (PMC9490952; doi:10.1186/s12915-022-01403-2)
Supplement: Supplementary file 1 — Additional file 1. Zipped folder with fasta and interactive html piRNA cluster information for the A. latifasciata genome. The nomenclature is as follows: number-pirna-cluster_sex_B-presence (f, female; m, male; 0b, without B chromosome; 1b, with B chromosome). [file 12915_2022_1403_MOESM1_ESM.zip › 109_m1b.html]

piRNA cluster 109\_m1b 66


Predicted piRNA cluster no. 109\_m1b
  

Show proTRAC run info
Hide proTRAC run info

/\  
                \_\_\_\_\_\_\_\_\_\_\_\_\_\_\_\_\_\_\_\_\_\_\_/\\_\_\_ /  \\_\_\_\_\_\_\_  
               I                      /  \  /    \      I  
               I     pro             /    \/      \     I  
               I        TRAC        /               \   I  
               I   \_\_\_\_\_\_\_\_\_\_\_\_\_\_\_\_/\_\_\_\_\_\_\_\_\_\_\_\_\_\_\_\_\_\\_ I  
               I   \              /                     I  
               I    \            /                      I  
               I     \  /\      /       V.2.4.2         I  
               I      \/  \    /                        I  
               I\_\_\_\_\_\_\_\_\_\_\_\  /\_\_\_\_\_\_\_\_\_\_\_\_\_\_\_\_\_\_\_\_\_\_\_\_\_I  
                            \/  
  
  
================================= proTRAC ====================================  
VERSION: .......... 2.4.2  
LAST MODIFIED: .... 11. May 2018  
  
Please cite:  
Rosenkranz D, Zischler H. proTRAC - a software for probabilistic piRNA cluster  
detection, visualization and analysis. 2012. BMC Bioinformatics 13:5.  
  
  
Contact:  
David Rosenkranz  
Institute of Organismic and Molecular Evolutionary Biology  
Dept. Anthropology, small RNA group  
Johannes Gutenberg University Mainz  
email: rosenkranz@uni-mainz.de  
  
You can find the latest proTRAC version at:  
http://sourceforge.net/projects/protrac/files  
http://www.smallRNAgroup-mainz.de/software  
==============================================================================  
  
PARAMETERS:  
Map file: ...............piwi-machos-1B.fa-collapse.map  
Genome file: ............../../../0B\_ala\_genome.fa  
RepeatMasker annotation: Alatifasciata-all0B-maryan-v2.fa\_corrected.out  
GeneSet:................./guest-storage/Data/annotation/Alatifasciata\_all0B\_maryan-v2\_out2017.gff  
  
Significant (p<=0.01) hit density will be calculated based  
on observed hit distribution.  
  
Sliding window size: ........................................ 5000 bp  
Sliding window increament: .................................. 1000 bp  
Normalize each hit by number of genomic hits: ............... yes  
Normalize each hit by number of sequence reads: ............. yes  
Normalize values (-> per million mapped reads): ............. yes  
Min. fraction of hits with 1T(U) or 10A: .................... 0.75  
Alternatively: Min. fraction of hits with 1T(U) and 10A: .... 0.5  
Min. fraction of hits with typical piRNA length: ............ 0.75  
Typical piRNA length: ....................................... 24-32 nt  
Min. size of a piRNA cluster: ............................... 1000 bp.  
Min. number of hits (absolute): ............................. 0  
Min. number of hits (normalized): ........................... 0  
Min. fraction of hits on the mainstrand: .................... 0.75  
Top fraction of mapped sequences (in terms of read counts): . 1%  
Top fraction accounts for max. n% of sequence reads: ........ 90%  
Min. fraction of hits on each arm of a bidirectional cluster: 0.05  
Output html file for each cluster: .......................... yes  
Output a summary table: ..................................... yes  
Output a FASTA file for each cluster (piRNA sequences): ..... yes  
Output a FASTA file comprising cluster sequences: ........... yes  
Output a GTF file for predicted piRNA clusters: ..............yes  
Search DNA motifs in clusters: .............................. yes  
Output flanking sequences: +/- .............................. 0 bp  
Output ~.pTi file: .......................................... no  
==============================================================================  
  
  
Genome size (without gaps): ............ 758543724 bp  
Gaps (N/X/-): .......................... 417479 bp  
Mapped reads: .......................... 26973943  
Non-identical sequences: ............... 6209225  
Genomic hits: .......................... 48438990  
Significant densitiy of mapped reads: .. 821.144211136946 reads/kb

Show proTRAC cluster info
Hide proTRAC cluster info

|  |  |
| --- | --- |
| Location | NODE\_283949\_length\_981\_cov\_75.131500 |
| Coordinates | 1-1047 |
| Size [bp] | 1047 |
| Sequence hit loci | 1625 |
| Mapped reads (normalized) | 9929.2 |
| Mapped reads (normalized) per kb | 9483.4 |
| Normalized reads with 1T (1U) | 83.9% |
| Normalized reads with 10A | 22.7% |
| Normalized reads with length 24-32 nt | 99.2% |
| Normalized reads on the main strand(s) | 93.6% |
| Predicted directionality | mono:plus |

100%

0%

1T (1U)  
reads

10A reads

24-32 nt  
reads

reads on mainstrand

**Either the amount of reads with 1T (1U) OR 10A has to exceed 75% (set with option: -1Tor10A)  
Alternatively the amount of reads with 1T (1U) AND 10A has to exceed 50% (set with option: -1Tand10A)  
Minimum amount of reads with preferred size is 75% (set with option: -pisize)  
Minimum amount of reads on the main strand(s) is 75% (set with option: -clstrand)**

Show read coverage
Hide read coverage

WHAT DO I SEE HERE?  
This chart shows the location of mapped sequence reads within a predicted piRNA cluster. The color refers to the number of genomic hits produced by the sequence read in question. A dark red bar indicates that this sequence read produces many other hits elsewhere in the genome. Many adjacent red or yellow bars can indicate the presence of a multi-copy element such as transposons or rRNA genes. A dark green bar indicates that this sequence read maps uniquely to this locus.

1 hit

2-5 hits

6-10 hits

11-20 hits

21-50 hits

51-100 hits

> 100 hits

NODE\_283949\_length\_981\_cov\_75.131500

1

1047

Gene Set

RepeatMasker

Mapped  
Reads

83.19

plus strand

minus strand

83.19

Region: NODE\_283949\_length\_981\_cov\_75.131500 1676-2. Max. coverage (+): 0.27. Max coverage (-): 0.02

Region: NODE\_283949\_length\_981\_cov\_75.131500 3-4. Max. coverage (+): 0.07. Max coverage (-): 0.04

Region: NODE\_283949\_length\_981\_cov\_75.131500 5-6. Max. coverage (+): 0.01. Max coverage (-): 0.07

Region: NODE\_283949\_length\_981\_cov\_75.131500 7-8. Max. coverage (+): 0.01. Max coverage (-): 0.06

Region: NODE\_283949\_length\_981\_cov\_75.131500 9-10. Max. coverage (+): 0.1. Max coverage (-): 0.05

Region: NODE\_283949\_length\_981\_cov\_75.131500 11-12. Max. coverage (+): 0.16. Max coverage (-): 0.07

Region: NODE\_283949\_length\_981\_cov\_75.131500 13-14. Max. coverage (+): 0.25. Max coverage (-): 0.04

Region: NODE\_283949\_length\_981\_cov\_75.131500 15-16. Max. coverage (+): 0.09. Max coverage (-): 0.09

Region: NODE\_283949\_length\_981\_cov\_75.131500 17-18. Max. coverage (+): 0.27. Max coverage (-): 0.57

Region: NODE\_283949\_length\_981\_cov\_75.131500 19-20. Max. coverage (+): 0.32. Max coverage (-): 0.23

Region: NODE\_283949\_length\_981\_cov\_75.131500 21-22. Max. coverage (+): 0.17. Max coverage (-): 0.23

Region: NODE\_283949\_length\_981\_cov\_75.131500 23-25. Max. coverage (+): 6.09. Max coverage (-): 0.1

Region: NODE\_283949\_length\_981\_cov\_75.131500 26-27. Max. coverage (+): 0.23. Max coverage (-): 0.1

Region: NODE\_283949\_length\_981\_cov\_75.131500 28-29. Max. coverage (+): 0.25. Max coverage (-): 0.02

Region: NODE\_283949\_length\_981\_cov\_75.131500 30-31. Max. coverage (+): 0.04. Max coverage (-): 0.01

Region: NODE\_283949\_length\_981\_cov\_75.131500 32-33. Max. coverage (+): 0.04. Max coverage (-): 0

Region: NODE\_283949\_length\_981\_cov\_75.131500 34-35. Max. coverage (+): 0.04. Max coverage (-): 0

Region: NODE\_283949\_length\_981\_cov\_75.131500 36-37. Max. coverage (+): 0. Max coverage (-): 0

Region: NODE\_283949\_length\_981\_cov\_75.131500 38-39. Max. coverage (+): 0.04. Max coverage (-): 0

Region: NODE\_283949\_length\_981\_cov\_75.131500 40-41. Max. coverage (+): 0.11. Max coverage (-): 0

Region: NODE\_283949\_length\_981\_cov\_75.131500 42-43. Max. coverage (+): 0.04. Max coverage (-): 0

Region: NODE\_283949\_length\_981\_cov\_75.131500 44-46. Max. coverage (+): 0.07. Max coverage (-): 0.04

Region: NODE\_283949\_length\_981\_cov\_75.131500 47-48. Max. coverage (+): 0.15. Max coverage (-): 0.04

Region: NODE\_283949\_length\_981\_cov\_75.131500 49-50. Max. coverage (+): 0. Max coverage (-): 0.19

Region: NODE\_283949\_length\_981\_cov\_75.131500 51-52. Max. coverage (+): 0. Max coverage (-): 0.48

Region: NODE\_283949\_length\_981\_cov\_75.131500 53-54. Max. coverage (+): 0. Max coverage (-): 0.52

Region: NODE\_283949\_length\_981\_cov\_75.131500 55-56. Max. coverage (+): 0.15. Max coverage (-): 0

Region: NODE\_283949\_length\_981\_cov\_75.131500 57-58. Max. coverage (+): 0.07. Max coverage (-): 0

Region: NODE\_283949\_length\_981\_cov\_75.131500 59-60. Max. coverage (+): 0.11. Max coverage (-): 0

Region: NODE\_283949\_length\_981\_cov\_75.131500 61-62. Max. coverage (+): 0.3. Max coverage (-): 0

Region: NODE\_283949\_length\_981\_cov\_75.131500 63-64. Max. coverage (+): 0.48. Max coverage (-): 0

Region: NODE\_283949\_length\_981\_cov\_75.131500 65-66. Max. coverage (+): 0.22. Max coverage (-): 0

Region: NODE\_283949\_length\_981\_cov\_75.131500 67-69. Max. coverage (+): 83.19. Max coverage (-): 0.15

Region: NODE\_283949\_length\_981\_cov\_75.131500 70-71. Max. coverage (+): 2.97. Max coverage (-): 0.04

Region: NODE\_283949\_length\_981\_cov\_75.131500 72-73. Max. coverage (+): 0.33. Max coverage (-): 0

Region: NODE\_283949\_length\_981\_cov\_75.131500 74-75. Max. coverage (+): 0. Max coverage (-): 0

Region: NODE\_283949\_length\_981\_cov\_75.131500 76-77. Max. coverage (+): 0. Max coverage (-): 0

Region: NODE\_283949\_length\_981\_cov\_75.131500 78-79. Max. coverage (+): 0. Max coverage (-): 0.19

Region: NODE\_283949\_length\_981\_cov\_75.131500 80-81. Max. coverage (+): 0. Max coverage (-): 0

Region: NODE\_283949\_length\_981\_cov\_75.131500 82-83. Max. coverage (+): 0.15. Max coverage (-): 0

Region: NODE\_283949\_length\_981\_cov\_75.131500 84-85. Max. coverage (+): 0.11. Max coverage (-): 0.15

Region: NODE\_283949\_length\_981\_cov\_75.131500 86-87. Max. coverage (+): 0.04. Max coverage (-): 0.07

Region: NODE\_283949\_length\_981\_cov\_75.131500 88-89. Max. coverage (+): 0.07. Max coverage (-): 0.04

Region: NODE\_283949\_length\_981\_cov\_75.131500 90-92. Max. coverage (+): 0.07. Max coverage (-): 0.04

Region: NODE\_283949\_length\_981\_cov\_75.131500 93-94. Max. coverage (+): 0.04. Max coverage (-): 0.04

Region: NODE\_283949\_length\_981\_cov\_75.131500 95-96. Max. coverage (+): 0.11. Max coverage (-): 0

Region: NODE\_283949\_length\_981\_cov\_75.131500 97-98. Max. coverage (+): 0.22. Max coverage (-): 0

Region: NODE\_283949\_length\_981\_cov\_75.131500 99-100. Max. coverage (+): 1.19. Max coverage (-): 0

Region: NODE\_283949\_length\_981\_cov\_75.131500 101-102. Max. coverage (+): 0. Max coverage (-): 0.04

Region: NODE\_283949\_length\_981\_cov\_75.131500 103-104. Max. coverage (+): 0.63. Max coverage (-): 0.07

Region: NODE\_283949\_length\_981\_cov\_75.131500 105-106. Max. coverage (+): 1.22. Max coverage (-): 0.04

Region: NODE\_283949\_length\_981\_cov\_75.131500 107-108. Max. coverage (+): 0.59. Max coverage (-): 0

Region: NODE\_283949\_length\_981\_cov\_75.131500 109-110. Max. coverage (+): 0. Max coverage (-): 0

Region: NODE\_283949\_length\_981\_cov\_75.131500 111-113. Max. coverage (+): 0. Max coverage (-): 0

Region: NODE\_283949\_length\_981\_cov\_75.131500 114-115. Max. coverage (+): 0. Max coverage (-): 0

Region: NODE\_283949\_length\_981\_cov\_75.131500 116-117. Max. coverage (+): 0. Max coverage (-): 0

Region: NODE\_283949\_length\_981\_cov\_75.131500 118-119. Max. coverage (+): 0. Max coverage (-): 0.04

Region: NODE\_283949\_length\_981\_cov\_75.131500 120-121. Max. coverage (+): 0.04. Max coverage (-): 0.04

Region: NODE\_283949\_length\_981\_cov\_75.131500 122-123. Max. coverage (+): 0.15. Max coverage (-): 0.07

Region: NODE\_283949\_length\_981\_cov\_75.131500 124-125. Max. coverage (+): 0.56. Max coverage (-): 0.07

Region: NODE\_283949\_length\_981\_cov\_75.131500 126-127. Max. coverage (+): 0.56. Max coverage (-): 0.15

Region: NODE\_283949\_length\_981\_cov\_75.131500 128-129. Max. coverage (+): 0.82. Max coverage (-): 0.3

Region: NODE\_283949\_length\_981\_cov\_75.131500 130-131. Max. coverage (+): 0.67. Max coverage (-): 0.3

Region: NODE\_283949\_length\_981\_cov\_75.131500 132-133. Max. coverage (+): 1.85. Max coverage (-): 0

Region: NODE\_283949\_length\_981\_cov\_75.131500 134-136. Max. coverage (+): 5.12. Max coverage (-): 0

Region: NODE\_283949\_length\_981\_cov\_75.131500 137-138. Max. coverage (+): 2.89. Max coverage (-): 0

Region: NODE\_283949\_length\_981\_cov\_75.131500 139-140. Max. coverage (+): 3.41. Max coverage (-): 0

Region: NODE\_283949\_length\_981\_cov\_75.131500 141-142. Max. coverage (+): 3.26. Max coverage (-): 0.04

Region: NODE\_283949\_length\_981\_cov\_75.131500 143-144. Max. coverage (+): 0.48. Max coverage (-): 0.04

Region: NODE\_283949\_length\_981\_cov\_75.131500 145-146. Max. coverage (+): 1.67. Max coverage (-): 0

Region: NODE\_283949\_length\_981\_cov\_75.131500 147-148. Max. coverage (+): 1.48. Max coverage (-): 0.04

Region: NODE\_283949\_length\_981\_cov\_75.131500 149-150. Max. coverage (+): 0.19. Max coverage (-): 0.04

Region: NODE\_283949\_length\_981\_cov\_75.131500 151-152. Max. coverage (+): 0.04. Max coverage (-): 0

Region: NODE\_283949\_length\_981\_cov\_75.131500 153-154. Max. coverage (+): 0.04. Max coverage (-): 0

Region: NODE\_283949\_length\_981\_cov\_75.131500 155-157. Max. coverage (+): 0. Max coverage (-): 0

Region: NODE\_283949\_length\_981\_cov\_75.131500 158-159. Max. coverage (+): 0.22. Max coverage (-): 0.11

Region: NODE\_283949\_length\_981\_cov\_75.131500 160-161. Max. coverage (+): 0.26. Max coverage (-): 0.11

Region: NODE\_283949\_length\_981\_cov\_75.131500 162-163. Max. coverage (+): 0.3. Max coverage (-): 0.04

Region: NODE\_283949\_length\_981\_cov\_75.131500 164-165. Max. coverage (+): 0.15. Max coverage (-): 0

Region: NODE\_283949\_length\_981\_cov\_75.131500 166-167. Max. coverage (+): 4.41. Max coverage (-): 0

Region: NODE\_283949\_length\_981\_cov\_75.131500 168-169. Max. coverage (+): 4.71. Max coverage (-): 0

Region: NODE\_283949\_length\_981\_cov\_75.131500 170-171. Max. coverage (+): 0.56. Max coverage (-): 0

Region: NODE\_283949\_length\_981\_cov\_75.131500 172-173. Max. coverage (+): 0.22. Max coverage (-): 0

Region: NODE\_283949\_length\_981\_cov\_75.131500 174-175. Max. coverage (+): 0.19. Max coverage (-): 0

Region: NODE\_283949\_length\_981\_cov\_75.131500 176-177. Max. coverage (+): 1.93. Max coverage (-): 0

Region: NODE\_283949\_length\_981\_cov\_75.131500 178-180. Max. coverage (+): 2.04. Max coverage (-): 0

Region: NODE\_283949\_length\_981\_cov\_75.131500 181-182. Max. coverage (+): 0.15. Max coverage (-): 0

Region: NODE\_283949\_length\_981\_cov\_75.131500 183-184. Max. coverage (+): 0. Max coverage (-): 0

Region: NODE\_283949\_length\_981\_cov\_75.131500 185-186. Max. coverage (+): 0.07. Max coverage (-): 0

Region: NODE\_283949\_length\_981\_cov\_75.131500 187-188. Max. coverage (+): 0.15. Max coverage (-): 0.04

Region: NODE\_283949\_length\_981\_cov\_75.131500 189-190. Max. coverage (+): 0.22. Max coverage (-): 0.04

Region: NODE\_283949\_length\_981\_cov\_75.131500 191-192. Max. coverage (+): 0.22. Max coverage (-): 0.07

Region: NODE\_283949\_length\_981\_cov\_75.131500 193-194. Max. coverage (+): 0.11. Max coverage (-): 0.04

Region: NODE\_283949\_length\_981\_cov\_75.131500 195-196. Max. coverage (+): 0. Max coverage (-): 0

Region: NODE\_283949\_length\_981\_cov\_75.131500 197-198. Max. coverage (+): 0. Max coverage (-): 0

Region: NODE\_283949\_length\_981\_cov\_75.131500 199-200. Max. coverage (+): 0.04. Max coverage (-): 0.04

Region: NODE\_283949\_length\_981\_cov\_75.131500 201-203. Max. coverage (+): 0.04. Max coverage (-): 0.04

Region: NODE\_283949\_length\_981\_cov\_75.131500 204-205. Max. coverage (+): 0.04. Max coverage (-): 0

Region: NODE\_283949\_length\_981\_cov\_75.131500 206-207. Max. coverage (+): 0. Max coverage (-): 0.07

Region: NODE\_283949\_length\_981\_cov\_75.131500 208-209. Max. coverage (+): 0. Max coverage (-): 0.07

Region: NODE\_283949\_length\_981\_cov\_75.131500 210-211. Max. coverage (+): 0.56. Max coverage (-): 0

Region: NODE\_283949\_length\_981\_cov\_75.131500 212-213. Max. coverage (+): 0.7. Max coverage (-): 0

Region: NODE\_283949\_length\_981\_cov\_75.131500 214-215. Max. coverage (+): 0.26. Max coverage (-): 0

Region: NODE\_283949\_length\_981\_cov\_75.131500 216-217. Max. coverage (+): 0.26. Max coverage (-): 0

Region: NODE\_283949\_length\_981\_cov\_75.131500 218-219. Max. coverage (+): 0.11. Max coverage (-): 0

Region: NODE\_283949\_length\_981\_cov\_75.131500 220-221. Max. coverage (+): 0.04. Max coverage (-): 0

Region: NODE\_283949\_length\_981\_cov\_75.131500 222-224. Max. coverage (+): 0.04. Max coverage (-): 0

Region: NODE\_283949\_length\_981\_cov\_75.131500 225-226. Max. coverage (+): 0.11. Max coverage (-): 0

Region: NODE\_283949\_length\_981\_cov\_75.131500 227-228. Max. coverage (+): 0.11. Max coverage (-): 0

Region: NODE\_283949\_length\_981\_cov\_75.131500 229-230. Max. coverage (+): 0.04. Max coverage (-): 0.04

Region: NODE\_283949\_length\_981\_cov\_75.131500 231-232. Max. coverage (+): 0.67. Max coverage (-): 0.04

Region: NODE\_283949\_length\_981\_cov\_75.131500 233-234. Max. coverage (+): 0.7. Max coverage (-): 0.04

Region: NODE\_283949\_length\_981\_cov\_75.131500 235-236. Max. coverage (+): 0.07. Max coverage (-): 0

Region: NODE\_283949\_length\_981\_cov\_75.131500 237-238. Max. coverage (+): 0.07. Max coverage (-): 0

Region: NODE\_283949\_length\_981\_cov\_75.131500 239-240. Max. coverage (+): 0.26. Max coverage (-): 0.04

Region: NODE\_283949\_length\_981\_cov\_75.131500 241-242. Max. coverage (+): 0.22. Max coverage (-): 0.11

Region: NODE\_283949\_length\_981\_cov\_75.131500 243-244. Max. coverage (+): 0.15. Max coverage (-): 0.07

Region: NODE\_283949\_length\_981\_cov\_75.131500 245-247. Max. coverage (+): 2.22. Max coverage (-): 0

Region: NODE\_283949\_length\_981\_cov\_75.131500 248-249. Max. coverage (+): 3.15. Max coverage (-): 0

Region: NODE\_283949\_length\_981\_cov\_75.131500 250-251. Max. coverage (+): 2.41. Max coverage (-): 0

Region: NODE\_283949\_length\_981\_cov\_75.131500 252-253. Max. coverage (+): 0.96. Max coverage (-): 0

Region: NODE\_283949\_length\_981\_cov\_75.131500 254-255. Max. coverage (+): 0.52. Max coverage (-): 0

Region: NODE\_283949\_length\_981\_cov\_75.131500 256-257. Max. coverage (+): 0.11. Max coverage (-): 0

Region: NODE\_283949\_length\_981\_cov\_75.131500 258-259. Max. coverage (+): 0. Max coverage (-): 0

Region: NODE\_283949\_length\_981\_cov\_75.131500 260-261. Max. coverage (+): 0. Max coverage (-): 0

Region: NODE\_283949\_length\_981\_cov\_75.131500 262-263. Max. coverage (+): 0.15. Max coverage (-): 0

Region: NODE\_283949\_length\_981\_cov\_75.131500 264-265. Max. coverage (+): 0.15. Max coverage (-): 0

Region: NODE\_283949\_length\_981\_cov\_75.131500 266-267. Max. coverage (+): 0. Max coverage (-): 0

Region: NODE\_283949\_length\_981\_cov\_75.131500 268-270. Max. coverage (+): 0. Max coverage (-): 0

Region: NODE\_283949\_length\_981\_cov\_75.131500 271-272. Max. coverage (+): 0. Max coverage (-): 0

Region: NODE\_283949\_length\_981\_cov\_75.131500 273-274. Max. coverage (+): 0.07. Max coverage (-): 0.04

Region: NODE\_283949\_length\_981\_cov\_75.131500 275-276. Max. coverage (+): 0.07. Max coverage (-): 0.15

Region: NODE\_283949\_length\_981\_cov\_75.131500 277-278. Max. coverage (+): 0.04. Max coverage (-): 0.56

Region: NODE\_283949\_length\_981\_cov\_75.131500 279-280. Max. coverage (+): 0.07. Max coverage (-): 0.78

Region: NODE\_283949\_length\_981\_cov\_75.131500 281-282. Max. coverage (+): 0.11. Max coverage (-): 1.41

Region: NODE\_283949\_length\_981\_cov\_75.131500 283-284. Max. coverage (+): 0.07. Max coverage (-): 0.89

Region: NODE\_283949\_length\_981\_cov\_75.131500 285-286. Max. coverage (+): 0.04. Max coverage (-): 0.33

Region: NODE\_283949\_length\_981\_cov\_75.131500 287-288. Max. coverage (+): 0. Max coverage (-): 0.22

Region: NODE\_283949\_length\_981\_cov\_75.131500 289-291. Max. coverage (+): 0. Max coverage (-): 0.22

Region: NODE\_283949\_length\_981\_cov\_75.131500 292-293. Max. coverage (+): 0. Max coverage (-): 0

Region: NODE\_283949\_length\_981\_cov\_75.131500 294-295. Max. coverage (+): 0.3. Max coverage (-): 0.04

Region: NODE\_283949\_length\_981\_cov\_75.131500 296-297. Max. coverage (+): 1.15. Max coverage (-): 0.04

Region: NODE\_283949\_length\_981\_cov\_75.131500 298-299. Max. coverage (+): 1.71. Max coverage (-): 0

Region: NODE\_283949\_length\_981\_cov\_75.131500 300-301. Max. coverage (+): 1.52. Max coverage (-): 0

Region: NODE\_283949\_length\_981\_cov\_75.131500 302-303. Max. coverage (+): 0.67. Max coverage (-): 0

Region: NODE\_283949\_length\_981\_cov\_75.131500 304-305. Max. coverage (+): 1.08. Max coverage (-): 0

Region: NODE\_283949\_length\_981\_cov\_75.131500 306-307. Max. coverage (+): 0.44. Max coverage (-): 0

Region: NODE\_283949\_length\_981\_cov\_75.131500 308-309. Max. coverage (+): 0.04. Max coverage (-): 0

Region: NODE\_283949\_length\_981\_cov\_75.131500 310-311. Max. coverage (+): 0. Max coverage (-): 0

Region: NODE\_283949\_length\_981\_cov\_75.131500 312-314. Max. coverage (+): 0. Max coverage (-): 0

Region: NODE\_283949\_length\_981\_cov\_75.131500 315-316. Max. coverage (+): 0. Max coverage (-): 0

Region: NODE\_283949\_length\_981\_cov\_75.131500 317-318. Max. coverage (+): 0. Max coverage (-): 0

Region: NODE\_283949\_length\_981\_cov\_75.131500 319-320. Max. coverage (+): 0. Max coverage (-): 0

Region: NODE\_283949\_length\_981\_cov\_75.131500 321-322. Max. coverage (+): 0. Max coverage (-): 0

Region: NODE\_283949\_length\_981\_cov\_75.131500 323-324. Max. coverage (+): 0. Max coverage (-): 0

Region: NODE\_283949\_length\_981\_cov\_75.131500 325-326. Max. coverage (+): 0. Max coverage (-): 0

Region: NODE\_283949\_length\_981\_cov\_75.131500 327-328. Max. coverage (+): 0.11. Max coverage (-): 0

Region: NODE\_283949\_length\_981\_cov\_75.131500 329-330. Max. coverage (+): 0.11. Max coverage (-): 0.04

Region: NODE\_283949\_length\_981\_cov\_75.131500 331-332. Max. coverage (+): 0.3. Max coverage (-): 0.07

Region: NODE\_283949\_length\_981\_cov\_75.131500 333-334. Max. coverage (+): 0.93. Max coverage (-): 0.04

Region: NODE\_283949\_length\_981\_cov\_75.131500 335-337. Max. coverage (+): 9.86. Max coverage (-): 0

Region: NODE\_283949\_length\_981\_cov\_75.131500 338-339. Max. coverage (+): 9.86. Max coverage (-): 0

Region: NODE\_283949\_length\_981\_cov\_75.131500 340-341. Max. coverage (+): 1.3. Max coverage (-): 0

Region: NODE\_283949\_length\_981\_cov\_75.131500 342-343. Max. coverage (+): 1.11. Max coverage (-): 0

Region: NODE\_283949\_length\_981\_cov\_75.131500 344-345. Max. coverage (+): 0.11. Max coverage (-): 0

Region: NODE\_283949\_length\_981\_cov\_75.131500 346-347. Max. coverage (+): 0.11. Max coverage (-): 0

Region: NODE\_283949\_length\_981\_cov\_75.131500 348-349. Max. coverage (+): 0.37. Max coverage (-): 0

Region: NODE\_283949\_length\_981\_cov\_75.131500 350-351. Max. coverage (+): 0.33. Max coverage (-): 0.04

Region: NODE\_283949\_length\_981\_cov\_75.131500 352-353. Max. coverage (+): 0.11. Max coverage (-): 0.11

Region: NODE\_283949\_length\_981\_cov\_75.131500 354-355. Max. coverage (+): 0.48. Max coverage (-): 0.26

Region: NODE\_283949\_length\_981\_cov\_75.131500 356-358. Max. coverage (+): 1.59. Max coverage (-): 0.19

Region: NODE\_283949\_length\_981\_cov\_75.131500 359-360. Max. coverage (+): 54.53. Max coverage (-): 0

Region: NODE\_283949\_length\_981\_cov\_75.131500 361-362. Max. coverage (+): 55.39. Max coverage (-): 0

Region: NODE\_283949\_length\_981\_cov\_75.131500 363-364. Max. coverage (+): 2.52. Max coverage (-): 0

Region: NODE\_283949\_length\_981\_cov\_75.131500 365-366. Max. coverage (+): 0.52. Max coverage (-): 0

Region: NODE\_283949\_length\_981\_cov\_75.131500 367-368. Max. coverage (+): 0.37. Max coverage (-): 0

Region: NODE\_283949\_length\_981\_cov\_75.131500 369-370. Max. coverage (+): 0.63. Max coverage (-): 0.07

Region: NODE\_283949\_length\_981\_cov\_75.131500 371-372. Max. coverage (+): 0.63. Max coverage (-): 0.07

Region: NODE\_283949\_length\_981\_cov\_75.131500 373-374. Max. coverage (+): 0.11. Max coverage (-): 0.48

Region: NODE\_283949\_length\_981\_cov\_75.131500 375-376. Max. coverage (+): 0.15. Max coverage (-): 0.52

Region: NODE\_283949\_length\_981\_cov\_75.131500 377-378. Max. coverage (+): 0.15. Max coverage (-): 0.26

Region: NODE\_283949\_length\_981\_cov\_75.131500 379-381. Max. coverage (+): 0.07. Max coverage (-): 0.22

Region: NODE\_283949\_length\_981\_cov\_75.131500 382-383. Max. coverage (+): 0.04. Max coverage (-): 0

Region: NODE\_283949\_length\_981\_cov\_75.131500 384-385. Max. coverage (+): 0.04. Max coverage (-): 1.04

Region: NODE\_283949\_length\_981\_cov\_75.131500 386-387. Max. coverage (+): 0.07. Max coverage (-): 1.04

Region: NODE\_283949\_length\_981\_cov\_75.131500 388-389. Max. coverage (+): 0.11. Max coverage (-): 1.74

Region: NODE\_283949\_length\_981\_cov\_75.131500 390-391. Max. coverage (+): 0.11. Max coverage (-): 1.63

Region: NODE\_283949\_length\_981\_cov\_75.131500 392-393. Max. coverage (+): 0.11. Max coverage (-): 0.07

Region: NODE\_283949\_length\_981\_cov\_75.131500 394-395. Max. coverage (+): 0.26. Max coverage (-): 0.07

Region: NODE\_283949\_length\_981\_cov\_75.131500 396-397. Max. coverage (+): 0.3. Max coverage (-): 0.07

Region: NODE\_283949\_length\_981\_cov\_75.131500 398-399. Max. coverage (+): 0.11. Max coverage (-): 0.04

Region: NODE\_283949\_length\_981\_cov\_75.131500 400-402. Max. coverage (+): 0.67. Max coverage (-): 0

Region: NODE\_283949\_length\_981\_cov\_75.131500 403-404. Max. coverage (+): 2.19. Max coverage (-): 0

Region: NODE\_283949\_length\_981\_cov\_75.131500 405-406. Max. coverage (+): 1.71. Max coverage (-): 0.04

Region: NODE\_283949\_length\_981\_cov\_75.131500 407-408. Max. coverage (+): 0.48. Max coverage (-): 0.04

Region: NODE\_283949\_length\_981\_cov\_75.131500 409-410. Max. coverage (+): 1.37. Max coverage (-): 0

Region: NODE\_283949\_length\_981\_cov\_75.131500 411-412. Max. coverage (+): 0.96. Max coverage (-): 0

Region: NODE\_283949\_length\_981\_cov\_75.131500 413-414. Max. coverage (+): 0.93. Max coverage (-): 0

Region: NODE\_283949\_length\_981\_cov\_75.131500 415-416. Max. coverage (+): 0.44. Max coverage (-): 0

Region: NODE\_283949\_length\_981\_cov\_75.131500 417-418. Max. coverage (+): 0.26. Max coverage (-): 0

Region: NODE\_283949\_length\_981\_cov\_75.131500 419-420. Max. coverage (+): 0.41. Max coverage (-): 0

Region: NODE\_283949\_length\_981\_cov\_75.131500 421-422. Max. coverage (+): 0.37. Max coverage (-): 0

Region: NODE\_283949\_length\_981\_cov\_75.131500 423-425. Max. coverage (+): 0.19. Max coverage (-): 0

Region: NODE\_283949\_length\_981\_cov\_75.131500 426-427. Max. coverage (+): 0.15. Max coverage (-): 0

Region: NODE\_283949\_length\_981\_cov\_75.131500 428-429. Max. coverage (+): 0.04. Max coverage (-): 0

Region: NODE\_283949\_length\_981\_cov\_75.131500 430-431. Max. coverage (+): 0. Max coverage (-): 0

Region: NODE\_283949\_length\_981\_cov\_75.131500 432-433. Max. coverage (+): 0. Max coverage (-): 0.07

Region: NODE\_283949\_length\_981\_cov\_75.131500 434-435. Max. coverage (+): 0.15. Max coverage (-): 0.11

Region: NODE\_283949\_length\_981\_cov\_75.131500 436-437. Max. coverage (+): 0.19. Max coverage (-): 0.04

Region: NODE\_283949\_length\_981\_cov\_75.131500 438-439. Max. coverage (+): 0.04. Max coverage (-): 0

Region: NODE\_283949\_length\_981\_cov\_75.131500 440-441. Max. coverage (+): 0. Max coverage (-): 0.04

Region: NODE\_283949\_length\_981\_cov\_75.131500 442-443. Max. coverage (+): 0.04. Max coverage (-): 0.04

Region: NODE\_283949\_length\_981\_cov\_75.131500 444-445. Max. coverage (+): 0.07. Max coverage (-): 0

Region: NODE\_283949\_length\_981\_cov\_75.131500 446-448. Max. coverage (+): 0.04. Max coverage (-): 0

Region: NODE\_283949\_length\_981\_cov\_75.131500 449-450. Max. coverage (+): 0.7. Max coverage (-): 0

Region: NODE\_283949\_length\_981\_cov\_75.131500 451-452. Max. coverage (+): 0.7. Max coverage (-): 0

Region: NODE\_283949\_length\_981\_cov\_75.131500 453-454. Max. coverage (+): 0.19. Max coverage (-): 0

Region: NODE\_283949\_length\_981\_cov\_75.131500 455-456. Max. coverage (+): 0.19. Max coverage (-): 0

Region: NODE\_283949\_length\_981\_cov\_75.131500 457-458. Max. coverage (+): 0.04. Max coverage (-): 0

Region: NODE\_283949\_length\_981\_cov\_75.131500 459-460. Max. coverage (+): 0.04. Max coverage (-): 0

Region: NODE\_283949\_length\_981\_cov\_75.131500 461-462. Max. coverage (+): 0.04. Max coverage (-): 0

Region: NODE\_283949\_length\_981\_cov\_75.131500 463-464. Max. coverage (+): 0.04. Max coverage (-): 0.04

Region: NODE\_283949\_length\_981\_cov\_75.131500 465-466. Max. coverage (+): 0.04. Max coverage (-): 0.04

Region: NODE\_283949\_length\_981\_cov\_75.131500 467-469. Max. coverage (+): 0. Max coverage (-): 0.04

Region: NODE\_283949\_length\_981\_cov\_75.131500 470-471. Max. coverage (+): 0. Max coverage (-): 0.04

Region: NODE\_283949\_length\_981\_cov\_75.131500 472-473. Max. coverage (+): 0. Max coverage (-): 0

Region: NODE\_283949\_length\_981\_cov\_75.131500 474-475. Max. coverage (+): 0. Max coverage (-): 0

Region: NODE\_283949\_length\_981\_cov\_75.131500 476-477. Max. coverage (+): 0. Max coverage (-): 0.04

Region: NODE\_283949\_length\_981\_cov\_75.131500 478-479. Max. coverage (+): 0.11. Max coverage (-): 0.04

Region: NODE\_283949\_length\_981\_cov\_75.131500 480-481. Max. coverage (+): 0.33. Max coverage (-): 0

Region: NODE\_283949\_length\_981\_cov\_75.131500 482-483. Max. coverage (+): 0.22. Max coverage (-): 0

Region: NODE\_283949\_length\_981\_cov\_75.131500 484-485. Max. coverage (+): 0.04. Max coverage (-): 0

Region: NODE\_283949\_length\_981\_cov\_75.131500 486-487. Max. coverage (+): 0. Max coverage (-): 0.07

Region: NODE\_283949\_length\_981\_cov\_75.131500 488-489. Max. coverage (+): 0. Max coverage (-): 0.07

Region: NODE\_283949\_length\_981\_cov\_75.131500 490-492. Max. coverage (+): 0.15. Max coverage (-): 0.26

Region: NODE\_283949\_length\_981\_cov\_75.131500 493-494. Max. coverage (+): 0.19. Max coverage (-): 0.07

Region: NODE\_283949\_length\_981\_cov\_75.131500 495-496. Max. coverage (+): 0.11. Max coverage (-): 0.44

Region: NODE\_283949\_length\_981\_cov\_75.131500 497-498. Max. coverage (+): 0.11. Max coverage (-): 0.67

Region: NODE\_283949\_length\_981\_cov\_75.131500 499-500. Max. coverage (+): 0.07. Max coverage (-): 1

Region: NODE\_283949\_length\_981\_cov\_75.131500 501-502. Max. coverage (+): 0.04. Max coverage (-): 0.59

Region: NODE\_283949\_length\_981\_cov\_75.131500 503-504. Max. coverage (+): 0. Max coverage (-): 0.04

Region: NODE\_283949\_length\_981\_cov\_75.131500 505-506. Max. coverage (+): 0.19. Max coverage (-): 0.07

Region: NODE\_283949\_length\_981\_cov\_75.131500 507-508. Max. coverage (+): 1.15. Max coverage (-): 0.07

Region: NODE\_283949\_length\_981\_cov\_75.131500 509-510. Max. coverage (+): 1.89. Max coverage (-): 0

Region: NODE\_283949\_length\_981\_cov\_75.131500 511-512. Max. coverage (+): 1.96. Max coverage (-): 0

Region: NODE\_283949\_length\_981\_cov\_75.131500 513-515. Max. coverage (+): 5.04. Max coverage (-): 0

Region: NODE\_283949\_length\_981\_cov\_75.131500 516-517. Max. coverage (+): 0.48. Max coverage (-): 0

Region: NODE\_283949\_length\_981\_cov\_75.131500 518-519. Max. coverage (+): 0.11. Max coverage (-): 0

Region: NODE\_283949\_length\_981\_cov\_75.131500 520-521. Max. coverage (+): 0.04. Max coverage (-): 0

Region: NODE\_283949\_length\_981\_cov\_75.131500 522-523. Max. coverage (+): 0. Max coverage (-): 0

Region: NODE\_283949\_length\_981\_cov\_75.131500 524-525. Max. coverage (+): 0. Max coverage (-): 0

Region: NODE\_283949\_length\_981\_cov\_75.131500 526-527. Max. coverage (+): 0. Max coverage (-): 0

Region: NODE\_283949\_length\_981\_cov\_75.131500 528-529. Max. coverage (+): 0. Max coverage (-): 0

Region: NODE\_283949\_length\_981\_cov\_75.131500 530-531. Max. coverage (+): 0. Max coverage (-): 0.19

Region: NODE\_283949\_length\_981\_cov\_75.131500 532-533. Max. coverage (+): 0.04. Max coverage (-): 0.19

Region: NODE\_283949\_length\_981\_cov\_75.131500 534-536. Max. coverage (+): 0.04. Max coverage (-): 0

Region: NODE\_283949\_length\_981\_cov\_75.131500 537-538. Max. coverage (+): 0.15. Max coverage (-): 0

Region: NODE\_283949\_length\_981\_cov\_75.131500 539-540. Max. coverage (+): 0.26. Max coverage (-): 0

Region: NODE\_283949\_length\_981\_cov\_75.131500 541-542. Max. coverage (+): 0.26. Max coverage (-): 0

Region: NODE\_283949\_length\_981\_cov\_75.131500 543-544. Max. coverage (+): 0.19. Max coverage (-): 0

Region: NODE\_283949\_length\_981\_cov\_75.131500 545-546. Max. coverage (+): 0.41. Max coverage (-): 0.04

Region: NODE\_283949\_length\_981\_cov\_75.131500 547-548. Max. coverage (+): 3.56. Max coverage (-): 0.04

Region: NODE\_283949\_length\_981\_cov\_75.131500 549-550. Max. coverage (+): 3.86. Max coverage (-): 0

Region: NODE\_283949\_length\_981\_cov\_75.131500 551-552. Max. coverage (+): 1.63. Max coverage (-): 0

Region: NODE\_283949\_length\_981\_cov\_75.131500 553-554. Max. coverage (+): 20.83. Max coverage (-): 0

Region: NODE\_283949\_length\_981\_cov\_75.131500 555-556. Max. coverage (+): 19.98. Max coverage (-): 0

Region: NODE\_283949\_length\_981\_cov\_75.131500 557-559. Max. coverage (+): 0.19. Max coverage (-): 0

Region: NODE\_283949\_length\_981\_cov\_75.131500 560-561. Max. coverage (+): 0.07. Max coverage (-): 0

Region: NODE\_283949\_length\_981\_cov\_75.131500 562-563. Max. coverage (+): 0. Max coverage (-): 0.04

Region: NODE\_283949\_length\_981\_cov\_75.131500 564-565. Max. coverage (+): 0. Max coverage (-): 0.04

Region: NODE\_283949\_length\_981\_cov\_75.131500 566-567. Max. coverage (+): 0.07. Max coverage (-): 0

Region: NODE\_283949\_length\_981\_cov\_75.131500 568-569. Max. coverage (+): 0.07. Max coverage (-): 0

Region: NODE\_283949\_length\_981\_cov\_75.131500 570-571. Max. coverage (+): 0. Max coverage (-): 0.04

Region: NODE\_283949\_length\_981\_cov\_75.131500 572-573. Max. coverage (+): 0. Max coverage (-): 0.04

Region: NODE\_283949\_length\_981\_cov\_75.131500 574-575. Max. coverage (+): 0. Max coverage (-): 0

Region: NODE\_283949\_length\_981\_cov\_75.131500 576-577. Max. coverage (+): 0. Max coverage (-): 0

Region: NODE\_283949\_length\_981\_cov\_75.131500 578-579. Max. coverage (+): 0. Max coverage (-): 0

Region: NODE\_283949\_length\_981\_cov\_75.131500 580-582. Max. coverage (+): 0. Max coverage (-): 0

Region: NODE\_283949\_length\_981\_cov\_75.131500 583-584. Max. coverage (+): 0.11. Max coverage (-): 0

Region: NODE\_283949\_length\_981\_cov\_75.131500 585-586. Max. coverage (+): 0.11. Max coverage (-): 0

Region: NODE\_283949\_length\_981\_cov\_75.131500 587-588. Max. coverage (+): 0. Max coverage (-): 0

Region: NODE\_283949\_length\_981\_cov\_75.131500 589-590. Max. coverage (+): 0. Max coverage (-): 0

Region: NODE\_283949\_length\_981\_cov\_75.131500 591-592. Max. coverage (+): 0. Max coverage (-): 0

Region: NODE\_283949\_length\_981\_cov\_75.131500 593-594. Max. coverage (+): 0.3. Max coverage (-): 0

Region: NODE\_283949\_length\_981\_cov\_75.131500 595-596. Max. coverage (+): 0.3. Max coverage (-): 0

Region: NODE\_283949\_length\_981\_cov\_75.131500 597-598. Max. coverage (+): 0.15. Max coverage (-): 0

Region: NODE\_283949\_length\_981\_cov\_75.131500 599-600. Max. coverage (+): 0.04. Max coverage (-): 0

Region: NODE\_283949\_length\_981\_cov\_75.131500 601-603. Max. coverage (+): 0.04. Max coverage (-): 0

Region: NODE\_283949\_length\_981\_cov\_75.131500 604-605. Max. coverage (+): 0.04. Max coverage (-): 0

Region: NODE\_283949\_length\_981\_cov\_75.131500 606-607. Max. coverage (+): 6.45. Max coverage (-): 0

Region: NODE\_283949\_length\_981\_cov\_75.131500 608-609. Max. coverage (+): 6.56. Max coverage (-): 0.04

Region: NODE\_283949\_length\_981\_cov\_75.131500 610-611. Max. coverage (+): 0.44. Max coverage (-): 0.04

Region: NODE\_283949\_length\_981\_cov\_75.131500 612-613. Max. coverage (+): 0.59. Max coverage (-): 0

Region: NODE\_283949\_length\_981\_cov\_75.131500 614-615. Max. coverage (+): 0.3. Max coverage (-): 0

Region: NODE\_283949\_length\_981\_cov\_75.131500 616-617. Max. coverage (+): 1.59. Max coverage (-): 0

Region: NODE\_283949\_length\_981\_cov\_75.131500 618-619. Max. coverage (+): 12.31. Max coverage (-): 0

Region: NODE\_283949\_length\_981\_cov\_75.131500 620-621. Max. coverage (+): 11.72. Max coverage (-): 0

Region: NODE\_283949\_length\_981\_cov\_75.131500 622-623. Max. coverage (+): 2.11. Max coverage (-): 0

Region: NODE\_283949\_length\_981\_cov\_75.131500 624-626. Max. coverage (+): 2.04. Max coverage (-): 0.11

Region: NODE\_283949\_length\_981\_cov\_75.131500 627-628. Max. coverage (+): 0.04. Max coverage (-): 0.07

Region: NODE\_283949\_length\_981\_cov\_75.131500 629-630. Max. coverage (+): 0. Max coverage (-): 0

Region: NODE\_283949\_length\_981\_cov\_75.131500 631-632. Max. coverage (+): 0. Max coverage (-): 0.04

Region: NODE\_283949\_length\_981\_cov\_75.131500 633-634. Max. coverage (+): 0. Max coverage (-): 0.11

Region: NODE\_283949\_length\_981\_cov\_75.131500 635-636. Max. coverage (+): 0.07. Max coverage (-): 0.15

Region: NODE\_283949\_length\_981\_cov\_75.131500 637-638. Max. coverage (+): 0.19. Max coverage (-): 0.26

Region: NODE\_283949\_length\_981\_cov\_75.131500 639-640. Max. coverage (+): 0.82. Max coverage (-): 0.19

Region: NODE\_283949\_length\_981\_cov\_75.131500 641-642. Max. coverage (+): 0.96. Max coverage (-): 0.11

Region: NODE\_283949\_length\_981\_cov\_75.131500 643-644. Max. coverage (+): 0.26. Max coverage (-): 0.11

Region: NODE\_283949\_length\_981\_cov\_75.131500 645-646. Max. coverage (+): 0.74. Max coverage (-): 0

Region: NODE\_283949\_length\_981\_cov\_75.131500 647-649. Max. coverage (+): 0.7. Max coverage (-): 0

Region: NODE\_283949\_length\_981\_cov\_75.131500 650-651. Max. coverage (+): 0.56. Max coverage (-): 0

Region: NODE\_283949\_length\_981\_cov\_75.131500 652-653. Max. coverage (+): 0.59. Max coverage (-): 0

Region: NODE\_283949\_length\_981\_cov\_75.131500 654-655. Max. coverage (+): 4.52. Max coverage (-): 0

Region: NODE\_283949\_length\_981\_cov\_75.131500 656-657. Max. coverage (+): 6.64. Max coverage (-): 0

Region: NODE\_283949\_length\_981\_cov\_75.131500 658-659. Max. coverage (+): 10.16. Max coverage (-): 0

Region: NODE\_283949\_length\_981\_cov\_75.131500 660-661. Max. coverage (+): 6.56. Max coverage (-): 0.04

Region: NODE\_283949\_length\_981\_cov\_75.131500 662-663. Max. coverage (+): 1. Max coverage (-): 0.04

Region: NODE\_283949\_length\_981\_cov\_75.131500 664-665. Max. coverage (+): 0.15. Max coverage (-): 0.04

Region: NODE\_283949\_length\_981\_cov\_75.131500 666-667. Max. coverage (+): 0.22. Max coverage (-): 0.04

Region: NODE\_283949\_length\_981\_cov\_75.131500 668-670. Max. coverage (+): 0.63. Max coverage (-): 0.19

Region: NODE\_283949\_length\_981\_cov\_75.131500 671-672. Max. coverage (+): 0.37. Max coverage (-): 0.19

Region: NODE\_283949\_length\_981\_cov\_75.131500 673-674. Max. coverage (+): 0.15. Max coverage (-): 0

Region: NODE\_283949\_length\_981\_cov\_75.131500 675-676. Max. coverage (+): 0.22. Max coverage (-): 0

Region: NODE\_283949\_length\_981\_cov\_75.131500 677-678. Max. coverage (+): 0.11. Max coverage (-): 0

Region: NODE\_283949\_length\_981\_cov\_75.131500 679-680. Max. coverage (+): 0. Max coverage (-): 0

Region: NODE\_283949\_length\_981\_cov\_75.131500 681-682. Max. coverage (+): 0. Max coverage (-): 0

Region: NODE\_283949\_length\_981\_cov\_75.131500 683-684. Max. coverage (+): 0. Max coverage (-): 0

Region: NODE\_283949\_length\_981\_cov\_75.131500 685-686. Max. coverage (+): 0.11. Max coverage (-): 0

Region: NODE\_283949\_length\_981\_cov\_75.131500 687-688. Max. coverage (+): 1.37. Max coverage (-): 0

Region: NODE\_283949\_length\_981\_cov\_75.131500 689-690. Max. coverage (+): 1.26. Max coverage (-): 0

Region: NODE\_283949\_length\_981\_cov\_75.131500 691-693. Max. coverage (+): 0.11. Max coverage (-): 0

Region: NODE\_283949\_length\_981\_cov\_75.131500 694-695. Max. coverage (+): 0.07. Max coverage (-): 0.04

Region: NODE\_283949\_length\_981\_cov\_75.131500 696-697. Max. coverage (+): 0.04. Max coverage (-): 0.04

Region: NODE\_283949\_length\_981\_cov\_75.131500 698-699. Max. coverage (+): 0.07. Max coverage (-): 0

Region: NODE\_283949\_length\_981\_cov\_75.131500 700-701. Max. coverage (+): 0.15. Max coverage (-): 0

Region: NODE\_283949\_length\_981\_cov\_75.131500 702-703. Max. coverage (+): 0.11. Max coverage (-): 0

Region: NODE\_283949\_length\_981\_cov\_75.131500 704-705. Max. coverage (+): 0.04. Max coverage (-): 0

Region: NODE\_283949\_length\_981\_cov\_75.131500 706-707. Max. coverage (+): 0.04. Max coverage (-): 0

Region: NODE\_283949\_length\_981\_cov\_75.131500 708-709. Max. coverage (+): 0.26. Max coverage (-): 0.04

Region: NODE\_283949\_length\_981\_cov\_75.131500 710-711. Max. coverage (+): 0.26. Max coverage (-): 0.04

Region: NODE\_283949\_length\_981\_cov\_75.131500 712-714. Max. coverage (+): 0.07. Max coverage (-): 0

Region: NODE\_283949\_length\_981\_cov\_75.131500 715-716. Max. coverage (+): 0.07. Max coverage (-): 0

Region: NODE\_283949\_length\_981\_cov\_75.131500 717-718. Max. coverage (+): 0.44. Max coverage (-): 0

Region: NODE\_283949\_length\_981\_cov\_75.131500 719-720. Max. coverage (+): 0.59. Max coverage (-): 0

Region: NODE\_283949\_length\_981\_cov\_75.131500 721-722. Max. coverage (+): 0.48. Max coverage (-): 0.04

Region: NODE\_283949\_length\_981\_cov\_75.131500 723-724. Max. coverage (+): 0.26. Max coverage (-): 0.04

Region: NODE\_283949\_length\_981\_cov\_75.131500 725-726. Max. coverage (+): 0. Max coverage (-): 0

Region: NODE\_283949\_length\_981\_cov\_75.131500 727-728. Max. coverage (+): 0. Max coverage (-): 0

Region: NODE\_283949\_length\_981\_cov\_75.131500 729-730. Max. coverage (+): 0. Max coverage (-): 0

Region: NODE\_283949\_length\_981\_cov\_75.131500 731-732. Max. coverage (+): 0. Max coverage (-): 0

Region: NODE\_283949\_length\_981\_cov\_75.131500 733-734. Max. coverage (+): 0. Max coverage (-): 0

Region: NODE\_283949\_length\_981\_cov\_75.131500 735-737. Max. coverage (+): 0.11. Max coverage (-): 0

Region: NODE\_283949\_length\_981\_cov\_75.131500 738-739. Max. coverage (+): 0.07. Max coverage (-): 0.04

Region: NODE\_283949\_length\_981\_cov\_75.131500 740-741. Max. coverage (+): 0. Max coverage (-): 0.04

Region: NODE\_283949\_length\_981\_cov\_75.131500 742-743. Max. coverage (+): 0.04. Max coverage (-): 0

Region: NODE\_283949\_length\_981\_cov\_75.131500 744-745. Max. coverage (+): 0.33. Max coverage (-): 0

Region: NODE\_283949\_length\_981\_cov\_75.131500 746-747. Max. coverage (+): 0.3. Max coverage (-): 0

Region: NODE\_283949\_length\_981\_cov\_75.131500 748-749. Max. coverage (+): 1.26. Max coverage (-): 0

Region: NODE\_283949\_length\_981\_cov\_75.131500 750-751. Max. coverage (+): 1.26. Max coverage (-): 0.04

Region: NODE\_283949\_length\_981\_cov\_75.131500 752-753. Max. coverage (+): 0.33. Max coverage (-): 0.04

Region: NODE\_283949\_length\_981\_cov\_75.131500 754-755. Max. coverage (+): 0.33. Max coverage (-): 0.04

Region: NODE\_283949\_length\_981\_cov\_75.131500 756-757. Max. coverage (+): 0.59. Max coverage (-): 0.04

Region: NODE\_283949\_length\_981\_cov\_75.131500 758-760. Max. coverage (+): 0.56. Max coverage (-): 0.04

Region: NODE\_283949\_length\_981\_cov\_75.131500 761-762. Max. coverage (+): 0. Max coverage (-): 0

Region: NODE\_283949\_length\_981\_cov\_75.131500 763-764. Max. coverage (+): 0. Max coverage (-): 0

Region: NODE\_283949\_length\_981\_cov\_75.131500 765-766. Max. coverage (+): 0.04. Max coverage (-): 0.04

Region: NODE\_283949\_length\_981\_cov\_75.131500 767-768. Max. coverage (+): 0.11. Max coverage (-): 0.04

Region: NODE\_283949\_length\_981\_cov\_75.131500 769-770. Max. coverage (+): 0.19. Max coverage (-): 0

Region: NODE\_283949\_length\_981\_cov\_75.131500 771-772. Max. coverage (+): 1.3. Max coverage (-): 0.15

Region: NODE\_283949\_length\_981\_cov\_75.131500 773-774. Max. coverage (+): 2.19. Max coverage (-): 0.33

Region: NODE\_283949\_length\_981\_cov\_75.131500 775-776. Max. coverage (+): 0.96. Max coverage (-): 0.44

Region: NODE\_283949\_length\_981\_cov\_75.131500 777-778. Max. coverage (+): 1.37. Max coverage (-): 0.26

Region: NODE\_283949\_length\_981\_cov\_75.131500 779-781. Max. coverage (+): 1.41. Max coverage (-): 0.07

Region: NODE\_283949\_length\_981\_cov\_75.131500 782-783. Max. coverage (+): 0.48. Max coverage (-): 0.7

Region: NODE\_283949\_length\_981\_cov\_75.131500 784-785. Max. coverage (+): 0.56. Max coverage (-): 0.93

Region: NODE\_283949\_length\_981\_cov\_75.131500 786-787. Max. coverage (+): 0.41. Max coverage (-): 0.26

Region: NODE\_283949\_length\_981\_cov\_75.131500 788-789. Max. coverage (+): 0.19. Max coverage (-): 0.26

Region: NODE\_283949\_length\_981\_cov\_75.131500 790-791. Max. coverage (+): 0.26. Max coverage (-): 0.37

Region: NODE\_283949\_length\_981\_cov\_75.131500 792-793. Max. coverage (+): 0.11. Max coverage (-): 1.19

Region: NODE\_283949\_length\_981\_cov\_75.131500 794-795. Max. coverage (+): 0.04. Max coverage (-): 1.04

Region: NODE\_283949\_length\_981\_cov\_75.131500 796-797. Max. coverage (+): 0. Max coverage (-): 0.59

Region: NODE\_283949\_length\_981\_cov\_75.131500 798-799. Max. coverage (+): 4.23. Max coverage (-): 0.44

Region: NODE\_283949\_length\_981\_cov\_75.131500 800-801. Max. coverage (+): 4.3. Max coverage (-): 0.22

Region: NODE\_283949\_length\_981\_cov\_75.131500 802-804. Max. coverage (+): 1.37. Max coverage (-): 0.15

Region: NODE\_283949\_length\_981\_cov\_75.131500 805-806. Max. coverage (+): 0.93. Max coverage (-): 0

Region: NODE\_283949\_length\_981\_cov\_75.131500 807-808. Max. coverage (+): 0.26. Max coverage (-): 0

Region: NODE\_283949\_length\_981\_cov\_75.131500 809-810. Max. coverage (+): 0.85. Max coverage (-): 0

Region: NODE\_283949\_length\_981\_cov\_75.131500 811-812. Max. coverage (+): 0.85. Max coverage (-): 0

Region: NODE\_283949\_length\_981\_cov\_75.131500 813-814. Max. coverage (+): 0.04. Max coverage (-): 0.04

Region: NODE\_283949\_length\_981\_cov\_75.131500 815-816. Max. coverage (+): 0.04. Max coverage (-): 0.04

Region: NODE\_283949\_length\_981\_cov\_75.131500 817-818. Max. coverage (+): 0. Max coverage (-): 0

Region: NODE\_283949\_length\_981\_cov\_75.131500 819-820. Max. coverage (+): 0. Max coverage (-): 0

Region: NODE\_283949\_length\_981\_cov\_75.131500 821-822. Max. coverage (+): 0.04. Max coverage (-): 0

Region: NODE\_283949\_length\_981\_cov\_75.131500 823-824. Max. coverage (+): 0.04. Max coverage (-): 0

Region: NODE\_283949\_length\_981\_cov\_75.131500 825-827. Max. coverage (+): 0. Max coverage (-): 0

Region: NODE\_283949\_length\_981\_cov\_75.131500 828-829. Max. coverage (+): 0.04. Max coverage (-): 0

Region: NODE\_283949\_length\_981\_cov\_75.131500 830-831. Max. coverage (+): 0.04. Max coverage (-): 0

Region: NODE\_283949\_length\_981\_cov\_75.131500 832-833. Max. coverage (+): 0. Max coverage (-): 0.04

Region: NODE\_283949\_length\_981\_cov\_75.131500 834-835. Max. coverage (+): 0. Max coverage (-): 0.04

Region: NODE\_283949\_length\_981\_cov\_75.131500 836-837. Max. coverage (+): 0. Max coverage (-): 0

Region: NODE\_283949\_length\_981\_cov\_75.131500 838-839. Max. coverage (+): 0.04. Max coverage (-): 0

Region: NODE\_283949\_length\_981\_cov\_75.131500 840-841. Max. coverage (+): 0.63. Max coverage (-): 0

Region: NODE\_283949\_length\_981\_cov\_75.131500 842-843. Max. coverage (+): 0.67. Max coverage (-): 0

Region: NODE\_283949\_length\_981\_cov\_75.131500 844-845. Max. coverage (+): 0.04. Max coverage (-): 0

Region: NODE\_283949\_length\_981\_cov\_75.131500 846-848. Max. coverage (+): 0.74. Max coverage (-): 0

Region: NODE\_283949\_length\_981\_cov\_75.131500 849-850. Max. coverage (+): 1. Max coverage (-): 0

Region: NODE\_283949\_length\_981\_cov\_75.131500 851-852. Max. coverage (+): 1.3. Max coverage (-): 0.04

Region: NODE\_283949\_length\_981\_cov\_75.131500 853-854. Max. coverage (+): 1.3. Max coverage (-): 0.04

Region: NODE\_283949\_length\_981\_cov\_75.131500 855-856. Max. coverage (+): 1.22. Max coverage (-): 0.07

Region: NODE\_283949\_length\_981\_cov\_75.131500 857-858. Max. coverage (+): 2.11. Max coverage (-): 0.04

Region: NODE\_283949\_length\_981\_cov\_75.131500 859-860. Max. coverage (+): 1.48. Max coverage (-): 0.04

Region: NODE\_283949\_length\_981\_cov\_75.131500 861-862. Max. coverage (+): 0.59. Max coverage (-): 0.04

Region: NODE\_283949\_length\_981\_cov\_75.131500 863-864. Max. coverage (+): 0.22. Max coverage (-): 0

Region: NODE\_283949\_length\_981\_cov\_75.131500 865-866. Max. coverage (+): 0.22. Max coverage (-): 0

Region: NODE\_283949\_length\_981\_cov\_75.131500 867-868. Max. coverage (+): 0.22. Max coverage (-): 0

Region: NODE\_283949\_length\_981\_cov\_75.131500 869-871. Max. coverage (+): 6.38. Max coverage (-): 0

Region: NODE\_283949\_length\_981\_cov\_75.131500 872-873. Max. coverage (+): 6.01. Max coverage (-): 0

Region: NODE\_283949\_length\_981\_cov\_75.131500 874-875. Max. coverage (+): 0.41. Max coverage (-): 0

Region: NODE\_283949\_length\_981\_cov\_75.131500 876-877. Max. coverage (+): 0.19. Max coverage (-): 0

Region: NODE\_283949\_length\_981\_cov\_75.131500 878-879. Max. coverage (+): 0.52. Max coverage (-): 0

Region: NODE\_283949\_length\_981\_cov\_75.131500 880-881. Max. coverage (+): 0.93. Max coverage (-): 0

Region: NODE\_283949\_length\_981\_cov\_75.131500 882-883. Max. coverage (+): 0.48. Max coverage (-): 0

Region: NODE\_283949\_length\_981\_cov\_75.131500 884-885. Max. coverage (+): 0.11. Max coverage (-): 0

Region: NODE\_283949\_length\_981\_cov\_75.131500 886-887. Max. coverage (+): 0.04. Max coverage (-): 0

Region: NODE\_283949\_length\_981\_cov\_75.131500 888-889. Max. coverage (+): 0. Max coverage (-): 0

Region: NODE\_283949\_length\_981\_cov\_75.131500 890-891. Max. coverage (+): 0.48. Max coverage (-): 0.04

Region: NODE\_283949\_length\_981\_cov\_75.131500 892-894. Max. coverage (+): 0.52. Max coverage (-): 0.11

Region: NODE\_283949\_length\_981\_cov\_75.131500 895-896. Max. coverage (+): 0.04. Max coverage (-): 0.07

Region: NODE\_283949\_length\_981\_cov\_75.131500 897-898. Max. coverage (+): 0. Max coverage (-): 0.78

Region: NODE\_283949\_length\_981\_cov\_75.131500 899-900. Max. coverage (+): 0.04. Max coverage (-): 0.93

Region: NODE\_283949\_length\_981\_cov\_75.131500 901-902. Max. coverage (+): 0.04. Max coverage (-): 2.15

Region: NODE\_283949\_length\_981\_cov\_75.131500 903-904. Max. coverage (+): 0. Max coverage (-): 2.34

Region: NODE\_283949\_length\_981\_cov\_75.131500 905-906. Max. coverage (+): 0.07. Max coverage (-): 0.26

Region: NODE\_283949\_length\_981\_cov\_75.131500 907-908. Max. coverage (+): 0.11. Max coverage (-): 0

Region: NODE\_283949\_length\_981\_cov\_75.131500 909-910. Max. coverage (+): 0.22. Max coverage (-): 0

Region: NODE\_283949\_length\_981\_cov\_75.131500 911-912. Max. coverage (+): 2.97. Max coverage (-): 0

Region: NODE\_283949\_length\_981\_cov\_75.131500 913-915. Max. coverage (+): 10.83. Max coverage (-): 0.04

Region: NODE\_283949\_length\_981\_cov\_75.131500 916-917. Max. coverage (+): 10.2. Max coverage (-): 0

Region: NODE\_283949\_length\_981\_cov\_75.131500 918-919. Max. coverage (+): 1.15. Max coverage (-): 0

Region: NODE\_283949\_length\_981\_cov\_75.131500 920-921. Max. coverage (+): 0.04. Max coverage (-): 0

Region: NODE\_283949\_length\_981\_cov\_75.131500 922-923. Max. coverage (+): 0.07. Max coverage (-): 0

Region: NODE\_283949\_length\_981\_cov\_75.131500 924-925. Max. coverage (+): 0.3. Max coverage (-): 0

Region: NODE\_283949\_length\_981\_cov\_75.131500 926-927. Max. coverage (+): 0.52. Max coverage (-): 0

Region: NODE\_283949\_length\_981\_cov\_75.131500 928-929. Max. coverage (+): 0.93. Max coverage (-): 0

Region: NODE\_283949\_length\_981\_cov\_75.131500 930-931. Max. coverage (+): 1.56. Max coverage (-): 0

Region: NODE\_283949\_length\_981\_cov\_75.131500 932-933. Max. coverage (+): 1.26. Max coverage (-): 0

Region: NODE\_283949\_length\_981\_cov\_75.131500 934-935. Max. coverage (+): 1. Max coverage (-): 0.07

Region: NODE\_283949\_length\_981\_cov\_75.131500 936-938. Max. coverage (+): 0.44. Max coverage (-): 0.07

Region: NODE\_283949\_length\_981\_cov\_75.131500 939-940. Max. coverage (+): 0. Max coverage (-): 0

Region: NODE\_283949\_length\_981\_cov\_75.131500 941-942. Max. coverage (+): 0. Max coverage (-): 0

Region: NODE\_283949\_length\_981\_cov\_75.131500 943-944. Max. coverage (+): 0. Max coverage (-): 0

Region: NODE\_283949\_length\_981\_cov\_75.131500 945-946. Max. coverage (+): 0. Max coverage (-): 0

Region: NODE\_283949\_length\_981\_cov\_75.131500 947-948. Max. coverage (+): 0. Max coverage (-): 0

Region: NODE\_283949\_length\_981\_cov\_75.131500 949-950. Max. coverage (+): 0.07. Max coverage (-): 0

Region: NODE\_283949\_length\_981\_cov\_75.131500 951-952. Max. coverage (+): 0.07. Max coverage (-): 0

Region: NODE\_283949\_length\_981\_cov\_75.131500 953-954. Max. coverage (+): 0. Max coverage (-): 0

Region: NODE\_283949\_length\_981\_cov\_75.131500 955-956. Max. coverage (+): 0. Max coverage (-): 0

Region: NODE\_283949\_length\_981\_cov\_75.131500 957-959. Max. coverage (+): 0. Max coverage (-): 0

Region: NODE\_283949\_length\_981\_cov\_75.131500 960-961. Max. coverage (+): 0. Max coverage (-): 0.07

Region: NODE\_283949\_length\_981\_cov\_75.131500 962-963. Max. coverage (+): 0. Max coverage (-): 0.26

Region: NODE\_283949\_length\_981\_cov\_75.131500 964-965. Max. coverage (+): 0. Max coverage (-): 0.22

Region: NODE\_283949\_length\_981\_cov\_75.131500 966-967. Max. coverage (+): 0. Max coverage (-): 0

Region: NODE\_283949\_length\_981\_cov\_75.131500 968-969. Max. coverage (+): 0.07. Max coverage (-): 0

Region: NODE\_283949\_length\_981\_cov\_75.131500 970-971. Max. coverage (+): 0.07. Max coverage (-): 0.11

Region: NODE\_283949\_length\_981\_cov\_75.131500 972-973. Max. coverage (+): 0.04. Max coverage (-): 0.19

Region: NODE\_283949\_length\_981\_cov\_75.131500 974-975. Max. coverage (+): 0.19. Max coverage (-): 0.07

Region: NODE\_283949\_length\_981\_cov\_75.131500 976-977. Max. coverage (+): 0.26. Max coverage (-): 0.11

Region: NODE\_283949\_length\_981\_cov\_75.131500 978-979. Max. coverage (+): 0.93. Max coverage (-): 0.04

Region: NODE\_283949\_length\_981\_cov\_75.131500 980-982. Max. coverage (+): 0.89. Max coverage (-): 0.04

Region: NODE\_283949\_length\_981\_cov\_75.131500 983-984. Max. coverage (+): 0.56. Max coverage (-): 0

Region: NODE\_283949\_length\_981\_cov\_75.131500 985-986. Max. coverage (+): 0.22. Max coverage (-): 0

Region: NODE\_283949\_length\_981\_cov\_75.131500 987-988. Max. coverage (+): 0.09. Max coverage (-): 0.02

Region: NODE\_283949\_length\_981\_cov\_75.131500 989-990. Max. coverage (+): 0.11. Max coverage (-): 0.02

Region: NODE\_283949\_length\_981\_cov\_75.131500 991-992. Max. coverage (+): 0.09. Max coverage (-): 0.04

Region: NODE\_283949\_length\_981\_cov\_75.131500 993-994. Max. coverage (+): 0. Max coverage (-): 0.04

Region: NODE\_283949\_length\_981\_cov\_75.131500 995-996. Max. coverage (+): 0. Max coverage (-): 0.19

Region: NODE\_283949\_length\_981\_cov\_75.131500 997-998. Max. coverage (+): 0. Max coverage (-): 0.17

Region: NODE\_283949\_length\_981\_cov\_75.131500 999-1000. Max. coverage (+): 0. Max coverage (-): 0

Region: NODE\_283949\_length\_981\_cov\_75.131500 1001-1002. Max. coverage (+): 0. Max coverage (-): 0.04

Region: NODE\_283949\_length\_981\_cov\_75.131500 1003-1005. Max. coverage (+): 0.04. Max coverage (-): 0.04

Region: NODE\_283949\_length\_981\_cov\_75.131500 1006-1007. Max. coverage (+): 0.04. Max coverage (-): 0

Region: NODE\_283949\_length\_981\_cov\_75.131500 1008-1009. Max. coverage (+): 0.04. Max coverage (-): 0

Region: NODE\_283949\_length\_981\_cov\_75.131500 1010-1011. Max. coverage (+): 0. Max coverage (-): 0

Region: NODE\_283949\_length\_981\_cov\_75.131500 1012-1013. Max. coverage (+): 0.02. Max coverage (-): 0

Region: NODE\_283949\_length\_981\_cov\_75.131500 1014-1015. Max. coverage (+): 0.13. Max coverage (-): 0.02

Region: NODE\_283949\_length\_981\_cov\_75.131500 1016-1017. Max. coverage (+): 0.2. Max coverage (-): 0.02

Region: NODE\_283949\_length\_981\_cov\_75.131500 1018-1019. Max. coverage (+): 0.2. Max coverage (-): 0

Region: NODE\_283949\_length\_981\_cov\_75.131500 1020-1021. Max. coverage (+): 0.11. Max coverage (-): 0

Region: NODE\_283949\_length\_981\_cov\_75.131500 1022-1023. Max. coverage (+): 0.02. Max coverage (-): 0

Region: NODE\_283949\_length\_981\_cov\_75.131500 1024-1026. Max. coverage (+): 0. Max coverage (-): 0

Region: NODE\_283949\_length\_981\_cov\_75.131500 1027-1028. Max. coverage (+): 0. Max coverage (-): 0

Region: NODE\_283949\_length\_981\_cov\_75.131500 1029-1030. Max. coverage (+): 0. Max coverage (-): 0

Region: NODE\_283949\_length\_981\_cov\_75.131500 1031-1032. Max. coverage (+): 0. Max coverage (-): 0

Region: NODE\_283949\_length\_981\_cov\_75.131500 1033-1034. Max. coverage (+): 0. Max coverage (-): 0

Region: NODE\_283949\_length\_981\_cov\_75.131500 1035-1036. Max. coverage (+): 0. Max coverage (-): 0

Region: NODE\_283949\_length\_981\_cov\_75.131500 1037-1038. Max. coverage (+): 0. Max coverage (-): 0

Region: NODE\_283949\_length\_981\_cov\_75.131500 1039-1040. Max. coverage (+): 0. Max coverage (-): 0

Region: NODE\_283949\_length\_981\_cov\_75.131500 1041-1042. Max. coverage (+): 0. Max coverage (-): 0

Region: NODE\_283949\_length\_981\_cov\_75.131500 1043-1044. Max. coverage (+): 0. Max coverage (-): 0

Region: NODE\_283949\_length\_981\_cov\_75.131500 1045-1046. Max. coverage (+): 0. Max coverage (-): 0

Region: NODE\_283949\_length\_981\_cov\_75.131500 1047-. Max. coverage (+): 0. Max coverage (-): 0

RepeatMasker Color Code

**+**

100-98% Identity

<98-95% Identity

<95-90% Identity

<90-85% Identity

<85-80% Identity

<80-75% Identity

<75-70% Identity

<70% Identity

**-**

Gene Set Color Code

**+**

Gene

Pseudogene

Other

**-**

Topology/Coverage Color Code

Coverage Plus Strand

Coverage Minus Strand

Mainstrand: Plus

Mainstrand: Minus

Complementary Strand

Flanking Region  
(if option -flank >0)

Gene Set Annotation  
  
RepeatMasker Annotation  

**1. AlRepD-182**: 7-114 (+), Divergence to consensus: 31.6%  
**2. AlRepE-2127**: 856-932 (+), Divergence to consensus: 39.2%  
**3. AlRepB-136**: 957-1045 (+), Divergence to consensus: 29.3%

  
Transcription Factor Binding Sites  

**POU5F1** (Sequence: TTTGCAT (-): 540)  
**FOXO3\_mmu** (Sequence: TCTAAACA (+): 567)  
**Sox5** (Sequence: AACAAT (-): 140)
