# Supplementary material for: First characterization of PIWI-interacting RNA clusters in a cichlid fish with a B chromosome
Source: BMC Biol. 2022 Sep 21;20:204. doi: 10.1186/s12915-022-01403-2 (PMC9490952; doi:10.1186/s12915-022-01403-2)
Supplement: Supplementary file 1 — Additional file 1. Zipped folder with fasta and interactive html piRNA cluster information for the A. latifasciata genome. The nomenclature is as follows: number-pirna-cluster_sex_B-presence (f, female; m, male; 0b, without B chromosome; 1b, with B chromosome). [file 12915_2022_1403_MOESM1_ESM.zip › 10_m0b.html]

piRNA cluster 10\_m0b 21


Predicted piRNA cluster no. 10\_m0b
  

Show proTRAC run info
Hide proTRAC run info

/\  
                \_\_\_\_\_\_\_\_\_\_\_\_\_\_\_\_\_\_\_\_\_\_\_/\\_\_\_ /  \\_\_\_\_\_\_\_  
               I                      /  \  /    \      I  
               I     pro             /    \/      \     I  
               I        TRAC        /               \   I  
               I   \_\_\_\_\_\_\_\_\_\_\_\_\_\_\_\_/\_\_\_\_\_\_\_\_\_\_\_\_\_\_\_\_\_\\_ I  
               I   \              /                     I  
               I    \            /                      I  
               I     \  /\      /       V.2.4.2         I  
               I      \/  \    /                        I  
               I\_\_\_\_\_\_\_\_\_\_\_\  /\_\_\_\_\_\_\_\_\_\_\_\_\_\_\_\_\_\_\_\_\_\_\_\_\_I  
                            \/  
  
  
================================= proTRAC ====================================  
VERSION: .......... 2.4.2  
LAST MODIFIED: .... 11. May 2018  
  
Please cite:  
Rosenkranz D, Zischler H. proTRAC - a software for probabilistic piRNA cluster  
detection, visualization and analysis. 2012. BMC Bioinformatics 13:5.  
  
  
Contact:  
David Rosenkranz  
Institute of Organismic and Molecular Evolutionary Biology  
Dept. Anthropology, small RNA group  
Johannes Gutenberg University Mainz  
email: rosenkranz@uni-mainz.de  
  
You can find the latest proTRAC version at:  
http://sourceforge.net/projects/protrac/files  
http://www.smallRNAgroup-mainz.de/software  
==============================================================================  
  
PARAMETERS:  
Map file: ...............piwi-machos-0B.fa-collapse.map  
Genome file: ............../../../0B\_ala\_genome.fa  
RepeatMasker annotation: Alatifasciata-all0B-maryan-v2.fa\_corrected.out  
GeneSet:................./guest-storage/Data/annotation/Alatifasciata\_all0B\_maryan-v2\_out2017.gff  
  
Significant (p<=0.01) hit density will be calculated based  
on observed hit distribution.  
  
Sliding window size: ........................................ 5000 bp  
Sliding window increament: .................................. 1000 bp  
Normalize each hit by number of genomic hits: ............... yes  
Normalize each hit by number of sequence reads: ............. yes  
Normalize values (-> per million mapped reads): ............. yes  
Min. fraction of hits with 1T(U) or 10A: .................... 0.75  
Alternatively: Min. fraction of hits with 1T(U) and 10A: .... 0.5  
Min. fraction of hits with typical piRNA length: ............ 0.75  
Typical piRNA length: ....................................... 24-32 nt  
Min. size of a piRNA cluster: ............................... 1000 bp.  
Min. number of hits (absolute): ............................. 0  
Min. number of hits (normalized): ........................... 0  
Min. fraction of hits on the mainstrand: .................... 0.75  
Top fraction of mapped sequences (in terms of read counts): . 1%  
Top fraction accounts for max. n% of sequence reads: ........ 90%  
Min. fraction of hits on each arm of a bidirectional cluster: 0.05  
Output html file for each cluster: .......................... yes  
Output a summary table: ..................................... yes  
Output a FASTA file for each cluster (piRNA sequences): ..... yes  
Output a FASTA file comprising cluster sequences: ........... yes  
Output a GTF file for predicted piRNA clusters: ..............yes  
Search DNA motifs in clusters: .............................. yes  
Output flanking sequences: +/- .............................. 0 bp  
Output ~.pTi file: .......................................... no  
==============================================================================  
  
  
Genome size (without gaps): ............ 758543724 bp  
Gaps (N/X/-): .......................... 417479 bp  
Mapped reads: .......................... 24765598  
Non-identical sequences: ............... 6158275  
Genomic hits: .......................... 53103584  
Significant densitiy of mapped reads: .. 763.098963422187 reads/kb

Show proTRAC cluster info
Hide proTRAC cluster info

|  |  |
| --- | --- |
| Location | NODE\_117098\_length\_959\_cov\_38.440041 |
| Coordinates | 4-1020 |
| Size [bp] | 1017 |
| Sequence hit loci | 2946 |
| Mapped reads (normalized) | 10078.5 |
| Mapped reads (normalized) per kb | 9910 |
| Normalized reads with 1T (1U) | 75.2% |
| Normalized reads with 10A | 23.3% |
| Normalized reads with length 24-32 nt | 98.6% |
| Normalized reads on the main strand(s) | 91.7% |
| Predicted directionality | mono:plus |

100%

0%

1T (1U)  
reads

10A reads

24-32 nt  
reads

reads on mainstrand

**Either the amount of reads with 1T (1U) OR 10A has to exceed 75% (set with option: -1Tor10A)  
Alternatively the amount of reads with 1T (1U) AND 10A has to exceed 50% (set with option: -1Tand10A)  
Minimum amount of reads with preferred size is 75% (set with option: -pisize)  
Minimum amount of reads on the main strand(s) is 75% (set with option: -clstrand)**

Show read coverage
Hide read coverage

WHAT DO I SEE HERE?  
This chart shows the location of mapped sequence reads within a predicted piRNA cluster. The color refers to the number of genomic hits produced by the sequence read in question. A dark red bar indicates that this sequence read produces many other hits elsewhere in the genome. Many adjacent red or yellow bars can indicate the presence of a multi-copy element such as transposons or rRNA genes. A dark green bar indicates that this sequence read maps uniquely to this locus.

1 hit

2-5 hits

6-10 hits

11-20 hits

21-50 hits

51-100 hits

> 100 hits

NODE\_117098\_length\_959\_cov\_38.440041

4

1020

Gene Set

RepeatMasker

Mapped  
Reads

26.81

plus strand

minus strand

26.81

Region: NODE\_117098\_length\_959\_cov\_38.440041 1943-5. Max. coverage (+): 0.01. Max coverage (-): 0

Region: NODE\_117098\_length\_959\_cov\_38.440041 6-7. Max. coverage (+): 0.03. Max coverage (-): 0

Region: NODE\_117098\_length\_959\_cov\_38.440041 8-9. Max. coverage (+): 0. Max coverage (-): 0

Region: NODE\_117098\_length\_959\_cov\_38.440041 10-11. Max. coverage (+): 0. Max coverage (-): 0.01

Region: NODE\_117098\_length\_959\_cov\_38.440041 12-13. Max. coverage (+): 0. Max coverage (-): 0

Region: NODE\_117098\_length\_959\_cov\_38.440041 14-15. Max. coverage (+): 0. Max coverage (-): 0

Region: NODE\_117098\_length\_959\_cov\_38.440041 16-17. Max. coverage (+): 0. Max coverage (-): 0.01

Region: NODE\_117098\_length\_959\_cov\_38.440041 18-19. Max. coverage (+): 0. Max coverage (-): 0.01

Region: NODE\_117098\_length\_959\_cov\_38.440041 20-21. Max. coverage (+): 0. Max coverage (-): 0.01

Region: NODE\_117098\_length\_959\_cov\_38.440041 22-23. Max. coverage (+): 0. Max coverage (-): 0.01

Region: NODE\_117098\_length\_959\_cov\_38.440041 24-25. Max. coverage (+): 0. Max coverage (-): 0.01

Region: NODE\_117098\_length\_959\_cov\_38.440041 26-27. Max. coverage (+): 0. Max coverage (-): 0

Region: NODE\_117098\_length\_959\_cov\_38.440041 28-29. Max. coverage (+): 0.01. Max coverage (-): 0

Region: NODE\_117098\_length\_959\_cov\_38.440041 30-31. Max. coverage (+): 0.01. Max coverage (-): 0

Region: NODE\_117098\_length\_959\_cov\_38.440041 32-33. Max. coverage (+): 0. Max coverage (-): 0

Region: NODE\_117098\_length\_959\_cov\_38.440041 34-35. Max. coverage (+): 0.01. Max coverage (-): 0

Region: NODE\_117098\_length\_959\_cov\_38.440041 36-37. Max. coverage (+): 0.01. Max coverage (-): 0

Region: NODE\_117098\_length\_959\_cov\_38.440041 38-39. Max. coverage (+): 0.01. Max coverage (-): 0

Region: NODE\_117098\_length\_959\_cov\_38.440041 40-41. Max. coverage (+): 0.03. Max coverage (-): 0

Region: NODE\_117098\_length\_959\_cov\_38.440041 42-43. Max. coverage (+): 0.03. Max coverage (-): 0

Region: NODE\_117098\_length\_959\_cov\_38.440041 44-45. Max. coverage (+): 0.06. Max coverage (-): 0

Region: NODE\_117098\_length\_959\_cov\_38.440041 46-47. Max. coverage (+): 0.06. Max coverage (-): 0.02

Region: NODE\_117098\_length\_959\_cov\_38.440041 48-49. Max. coverage (+): 0. Max coverage (-): 0.06

Region: NODE\_117098\_length\_959\_cov\_38.440041 50-51. Max. coverage (+): 0. Max coverage (-): 0

Region: NODE\_117098\_length\_959\_cov\_38.440041 52-53. Max. coverage (+): 0.02. Max coverage (-): 0.04

Region: NODE\_117098\_length\_959\_cov\_38.440041 54-55. Max. coverage (+): 0.16. Max coverage (-): 0.06

Region: NODE\_117098\_length\_959\_cov\_38.440041 56-57. Max. coverage (+): 0.32. Max coverage (-): 0.14

Region: NODE\_117098\_length\_959\_cov\_38.440041 58-59. Max. coverage (+): 1.9. Max coverage (-): 0.02

Region: NODE\_117098\_length\_959\_cov\_38.440041 60-61. Max. coverage (+): 1.98. Max coverage (-): 0.02

Region: NODE\_117098\_length\_959\_cov\_38.440041 62-64. Max. coverage (+): 0.1. Max coverage (-): 0.04

Region: NODE\_117098\_length\_959\_cov\_38.440041 65-66. Max. coverage (+): 0.89. Max coverage (-): 0.02

Region: NODE\_117098\_length\_959\_cov\_38.440041 67-68. Max. coverage (+): 0.63. Max coverage (-): 0

Region: NODE\_117098\_length\_959\_cov\_38.440041 69-70. Max. coverage (+): 1.35. Max coverage (-): 0

Region: NODE\_117098\_length\_959\_cov\_38.440041 71-72. Max. coverage (+): 0.73. Max coverage (-): 0

Region: NODE\_117098\_length\_959\_cov\_38.440041 73-74. Max. coverage (+): 0.36. Max coverage (-): 0

Region: NODE\_117098\_length\_959\_cov\_38.440041 75-76. Max. coverage (+): 0.06. Max coverage (-): 0.02

Region: NODE\_117098\_length\_959\_cov\_38.440041 77-78. Max. coverage (+): 0.1. Max coverage (-): 0.02

Region: NODE\_117098\_length\_959\_cov\_38.440041 79-80. Max. coverage (+): 0.08. Max coverage (-): 0

Region: NODE\_117098\_length\_959\_cov\_38.440041 81-82. Max. coverage (+): 0. Max coverage (-): 0

Region: NODE\_117098\_length\_959\_cov\_38.440041 83-84. Max. coverage (+): 0. Max coverage (-): 0

Region: NODE\_117098\_length\_959\_cov\_38.440041 85-86. Max. coverage (+): 0. Max coverage (-): 0

Region: NODE\_117098\_length\_959\_cov\_38.440041 87-88. Max. coverage (+): 0. Max coverage (-): 0

Region: NODE\_117098\_length\_959\_cov\_38.440041 89-90. Max. coverage (+): 0. Max coverage (-): 0

Region: NODE\_117098\_length\_959\_cov\_38.440041 91-92. Max. coverage (+): 0. Max coverage (-): 0

Region: NODE\_117098\_length\_959\_cov\_38.440041 93-94. Max. coverage (+): 0.01. Max coverage (-): 0

Region: NODE\_117098\_length\_959\_cov\_38.440041 95-96. Max. coverage (+): 0. Max coverage (-): 0

Region: NODE\_117098\_length\_959\_cov\_38.440041 97-98. Max. coverage (+): 0. Max coverage (-): 0

Region: NODE\_117098\_length\_959\_cov\_38.440041 99-100. Max. coverage (+): 0. Max coverage (-): 0.02

Region: NODE\_117098\_length\_959\_cov\_38.440041 101-102. Max. coverage (+): 0.04. Max coverage (-): 0.02

Region: NODE\_117098\_length\_959\_cov\_38.440041 103-104. Max. coverage (+): 0.06. Max coverage (-): 0.02

Region: NODE\_117098\_length\_959\_cov\_38.440041 105-106. Max. coverage (+): 0.06. Max coverage (-): 0.02

Region: NODE\_117098\_length\_959\_cov\_38.440041 107-108. Max. coverage (+): 0. Max coverage (-): 0

Region: NODE\_117098\_length\_959\_cov\_38.440041 109-110. Max. coverage (+): 0. Max coverage (-): 0

Region: NODE\_117098\_length\_959\_cov\_38.440041 111-112. Max. coverage (+): 0. Max coverage (-): 0.02

Region: NODE\_117098\_length\_959\_cov\_38.440041 113-114. Max. coverage (+): 0.61. Max coverage (-): 0.02

Region: NODE\_117098\_length\_959\_cov\_38.440041 115-116. Max. coverage (+): 0.85. Max coverage (-): 0

Region: NODE\_117098\_length\_959\_cov\_38.440041 117-118. Max. coverage (+): 0.24. Max coverage (-): 0

Region: NODE\_117098\_length\_959\_cov\_38.440041 119-120. Max. coverage (+): 0. Max coverage (-): 0

Region: NODE\_117098\_length\_959\_cov\_38.440041 121-122. Max. coverage (+): 0.02. Max coverage (-): 0.02

Region: NODE\_117098\_length\_959\_cov\_38.440041 123-125. Max. coverage (+): 0.04. Max coverage (-): 0.02

Region: NODE\_117098\_length\_959\_cov\_38.440041 126-127. Max. coverage (+): 0.02. Max coverage (-): 0

Region: NODE\_117098\_length\_959\_cov\_38.440041 128-129. Max. coverage (+): 0.04. Max coverage (-): 0

Region: NODE\_117098\_length\_959\_cov\_38.440041 130-131. Max. coverage (+): 0.14. Max coverage (-): 0

Region: NODE\_117098\_length\_959\_cov\_38.440041 132-133. Max. coverage (+): 0.14. Max coverage (-): 0

Region: NODE\_117098\_length\_959\_cov\_38.440041 134-135. Max. coverage (+): 0.08. Max coverage (-): 0.12

Region: NODE\_117098\_length\_959\_cov\_38.440041 136-137. Max. coverage (+): 0.04. Max coverage (-): 0.12

Region: NODE\_117098\_length\_959\_cov\_38.440041 138-139. Max. coverage (+): 0. Max coverage (-): 0

Region: NODE\_117098\_length\_959\_cov\_38.440041 140-141. Max. coverage (+): 0.08. Max coverage (-): 0.02

Region: NODE\_117098\_length\_959\_cov\_38.440041 142-143. Max. coverage (+): 0.2. Max coverage (-): 0.02

Region: NODE\_117098\_length\_959\_cov\_38.440041 144-145. Max. coverage (+): 0.2. Max coverage (-): 0.38

Region: NODE\_117098\_length\_959\_cov\_38.440041 146-147. Max. coverage (+): 0.32. Max coverage (-): 1.13

Region: NODE\_117098\_length\_959\_cov\_38.440041 148-149. Max. coverage (+): 0.38. Max coverage (-): 0.79

Region: NODE\_117098\_length\_959\_cov\_38.440041 150-151. Max. coverage (+): 0.44. Max coverage (-): 0.14

Region: NODE\_117098\_length\_959\_cov\_38.440041 152-153. Max. coverage (+): 2.83. Max coverage (-): 0

Region: NODE\_117098\_length\_959\_cov\_38.440041 154-155. Max. coverage (+): 2.69. Max coverage (-): 0.06

Region: NODE\_117098\_length\_959\_cov\_38.440041 156-157. Max. coverage (+): 0.18. Max coverage (-): 0.08

Region: NODE\_117098\_length\_959\_cov\_38.440041 158-159. Max. coverage (+): 0.04. Max coverage (-): 0.02

Region: NODE\_117098\_length\_959\_cov\_38.440041 160-161. Max. coverage (+): 0. Max coverage (-): 0.02

Region: NODE\_117098\_length\_959\_cov\_38.440041 162-163. Max. coverage (+): 0.02. Max coverage (-): 0.06

Region: NODE\_117098\_length\_959\_cov\_38.440041 164-165. Max. coverage (+): 0.08. Max coverage (-): 0.04

Region: NODE\_117098\_length\_959\_cov\_38.440041 166-167. Max. coverage (+): 0.12. Max coverage (-): 0.24

Region: NODE\_117098\_length\_959\_cov\_38.440041 168-169. Max. coverage (+): 0.1. Max coverage (-): 0.26

Region: NODE\_117098\_length\_959\_cov\_38.440041 170-171. Max. coverage (+): 0.06. Max coverage (-): 0.1

Region: NODE\_117098\_length\_959\_cov\_38.440041 172-173. Max. coverage (+): 0.08. Max coverage (-): 0.08

Region: NODE\_117098\_length\_959\_cov\_38.440041 174-175. Max. coverage (+): 0.08. Max coverage (-): 0

Region: NODE\_117098\_length\_959\_cov\_38.440041 176-177. Max. coverage (+): 0.06. Max coverage (-): 0

Region: NODE\_117098\_length\_959\_cov\_38.440041 178-179. Max. coverage (+): 0. Max coverage (-): 0

Region: NODE\_117098\_length\_959\_cov\_38.440041 180-181. Max. coverage (+): 0.06. Max coverage (-): 0.59

Region: NODE\_117098\_length\_959\_cov\_38.440041 182-184. Max. coverage (+): 1.21. Max coverage (-): 0.61

Region: NODE\_117098\_length\_959\_cov\_38.440041 185-186. Max. coverage (+): 3.92. Max coverage (-): 0.01

Region: NODE\_117098\_length\_959\_cov\_38.440041 187-188. Max. coverage (+): 3.29. Max coverage (-): 0.05

Region: NODE\_117098\_length\_959\_cov\_38.440041 189-190. Max. coverage (+): 0.57. Max coverage (-): 0.04

Region: NODE\_117098\_length\_959\_cov\_38.440041 191-192. Max. coverage (+): 0.44. Max coverage (-): 0.02

Region: NODE\_117098\_length\_959\_cov\_38.440041 193-194. Max. coverage (+): 2.47. Max coverage (-): 0.02

Region: NODE\_117098\_length\_959\_cov\_38.440041 195-196. Max. coverage (+): 2.88. Max coverage (-): 0

Region: NODE\_117098\_length\_959\_cov\_38.440041 197-198. Max. coverage (+): 1.14. Max coverage (-): 0

Region: NODE\_117098\_length\_959\_cov\_38.440041 199-200. Max. coverage (+): 0.98. Max coverage (-): 0.02

Region: NODE\_117098\_length\_959\_cov\_38.440041 201-202. Max. coverage (+): 1.35. Max coverage (-): 0.04

Region: NODE\_117098\_length\_959\_cov\_38.440041 203-204. Max. coverage (+): 1.19. Max coverage (-): 0.12

Region: NODE\_117098\_length\_959\_cov\_38.440041 205-206. Max. coverage (+): 0.06. Max coverage (-): 0.12

Region: NODE\_117098\_length\_959\_cov\_38.440041 207-208. Max. coverage (+): 1.88. Max coverage (-): 0.04

Region: NODE\_117098\_length\_959\_cov\_38.440041 209-210. Max. coverage (+): 1.88. Max coverage (-): 0.02

Region: NODE\_117098\_length\_959\_cov\_38.440041 211-212. Max. coverage (+): 0.12. Max coverage (-): 0.02

Region: NODE\_117098\_length\_959\_cov\_38.440041 213-214. Max. coverage (+): 0.12. Max coverage (-): 0.04

Region: NODE\_117098\_length\_959\_cov\_38.440041 215-216. Max. coverage (+): 0.32. Max coverage (-): 0.04

Region: NODE\_117098\_length\_959\_cov\_38.440041 217-218. Max. coverage (+): 0.24. Max coverage (-): 0

Region: NODE\_117098\_length\_959\_cov\_38.440041 219-220. Max. coverage (+): 3.67. Max coverage (-): 0.04

Region: NODE\_117098\_length\_959\_cov\_38.440041 221-222. Max. coverage (+): 3.59. Max coverage (-): 0.24

Region: NODE\_117098\_length\_959\_cov\_38.440041 223-224. Max. coverage (+): 0.36. Max coverage (-): 0.65

Region: NODE\_117098\_length\_959\_cov\_38.440041 225-226. Max. coverage (+): 1.86. Max coverage (-): 0.61

Region: NODE\_117098\_length\_959\_cov\_38.440041 227-228. Max. coverage (+): 1.57. Max coverage (-): 1.01

Region: NODE\_117098\_length\_959\_cov\_38.440041 229-230. Max. coverage (+): 0.77. Max coverage (-): 1.09

Region: NODE\_117098\_length\_959\_cov\_38.440041 231-232. Max. coverage (+): 0.57. Max coverage (-): 0.77

Region: NODE\_117098\_length\_959\_cov\_38.440041 233-234. Max. coverage (+): 0.32. Max coverage (-): 0.28

Region: NODE\_117098\_length\_959\_cov\_38.440041 235-236. Max. coverage (+): 0.08. Max coverage (-): 0.32

Region: NODE\_117098\_length\_959\_cov\_38.440041 237-238. Max. coverage (+): 0.26. Max coverage (-): 0.14

Region: NODE\_117098\_length\_959\_cov\_38.440041 239-240. Max. coverage (+): 0.26. Max coverage (-): 0.08

Region: NODE\_117098\_length\_959\_cov\_38.440041 241-242. Max. coverage (+): 0.34. Max coverage (-): 0.06

Region: NODE\_117098\_length\_959\_cov\_38.440041 243-245. Max. coverage (+): 0.44. Max coverage (-): 0.06

Region: NODE\_117098\_length\_959\_cov\_38.440041 246-247. Max. coverage (+): 0.42. Max coverage (-): 0

Region: NODE\_117098\_length\_959\_cov\_38.440041 248-249. Max. coverage (+): 0. Max coverage (-): 0

Region: NODE\_117098\_length\_959\_cov\_38.440041 250-251. Max. coverage (+): 0. Max coverage (-): 0

Region: NODE\_117098\_length\_959\_cov\_38.440041 252-253. Max. coverage (+): 0. Max coverage (-): 0

Region: NODE\_117098\_length\_959\_cov\_38.440041 254-255. Max. coverage (+): 0. Max coverage (-): 0

Region: NODE\_117098\_length\_959\_cov\_38.440041 256-257. Max. coverage (+): 0. Max coverage (-): 0

Region: NODE\_117098\_length\_959\_cov\_38.440041 258-259. Max. coverage (+): 0. Max coverage (-): 0

Region: NODE\_117098\_length\_959\_cov\_38.440041 260-261. Max. coverage (+): 0. Max coverage (-): 0

Region: NODE\_117098\_length\_959\_cov\_38.440041 262-263. Max. coverage (+): 0. Max coverage (-): 0

Region: NODE\_117098\_length\_959\_cov\_38.440041 264-265. Max. coverage (+): 0. Max coverage (-): 0

Region: NODE\_117098\_length\_959\_cov\_38.440041 266-267. Max. coverage (+): 0. Max coverage (-): 0

Region: NODE\_117098\_length\_959\_cov\_38.440041 268-269. Max. coverage (+): 0. Max coverage (-): 0.16

Region: NODE\_117098\_length\_959\_cov\_38.440041 270-271. Max. coverage (+): 0. Max coverage (-): 0.2

Region: NODE\_117098\_length\_959\_cov\_38.440041 272-273. Max. coverage (+): 0. Max coverage (-): 0.12

Region: NODE\_117098\_length\_959\_cov\_38.440041 274-275. Max. coverage (+): 0. Max coverage (-): 0.12

Region: NODE\_117098\_length\_959\_cov\_38.440041 276-277. Max. coverage (+): 0. Max coverage (-): 0.32

Region: NODE\_117098\_length\_959\_cov\_38.440041 278-279. Max. coverage (+): 0. Max coverage (-): 0.2

Region: NODE\_117098\_length\_959\_cov\_38.440041 280-281. Max. coverage (+): 0.24. Max coverage (-): 0

Region: NODE\_117098\_length\_959\_cov\_38.440041 282-283. Max. coverage (+): 0.93. Max coverage (-): 0

Region: NODE\_117098\_length\_959\_cov\_38.440041 284-285. Max. coverage (+): 10.86. Max coverage (-): 0.04

Region: NODE\_117098\_length\_959\_cov\_38.440041 286-287. Max. coverage (+): 10.7. Max coverage (-): 0.04

Region: NODE\_117098\_length\_959\_cov\_38.440041 288-289. Max. coverage (+): 5.61. Max coverage (-): 0

Region: NODE\_117098\_length\_959\_cov\_38.440041 290-291. Max. coverage (+): 5.41. Max coverage (-): 0

Region: NODE\_117098\_length\_959\_cov\_38.440041 292-293. Max. coverage (+): 0.4. Max coverage (-): 0

Region: NODE\_117098\_length\_959\_cov\_38.440041 294-295. Max. coverage (+): 0.2. Max coverage (-): 0

Region: NODE\_117098\_length\_959\_cov\_38.440041 296-297. Max. coverage (+): 0.24. Max coverage (-): 0

Region: NODE\_117098\_length\_959\_cov\_38.440041 298-299. Max. coverage (+): 0.32. Max coverage (-): 0.08

Region: NODE\_117098\_length\_959\_cov\_38.440041 300-301. Max. coverage (+): 0.16. Max coverage (-): 0.08

Region: NODE\_117098\_length\_959\_cov\_38.440041 302-304. Max. coverage (+): 0.97. Max coverage (-): 0.04

Region: NODE\_117098\_length\_959\_cov\_38.440041 305-306. Max. coverage (+): 4.32. Max coverage (-): 0.04

Region: NODE\_117098\_length\_959\_cov\_38.440041 307-308. Max. coverage (+): 12.36. Max coverage (-): 0

Region: NODE\_117098\_length\_959\_cov\_38.440041 309-310. Max. coverage (+): 13.73. Max coverage (-): 0

Region: NODE\_117098\_length\_959\_cov\_38.440041 311-312. Max. coverage (+): 11.47. Max coverage (-): 0.24

Region: NODE\_117098\_length\_959\_cov\_38.440041 313-314. Max. coverage (+): 1.17. Max coverage (-): 0.32

Region: NODE\_117098\_length\_959\_cov\_38.440041 315-316. Max. coverage (+): 1.37. Max coverage (-): 0.28

Region: NODE\_117098\_length\_959\_cov\_38.440041 317-318. Max. coverage (+): 1.21. Max coverage (-): 0.28

Region: NODE\_117098\_length\_959\_cov\_38.440041 319-320. Max. coverage (+): 0. Max coverage (-): 0.24

Region: NODE\_117098\_length\_959\_cov\_38.440041 321-322. Max. coverage (+): 0.28. Max coverage (-): 0.57

Region: NODE\_117098\_length\_959\_cov\_38.440041 323-324. Max. coverage (+): 0.52. Max coverage (-): 0.75

Region: NODE\_117098\_length\_959\_cov\_38.440041 325-326. Max. coverage (+): 0.41. Max coverage (-): 0.25

Region: NODE\_117098\_length\_959\_cov\_38.440041 327-328. Max. coverage (+): 0.4. Max coverage (-): 0.16

Region: NODE\_117098\_length\_959\_cov\_38.440041 329-330. Max. coverage (+): 2.15. Max coverage (-): 0.09

Region: NODE\_117098\_length\_959\_cov\_38.440041 331-332. Max. coverage (+): 11.65. Max coverage (-): 0.01

Region: NODE\_117098\_length\_959\_cov\_38.440041 333-334. Max. coverage (+): 9.99. Max coverage (-): 0.01

Region: NODE\_117098\_length\_959\_cov\_38.440041 335-336. Max. coverage (+): 3.13. Max coverage (-): 0.08

Region: NODE\_117098\_length\_959\_cov\_38.440041 337-338. Max. coverage (+): 3.21. Max coverage (-): 0.14

Region: NODE\_117098\_length\_959\_cov\_38.440041 339-340. Max. coverage (+): 0.48. Max coverage (-): 0.06

Region: NODE\_117098\_length\_959\_cov\_38.440041 341-342. Max. coverage (+): 1.27. Max coverage (-): 0.04

Region: NODE\_117098\_length\_959\_cov\_38.440041 343-344. Max. coverage (+): 0.97. Max coverage (-): 0.04

Region: NODE\_117098\_length\_959\_cov\_38.440041 345-346. Max. coverage (+): 0.69. Max coverage (-): 0.04

Region: NODE\_117098\_length\_959\_cov\_38.440041 347-348. Max. coverage (+): 1.45. Max coverage (-): 0.04

Region: NODE\_117098\_length\_959\_cov\_38.440041 349-350. Max. coverage (+): 1.17. Max coverage (-): 0.04

Region: NODE\_117098\_length\_959\_cov\_38.440041 351-352. Max. coverage (+): 0.32. Max coverage (-): 0.57

Region: NODE\_117098\_length\_959\_cov\_38.440041 353-354. Max. coverage (+): 0.2. Max coverage (-): 0.73

Region: NODE\_117098\_length\_959\_cov\_38.440041 355-356. Max. coverage (+): 0.32. Max coverage (-): 1.53

Region: NODE\_117098\_length\_959\_cov\_38.440041 357-358. Max. coverage (+): 0.93. Max coverage (-): 1.9

Region: NODE\_117098\_length\_959\_cov\_38.440041 359-360. Max. coverage (+): 9.57. Max coverage (-): 0.52

Region: NODE\_117098\_length\_959\_cov\_38.440041 361-363. Max. coverage (+): 10.42. Max coverage (-): 0.28

Region: NODE\_117098\_length\_959\_cov\_38.440041 364-365. Max. coverage (+): 0.44. Max coverage (-): 0.16

Region: NODE\_117098\_length\_959\_cov\_38.440041 366-367. Max. coverage (+): 0.4. Max coverage (-): 0.04

Region: NODE\_117098\_length\_959\_cov\_38.440041 368-369. Max. coverage (+): 0.32. Max coverage (-): 0.04

Region: NODE\_117098\_length\_959\_cov\_38.440041 370-371. Max. coverage (+): 0.16. Max coverage (-): 0.32

Region: NODE\_117098\_length\_959\_cov\_38.440041 372-373. Max. coverage (+): 0.89. Max coverage (-): 0.44

Region: NODE\_117098\_length\_959\_cov\_38.440041 374-375. Max. coverage (+): 0.97. Max coverage (-): 0.24

Region: NODE\_117098\_length\_959\_cov\_38.440041 376-377. Max. coverage (+): 0.4. Max coverage (-): 0.04

Region: NODE\_117098\_length\_959\_cov\_38.440041 378-379. Max. coverage (+): 7.53. Max coverage (-): 0.04

Region: NODE\_117098\_length\_959\_cov\_38.440041 380-381. Max. coverage (+): 8.42. Max coverage (-): 0.1

Region: NODE\_117098\_length\_959\_cov\_38.440041 382-383. Max. coverage (+): 1.84. Max coverage (-): 0.18

Region: NODE\_117098\_length\_959\_cov\_38.440041 384-385. Max. coverage (+): 0.73. Max coverage (-): 0.08

Region: NODE\_117098\_length\_959\_cov\_38.440041 386-387. Max. coverage (+): 19.4. Max coverage (-): 0

Region: NODE\_117098\_length\_959\_cov\_38.440041 388-389. Max. coverage (+): 24.77. Max coverage (-): 0

Region: NODE\_117098\_length\_959\_cov\_38.440041 390-391. Max. coverage (+): 5.94. Max coverage (-): 0

Region: NODE\_117098\_length\_959\_cov\_38.440041 392-393. Max. coverage (+): 0.59. Max coverage (-): 0

Region: NODE\_117098\_length\_959\_cov\_38.440041 394-395. Max. coverage (+): 0.02. Max coverage (-): 0

Region: NODE\_117098\_length\_959\_cov\_38.440041 396-397. Max. coverage (+): 0.36. Max coverage (-): 0

Region: NODE\_117098\_length\_959\_cov\_38.440041 398-399. Max. coverage (+): 0.36. Max coverage (-): 0

Region: NODE\_117098\_length\_959\_cov\_38.440041 400-401. Max. coverage (+): 0.04. Max coverage (-): 0.5

Region: NODE\_117098\_length\_959\_cov\_38.440041 402-403. Max. coverage (+): 0. Max coverage (-): 0.52

Region: NODE\_117098\_length\_959\_cov\_38.440041 404-405. Max. coverage (+): 0. Max coverage (-): 0.1

Region: NODE\_117098\_length\_959\_cov\_38.440041 406-407. Max. coverage (+): 0. Max coverage (-): 0.02

Region: NODE\_117098\_length\_959\_cov\_38.440041 408-409. Max. coverage (+): 0.08. Max coverage (-): 0.06

Region: NODE\_117098\_length\_959\_cov\_38.440041 410-411. Max. coverage (+): 0.14. Max coverage (-): 0.06

Region: NODE\_117098\_length\_959\_cov\_38.440041 412-413. Max. coverage (+): 0.28. Max coverage (-): 0

Region: NODE\_117098\_length\_959\_cov\_38.440041 414-415. Max. coverage (+): 0.3. Max coverage (-): 0

Region: NODE\_117098\_length\_959\_cov\_38.440041 416-417. Max. coverage (+): 0.12. Max coverage (-): 0

Region: NODE\_117098\_length\_959\_cov\_38.440041 418-419. Max. coverage (+): 0.55. Max coverage (-): 0.02

Region: NODE\_117098\_length\_959\_cov\_38.440041 420-421. Max. coverage (+): 0.48. Max coverage (-): 0.02

Region: NODE\_117098\_length\_959\_cov\_38.440041 422-424. Max. coverage (+): 1.11. Max coverage (-): 0.02

Region: NODE\_117098\_length\_959\_cov\_38.440041 425-426. Max. coverage (+): 6.62. Max coverage (-): 0.04

Region: NODE\_117098\_length\_959\_cov\_38.440041 427-428. Max. coverage (+): 6.14. Max coverage (-): 0.06

Region: NODE\_117098\_length\_959\_cov\_38.440041 429-430. Max. coverage (+): 0.24. Max coverage (-): 0.02

Region: NODE\_117098\_length\_959\_cov\_38.440041 431-432. Max. coverage (+): 0.06. Max coverage (-): 0

Region: NODE\_117098\_length\_959\_cov\_38.440041 433-434. Max. coverage (+): 0. Max coverage (-): 0

Region: NODE\_117098\_length\_959\_cov\_38.440041 435-436. Max. coverage (+): 0. Max coverage (-): 0

Region: NODE\_117098\_length\_959\_cov\_38.440041 437-438. Max. coverage (+): 0. Max coverage (-): 0

Region: NODE\_117098\_length\_959\_cov\_38.440041 439-440. Max. coverage (+): 0. Max coverage (-): 0

Region: NODE\_117098\_length\_959\_cov\_38.440041 441-442. Max. coverage (+): 0. Max coverage (-): 0

Region: NODE\_117098\_length\_959\_cov\_38.440041 443-444. Max. coverage (+): 0. Max coverage (-): 0

Region: NODE\_117098\_length\_959\_cov\_38.440041 445-446. Max. coverage (+): 0. Max coverage (-): 0

Region: NODE\_117098\_length\_959\_cov\_38.440041 447-448. Max. coverage (+): 0. Max coverage (-): 0

Region: NODE\_117098\_length\_959\_cov\_38.440041 449-450. Max. coverage (+): 0. Max coverage (-): 0

Region: NODE\_117098\_length\_959\_cov\_38.440041 451-452. Max. coverage (+): 0. Max coverage (-): 0

Region: NODE\_117098\_length\_959\_cov\_38.440041 453-454. Max. coverage (+): 0.42. Max coverage (-): 0.02

Region: NODE\_117098\_length\_959\_cov\_38.440041 455-456. Max. coverage (+): 4.12. Max coverage (-): 0.04

Region: NODE\_117098\_length\_959\_cov\_38.440041 457-458. Max. coverage (+): 3.88. Max coverage (-): 0.02

Region: NODE\_117098\_length\_959\_cov\_38.440041 459-460. Max. coverage (+): 15.28. Max coverage (-): 0.05

Region: NODE\_117098\_length\_959\_cov\_38.440041 461-462. Max. coverage (+): 15.06. Max coverage (-): 0.08

Region: NODE\_117098\_length\_959\_cov\_38.440041 463-464. Max. coverage (+): 0.12. Max coverage (-): 0.05

Region: NODE\_117098\_length\_959\_cov\_38.440041 465-466. Max. coverage (+): 0.38. Max coverage (-): 0

Region: NODE\_117098\_length\_959\_cov\_38.440041 467-468. Max. coverage (+): 0.49. Max coverage (-): 0

Region: NODE\_117098\_length\_959\_cov\_38.440041 469-470. Max. coverage (+): 0.76. Max coverage (-): 0.02

Region: NODE\_117098\_length\_959\_cov\_38.440041 471-472. Max. coverage (+): 1.71. Max coverage (-): 0.04

Region: NODE\_117098\_length\_959\_cov\_38.440041 473-474. Max. coverage (+): 2.17. Max coverage (-): 0.08

Region: NODE\_117098\_length\_959\_cov\_38.440041 475-476. Max. coverage (+): 0.71. Max coverage (-): 0.22

Region: NODE\_117098\_length\_959\_cov\_38.440041 477-478. Max. coverage (+): 1.29. Max coverage (-): 0.14

Region: NODE\_117098\_length\_959\_cov\_38.440041 479-480. Max. coverage (+): 1.39. Max coverage (-): 0

Region: NODE\_117098\_length\_959\_cov\_38.440041 481-483. Max. coverage (+): 0.14. Max coverage (-): 0

Region: NODE\_117098\_length\_959\_cov\_38.440041 484-485. Max. coverage (+): 0. Max coverage (-): 0

Region: NODE\_117098\_length\_959\_cov\_38.440041 486-487. Max. coverage (+): 0. Max coverage (-): 0

Region: NODE\_117098\_length\_959\_cov\_38.440041 488-489. Max. coverage (+): 0. Max coverage (-): 0

Region: NODE\_117098\_length\_959\_cov\_38.440041 490-491. Max. coverage (+): 0. Max coverage (-): 0

Region: NODE\_117098\_length\_959\_cov\_38.440041 492-493. Max. coverage (+): 0. Max coverage (-): 0

Region: NODE\_117098\_length\_959\_cov\_38.440041 494-495. Max. coverage (+): 0. Max coverage (-): 0

Region: NODE\_117098\_length\_959\_cov\_38.440041 496-497. Max. coverage (+): 0. Max coverage (-): 0

Region: NODE\_117098\_length\_959\_cov\_38.440041 498-499. Max. coverage (+): 0. Max coverage (-): 0

Region: NODE\_117098\_length\_959\_cov\_38.440041 500-501. Max. coverage (+): 0. Max coverage (-): 0

Region: NODE\_117098\_length\_959\_cov\_38.440041 502-503. Max. coverage (+): 0. Max coverage (-): 0

Region: NODE\_117098\_length\_959\_cov\_38.440041 504-505. Max. coverage (+): 0. Max coverage (-): 0

Region: NODE\_117098\_length\_959\_cov\_38.440041 506-507. Max. coverage (+): 0. Max coverage (-): 0

Region: NODE\_117098\_length\_959\_cov\_38.440041 508-509. Max. coverage (+): 0. Max coverage (-): 0

Region: NODE\_117098\_length\_959\_cov\_38.440041 510-511. Max. coverage (+): 0. Max coverage (-): 0

Region: NODE\_117098\_length\_959\_cov\_38.440041 512-513. Max. coverage (+): 0. Max coverage (-): 0

Region: NODE\_117098\_length\_959\_cov\_38.440041 514-515. Max. coverage (+): 0. Max coverage (-): 0

Region: NODE\_117098\_length\_959\_cov\_38.440041 516-517. Max. coverage (+): 0. Max coverage (-): 0

Region: NODE\_117098\_length\_959\_cov\_38.440041 518-519. Max. coverage (+): 0. Max coverage (-): 0

Region: NODE\_117098\_length\_959\_cov\_38.440041 520-521. Max. coverage (+): 0. Max coverage (-): 0

Region: NODE\_117098\_length\_959\_cov\_38.440041 522-523. Max. coverage (+): 0. Max coverage (-): 0.04

Region: NODE\_117098\_length\_959\_cov\_38.440041 524-525. Max. coverage (+): 0.1. Max coverage (-): 0.55

Region: NODE\_117098\_length\_959\_cov\_38.440041 526-527. Max. coverage (+): 6.18. Max coverage (-): 0.5

Region: NODE\_117098\_length\_959\_cov\_38.440041 528-529. Max. coverage (+): 6.16. Max coverage (-): 0

Region: NODE\_117098\_length\_959\_cov\_38.440041 530-531. Max. coverage (+): 1.07. Max coverage (-): 0

Region: NODE\_117098\_length\_959\_cov\_38.440041 532-533. Max. coverage (+): 1.41. Max coverage (-): 0

Region: NODE\_117098\_length\_959\_cov\_38.440041 534-535. Max. coverage (+): 2.08. Max coverage (-): 0

Region: NODE\_117098\_length\_959\_cov\_38.440041 536-537. Max. coverage (+): 2.26. Max coverage (-): 0.04

Region: NODE\_117098\_length\_959\_cov\_38.440041 538-539. Max. coverage (+): 2.28. Max coverage (-): 0.04

Region: NODE\_117098\_length\_959\_cov\_38.440041 540-541. Max. coverage (+): 1.66. Max coverage (-): 0.04

Region: NODE\_117098\_length\_959\_cov\_38.440041 542-544. Max. coverage (+): 0.16. Max coverage (-): 0.04

Region: NODE\_117098\_length\_959\_cov\_38.440041 545-546. Max. coverage (+): 0.22. Max coverage (-): 0.1

Region: NODE\_117098\_length\_959\_cov\_38.440041 547-548. Max. coverage (+): 0.18. Max coverage (-): 0.14

Region: NODE\_117098\_length\_959\_cov\_38.440041 549-550. Max. coverage (+): 0.18. Max coverage (-): 0.08

Region: NODE\_117098\_length\_959\_cov\_38.440041 551-552. Max. coverage (+): 0.2. Max coverage (-): 0.79

Region: NODE\_117098\_length\_959\_cov\_38.440041 553-554. Max. coverage (+): 0.02. Max coverage (-): 0.85

Region: NODE\_117098\_length\_959\_cov\_38.440041 555-556. Max. coverage (+): 0.69. Max coverage (-): 0.14

Region: NODE\_117098\_length\_959\_cov\_38.440041 557-558. Max. coverage (+): 1.41. Max coverage (-): 0.04

Region: NODE\_117098\_length\_959\_cov\_38.440041 559-560. Max. coverage (+): 1.15. Max coverage (-): 0.08

Region: NODE\_117098\_length\_959\_cov\_38.440041 561-562. Max. coverage (+): 0.52. Max coverage (-): 0.06

Region: NODE\_117098\_length\_959\_cov\_38.440041 563-564. Max. coverage (+): 0.26. Max coverage (-): 0.02

Region: NODE\_117098\_length\_959\_cov\_38.440041 565-566. Max. coverage (+): 0.57. Max coverage (-): 0.02

Region: NODE\_117098\_length\_959\_cov\_38.440041 567-568. Max. coverage (+): 0.67. Max coverage (-): 0

Region: NODE\_117098\_length\_959\_cov\_38.440041 569-570. Max. coverage (+): 0.24. Max coverage (-): 0.04

Region: NODE\_117098\_length\_959\_cov\_38.440041 571-572. Max. coverage (+): 0.02. Max coverage (-): 0.04

Region: NODE\_117098\_length\_959\_cov\_38.440041 573-574. Max. coverage (+): 0.02. Max coverage (-): 0

Region: NODE\_117098\_length\_959\_cov\_38.440041 575-576. Max. coverage (+): 0.04. Max coverage (-): 0

Region: NODE\_117098\_length\_959\_cov\_38.440041 577-578. Max. coverage (+): 0.28. Max coverage (-): 0

Region: NODE\_117098\_length\_959\_cov\_38.440041 579-580. Max. coverage (+): 0.28. Max coverage (-): 0.02

Region: NODE\_117098\_length\_959\_cov\_38.440041 581-582. Max. coverage (+): 0.42. Max coverage (-): 0.04

Region: NODE\_117098\_length\_959\_cov\_38.440041 583-584. Max. coverage (+): 0.75. Max coverage (-): 0.22

Region: NODE\_117098\_length\_959\_cov\_38.440041 585-586. Max. coverage (+): 0.83. Max coverage (-): 0.28

Region: NODE\_117098\_length\_959\_cov\_38.440041 587-588. Max. coverage (+): 0.46. Max coverage (-): 0.12

Region: NODE\_117098\_length\_959\_cov\_38.440041 589-590. Max. coverage (+): 0.42. Max coverage (-): 0.16

Region: NODE\_117098\_length\_959\_cov\_38.440041 591-592. Max. coverage (+): 0.16. Max coverage (-): 0.83

Region: NODE\_117098\_length\_959\_cov\_38.440041 593-594. Max. coverage (+): 0.12. Max coverage (-): 0.95

Region: NODE\_117098\_length\_959\_cov\_38.440041 595-596. Max. coverage (+): 0. Max coverage (-): 0.38

Region: NODE\_117098\_length\_959\_cov\_38.440041 597-598. Max. coverage (+): 0. Max coverage (-): 0.04

Region: NODE\_117098\_length\_959\_cov\_38.440041 599-600. Max. coverage (+): 0.2. Max coverage (-): 0.08

Region: NODE\_117098\_length\_959\_cov\_38.440041 601-603. Max. coverage (+): 6.74. Max coverage (-): 0.08

Region: NODE\_117098\_length\_959\_cov\_38.440041 604-605. Max. coverage (+): 6.58. Max coverage (-): 0.1

Region: NODE\_117098\_length\_959\_cov\_38.440041 606-607. Max. coverage (+): 0.06. Max coverage (-): 0.1

Region: NODE\_117098\_length\_959\_cov\_38.440041 608-609. Max. coverage (+): 7.11. Max coverage (-): 0.04

Region: NODE\_117098\_length\_959\_cov\_38.440041 610-611. Max. coverage (+): 7.11. Max coverage (-): 0.02

Region: NODE\_117098\_length\_959\_cov\_38.440041 612-613. Max. coverage (+): 0.06. Max coverage (-): 0.06

Region: NODE\_117098\_length\_959\_cov\_38.440041 614-615. Max. coverage (+): 0.34. Max coverage (-): 0.08

Region: NODE\_117098\_length\_959\_cov\_38.440041 616-617. Max. coverage (+): 0.32. Max coverage (-): 0.04

Region: NODE\_117098\_length\_959\_cov\_38.440041 618-619. Max. coverage (+): 0.28. Max coverage (-): 0.08

Region: NODE\_117098\_length\_959\_cov\_38.440041 620-621. Max. coverage (+): 0.28. Max coverage (-): 0.08

Region: NODE\_117098\_length\_959\_cov\_38.440041 622-623. Max. coverage (+): 0.08. Max coverage (-): 0.04

Region: NODE\_117098\_length\_959\_cov\_38.440041 624-625. Max. coverage (+): 0.02. Max coverage (-): 0.02

Region: NODE\_117098\_length\_959\_cov\_38.440041 626-627. Max. coverage (+): 0. Max coverage (-): 0.04

Region: NODE\_117098\_length\_959\_cov\_38.440041 628-629. Max. coverage (+): 0.02. Max coverage (-): 0.1

Region: NODE\_117098\_length\_959\_cov\_38.440041 630-631. Max. coverage (+): 0.1. Max coverage (-): 0.22

Region: NODE\_117098\_length\_959\_cov\_38.440041 632-633. Max. coverage (+): 0.2. Max coverage (-): 0.4

Region: NODE\_117098\_length\_959\_cov\_38.440041 634-635. Max. coverage (+): 0.14. Max coverage (-): 0.32

Region: NODE\_117098\_length\_959\_cov\_38.440041 636-637. Max. coverage (+): 0.5. Max coverage (-): 0.12

Region: NODE\_117098\_length\_959\_cov\_38.440041 638-639. Max. coverage (+): 0.61. Max coverage (-): 0.04

Region: NODE\_117098\_length\_959\_cov\_38.440041 640-641. Max. coverage (+): 0.16. Max coverage (-): 0.12

Region: NODE\_117098\_length\_959\_cov\_38.440041 642-643. Max. coverage (+): 0.73. Max coverage (-): 0.08

Region: NODE\_117098\_length\_959\_cov\_38.440041 644-645. Max. coverage (+): 0.67. Max coverage (-): 0.06

Region: NODE\_117098\_length\_959\_cov\_38.440041 646-647. Max. coverage (+): 0.61. Max coverage (-): 0.08

Region: NODE\_117098\_length\_959\_cov\_38.440041 648-649. Max. coverage (+): 0.36. Max coverage (-): 0.1

Region: NODE\_117098\_length\_959\_cov\_38.440041 650-651. Max. coverage (+): 4.76. Max coverage (-): 0.28

Region: NODE\_117098\_length\_959\_cov\_38.440041 652-653. Max. coverage (+): 5.96. Max coverage (-): 0.34

Region: NODE\_117098\_length\_959\_cov\_38.440041 654-655. Max. coverage (+): 2.18. Max coverage (-): 0.34

Region: NODE\_117098\_length\_959\_cov\_38.440041 656-657. Max. coverage (+): 0.14. Max coverage (-): 0.26

Region: NODE\_117098\_length\_959\_cov\_38.440041 658-659. Max. coverage (+): 0.12. Max coverage (-): 0.02

Region: NODE\_117098\_length\_959\_cov\_38.440041 660-661. Max. coverage (+): 0.59. Max coverage (-): 0.08

Region: NODE\_117098\_length\_959\_cov\_38.440041 662-664. Max. coverage (+): 0.52. Max coverage (-): 0.08

Region: NODE\_117098\_length\_959\_cov\_38.440041 665-666. Max. coverage (+): 0.08. Max coverage (-): 0.12

Region: NODE\_117098\_length\_959\_cov\_38.440041 667-668. Max. coverage (+): 1.13. Max coverage (-): 0.12

Region: NODE\_117098\_length\_959\_cov\_38.440041 669-670. Max. coverage (+): 1.05. Max coverage (-): 0.14

Region: NODE\_117098\_length\_959\_cov\_38.440041 671-672. Max. coverage (+): 0.26. Max coverage (-): 0.04

Region: NODE\_117098\_length\_959\_cov\_38.440041 673-674. Max. coverage (+): 0.87. Max coverage (-): 0.02

Region: NODE\_117098\_length\_959\_cov\_38.440041 675-676. Max. coverage (+): 0.81. Max coverage (-): 0.14

Region: NODE\_117098\_length\_959\_cov\_38.440041 677-678. Max. coverage (+): 0.2. Max coverage (-): 0.12

Region: NODE\_117098\_length\_959\_cov\_38.440041 679-680. Max. coverage (+): 0.67. Max coverage (-): 0.04

Region: NODE\_117098\_length\_959\_cov\_38.440041 681-682. Max. coverage (+): 1.03. Max coverage (-): 0

Region: NODE\_117098\_length\_959\_cov\_38.440041 683-684. Max. coverage (+): 4.46. Max coverage (-): 0.02

Region: NODE\_117098\_length\_959\_cov\_38.440041 685-686. Max. coverage (+): 4.2. Max coverage (-): 0.12

Region: NODE\_117098\_length\_959\_cov\_38.440041 687-688. Max. coverage (+): 1.11. Max coverage (-): 0.1

Region: NODE\_117098\_length\_959\_cov\_38.440041 689-690. Max. coverage (+): 0.95. Max coverage (-): 0.2

Region: NODE\_117098\_length\_959\_cov\_38.440041 691-692. Max. coverage (+): 0.95. Max coverage (-): 0.22

Region: NODE\_117098\_length\_959\_cov\_38.440041 693-694. Max. coverage (+): 0.89. Max coverage (-): 0.4

Region: NODE\_117098\_length\_959\_cov\_38.440041 695-696. Max. coverage (+): 0.1. Max coverage (-): 0.4

Region: NODE\_117098\_length\_959\_cov\_38.440041 697-698. Max. coverage (+): 0.34. Max coverage (-): 0.46

Region: NODE\_117098\_length\_959\_cov\_38.440041 699-700. Max. coverage (+): 4.46. Max coverage (-): 0.44

Region: NODE\_117098\_length\_959\_cov\_38.440041 701-702. Max. coverage (+): 7.75. Max coverage (-): 0.08

Region: NODE\_117098\_length\_959\_cov\_38.440041 703-704. Max. coverage (+): 8.62. Max coverage (-): 0.24

Region: NODE\_117098\_length\_959\_cov\_38.440041 705-706. Max. coverage (+): 8.56. Max coverage (-): 0.24

Region: NODE\_117098\_length\_959\_cov\_38.440041 707-708. Max. coverage (+): 10.05. Max coverage (-): 0.02

Region: NODE\_117098\_length\_959\_cov\_38.440041 709-710. Max. coverage (+): 5.43. Max coverage (-): 0.04

Region: NODE\_117098\_length\_959\_cov\_38.440041 711-712. Max. coverage (+): 3.21. Max coverage (-): 0.02

Region: NODE\_117098\_length\_959\_cov\_38.440041 713-714. Max. coverage (+): 0.18. Max coverage (-): 0.02

Region: NODE\_117098\_length\_959\_cov\_38.440041 715-716. Max. coverage (+): 0.04. Max coverage (-): 0.04

Region: NODE\_117098\_length\_959\_cov\_38.440041 717-718. Max. coverage (+): 0.2. Max coverage (-): 0.06

Region: NODE\_117098\_length\_959\_cov\_38.440041 719-720. Max. coverage (+): 0.24. Max coverage (-): 0.06

Region: NODE\_117098\_length\_959\_cov\_38.440041 721-723. Max. coverage (+): 0.16. Max coverage (-): 0.02

Region: NODE\_117098\_length\_959\_cov\_38.440041 724-725. Max. coverage (+): 0.48. Max coverage (-): 0

Region: NODE\_117098\_length\_959\_cov\_38.440041 726-727. Max. coverage (+): 0.63. Max coverage (-): 0

Region: NODE\_117098\_length\_959\_cov\_38.440041 728-729. Max. coverage (+): 0.2. Max coverage (-): 0

Region: NODE\_117098\_length\_959\_cov\_38.440041 730-731. Max. coverage (+): 0. Max coverage (-): 0

Region: NODE\_117098\_length\_959\_cov\_38.440041 732-733. Max. coverage (+): 0. Max coverage (-): 0

Region: NODE\_117098\_length\_959\_cov\_38.440041 734-735. Max. coverage (+): 0. Max coverage (-): 0

Region: NODE\_117098\_length\_959\_cov\_38.440041 736-737. Max. coverage (+): 0. Max coverage (-): 0

Region: NODE\_117098\_length\_959\_cov\_38.440041 738-739. Max. coverage (+): 0.02. Max coverage (-): 0

Region: NODE\_117098\_length\_959\_cov\_38.440041 740-741. Max. coverage (+): 0.04. Max coverage (-): 0

Region: NODE\_117098\_length\_959\_cov\_38.440041 742-743. Max. coverage (+): 0.02. Max coverage (-): 0

Region: NODE\_117098\_length\_959\_cov\_38.440041 744-745. Max. coverage (+): 0. Max coverage (-): 0

Region: NODE\_117098\_length\_959\_cov\_38.440041 746-747. Max. coverage (+): 0. Max coverage (-): 0

Region: NODE\_117098\_length\_959\_cov\_38.440041 748-749. Max. coverage (+): 3.78. Max coverage (-): 0.24

Region: NODE\_117098\_length\_959\_cov\_38.440041 750-751. Max. coverage (+): 4.74. Max coverage (-): 0.24

Region: NODE\_117098\_length\_959\_cov\_38.440041 752-753. Max. coverage (+): 5.17. Max coverage (-): 0.04

Region: NODE\_117098\_length\_959\_cov\_38.440041 754-755. Max. coverage (+): 12.19. Max coverage (-): 0

Region: NODE\_117098\_length\_959\_cov\_38.440041 756-757. Max. coverage (+): 8.16. Max coverage (-): 0

Region: NODE\_117098\_length\_959\_cov\_38.440041 758-759. Max. coverage (+): 0.3. Max coverage (-): 0

Region: NODE\_117098\_length\_959\_cov\_38.440041 760-761. Max. coverage (+): 0.06. Max coverage (-): 0

Region: NODE\_117098\_length\_959\_cov\_38.440041 762-763. Max. coverage (+): 0.08. Max coverage (-): 0.04

Region: NODE\_117098\_length\_959\_cov\_38.440041 764-765. Max. coverage (+): 0.02. Max coverage (-): 0.16

Region: NODE\_117098\_length\_959\_cov\_38.440041 766-767. Max. coverage (+): 0.08. Max coverage (-): 0.12

Region: NODE\_117098\_length\_959\_cov\_38.440041 768-769. Max. coverage (+): 0.12. Max coverage (-): 0.1

Region: NODE\_117098\_length\_959\_cov\_38.440041 770-771. Max. coverage (+): 0.85. Max coverage (-): 0.08

Region: NODE\_117098\_length\_959\_cov\_38.440041 772-773. Max. coverage (+): 0.85. Max coverage (-): 0.2

Region: NODE\_117098\_length\_959\_cov\_38.440041 774-775. Max. coverage (+): 0.46. Max coverage (-): 0.2

Region: NODE\_117098\_length\_959\_cov\_38.440041 776-777. Max. coverage (+): 0.79. Max coverage (-): 0.06

Region: NODE\_117098\_length\_959\_cov\_38.440041 778-779. Max. coverage (+): 0.57. Max coverage (-): 0.06

Region: NODE\_117098\_length\_959\_cov\_38.440041 780-782. Max. coverage (+): 0.63. Max coverage (-): 0.04

Region: NODE\_117098\_length\_959\_cov\_38.440041 783-784. Max. coverage (+): 1.33. Max coverage (-): 0.04

Region: NODE\_117098\_length\_959\_cov\_38.440041 785-786. Max. coverage (+): 0.97. Max coverage (-): 0.02

Region: NODE\_117098\_length\_959\_cov\_38.440041 787-788. Max. coverage (+): 5.43. Max coverage (-): 0

Region: NODE\_117098\_length\_959\_cov\_38.440041 789-790. Max. coverage (+): 5.13. Max coverage (-): 0

Region: NODE\_117098\_length\_959\_cov\_38.440041 791-792. Max. coverage (+): 0.48. Max coverage (-): 0

Region: NODE\_117098\_length\_959\_cov\_38.440041 793-794. Max. coverage (+): 1.09. Max coverage (-): 0

Region: NODE\_117098\_length\_959\_cov\_38.440041 795-796. Max. coverage (+): 0.67. Max coverage (-): 0

Region: NODE\_117098\_length\_959\_cov\_38.440041 797-798. Max. coverage (+): 0.1. Max coverage (-): 0

Region: NODE\_117098\_length\_959\_cov\_38.440041 799-800. Max. coverage (+): 0.18. Max coverage (-): 0.04

Region: NODE\_117098\_length\_959\_cov\_38.440041 801-802. Max. coverage (+): 1.21. Max coverage (-): 0.04

Region: NODE\_117098\_length\_959\_cov\_38.440041 803-804. Max. coverage (+): 7.85. Max coverage (-): 0.06

Region: NODE\_117098\_length\_959\_cov\_38.440041 805-806. Max. coverage (+): 6.86. Max coverage (-): 0.06

Region: NODE\_117098\_length\_959\_cov\_38.440041 807-808. Max. coverage (+): 0.1. Max coverage (-): 0.02

Region: NODE\_117098\_length\_959\_cov\_38.440041 809-810. Max. coverage (+): 0.1. Max coverage (-): 0.04

Region: NODE\_117098\_length\_959\_cov\_38.440041 811-812. Max. coverage (+): 0.1. Max coverage (-): 0.04

Region: NODE\_117098\_length\_959\_cov\_38.440041 813-814. Max. coverage (+): 0.06. Max coverage (-): 0.36

Region: NODE\_117098\_length\_959\_cov\_38.440041 815-816. Max. coverage (+): 0.02. Max coverage (-): 0.36

Region: NODE\_117098\_length\_959\_cov\_38.440041 817-818. Max. coverage (+): 0.14. Max coverage (-): 0.18

Region: NODE\_117098\_length\_959\_cov\_38.440041 819-820. Max. coverage (+): 0.28. Max coverage (-): 0.14

Region: NODE\_117098\_length\_959\_cov\_38.440041 821-822. Max. coverage (+): 0.46. Max coverage (-): 0.24

Region: NODE\_117098\_length\_959\_cov\_38.440041 823-824. Max. coverage (+): 0.32. Max coverage (-): 0.12

Region: NODE\_117098\_length\_959\_cov\_38.440041 825-826. Max. coverage (+): 0.04. Max coverage (-): 0.02

Region: NODE\_117098\_length\_959\_cov\_38.440041 827-828. Max. coverage (+): 0. Max coverage (-): 0

Region: NODE\_117098\_length\_959\_cov\_38.440041 829-830. Max. coverage (+): 0.1. Max coverage (-): 0.02

Region: NODE\_117098\_length\_959\_cov\_38.440041 831-832. Max. coverage (+): 0.5. Max coverage (-): 0.02

Region: NODE\_117098\_length\_959\_cov\_38.440041 833-834. Max. coverage (+): 0.95. Max coverage (-): 0

Region: NODE\_117098\_length\_959\_cov\_38.440041 835-836. Max. coverage (+): 6.36. Max coverage (-): 0

Region: NODE\_117098\_length\_959\_cov\_38.440041 837-838. Max. coverage (+): 6.9. Max coverage (-): 0

Region: NODE\_117098\_length\_959\_cov\_38.440041 839-840. Max. coverage (+): 6.84. Max coverage (-): 0

Region: NODE\_117098\_length\_959\_cov\_38.440041 841-843. Max. coverage (+): 1.7. Max coverage (-): 0.22

Region: NODE\_117098\_length\_959\_cov\_38.440041 844-845. Max. coverage (+): 26.67. Max coverage (-): 0.24

Region: NODE\_117098\_length\_959\_cov\_38.440041 846-847. Max. coverage (+): 26.81. Max coverage (-): 0.04

Region: NODE\_117098\_length\_959\_cov\_38.440041 848-849. Max. coverage (+): 0.81. Max coverage (-): 0.26

Region: NODE\_117098\_length\_959\_cov\_38.440041 850-851. Max. coverage (+): 0.46. Max coverage (-): 0.3

Region: NODE\_117098\_length\_959\_cov\_38.440041 852-853. Max. coverage (+): 0.79. Max coverage (-): 0.08

Region: NODE\_117098\_length\_959\_cov\_38.440041 854-855. Max. coverage (+): 0.46. Max coverage (-): 0.04

Region: NODE\_117098\_length\_959\_cov\_38.440041 856-857. Max. coverage (+): 0.14. Max coverage (-): 0

Region: NODE\_117098\_length\_959\_cov\_38.440041 858-859. Max. coverage (+): 0.16. Max coverage (-): 0.04

Region: NODE\_117098\_length\_959\_cov\_38.440041 860-861. Max. coverage (+): 0.06. Max coverage (-): 0.04

Region: NODE\_117098\_length\_959\_cov\_38.440041 862-863. Max. coverage (+): 0. Max coverage (-): 0.16

Region: NODE\_117098\_length\_959\_cov\_38.440041 864-865. Max. coverage (+): 0.06. Max coverage (-): 0.14

Region: NODE\_117098\_length\_959\_cov\_38.440041 866-867. Max. coverage (+): 0.06. Max coverage (-): 1.21

Region: NODE\_117098\_length\_959\_cov\_38.440041 868-869. Max. coverage (+): 0.04. Max coverage (-): 1.19

Region: NODE\_117098\_length\_959\_cov\_38.440041 870-871. Max. coverage (+): 0.04. Max coverage (-): 0.12

Region: NODE\_117098\_length\_959\_cov\_38.440041 872-873. Max. coverage (+): 0.04. Max coverage (-): 0.25

Region: NODE\_117098\_length\_959\_cov\_38.440041 874-875. Max. coverage (+): 3.32. Max coverage (-): 0.47

Region: NODE\_117098\_length\_959\_cov\_38.440041 876-877. Max. coverage (+): 3.34. Max coverage (-): 2.56

Region: NODE\_117098\_length\_959\_cov\_38.440041 878-879. Max. coverage (+): 0.83. Max coverage (-): 3.63

Region: NODE\_117098\_length\_959\_cov\_38.440041 880-881. Max. coverage (+): 1.98. Max coverage (-): 1.62

Region: NODE\_117098\_length\_959\_cov\_38.440041 882-883. Max. coverage (+): 1.82. Max coverage (-): 0.3

Region: NODE\_117098\_length\_959\_cov\_38.440041 884-885. Max. coverage (+): 0.19. Max coverage (-): 0.09

Region: NODE\_117098\_length\_959\_cov\_38.440041 886-887. Max. coverage (+): 0.13. Max coverage (-): 0.08

Region: NODE\_117098\_length\_959\_cov\_38.440041 888-889. Max. coverage (+): 0.35. Max coverage (-): 0.12

Region: NODE\_117098\_length\_959\_cov\_38.440041 890-891. Max. coverage (+): 0.98. Max coverage (-): 0.13

Region: NODE\_117098\_length\_959\_cov\_38.440041 892-893. Max. coverage (+): 3.59. Max coverage (-): 0.78

Region: NODE\_117098\_length\_959\_cov\_38.440041 894-895. Max. coverage (+): 4.25. Max coverage (-): 0.74

Region: NODE\_117098\_length\_959\_cov\_38.440041 896-897. Max. coverage (+): 0.79. Max coverage (-): 0.01

Region: NODE\_117098\_length\_959\_cov\_38.440041 898-899. Max. coverage (+): 0.07. Max coverage (-): 0.5

Region: NODE\_117098\_length\_959\_cov\_38.440041 900-902. Max. coverage (+): 0.79. Max coverage (-): 0.79

Region: NODE\_117098\_length\_959\_cov\_38.440041 903-904. Max. coverage (+): 0.75. Max coverage (-): 0.17

Region: NODE\_117098\_length\_959\_cov\_38.440041 905-906. Max. coverage (+): 0.08. Max coverage (-): 0.26

Region: NODE\_117098\_length\_959\_cov\_38.440041 907-908. Max. coverage (+): 0.68. Max coverage (-): 0.2

Region: NODE\_117098\_length\_959\_cov\_38.440041 909-910. Max. coverage (+): 0.62. Max coverage (-): 0.02

Region: NODE\_117098\_length\_959\_cov\_38.440041 911-912. Max. coverage (+): 0.34. Max coverage (-): 0.07

Region: NODE\_117098\_length\_959\_cov\_38.440041 913-914. Max. coverage (+): 0.41. Max coverage (-): 0.09

Region: NODE\_117098\_length\_959\_cov\_38.440041 915-916. Max. coverage (+): 1. Max coverage (-): 0.03

Region: NODE\_117098\_length\_959\_cov\_38.440041 917-918. Max. coverage (+): 2.03. Max coverage (-): 0.01

Region: NODE\_117098\_length\_959\_cov\_38.440041 919-920. Max. coverage (+): 14.26. Max coverage (-): 0.03

Region: NODE\_117098\_length\_959\_cov\_38.440041 921-922. Max. coverage (+): 12.9. Max coverage (-): 0.03

Region: NODE\_117098\_length\_959\_cov\_38.440041 923-924. Max. coverage (+): 0.98. Max coverage (-): 0.2

Region: NODE\_117098\_length\_959\_cov\_38.440041 925-926. Max. coverage (+): 1.07. Max coverage (-): 0.2

Region: NODE\_117098\_length\_959\_cov\_38.440041 927-928. Max. coverage (+): 1.11. Max coverage (-): 0.06

Region: NODE\_117098\_length\_959\_cov\_38.440041 929-930. Max. coverage (+): 0.18. Max coverage (-): 0.06

Region: NODE\_117098\_length\_959\_cov\_38.440041 931-932. Max. coverage (+): 0. Max coverage (-): 0

Region: NODE\_117098\_length\_959\_cov\_38.440041 933-934. Max. coverage (+): 0. Max coverage (-): 0

Region: NODE\_117098\_length\_959\_cov\_38.440041 935-936. Max. coverage (+): 0. Max coverage (-): 0

Region: NODE\_117098\_length\_959\_cov\_38.440041 937-938. Max. coverage (+): 0. Max coverage (-): 0

Region: NODE\_117098\_length\_959\_cov\_38.440041 939-940. Max. coverage (+): 0. Max coverage (-): 0

Region: NODE\_117098\_length\_959\_cov\_38.440041 941-942. Max. coverage (+): 0. Max coverage (-): 0

Region: NODE\_117098\_length\_959\_cov\_38.440041 943-944. Max. coverage (+): 0. Max coverage (-): 0

Region: NODE\_117098\_length\_959\_cov\_38.440041 945-946. Max. coverage (+): 0. Max coverage (-): 0

Region: NODE\_117098\_length\_959\_cov\_38.440041 947-948. Max. coverage (+): 0. Max coverage (-): 0

Region: NODE\_117098\_length\_959\_cov\_38.440041 949-950. Max. coverage (+): 0. Max coverage (-): 0

Region: NODE\_117098\_length\_959\_cov\_38.440041 951-952. Max. coverage (+): 0. Max coverage (-): 0

Region: NODE\_117098\_length\_959\_cov\_38.440041 953-954. Max. coverage (+): 0. Max coverage (-): 0

Region: NODE\_117098\_length\_959\_cov\_38.440041 955-956. Max. coverage (+): 0. Max coverage (-): 0

Region: NODE\_117098\_length\_959\_cov\_38.440041 957-958. Max. coverage (+): 0. Max coverage (-): 0

Region: NODE\_117098\_length\_959\_cov\_38.440041 959-960. Max. coverage (+): 0. Max coverage (-): 0

Region: NODE\_117098\_length\_959\_cov\_38.440041 961-963. Max. coverage (+): 0. Max coverage (-): 0

Region: NODE\_117098\_length\_959\_cov\_38.440041 964-965. Max. coverage (+): 0. Max coverage (-): 0

Region: NODE\_117098\_length\_959\_cov\_38.440041 966-967. Max. coverage (+): 0.01. Max coverage (-): 0

Region: NODE\_117098\_length\_959\_cov\_38.440041 968-969. Max. coverage (+): 0. Max coverage (-): 0

Region: NODE\_117098\_length\_959\_cov\_38.440041 970-971. Max. coverage (+): 0. Max coverage (-): 0

Region: NODE\_117098\_length\_959\_cov\_38.440041 972-973. Max. coverage (+): 0. Max coverage (-): 0

Region: NODE\_117098\_length\_959\_cov\_38.440041 974-975. Max. coverage (+): 0. Max coverage (-): 0

Region: NODE\_117098\_length\_959\_cov\_38.440041 976-977. Max. coverage (+): 0. Max coverage (-): 0

Region: NODE\_117098\_length\_959\_cov\_38.440041 978-979. Max. coverage (+): 0. Max coverage (-): 0

Region: NODE\_117098\_length\_959\_cov\_38.440041 980-981. Max. coverage (+): 0. Max coverage (-): 0

Region: NODE\_117098\_length\_959\_cov\_38.440041 982-983. Max. coverage (+): 0. Max coverage (-): 0

Region: NODE\_117098\_length\_959\_cov\_38.440041 984-985. Max. coverage (+): 0. Max coverage (-): 0

Region: NODE\_117098\_length\_959\_cov\_38.440041 986-987. Max. coverage (+): 0. Max coverage (-): 0

Region: NODE\_117098\_length\_959\_cov\_38.440041 988-989. Max. coverage (+): 0. Max coverage (-): 0

Region: NODE\_117098\_length\_959\_cov\_38.440041 990-991. Max. coverage (+): 0. Max coverage (-): 0

Region: NODE\_117098\_length\_959\_cov\_38.440041 992-993. Max. coverage (+): 0. Max coverage (-): 0

Region: NODE\_117098\_length\_959\_cov\_38.440041 994-995. Max. coverage (+): 0. Max coverage (-): 0

Region: NODE\_117098\_length\_959\_cov\_38.440041 996-997. Max. coverage (+): 0. Max coverage (-): 0

Region: NODE\_117098\_length\_959\_cov\_38.440041 998-999. Max. coverage (+): 0. Max coverage (-): 0

Region: NODE\_117098\_length\_959\_cov\_38.440041 1000-1001. Max. coverage (+): 0. Max coverage (-): 0

Region: NODE\_117098\_length\_959\_cov\_38.440041 1002-1003. Max. coverage (+): 0. Max coverage (-): 0

Region: NODE\_117098\_length\_959\_cov\_38.440041 1004-1005. Max. coverage (+): 0. Max coverage (-): 0

Region: NODE\_117098\_length\_959\_cov\_38.440041 1006-1007. Max. coverage (+): 0. Max coverage (-): 0

Region: NODE\_117098\_length\_959\_cov\_38.440041 1008-1009. Max. coverage (+): 0. Max coverage (-): 0

Region: NODE\_117098\_length\_959\_cov\_38.440041 1010-1011. Max. coverage (+): 0. Max coverage (-): 0

Region: NODE\_117098\_length\_959\_cov\_38.440041 1012-1013. Max. coverage (+): 0. Max coverage (-): 0

Region: NODE\_117098\_length\_959\_cov\_38.440041 1014-1015. Max. coverage (+): 0. Max coverage (-): 0

Region: NODE\_117098\_length\_959\_cov\_38.440041 1016-1017. Max. coverage (+): 0. Max coverage (-): 0

Region: NODE\_117098\_length\_959\_cov\_38.440041 1018-1019. Max. coverage (+): 0. Max coverage (-): 0

Region: NODE\_117098\_length\_959\_cov\_38.440041 1020-. Max. coverage (+): 0. Max coverage (-): 0

RepeatMasker Color Code

**+**

100-98% Identity

<98-95% Identity

<95-90% Identity

<90-85% Identity

<85-80% Identity

<80-75% Identity

<75-70% Identity

<70% Identity

**-**

Gene Set Color Code

**+**

Gene

Pseudogene

Other

**-**

Topology/Coverage Color Code

Coverage Plus Strand

Coverage Minus Strand

Mainstrand: Plus

Mainstrand: Minus

Complementary Strand

Flanking Region  
(if option -flank >0)

Gene Set Annotation  
  
RepeatMasker Annotation  

**1. AlRepE-2341**: 1-57 (-), Divergence to consensus: 23.2%  
**2. hAT-N97\_DR**: 964-1023 (+), Divergence to consensus: 0%

  
Transcription Factor Binding Sites  

**RHOXF1** (Sequence: GGATCA (-): 842)  
**RHOXF1** (Sequence: GGATTA (-): 962)  
**RHOXF1** (Sequence: TAAGCC (+): 143)  
**RHOXF1** (Sequence: TGAGCT (+): 360)  
**RHOXF1** (Sequence: TGATCT (+): 703)  
**RHOXF1** (Sequence: TAAGCT (+): 869)  
**RHOXF1** (Sequence: TGATCT (+): 922)  
**FOXO3\_mmu** (Sequence: TGTTTTGA (-): 253)
