# Supplementary material for: First characterization of PIWI-interacting RNA clusters in a cichlid fish with a B chromosome
Source: BMC Biol. 2022 Sep 21;20:204. doi: 10.1186/s12915-022-01403-2 (PMC9490952; doi:10.1186/s12915-022-01403-2)
Supplement: Supplementary file 1 — Additional file 1. Zipped folder with fasta and interactive html piRNA cluster information for the A. latifasciata genome. The nomenclature is as follows: number-pirna-cluster_sex_B-presence (f, female; m, male; 0b, without B chromosome; 1b, with B chromosome). [file 12915_2022_1403_MOESM1_ESM.zip › 10_m1b.html]

piRNA cluster 10\_m1b 26


Predicted piRNA cluster no. 10\_m1b
  

Show proTRAC run info
Hide proTRAC run info

/\  
                \_\_\_\_\_\_\_\_\_\_\_\_\_\_\_\_\_\_\_\_\_\_\_/\\_\_\_ /  \\_\_\_\_\_\_\_  
               I                      /  \  /    \      I  
               I     pro             /    \/      \     I  
               I        TRAC        /               \   I  
               I   \_\_\_\_\_\_\_\_\_\_\_\_\_\_\_\_/\_\_\_\_\_\_\_\_\_\_\_\_\_\_\_\_\_\\_ I  
               I   \              /                     I  
               I    \            /                      I  
               I     \  /\      /       V.2.4.2         I  
               I      \/  \    /                        I  
               I\_\_\_\_\_\_\_\_\_\_\_\  /\_\_\_\_\_\_\_\_\_\_\_\_\_\_\_\_\_\_\_\_\_\_\_\_\_I  
                            \/  
  
  
================================= proTRAC ====================================  
VERSION: .......... 2.4.2  
LAST MODIFIED: .... 11. May 2018  
  
Please cite:  
Rosenkranz D, Zischler H. proTRAC - a software for probabilistic piRNA cluster  
detection, visualization and analysis. 2012. BMC Bioinformatics 13:5.  
  
  
Contact:  
David Rosenkranz  
Institute of Organismic and Molecular Evolutionary Biology  
Dept. Anthropology, small RNA group  
Johannes Gutenberg University Mainz  
email: rosenkranz@uni-mainz.de  
  
You can find the latest proTRAC version at:  
http://sourceforge.net/projects/protrac/files  
http://www.smallRNAgroup-mainz.de/software  
==============================================================================  
  
PARAMETERS:  
Map file: ...............piwi-machos-1B.fa-collapse.map  
Genome file: ............../../../0B\_ala\_genome.fa  
RepeatMasker annotation: Alatifasciata-all0B-maryan-v2.fa\_corrected.out  
GeneSet:................./guest-storage/Data/annotation/Alatifasciata\_all0B\_maryan-v2\_out2017.gff  
  
Significant (p<=0.01) hit density will be calculated based  
on observed hit distribution.  
  
Sliding window size: ........................................ 5000 bp  
Sliding window increament: .................................. 1000 bp  
Normalize each hit by number of genomic hits: ............... yes  
Normalize each hit by number of sequence reads: ............. yes  
Normalize values (-> per million mapped reads): ............. yes  
Min. fraction of hits with 1T(U) or 10A: .................... 0.75  
Alternatively: Min. fraction of hits with 1T(U) and 10A: .... 0.5  
Min. fraction of hits with typical piRNA length: ............ 0.75  
Typical piRNA length: ....................................... 24-32 nt  
Min. size of a piRNA cluster: ............................... 1000 bp.  
Min. number of hits (absolute): ............................. 0  
Min. number of hits (normalized): ........................... 0  
Min. fraction of hits on the mainstrand: .................... 0.75  
Top fraction of mapped sequences (in terms of read counts): . 1%  
Top fraction accounts for max. n% of sequence reads: ........ 90%  
Min. fraction of hits on each arm of a bidirectional cluster: 0.05  
Output html file for each cluster: .......................... yes  
Output a summary table: ..................................... yes  
Output a FASTA file for each cluster (piRNA sequences): ..... yes  
Output a FASTA file comprising cluster sequences: ........... yes  
Output a GTF file for predicted piRNA clusters: ..............yes  
Search DNA motifs in clusters: .............................. yes  
Output flanking sequences: +/- .............................. 0 bp  
Output ~.pTi file: .......................................... no  
==============================================================================  
  
  
Genome size (without gaps): ............ 758543724 bp  
Gaps (N/X/-): .......................... 417479 bp  
Mapped reads: .......................... 26973943  
Non-identical sequences: ............... 6209225  
Genomic hits: .......................... 48438990  
Significant densitiy of mapped reads: .. 821.144211136946 reads/kb

Show proTRAC cluster info
Hide proTRAC cluster info

|  |  |
| --- | --- |
| Location | NODE\_117098\_length\_959\_cov\_38.440041 |
| Coordinates | 1-1020 |
| Size [bp] | 1020 |
| Sequence hit loci | 2786 |
| Mapped reads (normalized) | 9054.7 |
| Mapped reads (normalized) per kb | 8877.2 |
| Normalized reads with 1T (1U) | 77.1% |
| Normalized reads with 10A | 22.9% |
| Normalized reads with length 24-32 nt | 98.6% |
| Normalized reads on the main strand(s) | 93% |
| Predicted directionality | mono:plus |

100%

0%

1T (1U)  
reads

10A reads

24-32 nt  
reads

reads on mainstrand

**Either the amount of reads with 1T (1U) OR 10A has to exceed 75% (set with option: -1Tor10A)  
Alternatively the amount of reads with 1T (1U) AND 10A has to exceed 50% (set with option: -1Tand10A)  
Minimum amount of reads with preferred size is 75% (set with option: -pisize)  
Minimum amount of reads on the main strand(s) is 75% (set with option: -clstrand)**

Show read coverage
Hide read coverage

WHAT DO I SEE HERE?  
This chart shows the location of mapped sequence reads within a predicted piRNA cluster. The color refers to the number of genomic hits produced by the sequence read in question. A dark red bar indicates that this sequence read produces many other hits elsewhere in the genome. Many adjacent red or yellow bars can indicate the presence of a multi-copy element such as transposons or rRNA genes. A dark green bar indicates that this sequence read maps uniquely to this locus.

1 hit

2-5 hits

6-10 hits

11-20 hits

21-50 hits

51-100 hits

> 100 hits

NODE\_117098\_length\_959\_cov\_38.440041

1

1020

Gene Set

RepeatMasker

Mapped  
Reads

26.08

plus strand

minus strand

26.08

Region: NODE\_117098\_length\_959\_cov\_38.440041 5308-2. Max. coverage (+): 0.01. Max coverage (-): 0

Region: NODE\_117098\_length\_959\_cov\_38.440041 3-4. Max. coverage (+): 0.01. Max coverage (-): 0

Region: NODE\_117098\_length\_959\_cov\_38.440041 5-6. Max. coverage (+): 0.02. Max coverage (-): 0

Region: NODE\_117098\_length\_959\_cov\_38.440041 7-8. Max. coverage (+): 0. Max coverage (-): 0

Region: NODE\_117098\_length\_959\_cov\_38.440041 9-10. Max. coverage (+): 0. Max coverage (-): 0

Region: NODE\_117098\_length\_959\_cov\_38.440041 11-12. Max. coverage (+): 0. Max coverage (-): 0.01

Region: NODE\_117098\_length\_959\_cov\_38.440041 13-14. Max. coverage (+): 0. Max coverage (-): 0

Region: NODE\_117098\_length\_959\_cov\_38.440041 15-16. Max. coverage (+): 0. Max coverage (-): 0

Region: NODE\_117098\_length\_959\_cov\_38.440041 17-18. Max. coverage (+): 0.01. Max coverage (-): 0

Region: NODE\_117098\_length\_959\_cov\_38.440041 19-20. Max. coverage (+): 0.01. Max coverage (-): 0.01

Region: NODE\_117098\_length\_959\_cov\_38.440041 21-22. Max. coverage (+): 0. Max coverage (-): 0.01

Region: NODE\_117098\_length\_959\_cov\_38.440041 23-24. Max. coverage (+): 0. Max coverage (-): 0

Region: NODE\_117098\_length\_959\_cov\_38.440041 25-26. Max. coverage (+): 0. Max coverage (-): 0

Region: NODE\_117098\_length\_959\_cov\_38.440041 27-28. Max. coverage (+): 0. Max coverage (-): 0

Region: NODE\_117098\_length\_959\_cov\_38.440041 29-30. Max. coverage (+): 0.01. Max coverage (-): 0

Region: NODE\_117098\_length\_959\_cov\_38.440041 31-32. Max. coverage (+): 0.01. Max coverage (-): 0

Region: NODE\_117098\_length\_959\_cov\_38.440041 33-34. Max. coverage (+): 0.01. Max coverage (-): 0

Region: NODE\_117098\_length\_959\_cov\_38.440041 35-36. Max. coverage (+): 0.01. Max coverage (-): 0

Region: NODE\_117098\_length\_959\_cov\_38.440041 37-38. Max. coverage (+): 0.07. Max coverage (-): 0

Region: NODE\_117098\_length\_959\_cov\_38.440041 39-40. Max. coverage (+): 0.08. Max coverage (-): 0.01

Region: NODE\_117098\_length\_959\_cov\_38.440041 41-42. Max. coverage (+): 0. Max coverage (-): 0.01

Region: NODE\_117098\_length\_959\_cov\_38.440041 43-44. Max. coverage (+): 0. Max coverage (-): 0

Region: NODE\_117098\_length\_959\_cov\_38.440041 45-46. Max. coverage (+): 0.06. Max coverage (-): 0.02

Region: NODE\_117098\_length\_959\_cov\_38.440041 47-48. Max. coverage (+): 0.04. Max coverage (-): 0

Region: NODE\_117098\_length\_959\_cov\_38.440041 49-50. Max. coverage (+): 0. Max coverage (-): 0

Region: NODE\_117098\_length\_959\_cov\_38.440041 51-53. Max. coverage (+): 0. Max coverage (-): 0

Region: NODE\_117098\_length\_959\_cov\_38.440041 54-55. Max. coverage (+): 0.07. Max coverage (-): 0

Region: NODE\_117098\_length\_959\_cov\_38.440041 56-57. Max. coverage (+): 0.09. Max coverage (-): 0

Region: NODE\_117098\_length\_959\_cov\_38.440041 58-59. Max. coverage (+): 0.96. Max coverage (-): 0

Region: NODE\_117098\_length\_959\_cov\_38.440041 60-61. Max. coverage (+): 0.91. Max coverage (-): 0.04

Region: NODE\_117098\_length\_959\_cov\_38.440041 62-63. Max. coverage (+): 0.09. Max coverage (-): 0.04

Region: NODE\_117098\_length\_959\_cov\_38.440041 64-65. Max. coverage (+): 0.69. Max coverage (-): 0

Region: NODE\_117098\_length\_959\_cov\_38.440041 66-67. Max. coverage (+): 0.76. Max coverage (-): 0

Region: NODE\_117098\_length\_959\_cov\_38.440041 68-69. Max. coverage (+): 0.74. Max coverage (-): 0

Region: NODE\_117098\_length\_959\_cov\_38.440041 70-71. Max. coverage (+): 1.02. Max coverage (-): 0

Region: NODE\_117098\_length\_959\_cov\_38.440041 72-73. Max. coverage (+): 0.26. Max coverage (-): 0

Region: NODE\_117098\_length\_959\_cov\_38.440041 74-75. Max. coverage (+): 0.35. Max coverage (-): 0

Region: NODE\_117098\_length\_959\_cov\_38.440041 76-77. Max. coverage (+): 0.04. Max coverage (-): 0

Region: NODE\_117098\_length\_959\_cov\_38.440041 78-79. Max. coverage (+): 0.02. Max coverage (-): 0

Region: NODE\_117098\_length\_959\_cov\_38.440041 80-81. Max. coverage (+): 0. Max coverage (-): 0

Region: NODE\_117098\_length\_959\_cov\_38.440041 82-83. Max. coverage (+): 0. Max coverage (-): 0

Region: NODE\_117098\_length\_959\_cov\_38.440041 84-85. Max. coverage (+): 0. Max coverage (-): 0

Region: NODE\_117098\_length\_959\_cov\_38.440041 86-87. Max. coverage (+): 0. Max coverage (-): 0

Region: NODE\_117098\_length\_959\_cov\_38.440041 88-89. Max. coverage (+): 0. Max coverage (-): 0

Region: NODE\_117098\_length\_959\_cov\_38.440041 90-91. Max. coverage (+): 0.01. Max coverage (-): 0

Region: NODE\_117098\_length\_959\_cov\_38.440041 92-93. Max. coverage (+): 0.01. Max coverage (-): 0

Region: NODE\_117098\_length\_959\_cov\_38.440041 94-95. Max. coverage (+): 0.01. Max coverage (-): 0

Region: NODE\_117098\_length\_959\_cov\_38.440041 96-97. Max. coverage (+): 0.02. Max coverage (-): 0

Region: NODE\_117098\_length\_959\_cov\_38.440041 98-99. Max. coverage (+): 0. Max coverage (-): 0

Region: NODE\_117098\_length\_959\_cov\_38.440041 100-101. Max. coverage (+): 0. Max coverage (-): 0

Region: NODE\_117098\_length\_959\_cov\_38.440041 102-104. Max. coverage (+): 0. Max coverage (-): 0

Region: NODE\_117098\_length\_959\_cov\_38.440041 105-106. Max. coverage (+): 0. Max coverage (-): 0.04

Region: NODE\_117098\_length\_959\_cov\_38.440041 107-108. Max. coverage (+): 0. Max coverage (-): 0.04

Region: NODE\_117098\_length\_959\_cov\_38.440041 109-110. Max. coverage (+): 0.02. Max coverage (-): 0

Region: NODE\_117098\_length\_959\_cov\_38.440041 111-112. Max. coverage (+): 0.04. Max coverage (-): 0

Region: NODE\_117098\_length\_959\_cov\_38.440041 113-114. Max. coverage (+): 1.74. Max coverage (-): 0

Region: NODE\_117098\_length\_959\_cov\_38.440041 115-116. Max. coverage (+): 1.98. Max coverage (-): 0

Region: NODE\_117098\_length\_959\_cov\_38.440041 117-118. Max. coverage (+): 0.26. Max coverage (-): 0

Region: NODE\_117098\_length\_959\_cov\_38.440041 119-120. Max. coverage (+): 0.02. Max coverage (-): 0

Region: NODE\_117098\_length\_959\_cov\_38.440041 121-122. Max. coverage (+): 0.04. Max coverage (-): 0

Region: NODE\_117098\_length\_959\_cov\_38.440041 123-124. Max. coverage (+): 0.09. Max coverage (-): 0

Region: NODE\_117098\_length\_959\_cov\_38.440041 125-126. Max. coverage (+): 0.11. Max coverage (-): 0

Region: NODE\_117098\_length\_959\_cov\_38.440041 127-128. Max. coverage (+): 0.02. Max coverage (-): 0

Region: NODE\_117098\_length\_959\_cov\_38.440041 129-130. Max. coverage (+): 0.06. Max coverage (-): 0

Region: NODE\_117098\_length\_959\_cov\_38.440041 131-132. Max. coverage (+): 0.04. Max coverage (-): 0.02

Region: NODE\_117098\_length\_959\_cov\_38.440041 133-134. Max. coverage (+): 0.06. Max coverage (-): 0.02

Region: NODE\_117098\_length\_959\_cov\_38.440041 135-136. Max. coverage (+): 0.06. Max coverage (-): 0

Region: NODE\_117098\_length\_959\_cov\_38.440041 137-138. Max. coverage (+): 0. Max coverage (-): 0

Region: NODE\_117098\_length\_959\_cov\_38.440041 139-140. Max. coverage (+): 0.02. Max coverage (-): 0

Region: NODE\_117098\_length\_959\_cov\_38.440041 141-142. Max. coverage (+): 0.22. Max coverage (-): 0

Region: NODE\_117098\_length\_959\_cov\_38.440041 143-144. Max. coverage (+): 0.26. Max coverage (-): 0.35

Region: NODE\_117098\_length\_959\_cov\_38.440041 145-146. Max. coverage (+): 0.15. Max coverage (-): 0.83

Region: NODE\_117098\_length\_959\_cov\_38.440041 147-148. Max. coverage (+): 0.19. Max coverage (-): 0.52

Region: NODE\_117098\_length\_959\_cov\_38.440041 149-150. Max. coverage (+): 0.19. Max coverage (-): 0.07

Region: NODE\_117098\_length\_959\_cov\_38.440041 151-152. Max. coverage (+): 0.48. Max coverage (-): 0.02

Region: NODE\_117098\_length\_959\_cov\_38.440041 153-155. Max. coverage (+): 2.26. Max coverage (-): 0.02

Region: NODE\_117098\_length\_959\_cov\_38.440041 156-157. Max. coverage (+): 0.13. Max coverage (-): 0.04

Region: NODE\_117098\_length\_959\_cov\_38.440041 158-159. Max. coverage (+): 0.04. Max coverage (-): 0.02

Region: NODE\_117098\_length\_959\_cov\_38.440041 160-161. Max. coverage (+): 0.04. Max coverage (-): 0

Region: NODE\_117098\_length\_959\_cov\_38.440041 162-163. Max. coverage (+): 0. Max coverage (-): 0

Region: NODE\_117098\_length\_959\_cov\_38.440041 164-165. Max. coverage (+): 0.07. Max coverage (-): 0.02

Region: NODE\_117098\_length\_959\_cov\_38.440041 166-167. Max. coverage (+): 0.07. Max coverage (-): 0.07

Region: NODE\_117098\_length\_959\_cov\_38.440041 168-169. Max. coverage (+): 0.02. Max coverage (-): 0.06

Region: NODE\_117098\_length\_959\_cov\_38.440041 170-171. Max. coverage (+): 0.04. Max coverage (-): 0.07

Region: NODE\_117098\_length\_959\_cov\_38.440041 172-173. Max. coverage (+): 0.04. Max coverage (-): 0.07

Region: NODE\_117098\_length\_959\_cov\_38.440041 174-175. Max. coverage (+): 0.04. Max coverage (-): 0

Region: NODE\_117098\_length\_959\_cov\_38.440041 176-177. Max. coverage (+): 0.06. Max coverage (-): 0

Region: NODE\_117098\_length\_959\_cov\_38.440041 178-179. Max. coverage (+): 0.06. Max coverage (-): 0

Region: NODE\_117098\_length\_959\_cov\_38.440041 180-181. Max. coverage (+): 0. Max coverage (-): 0.33

Region: NODE\_117098\_length\_959\_cov\_38.440041 182-183. Max. coverage (+): 0.41. Max coverage (-): 0.35

Region: NODE\_117098\_length\_959\_cov\_38.440041 184-185. Max. coverage (+): 2.35. Max coverage (-): 0.04

Region: NODE\_117098\_length\_959\_cov\_38.440041 186-187. Max. coverage (+): 2.34. Max coverage (-): 0.07

Region: NODE\_117098\_length\_959\_cov\_38.440041 188-189. Max. coverage (+): 0.59. Max coverage (-): 0.09

Region: NODE\_117098\_length\_959\_cov\_38.440041 190-191. Max. coverage (+): 0.44. Max coverage (-): 0.02

Region: NODE\_117098\_length\_959\_cov\_38.440041 192-193. Max. coverage (+): 0.35. Max coverage (-): 0

Region: NODE\_117098\_length\_959\_cov\_38.440041 194-195. Max. coverage (+): 3.13. Max coverage (-): 0

Region: NODE\_117098\_length\_959\_cov\_38.440041 196-197. Max. coverage (+): 3.31. Max coverage (-): 0

Region: NODE\_117098\_length\_959\_cov\_38.440041 198-199. Max. coverage (+): 0.68. Max coverage (-): 0.04

Region: NODE\_117098\_length\_959\_cov\_38.440041 200-201. Max. coverage (+): 0.69. Max coverage (-): 0.04

Region: NODE\_117098\_length\_959\_cov\_38.440041 202-203. Max. coverage (+): 1.26. Max coverage (-): 0.02

Region: NODE\_117098\_length\_959\_cov\_38.440041 204-206. Max. coverage (+): 0.98. Max coverage (-): 0.04

Region: NODE\_117098\_length\_959\_cov\_38.440041 207-208. Max. coverage (+): 1.06. Max coverage (-): 0.02

Region: NODE\_117098\_length\_959\_cov\_38.440041 209-210. Max. coverage (+): 1.09. Max coverage (-): 0

Region: NODE\_117098\_length\_959\_cov\_38.440041 211-212. Max. coverage (+): 0.15. Max coverage (-): 0.02

Region: NODE\_117098\_length\_959\_cov\_38.440041 213-214. Max. coverage (+): 0.15. Max coverage (-): 0.02

Region: NODE\_117098\_length\_959\_cov\_38.440041 215-216. Max. coverage (+): 0.48. Max coverage (-): 0.04

Region: NODE\_117098\_length\_959\_cov\_38.440041 217-218. Max. coverage (+): 0.33. Max coverage (-): 0.04

Region: NODE\_117098\_length\_959\_cov\_38.440041 219-220. Max. coverage (+): 2.89. Max coverage (-): 0

Region: NODE\_117098\_length\_959\_cov\_38.440041 221-222. Max. coverage (+): 2.89. Max coverage (-): 0.11

Region: NODE\_117098\_length\_959\_cov\_38.440041 223-224. Max. coverage (+): 0.56. Max coverage (-): 0.22

Region: NODE\_117098\_length\_959\_cov\_38.440041 225-226. Max. coverage (+): 1.41. Max coverage (-): 0.26

Region: NODE\_117098\_length\_959\_cov\_38.440041 227-228. Max. coverage (+): 1. Max coverage (-): 0.3

Region: NODE\_117098\_length\_959\_cov\_38.440041 229-230. Max. coverage (+): 1. Max coverage (-): 0.48

Region: NODE\_117098\_length\_959\_cov\_38.440041 231-232. Max. coverage (+): 0.56. Max coverage (-): 0.41

Region: NODE\_117098\_length\_959\_cov\_38.440041 233-234. Max. coverage (+): 0.3. Max coverage (-): 0.04

Region: NODE\_117098\_length\_959\_cov\_38.440041 235-236. Max. coverage (+): 0.07. Max coverage (-): 0.07

Region: NODE\_117098\_length\_959\_cov\_38.440041 237-238. Max. coverage (+): 0.28. Max coverage (-): 0.11

Region: NODE\_117098\_length\_959\_cov\_38.440041 239-240. Max. coverage (+): 0.33. Max coverage (-): 0.07

Region: NODE\_117098\_length\_959\_cov\_38.440041 241-242. Max. coverage (+): 0.32. Max coverage (-): 0.09

Region: NODE\_117098\_length\_959\_cov\_38.440041 243-244. Max. coverage (+): 0.07. Max coverage (-): 0.09

Region: NODE\_117098\_length\_959\_cov\_38.440041 245-246. Max. coverage (+): 0.33. Max coverage (-): 0

Region: NODE\_117098\_length\_959\_cov\_38.440041 247-248. Max. coverage (+): 0.3. Max coverage (-): 0

Region: NODE\_117098\_length\_959\_cov\_38.440041 249-250. Max. coverage (+): 0. Max coverage (-): 0

Region: NODE\_117098\_length\_959\_cov\_38.440041 251-252. Max. coverage (+): 0. Max coverage (-): 0

Region: NODE\_117098\_length\_959\_cov\_38.440041 253-254. Max. coverage (+): 0. Max coverage (-): 0

Region: NODE\_117098\_length\_959\_cov\_38.440041 255-257. Max. coverage (+): 0. Max coverage (-): 0

Region: NODE\_117098\_length\_959\_cov\_38.440041 258-259. Max. coverage (+): 0. Max coverage (-): 0

Region: NODE\_117098\_length\_959\_cov\_38.440041 260-261. Max. coverage (+): 0. Max coverage (-): 0

Region: NODE\_117098\_length\_959\_cov\_38.440041 262-263. Max. coverage (+): 0. Max coverage (-): 0

Region: NODE\_117098\_length\_959\_cov\_38.440041 264-265. Max. coverage (+): 0. Max coverage (-): 0

Region: NODE\_117098\_length\_959\_cov\_38.440041 266-267. Max. coverage (+): 0. Max coverage (-): 0

Region: NODE\_117098\_length\_959\_cov\_38.440041 268-269. Max. coverage (+): 0. Max coverage (-): 0

Region: NODE\_117098\_length\_959\_cov\_38.440041 270-271. Max. coverage (+): 0. Max coverage (-): 0.15

Region: NODE\_117098\_length\_959\_cov\_38.440041 272-273. Max. coverage (+): 0. Max coverage (-): 0.19

Region: NODE\_117098\_length\_959\_cov\_38.440041 274-275. Max. coverage (+): 0. Max coverage (-): 0.44

Region: NODE\_117098\_length\_959\_cov\_38.440041 276-277. Max. coverage (+): 0. Max coverage (-): 0.41

Region: NODE\_117098\_length\_959\_cov\_38.440041 278-279. Max. coverage (+): 0. Max coverage (-): 0

Region: NODE\_117098\_length\_959\_cov\_38.440041 280-281. Max. coverage (+): 0.26. Max coverage (-): 0

Region: NODE\_117098\_length\_959\_cov\_38.440041 282-283. Max. coverage (+): 0.59. Max coverage (-): 0

Region: NODE\_117098\_length\_959\_cov\_38.440041 284-285. Max. coverage (+): 8.93. Max coverage (-): 0

Region: NODE\_117098\_length\_959\_cov\_38.440041 286-287. Max. coverage (+): 8.82. Max coverage (-): 0

Region: NODE\_117098\_length\_959\_cov\_38.440041 288-289. Max. coverage (+): 4.19. Max coverage (-): 0

Region: NODE\_117098\_length\_959\_cov\_38.440041 290-291. Max. coverage (+): 4.12. Max coverage (-): 0

Region: NODE\_117098\_length\_959\_cov\_38.440041 292-293. Max. coverage (+): 0.22. Max coverage (-): 0

Region: NODE\_117098\_length\_959\_cov\_38.440041 294-295. Max. coverage (+): 0.26. Max coverage (-): 0

Region: NODE\_117098\_length\_959\_cov\_38.440041 296-297. Max. coverage (+): 0.26. Max coverage (-): 0

Region: NODE\_117098\_length\_959\_cov\_38.440041 298-299. Max. coverage (+): 0.52. Max coverage (-): 0.04

Region: NODE\_117098\_length\_959\_cov\_38.440041 300-301. Max. coverage (+): 0.33. Max coverage (-): 0.04

Region: NODE\_117098\_length\_959\_cov\_38.440041 302-303. Max. coverage (+): 0.67. Max coverage (-): 0

Region: NODE\_117098\_length\_959\_cov\_38.440041 304-305. Max. coverage (+): 0.7. Max coverage (-): 0

Region: NODE\_117098\_length\_959\_cov\_38.440041 306-308. Max. coverage (+): 10.23. Max coverage (-): 0

Region: NODE\_117098\_length\_959\_cov\_38.440041 309-310. Max. coverage (+): 9.19. Max coverage (-): 0.04

Region: NODE\_117098\_length\_959\_cov\_38.440041 311-312. Max. coverage (+): 7.75. Max coverage (-): 0.22

Region: NODE\_117098\_length\_959\_cov\_38.440041 313-314. Max. coverage (+): 0.48. Max coverage (-): 0.26

Region: NODE\_117098\_length\_959\_cov\_38.440041 315-316. Max. coverage (+): 1.11. Max coverage (-): 0.07

Region: NODE\_117098\_length\_959\_cov\_38.440041 317-318. Max. coverage (+): 1.11. Max coverage (-): 0.13

Region: NODE\_117098\_length\_959\_cov\_38.440041 319-320. Max. coverage (+): 0.04. Max coverage (-): 0.09

Region: NODE\_117098\_length\_959\_cov\_38.440041 321-322. Max. coverage (+): 0.15. Max coverage (-): 0.33

Region: NODE\_117098\_length\_959\_cov\_38.440041 323-324. Max. coverage (+): 0.46. Max coverage (-): 0.35

Region: NODE\_117098\_length\_959\_cov\_38.440041 325-326. Max. coverage (+): 0.4. Max coverage (-): 0.08

Region: NODE\_117098\_length\_959\_cov\_38.440041 327-328. Max. coverage (+): 0.2. Max coverage (-): 0.1

Region: NODE\_117098\_length\_959\_cov\_38.440041 329-330. Max. coverage (+): 2.27. Max coverage (-): 0.06

Region: NODE\_117098\_length\_959\_cov\_38.440041 331-332. Max. coverage (+): 8.67. Max coverage (-): 0.01

Region: NODE\_117098\_length\_959\_cov\_38.440041 333-334. Max. coverage (+): 6.62. Max coverage (-): 0

Region: NODE\_117098\_length\_959\_cov\_38.440041 335-336. Max. coverage (+): 1.75. Max coverage (-): 0.02

Region: NODE\_117098\_length\_959\_cov\_38.440041 337-338. Max. coverage (+): 2.01. Max coverage (-): 0.13

Region: NODE\_117098\_length\_959\_cov\_38.440041 339-340. Max. coverage (+): 0.59. Max coverage (-): 0.11

Region: NODE\_117098\_length\_959\_cov\_38.440041 341-342. Max. coverage (+): 1.08. Max coverage (-): 0

Region: NODE\_117098\_length\_959\_cov\_38.440041 343-344. Max. coverage (+): 0.72. Max coverage (-): 0.04

Region: NODE\_117098\_length\_959\_cov\_38.440041 345-346. Max. coverage (+): 0.26. Max coverage (-): 0.04

Region: NODE\_117098\_length\_959\_cov\_38.440041 347-348. Max. coverage (+): 0.89. Max coverage (-): 0

Region: NODE\_117098\_length\_959\_cov\_38.440041 349-350. Max. coverage (+): 1.04. Max coverage (-): 0.04

Region: NODE\_117098\_length\_959\_cov\_38.440041 351-352. Max. coverage (+): 0.26. Max coverage (-): 0.63

Region: NODE\_117098\_length\_959\_cov\_38.440041 353-354. Max. coverage (+): 0.11. Max coverage (-): 0.67

Region: NODE\_117098\_length\_959\_cov\_38.440041 355-356. Max. coverage (+): 0.3. Max coverage (-): 2.26

Region: NODE\_117098\_length\_959\_cov\_38.440041 357-359. Max. coverage (+): 0.78. Max coverage (-): 2.74

Region: NODE\_117098\_length\_959\_cov\_38.440041 360-361. Max. coverage (+): 8.97. Max coverage (-): 0.52

Region: NODE\_117098\_length\_959\_cov\_38.440041 362-363. Max. coverage (+): 8.82. Max coverage (-): 0.52

Region: NODE\_117098\_length\_959\_cov\_38.440041 364-365. Max. coverage (+): 0.37. Max coverage (-): 0.33

Region: NODE\_117098\_length\_959\_cov\_38.440041 366-367. Max. coverage (+): 0.59. Max coverage (-): 0

Region: NODE\_117098\_length\_959\_cov\_38.440041 368-369. Max. coverage (+): 0.63. Max coverage (-): 0.04

Region: NODE\_117098\_length\_959\_cov\_38.440041 370-371. Max. coverage (+): 0.15. Max coverage (-): 0.07

Region: NODE\_117098\_length\_959\_cov\_38.440041 372-373. Max. coverage (+): 0.48. Max coverage (-): 0.11

Region: NODE\_117098\_length\_959\_cov\_38.440041 374-375. Max. coverage (+): 0.72. Max coverage (-): 0.11

Region: NODE\_117098\_length\_959\_cov\_38.440041 376-377. Max. coverage (+): 0.65. Max coverage (-): 0

Region: NODE\_117098\_length\_959\_cov\_38.440041 378-379. Max. coverage (+): 8.79. Max coverage (-): 0

Region: NODE\_117098\_length\_959\_cov\_38.440041 380-381. Max. coverage (+): 9.97. Max coverage (-): 0.13

Region: NODE\_117098\_length\_959\_cov\_38.440041 382-383. Max. coverage (+): 1.78. Max coverage (-): 0.13

Region: NODE\_117098\_length\_959\_cov\_38.440041 384-385. Max. coverage (+): 0.37. Max coverage (-): 0.04

Region: NODE\_117098\_length\_959\_cov\_38.440041 386-387. Max. coverage (+): 8.01. Max coverage (-): 0.04

Region: NODE\_117098\_length\_959\_cov\_38.440041 388-389. Max. coverage (+): 10.08. Max coverage (-): 0

Region: NODE\_117098\_length\_959\_cov\_38.440041 390-391. Max. coverage (+): 2.56. Max coverage (-): 0

Region: NODE\_117098\_length\_959\_cov\_38.440041 392-393. Max. coverage (+): 0.48. Max coverage (-): 0

Region: NODE\_117098\_length\_959\_cov\_38.440041 394-395. Max. coverage (+): 0.02. Max coverage (-): 0

Region: NODE\_117098\_length\_959\_cov\_38.440041 396-397. Max. coverage (+): 0.22. Max coverage (-): 0

Region: NODE\_117098\_length\_959\_cov\_38.440041 398-399. Max. coverage (+): 0.24. Max coverage (-): 0.04

Region: NODE\_117098\_length\_959\_cov\_38.440041 400-401. Max. coverage (+): 0.04. Max coverage (-): 0.33

Region: NODE\_117098\_length\_959\_cov\_38.440041 402-403. Max. coverage (+): 0. Max coverage (-): 0.3

Region: NODE\_117098\_length\_959\_cov\_38.440041 404-405. Max. coverage (+): 0. Max coverage (-): 0.17

Region: NODE\_117098\_length\_959\_cov\_38.440041 406-407. Max. coverage (+): 0.02. Max coverage (-): 0.11

Region: NODE\_117098\_length\_959\_cov\_38.440041 408-410. Max. coverage (+): 0.19. Max coverage (-): 0.02

Region: NODE\_117098\_length\_959\_cov\_38.440041 411-412. Max. coverage (+): 0.22. Max coverage (-): 0

Region: NODE\_117098\_length\_959\_cov\_38.440041 413-414. Max. coverage (+): 0.28. Max coverage (-): 0

Region: NODE\_117098\_length\_959\_cov\_38.440041 415-416. Max. coverage (+): 0.24. Max coverage (-): 0

Region: NODE\_117098\_length\_959\_cov\_38.440041 417-418. Max. coverage (+): 0.52. Max coverage (-): 0

Region: NODE\_117098\_length\_959\_cov\_38.440041 419-420. Max. coverage (+): 0.61. Max coverage (-): 0

Region: NODE\_117098\_length\_959\_cov\_38.440041 421-422. Max. coverage (+): 0.28. Max coverage (-): 0

Region: NODE\_117098\_length\_959\_cov\_38.440041 423-424. Max. coverage (+): 0.83. Max coverage (-): 0.02

Region: NODE\_117098\_length\_959\_cov\_38.440041 425-426. Max. coverage (+): 7.32. Max coverage (-): 0.06

Region: NODE\_117098\_length\_959\_cov\_38.440041 427-428. Max. coverage (+): 7.06. Max coverage (-): 0.06

Region: NODE\_117098\_length\_959\_cov\_38.440041 429-430. Max. coverage (+): 0.39. Max coverage (-): 0

Region: NODE\_117098\_length\_959\_cov\_38.440041 431-432. Max. coverage (+): 0.04. Max coverage (-): 0

Region: NODE\_117098\_length\_959\_cov\_38.440041 433-434. Max. coverage (+): 0. Max coverage (-): 0

Region: NODE\_117098\_length\_959\_cov\_38.440041 435-436. Max. coverage (+): 0. Max coverage (-): 0

Region: NODE\_117098\_length\_959\_cov\_38.440041 437-438. Max. coverage (+): 0. Max coverage (-): 0

Region: NODE\_117098\_length\_959\_cov\_38.440041 439-440. Max. coverage (+): 0. Max coverage (-): 0

Region: NODE\_117098\_length\_959\_cov\_38.440041 441-442. Max. coverage (+): 0. Max coverage (-): 0

Region: NODE\_117098\_length\_959\_cov\_38.440041 443-444. Max. coverage (+): 0. Max coverage (-): 0

Region: NODE\_117098\_length\_959\_cov\_38.440041 445-446. Max. coverage (+): 0. Max coverage (-): 0

Region: NODE\_117098\_length\_959\_cov\_38.440041 447-448. Max. coverage (+): 0. Max coverage (-): 0

Region: NODE\_117098\_length\_959\_cov\_38.440041 449-450. Max. coverage (+): 0. Max coverage (-): 0

Region: NODE\_117098\_length\_959\_cov\_38.440041 451-452. Max. coverage (+): 0. Max coverage (-): 0

Region: NODE\_117098\_length\_959\_cov\_38.440041 453-454. Max. coverage (+): 0.3. Max coverage (-): 0

Region: NODE\_117098\_length\_959\_cov\_38.440041 455-456. Max. coverage (+): 3.39. Max coverage (-): 0.07

Region: NODE\_117098\_length\_959\_cov\_38.440041 457-458. Max. coverage (+): 3.17. Max coverage (-): 0.09

Region: NODE\_117098\_length\_959\_cov\_38.440041 459-461. Max. coverage (+): 9.71. Max coverage (-): 0.02

Region: NODE\_117098\_length\_959\_cov\_38.440041 462-463. Max. coverage (+): 0.26. Max coverage (-): 0.06

Region: NODE\_117098\_length\_959\_cov\_38.440041 464-465. Max. coverage (+): 0.66. Max coverage (-): 0.06

Region: NODE\_117098\_length\_959\_cov\_38.440041 466-467. Max. coverage (+): 1.16. Max coverage (-): 0.01

Region: NODE\_117098\_length\_959\_cov\_38.440041 468-469. Max. coverage (+): 0.89. Max coverage (-): 0.06

Region: NODE\_117098\_length\_959\_cov\_38.440041 470-471. Max. coverage (+): 1.67. Max coverage (-): 0.06

Region: NODE\_117098\_length\_959\_cov\_38.440041 472-473. Max. coverage (+): 2.3. Max coverage (-): 0.02

Region: NODE\_117098\_length\_959\_cov\_38.440041 474-475. Max. coverage (+): 1.06. Max coverage (-): 0.11

Region: NODE\_117098\_length\_959\_cov\_38.440041 476-477. Max. coverage (+): 0.07. Max coverage (-): 0.09

Region: NODE\_117098\_length\_959\_cov\_38.440041 478-479. Max. coverage (+): 0.91. Max coverage (-): 0

Region: NODE\_117098\_length\_959\_cov\_38.440041 480-481. Max. coverage (+): 0.91. Max coverage (-): 0

Region: NODE\_117098\_length\_959\_cov\_38.440041 482-483. Max. coverage (+): 0.04. Max coverage (-): 0

Region: NODE\_117098\_length\_959\_cov\_38.440041 484-485. Max. coverage (+): 0.04. Max coverage (-): 0

Region: NODE\_117098\_length\_959\_cov\_38.440041 486-487. Max. coverage (+): 0. Max coverage (-): 0

Region: NODE\_117098\_length\_959\_cov\_38.440041 488-489. Max. coverage (+): 0. Max coverage (-): 0

Region: NODE\_117098\_length\_959\_cov\_38.440041 490-491. Max. coverage (+): 0. Max coverage (-): 0

Region: NODE\_117098\_length\_959\_cov\_38.440041 492-493. Max. coverage (+): 0. Max coverage (-): 0

Region: NODE\_117098\_length\_959\_cov\_38.440041 494-495. Max. coverage (+): 0. Max coverage (-): 0

Region: NODE\_117098\_length\_959\_cov\_38.440041 496-497. Max. coverage (+): 0. Max coverage (-): 0

Region: NODE\_117098\_length\_959\_cov\_38.440041 498-499. Max. coverage (+): 0. Max coverage (-): 0

Region: NODE\_117098\_length\_959\_cov\_38.440041 500-501. Max. coverage (+): 0. Max coverage (-): 0

Region: NODE\_117098\_length\_959\_cov\_38.440041 502-503. Max. coverage (+): 0. Max coverage (-): 0

Region: NODE\_117098\_length\_959\_cov\_38.440041 504-505. Max. coverage (+): 0. Max coverage (-): 0

Region: NODE\_117098\_length\_959\_cov\_38.440041 506-507. Max. coverage (+): 0. Max coverage (-): 0

Region: NODE\_117098\_length\_959\_cov\_38.440041 508-509. Max. coverage (+): 0. Max coverage (-): 0

Region: NODE\_117098\_length\_959\_cov\_38.440041 510-512. Max. coverage (+): 0. Max coverage (-): 0

Region: NODE\_117098\_length\_959\_cov\_38.440041 513-514. Max. coverage (+): 0. Max coverage (-): 0

Region: NODE\_117098\_length\_959\_cov\_38.440041 515-516. Max. coverage (+): 0. Max coverage (-): 0

Region: NODE\_117098\_length\_959\_cov\_38.440041 517-518. Max. coverage (+): 0. Max coverage (-): 0

Region: NODE\_117098\_length\_959\_cov\_38.440041 519-520. Max. coverage (+): 0. Max coverage (-): 0

Region: NODE\_117098\_length\_959\_cov\_38.440041 521-522. Max. coverage (+): 0. Max coverage (-): 0

Region: NODE\_117098\_length\_959\_cov\_38.440041 523-524. Max. coverage (+): 0.04. Max coverage (-): 0.17

Region: NODE\_117098\_length\_959\_cov\_38.440041 525-526. Max. coverage (+): 0.35. Max coverage (-): 0.28

Region: NODE\_117098\_length\_959\_cov\_38.440041 527-528. Max. coverage (+): 5.58. Max coverage (-): 0.11

Region: NODE\_117098\_length\_959\_cov\_38.440041 529-530. Max. coverage (+): 5.51. Max coverage (-): 0

Region: NODE\_117098\_length\_959\_cov\_38.440041 531-532. Max. coverage (+): 1.32. Max coverage (-): 0

Region: NODE\_117098\_length\_959\_cov\_38.440041 533-534. Max. coverage (+): 1.84. Max coverage (-): 0

Region: NODE\_117098\_length\_959\_cov\_38.440041 535-536. Max. coverage (+): 1.58. Max coverage (-): 0

Region: NODE\_117098\_length\_959\_cov\_38.440041 537-538. Max. coverage (+): 1.22. Max coverage (-): 0

Region: NODE\_117098\_length\_959\_cov\_38.440041 539-540. Max. coverage (+): 0.98. Max coverage (-): 0

Region: NODE\_117098\_length\_959\_cov\_38.440041 541-542. Max. coverage (+): 0.11. Max coverage (-): 0.04

Region: NODE\_117098\_length\_959\_cov\_38.440041 543-544. Max. coverage (+): 0.13. Max coverage (-): 0.06

Region: NODE\_117098\_length\_959\_cov\_38.440041 545-546. Max. coverage (+): 0.15. Max coverage (-): 0.02

Region: NODE\_117098\_length\_959\_cov\_38.440041 547-548. Max. coverage (+): 0.15. Max coverage (-): 0.02

Region: NODE\_117098\_length\_959\_cov\_38.440041 549-550. Max. coverage (+): 0.07. Max coverage (-): 0.04

Region: NODE\_117098\_length\_959\_cov\_38.440041 551-552. Max. coverage (+): 0.06. Max coverage (-): 0.46

Region: NODE\_117098\_length\_959\_cov\_38.440041 553-554. Max. coverage (+): 0. Max coverage (-): 0.57

Region: NODE\_117098\_length\_959\_cov\_38.440041 555-556. Max. coverage (+): 0.48. Max coverage (-): 0.19

Region: NODE\_117098\_length\_959\_cov\_38.440041 557-558. Max. coverage (+): 1.13. Max coverage (-): 0.11

Region: NODE\_117098\_length\_959\_cov\_38.440041 559-560. Max. coverage (+): 1.33. Max coverage (-): 0.02

Region: NODE\_117098\_length\_959\_cov\_38.440041 561-563. Max. coverage (+): 0.72. Max coverage (-): 0.04

Region: NODE\_117098\_length\_959\_cov\_38.440041 564-565. Max. coverage (+): 0.24. Max coverage (-): 0.09

Region: NODE\_117098\_length\_959\_cov\_38.440041 566-567. Max. coverage (+): 0.41. Max coverage (-): 0.15

Region: NODE\_117098\_length\_959\_cov\_38.440041 568-569. Max. coverage (+): 0.46. Max coverage (-): 0.09

Region: NODE\_117098\_length\_959\_cov\_38.440041 570-571. Max. coverage (+): 0.07. Max coverage (-): 0.02

Region: NODE\_117098\_length\_959\_cov\_38.440041 572-573. Max. coverage (+): 0.02. Max coverage (-): 0

Region: NODE\_117098\_length\_959\_cov\_38.440041 574-575. Max. coverage (+): 0. Max coverage (-): 0

Region: NODE\_117098\_length\_959\_cov\_38.440041 576-577. Max. coverage (+): 0.32. Max coverage (-): 0

Region: NODE\_117098\_length\_959\_cov\_38.440041 578-579. Max. coverage (+): 0.41. Max coverage (-): 0

Region: NODE\_117098\_length\_959\_cov\_38.440041 580-581. Max. coverage (+): 0.54. Max coverage (-): 0

Region: NODE\_117098\_length\_959\_cov\_38.440041 582-583. Max. coverage (+): 0.56. Max coverage (-): 0.02

Region: NODE\_117098\_length\_959\_cov\_38.440041 584-585. Max. coverage (+): 0.69. Max coverage (-): 0.13

Region: NODE\_117098\_length\_959\_cov\_38.440041 586-587. Max. coverage (+): 0.8. Max coverage (-): 0.22

Region: NODE\_117098\_length\_959\_cov\_38.440041 588-589. Max. coverage (+): 0.48. Max coverage (-): 0.26

Region: NODE\_117098\_length\_959\_cov\_38.440041 590-591. Max. coverage (+): 0.39. Max coverage (-): 0.22

Region: NODE\_117098\_length\_959\_cov\_38.440041 592-593. Max. coverage (+): 0.19. Max coverage (-): 0.41

Region: NODE\_117098\_length\_959\_cov\_38.440041 594-595. Max. coverage (+): 0.07. Max coverage (-): 0.5

Region: NODE\_117098\_length\_959\_cov\_38.440041 596-597. Max. coverage (+): 0.02. Max coverage (-): 0.13

Region: NODE\_117098\_length\_959\_cov\_38.440041 598-599. Max. coverage (+): 0.04. Max coverage (-): 0.04

Region: NODE\_117098\_length\_959\_cov\_38.440041 600-601. Max. coverage (+): 0.22. Max coverage (-): 0.04

Region: NODE\_117098\_length\_959\_cov\_38.440041 602-603. Max. coverage (+): 7.34. Max coverage (-): 0.11

Region: NODE\_117098\_length\_959\_cov\_38.440041 604-605. Max. coverage (+): 7.21. Max coverage (-): 0.15

Region: NODE\_117098\_length\_959\_cov\_38.440041 606-607. Max. coverage (+): 0.17. Max coverage (-): 0.07

Region: NODE\_117098\_length\_959\_cov\_38.440041 608-609. Max. coverage (+): 5.19. Max coverage (-): 0.04

Region: NODE\_117098\_length\_959\_cov\_38.440041 610-611. Max. coverage (+): 5.1. Max coverage (-): 0.02

Region: NODE\_117098\_length\_959\_cov\_38.440041 612-614. Max. coverage (+): 0.22. Max coverage (-): 0.04

Region: NODE\_117098\_length\_959\_cov\_38.440041 615-616. Max. coverage (+): 0.22. Max coverage (-): 0.06

Region: NODE\_117098\_length\_959\_cov\_38.440041 617-618. Max. coverage (+): 0.28. Max coverage (-): 0.02

Region: NODE\_117098\_length\_959\_cov\_38.440041 619-620. Max. coverage (+): 0.26. Max coverage (-): 0.02

Region: NODE\_117098\_length\_959\_cov\_38.440041 621-622. Max. coverage (+): 0.26. Max coverage (-): 0

Region: NODE\_117098\_length\_959\_cov\_38.440041 623-624. Max. coverage (+): 0.24. Max coverage (-): 0

Region: NODE\_117098\_length\_959\_cov\_38.440041 625-626. Max. coverage (+): 0.02. Max coverage (-): 0

Region: NODE\_117098\_length\_959\_cov\_38.440041 627-628. Max. coverage (+): 0. Max coverage (-): 0.09

Region: NODE\_117098\_length\_959\_cov\_38.440041 629-630. Max. coverage (+): 0.06. Max coverage (-): 0.09

Region: NODE\_117098\_length\_959\_cov\_38.440041 631-632. Max. coverage (+): 0.3. Max coverage (-): 0.17

Region: NODE\_117098\_length\_959\_cov\_38.440041 633-634. Max. coverage (+): 0.28. Max coverage (-): 0.2

Region: NODE\_117098\_length\_959\_cov\_38.440041 635-636. Max. coverage (+): 0.02. Max coverage (-): 0.09

Region: NODE\_117098\_length\_959\_cov\_38.440041 637-638. Max. coverage (+): 0.28. Max coverage (-): 0

Region: NODE\_117098\_length\_959\_cov\_38.440041 639-640. Max. coverage (+): 0.32. Max coverage (-): 0.15

Region: NODE\_117098\_length\_959\_cov\_38.440041 641-642. Max. coverage (+): 0.5. Max coverage (-): 0.15

Region: NODE\_117098\_length\_959\_cov\_38.440041 643-644. Max. coverage (+): 0.52. Max coverage (-): 0.02

Region: NODE\_117098\_length\_959\_cov\_38.440041 645-646. Max. coverage (+): 0.7. Max coverage (-): 0.02

Region: NODE\_117098\_length\_959\_cov\_38.440041 647-648. Max. coverage (+): 0.69. Max coverage (-): 0.06

Region: NODE\_117098\_length\_959\_cov\_38.440041 649-650. Max. coverage (+): 0.85. Max coverage (-): 0.2

Region: NODE\_117098\_length\_959\_cov\_38.440041 651-652. Max. coverage (+): 3.19. Max coverage (-): 0.3

Region: NODE\_117098\_length\_959\_cov\_38.440041 653-654. Max. coverage (+): 3.95. Max coverage (-): 0.22

Region: NODE\_117098\_length\_959\_cov\_38.440041 655-656. Max. coverage (+): 1.63. Max coverage (-): 0.22

Region: NODE\_117098\_length\_959\_cov\_38.440041 657-658. Max. coverage (+): 0.15. Max coverage (-): 0.07

Region: NODE\_117098\_length\_959\_cov\_38.440041 659-660. Max. coverage (+): 0.11. Max coverage (-): 0.09

Region: NODE\_117098\_length\_959\_cov\_38.440041 661-662. Max. coverage (+): 0.33. Max coverage (-): 0.07

Region: NODE\_117098\_length\_959\_cov\_38.440041 663-665. Max. coverage (+): 0.3. Max coverage (-): 0.15

Region: NODE\_117098\_length\_959\_cov\_38.440041 666-667. Max. coverage (+): 0.41. Max coverage (-): 0.15

Region: NODE\_117098\_length\_959\_cov\_38.440041 668-669. Max. coverage (+): 0.7. Max coverage (-): 0.02

Region: NODE\_117098\_length\_959\_cov\_38.440041 670-671. Max. coverage (+): 0.59. Max coverage (-): 0.04

Region: NODE\_117098\_length\_959\_cov\_38.440041 672-673. Max. coverage (+): 1.26. Max coverage (-): 0.02

Region: NODE\_117098\_length\_959\_cov\_38.440041 674-675. Max. coverage (+): 1.26. Max coverage (-): 0.11

Region: NODE\_117098\_length\_959\_cov\_38.440041 676-677. Max. coverage (+): 0.19. Max coverage (-): 0.13

Region: NODE\_117098\_length\_959\_cov\_38.440041 678-679. Max. coverage (+): 0.65. Max coverage (-): 0.02

Region: NODE\_117098\_length\_959\_cov\_38.440041 680-681. Max. coverage (+): 0.69. Max coverage (-): 0

Region: NODE\_117098\_length\_959\_cov\_38.440041 682-683. Max. coverage (+): 0.63. Max coverage (-): 0.02

Region: NODE\_117098\_length\_959\_cov\_38.440041 684-685. Max. coverage (+): 3.1. Max coverage (-): 0.07

Region: NODE\_117098\_length\_959\_cov\_38.440041 686-687. Max. coverage (+): 2.89. Max coverage (-): 0.07

Region: NODE\_117098\_length\_959\_cov\_38.440041 688-689. Max. coverage (+): 0.89. Max coverage (-): 0.07

Region: NODE\_117098\_length\_959\_cov\_38.440041 690-691. Max. coverage (+): 0.82. Max coverage (-): 0.11

Region: NODE\_117098\_length\_959\_cov\_38.440041 692-693. Max. coverage (+): 0.89. Max coverage (-): 0.15

Region: NODE\_117098\_length\_959\_cov\_38.440041 694-695. Max. coverage (+): 0.32. Max coverage (-): 0.3

Region: NODE\_117098\_length\_959\_cov\_38.440041 696-697. Max. coverage (+): 0.02. Max coverage (-): 0.22

Region: NODE\_117098\_length\_959\_cov\_38.440041 698-699. Max. coverage (+): 0.91. Max coverage (-): 0.22

Region: NODE\_117098\_length\_959\_cov\_38.440041 700-701. Max. coverage (+): 3.74. Max coverage (-): 0.07

Region: NODE\_117098\_length\_959\_cov\_38.440041 702-703. Max. coverage (+): 4.99. Max coverage (-): 0.07

Region: NODE\_117098\_length\_959\_cov\_38.440041 704-705. Max. coverage (+): 3.93. Max coverage (-): 0.06

Region: NODE\_117098\_length\_959\_cov\_38.440041 706-707. Max. coverage (+): 6.17. Max coverage (-): 0.02

Region: NODE\_117098\_length\_959\_cov\_38.440041 708-709. Max. coverage (+): 7.73. Max coverage (-): 0

Region: NODE\_117098\_length\_959\_cov\_38.440041 710-711. Max. coverage (+): 4.58. Max coverage (-): 0

Region: NODE\_117098\_length\_959\_cov\_38.440041 712-713. Max. coverage (+): 0.22. Max coverage (-): 0.02

Region: NODE\_117098\_length\_959\_cov\_38.440041 714-716. Max. coverage (+): 0.06. Max coverage (-): 0.02

Region: NODE\_117098\_length\_959\_cov\_38.440041 717-718. Max. coverage (+): 0.09. Max coverage (-): 0

Region: NODE\_117098\_length\_959\_cov\_38.440041 719-720. Max. coverage (+): 0.13. Max coverage (-): 0.02

Region: NODE\_117098\_length\_959\_cov\_38.440041 721-722. Max. coverage (+): 0.09. Max coverage (-): 0.02

Region: NODE\_117098\_length\_959\_cov\_38.440041 723-724. Max. coverage (+): 0.33. Max coverage (-): 0

Region: NODE\_117098\_length\_959\_cov\_38.440041 725-726. Max. coverage (+): 0.61. Max coverage (-): 0

Region: NODE\_117098\_length\_959\_cov\_38.440041 727-728. Max. coverage (+): 0.41. Max coverage (-): 0

Region: NODE\_117098\_length\_959\_cov\_38.440041 729-730. Max. coverage (+): 0.04. Max coverage (-): 0

Region: NODE\_117098\_length\_959\_cov\_38.440041 731-732. Max. coverage (+): 0. Max coverage (-): 0

Region: NODE\_117098\_length\_959\_cov\_38.440041 733-734. Max. coverage (+): 0. Max coverage (-): 0

Region: NODE\_117098\_length\_959\_cov\_38.440041 735-736. Max. coverage (+): 0. Max coverage (-): 0

Region: NODE\_117098\_length\_959\_cov\_38.440041 737-738. Max. coverage (+): 0. Max coverage (-): 0

Region: NODE\_117098\_length\_959\_cov\_38.440041 739-740. Max. coverage (+): 0. Max coverage (-): 0.02

Region: NODE\_117098\_length\_959\_cov\_38.440041 741-742. Max. coverage (+): 0. Max coverage (-): 0.02

Region: NODE\_117098\_length\_959\_cov\_38.440041 743-744. Max. coverage (+): 0. Max coverage (-): 0

Region: NODE\_117098\_length\_959\_cov\_38.440041 745-746. Max. coverage (+): 0. Max coverage (-): 0

Region: NODE\_117098\_length\_959\_cov\_38.440041 747-748. Max. coverage (+): 2.71. Max coverage (-): 0

Region: NODE\_117098\_length\_959\_cov\_38.440041 749-750. Max. coverage (+): 4.04. Max coverage (-): 0.11

Region: NODE\_117098\_length\_959\_cov\_38.440041 751-752. Max. coverage (+): 3.19. Max coverage (-): 0.15

Region: NODE\_117098\_length\_959\_cov\_38.440041 753-754. Max. coverage (+): 12.85. Max coverage (-): 0.06

Region: NODE\_117098\_length\_959\_cov\_38.440041 755-756. Max. coverage (+): 13.46. Max coverage (-): 0.02

Region: NODE\_117098\_length\_959\_cov\_38.440041 757-758. Max. coverage (+): 2.74. Max coverage (-): 0.02

Region: NODE\_117098\_length\_959\_cov\_38.440041 759-760. Max. coverage (+): 0.37. Max coverage (-): 0.02

Region: NODE\_117098\_length\_959\_cov\_38.440041 761-762. Max. coverage (+): 0.07. Max coverage (-): 0.04

Region: NODE\_117098\_length\_959\_cov\_38.440041 763-764. Max. coverage (+): 0.02. Max coverage (-): 0.37

Region: NODE\_117098\_length\_959\_cov\_38.440041 765-767. Max. coverage (+): 0.02. Max coverage (-): 0.35

Region: NODE\_117098\_length\_959\_cov\_38.440041 768-769. Max. coverage (+): 0.17. Max coverage (-): 0.06

Region: NODE\_117098\_length\_959\_cov\_38.440041 770-771. Max. coverage (+): 0.78. Max coverage (-): 0.06

Region: NODE\_117098\_length\_959\_cov\_38.440041 772-773. Max. coverage (+): 0.69. Max coverage (-): 0.17

Region: NODE\_117098\_length\_959\_cov\_38.440041 774-775. Max. coverage (+): 0.57. Max coverage (-): 0.15

Region: NODE\_117098\_length\_959\_cov\_38.440041 776-777. Max. coverage (+): 1.08. Max coverage (-): 0.09

Region: NODE\_117098\_length\_959\_cov\_38.440041 778-779. Max. coverage (+): 0.59. Max coverage (-): 0.06

Region: NODE\_117098\_length\_959\_cov\_38.440041 780-781. Max. coverage (+): 0.24. Max coverage (-): 0.02

Region: NODE\_117098\_length\_959\_cov\_38.440041 782-783. Max. coverage (+): 0.65. Max coverage (-): 0.04

Region: NODE\_117098\_length\_959\_cov\_38.440041 784-785. Max. coverage (+): 1.06. Max coverage (-): 0.07

Region: NODE\_117098\_length\_959\_cov\_38.440041 786-787. Max. coverage (+): 3.65. Max coverage (-): 0.06

Region: NODE\_117098\_length\_959\_cov\_38.440041 788-789. Max. coverage (+): 3.84. Max coverage (-): 0

Region: NODE\_117098\_length\_959\_cov\_38.440041 790-791. Max. coverage (+): 0.33. Max coverage (-): 0

Region: NODE\_117098\_length\_959\_cov\_38.440041 792-793. Max. coverage (+): 1. Max coverage (-): 0

Region: NODE\_117098\_length\_959\_cov\_38.440041 794-795. Max. coverage (+): 1.19. Max coverage (-): 0

Region: NODE\_117098\_length\_959\_cov\_38.440041 796-797. Max. coverage (+): 0.3. Max coverage (-): 0.02

Region: NODE\_117098\_length\_959\_cov\_38.440041 798-799. Max. coverage (+): 0.13. Max coverage (-): 0.04

Region: NODE\_117098\_length\_959\_cov\_38.440041 800-801. Max. coverage (+): 0.17. Max coverage (-): 0.02

Region: NODE\_117098\_length\_959\_cov\_38.440041 802-803. Max. coverage (+): 1.58. Max coverage (-): 0.04

Region: NODE\_117098\_length\_959\_cov\_38.440041 804-805. Max. coverage (+): 7.77. Max coverage (-): 0.04

Region: NODE\_117098\_length\_959\_cov\_38.440041 806-807. Max. coverage (+): 6.28. Max coverage (-): 0.07

Region: NODE\_117098\_length\_959\_cov\_38.440041 808-809. Max. coverage (+): 0.13. Max coverage (-): 0.09

Region: NODE\_117098\_length\_959\_cov\_38.440041 810-811. Max. coverage (+): 0.11. Max coverage (-): 0.02

Region: NODE\_117098\_length\_959\_cov\_38.440041 812-813. Max. coverage (+): 0.11. Max coverage (-): 0.24

Region: NODE\_117098\_length\_959\_cov\_38.440041 814-815. Max. coverage (+): 0.02. Max coverage (-): 0.28

Region: NODE\_117098\_length\_959\_cov\_38.440041 816-818. Max. coverage (+): 0.17. Max coverage (-): 0.13

Region: NODE\_117098\_length\_959\_cov\_38.440041 819-820. Max. coverage (+): 0.3. Max coverage (-): 0.11

Region: NODE\_117098\_length\_959\_cov\_38.440041 821-822. Max. coverage (+): 0.43. Max coverage (-): 0.24

Region: NODE\_117098\_length\_959\_cov\_38.440041 823-824. Max. coverage (+): 0.19. Max coverage (-): 0.17

Region: NODE\_117098\_length\_959\_cov\_38.440041 825-826. Max. coverage (+): 0. Max coverage (-): 0

Region: NODE\_117098\_length\_959\_cov\_38.440041 827-828. Max. coverage (+): 0. Max coverage (-): 0.01

Region: NODE\_117098\_length\_959\_cov\_38.440041 829-830. Max. coverage (+): 0.07. Max coverage (-): 0.03

Region: NODE\_117098\_length\_959\_cov\_38.440041 831-832. Max. coverage (+): 0.87. Max coverage (-): 0.04

Region: NODE\_117098\_length\_959\_cov\_38.440041 833-834. Max. coverage (+): 1.13. Max coverage (-): 0.04

Region: NODE\_117098\_length\_959\_cov\_38.440041 835-836. Max. coverage (+): 5.85. Max coverage (-): 0

Region: NODE\_117098\_length\_959\_cov\_38.440041 837-838. Max. coverage (+): 10.73. Max coverage (-): 0

Region: NODE\_117098\_length\_959\_cov\_38.440041 839-840. Max. coverage (+): 12.22. Max coverage (-): 0

Region: NODE\_117098\_length\_959\_cov\_38.440041 841-842. Max. coverage (+): 2.61. Max coverage (-): 0

Region: NODE\_117098\_length\_959\_cov\_38.440041 843-844. Max. coverage (+): 1.78. Max coverage (-): 0.13

Region: NODE\_117098\_length\_959\_cov\_38.440041 845-846. Max. coverage (+): 26.08. Max coverage (-): 0.15

Region: NODE\_117098\_length\_959\_cov\_38.440041 847-848. Max. coverage (+): 25.21. Max coverage (-): 0.17

Region: NODE\_117098\_length\_959\_cov\_38.440041 849-850. Max. coverage (+): 0.17. Max coverage (-): 0.22

Region: NODE\_117098\_length\_959\_cov\_38.440041 851-852. Max. coverage (+): 0.54. Max coverage (-): 0.09

Region: NODE\_117098\_length\_959\_cov\_38.440041 853-854. Max. coverage (+): 0.65. Max coverage (-): 0.02

Region: NODE\_117098\_length\_959\_cov\_38.440041 855-856. Max. coverage (+): 0.15. Max coverage (-): 0.02

Region: NODE\_117098\_length\_959\_cov\_38.440041 857-858. Max. coverage (+): 0.06. Max coverage (-): 0.07

Region: NODE\_117098\_length\_959\_cov\_38.440041 859-860. Max. coverage (+): 0.09. Max coverage (-): 0.07

Region: NODE\_117098\_length\_959\_cov\_38.440041 861-862. Max. coverage (+): 0.04. Max coverage (-): 0.02

Region: NODE\_117098\_length\_959\_cov\_38.440041 863-864. Max. coverage (+): 0. Max coverage (-): 0.04

Region: NODE\_117098\_length\_959\_cov\_38.440041 865-866. Max. coverage (+): 0.04. Max coverage (-): 0.57

Region: NODE\_117098\_length\_959\_cov\_38.440041 867-869. Max. coverage (+): 0.04. Max coverage (-): 0.65

Region: NODE\_117098\_length\_959\_cov\_38.440041 870-871. Max. coverage (+): 0.04. Max coverage (-): 0.04

Region: NODE\_117098\_length\_959\_cov\_38.440041 872-873. Max. coverage (+): 0.06. Max coverage (-): 0.07

Region: NODE\_117098\_length\_959\_cov\_38.440041 874-875. Max. coverage (+): 2.84. Max coverage (-): 0.45

Region: NODE\_117098\_length\_959\_cov\_38.440041 876-877. Max. coverage (+): 2.8. Max coverage (-): 1.74

Region: NODE\_117098\_length\_959\_cov\_38.440041 878-879. Max. coverage (+): 0.64. Max coverage (-): 1.87

Region: NODE\_117098\_length\_959\_cov\_38.440041 880-881. Max. coverage (+): 1.76. Max coverage (-): 0.71

Region: NODE\_117098\_length\_959\_cov\_38.440041 882-883. Max. coverage (+): 1.65. Max coverage (-): 0.21

Region: NODE\_117098\_length\_959\_cov\_38.440041 884-885. Max. coverage (+): 0.23. Max coverage (-): 0.06

Region: NODE\_117098\_length\_959\_cov\_38.440041 886-887. Max. coverage (+): 0.09. Max coverage (-): 0.04

Region: NODE\_117098\_length\_959\_cov\_38.440041 888-889. Max. coverage (+): 0.33. Max coverage (-): 0.16

Region: NODE\_117098\_length\_959\_cov\_38.440041 890-891. Max. coverage (+): 0.95. Max coverage (-): 0.16

Region: NODE\_117098\_length\_959\_cov\_38.440041 892-893. Max. coverage (+): 1.71. Max coverage (-): 0.46

Region: NODE\_117098\_length\_959\_cov\_38.440041 894-895. Max. coverage (+): 2.19. Max coverage (-): 0.48

Region: NODE\_117098\_length\_959\_cov\_38.440041 896-897. Max. coverage (+): 0.61. Max coverage (-): 0.05

Region: NODE\_117098\_length\_959\_cov\_38.440041 898-899. Max. coverage (+): 0.05. Max coverage (-): 0.44

Region: NODE\_117098\_length\_959\_cov\_38.440041 900-901. Max. coverage (+): 0.91. Max coverage (-): 0.61

Region: NODE\_117098\_length\_959\_cov\_38.440041 902-903. Max. coverage (+): 1.15. Max coverage (-): 0.17

Region: NODE\_117098\_length\_959\_cov\_38.440041 904-905. Max. coverage (+): 0.3. Max coverage (-): 0.16

Region: NODE\_117098\_length\_959\_cov\_38.440041 906-907. Max. coverage (+): 0.19. Max coverage (-): 0.1

Region: NODE\_117098\_length\_959\_cov\_38.440041 908-909. Max. coverage (+): 1.22. Max coverage (-): 0.08

Region: NODE\_117098\_length\_959\_cov\_38.440041 910-911. Max. coverage (+): 1.09. Max coverage (-): 0.01

Region: NODE\_117098\_length\_959\_cov\_38.440041 912-913. Max. coverage (+): 0.19. Max coverage (-): 0.05

Region: NODE\_117098\_length\_959\_cov\_38.440041 914-915. Max. coverage (+): 0.16. Max coverage (-): 0.05

Region: NODE\_117098\_length\_959\_cov\_38.440041 916-917. Max. coverage (+): 1.08. Max coverage (-): 0

Region: NODE\_117098\_length\_959\_cov\_38.440041 918-920. Max. coverage (+): 13.06. Max coverage (-): 0.04

Region: NODE\_117098\_length\_959\_cov\_38.440041 921-922. Max. coverage (+): 11.88. Max coverage (-): 0.02

Region: NODE\_117098\_length\_959\_cov\_38.440041 923-924. Max. coverage (+): 0.38. Max coverage (-): 0.28

Region: NODE\_117098\_length\_959\_cov\_38.440041 925-926. Max. coverage (+): 0.41. Max coverage (-): 0.28

Region: NODE\_117098\_length\_959\_cov\_38.440041 927-928. Max. coverage (+): 0.32. Max coverage (-): 0.17

Region: NODE\_117098\_length\_959\_cov\_38.440041 929-930. Max. coverage (+): 0.02. Max coverage (-): 0.17

Region: NODE\_117098\_length\_959\_cov\_38.440041 931-932. Max. coverage (+): 0. Max coverage (-): 0

Region: NODE\_117098\_length\_959\_cov\_38.440041 933-934. Max. coverage (+): 0. Max coverage (-): 0

Region: NODE\_117098\_length\_959\_cov\_38.440041 935-936. Max. coverage (+): 0. Max coverage (-): 0

Region: NODE\_117098\_length\_959\_cov\_38.440041 937-938. Max. coverage (+): 0. Max coverage (-): 0

Region: NODE\_117098\_length\_959\_cov\_38.440041 939-940. Max. coverage (+): 0. Max coverage (-): 0

Region: NODE\_117098\_length\_959\_cov\_38.440041 941-942. Max. coverage (+): 0. Max coverage (-): 0

Region: NODE\_117098\_length\_959\_cov\_38.440041 943-944. Max. coverage (+): 0. Max coverage (-): 0

Region: NODE\_117098\_length\_959\_cov\_38.440041 945-946. Max. coverage (+): 0. Max coverage (-): 0

Region: NODE\_117098\_length\_959\_cov\_38.440041 947-948. Max. coverage (+): 0. Max coverage (-): 0

Region: NODE\_117098\_length\_959\_cov\_38.440041 949-950. Max. coverage (+): 0. Max coverage (-): 0

Region: NODE\_117098\_length\_959\_cov\_38.440041 951-952. Max. coverage (+): 0. Max coverage (-): 0

Region: NODE\_117098\_length\_959\_cov\_38.440041 953-954. Max. coverage (+): 0. Max coverage (-): 0

Region: NODE\_117098\_length\_959\_cov\_38.440041 955-956. Max. coverage (+): 0. Max coverage (-): 0

Region: NODE\_117098\_length\_959\_cov\_38.440041 957-958. Max. coverage (+): 0. Max coverage (-): 0

Region: NODE\_117098\_length\_959\_cov\_38.440041 959-960. Max. coverage (+): 0. Max coverage (-): 0

Region: NODE\_117098\_length\_959\_cov\_38.440041 961-962. Max. coverage (+): 0. Max coverage (-): 0

Region: NODE\_117098\_length\_959\_cov\_38.440041 963-964. Max. coverage (+): 0. Max coverage (-): 0

Region: NODE\_117098\_length\_959\_cov\_38.440041 965-966. Max. coverage (+): 0.02. Max coverage (-): 0

Region: NODE\_117098\_length\_959\_cov\_38.440041 967-968. Max. coverage (+): 0.03. Max coverage (-): 0

Region: NODE\_117098\_length\_959\_cov\_38.440041 969-971. Max. coverage (+): 0.01. Max coverage (-): 0

Region: NODE\_117098\_length\_959\_cov\_38.440041 972-973. Max. coverage (+): 0.01. Max coverage (-): 0

Region: NODE\_117098\_length\_959\_cov\_38.440041 974-975. Max. coverage (+): 0. Max coverage (-): 0

Region: NODE\_117098\_length\_959\_cov\_38.440041 976-977. Max. coverage (+): 0. Max coverage (-): 0

Region: NODE\_117098\_length\_959\_cov\_38.440041 978-979. Max. coverage (+): 0. Max coverage (-): 0

Region: NODE\_117098\_length\_959\_cov\_38.440041 980-981. Max. coverage (+): 0. Max coverage (-): 0

Region: NODE\_117098\_length\_959\_cov\_38.440041 982-983. Max. coverage (+): 0. Max coverage (-): 0

Region: NODE\_117098\_length\_959\_cov\_38.440041 984-985. Max. coverage (+): 0. Max coverage (-): 0

Region: NODE\_117098\_length\_959\_cov\_38.440041 986-987. Max. coverage (+): 0. Max coverage (-): 0

Region: NODE\_117098\_length\_959\_cov\_38.440041 988-989. Max. coverage (+): 0. Max coverage (-): 0

Region: NODE\_117098\_length\_959\_cov\_38.440041 990-991. Max. coverage (+): 0. Max coverage (-): 0

Region: NODE\_117098\_length\_959\_cov\_38.440041 992-993. Max. coverage (+): 0. Max coverage (-): 0

Region: NODE\_117098\_length\_959\_cov\_38.440041 994-995. Max. coverage (+): 0. Max coverage (-): 0

Region: NODE\_117098\_length\_959\_cov\_38.440041 996-997. Max. coverage (+): 0. Max coverage (-): 0

Region: NODE\_117098\_length\_959\_cov\_38.440041 998-999. Max. coverage (+): 0. Max coverage (-): 0

Region: NODE\_117098\_length\_959\_cov\_38.440041 1000-1001. Max. coverage (+): 0. Max coverage (-): 0

Region: NODE\_117098\_length\_959\_cov\_38.440041 1002-1003. Max. coverage (+): 0. Max coverage (-): 0

Region: NODE\_117098\_length\_959\_cov\_38.440041 1004-1005. Max. coverage (+): 0. Max coverage (-): 0

Region: NODE\_117098\_length\_959\_cov\_38.440041 1006-1007. Max. coverage (+): 0. Max coverage (-): 0

Region: NODE\_117098\_length\_959\_cov\_38.440041 1008-1009. Max. coverage (+): 0. Max coverage (-): 0

Region: NODE\_117098\_length\_959\_cov\_38.440041 1010-1011. Max. coverage (+): 0. Max coverage (-): 0

Region: NODE\_117098\_length\_959\_cov\_38.440041 1012-1013. Max. coverage (+): 0. Max coverage (-): 0

Region: NODE\_117098\_length\_959\_cov\_38.440041 1014-1015. Max. coverage (+): 0. Max coverage (-): 0

Region: NODE\_117098\_length\_959\_cov\_38.440041 1016-1017. Max. coverage (+): 0. Max coverage (-): 0

Region: NODE\_117098\_length\_959\_cov\_38.440041 1018-1019. Max. coverage (+): 0. Max coverage (-): 0

Region: NODE\_117098\_length\_959\_cov\_38.440041 1020-. Max. coverage (+): 0. Max coverage (-): 0

RepeatMasker Color Code

**+**

100-98% Identity

<98-95% Identity

<95-90% Identity

<90-85% Identity

<85-80% Identity

<80-75% Identity

<75-70% Identity

<70% Identity

**-**

Gene Set Color Code

**+**

Gene

Pseudogene

Other

**-**

Topology/Coverage Color Code

Coverage Plus Strand

Coverage Minus Strand

Mainstrand: Plus

Mainstrand: Minus

Complementary Strand

Flanking Region  
(if option -flank >0)

Gene Set Annotation  
  
RepeatMasker Annotation  

**1. AlRepE-2341**: 1-57 (-), Divergence to consensus: 23.2%  
**2. hAT-N97\_DR**: 964-1023 (+), Divergence to consensus: 0%

  
Transcription Factor Binding Sites  

**RHOXF1** (Sequence: GGATCA (-): 842)  
**RHOXF1** (Sequence: GGATTA (-): 962)  
**RHOXF1** (Sequence: TAAGCC (+): 143)  
**RHOXF1** (Sequence: TGAGCT (+): 360)  
**RHOXF1** (Sequence: TGATCT (+): 703)  
**RHOXF1** (Sequence: TAAGCT (+): 869)  
**RHOXF1** (Sequence: TGATCT (+): 922)  
**FOXO3\_mmu** (Sequence: TGTTTTGA (-): 253)
