# Supplementary material for: First characterization of PIWI-interacting RNA clusters in a cichlid fish with a B chromosome
Source: BMC Biol. 2022 Sep 21;20:204. doi: 10.1186/s12915-022-01403-2 (PMC9490952; doi:10.1186/s12915-022-01403-2)
Supplement: Supplementary file 1 — Additional file 1. Zipped folder with fasta and interactive html piRNA cluster information for the A. latifasciata genome. The nomenclature is as follows: number-pirna-cluster_sex_B-presence (f, female; m, male; 0b, without B chromosome; 1b, with B chromosome). [file 12915_2022_1403_MOESM1_ESM.zip › 110_f0b.html]

piRNA cluster 110\_f0b 59


Predicted piRNA cluster no. 110\_f0b
  

Show proTRAC run info
Hide proTRAC run info

/\  
                \_\_\_\_\_\_\_\_\_\_\_\_\_\_\_\_\_\_\_\_\_\_\_/\\_\_\_ /  \\_\_\_\_\_\_\_  
               I                      /  \  /    \      I  
               I     pro             /    \/      \     I  
               I        TRAC        /               \   I  
               I   \_\_\_\_\_\_\_\_\_\_\_\_\_\_\_\_/\_\_\_\_\_\_\_\_\_\_\_\_\_\_\_\_\_\\_ I  
               I   \              /                     I  
               I    \            /                      I  
               I     \  /\      /       V.2.4.2         I  
               I      \/  \    /                        I  
               I\_\_\_\_\_\_\_\_\_\_\_\  /\_\_\_\_\_\_\_\_\_\_\_\_\_\_\_\_\_\_\_\_\_\_\_\_\_I  
                            \/  
  
  
================================= proTRAC ====================================  
VERSION: .......... 2.4.2  
LAST MODIFIED: .... 11. May 2018  
  
Please cite:  
Rosenkranz D, Zischler H. proTRAC - a software for probabilistic piRNA cluster  
detection, visualization and analysis. 2012. BMC Bioinformatics 13:5.  
  
  
Contact:  
David Rosenkranz  
Institute of Organismic and Molecular Evolutionary Biology  
Dept. Anthropology, small RNA group  
Johannes Gutenberg University Mainz  
email: rosenkranz@uni-mainz.de  
  
You can find the latest proTRAC version at:  
http://sourceforge.net/projects/protrac/files  
http://www.smallRNAgroup-mainz.de/software  
==============================================================================  
  
PARAMETERS:  
Map file: ...............piwi-femeas-0B.fa-collapse.map  
Genome file: ............../../../0B\_ala\_genome.fa  
RepeatMasker annotation: Alatifasciata-all0B-maryan-v2.fa\_corrected.out  
GeneSet:................./guest-storage/Data/annotation/Alatifasciata\_all0B\_maryan-v2\_out2017.gff  
  
Significant (p<=0.01) hit density will be calculated based  
on observed hit distribution.  
  
Sliding window size: ........................................ 5000 bp  
Sliding window increament: .................................. 1000 bp  
Normalize each hit by number of genomic hits: ............... yes  
Normalize each hit by number of sequence reads: ............. yes  
Normalize values (-> per million mapped reads): ............. yes  
Min. fraction of hits with 1T(U) or 10A: .................... 0.75  
Alternatively: Min. fraction of hits with 1T(U) and 10A: .... 0.5  
Min. fraction of hits with typical piRNA length: ............ 0.75  
Typical piRNA length: ....................................... 24-32 nt  
Min. size of a piRNA cluster: ............................... 1000 bp.  
Min. number of hits (absolute): ............................. 0  
Min. number of hits (normalized): ........................... 0  
Min. fraction of hits on the mainstrand: .................... 0.75  
Top fraction of mapped sequences (in terms of read counts): . 1%  
Top fraction accounts for max. n% of sequence reads: ........ 90%  
Min. fraction of hits on each arm of a bidirectional cluster: 0.05  
Output html file for each cluster: .......................... yes  
Output a summary table: ..................................... yes  
Output a FASTA file for each cluster (piRNA sequences): ..... yes  
Output a FASTA file comprising cluster sequences: ........... yes  
Output a GTF file for predicted piRNA clusters: ..............yes  
Search DNA motifs in clusters: .............................. yes  
Output flanking sequences: +/- .............................. 0 bp  
Output ~.pTi file: .......................................... no  
==============================================================================  
  
  
Genome size (without gaps): ............ 758543724 bp  
Gaps (N/X/-): .......................... 417479 bp  
Mapped reads: .......................... 13052187  
Non-identical sequences: ............... 3338911  
Genomic hits: .......................... 28737726  
Significant densitiy of mapped reads: .. 470.083249848448 reads/kb

Show proTRAC cluster info
Hide proTRAC cluster info

|  |  |
| --- | --- |
| Location | NODE\_288008\_length\_33935\_cov\_31.019037 |
| Coordinates | 7624-12427 |
| Size [bp] | 4804 |
| Sequence hit loci | 965 |
| Mapped reads (normalized) | 3251.7 |
| Mapped reads (normalized) per kb | 676.9 |
| Normalized reads with 1T (1U) | 90.9% |
| Normalized reads with 10A | 20.9% |
| Normalized reads with length 24-32 nt | 99.1% |
| Normalized reads on the main strand(s) | 97.5% |
| Predicted directionality | mono:plus |

100%

0%

1T (1U)  
reads

10A reads

24-32 nt  
reads

reads on mainstrand

**Either the amount of reads with 1T (1U) OR 10A has to exceed 75% (set with option: -1Tor10A)  
Alternatively the amount of reads with 1T (1U) AND 10A has to exceed 50% (set with option: -1Tand10A)  
Minimum amount of reads with preferred size is 75% (set with option: -pisize)  
Minimum amount of reads on the main strand(s) is 75% (set with option: -clstrand)**

Show read coverage
Hide read coverage

WHAT DO I SEE HERE?  
This chart shows the location of mapped sequence reads within a predicted piRNA cluster. The color refers to the number of genomic hits produced by the sequence read in question. A dark red bar indicates that this sequence read produces many other hits elsewhere in the genome. Many adjacent red or yellow bars can indicate the presence of a multi-copy element such as transposons or rRNA genes. A dark green bar indicates that this sequence read maps uniquely to this locus.

1 hit

2-5 hits

6-10 hits

11-20 hits

21-50 hits

51-100 hits

> 100 hits

NODE\_288008\_length\_33935\_cov\_31.019037

7624

12427

Gene Set

RepeatMasker

Mapped  
Reads

79.57

plus strand

minus strand

79.57

Region: NODE\_288008\_length\_33935\_cov\_31.019037 3023-7628. Max. coverage (+): 0.01. Max coverage (-): 0

Region: NODE\_288008\_length\_33935\_cov\_31.019037 7629-7638. Max. coverage (+): 0. Max coverage (-): 0

Region: NODE\_288008\_length\_33935\_cov\_31.019037 7639-7648. Max. coverage (+): 0. Max coverage (-): 0

Region: NODE\_288008\_length\_33935\_cov\_31.019037 7649-7657. Max. coverage (+): 0.16. Max coverage (-): 0

Region: NODE\_288008\_length\_33935\_cov\_31.019037 7658-7667. Max. coverage (+): 0.01. Max coverage (-): 0

Region: NODE\_288008\_length\_33935\_cov\_31.019037 7668-7676. Max. coverage (+): 0.08. Max coverage (-): 0

Region: NODE\_288008\_length\_33935\_cov\_31.019037 7677-7686. Max. coverage (+): 0.08. Max coverage (-): 0

Region: NODE\_288008\_length\_33935\_cov\_31.019037 7687-7696. Max. coverage (+): 0. Max coverage (-): 0

Region: NODE\_288008\_length\_33935\_cov\_31.019037 7697-7705. Max. coverage (+): 0. Max coverage (-): 0

Region: NODE\_288008\_length\_33935\_cov\_31.019037 7706-7715. Max. coverage (+): 0. Max coverage (-): 0

Region: NODE\_288008\_length\_33935\_cov\_31.019037 7716-7724. Max. coverage (+): 0.03. Max coverage (-): 0

Region: NODE\_288008\_length\_33935\_cov\_31.019037 7725-7734. Max. coverage (+): 0.03. Max coverage (-): 0

Region: NODE\_288008\_length\_33935\_cov\_31.019037 7735-7744. Max. coverage (+): 0. Max coverage (-): 0

Region: NODE\_288008\_length\_33935\_cov\_31.019037 7745-7753. Max. coverage (+): 0. Max coverage (-): 0

Region: NODE\_288008\_length\_33935\_cov\_31.019037 7754-7763. Max. coverage (+): 0. Max coverage (-): 0

Region: NODE\_288008\_length\_33935\_cov\_31.019037 7764-7772. Max. coverage (+): 0. Max coverage (-): 0

Region: NODE\_288008\_length\_33935\_cov\_31.019037 7773-7782. Max. coverage (+): 0.04. Max coverage (-): 0

Region: NODE\_288008\_length\_33935\_cov\_31.019037 7783-7792. Max. coverage (+): 0. Max coverage (-): 0

Region: NODE\_288008\_length\_33935\_cov\_31.019037 7793-7801. Max. coverage (+): 0. Max coverage (-): 0

Region: NODE\_288008\_length\_33935\_cov\_31.019037 7802-7811. Max. coverage (+): 0.06. Max coverage (-): 0

Region: NODE\_288008\_length\_33935\_cov\_31.019037 7812-7820. Max. coverage (+): 0. Max coverage (-): 0

Region: NODE\_288008\_length\_33935\_cov\_31.019037 7821-7830. Max. coverage (+): 0.15. Max coverage (-): 0

Region: NODE\_288008\_length\_33935\_cov\_31.019037 7831-7840. Max. coverage (+): 0.08. Max coverage (-): 0

Region: NODE\_288008\_length\_33935\_cov\_31.019037 7841-7849. Max. coverage (+): 0. Max coverage (-): 0

Region: NODE\_288008\_length\_33935\_cov\_31.019037 7850-7859. Max. coverage (+): 0.08. Max coverage (-): 0

Region: NODE\_288008\_length\_33935\_cov\_31.019037 7860-7869. Max. coverage (+): 0. Max coverage (-): 0

Region: NODE\_288008\_length\_33935\_cov\_31.019037 7870-7878. Max. coverage (+): 0. Max coverage (-): 0

Region: NODE\_288008\_length\_33935\_cov\_31.019037 7879-7888. Max. coverage (+): 0. Max coverage (-): 0

Region: NODE\_288008\_length\_33935\_cov\_31.019037 7889-7897. Max. coverage (+): 0.23. Max coverage (-): 0

Region: NODE\_288008\_length\_33935\_cov\_31.019037 7898-7907. Max. coverage (+): 0. Max coverage (-): 0

Region: NODE\_288008\_length\_33935\_cov\_31.019037 7908-7917. Max. coverage (+): 0. Max coverage (-): 0

Region: NODE\_288008\_length\_33935\_cov\_31.019037 7918-7926. Max. coverage (+): 0.66. Max coverage (-): 0

Region: NODE\_288008\_length\_33935\_cov\_31.019037 7927-7936. Max. coverage (+): 0.02. Max coverage (-): 0

Region: NODE\_288008\_length\_33935\_cov\_31.019037 7937-7945. Max. coverage (+): 0.08. Max coverage (-): 0

Region: NODE\_288008\_length\_33935\_cov\_31.019037 7946-7955. Max. coverage (+): 0.08. Max coverage (-): 0

Region: NODE\_288008\_length\_33935\_cov\_31.019037 7956-7965. Max. coverage (+): 0. Max coverage (-): 0

Region: NODE\_288008\_length\_33935\_cov\_31.019037 7966-7974. Max. coverage (+): 0.13. Max coverage (-): 0.04

Region: NODE\_288008\_length\_33935\_cov\_31.019037 7975-7984. Max. coverage (+): 0.08. Max coverage (-): 0.02

Region: NODE\_288008\_length\_33935\_cov\_31.019037 7985-7993. Max. coverage (+): 0.46. Max coverage (-): 0

Region: NODE\_288008\_length\_33935\_cov\_31.019037 7994-8003. Max. coverage (+): 0.06. Max coverage (-): 0.04

Region: NODE\_288008\_length\_33935\_cov\_31.019037 8004-8013. Max. coverage (+): 0.04. Max coverage (-): 0.04

Region: NODE\_288008\_length\_33935\_cov\_31.019037 8014-8022. Max. coverage (+): 0.61. Max coverage (-): 0

Region: NODE\_288008\_length\_33935\_cov\_31.019037 8023-8032. Max. coverage (+): 1.3. Max coverage (-): 0

Region: NODE\_288008\_length\_33935\_cov\_31.019037 8033-8041. Max. coverage (+): 0. Max coverage (-): 0

Region: NODE\_288008\_length\_33935\_cov\_31.019037 8042-8051. Max. coverage (+): 0. Max coverage (-): 0

Region: NODE\_288008\_length\_33935\_cov\_31.019037 8052-8061. Max. coverage (+): 0. Max coverage (-): 0

Region: NODE\_288008\_length\_33935\_cov\_31.019037 8062-8070. Max. coverage (+): 0. Max coverage (-): 0

Region: NODE\_288008\_length\_33935\_cov\_31.019037 8071-8080. Max. coverage (+): 2.98. Max coverage (-): 0

Region: NODE\_288008\_length\_33935\_cov\_31.019037 8081-8089. Max. coverage (+): 0.96. Max coverage (-): 0.15

Region: NODE\_288008\_length\_33935\_cov\_31.019037 8090-8099. Max. coverage (+): 0.08. Max coverage (-): 0.15

Region: NODE\_288008\_length\_33935\_cov\_31.019037 8100-8109. Max. coverage (+): 0.23. Max coverage (-): 0

Region: NODE\_288008\_length\_33935\_cov\_31.019037 8110-8118. Max. coverage (+): 0.23. Max coverage (-): 0

Region: NODE\_288008\_length\_33935\_cov\_31.019037 8119-8128. Max. coverage (+): 0. Max coverage (-): 0

Region: NODE\_288008\_length\_33935\_cov\_31.019037 8129-8138. Max. coverage (+): 0.08. Max coverage (-): 0

Region: NODE\_288008\_length\_33935\_cov\_31.019037 8139-8147. Max. coverage (+): 0.08. Max coverage (-): 0

Region: NODE\_288008\_length\_33935\_cov\_31.019037 8148-8157. Max. coverage (+): 0.08. Max coverage (-): 0

Region: NODE\_288008\_length\_33935\_cov\_31.019037 8158-8166. Max. coverage (+): 0. Max coverage (-): 0

Region: NODE\_288008\_length\_33935\_cov\_31.019037 8167-8176. Max. coverage (+): 0. Max coverage (-): 0

Region: NODE\_288008\_length\_33935\_cov\_31.019037 8177-8186. Max. coverage (+): 0. Max coverage (-): 0

Region: NODE\_288008\_length\_33935\_cov\_31.019037 8187-8195. Max. coverage (+): 0.23. Max coverage (-): 0

Region: NODE\_288008\_length\_33935\_cov\_31.019037 8196-8205. Max. coverage (+): 0.23. Max coverage (-): 0

Region: NODE\_288008\_length\_33935\_cov\_31.019037 8206-8214. Max. coverage (+): 0. Max coverage (-): 0

Region: NODE\_288008\_length\_33935\_cov\_31.019037 8215-8224. Max. coverage (+): 0. Max coverage (-): 0

Region: NODE\_288008\_length\_33935\_cov\_31.019037 8225-8234. Max. coverage (+): 0. Max coverage (-): 0

Region: NODE\_288008\_length\_33935\_cov\_31.019037 8235-8243. Max. coverage (+): 0. Max coverage (-): 0

Region: NODE\_288008\_length\_33935\_cov\_31.019037 8244-8253. Max. coverage (+): 0. Max coverage (-): 0

Region: NODE\_288008\_length\_33935\_cov\_31.019037 8254-8262. Max. coverage (+): 0. Max coverage (-): 0

Region: NODE\_288008\_length\_33935\_cov\_31.019037 8263-8272. Max. coverage (+): 0.38. Max coverage (-): 0

Region: NODE\_288008\_length\_33935\_cov\_31.019037 8273-8282. Max. coverage (+): 0. Max coverage (-): 0

Region: NODE\_288008\_length\_33935\_cov\_31.019037 8283-8291. Max. coverage (+): 0. Max coverage (-): 0

Region: NODE\_288008\_length\_33935\_cov\_31.019037 8292-8301. Max. coverage (+): 0. Max coverage (-): 0

Region: NODE\_288008\_length\_33935\_cov\_31.019037 8302-8310. Max. coverage (+): 0. Max coverage (-): 0

Region: NODE\_288008\_length\_33935\_cov\_31.019037 8311-8320. Max. coverage (+): 0. Max coverage (-): 0.04

Region: NODE\_288008\_length\_33935\_cov\_31.019037 8321-8330. Max. coverage (+): 0. Max coverage (-): 0.04

Region: NODE\_288008\_length\_33935\_cov\_31.019037 8331-8339. Max. coverage (+): 0. Max coverage (-): 0

Region: NODE\_288008\_length\_33935\_cov\_31.019037 8340-8349. Max. coverage (+): 0. Max coverage (-): 0

Region: NODE\_288008\_length\_33935\_cov\_31.019037 8350-8359. Max. coverage (+): 0. Max coverage (-): 0.03

Region: NODE\_288008\_length\_33935\_cov\_31.019037 8360-8368. Max. coverage (+): 0. Max coverage (-): 0.03

Region: NODE\_288008\_length\_33935\_cov\_31.019037 8369-8378. Max. coverage (+): 0.15. Max coverage (-): 0

Region: NODE\_288008\_length\_33935\_cov\_31.019037 8379-8387. Max. coverage (+): 0.15. Max coverage (-): 0

Region: NODE\_288008\_length\_33935\_cov\_31.019037 8388-8397. Max. coverage (+): 0. Max coverage (-): 0

Region: NODE\_288008\_length\_33935\_cov\_31.019037 8398-8407. Max. coverage (+): 0.27. Max coverage (-): 0.04

Region: NODE\_288008\_length\_33935\_cov\_31.019037 8408-8416. Max. coverage (+): 0.13. Max coverage (-): 0.08

Region: NODE\_288008\_length\_33935\_cov\_31.019037 8417-8426. Max. coverage (+): 0.15. Max coverage (-): 0.02

Region: NODE\_288008\_length\_33935\_cov\_31.019037 8427-8435. Max. coverage (+): 1.82. Max coverage (-): 0

Region: NODE\_288008\_length\_33935\_cov\_31.019037 8436-8445. Max. coverage (+): 0.03. Max coverage (-): 0

Region: NODE\_288008\_length\_33935\_cov\_31.019037 8446-8455. Max. coverage (+): 0. Max coverage (-): 0

Region: NODE\_288008\_length\_33935\_cov\_31.019037 8456-8464. Max. coverage (+): 0. Max coverage (-): 0

Region: NODE\_288008\_length\_33935\_cov\_31.019037 8465-8474. Max. coverage (+): 0.38. Max coverage (-): 0

Region: NODE\_288008\_length\_33935\_cov\_31.019037 8475-8483. Max. coverage (+): 0.38. Max coverage (-): 0

Region: NODE\_288008\_length\_33935\_cov\_31.019037 8484-8493. Max. coverage (+): 0.23. Max coverage (-): 0

Region: NODE\_288008\_length\_33935\_cov\_31.019037 8494-8503. Max. coverage (+): 0.04. Max coverage (-): 0

Region: NODE\_288008\_length\_33935\_cov\_31.019037 8504-8512. Max. coverage (+): 0. Max coverage (-): 0

Region: NODE\_288008\_length\_33935\_cov\_31.019037 8513-8522. Max. coverage (+): 0.04. Max coverage (-): 0

Region: NODE\_288008\_length\_33935\_cov\_31.019037 8523-8531. Max. coverage (+): 0.02. Max coverage (-): 0

Region: NODE\_288008\_length\_33935\_cov\_31.019037 8532-8541. Max. coverage (+): 0.06. Max coverage (-): 0.04

Region: NODE\_288008\_length\_33935\_cov\_31.019037 8542-8551. Max. coverage (+): 0.18. Max coverage (-): 0

Region: NODE\_288008\_length\_33935\_cov\_31.019037 8552-8560. Max. coverage (+): 0.92. Max coverage (-): 0

Region: NODE\_288008\_length\_33935\_cov\_31.019037 8561-8570. Max. coverage (+): 0.08. Max coverage (-): 0

Region: NODE\_288008\_length\_33935\_cov\_31.019037 8571-8579. Max. coverage (+): 0. Max coverage (-): 0

Region: NODE\_288008\_length\_33935\_cov\_31.019037 8580-8589. Max. coverage (+): 0.15. Max coverage (-): 0

Region: NODE\_288008\_length\_33935\_cov\_31.019037 8590-8599. Max. coverage (+): 0. Max coverage (-): 0

Region: NODE\_288008\_length\_33935\_cov\_31.019037 8600-8608. Max. coverage (+): 0. Max coverage (-): 0

Region: NODE\_288008\_length\_33935\_cov\_31.019037 8609-8618. Max. coverage (+): 0. Max coverage (-): 0

Region: NODE\_288008\_length\_33935\_cov\_31.019037 8619-8628. Max. coverage (+): 0.15. Max coverage (-): 0

Region: NODE\_288008\_length\_33935\_cov\_31.019037 8629-8637. Max. coverage (+): 0.15. Max coverage (-): 0

Region: NODE\_288008\_length\_33935\_cov\_31.019037 8638-8647. Max. coverage (+): 0. Max coverage (-): 0

Region: NODE\_288008\_length\_33935\_cov\_31.019037 8648-8656. Max. coverage (+): 0. Max coverage (-): 0

Region: NODE\_288008\_length\_33935\_cov\_31.019037 8657-8666. Max. coverage (+): 0. Max coverage (-): 0

Region: NODE\_288008\_length\_33935\_cov\_31.019037 8667-8676. Max. coverage (+): 0. Max coverage (-): 0

Region: NODE\_288008\_length\_33935\_cov\_31.019037 8677-8685. Max. coverage (+): 0. Max coverage (-): 0

Region: NODE\_288008\_length\_33935\_cov\_31.019037 8686-8695. Max. coverage (+): 0. Max coverage (-): 0

Region: NODE\_288008\_length\_33935\_cov\_31.019037 8696-8704. Max. coverage (+): 0. Max coverage (-): 0

Region: NODE\_288008\_length\_33935\_cov\_31.019037 8705-8714. Max. coverage (+): 0. Max coverage (-): 0

Region: NODE\_288008\_length\_33935\_cov\_31.019037 8715-8724. Max. coverage (+): 0. Max coverage (-): 0

Region: NODE\_288008\_length\_33935\_cov\_31.019037 8725-8733. Max. coverage (+): 0. Max coverage (-): 0

Region: NODE\_288008\_length\_33935\_cov\_31.019037 8734-8743. Max. coverage (+): 0.02. Max coverage (-): 0.02

Region: NODE\_288008\_length\_33935\_cov\_31.019037 8744-8752. Max. coverage (+): 0. Max coverage (-): 0

Region: NODE\_288008\_length\_33935\_cov\_31.019037 8753-8762. Max. coverage (+): 0.41. Max coverage (-): 0

Region: NODE\_288008\_length\_33935\_cov\_31.019037 8763-8772. Max. coverage (+): 0. Max coverage (-): 0

Region: NODE\_288008\_length\_33935\_cov\_31.019037 8773-8781. Max. coverage (+): 0. Max coverage (-): 0

Region: NODE\_288008\_length\_33935\_cov\_31.019037 8782-8791. Max. coverage (+): 0. Max coverage (-): 0

Region: NODE\_288008\_length\_33935\_cov\_31.019037 8792-8800. Max. coverage (+): 1. Max coverage (-): 0

Region: NODE\_288008\_length\_33935\_cov\_31.019037 8801-8810. Max. coverage (+): 0. Max coverage (-): 0

Region: NODE\_288008\_length\_33935\_cov\_31.019037 8811-8820. Max. coverage (+): 0. Max coverage (-): 0

Region: NODE\_288008\_length\_33935\_cov\_31.019037 8821-8829. Max. coverage (+): 0.08. Max coverage (-): 0

Region: NODE\_288008\_length\_33935\_cov\_31.019037 8830-8839. Max. coverage (+): 0.08. Max coverage (-): 0

Region: NODE\_288008\_length\_33935\_cov\_31.019037 8840-8849. Max. coverage (+): 0. Max coverage (-): 0

Region: NODE\_288008\_length\_33935\_cov\_31.019037 8850-8858. Max. coverage (+): 0. Max coverage (-): 0

Region: NODE\_288008\_length\_33935\_cov\_31.019037 8859-8868. Max. coverage (+): 0. Max coverage (-): 0.45

Region: NODE\_288008\_length\_33935\_cov\_31.019037 8869-8877. Max. coverage (+): 0. Max coverage (-): 0.03

Region: NODE\_288008\_length\_33935\_cov\_31.019037 8878-8887. Max. coverage (+): 0.02. Max coverage (-): 0

Region: NODE\_288008\_length\_33935\_cov\_31.019037 8888-8897. Max. coverage (+): 0. Max coverage (-): 0

Region: NODE\_288008\_length\_33935\_cov\_31.019037 8898-8906. Max. coverage (+): 0. Max coverage (-): 0

Region: NODE\_288008\_length\_33935\_cov\_31.019037 8907-8916. Max. coverage (+): 0. Max coverage (-): 0

Region: NODE\_288008\_length\_33935\_cov\_31.019037 8917-8925. Max. coverage (+): 0. Max coverage (-): 0

Region: NODE\_288008\_length\_33935\_cov\_31.019037 8926-8935. Max. coverage (+): 0. Max coverage (-): 0

Region: NODE\_288008\_length\_33935\_cov\_31.019037 8936-8945. Max. coverage (+): 0.03. Max coverage (-): 0

Region: NODE\_288008\_length\_33935\_cov\_31.019037 8946-8954. Max. coverage (+): 0. Max coverage (-): 0

Region: NODE\_288008\_length\_33935\_cov\_31.019037 8955-8964. Max. coverage (+): 0. Max coverage (-): 0

Region: NODE\_288008\_length\_33935\_cov\_31.019037 8965-8973. Max. coverage (+): 0.02. Max coverage (-): 0

Region: NODE\_288008\_length\_33935\_cov\_31.019037 8974-8983. Max. coverage (+): 0.02. Max coverage (-): 0

Region: NODE\_288008\_length\_33935\_cov\_31.019037 8984-8993. Max. coverage (+): 0. Max coverage (-): 0

Region: NODE\_288008\_length\_33935\_cov\_31.019037 8994-9002. Max. coverage (+): 0. Max coverage (-): 0.02

Region: NODE\_288008\_length\_33935\_cov\_31.019037 9003-9012. Max. coverage (+): 0.94. Max coverage (-): 0.02

Region: NODE\_288008\_length\_33935\_cov\_31.019037 9013-9021. Max. coverage (+): 0.28. Max coverage (-): 0.03

Region: NODE\_288008\_length\_33935\_cov\_31.019037 9022-9031. Max. coverage (+): 0.72. Max coverage (-): 0.05

Region: NODE\_288008\_length\_33935\_cov\_31.019037 9032-9041. Max. coverage (+): 0.13. Max coverage (-): 0

Region: NODE\_288008\_length\_33935\_cov\_31.019037 9042-9050. Max. coverage (+): 0. Max coverage (-): 0

Region: NODE\_288008\_length\_33935\_cov\_31.019037 9051-9060. Max. coverage (+): 0. Max coverage (-): 0

Region: NODE\_288008\_length\_33935\_cov\_31.019037 9061-9070. Max. coverage (+): 0. Max coverage (-): 0

Region: NODE\_288008\_length\_33935\_cov\_31.019037 9071-9079. Max. coverage (+): 0.31. Max coverage (-): 0

Region: NODE\_288008\_length\_33935\_cov\_31.019037 9080-9089. Max. coverage (+): 0. Max coverage (-): 0.08

Region: NODE\_288008\_length\_33935\_cov\_31.019037 9090-9098. Max. coverage (+): 0. Max coverage (-): 0.54

Region: NODE\_288008\_length\_33935\_cov\_31.019037 9099-9108. Max. coverage (+): 0. Max coverage (-): 0.15

Region: NODE\_288008\_length\_33935\_cov\_31.019037 9109-9118. Max. coverage (+): 0. Max coverage (-): 0

Region: NODE\_288008\_length\_33935\_cov\_31.019037 9119-9127. Max. coverage (+): 0. Max coverage (-): 0.08

Region: NODE\_288008\_length\_33935\_cov\_31.019037 9128-9137. Max. coverage (+): 0. Max coverage (-): 0

Region: NODE\_288008\_length\_33935\_cov\_31.019037 9138-9146. Max. coverage (+): 0. Max coverage (-): 0

Region: NODE\_288008\_length\_33935\_cov\_31.019037 9147-9156. Max. coverage (+): 0. Max coverage (-): 0

Region: NODE\_288008\_length\_33935\_cov\_31.019037 9157-9166. Max. coverage (+): 0.15. Max coverage (-): 0

Region: NODE\_288008\_length\_33935\_cov\_31.019037 9167-9175. Max. coverage (+): 0.15. Max coverage (-): 0

Region: NODE\_288008\_length\_33935\_cov\_31.019037 9176-9185. Max. coverage (+): 0.05. Max coverage (-): 0.26

Region: NODE\_288008\_length\_33935\_cov\_31.019037 9186-9194. Max. coverage (+): 0.05. Max coverage (-): 0.26

Region: NODE\_288008\_length\_33935\_cov\_31.019037 9195-9204. Max. coverage (+): 1.07. Max coverage (-): 0

Region: NODE\_288008\_length\_33935\_cov\_31.019037 9205-9214. Max. coverage (+): 1.05. Max coverage (-): 0

Region: NODE\_288008\_length\_33935\_cov\_31.019037 9215-9223. Max. coverage (+): 0. Max coverage (-): 0

Region: NODE\_288008\_length\_33935\_cov\_31.019037 9224-9233. Max. coverage (+): 0. Max coverage (-): 0

Region: NODE\_288008\_length\_33935\_cov\_31.019037 9234-9242. Max. coverage (+): 0. Max coverage (-): 0

Region: NODE\_288008\_length\_33935\_cov\_31.019037 9243-9252. Max. coverage (+): 0. Max coverage (-): 0

Region: NODE\_288008\_length\_33935\_cov\_31.019037 9253-9262. Max. coverage (+): 0.08. Max coverage (-): 0

Region: NODE\_288008\_length\_33935\_cov\_31.019037 9263-9271. Max. coverage (+): 0.38. Max coverage (-): 0

Region: NODE\_288008\_length\_33935\_cov\_31.019037 9272-9281. Max. coverage (+): 0. Max coverage (-): 0

Region: NODE\_288008\_length\_33935\_cov\_31.019037 9282-9290. Max. coverage (+): 0. Max coverage (-): 0

Region: NODE\_288008\_length\_33935\_cov\_31.019037 9291-9300. Max. coverage (+): 0. Max coverage (-): 0

Region: NODE\_288008\_length\_33935\_cov\_31.019037 9301-9310. Max. coverage (+): 0.15. Max coverage (-): 0

Region: NODE\_288008\_length\_33935\_cov\_31.019037 9311-9319. Max. coverage (+): 0.31. Max coverage (-): 0

Region: NODE\_288008\_length\_33935\_cov\_31.019037 9320-9329. Max. coverage (+): 0.03. Max coverage (-): 0.04

Region: NODE\_288008\_length\_33935\_cov\_31.019037 9330-9339. Max. coverage (+): 0.11. Max coverage (-): 0

Region: NODE\_288008\_length\_33935\_cov\_31.019037 9340-9348. Max. coverage (+): 0.1. Max coverage (-): 0.02

Region: NODE\_288008\_length\_33935\_cov\_31.019037 9349-9358. Max. coverage (+): 0.08. Max coverage (-): 0

Region: NODE\_288008\_length\_33935\_cov\_31.019037 9359-9367. Max. coverage (+): 0.08. Max coverage (-): 0

Region: NODE\_288008\_length\_33935\_cov\_31.019037 9368-9377. Max. coverage (+): 0.38. Max coverage (-): 0

Region: NODE\_288008\_length\_33935\_cov\_31.019037 9378-9387. Max. coverage (+): 0. Max coverage (-): 0

Region: NODE\_288008\_length\_33935\_cov\_31.019037 9388-9396. Max. coverage (+): 0. Max coverage (-): 0

Region: NODE\_288008\_length\_33935\_cov\_31.019037 9397-9406. Max. coverage (+): 0. Max coverage (-): 0

Region: NODE\_288008\_length\_33935\_cov\_31.019037 9407-9415. Max. coverage (+): 0.31. Max coverage (-): 0

Region: NODE\_288008\_length\_33935\_cov\_31.019037 9416-9425. Max. coverage (+): 0.28. Max coverage (-): 0

Region: NODE\_288008\_length\_33935\_cov\_31.019037 9426-9435. Max. coverage (+): 0.23. Max coverage (-): 0

Region: NODE\_288008\_length\_33935\_cov\_31.019037 9436-9444. Max. coverage (+): 0.41. Max coverage (-): 0

Region: NODE\_288008\_length\_33935\_cov\_31.019037 9445-9454. Max. coverage (+): 0.28. Max coverage (-): 0

Region: NODE\_288008\_length\_33935\_cov\_31.019037 9455-9463. Max. coverage (+): 0.03. Max coverage (-): 0

Region: NODE\_288008\_length\_33935\_cov\_31.019037 9464-9473. Max. coverage (+): 0.04. Max coverage (-): 0

Region: NODE\_288008\_length\_33935\_cov\_31.019037 9474-9483. Max. coverage (+): 0. Max coverage (-): 0

Region: NODE\_288008\_length\_33935\_cov\_31.019037 9484-9492. Max. coverage (+): 0. Max coverage (-): 0

Region: NODE\_288008\_length\_33935\_cov\_31.019037 9493-9502. Max. coverage (+): 0. Max coverage (-): 0.08

Region: NODE\_288008\_length\_33935\_cov\_31.019037 9503-9511. Max. coverage (+): 0.08. Max coverage (-): 0

Region: NODE\_288008\_length\_33935\_cov\_31.019037 9512-9521. Max. coverage (+): 0.08. Max coverage (-): 0

Region: NODE\_288008\_length\_33935\_cov\_31.019037 9522-9531. Max. coverage (+): 0. Max coverage (-): 0

Region: NODE\_288008\_length\_33935\_cov\_31.019037 9532-9540. Max. coverage (+): 0. Max coverage (-): 0

Region: NODE\_288008\_length\_33935\_cov\_31.019037 9541-9550. Max. coverage (+): 0.08. Max coverage (-): 0

Region: NODE\_288008\_length\_33935\_cov\_31.019037 9551-9560. Max. coverage (+): 0.05. Max coverage (-): 0.02

Region: NODE\_288008\_length\_33935\_cov\_31.019037 9561-9569. Max. coverage (+): 0. Max coverage (-): 0.08

Region: NODE\_288008\_length\_33935\_cov\_31.019037 9570-9579. Max. coverage (+): 0.08. Max coverage (-): 0

Region: NODE\_288008\_length\_33935\_cov\_31.019037 9580-9588. Max. coverage (+): 0.08. Max coverage (-): 0

Region: NODE\_288008\_length\_33935\_cov\_31.019037 9589-9598. Max. coverage (+): 0. Max coverage (-): 0

Region: NODE\_288008\_length\_33935\_cov\_31.019037 9599-9608. Max. coverage (+): 0. Max coverage (-): 0

Region: NODE\_288008\_length\_33935\_cov\_31.019037 9609-9617. Max. coverage (+): 10.71. Max coverage (-): 0.08

Region: NODE\_288008\_length\_33935\_cov\_31.019037 9618-9627. Max. coverage (+): 2.11. Max coverage (-): 0.03

Region: NODE\_288008\_length\_33935\_cov\_31.019037 9628-9636. Max. coverage (+): 0. Max coverage (-): 0

Region: NODE\_288008\_length\_33935\_cov\_31.019037 9637-9646. Max. coverage (+): 0. Max coverage (-): 0

Region: NODE\_288008\_length\_33935\_cov\_31.019037 9647-9656. Max. coverage (+): 0. Max coverage (-): 0

Region: NODE\_288008\_length\_33935\_cov\_31.019037 9657-9665. Max. coverage (+): 0. Max coverage (-): 0

Region: NODE\_288008\_length\_33935\_cov\_31.019037 9666-9675. Max. coverage (+): 0. Max coverage (-): 0

Region: NODE\_288008\_length\_33935\_cov\_31.019037 9676-9684. Max. coverage (+): 0.08. Max coverage (-): 0

Region: NODE\_288008\_length\_33935\_cov\_31.019037 9685-9694. Max. coverage (+): 0. Max coverage (-): 0

Region: NODE\_288008\_length\_33935\_cov\_31.019037 9695-9704. Max. coverage (+): 0. Max coverage (-): 0

Region: NODE\_288008\_length\_33935\_cov\_31.019037 9705-9713. Max. coverage (+): 0.08. Max coverage (-): 0

Region: NODE\_288008\_length\_33935\_cov\_31.019037 9714-9723. Max. coverage (+): 0. Max coverage (-): 0

Region: NODE\_288008\_length\_33935\_cov\_31.019037 9724-9732. Max. coverage (+): 0. Max coverage (-): 0

Region: NODE\_288008\_length\_33935\_cov\_31.019037 9733-9742. Max. coverage (+): 0.02. Max coverage (-): 0

Region: NODE\_288008\_length\_33935\_cov\_31.019037 9743-9752. Max. coverage (+): 0. Max coverage (-): 0

Region: NODE\_288008\_length\_33935\_cov\_31.019037 9753-9761. Max. coverage (+): 0. Max coverage (-): 0

Region: NODE\_288008\_length\_33935\_cov\_31.019037 9762-9771. Max. coverage (+): 0. Max coverage (-): 0

Region: NODE\_288008\_length\_33935\_cov\_31.019037 9772-9780. Max. coverage (+): 1. Max coverage (-): 0.08

Region: NODE\_288008\_length\_33935\_cov\_31.019037 9781-9790. Max. coverage (+): 0.84. Max coverage (-): 0

Region: NODE\_288008\_length\_33935\_cov\_31.019037 9791-9800. Max. coverage (+): 0. Max coverage (-): 0

Region: NODE\_288008\_length\_33935\_cov\_31.019037 9801-9809. Max. coverage (+): 0. Max coverage (-): 0.1

Region: NODE\_288008\_length\_33935\_cov\_31.019037 9810-9819. Max. coverage (+): 0.04. Max coverage (-): 0.1

Region: NODE\_288008\_length\_33935\_cov\_31.019037 9820-9829. Max. coverage (+): 0.11. Max coverage (-): 0

Region: NODE\_288008\_length\_33935\_cov\_31.019037 9830-9838. Max. coverage (+): 0. Max coverage (-): 0

Region: NODE\_288008\_length\_33935\_cov\_31.019037 9839-9848. Max. coverage (+): 0. Max coverage (-): 0

Region: NODE\_288008\_length\_33935\_cov\_31.019037 9849-9857. Max. coverage (+): 0. Max coverage (-): 0

Region: NODE\_288008\_length\_33935\_cov\_31.019037 9858-9867. Max. coverage (+): 0.08. Max coverage (-): 0

Region: NODE\_288008\_length\_33935\_cov\_31.019037 9868-9877. Max. coverage (+): 0.26. Max coverage (-): 0

Region: NODE\_288008\_length\_33935\_cov\_31.019037 9878-9886. Max. coverage (+): 0. Max coverage (-): 0

Region: NODE\_288008\_length\_33935\_cov\_31.019037 9887-9896. Max. coverage (+): 0.08. Max coverage (-): 0

Region: NODE\_288008\_length\_33935\_cov\_31.019037 9897-9905. Max. coverage (+): 0.08. Max coverage (-): 0

Region: NODE\_288008\_length\_33935\_cov\_31.019037 9906-9915. Max. coverage (+): 0.08. Max coverage (-): 0

Region: NODE\_288008\_length\_33935\_cov\_31.019037 9916-9925. Max. coverage (+): 0. Max coverage (-): 0.06

Region: NODE\_288008\_length\_33935\_cov\_31.019037 9926-9934. Max. coverage (+): 0. Max coverage (-): 0.06

Region: NODE\_288008\_length\_33935\_cov\_31.019037 9935-9944. Max. coverage (+): 7.21. Max coverage (-): 0

Region: NODE\_288008\_length\_33935\_cov\_31.019037 9945-9953. Max. coverage (+): 0.13. Max coverage (-): 0

Region: NODE\_288008\_length\_33935\_cov\_31.019037 9954-9963. Max. coverage (+): 0. Max coverage (-): 0

Region: NODE\_288008\_length\_33935\_cov\_31.019037 9964-9973. Max. coverage (+): 0.05. Max coverage (-): 0.34

Region: NODE\_288008\_length\_33935\_cov\_31.019037 9974-9982. Max. coverage (+): 41.49. Max coverage (-): 0.38

Region: NODE\_288008\_length\_33935\_cov\_31.019037 9983-9992. Max. coverage (+): 79.57. Max coverage (-): 0

Region: NODE\_288008\_length\_33935\_cov\_31.019037 9993-10001. Max. coverage (+): 48.87. Max coverage (-): 0

Region: NODE\_288008\_length\_33935\_cov\_31.019037 10002-10011. Max. coverage (+): 0.11. Max coverage (-): 0

Region: NODE\_288008\_length\_33935\_cov\_31.019037 10012-10021. Max. coverage (+): 0. Max coverage (-): 0

Region: NODE\_288008\_length\_33935\_cov\_31.019037 10022-10030. Max. coverage (+): 0. Max coverage (-): 0

Region: NODE\_288008\_length\_33935\_cov\_31.019037 10031-10040. Max. coverage (+): 0. Max coverage (-): 0

Region: NODE\_288008\_length\_33935\_cov\_31.019037 10041-10050. Max. coverage (+): 0.84. Max coverage (-): 0

Region: NODE\_288008\_length\_33935\_cov\_31.019037 10051-10059. Max. coverage (+): 0.13. Max coverage (-): 0.05

Region: NODE\_288008\_length\_33935\_cov\_31.019037 10060-10069. Max. coverage (+): 0. Max coverage (-): 0

Region: NODE\_288008\_length\_33935\_cov\_31.019037 10070-10078. Max. coverage (+): 0.08. Max coverage (-): 0

Region: NODE\_288008\_length\_33935\_cov\_31.019037 10079-10088. Max. coverage (+): 0. Max coverage (-): 0

Region: NODE\_288008\_length\_33935\_cov\_31.019037 10089-10098. Max. coverage (+): 0. Max coverage (-): 0

Region: NODE\_288008\_length\_33935\_cov\_31.019037 10099-10107. Max. coverage (+): 0.08. Max coverage (-): 0

Region: NODE\_288008\_length\_33935\_cov\_31.019037 10108-10117. Max. coverage (+): 0. Max coverage (-): 0

Region: NODE\_288008\_length\_33935\_cov\_31.019037 10118-10126. Max. coverage (+): 0. Max coverage (-): 0

Region: NODE\_288008\_length\_33935\_cov\_31.019037 10127-10136. Max. coverage (+): 0. Max coverage (-): 0

Region: NODE\_288008\_length\_33935\_cov\_31.019037 10137-10146. Max. coverage (+): 0.19. Max coverage (-): 0

Region: NODE\_288008\_length\_33935\_cov\_31.019037 10147-10155. Max. coverage (+): 0. Max coverage (-): 0

Region: NODE\_288008\_length\_33935\_cov\_31.019037 10156-10165. Max. coverage (+): 0. Max coverage (-): 0

Region: NODE\_288008\_length\_33935\_cov\_31.019037 10166-10174. Max. coverage (+): 0.08. Max coverage (-): 0

Region: NODE\_288008\_length\_33935\_cov\_31.019037 10175-10184. Max. coverage (+): 38.96. Max coverage (-): 0

Region: NODE\_288008\_length\_33935\_cov\_31.019037 10185-10194. Max. coverage (+): 0.45. Max coverage (-): 0.04

Region: NODE\_288008\_length\_33935\_cov\_31.019037 10195-10203. Max. coverage (+): 0.67. Max coverage (-): 0.02

Region: NODE\_288008\_length\_33935\_cov\_31.019037 10204-10213. Max. coverage (+): 0.64. Max coverage (-): 0.11

Region: NODE\_288008\_length\_33935\_cov\_31.019037 10214-10222. Max. coverage (+): 0.11. Max coverage (-): 0.04

Region: NODE\_288008\_length\_33935\_cov\_31.019037 10223-10232. Max. coverage (+): 4.75. Max coverage (-): 0.08

Region: NODE\_288008\_length\_33935\_cov\_31.019037 10233-10242. Max. coverage (+): 0.15. Max coverage (-): 0

Region: NODE\_288008\_length\_33935\_cov\_31.019037 10243-10251. Max. coverage (+): 1.03. Max coverage (-): 0.02

Region: NODE\_288008\_length\_33935\_cov\_31.019037 10252-10261. Max. coverage (+): 1.16. Max coverage (-): 0

Region: NODE\_288008\_length\_33935\_cov\_31.019037 10262-10271. Max. coverage (+): 0.08. Max coverage (-): 0

Region: NODE\_288008\_length\_33935\_cov\_31.019037 10272-10280. Max. coverage (+): 0. Max coverage (-): 0

Region: NODE\_288008\_length\_33935\_cov\_31.019037 10281-10290. Max. coverage (+): 0.04. Max coverage (-): 0.04

Region: NODE\_288008\_length\_33935\_cov\_31.019037 10291-10299. Max. coverage (+): 0.15. Max coverage (-): 0

Region: NODE\_288008\_length\_33935\_cov\_31.019037 10300-10309. Max. coverage (+): 7.36. Max coverage (-): 0

Region: NODE\_288008\_length\_33935\_cov\_31.019037 10310-10319. Max. coverage (+): 0. Max coverage (-): 0

Region: NODE\_288008\_length\_33935\_cov\_31.019037 10320-10328. Max. coverage (+): 0. Max coverage (-): 0

Region: NODE\_288008\_length\_33935\_cov\_31.019037 10329-10338. Max. coverage (+): 0.08. Max coverage (-): 0

Region: NODE\_288008\_length\_33935\_cov\_31.019037 10339-10347. Max. coverage (+): 0.1. Max coverage (-): 0.04

Region: NODE\_288008\_length\_33935\_cov\_31.019037 10348-10357. Max. coverage (+): 0.38. Max coverage (-): 0

Region: NODE\_288008\_length\_33935\_cov\_31.019037 10358-10367. Max. coverage (+): 0.38. Max coverage (-): 0

Region: NODE\_288008\_length\_33935\_cov\_31.019037 10368-10376. Max. coverage (+): 0. Max coverage (-): 0

Region: NODE\_288008\_length\_33935\_cov\_31.019037 10377-10386. Max. coverage (+): 0.08. Max coverage (-): 0

Region: NODE\_288008\_length\_33935\_cov\_31.019037 10387-10395. Max. coverage (+): 0. Max coverage (-): 0

Region: NODE\_288008\_length\_33935\_cov\_31.019037 10396-10405. Max. coverage (+): 0. Max coverage (-): 0

Region: NODE\_288008\_length\_33935\_cov\_31.019037 10406-10415. Max. coverage (+): 0. Max coverage (-): 1.61

Region: NODE\_288008\_length\_33935\_cov\_31.019037 10416-10424. Max. coverage (+): 0.04. Max coverage (-): 1.69

Region: NODE\_288008\_length\_33935\_cov\_31.019037 10425-10434. Max. coverage (+): 0.84. Max coverage (-): 0.04

Region: NODE\_288008\_length\_33935\_cov\_31.019037 10435-10443. Max. coverage (+): 0.92. Max coverage (-): 0

Region: NODE\_288008\_length\_33935\_cov\_31.019037 10444-10453. Max. coverage (+): 0.08. Max coverage (-): 0

Region: NODE\_288008\_length\_33935\_cov\_31.019037 10454-10463. Max. coverage (+): 0. Max coverage (-): 0

Region: NODE\_288008\_length\_33935\_cov\_31.019037 10464-10472. Max. coverage (+): 0.35. Max coverage (-): 0

Region: NODE\_288008\_length\_33935\_cov\_31.019037 10473-10482. Max. coverage (+): 0.41. Max coverage (-): 0.01

Region: NODE\_288008\_length\_33935\_cov\_31.019037 10483-10491. Max. coverage (+): 0.03. Max coverage (-): 0

Region: NODE\_288008\_length\_33935\_cov\_31.019037 10492-10501. Max. coverage (+): 0.01. Max coverage (-): 0

Region: NODE\_288008\_length\_33935\_cov\_31.019037 10502-10511. Max. coverage (+): 0.15. Max coverage (-): 0

Region: NODE\_288008\_length\_33935\_cov\_31.019037 10512-10520. Max. coverage (+): 0. Max coverage (-): 0

Region: NODE\_288008\_length\_33935\_cov\_31.019037 10521-10530. Max. coverage (+): 0.45. Max coverage (-): 0

Region: NODE\_288008\_length\_33935\_cov\_31.019037 10531-10540. Max. coverage (+): 0.01. Max coverage (-): 0

Region: NODE\_288008\_length\_33935\_cov\_31.019037 10541-10549. Max. coverage (+): 0. Max coverage (-): 0

Region: NODE\_288008\_length\_33935\_cov\_31.019037 10550-10559. Max. coverage (+): 0. Max coverage (-): 0

Region: NODE\_288008\_length\_33935\_cov\_31.019037 10560-10568. Max. coverage (+): 0.08. Max coverage (-): 0

Region: NODE\_288008\_length\_33935\_cov\_31.019037 10569-10578. Max. coverage (+): 0.08. Max coverage (-): 0

Region: NODE\_288008\_length\_33935\_cov\_31.019037 10579-10588. Max. coverage (+): 0. Max coverage (-): 0

Region: NODE\_288008\_length\_33935\_cov\_31.019037 10589-10597. Max. coverage (+): 0.01. Max coverage (-): 0.01

Region: NODE\_288008\_length\_33935\_cov\_31.019037 10598-10607. Max. coverage (+): 0.02. Max coverage (-): 0.01

Region: NODE\_288008\_length\_33935\_cov\_31.019037 10608-10616. Max. coverage (+): 0.56. Max coverage (-): 0

Region: NODE\_288008\_length\_33935\_cov\_31.019037 10617-10626. Max. coverage (+): 0.01. Max coverage (-): 0

Region: NODE\_288008\_length\_33935\_cov\_31.019037 10627-10636. Max. coverage (+): 0.08. Max coverage (-): 0

Region: NODE\_288008\_length\_33935\_cov\_31.019037 10637-10645. Max. coverage (+): 0.08. Max coverage (-): 0

Region: NODE\_288008\_length\_33935\_cov\_31.019037 10646-10655. Max. coverage (+): 0. Max coverage (-): 0

Region: NODE\_288008\_length\_33935\_cov\_31.019037 10656-10664. Max. coverage (+): 0. Max coverage (-): 0

Region: NODE\_288008\_length\_33935\_cov\_31.019037 10665-10674. Max. coverage (+): 0. Max coverage (-): 0

Region: NODE\_288008\_length\_33935\_cov\_31.019037 10675-10684. Max. coverage (+): 0. Max coverage (-): 0.04

Region: NODE\_288008\_length\_33935\_cov\_31.019037 10685-10693. Max. coverage (+): 0.05. Max coverage (-): 0.03

Region: NODE\_288008\_length\_33935\_cov\_31.019037 10694-10703. Max. coverage (+): 0.76. Max coverage (-): 0.01

Region: NODE\_288008\_length\_33935\_cov\_31.019037 10704-10712. Max. coverage (+): 0.76. Max coverage (-): 0

Region: NODE\_288008\_length\_33935\_cov\_31.019037 10713-10722. Max. coverage (+): 0. Max coverage (-): 0

Region: NODE\_288008\_length\_33935\_cov\_31.019037 10723-10732. Max. coverage (+): 0.04. Max coverage (-): 0

Region: NODE\_288008\_length\_33935\_cov\_31.019037 10733-10741. Max. coverage (+): 0.66. Max coverage (-): 0

Region: NODE\_288008\_length\_33935\_cov\_31.019037 10742-10751. Max. coverage (+): 0.26. Max coverage (-): 0

Region: NODE\_288008\_length\_33935\_cov\_31.019037 10752-10761. Max. coverage (+): 0. Max coverage (-): 0

Region: NODE\_288008\_length\_33935\_cov\_31.019037 10762-10770. Max. coverage (+): 0.23. Max coverage (-): 0

Region: NODE\_288008\_length\_33935\_cov\_31.019037 10771-10780. Max. coverage (+): 0.08. Max coverage (-): 0

Region: NODE\_288008\_length\_33935\_cov\_31.019037 10781-10789. Max. coverage (+): 0. Max coverage (-): 0

Region: NODE\_288008\_length\_33935\_cov\_31.019037 10790-10799. Max. coverage (+): 0. Max coverage (-): 0

Region: NODE\_288008\_length\_33935\_cov\_31.019037 10800-10809. Max. coverage (+): 0. Max coverage (-): 0

Region: NODE\_288008\_length\_33935\_cov\_31.019037 10810-10818. Max. coverage (+): 0. Max coverage (-): 0

Region: NODE\_288008\_length\_33935\_cov\_31.019037 10819-10828. Max. coverage (+): 0. Max coverage (-): 0

Region: NODE\_288008\_length\_33935\_cov\_31.019037 10829-10837. Max. coverage (+): 0.06. Max coverage (-): 0.02

Region: NODE\_288008\_length\_33935\_cov\_31.019037 10838-10847. Max. coverage (+): 0.03. Max coverage (-): 0

Region: NODE\_288008\_length\_33935\_cov\_31.019037 10848-10857. Max. coverage (+): 0. Max coverage (-): 0

Region: NODE\_288008\_length\_33935\_cov\_31.019037 10858-10866. Max. coverage (+): 0.57. Max coverage (-): 0

Region: NODE\_288008\_length\_33935\_cov\_31.019037 10867-10876. Max. coverage (+): 0. Max coverage (-): 0

Region: NODE\_288008\_length\_33935\_cov\_31.019037 10877-10885. Max. coverage (+): 0. Max coverage (-): 0

Region: NODE\_288008\_length\_33935\_cov\_31.019037 10886-10895. Max. coverage (+): 0. Max coverage (-): 0

Region: NODE\_288008\_length\_33935\_cov\_31.019037 10896-10905. Max. coverage (+): 0. Max coverage (-): 0

Region: NODE\_288008\_length\_33935\_cov\_31.019037 10906-10914. Max. coverage (+): 0.01. Max coverage (-): 0

Region: NODE\_288008\_length\_33935\_cov\_31.019037 10915-10924. Max. coverage (+): 0.01. Max coverage (-): 0

Region: NODE\_288008\_length\_33935\_cov\_31.019037 10925-10933. Max. coverage (+): 0.05. Max coverage (-): 0.01

Region: NODE\_288008\_length\_33935\_cov\_31.019037 10934-10943. Max. coverage (+): 0.02. Max coverage (-): 0.01

Region: NODE\_288008\_length\_33935\_cov\_31.019037 10944-10953. Max. coverage (+): 0.84. Max coverage (-): 0

Region: NODE\_288008\_length\_33935\_cov\_31.019037 10954-10962. Max. coverage (+): 0.84. Max coverage (-): 0

Region: NODE\_288008\_length\_33935\_cov\_31.019037 10963-10972. Max. coverage (+): 0. Max coverage (-): 0

Region: NODE\_288008\_length\_33935\_cov\_31.019037 10973-10981. Max. coverage (+): 0. Max coverage (-): 0

Region: NODE\_288008\_length\_33935\_cov\_31.019037 10982-10991. Max. coverage (+): 0.84. Max coverage (-): 0

Region: NODE\_288008\_length\_33935\_cov\_31.019037 10992-11001. Max. coverage (+): 1. Max coverage (-): 0

Region: NODE\_288008\_length\_33935\_cov\_31.019037 11002-11010. Max. coverage (+): 0. Max coverage (-): 0.23

Region: NODE\_288008\_length\_33935\_cov\_31.019037 11011-11020. Max. coverage (+): 0. Max coverage (-): 0.26

Region: NODE\_288008\_length\_33935\_cov\_31.019037 11021-11030. Max. coverage (+): 0.04. Max coverage (-): 0.04

Region: NODE\_288008\_length\_33935\_cov\_31.019037 11031-11039. Max. coverage (+): 0. Max coverage (-): 0.04

Region: NODE\_288008\_length\_33935\_cov\_31.019037 11040-11049. Max. coverage (+): 0.04. Max coverage (-): 0.02

Region: NODE\_288008\_length\_33935\_cov\_31.019037 11050-11058. Max. coverage (+): 0.06. Max coverage (-): 0.02

Region: NODE\_288008\_length\_33935\_cov\_31.019037 11059-11068. Max. coverage (+): 0. Max coverage (-): 0

Region: NODE\_288008\_length\_33935\_cov\_31.019037 11069-11078. Max. coverage (+): 0.02. Max coverage (-): 0.02

Region: NODE\_288008\_length\_33935\_cov\_31.019037 11079-11087. Max. coverage (+): 0.02. Max coverage (-): 0.15

Region: NODE\_288008\_length\_33935\_cov\_31.019037 11088-11097. Max. coverage (+): 0. Max coverage (-): 0

Region: NODE\_288008\_length\_33935\_cov\_31.019037 11098-11106. Max. coverage (+): 0. Max coverage (-): 0

Region: NODE\_288008\_length\_33935\_cov\_31.019037 11107-11116. Max. coverage (+): 0. Max coverage (-): 0

Region: NODE\_288008\_length\_33935\_cov\_31.019037 11117-11126. Max. coverage (+): 0. Max coverage (-): 0.02

Region: NODE\_288008\_length\_33935\_cov\_31.019037 11127-11135. Max. coverage (+): 0. Max coverage (-): 0.02

Region: NODE\_288008\_length\_33935\_cov\_31.019037 11136-11145. Max. coverage (+): 0. Max coverage (-): 0

Region: NODE\_288008\_length\_33935\_cov\_31.019037 11146-11154. Max. coverage (+): 0. Max coverage (-): 0

Region: NODE\_288008\_length\_33935\_cov\_31.019037 11155-11164. Max. coverage (+): 0.01. Max coverage (-): 0

Region: NODE\_288008\_length\_33935\_cov\_31.019037 11165-11174. Max. coverage (+): 0.36. Max coverage (-): 0

Region: NODE\_288008\_length\_33935\_cov\_31.019037 11175-11183. Max. coverage (+): 0.43. Max coverage (-): 0

Region: NODE\_288008\_length\_33935\_cov\_31.019037 11184-11193. Max. coverage (+): 0. Max coverage (-): 0

Region: NODE\_288008\_length\_33935\_cov\_31.019037 11194-11202. Max. coverage (+): 0. Max coverage (-): 0

Region: NODE\_288008\_length\_33935\_cov\_31.019037 11203-11212. Max. coverage (+): 0. Max coverage (-): 0

Region: NODE\_288008\_length\_33935\_cov\_31.019037 11213-11222. Max. coverage (+): 0. Max coverage (-): 0

Region: NODE\_288008\_length\_33935\_cov\_31.019037 11223-11231. Max. coverage (+): 0.06. Max coverage (-): 0

Region: NODE\_288008\_length\_33935\_cov\_31.019037 11232-11241. Max. coverage (+): 0. Max coverage (-): 0

Region: NODE\_288008\_length\_33935\_cov\_31.019037 11242-11251. Max. coverage (+): 0.08. Max coverage (-): 0

Region: NODE\_288008\_length\_33935\_cov\_31.019037 11252-11260. Max. coverage (+): 0.77. Max coverage (-): 0

Region: NODE\_288008\_length\_33935\_cov\_31.019037 11261-11270. Max. coverage (+): 0. Max coverage (-): 0

Region: NODE\_288008\_length\_33935\_cov\_31.019037 11271-11279. Max. coverage (+): 0. Max coverage (-): 0

Region: NODE\_288008\_length\_33935\_cov\_31.019037 11280-11289. Max. coverage (+): 0. Max coverage (-): 0

Region: NODE\_288008\_length\_33935\_cov\_31.019037 11290-11299. Max. coverage (+): 0. Max coverage (-): 0

Region: NODE\_288008\_length\_33935\_cov\_31.019037 11300-11308. Max. coverage (+): 0. Max coverage (-): 0

Region: NODE\_288008\_length\_33935\_cov\_31.019037 11309-11318. Max. coverage (+): 0. Max coverage (-): 0

Region: NODE\_288008\_length\_33935\_cov\_31.019037 11319-11327. Max. coverage (+): 0. Max coverage (-): 0

Region: NODE\_288008\_length\_33935\_cov\_31.019037 11328-11337. Max. coverage (+): 0. Max coverage (-): 0

Region: NODE\_288008\_length\_33935\_cov\_31.019037 11338-11347. Max. coverage (+): 0. Max coverage (-): 0

Region: NODE\_288008\_length\_33935\_cov\_31.019037 11348-11356. Max. coverage (+): 0. Max coverage (-): 0

Region: NODE\_288008\_length\_33935\_cov\_31.019037 11357-11366. Max. coverage (+): 0. Max coverage (-): 0

Region: NODE\_288008\_length\_33935\_cov\_31.019037 11367-11375. Max. coverage (+): 0. Max coverage (-): 0

Region: NODE\_288008\_length\_33935\_cov\_31.019037 11376-11385. Max. coverage (+): 0. Max coverage (-): 0

Region: NODE\_288008\_length\_33935\_cov\_31.019037 11386-11395. Max. coverage (+): 0. Max coverage (-): 0

Region: NODE\_288008\_length\_33935\_cov\_31.019037 11396-11404. Max. coverage (+): 0. Max coverage (-): 0

Region: NODE\_288008\_length\_33935\_cov\_31.019037 11405-11414. Max. coverage (+): 0. Max coverage (-): 0

Region: NODE\_288008\_length\_33935\_cov\_31.019037 11415-11423. Max. coverage (+): 0. Max coverage (-): 0

Region: NODE\_288008\_length\_33935\_cov\_31.019037 11424-11433. Max. coverage (+): 0. Max coverage (-): 0

Region: NODE\_288008\_length\_33935\_cov\_31.019037 11434-11443. Max. coverage (+): 0. Max coverage (-): 0

Region: NODE\_288008\_length\_33935\_cov\_31.019037 11444-11452. Max. coverage (+): 0. Max coverage (-): 0

Region: NODE\_288008\_length\_33935\_cov\_31.019037 11453-11462. Max. coverage (+): 0. Max coverage (-): 0

Region: NODE\_288008\_length\_33935\_cov\_31.019037 11463-11472. Max. coverage (+): 0. Max coverage (-): 0

Region: NODE\_288008\_length\_33935\_cov\_31.019037 11473-11481. Max. coverage (+): 0. Max coverage (-): 0

Region: NODE\_288008\_length\_33935\_cov\_31.019037 11482-11491. Max. coverage (+): 0. Max coverage (-): 0

Region: NODE\_288008\_length\_33935\_cov\_31.019037 11492-11500. Max. coverage (+): 0. Max coverage (-): 0

Region: NODE\_288008\_length\_33935\_cov\_31.019037 11501-11510. Max. coverage (+): 0. Max coverage (-): 0

Region: NODE\_288008\_length\_33935\_cov\_31.019037 11511-11520. Max. coverage (+): 0. Max coverage (-): 0

Region: NODE\_288008\_length\_33935\_cov\_31.019037 11521-11529. Max. coverage (+): 0. Max coverage (-): 0

Region: NODE\_288008\_length\_33935\_cov\_31.019037 11530-11539. Max. coverage (+): 0. Max coverage (-): 0

Region: NODE\_288008\_length\_33935\_cov\_31.019037 11540-11548. Max. coverage (+): 0. Max coverage (-): 0

Region: NODE\_288008\_length\_33935\_cov\_31.019037 11549-11558. Max. coverage (+): 0. Max coverage (-): 0

Region: NODE\_288008\_length\_33935\_cov\_31.019037 11559-11568. Max. coverage (+): 0. Max coverage (-): 0

Region: NODE\_288008\_length\_33935\_cov\_31.019037 11569-11577. Max. coverage (+): 0. Max coverage (-): 0

Region: NODE\_288008\_length\_33935\_cov\_31.019037 11578-11587. Max. coverage (+): 0. Max coverage (-): 0

Region: NODE\_288008\_length\_33935\_cov\_31.019037 11588-11596. Max. coverage (+): 0. Max coverage (-): 0

Region: NODE\_288008\_length\_33935\_cov\_31.019037 11597-11606. Max. coverage (+): 0. Max coverage (-): 0

Region: NODE\_288008\_length\_33935\_cov\_31.019037 11607-11616. Max. coverage (+): 0. Max coverage (-): 0

Region: NODE\_288008\_length\_33935\_cov\_31.019037 11617-11625. Max. coverage (+): 0. Max coverage (-): 0

Region: NODE\_288008\_length\_33935\_cov\_31.019037 11626-11635. Max. coverage (+): 0. Max coverage (-): 0

Region: NODE\_288008\_length\_33935\_cov\_31.019037 11636-11644. Max. coverage (+): 0. Max coverage (-): 0

Region: NODE\_288008\_length\_33935\_cov\_31.019037 11645-11654. Max. coverage (+): 0. Max coverage (-): 0

Region: NODE\_288008\_length\_33935\_cov\_31.019037 11655-11664. Max. coverage (+): 0. Max coverage (-): 0

Region: NODE\_288008\_length\_33935\_cov\_31.019037 11665-11673. Max. coverage (+): 0. Max coverage (-): 0

Region: NODE\_288008\_length\_33935\_cov\_31.019037 11674-11683. Max. coverage (+): 0. Max coverage (-): 0

Region: NODE\_288008\_length\_33935\_cov\_31.019037 11684-11692. Max. coverage (+): 0. Max coverage (-): 0

Region: NODE\_288008\_length\_33935\_cov\_31.019037 11693-11702. Max. coverage (+): 0. Max coverage (-): 0

Region: NODE\_288008\_length\_33935\_cov\_31.019037 11703-11712. Max. coverage (+): 0. Max coverage (-): 0

Region: NODE\_288008\_length\_33935\_cov\_31.019037 11713-11721. Max. coverage (+): 0. Max coverage (-): 0

Region: NODE\_288008\_length\_33935\_cov\_31.019037 11722-11731. Max. coverage (+): 0. Max coverage (-): 0

Region: NODE\_288008\_length\_33935\_cov\_31.019037 11732-11741. Max. coverage (+): 0. Max coverage (-): 0

Region: NODE\_288008\_length\_33935\_cov\_31.019037 11742-11750. Max. coverage (+): 0. Max coverage (-): 0

Region: NODE\_288008\_length\_33935\_cov\_31.019037 11751-11760. Max. coverage (+): 0. Max coverage (-): 0

Region: NODE\_288008\_length\_33935\_cov\_31.019037 11761-11769. Max. coverage (+): 0. Max coverage (-): 0

Region: NODE\_288008\_length\_33935\_cov\_31.019037 11770-11779. Max. coverage (+): 0. Max coverage (-): 0

Region: NODE\_288008\_length\_33935\_cov\_31.019037 11780-11789. Max. coverage (+): 0. Max coverage (-): 0

Region: NODE\_288008\_length\_33935\_cov\_31.019037 11790-11798. Max. coverage (+): 0. Max coverage (-): 0

Region: NODE\_288008\_length\_33935\_cov\_31.019037 11799-11808. Max. coverage (+): 0. Max coverage (-): 0

Region: NODE\_288008\_length\_33935\_cov\_31.019037 11809-11817. Max. coverage (+): 0. Max coverage (-): 0

Region: NODE\_288008\_length\_33935\_cov\_31.019037 11818-11827. Max. coverage (+): 0. Max coverage (-): 0

Region: NODE\_288008\_length\_33935\_cov\_31.019037 11828-11837. Max. coverage (+): 0. Max coverage (-): 0

Region: NODE\_288008\_length\_33935\_cov\_31.019037 11838-11846. Max. coverage (+): 0. Max coverage (-): 0

Region: NODE\_288008\_length\_33935\_cov\_31.019037 11847-11856. Max. coverage (+): 0. Max coverage (-): 0

Region: NODE\_288008\_length\_33935\_cov\_31.019037 11857-11865. Max. coverage (+): 0. Max coverage (-): 0

Region: NODE\_288008\_length\_33935\_cov\_31.019037 11866-11875. Max. coverage (+): 0. Max coverage (-): 0

Region: NODE\_288008\_length\_33935\_cov\_31.019037 11876-11885. Max. coverage (+): 0. Max coverage (-): 0

Region: NODE\_288008\_length\_33935\_cov\_31.019037 11886-11894. Max. coverage (+): 0. Max coverage (-): 0

Region: NODE\_288008\_length\_33935\_cov\_31.019037 11895-11904. Max. coverage (+): 0. Max coverage (-): 0

Region: NODE\_288008\_length\_33935\_cov\_31.019037 11905-11913. Max. coverage (+): 0. Max coverage (-): 0

Region: NODE\_288008\_length\_33935\_cov\_31.019037 11914-11923. Max. coverage (+): 0. Max coverage (-): 0

Region: NODE\_288008\_length\_33935\_cov\_31.019037 11924-11933. Max. coverage (+): 0. Max coverage (-): 0

Region: NODE\_288008\_length\_33935\_cov\_31.019037 11934-11942. Max. coverage (+): 0. Max coverage (-): 0

Region: NODE\_288008\_length\_33935\_cov\_31.019037 11943-11952. Max. coverage (+): 0. Max coverage (-): 0

Region: NODE\_288008\_length\_33935\_cov\_31.019037 11953-11962. Max. coverage (+): 0. Max coverage (-): 0

Region: NODE\_288008\_length\_33935\_cov\_31.019037 11963-11971. Max. coverage (+): 0. Max coverage (-): 0

Region: NODE\_288008\_length\_33935\_cov\_31.019037 11972-11981. Max. coverage (+): 0. Max coverage (-): 0

Region: NODE\_288008\_length\_33935\_cov\_31.019037 11982-11990. Max. coverage (+): 0. Max coverage (-): 0

Region: NODE\_288008\_length\_33935\_cov\_31.019037 11991-12000. Max. coverage (+): 0. Max coverage (-): 0

Region: NODE\_288008\_length\_33935\_cov\_31.019037 12001-12010. Max. coverage (+): 0. Max coverage (-): 0

Region: NODE\_288008\_length\_33935\_cov\_31.019037 12011-12019. Max. coverage (+): 0. Max coverage (-): 0

Region: NODE\_288008\_length\_33935\_cov\_31.019037 12020-12029. Max. coverage (+): 0. Max coverage (-): 0

Region: NODE\_288008\_length\_33935\_cov\_31.019037 12030-12038. Max. coverage (+): 0. Max coverage (-): 0

Region: NODE\_288008\_length\_33935\_cov\_31.019037 12039-12048. Max. coverage (+): 0. Max coverage (-): 0

Region: NODE\_288008\_length\_33935\_cov\_31.019037 12049-12058. Max. coverage (+): 0. Max coverage (-): 0

Region: NODE\_288008\_length\_33935\_cov\_31.019037 12059-12067. Max. coverage (+): 0. Max coverage (-): 0

Region: NODE\_288008\_length\_33935\_cov\_31.019037 12068-12077. Max. coverage (+): 0. Max coverage (-): 0

Region: NODE\_288008\_length\_33935\_cov\_31.019037 12078-12086. Max. coverage (+): 0.08. Max coverage (-): 0

Region: NODE\_288008\_length\_33935\_cov\_31.019037 12087-12096. Max. coverage (+): 0. Max coverage (-): 0

Region: NODE\_288008\_length\_33935\_cov\_31.019037 12097-12106. Max. coverage (+): 0.1. Max coverage (-): 0

Region: NODE\_288008\_length\_33935\_cov\_31.019037 12107-12115. Max. coverage (+): 0. Max coverage (-): 0

Region: NODE\_288008\_length\_33935\_cov\_31.019037 12116-12125. Max. coverage (+): 0. Max coverage (-): 0

Region: NODE\_288008\_length\_33935\_cov\_31.019037 12126-12134. Max. coverage (+): 0. Max coverage (-): 0

Region: NODE\_288008\_length\_33935\_cov\_31.019037 12135-12144. Max. coverage (+): 0. Max coverage (-): 0

Region: NODE\_288008\_length\_33935\_cov\_31.019037 12145-12154. Max. coverage (+): 0. Max coverage (-): 0

Region: NODE\_288008\_length\_33935\_cov\_31.019037 12155-12163. Max. coverage (+): 0. Max coverage (-): 0

Region: NODE\_288008\_length\_33935\_cov\_31.019037 12164-12173. Max. coverage (+): 0. Max coverage (-): 0

Region: NODE\_288008\_length\_33935\_cov\_31.019037 12174-12182. Max. coverage (+): 0. Max coverage (-): 0

Region: NODE\_288008\_length\_33935\_cov\_31.019037 12183-12192. Max. coverage (+): 0. Max coverage (-): 0

Region: NODE\_288008\_length\_33935\_cov\_31.019037 12193-12202. Max. coverage (+): 0. Max coverage (-): 0

Region: NODE\_288008\_length\_33935\_cov\_31.019037 12203-12211. Max. coverage (+): 0. Max coverage (-): 0

Region: NODE\_288008\_length\_33935\_cov\_31.019037 12212-12221. Max. coverage (+): 0. Max coverage (-): 0

Region: NODE\_288008\_length\_33935\_cov\_31.019037 12222-12231. Max. coverage (+): 0. Max coverage (-): 0

Region: NODE\_288008\_length\_33935\_cov\_31.019037 12232-12240. Max. coverage (+): 0. Max coverage (-): 0

Region: NODE\_288008\_length\_33935\_cov\_31.019037 12241-12250. Max. coverage (+): 0. Max coverage (-): 0

Region: NODE\_288008\_length\_33935\_cov\_31.019037 12251-12259. Max. coverage (+): 0. Max coverage (-): 0

Region: NODE\_288008\_length\_33935\_cov\_31.019037 12260-12269. Max. coverage (+): 0. Max coverage (-): 0

Region: NODE\_288008\_length\_33935\_cov\_31.019037 12270-12279. Max. coverage (+): 0. Max coverage (-): 0

Region: NODE\_288008\_length\_33935\_cov\_31.019037 12280-12288. Max. coverage (+): 0. Max coverage (-): 0

Region: NODE\_288008\_length\_33935\_cov\_31.019037 12289-12298. Max. coverage (+): 0. Max coverage (-): 0

Region: NODE\_288008\_length\_33935\_cov\_31.019037 12299-12307. Max. coverage (+): 0. Max coverage (-): 0

Region: NODE\_288008\_length\_33935\_cov\_31.019037 12308-12317. Max. coverage (+): 0.08. Max coverage (-): 0

Region: NODE\_288008\_length\_33935\_cov\_31.019037 12318-12327. Max. coverage (+): 0. Max coverage (-): 0

Region: NODE\_288008\_length\_33935\_cov\_31.019037 12328-12336. Max. coverage (+): 0. Max coverage (-): 0

Region: NODE\_288008\_length\_33935\_cov\_31.019037 12337-12346. Max. coverage (+): 0. Max coverage (-): 0

Region: NODE\_288008\_length\_33935\_cov\_31.019037 12347-12355. Max. coverage (+): 0. Max coverage (-): 0

Region: NODE\_288008\_length\_33935\_cov\_31.019037 12356-12365. Max. coverage (+): 0. Max coverage (-): 0

Region: NODE\_288008\_length\_33935\_cov\_31.019037 12366-12375. Max. coverage (+): 0. Max coverage (-): 0

Region: NODE\_288008\_length\_33935\_cov\_31.019037 12376-12384. Max. coverage (+): 0. Max coverage (-): 0

Region: NODE\_288008\_length\_33935\_cov\_31.019037 12385-12394. Max. coverage (+): 0. Max coverage (-): 0

Region: NODE\_288008\_length\_33935\_cov\_31.019037 12395-12403. Max. coverage (+): 0.08. Max coverage (-): 0

Region: NODE\_288008\_length\_33935\_cov\_31.019037 12404-12413. Max. coverage (+): 0. Max coverage (-): 0

Region: NODE\_288008\_length\_33935\_cov\_31.019037 12414-12423. Max. coverage (+): 0. Max coverage (-): 0

Region: NODE\_288008\_length\_33935\_cov\_31.019037 12424-. Max. coverage (+): 0. Max coverage (-): 0

RepeatMasker Color Code

**+**

100-98% Identity

<98-95% Identity

<95-90% Identity

<90-85% Identity

<85-80% Identity

<80-75% Identity

<75-70% Identity

<70% Identity

**-**

Gene Set Color Code

**+**

Gene

Pseudogene

Other

**-**

Topology/Coverage Color Code

Coverage Plus Strand

Coverage Minus Strand

Mainstrand: Plus

Mainstrand: Minus

Complementary Strand

Flanking Region  
(if option -flank >0)

Gene Set Annotation  
  
RepeatMasker Annotation  

**1. AlRepD-3099**: 7609-7956 (-), Divergence to consensus: 10.1%  
**2. Gypsy-36\_GA-I**: 8273-8764 (+), Divergence to consensus: 44%  
**3. Gypsy-36\_GA-I**: 8905-9442 (+), Divergence to consensus: 36%  
**4. Gypsy-36\_GA-I**: 9777-10600 (+), Divergence to consensus: 42.6%  
**5. Gypsy-36\_GA-I**: 10573-10654 (+), Divergence to consensus: 23.6%  
**6. Gypsy-36\_GA-I**: 10660-11200 (+), Divergence to consensus: 36.1%  
**7. AlRepD-3099**: 11209-11273 (-), Divergence to consensus: 7.7%  
**8. AlRepB-112**: 11693-11758 (+), Divergence to consensus: 28.8%  
**9. AlRepA-102**: 11770-11816 (+), Divergence to consensus: 10.6%  
**10. AlRepD-2509**: 11852-11912 (+), Divergence to consensus: 18.4%  
**11. AlRepB-103**: 11940-12539 (+), Divergence to consensus: 24%

  
Transcription Factor Binding Sites  

**RHOXF1** (Sequence: AGATTA (-): 8198)  
**RHOXF1** (Sequence: GGATCA (-): 8485)  
**RHOXF1** (Sequence: AGCTTA (-): 9094)  
**RHOXF1** (Sequence: AGCTTA (-): 9181)  
**RHOXF1** (Sequence: AGATCA (-): 9200)  
**RHOXF1** (Sequence: AGATTA (-): 10947)  
**RHOXF1** (Sequence: GGCTCA (-): 11016)  
**RHOXF1** (Sequence: AGATTA (-): 11769)  
**RHOXF1** (Sequence: AGATCA (-): 12042)  
**RHOXF1** (Sequence: AGCTCA (-): 12232)  
**RHOXF1** (Sequence: AGATTA (-): 12396)  
**RHOXF1** (Sequence: TGAGCC (+): 7926)  
**RHOXF1** (Sequence: TAAGCT (+): 7978)  
**RHOXF1** (Sequence: TAAGCC (+): 9670)  
**RHOXF1** (Sequence: TGAGCT (+): 10308)  
**RHOXF1** (Sequence: TGAGCC (+): 11257)  
**RHOXF1** (Sequence: TGATCT (+): 11521)  
**RHOXF1** (Sequence: TGATCC (+): 11705)  
**RHOXF1** (Sequence: TGATCT (+): 11732)  
**RHOXF1** (Sequence: TGATCC (+): 11890)  
**RHOXF1** (Sequence: TGATCT (+): 12149)  
**RFX4\_1** (Sequence: GTTGCCAGG (-): 8671)  
**FOXO1** (Sequence: GCTGTTTTC (+): 8463)  
**FOXO1** (Sequence: GTTGTTTTT (+): 11628)  
**FOXO3\_mmu** (Sequence: TGTTTTGC (-): 7632)  
**FOXO3\_mmu** (Sequence: TGTTTTGC (-): 8230)  
**FOXO3\_mmu** (Sequence: TGTTTTCA (-): 8465)  
**FOXO3\_mmu** (Sequence: TGTTTTGA (-): 9070)  
**Sox5** (Sequence: ATTGTT (+): 8103)  
**Sox5** (Sequence: ATTGTT (+): 8169)  
**Sox5** (Sequence: ATTGTT (+): 10237)  
**Sox5** (Sequence: ATTGTT (+): 11318)  
**SOX9** (Sequence: CCATTGTT (+): 11316)  
**FOXO3\_mmu** (Sequence: GCAAAACA (+): 10996)  
**FOXO3\_mmu** (Sequence: TCAAAACA (+): 11543)  
**Rhox11** (Sequence: TGCTGTTTT (+): 8462)  
**POU2F1** (Sequence: TATGTTAAT (+): 10951)
