# Supplementary material for: First characterization of PIWI-interacting RNA clusters in a cichlid fish with a B chromosome
Source: BMC Biol. 2022 Sep 21;20:204. doi: 10.1186/s12915-022-01403-2 (PMC9490952; doi:10.1186/s12915-022-01403-2)
Supplement: Supplementary file 1 — Additional file 1. Zipped folder with fasta and interactive html piRNA cluster information for the A. latifasciata genome. The nomenclature is as follows: number-pirna-cluster_sex_B-presence (f, female; m, male; 0b, without B chromosome; 1b, with B chromosome). [file 12915_2022_1403_MOESM1_ESM.zip › 110_f1b.html]

piRNA cluster 110\_f1b 55


Predicted piRNA cluster no. 110\_f1b
  

Show proTRAC run info
Hide proTRAC run info

/\  
                \_\_\_\_\_\_\_\_\_\_\_\_\_\_\_\_\_\_\_\_\_\_\_/\\_\_\_ /  \\_\_\_\_\_\_\_  
               I                      /  \  /    \      I  
               I     pro             /    \/      \     I  
               I        TRAC        /               \   I  
               I   \_\_\_\_\_\_\_\_\_\_\_\_\_\_\_\_/\_\_\_\_\_\_\_\_\_\_\_\_\_\_\_\_\_\\_ I  
               I   \              /                     I  
               I    \            /                      I  
               I     \  /\      /       V.2.4.2         I  
               I      \/  \    /                        I  
               I\_\_\_\_\_\_\_\_\_\_\_\  /\_\_\_\_\_\_\_\_\_\_\_\_\_\_\_\_\_\_\_\_\_\_\_\_\_I  
                            \/  
  
  
================================= proTRAC ====================================  
VERSION: .......... 2.4.2  
LAST MODIFIED: .... 11. May 2018  
  
Please cite:  
Rosenkranz D, Zischler H. proTRAC - a software for probabilistic piRNA cluster  
detection, visualization and analysis. 2012. BMC Bioinformatics 13:5.  
  
  
Contact:  
David Rosenkranz  
Institute of Organismic and Molecular Evolutionary Biology  
Dept. Anthropology, small RNA group  
Johannes Gutenberg University Mainz  
email: rosenkranz@uni-mainz.de  
  
You can find the latest proTRAC version at:  
http://sourceforge.net/projects/protrac/files  
http://www.smallRNAgroup-mainz.de/software  
==============================================================================  
  
PARAMETERS:  
Map file: ...............piwi-femeas-1B.fa-collapse.map  
Genome file: ............../../../0B\_ala\_genome.fa  
RepeatMasker annotation: Alatifasciata-all0B-maryan-v2.fa\_corrected.out  
GeneSet:................./guest-storage/Data/annotation/Alatifasciata\_all0B\_maryan-v2\_out2017.gff  
  
Significant (p<=0.01) hit density will be calculated based  
on observed hit distribution.  
  
Sliding window size: ........................................ 5000 bp  
Sliding window increament: .................................. 1000 bp  
Normalize each hit by number of genomic hits: ............... yes  
Normalize each hit by number of sequence reads: ............. yes  
Normalize values (-> per million mapped reads): ............. yes  
Min. fraction of hits with 1T(U) or 10A: .................... 0.75  
Alternatively: Min. fraction of hits with 1T(U) and 10A: .... 0.5  
Min. fraction of hits with typical piRNA length: ............ 0.75  
Typical piRNA length: ....................................... 24-32 nt  
Min. size of a piRNA cluster: ............................... 1000 bp.  
Min. number of hits (absolute): ............................. 0  
Min. number of hits (normalized): ........................... 0  
Min. fraction of hits on the mainstrand: .................... 0.75  
Top fraction of mapped sequences (in terms of read counts): . 1%  
Top fraction accounts for max. n% of sequence reads: ........ 90%  
Min. fraction of hits on each arm of a bidirectional cluster: 0.05  
Output html file for each cluster: .......................... yes  
Output a summary table: ..................................... yes  
Output a FASTA file for each cluster (piRNA sequences): ..... yes  
Output a FASTA file comprising cluster sequences: ........... yes  
Output a GTF file for predicted piRNA clusters: ..............yes  
Search DNA motifs in clusters: .............................. yes  
Output flanking sequences: +/- .............................. 0 bp  
Output ~.pTi file: .......................................... no  
==============================================================================  
  
  
Genome size (without gaps): ............ 758543724 bp  
Gaps (N/X/-): .......................... 417479 bp  
Mapped reads: .......................... 10641844  
Non-identical sequences: ............... 2832837  
Genomic hits: .......................... 26056853  
Significant densitiy of mapped reads: .. 368.713530323068 reads/kb

Show proTRAC cluster info
Hide proTRAC cluster info

|  |  |
| --- | --- |
| Location | NODE\_288008\_length\_33935\_cov\_31.019037 |
| Coordinates | 7628-13011 |
| Size [bp] | 5384 |
| Sequence hit loci | 910 |
| Mapped reads (normalized) | 2220.5 |
| Mapped reads (normalized) per kb | 412.4 |
| Normalized reads with 1T (1U) | 88.9% |
| Normalized reads with 10A | 23.4% |
| Normalized reads with length 24-32 nt | 99.6% |
| Normalized reads on the main strand(s) | 97.7% |
| Predicted directionality | mono:plus |

100%

0%

1T (1U)  
reads

10A reads

24-32 nt  
reads

reads on mainstrand

**Either the amount of reads with 1T (1U) OR 10A has to exceed 75% (set with option: -1Tor10A)  
Alternatively the amount of reads with 1T (1U) AND 10A has to exceed 50% (set with option: -1Tand10A)  
Minimum amount of reads with preferred size is 75% (set with option: -pisize)  
Minimum amount of reads on the main strand(s) is 75% (set with option: -clstrand)**

Show read coverage
Hide read coverage

WHAT DO I SEE HERE?  
This chart shows the location of mapped sequence reads within a predicted piRNA cluster. The color refers to the number of genomic hits produced by the sequence read in question. A dark red bar indicates that this sequence read produces many other hits elsewhere in the genome. Many adjacent red or yellow bars can indicate the presence of a multi-copy element such as transposons or rRNA genes. A dark green bar indicates that this sequence read maps uniquely to this locus.

1 hit

2-5 hits

6-10 hits

11-20 hits

21-50 hits

51-100 hits

> 100 hits

NODE\_288008\_length\_33935\_cov\_31.019037

7628

13011

Gene Set

RepeatMasker

Mapped  
Reads

82.94

plus strand

minus strand

82.94

Region: NODE\_288008\_length\_33935\_cov\_31.019037 3023-7633. Max. coverage (+): 0.01. Max coverage (-): 0

Region: NODE\_288008\_length\_33935\_cov\_31.019037 7634-7644. Max. coverage (+): 0. Max coverage (-): 0

Region: NODE\_288008\_length\_33935\_cov\_31.019037 7645-7654. Max. coverage (+): 0.19. Max coverage (-): 0

Region: NODE\_288008\_length\_33935\_cov\_31.019037 7655-7665. Max. coverage (+): 0.2. Max coverage (-): 0

Region: NODE\_288008\_length\_33935\_cov\_31.019037 7666-7676. Max. coverage (+): 0. Max coverage (-): 0

Region: NODE\_288008\_length\_33935\_cov\_31.019037 7677-7687. Max. coverage (+): 0. Max coverage (-): 0

Region: NODE\_288008\_length\_33935\_cov\_31.019037 7688-7697. Max. coverage (+): 0. Max coverage (-): 0

Region: NODE\_288008\_length\_33935\_cov\_31.019037 7698-7708. Max. coverage (+): 0. Max coverage (-): 0

Region: NODE\_288008\_length\_33935\_cov\_31.019037 7709-7719. Max. coverage (+): 0.01. Max coverage (-): 0

Region: NODE\_288008\_length\_33935\_cov\_31.019037 7720-7730. Max. coverage (+): 0.01. Max coverage (-): 0

Region: NODE\_288008\_length\_33935\_cov\_31.019037 7731-7741. Max. coverage (+): 0. Max coverage (-): 0

Region: NODE\_288008\_length\_33935\_cov\_31.019037 7742-7751. Max. coverage (+): 0. Max coverage (-): 0

Region: NODE\_288008\_length\_33935\_cov\_31.019037 7752-7762. Max. coverage (+): 0.09. Max coverage (-): 0

Region: NODE\_288008\_length\_33935\_cov\_31.019037 7763-7773. Max. coverage (+): 0.05. Max coverage (-): 0

Region: NODE\_288008\_length\_33935\_cov\_31.019037 7774-7784. Max. coverage (+): 0.14. Max coverage (-): 0

Region: NODE\_288008\_length\_33935\_cov\_31.019037 7785-7794. Max. coverage (+): 0.01. Max coverage (-): 0.02

Region: NODE\_288008\_length\_33935\_cov\_31.019037 7795-7805. Max. coverage (+): 0.02. Max coverage (-): 0

Region: NODE\_288008\_length\_33935\_cov\_31.019037 7806-7816. Max. coverage (+): 0.07. Max coverage (-): 0

Region: NODE\_288008\_length\_33935\_cov\_31.019037 7817-7827. Max. coverage (+): 0. Max coverage (-): 0

Region: NODE\_288008\_length\_33935\_cov\_31.019037 7828-7837. Max. coverage (+): 0.09. Max coverage (-): 0

Region: NODE\_288008\_length\_33935\_cov\_31.019037 7838-7848. Max. coverage (+): 0.09. Max coverage (-): 0

Region: NODE\_288008\_length\_33935\_cov\_31.019037 7849-7859. Max. coverage (+): 0. Max coverage (-): 0

Region: NODE\_288008\_length\_33935\_cov\_31.019037 7860-7870. Max. coverage (+): 0. Max coverage (-): 0

Region: NODE\_288008\_length\_33935\_cov\_31.019037 7871-7881. Max. coverage (+): 0. Max coverage (-): 0

Region: NODE\_288008\_length\_33935\_cov\_31.019037 7882-7891. Max. coverage (+): 0.09. Max coverage (-): 0

Region: NODE\_288008\_length\_33935\_cov\_31.019037 7892-7902. Max. coverage (+): 0.28. Max coverage (-): 0

Region: NODE\_288008\_length\_33935\_cov\_31.019037 7903-7913. Max. coverage (+): 0. Max coverage (-): 0

Region: NODE\_288008\_length\_33935\_cov\_31.019037 7914-7924. Max. coverage (+): 0.64. Max coverage (-): 0

Region: NODE\_288008\_length\_33935\_cov\_31.019037 7925-7934. Max. coverage (+): 0.09. Max coverage (-): 0

Region: NODE\_288008\_length\_33935\_cov\_31.019037 7935-7945. Max. coverage (+): 0. Max coverage (-): 0

Region: NODE\_288008\_length\_33935\_cov\_31.019037 7946-7956. Max. coverage (+): 0. Max coverage (-): 0

Region: NODE\_288008\_length\_33935\_cov\_31.019037 7957-7967. Max. coverage (+): 0.07. Max coverage (-): 0

Region: NODE\_288008\_length\_33935\_cov\_31.019037 7968-7977. Max. coverage (+): 0.23. Max coverage (-): 0

Region: NODE\_288008\_length\_33935\_cov\_31.019037 7978-7988. Max. coverage (+): 0.38. Max coverage (-): 0.02

Region: NODE\_288008\_length\_33935\_cov\_31.019037 7989-7999. Max. coverage (+): 0.31. Max coverage (-): 0.02

Region: NODE\_288008\_length\_33935\_cov\_31.019037 8000-8010. Max. coverage (+): 0. Max coverage (-): 0.09

Region: NODE\_288008\_length\_33935\_cov\_31.019037 8011-8021. Max. coverage (+): 0. Max coverage (-): 0

Region: NODE\_288008\_length\_33935\_cov\_31.019037 8022-8031. Max. coverage (+): 0.28. Max coverage (-): 0

Region: NODE\_288008\_length\_33935\_cov\_31.019037 8032-8042. Max. coverage (+): 0. Max coverage (-): 0

Region: NODE\_288008\_length\_33935\_cov\_31.019037 8043-8053. Max. coverage (+): 0. Max coverage (-): 0

Region: NODE\_288008\_length\_33935\_cov\_31.019037 8054-8064. Max. coverage (+): 0. Max coverage (-): 0

Region: NODE\_288008\_length\_33935\_cov\_31.019037 8065-8074. Max. coverage (+): 3.1. Max coverage (-): 0

Region: NODE\_288008\_length\_33935\_cov\_31.019037 8075-8085. Max. coverage (+): 3.92. Max coverage (-): 0

Region: NODE\_288008\_length\_33935\_cov\_31.019037 8086-8096. Max. coverage (+): 0.09. Max coverage (-): 0.14

Region: NODE\_288008\_length\_33935\_cov\_31.019037 8097-8107. Max. coverage (+): 0. Max coverage (-): 0

Region: NODE\_288008\_length\_33935\_cov\_31.019037 8108-8117. Max. coverage (+): 0. Max coverage (-): 0

Region: NODE\_288008\_length\_33935\_cov\_31.019037 8118-8128. Max. coverage (+): 0. Max coverage (-): 0

Region: NODE\_288008\_length\_33935\_cov\_31.019037 8129-8139. Max. coverage (+): 0. Max coverage (-): 0

Region: NODE\_288008\_length\_33935\_cov\_31.019037 8140-8150. Max. coverage (+): 0.09. Max coverage (-): 0

Region: NODE\_288008\_length\_33935\_cov\_31.019037 8151-8161. Max. coverage (+): 0. Max coverage (-): 0

Region: NODE\_288008\_length\_33935\_cov\_31.019037 8162-8171. Max. coverage (+): 0. Max coverage (-): 0

Region: NODE\_288008\_length\_33935\_cov\_31.019037 8172-8182. Max. coverage (+): 0. Max coverage (-): 0

Region: NODE\_288008\_length\_33935\_cov\_31.019037 8183-8193. Max. coverage (+): 0.09. Max coverage (-): 0

Region: NODE\_288008\_length\_33935\_cov\_31.019037 8194-8204. Max. coverage (+): 0. Max coverage (-): 0

Region: NODE\_288008\_length\_33935\_cov\_31.019037 8205-8214. Max. coverage (+): 0. Max coverage (-): 0

Region: NODE\_288008\_length\_33935\_cov\_31.019037 8215-8225. Max. coverage (+): 0.09. Max coverage (-): 0

Region: NODE\_288008\_length\_33935\_cov\_31.019037 8226-8236. Max. coverage (+): 0. Max coverage (-): 0

Region: NODE\_288008\_length\_33935\_cov\_31.019037 8237-8247. Max. coverage (+): 0. Max coverage (-): 0

Region: NODE\_288008\_length\_33935\_cov\_31.019037 8248-8257. Max. coverage (+): 0. Max coverage (-): 0

Region: NODE\_288008\_length\_33935\_cov\_31.019037 8258-8268. Max. coverage (+): 0.38. Max coverage (-): 0

Region: NODE\_288008\_length\_33935\_cov\_31.019037 8269-8279. Max. coverage (+): 0.19. Max coverage (-): 0

Region: NODE\_288008\_length\_33935\_cov\_31.019037 8280-8290. Max. coverage (+): 0. Max coverage (-): 0

Region: NODE\_288008\_length\_33935\_cov\_31.019037 8291-8300. Max. coverage (+): 0. Max coverage (-): 0

Region: NODE\_288008\_length\_33935\_cov\_31.019037 8301-8311. Max. coverage (+): 0.05. Max coverage (-): 0

Region: NODE\_288008\_length\_33935\_cov\_31.019037 8312-8322. Max. coverage (+): 0.05. Max coverage (-): 0.09

Region: NODE\_288008\_length\_33935\_cov\_31.019037 8323-8333. Max. coverage (+): 0. Max coverage (-): 0

Region: NODE\_288008\_length\_33935\_cov\_31.019037 8334-8344. Max. coverage (+): 0.09. Max coverage (-): 0

Region: NODE\_288008\_length\_33935\_cov\_31.019037 8345-8354. Max. coverage (+): 0. Max coverage (-): 0

Region: NODE\_288008\_length\_33935\_cov\_31.019037 8355-8365. Max. coverage (+): 0. Max coverage (-): 0.19

Region: NODE\_288008\_length\_33935\_cov\_31.019037 8366-8376. Max. coverage (+): 0.09. Max coverage (-): 0

Region: NODE\_288008\_length\_33935\_cov\_31.019037 8377-8387. Max. coverage (+): 0.28. Max coverage (-): 0

Region: NODE\_288008\_length\_33935\_cov\_31.019037 8388-8397. Max. coverage (+): 0.09. Max coverage (-): 0

Region: NODE\_288008\_length\_33935\_cov\_31.019037 8398-8408. Max. coverage (+): 0.35. Max coverage (-): 0.02

Region: NODE\_288008\_length\_33935\_cov\_31.019037 8409-8419. Max. coverage (+): 0.23. Max coverage (-): 0

Region: NODE\_288008\_length\_33935\_cov\_31.019037 8420-8430. Max. coverage (+): 1.88. Max coverage (-): 0

Region: NODE\_288008\_length\_33935\_cov\_31.019037 8431-8440. Max. coverage (+): 1.5. Max coverage (-): 0

Region: NODE\_288008\_length\_33935\_cov\_31.019037 8441-8451. Max. coverage (+): 0. Max coverage (-): 0

Region: NODE\_288008\_length\_33935\_cov\_31.019037 8452-8462. Max. coverage (+): 0. Max coverage (-): 0

Region: NODE\_288008\_length\_33935\_cov\_31.019037 8463-8473. Max. coverage (+): 0.19. Max coverage (-): 0

Region: NODE\_288008\_length\_33935\_cov\_31.019037 8474-8484. Max. coverage (+): 0.47. Max coverage (-): 0

Region: NODE\_288008\_length\_33935\_cov\_31.019037 8485-8494. Max. coverage (+): 0.28. Max coverage (-): 0

Region: NODE\_288008\_length\_33935\_cov\_31.019037 8495-8505. Max. coverage (+): 0. Max coverage (-): 0

Region: NODE\_288008\_length\_33935\_cov\_31.019037 8506-8516. Max. coverage (+): 0.06. Max coverage (-): 0.02

Region: NODE\_288008\_length\_33935\_cov\_31.019037 8517-8527. Max. coverage (+): 0.02. Max coverage (-): 0.02

Region: NODE\_288008\_length\_33935\_cov\_31.019037 8528-8537. Max. coverage (+): 0.05. Max coverage (-): 0.07

Region: NODE\_288008\_length\_33935\_cov\_31.019037 8538-8548. Max. coverage (+): 0.25. Max coverage (-): 0

Region: NODE\_288008\_length\_33935\_cov\_31.019037 8549-8559. Max. coverage (+): 0.56. Max coverage (-): 0

Region: NODE\_288008\_length\_33935\_cov\_31.019037 8560-8570. Max. coverage (+): 0.19. Max coverage (-): 0

Region: NODE\_288008\_length\_33935\_cov\_31.019037 8571-8580. Max. coverage (+): 0. Max coverage (-): 0

Region: NODE\_288008\_length\_33935\_cov\_31.019037 8581-8591. Max. coverage (+): 0.56. Max coverage (-): 0

Region: NODE\_288008\_length\_33935\_cov\_31.019037 8592-8602. Max. coverage (+): 0.09. Max coverage (-): 0

Region: NODE\_288008\_length\_33935\_cov\_31.019037 8603-8613. Max. coverage (+): 0. Max coverage (-): 0

Region: NODE\_288008\_length\_33935\_cov\_31.019037 8614-8624. Max. coverage (+): 0.09. Max coverage (-): 0

Region: NODE\_288008\_length\_33935\_cov\_31.019037 8625-8634. Max. coverage (+): 0. Max coverage (-): 0

Region: NODE\_288008\_length\_33935\_cov\_31.019037 8635-8645. Max. coverage (+): 0. Max coverage (-): 0

Region: NODE\_288008\_length\_33935\_cov\_31.019037 8646-8656. Max. coverage (+): 0. Max coverage (-): 0

Region: NODE\_288008\_length\_33935\_cov\_31.019037 8657-8667. Max. coverage (+): 0. Max coverage (-): 0

Region: NODE\_288008\_length\_33935\_cov\_31.019037 8668-8677. Max. coverage (+): 0. Max coverage (-): 0

Region: NODE\_288008\_length\_33935\_cov\_31.019037 8678-8688. Max. coverage (+): 0. Max coverage (-): 0

Region: NODE\_288008\_length\_33935\_cov\_31.019037 8689-8699. Max. coverage (+): 0. Max coverage (-): 0

Region: NODE\_288008\_length\_33935\_cov\_31.019037 8700-8710. Max. coverage (+): 0.09. Max coverage (-): 0

Region: NODE\_288008\_length\_33935\_cov\_31.019037 8711-8720. Max. coverage (+): 0. Max coverage (-): 0

Region: NODE\_288008\_length\_33935\_cov\_31.019037 8721-8731. Max. coverage (+): 0. Max coverage (-): 0

Region: NODE\_288008\_length\_33935\_cov\_31.019037 8732-8742. Max. coverage (+): 0. Max coverage (-): 0.05

Region: NODE\_288008\_length\_33935\_cov\_31.019037 8743-8753. Max. coverage (+): 0. Max coverage (-): 0.05

Region: NODE\_288008\_length\_33935\_cov\_31.019037 8754-8764. Max. coverage (+): 0.22. Max coverage (-): 0

Region: NODE\_288008\_length\_33935\_cov\_31.019037 8765-8774. Max. coverage (+): 0. Max coverage (-): 0

Region: NODE\_288008\_length\_33935\_cov\_31.019037 8775-8785. Max. coverage (+): 0. Max coverage (-): 0

Region: NODE\_288008\_length\_33935\_cov\_31.019037 8786-8796. Max. coverage (+): 0.85. Max coverage (-): 0

Region: NODE\_288008\_length\_33935\_cov\_31.019037 8797-8807. Max. coverage (+): 0. Max coverage (-): 0

Region: NODE\_288008\_length\_33935\_cov\_31.019037 8808-8817. Max. coverage (+): 0. Max coverage (-): 0

Region: NODE\_288008\_length\_33935\_cov\_31.019037 8818-8828. Max. coverage (+): 0. Max coverage (-): 0

Region: NODE\_288008\_length\_33935\_cov\_31.019037 8829-8839. Max. coverage (+): 0. Max coverage (-): 0

Region: NODE\_288008\_length\_33935\_cov\_31.019037 8840-8850. Max. coverage (+): 0.09. Max coverage (-): 0

Region: NODE\_288008\_length\_33935\_cov\_31.019037 8851-8860. Max. coverage (+): 0. Max coverage (-): 0

Region: NODE\_288008\_length\_33935\_cov\_31.019037 8861-8871. Max. coverage (+): 0. Max coverage (-): 0.52

Region: NODE\_288008\_length\_33935\_cov\_31.019037 8872-8882. Max. coverage (+): 0.11. Max coverage (-): 0.01

Region: NODE\_288008\_length\_33935\_cov\_31.019037 8883-8893. Max. coverage (+): 0.11. Max coverage (-): 0

Region: NODE\_288008\_length\_33935\_cov\_31.019037 8894-8904. Max. coverage (+): 0. Max coverage (-): 0

Region: NODE\_288008\_length\_33935\_cov\_31.019037 8905-8914. Max. coverage (+): 0. Max coverage (-): 0

Region: NODE\_288008\_length\_33935\_cov\_31.019037 8915-8925. Max. coverage (+): 0. Max coverage (-): 0

Region: NODE\_288008\_length\_33935\_cov\_31.019037 8926-8936. Max. coverage (+): 0. Max coverage (-): 0

Region: NODE\_288008\_length\_33935\_cov\_31.019037 8937-8947. Max. coverage (+): 0. Max coverage (-): 0

Region: NODE\_288008\_length\_33935\_cov\_31.019037 8948-8957. Max. coverage (+): 0. Max coverage (-): 0

Region: NODE\_288008\_length\_33935\_cov\_31.019037 8958-8968. Max. coverage (+): 0. Max coverage (-): 0

Region: NODE\_288008\_length\_33935\_cov\_31.019037 8969-8979. Max. coverage (+): 0.09. Max coverage (-): 0

Region: NODE\_288008\_length\_33935\_cov\_31.019037 8980-8990. Max. coverage (+): 0.09. Max coverage (-): 0

Region: NODE\_288008\_length\_33935\_cov\_31.019037 8991-9000. Max. coverage (+): 0. Max coverage (-): 0

Region: NODE\_288008\_length\_33935\_cov\_31.019037 9001-9011. Max. coverage (+): 0.68. Max coverage (-): 0.02

Region: NODE\_288008\_length\_33935\_cov\_31.019037 9012-9022. Max. coverage (+): 0.06. Max coverage (-): 0

Region: NODE\_288008\_length\_33935\_cov\_31.019037 9023-9033. Max. coverage (+): 1.16. Max coverage (-): 0.03

Region: NODE\_288008\_length\_33935\_cov\_31.019037 9034-9043. Max. coverage (+): 0.16. Max coverage (-): 0

Region: NODE\_288008\_length\_33935\_cov\_31.019037 9044-9054. Max. coverage (+): 0.09. Max coverage (-): 0

Region: NODE\_288008\_length\_33935\_cov\_31.019037 9055-9065. Max. coverage (+): 0. Max coverage (-): 0

Region: NODE\_288008\_length\_33935\_cov\_31.019037 9066-9076. Max. coverage (+): 0.56. Max coverage (-): 0

Region: NODE\_288008\_length\_33935\_cov\_31.019037 9077-9087. Max. coverage (+): 0.28. Max coverage (-): 0.09

Region: NODE\_288008\_length\_33935\_cov\_31.019037 9088-9097. Max. coverage (+): 0. Max coverage (-): 0.19

Region: NODE\_288008\_length\_33935\_cov\_31.019037 9098-9108. Max. coverage (+): 0. Max coverage (-): 0.06

Region: NODE\_288008\_length\_33935\_cov\_31.019037 9109-9119. Max. coverage (+): 0. Max coverage (-): 0

Region: NODE\_288008\_length\_33935\_cov\_31.019037 9120-9130. Max. coverage (+): 0. Max coverage (-): 0

Region: NODE\_288008\_length\_33935\_cov\_31.019037 9131-9140. Max. coverage (+): 0. Max coverage (-): 0

Region: NODE\_288008\_length\_33935\_cov\_31.019037 9141-9151. Max. coverage (+): 0. Max coverage (-): 0

Region: NODE\_288008\_length\_33935\_cov\_31.019037 9152-9162. Max. coverage (+): 0. Max coverage (-): 0

Region: NODE\_288008\_length\_33935\_cov\_31.019037 9163-9173. Max. coverage (+): 0.09. Max coverage (-): 0

Region: NODE\_288008\_length\_33935\_cov\_31.019037 9174-9183. Max. coverage (+): 0.03. Max coverage (-): 0.25

Region: NODE\_288008\_length\_33935\_cov\_31.019037 9184-9194. Max. coverage (+): 0.03. Max coverage (-): 0.16

Region: NODE\_288008\_length\_33935\_cov\_31.019037 9195-9205. Max. coverage (+): 1.63. Max coverage (-): 0

Region: NODE\_288008\_length\_33935\_cov\_31.019037 9206-9216. Max. coverage (+): 1.57. Max coverage (-): 0

Region: NODE\_288008\_length\_33935\_cov\_31.019037 9217-9227. Max. coverage (+): 0. Max coverage (-): 0

Region: NODE\_288008\_length\_33935\_cov\_31.019037 9228-9237. Max. coverage (+): 0. Max coverage (-): 0

Region: NODE\_288008\_length\_33935\_cov\_31.019037 9238-9248. Max. coverage (+): 0. Max coverage (-): 0

Region: NODE\_288008\_length\_33935\_cov\_31.019037 9249-9259. Max. coverage (+): 0. Max coverage (-): 0

Region: NODE\_288008\_length\_33935\_cov\_31.019037 9260-9270. Max. coverage (+): 0.66. Max coverage (-): 0

Region: NODE\_288008\_length\_33935\_cov\_31.019037 9271-9280. Max. coverage (+): 0. Max coverage (-): 0

Region: NODE\_288008\_length\_33935\_cov\_31.019037 9281-9291. Max. coverage (+): 0. Max coverage (-): 0

Region: NODE\_288008\_length\_33935\_cov\_31.019037 9292-9302. Max. coverage (+): 0. Max coverage (-): 0

Region: NODE\_288008\_length\_33935\_cov\_31.019037 9303-9313. Max. coverage (+): 0.28. Max coverage (-): 0

Region: NODE\_288008\_length\_33935\_cov\_31.019037 9314-9323. Max. coverage (+): 0.28. Max coverage (-): 0.03

Region: NODE\_288008\_length\_33935\_cov\_31.019037 9324-9334. Max. coverage (+): 0. Max coverage (-): 0.03

Region: NODE\_288008\_length\_33935\_cov\_31.019037 9335-9345. Max. coverage (+): 0.14. Max coverage (-): 0

Region: NODE\_288008\_length\_33935\_cov\_31.019037 9346-9356. Max. coverage (+): 0. Max coverage (-): 0

Region: NODE\_288008\_length\_33935\_cov\_31.019037 9357-9367. Max. coverage (+): 0. Max coverage (-): 0

Region: NODE\_288008\_length\_33935\_cov\_31.019037 9368-9377. Max. coverage (+): 0.19. Max coverage (-): 0

Region: NODE\_288008\_length\_33935\_cov\_31.019037 9378-9388. Max. coverage (+): 0.09. Max coverage (-): 0

Region: NODE\_288008\_length\_33935\_cov\_31.019037 9389-9399. Max. coverage (+): 0. Max coverage (-): 0.02

Region: NODE\_288008\_length\_33935\_cov\_31.019037 9400-9410. Max. coverage (+): 0.13. Max coverage (-): 0.02

Region: NODE\_288008\_length\_33935\_cov\_31.019037 9411-9420. Max. coverage (+): 0.6. Max coverage (-): 0

Region: NODE\_288008\_length\_33935\_cov\_31.019037 9421-9431. Max. coverage (+): 0.38. Max coverage (-): 0

Region: NODE\_288008\_length\_33935\_cov\_31.019037 9432-9442. Max. coverage (+): 0.19. Max coverage (-): 0.03

Region: NODE\_288008\_length\_33935\_cov\_31.019037 9443-9453. Max. coverage (+): 0.44. Max coverage (-): 0.03

Region: NODE\_288008\_length\_33935\_cov\_31.019037 9454-9463. Max. coverage (+): 0.06. Max coverage (-): 0

Region: NODE\_288008\_length\_33935\_cov\_31.019037 9464-9474. Max. coverage (+): 0.05. Max coverage (-): 0

Region: NODE\_288008\_length\_33935\_cov\_31.019037 9475-9485. Max. coverage (+): 0. Max coverage (-): 0

Region: NODE\_288008\_length\_33935\_cov\_31.019037 9486-9496. Max. coverage (+): 0.09. Max coverage (-): 0

Region: NODE\_288008\_length\_33935\_cov\_31.019037 9497-9507. Max. coverage (+): 0.09. Max coverage (-): 0

Region: NODE\_288008\_length\_33935\_cov\_31.019037 9508-9517. Max. coverage (+): 0.09. Max coverage (-): 0

Region: NODE\_288008\_length\_33935\_cov\_31.019037 9518-9528. Max. coverage (+): 0. Max coverage (-): 0

Region: NODE\_288008\_length\_33935\_cov\_31.019037 9529-9539. Max. coverage (+): 0. Max coverage (-): 0

Region: NODE\_288008\_length\_33935\_cov\_31.019037 9540-9550. Max. coverage (+): 0. Max coverage (-): 0

Region: NODE\_288008\_length\_33935\_cov\_31.019037 9551-9560. Max. coverage (+): 0.08. Max coverage (-): 0.02

Region: NODE\_288008\_length\_33935\_cov\_31.019037 9561-9571. Max. coverage (+): 0. Max coverage (-): 0

Region: NODE\_288008\_length\_33935\_cov\_31.019037 9572-9582. Max. coverage (+): 0. Max coverage (-): 0

Region: NODE\_288008\_length\_33935\_cov\_31.019037 9583-9593. Max. coverage (+): 0. Max coverage (-): 0

Region: NODE\_288008\_length\_33935\_cov\_31.019037 9594-9603. Max. coverage (+): 0. Max coverage (-): 0

Region: NODE\_288008\_length\_33935\_cov\_31.019037 9604-9614. Max. coverage (+): 10.8. Max coverage (-): 0

Region: NODE\_288008\_length\_33935\_cov\_31.019037 9615-9625. Max. coverage (+): 11.27. Max coverage (-): 0

Region: NODE\_288008\_length\_33935\_cov\_31.019037 9626-9636. Max. coverage (+): 0. Max coverage (-): 0

Region: NODE\_288008\_length\_33935\_cov\_31.019037 9637-9646. Max. coverage (+): 0. Max coverage (-): 0

Region: NODE\_288008\_length\_33935\_cov\_31.019037 9647-9657. Max. coverage (+): 0. Max coverage (-): 0

Region: NODE\_288008\_length\_33935\_cov\_31.019037 9658-9668. Max. coverage (+): 0. Max coverage (-): 0

Region: NODE\_288008\_length\_33935\_cov\_31.019037 9669-9679. Max. coverage (+): 0. Max coverage (-): 0

Region: NODE\_288008\_length\_33935\_cov\_31.019037 9680-9690. Max. coverage (+): 0. Max coverage (-): 0

Region: NODE\_288008\_length\_33935\_cov\_31.019037 9691-9700. Max. coverage (+): 0. Max coverage (-): 0

Region: NODE\_288008\_length\_33935\_cov\_31.019037 9701-9711. Max. coverage (+): 0.28. Max coverage (-): 0

Region: NODE\_288008\_length\_33935\_cov\_31.019037 9712-9722. Max. coverage (+): 0. Max coverage (-): 0

Region: NODE\_288008\_length\_33935\_cov\_31.019037 9723-9733. Max. coverage (+): 0. Max coverage (-): 0

Region: NODE\_288008\_length\_33935\_cov\_31.019037 9734-9743. Max. coverage (+): 0.12. Max coverage (-): 0

Region: NODE\_288008\_length\_33935\_cov\_31.019037 9744-9754. Max. coverage (+): 0. Max coverage (-): 0

Region: NODE\_288008\_length\_33935\_cov\_31.019037 9755-9765. Max. coverage (+): 0.19. Max coverage (-): 0

Region: NODE\_288008\_length\_33935\_cov\_31.019037 9766-9776. Max. coverage (+): 0.38. Max coverage (-): 0

Region: NODE\_288008\_length\_33935\_cov\_31.019037 9777-9786. Max. coverage (+): 0.47. Max coverage (-): 0

Region: NODE\_288008\_length\_33935\_cov\_31.019037 9787-9797. Max. coverage (+): 0. Max coverage (-): 0

Region: NODE\_288008\_length\_33935\_cov\_31.019037 9798-9808. Max. coverage (+): 0. Max coverage (-): 0.28

Region: NODE\_288008\_length\_33935\_cov\_31.019037 9809-9819. Max. coverage (+): 0. Max coverage (-): 0.31

Region: NODE\_288008\_length\_33935\_cov\_31.019037 9820-9830. Max. coverage (+): 0.38. Max coverage (-): 0

Region: NODE\_288008\_length\_33935\_cov\_31.019037 9831-9840. Max. coverage (+): 0. Max coverage (-): 0

Region: NODE\_288008\_length\_33935\_cov\_31.019037 9841-9851. Max. coverage (+): 0. Max coverage (-): 0

Region: NODE\_288008\_length\_33935\_cov\_31.019037 9852-9862. Max. coverage (+): 0. Max coverage (-): 0

Region: NODE\_288008\_length\_33935\_cov\_31.019037 9863-9873. Max. coverage (+): 0.03. Max coverage (-): 0.03

Region: NODE\_288008\_length\_33935\_cov\_31.019037 9874-9883. Max. coverage (+): 0.02. Max coverage (-): 0.03

Region: NODE\_288008\_length\_33935\_cov\_31.019037 9884-9894. Max. coverage (+): 0. Max coverage (-): 0

Region: NODE\_288008\_length\_33935\_cov\_31.019037 9895-9905. Max. coverage (+): 0.09. Max coverage (-): 0

Region: NODE\_288008\_length\_33935\_cov\_31.019037 9906-9916. Max. coverage (+): 0.09. Max coverage (-): 0

Region: NODE\_288008\_length\_33935\_cov\_31.019037 9917-9926. Max. coverage (+): 0.09. Max coverage (-): 0.12

Region: NODE\_288008\_length\_33935\_cov\_31.019037 9927-9937. Max. coverage (+): 0. Max coverage (-): 0.12

Region: NODE\_288008\_length\_33935\_cov\_31.019037 9938-9948. Max. coverage (+): 5.54. Max coverage (-): 0

Region: NODE\_288008\_length\_33935\_cov\_31.019037 9949-9959. Max. coverage (+): 0.06. Max coverage (-): 0

Region: NODE\_288008\_length\_33935\_cov\_31.019037 9960-9970. Max. coverage (+): 0.05. Max coverage (-): 0.05

Region: NODE\_288008\_length\_33935\_cov\_31.019037 9971-9980. Max. coverage (+): 0.74. Max coverage (-): 0.23

Region: NODE\_288008\_length\_33935\_cov\_31.019037 9981-9991. Max. coverage (+): 82.94. Max coverage (-): 0

Region: NODE\_288008\_length\_33935\_cov\_31.019037 9992-10002. Max. coverage (+): 70.88. Max coverage (-): 0.05

Region: NODE\_288008\_length\_33935\_cov\_31.019037 10003-10013. Max. coverage (+): 0. Max coverage (-): 0

Region: NODE\_288008\_length\_33935\_cov\_31.019037 10014-10023. Max. coverage (+): 0. Max coverage (-): 0

Region: NODE\_288008\_length\_33935\_cov\_31.019037 10024-10034. Max. coverage (+): 0. Max coverage (-): 0

Region: NODE\_288008\_length\_33935\_cov\_31.019037 10035-10045. Max. coverage (+): 0.94. Max coverage (-): 0

Region: NODE\_288008\_length\_33935\_cov\_31.019037 10046-10056. Max. coverage (+): 0.94. Max coverage (-): 0

Region: NODE\_288008\_length\_33935\_cov\_31.019037 10057-10066. Max. coverage (+): 0.09. Max coverage (-): 0

Region: NODE\_288008\_length\_33935\_cov\_31.019037 10067-10077. Max. coverage (+): 0.09. Max coverage (-): 0

Region: NODE\_288008\_length\_33935\_cov\_31.019037 10078-10088. Max. coverage (+): 0. Max coverage (-): 0

Region: NODE\_288008\_length\_33935\_cov\_31.019037 10089-10099. Max. coverage (+): 0. Max coverage (-): 0

Region: NODE\_288008\_length\_33935\_cov\_31.019037 10100-10110. Max. coverage (+): 0. Max coverage (-): 0

Region: NODE\_288008\_length\_33935\_cov\_31.019037 10111-10120. Max. coverage (+): 0. Max coverage (-): 0

Region: NODE\_288008\_length\_33935\_cov\_31.019037 10121-10131. Max. coverage (+): 0. Max coverage (-): 0

Region: NODE\_288008\_length\_33935\_cov\_31.019037 10132-10142. Max. coverage (+): 0.31. Max coverage (-): 0

Region: NODE\_288008\_length\_33935\_cov\_31.019037 10143-10153. Max. coverage (+): 0.16. Max coverage (-): 0

Region: NODE\_288008\_length\_33935\_cov\_31.019037 10154-10163. Max. coverage (+): 0. Max coverage (-): 0

Region: NODE\_288008\_length\_33935\_cov\_31.019037 10164-10174. Max. coverage (+): 0.05. Max coverage (-): 0

Region: NODE\_288008\_length\_33935\_cov\_31.019037 10175-10185. Max. coverage (+): 18.08. Max coverage (-): 0.02

Region: NODE\_288008\_length\_33935\_cov\_31.019037 10186-10196. Max. coverage (+): 0.32. Max coverage (-): 0.12

Region: NODE\_288008\_length\_33935\_cov\_31.019037 10197-10206. Max. coverage (+): 0.81. Max coverage (-): 0.15

Region: NODE\_288008\_length\_33935\_cov\_31.019037 10207-10217. Max. coverage (+): 0.66. Max coverage (-): 0.17

Region: NODE\_288008\_length\_33935\_cov\_31.019037 10218-10228. Max. coverage (+): 3.15. Max coverage (-): 0.09

Region: NODE\_288008\_length\_33935\_cov\_31.019037 10229-10239. Max. coverage (+): 0.28. Max coverage (-): 0.09

Region: NODE\_288008\_length\_33935\_cov\_31.019037 10240-10250. Max. coverage (+): 0.12. Max coverage (-): 0.05

Region: NODE\_288008\_length\_33935\_cov\_31.019037 10251-10260. Max. coverage (+): 1.59. Max coverage (-): 0.05

Region: NODE\_288008\_length\_33935\_cov\_31.019037 10261-10271. Max. coverage (+): 0. Max coverage (-): 0

Region: NODE\_288008\_length\_33935\_cov\_31.019037 10272-10282. Max. coverage (+): 0. Max coverage (-): 0

Region: NODE\_288008\_length\_33935\_cov\_31.019037 10283-10293. Max. coverage (+): 0.08. Max coverage (-): 0

Region: NODE\_288008\_length\_33935\_cov\_31.019037 10294-10303. Max. coverage (+): 0.54. Max coverage (-): 0

Region: NODE\_288008\_length\_33935\_cov\_31.019037 10304-10314. Max. coverage (+): 3.57. Max coverage (-): 0.02

Region: NODE\_288008\_length\_33935\_cov\_31.019037 10315-10325. Max. coverage (+): 0. Max coverage (-): 0

Region: NODE\_288008\_length\_33935\_cov\_31.019037 10326-10336. Max. coverage (+): 0. Max coverage (-): 0

Region: NODE\_288008\_length\_33935\_cov\_31.019037 10337-10346. Max. coverage (+): 0.12. Max coverage (-): 0.09

Region: NODE\_288008\_length\_33935\_cov\_31.019037 10347-10357. Max. coverage (+): 0. Max coverage (-): 0

Region: NODE\_288008\_length\_33935\_cov\_31.019037 10358-10368. Max. coverage (+): 0. Max coverage (-): 0

Region: NODE\_288008\_length\_33935\_cov\_31.019037 10369-10379. Max. coverage (+): 0. Max coverage (-): 0

Region: NODE\_288008\_length\_33935\_cov\_31.019037 10380-10389. Max. coverage (+): 0.09. Max coverage (-): 0

Region: NODE\_288008\_length\_33935\_cov\_31.019037 10390-10400. Max. coverage (+): 0.09. Max coverage (-): 0

Region: NODE\_288008\_length\_33935\_cov\_31.019037 10401-10411. Max. coverage (+): 0. Max coverage (-): 0

Region: NODE\_288008\_length\_33935\_cov\_31.019037 10412-10422. Max. coverage (+): 0. Max coverage (-): 0.42

Region: NODE\_288008\_length\_33935\_cov\_31.019037 10423-10433. Max. coverage (+): 0.38. Max coverage (-): 0.03

Region: NODE\_288008\_length\_33935\_cov\_31.019037 10434-10443. Max. coverage (+): 0.66. Max coverage (-): 0

Region: NODE\_288008\_length\_33935\_cov\_31.019037 10444-10454. Max. coverage (+): 0. Max coverage (-): 0

Region: NODE\_288008\_length\_33935\_cov\_31.019037 10455-10465. Max. coverage (+): 0. Max coverage (-): 0

Region: NODE\_288008\_length\_33935\_cov\_31.019037 10466-10476. Max. coverage (+): 0.47. Max coverage (-): 0.06

Region: NODE\_288008\_length\_33935\_cov\_31.019037 10477-10486. Max. coverage (+): 0.08. Max coverage (-): 0

Region: NODE\_288008\_length\_33935\_cov\_31.019037 10487-10497. Max. coverage (+): 0.01. Max coverage (-): 0

Region: NODE\_288008\_length\_33935\_cov\_31.019037 10498-10508. Max. coverage (+): 0.01. Max coverage (-): 0

Region: NODE\_288008\_length\_33935\_cov\_31.019037 10509-10519. Max. coverage (+): 0. Max coverage (-): 0

Region: NODE\_288008\_length\_33935\_cov\_31.019037 10520-10529. Max. coverage (+): 0.99. Max coverage (-): 0

Region: NODE\_288008\_length\_33935\_cov\_31.019037 10530-10540. Max. coverage (+): 0.99. Max coverage (-): 0

Region: NODE\_288008\_length\_33935\_cov\_31.019037 10541-10551. Max. coverage (+): 0. Max coverage (-): 0

Region: NODE\_288008\_length\_33935\_cov\_31.019037 10552-10562. Max. coverage (+): 0. Max coverage (-): 0

Region: NODE\_288008\_length\_33935\_cov\_31.019037 10563-10573. Max. coverage (+): 0. Max coverage (-): 0

Region: NODE\_288008\_length\_33935\_cov\_31.019037 10574-10583. Max. coverage (+): 0. Max coverage (-): 0

Region: NODE\_288008\_length\_33935\_cov\_31.019037 10584-10594. Max. coverage (+): 0.02. Max coverage (-): 0

Region: NODE\_288008\_length\_33935\_cov\_31.019037 10595-10605. Max. coverage (+): 0.02. Max coverage (-): 0.01

Region: NODE\_288008\_length\_33935\_cov\_31.019037 10606-10616. Max. coverage (+): 0.72. Max coverage (-): 0.05

Region: NODE\_288008\_length\_33935\_cov\_31.019037 10617-10626. Max. coverage (+): 0. Max coverage (-): 0.02

Region: NODE\_288008\_length\_33935\_cov\_31.019037 10627-10637. Max. coverage (+): 0. Max coverage (-): 0

Region: NODE\_288008\_length\_33935\_cov\_31.019037 10638-10648. Max. coverage (+): 0. Max coverage (-): 0

Region: NODE\_288008\_length\_33935\_cov\_31.019037 10649-10659. Max. coverage (+): 0. Max coverage (-): 0

Region: NODE\_288008\_length\_33935\_cov\_31.019037 10660-10669. Max. coverage (+): 0. Max coverage (-): 0

Region: NODE\_288008\_length\_33935\_cov\_31.019037 10670-10680. Max. coverage (+): 0. Max coverage (-): 0

Region: NODE\_288008\_length\_33935\_cov\_31.019037 10681-10691. Max. coverage (+): 0.03. Max coverage (-): 0

Region: NODE\_288008\_length\_33935\_cov\_31.019037 10692-10702. Max. coverage (+): 0.71. Max coverage (-): 0

Region: NODE\_288008\_length\_33935\_cov\_31.019037 10703-10713. Max. coverage (+): 0.71. Max coverage (-): 0

Region: NODE\_288008\_length\_33935\_cov\_31.019037 10714-10723. Max. coverage (+): 0. Max coverage (-): 0

Region: NODE\_288008\_length\_33935\_cov\_31.019037 10724-10734. Max. coverage (+): 1.55. Max coverage (-): 0

Region: NODE\_288008\_length\_33935\_cov\_31.019037 10735-10745. Max. coverage (+): 1.68. Max coverage (-): 0.03

Region: NODE\_288008\_length\_33935\_cov\_31.019037 10746-10756. Max. coverage (+): 0.03. Max coverage (-): 0

Region: NODE\_288008\_length\_33935\_cov\_31.019037 10757-10766. Max. coverage (+): 0.2. Max coverage (-): 0

Region: NODE\_288008\_length\_33935\_cov\_31.019037 10767-10777. Max. coverage (+): 0.2. Max coverage (-): 0

Region: NODE\_288008\_length\_33935\_cov\_31.019037 10778-10788. Max. coverage (+): 0. Max coverage (-): 0

Region: NODE\_288008\_length\_33935\_cov\_31.019037 10789-10799. Max. coverage (+): 0. Max coverage (-): 0

Region: NODE\_288008\_length\_33935\_cov\_31.019037 10800-10809. Max. coverage (+): 0. Max coverage (-): 0

Region: NODE\_288008\_length\_33935\_cov\_31.019037 10810-10820. Max. coverage (+): 0. Max coverage (-): 0

Region: NODE\_288008\_length\_33935\_cov\_31.019037 10821-10831. Max. coverage (+): 0.08. Max coverage (-): 0

Region: NODE\_288008\_length\_33935\_cov\_31.019037 10832-10842. Max. coverage (+): 0.09. Max coverage (-): 0

Region: NODE\_288008\_length\_33935\_cov\_31.019037 10843-10853. Max. coverage (+): 0. Max coverage (-): 0

Region: NODE\_288008\_length\_33935\_cov\_31.019037 10854-10863. Max. coverage (+): 0.23. Max coverage (-): 0.03

Region: NODE\_288008\_length\_33935\_cov\_31.019037 10864-10874. Max. coverage (+): 0.22. Max coverage (-): 0.03

Region: NODE\_288008\_length\_33935\_cov\_31.019037 10875-10885. Max. coverage (+): 0. Max coverage (-): 0

Region: NODE\_288008\_length\_33935\_cov\_31.019037 10886-10896. Max. coverage (+): 0. Max coverage (-): 0

Region: NODE\_288008\_length\_33935\_cov\_31.019037 10897-10906. Max. coverage (+): 0. Max coverage (-): 0

Region: NODE\_288008\_length\_33935\_cov\_31.019037 10907-10917. Max. coverage (+): 0. Max coverage (-): 0

Region: NODE\_288008\_length\_33935\_cov\_31.019037 10918-10928. Max. coverage (+): 0. Max coverage (-): 0.02

Region: NODE\_288008\_length\_33935\_cov\_31.019037 10929-10939. Max. coverage (+): 0.11. Max coverage (-): 0.01

Region: NODE\_288008\_length\_33935\_cov\_31.019037 10940-10949. Max. coverage (+): 0.09. Max coverage (-): 0

Region: NODE\_288008\_length\_33935\_cov\_31.019037 10950-10960. Max. coverage (+): 1.03. Max coverage (-): 0

Region: NODE\_288008\_length\_33935\_cov\_31.019037 10961-10971. Max. coverage (+): 0. Max coverage (-): 0

Region: NODE\_288008\_length\_33935\_cov\_31.019037 10972-10982. Max. coverage (+): 0.09. Max coverage (-): 0

Region: NODE\_288008\_length\_33935\_cov\_31.019037 10983-10992. Max. coverage (+): 0.38. Max coverage (-): 0

Region: NODE\_288008\_length\_33935\_cov\_31.019037 10993-11003. Max. coverage (+): 0.38. Max coverage (-): 0

Region: NODE\_288008\_length\_33935\_cov\_31.019037 11004-11014. Max. coverage (+): 0. Max coverage (-): 0.31

Region: NODE\_288008\_length\_33935\_cov\_31.019037 11015-11025. Max. coverage (+): 0.14. Max coverage (-): 0.06

Region: NODE\_288008\_length\_33935\_cov\_31.019037 11026-11036. Max. coverage (+): 0.14. Max coverage (-): 0

Region: NODE\_288008\_length\_33935\_cov\_31.019037 11037-11046. Max. coverage (+): 0.02. Max coverage (-): 0.02

Region: NODE\_288008\_length\_33935\_cov\_31.019037 11047-11057. Max. coverage (+): 0.05. Max coverage (-): 0.06

Region: NODE\_288008\_length\_33935\_cov\_31.019037 11058-11068. Max. coverage (+): 0. Max coverage (-): 0

Region: NODE\_288008\_length\_33935\_cov\_31.019037 11069-11079. Max. coverage (+): 0. Max coverage (-): 0

Region: NODE\_288008\_length\_33935\_cov\_31.019037 11080-11089. Max. coverage (+): 0.02. Max coverage (-): 0.08

Region: NODE\_288008\_length\_33935\_cov\_31.019037 11090-11100. Max. coverage (+): 0.02. Max coverage (-): 0

Region: NODE\_288008\_length\_33935\_cov\_31.019037 11101-11111. Max. coverage (+): 0. Max coverage (-): 0

Region: NODE\_288008\_length\_33935\_cov\_31.019037 11112-11122. Max. coverage (+): 0. Max coverage (-): 0

Region: NODE\_288008\_length\_33935\_cov\_31.019037 11123-11132. Max. coverage (+): 0. Max coverage (-): 0

Region: NODE\_288008\_length\_33935\_cov\_31.019037 11133-11143. Max. coverage (+): 0. Max coverage (-): 0

Region: NODE\_288008\_length\_33935\_cov\_31.019037 11144-11154. Max. coverage (+): 0. Max coverage (-): 0

Region: NODE\_288008\_length\_33935\_cov\_31.019037 11155-11165. Max. coverage (+): 0.16. Max coverage (-): 0

Region: NODE\_288008\_length\_33935\_cov\_31.019037 11166-11176. Max. coverage (+): 0.63. Max coverage (-): 0

Region: NODE\_288008\_length\_33935\_cov\_31.019037 11177-11186. Max. coverage (+): 0.6. Max coverage (-): 0

Region: NODE\_288008\_length\_33935\_cov\_31.019037 11187-11197. Max. coverage (+): 0. Max coverage (-): 0

Region: NODE\_288008\_length\_33935\_cov\_31.019037 11198-11208. Max. coverage (+): 0. Max coverage (-): 0

Region: NODE\_288008\_length\_33935\_cov\_31.019037 11209-11219. Max. coverage (+): 0. Max coverage (-): 0

Region: NODE\_288008\_length\_33935\_cov\_31.019037 11220-11229. Max. coverage (+): 0. Max coverage (-): 0

Region: NODE\_288008\_length\_33935\_cov\_31.019037 11230-11240. Max. coverage (+): 0.01. Max coverage (-): 0

Region: NODE\_288008\_length\_33935\_cov\_31.019037 11241-11251. Max. coverage (+): 0.01. Max coverage (-): 0

Region: NODE\_288008\_length\_33935\_cov\_31.019037 11252-11262. Max. coverage (+): 0.47. Max coverage (-): 0

Region: NODE\_288008\_length\_33935\_cov\_31.019037 11263-11272. Max. coverage (+): 0. Max coverage (-): 0

Region: NODE\_288008\_length\_33935\_cov\_31.019037 11273-11283. Max. coverage (+): 0. Max coverage (-): 0

Region: NODE\_288008\_length\_33935\_cov\_31.019037 11284-11294. Max. coverage (+): 0. Max coverage (-): 0

Region: NODE\_288008\_length\_33935\_cov\_31.019037 11295-11305. Max. coverage (+): 0. Max coverage (-): 0

Region: NODE\_288008\_length\_33935\_cov\_31.019037 11306-11316. Max. coverage (+): 0. Max coverage (-): 0

Region: NODE\_288008\_length\_33935\_cov\_31.019037 11317-11326. Max. coverage (+): 0. Max coverage (-): 0

Region: NODE\_288008\_length\_33935\_cov\_31.019037 11327-11337. Max. coverage (+): 0. Max coverage (-): 0

Region: NODE\_288008\_length\_33935\_cov\_31.019037 11338-11348. Max. coverage (+): 0. Max coverage (-): 0

Region: NODE\_288008\_length\_33935\_cov\_31.019037 11349-11359. Max. coverage (+): 0. Max coverage (-): 0

Region: NODE\_288008\_length\_33935\_cov\_31.019037 11360-11369. Max. coverage (+): 0. Max coverage (-): 0

Region: NODE\_288008\_length\_33935\_cov\_31.019037 11370-11380. Max. coverage (+): 0. Max coverage (-): 0

Region: NODE\_288008\_length\_33935\_cov\_31.019037 11381-11391. Max. coverage (+): 0. Max coverage (-): 0

Region: NODE\_288008\_length\_33935\_cov\_31.019037 11392-11402. Max. coverage (+): 0. Max coverage (-): 0

Region: NODE\_288008\_length\_33935\_cov\_31.019037 11403-11412. Max. coverage (+): 0. Max coverage (-): 0

Region: NODE\_288008\_length\_33935\_cov\_31.019037 11413-11423. Max. coverage (+): 0. Max coverage (-): 0

Region: NODE\_288008\_length\_33935\_cov\_31.019037 11424-11434. Max. coverage (+): 0. Max coverage (-): 0

Region: NODE\_288008\_length\_33935\_cov\_31.019037 11435-11445. Max. coverage (+): 0. Max coverage (-): 0

Region: NODE\_288008\_length\_33935\_cov\_31.019037 11446-11456. Max. coverage (+): 0. Max coverage (-): 0

Region: NODE\_288008\_length\_33935\_cov\_31.019037 11457-11466. Max. coverage (+): 0. Max coverage (-): 0

Region: NODE\_288008\_length\_33935\_cov\_31.019037 11467-11477. Max. coverage (+): 0. Max coverage (-): 0

Region: NODE\_288008\_length\_33935\_cov\_31.019037 11478-11488. Max. coverage (+): 0. Max coverage (-): 0

Region: NODE\_288008\_length\_33935\_cov\_31.019037 11489-11499. Max. coverage (+): 0. Max coverage (-): 0

Region: NODE\_288008\_length\_33935\_cov\_31.019037 11500-11509. Max. coverage (+): 0. Max coverage (-): 0

Region: NODE\_288008\_length\_33935\_cov\_31.019037 11510-11520. Max. coverage (+): 0. Max coverage (-): 0

Region: NODE\_288008\_length\_33935\_cov\_31.019037 11521-11531. Max. coverage (+): 0. Max coverage (-): 0

Region: NODE\_288008\_length\_33935\_cov\_31.019037 11532-11542. Max. coverage (+): 0. Max coverage (-): 0

Region: NODE\_288008\_length\_33935\_cov\_31.019037 11543-11552. Max. coverage (+): 0. Max coverage (-): 0

Region: NODE\_288008\_length\_33935\_cov\_31.019037 11553-11563. Max. coverage (+): 0. Max coverage (-): 0

Region: NODE\_288008\_length\_33935\_cov\_31.019037 11564-11574. Max. coverage (+): 0. Max coverage (-): 0

Region: NODE\_288008\_length\_33935\_cov\_31.019037 11575-11585. Max. coverage (+): 0. Max coverage (-): 0

Region: NODE\_288008\_length\_33935\_cov\_31.019037 11586-11596. Max. coverage (+): 0. Max coverage (-): 0

Region: NODE\_288008\_length\_33935\_cov\_31.019037 11597-11606. Max. coverage (+): 0. Max coverage (-): 0

Region: NODE\_288008\_length\_33935\_cov\_31.019037 11607-11617. Max. coverage (+): 0. Max coverage (-): 0

Region: NODE\_288008\_length\_33935\_cov\_31.019037 11618-11628. Max. coverage (+): 0. Max coverage (-): 0

Region: NODE\_288008\_length\_33935\_cov\_31.019037 11629-11639. Max. coverage (+): 0. Max coverage (-): 0

Region: NODE\_288008\_length\_33935\_cov\_31.019037 11640-11649. Max. coverage (+): 0. Max coverage (-): 0

Region: NODE\_288008\_length\_33935\_cov\_31.019037 11650-11660. Max. coverage (+): 0. Max coverage (-): 0

Region: NODE\_288008\_length\_33935\_cov\_31.019037 11661-11671. Max. coverage (+): 0. Max coverage (-): 0

Region: NODE\_288008\_length\_33935\_cov\_31.019037 11672-11682. Max. coverage (+): 0. Max coverage (-): 0

Region: NODE\_288008\_length\_33935\_cov\_31.019037 11683-11692. Max. coverage (+): 0. Max coverage (-): 0

Region: NODE\_288008\_length\_33935\_cov\_31.019037 11693-11703. Max. coverage (+): 0. Max coverage (-): 0

Region: NODE\_288008\_length\_33935\_cov\_31.019037 11704-11714. Max. coverage (+): 0. Max coverage (-): 0

Region: NODE\_288008\_length\_33935\_cov\_31.019037 11715-11725. Max. coverage (+): 0. Max coverage (-): 0

Region: NODE\_288008\_length\_33935\_cov\_31.019037 11726-11735. Max. coverage (+): 0. Max coverage (-): 0

Region: NODE\_288008\_length\_33935\_cov\_31.019037 11736-11746. Max. coverage (+): 0. Max coverage (-): 0

Region: NODE\_288008\_length\_33935\_cov\_31.019037 11747-11757. Max. coverage (+): 0. Max coverage (-): 0

Region: NODE\_288008\_length\_33935\_cov\_31.019037 11758-11768. Max. coverage (+): 0. Max coverage (-): 0

Region: NODE\_288008\_length\_33935\_cov\_31.019037 11769-11779. Max. coverage (+): 0. Max coverage (-): 0

Region: NODE\_288008\_length\_33935\_cov\_31.019037 11780-11789. Max. coverage (+): 0. Max coverage (-): 0

Region: NODE\_288008\_length\_33935\_cov\_31.019037 11790-11800. Max. coverage (+): 0. Max coverage (-): 0

Region: NODE\_288008\_length\_33935\_cov\_31.019037 11801-11811. Max. coverage (+): 0. Max coverage (-): 0

Region: NODE\_288008\_length\_33935\_cov\_31.019037 11812-11822. Max. coverage (+): 0. Max coverage (-): 0

Region: NODE\_288008\_length\_33935\_cov\_31.019037 11823-11832. Max. coverage (+): 0. Max coverage (-): 0

Region: NODE\_288008\_length\_33935\_cov\_31.019037 11833-11843. Max. coverage (+): 0. Max coverage (-): 0

Region: NODE\_288008\_length\_33935\_cov\_31.019037 11844-11854. Max. coverage (+): 0. Max coverage (-): 0

Region: NODE\_288008\_length\_33935\_cov\_31.019037 11855-11865. Max. coverage (+): 0. Max coverage (-): 0

Region: NODE\_288008\_length\_33935\_cov\_31.019037 11866-11875. Max. coverage (+): 0. Max coverage (-): 0

Region: NODE\_288008\_length\_33935\_cov\_31.019037 11876-11886. Max. coverage (+): 0. Max coverage (-): 0

Region: NODE\_288008\_length\_33935\_cov\_31.019037 11887-11897. Max. coverage (+): 0. Max coverage (-): 0

Region: NODE\_288008\_length\_33935\_cov\_31.019037 11898-11908. Max. coverage (+): 0. Max coverage (-): 0

Region: NODE\_288008\_length\_33935\_cov\_31.019037 11909-11919. Max. coverage (+): 0. Max coverage (-): 0

Region: NODE\_288008\_length\_33935\_cov\_31.019037 11920-11929. Max. coverage (+): 0. Max coverage (-): 0

Region: NODE\_288008\_length\_33935\_cov\_31.019037 11930-11940. Max. coverage (+): 0. Max coverage (-): 0

Region: NODE\_288008\_length\_33935\_cov\_31.019037 11941-11951. Max. coverage (+): 0. Max coverage (-): 0

Region: NODE\_288008\_length\_33935\_cov\_31.019037 11952-11962. Max. coverage (+): 0. Max coverage (-): 0

Region: NODE\_288008\_length\_33935\_cov\_31.019037 11963-11972. Max. coverage (+): 0. Max coverage (-): 0

Region: NODE\_288008\_length\_33935\_cov\_31.019037 11973-11983. Max. coverage (+): 0. Max coverage (-): 0

Region: NODE\_288008\_length\_33935\_cov\_31.019037 11984-11994. Max. coverage (+): 0. Max coverage (-): 0

Region: NODE\_288008\_length\_33935\_cov\_31.019037 11995-12005. Max. coverage (+): 0. Max coverage (-): 0

Region: NODE\_288008\_length\_33935\_cov\_31.019037 12006-12015. Max. coverage (+): 0. Max coverage (-): 0

Region: NODE\_288008\_length\_33935\_cov\_31.019037 12016-12026. Max. coverage (+): 0. Max coverage (-): 0

Region: NODE\_288008\_length\_33935\_cov\_31.019037 12027-12037. Max. coverage (+): 0. Max coverage (-): 0

Region: NODE\_288008\_length\_33935\_cov\_31.019037 12038-12048. Max. coverage (+): 0. Max coverage (-): 0

Region: NODE\_288008\_length\_33935\_cov\_31.019037 12049-12059. Max. coverage (+): 0. Max coverage (-): 0

Region: NODE\_288008\_length\_33935\_cov\_31.019037 12060-12069. Max. coverage (+): 0. Max coverage (-): 0

Region: NODE\_288008\_length\_33935\_cov\_31.019037 12070-12080. Max. coverage (+): 0. Max coverage (-): 0

Region: NODE\_288008\_length\_33935\_cov\_31.019037 12081-12091. Max. coverage (+): 0. Max coverage (-): 0

Region: NODE\_288008\_length\_33935\_cov\_31.019037 12092-12102. Max. coverage (+): 0. Max coverage (-): 0

Region: NODE\_288008\_length\_33935\_cov\_31.019037 12103-12112. Max. coverage (+): 0. Max coverage (-): 0

Region: NODE\_288008\_length\_33935\_cov\_31.019037 12113-12123. Max. coverage (+): 0. Max coverage (-): 0

Region: NODE\_288008\_length\_33935\_cov\_31.019037 12124-12134. Max. coverage (+): 0. Max coverage (-): 0

Region: NODE\_288008\_length\_33935\_cov\_31.019037 12135-12145. Max. coverage (+): 0. Max coverage (-): 0

Region: NODE\_288008\_length\_33935\_cov\_31.019037 12146-12155. Max. coverage (+): 0. Max coverage (-): 0

Region: NODE\_288008\_length\_33935\_cov\_31.019037 12156-12166. Max. coverage (+): 0. Max coverage (-): 0

Region: NODE\_288008\_length\_33935\_cov\_31.019037 12167-12177. Max. coverage (+): 0. Max coverage (-): 0

Region: NODE\_288008\_length\_33935\_cov\_31.019037 12178-12188. Max. coverage (+): 0. Max coverage (-): 0

Region: NODE\_288008\_length\_33935\_cov\_31.019037 12189-12199. Max. coverage (+): 0.09. Max coverage (-): 0

Region: NODE\_288008\_length\_33935\_cov\_31.019037 12200-12209. Max. coverage (+): 0. Max coverage (-): 0

Region: NODE\_288008\_length\_33935\_cov\_31.019037 12210-12220. Max. coverage (+): 0. Max coverage (-): 0

Region: NODE\_288008\_length\_33935\_cov\_31.019037 12221-12231. Max. coverage (+): 0. Max coverage (-): 0

Region: NODE\_288008\_length\_33935\_cov\_31.019037 12232-12242. Max. coverage (+): 0. Max coverage (-): 0

Region: NODE\_288008\_length\_33935\_cov\_31.019037 12243-12252. Max. coverage (+): 0. Max coverage (-): 0

Region: NODE\_288008\_length\_33935\_cov\_31.019037 12253-12263. Max. coverage (+): 0. Max coverage (-): 0

Region: NODE\_288008\_length\_33935\_cov\_31.019037 12264-12274. Max. coverage (+): 0. Max coverage (-): 0

Region: NODE\_288008\_length\_33935\_cov\_31.019037 12275-12285. Max. coverage (+): 0. Max coverage (-): 0

Region: NODE\_288008\_length\_33935\_cov\_31.019037 12286-12295. Max. coverage (+): 0. Max coverage (-): 0.03

Region: NODE\_288008\_length\_33935\_cov\_31.019037 12296-12306. Max. coverage (+): 0. Max coverage (-): 0.04

Region: NODE\_288008\_length\_33935\_cov\_31.019037 12307-12317. Max. coverage (+): 0. Max coverage (-): 0

Region: NODE\_288008\_length\_33935\_cov\_31.019037 12318-12328. Max. coverage (+): 0. Max coverage (-): 0

Region: NODE\_288008\_length\_33935\_cov\_31.019037 12329-12338. Max. coverage (+): 0. Max coverage (-): 0

Region: NODE\_288008\_length\_33935\_cov\_31.019037 12339-12349. Max. coverage (+): 0. Max coverage (-): 0

Region: NODE\_288008\_length\_33935\_cov\_31.019037 12350-12360. Max. coverage (+): 0. Max coverage (-): 0

Region: NODE\_288008\_length\_33935\_cov\_31.019037 12361-12371. Max. coverage (+): 0. Max coverage (-): 0

Region: NODE\_288008\_length\_33935\_cov\_31.019037 12372-12382. Max. coverage (+): 0. Max coverage (-): 0

Region: NODE\_288008\_length\_33935\_cov\_31.019037 12383-12392. Max. coverage (+): 0. Max coverage (-): 0

Region: NODE\_288008\_length\_33935\_cov\_31.019037 12393-12403. Max. coverage (+): 0. Max coverage (-): 0

Region: NODE\_288008\_length\_33935\_cov\_31.019037 12404-12414. Max. coverage (+): 0. Max coverage (-): 0

Region: NODE\_288008\_length\_33935\_cov\_31.019037 12415-12425. Max. coverage (+): 0. Max coverage (-): 0

Region: NODE\_288008\_length\_33935\_cov\_31.019037 12426-12435. Max. coverage (+): 0. Max coverage (-): 0

Region: NODE\_288008\_length\_33935\_cov\_31.019037 12436-12446. Max. coverage (+): 0. Max coverage (-): 0

Region: NODE\_288008\_length\_33935\_cov\_31.019037 12447-12457. Max. coverage (+): 0. Max coverage (-): 0

Region: NODE\_288008\_length\_33935\_cov\_31.019037 12458-12468. Max. coverage (+): 0. Max coverage (-): 0

Region: NODE\_288008\_length\_33935\_cov\_31.019037 12469-12478. Max. coverage (+): 0. Max coverage (-): 0

Region: NODE\_288008\_length\_33935\_cov\_31.019037 12479-12489. Max. coverage (+): 0. Max coverage (-): 0

Region: NODE\_288008\_length\_33935\_cov\_31.019037 12490-12500. Max. coverage (+): 0. Max coverage (-): 0

Region: NODE\_288008\_length\_33935\_cov\_31.019037 12501-12511. Max. coverage (+): 0. Max coverage (-): 0

Region: NODE\_288008\_length\_33935\_cov\_31.019037 12512-12522. Max. coverage (+): 0. Max coverage (-): 0

Region: NODE\_288008\_length\_33935\_cov\_31.019037 12523-12532. Max. coverage (+): 0. Max coverage (-): 0

Region: NODE\_288008\_length\_33935\_cov\_31.019037 12533-12543. Max. coverage (+): 0. Max coverage (-): 0

Region: NODE\_288008\_length\_33935\_cov\_31.019037 12544-12554. Max. coverage (+): 0. Max coverage (-): 0

Region: NODE\_288008\_length\_33935\_cov\_31.019037 12555-12565. Max. coverage (+): 0. Max coverage (-): 0

Region: NODE\_288008\_length\_33935\_cov\_31.019037 12566-12575. Max. coverage (+): 0. Max coverage (-): 0

Region: NODE\_288008\_length\_33935\_cov\_31.019037 12576-12586. Max. coverage (+): 0. Max coverage (-): 0

Region: NODE\_288008\_length\_33935\_cov\_31.019037 12587-12597. Max. coverage (+): 0. Max coverage (-): 0

Region: NODE\_288008\_length\_33935\_cov\_31.019037 12598-12608. Max. coverage (+): 0. Max coverage (-): 0

Region: NODE\_288008\_length\_33935\_cov\_31.019037 12609-12618. Max. coverage (+): 0. Max coverage (-): 0

Region: NODE\_288008\_length\_33935\_cov\_31.019037 12619-12629. Max. coverage (+): 0. Max coverage (-): 0

Region: NODE\_288008\_length\_33935\_cov\_31.019037 12630-12640. Max. coverage (+): 0. Max coverage (-): 0

Region: NODE\_288008\_length\_33935\_cov\_31.019037 12641-12651. Max. coverage (+): 0. Max coverage (-): 0

Region: NODE\_288008\_length\_33935\_cov\_31.019037 12652-12662. Max. coverage (+): 0. Max coverage (-): 0

Region: NODE\_288008\_length\_33935\_cov\_31.019037 12663-12672. Max. coverage (+): 0. Max coverage (-): 0

Region: NODE\_288008\_length\_33935\_cov\_31.019037 12673-12683. Max. coverage (+): 0. Max coverage (-): 0

Region: NODE\_288008\_length\_33935\_cov\_31.019037 12684-12694. Max. coverage (+): 0. Max coverage (-): 0

Region: NODE\_288008\_length\_33935\_cov\_31.019037 12695-12705. Max. coverage (+): 0. Max coverage (-): 0

Region: NODE\_288008\_length\_33935\_cov\_31.019037 12706-12715. Max. coverage (+): 0. Max coverage (-): 0

Region: NODE\_288008\_length\_33935\_cov\_31.019037 12716-12726. Max. coverage (+): 0. Max coverage (-): 0

Region: NODE\_288008\_length\_33935\_cov\_31.019037 12727-12737. Max. coverage (+): 0. Max coverage (-): 0

Region: NODE\_288008\_length\_33935\_cov\_31.019037 12738-12748. Max. coverage (+): 0. Max coverage (-): 0

Region: NODE\_288008\_length\_33935\_cov\_31.019037 12749-12758. Max. coverage (+): 0. Max coverage (-): 0

Region: NODE\_288008\_length\_33935\_cov\_31.019037 12759-12769. Max. coverage (+): 0. Max coverage (-): 0

Region: NODE\_288008\_length\_33935\_cov\_31.019037 12770-12780. Max. coverage (+): 0. Max coverage (-): 0

Region: NODE\_288008\_length\_33935\_cov\_31.019037 12781-12791. Max. coverage (+): 0. Max coverage (-): 0

Region: NODE\_288008\_length\_33935\_cov\_31.019037 12792-12802. Max. coverage (+): 0. Max coverage (-): 0

Region: NODE\_288008\_length\_33935\_cov\_31.019037 12803-12812. Max. coverage (+): 0. Max coverage (-): 0

Region: NODE\_288008\_length\_33935\_cov\_31.019037 12813-12823. Max. coverage (+): 0. Max coverage (-): 0

Region: NODE\_288008\_length\_33935\_cov\_31.019037 12824-12834. Max. coverage (+): 0. Max coverage (-): 0

Region: NODE\_288008\_length\_33935\_cov\_31.019037 12835-12845. Max. coverage (+): 0. Max coverage (-): 0

Region: NODE\_288008\_length\_33935\_cov\_31.019037 12846-12855. Max. coverage (+): 0. Max coverage (-): 0

Region: NODE\_288008\_length\_33935\_cov\_31.019037 12856-12866. Max. coverage (+): 0. Max coverage (-): 0

Region: NODE\_288008\_length\_33935\_cov\_31.019037 12867-12877. Max. coverage (+): 0. Max coverage (-): 0

Region: NODE\_288008\_length\_33935\_cov\_31.019037 12878-12888. Max. coverage (+): 0. Max coverage (-): 0

Region: NODE\_288008\_length\_33935\_cov\_31.019037 12889-12898. Max. coverage (+): 0.09. Max coverage (-): 0

Region: NODE\_288008\_length\_33935\_cov\_31.019037 12899-12909. Max. coverage (+): 0. Max coverage (-): 0

Region: NODE\_288008\_length\_33935\_cov\_31.019037 12910-12920. Max. coverage (+): 0. Max coverage (-): 0

Region: NODE\_288008\_length\_33935\_cov\_31.019037 12921-12931. Max. coverage (+): 0. Max coverage (-): 0

Region: NODE\_288008\_length\_33935\_cov\_31.019037 12932-12942. Max. coverage (+): 0.09. Max coverage (-): 0

Region: NODE\_288008\_length\_33935\_cov\_31.019037 12943-12952. Max. coverage (+): 0. Max coverage (-): 0

Region: NODE\_288008\_length\_33935\_cov\_31.019037 12953-12963. Max. coverage (+): 0. Max coverage (-): 0

Region: NODE\_288008\_length\_33935\_cov\_31.019037 12964-12974. Max. coverage (+): 0. Max coverage (-): 0

Region: NODE\_288008\_length\_33935\_cov\_31.019037 12975-12985. Max. coverage (+): 0. Max coverage (-): 0

Region: NODE\_288008\_length\_33935\_cov\_31.019037 12986-12995. Max. coverage (+): 0. Max coverage (-): 0

Region: NODE\_288008\_length\_33935\_cov\_31.019037 12996-13006. Max. coverage (+): 0. Max coverage (-): 0

Region: NODE\_288008\_length\_33935\_cov\_31.019037 13007-. Max. coverage (+): 0. Max coverage (-): 0

RepeatMasker Color Code

**+**

100-98% Identity

<98-95% Identity

<95-90% Identity

<90-85% Identity

<85-80% Identity

<80-75% Identity

<75-70% Identity

<70% Identity

**-**

Gene Set Color Code

**+**

Gene

Pseudogene

Other

**-**

Topology/Coverage Color Code

Coverage Plus Strand

Coverage Minus Strand

Mainstrand: Plus

Mainstrand: Minus

Complementary Strand

Flanking Region  
(if option -flank >0)

Gene Set Annotation  
  
RepeatMasker Annotation  

**1. AlRepD-3099**: 7609-7956 (-), Divergence to consensus: 10.1%  
**2. Gypsy-36\_GA-I**: 8273-8764 (+), Divergence to consensus: 44%  
**3. Gypsy-36\_GA-I**: 8905-9442 (+), Divergence to consensus: 36%  
**4. Gypsy-36\_GA-I**: 9777-10600 (+), Divergence to consensus: 42.6%  
**5. Gypsy-36\_GA-I**: 10573-10654 (+), Divergence to consensus: 23.6%  
**6. Gypsy-36\_GA-I**: 10660-11200 (+), Divergence to consensus: 36.1%  
**7. AlRepD-3099**: 11209-11273 (-), Divergence to consensus: 7.7%  
**8. AlRepB-112**: 11693-11758 (+), Divergence to consensus: 28.8%  
**9. AlRepA-102**: 11770-11816 (+), Divergence to consensus: 10.6%  
**10. AlRepD-2509**: 11852-11912 (+), Divergence to consensus: 18.4%  
**11. AlRepB-103**: 11940-12539 (+), Divergence to consensus: 24%  
**12. AlRepA-102**: 12638-13210 (+), Divergence to consensus: 34.6%

  
Transcription Factor Binding Sites  

**RFX4\_2** (Sequence: CCTGGATAC (+): 12511)  
**RHOXF1** (Sequence: AGATTA (-): 8198)  
**RHOXF1** (Sequence: GGATCA (-): 8485)  
**RHOXF1** (Sequence: AGCTTA (-): 9094)  
**RHOXF1** (Sequence: AGCTTA (-): 9181)  
**RHOXF1** (Sequence: AGATCA (-): 9200)  
**RHOXF1** (Sequence: AGATTA (-): 10947)  
**RHOXF1** (Sequence: GGCTCA (-): 11016)  
**RHOXF1** (Sequence: AGATTA (-): 11769)  
**RHOXF1** (Sequence: AGATCA (-): 12042)  
**RHOXF1** (Sequence: AGCTCA (-): 12232)  
**RHOXF1** (Sequence: AGATTA (-): 12396)  
**RHOXF1** (Sequence: GGCTCA (-): 12757)  
**RHOXF1** (Sequence: GGATTA (-): 12907)  
**RHOXF1** (Sequence: TGAGCC (+): 7926)  
**RHOXF1** (Sequence: TAAGCT (+): 7978)  
**RHOXF1** (Sequence: TAAGCC (+): 9670)  
**RHOXF1** (Sequence: TGAGCT (+): 10308)  
**RHOXF1** (Sequence: TGAGCC (+): 11257)  
**RHOXF1** (Sequence: TGATCT (+): 11521)  
**RHOXF1** (Sequence: TGATCC (+): 11705)  
**RHOXF1** (Sequence: TGATCT (+): 11732)  
**RHOXF1** (Sequence: TGATCC (+): 11890)  
**RHOXF1** (Sequence: TGATCT (+): 12149)  
**RHOXF1** (Sequence: TGAGCT (+): 12955)  
**RFX4\_1** (Sequence: GTTGCCAGG (-): 8671)  
**FOXO1** (Sequence: GCTGTTTTC (+): 8463)  
**FOXO1** (Sequence: GTTGTTTTT (+): 11628)  
**FOXO3\_mmu** (Sequence: TGTTTTGC (-): 7632)  
**FOXO3\_mmu** (Sequence: TGTTTTGC (-): 8230)  
**FOXO3\_mmu** (Sequence: TGTTTTCA (-): 8465)  
**FOXO3\_mmu** (Sequence: TGTTTTGA (-): 9070)  
**Sox5** (Sequence: ATTGTT (+): 8103)  
**Sox5** (Sequence: ATTGTT (+): 8169)  
**Sox5** (Sequence: ATTGTT (+): 10237)  
**Sox5** (Sequence: ATTGTT (+): 11318)  
**SOX9** (Sequence: CCATTGTT (+): 11316)  
**FOXO3\_mmu** (Sequence: GCAAAACA (+): 10996)  
**FOXO3\_mmu** (Sequence: TCAAAACA (+): 11543)  
**Rhox11** (Sequence: TGCTGTTTT (+): 8462)  
**POU2F1** (Sequence: TATGTTAAT (+): 10951)
