# Supplementary material for: First characterization of PIWI-interacting RNA clusters in a cichlid fish with a B chromosome
Source: BMC Biol. 2022 Sep 21;20:204. doi: 10.1186/s12915-022-01403-2 (PMC9490952; doi:10.1186/s12915-022-01403-2)
Supplement: Supplementary file 1 — Additional file 1. Zipped folder with fasta and interactive html piRNA cluster information for the A. latifasciata genome. The nomenclature is as follows: number-pirna-cluster_sex_B-presence (f, female; m, male; 0b, without B chromosome; 1b, with B chromosome). [file 12915_2022_1403_MOESM1_ESM.zip › 111_f0b.html]

piRNA cluster 111\_f0b 12


Predicted piRNA cluster no. 111\_f0b
  

Show proTRAC run info
Hide proTRAC run info

/\  
                \_\_\_\_\_\_\_\_\_\_\_\_\_\_\_\_\_\_\_\_\_\_\_/\\_\_\_ /  \\_\_\_\_\_\_\_  
               I                      /  \  /    \      I  
               I     pro             /    \/      \     I  
               I        TRAC        /               \   I  
               I   \_\_\_\_\_\_\_\_\_\_\_\_\_\_\_\_/\_\_\_\_\_\_\_\_\_\_\_\_\_\_\_\_\_\\_ I  
               I   \              /                     I  
               I    \            /                      I  
               I     \  /\      /       V.2.4.2         I  
               I      \/  \    /                        I  
               I\_\_\_\_\_\_\_\_\_\_\_\  /\_\_\_\_\_\_\_\_\_\_\_\_\_\_\_\_\_\_\_\_\_\_\_\_\_I  
                            \/  
  
  
================================= proTRAC ====================================  
VERSION: .......... 2.4.2  
LAST MODIFIED: .... 11. May 2018  
  
Please cite:  
Rosenkranz D, Zischler H. proTRAC - a software for probabilistic piRNA cluster  
detection, visualization and analysis. 2012. BMC Bioinformatics 13:5.  
  
  
Contact:  
David Rosenkranz  
Institute of Organismic and Molecular Evolutionary Biology  
Dept. Anthropology, small RNA group  
Johannes Gutenberg University Mainz  
email: rosenkranz@uni-mainz.de  
  
You can find the latest proTRAC version at:  
http://sourceforge.net/projects/protrac/files  
http://www.smallRNAgroup-mainz.de/software  
==============================================================================  
  
PARAMETERS:  
Map file: ...............piwi-femeas-0B.fa-collapse.map  
Genome file: ............../../../0B\_ala\_genome.fa  
RepeatMasker annotation: Alatifasciata-all0B-maryan-v2.fa\_corrected.out  
GeneSet:................./guest-storage/Data/annotation/Alatifasciata\_all0B\_maryan-v2\_out2017.gff  
  
Significant (p<=0.01) hit density will be calculated based  
on observed hit distribution.  
  
Sliding window size: ........................................ 5000 bp  
Sliding window increament: .................................. 1000 bp  
Normalize each hit by number of genomic hits: ............... yes  
Normalize each hit by number of sequence reads: ............. yes  
Normalize values (-> per million mapped reads): ............. yes  
Min. fraction of hits with 1T(U) or 10A: .................... 0.75  
Alternatively: Min. fraction of hits with 1T(U) and 10A: .... 0.5  
Min. fraction of hits with typical piRNA length: ............ 0.75  
Typical piRNA length: ....................................... 24-32 nt  
Min. size of a piRNA cluster: ............................... 1000 bp.  
Min. number of hits (absolute): ............................. 0  
Min. number of hits (normalized): ........................... 0  
Min. fraction of hits on the mainstrand: .................... 0.75  
Top fraction of mapped sequences (in terms of read counts): . 1%  
Top fraction accounts for max. n% of sequence reads: ........ 90%  
Min. fraction of hits on each arm of a bidirectional cluster: 0.05  
Output html file for each cluster: .......................... yes  
Output a summary table: ..................................... yes  
Output a FASTA file for each cluster (piRNA sequences): ..... yes  
Output a FASTA file comprising cluster sequences: ........... yes  
Output a GTF file for predicted piRNA clusters: ..............yes  
Search DNA motifs in clusters: .............................. yes  
Output flanking sequences: +/- .............................. 0 bp  
Output ~.pTi file: .......................................... no  
==============================================================================  
  
  
Genome size (without gaps): ............ 758543724 bp  
Gaps (N/X/-): .......................... 417479 bp  
Mapped reads: .......................... 13052187  
Non-identical sequences: ............... 3338911  
Genomic hits: .......................... 28737726  
Significant densitiy of mapped reads: .. 470.083249848448 reads/kb

Show proTRAC cluster info
Hide proTRAC cluster info

|  |  |
| --- | --- |
| Location | NODE\_28897\_length\_1748\_cov\_164.263153 |
| Coordinates | 4-1804 |
| Size [bp] | 1801 |
| Sequence hit loci | 682 |
| Mapped reads (normalized) | 2664.5 |
| Mapped reads (normalized) per kb | 1479.5 |
| Normalized reads with 1T (1U) | 82.8% |
| Normalized reads with 10A | 55.2% |
| Normalized reads with length 24-32 nt | 98.7% |
| Normalized reads on the main strand(s) | 85.3% |
| Predicted directionality | bi:plus-minus (split between 593 and 607) |

100%

0%

1T (1U)  
reads

10A reads

24-32 nt  
reads

reads on mainstrand

**Either the amount of reads with 1T (1U) OR 10A has to exceed 75% (set with option: -1Tor10A)  
Alternatively the amount of reads with 1T (1U) AND 10A has to exceed 50% (set with option: -1Tand10A)  
Minimum amount of reads with preferred size is 75% (set with option: -pisize)  
Minimum amount of reads on the main strand(s) is 75% (set with option: -clstrand)**

Show read coverage
Hide read coverage

WHAT DO I SEE HERE?  
This chart shows the location of mapped sequence reads within a predicted piRNA cluster. The color refers to the number of genomic hits produced by the sequence read in question. A dark red bar indicates that this sequence read produces many other hits elsewhere in the genome. Many adjacent red or yellow bars can indicate the presence of a multi-copy element such as transposons or rRNA genes. A dark green bar indicates that this sequence read maps uniquely to this locus.

1 hit

2-5 hits

6-10 hits

11-20 hits

21-50 hits

51-100 hits

> 100 hits

NODE\_28897\_length\_1748\_cov\_164.263153

4

1804

Gene Set

RepeatMasker

Mapped  
Reads

53.25

plus strand

minus strand

53.25

Region: NODE\_28897\_length\_1748\_cov\_164.263153 3835-5. Max. coverage (+): 0.03. Max coverage (-): 0.05

Region: NODE\_28897\_length\_1748\_cov\_164.263153 6-9. Max. coverage (+): 0. Max coverage (-): 0.03

Region: NODE\_28897\_length\_1748\_cov\_164.263153 10-13. Max. coverage (+): 0. Max coverage (-): 0

Region: NODE\_28897\_length\_1748\_cov\_164.263153 14-16. Max. coverage (+): 0.13. Max coverage (-): 0

Region: NODE\_28897\_length\_1748\_cov\_164.263153 17-20. Max. coverage (+): 0.1. Max coverage (-): 0

Region: NODE\_28897\_length\_1748\_cov\_164.263153 21-23. Max. coverage (+): 0.03. Max coverage (-): 0

Region: NODE\_28897\_length\_1748\_cov\_164.263153 24-27. Max. coverage (+): 0.18. Max coverage (-): 0

Region: NODE\_28897\_length\_1748\_cov\_164.263153 28-31. Max. coverage (+): 9.53. Max coverage (-): 0.05

Region: NODE\_28897\_length\_1748\_cov\_164.263153 32-34. Max. coverage (+): 0.33. Max coverage (-): 0.1

Region: NODE\_28897\_length\_1748\_cov\_164.263153 35-38. Max. coverage (+): 0.13. Max coverage (-): 0.08

Region: NODE\_28897\_length\_1748\_cov\_164.263153 39-41. Max. coverage (+): 0. Max coverage (-): 0

Region: NODE\_28897\_length\_1748\_cov\_164.263153 42-45. Max. coverage (+): 0. Max coverage (-): 0

Region: NODE\_28897\_length\_1748\_cov\_164.263153 46-49. Max. coverage (+): 0.23. Max coverage (-): 0

Region: NODE\_28897\_length\_1748\_cov\_164.263153 50-52. Max. coverage (+): 0. Max coverage (-): 0

Region: NODE\_28897\_length\_1748\_cov\_164.263153 53-56. Max. coverage (+): 0. Max coverage (-): 0

Region: NODE\_28897\_length\_1748\_cov\_164.263153 57-59. Max. coverage (+): 0. Max coverage (-): 0

Region: NODE\_28897\_length\_1748\_cov\_164.263153 60-63. Max. coverage (+): 0. Max coverage (-): 0

Region: NODE\_28897\_length\_1748\_cov\_164.263153 64-67. Max. coverage (+): 0. Max coverage (-): 0

Region: NODE\_28897\_length\_1748\_cov\_164.263153 68-70. Max. coverage (+): 0.15. Max coverage (-): 0

Region: NODE\_28897\_length\_1748\_cov\_164.263153 71-74. Max. coverage (+): 0. Max coverage (-): 0

Region: NODE\_28897\_length\_1748\_cov\_164.263153 75-77. Max. coverage (+): 0. Max coverage (-): 0

Region: NODE\_28897\_length\_1748\_cov\_164.263153 78-81. Max. coverage (+): 0. Max coverage (-): 0.08

Region: NODE\_28897\_length\_1748\_cov\_164.263153 82-85. Max. coverage (+): 0. Max coverage (-): 0.08

Region: NODE\_28897\_length\_1748\_cov\_164.263153 86-88. Max. coverage (+): 0. Max coverage (-): 0

Region: NODE\_28897\_length\_1748\_cov\_164.263153 89-92. Max. coverage (+): 0. Max coverage (-): 0

Region: NODE\_28897\_length\_1748\_cov\_164.263153 93-95. Max. coverage (+): 0. Max coverage (-): 0

Region: NODE\_28897\_length\_1748\_cov\_164.263153 96-99. Max. coverage (+): 0.23. Max coverage (-): 0

Region: NODE\_28897\_length\_1748\_cov\_164.263153 100-103. Max. coverage (+): 2.15. Max coverage (-): 0

Region: NODE\_28897\_length\_1748\_cov\_164.263153 104-106. Max. coverage (+): 0. Max coverage (-): 0

Region: NODE\_28897\_length\_1748\_cov\_164.263153 107-110. Max. coverage (+): 0. Max coverage (-): 0

Region: NODE\_28897\_length\_1748\_cov\_164.263153 111-113. Max. coverage (+): 0. Max coverage (-): 0

Region: NODE\_28897\_length\_1748\_cov\_164.263153 114-117. Max. coverage (+): 0. Max coverage (-): 0

Region: NODE\_28897\_length\_1748\_cov\_164.263153 118-121. Max. coverage (+): 0. Max coverage (-): 0

Region: NODE\_28897\_length\_1748\_cov\_164.263153 122-124. Max. coverage (+): 0. Max coverage (-): 0

Region: NODE\_28897\_length\_1748\_cov\_164.263153 125-128. Max. coverage (+): 0. Max coverage (-): 0

Region: NODE\_28897\_length\_1748\_cov\_164.263153 129-131. Max. coverage (+): 0. Max coverage (-): 0

Region: NODE\_28897\_length\_1748\_cov\_164.263153 132-135. Max. coverage (+): 0. Max coverage (-): 0

Region: NODE\_28897\_length\_1748\_cov\_164.263153 136-139. Max. coverage (+): 0. Max coverage (-): 0

Region: NODE\_28897\_length\_1748\_cov\_164.263153 140-142. Max. coverage (+): 0.38. Max coverage (-): 0

Region: NODE\_28897\_length\_1748\_cov\_164.263153 143-146. Max. coverage (+): 0.38. Max coverage (-): 0

Region: NODE\_28897\_length\_1748\_cov\_164.263153 147-149. Max. coverage (+): 2.45. Max coverage (-): 0.08

Region: NODE\_28897\_length\_1748\_cov\_164.263153 150-153. Max. coverage (+): 3.14. Max coverage (-): 0.08

Region: NODE\_28897\_length\_1748\_cov\_164.263153 154-157. Max. coverage (+): 0. Max coverage (-): 0

Region: NODE\_28897\_length\_1748\_cov\_164.263153 158-160. Max. coverage (+): 0. Max coverage (-): 0

Region: NODE\_28897\_length\_1748\_cov\_164.263153 161-164. Max. coverage (+): 0. Max coverage (-): 0

Region: NODE\_28897\_length\_1748\_cov\_164.263153 165-167. Max. coverage (+): 0.08. Max coverage (-): 0

Region: NODE\_28897\_length\_1748\_cov\_164.263153 168-171. Max. coverage (+): 24.21. Max coverage (-): 0

Region: NODE\_28897\_length\_1748\_cov\_164.263153 172-175. Max. coverage (+): 22.07. Max coverage (-): 0

Region: NODE\_28897\_length\_1748\_cov\_164.263153 176-178. Max. coverage (+): 0. Max coverage (-): 0

Region: NODE\_28897\_length\_1748\_cov\_164.263153 179-182. Max. coverage (+): 0. Max coverage (-): 0

Region: NODE\_28897\_length\_1748\_cov\_164.263153 183-185. Max. coverage (+): 0.15. Max coverage (-): 0

Region: NODE\_28897\_length\_1748\_cov\_164.263153 186-189. Max. coverage (+): 0.08. Max coverage (-): 0

Region: NODE\_28897\_length\_1748\_cov\_164.263153 190-193. Max. coverage (+): 0. Max coverage (-): 0

Region: NODE\_28897\_length\_1748\_cov\_164.263153 194-196. Max. coverage (+): 0. Max coverage (-): 0

Region: NODE\_28897\_length\_1748\_cov\_164.263153 197-200. Max. coverage (+): 0. Max coverage (-): 0

Region: NODE\_28897\_length\_1748\_cov\_164.263153 201-203. Max. coverage (+): 0.08. Max coverage (-): 0

Region: NODE\_28897\_length\_1748\_cov\_164.263153 204-207. Max. coverage (+): 0.08. Max coverage (-): 0

Region: NODE\_28897\_length\_1748\_cov\_164.263153 208-211. Max. coverage (+): 0.08. Max coverage (-): 0

Region: NODE\_28897\_length\_1748\_cov\_164.263153 212-214. Max. coverage (+): 0. Max coverage (-): 0

Region: NODE\_28897\_length\_1748\_cov\_164.263153 215-218. Max. coverage (+): 0. Max coverage (-): 0

Region: NODE\_28897\_length\_1748\_cov\_164.263153 219-221. Max. coverage (+): 0. Max coverage (-): 0

Region: NODE\_28897\_length\_1748\_cov\_164.263153 222-225. Max. coverage (+): 0. Max coverage (-): 0

Region: NODE\_28897\_length\_1748\_cov\_164.263153 226-229. Max. coverage (+): 0. Max coverage (-): 0

Region: NODE\_28897\_length\_1748\_cov\_164.263153 230-232. Max. coverage (+): 0. Max coverage (-): 0

Region: NODE\_28897\_length\_1748\_cov\_164.263153 233-236. Max. coverage (+): 0.08. Max coverage (-): 0

Region: NODE\_28897\_length\_1748\_cov\_164.263153 237-239. Max. coverage (+): 2.07. Max coverage (-): 0

Region: NODE\_28897\_length\_1748\_cov\_164.263153 240-243. Max. coverage (+): 2.15. Max coverage (-): 0

Region: NODE\_28897\_length\_1748\_cov\_164.263153 244-247. Max. coverage (+): 0. Max coverage (-): 0

Region: NODE\_28897\_length\_1748\_cov\_164.263153 248-250. Max. coverage (+): 0. Max coverage (-): 0

Region: NODE\_28897\_length\_1748\_cov\_164.263153 251-254. Max. coverage (+): 0. Max coverage (-): 0

Region: NODE\_28897\_length\_1748\_cov\_164.263153 255-257. Max. coverage (+): 0. Max coverage (-): 0

Region: NODE\_28897\_length\_1748\_cov\_164.263153 258-261. Max. coverage (+): 0. Max coverage (-): 0

Region: NODE\_28897\_length\_1748\_cov\_164.263153 262-265. Max. coverage (+): 0. Max coverage (-): 0.38

Region: NODE\_28897\_length\_1748\_cov\_164.263153 266-268. Max. coverage (+): 0. Max coverage (-): 0.54

Region: NODE\_28897\_length\_1748\_cov\_164.263153 269-272. Max. coverage (+): 0.08. Max coverage (-): 0

Region: NODE\_28897\_length\_1748\_cov\_164.263153 273-275. Max. coverage (+): 0. Max coverage (-): 0

Region: NODE\_28897\_length\_1748\_cov\_164.263153 276-279. Max. coverage (+): 0. Max coverage (-): 0

Region: NODE\_28897\_length\_1748\_cov\_164.263153 280-283. Max. coverage (+): 8.66. Max coverage (-): 0

Region: NODE\_28897\_length\_1748\_cov\_164.263153 284-286. Max. coverage (+): 8.66. Max coverage (-): 0

Region: NODE\_28897\_length\_1748\_cov\_164.263153 287-290. Max. coverage (+): 0. Max coverage (-): 0

Region: NODE\_28897\_length\_1748\_cov\_164.263153 291-293. Max. coverage (+): 0.08. Max coverage (-): 0.08

Region: NODE\_28897\_length\_1748\_cov\_164.263153 294-297. Max. coverage (+): 0. Max coverage (-): 0.15

Region: NODE\_28897\_length\_1748\_cov\_164.263153 298-301. Max. coverage (+): 0. Max coverage (-): 0

Region: NODE\_28897\_length\_1748\_cov\_164.263153 302-304. Max. coverage (+): 0. Max coverage (-): 0

Region: NODE\_28897\_length\_1748\_cov\_164.263153 305-308. Max. coverage (+): 0. Max coverage (-): 0

Region: NODE\_28897\_length\_1748\_cov\_164.263153 309-311. Max. coverage (+): 0.23. Max coverage (-): 0

Region: NODE\_28897\_length\_1748\_cov\_164.263153 312-315. Max. coverage (+): 0.54. Max coverage (-): 0

Region: NODE\_28897\_length\_1748\_cov\_164.263153 316-319. Max. coverage (+): 0.23. Max coverage (-): 0

Region: NODE\_28897\_length\_1748\_cov\_164.263153 320-322. Max. coverage (+): 0.08. Max coverage (-): 0.15

Region: NODE\_28897\_length\_1748\_cov\_164.263153 323-326. Max. coverage (+): 0. Max coverage (-): 0.23

Region: NODE\_28897\_length\_1748\_cov\_164.263153 327-329. Max. coverage (+): 0. Max coverage (-): 0

Region: NODE\_28897\_length\_1748\_cov\_164.263153 330-333. Max. coverage (+): 0. Max coverage (-): 0

Region: NODE\_28897\_length\_1748\_cov\_164.263153 334-337. Max. coverage (+): 0. Max coverage (-): 0.08

Region: NODE\_28897\_length\_1748\_cov\_164.263153 338-340. Max. coverage (+): 0. Max coverage (-): 0

Region: NODE\_28897\_length\_1748\_cov\_164.263153 341-344. Max. coverage (+): 9.5. Max coverage (-): 0

Region: NODE\_28897\_length\_1748\_cov\_164.263153 345-347. Max. coverage (+): 2.15. Max coverage (-): 0

Region: NODE\_28897\_length\_1748\_cov\_164.263153 348-351. Max. coverage (+): 0.31. Max coverage (-): 0

Region: NODE\_28897\_length\_1748\_cov\_164.263153 352-355. Max. coverage (+): 0. Max coverage (-): 0

Region: NODE\_28897\_length\_1748\_cov\_164.263153 356-358. Max. coverage (+): 0. Max coverage (-): 0

Region: NODE\_28897\_length\_1748\_cov\_164.263153 359-362. Max. coverage (+): 0. Max coverage (-): 0

Region: NODE\_28897\_length\_1748\_cov\_164.263153 363-366. Max. coverage (+): 0.08. Max coverage (-): 0.08

Region: NODE\_28897\_length\_1748\_cov\_164.263153 367-369. Max. coverage (+): 0.15. Max coverage (-): 0.08

Region: NODE\_28897\_length\_1748\_cov\_164.263153 370-373. Max. coverage (+): 0.08. Max coverage (-): 0

Region: NODE\_28897\_length\_1748\_cov\_164.263153 374-376. Max. coverage (+): 0.08. Max coverage (-): 0

Region: NODE\_28897\_length\_1748\_cov\_164.263153 377-380. Max. coverage (+): 0. Max coverage (-): 0.08

Region: NODE\_28897\_length\_1748\_cov\_164.263153 381-384. Max. coverage (+): 0. Max coverage (-): 0.23

Region: NODE\_28897\_length\_1748\_cov\_164.263153 385-387. Max. coverage (+): 0.08. Max coverage (-): 0.08

Region: NODE\_28897\_length\_1748\_cov\_164.263153 388-391. Max. coverage (+): 0. Max coverage (-): 0

Region: NODE\_28897\_length\_1748\_cov\_164.263153 392-394. Max. coverage (+): 0.08. Max coverage (-): 0

Region: NODE\_28897\_length\_1748\_cov\_164.263153 395-398. Max. coverage (+): 0.08. Max coverage (-): 0.08

Region: NODE\_28897\_length\_1748\_cov\_164.263153 399-402. Max. coverage (+): 1.61. Max coverage (-): 0

Region: NODE\_28897\_length\_1748\_cov\_164.263153 403-405. Max. coverage (+): 0.08. Max coverage (-): 0

Region: NODE\_28897\_length\_1748\_cov\_164.263153 406-409. Max. coverage (+): 0. Max coverage (-): 0

Region: NODE\_28897\_length\_1748\_cov\_164.263153 410-412. Max. coverage (+): 0.08. Max coverage (-): 0

Region: NODE\_28897\_length\_1748\_cov\_164.263153 413-416. Max. coverage (+): 0.08. Max coverage (-): 0

Region: NODE\_28897\_length\_1748\_cov\_164.263153 417-420. Max. coverage (+): 0. Max coverage (-): 0

Region: NODE\_28897\_length\_1748\_cov\_164.263153 421-423. Max. coverage (+): 0. Max coverage (-): 0

Region: NODE\_28897\_length\_1748\_cov\_164.263153 424-427. Max. coverage (+): 0. Max coverage (-): 0

Region: NODE\_28897\_length\_1748\_cov\_164.263153 428-430. Max. coverage (+): 0. Max coverage (-): 0

Region: NODE\_28897\_length\_1748\_cov\_164.263153 431-434. Max. coverage (+): 0.15. Max coverage (-): 0

Region: NODE\_28897\_length\_1748\_cov\_164.263153 435-438. Max. coverage (+): 0.23. Max coverage (-): 0

Region: NODE\_28897\_length\_1748\_cov\_164.263153 439-441. Max. coverage (+): 0.23. Max coverage (-): 0

Region: NODE\_28897\_length\_1748\_cov\_164.263153 442-445. Max. coverage (+): 0. Max coverage (-): 0

Region: NODE\_28897\_length\_1748\_cov\_164.263153 446-448. Max. coverage (+): 0. Max coverage (-): 0.23

Region: NODE\_28897\_length\_1748\_cov\_164.263153 449-452. Max. coverage (+): 0. Max coverage (-): 0.15

Region: NODE\_28897\_length\_1748\_cov\_164.263153 453-456. Max. coverage (+): 0. Max coverage (-): 0

Region: NODE\_28897\_length\_1748\_cov\_164.263153 457-459. Max. coverage (+): 0. Max coverage (-): 0

Region: NODE\_28897\_length\_1748\_cov\_164.263153 460-463. Max. coverage (+): 0. Max coverage (-): 0

Region: NODE\_28897\_length\_1748\_cov\_164.263153 464-466. Max. coverage (+): 3.06. Max coverage (-): 0

Region: NODE\_28897\_length\_1748\_cov\_164.263153 467-470. Max. coverage (+): 3.06. Max coverage (-): 0

Region: NODE\_28897\_length\_1748\_cov\_164.263153 471-474. Max. coverage (+): 0. Max coverage (-): 0

Region: NODE\_28897\_length\_1748\_cov\_164.263153 475-477. Max. coverage (+): 0. Max coverage (-): 1

Region: NODE\_28897\_length\_1748\_cov\_164.263153 478-481. Max. coverage (+): 0. Max coverage (-): 9.35

Region: NODE\_28897\_length\_1748\_cov\_164.263153 482-484. Max. coverage (+): 0. Max coverage (-): 1.92

Region: NODE\_28897\_length\_1748\_cov\_164.263153 485-488. Max. coverage (+): 0. Max coverage (-): 1

Region: NODE\_28897\_length\_1748\_cov\_164.263153 489-492. Max. coverage (+): 0.08. Max coverage (-): 0

Region: NODE\_28897\_length\_1748\_cov\_164.263153 493-495. Max. coverage (+): 0.23. Max coverage (-): 0

Region: NODE\_28897\_length\_1748\_cov\_164.263153 496-499. Max. coverage (+): 53.25. Max coverage (-): 0

Region: NODE\_28897\_length\_1748\_cov\_164.263153 500-502. Max. coverage (+): 49.72. Max coverage (-): 0

Region: NODE\_28897\_length\_1748\_cov\_164.263153 503-506. Max. coverage (+): 0. Max coverage (-): 0.15

Region: NODE\_28897\_length\_1748\_cov\_164.263153 507-510. Max. coverage (+): 0.08. Max coverage (-): 0.61

Region: NODE\_28897\_length\_1748\_cov\_164.263153 511-513. Max. coverage (+): 0.08. Max coverage (-): 0

Region: NODE\_28897\_length\_1748\_cov\_164.263153 514-517. Max. coverage (+): 0. Max coverage (-): 0

Region: NODE\_28897\_length\_1748\_cov\_164.263153 518-520. Max. coverage (+): 0. Max coverage (-): 0

Region: NODE\_28897\_length\_1748\_cov\_164.263153 521-524. Max. coverage (+): 0. Max coverage (-): 0

Region: NODE\_28897\_length\_1748\_cov\_164.263153 525-528. Max. coverage (+): 1.23. Max coverage (-): 0

Region: NODE\_28897\_length\_1748\_cov\_164.263153 529-531. Max. coverage (+): 0.46. Max coverage (-): 0

Region: NODE\_28897\_length\_1748\_cov\_164.263153 532-535. Max. coverage (+): 0.38. Max coverage (-): 0

Region: NODE\_28897\_length\_1748\_cov\_164.263153 536-538. Max. coverage (+): 0. Max coverage (-): 0.08

Region: NODE\_28897\_length\_1748\_cov\_164.263153 539-542. Max. coverage (+): 0. Max coverage (-): 0

Region: NODE\_28897\_length\_1748\_cov\_164.263153 543-546. Max. coverage (+): 0. Max coverage (-): 0

Region: NODE\_28897\_length\_1748\_cov\_164.263153 547-549. Max. coverage (+): 0.15. Max coverage (-): 0

Region: NODE\_28897\_length\_1748\_cov\_164.263153 550-553. Max. coverage (+): 0.38. Max coverage (-): 0

Region: NODE\_28897\_length\_1748\_cov\_164.263153 554-556. Max. coverage (+): 0.23. Max coverage (-): 0

Region: NODE\_28897\_length\_1748\_cov\_164.263153 557-560. Max. coverage (+): 0. Max coverage (-): 0

Region: NODE\_28897\_length\_1748\_cov\_164.263153 561-564. Max. coverage (+): 0.38. Max coverage (-): 0.08

Region: NODE\_28897\_length\_1748\_cov\_164.263153 565-567. Max. coverage (+): 0.38. Max coverage (-): 0.31

Region: NODE\_28897\_length\_1748\_cov\_164.263153 568-571. Max. coverage (+): 0.15. Max coverage (-): 0.31

Region: NODE\_28897\_length\_1748\_cov\_164.263153 572-574. Max. coverage (+): 0. Max coverage (-): 0

Region: NODE\_28897\_length\_1748\_cov\_164.263153 575-578. Max. coverage (+): 0. Max coverage (-): 0

Region: NODE\_28897\_length\_1748\_cov\_164.263153 579-582. Max. coverage (+): 0. Max coverage (-): 0

Region: NODE\_28897\_length\_1748\_cov\_164.263153 583-585. Max. coverage (+): 0.31. Max coverage (-): 0

Region: NODE\_28897\_length\_1748\_cov\_164.263153 586-589. Max. coverage (+): 0.31. Max coverage (-): 0

Region: NODE\_28897\_length\_1748\_cov\_164.263153 590-592. Max. coverage (+): 0. Max coverage (-): 0

Region: NODE\_28897\_length\_1748\_cov\_164.263153 593-596. Max. coverage (+): 0.08. Max coverage (-): 0

Region: NODE\_28897\_length\_1748\_cov\_164.263153 597-600. Max. coverage (+): 0. Max coverage (-): 0

Region: NODE\_28897\_length\_1748\_cov\_164.263153 601-603. Max. coverage (+): 0. Max coverage (-): 0

Region: NODE\_28897\_length\_1748\_cov\_164.263153 604-607. Max. coverage (+): 0. Max coverage (-): 0.08

Region: NODE\_28897\_length\_1748\_cov\_164.263153 608-610. Max. coverage (+): 0. Max coverage (-): 0.08

Region: NODE\_28897\_length\_1748\_cov\_164.263153 611-614. Max. coverage (+): 0. Max coverage (-): 0

Region: NODE\_28897\_length\_1748\_cov\_164.263153 615-618. Max. coverage (+): 0. Max coverage (-): 0.08

Region: NODE\_28897\_length\_1748\_cov\_164.263153 619-621. Max. coverage (+): 0. Max coverage (-): 0.15

Region: NODE\_28897\_length\_1748\_cov\_164.263153 622-625. Max. coverage (+): 0. Max coverage (-): 0.31

Region: NODE\_28897\_length\_1748\_cov\_164.263153 626-628. Max. coverage (+): 0.08. Max coverage (-): 0

Region: NODE\_28897\_length\_1748\_cov\_164.263153 629-632. Max. coverage (+): 0.15. Max coverage (-): 0

Region: NODE\_28897\_length\_1748\_cov\_164.263153 633-636. Max. coverage (+): 0. Max coverage (-): 0

Region: NODE\_28897\_length\_1748\_cov\_164.263153 637-639. Max. coverage (+): 0.08. Max coverage (-): 0.08

Region: NODE\_28897\_length\_1748\_cov\_164.263153 640-643. Max. coverage (+): 0.15. Max coverage (-): 0.08

Region: NODE\_28897\_length\_1748\_cov\_164.263153 644-646. Max. coverage (+): 0. Max coverage (-): 0.15

Region: NODE\_28897\_length\_1748\_cov\_164.263153 647-650. Max. coverage (+): 0. Max coverage (-): 0.23

Region: NODE\_28897\_length\_1748\_cov\_164.263153 651-654. Max. coverage (+): 0. Max coverage (-): 0.08

Region: NODE\_28897\_length\_1748\_cov\_164.263153 655-657. Max. coverage (+): 0. Max coverage (-): 0

Region: NODE\_28897\_length\_1748\_cov\_164.263153 658-661. Max. coverage (+): 0. Max coverage (-): 0

Region: NODE\_28897\_length\_1748\_cov\_164.263153 662-664. Max. coverage (+): 0. Max coverage (-): 0

Region: NODE\_28897\_length\_1748\_cov\_164.263153 665-668. Max. coverage (+): 0. Max coverage (-): 0

Region: NODE\_28897\_length\_1748\_cov\_164.263153 669-672. Max. coverage (+): 0.08. Max coverage (-): 0

Region: NODE\_28897\_length\_1748\_cov\_164.263153 673-675. Max. coverage (+): 0. Max coverage (-): 0

Region: NODE\_28897\_length\_1748\_cov\_164.263153 676-679. Max. coverage (+): 0.31. Max coverage (-): 0

Region: NODE\_28897\_length\_1748\_cov\_164.263153 680-682. Max. coverage (+): 0.15. Max coverage (-): 0.38

Region: NODE\_28897\_length\_1748\_cov\_164.263153 683-686. Max. coverage (+): 0. Max coverage (-): 0.54

Region: NODE\_28897\_length\_1748\_cov\_164.263153 687-690. Max. coverage (+): 0. Max coverage (-): 0

Region: NODE\_28897\_length\_1748\_cov\_164.263153 691-693. Max. coverage (+): 0. Max coverage (-): 0

Region: NODE\_28897\_length\_1748\_cov\_164.263153 694-697. Max. coverage (+): 0.08. Max coverage (-): 0

Region: NODE\_28897\_length\_1748\_cov\_164.263153 698-700. Max. coverage (+): 0. Max coverage (-): 0

Region: NODE\_28897\_length\_1748\_cov\_164.263153 701-704. Max. coverage (+): 0. Max coverage (-): 0

Region: NODE\_28897\_length\_1748\_cov\_164.263153 705-708. Max. coverage (+): 0.08. Max coverage (-): 0

Region: NODE\_28897\_length\_1748\_cov\_164.263153 709-711. Max. coverage (+): 0. Max coverage (-): 0

Region: NODE\_28897\_length\_1748\_cov\_164.263153 712-715. Max. coverage (+): 0.08. Max coverage (-): 0.15

Region: NODE\_28897\_length\_1748\_cov\_164.263153 716-718. Max. coverage (+): 0.08. Max coverage (-): 0.69

Region: NODE\_28897\_length\_1748\_cov\_164.263153 719-722. Max. coverage (+): 0. Max coverage (-): 0.69

Region: NODE\_28897\_length\_1748\_cov\_164.263153 723-726. Max. coverage (+): 0. Max coverage (-): 0.15

Region: NODE\_28897\_length\_1748\_cov\_164.263153 727-729. Max. coverage (+): 0. Max coverage (-): 0

Region: NODE\_28897\_length\_1748\_cov\_164.263153 730-733. Max. coverage (+): 0. Max coverage (-): 0

Region: NODE\_28897\_length\_1748\_cov\_164.263153 734-737. Max. coverage (+): 0.23. Max coverage (-): 0

Region: NODE\_28897\_length\_1748\_cov\_164.263153 738-740. Max. coverage (+): 0.15. Max coverage (-): 0

Region: NODE\_28897\_length\_1748\_cov\_164.263153 741-744. Max. coverage (+): 0.08. Max coverage (-): 0

Region: NODE\_28897\_length\_1748\_cov\_164.263153 745-747. Max. coverage (+): 0.15. Max coverage (-): 0

Region: NODE\_28897\_length\_1748\_cov\_164.263153 748-751. Max. coverage (+): 0.15. Max coverage (-): 0.08

Region: NODE\_28897\_length\_1748\_cov\_164.263153 752-755. Max. coverage (+): 0. Max coverage (-): 0.08

Region: NODE\_28897\_length\_1748\_cov\_164.263153 756-758. Max. coverage (+): 0. Max coverage (-): 0

Region: NODE\_28897\_length\_1748\_cov\_164.263153 759-762. Max. coverage (+): 0. Max coverage (-): 0

Region: NODE\_28897\_length\_1748\_cov\_164.263153 763-765. Max. coverage (+): 0. Max coverage (-): 0

Region: NODE\_28897\_length\_1748\_cov\_164.263153 766-769. Max. coverage (+): 0.38. Max coverage (-): 0

Region: NODE\_28897\_length\_1748\_cov\_164.263153 770-773. Max. coverage (+): 0. Max coverage (-): 0

Region: NODE\_28897\_length\_1748\_cov\_164.263153 774-776. Max. coverage (+): 0. Max coverage (-): 0

Region: NODE\_28897\_length\_1748\_cov\_164.263153 777-780. Max. coverage (+): 0.08. Max coverage (-): 0

Region: NODE\_28897\_length\_1748\_cov\_164.263153 781-783. Max. coverage (+): 0. Max coverage (-): 0

Region: NODE\_28897\_length\_1748\_cov\_164.263153 784-787. Max. coverage (+): 0. Max coverage (-): 0

Region: NODE\_28897\_length\_1748\_cov\_164.263153 788-791. Max. coverage (+): 0. Max coverage (-): 0

Region: NODE\_28897\_length\_1748\_cov\_164.263153 792-794. Max. coverage (+): 0. Max coverage (-): 0

Region: NODE\_28897\_length\_1748\_cov\_164.263153 795-798. Max. coverage (+): 0. Max coverage (-): 0

Region: NODE\_28897\_length\_1748\_cov\_164.263153 799-801. Max. coverage (+): 0. Max coverage (-): 0

Region: NODE\_28897\_length\_1748\_cov\_164.263153 802-805. Max. coverage (+): 0. Max coverage (-): 0.38

Region: NODE\_28897\_length\_1748\_cov\_164.263153 806-809. Max. coverage (+): 0. Max coverage (-): 0.46

Region: NODE\_28897\_length\_1748\_cov\_164.263153 810-812. Max. coverage (+): 0. Max coverage (-): 0

Region: NODE\_28897\_length\_1748\_cov\_164.263153 813-816. Max. coverage (+): 0. Max coverage (-): 0

Region: NODE\_28897\_length\_1748\_cov\_164.263153 817-819. Max. coverage (+): 0. Max coverage (-): 0

Region: NODE\_28897\_length\_1748\_cov\_164.263153 820-823. Max. coverage (+): 0. Max coverage (-): 0

Region: NODE\_28897\_length\_1748\_cov\_164.263153 824-827. Max. coverage (+): 0.31. Max coverage (-): 0

Region: NODE\_28897\_length\_1748\_cov\_164.263153 828-830. Max. coverage (+): 0. Max coverage (-): 0

Region: NODE\_28897\_length\_1748\_cov\_164.263153 831-834. Max. coverage (+): 0. Max coverage (-): 0.08

Region: NODE\_28897\_length\_1748\_cov\_164.263153 835-837. Max. coverage (+): 0. Max coverage (-): 0.15

Region: NODE\_28897\_length\_1748\_cov\_164.263153 838-841. Max. coverage (+): 0. Max coverage (-): 0.23

Region: NODE\_28897\_length\_1748\_cov\_164.263153 842-845. Max. coverage (+): 0. Max coverage (-): 0

Region: NODE\_28897\_length\_1748\_cov\_164.263153 846-848. Max. coverage (+): 0. Max coverage (-): 0

Region: NODE\_28897\_length\_1748\_cov\_164.263153 849-852. Max. coverage (+): 0. Max coverage (-): 0.15

Region: NODE\_28897\_length\_1748\_cov\_164.263153 853-855. Max. coverage (+): 0. Max coverage (-): 1.53

Region: NODE\_28897\_length\_1748\_cov\_164.263153 856-859. Max. coverage (+): 0. Max coverage (-): 0.08

Region: NODE\_28897\_length\_1748\_cov\_164.263153 860-863. Max. coverage (+): 0. Max coverage (-): 0

Region: NODE\_28897\_length\_1748\_cov\_164.263153 864-866. Max. coverage (+): 0. Max coverage (-): 0

Region: NODE\_28897\_length\_1748\_cov\_164.263153 867-870. Max. coverage (+): 0.08. Max coverage (-): 0.08

Region: NODE\_28897\_length\_1748\_cov\_164.263153 871-873. Max. coverage (+): 0.61. Max coverage (-): 0.08

Region: NODE\_28897\_length\_1748\_cov\_164.263153 874-877. Max. coverage (+): 0.61. Max coverage (-): 0

Region: NODE\_28897\_length\_1748\_cov\_164.263153 878-881. Max. coverage (+): 0. Max coverage (-): 0

Region: NODE\_28897\_length\_1748\_cov\_164.263153 882-884. Max. coverage (+): 0.08. Max coverage (-): 0.08

Region: NODE\_28897\_length\_1748\_cov\_164.263153 885-888. Max. coverage (+): 0.31. Max coverage (-): 0.08

Region: NODE\_28897\_length\_1748\_cov\_164.263153 889-891. Max. coverage (+): 0.31. Max coverage (-): 0.08

Region: NODE\_28897\_length\_1748\_cov\_164.263153 892-895. Max. coverage (+): 0. Max coverage (-): 0.08

Region: NODE\_28897\_length\_1748\_cov\_164.263153 896-899. Max. coverage (+): 0. Max coverage (-): 0

Region: NODE\_28897\_length\_1748\_cov\_164.263153 900-902. Max. coverage (+): 0.23. Max coverage (-): 0

Region: NODE\_28897\_length\_1748\_cov\_164.263153 903-906. Max. coverage (+): 0.08. Max coverage (-): 0

Region: NODE\_28897\_length\_1748\_cov\_164.263153 907-909. Max. coverage (+): 0. Max coverage (-): 0.15

Region: NODE\_28897\_length\_1748\_cov\_164.263153 910-913. Max. coverage (+): 0. Max coverage (-): 0.15

Region: NODE\_28897\_length\_1748\_cov\_164.263153 914-917. Max. coverage (+): 0. Max coverage (-): 0.15

Region: NODE\_28897\_length\_1748\_cov\_164.263153 918-920. Max. coverage (+): 0. Max coverage (-): 0.15

Region: NODE\_28897\_length\_1748\_cov\_164.263153 921-924. Max. coverage (+): 0. Max coverage (-): 0

Region: NODE\_28897\_length\_1748\_cov\_164.263153 925-927. Max. coverage (+): 0.31. Max coverage (-): 0

Region: NODE\_28897\_length\_1748\_cov\_164.263153 928-931. Max. coverage (+): 0.15. Max coverage (-): 0.23

Region: NODE\_28897\_length\_1748\_cov\_164.263153 932-935. Max. coverage (+): 0.46. Max coverage (-): 0.23

Region: NODE\_28897\_length\_1748\_cov\_164.263153 936-938. Max. coverage (+): 0.15. Max coverage (-): 0

Region: NODE\_28897\_length\_1748\_cov\_164.263153 939-942. Max. coverage (+): 0.08. Max coverage (-): 0

Region: NODE\_28897\_length\_1748\_cov\_164.263153 943-945. Max. coverage (+): 0.08. Max coverage (-): 0.08

Region: NODE\_28897\_length\_1748\_cov\_164.263153 946-949. Max. coverage (+): 0. Max coverage (-): 0.08

Region: NODE\_28897\_length\_1748\_cov\_164.263153 950-953. Max. coverage (+): 0. Max coverage (-): 0

Region: NODE\_28897\_length\_1748\_cov\_164.263153 954-956. Max. coverage (+): 0. Max coverage (-): 0

Region: NODE\_28897\_length\_1748\_cov\_164.263153 957-960. Max. coverage (+): 0. Max coverage (-): 0

Region: NODE\_28897\_length\_1748\_cov\_164.263153 961-963. Max. coverage (+): 0. Max coverage (-): 0

Region: NODE\_28897\_length\_1748\_cov\_164.263153 964-967. Max. coverage (+): 0. Max coverage (-): 0

Region: NODE\_28897\_length\_1748\_cov\_164.263153 968-971. Max. coverage (+): 0. Max coverage (-): 0

Region: NODE\_28897\_length\_1748\_cov\_164.263153 972-974. Max. coverage (+): 0. Max coverage (-): 0

Region: NODE\_28897\_length\_1748\_cov\_164.263153 975-978. Max. coverage (+): 0. Max coverage (-): 0

Region: NODE\_28897\_length\_1748\_cov\_164.263153 979-981. Max. coverage (+): 0. Max coverage (-): 0

Region: NODE\_28897\_length\_1748\_cov\_164.263153 982-985. Max. coverage (+): 0. Max coverage (-): 0

Region: NODE\_28897\_length\_1748\_cov\_164.263153 986-989. Max. coverage (+): 0. Max coverage (-): 0.08

Region: NODE\_28897\_length\_1748\_cov\_164.263153 990-992. Max. coverage (+): 0. Max coverage (-): 0

Region: NODE\_28897\_length\_1748\_cov\_164.263153 993-996. Max. coverage (+): 0. Max coverage (-): 0

Region: NODE\_28897\_length\_1748\_cov\_164.263153 997-999. Max. coverage (+): 0. Max coverage (-): 0

Region: NODE\_28897\_length\_1748\_cov\_164.263153 1000-1003. Max. coverage (+): 0. Max coverage (-): 0

Region: NODE\_28897\_length\_1748\_cov\_164.263153 1004-1007. Max. coverage (+): 0. Max coverage (-): 0

Region: NODE\_28897\_length\_1748\_cov\_164.263153 1008-1010. Max. coverage (+): 0. Max coverage (-): 0

Region: NODE\_28897\_length\_1748\_cov\_164.263153 1011-1014. Max. coverage (+): 0.08. Max coverage (-): 0.15

Region: NODE\_28897\_length\_1748\_cov\_164.263153 1015-1017. Max. coverage (+): 0.08. Max coverage (-): 0.15

Region: NODE\_28897\_length\_1748\_cov\_164.263153 1018-1021. Max. coverage (+): 0. Max coverage (-): 0

Region: NODE\_28897\_length\_1748\_cov\_164.263153 1022-1025. Max. coverage (+): 0. Max coverage (-): 0

Region: NODE\_28897\_length\_1748\_cov\_164.263153 1026-1028. Max. coverage (+): 0. Max coverage (-): 0

Region: NODE\_28897\_length\_1748\_cov\_164.263153 1029-1032. Max. coverage (+): 1.07. Max coverage (-): 0

Region: NODE\_28897\_length\_1748\_cov\_164.263153 1033-1035. Max. coverage (+): 1.07. Max coverage (-): 0.08

Region: NODE\_28897\_length\_1748\_cov\_164.263153 1036-1039. Max. coverage (+): 0. Max coverage (-): 0.08

Region: NODE\_28897\_length\_1748\_cov\_164.263153 1040-1043. Max. coverage (+): 0. Max coverage (-): 0.15

Region: NODE\_28897\_length\_1748\_cov\_164.263153 1044-1046. Max. coverage (+): 0.08. Max coverage (-): 0.15

Region: NODE\_28897\_length\_1748\_cov\_164.263153 1047-1050. Max. coverage (+): 0.08. Max coverage (-): 0.23

Region: NODE\_28897\_length\_1748\_cov\_164.263153 1051-1053. Max. coverage (+): 0. Max coverage (-): 0

Region: NODE\_28897\_length\_1748\_cov\_164.263153 1054-1057. Max. coverage (+): 0. Max coverage (-): 0

Region: NODE\_28897\_length\_1748\_cov\_164.263153 1058-1061. Max. coverage (+): 0. Max coverage (-): 0

Region: NODE\_28897\_length\_1748\_cov\_164.263153 1062-1064. Max. coverage (+): 0.23. Max coverage (-): 0

Region: NODE\_28897\_length\_1748\_cov\_164.263153 1065-1068. Max. coverage (+): 0.23. Max coverage (-): 0

Region: NODE\_28897\_length\_1748\_cov\_164.263153 1069-1071. Max. coverage (+): 0. Max coverage (-): 0

Region: NODE\_28897\_length\_1748\_cov\_164.263153 1072-1075. Max. coverage (+): 0. Max coverage (-): 0

Region: NODE\_28897\_length\_1748\_cov\_164.263153 1076-1079. Max. coverage (+): 0. Max coverage (-): 0

Region: NODE\_28897\_length\_1748\_cov\_164.263153 1080-1082. Max. coverage (+): 0. Max coverage (-): 0.23

Region: NODE\_28897\_length\_1748\_cov\_164.263153 1083-1086. Max. coverage (+): 0. Max coverage (-): 0.23

Region: NODE\_28897\_length\_1748\_cov\_164.263153 1087-1090. Max. coverage (+): 0. Max coverage (-): 0

Region: NODE\_28897\_length\_1748\_cov\_164.263153 1091-1093. Max. coverage (+): 0. Max coverage (-): 0

Region: NODE\_28897\_length\_1748\_cov\_164.263153 1094-1097. Max. coverage (+): 0. Max coverage (-): 0

Region: NODE\_28897\_length\_1748\_cov\_164.263153 1098-1100. Max. coverage (+): 0. Max coverage (-): 0

Region: NODE\_28897\_length\_1748\_cov\_164.263153 1101-1104. Max. coverage (+): 0. Max coverage (-): 0.15

Region: NODE\_28897\_length\_1748\_cov\_164.263153 1105-1108. Max. coverage (+): 0. Max coverage (-): 0.31

Region: NODE\_28897\_length\_1748\_cov\_164.263153 1109-1111. Max. coverage (+): 0. Max coverage (-): 0.31

Region: NODE\_28897\_length\_1748\_cov\_164.263153 1112-1115. Max. coverage (+): 0. Max coverage (-): 0.23

Region: NODE\_28897\_length\_1748\_cov\_164.263153 1116-1118. Max. coverage (+): 0. Max coverage (-): 0.15

Region: NODE\_28897\_length\_1748\_cov\_164.263153 1119-1122. Max. coverage (+): 0. Max coverage (-): 0

Region: NODE\_28897\_length\_1748\_cov\_164.263153 1123-1126. Max. coverage (+): 0. Max coverage (-): 0

Region: NODE\_28897\_length\_1748\_cov\_164.263153 1127-1129. Max. coverage (+): 0. Max coverage (-): 0

Region: NODE\_28897\_length\_1748\_cov\_164.263153 1130-1133. Max. coverage (+): 0.08. Max coverage (-): 0

Region: NODE\_28897\_length\_1748\_cov\_164.263153 1134-1136. Max. coverage (+): 0.08. Max coverage (-): 0

Region: NODE\_28897\_length\_1748\_cov\_164.263153 1137-1140. Max. coverage (+): 0. Max coverage (-): 0

Region: NODE\_28897\_length\_1748\_cov\_164.263153 1141-1144. Max. coverage (+): 0. Max coverage (-): 0

Region: NODE\_28897\_length\_1748\_cov\_164.263153 1145-1147. Max. coverage (+): 0. Max coverage (-): 0

Region: NODE\_28897\_length\_1748\_cov\_164.263153 1148-1151. Max. coverage (+): 0. Max coverage (-): 0.77

Region: NODE\_28897\_length\_1748\_cov\_164.263153 1152-1154. Max. coverage (+): 0. Max coverage (-): 0.38

Region: NODE\_28897\_length\_1748\_cov\_164.263153 1155-1158. Max. coverage (+): 0. Max coverage (-): 0.15

Region: NODE\_28897\_length\_1748\_cov\_164.263153 1159-1162. Max. coverage (+): 0. Max coverage (-): 0.31

Region: NODE\_28897\_length\_1748\_cov\_164.263153 1163-1165. Max. coverage (+): 0. Max coverage (-): 0

Region: NODE\_28897\_length\_1748\_cov\_164.263153 1166-1169. Max. coverage (+): 0. Max coverage (-): 0

Region: NODE\_28897\_length\_1748\_cov\_164.263153 1170-1172. Max. coverage (+): 0. Max coverage (-): 0

Region: NODE\_28897\_length\_1748\_cov\_164.263153 1173-1176. Max. coverage (+): 0. Max coverage (-): 0

Region: NODE\_28897\_length\_1748\_cov\_164.263153 1177-1180. Max. coverage (+): 0.15. Max coverage (-): 0

Region: NODE\_28897\_length\_1748\_cov\_164.263153 1181-1183. Max. coverage (+): 0.08. Max coverage (-): 0

Region: NODE\_28897\_length\_1748\_cov\_164.263153 1184-1187. Max. coverage (+): 0.23. Max coverage (-): 0

Region: NODE\_28897\_length\_1748\_cov\_164.263153 1188-1190. Max. coverage (+): 0.15. Max coverage (-): 0

Region: NODE\_28897\_length\_1748\_cov\_164.263153 1191-1194. Max. coverage (+): 0. Max coverage (-): 0.54

Region: NODE\_28897\_length\_1748\_cov\_164.263153 1195-1198. Max. coverage (+): 0. Max coverage (-): 1.3

Region: NODE\_28897\_length\_1748\_cov\_164.263153 1199-1201. Max. coverage (+): 0. Max coverage (-): 1.69

Region: NODE\_28897\_length\_1748\_cov\_164.263153 1202-1205. Max. coverage (+): 0. Max coverage (-): 0.46

Region: NODE\_28897\_length\_1748\_cov\_164.263153 1206-1208. Max. coverage (+): 0. Max coverage (-): 0

Region: NODE\_28897\_length\_1748\_cov\_164.263153 1209-1212. Max. coverage (+): 0. Max coverage (-): 0

Region: NODE\_28897\_length\_1748\_cov\_164.263153 1213-1216. Max. coverage (+): 0.31. Max coverage (-): 0

Region: NODE\_28897\_length\_1748\_cov\_164.263153 1217-1219. Max. coverage (+): 0.31. Max coverage (-): 0

Region: NODE\_28897\_length\_1748\_cov\_164.263153 1220-1223. Max. coverage (+): 0. Max coverage (-): 0

Region: NODE\_28897\_length\_1748\_cov\_164.263153 1224-1226. Max. coverage (+): 0. Max coverage (-): 0.08

Region: NODE\_28897\_length\_1748\_cov\_164.263153 1227-1230. Max. coverage (+): 0. Max coverage (-): 0.15

Region: NODE\_28897\_length\_1748\_cov\_164.263153 1231-1234. Max. coverage (+): 0. Max coverage (-): 0.23

Region: NODE\_28897\_length\_1748\_cov\_164.263153 1235-1237. Max. coverage (+): 0. Max coverage (-): 0.23

Region: NODE\_28897\_length\_1748\_cov\_164.263153 1238-1241. Max. coverage (+): 0. Max coverage (-): 0.15

Region: NODE\_28897\_length\_1748\_cov\_164.263153 1242-1244. Max. coverage (+): 0. Max coverage (-): 0.15

Region: NODE\_28897\_length\_1748\_cov\_164.263153 1245-1248. Max. coverage (+): 0. Max coverage (-): 0.08

Region: NODE\_28897\_length\_1748\_cov\_164.263153 1249-1252. Max. coverage (+): 0. Max coverage (-): 0

Region: NODE\_28897\_length\_1748\_cov\_164.263153 1253-1255. Max. coverage (+): 0.08. Max coverage (-): 0

Region: NODE\_28897\_length\_1748\_cov\_164.263153 1256-1259. Max. coverage (+): 0.08. Max coverage (-): 0

Region: NODE\_28897\_length\_1748\_cov\_164.263153 1260-1262. Max. coverage (+): 0. Max coverage (-): 0

Region: NODE\_28897\_length\_1748\_cov\_164.263153 1263-1266. Max. coverage (+): 0. Max coverage (-): 0

Region: NODE\_28897\_length\_1748\_cov\_164.263153 1267-1270. Max. coverage (+): 0. Max coverage (-): 0

Region: NODE\_28897\_length\_1748\_cov\_164.263153 1271-1273. Max. coverage (+): 0. Max coverage (-): 0.15

Region: NODE\_28897\_length\_1748\_cov\_164.263153 1274-1277. Max. coverage (+): 0. Max coverage (-): 1.46

Region: NODE\_28897\_length\_1748\_cov\_164.263153 1278-1280. Max. coverage (+): 0. Max coverage (-): 0.84

Region: NODE\_28897\_length\_1748\_cov\_164.263153 1281-1284. Max. coverage (+): 0. Max coverage (-): 0.31

Region: NODE\_28897\_length\_1748\_cov\_164.263153 1285-1288. Max. coverage (+): 0. Max coverage (-): 0.08

Region: NODE\_28897\_length\_1748\_cov\_164.263153 1289-1291. Max. coverage (+): 0. Max coverage (-): 0

Region: NODE\_28897\_length\_1748\_cov\_164.263153 1292-1295. Max. coverage (+): 0.38. Max coverage (-): 0

Region: NODE\_28897\_length\_1748\_cov\_164.263153 1296-1298. Max. coverage (+): 0.31. Max coverage (-): 0

Region: NODE\_28897\_length\_1748\_cov\_164.263153 1299-1302. Max. coverage (+): 0. Max coverage (-): 0

Region: NODE\_28897\_length\_1748\_cov\_164.263153 1303-1306. Max. coverage (+): 0. Max coverage (-): 0.08

Region: NODE\_28897\_length\_1748\_cov\_164.263153 1307-1309. Max. coverage (+): 0. Max coverage (-): 0.23

Region: NODE\_28897\_length\_1748\_cov\_164.263153 1310-1313. Max. coverage (+): 0. Max coverage (-): 0.15

Region: NODE\_28897\_length\_1748\_cov\_164.263153 1314-1316. Max. coverage (+): 0. Max coverage (-): 0.08

Region: NODE\_28897\_length\_1748\_cov\_164.263153 1317-1320. Max. coverage (+): 0. Max coverage (-): 0.15

Region: NODE\_28897\_length\_1748\_cov\_164.263153 1321-1324. Max. coverage (+): 0. Max coverage (-): 0.38

Region: NODE\_28897\_length\_1748\_cov\_164.263153 1325-1327. Max. coverage (+): 0. Max coverage (-): 0.54

Region: NODE\_28897\_length\_1748\_cov\_164.263153 1328-1331. Max. coverage (+): 0. Max coverage (-): 0.38

Region: NODE\_28897\_length\_1748\_cov\_164.263153 1332-1334. Max. coverage (+): 0. Max coverage (-): 0

Region: NODE\_28897\_length\_1748\_cov\_164.263153 1335-1338. Max. coverage (+): 0.08. Max coverage (-): 0

Region: NODE\_28897\_length\_1748\_cov\_164.263153 1339-1342. Max. coverage (+): 0.08. Max coverage (-): 0

Region: NODE\_28897\_length\_1748\_cov\_164.263153 1343-1345. Max. coverage (+): 0.08. Max coverage (-): 0.08

Region: NODE\_28897\_length\_1748\_cov\_164.263153 1346-1349. Max. coverage (+): 0.08. Max coverage (-): 0.69

Region: NODE\_28897\_length\_1748\_cov\_164.263153 1350-1352. Max. coverage (+): 0. Max coverage (-): 1.84

Region: NODE\_28897\_length\_1748\_cov\_164.263153 1353-1356. Max. coverage (+): 0. Max coverage (-): 1.53

Region: NODE\_28897\_length\_1748\_cov\_164.263153 1357-1360. Max. coverage (+): 0. Max coverage (-): 0

Region: NODE\_28897\_length\_1748\_cov\_164.263153 1361-1363. Max. coverage (+): 0. Max coverage (-): 0

Region: NODE\_28897\_length\_1748\_cov\_164.263153 1364-1367. Max. coverage (+): 0. Max coverage (-): 0

Region: NODE\_28897\_length\_1748\_cov\_164.263153 1368-1370. Max. coverage (+): 1.23. Max coverage (-): 0

Region: NODE\_28897\_length\_1748\_cov\_164.263153 1371-1374. Max. coverage (+): 1.23. Max coverage (-): 0

Region: NODE\_28897\_length\_1748\_cov\_164.263153 1375-1378. Max. coverage (+): 0. Max coverage (-): 0

Region: NODE\_28897\_length\_1748\_cov\_164.263153 1379-1381. Max. coverage (+): 0. Max coverage (-): 0.15

Region: NODE\_28897\_length\_1748\_cov\_164.263153 1382-1385. Max. coverage (+): 0. Max coverage (-): 0.15

Region: NODE\_28897\_length\_1748\_cov\_164.263153 1386-1388. Max. coverage (+): 0. Max coverage (-): 0.08

Region: NODE\_28897\_length\_1748\_cov\_164.263153 1389-1392. Max. coverage (+): 0. Max coverage (-): 13.02

Region: NODE\_28897\_length\_1748\_cov\_164.263153 1393-1396. Max. coverage (+): 0. Max coverage (-): 4.29

Region: NODE\_28897\_length\_1748\_cov\_164.263153 1397-1399. Max. coverage (+): 0. Max coverage (-): 0

Region: NODE\_28897\_length\_1748\_cov\_164.263153 1400-1403. Max. coverage (+): 0. Max coverage (-): 0

Region: NODE\_28897\_length\_1748\_cov\_164.263153 1404-1406. Max. coverage (+): 0. Max coverage (-): 0

Region: NODE\_28897\_length\_1748\_cov\_164.263153 1407-1410. Max. coverage (+): 0.27. Max coverage (-): 0

Region: NODE\_28897\_length\_1748\_cov\_164.263153 1411-1414. Max. coverage (+): 0.19. Max coverage (-): 0

Region: NODE\_28897\_length\_1748\_cov\_164.263153 1415-1417. Max. coverage (+): 0. Max coverage (-): 0

Region: NODE\_28897\_length\_1748\_cov\_164.263153 1418-1421. Max. coverage (+): 0. Max coverage (-): 0

Region: NODE\_28897\_length\_1748\_cov\_164.263153 1422-1424. Max. coverage (+): 0. Max coverage (-): 0

Region: NODE\_28897\_length\_1748\_cov\_164.263153 1425-1428. Max. coverage (+): 0.08. Max coverage (-): 0

Region: NODE\_28897\_length\_1748\_cov\_164.263153 1429-1432. Max. coverage (+): 0.08. Max coverage (-): 0

Region: NODE\_28897\_length\_1748\_cov\_164.263153 1433-1435. Max. coverage (+): 0. Max coverage (-): 0

Region: NODE\_28897\_length\_1748\_cov\_164.263153 1436-1439. Max. coverage (+): 0. Max coverage (-): 0.15

Region: NODE\_28897\_length\_1748\_cov\_164.263153 1440-1442. Max. coverage (+): 0. Max coverage (-): 0.23

Region: NODE\_28897\_length\_1748\_cov\_164.263153 1443-1446. Max. coverage (+): 0. Max coverage (-): 0.23

Region: NODE\_28897\_length\_1748\_cov\_164.263153 1447-1450. Max. coverage (+): 0. Max coverage (-): 0.15

Region: NODE\_28897\_length\_1748\_cov\_164.263153 1451-1453. Max. coverage (+): 0. Max coverage (-): 0.61

Region: NODE\_28897\_length\_1748\_cov\_164.263153 1454-1457. Max. coverage (+): 0. Max coverage (-): 0.61

Region: NODE\_28897\_length\_1748\_cov\_164.263153 1458-1461. Max. coverage (+): 0.23. Max coverage (-): 0.31

Region: NODE\_28897\_length\_1748\_cov\_164.263153 1462-1464. Max. coverage (+): 0.23. Max coverage (-): 0.23

Region: NODE\_28897\_length\_1748\_cov\_164.263153 1465-1468. Max. coverage (+): 0.23. Max coverage (-): 0

Region: NODE\_28897\_length\_1748\_cov\_164.263153 1469-1471. Max. coverage (+): 0.15. Max coverage (-): 0

Region: NODE\_28897\_length\_1748\_cov\_164.263153 1472-1475. Max. coverage (+): 0. Max coverage (-): 0.15

Region: NODE\_28897\_length\_1748\_cov\_164.263153 1476-1479. Max. coverage (+): 0. Max coverage (-): 0.15

Region: NODE\_28897\_length\_1748\_cov\_164.263153 1480-1482. Max. coverage (+): 0.08. Max coverage (-): 0

Region: NODE\_28897\_length\_1748\_cov\_164.263153 1483-1486. Max. coverage (+): 0.08. Max coverage (-): 0

Region: NODE\_28897\_length\_1748\_cov\_164.263153 1487-1489. Max. coverage (+): 0. Max coverage (-): 0

Region: NODE\_28897\_length\_1748\_cov\_164.263153 1490-1493. Max. coverage (+): 0. Max coverage (-): 0.23

Region: NODE\_28897\_length\_1748\_cov\_164.263153 1494-1497. Max. coverage (+): 0. Max coverage (-): 0.46

Region: NODE\_28897\_length\_1748\_cov\_164.263153 1498-1500. Max. coverage (+): 0. Max coverage (-): 0.31

Region: NODE\_28897\_length\_1748\_cov\_164.263153 1501-1504. Max. coverage (+): 0. Max coverage (-): 0.08

Region: NODE\_28897\_length\_1748\_cov\_164.263153 1505-1507. Max. coverage (+): 0. Max coverage (-): 0

Region: NODE\_28897\_length\_1748\_cov\_164.263153 1508-1511. Max. coverage (+): 0.31. Max coverage (-): 0.08

Region: NODE\_28897\_length\_1748\_cov\_164.263153 1512-1515. Max. coverage (+): 1.84. Max coverage (-): 0.08

Region: NODE\_28897\_length\_1748\_cov\_164.263153 1516-1518. Max. coverage (+): 1.38. Max coverage (-): 0

Region: NODE\_28897\_length\_1748\_cov\_164.263153 1519-1522. Max. coverage (+): 0.15. Max coverage (-): 0

Region: NODE\_28897\_length\_1748\_cov\_164.263153 1523-1525. Max. coverage (+): 0.15. Max coverage (-): 0.38

Region: NODE\_28897\_length\_1748\_cov\_164.263153 1526-1529. Max. coverage (+): 0. Max coverage (-): 0.61

Region: NODE\_28897\_length\_1748\_cov\_164.263153 1530-1533. Max. coverage (+): 0.08. Max coverage (-): 0

Region: NODE\_28897\_length\_1748\_cov\_164.263153 1534-1536. Max. coverage (+): 0.08. Max coverage (-): 0

Region: NODE\_28897\_length\_1748\_cov\_164.263153 1537-1540. Max. coverage (+): 0. Max coverage (-): 0

Region: NODE\_28897\_length\_1748\_cov\_164.263153 1541-1543. Max. coverage (+): 0. Max coverage (-): 0

Region: NODE\_28897\_length\_1748\_cov\_164.263153 1544-1547. Max. coverage (+): 0.77. Max coverage (-): 0

Region: NODE\_28897\_length\_1748\_cov\_164.263153 1548-1551. Max. coverage (+): 0.77. Max coverage (-): 0

Region: NODE\_28897\_length\_1748\_cov\_164.263153 1552-1554. Max. coverage (+): 0. Max coverage (-): 0

Region: NODE\_28897\_length\_1748\_cov\_164.263153 1555-1558. Max. coverage (+): 0. Max coverage (-): 0

Region: NODE\_28897\_length\_1748\_cov\_164.263153 1559-1561. Max. coverage (+): 0. Max coverage (-): 0

Region: NODE\_28897\_length\_1748\_cov\_164.263153 1562-1565. Max. coverage (+): 0. Max coverage (-): 0.15

Region: NODE\_28897\_length\_1748\_cov\_164.263153 1566-1569. Max. coverage (+): 0. Max coverage (-): 0.15

Region: NODE\_28897\_length\_1748\_cov\_164.263153 1570-1572. Max. coverage (+): 0. Max coverage (-): 1.92

Region: NODE\_28897\_length\_1748\_cov\_164.263153 1573-1576. Max. coverage (+): 0. Max coverage (-): 1.99

Region: NODE\_28897\_length\_1748\_cov\_164.263153 1577-1579. Max. coverage (+): 0. Max coverage (-): 0.31

Region: NODE\_28897\_length\_1748\_cov\_164.263153 1580-1583. Max. coverage (+): 0.08. Max coverage (-): 0

Region: NODE\_28897\_length\_1748\_cov\_164.263153 1584-1587. Max. coverage (+): 0.08. Max coverage (-): 0

Region: NODE\_28897\_length\_1748\_cov\_164.263153 1588-1590. Max. coverage (+): 0.77. Max coverage (-): 0

Region: NODE\_28897\_length\_1748\_cov\_164.263153 1591-1594. Max. coverage (+): 0.77. Max coverage (-): 0

Region: NODE\_28897\_length\_1748\_cov\_164.263153 1595-1597. Max. coverage (+): 0.23. Max coverage (-): 0

Region: NODE\_28897\_length\_1748\_cov\_164.263153 1598-1601. Max. coverage (+): 0.23. Max coverage (-): 0.15

Region: NODE\_28897\_length\_1748\_cov\_164.263153 1602-1605. Max. coverage (+): 0. Max coverage (-): 0.15

Region: NODE\_28897\_length\_1748\_cov\_164.263153 1606-1608. Max. coverage (+): 0. Max coverage (-): 0

Region: NODE\_28897\_length\_1748\_cov\_164.263153 1609-1612. Max. coverage (+): 0. Max coverage (-): 1.76

Region: NODE\_28897\_length\_1748\_cov\_164.263153 1613-1615. Max. coverage (+): 0. Max coverage (-): 1.61

Region: NODE\_28897\_length\_1748\_cov\_164.263153 1616-1619. Max. coverage (+): 0. Max coverage (-): 0.15

Region: NODE\_28897\_length\_1748\_cov\_164.263153 1620-1623. Max. coverage (+): 0. Max coverage (-): 0.15

Region: NODE\_28897\_length\_1748\_cov\_164.263153 1624-1626. Max. coverage (+): 0. Max coverage (-): 0

Region: NODE\_28897\_length\_1748\_cov\_164.263153 1627-1630. Max. coverage (+): 0.08. Max coverage (-): 0

Region: NODE\_28897\_length\_1748\_cov\_164.263153 1631-1633. Max. coverage (+): 0.08. Max coverage (-): 0

Region: NODE\_28897\_length\_1748\_cov\_164.263153 1634-1637. Max. coverage (+): 0. Max coverage (-): 0

Region: NODE\_28897\_length\_1748\_cov\_164.263153 1638-1641. Max. coverage (+): 0. Max coverage (-): 0

Region: NODE\_28897\_length\_1748\_cov\_164.263153 1642-1644. Max. coverage (+): 0. Max coverage (-): 0

Region: NODE\_28897\_length\_1748\_cov\_164.263153 1645-1648. Max. coverage (+): 0. Max coverage (-): 0

Region: NODE\_28897\_length\_1748\_cov\_164.263153 1649-1651. Max. coverage (+): 0. Max coverage (-): 0

Region: NODE\_28897\_length\_1748\_cov\_164.263153 1652-1655. Max. coverage (+): 0. Max coverage (-): 0.38

Region: NODE\_28897\_length\_1748\_cov\_164.263153 1656-1659. Max. coverage (+): 0. Max coverage (-): 2.3

Region: NODE\_28897\_length\_1748\_cov\_164.263153 1660-1662. Max. coverage (+): 0. Max coverage (-): 2.3

Region: NODE\_28897\_length\_1748\_cov\_164.263153 1663-1666. Max. coverage (+): 0. Max coverage (-): 2.83

Region: NODE\_28897\_length\_1748\_cov\_164.263153 1667-1669. Max. coverage (+): 0.08. Max coverage (-): 4.06

Region: NODE\_28897\_length\_1748\_cov\_164.263153 1670-1673. Max. coverage (+): 0.08. Max coverage (-): 1.3

Region: NODE\_28897\_length\_1748\_cov\_164.263153 1674-1677. Max. coverage (+): 0. Max coverage (-): 0.15

Region: NODE\_28897\_length\_1748\_cov\_164.263153 1678-1680. Max. coverage (+): 0. Max coverage (-): 0

Region: NODE\_28897\_length\_1748\_cov\_164.263153 1681-1684. Max. coverage (+): 0. Max coverage (-): 0

Region: NODE\_28897\_length\_1748\_cov\_164.263153 1685-1687. Max. coverage (+): 0.15. Max coverage (-): 0

Region: NODE\_28897\_length\_1748\_cov\_164.263153 1688-1691. Max. coverage (+): 0.23. Max coverage (-): 0

Region: NODE\_28897\_length\_1748\_cov\_164.263153 1692-1695. Max. coverage (+): 0.08. Max coverage (-): 0.08

Region: NODE\_28897\_length\_1748\_cov\_164.263153 1696-1698. Max. coverage (+): 0. Max coverage (-): 0.08

Region: NODE\_28897\_length\_1748\_cov\_164.263153 1699-1702. Max. coverage (+): 0. Max coverage (-): 0

Region: NODE\_28897\_length\_1748\_cov\_164.263153 1703-1705. Max. coverage (+): 0. Max coverage (-): 0

Region: NODE\_28897\_length\_1748\_cov\_164.263153 1706-1709. Max. coverage (+): 0. Max coverage (-): 0

Region: NODE\_28897\_length\_1748\_cov\_164.263153 1710-1713. Max. coverage (+): 0.15. Max coverage (-): 0.08

Region: NODE\_28897\_length\_1748\_cov\_164.263153 1714-1716. Max. coverage (+): 0.15. Max coverage (-): 0

Region: NODE\_28897\_length\_1748\_cov\_164.263153 1717-1720. Max. coverage (+): 0. Max coverage (-): 0

Region: NODE\_28897\_length\_1748\_cov\_164.263153 1721-1723. Max. coverage (+): 0.15. Max coverage (-): 0.08

Region: NODE\_28897\_length\_1748\_cov\_164.263153 1724-1727. Max. coverage (+): 0.15. Max coverage (-): 0.23

Region: NODE\_28897\_length\_1748\_cov\_164.263153 1728-1731. Max. coverage (+): 0. Max coverage (-): 0.23

Region: NODE\_28897\_length\_1748\_cov\_164.263153 1732-1734. Max. coverage (+): 0. Max coverage (-): 0.46

Region: NODE\_28897\_length\_1748\_cov\_164.263153 1735-1738. Max. coverage (+): 0. Max coverage (-): 0.23

Region: NODE\_28897\_length\_1748\_cov\_164.263153 1739-1741. Max. coverage (+): 0. Max coverage (-): 0

Region: NODE\_28897\_length\_1748\_cov\_164.263153 1742-1745. Max. coverage (+): 0. Max coverage (-): 0

Region: NODE\_28897\_length\_1748\_cov\_164.263153 1746-1749. Max. coverage (+): 0. Max coverage (-): 0

Region: NODE\_28897\_length\_1748\_cov\_164.263153 1750-1752. Max. coverage (+): 0. Max coverage (-): 0

Region: NODE\_28897\_length\_1748\_cov\_164.263153 1753-1756. Max. coverage (+): 0. Max coverage (-): 0

Region: NODE\_28897\_length\_1748\_cov\_164.263153 1757-1759. Max. coverage (+): 0. Max coverage (-): 0

Region: NODE\_28897\_length\_1748\_cov\_164.263153 1760-1763. Max. coverage (+): 0. Max coverage (-): 0

Region: NODE\_28897\_length\_1748\_cov\_164.263153 1764-1767. Max. coverage (+): 0.08. Max coverage (-): 0.15

Region: NODE\_28897\_length\_1748\_cov\_164.263153 1768-1770. Max. coverage (+): 0. Max coverage (-): 0.15

Region: NODE\_28897\_length\_1748\_cov\_164.263153 1771-1774. Max. coverage (+): 0. Max coverage (-): 0.23

Region: NODE\_28897\_length\_1748\_cov\_164.263153 1775-1777. Max. coverage (+): 0.08. Max coverage (-): 0.31

Region: NODE\_28897\_length\_1748\_cov\_164.263153 1778-1781. Max. coverage (+): 0.08. Max coverage (-): 0.08

Region: NODE\_28897\_length\_1748\_cov\_164.263153 1782-1785. Max. coverage (+): 0. Max coverage (-): 0

Region: NODE\_28897\_length\_1748\_cov\_164.263153 1786-1788. Max. coverage (+): 0. Max coverage (-): 0

Region: NODE\_28897\_length\_1748\_cov\_164.263153 1789-1792. Max. coverage (+): 0. Max coverage (-): 0

Region: NODE\_28897\_length\_1748\_cov\_164.263153 1793-1795. Max. coverage (+): 0. Max coverage (-): 0

Region: NODE\_28897\_length\_1748\_cov\_164.263153 1796-1799. Max. coverage (+): 0. Max coverage (-): 0

Region: NODE\_28897\_length\_1748\_cov\_164.263153 1800-1803. Max. coverage (+): 0. Max coverage (-): 0

Region: NODE\_28897\_length\_1748\_cov\_164.263153 1804-. Max. coverage (+): 0. Max coverage (-): 0

RepeatMasker Color Code

**+**

100-98% Identity

<98-95% Identity

<95-90% Identity

<90-85% Identity

<85-80% Identity

<80-75% Identity

<75-70% Identity

<70% Identity

**-**

Gene Set Color Code

**+**

Gene

Pseudogene

Other

**-**

Topology/Coverage Color Code

Coverage Plus Strand

Coverage Minus Strand

Mainstrand: Plus

Mainstrand: Minus

Complementary Strand

Flanking Region  
(if option -flank >0)

Gene Set Annotation  
  
RepeatMasker Annotation  

**1. Tx1-4B\_Crp**: 75-1131 (+), Divergence to consensus: 44.7%  
**2. L1-4B\_DR**: 336-1140 (+), Divergence to consensus: 42.7%

  
Transcription Factor Binding Sites  

**RHOXF1** (Sequence: AGATTA (-): 127)  
**RHOXF1** (Sequence: AGATTA (-): 243)  
**RHOXF1** (Sequence: GGATTA (-): 348)  
**RHOXF1** (Sequence: AGATTA (-): 536)  
**RHOXF1** (Sequence: AGATTA (-): 743)  
**RHOXF1** (Sequence: AGCTTA (-): 1059)  
**RHOXF1** (Sequence: GGATCA (-): 1107)  
**RHOXF1** (Sequence: AGCTTA (-): 1315)  
**RFX4\_2** (Sequence: GTATCTAGG (-): 1373)  
**Sox5** (Sequence: ATTGTT (+): 1601)  
**FOXO3\_mmu** (Sequence: GGAAAACA (+): 786)  
**Nobox** (Sequence: TAATTGCT (+): 559)  
**Rhox11** (Sequence: CGCTGTTAA (+): 1564)  
**Sox5** (Sequence: AACAAT (-): 790)  
**POU2F1** (Sequence: TATGCAAAT (+): 1548)  
**POU5F1** (Sequence: ATGCAAA (+): 1549)
