# Supplementary material for: First characterization of PIWI-interacting RNA clusters in a cichlid fish with a B chromosome
Source: BMC Biol. 2022 Sep 21;20:204. doi: 10.1186/s12915-022-01403-2 (PMC9490952; doi:10.1186/s12915-022-01403-2)
Supplement: Supplementary file 1 — Additional file 1. Zipped folder with fasta and interactive html piRNA cluster information for the A. latifasciata genome. The nomenclature is as follows: number-pirna-cluster_sex_B-presence (f, female; m, male; 0b, without B chromosome; 1b, with B chromosome). [file 12915_2022_1403_MOESM1_ESM.zip › 111_f1b.html]

piRNA cluster 111\_f1b 9


Predicted piRNA cluster no. 111\_f1b
  

Show proTRAC run info
Hide proTRAC run info

/\  
                \_\_\_\_\_\_\_\_\_\_\_\_\_\_\_\_\_\_\_\_\_\_\_/\\_\_\_ /  \\_\_\_\_\_\_\_  
               I                      /  \  /    \      I  
               I     pro             /    \/      \     I  
               I        TRAC        /               \   I  
               I   \_\_\_\_\_\_\_\_\_\_\_\_\_\_\_\_/\_\_\_\_\_\_\_\_\_\_\_\_\_\_\_\_\_\\_ I  
               I   \              /                     I  
               I    \            /                      I  
               I     \  /\      /       V.2.4.2         I  
               I      \/  \    /                        I  
               I\_\_\_\_\_\_\_\_\_\_\_\  /\_\_\_\_\_\_\_\_\_\_\_\_\_\_\_\_\_\_\_\_\_\_\_\_\_I  
                            \/  
  
  
================================= proTRAC ====================================  
VERSION: .......... 2.4.2  
LAST MODIFIED: .... 11. May 2018  
  
Please cite:  
Rosenkranz D, Zischler H. proTRAC - a software for probabilistic piRNA cluster  
detection, visualization and analysis. 2012. BMC Bioinformatics 13:5.  
  
  
Contact:  
David Rosenkranz  
Institute of Organismic and Molecular Evolutionary Biology  
Dept. Anthropology, small RNA group  
Johannes Gutenberg University Mainz  
email: rosenkranz@uni-mainz.de  
  
You can find the latest proTRAC version at:  
http://sourceforge.net/projects/protrac/files  
http://www.smallRNAgroup-mainz.de/software  
==============================================================================  
  
PARAMETERS:  
Map file: ...............piwi-femeas-1B.fa-collapse.map  
Genome file: ............../../../0B\_ala\_genome.fa  
RepeatMasker annotation: Alatifasciata-all0B-maryan-v2.fa\_corrected.out  
GeneSet:................./guest-storage/Data/annotation/Alatifasciata\_all0B\_maryan-v2\_out2017.gff  
  
Significant (p<=0.01) hit density will be calculated based  
on observed hit distribution.  
  
Sliding window size: ........................................ 5000 bp  
Sliding window increament: .................................. 1000 bp  
Normalize each hit by number of genomic hits: ............... yes  
Normalize each hit by number of sequence reads: ............. yes  
Normalize values (-> per million mapped reads): ............. yes  
Min. fraction of hits with 1T(U) or 10A: .................... 0.75  
Alternatively: Min. fraction of hits with 1T(U) and 10A: .... 0.5  
Min. fraction of hits with typical piRNA length: ............ 0.75  
Typical piRNA length: ....................................... 24-32 nt  
Min. size of a piRNA cluster: ............................... 1000 bp.  
Min. number of hits (absolute): ............................. 0  
Min. number of hits (normalized): ........................... 0  
Min. fraction of hits on the mainstrand: .................... 0.75  
Top fraction of mapped sequences (in terms of read counts): . 1%  
Top fraction accounts for max. n% of sequence reads: ........ 90%  
Min. fraction of hits on each arm of a bidirectional cluster: 0.05  
Output html file for each cluster: .......................... yes  
Output a summary table: ..................................... yes  
Output a FASTA file for each cluster (piRNA sequences): ..... yes  
Output a FASTA file comprising cluster sequences: ........... yes  
Output a GTF file for predicted piRNA clusters: ..............yes  
Search DNA motifs in clusters: .............................. yes  
Output flanking sequences: +/- .............................. 0 bp  
Output ~.pTi file: .......................................... no  
==============================================================================  
  
  
Genome size (without gaps): ............ 758543724 bp  
Gaps (N/X/-): .......................... 417479 bp  
Mapped reads: .......................... 10641844  
Non-identical sequences: ............... 2832837  
Genomic hits: .......................... 26056853  
Significant densitiy of mapped reads: .. 368.713530323068 reads/kb

Show proTRAC cluster info
Hide proTRAC cluster info

|  |  |
| --- | --- |
| Location | NODE\_28897\_length\_1748\_cov\_164.263153 |
| Coordinates | 6-1808 |
| Size [bp] | 1803 |
| Sequence hit loci | 617 |
| Mapped reads (normalized) | 1889 |
| Mapped reads (normalized) per kb | 1047.7 |
| Normalized reads with 1T (1U) | 80.1% |
| Normalized reads with 10A | 53.6% |
| Normalized reads with length 24-32 nt | 99.2% |
| Normalized reads on the main strand(s) | 80.3% |
| Predicted directionality | bi:plus-minus (split between 1548 and 1554) |

100%

0%

1T (1U)  
reads

10A reads

24-32 nt  
reads

reads on mainstrand

**Either the amount of reads with 1T (1U) OR 10A has to exceed 75% (set with option: -1Tor10A)  
Alternatively the amount of reads with 1T (1U) AND 10A has to exceed 50% (set with option: -1Tand10A)  
Minimum amount of reads with preferred size is 75% (set with option: -pisize)  
Minimum amount of reads on the main strand(s) is 75% (set with option: -clstrand)**

Show read coverage
Hide read coverage

WHAT DO I SEE HERE?  
This chart shows the location of mapped sequence reads within a predicted piRNA cluster. The color refers to the number of genomic hits produced by the sequence read in question. A dark red bar indicates that this sequence read produces many other hits elsewhere in the genome. Many adjacent red or yellow bars can indicate the presence of a multi-copy element such as transposons or rRNA genes. A dark green bar indicates that this sequence read maps uniquely to this locus.

1 hit

2-5 hits

6-10 hits

11-20 hits

21-50 hits

51-100 hits

> 100 hits

NODE\_28897\_length\_1748\_cov\_164.263153

6

1808

Gene Set

RepeatMasker

Mapped  
Reads

39.18

plus strand

minus strand

39.18

Region: NODE\_28897\_length\_1748\_cov\_164.263153 3829-7. Max. coverage (+): 0. Max coverage (-): 0.03

Region: NODE\_28897\_length\_1748\_cov\_164.263153 8-11. Max. coverage (+): 0.06. Max coverage (-): 0

Region: NODE\_28897\_length\_1748\_cov\_164.263153 12-15. Max. coverage (+): 0.06. Max coverage (-): 0

Region: NODE\_28897\_length\_1748\_cov\_164.263153 16-18. Max. coverage (+): 0.22. Max coverage (-): 0

Region: NODE\_28897\_length\_1748\_cov\_164.263153 19-22. Max. coverage (+): 0.09. Max coverage (-): 0

Region: NODE\_28897\_length\_1748\_cov\_164.263153 23-25. Max. coverage (+): 0.03. Max coverage (-): 0

Region: NODE\_28897\_length\_1748\_cov\_164.263153 26-29. Max. coverage (+): 0.06. Max coverage (-): 0.03

Region: NODE\_28897\_length\_1748\_cov\_164.263153 30-33. Max. coverage (+): 1.03. Max coverage (-): 0.06

Region: NODE\_28897\_length\_1748\_cov\_164.263153 34-36. Max. coverage (+): 0.06. Max coverage (-): 0.06

Region: NODE\_28897\_length\_1748\_cov\_164.263153 37-40. Max. coverage (+): 0.03. Max coverage (-): 0

Region: NODE\_28897\_length\_1748\_cov\_164.263153 41-43. Max. coverage (+): 0. Max coverage (-): 0

Region: NODE\_28897\_length\_1748\_cov\_164.263153 44-47. Max. coverage (+): 0.38. Max coverage (-): 0.09

Region: NODE\_28897\_length\_1748\_cov\_164.263153 48-51. Max. coverage (+): 0.09. Max coverage (-): 0

Region: NODE\_28897\_length\_1748\_cov\_164.263153 52-54. Max. coverage (+): 0. Max coverage (-): 0

Region: NODE\_28897\_length\_1748\_cov\_164.263153 55-58. Max. coverage (+): 0. Max coverage (-): 0

Region: NODE\_28897\_length\_1748\_cov\_164.263153 59-61. Max. coverage (+): 0. Max coverage (-): 0

Region: NODE\_28897\_length\_1748\_cov\_164.263153 62-65. Max. coverage (+): 0.09. Max coverage (-): 0

Region: NODE\_28897\_length\_1748\_cov\_164.263153 66-69. Max. coverage (+): 0. Max coverage (-): 0

Region: NODE\_28897\_length\_1748\_cov\_164.263153 70-72. Max. coverage (+): 0. Max coverage (-): 0

Region: NODE\_28897\_length\_1748\_cov\_164.263153 73-76. Max. coverage (+): 0. Max coverage (-): 0

Region: NODE\_28897\_length\_1748\_cov\_164.263153 77-79. Max. coverage (+): 0. Max coverage (-): 0.09

Region: NODE\_28897\_length\_1748\_cov\_164.263153 80-83. Max. coverage (+): 0. Max coverage (-): 0

Region: NODE\_28897\_length\_1748\_cov\_164.263153 84-87. Max. coverage (+): 0. Max coverage (-): 0

Region: NODE\_28897\_length\_1748\_cov\_164.263153 88-90. Max. coverage (+): 0. Max coverage (-): 0

Region: NODE\_28897\_length\_1748\_cov\_164.263153 91-94. Max. coverage (+): 0. Max coverage (-): 0

Region: NODE\_28897\_length\_1748\_cov\_164.263153 95-97. Max. coverage (+): 0. Max coverage (-): 0

Region: NODE\_28897\_length\_1748\_cov\_164.263153 98-101. Max. coverage (+): 1.03. Max coverage (-): 0

Region: NODE\_28897\_length\_1748\_cov\_164.263153 102-105. Max. coverage (+): 1.13. Max coverage (-): 0

Region: NODE\_28897\_length\_1748\_cov\_164.263153 106-108. Max. coverage (+): 0.09. Max coverage (-): 0.09

Region: NODE\_28897\_length\_1748\_cov\_164.263153 109-112. Max. coverage (+): 0.09. Max coverage (-): 0.09

Region: NODE\_28897\_length\_1748\_cov\_164.263153 113-115. Max. coverage (+): 0. Max coverage (-): 0

Region: NODE\_28897\_length\_1748\_cov\_164.263153 116-119. Max. coverage (+): 0. Max coverage (-): 0

Region: NODE\_28897\_length\_1748\_cov\_164.263153 120-123. Max. coverage (+): 0. Max coverage (-): 0

Region: NODE\_28897\_length\_1748\_cov\_164.263153 124-126. Max. coverage (+): 0. Max coverage (-): 0.09

Region: NODE\_28897\_length\_1748\_cov\_164.263153 127-130. Max. coverage (+): 0. Max coverage (-): 0

Region: NODE\_28897\_length\_1748\_cov\_164.263153 131-134. Max. coverage (+): 0.09. Max coverage (-): 0

Region: NODE\_28897\_length\_1748\_cov\_164.263153 135-137. Max. coverage (+): 0.09. Max coverage (-): 0

Region: NODE\_28897\_length\_1748\_cov\_164.263153 138-141. Max. coverage (+): 0.38. Max coverage (-): 0

Region: NODE\_28897\_length\_1748\_cov\_164.263153 142-144. Max. coverage (+): 0.19. Max coverage (-): 0

Region: NODE\_28897\_length\_1748\_cov\_164.263153 145-148. Max. coverage (+): 1.41. Max coverage (-): 0

Region: NODE\_28897\_length\_1748\_cov\_164.263153 149-152. Max. coverage (+): 1.6. Max coverage (-): 0

Region: NODE\_28897\_length\_1748\_cov\_164.263153 153-155. Max. coverage (+): 0. Max coverage (-): 0.09

Region: NODE\_28897\_length\_1748\_cov\_164.263153 156-159. Max. coverage (+): 0. Max coverage (-): 0

Region: NODE\_28897\_length\_1748\_cov\_164.263153 160-162. Max. coverage (+): 0. Max coverage (-): 0

Region: NODE\_28897\_length\_1748\_cov\_164.263153 163-166. Max. coverage (+): 0.09. Max coverage (-): 0

Region: NODE\_28897\_length\_1748\_cov\_164.263153 167-170. Max. coverage (+): 27.25. Max coverage (-): 0

Region: NODE\_28897\_length\_1748\_cov\_164.263153 171-173. Max. coverage (+): 23.02. Max coverage (-): 0

Region: NODE\_28897\_length\_1748\_cov\_164.263153 174-177. Max. coverage (+): 0.28. Max coverage (-): 0

Region: NODE\_28897\_length\_1748\_cov\_164.263153 178-180. Max. coverage (+): 0.19. Max coverage (-): 0

Region: NODE\_28897\_length\_1748\_cov\_164.263153 181-184. Max. coverage (+): 0. Max coverage (-): 0

Region: NODE\_28897\_length\_1748\_cov\_164.263153 185-188. Max. coverage (+): 0. Max coverage (-): 0

Region: NODE\_28897\_length\_1748\_cov\_164.263153 189-191. Max. coverage (+): 0. Max coverage (-): 0

Region: NODE\_28897\_length\_1748\_cov\_164.263153 192-195. Max. coverage (+): 0. Max coverage (-): 0

Region: NODE\_28897\_length\_1748\_cov\_164.263153 196-198. Max. coverage (+): 0. Max coverage (-): 0

Region: NODE\_28897\_length\_1748\_cov\_164.263153 199-202. Max. coverage (+): 0. Max coverage (-): 0

Region: NODE\_28897\_length\_1748\_cov\_164.263153 203-206. Max. coverage (+): 0.09. Max coverage (-): 0

Region: NODE\_28897\_length\_1748\_cov\_164.263153 207-209. Max. coverage (+): 0. Max coverage (-): 0

Region: NODE\_28897\_length\_1748\_cov\_164.263153 210-213. Max. coverage (+): 0.09. Max coverage (-): 0

Region: NODE\_28897\_length\_1748\_cov\_164.263153 214-216. Max. coverage (+): 0.09. Max coverage (-): 0

Region: NODE\_28897\_length\_1748\_cov\_164.263153 217-220. Max. coverage (+): 0.09. Max coverage (-): 0.09

Region: NODE\_28897\_length\_1748\_cov\_164.263153 221-224. Max. coverage (+): 0. Max coverage (-): 0.38

Region: NODE\_28897\_length\_1748\_cov\_164.263153 225-227. Max. coverage (+): 0. Max coverage (-): 0

Region: NODE\_28897\_length\_1748\_cov\_164.263153 228-231. Max. coverage (+): 0.09. Max coverage (-): 0

Region: NODE\_28897\_length\_1748\_cov\_164.263153 232-234. Max. coverage (+): 0.09. Max coverage (-): 0

Region: NODE\_28897\_length\_1748\_cov\_164.263153 235-238. Max. coverage (+): 0.28. Max coverage (-): 0

Region: NODE\_28897\_length\_1748\_cov\_164.263153 239-242. Max. coverage (+): 4.89. Max coverage (-): 0

Region: NODE\_28897\_length\_1748\_cov\_164.263153 243-245. Max. coverage (+): 0.38. Max coverage (-): 0

Region: NODE\_28897\_length\_1748\_cov\_164.263153 246-249. Max. coverage (+): 0. Max coverage (-): 0

Region: NODE\_28897\_length\_1748\_cov\_164.263153 250-253. Max. coverage (+): 0.09. Max coverage (-): 0

Region: NODE\_28897\_length\_1748\_cov\_164.263153 254-256. Max. coverage (+): 0. Max coverage (-): 0.09

Region: NODE\_28897\_length\_1748\_cov\_164.263153 257-260. Max. coverage (+): 0. Max coverage (-): 0.09

Region: NODE\_28897\_length\_1748\_cov\_164.263153 261-263. Max. coverage (+): 0. Max coverage (-): 0

Region: NODE\_28897\_length\_1748\_cov\_164.263153 264-267. Max. coverage (+): 0. Max coverage (-): 0.47

Region: NODE\_28897\_length\_1748\_cov\_164.263153 268-271. Max. coverage (+): 0. Max coverage (-): 0.19

Region: NODE\_28897\_length\_1748\_cov\_164.263153 272-274. Max. coverage (+): 0. Max coverage (-): 0

Region: NODE\_28897\_length\_1748\_cov\_164.263153 275-278. Max. coverage (+): 0.19. Max coverage (-): 0

Region: NODE\_28897\_length\_1748\_cov\_164.263153 279-281. Max. coverage (+): 0. Max coverage (-): 0

Region: NODE\_28897\_length\_1748\_cov\_164.263153 282-285. Max. coverage (+): 8.55. Max coverage (-): 0

Region: NODE\_28897\_length\_1748\_cov\_164.263153 286-289. Max. coverage (+): 0. Max coverage (-): 0

Region: NODE\_28897\_length\_1748\_cov\_164.263153 290-292. Max. coverage (+): 0. Max coverage (-): 0

Region: NODE\_28897\_length\_1748\_cov\_164.263153 293-296. Max. coverage (+): 0. Max coverage (-): 0

Region: NODE\_28897\_length\_1748\_cov\_164.263153 297-299. Max. coverage (+): 0. Max coverage (-): 0

Region: NODE\_28897\_length\_1748\_cov\_164.263153 300-303. Max. coverage (+): 0. Max coverage (-): 0

Region: NODE\_28897\_length\_1748\_cov\_164.263153 304-307. Max. coverage (+): 0. Max coverage (-): 0

Region: NODE\_28897\_length\_1748\_cov\_164.263153 308-310. Max. coverage (+): 0. Max coverage (-): 0

Region: NODE\_28897\_length\_1748\_cov\_164.263153 311-314. Max. coverage (+): 0. Max coverage (-): 0

Region: NODE\_28897\_length\_1748\_cov\_164.263153 315-317. Max. coverage (+): 0.19. Max coverage (-): 0

Region: NODE\_28897\_length\_1748\_cov\_164.263153 318-321. Max. coverage (+): 0.09. Max coverage (-): 0

Region: NODE\_28897\_length\_1748\_cov\_164.263153 322-325. Max. coverage (+): 0. Max coverage (-): 0

Region: NODE\_28897\_length\_1748\_cov\_164.263153 326-328. Max. coverage (+): 0. Max coverage (-): 0

Region: NODE\_28897\_length\_1748\_cov\_164.263153 329-332. Max. coverage (+): 0.09. Max coverage (-): 0

Region: NODE\_28897\_length\_1748\_cov\_164.263153 333-335. Max. coverage (+): 0.28. Max coverage (-): 0

Region: NODE\_28897\_length\_1748\_cov\_164.263153 336-339. Max. coverage (+): 0.28. Max coverage (-): 0

Region: NODE\_28897\_length\_1748\_cov\_164.263153 340-343. Max. coverage (+): 5.17. Max coverage (-): 0

Region: NODE\_28897\_length\_1748\_cov\_164.263153 344-346. Max. coverage (+): 4.98. Max coverage (-): 0

Region: NODE\_28897\_length\_1748\_cov\_164.263153 347-350. Max. coverage (+): 0.56. Max coverage (-): 0

Region: NODE\_28897\_length\_1748\_cov\_164.263153 351-353. Max. coverage (+): 0.09. Max coverage (-): 0

Region: NODE\_28897\_length\_1748\_cov\_164.263153 354-357. Max. coverage (+): 0.09. Max coverage (-): 0

Region: NODE\_28897\_length\_1748\_cov\_164.263153 358-361. Max. coverage (+): 0.09. Max coverage (-): 0

Region: NODE\_28897\_length\_1748\_cov\_164.263153 362-364. Max. coverage (+): 0.09. Max coverage (-): 0

Region: NODE\_28897\_length\_1748\_cov\_164.263153 365-368. Max. coverage (+): 0.47. Max coverage (-): 0

Region: NODE\_28897\_length\_1748\_cov\_164.263153 369-372. Max. coverage (+): 0.09. Max coverage (-): 0

Region: NODE\_28897\_length\_1748\_cov\_164.263153 373-375. Max. coverage (+): 0.19. Max coverage (-): 0.09

Region: NODE\_28897\_length\_1748\_cov\_164.263153 376-379. Max. coverage (+): 0.19. Max coverage (-): 0

Region: NODE\_28897\_length\_1748\_cov\_164.263153 380-382. Max. coverage (+): 0. Max coverage (-): 0

Region: NODE\_28897\_length\_1748\_cov\_164.263153 383-386. Max. coverage (+): 0.09. Max coverage (-): 0

Region: NODE\_28897\_length\_1748\_cov\_164.263153 387-390. Max. coverage (+): 0.19. Max coverage (-): 0

Region: NODE\_28897\_length\_1748\_cov\_164.263153 391-393. Max. coverage (+): 0.47. Max coverage (-): 0

Region: NODE\_28897\_length\_1748\_cov\_164.263153 394-397. Max. coverage (+): 0.66. Max coverage (-): 0

Region: NODE\_28897\_length\_1748\_cov\_164.263153 398-400. Max. coverage (+): 2.26. Max coverage (-): 0

Region: NODE\_28897\_length\_1748\_cov\_164.263153 401-404. Max. coverage (+): 2.16. Max coverage (-): 0

Region: NODE\_28897\_length\_1748\_cov\_164.263153 405-408. Max. coverage (+): 0. Max coverage (-): 0

Region: NODE\_28897\_length\_1748\_cov\_164.263153 409-411. Max. coverage (+): 0. Max coverage (-): 0

Region: NODE\_28897\_length\_1748\_cov\_164.263153 412-415. Max. coverage (+): 0. Max coverage (-): 0

Region: NODE\_28897\_length\_1748\_cov\_164.263153 416-418. Max. coverage (+): 0. Max coverage (-): 0

Region: NODE\_28897\_length\_1748\_cov\_164.263153 419-422. Max. coverage (+): 0. Max coverage (-): 0

Region: NODE\_28897\_length\_1748\_cov\_164.263153 423-426. Max. coverage (+): 0. Max coverage (-): 0

Region: NODE\_28897\_length\_1748\_cov\_164.263153 427-429. Max. coverage (+): 0. Max coverage (-): 0

Region: NODE\_28897\_length\_1748\_cov\_164.263153 430-433. Max. coverage (+): 0.38. Max coverage (-): 0

Region: NODE\_28897\_length\_1748\_cov\_164.263153 434-436. Max. coverage (+): 0.28. Max coverage (-): 0

Region: NODE\_28897\_length\_1748\_cov\_164.263153 437-440. Max. coverage (+): 0.47. Max coverage (-): 0

Region: NODE\_28897\_length\_1748\_cov\_164.263153 441-444. Max. coverage (+): 0.38. Max coverage (-): 0

Region: NODE\_28897\_length\_1748\_cov\_164.263153 445-447. Max. coverage (+): 0.09. Max coverage (-): 0

Region: NODE\_28897\_length\_1748\_cov\_164.263153 448-451. Max. coverage (+): 0. Max coverage (-): 0.09

Region: NODE\_28897\_length\_1748\_cov\_164.263153 452-454. Max. coverage (+): 0. Max coverage (-): 0.09

Region: NODE\_28897\_length\_1748\_cov\_164.263153 455-458. Max. coverage (+): 0. Max coverage (-): 0

Region: NODE\_28897\_length\_1748\_cov\_164.263153 459-462. Max. coverage (+): 0. Max coverage (-): 0

Region: NODE\_28897\_length\_1748\_cov\_164.263153 463-465. Max. coverage (+): 0. Max coverage (-): 0

Region: NODE\_28897\_length\_1748\_cov\_164.263153 466-469. Max. coverage (+): 3.38. Max coverage (-): 0

Region: NODE\_28897\_length\_1748\_cov\_164.263153 470-472. Max. coverage (+): 0. Max coverage (-): 0

Region: NODE\_28897\_length\_1748\_cov\_164.263153 473-476. Max. coverage (+): 0. Max coverage (-): 0.09

Region: NODE\_28897\_length\_1748\_cov\_164.263153 477-480. Max. coverage (+): 0. Max coverage (-): 7.71

Region: NODE\_28897\_length\_1748\_cov\_164.263153 481-483. Max. coverage (+): 0. Max coverage (-): 9.4

Region: NODE\_28897\_length\_1748\_cov\_164.263153 484-487. Max. coverage (+): 0.09. Max coverage (-): 1.13

Region: NODE\_28897\_length\_1748\_cov\_164.263153 488-491. Max. coverage (+): 0.19. Max coverage (-): 0

Region: NODE\_28897\_length\_1748\_cov\_164.263153 492-494. Max. coverage (+): 0.19. Max coverage (-): 0

Region: NODE\_28897\_length\_1748\_cov\_164.263153 495-498. Max. coverage (+): 34.11. Max coverage (-): 0

Region: NODE\_28897\_length\_1748\_cov\_164.263153 499-501. Max. coverage (+): 39.18. Max coverage (-): 0

Region: NODE\_28897\_length\_1748\_cov\_164.263153 502-505. Max. coverage (+): 0.09. Max coverage (-): 0

Region: NODE\_28897\_length\_1748\_cov\_164.263153 506-509. Max. coverage (+): 0. Max coverage (-): 0.09

Region: NODE\_28897\_length\_1748\_cov\_164.263153 510-512. Max. coverage (+): 0. Max coverage (-): 0.09

Region: NODE\_28897\_length\_1748\_cov\_164.263153 513-516. Max. coverage (+): 0. Max coverage (-): 0

Region: NODE\_28897\_length\_1748\_cov\_164.263153 517-519. Max. coverage (+): 0. Max coverage (-): 0

Region: NODE\_28897\_length\_1748\_cov\_164.263153 520-523. Max. coverage (+): 0. Max coverage (-): 0

Region: NODE\_28897\_length\_1748\_cov\_164.263153 524-527. Max. coverage (+): 1.5. Max coverage (-): 0

Region: NODE\_28897\_length\_1748\_cov\_164.263153 528-530. Max. coverage (+): 1.6. Max coverage (-): 0

Region: NODE\_28897\_length\_1748\_cov\_164.263153 531-534. Max. coverage (+): 0.38. Max coverage (-): 0

Region: NODE\_28897\_length\_1748\_cov\_164.263153 535-537. Max. coverage (+): 0. Max coverage (-): 0

Region: NODE\_28897\_length\_1748\_cov\_164.263153 538-541. Max. coverage (+): 0. Max coverage (-): 0

Region: NODE\_28897\_length\_1748\_cov\_164.263153 542-545. Max. coverage (+): 0. Max coverage (-): 0.19

Region: NODE\_28897\_length\_1748\_cov\_164.263153 546-548. Max. coverage (+): 0. Max coverage (-): 0.19

Region: NODE\_28897\_length\_1748\_cov\_164.263153 549-552. Max. coverage (+): 0.09. Max coverage (-): 0.28

Region: NODE\_28897\_length\_1748\_cov\_164.263153 553-555. Max. coverage (+): 0.19. Max coverage (-): 0.28

Region: NODE\_28897\_length\_1748\_cov\_164.263153 556-559. Max. coverage (+): 0.19. Max coverage (-): 0

Region: NODE\_28897\_length\_1748\_cov\_164.263153 560-563. Max. coverage (+): 0.56. Max coverage (-): 0

Region: NODE\_28897\_length\_1748\_cov\_164.263153 564-566. Max. coverage (+): 0.85. Max coverage (-): 0.09

Region: NODE\_28897\_length\_1748\_cov\_164.263153 567-570. Max. coverage (+): 0.75. Max coverage (-): 0.09

Region: NODE\_28897\_length\_1748\_cov\_164.263153 571-573. Max. coverage (+): 0.66. Max coverage (-): 0.09

Region: NODE\_28897\_length\_1748\_cov\_164.263153 574-577. Max. coverage (+): 0. Max coverage (-): 0

Region: NODE\_28897\_length\_1748\_cov\_164.263153 578-581. Max. coverage (+): 0.19. Max coverage (-): 0

Region: NODE\_28897\_length\_1748\_cov\_164.263153 582-584. Max. coverage (+): 0.19. Max coverage (-): 0

Region: NODE\_28897\_length\_1748\_cov\_164.263153 585-588. Max. coverage (+): 0.19. Max coverage (-): 0

Region: NODE\_28897\_length\_1748\_cov\_164.263153 589-591. Max. coverage (+): 0.19. Max coverage (-): 0

Region: NODE\_28897\_length\_1748\_cov\_164.263153 592-595. Max. coverage (+): 0.28. Max coverage (-): 0

Region: NODE\_28897\_length\_1748\_cov\_164.263153 596-599. Max. coverage (+): 0. Max coverage (-): 0

Region: NODE\_28897\_length\_1748\_cov\_164.263153 600-602. Max. coverage (+): 0.38. Max coverage (-): 0

Region: NODE\_28897\_length\_1748\_cov\_164.263153 603-606. Max. coverage (+): 0.66. Max coverage (-): 0

Region: NODE\_28897\_length\_1748\_cov\_164.263153 607-610. Max. coverage (+): 0.38. Max coverage (-): 0

Region: NODE\_28897\_length\_1748\_cov\_164.263153 611-613. Max. coverage (+): 0. Max coverage (-): 0

Region: NODE\_28897\_length\_1748\_cov\_164.263153 614-617. Max. coverage (+): 0. Max coverage (-): 0.09

Region: NODE\_28897\_length\_1748\_cov\_164.263153 618-620. Max. coverage (+): 0. Max coverage (-): 0.28

Region: NODE\_28897\_length\_1748\_cov\_164.263153 621-624. Max. coverage (+): 0. Max coverage (-): 0.56

Region: NODE\_28897\_length\_1748\_cov\_164.263153 625-628. Max. coverage (+): 0.19. Max coverage (-): 0

Region: NODE\_28897\_length\_1748\_cov\_164.263153 629-631. Max. coverage (+): 0.38. Max coverage (-): 0

Region: NODE\_28897\_length\_1748\_cov\_164.263153 632-635. Max. coverage (+): 0.19. Max coverage (-): 0

Region: NODE\_28897\_length\_1748\_cov\_164.263153 636-638. Max. coverage (+): 0. Max coverage (-): 0

Region: NODE\_28897\_length\_1748\_cov\_164.263153 639-642. Max. coverage (+): 0.19. Max coverage (-): 0

Region: NODE\_28897\_length\_1748\_cov\_164.263153 643-646. Max. coverage (+): 0. Max coverage (-): 0

Region: NODE\_28897\_length\_1748\_cov\_164.263153 647-649. Max. coverage (+): 0. Max coverage (-): 0.09

Region: NODE\_28897\_length\_1748\_cov\_164.263153 650-653. Max. coverage (+): 0. Max coverage (-): 0

Region: NODE\_28897\_length\_1748\_cov\_164.263153 654-656. Max. coverage (+): 0. Max coverage (-): 0

Region: NODE\_28897\_length\_1748\_cov\_164.263153 657-660. Max. coverage (+): 0.19. Max coverage (-): 0

Region: NODE\_28897\_length\_1748\_cov\_164.263153 661-664. Max. coverage (+): 0. Max coverage (-): 0

Region: NODE\_28897\_length\_1748\_cov\_164.263153 665-667. Max. coverage (+): 0. Max coverage (-): 0

Region: NODE\_28897\_length\_1748\_cov\_164.263153 668-671. Max. coverage (+): 0.09. Max coverage (-): 0

Region: NODE\_28897\_length\_1748\_cov\_164.263153 672-674. Max. coverage (+): 0.75. Max coverage (-): 0

Region: NODE\_28897\_length\_1748\_cov\_164.263153 675-678. Max. coverage (+): 0. Max coverage (-): 0

Region: NODE\_28897\_length\_1748\_cov\_164.263153 679-682. Max. coverage (+): 0. Max coverage (-): 0.19

Region: NODE\_28897\_length\_1748\_cov\_164.263153 683-685. Max. coverage (+): 0. Max coverage (-): 0.38

Region: NODE\_28897\_length\_1748\_cov\_164.263153 686-689. Max. coverage (+): 0. Max coverage (-): 0.09

Region: NODE\_28897\_length\_1748\_cov\_164.263153 690-692. Max. coverage (+): 0. Max coverage (-): 0

Region: NODE\_28897\_length\_1748\_cov\_164.263153 693-696. Max. coverage (+): 0. Max coverage (-): 0

Region: NODE\_28897\_length\_1748\_cov\_164.263153 697-700. Max. coverage (+): 0. Max coverage (-): 0

Region: NODE\_28897\_length\_1748\_cov\_164.263153 701-703. Max. coverage (+): 0.19. Max coverage (-): 0

Region: NODE\_28897\_length\_1748\_cov\_164.263153 704-707. Max. coverage (+): 0.19. Max coverage (-): 0

Region: NODE\_28897\_length\_1748\_cov\_164.263153 708-710. Max. coverage (+): 0. Max coverage (-): 0

Region: NODE\_28897\_length\_1748\_cov\_164.263153 711-714. Max. coverage (+): 0. Max coverage (-): 0.09

Region: NODE\_28897\_length\_1748\_cov\_164.263153 715-718. Max. coverage (+): 0. Max coverage (-): 0.75

Region: NODE\_28897\_length\_1748\_cov\_164.263153 719-721. Max. coverage (+): 0. Max coverage (-): 0.56

Region: NODE\_28897\_length\_1748\_cov\_164.263153 722-725. Max. coverage (+): 0. Max coverage (-): 0.09

Region: NODE\_28897\_length\_1748\_cov\_164.263153 726-729. Max. coverage (+): 0. Max coverage (-): 0.09

Region: NODE\_28897\_length\_1748\_cov\_164.263153 730-732. Max. coverage (+): 0. Max coverage (-): 0.09

Region: NODE\_28897\_length\_1748\_cov\_164.263153 733-736. Max. coverage (+): 0.19. Max coverage (-): 0

Region: NODE\_28897\_length\_1748\_cov\_164.263153 737-739. Max. coverage (+): 0.38. Max coverage (-): 0

Region: NODE\_28897\_length\_1748\_cov\_164.263153 740-743. Max. coverage (+): 0. Max coverage (-): 0

Region: NODE\_28897\_length\_1748\_cov\_164.263153 744-747. Max. coverage (+): 0.09. Max coverage (-): 0.09

Region: NODE\_28897\_length\_1748\_cov\_164.263153 748-750. Max. coverage (+): 0.09. Max coverage (-): 0.09

Region: NODE\_28897\_length\_1748\_cov\_164.263153 751-754. Max. coverage (+): 0. Max coverage (-): 0.09

Region: NODE\_28897\_length\_1748\_cov\_164.263153 755-757. Max. coverage (+): 0. Max coverage (-): 0

Region: NODE\_28897\_length\_1748\_cov\_164.263153 758-761. Max. coverage (+): 0. Max coverage (-): 0

Region: NODE\_28897\_length\_1748\_cov\_164.263153 762-765. Max. coverage (+): 0. Max coverage (-): 0

Region: NODE\_28897\_length\_1748\_cov\_164.263153 766-768. Max. coverage (+): 0.85. Max coverage (-): 0

Region: NODE\_28897\_length\_1748\_cov\_164.263153 769-772. Max. coverage (+): 0.09. Max coverage (-): 0

Region: NODE\_28897\_length\_1748\_cov\_164.263153 773-775. Max. coverage (+): 0. Max coverage (-): 0

Region: NODE\_28897\_length\_1748\_cov\_164.263153 776-779. Max. coverage (+): 0.09. Max coverage (-): 0

Region: NODE\_28897\_length\_1748\_cov\_164.263153 780-783. Max. coverage (+): 0.09. Max coverage (-): 0

Region: NODE\_28897\_length\_1748\_cov\_164.263153 784-786. Max. coverage (+): 0. Max coverage (-): 0

Region: NODE\_28897\_length\_1748\_cov\_164.263153 787-790. Max. coverage (+): 0. Max coverage (-): 0

Region: NODE\_28897\_length\_1748\_cov\_164.263153 791-793. Max. coverage (+): 0. Max coverage (-): 0

Region: NODE\_28897\_length\_1748\_cov\_164.263153 794-797. Max. coverage (+): 0. Max coverage (-): 0

Region: NODE\_28897\_length\_1748\_cov\_164.263153 798-801. Max. coverage (+): 0. Max coverage (-): 0

Region: NODE\_28897\_length\_1748\_cov\_164.263153 802-804. Max. coverage (+): 0. Max coverage (-): 0.28

Region: NODE\_28897\_length\_1748\_cov\_164.263153 805-808. Max. coverage (+): 0.09. Max coverage (-): 0.28

Region: NODE\_28897\_length\_1748\_cov\_164.263153 809-811. Max. coverage (+): 0.09. Max coverage (-): 0

Region: NODE\_28897\_length\_1748\_cov\_164.263153 812-815. Max. coverage (+): 0. Max coverage (-): 0

Region: NODE\_28897\_length\_1748\_cov\_164.263153 816-819. Max. coverage (+): 0. Max coverage (-): 0

Region: NODE\_28897\_length\_1748\_cov\_164.263153 820-822. Max. coverage (+): 0. Max coverage (-): 0

Region: NODE\_28897\_length\_1748\_cov\_164.263153 823-826. Max. coverage (+): 0.85. Max coverage (-): 0

Region: NODE\_28897\_length\_1748\_cov\_164.263153 827-829. Max. coverage (+): 0. Max coverage (-): 0

Region: NODE\_28897\_length\_1748\_cov\_164.263153 830-833. Max. coverage (+): 0. Max coverage (-): 0

Region: NODE\_28897\_length\_1748\_cov\_164.263153 834-837. Max. coverage (+): 0. Max coverage (-): 0.09

Region: NODE\_28897\_length\_1748\_cov\_164.263153 838-840. Max. coverage (+): 0. Max coverage (-): 0.09

Region: NODE\_28897\_length\_1748\_cov\_164.263153 841-844. Max. coverage (+): 0. Max coverage (-): 0

Region: NODE\_28897\_length\_1748\_cov\_164.263153 845-848. Max. coverage (+): 0. Max coverage (-): 0

Region: NODE\_28897\_length\_1748\_cov\_164.263153 849-851. Max. coverage (+): 0.09. Max coverage (-): 0

Region: NODE\_28897\_length\_1748\_cov\_164.263153 852-855. Max. coverage (+): 0.09. Max coverage (-): 0.56

Region: NODE\_28897\_length\_1748\_cov\_164.263153 856-858. Max. coverage (+): 0. Max coverage (-): 0.38

Region: NODE\_28897\_length\_1748\_cov\_164.263153 859-862. Max. coverage (+): 0.09. Max coverage (-): 0.09

Region: NODE\_28897\_length\_1748\_cov\_164.263153 863-866. Max. coverage (+): 0. Max coverage (-): 0.09

Region: NODE\_28897\_length\_1748\_cov\_164.263153 867-869. Max. coverage (+): 0. Max coverage (-): 0

Region: NODE\_28897\_length\_1748\_cov\_164.263153 870-873. Max. coverage (+): 1.03. Max coverage (-): 0

Region: NODE\_28897\_length\_1748\_cov\_164.263153 874-876. Max. coverage (+): 0.94. Max coverage (-): 0

Region: NODE\_28897\_length\_1748\_cov\_164.263153 877-880. Max. coverage (+): 0.09. Max coverage (-): 0

Region: NODE\_28897\_length\_1748\_cov\_164.263153 881-884. Max. coverage (+): 0. Max coverage (-): 0.09

Region: NODE\_28897\_length\_1748\_cov\_164.263153 885-887. Max. coverage (+): 0. Max coverage (-): 0.19

Region: NODE\_28897\_length\_1748\_cov\_164.263153 888-891. Max. coverage (+): 0.09. Max coverage (-): 0.09

Region: NODE\_28897\_length\_1748\_cov\_164.263153 892-894. Max. coverage (+): 0.09. Max coverage (-): 0.09

Region: NODE\_28897\_length\_1748\_cov\_164.263153 895-898. Max. coverage (+): 0. Max coverage (-): 0

Region: NODE\_28897\_length\_1748\_cov\_164.263153 899-902. Max. coverage (+): 0.38. Max coverage (-): 0

Region: NODE\_28897\_length\_1748\_cov\_164.263153 903-905. Max. coverage (+): 0.19. Max coverage (-): 0

Region: NODE\_28897\_length\_1748\_cov\_164.263153 906-909. Max. coverage (+): 0.19. Max coverage (-): 0

Region: NODE\_28897\_length\_1748\_cov\_164.263153 910-912. Max. coverage (+): 0. Max coverage (-): 0

Region: NODE\_28897\_length\_1748\_cov\_164.263153 913-916. Max. coverage (+): 0. Max coverage (-): 0

Region: NODE\_28897\_length\_1748\_cov\_164.263153 917-920. Max. coverage (+): 0. Max coverage (-): 0.09

Region: NODE\_28897\_length\_1748\_cov\_164.263153 921-923. Max. coverage (+): 0. Max coverage (-): 0.19

Region: NODE\_28897\_length\_1748\_cov\_164.263153 924-927. Max. coverage (+): 0.28. Max coverage (-): 0.19

Region: NODE\_28897\_length\_1748\_cov\_164.263153 928-930. Max. coverage (+): 0.19. Max coverage (-): 0

Region: NODE\_28897\_length\_1748\_cov\_164.263153 931-934. Max. coverage (+): 0.19. Max coverage (-): 0

Region: NODE\_28897\_length\_1748\_cov\_164.263153 935-938. Max. coverage (+): 0.47. Max coverage (-): 0

Region: NODE\_28897\_length\_1748\_cov\_164.263153 939-941. Max. coverage (+): 0.28. Max coverage (-): 0.09

Region: NODE\_28897\_length\_1748\_cov\_164.263153 942-945. Max. coverage (+): 0. Max coverage (-): 0.09

Region: NODE\_28897\_length\_1748\_cov\_164.263153 946-948. Max. coverage (+): 0. Max coverage (-): 0

Region: NODE\_28897\_length\_1748\_cov\_164.263153 949-952. Max. coverage (+): 0. Max coverage (-): 0

Region: NODE\_28897\_length\_1748\_cov\_164.263153 953-956. Max. coverage (+): 0. Max coverage (-): 0

Region: NODE\_28897\_length\_1748\_cov\_164.263153 957-959. Max. coverage (+): 0. Max coverage (-): 0

Region: NODE\_28897\_length\_1748\_cov\_164.263153 960-963. Max. coverage (+): 0.09. Max coverage (-): 0

Region: NODE\_28897\_length\_1748\_cov\_164.263153 964-966. Max. coverage (+): 0.09. Max coverage (-): 0

Region: NODE\_28897\_length\_1748\_cov\_164.263153 967-970. Max. coverage (+): 0. Max coverage (-): 0

Region: NODE\_28897\_length\_1748\_cov\_164.263153 971-974. Max. coverage (+): 0. Max coverage (-): 0

Region: NODE\_28897\_length\_1748\_cov\_164.263153 975-977. Max. coverage (+): 0. Max coverage (-): 0

Region: NODE\_28897\_length\_1748\_cov\_164.263153 978-981. Max. coverage (+): 0. Max coverage (-): 0

Region: NODE\_28897\_length\_1748\_cov\_164.263153 982-985. Max. coverage (+): 0. Max coverage (-): 0

Region: NODE\_28897\_length\_1748\_cov\_164.263153 986-988. Max. coverage (+): 0. Max coverage (-): 0

Region: NODE\_28897\_length\_1748\_cov\_164.263153 989-992. Max. coverage (+): 0. Max coverage (-): 0

Region: NODE\_28897\_length\_1748\_cov\_164.263153 993-995. Max. coverage (+): 0. Max coverage (-): 0

Region: NODE\_28897\_length\_1748\_cov\_164.263153 996-999. Max. coverage (+): 0. Max coverage (-): 0

Region: NODE\_28897\_length\_1748\_cov\_164.263153 1000-1003. Max. coverage (+): 0. Max coverage (-): 0

Region: NODE\_28897\_length\_1748\_cov\_164.263153 1004-1006. Max. coverage (+): 0. Max coverage (-): 0

Region: NODE\_28897\_length\_1748\_cov\_164.263153 1007-1010. Max. coverage (+): 0. Max coverage (-): 0.09

Region: NODE\_28897\_length\_1748\_cov\_164.263153 1011-1013. Max. coverage (+): 0. Max coverage (-): 0.28

Region: NODE\_28897\_length\_1748\_cov\_164.263153 1014-1017. Max. coverage (+): 0. Max coverage (-): 0.38

Region: NODE\_28897\_length\_1748\_cov\_164.263153 1018-1021. Max. coverage (+): 0. Max coverage (-): 0

Region: NODE\_28897\_length\_1748\_cov\_164.263153 1022-1024. Max. coverage (+): 0. Max coverage (-): 0

Region: NODE\_28897\_length\_1748\_cov\_164.263153 1025-1028. Max. coverage (+): 0.19. Max coverage (-): 0

Region: NODE\_28897\_length\_1748\_cov\_164.263153 1029-1031. Max. coverage (+): 1.79. Max coverage (-): 0

Region: NODE\_28897\_length\_1748\_cov\_164.263153 1032-1035. Max. coverage (+): 1.6. Max coverage (-): 0

Region: NODE\_28897\_length\_1748\_cov\_164.263153 1036-1039. Max. coverage (+): 0. Max coverage (-): 0

Region: NODE\_28897\_length\_1748\_cov\_164.263153 1040-1042. Max. coverage (+): 0. Max coverage (-): 0

Region: NODE\_28897\_length\_1748\_cov\_164.263153 1043-1046. Max. coverage (+): 0. Max coverage (-): 0.38

Region: NODE\_28897\_length\_1748\_cov\_164.263153 1047-1049. Max. coverage (+): 0. Max coverage (-): 0.75

Region: NODE\_28897\_length\_1748\_cov\_164.263153 1050-1053. Max. coverage (+): 0. Max coverage (-): 0.38

Region: NODE\_28897\_length\_1748\_cov\_164.263153 1054-1057. Max. coverage (+): 0. Max coverage (-): 0

Region: NODE\_28897\_length\_1748\_cov\_164.263153 1058-1060. Max. coverage (+): 0. Max coverage (-): 0

Region: NODE\_28897\_length\_1748\_cov\_164.263153 1061-1064. Max. coverage (+): 0.47. Max coverage (-): 0

Region: NODE\_28897\_length\_1748\_cov\_164.263153 1065-1067. Max. coverage (+): 0.66. Max coverage (-): 0

Region: NODE\_28897\_length\_1748\_cov\_164.263153 1068-1071. Max. coverage (+): 0.19. Max coverage (-): 0

Region: NODE\_28897\_length\_1748\_cov\_164.263153 1072-1075. Max. coverage (+): 0. Max coverage (-): 0

Region: NODE\_28897\_length\_1748\_cov\_164.263153 1076-1078. Max. coverage (+): 0. Max coverage (-): 0

Region: NODE\_28897\_length\_1748\_cov\_164.263153 1079-1082. Max. coverage (+): 0. Max coverage (-): 0.28

Region: NODE\_28897\_length\_1748\_cov\_164.263153 1083-1085. Max. coverage (+): 0. Max coverage (-): 0.28

Region: NODE\_28897\_length\_1748\_cov\_164.263153 1086-1089. Max. coverage (+): 0. Max coverage (-): 0.09

Region: NODE\_28897\_length\_1748\_cov\_164.263153 1090-1093. Max. coverage (+): 0. Max coverage (-): 0

Region: NODE\_28897\_length\_1748\_cov\_164.263153 1094-1096. Max. coverage (+): 0. Max coverage (-): 0

Region: NODE\_28897\_length\_1748\_cov\_164.263153 1097-1100. Max. coverage (+): 0.19. Max coverage (-): 0

Region: NODE\_28897\_length\_1748\_cov\_164.263153 1101-1104. Max. coverage (+): 0.19. Max coverage (-): 0

Region: NODE\_28897\_length\_1748\_cov\_164.263153 1105-1107. Max. coverage (+): 0.09. Max coverage (-): 0.19

Region: NODE\_28897\_length\_1748\_cov\_164.263153 1108-1111. Max. coverage (+): 0. Max coverage (-): 0.28

Region: NODE\_28897\_length\_1748\_cov\_164.263153 1112-1114. Max. coverage (+): 0. Max coverage (-): 0

Region: NODE\_28897\_length\_1748\_cov\_164.263153 1115-1118. Max. coverage (+): 0. Max coverage (-): 0

Region: NODE\_28897\_length\_1748\_cov\_164.263153 1119-1122. Max. coverage (+): 0.19. Max coverage (-): 0

Region: NODE\_28897\_length\_1748\_cov\_164.263153 1123-1125. Max. coverage (+): 0.19. Max coverage (-): 0

Region: NODE\_28897\_length\_1748\_cov\_164.263153 1126-1129. Max. coverage (+): 0. Max coverage (-): 0

Region: NODE\_28897\_length\_1748\_cov\_164.263153 1130-1132. Max. coverage (+): 0. Max coverage (-): 0

Region: NODE\_28897\_length\_1748\_cov\_164.263153 1133-1136. Max. coverage (+): 0. Max coverage (-): 0

Region: NODE\_28897\_length\_1748\_cov\_164.263153 1137-1140. Max. coverage (+): 0. Max coverage (-): 0

Region: NODE\_28897\_length\_1748\_cov\_164.263153 1141-1143. Max. coverage (+): 0. Max coverage (-): 0

Region: NODE\_28897\_length\_1748\_cov\_164.263153 1144-1147. Max. coverage (+): 0. Max coverage (-): 0

Region: NODE\_28897\_length\_1748\_cov\_164.263153 1148-1150. Max. coverage (+): 0. Max coverage (-): 0.38

Region: NODE\_28897\_length\_1748\_cov\_164.263153 1151-1154. Max. coverage (+): 0. Max coverage (-): 0.38

Region: NODE\_28897\_length\_1748\_cov\_164.263153 1155-1158. Max. coverage (+): 0.09. Max coverage (-): 0

Region: NODE\_28897\_length\_1748\_cov\_164.263153 1159-1161. Max. coverage (+): 0.09. Max coverage (-): 0

Region: NODE\_28897\_length\_1748\_cov\_164.263153 1162-1165. Max. coverage (+): 0. Max coverage (-): 0.09

Region: NODE\_28897\_length\_1748\_cov\_164.263153 1166-1168. Max. coverage (+): 0. Max coverage (-): 0.09

Region: NODE\_28897\_length\_1748\_cov\_164.263153 1169-1172. Max. coverage (+): 0. Max coverage (-): 0

Region: NODE\_28897\_length\_1748\_cov\_164.263153 1173-1176. Max. coverage (+): 0.09. Max coverage (-): 0

Region: NODE\_28897\_length\_1748\_cov\_164.263153 1177-1179. Max. coverage (+): 0.28. Max coverage (-): 0

Region: NODE\_28897\_length\_1748\_cov\_164.263153 1180-1183. Max. coverage (+): 0.19. Max coverage (-): 0

Region: NODE\_28897\_length\_1748\_cov\_164.263153 1184-1186. Max. coverage (+): 0.38. Max coverage (-): 0

Region: NODE\_28897\_length\_1748\_cov\_164.263153 1187-1190. Max. coverage (+): 1.13. Max coverage (-): 0

Region: NODE\_28897\_length\_1748\_cov\_164.263153 1191-1194. Max. coverage (+): 0. Max coverage (-): 0.09

Region: NODE\_28897\_length\_1748\_cov\_164.263153 1195-1197. Max. coverage (+): 0.09. Max coverage (-): 1.03

Region: NODE\_28897\_length\_1748\_cov\_164.263153 1198-1201. Max. coverage (+): 0.19. Max coverage (-): 2.07

Region: NODE\_28897\_length\_1748\_cov\_164.263153 1202-1204. Max. coverage (+): 0. Max coverage (-): 1.41

Region: NODE\_28897\_length\_1748\_cov\_164.263153 1205-1208. Max. coverage (+): 0. Max coverage (-): 0

Region: NODE\_28897\_length\_1748\_cov\_164.263153 1209-1212. Max. coverage (+): 0. Max coverage (-): 0

Region: NODE\_28897\_length\_1748\_cov\_164.263153 1213-1215. Max. coverage (+): 0.09. Max coverage (-): 0

Region: NODE\_28897\_length\_1748\_cov\_164.263153 1216-1219. Max. coverage (+): 0.38. Max coverage (-): 0

Region: NODE\_28897\_length\_1748\_cov\_164.263153 1220-1223. Max. coverage (+): 0.09. Max coverage (-): 0

Region: NODE\_28897\_length\_1748\_cov\_164.263153 1224-1226. Max. coverage (+): 0.09. Max coverage (-): 0

Region: NODE\_28897\_length\_1748\_cov\_164.263153 1227-1230. Max. coverage (+): 0.09. Max coverage (-): 0.09

Region: NODE\_28897\_length\_1748\_cov\_164.263153 1231-1233. Max. coverage (+): 0. Max coverage (-): 0.19

Region: NODE\_28897\_length\_1748\_cov\_164.263153 1234-1237. Max. coverage (+): 0. Max coverage (-): 0.28

Region: NODE\_28897\_length\_1748\_cov\_164.263153 1238-1241. Max. coverage (+): 0. Max coverage (-): 0

Region: NODE\_28897\_length\_1748\_cov\_164.263153 1242-1244. Max. coverage (+): 0. Max coverage (-): 0

Region: NODE\_28897\_length\_1748\_cov\_164.263153 1245-1248. Max. coverage (+): 0. Max coverage (-): 0.19

Region: NODE\_28897\_length\_1748\_cov\_164.263153 1249-1251. Max. coverage (+): 0. Max coverage (-): 0

Region: NODE\_28897\_length\_1748\_cov\_164.263153 1252-1255. Max. coverage (+): 0.28. Max coverage (-): 0

Region: NODE\_28897\_length\_1748\_cov\_164.263153 1256-1259. Max. coverage (+): 0.09. Max coverage (-): 0

Region: NODE\_28897\_length\_1748\_cov\_164.263153 1260-1262. Max. coverage (+): 0. Max coverage (-): 0

Region: NODE\_28897\_length\_1748\_cov\_164.263153 1263-1266. Max. coverage (+): 0. Max coverage (-): 0

Region: NODE\_28897\_length\_1748\_cov\_164.263153 1267-1269. Max. coverage (+): 0. Max coverage (-): 0.09

Region: NODE\_28897\_length\_1748\_cov\_164.263153 1270-1273. Max. coverage (+): 0. Max coverage (-): 0.28

Region: NODE\_28897\_length\_1748\_cov\_164.263153 1274-1277. Max. coverage (+): 0. Max coverage (-): 0.38

Region: NODE\_28897\_length\_1748\_cov\_164.263153 1278-1280. Max. coverage (+): 0. Max coverage (-): 0.38

Region: NODE\_28897\_length\_1748\_cov\_164.263153 1281-1284. Max. coverage (+): 0.09. Max coverage (-): 0.09

Region: NODE\_28897\_length\_1748\_cov\_164.263153 1285-1287. Max. coverage (+): 0.09. Max coverage (-): 0

Region: NODE\_28897\_length\_1748\_cov\_164.263153 1288-1291. Max. coverage (+): 0.09. Max coverage (-): 0.09

Region: NODE\_28897\_length\_1748\_cov\_164.263153 1292-1295. Max. coverage (+): 0.75. Max coverage (-): 0.09

Region: NODE\_28897\_length\_1748\_cov\_164.263153 1296-1298. Max. coverage (+): 0.47. Max coverage (-): 0.28

Region: NODE\_28897\_length\_1748\_cov\_164.263153 1299-1302. Max. coverage (+): 0. Max coverage (-): 0.28

Region: NODE\_28897\_length\_1748\_cov\_164.263153 1303-1305. Max. coverage (+): 0. Max coverage (-): 0.09

Region: NODE\_28897\_length\_1748\_cov\_164.263153 1306-1309. Max. coverage (+): 0. Max coverage (-): 0.19

Region: NODE\_28897\_length\_1748\_cov\_164.263153 1310-1313. Max. coverage (+): 0. Max coverage (-): 0

Region: NODE\_28897\_length\_1748\_cov\_164.263153 1314-1316. Max. coverage (+): 0.09. Max coverage (-): 0

Region: NODE\_28897\_length\_1748\_cov\_164.263153 1317-1320. Max. coverage (+): 0.09. Max coverage (-): 0

Region: NODE\_28897\_length\_1748\_cov\_164.263153 1321-1323. Max. coverage (+): 0. Max coverage (-): 0

Region: NODE\_28897\_length\_1748\_cov\_164.263153 1324-1327. Max. coverage (+): 0. Max coverage (-): 0.85

Region: NODE\_28897\_length\_1748\_cov\_164.263153 1328-1331. Max. coverage (+): 0. Max coverage (-): 1.03

Region: NODE\_28897\_length\_1748\_cov\_164.263153 1332-1334. Max. coverage (+): 0. Max coverage (-): 0.19

Region: NODE\_28897\_length\_1748\_cov\_164.263153 1335-1338. Max. coverage (+): 0. Max coverage (-): 0

Region: NODE\_28897\_length\_1748\_cov\_164.263153 1339-1342. Max. coverage (+): 0. Max coverage (-): 0

Region: NODE\_28897\_length\_1748\_cov\_164.263153 1343-1345. Max. coverage (+): 0. Max coverage (-): 0

Region: NODE\_28897\_length\_1748\_cov\_164.263153 1346-1349. Max. coverage (+): 0. Max coverage (-): 0.19

Region: NODE\_28897\_length\_1748\_cov\_164.263153 1350-1352. Max. coverage (+): 0. Max coverage (-): 0.38

Region: NODE\_28897\_length\_1748\_cov\_164.263153 1353-1356. Max. coverage (+): 0. Max coverage (-): 0.28

Region: NODE\_28897\_length\_1748\_cov\_164.263153 1357-1360. Max. coverage (+): 0. Max coverage (-): 0.19

Region: NODE\_28897\_length\_1748\_cov\_164.263153 1361-1363. Max. coverage (+): 0. Max coverage (-): 0

Region: NODE\_28897\_length\_1748\_cov\_164.263153 1364-1367. Max. coverage (+): 0. Max coverage (-): 0

Region: NODE\_28897\_length\_1748\_cov\_164.263153 1368-1370. Max. coverage (+): 1.41. Max coverage (-): 0

Region: NODE\_28897\_length\_1748\_cov\_164.263153 1371-1374. Max. coverage (+): 1.41. Max coverage (-): 0

Region: NODE\_28897\_length\_1748\_cov\_164.263153 1375-1378. Max. coverage (+): 0.09. Max coverage (-): 0

Region: NODE\_28897\_length\_1748\_cov\_164.263153 1379-1381. Max. coverage (+): 0.09. Max coverage (-): 0

Region: NODE\_28897\_length\_1748\_cov\_164.263153 1382-1385. Max. coverage (+): 0.09. Max coverage (-): 0

Region: NODE\_28897\_length\_1748\_cov\_164.263153 1386-1388. Max. coverage (+): 0.09. Max coverage (-): 0

Region: NODE\_28897\_length\_1748\_cov\_164.263153 1389-1392. Max. coverage (+): 0. Max coverage (-): 1.79

Region: NODE\_28897\_length\_1748\_cov\_164.263153 1393-1396. Max. coverage (+): 0. Max coverage (-): 0.75

Region: NODE\_28897\_length\_1748\_cov\_164.263153 1397-1399. Max. coverage (+): 0. Max coverage (-): 0

Region: NODE\_28897\_length\_1748\_cov\_164.263153 1400-1403. Max. coverage (+): 0. Max coverage (-): 0

Region: NODE\_28897\_length\_1748\_cov\_164.263153 1404-1406. Max. coverage (+): 0. Max coverage (-): 0

Region: NODE\_28897\_length\_1748\_cov\_164.263153 1407-1410. Max. coverage (+): 0.33. Max coverage (-): 0.05

Region: NODE\_28897\_length\_1748\_cov\_164.263153 1411-1414. Max. coverage (+): 0.23. Max coverage (-): 0.05

Region: NODE\_28897\_length\_1748\_cov\_164.263153 1415-1417. Max. coverage (+): 0. Max coverage (-): 0

Region: NODE\_28897\_length\_1748\_cov\_164.263153 1418-1421. Max. coverage (+): 0. Max coverage (-): 0

Region: NODE\_28897\_length\_1748\_cov\_164.263153 1422-1424. Max. coverage (+): 0. Max coverage (-): 0

Region: NODE\_28897\_length\_1748\_cov\_164.263153 1425-1428. Max. coverage (+): 0. Max coverage (-): 0

Region: NODE\_28897\_length\_1748\_cov\_164.263153 1429-1432. Max. coverage (+): 0. Max coverage (-): 0

Region: NODE\_28897\_length\_1748\_cov\_164.263153 1433-1435. Max. coverage (+): 0. Max coverage (-): 0

Region: NODE\_28897\_length\_1748\_cov\_164.263153 1436-1439. Max. coverage (+): 0.09. Max coverage (-): 0.19

Region: NODE\_28897\_length\_1748\_cov\_164.263153 1440-1442. Max. coverage (+): 0.09. Max coverage (-): 0.19

Region: NODE\_28897\_length\_1748\_cov\_164.263153 1443-1446. Max. coverage (+): 0. Max coverage (-): 0.19

Region: NODE\_28897\_length\_1748\_cov\_164.263153 1447-1450. Max. coverage (+): 0. Max coverage (-): 0.47

Region: NODE\_28897\_length\_1748\_cov\_164.263153 1451-1453. Max. coverage (+): 0. Max coverage (-): 0.94

Region: NODE\_28897\_length\_1748\_cov\_164.263153 1454-1457. Max. coverage (+): 0. Max coverage (-): 0.85

Region: NODE\_28897\_length\_1748\_cov\_164.263153 1458-1461. Max. coverage (+): 0. Max coverage (-): 0.09

Region: NODE\_28897\_length\_1748\_cov\_164.263153 1462-1464. Max. coverage (+): 0.19. Max coverage (-): 0.09

Region: NODE\_28897\_length\_1748\_cov\_164.263153 1465-1468. Max. coverage (+): 0.38. Max coverage (-): 0

Region: NODE\_28897\_length\_1748\_cov\_164.263153 1469-1471. Max. coverage (+): 0.09. Max coverage (-): 0.09

Region: NODE\_28897\_length\_1748\_cov\_164.263153 1472-1475. Max. coverage (+): 0. Max coverage (-): 0.09

Region: NODE\_28897\_length\_1748\_cov\_164.263153 1476-1479. Max. coverage (+): 0. Max coverage (-): 0.09

Region: NODE\_28897\_length\_1748\_cov\_164.263153 1480-1482. Max. coverage (+): 0. Max coverage (-): 0

Region: NODE\_28897\_length\_1748\_cov\_164.263153 1483-1486. Max. coverage (+): 0. Max coverage (-): 0

Region: NODE\_28897\_length\_1748\_cov\_164.263153 1487-1489. Max. coverage (+): 0. Max coverage (-): 0

Region: NODE\_28897\_length\_1748\_cov\_164.263153 1490-1493. Max. coverage (+): 0. Max coverage (-): 0

Region: NODE\_28897\_length\_1748\_cov\_164.263153 1494-1497. Max. coverage (+): 0. Max coverage (-): 0.19

Region: NODE\_28897\_length\_1748\_cov\_164.263153 1498-1500. Max. coverage (+): 0. Max coverage (-): 0.38

Region: NODE\_28897\_length\_1748\_cov\_164.263153 1501-1504. Max. coverage (+): 0. Max coverage (-): 0.19

Region: NODE\_28897\_length\_1748\_cov\_164.263153 1505-1507. Max. coverage (+): 0. Max coverage (-): 0

Region: NODE\_28897\_length\_1748\_cov\_164.263153 1508-1511. Max. coverage (+): 0.19. Max coverage (-): 0.09

Region: NODE\_28897\_length\_1748\_cov\_164.263153 1512-1515. Max. coverage (+): 2.26. Max coverage (-): 0.09

Region: NODE\_28897\_length\_1748\_cov\_164.263153 1516-1518. Max. coverage (+): 1.79. Max coverage (-): 0.09

Region: NODE\_28897\_length\_1748\_cov\_164.263153 1519-1522. Max. coverage (+): 0.28. Max coverage (-): 0

Region: NODE\_28897\_length\_1748\_cov\_164.263153 1523-1525. Max. coverage (+): 0.28. Max coverage (-): 0.09

Region: NODE\_28897\_length\_1748\_cov\_164.263153 1526-1529. Max. coverage (+): 0. Max coverage (-): 0.28

Region: NODE\_28897\_length\_1748\_cov\_164.263153 1530-1533. Max. coverage (+): 0. Max coverage (-): 0.28

Region: NODE\_28897\_length\_1748\_cov\_164.263153 1534-1536. Max. coverage (+): 0. Max coverage (-): 0.19

Region: NODE\_28897\_length\_1748\_cov\_164.263153 1537-1540. Max. coverage (+): 0. Max coverage (-): 0

Region: NODE\_28897\_length\_1748\_cov\_164.263153 1541-1543. Max. coverage (+): 0. Max coverage (-): 0

Region: NODE\_28897\_length\_1748\_cov\_164.263153 1544-1547. Max. coverage (+): 1.22. Max coverage (-): 0

Region: NODE\_28897\_length\_1748\_cov\_164.263153 1548-1551. Max. coverage (+): 1.32. Max coverage (-): 0

Region: NODE\_28897\_length\_1748\_cov\_164.263153 1552-1554. Max. coverage (+): 0. Max coverage (-): 0.09

Region: NODE\_28897\_length\_1748\_cov\_164.263153 1555-1558. Max. coverage (+): 0. Max coverage (-): 0.09

Region: NODE\_28897\_length\_1748\_cov\_164.263153 1559-1561. Max. coverage (+): 0. Max coverage (-): 0

Region: NODE\_28897\_length\_1748\_cov\_164.263153 1562-1565. Max. coverage (+): 0. Max coverage (-): 0.09

Region: NODE\_28897\_length\_1748\_cov\_164.263153 1566-1569. Max. coverage (+): 0. Max coverage (-): 0.09

Region: NODE\_28897\_length\_1748\_cov\_164.263153 1570-1572. Max. coverage (+): 0. Max coverage (-): 2.54

Region: NODE\_28897\_length\_1748\_cov\_164.263153 1573-1576. Max. coverage (+): 0. Max coverage (-): 3.19

Region: NODE\_28897\_length\_1748\_cov\_164.263153 1577-1580. Max. coverage (+): 0. Max coverage (-): 0.56

Region: NODE\_28897\_length\_1748\_cov\_164.263153 1581-1583. Max. coverage (+): 0. Max coverage (-): 0.09

Region: NODE\_28897\_length\_1748\_cov\_164.263153 1584-1587. Max. coverage (+): 0. Max coverage (-): 0.09

Region: NODE\_28897\_length\_1748\_cov\_164.263153 1588-1590. Max. coverage (+): 0.56. Max coverage (-): 0.09

Region: NODE\_28897\_length\_1748\_cov\_164.263153 1591-1594. Max. coverage (+): 0.56. Max coverage (-): 0.09

Region: NODE\_28897\_length\_1748\_cov\_164.263153 1595-1598. Max. coverage (+): 0.19. Max coverage (-): 0.09

Region: NODE\_28897\_length\_1748\_cov\_164.263153 1599-1601. Max. coverage (+): 0.09. Max coverage (-): 0.28

Region: NODE\_28897\_length\_1748\_cov\_164.263153 1602-1605. Max. coverage (+): 0.09. Max coverage (-): 0.19

Region: NODE\_28897\_length\_1748\_cov\_164.263153 1606-1608. Max. coverage (+): 0. Max coverage (-): 0

Region: NODE\_28897\_length\_1748\_cov\_164.263153 1609-1612. Max. coverage (+): 0. Max coverage (-): 0.85

Region: NODE\_28897\_length\_1748\_cov\_164.263153 1613-1616. Max. coverage (+): 0. Max coverage (-): 0.85

Region: NODE\_28897\_length\_1748\_cov\_164.263153 1617-1619. Max. coverage (+): 0. Max coverage (-): 0.09

Region: NODE\_28897\_length\_1748\_cov\_164.263153 1620-1623. Max. coverage (+): 0. Max coverage (-): 0.09

Region: NODE\_28897\_length\_1748\_cov\_164.263153 1624-1626. Max. coverage (+): 0. Max coverage (-): 0

Region: NODE\_28897\_length\_1748\_cov\_164.263153 1627-1630. Max. coverage (+): 0.09. Max coverage (-): 0

Region: NODE\_28897\_length\_1748\_cov\_164.263153 1631-1634. Max. coverage (+): 0.09. Max coverage (-): 0

Region: NODE\_28897\_length\_1748\_cov\_164.263153 1635-1637. Max. coverage (+): 0. Max coverage (-): 0

Region: NODE\_28897\_length\_1748\_cov\_164.263153 1638-1641. Max. coverage (+): 0. Max coverage (-): 0.09

Region: NODE\_28897\_length\_1748\_cov\_164.263153 1642-1644. Max. coverage (+): 0. Max coverage (-): 0.28

Region: NODE\_28897\_length\_1748\_cov\_164.263153 1645-1648. Max. coverage (+): 0. Max coverage (-): 0.28

Region: NODE\_28897\_length\_1748\_cov\_164.263153 1649-1652. Max. coverage (+): 0. Max coverage (-): 0.19

Region: NODE\_28897\_length\_1748\_cov\_164.263153 1653-1655. Max. coverage (+): 0. Max coverage (-): 0.38

Region: NODE\_28897\_length\_1748\_cov\_164.263153 1656-1659. Max. coverage (+): 0. Max coverage (-): 1.32

Region: NODE\_28897\_length\_1748\_cov\_164.263153 1660-1662. Max. coverage (+): 0. Max coverage (-): 1.22

Region: NODE\_28897\_length\_1748\_cov\_164.263153 1663-1666. Max. coverage (+): 0. Max coverage (-): 1.41

Region: NODE\_28897\_length\_1748\_cov\_164.263153 1667-1670. Max. coverage (+): 0.19. Max coverage (-): 1.97

Region: NODE\_28897\_length\_1748\_cov\_164.263153 1671-1673. Max. coverage (+): 0.09. Max coverage (-): 0.85

Region: NODE\_28897\_length\_1748\_cov\_164.263153 1674-1677. Max. coverage (+): 0.09. Max coverage (-): 0.09

Region: NODE\_28897\_length\_1748\_cov\_164.263153 1678-1680. Max. coverage (+): 0. Max coverage (-): 0

Region: NODE\_28897\_length\_1748\_cov\_164.263153 1681-1684. Max. coverage (+): 0. Max coverage (-): 0

Region: NODE\_28897\_length\_1748\_cov\_164.263153 1685-1688. Max. coverage (+): 0.09. Max coverage (-): 0

Region: NODE\_28897\_length\_1748\_cov\_164.263153 1689-1691. Max. coverage (+): 0.66. Max coverage (-): 0

Region: NODE\_28897\_length\_1748\_cov\_164.263153 1692-1695. Max. coverage (+): 0.56. Max coverage (-): 0

Region: NODE\_28897\_length\_1748\_cov\_164.263153 1696-1699. Max. coverage (+): 0. Max coverage (-): 0

Region: NODE\_28897\_length\_1748\_cov\_164.263153 1700-1702. Max. coverage (+): 0. Max coverage (-): 0

Region: NODE\_28897\_length\_1748\_cov\_164.263153 1703-1706. Max. coverage (+): 0. Max coverage (-): 0.19

Region: NODE\_28897\_length\_1748\_cov\_164.263153 1707-1709. Max. coverage (+): 0. Max coverage (-): 0.19

Region: NODE\_28897\_length\_1748\_cov\_164.263153 1710-1713. Max. coverage (+): 0.09. Max coverage (-): 0

Region: NODE\_28897\_length\_1748\_cov\_164.263153 1714-1717. Max. coverage (+): 0.28. Max coverage (-): 0

Region: NODE\_28897\_length\_1748\_cov\_164.263153 1718-1720. Max. coverage (+): 0.19. Max coverage (-): 0

Region: NODE\_28897\_length\_1748\_cov\_164.263153 1721-1724. Max. coverage (+): 0.38. Max coverage (-): 0

Region: NODE\_28897\_length\_1748\_cov\_164.263153 1725-1727. Max. coverage (+): 0.19. Max coverage (-): 0.28

Region: NODE\_28897\_length\_1748\_cov\_164.263153 1728-1731. Max. coverage (+): 0. Max coverage (-): 0.28

Region: NODE\_28897\_length\_1748\_cov\_164.263153 1732-1735. Max. coverage (+): 0. Max coverage (-): 0.19

Region: NODE\_28897\_length\_1748\_cov\_164.263153 1736-1738. Max. coverage (+): 0. Max coverage (-): 0.19

Region: NODE\_28897\_length\_1748\_cov\_164.263153 1739-1742. Max. coverage (+): 0.09. Max coverage (-): 0

Region: NODE\_28897\_length\_1748\_cov\_164.263153 1743-1745. Max. coverage (+): 0.09. Max coverage (-): 0

Region: NODE\_28897\_length\_1748\_cov\_164.263153 1746-1749. Max. coverage (+): 0.09. Max coverage (-): 0

Region: NODE\_28897\_length\_1748\_cov\_164.263153 1750-1753. Max. coverage (+): 0.19. Max coverage (-): 0

Region: NODE\_28897\_length\_1748\_cov\_164.263153 1754-1756. Max. coverage (+): 0.47. Max coverage (-): 0

Region: NODE\_28897\_length\_1748\_cov\_164.263153 1757-1760. Max. coverage (+): 0.47. Max coverage (-): 0.19

Region: NODE\_28897\_length\_1748\_cov\_164.263153 1761-1763. Max. coverage (+): 0.09. Max coverage (-): 0.19

Region: NODE\_28897\_length\_1748\_cov\_164.263153 1764-1767. Max. coverage (+): 0.09. Max coverage (-): 0

Region: NODE\_28897\_length\_1748\_cov\_164.263153 1768-1771. Max. coverage (+): 0. Max coverage (-): 0.09

Region: NODE\_28897\_length\_1748\_cov\_164.263153 1772-1774. Max. coverage (+): 0.09. Max coverage (-): 0.47

Region: NODE\_28897\_length\_1748\_cov\_164.263153 1775-1778. Max. coverage (+): 0.19. Max coverage (-): 0.47

Region: NODE\_28897\_length\_1748\_cov\_164.263153 1779-1781. Max. coverage (+): 0.09. Max coverage (-): 0.09

Region: NODE\_28897\_length\_1748\_cov\_164.263153 1782-1785. Max. coverage (+): 0. Max coverage (-): 0.09

Region: NODE\_28897\_length\_1748\_cov\_164.263153 1786-1789. Max. coverage (+): 0. Max coverage (-): 0

Region: NODE\_28897\_length\_1748\_cov\_164.263153 1790-1792. Max. coverage (+): 0. Max coverage (-): 0

Region: NODE\_28897\_length\_1748\_cov\_164.263153 1793-1796. Max. coverage (+): 0. Max coverage (-): 0

Region: NODE\_28897\_length\_1748\_cov\_164.263153 1797-1799. Max. coverage (+): 0. Max coverage (-): 0

Region: NODE\_28897\_length\_1748\_cov\_164.263153 1800-1803. Max. coverage (+): 0. Max coverage (-): 0

Region: NODE\_28897\_length\_1748\_cov\_164.263153 1804-1807. Max. coverage (+): 0. Max coverage (-): 0

Region: NODE\_28897\_length\_1748\_cov\_164.263153 1808-. Max. coverage (+): 0. Max coverage (-): 0

RepeatMasker Color Code

**+**

100-98% Identity

<98-95% Identity

<95-90% Identity

<90-85% Identity

<85-80% Identity

<80-75% Identity

<75-70% Identity

<70% Identity

**-**

Gene Set Color Code

**+**

Gene

Pseudogene

Other

**-**

Topology/Coverage Color Code

Coverage Plus Strand

Coverage Minus Strand

Mainstrand: Plus

Mainstrand: Minus

Complementary Strand

Flanking Region  
(if option -flank >0)

Gene Set Annotation  
  
RepeatMasker Annotation  

**1. Tx1-4B\_Crp**: 75-1131 (+), Divergence to consensus: 44.7%  
**2. L1-4B\_DR**: 336-1140 (+), Divergence to consensus: 42.7%

  
Transcription Factor Binding Sites  

**RHOXF1** (Sequence: AGATTA (-): 127)  
**RHOXF1** (Sequence: AGATTA (-): 243)  
**RHOXF1** (Sequence: GGATTA (-): 348)  
**RHOXF1** (Sequence: AGATTA (-): 536)  
**RHOXF1** (Sequence: AGATTA (-): 743)  
**RHOXF1** (Sequence: AGCTTA (-): 1059)  
**RHOXF1** (Sequence: GGATCA (-): 1107)  
**RHOXF1** (Sequence: AGCTTA (-): 1315)  
**RFX4\_2** (Sequence: GTATCTAGG (-): 1373)  
**Sox5** (Sequence: ATTGTT (+): 1601)  
**FOXO3\_mmu** (Sequence: GGAAAACA (+): 786)  
**Nobox** (Sequence: TAATTGCT (+): 559)  
**Rhox11** (Sequence: CGCTGTTAA (+): 1564)  
**Sox5** (Sequence: AACAAT (-): 790)  
**POU2F1** (Sequence: TATGCAAAT (+): 1548)  
**POU5F1** (Sequence: ATGCAAA (+): 1549)
