# Supplementary material for: First characterization of PIWI-interacting RNA clusters in a cichlid fish with a B chromosome
Source: BMC Biol. 2022 Sep 21;20:204. doi: 10.1186/s12915-022-01403-2 (PMC9490952; doi:10.1186/s12915-022-01403-2)
Supplement: Supplementary file 1 — Additional file 1. Zipped folder with fasta and interactive html piRNA cluster information for the A. latifasciata genome. The nomenclature is as follows: number-pirna-cluster_sex_B-presence (f, female; m, male; 0b, without B chromosome; 1b, with B chromosome). [file 12915_2022_1403_MOESM1_ESM.zip › 112_m1b.html]

piRNA cluster 112\_m1b 67


Predicted piRNA cluster no. 112\_m1b
  

Show proTRAC run info
Hide proTRAC run info

/\  
                \_\_\_\_\_\_\_\_\_\_\_\_\_\_\_\_\_\_\_\_\_\_\_/\\_\_\_ /  \\_\_\_\_\_\_\_  
               I                      /  \  /    \      I  
               I     pro             /    \/      \     I  
               I        TRAC        /               \   I  
               I   \_\_\_\_\_\_\_\_\_\_\_\_\_\_\_\_/\_\_\_\_\_\_\_\_\_\_\_\_\_\_\_\_\_\\_ I  
               I   \              /                     I  
               I    \            /                      I  
               I     \  /\      /       V.2.4.2         I  
               I      \/  \    /                        I  
               I\_\_\_\_\_\_\_\_\_\_\_\  /\_\_\_\_\_\_\_\_\_\_\_\_\_\_\_\_\_\_\_\_\_\_\_\_\_I  
                            \/  
  
  
================================= proTRAC ====================================  
VERSION: .......... 2.4.2  
LAST MODIFIED: .... 11. May 2018  
  
Please cite:  
Rosenkranz D, Zischler H. proTRAC - a software for probabilistic piRNA cluster  
detection, visualization and analysis. 2012. BMC Bioinformatics 13:5.  
  
  
Contact:  
David Rosenkranz  
Institute of Organismic and Molecular Evolutionary Biology  
Dept. Anthropology, small RNA group  
Johannes Gutenberg University Mainz  
email: rosenkranz@uni-mainz.de  
  
You can find the latest proTRAC version at:  
http://sourceforge.net/projects/protrac/files  
http://www.smallRNAgroup-mainz.de/software  
==============================================================================  
  
PARAMETERS:  
Map file: ...............piwi-machos-1B.fa-collapse.map  
Genome file: ............../../../0B\_ala\_genome.fa  
RepeatMasker annotation: Alatifasciata-all0B-maryan-v2.fa\_corrected.out  
GeneSet:................./guest-storage/Data/annotation/Alatifasciata\_all0B\_maryan-v2\_out2017.gff  
  
Significant (p<=0.01) hit density will be calculated based  
on observed hit distribution.  
  
Sliding window size: ........................................ 5000 bp  
Sliding window increament: .................................. 1000 bp  
Normalize each hit by number of genomic hits: ............... yes  
Normalize each hit by number of sequence reads: ............. yes  
Normalize values (-> per million mapped reads): ............. yes  
Min. fraction of hits with 1T(U) or 10A: .................... 0.75  
Alternatively: Min. fraction of hits with 1T(U) and 10A: .... 0.5  
Min. fraction of hits with typical piRNA length: ............ 0.75  
Typical piRNA length: ....................................... 24-32 nt  
Min. size of a piRNA cluster: ............................... 1000 bp.  
Min. number of hits (absolute): ............................. 0  
Min. number of hits (normalized): ........................... 0  
Min. fraction of hits on the mainstrand: .................... 0.75  
Top fraction of mapped sequences (in terms of read counts): . 1%  
Top fraction accounts for max. n% of sequence reads: ........ 90%  
Min. fraction of hits on each arm of a bidirectional cluster: 0.05  
Output html file for each cluster: .......................... yes  
Output a summary table: ..................................... yes  
Output a FASTA file for each cluster (piRNA sequences): ..... yes  
Output a FASTA file comprising cluster sequences: ........... yes  
Output a GTF file for predicted piRNA clusters: ..............yes  
Search DNA motifs in clusters: .............................. yes  
Output flanking sequences: +/- .............................. 0 bp  
Output ~.pTi file: .......................................... no  
==============================================================================  
  
  
Genome size (without gaps): ............ 758543724 bp  
Gaps (N/X/-): .......................... 417479 bp  
Mapped reads: .......................... 26973943  
Non-identical sequences: ............... 6209225  
Genomic hits: .......................... 48438990  
Significant densitiy of mapped reads: .. 821.144211136946 reads/kb

Show proTRAC cluster info
Hide proTRAC cluster info

|  |  |
| --- | --- |
| Location | NODE\_289404\_length\_16761\_cov\_29.864925 |
| Coordinates | 3008-12026 |
| Size [bp] | 9019 |
| Sequence hit loci | 4092 |
| Mapped reads (normalized) | 8542.2 |
| Mapped reads (normalized) per kb | 947.1 |
| Normalized reads with 1T (1U) | 76% |
| Normalized reads with 10A | 44.4% |
| Normalized reads with length 24-32 nt | 98.4% |
| Normalized reads on the main strand(s) | 84.8% |
| Predicted directionality | mono:minus |

100%

0%

1T (1U)  
reads

10A reads

24-32 nt  
reads

reads on mainstrand

**Either the amount of reads with 1T (1U) OR 10A has to exceed 75% (set with option: -1Tor10A)  
Alternatively the amount of reads with 1T (1U) AND 10A has to exceed 50% (set with option: -1Tand10A)  
Minimum amount of reads with preferred size is 75% (set with option: -pisize)  
Minimum amount of reads on the main strand(s) is 75% (set with option: -clstrand)**

Show read coverage
Hide read coverage

WHAT DO I SEE HERE?  
This chart shows the location of mapped sequence reads within a predicted piRNA cluster. The color refers to the number of genomic hits produced by the sequence read in question. A dark red bar indicates that this sequence read produces many other hits elsewhere in the genome. Many adjacent red or yellow bars can indicate the presence of a multi-copy element such as transposons or rRNA genes. A dark green bar indicates that this sequence read maps uniquely to this locus.

1 hit

2-5 hits

6-10 hits

11-20 hits

21-50 hits

51-100 hits

> 100 hits

NODE\_289404\_length\_16761\_cov\_29.864925

3008

12026

Gene Set

RepeatMasker

Mapped  
Reads

10.94

plus strand

minus strand

10.94

Region: NODE\_289404\_length\_16761\_cov\_29.864925 1048-3017. Max. coverage (+): 0. Max coverage (-): 0.04

Region: NODE\_289404\_length\_16761\_cov\_29.864925 3018-3035. Max. coverage (+): 0. Max coverage (-): 0.52

Region: NODE\_289404\_length\_16761\_cov\_29.864925 3036-3053. Max. coverage (+): 0. Max coverage (-): 0.04

Region: NODE\_289404\_length\_16761\_cov\_29.864925 3054-3071. Max. coverage (+): 0. Max coverage (-): 0.04

Region: NODE\_289404\_length\_16761\_cov\_29.864925 3072-3089. Max. coverage (+): 0. Max coverage (-): 0.19

Region: NODE\_289404\_length\_16761\_cov\_29.864925 3090-3107. Max. coverage (+): 0. Max coverage (-): 0.19

Region: NODE\_289404\_length\_16761\_cov\_29.864925 3108-3125. Max. coverage (+): 0. Max coverage (-): 0

Region: NODE\_289404\_length\_16761\_cov\_29.864925 3126-3143. Max. coverage (+): 0.11. Max coverage (-): 6.71

Region: NODE\_289404\_length\_16761\_cov\_29.864925 3144-3161. Max. coverage (+): 0.07. Max coverage (-): 1.3

Region: NODE\_289404\_length\_16761\_cov\_29.864925 3162-3179. Max. coverage (+): 0. Max coverage (-): 0

Region: NODE\_289404\_length\_16761\_cov\_29.864925 3180-3197. Max. coverage (+): 0. Max coverage (-): 0.04

Region: NODE\_289404\_length\_16761\_cov\_29.864925 3198-3215. Max. coverage (+): 0. Max coverage (-): 0.04

Region: NODE\_289404\_length\_16761\_cov\_29.864925 3216-3233. Max. coverage (+): 0. Max coverage (-): 0.96

Region: NODE\_289404\_length\_16761\_cov\_29.864925 3234-3251. Max. coverage (+): 0. Max coverage (-): 0.61

Region: NODE\_289404\_length\_16761\_cov\_29.864925 3252-3269. Max. coverage (+): 0.04. Max coverage (-): 0.02

Region: NODE\_289404\_length\_16761\_cov\_29.864925 3270-3287. Max. coverage (+): 0. Max coverage (-): 0.06

Region: NODE\_289404\_length\_16761\_cov\_29.864925 3288-3305. Max. coverage (+): 0.02. Max coverage (-): 0.04

Region: NODE\_289404\_length\_16761\_cov\_29.864925 3306-3323. Max. coverage (+): 0. Max coverage (-): 0

Region: NODE\_289404\_length\_16761\_cov\_29.864925 3324-3341. Max. coverage (+): 0. Max coverage (-): 0

Region: NODE\_289404\_length\_16761\_cov\_29.864925 3342-3359. Max. coverage (+): 0. Max coverage (-): 0.32

Region: NODE\_289404\_length\_16761\_cov\_29.864925 3360-3377. Max. coverage (+): 0. Max coverage (-): 0.11

Region: NODE\_289404\_length\_16761\_cov\_29.864925 3378-3395. Max. coverage (+): 0. Max coverage (-): 0

Region: NODE\_289404\_length\_16761\_cov\_29.864925 3396-3413. Max. coverage (+): 0. Max coverage (-): 0.02

Region: NODE\_289404\_length\_16761\_cov\_29.864925 3414-3431. Max. coverage (+): 0. Max coverage (-): 0.96

Region: NODE\_289404\_length\_16761\_cov\_29.864925 3432-3449. Max. coverage (+): 0. Max coverage (-): 0.69

Region: NODE\_289404\_length\_16761\_cov\_29.864925 3450-3467. Max. coverage (+): 0. Max coverage (-): 0.26

Region: NODE\_289404\_length\_16761\_cov\_29.864925 3468-3486. Max. coverage (+): 0. Max coverage (-): 0.06

Region: NODE\_289404\_length\_16761\_cov\_29.864925 3487-3504. Max. coverage (+): 0.02. Max coverage (-): 0.04

Region: NODE\_289404\_length\_16761\_cov\_29.864925 3505-3522. Max. coverage (+): 0. Max coverage (-): 0

Region: NODE\_289404\_length\_16761\_cov\_29.864925 3523-3540. Max. coverage (+): 0. Max coverage (-): 0.15

Region: NODE\_289404\_length\_16761\_cov\_29.864925 3541-3558. Max. coverage (+): 0. Max coverage (-): 0.32

Region: NODE\_289404\_length\_16761\_cov\_29.864925 3559-3576. Max. coverage (+): 0. Max coverage (-): 7.45

Region: NODE\_289404\_length\_16761\_cov\_29.864925 3577-3594. Max. coverage (+): 0. Max coverage (-): 0.33

Region: NODE\_289404\_length\_16761\_cov\_29.864925 3595-3612. Max. coverage (+): 0. Max coverage (-): 0.07

Region: NODE\_289404\_length\_16761\_cov\_29.864925 3613-3630. Max. coverage (+): 0. Max coverage (-): 0.19

Region: NODE\_289404\_length\_16761\_cov\_29.864925 3631-3648. Max. coverage (+): 0. Max coverage (-): 0.15

Region: NODE\_289404\_length\_16761\_cov\_29.864925 3649-3666. Max. coverage (+): 0. Max coverage (-): 0.96

Region: NODE\_289404\_length\_16761\_cov\_29.864925 3667-3684. Max. coverage (+): 0. Max coverage (-): 0

Region: NODE\_289404\_length\_16761\_cov\_29.864925 3685-3702. Max. coverage (+): 0. Max coverage (-): 0.04

Region: NODE\_289404\_length\_16761\_cov\_29.864925 3703-3720. Max. coverage (+): 0.04. Max coverage (-): 0.74

Region: NODE\_289404\_length\_16761\_cov\_29.864925 3721-3738. Max. coverage (+): 0.04. Max coverage (-): 0.56

Region: NODE\_289404\_length\_16761\_cov\_29.864925 3739-3756. Max. coverage (+): 0.04. Max coverage (-): 0.07

Region: NODE\_289404\_length\_16761\_cov\_29.864925 3757-3774. Max. coverage (+): 0. Max coverage (-): 0

Region: NODE\_289404\_length\_16761\_cov\_29.864925 3775-3792. Max. coverage (+): 0. Max coverage (-): 0

Region: NODE\_289404\_length\_16761\_cov\_29.864925 3793-3810. Max. coverage (+): 0. Max coverage (-): 0

Region: NODE\_289404\_length\_16761\_cov\_29.864925 3811-3828. Max. coverage (+): 0. Max coverage (-): 0

Region: NODE\_289404\_length\_16761\_cov\_29.864925 3829-3846. Max. coverage (+): 0. Max coverage (-): 0.7

Region: NODE\_289404\_length\_16761\_cov\_29.864925 3847-3864. Max. coverage (+): 0. Max coverage (-): 0.07

Region: NODE\_289404\_length\_16761\_cov\_29.864925 3865-3882. Max. coverage (+): 0.04. Max coverage (-): 0

Region: NODE\_289404\_length\_16761\_cov\_29.864925 3883-3900. Max. coverage (+): 0. Max coverage (-): 0

Region: NODE\_289404\_length\_16761\_cov\_29.864925 3901-3918. Max. coverage (+): 0. Max coverage (-): 0.11

Region: NODE\_289404\_length\_16761\_cov\_29.864925 3919-3936. Max. coverage (+): 0.04. Max coverage (-): 0.07

Region: NODE\_289404\_length\_16761\_cov\_29.864925 3937-3954. Max. coverage (+): 0.15. Max coverage (-): 0.07

Region: NODE\_289404\_length\_16761\_cov\_29.864925 3955-3973. Max. coverage (+): 0.07. Max coverage (-): 0.07

Region: NODE\_289404\_length\_16761\_cov\_29.864925 3974-3991. Max. coverage (+): 0.04. Max coverage (-): 5.3

Region: NODE\_289404\_length\_16761\_cov\_29.864925 3992-4009. Max. coverage (+): 0. Max coverage (-): 5.08

Region: NODE\_289404\_length\_16761\_cov\_29.864925 4010-4027. Max. coverage (+): 0.3. Max coverage (-): 0.52

Region: NODE\_289404\_length\_16761\_cov\_29.864925 4028-4045. Max. coverage (+): 0.04. Max coverage (-): 0.22

Region: NODE\_289404\_length\_16761\_cov\_29.864925 4046-4063. Max. coverage (+): 0.07. Max coverage (-): 0.07

Region: NODE\_289404\_length\_16761\_cov\_29.864925 4064-4081. Max. coverage (+): 0. Max coverage (-): 6.19

Region: NODE\_289404\_length\_16761\_cov\_29.864925 4082-4099. Max. coverage (+): 0. Max coverage (-): 0.04

Region: NODE\_289404\_length\_16761\_cov\_29.864925 4100-4117. Max. coverage (+): 0. Max coverage (-): 0.11

Region: NODE\_289404\_length\_16761\_cov\_29.864925 4118-4135. Max. coverage (+): 0. Max coverage (-): 0.11

Region: NODE\_289404\_length\_16761\_cov\_29.864925 4136-4153. Max. coverage (+): 0. Max coverage (-): 0.33

Region: NODE\_289404\_length\_16761\_cov\_29.864925 4154-4171. Max. coverage (+): 0. Max coverage (-): 0.07

Region: NODE\_289404\_length\_16761\_cov\_29.864925 4172-4189. Max. coverage (+): 0. Max coverage (-): 0.11

Region: NODE\_289404\_length\_16761\_cov\_29.864925 4190-4207. Max. coverage (+): 0. Max coverage (-): 0.3

Region: NODE\_289404\_length\_16761\_cov\_29.864925 4208-4225. Max. coverage (+): 0. Max coverage (-): 0.07

Region: NODE\_289404\_length\_16761\_cov\_29.864925 4226-4243. Max. coverage (+): 0.04. Max coverage (-): 0.48

Region: NODE\_289404\_length\_16761\_cov\_29.864925 4244-4261. Max. coverage (+): 0.04. Max coverage (-): 0.04

Region: NODE\_289404\_length\_16761\_cov\_29.864925 4262-4279. Max. coverage (+): 0. Max coverage (-): 0.04

Region: NODE\_289404\_length\_16761\_cov\_29.864925 4280-4297. Max. coverage (+): 0.04. Max coverage (-): 0.15

Region: NODE\_289404\_length\_16761\_cov\_29.864925 4298-4315. Max. coverage (+): 0. Max coverage (-): 0.04

Region: NODE\_289404\_length\_16761\_cov\_29.864925 4316-4333. Max. coverage (+): 0.04. Max coverage (-): 2.3

Region: NODE\_289404\_length\_16761\_cov\_29.864925 4334-4351. Max. coverage (+): 0. Max coverage (-): 0.59

Region: NODE\_289404\_length\_16761\_cov\_29.864925 4352-4369. Max. coverage (+): 0. Max coverage (-): 0.64

Region: NODE\_289404\_length\_16761\_cov\_29.864925 4370-4387. Max. coverage (+): 0.15. Max coverage (-): 0.15

Region: NODE\_289404\_length\_16761\_cov\_29.864925 4388-4405. Max. coverage (+): 0. Max coverage (-): 0.19

Region: NODE\_289404\_length\_16761\_cov\_29.864925 4406-4423. Max. coverage (+): 0. Max coverage (-): 0.15

Region: NODE\_289404\_length\_16761\_cov\_29.864925 4424-4442. Max. coverage (+): 0. Max coverage (-): 0.07

Region: NODE\_289404\_length\_16761\_cov\_29.864925 4443-4460. Max. coverage (+): 0. Max coverage (-): 0.04

Region: NODE\_289404\_length\_16761\_cov\_29.864925 4461-4478. Max. coverage (+): 0. Max coverage (-): 0.04

Region: NODE\_289404\_length\_16761\_cov\_29.864925 4479-4496. Max. coverage (+): 0.04. Max coverage (-): 0

Region: NODE\_289404\_length\_16761\_cov\_29.864925 4497-4514. Max. coverage (+): 0. Max coverage (-): 0.11

Region: NODE\_289404\_length\_16761\_cov\_29.864925 4515-4532. Max. coverage (+): 0. Max coverage (-): 0.07

Region: NODE\_289404\_length\_16761\_cov\_29.864925 4533-4550. Max. coverage (+): 0.04. Max coverage (-): 0.11

Region: NODE\_289404\_length\_16761\_cov\_29.864925 4551-4568. Max. coverage (+): 0. Max coverage (-): 0

Region: NODE\_289404\_length\_16761\_cov\_29.864925 4569-4586. Max. coverage (+): 0. Max coverage (-): 0

Region: NODE\_289404\_length\_16761\_cov\_29.864925 4587-4604. Max. coverage (+): 0. Max coverage (-): 0

Region: NODE\_289404\_length\_16761\_cov\_29.864925 4605-4622. Max. coverage (+): 0. Max coverage (-): 0.02

Region: NODE\_289404\_length\_16761\_cov\_29.864925 4623-4640. Max. coverage (+): 0. Max coverage (-): 0.51

Region: NODE\_289404\_length\_16761\_cov\_29.864925 4641-4658. Max. coverage (+): 0.04. Max coverage (-): 0

Region: NODE\_289404\_length\_16761\_cov\_29.864925 4659-4676. Max. coverage (+): 0. Max coverage (-): 0.02

Region: NODE\_289404\_length\_16761\_cov\_29.864925 4677-4694. Max. coverage (+): 0. Max coverage (-): 0.04

Region: NODE\_289404\_length\_16761\_cov\_29.864925 4695-4712. Max. coverage (+): 0. Max coverage (-): 0.13

Region: NODE\_289404\_length\_16761\_cov\_29.864925 4713-4730. Max. coverage (+): 0.07. Max coverage (-): 0.06

Region: NODE\_289404\_length\_16761\_cov\_29.864925 4731-4748. Max. coverage (+): 0.07. Max coverage (-): 0

Region: NODE\_289404\_length\_16761\_cov\_29.864925 4749-4766. Max. coverage (+): 0. Max coverage (-): 0

Region: NODE\_289404\_length\_16761\_cov\_29.864925 4767-4784. Max. coverage (+): 0. Max coverage (-): 0.25

Region: NODE\_289404\_length\_16761\_cov\_29.864925 4785-4802. Max. coverage (+): 0.02. Max coverage (-): 0.04

Region: NODE\_289404\_length\_16761\_cov\_29.864925 4803-4820. Max. coverage (+): 0. Max coverage (-): 0

Region: NODE\_289404\_length\_16761\_cov\_29.864925 4821-4838. Max. coverage (+): 0. Max coverage (-): 0.04

Region: NODE\_289404\_length\_16761\_cov\_29.864925 4839-4856. Max. coverage (+): 0.04. Max coverage (-): 0.04

Region: NODE\_289404\_length\_16761\_cov\_29.864925 4857-4874. Max. coverage (+): 0.48. Max coverage (-): 0.04

Region: NODE\_289404\_length\_16761\_cov\_29.864925 4875-4892. Max. coverage (+): 0. Max coverage (-): 0.3

Region: NODE\_289404\_length\_16761\_cov\_29.864925 4893-4911. Max. coverage (+): 0.07. Max coverage (-): 0.11

Region: NODE\_289404\_length\_16761\_cov\_29.864925 4912-4929. Max. coverage (+): 0. Max coverage (-): 0.19

Region: NODE\_289404\_length\_16761\_cov\_29.864925 4930-4947. Max. coverage (+): 0. Max coverage (-): 1.3

Region: NODE\_289404\_length\_16761\_cov\_29.864925 4948-4965. Max. coverage (+): 0.04. Max coverage (-): 0

Region: NODE\_289404\_length\_16761\_cov\_29.864925 4966-4983. Max. coverage (+): 0. Max coverage (-): 0.07

Region: NODE\_289404\_length\_16761\_cov\_29.864925 4984-5001. Max. coverage (+): 0. Max coverage (-): 0

Region: NODE\_289404\_length\_16761\_cov\_29.864925 5002-5019. Max. coverage (+): 0. Max coverage (-): 0.04

Region: NODE\_289404\_length\_16761\_cov\_29.864925 5020-5037. Max. coverage (+): 0. Max coverage (-): 0.04

Region: NODE\_289404\_length\_16761\_cov\_29.864925 5038-5055. Max. coverage (+): 0. Max coverage (-): 0

Region: NODE\_289404\_length\_16761\_cov\_29.864925 5056-5073. Max. coverage (+): 0.02. Max coverage (-): 0.13

Region: NODE\_289404\_length\_16761\_cov\_29.864925 5074-5091. Max. coverage (+): 0.07. Max coverage (-): 0

Region: NODE\_289404\_length\_16761\_cov\_29.864925 5092-5109. Max. coverage (+): 0.02. Max coverage (-): 0

Region: NODE\_289404\_length\_16761\_cov\_29.864925 5110-5127. Max. coverage (+): 0. Max coverage (-): 0

Region: NODE\_289404\_length\_16761\_cov\_29.864925 5128-5145. Max. coverage (+): 0. Max coverage (-): 0.26

Region: NODE\_289404\_length\_16761\_cov\_29.864925 5146-5163. Max. coverage (+): 0. Max coverage (-): 0.44

Region: NODE\_289404\_length\_16761\_cov\_29.864925 5164-5181. Max. coverage (+): 0.15. Max coverage (-): 0.26

Region: NODE\_289404\_length\_16761\_cov\_29.864925 5182-5199. Max. coverage (+): 0.07. Max coverage (-): 0

Region: NODE\_289404\_length\_16761\_cov\_29.864925 5200-5217. Max. coverage (+): 0.04. Max coverage (-): 0.07

Region: NODE\_289404\_length\_16761\_cov\_29.864925 5218-5235. Max. coverage (+): 0. Max coverage (-): 0.07

Region: NODE\_289404\_length\_16761\_cov\_29.864925 5236-5253. Max. coverage (+): 0.01. Max coverage (-): 0.89

Region: NODE\_289404\_length\_16761\_cov\_29.864925 5254-5271. Max. coverage (+): 0.46. Max coverage (-): 0.22

Region: NODE\_289404\_length\_16761\_cov\_29.864925 5272-5289. Max. coverage (+): 0.01. Max coverage (-): 0.06

Region: NODE\_289404\_length\_16761\_cov\_29.864925 5290-5307. Max. coverage (+): 0.28. Max coverage (-): 0.06

Region: NODE\_289404\_length\_16761\_cov\_29.864925 5308-5325. Max. coverage (+): 0. Max coverage (-): 1.17

Region: NODE\_289404\_length\_16761\_cov\_29.864925 5326-5343. Max. coverage (+): 0.11. Max coverage (-): 0.13

Region: NODE\_289404\_length\_16761\_cov\_29.864925 5344-5361. Max. coverage (+): 0.04. Max coverage (-): 1.74

Region: NODE\_289404\_length\_16761\_cov\_29.864925 5362-5379. Max. coverage (+): 0.01. Max coverage (-): 0.14

Region: NODE\_289404\_length\_16761\_cov\_29.864925 5380-5398. Max. coverage (+): 0. Max coverage (-): 0.37

Region: NODE\_289404\_length\_16761\_cov\_29.864925 5399-5416. Max. coverage (+): 0.17. Max coverage (-): 0.15

Region: NODE\_289404\_length\_16761\_cov\_29.864925 5417-5434. Max. coverage (+): 0. Max coverage (-): 0.37

Region: NODE\_289404\_length\_16761\_cov\_29.864925 5435-5452. Max. coverage (+): 0.07. Max coverage (-): 0.04

Region: NODE\_289404\_length\_16761\_cov\_29.864925 5453-5470. Max. coverage (+): 0. Max coverage (-): 1.33

Region: NODE\_289404\_length\_16761\_cov\_29.864925 5471-5488. Max. coverage (+): 0. Max coverage (-): 0.33

Region: NODE\_289404\_length\_16761\_cov\_29.864925 5489-5506. Max. coverage (+): 0. Max coverage (-): 0.11

Region: NODE\_289404\_length\_16761\_cov\_29.864925 5507-5524. Max. coverage (+): 0. Max coverage (-): 0

Region: NODE\_289404\_length\_16761\_cov\_29.864925 5525-5542. Max. coverage (+): 0. Max coverage (-): 1.96

Region: NODE\_289404\_length\_16761\_cov\_29.864925 5543-5560. Max. coverage (+): 0.04. Max coverage (-): 0.04

Region: NODE\_289404\_length\_16761\_cov\_29.864925 5561-5578. Max. coverage (+): 0. Max coverage (-): 0.04

Region: NODE\_289404\_length\_16761\_cov\_29.864925 5579-5596. Max. coverage (+): 0. Max coverage (-): 0.07

Region: NODE\_289404\_length\_16761\_cov\_29.864925 5597-5614. Max. coverage (+): 0.04. Max coverage (-): 0

Region: NODE\_289404\_length\_16761\_cov\_29.864925 5615-5632. Max. coverage (+): 0.04. Max coverage (-): 3.56

Region: NODE\_289404\_length\_16761\_cov\_29.864925 5633-5650. Max. coverage (+): 0.44. Max coverage (-): 1.67

Region: NODE\_289404\_length\_16761\_cov\_29.864925 5651-5668. Max. coverage (+): 0.19. Max coverage (-): 1.45

Region: NODE\_289404\_length\_16761\_cov\_29.864925 5669-5686. Max. coverage (+): 0.15. Max coverage (-): 0.33

Region: NODE\_289404\_length\_16761\_cov\_29.864925 5687-5704. Max. coverage (+): 0. Max coverage (-): 0.07

Region: NODE\_289404\_length\_16761\_cov\_29.864925 5705-5722. Max. coverage (+): 0. Max coverage (-): 0.15

Region: NODE\_289404\_length\_16761\_cov\_29.864925 5723-5740. Max. coverage (+): 0. Max coverage (-): 0.11

Region: NODE\_289404\_length\_16761\_cov\_29.864925 5741-5758. Max. coverage (+): 0. Max coverage (-): 0

Region: NODE\_289404\_length\_16761\_cov\_29.864925 5759-5776. Max. coverage (+): 0. Max coverage (-): 0.11

Region: NODE\_289404\_length\_16761\_cov\_29.864925 5777-5794. Max. coverage (+): 0. Max coverage (-): 0

Region: NODE\_289404\_length\_16761\_cov\_29.864925 5795-5812. Max. coverage (+): 0. Max coverage (-): 0.15

Region: NODE\_289404\_length\_16761\_cov\_29.864925 5813-5830. Max. coverage (+): 0.04. Max coverage (-): 0.04

Region: NODE\_289404\_length\_16761\_cov\_29.864925 5831-5848. Max. coverage (+): 0.04. Max coverage (-): 0.04

Region: NODE\_289404\_length\_16761\_cov\_29.864925 5849-5867. Max. coverage (+): 0.04. Max coverage (-): 4.89

Region: NODE\_289404\_length\_16761\_cov\_29.864925 5868-5885. Max. coverage (+): 0. Max coverage (-): 0.02

Region: NODE\_289404\_length\_16761\_cov\_29.864925 5886-5903. Max. coverage (+): 0. Max coverage (-): 0.37

Region: NODE\_289404\_length\_16761\_cov\_29.864925 5904-5921. Max. coverage (+): 0. Max coverage (-): 0.04

Region: NODE\_289404\_length\_16761\_cov\_29.864925 5922-5939. Max. coverage (+): 0. Max coverage (-): 0.15

Region: NODE\_289404\_length\_16761\_cov\_29.864925 5940-5957. Max. coverage (+): 0.04. Max coverage (-): 0.52

Region: NODE\_289404\_length\_16761\_cov\_29.864925 5958-5975. Max. coverage (+): 0.04. Max coverage (-): 0.04

Region: NODE\_289404\_length\_16761\_cov\_29.864925 5976-5993. Max. coverage (+): 0.11. Max coverage (-): 0.04

Region: NODE\_289404\_length\_16761\_cov\_29.864925 5994-6011. Max. coverage (+): 0. Max coverage (-): 0.04

Region: NODE\_289404\_length\_16761\_cov\_29.864925 6012-6029. Max. coverage (+): 0. Max coverage (-): 0.33

Region: NODE\_289404\_length\_16761\_cov\_29.864925 6030-6047. Max. coverage (+): 0.07. Max coverage (-): 0.15

Region: NODE\_289404\_length\_16761\_cov\_29.864925 6048-6065. Max. coverage (+): 0. Max coverage (-): 0.15

Region: NODE\_289404\_length\_16761\_cov\_29.864925 6066-6083. Max. coverage (+): 0.15. Max coverage (-): 0.22

Region: NODE\_289404\_length\_16761\_cov\_29.864925 6084-6101. Max. coverage (+): 0. Max coverage (-): 0.15

Region: NODE\_289404\_length\_16761\_cov\_29.864925 6102-6119. Max. coverage (+): 0.07. Max coverage (-): 0.22

Region: NODE\_289404\_length\_16761\_cov\_29.864925 6120-6137. Max. coverage (+): 0.04. Max coverage (-): 0.15

Region: NODE\_289404\_length\_16761\_cov\_29.864925 6138-6155. Max. coverage (+): 0.04. Max coverage (-): 0.22

Region: NODE\_289404\_length\_16761\_cov\_29.864925 6156-6173. Max. coverage (+): 0.04. Max coverage (-): 0.11

Region: NODE\_289404\_length\_16761\_cov\_29.864925 6174-6191. Max. coverage (+): 0. Max coverage (-): 0.44

Region: NODE\_289404\_length\_16761\_cov\_29.864925 6192-6209. Max. coverage (+): 0.07. Max coverage (-): 0.41

Region: NODE\_289404\_length\_16761\_cov\_29.864925 6210-6227. Max. coverage (+): 0.07. Max coverage (-): 0.22

Region: NODE\_289404\_length\_16761\_cov\_29.864925 6228-6245. Max. coverage (+): 0.04. Max coverage (-): 1.22

Region: NODE\_289404\_length\_16761\_cov\_29.864925 6246-6263. Max. coverage (+): 0.22. Max coverage (-): 0.19

Region: NODE\_289404\_length\_16761\_cov\_29.864925 6264-6281. Max. coverage (+): 0. Max coverage (-): 0.44

Region: NODE\_289404\_length\_16761\_cov\_29.864925 6282-6299. Max. coverage (+): 0. Max coverage (-): 0.41

Region: NODE\_289404\_length\_16761\_cov\_29.864925 6300-6317. Max. coverage (+): 0.11. Max coverage (-): 2.19

Region: NODE\_289404\_length\_16761\_cov\_29.864925 6318-6336. Max. coverage (+): 0.67. Max coverage (-): 0.82

Region: NODE\_289404\_length\_16761\_cov\_29.864925 6337-6354. Max. coverage (+): 0.7. Max coverage (-): 0.04

Region: NODE\_289404\_length\_16761\_cov\_29.864925 6355-6372. Max. coverage (+): 0.04. Max coverage (-): 0.04

Region: NODE\_289404\_length\_16761\_cov\_29.864925 6373-6390. Max. coverage (+): 0.11. Max coverage (-): 0.41

Region: NODE\_289404\_length\_16761\_cov\_29.864925 6391-6408. Max. coverage (+): 0.07. Max coverage (-): 0.3

Region: NODE\_289404\_length\_16761\_cov\_29.864925 6409-6426. Max. coverage (+): 0.07. Max coverage (-): 0.33

Region: NODE\_289404\_length\_16761\_cov\_29.864925 6427-6444. Max. coverage (+): 0. Max coverage (-): 0.04

Region: NODE\_289404\_length\_16761\_cov\_29.864925 6445-6462. Max. coverage (+): 0. Max coverage (-): 0.7

Region: NODE\_289404\_length\_16761\_cov\_29.864925 6463-6480. Max. coverage (+): 0.48. Max coverage (-): 0.82

Region: NODE\_289404\_length\_16761\_cov\_29.864925 6481-6498. Max. coverage (+): 0.44. Max coverage (-): 0.07

Region: NODE\_289404\_length\_16761\_cov\_29.864925 6499-6516. Max. coverage (+): 0. Max coverage (-): 0.33

Region: NODE\_289404\_length\_16761\_cov\_29.864925 6517-6534. Max. coverage (+): 0.04. Max coverage (-): 0.33

Region: NODE\_289404\_length\_16761\_cov\_29.864925 6535-6552. Max. coverage (+): 0. Max coverage (-): 0.04

Region: NODE\_289404\_length\_16761\_cov\_29.864925 6553-6570. Max. coverage (+): 0.19. Max coverage (-): 0.04

Region: NODE\_289404\_length\_16761\_cov\_29.864925 6571-6588. Max. coverage (+): 0.07. Max coverage (-): 0.3

Region: NODE\_289404\_length\_16761\_cov\_29.864925 6589-6606. Max. coverage (+): 0.33. Max coverage (-): 0.3

Region: NODE\_289404\_length\_16761\_cov\_29.864925 6607-6624. Max. coverage (+): 0.41. Max coverage (-): 0.22

Region: NODE\_289404\_length\_16761\_cov\_29.864925 6625-6642. Max. coverage (+): 0. Max coverage (-): 0.26

Region: NODE\_289404\_length\_16761\_cov\_29.864925 6643-6660. Max. coverage (+): 0.04. Max coverage (-): 0.15

Region: NODE\_289404\_length\_16761\_cov\_29.864925 6661-6678. Max. coverage (+): 0.04. Max coverage (-): 0.11

Region: NODE\_289404\_length\_16761\_cov\_29.864925 6679-6696. Max. coverage (+): 0. Max coverage (-): 1.11

Region: NODE\_289404\_length\_16761\_cov\_29.864925 6697-6714. Max. coverage (+): 0. Max coverage (-): 1.41

Region: NODE\_289404\_length\_16761\_cov\_29.864925 6715-6732. Max. coverage (+): 0.07. Max coverage (-): 0.19

Region: NODE\_289404\_length\_16761\_cov\_29.864925 6733-6750. Max. coverage (+): 0. Max coverage (-): 0.48

Region: NODE\_289404\_length\_16761\_cov\_29.864925 6751-6768. Max. coverage (+): 0.04. Max coverage (-): 0.07

Region: NODE\_289404\_length\_16761\_cov\_29.864925 6769-6786. Max. coverage (+): 0.04. Max coverage (-): 1.11

Region: NODE\_289404\_length\_16761\_cov\_29.864925 6787-6804. Max. coverage (+): 0.07. Max coverage (-): 0.15

Region: NODE\_289404\_length\_16761\_cov\_29.864925 6805-6823. Max. coverage (+): 0.04. Max coverage (-): 0.15

Region: NODE\_289404\_length\_16761\_cov\_29.864925 6824-6841. Max. coverage (+): 1.48. Max coverage (-): 0.04

Region: NODE\_289404\_length\_16761\_cov\_29.864925 6842-6859. Max. coverage (+): 0.04. Max coverage (-): 0.11

Region: NODE\_289404\_length\_16761\_cov\_29.864925 6860-6877. Max. coverage (+): 0.07. Max coverage (-): 0

Region: NODE\_289404\_length\_16761\_cov\_29.864925 6878-6895. Max. coverage (+): 0.06. Max coverage (-): 0.13

Region: NODE\_289404\_length\_16761\_cov\_29.864925 6896-6913. Max. coverage (+): 0.04. Max coverage (-): 0.13

Region: NODE\_289404\_length\_16761\_cov\_29.864925 6914-6931. Max. coverage (+): 0.04. Max coverage (-): 0.07

Region: NODE\_289404\_length\_16761\_cov\_29.864925 6932-6949. Max. coverage (+): 0.02. Max coverage (-): 0.19

Region: NODE\_289404\_length\_16761\_cov\_29.864925 6950-6967. Max. coverage (+): 0.02. Max coverage (-): 0.35

Region: NODE\_289404\_length\_16761\_cov\_29.864925 6968-6985. Max. coverage (+): 0. Max coverage (-): 1.96

Region: NODE\_289404\_length\_16761\_cov\_29.864925 6986-7003. Max. coverage (+): 0.05. Max coverage (-): 0.04

Region: NODE\_289404\_length\_16761\_cov\_29.864925 7004-7021. Max. coverage (+): 0. Max coverage (-): 0.06

Region: NODE\_289404\_length\_16761\_cov\_29.864925 7022-7039. Max. coverage (+): 0. Max coverage (-): 1.26

Region: NODE\_289404\_length\_16761\_cov\_29.864925 7040-7057. Max. coverage (+): 0.04. Max coverage (-): 1.71

Region: NODE\_289404\_length\_16761\_cov\_29.864925 7058-7075. Max. coverage (+): 0.04. Max coverage (-): 0.04

Region: NODE\_289404\_length\_16761\_cov\_29.864925 7076-7093. Max. coverage (+): 0.26. Max coverage (-): 0

Region: NODE\_289404\_length\_16761\_cov\_29.864925 7094-7111. Max. coverage (+): 0. Max coverage (-): 0.7

Region: NODE\_289404\_length\_16761\_cov\_29.864925 7112-7129. Max. coverage (+): 0.41. Max coverage (-): 0.07

Region: NODE\_289404\_length\_16761\_cov\_29.864925 7130-7147. Max. coverage (+): 0.41. Max coverage (-): 0.15

Region: NODE\_289404\_length\_16761\_cov\_29.864925 7148-7165. Max. coverage (+): 0.19. Max coverage (-): 0.04

Region: NODE\_289404\_length\_16761\_cov\_29.864925 7166-7183. Max. coverage (+): 0.26. Max coverage (-): 0.19

Region: NODE\_289404\_length\_16761\_cov\_29.864925 7184-7201. Max. coverage (+): 0. Max coverage (-): 0.33

Region: NODE\_289404\_length\_16761\_cov\_29.864925 7202-7219. Max. coverage (+): 0.04. Max coverage (-): 0.07

Region: NODE\_289404\_length\_16761\_cov\_29.864925 7220-7237. Max. coverage (+): 0. Max coverage (-): 0.07

Region: NODE\_289404\_length\_16761\_cov\_29.864925 7238-7255. Max. coverage (+): 0.04. Max coverage (-): 1.37

Region: NODE\_289404\_length\_16761\_cov\_29.864925 7256-7273. Max. coverage (+): 0.04. Max coverage (-): 3.34

Region: NODE\_289404\_length\_16761\_cov\_29.864925 7274-7292. Max. coverage (+): 0. Max coverage (-): 0.15

Region: NODE\_289404\_length\_16761\_cov\_29.864925 7293-7310. Max. coverage (+): 0.04. Max coverage (-): 0.48

Region: NODE\_289404\_length\_16761\_cov\_29.864925 7311-7328. Max. coverage (+): 0.19. Max coverage (-): 0.37

Region: NODE\_289404\_length\_16761\_cov\_29.864925 7329-7346. Max. coverage (+): 0.19. Max coverage (-): 0.63

Region: NODE\_289404\_length\_16761\_cov\_29.864925 7347-7364. Max. coverage (+): 0.11. Max coverage (-): 0.04

Region: NODE\_289404\_length\_16761\_cov\_29.864925 7365-7382. Max. coverage (+): 0. Max coverage (-): 0.04

Region: NODE\_289404\_length\_16761\_cov\_29.864925 7383-7400. Max. coverage (+): 0.04. Max coverage (-): 0.07

Region: NODE\_289404\_length\_16761\_cov\_29.864925 7401-7418. Max. coverage (+): 0. Max coverage (-): 0.07

Region: NODE\_289404\_length\_16761\_cov\_29.864925 7419-7436. Max. coverage (+): 0.15. Max coverage (-): 2.22

Region: NODE\_289404\_length\_16761\_cov\_29.864925 7437-7454. Max. coverage (+): 0.52. Max coverage (-): 0.63

Region: NODE\_289404\_length\_16761\_cov\_29.864925 7455-7472. Max. coverage (+): 0.11. Max coverage (-): 1.45

Region: NODE\_289404\_length\_16761\_cov\_29.864925 7473-7490. Max. coverage (+): 0. Max coverage (-): 1.45

Region: NODE\_289404\_length\_16761\_cov\_29.864925 7491-7508. Max. coverage (+): 0.11. Max coverage (-): 1.22

Region: NODE\_289404\_length\_16761\_cov\_29.864925 7509-7526. Max. coverage (+): 0. Max coverage (-): 1.15

Region: NODE\_289404\_length\_16761\_cov\_29.864925 7527-7544. Max. coverage (+): 0.19. Max coverage (-): 0.59

Region: NODE\_289404\_length\_16761\_cov\_29.864925 7545-7562. Max. coverage (+): 0.04. Max coverage (-): 1.37

Region: NODE\_289404\_length\_16761\_cov\_29.864925 7563-7580. Max. coverage (+): 0.11. Max coverage (-): 0.56

Region: NODE\_289404\_length\_16761\_cov\_29.864925 7581-7598. Max. coverage (+): 0.07. Max coverage (-): 0.48

Region: NODE\_289404\_length\_16761\_cov\_29.864925 7599-7616. Max. coverage (+): 0. Max coverage (-): 10.94

Region: NODE\_289404\_length\_16761\_cov\_29.864925 7617-7634. Max. coverage (+): 0.52. Max coverage (-): 1.41

Region: NODE\_289404\_length\_16761\_cov\_29.864925 7635-7652. Max. coverage (+): 0.15. Max coverage (-): 0.41

Region: NODE\_289404\_length\_16761\_cov\_29.864925 7653-7670. Max. coverage (+): 0. Max coverage (-): 0.11

Region: NODE\_289404\_length\_16761\_cov\_29.864925 7671-7688. Max. coverage (+): 0. Max coverage (-): 0

Region: NODE\_289404\_length\_16761\_cov\_29.864925 7689-7706. Max. coverage (+): 0.44. Max coverage (-): 0.15

Region: NODE\_289404\_length\_16761\_cov\_29.864925 7707-7724. Max. coverage (+): 0.04. Max coverage (-): 0.22

Region: NODE\_289404\_length\_16761\_cov\_29.864925 7725-7742. Max. coverage (+): 0.37. Max coverage (-): 0.48

Region: NODE\_289404\_length\_16761\_cov\_29.864925 7743-7761. Max. coverage (+): 0.04. Max coverage (-): 1.19

Region: NODE\_289404\_length\_16761\_cov\_29.864925 7762-7779. Max. coverage (+): 0. Max coverage (-): 0

Region: NODE\_289404\_length\_16761\_cov\_29.864925 7780-7797. Max. coverage (+): 0. Max coverage (-): 0

Region: NODE\_289404\_length\_16761\_cov\_29.864925 7798-7815. Max. coverage (+): 0. Max coverage (-): 0

Region: NODE\_289404\_length\_16761\_cov\_29.864925 7816-7833. Max. coverage (+): 0. Max coverage (-): 0.07

Region: NODE\_289404\_length\_16761\_cov\_29.864925 7834-7851. Max. coverage (+): 0. Max coverage (-): 0.04

Region: NODE\_289404\_length\_16761\_cov\_29.864925 7852-7869. Max. coverage (+): 0. Max coverage (-): 0.47

Region: NODE\_289404\_length\_16761\_cov\_29.864925 7870-7887. Max. coverage (+): 0.04. Max coverage (-): 0

Region: NODE\_289404\_length\_16761\_cov\_29.864925 7888-7905. Max. coverage (+): 0.04. Max coverage (-): 0.22

Region: NODE\_289404\_length\_16761\_cov\_29.864925 7906-7923. Max. coverage (+): 0. Max coverage (-): 0.07

Region: NODE\_289404\_length\_16761\_cov\_29.864925 7924-7941. Max. coverage (+): 0. Max coverage (-): 0.04

Region: NODE\_289404\_length\_16761\_cov\_29.864925 7942-7959. Max. coverage (+): 0. Max coverage (-): 1.04

Region: NODE\_289404\_length\_16761\_cov\_29.864925 7960-7977. Max. coverage (+): 0. Max coverage (-): 2.41

Region: NODE\_289404\_length\_16761\_cov\_29.864925 7978-7995. Max. coverage (+): 0.09. Max coverage (-): 0.41

Region: NODE\_289404\_length\_16761\_cov\_29.864925 7996-8013. Max. coverage (+): 0.04. Max coverage (-): 0.15

Region: NODE\_289404\_length\_16761\_cov\_29.864925 8014-8031. Max. coverage (+): 0. Max coverage (-): 0.59

Region: NODE\_289404\_length\_16761\_cov\_29.864925 8032-8049. Max. coverage (+): 0.07. Max coverage (-): 0.33

Region: NODE\_289404\_length\_16761\_cov\_29.864925 8050-8067. Max. coverage (+): 0.04. Max coverage (-): 0.33

Region: NODE\_289404\_length\_16761\_cov\_29.864925 8068-8085. Max. coverage (+): 0.04. Max coverage (-): 0.3

Region: NODE\_289404\_length\_16761\_cov\_29.864925 8086-8103. Max. coverage (+): 0.07. Max coverage (-): 0.19

Region: NODE\_289404\_length\_16761\_cov\_29.864925 8104-8121. Max. coverage (+): 0.22. Max coverage (-): 1.26

Region: NODE\_289404\_length\_16761\_cov\_29.864925 8122-8139. Max. coverage (+): 0.07. Max coverage (-): 0.48

Region: NODE\_289404\_length\_16761\_cov\_29.864925 8140-8157. Max. coverage (+): 0.32. Max coverage (-): 0.61

Region: NODE\_289404\_length\_16761\_cov\_29.864925 8158-8175. Max. coverage (+): 0.07. Max coverage (-): 0.19

Region: NODE\_289404\_length\_16761\_cov\_29.864925 8176-8193. Max. coverage (+): 0.07. Max coverage (-): 1.41

Region: NODE\_289404\_length\_16761\_cov\_29.864925 8194-8211. Max. coverage (+): 0.07. Max coverage (-): 0.11

Region: NODE\_289404\_length\_16761\_cov\_29.864925 8212-8230. Max. coverage (+): 0.07. Max coverage (-): 7.23

Region: NODE\_289404\_length\_16761\_cov\_29.864925 8231-8248. Max. coverage (+): 0.15. Max coverage (-): 1.96

Region: NODE\_289404\_length\_16761\_cov\_29.864925 8249-8266. Max. coverage (+): 0.11. Max coverage (-): 4.67

Region: NODE\_289404\_length\_16761\_cov\_29.864925 8267-8284. Max. coverage (+): 0.07. Max coverage (-): 0.56

Region: NODE\_289404\_length\_16761\_cov\_29.864925 8285-8302. Max. coverage (+): 0.07. Max coverage (-): 0.44

Region: NODE\_289404\_length\_16761\_cov\_29.864925 8303-8320. Max. coverage (+): 0.15. Max coverage (-): 0.19

Region: NODE\_289404\_length\_16761\_cov\_29.864925 8321-8338. Max. coverage (+): 0. Max coverage (-): 5.6

Region: NODE\_289404\_length\_16761\_cov\_29.864925 8339-8356. Max. coverage (+): 0.07. Max coverage (-): 0.56

Region: NODE\_289404\_length\_16761\_cov\_29.864925 8357-8374. Max. coverage (+): 0.07. Max coverage (-): 0

Region: NODE\_289404\_length\_16761\_cov\_29.864925 8375-8392. Max. coverage (+): 0.07. Max coverage (-): 2.89

Region: NODE\_289404\_length\_16761\_cov\_29.864925 8393-8410. Max. coverage (+): 0.12. Max coverage (-): 0.11

Region: NODE\_289404\_length\_16761\_cov\_29.864925 8411-8428. Max. coverage (+): 0.01. Max coverage (-): 0

Region: NODE\_289404\_length\_16761\_cov\_29.864925 8429-8446. Max. coverage (+): 0. Max coverage (-): 0.04

Region: NODE\_289404\_length\_16761\_cov\_29.864925 8447-8464. Max. coverage (+): 0. Max coverage (-): 0

Region: NODE\_289404\_length\_16761\_cov\_29.864925 8465-8482. Max. coverage (+): 0.04. Max coverage (-): 0.35

Region: NODE\_289404\_length\_16761\_cov\_29.864925 8483-8500. Max. coverage (+): 0.74. Max coverage (-): 0.04

Region: NODE\_289404\_length\_16761\_cov\_29.864925 8501-8518. Max. coverage (+): 0.07. Max coverage (-): 0.63

Region: NODE\_289404\_length\_16761\_cov\_29.864925 8519-8536. Max. coverage (+): 0.26. Max coverage (-): 0.41

Region: NODE\_289404\_length\_16761\_cov\_29.864925 8537-8554. Max. coverage (+): 0.19. Max coverage (-): 0.22

Region: NODE\_289404\_length\_16761\_cov\_29.864925 8555-8572. Max. coverage (+): 0.15. Max coverage (-): 0.26

Region: NODE\_289404\_length\_16761\_cov\_29.864925 8573-8590. Max. coverage (+): 0.26. Max coverage (-): 0.67

Region: NODE\_289404\_length\_16761\_cov\_29.864925 8591-8608. Max. coverage (+): 0. Max coverage (-): 1.63

Region: NODE\_289404\_length\_16761\_cov\_29.864925 8609-8626. Max. coverage (+): 0.04. Max coverage (-): 1.37

Region: NODE\_289404\_length\_16761\_cov\_29.864925 8627-8644. Max. coverage (+): 0.04. Max coverage (-): 0.19

Region: NODE\_289404\_length\_16761\_cov\_29.864925 8645-8662. Max. coverage (+): 0.04. Max coverage (-): 1.08

Region: NODE\_289404\_length\_16761\_cov\_29.864925 8663-8680. Max. coverage (+): 0.07. Max coverage (-): 0.04

Region: NODE\_289404\_length\_16761\_cov\_29.864925 8681-8698. Max. coverage (+): 0.04. Max coverage (-): 0

Region: NODE\_289404\_length\_16761\_cov\_29.864925 8699-8717. Max. coverage (+): 0.11. Max coverage (-): 0.19

Region: NODE\_289404\_length\_16761\_cov\_29.864925 8718-8735. Max. coverage (+): 0.04. Max coverage (-): 0.3

Region: NODE\_289404\_length\_16761\_cov\_29.864925 8736-8753. Max. coverage (+): 0.22. Max coverage (-): 0.22

Region: NODE\_289404\_length\_16761\_cov\_29.864925 8754-8771. Max. coverage (+): 0. Max coverage (-): 0.26

Region: NODE\_289404\_length\_16761\_cov\_29.864925 8772-8789. Max. coverage (+): 0.52. Max coverage (-): 0.37

Region: NODE\_289404\_length\_16761\_cov\_29.864925 8790-8807. Max. coverage (+): 0.48. Max coverage (-): 0.07

Region: NODE\_289404\_length\_16761\_cov\_29.864925 8808-8825. Max. coverage (+): 0.07. Max coverage (-): 3.19

Region: NODE\_289404\_length\_16761\_cov\_29.864925 8826-8843. Max. coverage (+): 0. Max coverage (-): 3.23

Region: NODE\_289404\_length\_16761\_cov\_29.864925 8844-8861. Max. coverage (+): 0.15. Max coverage (-): 0.52

Region: NODE\_289404\_length\_16761\_cov\_29.864925 8862-8879. Max. coverage (+): 0.04. Max coverage (-): 0.07

Region: NODE\_289404\_length\_16761\_cov\_29.864925 8880-8897. Max. coverage (+): 0. Max coverage (-): 0.48

Region: NODE\_289404\_length\_16761\_cov\_29.864925 8898-8915. Max. coverage (+): 0.04. Max coverage (-): 0.07

Region: NODE\_289404\_length\_16761\_cov\_29.864925 8916-8933. Max. coverage (+): 0. Max coverage (-): 0.04

Region: NODE\_289404\_length\_16761\_cov\_29.864925 8934-8951. Max. coverage (+): 0.07. Max coverage (-): 0.07

Region: NODE\_289404\_length\_16761\_cov\_29.864925 8952-8969. Max. coverage (+): 0.11. Max coverage (-): 0.07

Region: NODE\_289404\_length\_16761\_cov\_29.864925 8970-8987. Max. coverage (+): 0.04. Max coverage (-): 0.11

Region: NODE\_289404\_length\_16761\_cov\_29.864925 8988-9005. Max. coverage (+): 0.04. Max coverage (-): 0.19

Region: NODE\_289404\_length\_16761\_cov\_29.864925 9006-9023. Max. coverage (+): 0.22. Max coverage (-): 0.19

Region: NODE\_289404\_length\_16761\_cov\_29.864925 9024-9041. Max. coverage (+): 0.11. Max coverage (-): 0.07

Region: NODE\_289404\_length\_16761\_cov\_29.864925 9042-9059. Max. coverage (+): 0. Max coverage (-): 0.15

Region: NODE\_289404\_length\_16761\_cov\_29.864925 9060-9077. Max. coverage (+): 0.19. Max coverage (-): 4.82

Region: NODE\_289404\_length\_16761\_cov\_29.864925 9078-9095. Max. coverage (+): 0.15. Max coverage (-): 0.26

Region: NODE\_289404\_length\_16761\_cov\_29.864925 9096-9113. Max. coverage (+): 0.04. Max coverage (-): 0.22

Region: NODE\_289404\_length\_16761\_cov\_29.864925 9114-9131. Max. coverage (+): 0.22. Max coverage (-): 0.26

Region: NODE\_289404\_length\_16761\_cov\_29.864925 9132-9149. Max. coverage (+): 0.19. Max coverage (-): 0.26

Region: NODE\_289404\_length\_16761\_cov\_29.864925 9150-9167. Max. coverage (+): 0.07. Max coverage (-): 0.41

Region: NODE\_289404\_length\_16761\_cov\_29.864925 9168-9186. Max. coverage (+): 0.07. Max coverage (-): 0.82

Region: NODE\_289404\_length\_16761\_cov\_29.864925 9187-9204. Max. coverage (+): 0.04. Max coverage (-): 0.04

Region: NODE\_289404\_length\_16761\_cov\_29.864925 9205-9222. Max. coverage (+): 0. Max coverage (-): 0.48

Region: NODE\_289404\_length\_16761\_cov\_29.864925 9223-9240. Max. coverage (+): 0.07. Max coverage (-): 0.07

Region: NODE\_289404\_length\_16761\_cov\_29.864925 9241-9258. Max. coverage (+): 0. Max coverage (-): 0.22

Region: NODE\_289404\_length\_16761\_cov\_29.864925 9259-9276. Max. coverage (+): 0. Max coverage (-): 0.04

Region: NODE\_289404\_length\_16761\_cov\_29.864925 9277-9294. Max. coverage (+): 0.04. Max coverage (-): 0.07

Region: NODE\_289404\_length\_16761\_cov\_29.864925 9295-9312. Max. coverage (+): 0.11. Max coverage (-): 0

Region: NODE\_289404\_length\_16761\_cov\_29.864925 9313-9330. Max. coverage (+): 0.04. Max coverage (-): 0.04

Region: NODE\_289404\_length\_16761\_cov\_29.864925 9331-9348. Max. coverage (+): 0. Max coverage (-): 0.07

Region: NODE\_289404\_length\_16761\_cov\_29.864925 9349-9366. Max. coverage (+): 0.19. Max coverage (-): 1.15

Region: NODE\_289404\_length\_16761\_cov\_29.864925 9367-9384. Max. coverage (+): 1.78. Max coverage (-): 0.04

Region: NODE\_289404\_length\_16761\_cov\_29.864925 9385-9402. Max. coverage (+): 0.11. Max coverage (-): 0.07

Region: NODE\_289404\_length\_16761\_cov\_29.864925 9403-9420. Max. coverage (+): 0.14. Max coverage (-): 0

Region: NODE\_289404\_length\_16761\_cov\_29.864925 9421-9438. Max. coverage (+): 0.26. Max coverage (-): 0.07

Region: NODE\_289404\_length\_16761\_cov\_29.864925 9439-9456. Max. coverage (+): 0.15. Max coverage (-): 0.04

Region: NODE\_289404\_length\_16761\_cov\_29.864925 9457-9474. Max. coverage (+): 0. Max coverage (-): 0.04

Region: NODE\_289404\_length\_16761\_cov\_29.864925 9475-9492. Max. coverage (+): 0. Max coverage (-): 0.07

Region: NODE\_289404\_length\_16761\_cov\_29.864925 9493-9510. Max. coverage (+): 0. Max coverage (-): 0.59

Region: NODE\_289404\_length\_16761\_cov\_29.864925 9511-9528. Max. coverage (+): 0.04. Max coverage (-): 0.07

Region: NODE\_289404\_length\_16761\_cov\_29.864925 9529-9546. Max. coverage (+): 0. Max coverage (-): 0.37

Region: NODE\_289404\_length\_16761\_cov\_29.864925 9547-9564. Max. coverage (+): 0. Max coverage (-): 0.22

Region: NODE\_289404\_length\_16761\_cov\_29.864925 9565-9582. Max. coverage (+): 0.04. Max coverage (-): 0.19

Region: NODE\_289404\_length\_16761\_cov\_29.864925 9583-9600. Max. coverage (+): 0.04. Max coverage (-): 0.19

Region: NODE\_289404\_length\_16761\_cov\_29.864925 9601-9618. Max. coverage (+): 0. Max coverage (-): 0.11

Region: NODE\_289404\_length\_16761\_cov\_29.864925 9619-9636. Max. coverage (+): 0. Max coverage (-): 0.15

Region: NODE\_289404\_length\_16761\_cov\_29.864925 9637-9655. Max. coverage (+): 0. Max coverage (-): 0.85

Region: NODE\_289404\_length\_16761\_cov\_29.864925 9656-9673. Max. coverage (+): 0.15. Max coverage (-): 0.22

Region: NODE\_289404\_length\_16761\_cov\_29.864925 9674-9691. Max. coverage (+): 0. Max coverage (-): 0

Region: NODE\_289404\_length\_16761\_cov\_29.864925 9692-9709. Max. coverage (+): 0.04. Max coverage (-): 0.04

Region: NODE\_289404\_length\_16761\_cov\_29.864925 9710-9727. Max. coverage (+): 0.11. Max coverage (-): 0.11

Region: NODE\_289404\_length\_16761\_cov\_29.864925 9728-9745. Max. coverage (+): 0. Max coverage (-): 0.67

Region: NODE\_289404\_length\_16761\_cov\_29.864925 9746-9763. Max. coverage (+): 0.04. Max coverage (-): 0.04

Region: NODE\_289404\_length\_16761\_cov\_29.864925 9764-9781. Max. coverage (+): 4.15. Max coverage (-): 0.15

Region: NODE\_289404\_length\_16761\_cov\_29.864925 9782-9799. Max. coverage (+): 0.02. Max coverage (-): 0.11

Region: NODE\_289404\_length\_16761\_cov\_29.864925 9800-9817. Max. coverage (+): 0. Max coverage (-): 0.39

Region: NODE\_289404\_length\_16761\_cov\_29.864925 9818-9835. Max. coverage (+): 2.78. Max coverage (-): 0.13

Region: NODE\_289404\_length\_16761\_cov\_29.864925 9836-9853. Max. coverage (+): 0.32. Max coverage (-): 0.07

Region: NODE\_289404\_length\_16761\_cov\_29.864925 9854-9871. Max. coverage (+): 0.3. Max coverage (-): 2.04

Region: NODE\_289404\_length\_16761\_cov\_29.864925 9872-9889. Max. coverage (+): 0.19. Max coverage (-): 0.59

Region: NODE\_289404\_length\_16761\_cov\_29.864925 9890-9907. Max. coverage (+): 0.07. Max coverage (-): 0.07

Region: NODE\_289404\_length\_16761\_cov\_29.864925 9908-9925. Max. coverage (+): 0.04. Max coverage (-): 0.74

Region: NODE\_289404\_length\_16761\_cov\_29.864925 9926-9943. Max. coverage (+): 0.3. Max coverage (-): 0.22

Region: NODE\_289404\_length\_16761\_cov\_29.864925 9944-9961. Max. coverage (+): 0.22. Max coverage (-): 0.5

Region: NODE\_289404\_length\_16761\_cov\_29.864925 9962-9979. Max. coverage (+): 0.33. Max coverage (-): 3.95

Region: NODE\_289404\_length\_16761\_cov\_29.864925 9980-9997. Max. coverage (+): 0.07. Max coverage (-): 0.67

Region: NODE\_289404\_length\_16761\_cov\_29.864925 9998-10015. Max. coverage (+): 0.26. Max coverage (-): 0.22

Region: NODE\_289404\_length\_16761\_cov\_29.864925 10016-10033. Max. coverage (+): 0.11. Max coverage (-): 0.32

Region: NODE\_289404\_length\_16761\_cov\_29.864925 10034-10051. Max. coverage (+): 0.19. Max coverage (-): 0.41

Region: NODE\_289404\_length\_16761\_cov\_29.864925 10052-10069. Max. coverage (+): 0.22. Max coverage (-): 5.78

Region: NODE\_289404\_length\_16761\_cov\_29.864925 10070-10087. Max. coverage (+): 0.3. Max coverage (-): 0.04

Region: NODE\_289404\_length\_16761\_cov\_29.864925 10088-10105. Max. coverage (+): 0. Max coverage (-): 1.15

Region: NODE\_289404\_length\_16761\_cov\_29.864925 10106-10123. Max. coverage (+): 1.04. Max coverage (-): 1.52

Region: NODE\_289404\_length\_16761\_cov\_29.864925 10124-10142. Max. coverage (+): 1.04. Max coverage (-): 0.07

Region: NODE\_289404\_length\_16761\_cov\_29.864925 10143-10160. Max. coverage (+): 0.19. Max coverage (-): 0.56

Region: NODE\_289404\_length\_16761\_cov\_29.864925 10161-10178. Max. coverage (+): 0.19. Max coverage (-): 0.74

Region: NODE\_289404\_length\_16761\_cov\_29.864925 10179-10196. Max. coverage (+): 0.15. Max coverage (-): 0.7

Region: NODE\_289404\_length\_16761\_cov\_29.864925 10197-10214. Max. coverage (+): 0.33. Max coverage (-): 0.59

Region: NODE\_289404\_length\_16761\_cov\_29.864925 10215-10232. Max. coverage (+): 0.26. Max coverage (-): 0.15

Region: NODE\_289404\_length\_16761\_cov\_29.864925 10233-10250. Max. coverage (+): 0.15. Max coverage (-): 0.11

Region: NODE\_289404\_length\_16761\_cov\_29.864925 10251-10268. Max. coverage (+): 0.78. Max coverage (-): 0.15

Region: NODE\_289404\_length\_16761\_cov\_29.864925 10269-10286. Max. coverage (+): 0.37. Max coverage (-): 0

Region: NODE\_289404\_length\_16761\_cov\_29.864925 10287-10304. Max. coverage (+): 0. Max coverage (-): 0.22

Region: NODE\_289404\_length\_16761\_cov\_29.864925 10305-10322. Max. coverage (+): 0. Max coverage (-): 0.11

Region: NODE\_289404\_length\_16761\_cov\_29.864925 10323-10340. Max. coverage (+): 0. Max coverage (-): 0.11

Region: NODE\_289404\_length\_16761\_cov\_29.864925 10341-10358. Max. coverage (+): 0.04. Max coverage (-): 0.93

Region: NODE\_289404\_length\_16761\_cov\_29.864925 10359-10376. Max. coverage (+): 0.15. Max coverage (-): 0.89

Region: NODE\_289404\_length\_16761\_cov\_29.864925 10377-10394. Max. coverage (+): 0.41. Max coverage (-): 0.07

Region: NODE\_289404\_length\_16761\_cov\_29.864925 10395-10412. Max. coverage (+): 0.19. Max coverage (-): 0.85

Region: NODE\_289404\_length\_16761\_cov\_29.864925 10413-10430. Max. coverage (+): 0. Max coverage (-): 0.3

Region: NODE\_289404\_length\_16761\_cov\_29.864925 10431-10448. Max. coverage (+): 0.04. Max coverage (-): 0.07

Region: NODE\_289404\_length\_16761\_cov\_29.864925 10449-10466. Max. coverage (+): 0. Max coverage (-): 0.07

Region: NODE\_289404\_length\_16761\_cov\_29.864925 10467-10484. Max. coverage (+): 0.15. Max coverage (-): 0.11

Region: NODE\_289404\_length\_16761\_cov\_29.864925 10485-10502. Max. coverage (+): 0. Max coverage (-): 0.07

Region: NODE\_289404\_length\_16761\_cov\_29.864925 10503-10520. Max. coverage (+): 0. Max coverage (-): 0.19

Region: NODE\_289404\_length\_16761\_cov\_29.864925 10521-10538. Max. coverage (+): 0.15. Max coverage (-): 0.19

Region: NODE\_289404\_length\_16761\_cov\_29.864925 10539-10556. Max. coverage (+): 0.04. Max coverage (-): 0.37

Region: NODE\_289404\_length\_16761\_cov\_29.864925 10557-10574. Max. coverage (+): 0.06. Max coverage (-): 0.12

Region: NODE\_289404\_length\_16761\_cov\_29.864925 10575-10592. Max. coverage (+): 0. Max coverage (-): 0.15

Region: NODE\_289404\_length\_16761\_cov\_29.864925 10593-10611. Max. coverage (+): 0. Max coverage (-): 0.63

Region: NODE\_289404\_length\_16761\_cov\_29.864925 10612-10629. Max. coverage (+): 0.01. Max coverage (-): 0.29

Region: NODE\_289404\_length\_16761\_cov\_29.864925 10630-10647. Max. coverage (+): 0.01. Max coverage (-): 0.04

Region: NODE\_289404\_length\_16761\_cov\_29.864925 10648-10665. Max. coverage (+): 0.15. Max coverage (-): 0.67

Region: NODE\_289404\_length\_16761\_cov\_29.864925 10666-10683. Max. coverage (+): 0.04. Max coverage (-): 0.04

Region: NODE\_289404\_length\_16761\_cov\_29.864925 10684-10701. Max. coverage (+): 0. Max coverage (-): 0.06

Region: NODE\_289404\_length\_16761\_cov\_29.864925 10702-10719. Max. coverage (+): 0.01. Max coverage (-): 0.08

Region: NODE\_289404\_length\_16761\_cov\_29.864925 10720-10737. Max. coverage (+): 0.04. Max coverage (-): 0.33

Region: NODE\_289404\_length\_16761\_cov\_29.864925 10738-10755. Max. coverage (+): 0.15. Max coverage (-): 0.07

Region: NODE\_289404\_length\_16761\_cov\_29.864925 10756-10773. Max. coverage (+): 0.26. Max coverage (-): 0.04

Region: NODE\_289404\_length\_16761\_cov\_29.864925 10774-10791. Max. coverage (+): 0. Max coverage (-): 0.07

Region: NODE\_289404\_length\_16761\_cov\_29.864925 10792-10809. Max. coverage (+): 0.19. Max coverage (-): 0

Region: NODE\_289404\_length\_16761\_cov\_29.864925 10810-10827. Max. coverage (+): 0.07. Max coverage (-): 0

Region: NODE\_289404\_length\_16761\_cov\_29.864925 10828-10845. Max. coverage (+): 0.07. Max coverage (-): 0.11

Region: NODE\_289404\_length\_16761\_cov\_29.864925 10846-10863. Max. coverage (+): 0.04. Max coverage (-): 0.01

Region: NODE\_289404\_length\_16761\_cov\_29.864925 10864-10881. Max. coverage (+): 0. Max coverage (-): 0.15

Region: NODE\_289404\_length\_16761\_cov\_29.864925 10882-10899. Max. coverage (+): 0.04. Max coverage (-): 0.04

Region: NODE\_289404\_length\_16761\_cov\_29.864925 10900-10917. Max. coverage (+): 0.02. Max coverage (-): 0.28

Region: NODE\_289404\_length\_16761\_cov\_29.864925 10918-10935. Max. coverage (+): 0.11. Max coverage (-): 0.04

Region: NODE\_289404\_length\_16761\_cov\_29.864925 10936-10953. Max. coverage (+): 0.11. Max coverage (-): 0.04

Region: NODE\_289404\_length\_16761\_cov\_29.864925 10954-10971. Max. coverage (+): 0.04. Max coverage (-): 0.04

Region: NODE\_289404\_length\_16761\_cov\_29.864925 10972-10989. Max. coverage (+): 0.04. Max coverage (-): 0

Region: NODE\_289404\_length\_16761\_cov\_29.864925 10990-11007. Max. coverage (+): 0.04. Max coverage (-): 0

Region: NODE\_289404\_length\_16761\_cov\_29.864925 11008-11025. Max. coverage (+): 0.04. Max coverage (-): 0.04

Region: NODE\_289404\_length\_16761\_cov\_29.864925 11026-11043. Max. coverage (+): 0.04. Max coverage (-): 0

Region: NODE\_289404\_length\_16761\_cov\_29.864925 11044-11061. Max. coverage (+): 0. Max coverage (-): 0

Region: NODE\_289404\_length\_16761\_cov\_29.864925 11062-11080. Max. coverage (+): 0. Max coverage (-): 0

Region: NODE\_289404\_length\_16761\_cov\_29.864925 11081-11098. Max. coverage (+): 0. Max coverage (-): 0

Region: NODE\_289404\_length\_16761\_cov\_29.864925 11099-11116. Max. coverage (+): 0. Max coverage (-): 0

Region: NODE\_289404\_length\_16761\_cov\_29.864925 11117-11134. Max. coverage (+): 0. Max coverage (-): 0

Region: NODE\_289404\_length\_16761\_cov\_29.864925 11135-11152. Max. coverage (+): 0. Max coverage (-): 0

Region: NODE\_289404\_length\_16761\_cov\_29.864925 11153-11170. Max. coverage (+): 0.01. Max coverage (-): 0

Region: NODE\_289404\_length\_16761\_cov\_29.864925 11171-11188. Max. coverage (+): 0. Max coverage (-): 0

Region: NODE\_289404\_length\_16761\_cov\_29.864925 11189-11206. Max. coverage (+): 0.04. Max coverage (-): 0

Region: NODE\_289404\_length\_16761\_cov\_29.864925 11207-11224. Max. coverage (+): 0. Max coverage (-): 0

Region: NODE\_289404\_length\_16761\_cov\_29.864925 11225-11242. Max. coverage (+): 0. Max coverage (-): 0

Region: NODE\_289404\_length\_16761\_cov\_29.864925 11243-11260. Max. coverage (+): 0. Max coverage (-): 0

Region: NODE\_289404\_length\_16761\_cov\_29.864925 11261-11278. Max. coverage (+): 0. Max coverage (-): 0.02

Region: NODE\_289404\_length\_16761\_cov\_29.864925 11279-11296. Max. coverage (+): 0.05. Max coverage (-): 0.05

Region: NODE\_289404\_length\_16761\_cov\_29.864925 11297-11314. Max. coverage (+): 0.01. Max coverage (-): 0.06

Region: NODE\_289404\_length\_16761\_cov\_29.864925 11315-11332. Max. coverage (+): 0.02. Max coverage (-): 0.01

Region: NODE\_289404\_length\_16761\_cov\_29.864925 11333-11350. Max. coverage (+): 0. Max coverage (-): 0

Region: NODE\_289404\_length\_16761\_cov\_29.864925 11351-11368. Max. coverage (+): 0. Max coverage (-): 0.02

Region: NODE\_289404\_length\_16761\_cov\_29.864925 11369-11386. Max. coverage (+): 0. Max coverage (-): 0.01

Region: NODE\_289404\_length\_16761\_cov\_29.864925 11387-11404. Max. coverage (+): 0.01. Max coverage (-): 0.01

Region: NODE\_289404\_length\_16761\_cov\_29.864925 11405-11422. Max. coverage (+): 0.04. Max coverage (-): 0.02

Region: NODE\_289404\_length\_16761\_cov\_29.864925 11423-11440. Max. coverage (+): 0. Max coverage (-): 0

Region: NODE\_289404\_length\_16761\_cov\_29.864925 11441-11458. Max. coverage (+): 0. Max coverage (-): 0

Region: NODE\_289404\_length\_16761\_cov\_29.864925 11459-11476. Max. coverage (+): 0. Max coverage (-): 0

Region: NODE\_289404\_length\_16761\_cov\_29.864925 11477-11494. Max. coverage (+): 0. Max coverage (-): 0

Region: NODE\_289404\_length\_16761\_cov\_29.864925 11495-11512. Max. coverage (+): 0. Max coverage (-): 0

Region: NODE\_289404\_length\_16761\_cov\_29.864925 11513-11530. Max. coverage (+): 0.02. Max coverage (-): 0

Region: NODE\_289404\_length\_16761\_cov\_29.864925 11531-11548. Max. coverage (+): 0.01. Max coverage (-): 0

Region: NODE\_289404\_length\_16761\_cov\_29.864925 11549-11567. Max. coverage (+): 0. Max coverage (-): 0

Region: NODE\_289404\_length\_16761\_cov\_29.864925 11568-11585. Max. coverage (+): 0. Max coverage (-): 0

Region: NODE\_289404\_length\_16761\_cov\_29.864925 11586-11603. Max. coverage (+): 0. Max coverage (-): 0

Region: NODE\_289404\_length\_16761\_cov\_29.864925 11604-11621. Max. coverage (+): 0. Max coverage (-): 0.02

Region: NODE\_289404\_length\_16761\_cov\_29.864925 11622-11639. Max. coverage (+): 0. Max coverage (-): 0.02

Region: NODE\_289404\_length\_16761\_cov\_29.864925 11640-11657. Max. coverage (+): 0. Max coverage (-): 0.01

Region: NODE\_289404\_length\_16761\_cov\_29.864925 11658-11675. Max. coverage (+): 0.03. Max coverage (-): 0

Region: NODE\_289404\_length\_16761\_cov\_29.864925 11676-11693. Max. coverage (+): 0. Max coverage (-): 0

Region: NODE\_289404\_length\_16761\_cov\_29.864925 11694-11711. Max. coverage (+): 0. Max coverage (-): 0

Region: NODE\_289404\_length\_16761\_cov\_29.864925 11712-11729. Max. coverage (+): 0. Max coverage (-): 0

Region: NODE\_289404\_length\_16761\_cov\_29.864925 11730-11747. Max. coverage (+): 0. Max coverage (-): 0.02

Region: NODE\_289404\_length\_16761\_cov\_29.864925 11748-11765. Max. coverage (+): 0.01. Max coverage (-): 0

Region: NODE\_289404\_length\_16761\_cov\_29.864925 11766-11783. Max. coverage (+): 0. Max coverage (-): 0.02

Region: NODE\_289404\_length\_16761\_cov\_29.864925 11784-11801. Max. coverage (+): 0. Max coverage (-): 0.02

Region: NODE\_289404\_length\_16761\_cov\_29.864925 11802-11819. Max. coverage (+): 0. Max coverage (-): 0

Region: NODE\_289404\_length\_16761\_cov\_29.864925 11820-11837. Max. coverage (+): 0. Max coverage (-): 0

Region: NODE\_289404\_length\_16761\_cov\_29.864925 11838-11855. Max. coverage (+): 0. Max coverage (-): 0

Region: NODE\_289404\_length\_16761\_cov\_29.864925 11856-11873. Max. coverage (+): 0.04. Max coverage (-): 0

Region: NODE\_289404\_length\_16761\_cov\_29.864925 11874-11891. Max. coverage (+): 0.04. Max coverage (-): 0

Region: NODE\_289404\_length\_16761\_cov\_29.864925 11892-11909. Max. coverage (+): 0.33. Max coverage (-): 0

Region: NODE\_289404\_length\_16761\_cov\_29.864925 11910-11927. Max. coverage (+): 0.33. Max coverage (-): 0

Region: NODE\_289404\_length\_16761\_cov\_29.864925 11928-11945. Max. coverage (+): 0. Max coverage (-): 0.01

Region: NODE\_289404\_length\_16761\_cov\_29.864925 11946-11963. Max. coverage (+): 0. Max coverage (-): 0.01

Region: NODE\_289404\_length\_16761\_cov\_29.864925 11964-11981. Max. coverage (+): 0. Max coverage (-): 0

Region: NODE\_289404\_length\_16761\_cov\_29.864925 11982-11999. Max. coverage (+): 0. Max coverage (-): 0.01

Region: NODE\_289404\_length\_16761\_cov\_29.864925 12000-12017. Max. coverage (+): 0. Max coverage (-): 0.01

Region: NODE\_289404\_length\_16761\_cov\_29.864925 12018-. Max. coverage (+): 0. Max coverage (-): 0

RepeatMasker Color Code

**+**

100-98% Identity

<98-95% Identity

<95-90% Identity

<90-85% Identity

<85-80% Identity

<80-75% Identity

<75-70% Identity

<70% Identity

**-**

Gene Set Color Code

**+**

Gene

Pseudogene

Other

**-**

Topology/Coverage Color Code

Coverage Plus Strand

Coverage Minus Strand

Mainstrand: Plus

Mainstrand: Minus

Complementary Strand

Flanking Region  
(if option -flank >0)

Gene Set Annotation  

**1. unknown (unknownunknown) Tr:unknown**: 4369-4414 (+)  
**2. unknown (unknownunknown) Tr:unknown**: 4628-4948 (+)  
**3. unknown (unknownunknown) Tr:unknown**: 5150-5499 (+)

  
RepeatMasker Annotation  

**1. (TTAT)n**: 4587-4617 (+), Divergence to consensus: 17%  
**2. EnSpm-17\_HM**: 5573-5634 (+), Divergence to consensus: 22.6%  
**3. L1-11\_DR**: 5585-5655 (-), Divergence to consensus: 29.4%  
**4. TC1\_FR3**: 7332-7634 (+), Divergence to consensus: 31.2%  
**5. AlRepD-2046**: 7767-8071 (-), Divergence to consensus: 18%  
**6. AlRepD-2046**: 8128-8245 (-), Divergence to consensus: 34.1%  
**7. AlRepE-7493**: 8232-8842 (+), Divergence to consensus: 30.8%  
**8. EnSpm-N1\_CGi**: 9172-9301 (-), Divergence to consensus: 34.6%  
**9. L2-14\_DRe**: 10404-11208 (+), Divergence to consensus: 35.6%  
**10. DNA-8-18\_DR**: 11262-11370 (+), Divergence to consensus: 5.5%  
**11. DNA-8-18\_DR**: 11360-11494 (+), Divergence to consensus: 9.6%  
**12. AlRepB-127**: 11489-11633 (+), Divergence to consensus: 10.4%  
**13. AlRepB-127**: 11640-11802 (+), Divergence to consensus: 4.9%  
**14. AlRepC-532**: 11822-12322 (+), Divergence to consensus: 8.6%

  
Transcription Factor Binding Sites  

**RHOXF1** (Sequence: AGATTA (-): 3188)  
**RHOXF1** (Sequence: GGATTA (-): 4014)  
**RHOXF1** (Sequence: AGATCA (-): 4052)  
**RHOXF1** (Sequence: GGCTTA (-): 4268)  
**RHOXF1** (Sequence: AGATTA (-): 4467)  
**RHOXF1** (Sequence: AGCTTA (-): 4470)  
**RHOXF1** (Sequence: AGATTA (-): 4584)  
**RHOXF1** (Sequence: GGATTA (-): 4911)  
**RHOXF1** (Sequence: GGATTA (-): 5180)  
**RHOXF1** (Sequence: GGATCA (-): 5278)  
**RHOXF1** (Sequence: AGATCA (-): 6480)  
**RHOXF1** (Sequence: GGATCA (-): 7940)  
**RHOXF1** (Sequence: AGATCA (-): 8823)  
**RHOXF1** (Sequence: AGATTA (-): 8870)  
**RHOXF1** (Sequence: GGCTTA (-): 8974)  
**RHOXF1** (Sequence: AGCTCA (-): 10867)  
**RHOXF1** (Sequence: GGCTCA (-): 11636)  
**RHOXF1** (Sequence: TAAGCC (+): 3647)  
**RHOXF1** (Sequence: TGATCT (+): 3879)  
**RHOXF1** (Sequence: TAATCT (+): 4071)  
**RHOXF1** (Sequence: TAATCT (+): 4457)  
**RHOXF1** (Sequence: TAATCT (+): 6365)  
**RHOXF1** (Sequence: TAATCT (+): 6676)  
**RHOXF1** (Sequence: TGAGCT (+): 7223)  
**RHOXF1** (Sequence: TGATCC (+): 7490)  
**RHOXF1** (Sequence: TGATCT (+): 7647)  
**RHOXF1** (Sequence: TAAGCC (+): 8312)  
**RHOXF1** (Sequence: TGAGCT (+): 10840)  
**RHOXF1** (Sequence: TAAGCT (+): 11556)  
**Lhx8** (Sequence: TTAATTAA (-): 3335)  
**Lhx8** (Sequence: TTAATTAA (-): 3530)  
**Lhx8** (Sequence: TTAATTAA (-): 4564)  
**Gata4** (Sequence: GTTATCT (+): 7113)  
**Gata4** (Sequence: CTTATCT (+): 8348)  
**POU5F1** (Sequence: TTTGCAT (-): 7845)  
**POU5F1** (Sequence: TTTGCAT (-): 9552)  
**RFX4\_2** (Sequence: GTATCTACG (-): 5159)  
**RFX4\_2** (Sequence: GTAACTATG (-): 5228)  
**FOXO3\_hsa** (Sequence: GTAAACAA (+): 7175)  
**SOX9** (Sequence: AACAATGG (-): 5660)  
**SOX9** (Sequence: AACAATGG (-): 11452)  
**SOX9** (Sequence: AACAATAA (-): 11542)  
**FOXP1** (Sequence: GTAAACA (+): 7175)  
**FOXP1** (Sequence: GTAAACA (+): 9401)  
**FOXO1** (Sequence: CTTGTTTTT (+): 3096)  
**FOXO3\_mmu** (Sequence: TGTTTTCA (-): 5334)  
**FOXO3\_mmu** (Sequence: TGTTTTGC (-): 9748)  
**Sox5** (Sequence: ATTGTT (+): 7897)  
**Sox5** (Sequence: ATTGTT (+): 9274)  
**Sox5** (Sequence: ATTGTT (+): 11086)  
**Sox5** (Sequence: ATTGTT (+): 11114)  
**Sox5** (Sequence: ATTGTT (+): 11151)  
**Sox5** (Sequence: ATTGTT (+): 11751)  
**SOX9** (Sequence: TTATTGTT (+): 11084)  
**SOX9** (Sequence: TCATTGTT (+): 11112)  
**SOX9** (Sequence: CCATTGTT (+): 11749)  
**FOXO3\_mmu** (Sequence: GCAAAACA (+): 3662)  
**FOXO3\_mmu** (Sequence: TGTAAACA (+): 7174)  
**FOXO3\_mmu** (Sequence: TCAAAACA (+): 7683)  
**FOXO3\_mmu** (Sequence: TGTAAACA (+): 9400)  
**FOXO3\_mmu** (Sequence: TCAAAACA (+): 9762)  
**Nobox** (Sequence: GCCAATTA (-): 5439)  
**FOXO1** (Sequence: ATAAACAAG (-): 4849)  
**FOXO1** (Sequence: ATAAACAAC (-): 5003)  
**FOXO1** (Sequence: GTAAACAAG (-): 7175)  
**POU2F1** (Sequence: ATTAGCATA (-): 3174)  
**POU2F1** (Sequence: ATTTAAATA (-): 4431)  
**POU2F1** (Sequence: ATTAGCATA (-): 5520)  
**POU2F1** (Sequence: ATTTAAATA (-): 6543)  
**POU2F1** (Sequence: ATTTGCATA (-): 9551)  
**POU2F1** (Sequence: ATTAAAATA (-): 11337)  
**Rhox11** (Sequence: TGGTGTTTT (+): 3779)  
**Rhox11** (Sequence: TTAACACCA (-): 3282)  
**Rhox11** (Sequence: TTAACACCA (-): 3477)  
**Sox5** (Sequence: AACAAT (-): 4512)  
**Sox5** (Sequence: AACAAT (-): 5660)  
**Sox5** (Sequence: AACAAT (-): 11452)  
**Sox5** (Sequence: AACAAT (-): 11542)  
**POU2F1** (Sequence: TATTTTAAT (+): 11067)  
**POU5F1** (Sequence: ATGCAAA (+): 3584)
