# Supplementary material for: First characterization of PIWI-interacting RNA clusters in a cichlid fish with a B chromosome
Source: BMC Biol. 2022 Sep 21;20:204. doi: 10.1186/s12915-022-01403-2 (PMC9490952; doi:10.1186/s12915-022-01403-2)
Supplement: Supplementary file 1 — Additional file 1. Zipped folder with fasta and interactive html piRNA cluster information for the A. latifasciata genome. The nomenclature is as follows: number-pirna-cluster_sex_B-presence (f, female; m, male; 0b, without B chromosome; 1b, with B chromosome). [file 12915_2022_1403_MOESM1_ESM.zip › 113_f0b.html]

piRNA cluster 113\_f0b 60


Predicted piRNA cluster no. 113\_f0b
  

Show proTRAC run info
Hide proTRAC run info

/\  
                \_\_\_\_\_\_\_\_\_\_\_\_\_\_\_\_\_\_\_\_\_\_\_/\\_\_\_ /  \\_\_\_\_\_\_\_  
               I                      /  \  /    \      I  
               I     pro             /    \/      \     I  
               I        TRAC        /               \   I  
               I   \_\_\_\_\_\_\_\_\_\_\_\_\_\_\_\_/\_\_\_\_\_\_\_\_\_\_\_\_\_\_\_\_\_\\_ I  
               I   \              /                     I  
               I    \            /                      I  
               I     \  /\      /       V.2.4.2         I  
               I      \/  \    /                        I  
               I\_\_\_\_\_\_\_\_\_\_\_\  /\_\_\_\_\_\_\_\_\_\_\_\_\_\_\_\_\_\_\_\_\_\_\_\_\_I  
                            \/  
  
  
================================= proTRAC ====================================  
VERSION: .......... 2.4.2  
LAST MODIFIED: .... 11. May 2018  
  
Please cite:  
Rosenkranz D, Zischler H. proTRAC - a software for probabilistic piRNA cluster  
detection, visualization and analysis. 2012. BMC Bioinformatics 13:5.  
  
  
Contact:  
David Rosenkranz  
Institute of Organismic and Molecular Evolutionary Biology  
Dept. Anthropology, small RNA group  
Johannes Gutenberg University Mainz  
email: rosenkranz@uni-mainz.de  
  
You can find the latest proTRAC version at:  
http://sourceforge.net/projects/protrac/files  
http://www.smallRNAgroup-mainz.de/software  
==============================================================================  
  
PARAMETERS:  
Map file: ...............piwi-femeas-0B.fa-collapse.map  
Genome file: ............../../../0B\_ala\_genome.fa  
RepeatMasker annotation: Alatifasciata-all0B-maryan-v2.fa\_corrected.out  
GeneSet:................./guest-storage/Data/annotation/Alatifasciata\_all0B\_maryan-v2\_out2017.gff  
  
Significant (p<=0.01) hit density will be calculated based  
on observed hit distribution.  
  
Sliding window size: ........................................ 5000 bp  
Sliding window increament: .................................. 1000 bp  
Normalize each hit by number of genomic hits: ............... yes  
Normalize each hit by number of sequence reads: ............. yes  
Normalize values (-> per million mapped reads): ............. yes  
Min. fraction of hits with 1T(U) or 10A: .................... 0.75  
Alternatively: Min. fraction of hits with 1T(U) and 10A: .... 0.5  
Min. fraction of hits with typical piRNA length: ............ 0.75  
Typical piRNA length: ....................................... 24-32 nt  
Min. size of a piRNA cluster: ............................... 1000 bp.  
Min. number of hits (absolute): ............................. 0  
Min. number of hits (normalized): ........................... 0  
Min. fraction of hits on the mainstrand: .................... 0.75  
Top fraction of mapped sequences (in terms of read counts): . 1%  
Top fraction accounts for max. n% of sequence reads: ........ 90%  
Min. fraction of hits on each arm of a bidirectional cluster: 0.05  
Output html file for each cluster: .......................... yes  
Output a summary table: ..................................... yes  
Output a FASTA file for each cluster (piRNA sequences): ..... yes  
Output a FASTA file comprising cluster sequences: ........... yes  
Output a GTF file for predicted piRNA clusters: ..............yes  
Search DNA motifs in clusters: .............................. yes  
Output flanking sequences: +/- .............................. 0 bp  
Output ~.pTi file: .......................................... no  
==============================================================================  
  
  
Genome size (without gaps): ............ 758543724 bp  
Gaps (N/X/-): .......................... 417479 bp  
Mapped reads: .......................... 13052187  
Non-identical sequences: ............... 3338911  
Genomic hits: .......................... 28737726  
Significant densitiy of mapped reads: .. 470.083249848448 reads/kb

Show proTRAC cluster info
Hide proTRAC cluster info

|  |  |
| --- | --- |
| Location | NODE\_289545\_length\_2022\_cov\_31.587538 |
| Coordinates | 41-2085 |
| Size [bp] | 2045 |
| Sequence hit loci | 493 |
| Mapped reads (normalized) | 2431.5 |
| Mapped reads (normalized) per kb | 1189 |
| Normalized reads with 1T (1U) | 89.6% |
| Normalized reads with 10A | 70.8% |
| Normalized reads with length 24-32 nt | 99.5% |
| Normalized reads on the main strand(s) | 99.6% |
| Predicted directionality | mono:minus |

100%

0%

1T (1U)  
reads

10A reads

24-32 nt  
reads

reads on mainstrand

**Either the amount of reads with 1T (1U) OR 10A has to exceed 75% (set with option: -1Tor10A)  
Alternatively the amount of reads with 1T (1U) AND 10A has to exceed 50% (set with option: -1Tand10A)  
Minimum amount of reads with preferred size is 75% (set with option: -pisize)  
Minimum amount of reads on the main strand(s) is 75% (set with option: -clstrand)**

Show read coverage
Hide read coverage

WHAT DO I SEE HERE?  
This chart shows the location of mapped sequence reads within a predicted piRNA cluster. The color refers to the number of genomic hits produced by the sequence read in question. A dark red bar indicates that this sequence read produces many other hits elsewhere in the genome. Many adjacent red or yellow bars can indicate the presence of a multi-copy element such as transposons or rRNA genes. A dark green bar indicates that this sequence read maps uniquely to this locus.

1 hit

2-5 hits

6-10 hits

11-20 hits

21-50 hits

51-100 hits

> 100 hits

NODE\_289545\_length\_2022\_cov\_31.587538

41

2085

Gene Set

RepeatMasker

Mapped  
Reads

46.43

plus strand

minus strand

46.43

Region: NODE\_289545\_length\_2022\_cov\_31.587538 12425-43. Max. coverage (+): 0. Max coverage (-): 0.08

Region: NODE\_289545\_length\_2022\_cov\_31.587538 44-47. Max. coverage (+): 0. Max coverage (-): 0

Region: NODE\_289545\_length\_2022\_cov\_31.587538 48-51. Max. coverage (+): 0. Max coverage (-): 0

Region: NODE\_289545\_length\_2022\_cov\_31.587538 52-55. Max. coverage (+): 0. Max coverage (-): 0

Region: NODE\_289545\_length\_2022\_cov\_31.587538 56-59. Max. coverage (+): 0. Max coverage (-): 0

Region: NODE\_289545\_length\_2022\_cov\_31.587538 60-63. Max. coverage (+): 0.08. Max coverage (-): 0.08

Region: NODE\_289545\_length\_2022\_cov\_31.587538 64-67. Max. coverage (+): 0. Max coverage (-): 0.08

Region: NODE\_289545\_length\_2022\_cov\_31.587538 68-71. Max. coverage (+): 0. Max coverage (-): 0

Region: NODE\_289545\_length\_2022\_cov\_31.587538 72-75. Max. coverage (+): 0. Max coverage (-): 0

Region: NODE\_289545\_length\_2022\_cov\_31.587538 76-79. Max. coverage (+): 0. Max coverage (-): 0

Region: NODE\_289545\_length\_2022\_cov\_31.587538 80-83. Max. coverage (+): 0. Max coverage (-): 0

Region: NODE\_289545\_length\_2022\_cov\_31.587538 84-88. Max. coverage (+): 0. Max coverage (-): 0

Region: NODE\_289545\_length\_2022\_cov\_31.587538 89-92. Max. coverage (+): 0. Max coverage (-): 0.08

Region: NODE\_289545\_length\_2022\_cov\_31.587538 93-96. Max. coverage (+): 0. Max coverage (-): 0.15

Region: NODE\_289545\_length\_2022\_cov\_31.587538 97-100. Max. coverage (+): 0. Max coverage (-): 1.46

Region: NODE\_289545\_length\_2022\_cov\_31.587538 101-104. Max. coverage (+): 0. Max coverage (-): 0.23

Region: NODE\_289545\_length\_2022\_cov\_31.587538 105-108. Max. coverage (+): 0. Max coverage (-): 0

Region: NODE\_289545\_length\_2022\_cov\_31.587538 109-112. Max. coverage (+): 0. Max coverage (-): 0

Region: NODE\_289545\_length\_2022\_cov\_31.587538 113-116. Max. coverage (+): 0. Max coverage (-): 0

Region: NODE\_289545\_length\_2022\_cov\_31.587538 117-120. Max. coverage (+): 0. Max coverage (-): 0.04

Region: NODE\_289545\_length\_2022\_cov\_31.587538 121-124. Max. coverage (+): 0. Max coverage (-): 0.04

Region: NODE\_289545\_length\_2022\_cov\_31.587538 125-128. Max. coverage (+): 0. Max coverage (-): 0.01

Region: NODE\_289545\_length\_2022\_cov\_31.587538 129-133. Max. coverage (+): 0. Max coverage (-): 0

Region: NODE\_289545\_length\_2022\_cov\_31.587538 134-137. Max. coverage (+): 0. Max coverage (-): 0

Region: NODE\_289545\_length\_2022\_cov\_31.587538 138-141. Max. coverage (+): 0. Max coverage (-): 0

Region: NODE\_289545\_length\_2022\_cov\_31.587538 142-145. Max. coverage (+): 0. Max coverage (-): 0

Region: NODE\_289545\_length\_2022\_cov\_31.587538 146-149. Max. coverage (+): 0. Max coverage (-): 0.08

Region: NODE\_289545\_length\_2022\_cov\_31.587538 150-153. Max. coverage (+): 0. Max coverage (-): 0.08

Region: NODE\_289545\_length\_2022\_cov\_31.587538 154-157. Max. coverage (+): 0. Max coverage (-): 0

Region: NODE\_289545\_length\_2022\_cov\_31.587538 158-161. Max. coverage (+): 0. Max coverage (-): 0

Region: NODE\_289545\_length\_2022\_cov\_31.587538 162-165. Max. coverage (+): 0. Max coverage (-): 0

Region: NODE\_289545\_length\_2022\_cov\_31.587538 166-169. Max. coverage (+): 0. Max coverage (-): 0

Region: NODE\_289545\_length\_2022\_cov\_31.587538 170-173. Max. coverage (+): 0. Max coverage (-): 0

Region: NODE\_289545\_length\_2022\_cov\_31.587538 174-178. Max. coverage (+): 0. Max coverage (-): 0.02

Region: NODE\_289545\_length\_2022\_cov\_31.587538 179-182. Max. coverage (+): 0. Max coverage (-): 0.08

Region: NODE\_289545\_length\_2022\_cov\_31.587538 183-186. Max. coverage (+): 0. Max coverage (-): 0.04

Region: NODE\_289545\_length\_2022\_cov\_31.587538 187-190. Max. coverage (+): 0. Max coverage (-): 0

Region: NODE\_289545\_length\_2022\_cov\_31.587538 191-194. Max. coverage (+): 0. Max coverage (-): 0

Region: NODE\_289545\_length\_2022\_cov\_31.587538 195-198. Max. coverage (+): 0. Max coverage (-): 0.01

Region: NODE\_289545\_length\_2022\_cov\_31.587538 199-202. Max. coverage (+): 0. Max coverage (-): 0.08

Region: NODE\_289545\_length\_2022\_cov\_31.587538 203-206. Max. coverage (+): 0. Max coverage (-): 0.08

Region: NODE\_289545\_length\_2022\_cov\_31.587538 207-210. Max. coverage (+): 0. Max coverage (-): 0

Region: NODE\_289545\_length\_2022\_cov\_31.587538 211-214. Max. coverage (+): 0. Max coverage (-): 0

Region: NODE\_289545\_length\_2022\_cov\_31.587538 215-218. Max. coverage (+): 0. Max coverage (-): 0

Region: NODE\_289545\_length\_2022\_cov\_31.587538 219-223. Max. coverage (+): 0. Max coverage (-): 0

Region: NODE\_289545\_length\_2022\_cov\_31.587538 224-227. Max. coverage (+): 0. Max coverage (-): 0

Region: NODE\_289545\_length\_2022\_cov\_31.587538 228-231. Max. coverage (+): 0. Max coverage (-): 0

Region: NODE\_289545\_length\_2022\_cov\_31.587538 232-235. Max. coverage (+): 0. Max coverage (-): 1.84

Region: NODE\_289545\_length\_2022\_cov\_31.587538 236-239. Max. coverage (+): 0. Max coverage (-): 46.43

Region: NODE\_289545\_length\_2022\_cov\_31.587538 240-243. Max. coverage (+): 0. Max coverage (-): 42.83

Region: NODE\_289545\_length\_2022\_cov\_31.587538 244-247. Max. coverage (+): 0. Max coverage (-): 0.46

Region: NODE\_289545\_length\_2022\_cov\_31.587538 248-251. Max. coverage (+): 0. Max coverage (-): 1.23

Region: NODE\_289545\_length\_2022\_cov\_31.587538 252-255. Max. coverage (+): 0. Max coverage (-): 3.52

Region: NODE\_289545\_length\_2022\_cov\_31.587538 256-259. Max. coverage (+): 0. Max coverage (-): 1.46

Region: NODE\_289545\_length\_2022\_cov\_31.587538 260-263. Max. coverage (+): 0. Max coverage (-): 0

Region: NODE\_289545\_length\_2022\_cov\_31.587538 264-267. Max. coverage (+): 0. Max coverage (-): 0.34

Region: NODE\_289545\_length\_2022\_cov\_31.587538 268-272. Max. coverage (+): 0.05. Max coverage (-): 0.21

Region: NODE\_289545\_length\_2022\_cov\_31.587538 273-276. Max. coverage (+): 0.07. Max coverage (-): 0.02

Region: NODE\_289545\_length\_2022\_cov\_31.587538 277-280. Max. coverage (+): 0. Max coverage (-): 0.02

Region: NODE\_289545\_length\_2022\_cov\_31.587538 281-284. Max. coverage (+): 0. Max coverage (-): 0

Region: NODE\_289545\_length\_2022\_cov\_31.587538 285-288. Max. coverage (+): 0. Max coverage (-): 0

Region: NODE\_289545\_length\_2022\_cov\_31.587538 289-292. Max. coverage (+): 0. Max coverage (-): 0.08

Region: NODE\_289545\_length\_2022\_cov\_31.587538 293-296. Max. coverage (+): 0. Max coverage (-): 0.08

Region: NODE\_289545\_length\_2022\_cov\_31.587538 297-300. Max. coverage (+): 0. Max coverage (-): 0

Region: NODE\_289545\_length\_2022\_cov\_31.587538 301-304. Max. coverage (+): 0. Max coverage (-): 0

Region: NODE\_289545\_length\_2022\_cov\_31.587538 305-308. Max. coverage (+): 0. Max coverage (-): 0

Region: NODE\_289545\_length\_2022\_cov\_31.587538 309-312. Max. coverage (+): 0. Max coverage (-): 0

Region: NODE\_289545\_length\_2022\_cov\_31.587538 313-317. Max. coverage (+): 0. Max coverage (-): 0

Region: NODE\_289545\_length\_2022\_cov\_31.587538 318-321. Max. coverage (+): 0. Max coverage (-): 0

Region: NODE\_289545\_length\_2022\_cov\_31.587538 322-325. Max. coverage (+): 0. Max coverage (-): 0

Region: NODE\_289545\_length\_2022\_cov\_31.587538 326-329. Max. coverage (+): 0. Max coverage (-): 0

Region: NODE\_289545\_length\_2022\_cov\_31.587538 330-333. Max. coverage (+): 0. Max coverage (-): 0

Region: NODE\_289545\_length\_2022\_cov\_31.587538 334-337. Max. coverage (+): 0. Max coverage (-): 0

Region: NODE\_289545\_length\_2022\_cov\_31.587538 338-341. Max. coverage (+): 0. Max coverage (-): 0

Region: NODE\_289545\_length\_2022\_cov\_31.587538 342-345. Max. coverage (+): 0. Max coverage (-): 0

Region: NODE\_289545\_length\_2022\_cov\_31.587538 346-349. Max. coverage (+): 0. Max coverage (-): 0

Region: NODE\_289545\_length\_2022\_cov\_31.587538 350-353. Max. coverage (+): 0. Max coverage (-): 0

Region: NODE\_289545\_length\_2022\_cov\_31.587538 354-357. Max. coverage (+): 0. Max coverage (-): 0

Region: NODE\_289545\_length\_2022\_cov\_31.587538 358-362. Max. coverage (+): 0. Max coverage (-): 0

Region: NODE\_289545\_length\_2022\_cov\_31.587538 363-366. Max. coverage (+): 0. Max coverage (-): 0

Region: NODE\_289545\_length\_2022\_cov\_31.587538 367-370. Max. coverage (+): 0. Max coverage (-): 0

Region: NODE\_289545\_length\_2022\_cov\_31.587538 371-374. Max. coverage (+): 0. Max coverage (-): 0

Region: NODE\_289545\_length\_2022\_cov\_31.587538 375-378. Max. coverage (+): 0. Max coverage (-): 0

Region: NODE\_289545\_length\_2022\_cov\_31.587538 379-382. Max. coverage (+): 0. Max coverage (-): 0

Region: NODE\_289545\_length\_2022\_cov\_31.587538 383-386. Max. coverage (+): 0. Max coverage (-): 0

Region: NODE\_289545\_length\_2022\_cov\_31.587538 387-390. Max. coverage (+): 0. Max coverage (-): 0

Region: NODE\_289545\_length\_2022\_cov\_31.587538 391-394. Max. coverage (+): 0. Max coverage (-): 0

Region: NODE\_289545\_length\_2022\_cov\_31.587538 395-398. Max. coverage (+): 0. Max coverage (-): 0

Region: NODE\_289545\_length\_2022\_cov\_31.587538 399-402. Max. coverage (+): 0. Max coverage (-): 0

Region: NODE\_289545\_length\_2022\_cov\_31.587538 403-407. Max. coverage (+): 0. Max coverage (-): 0

Region: NODE\_289545\_length\_2022\_cov\_31.587538 408-411. Max. coverage (+): 0. Max coverage (-): 0

Region: NODE\_289545\_length\_2022\_cov\_31.587538 412-415. Max. coverage (+): 0. Max coverage (-): 0

Region: NODE\_289545\_length\_2022\_cov\_31.587538 416-419. Max. coverage (+): 0. Max coverage (-): 0

Region: NODE\_289545\_length\_2022\_cov\_31.587538 420-423. Max. coverage (+): 0. Max coverage (-): 0

Region: NODE\_289545\_length\_2022\_cov\_31.587538 424-427. Max. coverage (+): 0. Max coverage (-): 0

Region: NODE\_289545\_length\_2022\_cov\_31.587538 428-431. Max. coverage (+): 0. Max coverage (-): 0

Region: NODE\_289545\_length\_2022\_cov\_31.587538 432-435. Max. coverage (+): 0. Max coverage (-): 0

Region: NODE\_289545\_length\_2022\_cov\_31.587538 436-439. Max. coverage (+): 0. Max coverage (-): 0

Region: NODE\_289545\_length\_2022\_cov\_31.587538 440-443. Max. coverage (+): 0. Max coverage (-): 0

Region: NODE\_289545\_length\_2022\_cov\_31.587538 444-447. Max. coverage (+): 0. Max coverage (-): 0

Region: NODE\_289545\_length\_2022\_cov\_31.587538 448-452. Max. coverage (+): 0. Max coverage (-): 0

Region: NODE\_289545\_length\_2022\_cov\_31.587538 453-456. Max. coverage (+): 0. Max coverage (-): 0

Region: NODE\_289545\_length\_2022\_cov\_31.587538 457-460. Max. coverage (+): 0. Max coverage (-): 0

Region: NODE\_289545\_length\_2022\_cov\_31.587538 461-464. Max. coverage (+): 0. Max coverage (-): 0

Region: NODE\_289545\_length\_2022\_cov\_31.587538 465-468. Max. coverage (+): 0. Max coverage (-): 0

Region: NODE\_289545\_length\_2022\_cov\_31.587538 469-472. Max. coverage (+): 0. Max coverage (-): 0

Region: NODE\_289545\_length\_2022\_cov\_31.587538 473-476. Max. coverage (+): 0. Max coverage (-): 0

Region: NODE\_289545\_length\_2022\_cov\_31.587538 477-480. Max. coverage (+): 0. Max coverage (-): 0

Region: NODE\_289545\_length\_2022\_cov\_31.587538 481-484. Max. coverage (+): 0. Max coverage (-): 0

Region: NODE\_289545\_length\_2022\_cov\_31.587538 485-488. Max. coverage (+): 0. Max coverage (-): 0

Region: NODE\_289545\_length\_2022\_cov\_31.587538 489-492. Max. coverage (+): 0. Max coverage (-): 0

Region: NODE\_289545\_length\_2022\_cov\_31.587538 493-497. Max. coverage (+): 0. Max coverage (-): 0

Region: NODE\_289545\_length\_2022\_cov\_31.587538 498-501. Max. coverage (+): 0. Max coverage (-): 0

Region: NODE\_289545\_length\_2022\_cov\_31.587538 502-505. Max. coverage (+): 0. Max coverage (-): 0.38

Region: NODE\_289545\_length\_2022\_cov\_31.587538 506-509. Max. coverage (+): 0. Max coverage (-): 0

Region: NODE\_289545\_length\_2022\_cov\_31.587538 510-513. Max. coverage (+): 0. Max coverage (-): 0

Region: NODE\_289545\_length\_2022\_cov\_31.587538 514-517. Max. coverage (+): 0. Max coverage (-): 0

Region: NODE\_289545\_length\_2022\_cov\_31.587538 518-521. Max. coverage (+): 0. Max coverage (-): 0

Region: NODE\_289545\_length\_2022\_cov\_31.587538 522-525. Max. coverage (+): 0. Max coverage (-): 0

Region: NODE\_289545\_length\_2022\_cov\_31.587538 526-529. Max. coverage (+): 0. Max coverage (-): 0

Region: NODE\_289545\_length\_2022\_cov\_31.587538 530-533. Max. coverage (+): 0. Max coverage (-): 0.15

Region: NODE\_289545\_length\_2022\_cov\_31.587538 534-537. Max. coverage (+): 0. Max coverage (-): 0

Region: NODE\_289545\_length\_2022\_cov\_31.587538 538-542. Max. coverage (+): 0. Max coverage (-): 0

Region: NODE\_289545\_length\_2022\_cov\_31.587538 543-546. Max. coverage (+): 0. Max coverage (-): 0

Region: NODE\_289545\_length\_2022\_cov\_31.587538 547-550. Max. coverage (+): 0. Max coverage (-): 0

Region: NODE\_289545\_length\_2022\_cov\_31.587538 551-554. Max. coverage (+): 0. Max coverage (-): 0

Region: NODE\_289545\_length\_2022\_cov\_31.587538 555-558. Max. coverage (+): 0. Max coverage (-): 0.04

Region: NODE\_289545\_length\_2022\_cov\_31.587538 559-562. Max. coverage (+): 0. Max coverage (-): 0.04

Region: NODE\_289545\_length\_2022\_cov\_31.587538 563-566. Max. coverage (+): 0. Max coverage (-): 0

Region: NODE\_289545\_length\_2022\_cov\_31.587538 567-570. Max. coverage (+): 0. Max coverage (-): 0

Region: NODE\_289545\_length\_2022\_cov\_31.587538 571-574. Max. coverage (+): 0. Max coverage (-): 0

Region: NODE\_289545\_length\_2022\_cov\_31.587538 575-578. Max. coverage (+): 0. Max coverage (-): 0

Region: NODE\_289545\_length\_2022\_cov\_31.587538 579-582. Max. coverage (+): 0. Max coverage (-): 0

Region: NODE\_289545\_length\_2022\_cov\_31.587538 583-587. Max. coverage (+): 0. Max coverage (-): 0

Region: NODE\_289545\_length\_2022\_cov\_31.587538 588-591. Max. coverage (+): 0. Max coverage (-): 0.08

Region: NODE\_289545\_length\_2022\_cov\_31.587538 592-595. Max. coverage (+): 0. Max coverage (-): 0.08

Region: NODE\_289545\_length\_2022\_cov\_31.587538 596-599. Max. coverage (+): 0. Max coverage (-): 0

Region: NODE\_289545\_length\_2022\_cov\_31.587538 600-603. Max. coverage (+): 0. Max coverage (-): 0.08

Region: NODE\_289545\_length\_2022\_cov\_31.587538 604-607. Max. coverage (+): 0. Max coverage (-): 0

Region: NODE\_289545\_length\_2022\_cov\_31.587538 608-611. Max. coverage (+): 0. Max coverage (-): 0

Region: NODE\_289545\_length\_2022\_cov\_31.587538 612-615. Max. coverage (+): 0. Max coverage (-): 0

Region: NODE\_289545\_length\_2022\_cov\_31.587538 616-619. Max. coverage (+): 0. Max coverage (-): 0

Region: NODE\_289545\_length\_2022\_cov\_31.587538 620-623. Max. coverage (+): 0. Max coverage (-): 0

Region: NODE\_289545\_length\_2022\_cov\_31.587538 624-627. Max. coverage (+): 0. Max coverage (-): 0

Region: NODE\_289545\_length\_2022\_cov\_31.587538 628-632. Max. coverage (+): 0. Max coverage (-): 0.08

Region: NODE\_289545\_length\_2022\_cov\_31.587538 633-636. Max. coverage (+): 0. Max coverage (-): 0.08

Region: NODE\_289545\_length\_2022\_cov\_31.587538 637-640. Max. coverage (+): 0. Max coverage (-): 0.08

Region: NODE\_289545\_length\_2022\_cov\_31.587538 641-644. Max. coverage (+): 0. Max coverage (-): 0

Region: NODE\_289545\_length\_2022\_cov\_31.587538 645-648. Max. coverage (+): 0. Max coverage (-): 0.08

Region: NODE\_289545\_length\_2022\_cov\_31.587538 649-652. Max. coverage (+): 0. Max coverage (-): 0.08

Region: NODE\_289545\_length\_2022\_cov\_31.587538 653-656. Max. coverage (+): 0. Max coverage (-): 0

Region: NODE\_289545\_length\_2022\_cov\_31.587538 657-660. Max. coverage (+): 0. Max coverage (-): 0.54

Region: NODE\_289545\_length\_2022\_cov\_31.587538 661-664. Max. coverage (+): 0. Max coverage (-): 1.76

Region: NODE\_289545\_length\_2022\_cov\_31.587538 665-668. Max. coverage (+): 0. Max coverage (-): 4.83

Region: NODE\_289545\_length\_2022\_cov\_31.587538 669-672. Max. coverage (+): 0. Max coverage (-): 0.4

Region: NODE\_289545\_length\_2022\_cov\_31.587538 673-676. Max. coverage (+): 0. Max coverage (-): 0.56

Region: NODE\_289545\_length\_2022\_cov\_31.587538 677-681. Max. coverage (+): 0. Max coverage (-): 0

Region: NODE\_289545\_length\_2022\_cov\_31.587538 682-685. Max. coverage (+): 0. Max coverage (-): 0

Region: NODE\_289545\_length\_2022\_cov\_31.587538 686-689. Max. coverage (+): 0. Max coverage (-): 0

Region: NODE\_289545\_length\_2022\_cov\_31.587538 690-693. Max. coverage (+): 0. Max coverage (-): 0

Region: NODE\_289545\_length\_2022\_cov\_31.587538 694-697. Max. coverage (+): 0. Max coverage (-): 0

Region: NODE\_289545\_length\_2022\_cov\_31.587538 698-701. Max. coverage (+): 0. Max coverage (-): 0

Region: NODE\_289545\_length\_2022\_cov\_31.587538 702-705. Max. coverage (+): 0. Max coverage (-): 1

Region: NODE\_289545\_length\_2022\_cov\_31.587538 706-709. Max. coverage (+): 0. Max coverage (-): 1.38

Region: NODE\_289545\_length\_2022\_cov\_31.587538 710-713. Max. coverage (+): 0. Max coverage (-): 0.38

Region: NODE\_289545\_length\_2022\_cov\_31.587538 714-717. Max. coverage (+): 0. Max coverage (-): 0.15

Region: NODE\_289545\_length\_2022\_cov\_31.587538 718-721. Max. coverage (+): 0. Max coverage (-): 0.08

Region: NODE\_289545\_length\_2022\_cov\_31.587538 722-726. Max. coverage (+): 0. Max coverage (-): 0

Region: NODE\_289545\_length\_2022\_cov\_31.587538 727-730. Max. coverage (+): 0. Max coverage (-): 0

Region: NODE\_289545\_length\_2022\_cov\_31.587538 731-734. Max. coverage (+): 0. Max coverage (-): 0

Region: NODE\_289545\_length\_2022\_cov\_31.587538 735-738. Max. coverage (+): 0. Max coverage (-): 0

Region: NODE\_289545\_length\_2022\_cov\_31.587538 739-742. Max. coverage (+): 0. Max coverage (-): 0.08

Region: NODE\_289545\_length\_2022\_cov\_31.587538 743-746. Max. coverage (+): 0. Max coverage (-): 0.19

Region: NODE\_289545\_length\_2022\_cov\_31.587538 747-750. Max. coverage (+): 0. Max coverage (-): 0.15

Region: NODE\_289545\_length\_2022\_cov\_31.587538 751-754. Max. coverage (+): 0. Max coverage (-): 0.08

Region: NODE\_289545\_length\_2022\_cov\_31.587538 755-758. Max. coverage (+): 0. Max coverage (-): 0

Region: NODE\_289545\_length\_2022\_cov\_31.587538 759-762. Max. coverage (+): 0. Max coverage (-): 0.08

Region: NODE\_289545\_length\_2022\_cov\_31.587538 763-766. Max. coverage (+): 0. Max coverage (-): 0.08

Region: NODE\_289545\_length\_2022\_cov\_31.587538 767-771. Max. coverage (+): 0. Max coverage (-): 0

Region: NODE\_289545\_length\_2022\_cov\_31.587538 772-775. Max. coverage (+): 0. Max coverage (-): 0

Region: NODE\_289545\_length\_2022\_cov\_31.587538 776-779. Max. coverage (+): 0. Max coverage (-): 0

Region: NODE\_289545\_length\_2022\_cov\_31.587538 780-783. Max. coverage (+): 0. Max coverage (-): 0

Region: NODE\_289545\_length\_2022\_cov\_31.587538 784-787. Max. coverage (+): 0. Max coverage (-): 0

Region: NODE\_289545\_length\_2022\_cov\_31.587538 788-791. Max. coverage (+): 0. Max coverage (-): 0

Region: NODE\_289545\_length\_2022\_cov\_31.587538 792-795. Max. coverage (+): 0. Max coverage (-): 0

Region: NODE\_289545\_length\_2022\_cov\_31.587538 796-799. Max. coverage (+): 0. Max coverage (-): 0

Region: NODE\_289545\_length\_2022\_cov\_31.587538 800-803. Max. coverage (+): 0. Max coverage (-): 0

Region: NODE\_289545\_length\_2022\_cov\_31.587538 804-807. Max. coverage (+): 0. Max coverage (-): 0.46

Region: NODE\_289545\_length\_2022\_cov\_31.587538 808-811. Max. coverage (+): 0. Max coverage (-): 0.38

Region: NODE\_289545\_length\_2022\_cov\_31.587538 812-816. Max. coverage (+): 0. Max coverage (-): 0.08

Region: NODE\_289545\_length\_2022\_cov\_31.587538 817-820. Max. coverage (+): 0. Max coverage (-): 0

Region: NODE\_289545\_length\_2022\_cov\_31.587538 821-824. Max. coverage (+): 0. Max coverage (-): 0.08

Region: NODE\_289545\_length\_2022\_cov\_31.587538 825-828. Max. coverage (+): 0. Max coverage (-): 0.1

Region: NODE\_289545\_length\_2022\_cov\_31.587538 829-832. Max. coverage (+): 0. Max coverage (-): 0.1

Region: NODE\_289545\_length\_2022\_cov\_31.587538 833-836. Max. coverage (+): 0. Max coverage (-): 0.11

Region: NODE\_289545\_length\_2022\_cov\_31.587538 837-840. Max. coverage (+): 0. Max coverage (-): 0.02

Region: NODE\_289545\_length\_2022\_cov\_31.587538 841-844. Max. coverage (+): 0. Max coverage (-): 0.02

Region: NODE\_289545\_length\_2022\_cov\_31.587538 845-848. Max. coverage (+): 0. Max coverage (-): 0

Region: NODE\_289545\_length\_2022\_cov\_31.587538 849-852. Max. coverage (+): 0. Max coverage (-): 0

Region: NODE\_289545\_length\_2022\_cov\_31.587538 853-856. Max. coverage (+): 0. Max coverage (-): 0

Region: NODE\_289545\_length\_2022\_cov\_31.587538 857-861. Max. coverage (+): 0. Max coverage (-): 0.46

Region: NODE\_289545\_length\_2022\_cov\_31.587538 862-865. Max. coverage (+): 0. Max coverage (-): 0.08

Region: NODE\_289545\_length\_2022\_cov\_31.587538 866-869. Max. coverage (+): 0. Max coverage (-): 0.38

Region: NODE\_289545\_length\_2022\_cov\_31.587538 870-873. Max. coverage (+): 0. Max coverage (-): 1.23

Region: NODE\_289545\_length\_2022\_cov\_31.587538 874-877. Max. coverage (+): 0. Max coverage (-): 0.28

Region: NODE\_289545\_length\_2022\_cov\_31.587538 878-881. Max. coverage (+): 0. Max coverage (-): 0.11

Region: NODE\_289545\_length\_2022\_cov\_31.587538 882-885. Max. coverage (+): 0. Max coverage (-): 0

Region: NODE\_289545\_length\_2022\_cov\_31.587538 886-889. Max. coverage (+): 0. Max coverage (-): 0.08

Region: NODE\_289545\_length\_2022\_cov\_31.587538 890-893. Max. coverage (+): 0. Max coverage (-): 0.04

Region: NODE\_289545\_length\_2022\_cov\_31.587538 894-897. Max. coverage (+): 0. Max coverage (-): 0.19

Region: NODE\_289545\_length\_2022\_cov\_31.587538 898-901. Max. coverage (+): 0. Max coverage (-): 0.19

Region: NODE\_289545\_length\_2022\_cov\_31.587538 902-906. Max. coverage (+): 0. Max coverage (-): 0

Region: NODE\_289545\_length\_2022\_cov\_31.587538 907-910. Max. coverage (+): 0. Max coverage (-): 0

Region: NODE\_289545\_length\_2022\_cov\_31.587538 911-914. Max. coverage (+): 0. Max coverage (-): 0

Region: NODE\_289545\_length\_2022\_cov\_31.587538 915-918. Max. coverage (+): 0. Max coverage (-): 0

Region: NODE\_289545\_length\_2022\_cov\_31.587538 919-922. Max. coverage (+): 0. Max coverage (-): 0.31

Region: NODE\_289545\_length\_2022\_cov\_31.587538 923-926. Max. coverage (+): 0. Max coverage (-): 0.31

Region: NODE\_289545\_length\_2022\_cov\_31.587538 927-930. Max. coverage (+): 0. Max coverage (-): 0

Region: NODE\_289545\_length\_2022\_cov\_31.587538 931-934. Max. coverage (+): 0. Max coverage (-): 0.54

Region: NODE\_289545\_length\_2022\_cov\_31.587538 935-938. Max. coverage (+): 0. Max coverage (-): 0.38

Region: NODE\_289545\_length\_2022\_cov\_31.587538 939-942. Max. coverage (+): 0. Max coverage (-): 0.04

Region: NODE\_289545\_length\_2022\_cov\_31.587538 943-946. Max. coverage (+): 0. Max coverage (-): 0

Region: NODE\_289545\_length\_2022\_cov\_31.587538 947-951. Max. coverage (+): 0. Max coverage (-): 0.31

Region: NODE\_289545\_length\_2022\_cov\_31.587538 952-955. Max. coverage (+): 0. Max coverage (-): 0.31

Region: NODE\_289545\_length\_2022\_cov\_31.587538 956-959. Max. coverage (+): 0. Max coverage (-): 2.83

Region: NODE\_289545\_length\_2022\_cov\_31.587538 960-963. Max. coverage (+): 0. Max coverage (-): 0.15

Region: NODE\_289545\_length\_2022\_cov\_31.587538 964-967. Max. coverage (+): 0. Max coverage (-): 0.08

Region: NODE\_289545\_length\_2022\_cov\_31.587538 968-971. Max. coverage (+): 0. Max coverage (-): 0

Region: NODE\_289545\_length\_2022\_cov\_31.587538 972-975. Max. coverage (+): 0. Max coverage (-): 0

Region: NODE\_289545\_length\_2022\_cov\_31.587538 976-979. Max. coverage (+): 0. Max coverage (-): 0

Region: NODE\_289545\_length\_2022\_cov\_31.587538 980-983. Max. coverage (+): 0. Max coverage (-): 0

Region: NODE\_289545\_length\_2022\_cov\_31.587538 984-987. Max. coverage (+): 0. Max coverage (-): 0

Region: NODE\_289545\_length\_2022\_cov\_31.587538 988-991. Max. coverage (+): 0. Max coverage (-): 0

Region: NODE\_289545\_length\_2022\_cov\_31.587538 992-996. Max. coverage (+): 0. Max coverage (-): 0

Region: NODE\_289545\_length\_2022\_cov\_31.587538 997-1000. Max. coverage (+): 0. Max coverage (-): 0

Region: NODE\_289545\_length\_2022\_cov\_31.587538 1001-1004. Max. coverage (+): 0. Max coverage (-): 0

Region: NODE\_289545\_length\_2022\_cov\_31.587538 1005-1008. Max. coverage (+): 0. Max coverage (-): 0

Region: NODE\_289545\_length\_2022\_cov\_31.587538 1009-1012. Max. coverage (+): 0. Max coverage (-): 0

Region: NODE\_289545\_length\_2022\_cov\_31.587538 1013-1016. Max. coverage (+): 0. Max coverage (-): 0.01

Region: NODE\_289545\_length\_2022\_cov\_31.587538 1017-1020. Max. coverage (+): 0. Max coverage (-): 0.02

Region: NODE\_289545\_length\_2022\_cov\_31.587538 1021-1024. Max. coverage (+): 0. Max coverage (-): 0.02

Region: NODE\_289545\_length\_2022\_cov\_31.587538 1025-1028. Max. coverage (+): 0. Max coverage (-): 0

Region: NODE\_289545\_length\_2022\_cov\_31.587538 1029-1032. Max. coverage (+): 0. Max coverage (-): 0

Region: NODE\_289545\_length\_2022\_cov\_31.587538 1033-1036. Max. coverage (+): 0. Max coverage (-): 0

Region: NODE\_289545\_length\_2022\_cov\_31.587538 1037-1041. Max. coverage (+): 0. Max coverage (-): 0.09

Region: NODE\_289545\_length\_2022\_cov\_31.587538 1042-1045. Max. coverage (+): 0. Max coverage (-): 0.47

Region: NODE\_289545\_length\_2022\_cov\_31.587538 1046-1049. Max. coverage (+): 0. Max coverage (-): 0.48

Region: NODE\_289545\_length\_2022\_cov\_31.587538 1050-1053. Max. coverage (+): 0. Max coverage (-): 0.06

Region: NODE\_289545\_length\_2022\_cov\_31.587538 1054-1057. Max. coverage (+): 0. Max coverage (-): 0.33

Region: NODE\_289545\_length\_2022\_cov\_31.587538 1058-1061. Max. coverage (+): 0.02. Max coverage (-): 0.21

Region: NODE\_289545\_length\_2022\_cov\_31.587538 1062-1065. Max. coverage (+): 0.02. Max coverage (-): 0.03

Region: NODE\_289545\_length\_2022\_cov\_31.587538 1066-1069. Max. coverage (+): 0. Max coverage (-): 0

Region: NODE\_289545\_length\_2022\_cov\_31.587538 1070-1073. Max. coverage (+): 0. Max coverage (-): 0

Region: NODE\_289545\_length\_2022\_cov\_31.587538 1074-1077. Max. coverage (+): 0. Max coverage (-): 0

Region: NODE\_289545\_length\_2022\_cov\_31.587538 1078-1081. Max. coverage (+): 0. Max coverage (-): 0

Region: NODE\_289545\_length\_2022\_cov\_31.587538 1082-1085. Max. coverage (+): 0. Max coverage (-): 0

Region: NODE\_289545\_length\_2022\_cov\_31.587538 1086-1090. Max. coverage (+): 0. Max coverage (-): 0

Region: NODE\_289545\_length\_2022\_cov\_31.587538 1091-1094. Max. coverage (+): 0. Max coverage (-): 0.08

Region: NODE\_289545\_length\_2022\_cov\_31.587538 1095-1098. Max. coverage (+): 0. Max coverage (-): 0.08

Region: NODE\_289545\_length\_2022\_cov\_31.587538 1099-1102. Max. coverage (+): 0. Max coverage (-): 12.26

Region: NODE\_289545\_length\_2022\_cov\_31.587538 1103-1106. Max. coverage (+): 0. Max coverage (-): 0.15

Region: NODE\_289545\_length\_2022\_cov\_31.587538 1107-1110. Max. coverage (+): 0. Max coverage (-): 0.04

Region: NODE\_289545\_length\_2022\_cov\_31.587538 1111-1114. Max. coverage (+): 0. Max coverage (-): 0

Region: NODE\_289545\_length\_2022\_cov\_31.587538 1115-1118. Max. coverage (+): 0. Max coverage (-): 0.31

Region: NODE\_289545\_length\_2022\_cov\_31.587538 1119-1122. Max. coverage (+): 0. Max coverage (-): 0.31

Region: NODE\_289545\_length\_2022\_cov\_31.587538 1123-1126. Max. coverage (+): 0. Max coverage (-): 0.08

Region: NODE\_289545\_length\_2022\_cov\_31.587538 1127-1130. Max. coverage (+): 0. Max coverage (-): 0

Region: NODE\_289545\_length\_2022\_cov\_31.587538 1131-1135. Max. coverage (+): 0. Max coverage (-): 0

Region: NODE\_289545\_length\_2022\_cov\_31.587538 1136-1139. Max. coverage (+): 0. Max coverage (-): 0

Region: NODE\_289545\_length\_2022\_cov\_31.587538 1140-1143. Max. coverage (+): 0. Max coverage (-): 0

Region: NODE\_289545\_length\_2022\_cov\_31.587538 1144-1147. Max. coverage (+): 0. Max coverage (-): 0.15

Region: NODE\_289545\_length\_2022\_cov\_31.587538 1148-1151. Max. coverage (+): 0. Max coverage (-): 0.15

Region: NODE\_289545\_length\_2022\_cov\_31.587538 1152-1155. Max. coverage (+): 0. Max coverage (-): 0

Region: NODE\_289545\_length\_2022\_cov\_31.587538 1156-1159. Max. coverage (+): 0. Max coverage (-): 0

Region: NODE\_289545\_length\_2022\_cov\_31.587538 1160-1163. Max. coverage (+): 0. Max coverage (-): 0

Region: NODE\_289545\_length\_2022\_cov\_31.587538 1164-1167. Max. coverage (+): 0. Max coverage (-): 0

Region: NODE\_289545\_length\_2022\_cov\_31.587538 1168-1171. Max. coverage (+): 0. Max coverage (-): 0

Region: NODE\_289545\_length\_2022\_cov\_31.587538 1172-1175. Max. coverage (+): 0. Max coverage (-): 0

Region: NODE\_289545\_length\_2022\_cov\_31.587538 1176-1180. Max. coverage (+): 0. Max coverage (-): 0

Region: NODE\_289545\_length\_2022\_cov\_31.587538 1181-1184. Max. coverage (+): 0. Max coverage (-): 0

Region: NODE\_289545\_length\_2022\_cov\_31.587538 1185-1188. Max. coverage (+): 0. Max coverage (-): 0

Region: NODE\_289545\_length\_2022\_cov\_31.587538 1189-1192. Max. coverage (+): 0. Max coverage (-): 0

Region: NODE\_289545\_length\_2022\_cov\_31.587538 1193-1196. Max. coverage (+): 0. Max coverage (-): 0

Region: NODE\_289545\_length\_2022\_cov\_31.587538 1197-1200. Max. coverage (+): 0. Max coverage (-): 0

Region: NODE\_289545\_length\_2022\_cov\_31.587538 1201-1204. Max. coverage (+): 0. Max coverage (-): 0

Region: NODE\_289545\_length\_2022\_cov\_31.587538 1205-1208. Max. coverage (+): 0. Max coverage (-): 0

Region: NODE\_289545\_length\_2022\_cov\_31.587538 1209-1212. Max. coverage (+): 0. Max coverage (-): 0

Region: NODE\_289545\_length\_2022\_cov\_31.587538 1213-1216. Max. coverage (+): 0. Max coverage (-): 0.02

Region: NODE\_289545\_length\_2022\_cov\_31.587538 1217-1220. Max. coverage (+): 0. Max coverage (-): 0.02

Region: NODE\_289545\_length\_2022\_cov\_31.587538 1221-1225. Max. coverage (+): 0. Max coverage (-): 0

Region: NODE\_289545\_length\_2022\_cov\_31.587538 1226-1229. Max. coverage (+): 0. Max coverage (-): 0

Region: NODE\_289545\_length\_2022\_cov\_31.587538 1230-1233. Max. coverage (+): 0. Max coverage (-): 0

Region: NODE\_289545\_length\_2022\_cov\_31.587538 1234-1237. Max. coverage (+): 0. Max coverage (-): 0.08

Region: NODE\_289545\_length\_2022\_cov\_31.587538 1238-1241. Max. coverage (+): 0. Max coverage (-): 0.15

Region: NODE\_289545\_length\_2022\_cov\_31.587538 1242-1245. Max. coverage (+): 0. Max coverage (-): 0.15

Region: NODE\_289545\_length\_2022\_cov\_31.587538 1246-1249. Max. coverage (+): 0. Max coverage (-): 0

Region: NODE\_289545\_length\_2022\_cov\_31.587538 1250-1253. Max. coverage (+): 0. Max coverage (-): 0

Region: NODE\_289545\_length\_2022\_cov\_31.587538 1254-1257. Max. coverage (+): 0. Max coverage (-): 0

Region: NODE\_289545\_length\_2022\_cov\_31.587538 1258-1261. Max. coverage (+): 0. Max coverage (-): 0

Region: NODE\_289545\_length\_2022\_cov\_31.587538 1262-1265. Max. coverage (+): 0. Max coverage (-): 0

Region: NODE\_289545\_length\_2022\_cov\_31.587538 1266-1270. Max. coverage (+): 0. Max coverage (-): 0

Region: NODE\_289545\_length\_2022\_cov\_31.587538 1271-1274. Max. coverage (+): 0. Max coverage (-): 0

Region: NODE\_289545\_length\_2022\_cov\_31.587538 1275-1278. Max. coverage (+): 0. Max coverage (-): 0

Region: NODE\_289545\_length\_2022\_cov\_31.587538 1279-1282. Max. coverage (+): 0. Max coverage (-): 0.08

Region: NODE\_289545\_length\_2022\_cov\_31.587538 1283-1286. Max. coverage (+): 0. Max coverage (-): 0

Region: NODE\_289545\_length\_2022\_cov\_31.587538 1287-1290. Max. coverage (+): 0. Max coverage (-): 0

Region: NODE\_289545\_length\_2022\_cov\_31.587538 1291-1294. Max. coverage (+): 0. Max coverage (-): 0

Region: NODE\_289545\_length\_2022\_cov\_31.587538 1295-1298. Max. coverage (+): 0. Max coverage (-): 0

Region: NODE\_289545\_length\_2022\_cov\_31.587538 1299-1302. Max. coverage (+): 0. Max coverage (-): 0

Region: NODE\_289545\_length\_2022\_cov\_31.587538 1303-1306. Max. coverage (+): 0. Max coverage (-): 0

Region: NODE\_289545\_length\_2022\_cov\_31.587538 1307-1310. Max. coverage (+): 0. Max coverage (-): 1.46

Region: NODE\_289545\_length\_2022\_cov\_31.587538 1311-1315. Max. coverage (+): 0. Max coverage (-): 1.4

Region: NODE\_289545\_length\_2022\_cov\_31.587538 1316-1319. Max. coverage (+): 0. Max coverage (-): 0.33

Region: NODE\_289545\_length\_2022\_cov\_31.587538 1320-1323. Max. coverage (+): 0. Max coverage (-): 0

Region: NODE\_289545\_length\_2022\_cov\_31.587538 1324-1327. Max. coverage (+): 0. Max coverage (-): 0

Region: NODE\_289545\_length\_2022\_cov\_31.587538 1328-1331. Max. coverage (+): 0.08. Max coverage (-): 0

Region: NODE\_289545\_length\_2022\_cov\_31.587538 1332-1335. Max. coverage (+): 0. Max coverage (-): 0

Region: NODE\_289545\_length\_2022\_cov\_31.587538 1336-1339. Max. coverage (+): 0. Max coverage (-): 0.15

Region: NODE\_289545\_length\_2022\_cov\_31.587538 1340-1343. Max. coverage (+): 0. Max coverage (-): 0.23

Region: NODE\_289545\_length\_2022\_cov\_31.587538 1344-1347. Max. coverage (+): 0. Max coverage (-): 0.77

Region: NODE\_289545\_length\_2022\_cov\_31.587538 1348-1351. Max. coverage (+): 0. Max coverage (-): 0

Region: NODE\_289545\_length\_2022\_cov\_31.587538 1352-1355. Max. coverage (+): 0. Max coverage (-): 1.38

Region: NODE\_289545\_length\_2022\_cov\_31.587538 1356-1360. Max. coverage (+): 0. Max coverage (-): 2.83

Region: NODE\_289545\_length\_2022\_cov\_31.587538 1361-1364. Max. coverage (+): 0. Max coverage (-): 2.3

Region: NODE\_289545\_length\_2022\_cov\_31.587538 1365-1368. Max. coverage (+): 0. Max coverage (-): 0.23

Region: NODE\_289545\_length\_2022\_cov\_31.587538 1369-1372. Max. coverage (+): 0. Max coverage (-): 0.23

Region: NODE\_289545\_length\_2022\_cov\_31.587538 1373-1376. Max. coverage (+): 0. Max coverage (-): 0.31

Region: NODE\_289545\_length\_2022\_cov\_31.587538 1377-1380. Max. coverage (+): 0. Max coverage (-): 0

Region: NODE\_289545\_length\_2022\_cov\_31.587538 1381-1384. Max. coverage (+): 0. Max coverage (-): 0

Region: NODE\_289545\_length\_2022\_cov\_31.587538 1385-1388. Max. coverage (+): 0. Max coverage (-): 0

Region: NODE\_289545\_length\_2022\_cov\_31.587538 1389-1392. Max. coverage (+): 0.08. Max coverage (-): 0

Region: NODE\_289545\_length\_2022\_cov\_31.587538 1393-1396. Max. coverage (+): 0. Max coverage (-): 0

Region: NODE\_289545\_length\_2022\_cov\_31.587538 1397-1400. Max. coverage (+): 0. Max coverage (-): 0

Region: NODE\_289545\_length\_2022\_cov\_31.587538 1401-1405. Max. coverage (+): 0. Max coverage (-): 0.84

Region: NODE\_289545\_length\_2022\_cov\_31.587538 1406-1409. Max. coverage (+): 0. Max coverage (-): 34.32

Region: NODE\_289545\_length\_2022\_cov\_31.587538 1410-1413. Max. coverage (+): 0. Max coverage (-): 34.17

Region: NODE\_289545\_length\_2022\_cov\_31.587538 1414-1417. Max. coverage (+): 0. Max coverage (-): 0.08

Region: NODE\_289545\_length\_2022\_cov\_31.587538 1418-1421. Max. coverage (+): 0. Max coverage (-): 0

Region: NODE\_289545\_length\_2022\_cov\_31.587538 1422-1425. Max. coverage (+): 0. Max coverage (-): 0

Region: NODE\_289545\_length\_2022\_cov\_31.587538 1426-1429. Max. coverage (+): 0.08. Max coverage (-): 0

Region: NODE\_289545\_length\_2022\_cov\_31.587538 1430-1433. Max. coverage (+): 0. Max coverage (-): 0

Region: NODE\_289545\_length\_2022\_cov\_31.587538 1434-1437. Max. coverage (+): 0. Max coverage (-): 0.31

Region: NODE\_289545\_length\_2022\_cov\_31.587538 1438-1441. Max. coverage (+): 0. Max coverage (-): 0.15

Region: NODE\_289545\_length\_2022\_cov\_31.587538 1442-1445. Max. coverage (+): 0. Max coverage (-): 0.15

Region: NODE\_289545\_length\_2022\_cov\_31.587538 1446-1450. Max. coverage (+): 0. Max coverage (-): 0

Region: NODE\_289545\_length\_2022\_cov\_31.587538 1451-1454. Max. coverage (+): 0. Max coverage (-): 0

Region: NODE\_289545\_length\_2022\_cov\_31.587538 1455-1458. Max. coverage (+): 0. Max coverage (-): 0

Region: NODE\_289545\_length\_2022\_cov\_31.587538 1459-1462. Max. coverage (+): 0. Max coverage (-): 0

Region: NODE\_289545\_length\_2022\_cov\_31.587538 1463-1466. Max. coverage (+): 0. Max coverage (-): 0

Region: NODE\_289545\_length\_2022\_cov\_31.587538 1467-1470. Max. coverage (+): 0. Max coverage (-): 0.08

Region: NODE\_289545\_length\_2022\_cov\_31.587538 1471-1474. Max. coverage (+): 0. Max coverage (-): 4.6

Region: NODE\_289545\_length\_2022\_cov\_31.587538 1475-1478. Max. coverage (+): 0. Max coverage (-): 1.99

Region: NODE\_289545\_length\_2022\_cov\_31.587538 1479-1482. Max. coverage (+): 0. Max coverage (-): 0.23

Region: NODE\_289545\_length\_2022\_cov\_31.587538 1483-1486. Max. coverage (+): 0. Max coverage (-): 0

Region: NODE\_289545\_length\_2022\_cov\_31.587538 1487-1490. Max. coverage (+): 0. Max coverage (-): 0

Region: NODE\_289545\_length\_2022\_cov\_31.587538 1491-1494. Max. coverage (+): 0. Max coverage (-): 1.23

Region: NODE\_289545\_length\_2022\_cov\_31.587538 1495-1499. Max. coverage (+): 0. Max coverage (-): 1.23

Region: NODE\_289545\_length\_2022\_cov\_31.587538 1500-1503. Max. coverage (+): 0. Max coverage (-): 0

Region: NODE\_289545\_length\_2022\_cov\_31.587538 1504-1507. Max. coverage (+): 0. Max coverage (-): 0.46

Region: NODE\_289545\_length\_2022\_cov\_31.587538 1508-1511. Max. coverage (+): 0. Max coverage (-): 0.38

Region: NODE\_289545\_length\_2022\_cov\_31.587538 1512-1515. Max. coverage (+): 0. Max coverage (-): 0.88

Region: NODE\_289545\_length\_2022\_cov\_31.587538 1516-1519. Max. coverage (+): 0. Max coverage (-): 0.84

Region: NODE\_289545\_length\_2022\_cov\_31.587538 1520-1523. Max. coverage (+): 0. Max coverage (-): 0.11

Region: NODE\_289545\_length\_2022\_cov\_31.587538 1524-1527. Max. coverage (+): 0. Max coverage (-): 0.01

Region: NODE\_289545\_length\_2022\_cov\_31.587538 1528-1531. Max. coverage (+): 0. Max coverage (-): 0

Region: NODE\_289545\_length\_2022\_cov\_31.587538 1532-1535. Max. coverage (+): 0.08. Max coverage (-): 0

Region: NODE\_289545\_length\_2022\_cov\_31.587538 1536-1539. Max. coverage (+): 0.15. Max coverage (-): 0

Region: NODE\_289545\_length\_2022\_cov\_31.587538 1540-1544. Max. coverage (+): 0.08. Max coverage (-): 0

Region: NODE\_289545\_length\_2022\_cov\_31.587538 1545-1548. Max. coverage (+): 0. Max coverage (-): 0

Region: NODE\_289545\_length\_2022\_cov\_31.587538 1549-1552. Max. coverage (+): 0. Max coverage (-): 0

Region: NODE\_289545\_length\_2022\_cov\_31.587538 1553-1556. Max. coverage (+): 0. Max coverage (-): 0.31

Region: NODE\_289545\_length\_2022\_cov\_31.587538 1557-1560. Max. coverage (+): 0. Max coverage (-): 1.57

Region: NODE\_289545\_length\_2022\_cov\_31.587538 1561-1564. Max. coverage (+): 0. Max coverage (-): 2.07

Region: NODE\_289545\_length\_2022\_cov\_31.587538 1565-1568. Max. coverage (+): 0. Max coverage (-): 0.46

Region: NODE\_289545\_length\_2022\_cov\_31.587538 1569-1572. Max. coverage (+): 0. Max coverage (-): 0.11

Region: NODE\_289545\_length\_2022\_cov\_31.587538 1573-1576. Max. coverage (+): 0. Max coverage (-): 0.13

Region: NODE\_289545\_length\_2022\_cov\_31.587538 1577-1580. Max. coverage (+): 0. Max coverage (-): 0.02

Region: NODE\_289545\_length\_2022\_cov\_31.587538 1581-1584. Max. coverage (+): 0. Max coverage (-): 0.02

Region: NODE\_289545\_length\_2022\_cov\_31.587538 1585-1589. Max. coverage (+): 0. Max coverage (-): 0

Region: NODE\_289545\_length\_2022\_cov\_31.587538 1590-1593. Max. coverage (+): 0. Max coverage (-): 0

Region: NODE\_289545\_length\_2022\_cov\_31.587538 1594-1597. Max. coverage (+): 0. Max coverage (-): 0

Region: NODE\_289545\_length\_2022\_cov\_31.587538 1598-1601. Max. coverage (+): 0. Max coverage (-): 0

Region: NODE\_289545\_length\_2022\_cov\_31.587538 1602-1605. Max. coverage (+): 0. Max coverage (-): 0.33

Region: NODE\_289545\_length\_2022\_cov\_31.587538 1606-1609. Max. coverage (+): 0. Max coverage (-): 0.39

Region: NODE\_289545\_length\_2022\_cov\_31.587538 1610-1613. Max. coverage (+): 0. Max coverage (-): 0.02

Region: NODE\_289545\_length\_2022\_cov\_31.587538 1614-1617. Max. coverage (+): 0. Max coverage (-): 0

Region: NODE\_289545\_length\_2022\_cov\_31.587538 1618-1621. Max. coverage (+): 0. Max coverage (-): 0

Region: NODE\_289545\_length\_2022\_cov\_31.587538 1622-1625. Max. coverage (+): 0. Max coverage (-): 0.01

Region: NODE\_289545\_length\_2022\_cov\_31.587538 1626-1629. Max. coverage (+): 0. Max coverage (-): 0.01

Region: NODE\_289545\_length\_2022\_cov\_31.587538 1630-1634. Max. coverage (+): 0. Max coverage (-): 0

Region: NODE\_289545\_length\_2022\_cov\_31.587538 1635-1638. Max. coverage (+): 0. Max coverage (-): 0

Region: NODE\_289545\_length\_2022\_cov\_31.587538 1639-1642. Max. coverage (+): 0. Max coverage (-): 0

Region: NODE\_289545\_length\_2022\_cov\_31.587538 1643-1646. Max. coverage (+): 0. Max coverage (-): 0

Region: NODE\_289545\_length\_2022\_cov\_31.587538 1647-1650. Max. coverage (+): 0. Max coverage (-): 0

Region: NODE\_289545\_length\_2022\_cov\_31.587538 1651-1654. Max. coverage (+): 0. Max coverage (-): 0

Region: NODE\_289545\_length\_2022\_cov\_31.587538 1655-1658. Max. coverage (+): 0. Max coverage (-): 0

Region: NODE\_289545\_length\_2022\_cov\_31.587538 1659-1662. Max. coverage (+): 0. Max coverage (-): 0

Region: NODE\_289545\_length\_2022\_cov\_31.587538 1663-1666. Max. coverage (+): 0. Max coverage (-): 0

Region: NODE\_289545\_length\_2022\_cov\_31.587538 1667-1670. Max. coverage (+): 0. Max coverage (-): 0

Region: NODE\_289545\_length\_2022\_cov\_31.587538 1671-1674. Max. coverage (+): 0. Max coverage (-): 0

Region: NODE\_289545\_length\_2022\_cov\_31.587538 1675-1679. Max. coverage (+): 0. Max coverage (-): 2.49

Region: NODE\_289545\_length\_2022\_cov\_31.587538 1680-1683. Max. coverage (+): 0. Max coverage (-): 2.26

Region: NODE\_289545\_length\_2022\_cov\_31.587538 1684-1687. Max. coverage (+): 0. Max coverage (-): 0.31

Region: NODE\_289545\_length\_2022\_cov\_31.587538 1688-1691. Max. coverage (+): 0. Max coverage (-): 0.04

Region: NODE\_289545\_length\_2022\_cov\_31.587538 1692-1695. Max. coverage (+): 0. Max coverage (-): 0

Region: NODE\_289545\_length\_2022\_cov\_31.587538 1696-1699. Max. coverage (+): 0. Max coverage (-): 0

Region: NODE\_289545\_length\_2022\_cov\_31.587538 1700-1703. Max. coverage (+): 0. Max coverage (-): 0.08

Region: NODE\_289545\_length\_2022\_cov\_31.587538 1704-1707. Max. coverage (+): 0. Max coverage (-): 0

Region: NODE\_289545\_length\_2022\_cov\_31.587538 1708-1711. Max. coverage (+): 0. Max coverage (-): 0

Region: NODE\_289545\_length\_2022\_cov\_31.587538 1712-1715. Max. coverage (+): 0. Max coverage (-): 0

Region: NODE\_289545\_length\_2022\_cov\_31.587538 1716-1719. Max. coverage (+): 0. Max coverage (-): 0

Region: NODE\_289545\_length\_2022\_cov\_31.587538 1720-1724. Max. coverage (+): 0. Max coverage (-): 0

Region: NODE\_289545\_length\_2022\_cov\_31.587538 1725-1728. Max. coverage (+): 0. Max coverage (-): 0

Region: NODE\_289545\_length\_2022\_cov\_31.587538 1729-1732. Max. coverage (+): 0. Max coverage (-): 0

Region: NODE\_289545\_length\_2022\_cov\_31.587538 1733-1736. Max. coverage (+): 0. Max coverage (-): 0

Region: NODE\_289545\_length\_2022\_cov\_31.587538 1737-1740. Max. coverage (+): 0. Max coverage (-): 0

Region: NODE\_289545\_length\_2022\_cov\_31.587538 1741-1744. Max. coverage (+): 0. Max coverage (-): 0.38

Region: NODE\_289545\_length\_2022\_cov\_31.587538 1745-1748. Max. coverage (+): 0. Max coverage (-): 0.38

Region: NODE\_289545\_length\_2022\_cov\_31.587538 1749-1752. Max. coverage (+): 0. Max coverage (-): 0

Region: NODE\_289545\_length\_2022\_cov\_31.587538 1753-1756. Max. coverage (+): 0. Max coverage (-): 0

Region: NODE\_289545\_length\_2022\_cov\_31.587538 1757-1760. Max. coverage (+): 0. Max coverage (-): 0

Region: NODE\_289545\_length\_2022\_cov\_31.587538 1761-1764. Max. coverage (+): 0. Max coverage (-): 0.08

Region: NODE\_289545\_length\_2022\_cov\_31.587538 1765-1769. Max. coverage (+): 0. Max coverage (-): 0.08

Region: NODE\_289545\_length\_2022\_cov\_31.587538 1770-1773. Max. coverage (+): 0.08. Max coverage (-): 0

Region: NODE\_289545\_length\_2022\_cov\_31.587538 1774-1777. Max. coverage (+): 0.08. Max coverage (-): 0

Region: NODE\_289545\_length\_2022\_cov\_31.587538 1778-1781. Max. coverage (+): 0. Max coverage (-): 0

Region: NODE\_289545\_length\_2022\_cov\_31.587538 1782-1785. Max. coverage (+): 0. Max coverage (-): 0.08

Region: NODE\_289545\_length\_2022\_cov\_31.587538 1786-1789. Max. coverage (+): 0. Max coverage (-): 0.08

Region: NODE\_289545\_length\_2022\_cov\_31.587538 1790-1793. Max. coverage (+): 0. Max coverage (-): 2.6

Region: NODE\_289545\_length\_2022\_cov\_31.587538 1794-1797. Max. coverage (+): 0. Max coverage (-): 2.68

Region: NODE\_289545\_length\_2022\_cov\_31.587538 1798-1801. Max. coverage (+): 0. Max coverage (-): 0

Region: NODE\_289545\_length\_2022\_cov\_31.587538 1802-1805. Max. coverage (+): 0. Max coverage (-): 0

Region: NODE\_289545\_length\_2022\_cov\_31.587538 1806-1809. Max. coverage (+): 0. Max coverage (-): 0

Region: NODE\_289545\_length\_2022\_cov\_31.587538 1810-1814. Max. coverage (+): 0. Max coverage (-): 0

Region: NODE\_289545\_length\_2022\_cov\_31.587538 1815-1818. Max. coverage (+): 0. Max coverage (-): 0

Region: NODE\_289545\_length\_2022\_cov\_31.587538 1819-1822. Max. coverage (+): 0. Max coverage (-): 0

Region: NODE\_289545\_length\_2022\_cov\_31.587538 1823-1826. Max. coverage (+): 0. Max coverage (-): 0

Region: NODE\_289545\_length\_2022\_cov\_31.587538 1827-1830. Max. coverage (+): 0. Max coverage (-): 0

Region: NODE\_289545\_length\_2022\_cov\_31.587538 1831-1834. Max. coverage (+): 0. Max coverage (-): 0

Region: NODE\_289545\_length\_2022\_cov\_31.587538 1835-1838. Max. coverage (+): 0. Max coverage (-): 0

Region: NODE\_289545\_length\_2022\_cov\_31.587538 1839-1842. Max. coverage (+): 0. Max coverage (-): 0.31

Region: NODE\_289545\_length\_2022\_cov\_31.587538 1843-1846. Max. coverage (+): 0. Max coverage (-): 0.31

Region: NODE\_289545\_length\_2022\_cov\_31.587538 1847-1850. Max. coverage (+): 0. Max coverage (-): 0

Region: NODE\_289545\_length\_2022\_cov\_31.587538 1851-1854. Max. coverage (+): 0. Max coverage (-): 0.15

Region: NODE\_289545\_length\_2022\_cov\_31.587538 1855-1859. Max. coverage (+): 0. Max coverage (-): 0.15

Region: NODE\_289545\_length\_2022\_cov\_31.587538 1860-1863. Max. coverage (+): 0. Max coverage (-): 0

Region: NODE\_289545\_length\_2022\_cov\_31.587538 1864-1867. Max. coverage (+): 0. Max coverage (-): 0

Region: NODE\_289545\_length\_2022\_cov\_31.587538 1868-1871. Max. coverage (+): 0. Max coverage (-): 0

Region: NODE\_289545\_length\_2022\_cov\_31.587538 1872-1875. Max. coverage (+): 0. Max coverage (-): 0

Region: NODE\_289545\_length\_2022\_cov\_31.587538 1876-1879. Max. coverage (+): 0. Max coverage (-): 0

Region: NODE\_289545\_length\_2022\_cov\_31.587538 1880-1883. Max. coverage (+): 0. Max coverage (-): 0

Region: NODE\_289545\_length\_2022\_cov\_31.587538 1884-1887. Max. coverage (+): 0. Max coverage (-): 0

Region: NODE\_289545\_length\_2022\_cov\_31.587538 1888-1891. Max. coverage (+): 0. Max coverage (-): 0

Region: NODE\_289545\_length\_2022\_cov\_31.587538 1892-1895. Max. coverage (+): 0. Max coverage (-): 0

Region: NODE\_289545\_length\_2022\_cov\_31.587538 1896-1899. Max. coverage (+): 0. Max coverage (-): 0

Region: NODE\_289545\_length\_2022\_cov\_31.587538 1900-1903. Max. coverage (+): 0. Max coverage (-): 0

Region: NODE\_289545\_length\_2022\_cov\_31.587538 1904-1908. Max. coverage (+): 0. Max coverage (-): 0

Region: NODE\_289545\_length\_2022\_cov\_31.587538 1909-1912. Max. coverage (+): 0. Max coverage (-): 0

Region: NODE\_289545\_length\_2022\_cov\_31.587538 1913-1916. Max. coverage (+): 0. Max coverage (-): 0

Region: NODE\_289545\_length\_2022\_cov\_31.587538 1917-1920. Max. coverage (+): 0. Max coverage (-): 0.08

Region: NODE\_289545\_length\_2022\_cov\_31.587538 1921-1924. Max. coverage (+): 0. Max coverage (-): 0.19

Region: NODE\_289545\_length\_2022\_cov\_31.587538 1925-1928. Max. coverage (+): 0. Max coverage (-): 2.35

Region: NODE\_289545\_length\_2022\_cov\_31.587538 1929-1932. Max. coverage (+): 0. Max coverage (-): 2.37

Region: NODE\_289545\_length\_2022\_cov\_31.587538 1933-1936. Max. coverage (+): 0. Max coverage (-): 0.16

Region: NODE\_289545\_length\_2022\_cov\_31.587538 1937-1940. Max. coverage (+): 0. Max coverage (-): 0

Region: NODE\_289545\_length\_2022\_cov\_31.587538 1941-1944. Max. coverage (+): 0. Max coverage (-): 0

Region: NODE\_289545\_length\_2022\_cov\_31.587538 1945-1948. Max. coverage (+): 0. Max coverage (-): 0

Region: NODE\_289545\_length\_2022\_cov\_31.587538 1949-1953. Max. coverage (+): 0. Max coverage (-): 0

Region: NODE\_289545\_length\_2022\_cov\_31.587538 1954-1957. Max. coverage (+): 0. Max coverage (-): 0.08

Region: NODE\_289545\_length\_2022\_cov\_31.587538 1958-1961. Max. coverage (+): 0. Max coverage (-): 0.08

Region: NODE\_289545\_length\_2022\_cov\_31.587538 1962-1965. Max. coverage (+): 0. Max coverage (-): 0.08

Region: NODE\_289545\_length\_2022\_cov\_31.587538 1966-1969. Max. coverage (+): 0. Max coverage (-): 0

Region: NODE\_289545\_length\_2022\_cov\_31.587538 1970-1973. Max. coverage (+): 0. Max coverage (-): 0.08

Region: NODE\_289545\_length\_2022\_cov\_31.587538 1974-1977. Max. coverage (+): 0. Max coverage (-): 0.08

Region: NODE\_289545\_length\_2022\_cov\_31.587538 1978-1981. Max. coverage (+): 0. Max coverage (-): 0.08

Region: NODE\_289545\_length\_2022\_cov\_31.587538 1982-1985. Max. coverage (+): 0. Max coverage (-): 0

Region: NODE\_289545\_length\_2022\_cov\_31.587538 1986-1989. Max. coverage (+): 0. Max coverage (-): 0.08

Region: NODE\_289545\_length\_2022\_cov\_31.587538 1990-1993. Max. coverage (+): 0. Max coverage (-): 0.15

Region: NODE\_289545\_length\_2022\_cov\_31.587538 1994-1998. Max. coverage (+): 0. Max coverage (-): 0.54

Region: NODE\_289545\_length\_2022\_cov\_31.587538 1999-2002. Max. coverage (+): 0. Max coverage (-): 0.31

Region: NODE\_289545\_length\_2022\_cov\_31.587538 2003-2006. Max. coverage (+): 0. Max coverage (-): 0

Region: NODE\_289545\_length\_2022\_cov\_31.587538 2007-2010. Max. coverage (+): 0. Max coverage (-): 0.08

Region: NODE\_289545\_length\_2022\_cov\_31.587538 2011-2014. Max. coverage (+): 0. Max coverage (-): 0.54

Region: NODE\_289545\_length\_2022\_cov\_31.587538 2015-2018. Max. coverage (+): 0. Max coverage (-): 0.61

Region: NODE\_289545\_length\_2022\_cov\_31.587538 2019-2022. Max. coverage (+): 0. Max coverage (-): 0

Region: NODE\_289545\_length\_2022\_cov\_31.587538 2023-2026. Max. coverage (+): 0. Max coverage (-): 2.18

Region: NODE\_289545\_length\_2022\_cov\_31.587538 2027-2030. Max. coverage (+): 0. Max coverage (-): 2.26

Region: NODE\_289545\_length\_2022\_cov\_31.587538 2031-2034. Max. coverage (+): 0. Max coverage (-): 0.61

Region: NODE\_289545\_length\_2022\_cov\_31.587538 2035-2038. Max. coverage (+): 0. Max coverage (-): 0.88

Region: NODE\_289545\_length\_2022\_cov\_31.587538 2039-2043. Max. coverage (+): 0. Max coverage (-): 0.46

Region: NODE\_289545\_length\_2022\_cov\_31.587538 2044-2047. Max. coverage (+): 0.04. Max coverage (-): 0.33

Region: NODE\_289545\_length\_2022\_cov\_31.587538 2048-2051. Max. coverage (+): 0.04. Max coverage (-): 0.05

Region: NODE\_289545\_length\_2022\_cov\_31.587538 2052-2055. Max. coverage (+): 0. Max coverage (-): 0.01

Region: NODE\_289545\_length\_2022\_cov\_31.587538 2056-2059. Max. coverage (+): 0. Max coverage (-): 0.04

Region: NODE\_289545\_length\_2022\_cov\_31.587538 2060-2063. Max. coverage (+): 0. Max coverage (-): 0.04

Region: NODE\_289545\_length\_2022\_cov\_31.587538 2064-2067. Max. coverage (+): 0. Max coverage (-): 0

Region: NODE\_289545\_length\_2022\_cov\_31.587538 2068-2071. Max. coverage (+): 0. Max coverage (-): 0

Region: NODE\_289545\_length\_2022\_cov\_31.587538 2072-2075. Max. coverage (+): 0. Max coverage (-): 0

Region: NODE\_289545\_length\_2022\_cov\_31.587538 2076-2079. Max. coverage (+): 0. Max coverage (-): 0

Region: NODE\_289545\_length\_2022\_cov\_31.587538 2080-2083. Max. coverage (+): 0. Max coverage (-): 0

Region: NODE\_289545\_length\_2022\_cov\_31.587538 2084-. Max. coverage (+): 0. Max coverage (-): 0

RepeatMasker Color Code

**+**

100-98% Identity

<98-95% Identity

<95-90% Identity

<90-85% Identity

<85-80% Identity

<80-75% Identity

<75-70% Identity

<70% Identity

**-**

Gene Set Color Code

**+**

Gene

Pseudogene

Other

**-**

Topology/Coverage Color Code

Coverage Plus Strand

Coverage Minus Strand

Mainstrand: Plus

Mainstrand: Minus

Complementary Strand

Flanking Region  
(if option -flank >0)

Gene Set Annotation  
  
RepeatMasker Annotation  

**1. hAT-51\_HM**: 1-61 (+), Divergence to consensus: 21.3%  
**2. Dong\_FR**: 48-235 (+), Divergence to consensus: 16%  
**3. Merlin-1\_DR**: 62-78 (+), Divergence to consensus: 26.9%  
**4. Dong\_FR**: 240-339 (+), Divergence to consensus: 14%  
**5. AlRepD-1165**: 435-612 (-), Divergence to consensus: 32%  
**6. AlRepD-1491**: 645-1032 (-), Divergence to consensus: 31.2%  
**7. Daphne-27\_DRe**: 973-1981 (-), Divergence to consensus: 37.3%  
**8. Daphne-27\_DRe**: 2028-2074 (-), Divergence to consensus: 19.1%

  
Transcription Factor Binding Sites  

**RHOXF1** (Sequence: GGATCA (-): 51)  
**RHOXF1** (Sequence: AGCTCA (-): 1378)  
**RHOXF1** (Sequence: AGATTA (-): 1878)  
**RHOXF1** (Sequence: TGATCT (+): 176)  
**RHOXF1** (Sequence: TGATCC (+): 405)  
**RHOXF1** (Sequence: TAATCT (+): 1315)  
**RHOXF1** (Sequence: TGATCC (+): 1428)  
**RHOXF1** (Sequence: TGATCT (+): 2048)  
**FOXP1** (Sequence: GTAAACA (+): 533)  
**FOXO1** (Sequence: CCTGTTTTC (+): 964)  
**Sox5** (Sequence: ATTGTT (+): 1346)  
**SOX9** (Sequence: CTATTGTT (+): 1344)  
**FOXO3\_mmu** (Sequence: TCTAAACA (+): 1566)  
**FOXO1** (Sequence: ATAAACAAG (-): 832)  
**Rhox11** (Sequence: CGCTGTTTT (+): 1220)
