# Supplementary material for: First characterization of PIWI-interacting RNA clusters in a cichlid fish with a B chromosome
Source: BMC Biol. 2022 Sep 21;20:204. doi: 10.1186/s12915-022-01403-2 (PMC9490952; doi:10.1186/s12915-022-01403-2)
Supplement: Supplementary file 1 — Additional file 1. Zipped folder with fasta and interactive html piRNA cluster information for the A. latifasciata genome. The nomenclature is as follows: number-pirna-cluster_sex_B-presence (f, female; m, male; 0b, without B chromosome; 1b, with B chromosome). [file 12915_2022_1403_MOESM1_ESM.zip › 114_m1b.html]

piRNA cluster 114\_m1b 11


Predicted piRNA cluster no. 114\_m1b
  

Show proTRAC run info
Hide proTRAC run info

/\  
                \_\_\_\_\_\_\_\_\_\_\_\_\_\_\_\_\_\_\_\_\_\_\_/\\_\_\_ /  \\_\_\_\_\_\_\_  
               I                      /  \  /    \      I  
               I     pro             /    \/      \     I  
               I        TRAC        /               \   I  
               I   \_\_\_\_\_\_\_\_\_\_\_\_\_\_\_\_/\_\_\_\_\_\_\_\_\_\_\_\_\_\_\_\_\_\\_ I  
               I   \              /                     I  
               I    \            /                      I  
               I     \  /\      /       V.2.4.2         I  
               I      \/  \    /                        I  
               I\_\_\_\_\_\_\_\_\_\_\_\  /\_\_\_\_\_\_\_\_\_\_\_\_\_\_\_\_\_\_\_\_\_\_\_\_\_I  
                            \/  
  
  
================================= proTRAC ====================================  
VERSION: .......... 2.4.2  
LAST MODIFIED: .... 11. May 2018  
  
Please cite:  
Rosenkranz D, Zischler H. proTRAC - a software for probabilistic piRNA cluster  
detection, visualization and analysis. 2012. BMC Bioinformatics 13:5.  
  
  
Contact:  
David Rosenkranz  
Institute of Organismic and Molecular Evolutionary Biology  
Dept. Anthropology, small RNA group  
Johannes Gutenberg University Mainz  
email: rosenkranz@uni-mainz.de  
  
You can find the latest proTRAC version at:  
http://sourceforge.net/projects/protrac/files  
http://www.smallRNAgroup-mainz.de/software  
==============================================================================  
  
PARAMETERS:  
Map file: ...............piwi-machos-1B.fa-collapse.map  
Genome file: ............../../../0B\_ala\_genome.fa  
RepeatMasker annotation: Alatifasciata-all0B-maryan-v2.fa\_corrected.out  
GeneSet:................./guest-storage/Data/annotation/Alatifasciata\_all0B\_maryan-v2\_out2017.gff  
  
Significant (p<=0.01) hit density will be calculated based  
on observed hit distribution.  
  
Sliding window size: ........................................ 5000 bp  
Sliding window increament: .................................. 1000 bp  
Normalize each hit by number of genomic hits: ............... yes  
Normalize each hit by number of sequence reads: ............. yes  
Normalize values (-> per million mapped reads): ............. yes  
Min. fraction of hits with 1T(U) or 10A: .................... 0.75  
Alternatively: Min. fraction of hits with 1T(U) and 10A: .... 0.5  
Min. fraction of hits with typical piRNA length: ............ 0.75  
Typical piRNA length: ....................................... 24-32 nt  
Min. size of a piRNA cluster: ............................... 1000 bp.  
Min. number of hits (absolute): ............................. 0  
Min. number of hits (normalized): ........................... 0  
Min. fraction of hits on the mainstrand: .................... 0.75  
Top fraction of mapped sequences (in terms of read counts): . 1%  
Top fraction accounts for max. n% of sequence reads: ........ 90%  
Min. fraction of hits on each arm of a bidirectional cluster: 0.05  
Output html file for each cluster: .......................... yes  
Output a summary table: ..................................... yes  
Output a FASTA file for each cluster (piRNA sequences): ..... yes  
Output a FASTA file comprising cluster sequences: ........... yes  
Output a GTF file for predicted piRNA clusters: ..............yes  
Search DNA motifs in clusters: .............................. yes  
Output flanking sequences: +/- .............................. 0 bp  
Output ~.pTi file: .......................................... no  
==============================================================================  
  
  
Genome size (without gaps): ............ 758543724 bp  
Gaps (N/X/-): .......................... 417479 bp  
Mapped reads: .......................... 26973943  
Non-identical sequences: ............... 6209225  
Genomic hits: .......................... 48438990  
Significant densitiy of mapped reads: .. 821.144211136946 reads/kb

Show proTRAC cluster info
Hide proTRAC cluster info

|  |  |
| --- | --- |
| Location | NODE\_29094\_length\_1021\_cov\_98.511261 |
| Coordinates | 1-1078 |
| Size [bp] | 1078 |
| Sequence hit loci | 2933 |
| Mapped reads (normalized) | 22975 |
| Mapped reads (normalized) per kb | 21312.6 |
| Normalized reads with 1T (1U) | 75.9% |
| Normalized reads with 10A | 26.4% |
| Normalized reads with length 24-32 nt | 97.7% |
| Normalized reads on the main strand(s) | 90.4% |
| Predicted directionality | mono:plus |

100%

0%

1T (1U)  
reads

10A reads

24-32 nt  
reads

reads on mainstrand

**Either the amount of reads with 1T (1U) OR 10A has to exceed 75% (set with option: -1Tor10A)  
Alternatively the amount of reads with 1T (1U) AND 10A has to exceed 50% (set with option: -1Tand10A)  
Minimum amount of reads with preferred size is 75% (set with option: -pisize)  
Minimum amount of reads on the main strand(s) is 75% (set with option: -clstrand)**

Show read coverage
Hide read coverage

WHAT DO I SEE HERE?  
This chart shows the location of mapped sequence reads within a predicted piRNA cluster. The color refers to the number of genomic hits produced by the sequence read in question. A dark red bar indicates that this sequence read produces many other hits elsewhere in the genome. Many adjacent red or yellow bars can indicate the presence of a multi-copy element such as transposons or rRNA genes. A dark green bar indicates that this sequence read maps uniquely to this locus.

1 hit

2-5 hits

6-10 hits

11-20 hits

21-50 hits

51-100 hits

> 100 hits

NODE\_29094\_length\_1021\_cov\_98.511261

1

1078

Gene Set

RepeatMasker

Mapped  
Reads

101.13

plus strand

minus strand

101.13

Region: NODE\_29094\_length\_1021\_cov\_98.511261 1701-2. Max. coverage (+): 18.57. Max coverage (-): 0.02

Region: NODE\_29094\_length\_1021\_cov\_98.511261 3-4. Max. coverage (+): 6.6. Max coverage (-): 0

Region: NODE\_29094\_length\_1021\_cov\_98.511261 5-6. Max. coverage (+): 0.17. Max coverage (-): 0

Region: NODE\_29094\_length\_1021\_cov\_98.511261 7-8. Max. coverage (+): 0.19. Max coverage (-): 0.01

Region: NODE\_29094\_length\_1021\_cov\_98.511261 9-10. Max. coverage (+): 0.06. Max coverage (-): 0.01

Region: NODE\_29094\_length\_1021\_cov\_98.511261 11-12. Max. coverage (+): 0.15. Max coverage (-): 0.37

Region: NODE\_29094\_length\_1021\_cov\_98.511261 13-15. Max. coverage (+): 0.09. Max coverage (-): 0.38

Region: NODE\_29094\_length\_1021\_cov\_98.511261 16-17. Max. coverage (+): 0.05. Max coverage (-): 0.02

Region: NODE\_29094\_length\_1021\_cov\_98.511261 18-19. Max. coverage (+): 0.04. Max coverage (-): 0.01

Region: NODE\_29094\_length\_1021\_cov\_98.511261 20-21. Max. coverage (+): 0.05. Max coverage (-): 0.01

Region: NODE\_29094\_length\_1021\_cov\_98.511261 22-23. Max. coverage (+): 0.02. Max coverage (-): 0.04

Region: NODE\_29094\_length\_1021\_cov\_98.511261 24-25. Max. coverage (+): 0.07. Max coverage (-): 0.17

Region: NODE\_29094\_length\_1021\_cov\_98.511261 26-27. Max. coverage (+): 0.07. Max coverage (-): 0.37

Region: NODE\_29094\_length\_1021\_cov\_98.511261 28-30. Max. coverage (+): 0.41. Max coverage (-): 1.41

Region: NODE\_29094\_length\_1021\_cov\_98.511261 31-32. Max. coverage (+): 1.58. Max coverage (-): 1.39

Region: NODE\_29094\_length\_1021\_cov\_98.511261 33-34. Max. coverage (+): 1.67. Max coverage (-): 1

Region: NODE\_29094\_length\_1021\_cov\_98.511261 35-36. Max. coverage (+): 0.15. Max coverage (-): 0.09

Region: NODE\_29094\_length\_1021\_cov\_98.511261 37-38. Max. coverage (+): 0.13. Max coverage (-): 0.06

Region: NODE\_29094\_length\_1021\_cov\_98.511261 39-40. Max. coverage (+): 0.09. Max coverage (-): 0.09

Region: NODE\_29094\_length\_1021\_cov\_98.511261 41-43. Max. coverage (+): 0.36. Max coverage (-): 0.11

Region: NODE\_29094\_length\_1021\_cov\_98.511261 44-45. Max. coverage (+): 0.2. Max coverage (-): 0.02

Region: NODE\_29094\_length\_1021\_cov\_98.511261 46-47. Max. coverage (+): 0.13. Max coverage (-): 0.02

Region: NODE\_29094\_length\_1021\_cov\_98.511261 48-49. Max. coverage (+): 3.61. Max coverage (-): 0

Region: NODE\_29094\_length\_1021\_cov\_98.511261 50-51. Max. coverage (+): 0.15. Max coverage (-): 0.04

Region: NODE\_29094\_length\_1021\_cov\_98.511261 52-53. Max. coverage (+): 0. Max coverage (-): 0.04

Region: NODE\_29094\_length\_1021\_cov\_98.511261 54-55. Max. coverage (+): 0. Max coverage (-): 0.04

Region: NODE\_29094\_length\_1021\_cov\_98.511261 56-58. Max. coverage (+): 0.37. Max coverage (-): 16.5

Region: NODE\_29094\_length\_1021\_cov\_98.511261 59-60. Max. coverage (+): 1.26. Max coverage (-): 17.65

Region: NODE\_29094\_length\_1021\_cov\_98.511261 61-62. Max. coverage (+): 1.22. Max coverage (-): 2.67

Region: NODE\_29094\_length\_1021\_cov\_98.511261 63-64. Max. coverage (+): 0.11. Max coverage (-): 0.46

Region: NODE\_29094\_length\_1021\_cov\_98.511261 65-66. Max. coverage (+): 0.08. Max coverage (-): 0.42

Region: NODE\_29094\_length\_1021\_cov\_98.511261 67-68. Max. coverage (+): 0.07. Max coverage (-): 0.07

Region: NODE\_29094\_length\_1021\_cov\_98.511261 69-71. Max. coverage (+): 0. Max coverage (-): 0.05

Region: NODE\_29094\_length\_1021\_cov\_98.511261 72-73. Max. coverage (+): 0. Max coverage (-): 0

Region: NODE\_29094\_length\_1021\_cov\_98.511261 74-75. Max. coverage (+): 0.01. Max coverage (-): 0.01

Region: NODE\_29094\_length\_1021\_cov\_98.511261 76-77. Max. coverage (+): 0.01. Max coverage (-): 0

Region: NODE\_29094\_length\_1021\_cov\_98.511261 78-79. Max. coverage (+): 0. Max coverage (-): 0

Region: NODE\_29094\_length\_1021\_cov\_98.511261 80-81. Max. coverage (+): 0.57. Max coverage (-): 0

Region: NODE\_29094\_length\_1021\_cov\_98.511261 82-84. Max. coverage (+): 1.87. Max coverage (-): 0

Region: NODE\_29094\_length\_1021\_cov\_98.511261 85-86. Max. coverage (+): 0.51. Max coverage (-): 0.04

Region: NODE\_29094\_length\_1021\_cov\_98.511261 87-88. Max. coverage (+): 0.03. Max coverage (-): 0.04

Region: NODE\_29094\_length\_1021\_cov\_98.511261 89-90. Max. coverage (+): 0. Max coverage (-): 0

Region: NODE\_29094\_length\_1021\_cov\_98.511261 91-92. Max. coverage (+): 0.04. Max coverage (-): 0

Region: NODE\_29094\_length\_1021\_cov\_98.511261 93-94. Max. coverage (+): 0.26. Max coverage (-): 0.78

Region: NODE\_29094\_length\_1021\_cov\_98.511261 95-96. Max. coverage (+): 0.04. Max coverage (-): 0.59

Region: NODE\_29094\_length\_1021\_cov\_98.511261 97-99. Max. coverage (+): 0.04. Max coverage (-): 0.15

Region: NODE\_29094\_length\_1021\_cov\_98.511261 100-101. Max. coverage (+): 0. Max coverage (-): 0

Region: NODE\_29094\_length\_1021\_cov\_98.511261 102-103. Max. coverage (+): 0.11. Max coverage (-): 0.04

Region: NODE\_29094\_length\_1021\_cov\_98.511261 104-105. Max. coverage (+): 0.82. Max coverage (-): 0.04

Region: NODE\_29094\_length\_1021\_cov\_98.511261 106-107. Max. coverage (+): 0.7. Max coverage (-): 0

Region: NODE\_29094\_length\_1021\_cov\_98.511261 108-109. Max. coverage (+): 0. Max coverage (-): 0.04

Region: NODE\_29094\_length\_1021\_cov\_98.511261 110-112. Max. coverage (+): 0. Max coverage (-): 0.22

Region: NODE\_29094\_length\_1021\_cov\_98.511261 113-114. Max. coverage (+): 0.07. Max coverage (-): 0.63

Region: NODE\_29094\_length\_1021\_cov\_98.511261 115-116. Max. coverage (+): 0.11. Max coverage (-): 1.96

Region: NODE\_29094\_length\_1021\_cov\_98.511261 117-118. Max. coverage (+): 0.04. Max coverage (-): 2.95

Region: NODE\_29094\_length\_1021\_cov\_98.511261 119-120. Max. coverage (+): 0. Max coverage (-): 1.74

Region: NODE\_29094\_length\_1021\_cov\_98.511261 121-122. Max. coverage (+): 0. Max coverage (-): 0.43

Region: NODE\_29094\_length\_1021\_cov\_98.511261 123-124. Max. coverage (+): 0. Max coverage (-): 0.04

Region: NODE\_29094\_length\_1021\_cov\_98.511261 125-127. Max. coverage (+): 0.04. Max coverage (-): 0.06

Region: NODE\_29094\_length\_1021\_cov\_98.511261 128-129. Max. coverage (+): 0.06. Max coverage (-): 0.09

Region: NODE\_29094\_length\_1021\_cov\_98.511261 130-131. Max. coverage (+): 0.04. Max coverage (-): 0.11

Region: NODE\_29094\_length\_1021\_cov\_98.511261 132-133. Max. coverage (+): 0.24. Max coverage (-): 0.02

Region: NODE\_29094\_length\_1021\_cov\_98.511261 134-135. Max. coverage (+): 12.73. Max coverage (-): 0.15

Region: NODE\_29094\_length\_1021\_cov\_98.511261 136-137. Max. coverage (+): 12.86. Max coverage (-): 0.17

Region: NODE\_29094\_length\_1021\_cov\_98.511261 138-140. Max. coverage (+): 0.33. Max coverage (-): 0.59

Region: NODE\_29094\_length\_1021\_cov\_98.511261 141-142. Max. coverage (+): 0.33. Max coverage (-): 1.3

Region: NODE\_29094\_length\_1021\_cov\_98.511261 143-144. Max. coverage (+): 0.37. Max coverage (-): 13.57

Region: NODE\_29094\_length\_1021\_cov\_98.511261 145-146. Max. coverage (+): 0.22. Max coverage (-): 13.42

Region: NODE\_29094\_length\_1021\_cov\_98.511261 147-148. Max. coverage (+): 0.07. Max coverage (-): 1.45

Region: NODE\_29094\_length\_1021\_cov\_98.511261 149-150. Max. coverage (+): 0. Max coverage (-): 0.63

Region: NODE\_29094\_length\_1021\_cov\_98.511261 151-152. Max. coverage (+): 0.07. Max coverage (-): 0.19

Region: NODE\_29094\_length\_1021\_cov\_98.511261 153-155. Max. coverage (+): 0.11. Max coverage (-): 0.22

Region: NODE\_29094\_length\_1021\_cov\_98.511261 156-157. Max. coverage (+): 0.07. Max coverage (-): 0.11

Region: NODE\_29094\_length\_1021\_cov\_98.511261 158-159. Max. coverage (+): 0.07. Max coverage (-): 0.04

Region: NODE\_29094\_length\_1021\_cov\_98.511261 160-161. Max. coverage (+): 0.07. Max coverage (-): 0.04

Region: NODE\_29094\_length\_1021\_cov\_98.511261 162-163. Max. coverage (+): 6.38. Max coverage (-): 0

Region: NODE\_29094\_length\_1021\_cov\_98.511261 164-165. Max. coverage (+): 37.89. Max coverage (-): 0.19

Region: NODE\_29094\_length\_1021\_cov\_98.511261 166-168. Max. coverage (+): 37.59. Max coverage (-): 0.3

Region: NODE\_29094\_length\_1021\_cov\_98.511261 169-170. Max. coverage (+): 0.15. Max coverage (-): 0

Region: NODE\_29094\_length\_1021\_cov\_98.511261 171-172. Max. coverage (+): 0.04. Max coverage (-): 0.07

Region: NODE\_29094\_length\_1021\_cov\_98.511261 173-174. Max. coverage (+): 0.04. Max coverage (-): 0.26

Region: NODE\_29094\_length\_1021\_cov\_98.511261 175-176. Max. coverage (+): 0.85. Max coverage (-): 3.11

Region: NODE\_29094\_length\_1021\_cov\_98.511261 177-178. Max. coverage (+): 0.96. Max coverage (-): 3.48

Region: NODE\_29094\_length\_1021\_cov\_98.511261 179-181. Max. coverage (+): 1.89. Max coverage (-): 0.67

Region: NODE\_29094\_length\_1021\_cov\_98.511261 182-183. Max. coverage (+): 2.19. Max coverage (-): 0.44

Region: NODE\_29094\_length\_1021\_cov\_98.511261 184-185. Max. coverage (+): 0.63. Max coverage (-): 1.26

Region: NODE\_29094\_length\_1021\_cov\_98.511261 186-187. Max. coverage (+): 1.04. Max coverage (-): 2.67

Region: NODE\_29094\_length\_1021\_cov\_98.511261 188-189. Max. coverage (+): 1.52. Max coverage (-): 1.78

Region: NODE\_29094\_length\_1021\_cov\_98.511261 190-191. Max. coverage (+): 0.74. Max coverage (-): 0.63

Region: NODE\_29094\_length\_1021\_cov\_98.511261 192-193. Max. coverage (+): 0.19. Max coverage (-): 0.78

Region: NODE\_29094\_length\_1021\_cov\_98.511261 194-196. Max. coverage (+): 4.97. Max coverage (-): 0.7

Region: NODE\_29094\_length\_1021\_cov\_98.511261 197-198. Max. coverage (+): 5.71. Max coverage (-): 0.93

Region: NODE\_29094\_length\_1021\_cov\_98.511261 199-200. Max. coverage (+): 3.97. Max coverage (-): 0.89

Region: NODE\_29094\_length\_1021\_cov\_98.511261 201-202. Max. coverage (+): 17.09. Max coverage (-): 0.59

Region: NODE\_29094\_length\_1021\_cov\_98.511261 203-204. Max. coverage (+): 17.16. Max coverage (-): 0.59

Region: NODE\_29094\_length\_1021\_cov\_98.511261 205-206. Max. coverage (+): 14.38. Max coverage (-): 0.41

Region: NODE\_29094\_length\_1021\_cov\_98.511261 207-209. Max. coverage (+): 14.33. Max coverage (-): 0.07

Region: NODE\_29094\_length\_1021\_cov\_98.511261 210-211. Max. coverage (+): 0.44. Max coverage (-): 0.06

Region: NODE\_29094\_length\_1021\_cov\_98.511261 212-213. Max. coverage (+): 0.43. Max coverage (-): 0.19

Region: NODE\_29094\_length\_1021\_cov\_98.511261 214-215. Max. coverage (+): 0.46. Max coverage (-): 0.17

Region: NODE\_29094\_length\_1021\_cov\_98.511261 216-217. Max. coverage (+): 0.35. Max coverage (-): 0.13

Region: NODE\_29094\_length\_1021\_cov\_98.511261 218-219. Max. coverage (+): 6.75. Max coverage (-): 0.11

Region: NODE\_29094\_length\_1021\_cov\_98.511261 220-221. Max. coverage (+): 6.88. Max coverage (-): 0

Region: NODE\_29094\_length\_1021\_cov\_98.511261 222-224. Max. coverage (+): 2.56. Max coverage (-): 0.04

Region: NODE\_29094\_length\_1021\_cov\_98.511261 225-226. Max. coverage (+): 1.48. Max coverage (-): 0.06

Region: NODE\_29094\_length\_1021\_cov\_98.511261 227-228. Max. coverage (+): 6.52. Max coverage (-): 0.04

Region: NODE\_29094\_length\_1021\_cov\_98.511261 229-230. Max. coverage (+): 14.27. Max coverage (-): 0.15

Region: NODE\_29094\_length\_1021\_cov\_98.511261 231-232. Max. coverage (+): 8.64. Max coverage (-): 0.26

Region: NODE\_29094\_length\_1021\_cov\_98.511261 233-234. Max. coverage (+): 7.08. Max coverage (-): 0.15

Region: NODE\_29094\_length\_1021\_cov\_98.511261 235-237. Max. coverage (+): 7.67. Max coverage (-): 0

Region: NODE\_29094\_length\_1021\_cov\_98.511261 238-239. Max. coverage (+): 15.35. Max coverage (-): 0

Region: NODE\_29094\_length\_1021\_cov\_98.511261 240-241. Max. coverage (+): 19.8. Max coverage (-): 0

Region: NODE\_29094\_length\_1021\_cov\_98.511261 242-243. Max. coverage (+): 101.13. Max coverage (-): 0

Region: NODE\_29094\_length\_1021\_cov\_98.511261 244-245. Max. coverage (+): 96.06. Max coverage (-): 0

Region: NODE\_29094\_length\_1021\_cov\_98.511261 246-247. Max. coverage (+): 3.63. Max coverage (-): 0

Region: NODE\_29094\_length\_1021\_cov\_98.511261 248-250. Max. coverage (+): 52.83. Max coverage (-): 0

Region: NODE\_29094\_length\_1021\_cov\_98.511261 251-252. Max. coverage (+): 5.38. Max coverage (-): 0

Region: NODE\_29094\_length\_1021\_cov\_98.511261 253-254. Max. coverage (+): 0.07. Max coverage (-): 0

Region: NODE\_29094\_length\_1021\_cov\_98.511261 255-256. Max. coverage (+): 0. Max coverage (-): 0

Region: NODE\_29094\_length\_1021\_cov\_98.511261 257-258. Max. coverage (+): 0. Max coverage (-): 0

Region: NODE\_29094\_length\_1021\_cov\_98.511261 259-260. Max. coverage (+): 0. Max coverage (-): 0.11

Region: NODE\_29094\_length\_1021\_cov\_98.511261 261-262. Max. coverage (+): 0. Max coverage (-): 0.22

Region: NODE\_29094\_length\_1021\_cov\_98.511261 263-265. Max. coverage (+): 0.04. Max coverage (-): 0.63

Region: NODE\_29094\_length\_1021\_cov\_98.511261 266-267. Max. coverage (+): 0.33. Max coverage (-): 0.93

Region: NODE\_29094\_length\_1021\_cov\_98.511261 268-269. Max. coverage (+): 1.59. Max coverage (-): 1

Region: NODE\_29094\_length\_1021\_cov\_98.511261 270-271. Max. coverage (+): 5.82. Max coverage (-): 0.93

Region: NODE\_29094\_length\_1021\_cov\_98.511261 272-273. Max. coverage (+): 5.71. Max coverage (-): 0.52

Region: NODE\_29094\_length\_1021\_cov\_98.511261 274-275. Max. coverage (+): 1.78. Max coverage (-): 0.26

Region: NODE\_29094\_length\_1021\_cov\_98.511261 276-278. Max. coverage (+): 9.34. Max coverage (-): 0.33

Region: NODE\_29094\_length\_1021\_cov\_98.511261 279-280. Max. coverage (+): 5.86. Max coverage (-): 0.07

Region: NODE\_29094\_length\_1021\_cov\_98.511261 281-282. Max. coverage (+): 1.93. Max coverage (-): 0

Region: NODE\_29094\_length\_1021\_cov\_98.511261 283-284. Max. coverage (+): 0.7. Max coverage (-): 0.11

Region: NODE\_29094\_length\_1021\_cov\_98.511261 285-286. Max. coverage (+): 29.03. Max coverage (-): 0.11

Region: NODE\_29094\_length\_1021\_cov\_98.511261 287-288. Max. coverage (+): 36.74. Max coverage (-): 0.11

Region: NODE\_29094\_length\_1021\_cov\_98.511261 289-290. Max. coverage (+): 12.12. Max coverage (-): 0.11

Region: NODE\_29094\_length\_1021\_cov\_98.511261 291-293. Max. coverage (+): 6.15. Max coverage (-): 0

Region: NODE\_29094\_length\_1021\_cov\_98.511261 294-295. Max. coverage (+): 0. Max coverage (-): 0.02

Region: NODE\_29094\_length\_1021\_cov\_98.511261 296-297. Max. coverage (+): 0. Max coverage (-): 0.02

Region: NODE\_29094\_length\_1021\_cov\_98.511261 298-299. Max. coverage (+): 0.13. Max coverage (-): 0

Region: NODE\_29094\_length\_1021\_cov\_98.511261 300-301. Max. coverage (+): 0.13. Max coverage (-): 0

Region: NODE\_29094\_length\_1021\_cov\_98.511261 302-303. Max. coverage (+): 1.89. Max coverage (-): 0

Region: NODE\_29094\_length\_1021\_cov\_98.511261 304-306. Max. coverage (+): 1.89. Max coverage (-): 0.04

Region: NODE\_29094\_length\_1021\_cov\_98.511261 307-308. Max. coverage (+): 1.15. Max coverage (-): 0.04

Region: NODE\_29094\_length\_1021\_cov\_98.511261 309-310. Max. coverage (+): 0.19. Max coverage (-): 0

Region: NODE\_29094\_length\_1021\_cov\_98.511261 311-312. Max. coverage (+): 0. Max coverage (-): 0

Region: NODE\_29094\_length\_1021\_cov\_98.511261 313-314. Max. coverage (+): 1.19. Max coverage (-): 0.11

Region: NODE\_29094\_length\_1021\_cov\_98.511261 315-316. Max. coverage (+): 1.63. Max coverage (-): 0.33

Region: NODE\_29094\_length\_1021\_cov\_98.511261 317-319. Max. coverage (+): 1.3. Max coverage (-): 0.22

Region: NODE\_29094\_length\_1021\_cov\_98.511261 320-321. Max. coverage (+): 1.33. Max coverage (-): 0

Region: NODE\_29094\_length\_1021\_cov\_98.511261 322-323. Max. coverage (+): 0.56. Max coverage (-): 0

Region: NODE\_29094\_length\_1021\_cov\_98.511261 324-325. Max. coverage (+): 0.33. Max coverage (-): 0

Region: NODE\_29094\_length\_1021\_cov\_98.511261 326-327. Max. coverage (+): 0.52. Max coverage (-): 0

Region: NODE\_29094\_length\_1021\_cov\_98.511261 328-329. Max. coverage (+): 0.52. Max coverage (-): 0

Region: NODE\_29094\_length\_1021\_cov\_98.511261 330-331. Max. coverage (+): 1.19. Max coverage (-): 0

Region: NODE\_29094\_length\_1021\_cov\_98.511261 332-334. Max. coverage (+): 5.82. Max coverage (-): 0

Region: NODE\_29094\_length\_1021\_cov\_98.511261 335-336. Max. coverage (+): 0.11. Max coverage (-): 0

Region: NODE\_29094\_length\_1021\_cov\_98.511261 337-338. Max. coverage (+): 0.7. Max coverage (-): 0

Region: NODE\_29094\_length\_1021\_cov\_98.511261 339-340. Max. coverage (+): 0.78. Max coverage (-): 0

Region: NODE\_29094\_length\_1021\_cov\_98.511261 341-342. Max. coverage (+): 0.15. Max coverage (-): 0

Region: NODE\_29094\_length\_1021\_cov\_98.511261 343-344. Max. coverage (+): 0.3. Max coverage (-): 0

Region: NODE\_29094\_length\_1021\_cov\_98.511261 345-347. Max. coverage (+): 0.44. Max coverage (-): 0

Region: NODE\_29094\_length\_1021\_cov\_98.511261 348-349. Max. coverage (+): 0. Max coverage (-): 0

Region: NODE\_29094\_length\_1021\_cov\_98.511261 350-351. Max. coverage (+): 0. Max coverage (-): 0

Region: NODE\_29094\_length\_1021\_cov\_98.511261 352-353. Max. coverage (+): 0. Max coverage (-): 0

Region: NODE\_29094\_length\_1021\_cov\_98.511261 354-355. Max. coverage (+): 0. Max coverage (-): 0.04

Region: NODE\_29094\_length\_1021\_cov\_98.511261 356-357. Max. coverage (+): 0.07. Max coverage (-): 0.07

Region: NODE\_29094\_length\_1021\_cov\_98.511261 358-359. Max. coverage (+): 0.07. Max coverage (-): 0.07

Region: NODE\_29094\_length\_1021\_cov\_98.511261 360-362. Max. coverage (+): 0.04. Max coverage (-): 0.04

Region: NODE\_29094\_length\_1021\_cov\_98.511261 363-364. Max. coverage (+): 0. Max coverage (-): 0

Region: NODE\_29094\_length\_1021\_cov\_98.511261 365-366. Max. coverage (+): 0. Max coverage (-): 0

Region: NODE\_29094\_length\_1021\_cov\_98.511261 367-368. Max. coverage (+): 0.19. Max coverage (-): 0

Region: NODE\_29094\_length\_1021\_cov\_98.511261 369-370. Max. coverage (+): 0.19. Max coverage (-): 0.04

Region: NODE\_29094\_length\_1021\_cov\_98.511261 371-372. Max. coverage (+): 0.22. Max coverage (-): 0.04

Region: NODE\_29094\_length\_1021\_cov\_98.511261 373-375. Max. coverage (+): 1.04. Max coverage (-): 0

Region: NODE\_29094\_length\_1021\_cov\_98.511261 376-377. Max. coverage (+): 1.08. Max coverage (-): 0

Region: NODE\_29094\_length\_1021\_cov\_98.511261 378-379. Max. coverage (+): 0.22. Max coverage (-): 0

Region: NODE\_29094\_length\_1021\_cov\_98.511261 380-381. Max. coverage (+): 0.22. Max coverage (-): 0

Region: NODE\_29094\_length\_1021\_cov\_98.511261 382-383. Max. coverage (+): 0.04. Max coverage (-): 0

Region: NODE\_29094\_length\_1021\_cov\_98.511261 384-385. Max. coverage (+): 0. Max coverage (-): 0

Region: NODE\_29094\_length\_1021\_cov\_98.511261 386-388. Max. coverage (+): 0.3. Max coverage (-): 0

Region: NODE\_29094\_length\_1021\_cov\_98.511261 389-390. Max. coverage (+): 0.19. Max coverage (-): 0

Region: NODE\_29094\_length\_1021\_cov\_98.511261 391-392. Max. coverage (+): 0.26. Max coverage (-): 0

Region: NODE\_29094\_length\_1021\_cov\_98.511261 393-394. Max. coverage (+): 0.07. Max coverage (-): 0

Region: NODE\_29094\_length\_1021\_cov\_98.511261 395-396. Max. coverage (+): 0.04. Max coverage (-): 0

Region: NODE\_29094\_length\_1021\_cov\_98.511261 397-398. Max. coverage (+): 0.04. Max coverage (-): 0.04

Region: NODE\_29094\_length\_1021\_cov\_98.511261 399-400. Max. coverage (+): 0. Max coverage (-): 0.04

Region: NODE\_29094\_length\_1021\_cov\_98.511261 401-403. Max. coverage (+): 0.04. Max coverage (-): 0.04

Region: NODE\_29094\_length\_1021\_cov\_98.511261 404-405. Max. coverage (+): 0.04. Max coverage (-): 0.11

Region: NODE\_29094\_length\_1021\_cov\_98.511261 406-407. Max. coverage (+): 0.04. Max coverage (-): 0.11

Region: NODE\_29094\_length\_1021\_cov\_98.511261 408-409. Max. coverage (+): 0.04. Max coverage (-): 0

Region: NODE\_29094\_length\_1021\_cov\_98.511261 410-411. Max. coverage (+): 0.04. Max coverage (-): 0

Region: NODE\_29094\_length\_1021\_cov\_98.511261 412-413. Max. coverage (+): 0.04. Max coverage (-): 0

Region: NODE\_29094\_length\_1021\_cov\_98.511261 414-416. Max. coverage (+): 1.04. Max coverage (-): 0.19

Region: NODE\_29094\_length\_1021\_cov\_98.511261 417-418. Max. coverage (+): 3.11. Max coverage (-): 0.15

Region: NODE\_29094\_length\_1021\_cov\_98.511261 419-420. Max. coverage (+): 3.26. Max coverage (-): 0.04

Region: NODE\_29094\_length\_1021\_cov\_98.511261 421-422. Max. coverage (+): 0.74. Max coverage (-): 0

Region: NODE\_29094\_length\_1021\_cov\_98.511261 423-424. Max. coverage (+): 2.08. Max coverage (-): 0

Region: NODE\_29094\_length\_1021\_cov\_98.511261 425-426. Max. coverage (+): 2.11. Max coverage (-): 0

Region: NODE\_29094\_length\_1021\_cov\_98.511261 427-428. Max. coverage (+): 1.26. Max coverage (-): 0.07

Region: NODE\_29094\_length\_1021\_cov\_98.511261 429-431. Max. coverage (+): 15.16. Max coverage (-): 0.07

Region: NODE\_29094\_length\_1021\_cov\_98.511261 432-433. Max. coverage (+): 58.24. Max coverage (-): 0.04

Region: NODE\_29094\_length\_1021\_cov\_98.511261 434-435. Max. coverage (+): 45.45. Max coverage (-): 0.19

Region: NODE\_29094\_length\_1021\_cov\_98.511261 436-437. Max. coverage (+): 0.3. Max coverage (-): 0.19

Region: NODE\_29094\_length\_1021\_cov\_98.511261 438-439. Max. coverage (+): 0.15. Max coverage (-): 0

Region: NODE\_29094\_length\_1021\_cov\_98.511261 440-441. Max. coverage (+): 0.11. Max coverage (-): 0.04

Region: NODE\_29094\_length\_1021\_cov\_98.511261 442-444. Max. coverage (+): 0.04. Max coverage (-): 0.19

Region: NODE\_29094\_length\_1021\_cov\_98.511261 445-446. Max. coverage (+): 0.26. Max coverage (-): 0.19

Region: NODE\_29094\_length\_1021\_cov\_98.511261 447-448. Max. coverage (+): 0.44. Max coverage (-): 0.11

Region: NODE\_29094\_length\_1021\_cov\_98.511261 449-450. Max. coverage (+): 27.21. Max coverage (-): 0.33

Region: NODE\_29094\_length\_1021\_cov\_98.511261 451-452. Max. coverage (+): 27.29. Max coverage (-): 0.37

Region: NODE\_29094\_length\_1021\_cov\_98.511261 453-454. Max. coverage (+): 0.41. Max coverage (-): 0.07

Region: NODE\_29094\_length\_1021\_cov\_98.511261 455-456. Max. coverage (+): 0.22. Max coverage (-): 0

Region: NODE\_29094\_length\_1021\_cov\_98.511261 457-459. Max. coverage (+): 0.15. Max coverage (-): 0

Region: NODE\_29094\_length\_1021\_cov\_98.511261 460-461. Max. coverage (+): 2.93. Max coverage (-): 0.26

Region: NODE\_29094\_length\_1021\_cov\_98.511261 462-463. Max. coverage (+): 14.12. Max coverage (-): 0.44

Region: NODE\_29094\_length\_1021\_cov\_98.511261 464-465. Max. coverage (+): 14.98. Max coverage (-): 0.44

Region: NODE\_29094\_length\_1021\_cov\_98.511261 466-467. Max. coverage (+): 3.26. Max coverage (-): 0.04

Region: NODE\_29094\_length\_1021\_cov\_98.511261 468-469. Max. coverage (+): 1.82. Max coverage (-): 0.04

Region: NODE\_29094\_length\_1021\_cov\_98.511261 470-472. Max. coverage (+): 0.67. Max coverage (-): 0

Region: NODE\_29094\_length\_1021\_cov\_98.511261 473-474. Max. coverage (+): 0. Max coverage (-): 0.04

Region: NODE\_29094\_length\_1021\_cov\_98.511261 475-476. Max. coverage (+): 0. Max coverage (-): 0.07

Region: NODE\_29094\_length\_1021\_cov\_98.511261 477-478. Max. coverage (+): 0. Max coverage (-): 0.19

Region: NODE\_29094\_length\_1021\_cov\_98.511261 479-480. Max. coverage (+): 0.15. Max coverage (-): 0.11

Region: NODE\_29094\_length\_1021\_cov\_98.511261 481-482. Max. coverage (+): 0.19. Max coverage (-): 0.04

Region: NODE\_29094\_length\_1021\_cov\_98.511261 483-485. Max. coverage (+): 0.48. Max coverage (-): 0.04

Region: NODE\_29094\_length\_1021\_cov\_98.511261 486-487. Max. coverage (+): 0.59. Max coverage (-): 0.07

Region: NODE\_29094\_length\_1021\_cov\_98.511261 488-489. Max. coverage (+): 0.78. Max coverage (-): 0.04

Region: NODE\_29094\_length\_1021\_cov\_98.511261 490-491. Max. coverage (+): 0.44. Max coverage (-): 0

Region: NODE\_29094\_length\_1021\_cov\_98.511261 492-493. Max. coverage (+): 5.45. Max coverage (-): 0

Region: NODE\_29094\_length\_1021\_cov\_98.511261 494-495. Max. coverage (+): 5.93. Max coverage (-): 0

Region: NODE\_29094\_length\_1021\_cov\_98.511261 496-497. Max. coverage (+): 0.82. Max coverage (-): 0

Region: NODE\_29094\_length\_1021\_cov\_98.511261 498-500. Max. coverage (+): 16.68. Max coverage (-): 0

Region: NODE\_29094\_length\_1021\_cov\_98.511261 501-502. Max. coverage (+): 17.05. Max coverage (-): 0

Region: NODE\_29094\_length\_1021\_cov\_98.511261 503-504. Max. coverage (+): 4.41. Max coverage (-): 0.09

Region: NODE\_29094\_length\_1021\_cov\_98.511261 505-506. Max. coverage (+): 0.06. Max coverage (-): 0.09

Region: NODE\_29094\_length\_1021\_cov\_98.511261 507-508. Max. coverage (+): 0.04. Max coverage (-): 0.04

Region: NODE\_29094\_length\_1021\_cov\_98.511261 509-510. Max. coverage (+): 0. Max coverage (-): 0.2

Region: NODE\_29094\_length\_1021\_cov\_98.511261 511-513. Max. coverage (+): 0.07. Max coverage (-): 0.22

Region: NODE\_29094\_length\_1021\_cov\_98.511261 514-515. Max. coverage (+): 0.04. Max coverage (-): 0.26

Region: NODE\_29094\_length\_1021\_cov\_98.511261 516-517. Max. coverage (+): 0.04. Max coverage (-): 0.04

Region: NODE\_29094\_length\_1021\_cov\_98.511261 518-519. Max. coverage (+): 0. Max coverage (-): 0.04

Region: NODE\_29094\_length\_1021\_cov\_98.511261 520-521. Max. coverage (+): 0. Max coverage (-): 0.04

Region: NODE\_29094\_length\_1021\_cov\_98.511261 522-523. Max. coverage (+): 0.15. Max coverage (-): 0

Region: NODE\_29094\_length\_1021\_cov\_98.511261 524-525. Max. coverage (+): 0.41. Max coverage (-): 0

Region: NODE\_29094\_length\_1021\_cov\_98.511261 526-528. Max. coverage (+): 0.41. Max coverage (-): 0

Region: NODE\_29094\_length\_1021\_cov\_98.511261 529-530. Max. coverage (+): 0.48. Max coverage (-): 0

Region: NODE\_29094\_length\_1021\_cov\_98.511261 531-532. Max. coverage (+): 0.07. Max coverage (-): 0.07

Region: NODE\_29094\_length\_1021\_cov\_98.511261 533-534. Max. coverage (+): 0.04. Max coverage (-): 0.19

Region: NODE\_29094\_length\_1021\_cov\_98.511261 535-536. Max. coverage (+): 0. Max coverage (-): 0.22

Region: NODE\_29094\_length\_1021\_cov\_98.511261 537-538. Max. coverage (+): 0.37. Max coverage (-): 0.56

Region: NODE\_29094\_length\_1021\_cov\_98.511261 539-541. Max. coverage (+): 1.04. Max coverage (-): 0.52

Region: NODE\_29094\_length\_1021\_cov\_98.511261 542-543. Max. coverage (+): 0.74. Max coverage (-): 0.52

Region: NODE\_29094\_length\_1021\_cov\_98.511261 544-545. Max. coverage (+): 1.11. Max coverage (-): 0.44

Region: NODE\_29094\_length\_1021\_cov\_98.511261 546-547. Max. coverage (+): 1. Max coverage (-): 0

Region: NODE\_29094\_length\_1021\_cov\_98.511261 548-549. Max. coverage (+): 17.54. Max coverage (-): 0

Region: NODE\_29094\_length\_1021\_cov\_98.511261 550-551. Max. coverage (+): 17.65. Max coverage (-): 0

Region: NODE\_29094\_length\_1021\_cov\_98.511261 552-554. Max. coverage (+): 19.46. Max coverage (-): 0

Region: NODE\_29094\_length\_1021\_cov\_98.511261 555-556. Max. coverage (+): 20.35. Max coverage (-): 0

Region: NODE\_29094\_length\_1021\_cov\_98.511261 557-558. Max. coverage (+): 3.97. Max coverage (-): 0

Region: NODE\_29094\_length\_1021\_cov\_98.511261 559-560. Max. coverage (+): 3.37. Max coverage (-): 0

Region: NODE\_29094\_length\_1021\_cov\_98.511261 561-562. Max. coverage (+): 0.41. Max coverage (-): 0

Region: NODE\_29094\_length\_1021\_cov\_98.511261 563-564. Max. coverage (+): 0.04. Max coverage (-): 0

Region: NODE\_29094\_length\_1021\_cov\_98.511261 565-566. Max. coverage (+): 0. Max coverage (-): 0

Region: NODE\_29094\_length\_1021\_cov\_98.511261 567-569. Max. coverage (+): 0. Max coverage (-): 0.07

Region: NODE\_29094\_length\_1021\_cov\_98.511261 570-571. Max. coverage (+): 0. Max coverage (-): 0

Region: NODE\_29094\_length\_1021\_cov\_98.511261 572-573. Max. coverage (+): 0.22. Max coverage (-): 0.67

Region: NODE\_29094\_length\_1021\_cov\_98.511261 574-575. Max. coverage (+): 0.56. Max coverage (-): 0.67

Region: NODE\_29094\_length\_1021\_cov\_98.511261 576-577. Max. coverage (+): 0.33. Max coverage (-): 0.26

Region: NODE\_29094\_length\_1021\_cov\_98.511261 578-579. Max. coverage (+): 0. Max coverage (-): 0.26

Region: NODE\_29094\_length\_1021\_cov\_98.511261 580-582. Max. coverage (+): 0.04. Max coverage (-): 1.67

Region: NODE\_29094\_length\_1021\_cov\_98.511261 583-584. Max. coverage (+): 0.07. Max coverage (-): 0.89

Region: NODE\_29094\_length\_1021\_cov\_98.511261 585-586. Max. coverage (+): 0.07. Max coverage (-): 0.22

Region: NODE\_29094\_length\_1021\_cov\_98.511261 587-588. Max. coverage (+): 0. Max coverage (-): 0

Region: NODE\_29094\_length\_1021\_cov\_98.511261 589-590. Max. coverage (+): 0. Max coverage (-): 0

Region: NODE\_29094\_length\_1021\_cov\_98.511261 591-592. Max. coverage (+): 0. Max coverage (-): 0

Region: NODE\_29094\_length\_1021\_cov\_98.511261 593-594. Max. coverage (+): 0. Max coverage (-): 0.78

Region: NODE\_29094\_length\_1021\_cov\_98.511261 595-597. Max. coverage (+): 11.6. Max coverage (-): 0.78

Region: NODE\_29094\_length\_1021\_cov\_98.511261 598-599. Max. coverage (+): 11.49. Max coverage (-): 0.04

Region: NODE\_29094\_length\_1021\_cov\_98.511261 600-601. Max. coverage (+): 0.04. Max coverage (-): 0.04

Region: NODE\_29094\_length\_1021\_cov\_98.511261 602-603. Max. coverage (+): 0. Max coverage (-): 0.07

Region: NODE\_29094\_length\_1021\_cov\_98.511261 604-605. Max. coverage (+): 0. Max coverage (-): 0.15

Region: NODE\_29094\_length\_1021\_cov\_98.511261 606-607. Max. coverage (+): 0.04. Max coverage (-): 0.15

Region: NODE\_29094\_length\_1021\_cov\_98.511261 608-610. Max. coverage (+): 0.93. Max coverage (-): 0.07

Region: NODE\_29094\_length\_1021\_cov\_98.511261 611-612. Max. coverage (+): 0.11. Max coverage (-): 0.07

Region: NODE\_29094\_length\_1021\_cov\_98.511261 613-614. Max. coverage (+): 0. Max coverage (-): 0.04

Region: NODE\_29094\_length\_1021\_cov\_98.511261 615-616. Max. coverage (+): 0.15. Max coverage (-): 0.07

Region: NODE\_29094\_length\_1021\_cov\_98.511261 617-618. Max. coverage (+): 0.3. Max coverage (-): 0.07

Region: NODE\_29094\_length\_1021\_cov\_98.511261 619-620. Max. coverage (+): 0.67. Max coverage (-): 0

Region: NODE\_29094\_length\_1021\_cov\_98.511261 621-623. Max. coverage (+): 1.08. Max coverage (-): 0

Region: NODE\_29094\_length\_1021\_cov\_98.511261 624-625. Max. coverage (+): 0.7. Max coverage (-): 0

Region: NODE\_29094\_length\_1021\_cov\_98.511261 626-627. Max. coverage (+): 0.85. Max coverage (-): 0.11

Region: NODE\_29094\_length\_1021\_cov\_98.511261 628-629. Max. coverage (+): 1.41. Max coverage (-): 0.11

Region: NODE\_29094\_length\_1021\_cov\_98.511261 630-631. Max. coverage (+): 1.33. Max coverage (-): 0

Region: NODE\_29094\_length\_1021\_cov\_98.511261 632-633. Max. coverage (+): 0.26. Max coverage (-): 0.07

Region: NODE\_29094\_length\_1021\_cov\_98.511261 634-635. Max. coverage (+): 0.04. Max coverage (-): 0.11

Region: NODE\_29094\_length\_1021\_cov\_98.511261 636-638. Max. coverage (+): 0.11. Max coverage (-): 0.04

Region: NODE\_29094\_length\_1021\_cov\_98.511261 639-640. Max. coverage (+): 0.07. Max coverage (-): 0.19

Region: NODE\_29094\_length\_1021\_cov\_98.511261 641-642. Max. coverage (+): 0.52. Max coverage (-): 0.15

Region: NODE\_29094\_length\_1021\_cov\_98.511261 643-644. Max. coverage (+): 18.98. Max coverage (-): 0

Region: NODE\_29094\_length\_1021\_cov\_98.511261 645-646. Max. coverage (+): 18.54. Max coverage (-): 0.04

Region: NODE\_29094\_length\_1021\_cov\_98.511261 647-648. Max. coverage (+): 6.04. Max coverage (-): 0.04

Region: NODE\_29094\_length\_1021\_cov\_98.511261 649-651. Max. coverage (+): 2.97. Max coverage (-): 0

Region: NODE\_29094\_length\_1021\_cov\_98.511261 652-653. Max. coverage (+): 1.22. Max coverage (-): 0.15

Region: NODE\_29094\_length\_1021\_cov\_98.511261 654-655. Max. coverage (+): 1.74. Max coverage (-): 0.15

Region: NODE\_29094\_length\_1021\_cov\_98.511261 656-657. Max. coverage (+): 0.56. Max coverage (-): 0.07

Region: NODE\_29094\_length\_1021\_cov\_98.511261 658-659. Max. coverage (+): 0.67. Max coverage (-): 0.15

Region: NODE\_29094\_length\_1021\_cov\_98.511261 660-661. Max. coverage (+): 0.52. Max coverage (-): 0.07

Region: NODE\_29094\_length\_1021\_cov\_98.511261 662-663. Max. coverage (+): 0.07. Max coverage (-): 0.07

Region: NODE\_29094\_length\_1021\_cov\_98.511261 664-666. Max. coverage (+): 0.15. Max coverage (-): 0.3

Region: NODE\_29094\_length\_1021\_cov\_98.511261 667-668. Max. coverage (+): 0.07. Max coverage (-): 0.48

Region: NODE\_29094\_length\_1021\_cov\_98.511261 669-670. Max. coverage (+): 0.11. Max coverage (-): 0.67

Region: NODE\_29094\_length\_1021\_cov\_98.511261 671-672. Max. coverage (+): 0.11. Max coverage (-): 0.67

Region: NODE\_29094\_length\_1021\_cov\_98.511261 673-674. Max. coverage (+): 0.11. Max coverage (-): 0.3

Region: NODE\_29094\_length\_1021\_cov\_98.511261 675-676. Max. coverage (+): 0.22. Max coverage (-): 0.07

Region: NODE\_29094\_length\_1021\_cov\_98.511261 677-679. Max. coverage (+): 0.22. Max coverage (-): 0.11

Region: NODE\_29094\_length\_1021\_cov\_98.511261 680-681. Max. coverage (+): 0.33. Max coverage (-): 0.07

Region: NODE\_29094\_length\_1021\_cov\_98.511261 682-683. Max. coverage (+): 0.63. Max coverage (-): 0

Region: NODE\_29094\_length\_1021\_cov\_98.511261 684-685. Max. coverage (+): 0.41. Max coverage (-): 0

Region: NODE\_29094\_length\_1021\_cov\_98.511261 686-687. Max. coverage (+): 0.19. Max coverage (-): 0

Region: NODE\_29094\_length\_1021\_cov\_98.511261 688-689. Max. coverage (+): 0. Max coverage (-): 0

Region: NODE\_29094\_length\_1021\_cov\_98.511261 690-691. Max. coverage (+): 0.22. Max coverage (-): 0

Region: NODE\_29094\_length\_1021\_cov\_98.511261 692-694. Max. coverage (+): 0.26. Max coverage (-): 0.37

Region: NODE\_29094\_length\_1021\_cov\_98.511261 695-696. Max. coverage (+): 0.33. Max coverage (-): 1.26

Region: NODE\_29094\_length\_1021\_cov\_98.511261 697-698. Max. coverage (+): 0.26. Max coverage (-): 1.26

Region: NODE\_29094\_length\_1021\_cov\_98.511261 699-700. Max. coverage (+): 0.3. Max coverage (-): 1.04

Region: NODE\_29094\_length\_1021\_cov\_98.511261 701-702. Max. coverage (+): 1.78. Max coverage (-): 0.41

Region: NODE\_29094\_length\_1021\_cov\_98.511261 703-704. Max. coverage (+): 1.74. Max coverage (-): 0.07

Region: NODE\_29094\_length\_1021\_cov\_98.511261 705-707. Max. coverage (+): 0.3. Max coverage (-): 0.07

Region: NODE\_29094\_length\_1021\_cov\_98.511261 708-709. Max. coverage (+): 1.37. Max coverage (-): 0

Region: NODE\_29094\_length\_1021\_cov\_98.511261 710-711. Max. coverage (+): 1.3. Max coverage (-): 0

Region: NODE\_29094\_length\_1021\_cov\_98.511261 712-713. Max. coverage (+): 0.3. Max coverage (-): 0

Region: NODE\_29094\_length\_1021\_cov\_98.511261 714-715. Max. coverage (+): 1.15. Max coverage (-): 0

Region: NODE\_29094\_length\_1021\_cov\_98.511261 716-717. Max. coverage (+): 1.52. Max coverage (-): 0

Region: NODE\_29094\_length\_1021\_cov\_98.511261 718-720. Max. coverage (+): 0.59. Max coverage (-): 0.07

Region: NODE\_29094\_length\_1021\_cov\_98.511261 721-722. Max. coverage (+): 0.07. Max coverage (-): 0.07

Region: NODE\_29094\_length\_1021\_cov\_98.511261 723-724. Max. coverage (+): 0.02. Max coverage (-): 0.01

Region: NODE\_29094\_length\_1021\_cov\_98.511261 725-726. Max. coverage (+): 0.02. Max coverage (-): 0.01

Region: NODE\_29094\_length\_1021\_cov\_98.511261 727-728. Max. coverage (+): 0.02. Max coverage (-): 0

Region: NODE\_29094\_length\_1021\_cov\_98.511261 729-730. Max. coverage (+): 0.03. Max coverage (-): 0

Region: NODE\_29094\_length\_1021\_cov\_98.511261 731-732. Max. coverage (+): 0.02. Max coverage (-): 0

Region: NODE\_29094\_length\_1021\_cov\_98.511261 733-735. Max. coverage (+): 1.09. Max coverage (-): 0

Region: NODE\_29094\_length\_1021\_cov\_98.511261 736-737. Max. coverage (+): 1.25. Max coverage (-): 0

Region: NODE\_29094\_length\_1021\_cov\_98.511261 738-739. Max. coverage (+): 0.59. Max coverage (-): 0

Region: NODE\_29094\_length\_1021\_cov\_98.511261 740-741. Max. coverage (+): 0.52. Max coverage (-): 0.04

Region: NODE\_29094\_length\_1021\_cov\_98.511261 742-743. Max. coverage (+): 23.77. Max coverage (-): 0.07

Region: NODE\_29094\_length\_1021\_cov\_98.511261 744-745. Max. coverage (+): 25.07. Max coverage (-): 0.04

Region: NODE\_29094\_length\_1021\_cov\_98.511261 746-748. Max. coverage (+): 1.63. Max coverage (-): 0

Region: NODE\_29094\_length\_1021\_cov\_98.511261 749-750. Max. coverage (+): 1.48. Max coverage (-): 0.04

Region: NODE\_29094\_length\_1021\_cov\_98.511261 751-752. Max. coverage (+): 1.33. Max coverage (-): 0.04

Region: NODE\_29094\_length\_1021\_cov\_98.511261 753-754. Max. coverage (+): 0.3. Max coverage (-): 0.19

Region: NODE\_29094\_length\_1021\_cov\_98.511261 755-756. Max. coverage (+): 0.59. Max coverage (-): 0.19

Region: NODE\_29094\_length\_1021\_cov\_98.511261 757-758. Max. coverage (+): 0.85. Max coverage (-): 0.04

Region: NODE\_29094\_length\_1021\_cov\_98.511261 759-760. Max. coverage (+): 0.26. Max coverage (-): 0.11

Region: NODE\_29094\_length\_1021\_cov\_98.511261 761-763. Max. coverage (+): 0.11. Max coverage (-): 0.11

Region: NODE\_29094\_length\_1021\_cov\_98.511261 764-765. Max. coverage (+): 0.11. Max coverage (-): 0.22

Region: NODE\_29094\_length\_1021\_cov\_98.511261 766-767. Max. coverage (+): 0. Max coverage (-): 0.37

Region: NODE\_29094\_length\_1021\_cov\_98.511261 768-769. Max. coverage (+): 0.41. Max coverage (-): 0.22

Region: NODE\_29094\_length\_1021\_cov\_98.511261 770-771. Max. coverage (+): 0.41. Max coverage (-): 0.04

Region: NODE\_29094\_length\_1021\_cov\_98.511261 772-773. Max. coverage (+): 0.78. Max coverage (-): 0.04

Region: NODE\_29094\_length\_1021\_cov\_98.511261 774-776. Max. coverage (+): 0.7. Max coverage (-): 0

Region: NODE\_29094\_length\_1021\_cov\_98.511261 777-778. Max. coverage (+): 0.19. Max coverage (-): 0

Region: NODE\_29094\_length\_1021\_cov\_98.511261 779-780. Max. coverage (+): 0.15. Max coverage (-): 0

Region: NODE\_29094\_length\_1021\_cov\_98.511261 781-782. Max. coverage (+): 4.63. Max coverage (-): 0

Region: NODE\_29094\_length\_1021\_cov\_98.511261 783-784. Max. coverage (+): 4.75. Max coverage (-): 0.04

Region: NODE\_29094\_length\_1021\_cov\_98.511261 785-786. Max. coverage (+): 1.41. Max coverage (-): 0.07

Region: NODE\_29094\_length\_1021\_cov\_98.511261 787-789. Max. coverage (+): 2.22. Max coverage (-): 0.04

Region: NODE\_29094\_length\_1021\_cov\_98.511261 790-791. Max. coverage (+): 1. Max coverage (-): 0.04

Region: NODE\_29094\_length\_1021\_cov\_98.511261 792-793. Max. coverage (+): 3.71. Max coverage (-): 0.07

Region: NODE\_29094\_length\_1021\_cov\_98.511261 794-795. Max. coverage (+): 3.89. Max coverage (-): 0.11

Region: NODE\_29094\_length\_1021\_cov\_98.511261 796-797. Max. coverage (+): 0.85. Max coverage (-): 0.11

Region: NODE\_29094\_length\_1021\_cov\_98.511261 798-799. Max. coverage (+): 1.71. Max coverage (-): 0.22

Region: NODE\_29094\_length\_1021\_cov\_98.511261 800-801. Max. coverage (+): 1.04. Max coverage (-): 0.11

Region: NODE\_29094\_length\_1021\_cov\_98.511261 802-804. Max. coverage (+): 0.74. Max coverage (-): 0.11

Region: NODE\_29094\_length\_1021\_cov\_98.511261 805-806. Max. coverage (+): 0.44. Max coverage (-): 0

Region: NODE\_29094\_length\_1021\_cov\_98.511261 807-808. Max. coverage (+): 0.41. Max coverage (-): 0

Region: NODE\_29094\_length\_1021\_cov\_98.511261 809-810. Max. coverage (+): 0.67. Max coverage (-): 0

Region: NODE\_29094\_length\_1021\_cov\_98.511261 811-812. Max. coverage (+): 1.19. Max coverage (-): 0

Region: NODE\_29094\_length\_1021\_cov\_98.511261 813-814. Max. coverage (+): 8.79. Max coverage (-): 0.04

Region: NODE\_29094\_length\_1021\_cov\_98.511261 815-817. Max. coverage (+): 8.53. Max coverage (-): 0.07

Region: NODE\_29094\_length\_1021\_cov\_98.511261 818-819. Max. coverage (+): 0.56. Max coverage (-): 0.04

Region: NODE\_29094\_length\_1021\_cov\_98.511261 820-821. Max. coverage (+): 0.78. Max coverage (-): 0

Region: NODE\_29094\_length\_1021\_cov\_98.511261 822-823. Max. coverage (+): 2.63. Max coverage (-): 0

Region: NODE\_29094\_length\_1021\_cov\_98.511261 824-825. Max. coverage (+): 2.22. Max coverage (-): 0

Region: NODE\_29094\_length\_1021\_cov\_98.511261 826-827. Max. coverage (+): 0.22. Max coverage (-): 0

Region: NODE\_29094\_length\_1021\_cov\_98.511261 828-829. Max. coverage (+): 0.78. Max coverage (-): 0.04

Region: NODE\_29094\_length\_1021\_cov\_98.511261 830-832. Max. coverage (+): 0.85. Max coverage (-): 0.04

Region: NODE\_29094\_length\_1021\_cov\_98.511261 833-834. Max. coverage (+): 0.89. Max coverage (-): 0.04

Region: NODE\_29094\_length\_1021\_cov\_98.511261 835-836. Max. coverage (+): 0.15. Max coverage (-): 0.11

Region: NODE\_29094\_length\_1021\_cov\_98.511261 837-838. Max. coverage (+): 0.11. Max coverage (-): 0.11

Region: NODE\_29094\_length\_1021\_cov\_98.511261 839-840. Max. coverage (+): 0.07. Max coverage (-): 0.04

Region: NODE\_29094\_length\_1021\_cov\_98.511261 841-842. Max. coverage (+): 0.11. Max coverage (-): 0.33

Region: NODE\_29094\_length\_1021\_cov\_98.511261 843-845. Max. coverage (+): 0.22. Max coverage (-): 0.37

Region: NODE\_29094\_length\_1021\_cov\_98.511261 846-847. Max. coverage (+): 0.22. Max coverage (-): 0.15

Region: NODE\_29094\_length\_1021\_cov\_98.511261 848-849. Max. coverage (+): 2.04. Max coverage (-): 0.19

Region: NODE\_29094\_length\_1021\_cov\_98.511261 850-851. Max. coverage (+): 2.15. Max coverage (-): 0.04

Region: NODE\_29094\_length\_1021\_cov\_98.511261 852-853. Max. coverage (+): 0.63. Max coverage (-): 0

Region: NODE\_29094\_length\_1021\_cov\_98.511261 854-855. Max. coverage (+): 0.78. Max coverage (-): 0

Region: NODE\_29094\_length\_1021\_cov\_98.511261 856-858. Max. coverage (+): 1.96. Max coverage (-): 0

Region: NODE\_29094\_length\_1021\_cov\_98.511261 859-860. Max. coverage (+): 2. Max coverage (-): 0

Region: NODE\_29094\_length\_1021\_cov\_98.511261 861-862. Max. coverage (+): 1.15. Max coverage (-): 0

Region: NODE\_29094\_length\_1021\_cov\_98.511261 863-864. Max. coverage (+): 0.48. Max coverage (-): 0

Region: NODE\_29094\_length\_1021\_cov\_98.511261 865-866. Max. coverage (+): 0.17. Max coverage (-): 0

Region: NODE\_29094\_length\_1021\_cov\_98.511261 867-868. Max. coverage (+): 0.15. Max coverage (-): 0

Region: NODE\_29094\_length\_1021\_cov\_98.511261 869-870. Max. coverage (+): 2.69. Max coverage (-): 0

Region: NODE\_29094\_length\_1021\_cov\_98.511261 871-873. Max. coverage (+): 6.41. Max coverage (-): 0

Region: NODE\_29094\_length\_1021\_cov\_98.511261 874-875. Max. coverage (+): 0.85. Max coverage (-): 0.04

Region: NODE\_29094\_length\_1021\_cov\_98.511261 876-877. Max. coverage (+): 0.26. Max coverage (-): 0.04

Region: NODE\_29094\_length\_1021\_cov\_98.511261 878-879. Max. coverage (+): 2.08. Max coverage (-): 0

Region: NODE\_29094\_length\_1021\_cov\_98.511261 880-881. Max. coverage (+): 2.11. Max coverage (-): 0.04

Region: NODE\_29094\_length\_1021\_cov\_98.511261 882-883. Max. coverage (+): 0.19. Max coverage (-): 0.04

Region: NODE\_29094\_length\_1021\_cov\_98.511261 884-886. Max. coverage (+): 0.19. Max coverage (-): 0.04

Region: NODE\_29094\_length\_1021\_cov\_98.511261 887-888. Max. coverage (+): 1.71. Max coverage (-): 0.04

Region: NODE\_29094\_length\_1021\_cov\_98.511261 889-890. Max. coverage (+): 5.26. Max coverage (-): 0.04

Region: NODE\_29094\_length\_1021\_cov\_98.511261 891-892. Max. coverage (+): 3.67. Max coverage (-): 0

Region: NODE\_29094\_length\_1021\_cov\_98.511261 893-894. Max. coverage (+): 1.19. Max coverage (-): 0.04

Region: NODE\_29094\_length\_1021\_cov\_98.511261 895-896. Max. coverage (+): 0.93. Max coverage (-): 0.15

Region: NODE\_29094\_length\_1021\_cov\_98.511261 897-898. Max. coverage (+): 0.04. Max coverage (-): 0.11

Region: NODE\_29094\_length\_1021\_cov\_98.511261 899-901. Max. coverage (+): 1.33. Max coverage (-): 0

Region: NODE\_29094\_length\_1021\_cov\_98.511261 902-903. Max. coverage (+): 1.28. Max coverage (-): 0

Region: NODE\_29094\_length\_1021\_cov\_98.511261 904-905. Max. coverage (+): 0.02. Max coverage (-): 0

Region: NODE\_29094\_length\_1021\_cov\_98.511261 906-907. Max. coverage (+): 0. Max coverage (-): 0

Region: NODE\_29094\_length\_1021\_cov\_98.511261 908-909. Max. coverage (+): 0. Max coverage (-): 0

Region: NODE\_29094\_length\_1021\_cov\_98.511261 910-911. Max. coverage (+): 0. Max coverage (-): 0

Region: NODE\_29094\_length\_1021\_cov\_98.511261 912-914. Max. coverage (+): 0. Max coverage (-): 0.02

Region: NODE\_29094\_length\_1021\_cov\_98.511261 915-916. Max. coverage (+): 0. Max coverage (-): 0

Region: NODE\_29094\_length\_1021\_cov\_98.511261 917-918. Max. coverage (+): 0. Max coverage (-): 0

Region: NODE\_29094\_length\_1021\_cov\_98.511261 919-920. Max. coverage (+): 0. Max coverage (-): 0

Region: NODE\_29094\_length\_1021\_cov\_98.511261 921-922. Max. coverage (+): 0. Max coverage (-): 0

Region: NODE\_29094\_length\_1021\_cov\_98.511261 923-924. Max. coverage (+): 0. Max coverage (-): 0

Region: NODE\_29094\_length\_1021\_cov\_98.511261 925-927. Max. coverage (+): 0. Max coverage (-): 0

Region: NODE\_29094\_length\_1021\_cov\_98.511261 928-929. Max. coverage (+): 0. Max coverage (-): 0

Region: NODE\_29094\_length\_1021\_cov\_98.511261 930-931. Max. coverage (+): 0. Max coverage (-): 0.02

Region: NODE\_29094\_length\_1021\_cov\_98.511261 932-933. Max. coverage (+): 0. Max coverage (-): 0.06

Region: NODE\_29094\_length\_1021\_cov\_98.511261 934-935. Max. coverage (+): 0. Max coverage (-): 0.04

Region: NODE\_29094\_length\_1021\_cov\_98.511261 936-937. Max. coverage (+): 0. Max coverage (-): 0.04

Region: NODE\_29094\_length\_1021\_cov\_98.511261 938-939. Max. coverage (+): 0. Max coverage (-): 0

Region: NODE\_29094\_length\_1021\_cov\_98.511261 940-942. Max. coverage (+): 0.04. Max coverage (-): 0

Region: NODE\_29094\_length\_1021\_cov\_98.511261 943-944. Max. coverage (+): 0.04. Max coverage (-): 0

Region: NODE\_29094\_length\_1021\_cov\_98.511261 945-946. Max. coverage (+): 0. Max coverage (-): 0

Region: NODE\_29094\_length\_1021\_cov\_98.511261 947-948. Max. coverage (+): 0. Max coverage (-): 0.07

Region: NODE\_29094\_length\_1021\_cov\_98.511261 949-950. Max. coverage (+): 0. Max coverage (-): 0.11

Region: NODE\_29094\_length\_1021\_cov\_98.511261 951-952. Max. coverage (+): 0. Max coverage (-): 0.07

Region: NODE\_29094\_length\_1021\_cov\_98.511261 953-955. Max. coverage (+): 0.04. Max coverage (-): 0.04

Region: NODE\_29094\_length\_1021\_cov\_98.511261 956-957. Max. coverage (+): 0.04. Max coverage (-): 0

Region: NODE\_29094\_length\_1021\_cov\_98.511261 958-959. Max. coverage (+): 0. Max coverage (-): 0.04

Region: NODE\_29094\_length\_1021\_cov\_98.511261 960-961. Max. coverage (+): 0. Max coverage (-): 0.04

Region: NODE\_29094\_length\_1021\_cov\_98.511261 962-963. Max. coverage (+): 0. Max coverage (-): 0.15

Region: NODE\_29094\_length\_1021\_cov\_98.511261 964-965. Max. coverage (+): 0.07. Max coverage (-): 0.52

Region: NODE\_29094\_length\_1021\_cov\_98.511261 966-967. Max. coverage (+): 0.07. Max coverage (-): 0.48

Region: NODE\_29094\_length\_1021\_cov\_98.511261 968-970. Max. coverage (+): 0.74. Max coverage (-): 0.11

Region: NODE\_29094\_length\_1021\_cov\_98.511261 971-972. Max. coverage (+): 0.7. Max coverage (-): 0

Region: NODE\_29094\_length\_1021\_cov\_98.511261 973-974. Max. coverage (+): 1.3. Max coverage (-): 0

Region: NODE\_29094\_length\_1021\_cov\_98.511261 975-976. Max. coverage (+): 1.48. Max coverage (-): 0.04

Region: NODE\_29094\_length\_1021\_cov\_98.511261 977-978. Max. coverage (+): 0.56. Max coverage (-): 0.04

Region: NODE\_29094\_length\_1021\_cov\_98.511261 979-980. Max. coverage (+): 0.17. Max coverage (-): 0

Region: NODE\_29094\_length\_1021\_cov\_98.511261 981-983. Max. coverage (+): 0.22. Max coverage (-): 0

Region: NODE\_29094\_length\_1021\_cov\_98.511261 984-985. Max. coverage (+): 1.19. Max coverage (-): 0.02

Region: NODE\_29094\_length\_1021\_cov\_98.511261 986-987. Max. coverage (+): 1.19. Max coverage (-): 0.02

Region: NODE\_29094\_length\_1021\_cov\_98.511261 988-989. Max. coverage (+): 0.26. Max coverage (-): 0

Region: NODE\_29094\_length\_1021\_cov\_98.511261 990-991. Max. coverage (+): 0.24. Max coverage (-): 0

Region: NODE\_29094\_length\_1021\_cov\_98.511261 992-993. Max. coverage (+): 0.02. Max coverage (-): 0

Region: NODE\_29094\_length\_1021\_cov\_98.511261 994-995. Max. coverage (+): 0. Max coverage (-): 0

Region: NODE\_29094\_length\_1021\_cov\_98.511261 996-998. Max. coverage (+): 0.15. Max coverage (-): 0

Region: NODE\_29094\_length\_1021\_cov\_98.511261 999-1000. Max. coverage (+): 0.15. Max coverage (-): 0

Region: NODE\_29094\_length\_1021\_cov\_98.511261 1001-1002. Max. coverage (+): 0. Max coverage (-): 0

Region: NODE\_29094\_length\_1021\_cov\_98.511261 1003-1004. Max. coverage (+): 0. Max coverage (-): 0.02

Region: NODE\_29094\_length\_1021\_cov\_98.511261 1005-1006. Max. coverage (+): 0. Max coverage (-): 0.02

Region: NODE\_29094\_length\_1021\_cov\_98.511261 1007-1008. Max. coverage (+): 0. Max coverage (-): 0.02

Region: NODE\_29094\_length\_1021\_cov\_98.511261 1009-1011. Max. coverage (+): 0.04. Max coverage (-): 0

Region: NODE\_29094\_length\_1021\_cov\_98.511261 1012-1013. Max. coverage (+): 0.04. Max coverage (-): 0

Region: NODE\_29094\_length\_1021\_cov\_98.511261 1014-1015. Max. coverage (+): 0. Max coverage (-): 0

Region: NODE\_29094\_length\_1021\_cov\_98.511261 1016-1017. Max. coverage (+): 0. Max coverage (-): 0

Region: NODE\_29094\_length\_1021\_cov\_98.511261 1018-1019. Max. coverage (+): 0. Max coverage (-): 0

Region: NODE\_29094\_length\_1021\_cov\_98.511261 1020-1021. Max. coverage (+): 0. Max coverage (-): 0

Region: NODE\_29094\_length\_1021\_cov\_98.511261 1022-1024. Max. coverage (+): 0.04. Max coverage (-): 0

Region: NODE\_29094\_length\_1021\_cov\_98.511261 1025-1026. Max. coverage (+): 0.04. Max coverage (-): 0

Region: NODE\_29094\_length\_1021\_cov\_98.511261 1027-1028. Max. coverage (+): 0.04. Max coverage (-): 0

Region: NODE\_29094\_length\_1021\_cov\_98.511261 1029-1030. Max. coverage (+): 0.04. Max coverage (-): 0.11

Region: NODE\_29094\_length\_1021\_cov\_98.511261 1031-1032. Max. coverage (+): 0.04. Max coverage (-): 0.33

Region: NODE\_29094\_length\_1021\_cov\_98.511261 1033-1034. Max. coverage (+): 0.04. Max coverage (-): 0.37

Region: NODE\_29094\_length\_1021\_cov\_98.511261 1035-1036. Max. coverage (+): 0.04. Max coverage (-): 0.22

Region: NODE\_29094\_length\_1021\_cov\_98.511261 1037-1039. Max. coverage (+): 0.11. Max coverage (-): 0.26

Region: NODE\_29094\_length\_1021\_cov\_98.511261 1040-1041. Max. coverage (+): 0.15. Max coverage (-): 0.15

Region: NODE\_29094\_length\_1021\_cov\_98.511261 1042-1043. Max. coverage (+): 0.15. Max coverage (-): 0.15

Region: NODE\_29094\_length\_1021\_cov\_98.511261 1044-1045. Max. coverage (+): 0.19. Max coverage (-): 0.11

Region: NODE\_29094\_length\_1021\_cov\_98.511261 1046-1047. Max. coverage (+): 0.15. Max coverage (-): 0.04

Region: NODE\_29094\_length\_1021\_cov\_98.511261 1048-1049. Max. coverage (+): 0.11. Max coverage (-): 0

Region: NODE\_29094\_length\_1021\_cov\_98.511261 1050-1052. Max. coverage (+): 0.04. Max coverage (-): 0

Region: NODE\_29094\_length\_1021\_cov\_98.511261 1053-1054. Max. coverage (+): 0. Max coverage (-): 0.04

Region: NODE\_29094\_length\_1021\_cov\_98.511261 1055-1056. Max. coverage (+): 0. Max coverage (-): 0.04

Region: NODE\_29094\_length\_1021\_cov\_98.511261 1057-1058. Max. coverage (+): 0. Max coverage (-): 0

Region: NODE\_29094\_length\_1021\_cov\_98.511261 1059-1060. Max. coverage (+): 0. Max coverage (-): 0

Region: NODE\_29094\_length\_1021\_cov\_98.511261 1061-1062. Max. coverage (+): 0. Max coverage (-): 0

Region: NODE\_29094\_length\_1021\_cov\_98.511261 1063-1064. Max. coverage (+): 0. Max coverage (-): 0

Region: NODE\_29094\_length\_1021\_cov\_98.511261 1065-1067. Max. coverage (+): 0. Max coverage (-): 0

Region: NODE\_29094\_length\_1021\_cov\_98.511261 1068-1069. Max. coverage (+): 0. Max coverage (-): 0

Region: NODE\_29094\_length\_1021\_cov\_98.511261 1070-1071. Max. coverage (+): 0. Max coverage (-): 0

Region: NODE\_29094\_length\_1021\_cov\_98.511261 1072-1073. Max. coverage (+): 0. Max coverage (-): 0

Region: NODE\_29094\_length\_1021\_cov\_98.511261 1074-1075. Max. coverage (+): 0. Max coverage (-): 0

Region: NODE\_29094\_length\_1021\_cov\_98.511261 1076-1077. Max. coverage (+): 0. Max coverage (-): 0

Region: NODE\_29094\_length\_1021\_cov\_98.511261 1078-. Max. coverage (+): 0. Max coverage (-): 0

RepeatMasker Color Code

**+**

100-98% Identity

<98-95% Identity

<95-90% Identity

<90-85% Identity

<85-80% Identity

<80-75% Identity

<75-70% Identity

<70% Identity

**-**

Gene Set Color Code

**+**

Gene

Pseudogene

Other

**-**

Topology/Coverage Color Code

Coverage Plus Strand

Coverage Minus Strand

Mainstrand: Plus

Mainstrand: Minus

Complementary Strand

Flanking Region  
(if option -flank >0)

Gene Set Annotation  
  
RepeatMasker Annotation  
  
Transcription Factor Binding Sites  

**RFX4\_1** (Sequence: CCTAGCAAC (+): 373)  
**RHOXF1** (Sequence: AGATCA (-): 434)  
**RHOXF1** (Sequence: TAAGCC (+): 345)  
**POU5F1** (Sequence: TTTGCAT (-): 737)  
**FOXO3\_mmu** (Sequence: TGTTTTGC (-): 734)  
**FOXO1** (Sequence: AAAAACAAG (-): 1030)
