# Supplementary material for: First characterization of PIWI-interacting RNA clusters in a cichlid fish with a B chromosome
Source: BMC Biol. 2022 Sep 21;20:204. doi: 10.1186/s12915-022-01403-2 (PMC9490952; doi:10.1186/s12915-022-01403-2)
Supplement: Supplementary file 1 — Additional file 1. Zipped folder with fasta and interactive html piRNA cluster information for the A. latifasciata genome. The nomenclature is as follows: number-pirna-cluster_sex_B-presence (f, female; m, male; 0b, without B chromosome; 1b, with B chromosome). [file 12915_2022_1403_MOESM1_ESM.zip › 115_m0b.html]

piRNA cluster 115\_m0b 54


Predicted piRNA cluster no. 115\_m0b
  

Show proTRAC run info
Hide proTRAC run info

/\  
                \_\_\_\_\_\_\_\_\_\_\_\_\_\_\_\_\_\_\_\_\_\_\_/\\_\_\_ /  \\_\_\_\_\_\_\_  
               I                      /  \  /    \      I  
               I     pro             /    \/      \     I  
               I        TRAC        /               \   I  
               I   \_\_\_\_\_\_\_\_\_\_\_\_\_\_\_\_/\_\_\_\_\_\_\_\_\_\_\_\_\_\_\_\_\_\\_ I  
               I   \              /                     I  
               I    \            /                      I  
               I     \  /\      /       V.2.4.2         I  
               I      \/  \    /                        I  
               I\_\_\_\_\_\_\_\_\_\_\_\  /\_\_\_\_\_\_\_\_\_\_\_\_\_\_\_\_\_\_\_\_\_\_\_\_\_I  
                            \/  
  
  
================================= proTRAC ====================================  
VERSION: .......... 2.4.2  
LAST MODIFIED: .... 11. May 2018  
  
Please cite:  
Rosenkranz D, Zischler H. proTRAC - a software for probabilistic piRNA cluster  
detection, visualization and analysis. 2012. BMC Bioinformatics 13:5.  
  
  
Contact:  
David Rosenkranz  
Institute of Organismic and Molecular Evolutionary Biology  
Dept. Anthropology, small RNA group  
Johannes Gutenberg University Mainz  
email: rosenkranz@uni-mainz.de  
  
You can find the latest proTRAC version at:  
http://sourceforge.net/projects/protrac/files  
http://www.smallRNAgroup-mainz.de/software  
==============================================================================  
  
PARAMETERS:  
Map file: ...............piwi-machos-0B.fa-collapse.map  
Genome file: ............../../../0B\_ala\_genome.fa  
RepeatMasker annotation: Alatifasciata-all0B-maryan-v2.fa\_corrected.out  
GeneSet:................./guest-storage/Data/annotation/Alatifasciata\_all0B\_maryan-v2\_out2017.gff  
  
Significant (p<=0.01) hit density will be calculated based  
on observed hit distribution.  
  
Sliding window size: ........................................ 5000 bp  
Sliding window increament: .................................. 1000 bp  
Normalize each hit by number of genomic hits: ............... yes  
Normalize each hit by number of sequence reads: ............. yes  
Normalize values (-> per million mapped reads): ............. yes  
Min. fraction of hits with 1T(U) or 10A: .................... 0.75  
Alternatively: Min. fraction of hits with 1T(U) and 10A: .... 0.5  
Min. fraction of hits with typical piRNA length: ............ 0.75  
Typical piRNA length: ....................................... 24-32 nt  
Min. size of a piRNA cluster: ............................... 1000 bp.  
Min. number of hits (absolute): ............................. 0  
Min. number of hits (normalized): ........................... 0  
Min. fraction of hits on the mainstrand: .................... 0.75  
Top fraction of mapped sequences (in terms of read counts): . 1%  
Top fraction accounts for max. n% of sequence reads: ........ 90%  
Min. fraction of hits on each arm of a bidirectional cluster: 0.05  
Output html file for each cluster: .......................... yes  
Output a summary table: ..................................... yes  
Output a FASTA file for each cluster (piRNA sequences): ..... yes  
Output a FASTA file comprising cluster sequences: ........... yes  
Output a GTF file for predicted piRNA clusters: ..............yes  
Search DNA motifs in clusters: .............................. yes  
Output flanking sequences: +/- .............................. 0 bp  
Output ~.pTi file: .......................................... no  
==============================================================================  
  
  
Genome size (without gaps): ............ 758543724 bp  
Gaps (N/X/-): .......................... 417479 bp  
Mapped reads: .......................... 24765598  
Non-identical sequences: ............... 6158275  
Genomic hits: .......................... 53103584  
Significant densitiy of mapped reads: .. 763.098963422187 reads/kb

Show proTRAC cluster info
Hide proTRAC cluster info

|  |  |
| --- | --- |
| Location | NODE\_291749\_length\_38467\_cov\_32.565315 |
| Coordinates | 32001-39167 |
| Size [bp] | 7167 |
| Sequence hit loci | 2472 |
| Mapped reads (normalized) | 7126.4 |
| Mapped reads (normalized) per kb | 994.3 |
| Normalized reads with 1T (1U) | 75.7% |
| Normalized reads with 10A | 48.2% |
| Normalized reads with length 24-32 nt | 98.8% |
| Normalized reads on the main strand(s) | 86.4% |
| Predicted directionality | mono:plus |

100%

0%

1T (1U)  
reads

10A reads

24-32 nt  
reads

reads on mainstrand

**Either the amount of reads with 1T (1U) OR 10A has to exceed 75% (set with option: -1Tor10A)  
Alternatively the amount of reads with 1T (1U) AND 10A has to exceed 50% (set with option: -1Tand10A)  
Minimum amount of reads with preferred size is 75% (set with option: -pisize)  
Minimum amount of reads on the main strand(s) is 75% (set with option: -clstrand)**

Show read coverage
Hide read coverage

WHAT DO I SEE HERE?  
This chart shows the location of mapped sequence reads within a predicted piRNA cluster. The color refers to the number of genomic hits produced by the sequence read in question. A dark red bar indicates that this sequence read produces many other hits elsewhere in the genome. Many adjacent red or yellow bars can indicate the presence of a multi-copy element such as transposons or rRNA genes. A dark green bar indicates that this sequence read maps uniquely to this locus.

1 hit

2-5 hits

6-10 hits

11-20 hits

21-50 hits

51-100 hits

> 100 hits

NODE\_291749\_length\_38467\_cov\_32.565315

32001

39167

Gene Set

RepeatMasker

Mapped  
Reads

25.56

plus strand

minus strand

25.56

Region: NODE\_291749\_length\_38467\_cov\_32.565315 1048-32008. Max. coverage (+): 0. Max coverage (-): 0

Region: NODE\_291749\_length\_38467\_cov\_32.565315 32009-32022. Max. coverage (+): 0. Max coverage (-): 0.05

Region: NODE\_291749\_length\_38467\_cov\_32.565315 32023-32036. Max. coverage (+): 0. Max coverage (-): 0

Region: NODE\_291749\_length\_38467\_cov\_32.565315 32037-32051. Max. coverage (+): 0.01. Max coverage (-): 0.01

Region: NODE\_291749\_length\_38467\_cov\_32.565315 32052-32065. Max. coverage (+): 0. Max coverage (-): 0

Region: NODE\_291749\_length\_38467\_cov\_32.565315 32066-32079. Max. coverage (+): 0. Max coverage (-): 0.05

Region: NODE\_291749\_length\_38467\_cov\_32.565315 32080-32094. Max. coverage (+): 0. Max coverage (-): 0

Region: NODE\_291749\_length\_38467\_cov\_32.565315 32095-32108. Max. coverage (+): 0. Max coverage (-): 0

Region: NODE\_291749\_length\_38467\_cov\_32.565315 32109-32122. Max. coverage (+): 0. Max coverage (-): 0

Region: NODE\_291749\_length\_38467\_cov\_32.565315 32123-32137. Max. coverage (+): 0.01. Max coverage (-): 0

Region: NODE\_291749\_length\_38467\_cov\_32.565315 32138-32151. Max. coverage (+): 0. Max coverage (-): 0.01

Region: NODE\_291749\_length\_38467\_cov\_32.565315 32152-32165. Max. coverage (+): 0. Max coverage (-): 0

Region: NODE\_291749\_length\_38467\_cov\_32.565315 32166-32180. Max. coverage (+): 0. Max coverage (-): 0

Region: NODE\_291749\_length\_38467\_cov\_32.565315 32181-32194. Max. coverage (+): 0. Max coverage (-): 0

Region: NODE\_291749\_length\_38467\_cov\_32.565315 32195-32208. Max. coverage (+): 0. Max coverage (-): 0

Region: NODE\_291749\_length\_38467\_cov\_32.565315 32209-32223. Max. coverage (+): 0.01. Max coverage (-): 0

Region: NODE\_291749\_length\_38467\_cov\_32.565315 32224-32237. Max. coverage (+): 0. Max coverage (-): 0

Region: NODE\_291749\_length\_38467\_cov\_32.565315 32238-32251. Max. coverage (+): 0. Max coverage (-): 0

Region: NODE\_291749\_length\_38467\_cov\_32.565315 32252-32266. Max. coverage (+): 0. Max coverage (-): 0

Region: NODE\_291749\_length\_38467\_cov\_32.565315 32267-32280. Max. coverage (+): 0. Max coverage (-): 0

Region: NODE\_291749\_length\_38467\_cov\_32.565315 32281-32294. Max. coverage (+): 0. Max coverage (-): 0

Region: NODE\_291749\_length\_38467\_cov\_32.565315 32295-32309. Max. coverage (+): 0.03. Max coverage (-): 0

Region: NODE\_291749\_length\_38467\_cov\_32.565315 32310-32323. Max. coverage (+): 0. Max coverage (-): 0

Region: NODE\_291749\_length\_38467\_cov\_32.565315 32324-32337. Max. coverage (+): 0. Max coverage (-): 0

Region: NODE\_291749\_length\_38467\_cov\_32.565315 32338-32352. Max. coverage (+): 0. Max coverage (-): 0

Region: NODE\_291749\_length\_38467\_cov\_32.565315 32353-32366. Max. coverage (+): 0. Max coverage (-): 0

Region: NODE\_291749\_length\_38467\_cov\_32.565315 32367-32380. Max. coverage (+): 0. Max coverage (-): 0

Region: NODE\_291749\_length\_38467\_cov\_32.565315 32381-32395. Max. coverage (+): 0.02. Max coverage (-): 0

Region: NODE\_291749\_length\_38467\_cov\_32.565315 32396-32409. Max. coverage (+): 0. Max coverage (-): 0.04

Region: NODE\_291749\_length\_38467\_cov\_32.565315 32410-32423. Max. coverage (+): 0. Max coverage (-): 0

Region: NODE\_291749\_length\_38467\_cov\_32.565315 32424-32438. Max. coverage (+): 0. Max coverage (-): 0.01

Region: NODE\_291749\_length\_38467\_cov\_32.565315 32439-32452. Max. coverage (+): 0.01. Max coverage (-): 0.02

Region: NODE\_291749\_length\_38467\_cov\_32.565315 32453-32466. Max. coverage (+): 0. Max coverage (-): 0.04

Region: NODE\_291749\_length\_38467\_cov\_32.565315 32467-32481. Max. coverage (+): 0. Max coverage (-): 0

Region: NODE\_291749\_length\_38467\_cov\_32.565315 32482-32495. Max. coverage (+): 0. Max coverage (-): 0

Region: NODE\_291749\_length\_38467\_cov\_32.565315 32496-32509. Max. coverage (+): 0. Max coverage (-): 0.03

Region: NODE\_291749\_length\_38467\_cov\_32.565315 32510-32524. Max. coverage (+): 0. Max coverage (-): 0

Region: NODE\_291749\_length\_38467\_cov\_32.565315 32525-32538. Max. coverage (+): 0. Max coverage (-): 0

Region: NODE\_291749\_length\_38467\_cov\_32.565315 32539-32552. Max. coverage (+): 0. Max coverage (-): 0

Region: NODE\_291749\_length\_38467\_cov\_32.565315 32553-32567. Max. coverage (+): 0. Max coverage (-): 0

Region: NODE\_291749\_length\_38467\_cov\_32.565315 32568-32581. Max. coverage (+): 0. Max coverage (-): 0.01

Region: NODE\_291749\_length\_38467\_cov\_32.565315 32582-32595. Max. coverage (+): 0.01. Max coverage (-): 0

Region: NODE\_291749\_length\_38467\_cov\_32.565315 32596-32610. Max. coverage (+): 0.03. Max coverage (-): 0

Region: NODE\_291749\_length\_38467\_cov\_32.565315 32611-32624. Max. coverage (+): 0. Max coverage (-): 0

Region: NODE\_291749\_length\_38467\_cov\_32.565315 32625-32638. Max. coverage (+): 0. Max coverage (-): 0

Region: NODE\_291749\_length\_38467\_cov\_32.565315 32639-32653. Max. coverage (+): 0. Max coverage (-): 0

Region: NODE\_291749\_length\_38467\_cov\_32.565315 32654-32667. Max. coverage (+): 0. Max coverage (-): 0

Region: NODE\_291749\_length\_38467\_cov\_32.565315 32668-32681. Max. coverage (+): 0. Max coverage (-): 0

Region: NODE\_291749\_length\_38467\_cov\_32.565315 32682-32696. Max. coverage (+): 0. Max coverage (-): 0

Region: NODE\_291749\_length\_38467\_cov\_32.565315 32697-32710. Max. coverage (+): 0. Max coverage (-): 0

Region: NODE\_291749\_length\_38467\_cov\_32.565315 32711-32724. Max. coverage (+): 0. Max coverage (-): 0

Region: NODE\_291749\_length\_38467\_cov\_32.565315 32725-32739. Max. coverage (+): 0. Max coverage (-): 0

Region: NODE\_291749\_length\_38467\_cov\_32.565315 32740-32753. Max. coverage (+): 0. Max coverage (-): 0

Region: NODE\_291749\_length\_38467\_cov\_32.565315 32754-32767. Max. coverage (+): 0. Max coverage (-): 0

Region: NODE\_291749\_length\_38467\_cov\_32.565315 32768-32782. Max. coverage (+): 0. Max coverage (-): 0.04

Region: NODE\_291749\_length\_38467\_cov\_32.565315 32783-32796. Max. coverage (+): 0.01. Max coverage (-): 0.04

Region: NODE\_291749\_length\_38467\_cov\_32.565315 32797-32810. Max. coverage (+): 0. Max coverage (-): 0

Region: NODE\_291749\_length\_38467\_cov\_32.565315 32811-32825. Max. coverage (+): 0. Max coverage (-): 0.37

Region: NODE\_291749\_length\_38467\_cov\_32.565315 32826-32839. Max. coverage (+): 0. Max coverage (-): 0.2

Region: NODE\_291749\_length\_38467\_cov\_32.565315 32840-32853. Max. coverage (+): 0. Max coverage (-): 0

Region: NODE\_291749\_length\_38467\_cov\_32.565315 32854-32868. Max. coverage (+): 0. Max coverage (-): 0

Region: NODE\_291749\_length\_38467\_cov\_32.565315 32869-32882. Max. coverage (+): 0.03. Max coverage (-): 0

Region: NODE\_291749\_length\_38467\_cov\_32.565315 32883-32896. Max. coverage (+): 0.03. Max coverage (-): 0.02

Region: NODE\_291749\_length\_38467\_cov\_32.565315 32897-32911. Max. coverage (+): 0. Max coverage (-): 0.02

Region: NODE\_291749\_length\_38467\_cov\_32.565315 32912-32925. Max. coverage (+): 0.01. Max coverage (-): 0

Region: NODE\_291749\_length\_38467\_cov\_32.565315 32926-32939. Max. coverage (+): 0. Max coverage (-): 0.01

Region: NODE\_291749\_length\_38467\_cov\_32.565315 32940-32954. Max. coverage (+): 0. Max coverage (-): 0.4

Region: NODE\_291749\_length\_38467\_cov\_32.565315 32955-32968. Max. coverage (+): 0. Max coverage (-): 0

Region: NODE\_291749\_length\_38467\_cov\_32.565315 32969-32982. Max. coverage (+): 0. Max coverage (-): 0

Region: NODE\_291749\_length\_38467\_cov\_32.565315 32983-32997. Max. coverage (+): 0. Max coverage (-): 0

Region: NODE\_291749\_length\_38467\_cov\_32.565315 32998-33011. Max. coverage (+): 0. Max coverage (-): 0

Region: NODE\_291749\_length\_38467\_cov\_32.565315 33012-33025. Max. coverage (+): 0. Max coverage (-): 0

Region: NODE\_291749\_length\_38467\_cov\_32.565315 33026-33040. Max. coverage (+): 0. Max coverage (-): 0

Region: NODE\_291749\_length\_38467\_cov\_32.565315 33041-33054. Max. coverage (+): 0. Max coverage (-): 0

Region: NODE\_291749\_length\_38467\_cov\_32.565315 33055-33068. Max. coverage (+): 0. Max coverage (-): 0.04

Region: NODE\_291749\_length\_38467\_cov\_32.565315 33069-33083. Max. coverage (+): 0. Max coverage (-): 0

Region: NODE\_291749\_length\_38467\_cov\_32.565315 33084-33097. Max. coverage (+): 0. Max coverage (-): 0

Region: NODE\_291749\_length\_38467\_cov\_32.565315 33098-33111. Max. coverage (+): 0. Max coverage (-): 0

Region: NODE\_291749\_length\_38467\_cov\_32.565315 33112-33126. Max. coverage (+): 0.01. Max coverage (-): 0

Region: NODE\_291749\_length\_38467\_cov\_32.565315 33127-33140. Max. coverage (+): 0.01. Max coverage (-): 0.01

Region: NODE\_291749\_length\_38467\_cov\_32.565315 33141-33154. Max. coverage (+): 0. Max coverage (-): 0

Region: NODE\_291749\_length\_38467\_cov\_32.565315 33155-33169. Max. coverage (+): 0. Max coverage (-): 0

Region: NODE\_291749\_length\_38467\_cov\_32.565315 33170-33183. Max. coverage (+): 0. Max coverage (-): 0

Region: NODE\_291749\_length\_38467\_cov\_32.565315 33184-33197. Max. coverage (+): 0. Max coverage (-): 0.02

Region: NODE\_291749\_length\_38467\_cov\_32.565315 33198-33212. Max. coverage (+): 0.01. Max coverage (-): 0

Region: NODE\_291749\_length\_38467\_cov\_32.565315 33213-33226. Max. coverage (+): 0.12. Max coverage (-): 0

Region: NODE\_291749\_length\_38467\_cov\_32.565315 33227-33240. Max. coverage (+): 0.12. Max coverage (-): 0

Region: NODE\_291749\_length\_38467\_cov\_32.565315 33241-33255. Max. coverage (+): 0. Max coverage (-): 0.04

Region: NODE\_291749\_length\_38467\_cov\_32.565315 33256-33269. Max. coverage (+): 0.04. Max coverage (-): 0.04

Region: NODE\_291749\_length\_38467\_cov\_32.565315 33270-33283. Max. coverage (+): 0.08. Max coverage (-): 0

Region: NODE\_291749\_length\_38467\_cov\_32.565315 33284-33298. Max. coverage (+): 0. Max coverage (-): 0

Region: NODE\_291749\_length\_38467\_cov\_32.565315 33299-33312. Max. coverage (+): 0. Max coverage (-): 0

Region: NODE\_291749\_length\_38467\_cov\_32.565315 33313-33326. Max. coverage (+): 0.04. Max coverage (-): 0

Region: NODE\_291749\_length\_38467\_cov\_32.565315 33327-33341. Max. coverage (+): 0. Max coverage (-): 0

Region: NODE\_291749\_length\_38467\_cov\_32.565315 33342-33355. Max. coverage (+): 0. Max coverage (-): 0

Region: NODE\_291749\_length\_38467\_cov\_32.565315 33356-33369. Max. coverage (+): 0. Max coverage (-): 0

Region: NODE\_291749\_length\_38467\_cov\_32.565315 33370-33384. Max. coverage (+): 0. Max coverage (-): 0

Region: NODE\_291749\_length\_38467\_cov\_32.565315 33385-33398. Max. coverage (+): 0. Max coverage (-): 0

Region: NODE\_291749\_length\_38467\_cov\_32.565315 33399-33412. Max. coverage (+): 0. Max coverage (-): 0.04

Region: NODE\_291749\_length\_38467\_cov\_32.565315 33413-33427. Max. coverage (+): 0. Max coverage (-): 0.08

Region: NODE\_291749\_length\_38467\_cov\_32.565315 33428-33441. Max. coverage (+): 0. Max coverage (-): 0.04

Region: NODE\_291749\_length\_38467\_cov\_32.565315 33442-33455. Max. coverage (+): 0.08. Max coverage (-): 0

Region: NODE\_291749\_length\_38467\_cov\_32.565315 33456-33470. Max. coverage (+): 0.04. Max coverage (-): 0

Region: NODE\_291749\_length\_38467\_cov\_32.565315 33471-33484. Max. coverage (+): 0. Max coverage (-): 0

Region: NODE\_291749\_length\_38467\_cov\_32.565315 33485-33498. Max. coverage (+): 0.4. Max coverage (-): 0.04

Region: NODE\_291749\_length\_38467\_cov\_32.565315 33499-33513. Max. coverage (+): 0.04. Max coverage (-): 0.04

Region: NODE\_291749\_length\_38467\_cov\_32.565315 33514-33527. Max. coverage (+): 0. Max coverage (-): 0.04

Region: NODE\_291749\_length\_38467\_cov\_32.565315 33528-33541. Max. coverage (+): 0.04. Max coverage (-): 0.04

Region: NODE\_291749\_length\_38467\_cov\_32.565315 33542-33556. Max. coverage (+): 0.04. Max coverage (-): 0.04

Region: NODE\_291749\_length\_38467\_cov\_32.565315 33557-33570. Max. coverage (+): 0.08. Max coverage (-): 0.08

Region: NODE\_291749\_length\_38467\_cov\_32.565315 33571-33584. Max. coverage (+): 0.04. Max coverage (-): 0.04

Region: NODE\_291749\_length\_38467\_cov\_32.565315 33585-33599. Max. coverage (+): 0.04. Max coverage (-): 0

Region: NODE\_291749\_length\_38467\_cov\_32.565315 33600-33613. Max. coverage (+): 0.16. Max coverage (-): 0.2

Region: NODE\_291749\_length\_38467\_cov\_32.565315 33614-33627. Max. coverage (+): 0.08. Max coverage (-): 0.04

Region: NODE\_291749\_length\_38467\_cov\_32.565315 33628-33642. Max. coverage (+): 0.08. Max coverage (-): 0.04

Region: NODE\_291749\_length\_38467\_cov\_32.565315 33643-33656. Max. coverage (+): 0. Max coverage (-): 0.08

Region: NODE\_291749\_length\_38467\_cov\_32.565315 33657-33670. Max. coverage (+): 0.04. Max coverage (-): 0.04

Region: NODE\_291749\_length\_38467\_cov\_32.565315 33671-33685. Max. coverage (+): 0.77. Max coverage (-): 0.04

Region: NODE\_291749\_length\_38467\_cov\_32.565315 33686-33699. Max. coverage (+): 0.77. Max coverage (-): 0

Region: NODE\_291749\_length\_38467\_cov\_32.565315 33700-33713. Max. coverage (+): 0. Max coverage (-): 0.04

Region: NODE\_291749\_length\_38467\_cov\_32.565315 33714-33728. Max. coverage (+): 0.12. Max coverage (-): 0.24

Region: NODE\_291749\_length\_38467\_cov\_32.565315 33729-33742. Max. coverage (+): 0. Max coverage (-): 0.57

Region: NODE\_291749\_length\_38467\_cov\_32.565315 33743-33756. Max. coverage (+): 0.44. Max coverage (-): 0

Region: NODE\_291749\_length\_38467\_cov\_32.565315 33757-33771. Max. coverage (+): 0. Max coverage (-): 0.04

Region: NODE\_291749\_length\_38467\_cov\_32.565315 33772-33785. Max. coverage (+): 0.04. Max coverage (-): 0.04

Region: NODE\_291749\_length\_38467\_cov\_32.565315 33786-33799. Max. coverage (+): 0.04. Max coverage (-): 1.09

Region: NODE\_291749\_length\_38467\_cov\_32.565315 33800-33814. Max. coverage (+): 5.37. Max coverage (-): 0.57

Region: NODE\_291749\_length\_38467\_cov\_32.565315 33815-33828. Max. coverage (+): 2.91. Max coverage (-): 0.04

Region: NODE\_291749\_length\_38467\_cov\_32.565315 33829-33842. Max. coverage (+): 0. Max coverage (-): 0.04

Region: NODE\_291749\_length\_38467\_cov\_32.565315 33843-33857. Max. coverage (+): 0. Max coverage (-): 0.44

Region: NODE\_291749\_length\_38467\_cov\_32.565315 33858-33871. Max. coverage (+): 0. Max coverage (-): 0

Region: NODE\_291749\_length\_38467\_cov\_32.565315 33872-33885. Max. coverage (+): 0. Max coverage (-): 0.12

Region: NODE\_291749\_length\_38467\_cov\_32.565315 33886-33900. Max. coverage (+): 0.08. Max coverage (-): 0.04

Region: NODE\_291749\_length\_38467\_cov\_32.565315 33901-33914. Max. coverage (+): 0.08. Max coverage (-): 0.08

Region: NODE\_291749\_length\_38467\_cov\_32.565315 33915-33928. Max. coverage (+): 0.04. Max coverage (-): 0.08

Region: NODE\_291749\_length\_38467\_cov\_32.565315 33929-33943. Max. coverage (+): 0.81. Max coverage (-): 0

Region: NODE\_291749\_length\_38467\_cov\_32.565315 33944-33957. Max. coverage (+): 0.08. Max coverage (-): 0.04

Region: NODE\_291749\_length\_38467\_cov\_32.565315 33958-33971. Max. coverage (+): 0. Max coverage (-): 0.24

Region: NODE\_291749\_length\_38467\_cov\_32.565315 33972-33986. Max. coverage (+): 0. Max coverage (-): 0

Region: NODE\_291749\_length\_38467\_cov\_32.565315 33987-34000. Max. coverage (+): 0. Max coverage (-): 0

Region: NODE\_291749\_length\_38467\_cov\_32.565315 34001-34014. Max. coverage (+): 0. Max coverage (-): 0.04

Region: NODE\_291749\_length\_38467\_cov\_32.565315 34015-34029. Max. coverage (+): 0.32. Max coverage (-): 0.16

Region: NODE\_291749\_length\_38467\_cov\_32.565315 34030-34043. Max. coverage (+): 0.12. Max coverage (-): 0

Region: NODE\_291749\_length\_38467\_cov\_32.565315 34044-34057. Max. coverage (+): 0. Max coverage (-): 0

Region: NODE\_291749\_length\_38467\_cov\_32.565315 34058-34072. Max. coverage (+): 0. Max coverage (-): 0.04

Region: NODE\_291749\_length\_38467\_cov\_32.565315 34073-34086. Max. coverage (+): 0.04. Max coverage (-): 0

Region: NODE\_291749\_length\_38467\_cov\_32.565315 34087-34100. Max. coverage (+): 0.04. Max coverage (-): 0.04

Region: NODE\_291749\_length\_38467\_cov\_32.565315 34101-34115. Max. coverage (+): 0.24. Max coverage (-): 0.04

Region: NODE\_291749\_length\_38467\_cov\_32.565315 34116-34129. Max. coverage (+): 0.32. Max coverage (-): 0

Region: NODE\_291749\_length\_38467\_cov\_32.565315 34130-34143. Max. coverage (+): 0.04. Max coverage (-): 0.12

Region: NODE\_291749\_length\_38467\_cov\_32.565315 34144-34158. Max. coverage (+): 0.04. Max coverage (-): 0.4

Region: NODE\_291749\_length\_38467\_cov\_32.565315 34159-34172. Max. coverage (+): 0.04. Max coverage (-): 0

Region: NODE\_291749\_length\_38467\_cov\_32.565315 34173-34186. Max. coverage (+): 0.04. Max coverage (-): 0

Region: NODE\_291749\_length\_38467\_cov\_32.565315 34187-34201. Max. coverage (+): 0.08. Max coverage (-): 0

Region: NODE\_291749\_length\_38467\_cov\_32.565315 34202-34215. Max. coverage (+): 0. Max coverage (-): 0

Region: NODE\_291749\_length\_38467\_cov\_32.565315 34216-34229. Max. coverage (+): 0.04. Max coverage (-): 0

Region: NODE\_291749\_length\_38467\_cov\_32.565315 34230-34244. Max. coverage (+): 0. Max coverage (-): 0

Region: NODE\_291749\_length\_38467\_cov\_32.565315 34245-34258. Max. coverage (+): 0. Max coverage (-): 0.24

Region: NODE\_291749\_length\_38467\_cov\_32.565315 34259-34272. Max. coverage (+): 0. Max coverage (-): 0.02

Region: NODE\_291749\_length\_38467\_cov\_32.565315 34273-34287. Max. coverage (+): 0. Max coverage (-): 0

Region: NODE\_291749\_length\_38467\_cov\_32.565315 34288-34301. Max. coverage (+): 0.04. Max coverage (-): 0.04

Region: NODE\_291749\_length\_38467\_cov\_32.565315 34302-34315. Max. coverage (+): 0.04. Max coverage (-): 0.04

Region: NODE\_291749\_length\_38467\_cov\_32.565315 34316-34330. Max. coverage (+): 0. Max coverage (-): 0

Region: NODE\_291749\_length\_38467\_cov\_32.565315 34331-34344. Max. coverage (+): 0. Max coverage (-): 0

Region: NODE\_291749\_length\_38467\_cov\_32.565315 34345-34358. Max. coverage (+): 0.04. Max coverage (-): 0

Region: NODE\_291749\_length\_38467\_cov\_32.565315 34359-34373. Max. coverage (+): 0.77. Max coverage (-): 0.04

Region: NODE\_291749\_length\_38467\_cov\_32.565315 34374-34387. Max. coverage (+): 0.85. Max coverage (-): 0

Region: NODE\_291749\_length\_38467\_cov\_32.565315 34388-34401. Max. coverage (+): 0.2. Max coverage (-): 0

Region: NODE\_291749\_length\_38467\_cov\_32.565315 34402-34416. Max. coverage (+): 0.16. Max coverage (-): 0.04

Region: NODE\_291749\_length\_38467\_cov\_32.565315 34417-34430. Max. coverage (+): 0.22. Max coverage (-): 0

Region: NODE\_291749\_length\_38467\_cov\_32.565315 34431-34444. Max. coverage (+): 0.26. Max coverage (-): 0.06

Region: NODE\_291749\_length\_38467\_cov\_32.565315 34445-34459. Max. coverage (+): 0.16. Max coverage (-): 0.06

Region: NODE\_291749\_length\_38467\_cov\_32.565315 34460-34473. Max. coverage (+): 0.12. Max coverage (-): 0

Region: NODE\_291749\_length\_38467\_cov\_32.565315 34474-34487. Max. coverage (+): 0.04. Max coverage (-): 0

Region: NODE\_291749\_length\_38467\_cov\_32.565315 34488-34502. Max. coverage (+): 0.14. Max coverage (-): 0.2

Region: NODE\_291749\_length\_38467\_cov\_32.565315 34503-34516. Max. coverage (+): 0.16. Max coverage (-): 0.1

Region: NODE\_291749\_length\_38467\_cov\_32.565315 34517-34530. Max. coverage (+): 0.02. Max coverage (-): 0.06

Region: NODE\_291749\_length\_38467\_cov\_32.565315 34531-34545. Max. coverage (+): 0.02. Max coverage (-): 0.16

Region: NODE\_291749\_length\_38467\_cov\_32.565315 34546-34559. Max. coverage (+): 0.08. Max coverage (-): 0

Region: NODE\_291749\_length\_38467\_cov\_32.565315 34560-34573. Max. coverage (+): 0.04. Max coverage (-): 0.12

Region: NODE\_291749\_length\_38467\_cov\_32.565315 34574-34588. Max. coverage (+): 0.28. Max coverage (-): 0.08

Region: NODE\_291749\_length\_38467\_cov\_32.565315 34589-34602. Max. coverage (+): 0.57. Max coverage (-): 0

Region: NODE\_291749\_length\_38467\_cov\_32.565315 34603-34616. Max. coverage (+): 0. Max coverage (-): 0.04

Region: NODE\_291749\_length\_38467\_cov\_32.565315 34617-34631. Max. coverage (+): 0. Max coverage (-): 0

Region: NODE\_291749\_length\_38467\_cov\_32.565315 34632-34645. Max. coverage (+): 0. Max coverage (-): 0.04

Region: NODE\_291749\_length\_38467\_cov\_32.565315 34646-34659. Max. coverage (+): 0.36. Max coverage (-): 0.16

Region: NODE\_291749\_length\_38467\_cov\_32.565315 34660-34674. Max. coverage (+): 0.73. Max coverage (-): 0.2

Region: NODE\_291749\_length\_38467\_cov\_32.565315 34675-34688. Max. coverage (+): 0. Max coverage (-): 0.2

Region: NODE\_291749\_length\_38467\_cov\_32.565315 34689-34702. Max. coverage (+): 0.04. Max coverage (-): 0

Region: NODE\_291749\_length\_38467\_cov\_32.565315 34703-34717. Max. coverage (+): 0. Max coverage (-): 0

Region: NODE\_291749\_length\_38467\_cov\_32.565315 34718-34731. Max. coverage (+): 0. Max coverage (-): 0

Region: NODE\_291749\_length\_38467\_cov\_32.565315 34732-34745. Max. coverage (+): 0. Max coverage (-): 0

Region: NODE\_291749\_length\_38467\_cov\_32.565315 34746-34760. Max. coverage (+): 0.12. Max coverage (-): 0

Region: NODE\_291749\_length\_38467\_cov\_32.565315 34761-34774. Max. coverage (+): 0.12. Max coverage (-): 0

Region: NODE\_291749\_length\_38467\_cov\_32.565315 34775-34788. Max. coverage (+): 0.04. Max coverage (-): 0.04

Region: NODE\_291749\_length\_38467\_cov\_32.565315 34789-34803. Max. coverage (+): 0.28. Max coverage (-): 0.04

Region: NODE\_291749\_length\_38467\_cov\_32.565315 34804-34817. Max. coverage (+): 0.2. Max coverage (-): 0

Region: NODE\_291749\_length\_38467\_cov\_32.565315 34818-34831. Max. coverage (+): 0.08. Max coverage (-): 0.08

Region: NODE\_291749\_length\_38467\_cov\_32.565315 34832-34846. Max. coverage (+): 1.9. Max coverage (-): 0.04

Region: NODE\_291749\_length\_38467\_cov\_32.565315 34847-34860. Max. coverage (+): 2.5. Max coverage (-): 0.08

Region: NODE\_291749\_length\_38467\_cov\_32.565315 34861-34874. Max. coverage (+): 0.24. Max coverage (-): 0

Region: NODE\_291749\_length\_38467\_cov\_32.565315 34875-34889. Max. coverage (+): 0.04. Max coverage (-): 0.08

Region: NODE\_291749\_length\_38467\_cov\_32.565315 34890-34903. Max. coverage (+): 1.57. Max coverage (-): 0

Region: NODE\_291749\_length\_38467\_cov\_32.565315 34904-34917. Max. coverage (+): 0.32. Max coverage (-): 0

Region: NODE\_291749\_length\_38467\_cov\_32.565315 34918-34932. Max. coverage (+): 0.12. Max coverage (-): 0.04

Region: NODE\_291749\_length\_38467\_cov\_32.565315 34933-34946. Max. coverage (+): 2.38. Max coverage (-): 0

Region: NODE\_291749\_length\_38467\_cov\_32.565315 34947-34960. Max. coverage (+): 0.32. Max coverage (-): 0

Region: NODE\_291749\_length\_38467\_cov\_32.565315 34961-34975. Max. coverage (+): 0.04. Max coverage (-): 0.28

Region: NODE\_291749\_length\_38467\_cov\_32.565315 34976-34989. Max. coverage (+): 0.12. Max coverage (-): 0.04

Region: NODE\_291749\_length\_38467\_cov\_32.565315 34990-35003. Max. coverage (+): 0.2. Max coverage (-): 0.04

Region: NODE\_291749\_length\_38467\_cov\_32.565315 35004-35018. Max. coverage (+): 0. Max coverage (-): 0

Region: NODE\_291749\_length\_38467\_cov\_32.565315 35019-35032. Max. coverage (+): 0. Max coverage (-): 2.58

Region: NODE\_291749\_length\_38467\_cov\_32.565315 35033-35046. Max. coverage (+): 4.68. Max coverage (-): 0

Region: NODE\_291749\_length\_38467\_cov\_32.565315 35047-35061. Max. coverage (+): 0.04. Max coverage (-): 0.04

Region: NODE\_291749\_length\_38467\_cov\_32.565315 35062-35075. Max. coverage (+): 1.13. Max coverage (-): 0.08

Region: NODE\_291749\_length\_38467\_cov\_32.565315 35076-35089. Max. coverage (+): 1.29. Max coverage (-): 0.28

Region: NODE\_291749\_length\_38467\_cov\_32.565315 35090-35104. Max. coverage (+): 1.62. Max coverage (-): 0

Region: NODE\_291749\_length\_38467\_cov\_32.565315 35105-35118. Max. coverage (+): 0. Max coverage (-): 0.36

Region: NODE\_291749\_length\_38467\_cov\_32.565315 35119-35132. Max. coverage (+): 3.35. Max coverage (-): 0.04

Region: NODE\_291749\_length\_38467\_cov\_32.565315 35133-35147. Max. coverage (+): 1.13. Max coverage (-): 0.2

Region: NODE\_291749\_length\_38467\_cov\_32.565315 35148-35161. Max. coverage (+): 1.09. Max coverage (-): 0

Region: NODE\_291749\_length\_38467\_cov\_32.565315 35162-35175. Max. coverage (+): 0.08. Max coverage (-): 0

Region: NODE\_291749\_length\_38467\_cov\_32.565315 35176-35190. Max. coverage (+): 0.69. Max coverage (-): 0.08

Region: NODE\_291749\_length\_38467\_cov\_32.565315 35191-35204. Max. coverage (+): 0.93. Max coverage (-): 0.08

Region: NODE\_291749\_length\_38467\_cov\_32.565315 35205-35218. Max. coverage (+): 0.48. Max coverage (-): 0.04

Region: NODE\_291749\_length\_38467\_cov\_32.565315 35219-35233. Max. coverage (+): 5.57. Max coverage (-): 0.04

Region: NODE\_291749\_length\_38467\_cov\_32.565315 35234-35247. Max. coverage (+): 0.08. Max coverage (-): 0.44

Region: NODE\_291749\_length\_38467\_cov\_32.565315 35248-35261. Max. coverage (+): 0.08. Max coverage (-): 0.08

Region: NODE\_291749\_length\_38467\_cov\_32.565315 35262-35276. Max. coverage (+): 0.04. Max coverage (-): 0

Region: NODE\_291749\_length\_38467\_cov\_32.565315 35277-35290. Max. coverage (+): 0.44. Max coverage (-): 0

Region: NODE\_291749\_length\_38467\_cov\_32.565315 35291-35304. Max. coverage (+): 0.44. Max coverage (-): 0

Region: NODE\_291749\_length\_38467\_cov\_32.565315 35305-35319. Max. coverage (+): 1.09. Max coverage (-): 0

Region: NODE\_291749\_length\_38467\_cov\_32.565315 35320-35333. Max. coverage (+): 0.52. Max coverage (-): 0

Region: NODE\_291749\_length\_38467\_cov\_32.565315 35334-35347. Max. coverage (+): 0.04. Max coverage (-): 0.04

Region: NODE\_291749\_length\_38467\_cov\_32.565315 35348-35362. Max. coverage (+): 0. Max coverage (-): 0

Region: NODE\_291749\_length\_38467\_cov\_32.565315 35363-35376. Max. coverage (+): 0.2. Max coverage (-): 0

Region: NODE\_291749\_length\_38467\_cov\_32.565315 35377-35390. Max. coverage (+): 0.57. Max coverage (-): 0.16

Region: NODE\_291749\_length\_38467\_cov\_32.565315 35391-35405. Max. coverage (+): 0.44. Max coverage (-): 1.21

Region: NODE\_291749\_length\_38467\_cov\_32.565315 35406-35419. Max. coverage (+): 0.12. Max coverage (-): 1.01

Region: NODE\_291749\_length\_38467\_cov\_32.565315 35420-35433. Max. coverage (+): 0.04. Max coverage (-): 0

Region: NODE\_291749\_length\_38467\_cov\_32.565315 35434-35448. Max. coverage (+): 0.08. Max coverage (-): 0.12

Region: NODE\_291749\_length\_38467\_cov\_32.565315 35449-35462. Max. coverage (+): 1.05. Max coverage (-): 0.2

Region: NODE\_291749\_length\_38467\_cov\_32.565315 35463-35476. Max. coverage (+): 0.81. Max coverage (-): 0.08

Region: NODE\_291749\_length\_38467\_cov\_32.565315 35477-35491. Max. coverage (+): 0.48. Max coverage (-): 0

Region: NODE\_291749\_length\_38467\_cov\_32.565315 35492-35505. Max. coverage (+): 0.08. Max coverage (-): 0

Region: NODE\_291749\_length\_38467\_cov\_32.565315 35506-35519. Max. coverage (+): 0.12. Max coverage (-): 0

Region: NODE\_291749\_length\_38467\_cov\_32.565315 35520-35534. Max. coverage (+): 0. Max coverage (-): 0.12

Region: NODE\_291749\_length\_38467\_cov\_32.565315 35535-35548. Max. coverage (+): 0.2. Max coverage (-): 0

Region: NODE\_291749\_length\_38467\_cov\_32.565315 35549-35562. Max. coverage (+): 0.08. Max coverage (-): 0.24

Region: NODE\_291749\_length\_38467\_cov\_32.565315 35563-35577. Max. coverage (+): 0. Max coverage (-): 0

Region: NODE\_291749\_length\_38467\_cov\_32.565315 35578-35591. Max. coverage (+): 0. Max coverage (-): 0

Region: NODE\_291749\_length\_38467\_cov\_32.565315 35592-35606. Max. coverage (+): 0. Max coverage (-): 0

Region: NODE\_291749\_length\_38467\_cov\_32.565315 35607-35620. Max. coverage (+): 0. Max coverage (-): 0

Region: NODE\_291749\_length\_38467\_cov\_32.565315 35621-35634. Max. coverage (+): 0.04. Max coverage (-): 0

Region: NODE\_291749\_length\_38467\_cov\_32.565315 35635-35649. Max. coverage (+): 0.12. Max coverage (-): 0

Region: NODE\_291749\_length\_38467\_cov\_32.565315 35650-35663. Max. coverage (+): 0.2. Max coverage (-): 0.2

Region: NODE\_291749\_length\_38467\_cov\_32.565315 35664-35677. Max. coverage (+): 1.01. Max coverage (-): 0.04

Region: NODE\_291749\_length\_38467\_cov\_32.565315 35678-35692. Max. coverage (+): 0.97. Max coverage (-): 0

Region: NODE\_291749\_length\_38467\_cov\_32.565315 35693-35706. Max. coverage (+): 0. Max coverage (-): 0.04

Region: NODE\_291749\_length\_38467\_cov\_32.565315 35707-35720. Max. coverage (+): 0. Max coverage (-): 0.04

Region: NODE\_291749\_length\_38467\_cov\_32.565315 35721-35735. Max. coverage (+): 0.08. Max coverage (-): 0

Region: NODE\_291749\_length\_38467\_cov\_32.565315 35736-35749. Max. coverage (+): 0.08. Max coverage (-): 0

Region: NODE\_291749\_length\_38467\_cov\_32.565315 35750-35763. Max. coverage (+): 0. Max coverage (-): 0

Region: NODE\_291749\_length\_38467\_cov\_32.565315 35764-35778. Max. coverage (+): 0. Max coverage (-): 0

Region: NODE\_291749\_length\_38467\_cov\_32.565315 35779-35792. Max. coverage (+): 0. Max coverage (-): 0.04

Region: NODE\_291749\_length\_38467\_cov\_32.565315 35793-35806. Max. coverage (+): 0. Max coverage (-): 0.16

Region: NODE\_291749\_length\_38467\_cov\_32.565315 35807-35821. Max. coverage (+): 1.45. Max coverage (-): 0.04

Region: NODE\_291749\_length\_38467\_cov\_32.565315 35822-35835. Max. coverage (+): 0.16. Max coverage (-): 0

Region: NODE\_291749\_length\_38467\_cov\_32.565315 35836-35849. Max. coverage (+): 0. Max coverage (-): 0

Region: NODE\_291749\_length\_38467\_cov\_32.565315 35850-35864. Max. coverage (+): 0.08. Max coverage (-): 0.2

Region: NODE\_291749\_length\_38467\_cov\_32.565315 35865-35878. Max. coverage (+): 0.16. Max coverage (-): 0.2

Region: NODE\_291749\_length\_38467\_cov\_32.565315 35879-35892. Max. coverage (+): 25.56. Max coverage (-): 0.04

Region: NODE\_291749\_length\_38467\_cov\_32.565315 35893-35907. Max. coverage (+): 0.16. Max coverage (-): 0.24

Region: NODE\_291749\_length\_38467\_cov\_32.565315 35908-35921. Max. coverage (+): 1.29. Max coverage (-): 0.12

Region: NODE\_291749\_length\_38467\_cov\_32.565315 35922-35935. Max. coverage (+): 0.4. Max coverage (-): 0.32

Region: NODE\_291749\_length\_38467\_cov\_32.565315 35936-35950. Max. coverage (+): 0.32. Max coverage (-): 0.32

Region: NODE\_291749\_length\_38467\_cov\_32.565315 35951-35964. Max. coverage (+): 2.54. Max coverage (-): 0.04

Region: NODE\_291749\_length\_38467\_cov\_32.565315 35965-35978. Max. coverage (+): 0.2. Max coverage (-): 0.04

Region: NODE\_291749\_length\_38467\_cov\_32.565315 35979-35993. Max. coverage (+): 0.04. Max coverage (-): 0.52

Region: NODE\_291749\_length\_38467\_cov\_32.565315 35994-36007. Max. coverage (+): 0.04. Max coverage (-): 0.12

Region: NODE\_291749\_length\_38467\_cov\_32.565315 36008-36021. Max. coverage (+): 0.08. Max coverage (-): 0

Region: NODE\_291749\_length\_38467\_cov\_32.565315 36022-36036. Max. coverage (+): 0.12. Max coverage (-): 0.4

Region: NODE\_291749\_length\_38467\_cov\_32.565315 36037-36050. Max. coverage (+): 0.32. Max coverage (-): 0.08

Region: NODE\_291749\_length\_38467\_cov\_32.565315 36051-36064. Max. coverage (+): 0.04. Max coverage (-): 0

Region: NODE\_291749\_length\_38467\_cov\_32.565315 36065-36079. Max. coverage (+): 0.73. Max coverage (-): 0

Region: NODE\_291749\_length\_38467\_cov\_32.565315 36080-36093. Max. coverage (+): 1.53. Max coverage (-): 0.04

Region: NODE\_291749\_length\_38467\_cov\_32.565315 36094-36107. Max. coverage (+): 0.2. Max coverage (-): 0.48

Region: NODE\_291749\_length\_38467\_cov\_32.565315 36108-36122. Max. coverage (+): 0.44. Max coverage (-): 0.04

Region: NODE\_291749\_length\_38467\_cov\_32.565315 36123-36136. Max. coverage (+): 0.16. Max coverage (-): 0

Region: NODE\_291749\_length\_38467\_cov\_32.565315 36137-36150. Max. coverage (+): 0.08. Max coverage (-): 0.04

Region: NODE\_291749\_length\_38467\_cov\_32.565315 36151-36165. Max. coverage (+): 0.2. Max coverage (-): 0.24

Region: NODE\_291749\_length\_38467\_cov\_32.565315 36166-36179. Max. coverage (+): 0.04. Max coverage (-): 0

Region: NODE\_291749\_length\_38467\_cov\_32.565315 36180-36193. Max. coverage (+): 0.04. Max coverage (-): 0.02

Region: NODE\_291749\_length\_38467\_cov\_32.565315 36194-36208. Max. coverage (+): 1.59. Max coverage (-): 0.04

Region: NODE\_291749\_length\_38467\_cov\_32.565315 36209-36222. Max. coverage (+): 0.97. Max coverage (-): 0

Region: NODE\_291749\_length\_38467\_cov\_32.565315 36223-36236. Max. coverage (+): 0. Max coverage (-): 0

Region: NODE\_291749\_length\_38467\_cov\_32.565315 36237-36251. Max. coverage (+): 0. Max coverage (-): 0

Region: NODE\_291749\_length\_38467\_cov\_32.565315 36252-36265. Max. coverage (+): 0. Max coverage (-): 0

Region: NODE\_291749\_length\_38467\_cov\_32.565315 36266-36279. Max. coverage (+): 0. Max coverage (-): 0.08

Region: NODE\_291749\_length\_38467\_cov\_32.565315 36280-36294. Max. coverage (+): 0.02. Max coverage (-): 0.02

Region: NODE\_291749\_length\_38467\_cov\_32.565315 36295-36308. Max. coverage (+): 0. Max coverage (-): 0.69

Region: NODE\_291749\_length\_38467\_cov\_32.565315 36309-36322. Max. coverage (+): 3.59. Max coverage (-): 0.03

Region: NODE\_291749\_length\_38467\_cov\_32.565315 36323-36337. Max. coverage (+): 3.47. Max coverage (-): 0

Region: NODE\_291749\_length\_38467\_cov\_32.565315 36338-36351. Max. coverage (+): 0. Max coverage (-): 0.04

Region: NODE\_291749\_length\_38467\_cov\_32.565315 36352-36365. Max. coverage (+): 0.04. Max coverage (-): 0.04

Region: NODE\_291749\_length\_38467\_cov\_32.565315 36366-36380. Max. coverage (+): 0.1. Max coverage (-): 0.01

Region: NODE\_291749\_length\_38467\_cov\_32.565315 36381-36394. Max. coverage (+): 0. Max coverage (-): 0

Region: NODE\_291749\_length\_38467\_cov\_32.565315 36395-36408. Max. coverage (+): 0. Max coverage (-): 0.08

Region: NODE\_291749\_length\_38467\_cov\_32.565315 36409-36423. Max. coverage (+): 0.04. Max coverage (-): 0

Region: NODE\_291749\_length\_38467\_cov\_32.565315 36424-36437. Max. coverage (+): 0.16. Max coverage (-): 0.12

Region: NODE\_291749\_length\_38467\_cov\_32.565315 36438-36451. Max. coverage (+): 0.03. Max coverage (-): 0

Region: NODE\_291749\_length\_38467\_cov\_32.565315 36452-36466. Max. coverage (+): 0.01. Max coverage (-): 0

Region: NODE\_291749\_length\_38467\_cov\_32.565315 36467-36480. Max. coverage (+): 0.04. Max coverage (-): 0

Region: NODE\_291749\_length\_38467\_cov\_32.565315 36481-36494. Max. coverage (+): 0.08. Max coverage (-): 0

Region: NODE\_291749\_length\_38467\_cov\_32.565315 36495-36509. Max. coverage (+): 0. Max coverage (-): 0.04

Region: NODE\_291749\_length\_38467\_cov\_32.565315 36510-36523. Max. coverage (+): 0.2. Max coverage (-): 0.04

Region: NODE\_291749\_length\_38467\_cov\_32.565315 36524-36537. Max. coverage (+): 0.2. Max coverage (-): 0

Region: NODE\_291749\_length\_38467\_cov\_32.565315 36538-36552. Max. coverage (+): 0.08. Max coverage (-): 0

Region: NODE\_291749\_length\_38467\_cov\_32.565315 36553-36566. Max. coverage (+): 0. Max coverage (-): 0

Region: NODE\_291749\_length\_38467\_cov\_32.565315 36567-36580. Max. coverage (+): 0. Max coverage (-): 0.04

Region: NODE\_291749\_length\_38467\_cov\_32.565315 36581-36595. Max. coverage (+): 0.04. Max coverage (-): 0

Region: NODE\_291749\_length\_38467\_cov\_32.565315 36596-36609. Max. coverage (+): 0. Max coverage (-): 0

Region: NODE\_291749\_length\_38467\_cov\_32.565315 36610-36623. Max. coverage (+): 0.04. Max coverage (-): 0.08

Region: NODE\_291749\_length\_38467\_cov\_32.565315 36624-36638. Max. coverage (+): 0.2. Max coverage (-): 0.24

Region: NODE\_291749\_length\_38467\_cov\_32.565315 36639-36652. Max. coverage (+): 0.2. Max coverage (-): 0.04

Region: NODE\_291749\_length\_38467\_cov\_32.565315 36653-36666. Max. coverage (+): 0.44. Max coverage (-): 0.04

Region: NODE\_291749\_length\_38467\_cov\_32.565315 36667-36681. Max. coverage (+): 1.25. Max coverage (-): 0

Region: NODE\_291749\_length\_38467\_cov\_32.565315 36682-36695. Max. coverage (+): 1.13. Max coverage (-): 0.12

Region: NODE\_291749\_length\_38467\_cov\_32.565315 36696-36709. Max. coverage (+): 5.05. Max coverage (-): 0.04

Region: NODE\_291749\_length\_38467\_cov\_32.565315 36710-36724. Max. coverage (+): 0.04. Max coverage (-): 0.57

Region: NODE\_291749\_length\_38467\_cov\_32.565315 36725-36738. Max. coverage (+): 0.52. Max coverage (-): 0.24

Region: NODE\_291749\_length\_38467\_cov\_32.565315 36739-36752. Max. coverage (+): 0.52. Max coverage (-): 0

Region: NODE\_291749\_length\_38467\_cov\_32.565315 36753-36767. Max. coverage (+): 0.16. Max coverage (-): 0.24

Region: NODE\_291749\_length\_38467\_cov\_32.565315 36768-36781. Max. coverage (+): 0.81. Max coverage (-): 0

Region: NODE\_291749\_length\_38467\_cov\_32.565315 36782-36795. Max. coverage (+): 0.32. Max coverage (-): 0

Region: NODE\_291749\_length\_38467\_cov\_32.565315 36796-36810. Max. coverage (+): 0.16. Max coverage (-): 0.04

Region: NODE\_291749\_length\_38467\_cov\_32.565315 36811-36824. Max. coverage (+): 0.04. Max coverage (-): 0.04

Region: NODE\_291749\_length\_38467\_cov\_32.565315 36825-36838. Max. coverage (+): 0. Max coverage (-): 0.04

Region: NODE\_291749\_length\_38467\_cov\_32.565315 36839-36853. Max. coverage (+): 1.82. Max coverage (-): 0.04

Region: NODE\_291749\_length\_38467\_cov\_32.565315 36854-36867. Max. coverage (+): 0.28. Max coverage (-): 0.04

Region: NODE\_291749\_length\_38467\_cov\_32.565315 36868-36881. Max. coverage (+): 0.32. Max coverage (-): 0

Region: NODE\_291749\_length\_38467\_cov\_32.565315 36882-36896. Max. coverage (+): 0.12. Max coverage (-): 0.08

Region: NODE\_291749\_length\_38467\_cov\_32.565315 36897-36910. Max. coverage (+): 0.2. Max coverage (-): 0.04

Region: NODE\_291749\_length\_38467\_cov\_32.565315 36911-36924. Max. coverage (+): 0.04. Max coverage (-): 0.04

Region: NODE\_291749\_length\_38467\_cov\_32.565315 36925-36939. Max. coverage (+): 0. Max coverage (-): 0

Region: NODE\_291749\_length\_38467\_cov\_32.565315 36940-36953. Max. coverage (+): 5.94. Max coverage (-): 0.12

Region: NODE\_291749\_length\_38467\_cov\_32.565315 36954-36967. Max. coverage (+): 12.6. Max coverage (-): 0.04

Region: NODE\_291749\_length\_38467\_cov\_32.565315 36968-36982. Max. coverage (+): 0.12. Max coverage (-): 0.04

Region: NODE\_291749\_length\_38467\_cov\_32.565315 36983-36996. Max. coverage (+): 0.4. Max coverage (-): 0

Region: NODE\_291749\_length\_38467\_cov\_32.565315 36997-37010. Max. coverage (+): 0.04. Max coverage (-): 0.04

Region: NODE\_291749\_length\_38467\_cov\_32.565315 37011-37025. Max. coverage (+): 0. Max coverage (-): 0.04

Region: NODE\_291749\_length\_38467\_cov\_32.565315 37026-37039. Max. coverage (+): 0.32. Max coverage (-): 0

Region: NODE\_291749\_length\_38467\_cov\_32.565315 37040-37053. Max. coverage (+): 0. Max coverage (-): 0

Region: NODE\_291749\_length\_38467\_cov\_32.565315 37054-37068. Max. coverage (+): 0. Max coverage (-): 0.04

Region: NODE\_291749\_length\_38467\_cov\_32.565315 37069-37082. Max. coverage (+): 0.04. Max coverage (-): 0

Region: NODE\_291749\_length\_38467\_cov\_32.565315 37083-37096. Max. coverage (+): 0.2. Max coverage (-): 0.16

Region: NODE\_291749\_length\_38467\_cov\_32.565315 37097-37111. Max. coverage (+): 0.44. Max coverage (-): 0.16

Region: NODE\_291749\_length\_38467\_cov\_32.565315 37112-37125. Max. coverage (+): 2.34. Max coverage (-): 0

Region: NODE\_291749\_length\_38467\_cov\_32.565315 37126-37139. Max. coverage (+): 0. Max coverage (-): 0

Region: NODE\_291749\_length\_38467\_cov\_32.565315 37140-37154. Max. coverage (+): 0.36. Max coverage (-): 0

Region: NODE\_291749\_length\_38467\_cov\_32.565315 37155-37168. Max. coverage (+): 0.12. Max coverage (-): 0.04

Region: NODE\_291749\_length\_38467\_cov\_32.565315 37169-37182. Max. coverage (+): 0.24. Max coverage (-): 0

Region: NODE\_291749\_length\_38467\_cov\_32.565315 37183-37197. Max. coverage (+): 0.12. Max coverage (-): 0

Region: NODE\_291749\_length\_38467\_cov\_32.565315 37198-37211. Max. coverage (+): 5.33. Max coverage (-): 0

Region: NODE\_291749\_length\_38467\_cov\_32.565315 37212-37225. Max. coverage (+): 3.76. Max coverage (-): 0.04

Region: NODE\_291749\_length\_38467\_cov\_32.565315 37226-37240. Max. coverage (+): 0. Max coverage (-): 0

Region: NODE\_291749\_length\_38467\_cov\_32.565315 37241-37254. Max. coverage (+): 0. Max coverage (-): 0.12

Region: NODE\_291749\_length\_38467\_cov\_32.565315 37255-37268. Max. coverage (+): 0.16. Max coverage (-): 0.04

Region: NODE\_291749\_length\_38467\_cov\_32.565315 37269-37283. Max. coverage (+): 0.16. Max coverage (-): 0

Region: NODE\_291749\_length\_38467\_cov\_32.565315 37284-37297. Max. coverage (+): 0.04. Max coverage (-): 0.2

Region: NODE\_291749\_length\_38467\_cov\_32.565315 37298-37311. Max. coverage (+): 3.51. Max coverage (-): 0.24

Region: NODE\_291749\_length\_38467\_cov\_32.565315 37312-37326. Max. coverage (+): 11.63. Max coverage (-): 0.04

Region: NODE\_291749\_length\_38467\_cov\_32.565315 37327-37340. Max. coverage (+): 0.16. Max coverage (-): 0.28

Region: NODE\_291749\_length\_38467\_cov\_32.565315 37341-37354. Max. coverage (+): 0.08. Max coverage (-): 0

Region: NODE\_291749\_length\_38467\_cov\_32.565315 37355-37369. Max. coverage (+): 0.48. Max coverage (-): 0.04

Region: NODE\_291749\_length\_38467\_cov\_32.565315 37370-37383. Max. coverage (+): 0.65. Max coverage (-): 0.08

Region: NODE\_291749\_length\_38467\_cov\_32.565315 37384-37397. Max. coverage (+): 4.4. Max coverage (-): 0.04

Region: NODE\_291749\_length\_38467\_cov\_32.565315 37398-37412. Max. coverage (+): 4.2. Max coverage (-): 0.4

Region: NODE\_291749\_length\_38467\_cov\_32.565315 37413-37426. Max. coverage (+): 0.32. Max coverage (-): 0.28

Region: NODE\_291749\_length\_38467\_cov\_32.565315 37427-37440. Max. coverage (+): 0.28. Max coverage (-): 0

Region: NODE\_291749\_length\_38467\_cov\_32.565315 37441-37455. Max. coverage (+): 0. Max coverage (-): 0.12

Region: NODE\_291749\_length\_38467\_cov\_32.565315 37456-37469. Max. coverage (+): 0.08. Max coverage (-): 0.24

Region: NODE\_291749\_length\_38467\_cov\_32.565315 37470-37483. Max. coverage (+): 0.12. Max coverage (-): 0.12

Region: NODE\_291749\_length\_38467\_cov\_32.565315 37484-37498. Max. coverage (+): 0. Max coverage (-): 0.04

Region: NODE\_291749\_length\_38467\_cov\_32.565315 37499-37512. Max. coverage (+): 0.44. Max coverage (-): 0.08

Region: NODE\_291749\_length\_38467\_cov\_32.565315 37513-37526. Max. coverage (+): 11.23. Max coverage (-): 0.16

Region: NODE\_291749\_length\_38467\_cov\_32.565315 37527-37541. Max. coverage (+): 0.16. Max coverage (-): 0.04

Region: NODE\_291749\_length\_38467\_cov\_32.565315 37542-37555. Max. coverage (+): 0. Max coverage (-): 0.28

Region: NODE\_291749\_length\_38467\_cov\_32.565315 37556-37569. Max. coverage (+): 1.66. Max coverage (-): 0

Region: NODE\_291749\_length\_38467\_cov\_32.565315 37570-37584. Max. coverage (+): 0.36. Max coverage (-): 0.08

Region: NODE\_291749\_length\_38467\_cov\_32.565315 37585-37598. Max. coverage (+): 0.24. Max coverage (-): 0.08

Region: NODE\_291749\_length\_38467\_cov\_32.565315 37599-37612. Max. coverage (+): 0.93. Max coverage (-): 0.04

Region: NODE\_291749\_length\_38467\_cov\_32.565315 37613-37627. Max. coverage (+): 0.57. Max coverage (-): 0.04

Region: NODE\_291749\_length\_38467\_cov\_32.565315 37628-37641. Max. coverage (+): 0.61. Max coverage (-): 0.12

Region: NODE\_291749\_length\_38467\_cov\_32.565315 37642-37655. Max. coverage (+): 0.57. Max coverage (-): 0.2

Region: NODE\_291749\_length\_38467\_cov\_32.565315 37656-37670. Max. coverage (+): 1.21. Max coverage (-): 0.44

Region: NODE\_291749\_length\_38467\_cov\_32.565315 37671-37684. Max. coverage (+): 0.36. Max coverage (-): 0.28

Region: NODE\_291749\_length\_38467\_cov\_32.565315 37685-37698. Max. coverage (+): 0.08. Max coverage (-): 0.04

Region: NODE\_291749\_length\_38467\_cov\_32.565315 37699-37713. Max. coverage (+): 0.04. Max coverage (-): 0.57

Region: NODE\_291749\_length\_38467\_cov\_32.565315 37714-37727. Max. coverage (+): 2.38. Max coverage (-): 0

Region: NODE\_291749\_length\_38467\_cov\_32.565315 37728-37741. Max. coverage (+): 0.2. Max coverage (-): 0

Region: NODE\_291749\_length\_38467\_cov\_32.565315 37742-37756. Max. coverage (+): 0.08. Max coverage (-): 0.28

Region: NODE\_291749\_length\_38467\_cov\_32.565315 37757-37770. Max. coverage (+): 0.61. Max coverage (-): 0.08

Region: NODE\_291749\_length\_38467\_cov\_32.565315 37771-37784. Max. coverage (+): 0.08. Max coverage (-): 0

Region: NODE\_291749\_length\_38467\_cov\_32.565315 37785-37799. Max. coverage (+): 0.24. Max coverage (-): 0.16

Region: NODE\_291749\_length\_38467\_cov\_32.565315 37800-37813. Max. coverage (+): 1.09. Max coverage (-): 0.16

Region: NODE\_291749\_length\_38467\_cov\_32.565315 37814-37827. Max. coverage (+): 1.05. Max coverage (-): 0

Region: NODE\_291749\_length\_38467\_cov\_32.565315 37828-37842. Max. coverage (+): 0.08. Max coverage (-): 0.24

Region: NODE\_291749\_length\_38467\_cov\_32.565315 37843-37856. Max. coverage (+): 0.08. Max coverage (-): 0

Region: NODE\_291749\_length\_38467\_cov\_32.565315 37857-37870. Max. coverage (+): 0.04. Max coverage (-): 0.04

Region: NODE\_291749\_length\_38467\_cov\_32.565315 37871-37885. Max. coverage (+): 0.04. Max coverage (-): 0.04

Region: NODE\_291749\_length\_38467\_cov\_32.565315 37886-37899. Max. coverage (+): 0.57. Max coverage (-): 0

Region: NODE\_291749\_length\_38467\_cov\_32.565315 37900-37913. Max. coverage (+): 0.08. Max coverage (-): 0.04

Region: NODE\_291749\_length\_38467\_cov\_32.565315 37914-37928. Max. coverage (+): 0.12. Max coverage (-): 0.04

Region: NODE\_291749\_length\_38467\_cov\_32.565315 37929-37942. Max. coverage (+): 0. Max coverage (-): 0.04

Region: NODE\_291749\_length\_38467\_cov\_32.565315 37943-37956. Max. coverage (+): 0.28. Max coverage (-): 0.12

Region: NODE\_291749\_length\_38467\_cov\_32.565315 37957-37971. Max. coverage (+): 0.08. Max coverage (-): 0.2

Region: NODE\_291749\_length\_38467\_cov\_32.565315 37972-37985. Max. coverage (+): 0.61. Max coverage (-): 0.04

Region: NODE\_291749\_length\_38467\_cov\_32.565315 37986-37999. Max. coverage (+): 0.28. Max coverage (-): 0

Region: NODE\_291749\_length\_38467\_cov\_32.565315 38000-38014. Max. coverage (+): 0.28. Max coverage (-): 0.04

Region: NODE\_291749\_length\_38467\_cov\_32.565315 38015-38028. Max. coverage (+): 0.2. Max coverage (-): 0

Region: NODE\_291749\_length\_38467\_cov\_32.565315 38029-38042. Max. coverage (+): 0.04. Max coverage (-): 0.2

Region: NODE\_291749\_length\_38467\_cov\_32.565315 38043-38057. Max. coverage (+): 15.63. Max coverage (-): 0

Region: NODE\_291749\_length\_38467\_cov\_32.565315 38058-38071. Max. coverage (+): 0.65. Max coverage (-): 0

Region: NODE\_291749\_length\_38467\_cov\_32.565315 38072-38085. Max. coverage (+): 0.08. Max coverage (-): 0

Region: NODE\_291749\_length\_38467\_cov\_32.565315 38086-38100. Max. coverage (+): 0. Max coverage (-): 0.04

Region: NODE\_291749\_length\_38467\_cov\_32.565315 38101-38114. Max. coverage (+): 0. Max coverage (-): 0.04

Region: NODE\_291749\_length\_38467\_cov\_32.565315 38115-38128. Max. coverage (+): 0.04. Max coverage (-): 0

Region: NODE\_291749\_length\_38467\_cov\_32.565315 38129-38143. Max. coverage (+): 0.48. Max coverage (-): 0.08

Region: NODE\_291749\_length\_38467\_cov\_32.565315 38144-38157. Max. coverage (+): 0.73. Max coverage (-): 0.04

Region: NODE\_291749\_length\_38467\_cov\_32.565315 38158-38171. Max. coverage (+): 0.57. Max coverage (-): 0.32

Region: NODE\_291749\_length\_38467\_cov\_32.565315 38172-38186. Max. coverage (+): 0.36. Max coverage (-): 2.95

Region: NODE\_291749\_length\_38467\_cov\_32.565315 38187-38200. Max. coverage (+): 1.09. Max coverage (-): 0.08

Region: NODE\_291749\_length\_38467\_cov\_32.565315 38201-38214. Max. coverage (+): 0.24. Max coverage (-): 0

Region: NODE\_291749\_length\_38467\_cov\_32.565315 38215-38229. Max. coverage (+): 0.4. Max coverage (-): 0

Region: NODE\_291749\_length\_38467\_cov\_32.565315 38230-38243. Max. coverage (+): 0.16. Max coverage (-): 0

Region: NODE\_291749\_length\_38467\_cov\_32.565315 38244-38257. Max. coverage (+): 0.04. Max coverage (-): 0.08

Region: NODE\_291749\_length\_38467\_cov\_32.565315 38258-38272. Max. coverage (+): 0.12. Max coverage (-): 0

Region: NODE\_291749\_length\_38467\_cov\_32.565315 38273-38286. Max. coverage (+): 2.62. Max coverage (-): 0

Region: NODE\_291749\_length\_38467\_cov\_32.565315 38287-38300. Max. coverage (+): 0.77. Max coverage (-): 0.36

Region: NODE\_291749\_length\_38467\_cov\_32.565315 38301-38315. Max. coverage (+): 0.89. Max coverage (-): 0.08

Region: NODE\_291749\_length\_38467\_cov\_32.565315 38316-38329. Max. coverage (+): 0.93. Max coverage (-): 0.08

Region: NODE\_291749\_length\_38467\_cov\_32.565315 38330-38343. Max. coverage (+): 0.04. Max coverage (-): 0.52

Region: NODE\_291749\_length\_38467\_cov\_32.565315 38344-38358. Max. coverage (+): 22.13. Max coverage (-): 0.08

Region: NODE\_291749\_length\_38467\_cov\_32.565315 38359-38372. Max. coverage (+): 22.01. Max coverage (-): 0

Region: NODE\_291749\_length\_38467\_cov\_32.565315 38373-38386. Max. coverage (+): 0. Max coverage (-): 0.16

Region: NODE\_291749\_length\_38467\_cov\_32.565315 38387-38401. Max. coverage (+): 0.36. Max coverage (-): 0

Region: NODE\_291749\_length\_38467\_cov\_32.565315 38402-38415. Max. coverage (+): 0. Max coverage (-): 0

Region: NODE\_291749\_length\_38467\_cov\_32.565315 38416-38429. Max. coverage (+): 0.08. Max coverage (-): 0

Region: NODE\_291749\_length\_38467\_cov\_32.565315 38430-38444. Max. coverage (+): 0.12. Max coverage (-): 0

Region: NODE\_291749\_length\_38467\_cov\_32.565315 38445-38458. Max. coverage (+): 0.12. Max coverage (-): 0

Region: NODE\_291749\_length\_38467\_cov\_32.565315 38459-38472. Max. coverage (+): 0.08. Max coverage (-): 0

Region: NODE\_291749\_length\_38467\_cov\_32.565315 38473-38487. Max. coverage (+): 1.74. Max coverage (-): 0

Region: NODE\_291749\_length\_38467\_cov\_32.565315 38488-38501. Max. coverage (+): 1.94. Max coverage (-): 0

Region: NODE\_291749\_length\_38467\_cov\_32.565315 38502-38515. Max. coverage (+): 0. Max coverage (-): 0.2

Region: NODE\_291749\_length\_38467\_cov\_32.565315 38516-38530. Max. coverage (+): 0.48. Max coverage (-): 0

Region: NODE\_291749\_length\_38467\_cov\_32.565315 38531-38544. Max. coverage (+): 0. Max coverage (-): 0

Region: NODE\_291749\_length\_38467\_cov\_32.565315 38545-38558. Max. coverage (+): 0. Max coverage (-): 0

Region: NODE\_291749\_length\_38467\_cov\_32.565315 38559-38573. Max. coverage (+): 0.08. Max coverage (-): 0

Region: NODE\_291749\_length\_38467\_cov\_32.565315 38574-38587. Max. coverage (+): 0.12. Max coverage (-): 0.04

Region: NODE\_291749\_length\_38467\_cov\_32.565315 38588-38601. Max. coverage (+): 0.08. Max coverage (-): 0.04

Region: NODE\_291749\_length\_38467\_cov\_32.565315 38602-38616. Max. coverage (+): 1.21. Max coverage (-): 0.04

Region: NODE\_291749\_length\_38467\_cov\_32.565315 38617-38630. Max. coverage (+): 0. Max coverage (-): 0.08

Region: NODE\_291749\_length\_38467\_cov\_32.565315 38631-38644. Max. coverage (+): 0. Max coverage (-): 0

Region: NODE\_291749\_length\_38467\_cov\_32.565315 38645-38659. Max. coverage (+): 0.04. Max coverage (-): 0.12

Region: NODE\_291749\_length\_38467\_cov\_32.565315 38660-38673. Max. coverage (+): 0.04. Max coverage (-): 0.2

Region: NODE\_291749\_length\_38467\_cov\_32.565315 38674-38687. Max. coverage (+): 0.08. Max coverage (-): 0.08

Region: NODE\_291749\_length\_38467\_cov\_32.565315 38688-38702. Max. coverage (+): 0.2. Max coverage (-): 0.08

Region: NODE\_291749\_length\_38467\_cov\_32.565315 38703-38716. Max. coverage (+): 0.16. Max coverage (-): 0

Region: NODE\_291749\_length\_38467\_cov\_32.565315 38717-38730. Max. coverage (+): 0.16. Max coverage (-): 0.04

Region: NODE\_291749\_length\_38467\_cov\_32.565315 38731-38745. Max. coverage (+): 0.36. Max coverage (-): 0.12

Region: NODE\_291749\_length\_38467\_cov\_32.565315 38746-38759. Max. coverage (+): 1.21. Max coverage (-): 0.16

Region: NODE\_291749\_length\_38467\_cov\_32.565315 38760-38773. Max. coverage (+): 0.16. Max coverage (-): 0.04

Region: NODE\_291749\_length\_38467\_cov\_32.565315 38774-38788. Max. coverage (+): 0.04. Max coverage (-): 0

Region: NODE\_291749\_length\_38467\_cov\_32.565315 38789-38802. Max. coverage (+): 0. Max coverage (-): 1.29

Region: NODE\_291749\_length\_38467\_cov\_32.565315 38803-38816. Max. coverage (+): 0.04. Max coverage (-): 1.29

Region: NODE\_291749\_length\_38467\_cov\_32.565315 38817-38831. Max. coverage (+): 0.04. Max coverage (-): 0

Region: NODE\_291749\_length\_38467\_cov\_32.565315 38832-38845. Max. coverage (+): 0. Max coverage (-): 0

Region: NODE\_291749\_length\_38467\_cov\_32.565315 38846-38859. Max. coverage (+): 0.08. Max coverage (-): 0.12

Region: NODE\_291749\_length\_38467\_cov\_32.565315 38860-38874. Max. coverage (+): 0. Max coverage (-): 0.04

Region: NODE\_291749\_length\_38467\_cov\_32.565315 38875-38888. Max. coverage (+): 0.04. Max coverage (-): 0

Region: NODE\_291749\_length\_38467\_cov\_32.565315 38889-38902. Max. coverage (+): 0.85. Max coverage (-): 0

Region: NODE\_291749\_length\_38467\_cov\_32.565315 38903-38917. Max. coverage (+): 0.28. Max coverage (-): 0.04

Region: NODE\_291749\_length\_38467\_cov\_32.565315 38918-38931. Max. coverage (+): 0.24. Max coverage (-): 0

Region: NODE\_291749\_length\_38467\_cov\_32.565315 38932-38945. Max. coverage (+): 0. Max coverage (-): 0

Region: NODE\_291749\_length\_38467\_cov\_32.565315 38946-38960. Max. coverage (+): 0.04. Max coverage (-): 0.16

Region: NODE\_291749\_length\_38467\_cov\_32.565315 38961-38974. Max. coverage (+): 0.16. Max coverage (-): 0

Region: NODE\_291749\_length\_38467\_cov\_32.565315 38975-38988. Max. coverage (+): 0.2. Max coverage (-): 0

Region: NODE\_291749\_length\_38467\_cov\_32.565315 38989-39003. Max. coverage (+): 0.2. Max coverage (-): 0.08

Region: NODE\_291749\_length\_38467\_cov\_32.565315 39004-39017. Max. coverage (+): 0.04. Max coverage (-): 0

Region: NODE\_291749\_length\_38467\_cov\_32.565315 39018-39031. Max. coverage (+): 0. Max coverage (-): 0

Region: NODE\_291749\_length\_38467\_cov\_32.565315 39032-39046. Max. coverage (+): 0. Max coverage (-): 0

Region: NODE\_291749\_length\_38467\_cov\_32.565315 39047-39060. Max. coverage (+): 0. Max coverage (-): 0

Region: NODE\_291749\_length\_38467\_cov\_32.565315 39061-39074. Max. coverage (+): 0. Max coverage (-): 0

Region: NODE\_291749\_length\_38467\_cov\_32.565315 39075-39089. Max. coverage (+): 0. Max coverage (-): 0

Region: NODE\_291749\_length\_38467\_cov\_32.565315 39090-39103. Max. coverage (+): 0. Max coverage (-): 0

Region: NODE\_291749\_length\_38467\_cov\_32.565315 39104-39117. Max. coverage (+): 0. Max coverage (-): 0

Region: NODE\_291749\_length\_38467\_cov\_32.565315 39118-39132. Max. coverage (+): 0. Max coverage (-): 0.04

Region: NODE\_291749\_length\_38467\_cov\_32.565315 39133-39146. Max. coverage (+): 0.08. Max coverage (-): 0

Region: NODE\_291749\_length\_38467\_cov\_32.565315 39147-39160. Max. coverage (+): 0. Max coverage (-): 0

Region: NODE\_291749\_length\_38467\_cov\_32.565315 39161-. Max. coverage (+): 0. Max coverage (-): 0

RepeatMasker Color Code

**+**

100-98% Identity

<98-95% Identity

<95-90% Identity

<90-85% Identity

<85-80% Identity

<80-75% Identity

<75-70% Identity

<70% Identity

**-**

Gene Set Color Code

**+**

Gene

Pseudogene

Other

**-**

Topology/Coverage Color Code

Coverage Plus Strand

Coverage Minus Strand

Mainstrand: Plus

Mainstrand: Minus

Complementary Strand

Flanking Region  
(if option -flank >0)

Gene Set Annotation  
  
RepeatMasker Annotation  

**1. AlRepB-923**: 31984-32284 (+), Divergence to consensus: 5.7%  
**2. AlRepB-923**: 32284-33189 (+), Divergence to consensus: 7%  
**3. AlRepD-1165**: 33191-33238 (+), Divergence to consensus: 2.1%  
**4. AlRepD-779**: 33722-33935 (+), Divergence to consensus: 34.1%  
**5. AlRepE-294**: 33954-34018 (-), Divergence to consensus: 26.1%  
**6. AlRepB-180**: 35918-36654 (+), Divergence to consensus: 31.6%  
**7. AlRepB-180**: 36647-36711 (+), Divergence to consensus: 22.3%  
**8. AlRepC-631**: 36865-37018 (+), Divergence to consensus: 48.8%  
**9. (AGAC)n**: 37724-37797 (+), Divergence to consensus: 33.8%  
**10. (AAATT)n**: 38121-38148 (+), Divergence to consensus: 11.4%

  
Transcription Factor Binding Sites  

**RHOXF1** (Sequence: AGATTA (-): 32796)  
**RHOXF1** (Sequence: AGATTA (-): 33093)  
**RHOXF1** (Sequence: GGCTCA (-): 33364)  
**RHOXF1** (Sequence: AGCTTA (-): 33796)  
**RHOXF1** (Sequence: AGATCA (-): 34426)  
**RHOXF1** (Sequence: AGATTA (-): 35107)  
**RHOXF1** (Sequence: AGCTTA (-): 35671)  
**RHOXF1** (Sequence: AGATTA (-): 36437)  
**RHOXF1** (Sequence: GGATCA (-): 36881)  
**RHOXF1** (Sequence: AGATCA (-): 36931)  
**RHOXF1** (Sequence: GGATTA (-): 37401)  
**RHOXF1** (Sequence: AGATTA (-): 38048)  
**RHOXF1** (Sequence: TGATCC (+): 32130)  
**RHOXF1** (Sequence: TGAGCC (+): 32198)  
**RHOXF1** (Sequence: TAAGCC (+): 32298)  
**RHOXF1** (Sequence: TGATCC (+): 32552)  
**RHOXF1** (Sequence: TGATCT (+): 33348)  
**RHOXF1** (Sequence: TGAGCT (+): 33469)  
**RHOXF1** (Sequence: TGATCT (+): 33753)  
**RHOXF1** (Sequence: TGAGCT (+): 34662)  
**RHOXF1** (Sequence: TGAGCT (+): 35582)  
**RHOXF1** (Sequence: TGATCC (+): 35951)  
**RHOXF1** (Sequence: TGAGCT (+): 35980)  
**RHOXF1** (Sequence: TAAGCT (+): 36200)  
**RHOXF1** (Sequence: TAATCC (+): 36651)  
**RHOXF1** (Sequence: TAAGCT (+): 38539)  
**RHOXF1** (Sequence: TAATCT (+): 39035)  
**POU5F1** (Sequence: TTTGCAT (-): 36639)  
**FOXO3\_hsa** (Sequence: GTAAACAA (+): 32844)  
**SOX9** (Sequence: AACAATAA (-): 32847)  
**SOX9** (Sequence: AACAATGG (-): 35014)  
**FOXP1** (Sequence: GTAAACA (+): 32844)  
**FOXO1** (Sequence: GTTGTTTAT (+): 32608)  
**FOXO1** (Sequence: GCTGTTTTC (+): 37420)  
**FOXO3\_mmu** (Sequence: TGTTTTGC (-): 38452)  
**Sox5** (Sequence: ATTGTT (+): 33113)  
**Sox5** (Sequence: ATTGTT (+): 33862)  
**Sox5** (Sequence: ATTGTT (+): 33887)  
**Sox5** (Sequence: ATTGTT (+): 35292)  
**Sox5** (Sequence: ATTGTT (+): 36478)  
**Sox5** (Sequence: ATTGTT (+): 36743)  
**Sox5** (Sequence: ATTGTT (+): 37589)  
**FOXO3\_mmu** (Sequence: TGTAAACA (+): 32843)  
**FOXO3\_mmu** (Sequence: TGAAAACA (+): 38371)  
**FOXO3\_mmu** (Sequence: TCTAAACA (+): 38996)  
**FOXO1** (Sequence: AAAAACAAC (-): 32272)  
**FOXO1** (Sequence: AAAAACAAC (-): 32689)  
**FOXO1** (Sequence: GAAAACAAC (-): 37561)  
**Nobox** (Sequence: TAATTAGT (+): 35257)  
**POU2F1** (Sequence: ATTTACATA (-): 33676)  
**POU2F1** (Sequence: ATTAAAATA (-): 39028)  
**Rhox11** (Sequence: TGCTGTAAA (+): 33690)  
**Rhox11** (Sequence: TTAACACCA (-): 38051)  
**Sox5** (Sequence: AACAAT (-): 32847)  
**Sox5** (Sequence: AACAAT (-): 34740)  
**Sox5** (Sequence: AACAAT (-): 35014)  
**Sox5** (Sequence: AACAAT (-): 38203)  
**POU5F1** (Sequence: ATGCAAA (+): 33454)  
**POU5F1** (Sequence: ATGCAAA (+): 37240)
