# Supplementary material for: First characterization of PIWI-interacting RNA clusters in a cichlid fish with a B chromosome
Source: BMC Biol. 2022 Sep 21;20:204. doi: 10.1186/s12915-022-01403-2 (PMC9490952; doi:10.1186/s12915-022-01403-2)
Supplement: Supplementary file 1 — Additional file 1. Zipped folder with fasta and interactive html piRNA cluster information for the A. latifasciata genome. The nomenclature is as follows: number-pirna-cluster_sex_B-presence (f, female; m, male; 0b, without B chromosome; 1b, with B chromosome). [file 12915_2022_1403_MOESM1_ESM.zip › 116_m0b.html]

piRNA cluster 116\_m0b 55


Predicted piRNA cluster no. 116\_m0b
  

Show proTRAC run info
Hide proTRAC run info

/\  
                \_\_\_\_\_\_\_\_\_\_\_\_\_\_\_\_\_\_\_\_\_\_\_/\\_\_\_ /  \\_\_\_\_\_\_\_  
               I                      /  \  /    \      I  
               I     pro             /    \/      \     I  
               I        TRAC        /               \   I  
               I   \_\_\_\_\_\_\_\_\_\_\_\_\_\_\_\_/\_\_\_\_\_\_\_\_\_\_\_\_\_\_\_\_\_\\_ I  
               I   \              /                     I  
               I    \            /                      I  
               I     \  /\      /       V.2.4.2         I  
               I      \/  \    /                        I  
               I\_\_\_\_\_\_\_\_\_\_\_\  /\_\_\_\_\_\_\_\_\_\_\_\_\_\_\_\_\_\_\_\_\_\_\_\_\_I  
                            \/  
  
  
================================= proTRAC ====================================  
VERSION: .......... 2.4.2  
LAST MODIFIED: .... 11. May 2018  
  
Please cite:  
Rosenkranz D, Zischler H. proTRAC - a software for probabilistic piRNA cluster  
detection, visualization and analysis. 2012. BMC Bioinformatics 13:5.  
  
  
Contact:  
David Rosenkranz  
Institute of Organismic and Molecular Evolutionary Biology  
Dept. Anthropology, small RNA group  
Johannes Gutenberg University Mainz  
email: rosenkranz@uni-mainz.de  
  
You can find the latest proTRAC version at:  
http://sourceforge.net/projects/protrac/files  
http://www.smallRNAgroup-mainz.de/software  
==============================================================================  
  
PARAMETERS:  
Map file: ...............piwi-machos-0B.fa-collapse.map  
Genome file: ............../../../0B\_ala\_genome.fa  
RepeatMasker annotation: Alatifasciata-all0B-maryan-v2.fa\_corrected.out  
GeneSet:................./guest-storage/Data/annotation/Alatifasciata\_all0B\_maryan-v2\_out2017.gff  
  
Significant (p<=0.01) hit density will be calculated based  
on observed hit distribution.  
  
Sliding window size: ........................................ 5000 bp  
Sliding window increament: .................................. 1000 bp  
Normalize each hit by number of genomic hits: ............... yes  
Normalize each hit by number of sequence reads: ............. yes  
Normalize values (-> per million mapped reads): ............. yes  
Min. fraction of hits with 1T(U) or 10A: .................... 0.75  
Alternatively: Min. fraction of hits with 1T(U) and 10A: .... 0.5  
Min. fraction of hits with typical piRNA length: ............ 0.75  
Typical piRNA length: ....................................... 24-32 nt  
Min. size of a piRNA cluster: ............................... 1000 bp.  
Min. number of hits (absolute): ............................. 0  
Min. number of hits (normalized): ........................... 0  
Min. fraction of hits on the mainstrand: .................... 0.75  
Top fraction of mapped sequences (in terms of read counts): . 1%  
Top fraction accounts for max. n% of sequence reads: ........ 90%  
Min. fraction of hits on each arm of a bidirectional cluster: 0.05  
Output html file for each cluster: .......................... yes  
Output a summary table: ..................................... yes  
Output a FASTA file for each cluster (piRNA sequences): ..... yes  
Output a FASTA file comprising cluster sequences: ........... yes  
Output a GTF file for predicted piRNA clusters: ..............yes  
Search DNA motifs in clusters: .............................. yes  
Output flanking sequences: +/- .............................. 0 bp  
Output ~.pTi file: .......................................... no  
==============================================================================  
  
  
Genome size (without gaps): ............ 758543724 bp  
Gaps (N/X/-): .......................... 417479 bp  
Mapped reads: .......................... 24765598  
Non-identical sequences: ............... 6158275  
Genomic hits: .......................... 53103584  
Significant densitiy of mapped reads: .. 763.098963422187 reads/kb

Show proTRAC cluster info
Hide proTRAC cluster info

|  |  |
| --- | --- |
| Location | NODE\_296637\_length\_14495\_cov\_30.429251 |
| Coordinates | 9005-14620 |
| Size [bp] | 5616 |
| Sequence hit loci | 2704 |
| Mapped reads (normalized) | 4762.9 |
| Mapped reads (normalized) per kb | 848.1 |
| Normalized reads with 1T (1U) | 76.4% |
| Normalized reads with 10A | 34.9% |
| Normalized reads with length 24-32 nt | 99.1% |
| Normalized reads on the main strand(s) | 85.7% |
| Predicted directionality | mono:minus |

100%

0%

1T (1U)  
reads

10A reads

24-32 nt  
reads

reads on mainstrand

**Either the amount of reads with 1T (1U) OR 10A has to exceed 75% (set with option: -1Tor10A)  
Alternatively the amount of reads with 1T (1U) AND 10A has to exceed 50% (set with option: -1Tand10A)  
Minimum amount of reads with preferred size is 75% (set with option: -pisize)  
Minimum amount of reads on the main strand(s) is 75% (set with option: -clstrand)**

Show read coverage
Hide read coverage

WHAT DO I SEE HERE?  
This chart shows the location of mapped sequence reads within a predicted piRNA cluster. The color refers to the number of genomic hits produced by the sequence read in question. A dark red bar indicates that this sequence read produces many other hits elsewhere in the genome. Many adjacent red or yellow bars can indicate the presence of a multi-copy element such as transposons or rRNA genes. A dark green bar indicates that this sequence read maps uniquely to this locus.

1 hit

2-5 hits

6-10 hits

11-20 hits

21-50 hits

51-100 hits

> 100 hits

NODE\_296637\_length\_14495\_cov\_30.429251

9005

14620

Gene Set

RepeatMasker

Mapped  
Reads

12.03

plus strand

minus strand

12.03

Region: NODE\_296637\_length\_14495\_cov\_30.429251 39162-9010. Max. coverage (+): 0. Max coverage (-): 0

Region: NODE\_296637\_length\_14495\_cov\_30.429251 9011-9021. Max. coverage (+): 0. Max coverage (-): 0

Region: NODE\_296637\_length\_14495\_cov\_30.429251 9022-9033. Max. coverage (+): 0. Max coverage (-): 0

Region: NODE\_296637\_length\_14495\_cov\_30.429251 9034-9044. Max. coverage (+): 0. Max coverage (-): 0.01

Region: NODE\_296637\_length\_14495\_cov\_30.429251 9045-9055. Max. coverage (+): 0. Max coverage (-): 0.01

Region: NODE\_296637\_length\_14495\_cov\_30.429251 9056-9066. Max. coverage (+): 0. Max coverage (-): 0

Region: NODE\_296637\_length\_14495\_cov\_30.429251 9067-9078. Max. coverage (+): 0. Max coverage (-): 0

Region: NODE\_296637\_length\_14495\_cov\_30.429251 9079-9089. Max. coverage (+): 0.02. Max coverage (-): 0.02

Region: NODE\_296637\_length\_14495\_cov\_30.429251 9090-9100. Max. coverage (+): 0.01. Max coverage (-): 0.07

Region: NODE\_296637\_length\_14495\_cov\_30.429251 9101-9111. Max. coverage (+): 0. Max coverage (-): 0.01

Region: NODE\_296637\_length\_14495\_cov\_30.429251 9112-9122. Max. coverage (+): 0.01. Max coverage (-): 0

Region: NODE\_296637\_length\_14495\_cov\_30.429251 9123-9134. Max. coverage (+): 0. Max coverage (-): 0

Region: NODE\_296637\_length\_14495\_cov\_30.429251 9135-9145. Max. coverage (+): 0. Max coverage (-): 0.04

Region: NODE\_296637\_length\_14495\_cov\_30.429251 9146-9156. Max. coverage (+): 0. Max coverage (-): 0

Region: NODE\_296637\_length\_14495\_cov\_30.429251 9157-9167. Max. coverage (+): 0. Max coverage (-): 0.14

Region: NODE\_296637\_length\_14495\_cov\_30.429251 9168-9179. Max. coverage (+): 0.01. Max coverage (-): 0.14

Region: NODE\_296637\_length\_14495\_cov\_30.429251 9180-9190. Max. coverage (+): 0.01. Max coverage (-): 0

Region: NODE\_296637\_length\_14495\_cov\_30.429251 9191-9201. Max. coverage (+): 0. Max coverage (-): 0

Region: NODE\_296637\_length\_14495\_cov\_30.429251 9202-9212. Max. coverage (+): 0. Max coverage (-): 0

Region: NODE\_296637\_length\_14495\_cov\_30.429251 9213-9224. Max. coverage (+): 0. Max coverage (-): 0.04

Region: NODE\_296637\_length\_14495\_cov\_30.429251 9225-9235. Max. coverage (+): 0. Max coverage (-): 0.01

Region: NODE\_296637\_length\_14495\_cov\_30.429251 9236-9246. Max. coverage (+): 0.06. Max coverage (-): 0

Region: NODE\_296637\_length\_14495\_cov\_30.429251 9247-9257. Max. coverage (+): 0.13. Max coverage (-): 0

Region: NODE\_296637\_length\_14495\_cov\_30.429251 9258-9268. Max. coverage (+): 0. Max coverage (-): 0.02

Region: NODE\_296637\_length\_14495\_cov\_30.429251 9269-9280. Max. coverage (+): 0.06. Max coverage (-): 0.02

Region: NODE\_296637\_length\_14495\_cov\_30.429251 9281-9291. Max. coverage (+): 0.07. Max coverage (-): 0

Region: NODE\_296637\_length\_14495\_cov\_30.429251 9292-9302. Max. coverage (+): 0.01. Max coverage (-): 0

Region: NODE\_296637\_length\_14495\_cov\_30.429251 9303-9313. Max. coverage (+): 0. Max coverage (-): 0.02

Region: NODE\_296637\_length\_14495\_cov\_30.429251 9314-9325. Max. coverage (+): 0.06. Max coverage (-): 0

Region: NODE\_296637\_length\_14495\_cov\_30.429251 9326-9336. Max. coverage (+): 0.02. Max coverage (-): 0

Region: NODE\_296637\_length\_14495\_cov\_30.429251 9337-9347. Max. coverage (+): 0.02. Max coverage (-): 0

Region: NODE\_296637\_length\_14495\_cov\_30.429251 9348-9358. Max. coverage (+): 0. Max coverage (-): 0

Region: NODE\_296637\_length\_14495\_cov\_30.429251 9359-9370. Max. coverage (+): 0. Max coverage (-): 0

Region: NODE\_296637\_length\_14495\_cov\_30.429251 9371-9381. Max. coverage (+): 0. Max coverage (-): 0

Region: NODE\_296637\_length\_14495\_cov\_30.429251 9382-9392. Max. coverage (+): 0. Max coverage (-): 0.01

Region: NODE\_296637\_length\_14495\_cov\_30.429251 9393-9403. Max. coverage (+): 0.02. Max coverage (-): 0.01

Region: NODE\_296637\_length\_14495\_cov\_30.429251 9404-9414. Max. coverage (+): 0.01. Max coverage (-): 0

Region: NODE\_296637\_length\_14495\_cov\_30.429251 9415-9426. Max. coverage (+): 0. Max coverage (-): 0

Region: NODE\_296637\_length\_14495\_cov\_30.429251 9427-9437. Max. coverage (+): 0. Max coverage (-): 0

Region: NODE\_296637\_length\_14495\_cov\_30.429251 9438-9448. Max. coverage (+): 0. Max coverage (-): 0

Region: NODE\_296637\_length\_14495\_cov\_30.429251 9449-9459. Max. coverage (+): 0.07. Max coverage (-): 0

Region: NODE\_296637\_length\_14495\_cov\_30.429251 9460-9471. Max. coverage (+): 0. Max coverage (-): 0

Region: NODE\_296637\_length\_14495\_cov\_30.429251 9472-9482. Max. coverage (+): 0. Max coverage (-): 0

Region: NODE\_296637\_length\_14495\_cov\_30.429251 9483-9493. Max. coverage (+): 0.03. Max coverage (-): 0

Region: NODE\_296637\_length\_14495\_cov\_30.429251 9494-9504. Max. coverage (+): 0.03. Max coverage (-): 0

Region: NODE\_296637\_length\_14495\_cov\_30.429251 9505-9516. Max. coverage (+): 0.03. Max coverage (-): 0

Region: NODE\_296637\_length\_14495\_cov\_30.429251 9517-9527. Max. coverage (+): 0.02. Max coverage (-): 0.02

Region: NODE\_296637\_length\_14495\_cov\_30.429251 9528-9538. Max. coverage (+): 0.01. Max coverage (-): 0.11

Region: NODE\_296637\_length\_14495\_cov\_30.429251 9539-9549. Max. coverage (+): 0.09. Max coverage (-): 0.03

Region: NODE\_296637\_length\_14495\_cov\_30.429251 9550-9560. Max. coverage (+): 0.09. Max coverage (-): 0.02

Region: NODE\_296637\_length\_14495\_cov\_30.429251 9561-9572. Max. coverage (+): 0. Max coverage (-): 0.01

Region: NODE\_296637\_length\_14495\_cov\_30.429251 9573-9583. Max. coverage (+): 0.04. Max coverage (-): 0.06

Region: NODE\_296637\_length\_14495\_cov\_30.429251 9584-9594. Max. coverage (+): 0.03. Max coverage (-): 0.01

Region: NODE\_296637\_length\_14495\_cov\_30.429251 9595-9605. Max. coverage (+): 0. Max coverage (-): 0

Region: NODE\_296637\_length\_14495\_cov\_30.429251 9606-9617. Max. coverage (+): 0. Max coverage (-): 0

Region: NODE\_296637\_length\_14495\_cov\_30.429251 9618-9628. Max. coverage (+): 0.07. Max coverage (-): 0

Region: NODE\_296637\_length\_14495\_cov\_30.429251 9629-9639. Max. coverage (+): 0.03. Max coverage (-): 0

Region: NODE\_296637\_length\_14495\_cov\_30.429251 9640-9650. Max. coverage (+): 0. Max coverage (-): 0.08

Region: NODE\_296637\_length\_14495\_cov\_30.429251 9651-9662. Max. coverage (+): 0. Max coverage (-): 0

Region: NODE\_296637\_length\_14495\_cov\_30.429251 9663-9673. Max. coverage (+): 0. Max coverage (-): 0

Region: NODE\_296637\_length\_14495\_cov\_30.429251 9674-9684. Max. coverage (+): 0.01. Max coverage (-): 0.02

Region: NODE\_296637\_length\_14495\_cov\_30.429251 9685-9695. Max. coverage (+): 0.02. Max coverage (-): 0

Region: NODE\_296637\_length\_14495\_cov\_30.429251 9696-9706. Max. coverage (+): 0. Max coverage (-): 0

Region: NODE\_296637\_length\_14495\_cov\_30.429251 9707-9718. Max. coverage (+): 0. Max coverage (-): 0

Region: NODE\_296637\_length\_14495\_cov\_30.429251 9719-9729. Max. coverage (+): 0. Max coverage (-): 0

Region: NODE\_296637\_length\_14495\_cov\_30.429251 9730-9740. Max. coverage (+): 0.03. Max coverage (-): 0.02

Region: NODE\_296637\_length\_14495\_cov\_30.429251 9741-9751. Max. coverage (+): 0.07. Max coverage (-): 0.02

Region: NODE\_296637\_length\_14495\_cov\_30.429251 9752-9763. Max. coverage (+): 0. Max coverage (-): 0.01

Region: NODE\_296637\_length\_14495\_cov\_30.429251 9764-9774. Max. coverage (+): 0. Max coverage (-): 0.39

Region: NODE\_296637\_length\_14495\_cov\_30.429251 9775-9785. Max. coverage (+): 0.08. Max coverage (-): 0.04

Region: NODE\_296637\_length\_14495\_cov\_30.429251 9786-9796. Max. coverage (+): 0.08. Max coverage (-): 0.06

Region: NODE\_296637\_length\_14495\_cov\_30.429251 9797-9808. Max. coverage (+): 0. Max coverage (-): 0.06

Region: NODE\_296637\_length\_14495\_cov\_30.429251 9809-9819. Max. coverage (+): 0.01. Max coverage (-): 0.04

Region: NODE\_296637\_length\_14495\_cov\_30.429251 9820-9830. Max. coverage (+): 0. Max coverage (-): 0

Region: NODE\_296637\_length\_14495\_cov\_30.429251 9831-9841. Max. coverage (+): 0. Max coverage (-): 0

Region: NODE\_296637\_length\_14495\_cov\_30.429251 9842-9853. Max. coverage (+): 0.16. Max coverage (-): 0.04

Region: NODE\_296637\_length\_14495\_cov\_30.429251 9854-9864. Max. coverage (+): 0. Max coverage (-): 0.04

Region: NODE\_296637\_length\_14495\_cov\_30.429251 9865-9875. Max. coverage (+): 0.04. Max coverage (-): 0

Region: NODE\_296637\_length\_14495\_cov\_30.429251 9876-9886. Max. coverage (+): 0.05. Max coverage (-): 0

Region: NODE\_296637\_length\_14495\_cov\_30.429251 9887-9897. Max. coverage (+): 0.02. Max coverage (-): 0.02

Region: NODE\_296637\_length\_14495\_cov\_30.429251 9898-9909. Max. coverage (+): 0. Max coverage (-): 0.11

Region: NODE\_296637\_length\_14495\_cov\_30.429251 9910-9920. Max. coverage (+): 0.06. Max coverage (-): 0

Region: NODE\_296637\_length\_14495\_cov\_30.429251 9921-9931. Max. coverage (+): 0. Max coverage (-): 0.04

Region: NODE\_296637\_length\_14495\_cov\_30.429251 9932-9942. Max. coverage (+): 0. Max coverage (-): 0

Region: NODE\_296637\_length\_14495\_cov\_30.429251 9943-9954. Max. coverage (+): 0. Max coverage (-): 0.04

Region: NODE\_296637\_length\_14495\_cov\_30.429251 9955-9965. Max. coverage (+): 0.04. Max coverage (-): 0.08

Region: NODE\_296637\_length\_14495\_cov\_30.429251 9966-9976. Max. coverage (+): 0.04. Max coverage (-): 0.04

Region: NODE\_296637\_length\_14495\_cov\_30.429251 9977-9987. Max. coverage (+): 0. Max coverage (-): 0

Region: NODE\_296637\_length\_14495\_cov\_30.429251 9988-9999. Max. coverage (+): 0. Max coverage (-): 0

Region: NODE\_296637\_length\_14495\_cov\_30.429251 10000-10010. Max. coverage (+): 0. Max coverage (-): 0.12

Region: NODE\_296637\_length\_14495\_cov\_30.429251 10011-10021. Max. coverage (+): 0. Max coverage (-): 0

Region: NODE\_296637\_length\_14495\_cov\_30.429251 10022-10032. Max. coverage (+): 0. Max coverage (-): 0.12

Region: NODE\_296637\_length\_14495\_cov\_30.429251 10033-10043. Max. coverage (+): 0. Max coverage (-): 0

Region: NODE\_296637\_length\_14495\_cov\_30.429251 10044-10055. Max. coverage (+): 0. Max coverage (-): 0.24

Region: NODE\_296637\_length\_14495\_cov\_30.429251 10056-10066. Max. coverage (+): 0. Max coverage (-): 0.2

Region: NODE\_296637\_length\_14495\_cov\_30.429251 10067-10077. Max. coverage (+): 0.04. Max coverage (-): 0.36

Region: NODE\_296637\_length\_14495\_cov\_30.429251 10078-10088. Max. coverage (+): 0. Max coverage (-): 0.24

Region: NODE\_296637\_length\_14495\_cov\_30.429251 10089-10100. Max. coverage (+): 0. Max coverage (-): 0.2

Region: NODE\_296637\_length\_14495\_cov\_30.429251 10101-10111. Max. coverage (+): 0. Max coverage (-): 0.08

Region: NODE\_296637\_length\_14495\_cov\_30.429251 10112-10122. Max. coverage (+): 0.12. Max coverage (-): 0

Region: NODE\_296637\_length\_14495\_cov\_30.429251 10123-10133. Max. coverage (+): 0. Max coverage (-): 0.04

Region: NODE\_296637\_length\_14495\_cov\_30.429251 10134-10145. Max. coverage (+): 0. Max coverage (-): 0.36

Region: NODE\_296637\_length\_14495\_cov\_30.429251 10146-10156. Max. coverage (+): 0. Max coverage (-): 0

Region: NODE\_296637\_length\_14495\_cov\_30.429251 10157-10167. Max. coverage (+): 0. Max coverage (-): 0

Region: NODE\_296637\_length\_14495\_cov\_30.429251 10168-10178. Max. coverage (+): 0. Max coverage (-): 0

Region: NODE\_296637\_length\_14495\_cov\_30.429251 10179-10189. Max. coverage (+): 0. Max coverage (-): 0

Region: NODE\_296637\_length\_14495\_cov\_30.429251 10190-10201. Max. coverage (+): 0. Max coverage (-): 0.24

Region: NODE\_296637\_length\_14495\_cov\_30.429251 10202-10212. Max. coverage (+): 0.52. Max coverage (-): 0.2

Region: NODE\_296637\_length\_14495\_cov\_30.429251 10213-10223. Max. coverage (+): 0.08. Max coverage (-): 0

Region: NODE\_296637\_length\_14495\_cov\_30.429251 10224-10234. Max. coverage (+): 0. Max coverage (-): 0

Region: NODE\_296637\_length\_14495\_cov\_30.429251 10235-10246. Max. coverage (+): 0. Max coverage (-): 0.08

Region: NODE\_296637\_length\_14495\_cov\_30.429251 10247-10257. Max. coverage (+): 0. Max coverage (-): 3.39

Region: NODE\_296637\_length\_14495\_cov\_30.429251 10258-10268. Max. coverage (+): 0.24. Max coverage (-): 0.85

Region: NODE\_296637\_length\_14495\_cov\_30.429251 10269-10279. Max. coverage (+): 0.36. Max coverage (-): 0

Region: NODE\_296637\_length\_14495\_cov\_30.429251 10280-10291. Max. coverage (+): 0. Max coverage (-): 0

Region: NODE\_296637\_length\_14495\_cov\_30.429251 10292-10302. Max. coverage (+): 0. Max coverage (-): 0

Region: NODE\_296637\_length\_14495\_cov\_30.429251 10303-10313. Max. coverage (+): 0.08. Max coverage (-): 0.2

Region: NODE\_296637\_length\_14495\_cov\_30.429251 10314-10324. Max. coverage (+): 0.04. Max coverage (-): 0.04

Region: NODE\_296637\_length\_14495\_cov\_30.429251 10325-10335. Max. coverage (+): 0.04. Max coverage (-): 0

Region: NODE\_296637\_length\_14495\_cov\_30.429251 10336-10347. Max. coverage (+): 0. Max coverage (-): 0

Region: NODE\_296637\_length\_14495\_cov\_30.429251 10348-10358. Max. coverage (+): 0.04. Max coverage (-): 0.08

Region: NODE\_296637\_length\_14495\_cov\_30.429251 10359-10369. Max. coverage (+): 0.04. Max coverage (-): 0.69

Region: NODE\_296637\_length\_14495\_cov\_30.429251 10370-10380. Max. coverage (+): 0.04. Max coverage (-): 0.97

Region: NODE\_296637\_length\_14495\_cov\_30.429251 10381-10392. Max. coverage (+): 0.08. Max coverage (-): 1.33

Region: NODE\_296637\_length\_14495\_cov\_30.429251 10393-10403. Max. coverage (+): 0.12. Max coverage (-): 0

Region: NODE\_296637\_length\_14495\_cov\_30.429251 10404-10414. Max. coverage (+): 0.08. Max coverage (-): 0

Region: NODE\_296637\_length\_14495\_cov\_30.429251 10415-10425. Max. coverage (+): 0.04. Max coverage (-): 0

Region: NODE\_296637\_length\_14495\_cov\_30.429251 10426-10437. Max. coverage (+): 0.08. Max coverage (-): 0

Region: NODE\_296637\_length\_14495\_cov\_30.429251 10438-10448. Max. coverage (+): 0. Max coverage (-): 0.16

Region: NODE\_296637\_length\_14495\_cov\_30.429251 10449-10459. Max. coverage (+): 0. Max coverage (-): 0.12

Region: NODE\_296637\_length\_14495\_cov\_30.429251 10460-10470. Max. coverage (+): 0.24. Max coverage (-): 0

Region: NODE\_296637\_length\_14495\_cov\_30.429251 10471-10482. Max. coverage (+): 0.08. Max coverage (-): 0.08

Region: NODE\_296637\_length\_14495\_cov\_30.429251 10483-10493. Max. coverage (+): 0. Max coverage (-): 1.86

Region: NODE\_296637\_length\_14495\_cov\_30.429251 10494-10504. Max. coverage (+): 0.69. Max coverage (-): 0.04

Region: NODE\_296637\_length\_14495\_cov\_30.429251 10505-10515. Max. coverage (+): 0.65. Max coverage (-): 0

Region: NODE\_296637\_length\_14495\_cov\_30.429251 10516-10526. Max. coverage (+): 0. Max coverage (-): 0.57

Region: NODE\_296637\_length\_14495\_cov\_30.429251 10527-10538. Max. coverage (+): 0.04. Max coverage (-): 0.61

Region: NODE\_296637\_length\_14495\_cov\_30.429251 10539-10549. Max. coverage (+): 0.08. Max coverage (-): 0

Region: NODE\_296637\_length\_14495\_cov\_30.429251 10550-10560. Max. coverage (+): 0.04. Max coverage (-): 0.04

Region: NODE\_296637\_length\_14495\_cov\_30.429251 10561-10571. Max. coverage (+): 0.16. Max coverage (-): 0.24

Region: NODE\_296637\_length\_14495\_cov\_30.429251 10572-10583. Max. coverage (+): 0. Max coverage (-): 0.57

Region: NODE\_296637\_length\_14495\_cov\_30.429251 10584-10594. Max. coverage (+): 0. Max coverage (-): 0.2

Region: NODE\_296637\_length\_14495\_cov\_30.429251 10595-10605. Max. coverage (+): 0. Max coverage (-): 0

Region: NODE\_296637\_length\_14495\_cov\_30.429251 10606-10616. Max. coverage (+): 0. Max coverage (-): 0

Region: NODE\_296637\_length\_14495\_cov\_30.429251 10617-10628. Max. coverage (+): 0. Max coverage (-): 0.08

Region: NODE\_296637\_length\_14495\_cov\_30.429251 10629-10639. Max. coverage (+): 0. Max coverage (-): 0.08

Region: NODE\_296637\_length\_14495\_cov\_30.429251 10640-10650. Max. coverage (+): 0. Max coverage (-): 0.16

Region: NODE\_296637\_length\_14495\_cov\_30.429251 10651-10661. Max. coverage (+): 0.04. Max coverage (-): 0.85

Region: NODE\_296637\_length\_14495\_cov\_30.429251 10662-10672. Max. coverage (+): 0.48. Max coverage (-): 0.04

Region: NODE\_296637\_length\_14495\_cov\_30.429251 10673-10684. Max. coverage (+): 0.48. Max coverage (-): 0.12

Region: NODE\_296637\_length\_14495\_cov\_30.429251 10685-10695. Max. coverage (+): 0.04. Max coverage (-): 0.2

Region: NODE\_296637\_length\_14495\_cov\_30.429251 10696-10706. Max. coverage (+): 0. Max coverage (-): 0.24

Region: NODE\_296637\_length\_14495\_cov\_30.429251 10707-10717. Max. coverage (+): 0.04. Max coverage (-): 0.36

Region: NODE\_296637\_length\_14495\_cov\_30.429251 10718-10729. Max. coverage (+): 0.12. Max coverage (-): 0.08

Region: NODE\_296637\_length\_14495\_cov\_30.429251 10730-10740. Max. coverage (+): 0.12. Max coverage (-): 0.65

Region: NODE\_296637\_length\_14495\_cov\_30.429251 10741-10751. Max. coverage (+): 0.08. Max coverage (-): 0.81

Region: NODE\_296637\_length\_14495\_cov\_30.429251 10752-10762. Max. coverage (+): 0.52. Max coverage (-): 0.08

Region: NODE\_296637\_length\_14495\_cov\_30.429251 10763-10774. Max. coverage (+): 0.48. Max coverage (-): 0.69

Region: NODE\_296637\_length\_14495\_cov\_30.429251 10775-10785. Max. coverage (+): 0. Max coverage (-): 0.65

Region: NODE\_296637\_length\_14495\_cov\_30.429251 10786-10796. Max. coverage (+): 0. Max coverage (-): 0.4

Region: NODE\_296637\_length\_14495\_cov\_30.429251 10797-10807. Max. coverage (+): 0.04. Max coverage (-): 0.08

Region: NODE\_296637\_length\_14495\_cov\_30.429251 10808-10818. Max. coverage (+): 0.04. Max coverage (-): 0.08

Region: NODE\_296637\_length\_14495\_cov\_30.429251 10819-10830. Max. coverage (+): 0.04. Max coverage (-): 0

Region: NODE\_296637\_length\_14495\_cov\_30.429251 10831-10841. Max. coverage (+): 0.2. Max coverage (-): 1.62

Region: NODE\_296637\_length\_14495\_cov\_30.429251 10842-10852. Max. coverage (+): 0. Max coverage (-): 0.52

Region: NODE\_296637\_length\_14495\_cov\_30.429251 10853-10863. Max. coverage (+): 0. Max coverage (-): 1.57

Region: NODE\_296637\_length\_14495\_cov\_30.429251 10864-10875. Max. coverage (+): 0.08. Max coverage (-): 2.71

Region: NODE\_296637\_length\_14495\_cov\_30.429251 10876-10886. Max. coverage (+): 0.12. Max coverage (-): 0.36

Region: NODE\_296637\_length\_14495\_cov\_30.429251 10887-10897. Max. coverage (+): 0. Max coverage (-): 2.54

Region: NODE\_296637\_length\_14495\_cov\_30.429251 10898-10908. Max. coverage (+): 0.04. Max coverage (-): 2.5

Region: NODE\_296637\_length\_14495\_cov\_30.429251 10909-10920. Max. coverage (+): 0.04. Max coverage (-): 0.12

Region: NODE\_296637\_length\_14495\_cov\_30.429251 10921-10931. Max. coverage (+): 0.04. Max coverage (-): 0.16

Region: NODE\_296637\_length\_14495\_cov\_30.429251 10932-10942. Max. coverage (+): 0. Max coverage (-): 0.08

Region: NODE\_296637\_length\_14495\_cov\_30.429251 10943-10953. Max. coverage (+): 0. Max coverage (-): 0.65

Region: NODE\_296637\_length\_14495\_cov\_30.429251 10954-10964. Max. coverage (+): 0.08. Max coverage (-): 0.12

Region: NODE\_296637\_length\_14495\_cov\_30.429251 10965-10976. Max. coverage (+): 0.08. Max coverage (-): 0

Region: NODE\_296637\_length\_14495\_cov\_30.429251 10977-10987. Max. coverage (+): 0. Max coverage (-): 0

Region: NODE\_296637\_length\_14495\_cov\_30.429251 10988-10998. Max. coverage (+): 0. Max coverage (-): 0.04

Region: NODE\_296637\_length\_14495\_cov\_30.429251 10999-11009. Max. coverage (+): 0. Max coverage (-): 0.04

Region: NODE\_296637\_length\_14495\_cov\_30.429251 11010-11021. Max. coverage (+): 0. Max coverage (-): 0.04

Region: NODE\_296637\_length\_14495\_cov\_30.429251 11022-11032. Max. coverage (+): 0. Max coverage (-): 0.97

Region: NODE\_296637\_length\_14495\_cov\_30.429251 11033-11043. Max. coverage (+): 0. Max coverage (-): 0.89

Region: NODE\_296637\_length\_14495\_cov\_30.429251 11044-11054. Max. coverage (+): 0.04. Max coverage (-): 0.48

Region: NODE\_296637\_length\_14495\_cov\_30.429251 11055-11066. Max. coverage (+): 0. Max coverage (-): 0.57

Region: NODE\_296637\_length\_14495\_cov\_30.429251 11067-11077. Max. coverage (+): 0. Max coverage (-): 0.08

Region: NODE\_296637\_length\_14495\_cov\_30.429251 11078-11088. Max. coverage (+): 0. Max coverage (-): 0.08

Region: NODE\_296637\_length\_14495\_cov\_30.429251 11089-11099. Max. coverage (+): 0. Max coverage (-): 0

Region: NODE\_296637\_length\_14495\_cov\_30.429251 11100-11110. Max. coverage (+): 0. Max coverage (-): 0.48

Region: NODE\_296637\_length\_14495\_cov\_30.429251 11111-11122. Max. coverage (+): 0. Max coverage (-): 0.48

Region: NODE\_296637\_length\_14495\_cov\_30.429251 11123-11133. Max. coverage (+): 0. Max coverage (-): 0

Region: NODE\_296637\_length\_14495\_cov\_30.429251 11134-11144. Max. coverage (+): 0. Max coverage (-): 0

Region: NODE\_296637\_length\_14495\_cov\_30.429251 11145-11155. Max. coverage (+): 0. Max coverage (-): 0

Region: NODE\_296637\_length\_14495\_cov\_30.429251 11156-11167. Max. coverage (+): 0. Max coverage (-): 0

Region: NODE\_296637\_length\_14495\_cov\_30.429251 11168-11178. Max. coverage (+): 0. Max coverage (-): 0

Region: NODE\_296637\_length\_14495\_cov\_30.429251 11179-11189. Max. coverage (+): 0.04. Max coverage (-): 0.12

Region: NODE\_296637\_length\_14495\_cov\_30.429251 11190-11200. Max. coverage (+): 0. Max coverage (-): 0.65

Region: NODE\_296637\_length\_14495\_cov\_30.429251 11201-11212. Max. coverage (+): 0.32. Max coverage (-): 0.36

Region: NODE\_296637\_length\_14495\_cov\_30.429251 11213-11223. Max. coverage (+): 0.32. Max coverage (-): 0

Region: NODE\_296637\_length\_14495\_cov\_30.429251 11224-11234. Max. coverage (+): 0. Max coverage (-): 0

Region: NODE\_296637\_length\_14495\_cov\_30.429251 11235-11245. Max. coverage (+): 0.08. Max coverage (-): 0.24

Region: NODE\_296637\_length\_14495\_cov\_30.429251 11246-11257. Max. coverage (+): 0.12. Max coverage (-): 7.31

Region: NODE\_296637\_length\_14495\_cov\_30.429251 11258-11268. Max. coverage (+): 0.44. Max coverage (-): 0.12

Region: NODE\_296637\_length\_14495\_cov\_30.429251 11269-11279. Max. coverage (+): 0.04. Max coverage (-): 0.77

Region: NODE\_296637\_length\_14495\_cov\_30.429251 11280-11290. Max. coverage (+): 0. Max coverage (-): 0.77

Region: NODE\_296637\_length\_14495\_cov\_30.429251 11291-11301. Max. coverage (+): 0. Max coverage (-): 6.62

Region: NODE\_296637\_length\_14495\_cov\_30.429251 11302-11313. Max. coverage (+): 0. Max coverage (-): 8.28

Region: NODE\_296637\_length\_14495\_cov\_30.429251 11314-11324. Max. coverage (+): 0.16. Max coverage (-): 0.48

Region: NODE\_296637\_length\_14495\_cov\_30.429251 11325-11335. Max. coverage (+): 0.28. Max coverage (-): 0.12

Region: NODE\_296637\_length\_14495\_cov\_30.429251 11336-11346. Max. coverage (+): 0. Max coverage (-): 0

Region: NODE\_296637\_length\_14495\_cov\_30.429251 11347-11358. Max. coverage (+): 0. Max coverage (-): 0.04

Region: NODE\_296637\_length\_14495\_cov\_30.429251 11359-11369. Max. coverage (+): 0. Max coverage (-): 2.22

Region: NODE\_296637\_length\_14495\_cov\_30.429251 11370-11380. Max. coverage (+): 0. Max coverage (-): 1.98

Region: NODE\_296637\_length\_14495\_cov\_30.429251 11381-11391. Max. coverage (+): 0.04. Max coverage (-): 0.16

Region: NODE\_296637\_length\_14495\_cov\_30.429251 11392-11403. Max. coverage (+): 0. Max coverage (-): 0

Region: NODE\_296637\_length\_14495\_cov\_30.429251 11404-11414. Max. coverage (+): 0. Max coverage (-): 2.02

Region: NODE\_296637\_length\_14495\_cov\_30.429251 11415-11425. Max. coverage (+): 0.04. Max coverage (-): 2.54

Region: NODE\_296637\_length\_14495\_cov\_30.429251 11426-11436. Max. coverage (+): 0.12. Max coverage (-): 0

Region: NODE\_296637\_length\_14495\_cov\_30.429251 11437-11447. Max. coverage (+): 0.04. Max coverage (-): 0.12

Region: NODE\_296637\_length\_14495\_cov\_30.429251 11448-11459. Max. coverage (+): 0.16. Max coverage (-): 0.2

Region: NODE\_296637\_length\_14495\_cov\_30.429251 11460-11470. Max. coverage (+): 0.08. Max coverage (-): 0.36

Region: NODE\_296637\_length\_14495\_cov\_30.429251 11471-11481. Max. coverage (+): 0.12. Max coverage (-): 0.36

Region: NODE\_296637\_length\_14495\_cov\_30.429251 11482-11492. Max. coverage (+): 0.08. Max coverage (-): 0

Region: NODE\_296637\_length\_14495\_cov\_30.429251 11493-11504. Max. coverage (+): 0. Max coverage (-): 0.12

Region: NODE\_296637\_length\_14495\_cov\_30.429251 11505-11515. Max. coverage (+): 0. Max coverage (-): 0.16

Region: NODE\_296637\_length\_14495\_cov\_30.429251 11516-11526. Max. coverage (+): 0.77. Max coverage (-): 0.04

Region: NODE\_296637\_length\_14495\_cov\_30.429251 11527-11537. Max. coverage (+): 0.48. Max coverage (-): 0.04

Region: NODE\_296637\_length\_14495\_cov\_30.429251 11538-11549. Max. coverage (+): 0.04. Max coverage (-): 0

Region: NODE\_296637\_length\_14495\_cov\_30.429251 11550-11560. Max. coverage (+): 0. Max coverage (-): 0

Region: NODE\_296637\_length\_14495\_cov\_30.429251 11561-11571. Max. coverage (+): 0. Max coverage (-): 0.08

Region: NODE\_296637\_length\_14495\_cov\_30.429251 11572-11582. Max. coverage (+): 0. Max coverage (-): 0.04

Region: NODE\_296637\_length\_14495\_cov\_30.429251 11583-11593. Max. coverage (+): 0. Max coverage (-): 0.08

Region: NODE\_296637\_length\_14495\_cov\_30.429251 11594-11605. Max. coverage (+): 0. Max coverage (-): 0

Region: NODE\_296637\_length\_14495\_cov\_30.429251 11606-11616. Max. coverage (+): 0.04. Max coverage (-): 0

Region: NODE\_296637\_length\_14495\_cov\_30.429251 11617-11627. Max. coverage (+): 0. Max coverage (-): 0.24

Region: NODE\_296637\_length\_14495\_cov\_30.429251 11628-11638. Max. coverage (+): 0.08. Max coverage (-): 0.36

Region: NODE\_296637\_length\_14495\_cov\_30.429251 11639-11650. Max. coverage (+): 0.32. Max coverage (-): 0.08

Region: NODE\_296637\_length\_14495\_cov\_30.429251 11651-11661. Max. coverage (+): 0.32. Max coverage (-): 0.73

Region: NODE\_296637\_length\_14495\_cov\_30.429251 11662-11672. Max. coverage (+): 0. Max coverage (-): 0.36

Region: NODE\_296637\_length\_14495\_cov\_30.429251 11673-11683. Max. coverage (+): 0.32. Max coverage (-): 0.16

Region: NODE\_296637\_length\_14495\_cov\_30.429251 11684-11695. Max. coverage (+): 0.16. Max coverage (-): 0.12

Region: NODE\_296637\_length\_14495\_cov\_30.429251 11696-11706. Max. coverage (+): 0.08. Max coverage (-): 0.36

Region: NODE\_296637\_length\_14495\_cov\_30.429251 11707-11717. Max. coverage (+): 0. Max coverage (-): 0.69

Region: NODE\_296637\_length\_14495\_cov\_30.429251 11718-11728. Max. coverage (+): 0.12. Max coverage (-): 0.08

Region: NODE\_296637\_length\_14495\_cov\_30.429251 11729-11739. Max. coverage (+): 0.12. Max coverage (-): 0.12

Region: NODE\_296637\_length\_14495\_cov\_30.429251 11740-11751. Max. coverage (+): 0. Max coverage (-): 0.16

Region: NODE\_296637\_length\_14495\_cov\_30.429251 11752-11762. Max. coverage (+): 0.04. Max coverage (-): 0.04

Region: NODE\_296637\_length\_14495\_cov\_30.429251 11763-11773. Max. coverage (+): 0.04. Max coverage (-): 1.01

Region: NODE\_296637\_length\_14495\_cov\_30.429251 11774-11784. Max. coverage (+): 0. Max coverage (-): 1.01

Region: NODE\_296637\_length\_14495\_cov\_30.429251 11785-11796. Max. coverage (+): 0. Max coverage (-): 0.48

Region: NODE\_296637\_length\_14495\_cov\_30.429251 11797-11807. Max. coverage (+): 0.04. Max coverage (-): 0.32

Region: NODE\_296637\_length\_14495\_cov\_30.429251 11808-11818. Max. coverage (+): 0.04. Max coverage (-): 5.05

Region: NODE\_296637\_length\_14495\_cov\_30.429251 11819-11829. Max. coverage (+): 0.04. Max coverage (-): 0.12

Region: NODE\_296637\_length\_14495\_cov\_30.429251 11830-11841. Max. coverage (+): 0.04. Max coverage (-): 0

Region: NODE\_296637\_length\_14495\_cov\_30.429251 11842-11852. Max. coverage (+): 0.04. Max coverage (-): 0.4

Region: NODE\_296637\_length\_14495\_cov\_30.429251 11853-11863. Max. coverage (+): 0.04. Max coverage (-): 0.44

Region: NODE\_296637\_length\_14495\_cov\_30.429251 11864-11874. Max. coverage (+): 0.4. Max coverage (-): 0.48

Region: NODE\_296637\_length\_14495\_cov\_30.429251 11875-11886. Max. coverage (+): 0. Max coverage (-): 2.42

Region: NODE\_296637\_length\_14495\_cov\_30.429251 11887-11897. Max. coverage (+): 0.12. Max coverage (-): 0.04

Region: NODE\_296637\_length\_14495\_cov\_30.429251 11898-11908. Max. coverage (+): 0.12. Max coverage (-): 0

Region: NODE\_296637\_length\_14495\_cov\_30.429251 11909-11919. Max. coverage (+): 0.04. Max coverage (-): 0

Region: NODE\_296637\_length\_14495\_cov\_30.429251 11920-11930. Max. coverage (+): 0. Max coverage (-): 0.32

Region: NODE\_296637\_length\_14495\_cov\_30.429251 11931-11942. Max. coverage (+): 0. Max coverage (-): 0.32

Region: NODE\_296637\_length\_14495\_cov\_30.429251 11943-11953. Max. coverage (+): 0. Max coverage (-): 0.08

Region: NODE\_296637\_length\_14495\_cov\_30.429251 11954-11964. Max. coverage (+): 0.04. Max coverage (-): 0

Region: NODE\_296637\_length\_14495\_cov\_30.429251 11965-11975. Max. coverage (+): 0.04. Max coverage (-): 1.57

Region: NODE\_296637\_length\_14495\_cov\_30.429251 11976-11987. Max. coverage (+): 0. Max coverage (-): 0.69

Region: NODE\_296637\_length\_14495\_cov\_30.429251 11988-11998. Max. coverage (+): 0. Max coverage (-): 0

Region: NODE\_296637\_length\_14495\_cov\_30.429251 11999-12009. Max. coverage (+): 0. Max coverage (-): 0.04

Region: NODE\_296637\_length\_14495\_cov\_30.429251 12010-12020. Max. coverage (+): 0. Max coverage (-): 0.04

Region: NODE\_296637\_length\_14495\_cov\_30.429251 12021-12032. Max. coverage (+): 0. Max coverage (-): 0.04

Region: NODE\_296637\_length\_14495\_cov\_30.429251 12033-12043. Max. coverage (+): 0. Max coverage (-): 0.08

Region: NODE\_296637\_length\_14495\_cov\_30.429251 12044-12054. Max. coverage (+): 0. Max coverage (-): 0.04

Region: NODE\_296637\_length\_14495\_cov\_30.429251 12055-12065. Max. coverage (+): 0. Max coverage (-): 0.04

Region: NODE\_296637\_length\_14495\_cov\_30.429251 12066-12076. Max. coverage (+): 0. Max coverage (-): 0

Region: NODE\_296637\_length\_14495\_cov\_30.429251 12077-12088. Max. coverage (+): 0.04. Max coverage (-): 0.08

Region: NODE\_296637\_length\_14495\_cov\_30.429251 12089-12099. Max. coverage (+): 0.04. Max coverage (-): 0.08

Region: NODE\_296637\_length\_14495\_cov\_30.429251 12100-12110. Max. coverage (+): 0. Max coverage (-): 0.08

Region: NODE\_296637\_length\_14495\_cov\_30.429251 12111-12121. Max. coverage (+): 0. Max coverage (-): 0.12

Region: NODE\_296637\_length\_14495\_cov\_30.429251 12122-12133. Max. coverage (+): 0.08. Max coverage (-): 0.12

Region: NODE\_296637\_length\_14495\_cov\_30.429251 12134-12144. Max. coverage (+): 0. Max coverage (-): 0.04

Region: NODE\_296637\_length\_14495\_cov\_30.429251 12145-12155. Max. coverage (+): 0.04. Max coverage (-): 0.04

Region: NODE\_296637\_length\_14495\_cov\_30.429251 12156-12166. Max. coverage (+): 0. Max coverage (-): 0

Region: NODE\_296637\_length\_14495\_cov\_30.429251 12167-12178. Max. coverage (+): 0. Max coverage (-): 0

Region: NODE\_296637\_length\_14495\_cov\_30.429251 12179-12189. Max. coverage (+): 0. Max coverage (-): 0.08

Region: NODE\_296637\_length\_14495\_cov\_30.429251 12190-12200. Max. coverage (+): 0. Max coverage (-): 0.57

Region: NODE\_296637\_length\_14495\_cov\_30.429251 12201-12211. Max. coverage (+): 0.04. Max coverage (-): 3.07

Region: NODE\_296637\_length\_14495\_cov\_30.429251 12212-12222. Max. coverage (+): 0.12. Max coverage (-): 2.02

Region: NODE\_296637\_length\_14495\_cov\_30.429251 12223-12234. Max. coverage (+): 0.16. Max coverage (-): 0.48

Region: NODE\_296637\_length\_14495\_cov\_30.429251 12235-12245. Max. coverage (+): 0. Max coverage (-): 0.2

Region: NODE\_296637\_length\_14495\_cov\_30.429251 12246-12256. Max. coverage (+): 0.16. Max coverage (-): 0

Region: NODE\_296637\_length\_14495\_cov\_30.429251 12257-12267. Max. coverage (+): 0.16. Max coverage (-): 0.12

Region: NODE\_296637\_length\_14495\_cov\_30.429251 12268-12279. Max. coverage (+): 0. Max coverage (-): 0.12

Region: NODE\_296637\_length\_14495\_cov\_30.429251 12280-12290. Max. coverage (+): 0.04. Max coverage (-): 0

Region: NODE\_296637\_length\_14495\_cov\_30.429251 12291-12301. Max. coverage (+): 0.24. Max coverage (-): 0.04

Region: NODE\_296637\_length\_14495\_cov\_30.429251 12302-12312. Max. coverage (+): 0.08. Max coverage (-): 2.34

Region: NODE\_296637\_length\_14495\_cov\_30.429251 12313-12324. Max. coverage (+): 0.04. Max coverage (-): 0.28

Region: NODE\_296637\_length\_14495\_cov\_30.429251 12325-12335. Max. coverage (+): 0.57. Max coverage (-): 0.12

Region: NODE\_296637\_length\_14495\_cov\_30.429251 12336-12346. Max. coverage (+): 0.16. Max coverage (-): 0.65

Region: NODE\_296637\_length\_14495\_cov\_30.429251 12347-12357. Max. coverage (+): 0. Max coverage (-): 0.04

Region: NODE\_296637\_length\_14495\_cov\_30.429251 12358-12368. Max. coverage (+): 0.48. Max coverage (-): 0.4

Region: NODE\_296637\_length\_14495\_cov\_30.429251 12369-12380. Max. coverage (+): 0.44. Max coverage (-): 2.22

Region: NODE\_296637\_length\_14495\_cov\_30.429251 12381-12391. Max. coverage (+): 0. Max coverage (-): 0.69

Region: NODE\_296637\_length\_14495\_cov\_30.429251 12392-12402. Max. coverage (+): 0. Max coverage (-): 0.89

Region: NODE\_296637\_length\_14495\_cov\_30.429251 12403-12413. Max. coverage (+): 0. Max coverage (-): 0.77

Region: NODE\_296637\_length\_14495\_cov\_30.429251 12414-12425. Max. coverage (+): 0. Max coverage (-): 0.65

Region: NODE\_296637\_length\_14495\_cov\_30.429251 12426-12436. Max. coverage (+): 0. Max coverage (-): 0.08

Region: NODE\_296637\_length\_14495\_cov\_30.429251 12437-12447. Max. coverage (+): 0.04. Max coverage (-): 0.04

Region: NODE\_296637\_length\_14495\_cov\_30.429251 12448-12458. Max. coverage (+): 0.04. Max coverage (-): 0.08

Region: NODE\_296637\_length\_14495\_cov\_30.429251 12459-12470. Max. coverage (+): 0.4. Max coverage (-): 0.16

Region: NODE\_296637\_length\_14495\_cov\_30.429251 12471-12481. Max. coverage (+): 0.4. Max coverage (-): 0.16

Region: NODE\_296637\_length\_14495\_cov\_30.429251 12482-12492. Max. coverage (+): 0. Max coverage (-): 0.12

Region: NODE\_296637\_length\_14495\_cov\_30.429251 12493-12503. Max. coverage (+): 0. Max coverage (-): 0.12

Region: NODE\_296637\_length\_14495\_cov\_30.429251 12504-12514. Max. coverage (+): 0. Max coverage (-): 0.2

Region: NODE\_296637\_length\_14495\_cov\_30.429251 12515-12526. Max. coverage (+): 0.04. Max coverage (-): 0

Region: NODE\_296637\_length\_14495\_cov\_30.429251 12527-12537. Max. coverage (+): 0.04. Max coverage (-): 0

Region: NODE\_296637\_length\_14495\_cov\_30.429251 12538-12548. Max. coverage (+): 0.04. Max coverage (-): 0.08

Region: NODE\_296637\_length\_14495\_cov\_30.429251 12549-12559. Max. coverage (+): 0. Max coverage (-): 0

Region: NODE\_296637\_length\_14495\_cov\_30.429251 12560-12571. Max. coverage (+): 0. Max coverage (-): 0.08

Region: NODE\_296637\_length\_14495\_cov\_30.429251 12572-12582. Max. coverage (+): 0. Max coverage (-): 0.48

Region: NODE\_296637\_length\_14495\_cov\_30.429251 12583-12593. Max. coverage (+): 0.04. Max coverage (-): 6.99

Region: NODE\_296637\_length\_14495\_cov\_30.429251 12594-12604. Max. coverage (+): 0.04. Max coverage (-): 9.53

Region: NODE\_296637\_length\_14495\_cov\_30.429251 12605-12616. Max. coverage (+): 1.37. Max coverage (-): 0.04

Region: NODE\_296637\_length\_14495\_cov\_30.429251 12617-12627. Max. coverage (+): 1.01. Max coverage (-): 0

Region: NODE\_296637\_length\_14495\_cov\_30.429251 12628-12638. Max. coverage (+): 0. Max coverage (-): 0.28

Region: NODE\_296637\_length\_14495\_cov\_30.429251 12639-12649. Max. coverage (+): 0. Max coverage (-): 0.04

Region: NODE\_296637\_length\_14495\_cov\_30.429251 12650-12661. Max. coverage (+): 0.04. Max coverage (-): 0.48

Region: NODE\_296637\_length\_14495\_cov\_30.429251 12662-12672. Max. coverage (+): 0.04. Max coverage (-): 0.2

Region: NODE\_296637\_length\_14495\_cov\_30.429251 12673-12683. Max. coverage (+): 0.04. Max coverage (-): 0.08

Region: NODE\_296637\_length\_14495\_cov\_30.429251 12684-12694. Max. coverage (+): 0. Max coverage (-): 0.24

Region: NODE\_296637\_length\_14495\_cov\_30.429251 12695-12705. Max. coverage (+): 0. Max coverage (-): 0.08

Region: NODE\_296637\_length\_14495\_cov\_30.429251 12706-12717. Max. coverage (+): 0. Max coverage (-): 0

Region: NODE\_296637\_length\_14495\_cov\_30.429251 12718-12728. Max. coverage (+): 0. Max coverage (-): 2.38

Region: NODE\_296637\_length\_14495\_cov\_30.429251 12729-12739. Max. coverage (+): 0. Max coverage (-): 2.38

Region: NODE\_296637\_length\_14495\_cov\_30.429251 12740-12750. Max. coverage (+): 0. Max coverage (-): 0

Region: NODE\_296637\_length\_14495\_cov\_30.429251 12751-12762. Max. coverage (+): 0. Max coverage (-): 0

Region: NODE\_296637\_length\_14495\_cov\_30.429251 12763-12773. Max. coverage (+): 0. Max coverage (-): 0.08

Region: NODE\_296637\_length\_14495\_cov\_30.429251 12774-12784. Max. coverage (+): 0. Max coverage (-): 0

Region: NODE\_296637\_length\_14495\_cov\_30.429251 12785-12795. Max. coverage (+): 0. Max coverage (-): 0

Region: NODE\_296637\_length\_14495\_cov\_30.429251 12796-12807. Max. coverage (+): 0. Max coverage (-): 0.04

Region: NODE\_296637\_length\_14495\_cov\_30.429251 12808-12818. Max. coverage (+): 0. Max coverage (-): 0

Region: NODE\_296637\_length\_14495\_cov\_30.429251 12819-12829. Max. coverage (+): 0. Max coverage (-): 0

Region: NODE\_296637\_length\_14495\_cov\_30.429251 12830-12840. Max. coverage (+): 0. Max coverage (-): 0

Region: NODE\_296637\_length\_14495\_cov\_30.429251 12841-12851. Max. coverage (+): 0. Max coverage (-): 0

Region: NODE\_296637\_length\_14495\_cov\_30.429251 12852-12863. Max. coverage (+): 0. Max coverage (-): 0.16

Region: NODE\_296637\_length\_14495\_cov\_30.429251 12864-12874. Max. coverage (+): 0. Max coverage (-): 0.08

Region: NODE\_296637\_length\_14495\_cov\_30.429251 12875-12885. Max. coverage (+): 0.04. Max coverage (-): 0.57

Region: NODE\_296637\_length\_14495\_cov\_30.429251 12886-12896. Max. coverage (+): 0.04. Max coverage (-): 1.25

Region: NODE\_296637\_length\_14495\_cov\_30.429251 12897-12908. Max. coverage (+): 0.12. Max coverage (-): 0.08

Region: NODE\_296637\_length\_14495\_cov\_30.429251 12909-12919. Max. coverage (+): 0.08. Max coverage (-): 0.16

Region: NODE\_296637\_length\_14495\_cov\_30.429251 12920-12930. Max. coverage (+): 0.48. Max coverage (-): 0.08

Region: NODE\_296637\_length\_14495\_cov\_30.429251 12931-12941. Max. coverage (+): 0.48. Max coverage (-): 0.52

Region: NODE\_296637\_length\_14495\_cov\_30.429251 12942-12953. Max. coverage (+): 0.04. Max coverage (-): 0.61

Region: NODE\_296637\_length\_14495\_cov\_30.429251 12954-12964. Max. coverage (+): 1.37. Max coverage (-): 0.36

Region: NODE\_296637\_length\_14495\_cov\_30.429251 12965-12975. Max. coverage (+): 0.57. Max coverage (-): 0.04

Region: NODE\_296637\_length\_14495\_cov\_30.429251 12976-12986. Max. coverage (+): 0.52. Max coverage (-): 0.04

Region: NODE\_296637\_length\_14495\_cov\_30.429251 12987-12997. Max. coverage (+): 0. Max coverage (-): 0.04

Region: NODE\_296637\_length\_14495\_cov\_30.429251 12998-13009. Max. coverage (+): 0.12. Max coverage (-): 0.24

Region: NODE\_296637\_length\_14495\_cov\_30.429251 13010-13020. Max. coverage (+): 0.08. Max coverage (-): 0.2

Region: NODE\_296637\_length\_14495\_cov\_30.429251 13021-13031. Max. coverage (+): 0. Max coverage (-): 0.2

Region: NODE\_296637\_length\_14495\_cov\_30.429251 13032-13042. Max. coverage (+): 0. Max coverage (-): 0

Region: NODE\_296637\_length\_14495\_cov\_30.429251 13043-13054. Max. coverage (+): 0. Max coverage (-): 0

Region: NODE\_296637\_length\_14495\_cov\_30.429251 13055-13065. Max. coverage (+): 0. Max coverage (-): 0

Region: NODE\_296637\_length\_14495\_cov\_30.429251 13066-13076. Max. coverage (+): 0. Max coverage (-): 0

Region: NODE\_296637\_length\_14495\_cov\_30.429251 13077-13087. Max. coverage (+): 0.04. Max coverage (-): 0.2

Region: NODE\_296637\_length\_14495\_cov\_30.429251 13088-13099. Max. coverage (+): 0.04. Max coverage (-): 0.44

Region: NODE\_296637\_length\_14495\_cov\_30.429251 13100-13110. Max. coverage (+): 0. Max coverage (-): 0.36

Region: NODE\_296637\_length\_14495\_cov\_30.429251 13111-13121. Max. coverage (+): 1.94. Max coverage (-): 0

Region: NODE\_296637\_length\_14495\_cov\_30.429251 13122-13132. Max. coverage (+): 1.86. Max coverage (-): 0.04

Region: NODE\_296637\_length\_14495\_cov\_30.429251 13133-13143. Max. coverage (+): 0. Max coverage (-): 0

Region: NODE\_296637\_length\_14495\_cov\_30.429251 13144-13155. Max. coverage (+): 0. Max coverage (-): 0

Region: NODE\_296637\_length\_14495\_cov\_30.429251 13156-13166. Max. coverage (+): 0. Max coverage (-): 0

Region: NODE\_296637\_length\_14495\_cov\_30.429251 13167-13177. Max. coverage (+): 0. Max coverage (-): 0.89

Region: NODE\_296637\_length\_14495\_cov\_30.429251 13178-13188. Max. coverage (+): 0. Max coverage (-): 1.74

Region: NODE\_296637\_length\_14495\_cov\_30.429251 13189-13200. Max. coverage (+): 0. Max coverage (-): 0.04

Region: NODE\_296637\_length\_14495\_cov\_30.429251 13201-13211. Max. coverage (+): 0.04. Max coverage (-): 0.2

Region: NODE\_296637\_length\_14495\_cov\_30.429251 13212-13222. Max. coverage (+): 0. Max coverage (-): 0.08

Region: NODE\_296637\_length\_14495\_cov\_30.429251 13223-13233. Max. coverage (+): 0. Max coverage (-): 0.24

Region: NODE\_296637\_length\_14495\_cov\_30.429251 13234-13245. Max. coverage (+): 0. Max coverage (-): 0.12

Region: NODE\_296637\_length\_14495\_cov\_30.429251 13246-13256. Max. coverage (+): 0. Max coverage (-): 0.52

Region: NODE\_296637\_length\_14495\_cov\_30.429251 13257-13267. Max. coverage (+): 0.04. Max coverage (-): 0.48

Region: NODE\_296637\_length\_14495\_cov\_30.429251 13268-13278. Max. coverage (+): 0.12. Max coverage (-): 0.04

Region: NODE\_296637\_length\_14495\_cov\_30.429251 13279-13290. Max. coverage (+): 0. Max coverage (-): 10.54

Region: NODE\_296637\_length\_14495\_cov\_30.429251 13291-13301. Max. coverage (+): 0. Max coverage (-): 12.03

Region: NODE\_296637\_length\_14495\_cov\_30.429251 13302-13312. Max. coverage (+): 0.16. Max coverage (-): 0.24

Region: NODE\_296637\_length\_14495\_cov\_30.429251 13313-13323. Max. coverage (+): 0.04. Max coverage (-): 0.32

Region: NODE\_296637\_length\_14495\_cov\_30.429251 13324-13334. Max. coverage (+): 0. Max coverage (-): 0.08

Region: NODE\_296637\_length\_14495\_cov\_30.429251 13335-13346. Max. coverage (+): 0. Max coverage (-): 0.12

Region: NODE\_296637\_length\_14495\_cov\_30.429251 13347-13357. Max. coverage (+): 0.04. Max coverage (-): 0.2

Region: NODE\_296637\_length\_14495\_cov\_30.429251 13358-13368. Max. coverage (+): 0. Max coverage (-): 0.08

Region: NODE\_296637\_length\_14495\_cov\_30.429251 13369-13379. Max. coverage (+): 0. Max coverage (-): 0

Region: NODE\_296637\_length\_14495\_cov\_30.429251 13380-13391. Max. coverage (+): 0.08. Max coverage (-): 0.16

Region: NODE\_296637\_length\_14495\_cov\_30.429251 13392-13402. Max. coverage (+): 0.08. Max coverage (-): 0.2

Region: NODE\_296637\_length\_14495\_cov\_30.429251 13403-13413. Max. coverage (+): 0.28. Max coverage (-): 0

Region: NODE\_296637\_length\_14495\_cov\_30.429251 13414-13424. Max. coverage (+): 0.12. Max coverage (-): 0.04

Region: NODE\_296637\_length\_14495\_cov\_30.429251 13425-13436. Max. coverage (+): 0. Max coverage (-): 1.05

Region: NODE\_296637\_length\_14495\_cov\_30.429251 13437-13447. Max. coverage (+): 0. Max coverage (-): 0.08

Region: NODE\_296637\_length\_14495\_cov\_30.429251 13448-13458. Max. coverage (+): 0.2. Max coverage (-): 0

Region: NODE\_296637\_length\_14495\_cov\_30.429251 13459-13469. Max. coverage (+): 0. Max coverage (-): 0.2

Region: NODE\_296637\_length\_14495\_cov\_30.429251 13470-13480. Max. coverage (+): 0.52. Max coverage (-): 0.24

Region: NODE\_296637\_length\_14495\_cov\_30.429251 13481-13492. Max. coverage (+): 0.44. Max coverage (-): 0.28

Region: NODE\_296637\_length\_14495\_cov\_30.429251 13493-13503. Max. coverage (+): 0.04. Max coverage (-): 1.21

Region: NODE\_296637\_length\_14495\_cov\_30.429251 13504-13514. Max. coverage (+): 0.16. Max coverage (-): 0.28

Region: NODE\_296637\_length\_14495\_cov\_30.429251 13515-13525. Max. coverage (+): 0.02. Max coverage (-): 0.28

Region: NODE\_296637\_length\_14495\_cov\_30.429251 13526-13537. Max. coverage (+): 0.04. Max coverage (-): 0.04

Region: NODE\_296637\_length\_14495\_cov\_30.429251 13538-13548. Max. coverage (+): 0.04. Max coverage (-): 0.24

Region: NODE\_296637\_length\_14495\_cov\_30.429251 13549-13559. Max. coverage (+): 0.12. Max coverage (-): 0.12

Region: NODE\_296637\_length\_14495\_cov\_30.429251 13560-13570. Max. coverage (+): 0.2. Max coverage (-): 0.04

Region: NODE\_296637\_length\_14495\_cov\_30.429251 13571-13582. Max. coverage (+): 0.04. Max coverage (-): 1.01

Region: NODE\_296637\_length\_14495\_cov\_30.429251 13583-13593. Max. coverage (+): 0.36. Max coverage (-): 0.28

Region: NODE\_296637\_length\_14495\_cov\_30.429251 13594-13604. Max. coverage (+): 0.61. Max coverage (-): 0.08

Region: NODE\_296637\_length\_14495\_cov\_30.429251 13605-13615. Max. coverage (+): 0. Max coverage (-): 0.24

Region: NODE\_296637\_length\_14495\_cov\_30.429251 13616-13626. Max. coverage (+): 0. Max coverage (-): 0.12

Region: NODE\_296637\_length\_14495\_cov\_30.429251 13627-13638. Max. coverage (+): 0. Max coverage (-): 0.08

Region: NODE\_296637\_length\_14495\_cov\_30.429251 13639-13649. Max. coverage (+): 0. Max coverage (-): 0.08

Region: NODE\_296637\_length\_14495\_cov\_30.429251 13650-13660. Max. coverage (+): 0. Max coverage (-): 0

Region: NODE\_296637\_length\_14495\_cov\_30.429251 13661-13671. Max. coverage (+): 0. Max coverage (-): 0.12

Region: NODE\_296637\_length\_14495\_cov\_30.429251 13672-13683. Max. coverage (+): 0. Max coverage (-): 0.04

Region: NODE\_296637\_length\_14495\_cov\_30.429251 13684-13694. Max. coverage (+): 0.12. Max coverage (-): 0.93

Region: NODE\_296637\_length\_14495\_cov\_30.429251 13695-13705. Max. coverage (+): 0.04. Max coverage (-): 0.24

Region: NODE\_296637\_length\_14495\_cov\_30.429251 13706-13716. Max. coverage (+): 0.04. Max coverage (-): 0.12

Region: NODE\_296637\_length\_14495\_cov\_30.429251 13717-13728. Max. coverage (+): 0.04. Max coverage (-): 1.01

Region: NODE\_296637\_length\_14495\_cov\_30.429251 13729-13739. Max. coverage (+): 0. Max coverage (-): 0.48

Region: NODE\_296637\_length\_14495\_cov\_30.429251 13740-13750. Max. coverage (+): 0.12. Max coverage (-): 0.04

Region: NODE\_296637\_length\_14495\_cov\_30.429251 13751-13761. Max. coverage (+): 0. Max coverage (-): 0.16

Region: NODE\_296637\_length\_14495\_cov\_30.429251 13762-13772. Max. coverage (+): 0.04. Max coverage (-): 0.2

Region: NODE\_296637\_length\_14495\_cov\_30.429251 13773-13784. Max. coverage (+): 0.08. Max coverage (-): 0.16

Region: NODE\_296637\_length\_14495\_cov\_30.429251 13785-13795. Max. coverage (+): 0.04. Max coverage (-): 0.16

Region: NODE\_296637\_length\_14495\_cov\_30.429251 13796-13806. Max. coverage (+): 0.04. Max coverage (-): 0.44

Region: NODE\_296637\_length\_14495\_cov\_30.429251 13807-13817. Max. coverage (+): 0. Max coverage (-): 0.36

Region: NODE\_296637\_length\_14495\_cov\_30.429251 13818-13829. Max. coverage (+): 0.04. Max coverage (-): 0.04

Region: NODE\_296637\_length\_14495\_cov\_30.429251 13830-13840. Max. coverage (+): 0. Max coverage (-): 0

Region: NODE\_296637\_length\_14495\_cov\_30.429251 13841-13851. Max. coverage (+): 0.04. Max coverage (-): 0.08

Region: NODE\_296637\_length\_14495\_cov\_30.429251 13852-13862. Max. coverage (+): 0.04. Max coverage (-): 0.16

Region: NODE\_296637\_length\_14495\_cov\_30.429251 13863-13874. Max. coverage (+): 0.36. Max coverage (-): 0.16

Region: NODE\_296637\_length\_14495\_cov\_30.429251 13875-13885. Max. coverage (+): 0.32. Max coverage (-): 0.04

Region: NODE\_296637\_length\_14495\_cov\_30.429251 13886-13896. Max. coverage (+): 0. Max coverage (-): 0.12

Region: NODE\_296637\_length\_14495\_cov\_30.429251 13897-13907. Max. coverage (+): 0. Max coverage (-): 0

Region: NODE\_296637\_length\_14495\_cov\_30.429251 13908-13918. Max. coverage (+): 0. Max coverage (-): 0.12

Region: NODE\_296637\_length\_14495\_cov\_30.429251 13919-13930. Max. coverage (+): 0. Max coverage (-): 0

Region: NODE\_296637\_length\_14495\_cov\_30.429251 13931-13941. Max. coverage (+): 0. Max coverage (-): 0.36

Region: NODE\_296637\_length\_14495\_cov\_30.429251 13942-13952. Max. coverage (+): 0. Max coverage (-): 0.24

Region: NODE\_296637\_length\_14495\_cov\_30.429251 13953-13963. Max. coverage (+): 0.04. Max coverage (-): 0

Region: NODE\_296637\_length\_14495\_cov\_30.429251 13964-13975. Max. coverage (+): 0.04. Max coverage (-): 0

Region: NODE\_296637\_length\_14495\_cov\_30.429251 13976-13986. Max. coverage (+): 0. Max coverage (-): 0

Region: NODE\_296637\_length\_14495\_cov\_30.429251 13987-13997. Max. coverage (+): 0. Max coverage (-): 0

Region: NODE\_296637\_length\_14495\_cov\_30.429251 13998-14008. Max. coverage (+): 0. Max coverage (-): 0

Region: NODE\_296637\_length\_14495\_cov\_30.429251 14009-14020. Max. coverage (+): 0. Max coverage (-): 0.04

Region: NODE\_296637\_length\_14495\_cov\_30.429251 14021-14031. Max. coverage (+): 0. Max coverage (-): 0.04

Region: NODE\_296637\_length\_14495\_cov\_30.429251 14032-14042. Max. coverage (+): 0. Max coverage (-): 0

Region: NODE\_296637\_length\_14495\_cov\_30.429251 14043-14053. Max. coverage (+): 0.04. Max coverage (-): 0

Region: NODE\_296637\_length\_14495\_cov\_30.429251 14054-14065. Max. coverage (+): 0. Max coverage (-): 0.08

Region: NODE\_296637\_length\_14495\_cov\_30.429251 14066-14076. Max. coverage (+): 0. Max coverage (-): 0

Region: NODE\_296637\_length\_14495\_cov\_30.429251 14077-14087. Max. coverage (+): 0.04. Max coverage (-): 0.04

Region: NODE\_296637\_length\_14495\_cov\_30.429251 14088-14098. Max. coverage (+): 0.02. Max coverage (-): 0.04

Region: NODE\_296637\_length\_14495\_cov\_30.429251 14099-14109. Max. coverage (+): 0. Max coverage (-): 0

Region: NODE\_296637\_length\_14495\_cov\_30.429251 14110-14121. Max. coverage (+): 0. Max coverage (-): 0

Region: NODE\_296637\_length\_14495\_cov\_30.429251 14122-14132. Max. coverage (+): 0. Max coverage (-): 0.16

Region: NODE\_296637\_length\_14495\_cov\_30.429251 14133-14143. Max. coverage (+): 0. Max coverage (-): 0

Region: NODE\_296637\_length\_14495\_cov\_30.429251 14144-14154. Max. coverage (+): 0. Max coverage (-): 0

Region: NODE\_296637\_length\_14495\_cov\_30.429251 14155-14166. Max. coverage (+): 0. Max coverage (-): 0

Region: NODE\_296637\_length\_14495\_cov\_30.429251 14167-14177. Max. coverage (+): 0. Max coverage (-): 0

Region: NODE\_296637\_length\_14495\_cov\_30.429251 14178-14188. Max. coverage (+): 0. Max coverage (-): 0

Region: NODE\_296637\_length\_14495\_cov\_30.429251 14189-14199. Max. coverage (+): 0. Max coverage (-): 0

Region: NODE\_296637\_length\_14495\_cov\_30.429251 14200-14211. Max. coverage (+): 0. Max coverage (-): 0

Region: NODE\_296637\_length\_14495\_cov\_30.429251 14212-14222. Max. coverage (+): 0. Max coverage (-): 0

Region: NODE\_296637\_length\_14495\_cov\_30.429251 14223-14233. Max. coverage (+): 0. Max coverage (-): 0.05

Region: NODE\_296637\_length\_14495\_cov\_30.429251 14234-14244. Max. coverage (+): 0.01. Max coverage (-): 0.01

Region: NODE\_296637\_length\_14495\_cov\_30.429251 14245-14255. Max. coverage (+): 0.4. Max coverage (-): 0.01

Region: NODE\_296637\_length\_14495\_cov\_30.429251 14256-14267. Max. coverage (+): 0. Max coverage (-): 0.04

Region: NODE\_296637\_length\_14495\_cov\_30.429251 14268-14278. Max. coverage (+): 0.01. Max coverage (-): 0

Region: NODE\_296637\_length\_14495\_cov\_30.429251 14279-14289. Max. coverage (+): 0.01. Max coverage (-): 0.01

Region: NODE\_296637\_length\_14495\_cov\_30.429251 14290-14300. Max. coverage (+): 0.08. Max coverage (-): 0

Region: NODE\_296637\_length\_14495\_cov\_30.429251 14301-14312. Max. coverage (+): 0. Max coverage (-): 0

Region: NODE\_296637\_length\_14495\_cov\_30.429251 14313-14323. Max. coverage (+): 0. Max coverage (-): 0

Region: NODE\_296637\_length\_14495\_cov\_30.429251 14324-14334. Max. coverage (+): 0. Max coverage (-): 0.04

Region: NODE\_296637\_length\_14495\_cov\_30.429251 14335-14345. Max. coverage (+): 0. Max coverage (-): 0.04

Region: NODE\_296637\_length\_14495\_cov\_30.429251 14346-14357. Max. coverage (+): 0.08. Max coverage (-): 0

Region: NODE\_296637\_length\_14495\_cov\_30.429251 14358-14368. Max. coverage (+): 0.04. Max coverage (-): 0.04

Region: NODE\_296637\_length\_14495\_cov\_30.429251 14369-14379. Max. coverage (+): 0. Max coverage (-): 0.11

Region: NODE\_296637\_length\_14495\_cov\_30.429251 14380-14390. Max. coverage (+): 0. Max coverage (-): 0.05

Region: NODE\_296637\_length\_14495\_cov\_30.429251 14391-14401. Max. coverage (+): 0.03. Max coverage (-): 0.01

Region: NODE\_296637\_length\_14495\_cov\_30.429251 14402-14413. Max. coverage (+): 0.01. Max coverage (-): 0.01

Region: NODE\_296637\_length\_14495\_cov\_30.429251 14414-14424. Max. coverage (+): 0. Max coverage (-): 0

Region: NODE\_296637\_length\_14495\_cov\_30.429251 14425-14435. Max. coverage (+): 0. Max coverage (-): 0

Region: NODE\_296637\_length\_14495\_cov\_30.429251 14436-14446. Max. coverage (+): 0. Max coverage (-): 0

Region: NODE\_296637\_length\_14495\_cov\_30.429251 14447-14458. Max. coverage (+): 0. Max coverage (-): 0

Region: NODE\_296637\_length\_14495\_cov\_30.429251 14459-14469. Max. coverage (+): 0. Max coverage (-): 0

Region: NODE\_296637\_length\_14495\_cov\_30.429251 14470-14480. Max. coverage (+): 0. Max coverage (-): 0

Region: NODE\_296637\_length\_14495\_cov\_30.429251 14481-14491. Max. coverage (+): 0. Max coverage (-): 0

Region: NODE\_296637\_length\_14495\_cov\_30.429251 14492-14503. Max. coverage (+): 0. Max coverage (-): 0

Region: NODE\_296637\_length\_14495\_cov\_30.429251 14504-14514. Max. coverage (+): 0. Max coverage (-): 0

Region: NODE\_296637\_length\_14495\_cov\_30.429251 14515-14525. Max. coverage (+): 0. Max coverage (-): 0

Region: NODE\_296637\_length\_14495\_cov\_30.429251 14526-14536. Max. coverage (+): 0. Max coverage (-): 0

Region: NODE\_296637\_length\_14495\_cov\_30.429251 14537-14547. Max. coverage (+): 0. Max coverage (-): 0

Region: NODE\_296637\_length\_14495\_cov\_30.429251 14548-14559. Max. coverage (+): 0.01. Max coverage (-): 0

Region: NODE\_296637\_length\_14495\_cov\_30.429251 14560-14570. Max. coverage (+): 0. Max coverage (-): 0

Region: NODE\_296637\_length\_14495\_cov\_30.429251 14571-14581. Max. coverage (+): 0. Max coverage (-): 0

Region: NODE\_296637\_length\_14495\_cov\_30.429251 14582-14592. Max. coverage (+): 0. Max coverage (-): 0

Region: NODE\_296637\_length\_14495\_cov\_30.429251 14593-14604. Max. coverage (+): 0. Max coverage (-): 0

Region: NODE\_296637\_length\_14495\_cov\_30.429251 14605-14615. Max. coverage (+): 0. Max coverage (-): 0

Region: NODE\_296637\_length\_14495\_cov\_30.429251 14616-. Max. coverage (+): 0. Max coverage (-): 0

RepeatMasker Color Code

**+**

100-98% Identity

<98-95% Identity

<95-90% Identity

<90-85% Identity

<85-80% Identity

<80-75% Identity

<75-70% Identity

<70% Identity

**-**

Gene Set Color Code

**+**

Gene

Pseudogene

Other

**-**

Topology/Coverage Color Code

Coverage Plus Strand

Coverage Minus Strand

Mainstrand: Plus

Mainstrand: Minus

Complementary Strand

Flanking Region  
(if option -flank >0)

Gene Set Annotation  
  
RepeatMasker Annotation  

**1. AlRepD-4565**: 8831-9135 (-), Divergence to consensus: 8.2%  
**2. REX1-9\_DR**: 9136-9388 (-), Divergence to consensus: 31.9%  
**3. REX1-3\_AFC**: 9384-9655 (-), Divergence to consensus: 28.4%  
**4. Mariner-11\_DR**: 9664-9727 (+), Divergence to consensus: 0%  
**5. REX1-9\_DR**: 9729-9953 (-), Divergence to consensus: 34.6%  
**6. AlRepA-115**: 13131-13205 (-), Divergence to consensus: 20%  
**7. TC1DR3**: 13493-13623 (+), Divergence to consensus: 41.1%  
**8. TC1DR3**: 13696-13919 (+), Divergence to consensus: 32.6%  
**9. (TGTAT)n**: 13981-14011 (+), Divergence to consensus: 16.4%  
**10. AlRepC-326**: 14051-14234 (+), Divergence to consensus: 18%  
**11. AlRepB-420**: 14234-14621 (+), Divergence to consensus: 20%

  
Transcription Factor Binding Sites  

**RHOXF1** (Sequence: AGATCA (-): 9213)  
**RHOXF1** (Sequence: GGATCA (-): 9507)  
**RHOXF1** (Sequence: GGATCA (-): 9880)  
**RHOXF1** (Sequence: GGATCA (-): 10152)  
**RHOXF1** (Sequence: GGATTA (-): 11352)  
**RHOXF1** (Sequence: AGCTCA (-): 11366)  
**RHOXF1** (Sequence: GGCTCA (-): 11797)  
**RHOXF1** (Sequence: AGCTTA (-): 12588)  
**RHOXF1** (Sequence: GGATTA (-): 12998)  
**RHOXF1** (Sequence: AGCTTA (-): 13167)  
**RHOXF1** (Sequence: AGCTTA (-): 13434)  
**RHOXF1** (Sequence: AGATCA (-): 13588)  
**RHOXF1** (Sequence: TGATCC (+): 9321)  
**RHOXF1** (Sequence: TAATCC (+): 9665)  
**RHOXF1** (Sequence: TGATCT (+): 9704)  
**RHOXF1** (Sequence: TGATCC (+): 9785)  
**RHOXF1** (Sequence: TGAGCC (+): 10248)  
**RHOXF1** (Sequence: TAAGCT (+): 11013)  
**RHOXF1** (Sequence: TAATCC (+): 11640)  
**RHOXF1** (Sequence: TGAGCT (+): 11752)  
**RHOXF1** (Sequence: TAAGCT (+): 12189)  
**RHOXF1** (Sequence: TAATCT (+): 12284)  
**RHOXF1** (Sequence: TAAGCT (+): 12586)  
**RHOXF1** (Sequence: TAATCC (+): 14231)  
**Gata4** (Sequence: CTTATCT (+): 11584)  
**RFX4\_1** (Sequence: GTTGCCATG (-): 10819)  
**SOX9** (Sequence: AACAATGA (-): 13365)  
**FOXO3\_mmu** (Sequence: TGTTTTCA (-): 10214)  
**FOXO3\_mmu** (Sequence: TGTTTTGC (-): 10565)  
**Sox5** (Sequence: ATTGTT (+): 13559)  
**Sox5** (Sequence: ATTGTT (+): 13969)  
**FIGLA** (Sequence: ACCAGGTGTA (-): 14382)  
**A-MYB** (Sequence: CCAACTGCCA (-): 14189)  
**FOXO3\_mmu** (Sequence: GGAAAACA (+): 12534)  
**FOXO3\_mmu** (Sequence: TGAAAACA (+): 12562)  
**Nobox** (Sequence: ACCAATTA (-): 12784)  
**Nobox** (Sequence: TAATTGCT (+): 10430)  
**POU2F1** (Sequence: ATTTAAATA (-): 12913)  
**Rhox11** (Sequence: TTAACACCA (-): 9976)  
**Rhox11** (Sequence: AATACAGCA (-): 11323)  
**Rhox11** (Sequence: TAAACAGCA (-): 14605)  
**Gata4** (Sequence: AGATAAC (-): 12964)  
**Sox5** (Sequence: AACAAT (-): 12091)  
**Sox5** (Sequence: AACAAT (-): 13365)  
**Sox5** (Sequence: AACAAT (-): 14391)  
**POU2F1** (Sequence: TATGCAAAT (+): 10088)  
**POU2F1** (Sequence: TATTTTAAT (+): 12640)  
**POU5F1** (Sequence: ATGCAAA (+): 10089)  
**POU5F1** (Sequence: ATGCAAA (+): 13267)
