# Supplementary material for: First characterization of PIWI-interacting RNA clusters in a cichlid fish with a B chromosome
Source: BMC Biol. 2022 Sep 21;20:204. doi: 10.1186/s12915-022-01403-2 (PMC9490952; doi:10.1186/s12915-022-01403-2)
Supplement: Supplementary file 1 — Additional file 1. Zipped folder with fasta and interactive html piRNA cluster information for the A. latifasciata genome. The nomenclature is as follows: number-pirna-cluster_sex_B-presence (f, female; m, male; 0b, without B chromosome; 1b, with B chromosome). [file 12915_2022_1403_MOESM1_ESM.zip › 117_m1b.html]

piRNA cluster 117\_m1b 68


Predicted piRNA cluster no. 117\_m1b
  

Show proTRAC run info
Hide proTRAC run info

/\  
                \_\_\_\_\_\_\_\_\_\_\_\_\_\_\_\_\_\_\_\_\_\_\_/\\_\_\_ /  \\_\_\_\_\_\_\_  
               I                      /  \  /    \      I  
               I     pro             /    \/      \     I  
               I        TRAC        /               \   I  
               I   \_\_\_\_\_\_\_\_\_\_\_\_\_\_\_\_/\_\_\_\_\_\_\_\_\_\_\_\_\_\_\_\_\_\\_ I  
               I   \              /                     I  
               I    \            /                      I  
               I     \  /\      /       V.2.4.2         I  
               I      \/  \    /                        I  
               I\_\_\_\_\_\_\_\_\_\_\_\  /\_\_\_\_\_\_\_\_\_\_\_\_\_\_\_\_\_\_\_\_\_\_\_\_\_I  
                            \/  
  
  
================================= proTRAC ====================================  
VERSION: .......... 2.4.2  
LAST MODIFIED: .... 11. May 2018  
  
Please cite:  
Rosenkranz D, Zischler H. proTRAC - a software for probabilistic piRNA cluster  
detection, visualization and analysis. 2012. BMC Bioinformatics 13:5.  
  
  
Contact:  
David Rosenkranz  
Institute of Organismic and Molecular Evolutionary Biology  
Dept. Anthropology, small RNA group  
Johannes Gutenberg University Mainz  
email: rosenkranz@uni-mainz.de  
  
You can find the latest proTRAC version at:  
http://sourceforge.net/projects/protrac/files  
http://www.smallRNAgroup-mainz.de/software  
==============================================================================  
  
PARAMETERS:  
Map file: ...............piwi-machos-1B.fa-collapse.map  
Genome file: ............../../../0B\_ala\_genome.fa  
RepeatMasker annotation: Alatifasciata-all0B-maryan-v2.fa\_corrected.out  
GeneSet:................./guest-storage/Data/annotation/Alatifasciata\_all0B\_maryan-v2\_out2017.gff  
  
Significant (p<=0.01) hit density will be calculated based  
on observed hit distribution.  
  
Sliding window size: ........................................ 5000 bp  
Sliding window increament: .................................. 1000 bp  
Normalize each hit by number of genomic hits: ............... yes  
Normalize each hit by number of sequence reads: ............. yes  
Normalize values (-> per million mapped reads): ............. yes  
Min. fraction of hits with 1T(U) or 10A: .................... 0.75  
Alternatively: Min. fraction of hits with 1T(U) and 10A: .... 0.5  
Min. fraction of hits with typical piRNA length: ............ 0.75  
Typical piRNA length: ....................................... 24-32 nt  
Min. size of a piRNA cluster: ............................... 1000 bp.  
Min. number of hits (absolute): ............................. 0  
Min. number of hits (normalized): ........................... 0  
Min. fraction of hits on the mainstrand: .................... 0.75  
Top fraction of mapped sequences (in terms of read counts): . 1%  
Top fraction accounts for max. n% of sequence reads: ........ 90%  
Min. fraction of hits on each arm of a bidirectional cluster: 0.05  
Output html file for each cluster: .......................... yes  
Output a summary table: ..................................... yes  
Output a FASTA file for each cluster (piRNA sequences): ..... yes  
Output a FASTA file comprising cluster sequences: ........... yes  
Output a GTF file for predicted piRNA clusters: ..............yes  
Search DNA motifs in clusters: .............................. yes  
Output flanking sequences: +/- .............................. 0 bp  
Output ~.pTi file: .......................................... no  
==============================================================================  
  
  
Genome size (without gaps): ............ 758543724 bp  
Gaps (N/X/-): .......................... 417479 bp  
Mapped reads: .......................... 26973943  
Non-identical sequences: ............... 6209225  
Genomic hits: .......................... 48438990  
Significant densitiy of mapped reads: .. 821.144211136946 reads/kb

Show proTRAC cluster info
Hide proTRAC cluster info

|  |  |
| --- | --- |
| Location | NODE\_298130\_length\_4886\_cov\_17.340155 |
| Coordinates | 7-5018 |
| Size [bp] | 5012 |
| Sequence hit loci | 8082 |
| Mapped reads (normalized) | 30320.4 |
| Mapped reads (normalized) per kb | 6049.6 |
| Normalized reads with 1T (1U) | 78.6% |
| Normalized reads with 10A | 46.2% |
| Normalized reads with length 24-32 nt | 99% |
| Normalized reads on the main strand(s) | 96.6% |
| Predicted directionality | mono:minus |

100%

0%

1T (1U)  
reads

10A reads

24-32 nt  
reads

reads on mainstrand

**Either the amount of reads with 1T (1U) OR 10A has to exceed 75% (set with option: -1Tor10A)  
Alternatively the amount of reads with 1T (1U) AND 10A has to exceed 50% (set with option: -1Tand10A)  
Minimum amount of reads with preferred size is 75% (set with option: -pisize)  
Minimum amount of reads on the main strand(s) is 75% (set with option: -clstrand)**

Show read coverage
Hide read coverage

WHAT DO I SEE HERE?  
This chart shows the location of mapped sequence reads within a predicted piRNA cluster. The color refers to the number of genomic hits produced by the sequence read in question. A dark red bar indicates that this sequence read produces many other hits elsewhere in the genome. Many adjacent red or yellow bars can indicate the presence of a multi-copy element such as transposons or rRNA genes. A dark green bar indicates that this sequence read maps uniquely to this locus.

1 hit

2-5 hits

6-10 hits

11-20 hits

21-50 hits

51-100 hits

> 100 hits

NODE\_298130\_length\_4886\_cov\_17.340155

7

5018

Gene Set

RepeatMasker

Mapped  
Reads

156.42

plus strand

minus strand

156.42

Region: NODE\_298130\_length\_4886\_cov\_17.340155 12019-12. Max. coverage (+): 0. Max coverage (-): 0

Region: NODE\_298130\_length\_4886\_cov\_17.340155 13-22. Max. coverage (+): 0. Max coverage (-): 0

Region: NODE\_298130\_length\_4886\_cov\_17.340155 23-32. Max. coverage (+): 0. Max coverage (-): 0.01

Region: NODE\_298130\_length\_4886\_cov\_17.340155 33-42. Max. coverage (+): 0. Max coverage (-): 0.01

Region: NODE\_298130\_length\_4886\_cov\_17.340155 43-52. Max. coverage (+): 0. Max coverage (-): 0

Region: NODE\_298130\_length\_4886\_cov\_17.340155 53-62. Max. coverage (+): 0. Max coverage (-): 0.02

Region: NODE\_298130\_length\_4886\_cov\_17.340155 63-72. Max. coverage (+): 0. Max coverage (-): 0.06

Region: NODE\_298130\_length\_4886\_cov\_17.340155 73-82. Max. coverage (+): 0. Max coverage (-): 0.04

Region: NODE\_298130\_length\_4886\_cov\_17.340155 83-92. Max. coverage (+): 0.04. Max coverage (-): 0.22

Region: NODE\_298130\_length\_4886\_cov\_17.340155 93-102. Max. coverage (+): 0. Max coverage (-): 2.08

Region: NODE\_298130\_length\_4886\_cov\_17.340155 103-112. Max. coverage (+): 0. Max coverage (-): 1.73

Region: NODE\_298130\_length\_4886\_cov\_17.340155 113-122. Max. coverage (+): 0.04. Max coverage (-): 0

Region: NODE\_298130\_length\_4886\_cov\_17.340155 123-132. Max. coverage (+): 0.04. Max coverage (-): 0

Region: NODE\_298130\_length\_4886\_cov\_17.340155 133-142. Max. coverage (+): 0. Max coverage (-): 1.45

Region: NODE\_298130\_length\_4886\_cov\_17.340155 143-152. Max. coverage (+): 0. Max coverage (-): 1.3

Region: NODE\_298130\_length\_4886\_cov\_17.340155 153-162. Max. coverage (+): 0.04. Max coverage (-): 1.35

Region: NODE\_298130\_length\_4886\_cov\_17.340155 163-172. Max. coverage (+): 0.04. Max coverage (-): 0.11

Region: NODE\_298130\_length\_4886\_cov\_17.340155 173-182. Max. coverage (+): 0. Max coverage (-): 0.07

Region: NODE\_298130\_length\_4886\_cov\_17.340155 183-192. Max. coverage (+): 0. Max coverage (-): 0.04

Region: NODE\_298130\_length\_4886\_cov\_17.340155 193-202. Max. coverage (+): 0. Max coverage (-): 0.24

Region: NODE\_298130\_length\_4886\_cov\_17.340155 203-212. Max. coverage (+): 0. Max coverage (-): 0.32

Region: NODE\_298130\_length\_4886\_cov\_17.340155 213-222. Max. coverage (+): 0.07. Max coverage (-): 0.46

Region: NODE\_298130\_length\_4886\_cov\_17.340155 223-232. Max. coverage (+): 0.05. Max coverage (-): 0.05

Region: NODE\_298130\_length\_4886\_cov\_17.340155 233-242. Max. coverage (+): 0.17. Max coverage (-): 0.13

Region: NODE\_298130\_length\_4886\_cov\_17.340155 243-252. Max. coverage (+): 0.02. Max coverage (-): 0.44

Region: NODE\_298130\_length\_4886\_cov\_17.340155 253-262. Max. coverage (+): 0. Max coverage (-): 0.09

Region: NODE\_298130\_length\_4886\_cov\_17.340155 263-272. Max. coverage (+): 0. Max coverage (-): 0.19

Region: NODE\_298130\_length\_4886\_cov\_17.340155 273-282. Max. coverage (+): 0.09. Max coverage (-): 0.11

Region: NODE\_298130\_length\_4886\_cov\_17.340155 283-292. Max. coverage (+): 0.09. Max coverage (-): 0.02

Region: NODE\_298130\_length\_4886\_cov\_17.340155 293-302. Max. coverage (+): 0.01. Max coverage (-): 0.23

Region: NODE\_298130\_length\_4886\_cov\_17.340155 303-312. Max. coverage (+): 0.01. Max coverage (-): 1.15

Region: NODE\_298130\_length\_4886\_cov\_17.340155 313-322. Max. coverage (+): 0.02. Max coverage (-): 0.04

Region: NODE\_298130\_length\_4886\_cov\_17.340155 323-332. Max. coverage (+): 0.15. Max coverage (-): 0

Region: NODE\_298130\_length\_4886\_cov\_17.340155 333-342. Max. coverage (+): 0. Max coverage (-): 0

Region: NODE\_298130\_length\_4886\_cov\_17.340155 343-352. Max. coverage (+): 0. Max coverage (-): 0.04

Region: NODE\_298130\_length\_4886\_cov\_17.340155 353-362. Max. coverage (+): 0. Max coverage (-): 0.06

Region: NODE\_298130\_length\_4886\_cov\_17.340155 363-372. Max. coverage (+): 0.01. Max coverage (-): 0.64

Region: NODE\_298130\_length\_4886\_cov\_17.340155 373-382. Max. coverage (+): 0.01. Max coverage (-): 1.41

Region: NODE\_298130\_length\_4886\_cov\_17.340155 383-392. Max. coverage (+): 0.04. Max coverage (-): 3.41

Region: NODE\_298130\_length\_4886\_cov\_17.340155 393-402. Max. coverage (+): 0. Max coverage (-): 0.04

Region: NODE\_298130\_length\_4886\_cov\_17.340155 403-412. Max. coverage (+): 0. Max coverage (-): 0.82

Region: NODE\_298130\_length\_4886\_cov\_17.340155 413-422. Max. coverage (+): 0. Max coverage (-): 0.19

Region: NODE\_298130\_length\_4886\_cov\_17.340155 423-433. Max. coverage (+): 0.02. Max coverage (-): 0.19

Region: NODE\_298130\_length\_4886\_cov\_17.340155 434-443. Max. coverage (+): 0.02. Max coverage (-): 0.04

Region: NODE\_298130\_length\_4886\_cov\_17.340155 444-453. Max. coverage (+): 0. Max coverage (-): 0.26

Region: NODE\_298130\_length\_4886\_cov\_17.340155 454-463. Max. coverage (+): 0. Max coverage (-): 0.28

Region: NODE\_298130\_length\_4886\_cov\_17.340155 464-473. Max. coverage (+): 0.04. Max coverage (-): 0.04

Region: NODE\_298130\_length\_4886\_cov\_17.340155 474-483. Max. coverage (+): 0.2. Max coverage (-): 5.49

Region: NODE\_298130\_length\_4886\_cov\_17.340155 484-493. Max. coverage (+): 0.02. Max coverage (-): 1.31

Region: NODE\_298130\_length\_4886\_cov\_17.340155 494-503. Max. coverage (+): 0.02. Max coverage (-): 0.06

Region: NODE\_298130\_length\_4886\_cov\_17.340155 504-513. Max. coverage (+): 0. Max coverage (-): 0.24

Region: NODE\_298130\_length\_4886\_cov\_17.340155 514-523. Max. coverage (+): 0. Max coverage (-): 1.89

Region: NODE\_298130\_length\_4886\_cov\_17.340155 524-533. Max. coverage (+): 0.06. Max coverage (-): 0.6

Region: NODE\_298130\_length\_4886\_cov\_17.340155 534-543. Max. coverage (+): 0.02. Max coverage (-): 0.19

Region: NODE\_298130\_length\_4886\_cov\_17.340155 544-553. Max. coverage (+): 0.01. Max coverage (-): 0.78

Region: NODE\_298130\_length\_4886\_cov\_17.340155 554-563. Max. coverage (+): 0. Max coverage (-): 0.01

Region: NODE\_298130\_length\_4886\_cov\_17.340155 564-573. Max. coverage (+): 0.04. Max coverage (-): 0.04

Region: NODE\_298130\_length\_4886\_cov\_17.340155 574-583. Max. coverage (+): 0.04. Max coverage (-): 0.95

Region: NODE\_298130\_length\_4886\_cov\_17.340155 584-593. Max. coverage (+): 0.09. Max coverage (-): 0.95

Region: NODE\_298130\_length\_4886\_cov\_17.340155 594-603. Max. coverage (+): 0.02. Max coverage (-): 3.37

Region: NODE\_298130\_length\_4886\_cov\_17.340155 604-613. Max. coverage (+): 0.02. Max coverage (-): 18.94

Region: NODE\_298130\_length\_4886\_cov\_17.340155 614-623. Max. coverage (+): 0.3. Max coverage (-): 0.37

Region: NODE\_298130\_length\_4886\_cov\_17.340155 624-633. Max. coverage (+): 0.56. Max coverage (-): 0.61

Region: NODE\_298130\_length\_4886\_cov\_17.340155 634-643. Max. coverage (+): 0.13. Max coverage (-): 4.8

Region: NODE\_298130\_length\_4886\_cov\_17.340155 644-653. Max. coverage (+): 0.09. Max coverage (-): 0.37

Region: NODE\_298130\_length\_4886\_cov\_17.340155 654-663. Max. coverage (+): 0.09. Max coverage (-): 0.33

Region: NODE\_298130\_length\_4886\_cov\_17.340155 664-673. Max. coverage (+): 0. Max coverage (-): 0

Region: NODE\_298130\_length\_4886\_cov\_17.340155 674-683. Max. coverage (+): 0. Max coverage (-): 0.17

Region: NODE\_298130\_length\_4886\_cov\_17.340155 684-693. Max. coverage (+): 0. Max coverage (-): 0.06

Region: NODE\_298130\_length\_4886\_cov\_17.340155 694-703. Max. coverage (+): 0. Max coverage (-): 0.19

Region: NODE\_298130\_length\_4886\_cov\_17.340155 704-713. Max. coverage (+): 0.02. Max coverage (-): 0.67

Region: NODE\_298130\_length\_4886\_cov\_17.340155 714-723. Max. coverage (+): 0.19. Max coverage (-): 0.33

Region: NODE\_298130\_length\_4886\_cov\_17.340155 724-733. Max. coverage (+): 0.09. Max coverage (-): 0.02

Region: NODE\_298130\_length\_4886\_cov\_17.340155 734-743. Max. coverage (+): 0. Max coverage (-): 0

Region: NODE\_298130\_length\_4886\_cov\_17.340155 744-753. Max. coverage (+): 0. Max coverage (-): 0

Region: NODE\_298130\_length\_4886\_cov\_17.340155 754-763. Max. coverage (+): 0.06. Max coverage (-): 0.13

Region: NODE\_298130\_length\_4886\_cov\_17.340155 764-773. Max. coverage (+): 0.69. Max coverage (-): 0.09

Region: NODE\_298130\_length\_4886\_cov\_17.340155 774-783. Max. coverage (+): 0.28. Max coverage (-): 1.96

Region: NODE\_298130\_length\_4886\_cov\_17.340155 784-793. Max. coverage (+): 0.09. Max coverage (-): 1.17

Region: NODE\_298130\_length\_4886\_cov\_17.340155 794-803. Max. coverage (+): 0.15. Max coverage (-): 0.07

Region: NODE\_298130\_length\_4886\_cov\_17.340155 804-813. Max. coverage (+): 0.8. Max coverage (-): 0.15

Region: NODE\_298130\_length\_4886\_cov\_17.340155 814-823. Max. coverage (+): 0.24. Max coverage (-): 1.46

Region: NODE\_298130\_length\_4886\_cov\_17.340155 824-833. Max. coverage (+): 0.11. Max coverage (-): 0.95

Region: NODE\_298130\_length\_4886\_cov\_17.340155 834-844. Max. coverage (+): 0.11. Max coverage (-): 1.93

Region: NODE\_298130\_length\_4886\_cov\_17.340155 845-854. Max. coverage (+): 0.02. Max coverage (-): 83.28

Region: NODE\_298130\_length\_4886\_cov\_17.340155 855-864. Max. coverage (+): 0.09. Max coverage (-): 0.74

Region: NODE\_298130\_length\_4886\_cov\_17.340155 865-874. Max. coverage (+): 0. Max coverage (-): 0.02

Region: NODE\_298130\_length\_4886\_cov\_17.340155 875-884. Max. coverage (+): 0.02. Max coverage (-): 0.07

Region: NODE\_298130\_length\_4886\_cov\_17.340155 885-894. Max. coverage (+): 0. Max coverage (-): 2.3

Region: NODE\_298130\_length\_4886\_cov\_17.340155 895-904. Max. coverage (+): 0.06. Max coverage (-): 0.39

Region: NODE\_298130\_length\_4886\_cov\_17.340155 905-914. Max. coverage (+): 0.02. Max coverage (-): 1.74

Region: NODE\_298130\_length\_4886\_cov\_17.340155 915-924. Max. coverage (+): 0. Max coverage (-): 1.71

Region: NODE\_298130\_length\_4886\_cov\_17.340155 925-934. Max. coverage (+): 1.82. Max coverage (-): 2.19

Region: NODE\_298130\_length\_4886\_cov\_17.340155 935-944. Max. coverage (+): 0. Max coverage (-): 6.82

Region: NODE\_298130\_length\_4886\_cov\_17.340155 945-954. Max. coverage (+): 0.04. Max coverage (-): 1.54

Region: NODE\_298130\_length\_4886\_cov\_17.340155 955-964. Max. coverage (+): 0.04. Max coverage (-): 12.79

Region: NODE\_298130\_length\_4886\_cov\_17.340155 965-974. Max. coverage (+): 0. Max coverage (-): 4.89

Region: NODE\_298130\_length\_4886\_cov\_17.340155 975-984. Max. coverage (+): 0.04. Max coverage (-): 0.04

Region: NODE\_298130\_length\_4886\_cov\_17.340155 985-994. Max. coverage (+): 0.02. Max coverage (-): 0.04

Region: NODE\_298130\_length\_4886\_cov\_17.340155 995-1004. Max. coverage (+): 0.15. Max coverage (-): 0.85

Region: NODE\_298130\_length\_4886\_cov\_17.340155 1005-1014. Max. coverage (+): 0.13. Max coverage (-): 5.64

Region: NODE\_298130\_length\_4886\_cov\_17.340155 1015-1024. Max. coverage (+): 0.02. Max coverage (-): 0.63

Region: NODE\_298130\_length\_4886\_cov\_17.340155 1025-1034. Max. coverage (+): 0.02. Max coverage (-): 0.33

Region: NODE\_298130\_length\_4886\_cov\_17.340155 1035-1044. Max. coverage (+): 0.02. Max coverage (-): 0.13

Region: NODE\_298130\_length\_4886\_cov\_17.340155 1045-1054. Max. coverage (+): 0.09. Max coverage (-): 0.15

Region: NODE\_298130\_length\_4886\_cov\_17.340155 1055-1064. Max. coverage (+): 0. Max coverage (-): 31.96

Region: NODE\_298130\_length\_4886\_cov\_17.340155 1065-1074. Max. coverage (+): 0. Max coverage (-): 25.65

Region: NODE\_298130\_length\_4886\_cov\_17.340155 1075-1084. Max. coverage (+): 0.26. Max coverage (-): 3

Region: NODE\_298130\_length\_4886\_cov\_17.340155 1085-1094. Max. coverage (+): 0.22. Max coverage (-): 0.07

Region: NODE\_298130\_length\_4886\_cov\_17.340155 1095-1104. Max. coverage (+): 0.02. Max coverage (-): 2.15

Region: NODE\_298130\_length\_4886\_cov\_17.340155 1105-1114. Max. coverage (+): 0.02. Max coverage (-): 4.52

Region: NODE\_298130\_length\_4886\_cov\_17.340155 1115-1124. Max. coverage (+): 0.59. Max coverage (-): 0.91

Region: NODE\_298130\_length\_4886\_cov\_17.340155 1125-1134. Max. coverage (+): 0.84. Max coverage (-): 0.41

Region: NODE\_298130\_length\_4886\_cov\_17.340155 1135-1144. Max. coverage (+): 0. Max coverage (-): 2.48

Region: NODE\_298130\_length\_4886\_cov\_17.340155 1145-1154. Max. coverage (+): 0. Max coverage (-): 6.23

Region: NODE\_298130\_length\_4886\_cov\_17.340155 1155-1164. Max. coverage (+): 0. Max coverage (-): 0.22

Region: NODE\_298130\_length\_4886\_cov\_17.340155 1165-1174. Max. coverage (+): 0. Max coverage (-): 1.39

Region: NODE\_298130\_length\_4886\_cov\_17.340155 1175-1184. Max. coverage (+): 0. Max coverage (-): 0.87

Region: NODE\_298130\_length\_4886\_cov\_17.340155 1185-1194. Max. coverage (+): 0. Max coverage (-): 0.33

Region: NODE\_298130\_length\_4886\_cov\_17.340155 1195-1204. Max. coverage (+): 0. Max coverage (-): 0.98

Region: NODE\_298130\_length\_4886\_cov\_17.340155 1205-1214. Max. coverage (+): 0. Max coverage (-): 1.02

Region: NODE\_298130\_length\_4886\_cov\_17.340155 1215-1224. Max. coverage (+): 0. Max coverage (-): 0.11

Region: NODE\_298130\_length\_4886\_cov\_17.340155 1225-1234. Max. coverage (+): 0. Max coverage (-): 0.52

Region: NODE\_298130\_length\_4886\_cov\_17.340155 1235-1244. Max. coverage (+): 0.01. Max coverage (-): 0.11

Region: NODE\_298130\_length\_4886\_cov\_17.340155 1245-1254. Max. coverage (+): 0. Max coverage (-): 0.27

Region: NODE\_298130\_length\_4886\_cov\_17.340155 1255-1265. Max. coverage (+): 0. Max coverage (-): 0.63

Region: NODE\_298130\_length\_4886\_cov\_17.340155 1266-1275. Max. coverage (+): 0. Max coverage (-): 0.67

Region: NODE\_298130\_length\_4886\_cov\_17.340155 1276-1285. Max. coverage (+): 0.04. Max coverage (-): 0.54

Region: NODE\_298130\_length\_4886\_cov\_17.340155 1286-1295. Max. coverage (+): 0. Max coverage (-): 5

Region: NODE\_298130\_length\_4886\_cov\_17.340155 1296-1305. Max. coverage (+): 0.07. Max coverage (-): 0.41

Region: NODE\_298130\_length\_4886\_cov\_17.340155 1306-1315. Max. coverage (+): 0.22. Max coverage (-): 0.37

Region: NODE\_298130\_length\_4886\_cov\_17.340155 1316-1325. Max. coverage (+): 0.07. Max coverage (-): 12.6

Region: NODE\_298130\_length\_4886\_cov\_17.340155 1326-1335. Max. coverage (+): 0.05. Max coverage (-): 9.12

Region: NODE\_298130\_length\_4886\_cov\_17.340155 1336-1345. Max. coverage (+): 0.06. Max coverage (-): 4.75

Region: NODE\_298130\_length\_4886\_cov\_17.340155 1346-1355. Max. coverage (+): 0.05. Max coverage (-): 16.65

Region: NODE\_298130\_length\_4886\_cov\_17.340155 1356-1365. Max. coverage (+): 0.26. Max coverage (-): 3.5

Region: NODE\_298130\_length\_4886\_cov\_17.340155 1366-1375. Max. coverage (+): 0.02. Max coverage (-): 0.17

Region: NODE\_298130\_length\_4886\_cov\_17.340155 1376-1385. Max. coverage (+): 0.06. Max coverage (-): 3.38

Region: NODE\_298130\_length\_4886\_cov\_17.340155 1386-1395. Max. coverage (+): 0.15. Max coverage (-): 5.73

Region: NODE\_298130\_length\_4886\_cov\_17.340155 1396-1405. Max. coverage (+): 0.15. Max coverage (-): 1.29

Region: NODE\_298130\_length\_4886\_cov\_17.340155 1406-1415. Max. coverage (+): 0.17. Max coverage (-): 0.46

Region: NODE\_298130\_length\_4886\_cov\_17.340155 1416-1425. Max. coverage (+): 0.06. Max coverage (-): 0.19

Region: NODE\_298130\_length\_4886\_cov\_17.340155 1426-1435. Max. coverage (+): 0.15. Max coverage (-): 0.11

Region: NODE\_298130\_length\_4886\_cov\_17.340155 1436-1445. Max. coverage (+): 0.01. Max coverage (-): 0.17

Region: NODE\_298130\_length\_4886\_cov\_17.340155 1446-1455. Max. coverage (+): 0.01. Max coverage (-): 0.91

Region: NODE\_298130\_length\_4886\_cov\_17.340155 1456-1465. Max. coverage (+): 0.13. Max coverage (-): 0.89

Region: NODE\_298130\_length\_4886\_cov\_17.340155 1466-1475. Max. coverage (+): 0.01. Max coverage (-): 0.26

Region: NODE\_298130\_length\_4886\_cov\_17.340155 1476-1485. Max. coverage (+): 0. Max coverage (-): 0.11

Region: NODE\_298130\_length\_4886\_cov\_17.340155 1486-1495. Max. coverage (+): 0. Max coverage (-): 0.04

Region: NODE\_298130\_length\_4886\_cov\_17.340155 1496-1505. Max. coverage (+): 0. Max coverage (-): 0.07

Region: NODE\_298130\_length\_4886\_cov\_17.340155 1506-1515. Max. coverage (+): 0. Max coverage (-): 0.3

Region: NODE\_298130\_length\_4886\_cov\_17.340155 1516-1525. Max. coverage (+): 0.01. Max coverage (-): 0.09

Region: NODE\_298130\_length\_4886\_cov\_17.340155 1526-1535. Max. coverage (+): 0.01. Max coverage (-): 0

Region: NODE\_298130\_length\_4886\_cov\_17.340155 1536-1545. Max. coverage (+): 0.03. Max coverage (-): 0.12

Region: NODE\_298130\_length\_4886\_cov\_17.340155 1546-1555. Max. coverage (+): 0.02. Max coverage (-): 0.09

Region: NODE\_298130\_length\_4886\_cov\_17.340155 1556-1565. Max. coverage (+): 0.02. Max coverage (-): 0.43

Region: NODE\_298130\_length\_4886\_cov\_17.340155 1566-1575. Max. coverage (+): 0. Max coverage (-): 0.02

Region: NODE\_298130\_length\_4886\_cov\_17.340155 1576-1585. Max. coverage (+): 0.01. Max coverage (-): 0.01

Region: NODE\_298130\_length\_4886\_cov\_17.340155 1586-1595. Max. coverage (+): 0. Max coverage (-): 0.02

Region: NODE\_298130\_length\_4886\_cov\_17.340155 1596-1605. Max. coverage (+): 0. Max coverage (-): 0.01

Region: NODE\_298130\_length\_4886\_cov\_17.340155 1606-1615. Max. coverage (+): 0. Max coverage (-): 0.02

Region: NODE\_298130\_length\_4886\_cov\_17.340155 1616-1625. Max. coverage (+): 0.02. Max coverage (-): 0.07

Region: NODE\_298130\_length\_4886\_cov\_17.340155 1626-1635. Max. coverage (+): 0.1. Max coverage (-): 0.16

Region: NODE\_298130\_length\_4886\_cov\_17.340155 1636-1645. Max. coverage (+): 0.12. Max coverage (-): 0.08

Region: NODE\_298130\_length\_4886\_cov\_17.340155 1646-1655. Max. coverage (+): 0.04. Max coverage (-): 0.33

Region: NODE\_298130\_length\_4886\_cov\_17.340155 1656-1665. Max. coverage (+): 0.01. Max coverage (-): 1

Region: NODE\_298130\_length\_4886\_cov\_17.340155 1666-1675. Max. coverage (+): 0.01. Max coverage (-): 0.41

Region: NODE\_298130\_length\_4886\_cov\_17.340155 1676-1686. Max. coverage (+): 0.07. Max coverage (-): 0.17

Region: NODE\_298130\_length\_4886\_cov\_17.340155 1687-1696. Max. coverage (+): 0.04. Max coverage (-): 2.34

Region: NODE\_298130\_length\_4886\_cov\_17.340155 1697-1706. Max. coverage (+): 0. Max coverage (-): 156.42

Region: NODE\_298130\_length\_4886\_cov\_17.340155 1707-1716. Max. coverage (+): 0.15. Max coverage (-): 3.23

Region: NODE\_298130\_length\_4886\_cov\_17.340155 1717-1726. Max. coverage (+): 0.7. Max coverage (-): 0.07

Region: NODE\_298130\_length\_4886\_cov\_17.340155 1727-1736. Max. coverage (+): 0.22. Max coverage (-): 0.92

Region: NODE\_298130\_length\_4886\_cov\_17.340155 1737-1746. Max. coverage (+): 0.02. Max coverage (-): 16.55

Region: NODE\_298130\_length\_4886\_cov\_17.340155 1747-1756. Max. coverage (+): 0.06. Max coverage (-): 10.79

Region: NODE\_298130\_length\_4886\_cov\_17.340155 1757-1766. Max. coverage (+): 0.33. Max coverage (-): 0.63

Region: NODE\_298130\_length\_4886\_cov\_17.340155 1767-1776. Max. coverage (+): 0.07. Max coverage (-): 6.14

Region: NODE\_298130\_length\_4886\_cov\_17.340155 1777-1786. Max. coverage (+): 0. Max coverage (-): 6.96

Region: NODE\_298130\_length\_4886\_cov\_17.340155 1787-1796. Max. coverage (+): 0. Max coverage (-): 1.82

Region: NODE\_298130\_length\_4886\_cov\_17.340155 1797-1806. Max. coverage (+): 0.01. Max coverage (-): 1.14

Region: NODE\_298130\_length\_4886\_cov\_17.340155 1807-1816. Max. coverage (+): 0.07. Max coverage (-): 1.4

Region: NODE\_298130\_length\_4886\_cov\_17.340155 1817-1826. Max. coverage (+): 0.01. Max coverage (-): 0.32

Region: NODE\_298130\_length\_4886\_cov\_17.340155 1827-1836. Max. coverage (+): 0.02. Max coverage (-): 1.58

Region: NODE\_298130\_length\_4886\_cov\_17.340155 1837-1846. Max. coverage (+): 0.19. Max coverage (-): 0.61

Region: NODE\_298130\_length\_4886\_cov\_17.340155 1847-1856. Max. coverage (+): 0.06. Max coverage (-): 0.09

Region: NODE\_298130\_length\_4886\_cov\_17.340155 1857-1866. Max. coverage (+): 0. Max coverage (-): 0

Region: NODE\_298130\_length\_4886\_cov\_17.340155 1867-1876. Max. coverage (+): 0.01. Max coverage (-): 0

Region: NODE\_298130\_length\_4886\_cov\_17.340155 1877-1886. Max. coverage (+): 0.11. Max coverage (-): 0

Region: NODE\_298130\_length\_4886\_cov\_17.340155 1887-1896. Max. coverage (+): 0.07. Max coverage (-): 0

Region: NODE\_298130\_length\_4886\_cov\_17.340155 1897-1906. Max. coverage (+): 0.04. Max coverage (-): 0.04

Region: NODE\_298130\_length\_4886\_cov\_17.340155 1907-1916. Max. coverage (+): 0.04. Max coverage (-): 0.04

Region: NODE\_298130\_length\_4886\_cov\_17.340155 1917-1926. Max. coverage (+): 0. Max coverage (-): 0.07

Region: NODE\_298130\_length\_4886\_cov\_17.340155 1927-1936. Max. coverage (+): 0. Max coverage (-): 0.04

Region: NODE\_298130\_length\_4886\_cov\_17.340155 1937-1946. Max. coverage (+): 0.04. Max coverage (-): 0.06

Region: NODE\_298130\_length\_4886\_cov\_17.340155 1947-1956. Max. coverage (+): 0. Max coverage (-): 0.04

Region: NODE\_298130\_length\_4886\_cov\_17.340155 1957-1966. Max. coverage (+): 0.05. Max coverage (-): 0.01

Region: NODE\_298130\_length\_4886\_cov\_17.340155 1967-1976. Max. coverage (+): 0. Max coverage (-): 0

Region: NODE\_298130\_length\_4886\_cov\_17.340155 1977-1986. Max. coverage (+): 0.04. Max coverage (-): 0.1

Region: NODE\_298130\_length\_4886\_cov\_17.340155 1987-1996. Max. coverage (+): 0. Max coverage (-): 0.16

Region: NODE\_298130\_length\_4886\_cov\_17.340155 1997-2006. Max. coverage (+): 0. Max coverage (-): 0

Region: NODE\_298130\_length\_4886\_cov\_17.340155 2007-2016. Max. coverage (+): 0.03. Max coverage (-): 0.03

Region: NODE\_298130\_length\_4886\_cov\_17.340155 2017-2026. Max. coverage (+): 0. Max coverage (-): 0.02

Region: NODE\_298130\_length\_4886\_cov\_17.340155 2027-2036. Max. coverage (+): 0.12. Max coverage (-): 0.01

Region: NODE\_298130\_length\_4886\_cov\_17.340155 2037-2046. Max. coverage (+): 0.11. Max coverage (-): 0.17

Region: NODE\_298130\_length\_4886\_cov\_17.340155 2047-2056. Max. coverage (+): 0.11. Max coverage (-): 0.26

Region: NODE\_298130\_length\_4886\_cov\_17.340155 2057-2066. Max. coverage (+): 0.02. Max coverage (-): 0.01

Region: NODE\_298130\_length\_4886\_cov\_17.340155 2067-2076. Max. coverage (+): 0.02. Max coverage (-): 0

Region: NODE\_298130\_length\_4886\_cov\_17.340155 2077-2086. Max. coverage (+): 0. Max coverage (-): 0.37

Region: NODE\_298130\_length\_4886\_cov\_17.340155 2087-2097. Max. coverage (+): 0.11. Max coverage (-): 0.56

Region: NODE\_298130\_length\_4886\_cov\_17.340155 2098-2107. Max. coverage (+): 0.43. Max coverage (-): 0

Region: NODE\_298130\_length\_4886\_cov\_17.340155 2108-2117. Max. coverage (+): 0. Max coverage (-): 0.04

Region: NODE\_298130\_length\_4886\_cov\_17.340155 2118-2127. Max. coverage (+): 0. Max coverage (-): 0.83

Region: NODE\_298130\_length\_4886\_cov\_17.340155 2128-2137. Max. coverage (+): 0.01. Max coverage (-): 0.7

Region: NODE\_298130\_length\_4886\_cov\_17.340155 2138-2147. Max. coverage (+): 0.09. Max coverage (-): 0.04

Region: NODE\_298130\_length\_4886\_cov\_17.340155 2148-2157. Max. coverage (+): 0.01. Max coverage (-): 0

Region: NODE\_298130\_length\_4886\_cov\_17.340155 2158-2167. Max. coverage (+): 0. Max coverage (-): 0.02

Region: NODE\_298130\_length\_4886\_cov\_17.340155 2168-2177. Max. coverage (+): 0. Max coverage (-): 0.19

Region: NODE\_298130\_length\_4886\_cov\_17.340155 2178-2187. Max. coverage (+): 0.01. Max coverage (-): 0.55

Region: NODE\_298130\_length\_4886\_cov\_17.340155 2188-2197. Max. coverage (+): 0.09. Max coverage (-): 0.06

Region: NODE\_298130\_length\_4886\_cov\_17.340155 2198-2207. Max. coverage (+): 0.04. Max coverage (-): 0.22

Region: NODE\_298130\_length\_4886\_cov\_17.340155 2208-2217. Max. coverage (+): 0.01. Max coverage (-): 0.16

Region: NODE\_298130\_length\_4886\_cov\_17.340155 2218-2227. Max. coverage (+): 0. Max coverage (-): 0

Region: NODE\_298130\_length\_4886\_cov\_17.340155 2228-2237. Max. coverage (+): 0. Max coverage (-): 0.03

Region: NODE\_298130\_length\_4886\_cov\_17.340155 2238-2247. Max. coverage (+): 0. Max coverage (-): 0.04

Region: NODE\_298130\_length\_4886\_cov\_17.340155 2248-2257. Max. coverage (+): 0.01. Max coverage (-): 0

Region: NODE\_298130\_length\_4886\_cov\_17.340155 2258-2267. Max. coverage (+): 0.01. Max coverage (-): 0

Region: NODE\_298130\_length\_4886\_cov\_17.340155 2268-2277. Max. coverage (+): 0. Max coverage (-): 0.06

Region: NODE\_298130\_length\_4886\_cov\_17.340155 2278-2287. Max. coverage (+): 0. Max coverage (-): 0.08

Region: NODE\_298130\_length\_4886\_cov\_17.340155 2288-2297. Max. coverage (+): 0.05. Max coverage (-): 0.03

Region: NODE\_298130\_length\_4886\_cov\_17.340155 2298-2307. Max. coverage (+): 0.01. Max coverage (-): 0.31

Region: NODE\_298130\_length\_4886\_cov\_17.340155 2308-2317. Max. coverage (+): 0. Max coverage (-): 0

Region: NODE\_298130\_length\_4886\_cov\_17.340155 2318-2327. Max. coverage (+): 0. Max coverage (-): 0

Region: NODE\_298130\_length\_4886\_cov\_17.340155 2328-2337. Max. coverage (+): 0. Max coverage (-): 0

Region: NODE\_298130\_length\_4886\_cov\_17.340155 2338-2347. Max. coverage (+): 0. Max coverage (-): 0

Region: NODE\_298130\_length\_4886\_cov\_17.340155 2348-2357. Max. coverage (+): 0. Max coverage (-): 0

Region: NODE\_298130\_length\_4886\_cov\_17.340155 2358-2367. Max. coverage (+): 0. Max coverage (-): 0.02

Region: NODE\_298130\_length\_4886\_cov\_17.340155 2368-2377. Max. coverage (+): 0.01. Max coverage (-): 0.02

Region: NODE\_298130\_length\_4886\_cov\_17.340155 2378-2387. Max. coverage (+): 0.01. Max coverage (-): 0.01

Region: NODE\_298130\_length\_4886\_cov\_17.340155 2388-2397. Max. coverage (+): 0.01. Max coverage (-): 0

Region: NODE\_298130\_length\_4886\_cov\_17.340155 2398-2407. Max. coverage (+): 0. Max coverage (-): 0

Region: NODE\_298130\_length\_4886\_cov\_17.340155 2408-2417. Max. coverage (+): 0. Max coverage (-): 0

Region: NODE\_298130\_length\_4886\_cov\_17.340155 2418-2427. Max. coverage (+): 0. Max coverage (-): 0

Region: NODE\_298130\_length\_4886\_cov\_17.340155 2428-2437. Max. coverage (+): 0. Max coverage (-): 0

Region: NODE\_298130\_length\_4886\_cov\_17.340155 2438-2447. Max. coverage (+): 0. Max coverage (-): 0

Region: NODE\_298130\_length\_4886\_cov\_17.340155 2448-2457. Max. coverage (+): 0. Max coverage (-): 0

Region: NODE\_298130\_length\_4886\_cov\_17.340155 2458-2467. Max. coverage (+): 0. Max coverage (-): 0

Region: NODE\_298130\_length\_4886\_cov\_17.340155 2468-2477. Max. coverage (+): 0. Max coverage (-): 0

Region: NODE\_298130\_length\_4886\_cov\_17.340155 2478-2487. Max. coverage (+): 0. Max coverage (-): 0

Region: NODE\_298130\_length\_4886\_cov\_17.340155 2488-2497. Max. coverage (+): 0. Max coverage (-): 0

Region: NODE\_298130\_length\_4886\_cov\_17.340155 2498-2507. Max. coverage (+): 0. Max coverage (-): 0

Region: NODE\_298130\_length\_4886\_cov\_17.340155 2508-2518. Max. coverage (+): 0. Max coverage (-): 0

Region: NODE\_298130\_length\_4886\_cov\_17.340155 2519-2528. Max. coverage (+): 0. Max coverage (-): 0

Region: NODE\_298130\_length\_4886\_cov\_17.340155 2529-2538. Max. coverage (+): 0. Max coverage (-): 0

Region: NODE\_298130\_length\_4886\_cov\_17.340155 2539-2548. Max. coverage (+): 0. Max coverage (-): 0

Region: NODE\_298130\_length\_4886\_cov\_17.340155 2549-2558. Max. coverage (+): 0. Max coverage (-): 0

Region: NODE\_298130\_length\_4886\_cov\_17.340155 2559-2568. Max. coverage (+): 0. Max coverage (-): 0.32

Region: NODE\_298130\_length\_4886\_cov\_17.340155 2569-2578. Max. coverage (+): 0. Max coverage (-): 0.43

Region: NODE\_298130\_length\_4886\_cov\_17.340155 2579-2588. Max. coverage (+): 0. Max coverage (-): 0

Region: NODE\_298130\_length\_4886\_cov\_17.340155 2589-2598. Max. coverage (+): 0. Max coverage (-): 0

Region: NODE\_298130\_length\_4886\_cov\_17.340155 2599-2608. Max. coverage (+): 0. Max coverage (-): 0.01

Region: NODE\_298130\_length\_4886\_cov\_17.340155 2609-2618. Max. coverage (+): 0. Max coverage (-): 0.01

Region: NODE\_298130\_length\_4886\_cov\_17.340155 2619-2628. Max. coverage (+): 0. Max coverage (-): 0.03

Region: NODE\_298130\_length\_4886\_cov\_17.340155 2629-2638. Max. coverage (+): 0.02. Max coverage (-): 0.16

Region: NODE\_298130\_length\_4886\_cov\_17.340155 2639-2648. Max. coverage (+): 0.12. Max coverage (-): 0.08

Region: NODE\_298130\_length\_4886\_cov\_17.340155 2649-2658. Max. coverage (+): 0.04. Max coverage (-): 0.33

Region: NODE\_298130\_length\_4886\_cov\_17.340155 2659-2668. Max. coverage (+): 0.01. Max coverage (-): 1

Region: NODE\_298130\_length\_4886\_cov\_17.340155 2669-2678. Max. coverage (+): 0.01. Max coverage (-): 0.41

Region: NODE\_298130\_length\_4886\_cov\_17.340155 2679-2688. Max. coverage (+): 0.07. Max coverage (-): 0.17

Region: NODE\_298130\_length\_4886\_cov\_17.340155 2689-2698. Max. coverage (+): 0.02. Max coverage (-): 0.01

Region: NODE\_298130\_length\_4886\_cov\_17.340155 2699-2708. Max. coverage (+): 0. Max coverage (-): 3.91

Region: NODE\_298130\_length\_4886\_cov\_17.340155 2709-2718. Max. coverage (+): 0.06. Max coverage (-): 0.96

Region: NODE\_298130\_length\_4886\_cov\_17.340155 2719-2728. Max. coverage (+): 0.7. Max coverage (-): 0.07

Region: NODE\_298130\_length\_4886\_cov\_17.340155 2729-2738. Max. coverage (+): 0.22. Max coverage (-): 0.92

Region: NODE\_298130\_length\_4886\_cov\_17.340155 2739-2748. Max. coverage (+): 0.01. Max coverage (-): 0.63

Region: NODE\_298130\_length\_4886\_cov\_17.340155 2749-2758. Max. coverage (+): 0.15. Max coverage (-): 0

Region: NODE\_298130\_length\_4886\_cov\_17.340155 2759-2768. Max. coverage (+): 0.07. Max coverage (-): 0

Region: NODE\_298130\_length\_4886\_cov\_17.340155 2769-2778. Max. coverage (+): 0.11. Max coverage (-): 0.06

Region: NODE\_298130\_length\_4886\_cov\_17.340155 2779-2788. Max. coverage (+): 0. Max coverage (-): 1.07

Region: NODE\_298130\_length\_4886\_cov\_17.340155 2789-2798. Max. coverage (+): 0. Max coverage (-): 1.82

Region: NODE\_298130\_length\_4886\_cov\_17.340155 2799-2808. Max. coverage (+): 0.01. Max coverage (-): 1.36

Region: NODE\_298130\_length\_4886\_cov\_17.340155 2809-2818. Max. coverage (+): 0.07. Max coverage (-): 1.4

Region: NODE\_298130\_length\_4886\_cov\_17.340155 2819-2828. Max. coverage (+): 0. Max coverage (-): 0.09

Region: NODE\_298130\_length\_4886\_cov\_17.340155 2829-2838. Max. coverage (+): 0.02. Max coverage (-): 1.3

Region: NODE\_298130\_length\_4886\_cov\_17.340155 2839-2848. Max. coverage (+): 0.19. Max coverage (-): 1.58

Region: NODE\_298130\_length\_4886\_cov\_17.340155 2849-2858. Max. coverage (+): 0.09. Max coverage (-): 0.22

Region: NODE\_298130\_length\_4886\_cov\_17.340155 2859-2868. Max. coverage (+): 0.02. Max coverage (-): 0.04

Region: NODE\_298130\_length\_4886\_cov\_17.340155 2869-2878. Max. coverage (+): 0.01. Max coverage (-): 0.12

Region: NODE\_298130\_length\_4886\_cov\_17.340155 2879-2888. Max. coverage (+): 0.03. Max coverage (-): 0.55

Region: NODE\_298130\_length\_4886\_cov\_17.340155 2889-2898. Max. coverage (+): 0.67. Max coverage (-): 0.11

Region: NODE\_298130\_length\_4886\_cov\_17.340155 2899-2908. Max. coverage (+): 0.04. Max coverage (-): 0.05

Region: NODE\_298130\_length\_4886\_cov\_17.340155 2909-2918. Max. coverage (+): 0.02. Max coverage (-): 0

Region: NODE\_298130\_length\_4886\_cov\_17.340155 2919-2928. Max. coverage (+): 0.03. Max coverage (-): 0.12

Region: NODE\_298130\_length\_4886\_cov\_17.340155 2929-2939. Max. coverage (+): 0.06. Max coverage (-): 0.13

Region: NODE\_298130\_length\_4886\_cov\_17.340155 2940-2949. Max. coverage (+): 0.01. Max coverage (-): 1.32

Region: NODE\_298130\_length\_4886\_cov\_17.340155 2950-2959. Max. coverage (+): 0.01. Max coverage (-): 0.58

Region: NODE\_298130\_length\_4886\_cov\_17.340155 2960-2969. Max. coverage (+): 0.03. Max coverage (-): 1.66

Region: NODE\_298130\_length\_4886\_cov\_17.340155 2970-2979. Max. coverage (+): 0.02. Max coverage (-): 41.93

Region: NODE\_298130\_length\_4886\_cov\_17.340155 2980-2989. Max. coverage (+): 0. Max coverage (-): 6.27

Region: NODE\_298130\_length\_4886\_cov\_17.340155 2990-2999. Max. coverage (+): 1.39. Max coverage (-): 0.85

Region: NODE\_298130\_length\_4886\_cov\_17.340155 3000-3009. Max. coverage (+): 0.76. Max coverage (-): 2.34

Region: NODE\_298130\_length\_4886\_cov\_17.340155 3010-3019. Max. coverage (+): 0.04. Max coverage (-): 2.11

Region: NODE\_298130\_length\_4886\_cov\_17.340155 3020-3029. Max. coverage (+): 0. Max coverage (-): 0.33

Region: NODE\_298130\_length\_4886\_cov\_17.340155 3030-3039. Max. coverage (+): 0. Max coverage (-): 3.05

Region: NODE\_298130\_length\_4886\_cov\_17.340155 3040-3049. Max. coverage (+): 0.17. Max coverage (-): 0.16

Region: NODE\_298130\_length\_4886\_cov\_17.340155 3050-3059. Max. coverage (+): 0.17. Max coverage (-): 0.15

Region: NODE\_298130\_length\_4886\_cov\_17.340155 3060-3069. Max. coverage (+): 0.04. Max coverage (-): 0.09

Region: NODE\_298130\_length\_4886\_cov\_17.340155 3070-3079. Max. coverage (+): 0.02. Max coverage (-): 0.22

Region: NODE\_298130\_length\_4886\_cov\_17.340155 3080-3089. Max. coverage (+): 0.09. Max coverage (-): 0.42

Region: NODE\_298130\_length\_4886\_cov\_17.340155 3090-3099. Max. coverage (+): 0.04. Max coverage (-): 14.75

Region: NODE\_298130\_length\_4886\_cov\_17.340155 3100-3109. Max. coverage (+): 0.13. Max coverage (-): 37.85

Region: NODE\_298130\_length\_4886\_cov\_17.340155 3110-3119. Max. coverage (+): 0.03. Max coverage (-): 2.44

Region: NODE\_298130\_length\_4886\_cov\_17.340155 3120-3129. Max. coverage (+): 0.04. Max coverage (-): 0.15

Region: NODE\_298130\_length\_4886\_cov\_17.340155 3130-3139. Max. coverage (+): 0.03. Max coverage (-): 0.09

Region: NODE\_298130\_length\_4886\_cov\_17.340155 3140-3149. Max. coverage (+): 0.11. Max coverage (-): 0.04

Region: NODE\_298130\_length\_4886\_cov\_17.340155 3150-3159. Max. coverage (+): 0.05. Max coverage (-): 0.02

Region: NODE\_298130\_length\_4886\_cov\_17.340155 3160-3169. Max. coverage (+): 0.07. Max coverage (-): 0.06

Region: NODE\_298130\_length\_4886\_cov\_17.340155 3170-3179. Max. coverage (+): 0.02. Max coverage (-): 0.11

Region: NODE\_298130\_length\_4886\_cov\_17.340155 3180-3189. Max. coverage (+): 0.01. Max coverage (-): 1.15

Region: NODE\_298130\_length\_4886\_cov\_17.340155 3190-3199. Max. coverage (+): 0. Max coverage (-): 1.41

Region: NODE\_298130\_length\_4886\_cov\_17.340155 3200-3209. Max. coverage (+): 0.02. Max coverage (-): 0.13

Region: NODE\_298130\_length\_4886\_cov\_17.340155 3210-3219. Max. coverage (+): 0.07. Max coverage (-): 0.46

Region: NODE\_298130\_length\_4886\_cov\_17.340155 3220-3229. Max. coverage (+): 0.04. Max coverage (-): 0.9

Region: NODE\_298130\_length\_4886\_cov\_17.340155 3230-3239. Max. coverage (+): 0.04. Max coverage (-): 1

Region: NODE\_298130\_length\_4886\_cov\_17.340155 3240-3249. Max. coverage (+): 0.06. Max coverage (-): 7.84

Region: NODE\_298130\_length\_4886\_cov\_17.340155 3250-3259. Max. coverage (+): 0.07. Max coverage (-): 3.43

Region: NODE\_298130\_length\_4886\_cov\_17.340155 3260-3269. Max. coverage (+): 0.15. Max coverage (-): 1.59

Region: NODE\_298130\_length\_4886\_cov\_17.340155 3270-3279. Max. coverage (+): 0.02. Max coverage (-): 2.64

Region: NODE\_298130\_length\_4886\_cov\_17.340155 3280-3289. Max. coverage (+): 0.02. Max coverage (-): 1.8

Region: NODE\_298130\_length\_4886\_cov\_17.340155 3290-3299. Max. coverage (+): 0.22. Max coverage (-): 0.07

Region: NODE\_298130\_length\_4886\_cov\_17.340155 3300-3309. Max. coverage (+): 0. Max coverage (-): 1.82

Region: NODE\_298130\_length\_4886\_cov\_17.340155 3310-3319. Max. coverage (+): 0.02. Max coverage (-): 1.04

Region: NODE\_298130\_length\_4886\_cov\_17.340155 3320-3329. Max. coverage (+): 3.61. Max coverage (-): 0.33

Region: NODE\_298130\_length\_4886\_cov\_17.340155 3330-3339. Max. coverage (+): 0.52. Max coverage (-): 0.3

Region: NODE\_298130\_length\_4886\_cov\_17.340155 3340-3350. Max. coverage (+): 0. Max coverage (-): 2.41

Region: NODE\_298130\_length\_4886\_cov\_17.340155 3351-3360. Max. coverage (+): 0.11. Max coverage (-): 1.78

Region: NODE\_298130\_length\_4886\_cov\_17.340155 3361-3370. Max. coverage (+): 0.11. Max coverage (-): 5.26

Region: NODE\_298130\_length\_4886\_cov\_17.340155 3371-3380. Max. coverage (+): 0.11. Max coverage (-): 4.67

Region: NODE\_298130\_length\_4886\_cov\_17.340155 3381-3390. Max. coverage (+): 0.11. Max coverage (-): 1.08

Region: NODE\_298130\_length\_4886\_cov\_17.340155 3391-3400. Max. coverage (+): 0. Max coverage (-): 5.08

Region: NODE\_298130\_length\_4886\_cov\_17.340155 3401-3410. Max. coverage (+): 0. Max coverage (-): 0.32

Region: NODE\_298130\_length\_4886\_cov\_17.340155 3411-3420. Max. coverage (+): 0.04. Max coverage (-): 0.52

Region: NODE\_298130\_length\_4886\_cov\_17.340155 3421-3430. Max. coverage (+): 0.04. Max coverage (-): 0.44

Region: NODE\_298130\_length\_4886\_cov\_17.340155 3431-3440. Max. coverage (+): 0.13. Max coverage (-): 0.11

Region: NODE\_298130\_length\_4886\_cov\_17.340155 3441-3450. Max. coverage (+): 0.09. Max coverage (-): 0.15

Region: NODE\_298130\_length\_4886\_cov\_17.340155 3451-3460. Max. coverage (+): 0.04. Max coverage (-): 0.22

Region: NODE\_298130\_length\_4886\_cov\_17.340155 3461-3470. Max. coverage (+): 0.07. Max coverage (-): 0.13

Region: NODE\_298130\_length\_4886\_cov\_17.340155 3471-3480. Max. coverage (+): 0.06. Max coverage (-): 0.06

Region: NODE\_298130\_length\_4886\_cov\_17.340155 3481-3490. Max. coverage (+): 0. Max coverage (-): 0.02

Region: NODE\_298130\_length\_4886\_cov\_17.340155 3491-3500. Max. coverage (+): 0. Max coverage (-): 0.32

Region: NODE\_298130\_length\_4886\_cov\_17.340155 3501-3510. Max. coverage (+): 0.01. Max coverage (-): 1.76

Region: NODE\_298130\_length\_4886\_cov\_17.340155 3511-3520. Max. coverage (+): 0.02. Max coverage (-): 2.67

Region: NODE\_298130\_length\_4886\_cov\_17.340155 3521-3530. Max. coverage (+): 0.04. Max coverage (-): 1.73

Region: NODE\_298130\_length\_4886\_cov\_17.340155 3531-3540. Max. coverage (+): 0.07. Max coverage (-): 2.3

Region: NODE\_298130\_length\_4886\_cov\_17.340155 3541-3550. Max. coverage (+): 0.09. Max coverage (-): 3.52

Region: NODE\_298130\_length\_4886\_cov\_17.340155 3551-3560. Max. coverage (+): 0.04. Max coverage (-): 23.13

Region: NODE\_298130\_length\_4886\_cov\_17.340155 3561-3570. Max. coverage (+): 0.04. Max coverage (-): 23.65

Region: NODE\_298130\_length\_4886\_cov\_17.340155 3571-3580. Max. coverage (+): 0.19. Max coverage (-): 2.3

Region: NODE\_298130\_length\_4886\_cov\_17.340155 3581-3590. Max. coverage (+): 0.07. Max coverage (-): 2.82

Region: NODE\_298130\_length\_4886\_cov\_17.340155 3591-3600. Max. coverage (+): 0.15. Max coverage (-): 1.26

Region: NODE\_298130\_length\_4886\_cov\_17.340155 3601-3610. Max. coverage (+): 0.15. Max coverage (-): 2.56

Region: NODE\_298130\_length\_4886\_cov\_17.340155 3611-3620. Max. coverage (+): 0.06. Max coverage (-): 1.8

Region: NODE\_298130\_length\_4886\_cov\_17.340155 3621-3630. Max. coverage (+): 0.26. Max coverage (-): 1.19

Region: NODE\_298130\_length\_4886\_cov\_17.340155 3631-3640. Max. coverage (+): 0. Max coverage (-): 1.52

Region: NODE\_298130\_length\_4886\_cov\_17.340155 3641-3650. Max. coverage (+): 0.19. Max coverage (-): 1.82

Region: NODE\_298130\_length\_4886\_cov\_17.340155 3651-3660. Max. coverage (+): 0.26. Max coverage (-): 19.61

Region: NODE\_298130\_length\_4886\_cov\_17.340155 3661-3670. Max. coverage (+): 0.78. Max coverage (-): 10.01

Region: NODE\_298130\_length\_4886\_cov\_17.340155 3671-3680. Max. coverage (+): 1.04. Max coverage (-): 4.04

Region: NODE\_298130\_length\_4886\_cov\_17.340155 3681-3690. Max. coverage (+): 0.04. Max coverage (-): 3.93

Region: NODE\_298130\_length\_4886\_cov\_17.340155 3691-3700. Max. coverage (+): 0.02. Max coverage (-): 4.33

Region: NODE\_298130\_length\_4886\_cov\_17.340155 3701-3710. Max. coverage (+): 0.01. Max coverage (-): 7.61

Region: NODE\_298130\_length\_4886\_cov\_17.340155 3711-3720. Max. coverage (+): 0.14. Max coverage (-): 0.57

Region: NODE\_298130\_length\_4886\_cov\_17.340155 3721-3730. Max. coverage (+): 0.04. Max coverage (-): 0.01

Region: NODE\_298130\_length\_4886\_cov\_17.340155 3731-3740. Max. coverage (+): 0.05. Max coverage (-): 0.2

Region: NODE\_298130\_length\_4886\_cov\_17.340155 3741-3750. Max. coverage (+): 0.06. Max coverage (-): 5.51

Region: NODE\_298130\_length\_4886\_cov\_17.340155 3751-3760. Max. coverage (+): 0.04. Max coverage (-): 5.32

Region: NODE\_298130\_length\_4886\_cov\_17.340155 3761-3771. Max. coverage (+): 0.17. Max coverage (-): 0.32

Region: NODE\_298130\_length\_4886\_cov\_17.340155 3772-3781. Max. coverage (+): 0.17. Max coverage (-): 1.15

Region: NODE\_298130\_length\_4886\_cov\_17.340155 3782-3791. Max. coverage (+): 0.01. Max coverage (-): 3.88

Region: NODE\_298130\_length\_4886\_cov\_17.340155 3792-3801. Max. coverage (+): 0.24. Max coverage (-): 0.81

Region: NODE\_298130\_length\_4886\_cov\_17.340155 3802-3811. Max. coverage (+): 0.26. Max coverage (-): 0.06

Region: NODE\_298130\_length\_4886\_cov\_17.340155 3812-3821. Max. coverage (+): 0.01. Max coverage (-): 0.75

Region: NODE\_298130\_length\_4886\_cov\_17.340155 3822-3831. Max. coverage (+): 0. Max coverage (-): 0.43

Region: NODE\_298130\_length\_4886\_cov\_17.340155 3832-3841. Max. coverage (+): 0.06. Max coverage (-): 0.94

Region: NODE\_298130\_length\_4886\_cov\_17.340155 3842-3851. Max. coverage (+): 0. Max coverage (-): 3.45

Region: NODE\_298130\_length\_4886\_cov\_17.340155 3852-3861. Max. coverage (+): 0.07. Max coverage (-): 2.48

Region: NODE\_298130\_length\_4886\_cov\_17.340155 3862-3871. Max. coverage (+): 0.19. Max coverage (-): 1

Region: NODE\_298130\_length\_4886\_cov\_17.340155 3872-3881. Max. coverage (+): 0.21. Max coverage (-): 0.1

Region: NODE\_298130\_length\_4886\_cov\_17.340155 3882-3891. Max. coverage (+): 0.13. Max coverage (-): 0.75

Region: NODE\_298130\_length\_4886\_cov\_17.340155 3892-3901. Max. coverage (+): 0.01. Max coverage (-): 0.14

Region: NODE\_298130\_length\_4886\_cov\_17.340155 3902-3911. Max. coverage (+): 0. Max coverage (-): 0.22

Region: NODE\_298130\_length\_4886\_cov\_17.340155 3912-3921. Max. coverage (+): 0. Max coverage (-): 0.67

Region: NODE\_298130\_length\_4886\_cov\_17.340155 3922-3931. Max. coverage (+): 0. Max coverage (-): 0.22

Region: NODE\_298130\_length\_4886\_cov\_17.340155 3932-3941. Max. coverage (+): 0.01. Max coverage (-): 0.07

Region: NODE\_298130\_length\_4886\_cov\_17.340155 3942-3951. Max. coverage (+): 0.04. Max coverage (-): 10.42

Region: NODE\_298130\_length\_4886\_cov\_17.340155 3952-3961. Max. coverage (+): 0. Max coverage (-): 15.9

Region: NODE\_298130\_length\_4886\_cov\_17.340155 3962-3971. Max. coverage (+): 0.1. Max coverage (-): 0.16

Region: NODE\_298130\_length\_4886\_cov\_17.340155 3972-3981. Max. coverage (+): 0.02. Max coverage (-): 1.18

Region: NODE\_298130\_length\_4886\_cov\_17.340155 3982-3991. Max. coverage (+): 0.07. Max coverage (-): 1.04

Region: NODE\_298130\_length\_4886\_cov\_17.340155 3992-4001. Max. coverage (+): 0.07. Max coverage (-): 0.7

Region: NODE\_298130\_length\_4886\_cov\_17.340155 4002-4011. Max. coverage (+): 0. Max coverage (-): 0.52

Region: NODE\_298130\_length\_4886\_cov\_17.340155 4012-4021. Max. coverage (+): 0. Max coverage (-): 0.04

Region: NODE\_298130\_length\_4886\_cov\_17.340155 4022-4031. Max. coverage (+): 0.04. Max coverage (-): 0.15

Region: NODE\_298130\_length\_4886\_cov\_17.340155 4032-4041. Max. coverage (+): 0.04. Max coverage (-): 5.75

Region: NODE\_298130\_length\_4886\_cov\_17.340155 4042-4051. Max. coverage (+): 0. Max coverage (-): 0.59

Region: NODE\_298130\_length\_4886\_cov\_17.340155 4052-4061. Max. coverage (+): 0.04. Max coverage (-): 0.19

Region: NODE\_298130\_length\_4886\_cov\_17.340155 4062-4071. Max. coverage (+): 0.04. Max coverage (-): 0.63

Region: NODE\_298130\_length\_4886\_cov\_17.340155 4072-4081. Max. coverage (+): 0. Max coverage (-): 4.97

Region: NODE\_298130\_length\_4886\_cov\_17.340155 4082-4091. Max. coverage (+): 0. Max coverage (-): 2

Region: NODE\_298130\_length\_4886\_cov\_17.340155 4092-4101. Max. coverage (+): 0.01. Max coverage (-): 0.11

Region: NODE\_298130\_length\_4886\_cov\_17.340155 4102-4111. Max. coverage (+): 0.04. Max coverage (-): 0.01

Region: NODE\_298130\_length\_4886\_cov\_17.340155 4112-4121. Max. coverage (+): 0. Max coverage (-): 0.03

Region: NODE\_298130\_length\_4886\_cov\_17.340155 4122-4131. Max. coverage (+): 0.01. Max coverage (-): 0.02

Region: NODE\_298130\_length\_4886\_cov\_17.340155 4132-4141. Max. coverage (+): 0.01. Max coverage (-): 0.01

Region: NODE\_298130\_length\_4886\_cov\_17.340155 4142-4151. Max. coverage (+): 0.01. Max coverage (-): 6.71

Region: NODE\_298130\_length\_4886\_cov\_17.340155 4152-4161. Max. coverage (+): 0. Max coverage (-): 1.37

Region: NODE\_298130\_length\_4886\_cov\_17.340155 4162-4171. Max. coverage (+): 0. Max coverage (-): 0

Region: NODE\_298130\_length\_4886\_cov\_17.340155 4172-4181. Max. coverage (+): 0. Max coverage (-): 0.74

Region: NODE\_298130\_length\_4886\_cov\_17.340155 4182-4192. Max. coverage (+): 0. Max coverage (-): 8.79

Region: NODE\_298130\_length\_4886\_cov\_17.340155 4193-4202. Max. coverage (+): 0. Max coverage (-): 8.93

Region: NODE\_298130\_length\_4886\_cov\_17.340155 4203-4212. Max. coverage (+): 0.07. Max coverage (-): 0.07

Region: NODE\_298130\_length\_4886\_cov\_17.340155 4213-4222. Max. coverage (+): 0.07. Max coverage (-): 0.22

Region: NODE\_298130\_length\_4886\_cov\_17.340155 4223-4232. Max. coverage (+): 0. Max coverage (-): 0

Region: NODE\_298130\_length\_4886\_cov\_17.340155 4233-4242. Max. coverage (+): 0. Max coverage (-): 0.3

Region: NODE\_298130\_length\_4886\_cov\_17.340155 4243-4252. Max. coverage (+): 0. Max coverage (-): 0.36

Region: NODE\_298130\_length\_4886\_cov\_17.340155 4253-4262. Max. coverage (+): 0.1. Max coverage (-): 0.45

Region: NODE\_298130\_length\_4886\_cov\_17.340155 4263-4272. Max. coverage (+): 0.1. Max coverage (-): 0.38

Region: NODE\_298130\_length\_4886\_cov\_17.340155 4273-4282. Max. coverage (+): 0. Max coverage (-): 0.78

Region: NODE\_298130\_length\_4886\_cov\_17.340155 4283-4292. Max. coverage (+): 0. Max coverage (-): 0.63

Region: NODE\_298130\_length\_4886\_cov\_17.340155 4293-4302. Max. coverage (+): 0.18. Max coverage (-): 0.05

Region: NODE\_298130\_length\_4886\_cov\_17.340155 4303-4312. Max. coverage (+): 0.01. Max coverage (-): 0.01

Region: NODE\_298130\_length\_4886\_cov\_17.340155 4313-4322. Max. coverage (+): 0. Max coverage (-): 0.15

Region: NODE\_298130\_length\_4886\_cov\_17.340155 4323-4332. Max. coverage (+): 0.07. Max coverage (-): 0.04

Region: NODE\_298130\_length\_4886\_cov\_17.340155 4333-4342. Max. coverage (+): 0. Max coverage (-): 0.07

Region: NODE\_298130\_length\_4886\_cov\_17.340155 4343-4352. Max. coverage (+): 0.07. Max coverage (-): 0.63

Region: NODE\_298130\_length\_4886\_cov\_17.340155 4353-4362. Max. coverage (+): 0.06. Max coverage (-): 0.63

Region: NODE\_298130\_length\_4886\_cov\_17.340155 4363-4372. Max. coverage (+): 0.08. Max coverage (-): 0.01

Region: NODE\_298130\_length\_4886\_cov\_17.340155 4373-4382. Max. coverage (+): 0.06. Max coverage (-): 0.02

Region: NODE\_298130\_length\_4886\_cov\_17.340155 4383-4392. Max. coverage (+): 0.03. Max coverage (-): 0.13

Region: NODE\_298130\_length\_4886\_cov\_17.340155 4393-4402. Max. coverage (+): 0. Max coverage (-): 0.03

Region: NODE\_298130\_length\_4886\_cov\_17.340155 4403-4412. Max. coverage (+): 0. Max coverage (-): 0.11

Region: NODE\_298130\_length\_4886\_cov\_17.340155 4413-4422. Max. coverage (+): 0.01. Max coverage (-): 0.14

Region: NODE\_298130\_length\_4886\_cov\_17.340155 4423-4432. Max. coverage (+): 0. Max coverage (-): 0.07

Region: NODE\_298130\_length\_4886\_cov\_17.340155 4433-4442. Max. coverage (+): 0. Max coverage (-): 0.04

Region: NODE\_298130\_length\_4886\_cov\_17.340155 4443-4452. Max. coverage (+): 0. Max coverage (-): 0

Region: NODE\_298130\_length\_4886\_cov\_17.340155 4453-4462. Max. coverage (+): 0.04. Max coverage (-): 0

Region: NODE\_298130\_length\_4886\_cov\_17.340155 4463-4472. Max. coverage (+): 0.04. Max coverage (-): 0.04

Region: NODE\_298130\_length\_4886\_cov\_17.340155 4473-4482. Max. coverage (+): 0.04. Max coverage (-): 0.24

Region: NODE\_298130\_length\_4886\_cov\_17.340155 4483-4492. Max. coverage (+): 0.04. Max coverage (-): 0.24

Region: NODE\_298130\_length\_4886\_cov\_17.340155 4493-4502. Max. coverage (+): 0.26. Max coverage (-): 1

Region: NODE\_298130\_length\_4886\_cov\_17.340155 4503-4512. Max. coverage (+): 0.3. Max coverage (-): 3.67

Region: NODE\_298130\_length\_4886\_cov\_17.340155 4513-4522. Max. coverage (+): 0.15. Max coverage (-): 0.41

Region: NODE\_298130\_length\_4886\_cov\_17.340155 4523-4532. Max. coverage (+): 0.1. Max coverage (-): 0.39

Region: NODE\_298130\_length\_4886\_cov\_17.340155 4533-4542. Max. coverage (+): 0.06. Max coverage (-): 2.48

Region: NODE\_298130\_length\_4886\_cov\_17.340155 4543-4552. Max. coverage (+): 0. Max coverage (-): 0.18

Region: NODE\_298130\_length\_4886\_cov\_17.340155 4553-4562. Max. coverage (+): 0. Max coverage (-): 0.15

Region: NODE\_298130\_length\_4886\_cov\_17.340155 4563-4572. Max. coverage (+): 0. Max coverage (-): 0.15

Region: NODE\_298130\_length\_4886\_cov\_17.340155 4573-4582. Max. coverage (+): 0.04. Max coverage (-): 5.58

Region: NODE\_298130\_length\_4886\_cov\_17.340155 4583-4592. Max. coverage (+): 0.11. Max coverage (-): 4.25

Region: NODE\_298130\_length\_4886\_cov\_17.340155 4593-4603. Max. coverage (+): 0.02. Max coverage (-): 0.01

Region: NODE\_298130\_length\_4886\_cov\_17.340155 4604-4613. Max. coverage (+): 0. Max coverage (-): 1.63

Region: NODE\_298130\_length\_4886\_cov\_17.340155 4614-4623. Max. coverage (+): 0. Max coverage (-): 0.63

Region: NODE\_298130\_length\_4886\_cov\_17.340155 4624-4633. Max. coverage (+): 0.15. Max coverage (-): 0.11

Region: NODE\_298130\_length\_4886\_cov\_17.340155 4634-4643. Max. coverage (+): 0.04. Max coverage (-): 0.11

Region: NODE\_298130\_length\_4886\_cov\_17.340155 4644-4653. Max. coverage (+): 0.01. Max coverage (-): 1.59

Region: NODE\_298130\_length\_4886\_cov\_17.340155 4654-4663. Max. coverage (+): 0.06. Max coverage (-): 0.21

Region: NODE\_298130\_length\_4886\_cov\_17.340155 4664-4673. Max. coverage (+): 0.81. Max coverage (-): 0.36

Region: NODE\_298130\_length\_4886\_cov\_17.340155 4674-4683. Max. coverage (+): 0. Max coverage (-): 0.21

Region: NODE\_298130\_length\_4886\_cov\_17.340155 4684-4693. Max. coverage (+): 0. Max coverage (-): 0.04

Region: NODE\_298130\_length\_4886\_cov\_17.340155 4694-4703. Max. coverage (+): 0.04. Max coverage (-): 0.07

Region: NODE\_298130\_length\_4886\_cov\_17.340155 4704-4713. Max. coverage (+): 0.03. Max coverage (-): 0.5

Region: NODE\_298130\_length\_4886\_cov\_17.340155 4714-4723. Max. coverage (+): 0.01. Max coverage (-): 0.53

Region: NODE\_298130\_length\_4886\_cov\_17.340155 4724-4733. Max. coverage (+): 0.04. Max coverage (-): 0.54

Region: NODE\_298130\_length\_4886\_cov\_17.340155 4734-4743. Max. coverage (+): 0.04. Max coverage (-): 0.09

Region: NODE\_298130\_length\_4886\_cov\_17.340155 4744-4753. Max. coverage (+): 1.19. Max coverage (-): 0.41

Region: NODE\_298130\_length\_4886\_cov\_17.340155 4754-4763. Max. coverage (+): 0.11. Max coverage (-): 7.27

Region: NODE\_298130\_length\_4886\_cov\_17.340155 4764-4773. Max. coverage (+): 0.04. Max coverage (-): 0.48

Region: NODE\_298130\_length\_4886\_cov\_17.340155 4774-4783. Max. coverage (+): 0. Max coverage (-): 0.03

Region: NODE\_298130\_length\_4886\_cov\_17.340155 4784-4793. Max. coverage (+): 0. Max coverage (-): 0.03

Region: NODE\_298130\_length\_4886\_cov\_17.340155 4794-4803. Max. coverage (+): 0.01. Max coverage (-): 0.02

Region: NODE\_298130\_length\_4886\_cov\_17.340155 4804-4813. Max. coverage (+): 0.01. Max coverage (-): 0.07

Region: NODE\_298130\_length\_4886\_cov\_17.340155 4814-4823. Max. coverage (+): 0.04. Max coverage (-): 0.01

Region: NODE\_298130\_length\_4886\_cov\_17.340155 4824-4833. Max. coverage (+): 0.02. Max coverage (-): 0.01

Region: NODE\_298130\_length\_4886\_cov\_17.340155 4834-4843. Max. coverage (+): 0.02. Max coverage (-): 0

Region: NODE\_298130\_length\_4886\_cov\_17.340155 4844-4853. Max. coverage (+): 0.02. Max coverage (-): 0.03

Region: NODE\_298130\_length\_4886\_cov\_17.340155 4854-4863. Max. coverage (+): 0.01. Max coverage (-): 0.02

Region: NODE\_298130\_length\_4886\_cov\_17.340155 4864-4873. Max. coverage (+): 0.03. Max coverage (-): 0.21

Region: NODE\_298130\_length\_4886\_cov\_17.340155 4874-4883. Max. coverage (+): 0.03. Max coverage (-): 0.47

Region: NODE\_298130\_length\_4886\_cov\_17.340155 4884-4893. Max. coverage (+): 0.01. Max coverage (-): 0.04

Region: NODE\_298130\_length\_4886\_cov\_17.340155 4894-4903. Max. coverage (+): 0. Max coverage (-): 0.05

Region: NODE\_298130\_length\_4886\_cov\_17.340155 4904-4913. Max. coverage (+): 0.11. Max coverage (-): 0.18

Region: NODE\_298130\_length\_4886\_cov\_17.340155 4914-4923. Max. coverage (+): 0.19. Max coverage (-): 0.15

Region: NODE\_298130\_length\_4886\_cov\_17.340155 4924-4933. Max. coverage (+): 0.07. Max coverage (-): 0.11

Region: NODE\_298130\_length\_4886\_cov\_17.340155 4934-4943. Max. coverage (+): 0.19. Max coverage (-): 0.04

Region: NODE\_298130\_length\_4886\_cov\_17.340155 4944-4953. Max. coverage (+): 0.07. Max coverage (-): 0

Region: NODE\_298130\_length\_4886\_cov\_17.340155 4954-4963. Max. coverage (+): 0.01. Max coverage (-): 0.01

Region: NODE\_298130\_length\_4886\_cov\_17.340155 4964-4973. Max. coverage (+): 0. Max coverage (-): 0.01

Region: NODE\_298130\_length\_4886\_cov\_17.340155 4974-4983. Max. coverage (+): 0. Max coverage (-): 0

Region: NODE\_298130\_length\_4886\_cov\_17.340155 4984-4993. Max. coverage (+): 0. Max coverage (-): 0

Region: NODE\_298130\_length\_4886\_cov\_17.340155 4994-5003. Max. coverage (+): 0. Max coverage (-): 0

Region: NODE\_298130\_length\_4886\_cov\_17.340155 5004-5013. Max. coverage (+): 0. Max coverage (-): 0

Region: NODE\_298130\_length\_4886\_cov\_17.340155 5014-. Max. coverage (+): 0. Max coverage (-): 0

RepeatMasker Color Code

**+**

100-98% Identity

<98-95% Identity

<95-90% Identity

<90-85% Identity

<85-80% Identity

<80-75% Identity

<75-70% Identity

<70% Identity

**-**

Gene Set Color Code

**+**

Gene

Pseudogene

Other

**-**

Topology/Coverage Color Code

Coverage Plus Strand

Coverage Minus Strand

Mainstrand: Plus

Mainstrand: Minus

Complementary Strand

Flanking Region  
(if option -flank >0)

Gene Set Annotation  

**1. unknown (unknownunknown) Tr:unknown**: 522-657 (+)  
**2. unknown (unknownunknown) Tr:unknown**: 757-827 (+)  
**3. unknown (unknownunknown) Tr:unknown**: 1316-1465 (+)  
**4. unknown (unknownunknown) Tr:unknown**: 2705-2805 (+)  
**5. unknown (unknownunknown) Tr:unknown**: 2989-3154 (+)  
**6. unknown (unknownunknown) Tr:unknown**: 3253-4023 (+)

  
RepeatMasker Annotation  

**1. AlRepB-420**: 2-104 (+), Divergence to consensus: 9.7%  
**2. (ATTT)n**: 342-366 (+), Divergence to consensus: 13.1%  
**3. G-rich**: 1883-1928 (+), Divergence to consensus: 24.3%  
**4. AlRepD-29**: 1978-2453 (+), Divergence to consensus: 16.9%  
**5. (TCAAT)n**: 2454-2479 (+), Divergence to consensus: 0%  
**6. AlRepD-2251**: 2480-2541 (+), Divergence to consensus: 12.9%  
**7. AlRepD-1165**: 2542-2579 (-), Divergence to consensus: 17.3%  
**8. (TCC)n**: 3460-3527 (+), Divergence to consensus: 31%  
**9. EnSpm-3\_DR**: 4582-4767 (-), Divergence to consensus: 38.3%  
**10. AlRepD-727**: 4959-5018 (+), Divergence to consensus: 0%

  
Transcription Factor Binding Sites  

**RHOXF1** (Sequence: AGCTCA (-): 1637)  
**RHOXF1** (Sequence: GGCTTA (-): 1832)  
**RHOXF1** (Sequence: AGCTCA (-): 2643)  
**RHOXF1** (Sequence: GGCTTA (-): 2838)  
**RHOXF1** (Sequence: GGATCA (-): 3612)  
**RHOXF1** (Sequence: GGATTA (-): 3811)  
**RHOXF1** (Sequence: GGCTCA (-): 3953)  
**RHOXF1** (Sequence: GGATCA (-): 3980)  
**RHOXF1** (Sequence: GGATCA (-): 4084)  
**RHOXF1** (Sequence: AGATTA (-): 4146)  
**RHOXF1** (Sequence: AGATCA (-): 4667)  
**RHOXF1** (Sequence: GGCTCA (-): 4670)  
**RHOXF1** (Sequence: TGATCC (+): 3075)  
**RHOXF1** (Sequence: TAAGCC (+): 3371)  
**RHOXF1** (Sequence: TGAGCC (+): 4391)  
**POU5F1** (Sequence: TTTGCAT (-): 1274)  
**POU5F1** (Sequence: TTTGCAT (-): 4850)  
**FOXO3\_hsa** (Sequence: GTAAACAT (+): 1804)  
**FOXO3\_hsa** (Sequence: GTAAACAT (+): 2810)  
**SOX9** (Sequence: AACAATGG (-): 4696)  
**FOXP1** (Sequence: GTAAACA (+): 1804)  
**FOXP1** (Sequence: GTAAACA (+): 2810)  
**FOXO1** (Sequence: GTTGTTTTT (+): 3187)  
**FOXO3\_mmu** (Sequence: TGTTTACA (-): 4138)  
**FOXO3\_mmu** (Sequence: TGTTTTGC (-): 4248)  
**FOXO3\_mmu** (Sequence: TGTTTACA (-): 4317)  
**FOXO3\_mmu** (Sequence: TGTTTTGA (-): 4381)  
**Sox5** (Sequence: ATTGTT (+): 537)  
**Sox5** (Sequence: ATTGTT (+): 4180)  
**SOX9** (Sequence: TTATTGTT (+): 4178)  
**FOXO3\_mmu** (Sequence: TCAAAACA (+): 4527)  
**Nobox** (Sequence: AGTAATTA (-): 430)  
**FOXO1** (Sequence: ATAAACAAC (-): 1527)  
**FOXO3\_hsa** (Sequence: ATGTTTAC (-): 4316)  
**FOXP1** (Sequence: TGTTTAC (-): 4138)  
**FOXP1** (Sequence: TGTTTAC (-): 4317)  
**POU2F1** (Sequence: ATTTAAATA (-): 2335)  
**POU2F1** (Sequence: ATTTGCATA (-): 4849)  
**Sox5** (Sequence: AACAAT (-): 96)  
**Sox5** (Sequence: AACAAT (-): 4696)  
**POU2F1** (Sequence: TATTTAAAT (+): 2334)  
**POU2F1** (Sequence: TATTTTAAT (+): 2354)  
**POU5F1** (Sequence: ATGCAAA (+): 1556)  
**POU5F1** (Sequence: ATGCAAA (+): 4790)
