# Supplementary material for: First characterization of PIWI-interacting RNA clusters in a cichlid fish with a B chromosome
Source: BMC Biol. 2022 Sep 21;20:204. doi: 10.1186/s12915-022-01403-2 (PMC9490952; doi:10.1186/s12915-022-01403-2)
Supplement: Supplementary file 1 — Additional file 1. Zipped folder with fasta and interactive html piRNA cluster information for the A. latifasciata genome. The nomenclature is as follows: number-pirna-cluster_sex_B-presence (f, female; m, male; 0b, without B chromosome; 1b, with B chromosome). [file 12915_2022_1403_MOESM1_ESM.zip › 118_f1b.html]

piRNA cluster 118\_f1b 56


Predicted piRNA cluster no. 118\_f1b
  

Show proTRAC run info
Hide proTRAC run info

/\  
                \_\_\_\_\_\_\_\_\_\_\_\_\_\_\_\_\_\_\_\_\_\_\_/\\_\_\_ /  \\_\_\_\_\_\_\_  
               I                      /  \  /    \      I  
               I     pro             /    \/      \     I  
               I        TRAC        /               \   I  
               I   \_\_\_\_\_\_\_\_\_\_\_\_\_\_\_\_/\_\_\_\_\_\_\_\_\_\_\_\_\_\_\_\_\_\\_ I  
               I   \              /                     I  
               I    \            /                      I  
               I     \  /\      /       V.2.4.2         I  
               I      \/  \    /                        I  
               I\_\_\_\_\_\_\_\_\_\_\_\  /\_\_\_\_\_\_\_\_\_\_\_\_\_\_\_\_\_\_\_\_\_\_\_\_\_I  
                            \/  
  
  
================================= proTRAC ====================================  
VERSION: .......... 2.4.2  
LAST MODIFIED: .... 11. May 2018  
  
Please cite:  
Rosenkranz D, Zischler H. proTRAC - a software for probabilistic piRNA cluster  
detection, visualization and analysis. 2012. BMC Bioinformatics 13:5.  
  
  
Contact:  
David Rosenkranz  
Institute of Organismic and Molecular Evolutionary Biology  
Dept. Anthropology, small RNA group  
Johannes Gutenberg University Mainz  
email: rosenkranz@uni-mainz.de  
  
You can find the latest proTRAC version at:  
http://sourceforge.net/projects/protrac/files  
http://www.smallRNAgroup-mainz.de/software  
==============================================================================  
  
PARAMETERS:  
Map file: ...............piwi-femeas-1B.fa-collapse.map  
Genome file: ............../../../0B\_ala\_genome.fa  
RepeatMasker annotation: Alatifasciata-all0B-maryan-v2.fa\_corrected.out  
GeneSet:................./guest-storage/Data/annotation/Alatifasciata\_all0B\_maryan-v2\_out2017.gff  
  
Significant (p<=0.01) hit density will be calculated based  
on observed hit distribution.  
  
Sliding window size: ........................................ 5000 bp  
Sliding window increament: .................................. 1000 bp  
Normalize each hit by number of genomic hits: ............... yes  
Normalize each hit by number of sequence reads: ............. yes  
Normalize values (-> per million mapped reads): ............. yes  
Min. fraction of hits with 1T(U) or 10A: .................... 0.75  
Alternatively: Min. fraction of hits with 1T(U) and 10A: .... 0.5  
Min. fraction of hits with typical piRNA length: ............ 0.75  
Typical piRNA length: ....................................... 24-32 nt  
Min. size of a piRNA cluster: ............................... 1000 bp.  
Min. number of hits (absolute): ............................. 0  
Min. number of hits (normalized): ........................... 0  
Min. fraction of hits on the mainstrand: .................... 0.75  
Top fraction of mapped sequences (in terms of read counts): . 1%  
Top fraction accounts for max. n% of sequence reads: ........ 90%  
Min. fraction of hits on each arm of a bidirectional cluster: 0.05  
Output html file for each cluster: .......................... yes  
Output a summary table: ..................................... yes  
Output a FASTA file for each cluster (piRNA sequences): ..... yes  
Output a FASTA file comprising cluster sequences: ........... yes  
Output a GTF file for predicted piRNA clusters: ..............yes  
Search DNA motifs in clusters: .............................. yes  
Output flanking sequences: +/- .............................. 0 bp  
Output ~.pTi file: .......................................... no  
==============================================================================  
  
  
Genome size (without gaps): ............ 758543724 bp  
Gaps (N/X/-): .......................... 417479 bp  
Mapped reads: .......................... 10641844  
Non-identical sequences: ............... 2832837  
Genomic hits: .......................... 26056853  
Significant densitiy of mapped reads: .. 368.713530323068 reads/kb

Show proTRAC cluster info
Hide proTRAC cluster info

|  |  |
| --- | --- |
| Location | NODE\_302677\_length\_8241\_cov\_21.205557 |
| Coordinates | 5-6004 |
| Size [bp] | 6000 |
| Sequence hit loci | 1606 |
| Mapped reads (normalized) | 5914.3 |
| Mapped reads (normalized) per kb | 985.7 |
| Normalized reads with 1T (1U) | 94% |
| Normalized reads with 10A | 19.5% |
| Normalized reads with length 24-32 nt | 99.5% |
| Normalized reads on the main strand(s) | 96% |
| Predicted directionality | mono:plus |

100%

0%

1T (1U)  
reads

10A reads

24-32 nt  
reads

reads on mainstrand

**Either the amount of reads with 1T (1U) OR 10A has to exceed 75% (set with option: -1Tor10A)  
Alternatively the amount of reads with 1T (1U) AND 10A has to exceed 50% (set with option: -1Tand10A)  
Minimum amount of reads with preferred size is 75% (set with option: -pisize)  
Minimum amount of reads on the main strand(s) is 75% (set with option: -clstrand)**

Show read coverage
Hide read coverage

WHAT DO I SEE HERE?  
This chart shows the location of mapped sequence reads within a predicted piRNA cluster. The color refers to the number of genomic hits produced by the sequence read in question. A dark red bar indicates that this sequence read produces many other hits elsewhere in the genome. Many adjacent red or yellow bars can indicate the presence of a multi-copy element such as transposons or rRNA genes. A dark green bar indicates that this sequence read maps uniquely to this locus.

1 hit

2-5 hits

6-10 hits

11-20 hits

21-50 hits

51-100 hits

> 100 hits

NODE\_302677\_length\_8241\_cov\_21.205557

5

6004

Gene Set

RepeatMasker

Mapped  
Reads

219.89

plus strand

minus strand

219.89

Region: NODE\_302677\_length\_8241\_cov\_21.205557 13008-10. Max. coverage (+): 0. Max coverage (-): 0

Region: NODE\_302677\_length\_8241\_cov\_21.205557 11-22. Max. coverage (+): 0.02. Max coverage (-): 0

Region: NODE\_302677\_length\_8241\_cov\_21.205557 23-34. Max. coverage (+): 0. Max coverage (-): 0

Region: NODE\_302677\_length\_8241\_cov\_21.205557 35-46. Max. coverage (+): 0. Max coverage (-): 0

Region: NODE\_302677\_length\_8241\_cov\_21.205557 47-58. Max. coverage (+): 0.01. Max coverage (-): 0

Region: NODE\_302677\_length\_8241\_cov\_21.205557 59-70. Max. coverage (+): 0. Max coverage (-): 0

Region: NODE\_302677\_length\_8241\_cov\_21.205557 71-82. Max. coverage (+): 0. Max coverage (-): 0.01

Region: NODE\_302677\_length\_8241\_cov\_21.205557 83-94. Max. coverage (+): 0. Max coverage (-): 0

Region: NODE\_302677\_length\_8241\_cov\_21.205557 95-106. Max. coverage (+): 0. Max coverage (-): 0

Region: NODE\_302677\_length\_8241\_cov\_21.205557 107-118. Max. coverage (+): 0. Max coverage (-): 0.01

Region: NODE\_302677\_length\_8241\_cov\_21.205557 119-130. Max. coverage (+): 0. Max coverage (-): 0

Region: NODE\_302677\_length\_8241\_cov\_21.205557 131-142. Max. coverage (+): 0. Max coverage (-): 0

Region: NODE\_302677\_length\_8241\_cov\_21.205557 143-154. Max. coverage (+): 0.03. Max coverage (-): 0

Region: NODE\_302677\_length\_8241\_cov\_21.205557 155-166. Max. coverage (+): 0.03. Max coverage (-): 0.01

Region: NODE\_302677\_length\_8241\_cov\_21.205557 167-178. Max. coverage (+): 0.01. Max coverage (-): 0.03

Region: NODE\_302677\_length\_8241\_cov\_21.205557 179-190. Max. coverage (+): 0.01. Max coverage (-): 0.01

Region: NODE\_302677\_length\_8241\_cov\_21.205557 191-202. Max. coverage (+): 0. Max coverage (-): 0.01

Region: NODE\_302677\_length\_8241\_cov\_21.205557 203-214. Max. coverage (+): 0.01. Max coverage (-): 0

Region: NODE\_302677\_length\_8241\_cov\_21.205557 215-226. Max. coverage (+): 0. Max coverage (-): 0

Region: NODE\_302677\_length\_8241\_cov\_21.205557 227-238. Max. coverage (+): 0. Max coverage (-): 0

Region: NODE\_302677\_length\_8241\_cov\_21.205557 239-250. Max. coverage (+): 0. Max coverage (-): 0

Region: NODE\_302677\_length\_8241\_cov\_21.205557 251-262. Max. coverage (+): 0. Max coverage (-): 0.03

Region: NODE\_302677\_length\_8241\_cov\_21.205557 263-274. Max. coverage (+): 0. Max coverage (-): 0.06

Region: NODE\_302677\_length\_8241\_cov\_21.205557 275-286. Max. coverage (+): 0.17. Max coverage (-): 0

Region: NODE\_302677\_length\_8241\_cov\_21.205557 287-298. Max. coverage (+): 0.17. Max coverage (-): 0

Region: NODE\_302677\_length\_8241\_cov\_21.205557 299-310. Max. coverage (+): 0. Max coverage (-): 0

Region: NODE\_302677\_length\_8241\_cov\_21.205557 311-322. Max. coverage (+): 0. Max coverage (-): 0

Region: NODE\_302677\_length\_8241\_cov\_21.205557 323-334. Max. coverage (+): 0.02. Max coverage (-): 0

Region: NODE\_302677\_length\_8241\_cov\_21.205557 335-346. Max. coverage (+): 0. Max coverage (-): 0

Region: NODE\_302677\_length\_8241\_cov\_21.205557 347-358. Max. coverage (+): 0. Max coverage (-): 0

Region: NODE\_302677\_length\_8241\_cov\_21.205557 359-370. Max. coverage (+): 0. Max coverage (-): 0

Region: NODE\_302677\_length\_8241\_cov\_21.205557 371-382. Max. coverage (+): 0.19. Max coverage (-): 0.03

Region: NODE\_302677\_length\_8241\_cov\_21.205557 383-394. Max. coverage (+): 0.19. Max coverage (-): 0.63

Region: NODE\_302677\_length\_8241\_cov\_21.205557 395-406. Max. coverage (+): 5.26. Max coverage (-): 0.17

Region: NODE\_302677\_length\_8241\_cov\_21.205557 407-418. Max. coverage (+): 0.61. Max coverage (-): 0.19

Region: NODE\_302677\_length\_8241\_cov\_21.205557 419-430. Max. coverage (+): 0. Max coverage (-): 0.19

Region: NODE\_302677\_length\_8241\_cov\_21.205557 431-442. Max. coverage (+): 2.07. Max coverage (-): 0

Region: NODE\_302677\_length\_8241\_cov\_21.205557 443-454. Max. coverage (+): 0. Max coverage (-): 0

Region: NODE\_302677\_length\_8241\_cov\_21.205557 455-466. Max. coverage (+): 0. Max coverage (-): 0

Region: NODE\_302677\_length\_8241\_cov\_21.205557 467-478. Max. coverage (+): 0.33. Max coverage (-): 0

Region: NODE\_302677\_length\_8241\_cov\_21.205557 479-490. Max. coverage (+): 1.03. Max coverage (-): 0

Region: NODE\_302677\_length\_8241\_cov\_21.205557 491-502. Max. coverage (+): 0. Max coverage (-): 0

Region: NODE\_302677\_length\_8241\_cov\_21.205557 503-514. Max. coverage (+): 0. Max coverage (-): 0

Region: NODE\_302677\_length\_8241\_cov\_21.205557 515-526. Max. coverage (+): 0.06. Max coverage (-): 0

Region: NODE\_302677\_length\_8241\_cov\_21.205557 527-538. Max. coverage (+): 0. Max coverage (-): 0

Region: NODE\_302677\_length\_8241\_cov\_21.205557 539-550. Max. coverage (+): 0.38. Max coverage (-): 0

Region: NODE\_302677\_length\_8241\_cov\_21.205557 551-562. Max. coverage (+): 0.47. Max coverage (-): 0.05

Region: NODE\_302677\_length\_8241\_cov\_21.205557 563-574. Max. coverage (+): 0. Max coverage (-): 0

Region: NODE\_302677\_length\_8241\_cov\_21.205557 575-586. Max. coverage (+): 0. Max coverage (-): 0

Region: NODE\_302677\_length\_8241\_cov\_21.205557 587-598. Max. coverage (+): 0. Max coverage (-): 0

Region: NODE\_302677\_length\_8241\_cov\_21.205557 599-610. Max. coverage (+): 0.09. Max coverage (-): 0

Region: NODE\_302677\_length\_8241\_cov\_21.205557 611-622. Max. coverage (+): 0.03. Max coverage (-): 0

Region: NODE\_302677\_length\_8241\_cov\_21.205557 623-634. Max. coverage (+): 0. Max coverage (-): 0

Region: NODE\_302677\_length\_8241\_cov\_21.205557 635-646. Max. coverage (+): 0.09. Max coverage (-): 0.28

Region: NODE\_302677\_length\_8241\_cov\_21.205557 647-658. Max. coverage (+): 0.23. Max coverage (-): 0

Region: NODE\_302677\_length\_8241\_cov\_21.205557 659-670. Max. coverage (+): 0.23. Max coverage (-): 0

Region: NODE\_302677\_length\_8241\_cov\_21.205557 671-682. Max. coverage (+): 0.05. Max coverage (-): 0.05

Region: NODE\_302677\_length\_8241\_cov\_21.205557 683-694. Max. coverage (+): 0. Max coverage (-): 0

Region: NODE\_302677\_length\_8241\_cov\_21.205557 695-706. Max. coverage (+): 0. Max coverage (-): 0

Region: NODE\_302677\_length\_8241\_cov\_21.205557 707-718. Max. coverage (+): 0. Max coverage (-): 0.03

Region: NODE\_302677\_length\_8241\_cov\_21.205557 719-730. Max. coverage (+): 1.33. Max coverage (-): 0.02

Region: NODE\_302677\_length\_8241\_cov\_21.205557 731-742. Max. coverage (+): 1.32. Max coverage (-): 0.15

Region: NODE\_302677\_length\_8241\_cov\_21.205557 743-754. Max. coverage (+): 0.02. Max coverage (-): 0

Region: NODE\_302677\_length\_8241\_cov\_21.205557 755-766. Max. coverage (+): 0. Max coverage (-): 0

Region: NODE\_302677\_length\_8241\_cov\_21.205557 767-778. Max. coverage (+): 0.37. Max coverage (-): 0.06

Region: NODE\_302677\_length\_8241\_cov\_21.205557 779-790. Max. coverage (+): 3.57. Max coverage (-): 0.03

Region: NODE\_302677\_length\_8241\_cov\_21.205557 791-802. Max. coverage (+): 12.94. Max coverage (-): 0.03

Region: NODE\_302677\_length\_8241\_cov\_21.205557 803-814. Max. coverage (+): 0.09. Max coverage (-): 0.09

Region: NODE\_302677\_length\_8241\_cov\_21.205557 815-826. Max. coverage (+): 0.23. Max coverage (-): 0.05

Region: NODE\_302677\_length\_8241\_cov\_21.205557 827-838. Max. coverage (+): 164.54. Max coverage (-): 0.05

Region: NODE\_302677\_length\_8241\_cov\_21.205557 839-850. Max. coverage (+): 0.05. Max coverage (-): 0

Region: NODE\_302677\_length\_8241\_cov\_21.205557 851-862. Max. coverage (+): 0.42. Max coverage (-): 0

Region: NODE\_302677\_length\_8241\_cov\_21.205557 863-874. Max. coverage (+): 1.08. Max coverage (-): 2.35

Region: NODE\_302677\_length\_8241\_cov\_21.205557 875-886. Max. coverage (+): 0.14. Max coverage (-): 0.05

Region: NODE\_302677\_length\_8241\_cov\_21.205557 887-898. Max. coverage (+): 23.94. Max coverage (-): 0.02

Region: NODE\_302677\_length\_8241\_cov\_21.205557 899-910. Max. coverage (+): 0. Max coverage (-): 0

Region: NODE\_302677\_length\_8241\_cov\_21.205557 911-922. Max. coverage (+): 0. Max coverage (-): 0

Region: NODE\_302677\_length\_8241\_cov\_21.205557 923-934. Max. coverage (+): 0. Max coverage (-): 0

Region: NODE\_302677\_length\_8241\_cov\_21.205557 935-946. Max. coverage (+): 0. Max coverage (-): 0

Region: NODE\_302677\_length\_8241\_cov\_21.205557 947-958. Max. coverage (+): 0. Max coverage (-): 0

Region: NODE\_302677\_length\_8241\_cov\_21.205557 959-970. Max. coverage (+): 0.06. Max coverage (-): 0

Region: NODE\_302677\_length\_8241\_cov\_21.205557 971-982. Max. coverage (+): 0. Max coverage (-): 0.14

Region: NODE\_302677\_length\_8241\_cov\_21.205557 983-994. Max. coverage (+): 0. Max coverage (-): 0

Region: NODE\_302677\_length\_8241\_cov\_21.205557 995-1006. Max. coverage (+): 0. Max coverage (-): 0

Region: NODE\_302677\_length\_8241\_cov\_21.205557 1007-1018. Max. coverage (+): 2.18. Max coverage (-): 0

Region: NODE\_302677\_length\_8241\_cov\_21.205557 1019-1030. Max. coverage (+): 2.42. Max coverage (-): 0

Region: NODE\_302677\_length\_8241\_cov\_21.205557 1031-1042. Max. coverage (+): 0.14. Max coverage (-): 0

Region: NODE\_302677\_length\_8241\_cov\_21.205557 1043-1054. Max. coverage (+): 0.09. Max coverage (-): 0.56

Region: NODE\_302677\_length\_8241\_cov\_21.205557 1055-1066. Max. coverage (+): 0.94. Max coverage (-): 0.38

Region: NODE\_302677\_length\_8241\_cov\_21.205557 1067-1078. Max. coverage (+): 219.89. Max coverage (-): 0

Region: NODE\_302677\_length\_8241\_cov\_21.205557 1079-1090. Max. coverage (+): 0.05. Max coverage (-): 0.02

Region: NODE\_302677\_length\_8241\_cov\_21.205557 1091-1102. Max. coverage (+): 0.09. Max coverage (-): 3.43

Region: NODE\_302677\_length\_8241\_cov\_21.205557 1103-1114. Max. coverage (+): 0.17. Max coverage (-): 0.05

Region: NODE\_302677\_length\_8241\_cov\_21.205557 1115-1126. Max. coverage (+): 0.09. Max coverage (-): 0

Region: NODE\_302677\_length\_8241\_cov\_21.205557 1127-1138. Max. coverage (+): 0.05. Max coverage (-): 0.16

Region: NODE\_302677\_length\_8241\_cov\_21.205557 1139-1150. Max. coverage (+): 0. Max coverage (-): 0

Region: NODE\_302677\_length\_8241\_cov\_21.205557 1151-1162. Max. coverage (+): 0. Max coverage (-): 0

Region: NODE\_302677\_length\_8241\_cov\_21.205557 1163-1174. Max. coverage (+): 0.05. Max coverage (-): 0

Region: NODE\_302677\_length\_8241\_cov\_21.205557 1175-1186. Max. coverage (+): 0.05. Max coverage (-): 0.09

Region: NODE\_302677\_length\_8241\_cov\_21.205557 1187-1198. Max. coverage (+): 0.11. Max coverage (-): 0.35

Region: NODE\_302677\_length\_8241\_cov\_21.205557 1199-1210. Max. coverage (+): 0. Max coverage (-): 0

Region: NODE\_302677\_length\_8241\_cov\_21.205557 1211-1222. Max. coverage (+): 0. Max coverage (-): 0

Region: NODE\_302677\_length\_8241\_cov\_21.205557 1223-1234. Max. coverage (+): 0.06. Max coverage (-): 0.64

Region: NODE\_302677\_length\_8241\_cov\_21.205557 1235-1246. Max. coverage (+): 0.52. Max coverage (-): 0.03

Region: NODE\_302677\_length\_8241\_cov\_21.205557 1247-1258. Max. coverage (+): 2.59. Max coverage (-): 0.03

Region: NODE\_302677\_length\_8241\_cov\_21.205557 1259-1270. Max. coverage (+): 0.03. Max coverage (-): 0.03

Region: NODE\_302677\_length\_8241\_cov\_21.205557 1271-1282. Max. coverage (+): 0. Max coverage (-): 0

Region: NODE\_302677\_length\_8241\_cov\_21.205557 1283-1294. Max. coverage (+): 0.02. Max coverage (-): 0.09

Region: NODE\_302677\_length\_8241\_cov\_21.205557 1295-1306. Max. coverage (+): 0.24. Max coverage (-): 0.07

Region: NODE\_302677\_length\_8241\_cov\_21.205557 1307-1318. Max. coverage (+): 0.09. Max coverage (-): 0

Region: NODE\_302677\_length\_8241\_cov\_21.205557 1319-1330. Max. coverage (+): 0. Max coverage (-): 0

Region: NODE\_302677\_length\_8241\_cov\_21.205557 1331-1342. Max. coverage (+): 0.05. Max coverage (-): 0

Region: NODE\_302677\_length\_8241\_cov\_21.205557 1343-1354. Max. coverage (+): 0. Max coverage (-): 0.01

Region: NODE\_302677\_length\_8241\_cov\_21.205557 1355-1366. Max. coverage (+): 0. Max coverage (-): 0.12

Region: NODE\_302677\_length\_8241\_cov\_21.205557 1367-1378. Max. coverage (+): 0.38. Max coverage (-): 0

Region: NODE\_302677\_length\_8241\_cov\_21.205557 1379-1390. Max. coverage (+): 0. Max coverage (-): 0

Region: NODE\_302677\_length\_8241\_cov\_21.205557 1391-1402. Max. coverage (+): 0. Max coverage (-): 0

Region: NODE\_302677\_length\_8241\_cov\_21.205557 1403-1414. Max. coverage (+): 0. Max coverage (-): 0.01

Region: NODE\_302677\_length\_8241\_cov\_21.205557 1415-1426. Max. coverage (+): 0. Max coverage (-): 0.29

Region: NODE\_302677\_length\_8241\_cov\_21.205557 1427-1438. Max. coverage (+): 0.07. Max coverage (-): 0

Region: NODE\_302677\_length\_8241\_cov\_21.205557 1439-1450. Max. coverage (+): 0.08. Max coverage (-): 0

Region: NODE\_302677\_length\_8241\_cov\_21.205557 1451-1462. Max. coverage (+): 0. Max coverage (-): 0

Region: NODE\_302677\_length\_8241\_cov\_21.205557 1463-1474. Max. coverage (+): 0. Max coverage (-): 0

Region: NODE\_302677\_length\_8241\_cov\_21.205557 1475-1486. Max. coverage (+): 0. Max coverage (-): 0.12

Region: NODE\_302677\_length\_8241\_cov\_21.205557 1487-1498. Max. coverage (+): 0. Max coverage (-): 0.11

Region: NODE\_302677\_length\_8241\_cov\_21.205557 1499-1510. Max. coverage (+): 0.08. Max coverage (-): 0

Region: NODE\_302677\_length\_8241\_cov\_21.205557 1511-1522. Max. coverage (+): 0. Max coverage (-): 0

Region: NODE\_302677\_length\_8241\_cov\_21.205557 1523-1534. Max. coverage (+): 0. Max coverage (-): 0

Region: NODE\_302677\_length\_8241\_cov\_21.205557 1535-1546. Max. coverage (+): 0.03. Max coverage (-): 0

Region: NODE\_302677\_length\_8241\_cov\_21.205557 1547-1558. Max. coverage (+): 0. Max coverage (-): 0

Region: NODE\_302677\_length\_8241\_cov\_21.205557 1559-1570. Max. coverage (+): 0. Max coverage (-): 0

Region: NODE\_302677\_length\_8241\_cov\_21.205557 1571-1582. Max. coverage (+): 0.01. Max coverage (-): 0

Region: NODE\_302677\_length\_8241\_cov\_21.205557 1583-1594. Max. coverage (+): 0. Max coverage (-): 0

Region: NODE\_302677\_length\_8241\_cov\_21.205557 1595-1606. Max. coverage (+): 0.01. Max coverage (-): 0

Region: NODE\_302677\_length\_8241\_cov\_21.205557 1607-1618. Max. coverage (+): 0.01. Max coverage (-): 0

Region: NODE\_302677\_length\_8241\_cov\_21.205557 1619-1630. Max. coverage (+): 0. Max coverage (-): 0

Region: NODE\_302677\_length\_8241\_cov\_21.205557 1631-1642. Max. coverage (+): 0.03. Max coverage (-): 0

Region: NODE\_302677\_length\_8241\_cov\_21.205557 1643-1654. Max. coverage (+): 0. Max coverage (-): 0

Region: NODE\_302677\_length\_8241\_cov\_21.205557 1655-1666. Max. coverage (+): 0. Max coverage (-): 0

Region: NODE\_302677\_length\_8241\_cov\_21.205557 1667-1678. Max. coverage (+): 0. Max coverage (-): 0

Region: NODE\_302677\_length\_8241\_cov\_21.205557 1679-1690. Max. coverage (+): 0. Max coverage (-): 0

Region: NODE\_302677\_length\_8241\_cov\_21.205557 1691-1702. Max. coverage (+): 0. Max coverage (-): 0

Region: NODE\_302677\_length\_8241\_cov\_21.205557 1703-1714. Max. coverage (+): 0. Max coverage (-): 0

Region: NODE\_302677\_length\_8241\_cov\_21.205557 1715-1726. Max. coverage (+): 0. Max coverage (-): 0

Region: NODE\_302677\_length\_8241\_cov\_21.205557 1727-1738. Max. coverage (+): 0. Max coverage (-): 0

Region: NODE\_302677\_length\_8241\_cov\_21.205557 1739-1750. Max. coverage (+): 0. Max coverage (-): 0

Region: NODE\_302677\_length\_8241\_cov\_21.205557 1751-1762. Max. coverage (+): 0. Max coverage (-): 0

Region: NODE\_302677\_length\_8241\_cov\_21.205557 1763-1774. Max. coverage (+): 0. Max coverage (-): 0

Region: NODE\_302677\_length\_8241\_cov\_21.205557 1775-1786. Max. coverage (+): 0. Max coverage (-): 0

Region: NODE\_302677\_length\_8241\_cov\_21.205557 1787-1798. Max. coverage (+): 0. Max coverage (-): 0

Region: NODE\_302677\_length\_8241\_cov\_21.205557 1799-1810. Max. coverage (+): 0. Max coverage (-): 0

Region: NODE\_302677\_length\_8241\_cov\_21.205557 1811-1822. Max. coverage (+): 0. Max coverage (-): 0

Region: NODE\_302677\_length\_8241\_cov\_21.205557 1823-1834. Max. coverage (+): 0. Max coverage (-): 0

Region: NODE\_302677\_length\_8241\_cov\_21.205557 1835-1846. Max. coverage (+): 0. Max coverage (-): 0

Region: NODE\_302677\_length\_8241\_cov\_21.205557 1847-1858. Max. coverage (+): 0. Max coverage (-): 0

Region: NODE\_302677\_length\_8241\_cov\_21.205557 1859-1870. Max. coverage (+): 0. Max coverage (-): 0

Region: NODE\_302677\_length\_8241\_cov\_21.205557 1871-1882. Max. coverage (+): 0. Max coverage (-): 0

Region: NODE\_302677\_length\_8241\_cov\_21.205557 1883-1894. Max. coverage (+): 0. Max coverage (-): 0

Region: NODE\_302677\_length\_8241\_cov\_21.205557 1895-1906. Max. coverage (+): 0.06. Max coverage (-): 0

Region: NODE\_302677\_length\_8241\_cov\_21.205557 1907-1918. Max. coverage (+): 0. Max coverage (-): 0

Region: NODE\_302677\_length\_8241\_cov\_21.205557 1919-1930. Max. coverage (+): 0. Max coverage (-): 0.01

Region: NODE\_302677\_length\_8241\_cov\_21.205557 1931-1942. Max. coverage (+): 0. Max coverage (-): 0

Region: NODE\_302677\_length\_8241\_cov\_21.205557 1943-1954. Max. coverage (+): 0. Max coverage (-): 0

Region: NODE\_302677\_length\_8241\_cov\_21.205557 1955-1966. Max. coverage (+): 0. Max coverage (-): 0

Region: NODE\_302677\_length\_8241\_cov\_21.205557 1967-1978. Max. coverage (+): 0. Max coverage (-): 0

Region: NODE\_302677\_length\_8241\_cov\_21.205557 1979-1990. Max. coverage (+): 0. Max coverage (-): 0.09

Region: NODE\_302677\_length\_8241\_cov\_21.205557 1991-2002. Max. coverage (+): 0. Max coverage (-): 0

Region: NODE\_302677\_length\_8241\_cov\_21.205557 2003-2014. Max. coverage (+): 0. Max coverage (-): 0

Region: NODE\_302677\_length\_8241\_cov\_21.205557 2015-2026. Max. coverage (+): 0. Max coverage (-): 0

Region: NODE\_302677\_length\_8241\_cov\_21.205557 2027-2038. Max. coverage (+): 0.09. Max coverage (-): 0

Region: NODE\_302677\_length\_8241\_cov\_21.205557 2039-2050. Max. coverage (+): 0. Max coverage (-): 0

Region: NODE\_302677\_length\_8241\_cov\_21.205557 2051-2062. Max. coverage (+): 0. Max coverage (-): 0

Region: NODE\_302677\_length\_8241\_cov\_21.205557 2063-2074. Max. coverage (+): 0. Max coverage (-): 0

Region: NODE\_302677\_length\_8241\_cov\_21.205557 2075-2086. Max. coverage (+): 0. Max coverage (-): 0.09

Region: NODE\_302677\_length\_8241\_cov\_21.205557 2087-2098. Max. coverage (+): 0. Max coverage (-): 0.09

Region: NODE\_302677\_length\_8241\_cov\_21.205557 2099-2110. Max. coverage (+): 0.09. Max coverage (-): 0.09

Region: NODE\_302677\_length\_8241\_cov\_21.205557 2111-2122. Max. coverage (+): 0.09. Max coverage (-): 0

Region: NODE\_302677\_length\_8241\_cov\_21.205557 2123-2134. Max. coverage (+): 0.09. Max coverage (-): 0

Region: NODE\_302677\_length\_8241\_cov\_21.205557 2135-2146. Max. coverage (+): 0. Max coverage (-): 0.09

Region: NODE\_302677\_length\_8241\_cov\_21.205557 2147-2158. Max. coverage (+): 0. Max coverage (-): 0.09

Region: NODE\_302677\_length\_8241\_cov\_21.205557 2159-2170. Max. coverage (+): 0. Max coverage (-): 0

Region: NODE\_302677\_length\_8241\_cov\_21.205557 2171-2182. Max. coverage (+): 0. Max coverage (-): 0.38

Region: NODE\_302677\_length\_8241\_cov\_21.205557 2183-2194. Max. coverage (+): 0. Max coverage (-): 0.03

Region: NODE\_302677\_length\_8241\_cov\_21.205557 2195-2206. Max. coverage (+): 0. Max coverage (-): 0

Region: NODE\_302677\_length\_8241\_cov\_21.205557 2207-2218. Max. coverage (+): 0. Max coverage (-): 0

Region: NODE\_302677\_length\_8241\_cov\_21.205557 2219-2230. Max. coverage (+): 0. Max coverage (-): 0.04

Region: NODE\_302677\_length\_8241\_cov\_21.205557 2231-2242. Max. coverage (+): 0.49. Max coverage (-): 0

Region: NODE\_302677\_length\_8241\_cov\_21.205557 2243-2254. Max. coverage (+): 0. Max coverage (-): 0

Region: NODE\_302677\_length\_8241\_cov\_21.205557 2255-2266. Max. coverage (+): 0. Max coverage (-): 0

Region: NODE\_302677\_length\_8241\_cov\_21.205557 2267-2278. Max. coverage (+): 0.19. Max coverage (-): 0

Region: NODE\_302677\_length\_8241\_cov\_21.205557 2279-2290. Max. coverage (+): 0.09. Max coverage (-): 0

Region: NODE\_302677\_length\_8241\_cov\_21.205557 2291-2302. Max. coverage (+): 0.38. Max coverage (-): 0

Region: NODE\_302677\_length\_8241\_cov\_21.205557 2303-2314. Max. coverage (+): 0.38. Max coverage (-): 0

Region: NODE\_302677\_length\_8241\_cov\_21.205557 2315-2326. Max. coverage (+): 0.09. Max coverage (-): 0

Region: NODE\_302677\_length\_8241\_cov\_21.205557 2327-2338. Max. coverage (+): 0. Max coverage (-): 0

Region: NODE\_302677\_length\_8241\_cov\_21.205557 2339-2350. Max. coverage (+): 0. Max coverage (-): 0

Region: NODE\_302677\_length\_8241\_cov\_21.205557 2351-2362. Max. coverage (+): 0.09. Max coverage (-): 0

Region: NODE\_302677\_length\_8241\_cov\_21.205557 2363-2374. Max. coverage (+): 0. Max coverage (-): 0

Region: NODE\_302677\_length\_8241\_cov\_21.205557 2375-2386. Max. coverage (+): 0.09. Max coverage (-): 0

Region: NODE\_302677\_length\_8241\_cov\_21.205557 2387-2398. Max. coverage (+): 0. Max coverage (-): 0

Region: NODE\_302677\_length\_8241\_cov\_21.205557 2399-2410. Max. coverage (+): 0. Max coverage (-): 0

Region: NODE\_302677\_length\_8241\_cov\_21.205557 2411-2422. Max. coverage (+): 0.02. Max coverage (-): 0.03

Region: NODE\_302677\_length\_8241\_cov\_21.205557 2423-2434. Max. coverage (+): 0.02. Max coverage (-): 0.57

Region: NODE\_302677\_length\_8241\_cov\_21.205557 2435-2446. Max. coverage (+): 0. Max coverage (-): 0.26

Region: NODE\_302677\_length\_8241\_cov\_21.205557 2447-2458. Max. coverage (+): 0. Max coverage (-): 0

Region: NODE\_302677\_length\_8241\_cov\_21.205557 2459-2470. Max. coverage (+): 0.09. Max coverage (-): 0.38

Region: NODE\_302677\_length\_8241\_cov\_21.205557 2471-2482. Max. coverage (+): 0.09. Max coverage (-): 0.28

Region: NODE\_302677\_length\_8241\_cov\_21.205557 2483-2494. Max. coverage (+): 0.02. Max coverage (-): 0

Region: NODE\_302677\_length\_8241\_cov\_21.205557 2495-2506. Max. coverage (+): 0. Max coverage (-): 0

Region: NODE\_302677\_length\_8241\_cov\_21.205557 2507-2518. Max. coverage (+): 0. Max coverage (-): 0

Region: NODE\_302677\_length\_8241\_cov\_21.205557 2519-2530. Max. coverage (+): 0. Max coverage (-): 0

Region: NODE\_302677\_length\_8241\_cov\_21.205557 2531-2542. Max. coverage (+): 0. Max coverage (-): 0

Region: NODE\_302677\_length\_8241\_cov\_21.205557 2543-2554. Max. coverage (+): 0. Max coverage (-): 0

Region: NODE\_302677\_length\_8241\_cov\_21.205557 2555-2566. Max. coverage (+): 0. Max coverage (-): 0.09

Region: NODE\_302677\_length\_8241\_cov\_21.205557 2567-2578. Max. coverage (+): 0.09. Max coverage (-): 0

Region: NODE\_302677\_length\_8241\_cov\_21.205557 2579-2590. Max. coverage (+): 0.01. Max coverage (-): 0.02

Region: NODE\_302677\_length\_8241\_cov\_21.205557 2591-2602. Max. coverage (+): 0.01. Max coverage (-): 0.03

Region: NODE\_302677\_length\_8241\_cov\_21.205557 2603-2614. Max. coverage (+): 0.02. Max coverage (-): 0

Region: NODE\_302677\_length\_8241\_cov\_21.205557 2615-2626. Max. coverage (+): 0. Max coverage (-): 0.06

Region: NODE\_302677\_length\_8241\_cov\_21.205557 2627-2638. Max. coverage (+): 0.01. Max coverage (-): 0.06

Region: NODE\_302677\_length\_8241\_cov\_21.205557 2639-2650. Max. coverage (+): 0.25. Max coverage (-): 0

Region: NODE\_302677\_length\_8241\_cov\_21.205557 2651-2662. Max. coverage (+): 0.01. Max coverage (-): 0.03

Region: NODE\_302677\_length\_8241\_cov\_21.205557 2663-2674. Max. coverage (+): 0.01. Max coverage (-): 0.01

Region: NODE\_302677\_length\_8241\_cov\_21.205557 2675-2686. Max. coverage (+): 0. Max coverage (-): 0

Region: NODE\_302677\_length\_8241\_cov\_21.205557 2687-2698. Max. coverage (+): 0. Max coverage (-): 0

Region: NODE\_302677\_length\_8241\_cov\_21.205557 2699-2710. Max. coverage (+): 0. Max coverage (-): 0

Region: NODE\_302677\_length\_8241\_cov\_21.205557 2711-2722. Max. coverage (+): 0. Max coverage (-): 0

Region: NODE\_302677\_length\_8241\_cov\_21.205557 2723-2734. Max. coverage (+): 0. Max coverage (-): 0

Region: NODE\_302677\_length\_8241\_cov\_21.205557 2735-2746. Max. coverage (+): 0. Max coverage (-): 0

Region: NODE\_302677\_length\_8241\_cov\_21.205557 2747-2758. Max. coverage (+): 0. Max coverage (-): 0

Region: NODE\_302677\_length\_8241\_cov\_21.205557 2759-2770. Max. coverage (+): 0. Max coverage (-): 0.04

Region: NODE\_302677\_length\_8241\_cov\_21.205557 2771-2782. Max. coverage (+): 0. Max coverage (-): 0.03

Region: NODE\_302677\_length\_8241\_cov\_21.205557 2783-2794. Max. coverage (+): 0.14. Max coverage (-): 0

Region: NODE\_302677\_length\_8241\_cov\_21.205557 2795-2806. Max. coverage (+): 0. Max coverage (-): 0

Region: NODE\_302677\_length\_8241\_cov\_21.205557 2807-2818. Max. coverage (+): 0. Max coverage (-): 0

Region: NODE\_302677\_length\_8241\_cov\_21.205557 2819-2830. Max. coverage (+): 0. Max coverage (-): 0

Region: NODE\_302677\_length\_8241\_cov\_21.205557 2831-2842. Max. coverage (+): 0. Max coverage (-): 0

Region: NODE\_302677\_length\_8241\_cov\_21.205557 2843-2854. Max. coverage (+): 0. Max coverage (-): 0

Region: NODE\_302677\_length\_8241\_cov\_21.205557 2855-2866. Max. coverage (+): 0.61. Max coverage (-): 0

Region: NODE\_302677\_length\_8241\_cov\_21.205557 2867-2878. Max. coverage (+): 0.47. Max coverage (-): 0

Region: NODE\_302677\_length\_8241\_cov\_21.205557 2879-2890. Max. coverage (+): 0. Max coverage (-): 0

Region: NODE\_302677\_length\_8241\_cov\_21.205557 2891-2902. Max. coverage (+): 0.03. Max coverage (-): 0

Region: NODE\_302677\_length\_8241\_cov\_21.205557 2903-2914. Max. coverage (+): 0. Max coverage (-): 0

Region: NODE\_302677\_length\_8241\_cov\_21.205557 2915-2926. Max. coverage (+): 0. Max coverage (-): 0

Region: NODE\_302677\_length\_8241\_cov\_21.205557 2927-2938. Max. coverage (+): 0.05. Max coverage (-): 0

Region: NODE\_302677\_length\_8241\_cov\_21.205557 2939-2950. Max. coverage (+): 0. Max coverage (-): 0

Region: NODE\_302677\_length\_8241\_cov\_21.205557 2951-2962. Max. coverage (+): 0. Max coverage (-): 0

Region: NODE\_302677\_length\_8241\_cov\_21.205557 2963-2974. Max. coverage (+): 0. Max coverage (-): 0

Region: NODE\_302677\_length\_8241\_cov\_21.205557 2975-2986. Max. coverage (+): 0. Max coverage (-): 0

Region: NODE\_302677\_length\_8241\_cov\_21.205557 2987-2998. Max. coverage (+): 0. Max coverage (-): 0

Region: NODE\_302677\_length\_8241\_cov\_21.205557 2999-3010. Max. coverage (+): 0. Max coverage (-): 0

Region: NODE\_302677\_length\_8241\_cov\_21.205557 3011-3022. Max. coverage (+): 0.05. Max coverage (-): 0

Region: NODE\_302677\_length\_8241\_cov\_21.205557 3023-3034. Max. coverage (+): 0.08. Max coverage (-): 0

Region: NODE\_302677\_length\_8241\_cov\_21.205557 3035-3046. Max. coverage (+): 0.06. Max coverage (-): 0.02

Region: NODE\_302677\_length\_8241\_cov\_21.205557 3047-3058. Max. coverage (+): 0. Max coverage (-): 0.34

Region: NODE\_302677\_length\_8241\_cov\_21.205557 3059-3070. Max. coverage (+): 0.24. Max coverage (-): 0.03

Region: NODE\_302677\_length\_8241\_cov\_21.205557 3071-3082. Max. coverage (+): 0.26. Max coverage (-): 0.17

Region: NODE\_302677\_length\_8241\_cov\_21.205557 3083-3094. Max. coverage (+): 0. Max coverage (-): 0.18

Region: NODE\_302677\_length\_8241\_cov\_21.205557 3095-3106. Max. coverage (+): 0. Max coverage (-): 0

Region: NODE\_302677\_length\_8241\_cov\_21.205557 3107-3118. Max. coverage (+): 0. Max coverage (-): 0.09

Region: NODE\_302677\_length\_8241\_cov\_21.205557 3119-3130. Max. coverage (+): 0. Max coverage (-): 0

Region: NODE\_302677\_length\_8241\_cov\_21.205557 3131-3142. Max. coverage (+): 0. Max coverage (-): 0

Region: NODE\_302677\_length\_8241\_cov\_21.205557 3143-3154. Max. coverage (+): 0. Max coverage (-): 0

Region: NODE\_302677\_length\_8241\_cov\_21.205557 3155-3166. Max. coverage (+): 0.28. Max coverage (-): 0

Region: NODE\_302677\_length\_8241\_cov\_21.205557 3167-3178. Max. coverage (+): 0.19. Max coverage (-): 0

Region: NODE\_302677\_length\_8241\_cov\_21.205557 3179-3190. Max. coverage (+): 0. Max coverage (-): 0.19

Region: NODE\_302677\_length\_8241\_cov\_21.205557 3191-3202. Max. coverage (+): 0.02. Max coverage (-): 0.19

Region: NODE\_302677\_length\_8241\_cov\_21.205557 3203-3214. Max. coverage (+): 0.31. Max coverage (-): 0.01

Region: NODE\_302677\_length\_8241\_cov\_21.205557 3215-3226. Max. coverage (+): 0.01. Max coverage (-): 0.14

Region: NODE\_302677\_length\_8241\_cov\_21.205557 3227-3238. Max. coverage (+): 0.34. Max coverage (-): 0.14

Region: NODE\_302677\_length\_8241\_cov\_21.205557 3239-3250. Max. coverage (+): 0.38. Max coverage (-): 0.19

Region: NODE\_302677\_length\_8241\_cov\_21.205557 3251-3262. Max. coverage (+): 0.38. Max coverage (-): 0

Region: NODE\_302677\_length\_8241\_cov\_21.205557 3263-3274. Max. coverage (+): 0.09. Max coverage (-): 0.09

Region: NODE\_302677\_length\_8241\_cov\_21.205557 3275-3286. Max. coverage (+): 0. Max coverage (-): 0.09

Region: NODE\_302677\_length\_8241\_cov\_21.205557 3287-3298. Max. coverage (+): 46.8. Max coverage (-): 0

Region: NODE\_302677\_length\_8241\_cov\_21.205557 3299-3310. Max. coverage (+): 0.09. Max coverage (-): 0

Region: NODE\_302677\_length\_8241\_cov\_21.205557 3311-3322. Max. coverage (+): 0. Max coverage (-): 0.23

Region: NODE\_302677\_length\_8241\_cov\_21.205557 3323-3334. Max. coverage (+): 0. Max coverage (-): 0

Region: NODE\_302677\_length\_8241\_cov\_21.205557 3335-3346. Max. coverage (+): 0. Max coverage (-): 0

Region: NODE\_302677\_length\_8241\_cov\_21.205557 3347-3358. Max. coverage (+): 0. Max coverage (-): 0.09

Region: NODE\_302677\_length\_8241\_cov\_21.205557 3359-3370. Max. coverage (+): 0.19. Max coverage (-): 0

Region: NODE\_302677\_length\_8241\_cov\_21.205557 3371-3382. Max. coverage (+): 0.19. Max coverage (-): 0

Region: NODE\_302677\_length\_8241\_cov\_21.205557 3383-3394. Max. coverage (+): 0. Max coverage (-): 0

Region: NODE\_302677\_length\_8241\_cov\_21.205557 3395-3406. Max. coverage (+): 0.09. Max coverage (-): 0

Region: NODE\_302677\_length\_8241\_cov\_21.205557 3407-3418. Max. coverage (+): 0. Max coverage (-): 0

Region: NODE\_302677\_length\_8241\_cov\_21.205557 3419-3430. Max. coverage (+): 0. Max coverage (-): 0

Region: NODE\_302677\_length\_8241\_cov\_21.205557 3431-3442. Max. coverage (+): 0.09. Max coverage (-): 0

Region: NODE\_302677\_length\_8241\_cov\_21.205557 3443-3454. Max. coverage (+): 0. Max coverage (-): 0.09

Region: NODE\_302677\_length\_8241\_cov\_21.205557 3455-3466. Max. coverage (+): 0.29. Max coverage (-): 0.13

Region: NODE\_302677\_length\_8241\_cov\_21.205557 3467-3478. Max. coverage (+): 1.34. Max coverage (-): 0

Region: NODE\_302677\_length\_8241\_cov\_21.205557 3479-3490. Max. coverage (+): 0. Max coverage (-): 0

Region: NODE\_302677\_length\_8241\_cov\_21.205557 3491-3502. Max. coverage (+): 0.09. Max coverage (-): 0

Region: NODE\_302677\_length\_8241\_cov\_21.205557 3503-3514. Max. coverage (+): 0.09. Max coverage (-): 0.38

Region: NODE\_302677\_length\_8241\_cov\_21.205557 3515-3526. Max. coverage (+): 0. Max coverage (-): 0.38

Region: NODE\_302677\_length\_8241\_cov\_21.205557 3527-3538. Max. coverage (+): 0.09. Max coverage (-): 0

Region: NODE\_302677\_length\_8241\_cov\_21.205557 3539-3550. Max. coverage (+): 0. Max coverage (-): 0.09

Region: NODE\_302677\_length\_8241\_cov\_21.205557 3551-3562. Max. coverage (+): 0. Max coverage (-): 0

Region: NODE\_302677\_length\_8241\_cov\_21.205557 3563-3574. Max. coverage (+): 0. Max coverage (-): 0

Region: NODE\_302677\_length\_8241\_cov\_21.205557 3575-3586. Max. coverage (+): 0. Max coverage (-): 0

Region: NODE\_302677\_length\_8241\_cov\_21.205557 3587-3598. Max. coverage (+): 0.01. Max coverage (-): 0

Region: NODE\_302677\_length\_8241\_cov\_21.205557 3599-3610. Max. coverage (+): 0.42. Max coverage (-): 0

Region: NODE\_302677\_length\_8241\_cov\_21.205557 3611-3622. Max. coverage (+): 0.01. Max coverage (-): 0.09

Region: NODE\_302677\_length\_8241\_cov\_21.205557 3623-3634. Max. coverage (+): 0.09. Max coverage (-): 0.09

Region: NODE\_302677\_length\_8241\_cov\_21.205557 3635-3646. Max. coverage (+): 0.09. Max coverage (-): 0

Region: NODE\_302677\_length\_8241\_cov\_21.205557 3647-3658. Max. coverage (+): 0.47. Max coverage (-): 0.05

Region: NODE\_302677\_length\_8241\_cov\_21.205557 3659-3670. Max. coverage (+): 0.47. Max coverage (-): 0

Region: NODE\_302677\_length\_8241\_cov\_21.205557 3671-3682. Max. coverage (+): 0. Max coverage (-): 0.19

Region: NODE\_302677\_length\_8241\_cov\_21.205557 3683-3694. Max. coverage (+): 0.09. Max coverage (-): 0

Region: NODE\_302677\_length\_8241\_cov\_21.205557 3695-3706. Max. coverage (+): 0.28. Max coverage (-): 0

Region: NODE\_302677\_length\_8241\_cov\_21.205557 3707-3718. Max. coverage (+): 0. Max coverage (-): 0

Region: NODE\_302677\_length\_8241\_cov\_21.205557 3719-3730. Max. coverage (+): 0. Max coverage (-): 0

Region: NODE\_302677\_length\_8241\_cov\_21.205557 3731-3742. Max. coverage (+): 0. Max coverage (-): 0

Region: NODE\_302677\_length\_8241\_cov\_21.205557 3743-3754. Max. coverage (+): 0.09. Max coverage (-): 0

Region: NODE\_302677\_length\_8241\_cov\_21.205557 3755-3766. Max. coverage (+): 0. Max coverage (-): 0

Region: NODE\_302677\_length\_8241\_cov\_21.205557 3767-3778. Max. coverage (+): 0. Max coverage (-): 0

Region: NODE\_302677\_length\_8241\_cov\_21.205557 3779-3790. Max. coverage (+): 0. Max coverage (-): 0

Region: NODE\_302677\_length\_8241\_cov\_21.205557 3791-3802. Max. coverage (+): 0. Max coverage (-): 0.19

Region: NODE\_302677\_length\_8241\_cov\_21.205557 3803-3814. Max. coverage (+): 0.09. Max coverage (-): 0

Region: NODE\_302677\_length\_8241\_cov\_21.205557 3815-3826. Max. coverage (+): 0.09. Max coverage (-): 0

Region: NODE\_302677\_length\_8241\_cov\_21.205557 3827-3838. Max. coverage (+): 0.09. Max coverage (-): 0

Region: NODE\_302677\_length\_8241\_cov\_21.205557 3839-3850. Max. coverage (+): 0.09. Max coverage (-): 0

Region: NODE\_302677\_length\_8241\_cov\_21.205557 3851-3862. Max. coverage (+): 0. Max coverage (-): 0

Region: NODE\_302677\_length\_8241\_cov\_21.205557 3863-3874. Max. coverage (+): 0. Max coverage (-): 0

Region: NODE\_302677\_length\_8241\_cov\_21.205557 3875-3886. Max. coverage (+): 0. Max coverage (-): 0

Region: NODE\_302677\_length\_8241\_cov\_21.205557 3887-3898. Max. coverage (+): 0. Max coverage (-): 0

Region: NODE\_302677\_length\_8241\_cov\_21.205557 3899-3910. Max. coverage (+): 0. Max coverage (-): 0

Region: NODE\_302677\_length\_8241\_cov\_21.205557 3911-3922. Max. coverage (+): 0. Max coverage (-): 0

Region: NODE\_302677\_length\_8241\_cov\_21.205557 3923-3934. Max. coverage (+): 0. Max coverage (-): 0

Region: NODE\_302677\_length\_8241\_cov\_21.205557 3935-3946. Max. coverage (+): 0.28. Max coverage (-): 0

Region: NODE\_302677\_length\_8241\_cov\_21.205557 3947-3958. Max. coverage (+): 0.19. Max coverage (-): 0.38

Region: NODE\_302677\_length\_8241\_cov\_21.205557 3959-3970. Max. coverage (+): 0. Max coverage (-): 1.41

Region: NODE\_302677\_length\_8241\_cov\_21.205557 3971-3982. Max. coverage (+): 0.56. Max coverage (-): 0

Region: NODE\_302677\_length\_8241\_cov\_21.205557 3983-3994. Max. coverage (+): 0.09. Max coverage (-): 0.09

Region: NODE\_302677\_length\_8241\_cov\_21.205557 3995-4006. Max. coverage (+): 0.94. Max coverage (-): 0

Region: NODE\_302677\_length\_8241\_cov\_21.205557 4007-4018. Max. coverage (+): 0.85. Max coverage (-): 0

Region: NODE\_302677\_length\_8241\_cov\_21.205557 4019-4030. Max. coverage (+): 0. Max coverage (-): 0

Region: NODE\_302677\_length\_8241\_cov\_21.205557 4031-4042. Max. coverage (+): 0. Max coverage (-): 0

Region: NODE\_302677\_length\_8241\_cov\_21.205557 4043-4054. Max. coverage (+): 0. Max coverage (-): 0.09

Region: NODE\_302677\_length\_8241\_cov\_21.205557 4055-4066. Max. coverage (+): 0. Max coverage (-): 0

Region: NODE\_302677\_length\_8241\_cov\_21.205557 4067-4078. Max. coverage (+): 0.09. Max coverage (-): 0

Region: NODE\_302677\_length\_8241\_cov\_21.205557 4079-4090. Max. coverage (+): 0.09. Max coverage (-): 0

Region: NODE\_302677\_length\_8241\_cov\_21.205557 4091-4102. Max. coverage (+): 0. Max coverage (-): 0.09

Region: NODE\_302677\_length\_8241\_cov\_21.205557 4103-4114. Max. coverage (+): 0.09. Max coverage (-): 0

Region: NODE\_302677\_length\_8241\_cov\_21.205557 4115-4126. Max. coverage (+): 0.56. Max coverage (-): 0

Region: NODE\_302677\_length\_8241\_cov\_21.205557 4127-4138. Max. coverage (+): 0. Max coverage (-): 0

Region: NODE\_302677\_length\_8241\_cov\_21.205557 4139-4150. Max. coverage (+): 0. Max coverage (-): 0.09

Region: NODE\_302677\_length\_8241\_cov\_21.205557 4151-4162. Max. coverage (+): 1.6. Max coverage (-): 0

Region: NODE\_302677\_length\_8241\_cov\_21.205557 4163-4174. Max. coverage (+): 1.5. Max coverage (-): 0

Region: NODE\_302677\_length\_8241\_cov\_21.205557 4175-4186. Max. coverage (+): 0. Max coverage (-): 0.09

Region: NODE\_302677\_length\_8241\_cov\_21.205557 4187-4198. Max. coverage (+): 0. Max coverage (-): 0.09

Region: NODE\_302677\_length\_8241\_cov\_21.205557 4199-4210. Max. coverage (+): 0.19. Max coverage (-): 0.09

Region: NODE\_302677\_length\_8241\_cov\_21.205557 4211-4222. Max. coverage (+): 0.19. Max coverage (-): 0

Region: NODE\_302677\_length\_8241\_cov\_21.205557 4223-4234. Max. coverage (+): 0. Max coverage (-): 0.09

Region: NODE\_302677\_length\_8241\_cov\_21.205557 4235-4246. Max. coverage (+): 0.5. Max coverage (-): 0

Region: NODE\_302677\_length\_8241\_cov\_21.205557 4247-4258. Max. coverage (+): 0.45. Max coverage (-): 0

Region: NODE\_302677\_length\_8241\_cov\_21.205557 4259-4270. Max. coverage (+): 0. Max coverage (-): 0

Region: NODE\_302677\_length\_8241\_cov\_21.205557 4271-4282. Max. coverage (+): 0.09. Max coverage (-): 0

Region: NODE\_302677\_length\_8241\_cov\_21.205557 4283-4294. Max. coverage (+): 0.09. Max coverage (-): 0

Region: NODE\_302677\_length\_8241\_cov\_21.205557 4295-4306. Max. coverage (+): 0. Max coverage (-): 0.02

Region: NODE\_302677\_length\_8241\_cov\_21.205557 4307-4318. Max. coverage (+): 0.47. Max coverage (-): 0

Region: NODE\_302677\_length\_8241\_cov\_21.205557 4319-4330. Max. coverage (+): 0.47. Max coverage (-): 0

Region: NODE\_302677\_length\_8241\_cov\_21.205557 4331-4342. Max. coverage (+): 0.09. Max coverage (-): 0

Region: NODE\_302677\_length\_8241\_cov\_21.205557 4343-4354. Max. coverage (+): 0. Max coverage (-): 0.19

Region: NODE\_302677\_length\_8241\_cov\_21.205557 4355-4366. Max. coverage (+): 0. Max coverage (-): 0.19

Region: NODE\_302677\_length\_8241\_cov\_21.205557 4367-4378. Max. coverage (+): 0. Max coverage (-): 0

Region: NODE\_302677\_length\_8241\_cov\_21.205557 4379-4390. Max. coverage (+): 0. Max coverage (-): 0

Region: NODE\_302677\_length\_8241\_cov\_21.205557 4391-4402. Max. coverage (+): 0. Max coverage (-): 0.09

Region: NODE\_302677\_length\_8241\_cov\_21.205557 4403-4414. Max. coverage (+): 0. Max coverage (-): 0

Region: NODE\_302677\_length\_8241\_cov\_21.205557 4415-4426. Max. coverage (+): 0. Max coverage (-): 0.19

Region: NODE\_302677\_length\_8241\_cov\_21.205557 4427-4438. Max. coverage (+): 0. Max coverage (-): 0.19

Region: NODE\_302677\_length\_8241\_cov\_21.205557 4439-4450. Max. coverage (+): 0. Max coverage (-): 0

Region: NODE\_302677\_length\_8241\_cov\_21.205557 4451-4462. Max. coverage (+): 0. Max coverage (-): 0

Region: NODE\_302677\_length\_8241\_cov\_21.205557 4463-4474. Max. coverage (+): 0. Max coverage (-): 0

Region: NODE\_302677\_length\_8241\_cov\_21.205557 4475-4486. Max. coverage (+): 0. Max coverage (-): 0

Region: NODE\_302677\_length\_8241\_cov\_21.205557 4487-4498. Max. coverage (+): 0. Max coverage (-): 0

Region: NODE\_302677\_length\_8241\_cov\_21.205557 4499-4510. Max. coverage (+): 0. Max coverage (-): 0

Region: NODE\_302677\_length\_8241\_cov\_21.205557 4511-4522. Max. coverage (+): 0.09. Max coverage (-): 0

Region: NODE\_302677\_length\_8241\_cov\_21.205557 4523-4534. Max. coverage (+): 0. Max coverage (-): 0

Region: NODE\_302677\_length\_8241\_cov\_21.205557 4535-4546. Max. coverage (+): 0. Max coverage (-): 0

Region: NODE\_302677\_length\_8241\_cov\_21.205557 4547-4558. Max. coverage (+): 0.05. Max coverage (-): 0

Region: NODE\_302677\_length\_8241\_cov\_21.205557 4559-4570. Max. coverage (+): 0. Max coverage (-): 0

Region: NODE\_302677\_length\_8241\_cov\_21.205557 4571-4582. Max. coverage (+): 0. Max coverage (-): 0

Region: NODE\_302677\_length\_8241\_cov\_21.205557 4583-4594. Max. coverage (+): 0. Max coverage (-): 0

Region: NODE\_302677\_length\_8241\_cov\_21.205557 4595-4606. Max. coverage (+): 0.38. Max coverage (-): 0

Region: NODE\_302677\_length\_8241\_cov\_21.205557 4607-4618. Max. coverage (+): 0. Max coverage (-): 0

Region: NODE\_302677\_length\_8241\_cov\_21.205557 4619-4630. Max. coverage (+): 0. Max coverage (-): 0

Region: NODE\_302677\_length\_8241\_cov\_21.205557 4631-4642. Max. coverage (+): 0. Max coverage (-): 0

Region: NODE\_302677\_length\_8241\_cov\_21.205557 4643-4654. Max. coverage (+): 0. Max coverage (-): 0

Region: NODE\_302677\_length\_8241\_cov\_21.205557 4655-4666. Max. coverage (+): 0. Max coverage (-): 0

Region: NODE\_302677\_length\_8241\_cov\_21.205557 4667-4678. Max. coverage (+): 0. Max coverage (-): 0

Region: NODE\_302677\_length\_8241\_cov\_21.205557 4679-4690. Max. coverage (+): 0. Max coverage (-): 0

Region: NODE\_302677\_length\_8241\_cov\_21.205557 4691-4702. Max. coverage (+): 0. Max coverage (-): 0

Region: NODE\_302677\_length\_8241\_cov\_21.205557 4703-4714. Max. coverage (+): 0.28. Max coverage (-): 0

Region: NODE\_302677\_length\_8241\_cov\_21.205557 4715-4726. Max. coverage (+): 0.56. Max coverage (-): 0

Region: NODE\_302677\_length\_8241\_cov\_21.205557 4727-4738. Max. coverage (+): 0. Max coverage (-): 0

Region: NODE\_302677\_length\_8241\_cov\_21.205557 4739-4750. Max. coverage (+): 0.31. Max coverage (-): 0

Region: NODE\_302677\_length\_8241\_cov\_21.205557 4751-4762. Max. coverage (+): 0.26. Max coverage (-): 0

Region: NODE\_302677\_length\_8241\_cov\_21.205557 4763-4774. Max. coverage (+): 0.05. Max coverage (-): 0

Region: NODE\_302677\_length\_8241\_cov\_21.205557 4775-4786. Max. coverage (+): 0. Max coverage (-): 0

Region: NODE\_302677\_length\_8241\_cov\_21.205557 4787-4798. Max. coverage (+): 0. Max coverage (-): 0

Region: NODE\_302677\_length\_8241\_cov\_21.205557 4799-4810. Max. coverage (+): 0. Max coverage (-): 0.09

Region: NODE\_302677\_length\_8241\_cov\_21.205557 4811-4822. Max. coverage (+): 0. Max coverage (-): 0

Region: NODE\_302677\_length\_8241\_cov\_21.205557 4823-4834. Max. coverage (+): 0. Max coverage (-): 0

Region: NODE\_302677\_length\_8241\_cov\_21.205557 4835-4846. Max. coverage (+): 0. Max coverage (-): 0

Region: NODE\_302677\_length\_8241\_cov\_21.205557 4847-4858. Max. coverage (+): 0. Max coverage (-): 0.09

Region: NODE\_302677\_length\_8241\_cov\_21.205557 4859-4870. Max. coverage (+): 0. Max coverage (-): 0

Region: NODE\_302677\_length\_8241\_cov\_21.205557 4871-4882. Max. coverage (+): 0. Max coverage (-): 0

Region: NODE\_302677\_length\_8241\_cov\_21.205557 4883-4894. Max. coverage (+): 0. Max coverage (-): 0

Region: NODE\_302677\_length\_8241\_cov\_21.205557 4895-4906. Max. coverage (+): 0. Max coverage (-): 0

Region: NODE\_302677\_length\_8241\_cov\_21.205557 4907-4918. Max. coverage (+): 0. Max coverage (-): 0

Region: NODE\_302677\_length\_8241\_cov\_21.205557 4919-4930. Max. coverage (+): 0. Max coverage (-): 0

Region: NODE\_302677\_length\_8241\_cov\_21.205557 4931-4942. Max. coverage (+): 0. Max coverage (-): 0

Region: NODE\_302677\_length\_8241\_cov\_21.205557 4943-4954. Max. coverage (+): 0. Max coverage (-): 0

Region: NODE\_302677\_length\_8241\_cov\_21.205557 4955-4966. Max. coverage (+): 0. Max coverage (-): 0

Region: NODE\_302677\_length\_8241\_cov\_21.205557 4967-4978. Max. coverage (+): 0. Max coverage (-): 0

Region: NODE\_302677\_length\_8241\_cov\_21.205557 4979-4990. Max. coverage (+): 0. Max coverage (-): 0.09

Region: NODE\_302677\_length\_8241\_cov\_21.205557 4991-5002. Max. coverage (+): 0. Max coverage (-): 0

Region: NODE\_302677\_length\_8241\_cov\_21.205557 5003-5014. Max. coverage (+): 0. Max coverage (-): 0.15

Region: NODE\_302677\_length\_8241\_cov\_21.205557 5015-5026. Max. coverage (+): 0.02. Max coverage (-): 0.17

Region: NODE\_302677\_length\_8241\_cov\_21.205557 5027-5038. Max. coverage (+): 0. Max coverage (-): 0

Region: NODE\_302677\_length\_8241\_cov\_21.205557 5039-5050. Max. coverage (+): 0.19. Max coverage (-): 0

Region: NODE\_302677\_length\_8241\_cov\_21.205557 5051-5062. Max. coverage (+): 0. Max coverage (-): 0

Region: NODE\_302677\_length\_8241\_cov\_21.205557 5063-5074. Max. coverage (+): 0.09. Max coverage (-): 0

Region: NODE\_302677\_length\_8241\_cov\_21.205557 5075-5086. Max. coverage (+): 0. Max coverage (-): 0

Region: NODE\_302677\_length\_8241\_cov\_21.205557 5087-5098. Max. coverage (+): 0. Max coverage (-): 0

Region: NODE\_302677\_length\_8241\_cov\_21.205557 5099-5110. Max. coverage (+): 0. Max coverage (-): 0

Region: NODE\_302677\_length\_8241\_cov\_21.205557 5111-5122. Max. coverage (+): 0. Max coverage (-): 0

Region: NODE\_302677\_length\_8241\_cov\_21.205557 5123-5134. Max. coverage (+): 0. Max coverage (-): 0

Region: NODE\_302677\_length\_8241\_cov\_21.205557 5135-5146. Max. coverage (+): 0. Max coverage (-): 0

Region: NODE\_302677\_length\_8241\_cov\_21.205557 5147-5158. Max. coverage (+): 0.19. Max coverage (-): 0

Region: NODE\_302677\_length\_8241\_cov\_21.205557 5159-5170. Max. coverage (+): 0.19. Max coverage (-): 0

Region: NODE\_302677\_length\_8241\_cov\_21.205557 5171-5182. Max. coverage (+): 0. Max coverage (-): 0

Region: NODE\_302677\_length\_8241\_cov\_21.205557 5183-5194. Max. coverage (+): 0. Max coverage (-): 0

Region: NODE\_302677\_length\_8241\_cov\_21.205557 5195-5206. Max. coverage (+): 0.19. Max coverage (-): 0

Region: NODE\_302677\_length\_8241\_cov\_21.205557 5207-5218. Max. coverage (+): 0. Max coverage (-): 0

Region: NODE\_302677\_length\_8241\_cov\_21.205557 5219-5230. Max. coverage (+): 0. Max coverage (-): 0

Region: NODE\_302677\_length\_8241\_cov\_21.205557 5231-5242. Max. coverage (+): 0. Max coverage (-): 0

Region: NODE\_302677\_length\_8241\_cov\_21.205557 5243-5254. Max. coverage (+): 0.09. Max coverage (-): 0

Region: NODE\_302677\_length\_8241\_cov\_21.205557 5255-5266. Max. coverage (+): 0. Max coverage (-): 0

Region: NODE\_302677\_length\_8241\_cov\_21.205557 5267-5278. Max. coverage (+): 0. Max coverage (-): 0

Region: NODE\_302677\_length\_8241\_cov\_21.205557 5279-5290. Max. coverage (+): 0. Max coverage (-): 0

Region: NODE\_302677\_length\_8241\_cov\_21.205557 5291-5302. Max. coverage (+): 0. Max coverage (-): 0

Region: NODE\_302677\_length\_8241\_cov\_21.205557 5303-5314. Max. coverage (+): 0. Max coverage (-): 0

Region: NODE\_302677\_length\_8241\_cov\_21.205557 5315-5326. Max. coverage (+): 0.09. Max coverage (-): 0

Region: NODE\_302677\_length\_8241\_cov\_21.205557 5327-5338. Max. coverage (+): 0.09. Max coverage (-): 0

Region: NODE\_302677\_length\_8241\_cov\_21.205557 5339-5350. Max. coverage (+): 0. Max coverage (-): 0

Region: NODE\_302677\_length\_8241\_cov\_21.205557 5351-5362. Max. coverage (+): 0. Max coverage (-): 0

Region: NODE\_302677\_length\_8241\_cov\_21.205557 5363-5374. Max. coverage (+): 0. Max coverage (-): 0

Region: NODE\_302677\_length\_8241\_cov\_21.205557 5375-5386. Max. coverage (+): 0. Max coverage (-): 0

Region: NODE\_302677\_length\_8241\_cov\_21.205557 5387-5398. Max. coverage (+): 0. Max coverage (-): 0

Region: NODE\_302677\_length\_8241\_cov\_21.205557 5399-5410. Max. coverage (+): 0.09. Max coverage (-): 0

Region: NODE\_302677\_length\_8241\_cov\_21.205557 5411-5422. Max. coverage (+): 0. Max coverage (-): 0

Region: NODE\_302677\_length\_8241\_cov\_21.205557 5423-5434. Max. coverage (+): 0. Max coverage (-): 0

Region: NODE\_302677\_length\_8241\_cov\_21.205557 5435-5446. Max. coverage (+): 0. Max coverage (-): 0

Region: NODE\_302677\_length\_8241\_cov\_21.205557 5447-5458. Max. coverage (+): 0.09. Max coverage (-): 0

Region: NODE\_302677\_length\_8241\_cov\_21.205557 5459-5470. Max. coverage (+): 0.09. Max coverage (-): 0

Region: NODE\_302677\_length\_8241\_cov\_21.205557 5471-5482. Max. coverage (+): 0. Max coverage (-): 0

Region: NODE\_302677\_length\_8241\_cov\_21.205557 5483-5494. Max. coverage (+): 0. Max coverage (-): 0

Region: NODE\_302677\_length\_8241\_cov\_21.205557 5495-5506. Max. coverage (+): 0. Max coverage (-): 0

Region: NODE\_302677\_length\_8241\_cov\_21.205557 5507-5518. Max. coverage (+): 0. Max coverage (-): 0

Region: NODE\_302677\_length\_8241\_cov\_21.205557 5519-5530. Max. coverage (+): 0. Max coverage (-): 0

Region: NODE\_302677\_length\_8241\_cov\_21.205557 5531-5542. Max. coverage (+): 0. Max coverage (-): 0

Region: NODE\_302677\_length\_8241\_cov\_21.205557 5543-5554. Max. coverage (+): 0. Max coverage (-): 0

Region: NODE\_302677\_length\_8241\_cov\_21.205557 5555-5566. Max. coverage (+): 0. Max coverage (-): 0

Region: NODE\_302677\_length\_8241\_cov\_21.205557 5567-5578. Max. coverage (+): 0. Max coverage (-): 0

Region: NODE\_302677\_length\_8241\_cov\_21.205557 5579-5590. Max. coverage (+): 0. Max coverage (-): 0

Region: NODE\_302677\_length\_8241\_cov\_21.205557 5591-5602. Max. coverage (+): 0. Max coverage (-): 0

Region: NODE\_302677\_length\_8241\_cov\_21.205557 5603-5614. Max. coverage (+): 0. Max coverage (-): 0

Region: NODE\_302677\_length\_8241\_cov\_21.205557 5615-5626. Max. coverage (+): 0. Max coverage (-): 0.09

Region: NODE\_302677\_length\_8241\_cov\_21.205557 5627-5638. Max. coverage (+): 0. Max coverage (-): 0

Region: NODE\_302677\_length\_8241\_cov\_21.205557 5639-5650. Max. coverage (+): 0. Max coverage (-): 0

Region: NODE\_302677\_length\_8241\_cov\_21.205557 5651-5662. Max. coverage (+): 0. Max coverage (-): 0

Region: NODE\_302677\_length\_8241\_cov\_21.205557 5663-5674. Max. coverage (+): 0. Max coverage (-): 0.09

Region: NODE\_302677\_length\_8241\_cov\_21.205557 5675-5686. Max. coverage (+): 0. Max coverage (-): 0

Region: NODE\_302677\_length\_8241\_cov\_21.205557 5687-5698. Max. coverage (+): 0. Max coverage (-): 0

Region: NODE\_302677\_length\_8241\_cov\_21.205557 5699-5710. Max. coverage (+): 0. Max coverage (-): 0

Region: NODE\_302677\_length\_8241\_cov\_21.205557 5711-5722. Max. coverage (+): 0. Max coverage (-): 0

Region: NODE\_302677\_length\_8241\_cov\_21.205557 5723-5734. Max. coverage (+): 0. Max coverage (-): 0

Region: NODE\_302677\_length\_8241\_cov\_21.205557 5735-5746. Max. coverage (+): 0. Max coverage (-): 0

Region: NODE\_302677\_length\_8241\_cov\_21.205557 5747-5758. Max. coverage (+): 0.09. Max coverage (-): 0

Region: NODE\_302677\_length\_8241\_cov\_21.205557 5759-5770. Max. coverage (+): 0.09. Max coverage (-): 0.47

Region: NODE\_302677\_length\_8241\_cov\_21.205557 5771-5782. Max. coverage (+): 0.09. Max coverage (-): 0.47

Region: NODE\_302677\_length\_8241\_cov\_21.205557 5783-5794. Max. coverage (+): 1.08. Max coverage (-): 0

Region: NODE\_302677\_length\_8241\_cov\_21.205557 5795-5806. Max. coverage (+): 0.09. Max coverage (-): 0

Region: NODE\_302677\_length\_8241\_cov\_21.205557 5807-5818. Max. coverage (+): 0. Max coverage (-): 0

Region: NODE\_302677\_length\_8241\_cov\_21.205557 5819-5830. Max. coverage (+): 0. Max coverage (-): 0

Region: NODE\_302677\_length\_8241\_cov\_21.205557 5831-5842. Max. coverage (+): 0. Max coverage (-): 0.09

Region: NODE\_302677\_length\_8241\_cov\_21.205557 5843-5854. Max. coverage (+): 0.09. Max coverage (-): 0.09

Region: NODE\_302677\_length\_8241\_cov\_21.205557 5855-5866. Max. coverage (+): 0. Max coverage (-): 0

Region: NODE\_302677\_length\_8241\_cov\_21.205557 5867-5878. Max. coverage (+): 0.09. Max coverage (-): 0

Region: NODE\_302677\_length\_8241\_cov\_21.205557 5879-5890. Max. coverage (+): 0. Max coverage (-): 0

Region: NODE\_302677\_length\_8241\_cov\_21.205557 5891-5902. Max. coverage (+): 0.09. Max coverage (-): 0

Region: NODE\_302677\_length\_8241\_cov\_21.205557 5903-5914. Max. coverage (+): 0. Max coverage (-): 0

Region: NODE\_302677\_length\_8241\_cov\_21.205557 5915-5926. Max. coverage (+): 0. Max coverage (-): 0

Region: NODE\_302677\_length\_8241\_cov\_21.205557 5927-5938. Max. coverage (+): 0. Max coverage (-): 0

Region: NODE\_302677\_length\_8241\_cov\_21.205557 5939-5950. Max. coverage (+): 0. Max coverage (-): 0

Region: NODE\_302677\_length\_8241\_cov\_21.205557 5951-5962. Max. coverage (+): 0. Max coverage (-): 0.19

Region: NODE\_302677\_length\_8241\_cov\_21.205557 5963-5974. Max. coverage (+): 0. Max coverage (-): 0

Region: NODE\_302677\_length\_8241\_cov\_21.205557 5975-5986. Max. coverage (+): 2.54. Max coverage (-): 0

Region: NODE\_302677\_length\_8241\_cov\_21.205557 5987-5998. Max. coverage (+): 0. Max coverage (-): 0

Region: NODE\_302677\_length\_8241\_cov\_21.205557 5999-. Max. coverage (+): 0. Max coverage (-): 0

RepeatMasker Color Code

**+**

100-98% Identity

<98-95% Identity

<95-90% Identity

<90-85% Identity

<85-80% Identity

<80-75% Identity

<75-70% Identity

<70% Identity

**-**

Gene Set Color Code

**+**

Gene

Pseudogene

Other

**-**

Topology/Coverage Color Code

Coverage Plus Strand

Coverage Minus Strand

Mainstrand: Plus

Mainstrand: Minus

Complementary Strand

Flanking Region  
(if option -flank >0)

Gene Set Annotation  
  
RepeatMasker Annotation  

**1. AlRepE-1560**: 1-261 (+), Divergence to consensus: 3.5%  
**2. DNA7-N1\_DR**: 262-313 (-), Divergence to consensus: 22.9%  
**3. BEL5-I\_DR**: 440-1327 (+), Divergence to consensus: 29.1%  
**4. SINE\_AFC**: 1339-1404 (+), Divergence to consensus: 1.5%  
**5. SINE\_AFC**: 1403-1469 (+), Divergence to consensus: 3%  
**6. SINE\_AFC**: 1467-1602 (+), Divergence to consensus: 4.4%  
**7. AlRepA-24**: 1600-1736 (+), Divergence to consensus: 11.7%  
**8. AlRepA-24**: 1728-1802 (+), Divergence to consensus: 4%  
**9. AlRepA-24**: 1791-1974 (+), Divergence to consensus: 9.3%  
**10. L2-43\_DR**: 2562-2825 (+), Divergence to consensus: 45.8%  
**11. AlRepE-528**: 5074-5626 (+), Divergence to consensus: 29.3%  
**12. Ronin2\_I-int**: 5699-7106 (-), Divergence to consensus: 23.3%

  
Transcription Factor Binding Sites  

**RHOXF1** (Sequence: GGCTCA (-): 1349)  
**RHOXF1** (Sequence: GGCTCA (-): 1413)  
**RHOXF1** (Sequence: GGCTCA (-): 1476)  
**RHOXF1** (Sequence: AGCTCA (-): 2160)  
**RHOXF1** (Sequence: AGATCA (-): 3636)  
**RHOXF1** (Sequence: GGCTCA (-): 4100)  
**RHOXF1** (Sequence: AGCTTA (-): 4220)  
**RHOXF1** (Sequence: AGATCA (-): 4809)  
**RHOXF1** (Sequence: GGCTCA (-): 5223)  
**RHOXF1** (Sequence: TAATCC (+): 551)  
**RHOXF1** (Sequence: TGATCC (+): 1116)  
**RHOXF1** (Sequence: TAAGCC (+): 1254)  
**RHOXF1** (Sequence: TAATCT (+): 2293)  
**RHOXF1** (Sequence: TGATCT (+): 2578)  
**RHOXF1** (Sequence: TGATCC (+): 2882)  
**RHOXF1** (Sequence: TGATCT (+): 5339)  
**RHOXF1** (Sequence: TAATCT (+): 5918)  
**Gata4** (Sequence: CTTATCT (+): 2735)  
**POU5F1** (Sequence: TTTGCAT (-): 3430)  
**RFX4\_2** (Sequence: GTAACCACG (-): 5364)  
**FOXO3\_hsa** (Sequence: GTAAACAT (+): 3947)  
**A-MYB** (Sequence: TGACAGTTGG (+): 4374)  
**FOXP1** (Sequence: GTAAACA (+): 3947)  
**FOXO1** (Sequence: CCTGTTTTT (+): 72)  
**FOXO1** (Sequence: GTTGTTTAC (+): 87)  
**FOXO3\_mmu** (Sequence: TGTTTACA (-): 89)  
**FOXO3\_mmu** (Sequence: TGTTTTGC (-): 3427)  
**Sox5** (Sequence: ATTGTT (+): 3812)  
**Sox5** (Sequence: ATTGTT (+): 5774)  
**FOXO3\_mmu** (Sequence: TCAAAACA (+): 3903)  
**FOXO3\_mmu** (Sequence: TGTAAACA (+): 3946)  
**FOXO3\_mmu** (Sequence: GCTAAACA (+): 4517)  
**Nobox** (Sequence: GGTAATTA (-): 4362)  
**Nobox** (Sequence: AGCAATTA (-): 4441)  
**FOXO1** (Sequence: AAAAACAAG (-): 4571)  
**FOXO1** (Sequence: ATAAACAGG (-): 5530)  
**FOXO1** (Sequence: GAAAACAAC (-): 5582)  
**FOXO3\_hsa** (Sequence: TTGTTTAC (-): 88)  
**FOXP1** (Sequence: TGTTTAC (-): 89)  
**Nobox** (Sequence: TAATTGCT (+): 4290)  
**Rhox11** (Sequence: TGCTGTAAT (+): 916)  
**Rhox11** (Sequence: ATAACAGCG (-): 674)  
**Sox5** (Sequence: AACAAT (-): 49)  
**Sox5** (Sequence: AACAAT (-): 701)  
**Sox5** (Sequence: AACAAT (-): 3315)  
**Sox5** (Sequence: AACAAT (-): 5272)  
**POU2F1** (Sequence: TATTTTAAT (+): 4012)  
**POU2F1** (Sequence: TATGTAAAT (+): 4506)  
**POU5F1** (Sequence: ATGCAAA (+): 441)  
**POU5F1** (Sequence: ATGCAAA (+): 719)
