# Supplementary material for: First characterization of PIWI-interacting RNA clusters in a cichlid fish with a B chromosome
Source: BMC Biol. 2022 Sep 21;20:204. doi: 10.1186/s12915-022-01403-2 (PMC9490952; doi:10.1186/s12915-022-01403-2)
Supplement: Supplementary file 1 — Additional file 1. Zipped folder with fasta and interactive html piRNA cluster information for the A. latifasciata genome. The nomenclature is as follows: number-pirna-cluster_sex_B-presence (f, female; m, male; 0b, without B chromosome; 1b, with B chromosome). [file 12915_2022_1403_MOESM1_ESM.zip › 119_m0b.html]

piRNA cluster 119\_m0b 56


Predicted piRNA cluster no. 119\_m0b
  

Show proTRAC run info
Hide proTRAC run info

/\  
                \_\_\_\_\_\_\_\_\_\_\_\_\_\_\_\_\_\_\_\_\_\_\_/\\_\_\_ /  \\_\_\_\_\_\_\_  
               I                      /  \  /    \      I  
               I     pro             /    \/      \     I  
               I        TRAC        /               \   I  
               I   \_\_\_\_\_\_\_\_\_\_\_\_\_\_\_\_/\_\_\_\_\_\_\_\_\_\_\_\_\_\_\_\_\_\\_ I  
               I   \              /                     I  
               I    \            /                      I  
               I     \  /\      /       V.2.4.2         I  
               I      \/  \    /                        I  
               I\_\_\_\_\_\_\_\_\_\_\_\  /\_\_\_\_\_\_\_\_\_\_\_\_\_\_\_\_\_\_\_\_\_\_\_\_\_I  
                            \/  
  
  
================================= proTRAC ====================================  
VERSION: .......... 2.4.2  
LAST MODIFIED: .... 11. May 2018  
  
Please cite:  
Rosenkranz D, Zischler H. proTRAC - a software for probabilistic piRNA cluster  
detection, visualization and analysis. 2012. BMC Bioinformatics 13:5.  
  
  
Contact:  
David Rosenkranz  
Institute of Organismic and Molecular Evolutionary Biology  
Dept. Anthropology, small RNA group  
Johannes Gutenberg University Mainz  
email: rosenkranz@uni-mainz.de  
  
You can find the latest proTRAC version at:  
http://sourceforge.net/projects/protrac/files  
http://www.smallRNAgroup-mainz.de/software  
==============================================================================  
  
PARAMETERS:  
Map file: ...............piwi-machos-0B.fa-collapse.map  
Genome file: ............../../../0B\_ala\_genome.fa  
RepeatMasker annotation: Alatifasciata-all0B-maryan-v2.fa\_corrected.out  
GeneSet:................./guest-storage/Data/annotation/Alatifasciata\_all0B\_maryan-v2\_out2017.gff  
  
Significant (p<=0.01) hit density will be calculated based  
on observed hit distribution.  
  
Sliding window size: ........................................ 5000 bp  
Sliding window increament: .................................. 1000 bp  
Normalize each hit by number of genomic hits: ............... yes  
Normalize each hit by number of sequence reads: ............. yes  
Normalize values (-> per million mapped reads): ............. yes  
Min. fraction of hits with 1T(U) or 10A: .................... 0.75  
Alternatively: Min. fraction of hits with 1T(U) and 10A: .... 0.5  
Min. fraction of hits with typical piRNA length: ............ 0.75  
Typical piRNA length: ....................................... 24-32 nt  
Min. size of a piRNA cluster: ............................... 1000 bp.  
Min. number of hits (absolute): ............................. 0  
Min. number of hits (normalized): ........................... 0  
Min. fraction of hits on the mainstrand: .................... 0.75  
Top fraction of mapped sequences (in terms of read counts): . 1%  
Top fraction accounts for max. n% of sequence reads: ........ 90%  
Min. fraction of hits on each arm of a bidirectional cluster: 0.05  
Output html file for each cluster: .......................... yes  
Output a summary table: ..................................... yes  
Output a FASTA file for each cluster (piRNA sequences): ..... yes  
Output a FASTA file comprising cluster sequences: ........... yes  
Output a GTF file for predicted piRNA clusters: ..............yes  
Search DNA motifs in clusters: .............................. yes  
Output flanking sequences: +/- .............................. 0 bp  
Output ~.pTi file: .......................................... no  
==============================================================================  
  
  
Genome size (without gaps): ............ 758543724 bp  
Gaps (N/X/-): .......................... 417479 bp  
Mapped reads: .......................... 24765598  
Non-identical sequences: ............... 6158275  
Genomic hits: .......................... 53103584  
Significant densitiy of mapped reads: .. 763.098963422187 reads/kb

Show proTRAC cluster info
Hide proTRAC cluster info

|  |  |
| --- | --- |
| Location | NODE\_304263\_length\_4982\_cov\_28.985548 |
| Coordinates | 1-5046 |
| Size [bp] | 5046 |
| Sequence hit loci | 3924 |
| Mapped reads (normalized) | 18289 |
| Mapped reads (normalized) per kb | 3624.5 |
| Normalized reads with 1T (1U) | 82.2% |
| Normalized reads with 10A | 43.7% |
| Normalized reads with length 24-32 nt | 99.2% |
| Normalized reads on the main strand(s) | 94.4% |
| Predicted directionality | mono:minus |

100%

0%

1T (1U)  
reads

10A reads

24-32 nt  
reads

reads on mainstrand

**Either the amount of reads with 1T (1U) OR 10A has to exceed 75% (set with option: -1Tor10A)  
Alternatively the amount of reads with 1T (1U) AND 10A has to exceed 50% (set with option: -1Tand10A)  
Minimum amount of reads with preferred size is 75% (set with option: -pisize)  
Minimum amount of reads on the main strand(s) is 75% (set with option: -clstrand)**

Show read coverage
Hide read coverage

WHAT DO I SEE HERE?  
This chart shows the location of mapped sequence reads within a predicted piRNA cluster. The color refers to the number of genomic hits produced by the sequence read in question. A dark red bar indicates that this sequence read produces many other hits elsewhere in the genome. Many adjacent red or yellow bars can indicate the presence of a multi-copy element such as transposons or rRNA genes. A dark green bar indicates that this sequence read maps uniquely to this locus.

1 hit

2-5 hits

6-10 hits

11-20 hits

21-50 hits

51-100 hits

> 100 hits

NODE\_304263\_length\_4982\_cov\_28.985548

1

5046

Gene Set

RepeatMasker

Mapped  
Reads

89.03

plus strand

minus strand

89.03

Region: NODE\_304263\_length\_4982\_cov\_28.985548 14617-6. Max. coverage (+): 0. Max coverage (-): 0.02

Region: NODE\_304263\_length\_4982\_cov\_28.985548 7-16. Max. coverage (+): 0. Max coverage (-): 0.52

Region: NODE\_304263\_length\_4982\_cov\_28.985548 17-26. Max. coverage (+): 0.01. Max coverage (-): 0

Region: NODE\_304263\_length\_4982\_cov\_28.985548 27-36. Max. coverage (+): 0.01. Max coverage (-): 0

Region: NODE\_304263\_length\_4982\_cov\_28.985548 37-46. Max. coverage (+): 0. Max coverage (-): 0

Region: NODE\_304263\_length\_4982\_cov\_28.985548 47-56. Max. coverage (+): 0. Max coverage (-): 0

Region: NODE\_304263\_length\_4982\_cov\_28.985548 57-66. Max. coverage (+): 0. Max coverage (-): 0

Region: NODE\_304263\_length\_4982\_cov\_28.985548 67-76. Max. coverage (+): 0. Max coverage (-): 0.04

Region: NODE\_304263\_length\_4982\_cov\_28.985548 77-86. Max. coverage (+): 0. Max coverage (-): 0

Region: NODE\_304263\_length\_4982\_cov\_28.985548 87-96. Max. coverage (+): 0. Max coverage (-): 0.16

Region: NODE\_304263\_length\_4982\_cov\_28.985548 97-106. Max. coverage (+): 0.12. Max coverage (-): 0.08

Region: NODE\_304263\_length\_4982\_cov\_28.985548 107-117. Max. coverage (+): 0.12. Max coverage (-): 0

Region: NODE\_304263\_length\_4982\_cov\_28.985548 118-127. Max. coverage (+): 0. Max coverage (-): 0.04

Region: NODE\_304263\_length\_4982\_cov\_28.985548 128-137. Max. coverage (+): 0. Max coverage (-): 0.36

Region: NODE\_304263\_length\_4982\_cov\_28.985548 138-147. Max. coverage (+): 0. Max coverage (-): 0.12

Region: NODE\_304263\_length\_4982\_cov\_28.985548 148-157. Max. coverage (+): 0. Max coverage (-): 0.12

Region: NODE\_304263\_length\_4982\_cov\_28.985548 158-167. Max. coverage (+): 0. Max coverage (-): 0.04

Region: NODE\_304263\_length\_4982\_cov\_28.985548 168-177. Max. coverage (+): 0. Max coverage (-): 0.04

Region: NODE\_304263\_length\_4982\_cov\_28.985548 178-187. Max. coverage (+): 0. Max coverage (-): 0

Region: NODE\_304263\_length\_4982\_cov\_28.985548 188-197. Max. coverage (+): 0. Max coverage (-): 0

Region: NODE\_304263\_length\_4982\_cov\_28.985548 198-207. Max. coverage (+): 0. Max coverage (-): 0.24

Region: NODE\_304263\_length\_4982\_cov\_28.985548 208-217. Max. coverage (+): 0. Max coverage (-): 1.13

Region: NODE\_304263\_length\_4982\_cov\_28.985548 218-228. Max. coverage (+): 0. Max coverage (-): 0

Region: NODE\_304263\_length\_4982\_cov\_28.985548 229-238. Max. coverage (+): 0.08. Max coverage (-): 0

Region: NODE\_304263\_length\_4982\_cov\_28.985548 239-248. Max. coverage (+): 0.04. Max coverage (-): 0

Region: NODE\_304263\_length\_4982\_cov\_28.985548 249-258. Max. coverage (+): 0. Max coverage (-): 0.08

Region: NODE\_304263\_length\_4982\_cov\_28.985548 259-268. Max. coverage (+): 0. Max coverage (-): 0.12

Region: NODE\_304263\_length\_4982\_cov\_28.985548 269-278. Max. coverage (+): 0. Max coverage (-): 0.12

Region: NODE\_304263\_length\_4982\_cov\_28.985548 279-288. Max. coverage (+): 0. Max coverage (-): 0.04

Region: NODE\_304263\_length\_4982\_cov\_28.985548 289-298. Max. coverage (+): 0. Max coverage (-): 0

Region: NODE\_304263\_length\_4982\_cov\_28.985548 299-308. Max. coverage (+): 0. Max coverage (-): 0.08

Region: NODE\_304263\_length\_4982\_cov\_28.985548 309-318. Max. coverage (+): 0. Max coverage (-): 0.12

Region: NODE\_304263\_length\_4982\_cov\_28.985548 319-328. Max. coverage (+): 0. Max coverage (-): 0.12

Region: NODE\_304263\_length\_4982\_cov\_28.985548 329-339. Max. coverage (+): 0. Max coverage (-): 0.04

Region: NODE\_304263\_length\_4982\_cov\_28.985548 340-349. Max. coverage (+): 0. Max coverage (-): 0

Region: NODE\_304263\_length\_4982\_cov\_28.985548 350-359. Max. coverage (+): 0. Max coverage (-): 0

Region: NODE\_304263\_length\_4982\_cov\_28.985548 360-369. Max. coverage (+): 0.04. Max coverage (-): 0.08

Region: NODE\_304263\_length\_4982\_cov\_28.985548 370-379. Max. coverage (+): 0.08. Max coverage (-): 0.2

Region: NODE\_304263\_length\_4982\_cov\_28.985548 380-389. Max. coverage (+): 0. Max coverage (-): 0.73

Region: NODE\_304263\_length\_4982\_cov\_28.985548 390-399. Max. coverage (+): 0. Max coverage (-): 0.2

Region: NODE\_304263\_length\_4982\_cov\_28.985548 400-409. Max. coverage (+): 0. Max coverage (-): 0.04

Region: NODE\_304263\_length\_4982\_cov\_28.985548 410-419. Max. coverage (+): 0. Max coverage (-): 0

Region: NODE\_304263\_length\_4982\_cov\_28.985548 420-429. Max. coverage (+): 0. Max coverage (-): 0.08

Region: NODE\_304263\_length\_4982\_cov\_28.985548 430-440. Max. coverage (+): 0. Max coverage (-): 0.08

Region: NODE\_304263\_length\_4982\_cov\_28.985548 441-450. Max. coverage (+): 0.08. Max coverage (-): 0

Region: NODE\_304263\_length\_4982\_cov\_28.985548 451-460. Max. coverage (+): 0.16. Max coverage (-): 0

Region: NODE\_304263\_length\_4982\_cov\_28.985548 461-470. Max. coverage (+): 0.08. Max coverage (-): 0

Region: NODE\_304263\_length\_4982\_cov\_28.985548 471-480. Max. coverage (+): 0.04. Max coverage (-): 0

Region: NODE\_304263\_length\_4982\_cov\_28.985548 481-490. Max. coverage (+): 0. Max coverage (-): 0.04

Region: NODE\_304263\_length\_4982\_cov\_28.985548 491-500. Max. coverage (+): 0. Max coverage (-): 0.04

Region: NODE\_304263\_length\_4982\_cov\_28.985548 501-510. Max. coverage (+): 0.04. Max coverage (-): 0.24

Region: NODE\_304263\_length\_4982\_cov\_28.985548 511-520. Max. coverage (+): 0. Max coverage (-): 0

Region: NODE\_304263\_length\_4982\_cov\_28.985548 521-530. Max. coverage (+): 0. Max coverage (-): 0.4

Region: NODE\_304263\_length\_4982\_cov\_28.985548 531-540. Max. coverage (+): 0.04. Max coverage (-): 0.16

Region: NODE\_304263\_length\_4982\_cov\_28.985548 541-551. Max. coverage (+): 0.08. Max coverage (-): 0.08

Region: NODE\_304263\_length\_4982\_cov\_28.985548 552-561. Max. coverage (+): 0. Max coverage (-): 0

Region: NODE\_304263\_length\_4982\_cov\_28.985548 562-571. Max. coverage (+): 0. Max coverage (-): 0.4

Region: NODE\_304263\_length\_4982\_cov\_28.985548 572-581. Max. coverage (+): 0. Max coverage (-): 0.08

Region: NODE\_304263\_length\_4982\_cov\_28.985548 582-591. Max. coverage (+): 0. Max coverage (-): 0.04

Region: NODE\_304263\_length\_4982\_cov\_28.985548 592-601. Max. coverage (+): 0. Max coverage (-): 0.12

Region: NODE\_304263\_length\_4982\_cov\_28.985548 602-611. Max. coverage (+): 0. Max coverage (-): 0

Region: NODE\_304263\_length\_4982\_cov\_28.985548 612-621. Max. coverage (+): 0.04. Max coverage (-): 0

Region: NODE\_304263\_length\_4982\_cov\_28.985548 622-631. Max. coverage (+): 0.04. Max coverage (-): 0.04

Region: NODE\_304263\_length\_4982\_cov\_28.985548 632-641. Max. coverage (+): 0.08. Max coverage (-): 0.12

Region: NODE\_304263\_length\_4982\_cov\_28.985548 642-651. Max. coverage (+): 0. Max coverage (-): 0.48

Region: NODE\_304263\_length\_4982\_cov\_28.985548 652-662. Max. coverage (+): 0.04. Max coverage (-): 0.36

Region: NODE\_304263\_length\_4982\_cov\_28.985548 663-672. Max. coverage (+): 0. Max coverage (-): 0

Region: NODE\_304263\_length\_4982\_cov\_28.985548 673-682. Max. coverage (+): 0.04. Max coverage (-): 0.2

Region: NODE\_304263\_length\_4982\_cov\_28.985548 683-692. Max. coverage (+): 0. Max coverage (-): 4.08

Region: NODE\_304263\_length\_4982\_cov\_28.985548 693-702. Max. coverage (+): 0. Max coverage (-): 4.4

Region: NODE\_304263\_length\_4982\_cov\_28.985548 703-712. Max. coverage (+): 0.08. Max coverage (-): 2.14

Region: NODE\_304263\_length\_4982\_cov\_28.985548 713-722. Max. coverage (+): 0.04. Max coverage (-): 0.04

Region: NODE\_304263\_length\_4982\_cov\_28.985548 723-732. Max. coverage (+): 0. Max coverage (-): 0

Region: NODE\_304263\_length\_4982\_cov\_28.985548 733-742. Max. coverage (+): 0.04. Max coverage (-): 0.16

Region: NODE\_304263\_length\_4982\_cov\_28.985548 743-752. Max. coverage (+): 0.08. Max coverage (-): 0.12

Region: NODE\_304263\_length\_4982\_cov\_28.985548 753-762. Max. coverage (+): 0.04. Max coverage (-): 0.77

Region: NODE\_304263\_length\_4982\_cov\_28.985548 763-773. Max. coverage (+): 0. Max coverage (-): 3.67

Region: NODE\_304263\_length\_4982\_cov\_28.985548 774-783. Max. coverage (+): 0.4. Max coverage (-): 0.24

Region: NODE\_304263\_length\_4982\_cov\_28.985548 784-793. Max. coverage (+): 0.4. Max coverage (-): 0

Region: NODE\_304263\_length\_4982\_cov\_28.985548 794-803. Max. coverage (+): 0. Max coverage (-): 0.73

Region: NODE\_304263\_length\_4982\_cov\_28.985548 804-813. Max. coverage (+): 0.04. Max coverage (-): 0.32

Region: NODE\_304263\_length\_4982\_cov\_28.985548 814-823. Max. coverage (+): 0.04. Max coverage (-): 0.12

Region: NODE\_304263\_length\_4982\_cov\_28.985548 824-833. Max. coverage (+): 0.04. Max coverage (-): 0.32

Region: NODE\_304263\_length\_4982\_cov\_28.985548 834-843. Max. coverage (+): 0.08. Max coverage (-): 0.32

Region: NODE\_304263\_length\_4982\_cov\_28.985548 844-853. Max. coverage (+): 0.2. Max coverage (-): 0.04

Region: NODE\_304263\_length\_4982\_cov\_28.985548 854-863. Max. coverage (+): 0.04. Max coverage (-): 0.04

Region: NODE\_304263\_length\_4982\_cov\_28.985548 864-873. Max. coverage (+): 0. Max coverage (-): 0.2

Region: NODE\_304263\_length\_4982\_cov\_28.985548 874-884. Max. coverage (+): 0. Max coverage (-): 0.16

Region: NODE\_304263\_length\_4982\_cov\_28.985548 885-894. Max. coverage (+): 0. Max coverage (-): 0.44

Region: NODE\_304263\_length\_4982\_cov\_28.985548 895-904. Max. coverage (+): 0. Max coverage (-): 0.08

Region: NODE\_304263\_length\_4982\_cov\_28.985548 905-914. Max. coverage (+): 0.04. Max coverage (-): 0.04

Region: NODE\_304263\_length\_4982\_cov\_28.985548 915-924. Max. coverage (+): 0.04. Max coverage (-): 0.04

Region: NODE\_304263\_length\_4982\_cov\_28.985548 925-934. Max. coverage (+): 0. Max coverage (-): 0

Region: NODE\_304263\_length\_4982\_cov\_28.985548 935-944. Max. coverage (+): 0.04. Max coverage (-): 1.17

Region: NODE\_304263\_length\_4982\_cov\_28.985548 945-954. Max. coverage (+): 0. Max coverage (-): 1.17

Region: NODE\_304263\_length\_4982\_cov\_28.985548 955-964. Max. coverage (+): 0.08. Max coverage (-): 0.52

Region: NODE\_304263\_length\_4982\_cov\_28.985548 965-974. Max. coverage (+): 0.16. Max coverage (-): 0.24

Region: NODE\_304263\_length\_4982\_cov\_28.985548 975-984. Max. coverage (+): 0. Max coverage (-): 0.2

Region: NODE\_304263\_length\_4982\_cov\_28.985548 985-995. Max. coverage (+): 0. Max coverage (-): 0.08

Region: NODE\_304263\_length\_4982\_cov\_28.985548 996-1005. Max. coverage (+): 0.04. Max coverage (-): 0.04

Region: NODE\_304263\_length\_4982\_cov\_28.985548 1006-1015. Max. coverage (+): 0. Max coverage (-): 0.04

Region: NODE\_304263\_length\_4982\_cov\_28.985548 1016-1025. Max. coverage (+): 0.12. Max coverage (-): 0.61

Region: NODE\_304263\_length\_4982\_cov\_28.985548 1026-1035. Max. coverage (+): 0.12. Max coverage (-): 0.65

Region: NODE\_304263\_length\_4982\_cov\_28.985548 1036-1045. Max. coverage (+): 0.16. Max coverage (-): 0.16

Region: NODE\_304263\_length\_4982\_cov\_28.985548 1046-1055. Max. coverage (+): 0.04. Max coverage (-): 0.24

Region: NODE\_304263\_length\_4982\_cov\_28.985548 1056-1065. Max. coverage (+): 0. Max coverage (-): 0.24

Region: NODE\_304263\_length\_4982\_cov\_28.985548 1066-1075. Max. coverage (+): 0. Max coverage (-): 0.04

Region: NODE\_304263\_length\_4982\_cov\_28.985548 1076-1085. Max. coverage (+): 0.04. Max coverage (-): 2.14

Region: NODE\_304263\_length\_4982\_cov\_28.985548 1086-1095. Max. coverage (+): 0. Max coverage (-): 0.16

Region: NODE\_304263\_length\_4982\_cov\_28.985548 1096-1106. Max. coverage (+): 0.04. Max coverage (-): 0

Region: NODE\_304263\_length\_4982\_cov\_28.985548 1107-1116. Max. coverage (+): 0.04. Max coverage (-): 0.04

Region: NODE\_304263\_length\_4982\_cov\_28.985548 1117-1126. Max. coverage (+): 0.04. Max coverage (-): 0.04

Region: NODE\_304263\_length\_4982\_cov\_28.985548 1127-1136. Max. coverage (+): 0. Max coverage (-): 0.04

Region: NODE\_304263\_length\_4982\_cov\_28.985548 1137-1146. Max. coverage (+): 0.08. Max coverage (-): 0.08

Region: NODE\_304263\_length\_4982\_cov\_28.985548 1147-1156. Max. coverage (+): 0. Max coverage (-): 0.08

Region: NODE\_304263\_length\_4982\_cov\_28.985548 1157-1166. Max. coverage (+): 0. Max coverage (-): 0.16

Region: NODE\_304263\_length\_4982\_cov\_28.985548 1167-1176. Max. coverage (+): 0. Max coverage (-): 0

Region: NODE\_304263\_length\_4982\_cov\_28.985548 1177-1186. Max. coverage (+): 0. Max coverage (-): 0

Region: NODE\_304263\_length\_4982\_cov\_28.985548 1187-1196. Max. coverage (+): 0. Max coverage (-): 0.04

Region: NODE\_304263\_length\_4982\_cov\_28.985548 1197-1206. Max. coverage (+): 0. Max coverage (-): 0.16

Region: NODE\_304263\_length\_4982\_cov\_28.985548 1207-1217. Max. coverage (+): 0.04. Max coverage (-): 0.04

Region: NODE\_304263\_length\_4982\_cov\_28.985548 1218-1227. Max. coverage (+): 0. Max coverage (-): 0.61

Region: NODE\_304263\_length\_4982\_cov\_28.985548 1228-1237. Max. coverage (+): 0. Max coverage (-): 0.65

Region: NODE\_304263\_length\_4982\_cov\_28.985548 1238-1247. Max. coverage (+): 0. Max coverage (-): 0.04

Region: NODE\_304263\_length\_4982\_cov\_28.985548 1248-1257. Max. coverage (+): 0. Max coverage (-): 0.04

Region: NODE\_304263\_length\_4982\_cov\_28.985548 1258-1267. Max. coverage (+): 0.04. Max coverage (-): 2.1

Region: NODE\_304263\_length\_4982\_cov\_28.985548 1268-1277. Max. coverage (+): 0. Max coverage (-): 2.14

Region: NODE\_304263\_length\_4982\_cov\_28.985548 1278-1287. Max. coverage (+): 0.2. Max coverage (-): 0.08

Region: NODE\_304263\_length\_4982\_cov\_28.985548 1288-1297. Max. coverage (+): 0.04. Max coverage (-): 0.04

Region: NODE\_304263\_length\_4982\_cov\_28.985548 1298-1307. Max. coverage (+): 0. Max coverage (-): 0.04

Region: NODE\_304263\_length\_4982\_cov\_28.985548 1308-1318. Max. coverage (+): 0.04. Max coverage (-): 0.16

Region: NODE\_304263\_length\_4982\_cov\_28.985548 1319-1328. Max. coverage (+): 0.04. Max coverage (-): 34.24

Region: NODE\_304263\_length\_4982\_cov\_28.985548 1329-1338. Max. coverage (+): 0. Max coverage (-): 5.21

Region: NODE\_304263\_length\_4982\_cov\_28.985548 1339-1348. Max. coverage (+): 1.09. Max coverage (-): 0.2

Region: NODE\_304263\_length\_4982\_cov\_28.985548 1349-1358. Max. coverage (+): 0.16. Max coverage (-): 0.2

Region: NODE\_304263\_length\_4982\_cov\_28.985548 1359-1368. Max. coverage (+): 0.04. Max coverage (-): 0.04

Region: NODE\_304263\_length\_4982\_cov\_28.985548 1369-1378. Max. coverage (+): 0.08. Max coverage (-): 0.24

Region: NODE\_304263\_length\_4982\_cov\_28.985548 1379-1388. Max. coverage (+): 0.04. Max coverage (-): 0.04

Region: NODE\_304263\_length\_4982\_cov\_28.985548 1389-1398. Max. coverage (+): 0.04. Max coverage (-): 0.16

Region: NODE\_304263\_length\_4982\_cov\_28.985548 1399-1408. Max. coverage (+): 0.12. Max coverage (-): 0.24

Region: NODE\_304263\_length\_4982\_cov\_28.985548 1409-1418. Max. coverage (+): 0. Max coverage (-): 0.28

Region: NODE\_304263\_length\_4982\_cov\_28.985548 1419-1429. Max. coverage (+): 0.04. Max coverage (-): 0.16

Region: NODE\_304263\_length\_4982\_cov\_28.985548 1430-1439. Max. coverage (+): 0. Max coverage (-): 0.2

Region: NODE\_304263\_length\_4982\_cov\_28.985548 1440-1449. Max. coverage (+): 0. Max coverage (-): 0.08

Region: NODE\_304263\_length\_4982\_cov\_28.985548 1450-1459. Max. coverage (+): 0.04. Max coverage (-): 0.12

Region: NODE\_304263\_length\_4982\_cov\_28.985548 1460-1469. Max. coverage (+): 0. Max coverage (-): 3.11

Region: NODE\_304263\_length\_4982\_cov\_28.985548 1470-1479. Max. coverage (+): 0. Max coverage (-): 1.41

Region: NODE\_304263\_length\_4982\_cov\_28.985548 1480-1489. Max. coverage (+): 0. Max coverage (-): 0.08

Region: NODE\_304263\_length\_4982\_cov\_28.985548 1490-1499. Max. coverage (+): 0. Max coverage (-): 0.08

Region: NODE\_304263\_length\_4982\_cov\_28.985548 1500-1509. Max. coverage (+): 0. Max coverage (-): 0.08

Region: NODE\_304263\_length\_4982\_cov\_28.985548 1510-1519. Max. coverage (+): 0. Max coverage (-): 1.9

Region: NODE\_304263\_length\_4982\_cov\_28.985548 1520-1529. Max. coverage (+): 0.04. Max coverage (-): 0.24

Region: NODE\_304263\_length\_4982\_cov\_28.985548 1530-1540. Max. coverage (+): 0.04. Max coverage (-): 0.04

Region: NODE\_304263\_length\_4982\_cov\_28.985548 1541-1550. Max. coverage (+): 0.04. Max coverage (-): 0.4

Region: NODE\_304263\_length\_4982\_cov\_28.985548 1551-1560. Max. coverage (+): 0.04. Max coverage (-): 0.16

Region: NODE\_304263\_length\_4982\_cov\_28.985548 1561-1570. Max. coverage (+): 0.04. Max coverage (-): 0

Region: NODE\_304263\_length\_4982\_cov\_28.985548 1571-1580. Max. coverage (+): 0. Max coverage (-): 2.18

Region: NODE\_304263\_length\_4982\_cov\_28.985548 1581-1590. Max. coverage (+): 0.36. Max coverage (-): 0.48

Region: NODE\_304263\_length\_4982\_cov\_28.985548 1591-1600. Max. coverage (+): 0.16. Max coverage (-): 0

Region: NODE\_304263\_length\_4982\_cov\_28.985548 1601-1610. Max. coverage (+): 0. Max coverage (-): 0.65

Region: NODE\_304263\_length\_4982\_cov\_28.985548 1611-1620. Max. coverage (+): 0. Max coverage (-): 1.9

Region: NODE\_304263\_length\_4982\_cov\_28.985548 1621-1630. Max. coverage (+): 0. Max coverage (-): 0.32

Region: NODE\_304263\_length\_4982\_cov\_28.985548 1631-1640. Max. coverage (+): 0.28. Max coverage (-): 0.4

Region: NODE\_304263\_length\_4982\_cov\_28.985548 1641-1651. Max. coverage (+): 0.04. Max coverage (-): 0.44

Region: NODE\_304263\_length\_4982\_cov\_28.985548 1652-1661. Max. coverage (+): 0. Max coverage (-): 2.02

Region: NODE\_304263\_length\_4982\_cov\_28.985548 1662-1671. Max. coverage (+): 0. Max coverage (-): 0.52

Region: NODE\_304263\_length\_4982\_cov\_28.985548 1672-1681. Max. coverage (+): 0. Max coverage (-): 0.44

Region: NODE\_304263\_length\_4982\_cov\_28.985548 1682-1691. Max. coverage (+): 0. Max coverage (-): 0

Region: NODE\_304263\_length\_4982\_cov\_28.985548 1692-1701. Max. coverage (+): 0.04. Max coverage (-): 0

Region: NODE\_304263\_length\_4982\_cov\_28.985548 1702-1711. Max. coverage (+): 0. Max coverage (-): 0.24

Region: NODE\_304263\_length\_4982\_cov\_28.985548 1712-1721. Max. coverage (+): 0. Max coverage (-): 1.45

Region: NODE\_304263\_length\_4982\_cov\_28.985548 1722-1731. Max. coverage (+): 0. Max coverage (-): 0.32

Region: NODE\_304263\_length\_4982\_cov\_28.985548 1732-1741. Max. coverage (+): 0.04. Max coverage (-): 0.28

Region: NODE\_304263\_length\_4982\_cov\_28.985548 1742-1751. Max. coverage (+): 0.04. Max coverage (-): 0.2

Region: NODE\_304263\_length\_4982\_cov\_28.985548 1752-1762. Max. coverage (+): 0.04. Max coverage (-): 0.16

Region: NODE\_304263\_length\_4982\_cov\_28.985548 1763-1772. Max. coverage (+): 0. Max coverage (-): 1.49

Region: NODE\_304263\_length\_4982\_cov\_28.985548 1773-1782. Max. coverage (+): 0.04. Max coverage (-): 0.16

Region: NODE\_304263\_length\_4982\_cov\_28.985548 1783-1792. Max. coverage (+): 0.12. Max coverage (-): 0.12

Region: NODE\_304263\_length\_4982\_cov\_28.985548 1793-1802. Max. coverage (+): 0. Max coverage (-): 1.78

Region: NODE\_304263\_length\_4982\_cov\_28.985548 1803-1812. Max. coverage (+): 0. Max coverage (-): 1.78

Region: NODE\_304263\_length\_4982\_cov\_28.985548 1813-1822. Max. coverage (+): 0.04. Max coverage (-): 1.49

Region: NODE\_304263\_length\_4982\_cov\_28.985548 1823-1832. Max. coverage (+): 0.4. Max coverage (-): 0

Region: NODE\_304263\_length\_4982\_cov\_28.985548 1833-1842. Max. coverage (+): 0.4. Max coverage (-): 1.33

Region: NODE\_304263\_length\_4982\_cov\_28.985548 1843-1852. Max. coverage (+): 0. Max coverage (-): 1.29

Region: NODE\_304263\_length\_4982\_cov\_28.985548 1853-1862. Max. coverage (+): 0.04. Max coverage (-): 0.08

Region: NODE\_304263\_length\_4982\_cov\_28.985548 1863-1873. Max. coverage (+): 0. Max coverage (-): 0

Region: NODE\_304263\_length\_4982\_cov\_28.985548 1874-1883. Max. coverage (+): 0. Max coverage (-): 0.12

Region: NODE\_304263\_length\_4982\_cov\_28.985548 1884-1893. Max. coverage (+): 0.04. Max coverage (-): 0.08

Region: NODE\_304263\_length\_4982\_cov\_28.985548 1894-1903. Max. coverage (+): 0.08. Max coverage (-): 0.16

Region: NODE\_304263\_length\_4982\_cov\_28.985548 1904-1913. Max. coverage (+): 1.37. Max coverage (-): 0

Region: NODE\_304263\_length\_4982\_cov\_28.985548 1914-1923. Max. coverage (+): 0. Max coverage (-): 0

Region: NODE\_304263\_length\_4982\_cov\_28.985548 1924-1933. Max. coverage (+): 0. Max coverage (-): 0.04

Region: NODE\_304263\_length\_4982\_cov\_28.985548 1934-1943. Max. coverage (+): 0. Max coverage (-): 0.08

Region: NODE\_304263\_length\_4982\_cov\_28.985548 1944-1953. Max. coverage (+): 0. Max coverage (-): 0.08

Region: NODE\_304263\_length\_4982\_cov\_28.985548 1954-1963. Max. coverage (+): 0. Max coverage (-): 0.2

Region: NODE\_304263\_length\_4982\_cov\_28.985548 1964-1973. Max. coverage (+): 0.04. Max coverage (-): 0.12

Region: NODE\_304263\_length\_4982\_cov\_28.985548 1974-1984. Max. coverage (+): 0. Max coverage (-): 0.52

Region: NODE\_304263\_length\_4982\_cov\_28.985548 1985-1994. Max. coverage (+): 0. Max coverage (-): 0.2

Region: NODE\_304263\_length\_4982\_cov\_28.985548 1995-2004. Max. coverage (+): 0. Max coverage (-): 0.4

Region: NODE\_304263\_length\_4982\_cov\_28.985548 2005-2014. Max. coverage (+): 0.04. Max coverage (-): 0.32

Region: NODE\_304263\_length\_4982\_cov\_28.985548 2015-2024. Max. coverage (+): 0. Max coverage (-): 0.12

Region: NODE\_304263\_length\_4982\_cov\_28.985548 2025-2034. Max. coverage (+): 0. Max coverage (-): 0.4

Region: NODE\_304263\_length\_4982\_cov\_28.985548 2035-2044. Max. coverage (+): 0.12. Max coverage (-): 0.52

Region: NODE\_304263\_length\_4982\_cov\_28.985548 2045-2054. Max. coverage (+): 0.12. Max coverage (-): 0

Region: NODE\_304263\_length\_4982\_cov\_28.985548 2055-2064. Max. coverage (+): 0.16. Max coverage (-): 1.53

Region: NODE\_304263\_length\_4982\_cov\_28.985548 2065-2074. Max. coverage (+): 0. Max coverage (-): 0.28

Region: NODE\_304263\_length\_4982\_cov\_28.985548 2075-2084. Max. coverage (+): 0.04. Max coverage (-): 0.48

Region: NODE\_304263\_length\_4982\_cov\_28.985548 2085-2095. Max. coverage (+): 0.04. Max coverage (-): 0

Region: NODE\_304263\_length\_4982\_cov\_28.985548 2096-2105. Max. coverage (+): 0. Max coverage (-): 0.12

Region: NODE\_304263\_length\_4982\_cov\_28.985548 2106-2115. Max. coverage (+): 0. Max coverage (-): 0.61

Region: NODE\_304263\_length\_4982\_cov\_28.985548 2116-2125. Max. coverage (+): 0. Max coverage (-): 0.57

Region: NODE\_304263\_length\_4982\_cov\_28.985548 2126-2135. Max. coverage (+): 0. Max coverage (-): 0.04

Region: NODE\_304263\_length\_4982\_cov\_28.985548 2136-2145. Max. coverage (+): 0. Max coverage (-): 0

Region: NODE\_304263\_length\_4982\_cov\_28.985548 2146-2155. Max. coverage (+): 0. Max coverage (-): 0.04

Region: NODE\_304263\_length\_4982\_cov\_28.985548 2156-2165. Max. coverage (+): 0. Max coverage (-): 0.04

Region: NODE\_304263\_length\_4982\_cov\_28.985548 2166-2175. Max. coverage (+): 0. Max coverage (-): 0.36

Region: NODE\_304263\_length\_4982\_cov\_28.985548 2176-2185. Max. coverage (+): 0. Max coverage (-): 0.44

Region: NODE\_304263\_length\_4982\_cov\_28.985548 2186-2196. Max. coverage (+): 0.04. Max coverage (-): 2.14

Region: NODE\_304263\_length\_4982\_cov\_28.985548 2197-2206. Max. coverage (+): 0.16. Max coverage (-): 0.89

Region: NODE\_304263\_length\_4982\_cov\_28.985548 2207-2216. Max. coverage (+): 0.04. Max coverage (-): 0.48

Region: NODE\_304263\_length\_4982\_cov\_28.985548 2217-2226. Max. coverage (+): 0.08. Max coverage (-): 0.08

Region: NODE\_304263\_length\_4982\_cov\_28.985548 2227-2236. Max. coverage (+): 0. Max coverage (-): 0.52

Region: NODE\_304263\_length\_4982\_cov\_28.985548 2237-2246. Max. coverage (+): 0. Max coverage (-): 0.69

Region: NODE\_304263\_length\_4982\_cov\_28.985548 2247-2256. Max. coverage (+): 0.08. Max coverage (-): 0

Region: NODE\_304263\_length\_4982\_cov\_28.985548 2257-2266. Max. coverage (+): 0.08. Max coverage (-): 0

Region: NODE\_304263\_length\_4982\_cov\_28.985548 2267-2276. Max. coverage (+): 0. Max coverage (-): 0.12

Region: NODE\_304263\_length\_4982\_cov\_28.985548 2277-2286. Max. coverage (+): 0. Max coverage (-): 0.08

Region: NODE\_304263\_length\_4982\_cov\_28.985548 2287-2296. Max. coverage (+): 0.04. Max coverage (-): 0.48

Region: NODE\_304263\_length\_4982\_cov\_28.985548 2297-2307. Max. coverage (+): 0.16. Max coverage (-): 1.05

Region: NODE\_304263\_length\_4982\_cov\_28.985548 2308-2317. Max. coverage (+): 0.32. Max coverage (-): 2.58

Region: NODE\_304263\_length\_4982\_cov\_28.985548 2318-2327. Max. coverage (+): 0.16. Max coverage (-): 0.4

Region: NODE\_304263\_length\_4982\_cov\_28.985548 2328-2337. Max. coverage (+): 0.16. Max coverage (-): 0.12

Region: NODE\_304263\_length\_4982\_cov\_28.985548 2338-2347. Max. coverage (+): 0. Max coverage (-): 0.24

Region: NODE\_304263\_length\_4982\_cov\_28.985548 2348-2357. Max. coverage (+): 0.04. Max coverage (-): 1.01

Region: NODE\_304263\_length\_4982\_cov\_28.985548 2358-2367. Max. coverage (+): 0.04. Max coverage (-): 1.78

Region: NODE\_304263\_length\_4982\_cov\_28.985548 2368-2377. Max. coverage (+): 0.08. Max coverage (-): 1.82

Region: NODE\_304263\_length\_4982\_cov\_28.985548 2378-2387. Max. coverage (+): 0.04. Max coverage (-): 0.04

Region: NODE\_304263\_length\_4982\_cov\_28.985548 2388-2397. Max. coverage (+): 0.04. Max coverage (-): 0.89

Region: NODE\_304263\_length\_4982\_cov\_28.985548 2398-2407. Max. coverage (+): 0. Max coverage (-): 0.2

Region: NODE\_304263\_length\_4982\_cov\_28.985548 2408-2418. Max. coverage (+): 0.24. Max coverage (-): 0.24

Region: NODE\_304263\_length\_4982\_cov\_28.985548 2419-2428. Max. coverage (+): 0.24. Max coverage (-): 0.24

Region: NODE\_304263\_length\_4982\_cov\_28.985548 2429-2438. Max. coverage (+): 0. Max coverage (-): 0.28

Region: NODE\_304263\_length\_4982\_cov\_28.985548 2439-2448. Max. coverage (+): 0.24. Max coverage (-): 0.2

Region: NODE\_304263\_length\_4982\_cov\_28.985548 2449-2458. Max. coverage (+): 0.16. Max coverage (-): 0.16

Region: NODE\_304263\_length\_4982\_cov\_28.985548 2459-2468. Max. coverage (+): 0. Max coverage (-): 0.48

Region: NODE\_304263\_length\_4982\_cov\_28.985548 2469-2478. Max. coverage (+): 0.04. Max coverage (-): 0.12

Region: NODE\_304263\_length\_4982\_cov\_28.985548 2479-2488. Max. coverage (+): 0.16. Max coverage (-): 0.04

Region: NODE\_304263\_length\_4982\_cov\_28.985548 2489-2498. Max. coverage (+): 0.16. Max coverage (-): 0.16

Region: NODE\_304263\_length\_4982\_cov\_28.985548 2499-2508. Max. coverage (+): 0. Max coverage (-): 0.04

Region: NODE\_304263\_length\_4982\_cov\_28.985548 2509-2518. Max. coverage (+): 0. Max coverage (-): 0.04

Region: NODE\_304263\_length\_4982\_cov\_28.985548 2519-2529. Max. coverage (+): 0. Max coverage (-): 0.12

Region: NODE\_304263\_length\_4982\_cov\_28.985548 2530-2539. Max. coverage (+): 0. Max coverage (-): 0.08

Region: NODE\_304263\_length\_4982\_cov\_28.985548 2540-2549. Max. coverage (+): 0. Max coverage (-): 0.04

Region: NODE\_304263\_length\_4982\_cov\_28.985548 2550-2559. Max. coverage (+): 0.04. Max coverage (-): 2.42

Region: NODE\_304263\_length\_4982\_cov\_28.985548 2560-2569. Max. coverage (+): 0.04. Max coverage (-): 2.1

Region: NODE\_304263\_length\_4982\_cov\_28.985548 2570-2579. Max. coverage (+): 0. Max coverage (-): 1.37

Region: NODE\_304263\_length\_4982\_cov\_28.985548 2580-2589. Max. coverage (+): 0.04. Max coverage (-): 4.32

Region: NODE\_304263\_length\_4982\_cov\_28.985548 2590-2599. Max. coverage (+): 0.12. Max coverage (-): 6.02

Region: NODE\_304263\_length\_4982\_cov\_28.985548 2600-2609. Max. coverage (+): 0. Max coverage (-): 3.71

Region: NODE\_304263\_length\_4982\_cov\_28.985548 2610-2619. Max. coverage (+): 0.04. Max coverage (-): 3.23

Region: NODE\_304263\_length\_4982\_cov\_28.985548 2620-2629. Max. coverage (+): 0. Max coverage (-): 3.27

Region: NODE\_304263\_length\_4982\_cov\_28.985548 2630-2640. Max. coverage (+): 0.12. Max coverage (-): 0.69

Region: NODE\_304263\_length\_4982\_cov\_28.985548 2641-2650. Max. coverage (+): 0.16. Max coverage (-): 1.29

Region: NODE\_304263\_length\_4982\_cov\_28.985548 2651-2660. Max. coverage (+): 0. Max coverage (-): 1.29

Region: NODE\_304263\_length\_4982\_cov\_28.985548 2661-2670. Max. coverage (+): 0. Max coverage (-): 0.04

Region: NODE\_304263\_length\_4982\_cov\_28.985548 2671-2680. Max. coverage (+): 0. Max coverage (-): 0

Region: NODE\_304263\_length\_4982\_cov\_28.985548 2681-2690. Max. coverage (+): 0. Max coverage (-): 0.04

Region: NODE\_304263\_length\_4982\_cov\_28.985548 2691-2700. Max. coverage (+): 0. Max coverage (-): 0.04

Region: NODE\_304263\_length\_4982\_cov\_28.985548 2701-2710. Max. coverage (+): 0. Max coverage (-): 1.74

Region: NODE\_304263\_length\_4982\_cov\_28.985548 2711-2720. Max. coverage (+): 0. Max coverage (-): 0.81

Region: NODE\_304263\_length\_4982\_cov\_28.985548 2721-2730. Max. coverage (+): 0. Max coverage (-): 0.44

Region: NODE\_304263\_length\_4982\_cov\_28.985548 2731-2740. Max. coverage (+): 0.2. Max coverage (-): 0.44

Region: NODE\_304263\_length\_4982\_cov\_28.985548 2741-2751. Max. coverage (+): 0.12. Max coverage (-): 0.16

Region: NODE\_304263\_length\_4982\_cov\_28.985548 2752-2761. Max. coverage (+): 0.04. Max coverage (-): 0.24

Region: NODE\_304263\_length\_4982\_cov\_28.985548 2762-2771. Max. coverage (+): 0.04. Max coverage (-): 1.21

Region: NODE\_304263\_length\_4982\_cov\_28.985548 2772-2781. Max. coverage (+): 0. Max coverage (-): 0.04

Region: NODE\_304263\_length\_4982\_cov\_28.985548 2782-2791. Max. coverage (+): 0.12. Max coverage (-): 0.2

Region: NODE\_304263\_length\_4982\_cov\_28.985548 2792-2801. Max. coverage (+): 0. Max coverage (-): 0.16

Region: NODE\_304263\_length\_4982\_cov\_28.985548 2802-2811. Max. coverage (+): 0.04. Max coverage (-): 0.08

Region: NODE\_304263\_length\_4982\_cov\_28.985548 2812-2821. Max. coverage (+): 0. Max coverage (-): 0.04

Region: NODE\_304263\_length\_4982\_cov\_28.985548 2822-2831. Max. coverage (+): 0. Max coverage (-): 0.28

Region: NODE\_304263\_length\_4982\_cov\_28.985548 2832-2841. Max. coverage (+): 0.08. Max coverage (-): 0.32

Region: NODE\_304263\_length\_4982\_cov\_28.985548 2842-2851. Max. coverage (+): 0.04. Max coverage (-): 0.24

Region: NODE\_304263\_length\_4982\_cov\_28.985548 2852-2862. Max. coverage (+): 0.2. Max coverage (-): 3.76

Region: NODE\_304263\_length\_4982\_cov\_28.985548 2863-2872. Max. coverage (+): 0.04. Max coverage (-): 0.2

Region: NODE\_304263\_length\_4982\_cov\_28.985548 2873-2882. Max. coverage (+): 0.08. Max coverage (-): 0.16

Region: NODE\_304263\_length\_4982\_cov\_28.985548 2883-2892. Max. coverage (+): 0.2. Max coverage (-): 0.4

Region: NODE\_304263\_length\_4982\_cov\_28.985548 2893-2902. Max. coverage (+): 0. Max coverage (-): 0.24

Region: NODE\_304263\_length\_4982\_cov\_28.985548 2903-2912. Max. coverage (+): 0.08. Max coverage (-): 0.16

Region: NODE\_304263\_length\_4982\_cov\_28.985548 2913-2922. Max. coverage (+): 0. Max coverage (-): 0

Region: NODE\_304263\_length\_4982\_cov\_28.985548 2923-2932. Max. coverage (+): 0. Max coverage (-): 0.12

Region: NODE\_304263\_length\_4982\_cov\_28.985548 2933-2942. Max. coverage (+): 0. Max coverage (-): 1.78

Region: NODE\_304263\_length\_4982\_cov\_28.985548 2943-2952. Max. coverage (+): 0.2. Max coverage (-): 0

Region: NODE\_304263\_length\_4982\_cov\_28.985548 2953-2963. Max. coverage (+): 0.28. Max coverage (-): 0

Region: NODE\_304263\_length\_4982\_cov\_28.985548 2964-2973. Max. coverage (+): 0. Max coverage (-): 0

Region: NODE\_304263\_length\_4982\_cov\_28.985548 2974-2983. Max. coverage (+): 0. Max coverage (-): 0.04

Region: NODE\_304263\_length\_4982\_cov\_28.985548 2984-2993. Max. coverage (+): 0. Max coverage (-): 0

Region: NODE\_304263\_length\_4982\_cov\_28.985548 2994-3003. Max. coverage (+): 0.12. Max coverage (-): 0.44

Region: NODE\_304263\_length\_4982\_cov\_28.985548 3004-3013. Max. coverage (+): 0.08. Max coverage (-): 2.3

Region: NODE\_304263\_length\_4982\_cov\_28.985548 3014-3023. Max. coverage (+): 0.08. Max coverage (-): 0.12

Region: NODE\_304263\_length\_4982\_cov\_28.985548 3024-3033. Max. coverage (+): 0.08. Max coverage (-): 1.09

Region: NODE\_304263\_length\_4982\_cov\_28.985548 3034-3043. Max. coverage (+): 0. Max coverage (-): 3.92

Region: NODE\_304263\_length\_4982\_cov\_28.985548 3044-3053. Max. coverage (+): 0.2. Max coverage (-): 0.04

Region: NODE\_304263\_length\_4982\_cov\_28.985548 3054-3063. Max. coverage (+): 0.28. Max coverage (-): 0.32

Region: NODE\_304263\_length\_4982\_cov\_28.985548 3064-3074. Max. coverage (+): 0. Max coverage (-): 0.65

Region: NODE\_304263\_length\_4982\_cov\_28.985548 3075-3084. Max. coverage (+): 0. Max coverage (-): 0.52

Region: NODE\_304263\_length\_4982\_cov\_28.985548 3085-3094. Max. coverage (+): 0.12. Max coverage (-): 2.91

Region: NODE\_304263\_length\_4982\_cov\_28.985548 3095-3104. Max. coverage (+): 0.36. Max coverage (-): 0

Region: NODE\_304263\_length\_4982\_cov\_28.985548 3105-3114. Max. coverage (+): 0.04. Max coverage (-): 0.65

Region: NODE\_304263\_length\_4982\_cov\_28.985548 3115-3124. Max. coverage (+): 0.04. Max coverage (-): 0.36

Region: NODE\_304263\_length\_4982\_cov\_28.985548 3125-3134. Max. coverage (+): 0.12. Max coverage (-): 0.12

Region: NODE\_304263\_length\_4982\_cov\_28.985548 3135-3144. Max. coverage (+): 0. Max coverage (-): 0

Region: NODE\_304263\_length\_4982\_cov\_28.985548 3145-3154. Max. coverage (+): 0. Max coverage (-): 0.36

Region: NODE\_304263\_length\_4982\_cov\_28.985548 3155-3164. Max. coverage (+): 0. Max coverage (-): 0

Region: NODE\_304263\_length\_4982\_cov\_28.985548 3165-3174. Max. coverage (+): 0. Max coverage (-): 4.48

Region: NODE\_304263\_length\_4982\_cov\_28.985548 3175-3185. Max. coverage (+): 0. Max coverage (-): 89.03

Region: NODE\_304263\_length\_4982\_cov\_28.985548 3186-3195. Max. coverage (+): 0.12. Max coverage (-): 6.26

Region: NODE\_304263\_length\_4982\_cov\_28.985548 3196-3205. Max. coverage (+): 0.12. Max coverage (-): 1.41

Region: NODE\_304263\_length\_4982\_cov\_28.985548 3206-3215. Max. coverage (+): 0.04. Max coverage (-): 0.77

Region: NODE\_304263\_length\_4982\_cov\_28.985548 3216-3225. Max. coverage (+): 0.04. Max coverage (-): 5.65

Region: NODE\_304263\_length\_4982\_cov\_28.985548 3226-3235. Max. coverage (+): 0. Max coverage (-): 6.34

Region: NODE\_304263\_length\_4982\_cov\_28.985548 3236-3245. Max. coverage (+): 0.12. Max coverage (-): 0

Region: NODE\_304263\_length\_4982\_cov\_28.985548 3246-3255. Max. coverage (+): 0.04. Max coverage (-): 0.08

Region: NODE\_304263\_length\_4982\_cov\_28.985548 3256-3265. Max. coverage (+): 0.04. Max coverage (-): 1.17

Region: NODE\_304263\_length\_4982\_cov\_28.985548 3266-3275. Max. coverage (+): 0.2. Max coverage (-): 3.23

Region: NODE\_304263\_length\_4982\_cov\_28.985548 3276-3285. Max. coverage (+): 0.04. Max coverage (-): 0.32

Region: NODE\_304263\_length\_4982\_cov\_28.985548 3286-3296. Max. coverage (+): 0.24. Max coverage (-): 0.44

Region: NODE\_304263\_length\_4982\_cov\_28.985548 3297-3306. Max. coverage (+): 0.04. Max coverage (-): 16.23

Region: NODE\_304263\_length\_4982\_cov\_28.985548 3307-3316. Max. coverage (+): 0. Max coverage (-): 1.82

Region: NODE\_304263\_length\_4982\_cov\_28.985548 3317-3326. Max. coverage (+): 0.4. Max coverage (-): 1.74

Region: NODE\_304263\_length\_4982\_cov\_28.985548 3327-3336. Max. coverage (+): 0. Max coverage (-): 0

Region: NODE\_304263\_length\_4982\_cov\_28.985548 3337-3346. Max. coverage (+): 0. Max coverage (-): 0.16

Region: NODE\_304263\_length\_4982\_cov\_28.985548 3347-3356. Max. coverage (+): 0.04. Max coverage (-): 2.75

Region: NODE\_304263\_length\_4982\_cov\_28.985548 3357-3366. Max. coverage (+): 0. Max coverage (-): 1.86

Region: NODE\_304263\_length\_4982\_cov\_28.985548 3367-3376. Max. coverage (+): 0.08. Max coverage (-): 0.24

Region: NODE\_304263\_length\_4982\_cov\_28.985548 3377-3386. Max. coverage (+): 0.08. Max coverage (-): 0.28

Region: NODE\_304263\_length\_4982\_cov\_28.985548 3387-3396. Max. coverage (+): 0. Max coverage (-): 0.16

Region: NODE\_304263\_length\_4982\_cov\_28.985548 3397-3407. Max. coverage (+): 0. Max coverage (-): 0.52

Region: NODE\_304263\_length\_4982\_cov\_28.985548 3408-3417. Max. coverage (+): 0.08. Max coverage (-): 4.08

Region: NODE\_304263\_length\_4982\_cov\_28.985548 3418-3427. Max. coverage (+): 2.06. Max coverage (-): 0.32

Region: NODE\_304263\_length\_4982\_cov\_28.985548 3428-3437. Max. coverage (+): 1.17. Max coverage (-): 0.28

Region: NODE\_304263\_length\_4982\_cov\_28.985548 3438-3447. Max. coverage (+): 0. Max coverage (-): 0.36

Region: NODE\_304263\_length\_4982\_cov\_28.985548 3448-3457. Max. coverage (+): 0. Max coverage (-): 0.48

Region: NODE\_304263\_length\_4982\_cov\_28.985548 3458-3467. Max. coverage (+): 0.08. Max coverage (-): 30.41

Region: NODE\_304263\_length\_4982\_cov\_28.985548 3468-3477. Max. coverage (+): 0. Max coverage (-): 0.24

Region: NODE\_304263\_length\_4982\_cov\_28.985548 3478-3487. Max. coverage (+): 0.16. Max coverage (-): 0.44

Region: NODE\_304263\_length\_4982\_cov\_28.985548 3488-3497. Max. coverage (+): 0.04. Max coverage (-): 1.45

Region: NODE\_304263\_length\_4982\_cov\_28.985548 3498-3507. Max. coverage (+): 0. Max coverage (-): 0.97

Region: NODE\_304263\_length\_4982\_cov\_28.985548 3508-3518. Max. coverage (+): 1.29. Max coverage (-): 0.73

Region: NODE\_304263\_length\_4982\_cov\_28.985548 3519-3528. Max. coverage (+): 0. Max coverage (-): 0.36

Region: NODE\_304263\_length\_4982\_cov\_28.985548 3529-3538. Max. coverage (+): 0. Max coverage (-): 34.97

Region: NODE\_304263\_length\_4982\_cov\_28.985548 3539-3548. Max. coverage (+): 0. Max coverage (-): 35.78

Region: NODE\_304263\_length\_4982\_cov\_28.985548 3549-3558. Max. coverage (+): 0.52. Max coverage (-): 0.16

Region: NODE\_304263\_length\_4982\_cov\_28.985548 3559-3568. Max. coverage (+): 0.52. Max coverage (-): 0.08

Region: NODE\_304263\_length\_4982\_cov\_28.985548 3569-3578. Max. coverage (+): 0. Max coverage (-): 0.61

Region: NODE\_304263\_length\_4982\_cov\_28.985548 3579-3588. Max. coverage (+): 0. Max coverage (-): 1.13

Region: NODE\_304263\_length\_4982\_cov\_28.985548 3589-3598. Max. coverage (+): 0.61. Max coverage (-): 0.04

Region: NODE\_304263\_length\_4982\_cov\_28.985548 3599-3608. Max. coverage (+): 0.61. Max coverage (-): 0

Region: NODE\_304263\_length\_4982\_cov\_28.985548 3609-3618. Max. coverage (+): 0.04. Max coverage (-): 0.12

Region: NODE\_304263\_length\_4982\_cov\_28.985548 3619-3629. Max. coverage (+): 0. Max coverage (-): 0.12

Region: NODE\_304263\_length\_4982\_cov\_28.985548 3630-3639. Max. coverage (+): 0.12. Max coverage (-): 2.1

Region: NODE\_304263\_length\_4982\_cov\_28.985548 3640-3649. Max. coverage (+): 0.16. Max coverage (-): 2.26

Region: NODE\_304263\_length\_4982\_cov\_28.985548 3650-3659. Max. coverage (+): 0.12. Max coverage (-): 0.36

Region: NODE\_304263\_length\_4982\_cov\_28.985548 3660-3669. Max. coverage (+): 0. Max coverage (-): 0.44

Region: NODE\_304263\_length\_4982\_cov\_28.985548 3670-3679. Max. coverage (+): 0. Max coverage (-): 0.12

Region: NODE\_304263\_length\_4982\_cov\_28.985548 3680-3689. Max. coverage (+): 0. Max coverage (-): 0.32

Region: NODE\_304263\_length\_4982\_cov\_28.985548 3690-3699. Max. coverage (+): 0. Max coverage (-): 11.95

Region: NODE\_304263\_length\_4982\_cov\_28.985548 3700-3709. Max. coverage (+): 0. Max coverage (-): 2.83

Region: NODE\_304263\_length\_4982\_cov\_28.985548 3710-3719. Max. coverage (+): 0.73. Max coverage (-): 1.7

Region: NODE\_304263\_length\_4982\_cov\_28.985548 3720-3729. Max. coverage (+): 0. Max coverage (-): 0.2

Region: NODE\_304263\_length\_4982\_cov\_28.985548 3730-3740. Max. coverage (+): 0.04. Max coverage (-): 2.83

Region: NODE\_304263\_length\_4982\_cov\_28.985548 3741-3750. Max. coverage (+): 0. Max coverage (-): 5.57

Region: NODE\_304263\_length\_4982\_cov\_28.985548 3751-3760. Max. coverage (+): 0. Max coverage (-): 1.49

Region: NODE\_304263\_length\_4982\_cov\_28.985548 3761-3770. Max. coverage (+): 0.4. Max coverage (-): 0.24

Region: NODE\_304263\_length\_4982\_cov\_28.985548 3771-3780. Max. coverage (+): 0.04. Max coverage (-): 1.82

Region: NODE\_304263\_length\_4982\_cov\_28.985548 3781-3790. Max. coverage (+): 0. Max coverage (-): 4.36

Region: NODE\_304263\_length\_4982\_cov\_28.985548 3791-3800. Max. coverage (+): 0. Max coverage (-): 0.28

Region: NODE\_304263\_length\_4982\_cov\_28.985548 3801-3810. Max. coverage (+): 0. Max coverage (-): 0.4

Region: NODE\_304263\_length\_4982\_cov\_28.985548 3811-3820. Max. coverage (+): 0. Max coverage (-): 1.09

Region: NODE\_304263\_length\_4982\_cov\_28.985548 3821-3830. Max. coverage (+): 0. Max coverage (-): 0.16

Region: NODE\_304263\_length\_4982\_cov\_28.985548 3831-3841. Max. coverage (+): 0. Max coverage (-): 3.8

Region: NODE\_304263\_length\_4982\_cov\_28.985548 3842-3851. Max. coverage (+): 0.81. Max coverage (-): 1.09

Region: NODE\_304263\_length\_4982\_cov\_28.985548 3852-3861. Max. coverage (+): 0.81. Max coverage (-): 8.28

Region: NODE\_304263\_length\_4982\_cov\_28.985548 3862-3871. Max. coverage (+): 0.04. Max coverage (-): 3.19

Region: NODE\_304263\_length\_4982\_cov\_28.985548 3872-3881. Max. coverage (+): 0.08. Max coverage (-): 0.12

Region: NODE\_304263\_length\_4982\_cov\_28.985548 3882-3891. Max. coverage (+): 0.08. Max coverage (-): 2.58

Region: NODE\_304263\_length\_4982\_cov\_28.985548 3892-3901. Max. coverage (+): 0.08. Max coverage (-): 2.46

Region: NODE\_304263\_length\_4982\_cov\_28.985548 3902-3911. Max. coverage (+): 0.77. Max coverage (-): 0.24

Region: NODE\_304263\_length\_4982\_cov\_28.985548 3912-3921. Max. coverage (+): 0.08. Max coverage (-): 0.2

Region: NODE\_304263\_length\_4982\_cov\_28.985548 3922-3931. Max. coverage (+): 0. Max coverage (-): 1.82

Region: NODE\_304263\_length\_4982\_cov\_28.985548 3932-3941. Max. coverage (+): 0. Max coverage (-): 0.4

Region: NODE\_304263\_length\_4982\_cov\_28.985548 3942-3952. Max. coverage (+): 0.2. Max coverage (-): 2.71

Region: NODE\_304263\_length\_4982\_cov\_28.985548 3953-3962. Max. coverage (+): 0.04. Max coverage (-): 1.05

Region: NODE\_304263\_length\_4982\_cov\_28.985548 3963-3972. Max. coverage (+): 0.08. Max coverage (-): 0.2

Region: NODE\_304263\_length\_4982\_cov\_28.985548 3973-3982. Max. coverage (+): 0.04. Max coverage (-): 0.4

Region: NODE\_304263\_length\_4982\_cov\_28.985548 3983-3992. Max. coverage (+): 0. Max coverage (-): 0.4

Region: NODE\_304263\_length\_4982\_cov\_28.985548 3993-4002. Max. coverage (+): 0. Max coverage (-): 0.16

Region: NODE\_304263\_length\_4982\_cov\_28.985548 4003-4012. Max. coverage (+): 0.04. Max coverage (-): 3.55

Region: NODE\_304263\_length\_4982\_cov\_28.985548 4013-4022. Max. coverage (+): 0.08. Max coverage (-): 0.04

Region: NODE\_304263\_length\_4982\_cov\_28.985548 4023-4032. Max. coverage (+): 0.85. Max coverage (-): 0.12

Region: NODE\_304263\_length\_4982\_cov\_28.985548 4033-4042. Max. coverage (+): 0. Max coverage (-): 7.11

Region: NODE\_304263\_length\_4982\_cov\_28.985548 4043-4052. Max. coverage (+): 0.12. Max coverage (-): 0.32

Region: NODE\_304263\_length\_4982\_cov\_28.985548 4053-4063. Max. coverage (+): 0.2. Max coverage (-): 1.94

Region: NODE\_304263\_length\_4982\_cov\_28.985548 4064-4073. Max. coverage (+): 0.08. Max coverage (-): 2.22

Region: NODE\_304263\_length\_4982\_cov\_28.985548 4074-4083. Max. coverage (+): 0.08. Max coverage (-): 0.08

Region: NODE\_304263\_length\_4982\_cov\_28.985548 4084-4093. Max. coverage (+): 0.08. Max coverage (-): 0.08

Region: NODE\_304263\_length\_4982\_cov\_28.985548 4094-4103. Max. coverage (+): 0. Max coverage (-): 0.44

Region: NODE\_304263\_length\_4982\_cov\_28.985548 4104-4113. Max. coverage (+): 0. Max coverage (-): 2.18

Region: NODE\_304263\_length\_4982\_cov\_28.985548 4114-4123. Max. coverage (+): 0.04. Max coverage (-): 1.25

Region: NODE\_304263\_length\_4982\_cov\_28.985548 4124-4133. Max. coverage (+): 0.36. Max coverage (-): 0.16

Region: NODE\_304263\_length\_4982\_cov\_28.985548 4134-4143. Max. coverage (+): 0.08. Max coverage (-): 0.24

Region: NODE\_304263\_length\_4982\_cov\_28.985548 4144-4153. Max. coverage (+): 0. Max coverage (-): 1.21

Region: NODE\_304263\_length\_4982\_cov\_28.985548 4154-4163. Max. coverage (+): 0. Max coverage (-): 1.82

Region: NODE\_304263\_length\_4982\_cov\_28.985548 4164-4174. Max. coverage (+): 0.08. Max coverage (-): 1.94

Region: NODE\_304263\_length\_4982\_cov\_28.985548 4175-4184. Max. coverage (+): 0.16. Max coverage (-): 1.17

Region: NODE\_304263\_length\_4982\_cov\_28.985548 4185-4194. Max. coverage (+): 0. Max coverage (-): 9.09

Region: NODE\_304263\_length\_4982\_cov\_28.985548 4195-4204. Max. coverage (+): 0. Max coverage (-): 1.37

Region: NODE\_304263\_length\_4982\_cov\_28.985548 4205-4214. Max. coverage (+): 3.84. Max coverage (-): 0.12

Region: NODE\_304263\_length\_4982\_cov\_28.985548 4215-4224. Max. coverage (+): 0.16. Max coverage (-): 0.16

Region: NODE\_304263\_length\_4982\_cov\_28.985548 4225-4234. Max. coverage (+): 0. Max coverage (-): 1.45

Region: NODE\_304263\_length\_4982\_cov\_28.985548 4235-4244. Max. coverage (+): 0.04. Max coverage (-): 0.08

Region: NODE\_304263\_length\_4982\_cov\_28.985548 4245-4254. Max. coverage (+): 0. Max coverage (-): 3.11

Region: NODE\_304263\_length\_4982\_cov\_28.985548 4255-4264. Max. coverage (+): 0. Max coverage (-): 5.05

Region: NODE\_304263\_length\_4982\_cov\_28.985548 4265-4274. Max. coverage (+): 0. Max coverage (-): 0.08

Region: NODE\_304263\_length\_4982\_cov\_28.985548 4275-4285. Max. coverage (+): 0. Max coverage (-): 0.57

Region: NODE\_304263\_length\_4982\_cov\_28.985548 4286-4295. Max. coverage (+): 0.57. Max coverage (-): 0.48

Region: NODE\_304263\_length\_4982\_cov\_28.985548 4296-4305. Max. coverage (+): 1.57. Max coverage (-): 0.24

Region: NODE\_304263\_length\_4982\_cov\_28.985548 4306-4315. Max. coverage (+): 0. Max coverage (-): 3.31

Region: NODE\_304263\_length\_4982\_cov\_28.985548 4316-4325. Max. coverage (+): 0.04. Max coverage (-): 2.58

Region: NODE\_304263\_length\_4982\_cov\_28.985548 4326-4335. Max. coverage (+): 0.36. Max coverage (-): 0

Region: NODE\_304263\_length\_4982\_cov\_28.985548 4336-4345. Max. coverage (+): 0.08. Max coverage (-): 1.66

Region: NODE\_304263\_length\_4982\_cov\_28.985548 4346-4355. Max. coverage (+): 0.08. Max coverage (-): 1.41

Region: NODE\_304263\_length\_4982\_cov\_28.985548 4356-4365. Max. coverage (+): 0.57. Max coverage (-): 0.57

Region: NODE\_304263\_length\_4982\_cov\_28.985548 4366-4375. Max. coverage (+): 0. Max coverage (-): 0.16

Region: NODE\_304263\_length\_4982\_cov\_28.985548 4376-4385. Max. coverage (+): 0. Max coverage (-): 0.04

Region: NODE\_304263\_length\_4982\_cov\_28.985548 4386-4396. Max. coverage (+): 0. Max coverage (-): 2.22

Region: NODE\_304263\_length\_4982\_cov\_28.985548 4397-4406. Max. coverage (+): 0. Max coverage (-): 1.49

Region: NODE\_304263\_length\_4982\_cov\_28.985548 4407-4416. Max. coverage (+): 0.28. Max coverage (-): 0.28

Region: NODE\_304263\_length\_4982\_cov\_28.985548 4417-4426. Max. coverage (+): 0. Max coverage (-): 0

Region: NODE\_304263\_length\_4982\_cov\_28.985548 4427-4436. Max. coverage (+): 0. Max coverage (-): 18.65

Region: NODE\_304263\_length\_4982\_cov\_28.985548 4437-4446. Max. coverage (+): 0.08. Max coverage (-): 18.82

Region: NODE\_304263\_length\_4982\_cov\_28.985548 4447-4456. Max. coverage (+): 0.16. Max coverage (-): 0.4

Region: NODE\_304263\_length\_4982\_cov\_28.985548 4457-4466. Max. coverage (+): 0.2. Max coverage (-): 0.04

Region: NODE\_304263\_length\_4982\_cov\_28.985548 4467-4476. Max. coverage (+): 0. Max coverage (-): 6.42

Region: NODE\_304263\_length\_4982\_cov\_28.985548 4477-4486. Max. coverage (+): 0. Max coverage (-): 4.97

Region: NODE\_304263\_length\_4982\_cov\_28.985548 4487-4496. Max. coverage (+): 0. Max coverage (-): 0.32

Region: NODE\_304263\_length\_4982\_cov\_28.985548 4497-4507. Max. coverage (+): 0. Max coverage (-): 0.12

Region: NODE\_304263\_length\_4982\_cov\_28.985548 4508-4517. Max. coverage (+): 0.08. Max coverage (-): 0

Region: NODE\_304263\_length\_4982\_cov\_28.985548 4518-4527. Max. coverage (+): 0. Max coverage (-): 0.81

Region: NODE\_304263\_length\_4982\_cov\_28.985548 4528-4537. Max. coverage (+): 0.12. Max coverage (-): 0.52

Region: NODE\_304263\_length\_4982\_cov\_28.985548 4538-4547. Max. coverage (+): 0.24. Max coverage (-): 0.61

Region: NODE\_304263\_length\_4982\_cov\_28.985548 4548-4557. Max. coverage (+): 0.04. Max coverage (-): 0.24

Region: NODE\_304263\_length\_4982\_cov\_28.985548 4558-4567. Max. coverage (+): 0. Max coverage (-): 0.93

Region: NODE\_304263\_length\_4982\_cov\_28.985548 4568-4577. Max. coverage (+): 0.08. Max coverage (-): 0.36

Region: NODE\_304263\_length\_4982\_cov\_28.985548 4578-4587. Max. coverage (+): 0.08. Max coverage (-): 0.04

Region: NODE\_304263\_length\_4982\_cov\_28.985548 4588-4597. Max. coverage (+): 0.08. Max coverage (-): 0.04

Region: NODE\_304263\_length\_4982\_cov\_28.985548 4598-4607. Max. coverage (+): 0. Max coverage (-): 0.97

Region: NODE\_304263\_length\_4982\_cov\_28.985548 4608-4618. Max. coverage (+): 0. Max coverage (-): 0.77

Region: NODE\_304263\_length\_4982\_cov\_28.985548 4619-4628. Max. coverage (+): 0.2. Max coverage (-): 0.08

Region: NODE\_304263\_length\_4982\_cov\_28.985548 4629-4638. Max. coverage (+): 0.16. Max coverage (-): 0.4

Region: NODE\_304263\_length\_4982\_cov\_28.985548 4639-4648. Max. coverage (+): 0. Max coverage (-): 1.94

Region: NODE\_304263\_length\_4982\_cov\_28.985548 4649-4658. Max. coverage (+): 0.04. Max coverage (-): 0.32

Region: NODE\_304263\_length\_4982\_cov\_28.985548 4659-4668. Max. coverage (+): 0.04. Max coverage (-): 0.28

Region: NODE\_304263\_length\_4982\_cov\_28.985548 4669-4678. Max. coverage (+): 0. Max coverage (-): 3.67

Region: NODE\_304263\_length\_4982\_cov\_28.985548 4679-4688. Max. coverage (+): 0.04. Max coverage (-): 4.24

Region: NODE\_304263\_length\_4982\_cov\_28.985548 4689-4698. Max. coverage (+): 0.36. Max coverage (-): 0.2

Region: NODE\_304263\_length\_4982\_cov\_28.985548 4699-4708. Max. coverage (+): 0.04. Max coverage (-): 0

Region: NODE\_304263\_length\_4982\_cov\_28.985548 4709-4719. Max. coverage (+): 0. Max coverage (-): 0.04

Region: NODE\_304263\_length\_4982\_cov\_28.985548 4720-4729. Max. coverage (+): 0. Max coverage (-): 26.21

Region: NODE\_304263\_length\_4982\_cov\_28.985548 4730-4739. Max. coverage (+): 0.08. Max coverage (-): 0.57

Region: NODE\_304263\_length\_4982\_cov\_28.985548 4740-4749. Max. coverage (+): 0.48. Max coverage (-): 0.24

Region: NODE\_304263\_length\_4982\_cov\_28.985548 4750-4759. Max. coverage (+): 0. Max coverage (-): 0.16

Region: NODE\_304263\_length\_4982\_cov\_28.985548 4760-4769. Max. coverage (+): 0. Max coverage (-): 0.08

Region: NODE\_304263\_length\_4982\_cov\_28.985548 4770-4779. Max. coverage (+): 0. Max coverage (-): 0.44

Region: NODE\_304263\_length\_4982\_cov\_28.985548 4780-4789. Max. coverage (+): 0. Max coverage (-): 0.08

Region: NODE\_304263\_length\_4982\_cov\_28.985548 4790-4799. Max. coverage (+): 0.04. Max coverage (-): 0.16

Region: NODE\_304263\_length\_4982\_cov\_28.985548 4800-4809. Max. coverage (+): 0. Max coverage (-): 0.24

Region: NODE\_304263\_length\_4982\_cov\_28.985548 4810-4819. Max. coverage (+): 0. Max coverage (-): 0.2

Region: NODE\_304263\_length\_4982\_cov\_28.985548 4820-4830. Max. coverage (+): 0. Max coverage (-): 3.27

Region: NODE\_304263\_length\_4982\_cov\_28.985548 4831-4840. Max. coverage (+): 0.04. Max coverage (-): 3.55

Region: NODE\_304263\_length\_4982\_cov\_28.985548 4841-4850. Max. coverage (+): 0. Max coverage (-): 12.64

Region: NODE\_304263\_length\_4982\_cov\_28.985548 4851-4860. Max. coverage (+): 0.04. Max coverage (-): 1.21

Region: NODE\_304263\_length\_4982\_cov\_28.985548 4861-4870. Max. coverage (+): 0.52. Max coverage (-): 1.53

Region: NODE\_304263\_length\_4982\_cov\_28.985548 4871-4880. Max. coverage (+): 0.24. Max coverage (-): 1.49

Region: NODE\_304263\_length\_4982\_cov\_28.985548 4881-4890. Max. coverage (+): 0.12. Max coverage (-): 0.08

Region: NODE\_304263\_length\_4982\_cov\_28.985548 4891-4900. Max. coverage (+): 0. Max coverage (-): 0.4

Region: NODE\_304263\_length\_4982\_cov\_28.985548 4901-4910. Max. coverage (+): 0. Max coverage (-): 0.69

Region: NODE\_304263\_length\_4982\_cov\_28.985548 4911-4920. Max. coverage (+): 0.32. Max coverage (-): 0

Region: NODE\_304263\_length\_4982\_cov\_28.985548 4921-4930. Max. coverage (+): 0. Max coverage (-): 0.12

Region: NODE\_304263\_length\_4982\_cov\_28.985548 4931-4941. Max. coverage (+): 0.04. Max coverage (-): 1.86

Region: NODE\_304263\_length\_4982\_cov\_28.985548 4942-4951. Max. coverage (+): 0.04. Max coverage (-): 0.4

Region: NODE\_304263\_length\_4982\_cov\_28.985548 4952-4961. Max. coverage (+): 0.04. Max coverage (-): 0.12

Region: NODE\_304263\_length\_4982\_cov\_28.985548 4962-4971. Max. coverage (+): 0. Max coverage (-): 0

Region: NODE\_304263\_length\_4982\_cov\_28.985548 4972-4981. Max. coverage (+): 0. Max coverage (-): 0

Region: NODE\_304263\_length\_4982\_cov\_28.985548 4982-4991. Max. coverage (+): 0. Max coverage (-): 10.5

Region: NODE\_304263\_length\_4982\_cov\_28.985548 4992-5001. Max. coverage (+): 0.04. Max coverage (-): 15.22

Region: NODE\_304263\_length\_4982\_cov\_28.985548 5002-5011. Max. coverage (+): 0.16. Max coverage (-): 1.45

Region: NODE\_304263\_length\_4982\_cov\_28.985548 5012-5021. Max. coverage (+): 0. Max coverage (-): 0.61

Region: NODE\_304263\_length\_4982\_cov\_28.985548 5022-5031. Max. coverage (+): 0. Max coverage (-): 0.12

Region: NODE\_304263\_length\_4982\_cov\_28.985548 5032-5041. Max. coverage (+): 0. Max coverage (-): 0

Region: NODE\_304263\_length\_4982\_cov\_28.985548 5042-. Max. coverage (+): 0. Max coverage (-): 0

RepeatMasker Color Code

**+**

100-98% Identity

<98-95% Identity

<95-90% Identity

<90-85% Identity

<85-80% Identity

<80-75% Identity

<75-70% Identity

<70% Identity

**-**

Gene Set Color Code

**+**

Gene

Pseudogene

Other

**-**

Topology/Coverage Color Code

Coverage Plus Strand

Coverage Minus Strand

Mainstrand: Plus

Mainstrand: Minus

Complementary Strand

Flanking Region  
(if option -flank >0)

Gene Set Annotation  
  
RepeatMasker Annotation  

**1. AlRepC-13**: 50-172 (+), Divergence to consensus: 26.8%  
**2. TE-X-4\_DR**: 301-342 (+), Divergence to consensus: 19.1%  
**3. AlRepB-103**: 398-807 (-), Divergence to consensus: 40.5%  
**4. Expander2**: 820-890 (-), Divergence to consensus: 32.5%  
**5. AlRepE-1213**: 977-1025 (-), Divergence to consensus: 28.5%  
**6. A-rich**: 1933-1986 (+), Divergence to consensus: 32.7%  
**7. AlRepD-2057**: 2634-2693 (+), Divergence to consensus: 21.7%  
**8. (AACATTT)n**: 2982-3018 (+), Divergence to consensus: 24.5%  
**9. I\_Ele34**: 4567-4618 (+), Divergence to consensus: 34.6%

  
Transcription Factor Binding Sites  

**RHOXF1** (Sequence: AGCTCA (-): 442)  
**RHOXF1** (Sequence: AGCTTA (-): 659)  
**RHOXF1** (Sequence: AGATCA (-): 766)  
**RHOXF1** (Sequence: AGCTCA (-): 1577)  
**RHOXF1** (Sequence: AGCTCA (-): 2076)  
**RHOXF1** (Sequence: AGCTTA (-): 2123)  
**RHOXF1** (Sequence: GGCTCA (-): 2188)  
**RHOXF1** (Sequence: TAATCT (+): 493)  
**RHOXF1** (Sequence: TGAGCT (+): 2121)  
**RHOXF1** (Sequence: TAATCT (+): 2513)  
**RHOXF1** (Sequence: TAATCT (+): 2942)  
**RHOXF1** (Sequence: TGAGCC (+): 3034)  
**RHOXF1** (Sequence: TGATCT (+): 3132)  
**RHOXF1** (Sequence: TGATCT (+): 3780)  
**RHOXF1** (Sequence: TGATCT (+): 3946)  
**RHOXF1** (Sequence: TGAGCT (+): 4240)  
**RHOXF1** (Sequence: TAATCT (+): 4515)  
**Lhx8** (Sequence: TTAATTAA (-): 2102)  
**SOX9** (Sequence: AACAATGA (-): 456)  
**FOXO3\_mmu** (Sequence: TGTTTTGA (-): 3411)  
**Sox5** (Sequence: ATTGTT (+): 2840)  
**FIGLA** (Sequence: AACACCTGGA (-): 783)  
**SOX9** (Sequence: TTATTGTT (+): 2838)  
**FOXO3\_mmu** (Sequence: TGAAAACA (+): 1599)  
**FOXO3\_mmu** (Sequence: GCAAAACA (+): 1623)  
**FOXO3\_mmu** (Sequence: GCAAAACA (+): 2440)  
**FOXO3\_mmu** (Sequence: TGAAAACA (+): 2531)  
**FOXO3\_mmu** (Sequence: TGAAAACA (+): 2599)  
**FOXO1** (Sequence: GAAAACAGG (-): 1600)  
**FOXO1** (Sequence: AAAAACAAC (-): 1976)  
**FOXO1** (Sequence: GAAAACAGG (-): 2532)  
**FOXO1** (Sequence: GAAAACAGC (-): 2600)  
**FOXO1** (Sequence: AAAAACAAC (-): 3381)  
**Gata4** (Sequence: AGATAAC (-): 2418)  
**Sox5** (Sequence: AACAAT (-): 456)  
**Sox5** (Sequence: AACAAT (-): 908)  
**Sox5** (Sequence: AACAAT (-): 1963)  
**Sox5** (Sequence: AACAAT (-): 1968)  
**Sox5** (Sequence: AACAAT (-): 2548)  
**Sox5** (Sequence: AACAAT (-): 2736)  
**POU5F1** (Sequence: ATGCAAA (+): 2522)
