# Supplementary material for: First characterization of PIWI-interacting RNA clusters in a cichlid fish with a B chromosome
Source: BMC Biol. 2022 Sep 21;20:204. doi: 10.1186/s12915-022-01403-2 (PMC9490952; doi:10.1186/s12915-022-01403-2)
Supplement: Supplementary file 1 — Additional file 1. Zipped folder with fasta and interactive html piRNA cluster information for the A. latifasciata genome. The nomenclature is as follows: number-pirna-cluster_sex_B-presence (f, female; m, male; 0b, without B chromosome; 1b, with B chromosome). [file 12915_2022_1403_MOESM1_ESM.zip › 119_m1b.html]

piRNA cluster 119\_m1b 69


Predicted piRNA cluster no. 119\_m1b
  

Show proTRAC run info
Hide proTRAC run info

/\  
                \_\_\_\_\_\_\_\_\_\_\_\_\_\_\_\_\_\_\_\_\_\_\_/\\_\_\_ /  \\_\_\_\_\_\_\_  
               I                      /  \  /    \      I  
               I     pro             /    \/      \     I  
               I        TRAC        /               \   I  
               I   \_\_\_\_\_\_\_\_\_\_\_\_\_\_\_\_/\_\_\_\_\_\_\_\_\_\_\_\_\_\_\_\_\_\\_ I  
               I   \              /                     I  
               I    \            /                      I  
               I     \  /\      /       V.2.4.2         I  
               I      \/  \    /                        I  
               I\_\_\_\_\_\_\_\_\_\_\_\  /\_\_\_\_\_\_\_\_\_\_\_\_\_\_\_\_\_\_\_\_\_\_\_\_\_I  
                            \/  
  
  
================================= proTRAC ====================================  
VERSION: .......... 2.4.2  
LAST MODIFIED: .... 11. May 2018  
  
Please cite:  
Rosenkranz D, Zischler H. proTRAC - a software for probabilistic piRNA cluster  
detection, visualization and analysis. 2012. BMC Bioinformatics 13:5.  
  
  
Contact:  
David Rosenkranz  
Institute of Organismic and Molecular Evolutionary Biology  
Dept. Anthropology, small RNA group  
Johannes Gutenberg University Mainz  
email: rosenkranz@uni-mainz.de  
  
You can find the latest proTRAC version at:  
http://sourceforge.net/projects/protrac/files  
http://www.smallRNAgroup-mainz.de/software  
==============================================================================  
  
PARAMETERS:  
Map file: ...............piwi-machos-1B.fa-collapse.map  
Genome file: ............../../../0B\_ala\_genome.fa  
RepeatMasker annotation: Alatifasciata-all0B-maryan-v2.fa\_corrected.out  
GeneSet:................./guest-storage/Data/annotation/Alatifasciata\_all0B\_maryan-v2\_out2017.gff  
  
Significant (p<=0.01) hit density will be calculated based  
on observed hit distribution.  
  
Sliding window size: ........................................ 5000 bp  
Sliding window increament: .................................. 1000 bp  
Normalize each hit by number of genomic hits: ............... yes  
Normalize each hit by number of sequence reads: ............. yes  
Normalize values (-> per million mapped reads): ............. yes  
Min. fraction of hits with 1T(U) or 10A: .................... 0.75  
Alternatively: Min. fraction of hits with 1T(U) and 10A: .... 0.5  
Min. fraction of hits with typical piRNA length: ............ 0.75  
Typical piRNA length: ....................................... 24-32 nt  
Min. size of a piRNA cluster: ............................... 1000 bp.  
Min. number of hits (absolute): ............................. 0  
Min. number of hits (normalized): ........................... 0  
Min. fraction of hits on the mainstrand: .................... 0.75  
Top fraction of mapped sequences (in terms of read counts): . 1%  
Top fraction accounts for max. n% of sequence reads: ........ 90%  
Min. fraction of hits on each arm of a bidirectional cluster: 0.05  
Output html file for each cluster: .......................... yes  
Output a summary table: ..................................... yes  
Output a FASTA file for each cluster (piRNA sequences): ..... yes  
Output a FASTA file comprising cluster sequences: ........... yes  
Output a GTF file for predicted piRNA clusters: ..............yes  
Search DNA motifs in clusters: .............................. yes  
Output flanking sequences: +/- .............................. 0 bp  
Output ~.pTi file: .......................................... no  
==============================================================================  
  
  
Genome size (without gaps): ............ 758543724 bp  
Gaps (N/X/-): .......................... 417479 bp  
Mapped reads: .......................... 26973943  
Non-identical sequences: ............... 6209225  
Genomic hits: .......................... 48438990  
Significant densitiy of mapped reads: .. 821.144211136946 reads/kb

Show proTRAC cluster info
Hide proTRAC cluster info

|  |  |
| --- | --- |
| Location | NODE\_304263\_length\_4982\_cov\_28.985548 |
| Coordinates | 1-5045 |
| Size [bp] | 5045 |
| Sequence hit loci | 4858 |
| Mapped reads (normalized) | 27617.9 |
| Mapped reads (normalized) per kb | 5474.3 |
| Normalized reads with 1T (1U) | 82.3% |
| Normalized reads with 10A | 40.3% |
| Normalized reads with length 24-32 nt | 99.2% |
| Normalized reads on the main strand(s) | 94.2% |
| Predicted directionality | mono:minus |

100%

0%

1T (1U)  
reads

10A reads

24-32 nt  
reads

reads on mainstrand

**Either the amount of reads with 1T (1U) OR 10A has to exceed 75% (set with option: -1Tor10A)  
Alternatively the amount of reads with 1T (1U) AND 10A has to exceed 50% (set with option: -1Tand10A)  
Minimum amount of reads with preferred size is 75% (set with option: -pisize)  
Minimum amount of reads on the main strand(s) is 75% (set with option: -clstrand)**

Show read coverage
Hide read coverage

WHAT DO I SEE HERE?  
This chart shows the location of mapped sequence reads within a predicted piRNA cluster. The color refers to the number of genomic hits produced by the sequence read in question. A dark red bar indicates that this sequence read produces many other hits elsewhere in the genome. Many adjacent red or yellow bars can indicate the presence of a multi-copy element such as transposons or rRNA genes. A dark green bar indicates that this sequence read maps uniquely to this locus.

1 hit

2-5 hits

6-10 hits

11-20 hits

21-50 hits

51-100 hits

> 100 hits

NODE\_304263\_length\_4982\_cov\_28.985548

1

5045

Gene Set

RepeatMasker

Mapped  
Reads

109.18

plus strand

minus strand

109.18

Region: NODE\_304263\_length\_4982\_cov\_28.985548 5015-6. Max. coverage (+): 0. Max coverage (-): 0.03

Region: NODE\_304263\_length\_4982\_cov\_28.985548 7-16. Max. coverage (+): 0. Max coverage (-): 0.69

Region: NODE\_304263\_length\_4982\_cov\_28.985548 17-26. Max. coverage (+): 0. Max coverage (-): 0

Region: NODE\_304263\_length\_4982\_cov\_28.985548 27-36. Max. coverage (+): 0. Max coverage (-): 0

Region: NODE\_304263\_length\_4982\_cov\_28.985548 37-46. Max. coverage (+): 0. Max coverage (-): 0

Region: NODE\_304263\_length\_4982\_cov\_28.985548 47-56. Max. coverage (+): 0. Max coverage (-): 0.04

Region: NODE\_304263\_length\_4982\_cov\_28.985548 57-66. Max. coverage (+): 0. Max coverage (-): 0.04

Region: NODE\_304263\_length\_4982\_cov\_28.985548 67-76. Max. coverage (+): 0. Max coverage (-): 0.04

Region: NODE\_304263\_length\_4982\_cov\_28.985548 77-86. Max. coverage (+): 0.04. Max coverage (-): 0.07

Region: NODE\_304263\_length\_4982\_cov\_28.985548 87-96. Max. coverage (+): 0. Max coverage (-): 0.15

Region: NODE\_304263\_length\_4982\_cov\_28.985548 97-106. Max. coverage (+): 0.04. Max coverage (-): 0.07

Region: NODE\_304263\_length\_4982\_cov\_28.985548 107-117. Max. coverage (+): 0.04. Max coverage (-): 0.04

Region: NODE\_304263\_length\_4982\_cov\_28.985548 118-127. Max. coverage (+): 0. Max coverage (-): 0.04

Region: NODE\_304263\_length\_4982\_cov\_28.985548 128-137. Max. coverage (+): 0. Max coverage (-): 0.7

Region: NODE\_304263\_length\_4982\_cov\_28.985548 138-147. Max. coverage (+): 0. Max coverage (-): 0.11

Region: NODE\_304263\_length\_4982\_cov\_28.985548 148-157. Max. coverage (+): 0. Max coverage (-): 0.04

Region: NODE\_304263\_length\_4982\_cov\_28.985548 158-167. Max. coverage (+): 0. Max coverage (-): 0.04

Region: NODE\_304263\_length\_4982\_cov\_28.985548 168-177. Max. coverage (+): 0. Max coverage (-): 0.07

Region: NODE\_304263\_length\_4982\_cov\_28.985548 178-187. Max. coverage (+): 0. Max coverage (-): 0

Region: NODE\_304263\_length\_4982\_cov\_28.985548 188-197. Max. coverage (+): 0.07. Max coverage (-): 0.04

Region: NODE\_304263\_length\_4982\_cov\_28.985548 198-207. Max. coverage (+): 0. Max coverage (-): 0.37

Region: NODE\_304263\_length\_4982\_cov\_28.985548 208-217. Max. coverage (+): 0. Max coverage (-): 1.59

Region: NODE\_304263\_length\_4982\_cov\_28.985548 218-228. Max. coverage (+): 0. Max coverage (-): 0

Region: NODE\_304263\_length\_4982\_cov\_28.985548 229-238. Max. coverage (+): 0. Max coverage (-): 0.07

Region: NODE\_304263\_length\_4982\_cov\_28.985548 239-248. Max. coverage (+): 0. Max coverage (-): 0.07

Region: NODE\_304263\_length\_4982\_cov\_28.985548 249-258. Max. coverage (+): 0. Max coverage (-): 0.15

Region: NODE\_304263\_length\_4982\_cov\_28.985548 259-268. Max. coverage (+): 0.04. Max coverage (-): 0.3

Region: NODE\_304263\_length\_4982\_cov\_28.985548 269-278. Max. coverage (+): 0. Max coverage (-): 0.3

Region: NODE\_304263\_length\_4982\_cov\_28.985548 279-288. Max. coverage (+): 0. Max coverage (-): 0

Region: NODE\_304263\_length\_4982\_cov\_28.985548 289-298. Max. coverage (+): 0.04. Max coverage (-): 0.04

Region: NODE\_304263\_length\_4982\_cov\_28.985548 299-308. Max. coverage (+): 0. Max coverage (-): 0.22

Region: NODE\_304263\_length\_4982\_cov\_28.985548 309-318. Max. coverage (+): 0. Max coverage (-): 0.07

Region: NODE\_304263\_length\_4982\_cov\_28.985548 319-328. Max. coverage (+): 0. Max coverage (-): 0.15

Region: NODE\_304263\_length\_4982\_cov\_28.985548 329-339. Max. coverage (+): 0. Max coverage (-): 0

Region: NODE\_304263\_length\_4982\_cov\_28.985548 340-349. Max. coverage (+): 0. Max coverage (-): 0

Region: NODE\_304263\_length\_4982\_cov\_28.985548 350-359. Max. coverage (+): 0. Max coverage (-): 0.04

Region: NODE\_304263\_length\_4982\_cov\_28.985548 360-369. Max. coverage (+): 0. Max coverage (-): 0.04

Region: NODE\_304263\_length\_4982\_cov\_28.985548 370-379. Max. coverage (+): 0. Max coverage (-): 0.33

Region: NODE\_304263\_length\_4982\_cov\_28.985548 380-389. Max. coverage (+): 0.07. Max coverage (-): 0.56

Region: NODE\_304263\_length\_4982\_cov\_28.985548 390-399. Max. coverage (+): 0. Max coverage (-): 0.26

Region: NODE\_304263\_length\_4982\_cov\_28.985548 400-409. Max. coverage (+): 0. Max coverage (-): 0

Region: NODE\_304263\_length\_4982\_cov\_28.985548 410-419. Max. coverage (+): 0.04. Max coverage (-): 0

Region: NODE\_304263\_length\_4982\_cov\_28.985548 420-429. Max. coverage (+): 0. Max coverage (-): 0.11

Region: NODE\_304263\_length\_4982\_cov\_28.985548 430-439. Max. coverage (+): 0. Max coverage (-): 0

Region: NODE\_304263\_length\_4982\_cov\_28.985548 440-450. Max. coverage (+): 0.15. Max coverage (-): 0.04

Region: NODE\_304263\_length\_4982\_cov\_28.985548 451-460. Max. coverage (+): 0.07. Max coverage (-): 0

Region: NODE\_304263\_length\_4982\_cov\_28.985548 461-470. Max. coverage (+): 0. Max coverage (-): 0.04

Region: NODE\_304263\_length\_4982\_cov\_28.985548 471-480. Max. coverage (+): 0. Max coverage (-): 0.04

Region: NODE\_304263\_length\_4982\_cov\_28.985548 481-490. Max. coverage (+): 0. Max coverage (-): 0.07

Region: NODE\_304263\_length\_4982\_cov\_28.985548 491-500. Max. coverage (+): 0.04. Max coverage (-): 0.04

Region: NODE\_304263\_length\_4982\_cov\_28.985548 501-510. Max. coverage (+): 0. Max coverage (-): 0.26

Region: NODE\_304263\_length\_4982\_cov\_28.985548 511-520. Max. coverage (+): 0.04. Max coverage (-): 0.04

Region: NODE\_304263\_length\_4982\_cov\_28.985548 521-530. Max. coverage (+): 0. Max coverage (-): 0.33

Region: NODE\_304263\_length\_4982\_cov\_28.985548 531-540. Max. coverage (+): 0. Max coverage (-): 0.12

Region: NODE\_304263\_length\_4982\_cov\_28.985548 541-550. Max. coverage (+): 0.04. Max coverage (-): 0.08

Region: NODE\_304263\_length\_4982\_cov\_28.985548 551-560. Max. coverage (+): 0. Max coverage (-): 0

Region: NODE\_304263\_length\_4982\_cov\_28.985548 561-571. Max. coverage (+): 0. Max coverage (-): 0.22

Region: NODE\_304263\_length\_4982\_cov\_28.985548 572-581. Max. coverage (+): 0.07. Max coverage (-): 0.04

Region: NODE\_304263\_length\_4982\_cov\_28.985548 582-591. Max. coverage (+): 0. Max coverage (-): 0

Region: NODE\_304263\_length\_4982\_cov\_28.985548 592-601. Max. coverage (+): 0. Max coverage (-): 0.04

Region: NODE\_304263\_length\_4982\_cov\_28.985548 602-611. Max. coverage (+): 0. Max coverage (-): 0.07

Region: NODE\_304263\_length\_4982\_cov\_28.985548 612-621. Max. coverage (+): 0. Max coverage (-): 0.07

Region: NODE\_304263\_length\_4982\_cov\_28.985548 622-631. Max. coverage (+): 0.04. Max coverage (-): 0.07

Region: NODE\_304263\_length\_4982\_cov\_28.985548 632-641. Max. coverage (+): 0.07. Max coverage (-): 0.33

Region: NODE\_304263\_length\_4982\_cov\_28.985548 642-651. Max. coverage (+): 0.04. Max coverage (-): 0.41

Region: NODE\_304263\_length\_4982\_cov\_28.985548 652-661. Max. coverage (+): 0.15. Max coverage (-): 0.11

Region: NODE\_304263\_length\_4982\_cov\_28.985548 662-671. Max. coverage (+): 0.11. Max coverage (-): 0.04

Region: NODE\_304263\_length\_4982\_cov\_28.985548 672-682. Max. coverage (+): 0. Max coverage (-): 0.3

Region: NODE\_304263\_length\_4982\_cov\_28.985548 683-692. Max. coverage (+): 0.04. Max coverage (-): 3.04

Region: NODE\_304263\_length\_4982\_cov\_28.985548 693-702. Max. coverage (+): 0.04. Max coverage (-): 12.72

Region: NODE\_304263\_length\_4982\_cov\_28.985548 703-712. Max. coverage (+): 0.04. Max coverage (-): 3.19

Region: NODE\_304263\_length\_4982\_cov\_28.985548 713-722. Max. coverage (+): 0.04. Max coverage (-): 0.04

Region: NODE\_304263\_length\_4982\_cov\_28.985548 723-732. Max. coverage (+): 0. Max coverage (-): 0.04

Region: NODE\_304263\_length\_4982\_cov\_28.985548 733-742. Max. coverage (+): 0. Max coverage (-): 0.11

Region: NODE\_304263\_length\_4982\_cov\_28.985548 743-752. Max. coverage (+): 0.07. Max coverage (-): 0.3

Region: NODE\_304263\_length\_4982\_cov\_28.985548 753-762. Max. coverage (+): 0.04. Max coverage (-): 1.78

Region: NODE\_304263\_length\_4982\_cov\_28.985548 763-772. Max. coverage (+): 0. Max coverage (-): 5.12

Region: NODE\_304263\_length\_4982\_cov\_28.985548 773-782. Max. coverage (+): 0.04. Max coverage (-): 0.19

Region: NODE\_304263\_length\_4982\_cov\_28.985548 783-793. Max. coverage (+): 0.48. Max coverage (-): 0

Region: NODE\_304263\_length\_4982\_cov\_28.985548 794-803. Max. coverage (+): 0. Max coverage (-): 0.26

Region: NODE\_304263\_length\_4982\_cov\_28.985548 804-813. Max. coverage (+): 0. Max coverage (-): 0.19

Region: NODE\_304263\_length\_4982\_cov\_28.985548 814-823. Max. coverage (+): 0.07. Max coverage (-): 0.11

Region: NODE\_304263\_length\_4982\_cov\_28.985548 824-833. Max. coverage (+): 0. Max coverage (-): 2.63

Region: NODE\_304263\_length\_4982\_cov\_28.985548 834-843. Max. coverage (+): 0.04. Max coverage (-): 0.41

Region: NODE\_304263\_length\_4982\_cov\_28.985548 844-853. Max. coverage (+): 0.04. Max coverage (-): 0.15

Region: NODE\_304263\_length\_4982\_cov\_28.985548 854-863. Max. coverage (+): 0. Max coverage (-): 0.19

Region: NODE\_304263\_length\_4982\_cov\_28.985548 864-873. Max. coverage (+): 0. Max coverage (-): 0.07

Region: NODE\_304263\_length\_4982\_cov\_28.985548 874-883. Max. coverage (+): 0. Max coverage (-): 0.67

Region: NODE\_304263\_length\_4982\_cov\_28.985548 884-893. Max. coverage (+): 0. Max coverage (-): 0.56

Region: NODE\_304263\_length\_4982\_cov\_28.985548 894-904. Max. coverage (+): 0. Max coverage (-): 0

Region: NODE\_304263\_length\_4982\_cov\_28.985548 905-914. Max. coverage (+): 0. Max coverage (-): 0.04

Region: NODE\_304263\_length\_4982\_cov\_28.985548 915-924. Max. coverage (+): 0.04. Max coverage (-): 0.07

Region: NODE\_304263\_length\_4982\_cov\_28.985548 925-934. Max. coverage (+): 0.04. Max coverage (-): 0.04

Region: NODE\_304263\_length\_4982\_cov\_28.985548 935-944. Max. coverage (+): 0. Max coverage (-): 2.52

Region: NODE\_304263\_length\_4982\_cov\_28.985548 945-954. Max. coverage (+): 0. Max coverage (-): 2.52

Region: NODE\_304263\_length\_4982\_cov\_28.985548 955-964. Max. coverage (+): 0.04. Max coverage (-): 0.78

Region: NODE\_304263\_length\_4982\_cov\_28.985548 965-974. Max. coverage (+): 0.22. Max coverage (-): 0.26

Region: NODE\_304263\_length\_4982\_cov\_28.985548 975-984. Max. coverage (+): 0. Max coverage (-): 0.07

Region: NODE\_304263\_length\_4982\_cov\_28.985548 985-994. Max. coverage (+): 0.04. Max coverage (-): 0.52

Region: NODE\_304263\_length\_4982\_cov\_28.985548 995-1004. Max. coverage (+): 0.07. Max coverage (-): 0.15

Region: NODE\_304263\_length\_4982\_cov\_28.985548 1005-1015. Max. coverage (+): 0. Max coverage (-): 0.19

Region: NODE\_304263\_length\_4982\_cov\_28.985548 1016-1025. Max. coverage (+): 0.15. Max coverage (-): 0.67

Region: NODE\_304263\_length\_4982\_cov\_28.985548 1026-1035. Max. coverage (+): 0.15. Max coverage (-): 0.74

Region: NODE\_304263\_length\_4982\_cov\_28.985548 1036-1045. Max. coverage (+): 0.22. Max coverage (-): 0.93

Region: NODE\_304263\_length\_4982\_cov\_28.985548 1046-1055. Max. coverage (+): 0. Max coverage (-): 0.3

Region: NODE\_304263\_length\_4982\_cov\_28.985548 1056-1065. Max. coverage (+): 0.04. Max coverage (-): 0.33

Region: NODE\_304263\_length\_4982\_cov\_28.985548 1066-1075. Max. coverage (+): 0.04. Max coverage (-): 0.19

Region: NODE\_304263\_length\_4982\_cov\_28.985548 1076-1085. Max. coverage (+): 0.04. Max coverage (-): 5.08

Region: NODE\_304263\_length\_4982\_cov\_28.985548 1086-1095. Max. coverage (+): 0. Max coverage (-): 0.11

Region: NODE\_304263\_length\_4982\_cov\_28.985548 1096-1105. Max. coverage (+): 0. Max coverage (-): 0.11

Region: NODE\_304263\_length\_4982\_cov\_28.985548 1106-1115. Max. coverage (+): 0.04. Max coverage (-): 0.04

Region: NODE\_304263\_length\_4982\_cov\_28.985548 1116-1126. Max. coverage (+): 0.04. Max coverage (-): 0.07

Region: NODE\_304263\_length\_4982\_cov\_28.985548 1127-1136. Max. coverage (+): 0.04. Max coverage (-): 0.04

Region: NODE\_304263\_length\_4982\_cov\_28.985548 1137-1146. Max. coverage (+): 0. Max coverage (-): 0.04

Region: NODE\_304263\_length\_4982\_cov\_28.985548 1147-1156. Max. coverage (+): 0.04. Max coverage (-): 0

Region: NODE\_304263\_length\_4982\_cov\_28.985548 1157-1166. Max. coverage (+): 0.04. Max coverage (-): 0.19

Region: NODE\_304263\_length\_4982\_cov\_28.985548 1167-1176. Max. coverage (+): 0. Max coverage (-): 0

Region: NODE\_304263\_length\_4982\_cov\_28.985548 1177-1186. Max. coverage (+): 0.04. Max coverage (-): 0

Region: NODE\_304263\_length\_4982\_cov\_28.985548 1187-1196. Max. coverage (+): 0.04. Max coverage (-): 0.19

Region: NODE\_304263\_length\_4982\_cov\_28.985548 1197-1206. Max. coverage (+): 0. Max coverage (-): 0.15

Region: NODE\_304263\_length\_4982\_cov\_28.985548 1207-1216. Max. coverage (+): 0. Max coverage (-): 0.07

Region: NODE\_304263\_length\_4982\_cov\_28.985548 1217-1226. Max. coverage (+): 0. Max coverage (-): 1.26

Region: NODE\_304263\_length\_4982\_cov\_28.985548 1227-1237. Max. coverage (+): 0. Max coverage (-): 1.19

Region: NODE\_304263\_length\_4982\_cov\_28.985548 1238-1247. Max. coverage (+): 0.11. Max coverage (-): 0.07

Region: NODE\_304263\_length\_4982\_cov\_28.985548 1248-1257. Max. coverage (+): 0. Max coverage (-): 0.04

Region: NODE\_304263\_length\_4982\_cov\_28.985548 1258-1267. Max. coverage (+): 0. Max coverage (-): 1.82

Region: NODE\_304263\_length\_4982\_cov\_28.985548 1268-1277. Max. coverage (+): 0. Max coverage (-): 1.82

Region: NODE\_304263\_length\_4982\_cov\_28.985548 1278-1287. Max. coverage (+): 0.96. Max coverage (-): 0.19

Region: NODE\_304263\_length\_4982\_cov\_28.985548 1288-1297. Max. coverage (+): 0.04. Max coverage (-): 0.04

Region: NODE\_304263\_length\_4982\_cov\_28.985548 1298-1307. Max. coverage (+): 0. Max coverage (-): 0.04

Region: NODE\_304263\_length\_4982\_cov\_28.985548 1308-1317. Max. coverage (+): 0.07. Max coverage (-): 0.07

Region: NODE\_304263\_length\_4982\_cov\_28.985548 1318-1327. Max. coverage (+): 0.07. Max coverage (-): 109.18

Region: NODE\_304263\_length\_4982\_cov\_28.985548 1328-1337. Max. coverage (+): 0. Max coverage (-): 12.05

Region: NODE\_304263\_length\_4982\_cov\_28.985548 1338-1348. Max. coverage (+): 1.82. Max coverage (-): 0.3

Region: NODE\_304263\_length\_4982\_cov\_28.985548 1349-1358. Max. coverage (+): 0.07. Max coverage (-): 0.19

Region: NODE\_304263\_length\_4982\_cov\_28.985548 1359-1368. Max. coverage (+): 0. Max coverage (-): 0.11

Region: NODE\_304263\_length\_4982\_cov\_28.985548 1369-1378. Max. coverage (+): 0.07. Max coverage (-): 0.44

Region: NODE\_304263\_length\_4982\_cov\_28.985548 1379-1388. Max. coverage (+): 0. Max coverage (-): 0.11

Region: NODE\_304263\_length\_4982\_cov\_28.985548 1389-1398. Max. coverage (+): 0.11. Max coverage (-): 0.41

Region: NODE\_304263\_length\_4982\_cov\_28.985548 1399-1408. Max. coverage (+): 0.11. Max coverage (-): 0.48

Region: NODE\_304263\_length\_4982\_cov\_28.985548 1409-1418. Max. coverage (+): 0.04. Max coverage (-): 0.41

Region: NODE\_304263\_length\_4982\_cov\_28.985548 1419-1428. Max. coverage (+): 0. Max coverage (-): 0.07

Region: NODE\_304263\_length\_4982\_cov\_28.985548 1429-1438. Max. coverage (+): 0.07. Max coverage (-): 0.15

Region: NODE\_304263\_length\_4982\_cov\_28.985548 1439-1448. Max. coverage (+): 0. Max coverage (-): 0.26

Region: NODE\_304263\_length\_4982\_cov\_28.985548 1449-1459. Max. coverage (+): 0.07. Max coverage (-): 0.41

Region: NODE\_304263\_length\_4982\_cov\_28.985548 1460-1469. Max. coverage (+): 0. Max coverage (-): 4.71

Region: NODE\_304263\_length\_4982\_cov\_28.985548 1470-1479. Max. coverage (+): 0. Max coverage (-): 2.71

Region: NODE\_304263\_length\_4982\_cov\_28.985548 1480-1489. Max. coverage (+): 0.04. Max coverage (-): 0.19

Region: NODE\_304263\_length\_4982\_cov\_28.985548 1490-1499. Max. coverage (+): 0.04. Max coverage (-): 0.04

Region: NODE\_304263\_length\_4982\_cov\_28.985548 1500-1509. Max. coverage (+): 0. Max coverage (-): 0.11

Region: NODE\_304263\_length\_4982\_cov\_28.985548 1510-1519. Max. coverage (+): 0.04. Max coverage (-): 4.37

Region: NODE\_304263\_length\_4982\_cov\_28.985548 1520-1529. Max. coverage (+): 0. Max coverage (-): 0.48

Region: NODE\_304263\_length\_4982\_cov\_28.985548 1530-1539. Max. coverage (+): 0.15. Max coverage (-): 0.15

Region: NODE\_304263\_length\_4982\_cov\_28.985548 1540-1549. Max. coverage (+): 0. Max coverage (-): 0.85

Region: NODE\_304263\_length\_4982\_cov\_28.985548 1550-1559. Max. coverage (+): 0. Max coverage (-): 0.56

Region: NODE\_304263\_length\_4982\_cov\_28.985548 1560-1569. Max. coverage (+): 0.26. Max coverage (-): 0.07

Region: NODE\_304263\_length\_4982\_cov\_28.985548 1570-1580. Max. coverage (+): 0. Max coverage (-): 4.6

Region: NODE\_304263\_length\_4982\_cov\_28.985548 1581-1590. Max. coverage (+): 0.26. Max coverage (-): 1.19

Region: NODE\_304263\_length\_4982\_cov\_28.985548 1591-1600. Max. coverage (+): 0.19. Max coverage (-): 0.07

Region: NODE\_304263\_length\_4982\_cov\_28.985548 1601-1610. Max. coverage (+): 0. Max coverage (-): 0.63

Region: NODE\_304263\_length\_4982\_cov\_28.985548 1611-1620. Max. coverage (+): 0. Max coverage (-): 2.56

Region: NODE\_304263\_length\_4982\_cov\_28.985548 1621-1630. Max. coverage (+): 0.04. Max coverage (-): 1.26

Region: NODE\_304263\_length\_4982\_cov\_28.985548 1631-1640. Max. coverage (+): 0.07. Max coverage (-): 0.15

Region: NODE\_304263\_length\_4982\_cov\_28.985548 1641-1650. Max. coverage (+): 0. Max coverage (-): 0.59

Region: NODE\_304263\_length\_4982\_cov\_28.985548 1651-1660. Max. coverage (+): 0.04. Max coverage (-): 2.08

Region: NODE\_304263\_length\_4982\_cov\_28.985548 1661-1670. Max. coverage (+): 0. Max coverage (-): 0.26

Region: NODE\_304263\_length\_4982\_cov\_28.985548 1671-1680. Max. coverage (+): 0. Max coverage (-): 1.15

Region: NODE\_304263\_length\_4982\_cov\_28.985548 1681-1691. Max. coverage (+): 0. Max coverage (-): 0.26

Region: NODE\_304263\_length\_4982\_cov\_28.985548 1692-1701. Max. coverage (+): 0.04. Max coverage (-): 0

Region: NODE\_304263\_length\_4982\_cov\_28.985548 1702-1711. Max. coverage (+): 0. Max coverage (-): 0.33

Region: NODE\_304263\_length\_4982\_cov\_28.985548 1712-1721. Max. coverage (+): 0. Max coverage (-): 2.11

Region: NODE\_304263\_length\_4982\_cov\_28.985548 1722-1731. Max. coverage (+): 0. Max coverage (-): 0.78

Region: NODE\_304263\_length\_4982\_cov\_28.985548 1732-1741. Max. coverage (+): 0.07. Max coverage (-): 0.07

Region: NODE\_304263\_length\_4982\_cov\_28.985548 1742-1751. Max. coverage (+): 0.07. Max coverage (-): 0.04

Region: NODE\_304263\_length\_4982\_cov\_28.985548 1752-1761. Max. coverage (+): 0.15. Max coverage (-): 0.11

Region: NODE\_304263\_length\_4982\_cov\_28.985548 1762-1771. Max. coverage (+): 0. Max coverage (-): 1.93

Region: NODE\_304263\_length\_4982\_cov\_28.985548 1772-1781. Max. coverage (+): 0. Max coverage (-): 0.59

Region: NODE\_304263\_length\_4982\_cov\_28.985548 1782-1791. Max. coverage (+): 0.19. Max coverage (-): 0.3

Region: NODE\_304263\_length\_4982\_cov\_28.985548 1792-1802. Max. coverage (+): 0. Max coverage (-): 1.67

Region: NODE\_304263\_length\_4982\_cov\_28.985548 1803-1812. Max. coverage (+): 0. Max coverage (-): 1.82

Region: NODE\_304263\_length\_4982\_cov\_28.985548 1813-1822. Max. coverage (+): 0.15. Max coverage (-): 2.11

Region: NODE\_304263\_length\_4982\_cov\_28.985548 1823-1832. Max. coverage (+): 0.78. Max coverage (-): 0.04

Region: NODE\_304263\_length\_4982\_cov\_28.985548 1833-1842. Max. coverage (+): 0.78. Max coverage (-): 2.45

Region: NODE\_304263\_length\_4982\_cov\_28.985548 1843-1852. Max. coverage (+): 0. Max coverage (-): 2.45

Region: NODE\_304263\_length\_4982\_cov\_28.985548 1853-1862. Max. coverage (+): 0. Max coverage (-): 0.11

Region: NODE\_304263\_length\_4982\_cov\_28.985548 1863-1872. Max. coverage (+): 0.04. Max coverage (-): 0.07

Region: NODE\_304263\_length\_4982\_cov\_28.985548 1873-1882. Max. coverage (+): 0. Max coverage (-): 0.04

Region: NODE\_304263\_length\_4982\_cov\_28.985548 1883-1892. Max. coverage (+): 0. Max coverage (-): 0.15

Region: NODE\_304263\_length\_4982\_cov\_28.985548 1893-1902. Max. coverage (+): 0.07. Max coverage (-): 0.41

Region: NODE\_304263\_length\_4982\_cov\_28.985548 1903-1913. Max. coverage (+): 3.37. Max coverage (-): 0

Region: NODE\_304263\_length\_4982\_cov\_28.985548 1914-1923. Max. coverage (+): 0. Max coverage (-): 0

Region: NODE\_304263\_length\_4982\_cov\_28.985548 1924-1933. Max. coverage (+): 0. Max coverage (-): 0

Region: NODE\_304263\_length\_4982\_cov\_28.985548 1934-1943. Max. coverage (+): 0. Max coverage (-): 0.07

Region: NODE\_304263\_length\_4982\_cov\_28.985548 1944-1953. Max. coverage (+): 0. Max coverage (-): 0.15

Region: NODE\_304263\_length\_4982\_cov\_28.985548 1954-1963. Max. coverage (+): 0. Max coverage (-): 0.7

Region: NODE\_304263\_length\_4982\_cov\_28.985548 1964-1973. Max. coverage (+): 0.04. Max coverage (-): 0.3

Region: NODE\_304263\_length\_4982\_cov\_28.985548 1974-1983. Max. coverage (+): 0.04. Max coverage (-): 0.63

Region: NODE\_304263\_length\_4982\_cov\_28.985548 1984-1993. Max. coverage (+): 0. Max coverage (-): 0.3

Region: NODE\_304263\_length\_4982\_cov\_28.985548 1994-2003. Max. coverage (+): 0. Max coverage (-): 0.89

Region: NODE\_304263\_length\_4982\_cov\_28.985548 2004-2013. Max. coverage (+): 0. Max coverage (-): 0.74

Region: NODE\_304263\_length\_4982\_cov\_28.985548 2014-2024. Max. coverage (+): 0. Max coverage (-): 0.15

Region: NODE\_304263\_length\_4982\_cov\_28.985548 2025-2034. Max. coverage (+): 0.04. Max coverage (-): 0.52

Region: NODE\_304263\_length\_4982\_cov\_28.985548 2035-2044. Max. coverage (+): 0.48. Max coverage (-): 0.67

Region: NODE\_304263\_length\_4982\_cov\_28.985548 2045-2054. Max. coverage (+): 0.11. Max coverage (-): 0.04

Region: NODE\_304263\_length\_4982\_cov\_28.985548 2055-2064. Max. coverage (+): 0.07. Max coverage (-): 3.48

Region: NODE\_304263\_length\_4982\_cov\_28.985548 2065-2074. Max. coverage (+): 0. Max coverage (-): 0.7

Region: NODE\_304263\_length\_4982\_cov\_28.985548 2075-2084. Max. coverage (+): 0.11. Max coverage (-): 0.52

Region: NODE\_304263\_length\_4982\_cov\_28.985548 2085-2094. Max. coverage (+): 0. Max coverage (-): 0.07

Region: NODE\_304263\_length\_4982\_cov\_28.985548 2095-2104. Max. coverage (+): 0. Max coverage (-): 0.15

Region: NODE\_304263\_length\_4982\_cov\_28.985548 2105-2114. Max. coverage (+): 0. Max coverage (-): 0.85

Region: NODE\_304263\_length\_4982\_cov\_28.985548 2115-2124. Max. coverage (+): 0. Max coverage (-): 0.89

Region: NODE\_304263\_length\_4982\_cov\_28.985548 2125-2135. Max. coverage (+): 0. Max coverage (-): 0.04

Region: NODE\_304263\_length\_4982\_cov\_28.985548 2136-2145. Max. coverage (+): 0. Max coverage (-): 0

Region: NODE\_304263\_length\_4982\_cov\_28.985548 2146-2155. Max. coverage (+): 0. Max coverage (-): 0.07

Region: NODE\_304263\_length\_4982\_cov\_28.985548 2156-2165. Max. coverage (+): 0. Max coverage (-): 0.19

Region: NODE\_304263\_length\_4982\_cov\_28.985548 2166-2175. Max. coverage (+): 0. Max coverage (-): 0.37

Region: NODE\_304263\_length\_4982\_cov\_28.985548 2176-2185. Max. coverage (+): 0. Max coverage (-): 0.7

Region: NODE\_304263\_length\_4982\_cov\_28.985548 2186-2195. Max. coverage (+): 0.26. Max coverage (-): 4.52

Region: NODE\_304263\_length\_4982\_cov\_28.985548 2196-2205. Max. coverage (+): 0.19. Max coverage (-): 3.89

Region: NODE\_304263\_length\_4982\_cov\_28.985548 2206-2215. Max. coverage (+): 0.15. Max coverage (-): 0.63

Region: NODE\_304263\_length\_4982\_cov\_28.985548 2216-2225. Max. coverage (+): 0.11. Max coverage (-): 0.37

Region: NODE\_304263\_length\_4982\_cov\_28.985548 2226-2235. Max. coverage (+): 0.04. Max coverage (-): 1.04

Region: NODE\_304263\_length\_4982\_cov\_28.985548 2236-2246. Max. coverage (+): 0. Max coverage (-): 1.93

Region: NODE\_304263\_length\_4982\_cov\_28.985548 2247-2256. Max. coverage (+): 0.15. Max coverage (-): 0

Region: NODE\_304263\_length\_4982\_cov\_28.985548 2257-2266. Max. coverage (+): 0.15. Max coverage (-): 0

Region: NODE\_304263\_length\_4982\_cov\_28.985548 2267-2276. Max. coverage (+): 0. Max coverage (-): 0.3

Region: NODE\_304263\_length\_4982\_cov\_28.985548 2277-2286. Max. coverage (+): 0. Max coverage (-): 0.07

Region: NODE\_304263\_length\_4982\_cov\_28.985548 2287-2296. Max. coverage (+): 0.04. Max coverage (-): 0.93

Region: NODE\_304263\_length\_4982\_cov\_28.985548 2297-2306. Max. coverage (+): 0.07. Max coverage (-): 0.89

Region: NODE\_304263\_length\_4982\_cov\_28.985548 2307-2316. Max. coverage (+): 0.52. Max coverage (-): 4.63

Region: NODE\_304263\_length\_4982\_cov\_28.985548 2317-2326. Max. coverage (+): 0.33. Max coverage (-): 1.71

Region: NODE\_304263\_length\_4982\_cov\_28.985548 2327-2336. Max. coverage (+): 0.11. Max coverage (-): 0.04

Region: NODE\_304263\_length\_4982\_cov\_28.985548 2337-2346. Max. coverage (+): 0.04. Max coverage (-): 0.48

Region: NODE\_304263\_length\_4982\_cov\_28.985548 2347-2357. Max. coverage (+): 0.04. Max coverage (-): 2.19

Region: NODE\_304263\_length\_4982\_cov\_28.985548 2358-2367. Max. coverage (+): 0.04. Max coverage (-): 4.37

Region: NODE\_304263\_length\_4982\_cov\_28.985548 2368-2377. Max. coverage (+): 0.04. Max coverage (-): 4.12

Region: NODE\_304263\_length\_4982\_cov\_28.985548 2378-2387. Max. coverage (+): 0.33. Max coverage (-): 0.26

Region: NODE\_304263\_length\_4982\_cov\_28.985548 2388-2397. Max. coverage (+): 0.11. Max coverage (-): 1.48

Region: NODE\_304263\_length\_4982\_cov\_28.985548 2398-2407. Max. coverage (+): 0. Max coverage (-): 0.48

Region: NODE\_304263\_length\_4982\_cov\_28.985548 2408-2417. Max. coverage (+): 0.11. Max coverage (-): 0.44

Region: NODE\_304263\_length\_4982\_cov\_28.985548 2418-2427. Max. coverage (+): 0.11. Max coverage (-): 0.07

Region: NODE\_304263\_length\_4982\_cov\_28.985548 2428-2437. Max. coverage (+): 0.04. Max coverage (-): 0.22

Region: NODE\_304263\_length\_4982\_cov\_28.985548 2438-2447. Max. coverage (+): 0.22. Max coverage (-): 0.22

Region: NODE\_304263\_length\_4982\_cov\_28.985548 2448-2457. Max. coverage (+): 0.11. Max coverage (-): 0.19

Region: NODE\_304263\_length\_4982\_cov\_28.985548 2458-2468. Max. coverage (+): 0. Max coverage (-): 0.89

Region: NODE\_304263\_length\_4982\_cov\_28.985548 2469-2478. Max. coverage (+): 0.07. Max coverage (-): 0.37

Region: NODE\_304263\_length\_4982\_cov\_28.985548 2479-2488. Max. coverage (+): 0.07. Max coverage (-): 0

Region: NODE\_304263\_length\_4982\_cov\_28.985548 2489-2498. Max. coverage (+): 0.11. Max coverage (-): 0

Region: NODE\_304263\_length\_4982\_cov\_28.985548 2499-2508. Max. coverage (+): 0. Max coverage (-): 0.07

Region: NODE\_304263\_length\_4982\_cov\_28.985548 2509-2518. Max. coverage (+): 0. Max coverage (-): 0.07

Region: NODE\_304263\_length\_4982\_cov\_28.985548 2519-2528. Max. coverage (+): 0. Max coverage (-): 0.07

Region: NODE\_304263\_length\_4982\_cov\_28.985548 2529-2538. Max. coverage (+): 0.04. Max coverage (-): 0.07

Region: NODE\_304263\_length\_4982\_cov\_28.985548 2539-2548. Max. coverage (+): 0. Max coverage (-): 0

Region: NODE\_304263\_length\_4982\_cov\_28.985548 2549-2558. Max. coverage (+): 0. Max coverage (-): 1.93

Region: NODE\_304263\_length\_4982\_cov\_28.985548 2559-2568. Max. coverage (+): 0. Max coverage (-): 1.82

Region: NODE\_304263\_length\_4982\_cov\_28.985548 2569-2578. Max. coverage (+): 0. Max coverage (-): 2.93

Region: NODE\_304263\_length\_4982\_cov\_28.985548 2579-2589. Max. coverage (+): 0.26. Max coverage (-): 6.23

Region: NODE\_304263\_length\_4982\_cov\_28.985548 2590-2599. Max. coverage (+): 0.22. Max coverage (-): 9.79

Region: NODE\_304263\_length\_4982\_cov\_28.985548 2600-2609. Max. coverage (+): 0. Max coverage (-): 6.12

Region: NODE\_304263\_length\_4982\_cov\_28.985548 2610-2619. Max. coverage (+): 0.04. Max coverage (-): 4.78

Region: NODE\_304263\_length\_4982\_cov\_28.985548 2620-2629. Max. coverage (+): 0.07. Max coverage (-): 4.45

Region: NODE\_304263\_length\_4982\_cov\_28.985548 2630-2639. Max. coverage (+): 0.33. Max coverage (-): 0.85

Region: NODE\_304263\_length\_4982\_cov\_28.985548 2640-2649. Max. coverage (+): 0.33. Max coverage (-): 2.15

Region: NODE\_304263\_length\_4982\_cov\_28.985548 2650-2659. Max. coverage (+): 0.04. Max coverage (-): 2.22

Region: NODE\_304263\_length\_4982\_cov\_28.985548 2660-2669. Max. coverage (+): 0. Max coverage (-): 0.07

Region: NODE\_304263\_length\_4982\_cov\_28.985548 2670-2679. Max. coverage (+): 0. Max coverage (-): 0

Region: NODE\_304263\_length\_4982\_cov\_28.985548 2680-2689. Max. coverage (+): 0. Max coverage (-): 0.07

Region: NODE\_304263\_length\_4982\_cov\_28.985548 2690-2700. Max. coverage (+): 0.15. Max coverage (-): 0.07

Region: NODE\_304263\_length\_4982\_cov\_28.985548 2701-2710. Max. coverage (+): 0.19. Max coverage (-): 0.15

Region: NODE\_304263\_length\_4982\_cov\_28.985548 2711-2720. Max. coverage (+): 0. Max coverage (-): 1.08

Region: NODE\_304263\_length\_4982\_cov\_28.985548 2721-2730. Max. coverage (+): 0.04. Max coverage (-): 0.11

Region: NODE\_304263\_length\_4982\_cov\_28.985548 2731-2740. Max. coverage (+): 0.26. Max coverage (-): 0.19

Region: NODE\_304263\_length\_4982\_cov\_28.985548 2741-2750. Max. coverage (+): 0.07. Max coverage (-): 0.15

Region: NODE\_304263\_length\_4982\_cov\_28.985548 2751-2760. Max. coverage (+): 0. Max coverage (-): 0.19

Region: NODE\_304263\_length\_4982\_cov\_28.985548 2761-2770. Max. coverage (+): 0.04. Max coverage (-): 1.93

Region: NODE\_304263\_length\_4982\_cov\_28.985548 2771-2780. Max. coverage (+): 0.04. Max coverage (-): 0.07

Region: NODE\_304263\_length\_4982\_cov\_28.985548 2781-2790. Max. coverage (+): 0.33. Max coverage (-): 0.19

Region: NODE\_304263\_length\_4982\_cov\_28.985548 2791-2800. Max. coverage (+): 0.04. Max coverage (-): 0.26

Region: NODE\_304263\_length\_4982\_cov\_28.985548 2801-2811. Max. coverage (+): 0.04. Max coverage (-): 0.15

Region: NODE\_304263\_length\_4982\_cov\_28.985548 2812-2821. Max. coverage (+): 0. Max coverage (-): 0.11

Region: NODE\_304263\_length\_4982\_cov\_28.985548 2822-2831. Max. coverage (+): 0.04. Max coverage (-): 0.56

Region: NODE\_304263\_length\_4982\_cov\_28.985548 2832-2841. Max. coverage (+): 0.11. Max coverage (-): 0.63

Region: NODE\_304263\_length\_4982\_cov\_28.985548 2842-2851. Max. coverage (+): 0.11. Max coverage (-): 0.67

Region: NODE\_304263\_length\_4982\_cov\_28.985548 2852-2861. Max. coverage (+): 0.3. Max coverage (-): 7.45

Region: NODE\_304263\_length\_4982\_cov\_28.985548 2862-2871. Max. coverage (+): 0.11. Max coverage (-): 0.41

Region: NODE\_304263\_length\_4982\_cov\_28.985548 2872-2881. Max. coverage (+): 0.11. Max coverage (-): 0.07

Region: NODE\_304263\_length\_4982\_cov\_28.985548 2882-2891. Max. coverage (+): 0.07. Max coverage (-): 0.44

Region: NODE\_304263\_length\_4982\_cov\_28.985548 2892-2901. Max. coverage (+): 0.04. Max coverage (-): 0.63

Region: NODE\_304263\_length\_4982\_cov\_28.985548 2902-2911. Max. coverage (+): 0.19. Max coverage (-): 0.19

Region: NODE\_304263\_length\_4982\_cov\_28.985548 2912-2922. Max. coverage (+): 0. Max coverage (-): 0

Region: NODE\_304263\_length\_4982\_cov\_28.985548 2923-2932. Max. coverage (+): 0. Max coverage (-): 0.22

Region: NODE\_304263\_length\_4982\_cov\_28.985548 2933-2942. Max. coverage (+): 0. Max coverage (-): 5.56

Region: NODE\_304263\_length\_4982\_cov\_28.985548 2943-2952. Max. coverage (+): 0.44. Max coverage (-): 0

Region: NODE\_304263\_length\_4982\_cov\_28.985548 2953-2962. Max. coverage (+): 0.56. Max coverage (-): 0

Region: NODE\_304263\_length\_4982\_cov\_28.985548 2963-2972. Max. coverage (+): 0. Max coverage (-): 0

Region: NODE\_304263\_length\_4982\_cov\_28.985548 2973-2982. Max. coverage (+): 0. Max coverage (-): 0.07

Region: NODE\_304263\_length\_4982\_cov\_28.985548 2983-2992. Max. coverage (+): 0.04. Max coverage (-): 0.04

Region: NODE\_304263\_length\_4982\_cov\_28.985548 2993-3002. Max. coverage (+): 0.07. Max coverage (-): 0.41

Region: NODE\_304263\_length\_4982\_cov\_28.985548 3003-3012. Max. coverage (+): 0. Max coverage (-): 4.78

Region: NODE\_304263\_length\_4982\_cov\_28.985548 3013-3022. Max. coverage (+): 0. Max coverage (-): 0.11

Region: NODE\_304263\_length\_4982\_cov\_28.985548 3023-3033. Max. coverage (+): 0.07. Max coverage (-): 1.89

Region: NODE\_304263\_length\_4982\_cov\_28.985548 3034-3043. Max. coverage (+): 0. Max coverage (-): 8.79

Region: NODE\_304263\_length\_4982\_cov\_28.985548 3044-3053. Max. coverage (+): 0.04. Max coverage (-): 0.15

Region: NODE\_304263\_length\_4982\_cov\_28.985548 3054-3063. Max. coverage (+): 0.67. Max coverage (-): 0.37

Region: NODE\_304263\_length\_4982\_cov\_28.985548 3064-3073. Max. coverage (+): 0.04. Max coverage (-): 1.22

Region: NODE\_304263\_length\_4982\_cov\_28.985548 3074-3083. Max. coverage (+): 0.19. Max coverage (-): 1.22

Region: NODE\_304263\_length\_4982\_cov\_28.985548 3084-3093. Max. coverage (+): 0.19. Max coverage (-): 3.97

Region: NODE\_304263\_length\_4982\_cov\_28.985548 3094-3103. Max. coverage (+): 0.26. Max coverage (-): 0

Region: NODE\_304263\_length\_4982\_cov\_28.985548 3104-3113. Max. coverage (+): 0. Max coverage (-): 1.89

Region: NODE\_304263\_length\_4982\_cov\_28.985548 3114-3123. Max. coverage (+): 0.04. Max coverage (-): 1.22

Region: NODE\_304263\_length\_4982\_cov\_28.985548 3124-3133. Max. coverage (+): 0.11. Max coverage (-): 0.15

Region: NODE\_304263\_length\_4982\_cov\_28.985548 3134-3144. Max. coverage (+): 0. Max coverage (-): 0.19

Region: NODE\_304263\_length\_4982\_cov\_28.985548 3145-3154. Max. coverage (+): 0. Max coverage (-): 0.48

Region: NODE\_304263\_length\_4982\_cov\_28.985548 3155-3164. Max. coverage (+): 0.04. Max coverage (-): 0

Region: NODE\_304263\_length\_4982\_cov\_28.985548 3165-3174. Max. coverage (+): 0.11. Max coverage (-): 9.38

Region: NODE\_304263\_length\_4982\_cov\_28.985548 3175-3184. Max. coverage (+): 0.11. Max coverage (-): 84.38

Region: NODE\_304263\_length\_4982\_cov\_28.985548 3185-3194. Max. coverage (+): 0.04. Max coverage (-): 7.49

Region: NODE\_304263\_length\_4982\_cov\_28.985548 3195-3204. Max. coverage (+): 0.04. Max coverage (-): 0.89

Region: NODE\_304263\_length\_4982\_cov\_28.985548 3205-3214. Max. coverage (+): 0. Max coverage (-): 0.44

Region: NODE\_304263\_length\_4982\_cov\_28.985548 3215-3224. Max. coverage (+): 0. Max coverage (-): 4.08

Region: NODE\_304263\_length\_4982\_cov\_28.985548 3225-3234. Max. coverage (+): 0. Max coverage (-): 1.85

Region: NODE\_304263\_length\_4982\_cov\_28.985548 3235-3244. Max. coverage (+): 0.04. Max coverage (-): 0

Region: NODE\_304263\_length\_4982\_cov\_28.985548 3245-3255. Max. coverage (+): 0. Max coverage (-): 0.07

Region: NODE\_304263\_length\_4982\_cov\_28.985548 3256-3265. Max. coverage (+): 0.04. Max coverage (-): 1.22

Region: NODE\_304263\_length\_4982\_cov\_28.985548 3266-3275. Max. coverage (+): 0.59. Max coverage (-): 3.63

Region: NODE\_304263\_length\_4982\_cov\_28.985548 3276-3285. Max. coverage (+): 0.07. Max coverage (-): 0.52

Region: NODE\_304263\_length\_4982\_cov\_28.985548 3286-3295. Max. coverage (+): 0.22. Max coverage (-): 1.04

Region: NODE\_304263\_length\_4982\_cov\_28.985548 3296-3305. Max. coverage (+): 0.11. Max coverage (-): 17.5

Region: NODE\_304263\_length\_4982\_cov\_28.985548 3306-3315. Max. coverage (+): 0.11. Max coverage (-): 17.09

Region: NODE\_304263\_length\_4982\_cov\_28.985548 3316-3325. Max. coverage (+): 0.3. Max coverage (-): 2.37

Region: NODE\_304263\_length\_4982\_cov\_28.985548 3326-3335. Max. coverage (+): 0.04. Max coverage (-): 0.04

Region: NODE\_304263\_length\_4982\_cov\_28.985548 3336-3345. Max. coverage (+): 0.04. Max coverage (-): 0.26

Region: NODE\_304263\_length\_4982\_cov\_28.985548 3346-3355. Max. coverage (+): 0.04. Max coverage (-): 2.04

Region: NODE\_304263\_length\_4982\_cov\_28.985548 3356-3366. Max. coverage (+): 0. Max coverage (-): 1.19

Region: NODE\_304263\_length\_4982\_cov\_28.985548 3367-3376. Max. coverage (+): 0.37. Max coverage (-): 0.48

Region: NODE\_304263\_length\_4982\_cov\_28.985548 3377-3386. Max. coverage (+): 0.15. Max coverage (-): 0.59

Region: NODE\_304263\_length\_4982\_cov\_28.985548 3387-3396. Max. coverage (+): 0. Max coverage (-): 0.15

Region: NODE\_304263\_length\_4982\_cov\_28.985548 3397-3406. Max. coverage (+): 0.04. Max coverage (-): 1.04

Region: NODE\_304263\_length\_4982\_cov\_28.985548 3407-3416. Max. coverage (+): 0.04. Max coverage (-): 9.94

Region: NODE\_304263\_length\_4982\_cov\_28.985548 3417-3426. Max. coverage (+): 4.56. Max coverage (-): 0.41

Region: NODE\_304263\_length\_4982\_cov\_28.985548 3427-3436. Max. coverage (+): 3.41. Max coverage (-): 0.48

Region: NODE\_304263\_length\_4982\_cov\_28.985548 3437-3446. Max. coverage (+): 0. Max coverage (-): 0.41

Region: NODE\_304263\_length\_4982\_cov\_28.985548 3447-3456. Max. coverage (+): 0.04. Max coverage (-): 1.3

Region: NODE\_304263\_length\_4982\_cov\_28.985548 3457-3466. Max. coverage (+): 0. Max coverage (-): 55.83

Region: NODE\_304263\_length\_4982\_cov\_28.985548 3467-3477. Max. coverage (+): 0.04. Max coverage (-): 5.26

Region: NODE\_304263\_length\_4982\_cov\_28.985548 3478-3487. Max. coverage (+): 0.11. Max coverage (-): 0.22

Region: NODE\_304263\_length\_4982\_cov\_28.985548 3488-3497. Max. coverage (+): 0. Max coverage (-): 2.97

Region: NODE\_304263\_length\_4982\_cov\_28.985548 3498-3507. Max. coverage (+): 0.07. Max coverage (-): 2

Region: NODE\_304263\_length\_4982\_cov\_28.985548 3508-3517. Max. coverage (+): 1.08. Max coverage (-): 1.04

Region: NODE\_304263\_length\_4982\_cov\_28.985548 3518-3527. Max. coverage (+): 0. Max coverage (-): 0

Region: NODE\_304263\_length\_4982\_cov\_28.985548 3528-3537. Max. coverage (+): 0. Max coverage (-): 36.44

Region: NODE\_304263\_length\_4982\_cov\_28.985548 3538-3547. Max. coverage (+): 0. Max coverage (-): 37.33

Region: NODE\_304263\_length\_4982\_cov\_28.985548 3548-3557. Max. coverage (+): 0.37. Max coverage (-): 0.3

Region: NODE\_304263\_length\_4982\_cov\_28.985548 3558-3567. Max. coverage (+): 0.33. Max coverage (-): 0.04

Region: NODE\_304263\_length\_4982\_cov\_28.985548 3568-3577. Max. coverage (+): 0.22. Max coverage (-): 0.33

Region: NODE\_304263\_length\_4982\_cov\_28.985548 3578-3587. Max. coverage (+): 0. Max coverage (-): 2.93

Region: NODE\_304263\_length\_4982\_cov\_28.985548 3588-3598. Max. coverage (+): 1. Max coverage (-): 0.07

Region: NODE\_304263\_length\_4982\_cov\_28.985548 3599-3608. Max. coverage (+): 1. Max coverage (-): 0.07

Region: NODE\_304263\_length\_4982\_cov\_28.985548 3609-3618. Max. coverage (+): 0. Max coverage (-): 0.19

Region: NODE\_304263\_length\_4982\_cov\_28.985548 3619-3628. Max. coverage (+): 0.04. Max coverage (-): 0.11

Region: NODE\_304263\_length\_4982\_cov\_28.985548 3629-3638. Max. coverage (+): 0.22. Max coverage (-): 3.45

Region: NODE\_304263\_length\_4982\_cov\_28.985548 3639-3648. Max. coverage (+): 0.26. Max coverage (-): 4

Region: NODE\_304263\_length\_4982\_cov\_28.985548 3649-3658. Max. coverage (+): 0.41. Max coverage (-): 0.59

Region: NODE\_304263\_length\_4982\_cov\_28.985548 3659-3668. Max. coverage (+): 0.33. Max coverage (-): 0.37

Region: NODE\_304263\_length\_4982\_cov\_28.985548 3669-3678. Max. coverage (+): 0. Max coverage (-): 0.19

Region: NODE\_304263\_length\_4982\_cov\_28.985548 3679-3688. Max. coverage (+): 0. Max coverage (-): 0.41

Region: NODE\_304263\_length\_4982\_cov\_28.985548 3689-3698. Max. coverage (+): 0. Max coverage (-): 12.09

Region: NODE\_304263\_length\_4982\_cov\_28.985548 3699-3709. Max. coverage (+): 0.19. Max coverage (-): 13.61

Region: NODE\_304263\_length\_4982\_cov\_28.985548 3710-3719. Max. coverage (+): 0.93. Max coverage (-): 2

Region: NODE\_304263\_length\_4982\_cov\_28.985548 3720-3729. Max. coverage (+): 0.04. Max coverage (-): 0.33

Region: NODE\_304263\_length\_4982\_cov\_28.985548 3730-3739. Max. coverage (+): 0. Max coverage (-): 7.16

Region: NODE\_304263\_length\_4982\_cov\_28.985548 3740-3749. Max. coverage (+): 0. Max coverage (-): 6.41

Region: NODE\_304263\_length\_4982\_cov\_28.985548 3750-3759. Max. coverage (+): 0.04. Max coverage (-): 1.96

Region: NODE\_304263\_length\_4982\_cov\_28.985548 3760-3769. Max. coverage (+): 0.19. Max coverage (-): 0.41

Region: NODE\_304263\_length\_4982\_cov\_28.985548 3770-3779. Max. coverage (+): 0.04. Max coverage (-): 2.04

Region: NODE\_304263\_length\_4982\_cov\_28.985548 3780-3789. Max. coverage (+): 0. Max coverage (-): 5.08

Region: NODE\_304263\_length\_4982\_cov\_28.985548 3790-3799. Max. coverage (+): 0.04. Max coverage (-): 0.33

Region: NODE\_304263\_length\_4982\_cov\_28.985548 3800-3809. Max. coverage (+): 0.04. Max coverage (-): 0.33

Region: NODE\_304263\_length\_4982\_cov\_28.985548 3810-3820. Max. coverage (+): 0. Max coverage (-): 0.56

Region: NODE\_304263\_length\_4982\_cov\_28.985548 3821-3830. Max. coverage (+): 0. Max coverage (-): 0.44

Region: NODE\_304263\_length\_4982\_cov\_28.985548 3831-3840. Max. coverage (+): 0. Max coverage (-): 6.3

Region: NODE\_304263\_length\_4982\_cov\_28.985548 3841-3850. Max. coverage (+): 0.74. Max coverage (-): 0.59

Region: NODE\_304263\_length\_4982\_cov\_28.985548 3851-3860. Max. coverage (+): 1.19. Max coverage (-): 10.97

Region: NODE\_304263\_length\_4982\_cov\_28.985548 3861-3870. Max. coverage (+): 0.11. Max coverage (-): 1.93

Region: NODE\_304263\_length\_4982\_cov\_28.985548 3871-3880. Max. coverage (+): 0. Max coverage (-): 0.07

Region: NODE\_304263\_length\_4982\_cov\_28.985548 3881-3890. Max. coverage (+): 0.04. Max coverage (-): 2

Region: NODE\_304263\_length\_4982\_cov\_28.985548 3891-3900. Max. coverage (+): 0.33. Max coverage (-): 1.78

Region: NODE\_304263\_length\_4982\_cov\_28.985548 3901-3910. Max. coverage (+): 3.52. Max coverage (-): 0.19

Region: NODE\_304263\_length\_4982\_cov\_28.985548 3911-3920. Max. coverage (+): 0.07. Max coverage (-): 1.33

Region: NODE\_304263\_length\_4982\_cov\_28.985548 3921-3931. Max. coverage (+): 0. Max coverage (-): 1.45

Region: NODE\_304263\_length\_4982\_cov\_28.985548 3932-3941. Max. coverage (+): 0. Max coverage (-): 0.19

Region: NODE\_304263\_length\_4982\_cov\_28.985548 3942-3951. Max. coverage (+): 0. Max coverage (-): 4.67

Region: NODE\_304263\_length\_4982\_cov\_28.985548 3952-3961. Max. coverage (+): 0.04. Max coverage (-): 0.82

Region: NODE\_304263\_length\_4982\_cov\_28.985548 3962-3971. Max. coverage (+): 0.26. Max coverage (-): 0.19

Region: NODE\_304263\_length\_4982\_cov\_28.985548 3972-3981. Max. coverage (+): 0. Max coverage (-): 0.48

Region: NODE\_304263\_length\_4982\_cov\_28.985548 3982-3991. Max. coverage (+): 0. Max coverage (-): 0.15

Region: NODE\_304263\_length\_4982\_cov\_28.985548 3992-4001. Max. coverage (+): 0.04. Max coverage (-): 0.19

Region: NODE\_304263\_length\_4982\_cov\_28.985548 4002-4011. Max. coverage (+): 0.04. Max coverage (-): 4.6

Region: NODE\_304263\_length\_4982\_cov\_28.985548 4012-4021. Max. coverage (+): 0.22. Max coverage (-): 0.63

Region: NODE\_304263\_length\_4982\_cov\_28.985548 4022-4031. Max. coverage (+): 1.96. Max coverage (-): 0.04

Region: NODE\_304263\_length\_4982\_cov\_28.985548 4032-4042. Max. coverage (+): 0. Max coverage (-): 8.64

Region: NODE\_304263\_length\_4982\_cov\_28.985548 4043-4052. Max. coverage (+): 0.11. Max coverage (-): 0.19

Region: NODE\_304263\_length\_4982\_cov\_28.985548 4053-4062. Max. coverage (+): 0.22. Max coverage (-): 0.52

Region: NODE\_304263\_length\_4982\_cov\_28.985548 4063-4072. Max. coverage (+): 0. Max coverage (-): 3.93

Region: NODE\_304263\_length\_4982\_cov\_28.985548 4073-4082. Max. coverage (+): 0. Max coverage (-): 0.04

Region: NODE\_304263\_length\_4982\_cov\_28.985548 4083-4092. Max. coverage (+): 0.22. Max coverage (-): 0.11

Region: NODE\_304263\_length\_4982\_cov\_28.985548 4093-4102. Max. coverage (+): 0. Max coverage (-): 0.7

Region: NODE\_304263\_length\_4982\_cov\_28.985548 4103-4112. Max. coverage (+): 0. Max coverage (-): 2.37

Region: NODE\_304263\_length\_4982\_cov\_28.985548 4113-4122. Max. coverage (+): 0. Max coverage (-): 1.33

Region: NODE\_304263\_length\_4982\_cov\_28.985548 4123-4132. Max. coverage (+): 0.26. Max coverage (-): 0.07

Region: NODE\_304263\_length\_4982\_cov\_28.985548 4133-4142. Max. coverage (+): 0.04. Max coverage (-): 0.15

Region: NODE\_304263\_length\_4982\_cov\_28.985548 4143-4153. Max. coverage (+): 0. Max coverage (-): 1.59

Region: NODE\_304263\_length\_4982\_cov\_28.985548 4154-4163. Max. coverage (+): 0.04. Max coverage (-): 2.56

Region: NODE\_304263\_length\_4982\_cov\_28.985548 4164-4173. Max. coverage (+): 0.07. Max coverage (-): 2.15

Region: NODE\_304263\_length\_4982\_cov\_28.985548 4174-4183. Max. coverage (+): 0.04. Max coverage (-): 2.15

Region: NODE\_304263\_length\_4982\_cov\_28.985548 4184-4193. Max. coverage (+): 0. Max coverage (-): 11.2

Region: NODE\_304263\_length\_4982\_cov\_28.985548 4194-4203. Max. coverage (+): 0.04. Max coverage (-): 1.96

Region: NODE\_304263\_length\_4982\_cov\_28.985548 4204-4213. Max. coverage (+): 4.08. Max coverage (-): 0.15

Region: NODE\_304263\_length\_4982\_cov\_28.985548 4214-4223. Max. coverage (+): 0.3. Max coverage (-): 0.07

Region: NODE\_304263\_length\_4982\_cov\_28.985548 4224-4233. Max. coverage (+): 0. Max coverage (-): 2.3

Region: NODE\_304263\_length\_4982\_cov\_28.985548 4234-4243. Max. coverage (+): 0.04. Max coverage (-): 0.26

Region: NODE\_304263\_length\_4982\_cov\_28.985548 4244-4253. Max. coverage (+): 0.19. Max coverage (-): 2.89

Region: NODE\_304263\_length\_4982\_cov\_28.985548 4254-4264. Max. coverage (+): 0.15. Max coverage (-): 5.41

Region: NODE\_304263\_length\_4982\_cov\_28.985548 4265-4274. Max. coverage (+): 0.11. Max coverage (-): 0.3

Region: NODE\_304263\_length\_4982\_cov\_28.985548 4275-4284. Max. coverage (+): 0.15. Max coverage (-): 0.85

Region: NODE\_304263\_length\_4982\_cov\_28.985548 4285-4294. Max. coverage (+): 0.41. Max coverage (-): 0.93

Region: NODE\_304263\_length\_4982\_cov\_28.985548 4295-4304. Max. coverage (+): 2.04. Max coverage (-): 0.22

Region: NODE\_304263\_length\_4982\_cov\_28.985548 4305-4314. Max. coverage (+): 0.04. Max coverage (-): 2.22

Region: NODE\_304263\_length\_4982\_cov\_28.985548 4315-4324. Max. coverage (+): 0.04. Max coverage (-): 1.89

Region: NODE\_304263\_length\_4982\_cov\_28.985548 4325-4334. Max. coverage (+): 0.15. Max coverage (-): 0.07

Region: NODE\_304263\_length\_4982\_cov\_28.985548 4335-4344. Max. coverage (+): 0. Max coverage (-): 0.82

Region: NODE\_304263\_length\_4982\_cov\_28.985548 4345-4354. Max. coverage (+): 0.15. Max coverage (-): 1.08

Region: NODE\_304263\_length\_4982\_cov\_28.985548 4355-4364. Max. coverage (+): 0.48. Max coverage (-): 0.96

Region: NODE\_304263\_length\_4982\_cov\_28.985548 4365-4375. Max. coverage (+): 0.48. Max coverage (-): 0.07

Region: NODE\_304263\_length\_4982\_cov\_28.985548 4376-4385. Max. coverage (+): 0.04. Max coverage (-): 0.11

Region: NODE\_304263\_length\_4982\_cov\_28.985548 4386-4395. Max. coverage (+): 0.04. Max coverage (-): 2.89

Region: NODE\_304263\_length\_4982\_cov\_28.985548 4396-4405. Max. coverage (+): 0.04. Max coverage (-): 1.19

Region: NODE\_304263\_length\_4982\_cov\_28.985548 4406-4415. Max. coverage (+): 0.11. Max coverage (-): 0.19

Region: NODE\_304263\_length\_4982\_cov\_28.985548 4416-4425. Max. coverage (+): 0. Max coverage (-): 0.15

Region: NODE\_304263\_length\_4982\_cov\_28.985548 4426-4435. Max. coverage (+): 0. Max coverage (-): 1.33

Region: NODE\_304263\_length\_4982\_cov\_28.985548 4436-4445. Max. coverage (+): 0. Max coverage (-): 21.47

Region: NODE\_304263\_length\_4982\_cov\_28.985548 4446-4455. Max. coverage (+): 0.04. Max coverage (-): 0.52

Region: NODE\_304263\_length\_4982\_cov\_28.985548 4456-4465. Max. coverage (+): 0.07. Max coverage (-): 0.19

Region: NODE\_304263\_length\_4982\_cov\_28.985548 4466-4475. Max. coverage (+): 0.04. Max coverage (-): 6.56

Region: NODE\_304263\_length\_4982\_cov\_28.985548 4476-4486. Max. coverage (+): 0. Max coverage (-): 7.27

Region: NODE\_304263\_length\_4982\_cov\_28.985548 4487-4496. Max. coverage (+): 0.04. Max coverage (-): 0.07

Region: NODE\_304263\_length\_4982\_cov\_28.985548 4497-4506. Max. coverage (+): 0.04. Max coverage (-): 0.04

Region: NODE\_304263\_length\_4982\_cov\_28.985548 4507-4516. Max. coverage (+): 0.07. Max coverage (-): 0.07

Region: NODE\_304263\_length\_4982\_cov\_28.985548 4517-4526. Max. coverage (+): 0. Max coverage (-): 0.7

Region: NODE\_304263\_length\_4982\_cov\_28.985548 4527-4536. Max. coverage (+): 0.04. Max coverage (-): 0.78

Region: NODE\_304263\_length\_4982\_cov\_28.985548 4537-4546. Max. coverage (+): 0.37. Max coverage (-): 0.59

Region: NODE\_304263\_length\_4982\_cov\_28.985548 4547-4556. Max. coverage (+): 0.37. Max coverage (-): 0.19

Region: NODE\_304263\_length\_4982\_cov\_28.985548 4557-4566. Max. coverage (+): 0. Max coverage (-): 0.96

Region: NODE\_304263\_length\_4982\_cov\_28.985548 4567-4576. Max. coverage (+): 0. Max coverage (-): 0.7

Region: NODE\_304263\_length\_4982\_cov\_28.985548 4577-4586. Max. coverage (+): 0.19. Max coverage (-): 0

Region: NODE\_304263\_length\_4982\_cov\_28.985548 4587-4596. Max. coverage (+): 0.04. Max coverage (-): 0.19

Region: NODE\_304263\_length\_4982\_cov\_28.985548 4597-4607. Max. coverage (+): 0. Max coverage (-): 1.48

Region: NODE\_304263\_length\_4982\_cov\_28.985548 4608-4617. Max. coverage (+): 0.19. Max coverage (-): 0.78

Region: NODE\_304263\_length\_4982\_cov\_28.985548 4618-4627. Max. coverage (+): 0.19. Max coverage (-): 0.26

Region: NODE\_304263\_length\_4982\_cov\_28.985548 4628-4637. Max. coverage (+): 0.07. Max coverage (-): 0.67

Region: NODE\_304263\_length\_4982\_cov\_28.985548 4638-4647. Max. coverage (+): 0. Max coverage (-): 2.08

Region: NODE\_304263\_length\_4982\_cov\_28.985548 4648-4657. Max. coverage (+): 0.04. Max coverage (-): 0.78

Region: NODE\_304263\_length\_4982\_cov\_28.985548 4658-4667. Max. coverage (+): 0.04. Max coverage (-): 0.41

Region: NODE\_304263\_length\_4982\_cov\_28.985548 4668-4677. Max. coverage (+): 0.04. Max coverage (-): 4.6

Region: NODE\_304263\_length\_4982\_cov\_28.985548 4678-4687. Max. coverage (+): 0. Max coverage (-): 6.12

Region: NODE\_304263\_length\_4982\_cov\_28.985548 4688-4697. Max. coverage (+): 0.11. Max coverage (-): 0.22

Region: NODE\_304263\_length\_4982\_cov\_28.985548 4698-4707. Max. coverage (+): 0.04. Max coverage (-): 0

Region: NODE\_304263\_length\_4982\_cov\_28.985548 4708-4718. Max. coverage (+): 0. Max coverage (-): 0

Region: NODE\_304263\_length\_4982\_cov\_28.985548 4719-4728. Max. coverage (+): 0. Max coverage (-): 31.29

Region: NODE\_304263\_length\_4982\_cov\_28.985548 4729-4738. Max. coverage (+): 0. Max coverage (-): 2.82

Region: NODE\_304263\_length\_4982\_cov\_28.985548 4739-4748. Max. coverage (+): 0.37. Max coverage (-): 0.26

Region: NODE\_304263\_length\_4982\_cov\_28.985548 4749-4758. Max. coverage (+): 0.04. Max coverage (-): 0.26

Region: NODE\_304263\_length\_4982\_cov\_28.985548 4759-4768. Max. coverage (+): 0.04. Max coverage (-): 0.7

Region: NODE\_304263\_length\_4982\_cov\_28.985548 4769-4778. Max. coverage (+): 0. Max coverage (-): 1

Region: NODE\_304263\_length\_4982\_cov\_28.985548 4779-4788. Max. coverage (+): 0. Max coverage (-): 0.15

Region: NODE\_304263\_length\_4982\_cov\_28.985548 4789-4798. Max. coverage (+): 0. Max coverage (-): 0.56

Region: NODE\_304263\_length\_4982\_cov\_28.985548 4799-4808. Max. coverage (+): 0. Max coverage (-): 0.44

Region: NODE\_304263\_length\_4982\_cov\_28.985548 4809-4818. Max. coverage (+): 0. Max coverage (-): 0.22

Region: NODE\_304263\_length\_4982\_cov\_28.985548 4819-4829. Max. coverage (+): 0.04. Max coverage (-): 1.45

Region: NODE\_304263\_length\_4982\_cov\_28.985548 4830-4839. Max. coverage (+): 0.04. Max coverage (-): 5

Region: NODE\_304263\_length\_4982\_cov\_28.985548 4840-4849. Max. coverage (+): 0.04. Max coverage (-): 6.27

Region: NODE\_304263\_length\_4982\_cov\_28.985548 4850-4859. Max. coverage (+): 0. Max coverage (-): 5.15

Region: NODE\_304263\_length\_4982\_cov\_28.985548 4860-4869. Max. coverage (+): 2.78. Max coverage (-): 1

Region: NODE\_304263\_length\_4982\_cov\_28.985548 4870-4879. Max. coverage (+): 0.19. Max coverage (-): 1.41

Region: NODE\_304263\_length\_4982\_cov\_28.985548 4880-4889. Max. coverage (+): 0.04. Max coverage (-): 0.07

Region: NODE\_304263\_length\_4982\_cov\_28.985548 4890-4899. Max. coverage (+): 0.04. Max coverage (-): 0.44

Region: NODE\_304263\_length\_4982\_cov\_28.985548 4900-4909. Max. coverage (+): 0. Max coverage (-): 0.7

Region: NODE\_304263\_length\_4982\_cov\_28.985548 4910-4919. Max. coverage (+): 0.37. Max coverage (-): 0

Region: NODE\_304263\_length\_4982\_cov\_28.985548 4920-4929. Max. coverage (+): 0. Max coverage (-): 0.04

Region: NODE\_304263\_length\_4982\_cov\_28.985548 4930-4940. Max. coverage (+): 0. Max coverage (-): 2.71

Region: NODE\_304263\_length\_4982\_cov\_28.985548 4941-4950. Max. coverage (+): 0. Max coverage (-): 0.82

Region: NODE\_304263\_length\_4982\_cov\_28.985548 4951-4960. Max. coverage (+): 0. Max coverage (-): 0.3

Region: NODE\_304263\_length\_4982\_cov\_28.985548 4961-4970. Max. coverage (+): 0. Max coverage (-): 0.04

Region: NODE\_304263\_length\_4982\_cov\_28.985548 4971-4980. Max. coverage (+): 0. Max coverage (-): 0.04

Region: NODE\_304263\_length\_4982\_cov\_28.985548 4981-4990. Max. coverage (+): 0. Max coverage (-): 8.86

Region: NODE\_304263\_length\_4982\_cov\_28.985548 4991-5000. Max. coverage (+): 0. Max coverage (-): 14.83

Region: NODE\_304263\_length\_4982\_cov\_28.985548 5001-5010. Max. coverage (+): 0.11. Max coverage (-): 3.3

Region: NODE\_304263\_length\_4982\_cov\_28.985548 5011-5020. Max. coverage (+): 0.07. Max coverage (-): 0.44

Region: NODE\_304263\_length\_4982\_cov\_28.985548 5021-5030. Max. coverage (+): 0.07. Max coverage (-): 0.15

Region: NODE\_304263\_length\_4982\_cov\_28.985548 5031-5040. Max. coverage (+): 0. Max coverage (-): 0

Region: NODE\_304263\_length\_4982\_cov\_28.985548 5041-. Max. coverage (+): 0. Max coverage (-): 0

RepeatMasker Color Code

**+**

100-98% Identity

<98-95% Identity

<95-90% Identity

<90-85% Identity

<85-80% Identity

<80-75% Identity

<75-70% Identity

<70% Identity

**-**

Gene Set Color Code

**+**

Gene

Pseudogene

Other

**-**

Topology/Coverage Color Code

Coverage Plus Strand

Coverage Minus Strand

Mainstrand: Plus

Mainstrand: Minus

Complementary Strand

Flanking Region  
(if option -flank >0)

Gene Set Annotation  
  
RepeatMasker Annotation  

**1. AlRepC-13**: 50-172 (+), Divergence to consensus: 26.8%  
**2. TE-X-4\_DR**: 301-342 (+), Divergence to consensus: 19.1%  
**3. AlRepB-103**: 398-807 (-), Divergence to consensus: 40.5%  
**4. Expander2**: 820-890 (-), Divergence to consensus: 32.5%  
**5. AlRepE-1213**: 977-1025 (-), Divergence to consensus: 28.5%  
**6. A-rich**: 1933-1986 (+), Divergence to consensus: 32.7%  
**7. AlRepD-2057**: 2634-2693 (+), Divergence to consensus: 21.7%  
**8. (AACATTT)n**: 2982-3018 (+), Divergence to consensus: 24.5%  
**9. I\_Ele34**: 4567-4618 (+), Divergence to consensus: 34.6%

  
Transcription Factor Binding Sites  

**RHOXF1** (Sequence: AGCTCA (-): 442)  
**RHOXF1** (Sequence: AGCTTA (-): 659)  
**RHOXF1** (Sequence: AGATCA (-): 766)  
**RHOXF1** (Sequence: AGCTCA (-): 1577)  
**RHOXF1** (Sequence: AGCTCA (-): 2076)  
**RHOXF1** (Sequence: AGCTTA (-): 2123)  
**RHOXF1** (Sequence: GGCTCA (-): 2188)  
**RHOXF1** (Sequence: TAATCT (+): 493)  
**RHOXF1** (Sequence: TGAGCT (+): 2121)  
**RHOXF1** (Sequence: TAATCT (+): 2513)  
**RHOXF1** (Sequence: TAATCT (+): 2942)  
**RHOXF1** (Sequence: TGAGCC (+): 3034)  
**RHOXF1** (Sequence: TGATCT (+): 3132)  
**RHOXF1** (Sequence: TGATCT (+): 3780)  
**RHOXF1** (Sequence: TGATCT (+): 3946)  
**RHOXF1** (Sequence: TGAGCT (+): 4240)  
**RHOXF1** (Sequence: TAATCT (+): 4515)  
**Lhx8** (Sequence: TTAATTAA (-): 2102)  
**SOX9** (Sequence: AACAATGA (-): 456)  
**FOXO3\_mmu** (Sequence: TGTTTTGA (-): 3411)  
**Sox5** (Sequence: ATTGTT (+): 2840)  
**FIGLA** (Sequence: AACACCTGGA (-): 783)  
**SOX9** (Sequence: TTATTGTT (+): 2838)  
**FOXO3\_mmu** (Sequence: TGAAAACA (+): 1599)  
**FOXO3\_mmu** (Sequence: GCAAAACA (+): 1623)  
**FOXO3\_mmu** (Sequence: GCAAAACA (+): 2440)  
**FOXO3\_mmu** (Sequence: TGAAAACA (+): 2531)  
**FOXO3\_mmu** (Sequence: TGAAAACA (+): 2599)  
**FOXO1** (Sequence: GAAAACAGG (-): 1600)  
**FOXO1** (Sequence: AAAAACAAC (-): 1976)  
**FOXO1** (Sequence: GAAAACAGG (-): 2532)  
**FOXO1** (Sequence: GAAAACAGC (-): 2600)  
**FOXO1** (Sequence: AAAAACAAC (-): 3381)  
**Gata4** (Sequence: AGATAAC (-): 2418)  
**Sox5** (Sequence: AACAAT (-): 456)  
**Sox5** (Sequence: AACAAT (-): 908)  
**Sox5** (Sequence: AACAAT (-): 1963)  
**Sox5** (Sequence: AACAAT (-): 1968)  
**Sox5** (Sequence: AACAAT (-): 2548)  
**Sox5** (Sequence: AACAAT (-): 2736)  
**POU5F1** (Sequence: ATGCAAA (+): 2522)
