# Supplementary material for: First characterization of PIWI-interacting RNA clusters in a cichlid fish with a B chromosome
Source: BMC Biol. 2022 Sep 21;20:204. doi: 10.1186/s12915-022-01403-2 (PMC9490952; doi:10.1186/s12915-022-01403-2)
Supplement: Supplementary file 1 — Additional file 1. Zipped folder with fasta and interactive html piRNA cluster information for the A. latifasciata genome. The nomenclature is as follows: number-pirna-cluster_sex_B-presence (f, female; m, male; 0b, without B chromosome; 1b, with B chromosome). [file 12915_2022_1403_MOESM1_ESM.zip › 11_f0b.html]

piRNA cluster 11\_f0b 26


Predicted piRNA cluster no. 11\_f0b
  

Show proTRAC run info
Hide proTRAC run info

/\  
                \_\_\_\_\_\_\_\_\_\_\_\_\_\_\_\_\_\_\_\_\_\_\_/\\_\_\_ /  \\_\_\_\_\_\_\_  
               I                      /  \  /    \      I  
               I     pro             /    \/      \     I  
               I        TRAC        /               \   I  
               I   \_\_\_\_\_\_\_\_\_\_\_\_\_\_\_\_/\_\_\_\_\_\_\_\_\_\_\_\_\_\_\_\_\_\\_ I  
               I   \              /                     I  
               I    \            /                      I  
               I     \  /\      /       V.2.4.2         I  
               I      \/  \    /                        I  
               I\_\_\_\_\_\_\_\_\_\_\_\  /\_\_\_\_\_\_\_\_\_\_\_\_\_\_\_\_\_\_\_\_\_\_\_\_\_I  
                            \/  
  
  
================================= proTRAC ====================================  
VERSION: .......... 2.4.2  
LAST MODIFIED: .... 11. May 2018  
  
Please cite:  
Rosenkranz D, Zischler H. proTRAC - a software for probabilistic piRNA cluster  
detection, visualization and analysis. 2012. BMC Bioinformatics 13:5.  
  
  
Contact:  
David Rosenkranz  
Institute of Organismic and Molecular Evolutionary Biology  
Dept. Anthropology, small RNA group  
Johannes Gutenberg University Mainz  
email: rosenkranz@uni-mainz.de  
  
You can find the latest proTRAC version at:  
http://sourceforge.net/projects/protrac/files  
http://www.smallRNAgroup-mainz.de/software  
==============================================================================  
  
PARAMETERS:  
Map file: ...............piwi-femeas-0B.fa-collapse.map  
Genome file: ............../../../0B\_ala\_genome.fa  
RepeatMasker annotation: Alatifasciata-all0B-maryan-v2.fa\_corrected.out  
GeneSet:................./guest-storage/Data/annotation/Alatifasciata\_all0B\_maryan-v2\_out2017.gff  
  
Significant (p<=0.01) hit density will be calculated based  
on observed hit distribution.  
  
Sliding window size: ........................................ 5000 bp  
Sliding window increament: .................................. 1000 bp  
Normalize each hit by number of genomic hits: ............... yes  
Normalize each hit by number of sequence reads: ............. yes  
Normalize values (-> per million mapped reads): ............. yes  
Min. fraction of hits with 1T(U) or 10A: .................... 0.75  
Alternatively: Min. fraction of hits with 1T(U) and 10A: .... 0.5  
Min. fraction of hits with typical piRNA length: ............ 0.75  
Typical piRNA length: ....................................... 24-32 nt  
Min. size of a piRNA cluster: ............................... 1000 bp.  
Min. number of hits (absolute): ............................. 0  
Min. number of hits (normalized): ........................... 0  
Min. fraction of hits on the mainstrand: .................... 0.75  
Top fraction of mapped sequences (in terms of read counts): . 1%  
Top fraction accounts for max. n% of sequence reads: ........ 90%  
Min. fraction of hits on each arm of a bidirectional cluster: 0.05  
Output html file for each cluster: .......................... yes  
Output a summary table: ..................................... yes  
Output a FASTA file for each cluster (piRNA sequences): ..... yes  
Output a FASTA file comprising cluster sequences: ........... yes  
Output a GTF file for predicted piRNA clusters: ..............yes  
Search DNA motifs in clusters: .............................. yes  
Output flanking sequences: +/- .............................. 0 bp  
Output ~.pTi file: .......................................... no  
==============================================================================  
  
  
Genome size (without gaps): ............ 758543724 bp  
Gaps (N/X/-): .......................... 417479 bp  
Mapped reads: .......................... 13052187  
Non-identical sequences: ............... 3338911  
Genomic hits: .......................... 28737726  
Significant densitiy of mapped reads: .. 470.083249848448 reads/kb

Show proTRAC cluster info
Hide proTRAC cluster info

|  |  |
| --- | --- |
| Location | NODE\_117571\_length\_15591\_cov\_25.186710 |
| Coordinates | 5029-15783 |
| Size [bp] | 10755 |
| Sequence hit loci | 2288 |
| Mapped reads (normalized) | 6493.8 |
| Mapped reads (normalized) per kb | 603.8 |
| Normalized reads with 1T (1U) | 79.5% |
| Normalized reads with 10A | 32.6% |
| Normalized reads with length 24-32 nt | 98.9% |
| Normalized reads on the main strand(s) | 89.7% |
| Predicted directionality | bi:plus-minus (split between 9704 and 9707) |

100%

0%

1T (1U)  
reads

10A reads

24-32 nt  
reads

reads on mainstrand

**Either the amount of reads with 1T (1U) OR 10A has to exceed 75% (set with option: -1Tor10A)  
Alternatively the amount of reads with 1T (1U) AND 10A has to exceed 50% (set with option: -1Tand10A)  
Minimum amount of reads with preferred size is 75% (set with option: -pisize)  
Minimum amount of reads on the main strand(s) is 75% (set with option: -clstrand)**

Show read coverage
Hide read coverage

WHAT DO I SEE HERE?  
This chart shows the location of mapped sequence reads within a predicted piRNA cluster. The color refers to the number of genomic hits produced by the sequence read in question. A dark red bar indicates that this sequence read produces many other hits elsewhere in the genome. Many adjacent red or yellow bars can indicate the presence of a multi-copy element such as transposons or rRNA genes. A dark green bar indicates that this sequence read maps uniquely to this locus.

1 hit

2-5 hits

6-10 hits

11-20 hits

21-50 hits

51-100 hits

> 100 hits

NODE\_117571\_length\_15591\_cov\_25.186710

5029

15783

Gene Set

RepeatMasker

Mapped  
Reads

59.38

plus strand

minus strand

59.38

Region: NODE\_117571\_length\_15591\_cov\_25.186710 11638-5039. Max. coverage (+): 0.08. Max coverage (-): 0

Region: NODE\_117571\_length\_15591\_cov\_25.186710 5040-5061. Max. coverage (+): 0. Max coverage (-): 0

Region: NODE\_117571\_length\_15591\_cov\_25.186710 5062-5082. Max. coverage (+): 0. Max coverage (-): 0

Region: NODE\_117571\_length\_15591\_cov\_25.186710 5083-5104. Max. coverage (+): 0. Max coverage (-): 0

Region: NODE\_117571\_length\_15591\_cov\_25.186710 5105-5125. Max. coverage (+): 0. Max coverage (-): 0

Region: NODE\_117571\_length\_15591\_cov\_25.186710 5126-5147. Max. coverage (+): 0.15. Max coverage (-): 0.08

Region: NODE\_117571\_length\_15591\_cov\_25.186710 5148-5168. Max. coverage (+): 0.23. Max coverage (-): 0

Region: NODE\_117571\_length\_15591\_cov\_25.186710 5169-5190. Max. coverage (+): 0.08. Max coverage (-): 0

Region: NODE\_117571\_length\_15591\_cov\_25.186710 5191-5211. Max. coverage (+): 0.08. Max coverage (-): 0

Region: NODE\_117571\_length\_15591\_cov\_25.186710 5212-5233. Max. coverage (+): 0. Max coverage (-): 0

Region: NODE\_117571\_length\_15591\_cov\_25.186710 5234-5254. Max. coverage (+): 0. Max coverage (-): 0.23

Region: NODE\_117571\_length\_15591\_cov\_25.186710 5255-5276. Max. coverage (+): 0. Max coverage (-): 0.23

Region: NODE\_117571\_length\_15591\_cov\_25.186710 5277-5297. Max. coverage (+): 0. Max coverage (-): 0

Region: NODE\_117571\_length\_15591\_cov\_25.186710 5298-5319. Max. coverage (+): 0.08. Max coverage (-): 0.46

Region: NODE\_117571\_length\_15591\_cov\_25.186710 5320-5340. Max. coverage (+): 0.08. Max coverage (-): 0.46

Region: NODE\_117571\_length\_15591\_cov\_25.186710 5341-5362. Max. coverage (+): 0. Max coverage (-): 0

Region: NODE\_117571\_length\_15591\_cov\_25.186710 5363-5383. Max. coverage (+): 0. Max coverage (-): 0

Region: NODE\_117571\_length\_15591\_cov\_25.186710 5384-5405. Max. coverage (+): 0.08. Max coverage (-): 0

Region: NODE\_117571\_length\_15591\_cov\_25.186710 5406-5426. Max. coverage (+): 0.15. Max coverage (-): 0

Region: NODE\_117571\_length\_15591\_cov\_25.186710 5427-5448. Max. coverage (+): 0. Max coverage (-): 0

Region: NODE\_117571\_length\_15591\_cov\_25.186710 5449-5469. Max. coverage (+): 0.08. Max coverage (-): 0

Region: NODE\_117571\_length\_15591\_cov\_25.186710 5470-5491. Max. coverage (+): 0. Max coverage (-): 0

Region: NODE\_117571\_length\_15591\_cov\_25.186710 5492-5512. Max. coverage (+): 0. Max coverage (-): 0

Region: NODE\_117571\_length\_15591\_cov\_25.186710 5513-5534. Max. coverage (+): 0. Max coverage (-): 0

Region: NODE\_117571\_length\_15591\_cov\_25.186710 5535-5555. Max. coverage (+): 0.15. Max coverage (-): 0

Region: NODE\_117571\_length\_15591\_cov\_25.186710 5556-5577. Max. coverage (+): 2.07. Max coverage (-): 0

Region: NODE\_117571\_length\_15591\_cov\_25.186710 5578-5599. Max. coverage (+): 0.08. Max coverage (-): 0

Region: NODE\_117571\_length\_15591\_cov\_25.186710 5600-5620. Max. coverage (+): 0.01. Max coverage (-): 0

Region: NODE\_117571\_length\_15591\_cov\_25.186710 5621-5642. Max. coverage (+): 0.23. Max coverage (-): 0

Region: NODE\_117571\_length\_15591\_cov\_25.186710 5643-5663. Max. coverage (+): 0. Max coverage (-): 0

Region: NODE\_117571\_length\_15591\_cov\_25.186710 5664-5685. Max. coverage (+): 0.08. Max coverage (-): 0

Region: NODE\_117571\_length\_15591\_cov\_25.186710 5686-5706. Max. coverage (+): 0. Max coverage (-): 0

Region: NODE\_117571\_length\_15591\_cov\_25.186710 5707-5728. Max. coverage (+): 0. Max coverage (-): 0

Region: NODE\_117571\_length\_15591\_cov\_25.186710 5729-5749. Max. coverage (+): 0. Max coverage (-): 0.15

Region: NODE\_117571\_length\_15591\_cov\_25.186710 5750-5771. Max. coverage (+): 0. Max coverage (-): 0

Region: NODE\_117571\_length\_15591\_cov\_25.186710 5772-5792. Max. coverage (+): 0.08. Max coverage (-): 0

Region: NODE\_117571\_length\_15591\_cov\_25.186710 5793-5814. Max. coverage (+): 0. Max coverage (-): 0.08

Region: NODE\_117571\_length\_15591\_cov\_25.186710 5815-5835. Max. coverage (+): 0.08. Max coverage (-): 0

Region: NODE\_117571\_length\_15591\_cov\_25.186710 5836-5857. Max. coverage (+): 0.31. Max coverage (-): 0.38

Region: NODE\_117571\_length\_15591\_cov\_25.186710 5858-5878. Max. coverage (+): 0.84. Max coverage (-): 0.08

Region: NODE\_117571\_length\_15591\_cov\_25.186710 5879-5900. Max. coverage (+): 0.15. Max coverage (-): 0

Region: NODE\_117571\_length\_15591\_cov\_25.186710 5901-5921. Max. coverage (+): 0.15. Max coverage (-): 0

Region: NODE\_117571\_length\_15591\_cov\_25.186710 5922-5943. Max. coverage (+): 1.84. Max coverage (-): 0

Region: NODE\_117571\_length\_15591\_cov\_25.186710 5944-5964. Max. coverage (+): 1.3. Max coverage (-): 0

Region: NODE\_117571\_length\_15591\_cov\_25.186710 5965-5986. Max. coverage (+): 0.08. Max coverage (-): 0

Region: NODE\_117571\_length\_15591\_cov\_25.186710 5987-6007. Max. coverage (+): 0.46. Max coverage (-): 0

Region: NODE\_117571\_length\_15591\_cov\_25.186710 6008-6029. Max. coverage (+): 0. Max coverage (-): 0

Region: NODE\_117571\_length\_15591\_cov\_25.186710 6030-6050. Max. coverage (+): 0.61. Max coverage (-): 0

Region: NODE\_117571\_length\_15591\_cov\_25.186710 6051-6072. Max. coverage (+): 0.15. Max coverage (-): 0.15

Region: NODE\_117571\_length\_15591\_cov\_25.186710 6073-6093. Max. coverage (+): 0.15. Max coverage (-): 0

Region: NODE\_117571\_length\_15591\_cov\_25.186710 6094-6115. Max. coverage (+): 1.46. Max coverage (-): 0.23

Region: NODE\_117571\_length\_15591\_cov\_25.186710 6116-6136. Max. coverage (+): 0.31. Max coverage (-): 0.15

Region: NODE\_117571\_length\_15591\_cov\_25.186710 6137-6158. Max. coverage (+): 0.23. Max coverage (-): 0

Region: NODE\_117571\_length\_15591\_cov\_25.186710 6159-6179. Max. coverage (+): 0.23. Max coverage (-): 0.15

Region: NODE\_117571\_length\_15591\_cov\_25.186710 6180-6201. Max. coverage (+): 0.54. Max coverage (-): 0.15

Region: NODE\_117571\_length\_15591\_cov\_25.186710 6202-6222. Max. coverage (+): 0.46. Max coverage (-): 0

Region: NODE\_117571\_length\_15591\_cov\_25.186710 6223-6244. Max. coverage (+): 0.46. Max coverage (-): 0

Region: NODE\_117571\_length\_15591\_cov\_25.186710 6245-6265. Max. coverage (+): 0. Max coverage (-): 0.08

Region: NODE\_117571\_length\_15591\_cov\_25.186710 6266-6287. Max. coverage (+): 0. Max coverage (-): 0

Region: NODE\_117571\_length\_15591\_cov\_25.186710 6288-6308. Max. coverage (+): 1.92. Max coverage (-): 0

Region: NODE\_117571\_length\_15591\_cov\_25.186710 6309-6330. Max. coverage (+): 0.08. Max coverage (-): 0

Region: NODE\_117571\_length\_15591\_cov\_25.186710 6331-6351. Max. coverage (+): 0.38. Max coverage (-): 0.23

Region: NODE\_117571\_length\_15591\_cov\_25.186710 6352-6373. Max. coverage (+): 0.15. Max coverage (-): 0

Region: NODE\_117571\_length\_15591\_cov\_25.186710 6374-6394. Max. coverage (+): 0. Max coverage (-): 0.08

Region: NODE\_117571\_length\_15591\_cov\_25.186710 6395-6416. Max. coverage (+): 1.23. Max coverage (-): 0

Region: NODE\_117571\_length\_15591\_cov\_25.186710 6417-6437. Max. coverage (+): 0.31. Max coverage (-): 0.15

Region: NODE\_117571\_length\_15591\_cov\_25.186710 6438-6459. Max. coverage (+): 0.84. Max coverage (-): 0.08

Region: NODE\_117571\_length\_15591\_cov\_25.186710 6460-6480. Max. coverage (+): 0. Max coverage (-): 0.08

Region: NODE\_117571\_length\_15591\_cov\_25.186710 6481-6502. Max. coverage (+): 0.23. Max coverage (-): 0.08

Region: NODE\_117571\_length\_15591\_cov\_25.186710 6503-6523. Max. coverage (+): 0.23. Max coverage (-): 0

Region: NODE\_117571\_length\_15591\_cov\_25.186710 6524-6545. Max. coverage (+): 1.46. Max coverage (-): 0

Region: NODE\_117571\_length\_15591\_cov\_25.186710 6546-6566. Max. coverage (+): 0.23. Max coverage (-): 0.08

Region: NODE\_117571\_length\_15591\_cov\_25.186710 6567-6588. Max. coverage (+): 0.08. Max coverage (-): 0.23

Region: NODE\_117571\_length\_15591\_cov\_25.186710 6589-6609. Max. coverage (+): 0.92. Max coverage (-): 0

Region: NODE\_117571\_length\_15591\_cov\_25.186710 6610-6631. Max. coverage (+): 0.15. Max coverage (-): 0.08

Region: NODE\_117571\_length\_15591\_cov\_25.186710 6632-6653. Max. coverage (+): 0. Max coverage (-): 0

Region: NODE\_117571\_length\_15591\_cov\_25.186710 6654-6674. Max. coverage (+): 0.46. Max coverage (-): 0

Region: NODE\_117571\_length\_15591\_cov\_25.186710 6675-6696. Max. coverage (+): 0.69. Max coverage (-): 0

Region: NODE\_117571\_length\_15591\_cov\_25.186710 6697-6717. Max. coverage (+): 0.08. Max coverage (-): 0.15

Region: NODE\_117571\_length\_15591\_cov\_25.186710 6718-6739. Max. coverage (+): 0.61. Max coverage (-): 0

Region: NODE\_117571\_length\_15591\_cov\_25.186710 6740-6760. Max. coverage (+): 0.38. Max coverage (-): 0

Region: NODE\_117571\_length\_15591\_cov\_25.186710 6761-6782. Max. coverage (+): 0. Max coverage (-): 0

Region: NODE\_117571\_length\_15591\_cov\_25.186710 6783-6803. Max. coverage (+): 0. Max coverage (-): 0.15

Region: NODE\_117571\_length\_15591\_cov\_25.186710 6804-6825. Max. coverage (+): 0.69. Max coverage (-): 0

Region: NODE\_117571\_length\_15591\_cov\_25.186710 6826-6846. Max. coverage (+): 0.31. Max coverage (-): 0.08

Region: NODE\_117571\_length\_15591\_cov\_25.186710 6847-6868. Max. coverage (+): 0. Max coverage (-): 0

Region: NODE\_117571\_length\_15591\_cov\_25.186710 6869-6889. Max. coverage (+): 0.08. Max coverage (-): 0.61

Region: NODE\_117571\_length\_15591\_cov\_25.186710 6890-6911. Max. coverage (+): 0.92. Max coverage (-): 0.61

Region: NODE\_117571\_length\_15591\_cov\_25.186710 6912-6932. Max. coverage (+): 0. Max coverage (-): 0

Region: NODE\_117571\_length\_15591\_cov\_25.186710 6933-6954. Max. coverage (+): 0.15. Max coverage (-): 0

Region: NODE\_117571\_length\_15591\_cov\_25.186710 6955-6975. Max. coverage (+): 0. Max coverage (-): 0.02

Region: NODE\_117571\_length\_15591\_cov\_25.186710 6976-6997. Max. coverage (+): 0. Max coverage (-): 0.08

Region: NODE\_117571\_length\_15591\_cov\_25.186710 6998-7018. Max. coverage (+): 0.38. Max coverage (-): 0.23

Region: NODE\_117571\_length\_15591\_cov\_25.186710 7019-7040. Max. coverage (+): 0.15. Max coverage (-): 0

Region: NODE\_117571\_length\_15591\_cov\_25.186710 7041-7061. Max. coverage (+): 0.08. Max coverage (-): 0

Region: NODE\_117571\_length\_15591\_cov\_25.186710 7062-7083. Max. coverage (+): 1.23. Max coverage (-): 0

Region: NODE\_117571\_length\_15591\_cov\_25.186710 7084-7104. Max. coverage (+): 1.15. Max coverage (-): 0.08

Region: NODE\_117571\_length\_15591\_cov\_25.186710 7105-7126. Max. coverage (+): 0. Max coverage (-): 0.08

Region: NODE\_117571\_length\_15591\_cov\_25.186710 7127-7147. Max. coverage (+): 0.23. Max coverage (-): 0

Region: NODE\_117571\_length\_15591\_cov\_25.186710 7148-7169. Max. coverage (+): 0.23. Max coverage (-): 0.08

Region: NODE\_117571\_length\_15591\_cov\_25.186710 7170-7190. Max. coverage (+): 0.23. Max coverage (-): 0.15

Region: NODE\_117571\_length\_15591\_cov\_25.186710 7191-7212. Max. coverage (+): 0. Max coverage (-): 0

Region: NODE\_117571\_length\_15591\_cov\_25.186710 7213-7233. Max. coverage (+): 2.38. Max coverage (-): 0.38

Region: NODE\_117571\_length\_15591\_cov\_25.186710 7234-7255. Max. coverage (+): 22.6. Max coverage (-): 0

Region: NODE\_117571\_length\_15591\_cov\_25.186710 7256-7276. Max. coverage (+): 0.38. Max coverage (-): 0

Region: NODE\_117571\_length\_15591\_cov\_25.186710 7277-7298. Max. coverage (+): 12.11. Max coverage (-): 0

Region: NODE\_117571\_length\_15591\_cov\_25.186710 7299-7319. Max. coverage (+): 0.08. Max coverage (-): 0

Region: NODE\_117571\_length\_15591\_cov\_25.186710 7320-7341. Max. coverage (+): 1.07. Max coverage (-): 0.23

Region: NODE\_117571\_length\_15591\_cov\_25.186710 7342-7362. Max. coverage (+): 0. Max coverage (-): 0.23

Region: NODE\_117571\_length\_15591\_cov\_25.186710 7363-7384. Max. coverage (+): 0.38. Max coverage (-): 0.61

Region: NODE\_117571\_length\_15591\_cov\_25.186710 7385-7405. Max. coverage (+): 0. Max coverage (-): 0

Region: NODE\_117571\_length\_15591\_cov\_25.186710 7406-7427. Max. coverage (+): 2.45. Max coverage (-): 0

Region: NODE\_117571\_length\_15591\_cov\_25.186710 7428-7448. Max. coverage (+): 2.45. Max coverage (-): 0

Region: NODE\_117571\_length\_15591\_cov\_25.186710 7449-7470. Max. coverage (+): 0.23. Max coverage (-): 0.08

Region: NODE\_117571\_length\_15591\_cov\_25.186710 7471-7491. Max. coverage (+): 0.23. Max coverage (-): 0.08

Region: NODE\_117571\_length\_15591\_cov\_25.186710 7492-7513. Max. coverage (+): 0. Max coverage (-): 0

Region: NODE\_117571\_length\_15591\_cov\_25.186710 7514-7534. Max. coverage (+): 0.38. Max coverage (-): 0

Region: NODE\_117571\_length\_15591\_cov\_25.186710 7535-7556. Max. coverage (+): 0.15. Max coverage (-): 0.15

Region: NODE\_117571\_length\_15591\_cov\_25.186710 7557-7577. Max. coverage (+): 0. Max coverage (-): 0.08

Region: NODE\_117571\_length\_15591\_cov\_25.186710 7578-7599. Max. coverage (+): 0.54. Max coverage (-): 0

Region: NODE\_117571\_length\_15591\_cov\_25.186710 7600-7620. Max. coverage (+): 0.08. Max coverage (-): 0

Region: NODE\_117571\_length\_15591\_cov\_25.186710 7621-7642. Max. coverage (+): 1. Max coverage (-): 0

Region: NODE\_117571\_length\_15591\_cov\_25.186710 7643-7663. Max. coverage (+): 0.38. Max coverage (-): 0

Region: NODE\_117571\_length\_15591\_cov\_25.186710 7664-7685. Max. coverage (+): 0. Max coverage (-): 0

Region: NODE\_117571\_length\_15591\_cov\_25.186710 7686-7706. Max. coverage (+): 0. Max coverage (-): 0.08

Region: NODE\_117571\_length\_15591\_cov\_25.186710 7707-7728. Max. coverage (+): 3.22. Max coverage (-): 0

Region: NODE\_117571\_length\_15591\_cov\_25.186710 7729-7750. Max. coverage (+): 0. Max coverage (-): 0.15

Region: NODE\_117571\_length\_15591\_cov\_25.186710 7751-7771. Max. coverage (+): 0.31. Max coverage (-): 0.08

Region: NODE\_117571\_length\_15591\_cov\_25.186710 7772-7793. Max. coverage (+): 0.23. Max coverage (-): 0.23

Region: NODE\_117571\_length\_15591\_cov\_25.186710 7794-7814. Max. coverage (+): 2.07. Max coverage (-): 0

Region: NODE\_117571\_length\_15591\_cov\_25.186710 7815-7836. Max. coverage (+): 0.08. Max coverage (-): 0.15

Region: NODE\_117571\_length\_15591\_cov\_25.186710 7837-7857. Max. coverage (+): 0.08. Max coverage (-): 0

Region: NODE\_117571\_length\_15591\_cov\_25.186710 7858-7879. Max. coverage (+): 0. Max coverage (-): 0

Region: NODE\_117571\_length\_15591\_cov\_25.186710 7880-7900. Max. coverage (+): 0.23. Max coverage (-): 0

Region: NODE\_117571\_length\_15591\_cov\_25.186710 7901-7922. Max. coverage (+): 0. Max coverage (-): 0

Region: NODE\_117571\_length\_15591\_cov\_25.186710 7923-7943. Max. coverage (+): 0. Max coverage (-): 0

Region: NODE\_117571\_length\_15591\_cov\_25.186710 7944-7965. Max. coverage (+): 0. Max coverage (-): 0

Region: NODE\_117571\_length\_15591\_cov\_25.186710 7966-7986. Max. coverage (+): 0.15. Max coverage (-): 0

Region: NODE\_117571\_length\_15591\_cov\_25.186710 7987-8008. Max. coverage (+): 0. Max coverage (-): 0.08

Region: NODE\_117571\_length\_15591\_cov\_25.186710 8009-8029. Max. coverage (+): 0.15. Max coverage (-): 0

Region: NODE\_117571\_length\_15591\_cov\_25.186710 8030-8051. Max. coverage (+): 0.08. Max coverage (-): 0.23

Region: NODE\_117571\_length\_15591\_cov\_25.186710 8052-8072. Max. coverage (+): 1.92. Max coverage (-): 0

Region: NODE\_117571\_length\_15591\_cov\_25.186710 8073-8094. Max. coverage (+): 1.38. Max coverage (-): 0.08

Region: NODE\_117571\_length\_15591\_cov\_25.186710 8095-8115. Max. coverage (+): 1.38. Max coverage (-): 0

Region: NODE\_117571\_length\_15591\_cov\_25.186710 8116-8137. Max. coverage (+): 0.69. Max coverage (-): 0

Region: NODE\_117571\_length\_15591\_cov\_25.186710 8138-8158. Max. coverage (+): 0.08. Max coverage (-): 0.08

Region: NODE\_117571\_length\_15591\_cov\_25.186710 8159-8180. Max. coverage (+): 8.27. Max coverage (-): 0.08

Region: NODE\_117571\_length\_15591\_cov\_25.186710 8181-8201. Max. coverage (+): 0.08. Max coverage (-): 0.46

Region: NODE\_117571\_length\_15591\_cov\_25.186710 8202-8223. Max. coverage (+): 0.15. Max coverage (-): 0.61

Region: NODE\_117571\_length\_15591\_cov\_25.186710 8224-8244. Max. coverage (+): 0.38. Max coverage (-): 0

Region: NODE\_117571\_length\_15591\_cov\_25.186710 8245-8266. Max. coverage (+): 0.69. Max coverage (-): 0

Region: NODE\_117571\_length\_15591\_cov\_25.186710 8267-8287. Max. coverage (+): 0. Max coverage (-): 0

Region: NODE\_117571\_length\_15591\_cov\_25.186710 8288-8309. Max. coverage (+): 0. Max coverage (-): 0

Region: NODE\_117571\_length\_15591\_cov\_25.186710 8310-8330. Max. coverage (+): 0. Max coverage (-): 0

Region: NODE\_117571\_length\_15591\_cov\_25.186710 8331-8352. Max. coverage (+): 0.23. Max coverage (-): 0

Region: NODE\_117571\_length\_15591\_cov\_25.186710 8353-8373. Max. coverage (+): 0.08. Max coverage (-): 0.08

Region: NODE\_117571\_length\_15591\_cov\_25.186710 8374-8395. Max. coverage (+): 0. Max coverage (-): 0.08

Region: NODE\_117571\_length\_15591\_cov\_25.186710 8396-8416. Max. coverage (+): 1.84. Max coverage (-): 0.15

Region: NODE\_117571\_length\_15591\_cov\_25.186710 8417-8438. Max. coverage (+): 1.99. Max coverage (-): 0

Region: NODE\_117571\_length\_15591\_cov\_25.186710 8439-8459. Max. coverage (+): 0. Max coverage (-): 0

Region: NODE\_117571\_length\_15591\_cov\_25.186710 8460-8481. Max. coverage (+): 39.76. Max coverage (-): 0

Region: NODE\_117571\_length\_15591\_cov\_25.186710 8482-8502. Max. coverage (+): 0. Max coverage (-): 0

Region: NODE\_117571\_length\_15591\_cov\_25.186710 8503-8524. Max. coverage (+): 0.38. Max coverage (-): 0

Region: NODE\_117571\_length\_15591\_cov\_25.186710 8525-8545. Max. coverage (+): 0.08. Max coverage (-): 0

Region: NODE\_117571\_length\_15591\_cov\_25.186710 8546-8567. Max. coverage (+): 0. Max coverage (-): 0.08

Region: NODE\_117571\_length\_15591\_cov\_25.186710 8568-8588. Max. coverage (+): 0.08. Max coverage (-): 0

Region: NODE\_117571\_length\_15591\_cov\_25.186710 8589-8610. Max. coverage (+): 0.38. Max coverage (-): 0

Region: NODE\_117571\_length\_15591\_cov\_25.186710 8611-8631. Max. coverage (+): 0.84. Max coverage (-): 0.23

Region: NODE\_117571\_length\_15591\_cov\_25.186710 8632-8653. Max. coverage (+): 0.15. Max coverage (-): 0.08

Region: NODE\_117571\_length\_15591\_cov\_25.186710 8654-8674. Max. coverage (+): 0.08. Max coverage (-): 0.46

Region: NODE\_117571\_length\_15591\_cov\_25.186710 8675-8696. Max. coverage (+): 0.54. Max coverage (-): 0

Region: NODE\_117571\_length\_15591\_cov\_25.186710 8697-8717. Max. coverage (+): 0. Max coverage (-): 0

Region: NODE\_117571\_length\_15591\_cov\_25.186710 8718-8739. Max. coverage (+): 0.69. Max coverage (-): 0

Region: NODE\_117571\_length\_15591\_cov\_25.186710 8740-8760. Max. coverage (+): 0.08. Max coverage (-): 0

Region: NODE\_117571\_length\_15591\_cov\_25.186710 8761-8782. Max. coverage (+): 0.23. Max coverage (-): 0.08

Region: NODE\_117571\_length\_15591\_cov\_25.186710 8783-8804. Max. coverage (+): 3.29. Max coverage (-): 0.38

Region: NODE\_117571\_length\_15591\_cov\_25.186710 8805-8825. Max. coverage (+): 1.61. Max coverage (-): 0.23

Region: NODE\_117571\_length\_15591\_cov\_25.186710 8826-8847. Max. coverage (+): 0.92. Max coverage (-): 0.08

Region: NODE\_117571\_length\_15591\_cov\_25.186710 8848-8868. Max. coverage (+): 0.31. Max coverage (-): 0.08

Region: NODE\_117571\_length\_15591\_cov\_25.186710 8869-8890. Max. coverage (+): 0. Max coverage (-): 0

Region: NODE\_117571\_length\_15591\_cov\_25.186710 8891-8911. Max. coverage (+): 0.38. Max coverage (-): 0

Region: NODE\_117571\_length\_15591\_cov\_25.186710 8912-8933. Max. coverage (+): 0.15. Max coverage (-): 0

Region: NODE\_117571\_length\_15591\_cov\_25.186710 8934-8954. Max. coverage (+): 0. Max coverage (-): 0

Region: NODE\_117571\_length\_15591\_cov\_25.186710 8955-8976. Max. coverage (+): 0.08. Max coverage (-): 0

Region: NODE\_117571\_length\_15591\_cov\_25.186710 8977-8997. Max. coverage (+): 0. Max coverage (-): 0.15

Region: NODE\_117571\_length\_15591\_cov\_25.186710 8998-9019. Max. coverage (+): 0.31. Max coverage (-): 0

Region: NODE\_117571\_length\_15591\_cov\_25.186710 9020-9040. Max. coverage (+): 0. Max coverage (-): 0.46

Region: NODE\_117571\_length\_15591\_cov\_25.186710 9041-9062. Max. coverage (+): 1.15. Max coverage (-): 0.31

Region: NODE\_117571\_length\_15591\_cov\_25.186710 9063-9083. Max. coverage (+): 0.08. Max coverage (-): 0.15

Region: NODE\_117571\_length\_15591\_cov\_25.186710 9084-9105. Max. coverage (+): 0.08. Max coverage (-): 0

Region: NODE\_117571\_length\_15591\_cov\_25.186710 9106-9126. Max. coverage (+): 2.6. Max coverage (-): 0

Region: NODE\_117571\_length\_15591\_cov\_25.186710 9127-9148. Max. coverage (+): 2.6. Max coverage (-): 0

Region: NODE\_117571\_length\_15591\_cov\_25.186710 9149-9169. Max. coverage (+): 0. Max coverage (-): 0

Region: NODE\_117571\_length\_15591\_cov\_25.186710 9170-9191. Max. coverage (+): 0.23. Max coverage (-): 0.08

Region: NODE\_117571\_length\_15591\_cov\_25.186710 9192-9212. Max. coverage (+): 0.61. Max coverage (-): 0.08

Region: NODE\_117571\_length\_15591\_cov\_25.186710 9213-9234. Max. coverage (+): 0. Max coverage (-): 0.08

Region: NODE\_117571\_length\_15591\_cov\_25.186710 9235-9255. Max. coverage (+): 1.23. Max coverage (-): 0

Region: NODE\_117571\_length\_15591\_cov\_25.186710 9256-9277. Max. coverage (+): 0.15. Max coverage (-): 0

Region: NODE\_117571\_length\_15591\_cov\_25.186710 9278-9298. Max. coverage (+): 3.75. Max coverage (-): 1

Region: NODE\_117571\_length\_15591\_cov\_25.186710 9299-9320. Max. coverage (+): 0.84. Max coverage (-): 0.08

Region: NODE\_117571\_length\_15591\_cov\_25.186710 9321-9341. Max. coverage (+): 0.31. Max coverage (-): 0.15

Region: NODE\_117571\_length\_15591\_cov\_25.186710 9342-9363. Max. coverage (+): 0.46. Max coverage (-): 0

Region: NODE\_117571\_length\_15591\_cov\_25.186710 9364-9384. Max. coverage (+): 0.08. Max coverage (-): 0.23

Region: NODE\_117571\_length\_15591\_cov\_25.186710 9385-9406. Max. coverage (+): 0.31. Max coverage (-): 0.08

Region: NODE\_117571\_length\_15591\_cov\_25.186710 9407-9427. Max. coverage (+): 0. Max coverage (-): 0

Region: NODE\_117571\_length\_15591\_cov\_25.186710 9428-9449. Max. coverage (+): 0.23. Max coverage (-): 0

Region: NODE\_117571\_length\_15591\_cov\_25.186710 9450-9470. Max. coverage (+): 0.23. Max coverage (-): 0

Region: NODE\_117571\_length\_15591\_cov\_25.186710 9471-9492. Max. coverage (+): 0.77. Max coverage (-): 0

Region: NODE\_117571\_length\_15591\_cov\_25.186710 9493-9513. Max. coverage (+): 0.77. Max coverage (-): 0

Region: NODE\_117571\_length\_15591\_cov\_25.186710 9514-9535. Max. coverage (+): 0.08. Max coverage (-): 0.08

Region: NODE\_117571\_length\_15591\_cov\_25.186710 9536-9556. Max. coverage (+): 0.15. Max coverage (-): 0

Region: NODE\_117571\_length\_15591\_cov\_25.186710 9557-9578. Max. coverage (+): 0.23. Max coverage (-): 0

Region: NODE\_117571\_length\_15591\_cov\_25.186710 9579-9599. Max. coverage (+): 0.15. Max coverage (-): 0

Region: NODE\_117571\_length\_15591\_cov\_25.186710 9600-9621. Max. coverage (+): 6.67. Max coverage (-): 0

Region: NODE\_117571\_length\_15591\_cov\_25.186710 9622-9642. Max. coverage (+): 0. Max coverage (-): 0

Region: NODE\_117571\_length\_15591\_cov\_25.186710 9643-9664. Max. coverage (+): 0.23. Max coverage (-): 0

Region: NODE\_117571\_length\_15591\_cov\_25.186710 9665-9685. Max. coverage (+): 0.23. Max coverage (-): 0.01

Region: NODE\_117571\_length\_15591\_cov\_25.186710 9686-9707. Max. coverage (+): 0.03. Max coverage (-): 0.01

Region: NODE\_117571\_length\_15591\_cov\_25.186710 9708-9728. Max. coverage (+): 0. Max coverage (-): 0.01

Region: NODE\_117571\_length\_15591\_cov\_25.186710 9729-9750. Max. coverage (+): 0. Max coverage (-): 0

Region: NODE\_117571\_length\_15591\_cov\_25.186710 9751-9771. Max. coverage (+): 0. Max coverage (-): 0

Region: NODE\_117571\_length\_15591\_cov\_25.186710 9772-9793. Max. coverage (+): 0. Max coverage (-): 0.08

Region: NODE\_117571\_length\_15591\_cov\_25.186710 9794-9814. Max. coverage (+): 0. Max coverage (-): 0.1

Region: NODE\_117571\_length\_15591\_cov\_25.186710 9815-9836. Max. coverage (+): 0.01. Max coverage (-): 0

Region: NODE\_117571\_length\_15591\_cov\_25.186710 9837-9857. Max. coverage (+): 0. Max coverage (-): 0.04

Region: NODE\_117571\_length\_15591\_cov\_25.186710 9858-9879. Max. coverage (+): 0.02. Max coverage (-): 0

Region: NODE\_117571\_length\_15591\_cov\_25.186710 9880-9901. Max. coverage (+): 0. Max coverage (-): 0.94

Region: NODE\_117571\_length\_15591\_cov\_25.186710 9902-9922. Max. coverage (+): 0.01. Max coverage (-): 0.2

Region: NODE\_117571\_length\_15591\_cov\_25.186710 9923-9944. Max. coverage (+): 0. Max coverage (-): 0

Region: NODE\_117571\_length\_15591\_cov\_25.186710 9945-9965. Max. coverage (+): 0. Max coverage (-): 0

Region: NODE\_117571\_length\_15591\_cov\_25.186710 9966-9987. Max. coverage (+): 0. Max coverage (-): 0

Region: NODE\_117571\_length\_15591\_cov\_25.186710 9988-10008. Max. coverage (+): 0. Max coverage (-): 0.08

Region: NODE\_117571\_length\_15591\_cov\_25.186710 10009-10030. Max. coverage (+): 0. Max coverage (-): 0.08

Region: NODE\_117571\_length\_15591\_cov\_25.186710 10031-10051. Max. coverage (+): 0. Max coverage (-): 0

Region: NODE\_117571\_length\_15591\_cov\_25.186710 10052-10073. Max. coverage (+): 0. Max coverage (-): 0.46

Region: NODE\_117571\_length\_15591\_cov\_25.186710 10074-10094. Max. coverage (+): 0. Max coverage (-): 0.61

Region: NODE\_117571\_length\_15591\_cov\_25.186710 10095-10116. Max. coverage (+): 0. Max coverage (-): 0.15

Region: NODE\_117571\_length\_15591\_cov\_25.186710 10117-10137. Max. coverage (+): 0. Max coverage (-): 0.38

Region: NODE\_117571\_length\_15591\_cov\_25.186710 10138-10159. Max. coverage (+): 0. Max coverage (-): 0.08

Region: NODE\_117571\_length\_15591\_cov\_25.186710 10160-10180. Max. coverage (+): 0.08. Max coverage (-): 0

Region: NODE\_117571\_length\_15591\_cov\_25.186710 10181-10202. Max. coverage (+): 0. Max coverage (-): 0

Region: NODE\_117571\_length\_15591\_cov\_25.186710 10203-10223. Max. coverage (+): 0.08. Max coverage (-): 0.61

Region: NODE\_117571\_length\_15591\_cov\_25.186710 10224-10245. Max. coverage (+): 0.08. Max coverage (-): 0.61

Region: NODE\_117571\_length\_15591\_cov\_25.186710 10246-10266. Max. coverage (+): 0. Max coverage (-): 0.69

Region: NODE\_117571\_length\_15591\_cov\_25.186710 10267-10288. Max. coverage (+): 0.08. Max coverage (-): 0.31

Region: NODE\_117571\_length\_15591\_cov\_25.186710 10289-10309. Max. coverage (+): 0.15. Max coverage (-): 0.31

Region: NODE\_117571\_length\_15591\_cov\_25.186710 10310-10331. Max. coverage (+): 0. Max coverage (-): 1.07

Region: NODE\_117571\_length\_15591\_cov\_25.186710 10332-10352. Max. coverage (+): 0.61. Max coverage (-): 3.22

Region: NODE\_117571\_length\_15591\_cov\_25.186710 10353-10374. Max. coverage (+): 0.46. Max coverage (-): 0

Region: NODE\_117571\_length\_15591\_cov\_25.186710 10375-10395. Max. coverage (+): 0.23. Max coverage (-): 0.08

Region: NODE\_117571\_length\_15591\_cov\_25.186710 10396-10417. Max. coverage (+): 0. Max coverage (-): 1.07

Region: NODE\_117571\_length\_15591\_cov\_25.186710 10418-10438. Max. coverage (+): 0. Max coverage (-): 0.38

Region: NODE\_117571\_length\_15591\_cov\_25.186710 10439-10460. Max. coverage (+): 0. Max coverage (-): 0.08

Region: NODE\_117571\_length\_15591\_cov\_25.186710 10461-10481. Max. coverage (+): 0.69. Max coverage (-): 0.15

Region: NODE\_117571\_length\_15591\_cov\_25.186710 10482-10503. Max. coverage (+): 0.84. Max coverage (-): 0.08

Region: NODE\_117571\_length\_15591\_cov\_25.186710 10504-10524. Max. coverage (+): 0.38. Max coverage (-): 0

Region: NODE\_117571\_length\_15591\_cov\_25.186710 10525-10546. Max. coverage (+): 0. Max coverage (-): 1.69

Region: NODE\_117571\_length\_15591\_cov\_25.186710 10547-10567. Max. coverage (+): 0.31. Max coverage (-): 1.76

Region: NODE\_117571\_length\_15591\_cov\_25.186710 10568-10589. Max. coverage (+): 0.08. Max coverage (-): 0.31

Region: NODE\_117571\_length\_15591\_cov\_25.186710 10590-10610. Max. coverage (+): 0.08. Max coverage (-): 0.08

Region: NODE\_117571\_length\_15591\_cov\_25.186710 10611-10632. Max. coverage (+): 0.08. Max coverage (-): 0

Region: NODE\_117571\_length\_15591\_cov\_25.186710 10633-10653. Max. coverage (+): 0. Max coverage (-): 1.76

Region: NODE\_117571\_length\_15591\_cov\_25.186710 10654-10675. Max. coverage (+): 0.31. Max coverage (-): 0.31

Region: NODE\_117571\_length\_15591\_cov\_25.186710 10676-10696. Max. coverage (+): 0.08. Max coverage (-): 1.92

Region: NODE\_117571\_length\_15591\_cov\_25.186710 10697-10718. Max. coverage (+): 0.08. Max coverage (-): 0.23

Region: NODE\_117571\_length\_15591\_cov\_25.186710 10719-10739. Max. coverage (+): 0.61. Max coverage (-): 1.84

Region: NODE\_117571\_length\_15591\_cov\_25.186710 10740-10761. Max. coverage (+): 0.61. Max coverage (-): 0

Region: NODE\_117571\_length\_15591\_cov\_25.186710 10762-10782. Max. coverage (+): 0. Max coverage (-): 0.08

Region: NODE\_117571\_length\_15591\_cov\_25.186710 10783-10804. Max. coverage (+): 0. Max coverage (-): 0.38

Region: NODE\_117571\_length\_15591\_cov\_25.186710 10805-10825. Max. coverage (+): 0. Max coverage (-): 0

Region: NODE\_117571\_length\_15591\_cov\_25.186710 10826-10847. Max. coverage (+): 0. Max coverage (-): 1.07

Region: NODE\_117571\_length\_15591\_cov\_25.186710 10848-10868. Max. coverage (+): 0.08. Max coverage (-): 0

Region: NODE\_117571\_length\_15591\_cov\_25.186710 10869-10890. Max. coverage (+): 0.08. Max coverage (-): 0.08

Region: NODE\_117571\_length\_15591\_cov\_25.186710 10891-10911. Max. coverage (+): 0.08. Max coverage (-): 0.08

Region: NODE\_117571\_length\_15591\_cov\_25.186710 10912-10933. Max. coverage (+): 7.81. Max coverage (-): 0.23

Region: NODE\_117571\_length\_15591\_cov\_25.186710 10934-10955. Max. coverage (+): 0. Max coverage (-): 0.23

Region: NODE\_117571\_length\_15591\_cov\_25.186710 10956-10976. Max. coverage (+): 0. Max coverage (-): 0.23

Region: NODE\_117571\_length\_15591\_cov\_25.186710 10977-10998. Max. coverage (+): 0. Max coverage (-): 0.08

Region: NODE\_117571\_length\_15591\_cov\_25.186710 10999-11019. Max. coverage (+): 0. Max coverage (-): 0.54

Region: NODE\_117571\_length\_15591\_cov\_25.186710 11020-11041. Max. coverage (+): 0.15. Max coverage (-): 0

Region: NODE\_117571\_length\_15591\_cov\_25.186710 11042-11062. Max. coverage (+): 0.31. Max coverage (-): 0

Region: NODE\_117571\_length\_15591\_cov\_25.186710 11063-11084. Max. coverage (+): 0. Max coverage (-): 0.15

Region: NODE\_117571\_length\_15591\_cov\_25.186710 11085-11105. Max. coverage (+): 0.15. Max coverage (-): 0.92

Region: NODE\_117571\_length\_15591\_cov\_25.186710 11106-11127. Max. coverage (+): 0.23. Max coverage (-): 0.15

Region: NODE\_117571\_length\_15591\_cov\_25.186710 11128-11148. Max. coverage (+): 0. Max coverage (-): 0

Region: NODE\_117571\_length\_15591\_cov\_25.186710 11149-11170. Max. coverage (+): 0. Max coverage (-): 3.45

Region: NODE\_117571\_length\_15591\_cov\_25.186710 11171-11191. Max. coverage (+): 0.23. Max coverage (-): 3.52

Region: NODE\_117571\_length\_15591\_cov\_25.186710 11192-11213. Max. coverage (+): 0.23. Max coverage (-): 0

Region: NODE\_117571\_length\_15591\_cov\_25.186710 11214-11234. Max. coverage (+): 0.08. Max coverage (-): 0

Region: NODE\_117571\_length\_15591\_cov\_25.186710 11235-11256. Max. coverage (+): 0. Max coverage (-): 1.99

Region: NODE\_117571\_length\_15591\_cov\_25.186710 11257-11277. Max. coverage (+): 0. Max coverage (-): 0.08

Region: NODE\_117571\_length\_15591\_cov\_25.186710 11278-11299. Max. coverage (+): 0.08. Max coverage (-): 0.23

Region: NODE\_117571\_length\_15591\_cov\_25.186710 11300-11320. Max. coverage (+): 0.15. Max coverage (-): 0.23

Region: NODE\_117571\_length\_15591\_cov\_25.186710 11321-11342. Max. coverage (+): 0.15. Max coverage (-): 0.61

Region: NODE\_117571\_length\_15591\_cov\_25.186710 11343-11363. Max. coverage (+): 0.31. Max coverage (-): 0.61

Region: NODE\_117571\_length\_15591\_cov\_25.186710 11364-11385. Max. coverage (+): 0.38. Max coverage (-): 3.29

Region: NODE\_117571\_length\_15591\_cov\_25.186710 11386-11406. Max. coverage (+): 0. Max coverage (-): 0.15

Region: NODE\_117571\_length\_15591\_cov\_25.186710 11407-11428. Max. coverage (+): 0. Max coverage (-): 0

Region: NODE\_117571\_length\_15591\_cov\_25.186710 11429-11449. Max. coverage (+): 0. Max coverage (-): 0.84

Region: NODE\_117571\_length\_15591\_cov\_25.186710 11450-11471. Max. coverage (+): 0.46. Max coverage (-): 0

Region: NODE\_117571\_length\_15591\_cov\_25.186710 11472-11492. Max. coverage (+): 0. Max coverage (-): 0.15

Region: NODE\_117571\_length\_15591\_cov\_25.186710 11493-11514. Max. coverage (+): 0. Max coverage (-): 0.77

Region: NODE\_117571\_length\_15591\_cov\_25.186710 11515-11535. Max. coverage (+): 0. Max coverage (-): 0.92

Region: NODE\_117571\_length\_15591\_cov\_25.186710 11536-11557. Max. coverage (+): 0.54. Max coverage (-): 1.46

Region: NODE\_117571\_length\_15591\_cov\_25.186710 11558-11578. Max. coverage (+): 0.54. Max coverage (-): 0

Region: NODE\_117571\_length\_15591\_cov\_25.186710 11579-11600. Max. coverage (+): 0.15. Max coverage (-): 0.15

Region: NODE\_117571\_length\_15591\_cov\_25.186710 11601-11621. Max. coverage (+): 0. Max coverage (-): 0.15

Region: NODE\_117571\_length\_15591\_cov\_25.186710 11622-11643. Max. coverage (+): 0.08. Max coverage (-): 0

Region: NODE\_117571\_length\_15591\_cov\_25.186710 11644-11664. Max. coverage (+): 0. Max coverage (-): 0.08

Region: NODE\_117571\_length\_15591\_cov\_25.186710 11665-11686. Max. coverage (+): 0.15. Max coverage (-): 0.08

Region: NODE\_117571\_length\_15591\_cov\_25.186710 11687-11707. Max. coverage (+): 0. Max coverage (-): 0.54

Region: NODE\_117571\_length\_15591\_cov\_25.186710 11708-11729. Max. coverage (+): 1.3. Max coverage (-): 7.43

Region: NODE\_117571\_length\_15591\_cov\_25.186710 11730-11750. Max. coverage (+): 0.08. Max coverage (-): 0.31

Region: NODE\_117571\_length\_15591\_cov\_25.186710 11751-11772. Max. coverage (+): 0. Max coverage (-): 0.31

Region: NODE\_117571\_length\_15591\_cov\_25.186710 11773-11793. Max. coverage (+): 0. Max coverage (-): 0.23

Region: NODE\_117571\_length\_15591\_cov\_25.186710 11794-11815. Max. coverage (+): 0. Max coverage (-): 0

Region: NODE\_117571\_length\_15591\_cov\_25.186710 11816-11836. Max. coverage (+): 0. Max coverage (-): 0

Region: NODE\_117571\_length\_15591\_cov\_25.186710 11837-11858. Max. coverage (+): 0. Max coverage (-): 0

Region: NODE\_117571\_length\_15591\_cov\_25.186710 11859-11879. Max. coverage (+): 0.08. Max coverage (-): 0.08

Region: NODE\_117571\_length\_15591\_cov\_25.186710 11880-11901. Max. coverage (+): 0. Max coverage (-): 0.08

Region: NODE\_117571\_length\_15591\_cov\_25.186710 11902-11922. Max. coverage (+): 0. Max coverage (-): 0

Region: NODE\_117571\_length\_15591\_cov\_25.186710 11923-11944. Max. coverage (+): 0. Max coverage (-): 0.08

Region: NODE\_117571\_length\_15591\_cov\_25.186710 11945-11965. Max. coverage (+): 0.08. Max coverage (-): 0.08

Region: NODE\_117571\_length\_15591\_cov\_25.186710 11966-11987. Max. coverage (+): 0. Max coverage (-): 0

Region: NODE\_117571\_length\_15591\_cov\_25.186710 11988-12008. Max. coverage (+): 0. Max coverage (-): 0

Region: NODE\_117571\_length\_15591\_cov\_25.186710 12009-12030. Max. coverage (+): 0. Max coverage (-): 0.08

Region: NODE\_117571\_length\_15591\_cov\_25.186710 12031-12052. Max. coverage (+): 0. Max coverage (-): 1.84

Region: NODE\_117571\_length\_15591\_cov\_25.186710 12053-12073. Max. coverage (+): 0. Max coverage (-): 0

Region: NODE\_117571\_length\_15591\_cov\_25.186710 12074-12095. Max. coverage (+): 0.08. Max coverage (-): 0.54

Region: NODE\_117571\_length\_15591\_cov\_25.186710 12096-12116. Max. coverage (+): 0. Max coverage (-): 0.08

Region: NODE\_117571\_length\_15591\_cov\_25.186710 12117-12138. Max. coverage (+): 0. Max coverage (-): 0.15

Region: NODE\_117571\_length\_15591\_cov\_25.186710 12139-12159. Max. coverage (+): 0.08. Max coverage (-): 0.31

Region: NODE\_117571\_length\_15591\_cov\_25.186710 12160-12181. Max. coverage (+): 0.08. Max coverage (-): 0.54

Region: NODE\_117571\_length\_15591\_cov\_25.186710 12182-12202. Max. coverage (+): 0. Max coverage (-): 0.46

Region: NODE\_117571\_length\_15591\_cov\_25.186710 12203-12224. Max. coverage (+): 0.15. Max coverage (-): 0

Region: NODE\_117571\_length\_15591\_cov\_25.186710 12225-12245. Max. coverage (+): 0.08. Max coverage (-): 0.77

Region: NODE\_117571\_length\_15591\_cov\_25.186710 12246-12267. Max. coverage (+): 0. Max coverage (-): 0.15

Region: NODE\_117571\_length\_15591\_cov\_25.186710 12268-12288. Max. coverage (+): 0. Max coverage (-): 0.08

Region: NODE\_117571\_length\_15591\_cov\_25.186710 12289-12310. Max. coverage (+): 0.08. Max coverage (-): 0.38

Region: NODE\_117571\_length\_15591\_cov\_25.186710 12311-12331. Max. coverage (+): 0.08. Max coverage (-): 6.9

Region: NODE\_117571\_length\_15591\_cov\_25.186710 12332-12353. Max. coverage (+): 0. Max coverage (-): 1

Region: NODE\_117571\_length\_15591\_cov\_25.186710 12354-12374. Max. coverage (+): 0. Max coverage (-): 0.08

Region: NODE\_117571\_length\_15591\_cov\_25.186710 12375-12396. Max. coverage (+): 0. Max coverage (-): 0.61

Region: NODE\_117571\_length\_15591\_cov\_25.186710 12397-12417. Max. coverage (+): 0.31. Max coverage (-): 0

Region: NODE\_117571\_length\_15591\_cov\_25.186710 12418-12439. Max. coverage (+): 0. Max coverage (-): 1.07

Region: NODE\_117571\_length\_15591\_cov\_25.186710 12440-12460. Max. coverage (+): 0.15. Max coverage (-): 0

Region: NODE\_117571\_length\_15591\_cov\_25.186710 12461-12482. Max. coverage (+): 0. Max coverage (-): 1.15

Region: NODE\_117571\_length\_15591\_cov\_25.186710 12483-12503. Max. coverage (+): 0.31. Max coverage (-): 1.23

Region: NODE\_117571\_length\_15591\_cov\_25.186710 12504-12525. Max. coverage (+): 0.31. Max coverage (-): 0.15

Region: NODE\_117571\_length\_15591\_cov\_25.186710 12526-12546. Max. coverage (+): 0.15. Max coverage (-): 0.38

Region: NODE\_117571\_length\_15591\_cov\_25.186710 12547-12568. Max. coverage (+): 0.08. Max coverage (-): 0.23

Region: NODE\_117571\_length\_15591\_cov\_25.186710 12569-12589. Max. coverage (+): 0. Max coverage (-): 0

Region: NODE\_117571\_length\_15591\_cov\_25.186710 12590-12611. Max. coverage (+): 0.08. Max coverage (-): 59.38

Region: NODE\_117571\_length\_15591\_cov\_25.186710 12612-12632. Max. coverage (+): 0.08. Max coverage (-): 59.3

Region: NODE\_117571\_length\_15591\_cov\_25.186710 12633-12654. Max. coverage (+): 0.08. Max coverage (-): 0.08

Region: NODE\_117571\_length\_15591\_cov\_25.186710 12655-12675. Max. coverage (+): 0.08. Max coverage (-): 0.46

Region: NODE\_117571\_length\_15591\_cov\_25.186710 12676-12697. Max. coverage (+): 0.15. Max coverage (-): 0.54

Region: NODE\_117571\_length\_15591\_cov\_25.186710 12698-12718. Max. coverage (+): 0.38. Max coverage (-): 0.23

Region: NODE\_117571\_length\_15591\_cov\_25.186710 12719-12740. Max. coverage (+): 0.08. Max coverage (-): 1.46

Region: NODE\_117571\_length\_15591\_cov\_25.186710 12741-12761. Max. coverage (+): 0. Max coverage (-): 1.61

Region: NODE\_117571\_length\_15591\_cov\_25.186710 12762-12783. Max. coverage (+): 0.38. Max coverage (-): 0.08

Region: NODE\_117571\_length\_15591\_cov\_25.186710 12784-12804. Max. coverage (+): 0. Max coverage (-): 0.38

Region: NODE\_117571\_length\_15591\_cov\_25.186710 12805-12826. Max. coverage (+): 0.23. Max coverage (-): 2.53

Region: NODE\_117571\_length\_15591\_cov\_25.186710 12827-12847. Max. coverage (+): 0. Max coverage (-): 2.3

Region: NODE\_117571\_length\_15591\_cov\_25.186710 12848-12869. Max. coverage (+): 0. Max coverage (-): 0.69

Region: NODE\_117571\_length\_15591\_cov\_25.186710 12870-12890. Max. coverage (+): 0. Max coverage (-): 0.15

Region: NODE\_117571\_length\_15591\_cov\_25.186710 12891-12912. Max. coverage (+): 0. Max coverage (-): 1

Region: NODE\_117571\_length\_15591\_cov\_25.186710 12913-12933. Max. coverage (+): 0. Max coverage (-): 1.07

Region: NODE\_117571\_length\_15591\_cov\_25.186710 12934-12955. Max. coverage (+): 0.61. Max coverage (-): 1.07

Region: NODE\_117571\_length\_15591\_cov\_25.186710 12956-12976. Max. coverage (+): 0.61. Max coverage (-): 0.08

Region: NODE\_117571\_length\_15591\_cov\_25.186710 12977-12998. Max. coverage (+): 0. Max coverage (-): 0.15

Region: NODE\_117571\_length\_15591\_cov\_25.186710 12999-13019. Max. coverage (+): 0. Max coverage (-): 0.15

Region: NODE\_117571\_length\_15591\_cov\_25.186710 13020-13041. Max. coverage (+): 0.54. Max coverage (-): 0.08

Region: NODE\_117571\_length\_15591\_cov\_25.186710 13042-13062. Max. coverage (+): 0.15. Max coverage (-): 2.91

Region: NODE\_117571\_length\_15591\_cov\_25.186710 13063-13084. Max. coverage (+): 2.6. Max coverage (-): 0.23

Region: NODE\_117571\_length\_15591\_cov\_25.186710 13085-13106. Max. coverage (+): 0. Max coverage (-): 1.46

Region: NODE\_117571\_length\_15591\_cov\_25.186710 13107-13127. Max. coverage (+): 0.08. Max coverage (-): 0.08

Region: NODE\_117571\_length\_15591\_cov\_25.186710 13128-13149. Max. coverage (+): 0.15. Max coverage (-): 2.45

Region: NODE\_117571\_length\_15591\_cov\_25.186710 13150-13170. Max. coverage (+): 0.31. Max coverage (-): 0.15

Region: NODE\_117571\_length\_15591\_cov\_25.186710 13171-13192. Max. coverage (+): 0.15. Max coverage (-): 0.84

Region: NODE\_117571\_length\_15591\_cov\_25.186710 13193-13213. Max. coverage (+): 0. Max coverage (-): 0

Region: NODE\_117571\_length\_15591\_cov\_25.186710 13214-13235. Max. coverage (+): 0. Max coverage (-): 0.61

Region: NODE\_117571\_length\_15591\_cov\_25.186710 13236-13256. Max. coverage (+): 0. Max coverage (-): 0.08

Region: NODE\_117571\_length\_15591\_cov\_25.186710 13257-13278. Max. coverage (+): 0. Max coverage (-): 0

Region: NODE\_117571\_length\_15591\_cov\_25.186710 13279-13299. Max. coverage (+): 0. Max coverage (-): 0.08

Region: NODE\_117571\_length\_15591\_cov\_25.186710 13300-13321. Max. coverage (+): 0. Max coverage (-): 0.31

Region: NODE\_117571\_length\_15591\_cov\_25.186710 13322-13342. Max. coverage (+): 0.08. Max coverage (-): 0.31

Region: NODE\_117571\_length\_15591\_cov\_25.186710 13343-13364. Max. coverage (+): 0.08. Max coverage (-): 0.15

Region: NODE\_117571\_length\_15591\_cov\_25.186710 13365-13385. Max. coverage (+): 0. Max coverage (-): 0.15

Region: NODE\_117571\_length\_15591\_cov\_25.186710 13386-13407. Max. coverage (+): 0. Max coverage (-): 0

Region: NODE\_117571\_length\_15591\_cov\_25.186710 13408-13428. Max. coverage (+): 0. Max coverage (-): 0

Region: NODE\_117571\_length\_15591\_cov\_25.186710 13429-13450. Max. coverage (+): 0. Max coverage (-): 0

Region: NODE\_117571\_length\_15591\_cov\_25.186710 13451-13471. Max. coverage (+): 0. Max coverage (-): 0.01

Region: NODE\_117571\_length\_15591\_cov\_25.186710 13472-13493. Max. coverage (+): 0. Max coverage (-): 0.54

Region: NODE\_117571\_length\_15591\_cov\_25.186710 13494-13514. Max. coverage (+): 0. Max coverage (-): 0.08

Region: NODE\_117571\_length\_15591\_cov\_25.186710 13515-13536. Max. coverage (+): 0. Max coverage (-): 0

Region: NODE\_117571\_length\_15591\_cov\_25.186710 13537-13557. Max. coverage (+): 0. Max coverage (-): 0.08

Region: NODE\_117571\_length\_15591\_cov\_25.186710 13558-13579. Max. coverage (+): 0. Max coverage (-): 0.38

Region: NODE\_117571\_length\_15591\_cov\_25.186710 13580-13600. Max. coverage (+): 0.23. Max coverage (-): 0.46

Region: NODE\_117571\_length\_15591\_cov\_25.186710 13601-13622. Max. coverage (+): 0.46. Max coverage (-): 0.15

Region: NODE\_117571\_length\_15591\_cov\_25.186710 13623-13643. Max. coverage (+): 0.54. Max coverage (-): 0.08

Region: NODE\_117571\_length\_15591\_cov\_25.186710 13644-13665. Max. coverage (+): 0.08. Max coverage (-): 0

Region: NODE\_117571\_length\_15591\_cov\_25.186710 13666-13686. Max. coverage (+): 0. Max coverage (-): 0.23

Region: NODE\_117571\_length\_15591\_cov\_25.186710 13687-13708. Max. coverage (+): 0. Max coverage (-): 0.08

Region: NODE\_117571\_length\_15591\_cov\_25.186710 13709-13729. Max. coverage (+): 0. Max coverage (-): 0

Region: NODE\_117571\_length\_15591\_cov\_25.186710 13730-13751. Max. coverage (+): 0. Max coverage (-): 0.31

Region: NODE\_117571\_length\_15591\_cov\_25.186710 13752-13772. Max. coverage (+): 0.92. Max coverage (-): 0.31

Region: NODE\_117571\_length\_15591\_cov\_25.186710 13773-13794. Max. coverage (+): 0.23. Max coverage (-): 0.46

Region: NODE\_117571\_length\_15591\_cov\_25.186710 13795-13815. Max. coverage (+): 0. Max coverage (-): 0

Region: NODE\_117571\_length\_15591\_cov\_25.186710 13816-13837. Max. coverage (+): 0.08. Max coverage (-): 0.15

Region: NODE\_117571\_length\_15591\_cov\_25.186710 13838-13858. Max. coverage (+): 0. Max coverage (-): 0

Region: NODE\_117571\_length\_15591\_cov\_25.186710 13859-13880. Max. coverage (+): 0. Max coverage (-): 0.23

Region: NODE\_117571\_length\_15591\_cov\_25.186710 13881-13901. Max. coverage (+): 0. Max coverage (-): 0.08

Region: NODE\_117571\_length\_15591\_cov\_25.186710 13902-13923. Max. coverage (+): 0.23. Max coverage (-): 0.31

Region: NODE\_117571\_length\_15591\_cov\_25.186710 13924-13944. Max. coverage (+): 1.07. Max coverage (-): 0

Region: NODE\_117571\_length\_15591\_cov\_25.186710 13945-13966. Max. coverage (+): 0.08. Max coverage (-): 3.29

Region: NODE\_117571\_length\_15591\_cov\_25.186710 13967-13987. Max. coverage (+): 0.38. Max coverage (-): 0.08

Region: NODE\_117571\_length\_15591\_cov\_25.186710 13988-14009. Max. coverage (+): 0.15. Max coverage (-): 1.07

Region: NODE\_117571\_length\_15591\_cov\_25.186710 14010-14030. Max. coverage (+): 0.08. Max coverage (-): 0.69

Region: NODE\_117571\_length\_15591\_cov\_25.186710 14031-14052. Max. coverage (+): 0. Max coverage (-): 0.92

Region: NODE\_117571\_length\_15591\_cov\_25.186710 14053-14073. Max. coverage (+): 0. Max coverage (-): 0.38

Region: NODE\_117571\_length\_15591\_cov\_25.186710 14074-14095. Max. coverage (+): 0. Max coverage (-): 0.23

Region: NODE\_117571\_length\_15591\_cov\_25.186710 14096-14116. Max. coverage (+): 0. Max coverage (-): 0.38

Region: NODE\_117571\_length\_15591\_cov\_25.186710 14117-14138. Max. coverage (+): 0.08. Max coverage (-): 0.31

Region: NODE\_117571\_length\_15591\_cov\_25.186710 14139-14159. Max. coverage (+): 0.08. Max coverage (-): 1.15

Region: NODE\_117571\_length\_15591\_cov\_25.186710 14160-14181. Max. coverage (+): 0.15. Max coverage (-): 1.69

Region: NODE\_117571\_length\_15591\_cov\_25.186710 14182-14203. Max. coverage (+): 0.15. Max coverage (-): 1.15

Region: NODE\_117571\_length\_15591\_cov\_25.186710 14204-14224. Max. coverage (+): 0. Max coverage (-): 21.15

Region: NODE\_117571\_length\_15591\_cov\_25.186710 14225-14246. Max. coverage (+): 0.23. Max coverage (-): 0.08

Region: NODE\_117571\_length\_15591\_cov\_25.186710 14247-14267. Max. coverage (+): 0. Max coverage (-): 0

Region: NODE\_117571\_length\_15591\_cov\_25.186710 14268-14289. Max. coverage (+): 0.15. Max coverage (-): 0.08

Region: NODE\_117571\_length\_15591\_cov\_25.186710 14290-14310. Max. coverage (+): 0.08. Max coverage (-): 17.62

Region: NODE\_117571\_length\_15591\_cov\_25.186710 14311-14332. Max. coverage (+): 0.15. Max coverage (-): 2.07

Region: NODE\_117571\_length\_15591\_cov\_25.186710 14333-14353. Max. coverage (+): 0.15. Max coverage (-): 1.84

Region: NODE\_117571\_length\_15591\_cov\_25.186710 14354-14375. Max. coverage (+): 0.38. Max coverage (-): 0.23

Region: NODE\_117571\_length\_15591\_cov\_25.186710 14376-14396. Max. coverage (+): 0. Max coverage (-): 0.46

Region: NODE\_117571\_length\_15591\_cov\_25.186710 14397-14418. Max. coverage (+): 0.08. Max coverage (-): 0

Region: NODE\_117571\_length\_15591\_cov\_25.186710 14419-14439. Max. coverage (+): 0. Max coverage (-): 0.31

Region: NODE\_117571\_length\_15591\_cov\_25.186710 14440-14461. Max. coverage (+): 0. Max coverage (-): 0.77

Region: NODE\_117571\_length\_15591\_cov\_25.186710 14462-14482. Max. coverage (+): 0. Max coverage (-): 1

Region: NODE\_117571\_length\_15591\_cov\_25.186710 14483-14504. Max. coverage (+): 0. Max coverage (-): 0

Region: NODE\_117571\_length\_15591\_cov\_25.186710 14505-14525. Max. coverage (+): 0. Max coverage (-): 0

Region: NODE\_117571\_length\_15591\_cov\_25.186710 14526-14547. Max. coverage (+): 0. Max coverage (-): 0.04

Region: NODE\_117571\_length\_15591\_cov\_25.186710 14548-14568. Max. coverage (+): 0. Max coverage (-): 0.08

Region: NODE\_117571\_length\_15591\_cov\_25.186710 14569-14590. Max. coverage (+): 0. Max coverage (-): 0.15

Region: NODE\_117571\_length\_15591\_cov\_25.186710 14591-14611. Max. coverage (+): 0.08. Max coverage (-): 0

Region: NODE\_117571\_length\_15591\_cov\_25.186710 14612-14633. Max. coverage (+): 0. Max coverage (-): 0.02

Region: NODE\_117571\_length\_15591\_cov\_25.186710 14634-14654. Max. coverage (+): 0. Max coverage (-): 0

Region: NODE\_117571\_length\_15591\_cov\_25.186710 14655-14676. Max. coverage (+): 0. Max coverage (-): 1.46

Region: NODE\_117571\_length\_15591\_cov\_25.186710 14677-14697. Max. coverage (+): 0.08. Max coverage (-): 0

Region: NODE\_117571\_length\_15591\_cov\_25.186710 14698-14719. Max. coverage (+): 0. Max coverage (-): 6.51

Region: NODE\_117571\_length\_15591\_cov\_25.186710 14720-14740. Max. coverage (+): 0. Max coverage (-): 0

Region: NODE\_117571\_length\_15591\_cov\_25.186710 14741-14762. Max. coverage (+): 0. Max coverage (-): 1.84

Region: NODE\_117571\_length\_15591\_cov\_25.186710 14763-14783. Max. coverage (+): 0. Max coverage (-): 9.65

Region: NODE\_117571\_length\_15591\_cov\_25.186710 14784-14805. Max. coverage (+): 0.15. Max coverage (-): 0.77

Region: NODE\_117571\_length\_15591\_cov\_25.186710 14806-14826. Max. coverage (+): 0.15. Max coverage (-): 1.46

Region: NODE\_117571\_length\_15591\_cov\_25.186710 14827-14848. Max. coverage (+): 0. Max coverage (-): 0.84

Region: NODE\_117571\_length\_15591\_cov\_25.186710 14849-14869. Max. coverage (+): 0.31. Max coverage (-): 0.77

Region: NODE\_117571\_length\_15591\_cov\_25.186710 14870-14891. Max. coverage (+): 0.08. Max coverage (-): 0.46

Region: NODE\_117571\_length\_15591\_cov\_25.186710 14892-14912. Max. coverage (+): 0. Max coverage (-): 0.15

Region: NODE\_117571\_length\_15591\_cov\_25.186710 14913-14934. Max. coverage (+): 0.08. Max coverage (-): 4.29

Region: NODE\_117571\_length\_15591\_cov\_25.186710 14935-14955. Max. coverage (+): 0.04. Max coverage (-): 1.92

Region: NODE\_117571\_length\_15591\_cov\_25.186710 14956-14977. Max. coverage (+): 0. Max coverage (-): 0.01

Region: NODE\_117571\_length\_15591\_cov\_25.186710 14978-14998. Max. coverage (+): 0. Max coverage (-): 0

Region: NODE\_117571\_length\_15591\_cov\_25.186710 14999-15020. Max. coverage (+): 0.15. Max coverage (-): 0

Region: NODE\_117571\_length\_15591\_cov\_25.186710 15021-15041. Max. coverage (+): 0.15. Max coverage (-): 0

Region: NODE\_117571\_length\_15591\_cov\_25.186710 15042-15063. Max. coverage (+): 0. Max coverage (-): 0.15

Region: NODE\_117571\_length\_15591\_cov\_25.186710 15064-15084. Max. coverage (+): 0.08. Max coverage (-): 0.09

Region: NODE\_117571\_length\_15591\_cov\_25.186710 15085-15106. Max. coverage (+): 0.08. Max coverage (-): 0.23

Region: NODE\_117571\_length\_15591\_cov\_25.186710 15107-15127. Max. coverage (+): 0. Max coverage (-): 0.23

Region: NODE\_117571\_length\_15591\_cov\_25.186710 15128-15149. Max. coverage (+): 0.08. Max coverage (-): 0

Region: NODE\_117571\_length\_15591\_cov\_25.186710 15150-15170. Max. coverage (+): 0. Max coverage (-): 0.27

Region: NODE\_117571\_length\_15591\_cov\_25.186710 15171-15192. Max. coverage (+): 0.08. Max coverage (-): 0.27

Region: NODE\_117571\_length\_15591\_cov\_25.186710 15193-15213. Max. coverage (+): 0.08. Max coverage (-): 0.08

Region: NODE\_117571\_length\_15591\_cov\_25.186710 15214-15235. Max. coverage (+): 0. Max coverage (-): 0.08

Region: NODE\_117571\_length\_15591\_cov\_25.186710 15236-15257. Max. coverage (+): 0. Max coverage (-): 0.08

Region: NODE\_117571\_length\_15591\_cov\_25.186710 15258-15278. Max. coverage (+): 0. Max coverage (-): 0

Region: NODE\_117571\_length\_15591\_cov\_25.186710 15279-15300. Max. coverage (+): 0.08. Max coverage (-): 0

Region: NODE\_117571\_length\_15591\_cov\_25.186710 15301-15321. Max. coverage (+): 0. Max coverage (-): 0

Region: NODE\_117571\_length\_15591\_cov\_25.186710 15322-15343. Max. coverage (+): 0.02. Max coverage (-): 0.23

Region: NODE\_117571\_length\_15591\_cov\_25.186710 15344-15364. Max. coverage (+): 0.02. Max coverage (-): 0.22

Region: NODE\_117571\_length\_15591\_cov\_25.186710 15365-15386. Max. coverage (+): 0. Max coverage (-): 2.01

Region: NODE\_117571\_length\_15591\_cov\_25.186710 15387-15407. Max. coverage (+): 0.15. Max coverage (-): 0

Region: NODE\_117571\_length\_15591\_cov\_25.186710 15408-15429. Max. coverage (+): 0. Max coverage (-): 0.27

Region: NODE\_117571\_length\_15591\_cov\_25.186710 15430-15450. Max. coverage (+): 0.06. Max coverage (-): 0

Region: NODE\_117571\_length\_15591\_cov\_25.186710 15451-15472. Max. coverage (+): 0. Max coverage (-): 0

Region: NODE\_117571\_length\_15591\_cov\_25.186710 15473-15493. Max. coverage (+): 0.05. Max coverage (-): 0

Region: NODE\_117571\_length\_15591\_cov\_25.186710 15494-15515. Max. coverage (+): 0.05. Max coverage (-): 0

Region: NODE\_117571\_length\_15591\_cov\_25.186710 15516-15536. Max. coverage (+): 0. Max coverage (-): 0

Region: NODE\_117571\_length\_15591\_cov\_25.186710 15537-15558. Max. coverage (+): 0. Max coverage (-): 0

Region: NODE\_117571\_length\_15591\_cov\_25.186710 15559-15579. Max. coverage (+): 0. Max coverage (-): 0

Region: NODE\_117571\_length\_15591\_cov\_25.186710 15580-15601. Max. coverage (+): 0. Max coverage (-): 0

Region: NODE\_117571\_length\_15591\_cov\_25.186710 15602-15622. Max. coverage (+): 0. Max coverage (-): 0

Region: NODE\_117571\_length\_15591\_cov\_25.186710 15623-15644. Max. coverage (+): 0. Max coverage (-): 0

Region: NODE\_117571\_length\_15591\_cov\_25.186710 15645-15665. Max. coverage (+): 0. Max coverage (-): 0

Region: NODE\_117571\_length\_15591\_cov\_25.186710 15666-15687. Max. coverage (+): 0.04. Max coverage (-): 0

Region: NODE\_117571\_length\_15591\_cov\_25.186710 15688-15708. Max. coverage (+): 0. Max coverage (-): 0

Region: NODE\_117571\_length\_15591\_cov\_25.186710 15709-15730. Max. coverage (+): 0. Max coverage (-): 0

Region: NODE\_117571\_length\_15591\_cov\_25.186710 15731-15751. Max. coverage (+): 0. Max coverage (-): 0

Region: NODE\_117571\_length\_15591\_cov\_25.186710 15752-15773. Max. coverage (+): 0. Max coverage (-): 0.01

Region: NODE\_117571\_length\_15591\_cov\_25.186710 15774-. Max. coverage (+): 0. Max coverage (-): 0

RepeatMasker Color Code

**+**

100-98% Identity

<98-95% Identity

<95-90% Identity

<90-85% Identity

<85-80% Identity

<80-75% Identity

<75-70% Identity

<70% Identity

**-**

Gene Set Color Code

**+**

Gene

Pseudogene

Other

**-**

Topology/Coverage Color Code

Coverage Plus Strand

Coverage Minus Strand

Mainstrand: Plus

Mainstrand: Minus

Complementary Strand

Flanking Region  
(if option -flank >0)

Gene Set Annotation  
  
RepeatMasker Annotation  

**1. AlRepD-1165**: 5601-5706 (-), Divergence to consensus: 24%  
**2. AlRepC-373**: 5961-6170 (+), Divergence to consensus: 33.6%  
**3. AlRepC-905**: 6120-6246 (-), Divergence to consensus: 41.3%  
**4. (TTTG)n**: 6971-7006 (+), Divergence to consensus: 11.2%  
**5. Samurai\_I-int**: 8814-9228 (-), Divergence to consensus: 30.9%  
**6. AlRepE-2838**: 9584-9679 (-), Divergence to consensus: 41.7%  
**7. AlRepC-1442**: 9682-9782 (+), Divergence to consensus: 7.3%  
**8. AlRepC-1442**: 9777-10016 (+), Divergence to consensus: 5.9%  
**9. (T)n**: 11984-12018 (+), Divergence to consensus: 14.9%  
**10. AlRepD-1165**: 13401-13528 (-), Divergence to consensus: 19.5%  
**11. AlRepB-103**: 13637-13708 (-), Divergence to consensus: 27.7%  
**12. AlRepA-51**: 13867-13942 (-), Divergence to consensus: 43.1%  
**13. AlRepE-65**: 14495-14535 (+), Divergence to consensus: 12.5%  
**14. AlRepA-115**: 14511-14618 (+), Divergence to consensus: 36.3%  
**15. AlRepC-754**: 14619-14687 (+), Divergence to consensus: 17.9%  
**16. AlRepB-420**: 15429-15729 (+), Divergence to consensus: 16.3%  
**17. hAT-N93\_DR**: 15731-15788 (+), Divergence to consensus: 0%

  
Transcription Factor Binding Sites  

**RFX4\_2** (Sequence: CTTGGTTAC (+): 15066)  
**RHOXF1** (Sequence: AGCTTA (-): 5334)  
**RHOXF1** (Sequence: AGATTA (-): 5423)  
**RHOXF1** (Sequence: AGCTCA (-): 5901)  
**RHOXF1** (Sequence: AGCTCA (-): 8165)  
**RHOXF1** (Sequence: AGATCA (-): 8177)  
**RHOXF1** (Sequence: GGCTCA (-): 8861)  
**RHOXF1** (Sequence: AGCTCA (-): 8953)  
**RHOXF1** (Sequence: GGATCA (-): 9380)  
**RHOXF1** (Sequence: GGATCA (-): 10234)  
**RHOXF1** (Sequence: GGATCA (-): 10357)  
**RHOXF1** (Sequence: AGATTA (-): 10643)  
**RHOXF1** (Sequence: GGATTA (-): 11366)  
**RHOXF1** (Sequence: AGATTA (-): 11779)  
**RHOXF1** (Sequence: AGCTTA (-): 12032)  
**RHOXF1** (Sequence: AGATTA (-): 12099)  
**RHOXF1** (Sequence: AGCTTA (-): 12796)  
**RHOXF1** (Sequence: GGATCA (-): 12984)  
**RHOXF1** (Sequence: AGATCA (-): 13222)  
**RHOXF1** (Sequence: GGATTA (-): 13382)  
**RHOXF1** (Sequence: AGCTCA (-): 14477)  
**RHOXF1** (Sequence: TAATCC (+): 5338)  
**RHOXF1** (Sequence: TGATCT (+): 5786)  
**RHOXF1** (Sequence: TGATCT (+): 6126)  
**RHOXF1** (Sequence: TAATCT (+): 6145)  
**RHOXF1** (Sequence: TAATCC (+): 6342)  
**RHOXF1** (Sequence: TAATCT (+): 6686)  
**RHOXF1** (Sequence: TAAGCT (+): 6927)  
**RHOXF1** (Sequence: TAATCT (+): 7048)  
**RHOXF1** (Sequence: TAAGCT (+): 7557)  
**RHOXF1** (Sequence: TAATCC (+): 9312)  
**RHOXF1** (Sequence: TGAGCT (+): 9846)  
**RHOXF1** (Sequence: TGAGCT (+): 10577)  
**RHOXF1** (Sequence: TGAGCC (+): 10676)  
**RHOXF1** (Sequence: TGAGCT (+): 11304)  
**RHOXF1** (Sequence: TGAGCT (+): 12177)  
**RHOXF1** (Sequence: TGAGCT (+): 12811)  
**RHOXF1** (Sequence: TAATCC (+): 12907)  
**RHOXF1** (Sequence: TGATCC (+): 12976)  
**RHOXF1** (Sequence: TGAGCC (+): 13046)  
**RHOXF1** (Sequence: TGAGCC (+): 13323)  
**RHOXF1** (Sequence: TGATCC (+): 13890)  
**RHOXF1** (Sequence: TAATCC (+): 14044)  
**RHOXF1** (Sequence: TGAGCT (+): 14281)  
**Lhx8** (Sequence: CTAATTAG (-): 5533)  
**Lhx8** (Sequence: TTAATTAA (-): 13712)  
**Gata4** (Sequence: GTTATCT (+): 12859)  
**POU5F1** (Sequence: TTTGCAT (-): 10527)  
**POU5F1** (Sequence: TTTGCAT (-): 12571)  
**POU5F1** (Sequence: TTTGCAT (-): 13867)  
**RFX4\_2** (Sequence: GTAACTACG (-): 15769)  
**SOX9** (Sequence: AACAATAA (-): 5701)  
**SOX9** (Sequence: AACAATGA (-): 13502)  
**SOX9** (Sequence: AACAATAG (-): 13805)  
**SOX9** (Sequence: AACAATAA (-): 13825)  
**FOXP1** (Sequence: GTAAACA (+): 11447)  
**FOXO1** (Sequence: CTTGTTTAT (+): 5148)  
**FOXO1** (Sequence: GCTGTTTAT (+): 7353)  
**FOXO1** (Sequence: GCTGTTTTT (+): 14284)  
**FOXO3\_mmu** (Sequence: TGTTTTCA (-): 5282)  
**FOXO3\_mmu** (Sequence: TGTTTTCA (-): 8398)  
**FOXO3\_mmu** (Sequence: TGTTTTGA (-): 9051)  
**Sox5** (Sequence: ATTGTT (+): 7011)  
**Sox5** (Sequence: ATTGTT (+): 8680)  
**Sox5** (Sequence: ATTGTT (+): 11988)  
**Sox5** (Sequence: ATTGTT (+): 15433)  
**FIGLA** (Sequence: ACCAGCTGGT (-): 9027)  
**FIGLA** (Sequence: ACCAGGTGGA (-): 15577)  
**FOXO3\_mmu** (Sequence: TCAAAACA (+): 7974)  
**FOXO3\_mmu** (Sequence: GGAAAACA (+): 10300)  
**FOXO3\_mmu** (Sequence: TGAAAACA (+): 12485)  
**FOXO3\_mmu** (Sequence: TGAAAACA (+): 12770)  
**Nobox** (Sequence: GCTAATTA (-): 5532)  
**Nobox** (Sequence: AGCAATTA (-): 9686)  
**FOXO1** (Sequence: GAAAACAGC (-): 12486)  
**FOXO1** (Sequence: AAAAACAGG (-): 12500)  
**FOXO1** (Sequence: GAAAACAAC (-): 12771)  
**Nobox** (Sequence: TAATTAGC (+): 5534)  
**POU2F1** (Sequence: ATTAAAATA (-): 6673)  
**Rhox11** (Sequence: TGCTGTTAT (+): 6843)  
**Rhox11** (Sequence: TGCTGTATA (+): 7196)  
**Rhox11** (Sequence: TGGTGTATT (+): 10818)  
**Rhox11** (Sequence: TGGTGTTTT (+): 10915)  
**Rhox11** (Sequence: TATACAGCG (-): 10986)  
**Rhox11** (Sequence: TTTACACCA (-): 12641)  
**Gata4** (Sequence: AGATAAG (-): 5558)  
**Sox5** (Sequence: AACAAT (-): 5701)  
**Sox5** (Sequence: AACAAT (-): 9430)  
**Sox5** (Sequence: AACAAT (-): 9946)  
**Sox5** (Sequence: AACAAT (-): 11017)  
**Sox5** (Sequence: AACAAT (-): 13502)  
**Sox5** (Sequence: AACAAT (-): 13805)  
**Sox5** (Sequence: AACAAT (-): 13825)  
**Sox5** (Sequence: AACAAT (-): 14327)  
**Sox5** (Sequence: AACAAT (-): 14501)  
**Sox5** (Sequence: AACAAT (-): 14685)  
**Sox5** (Sequence: AACAAT (-): 15586)  
**POU2F1** (Sequence: TATTTTAAT (+): 5218)  
**POU2F1** (Sequence: TATTTTAAT (+): 12211)  
**POU2F1** (Sequence: TATGCAAAT (+): 15140)  
**POU5F1** (Sequence: ATGCAAA (+): 9661)  
**POU5F1** (Sequence: ATGCAAA (+): 14680)  
**POU5F1** (Sequence: ATGCAAA (+): 15141)
