# Supplementary material for: First characterization of PIWI-interacting RNA clusters in a cichlid fish with a B chromosome
Source: BMC Biol. 2022 Sep 21;20:204. doi: 10.1186/s12915-022-01403-2 (PMC9490952; doi:10.1186/s12915-022-01403-2)
Supplement: Supplementary file 1 — Additional file 1. Zipped folder with fasta and interactive html piRNA cluster information for the A. latifasciata genome. The nomenclature is as follows: number-pirna-cluster_sex_B-presence (f, female; m, male; 0b, without B chromosome; 1b, with B chromosome). [file 12915_2022_1403_MOESM1_ESM.zip › 11_f1b.html]

piRNA cluster 11\_f1b 20


Predicted piRNA cluster no. 11\_f1b
  

Show proTRAC run info
Hide proTRAC run info

/\  
                \_\_\_\_\_\_\_\_\_\_\_\_\_\_\_\_\_\_\_\_\_\_\_/\\_\_\_ /  \\_\_\_\_\_\_\_  
               I                      /  \  /    \      I  
               I     pro             /    \/      \     I  
               I        TRAC        /               \   I  
               I   \_\_\_\_\_\_\_\_\_\_\_\_\_\_\_\_/\_\_\_\_\_\_\_\_\_\_\_\_\_\_\_\_\_\\_ I  
               I   \              /                     I  
               I    \            /                      I  
               I     \  /\      /       V.2.4.2         I  
               I      \/  \    /                        I  
               I\_\_\_\_\_\_\_\_\_\_\_\  /\_\_\_\_\_\_\_\_\_\_\_\_\_\_\_\_\_\_\_\_\_\_\_\_\_I  
                            \/  
  
  
================================= proTRAC ====================================  
VERSION: .......... 2.4.2  
LAST MODIFIED: .... 11. May 2018  
  
Please cite:  
Rosenkranz D, Zischler H. proTRAC - a software for probabilistic piRNA cluster  
detection, visualization and analysis. 2012. BMC Bioinformatics 13:5.  
  
  
Contact:  
David Rosenkranz  
Institute of Organismic and Molecular Evolutionary Biology  
Dept. Anthropology, small RNA group  
Johannes Gutenberg University Mainz  
email: rosenkranz@uni-mainz.de  
  
You can find the latest proTRAC version at:  
http://sourceforge.net/projects/protrac/files  
http://www.smallRNAgroup-mainz.de/software  
==============================================================================  
  
PARAMETERS:  
Map file: ...............piwi-femeas-1B.fa-collapse.map  
Genome file: ............../../../0B\_ala\_genome.fa  
RepeatMasker annotation: Alatifasciata-all0B-maryan-v2.fa\_corrected.out  
GeneSet:................./guest-storage/Data/annotation/Alatifasciata\_all0B\_maryan-v2\_out2017.gff  
  
Significant (p<=0.01) hit density will be calculated based  
on observed hit distribution.  
  
Sliding window size: ........................................ 5000 bp  
Sliding window increament: .................................. 1000 bp  
Normalize each hit by number of genomic hits: ............... yes  
Normalize each hit by number of sequence reads: ............. yes  
Normalize values (-> per million mapped reads): ............. yes  
Min. fraction of hits with 1T(U) or 10A: .................... 0.75  
Alternatively: Min. fraction of hits with 1T(U) and 10A: .... 0.5  
Min. fraction of hits with typical piRNA length: ............ 0.75  
Typical piRNA length: ....................................... 24-32 nt  
Min. size of a piRNA cluster: ............................... 1000 bp.  
Min. number of hits (absolute): ............................. 0  
Min. number of hits (normalized): ........................... 0  
Min. fraction of hits on the mainstrand: .................... 0.75  
Top fraction of mapped sequences (in terms of read counts): . 1%  
Top fraction accounts for max. n% of sequence reads: ........ 90%  
Min. fraction of hits on each arm of a bidirectional cluster: 0.05  
Output html file for each cluster: .......................... yes  
Output a summary table: ..................................... yes  
Output a FASTA file for each cluster (piRNA sequences): ..... yes  
Output a FASTA file comprising cluster sequences: ........... yes  
Output a GTF file for predicted piRNA clusters: ..............yes  
Search DNA motifs in clusters: .............................. yes  
Output flanking sequences: +/- .............................. 0 bp  
Output ~.pTi file: .......................................... no  
==============================================================================  
  
  
Genome size (without gaps): ............ 758543724 bp  
Gaps (N/X/-): .......................... 417479 bp  
Mapped reads: .......................... 10641844  
Non-identical sequences: ............... 2832837  
Genomic hits: .......................... 26056853  
Significant densitiy of mapped reads: .. 368.713530323068 reads/kb

Show proTRAC cluster info
Hide proTRAC cluster info

|  |  |
| --- | --- |
| Location | NODE\_117571\_length\_15591\_cov\_25.186710 |
| Coordinates | 6008-15786 |
| Size [bp] | 9779 |
| Sequence hit loci | 1956 |
| Mapped reads (normalized) | 4472.2 |
| Mapped reads (normalized) per kb | 457.3 |
| Normalized reads with 1T (1U) | 79.8% |
| Normalized reads with 10A | 32.2% |
| Normalized reads with length 24-32 nt | 99.2% |
| Normalized reads on the main strand(s) | 89.2% |
| Predicted directionality | bi:plus-minus (split between 9668 and 9669) |

100%

0%

1T (1U)  
reads

10A reads

24-32 nt  
reads

reads on mainstrand

**Either the amount of reads with 1T (1U) OR 10A has to exceed 75% (set with option: -1Tor10A)  
Alternatively the amount of reads with 1T (1U) AND 10A has to exceed 50% (set with option: -1Tand10A)  
Minimum amount of reads with preferred size is 75% (set with option: -pisize)  
Minimum amount of reads on the main strand(s) is 75% (set with option: -clstrand)**

Show read coverage
Hide read coverage

WHAT DO I SEE HERE?  
This chart shows the location of mapped sequence reads within a predicted piRNA cluster. The color refers to the number of genomic hits produced by the sequence read in question. A dark red bar indicates that this sequence read produces many other hits elsewhere in the genome. Many adjacent red or yellow bars can indicate the presence of a multi-copy element such as transposons or rRNA genes. A dark green bar indicates that this sequence read maps uniquely to this locus.

1 hit

2-5 hits

6-10 hits

11-20 hits

21-50 hits

51-100 hits

> 100 hits

NODE\_117571\_length\_15591\_cov\_25.186710

6008

15786

Gene Set

RepeatMasker

Mapped  
Reads

48.49

plus strand

minus strand

48.49

Region: NODE\_117571\_length\_15591\_cov\_25.186710 2955-6017. Max. coverage (+): 0.09. Max coverage (-): 0.09

Region: NODE\_117571\_length\_15591\_cov\_25.186710 6018-6037. Max. coverage (+): 0.19. Max coverage (-): 0

Region: NODE\_117571\_length\_15591\_cov\_25.186710 6038-6056. Max. coverage (+): 0.19. Max coverage (-): 0

Region: NODE\_117571\_length\_15591\_cov\_25.186710 6057-6076. Max. coverage (+): 0.56. Max coverage (-): 0.28

Region: NODE\_117571\_length\_15591\_cov\_25.186710 6077-6096. Max. coverage (+): 0.47. Max coverage (-): 0.09

Region: NODE\_117571\_length\_15591\_cov\_25.186710 6097-6115. Max. coverage (+): 1.41. Max coverage (-): 0.09

Region: NODE\_117571\_length\_15591\_cov\_25.186710 6116-6135. Max. coverage (+): 0.66. Max coverage (-): 0.09

Region: NODE\_117571\_length\_15591\_cov\_25.186710 6136-6154. Max. coverage (+): 0.66. Max coverage (-): 0

Region: NODE\_117571\_length\_15591\_cov\_25.186710 6155-6174. Max. coverage (+): 0. Max coverage (-): 0

Region: NODE\_117571\_length\_15591\_cov\_25.186710 6175-6193. Max. coverage (+): 0.47. Max coverage (-): 0.09

Region: NODE\_117571\_length\_15591\_cov\_25.186710 6194-6213. Max. coverage (+): 0. Max coverage (-): 0

Region: NODE\_117571\_length\_15591\_cov\_25.186710 6214-6232. Max. coverage (+): 0.38. Max coverage (-): 0

Region: NODE\_117571\_length\_15591\_cov\_25.186710 6233-6252. Max. coverage (+): 0. Max coverage (-): 0.09

Region: NODE\_117571\_length\_15591\_cov\_25.186710 6253-6272. Max. coverage (+): 0.09. Max coverage (-): 0

Region: NODE\_117571\_length\_15591\_cov\_25.186710 6273-6291. Max. coverage (+): 0.09. Max coverage (-): 0

Region: NODE\_117571\_length\_15591\_cov\_25.186710 6292-6311. Max. coverage (+): 2.07. Max coverage (-): 0

Region: NODE\_117571\_length\_15591\_cov\_25.186710 6312-6330. Max. coverage (+): 0.09. Max coverage (-): 0

Region: NODE\_117571\_length\_15591\_cov\_25.186710 6331-6350. Max. coverage (+): 0.85. Max coverage (-): 0

Region: NODE\_117571\_length\_15591\_cov\_25.186710 6351-6369. Max. coverage (+): 0.19. Max coverage (-): 0

Region: NODE\_117571\_length\_15591\_cov\_25.186710 6370-6389. Max. coverage (+): 0.09. Max coverage (-): 0

Region: NODE\_117571\_length\_15591\_cov\_25.186710 6390-6408. Max. coverage (+): 0.75. Max coverage (-): 0.19

Region: NODE\_117571\_length\_15591\_cov\_25.186710 6409-6428. Max. coverage (+): 0.75. Max coverage (-): 0

Region: NODE\_117571\_length\_15591\_cov\_25.186710 6429-6448. Max. coverage (+): 0. Max coverage (-): 0

Region: NODE\_117571\_length\_15591\_cov\_25.186710 6449-6467. Max. coverage (+): 1.32. Max coverage (-): 0

Region: NODE\_117571\_length\_15591\_cov\_25.186710 6468-6487. Max. coverage (+): 0. Max coverage (-): 0.09

Region: NODE\_117571\_length\_15591\_cov\_25.186710 6488-6506. Max. coverage (+): 0.19. Max coverage (-): 0

Region: NODE\_117571\_length\_15591\_cov\_25.186710 6507-6526. Max. coverage (+): 0. Max coverage (-): 0

Region: NODE\_117571\_length\_15591\_cov\_25.186710 6527-6545. Max. coverage (+): 1.03. Max coverage (-): 0

Region: NODE\_117571\_length\_15591\_cov\_25.186710 6546-6565. Max. coverage (+): 0.09. Max coverage (-): 0

Region: NODE\_117571\_length\_15591\_cov\_25.186710 6566-6584. Max. coverage (+): 0.19. Max coverage (-): 0

Region: NODE\_117571\_length\_15591\_cov\_25.186710 6585-6604. Max. coverage (+): 1.41. Max coverage (-): 0

Region: NODE\_117571\_length\_15591\_cov\_25.186710 6605-6624. Max. coverage (+): 0. Max coverage (-): 0.09

Region: NODE\_117571\_length\_15591\_cov\_25.186710 6625-6643. Max. coverage (+): 0.28. Max coverage (-): 0.09

Region: NODE\_117571\_length\_15591\_cov\_25.186710 6644-6663. Max. coverage (+): 0. Max coverage (-): 0

Region: NODE\_117571\_length\_15591\_cov\_25.186710 6664-6682. Max. coverage (+): 0.47. Max coverage (-): 0.09

Region: NODE\_117571\_length\_15591\_cov\_25.186710 6683-6702. Max. coverage (+): 0.09. Max coverage (-): 0

Region: NODE\_117571\_length\_15591\_cov\_25.186710 6703-6721. Max. coverage (+): 0.47. Max coverage (-): 0

Region: NODE\_117571\_length\_15591\_cov\_25.186710 6722-6741. Max. coverage (+): 0.47. Max coverage (-): 0.09

Region: NODE\_117571\_length\_15591\_cov\_25.186710 6742-6760. Max. coverage (+): 0.19. Max coverage (-): 0.09

Region: NODE\_117571\_length\_15591\_cov\_25.186710 6761-6780. Max. coverage (+): 0. Max coverage (-): 0

Region: NODE\_117571\_length\_15591\_cov\_25.186710 6781-6800. Max. coverage (+): 0. Max coverage (-): 0.09

Region: NODE\_117571\_length\_15591\_cov\_25.186710 6801-6819. Max. coverage (+): 0.47. Max coverage (-): 0.09

Region: NODE\_117571\_length\_15591\_cov\_25.186710 6820-6839. Max. coverage (+): 0. Max coverage (-): 0.09

Region: NODE\_117571\_length\_15591\_cov\_25.186710 6840-6858. Max. coverage (+): 0.19. Max coverage (-): 0

Region: NODE\_117571\_length\_15591\_cov\_25.186710 6859-6878. Max. coverage (+): 0. Max coverage (-): 0

Region: NODE\_117571\_length\_15591\_cov\_25.186710 6879-6897. Max. coverage (+): 0. Max coverage (-): 0.85

Region: NODE\_117571\_length\_15591\_cov\_25.186710 6898-6917. Max. coverage (+): 0.47. Max coverage (-): 0

Region: NODE\_117571\_length\_15591\_cov\_25.186710 6918-6937. Max. coverage (+): 0. Max coverage (-): 0

Region: NODE\_117571\_length\_15591\_cov\_25.186710 6938-6956. Max. coverage (+): 0.09. Max coverage (-): 0.09

Region: NODE\_117571\_length\_15591\_cov\_25.186710 6957-6976. Max. coverage (+): 0. Max coverage (-): 0.09

Region: NODE\_117571\_length\_15591\_cov\_25.186710 6977-6995. Max. coverage (+): 0. Max coverage (-): 0

Region: NODE\_117571\_length\_15591\_cov\_25.186710 6996-7015. Max. coverage (+): 0.28. Max coverage (-): 0.09

Region: NODE\_117571\_length\_15591\_cov\_25.186710 7016-7034. Max. coverage (+): 0.28. Max coverage (-): 0

Region: NODE\_117571\_length\_15591\_cov\_25.186710 7035-7054. Max. coverage (+): 0. Max coverage (-): 0

Region: NODE\_117571\_length\_15591\_cov\_25.186710 7055-7073. Max. coverage (+): 0. Max coverage (-): 0

Region: NODE\_117571\_length\_15591\_cov\_25.186710 7074-7093. Max. coverage (+): 1.13. Max coverage (-): 0

Region: NODE\_117571\_length\_15591\_cov\_25.186710 7094-7113. Max. coverage (+): 0.56. Max coverage (-): 0

Region: NODE\_117571\_length\_15591\_cov\_25.186710 7114-7132. Max. coverage (+): 0. Max coverage (-): 0.09

Region: NODE\_117571\_length\_15591\_cov\_25.186710 7133-7152. Max. coverage (+): 0.19. Max coverage (-): 0

Region: NODE\_117571\_length\_15591\_cov\_25.186710 7153-7171. Max. coverage (+): 0.56. Max coverage (-): 0.09

Region: NODE\_117571\_length\_15591\_cov\_25.186710 7172-7191. Max. coverage (+): 0.38. Max coverage (-): 0.09

Region: NODE\_117571\_length\_15591\_cov\_25.186710 7192-7210. Max. coverage (+): 0.09. Max coverage (-): 0

Region: NODE\_117571\_length\_15591\_cov\_25.186710 7211-7230. Max. coverage (+): 0.28. Max coverage (-): 0.66

Region: NODE\_117571\_length\_15591\_cov\_25.186710 7231-7249. Max. coverage (+): 14.94. Max coverage (-): 0

Region: NODE\_117571\_length\_15591\_cov\_25.186710 7250-7269. Max. coverage (+): 0. Max coverage (-): 0.19

Region: NODE\_117571\_length\_15591\_cov\_25.186710 7270-7289. Max. coverage (+): 9.4. Max coverage (-): 0

Region: NODE\_117571\_length\_15591\_cov\_25.186710 7290-7308. Max. coverage (+): 0. Max coverage (-): 0

Region: NODE\_117571\_length\_15591\_cov\_25.186710 7309-7328. Max. coverage (+): 0.19. Max coverage (-): 0.09

Region: NODE\_117571\_length\_15591\_cov\_25.186710 7329-7347. Max. coverage (+): 0.47. Max coverage (-): 0.28

Region: NODE\_117571\_length\_15591\_cov\_25.186710 7348-7367. Max. coverage (+): 0.09. Max coverage (-): 0.38

Region: NODE\_117571\_length\_15591\_cov\_25.186710 7368-7386. Max. coverage (+): 0.38. Max coverage (-): 0.09

Region: NODE\_117571\_length\_15591\_cov\_25.186710 7387-7406. Max. coverage (+): 0. Max coverage (-): 0.19

Region: NODE\_117571\_length\_15591\_cov\_25.186710 7407-7425. Max. coverage (+): 0. Max coverage (-): 0.28

Region: NODE\_117571\_length\_15591\_cov\_25.186710 7426-7445. Max. coverage (+): 1.88. Max coverage (-): 0

Region: NODE\_117571\_length\_15591\_cov\_25.186710 7446-7465. Max. coverage (+): 0.09. Max coverage (-): 0

Region: NODE\_117571\_length\_15591\_cov\_25.186710 7466-7484. Max. coverage (+): 0.19. Max coverage (-): 0.09

Region: NODE\_117571\_length\_15591\_cov\_25.186710 7485-7504. Max. coverage (+): 0.28. Max coverage (-): 0

Region: NODE\_117571\_length\_15591\_cov\_25.186710 7505-7523. Max. coverage (+): 0.75. Max coverage (-): 0

Region: NODE\_117571\_length\_15591\_cov\_25.186710 7524-7543. Max. coverage (+): 0.75. Max coverage (-): 0.19

Region: NODE\_117571\_length\_15591\_cov\_25.186710 7544-7562. Max. coverage (+): 0.09. Max coverage (-): 0

Region: NODE\_117571\_length\_15591\_cov\_25.186710 7563-7582. Max. coverage (+): 0. Max coverage (-): 0.09

Region: NODE\_117571\_length\_15591\_cov\_25.186710 7583-7601. Max. coverage (+): 0.09. Max coverage (-): 0

Region: NODE\_117571\_length\_15591\_cov\_25.186710 7602-7621. Max. coverage (+): 0. Max coverage (-): 0

Region: NODE\_117571\_length\_15591\_cov\_25.186710 7622-7641. Max. coverage (+): 3.29. Max coverage (-): 0

Region: NODE\_117571\_length\_15591\_cov\_25.186710 7642-7660. Max. coverage (+): 0.38. Max coverage (-): 0.09

Region: NODE\_117571\_length\_15591\_cov\_25.186710 7661-7680. Max. coverage (+): 0.09. Max coverage (-): 0

Region: NODE\_117571\_length\_15591\_cov\_25.186710 7681-7699. Max. coverage (+): 0. Max coverage (-): 0.19

Region: NODE\_117571\_length\_15591\_cov\_25.186710 7700-7719. Max. coverage (+): 1.69. Max coverage (-): 0.38

Region: NODE\_117571\_length\_15591\_cov\_25.186710 7720-7738. Max. coverage (+): 1.69. Max coverage (-): 0.19

Region: NODE\_117571\_length\_15591\_cov\_25.186710 7739-7758. Max. coverage (+): 0.28. Max coverage (-): 0.09

Region: NODE\_117571\_length\_15591\_cov\_25.186710 7759-7777. Max. coverage (+): 0.28. Max coverage (-): 0

Region: NODE\_117571\_length\_15591\_cov\_25.186710 7778-7797. Max. coverage (+): 0. Max coverage (-): 0.09

Region: NODE\_117571\_length\_15591\_cov\_25.186710 7798-7817. Max. coverage (+): 1.5. Max coverage (-): 0

Region: NODE\_117571\_length\_15591\_cov\_25.186710 7818-7836. Max. coverage (+): 0. Max coverage (-): 0.19

Region: NODE\_117571\_length\_15591\_cov\_25.186710 7837-7856. Max. coverage (+): 0.09. Max coverage (-): 0

Region: NODE\_117571\_length\_15591\_cov\_25.186710 7857-7875. Max. coverage (+): 0. Max coverage (-): 0

Region: NODE\_117571\_length\_15591\_cov\_25.186710 7876-7895. Max. coverage (+): 0. Max coverage (-): 0

Region: NODE\_117571\_length\_15591\_cov\_25.186710 7896-7914. Max. coverage (+): 0. Max coverage (-): 0

Region: NODE\_117571\_length\_15591\_cov\_25.186710 7915-7934. Max. coverage (+): 0.09. Max coverage (-): 0

Region: NODE\_117571\_length\_15591\_cov\_25.186710 7935-7954. Max. coverage (+): 0. Max coverage (-): 0

Region: NODE\_117571\_length\_15591\_cov\_25.186710 7955-7973. Max. coverage (+): 0. Max coverage (-): 0

Region: NODE\_117571\_length\_15591\_cov\_25.186710 7974-7993. Max. coverage (+): 0.09. Max coverage (-): 0

Region: NODE\_117571\_length\_15591\_cov\_25.186710 7994-8012. Max. coverage (+): 0. Max coverage (-): 0

Region: NODE\_117571\_length\_15591\_cov\_25.186710 8013-8032. Max. coverage (+): 0.09. Max coverage (-): 0

Region: NODE\_117571\_length\_15591\_cov\_25.186710 8033-8051. Max. coverage (+): 0.38. Max coverage (-): 0

Region: NODE\_117571\_length\_15591\_cov\_25.186710 8052-8071. Max. coverage (+): 1.79. Max coverage (-): 0

Region: NODE\_117571\_length\_15591\_cov\_25.186710 8072-8090. Max. coverage (+): 0.47. Max coverage (-): 0.28

Region: NODE\_117571\_length\_15591\_cov\_25.186710 8091-8110. Max. coverage (+): 0.85. Max coverage (-): 0

Region: NODE\_117571\_length\_15591\_cov\_25.186710 8111-8130. Max. coverage (+): 0.28. Max coverage (-): 0

Region: NODE\_117571\_length\_15591\_cov\_25.186710 8131-8149. Max. coverage (+): 0. Max coverage (-): 0

Region: NODE\_117571\_length\_15591\_cov\_25.186710 8150-8169. Max. coverage (+): 0. Max coverage (-): 0.28

Region: NODE\_117571\_length\_15591\_cov\_25.186710 8170-8188. Max. coverage (+): 5.17. Max coverage (-): 0

Region: NODE\_117571\_length\_15591\_cov\_25.186710 8189-8208. Max. coverage (+): 0.28. Max coverage (-): 0

Region: NODE\_117571\_length\_15591\_cov\_25.186710 8209-8227. Max. coverage (+): 0.09. Max coverage (-): 0.38

Region: NODE\_117571\_length\_15591\_cov\_25.186710 8228-8247. Max. coverage (+): 0.09. Max coverage (-): 0.09

Region: NODE\_117571\_length\_15591\_cov\_25.186710 8248-8266. Max. coverage (+): 0.85. Max coverage (-): 0

Region: NODE\_117571\_length\_15591\_cov\_25.186710 8267-8286. Max. coverage (+): 0. Max coverage (-): 0

Region: NODE\_117571\_length\_15591\_cov\_25.186710 8287-8306. Max. coverage (+): 0. Max coverage (-): 0

Region: NODE\_117571\_length\_15591\_cov\_25.186710 8307-8325. Max. coverage (+): 0. Max coverage (-): 0

Region: NODE\_117571\_length\_15591\_cov\_25.186710 8326-8345. Max. coverage (+): 0.09. Max coverage (-): 0

Region: NODE\_117571\_length\_15591\_cov\_25.186710 8346-8364. Max. coverage (+): 0.09. Max coverage (-): 0.09

Region: NODE\_117571\_length\_15591\_cov\_25.186710 8365-8384. Max. coverage (+): 0. Max coverage (-): 0

Region: NODE\_117571\_length\_15591\_cov\_25.186710 8385-8403. Max. coverage (+): 0. Max coverage (-): 0

Region: NODE\_117571\_length\_15591\_cov\_25.186710 8404-8423. Max. coverage (+): 1.97. Max coverage (-): 0.09

Region: NODE\_117571\_length\_15591\_cov\_25.186710 8424-8442. Max. coverage (+): 0. Max coverage (-): 0

Region: NODE\_117571\_length\_15591\_cov\_25.186710 8443-8462. Max. coverage (+): 22.08. Max coverage (-): 0.09

Region: NODE\_117571\_length\_15591\_cov\_25.186710 8463-8482. Max. coverage (+): 22.27. Max coverage (-): 0

Region: NODE\_117571\_length\_15591\_cov\_25.186710 8483-8501. Max. coverage (+): 0. Max coverage (-): 0

Region: NODE\_117571\_length\_15591\_cov\_25.186710 8502-8521. Max. coverage (+): 0.19. Max coverage (-): 0

Region: NODE\_117571\_length\_15591\_cov\_25.186710 8522-8540. Max. coverage (+): 0.19. Max coverage (-): 0

Region: NODE\_117571\_length\_15591\_cov\_25.186710 8541-8560. Max. coverage (+): 0.19. Max coverage (-): 0.09

Region: NODE\_117571\_length\_15591\_cov\_25.186710 8561-8579. Max. coverage (+): 0.19. Max coverage (-): 0

Region: NODE\_117571\_length\_15591\_cov\_25.186710 8580-8599. Max. coverage (+): 0. Max coverage (-): 0

Region: NODE\_117571\_length\_15591\_cov\_25.186710 8600-8618. Max. coverage (+): 0.85. Max coverage (-): 0

Region: NODE\_117571\_length\_15591\_cov\_25.186710 8619-8638. Max. coverage (+): 0. Max coverage (-): 0.19

Region: NODE\_117571\_length\_15591\_cov\_25.186710 8639-8658. Max. coverage (+): 0. Max coverage (-): 0

Region: NODE\_117571\_length\_15591\_cov\_25.186710 8659-8677. Max. coverage (+): 0. Max coverage (-): 0.28

Region: NODE\_117571\_length\_15591\_cov\_25.186710 8678-8697. Max. coverage (+): 0.85. Max coverage (-): 0

Region: NODE\_117571\_length\_15591\_cov\_25.186710 8698-8716. Max. coverage (+): 0. Max coverage (-): 0

Region: NODE\_117571\_length\_15591\_cov\_25.186710 8717-8736. Max. coverage (+): 0.38. Max coverage (-): 0

Region: NODE\_117571\_length\_15591\_cov\_25.186710 8737-8755. Max. coverage (+): 0. Max coverage (-): 0.19

Region: NODE\_117571\_length\_15591\_cov\_25.186710 8756-8775. Max. coverage (+): 0.09. Max coverage (-): 0.09

Region: NODE\_117571\_length\_15591\_cov\_25.186710 8776-8795. Max. coverage (+): 2.26. Max coverage (-): 0

Region: NODE\_117571\_length\_15591\_cov\_25.186710 8796-8814. Max. coverage (+): 0.09. Max coverage (-): 0.28

Region: NODE\_117571\_length\_15591\_cov\_25.186710 8815-8834. Max. coverage (+): 1.5. Max coverage (-): 0

Region: NODE\_117571\_length\_15591\_cov\_25.186710 8835-8853. Max. coverage (+): 0. Max coverage (-): 0

Region: NODE\_117571\_length\_15591\_cov\_25.186710 8854-8873. Max. coverage (+): 0.56. Max coverage (-): 0.09

Region: NODE\_117571\_length\_15591\_cov\_25.186710 8874-8892. Max. coverage (+): 0.56. Max coverage (-): 0.09

Region: NODE\_117571\_length\_15591\_cov\_25.186710 8893-8912. Max. coverage (+): 0.66. Max coverage (-): 0

Region: NODE\_117571\_length\_15591\_cov\_25.186710 8913-8931. Max. coverage (+): 0.19. Max coverage (-): 0

Region: NODE\_117571\_length\_15591\_cov\_25.186710 8932-8951. Max. coverage (+): 0.19. Max coverage (-): 0

Region: NODE\_117571\_length\_15591\_cov\_25.186710 8952-8971. Max. coverage (+): 0.38. Max coverage (-): 0

Region: NODE\_117571\_length\_15591\_cov\_25.186710 8972-8990. Max. coverage (+): 0.28. Max coverage (-): 0.19

Region: NODE\_117571\_length\_15591\_cov\_25.186710 8991-9010. Max. coverage (+): 0.75. Max coverage (-): 0.19

Region: NODE\_117571\_length\_15591\_cov\_25.186710 9011-9029. Max. coverage (+): 0.47. Max coverage (-): 0.19

Region: NODE\_117571\_length\_15591\_cov\_25.186710 9030-9049. Max. coverage (+): 0.56. Max coverage (-): 0.75

Region: NODE\_117571\_length\_15591\_cov\_25.186710 9050-9068. Max. coverage (+): 0. Max coverage (-): 0.19

Region: NODE\_117571\_length\_15591\_cov\_25.186710 9069-9088. Max. coverage (+): 0.19. Max coverage (-): 0

Region: NODE\_117571\_length\_15591\_cov\_25.186710 9089-9107. Max. coverage (+): 0.09. Max coverage (-): 0.19

Region: NODE\_117571\_length\_15591\_cov\_25.186710 9108-9127. Max. coverage (+): 2.73. Max coverage (-): 0.19

Region: NODE\_117571\_length\_15591\_cov\_25.186710 9128-9147. Max. coverage (+): 2.35. Max coverage (-): 0

Region: NODE\_117571\_length\_15591\_cov\_25.186710 9148-9166. Max. coverage (+): 0.09. Max coverage (-): 0

Region: NODE\_117571\_length\_15591\_cov\_25.186710 9167-9186. Max. coverage (+): 0.09. Max coverage (-): 0

Region: NODE\_117571\_length\_15591\_cov\_25.186710 9187-9205. Max. coverage (+): 0. Max coverage (-): 0.09

Region: NODE\_117571\_length\_15591\_cov\_25.186710 9206-9225. Max. coverage (+): 0.94. Max coverage (-): 0

Region: NODE\_117571\_length\_15591\_cov\_25.186710 9226-9244. Max. coverage (+): 0.19. Max coverage (-): 0

Region: NODE\_117571\_length\_15591\_cov\_25.186710 9245-9264. Max. coverage (+): 0.75. Max coverage (-): 0

Region: NODE\_117571\_length\_15591\_cov\_25.186710 9265-9283. Max. coverage (+): 2.07. Max coverage (-): 0

Region: NODE\_117571\_length\_15591\_cov\_25.186710 9284-9303. Max. coverage (+): 1.97. Max coverage (-): 1.69

Region: NODE\_117571\_length\_15591\_cov\_25.186710 9304-9323. Max. coverage (+): 1.03. Max coverage (-): 0

Region: NODE\_117571\_length\_15591\_cov\_25.186710 9324-9342. Max. coverage (+): 0.19. Max coverage (-): 0

Region: NODE\_117571\_length\_15591\_cov\_25.186710 9343-9362. Max. coverage (+): 0.19. Max coverage (-): 0

Region: NODE\_117571\_length\_15591\_cov\_25.186710 9363-9381. Max. coverage (+): 0.09. Max coverage (-): 0

Region: NODE\_117571\_length\_15591\_cov\_25.186710 9382-9401. Max. coverage (+): 0.28. Max coverage (-): 0

Region: NODE\_117571\_length\_15591\_cov\_25.186710 9402-9420. Max. coverage (+): 0.09. Max coverage (-): 0

Region: NODE\_117571\_length\_15591\_cov\_25.186710 9421-9440. Max. coverage (+): 0.19. Max coverage (-): 0

Region: NODE\_117571\_length\_15591\_cov\_25.186710 9441-9459. Max. coverage (+): 0.09. Max coverage (-): 0

Region: NODE\_117571\_length\_15591\_cov\_25.186710 9460-9479. Max. coverage (+): 0. Max coverage (-): 0

Region: NODE\_117571\_length\_15591\_cov\_25.186710 9480-9499. Max. coverage (+): 0.38. Max coverage (-): 0

Region: NODE\_117571\_length\_15591\_cov\_25.186710 9500-9518. Max. coverage (+): 0.19. Max coverage (-): 0

Region: NODE\_117571\_length\_15591\_cov\_25.186710 9519-9538. Max. coverage (+): 0.09. Max coverage (-): 0

Region: NODE\_117571\_length\_15591\_cov\_25.186710 9539-9557. Max. coverage (+): 0.19. Max coverage (-): 0

Region: NODE\_117571\_length\_15591\_cov\_25.186710 9558-9577. Max. coverage (+): 0. Max coverage (-): 0

Region: NODE\_117571\_length\_15591\_cov\_25.186710 9578-9596. Max. coverage (+): 0.09. Max coverage (-): 0

Region: NODE\_117571\_length\_15591\_cov\_25.186710 9597-9616. Max. coverage (+): 3.1. Max coverage (-): 0

Region: NODE\_117571\_length\_15591\_cov\_25.186710 9617-9636. Max. coverage (+): 0. Max coverage (-): 0

Region: NODE\_117571\_length\_15591\_cov\_25.186710 9637-9655. Max. coverage (+): 0.19. Max coverage (-): 0

Region: NODE\_117571\_length\_15591\_cov\_25.186710 9656-9675. Max. coverage (+): 0.38. Max coverage (-): 0.09

Region: NODE\_117571\_length\_15591\_cov\_25.186710 9676-9694. Max. coverage (+): 0.01. Max coverage (-): 0.02

Region: NODE\_117571\_length\_15591\_cov\_25.186710 9695-9714. Max. coverage (+): 0. Max coverage (-): 0

Region: NODE\_117571\_length\_15591\_cov\_25.186710 9715-9733. Max. coverage (+): 0. Max coverage (-): 0.02

Region: NODE\_117571\_length\_15591\_cov\_25.186710 9734-9753. Max. coverage (+): 0. Max coverage (-): 0

Region: NODE\_117571\_length\_15591\_cov\_25.186710 9754-9772. Max. coverage (+): 0. Max coverage (-): 0

Region: NODE\_117571\_length\_15591\_cov\_25.186710 9773-9792. Max. coverage (+): 0. Max coverage (-): 0.03

Region: NODE\_117571\_length\_15591\_cov\_25.186710 9793-9812. Max. coverage (+): 0.01. Max coverage (-): 0.06

Region: NODE\_117571\_length\_15591\_cov\_25.186710 9813-9831. Max. coverage (+): 0.01. Max coverage (-): 0

Region: NODE\_117571\_length\_15591\_cov\_25.186710 9832-9851. Max. coverage (+): 0. Max coverage (-): 0.09

Region: NODE\_117571\_length\_15591\_cov\_25.186710 9852-9870. Max. coverage (+): 0.02. Max coverage (-): 0

Region: NODE\_117571\_length\_15591\_cov\_25.186710 9871-9890. Max. coverage (+): 0. Max coverage (-): 0

Region: NODE\_117571\_length\_15591\_cov\_25.186710 9891-9909. Max. coverage (+): 0. Max coverage (-): 1.35

Region: NODE\_117571\_length\_15591\_cov\_25.186710 9910-9929. Max. coverage (+): 0.01. Max coverage (-): 0

Region: NODE\_117571\_length\_15591\_cov\_25.186710 9930-9948. Max. coverage (+): 0. Max coverage (-): 0

Region: NODE\_117571\_length\_15591\_cov\_25.186710 9949-9968. Max. coverage (+): 0. Max coverage (-): 0

Region: NODE\_117571\_length\_15591\_cov\_25.186710 9969-9988. Max. coverage (+): 0. Max coverage (-): 0

Region: NODE\_117571\_length\_15591\_cov\_25.186710 9989-10007. Max. coverage (+): 0. Max coverage (-): 0.09

Region: NODE\_117571\_length\_15591\_cov\_25.186710 10008-10027. Max. coverage (+): 0. Max coverage (-): 0.09

Region: NODE\_117571\_length\_15591\_cov\_25.186710 10028-10046. Max. coverage (+): 0. Max coverage (-): 0.09

Region: NODE\_117571\_length\_15591\_cov\_25.186710 10047-10066. Max. coverage (+): 0. Max coverage (-): 0.09

Region: NODE\_117571\_length\_15591\_cov\_25.186710 10067-10085. Max. coverage (+): 0. Max coverage (-): 0.56

Region: NODE\_117571\_length\_15591\_cov\_25.186710 10086-10105. Max. coverage (+): 0.09. Max coverage (-): 0

Region: NODE\_117571\_length\_15591\_cov\_25.186710 10106-10124. Max. coverage (+): 0. Max coverage (-): 0.47

Region: NODE\_117571\_length\_15591\_cov\_25.186710 10125-10144. Max. coverage (+): 0. Max coverage (-): 0.09

Region: NODE\_117571\_length\_15591\_cov\_25.186710 10145-10164. Max. coverage (+): 0. Max coverage (-): 0.19

Region: NODE\_117571\_length\_15591\_cov\_25.186710 10165-10183. Max. coverage (+): 0.09. Max coverage (-): 0

Region: NODE\_117571\_length\_15591\_cov\_25.186710 10184-10203. Max. coverage (+): 0. Max coverage (-): 0.09

Region: NODE\_117571\_length\_15591\_cov\_25.186710 10204-10222. Max. coverage (+): 0. Max coverage (-): 0.38

Region: NODE\_117571\_length\_15591\_cov\_25.186710 10223-10242. Max. coverage (+): 0. Max coverage (-): 0.56

Region: NODE\_117571\_length\_15591\_cov\_25.186710 10243-10261. Max. coverage (+): 0. Max coverage (-): 1.6

Region: NODE\_117571\_length\_15591\_cov\_25.186710 10262-10281. Max. coverage (+): 0.09. Max coverage (-): 0.28

Region: NODE\_117571\_length\_15591\_cov\_25.186710 10282-10300. Max. coverage (+): 0.19. Max coverage (-): 0.47

Region: NODE\_117571\_length\_15591\_cov\_25.186710 10301-10320. Max. coverage (+): 0.19. Max coverage (-): 0.19

Region: NODE\_117571\_length\_15591\_cov\_25.186710 10321-10340. Max. coverage (+): 0.19. Max coverage (-): 3.57

Region: NODE\_117571\_length\_15591\_cov\_25.186710 10341-10359. Max. coverage (+): 0.56. Max coverage (-): 1.5

Region: NODE\_117571\_length\_15591\_cov\_25.186710 10360-10379. Max. coverage (+): 0.19. Max coverage (-): 0.47

Region: NODE\_117571\_length\_15591\_cov\_25.186710 10380-10398. Max. coverage (+): 0.19. Max coverage (-): 1.03

Region: NODE\_117571\_length\_15591\_cov\_25.186710 10399-10418. Max. coverage (+): 0.09. Max coverage (-): 1.03

Region: NODE\_117571\_length\_15591\_cov\_25.186710 10419-10437. Max. coverage (+): 0.19. Max coverage (-): 0.38

Region: NODE\_117571\_length\_15591\_cov\_25.186710 10438-10457. Max. coverage (+): 0. Max coverage (-): 0.19

Region: NODE\_117571\_length\_15591\_cov\_25.186710 10458-10477. Max. coverage (+): 0.09. Max coverage (-): 0.47

Region: NODE\_117571\_length\_15591\_cov\_25.186710 10478-10496. Max. coverage (+): 0.28. Max coverage (-): 0.09

Region: NODE\_117571\_length\_15591\_cov\_25.186710 10497-10516. Max. coverage (+): 0. Max coverage (-): 0.28

Region: NODE\_117571\_length\_15591\_cov\_25.186710 10517-10535. Max. coverage (+): 0.85. Max coverage (-): 0

Region: NODE\_117571\_length\_15591\_cov\_25.186710 10536-10555. Max. coverage (+): 0.09. Max coverage (-): 1.5

Region: NODE\_117571\_length\_15591\_cov\_25.186710 10556-10574. Max. coverage (+): 0.47. Max coverage (-): 0.38

Region: NODE\_117571\_length\_15591\_cov\_25.186710 10575-10594. Max. coverage (+): 0. Max coverage (-): 0.38

Region: NODE\_117571\_length\_15591\_cov\_25.186710 10595-10613. Max. coverage (+): 0. Max coverage (-): 0.28

Region: NODE\_117571\_length\_15591\_cov\_25.186710 10614-10633. Max. coverage (+): 0.19. Max coverage (-): 0

Region: NODE\_117571\_length\_15591\_cov\_25.186710 10634-10653. Max. coverage (+): 0. Max coverage (-): 0.85

Region: NODE\_117571\_length\_15591\_cov\_25.186710 10654-10672. Max. coverage (+): 0.28. Max coverage (-): 0.28

Region: NODE\_117571\_length\_15591\_cov\_25.186710 10673-10692. Max. coverage (+): 0. Max coverage (-): 2.91

Region: NODE\_117571\_length\_15591\_cov\_25.186710 10693-10711. Max. coverage (+): 0. Max coverage (-): 0.09

Region: NODE\_117571\_length\_15591\_cov\_25.186710 10712-10731. Max. coverage (+): 0. Max coverage (-): 2.07

Region: NODE\_117571\_length\_15591\_cov\_25.186710 10732-10750. Max. coverage (+): 0.75. Max coverage (-): 0.09

Region: NODE\_117571\_length\_15591\_cov\_25.186710 10751-10770. Max. coverage (+): 0. Max coverage (-): 0.19

Region: NODE\_117571\_length\_15591\_cov\_25.186710 10771-10789. Max. coverage (+): 0. Max coverage (-): 0.28

Region: NODE\_117571\_length\_15591\_cov\_25.186710 10790-10809. Max. coverage (+): 0. Max coverage (-): 0.28

Region: NODE\_117571\_length\_15591\_cov\_25.186710 10810-10829. Max. coverage (+): 0. Max coverage (-): 0.85

Region: NODE\_117571\_length\_15591\_cov\_25.186710 10830-10848. Max. coverage (+): 0. Max coverage (-): 0.94

Region: NODE\_117571\_length\_15591\_cov\_25.186710 10849-10868. Max. coverage (+): 0.19. Max coverage (-): 0.09

Region: NODE\_117571\_length\_15591\_cov\_25.186710 10869-10887. Max. coverage (+): 0. Max coverage (-): 0.19

Region: NODE\_117571\_length\_15591\_cov\_25.186710 10888-10907. Max. coverage (+): 0. Max coverage (-): 0.19

Region: NODE\_117571\_length\_15591\_cov\_25.186710 10908-10926. Max. coverage (+): 2.26. Max coverage (-): 0.09

Region: NODE\_117571\_length\_15591\_cov\_25.186710 10927-10946. Max. coverage (+): 2.91. Max coverage (-): 0

Region: NODE\_117571\_length\_15591\_cov\_25.186710 10947-10965. Max. coverage (+): 0. Max coverage (-): 0.28

Region: NODE\_117571\_length\_15591\_cov\_25.186710 10966-10985. Max. coverage (+): 0. Max coverage (-): 0.09

Region: NODE\_117571\_length\_15591\_cov\_25.186710 10986-11005. Max. coverage (+): 0. Max coverage (-): 0.38

Region: NODE\_117571\_length\_15591\_cov\_25.186710 11006-11024. Max. coverage (+): 0.28. Max coverage (-): 0.75

Region: NODE\_117571\_length\_15591\_cov\_25.186710 11025-11044. Max. coverage (+): 0.28. Max coverage (-): 0.09

Region: NODE\_117571\_length\_15591\_cov\_25.186710 11045-11063. Max. coverage (+): 0.19. Max coverage (-): 0

Region: NODE\_117571\_length\_15591\_cov\_25.186710 11064-11083. Max. coverage (+): 0. Max coverage (-): 0.38

Region: NODE\_117571\_length\_15591\_cov\_25.186710 11084-11102. Max. coverage (+): 0.19. Max coverage (-): 1.13

Region: NODE\_117571\_length\_15591\_cov\_25.186710 11103-11122. Max. coverage (+): 0.38. Max coverage (-): 1.13

Region: NODE\_117571\_length\_15591\_cov\_25.186710 11123-11141. Max. coverage (+): 0.38. Max coverage (-): 0

Region: NODE\_117571\_length\_15591\_cov\_25.186710 11142-11161. Max. coverage (+): 0. Max coverage (-): 0

Region: NODE\_117571\_length\_15591\_cov\_25.186710 11162-11181. Max. coverage (+): 0. Max coverage (-): 2.44

Region: NODE\_117571\_length\_15591\_cov\_25.186710 11182-11200. Max. coverage (+): 0.19. Max coverage (-): 0.09

Region: NODE\_117571\_length\_15591\_cov\_25.186710 11201-11220. Max. coverage (+): 0. Max coverage (-): 0

Region: NODE\_117571\_length\_15591\_cov\_25.186710 11221-11239. Max. coverage (+): 0. Max coverage (-): 0.19

Region: NODE\_117571\_length\_15591\_cov\_25.186710 11240-11259. Max. coverage (+): 0. Max coverage (-): 2.35

Region: NODE\_117571\_length\_15591\_cov\_25.186710 11260-11278. Max. coverage (+): 0. Max coverage (-): 0.47

Region: NODE\_117571\_length\_15591\_cov\_25.186710 11279-11298. Max. coverage (+): 0. Max coverage (-): 0.19

Region: NODE\_117571\_length\_15591\_cov\_25.186710 11299-11317. Max. coverage (+): 0. Max coverage (-): 0.19

Region: NODE\_117571\_length\_15591\_cov\_25.186710 11318-11337. Max. coverage (+): 0. Max coverage (-): 0

Region: NODE\_117571\_length\_15591\_cov\_25.186710 11338-11357. Max. coverage (+): 0. Max coverage (-): 0.38

Region: NODE\_117571\_length\_15591\_cov\_25.186710 11358-11376. Max. coverage (+): 0.47. Max coverage (-): 0

Region: NODE\_117571\_length\_15591\_cov\_25.186710 11377-11396. Max. coverage (+): 0.09. Max coverage (-): 2.54

Region: NODE\_117571\_length\_15591\_cov\_25.186710 11397-11415. Max. coverage (+): 0. Max coverage (-): 0.19

Region: NODE\_117571\_length\_15591\_cov\_25.186710 11416-11435. Max. coverage (+): 0. Max coverage (-): 1.22

Region: NODE\_117571\_length\_15591\_cov\_25.186710 11436-11454. Max. coverage (+): 1.13. Max coverage (-): 1.32

Region: NODE\_117571\_length\_15591\_cov\_25.186710 11455-11474. Max. coverage (+): 1.13. Max coverage (-): 0.09

Region: NODE\_117571\_length\_15591\_cov\_25.186710 11475-11494. Max. coverage (+): 0. Max coverage (-): 0.85

Region: NODE\_117571\_length\_15591\_cov\_25.186710 11495-11513. Max. coverage (+): 0. Max coverage (-): 1.88

Region: NODE\_117571\_length\_15591\_cov\_25.186710 11514-11533. Max. coverage (+): 0. Max coverage (-): 0

Region: NODE\_117571\_length\_15591\_cov\_25.186710 11534-11552. Max. coverage (+): 0. Max coverage (-): 0.94

Region: NODE\_117571\_length\_15591\_cov\_25.186710 11553-11572. Max. coverage (+): 0.56. Max coverage (-): 0.09

Region: NODE\_117571\_length\_15591\_cov\_25.186710 11573-11591. Max. coverage (+): 0. Max coverage (-): 0.28

Region: NODE\_117571\_length\_15591\_cov\_25.186710 11592-11611. Max. coverage (+): 0.19. Max coverage (-): 0.09

Region: NODE\_117571\_length\_15591\_cov\_25.186710 11612-11630. Max. coverage (+): 0.09. Max coverage (-): 0.19

Region: NODE\_117571\_length\_15591\_cov\_25.186710 11631-11650. Max. coverage (+): 0.28. Max coverage (-): 0.19

Region: NODE\_117571\_length\_15591\_cov\_25.186710 11651-11670. Max. coverage (+): 0. Max coverage (-): 0.19

Region: NODE\_117571\_length\_15591\_cov\_25.186710 11671-11689. Max. coverage (+): 0.09. Max coverage (-): 0.19

Region: NODE\_117571\_length\_15591\_cov\_25.186710 11690-11709. Max. coverage (+): 0. Max coverage (-): 1.41

Region: NODE\_117571\_length\_15591\_cov\_25.186710 11710-11728. Max. coverage (+): 0.28. Max coverage (-): 4.13

Region: NODE\_117571\_length\_15591\_cov\_25.186710 11729-11748. Max. coverage (+): 0. Max coverage (-): 4.13

Region: NODE\_117571\_length\_15591\_cov\_25.186710 11749-11767. Max. coverage (+): 0. Max coverage (-): 0.47

Region: NODE\_117571\_length\_15591\_cov\_25.186710 11768-11787. Max. coverage (+): 0. Max coverage (-): 0.09

Region: NODE\_117571\_length\_15591\_cov\_25.186710 11788-11806. Max. coverage (+): 0. Max coverage (-): 0

Region: NODE\_117571\_length\_15591\_cov\_25.186710 11807-11826. Max. coverage (+): 0. Max coverage (-): 0

Region: NODE\_117571\_length\_15591\_cov\_25.186710 11827-11846. Max. coverage (+): 0. Max coverage (-): 0

Region: NODE\_117571\_length\_15591\_cov\_25.186710 11847-11865. Max. coverage (+): 0. Max coverage (-): 0

Region: NODE\_117571\_length\_15591\_cov\_25.186710 11866-11885. Max. coverage (+): 0. Max coverage (-): 0

Region: NODE\_117571\_length\_15591\_cov\_25.186710 11886-11904. Max. coverage (+): 0.09. Max coverage (-): 0.09

Region: NODE\_117571\_length\_15591\_cov\_25.186710 11905-11924. Max. coverage (+): 0. Max coverage (-): 0

Region: NODE\_117571\_length\_15591\_cov\_25.186710 11925-11943. Max. coverage (+): 0. Max coverage (-): 0.09

Region: NODE\_117571\_length\_15591\_cov\_25.186710 11944-11963. Max. coverage (+): 0.09. Max coverage (-): 0.19

Region: NODE\_117571\_length\_15591\_cov\_25.186710 11964-11982. Max. coverage (+): 0. Max coverage (-): 0.09

Region: NODE\_117571\_length\_15591\_cov\_25.186710 11983-12002. Max. coverage (+): 0. Max coverage (-): 0

Region: NODE\_117571\_length\_15591\_cov\_25.186710 12003-12022. Max. coverage (+): 0. Max coverage (-): 0.09

Region: NODE\_117571\_length\_15591\_cov\_25.186710 12023-12041. Max. coverage (+): 0. Max coverage (-): 2.26

Region: NODE\_117571\_length\_15591\_cov\_25.186710 12042-12061. Max. coverage (+): 0. Max coverage (-): 0.19

Region: NODE\_117571\_length\_15591\_cov\_25.186710 12062-12080. Max. coverage (+): 0. Max coverage (-): 0.09

Region: NODE\_117571\_length\_15591\_cov\_25.186710 12081-12100. Max. coverage (+): 0. Max coverage (-): 0.75

Region: NODE\_117571\_length\_15591\_cov\_25.186710 12101-12119. Max. coverage (+): 0. Max coverage (-): 0.19

Region: NODE\_117571\_length\_15591\_cov\_25.186710 12120-12139. Max. coverage (+): 0. Max coverage (-): 0

Region: NODE\_117571\_length\_15591\_cov\_25.186710 12140-12158. Max. coverage (+): 0. Max coverage (-): 0.66

Region: NODE\_117571\_length\_15591\_cov\_25.186710 12159-12178. Max. coverage (+): 0. Max coverage (-): 0.94

Region: NODE\_117571\_length\_15591\_cov\_25.186710 12179-12198. Max. coverage (+): 0. Max coverage (-): 0.47

Region: NODE\_117571\_length\_15591\_cov\_25.186710 12199-12217. Max. coverage (+): 0. Max coverage (-): 0

Region: NODE\_117571\_length\_15591\_cov\_25.186710 12218-12237. Max. coverage (+): 0.09. Max coverage (-): 1.32

Region: NODE\_117571\_length\_15591\_cov\_25.186710 12238-12256. Max. coverage (+): 0. Max coverage (-): 0.09

Region: NODE\_117571\_length\_15591\_cov\_25.186710 12257-12276. Max. coverage (+): 0. Max coverage (-): 0

Region: NODE\_117571\_length\_15591\_cov\_25.186710 12277-12295. Max. coverage (+): 0. Max coverage (-): 0

Region: NODE\_117571\_length\_15591\_cov\_25.186710 12296-12315. Max. coverage (+): 0.09. Max coverage (-): 7.05

Region: NODE\_117571\_length\_15591\_cov\_25.186710 12316-12335. Max. coverage (+): 0.09. Max coverage (-): 6.95

Region: NODE\_117571\_length\_15591\_cov\_25.186710 12336-12354. Max. coverage (+): 0. Max coverage (-): 0.66

Region: NODE\_117571\_length\_15591\_cov\_25.186710 12355-12374. Max. coverage (+): 0. Max coverage (-): 0.38

Region: NODE\_117571\_length\_15591\_cov\_25.186710 12375-12393. Max. coverage (+): 0.09. Max coverage (-): 0.85

Region: NODE\_117571\_length\_15591\_cov\_25.186710 12394-12413. Max. coverage (+): 0.19. Max coverage (-): 0.38

Region: NODE\_117571\_length\_15591\_cov\_25.186710 12414-12432. Max. coverage (+): 0.09. Max coverage (-): 0.19

Region: NODE\_117571\_length\_15591\_cov\_25.186710 12433-12452. Max. coverage (+): 0. Max coverage (-): 1.22

Region: NODE\_117571\_length\_15591\_cov\_25.186710 12453-12471. Max. coverage (+): 0. Max coverage (-): 0.09

Region: NODE\_117571\_length\_15591\_cov\_25.186710 12472-12491. Max. coverage (+): 0. Max coverage (-): 1.22

Region: NODE\_117571\_length\_15591\_cov\_25.186710 12492-12511. Max. coverage (+): 0.19. Max coverage (-): 0.28

Region: NODE\_117571\_length\_15591\_cov\_25.186710 12512-12530. Max. coverage (+): 0.28. Max coverage (-): 0.19

Region: NODE\_117571\_length\_15591\_cov\_25.186710 12531-12550. Max. coverage (+): 0.19. Max coverage (-): 0.28

Region: NODE\_117571\_length\_15591\_cov\_25.186710 12551-12569. Max. coverage (+): 0. Max coverage (-): 0.19

Region: NODE\_117571\_length\_15591\_cov\_25.186710 12570-12589. Max. coverage (+): 0. Max coverage (-): 0

Region: NODE\_117571\_length\_15591\_cov\_25.186710 12590-12608. Max. coverage (+): 0.19. Max coverage (-): 0.47

Region: NODE\_117571\_length\_15591\_cov\_25.186710 12609-12628. Max. coverage (+): 0.19. Max coverage (-): 48.49

Region: NODE\_117571\_length\_15591\_cov\_25.186710 12629-12647. Max. coverage (+): 0.09. Max coverage (-): 0.19

Region: NODE\_117571\_length\_15591\_cov\_25.186710 12648-12667. Max. coverage (+): 0. Max coverage (-): 0.38

Region: NODE\_117571\_length\_15591\_cov\_25.186710 12668-12687. Max. coverage (+): 0. Max coverage (-): 0.75

Region: NODE\_117571\_length\_15591\_cov\_25.186710 12688-12706. Max. coverage (+): 0.19. Max coverage (-): 1.88

Region: NODE\_117571\_length\_15591\_cov\_25.186710 12707-12726. Max. coverage (+): 0.85. Max coverage (-): 0.85

Region: NODE\_117571\_length\_15591\_cov\_25.186710 12727-12745. Max. coverage (+): 0. Max coverage (-): 0.09

Region: NODE\_117571\_length\_15591\_cov\_25.186710 12746-12765. Max. coverage (+): 0. Max coverage (-): 1.88

Region: NODE\_117571\_length\_15591\_cov\_25.186710 12766-12784. Max. coverage (+): 0.38. Max coverage (-): 0

Region: NODE\_117571\_length\_15591\_cov\_25.186710 12785-12804. Max. coverage (+): 0. Max coverage (-): 0.66

Region: NODE\_117571\_length\_15591\_cov\_25.186710 12805-12823. Max. coverage (+): 0.28. Max coverage (-): 3.29

Region: NODE\_117571\_length\_15591\_cov\_25.186710 12824-12843. Max. coverage (+): 0.56. Max coverage (-): 1.6

Region: NODE\_117571\_length\_15591\_cov\_25.186710 12844-12863. Max. coverage (+): 0.09. Max coverage (-): 1.22

Region: NODE\_117571\_length\_15591\_cov\_25.186710 12864-12882. Max. coverage (+): 0. Max coverage (-): 1.22

Region: NODE\_117571\_length\_15591\_cov\_25.186710 12883-12902. Max. coverage (+): 0. Max coverage (-): 0.38

Region: NODE\_117571\_length\_15591\_cov\_25.186710 12903-12921. Max. coverage (+): 0. Max coverage (-): 0

Region: NODE\_117571\_length\_15591\_cov\_25.186710 12922-12941. Max. coverage (+): 0. Max coverage (-): 1.6

Region: NODE\_117571\_length\_15591\_cov\_25.186710 12942-12960. Max. coverage (+): 0.85. Max coverage (-): 0

Region: NODE\_117571\_length\_15591\_cov\_25.186710 12961-12980. Max. coverage (+): 0. Max coverage (-): 0.38

Region: NODE\_117571\_length\_15591\_cov\_25.186710 12981-12999. Max. coverage (+): 0.75. Max coverage (-): 0.38

Region: NODE\_117571\_length\_15591\_cov\_25.186710 13000-13019. Max. coverage (+): 0. Max coverage (-): 0.28

Region: NODE\_117571\_length\_15591\_cov\_25.186710 13020-13039. Max. coverage (+): 0.85. Max coverage (-): 0

Region: NODE\_117571\_length\_15591\_cov\_25.186710 13040-13058. Max. coverage (+): 0. Max coverage (-): 3.1

Region: NODE\_117571\_length\_15591\_cov\_25.186710 13059-13078. Max. coverage (+): 1.69. Max coverage (-): 1.5

Region: NODE\_117571\_length\_15591\_cov\_25.186710 13079-13097. Max. coverage (+): 0.19. Max coverage (-): 2.07

Region: NODE\_117571\_length\_15591\_cov\_25.186710 13098-13117. Max. coverage (+): 0.09. Max coverage (-): 0.19

Region: NODE\_117571\_length\_15591\_cov\_25.186710 13118-13136. Max. coverage (+): 0. Max coverage (-): 3.01

Region: NODE\_117571\_length\_15591\_cov\_25.186710 13137-13156. Max. coverage (+): 0.19. Max coverage (-): 0.09

Region: NODE\_117571\_length\_15591\_cov\_25.186710 13157-13176. Max. coverage (+): 0. Max coverage (-): 0.56

Region: NODE\_117571\_length\_15591\_cov\_25.186710 13177-13195. Max. coverage (+): 0. Max coverage (-): 1.03

Region: NODE\_117571\_length\_15591\_cov\_25.186710 13196-13215. Max. coverage (+): 0. Max coverage (-): 0

Region: NODE\_117571\_length\_15591\_cov\_25.186710 13216-13234. Max. coverage (+): 0. Max coverage (-): 0.28

Region: NODE\_117571\_length\_15591\_cov\_25.186710 13235-13254. Max. coverage (+): 0. Max coverage (-): 0

Region: NODE\_117571\_length\_15591\_cov\_25.186710 13255-13273. Max. coverage (+): 0. Max coverage (-): 0

Region: NODE\_117571\_length\_15591\_cov\_25.186710 13274-13293. Max. coverage (+): 0.09. Max coverage (-): 0

Region: NODE\_117571\_length\_15591\_cov\_25.186710 13294-13312. Max. coverage (+): 0. Max coverage (-): 0

Region: NODE\_117571\_length\_15591\_cov\_25.186710 13313-13332. Max. coverage (+): 0. Max coverage (-): 0.66

Region: NODE\_117571\_length\_15591\_cov\_25.186710 13333-13352. Max. coverage (+): 0. Max coverage (-): 0.28

Region: NODE\_117571\_length\_15591\_cov\_25.186710 13353-13371. Max. coverage (+): 0.09. Max coverage (-): 0

Region: NODE\_117571\_length\_15591\_cov\_25.186710 13372-13391. Max. coverage (+): 0. Max coverage (-): 0

Region: NODE\_117571\_length\_15591\_cov\_25.186710 13392-13410. Max. coverage (+): 0. Max coverage (-): 0.09

Region: NODE\_117571\_length\_15591\_cov\_25.186710 13411-13430. Max. coverage (+): 0. Max coverage (-): 0.19

Region: NODE\_117571\_length\_15591\_cov\_25.186710 13431-13449. Max. coverage (+): 0. Max coverage (-): 0.12

Region: NODE\_117571\_length\_15591\_cov\_25.186710 13450-13469. Max. coverage (+): 0. Max coverage (-): 0

Region: NODE\_117571\_length\_15591\_cov\_25.186710 13470-13488. Max. coverage (+): 0. Max coverage (-): 0.47

Region: NODE\_117571\_length\_15591\_cov\_25.186710 13489-13508. Max. coverage (+): 0.09. Max coverage (-): 0

Region: NODE\_117571\_length\_15591\_cov\_25.186710 13509-13528. Max. coverage (+): 0.09. Max coverage (-): 0

Region: NODE\_117571\_length\_15591\_cov\_25.186710 13529-13547. Max. coverage (+): 0. Max coverage (-): 0

Region: NODE\_117571\_length\_15591\_cov\_25.186710 13548-13567. Max. coverage (+): 0. Max coverage (-): 0.75

Region: NODE\_117571\_length\_15591\_cov\_25.186710 13568-13586. Max. coverage (+): 0.19. Max coverage (-): 0.38

Region: NODE\_117571\_length\_15591\_cov\_25.186710 13587-13606. Max. coverage (+): 0. Max coverage (-): 0.47

Region: NODE\_117571\_length\_15591\_cov\_25.186710 13607-13625. Max. coverage (+): 0.28. Max coverage (-): 0.38

Region: NODE\_117571\_length\_15591\_cov\_25.186710 13626-13645. Max. coverage (+): 0.19. Max coverage (-): 0.19

Region: NODE\_117571\_length\_15591\_cov\_25.186710 13646-13664. Max. coverage (+): 0. Max coverage (-): 0.19

Region: NODE\_117571\_length\_15591\_cov\_25.186710 13665-13684. Max. coverage (+): 0. Max coverage (-): 0.94

Region: NODE\_117571\_length\_15591\_cov\_25.186710 13685-13704. Max. coverage (+): 0. Max coverage (-): 0.38

Region: NODE\_117571\_length\_15591\_cov\_25.186710 13705-13723. Max. coverage (+): 0. Max coverage (-): 0.09

Region: NODE\_117571\_length\_15591\_cov\_25.186710 13724-13743. Max. coverage (+): 0. Max coverage (-): 0

Region: NODE\_117571\_length\_15591\_cov\_25.186710 13744-13762. Max. coverage (+): 0. Max coverage (-): 0.56

Region: NODE\_117571\_length\_15591\_cov\_25.186710 13763-13782. Max. coverage (+): 1.32. Max coverage (-): 0.19

Region: NODE\_117571\_length\_15591\_cov\_25.186710 13783-13801. Max. coverage (+): 0. Max coverage (-): 0.19

Region: NODE\_117571\_length\_15591\_cov\_25.186710 13802-13821. Max. coverage (+): 0. Max coverage (-): 0.09

Region: NODE\_117571\_length\_15591\_cov\_25.186710 13822-13840. Max. coverage (+): 0. Max coverage (-): 0.09

Region: NODE\_117571\_length\_15591\_cov\_25.186710 13841-13860. Max. coverage (+): 0. Max coverage (-): 0.09

Region: NODE\_117571\_length\_15591\_cov\_25.186710 13861-13880. Max. coverage (+): 0. Max coverage (-): 0.19

Region: NODE\_117571\_length\_15591\_cov\_25.186710 13881-13899. Max. coverage (+): 0. Max coverage (-): 0.19

Region: NODE\_117571\_length\_15591\_cov\_25.186710 13900-13919. Max. coverage (+): 0. Max coverage (-): 0.09

Region: NODE\_117571\_length\_15591\_cov\_25.186710 13920-13938. Max. coverage (+): 1.22. Max coverage (-): 0.09

Region: NODE\_117571\_length\_15591\_cov\_25.186710 13939-13958. Max. coverage (+): 0. Max coverage (-): 3.66

Region: NODE\_117571\_length\_15591\_cov\_25.186710 13959-13977. Max. coverage (+): 0. Max coverage (-): 1.32

Region: NODE\_117571\_length\_15591\_cov\_25.186710 13978-13997. Max. coverage (+): 0. Max coverage (-): 0.38

Region: NODE\_117571\_length\_15591\_cov\_25.186710 13998-14017. Max. coverage (+): 0.09. Max coverage (-): 0.75

Region: NODE\_117571\_length\_15591\_cov\_25.186710 14018-14036. Max. coverage (+): 0. Max coverage (-): 2.16

Region: NODE\_117571\_length\_15591\_cov\_25.186710 14037-14056. Max. coverage (+): 0. Max coverage (-): 2.16

Region: NODE\_117571\_length\_15591\_cov\_25.186710 14057-14075. Max. coverage (+): 0. Max coverage (-): 0.28

Region: NODE\_117571\_length\_15591\_cov\_25.186710 14076-14095. Max. coverage (+): 0.09. Max coverage (-): 0.19

Region: NODE\_117571\_length\_15591\_cov\_25.186710 14096-14114. Max. coverage (+): 0. Max coverage (-): 0.38

Region: NODE\_117571\_length\_15591\_cov\_25.186710 14115-14134. Max. coverage (+): 0. Max coverage (-): 0

Region: NODE\_117571\_length\_15591\_cov\_25.186710 14135-14153. Max. coverage (+): 0.47. Max coverage (-): 0.38

Region: NODE\_117571\_length\_15591\_cov\_25.186710 14154-14173. Max. coverage (+): 0.47. Max coverage (-): 1.03

Region: NODE\_117571\_length\_15591\_cov\_25.186710 14174-14193. Max. coverage (+): 0.56. Max coverage (-): 1.97

Region: NODE\_117571\_length\_15591\_cov\_25.186710 14194-14212. Max. coverage (+): 0.09. Max coverage (-): 9.68

Region: NODE\_117571\_length\_15591\_cov\_25.186710 14213-14232. Max. coverage (+): 0.56. Max coverage (-): 10.9

Region: NODE\_117571\_length\_15591\_cov\_25.186710 14233-14251. Max. coverage (+): 0.56. Max coverage (-): 0

Region: NODE\_117571\_length\_15591\_cov\_25.186710 14252-14271. Max. coverage (+): 0. Max coverage (-): 0

Region: NODE\_117571\_length\_15591\_cov\_25.186710 14272-14290. Max. coverage (+): 0.09. Max coverage (-): 0

Region: NODE\_117571\_length\_15591\_cov\_25.186710 14291-14310. Max. coverage (+): 0.09. Max coverage (-): 9.4

Region: NODE\_117571\_length\_15591\_cov\_25.186710 14311-14329. Max. coverage (+): 0.28. Max coverage (-): 1.79

Region: NODE\_117571\_length\_15591\_cov\_25.186710 14330-14349. Max. coverage (+): 0. Max coverage (-): 2.16

Region: NODE\_117571\_length\_15591\_cov\_25.186710 14350-14369. Max. coverage (+): 0. Max coverage (-): 0.09

Region: NODE\_117571\_length\_15591\_cov\_25.186710 14370-14388. Max. coverage (+): 0. Max coverage (-): 0.75

Region: NODE\_117571\_length\_15591\_cov\_25.186710 14389-14408. Max. coverage (+): 0.19. Max coverage (-): 0.19

Region: NODE\_117571\_length\_15591\_cov\_25.186710 14409-14427. Max. coverage (+): 0.19. Max coverage (-): 0.09

Region: NODE\_117571\_length\_15591\_cov\_25.186710 14428-14447. Max. coverage (+): 0.09. Max coverage (-): 0.19

Region: NODE\_117571\_length\_15591\_cov\_25.186710 14448-14466. Max. coverage (+): 0.09. Max coverage (-): 1.32

Region: NODE\_117571\_length\_15591\_cov\_25.186710 14467-14486. Max. coverage (+): 0. Max coverage (-): 0.85

Region: NODE\_117571\_length\_15591\_cov\_25.186710 14487-14505. Max. coverage (+): 0. Max coverage (-): 0

Region: NODE\_117571\_length\_15591\_cov\_25.186710 14506-14525. Max. coverage (+): 0. Max coverage (-): 0.19

Region: NODE\_117571\_length\_15591\_cov\_25.186710 14526-14545. Max. coverage (+): 0.09. Max coverage (-): 0.47

Region: NODE\_117571\_length\_15591\_cov\_25.186710 14546-14564. Max. coverage (+): 0. Max coverage (-): 0.28

Region: NODE\_117571\_length\_15591\_cov\_25.186710 14565-14584. Max. coverage (+): 0. Max coverage (-): 0.09

Region: NODE\_117571\_length\_15591\_cov\_25.186710 14585-14603. Max. coverage (+): 0. Max coverage (-): 0

Region: NODE\_117571\_length\_15591\_cov\_25.186710 14604-14623. Max. coverage (+): 0. Max coverage (-): 0

Region: NODE\_117571\_length\_15591\_cov\_25.186710 14624-14642. Max. coverage (+): 0. Max coverage (-): 0.09

Region: NODE\_117571\_length\_15591\_cov\_25.186710 14643-14662. Max. coverage (+): 0. Max coverage (-): 1.41

Region: NODE\_117571\_length\_15591\_cov\_25.186710 14663-14681. Max. coverage (+): 0.19. Max coverage (-): 1.69

Region: NODE\_117571\_length\_15591\_cov\_25.186710 14682-14701. Max. coverage (+): 0.19. Max coverage (-): 0.09

Region: NODE\_117571\_length\_15591\_cov\_25.186710 14702-14721. Max. coverage (+): 0. Max coverage (-): 5.54

Region: NODE\_117571\_length\_15591\_cov\_25.186710 14722-14740. Max. coverage (+): 0. Max coverage (-): 0

Region: NODE\_117571\_length\_15591\_cov\_25.186710 14741-14760. Max. coverage (+): 0. Max coverage (-): 3.38

Region: NODE\_117571\_length\_15591\_cov\_25.186710 14761-14779. Max. coverage (+): 0. Max coverage (-): 5.17

Region: NODE\_117571\_length\_15591\_cov\_25.186710 14780-14799. Max. coverage (+): 0.19. Max coverage (-): 0.19

Region: NODE\_117571\_length\_15591\_cov\_25.186710 14800-14818. Max. coverage (+): 0. Max coverage (-): 1.79

Region: NODE\_117571\_length\_15591\_cov\_25.186710 14819-14838. Max. coverage (+): 0.09. Max coverage (-): 0.66

Region: NODE\_117571\_length\_15591\_cov\_25.186710 14839-14857. Max. coverage (+): 0. Max coverage (-): 0.66

Region: NODE\_117571\_length\_15591\_cov\_25.186710 14858-14877. Max. coverage (+): 0.66. Max coverage (-): 0.56

Region: NODE\_117571\_length\_15591\_cov\_25.186710 14878-14897. Max. coverage (+): 0. Max coverage (-): 0.47

Region: NODE\_117571\_length\_15591\_cov\_25.186710 14898-14916. Max. coverage (+): 0. Max coverage (-): 0.38

Region: NODE\_117571\_length\_15591\_cov\_25.186710 14917-14936. Max. coverage (+): 0. Max coverage (-): 2.88

Region: NODE\_117571\_length\_15591\_cov\_25.186710 14937-14955. Max. coverage (+): 0. Max coverage (-): 0.28

Region: NODE\_117571\_length\_15591\_cov\_25.186710 14956-14975. Max. coverage (+): 0. Max coverage (-): 0.09

Region: NODE\_117571\_length\_15591\_cov\_25.186710 14976-14994. Max. coverage (+): 0. Max coverage (-): 0

Region: NODE\_117571\_length\_15591\_cov\_25.186710 14995-15014. Max. coverage (+): 0. Max coverage (-): 0.02

Region: NODE\_117571\_length\_15591\_cov\_25.186710 15015-15034. Max. coverage (+): 0. Max coverage (-): 0.02

Region: NODE\_117571\_length\_15591\_cov\_25.186710 15035-15053. Max. coverage (+): 0. Max coverage (-): 0.14

Region: NODE\_117571\_length\_15591\_cov\_25.186710 15054-15073. Max. coverage (+): 0.09. Max coverage (-): 0.14

Region: NODE\_117571\_length\_15591\_cov\_25.186710 15074-15092. Max. coverage (+): 0.09. Max coverage (-): 0

Region: NODE\_117571\_length\_15591\_cov\_25.186710 15093-15112. Max. coverage (+): 0. Max coverage (-): 0.75

Region: NODE\_117571\_length\_15591\_cov\_25.186710 15113-15131. Max. coverage (+): 0. Max coverage (-): 0.19

Region: NODE\_117571\_length\_15591\_cov\_25.186710 15132-15151. Max. coverage (+): 0.09. Max coverage (-): 0

Region: NODE\_117571\_length\_15591\_cov\_25.186710 15152-15170. Max. coverage (+): 0. Max coverage (-): 0.38

Region: NODE\_117571\_length\_15591\_cov\_25.186710 15171-15190. Max. coverage (+): 0. Max coverage (-): 0.38

Region: NODE\_117571\_length\_15591\_cov\_25.186710 15191-15210. Max. coverage (+): 0. Max coverage (-): 0.09

Region: NODE\_117571\_length\_15591\_cov\_25.186710 15211-15229. Max. coverage (+): 0. Max coverage (-): 0.28

Region: NODE\_117571\_length\_15591\_cov\_25.186710 15230-15249. Max. coverage (+): 0. Max coverage (-): 0

Region: NODE\_117571\_length\_15591\_cov\_25.186710 15250-15268. Max. coverage (+): 0. Max coverage (-): 0.28

Region: NODE\_117571\_length\_15591\_cov\_25.186710 15269-15288. Max. coverage (+): 0. Max coverage (-): 0.09

Region: NODE\_117571\_length\_15591\_cov\_25.186710 15289-15307. Max. coverage (+): 0.05. Max coverage (-): 0

Region: NODE\_117571\_length\_15591\_cov\_25.186710 15308-15327. Max. coverage (+): 0. Max coverage (-): 0.47

Region: NODE\_117571\_length\_15591\_cov\_25.186710 15328-15346. Max. coverage (+): 0.03. Max coverage (-): 0.38

Region: NODE\_117571\_length\_15591\_cov\_25.186710 15347-15366. Max. coverage (+): 0.05. Max coverage (-): 2.13

Region: NODE\_117571\_length\_15591\_cov\_25.186710 15367-15386. Max. coverage (+): 0.09. Max coverage (-): 2.13

Region: NODE\_117571\_length\_15591\_cov\_25.186710 15387-15405. Max. coverage (+): 0.09. Max coverage (-): 0.09

Region: NODE\_117571\_length\_15591\_cov\_25.186710 15406-15425. Max. coverage (+): 0. Max coverage (-): 0.27

Region: NODE\_117571\_length\_15591\_cov\_25.186710 15426-15444. Max. coverage (+): 0.05. Max coverage (-): 0.18

Region: NODE\_117571\_length\_15591\_cov\_25.186710 15445-15464. Max. coverage (+): 0.05. Max coverage (-): 0

Region: NODE\_117571\_length\_15591\_cov\_25.186710 15465-15483. Max. coverage (+): 0. Max coverage (-): 0.01

Region: NODE\_117571\_length\_15591\_cov\_25.186710 15484-15503. Max. coverage (+): 0.04. Max coverage (-): 0.01

Region: NODE\_117571\_length\_15591\_cov\_25.186710 15504-15522. Max. coverage (+): 0.01. Max coverage (-): 0

Region: NODE\_117571\_length\_15591\_cov\_25.186710 15523-15542. Max. coverage (+): 0. Max coverage (-): 0

Region: NODE\_117571\_length\_15591\_cov\_25.186710 15543-15562. Max. coverage (+): 0. Max coverage (-): 0

Region: NODE\_117571\_length\_15591\_cov\_25.186710 15563-15581. Max. coverage (+): 0. Max coverage (-): 0

Region: NODE\_117571\_length\_15591\_cov\_25.186710 15582-15601. Max. coverage (+): 0. Max coverage (-): 0

Region: NODE\_117571\_length\_15591\_cov\_25.186710 15602-15620. Max. coverage (+): 0. Max coverage (-): 0

Region: NODE\_117571\_length\_15591\_cov\_25.186710 15621-15640. Max. coverage (+): 0. Max coverage (-): 0

Region: NODE\_117571\_length\_15591\_cov\_25.186710 15641-15659. Max. coverage (+): 0. Max coverage (-): 0

Region: NODE\_117571\_length\_15591\_cov\_25.186710 15660-15679. Max. coverage (+): 0. Max coverage (-): 0

Region: NODE\_117571\_length\_15591\_cov\_25.186710 15680-15698. Max. coverage (+): 0. Max coverage (-): 0

Region: NODE\_117571\_length\_15591\_cov\_25.186710 15699-15718. Max. coverage (+): 0.09. Max coverage (-): 0

Region: NODE\_117571\_length\_15591\_cov\_25.186710 15719-15738. Max. coverage (+): 0. Max coverage (-): 0

Region: NODE\_117571\_length\_15591\_cov\_25.186710 15739-15757. Max. coverage (+): 0. Max coverage (-): 0

Region: NODE\_117571\_length\_15591\_cov\_25.186710 15758-15777. Max. coverage (+): 0. Max coverage (-): 0

Region: NODE\_117571\_length\_15591\_cov\_25.186710 15778-. Max. coverage (+): 0. Max coverage (-): 0

RepeatMasker Color Code

**+**

100-98% Identity

<98-95% Identity

<95-90% Identity

<90-85% Identity

<85-80% Identity

<80-75% Identity

<75-70% Identity

<70% Identity

**-**

Gene Set Color Code

**+**

Gene

Pseudogene

Other

**-**

Topology/Coverage Color Code

Coverage Plus Strand

Coverage Minus Strand

Mainstrand: Plus

Mainstrand: Minus

Complementary Strand

Flanking Region  
(if option -flank >0)

Gene Set Annotation  
  
RepeatMasker Annotation  

**1. AlRepC-373**: 5961-6170 (+), Divergence to consensus: 33.6%  
**2. AlRepC-905**: 6120-6246 (-), Divergence to consensus: 41.3%  
**3. (TTTG)n**: 6971-7006 (+), Divergence to consensus: 11.2%  
**4. Samurai\_I-int**: 8814-9228 (-), Divergence to consensus: 30.9%  
**5. AlRepE-2838**: 9584-9679 (-), Divergence to consensus: 41.7%  
**6. AlRepC-1442**: 9682-9782 (+), Divergence to consensus: 7.3%  
**7. AlRepC-1442**: 9777-10016 (+), Divergence to consensus: 5.9%  
**8. (T)n**: 11984-12018 (+), Divergence to consensus: 14.9%  
**9. AlRepD-1165**: 13401-13528 (-), Divergence to consensus: 19.5%  
**10. AlRepB-103**: 13637-13708 (-), Divergence to consensus: 27.7%  
**11. AlRepA-51**: 13867-13942 (-), Divergence to consensus: 43.1%  
**12. AlRepE-65**: 14495-14535 (+), Divergence to consensus: 12.5%  
**13. AlRepA-115**: 14511-14618 (+), Divergence to consensus: 36.3%  
**14. AlRepC-754**: 14619-14687 (+), Divergence to consensus: 17.9%  
**15. AlRepB-420**: 15429-15729 (+), Divergence to consensus: 16.3%  
**16. hAT-N93\_DR**: 15731-15788 (+), Divergence to consensus: 0%

  
Transcription Factor Binding Sites  

**RFX4\_2** (Sequence: CTTGGTTAC (+): 15066)  
**RHOXF1** (Sequence: AGCTCA (-): 8165)  
**RHOXF1** (Sequence: AGATCA (-): 8177)  
**RHOXF1** (Sequence: GGCTCA (-): 8861)  
**RHOXF1** (Sequence: AGCTCA (-): 8953)  
**RHOXF1** (Sequence: GGATCA (-): 9380)  
**RHOXF1** (Sequence: GGATCA (-): 10234)  
**RHOXF1** (Sequence: GGATCA (-): 10357)  
**RHOXF1** (Sequence: AGATTA (-): 10643)  
**RHOXF1** (Sequence: GGATTA (-): 11366)  
**RHOXF1** (Sequence: AGATTA (-): 11779)  
**RHOXF1** (Sequence: AGCTTA (-): 12032)  
**RHOXF1** (Sequence: AGATTA (-): 12099)  
**RHOXF1** (Sequence: AGCTTA (-): 12796)  
**RHOXF1** (Sequence: GGATCA (-): 12984)  
**RHOXF1** (Sequence: AGATCA (-): 13222)  
**RHOXF1** (Sequence: GGATTA (-): 13382)  
**RHOXF1** (Sequence: AGCTCA (-): 14477)  
**RHOXF1** (Sequence: TGATCT (+): 6126)  
**RHOXF1** (Sequence: TAATCT (+): 6145)  
**RHOXF1** (Sequence: TAATCC (+): 6342)  
**RHOXF1** (Sequence: TAATCT (+): 6686)  
**RHOXF1** (Sequence: TAAGCT (+): 6927)  
**RHOXF1** (Sequence: TAATCT (+): 7048)  
**RHOXF1** (Sequence: TAAGCT (+): 7557)  
**RHOXF1** (Sequence: TAATCC (+): 9312)  
**RHOXF1** (Sequence: TGAGCT (+): 9846)  
**RHOXF1** (Sequence: TGAGCT (+): 10577)  
**RHOXF1** (Sequence: TGAGCC (+): 10676)  
**RHOXF1** (Sequence: TGAGCT (+): 11304)  
**RHOXF1** (Sequence: TGAGCT (+): 12177)  
**RHOXF1** (Sequence: TGAGCT (+): 12811)  
**RHOXF1** (Sequence: TAATCC (+): 12907)  
**RHOXF1** (Sequence: TGATCC (+): 12976)  
**RHOXF1** (Sequence: TGAGCC (+): 13046)  
**RHOXF1** (Sequence: TGAGCC (+): 13323)  
**RHOXF1** (Sequence: TGATCC (+): 13890)  
**RHOXF1** (Sequence: TAATCC (+): 14044)  
**RHOXF1** (Sequence: TGAGCT (+): 14281)  
**Lhx8** (Sequence: TTAATTAA (-): 13712)  
**Gata4** (Sequence: GTTATCT (+): 12859)  
**POU5F1** (Sequence: TTTGCAT (-): 10527)  
**POU5F1** (Sequence: TTTGCAT (-): 12571)  
**POU5F1** (Sequence: TTTGCAT (-): 13867)  
**RFX4\_2** (Sequence: GTAACTACG (-): 15769)  
**SOX9** (Sequence: AACAATGA (-): 13502)  
**SOX9** (Sequence: AACAATAG (-): 13805)  
**SOX9** (Sequence: AACAATAA (-): 13825)  
**FOXP1** (Sequence: GTAAACA (+): 11447)  
**FOXO1** (Sequence: GCTGTTTAT (+): 7353)  
**FOXO1** (Sequence: GCTGTTTTT (+): 14284)  
**FOXO3\_mmu** (Sequence: TGTTTTCA (-): 8398)  
**FOXO3\_mmu** (Sequence: TGTTTTGA (-): 9051)  
**Sox5** (Sequence: ATTGTT (+): 7011)  
**Sox5** (Sequence: ATTGTT (+): 8680)  
**Sox5** (Sequence: ATTGTT (+): 11988)  
**Sox5** (Sequence: ATTGTT (+): 15433)  
**FIGLA** (Sequence: ACCAGCTGGT (-): 9027)  
**FIGLA** (Sequence: ACCAGGTGGA (-): 15577)  
**FOXO3\_mmu** (Sequence: TCAAAACA (+): 7974)  
**FOXO3\_mmu** (Sequence: GGAAAACA (+): 10300)  
**FOXO3\_mmu** (Sequence: TGAAAACA (+): 12485)  
**FOXO3\_mmu** (Sequence: TGAAAACA (+): 12770)  
**Nobox** (Sequence: AGCAATTA (-): 9686)  
**FOXO1** (Sequence: GAAAACAGC (-): 12486)  
**FOXO1** (Sequence: AAAAACAGG (-): 12500)  
**FOXO1** (Sequence: GAAAACAAC (-): 12771)  
**POU2F1** (Sequence: ATTAAAATA (-): 6673)  
**Rhox11** (Sequence: TGCTGTTAT (+): 6843)  
**Rhox11** (Sequence: TGCTGTATA (+): 7196)  
**Rhox11** (Sequence: TGGTGTATT (+): 10818)  
**Rhox11** (Sequence: TGGTGTTTT (+): 10915)  
**Rhox11** (Sequence: TATACAGCG (-): 10986)  
**Rhox11** (Sequence: TTTACACCA (-): 12641)  
**Sox5** (Sequence: AACAAT (-): 9430)  
**Sox5** (Sequence: AACAAT (-): 9946)  
**Sox5** (Sequence: AACAAT (-): 11017)  
**Sox5** (Sequence: AACAAT (-): 13502)  
**Sox5** (Sequence: AACAAT (-): 13805)  
**Sox5** (Sequence: AACAAT (-): 13825)  
**Sox5** (Sequence: AACAAT (-): 14327)  
**Sox5** (Sequence: AACAAT (-): 14501)  
**Sox5** (Sequence: AACAAT (-): 14685)  
**Sox5** (Sequence: AACAAT (-): 15586)  
**POU2F1** (Sequence: TATTTTAAT (+): 12211)  
**POU2F1** (Sequence: TATGCAAAT (+): 15140)  
**POU5F1** (Sequence: ATGCAAA (+): 9661)  
**POU5F1** (Sequence: ATGCAAA (+): 14680)  
**POU5F1** (Sequence: ATGCAAA (+): 15141)
