# Supplementary material for: First characterization of PIWI-interacting RNA clusters in a cichlid fish with a B chromosome
Source: BMC Biol. 2022 Sep 21;20:204. doi: 10.1186/s12915-022-01403-2 (PMC9490952; doi:10.1186/s12915-022-01403-2)
Supplement: Supplementary file 1 — Additional file 1. Zipped folder with fasta and interactive html piRNA cluster information for the A. latifasciata genome. The nomenclature is as follows: number-pirna-cluster_sex_B-presence (f, female; m, male; 0b, without B chromosome; 1b, with B chromosome). [file 12915_2022_1403_MOESM1_ESM.zip › 120_f1b.html]

piRNA cluster 120\_f1b 57


Predicted piRNA cluster no. 120\_f1b
  

Show proTRAC run info
Hide proTRAC run info

/\  
                \_\_\_\_\_\_\_\_\_\_\_\_\_\_\_\_\_\_\_\_\_\_\_/\\_\_\_ /  \\_\_\_\_\_\_\_  
               I                      /  \  /    \      I  
               I     pro             /    \/      \     I  
               I        TRAC        /               \   I  
               I   \_\_\_\_\_\_\_\_\_\_\_\_\_\_\_\_/\_\_\_\_\_\_\_\_\_\_\_\_\_\_\_\_\_\\_ I  
               I   \              /                     I  
               I    \            /                      I  
               I     \  /\      /       V.2.4.2         I  
               I      \/  \    /                        I  
               I\_\_\_\_\_\_\_\_\_\_\_\  /\_\_\_\_\_\_\_\_\_\_\_\_\_\_\_\_\_\_\_\_\_\_\_\_\_I  
                            \/  
  
  
================================= proTRAC ====================================  
VERSION: .......... 2.4.2  
LAST MODIFIED: .... 11. May 2018  
  
Please cite:  
Rosenkranz D, Zischler H. proTRAC - a software for probabilistic piRNA cluster  
detection, visualization and analysis. 2012. BMC Bioinformatics 13:5.  
  
  
Contact:  
David Rosenkranz  
Institute of Organismic and Molecular Evolutionary Biology  
Dept. Anthropology, small RNA group  
Johannes Gutenberg University Mainz  
email: rosenkranz@uni-mainz.de  
  
You can find the latest proTRAC version at:  
http://sourceforge.net/projects/protrac/files  
http://www.smallRNAgroup-mainz.de/software  
==============================================================================  
  
PARAMETERS:  
Map file: ...............piwi-femeas-1B.fa-collapse.map  
Genome file: ............../../../0B\_ala\_genome.fa  
RepeatMasker annotation: Alatifasciata-all0B-maryan-v2.fa\_corrected.out  
GeneSet:................./guest-storage/Data/annotation/Alatifasciata\_all0B\_maryan-v2\_out2017.gff  
  
Significant (p<=0.01) hit density will be calculated based  
on observed hit distribution.  
  
Sliding window size: ........................................ 5000 bp  
Sliding window increament: .................................. 1000 bp  
Normalize each hit by number of genomic hits: ............... yes  
Normalize each hit by number of sequence reads: ............. yes  
Normalize values (-> per million mapped reads): ............. yes  
Min. fraction of hits with 1T(U) or 10A: .................... 0.75  
Alternatively: Min. fraction of hits with 1T(U) and 10A: .... 0.5  
Min. fraction of hits with typical piRNA length: ............ 0.75  
Typical piRNA length: ....................................... 24-32 nt  
Min. size of a piRNA cluster: ............................... 1000 bp.  
Min. number of hits (absolute): ............................. 0  
Min. number of hits (normalized): ........................... 0  
Min. fraction of hits on the mainstrand: .................... 0.75  
Top fraction of mapped sequences (in terms of read counts): . 1%  
Top fraction accounts for max. n% of sequence reads: ........ 90%  
Min. fraction of hits on each arm of a bidirectional cluster: 0.05  
Output html file for each cluster: .......................... yes  
Output a summary table: ..................................... yes  
Output a FASTA file for each cluster (piRNA sequences): ..... yes  
Output a FASTA file comprising cluster sequences: ........... yes  
Output a GTF file for predicted piRNA clusters: ..............yes  
Search DNA motifs in clusters: .............................. yes  
Output flanking sequences: +/- .............................. 0 bp  
Output ~.pTi file: .......................................... no  
==============================================================================  
  
  
Genome size (without gaps): ............ 758543724 bp  
Gaps (N/X/-): .......................... 417479 bp  
Mapped reads: .......................... 10641844  
Non-identical sequences: ............... 2832837  
Genomic hits: .......................... 26056853  
Significant densitiy of mapped reads: .. 368.713530323068 reads/kb

Show proTRAC cluster info
Hide proTRAC cluster info

|  |  |
| --- | --- |
| Location | NODE\_306037\_length\_18339\_cov\_27.068979 |
| Coordinates | 12039-13938 |
| Size [bp] | 1900 |
| Sequence hit loci | 84 |
| Mapped reads (normalized) | 3875.2 |
| Mapped reads (normalized) per kb | 2039.6 |
| Normalized reads with 1T (1U) | 77.4% |
| Normalized reads with 10A | 18.1% |
| Normalized reads with length 24-32 nt | 94.2% |
| Normalized reads on the main strand(s) | 100% |
| Predicted directionality | mono:plus |

100%

0%

1T (1U)  
reads

10A reads

24-32 nt  
reads

reads on mainstrand

**Either the amount of reads with 1T (1U) OR 10A has to exceed 75% (set with option: -1Tor10A)  
Alternatively the amount of reads with 1T (1U) AND 10A has to exceed 50% (set with option: -1Tand10A)  
Minimum amount of reads with preferred size is 75% (set with option: -pisize)  
Minimum amount of reads on the main strand(s) is 75% (set with option: -clstrand)**

Show read coverage
Hide read coverage

WHAT DO I SEE HERE?  
This chart shows the location of mapped sequence reads within a predicted piRNA cluster. The color refers to the number of genomic hits produced by the sequence read in question. A dark red bar indicates that this sequence read produces many other hits elsewhere in the genome. Many adjacent red or yellow bars can indicate the presence of a multi-copy element such as transposons or rRNA genes. A dark green bar indicates that this sequence read maps uniquely to this locus.

1 hit

2-5 hits

6-10 hits

11-20 hits

21-50 hits

51-100 hits

> 100 hits

NODE\_306037\_length\_18339\_cov\_27.068979

12039

13938

Gene Set

RepeatMasker

Mapped  
Reads

286.7

plus strand

minus strand

286.7

Region: NODE\_306037\_length\_18339\_cov\_27.068979 6000-12040. Max. coverage (+): 0.01. Max coverage (-): 0

Region: NODE\_306037\_length\_18339\_cov\_27.068979 12041-12044. Max. coverage (+): 0.01. Max coverage (-): 0

Region: NODE\_306037\_length\_18339\_cov\_27.068979 12045-12048. Max. coverage (+): 0. Max coverage (-): 0

Region: NODE\_306037\_length\_18339\_cov\_27.068979 12049-12052. Max. coverage (+): 0. Max coverage (-): 0

Region: NODE\_306037\_length\_18339\_cov\_27.068979 12053-12056. Max. coverage (+): 0. Max coverage (-): 0

Region: NODE\_306037\_length\_18339\_cov\_27.068979 12057-12059. Max. coverage (+): 0. Max coverage (-): 0

Region: NODE\_306037\_length\_18339\_cov\_27.068979 12060-12063. Max. coverage (+): 0. Max coverage (-): 0

Region: NODE\_306037\_length\_18339\_cov\_27.068979 12064-12067. Max. coverage (+): 0. Max coverage (-): 0

Region: NODE\_306037\_length\_18339\_cov\_27.068979 12068-12071. Max. coverage (+): 0. Max coverage (-): 0

Region: NODE\_306037\_length\_18339\_cov\_27.068979 12072-12075. Max. coverage (+): 0. Max coverage (-): 0

Region: NODE\_306037\_length\_18339\_cov\_27.068979 12076-12078. Max. coverage (+): 0. Max coverage (-): 0

Region: NODE\_306037\_length\_18339\_cov\_27.068979 12079-12082. Max. coverage (+): 0. Max coverage (-): 0

Region: NODE\_306037\_length\_18339\_cov\_27.068979 12083-12086. Max. coverage (+): 0. Max coverage (-): 0

Region: NODE\_306037\_length\_18339\_cov\_27.068979 12087-12090. Max. coverage (+): 0. Max coverage (-): 0

Region: NODE\_306037\_length\_18339\_cov\_27.068979 12091-12094. Max. coverage (+): 0. Max coverage (-): 0

Region: NODE\_306037\_length\_18339\_cov\_27.068979 12095-12097. Max. coverage (+): 0. Max coverage (-): 0

Region: NODE\_306037\_length\_18339\_cov\_27.068979 12098-12101. Max. coverage (+): 0. Max coverage (-): 0

Region: NODE\_306037\_length\_18339\_cov\_27.068979 12102-12105. Max. coverage (+): 0. Max coverage (-): 0

Region: NODE\_306037\_length\_18339\_cov\_27.068979 12106-12109. Max. coverage (+): 0. Max coverage (-): 0

Region: NODE\_306037\_length\_18339\_cov\_27.068979 12110-12113. Max. coverage (+): 0. Max coverage (-): 0

Region: NODE\_306037\_length\_18339\_cov\_27.068979 12114-12116. Max. coverage (+): 0. Max coverage (-): 0

Region: NODE\_306037\_length\_18339\_cov\_27.068979 12117-12120. Max. coverage (+): 0. Max coverage (-): 0

Region: NODE\_306037\_length\_18339\_cov\_27.068979 12121-12124. Max. coverage (+): 0. Max coverage (-): 0

Region: NODE\_306037\_length\_18339\_cov\_27.068979 12125-12128. Max. coverage (+): 0. Max coverage (-): 0

Region: NODE\_306037\_length\_18339\_cov\_27.068979 12129-12132. Max. coverage (+): 0. Max coverage (-): 0

Region: NODE\_306037\_length\_18339\_cov\_27.068979 12133-12135. Max. coverage (+): 0. Max coverage (-): 0

Region: NODE\_306037\_length\_18339\_cov\_27.068979 12136-12139. Max. coverage (+): 0. Max coverage (-): 0

Region: NODE\_306037\_length\_18339\_cov\_27.068979 12140-12143. Max. coverage (+): 0. Max coverage (-): 0

Region: NODE\_306037\_length\_18339\_cov\_27.068979 12144-12147. Max. coverage (+): 0. Max coverage (-): 0

Region: NODE\_306037\_length\_18339\_cov\_27.068979 12148-12151. Max. coverage (+): 0. Max coverage (-): 0

Region: NODE\_306037\_length\_18339\_cov\_27.068979 12152-12154. Max. coverage (+): 0. Max coverage (-): 0

Region: NODE\_306037\_length\_18339\_cov\_27.068979 12155-12158. Max. coverage (+): 0. Max coverage (-): 0

Region: NODE\_306037\_length\_18339\_cov\_27.068979 12159-12162. Max. coverage (+): 0. Max coverage (-): 0

Region: NODE\_306037\_length\_18339\_cov\_27.068979 12163-12166. Max. coverage (+): 0. Max coverage (-): 0

Region: NODE\_306037\_length\_18339\_cov\_27.068979 12167-12170. Max. coverage (+): 0. Max coverage (-): 0

Region: NODE\_306037\_length\_18339\_cov\_27.068979 12171-12173. Max. coverage (+): 0. Max coverage (-): 0

Region: NODE\_306037\_length\_18339\_cov\_27.068979 12174-12177. Max. coverage (+): 0. Max coverage (-): 0

Region: NODE\_306037\_length\_18339\_cov\_27.068979 12178-12181. Max. coverage (+): 0. Max coverage (-): 0

Region: NODE\_306037\_length\_18339\_cov\_27.068979 12182-12185. Max. coverage (+): 0. Max coverage (-): 0

Region: NODE\_306037\_length\_18339\_cov\_27.068979 12186-12189. Max. coverage (+): 0. Max coverage (-): 0

Region: NODE\_306037\_length\_18339\_cov\_27.068979 12190-12192. Max. coverage (+): 0.01. Max coverage (-): 0

Region: NODE\_306037\_length\_18339\_cov\_27.068979 12193-12196. Max. coverage (+): 0.01. Max coverage (-): 0

Region: NODE\_306037\_length\_18339\_cov\_27.068979 12197-12200. Max. coverage (+): 0. Max coverage (-): 0

Region: NODE\_306037\_length\_18339\_cov\_27.068979 12201-12204. Max. coverage (+): 0. Max coverage (-): 0

Region: NODE\_306037\_length\_18339\_cov\_27.068979 12205-12208. Max. coverage (+): 0. Max coverage (-): 0

Region: NODE\_306037\_length\_18339\_cov\_27.068979 12209-12211. Max. coverage (+): 0. Max coverage (-): 0

Region: NODE\_306037\_length\_18339\_cov\_27.068979 12212-12215. Max. coverage (+): 0. Max coverage (-): 0

Region: NODE\_306037\_length\_18339\_cov\_27.068979 12216-12219. Max. coverage (+): 0. Max coverage (-): 0

Region: NODE\_306037\_length\_18339\_cov\_27.068979 12220-12223. Max. coverage (+): 0. Max coverage (-): 0

Region: NODE\_306037\_length\_18339\_cov\_27.068979 12224-12227. Max. coverage (+): 0. Max coverage (-): 0

Region: NODE\_306037\_length\_18339\_cov\_27.068979 12228-12230. Max. coverage (+): 0. Max coverage (-): 0

Region: NODE\_306037\_length\_18339\_cov\_27.068979 12231-12234. Max. coverage (+): 0. Max coverage (-): 0

Region: NODE\_306037\_length\_18339\_cov\_27.068979 12235-12238. Max. coverage (+): 0. Max coverage (-): 0

Region: NODE\_306037\_length\_18339\_cov\_27.068979 12239-12242. Max. coverage (+): 0. Max coverage (-): 0

Region: NODE\_306037\_length\_18339\_cov\_27.068979 12243-12246. Max. coverage (+): 0. Max coverage (-): 0

Region: NODE\_306037\_length\_18339\_cov\_27.068979 12247-12249. Max. coverage (+): 0. Max coverage (-): 0

Region: NODE\_306037\_length\_18339\_cov\_27.068979 12250-12253. Max. coverage (+): 0. Max coverage (-): 0

Region: NODE\_306037\_length\_18339\_cov\_27.068979 12254-12257. Max. coverage (+): 0. Max coverage (-): 0

Region: NODE\_306037\_length\_18339\_cov\_27.068979 12258-12261. Max. coverage (+): 0. Max coverage (-): 0

Region: NODE\_306037\_length\_18339\_cov\_27.068979 12262-12265. Max. coverage (+): 0. Max coverage (-): 0

Region: NODE\_306037\_length\_18339\_cov\_27.068979 12266-12268. Max. coverage (+): 0. Max coverage (-): 0

Region: NODE\_306037\_length\_18339\_cov\_27.068979 12269-12272. Max. coverage (+): 0. Max coverage (-): 0

Region: NODE\_306037\_length\_18339\_cov\_27.068979 12273-12276. Max. coverage (+): 0. Max coverage (-): 0

Region: NODE\_306037\_length\_18339\_cov\_27.068979 12277-12280. Max. coverage (+): 0. Max coverage (-): 0

Region: NODE\_306037\_length\_18339\_cov\_27.068979 12281-12284. Max. coverage (+): 0. Max coverage (-): 0

Region: NODE\_306037\_length\_18339\_cov\_27.068979 12285-12287. Max. coverage (+): 0. Max coverage (-): 0

Region: NODE\_306037\_length\_18339\_cov\_27.068979 12288-12291. Max. coverage (+): 0. Max coverage (-): 0

Region: NODE\_306037\_length\_18339\_cov\_27.068979 12292-12295. Max. coverage (+): 0. Max coverage (-): 0

Region: NODE\_306037\_length\_18339\_cov\_27.068979 12296-12299. Max. coverage (+): 0. Max coverage (-): 0

Region: NODE\_306037\_length\_18339\_cov\_27.068979 12300-12303. Max. coverage (+): 0. Max coverage (-): 0

Region: NODE\_306037\_length\_18339\_cov\_27.068979 12304-12306. Max. coverage (+): 0. Max coverage (-): 0

Region: NODE\_306037\_length\_18339\_cov\_27.068979 12307-12310. Max. coverage (+): 0. Max coverage (-): 0

Region: NODE\_306037\_length\_18339\_cov\_27.068979 12311-12314. Max. coverage (+): 0. Max coverage (-): 0

Region: NODE\_306037\_length\_18339\_cov\_27.068979 12315-12318. Max. coverage (+): 0. Max coverage (-): 0

Region: NODE\_306037\_length\_18339\_cov\_27.068979 12319-12322. Max. coverage (+): 0. Max coverage (-): 0

Region: NODE\_306037\_length\_18339\_cov\_27.068979 12323-12325. Max. coverage (+): 0. Max coverage (-): 0

Region: NODE\_306037\_length\_18339\_cov\_27.068979 12326-12329. Max. coverage (+): 0. Max coverage (-): 0

Region: NODE\_306037\_length\_18339\_cov\_27.068979 12330-12333. Max. coverage (+): 0. Max coverage (-): 0

Region: NODE\_306037\_length\_18339\_cov\_27.068979 12334-12337. Max. coverage (+): 0. Max coverage (-): 0

Region: NODE\_306037\_length\_18339\_cov\_27.068979 12338-12341. Max. coverage (+): 0. Max coverage (-): 0

Region: NODE\_306037\_length\_18339\_cov\_27.068979 12342-12344. Max. coverage (+): 0. Max coverage (-): 0

Region: NODE\_306037\_length\_18339\_cov\_27.068979 12345-12348. Max. coverage (+): 0. Max coverage (-): 0

Region: NODE\_306037\_length\_18339\_cov\_27.068979 12349-12352. Max. coverage (+): 0. Max coverage (-): 0

Region: NODE\_306037\_length\_18339\_cov\_27.068979 12353-12356. Max. coverage (+): 0. Max coverage (-): 0

Region: NODE\_306037\_length\_18339\_cov\_27.068979 12357-12360. Max. coverage (+): 0. Max coverage (-): 0

Region: NODE\_306037\_length\_18339\_cov\_27.068979 12361-12363. Max. coverage (+): 0. Max coverage (-): 0

Region: NODE\_306037\_length\_18339\_cov\_27.068979 12364-12367. Max. coverage (+): 0. Max coverage (-): 0

Region: NODE\_306037\_length\_18339\_cov\_27.068979 12368-12371. Max. coverage (+): 0. Max coverage (-): 0

Region: NODE\_306037\_length\_18339\_cov\_27.068979 12372-12375. Max. coverage (+): 0. Max coverage (-): 0

Region: NODE\_306037\_length\_18339\_cov\_27.068979 12376-12379. Max. coverage (+): 0. Max coverage (-): 0

Region: NODE\_306037\_length\_18339\_cov\_27.068979 12380-12382. Max. coverage (+): 0. Max coverage (-): 0

Region: NODE\_306037\_length\_18339\_cov\_27.068979 12383-12386. Max. coverage (+): 0. Max coverage (-): 0

Region: NODE\_306037\_length\_18339\_cov\_27.068979 12387-12390. Max. coverage (+): 0. Max coverage (-): 0

Region: NODE\_306037\_length\_18339\_cov\_27.068979 12391-12394. Max. coverage (+): 0. Max coverage (-): 0

Region: NODE\_306037\_length\_18339\_cov\_27.068979 12395-12398. Max. coverage (+): 0. Max coverage (-): 0

Region: NODE\_306037\_length\_18339\_cov\_27.068979 12399-12401. Max. coverage (+): 0. Max coverage (-): 0

Region: NODE\_306037\_length\_18339\_cov\_27.068979 12402-12405. Max. coverage (+): 0. Max coverage (-): 0

Region: NODE\_306037\_length\_18339\_cov\_27.068979 12406-12409. Max. coverage (+): 0. Max coverage (-): 0

Region: NODE\_306037\_length\_18339\_cov\_27.068979 12410-12413. Max. coverage (+): 0. Max coverage (-): 0

Region: NODE\_306037\_length\_18339\_cov\_27.068979 12414-12417. Max. coverage (+): 0. Max coverage (-): 0

Region: NODE\_306037\_length\_18339\_cov\_27.068979 12418-12420. Max. coverage (+): 0. Max coverage (-): 0

Region: NODE\_306037\_length\_18339\_cov\_27.068979 12421-12424. Max. coverage (+): 0. Max coverage (-): 0

Region: NODE\_306037\_length\_18339\_cov\_27.068979 12425-12428. Max. coverage (+): 0. Max coverage (-): 0

Region: NODE\_306037\_length\_18339\_cov\_27.068979 12429-12432. Max. coverage (+): 0. Max coverage (-): 0

Region: NODE\_306037\_length\_18339\_cov\_27.068979 12433-12436. Max. coverage (+): 0. Max coverage (-): 0

Region: NODE\_306037\_length\_18339\_cov\_27.068979 12437-12439. Max. coverage (+): 0. Max coverage (-): 0

Region: NODE\_306037\_length\_18339\_cov\_27.068979 12440-12443. Max. coverage (+): 0. Max coverage (-): 0

Region: NODE\_306037\_length\_18339\_cov\_27.068979 12444-12447. Max. coverage (+): 0. Max coverage (-): 0

Region: NODE\_306037\_length\_18339\_cov\_27.068979 12448-12451. Max. coverage (+): 0. Max coverage (-): 0

Region: NODE\_306037\_length\_18339\_cov\_27.068979 12452-12455. Max. coverage (+): 0. Max coverage (-): 0

Region: NODE\_306037\_length\_18339\_cov\_27.068979 12456-12458. Max. coverage (+): 0. Max coverage (-): 0

Region: NODE\_306037\_length\_18339\_cov\_27.068979 12459-12462. Max. coverage (+): 0. Max coverage (-): 0

Region: NODE\_306037\_length\_18339\_cov\_27.068979 12463-12466. Max. coverage (+): 0. Max coverage (-): 0

Region: NODE\_306037\_length\_18339\_cov\_27.068979 12467-12470. Max. coverage (+): 0. Max coverage (-): 0

Region: NODE\_306037\_length\_18339\_cov\_27.068979 12471-12474. Max. coverage (+): 0. Max coverage (-): 0

Region: NODE\_306037\_length\_18339\_cov\_27.068979 12475-12477. Max. coverage (+): 0. Max coverage (-): 0

Region: NODE\_306037\_length\_18339\_cov\_27.068979 12478-12481. Max. coverage (+): 0. Max coverage (-): 0

Region: NODE\_306037\_length\_18339\_cov\_27.068979 12482-12485. Max. coverage (+): 0. Max coverage (-): 0

Region: NODE\_306037\_length\_18339\_cov\_27.068979 12486-12489. Max. coverage (+): 0. Max coverage (-): 0

Region: NODE\_306037\_length\_18339\_cov\_27.068979 12490-12493. Max. coverage (+): 0. Max coverage (-): 0

Region: NODE\_306037\_length\_18339\_cov\_27.068979 12494-12496. Max. coverage (+): 0. Max coverage (-): 0

Region: NODE\_306037\_length\_18339\_cov\_27.068979 12497-12500. Max. coverage (+): 0. Max coverage (-): 0

Region: NODE\_306037\_length\_18339\_cov\_27.068979 12501-12504. Max. coverage (+): 0. Max coverage (-): 0

Region: NODE\_306037\_length\_18339\_cov\_27.068979 12505-12508. Max. coverage (+): 0. Max coverage (-): 0

Region: NODE\_306037\_length\_18339\_cov\_27.068979 12509-12512. Max. coverage (+): 0. Max coverage (-): 0

Region: NODE\_306037\_length\_18339\_cov\_27.068979 12513-12515. Max. coverage (+): 0. Max coverage (-): 0

Region: NODE\_306037\_length\_18339\_cov\_27.068979 12516-12519. Max. coverage (+): 0. Max coverage (-): 0

Region: NODE\_306037\_length\_18339\_cov\_27.068979 12520-12523. Max. coverage (+): 0. Max coverage (-): 0

Region: NODE\_306037\_length\_18339\_cov\_27.068979 12524-12527. Max. coverage (+): 0. Max coverage (-): 0

Region: NODE\_306037\_length\_18339\_cov\_27.068979 12528-12531. Max. coverage (+): 0. Max coverage (-): 0

Region: NODE\_306037\_length\_18339\_cov\_27.068979 12532-12534. Max. coverage (+): 0. Max coverage (-): 0

Region: NODE\_306037\_length\_18339\_cov\_27.068979 12535-12538. Max. coverage (+): 0. Max coverage (-): 0

Region: NODE\_306037\_length\_18339\_cov\_27.068979 12539-12542. Max. coverage (+): 0. Max coverage (-): 0

Region: NODE\_306037\_length\_18339\_cov\_27.068979 12543-12546. Max. coverage (+): 0. Max coverage (-): 0

Region: NODE\_306037\_length\_18339\_cov\_27.068979 12547-12550. Max. coverage (+): 0. Max coverage (-): 0

Region: NODE\_306037\_length\_18339\_cov\_27.068979 12551-12553. Max. coverage (+): 0. Max coverage (-): 0

Region: NODE\_306037\_length\_18339\_cov\_27.068979 12554-12557. Max. coverage (+): 0. Max coverage (-): 0

Region: NODE\_306037\_length\_18339\_cov\_27.068979 12558-12561. Max. coverage (+): 0. Max coverage (-): 0

Region: NODE\_306037\_length\_18339\_cov\_27.068979 12562-12565. Max. coverage (+): 0. Max coverage (-): 0

Region: NODE\_306037\_length\_18339\_cov\_27.068979 12566-12569. Max. coverage (+): 0. Max coverage (-): 0

Region: NODE\_306037\_length\_18339\_cov\_27.068979 12570-12572. Max. coverage (+): 0. Max coverage (-): 0

Region: NODE\_306037\_length\_18339\_cov\_27.068979 12573-12576. Max. coverage (+): 0. Max coverage (-): 0

Region: NODE\_306037\_length\_18339\_cov\_27.068979 12577-12580. Max. coverage (+): 0. Max coverage (-): 0

Region: NODE\_306037\_length\_18339\_cov\_27.068979 12581-12584. Max. coverage (+): 0. Max coverage (-): 0

Region: NODE\_306037\_length\_18339\_cov\_27.068979 12585-12588. Max. coverage (+): 0. Max coverage (-): 0

Region: NODE\_306037\_length\_18339\_cov\_27.068979 12589-12591. Max. coverage (+): 0. Max coverage (-): 0

Region: NODE\_306037\_length\_18339\_cov\_27.068979 12592-12595. Max. coverage (+): 0. Max coverage (-): 0

Region: NODE\_306037\_length\_18339\_cov\_27.068979 12596-12599. Max. coverage (+): 0. Max coverage (-): 0

Region: NODE\_306037\_length\_18339\_cov\_27.068979 12600-12603. Max. coverage (+): 0. Max coverage (-): 0

Region: NODE\_306037\_length\_18339\_cov\_27.068979 12604-12607. Max. coverage (+): 0. Max coverage (-): 0

Region: NODE\_306037\_length\_18339\_cov\_27.068979 12608-12610. Max. coverage (+): 0. Max coverage (-): 0

Region: NODE\_306037\_length\_18339\_cov\_27.068979 12611-12614. Max. coverage (+): 0. Max coverage (-): 0

Region: NODE\_306037\_length\_18339\_cov\_27.068979 12615-12618. Max. coverage (+): 0. Max coverage (-): 0

Region: NODE\_306037\_length\_18339\_cov\_27.068979 12619-12622. Max. coverage (+): 0. Max coverage (-): 0

Region: NODE\_306037\_length\_18339\_cov\_27.068979 12623-12626. Max. coverage (+): 0. Max coverage (-): 0

Region: NODE\_306037\_length\_18339\_cov\_27.068979 12627-12629. Max. coverage (+): 0. Max coverage (-): 0

Region: NODE\_306037\_length\_18339\_cov\_27.068979 12630-12633. Max. coverage (+): 0. Max coverage (-): 0

Region: NODE\_306037\_length\_18339\_cov\_27.068979 12634-12637. Max. coverage (+): 0. Max coverage (-): 0

Region: NODE\_306037\_length\_18339\_cov\_27.068979 12638-12641. Max. coverage (+): 0. Max coverage (-): 0

Region: NODE\_306037\_length\_18339\_cov\_27.068979 12642-12645. Max. coverage (+): 0. Max coverage (-): 0

Region: NODE\_306037\_length\_18339\_cov\_27.068979 12646-12648. Max. coverage (+): 0. Max coverage (-): 0

Region: NODE\_306037\_length\_18339\_cov\_27.068979 12649-12652. Max. coverage (+): 0. Max coverage (-): 0

Region: NODE\_306037\_length\_18339\_cov\_27.068979 12653-12656. Max. coverage (+): 0. Max coverage (-): 0

Region: NODE\_306037\_length\_18339\_cov\_27.068979 12657-12660. Max. coverage (+): 0. Max coverage (-): 0

Region: NODE\_306037\_length\_18339\_cov\_27.068979 12661-12664. Max. coverage (+): 0. Max coverage (-): 0

Region: NODE\_306037\_length\_18339\_cov\_27.068979 12665-12667. Max. coverage (+): 0. Max coverage (-): 0

Region: NODE\_306037\_length\_18339\_cov\_27.068979 12668-12671. Max. coverage (+): 0. Max coverage (-): 0

Region: NODE\_306037\_length\_18339\_cov\_27.068979 12672-12675. Max. coverage (+): 0. Max coverage (-): 0

Region: NODE\_306037\_length\_18339\_cov\_27.068979 12676-12679. Max. coverage (+): 0. Max coverage (-): 0

Region: NODE\_306037\_length\_18339\_cov\_27.068979 12680-12683. Max. coverage (+): 0. Max coverage (-): 0

Region: NODE\_306037\_length\_18339\_cov\_27.068979 12684-12686. Max. coverage (+): 0. Max coverage (-): 0

Region: NODE\_306037\_length\_18339\_cov\_27.068979 12687-12690. Max. coverage (+): 0. Max coverage (-): 0

Region: NODE\_306037\_length\_18339\_cov\_27.068979 12691-12694. Max. coverage (+): 0. Max coverage (-): 0

Region: NODE\_306037\_length\_18339\_cov\_27.068979 12695-12698. Max. coverage (+): 0. Max coverage (-): 0

Region: NODE\_306037\_length\_18339\_cov\_27.068979 12699-12702. Max. coverage (+): 0. Max coverage (-): 0

Region: NODE\_306037\_length\_18339\_cov\_27.068979 12703-12705. Max. coverage (+): 0. Max coverage (-): 0

Region: NODE\_306037\_length\_18339\_cov\_27.068979 12706-12709. Max. coverage (+): 0. Max coverage (-): 0

Region: NODE\_306037\_length\_18339\_cov\_27.068979 12710-12713. Max. coverage (+): 0. Max coverage (-): 0

Region: NODE\_306037\_length\_18339\_cov\_27.068979 12714-12717. Max. coverage (+): 0. Max coverage (-): 0

Region: NODE\_306037\_length\_18339\_cov\_27.068979 12718-12721. Max. coverage (+): 0. Max coverage (-): 0

Region: NODE\_306037\_length\_18339\_cov\_27.068979 12722-12724. Max. coverage (+): 0. Max coverage (-): 0

Region: NODE\_306037\_length\_18339\_cov\_27.068979 12725-12728. Max. coverage (+): 0. Max coverage (-): 0

Region: NODE\_306037\_length\_18339\_cov\_27.068979 12729-12732. Max. coverage (+): 0. Max coverage (-): 0

Region: NODE\_306037\_length\_18339\_cov\_27.068979 12733-12736. Max. coverage (+): 0. Max coverage (-): 0

Region: NODE\_306037\_length\_18339\_cov\_27.068979 12737-12740. Max. coverage (+): 0. Max coverage (-): 0

Region: NODE\_306037\_length\_18339\_cov\_27.068979 12741-12743. Max. coverage (+): 0. Max coverage (-): 0

Region: NODE\_306037\_length\_18339\_cov\_27.068979 12744-12747. Max. coverage (+): 0. Max coverage (-): 0

Region: NODE\_306037\_length\_18339\_cov\_27.068979 12748-12751. Max. coverage (+): 0. Max coverage (-): 0

Region: NODE\_306037\_length\_18339\_cov\_27.068979 12752-12755. Max. coverage (+): 0. Max coverage (-): 0

Region: NODE\_306037\_length\_18339\_cov\_27.068979 12756-12759. Max. coverage (+): 0. Max coverage (-): 0

Region: NODE\_306037\_length\_18339\_cov\_27.068979 12760-12762. Max. coverage (+): 0. Max coverage (-): 0

Region: NODE\_306037\_length\_18339\_cov\_27.068979 12763-12766. Max. coverage (+): 0. Max coverage (-): 0

Region: NODE\_306037\_length\_18339\_cov\_27.068979 12767-12770. Max. coverage (+): 0. Max coverage (-): 0

Region: NODE\_306037\_length\_18339\_cov\_27.068979 12771-12774. Max. coverage (+): 0. Max coverage (-): 0

Region: NODE\_306037\_length\_18339\_cov\_27.068979 12775-12778. Max. coverage (+): 0. Max coverage (-): 0

Region: NODE\_306037\_length\_18339\_cov\_27.068979 12779-12781. Max. coverage (+): 0. Max coverage (-): 0

Region: NODE\_306037\_length\_18339\_cov\_27.068979 12782-12785. Max. coverage (+): 0. Max coverage (-): 0

Region: NODE\_306037\_length\_18339\_cov\_27.068979 12786-12789. Max. coverage (+): 0. Max coverage (-): 0

Region: NODE\_306037\_length\_18339\_cov\_27.068979 12790-12793. Max. coverage (+): 0. Max coverage (-): 0

Region: NODE\_306037\_length\_18339\_cov\_27.068979 12794-12797. Max. coverage (+): 0. Max coverage (-): 0

Region: NODE\_306037\_length\_18339\_cov\_27.068979 12798-12800. Max. coverage (+): 0. Max coverage (-): 0

Region: NODE\_306037\_length\_18339\_cov\_27.068979 12801-12804. Max. coverage (+): 0. Max coverage (-): 0

Region: NODE\_306037\_length\_18339\_cov\_27.068979 12805-12808. Max. coverage (+): 0. Max coverage (-): 0

Region: NODE\_306037\_length\_18339\_cov\_27.068979 12809-12812. Max. coverage (+): 0. Max coverage (-): 0

Region: NODE\_306037\_length\_18339\_cov\_27.068979 12813-12816. Max. coverage (+): 0. Max coverage (-): 0

Region: NODE\_306037\_length\_18339\_cov\_27.068979 12817-12819. Max. coverage (+): 0. Max coverage (-): 0

Region: NODE\_306037\_length\_18339\_cov\_27.068979 12820-12823. Max. coverage (+): 0. Max coverage (-): 0

Region: NODE\_306037\_length\_18339\_cov\_27.068979 12824-12827. Max. coverage (+): 0. Max coverage (-): 0

Region: NODE\_306037\_length\_18339\_cov\_27.068979 12828-12831. Max. coverage (+): 0. Max coverage (-): 0

Region: NODE\_306037\_length\_18339\_cov\_27.068979 12832-12835. Max. coverage (+): 0. Max coverage (-): 0

Region: NODE\_306037\_length\_18339\_cov\_27.068979 12836-12838. Max. coverage (+): 0. Max coverage (-): 0

Region: NODE\_306037\_length\_18339\_cov\_27.068979 12839-12842. Max. coverage (+): 0. Max coverage (-): 0

Region: NODE\_306037\_length\_18339\_cov\_27.068979 12843-12846. Max. coverage (+): 0. Max coverage (-): 0

Region: NODE\_306037\_length\_18339\_cov\_27.068979 12847-12850. Max. coverage (+): 0. Max coverage (-): 0

Region: NODE\_306037\_length\_18339\_cov\_27.068979 12851-12854. Max. coverage (+): 0. Max coverage (-): 0

Region: NODE\_306037\_length\_18339\_cov\_27.068979 12855-12857. Max. coverage (+): 0. Max coverage (-): 0

Region: NODE\_306037\_length\_18339\_cov\_27.068979 12858-12861. Max. coverage (+): 0. Max coverage (-): 0

Region: NODE\_306037\_length\_18339\_cov\_27.068979 12862-12865. Max. coverage (+): 0. Max coverage (-): 0

Region: NODE\_306037\_length\_18339\_cov\_27.068979 12866-12869. Max. coverage (+): 0. Max coverage (-): 0

Region: NODE\_306037\_length\_18339\_cov\_27.068979 12870-12873. Max. coverage (+): 0. Max coverage (-): 0

Region: NODE\_306037\_length\_18339\_cov\_27.068979 12874-12876. Max. coverage (+): 0. Max coverage (-): 0

Region: NODE\_306037\_length\_18339\_cov\_27.068979 12877-12880. Max. coverage (+): 0. Max coverage (-): 0

Region: NODE\_306037\_length\_18339\_cov\_27.068979 12881-12884. Max. coverage (+): 0. Max coverage (-): 0

Region: NODE\_306037\_length\_18339\_cov\_27.068979 12885-12888. Max. coverage (+): 0. Max coverage (-): 0

Region: NODE\_306037\_length\_18339\_cov\_27.068979 12889-12892. Max. coverage (+): 0. Max coverage (-): 0

Region: NODE\_306037\_length\_18339\_cov\_27.068979 12893-12895. Max. coverage (+): 0. Max coverage (-): 0

Region: NODE\_306037\_length\_18339\_cov\_27.068979 12896-12899. Max. coverage (+): 0. Max coverage (-): 0

Region: NODE\_306037\_length\_18339\_cov\_27.068979 12900-12903. Max. coverage (+): 0. Max coverage (-): 0

Region: NODE\_306037\_length\_18339\_cov\_27.068979 12904-12907. Max. coverage (+): 0. Max coverage (-): 0

Region: NODE\_306037\_length\_18339\_cov\_27.068979 12908-12911. Max. coverage (+): 0. Max coverage (-): 0

Region: NODE\_306037\_length\_18339\_cov\_27.068979 12912-12914. Max. coverage (+): 0. Max coverage (-): 0

Region: NODE\_306037\_length\_18339\_cov\_27.068979 12915-12918. Max. coverage (+): 0. Max coverage (-): 0

Region: NODE\_306037\_length\_18339\_cov\_27.068979 12919-12922. Max. coverage (+): 0. Max coverage (-): 0

Region: NODE\_306037\_length\_18339\_cov\_27.068979 12923-12926. Max. coverage (+): 0. Max coverage (-): 0

Region: NODE\_306037\_length\_18339\_cov\_27.068979 12927-12930. Max. coverage (+): 0. Max coverage (-): 0

Region: NODE\_306037\_length\_18339\_cov\_27.068979 12931-12933. Max. coverage (+): 0. Max coverage (-): 0

Region: NODE\_306037\_length\_18339\_cov\_27.068979 12934-12937. Max. coverage (+): 0. Max coverage (-): 0

Region: NODE\_306037\_length\_18339\_cov\_27.068979 12938-12941. Max. coverage (+): 0. Max coverage (-): 0

Region: NODE\_306037\_length\_18339\_cov\_27.068979 12942-12945. Max. coverage (+): 0. Max coverage (-): 0

Region: NODE\_306037\_length\_18339\_cov\_27.068979 12946-12949. Max. coverage (+): 0. Max coverage (-): 0

Region: NODE\_306037\_length\_18339\_cov\_27.068979 12950-12952. Max. coverage (+): 0. Max coverage (-): 0

Region: NODE\_306037\_length\_18339\_cov\_27.068979 12953-12956. Max. coverage (+): 0. Max coverage (-): 0

Region: NODE\_306037\_length\_18339\_cov\_27.068979 12957-12960. Max. coverage (+): 0. Max coverage (-): 0

Region: NODE\_306037\_length\_18339\_cov\_27.068979 12961-12964. Max. coverage (+): 0. Max coverage (-): 0

Region: NODE\_306037\_length\_18339\_cov\_27.068979 12965-12968. Max. coverage (+): 0. Max coverage (-): 0

Region: NODE\_306037\_length\_18339\_cov\_27.068979 12969-12971. Max. coverage (+): 0. Max coverage (-): 0

Region: NODE\_306037\_length\_18339\_cov\_27.068979 12972-12975. Max. coverage (+): 0. Max coverage (-): 0

Region: NODE\_306037\_length\_18339\_cov\_27.068979 12976-12979. Max. coverage (+): 0. Max coverage (-): 0

Region: NODE\_306037\_length\_18339\_cov\_27.068979 12980-12983. Max. coverage (+): 0. Max coverage (-): 0

Region: NODE\_306037\_length\_18339\_cov\_27.068979 12984-12987. Max. coverage (+): 0. Max coverage (-): 0

Region: NODE\_306037\_length\_18339\_cov\_27.068979 12988-12990. Max. coverage (+): 0. Max coverage (-): 0

Region: NODE\_306037\_length\_18339\_cov\_27.068979 12991-12994. Max. coverage (+): 0. Max coverage (-): 0

Region: NODE\_306037\_length\_18339\_cov\_27.068979 12995-12998. Max. coverage (+): 0. Max coverage (-): 0

Region: NODE\_306037\_length\_18339\_cov\_27.068979 12999-13002. Max. coverage (+): 0. Max coverage (-): 0

Region: NODE\_306037\_length\_18339\_cov\_27.068979 13003-13006. Max. coverage (+): 0. Max coverage (-): 0

Region: NODE\_306037\_length\_18339\_cov\_27.068979 13007-13009. Max. coverage (+): 0. Max coverage (-): 0

Region: NODE\_306037\_length\_18339\_cov\_27.068979 13010-13013. Max. coverage (+): 0. Max coverage (-): 0

Region: NODE\_306037\_length\_18339\_cov\_27.068979 13014-13017. Max. coverage (+): 0. Max coverage (-): 0

Region: NODE\_306037\_length\_18339\_cov\_27.068979 13018-13021. Max. coverage (+): 0. Max coverage (-): 0

Region: NODE\_306037\_length\_18339\_cov\_27.068979 13022-13025. Max. coverage (+): 0. Max coverage (-): 0

Region: NODE\_306037\_length\_18339\_cov\_27.068979 13026-13028. Max. coverage (+): 0. Max coverage (-): 0

Region: NODE\_306037\_length\_18339\_cov\_27.068979 13029-13032. Max. coverage (+): 0. Max coverage (-): 0

Region: NODE\_306037\_length\_18339\_cov\_27.068979 13033-13036. Max. coverage (+): 0. Max coverage (-): 0

Region: NODE\_306037\_length\_18339\_cov\_27.068979 13037-13040. Max. coverage (+): 0. Max coverage (-): 0

Region: NODE\_306037\_length\_18339\_cov\_27.068979 13041-13044. Max. coverage (+): 0. Max coverage (-): 0

Region: NODE\_306037\_length\_18339\_cov\_27.068979 13045-13047. Max. coverage (+): 0. Max coverage (-): 0

Region: NODE\_306037\_length\_18339\_cov\_27.068979 13048-13051. Max. coverage (+): 0. Max coverage (-): 0

Region: NODE\_306037\_length\_18339\_cov\_27.068979 13052-13055. Max. coverage (+): 0. Max coverage (-): 0

Region: NODE\_306037\_length\_18339\_cov\_27.068979 13056-13059. Max. coverage (+): 0. Max coverage (-): 0

Region: NODE\_306037\_length\_18339\_cov\_27.068979 13060-13063. Max. coverage (+): 0. Max coverage (-): 0

Region: NODE\_306037\_length\_18339\_cov\_27.068979 13064-13066. Max. coverage (+): 0. Max coverage (-): 0

Region: NODE\_306037\_length\_18339\_cov\_27.068979 13067-13070. Max. coverage (+): 0. Max coverage (-): 0

Region: NODE\_306037\_length\_18339\_cov\_27.068979 13071-13074. Max. coverage (+): 0. Max coverage (-): 0

Region: NODE\_306037\_length\_18339\_cov\_27.068979 13075-13078. Max. coverage (+): 0. Max coverage (-): 0

Region: NODE\_306037\_length\_18339\_cov\_27.068979 13079-13082. Max. coverage (+): 0. Max coverage (-): 0

Region: NODE\_306037\_length\_18339\_cov\_27.068979 13083-13085. Max. coverage (+): 0. Max coverage (-): 0

Region: NODE\_306037\_length\_18339\_cov\_27.068979 13086-13089. Max. coverage (+): 0. Max coverage (-): 0

Region: NODE\_306037\_length\_18339\_cov\_27.068979 13090-13093. Max. coverage (+): 0. Max coverage (-): 0

Region: NODE\_306037\_length\_18339\_cov\_27.068979 13094-13097. Max. coverage (+): 0. Max coverage (-): 0

Region: NODE\_306037\_length\_18339\_cov\_27.068979 13098-13101. Max. coverage (+): 0. Max coverage (-): 0

Region: NODE\_306037\_length\_18339\_cov\_27.068979 13102-13104. Max. coverage (+): 0. Max coverage (-): 0

Region: NODE\_306037\_length\_18339\_cov\_27.068979 13105-13108. Max. coverage (+): 0. Max coverage (-): 0

Region: NODE\_306037\_length\_18339\_cov\_27.068979 13109-13112. Max. coverage (+): 0. Max coverage (-): 0

Region: NODE\_306037\_length\_18339\_cov\_27.068979 13113-13116. Max. coverage (+): 0. Max coverage (-): 0

Region: NODE\_306037\_length\_18339\_cov\_27.068979 13117-13120. Max. coverage (+): 0. Max coverage (-): 0

Region: NODE\_306037\_length\_18339\_cov\_27.068979 13121-13123. Max. coverage (+): 0. Max coverage (-): 0

Region: NODE\_306037\_length\_18339\_cov\_27.068979 13124-13127. Max. coverage (+): 0. Max coverage (-): 0

Region: NODE\_306037\_length\_18339\_cov\_27.068979 13128-13131. Max. coverage (+): 0. Max coverage (-): 0

Region: NODE\_306037\_length\_18339\_cov\_27.068979 13132-13135. Max. coverage (+): 0. Max coverage (-): 0

Region: NODE\_306037\_length\_18339\_cov\_27.068979 13136-13139. Max. coverage (+): 0. Max coverage (-): 0

Region: NODE\_306037\_length\_18339\_cov\_27.068979 13140-13142. Max. coverage (+): 0. Max coverage (-): 0

Region: NODE\_306037\_length\_18339\_cov\_27.068979 13143-13146. Max. coverage (+): 0. Max coverage (-): 0

Region: NODE\_306037\_length\_18339\_cov\_27.068979 13147-13150. Max. coverage (+): 0. Max coverage (-): 0

Region: NODE\_306037\_length\_18339\_cov\_27.068979 13151-13154. Max. coverage (+): 0. Max coverage (-): 0

Region: NODE\_306037\_length\_18339\_cov\_27.068979 13155-13158. Max. coverage (+): 0. Max coverage (-): 0

Region: NODE\_306037\_length\_18339\_cov\_27.068979 13159-13161. Max. coverage (+): 0. Max coverage (-): 0

Region: NODE\_306037\_length\_18339\_cov\_27.068979 13162-13165. Max. coverage (+): 0. Max coverage (-): 0

Region: NODE\_306037\_length\_18339\_cov\_27.068979 13166-13169. Max. coverage (+): 0. Max coverage (-): 0

Region: NODE\_306037\_length\_18339\_cov\_27.068979 13170-13173. Max. coverage (+): 0. Max coverage (-): 0

Region: NODE\_306037\_length\_18339\_cov\_27.068979 13174-13177. Max. coverage (+): 0. Max coverage (-): 0

Region: NODE\_306037\_length\_18339\_cov\_27.068979 13178-13180. Max. coverage (+): 0. Max coverage (-): 0

Region: NODE\_306037\_length\_18339\_cov\_27.068979 13181-13184. Max. coverage (+): 0. Max coverage (-): 0

Region: NODE\_306037\_length\_18339\_cov\_27.068979 13185-13188. Max. coverage (+): 0. Max coverage (-): 0

Region: NODE\_306037\_length\_18339\_cov\_27.068979 13189-13192. Max. coverage (+): 0. Max coverage (-): 0

Region: NODE\_306037\_length\_18339\_cov\_27.068979 13193-13196. Max. coverage (+): 0. Max coverage (-): 0

Region: NODE\_306037\_length\_18339\_cov\_27.068979 13197-13199. Max. coverage (+): 0. Max coverage (-): 0

Region: NODE\_306037\_length\_18339\_cov\_27.068979 13200-13203. Max. coverage (+): 0. Max coverage (-): 0

Region: NODE\_306037\_length\_18339\_cov\_27.068979 13204-13207. Max. coverage (+): 0. Max coverage (-): 0

Region: NODE\_306037\_length\_18339\_cov\_27.068979 13208-13211. Max. coverage (+): 0. Max coverage (-): 0

Region: NODE\_306037\_length\_18339\_cov\_27.068979 13212-13215. Max. coverage (+): 0. Max coverage (-): 0

Region: NODE\_306037\_length\_18339\_cov\_27.068979 13216-13218. Max. coverage (+): 0. Max coverage (-): 0

Region: NODE\_306037\_length\_18339\_cov\_27.068979 13219-13222. Max. coverage (+): 0. Max coverage (-): 0

Region: NODE\_306037\_length\_18339\_cov\_27.068979 13223-13226. Max. coverage (+): 0. Max coverage (-): 0

Region: NODE\_306037\_length\_18339\_cov\_27.068979 13227-13230. Max. coverage (+): 0. Max coverage (-): 0

Region: NODE\_306037\_length\_18339\_cov\_27.068979 13231-13234. Max. coverage (+): 0. Max coverage (-): 0

Region: NODE\_306037\_length\_18339\_cov\_27.068979 13235-13237. Max. coverage (+): 0. Max coverage (-): 0

Region: NODE\_306037\_length\_18339\_cov\_27.068979 13238-13241. Max. coverage (+): 0. Max coverage (-): 0

Region: NODE\_306037\_length\_18339\_cov\_27.068979 13242-13245. Max. coverage (+): 0. Max coverage (-): 0

Region: NODE\_306037\_length\_18339\_cov\_27.068979 13246-13249. Max. coverage (+): 0. Max coverage (-): 0

Region: NODE\_306037\_length\_18339\_cov\_27.068979 13250-13253. Max. coverage (+): 0. Max coverage (-): 0

Region: NODE\_306037\_length\_18339\_cov\_27.068979 13254-13256. Max. coverage (+): 0. Max coverage (-): 0

Region: NODE\_306037\_length\_18339\_cov\_27.068979 13257-13260. Max. coverage (+): 0. Max coverage (-): 0

Region: NODE\_306037\_length\_18339\_cov\_27.068979 13261-13264. Max. coverage (+): 0. Max coverage (-): 0

Region: NODE\_306037\_length\_18339\_cov\_27.068979 13265-13268. Max. coverage (+): 0. Max coverage (-): 0

Region: NODE\_306037\_length\_18339\_cov\_27.068979 13269-13272. Max. coverage (+): 0. Max coverage (-): 0

Region: NODE\_306037\_length\_18339\_cov\_27.068979 13273-13275. Max. coverage (+): 0. Max coverage (-): 0

Region: NODE\_306037\_length\_18339\_cov\_27.068979 13276-13279. Max. coverage (+): 0. Max coverage (-): 0

Region: NODE\_306037\_length\_18339\_cov\_27.068979 13280-13283. Max. coverage (+): 0. Max coverage (-): 0

Region: NODE\_306037\_length\_18339\_cov\_27.068979 13284-13287. Max. coverage (+): 0. Max coverage (-): 0

Region: NODE\_306037\_length\_18339\_cov\_27.068979 13288-13291. Max. coverage (+): 0. Max coverage (-): 0

Region: NODE\_306037\_length\_18339\_cov\_27.068979 13292-13294. Max. coverage (+): 0. Max coverage (-): 0

Region: NODE\_306037\_length\_18339\_cov\_27.068979 13295-13298. Max. coverage (+): 0. Max coverage (-): 0

Region: NODE\_306037\_length\_18339\_cov\_27.068979 13299-13302. Max. coverage (+): 0. Max coverage (-): 0

Region: NODE\_306037\_length\_18339\_cov\_27.068979 13303-13306. Max. coverage (+): 0. Max coverage (-): 0

Region: NODE\_306037\_length\_18339\_cov\_27.068979 13307-13310. Max. coverage (+): 0. Max coverage (-): 0

Region: NODE\_306037\_length\_18339\_cov\_27.068979 13311-13313. Max. coverage (+): 0. Max coverage (-): 0

Region: NODE\_306037\_length\_18339\_cov\_27.068979 13314-13317. Max. coverage (+): 0. Max coverage (-): 0

Region: NODE\_306037\_length\_18339\_cov\_27.068979 13318-13321. Max. coverage (+): 0. Max coverage (-): 0

Region: NODE\_306037\_length\_18339\_cov\_27.068979 13322-13325. Max. coverage (+): 0. Max coverage (-): 0

Region: NODE\_306037\_length\_18339\_cov\_27.068979 13326-13329. Max. coverage (+): 0. Max coverage (-): 0

Region: NODE\_306037\_length\_18339\_cov\_27.068979 13330-13332. Max. coverage (+): 0. Max coverage (-): 0

Region: NODE\_306037\_length\_18339\_cov\_27.068979 13333-13336. Max. coverage (+): 0. Max coverage (-): 0

Region: NODE\_306037\_length\_18339\_cov\_27.068979 13337-13340. Max. coverage (+): 0. Max coverage (-): 0

Region: NODE\_306037\_length\_18339\_cov\_27.068979 13341-13344. Max. coverage (+): 0. Max coverage (-): 0

Region: NODE\_306037\_length\_18339\_cov\_27.068979 13345-13348. Max. coverage (+): 0. Max coverage (-): 0

Region: NODE\_306037\_length\_18339\_cov\_27.068979 13349-13351. Max. coverage (+): 0. Max coverage (-): 0

Region: NODE\_306037\_length\_18339\_cov\_27.068979 13352-13355. Max. coverage (+): 0. Max coverage (-): 0

Region: NODE\_306037\_length\_18339\_cov\_27.068979 13356-13359. Max. coverage (+): 0. Max coverage (-): 0

Region: NODE\_306037\_length\_18339\_cov\_27.068979 13360-13363. Max. coverage (+): 0. Max coverage (-): 0

Region: NODE\_306037\_length\_18339\_cov\_27.068979 13364-13367. Max. coverage (+): 0. Max coverage (-): 0

Region: NODE\_306037\_length\_18339\_cov\_27.068979 13368-13370. Max. coverage (+): 0. Max coverage (-): 0

Region: NODE\_306037\_length\_18339\_cov\_27.068979 13371-13374. Max. coverage (+): 0. Max coverage (-): 0

Region: NODE\_306037\_length\_18339\_cov\_27.068979 13375-13378. Max. coverage (+): 0. Max coverage (-): 0

Region: NODE\_306037\_length\_18339\_cov\_27.068979 13379-13382. Max. coverage (+): 0. Max coverage (-): 0

Region: NODE\_306037\_length\_18339\_cov\_27.068979 13383-13386. Max. coverage (+): 0. Max coverage (-): 0

Region: NODE\_306037\_length\_18339\_cov\_27.068979 13387-13389. Max. coverage (+): 0. Max coverage (-): 0

Region: NODE\_306037\_length\_18339\_cov\_27.068979 13390-13393. Max. coverage (+): 0. Max coverage (-): 0

Region: NODE\_306037\_length\_18339\_cov\_27.068979 13394-13397. Max. coverage (+): 0. Max coverage (-): 0

Region: NODE\_306037\_length\_18339\_cov\_27.068979 13398-13401. Max. coverage (+): 0. Max coverage (-): 0

Region: NODE\_306037\_length\_18339\_cov\_27.068979 13402-13405. Max. coverage (+): 0. Max coverage (-): 0

Region: NODE\_306037\_length\_18339\_cov\_27.068979 13406-13408. Max. coverage (+): 0. Max coverage (-): 0

Region: NODE\_306037\_length\_18339\_cov\_27.068979 13409-13412. Max. coverage (+): 0. Max coverage (-): 0

Region: NODE\_306037\_length\_18339\_cov\_27.068979 13413-13416. Max. coverage (+): 0. Max coverage (-): 0

Region: NODE\_306037\_length\_18339\_cov\_27.068979 13417-13420. Max. coverage (+): 0. Max coverage (-): 0

Region: NODE\_306037\_length\_18339\_cov\_27.068979 13421-13424. Max. coverage (+): 0. Max coverage (-): 0

Region: NODE\_306037\_length\_18339\_cov\_27.068979 13425-13427. Max. coverage (+): 0. Max coverage (-): 0

Region: NODE\_306037\_length\_18339\_cov\_27.068979 13428-13431. Max. coverage (+): 0. Max coverage (-): 0

Region: NODE\_306037\_length\_18339\_cov\_27.068979 13432-13435. Max. coverage (+): 0. Max coverage (-): 0

Region: NODE\_306037\_length\_18339\_cov\_27.068979 13436-13439. Max. coverage (+): 0. Max coverage (-): 0

Region: NODE\_306037\_length\_18339\_cov\_27.068979 13440-13443. Max. coverage (+): 0. Max coverage (-): 0

Region: NODE\_306037\_length\_18339\_cov\_27.068979 13444-13446. Max. coverage (+): 0. Max coverage (-): 0

Region: NODE\_306037\_length\_18339\_cov\_27.068979 13447-13450. Max. coverage (+): 0. Max coverage (-): 0

Region: NODE\_306037\_length\_18339\_cov\_27.068979 13451-13454. Max. coverage (+): 0. Max coverage (-): 0

Region: NODE\_306037\_length\_18339\_cov\_27.068979 13455-13458. Max. coverage (+): 0. Max coverage (-): 0

Region: NODE\_306037\_length\_18339\_cov\_27.068979 13459-13462. Max. coverage (+): 0. Max coverage (-): 0

Region: NODE\_306037\_length\_18339\_cov\_27.068979 13463-13465. Max. coverage (+): 0. Max coverage (-): 0

Region: NODE\_306037\_length\_18339\_cov\_27.068979 13466-13469. Max. coverage (+): 0. Max coverage (-): 0

Region: NODE\_306037\_length\_18339\_cov\_27.068979 13470-13473. Max. coverage (+): 0. Max coverage (-): 0

Region: NODE\_306037\_length\_18339\_cov\_27.068979 13474-13477. Max. coverage (+): 0. Max coverage (-): 0

Region: NODE\_306037\_length\_18339\_cov\_27.068979 13478-13481. Max. coverage (+): 0. Max coverage (-): 0

Region: NODE\_306037\_length\_18339\_cov\_27.068979 13482-13484. Max. coverage (+): 0. Max coverage (-): 0

Region: NODE\_306037\_length\_18339\_cov\_27.068979 13485-13488. Max. coverage (+): 0. Max coverage (-): 0

Region: NODE\_306037\_length\_18339\_cov\_27.068979 13489-13492. Max. coverage (+): 0. Max coverage (-): 0

Region: NODE\_306037\_length\_18339\_cov\_27.068979 13493-13496. Max. coverage (+): 0. Max coverage (-): 0

Region: NODE\_306037\_length\_18339\_cov\_27.068979 13497-13500. Max. coverage (+): 0. Max coverage (-): 0

Region: NODE\_306037\_length\_18339\_cov\_27.068979 13501-13503. Max. coverage (+): 0. Max coverage (-): 0

Region: NODE\_306037\_length\_18339\_cov\_27.068979 13504-13507. Max. coverage (+): 0. Max coverage (-): 0

Region: NODE\_306037\_length\_18339\_cov\_27.068979 13508-13511. Max. coverage (+): 0. Max coverage (-): 0

Region: NODE\_306037\_length\_18339\_cov\_27.068979 13512-13515. Max. coverage (+): 0. Max coverage (-): 0

Region: NODE\_306037\_length\_18339\_cov\_27.068979 13516-13519. Max. coverage (+): 0. Max coverage (-): 0

Region: NODE\_306037\_length\_18339\_cov\_27.068979 13520-13522. Max. coverage (+): 0. Max coverage (-): 0

Region: NODE\_306037\_length\_18339\_cov\_27.068979 13523-13526. Max. coverage (+): 0. Max coverage (-): 0

Region: NODE\_306037\_length\_18339\_cov\_27.068979 13527-13530. Max. coverage (+): 0. Max coverage (-): 0

Region: NODE\_306037\_length\_18339\_cov\_27.068979 13531-13534. Max. coverage (+): 0. Max coverage (-): 0

Region: NODE\_306037\_length\_18339\_cov\_27.068979 13535-13538. Max. coverage (+): 0. Max coverage (-): 0

Region: NODE\_306037\_length\_18339\_cov\_27.068979 13539-13541. Max. coverage (+): 0. Max coverage (-): 0

Region: NODE\_306037\_length\_18339\_cov\_27.068979 13542-13545. Max. coverage (+): 0. Max coverage (-): 0

Region: NODE\_306037\_length\_18339\_cov\_27.068979 13546-13549. Max. coverage (+): 0. Max coverage (-): 0

Region: NODE\_306037\_length\_18339\_cov\_27.068979 13550-13553. Max. coverage (+): 0. Max coverage (-): 0

Region: NODE\_306037\_length\_18339\_cov\_27.068979 13554-13557. Max. coverage (+): 0. Max coverage (-): 0

Region: NODE\_306037\_length\_18339\_cov\_27.068979 13558-13560. Max. coverage (+): 0. Max coverage (-): 0

Region: NODE\_306037\_length\_18339\_cov\_27.068979 13561-13564. Max. coverage (+): 0. Max coverage (-): 0

Region: NODE\_306037\_length\_18339\_cov\_27.068979 13565-13568. Max. coverage (+): 0. Max coverage (-): 0

Region: NODE\_306037\_length\_18339\_cov\_27.068979 13569-13572. Max. coverage (+): 0. Max coverage (-): 0

Region: NODE\_306037\_length\_18339\_cov\_27.068979 13573-13576. Max. coverage (+): 0. Max coverage (-): 0

Region: NODE\_306037\_length\_18339\_cov\_27.068979 13577-13579. Max. coverage (+): 0. Max coverage (-): 0

Region: NODE\_306037\_length\_18339\_cov\_27.068979 13580-13583. Max. coverage (+): 0. Max coverage (-): 0

Region: NODE\_306037\_length\_18339\_cov\_27.068979 13584-13587. Max. coverage (+): 0. Max coverage (-): 0

Region: NODE\_306037\_length\_18339\_cov\_27.068979 13588-13591. Max. coverage (+): 0. Max coverage (-): 0

Region: NODE\_306037\_length\_18339\_cov\_27.068979 13592-13595. Max. coverage (+): 0. Max coverage (-): 0

Region: NODE\_306037\_length\_18339\_cov\_27.068979 13596-13598. Max. coverage (+): 0. Max coverage (-): 0

Region: NODE\_306037\_length\_18339\_cov\_27.068979 13599-13602. Max. coverage (+): 0. Max coverage (-): 0

Region: NODE\_306037\_length\_18339\_cov\_27.068979 13603-13606. Max. coverage (+): 0. Max coverage (-): 0

Region: NODE\_306037\_length\_18339\_cov\_27.068979 13607-13610. Max. coverage (+): 0. Max coverage (-): 0

Region: NODE\_306037\_length\_18339\_cov\_27.068979 13611-13614. Max. coverage (+): 0. Max coverage (-): 0

Region: NODE\_306037\_length\_18339\_cov\_27.068979 13615-13617. Max. coverage (+): 0. Max coverage (-): 0

Region: NODE\_306037\_length\_18339\_cov\_27.068979 13618-13621. Max. coverage (+): 0. Max coverage (-): 0

Region: NODE\_306037\_length\_18339\_cov\_27.068979 13622-13625. Max. coverage (+): 0. Max coverage (-): 0

Region: NODE\_306037\_length\_18339\_cov\_27.068979 13626-13629. Max. coverage (+): 0. Max coverage (-): 0

Region: NODE\_306037\_length\_18339\_cov\_27.068979 13630-13633. Max. coverage (+): 0. Max coverage (-): 0

Region: NODE\_306037\_length\_18339\_cov\_27.068979 13634-13636. Max. coverage (+): 0. Max coverage (-): 0

Region: NODE\_306037\_length\_18339\_cov\_27.068979 13637-13640. Max. coverage (+): 0. Max coverage (-): 0

Region: NODE\_306037\_length\_18339\_cov\_27.068979 13641-13644. Max. coverage (+): 0. Max coverage (-): 0

Region: NODE\_306037\_length\_18339\_cov\_27.068979 13645-13648. Max. coverage (+): 0. Max coverage (-): 0

Region: NODE\_306037\_length\_18339\_cov\_27.068979 13649-13652. Max. coverage (+): 0. Max coverage (-): 0

Region: NODE\_306037\_length\_18339\_cov\_27.068979 13653-13655. Max. coverage (+): 0. Max coverage (-): 0

Region: NODE\_306037\_length\_18339\_cov\_27.068979 13656-13659. Max. coverage (+): 0. Max coverage (-): 0

Region: NODE\_306037\_length\_18339\_cov\_27.068979 13660-13663. Max. coverage (+): 0. Max coverage (-): 0

Region: NODE\_306037\_length\_18339\_cov\_27.068979 13664-13667. Max. coverage (+): 0. Max coverage (-): 0

Region: NODE\_306037\_length\_18339\_cov\_27.068979 13668-13671. Max. coverage (+): 0. Max coverage (-): 0

Region: NODE\_306037\_length\_18339\_cov\_27.068979 13672-13674. Max. coverage (+): 0. Max coverage (-): 0

Region: NODE\_306037\_length\_18339\_cov\_27.068979 13675-13678. Max. coverage (+): 0. Max coverage (-): 0

Region: NODE\_306037\_length\_18339\_cov\_27.068979 13679-13682. Max. coverage (+): 0. Max coverage (-): 0

Region: NODE\_306037\_length\_18339\_cov\_27.068979 13683-13686. Max. coverage (+): 0. Max coverage (-): 0

Region: NODE\_306037\_length\_18339\_cov\_27.068979 13687-13690. Max. coverage (+): 0. Max coverage (-): 0

Region: NODE\_306037\_length\_18339\_cov\_27.068979 13691-13693. Max. coverage (+): 0. Max coverage (-): 0

Region: NODE\_306037\_length\_18339\_cov\_27.068979 13694-13697. Max. coverage (+): 0. Max coverage (-): 0

Region: NODE\_306037\_length\_18339\_cov\_27.068979 13698-13701. Max. coverage (+): 0. Max coverage (-): 0

Region: NODE\_306037\_length\_18339\_cov\_27.068979 13702-13705. Max. coverage (+): 0. Max coverage (-): 0

Region: NODE\_306037\_length\_18339\_cov\_27.068979 13706-13709. Max. coverage (+): 0. Max coverage (-): 0

Region: NODE\_306037\_length\_18339\_cov\_27.068979 13710-13712. Max. coverage (+): 0. Max coverage (-): 0

Region: NODE\_306037\_length\_18339\_cov\_27.068979 13713-13716. Max. coverage (+): 0. Max coverage (-): 0

Region: NODE\_306037\_length\_18339\_cov\_27.068979 13717-13720. Max. coverage (+): 0. Max coverage (-): 0

Region: NODE\_306037\_length\_18339\_cov\_27.068979 13721-13724. Max. coverage (+): 0. Max coverage (-): 0

Region: NODE\_306037\_length\_18339\_cov\_27.068979 13725-13728. Max. coverage (+): 0. Max coverage (-): 0

Region: NODE\_306037\_length\_18339\_cov\_27.068979 13729-13731. Max. coverage (+): 0. Max coverage (-): 0

Region: NODE\_306037\_length\_18339\_cov\_27.068979 13732-13735. Max. coverage (+): 0. Max coverage (-): 0

Region: NODE\_306037\_length\_18339\_cov\_27.068979 13736-13739. Max. coverage (+): 0. Max coverage (-): 0

Region: NODE\_306037\_length\_18339\_cov\_27.068979 13740-13743. Max. coverage (+): 0. Max coverage (-): 0

Region: NODE\_306037\_length\_18339\_cov\_27.068979 13744-13747. Max. coverage (+): 0. Max coverage (-): 0

Region: NODE\_306037\_length\_18339\_cov\_27.068979 13748-13750. Max. coverage (+): 0. Max coverage (-): 0

Region: NODE\_306037\_length\_18339\_cov\_27.068979 13751-13754. Max. coverage (+): 0. Max coverage (-): 0

Region: NODE\_306037\_length\_18339\_cov\_27.068979 13755-13758. Max. coverage (+): 0. Max coverage (-): 0

Region: NODE\_306037\_length\_18339\_cov\_27.068979 13759-13762. Max. coverage (+): 0. Max coverage (-): 0

Region: NODE\_306037\_length\_18339\_cov\_27.068979 13763-13766. Max. coverage (+): 0. Max coverage (-): 0

Region: NODE\_306037\_length\_18339\_cov\_27.068979 13767-13769. Max. coverage (+): 0. Max coverage (-): 0

Region: NODE\_306037\_length\_18339\_cov\_27.068979 13770-13773. Max. coverage (+): 0. Max coverage (-): 0

Region: NODE\_306037\_length\_18339\_cov\_27.068979 13774-13777. Max. coverage (+): 0. Max coverage (-): 0

Region: NODE\_306037\_length\_18339\_cov\_27.068979 13778-13781. Max. coverage (+): 0. Max coverage (-): 0

Region: NODE\_306037\_length\_18339\_cov\_27.068979 13782-13785. Max. coverage (+): 0.19. Max coverage (-): 0

Region: NODE\_306037\_length\_18339\_cov\_27.068979 13786-13788. Max. coverage (+): 0.19. Max coverage (-): 0

Region: NODE\_306037\_length\_18339\_cov\_27.068979 13789-13792. Max. coverage (+): 0.09. Max coverage (-): 0

Region: NODE\_306037\_length\_18339\_cov\_27.068979 13793-13796. Max. coverage (+): 0. Max coverage (-): 0

Region: NODE\_306037\_length\_18339\_cov\_27.068979 13797-13800. Max. coverage (+): 0. Max coverage (-): 0

Region: NODE\_306037\_length\_18339\_cov\_27.068979 13801-13804. Max. coverage (+): 0. Max coverage (-): 0

Region: NODE\_306037\_length\_18339\_cov\_27.068979 13805-13807. Max. coverage (+): 0. Max coverage (-): 0

Region: NODE\_306037\_length\_18339\_cov\_27.068979 13808-13811. Max. coverage (+): 0. Max coverage (-): 0

Region: NODE\_306037\_length\_18339\_cov\_27.068979 13812-13815. Max. coverage (+): 0. Max coverage (-): 0

Region: NODE\_306037\_length\_18339\_cov\_27.068979 13816-13819. Max. coverage (+): 0. Max coverage (-): 0

Region: NODE\_306037\_length\_18339\_cov\_27.068979 13820-13823. Max. coverage (+): 0. Max coverage (-): 0

Region: NODE\_306037\_length\_18339\_cov\_27.068979 13824-13826. Max. coverage (+): 0. Max coverage (-): 0

Region: NODE\_306037\_length\_18339\_cov\_27.068979 13827-13830. Max. coverage (+): 0. Max coverage (-): 0

Region: NODE\_306037\_length\_18339\_cov\_27.068979 13831-13834. Max. coverage (+): 0. Max coverage (-): 0

Region: NODE\_306037\_length\_18339\_cov\_27.068979 13835-13838. Max. coverage (+): 0. Max coverage (-): 0

Region: NODE\_306037\_length\_18339\_cov\_27.068979 13839-13842. Max. coverage (+): 0. Max coverage (-): 0

Region: NODE\_306037\_length\_18339\_cov\_27.068979 13843-13845. Max. coverage (+): 0. Max coverage (-): 0

Region: NODE\_306037\_length\_18339\_cov\_27.068979 13846-13849. Max. coverage (+): 0. Max coverage (-): 0

Region: NODE\_306037\_length\_18339\_cov\_27.068979 13850-13853. Max. coverage (+): 0. Max coverage (-): 0

Region: NODE\_306037\_length\_18339\_cov\_27.068979 13854-13857. Max. coverage (+): 0. Max coverage (-): 0

Region: NODE\_306037\_length\_18339\_cov\_27.068979 13858-13861. Max. coverage (+): 0. Max coverage (-): 0

Region: NODE\_306037\_length\_18339\_cov\_27.068979 13862-13864. Max. coverage (+): 0. Max coverage (-): 0

Region: NODE\_306037\_length\_18339\_cov\_27.068979 13865-13868. Max. coverage (+): 283.5. Max coverage (-): 0

Region: NODE\_306037\_length\_18339\_cov\_27.068979 13869-13872. Max. coverage (+): 286.7. Max coverage (-): 0

Region: NODE\_306037\_length\_18339\_cov\_27.068979 13873-13876. Max. coverage (+): 3.29. Max coverage (-): 0

Region: NODE\_306037\_length\_18339\_cov\_27.068979 13877-13880. Max. coverage (+): 0. Max coverage (-): 0

Region: NODE\_306037\_length\_18339\_cov\_27.068979 13881-13883. Max. coverage (+): 0. Max coverage (-): 0

Region: NODE\_306037\_length\_18339\_cov\_27.068979 13884-13887. Max. coverage (+): 0.09. Max coverage (-): 0

Region: NODE\_306037\_length\_18339\_cov\_27.068979 13888-13891. Max. coverage (+): 0.09. Max coverage (-): 0

Region: NODE\_306037\_length\_18339\_cov\_27.068979 13892-13895. Max. coverage (+): 0. Max coverage (-): 0

Region: NODE\_306037\_length\_18339\_cov\_27.068979 13896-13899. Max. coverage (+): 0. Max coverage (-): 0

Region: NODE\_306037\_length\_18339\_cov\_27.068979 13900-13902. Max. coverage (+): 6.39. Max coverage (-): 0

Region: NODE\_306037\_length\_18339\_cov\_27.068979 13903-13906. Max. coverage (+): 70.01. Max coverage (-): 0

Region: NODE\_306037\_length\_18339\_cov\_27.068979 13907-13910. Max. coverage (+): 64.93. Max coverage (-): 0

Region: NODE\_306037\_length\_18339\_cov\_27.068979 13911-13914. Max. coverage (+): 4.7. Max coverage (-): 0

Region: NODE\_306037\_length\_18339\_cov\_27.068979 13915-13918. Max. coverage (+): 3.85. Max coverage (-): 0

Region: NODE\_306037\_length\_18339\_cov\_27.068979 13919-13921. Max. coverage (+): 0. Max coverage (-): 0

Region: NODE\_306037\_length\_18339\_cov\_27.068979 13922-13925. Max. coverage (+): 0. Max coverage (-): 0

Region: NODE\_306037\_length\_18339\_cov\_27.068979 13926-13929. Max. coverage (+): 0. Max coverage (-): 0

Region: NODE\_306037\_length\_18339\_cov\_27.068979 13930-13933. Max. coverage (+): 0. Max coverage (-): 0

Region: NODE\_306037\_length\_18339\_cov\_27.068979 13934-13937. Max. coverage (+): 0. Max coverage (-): 0

Region: NODE\_306037\_length\_18339\_cov\_27.068979 13938-. Max. coverage (+): 0. Max coverage (-): 0

RepeatMasker Color Code

**+**

100-98% Identity

<98-95% Identity

<95-90% Identity

<90-85% Identity

<85-80% Identity

<80-75% Identity

<75-70% Identity

<70% Identity

**-**

Gene Set Color Code

**+**

Gene

Pseudogene

Other

**-**

Topology/Coverage Color Code

Coverage Plus Strand

Coverage Minus Strand

Mainstrand: Plus

Mainstrand: Minus

Complementary Strand

Flanking Region  
(if option -flank >0)

Gene Set Annotation  

**1. unknown (unknownunknown) Tr:unknown**: 13414-13825 (+)  
**2. unknown (unknownunknown) Tr:unknown UTR**: 13414-13785 (+)

  
RepeatMasker Annotation  

**1. DNA-TA-5\_DR**: 11898-12076 (-), Divergence to consensus: 9.6%  
**2. AlRepD-337**: 12077-12157 (+), Divergence to consensus: 13.6%  
**3. Mariner-N14\_DR**: 12174-12438 (-), Divergence to consensus: 33.2%  
**4. DNA-8-18\_DR**: 12454-12633 (-), Divergence to consensus: 37.4%  
**5. AlRepC-132**: 12628-12739 (+), Divergence to consensus: 21.5%  
**6. AlRepC-1282**: 13081-13172 (+), Divergence to consensus: 23.9%

  
Transcription Factor Binding Sites  

**RHOXF1** (Sequence: AGATCA (-): 12050)  
**RHOXF1** (Sequence: AGCTTA (-): 12730)  
**RHOXF1** (Sequence: AGCTCA (-): 12775)  
**RHOXF1** (Sequence: TAATCT (+): 12158)  
**RHOXF1** (Sequence: TGAGCT (+): 12420)  
**RHOXF1** (Sequence: TGATCC (+): 12584)  
**RHOXF1** (Sequence: TGAGCT (+): 13930)  
**RFX4\_2** (Sequence: GTAACCACG (-): 13201)  
**FOXO1** (Sequence: CCTGTTTTC (+): 12059)  
**FOXO1** (Sequence: GTTGTTTAT (+): 12275)  
**FOXO3\_mmu** (Sequence: TGTTTTCA (-): 12061)  
**Sox5** (Sequence: ATTGTT (+): 12272)  
**FOXO3\_mmu** (Sequence: TGAAAACA (+): 13907)  
**Nobox** (Sequence: GGCAATTA (-): 12978)  
**FOXP1** (Sequence: TGTTTAC (-): 13180)  
**POU2F1** (Sequence: ATTAAAATA (-): 12916)  
**POU2F1** (Sequence: TATGTAAAT (+): 12319)  
**POU5F1** (Sequence: ATGCAAA (+): 13010)
