# Supplementary material for: First characterization of PIWI-interacting RNA clusters in a cichlid fish with a B chromosome
Source: BMC Biol. 2022 Sep 21;20:204. doi: 10.1186/s12915-022-01403-2 (PMC9490952; doi:10.1186/s12915-022-01403-2)
Supplement: Supplementary file 1 — Additional file 1. Zipped folder with fasta and interactive html piRNA cluster information for the A. latifasciata genome. The nomenclature is as follows: number-pirna-cluster_sex_B-presence (f, female; m, male; 0b, without B chromosome; 1b, with B chromosome). [file 12915_2022_1403_MOESM1_ESM.zip › 121_f0b.html]

piRNA cluster 121\_f0b 62


Predicted piRNA cluster no. 121\_f0b
  

Show proTRAC run info
Hide proTRAC run info

/\  
                \_\_\_\_\_\_\_\_\_\_\_\_\_\_\_\_\_\_\_\_\_\_\_/\\_\_\_ /  \\_\_\_\_\_\_\_  
               I                      /  \  /    \      I  
               I     pro             /    \/      \     I  
               I        TRAC        /               \   I  
               I   \_\_\_\_\_\_\_\_\_\_\_\_\_\_\_\_/\_\_\_\_\_\_\_\_\_\_\_\_\_\_\_\_\_\\_ I  
               I   \              /                     I  
               I    \            /                      I  
               I     \  /\      /       V.2.4.2         I  
               I      \/  \    /                        I  
               I\_\_\_\_\_\_\_\_\_\_\_\  /\_\_\_\_\_\_\_\_\_\_\_\_\_\_\_\_\_\_\_\_\_\_\_\_\_I  
                            \/  
  
  
================================= proTRAC ====================================  
VERSION: .......... 2.4.2  
LAST MODIFIED: .... 11. May 2018  
  
Please cite:  
Rosenkranz D, Zischler H. proTRAC - a software for probabilistic piRNA cluster  
detection, visualization and analysis. 2012. BMC Bioinformatics 13:5.  
  
  
Contact:  
David Rosenkranz  
Institute of Organismic and Molecular Evolutionary Biology  
Dept. Anthropology, small RNA group  
Johannes Gutenberg University Mainz  
email: rosenkranz@uni-mainz.de  
  
You can find the latest proTRAC version at:  
http://sourceforge.net/projects/protrac/files  
http://www.smallRNAgroup-mainz.de/software  
==============================================================================  
  
PARAMETERS:  
Map file: ...............piwi-femeas-0B.fa-collapse.map  
Genome file: ............../../../0B\_ala\_genome.fa  
RepeatMasker annotation: Alatifasciata-all0B-maryan-v2.fa\_corrected.out  
GeneSet:................./guest-storage/Data/annotation/Alatifasciata\_all0B\_maryan-v2\_out2017.gff  
  
Significant (p<=0.01) hit density will be calculated based  
on observed hit distribution.  
  
Sliding window size: ........................................ 5000 bp  
Sliding window increament: .................................. 1000 bp  
Normalize each hit by number of genomic hits: ............... yes  
Normalize each hit by number of sequence reads: ............. yes  
Normalize values (-> per million mapped reads): ............. yes  
Min. fraction of hits with 1T(U) or 10A: .................... 0.75  
Alternatively: Min. fraction of hits with 1T(U) and 10A: .... 0.5  
Min. fraction of hits with typical piRNA length: ............ 0.75  
Typical piRNA length: ....................................... 24-32 nt  
Min. size of a piRNA cluster: ............................... 1000 bp.  
Min. number of hits (absolute): ............................. 0  
Min. number of hits (normalized): ........................... 0  
Min. fraction of hits on the mainstrand: .................... 0.75  
Top fraction of mapped sequences (in terms of read counts): . 1%  
Top fraction accounts for max. n% of sequence reads: ........ 90%  
Min. fraction of hits on each arm of a bidirectional cluster: 0.05  
Output html file for each cluster: .......................... yes  
Output a summary table: ..................................... yes  
Output a FASTA file for each cluster (piRNA sequences): ..... yes  
Output a FASTA file comprising cluster sequences: ........... yes  
Output a GTF file for predicted piRNA clusters: ..............yes  
Search DNA motifs in clusters: .............................. yes  
Output flanking sequences: +/- .............................. 0 bp  
Output ~.pTi file: .......................................... no  
==============================================================================  
  
  
Genome size (without gaps): ............ 758543724 bp  
Gaps (N/X/-): .......................... 417479 bp  
Mapped reads: .......................... 13052187  
Non-identical sequences: ............... 3338911  
Genomic hits: .......................... 28737726  
Significant densitiy of mapped reads: .. 470.083249848448 reads/kb

Show proTRAC cluster info
Hide proTRAC cluster info

|  |  |
| --- | --- |
| Location | NODE\_312079\_length\_70626\_cov\_31.911549 |
| Coordinates | 48101-66277 |
| Size [bp] | 18177 |
| Sequence hit loci | 2984 |
| Mapped reads (normalized) | 11379.4 |
| Mapped reads (normalized) per kb | 626 |
| Normalized reads with 1T (1U) | 81.1% |
| Normalized reads with 10A | 43.5% |
| Normalized reads with length 24-32 nt | 97.1% |
| Normalized reads on the main strand(s) | 90.3% |
| Predicted directionality | bi:minus-plus (split between 53270 and 53285) |

100%

0%

1T (1U)  
reads

10A reads

24-32 nt  
reads

reads on mainstrand

**Either the amount of reads with 1T (1U) OR 10A has to exceed 75% (set with option: -1Tor10A)  
Alternatively the amount of reads with 1T (1U) AND 10A has to exceed 50% (set with option: -1Tand10A)  
Minimum amount of reads with preferred size is 75% (set with option: -pisize)  
Minimum amount of reads on the main strand(s) is 75% (set with option: -clstrand)**

Show read coverage
Hide read coverage

WHAT DO I SEE HERE?  
This chart shows the location of mapped sequence reads within a predicted piRNA cluster. The color refers to the number of genomic hits produced by the sequence read in question. A dark red bar indicates that this sequence read produces many other hits elsewhere in the genome. Many adjacent red or yellow bars can indicate the presence of a multi-copy element such as transposons or rRNA genes. A dark green bar indicates that this sequence read maps uniquely to this locus.

1 hit

2-5 hits

6-10 hits

11-20 hits

21-50 hits

51-100 hits

> 100 hits

NODE\_312079\_length\_70626\_cov\_31.911549

48101

66277

Gene Set

RepeatMasker

Mapped  
Reads

55.93

plus strand

minus strand

55.93

Region: NODE\_312079\_length\_70626\_cov\_31.911549 6000-48119. Max. coverage (+): 0. Max coverage (-): 0.08

Region: NODE\_312079\_length\_70626\_cov\_31.911549 48120-48155. Max. coverage (+): 0. Max coverage (-): 0

Region: NODE\_312079\_length\_70626\_cov\_31.911549 48156-48191. Max. coverage (+): 0. Max coverage (-): 0

Region: NODE\_312079\_length\_70626\_cov\_31.911549 48192-48228. Max. coverage (+): 0. Max coverage (-): 0

Region: NODE\_312079\_length\_70626\_cov\_31.911549 48229-48264. Max. coverage (+): 0. Max coverage (-): 0

Region: NODE\_312079\_length\_70626\_cov\_31.911549 48265-48300. Max. coverage (+): 0. Max coverage (-): 0

Region: NODE\_312079\_length\_70626\_cov\_31.911549 48301-48337. Max. coverage (+): 0. Max coverage (-): 0

Region: NODE\_312079\_length\_70626\_cov\_31.911549 48338-48373. Max. coverage (+): 0. Max coverage (-): 0

Region: NODE\_312079\_length\_70626\_cov\_31.911549 48374-48410. Max. coverage (+): 0. Max coverage (-): 0

Region: NODE\_312079\_length\_70626\_cov\_31.911549 48411-48446. Max. coverage (+): 0. Max coverage (-): 0

Region: NODE\_312079\_length\_70626\_cov\_31.911549 48447-48482. Max. coverage (+): 0. Max coverage (-): 0

Region: NODE\_312079\_length\_70626\_cov\_31.911549 48483-48519. Max. coverage (+): 0. Max coverage (-): 0

Region: NODE\_312079\_length\_70626\_cov\_31.911549 48520-48555. Max. coverage (+): 0. Max coverage (-): 0

Region: NODE\_312079\_length\_70626\_cov\_31.911549 48556-48591. Max. coverage (+): 0.08. Max coverage (-): 0

Region: NODE\_312079\_length\_70626\_cov\_31.911549 48592-48628. Max. coverage (+): 0. Max coverage (-): 0

Region: NODE\_312079\_length\_70626\_cov\_31.911549 48629-48664. Max. coverage (+): 0. Max coverage (-): 0

Region: NODE\_312079\_length\_70626\_cov\_31.911549 48665-48700. Max. coverage (+): 0. Max coverage (-): 0

Region: NODE\_312079\_length\_70626\_cov\_31.911549 48701-48737. Max. coverage (+): 0. Max coverage (-): 0

Region: NODE\_312079\_length\_70626\_cov\_31.911549 48738-48773. Max. coverage (+): 0. Max coverage (-): 0

Region: NODE\_312079\_length\_70626\_cov\_31.911549 48774-48809. Max. coverage (+): 0. Max coverage (-): 0

Region: NODE\_312079\_length\_70626\_cov\_31.911549 48810-48846. Max. coverage (+): 0.01. Max coverage (-): 0.01

Region: NODE\_312079\_length\_70626\_cov\_31.911549 48847-48882. Max. coverage (+): 0. Max coverage (-): 0.01

Region: NODE\_312079\_length\_70626\_cov\_31.911549 48883-48918. Max. coverage (+): 0. Max coverage (-): 0

Region: NODE\_312079\_length\_70626\_cov\_31.911549 48919-48955. Max. coverage (+): 0. Max coverage (-): 0

Region: NODE\_312079\_length\_70626\_cov\_31.911549 48956-48991. Max. coverage (+): 0. Max coverage (-): 0

Region: NODE\_312079\_length\_70626\_cov\_31.911549 48992-49028. Max. coverage (+): 0. Max coverage (-): 0

Region: NODE\_312079\_length\_70626\_cov\_31.911549 49029-49064. Max. coverage (+): 0.02. Max coverage (-): 0

Region: NODE\_312079\_length\_70626\_cov\_31.911549 49065-49100. Max. coverage (+): 0.01. Max coverage (-): 0.01

Region: NODE\_312079\_length\_70626\_cov\_31.911549 49101-49137. Max. coverage (+): 0. Max coverage (-): 0.15

Region: NODE\_312079\_length\_70626\_cov\_31.911549 49138-49173. Max. coverage (+): 0.08. Max coverage (-): 0

Region: NODE\_312079\_length\_70626\_cov\_31.911549 49174-49209. Max. coverage (+): 0. Max coverage (-): 0

Region: NODE\_312079\_length\_70626\_cov\_31.911549 49210-49246. Max. coverage (+): 0. Max coverage (-): 0.15

Region: NODE\_312079\_length\_70626\_cov\_31.911549 49247-49282. Max. coverage (+): 0. Max coverage (-): 0.15

Region: NODE\_312079\_length\_70626\_cov\_31.911549 49283-49318. Max. coverage (+): 0. Max coverage (-): 0.77

Region: NODE\_312079\_length\_70626\_cov\_31.911549 49319-49355. Max. coverage (+): 0. Max coverage (-): 0.08

Region: NODE\_312079\_length\_70626\_cov\_31.911549 49356-49391. Max. coverage (+): 0. Max coverage (-): 0.54

Region: NODE\_312079\_length\_70626\_cov\_31.911549 49392-49427. Max. coverage (+): 0. Max coverage (-): 0.08

Region: NODE\_312079\_length\_70626\_cov\_31.911549 49428-49464. Max. coverage (+): 0. Max coverage (-): 0.08

Region: NODE\_312079\_length\_70626\_cov\_31.911549 49465-49500. Max. coverage (+): 0. Max coverage (-): 0.08

Region: NODE\_312079\_length\_70626\_cov\_31.911549 49501-49536. Max. coverage (+): 0. Max coverage (-): 1.15

Region: NODE\_312079\_length\_70626\_cov\_31.911549 49537-49573. Max. coverage (+): 0.23. Max coverage (-): 0.69

Region: NODE\_312079\_length\_70626\_cov\_31.911549 49574-49609. Max. coverage (+): 0.84. Max coverage (-): 0.15

Region: NODE\_312079\_length\_70626\_cov\_31.911549 49610-49646. Max. coverage (+): 0.08. Max coverage (-): 0.61

Region: NODE\_312079\_length\_70626\_cov\_31.911549 49647-49682. Max. coverage (+): 0. Max coverage (-): 0.31

Region: NODE\_312079\_length\_70626\_cov\_31.911549 49683-49718. Max. coverage (+): 0. Max coverage (-): 0.61

Region: NODE\_312079\_length\_70626\_cov\_31.911549 49719-49755. Max. coverage (+): 0.08. Max coverage (-): 0.84

Region: NODE\_312079\_length\_70626\_cov\_31.911549 49756-49791. Max. coverage (+): 0.08. Max coverage (-): 0.15

Region: NODE\_312079\_length\_70626\_cov\_31.911549 49792-49827. Max. coverage (+): 0. Max coverage (-): 0.15

Region: NODE\_312079\_length\_70626\_cov\_31.911549 49828-49864. Max. coverage (+): 0. Max coverage (-): 0

Region: NODE\_312079\_length\_70626\_cov\_31.911549 49865-49900. Max. coverage (+): 0. Max coverage (-): 0.08

Region: NODE\_312079\_length\_70626\_cov\_31.911549 49901-49936. Max. coverage (+): 0.15. Max coverage (-): 0.46

Region: NODE\_312079\_length\_70626\_cov\_31.911549 49937-49973. Max. coverage (+): 0.15. Max coverage (-): 0.08

Region: NODE\_312079\_length\_70626\_cov\_31.911549 49974-50009. Max. coverage (+): 0.08. Max coverage (-): 0.31

Region: NODE\_312079\_length\_70626\_cov\_31.911549 50010-50045. Max. coverage (+): 8.12. Max coverage (-): 0.31

Region: NODE\_312079\_length\_70626\_cov\_31.911549 50046-50082. Max. coverage (+): 0.31. Max coverage (-): 0.31

Region: NODE\_312079\_length\_70626\_cov\_31.911549 50083-50118. Max. coverage (+): 0. Max coverage (-): 0.08

Region: NODE\_312079\_length\_70626\_cov\_31.911549 50119-50155. Max. coverage (+): 0.31. Max coverage (-): 0.08

Region: NODE\_312079\_length\_70626\_cov\_31.911549 50156-50191. Max. coverage (+): 0.08. Max coverage (-): 1.3

Region: NODE\_312079\_length\_70626\_cov\_31.911549 50192-50227. Max. coverage (+): 0.31. Max coverage (-): 1.92

Region: NODE\_312079\_length\_70626\_cov\_31.911549 50228-50264. Max. coverage (+): 0. Max coverage (-): 0.15

Region: NODE\_312079\_length\_70626\_cov\_31.911549 50265-50300. Max. coverage (+): 0.15. Max coverage (-): 0.08

Region: NODE\_312079\_length\_70626\_cov\_31.911549 50301-50336. Max. coverage (+): 0.15. Max coverage (-): 0.23

Region: NODE\_312079\_length\_70626\_cov\_31.911549 50337-50373. Max. coverage (+): 0.08. Max coverage (-): 0.23

Region: NODE\_312079\_length\_70626\_cov\_31.911549 50374-50409. Max. coverage (+): 0. Max coverage (-): 0.69

Region: NODE\_312079\_length\_70626\_cov\_31.911549 50410-50445. Max. coverage (+): 0.69. Max coverage (-): 6.28

Region: NODE\_312079\_length\_70626\_cov\_31.911549 50446-50482. Max. coverage (+): 0. Max coverage (-): 2.91

Region: NODE\_312079\_length\_70626\_cov\_31.911549 50483-50518. Max. coverage (+): 0. Max coverage (-): 0.38

Region: NODE\_312079\_length\_70626\_cov\_31.911549 50519-50554. Max. coverage (+): 0.08. Max coverage (-): 0.23

Region: NODE\_312079\_length\_70626\_cov\_31.911549 50555-50591. Max. coverage (+): 0.08. Max coverage (-): 13.1

Region: NODE\_312079\_length\_70626\_cov\_31.911549 50592-50627. Max. coverage (+): 0. Max coverage (-): 0

Region: NODE\_312079\_length\_70626\_cov\_31.911549 50628-50663. Max. coverage (+): 0.08. Max coverage (-): 0.38

Region: NODE\_312079\_length\_70626\_cov\_31.911549 50664-50700. Max. coverage (+): 0. Max coverage (-): 0.61

Region: NODE\_312079\_length\_70626\_cov\_31.911549 50701-50736. Max. coverage (+): 0.15. Max coverage (-): 9.73

Region: NODE\_312079\_length\_70626\_cov\_31.911549 50737-50773. Max. coverage (+): 0.08. Max coverage (-): 0.31

Region: NODE\_312079\_length\_70626\_cov\_31.911549 50774-50809. Max. coverage (+): 0.38. Max coverage (-): 1.46

Region: NODE\_312079\_length\_70626\_cov\_31.911549 50810-50845. Max. coverage (+): 0. Max coverage (-): 0.69

Region: NODE\_312079\_length\_70626\_cov\_31.911549 50846-50882. Max. coverage (+): 0.23. Max coverage (-): 0.61

Region: NODE\_312079\_length\_70626\_cov\_31.911549 50883-50918. Max. coverage (+): 1.07. Max coverage (-): 0.61

Region: NODE\_312079\_length\_70626\_cov\_31.911549 50919-50954. Max. coverage (+): 0.15. Max coverage (-): 0.61

Region: NODE\_312079\_length\_70626\_cov\_31.911549 50955-50991. Max. coverage (+): 0. Max coverage (-): 5.06

Region: NODE\_312079\_length\_70626\_cov\_31.911549 50992-51027. Max. coverage (+): 0.15. Max coverage (-): 0.46

Region: NODE\_312079\_length\_70626\_cov\_31.911549 51028-51063. Max. coverage (+): 0.31. Max coverage (-): 5.98

Region: NODE\_312079\_length\_70626\_cov\_31.911549 51064-51100. Max. coverage (+): 2.45. Max coverage (-): 6.97

Region: NODE\_312079\_length\_70626\_cov\_31.911549 51101-51136. Max. coverage (+): 0.08. Max coverage (-): 0.38

Region: NODE\_312079\_length\_70626\_cov\_31.911549 51137-51172. Max. coverage (+): 0. Max coverage (-): 0.23

Region: NODE\_312079\_length\_70626\_cov\_31.911549 51173-51209. Max. coverage (+): 0.15. Max coverage (-): 43.21

Region: NODE\_312079\_length\_70626\_cov\_31.911549 51210-51245. Max. coverage (+): 0. Max coverage (-): 0.69

Region: NODE\_312079\_length\_70626\_cov\_31.911549 51246-51281. Max. coverage (+): 0. Max coverage (-): 0.77

Region: NODE\_312079\_length\_70626\_cov\_31.911549 51282-51318. Max. coverage (+): 0.08. Max coverage (-): 0.15

Region: NODE\_312079\_length\_70626\_cov\_31.911549 51319-51354. Max. coverage (+): 0.46. Max coverage (-): 1.53

Region: NODE\_312079\_length\_70626\_cov\_31.911549 51355-51391. Max. coverage (+): 0.15. Max coverage (-): 0.23

Region: NODE\_312079\_length\_70626\_cov\_31.911549 51392-51427. Max. coverage (+): 0.31. Max coverage (-): 0.23

Region: NODE\_312079\_length\_70626\_cov\_31.911549 51428-51463. Max. coverage (+): 0.15. Max coverage (-): 0.46

Region: NODE\_312079\_length\_70626\_cov\_31.911549 51464-51500. Max. coverage (+): 0.38. Max coverage (-): 0.61

Region: NODE\_312079\_length\_70626\_cov\_31.911549 51501-51536. Max. coverage (+): 3.06. Max coverage (-): 0.23

Region: NODE\_312079\_length\_70626\_cov\_31.911549 51537-51572. Max. coverage (+): 3.06. Max coverage (-): 0.46

Region: NODE\_312079\_length\_70626\_cov\_31.911549 51573-51609. Max. coverage (+): 0. Max coverage (-): 0.54

Region: NODE\_312079\_length\_70626\_cov\_31.911549 51610-51645. Max. coverage (+): 0.08. Max coverage (-): 0.15

Region: NODE\_312079\_length\_70626\_cov\_31.911549 51646-51681. Max. coverage (+): 0. Max coverage (-): 0

Region: NODE\_312079\_length\_70626\_cov\_31.911549 51682-51718. Max. coverage (+): 0. Max coverage (-): 0

Region: NODE\_312079\_length\_70626\_cov\_31.911549 51719-51754. Max. coverage (+): 0.08. Max coverage (-): 0.08

Region: NODE\_312079\_length\_70626\_cov\_31.911549 51755-51790. Max. coverage (+): 0.08. Max coverage (-): 0.08

Region: NODE\_312079\_length\_70626\_cov\_31.911549 51791-51827. Max. coverage (+): 0.08. Max coverage (-): 0.08

Region: NODE\_312079\_length\_70626\_cov\_31.911549 51828-51863. Max. coverage (+): 0. Max coverage (-): 0.23

Region: NODE\_312079\_length\_70626\_cov\_31.911549 51864-51899. Max. coverage (+): 0.08. Max coverage (-): 0.08

Region: NODE\_312079\_length\_70626\_cov\_31.911549 51900-51936. Max. coverage (+): 0.08. Max coverage (-): 0.15

Region: NODE\_312079\_length\_70626\_cov\_31.911549 51937-51972. Max. coverage (+): 0. Max coverage (-): 2.15

Region: NODE\_312079\_length\_70626\_cov\_31.911549 51973-52009. Max. coverage (+): 0. Max coverage (-): 0.77

Region: NODE\_312079\_length\_70626\_cov\_31.911549 52010-52045. Max. coverage (+): 0. Max coverage (-): 0.08

Region: NODE\_312079\_length\_70626\_cov\_31.911549 52046-52081. Max. coverage (+): 0.08. Max coverage (-): 0.77

Region: NODE\_312079\_length\_70626\_cov\_31.911549 52082-52118. Max. coverage (+): 0.08. Max coverage (-): 0

Region: NODE\_312079\_length\_70626\_cov\_31.911549 52119-52154. Max. coverage (+): 0. Max coverage (-): 1.92

Region: NODE\_312079\_length\_70626\_cov\_31.911549 52155-52190. Max. coverage (+): 0.15. Max coverage (-): 0.61

Region: NODE\_312079\_length\_70626\_cov\_31.911549 52191-52227. Max. coverage (+): 0. Max coverage (-): 0.08

Region: NODE\_312079\_length\_70626\_cov\_31.911549 52228-52263. Max. coverage (+): 0. Max coverage (-): 0.23

Region: NODE\_312079\_length\_70626\_cov\_31.911549 52264-52299. Max. coverage (+): 0. Max coverage (-): 0.54

Region: NODE\_312079\_length\_70626\_cov\_31.911549 52300-52336. Max. coverage (+): 0.15. Max coverage (-): 0.77

Region: NODE\_312079\_length\_70626\_cov\_31.911549 52337-52372. Max. coverage (+): 0.08. Max coverage (-): 4.83

Region: NODE\_312079\_length\_70626\_cov\_31.911549 52373-52408. Max. coverage (+): 0.08. Max coverage (-): 0.23

Region: NODE\_312079\_length\_70626\_cov\_31.911549 52409-52445. Max. coverage (+): 0.61. Max coverage (-): 0.46

Region: NODE\_312079\_length\_70626\_cov\_31.911549 52446-52481. Max. coverage (+): 0.23. Max coverage (-): 0.23

Region: NODE\_312079\_length\_70626\_cov\_31.911549 52482-52518. Max. coverage (+): 0.31. Max coverage (-): 0.08

Region: NODE\_312079\_length\_70626\_cov\_31.911549 52519-52554. Max. coverage (+): 0.15. Max coverage (-): 0.23

Region: NODE\_312079\_length\_70626\_cov\_31.911549 52555-52590. Max. coverage (+): 0. Max coverage (-): 0.15

Region: NODE\_312079\_length\_70626\_cov\_31.911549 52591-52627. Max. coverage (+): 0.08. Max coverage (-): 0.69

Region: NODE\_312079\_length\_70626\_cov\_31.911549 52628-52663. Max. coverage (+): 0. Max coverage (-): 3.14

Region: NODE\_312079\_length\_70626\_cov\_31.911549 52664-52699. Max. coverage (+): 0.08. Max coverage (-): 0

Region: NODE\_312079\_length\_70626\_cov\_31.911549 52700-52736. Max. coverage (+): 0.08. Max coverage (-): 0.31

Region: NODE\_312079\_length\_70626\_cov\_31.911549 52737-52772. Max. coverage (+): 0.69. Max coverage (-): 0.38

Region: NODE\_312079\_length\_70626\_cov\_31.911549 52773-52808. Max. coverage (+): 0.08. Max coverage (-): 0.46

Region: NODE\_312079\_length\_70626\_cov\_31.911549 52809-52845. Max. coverage (+): 0.08. Max coverage (-): 0.84

Region: NODE\_312079\_length\_70626\_cov\_31.911549 52846-52881. Max. coverage (+): 12.64. Max coverage (-): 1.84

Region: NODE\_312079\_length\_70626\_cov\_31.911549 52882-52917. Max. coverage (+): 11.42. Max coverage (-): 0

Region: NODE\_312079\_length\_70626\_cov\_31.911549 52918-52954. Max. coverage (+): 0.23. Max coverage (-): 0.15

Region: NODE\_312079\_length\_70626\_cov\_31.911549 52955-52990. Max. coverage (+): 0.08. Max coverage (-): 8.43

Region: NODE\_312079\_length\_70626\_cov\_31.911549 52991-53026. Max. coverage (+): 0.08. Max coverage (-): 4.67

Region: NODE\_312079\_length\_70626\_cov\_31.911549 53027-53063. Max. coverage (+): 0.08. Max coverage (-): 0.69

Region: NODE\_312079\_length\_70626\_cov\_31.911549 53064-53099. Max. coverage (+): 0.23. Max coverage (-): 0.46

Region: NODE\_312079\_length\_70626\_cov\_31.911549 53100-53136. Max. coverage (+): 0. Max coverage (-): 0.31

Region: NODE\_312079\_length\_70626\_cov\_31.911549 53137-53172. Max. coverage (+): 0.08. Max coverage (-): 0.23

Region: NODE\_312079\_length\_70626\_cov\_31.911549 53173-53208. Max. coverage (+): 0.08. Max coverage (-): 0.46

Region: NODE\_312079\_length\_70626\_cov\_31.911549 53209-53245. Max. coverage (+): 0.08. Max coverage (-): 0.23

Region: NODE\_312079\_length\_70626\_cov\_31.911549 53246-53281. Max. coverage (+): 0.08. Max coverage (-): 1.38

Region: NODE\_312079\_length\_70626\_cov\_31.911549 53282-53317. Max. coverage (+): 0.46. Max coverage (-): 0

Region: NODE\_312079\_length\_70626\_cov\_31.911549 53318-53354. Max. coverage (+): 0. Max coverage (-): 0

Region: NODE\_312079\_length\_70626\_cov\_31.911549 53355-53390. Max. coverage (+): 0. Max coverage (-): 0

Region: NODE\_312079\_length\_70626\_cov\_31.911549 53391-53426. Max. coverage (+): 0. Max coverage (-): 0.08

Region: NODE\_312079\_length\_70626\_cov\_31.911549 53427-53463. Max. coverage (+): 0. Max coverage (-): 0.08

Region: NODE\_312079\_length\_70626\_cov\_31.911549 53464-53499. Max. coverage (+): 0. Max coverage (-): 0.08

Region: NODE\_312079\_length\_70626\_cov\_31.911549 53500-53535. Max. coverage (+): 0. Max coverage (-): 0

Region: NODE\_312079\_length\_70626\_cov\_31.911549 53536-53572. Max. coverage (+): 0. Max coverage (-): 0

Region: NODE\_312079\_length\_70626\_cov\_31.911549 53573-53608. Max. coverage (+): 0. Max coverage (-): 0.08

Region: NODE\_312079\_length\_70626\_cov\_31.911549 53609-53644. Max. coverage (+): 0.08. Max coverage (-): 0

Region: NODE\_312079\_length\_70626\_cov\_31.911549 53645-53681. Max. coverage (+): 0.02. Max coverage (-): 0

Region: NODE\_312079\_length\_70626\_cov\_31.911549 53682-53717. Max. coverage (+): 0. Max coverage (-): 0

Region: NODE\_312079\_length\_70626\_cov\_31.911549 53718-53754. Max. coverage (+): 0.01. Max coverage (-): 0

Region: NODE\_312079\_length\_70626\_cov\_31.911549 53755-53790. Max. coverage (+): 0. Max coverage (-): 0.04

Region: NODE\_312079\_length\_70626\_cov\_31.911549 53791-53826. Max. coverage (+): 0.01. Max coverage (-): 0

Region: NODE\_312079\_length\_70626\_cov\_31.911549 53827-53863. Max. coverage (+): 0.01. Max coverage (-): 0

Region: NODE\_312079\_length\_70626\_cov\_31.911549 53864-53899. Max. coverage (+): 0. Max coverage (-): 0

Region: NODE\_312079\_length\_70626\_cov\_31.911549 53900-53935. Max. coverage (+): 0. Max coverage (-): 0

Region: NODE\_312079\_length\_70626\_cov\_31.911549 53936-53972. Max. coverage (+): 0. Max coverage (-): 0

Region: NODE\_312079\_length\_70626\_cov\_31.911549 53973-54008. Max. coverage (+): 0. Max coverage (-): 0

Region: NODE\_312079\_length\_70626\_cov\_31.911549 54009-54044. Max. coverage (+): 0. Max coverage (-): 0

Region: NODE\_312079\_length\_70626\_cov\_31.911549 54045-54081. Max. coverage (+): 0. Max coverage (-): 0

Region: NODE\_312079\_length\_70626\_cov\_31.911549 54082-54117. Max. coverage (+): 0. Max coverage (-): 0

Region: NODE\_312079\_length\_70626\_cov\_31.911549 54118-54153. Max. coverage (+): 0. Max coverage (-): 0

Region: NODE\_312079\_length\_70626\_cov\_31.911549 54154-54190. Max. coverage (+): 0. Max coverage (-): 0

Region: NODE\_312079\_length\_70626\_cov\_31.911549 54191-54226. Max. coverage (+): 0. Max coverage (-): 0

Region: NODE\_312079\_length\_70626\_cov\_31.911549 54227-54263. Max. coverage (+): 0. Max coverage (-): 0

Region: NODE\_312079\_length\_70626\_cov\_31.911549 54264-54299. Max. coverage (+): 0. Max coverage (-): 0

Region: NODE\_312079\_length\_70626\_cov\_31.911549 54300-54335. Max. coverage (+): 0. Max coverage (-): 0

Region: NODE\_312079\_length\_70626\_cov\_31.911549 54336-54372. Max. coverage (+): 0. Max coverage (-): 0

Region: NODE\_312079\_length\_70626\_cov\_31.911549 54373-54408. Max. coverage (+): 0. Max coverage (-): 0

Region: NODE\_312079\_length\_70626\_cov\_31.911549 54409-54444. Max. coverage (+): 0. Max coverage (-): 0

Region: NODE\_312079\_length\_70626\_cov\_31.911549 54445-54481. Max. coverage (+): 0. Max coverage (-): 0

Region: NODE\_312079\_length\_70626\_cov\_31.911549 54482-54517. Max. coverage (+): 0. Max coverage (-): 0

Region: NODE\_312079\_length\_70626\_cov\_31.911549 54518-54553. Max. coverage (+): 0. Max coverage (-): 0

Region: NODE\_312079\_length\_70626\_cov\_31.911549 54554-54590. Max. coverage (+): 0. Max coverage (-): 0

Region: NODE\_312079\_length\_70626\_cov\_31.911549 54591-54626. Max. coverage (+): 0. Max coverage (-): 0

Region: NODE\_312079\_length\_70626\_cov\_31.911549 54627-54662. Max. coverage (+): 0. Max coverage (-): 0

Region: NODE\_312079\_length\_70626\_cov\_31.911549 54663-54699. Max. coverage (+): 0. Max coverage (-): 0

Region: NODE\_312079\_length\_70626\_cov\_31.911549 54700-54735. Max. coverage (+): 0. Max coverage (-): 0

Region: NODE\_312079\_length\_70626\_cov\_31.911549 54736-54771. Max. coverage (+): 0. Max coverage (-): 0

Region: NODE\_312079\_length\_70626\_cov\_31.911549 54772-54808. Max. coverage (+): 0. Max coverage (-): 0

Region: NODE\_312079\_length\_70626\_cov\_31.911549 54809-54844. Max. coverage (+): 0. Max coverage (-): 0

Region: NODE\_312079\_length\_70626\_cov\_31.911549 54845-54881. Max. coverage (+): 0. Max coverage (-): 0

Region: NODE\_312079\_length\_70626\_cov\_31.911549 54882-54917. Max. coverage (+): 0.08. Max coverage (-): 0

Region: NODE\_312079\_length\_70626\_cov\_31.911549 54918-54953. Max. coverage (+): 0. Max coverage (-): 0

Region: NODE\_312079\_length\_70626\_cov\_31.911549 54954-54990. Max. coverage (+): 0. Max coverage (-): 0

Region: NODE\_312079\_length\_70626\_cov\_31.911549 54991-55026. Max. coverage (+): 0. Max coverage (-): 0

Region: NODE\_312079\_length\_70626\_cov\_31.911549 55027-55062. Max. coverage (+): 0. Max coverage (-): 0

Region: NODE\_312079\_length\_70626\_cov\_31.911549 55063-55099. Max. coverage (+): 0. Max coverage (-): 0

Region: NODE\_312079\_length\_70626\_cov\_31.911549 55100-55135. Max. coverage (+): 0. Max coverage (-): 0

Region: NODE\_312079\_length\_70626\_cov\_31.911549 55136-55171. Max. coverage (+): 0. Max coverage (-): 0

Region: NODE\_312079\_length\_70626\_cov\_31.911549 55172-55208. Max. coverage (+): 0. Max coverage (-): 0

Region: NODE\_312079\_length\_70626\_cov\_31.911549 55209-55244. Max. coverage (+): 0. Max coverage (-): 0

Region: NODE\_312079\_length\_70626\_cov\_31.911549 55245-55280. Max. coverage (+): 0. Max coverage (-): 0

Region: NODE\_312079\_length\_70626\_cov\_31.911549 55281-55317. Max. coverage (+): 0. Max coverage (-): 0

Region: NODE\_312079\_length\_70626\_cov\_31.911549 55318-55353. Max. coverage (+): 0. Max coverage (-): 0

Region: NODE\_312079\_length\_70626\_cov\_31.911549 55354-55389. Max. coverage (+): 0. Max coverage (-): 0

Region: NODE\_312079\_length\_70626\_cov\_31.911549 55390-55426. Max. coverage (+): 0. Max coverage (-): 0

Region: NODE\_312079\_length\_70626\_cov\_31.911549 55427-55462. Max. coverage (+): 0. Max coverage (-): 0

Region: NODE\_312079\_length\_70626\_cov\_31.911549 55463-55499. Max. coverage (+): 0. Max coverage (-): 0

Region: NODE\_312079\_length\_70626\_cov\_31.911549 55500-55535. Max. coverage (+): 0. Max coverage (-): 0

Region: NODE\_312079\_length\_70626\_cov\_31.911549 55536-55571. Max. coverage (+): 0. Max coverage (-): 0

Region: NODE\_312079\_length\_70626\_cov\_31.911549 55572-55608. Max. coverage (+): 0. Max coverage (-): 0

Region: NODE\_312079\_length\_70626\_cov\_31.911549 55609-55644. Max. coverage (+): 0. Max coverage (-): 0

Region: NODE\_312079\_length\_70626\_cov\_31.911549 55645-55680. Max. coverage (+): 0. Max coverage (-): 0

Region: NODE\_312079\_length\_70626\_cov\_31.911549 55681-55717. Max. coverage (+): 0. Max coverage (-): 0

Region: NODE\_312079\_length\_70626\_cov\_31.911549 55718-55753. Max. coverage (+): 0. Max coverage (-): 0

Region: NODE\_312079\_length\_70626\_cov\_31.911549 55754-55789. Max. coverage (+): 0.05. Max coverage (-): 0

Region: NODE\_312079\_length\_70626\_cov\_31.911549 55790-55826. Max. coverage (+): 0.03. Max coverage (-): 0

Region: NODE\_312079\_length\_70626\_cov\_31.911549 55827-55862. Max. coverage (+): 0.03. Max coverage (-): 0

Region: NODE\_312079\_length\_70626\_cov\_31.911549 55863-55898. Max. coverage (+): 0.01. Max coverage (-): 0

Region: NODE\_312079\_length\_70626\_cov\_31.911549 55899-55935. Max. coverage (+): 0. Max coverage (-): 0

Region: NODE\_312079\_length\_70626\_cov\_31.911549 55936-55971. Max. coverage (+): 0. Max coverage (-): 0

Region: NODE\_312079\_length\_70626\_cov\_31.911549 55972-56007. Max. coverage (+): 0. Max coverage (-): 0

Region: NODE\_312079\_length\_70626\_cov\_31.911549 56008-56044. Max. coverage (+): 0. Max coverage (-): 0

Region: NODE\_312079\_length\_70626\_cov\_31.911549 56045-56080. Max. coverage (+): 0. Max coverage (-): 0

Region: NODE\_312079\_length\_70626\_cov\_31.911549 56081-56117. Max. coverage (+): 0. Max coverage (-): 0.08

Region: NODE\_312079\_length\_70626\_cov\_31.911549 56118-56153. Max. coverage (+): 0. Max coverage (-): 0

Region: NODE\_312079\_length\_70626\_cov\_31.911549 56154-56189. Max. coverage (+): 0. Max coverage (-): 0

Region: NODE\_312079\_length\_70626\_cov\_31.911549 56190-56226. Max. coverage (+): 0.08. Max coverage (-): 0

Region: NODE\_312079\_length\_70626\_cov\_31.911549 56227-56262. Max. coverage (+): 0.08. Max coverage (-): 0

Region: NODE\_312079\_length\_70626\_cov\_31.911549 56263-56298. Max. coverage (+): 0. Max coverage (-): 0

Region: NODE\_312079\_length\_70626\_cov\_31.911549 56299-56335. Max. coverage (+): 0. Max coverage (-): 0

Region: NODE\_312079\_length\_70626\_cov\_31.911549 56336-56371. Max. coverage (+): 0.77. Max coverage (-): 0

Region: NODE\_312079\_length\_70626\_cov\_31.911549 56372-56407. Max. coverage (+): 0. Max coverage (-): 0

Region: NODE\_312079\_length\_70626\_cov\_31.911549 56408-56444. Max. coverage (+): 0.08. Max coverage (-): 0

Region: NODE\_312079\_length\_70626\_cov\_31.911549 56445-56480. Max. coverage (+): 0.31. Max coverage (-): 0.08

Region: NODE\_312079\_length\_70626\_cov\_31.911549 56481-56516. Max. coverage (+): 0.46. Max coverage (-): 0.08

Region: NODE\_312079\_length\_70626\_cov\_31.911549 56517-56553. Max. coverage (+): 0.15. Max coverage (-): 0

Region: NODE\_312079\_length\_70626\_cov\_31.911549 56554-56589. Max. coverage (+): 2.45. Max coverage (-): 0

Region: NODE\_312079\_length\_70626\_cov\_31.911549 56590-56626. Max. coverage (+): 0.23. Max coverage (-): 0

Region: NODE\_312079\_length\_70626\_cov\_31.911549 56627-56662. Max. coverage (+): 1. Max coverage (-): 0

Region: NODE\_312079\_length\_70626\_cov\_31.911549 56663-56698. Max. coverage (+): 0.08. Max coverage (-): 0.15

Region: NODE\_312079\_length\_70626\_cov\_31.911549 56699-56735. Max. coverage (+): 5.98. Max coverage (-): 0

Region: NODE\_312079\_length\_70626\_cov\_31.911549 56736-56771. Max. coverage (+): 6.05. Max coverage (-): 0.23

Region: NODE\_312079\_length\_70626\_cov\_31.911549 56772-56807. Max. coverage (+): 3.29. Max coverage (-): 0

Region: NODE\_312079\_length\_70626\_cov\_31.911549 56808-56844. Max. coverage (+): 1. Max coverage (-): 0

Region: NODE\_312079\_length\_70626\_cov\_31.911549 56845-56880. Max. coverage (+): 0.38. Max coverage (-): 0

Region: NODE\_312079\_length\_70626\_cov\_31.911549 56881-56916. Max. coverage (+): 3.14. Max coverage (-): 0

Region: NODE\_312079\_length\_70626\_cov\_31.911549 56917-56953. Max. coverage (+): 3.22. Max coverage (-): 0.08

Region: NODE\_312079\_length\_70626\_cov\_31.911549 56954-56989. Max. coverage (+): 17.47. Max coverage (-): 0.38

Region: NODE\_312079\_length\_70626\_cov\_31.911549 56990-57025. Max. coverage (+): 0.69. Max coverage (-): 4.83

Region: NODE\_312079\_length\_70626\_cov\_31.911549 57026-57062. Max. coverage (+): 1.92. Max coverage (-): 5.44

Region: NODE\_312079\_length\_70626\_cov\_31.911549 57063-57098. Max. coverage (+): 0.15. Max coverage (-): 0.15

Region: NODE\_312079\_length\_70626\_cov\_31.911549 57099-57134. Max. coverage (+): 2.38. Max coverage (-): 0

Region: NODE\_312079\_length\_70626\_cov\_31.911549 57135-57171. Max. coverage (+): 1.23. Max coverage (-): 0

Region: NODE\_312079\_length\_70626\_cov\_31.911549 57172-57207. Max. coverage (+): 1.92. Max coverage (-): 0.08

Region: NODE\_312079\_length\_70626\_cov\_31.911549 57208-57244. Max. coverage (+): 0.38. Max coverage (-): 0

Region: NODE\_312079\_length\_70626\_cov\_31.911549 57245-57280. Max. coverage (+): 0.15. Max coverage (-): 0

Region: NODE\_312079\_length\_70626\_cov\_31.911549 57281-57316. Max. coverage (+): 0.15. Max coverage (-): 0

Region: NODE\_312079\_length\_70626\_cov\_31.911549 57317-57353. Max. coverage (+): 0. Max coverage (-): 0

Region: NODE\_312079\_length\_70626\_cov\_31.911549 57354-57389. Max. coverage (+): 0. Max coverage (-): 0

Region: NODE\_312079\_length\_70626\_cov\_31.911549 57390-57425. Max. coverage (+): 0. Max coverage (-): 0

Region: NODE\_312079\_length\_70626\_cov\_31.911549 57426-57462. Max. coverage (+): 0.15. Max coverage (-): 0

Region: NODE\_312079\_length\_70626\_cov\_31.911549 57463-57498. Max. coverage (+): 0.15. Max coverage (-): 0

Region: NODE\_312079\_length\_70626\_cov\_31.911549 57499-57534. Max. coverage (+): 16.09. Max coverage (-): 0.15

Region: NODE\_312079\_length\_70626\_cov\_31.911549 57535-57571. Max. coverage (+): 0.08. Max coverage (-): 0.61

Region: NODE\_312079\_length\_70626\_cov\_31.911549 57572-57607. Max. coverage (+): 0.31. Max coverage (-): 0

Region: NODE\_312079\_length\_70626\_cov\_31.911549 57608-57643. Max. coverage (+): 0.46. Max coverage (-): 0

Region: NODE\_312079\_length\_70626\_cov\_31.911549 57644-57680. Max. coverage (+): 0.23. Max coverage (-): 0.31

Region: NODE\_312079\_length\_70626\_cov\_31.911549 57681-57716. Max. coverage (+): 0.15. Max coverage (-): 0.23

Region: NODE\_312079\_length\_70626\_cov\_31.911549 57717-57752. Max. coverage (+): 0.31. Max coverage (-): 0.08

Region: NODE\_312079\_length\_70626\_cov\_31.911549 57753-57789. Max. coverage (+): 3.45. Max coverage (-): 0.08

Region: NODE\_312079\_length\_70626\_cov\_31.911549 57790-57825. Max. coverage (+): 2.22. Max coverage (-): 0

Region: NODE\_312079\_length\_70626\_cov\_31.911549 57826-57862. Max. coverage (+): 1.99. Max coverage (-): 0.23

Region: NODE\_312079\_length\_70626\_cov\_31.911549 57863-57898. Max. coverage (+): 0.84. Max coverage (-): 0.08

Region: NODE\_312079\_length\_70626\_cov\_31.911549 57899-57934. Max. coverage (+): 0. Max coverage (-): 0.08

Region: NODE\_312079\_length\_70626\_cov\_31.911549 57935-57971. Max. coverage (+): 0.38. Max coverage (-): 0.15

Region: NODE\_312079\_length\_70626\_cov\_31.911549 57972-58007. Max. coverage (+): 0.46. Max coverage (-): 0.08

Region: NODE\_312079\_length\_70626\_cov\_31.911549 58008-58043. Max. coverage (+): 0.08. Max coverage (-): 0.08

Region: NODE\_312079\_length\_70626\_cov\_31.911549 58044-58080. Max. coverage (+): 0.31. Max coverage (-): 0

Region: NODE\_312079\_length\_70626\_cov\_31.911549 58081-58116. Max. coverage (+): 0.23. Max coverage (-): 0

Region: NODE\_312079\_length\_70626\_cov\_31.911549 58117-58152. Max. coverage (+): 0. Max coverage (-): 0

Region: NODE\_312079\_length\_70626\_cov\_31.911549 58153-58189. Max. coverage (+): 1.84. Max coverage (-): 0.15

Region: NODE\_312079\_length\_70626\_cov\_31.911549 58190-58225. Max. coverage (+): 0.15. Max coverage (-): 0

Region: NODE\_312079\_length\_70626\_cov\_31.911549 58226-58261. Max. coverage (+): 0.46. Max coverage (-): 0

Region: NODE\_312079\_length\_70626\_cov\_31.911549 58262-58298. Max. coverage (+): 0.46. Max coverage (-): 0

Region: NODE\_312079\_length\_70626\_cov\_31.911549 58299-58334. Max. coverage (+): 0.77. Max coverage (-): 0

Region: NODE\_312079\_length\_70626\_cov\_31.911549 58335-58371. Max. coverage (+): 0.31. Max coverage (-): 0.08

Region: NODE\_312079\_length\_70626\_cov\_31.911549 58372-58407. Max. coverage (+): 0.01. Max coverage (-): 0

Region: NODE\_312079\_length\_70626\_cov\_31.911549 58408-58443. Max. coverage (+): 0.01. Max coverage (-): 0

Region: NODE\_312079\_length\_70626\_cov\_31.911549 58444-58480. Max. coverage (+): 0. Max coverage (-): 0

Region: NODE\_312079\_length\_70626\_cov\_31.911549 58481-58516. Max. coverage (+): 0. Max coverage (-): 0

Region: NODE\_312079\_length\_70626\_cov\_31.911549 58517-58552. Max. coverage (+): 0.08. Max coverage (-): 0

Region: NODE\_312079\_length\_70626\_cov\_31.911549 58553-58589. Max. coverage (+): 0. Max coverage (-): 0

Region: NODE\_312079\_length\_70626\_cov\_31.911549 58590-58625. Max. coverage (+): 0.23. Max coverage (-): 0

Region: NODE\_312079\_length\_70626\_cov\_31.911549 58626-58661. Max. coverage (+): 0. Max coverage (-): 0

Region: NODE\_312079\_length\_70626\_cov\_31.911549 58662-58698. Max. coverage (+): 0. Max coverage (-): 0

Region: NODE\_312079\_length\_70626\_cov\_31.911549 58699-58734. Max. coverage (+): 0.23. Max coverage (-): 0

Region: NODE\_312079\_length\_70626\_cov\_31.911549 58735-58770. Max. coverage (+): 0.54. Max coverage (-): 0

Region: NODE\_312079\_length\_70626\_cov\_31.911549 58771-58807. Max. coverage (+): 0.15. Max coverage (-): 0

Region: NODE\_312079\_length\_70626\_cov\_31.911549 58808-58843. Max. coverage (+): 0. Max coverage (-): 0

Region: NODE\_312079\_length\_70626\_cov\_31.911549 58844-58879. Max. coverage (+): 0.31. Max coverage (-): 0.08

Region: NODE\_312079\_length\_70626\_cov\_31.911549 58880-58916. Max. coverage (+): 0.23. Max coverage (-): 0.08

Region: NODE\_312079\_length\_70626\_cov\_31.911549 58917-58952. Max. coverage (+): 0.08. Max coverage (-): 0.08

Region: NODE\_312079\_length\_70626\_cov\_31.911549 58953-58989. Max. coverage (+): 0.15. Max coverage (-): 0.08

Region: NODE\_312079\_length\_70626\_cov\_31.911549 58990-59025. Max. coverage (+): 0.15. Max coverage (-): 0.15

Region: NODE\_312079\_length\_70626\_cov\_31.911549 59026-59061. Max. coverage (+): 0.92. Max coverage (-): 0

Region: NODE\_312079\_length\_70626\_cov\_31.911549 59062-59098. Max. coverage (+): 2.45. Max coverage (-): 0.23

Region: NODE\_312079\_length\_70626\_cov\_31.911549 59099-59134. Max. coverage (+): 1.61. Max coverage (-): 0.15

Region: NODE\_312079\_length\_70626\_cov\_31.911549 59135-59170. Max. coverage (+): 0.23. Max coverage (-): 0.08

Region: NODE\_312079\_length\_70626\_cov\_31.911549 59171-59207. Max. coverage (+): 0.08. Max coverage (-): 4.21

Region: NODE\_312079\_length\_70626\_cov\_31.911549 59208-59243. Max. coverage (+): 7.28. Max coverage (-): 0

Region: NODE\_312079\_length\_70626\_cov\_31.911549 59244-59279. Max. coverage (+): 0.38. Max coverage (-): 0.08

Region: NODE\_312079\_length\_70626\_cov\_31.911549 59280-59316. Max. coverage (+): 0. Max coverage (-): 0.08

Region: NODE\_312079\_length\_70626\_cov\_31.911549 59317-59352. Max. coverage (+): 0.38. Max coverage (-): 0.31

Region: NODE\_312079\_length\_70626\_cov\_31.911549 59353-59388. Max. coverage (+): 0.08. Max coverage (-): 0.08

Region: NODE\_312079\_length\_70626\_cov\_31.911549 59389-59425. Max. coverage (+): 0. Max coverage (-): 0

Region: NODE\_312079\_length\_70626\_cov\_31.911549 59426-59461. Max. coverage (+): 0.54. Max coverage (-): 0

Region: NODE\_312079\_length\_70626\_cov\_31.911549 59462-59497. Max. coverage (+): 50.87. Max coverage (-): 0.31

Region: NODE\_312079\_length\_70626\_cov\_31.911549 59498-59534. Max. coverage (+): 0.31. Max coverage (-): 0

Region: NODE\_312079\_length\_70626\_cov\_31.911549 59535-59570. Max. coverage (+): 10.11. Max coverage (-): 0.08

Region: NODE\_312079\_length\_70626\_cov\_31.911549 59571-59607. Max. coverage (+): 0.54. Max coverage (-): 0.08

Region: NODE\_312079\_length\_70626\_cov\_31.911549 59608-59643. Max. coverage (+): 0.77. Max coverage (-): 0

Region: NODE\_312079\_length\_70626\_cov\_31.911549 59644-59679. Max. coverage (+): 0.15. Max coverage (-): 0

Region: NODE\_312079\_length\_70626\_cov\_31.911549 59680-59716. Max. coverage (+): 1. Max coverage (-): 0.46

Region: NODE\_312079\_length\_70626\_cov\_31.911549 59717-59752. Max. coverage (+): 0.31. Max coverage (-): 0.23

Region: NODE\_312079\_length\_70626\_cov\_31.911549 59753-59788. Max. coverage (+): 2.38. Max coverage (-): 0.08

Region: NODE\_312079\_length\_70626\_cov\_31.911549 59789-59825. Max. coverage (+): 1.07. Max coverage (-): 0.08

Region: NODE\_312079\_length\_70626\_cov\_31.911549 59826-59861. Max. coverage (+): 0.69. Max coverage (-): 0.15

Region: NODE\_312079\_length\_70626\_cov\_31.911549 59862-59897. Max. coverage (+): 0.38. Max coverage (-): 0.15

Region: NODE\_312079\_length\_70626\_cov\_31.911549 59898-59934. Max. coverage (+): 0.54. Max coverage (-): 0.15

Region: NODE\_312079\_length\_70626\_cov\_31.911549 59935-59970. Max. coverage (+): 0.54. Max coverage (-): 0.08

Region: NODE\_312079\_length\_70626\_cov\_31.911549 59971-60006. Max. coverage (+): 0.15. Max coverage (-): 0

Region: NODE\_312079\_length\_70626\_cov\_31.911549 60007-60043. Max. coverage (+): 1.07. Max coverage (-): 0.15

Region: NODE\_312079\_length\_70626\_cov\_31.911549 60044-60079. Max. coverage (+): 1.38. Max coverage (-): 0

Region: NODE\_312079\_length\_70626\_cov\_31.911549 60080-60115. Max. coverage (+): 0.31. Max coverage (-): 0

Region: NODE\_312079\_length\_70626\_cov\_31.911549 60116-60152. Max. coverage (+): 0. Max coverage (-): 0.23

Region: NODE\_312079\_length\_70626\_cov\_31.911549 60153-60188. Max. coverage (+): 26.97. Max coverage (-): 0.31

Region: NODE\_312079\_length\_70626\_cov\_31.911549 60189-60225. Max. coverage (+): 0.23. Max coverage (-): 0.15

Region: NODE\_312079\_length\_70626\_cov\_31.911549 60226-60261. Max. coverage (+): 0.38. Max coverage (-): 0.08

Region: NODE\_312079\_length\_70626\_cov\_31.911549 60262-60297. Max. coverage (+): 21.61. Max coverage (-): 0.77

Region: NODE\_312079\_length\_70626\_cov\_31.911549 60298-60334. Max. coverage (+): 0.61. Max coverage (-): 0.15

Region: NODE\_312079\_length\_70626\_cov\_31.911549 60335-60370. Max. coverage (+): 2.22. Max coverage (-): 0.08

Region: NODE\_312079\_length\_70626\_cov\_31.911549 60371-60406. Max. coverage (+): 4.06. Max coverage (-): 0.31

Region: NODE\_312079\_length\_70626\_cov\_31.911549 60407-60443. Max. coverage (+): 0.54. Max coverage (-): 0.23

Region: NODE\_312079\_length\_70626\_cov\_31.911549 60444-60479. Max. coverage (+): 3.37. Max coverage (-): 0.08

Region: NODE\_312079\_length\_70626\_cov\_31.911549 60480-60515. Max. coverage (+): 9.73. Max coverage (-): 0.08

Region: NODE\_312079\_length\_70626\_cov\_31.911549 60516-60552. Max. coverage (+): 3.68. Max coverage (-): 0

Region: NODE\_312079\_length\_70626\_cov\_31.911549 60553-60588. Max. coverage (+): 0.08. Max coverage (-): 0.08

Region: NODE\_312079\_length\_70626\_cov\_31.911549 60589-60624. Max. coverage (+): 4.29. Max coverage (-): 0.08

Region: NODE\_312079\_length\_70626\_cov\_31.911549 60625-60661. Max. coverage (+): 0.15. Max coverage (-): 0

Region: NODE\_312079\_length\_70626\_cov\_31.911549 60662-60697. Max. coverage (+): 0.15. Max coverage (-): 0

Region: NODE\_312079\_length\_70626\_cov\_31.911549 60698-60734. Max. coverage (+): 2.45. Max coverage (-): 0.08

Region: NODE\_312079\_length\_70626\_cov\_31.911549 60735-60770. Max. coverage (+): 0.31. Max coverage (-): 0.08

Region: NODE\_312079\_length\_70626\_cov\_31.911549 60771-60806. Max. coverage (+): 0.31. Max coverage (-): 0

Region: NODE\_312079\_length\_70626\_cov\_31.911549 60807-60843. Max. coverage (+): 1.07. Max coverage (-): 0

Region: NODE\_312079\_length\_70626\_cov\_31.911549 60844-60879. Max. coverage (+): 18.69. Max coverage (-): 0

Region: NODE\_312079\_length\_70626\_cov\_31.911549 60880-60915. Max. coverage (+): 4.9. Max coverage (-): 0

Region: NODE\_312079\_length\_70626\_cov\_31.911549 60916-60952. Max. coverage (+): 0.15. Max coverage (-): 0

Region: NODE\_312079\_length\_70626\_cov\_31.911549 60953-60988. Max. coverage (+): 0.15. Max coverage (-): 0

Region: NODE\_312079\_length\_70626\_cov\_31.911549 60989-61024. Max. coverage (+): 0.84. Max coverage (-): 0

Region: NODE\_312079\_length\_70626\_cov\_31.911549 61025-61061. Max. coverage (+): 1.76. Max coverage (-): 0.23

Region: NODE\_312079\_length\_70626\_cov\_31.911549 61062-61097. Max. coverage (+): 0.08. Max coverage (-): 0.23

Region: NODE\_312079\_length\_70626\_cov\_31.911549 61098-61133. Max. coverage (+): 9.35. Max coverage (-): 0.31

Region: NODE\_312079\_length\_70626\_cov\_31.911549 61134-61170. Max. coverage (+): 0.23. Max coverage (-): 0.15

Region: NODE\_312079\_length\_70626\_cov\_31.911549 61171-61206. Max. coverage (+): 0.38. Max coverage (-): 0.23

Region: NODE\_312079\_length\_70626\_cov\_31.911549 61207-61242. Max. coverage (+): 1.61. Max coverage (-): 0

Region: NODE\_312079\_length\_70626\_cov\_31.911549 61243-61279. Max. coverage (+): 0.15. Max coverage (-): 0.08

Region: NODE\_312079\_length\_70626\_cov\_31.911549 61280-61315. Max. coverage (+): 0.08. Max coverage (-): 0

Region: NODE\_312079\_length\_70626\_cov\_31.911549 61316-61352. Max. coverage (+): 0.61. Max coverage (-): 0

Region: NODE\_312079\_length\_70626\_cov\_31.911549 61353-61388. Max. coverage (+): 0.08. Max coverage (-): 0

Region: NODE\_312079\_length\_70626\_cov\_31.911549 61389-61424. Max. coverage (+): 0. Max coverage (-): 0

Region: NODE\_312079\_length\_70626\_cov\_31.911549 61425-61461. Max. coverage (+): 0.15. Max coverage (-): 1

Region: NODE\_312079\_length\_70626\_cov\_31.911549 61462-61497. Max. coverage (+): 0. Max coverage (-): 0

Region: NODE\_312079\_length\_70626\_cov\_31.911549 61498-61533. Max. coverage (+): 1.07. Max coverage (-): 0

Region: NODE\_312079\_length\_70626\_cov\_31.911549 61534-61570. Max. coverage (+): 0.15. Max coverage (-): 0.08

Region: NODE\_312079\_length\_70626\_cov\_31.911549 61571-61606. Max. coverage (+): 0.08. Max coverage (-): 0.08

Region: NODE\_312079\_length\_70626\_cov\_31.911549 61607-61642. Max. coverage (+): 0.46. Max coverage (-): 0.46

Region: NODE\_312079\_length\_70626\_cov\_31.911549 61643-61679. Max. coverage (+): 2.6. Max coverage (-): 0.69

Region: NODE\_312079\_length\_70626\_cov\_31.911549 61680-61715. Max. coverage (+): 0.08. Max coverage (-): 0

Region: NODE\_312079\_length\_70626\_cov\_31.911549 61716-61751. Max. coverage (+): 1.92. Max coverage (-): 0.08

Region: NODE\_312079\_length\_70626\_cov\_31.911549 61752-61788. Max. coverage (+): 39.61. Max coverage (-): 0

Region: NODE\_312079\_length\_70626\_cov\_31.911549 61789-61824. Max. coverage (+): 0.31. Max coverage (-): 0

Region: NODE\_312079\_length\_70626\_cov\_31.911549 61825-61860. Max. coverage (+): 0.15. Max coverage (-): 0.15

Region: NODE\_312079\_length\_70626\_cov\_31.911549 61861-61897. Max. coverage (+): 7.43. Max coverage (-): 0.38

Region: NODE\_312079\_length\_70626\_cov\_31.911549 61898-61933. Max. coverage (+): 0.46. Max coverage (-): 0

Region: NODE\_312079\_length\_70626\_cov\_31.911549 61934-61970. Max. coverage (+): 1.15. Max coverage (-): 0.08

Region: NODE\_312079\_length\_70626\_cov\_31.911549 61971-62006. Max. coverage (+): 0.54. Max coverage (-): 0

Region: NODE\_312079\_length\_70626\_cov\_31.911549 62007-62042. Max. coverage (+): 1.61. Max coverage (-): 0

Region: NODE\_312079\_length\_70626\_cov\_31.911549 62043-62079. Max. coverage (+): 0.61. Max coverage (-): 0

Region: NODE\_312079\_length\_70626\_cov\_31.911549 62080-62115. Max. coverage (+): 0.38. Max coverage (-): 0

Region: NODE\_312079\_length\_70626\_cov\_31.911549 62116-62151. Max. coverage (+): 0. Max coverage (-): 0.08

Region: NODE\_312079\_length\_70626\_cov\_31.911549 62152-62188. Max. coverage (+): 0.46. Max coverage (-): 0

Region: NODE\_312079\_length\_70626\_cov\_31.911549 62189-62224. Max. coverage (+): 3.29. Max coverage (-): 0.15

Region: NODE\_312079\_length\_70626\_cov\_31.911549 62225-62260. Max. coverage (+): 3.91. Max coverage (-): 0.08

Region: NODE\_312079\_length\_70626\_cov\_31.911549 62261-62297. Max. coverage (+): 2.07. Max coverage (-): 0.08

Region: NODE\_312079\_length\_70626\_cov\_31.911549 62298-62333. Max. coverage (+): 1.07. Max coverage (-): 0.08

Region: NODE\_312079\_length\_70626\_cov\_31.911549 62334-62369. Max. coverage (+): 2.53. Max coverage (-): 1.99

Region: NODE\_312079\_length\_70626\_cov\_31.911549 62370-62406. Max. coverage (+): 0.23. Max coverage (-): 0.08

Region: NODE\_312079\_length\_70626\_cov\_31.911549 62407-62442. Max. coverage (+): 5.67. Max coverage (-): 0.31

Region: NODE\_312079\_length\_70626\_cov\_31.911549 62443-62479. Max. coverage (+): 0.08. Max coverage (-): 0.31

Region: NODE\_312079\_length\_70626\_cov\_31.911549 62480-62515. Max. coverage (+): 1.38. Max coverage (-): 0.38

Region: NODE\_312079\_length\_70626\_cov\_31.911549 62516-62551. Max. coverage (+): 0. Max coverage (-): 0.08

Region: NODE\_312079\_length\_70626\_cov\_31.911549 62552-62588. Max. coverage (+): 0.31. Max coverage (-): 0.31

Region: NODE\_312079\_length\_70626\_cov\_31.911549 62589-62624. Max. coverage (+): 0.08. Max coverage (-): 0

Region: NODE\_312079\_length\_70626\_cov\_31.911549 62625-62660. Max. coverage (+): 0. Max coverage (-): 0.08

Region: NODE\_312079\_length\_70626\_cov\_31.911549 62661-62697. Max. coverage (+): 0. Max coverage (-): 0.46

Region: NODE\_312079\_length\_70626\_cov\_31.911549 62698-62733. Max. coverage (+): 0. Max coverage (-): 0

Region: NODE\_312079\_length\_70626\_cov\_31.911549 62734-62769. Max. coverage (+): 0.69. Max coverage (-): 0

Region: NODE\_312079\_length\_70626\_cov\_31.911549 62770-62806. Max. coverage (+): 0.31. Max coverage (-): 0

Region: NODE\_312079\_length\_70626\_cov\_31.911549 62807-62842. Max. coverage (+): 4.98. Max coverage (-): 0.08

Region: NODE\_312079\_length\_70626\_cov\_31.911549 62843-62878. Max. coverage (+): 3.45. Max coverage (-): 0.38

Region: NODE\_312079\_length\_70626\_cov\_31.911549 62879-62915. Max. coverage (+): 0.46. Max coverage (-): 0.08

Region: NODE\_312079\_length\_70626\_cov\_31.911549 62916-62951. Max. coverage (+): 0. Max coverage (-): 2.6

Region: NODE\_312079\_length\_70626\_cov\_31.911549 62952-62987. Max. coverage (+): 1.3. Max coverage (-): 3.68

Region: NODE\_312079\_length\_70626\_cov\_31.911549 62988-63024. Max. coverage (+): 0.31. Max coverage (-): 0

Region: NODE\_312079\_length\_70626\_cov\_31.911549 63025-63060. Max. coverage (+): 0.08. Max coverage (-): 0

Region: NODE\_312079\_length\_70626\_cov\_31.911549 63061-63097. Max. coverage (+): 2.07. Max coverage (-): 0.15

Region: NODE\_312079\_length\_70626\_cov\_31.911549 63098-63133. Max. coverage (+): 0.08. Max coverage (-): 0

Region: NODE\_312079\_length\_70626\_cov\_31.911549 63134-63169. Max. coverage (+): 0.61. Max coverage (-): 0.08

Region: NODE\_312079\_length\_70626\_cov\_31.911549 63170-63206. Max. coverage (+): 0.23. Max coverage (-): 0.15

Region: NODE\_312079\_length\_70626\_cov\_31.911549 63207-63242. Max. coverage (+): 10.65. Max coverage (-): 1.84

Region: NODE\_312079\_length\_70626\_cov\_31.911549 63243-63278. Max. coverage (+): 1.07. Max coverage (-): 0.08

Region: NODE\_312079\_length\_70626\_cov\_31.911549 63279-63315. Max. coverage (+): 1.15. Max coverage (-): 0.08

Region: NODE\_312079\_length\_70626\_cov\_31.911549 63316-63351. Max. coverage (+): 0.23. Max coverage (-): 0.08

Region: NODE\_312079\_length\_70626\_cov\_31.911549 63352-63387. Max. coverage (+): 3.98. Max coverage (-): 0

Region: NODE\_312079\_length\_70626\_cov\_31.911549 63388-63424. Max. coverage (+): 0.08. Max coverage (-): 0

Region: NODE\_312079\_length\_70626\_cov\_31.911549 63425-63460. Max. coverage (+): 0.23. Max coverage (-): 0

Region: NODE\_312079\_length\_70626\_cov\_31.911549 63461-63496. Max. coverage (+): 0. Max coverage (-): 0

Region: NODE\_312079\_length\_70626\_cov\_31.911549 63497-63533. Max. coverage (+): 0.08. Max coverage (-): 0.08

Region: NODE\_312079\_length\_70626\_cov\_31.911549 63534-63569. Max. coverage (+): 0.84. Max coverage (-): 0.08

Region: NODE\_312079\_length\_70626\_cov\_31.911549 63570-63605. Max. coverage (+): 0.08. Max coverage (-): 0.38

Region: NODE\_312079\_length\_70626\_cov\_31.911549 63606-63642. Max. coverage (+): 4.29. Max coverage (-): 0.31

Region: NODE\_312079\_length\_70626\_cov\_31.911549 63643-63678. Max. coverage (+): 0. Max coverage (-): 0

Region: NODE\_312079\_length\_70626\_cov\_31.911549 63679-63715. Max. coverage (+): 0.92. Max coverage (-): 0.08

Region: NODE\_312079\_length\_70626\_cov\_31.911549 63716-63751. Max. coverage (+): 0.23. Max coverage (-): 0

Region: NODE\_312079\_length\_70626\_cov\_31.911549 63752-63787. Max. coverage (+): 0.08. Max coverage (-): 0.15

Region: NODE\_312079\_length\_70626\_cov\_31.911549 63788-63824. Max. coverage (+): 0.08. Max coverage (-): 0.23

Region: NODE\_312079\_length\_70626\_cov\_31.911549 63825-63860. Max. coverage (+): 0.46. Max coverage (-): 0.23

Region: NODE\_312079\_length\_70626\_cov\_31.911549 63861-63896. Max. coverage (+): 0.46. Max coverage (-): 0.23

Region: NODE\_312079\_length\_70626\_cov\_31.911549 63897-63933. Max. coverage (+): 0.77. Max coverage (-): 0

Region: NODE\_312079\_length\_70626\_cov\_31.911549 63934-63969. Max. coverage (+): 0.15. Max coverage (-): 0

Region: NODE\_312079\_length\_70626\_cov\_31.911549 63970-64005. Max. coverage (+): 1.69. Max coverage (-): 0

Region: NODE\_312079\_length\_70626\_cov\_31.911549 64006-64042. Max. coverage (+): 0.38. Max coverage (-): 0.08

Region: NODE\_312079\_length\_70626\_cov\_31.911549 64043-64078. Max. coverage (+): 0.38. Max coverage (-): 0.08

Region: NODE\_312079\_length\_70626\_cov\_31.911549 64079-64114. Max. coverage (+): 0. Max coverage (-): 0.54

Region: NODE\_312079\_length\_70626\_cov\_31.911549 64115-64151. Max. coverage (+): 1.76. Max coverage (-): 0.08

Region: NODE\_312079\_length\_70626\_cov\_31.911549 64152-64187. Max. coverage (+): 20.99. Max coverage (-): 0

Region: NODE\_312079\_length\_70626\_cov\_31.911549 64188-64223. Max. coverage (+): 1.61. Max coverage (-): 0

Region: NODE\_312079\_length\_70626\_cov\_31.911549 64224-64260. Max. coverage (+): 1.61. Max coverage (-): 0

Region: NODE\_312079\_length\_70626\_cov\_31.911549 64261-64296. Max. coverage (+): 0.08. Max coverage (-): 0

Region: NODE\_312079\_length\_70626\_cov\_31.911549 64297-64333. Max. coverage (+): 0.23. Max coverage (-): 0

Region: NODE\_312079\_length\_70626\_cov\_31.911549 64334-64369. Max. coverage (+): 1.07. Max coverage (-): 0

Region: NODE\_312079\_length\_70626\_cov\_31.911549 64370-64405. Max. coverage (+): 0.11. Max coverage (-): 0.08

Region: NODE\_312079\_length\_70626\_cov\_31.911549 64406-64442. Max. coverage (+): 55.93. Max coverage (-): 0

Region: NODE\_312079\_length\_70626\_cov\_31.911549 64443-64478. Max. coverage (+): 1. Max coverage (-): 0

Region: NODE\_312079\_length\_70626\_cov\_31.911549 64479-64514. Max. coverage (+): 0.84. Max coverage (-): 0.31

Region: NODE\_312079\_length\_70626\_cov\_31.911549 64515-64551. Max. coverage (+): 0. Max coverage (-): 0.15

Region: NODE\_312079\_length\_70626\_cov\_31.911549 64552-64587. Max. coverage (+): 19.84. Max coverage (-): 0.08

Region: NODE\_312079\_length\_70626\_cov\_31.911549 64588-64623. Max. coverage (+): 0.54. Max coverage (-): 0.08

Region: NODE\_312079\_length\_70626\_cov\_31.911549 64624-64660. Max. coverage (+): 1.38. Max coverage (-): 0.08

Region: NODE\_312079\_length\_70626\_cov\_31.911549 64661-64696. Max. coverage (+): 0.31. Max coverage (-): 0

Region: NODE\_312079\_length\_70626\_cov\_31.911549 64697-64732. Max. coverage (+): 0.54. Max coverage (-): 0

Region: NODE\_312079\_length\_70626\_cov\_31.911549 64733-64769. Max. coverage (+): 0.15. Max coverage (-): 0

Region: NODE\_312079\_length\_70626\_cov\_31.911549 64770-64805. Max. coverage (+): 0. Max coverage (-): 0

Region: NODE\_312079\_length\_70626\_cov\_31.911549 64806-64842. Max. coverage (+): 0.03. Max coverage (-): 0

Region: NODE\_312079\_length\_70626\_cov\_31.911549 64843-64878. Max. coverage (+): 0. Max coverage (-): 0

Region: NODE\_312079\_length\_70626\_cov\_31.911549 64879-64914. Max. coverage (+): 0. Max coverage (-): 0

Region: NODE\_312079\_length\_70626\_cov\_31.911549 64915-64951. Max. coverage (+): 0. Max coverage (-): 0

Region: NODE\_312079\_length\_70626\_cov\_31.911549 64952-64987. Max. coverage (+): 0. Max coverage (-): 0

Region: NODE\_312079\_length\_70626\_cov\_31.911549 64988-65023. Max. coverage (+): 0. Max coverage (-): 0

Region: NODE\_312079\_length\_70626\_cov\_31.911549 65024-65060. Max. coverage (+): 0. Max coverage (-): 0

Region: NODE\_312079\_length\_70626\_cov\_31.911549 65061-65096. Max. coverage (+): 0. Max coverage (-): 0

Region: NODE\_312079\_length\_70626\_cov\_31.911549 65097-65132. Max. coverage (+): 0. Max coverage (-): 0

Region: NODE\_312079\_length\_70626\_cov\_31.911549 65133-65169. Max. coverage (+): 0. Max coverage (-): 0

Region: NODE\_312079\_length\_70626\_cov\_31.911549 65170-65205. Max. coverage (+): 0. Max coverage (-): 0

Region: NODE\_312079\_length\_70626\_cov\_31.911549 65206-65241. Max. coverage (+): 0. Max coverage (-): 0

Region: NODE\_312079\_length\_70626\_cov\_31.911549 65242-65278. Max. coverage (+): 0. Max coverage (-): 0

Region: NODE\_312079\_length\_70626\_cov\_31.911549 65279-65314. Max. coverage (+): 0. Max coverage (-): 0

Region: NODE\_312079\_length\_70626\_cov\_31.911549 65315-65350. Max. coverage (+): 0. Max coverage (-): 0

Region: NODE\_312079\_length\_70626\_cov\_31.911549 65351-65387. Max. coverage (+): 0. Max coverage (-): 0

Region: NODE\_312079\_length\_70626\_cov\_31.911549 65388-65423. Max. coverage (+): 0. Max coverage (-): 0

Region: NODE\_312079\_length\_70626\_cov\_31.911549 65424-65460. Max. coverage (+): 0. Max coverage (-): 0

Region: NODE\_312079\_length\_70626\_cov\_31.911549 65461-65496. Max. coverage (+): 0. Max coverage (-): 0

Region: NODE\_312079\_length\_70626\_cov\_31.911549 65497-65532. Max. coverage (+): 0. Max coverage (-): 0

Region: NODE\_312079\_length\_70626\_cov\_31.911549 65533-65569. Max. coverage (+): 0. Max coverage (-): 0

Region: NODE\_312079\_length\_70626\_cov\_31.911549 65570-65605. Max. coverage (+): 0. Max coverage (-): 0

Region: NODE\_312079\_length\_70626\_cov\_31.911549 65606-65641. Max. coverage (+): 0. Max coverage (-): 0

Region: NODE\_312079\_length\_70626\_cov\_31.911549 65642-65678. Max. coverage (+): 0. Max coverage (-): 0.08

Region: NODE\_312079\_length\_70626\_cov\_31.911549 65679-65714. Max. coverage (+): 0. Max coverage (-): 0

Region: NODE\_312079\_length\_70626\_cov\_31.911549 65715-65750. Max. coverage (+): 0. Max coverage (-): 0

Region: NODE\_312079\_length\_70626\_cov\_31.911549 65751-65787. Max. coverage (+): 0. Max coverage (-): 0

Region: NODE\_312079\_length\_70626\_cov\_31.911549 65788-65823. Max. coverage (+): 0. Max coverage (-): 0

Region: NODE\_312079\_length\_70626\_cov\_31.911549 65824-65859. Max. coverage (+): 0. Max coverage (-): 0

Region: NODE\_312079\_length\_70626\_cov\_31.911549 65860-65896. Max. coverage (+): 0. Max coverage (-): 0

Region: NODE\_312079\_length\_70626\_cov\_31.911549 65897-65932. Max. coverage (+): 0. Max coverage (-): 0

Region: NODE\_312079\_length\_70626\_cov\_31.911549 65933-65968. Max. coverage (+): 0. Max coverage (-): 0

Region: NODE\_312079\_length\_70626\_cov\_31.911549 65969-66005. Max. coverage (+): 0. Max coverage (-): 0

Region: NODE\_312079\_length\_70626\_cov\_31.911549 66006-66041. Max. coverage (+): 0. Max coverage (-): 0

Region: NODE\_312079\_length\_70626\_cov\_31.911549 66042-66078. Max. coverage (+): 0. Max coverage (-): 0

Region: NODE\_312079\_length\_70626\_cov\_31.911549 66079-66114. Max. coverage (+): 0. Max coverage (-): 0

Region: NODE\_312079\_length\_70626\_cov\_31.911549 66115-66150. Max. coverage (+): 0. Max coverage (-): 0

Region: NODE\_312079\_length\_70626\_cov\_31.911549 66151-66187. Max. coverage (+): 0. Max coverage (-): 0

Region: NODE\_312079\_length\_70626\_cov\_31.911549 66188-66223. Max. coverage (+): 0. Max coverage (-): 0

Region: NODE\_312079\_length\_70626\_cov\_31.911549 66224-66259. Max. coverage (+): 0. Max coverage (-): 0.08

Region: NODE\_312079\_length\_70626\_cov\_31.911549 66260-. Max. coverage (+): 0. Max coverage (-): 0

RepeatMasker Color Code

**+**

100-98% Identity

<98-95% Identity

<95-90% Identity

<90-85% Identity

<85-80% Identity

<80-75% Identity

<75-70% Identity

<70% Identity

**-**

Gene Set Color Code

**+**

Gene

Pseudogene

Other

**-**

Topology/Coverage Color Code

Coverage Plus Strand

Coverage Minus Strand

Mainstrand: Plus

Mainstrand: Minus

Complementary Strand

Flanking Region  
(if option -flank >0)

Gene Set Annotation  

**1. unknown (unknownunknown) Tr:unknown**: 65369-66138 (-)  
**2. unknown (unknownunknown) Tr:unknown**: 66235-66281 (-)  
**3. unknown (unknownunknown) Tr:unknown UTR**: 65369-65599 (-)

  
RepeatMasker Annotation  

**1. AlRepC-136**: 48775-48887 (+), Divergence to consensus: 6.3%  
**2. AlRepB-2**: 48874-49130 (+), Divergence to consensus: 15.5%  
**3. Penelope-1\_AFC**: 51503-51545 (+), Divergence to consensus: 13.9%  
**4. REX1-1\_AFC**: 53635-53855 (+), Divergence to consensus: 8.8%  
**5. A-rich**: 55236-55267 (+), Divergence to consensus: 16.9%  
**6. AlRepB-1625**: 55750-55947 (-), Divergence to consensus: 13.2%  
**7. AlRepD-4675**: 55953-56014 (-), Divergence to consensus: 1.6%  
**8. Kolobok-2\_XT**: 58381-58424 (-), Divergence to consensus: 13.6%  
**9. AlRepB-438**: 58384-58678 (-), Divergence to consensus: 18.6%  
**10. Kolobok-2\_XT**: 58630-58682 (-), Divergence to consensus: 19.2%  
**11. TC1\_FR3**: 59601-59783 (-), Divergence to consensus: 28.5%  
**12. (TAGCTT)n**: 59921-59961 (+), Divergence to consensus: 20.4%  
**13. AlRepD-5020**: 62546-62959 (-), Divergence to consensus: 38.3%  
**14. AlRepE-1134**: 63069-63243 (-), Divergence to consensus: 38%  
**15. AlRepD-1895**: 63282-63336 (+), Divergence to consensus: 18.6%  
**16. AlRepA-4**: 64070-64295 (+), Divergence to consensus: 29.3%  
**17. AlRepA-4**: 64408-64557 (+), Divergence to consensus: 38.6%  
**18. Tc1-2\_FR**: 64752-64806 (+), Divergence to consensus: 11%  
**19. Tc1-2\_FR**: 64807-64848 (-), Divergence to consensus: 2.4%  
**20. AlRepB-13**: 65037-65100 (+), Divergence to consensus: 24.2%  
**21. AlRepE-3032**: 65129-65211 (-), Divergence to consensus: 35.6%  
**22. AlRepE-3032**: 65216-65296 (-), Divergence to consensus: 21.1%  
**23. (CAA)n**: 65297-65321 (+), Divergence to consensus: 12.4%  
**24. AlRepC-580**: 65611-66142 (+), Divergence to consensus: 29.7%  
**25. AlRepC-580**: 66225-66328 (+), Divergence to consensus: 34.4%

  
Transcription Factor Binding Sites  

**RHOXF1** (Sequence: AGATTA (-): 48723)  
**RHOXF1** (Sequence: AGATCA (-): 49206)  
**RHOXF1** (Sequence: GGCTTA (-): 49673)  
**RHOXF1** (Sequence: AGATCA (-): 50444)  
**RHOXF1** (Sequence: AGATCA (-): 51400)  
**RHOXF1** (Sequence: AGATTA (-): 52974)  
**RHOXF1** (Sequence: GGCTCA (-): 53625)  
**RHOXF1** (Sequence: AGATTA (-): 54742)  
**RHOXF1** (Sequence: GGATTA (-): 55063)  
**RHOXF1** (Sequence: GGCTTA (-): 56243)  
**RHOXF1** (Sequence: AGCTTA (-): 56989)  
**RHOXF1** (Sequence: AGCTTA (-): 57604)  
**RHOXF1** (Sequence: GGATTA (-): 57921)  
**RHOXF1** (Sequence: AGCTTA (-): 58919)  
**RHOXF1** (Sequence: AGATCA (-): 58921)  
**RHOXF1** (Sequence: AGATCA (-): 59452)  
**RHOXF1** (Sequence: AGATCA (-): 59465)  
**RHOXF1** (Sequence: AGCTCA (-): 59751)  
**RHOXF1** (Sequence: AGATCA (-): 59867)  
**RHOXF1** (Sequence: AGATTA (-): 60359)  
**RHOXF1** (Sequence: GGATCA (-): 60806)  
**RHOXF1** (Sequence: AGCTCA (-): 60858)  
**RHOXF1** (Sequence: AGATTA (-): 61136)  
**RHOXF1** (Sequence: AGCTCA (-): 61232)  
**RHOXF1** (Sequence: AGATCA (-): 61267)  
**RHOXF1** (Sequence: GGCTCA (-): 61610)  
**RHOXF1** (Sequence: AGATTA (-): 61667)  
**RHOXF1** (Sequence: AGATTA (-): 63254)  
**RHOXF1** (Sequence: AGATCA (-): 64071)  
**RHOXF1** (Sequence: AGATTA (-): 64527)  
**RHOXF1** (Sequence: GGATTA (-): 65257)  
**RHOXF1** (Sequence: AGATTA (-): 65597)  
**RHOXF1** (Sequence: GGATTA (-): 65599)  
**RHOXF1** (Sequence: TGAGCT (+): 48263)  
**RHOXF1** (Sequence: TGATCT (+): 48316)  
**RHOXF1** (Sequence: TGATCT (+): 48466)  
**RHOXF1** (Sequence: TAATCC (+): 48707)  
**RHOXF1** (Sequence: TAATCC (+): 48863)  
**RHOXF1** (Sequence: TAATCT (+): 50194)  
**RHOXF1** (Sequence: TGATCT (+): 50573)  
**RHOXF1** (Sequence: TGAGCC (+): 50874)  
**RHOXF1** (Sequence: TGATCT (+): 51728)  
**RHOXF1** (Sequence: TGATCT (+): 51994)  
**RHOXF1** (Sequence: TGAGCT (+): 52771)  
**RHOXF1** (Sequence: TAATCC (+): 53021)  
**RHOXF1** (Sequence: TGATCT (+): 53431)  
**RHOXF1** (Sequence: TAATCT (+): 54051)  
**RHOXF1** (Sequence: TAATCC (+): 54057)  
**RHOXF1** (Sequence: TAATCT (+): 54464)  
**RHOXF1** (Sequence: TAAGCT (+): 55205)  
**RHOXF1** (Sequence: TGATCT (+): 55553)  
**RHOXF1** (Sequence: TAATCT (+): 55634)  
**RHOXF1** (Sequence: TAAGCC (+): 55931)  
**RHOXF1** (Sequence: TGATCT (+): 57130)  
**RHOXF1** (Sequence: TAAGCT (+): 57602)  
**RHOXF1** (Sequence: TAAGCC (+): 57807)  
**RHOXF1** (Sequence: TAATCT (+): 60727)  
**RHOXF1** (Sequence: TGAGCT (+): 60929)  
**RHOXF1** (Sequence: TGAGCC (+): 61078)  
**RHOXF1** (Sequence: TGAGCT (+): 61253)  
**RHOXF1** (Sequence: TGATCC (+): 61736)  
**RHOXF1** (Sequence: TAATCT (+): 62644)  
**RHOXF1** (Sequence: TAAGCC (+): 62890)  
**RHOXF1** (Sequence: TGATCT (+): 63009)  
**RHOXF1** (Sequence: TGAGCT (+): 63096)  
**RHOXF1** (Sequence: TGAGCC (+): 64536)  
**Lhx8** (Sequence: CTAATTAG (-): 48523)  
**Lhx8** (Sequence: TTAATTAG (-): 53574)  
**Lhx8** (Sequence: TTAATTAA (-): 56181)  
**Lhx8** (Sequence: TTAATTAA (-): 56752)  
**Lhx8** (Sequence: TTAATTAG (-): 65371)  
**Gata4** (Sequence: CTTATCT (+): 53174)  
**Gata4** (Sequence: GTTATCT (+): 62969)  
**POU5F1** (Sequence: TTTGCAT (-): 61418)  
**FOXO3\_hsa** (Sequence: GTAAACAA (+): 50467)  
**FOXO3\_hsa** (Sequence: GTAAACAT (+): 54294)  
**SOX9** (Sequence: AACAATAG (-): 52108)  
**SOX9** (Sequence: AACAATGA (-): 53204)  
**SOX9** (Sequence: AACAATAA (-): 55880)  
**FOXP1** (Sequence: GTAAACA (+): 50467)  
**FOXP1** (Sequence: GTAAACA (+): 54294)  
**FOXO1** (Sequence: CTTGTTTAT (+): 51586)  
**FOXO1** (Sequence: CTTGTTTTT (+): 55194)  
**FOXO1** (Sequence: GCTGTTTTT (+): 57253)  
**FOXO1** (Sequence: CTTGTTTAT (+): 58800)  
**FOXO1** (Sequence: GTTGTTTAC (+): 65327)  
**FOXO3\_mmu** (Sequence: TGTTTTGC (-): 49410)  
**FOXO3\_mmu** (Sequence: TGTTTTCC (-): 50243)  
**FOXO3\_mmu** (Sequence: TGTTTTCC (-): 50983)  
**FOXO3\_mmu** (Sequence: TGTTTACA (-): 52703)  
**FOXO3\_mmu** (Sequence: TGTTTTCA (-): 53607)  
**FOXO3\_mmu** (Sequence: TGTTTTGC (-): 56926)  
**FOXO3\_mmu** (Sequence: TGTTTTGA (-): 57165)  
**FOXO3\_mmu** (Sequence: TGTTTACA (-): 57723)  
**FOXO3\_mmu** (Sequence: TGTTTAGA (-): 59040)  
**FOXO3\_mmu** (Sequence: TGTTTTGA (-): 62152)  
**FOXO3\_mmu** (Sequence: TGTTTTCA (-): 62541)  
**Sox5** (Sequence: ATTGTT (+): 48701)  
**Sox5** (Sequence: ATTGTT (+): 52525)  
**Sox5** (Sequence: ATTGTT (+): 56584)  
**Sox5** (Sequence: ATTGTT (+): 56800)  
**Sox5** (Sequence: ATTGTT (+): 57586)  
**Sox5** (Sequence: ATTGTT (+): 57588)  
**Sox5** (Sequence: ATTGTT (+): 59562)  
**Sox5** (Sequence: ATTGTT (+): 63194)  
**Sox5** (Sequence: ATTGTT (+): 63467)  
**Sox5** (Sequence: ATTGTT (+): 64714)  
**FIGLA** (Sequence: TACAGCTGGT (-): 50475)  
**FIGLA** (Sequence: TACAGCTGGA (-): 52139)  
**FIGLA** (Sequence: AACACCTGGA (-): 53227)  
**FIGLA** (Sequence: ACCACCTGTA (-): 54307)  
**FIGLA** (Sequence: TCCAGCTGTA (-): 62345)  
**FOXO3\_mmu** (Sequence: TGAAAACA (+): 49715)  
**FOXO3\_mmu** (Sequence: GGAAAACA (+): 51362)  
**FOXO3\_mmu** (Sequence: TGTAAACA (+): 54293)  
**FOXO3\_mmu** (Sequence: TGAAAACA (+): 55813)  
**FOXO3\_mmu** (Sequence: GGAAAACA (+): 63920)  
**FOXO3\_mmu** (Sequence: TGAAAACA (+): 65285)  
**Nobox** (Sequence: GCTAATTA (-): 48522)  
**FOXO1** (Sequence: AAAAACAGC (-): 48653)  
**FOXO1** (Sequence: AAAAACAAC (-): 62753)  
**FOXO1** (Sequence: ATAAACAGC (-): 64953)  
**FOXO1** (Sequence: GAAAACAAC (-): 65286)  
**FOXO3\_hsa** (Sequence: ATGTTTAC (-): 52702)  
**FOXO3\_hsa** (Sequence: ATGTTTAC (-): 57722)  
**FOXO3\_hsa** (Sequence: TTGTTTAC (-): 65328)  
**FOXP1** (Sequence: TGTTTAC (-): 52703)  
**FOXP1** (Sequence: TGTTTAC (-): 57723)  
**FOXP1** (Sequence: TGTTTAC (-): 65329)  
**Nobox** (Sequence: TAATTAGC (+): 53575)  
**Nobox** (Sequence: TAATTGCT (+): 59882)  
**Nobox** (Sequence: TAATTAGC (+): 65372)  
**POU2F1** (Sequence: ATTAAAATA (-): 60583)  
**POU2F1** (Sequence: ATTAAAATA (-): 60825)  
**POU2F1** (Sequence: ATTAAAATA (-): 65405)  
**Rhox11** (Sequence: TGCTGTAAT (+): 50365)  
**Rhox11** (Sequence: CGCTGTTTT (+): 57252)  
**Rhox11** (Sequence: TGCTGTAAA (+): 58672)  
**Rhox11** (Sequence: TTAACACCA (-): 49893)  
**Rhox11** (Sequence: AAAACACCA (-): 54863)  
**Rhox11** (Sequence: AAAACACCA (-): 65230)  
**Rhox11** (Sequence: AATACAGCA (-): 66168)  
**Gata4** (Sequence: AGATAAC (-): 49556)  
**Gata4** (Sequence: AGATAAC (-): 51421)  
**Sox5** (Sequence: AACAAT (-): 49560)  
**Sox5** (Sequence: AACAAT (-): 50032)  
**Sox5** (Sequence: AACAAT (-): 50058)  
**Sox5** (Sequence: AACAAT (-): 50470)  
**Sox5** (Sequence: AACAAT (-): 51279)  
**Sox5** (Sequence: AACAAT (-): 51488)  
**Sox5** (Sequence: AACAAT (-): 51967)  
**Sox5** (Sequence: AACAAT (-): 52108)  
**Sox5** (Sequence: AACAAT (-): 52763)  
**Sox5** (Sequence: AACAAT (-): 53204)  
**Sox5** (Sequence: AACAAT (-): 55111)  
**Sox5** (Sequence: AACAAT (-): 55530)  
**Sox5** (Sequence: AACAAT (-): 55880)  
**Sox5** (Sequence: AACAAT (-): 56079)  
**Sox5** (Sequence: AACAAT (-): 56719)  
**Sox5** (Sequence: AACAAT (-): 57038)  
**Sox5** (Sequence: AACAAT (-): 61906)  
**Sox5** (Sequence: AACAAT (-): 63239)  
**Sox5** (Sequence: AACAAT (-): 63924)  
**Sox5** (Sequence: AACAAT (-): 64274)  
**POU2F1** (Sequence: TATGTAAAT (+): 50071)  
**POU2F1** (Sequence: TATTTTAAT (+): 53570)  
**POU2F1** (Sequence: TATTTAAAT (+): 64179)  
**POU5F1** (Sequence: ATGCAAA (+): 51795)  
**POU5F1** (Sequence: ATGCAAA (+): 55534)  
**POU5F1** (Sequence: ATGCAAA (+): 63539)
