# Supplementary material for: First characterization of PIWI-interacting RNA clusters in a cichlid fish with a B chromosome
Source: BMC Biol. 2022 Sep 21;20:204. doi: 10.1186/s12915-022-01403-2 (PMC9490952; doi:10.1186/s12915-022-01403-2)
Supplement: Supplementary file 1 — Additional file 1. Zipped folder with fasta and interactive html piRNA cluster information for the A. latifasciata genome. The nomenclature is as follows: number-pirna-cluster_sex_B-presence (f, female; m, male; 0b, without B chromosome; 1b, with B chromosome). [file 12915_2022_1403_MOESM1_ESM.zip › 121_f1b.html]

piRNA cluster 121\_f1b 58


Predicted piRNA cluster no. 121\_f1b
  

Show proTRAC run info
Hide proTRAC run info

/\  
                \_\_\_\_\_\_\_\_\_\_\_\_\_\_\_\_\_\_\_\_\_\_\_/\\_\_\_ /  \\_\_\_\_\_\_\_  
               I                      /  \  /    \      I  
               I     pro             /    \/      \     I  
               I        TRAC        /               \   I  
               I   \_\_\_\_\_\_\_\_\_\_\_\_\_\_\_\_/\_\_\_\_\_\_\_\_\_\_\_\_\_\_\_\_\_\\_ I  
               I   \              /                     I  
               I    \            /                      I  
               I     \  /\      /       V.2.4.2         I  
               I      \/  \    /                        I  
               I\_\_\_\_\_\_\_\_\_\_\_\  /\_\_\_\_\_\_\_\_\_\_\_\_\_\_\_\_\_\_\_\_\_\_\_\_\_I  
                            \/  
  
  
================================= proTRAC ====================================  
VERSION: .......... 2.4.2  
LAST MODIFIED: .... 11. May 2018  
  
Please cite:  
Rosenkranz D, Zischler H. proTRAC - a software for probabilistic piRNA cluster  
detection, visualization and analysis. 2012. BMC Bioinformatics 13:5.  
  
  
Contact:  
David Rosenkranz  
Institute of Organismic and Molecular Evolutionary Biology  
Dept. Anthropology, small RNA group  
Johannes Gutenberg University Mainz  
email: rosenkranz@uni-mainz.de  
  
You can find the latest proTRAC version at:  
http://sourceforge.net/projects/protrac/files  
http://www.smallRNAgroup-mainz.de/software  
==============================================================================  
  
PARAMETERS:  
Map file: ...............piwi-femeas-1B.fa-collapse.map  
Genome file: ............../../../0B\_ala\_genome.fa  
RepeatMasker annotation: Alatifasciata-all0B-maryan-v2.fa\_corrected.out  
GeneSet:................./guest-storage/Data/annotation/Alatifasciata\_all0B\_maryan-v2\_out2017.gff  
  
Significant (p<=0.01) hit density will be calculated based  
on observed hit distribution.  
  
Sliding window size: ........................................ 5000 bp  
Sliding window increament: .................................. 1000 bp  
Normalize each hit by number of genomic hits: ............... yes  
Normalize each hit by number of sequence reads: ............. yes  
Normalize values (-> per million mapped reads): ............. yes  
Min. fraction of hits with 1T(U) or 10A: .................... 0.75  
Alternatively: Min. fraction of hits with 1T(U) and 10A: .... 0.5  
Min. fraction of hits with typical piRNA length: ............ 0.75  
Typical piRNA length: ....................................... 24-32 nt  
Min. size of a piRNA cluster: ............................... 1000 bp.  
Min. number of hits (absolute): ............................. 0  
Min. number of hits (normalized): ........................... 0  
Min. fraction of hits on the mainstrand: .................... 0.75  
Top fraction of mapped sequences (in terms of read counts): . 1%  
Top fraction accounts for max. n% of sequence reads: ........ 90%  
Min. fraction of hits on each arm of a bidirectional cluster: 0.05  
Output html file for each cluster: .......................... yes  
Output a summary table: ..................................... yes  
Output a FASTA file for each cluster (piRNA sequences): ..... yes  
Output a FASTA file comprising cluster sequences: ........... yes  
Output a GTF file for predicted piRNA clusters: ..............yes  
Search DNA motifs in clusters: .............................. yes  
Output flanking sequences: +/- .............................. 0 bp  
Output ~.pTi file: .......................................... no  
==============================================================================  
  
  
Genome size (without gaps): ............ 758543724 bp  
Gaps (N/X/-): .......................... 417479 bp  
Mapped reads: .......................... 10641844  
Non-identical sequences: ............... 2832837  
Genomic hits: .......................... 26056853  
Significant densitiy of mapped reads: .. 368.713530323068 reads/kb

Show proTRAC cluster info
Hide proTRAC cluster info

|  |  |
| --- | --- |
| Location | NODE\_312079\_length\_70626\_cov\_31.911549 |
| Coordinates | 56146-64841 |
| Size [bp] | 8696 |
| Sequence hit loci | 1511 |
| Mapped reads (normalized) | 3751 |
| Mapped reads (normalized) per kb | 431.3 |
| Normalized reads with 1T (1U) | 79.6% |
| Normalized reads with 10A | 43.8% |
| Normalized reads with length 24-32 nt | 98.7% |
| Normalized reads on the main strand(s) | 91.3% |
| Predicted directionality | mono:plus |

100%

0%

1T (1U)  
reads

10A reads

24-32 nt  
reads

reads on mainstrand

**Either the amount of reads with 1T (1U) OR 10A has to exceed 75% (set with option: -1Tor10A)  
Alternatively the amount of reads with 1T (1U) AND 10A has to exceed 50% (set with option: -1Tand10A)  
Minimum amount of reads with preferred size is 75% (set with option: -pisize)  
Minimum amount of reads on the main strand(s) is 75% (set with option: -clstrand)**

Show read coverage
Hide read coverage

WHAT DO I SEE HERE?  
This chart shows the location of mapped sequence reads within a predicted piRNA cluster. The color refers to the number of genomic hits produced by the sequence read in question. A dark red bar indicates that this sequence read produces many other hits elsewhere in the genome. Many adjacent red or yellow bars can indicate the presence of a multi-copy element such as transposons or rRNA genes. A dark green bar indicates that this sequence read maps uniquely to this locus.

1 hit

2-5 hits

6-10 hits

11-20 hits

21-50 hits

51-100 hits

> 100 hits

NODE\_312079\_length\_70626\_cov\_31.911549

56146

64841

Gene Set

RepeatMasker

Mapped  
Reads

17.95

plus strand

minus strand

17.95

Region: NODE\_312079\_length\_70626\_cov\_31.911549 13939-56154. Max. coverage (+): 0.09. Max coverage (-): 0

Region: NODE\_312079\_length\_70626\_cov\_31.911549 56155-56172. Max. coverage (+): 0. Max coverage (-): 0

Region: NODE\_312079\_length\_70626\_cov\_31.911549 56173-56189. Max. coverage (+): 0.09. Max coverage (-): 0

Region: NODE\_312079\_length\_70626\_cov\_31.911549 56190-56206. Max. coverage (+): 0.09. Max coverage (-): 0

Region: NODE\_312079\_length\_70626\_cov\_31.911549 56207-56224. Max. coverage (+): 0.19. Max coverage (-): 0

Region: NODE\_312079\_length\_70626\_cov\_31.911549 56225-56241. Max. coverage (+): 0. Max coverage (-): 0

Region: NODE\_312079\_length\_70626\_cov\_31.911549 56242-56259. Max. coverage (+): 0. Max coverage (-): 0

Region: NODE\_312079\_length\_70626\_cov\_31.911549 56260-56276. Max. coverage (+): 0. Max coverage (-): 0

Region: NODE\_312079\_length\_70626\_cov\_31.911549 56277-56293. Max. coverage (+): 0. Max coverage (-): 0

Region: NODE\_312079\_length\_70626\_cov\_31.911549 56294-56311. Max. coverage (+): 0. Max coverage (-): 0

Region: NODE\_312079\_length\_70626\_cov\_31.911549 56312-56328. Max. coverage (+): 0. Max coverage (-): 0

Region: NODE\_312079\_length\_70626\_cov\_31.911549 56329-56346. Max. coverage (+): 0.09. Max coverage (-): 0

Region: NODE\_312079\_length\_70626\_cov\_31.911549 56347-56363. Max. coverage (+): 0.94. Max coverage (-): 0

Region: NODE\_312079\_length\_70626\_cov\_31.911549 56364-56380. Max. coverage (+): 0. Max coverage (-): 0

Region: NODE\_312079\_length\_70626\_cov\_31.911549 56381-56398. Max. coverage (+): 0. Max coverage (-): 0

Region: NODE\_312079\_length\_70626\_cov\_31.911549 56399-56415. Max. coverage (+): 0. Max coverage (-): 0

Region: NODE\_312079\_length\_70626\_cov\_31.911549 56416-56432. Max. coverage (+): 0. Max coverage (-): 0

Region: NODE\_312079\_length\_70626\_cov\_31.911549 56433-56450. Max. coverage (+): 0. Max coverage (-): 0

Region: NODE\_312079\_length\_70626\_cov\_31.911549 56451-56467. Max. coverage (+): 0. Max coverage (-): 0

Region: NODE\_312079\_length\_70626\_cov\_31.911549 56468-56485. Max. coverage (+): 0. Max coverage (-): 0

Region: NODE\_312079\_length\_70626\_cov\_31.911549 56486-56502. Max. coverage (+): 0. Max coverage (-): 0

Region: NODE\_312079\_length\_70626\_cov\_31.911549 56503-56519. Max. coverage (+): 0. Max coverage (-): 0

Region: NODE\_312079\_length\_70626\_cov\_31.911549 56520-56537. Max. coverage (+): 0. Max coverage (-): 0

Region: NODE\_312079\_length\_70626\_cov\_31.911549 56538-56554. Max. coverage (+): 0. Max coverage (-): 0.09

Region: NODE\_312079\_length\_70626\_cov\_31.911549 56555-56572. Max. coverage (+): 0. Max coverage (-): 0

Region: NODE\_312079\_length\_70626\_cov\_31.911549 56573-56589. Max. coverage (+): 0.47. Max coverage (-): 0.09

Region: NODE\_312079\_length\_70626\_cov\_31.911549 56590-56606. Max. coverage (+): 0. Max coverage (-): 0

Region: NODE\_312079\_length\_70626\_cov\_31.911549 56607-56624. Max. coverage (+): 0. Max coverage (-): 0

Region: NODE\_312079\_length\_70626\_cov\_31.911549 56625-56641. Max. coverage (+): 0.19. Max coverage (-): 0

Region: NODE\_312079\_length\_70626\_cov\_31.911549 56642-56659. Max. coverage (+): 0.19. Max coverage (-): 0

Region: NODE\_312079\_length\_70626\_cov\_31.911549 56660-56676. Max. coverage (+): 0.09. Max coverage (-): 0.09

Region: NODE\_312079\_length\_70626\_cov\_31.911549 56677-56693. Max. coverage (+): 0. Max coverage (-): 0

Region: NODE\_312079\_length\_70626\_cov\_31.911549 56694-56711. Max. coverage (+): 0.09. Max coverage (-): 0

Region: NODE\_312079\_length\_70626\_cov\_31.911549 56712-56728. Max. coverage (+): 5.64. Max coverage (-): 0

Region: NODE\_312079\_length\_70626\_cov\_31.911549 56729-56746. Max. coverage (+): 0. Max coverage (-): 0

Region: NODE\_312079\_length\_70626\_cov\_31.911549 56747-56763. Max. coverage (+): 0. Max coverage (-): 0

Region: NODE\_312079\_length\_70626\_cov\_31.911549 56764-56780. Max. coverage (+): 3.29. Max coverage (-): 0

Region: NODE\_312079\_length\_70626\_cov\_31.911549 56781-56798. Max. coverage (+): 0.09. Max coverage (-): 0

Region: NODE\_312079\_length\_70626\_cov\_31.911549 56799-56815. Max. coverage (+): 1.69. Max coverage (-): 0

Region: NODE\_312079\_length\_70626\_cov\_31.911549 56816-56832. Max. coverage (+): 0.28. Max coverage (-): 0

Region: NODE\_312079\_length\_70626\_cov\_31.911549 56833-56850. Max. coverage (+): 0.28. Max coverage (-): 0

Region: NODE\_312079\_length\_70626\_cov\_31.911549 56851-56867. Max. coverage (+): 0.19. Max coverage (-): 0.19

Region: NODE\_312079\_length\_70626\_cov\_31.911549 56868-56885. Max. coverage (+): 0.19. Max coverage (-): 0

Region: NODE\_312079\_length\_70626\_cov\_31.911549 56886-56902. Max. coverage (+): 0.19. Max coverage (-): 0.09

Region: NODE\_312079\_length\_70626\_cov\_31.911549 56903-56919. Max. coverage (+): 1.32. Max coverage (-): 0

Region: NODE\_312079\_length\_70626\_cov\_31.911549 56920-56937. Max. coverage (+): 1.32. Max coverage (-): 0

Region: NODE\_312079\_length\_70626\_cov\_31.911549 56938-56954. Max. coverage (+): 0.28. Max coverage (-): 0

Region: NODE\_312079\_length\_70626\_cov\_31.911549 56955-56972. Max. coverage (+): 0. Max coverage (-): 0

Region: NODE\_312079\_length\_70626\_cov\_31.911549 56973-56989. Max. coverage (+): 4.6. Max coverage (-): 0

Region: NODE\_312079\_length\_70626\_cov\_31.911549 56990-57006. Max. coverage (+): 0.28. Max coverage (-): 0

Region: NODE\_312079\_length\_70626\_cov\_31.911549 57007-57024. Max. coverage (+): 0.19. Max coverage (-): 0.09

Region: NODE\_312079\_length\_70626\_cov\_31.911549 57025-57041. Max. coverage (+): 0.28. Max coverage (-): 2.54

Region: NODE\_312079\_length\_70626\_cov\_31.911549 57042-57059. Max. coverage (+): 1.5. Max coverage (-): 0

Region: NODE\_312079\_length\_70626\_cov\_31.911549 57060-57076. Max. coverage (+): 0.09. Max coverage (-): 0

Region: NODE\_312079\_length\_70626\_cov\_31.911549 57077-57093. Max. coverage (+): 0.19. Max coverage (-): 0

Region: NODE\_312079\_length\_70626\_cov\_31.911549 57094-57111. Max. coverage (+): 0. Max coverage (-): 0

Region: NODE\_312079\_length\_70626\_cov\_31.911549 57112-57128. Max. coverage (+): 1.22. Max coverage (-): 0

Region: NODE\_312079\_length\_70626\_cov\_31.911549 57129-57146. Max. coverage (+): 1.22. Max coverage (-): 0

Region: NODE\_312079\_length\_70626\_cov\_31.911549 57147-57163. Max. coverage (+): 0.66. Max coverage (-): 0

Region: NODE\_312079\_length\_70626\_cov\_31.911549 57164-57180. Max. coverage (+): 0.56. Max coverage (-): 0.09

Region: NODE\_312079\_length\_70626\_cov\_31.911549 57181-57198. Max. coverage (+): 0.56. Max coverage (-): 0

Region: NODE\_312079\_length\_70626\_cov\_31.911549 57199-57215. Max. coverage (+): 0. Max coverage (-): 0

Region: NODE\_312079\_length\_70626\_cov\_31.911549 57216-57232. Max. coverage (+): 0.09. Max coverage (-): 0

Region: NODE\_312079\_length\_70626\_cov\_31.911549 57233-57250. Max. coverage (+): 0.09. Max coverage (-): 0

Region: NODE\_312079\_length\_70626\_cov\_31.911549 57251-57267. Max. coverage (+): 0.09. Max coverage (-): 0

Region: NODE\_312079\_length\_70626\_cov\_31.911549 57268-57285. Max. coverage (+): 0.09. Max coverage (-): 0

Region: NODE\_312079\_length\_70626\_cov\_31.911549 57286-57302. Max. coverage (+): 0. Max coverage (-): 0

Region: NODE\_312079\_length\_70626\_cov\_31.911549 57303-57319. Max. coverage (+): 0. Max coverage (-): 0

Region: NODE\_312079\_length\_70626\_cov\_31.911549 57320-57337. Max. coverage (+): 0. Max coverage (-): 0

Region: NODE\_312079\_length\_70626\_cov\_31.911549 57338-57354. Max. coverage (+): 0. Max coverage (-): 0

Region: NODE\_312079\_length\_70626\_cov\_31.911549 57355-57372. Max. coverage (+): 0. Max coverage (-): 0

Region: NODE\_312079\_length\_70626\_cov\_31.911549 57373-57389. Max. coverage (+): 0. Max coverage (-): 0

Region: NODE\_312079\_length\_70626\_cov\_31.911549 57390-57406. Max. coverage (+): 0.09. Max coverage (-): 0

Region: NODE\_312079\_length\_70626\_cov\_31.911549 57407-57424. Max. coverage (+): 0.09. Max coverage (-): 0

Region: NODE\_312079\_length\_70626\_cov\_31.911549 57425-57441. Max. coverage (+): 0.09. Max coverage (-): 0

Region: NODE\_312079\_length\_70626\_cov\_31.911549 57442-57459. Max. coverage (+): 0. Max coverage (-): 0

Region: NODE\_312079\_length\_70626\_cov\_31.911549 57460-57476. Max. coverage (+): 0.09. Max coverage (-): 0

Region: NODE\_312079\_length\_70626\_cov\_31.911549 57477-57493. Max. coverage (+): 0. Max coverage (-): 0.09

Region: NODE\_312079\_length\_70626\_cov\_31.911549 57494-57511. Max. coverage (+): 0.09. Max coverage (-): 0

Region: NODE\_312079\_length\_70626\_cov\_31.911549 57512-57528. Max. coverage (+): 15.22. Max coverage (-): 0

Region: NODE\_312079\_length\_70626\_cov\_31.911549 57529-57546. Max. coverage (+): 0.28. Max coverage (-): 0

Region: NODE\_312079\_length\_70626\_cov\_31.911549 57547-57563. Max. coverage (+): 0.09. Max coverage (-): 0.19

Region: NODE\_312079\_length\_70626\_cov\_31.911549 57564-57580. Max. coverage (+): 0.47. Max coverage (-): 0.19

Region: NODE\_312079\_length\_70626\_cov\_31.911549 57581-57598. Max. coverage (+): 0.56. Max coverage (-): 0

Region: NODE\_312079\_length\_70626\_cov\_31.911549 57599-57615. Max. coverage (+): 0. Max coverage (-): 0

Region: NODE\_312079\_length\_70626\_cov\_31.911549 57616-57633. Max. coverage (+): 0.19. Max coverage (-): 0

Region: NODE\_312079\_length\_70626\_cov\_31.911549 57634-57650. Max. coverage (+): 1.32. Max coverage (-): 0

Region: NODE\_312079\_length\_70626\_cov\_31.911549 57651-57667. Max. coverage (+): 0.28. Max coverage (-): 0.09

Region: NODE\_312079\_length\_70626\_cov\_31.911549 57668-57685. Max. coverage (+): 0.38. Max coverage (-): 0.09

Region: NODE\_312079\_length\_70626\_cov\_31.911549 57686-57702. Max. coverage (+): 0.75. Max coverage (-): 0

Region: NODE\_312079\_length\_70626\_cov\_31.911549 57703-57719. Max. coverage (+): 0.19. Max coverage (-): 0.28

Region: NODE\_312079\_length\_70626\_cov\_31.911549 57720-57737. Max. coverage (+): 0.47. Max coverage (-): 0

Region: NODE\_312079\_length\_70626\_cov\_31.911549 57738-57754. Max. coverage (+): 0.09. Max coverage (-): 0.28

Region: NODE\_312079\_length\_70626\_cov\_31.911549 57755-57772. Max. coverage (+): 0.66. Max coverage (-): 0

Region: NODE\_312079\_length\_70626\_cov\_31.911549 57773-57789. Max. coverage (+): 0.19. Max coverage (-): 0

Region: NODE\_312079\_length\_70626\_cov\_31.911549 57790-57806. Max. coverage (+): 0.75. Max coverage (-): 0

Region: NODE\_312079\_length\_70626\_cov\_31.911549 57807-57824. Max. coverage (+): 2.63. Max coverage (-): 0

Region: NODE\_312079\_length\_70626\_cov\_31.911549 57825-57841. Max. coverage (+): 0. Max coverage (-): 0.19

Region: NODE\_312079\_length\_70626\_cov\_31.911549 57842-57859. Max. coverage (+): 3.95. Max coverage (-): 0

Region: NODE\_312079\_length\_70626\_cov\_31.911549 57860-57876. Max. coverage (+): 0. Max coverage (-): 0

Region: NODE\_312079\_length\_70626\_cov\_31.911549 57877-57893. Max. coverage (+): 0.56. Max coverage (-): 0

Region: NODE\_312079\_length\_70626\_cov\_31.911549 57894-57911. Max. coverage (+): 0.09. Max coverage (-): 0

Region: NODE\_312079\_length\_70626\_cov\_31.911549 57912-57928. Max. coverage (+): 0. Max coverage (-): 0.09

Region: NODE\_312079\_length\_70626\_cov\_31.911549 57929-57946. Max. coverage (+): 0. Max coverage (-): 0

Region: NODE\_312079\_length\_70626\_cov\_31.911549 57947-57963. Max. coverage (+): 0.47. Max coverage (-): 0

Region: NODE\_312079\_length\_70626\_cov\_31.911549 57964-57980. Max. coverage (+): 0. Max coverage (-): 0.19

Region: NODE\_312079\_length\_70626\_cov\_31.911549 57981-57998. Max. coverage (+): 0.19. Max coverage (-): 0

Region: NODE\_312079\_length\_70626\_cov\_31.911549 57999-58015. Max. coverage (+): 0. Max coverage (-): 0.56

Region: NODE\_312079\_length\_70626\_cov\_31.911549 58016-58033. Max. coverage (+): 0.19. Max coverage (-): 0.19

Region: NODE\_312079\_length\_70626\_cov\_31.911549 58034-58050. Max. coverage (+): 0.09. Max coverage (-): 0.09

Region: NODE\_312079\_length\_70626\_cov\_31.911549 58051-58067. Max. coverage (+): 0. Max coverage (-): 0.09

Region: NODE\_312079\_length\_70626\_cov\_31.911549 58068-58085. Max. coverage (+): 0.09. Max coverage (-): 0.09

Region: NODE\_312079\_length\_70626\_cov\_31.911549 58086-58102. Max. coverage (+): 0. Max coverage (-): 0

Region: NODE\_312079\_length\_70626\_cov\_31.911549 58103-58119. Max. coverage (+): 0. Max coverage (-): 0

Region: NODE\_312079\_length\_70626\_cov\_31.911549 58120-58137. Max. coverage (+): 0. Max coverage (-): 0

Region: NODE\_312079\_length\_70626\_cov\_31.911549 58138-58154. Max. coverage (+): 0.19. Max coverage (-): 0.09

Region: NODE\_312079\_length\_70626\_cov\_31.911549 58155-58172. Max. coverage (+): 0.09. Max coverage (-): 0.19

Region: NODE\_312079\_length\_70626\_cov\_31.911549 58173-58189. Max. coverage (+): 0.47. Max coverage (-): 0

Region: NODE\_312079\_length\_70626\_cov\_31.911549 58190-58206. Max. coverage (+): 0.19. Max coverage (-): 0

Region: NODE\_312079\_length\_70626\_cov\_31.911549 58207-58224. Max. coverage (+): 0.19. Max coverage (-): 0

Region: NODE\_312079\_length\_70626\_cov\_31.911549 58225-58241. Max. coverage (+): 0. Max coverage (-): 0

Region: NODE\_312079\_length\_70626\_cov\_31.911549 58242-58259. Max. coverage (+): 0.38. Max coverage (-): 0

Region: NODE\_312079\_length\_70626\_cov\_31.911549 58260-58276. Max. coverage (+): 0.47. Max coverage (-): 0

Region: NODE\_312079\_length\_70626\_cov\_31.911549 58277-58293. Max. coverage (+): 0.19. Max coverage (-): 0

Region: NODE\_312079\_length\_70626\_cov\_31.911549 58294-58311. Max. coverage (+): 0.19. Max coverage (-): 0

Region: NODE\_312079\_length\_70626\_cov\_31.911549 58312-58328. Max. coverage (+): 0.19. Max coverage (-): 0.19

Region: NODE\_312079\_length\_70626\_cov\_31.911549 58329-58346. Max. coverage (+): 0.56. Max coverage (-): 0.09

Region: NODE\_312079\_length\_70626\_cov\_31.911549 58347-58363. Max. coverage (+): 0. Max coverage (-): 0

Region: NODE\_312079\_length\_70626\_cov\_31.911549 58364-58380. Max. coverage (+): 0.05. Max coverage (-): 0

Region: NODE\_312079\_length\_70626\_cov\_31.911549 58381-58398. Max. coverage (+): 0.05. Max coverage (-): 0

Region: NODE\_312079\_length\_70626\_cov\_31.911549 58399-58415. Max. coverage (+): 0. Max coverage (-): 0

Region: NODE\_312079\_length\_70626\_cov\_31.911549 58416-58433. Max. coverage (+): 0. Max coverage (-): 0

Region: NODE\_312079\_length\_70626\_cov\_31.911549 58434-58450. Max. coverage (+): 0. Max coverage (-): 0

Region: NODE\_312079\_length\_70626\_cov\_31.911549 58451-58467. Max. coverage (+): 0. Max coverage (-): 0

Region: NODE\_312079\_length\_70626\_cov\_31.911549 58468-58485. Max. coverage (+): 0. Max coverage (-): 0.01

Region: NODE\_312079\_length\_70626\_cov\_31.911549 58486-58502. Max. coverage (+): 0. Max coverage (-): 0.01

Region: NODE\_312079\_length\_70626\_cov\_31.911549 58503-58520. Max. coverage (+): 0. Max coverage (-): 0

Region: NODE\_312079\_length\_70626\_cov\_31.911549 58521-58537. Max. coverage (+): 0. Max coverage (-): 0

Region: NODE\_312079\_length\_70626\_cov\_31.911549 58538-58554. Max. coverage (+): 0. Max coverage (-): 0

Region: NODE\_312079\_length\_70626\_cov\_31.911549 58555-58572. Max. coverage (+): 0. Max coverage (-): 0

Region: NODE\_312079\_length\_70626\_cov\_31.911549 58573-58589. Max. coverage (+): 0.14. Max coverage (-): 0

Region: NODE\_312079\_length\_70626\_cov\_31.911549 58590-58606. Max. coverage (+): 0.71. Max coverage (-): 0

Region: NODE\_312079\_length\_70626\_cov\_31.911549 58607-58624. Max. coverage (+): 0. Max coverage (-): 0

Region: NODE\_312079\_length\_70626\_cov\_31.911549 58625-58641. Max. coverage (+): 0. Max coverage (-): 0

Region: NODE\_312079\_length\_70626\_cov\_31.911549 58642-58659. Max. coverage (+): 0. Max coverage (-): 0

Region: NODE\_312079\_length\_70626\_cov\_31.911549 58660-58676. Max. coverage (+): 0. Max coverage (-): 0

Region: NODE\_312079\_length\_70626\_cov\_31.911549 58677-58693. Max. coverage (+): 0. Max coverage (-): 0

Region: NODE\_312079\_length\_70626\_cov\_31.911549 58694-58711. Max. coverage (+): 0.09. Max coverage (-): 0

Region: NODE\_312079\_length\_70626\_cov\_31.911549 58712-58728. Max. coverage (+): 0.09. Max coverage (-): 0

Region: NODE\_312079\_length\_70626\_cov\_31.911549 58729-58746. Max. coverage (+): 0.09. Max coverage (-): 0

Region: NODE\_312079\_length\_70626\_cov\_31.911549 58747-58763. Max. coverage (+): 0.09. Max coverage (-): 0

Region: NODE\_312079\_length\_70626\_cov\_31.911549 58764-58780. Max. coverage (+): 0.09. Max coverage (-): 0

Region: NODE\_312079\_length\_70626\_cov\_31.911549 58781-58798. Max. coverage (+): 0. Max coverage (-): 0

Region: NODE\_312079\_length\_70626\_cov\_31.911549 58799-58815. Max. coverage (+): 0. Max coverage (-): 0

Region: NODE\_312079\_length\_70626\_cov\_31.911549 58816-58833. Max. coverage (+): 0. Max coverage (-): 0

Region: NODE\_312079\_length\_70626\_cov\_31.911549 58834-58850. Max. coverage (+): 0.28. Max coverage (-): 0

Region: NODE\_312079\_length\_70626\_cov\_31.911549 58851-58867. Max. coverage (+): 0.09. Max coverage (-): 0

Region: NODE\_312079\_length\_70626\_cov\_31.911549 58868-58885. Max. coverage (+): 0.28. Max coverage (-): 0.19

Region: NODE\_312079\_length\_70626\_cov\_31.911549 58886-58902. Max. coverage (+): 0.38. Max coverage (-): 0

Region: NODE\_312079\_length\_70626\_cov\_31.911549 58903-58920. Max. coverage (+): 0.09. Max coverage (-): 0

Region: NODE\_312079\_length\_70626\_cov\_31.911549 58921-58937. Max. coverage (+): 0. Max coverage (-): 0.19

Region: NODE\_312079\_length\_70626\_cov\_31.911549 58938-58954. Max. coverage (+): 0.38. Max coverage (-): 0

Region: NODE\_312079\_length\_70626\_cov\_31.911549 58955-58972. Max. coverage (+): 0. Max coverage (-): 0

Region: NODE\_312079\_length\_70626\_cov\_31.911549 58973-58989. Max. coverage (+): 0.28. Max coverage (-): 0.09

Region: NODE\_312079\_length\_70626\_cov\_31.911549 58990-59006. Max. coverage (+): 0.09. Max coverage (-): 0.09

Region: NODE\_312079\_length\_70626\_cov\_31.911549 59007-59024. Max. coverage (+): 0.19. Max coverage (-): 0

Region: NODE\_312079\_length\_70626\_cov\_31.911549 59025-59041. Max. coverage (+): 0.38. Max coverage (-): 0

Region: NODE\_312079\_length\_70626\_cov\_31.911549 59042-59059. Max. coverage (+): 0.38. Max coverage (-): 0

Region: NODE\_312079\_length\_70626\_cov\_31.911549 59060-59076. Max. coverage (+): 1.79. Max coverage (-): 0

Region: NODE\_312079\_length\_70626\_cov\_31.911549 59077-59093. Max. coverage (+): 0. Max coverage (-): 0.09

Region: NODE\_312079\_length\_70626\_cov\_31.911549 59094-59111. Max. coverage (+): 0.09. Max coverage (-): 0.09

Region: NODE\_312079\_length\_70626\_cov\_31.911549 59112-59128. Max. coverage (+): 3.1. Max coverage (-): 0

Region: NODE\_312079\_length\_70626\_cov\_31.911549 59129-59146. Max. coverage (+): 0. Max coverage (-): 0

Region: NODE\_312079\_length\_70626\_cov\_31.911549 59147-59163. Max. coverage (+): 0.19. Max coverage (-): 0

Region: NODE\_312079\_length\_70626\_cov\_31.911549 59164-59180. Max. coverage (+): 0.09. Max coverage (-): 0.09

Region: NODE\_312079\_length\_70626\_cov\_31.911549 59181-59198. Max. coverage (+): 0.19. Max coverage (-): 0.19

Region: NODE\_312079\_length\_70626\_cov\_31.911549 59199-59215. Max. coverage (+): 0.09. Max coverage (-): 1.22

Region: NODE\_312079\_length\_70626\_cov\_31.911549 59216-59233. Max. coverage (+): 6.67. Max coverage (-): 0.09

Region: NODE\_312079\_length\_70626\_cov\_31.911549 59234-59250. Max. coverage (+): 0.09. Max coverage (-): 0.09

Region: NODE\_312079\_length\_70626\_cov\_31.911549 59251-59267. Max. coverage (+): 1.03. Max coverage (-): 0

Region: NODE\_312079\_length\_70626\_cov\_31.911549 59268-59285. Max. coverage (+): 0.28. Max coverage (-): 0.19

Region: NODE\_312079\_length\_70626\_cov\_31.911549 59286-59302. Max. coverage (+): 0. Max coverage (-): 0

Region: NODE\_312079\_length\_70626\_cov\_31.911549 59303-59320. Max. coverage (+): 0. Max coverage (-): 0

Region: NODE\_312079\_length\_70626\_cov\_31.911549 59321-59337. Max. coverage (+): 0. Max coverage (-): 0.09

Region: NODE\_312079\_length\_70626\_cov\_31.911549 59338-59354. Max. coverage (+): 0.28. Max coverage (-): 0

Region: NODE\_312079\_length\_70626\_cov\_31.911549 59355-59372. Max. coverage (+): 0.09. Max coverage (-): 0

Region: NODE\_312079\_length\_70626\_cov\_31.911549 59373-59389. Max. coverage (+): 0. Max coverage (-): 0.28

Region: NODE\_312079\_length\_70626\_cov\_31.911549 59390-59406. Max. coverage (+): 0. Max coverage (-): 0

Region: NODE\_312079\_length\_70626\_cov\_31.911549 59407-59424. Max. coverage (+): 0. Max coverage (-): 0

Region: NODE\_312079\_length\_70626\_cov\_31.911549 59425-59441. Max. coverage (+): 0.75. Max coverage (-): 0.09

Region: NODE\_312079\_length\_70626\_cov\_31.911549 59442-59459. Max. coverage (+): 0.09. Max coverage (-): 0

Region: NODE\_312079\_length\_70626\_cov\_31.911549 59460-59476. Max. coverage (+): 17.95. Max coverage (-): 0.19

Region: NODE\_312079\_length\_70626\_cov\_31.911549 59477-59493. Max. coverage (+): 0. Max coverage (-): 0.38

Region: NODE\_312079\_length\_70626\_cov\_31.911549 59494-59511. Max. coverage (+): 0. Max coverage (-): 0

Region: NODE\_312079\_length\_70626\_cov\_31.911549 59512-59528. Max. coverage (+): 0. Max coverage (-): 0.09

Region: NODE\_312079\_length\_70626\_cov\_31.911549 59529-59546. Max. coverage (+): 2.07. Max coverage (-): 0.09

Region: NODE\_312079\_length\_70626\_cov\_31.911549 59547-59563. Max. coverage (+): 2.82. Max coverage (-): 0.09

Region: NODE\_312079\_length\_70626\_cov\_31.911549 59564-59580. Max. coverage (+): 0.09. Max coverage (-): 0.38

Region: NODE\_312079\_length\_70626\_cov\_31.911549 59581-59598. Max. coverage (+): 0.38. Max coverage (-): 0

Region: NODE\_312079\_length\_70626\_cov\_31.911549 59599-59615. Max. coverage (+): 0. Max coverage (-): 0

Region: NODE\_312079\_length\_70626\_cov\_31.911549 59616-59633. Max. coverage (+): 0. Max coverage (-): 0

Region: NODE\_312079\_length\_70626\_cov\_31.911549 59634-59650. Max. coverage (+): 0.47. Max coverage (-): 0

Region: NODE\_312079\_length\_70626\_cov\_31.911549 59651-59667. Max. coverage (+): 0. Max coverage (-): 0

Region: NODE\_312079\_length\_70626\_cov\_31.911549 59668-59685. Max. coverage (+): 0.09. Max coverage (-): 0

Region: NODE\_312079\_length\_70626\_cov\_31.911549 59686-59702. Max. coverage (+): 0.19. Max coverage (-): 0.19

Region: NODE\_312079\_length\_70626\_cov\_31.911549 59703-59720. Max. coverage (+): 0.56. Max coverage (-): 0.19

Region: NODE\_312079\_length\_70626\_cov\_31.911549 59721-59737. Max. coverage (+): 0.09. Max coverage (-): 0.09

Region: NODE\_312079\_length\_70626\_cov\_31.911549 59738-59754. Max. coverage (+): 0.47. Max coverage (-): 0

Region: NODE\_312079\_length\_70626\_cov\_31.911549 59755-59772. Max. coverage (+): 1.03. Max coverage (-): 0

Region: NODE\_312079\_length\_70626\_cov\_31.911549 59773-59789. Max. coverage (+): 0.94. Max coverage (-): 0

Region: NODE\_312079\_length\_70626\_cov\_31.911549 59790-59807. Max. coverage (+): 1.32. Max coverage (-): 0

Region: NODE\_312079\_length\_70626\_cov\_31.911549 59808-59824. Max. coverage (+): 0.28. Max coverage (-): 0

Region: NODE\_312079\_length\_70626\_cov\_31.911549 59825-59841. Max. coverage (+): 0.28. Max coverage (-): 0.09

Region: NODE\_312079\_length\_70626\_cov\_31.911549 59842-59859. Max. coverage (+): 0.94. Max coverage (-): 0

Region: NODE\_312079\_length\_70626\_cov\_31.911549 59860-59876. Max. coverage (+): 0.66. Max coverage (-): 0.09

Region: NODE\_312079\_length\_70626\_cov\_31.911549 59877-59893. Max. coverage (+): 0.19. Max coverage (-): 0

Region: NODE\_312079\_length\_70626\_cov\_31.911549 59894-59911. Max. coverage (+): 0. Max coverage (-): 0

Region: NODE\_312079\_length\_70626\_cov\_31.911549 59912-59928. Max. coverage (+): 0.09. Max coverage (-): 0

Region: NODE\_312079\_length\_70626\_cov\_31.911549 59929-59946. Max. coverage (+): 0.56. Max coverage (-): 0

Region: NODE\_312079\_length\_70626\_cov\_31.911549 59947-59963. Max. coverage (+): 0. Max coverage (-): 0.09

Region: NODE\_312079\_length\_70626\_cov\_31.911549 59964-59980. Max. coverage (+): 0. Max coverage (-): 0

Region: NODE\_312079\_length\_70626\_cov\_31.911549 59981-59998. Max. coverage (+): 0. Max coverage (-): 0

Region: NODE\_312079\_length\_70626\_cov\_31.911549 59999-60015. Max. coverage (+): 0. Max coverage (-): 0

Region: NODE\_312079\_length\_70626\_cov\_31.911549 60016-60033. Max. coverage (+): 0.09. Max coverage (-): 0.19

Region: NODE\_312079\_length\_70626\_cov\_31.911549 60034-60050. Max. coverage (+): 1.13. Max coverage (-): 0.19

Region: NODE\_312079\_length\_70626\_cov\_31.911549 60051-60067. Max. coverage (+): 0.38. Max coverage (-): 0

Region: NODE\_312079\_length\_70626\_cov\_31.911549 60068-60085. Max. coverage (+): 0. Max coverage (-): 0

Region: NODE\_312079\_length\_70626\_cov\_31.911549 60086-60102. Max. coverage (+): 0. Max coverage (-): 0

Region: NODE\_312079\_length\_70626\_cov\_31.911549 60103-60120. Max. coverage (+): 0.09. Max coverage (-): 0

Region: NODE\_312079\_length\_70626\_cov\_31.911549 60121-60137. Max. coverage (+): 0. Max coverage (-): 0

Region: NODE\_312079\_length\_70626\_cov\_31.911549 60138-60154. Max. coverage (+): 0.19. Max coverage (-): 0.47

Region: NODE\_312079\_length\_70626\_cov\_31.911549 60155-60172. Max. coverage (+): 17.38. Max coverage (-): 0

Region: NODE\_312079\_length\_70626\_cov\_31.911549 60173-60189. Max. coverage (+): 0. Max coverage (-): 0

Region: NODE\_312079\_length\_70626\_cov\_31.911549 60190-60207. Max. coverage (+): 0. Max coverage (-): 0.09

Region: NODE\_312079\_length\_70626\_cov\_31.911549 60208-60224. Max. coverage (+): 0. Max coverage (-): 0

Region: NODE\_312079\_length\_70626\_cov\_31.911549 60225-60241. Max. coverage (+): 0.19. Max coverage (-): 0

Region: NODE\_312079\_length\_70626\_cov\_31.911549 60242-60259. Max. coverage (+): 0.28. Max coverage (-): 0

Region: NODE\_312079\_length\_70626\_cov\_31.911549 60260-60276. Max. coverage (+): 0.85. Max coverage (-): 0.09

Region: NODE\_312079\_length\_70626\_cov\_31.911549 60277-60293. Max. coverage (+): 17.57. Max coverage (-): 0.19

Region: NODE\_312079\_length\_70626\_cov\_31.911549 60294-60311. Max. coverage (+): 0.38. Max coverage (-): 0.19

Region: NODE\_312079\_length\_70626\_cov\_31.911549 60312-60328. Max. coverage (+): 0.38. Max coverage (-): 0

Region: NODE\_312079\_length\_70626\_cov\_31.911549 60329-60346. Max. coverage (+): 1.13. Max coverage (-): 0

Region: NODE\_312079\_length\_70626\_cov\_31.911549 60347-60363. Max. coverage (+): 0.94. Max coverage (-): 0

Region: NODE\_312079\_length\_70626\_cov\_31.911549 60364-60380. Max. coverage (+): 0. Max coverage (-): 0

Region: NODE\_312079\_length\_70626\_cov\_31.911549 60381-60398. Max. coverage (+): 1.03. Max coverage (-): 0.09

Region: NODE\_312079\_length\_70626\_cov\_31.911549 60399-60415. Max. coverage (+): 1.22. Max coverage (-): 0

Region: NODE\_312079\_length\_70626\_cov\_31.911549 60416-60433. Max. coverage (+): 0. Max coverage (-): 0.09

Region: NODE\_312079\_length\_70626\_cov\_31.911549 60434-60450. Max. coverage (+): 0. Max coverage (-): 0

Region: NODE\_312079\_length\_70626\_cov\_31.911549 60451-60467. Max. coverage (+): 0.19. Max coverage (-): 0

Region: NODE\_312079\_length\_70626\_cov\_31.911549 60468-60485. Max. coverage (+): 1.03. Max coverage (-): 0

Region: NODE\_312079\_length\_70626\_cov\_31.911549 60486-60502. Max. coverage (+): 0.28. Max coverage (-): 0

Region: NODE\_312079\_length\_70626\_cov\_31.911549 60503-60520. Max. coverage (+): 3.01. Max coverage (-): 0

Region: NODE\_312079\_length\_70626\_cov\_31.911549 60521-60537. Max. coverage (+): 0. Max coverage (-): 0

Region: NODE\_312079\_length\_70626\_cov\_31.911549 60538-60554. Max. coverage (+): 0.75. Max coverage (-): 0

Region: NODE\_312079\_length\_70626\_cov\_31.911549 60555-60572. Max. coverage (+): 0.19. Max coverage (-): 0

Region: NODE\_312079\_length\_70626\_cov\_31.911549 60573-60589. Max. coverage (+): 0. Max coverage (-): 0.19

Region: NODE\_312079\_length\_70626\_cov\_31.911549 60590-60607. Max. coverage (+): 1.6. Max coverage (-): 0

Region: NODE\_312079\_length\_70626\_cov\_31.911549 60608-60624. Max. coverage (+): 0. Max coverage (-): 0.09

Region: NODE\_312079\_length\_70626\_cov\_31.911549 60625-60641. Max. coverage (+): 0.19. Max coverage (-): 0

Region: NODE\_312079\_length\_70626\_cov\_31.911549 60642-60659. Max. coverage (+): 0. Max coverage (-): 0

Region: NODE\_312079\_length\_70626\_cov\_31.911549 60660-60676. Max. coverage (+): 0.75. Max coverage (-): 0

Region: NODE\_312079\_length\_70626\_cov\_31.911549 60677-60694. Max. coverage (+): 2.26. Max coverage (-): 0

Region: NODE\_312079\_length\_70626\_cov\_31.911549 60695-60711. Max. coverage (+): 1.79. Max coverage (-): 0

Region: NODE\_312079\_length\_70626\_cov\_31.911549 60712-60728. Max. coverage (+): 0.38. Max coverage (-): 0

Region: NODE\_312079\_length\_70626\_cov\_31.911549 60729-60746. Max. coverage (+): 1.03. Max coverage (-): 0

Region: NODE\_312079\_length\_70626\_cov\_31.911549 60747-60763. Max. coverage (+): 0.19. Max coverage (-): 0

Region: NODE\_312079\_length\_70626\_cov\_31.911549 60764-60780. Max. coverage (+): 0.19. Max coverage (-): 0.09

Region: NODE\_312079\_length\_70626\_cov\_31.911549 60781-60798. Max. coverage (+): 0.28. Max coverage (-): 0.09

Region: NODE\_312079\_length\_70626\_cov\_31.911549 60799-60815. Max. coverage (+): 0.09. Max coverage (-): 0

Region: NODE\_312079\_length\_70626\_cov\_31.911549 60816-60833. Max. coverage (+): 0. Max coverage (-): 0

Region: NODE\_312079\_length\_70626\_cov\_31.911549 60834-60850. Max. coverage (+): 0.56. Max coverage (-): 0

Region: NODE\_312079\_length\_70626\_cov\_31.911549 60851-60867. Max. coverage (+): 4.51. Max coverage (-): 0

Region: NODE\_312079\_length\_70626\_cov\_31.911549 60868-60885. Max. coverage (+): 0.94. Max coverage (-): 0

Region: NODE\_312079\_length\_70626\_cov\_31.911549 60886-60902. Max. coverage (+): 1.13. Max coverage (-): 0

Region: NODE\_312079\_length\_70626\_cov\_31.911549 60903-60920. Max. coverage (+): 0. Max coverage (-): 0

Region: NODE\_312079\_length\_70626\_cov\_31.911549 60921-60937. Max. coverage (+): 0.19. Max coverage (-): 0

Region: NODE\_312079\_length\_70626\_cov\_31.911549 60938-60954. Max. coverage (+): 0. Max coverage (-): 0

Region: NODE\_312079\_length\_70626\_cov\_31.911549 60955-60972. Max. coverage (+): 0.09. Max coverage (-): 0

Region: NODE\_312079\_length\_70626\_cov\_31.911549 60973-60989. Max. coverage (+): 0.09. Max coverage (-): 0

Region: NODE\_312079\_length\_70626\_cov\_31.911549 60990-61007. Max. coverage (+): 0.47. Max coverage (-): 0

Region: NODE\_312079\_length\_70626\_cov\_31.911549 61008-61024. Max. coverage (+): 0.56. Max coverage (-): 0

Region: NODE\_312079\_length\_70626\_cov\_31.911549 61025-61041. Max. coverage (+): 0. Max coverage (-): 0

Region: NODE\_312079\_length\_70626\_cov\_31.911549 61042-61059. Max. coverage (+): 0.94. Max coverage (-): 0.09

Region: NODE\_312079\_length\_70626\_cov\_31.911549 61060-61076. Max. coverage (+): 0.09. Max coverage (-): 0.09

Region: NODE\_312079\_length\_70626\_cov\_31.911549 61077-61094. Max. coverage (+): 0. Max coverage (-): 0.09

Region: NODE\_312079\_length\_70626\_cov\_31.911549 61095-61111. Max. coverage (+): 0. Max coverage (-): 0.47

Region: NODE\_312079\_length\_70626\_cov\_31.911549 61112-61128. Max. coverage (+): 7.52. Max coverage (-): 0.19

Region: NODE\_312079\_length\_70626\_cov\_31.911549 61129-61146. Max. coverage (+): 0.09. Max coverage (-): 0.19

Region: NODE\_312079\_length\_70626\_cov\_31.911549 61147-61163. Max. coverage (+): 0.09. Max coverage (-): 0.09

Region: NODE\_312079\_length\_70626\_cov\_31.911549 61164-61180. Max. coverage (+): 0.09. Max coverage (-): 0.19

Region: NODE\_312079\_length\_70626\_cov\_31.911549 61181-61198. Max. coverage (+): 0.47. Max coverage (-): 0.47

Region: NODE\_312079\_length\_70626\_cov\_31.911549 61199-61215. Max. coverage (+): 0.09. Max coverage (-): 0

Region: NODE\_312079\_length\_70626\_cov\_31.911549 61216-61233. Max. coverage (+): 0.66. Max coverage (-): 0

Region: NODE\_312079\_length\_70626\_cov\_31.911549 61234-61250. Max. coverage (+): 0.09. Max coverage (-): 0

Region: NODE\_312079\_length\_70626\_cov\_31.911549 61251-61267. Max. coverage (+): 0. Max coverage (-): 0.09

Region: NODE\_312079\_length\_70626\_cov\_31.911549 61268-61285. Max. coverage (+): 0.09. Max coverage (-): 0

Region: NODE\_312079\_length\_70626\_cov\_31.911549 61286-61302. Max. coverage (+): 0. Max coverage (-): 0

Region: NODE\_312079\_length\_70626\_cov\_31.911549 61303-61320. Max. coverage (+): 1.41. Max coverage (-): 0

Region: NODE\_312079\_length\_70626\_cov\_31.911549 61321-61337. Max. coverage (+): 1.32. Max coverage (-): 0

Region: NODE\_312079\_length\_70626\_cov\_31.911549 61338-61354. Max. coverage (+): 0.09. Max coverage (-): 0

Region: NODE\_312079\_length\_70626\_cov\_31.911549 61355-61372. Max. coverage (+): 0.09. Max coverage (-): 0

Region: NODE\_312079\_length\_70626\_cov\_31.911549 61373-61389. Max. coverage (+): 0.19. Max coverage (-): 0.09

Region: NODE\_312079\_length\_70626\_cov\_31.911549 61390-61407. Max. coverage (+): 0. Max coverage (-): 0

Region: NODE\_312079\_length\_70626\_cov\_31.911549 61408-61424. Max. coverage (+): 0. Max coverage (-): 0.09

Region: NODE\_312079\_length\_70626\_cov\_31.911549 61425-61441. Max. coverage (+): 0.09. Max coverage (-): 0.85

Region: NODE\_312079\_length\_70626\_cov\_31.911549 61442-61459. Max. coverage (+): 0.09. Max coverage (-): 0

Region: NODE\_312079\_length\_70626\_cov\_31.911549 61460-61476. Max. coverage (+): 0. Max coverage (-): 0.09

Region: NODE\_312079\_length\_70626\_cov\_31.911549 61477-61494. Max. coverage (+): 0.09. Max coverage (-): 0.38

Region: NODE\_312079\_length\_70626\_cov\_31.911549 61495-61511. Max. coverage (+): 0. Max coverage (-): 0.38

Region: NODE\_312079\_length\_70626\_cov\_31.911549 61512-61528. Max. coverage (+): 0.75. Max coverage (-): 0

Region: NODE\_312079\_length\_70626\_cov\_31.911549 61529-61546. Max. coverage (+): 0.56. Max coverage (-): 0

Region: NODE\_312079\_length\_70626\_cov\_31.911549 61547-61563. Max. coverage (+): 0. Max coverage (-): 0

Region: NODE\_312079\_length\_70626\_cov\_31.911549 61564-61580. Max. coverage (+): 0.09. Max coverage (-): 0

Region: NODE\_312079\_length\_70626\_cov\_31.911549 61581-61598. Max. coverage (+): 0.09. Max coverage (-): 0

Region: NODE\_312079\_length\_70626\_cov\_31.911549 61599-61615. Max. coverage (+): 0. Max coverage (-): 0

Region: NODE\_312079\_length\_70626\_cov\_31.911549 61616-61633. Max. coverage (+): 0.47. Max coverage (-): 0

Region: NODE\_312079\_length\_70626\_cov\_31.911549 61634-61650. Max. coverage (+): 0.09. Max coverage (-): 0.56

Region: NODE\_312079\_length\_70626\_cov\_31.911549 61651-61667. Max. coverage (+): 1.03. Max coverage (-): 0

Region: NODE\_312079\_length\_70626\_cov\_31.911549 61668-61685. Max. coverage (+): 0.19. Max coverage (-): 0

Region: NODE\_312079\_length\_70626\_cov\_31.911549 61686-61702. Max. coverage (+): 0.28. Max coverage (-): 0

Region: NODE\_312079\_length\_70626\_cov\_31.911549 61703-61720. Max. coverage (+): 0. Max coverage (-): 0

Region: NODE\_312079\_length\_70626\_cov\_31.911549 61721-61737. Max. coverage (+): 1.03. Max coverage (-): 0

Region: NODE\_312079\_length\_70626\_cov\_31.911549 61738-61754. Max. coverage (+): 0. Max coverage (-): 0.09

Region: NODE\_312079\_length\_70626\_cov\_31.911549 61755-61772. Max. coverage (+): 9.4. Max coverage (-): 0

Region: NODE\_312079\_length\_70626\_cov\_31.911549 61773-61789. Max. coverage (+): 0. Max coverage (-): 0

Region: NODE\_312079\_length\_70626\_cov\_31.911549 61790-61807. Max. coverage (+): 0.66. Max coverage (-): 0

Region: NODE\_312079\_length\_70626\_cov\_31.911549 61808-61824. Max. coverage (+): 0.28. Max coverage (-): 0

Region: NODE\_312079\_length\_70626\_cov\_31.911549 61825-61841. Max. coverage (+): 0. Max coverage (-): 0.09

Region: NODE\_312079\_length\_70626\_cov\_31.911549 61842-61859. Max. coverage (+): 0.09. Max coverage (-): 0.09

Region: NODE\_312079\_length\_70626\_cov\_31.911549 61860-61876. Max. coverage (+): 3.85. Max coverage (-): 0.75

Region: NODE\_312079\_length\_70626\_cov\_31.911549 61877-61894. Max. coverage (+): 3.57. Max coverage (-): 0.28

Region: NODE\_312079\_length\_70626\_cov\_31.911549 61895-61911. Max. coverage (+): 0.28. Max coverage (-): 0

Region: NODE\_312079\_length\_70626\_cov\_31.911549 61912-61928. Max. coverage (+): 0.28. Max coverage (-): 0

Region: NODE\_312079\_length\_70626\_cov\_31.911549 61929-61946. Max. coverage (+): 0. Max coverage (-): 0

Region: NODE\_312079\_length\_70626\_cov\_31.911549 61947-61963. Max. coverage (+): 1.22. Max coverage (-): 0

Region: NODE\_312079\_length\_70626\_cov\_31.911549 61964-61981. Max. coverage (+): 0.28. Max coverage (-): 0

Region: NODE\_312079\_length\_70626\_cov\_31.911549 61982-61998. Max. coverage (+): 0.47. Max coverage (-): 0

Region: NODE\_312079\_length\_70626\_cov\_31.911549 61999-62015. Max. coverage (+): 0.47. Max coverage (-): 0

Region: NODE\_312079\_length\_70626\_cov\_31.911549 62016-62033. Max. coverage (+): 1.13. Max coverage (-): 0.09

Region: NODE\_312079\_length\_70626\_cov\_31.911549 62034-62050. Max. coverage (+): 1.13. Max coverage (-): 0.09

Region: NODE\_312079\_length\_70626\_cov\_31.911549 62051-62067. Max. coverage (+): 0.56. Max coverage (-): 0

Region: NODE\_312079\_length\_70626\_cov\_31.911549 62068-62085. Max. coverage (+): 0.38. Max coverage (-): 0

Region: NODE\_312079\_length\_70626\_cov\_31.911549 62086-62102. Max. coverage (+): 0. Max coverage (-): 0.09

Region: NODE\_312079\_length\_70626\_cov\_31.911549 62103-62120. Max. coverage (+): 0. Max coverage (-): 0

Region: NODE\_312079\_length\_70626\_cov\_31.911549 62121-62137. Max. coverage (+): 0. Max coverage (-): 0

Region: NODE\_312079\_length\_70626\_cov\_31.911549 62138-62154. Max. coverage (+): 0. Max coverage (-): 0

Region: NODE\_312079\_length\_70626\_cov\_31.911549 62155-62172. Max. coverage (+): 0.09. Max coverage (-): 0

Region: NODE\_312079\_length\_70626\_cov\_31.911549 62173-62189. Max. coverage (+): 0.09. Max coverage (-): 0

Region: NODE\_312079\_length\_70626\_cov\_31.911549 62190-62207. Max. coverage (+): 0.19. Max coverage (-): 0.09

Region: NODE\_312079\_length\_70626\_cov\_31.911549 62208-62224. Max. coverage (+): 1.5. Max coverage (-): 0.09

Region: NODE\_312079\_length\_70626\_cov\_31.911549 62225-62241. Max. coverage (+): 4.23. Max coverage (-): 0.09

Region: NODE\_312079\_length\_70626\_cov\_31.911549 62242-62259. Max. coverage (+): 1.03. Max coverage (-): 0.09

Region: NODE\_312079\_length\_70626\_cov\_31.911549 62260-62276. Max. coverage (+): 0.75. Max coverage (-): 0

Region: NODE\_312079\_length\_70626\_cov\_31.911549 62277-62294. Max. coverage (+): 1.5. Max coverage (-): 0

Region: NODE\_312079\_length\_70626\_cov\_31.911549 62295-62311. Max. coverage (+): 0.66. Max coverage (-): 0

Region: NODE\_312079\_length\_70626\_cov\_31.911549 62312-62328. Max. coverage (+): 0.09. Max coverage (-): 0.19

Region: NODE\_312079\_length\_70626\_cov\_31.911549 62329-62346. Max. coverage (+): 1.32. Max coverage (-): 0.19

Region: NODE\_312079\_length\_70626\_cov\_31.911549 62347-62363. Max. coverage (+): 0.38. Max coverage (-): 3.1

Region: NODE\_312079\_length\_70626\_cov\_31.911549 62364-62381. Max. coverage (+): 0.09. Max coverage (-): 0

Region: NODE\_312079\_length\_70626\_cov\_31.911549 62382-62398. Max. coverage (+): 0.19. Max coverage (-): 0.09

Region: NODE\_312079\_length\_70626\_cov\_31.911549 62399-62415. Max. coverage (+): 4.79. Max coverage (-): 0.09

Region: NODE\_312079\_length\_70626\_cov\_31.911549 62416-62433. Max. coverage (+): 0.09. Max coverage (-): 0.09

Region: NODE\_312079\_length\_70626\_cov\_31.911549 62434-62450. Max. coverage (+): 0.09. Max coverage (-): 0

Region: NODE\_312079\_length\_70626\_cov\_31.911549 62451-62467. Max. coverage (+): 0.09. Max coverage (-): 0

Region: NODE\_312079\_length\_70626\_cov\_31.911549 62468-62485. Max. coverage (+): 0.38. Max coverage (-): 0.09

Region: NODE\_312079\_length\_70626\_cov\_31.911549 62486-62502. Max. coverage (+): 1.22. Max coverage (-): 0.09

Region: NODE\_312079\_length\_70626\_cov\_31.911549 62503-62520. Max. coverage (+): 0.09. Max coverage (-): 0.19

Region: NODE\_312079\_length\_70626\_cov\_31.911549 62521-62537. Max. coverage (+): 0. Max coverage (-): 0

Region: NODE\_312079\_length\_70626\_cov\_31.911549 62538-62554. Max. coverage (+): 0.09. Max coverage (-): 0

Region: NODE\_312079\_length\_70626\_cov\_31.911549 62555-62572. Max. coverage (+): 0.09. Max coverage (-): 0.28

Region: NODE\_312079\_length\_70626\_cov\_31.911549 62573-62589. Max. coverage (+): 0.19. Max coverage (-): 0.09

Region: NODE\_312079\_length\_70626\_cov\_31.911549 62590-62607. Max. coverage (+): 0.09. Max coverage (-): 0

Region: NODE\_312079\_length\_70626\_cov\_31.911549 62608-62624. Max. coverage (+): 0. Max coverage (-): 0

Region: NODE\_312079\_length\_70626\_cov\_31.911549 62625-62641. Max. coverage (+): 0. Max coverage (-): 0

Region: NODE\_312079\_length\_70626\_cov\_31.911549 62642-62659. Max. coverage (+): 0.09. Max coverage (-): 0

Region: NODE\_312079\_length\_70626\_cov\_31.911549 62660-62676. Max. coverage (+): 0. Max coverage (-): 0

Region: NODE\_312079\_length\_70626\_cov\_31.911549 62677-62694. Max. coverage (+): 0.19. Max coverage (-): 0

Region: NODE\_312079\_length\_70626\_cov\_31.911549 62695-62711. Max. coverage (+): 0. Max coverage (-): 0

Region: NODE\_312079\_length\_70626\_cov\_31.911549 62712-62728. Max. coverage (+): 0. Max coverage (-): 0

Region: NODE\_312079\_length\_70626\_cov\_31.911549 62729-62746. Max. coverage (+): 0. Max coverage (-): 0

Region: NODE\_312079\_length\_70626\_cov\_31.911549 62747-62763. Max. coverage (+): 0.09. Max coverage (-): 0

Region: NODE\_312079\_length\_70626\_cov\_31.911549 62764-62781. Max. coverage (+): 0.47. Max coverage (-): 0

Region: NODE\_312079\_length\_70626\_cov\_31.911549 62782-62798. Max. coverage (+): 0. Max coverage (-): 0.09

Region: NODE\_312079\_length\_70626\_cov\_31.911549 62799-62815. Max. coverage (+): 0.19. Max coverage (-): 0

Region: NODE\_312079\_length\_70626\_cov\_31.911549 62816-62833. Max. coverage (+): 0.47. Max coverage (-): 0.09

Region: NODE\_312079\_length\_70626\_cov\_31.911549 62834-62850. Max. coverage (+): 0.09. Max coverage (-): 0.66

Region: NODE\_312079\_length\_70626\_cov\_31.911549 62851-62868. Max. coverage (+): 1.32. Max coverage (-): 0.66

Region: NODE\_312079\_length\_70626\_cov\_31.911549 62869-62885. Max. coverage (+): 1.13. Max coverage (-): 0

Region: NODE\_312079\_length\_70626\_cov\_31.911549 62886-62902. Max. coverage (+): 0.94. Max coverage (-): 0

Region: NODE\_312079\_length\_70626\_cov\_31.911549 62903-62920. Max. coverage (+): 0.38. Max coverage (-): 0

Region: NODE\_312079\_length\_70626\_cov\_31.911549 62921-62937. Max. coverage (+): 0.38. Max coverage (-): 0.19

Region: NODE\_312079\_length\_70626\_cov\_31.911549 62938-62954. Max. coverage (+): 0. Max coverage (-): 1.5

Region: NODE\_312079\_length\_70626\_cov\_31.911549 62955-62972. Max. coverage (+): 0. Max coverage (-): 1.6

Region: NODE\_312079\_length\_70626\_cov\_31.911549 62973-62989. Max. coverage (+): 0.94. Max coverage (-): 0

Region: NODE\_312079\_length\_70626\_cov\_31.911549 62990-63007. Max. coverage (+): 0.19. Max coverage (-): 0

Region: NODE\_312079\_length\_70626\_cov\_31.911549 63008-63024. Max. coverage (+): 0.09. Max coverage (-): 0

Region: NODE\_312079\_length\_70626\_cov\_31.911549 63025-63041. Max. coverage (+): 0. Max coverage (-): 0.09

Region: NODE\_312079\_length\_70626\_cov\_31.911549 63042-63059. Max. coverage (+): 0.09. Max coverage (-): 0

Region: NODE\_312079\_length\_70626\_cov\_31.911549 63060-63076. Max. coverage (+): 0.75. Max coverage (-): 0

Region: NODE\_312079\_length\_70626\_cov\_31.911549 63077-63094. Max. coverage (+): 0.47. Max coverage (-): 0

Region: NODE\_312079\_length\_70626\_cov\_31.911549 63095-63111. Max. coverage (+): 0.09. Max coverage (-): 0

Region: NODE\_312079\_length\_70626\_cov\_31.911549 63112-63128. Max. coverage (+): 0.09. Max coverage (-): 0

Region: NODE\_312079\_length\_70626\_cov\_31.911549 63129-63146. Max. coverage (+): 0. Max coverage (-): 0

Region: NODE\_312079\_length\_70626\_cov\_31.911549 63147-63163. Max. coverage (+): 0.85. Max coverage (-): 0

Region: NODE\_312079\_length\_70626\_cov\_31.911549 63164-63181. Max. coverage (+): 0.09. Max coverage (-): 0

Region: NODE\_312079\_length\_70626\_cov\_31.911549 63182-63198. Max. coverage (+): 0.09. Max coverage (-): 0

Region: NODE\_312079\_length\_70626\_cov\_31.911549 63199-63215. Max. coverage (+): 3.1. Max coverage (-): 0.09

Region: NODE\_312079\_length\_70626\_cov\_31.911549 63216-63233. Max. coverage (+): 6.01. Max coverage (-): 1.32

Region: NODE\_312079\_length\_70626\_cov\_31.911549 63234-63250. Max. coverage (+): 0.28. Max coverage (-): 1.32

Region: NODE\_312079\_length\_70626\_cov\_31.911549 63251-63268. Max. coverage (+): 0.94. Max coverage (-): 0

Region: NODE\_312079\_length\_70626\_cov\_31.911549 63269-63285. Max. coverage (+): 0.47. Max coverage (-): 0

Region: NODE\_312079\_length\_70626\_cov\_31.911549 63286-63302. Max. coverage (+): 0.47. Max coverage (-): 0

Region: NODE\_312079\_length\_70626\_cov\_31.911549 63303-63320. Max. coverage (+): 0.19. Max coverage (-): 0

Region: NODE\_312079\_length\_70626\_cov\_31.911549 63321-63337. Max. coverage (+): 0. Max coverage (-): 0

Region: NODE\_312079\_length\_70626\_cov\_31.911549 63338-63354. Max. coverage (+): 0.38. Max coverage (-): 0.09

Region: NODE\_312079\_length\_70626\_cov\_31.911549 63355-63372. Max. coverage (+): 2.35. Max coverage (-): 0.09

Region: NODE\_312079\_length\_70626\_cov\_31.911549 63373-63389. Max. coverage (+): 0.85. Max coverage (-): 0

Region: NODE\_312079\_length\_70626\_cov\_31.911549 63390-63407. Max. coverage (+): 0. Max coverage (-): 0

Region: NODE\_312079\_length\_70626\_cov\_31.911549 63408-63424. Max. coverage (+): 0.09. Max coverage (-): 0

Region: NODE\_312079\_length\_70626\_cov\_31.911549 63425-63441. Max. coverage (+): 0.19. Max coverage (-): 0

Region: NODE\_312079\_length\_70626\_cov\_31.911549 63442-63459. Max. coverage (+): 0. Max coverage (-): 0

Region: NODE\_312079\_length\_70626\_cov\_31.911549 63460-63476. Max. coverage (+): 0. Max coverage (-): 0

Region: NODE\_312079\_length\_70626\_cov\_31.911549 63477-63494. Max. coverage (+): 0. Max coverage (-): 0

Region: NODE\_312079\_length\_70626\_cov\_31.911549 63495-63511. Max. coverage (+): 0. Max coverage (-): 0

Region: NODE\_312079\_length\_70626\_cov\_31.911549 63512-63528. Max. coverage (+): 0. Max coverage (-): 0

Region: NODE\_312079\_length\_70626\_cov\_31.911549 63529-63546. Max. coverage (+): 0.75. Max coverage (-): 0

Region: NODE\_312079\_length\_70626\_cov\_31.911549 63547-63563. Max. coverage (+): 1.03. Max coverage (-): 0

Region: NODE\_312079\_length\_70626\_cov\_31.911549 63564-63581. Max. coverage (+): 0. Max coverage (-): 0

Region: NODE\_312079\_length\_70626\_cov\_31.911549 63582-63598. Max. coverage (+): 0. Max coverage (-): 0

Region: NODE\_312079\_length\_70626\_cov\_31.911549 63599-63615. Max. coverage (+): 0. Max coverage (-): 0.47

Region: NODE\_312079\_length\_70626\_cov\_31.911549 63616-63633. Max. coverage (+): 3.48. Max coverage (-): 0

Region: NODE\_312079\_length\_70626\_cov\_31.911549 63634-63650. Max. coverage (+): 0.28. Max coverage (-): 0.09

Region: NODE\_312079\_length\_70626\_cov\_31.911549 63651-63668. Max. coverage (+): 0.09. Max coverage (-): 0

Region: NODE\_312079\_length\_70626\_cov\_31.911549 63669-63685. Max. coverage (+): 0.19. Max coverage (-): 0

Region: NODE\_312079\_length\_70626\_cov\_31.911549 63686-63702. Max. coverage (+): 0.47. Max coverage (-): 0.09

Region: NODE\_312079\_length\_70626\_cov\_31.911549 63703-63720. Max. coverage (+): 0. Max coverage (-): 0

Region: NODE\_312079\_length\_70626\_cov\_31.911549 63721-63737. Max. coverage (+): 0. Max coverage (-): 0

Region: NODE\_312079\_length\_70626\_cov\_31.911549 63738-63754. Max. coverage (+): 0. Max coverage (-): 0

Region: NODE\_312079\_length\_70626\_cov\_31.911549 63755-63772. Max. coverage (+): 0.09. Max coverage (-): 0.09

Region: NODE\_312079\_length\_70626\_cov\_31.911549 63773-63789. Max. coverage (+): 0.09. Max coverage (-): 0

Region: NODE\_312079\_length\_70626\_cov\_31.911549 63790-63807. Max. coverage (+): 0.28. Max coverage (-): 0.19

Region: NODE\_312079\_length\_70626\_cov\_31.911549 63808-63824. Max. coverage (+): 0.09. Max coverage (-): 0.09

Region: NODE\_312079\_length\_70626\_cov\_31.911549 63825-63841. Max. coverage (+): 0.19. Max coverage (-): 0.09

Region: NODE\_312079\_length\_70626\_cov\_31.911549 63842-63859. Max. coverage (+): 0.19. Max coverage (-): 0

Region: NODE\_312079\_length\_70626\_cov\_31.911549 63860-63876. Max. coverage (+): 0.56. Max coverage (-): 0

Region: NODE\_312079\_length\_70626\_cov\_31.911549 63877-63894. Max. coverage (+): 0.19. Max coverage (-): 0.09

Region: NODE\_312079\_length\_70626\_cov\_31.911549 63895-63911. Max. coverage (+): 0.09. Max coverage (-): 0

Region: NODE\_312079\_length\_70626\_cov\_31.911549 63912-63928. Max. coverage (+): 0.19. Max coverage (-): 0

Region: NODE\_312079\_length\_70626\_cov\_31.911549 63929-63946. Max. coverage (+): 0.75. Max coverage (-): 0

Region: NODE\_312079\_length\_70626\_cov\_31.911549 63947-63963. Max. coverage (+): 0.19. Max coverage (-): 0

Region: NODE\_312079\_length\_70626\_cov\_31.911549 63964-63981. Max. coverage (+): 0.56. Max coverage (-): 0

Region: NODE\_312079\_length\_70626\_cov\_31.911549 63982-63998. Max. coverage (+): 0. Max coverage (-): 0.09

Region: NODE\_312079\_length\_70626\_cov\_31.911549 63999-64015. Max. coverage (+): 0.28. Max coverage (-): 0

Region: NODE\_312079\_length\_70626\_cov\_31.911549 64016-64033. Max. coverage (+): 0.09. Max coverage (-): 0

Region: NODE\_312079\_length\_70626\_cov\_31.911549 64034-64050. Max. coverage (+): 0. Max coverage (-): 0

Region: NODE\_312079\_length\_70626\_cov\_31.911549 64051-64068. Max. coverage (+): 0.38. Max coverage (-): 0.09

Region: NODE\_312079\_length\_70626\_cov\_31.911549 64069-64085. Max. coverage (+): 0.94. Max coverage (-): 0.09

Region: NODE\_312079\_length\_70626\_cov\_31.911549 64086-64102. Max. coverage (+): 0.19. Max coverage (-): 0.09

Region: NODE\_312079\_length\_70626\_cov\_31.911549 64103-64120. Max. coverage (+): 0.94. Max coverage (-): 0.09

Region: NODE\_312079\_length\_70626\_cov\_31.911549 64121-64137. Max. coverage (+): 0.85. Max coverage (-): 0

Region: NODE\_312079\_length\_70626\_cov\_31.911549 64138-64155. Max. coverage (+): 0.19. Max coverage (-): 0

Region: NODE\_312079\_length\_70626\_cov\_31.911549 64156-64172. Max. coverage (+): 9.58. Max coverage (-): 0

Region: NODE\_312079\_length\_70626\_cov\_31.911549 64173-64189. Max. coverage (+): 0. Max coverage (-): 0

Region: NODE\_312079\_length\_70626\_cov\_31.911549 64190-64207. Max. coverage (+): 0.19. Max coverage (-): 0

Region: NODE\_312079\_length\_70626\_cov\_31.911549 64208-64224. Max. coverage (+): 0.28. Max coverage (-): 0

Region: NODE\_312079\_length\_70626\_cov\_31.911549 64225-64241. Max. coverage (+): 0.28. Max coverage (-): 0

Region: NODE\_312079\_length\_70626\_cov\_31.911549 64242-64259. Max. coverage (+): 0.09. Max coverage (-): 0

Region: NODE\_312079\_length\_70626\_cov\_31.911549 64260-64276. Max. coverage (+): 0.09. Max coverage (-): 0

Region: NODE\_312079\_length\_70626\_cov\_31.911549 64277-64294. Max. coverage (+): 0. Max coverage (-): 0.09

Region: NODE\_312079\_length\_70626\_cov\_31.911549 64295-64311. Max. coverage (+): 0. Max coverage (-): 0.09

Region: NODE\_312079\_length\_70626\_cov\_31.911549 64312-64328. Max. coverage (+): 0.09. Max coverage (-): 0

Region: NODE\_312079\_length\_70626\_cov\_31.911549 64329-64346. Max. coverage (+): 0. Max coverage (-): 0

Region: NODE\_312079\_length\_70626\_cov\_31.911549 64347-64363. Max. coverage (+): 1.5. Max coverage (-): 0.09

Region: NODE\_312079\_length\_70626\_cov\_31.911549 64364-64381. Max. coverage (+): 0. Max coverage (-): 0

Region: NODE\_312079\_length\_70626\_cov\_31.911549 64382-64398. Max. coverage (+): 0. Max coverage (-): 0

Region: NODE\_312079\_length\_70626\_cov\_31.911549 64399-64415. Max. coverage (+): 0.75. Max coverage (-): 0.28

Region: NODE\_312079\_length\_70626\_cov\_31.911549 64416-64433. Max. coverage (+): 12.87. Max coverage (-): 0

Region: NODE\_312079\_length\_70626\_cov\_31.911549 64434-64450. Max. coverage (+): 0.19. Max coverage (-): 0

Region: NODE\_312079\_length\_70626\_cov\_31.911549 64451-64468. Max. coverage (+): 0.38. Max coverage (-): 0

Region: NODE\_312079\_length\_70626\_cov\_31.911549 64469-64485. Max. coverage (+): 0. Max coverage (-): 0

Region: NODE\_312079\_length\_70626\_cov\_31.911549 64486-64502. Max. coverage (+): 0.09. Max coverage (-): 0.28

Region: NODE\_312079\_length\_70626\_cov\_31.911549 64503-64520. Max. coverage (+): 0.19. Max coverage (-): 0.28

Region: NODE\_312079\_length\_70626\_cov\_31.911549 64521-64537. Max. coverage (+): 0.19. Max coverage (-): 0

Region: NODE\_312079\_length\_70626\_cov\_31.911549 64538-64555. Max. coverage (+): 0.09. Max coverage (-): 0.09

Region: NODE\_312079\_length\_70626\_cov\_31.911549 64556-64572. Max. coverage (+): 9.3. Max coverage (-): 0

Region: NODE\_312079\_length\_70626\_cov\_31.911549 64573-64589. Max. coverage (+): 0.19. Max coverage (-): 0.66

Region: NODE\_312079\_length\_70626\_cov\_31.911549 64590-64607. Max. coverage (+): 0.38. Max coverage (-): 0

Region: NODE\_312079\_length\_70626\_cov\_31.911549 64608-64624. Max. coverage (+): 0.19. Max coverage (-): 0

Region: NODE\_312079\_length\_70626\_cov\_31.911549 64625-64641. Max. coverage (+): 0.09. Max coverage (-): 0

Region: NODE\_312079\_length\_70626\_cov\_31.911549 64642-64659. Max. coverage (+): 0.38. Max coverage (-): 0

Region: NODE\_312079\_length\_70626\_cov\_31.911549 64660-64676. Max. coverage (+): 0. Max coverage (-): 0

Region: NODE\_312079\_length\_70626\_cov\_31.911549 64677-64694. Max. coverage (+): 0. Max coverage (-): 0

Region: NODE\_312079\_length\_70626\_cov\_31.911549 64695-64711. Max. coverage (+): 0. Max coverage (-): 0

Region: NODE\_312079\_length\_70626\_cov\_31.911549 64712-64728. Max. coverage (+): 0.28. Max coverage (-): 0

Region: NODE\_312079\_length\_70626\_cov\_31.911549 64729-64746. Max. coverage (+): 0.28. Max coverage (-): 0

Region: NODE\_312079\_length\_70626\_cov\_31.911549 64747-64763. Max. coverage (+): 0. Max coverage (-): 0

Region: NODE\_312079\_length\_70626\_cov\_31.911549 64764-64781. Max. coverage (+): 0. Max coverage (-): 0

Region: NODE\_312079\_length\_70626\_cov\_31.911549 64782-64798. Max. coverage (+): 0. Max coverage (-): 0

Region: NODE\_312079\_length\_70626\_cov\_31.911549 64799-64815. Max. coverage (+): 0.03. Max coverage (-): 0

Region: NODE\_312079\_length\_70626\_cov\_31.911549 64816-64833. Max. coverage (+): 0. Max coverage (-): 0

Region: NODE\_312079\_length\_70626\_cov\_31.911549 64834-. Max. coverage (+): 0. Max coverage (-): 0

RepeatMasker Color Code

**+**

100-98% Identity

<98-95% Identity

<95-90% Identity

<90-85% Identity

<85-80% Identity

<80-75% Identity

<75-70% Identity

<70% Identity

**-**

Gene Set Color Code

**+**

Gene

Pseudogene

Other

**-**

Topology/Coverage Color Code

Coverage Plus Strand

Coverage Minus Strand

Mainstrand: Plus

Mainstrand: Minus

Complementary Strand

Flanking Region  
(if option -flank >0)

Gene Set Annotation  
  
RepeatMasker Annotation  

**1. Kolobok-2\_XT**: 58381-58424 (-), Divergence to consensus: 13.6%  
**2. AlRepB-438**: 58384-58678 (-), Divergence to consensus: 18.6%  
**3. Kolobok-2\_XT**: 58630-58682 (-), Divergence to consensus: 19.2%  
**4. TC1\_FR3**: 59601-59783 (-), Divergence to consensus: 28.5%  
**5. (TAGCTT)n**: 59921-59961 (+), Divergence to consensus: 20.4%  
**6. AlRepD-5020**: 62546-62959 (-), Divergence to consensus: 38.3%  
**7. AlRepE-1134**: 63069-63243 (-), Divergence to consensus: 38%  
**8. AlRepD-1895**: 63282-63336 (+), Divergence to consensus: 18.6%  
**9. AlRepA-4**: 64070-64295 (+), Divergence to consensus: 29.3%  
**10. AlRepA-4**: 64408-64557 (+), Divergence to consensus: 38.6%  
**11. Tc1-2\_FR**: 64752-64806 (+), Divergence to consensus: 11%  
**12. Tc1-2\_FR**: 64807-64848 (-), Divergence to consensus: 2.4%

  
Transcription Factor Binding Sites  

**RHOXF1** (Sequence: GGCTTA (-): 56243)  
**RHOXF1** (Sequence: AGCTTA (-): 56989)  
**RHOXF1** (Sequence: AGCTTA (-): 57604)  
**RHOXF1** (Sequence: GGATTA (-): 57921)  
**RHOXF1** (Sequence: AGCTTA (-): 58919)  
**RHOXF1** (Sequence: AGATCA (-): 58921)  
**RHOXF1** (Sequence: AGATCA (-): 59452)  
**RHOXF1** (Sequence: AGATCA (-): 59465)  
**RHOXF1** (Sequence: AGCTCA (-): 59751)  
**RHOXF1** (Sequence: AGATCA (-): 59867)  
**RHOXF1** (Sequence: AGATTA (-): 60359)  
**RHOXF1** (Sequence: GGATCA (-): 60806)  
**RHOXF1** (Sequence: AGCTCA (-): 60858)  
**RHOXF1** (Sequence: AGATTA (-): 61136)  
**RHOXF1** (Sequence: AGCTCA (-): 61232)  
**RHOXF1** (Sequence: AGATCA (-): 61267)  
**RHOXF1** (Sequence: GGCTCA (-): 61610)  
**RHOXF1** (Sequence: AGATTA (-): 61667)  
**RHOXF1** (Sequence: AGATTA (-): 63254)  
**RHOXF1** (Sequence: AGATCA (-): 64071)  
**RHOXF1** (Sequence: AGATTA (-): 64527)  
**RHOXF1** (Sequence: TGATCT (+): 57130)  
**RHOXF1** (Sequence: TAAGCT (+): 57602)  
**RHOXF1** (Sequence: TAAGCC (+): 57807)  
**RHOXF1** (Sequence: TAATCT (+): 60727)  
**RHOXF1** (Sequence: TGAGCT (+): 60929)  
**RHOXF1** (Sequence: TGAGCC (+): 61078)  
**RHOXF1** (Sequence: TGAGCT (+): 61253)  
**RHOXF1** (Sequence: TGATCC (+): 61736)  
**RHOXF1** (Sequence: TAATCT (+): 62644)  
**RHOXF1** (Sequence: TAAGCC (+): 62890)  
**RHOXF1** (Sequence: TGATCT (+): 63009)  
**RHOXF1** (Sequence: TGAGCT (+): 63096)  
**RHOXF1** (Sequence: TGAGCC (+): 64536)  
**Lhx8** (Sequence: TTAATTAA (-): 56181)  
**Lhx8** (Sequence: TTAATTAA (-): 56752)  
**Gata4** (Sequence: GTTATCT (+): 62969)  
**POU5F1** (Sequence: TTTGCAT (-): 61418)  
**FOXO1** (Sequence: GCTGTTTTT (+): 57253)  
**FOXO1** (Sequence: CTTGTTTAT (+): 58800)  
**FOXO3\_mmu** (Sequence: TGTTTTGC (-): 56926)  
**FOXO3\_mmu** (Sequence: TGTTTTGA (-): 57165)  
**FOXO3\_mmu** (Sequence: TGTTTACA (-): 57723)  
**FOXO3\_mmu** (Sequence: TGTTTAGA (-): 59040)  
**FOXO3\_mmu** (Sequence: TGTTTTGA (-): 62152)  
**FOXO3\_mmu** (Sequence: TGTTTTCA (-): 62541)  
**Sox5** (Sequence: ATTGTT (+): 56584)  
**Sox5** (Sequence: ATTGTT (+): 56800)  
**Sox5** (Sequence: ATTGTT (+): 57586)  
**Sox5** (Sequence: ATTGTT (+): 57588)  
**Sox5** (Sequence: ATTGTT (+): 59562)  
**Sox5** (Sequence: ATTGTT (+): 63194)  
**Sox5** (Sequence: ATTGTT (+): 63467)  
**Sox5** (Sequence: ATTGTT (+): 64714)  
**FIGLA** (Sequence: TCCAGCTGTA (-): 62345)  
**FOXO3\_mmu** (Sequence: GGAAAACA (+): 63920)  
**FOXO1** (Sequence: AAAAACAAC (-): 62753)  
**FOXO3\_hsa** (Sequence: ATGTTTAC (-): 57722)  
**FOXP1** (Sequence: TGTTTAC (-): 57723)  
**Nobox** (Sequence: TAATTGCT (+): 59882)  
**POU2F1** (Sequence: ATTAAAATA (-): 60583)  
**POU2F1** (Sequence: ATTAAAATA (-): 60825)  
**Rhox11** (Sequence: CGCTGTTTT (+): 57252)  
**Rhox11** (Sequence: TGCTGTAAA (+): 58672)  
**Sox5** (Sequence: AACAAT (-): 56719)  
**Sox5** (Sequence: AACAAT (-): 57038)  
**Sox5** (Sequence: AACAAT (-): 61906)  
**Sox5** (Sequence: AACAAT (-): 63239)  
**Sox5** (Sequence: AACAAT (-): 63924)  
**Sox5** (Sequence: AACAAT (-): 64274)  
**POU2F1** (Sequence: TATTTAAAT (+): 64179)  
**POU5F1** (Sequence: ATGCAAA (+): 63539)
