# Supplementary material for: First characterization of PIWI-interacting RNA clusters in a cichlid fish with a B chromosome
Source: BMC Biol. 2022 Sep 21;20:204. doi: 10.1186/s12915-022-01403-2 (PMC9490952; doi:10.1186/s12915-022-01403-2)
Supplement: Supplementary file 1 — Additional file 1. Zipped folder with fasta and interactive html piRNA cluster information for the A. latifasciata genome. The nomenclature is as follows: number-pirna-cluster_sex_B-presence (f, female; m, male; 0b, without B chromosome; 1b, with B chromosome). [file 12915_2022_1403_MOESM1_ESM.zip › 121_m0b.html]

piRNA cluster 121\_m0b 57


Predicted piRNA cluster no. 121\_m0b
  

Show proTRAC run info
Hide proTRAC run info

/\  
                \_\_\_\_\_\_\_\_\_\_\_\_\_\_\_\_\_\_\_\_\_\_\_/\\_\_\_ /  \\_\_\_\_\_\_\_  
               I                      /  \  /    \      I  
               I     pro             /    \/      \     I  
               I        TRAC        /               \   I  
               I   \_\_\_\_\_\_\_\_\_\_\_\_\_\_\_\_/\_\_\_\_\_\_\_\_\_\_\_\_\_\_\_\_\_\\_ I  
               I   \              /                     I  
               I    \            /                      I  
               I     \  /\      /       V.2.4.2         I  
               I      \/  \    /                        I  
               I\_\_\_\_\_\_\_\_\_\_\_\  /\_\_\_\_\_\_\_\_\_\_\_\_\_\_\_\_\_\_\_\_\_\_\_\_\_I  
                            \/  
  
  
================================= proTRAC ====================================  
VERSION: .......... 2.4.2  
LAST MODIFIED: .... 11. May 2018  
  
Please cite:  
Rosenkranz D, Zischler H. proTRAC - a software for probabilistic piRNA cluster  
detection, visualization and analysis. 2012. BMC Bioinformatics 13:5.  
  
  
Contact:  
David Rosenkranz  
Institute of Organismic and Molecular Evolutionary Biology  
Dept. Anthropology, small RNA group  
Johannes Gutenberg University Mainz  
email: rosenkranz@uni-mainz.de  
  
You can find the latest proTRAC version at:  
http://sourceforge.net/projects/protrac/files  
http://www.smallRNAgroup-mainz.de/software  
==============================================================================  
  
PARAMETERS:  
Map file: ...............piwi-machos-0B.fa-collapse.map  
Genome file: ............../../../0B\_ala\_genome.fa  
RepeatMasker annotation: Alatifasciata-all0B-maryan-v2.fa\_corrected.out  
GeneSet:................./guest-storage/Data/annotation/Alatifasciata\_all0B\_maryan-v2\_out2017.gff  
  
Significant (p<=0.01) hit density will be calculated based  
on observed hit distribution.  
  
Sliding window size: ........................................ 5000 bp  
Sliding window increament: .................................. 1000 bp  
Normalize each hit by number of genomic hits: ............... yes  
Normalize each hit by number of sequence reads: ............. yes  
Normalize values (-> per million mapped reads): ............. yes  
Min. fraction of hits with 1T(U) or 10A: .................... 0.75  
Alternatively: Min. fraction of hits with 1T(U) and 10A: .... 0.5  
Min. fraction of hits with typical piRNA length: ............ 0.75  
Typical piRNA length: ....................................... 24-32 nt  
Min. size of a piRNA cluster: ............................... 1000 bp.  
Min. number of hits (absolute): ............................. 0  
Min. number of hits (normalized): ........................... 0  
Min. fraction of hits on the mainstrand: .................... 0.75  
Top fraction of mapped sequences (in terms of read counts): . 1%  
Top fraction accounts for max. n% of sequence reads: ........ 90%  
Min. fraction of hits on each arm of a bidirectional cluster: 0.05  
Output html file for each cluster: .......................... yes  
Output a summary table: ..................................... yes  
Output a FASTA file for each cluster (piRNA sequences): ..... yes  
Output a FASTA file comprising cluster sequences: ........... yes  
Output a GTF file for predicted piRNA clusters: ..............yes  
Search DNA motifs in clusters: .............................. yes  
Output flanking sequences: +/- .............................. 0 bp  
Output ~.pTi file: .......................................... no  
==============================================================================  
  
  
Genome size (without gaps): ............ 758543724 bp  
Gaps (N/X/-): .......................... 417479 bp  
Mapped reads: .......................... 24765598  
Non-identical sequences: ............... 6158275  
Genomic hits: .......................... 53103584  
Significant densitiy of mapped reads: .. 763.098963422187 reads/kb

Show proTRAC cluster info
Hide proTRAC cluster info

|  |  |
| --- | --- |
| Location | NODE\_312079\_length\_70626\_cov\_31.911549 |
| Coordinates | 46658-65990 |
| Size [bp] | 19333 |
| Sequence hit loci | 8466 |
| Mapped reads (normalized) | 34747.2 |
| Mapped reads (normalized) per kb | 1797.3 |
| Normalized reads with 1T (1U) | 75.6% |
| Normalized reads with 10A | 42% |
| Normalized reads with length 24-32 nt | 98.9% |
| Normalized reads on the main strand(s) | 85.9% |
| Predicted directionality | bi:minus-plus (split between 53605 and 53621) |

100%

0%

1T (1U)  
reads

10A reads

24-32 nt  
reads

reads on mainstrand

**Either the amount of reads with 1T (1U) OR 10A has to exceed 75% (set with option: -1Tor10A)  
Alternatively the amount of reads with 1T (1U) AND 10A has to exceed 50% (set with option: -1Tand10A)  
Minimum amount of reads with preferred size is 75% (set with option: -pisize)  
Minimum amount of reads on the main strand(s) is 75% (set with option: -clstrand)**

Show read coverage
Hide read coverage

WHAT DO I SEE HERE?  
This chart shows the location of mapped sequence reads within a predicted piRNA cluster. The color refers to the number of genomic hits produced by the sequence read in question. A dark red bar indicates that this sequence read produces many other hits elsewhere in the genome. Many adjacent red or yellow bars can indicate the presence of a multi-copy element such as transposons or rRNA genes. A dark green bar indicates that this sequence read maps uniquely to this locus.

1 hit

2-5 hits

6-10 hits

11-20 hits

21-50 hits

51-100 hits

> 100 hits

NODE\_312079\_length\_70626\_cov\_31.911549

46658

65990

Gene Set

RepeatMasker

Mapped  
Reads

73.49

plus strand

minus strand

73.49

Region: NODE\_312079\_length\_70626\_cov\_31.911549 5043-46677. Max. coverage (+): 0.04. Max coverage (-): 0

Region: NODE\_312079\_length\_70626\_cov\_31.911549 46678-46715. Max. coverage (+): 0. Max coverage (-): 0

Region: NODE\_312079\_length\_70626\_cov\_31.911549 46716-46754. Max. coverage (+): 0. Max coverage (-): 0

Region: NODE\_312079\_length\_70626\_cov\_31.911549 46755-46793. Max. coverage (+): 0. Max coverage (-): 0

Region: NODE\_312079\_length\_70626\_cov\_31.911549 46794-46831. Max. coverage (+): 0. Max coverage (-): 0

Region: NODE\_312079\_length\_70626\_cov\_31.911549 46832-46870. Max. coverage (+): 0. Max coverage (-): 0

Region: NODE\_312079\_length\_70626\_cov\_31.911549 46871-46909. Max. coverage (+): 0. Max coverage (-): 0

Region: NODE\_312079\_length\_70626\_cov\_31.911549 46910-46947. Max. coverage (+): 0. Max coverage (-): 0

Region: NODE\_312079\_length\_70626\_cov\_31.911549 46948-46986. Max. coverage (+): 0. Max coverage (-): 0

Region: NODE\_312079\_length\_70626\_cov\_31.911549 46987-47025. Max. coverage (+): 0. Max coverage (-): 0

Region: NODE\_312079\_length\_70626\_cov\_31.911549 47026-47063. Max. coverage (+): 0. Max coverage (-): 0

Region: NODE\_312079\_length\_70626\_cov\_31.911549 47064-47102. Max. coverage (+): 0. Max coverage (-): 0

Region: NODE\_312079\_length\_70626\_cov\_31.911549 47103-47141. Max. coverage (+): 0. Max coverage (-): 0

Region: NODE\_312079\_length\_70626\_cov\_31.911549 47142-47179. Max. coverage (+): 0. Max coverage (-): 0

Region: NODE\_312079\_length\_70626\_cov\_31.911549 47180-47218. Max. coverage (+): 0. Max coverage (-): 0

Region: NODE\_312079\_length\_70626\_cov\_31.911549 47219-47257. Max. coverage (+): 0. Max coverage (-): 0

Region: NODE\_312079\_length\_70626\_cov\_31.911549 47258-47295. Max. coverage (+): 0. Max coverage (-): 0

Region: NODE\_312079\_length\_70626\_cov\_31.911549 47296-47334. Max. coverage (+): 0. Max coverage (-): 0

Region: NODE\_312079\_length\_70626\_cov\_31.911549 47335-47373. Max. coverage (+): 0. Max coverage (-): 0

Region: NODE\_312079\_length\_70626\_cov\_31.911549 47374-47411. Max. coverage (+): 0. Max coverage (-): 0

Region: NODE\_312079\_length\_70626\_cov\_31.911549 47412-47450. Max. coverage (+): 0. Max coverage (-): 0

Region: NODE\_312079\_length\_70626\_cov\_31.911549 47451-47489. Max. coverage (+): 0. Max coverage (-): 0

Region: NODE\_312079\_length\_70626\_cov\_31.911549 47490-47527. Max. coverage (+): 0. Max coverage (-): 0

Region: NODE\_312079\_length\_70626\_cov\_31.911549 47528-47566. Max. coverage (+): 0. Max coverage (-): 0

Region: NODE\_312079\_length\_70626\_cov\_31.911549 47567-47605. Max. coverage (+): 0. Max coverage (-): 0

Region: NODE\_312079\_length\_70626\_cov\_31.911549 47606-47643. Max. coverage (+): 0. Max coverage (-): 0

Region: NODE\_312079\_length\_70626\_cov\_31.911549 47644-47682. Max. coverage (+): 0. Max coverage (-): 0

Region: NODE\_312079\_length\_70626\_cov\_31.911549 47683-47721. Max. coverage (+): 0. Max coverage (-): 0

Region: NODE\_312079\_length\_70626\_cov\_31.911549 47722-47759. Max. coverage (+): 0. Max coverage (-): 0

Region: NODE\_312079\_length\_70626\_cov\_31.911549 47760-47798. Max. coverage (+): 0. Max coverage (-): 0

Region: NODE\_312079\_length\_70626\_cov\_31.911549 47799-47837. Max. coverage (+): 0.08. Max coverage (-): 0

Region: NODE\_312079\_length\_70626\_cov\_31.911549 47838-47875. Max. coverage (+): 0. Max coverage (-): 0

Region: NODE\_312079\_length\_70626\_cov\_31.911549 47876-47914. Max. coverage (+): 0. Max coverage (-): 0

Region: NODE\_312079\_length\_70626\_cov\_31.911549 47915-47953. Max. coverage (+): 0. Max coverage (-): 0

Region: NODE\_312079\_length\_70626\_cov\_31.911549 47954-47991. Max. coverage (+): 0. Max coverage (-): 0

Region: NODE\_312079\_length\_70626\_cov\_31.911549 47992-48030. Max. coverage (+): 0. Max coverage (-): 0

Region: NODE\_312079\_length\_70626\_cov\_31.911549 48031-48069. Max. coverage (+): 0. Max coverage (-): 0

Region: NODE\_312079\_length\_70626\_cov\_31.911549 48070-48107. Max. coverage (+): 0. Max coverage (-): 0

Region: NODE\_312079\_length\_70626\_cov\_31.911549 48108-48146. Max. coverage (+): 0. Max coverage (-): 0.04

Region: NODE\_312079\_length\_70626\_cov\_31.911549 48147-48185. Max. coverage (+): 0. Max coverage (-): 0

Region: NODE\_312079\_length\_70626\_cov\_31.911549 48186-48223. Max. coverage (+): 0. Max coverage (-): 0

Region: NODE\_312079\_length\_70626\_cov\_31.911549 48224-48262. Max. coverage (+): 0. Max coverage (-): 0

Region: NODE\_312079\_length\_70626\_cov\_31.911549 48263-48301. Max. coverage (+): 0. Max coverage (-): 0

Region: NODE\_312079\_length\_70626\_cov\_31.911549 48302-48339. Max. coverage (+): 0. Max coverage (-): 0

Region: NODE\_312079\_length\_70626\_cov\_31.911549 48340-48378. Max. coverage (+): 0. Max coverage (-): 0

Region: NODE\_312079\_length\_70626\_cov\_31.911549 48379-48417. Max. coverage (+): 0. Max coverage (-): 0

Region: NODE\_312079\_length\_70626\_cov\_31.911549 48418-48455. Max. coverage (+): 0. Max coverage (-): 0

Region: NODE\_312079\_length\_70626\_cov\_31.911549 48456-48494. Max. coverage (+): 0. Max coverage (-): 0

Region: NODE\_312079\_length\_70626\_cov\_31.911549 48495-48533. Max. coverage (+): 0. Max coverage (-): 0

Region: NODE\_312079\_length\_70626\_cov\_31.911549 48534-48571. Max. coverage (+): 0. Max coverage (-): 0

Region: NODE\_312079\_length\_70626\_cov\_31.911549 48572-48610. Max. coverage (+): 0. Max coverage (-): 0

Region: NODE\_312079\_length\_70626\_cov\_31.911549 48611-48649. Max. coverage (+): 0. Max coverage (-): 0

Region: NODE\_312079\_length\_70626\_cov\_31.911549 48650-48687. Max. coverage (+): 0. Max coverage (-): 0

Region: NODE\_312079\_length\_70626\_cov\_31.911549 48688-48726. Max. coverage (+): 0. Max coverage (-): 0

Region: NODE\_312079\_length\_70626\_cov\_31.911549 48727-48765. Max. coverage (+): 0. Max coverage (-): 0.04

Region: NODE\_312079\_length\_70626\_cov\_31.911549 48766-48803. Max. coverage (+): 0. Max coverage (-): 0.02

Region: NODE\_312079\_length\_70626\_cov\_31.911549 48804-48842. Max. coverage (+): 0. Max coverage (-): 0.01

Region: NODE\_312079\_length\_70626\_cov\_31.911549 48843-48881. Max. coverage (+): 0. Max coverage (-): 0.02

Region: NODE\_312079\_length\_70626\_cov\_31.911549 48882-48919. Max. coverage (+): 0. Max coverage (-): 0

Region: NODE\_312079\_length\_70626\_cov\_31.911549 48920-48958. Max. coverage (+): 0. Max coverage (-): 0.01

Region: NODE\_312079\_length\_70626\_cov\_31.911549 48959-48997. Max. coverage (+): 0. Max coverage (-): 0

Region: NODE\_312079\_length\_70626\_cov\_31.911549 48998-49035. Max. coverage (+): 0. Max coverage (-): 0.01

Region: NODE\_312079\_length\_70626\_cov\_31.911549 49036-49074. Max. coverage (+): 0.03. Max coverage (-): 0.01

Region: NODE\_312079\_length\_70626\_cov\_31.911549 49075-49113. Max. coverage (+): 0.02. Max coverage (-): 0.03

Region: NODE\_312079\_length\_70626\_cov\_31.911549 49114-49151. Max. coverage (+): 0.04. Max coverage (-): 0.08

Region: NODE\_312079\_length\_70626\_cov\_31.911549 49152-49190. Max. coverage (+): 0. Max coverage (-): 0.12

Region: NODE\_312079\_length\_70626\_cov\_31.911549 49191-49229. Max. coverage (+): 0.08. Max coverage (-): 0.04

Region: NODE\_312079\_length\_70626\_cov\_31.911549 49230-49267. Max. coverage (+): 0.93. Max coverage (-): 0.93

Region: NODE\_312079\_length\_70626\_cov\_31.911549 49268-49306. Max. coverage (+): 0. Max coverage (-): 1.98

Region: NODE\_312079\_length\_70626\_cov\_31.911549 49307-49345. Max. coverage (+): 0.04. Max coverage (-): 0.48

Region: NODE\_312079\_length\_70626\_cov\_31.911549 49346-49383. Max. coverage (+): 0. Max coverage (-): 3.11

Region: NODE\_312079\_length\_70626\_cov\_31.911549 49384-49422. Max. coverage (+): 0.12. Max coverage (-): 0.2

Region: NODE\_312079\_length\_70626\_cov\_31.911549 49423-49461. Max. coverage (+): 0.16. Max coverage (-): 0.16

Region: NODE\_312079\_length\_70626\_cov\_31.911549 49462-49499. Max. coverage (+): 0.04. Max coverage (-): 0.32

Region: NODE\_312079\_length\_70626\_cov\_31.911549 49500-49538. Max. coverage (+): 0.04. Max coverage (-): 2.62

Region: NODE\_312079\_length\_70626\_cov\_31.911549 49539-49577. Max. coverage (+): 0.36. Max coverage (-): 0.85

Region: NODE\_312079\_length\_70626\_cov\_31.911549 49578-49615. Max. coverage (+): 0.77. Max coverage (-): 1.62

Region: NODE\_312079\_length\_70626\_cov\_31.911549 49616-49654. Max. coverage (+): 0. Max coverage (-): 1.41

Region: NODE\_312079\_length\_70626\_cov\_31.911549 49655-49693. Max. coverage (+): 0.2. Max coverage (-): 3.23

Region: NODE\_312079\_length\_70626\_cov\_31.911549 49694-49731. Max. coverage (+): 0.16. Max coverage (-): 0

Region: NODE\_312079\_length\_70626\_cov\_31.911549 49732-49770. Max. coverage (+): 1.66. Max coverage (-): 8.4

Region: NODE\_312079\_length\_70626\_cov\_31.911549 49771-49809. Max. coverage (+): 0.2. Max coverage (-): 0.44

Region: NODE\_312079\_length\_70626\_cov\_31.911549 49810-49847. Max. coverage (+): 0.12. Max coverage (-): 0.12

Region: NODE\_312079\_length\_70626\_cov\_31.911549 49848-49886. Max. coverage (+): 0. Max coverage (-): 0.08

Region: NODE\_312079\_length\_70626\_cov\_31.911549 49887-49925. Max. coverage (+): 0.48. Max coverage (-): 0.57

Region: NODE\_312079\_length\_70626\_cov\_31.911549 49926-49963. Max. coverage (+): 0.12. Max coverage (-): 0.2

Region: NODE\_312079\_length\_70626\_cov\_31.911549 49964-50002. Max. coverage (+): 0.04. Max coverage (-): 0.69

Region: NODE\_312079\_length\_70626\_cov\_31.911549 50003-50041. Max. coverage (+): 3.8. Max coverage (-): 0.81

Region: NODE\_312079\_length\_70626\_cov\_31.911549 50042-50079. Max. coverage (+): 0.52. Max coverage (-): 2.26

Region: NODE\_312079\_length\_70626\_cov\_31.911549 50080-50118. Max. coverage (+): 0.04. Max coverage (-): 2.26

Region: NODE\_312079\_length\_70626\_cov\_31.911549 50119-50157. Max. coverage (+): 0.24. Max coverage (-): 0.24

Region: NODE\_312079\_length\_70626\_cov\_31.911549 50158-50195. Max. coverage (+): 0.69. Max coverage (-): 16.11

Region: NODE\_312079\_length\_70626\_cov\_31.911549 50196-50234. Max. coverage (+): 0.44. Max coverage (-): 19.42

Region: NODE\_312079\_length\_70626\_cov\_31.911549 50235-50273. Max. coverage (+): 0.44. Max coverage (-): 0.28

Region: NODE\_312079\_length\_70626\_cov\_31.911549 50274-50311. Max. coverage (+): 0.28. Max coverage (-): 0.77

Region: NODE\_312079\_length\_70626\_cov\_31.911549 50312-50350. Max. coverage (+): 0.28. Max coverage (-): 0.93

Region: NODE\_312079\_length\_70626\_cov\_31.911549 50351-50389. Max. coverage (+): 0.52. Max coverage (-): 0.73

Region: NODE\_312079\_length\_70626\_cov\_31.911549 50390-50427. Max. coverage (+): 0.12. Max coverage (-): 4.85

Region: NODE\_312079\_length\_70626\_cov\_31.911549 50428-50466. Max. coverage (+): 1.21. Max coverage (-): 2.3

Region: NODE\_312079\_length\_70626\_cov\_31.911549 50467-50505. Max. coverage (+): 0.16. Max coverage (-): 1.78

Region: NODE\_312079\_length\_70626\_cov\_31.911549 50506-50543. Max. coverage (+): 0.32. Max coverage (-): 0.2

Region: NODE\_312079\_length\_70626\_cov\_31.911549 50544-50582. Max. coverage (+): 0.16. Max coverage (-): 13.97

Region: NODE\_312079\_length\_70626\_cov\_31.911549 50583-50621. Max. coverage (+): 0.24. Max coverage (-): 0.16

Region: NODE\_312079\_length\_70626\_cov\_31.911549 50622-50659. Max. coverage (+): 1.29. Max coverage (-): 0.57

Region: NODE\_312079\_length\_70626\_cov\_31.911549 50660-50698. Max. coverage (+): 1.17. Max coverage (-): 0.24

Region: NODE\_312079\_length\_70626\_cov\_31.911549 50699-50737. Max. coverage (+): 0.89. Max coverage (-): 12.48

Region: NODE\_312079\_length\_70626\_cov\_31.911549 50738-50775. Max. coverage (+): 0.44. Max coverage (-): 4

Region: NODE\_312079\_length\_70626\_cov\_31.911549 50776-50814. Max. coverage (+): 0.32. Max coverage (-): 4.85

Region: NODE\_312079\_length\_70626\_cov\_31.911549 50815-50853. Max. coverage (+): 0.32. Max coverage (-): 1.13

Region: NODE\_312079\_length\_70626\_cov\_31.911549 50854-50891. Max. coverage (+): 3.23. Max coverage (-): 2.26

Region: NODE\_312079\_length\_70626\_cov\_31.911549 50892-50930. Max. coverage (+): 0.2. Max coverage (-): 1.29

Region: NODE\_312079\_length\_70626\_cov\_31.911549 50931-50969. Max. coverage (+): 0.16. Max coverage (-): 0.69

Region: NODE\_312079\_length\_70626\_cov\_31.911549 50970-51007. Max. coverage (+): 0.52. Max coverage (-): 3.63

Region: NODE\_312079\_length\_70626\_cov\_31.911549 51008-51046. Max. coverage (+): 0.44. Max coverage (-): 0.69

Region: NODE\_312079\_length\_70626\_cov\_31.911549 51047-51085. Max. coverage (+): 1.13. Max coverage (-): 19.14

Region: NODE\_312079\_length\_70626\_cov\_31.911549 51086-51123. Max. coverage (+): 0.36. Max coverage (-): 0.48

Region: NODE\_312079\_length\_70626\_cov\_31.911549 51124-51162. Max. coverage (+): 0.08. Max coverage (-): 0.57

Region: NODE\_312079\_length\_70626\_cov\_31.911549 51163-51201. Max. coverage (+): 4.16. Max coverage (-): 73.45

Region: NODE\_312079\_length\_70626\_cov\_31.911549 51202-51239. Max. coverage (+): 0.12. Max coverage (-): 1.09

Region: NODE\_312079\_length\_70626\_cov\_31.911549 51240-51278. Max. coverage (+): 0. Max coverage (-): 1.09

Region: NODE\_312079\_length\_70626\_cov\_31.911549 51279-51317. Max. coverage (+): 0.04. Max coverage (-): 0.24

Region: NODE\_312079\_length\_70626\_cov\_31.911549 51318-51355. Max. coverage (+): 0.48. Max coverage (-): 5.65

Region: NODE\_312079\_length\_70626\_cov\_31.911549 51356-51394. Max. coverage (+): 0.44. Max coverage (-): 0.89

Region: NODE\_312079\_length\_70626\_cov\_31.911549 51395-51433. Max. coverage (+): 5.81. Max coverage (-): 3.27

Region: NODE\_312079\_length\_70626\_cov\_31.911549 51434-51471. Max. coverage (+): 0.2. Max coverage (-): 1.17

Region: NODE\_312079\_length\_70626\_cov\_31.911549 51472-51510. Max. coverage (+): 0.61. Max coverage (-): 0.52

Region: NODE\_312079\_length\_70626\_cov\_31.911549 51511-51549. Max. coverage (+): 2.66. Max coverage (-): 0.69

Region: NODE\_312079\_length\_70626\_cov\_31.911549 51550-51587. Max. coverage (+): 0.04. Max coverage (-): 1.29

Region: NODE\_312079\_length\_70626\_cov\_31.911549 51588-51626. Max. coverage (+): 0.2. Max coverage (-): 0.44

Region: NODE\_312079\_length\_70626\_cov\_31.911549 51627-51665. Max. coverage (+): 0.28. Max coverage (-): 0.2

Region: NODE\_312079\_length\_70626\_cov\_31.911549 51666-51703. Max. coverage (+): 0.52. Max coverage (-): 0.04

Region: NODE\_312079\_length\_70626\_cov\_31.911549 51704-51742. Max. coverage (+): 0.24. Max coverage (-): 0.16

Region: NODE\_312079\_length\_70626\_cov\_31.911549 51743-51781. Max. coverage (+): 0.28. Max coverage (-): 0.89

Region: NODE\_312079\_length\_70626\_cov\_31.911549 51782-51819. Max. coverage (+): 0.93. Max coverage (-): 0.04

Region: NODE\_312079\_length\_70626\_cov\_31.911549 51820-51858. Max. coverage (+): 0.04. Max coverage (-): 0.65

Region: NODE\_312079\_length\_70626\_cov\_31.911549 51859-51897. Max. coverage (+): 0.24. Max coverage (-): 1.25

Region: NODE\_312079\_length\_70626\_cov\_31.911549 51898-51935. Max. coverage (+): 0.28. Max coverage (-): 1.25

Region: NODE\_312079\_length\_70626\_cov\_31.911549 51936-51974. Max. coverage (+): 0.69. Max coverage (-): 1.41

Region: NODE\_312079\_length\_70626\_cov\_31.911549 51975-52013. Max. coverage (+): 1.25. Max coverage (-): 6.7

Region: NODE\_312079\_length\_70626\_cov\_31.911549 52014-52051. Max. coverage (+): 0.04. Max coverage (-): 0.2

Region: NODE\_312079\_length\_70626\_cov\_31.911549 52052-52090. Max. coverage (+): 0.48. Max coverage (-): 2.02

Region: NODE\_312079\_length\_70626\_cov\_31.911549 52091-52129. Max. coverage (+): 0.69. Max coverage (-): 0.16

Region: NODE\_312079\_length\_70626\_cov\_31.911549 52130-52167. Max. coverage (+): 0.16. Max coverage (-): 4

Region: NODE\_312079\_length\_70626\_cov\_31.911549 52168-52206. Max. coverage (+): 0.04. Max coverage (-): 0.65

Region: NODE\_312079\_length\_70626\_cov\_31.911549 52207-52245. Max. coverage (+): 0. Max coverage (-): 0.77

Region: NODE\_312079\_length\_70626\_cov\_31.911549 52246-52283. Max. coverage (+): 0.04. Max coverage (-): 0.2

Region: NODE\_312079\_length\_70626\_cov\_31.911549 52284-52322. Max. coverage (+): 0.89. Max coverage (-): 2.62

Region: NODE\_312079\_length\_70626\_cov\_31.911549 52323-52361. Max. coverage (+): 1.29. Max coverage (-): 2.46

Region: NODE\_312079\_length\_70626\_cov\_31.911549 52362-52399. Max. coverage (+): 0.48. Max coverage (-): 5.85

Region: NODE\_312079\_length\_70626\_cov\_31.911549 52400-52438. Max. coverage (+): 0.57. Max coverage (-): 1.9

Region: NODE\_312079\_length\_70626\_cov\_31.911549 52439-52477. Max. coverage (+): 0.61. Max coverage (-): 2.5

Region: NODE\_312079\_length\_70626\_cov\_31.911549 52478-52515. Max. coverage (+): 0.61. Max coverage (-): 1.01

Region: NODE\_312079\_length\_70626\_cov\_31.911549 52516-52554. Max. coverage (+): 0.44. Max coverage (-): 0.89

Region: NODE\_312079\_length\_70626\_cov\_31.911549 52555-52593. Max. coverage (+): 0.08. Max coverage (-): 0.61

Region: NODE\_312079\_length\_70626\_cov\_31.911549 52594-52631. Max. coverage (+): 0.24. Max coverage (-): 1.78

Region: NODE\_312079\_length\_70626\_cov\_31.911549 52632-52670. Max. coverage (+): 0.28. Max coverage (-): 3.11

Region: NODE\_312079\_length\_70626\_cov\_31.911549 52671-52709. Max. coverage (+): 0.08. Max coverage (-): 0.36

Region: NODE\_312079\_length\_70626\_cov\_31.911549 52710-52747. Max. coverage (+): 0.4. Max coverage (-): 2.1

Region: NODE\_312079\_length\_70626\_cov\_31.911549 52748-52786. Max. coverage (+): 1.13. Max coverage (-): 2.02

Region: NODE\_312079\_length\_70626\_cov\_31.911549 52787-52825. Max. coverage (+): 0.08. Max coverage (-): 1.94

Region: NODE\_312079\_length\_70626\_cov\_31.911549 52826-52863. Max. coverage (+): 0.04. Max coverage (-): 1.57

Region: NODE\_312079\_length\_70626\_cov\_31.911549 52864-52902. Max. coverage (+): 13.37. Max coverage (-): 0.12

Region: NODE\_312079\_length\_70626\_cov\_31.911549 52903-52941. Max. coverage (+): 0.32. Max coverage (-): 0.24

Region: NODE\_312079\_length\_70626\_cov\_31.911549 52942-52979. Max. coverage (+): 0.44. Max coverage (-): 9.97

Region: NODE\_312079\_length\_70626\_cov\_31.911549 52980-53018. Max. coverage (+): 0.08. Max coverage (-): 2.71

Region: NODE\_312079\_length\_70626\_cov\_31.911549 53019-53057. Max. coverage (+): 0.16. Max coverage (-): 0.77

Region: NODE\_312079\_length\_70626\_cov\_31.911549 53058-53095. Max. coverage (+): 0.2. Max coverage (-): 1.01

Region: NODE\_312079\_length\_70626\_cov\_31.911549 53096-53134. Max. coverage (+): 0.04. Max coverage (-): 1.05

Region: NODE\_312079\_length\_70626\_cov\_31.911549 53135-53173. Max. coverage (+): 0.44. Max coverage (-): 0.4

Region: NODE\_312079\_length\_70626\_cov\_31.911549 53174-53211. Max. coverage (+): 0.08. Max coverage (-): 0.28

Region: NODE\_312079\_length\_70626\_cov\_31.911549 53212-53250. Max. coverage (+): 0.04. Max coverage (-): 0.28

Region: NODE\_312079\_length\_70626\_cov\_31.911549 53251-53289. Max. coverage (+): 0.2. Max coverage (-): 1.74

Region: NODE\_312079\_length\_70626\_cov\_31.911549 53290-53327. Max. coverage (+): 0.08. Max coverage (-): 0.12

Region: NODE\_312079\_length\_70626\_cov\_31.911549 53328-53366. Max. coverage (+): 0. Max coverage (-): 0.28

Region: NODE\_312079\_length\_70626\_cov\_31.911549 53367-53405. Max. coverage (+): 0.04. Max coverage (-): 0.08

Region: NODE\_312079\_length\_70626\_cov\_31.911549 53406-53443. Max. coverage (+): 0. Max coverage (-): 0

Region: NODE\_312079\_length\_70626\_cov\_31.911549 53444-53482. Max. coverage (+): 0.04. Max coverage (-): 0.36

Region: NODE\_312079\_length\_70626\_cov\_31.911549 53483-53521. Max. coverage (+): 0. Max coverage (-): 0.12

Region: NODE\_312079\_length\_70626\_cov\_31.911549 53522-53559. Max. coverage (+): 0. Max coverage (-): 0.04

Region: NODE\_312079\_length\_70626\_cov\_31.911549 53560-53598. Max. coverage (+): 0. Max coverage (-): 0

Region: NODE\_312079\_length\_70626\_cov\_31.911549 53599-53637. Max. coverage (+): 0.08. Max coverage (-): 0.04

Region: NODE\_312079\_length\_70626\_cov\_31.911549 53638-53675. Max. coverage (+): 0.02. Max coverage (-): 0

Region: NODE\_312079\_length\_70626\_cov\_31.911549 53676-53714. Max. coverage (+): 0. Max coverage (-): 0

Region: NODE\_312079\_length\_70626\_cov\_31.911549 53715-53753. Max. coverage (+): 0.01. Max coverage (-): 0

Region: NODE\_312079\_length\_70626\_cov\_31.911549 53754-53791. Max. coverage (+): 0.01. Max coverage (-): 0.01

Region: NODE\_312079\_length\_70626\_cov\_31.911549 53792-53830. Max. coverage (+): 0.03. Max coverage (-): 0

Region: NODE\_312079\_length\_70626\_cov\_31.911549 53831-53869. Max. coverage (+): 0. Max coverage (-): 0

Region: NODE\_312079\_length\_70626\_cov\_31.911549 53870-53907. Max. coverage (+): 0. Max coverage (-): 0

Region: NODE\_312079\_length\_70626\_cov\_31.911549 53908-53946. Max. coverage (+): 0. Max coverage (-): 0

Region: NODE\_312079\_length\_70626\_cov\_31.911549 53947-53985. Max. coverage (+): 0. Max coverage (-): 0

Region: NODE\_312079\_length\_70626\_cov\_31.911549 53986-54023. Max. coverage (+): 0. Max coverage (-): 0

Region: NODE\_312079\_length\_70626\_cov\_31.911549 54024-54062. Max. coverage (+): 0. Max coverage (-): 0

Region: NODE\_312079\_length\_70626\_cov\_31.911549 54063-54101. Max. coverage (+): 0. Max coverage (-): 0

Region: NODE\_312079\_length\_70626\_cov\_31.911549 54102-54139. Max. coverage (+): 0. Max coverage (-): 0

Region: NODE\_312079\_length\_70626\_cov\_31.911549 54140-54178. Max. coverage (+): 0. Max coverage (-): 0

Region: NODE\_312079\_length\_70626\_cov\_31.911549 54179-54217. Max. coverage (+): 0. Max coverage (-): 0

Region: NODE\_312079\_length\_70626\_cov\_31.911549 54218-54255. Max. coverage (+): 0. Max coverage (-): 0

Region: NODE\_312079\_length\_70626\_cov\_31.911549 54256-54294. Max. coverage (+): 0. Max coverage (-): 0

Region: NODE\_312079\_length\_70626\_cov\_31.911549 54295-54333. Max. coverage (+): 0. Max coverage (-): 0

Region: NODE\_312079\_length\_70626\_cov\_31.911549 54334-54371. Max. coverage (+): 0.04. Max coverage (-): 0

Region: NODE\_312079\_length\_70626\_cov\_31.911549 54372-54410. Max. coverage (+): 0. Max coverage (-): 0

Region: NODE\_312079\_length\_70626\_cov\_31.911549 54411-54449. Max. coverage (+): 0.04. Max coverage (-): 0

Region: NODE\_312079\_length\_70626\_cov\_31.911549 54450-54487. Max. coverage (+): 0. Max coverage (-): 0

Region: NODE\_312079\_length\_70626\_cov\_31.911549 54488-54526. Max. coverage (+): 0. Max coverage (-): 0

Region: NODE\_312079\_length\_70626\_cov\_31.911549 54527-54565. Max. coverage (+): 0. Max coverage (-): 0

Region: NODE\_312079\_length\_70626\_cov\_31.911549 54566-54603. Max. coverage (+): 0. Max coverage (-): 0

Region: NODE\_312079\_length\_70626\_cov\_31.911549 54604-54642. Max. coverage (+): 0. Max coverage (-): 0

Region: NODE\_312079\_length\_70626\_cov\_31.911549 54643-54681. Max. coverage (+): 0. Max coverage (-): 0

Region: NODE\_312079\_length\_70626\_cov\_31.911549 54682-54719. Max. coverage (+): 0. Max coverage (-): 0

Region: NODE\_312079\_length\_70626\_cov\_31.911549 54720-54758. Max. coverage (+): 0.04. Max coverage (-): 0

Region: NODE\_312079\_length\_70626\_cov\_31.911549 54759-54797. Max. coverage (+): 0. Max coverage (-): 0

Region: NODE\_312079\_length\_70626\_cov\_31.911549 54798-54835. Max. coverage (+): 0. Max coverage (-): 0.04

Region: NODE\_312079\_length\_70626\_cov\_31.911549 54836-54874. Max. coverage (+): 0. Max coverage (-): 0

Region: NODE\_312079\_length\_70626\_cov\_31.911549 54875-54913. Max. coverage (+): 0.08. Max coverage (-): 0

Region: NODE\_312079\_length\_70626\_cov\_31.911549 54914-54951. Max. coverage (+): 0.04. Max coverage (-): 0.04

Region: NODE\_312079\_length\_70626\_cov\_31.911549 54952-54990. Max. coverage (+): 0.08. Max coverage (-): 0

Region: NODE\_312079\_length\_70626\_cov\_31.911549 54991-55029. Max. coverage (+): 0. Max coverage (-): 0

Region: NODE\_312079\_length\_70626\_cov\_31.911549 55030-55067. Max. coverage (+): 0. Max coverage (-): 0

Region: NODE\_312079\_length\_70626\_cov\_31.911549 55068-55106. Max. coverage (+): 0.04. Max coverage (-): 0

Region: NODE\_312079\_length\_70626\_cov\_31.911549 55107-55145. Max. coverage (+): 0.04. Max coverage (-): 0

Region: NODE\_312079\_length\_70626\_cov\_31.911549 55146-55183. Max. coverage (+): 0. Max coverage (-): 0

Region: NODE\_312079\_length\_70626\_cov\_31.911549 55184-55222. Max. coverage (+): 0.04. Max coverage (-): 0

Region: NODE\_312079\_length\_70626\_cov\_31.911549 55223-55261. Max. coverage (+): 0. Max coverage (-): 0

Region: NODE\_312079\_length\_70626\_cov\_31.911549 55262-55299. Max. coverage (+): 0. Max coverage (-): 0

Region: NODE\_312079\_length\_70626\_cov\_31.911549 55300-55338. Max. coverage (+): 0. Max coverage (-): 0

Region: NODE\_312079\_length\_70626\_cov\_31.911549 55339-55377. Max. coverage (+): 0.04. Max coverage (-): 0

Region: NODE\_312079\_length\_70626\_cov\_31.911549 55378-55415. Max. coverage (+): 0. Max coverage (-): 0

Region: NODE\_312079\_length\_70626\_cov\_31.911549 55416-55454. Max. coverage (+): 0. Max coverage (-): 0

Region: NODE\_312079\_length\_70626\_cov\_31.911549 55455-55493. Max. coverage (+): 0.04. Max coverage (-): 0

Region: NODE\_312079\_length\_70626\_cov\_31.911549 55494-55531. Max. coverage (+): 0. Max coverage (-): 0

Region: NODE\_312079\_length\_70626\_cov\_31.911549 55532-55570. Max. coverage (+): 0. Max coverage (-): 0

Region: NODE\_312079\_length\_70626\_cov\_31.911549 55571-55609. Max. coverage (+): 0. Max coverage (-): 0

Region: NODE\_312079\_length\_70626\_cov\_31.911549 55610-55647. Max. coverage (+): 0. Max coverage (-): 0.04

Region: NODE\_312079\_length\_70626\_cov\_31.911549 55648-55686. Max. coverage (+): 0. Max coverage (-): 0

Region: NODE\_312079\_length\_70626\_cov\_31.911549 55687-55725. Max. coverage (+): 0.04. Max coverage (-): 0

Region: NODE\_312079\_length\_70626\_cov\_31.911549 55726-55763. Max. coverage (+): 0. Max coverage (-): 0.01

Region: NODE\_312079\_length\_70626\_cov\_31.911549 55764-55802. Max. coverage (+): 0.1. Max coverage (-): 0.01

Region: NODE\_312079\_length\_70626\_cov\_31.911549 55803-55841. Max. coverage (+): 0.01. Max coverage (-): 0.01

Region: NODE\_312079\_length\_70626\_cov\_31.911549 55842-55879. Max. coverage (+): 0. Max coverage (-): 0

Region: NODE\_312079\_length\_70626\_cov\_31.911549 55880-55918. Max. coverage (+): 0. Max coverage (-): 0

Region: NODE\_312079\_length\_70626\_cov\_31.911549 55919-55957. Max. coverage (+): 0.05. Max coverage (-): 0

Region: NODE\_312079\_length\_70626\_cov\_31.911549 55958-55995. Max. coverage (+): 0. Max coverage (-): 0

Region: NODE\_312079\_length\_70626\_cov\_31.911549 55996-56034. Max. coverage (+): 0.08. Max coverage (-): 0.04

Region: NODE\_312079\_length\_70626\_cov\_31.911549 56035-56073. Max. coverage (+): 0.08. Max coverage (-): 0.08

Region: NODE\_312079\_length\_70626\_cov\_31.911549 56074-56111. Max. coverage (+): 0. Max coverage (-): 0.08

Region: NODE\_312079\_length\_70626\_cov\_31.911549 56112-56150. Max. coverage (+): 0.28. Max coverage (-): 0.04

Region: NODE\_312079\_length\_70626\_cov\_31.911549 56151-56189. Max. coverage (+): 0.16. Max coverage (-): 0

Region: NODE\_312079\_length\_70626\_cov\_31.911549 56190-56227. Max. coverage (+): 0.36. Max coverage (-): 0

Region: NODE\_312079\_length\_70626\_cov\_31.911549 56228-56266. Max. coverage (+): 0.04. Max coverage (-): 0

Region: NODE\_312079\_length\_70626\_cov\_31.911549 56267-56305. Max. coverage (+): 0.04. Max coverage (-): 0.04

Region: NODE\_312079\_length\_70626\_cov\_31.911549 56306-56343. Max. coverage (+): 0.32. Max coverage (-): 0

Region: NODE\_312079\_length\_70626\_cov\_31.911549 56344-56382. Max. coverage (+): 3.11. Max coverage (-): 0.48

Region: NODE\_312079\_length\_70626\_cov\_31.911549 56383-56421. Max. coverage (+): 0.28. Max coverage (-): 0.48

Region: NODE\_312079\_length\_70626\_cov\_31.911549 56422-56459. Max. coverage (+): 0.2. Max coverage (-): 0.04

Region: NODE\_312079\_length\_70626\_cov\_31.911549 56460-56498. Max. coverage (+): 0.2. Max coverage (-): 0

Region: NODE\_312079\_length\_70626\_cov\_31.911549 56499-56537. Max. coverage (+): 0. Max coverage (-): 0.12

Region: NODE\_312079\_length\_70626\_cov\_31.911549 56538-56575. Max. coverage (+): 0.24. Max coverage (-): 0.12

Region: NODE\_312079\_length\_70626\_cov\_31.911549 56576-56614. Max. coverage (+): 4.6. Max coverage (-): 0

Region: NODE\_312079\_length\_70626\_cov\_31.911549 56615-56653. Max. coverage (+): 0.24. Max coverage (-): 0

Region: NODE\_312079\_length\_70626\_cov\_31.911549 56654-56691. Max. coverage (+): 0.16. Max coverage (-): 0.32

Region: NODE\_312079\_length\_70626\_cov\_31.911549 56692-56730. Max. coverage (+): 16.64. Max coverage (-): 0.08

Region: NODE\_312079\_length\_70626\_cov\_31.911549 56731-56769. Max. coverage (+): 3.8. Max coverage (-): 0.52

Region: NODE\_312079\_length\_70626\_cov\_31.911549 56770-56807. Max. coverage (+): 3.76. Max coverage (-): 0.24

Region: NODE\_312079\_length\_70626\_cov\_31.911549 56808-56846. Max. coverage (+): 1.66. Max coverage (-): 0.12

Region: NODE\_312079\_length\_70626\_cov\_31.911549 56847-56885. Max. coverage (+): 0.69. Max coverage (-): 0.28

Region: NODE\_312079\_length\_70626\_cov\_31.911549 56886-56923. Max. coverage (+): 5.98. Max coverage (-): 0.2

Region: NODE\_312079\_length\_70626\_cov\_31.911549 56924-56962. Max. coverage (+): 0.24. Max coverage (-): 0.2

Region: NODE\_312079\_length\_70626\_cov\_31.911549 56963-57001. Max. coverage (+): 9.61. Max coverage (-): 0.4

Region: NODE\_312079\_length\_70626\_cov\_31.911549 57002-57039. Max. coverage (+): 6.99. Max coverage (-): 12.68

Region: NODE\_312079\_length\_70626\_cov\_31.911549 57040-57078. Max. coverage (+): 2.02. Max coverage (-): 0.32

Region: NODE\_312079\_length\_70626\_cov\_31.911549 57079-57117. Max. coverage (+): 0.69. Max coverage (-): 0.04

Region: NODE\_312079\_length\_70626\_cov\_31.911549 57118-57155. Max. coverage (+): 7.39. Max coverage (-): 0.32

Region: NODE\_312079\_length\_70626\_cov\_31.911549 57156-57194. Max. coverage (+): 5.45. Max coverage (-): 0.65

Region: NODE\_312079\_length\_70626\_cov\_31.911549 57195-57233. Max. coverage (+): 2.18. Max coverage (-): 0.16

Region: NODE\_312079\_length\_70626\_cov\_31.911549 57234-57271. Max. coverage (+): 0.08. Max coverage (-): 0.04

Region: NODE\_312079\_length\_70626\_cov\_31.911549 57272-57310. Max. coverage (+): 1.21. Max coverage (-): 0

Region: NODE\_312079\_length\_70626\_cov\_31.911549 57311-57349. Max. coverage (+): 0. Max coverage (-): 0

Region: NODE\_312079\_length\_70626\_cov\_31.911549 57350-57387. Max. coverage (+): 0. Max coverage (-): 0

Region: NODE\_312079\_length\_70626\_cov\_31.911549 57388-57426. Max. coverage (+): 0.12. Max coverage (-): 0

Region: NODE\_312079\_length\_70626\_cov\_31.911549 57427-57465. Max. coverage (+): 0.04. Max coverage (-): 0.04

Region: NODE\_312079\_length\_70626\_cov\_31.911549 57466-57503. Max. coverage (+): 0.77. Max coverage (-): 0.36

Region: NODE\_312079\_length\_70626\_cov\_31.911549 57504-57542. Max. coverage (+): 34.85. Max coverage (-): 0.57

Region: NODE\_312079\_length\_70626\_cov\_31.911549 57543-57581. Max. coverage (+): 1.82. Max coverage (-): 7.83

Region: NODE\_312079\_length\_70626\_cov\_31.911549 57582-57619. Max. coverage (+): 0.24. Max coverage (-): 0.12

Region: NODE\_312079\_length\_70626\_cov\_31.911549 57620-57658. Max. coverage (+): 6.38. Max coverage (-): 0.12

Region: NODE\_312079\_length\_70626\_cov\_31.911549 57659-57697. Max. coverage (+): 3.51. Max coverage (-): 0.16

Region: NODE\_312079\_length\_70626\_cov\_31.911549 57698-57735. Max. coverage (+): 3.59. Max coverage (-): 1.33

Region: NODE\_312079\_length\_70626\_cov\_31.911549 57736-57774. Max. coverage (+): 8.08. Max coverage (-): 1.09

Region: NODE\_312079\_length\_70626\_cov\_31.911549 57775-57813. Max. coverage (+): 10.01. Max coverage (-): 0.16

Region: NODE\_312079\_length\_70626\_cov\_31.911549 57814-57851. Max. coverage (+): 22.45. Max coverage (-): 0.81

Region: NODE\_312079\_length\_70626\_cov\_31.911549 57852-57890. Max. coverage (+): 3.51. Max coverage (-): 0.36

Region: NODE\_312079\_length\_70626\_cov\_31.911549 57891-57929. Max. coverage (+): 0.73. Max coverage (-): 0.36

Region: NODE\_312079\_length\_70626\_cov\_31.911549 57930-57967. Max. coverage (+): 0.73. Max coverage (-): 0.08

Region: NODE\_312079\_length\_70626\_cov\_31.911549 57968-58006. Max. coverage (+): 0.4. Max coverage (-): 0.65

Region: NODE\_312079\_length\_70626\_cov\_31.911549 58007-58045. Max. coverage (+): 0.36. Max coverage (-): 1.62

Region: NODE\_312079\_length\_70626\_cov\_31.911549 58046-58083. Max. coverage (+): 0.69. Max coverage (-): 0.08

Region: NODE\_312079\_length\_70626\_cov\_31.911549 58084-58122. Max. coverage (+): 0.08. Max coverage (-): 0.04

Region: NODE\_312079\_length\_70626\_cov\_31.911549 58123-58161. Max. coverage (+): 0.73. Max coverage (-): 0.57

Region: NODE\_312079\_length\_70626\_cov\_31.911549 58162-58199. Max. coverage (+): 2.1. Max coverage (-): 0.12

Region: NODE\_312079\_length\_70626\_cov\_31.911549 58200-58238. Max. coverage (+): 1.78. Max coverage (-): 0.08

Region: NODE\_312079\_length\_70626\_cov\_31.911549 58239-58277. Max. coverage (+): 0.69. Max coverage (-): 0.08

Region: NODE\_312079\_length\_70626\_cov\_31.911549 58278-58315. Max. coverage (+): 0.52. Max coverage (-): 0.2

Region: NODE\_312079\_length\_70626\_cov\_31.911549 58316-58354. Max. coverage (+): 0.36. Max coverage (-): 0.16

Region: NODE\_312079\_length\_70626\_cov\_31.911549 58355-58393. Max. coverage (+): 0.12. Max coverage (-): 0.08

Region: NODE\_312079\_length\_70626\_cov\_31.911549 58394-58431. Max. coverage (+): 0.04. Max coverage (-): 0.04

Region: NODE\_312079\_length\_70626\_cov\_31.911549 58432-58470. Max. coverage (+): 0.04. Max coverage (-): 0.08

Region: NODE\_312079\_length\_70626\_cov\_31.911549 58471-58509. Max. coverage (+): 0.02. Max coverage (-): 0

Region: NODE\_312079\_length\_70626\_cov\_31.911549 58510-58547. Max. coverage (+): 0.32. Max coverage (-): 0

Region: NODE\_312079\_length\_70626\_cov\_31.911549 58548-58586. Max. coverage (+): 0.04. Max coverage (-): 0.04

Region: NODE\_312079\_length\_70626\_cov\_31.911549 58587-58625. Max. coverage (+): 2.24. Max coverage (-): 0

Region: NODE\_312079\_length\_70626\_cov\_31.911549 58626-58663. Max. coverage (+): 0.08. Max coverage (-): 0.02

Region: NODE\_312079\_length\_70626\_cov\_31.911549 58664-58702. Max. coverage (+): 0.24. Max coverage (-): 0

Region: NODE\_312079\_length\_70626\_cov\_31.911549 58703-58741. Max. coverage (+): 0.36. Max coverage (-): 0

Region: NODE\_312079\_length\_70626\_cov\_31.911549 58742-58779. Max. coverage (+): 0.97. Max coverage (-): 0.16

Region: NODE\_312079\_length\_70626\_cov\_31.911549 58780-58818. Max. coverage (+): 0.08. Max coverage (-): 0.04

Region: NODE\_312079\_length\_70626\_cov\_31.911549 58819-58857. Max. coverage (+): 1.45. Max coverage (-): 0.12

Region: NODE\_312079\_length\_70626\_cov\_31.911549 58858-58895. Max. coverage (+): 0.65. Max coverage (-): 0.16

Region: NODE\_312079\_length\_70626\_cov\_31.911549 58896-58934. Max. coverage (+): 0.57. Max coverage (-): 0.44

Region: NODE\_312079\_length\_70626\_cov\_31.911549 58935-58973. Max. coverage (+): 0.69. Max coverage (-): 0.08

Region: NODE\_312079\_length\_70626\_cov\_31.911549 58974-59011. Max. coverage (+): 0.28. Max coverage (-): 0.4

Region: NODE\_312079\_length\_70626\_cov\_31.911549 59012-59050. Max. coverage (+): 1.09. Max coverage (-): 0.12

Region: NODE\_312079\_length\_70626\_cov\_31.911549 59051-59089. Max. coverage (+): 21.52. Max coverage (-): 0.12

Region: NODE\_312079\_length\_70626\_cov\_31.911549 59090-59127. Max. coverage (+): 3.39. Max coverage (-): 0.28

Region: NODE\_312079\_length\_70626\_cov\_31.911549 59128-59166. Max. coverage (+): 0.4. Max coverage (-): 0.08

Region: NODE\_312079\_length\_70626\_cov\_31.911549 59167-59205. Max. coverage (+): 0.4. Max coverage (-): 3.03

Region: NODE\_312079\_length\_70626\_cov\_31.911549 59206-59243. Max. coverage (+): 24.15. Max coverage (-): 0.65

Region: NODE\_312079\_length\_70626\_cov\_31.911549 59244-59282. Max. coverage (+): 2.14. Max coverage (-): 0.65

Region: NODE\_312079\_length\_70626\_cov\_31.911549 59283-59321. Max. coverage (+): 0.08. Max coverage (-): 0.12

Region: NODE\_312079\_length\_70626\_cov\_31.911549 59322-59359. Max. coverage (+): 0.52. Max coverage (-): 0.12

Region: NODE\_312079\_length\_70626\_cov\_31.911549 59360-59398. Max. coverage (+): 0.08. Max coverage (-): 0.12

Region: NODE\_312079\_length\_70626\_cov\_31.911549 59399-59437. Max. coverage (+): 1.62. Max coverage (-): 0.08

Region: NODE\_312079\_length\_70626\_cov\_31.911549 59438-59475. Max. coverage (+): 38.84. Max coverage (-): 0.28

Region: NODE\_312079\_length\_70626\_cov\_31.911549 59476-59514. Max. coverage (+): 0.65. Max coverage (-): 0.52

Region: NODE\_312079\_length\_70626\_cov\_31.911549 59515-59553. Max. coverage (+): 10.3. Max coverage (-): 0.04

Region: NODE\_312079\_length\_70626\_cov\_31.911549 59554-59591. Max. coverage (+): 0.48. Max coverage (-): 0.4

Region: NODE\_312079\_length\_70626\_cov\_31.911549 59592-59630. Max. coverage (+): 0.2. Max coverage (-): 0.12

Region: NODE\_312079\_length\_70626\_cov\_31.911549 59631-59669. Max. coverage (+): 1.01. Max coverage (-): 0.04

Region: NODE\_312079\_length\_70626\_cov\_31.911549 59670-59707. Max. coverage (+): 5.29. Max coverage (-): 0.36

Region: NODE\_312079\_length\_70626\_cov\_31.911549 59708-59746. Max. coverage (+): 1.09. Max coverage (-): 0.36

Region: NODE\_312079\_length\_70626\_cov\_31.911549 59747-59785. Max. coverage (+): 1.33. Max coverage (-): 0.28

Region: NODE\_312079\_length\_70626\_cov\_31.911549 59786-59823. Max. coverage (+): 1.74. Max coverage (-): 0.48

Region: NODE\_312079\_length\_70626\_cov\_31.911549 59824-59862. Max. coverage (+): 4.28. Max coverage (-): 0.2

Region: NODE\_312079\_length\_70626\_cov\_31.911549 59863-59901. Max. coverage (+): 1.62. Max coverage (-): 0.24

Region: NODE\_312079\_length\_70626\_cov\_31.911549 59902-59939. Max. coverage (+): 0.44. Max coverage (-): 0

Region: NODE\_312079\_length\_70626\_cov\_31.911549 59940-59978. Max. coverage (+): 0.04. Max coverage (-): 0.04

Region: NODE\_312079\_length\_70626\_cov\_31.911549 59979-60017. Max. coverage (+): 0.32. Max coverage (-): 0

Region: NODE\_312079\_length\_70626\_cov\_31.911549 60018-60055. Max. coverage (+): 6.34. Max coverage (-): 0.36

Region: NODE\_312079\_length\_70626\_cov\_31.911549 60056-60094. Max. coverage (+): 0.28. Max coverage (-): 0.04

Region: NODE\_312079\_length\_70626\_cov\_31.911549 60095-60133. Max. coverage (+): 0.2. Max coverage (-): 0.04

Region: NODE\_312079\_length\_70626\_cov\_31.911549 60134-60171. Max. coverage (+): 73.49. Max coverage (-): 0.2

Region: NODE\_312079\_length\_70626\_cov\_31.911549 60172-60210. Max. coverage (+): 0.12. Max coverage (-): 0.28

Region: NODE\_312079\_length\_70626\_cov\_31.911549 60211-60249. Max. coverage (+): 1.33. Max coverage (-): 0.04

Region: NODE\_312079\_length\_70626\_cov\_31.911549 60250-60287. Max. coverage (+): 24.75. Max coverage (-): 0.2

Region: NODE\_312079\_length\_70626\_cov\_31.911549 60288-60326. Max. coverage (+): 15.55. Max coverage (-): 0.2

Region: NODE\_312079\_length\_70626\_cov\_31.911549 60327-60365. Max. coverage (+): 3.92. Max coverage (-): 0.2

Region: NODE\_312079\_length\_70626\_cov\_31.911549 60366-60403. Max. coverage (+): 4.08. Max coverage (-): 0.28

Region: NODE\_312079\_length\_70626\_cov\_31.911549 60404-60442. Max. coverage (+): 4.08. Max coverage (-): 0.36

Region: NODE\_312079\_length\_70626\_cov\_31.911549 60443-60481. Max. coverage (+): 4.04. Max coverage (-): 0.12

Region: NODE\_312079\_length\_70626\_cov\_31.911549 60482-60519. Max. coverage (+): 7.19. Max coverage (-): 0.24

Region: NODE\_312079\_length\_70626\_cov\_31.911549 60520-60558. Max. coverage (+): 0.89. Max coverage (-): 0.08

Region: NODE\_312079\_length\_70626\_cov\_31.911549 60559-60597. Max. coverage (+): 5.69. Max coverage (-): 0.69

Region: NODE\_312079\_length\_70626\_cov\_31.911549 60598-60635. Max. coverage (+): 5.53. Max coverage (-): 0.44

Region: NODE\_312079\_length\_70626\_cov\_31.911549 60636-60674. Max. coverage (+): 1.25. Max coverage (-): 0.04

Region: NODE\_312079\_length\_70626\_cov\_31.911549 60675-60713. Max. coverage (+): 1.62. Max coverage (-): 0.16

Region: NODE\_312079\_length\_70626\_cov\_31.911549 60714-60751. Max. coverage (+): 4.89. Max coverage (-): 0.32

Region: NODE\_312079\_length\_70626\_cov\_31.911549 60752-60790. Max. coverage (+): 0.32. Max coverage (-): 0.24

Region: NODE\_312079\_length\_70626\_cov\_31.911549 60791-60829. Max. coverage (+): 0.32. Max coverage (-): 0.08

Region: NODE\_312079\_length\_70626\_cov\_31.911549 60830-60867. Max. coverage (+): 11.43. Max coverage (-): 0.2

Region: NODE\_312079\_length\_70626\_cov\_31.911549 60868-60906. Max. coverage (+): 2.83. Max coverage (-): 0.04

Region: NODE\_312079\_length\_70626\_cov\_31.911549 60907-60945. Max. coverage (+): 0.08. Max coverage (-): 0

Region: NODE\_312079\_length\_70626\_cov\_31.911549 60946-60983. Max. coverage (+): 0.81. Max coverage (-): 0.24

Region: NODE\_312079\_length\_70626\_cov\_31.911549 60984-61022. Max. coverage (+): 0.52. Max coverage (-): 0.04

Region: NODE\_312079\_length\_70626\_cov\_31.911549 61023-61061. Max. coverage (+): 4.04. Max coverage (-): 0.32

Region: NODE\_312079\_length\_70626\_cov\_31.911549 61062-61099. Max. coverage (+): 0.85. Max coverage (-): 1.82

Region: NODE\_312079\_length\_70626\_cov\_31.911549 61100-61138. Max. coverage (+): 12.68. Max coverage (-): 1.62

Region: NODE\_312079\_length\_70626\_cov\_31.911549 61139-61177. Max. coverage (+): 0.24. Max coverage (-): 0.48

Region: NODE\_312079\_length\_70626\_cov\_31.911549 61178-61215. Max. coverage (+): 1.29. Max coverage (-): 0.73

Region: NODE\_312079\_length\_70626\_cov\_31.911549 61216-61254. Max. coverage (+): 2.34. Max coverage (-): 0.48

Region: NODE\_312079\_length\_70626\_cov\_31.911549 61255-61293. Max. coverage (+): 0.2. Max coverage (-): 2.38

Region: NODE\_312079\_length\_70626\_cov\_31.911549 61294-61331. Max. coverage (+): 3.63. Max coverage (-): 0.08

Region: NODE\_312079\_length\_70626\_cov\_31.911549 61332-61370. Max. coverage (+): 2.54. Max coverage (-): 0.04

Region: NODE\_312079\_length\_70626\_cov\_31.911549 61371-61409. Max. coverage (+): 0.44. Max coverage (-): 0.08

Region: NODE\_312079\_length\_70626\_cov\_31.911549 61410-61447. Max. coverage (+): 0.24. Max coverage (-): 3.92

Region: NODE\_312079\_length\_70626\_cov\_31.911549 61448-61486. Max. coverage (+): 0.44. Max coverage (-): 0

Region: NODE\_312079\_length\_70626\_cov\_31.911549 61487-61525. Max. coverage (+): 1.01. Max coverage (-): 0.93

Region: NODE\_312079\_length\_70626\_cov\_31.911549 61526-61563. Max. coverage (+): 1.01. Max coverage (-): 0.57

Region: NODE\_312079\_length\_70626\_cov\_31.911549 61564-61602. Max. coverage (+): 1.01. Max coverage (-): 0.08

Region: NODE\_312079\_length\_70626\_cov\_31.911549 61603-61641. Max. coverage (+): 0.48. Max coverage (-): 0.32

Region: NODE\_312079\_length\_70626\_cov\_31.911549 61642-61679. Max. coverage (+): 4.64. Max coverage (-): 1.78

Region: NODE\_312079\_length\_70626\_cov\_31.911549 61680-61718. Max. coverage (+): 0.16. Max coverage (-): 0.32

Region: NODE\_312079\_length\_70626\_cov\_31.911549 61719-61757. Max. coverage (+): 25.12. Max coverage (-): 0.32

Region: NODE\_312079\_length\_70626\_cov\_31.911549 61758-61795. Max. coverage (+): 27.98. Max coverage (-): 0.28

Region: NODE\_312079\_length\_70626\_cov\_31.911549 61796-61834. Max. coverage (+): 16.23. Max coverage (-): 0.4

Region: NODE\_312079\_length\_70626\_cov\_31.911549 61835-61873. Max. coverage (+): 9.09. Max coverage (-): 1.66

Region: NODE\_312079\_length\_70626\_cov\_31.911549 61874-61911. Max. coverage (+): 6.66. Max coverage (-): 1.74

Region: NODE\_312079\_length\_70626\_cov\_31.911549 61912-61950. Max. coverage (+): 0.48. Max coverage (-): 0.04

Region: NODE\_312079\_length\_70626\_cov\_31.911549 61951-61989. Max. coverage (+): 6.74. Max coverage (-): 0.16

Region: NODE\_312079\_length\_70626\_cov\_31.911549 61990-62027. Max. coverage (+): 2.83. Max coverage (-): 0.04

Region: NODE\_312079\_length\_70626\_cov\_31.911549 62028-62066. Max. coverage (+): 1.74. Max coverage (-): 1.13

Region: NODE\_312079\_length\_70626\_cov\_31.911549 62067-62105. Max. coverage (+): 0.85. Max coverage (-): 0.85

Region: NODE\_312079\_length\_70626\_cov\_31.911549 62106-62143. Max. coverage (+): 0.89. Max coverage (-): 0.32

Region: NODE\_312079\_length\_70626\_cov\_31.911549 62144-62182. Max. coverage (+): 0.61. Max coverage (-): 0.08

Region: NODE\_312079\_length\_70626\_cov\_31.911549 62183-62221. Max. coverage (+): 4.04. Max coverage (-): 0.24

Region: NODE\_312079\_length\_70626\_cov\_31.911549 62222-62259. Max. coverage (+): 3.76. Max coverage (-): 1.29

Region: NODE\_312079\_length\_70626\_cov\_31.911549 62260-62298. Max. coverage (+): 1.62. Max coverage (-): 0.48

Region: NODE\_312079\_length\_70626\_cov\_31.911549 62299-62337. Max. coverage (+): 5.61. Max coverage (-): 0.16

Region: NODE\_312079\_length\_70626\_cov\_31.911549 62338-62375. Max. coverage (+): 5.9. Max coverage (-): 7.75

Region: NODE\_312079\_length\_70626\_cov\_31.911549 62376-62414. Max. coverage (+): 8.8. Max coverage (-): 0.61

Region: NODE\_312079\_length\_70626\_cov\_31.911549 62415-62453. Max. coverage (+): 3.31. Max coverage (-): 0.36

Region: NODE\_312079\_length\_70626\_cov\_31.911549 62454-62491. Max. coverage (+): 0.36. Max coverage (-): 0.4

Region: NODE\_312079\_length\_70626\_cov\_31.911549 62492-62530. Max. coverage (+): 0.89. Max coverage (-): 0.08

Region: NODE\_312079\_length\_70626\_cov\_31.911549 62531-62569. Max. coverage (+): 0.32. Max coverage (-): 1.41

Region: NODE\_312079\_length\_70626\_cov\_31.911549 62570-62607. Max. coverage (+): 0.28. Max coverage (-): 0.65

Region: NODE\_312079\_length\_70626\_cov\_31.911549 62608-62646. Max. coverage (+): 0.08. Max coverage (-): 0.08

Region: NODE\_312079\_length\_70626\_cov\_31.911549 62647-62685. Max. coverage (+): 0.08. Max coverage (-): 0.52

Region: NODE\_312079\_length\_70626\_cov\_31.911549 62686-62723. Max. coverage (+): 0.2. Max coverage (-): 0.08

Region: NODE\_312079\_length\_70626\_cov\_31.911549 62724-62762. Max. coverage (+): 0.08. Max coverage (-): 0

Region: NODE\_312079\_length\_70626\_cov\_31.911549 62763-62801. Max. coverage (+): 0.2. Max coverage (-): 0.08

Region: NODE\_312079\_length\_70626\_cov\_31.911549 62802-62839. Max. coverage (+): 3.67. Max coverage (-): 0.04

Region: NODE\_312079\_length\_70626\_cov\_31.911549 62840-62878. Max. coverage (+): 1.7. Max coverage (-): 0.77

Region: NODE\_312079\_length\_70626\_cov\_31.911549 62879-62917. Max. coverage (+): 3.43. Max coverage (-): 0.12

Region: NODE\_312079\_length\_70626\_cov\_31.911549 62918-62955. Max. coverage (+): 0.48. Max coverage (-): 2.54

Region: NODE\_312079\_length\_70626\_cov\_31.911549 62956-62994. Max. coverage (+): 0.65. Max coverage (-): 1.45

Region: NODE\_312079\_length\_70626\_cov\_31.911549 62995-63033. Max. coverage (+): 0.52. Max coverage (-): 0.4

Region: NODE\_312079\_length\_70626\_cov\_31.911549 63034-63071. Max. coverage (+): 1.09. Max coverage (-): 0.04

Region: NODE\_312079\_length\_70626\_cov\_31.911549 63072-63110. Max. coverage (+): 1.37. Max coverage (-): 0.12

Region: NODE\_312079\_length\_70626\_cov\_31.911549 63111-63149. Max. coverage (+): 0.32. Max coverage (-): 0.12

Region: NODE\_312079\_length\_70626\_cov\_31.911549 63150-63187. Max. coverage (+): 1.13. Max coverage (-): 0.04

Region: NODE\_312079\_length\_70626\_cov\_31.911549 63188-63226. Max. coverage (+): 7.43. Max coverage (-): 0.08

Region: NODE\_312079\_length\_70626\_cov\_31.911549 63227-63265. Max. coverage (+): 0.36. Max coverage (-): 1.13

Region: NODE\_312079\_length\_70626\_cov\_31.911549 63266-63303. Max. coverage (+): 1.05. Max coverage (-): 0.04

Region: NODE\_312079\_length\_70626\_cov\_31.911549 63304-63342. Max. coverage (+): 0.28. Max coverage (-): 0.04

Region: NODE\_312079\_length\_70626\_cov\_31.911549 63343-63381. Max. coverage (+): 1.78. Max coverage (-): 0.04

Region: NODE\_312079\_length\_70626\_cov\_31.911549 63382-63419. Max. coverage (+): 0.08. Max coverage (-): 0

Region: NODE\_312079\_length\_70626\_cov\_31.911549 63420-63458. Max. coverage (+): 0.32. Max coverage (-): 0

Region: NODE\_312079\_length\_70626\_cov\_31.911549 63459-63497. Max. coverage (+): 0. Max coverage (-): 0

Region: NODE\_312079\_length\_70626\_cov\_31.911549 63498-63535. Max. coverage (+): 0.44. Max coverage (-): 0.08

Region: NODE\_312079\_length\_70626\_cov\_31.911549 63536-63574. Max. coverage (+): 0.65. Max coverage (-): 0

Region: NODE\_312079\_length\_70626\_cov\_31.911549 63575-63613. Max. coverage (+): 0.12. Max coverage (-): 0.12

Region: NODE\_312079\_length\_70626\_cov\_31.911549 63614-63651. Max. coverage (+): 0.69. Max coverage (-): 0.08

Region: NODE\_312079\_length\_70626\_cov\_31.911549 63652-63690. Max. coverage (+): 0.4. Max coverage (-): 0.04

Region: NODE\_312079\_length\_70626\_cov\_31.911549 63691-63729. Max. coverage (+): 0.32. Max coverage (-): 0.04

Region: NODE\_312079\_length\_70626\_cov\_31.911549 63730-63767. Max. coverage (+): 0.08. Max coverage (-): 0.04

Region: NODE\_312079\_length\_70626\_cov\_31.911549 63768-63806. Max. coverage (+): 0.16. Max coverage (-): 0.08

Region: NODE\_312079\_length\_70626\_cov\_31.911549 63807-63845. Max. coverage (+): 0.16. Max coverage (-): 0.08

Region: NODE\_312079\_length\_70626\_cov\_31.911549 63846-63883. Max. coverage (+): 0.24. Max coverage (-): 0.04

Region: NODE\_312079\_length\_70626\_cov\_31.911549 63884-63922. Max. coverage (+): 0.16. Max coverage (-): 0.08

Region: NODE\_312079\_length\_70626\_cov\_31.911549 63923-63961. Max. coverage (+): 0.44. Max coverage (-): 0

Region: NODE\_312079\_length\_70626\_cov\_31.911549 63962-63999. Max. coverage (+): 0.16. Max coverage (-): 0

Region: NODE\_312079\_length\_70626\_cov\_31.911549 64000-64038. Max. coverage (+): 0.08. Max coverage (-): 0.04

Region: NODE\_312079\_length\_70626\_cov\_31.911549 64039-64077. Max. coverage (+): 0.04. Max coverage (-): 0.16

Region: NODE\_312079\_length\_70626\_cov\_31.911549 64078-64115. Max. coverage (+): 0.08. Max coverage (-): 0.08

Region: NODE\_312079\_length\_70626\_cov\_31.911549 64116-64154. Max. coverage (+): 0.16. Max coverage (-): 0

Region: NODE\_312079\_length\_70626\_cov\_31.911549 64155-64193. Max. coverage (+): 0.2. Max coverage (-): 0

Region: NODE\_312079\_length\_70626\_cov\_31.911549 64194-64231. Max. coverage (+): 0.16. Max coverage (-): 0

Region: NODE\_312079\_length\_70626\_cov\_31.911549 64232-64270. Max. coverage (+): 0.04. Max coverage (-): 0

Region: NODE\_312079\_length\_70626\_cov\_31.911549 64271-64309. Max. coverage (+): 0.04. Max coverage (-): 0

Region: NODE\_312079\_length\_70626\_cov\_31.911549 64310-64347. Max. coverage (+): 0.04. Max coverage (-): 0

Region: NODE\_312079\_length\_70626\_cov\_31.911549 64348-64386. Max. coverage (+): 0.12. Max coverage (-): 0.08

Region: NODE\_312079\_length\_70626\_cov\_31.911549 64387-64425. Max. coverage (+): 1.13. Max coverage (-): 0

Region: NODE\_312079\_length\_70626\_cov\_31.911549 64426-64463. Max. coverage (+): 1.21. Max coverage (-): 0

Region: NODE\_312079\_length\_70626\_cov\_31.911549 64464-64502. Max. coverage (+): 0.12. Max coverage (-): 0.04

Region: NODE\_312079\_length\_70626\_cov\_31.911549 64503-64541. Max. coverage (+): 0.04. Max coverage (-): 0.08

Region: NODE\_312079\_length\_70626\_cov\_31.911549 64542-64579. Max. coverage (+): 1.33. Max coverage (-): 0.04

Region: NODE\_312079\_length\_70626\_cov\_31.911549 64580-64618. Max. coverage (+): 0. Max coverage (-): 0

Region: NODE\_312079\_length\_70626\_cov\_31.911549 64619-64657. Max. coverage (+): 0.08. Max coverage (-): 0

Region: NODE\_312079\_length\_70626\_cov\_31.911549 64658-64695. Max. coverage (+): 0. Max coverage (-): 0.04

Region: NODE\_312079\_length\_70626\_cov\_31.911549 64696-64734. Max. coverage (+): 0.04. Max coverage (-): 0

Region: NODE\_312079\_length\_70626\_cov\_31.911549 64735-64773. Max. coverage (+): 0. Max coverage (-): 0

Region: NODE\_312079\_length\_70626\_cov\_31.911549 64774-64811. Max. coverage (+): 0.02. Max coverage (-): 0

Region: NODE\_312079\_length\_70626\_cov\_31.911549 64812-64850. Max. coverage (+): 0.02. Max coverage (-): 0

Region: NODE\_312079\_length\_70626\_cov\_31.911549 64851-64889. Max. coverage (+): 0. Max coverage (-): 0

Region: NODE\_312079\_length\_70626\_cov\_31.911549 64890-64927. Max. coverage (+): 0. Max coverage (-): 0

Region: NODE\_312079\_length\_70626\_cov\_31.911549 64928-64966. Max. coverage (+): 0. Max coverage (-): 0

Region: NODE\_312079\_length\_70626\_cov\_31.911549 64967-65005. Max. coverage (+): 0. Max coverage (-): 0

Region: NODE\_312079\_length\_70626\_cov\_31.911549 65006-65043. Max. coverage (+): 0. Max coverage (-): 0

Region: NODE\_312079\_length\_70626\_cov\_31.911549 65044-65082. Max. coverage (+): 0. Max coverage (-): 0

Region: NODE\_312079\_length\_70626\_cov\_31.911549 65083-65121. Max. coverage (+): 0. Max coverage (-): 0

Region: NODE\_312079\_length\_70626\_cov\_31.911549 65122-65159. Max. coverage (+): 0. Max coverage (-): 0

Region: NODE\_312079\_length\_70626\_cov\_31.911549 65160-65198. Max. coverage (+): 0. Max coverage (-): 0

Region: NODE\_312079\_length\_70626\_cov\_31.911549 65199-65237. Max. coverage (+): 0. Max coverage (-): 0

Region: NODE\_312079\_length\_70626\_cov\_31.911549 65238-65275. Max. coverage (+): 0. Max coverage (-): 0

Region: NODE\_312079\_length\_70626\_cov\_31.911549 65276-65314. Max. coverage (+): 0. Max coverage (-): 0

Region: NODE\_312079\_length\_70626\_cov\_31.911549 65315-65353. Max. coverage (+): 0. Max coverage (-): 0

Region: NODE\_312079\_length\_70626\_cov\_31.911549 65354-65391. Max. coverage (+): 0. Max coverage (-): 0

Region: NODE\_312079\_length\_70626\_cov\_31.911549 65392-65430. Max. coverage (+): 0. Max coverage (-): 0

Region: NODE\_312079\_length\_70626\_cov\_31.911549 65431-65469. Max. coverage (+): 0. Max coverage (-): 0

Region: NODE\_312079\_length\_70626\_cov\_31.911549 65470-65507. Max. coverage (+): 0. Max coverage (-): 0

Region: NODE\_312079\_length\_70626\_cov\_31.911549 65508-65546. Max. coverage (+): 0. Max coverage (-): 0

Region: NODE\_312079\_length\_70626\_cov\_31.911549 65547-65585. Max. coverage (+): 0. Max coverage (-): 0

Region: NODE\_312079\_length\_70626\_cov\_31.911549 65586-65623. Max. coverage (+): 0. Max coverage (-): 0

Region: NODE\_312079\_length\_70626\_cov\_31.911549 65624-65662. Max. coverage (+): 0. Max coverage (-): 0

Region: NODE\_312079\_length\_70626\_cov\_31.911549 65663-65701. Max. coverage (+): 0. Max coverage (-): 0

Region: NODE\_312079\_length\_70626\_cov\_31.911549 65702-65739. Max. coverage (+): 0. Max coverage (-): 0

Region: NODE\_312079\_length\_70626\_cov\_31.911549 65740-65778. Max. coverage (+): 0. Max coverage (-): 0

Region: NODE\_312079\_length\_70626\_cov\_31.911549 65779-65817. Max. coverage (+): 0. Max coverage (-): 0

Region: NODE\_312079\_length\_70626\_cov\_31.911549 65818-65855. Max. coverage (+): 0. Max coverage (-): 0

Region: NODE\_312079\_length\_70626\_cov\_31.911549 65856-65894. Max. coverage (+): 0. Max coverage (-): 0

Region: NODE\_312079\_length\_70626\_cov\_31.911549 65895-65933. Max. coverage (+): 0. Max coverage (-): 0

Region: NODE\_312079\_length\_70626\_cov\_31.911549 65934-65971. Max. coverage (+): 0.04. Max coverage (-): 0

Region: NODE\_312079\_length\_70626\_cov\_31.911549 65972-. Max. coverage (+): 0. Max coverage (-): 0

RepeatMasker Color Code

**+**

100-98% Identity

<98-95% Identity

<95-90% Identity

<90-85% Identity

<85-80% Identity

<80-75% Identity

<75-70% Identity

<70% Identity

**-**

Gene Set Color Code

**+**

Gene

Pseudogene

Other

**-**

Topology/Coverage Color Code

Coverage Plus Strand

Coverage Minus Strand

Mainstrand: Plus

Mainstrand: Minus

Complementary Strand

Flanking Region  
(if option -flank >0)

Gene Set Annotation  

**1. unknown (unknownunknown) Tr:unknown**: 65369-66138 (-)  
**2. unknown (unknownunknown) Tr:unknown UTR**: 65369-65599 (-)

  
RepeatMasker Annotation  

**1. AlRepC-136**: 48775-48887 (+), Divergence to consensus: 6.3%  
**2. AlRepB-2**: 48874-49130 (+), Divergence to consensus: 15.5%  
**3. Penelope-1\_AFC**: 51503-51545 (+), Divergence to consensus: 13.9%  
**4. REX1-1\_AFC**: 53635-53855 (+), Divergence to consensus: 8.8%  
**5. A-rich**: 55236-55267 (+), Divergence to consensus: 16.9%  
**6. AlRepB-1625**: 55750-55947 (-), Divergence to consensus: 13.2%  
**7. AlRepD-4675**: 55953-56014 (-), Divergence to consensus: 1.6%  
**8. Kolobok-2\_XT**: 58381-58424 (-), Divergence to consensus: 13.6%  
**9. AlRepB-438**: 58384-58678 (-), Divergence to consensus: 18.6%  
**10. Kolobok-2\_XT**: 58630-58682 (-), Divergence to consensus: 19.2%  
**11. TC1\_FR3**: 59601-59783 (-), Divergence to consensus: 28.5%  
**12. (TAGCTT)n**: 59921-59961 (+), Divergence to consensus: 20.4%  
**13. AlRepD-5020**: 62546-62959 (-), Divergence to consensus: 38.3%  
**14. AlRepE-1134**: 63069-63243 (-), Divergence to consensus: 38%  
**15. AlRepD-1895**: 63282-63336 (+), Divergence to consensus: 18.6%  
**16. AlRepA-4**: 64070-64295 (+), Divergence to consensus: 29.3%  
**17. AlRepA-4**: 64408-64557 (+), Divergence to consensus: 38.6%  
**18. Tc1-2\_FR**: 64752-64806 (+), Divergence to consensus: 11%  
**19. Tc1-2\_FR**: 64807-64848 (-), Divergence to consensus: 2.4%  
**20. AlRepB-13**: 65037-65100 (+), Divergence to consensus: 24.2%  
**21. AlRepE-3032**: 65129-65211 (-), Divergence to consensus: 35.6%  
**22. AlRepE-3032**: 65216-65296 (-), Divergence to consensus: 21.1%  
**23. (CAA)n**: 65297-65321 (+), Divergence to consensus: 12.4%  
**24. AlRepC-580**: 65611-66142 (+), Divergence to consensus: 29.7%

  
Transcription Factor Binding Sites  

**RHOXF1** (Sequence: AGCTCA (-): 46823)  
**RHOXF1** (Sequence: GGCTTA (-): 47517)  
**RHOXF1** (Sequence: AGATTA (-): 48723)  
**RHOXF1** (Sequence: AGATCA (-): 49206)  
**RHOXF1** (Sequence: GGCTTA (-): 49673)  
**RHOXF1** (Sequence: AGATCA (-): 50444)  
**RHOXF1** (Sequence: AGATCA (-): 51400)  
**RHOXF1** (Sequence: AGATTA (-): 52974)  
**RHOXF1** (Sequence: GGCTCA (-): 53625)  
**RHOXF1** (Sequence: AGATTA (-): 54742)  
**RHOXF1** (Sequence: GGATTA (-): 55063)  
**RHOXF1** (Sequence: GGCTTA (-): 56243)  
**RHOXF1** (Sequence: AGCTTA (-): 56989)  
**RHOXF1** (Sequence: AGCTTA (-): 57604)  
**RHOXF1** (Sequence: GGATTA (-): 57921)  
**RHOXF1** (Sequence: AGCTTA (-): 58919)  
**RHOXF1** (Sequence: AGATCA (-): 58921)  
**RHOXF1** (Sequence: AGATCA (-): 59452)  
**RHOXF1** (Sequence: AGATCA (-): 59465)  
**RHOXF1** (Sequence: AGCTCA (-): 59751)  
**RHOXF1** (Sequence: AGATCA (-): 59867)  
**RHOXF1** (Sequence: AGATTA (-): 60359)  
**RHOXF1** (Sequence: GGATCA (-): 60806)  
**RHOXF1** (Sequence: AGCTCA (-): 60858)  
**RHOXF1** (Sequence: AGATTA (-): 61136)  
**RHOXF1** (Sequence: AGCTCA (-): 61232)  
**RHOXF1** (Sequence: AGATCA (-): 61267)  
**RHOXF1** (Sequence: GGCTCA (-): 61610)  
**RHOXF1** (Sequence: AGATTA (-): 61667)  
**RHOXF1** (Sequence: AGATTA (-): 63254)  
**RHOXF1** (Sequence: AGATCA (-): 64071)  
**RHOXF1** (Sequence: AGATTA (-): 64527)  
**RHOXF1** (Sequence: GGATTA (-): 65257)  
**RHOXF1** (Sequence: AGATTA (-): 65597)  
**RHOXF1** (Sequence: GGATTA (-): 65599)  
**RHOXF1** (Sequence: TGAGCT (+): 48263)  
**RHOXF1** (Sequence: TGATCT (+): 48316)  
**RHOXF1** (Sequence: TGATCT (+): 48466)  
**RHOXF1** (Sequence: TAATCC (+): 48707)  
**RHOXF1** (Sequence: TAATCC (+): 48863)  
**RHOXF1** (Sequence: TAATCT (+): 50194)  
**RHOXF1** (Sequence: TGATCT (+): 50573)  
**RHOXF1** (Sequence: TGAGCC (+): 50874)  
**RHOXF1** (Sequence: TGATCT (+): 51728)  
**RHOXF1** (Sequence: TGATCT (+): 51994)  
**RHOXF1** (Sequence: TGAGCT (+): 52771)  
**RHOXF1** (Sequence: TAATCC (+): 53021)  
**RHOXF1** (Sequence: TGATCT (+): 53431)  
**RHOXF1** (Sequence: TAATCT (+): 54051)  
**RHOXF1** (Sequence: TAATCC (+): 54057)  
**RHOXF1** (Sequence: TAATCT (+): 54464)  
**RHOXF1** (Sequence: TAAGCT (+): 55205)  
**RHOXF1** (Sequence: TGATCT (+): 55553)  
**RHOXF1** (Sequence: TAATCT (+): 55634)  
**RHOXF1** (Sequence: TAAGCC (+): 55931)  
**RHOXF1** (Sequence: TGATCT (+): 57130)  
**RHOXF1** (Sequence: TAAGCT (+): 57602)  
**RHOXF1** (Sequence: TAAGCC (+): 57807)  
**RHOXF1** (Sequence: TAATCT (+): 60727)  
**RHOXF1** (Sequence: TGAGCT (+): 60929)  
**RHOXF1** (Sequence: TGAGCC (+): 61078)  
**RHOXF1** (Sequence: TGAGCT (+): 61253)  
**RHOXF1** (Sequence: TGATCC (+): 61736)  
**RHOXF1** (Sequence: TAATCT (+): 62644)  
**RHOXF1** (Sequence: TAAGCC (+): 62890)  
**RHOXF1** (Sequence: TGATCT (+): 63009)  
**RHOXF1** (Sequence: TGAGCT (+): 63096)  
**RHOXF1** (Sequence: TGAGCC (+): 64536)  
**Lhx8** (Sequence: CTAATTAA (-): 46875)  
**Lhx8** (Sequence: CTAATTAA (-): 47761)  
**Lhx8** (Sequence: CTAATTAG (-): 48523)  
**Lhx8** (Sequence: TTAATTAG (-): 53574)  
**Lhx8** (Sequence: TTAATTAA (-): 56181)  
**Lhx8** (Sequence: TTAATTAA (-): 56752)  
**Lhx8** (Sequence: TTAATTAG (-): 65371)  
**Gata4** (Sequence: CTTATCT (+): 53174)  
**Gata4** (Sequence: GTTATCT (+): 62969)  
**POU5F1** (Sequence: TTTGCAT (-): 47800)  
**POU5F1** (Sequence: TTTGCAT (-): 61418)  
**FOXO3\_hsa** (Sequence: GTAAACAA (+): 50467)  
**FOXO3\_hsa** (Sequence: GTAAACAT (+): 54294)  
**SOX9** (Sequence: AACAATAG (-): 52108)  
**SOX9** (Sequence: AACAATGA (-): 53204)  
**SOX9** (Sequence: AACAATAA (-): 55880)  
**FOXP1** (Sequence: GTAAACA (+): 50467)  
**FOXP1** (Sequence: GTAAACA (+): 54294)  
**FOXO1** (Sequence: CTTGTTTAT (+): 51586)  
**FOXO1** (Sequence: CTTGTTTTT (+): 55194)  
**FOXO1** (Sequence: GCTGTTTTT (+): 57253)  
**FOXO1** (Sequence: CTTGTTTAT (+): 58800)  
**FOXO1** (Sequence: GTTGTTTAC (+): 65327)  
**FOXO3\_mmu** (Sequence: TGTTTTGC (-): 49410)  
**FOXO3\_mmu** (Sequence: TGTTTTCC (-): 50243)  
**FOXO3\_mmu** (Sequence: TGTTTTCC (-): 50983)  
**FOXO3\_mmu** (Sequence: TGTTTACA (-): 52703)  
**FOXO3\_mmu** (Sequence: TGTTTTCA (-): 53607)  
**FOXO3\_mmu** (Sequence: TGTTTTGC (-): 56926)  
**FOXO3\_mmu** (Sequence: TGTTTTGA (-): 57165)  
**FOXO3\_mmu** (Sequence: TGTTTACA (-): 57723)  
**FOXO3\_mmu** (Sequence: TGTTTAGA (-): 59040)  
**FOXO3\_mmu** (Sequence: TGTTTTGA (-): 62152)  
**FOXO3\_mmu** (Sequence: TGTTTTCA (-): 62541)  
**Sox5** (Sequence: ATTGTT (+): 46866)  
**Sox5** (Sequence: ATTGTT (+): 48701)  
**Sox5** (Sequence: ATTGTT (+): 52525)  
**Sox5** (Sequence: ATTGTT (+): 56584)  
**Sox5** (Sequence: ATTGTT (+): 56800)  
**Sox5** (Sequence: ATTGTT (+): 57586)  
**Sox5** (Sequence: ATTGTT (+): 57588)  
**Sox5** (Sequence: ATTGTT (+): 59562)  
**Sox5** (Sequence: ATTGTT (+): 63194)  
**Sox5** (Sequence: ATTGTT (+): 63467)  
**Sox5** (Sequence: ATTGTT (+): 64714)  
**FIGLA** (Sequence: TACAGCTGGT (-): 50475)  
**FIGLA** (Sequence: TACAGCTGGA (-): 52139)  
**FIGLA** (Sequence: AACACCTGGA (-): 53227)  
**FIGLA** (Sequence: ACCACCTGTA (-): 54307)  
**FIGLA** (Sequence: TCCAGCTGTA (-): 62345)  
**FOXO3\_mmu** (Sequence: TGAAAACA (+): 49715)  
**FOXO3\_mmu** (Sequence: GGAAAACA (+): 51362)  
**FOXO3\_mmu** (Sequence: TGTAAACA (+): 54293)  
**FOXO3\_mmu** (Sequence: TGAAAACA (+): 55813)  
**FOXO3\_mmu** (Sequence: GGAAAACA (+): 63920)  
**FOXO3\_mmu** (Sequence: TGAAAACA (+): 65285)  
**Nobox** (Sequence: GCTAATTA (-): 48522)  
**FOXO1** (Sequence: AAAAACAGC (-): 48653)  
**FOXO1** (Sequence: AAAAACAAC (-): 62753)  
**FOXO1** (Sequence: ATAAACAGC (-): 64953)  
**FOXO1** (Sequence: GAAAACAAC (-): 65286)  
**FOXO3\_hsa** (Sequence: ATGTTTAC (-): 52702)  
**FOXO3\_hsa** (Sequence: ATGTTTAC (-): 57722)  
**FOXO3\_hsa** (Sequence: TTGTTTAC (-): 65328)  
**FOXP1** (Sequence: TGTTTAC (-): 52703)  
**FOXP1** (Sequence: TGTTTAC (-): 57723)  
**FOXP1** (Sequence: TGTTTAC (-): 65329)  
**Nobox** (Sequence: TAATTAGC (+): 53575)  
**Nobox** (Sequence: TAATTGCT (+): 59882)  
**Nobox** (Sequence: TAATTAGC (+): 65372)  
**POU2F1** (Sequence: ATTAAAATA (-): 60583)  
**POU2F1** (Sequence: ATTAAAATA (-): 60825)  
**POU2F1** (Sequence: ATTAAAATA (-): 65405)  
**Rhox11** (Sequence: TGCTGTATT (+): 47659)  
**Rhox11** (Sequence: TGCTGTAAT (+): 50365)  
**Rhox11** (Sequence: CGCTGTTTT (+): 57252)  
**Rhox11** (Sequence: TGCTGTAAA (+): 58672)  
**Rhox11** (Sequence: TTAACACCA (-): 49893)  
**Rhox11** (Sequence: AAAACACCA (-): 54863)  
**Rhox11** (Sequence: AAAACACCA (-): 65230)  
**Gata4** (Sequence: AGATAAC (-): 49556)  
**Gata4** (Sequence: AGATAAC (-): 51421)  
**Sox5** (Sequence: AACAAT (-): 49560)  
**Sox5** (Sequence: AACAAT (-): 50032)  
**Sox5** (Sequence: AACAAT (-): 50058)  
**Sox5** (Sequence: AACAAT (-): 50470)  
**Sox5** (Sequence: AACAAT (-): 51279)  
**Sox5** (Sequence: AACAAT (-): 51488)  
**Sox5** (Sequence: AACAAT (-): 51967)  
**Sox5** (Sequence: AACAAT (-): 52108)  
**Sox5** (Sequence: AACAAT (-): 52763)  
**Sox5** (Sequence: AACAAT (-): 53204)  
**Sox5** (Sequence: AACAAT (-): 55111)  
**Sox5** (Sequence: AACAAT (-): 55530)  
**Sox5** (Sequence: AACAAT (-): 55880)  
**Sox5** (Sequence: AACAAT (-): 56079)  
**Sox5** (Sequence: AACAAT (-): 56719)  
**Sox5** (Sequence: AACAAT (-): 57038)  
**Sox5** (Sequence: AACAAT (-): 61906)  
**Sox5** (Sequence: AACAAT (-): 63239)  
**Sox5** (Sequence: AACAAT (-): 63924)  
**Sox5** (Sequence: AACAAT (-): 64274)  
**POU2F1** (Sequence: TATGTAAAT (+): 50071)  
**POU2F1** (Sequence: TATTTTAAT (+): 53570)  
**POU2F1** (Sequence: TATTTAAAT (+): 64179)  
**POU5F1** (Sequence: ATGCAAA (+): 51795)  
**POU5F1** (Sequence: ATGCAAA (+): 55534)  
**POU5F1** (Sequence: ATGCAAA (+): 63539)
