# Supplementary material for: First characterization of PIWI-interacting RNA clusters in a cichlid fish with a B chromosome
Source: BMC Biol. 2022 Sep 21;20:204. doi: 10.1186/s12915-022-01403-2 (PMC9490952; doi:10.1186/s12915-022-01403-2)
Supplement: Supplementary file 1 — Additional file 1. Zipped folder with fasta and interactive html piRNA cluster information for the A. latifasciata genome. The nomenclature is as follows: number-pirna-cluster_sex_B-presence (f, female; m, male; 0b, without B chromosome; 1b, with B chromosome). [file 12915_2022_1403_MOESM1_ESM.zip › 121_m1b.html]

piRNA cluster 121\_m1b 70


Predicted piRNA cluster no. 121\_m1b
  

Show proTRAC run info
Hide proTRAC run info

/\  
                \_\_\_\_\_\_\_\_\_\_\_\_\_\_\_\_\_\_\_\_\_\_\_/\\_\_\_ /  \\_\_\_\_\_\_\_  
               I                      /  \  /    \      I  
               I     pro             /    \/      \     I  
               I        TRAC        /               \   I  
               I   \_\_\_\_\_\_\_\_\_\_\_\_\_\_\_\_/\_\_\_\_\_\_\_\_\_\_\_\_\_\_\_\_\_\\_ I  
               I   \              /                     I  
               I    \            /                      I  
               I     \  /\      /       V.2.4.2         I  
               I      \/  \    /                        I  
               I\_\_\_\_\_\_\_\_\_\_\_\  /\_\_\_\_\_\_\_\_\_\_\_\_\_\_\_\_\_\_\_\_\_\_\_\_\_I  
                            \/  
  
  
================================= proTRAC ====================================  
VERSION: .......... 2.4.2  
LAST MODIFIED: .... 11. May 2018  
  
Please cite:  
Rosenkranz D, Zischler H. proTRAC - a software for probabilistic piRNA cluster  
detection, visualization and analysis. 2012. BMC Bioinformatics 13:5.  
  
  
Contact:  
David Rosenkranz  
Institute of Organismic and Molecular Evolutionary Biology  
Dept. Anthropology, small RNA group  
Johannes Gutenberg University Mainz  
email: rosenkranz@uni-mainz.de  
  
You can find the latest proTRAC version at:  
http://sourceforge.net/projects/protrac/files  
http://www.smallRNAgroup-mainz.de/software  
==============================================================================  
  
PARAMETERS:  
Map file: ...............piwi-machos-1B.fa-collapse.map  
Genome file: ............../../../0B\_ala\_genome.fa  
RepeatMasker annotation: Alatifasciata-all0B-maryan-v2.fa\_corrected.out  
GeneSet:................./guest-storage/Data/annotation/Alatifasciata\_all0B\_maryan-v2\_out2017.gff  
  
Significant (p<=0.01) hit density will be calculated based  
on observed hit distribution.  
  
Sliding window size: ........................................ 5000 bp  
Sliding window increament: .................................. 1000 bp  
Normalize each hit by number of genomic hits: ............... yes  
Normalize each hit by number of sequence reads: ............. yes  
Normalize values (-> per million mapped reads): ............. yes  
Min. fraction of hits with 1T(U) or 10A: .................... 0.75  
Alternatively: Min. fraction of hits with 1T(U) and 10A: .... 0.5  
Min. fraction of hits with typical piRNA length: ............ 0.75  
Typical piRNA length: ....................................... 24-32 nt  
Min. size of a piRNA cluster: ............................... 1000 bp.  
Min. number of hits (absolute): ............................. 0  
Min. number of hits (normalized): ........................... 0  
Min. fraction of hits on the mainstrand: .................... 0.75  
Top fraction of mapped sequences (in terms of read counts): . 1%  
Top fraction accounts for max. n% of sequence reads: ........ 90%  
Min. fraction of hits on each arm of a bidirectional cluster: 0.05  
Output html file for each cluster: .......................... yes  
Output a summary table: ..................................... yes  
Output a FASTA file for each cluster (piRNA sequences): ..... yes  
Output a FASTA file comprising cluster sequences: ........... yes  
Output a GTF file for predicted piRNA clusters: ..............yes  
Search DNA motifs in clusters: .............................. yes  
Output flanking sequences: +/- .............................. 0 bp  
Output ~.pTi file: .......................................... no  
==============================================================================  
  
  
Genome size (without gaps): ............ 758543724 bp  
Gaps (N/X/-): .......................... 417479 bp  
Mapped reads: .......................... 26973943  
Non-identical sequences: ............... 6209225  
Genomic hits: .......................... 48438990  
Significant densitiy of mapped reads: .. 821.144211136946 reads/kb

Show proTRAC cluster info
Hide proTRAC cluster info

|  |  |
| --- | --- |
| Location | NODE\_312079\_length\_70626\_cov\_31.911549 |
| Coordinates | 46417-64841 |
| Size [bp] | 18425 |
| Sequence hit loci | 8416 |
| Mapped reads (normalized) | 39534.7 |
| Mapped reads (normalized) per kb | 2145.7 |
| Normalized reads with 1T (1U) | 80.4% |
| Normalized reads with 10A | 43.7% |
| Normalized reads with length 24-32 nt | 98% |
| Normalized reads on the main strand(s) | 89.9% |
| Predicted directionality | bi:minus-plus (split between 54939 and 55013) |

100%

0%

1T (1U)  
reads

10A reads

24-32 nt  
reads

reads on mainstrand

**Either the amount of reads with 1T (1U) OR 10A has to exceed 75% (set with option: -1Tor10A)  
Alternatively the amount of reads with 1T (1U) AND 10A has to exceed 50% (set with option: -1Tand10A)  
Minimum amount of reads with preferred size is 75% (set with option: -pisize)  
Minimum amount of reads on the main strand(s) is 75% (set with option: -clstrand)**

Show read coverage
Hide read coverage

WHAT DO I SEE HERE?  
This chart shows the location of mapped sequence reads within a predicted piRNA cluster. The color refers to the number of genomic hits produced by the sequence read in question. A dark red bar indicates that this sequence read produces many other hits elsewhere in the genome. Many adjacent red or yellow bars can indicate the presence of a multi-copy element such as transposons or rRNA genes. A dark green bar indicates that this sequence read maps uniquely to this locus.

1 hit

2-5 hits

6-10 hits

11-20 hits

21-50 hits

51-100 hits

> 100 hits

NODE\_312079\_length\_70626\_cov\_31.911549

46417

64841

Gene Set

RepeatMasker

Mapped  
Reads

103.03

plus strand

minus strand

103.03

Region: NODE\_312079\_length\_70626\_cov\_31.911549 5042-46435. Max. coverage (+): 0. Max coverage (-): 0.04

Region: NODE\_312079\_length\_70626\_cov\_31.911549 46436-46472. Max. coverage (+): 0. Max coverage (-): 0

Region: NODE\_312079\_length\_70626\_cov\_31.911549 46473-46509. Max. coverage (+): 0. Max coverage (-): 0

Region: NODE\_312079\_length\_70626\_cov\_31.911549 46510-46545. Max. coverage (+): 0. Max coverage (-): 0

Region: NODE\_312079\_length\_70626\_cov\_31.911549 46546-46582. Max. coverage (+): 0. Max coverage (-): 0

Region: NODE\_312079\_length\_70626\_cov\_31.911549 46583-46619. Max. coverage (+): 0. Max coverage (-): 0

Region: NODE\_312079\_length\_70626\_cov\_31.911549 46620-46656. Max. coverage (+): 0. Max coverage (-): 0

Region: NODE\_312079\_length\_70626\_cov\_31.911549 46657-46693. Max. coverage (+): 0. Max coverage (-): 0

Region: NODE\_312079\_length\_70626\_cov\_31.911549 46694-46730. Max. coverage (+): 0. Max coverage (-): 0

Region: NODE\_312079\_length\_70626\_cov\_31.911549 46731-46767. Max. coverage (+): 0. Max coverage (-): 0

Region: NODE\_312079\_length\_70626\_cov\_31.911549 46768-46803. Max. coverage (+): 0. Max coverage (-): 0

Region: NODE\_312079\_length\_70626\_cov\_31.911549 46804-46840. Max. coverage (+): 0. Max coverage (-): 0

Region: NODE\_312079\_length\_70626\_cov\_31.911549 46841-46877. Max. coverage (+): 0. Max coverage (-): 0

Region: NODE\_312079\_length\_70626\_cov\_31.911549 46878-46914. Max. coverage (+): 0. Max coverage (-): 0

Region: NODE\_312079\_length\_70626\_cov\_31.911549 46915-46951. Max. coverage (+): 0. Max coverage (-): 0

Region: NODE\_312079\_length\_70626\_cov\_31.911549 46952-46988. Max. coverage (+): 0. Max coverage (-): 0

Region: NODE\_312079\_length\_70626\_cov\_31.911549 46989-47025. Max. coverage (+): 0. Max coverage (-): 0

Region: NODE\_312079\_length\_70626\_cov\_31.911549 47026-47061. Max. coverage (+): 0. Max coverage (-): 0

Region: NODE\_312079\_length\_70626\_cov\_31.911549 47062-47098. Max. coverage (+): 0. Max coverage (-): 0

Region: NODE\_312079\_length\_70626\_cov\_31.911549 47099-47135. Max. coverage (+): 0. Max coverage (-): 0.04

Region: NODE\_312079\_length\_70626\_cov\_31.911549 47136-47172. Max. coverage (+): 0. Max coverage (-): 0.04

Region: NODE\_312079\_length\_70626\_cov\_31.911549 47173-47209. Max. coverage (+): 0. Max coverage (-): 0

Region: NODE\_312079\_length\_70626\_cov\_31.911549 47210-47246. Max. coverage (+): 0. Max coverage (-): 0

Region: NODE\_312079\_length\_70626\_cov\_31.911549 47247-47282. Max. coverage (+): 0. Max coverage (-): 0

Region: NODE\_312079\_length\_70626\_cov\_31.911549 47283-47319. Max. coverage (+): 0. Max coverage (-): 0

Region: NODE\_312079\_length\_70626\_cov\_31.911549 47320-47356. Max. coverage (+): 0. Max coverage (-): 0

Region: NODE\_312079\_length\_70626\_cov\_31.911549 47357-47393. Max. coverage (+): 0. Max coverage (-): 0

Region: NODE\_312079\_length\_70626\_cov\_31.911549 47394-47430. Max. coverage (+): 0. Max coverage (-): 0

Region: NODE\_312079\_length\_70626\_cov\_31.911549 47431-47467. Max. coverage (+): 0. Max coverage (-): 0

Region: NODE\_312079\_length\_70626\_cov\_31.911549 47468-47504. Max. coverage (+): 0. Max coverage (-): 0

Region: NODE\_312079\_length\_70626\_cov\_31.911549 47505-47540. Max. coverage (+): 0. Max coverage (-): 0

Region: NODE\_312079\_length\_70626\_cov\_31.911549 47541-47577. Max. coverage (+): 0. Max coverage (-): 0

Region: NODE\_312079\_length\_70626\_cov\_31.911549 47578-47614. Max. coverage (+): 0. Max coverage (-): 0

Region: NODE\_312079\_length\_70626\_cov\_31.911549 47615-47651. Max. coverage (+): 0. Max coverage (-): 0

Region: NODE\_312079\_length\_70626\_cov\_31.911549 47652-47688. Max. coverage (+): 0. Max coverage (-): 0

Region: NODE\_312079\_length\_70626\_cov\_31.911549 47689-47725. Max. coverage (+): 0. Max coverage (-): 0

Region: NODE\_312079\_length\_70626\_cov\_31.911549 47726-47762. Max. coverage (+): 0. Max coverage (-): 0

Region: NODE\_312079\_length\_70626\_cov\_31.911549 47763-47798. Max. coverage (+): 0. Max coverage (-): 0

Region: NODE\_312079\_length\_70626\_cov\_31.911549 47799-47835. Max. coverage (+): 0. Max coverage (-): 0

Region: NODE\_312079\_length\_70626\_cov\_31.911549 47836-47872. Max. coverage (+): 0. Max coverage (-): 0

Region: NODE\_312079\_length\_70626\_cov\_31.911549 47873-47909. Max. coverage (+): 0. Max coverage (-): 0

Region: NODE\_312079\_length\_70626\_cov\_31.911549 47910-47946. Max. coverage (+): 0. Max coverage (-): 0

Region: NODE\_312079\_length\_70626\_cov\_31.911549 47947-47983. Max. coverage (+): 0. Max coverage (-): 0

Region: NODE\_312079\_length\_70626\_cov\_31.911549 47984-48019. Max. coverage (+): 0. Max coverage (-): 0

Region: NODE\_312079\_length\_70626\_cov\_31.911549 48020-48056. Max. coverage (+): 0. Max coverage (-): 0

Region: NODE\_312079\_length\_70626\_cov\_31.911549 48057-48093. Max. coverage (+): 0. Max coverage (-): 0

Region: NODE\_312079\_length\_70626\_cov\_31.911549 48094-48130. Max. coverage (+): 0. Max coverage (-): 0

Region: NODE\_312079\_length\_70626\_cov\_31.911549 48131-48167. Max. coverage (+): 0. Max coverage (-): 0

Region: NODE\_312079\_length\_70626\_cov\_31.911549 48168-48204. Max. coverage (+): 0. Max coverage (-): 0

Region: NODE\_312079\_length\_70626\_cov\_31.911549 48205-48241. Max. coverage (+): 0. Max coverage (-): 0

Region: NODE\_312079\_length\_70626\_cov\_31.911549 48242-48277. Max. coverage (+): 0. Max coverage (-): 0

Region: NODE\_312079\_length\_70626\_cov\_31.911549 48278-48314. Max. coverage (+): 0.04. Max coverage (-): 0

Region: NODE\_312079\_length\_70626\_cov\_31.911549 48315-48351. Max. coverage (+): 0. Max coverage (-): 0

Region: NODE\_312079\_length\_70626\_cov\_31.911549 48352-48388. Max. coverage (+): 0. Max coverage (-): 0

Region: NODE\_312079\_length\_70626\_cov\_31.911549 48389-48425. Max. coverage (+): 0. Max coverage (-): 0

Region: NODE\_312079\_length\_70626\_cov\_31.911549 48426-48462. Max. coverage (+): 0.04. Max coverage (-): 0

Region: NODE\_312079\_length\_70626\_cov\_31.911549 48463-48499. Max. coverage (+): 0. Max coverage (-): 0

Region: NODE\_312079\_length\_70626\_cov\_31.911549 48500-48535. Max. coverage (+): 0.04. Max coverage (-): 0

Region: NODE\_312079\_length\_70626\_cov\_31.911549 48536-48572. Max. coverage (+): 0. Max coverage (-): 0

Region: NODE\_312079\_length\_70626\_cov\_31.911549 48573-48609. Max. coverage (+): 0. Max coverage (-): 0

Region: NODE\_312079\_length\_70626\_cov\_31.911549 48610-48646. Max. coverage (+): 0. Max coverage (-): 0

Region: NODE\_312079\_length\_70626\_cov\_31.911549 48647-48683. Max. coverage (+): 0. Max coverage (-): 0

Region: NODE\_312079\_length\_70626\_cov\_31.911549 48684-48720. Max. coverage (+): 0. Max coverage (-): 0

Region: NODE\_312079\_length\_70626\_cov\_31.911549 48721-48756. Max. coverage (+): 0. Max coverage (-): 0.07

Region: NODE\_312079\_length\_70626\_cov\_31.911549 48757-48793. Max. coverage (+): 0. Max coverage (-): 0.04

Region: NODE\_312079\_length\_70626\_cov\_31.911549 48794-48830. Max. coverage (+): 0.01. Max coverage (-): 0

Region: NODE\_312079\_length\_70626\_cov\_31.911549 48831-48867. Max. coverage (+): 0. Max coverage (-): 0.02

Region: NODE\_312079\_length\_70626\_cov\_31.911549 48868-48904. Max. coverage (+): 0. Max coverage (-): 0

Region: NODE\_312079\_length\_70626\_cov\_31.911549 48905-48941. Max. coverage (+): 0.04. Max coverage (-): 0

Region: NODE\_312079\_length\_70626\_cov\_31.911549 48942-48978. Max. coverage (+): 0.04. Max coverage (-): 0

Region: NODE\_312079\_length\_70626\_cov\_31.911549 48979-49014. Max. coverage (+): 0.04. Max coverage (-): 0

Region: NODE\_312079\_length\_70626\_cov\_31.911549 49015-49051. Max. coverage (+): 0. Max coverage (-): 0.01

Region: NODE\_312079\_length\_70626\_cov\_31.911549 49052-49088. Max. coverage (+): 0.02. Max coverage (-): 0

Region: NODE\_312079\_length\_70626\_cov\_31.911549 49089-49125. Max. coverage (+): 0. Max coverage (-): 0.07

Region: NODE\_312079\_length\_70626\_cov\_31.911549 49126-49162. Max. coverage (+): 0.04. Max coverage (-): 0.04

Region: NODE\_312079\_length\_70626\_cov\_31.911549 49163-49199. Max. coverage (+): 0. Max coverage (-): 0.04

Region: NODE\_312079\_length\_70626\_cov\_31.911549 49200-49236. Max. coverage (+): 0.04. Max coverage (-): 0.85

Region: NODE\_312079\_length\_70626\_cov\_31.911549 49237-49272. Max. coverage (+): 0.41. Max coverage (-): 1.04

Region: NODE\_312079\_length\_70626\_cov\_31.911549 49273-49309. Max. coverage (+): 0. Max coverage (-): 1.37

Region: NODE\_312079\_length\_70626\_cov\_31.911549 49310-49346. Max. coverage (+): 0.04. Max coverage (-): 0.3

Region: NODE\_312079\_length\_70626\_cov\_31.911549 49347-49383. Max. coverage (+): 0.04. Max coverage (-): 3.3

Region: NODE\_312079\_length\_70626\_cov\_31.911549 49384-49420. Max. coverage (+): 0.11. Max coverage (-): 0.19

Region: NODE\_312079\_length\_70626\_cov\_31.911549 49421-49457. Max. coverage (+): 0.04. Max coverage (-): 0.22

Region: NODE\_312079\_length\_70626\_cov\_31.911549 49458-49493. Max. coverage (+): 0. Max coverage (-): 0.37

Region: NODE\_312079\_length\_70626\_cov\_31.911549 49494-49530. Max. coverage (+): 0.22. Max coverage (-): 0.52

Region: NODE\_312079\_length\_70626\_cov\_31.911549 49531-49567. Max. coverage (+): 0.48. Max coverage (-): 1.19

Region: NODE\_312079\_length\_70626\_cov\_31.911549 49568-49604. Max. coverage (+): 0.56. Max coverage (-): 0.33

Region: NODE\_312079\_length\_70626\_cov\_31.911549 49605-49641. Max. coverage (+): 0.04. Max coverage (-): 1.41

Region: NODE\_312079\_length\_70626\_cov\_31.911549 49642-49678. Max. coverage (+): 0.19. Max coverage (-): 0.26

Region: NODE\_312079\_length\_70626\_cov\_31.911549 49679-49715. Max. coverage (+): 0.15. Max coverage (-): 2.22

Region: NODE\_312079\_length\_70626\_cov\_31.911549 49716-49751. Max. coverage (+): 1.19. Max coverage (-): 10.16

Region: NODE\_312079\_length\_70626\_cov\_31.911549 49752-49788. Max. coverage (+): 0.85. Max coverage (-): 0.15

Region: NODE\_312079\_length\_70626\_cov\_31.911549 49789-49825. Max. coverage (+): 0.07. Max coverage (-): 0.93

Region: NODE\_312079\_length\_70626\_cov\_31.911549 49826-49862. Max. coverage (+): 0.04. Max coverage (-): 0.07

Region: NODE\_312079\_length\_70626\_cov\_31.911549 49863-49899. Max. coverage (+): 0.07. Max coverage (-): 0.3

Region: NODE\_312079\_length\_70626\_cov\_31.911549 49900-49936. Max. coverage (+): 0.7. Max coverage (-): 0.78

Region: NODE\_312079\_length\_70626\_cov\_31.911549 49937-49973. Max. coverage (+): 0.07. Max coverage (-): 0.07

Region: NODE\_312079\_length\_70626\_cov\_31.911549 49974-50009. Max. coverage (+): 0.07. Max coverage (-): 1.93

Region: NODE\_312079\_length\_70626\_cov\_31.911549 50010-50046. Max. coverage (+): 2.52. Max coverage (-): 0.93

Region: NODE\_312079\_length\_70626\_cov\_31.911549 50047-50083. Max. coverage (+): 0.41. Max coverage (-): 1.74

Region: NODE\_312079\_length\_70626\_cov\_31.911549 50084-50120. Max. coverage (+): 0.11. Max coverage (-): 0.41

Region: NODE\_312079\_length\_70626\_cov\_31.911549 50121-50157. Max. coverage (+): 0.3. Max coverage (-): 0.63

Region: NODE\_312079\_length\_70626\_cov\_31.911549 50158-50194. Max. coverage (+): 0.37. Max coverage (-): 12.75

Region: NODE\_312079\_length\_70626\_cov\_31.911549 50195-50230. Max. coverage (+): 0.96. Max coverage (-): 14.12

Region: NODE\_312079\_length\_70626\_cov\_31.911549 50231-50267. Max. coverage (+): 0.19. Max coverage (-): 1.48

Region: NODE\_312079\_length\_70626\_cov\_31.911549 50268-50304. Max. coverage (+): 0.33. Max coverage (-): 0.41

Region: NODE\_312079\_length\_70626\_cov\_31.911549 50305-50341. Max. coverage (+): 0.11. Max coverage (-): 0.89

Region: NODE\_312079\_length\_70626\_cov\_31.911549 50342-50378. Max. coverage (+): 0.63. Max coverage (-): 1.11

Region: NODE\_312079\_length\_70626\_cov\_31.911549 50379-50415. Max. coverage (+): 0.22. Max coverage (-): 6.45

Region: NODE\_312079\_length\_70626\_cov\_31.911549 50416-50452. Max. coverage (+): 1.04. Max coverage (-): 6.08

Region: NODE\_312079\_length\_70626\_cov\_31.911549 50453-50488. Max. coverage (+): 0.3. Max coverage (-): 2.08

Region: NODE\_312079\_length\_70626\_cov\_31.911549 50489-50525. Max. coverage (+): 0.11. Max coverage (-): 0.33

Region: NODE\_312079\_length\_70626\_cov\_31.911549 50526-50562. Max. coverage (+): 0.19. Max coverage (-): 4.49

Region: NODE\_312079\_length\_70626\_cov\_31.911549 50563-50599. Max. coverage (+): 0.19. Max coverage (-): 9.79

Region: NODE\_312079\_length\_70626\_cov\_31.911549 50600-50636. Max. coverage (+): 0.33. Max coverage (-): 0.26

Region: NODE\_312079\_length\_70626\_cov\_31.911549 50637-50673. Max. coverage (+): 0.56. Max coverage (-): 0.93

Region: NODE\_312079\_length\_70626\_cov\_31.911549 50674-50710. Max. coverage (+): 0.04. Max coverage (-): 7.27

Region: NODE\_312079\_length\_70626\_cov\_31.911549 50711-50746. Max. coverage (+): 0.7. Max coverage (-): 0.93

Region: NODE\_312079\_length\_70626\_cov\_31.911549 50747-50783. Max. coverage (+): 0.33. Max coverage (-): 6.04

Region: NODE\_312079\_length\_70626\_cov\_31.911549 50784-50820. Max. coverage (+): 0.33. Max coverage (-): 0.89

Region: NODE\_312079\_length\_70626\_cov\_31.911549 50821-50857. Max. coverage (+): 0.41. Max coverage (-): 0.63

Region: NODE\_312079\_length\_70626\_cov\_31.911549 50858-50894. Max. coverage (+): 2.04. Max coverage (-): 1.48

Region: NODE\_312079\_length\_70626\_cov\_31.911549 50895-50931. Max. coverage (+): 0.3. Max coverage (-): 2.08

Region: NODE\_312079\_length\_70626\_cov\_31.911549 50932-50967. Max. coverage (+): 0.07. Max coverage (-): 0.37

Region: NODE\_312079\_length\_70626\_cov\_31.911549 50968-51004. Max. coverage (+): 0.26. Max coverage (-): 5.3

Region: NODE\_312079\_length\_70626\_cov\_31.911549 51005-51041. Max. coverage (+): 0.33. Max coverage (-): 0.96

Region: NODE\_312079\_length\_70626\_cov\_31.911549 51042-51078. Max. coverage (+): 0.26. Max coverage (-): 8.71

Region: NODE\_312079\_length\_70626\_cov\_31.911549 51079-51115. Max. coverage (+): 2.37. Max coverage (-): 20.43

Region: NODE\_312079\_length\_70626\_cov\_31.911549 51116-51152. Max. coverage (+): 0.11. Max coverage (-): 1.26

Region: NODE\_312079\_length\_70626\_cov\_31.911549 51153-51189. Max. coverage (+): 0.22. Max coverage (-): 103.03

Region: NODE\_312079\_length\_70626\_cov\_31.911549 51190-51225. Max. coverage (+): 3.23. Max coverage (-): 3.56

Region: NODE\_312079\_length\_70626\_cov\_31.911549 51226-51262. Max. coverage (+): 0.04. Max coverage (-): 0.96

Region: NODE\_312079\_length\_70626\_cov\_31.911549 51263-51299. Max. coverage (+): 0.04. Max coverage (-): 1.08

Region: NODE\_312079\_length\_70626\_cov\_31.911549 51300-51336. Max. coverage (+): 2. Max coverage (-): 1.93

Region: NODE\_312079\_length\_70626\_cov\_31.911549 51337-51373. Max. coverage (+): 0.15. Max coverage (-): 6.52

Region: NODE\_312079\_length\_70626\_cov\_31.911549 51374-51410. Max. coverage (+): 0.15. Max coverage (-): 1.59

Region: NODE\_312079\_length\_70626\_cov\_31.911549 51411-51447. Max. coverage (+): 0.89. Max coverage (-): 0.89

Region: NODE\_312079\_length\_70626\_cov\_31.911549 51448-51483. Max. coverage (+): 0.37. Max coverage (-): 1.41

Region: NODE\_312079\_length\_70626\_cov\_31.911549 51484-51520. Max. coverage (+): 0.3. Max coverage (-): 0.63

Region: NODE\_312079\_length\_70626\_cov\_31.911549 51521-51557. Max. coverage (+): 1.74. Max coverage (-): 1

Region: NODE\_312079\_length\_70626\_cov\_31.911549 51558-51594. Max. coverage (+): 0.3. Max coverage (-): 1.52

Region: NODE\_312079\_length\_70626\_cov\_31.911549 51595-51631. Max. coverage (+): 0.56. Max coverage (-): 0.22

Region: NODE\_312079\_length\_70626\_cov\_31.911549 51632-51668. Max. coverage (+): 0.37. Max coverage (-): 0.11

Region: NODE\_312079\_length\_70626\_cov\_31.911549 51669-51704. Max. coverage (+): 0.7. Max coverage (-): 0.07

Region: NODE\_312079\_length\_70626\_cov\_31.911549 51705-51741. Max. coverage (+): 0.07. Max coverage (-): 0.19

Region: NODE\_312079\_length\_70626\_cov\_31.911549 51742-51778. Max. coverage (+): 0.07. Max coverage (-): 0.37

Region: NODE\_312079\_length\_70626\_cov\_31.911549 51779-51815. Max. coverage (+): 1.04. Max coverage (-): 0.11

Region: NODE\_312079\_length\_70626\_cov\_31.911549 51816-51852. Max. coverage (+): 0. Max coverage (-): 0.26

Region: NODE\_312079\_length\_70626\_cov\_31.911549 51853-51889. Max. coverage (+): 0.04. Max coverage (-): 0.78

Region: NODE\_312079\_length\_70626\_cov\_31.911549 51890-51926. Max. coverage (+): 0.41. Max coverage (-): 1.3

Region: NODE\_312079\_length\_70626\_cov\_31.911549 51927-51962. Max. coverage (+): 0.89. Max coverage (-): 2.45

Region: NODE\_312079\_length\_70626\_cov\_31.911549 51963-51999. Max. coverage (+): 0.7. Max coverage (-): 9.05

Region: NODE\_312079\_length\_70626\_cov\_31.911549 52000-52036. Max. coverage (+): 1.33. Max coverage (-): 0.07

Region: NODE\_312079\_length\_70626\_cov\_31.911549 52037-52073. Max. coverage (+): 0.52. Max coverage (-): 2.26

Region: NODE\_312079\_length\_70626\_cov\_31.911549 52074-52110. Max. coverage (+): 0.85. Max coverage (-): 0.15

Region: NODE\_312079\_length\_70626\_cov\_31.911549 52111-52147. Max. coverage (+): 0.78. Max coverage (-): 4.93

Region: NODE\_312079\_length\_70626\_cov\_31.911549 52148-52184. Max. coverage (+): 0.15. Max coverage (-): 1.11

Region: NODE\_312079\_length\_70626\_cov\_31.911549 52185-52220. Max. coverage (+): 0. Max coverage (-): 1.22

Region: NODE\_312079\_length\_70626\_cov\_31.911549 52221-52257. Max. coverage (+): 0. Max coverage (-): 0.78

Region: NODE\_312079\_length\_70626\_cov\_31.911549 52258-52294. Max. coverage (+): 0. Max coverage (-): 0.3

Region: NODE\_312079\_length\_70626\_cov\_31.911549 52295-52331. Max. coverage (+): 0.56. Max coverage (-): 1.78

Region: NODE\_312079\_length\_70626\_cov\_31.911549 52332-52368. Max. coverage (+): 1.52. Max coverage (-): 4.3

Region: NODE\_312079\_length\_70626\_cov\_31.911549 52369-52405. Max. coverage (+): 0.56. Max coverage (-): 3.26

Region: NODE\_312079\_length\_70626\_cov\_31.911549 52406-52441. Max. coverage (+): 0.63. Max coverage (-): 1.56

Region: NODE\_312079\_length\_70626\_cov\_31.911549 52442-52478. Max. coverage (+): 0.59. Max coverage (-): 1.45

Region: NODE\_312079\_length\_70626\_cov\_31.911549 52479-52515. Max. coverage (+): 0.37. Max coverage (-): 0.78

Region: NODE\_312079\_length\_70626\_cov\_31.911549 52516-52552. Max. coverage (+): 0.37. Max coverage (-): 0.41

Region: NODE\_312079\_length\_70626\_cov\_31.911549 52553-52589. Max. coverage (+): 0.07. Max coverage (-): 0.44

Region: NODE\_312079\_length\_70626\_cov\_31.911549 52590-52626. Max. coverage (+): 0.15. Max coverage (-): 1.26

Region: NODE\_312079\_length\_70626\_cov\_31.911549 52627-52663. Max. coverage (+): 0.3. Max coverage (-): 3.04

Region: NODE\_312079\_length\_70626\_cov\_31.911549 52664-52699. Max. coverage (+): 0.15. Max coverage (-): 0.07

Region: NODE\_312079\_length\_70626\_cov\_31.911549 52700-52736. Max. coverage (+): 0.19. Max coverage (-): 0.7

Region: NODE\_312079\_length\_70626\_cov\_31.911549 52737-52773. Max. coverage (+): 2.04. Max coverage (-): 3.11

Region: NODE\_312079\_length\_70626\_cov\_31.911549 52774-52810. Max. coverage (+): 0. Max coverage (-): 2.85

Region: NODE\_312079\_length\_70626\_cov\_31.911549 52811-52847. Max. coverage (+): 0.04. Max coverage (-): 0.3

Region: NODE\_312079\_length\_70626\_cov\_31.911549 52848-52884. Max. coverage (+): 6.27. Max coverage (-): 1.56

Region: NODE\_312079\_length\_70626\_cov\_31.911549 52885-52921. Max. coverage (+): 0.33. Max coverage (-): 0.19

Region: NODE\_312079\_length\_70626\_cov\_31.911549 52922-52957. Max. coverage (+): 0.33. Max coverage (-): 0.04

Region: NODE\_312079\_length\_70626\_cov\_31.911549 52958-52994. Max. coverage (+): 0.11. Max coverage (-): 10.53

Region: NODE\_312079\_length\_70626\_cov\_31.911549 52995-53031. Max. coverage (+): 0.15. Max coverage (-): 2.11

Region: NODE\_312079\_length\_70626\_cov\_31.911549 53032-53068. Max. coverage (+): 0.15. Max coverage (-): 0.56

Region: NODE\_312079\_length\_70626\_cov\_31.911549 53069-53105. Max. coverage (+): 0.07. Max coverage (-): 0.78

Region: NODE\_312079\_length\_70626\_cov\_31.911549 53106-53142. Max. coverage (+): 0.15. Max coverage (-): 0.41

Region: NODE\_312079\_length\_70626\_cov\_31.911549 53143-53178. Max. coverage (+): 0.07. Max coverage (-): 0.41

Region: NODE\_312079\_length\_70626\_cov\_31.911549 53179-53215. Max. coverage (+): 0.04. Max coverage (-): 0.59

Region: NODE\_312079\_length\_70626\_cov\_31.911549 53216-53252. Max. coverage (+): 0. Max coverage (-): 0.41

Region: NODE\_312079\_length\_70626\_cov\_31.911549 53253-53289. Max. coverage (+): 0.19. Max coverage (-): 1.33

Region: NODE\_312079\_length\_70626\_cov\_31.911549 53290-53326. Max. coverage (+): 0.04. Max coverage (-): 0.15

Region: NODE\_312079\_length\_70626\_cov\_31.911549 53327-53363. Max. coverage (+): 0. Max coverage (-): 0.15

Region: NODE\_312079\_length\_70626\_cov\_31.911549 53364-53400. Max. coverage (+): 0.04. Max coverage (-): 0.04

Region: NODE\_312079\_length\_70626\_cov\_31.911549 53401-53436. Max. coverage (+): 0.04. Max coverage (-): 0.04

Region: NODE\_312079\_length\_70626\_cov\_31.911549 53437-53473. Max. coverage (+): 0.04. Max coverage (-): 0.22

Region: NODE\_312079\_length\_70626\_cov\_31.911549 53474-53510. Max. coverage (+): 0. Max coverage (-): 0.04

Region: NODE\_312079\_length\_70626\_cov\_31.911549 53511-53547. Max. coverage (+): 0.04. Max coverage (-): 0

Region: NODE\_312079\_length\_70626\_cov\_31.911549 53548-53584. Max. coverage (+): 0.04. Max coverage (-): 0.04

Region: NODE\_312079\_length\_70626\_cov\_31.911549 53585-53621. Max. coverage (+): 0.04. Max coverage (-): 0.07

Region: NODE\_312079\_length\_70626\_cov\_31.911549 53622-53658. Max. coverage (+): 0.02. Max coverage (-): 0

Region: NODE\_312079\_length\_70626\_cov\_31.911549 53659-53694. Max. coverage (+): 0. Max coverage (-): 0

Region: NODE\_312079\_length\_70626\_cov\_31.911549 53695-53731. Max. coverage (+): 0. Max coverage (-): 0

Region: NODE\_312079\_length\_70626\_cov\_31.911549 53732-53768. Max. coverage (+): 0. Max coverage (-): 0

Region: NODE\_312079\_length\_70626\_cov\_31.911549 53769-53805. Max. coverage (+): 0.01. Max coverage (-): 0.01

Region: NODE\_312079\_length\_70626\_cov\_31.911549 53806-53842. Max. coverage (+): 0.02. Max coverage (-): 0

Region: NODE\_312079\_length\_70626\_cov\_31.911549 53843-53879. Max. coverage (+): 0.04. Max coverage (-): 0

Region: NODE\_312079\_length\_70626\_cov\_31.911549 53880-53915. Max. coverage (+): 0. Max coverage (-): 0

Region: NODE\_312079\_length\_70626\_cov\_31.911549 53916-53952. Max. coverage (+): 0. Max coverage (-): 0

Region: NODE\_312079\_length\_70626\_cov\_31.911549 53953-53989. Max. coverage (+): 0. Max coverage (-): 0.04

Region: NODE\_312079\_length\_70626\_cov\_31.911549 53990-54026. Max. coverage (+): 0. Max coverage (-): 0

Region: NODE\_312079\_length\_70626\_cov\_31.911549 54027-54063. Max. coverage (+): 0. Max coverage (-): 0

Region: NODE\_312079\_length\_70626\_cov\_31.911549 54064-54100. Max. coverage (+): 0. Max coverage (-): 0

Region: NODE\_312079\_length\_70626\_cov\_31.911549 54101-54137. Max. coverage (+): 0. Max coverage (-): 0

Region: NODE\_312079\_length\_70626\_cov\_31.911549 54138-54173. Max. coverage (+): 0. Max coverage (-): 0

Region: NODE\_312079\_length\_70626\_cov\_31.911549 54174-54210. Max. coverage (+): 0. Max coverage (-): 0

Region: NODE\_312079\_length\_70626\_cov\_31.911549 54211-54247. Max. coverage (+): 0. Max coverage (-): 0

Region: NODE\_312079\_length\_70626\_cov\_31.911549 54248-54284. Max. coverage (+): 0. Max coverage (-): 0.04

Region: NODE\_312079\_length\_70626\_cov\_31.911549 54285-54321. Max. coverage (+): 0. Max coverage (-): 0

Region: NODE\_312079\_length\_70626\_cov\_31.911549 54322-54358. Max. coverage (+): 0. Max coverage (-): 0.04

Region: NODE\_312079\_length\_70626\_cov\_31.911549 54359-54395. Max. coverage (+): 0. Max coverage (-): 0

Region: NODE\_312079\_length\_70626\_cov\_31.911549 54396-54431. Max. coverage (+): 0. Max coverage (-): 0

Region: NODE\_312079\_length\_70626\_cov\_31.911549 54432-54468. Max. coverage (+): 0. Max coverage (-): 0

Region: NODE\_312079\_length\_70626\_cov\_31.911549 54469-54505. Max. coverage (+): 0. Max coverage (-): 0

Region: NODE\_312079\_length\_70626\_cov\_31.911549 54506-54542. Max. coverage (+): 0. Max coverage (-): 0

Region: NODE\_312079\_length\_70626\_cov\_31.911549 54543-54579. Max. coverage (+): 0. Max coverage (-): 0

Region: NODE\_312079\_length\_70626\_cov\_31.911549 54580-54616. Max. coverage (+): 0.04. Max coverage (-): 0

Region: NODE\_312079\_length\_70626\_cov\_31.911549 54617-54652. Max. coverage (+): 0.04. Max coverage (-): 0.04

Region: NODE\_312079\_length\_70626\_cov\_31.911549 54653-54689. Max. coverage (+): 0. Max coverage (-): 0.04

Region: NODE\_312079\_length\_70626\_cov\_31.911549 54690-54726. Max. coverage (+): 0. Max coverage (-): 0.07

Region: NODE\_312079\_length\_70626\_cov\_31.911549 54727-54763. Max. coverage (+): 0. Max coverage (-): 0

Region: NODE\_312079\_length\_70626\_cov\_31.911549 54764-54800. Max. coverage (+): 0. Max coverage (-): 0

Region: NODE\_312079\_length\_70626\_cov\_31.911549 54801-54837. Max. coverage (+): 0. Max coverage (-): 0

Region: NODE\_312079\_length\_70626\_cov\_31.911549 54838-54874. Max. coverage (+): 0. Max coverage (-): 0

Region: NODE\_312079\_length\_70626\_cov\_31.911549 54875-54910. Max. coverage (+): 0. Max coverage (-): 0

Region: NODE\_312079\_length\_70626\_cov\_31.911549 54911-54947. Max. coverage (+): 0. Max coverage (-): 0.04

Region: NODE\_312079\_length\_70626\_cov\_31.911549 54948-54984. Max. coverage (+): 0. Max coverage (-): 0

Region: NODE\_312079\_length\_70626\_cov\_31.911549 54985-55021. Max. coverage (+): 0.04. Max coverage (-): 0

Region: NODE\_312079\_length\_70626\_cov\_31.911549 55022-55058. Max. coverage (+): 0. Max coverage (-): 0

Region: NODE\_312079\_length\_70626\_cov\_31.911549 55059-55095. Max. coverage (+): 0. Max coverage (-): 0

Region: NODE\_312079\_length\_70626\_cov\_31.911549 55096-55132. Max. coverage (+): 0. Max coverage (-): 0

Region: NODE\_312079\_length\_70626\_cov\_31.911549 55133-55168. Max. coverage (+): 0. Max coverage (-): 0

Region: NODE\_312079\_length\_70626\_cov\_31.911549 55169-55205. Max. coverage (+): 0. Max coverage (-): 0

Region: NODE\_312079\_length\_70626\_cov\_31.911549 55206-55242. Max. coverage (+): 0. Max coverage (-): 0

Region: NODE\_312079\_length\_70626\_cov\_31.911549 55243-55279. Max. coverage (+): 0. Max coverage (-): 0

Region: NODE\_312079\_length\_70626\_cov\_31.911549 55280-55316. Max. coverage (+): 0. Max coverage (-): 0

Region: NODE\_312079\_length\_70626\_cov\_31.911549 55317-55353. Max. coverage (+): 0.04. Max coverage (-): 0

Region: NODE\_312079\_length\_70626\_cov\_31.911549 55354-55389. Max. coverage (+): 0. Max coverage (-): 0

Region: NODE\_312079\_length\_70626\_cov\_31.911549 55390-55426. Max. coverage (+): 0. Max coverage (-): 0

Region: NODE\_312079\_length\_70626\_cov\_31.911549 55427-55463. Max. coverage (+): 0. Max coverage (-): 0

Region: NODE\_312079\_length\_70626\_cov\_31.911549 55464-55500. Max. coverage (+): 0. Max coverage (-): 0

Region: NODE\_312079\_length\_70626\_cov\_31.911549 55501-55537. Max. coverage (+): 0. Max coverage (-): 0

Region: NODE\_312079\_length\_70626\_cov\_31.911549 55538-55574. Max. coverage (+): 0. Max coverage (-): 0

Region: NODE\_312079\_length\_70626\_cov\_31.911549 55575-55611. Max. coverage (+): 0. Max coverage (-): 0.04

Region: NODE\_312079\_length\_70626\_cov\_31.911549 55612-55647. Max. coverage (+): 0. Max coverage (-): 0

Region: NODE\_312079\_length\_70626\_cov\_31.911549 55648-55684. Max. coverage (+): 0. Max coverage (-): 0

Region: NODE\_312079\_length\_70626\_cov\_31.911549 55685-55721. Max. coverage (+): 0. Max coverage (-): 0

Region: NODE\_312079\_length\_70626\_cov\_31.911549 55722-55758. Max. coverage (+): 0.07. Max coverage (-): 0

Region: NODE\_312079\_length\_70626\_cov\_31.911549 55759-55795. Max. coverage (+): 0.08. Max coverage (-): 0.03

Region: NODE\_312079\_length\_70626\_cov\_31.911549 55796-55832. Max. coverage (+): 0. Max coverage (-): 0.01

Region: NODE\_312079\_length\_70626\_cov\_31.911549 55833-55869. Max. coverage (+): 0. Max coverage (-): 0

Region: NODE\_312079\_length\_70626\_cov\_31.911549 55870-55905. Max. coverage (+): 0.01. Max coverage (-): 0

Region: NODE\_312079\_length\_70626\_cov\_31.911549 55906-55942. Max. coverage (+): 0. Max coverage (-): 0

Region: NODE\_312079\_length\_70626\_cov\_31.911549 55943-55979. Max. coverage (+): 0.01. Max coverage (-): 0

Region: NODE\_312079\_length\_70626\_cov\_31.911549 55980-56016. Max. coverage (+): 0.11. Max coverage (-): 0

Region: NODE\_312079\_length\_70626\_cov\_31.911549 56017-56053. Max. coverage (+): 0.04. Max coverage (-): 0.04

Region: NODE\_312079\_length\_70626\_cov\_31.911549 56054-56090. Max. coverage (+): 0.04. Max coverage (-): 0.04

Region: NODE\_312079\_length\_70626\_cov\_31.911549 56091-56126. Max. coverage (+): 0.22. Max coverage (-): 0.04

Region: NODE\_312079\_length\_70626\_cov\_31.911549 56127-56163. Max. coverage (+): 0.19. Max coverage (-): 0.04

Region: NODE\_312079\_length\_70626\_cov\_31.911549 56164-56200. Max. coverage (+): 0.56. Max coverage (-): 0

Region: NODE\_312079\_length\_70626\_cov\_31.911549 56201-56237. Max. coverage (+): 0.78. Max coverage (-): 0.04

Region: NODE\_312079\_length\_70626\_cov\_31.911549 56238-56274. Max. coverage (+): 0.15. Max coverage (-): 0.07

Region: NODE\_312079\_length\_70626\_cov\_31.911549 56275-56311. Max. coverage (+): 0.07. Max coverage (-): 0.04

Region: NODE\_312079\_length\_70626\_cov\_31.911549 56312-56348. Max. coverage (+): 6.12. Max coverage (-): 0.04

Region: NODE\_312079\_length\_70626\_cov\_31.911549 56349-56384. Max. coverage (+): 5.93. Max coverage (-): 1.41

Region: NODE\_312079\_length\_70626\_cov\_31.911549 56385-56421. Max. coverage (+): 0.15. Max coverage (-): 0.7

Region: NODE\_312079\_length\_70626\_cov\_31.911549 56422-56458. Max. coverage (+): 0.11. Max coverage (-): 0.07

Region: NODE\_312079\_length\_70626\_cov\_31.911549 56459-56495. Max. coverage (+): 0.3. Max coverage (-): 0

Region: NODE\_312079\_length\_70626\_cov\_31.911549 56496-56532. Max. coverage (+): 0.07. Max coverage (-): 0.59

Region: NODE\_312079\_length\_70626\_cov\_31.911549 56533-56569. Max. coverage (+): 0.44. Max coverage (-): 0.3

Region: NODE\_312079\_length\_70626\_cov\_31.911549 56570-56606. Max. coverage (+): 10.68. Max coverage (-): 0.07

Region: NODE\_312079\_length\_70626\_cov\_31.911549 56607-56642. Max. coverage (+): 0.37. Max coverage (-): 0.04

Region: NODE\_312079\_length\_70626\_cov\_31.911549 56643-56679. Max. coverage (+): 0.33. Max coverage (-): 0.26

Region: NODE\_312079\_length\_70626\_cov\_31.911549 56680-56716. Max. coverage (+): 12.86. Max coverage (-): 0.04

Region: NODE\_312079\_length\_70626\_cov\_31.911549 56717-56753. Max. coverage (+): 12.9. Max coverage (-): 0.93

Region: NODE\_312079\_length\_70626\_cov\_31.911549 56754-56790. Max. coverage (+): 6.9. Max coverage (-): 0.26

Region: NODE\_312079\_length\_70626\_cov\_31.911549 56791-56827. Max. coverage (+): 7.19. Max coverage (-): 0.26

Region: NODE\_312079\_length\_70626\_cov\_31.911549 56828-56863. Max. coverage (+): 0.96. Max coverage (-): 0

Region: NODE\_312079\_length\_70626\_cov\_31.911549 56864-56900. Max. coverage (+): 0.89. Max coverage (-): 0.33

Region: NODE\_312079\_length\_70626\_cov\_31.911549 56901-56937. Max. coverage (+): 9.6. Max coverage (-): 0.26

Region: NODE\_312079\_length\_70626\_cov\_31.911549 56938-56974. Max. coverage (+): 0.19. Max coverage (-): 0.48

Region: NODE\_312079\_length\_70626\_cov\_31.911549 56975-57011. Max. coverage (+): 19.8. Max coverage (-): 0.48

Region: NODE\_312079\_length\_70626\_cov\_31.911549 57012-57048. Max. coverage (+): 2.15. Max coverage (-): 10.9

Region: NODE\_312079\_length\_70626\_cov\_31.911549 57049-57085. Max. coverage (+): 0.89. Max coverage (-): 0.44

Region: NODE\_312079\_length\_70626\_cov\_31.911549 57086-57121. Max. coverage (+): 0.48. Max coverage (-): 0.04

Region: NODE\_312079\_length\_70626\_cov\_31.911549 57122-57158. Max. coverage (+): 8.08. Max coverage (-): 0.07

Region: NODE\_312079\_length\_70626\_cov\_31.911549 57159-57195. Max. coverage (+): 5.56. Max coverage (-): 0.74

Region: NODE\_312079\_length\_70626\_cov\_31.911549 57196-57232. Max. coverage (+): 1.89. Max coverage (-): 0.04

Region: NODE\_312079\_length\_70626\_cov\_31.911549 57233-57269. Max. coverage (+): 0.11. Max coverage (-): 0.04

Region: NODE\_312079\_length\_70626\_cov\_31.911549 57270-57306. Max. coverage (+): 0.89. Max coverage (-): 0

Region: NODE\_312079\_length\_70626\_cov\_31.911549 57307-57343. Max. coverage (+): 0. Max coverage (-): 0

Region: NODE\_312079\_length\_70626\_cov\_31.911549 57344-57379. Max. coverage (+): 0. Max coverage (-): 0

Region: NODE\_312079\_length\_70626\_cov\_31.911549 57380-57416. Max. coverage (+): 0.07. Max coverage (-): 0

Region: NODE\_312079\_length\_70626\_cov\_31.911549 57417-57453. Max. coverage (+): 0.19. Max coverage (-): 0.07

Region: NODE\_312079\_length\_70626\_cov\_31.911549 57454-57490. Max. coverage (+): 0.07. Max coverage (-): 0.04

Region: NODE\_312079\_length\_70626\_cov\_31.911549 57491-57527. Max. coverage (+): 94.2. Max coverage (-): 0.15

Region: NODE\_312079\_length\_70626\_cov\_31.911549 57528-57564. Max. coverage (+): 1.89. Max coverage (-): 5.3

Region: NODE\_312079\_length\_70626\_cov\_31.911549 57565-57600. Max. coverage (+): 2.45. Max coverage (-): 5.3

Region: NODE\_312079\_length\_70626\_cov\_31.911549 57601-57637. Max. coverage (+): 3.82. Max coverage (-): 0.22

Region: NODE\_312079\_length\_70626\_cov\_31.911549 57638-57674. Max. coverage (+): 4.23. Max coverage (-): 0.48

Region: NODE\_312079\_length\_70626\_cov\_31.911549 57675-57711. Max. coverage (+): 2.97. Max coverage (-): 0.63

Region: NODE\_312079\_length\_70626\_cov\_31.911549 57712-57748. Max. coverage (+): 0.85. Max coverage (-): 0.63

Region: NODE\_312079\_length\_70626\_cov\_31.911549 57749-57785. Max. coverage (+): 2.97. Max coverage (-): 1.22

Region: NODE\_312079\_length\_70626\_cov\_31.911549 57786-57822. Max. coverage (+): 7.9. Max coverage (-): 0.04

Region: NODE\_312079\_length\_70626\_cov\_31.911549 57823-57858. Max. coverage (+): 30.51. Max coverage (-): 0.33

Region: NODE\_312079\_length\_70626\_cov\_31.911549 57859-57895. Max. coverage (+): 4. Max coverage (-): 0.19

Region: NODE\_312079\_length\_70626\_cov\_31.911549 57896-57932. Max. coverage (+): 0.67. Max coverage (-): 0.22

Region: NODE\_312079\_length\_70626\_cov\_31.911549 57933-57969. Max. coverage (+): 0.85. Max coverage (-): 0.15

Region: NODE\_312079\_length\_70626\_cov\_31.911549 57970-58006. Max. coverage (+): 0.56. Max coverage (-): 0.44

Region: NODE\_312079\_length\_70626\_cov\_31.911549 58007-58043. Max. coverage (+): 0.41. Max coverage (-): 1.04

Region: NODE\_312079\_length\_70626\_cov\_31.911549 58044-58080. Max. coverage (+): 0.67. Max coverage (-): 0.04

Region: NODE\_312079\_length\_70626\_cov\_31.911549 58081-58116. Max. coverage (+): 0.44. Max coverage (-): 0.04

Region: NODE\_312079\_length\_70626\_cov\_31.911549 58117-58153. Max. coverage (+): 1. Max coverage (-): 0.07

Region: NODE\_312079\_length\_70626\_cov\_31.911549 58154-58190. Max. coverage (+): 1.89. Max coverage (-): 0.19

Region: NODE\_312079\_length\_70626\_cov\_31.911549 58191-58227. Max. coverage (+): 1.45. Max coverage (-): 0.11

Region: NODE\_312079\_length\_70626\_cov\_31.911549 58228-58264. Max. coverage (+): 0.52. Max coverage (-): 0.04

Region: NODE\_312079\_length\_70626\_cov\_31.911549 58265-58301. Max. coverage (+): 0.19. Max coverage (-): 0.07

Region: NODE\_312079\_length\_70626\_cov\_31.911549 58302-58337. Max. coverage (+): 0.44. Max coverage (-): 0.19

Region: NODE\_312079\_length\_70626\_cov\_31.911549 58338-58374. Max. coverage (+): 0.33. Max coverage (-): 0.11

Region: NODE\_312079\_length\_70626\_cov\_31.911549 58375-58411. Max. coverage (+): 0.04. Max coverage (-): 0

Region: NODE\_312079\_length\_70626\_cov\_31.911549 58412-58448. Max. coverage (+): 0. Max coverage (-): 0.04

Region: NODE\_312079\_length\_70626\_cov\_31.911549 58449-58485. Max. coverage (+): 0.01. Max coverage (-): 0

Region: NODE\_312079\_length\_70626\_cov\_31.911549 58486-58522. Max. coverage (+): 0.02. Max coverage (-): 0

Region: NODE\_312079\_length\_70626\_cov\_31.911549 58523-58559. Max. coverage (+): 0.15. Max coverage (-): 0

Region: NODE\_312079\_length\_70626\_cov\_31.911549 58560-58595. Max. coverage (+): 1.46. Max coverage (-): 0.04

Region: NODE\_312079\_length\_70626\_cov\_31.911549 58596-58632. Max. coverage (+): 0.48. Max coverage (-): 0.04

Region: NODE\_312079\_length\_70626\_cov\_31.911549 58633-58669. Max. coverage (+): 0.01. Max coverage (-): 0

Region: NODE\_312079\_length\_70626\_cov\_31.911549 58670-58706. Max. coverage (+): 0.07. Max coverage (-): 0

Region: NODE\_312079\_length\_70626\_cov\_31.911549 58707-58743. Max. coverage (+): 0.3. Max coverage (-): 0.04

Region: NODE\_312079\_length\_70626\_cov\_31.911549 58744-58780. Max. coverage (+): 0.82. Max coverage (-): 0.11

Region: NODE\_312079\_length\_70626\_cov\_31.911549 58781-58817. Max. coverage (+): 0. Max coverage (-): 0

Region: NODE\_312079\_length\_70626\_cov\_31.911549 58818-58853. Max. coverage (+): 1.3. Max coverage (-): 0.04

Region: NODE\_312079\_length\_70626\_cov\_31.911549 58854-58890. Max. coverage (+): 1. Max coverage (-): 0.11

Region: NODE\_312079\_length\_70626\_cov\_31.911549 58891-58927. Max. coverage (+): 1.04. Max coverage (-): 0.63

Region: NODE\_312079\_length\_70626\_cov\_31.911549 58928-58964. Max. coverage (+): 1.19. Max coverage (-): 0.82

Region: NODE\_312079\_length\_70626\_cov\_31.911549 58965-59001. Max. coverage (+): 0.33. Max coverage (-): 0.07

Region: NODE\_312079\_length\_70626\_cov\_31.911549 59002-59038. Max. coverage (+): 1.82. Max coverage (-): 0.11

Region: NODE\_312079\_length\_70626\_cov\_31.911549 59039-59074. Max. coverage (+): 21.47. Max coverage (-): 0.26

Region: NODE\_312079\_length\_70626\_cov\_31.911549 59075-59111. Max. coverage (+): 0.3. Max coverage (-): 0.37

Region: NODE\_312079\_length\_70626\_cov\_31.911549 59112-59148. Max. coverage (+): 5.56. Max coverage (-): 0.07

Region: NODE\_312079\_length\_70626\_cov\_31.911549 59149-59185. Max. coverage (+): 0.48. Max coverage (-): 0.15

Region: NODE\_312079\_length\_70626\_cov\_31.911549 59186-59222. Max. coverage (+): 26.8. Max coverage (-): 3.52

Region: NODE\_312079\_length\_70626\_cov\_31.911549 59223-59259. Max. coverage (+): 0.85. Max coverage (-): 0.3

Region: NODE\_312079\_length\_70626\_cov\_31.911549 59260-59296. Max. coverage (+): 0.63. Max coverage (-): 0.63

Region: NODE\_312079\_length\_70626\_cov\_31.911549 59297-59332. Max. coverage (+): 0.22. Max coverage (-): 0.15

Region: NODE\_312079\_length\_70626\_cov\_31.911549 59333-59369. Max. coverage (+): 0.7. Max coverage (-): 0.15

Region: NODE\_312079\_length\_70626\_cov\_31.911549 59370-59406. Max. coverage (+): 0.11. Max coverage (-): 0.22

Region: NODE\_312079\_length\_70626\_cov\_31.911549 59407-59443. Max. coverage (+): 1.71. Max coverage (-): 0.04

Region: NODE\_312079\_length\_70626\_cov\_31.911549 59444-59480. Max. coverage (+): 43.93. Max coverage (-): 0.3

Region: NODE\_312079\_length\_70626\_cov\_31.911549 59481-59517. Max. coverage (+): 0.22. Max coverage (-): 0.63

Region: NODE\_312079\_length\_70626\_cov\_31.911549 59518-59554. Max. coverage (+): 13.57. Max coverage (-): 0.11

Region: NODE\_312079\_length\_70626\_cov\_31.911549 59555-59590. Max. coverage (+): 0.85. Max coverage (-): 0.26

Region: NODE\_312079\_length\_70626\_cov\_31.911549 59591-59627. Max. coverage (+): 0.89. Max coverage (-): 0.11

Region: NODE\_312079\_length\_70626\_cov\_31.911549 59628-59664. Max. coverage (+): 1.3. Max coverage (-): 0.07

Region: NODE\_312079\_length\_70626\_cov\_31.911549 59665-59701. Max. coverage (+): 2.63. Max coverage (-): 0.37

Region: NODE\_312079\_length\_70626\_cov\_31.911549 59702-59738. Max. coverage (+): 1.48. Max coverage (-): 0.33

Region: NODE\_312079\_length\_70626\_cov\_31.911549 59739-59775. Max. coverage (+): 1.41. Max coverage (-): 0.19

Region: NODE\_312079\_length\_70626\_cov\_31.911549 59776-59811. Max. coverage (+): 1.93. Max coverage (-): 0.15

Region: NODE\_312079\_length\_70626\_cov\_31.911549 59812-59848. Max. coverage (+): 1.33. Max coverage (-): 0.15

Region: NODE\_312079\_length\_70626\_cov\_31.911549 59849-59885. Max. coverage (+): 4.75. Max coverage (-): 0.22

Region: NODE\_312079\_length\_70626\_cov\_31.911549 59886-59922. Max. coverage (+): 0.11. Max coverage (-): 0

Region: NODE\_312079\_length\_70626\_cov\_31.911549 59923-59959. Max. coverage (+): 1.26. Max coverage (-): 0.07

Region: NODE\_312079\_length\_70626\_cov\_31.911549 59960-59996. Max. coverage (+): 0.19. Max coverage (-): 0.07

Region: NODE\_312079\_length\_70626\_cov\_31.911549 59997-60033. Max. coverage (+): 0.15. Max coverage (-): 0.15

Region: NODE\_312079\_length\_70626\_cov\_31.911549 60034-60069. Max. coverage (+): 3.71. Max coverage (-): 0.22

Region: NODE\_312079\_length\_70626\_cov\_31.911549 60070-60106. Max. coverage (+): 0.67. Max coverage (-): 0.04

Region: NODE\_312079\_length\_70626\_cov\_31.911549 60107-60143. Max. coverage (+): 0.63. Max coverage (-): 0.04

Region: NODE\_312079\_length\_70626\_cov\_31.911549 60144-60180. Max. coverage (+): 102.51. Max coverage (-): 0.26

Region: NODE\_312079\_length\_70626\_cov\_31.911549 60181-60217. Max. coverage (+): 0.11. Max coverage (-): 0.3

Region: NODE\_312079\_length\_70626\_cov\_31.911549 60218-60254. Max. coverage (+): 1.78. Max coverage (-): 0.04

Region: NODE\_312079\_length\_70626\_cov\_31.911549 60255-60291. Max. coverage (+): 16.5. Max coverage (-): 0.7

Region: NODE\_312079\_length\_70626\_cov\_31.911549 60292-60327. Max. coverage (+): 1.37. Max coverage (-): 0.15

Region: NODE\_312079\_length\_70626\_cov\_31.911549 60328-60364. Max. coverage (+): 3.37. Max coverage (-): 0.26

Region: NODE\_312079\_length\_70626\_cov\_31.911549 60365-60401. Max. coverage (+): 4.23. Max coverage (-): 0.19

Region: NODE\_312079\_length\_70626\_cov\_31.911549 60402-60438. Max. coverage (+): 4.34. Max coverage (-): 0.15

Region: NODE\_312079\_length\_70626\_cov\_31.911549 60439-60475. Max. coverage (+): 4.97. Max coverage (-): 0.04

Region: NODE\_312079\_length\_70626\_cov\_31.911549 60476-60512. Max. coverage (+): 8.53. Max coverage (-): 0.33

Region: NODE\_312079\_length\_70626\_cov\_31.911549 60513-60548. Max. coverage (+): 8.42. Max coverage (-): 0.07

Region: NODE\_312079\_length\_70626\_cov\_31.911549 60549-60585. Max. coverage (+): 0.63. Max coverage (-): 0.48

Region: NODE\_312079\_length\_70626\_cov\_31.911549 60586-60622. Max. coverage (+): 4.41. Max coverage (-): 0.19

Region: NODE\_312079\_length\_70626\_cov\_31.911549 60623-60659. Max. coverage (+): 0.85. Max coverage (-): 0.04

Region: NODE\_312079\_length\_70626\_cov\_31.911549 60660-60696. Max. coverage (+): 3.37. Max coverage (-): 0.07

Region: NODE\_312079\_length\_70626\_cov\_31.911549 60697-60733. Max. coverage (+): 0.67. Max coverage (-): 0.04

Region: NODE\_312079\_length\_70626\_cov\_31.911549 60734-60770. Max. coverage (+): 1. Max coverage (-): 0.04

Region: NODE\_312079\_length\_70626\_cov\_31.911549 60771-60806. Max. coverage (+): 0.59. Max coverage (-): 0.07

Region: NODE\_312079\_length\_70626\_cov\_31.911549 60807-60843. Max. coverage (+): 2.63. Max coverage (-): 0.26

Region: NODE\_312079\_length\_70626\_cov\_31.911549 60844-60880. Max. coverage (+): 13.05. Max coverage (-): 0.04

Region: NODE\_312079\_length\_70626\_cov\_31.911549 60881-60917. Max. coverage (+): 2.34. Max coverage (-): 0

Region: NODE\_312079\_length\_70626\_cov\_31.911549 60918-60954. Max. coverage (+): 0.26. Max coverage (-): 0.22

Region: NODE\_312079\_length\_70626\_cov\_31.911549 60955-60991. Max. coverage (+): 0.44. Max coverage (-): 0.04

Region: NODE\_312079\_length\_70626\_cov\_31.911549 60992-61028. Max. coverage (+): 0.48. Max coverage (-): 0.15

Region: NODE\_312079\_length\_70626\_cov\_31.911549 61029-61064. Max. coverage (+): 4.67. Max coverage (-): 0.33

Region: NODE\_312079\_length\_70626\_cov\_31.911549 61065-61101. Max. coverage (+): 0.22. Max coverage (-): 1

Region: NODE\_312079\_length\_70626\_cov\_31.911549 61102-61138. Max. coverage (+): 14.42. Max coverage (-): 0.56

Region: NODE\_312079\_length\_70626\_cov\_31.911549 61139-61175. Max. coverage (+): 0.33. Max coverage (-): 0.07

Region: NODE\_312079\_length\_70626\_cov\_31.911549 61176-61212. Max. coverage (+): 1.11. Max coverage (-): 0.19

Region: NODE\_312079\_length\_70626\_cov\_31.911549 61213-61249. Max. coverage (+): 2.41. Max coverage (-): 0.04

Region: NODE\_312079\_length\_70626\_cov\_31.911549 61250-61285. Max. coverage (+): 0.11. Max coverage (-): 0.59

Region: NODE\_312079\_length\_70626\_cov\_31.911549 61286-61322. Max. coverage (+): 3.82. Max coverage (-): 0.04

Region: NODE\_312079\_length\_70626\_cov\_31.911549 61323-61359. Max. coverage (+): 2.6. Max coverage (-): 0

Region: NODE\_312079\_length\_70626\_cov\_31.911549 61360-61396. Max. coverage (+): 0.41. Max coverage (-): 0

Region: NODE\_312079\_length\_70626\_cov\_31.911549 61397-61433. Max. coverage (+): 0. Max coverage (-): 0.96

Region: NODE\_312079\_length\_70626\_cov\_31.911549 61434-61470. Max. coverage (+): 0.41. Max coverage (-): 0.41

Region: NODE\_312079\_length\_70626\_cov\_31.911549 61471-61507. Max. coverage (+): 0.07. Max coverage (-): 0.26

Region: NODE\_312079\_length\_70626\_cov\_31.911549 61508-61543. Max. coverage (+): 1.33. Max coverage (-): 0

Region: NODE\_312079\_length\_70626\_cov\_31.911549 61544-61580. Max. coverage (+): 0.19. Max coverage (-): 0.7

Region: NODE\_312079\_length\_70626\_cov\_31.911549 61581-61617. Max. coverage (+): 0.26. Max coverage (-): 0.22

Region: NODE\_312079\_length\_70626\_cov\_31.911549 61618-61654. Max. coverage (+): 0.67. Max coverage (-): 1.11

Region: NODE\_312079\_length\_70626\_cov\_31.911549 61655-61691. Max. coverage (+): 5.71. Max coverage (-): 0.04

Region: NODE\_312079\_length\_70626\_cov\_31.911549 61692-61728. Max. coverage (+): 2.97. Max coverage (-): 0

Region: NODE\_312079\_length\_70626\_cov\_31.911549 61729-61765. Max. coverage (+): 28.95. Max coverage (-): 0.22

Region: NODE\_312079\_length\_70626\_cov\_31.911549 61766-61801. Max. coverage (+): 0.67. Max coverage (-): 0.15

Region: NODE\_312079\_length\_70626\_cov\_31.911549 61802-61838. Max. coverage (+): 0.19. Max coverage (-): 0.22

Region: NODE\_312079\_length\_70626\_cov\_31.911549 61839-61875. Max. coverage (+): 4.41. Max coverage (-): 1.78

Region: NODE\_312079\_length\_70626\_cov\_31.911549 61876-61912. Max. coverage (+): 14.38. Max coverage (-): 1.63

Region: NODE\_312079\_length\_70626\_cov\_31.911549 61913-61949. Max. coverage (+): 0.3. Max coverage (-): 0

Region: NODE\_312079\_length\_70626\_cov\_31.911549 61950-61986. Max. coverage (+): 6.56. Max coverage (-): 0.11

Region: NODE\_312079\_length\_70626\_cov\_31.911549 61987-62022. Max. coverage (+): 2.08. Max coverage (-): 0

Region: NODE\_312079\_length\_70626\_cov\_31.911549 62023-62059. Max. coverage (+): 1.22. Max coverage (-): 0

Region: NODE\_312079\_length\_70626\_cov\_31.911549 62060-62096. Max. coverage (+): 0.63. Max coverage (-): 0.04

Region: NODE\_312079\_length\_70626\_cov\_31.911549 62097-62133. Max. coverage (+): 0.15. Max coverage (-): 0.04

Region: NODE\_312079\_length\_70626\_cov\_31.911549 62134-62170. Max. coverage (+): 0.37. Max coverage (-): 0.04

Region: NODE\_312079\_length\_70626\_cov\_31.911549 62171-62207. Max. coverage (+): 0.93. Max coverage (-): 0.15

Region: NODE\_312079\_length\_70626\_cov\_31.911549 62208-62244. Max. coverage (+): 8.42. Max coverage (-): 1.15

Region: NODE\_312079\_length\_70626\_cov\_31.911549 62245-62280. Max. coverage (+): 1.59. Max coverage (-): 0.26

Region: NODE\_312079\_length\_70626\_cov\_31.911549 62281-62317. Max. coverage (+): 3.26. Max coverage (-): 0.33

Region: NODE\_312079\_length\_70626\_cov\_31.911549 62318-62354. Max. coverage (+): 4.12. Max coverage (-): 4.86

Region: NODE\_312079\_length\_70626\_cov\_31.911549 62355-62391. Max. coverage (+): 0.59. Max coverage (-): 0.41

Region: NODE\_312079\_length\_70626\_cov\_31.911549 62392-62428. Max. coverage (+): 9.27. Max coverage (-): 0.7

Region: NODE\_312079\_length\_70626\_cov\_31.911549 62429-62465. Max. coverage (+): 0.15. Max coverage (-): 0.22

Region: NODE\_312079\_length\_70626\_cov\_31.911549 62466-62502. Max. coverage (+): 0.63. Max coverage (-): 0.11

Region: NODE\_312079\_length\_70626\_cov\_31.911549 62503-62538. Max. coverage (+): 0.67. Max coverage (-): 0.11

Region: NODE\_312079\_length\_70626\_cov\_31.911549 62539-62575. Max. coverage (+): 0.37. Max coverage (-): 0.26

Region: NODE\_312079\_length\_70626\_cov\_31.911549 62576-62612. Max. coverage (+): 0.44. Max coverage (-): 0.11

Region: NODE\_312079\_length\_70626\_cov\_31.911549 62613-62649. Max. coverage (+): 0. Max coverage (-): 0

Region: NODE\_312079\_length\_70626\_cov\_31.911549 62650-62686. Max. coverage (+): 0.33. Max coverage (-): 0.67

Region: NODE\_312079\_length\_70626\_cov\_31.911549 62687-62723. Max. coverage (+): 0.22. Max coverage (-): 0.04

Region: NODE\_312079\_length\_70626\_cov\_31.911549 62724-62759. Max. coverage (+): 0.22. Max coverage (-): 0.04

Region: NODE\_312079\_length\_70626\_cov\_31.911549 62760-62796. Max. coverage (+): 0.22. Max coverage (-): 0.07

Region: NODE\_312079\_length\_70626\_cov\_31.911549 62797-62833. Max. coverage (+): 1.41. Max coverage (-): 0.07

Region: NODE\_312079\_length\_70626\_cov\_31.911549 62834-62870. Max. coverage (+): 2.82. Max coverage (-): 0.7

Region: NODE\_312079\_length\_70626\_cov\_31.911549 62871-62907. Max. coverage (+): 2.11. Max coverage (-): 0.19

Region: NODE\_312079\_length\_70626\_cov\_31.911549 62908-62944. Max. coverage (+): 0.22. Max coverage (-): 0.37

Region: NODE\_312079\_length\_70626\_cov\_31.911549 62945-62981. Max. coverage (+): 0.82. Max coverage (-): 1.37

Region: NODE\_312079\_length\_70626\_cov\_31.911549 62982-63017. Max. coverage (+): 0.7. Max coverage (-): 0.04

Region: NODE\_312079\_length\_70626\_cov\_31.911549 63018-63054. Max. coverage (+): 0.15. Max coverage (-): 0.22

Region: NODE\_312079\_length\_70626\_cov\_31.911549 63055-63091. Max. coverage (+): 1.56. Max coverage (-): 0.15

Region: NODE\_312079\_length\_70626\_cov\_31.911549 63092-63128. Max. coverage (+): 0.15. Max coverage (-): 0

Region: NODE\_312079\_length\_70626\_cov\_31.911549 63129-63165. Max. coverage (+): 0.85. Max coverage (-): 0

Region: NODE\_312079\_length\_70626\_cov\_31.911549 63166-63202. Max. coverage (+): 0.15. Max coverage (-): 0.04

Region: NODE\_312079\_length\_70626\_cov\_31.911549 63203-63239. Max. coverage (+): 6.6. Max coverage (-): 1.08

Region: NODE\_312079\_length\_70626\_cov\_31.911549 63240-63275. Max. coverage (+): 0.37. Max coverage (-): 0.07

Region: NODE\_312079\_length\_70626\_cov\_31.911549 63276-63312. Max. coverage (+): 1.15. Max coverage (-): 0.07

Region: NODE\_312079\_length\_70626\_cov\_31.911549 63313-63349. Max. coverage (+): 0.07. Max coverage (-): 0.15

Region: NODE\_312079\_length\_70626\_cov\_31.911549 63350-63386. Max. coverage (+): 4.15. Max coverage (-): 0.15

Region: NODE\_312079\_length\_70626\_cov\_31.911549 63387-63423. Max. coverage (+): 0.15. Max coverage (-): 0

Region: NODE\_312079\_length\_70626\_cov\_31.911549 63424-63460. Max. coverage (+): 0.33. Max coverage (-): 0

Region: NODE\_312079\_length\_70626\_cov\_31.911549 63461-63496. Max. coverage (+): 0. Max coverage (-): 0

Region: NODE\_312079\_length\_70626\_cov\_31.911549 63497-63533. Max. coverage (+): 0.11. Max coverage (-): 0

Region: NODE\_312079\_length\_70626\_cov\_31.911549 63534-63570. Max. coverage (+): 0.41. Max coverage (-): 0.04

Region: NODE\_312079\_length\_70626\_cov\_31.911549 63571-63607. Max. coverage (+): 0.07. Max coverage (-): 0.22

Region: NODE\_312079\_length\_70626\_cov\_31.911549 63608-63644. Max. coverage (+): 1.08. Max coverage (-): 0.11

Region: NODE\_312079\_length\_70626\_cov\_31.911549 63645-63681. Max. coverage (+): 0.11. Max coverage (-): 0

Region: NODE\_312079\_length\_70626\_cov\_31.911549 63682-63718. Max. coverage (+): 0.3. Max coverage (-): 0.11

Region: NODE\_312079\_length\_70626\_cov\_31.911549 63719-63754. Max. coverage (+): 0.07. Max coverage (-): 0

Region: NODE\_312079\_length\_70626\_cov\_31.911549 63755-63791. Max. coverage (+): 0.07. Max coverage (-): 0.07

Region: NODE\_312079\_length\_70626\_cov\_31.911549 63792-63828. Max. coverage (+): 0.22. Max coverage (-): 0.22

Region: NODE\_312079\_length\_70626\_cov\_31.911549 63829-63865. Max. coverage (+): 0.15. Max coverage (-): 0.04

Region: NODE\_312079\_length\_70626\_cov\_31.911549 63866-63902. Max. coverage (+): 0.3. Max coverage (-): 0.04

Region: NODE\_312079\_length\_70626\_cov\_31.911549 63903-63939. Max. coverage (+): 0.67. Max coverage (-): 0

Region: NODE\_312079\_length\_70626\_cov\_31.911549 63940-63976. Max. coverage (+): 0.33. Max coverage (-): 0

Region: NODE\_312079\_length\_70626\_cov\_31.911549 63977-64012. Max. coverage (+): 0.33. Max coverage (-): 0.07

Region: NODE\_312079\_length\_70626\_cov\_31.911549 64013-64049. Max. coverage (+): 0.07. Max coverage (-): 0.04

Region: NODE\_312079\_length\_70626\_cov\_31.911549 64050-64086. Max. coverage (+): 0.19. Max coverage (-): 0.15

Region: NODE\_312079\_length\_70626\_cov\_31.911549 64087-64123. Max. coverage (+): 0.15. Max coverage (-): 0.07

Region: NODE\_312079\_length\_70626\_cov\_31.911549 64124-64160. Max. coverage (+): 0.56. Max coverage (-): 0

Region: NODE\_312079\_length\_70626\_cov\_31.911549 64161-64197. Max. coverage (+): 0.63. Max coverage (-): 0.04

Region: NODE\_312079\_length\_70626\_cov\_31.911549 64198-64233. Max. coverage (+): 0.07. Max coverage (-): 0.04

Region: NODE\_312079\_length\_70626\_cov\_31.911549 64234-64270. Max. coverage (+): 0.19. Max coverage (-): 0.04

Region: NODE\_312079\_length\_70626\_cov\_31.911549 64271-64307. Max. coverage (+): 0. Max coverage (-): 0

Region: NODE\_312079\_length\_70626\_cov\_31.911549 64308-64344. Max. coverage (+): 0. Max coverage (-): 0

Region: NODE\_312079\_length\_70626\_cov\_31.911549 64345-64381. Max. coverage (+): 0.15. Max coverage (-): 0

Region: NODE\_312079\_length\_70626\_cov\_31.911549 64382-64418. Max. coverage (+): 0.19. Max coverage (-): 0

Region: NODE\_312079\_length\_70626\_cov\_31.911549 64419-64455. Max. coverage (+): 1.22. Max coverage (-): 0.04

Region: NODE\_312079\_length\_70626\_cov\_31.911549 64456-64491. Max. coverage (+): 0.04. Max coverage (-): 0

Region: NODE\_312079\_length\_70626\_cov\_31.911549 64492-64528. Max. coverage (+): 0. Max coverage (-): 0

Region: NODE\_312079\_length\_70626\_cov\_31.911549 64529-64565. Max. coverage (+): 1.11. Max coverage (-): 0

Region: NODE\_312079\_length\_70626\_cov\_31.911549 64566-64602. Max. coverage (+): 0.44. Max coverage (-): 0.04

Region: NODE\_312079\_length\_70626\_cov\_31.911549 64603-64639. Max. coverage (+): 0.04. Max coverage (-): 0.04

Region: NODE\_312079\_length\_70626\_cov\_31.911549 64640-64676. Max. coverage (+): 0.11. Max coverage (-): 0

Region: NODE\_312079\_length\_70626\_cov\_31.911549 64677-64713. Max. coverage (+): 0. Max coverage (-): 0.04

Region: NODE\_312079\_length\_70626\_cov\_31.911549 64714-64749. Max. coverage (+): 0. Max coverage (-): 0.04

Region: NODE\_312079\_length\_70626\_cov\_31.911549 64750-64786. Max. coverage (+): 0. Max coverage (-): 0

Region: NODE\_312079\_length\_70626\_cov\_31.911549 64787-64823. Max. coverage (+): 0.01. Max coverage (-): 0

Region: NODE\_312079\_length\_70626\_cov\_31.911549 64824-. Max. coverage (+): 0. Max coverage (-): 0

RepeatMasker Color Code

**+**

100-98% Identity

<98-95% Identity

<95-90% Identity

<90-85% Identity

<85-80% Identity

<80-75% Identity

<75-70% Identity

<70% Identity

**-**

Gene Set Color Code

**+**

Gene

Pseudogene

Other

**-**

Topology/Coverage Color Code

Coverage Plus Strand

Coverage Minus Strand

Mainstrand: Plus

Mainstrand: Minus

Complementary Strand

Flanking Region  
(if option -flank >0)

Gene Set Annotation  
  
RepeatMasker Annotation  

**1. AlRepC-136**: 48775-48887 (+), Divergence to consensus: 6.3%  
**2. AlRepB-2**: 48874-49130 (+), Divergence to consensus: 15.5%  
**3. Penelope-1\_AFC**: 51503-51545 (+), Divergence to consensus: 13.9%  
**4. REX1-1\_AFC**: 53635-53855 (+), Divergence to consensus: 8.8%  
**5. A-rich**: 55236-55267 (+), Divergence to consensus: 16.9%  
**6. AlRepB-1625**: 55750-55947 (-), Divergence to consensus: 13.2%  
**7. AlRepD-4675**: 55953-56014 (-), Divergence to consensus: 1.6%  
**8. Kolobok-2\_XT**: 58381-58424 (-), Divergence to consensus: 13.6%  
**9. AlRepB-438**: 58384-58678 (-), Divergence to consensus: 18.6%  
**10. Kolobok-2\_XT**: 58630-58682 (-), Divergence to consensus: 19.2%  
**11. TC1\_FR3**: 59601-59783 (-), Divergence to consensus: 28.5%  
**12. (TAGCTT)n**: 59921-59961 (+), Divergence to consensus: 20.4%  
**13. AlRepD-5020**: 62546-62959 (-), Divergence to consensus: 38.3%  
**14. AlRepE-1134**: 63069-63243 (-), Divergence to consensus: 38%  
**15. AlRepD-1895**: 63282-63336 (+), Divergence to consensus: 18.6%  
**16. AlRepA-4**: 64070-64295 (+), Divergence to consensus: 29.3%  
**17. AlRepA-4**: 64408-64557 (+), Divergence to consensus: 38.6%  
**18. Tc1-2\_FR**: 64752-64806 (+), Divergence to consensus: 11%  
**19. Tc1-2\_FR**: 64807-64848 (-), Divergence to consensus: 2.4%

  
Transcription Factor Binding Sites  

**RHOXF1** (Sequence: AGCTCA (-): 46494)  
**RHOXF1** (Sequence: AGCTTA (-): 46595)  
**RHOXF1** (Sequence: AGCTCA (-): 46823)  
**RHOXF1** (Sequence: GGCTTA (-): 47517)  
**RHOXF1** (Sequence: AGATTA (-): 48723)  
**RHOXF1** (Sequence: AGATCA (-): 49206)  
**RHOXF1** (Sequence: GGCTTA (-): 49673)  
**RHOXF1** (Sequence: AGATCA (-): 50444)  
**RHOXF1** (Sequence: AGATCA (-): 51400)  
**RHOXF1** (Sequence: AGATTA (-): 52974)  
**RHOXF1** (Sequence: GGCTCA (-): 53625)  
**RHOXF1** (Sequence: AGATTA (-): 54742)  
**RHOXF1** (Sequence: GGATTA (-): 55063)  
**RHOXF1** (Sequence: GGCTTA (-): 56243)  
**RHOXF1** (Sequence: AGCTTA (-): 56989)  
**RHOXF1** (Sequence: AGCTTA (-): 57604)  
**RHOXF1** (Sequence: GGATTA (-): 57921)  
**RHOXF1** (Sequence: AGCTTA (-): 58919)  
**RHOXF1** (Sequence: AGATCA (-): 58921)  
**RHOXF1** (Sequence: AGATCA (-): 59452)  
**RHOXF1** (Sequence: AGATCA (-): 59465)  
**RHOXF1** (Sequence: AGCTCA (-): 59751)  
**RHOXF1** (Sequence: AGATCA (-): 59867)  
**RHOXF1** (Sequence: AGATTA (-): 60359)  
**RHOXF1** (Sequence: GGATCA (-): 60806)  
**RHOXF1** (Sequence: AGCTCA (-): 60858)  
**RHOXF1** (Sequence: AGATTA (-): 61136)  
**RHOXF1** (Sequence: AGCTCA (-): 61232)  
**RHOXF1** (Sequence: AGATCA (-): 61267)  
**RHOXF1** (Sequence: GGCTCA (-): 61610)  
**RHOXF1** (Sequence: AGATTA (-): 61667)  
**RHOXF1** (Sequence: AGATTA (-): 63254)  
**RHOXF1** (Sequence: AGATCA (-): 64071)  
**RHOXF1** (Sequence: AGATTA (-): 64527)  
**RHOXF1** (Sequence: TGAGCT (+): 48263)  
**RHOXF1** (Sequence: TGATCT (+): 48316)  
**RHOXF1** (Sequence: TGATCT (+): 48466)  
**RHOXF1** (Sequence: TAATCC (+): 48707)  
**RHOXF1** (Sequence: TAATCC (+): 48863)  
**RHOXF1** (Sequence: TAATCT (+): 50194)  
**RHOXF1** (Sequence: TGATCT (+): 50573)  
**RHOXF1** (Sequence: TGAGCC (+): 50874)  
**RHOXF1** (Sequence: TGATCT (+): 51728)  
**RHOXF1** (Sequence: TGATCT (+): 51994)  
**RHOXF1** (Sequence: TGAGCT (+): 52771)  
**RHOXF1** (Sequence: TAATCC (+): 53021)  
**RHOXF1** (Sequence: TGATCT (+): 53431)  
**RHOXF1** (Sequence: TAATCT (+): 54051)  
**RHOXF1** (Sequence: TAATCC (+): 54057)  
**RHOXF1** (Sequence: TAATCT (+): 54464)  
**RHOXF1** (Sequence: TAAGCT (+): 55205)  
**RHOXF1** (Sequence: TGATCT (+): 55553)  
**RHOXF1** (Sequence: TAATCT (+): 55634)  
**RHOXF1** (Sequence: TAAGCC (+): 55931)  
**RHOXF1** (Sequence: TGATCT (+): 57130)  
**RHOXF1** (Sequence: TAAGCT (+): 57602)  
**RHOXF1** (Sequence: TAAGCC (+): 57807)  
**RHOXF1** (Sequence: TAATCT (+): 60727)  
**RHOXF1** (Sequence: TGAGCT (+): 60929)  
**RHOXF1** (Sequence: TGAGCC (+): 61078)  
**RHOXF1** (Sequence: TGAGCT (+): 61253)  
**RHOXF1** (Sequence: TGATCC (+): 61736)  
**RHOXF1** (Sequence: TAATCT (+): 62644)  
**RHOXF1** (Sequence: TAAGCC (+): 62890)  
**RHOXF1** (Sequence: TGATCT (+): 63009)  
**RHOXF1** (Sequence: TGAGCT (+): 63096)  
**RHOXF1** (Sequence: TGAGCC (+): 64536)  
**Lhx8** (Sequence: CTAATTAA (-): 46875)  
**Lhx8** (Sequence: CTAATTAA (-): 47761)  
**Lhx8** (Sequence: CTAATTAG (-): 48523)  
**Lhx8** (Sequence: TTAATTAG (-): 53574)  
**Lhx8** (Sequence: TTAATTAA (-): 56181)  
**Lhx8** (Sequence: TTAATTAA (-): 56752)  
**Gata4** (Sequence: CTTATCT (+): 53174)  
**Gata4** (Sequence: GTTATCT (+): 62969)  
**POU5F1** (Sequence: TTTGCAT (-): 47800)  
**POU5F1** (Sequence: TTTGCAT (-): 61418)  
**FOXO3\_hsa** (Sequence: GTAAACAA (+): 50467)  
**FOXO3\_hsa** (Sequence: GTAAACAT (+): 54294)  
**SOX9** (Sequence: AACAATAG (-): 52108)  
**SOX9** (Sequence: AACAATGA (-): 53204)  
**SOX9** (Sequence: AACAATAA (-): 55880)  
**FOXP1** (Sequence: GTAAACA (+): 50467)  
**FOXP1** (Sequence: GTAAACA (+): 54294)  
**FOXO1** (Sequence: CTTGTTTAT (+): 51586)  
**FOXO1** (Sequence: CTTGTTTTT (+): 55194)  
**FOXO1** (Sequence: GCTGTTTTT (+): 57253)  
**FOXO1** (Sequence: CTTGTTTAT (+): 58800)  
**FOXO3\_mmu** (Sequence: TGTTTTGC (-): 49410)  
**FOXO3\_mmu** (Sequence: TGTTTTCC (-): 50243)  
**FOXO3\_mmu** (Sequence: TGTTTTCC (-): 50983)  
**FOXO3\_mmu** (Sequence: TGTTTACA (-): 52703)  
**FOXO3\_mmu** (Sequence: TGTTTTCA (-): 53607)  
**FOXO3\_mmu** (Sequence: TGTTTTGC (-): 56926)  
**FOXO3\_mmu** (Sequence: TGTTTTGA (-): 57165)  
**FOXO3\_mmu** (Sequence: TGTTTACA (-): 57723)  
**FOXO3\_mmu** (Sequence: TGTTTAGA (-): 59040)  
**FOXO3\_mmu** (Sequence: TGTTTTGA (-): 62152)  
**FOXO3\_mmu** (Sequence: TGTTTTCA (-): 62541)  
**Sox5** (Sequence: ATTGTT (+): 46648)  
**Sox5** (Sequence: ATTGTT (+): 46866)  
**Sox5** (Sequence: ATTGTT (+): 48701)  
**Sox5** (Sequence: ATTGTT (+): 52525)  
**Sox5** (Sequence: ATTGTT (+): 56584)  
**Sox5** (Sequence: ATTGTT (+): 56800)  
**Sox5** (Sequence: ATTGTT (+): 57586)  
**Sox5** (Sequence: ATTGTT (+): 57588)  
**Sox5** (Sequence: ATTGTT (+): 59562)  
**Sox5** (Sequence: ATTGTT (+): 63194)  
**Sox5** (Sequence: ATTGTT (+): 63467)  
**Sox5** (Sequence: ATTGTT (+): 64714)  
**FIGLA** (Sequence: TACAGCTGGT (-): 50475)  
**FIGLA** (Sequence: TACAGCTGGA (-): 52139)  
**FIGLA** (Sequence: AACACCTGGA (-): 53227)  
**FIGLA** (Sequence: ACCACCTGTA (-): 54307)  
**FIGLA** (Sequence: TCCAGCTGTA (-): 62345)  
**FOXO3\_mmu** (Sequence: TGAAAACA (+): 49715)  
**FOXO3\_mmu** (Sequence: GGAAAACA (+): 51362)  
**FOXO3\_mmu** (Sequence: TGTAAACA (+): 54293)  
**FOXO3\_mmu** (Sequence: TGAAAACA (+): 55813)  
**FOXO3\_mmu** (Sequence: GGAAAACA (+): 63920)  
**Nobox** (Sequence: GCTAATTA (-): 48522)  
**FOXO1** (Sequence: AAAAACAGC (-): 48653)  
**FOXO1** (Sequence: AAAAACAAC (-): 62753)  
**FOXO3\_hsa** (Sequence: ATGTTTAC (-): 52702)  
**FOXO3\_hsa** (Sequence: ATGTTTAC (-): 57722)  
**FOXP1** (Sequence: TGTTTAC (-): 52703)  
**FOXP1** (Sequence: TGTTTAC (-): 57723)  
**Nobox** (Sequence: TAATTAGC (+): 53575)  
**Nobox** (Sequence: TAATTGCT (+): 59882)  
**POU2F1** (Sequence: ATTAAAATA (-): 60583)  
**POU2F1** (Sequence: ATTAAAATA (-): 60825)  
**Rhox11** (Sequence: TGCTGTATT (+): 47659)  
**Rhox11** (Sequence: TGCTGTAAT (+): 50365)  
**Rhox11** (Sequence: CGCTGTTTT (+): 57252)  
**Rhox11** (Sequence: TGCTGTAAA (+): 58672)  
**Rhox11** (Sequence: TTAACACCA (-): 49893)  
**Rhox11** (Sequence: AAAACACCA (-): 54863)  
**Gata4** (Sequence: AGATAAC (-): 49556)  
**Gata4** (Sequence: AGATAAC (-): 51421)  
**Sox5** (Sequence: AACAAT (-): 49560)  
**Sox5** (Sequence: AACAAT (-): 50032)  
**Sox5** (Sequence: AACAAT (-): 50058)  
**Sox5** (Sequence: AACAAT (-): 50470)  
**Sox5** (Sequence: AACAAT (-): 51279)  
**Sox5** (Sequence: AACAAT (-): 51488)  
**Sox5** (Sequence: AACAAT (-): 51967)  
**Sox5** (Sequence: AACAAT (-): 52108)  
**Sox5** (Sequence: AACAAT (-): 52763)  
**Sox5** (Sequence: AACAAT (-): 53204)  
**Sox5** (Sequence: AACAAT (-): 55111)  
**Sox5** (Sequence: AACAAT (-): 55530)  
**Sox5** (Sequence: AACAAT (-): 55880)  
**Sox5** (Sequence: AACAAT (-): 56079)  
**Sox5** (Sequence: AACAAT (-): 56719)  
**Sox5** (Sequence: AACAAT (-): 57038)  
**Sox5** (Sequence: AACAAT (-): 61906)  
**Sox5** (Sequence: AACAAT (-): 63239)  
**Sox5** (Sequence: AACAAT (-): 63924)  
**Sox5** (Sequence: AACAAT (-): 64274)  
**POU2F1** (Sequence: TATGTAAAT (+): 50071)  
**POU2F1** (Sequence: TATTTTAAT (+): 53570)  
**POU2F1** (Sequence: TATTTAAAT (+): 64179)  
**POU5F1** (Sequence: ATGCAAA (+): 51795)  
**POU5F1** (Sequence: ATGCAAA (+): 55534)  
**POU5F1** (Sequence: ATGCAAA (+): 63539)
