# Supplementary material for: First characterization of PIWI-interacting RNA clusters in a cichlid fish with a B chromosome
Source: BMC Biol. 2022 Sep 21;20:204. doi: 10.1186/s12915-022-01403-2 (PMC9490952; doi:10.1186/s12915-022-01403-2)
Supplement: Supplementary file 1 — Additional file 1. Zipped folder with fasta and interactive html piRNA cluster information for the A. latifasciata genome. The nomenclature is as follows: number-pirna-cluster_sex_B-presence (f, female; m, male; 0b, without B chromosome; 1b, with B chromosome). [file 12915_2022_1403_MOESM1_ESM.zip › 122_m0b.html]

piRNA cluster 122\_m0b 58


Predicted piRNA cluster no. 122\_m0b
  

Show proTRAC run info
Hide proTRAC run info

/\  
                \_\_\_\_\_\_\_\_\_\_\_\_\_\_\_\_\_\_\_\_\_\_\_/\\_\_\_ /  \\_\_\_\_\_\_\_  
               I                      /  \  /    \      I  
               I     pro             /    \/      \     I  
               I        TRAC        /               \   I  
               I   \_\_\_\_\_\_\_\_\_\_\_\_\_\_\_\_/\_\_\_\_\_\_\_\_\_\_\_\_\_\_\_\_\_\\_ I  
               I   \              /                     I  
               I    \            /                      I  
               I     \  /\      /       V.2.4.2         I  
               I      \/  \    /                        I  
               I\_\_\_\_\_\_\_\_\_\_\_\  /\_\_\_\_\_\_\_\_\_\_\_\_\_\_\_\_\_\_\_\_\_\_\_\_\_I  
                            \/  
  
  
================================= proTRAC ====================================  
VERSION: .......... 2.4.2  
LAST MODIFIED: .... 11. May 2018  
  
Please cite:  
Rosenkranz D, Zischler H. proTRAC - a software for probabilistic piRNA cluster  
detection, visualization and analysis. 2012. BMC Bioinformatics 13:5.  
  
  
Contact:  
David Rosenkranz  
Institute of Organismic and Molecular Evolutionary Biology  
Dept. Anthropology, small RNA group  
Johannes Gutenberg University Mainz  
email: rosenkranz@uni-mainz.de  
  
You can find the latest proTRAC version at:  
http://sourceforge.net/projects/protrac/files  
http://www.smallRNAgroup-mainz.de/software  
==============================================================================  
  
PARAMETERS:  
Map file: ...............piwi-machos-0B.fa-collapse.map  
Genome file: ............../../../0B\_ala\_genome.fa  
RepeatMasker annotation: Alatifasciata-all0B-maryan-v2.fa\_corrected.out  
GeneSet:................./guest-storage/Data/annotation/Alatifasciata\_all0B\_maryan-v2\_out2017.gff  
  
Significant (p<=0.01) hit density will be calculated based  
on observed hit distribution.  
  
Sliding window size: ........................................ 5000 bp  
Sliding window increament: .................................. 1000 bp  
Normalize each hit by number of genomic hits: ............... yes  
Normalize each hit by number of sequence reads: ............. yes  
Normalize values (-> per million mapped reads): ............. yes  
Min. fraction of hits with 1T(U) or 10A: .................... 0.75  
Alternatively: Min. fraction of hits with 1T(U) and 10A: .... 0.5  
Min. fraction of hits with typical piRNA length: ............ 0.75  
Typical piRNA length: ....................................... 24-32 nt  
Min. size of a piRNA cluster: ............................... 1000 bp.  
Min. number of hits (absolute): ............................. 0  
Min. number of hits (normalized): ........................... 0  
Min. fraction of hits on the mainstrand: .................... 0.75  
Top fraction of mapped sequences (in terms of read counts): . 1%  
Top fraction accounts for max. n% of sequence reads: ........ 90%  
Min. fraction of hits on each arm of a bidirectional cluster: 0.05  
Output html file for each cluster: .......................... yes  
Output a summary table: ..................................... yes  
Output a FASTA file for each cluster (piRNA sequences): ..... yes  
Output a FASTA file comprising cluster sequences: ........... yes  
Output a GTF file for predicted piRNA clusters: ..............yes  
Search DNA motifs in clusters: .............................. yes  
Output flanking sequences: +/- .............................. 0 bp  
Output ~.pTi file: .......................................... no  
==============================================================================  
  
  
Genome size (without gaps): ............ 758543724 bp  
Gaps (N/X/-): .......................... 417479 bp  
Mapped reads: .......................... 24765598  
Non-identical sequences: ............... 6158275  
Genomic hits: .......................... 53103584  
Significant densitiy of mapped reads: .. 763.098963422187 reads/kb

Show proTRAC cluster info
Hide proTRAC cluster info

|  |  |
| --- | --- |
| Location | NODE\_315754\_length\_34971\_cov\_28.695518 |
| Coordinates | 23238-30023 |
| Size [bp] | 6786 |
| Sequence hit loci | 1770 |
| Mapped reads (normalized) | 4197.3 |
| Mapped reads (normalized) per kb | 618.5 |
| Normalized reads with 1T (1U) | 82.6% |
| Normalized reads with 10A | 39% |
| Normalized reads with length 24-32 nt | 98.9% |
| Normalized reads on the main strand(s) | 90.1% |
| Predicted directionality | mono:plus |

100%

0%

1T (1U)  
reads

10A reads

24-32 nt  
reads

reads on mainstrand

**Either the amount of reads with 1T (1U) OR 10A has to exceed 75% (set with option: -1Tor10A)  
Alternatively the amount of reads with 1T (1U) AND 10A has to exceed 50% (set with option: -1Tand10A)  
Minimum amount of reads with preferred size is 75% (set with option: -pisize)  
Minimum amount of reads on the main strand(s) is 75% (set with option: -clstrand)**

Show read coverage
Hide read coverage

WHAT DO I SEE HERE?  
This chart shows the location of mapped sequence reads within a predicted piRNA cluster. The color refers to the number of genomic hits produced by the sequence read in question. A dark red bar indicates that this sequence read produces many other hits elsewhere in the genome. Many adjacent red or yellow bars can indicate the presence of a multi-copy element such as transposons or rRNA genes. A dark green bar indicates that this sequence read maps uniquely to this locus.

1 hit

2-5 hits

6-10 hits

11-20 hits

21-50 hits

51-100 hits

> 100 hits

NODE\_315754\_length\_34971\_cov\_28.695518

23238

30023

Gene Set

RepeatMasker

Mapped  
Reads

50.96

plus strand

minus strand

50.96

Region: NODE\_315754\_length\_34971\_cov\_28.695518 65973-23244. Max. coverage (+): 0. Max coverage (-): 0.04

Region: NODE\_315754\_length\_34971\_cov\_28.695518 23245-23258. Max. coverage (+): 0. Max coverage (-): 0

Region: NODE\_315754\_length\_34971\_cov\_28.695518 23259-23271. Max. coverage (+): 0. Max coverage (-): 0

Region: NODE\_315754\_length\_34971\_cov\_28.695518 23272-23285. Max. coverage (+): 0. Max coverage (-): 0

Region: NODE\_315754\_length\_34971\_cov\_28.695518 23286-23299. Max. coverage (+): 0. Max coverage (-): 0

Region: NODE\_315754\_length\_34971\_cov\_28.695518 23300-23312. Max. coverage (+): 0. Max coverage (-): 0

Region: NODE\_315754\_length\_34971\_cov\_28.695518 23313-23326. Max. coverage (+): 0. Max coverage (-): 0

Region: NODE\_315754\_length\_34971\_cov\_28.695518 23327-23339. Max. coverage (+): 0. Max coverage (-): 0

Region: NODE\_315754\_length\_34971\_cov\_28.695518 23340-23353. Max. coverage (+): 0. Max coverage (-): 0

Region: NODE\_315754\_length\_34971\_cov\_28.695518 23354-23366. Max. coverage (+): 0. Max coverage (-): 0

Region: NODE\_315754\_length\_34971\_cov\_28.695518 23367-23380. Max. coverage (+): 0. Max coverage (-): 0

Region: NODE\_315754\_length\_34971\_cov\_28.695518 23381-23394. Max. coverage (+): 0. Max coverage (-): 0

Region: NODE\_315754\_length\_34971\_cov\_28.695518 23395-23407. Max. coverage (+): 0. Max coverage (-): 0

Region: NODE\_315754\_length\_34971\_cov\_28.695518 23408-23421. Max. coverage (+): 0. Max coverage (-): 0

Region: NODE\_315754\_length\_34971\_cov\_28.695518 23422-23434. Max. coverage (+): 0. Max coverage (-): 0

Region: NODE\_315754\_length\_34971\_cov\_28.695518 23435-23448. Max. coverage (+): 0. Max coverage (-): 0

Region: NODE\_315754\_length\_34971\_cov\_28.695518 23449-23461. Max. coverage (+): 0. Max coverage (-): 0

Region: NODE\_315754\_length\_34971\_cov\_28.695518 23462-23475. Max. coverage (+): 0. Max coverage (-): 0

Region: NODE\_315754\_length\_34971\_cov\_28.695518 23476-23489. Max. coverage (+): 0. Max coverage (-): 0

Region: NODE\_315754\_length\_34971\_cov\_28.695518 23490-23502. Max. coverage (+): 0. Max coverage (-): 0

Region: NODE\_315754\_length\_34971\_cov\_28.695518 23503-23516. Max. coverage (+): 0. Max coverage (-): 0

Region: NODE\_315754\_length\_34971\_cov\_28.695518 23517-23529. Max. coverage (+): 0. Max coverage (-): 0

Region: NODE\_315754\_length\_34971\_cov\_28.695518 23530-23543. Max. coverage (+): 0. Max coverage (-): 0

Region: NODE\_315754\_length\_34971\_cov\_28.695518 23544-23556. Max. coverage (+): 0. Max coverage (-): 0

Region: NODE\_315754\_length\_34971\_cov\_28.695518 23557-23570. Max. coverage (+): 0. Max coverage (-): 0

Region: NODE\_315754\_length\_34971\_cov\_28.695518 23571-23584. Max. coverage (+): 0. Max coverage (-): 0

Region: NODE\_315754\_length\_34971\_cov\_28.695518 23585-23597. Max. coverage (+): 0.01. Max coverage (-): 0

Region: NODE\_315754\_length\_34971\_cov\_28.695518 23598-23611. Max. coverage (+): 0. Max coverage (-): 0.02

Region: NODE\_315754\_length\_34971\_cov\_28.695518 23612-23624. Max. coverage (+): 0.02. Max coverage (-): 0

Region: NODE\_315754\_length\_34971\_cov\_28.695518 23625-23638. Max. coverage (+): 0. Max coverage (-): 0.01

Region: NODE\_315754\_length\_34971\_cov\_28.695518 23639-23651. Max. coverage (+): 0. Max coverage (-): 0.01

Region: NODE\_315754\_length\_34971\_cov\_28.695518 23652-23665. Max. coverage (+): 0.04. Max coverage (-): 0

Region: NODE\_315754\_length\_34971\_cov\_28.695518 23666-23679. Max. coverage (+): 0. Max coverage (-): 0

Region: NODE\_315754\_length\_34971\_cov\_28.695518 23680-23692. Max. coverage (+): 0. Max coverage (-): 0

Region: NODE\_315754\_length\_34971\_cov\_28.695518 23693-23706. Max. coverage (+): 0. Max coverage (-): 0.01

Region: NODE\_315754\_length\_34971\_cov\_28.695518 23707-23719. Max. coverage (+): 0. Max coverage (-): 0.01

Region: NODE\_315754\_length\_34971\_cov\_28.695518 23720-23733. Max. coverage (+): 0. Max coverage (-): 0

Region: NODE\_315754\_length\_34971\_cov\_28.695518 23734-23746. Max. coverage (+): 0. Max coverage (-): 0.01

Region: NODE\_315754\_length\_34971\_cov\_28.695518 23747-23760. Max. coverage (+): 0. Max coverage (-): 0.01

Region: NODE\_315754\_length\_34971\_cov\_28.695518 23761-23774. Max. coverage (+): 0. Max coverage (-): 0

Region: NODE\_315754\_length\_34971\_cov\_28.695518 23775-23787. Max. coverage (+): 0. Max coverage (-): 0.24

Region: NODE\_315754\_length\_34971\_cov\_28.695518 23788-23801. Max. coverage (+): 0.69. Max coverage (-): 0

Region: NODE\_315754\_length\_34971\_cov\_28.695518 23802-23814. Max. coverage (+): 0.04. Max coverage (-): 0

Region: NODE\_315754\_length\_34971\_cov\_28.695518 23815-23828. Max. coverage (+): 0. Max coverage (-): 0

Region: NODE\_315754\_length\_34971\_cov\_28.695518 23829-23841. Max. coverage (+): 0.02. Max coverage (-): 0

Region: NODE\_315754\_length\_34971\_cov\_28.695518 23842-23855. Max. coverage (+): 0. Max coverage (-): 0

Region: NODE\_315754\_length\_34971\_cov\_28.695518 23856-23869. Max. coverage (+): 0. Max coverage (-): 0.01

Region: NODE\_315754\_length\_34971\_cov\_28.695518 23870-23882. Max. coverage (+): 0. Max coverage (-): 0

Region: NODE\_315754\_length\_34971\_cov\_28.695518 23883-23896. Max. coverage (+): 0. Max coverage (-): 0.05

Region: NODE\_315754\_length\_34971\_cov\_28.695518 23897-23909. Max. coverage (+): 0.04. Max coverage (-): 0

Region: NODE\_315754\_length\_34971\_cov\_28.695518 23910-23923. Max. coverage (+): 0.04. Max coverage (-): 0

Region: NODE\_315754\_length\_34971\_cov\_28.695518 23924-23936. Max. coverage (+): 0. Max coverage (-): 0

Region: NODE\_315754\_length\_34971\_cov\_28.695518 23937-23950. Max. coverage (+): 0. Max coverage (-): 0.04

Region: NODE\_315754\_length\_34971\_cov\_28.695518 23951-23964. Max. coverage (+): 0. Max coverage (-): 0

Region: NODE\_315754\_length\_34971\_cov\_28.695518 23965-23977. Max. coverage (+): 0. Max coverage (-): 0

Region: NODE\_315754\_length\_34971\_cov\_28.695518 23978-23991. Max. coverage (+): 0. Max coverage (-): 0

Region: NODE\_315754\_length\_34971\_cov\_28.695518 23992-24004. Max. coverage (+): 0. Max coverage (-): 0.08

Region: NODE\_315754\_length\_34971\_cov\_28.695518 24005-24018. Max. coverage (+): 0. Max coverage (-): 0

Region: NODE\_315754\_length\_34971\_cov\_28.695518 24019-24031. Max. coverage (+): 0. Max coverage (-): 0

Region: NODE\_315754\_length\_34971\_cov\_28.695518 24032-24045. Max. coverage (+): 0. Max coverage (-): 0.06

Region: NODE\_315754\_length\_34971\_cov\_28.695518 24046-24059. Max. coverage (+): 0. Max coverage (-): 0

Region: NODE\_315754\_length\_34971\_cov\_28.695518 24060-24072. Max. coverage (+): 0. Max coverage (-): 0

Region: NODE\_315754\_length\_34971\_cov\_28.695518 24073-24086. Max. coverage (+): 0. Max coverage (-): 0

Region: NODE\_315754\_length\_34971\_cov\_28.695518 24087-24099. Max. coverage (+): 0.01. Max coverage (-): 0.01

Region: NODE\_315754\_length\_34971\_cov\_28.695518 24100-24113. Max. coverage (+): 0.01. Max coverage (-): 0.01

Region: NODE\_315754\_length\_34971\_cov\_28.695518 24114-24126. Max. coverage (+): 0. Max coverage (-): 0.01

Region: NODE\_315754\_length\_34971\_cov\_28.695518 24127-24140. Max. coverage (+): 0. Max coverage (-): 0

Region: NODE\_315754\_length\_34971\_cov\_28.695518 24141-24154. Max. coverage (+): 0. Max coverage (-): 0.01

Region: NODE\_315754\_length\_34971\_cov\_28.695518 24155-24167. Max. coverage (+): 0. Max coverage (-): 0

Region: NODE\_315754\_length\_34971\_cov\_28.695518 24168-24181. Max. coverage (+): 0. Max coverage (-): 0

Region: NODE\_315754\_length\_34971\_cov\_28.695518 24182-24194. Max. coverage (+): 0. Max coverage (-): 0

Region: NODE\_315754\_length\_34971\_cov\_28.695518 24195-24208. Max. coverage (+): 0.05. Max coverage (-): 0

Region: NODE\_315754\_length\_34971\_cov\_28.695518 24209-24221. Max. coverage (+): 0.06. Max coverage (-): 0.02

Region: NODE\_315754\_length\_34971\_cov\_28.695518 24222-24235. Max. coverage (+): 0.16. Max coverage (-): 0.01

Region: NODE\_315754\_length\_34971\_cov\_28.695518 24236-24249. Max. coverage (+): 0.17. Max coverage (-): 0

Region: NODE\_315754\_length\_34971\_cov\_28.695518 24250-24262. Max. coverage (+): 0.01. Max coverage (-): 0.02

Region: NODE\_315754\_length\_34971\_cov\_28.695518 24263-24276. Max. coverage (+): 0.01. Max coverage (-): 0.02

Region: NODE\_315754\_length\_34971\_cov\_28.695518 24277-24289. Max. coverage (+): 0. Max coverage (-): 0

Region: NODE\_315754\_length\_34971\_cov\_28.695518 24290-24303. Max. coverage (+): 0.01. Max coverage (-): 0.05

Region: NODE\_315754\_length\_34971\_cov\_28.695518 24304-24316. Max. coverage (+): 0.01. Max coverage (-): 0

Region: NODE\_315754\_length\_34971\_cov\_28.695518 24317-24330. Max. coverage (+): 0. Max coverage (-): 0

Region: NODE\_315754\_length\_34971\_cov\_28.695518 24331-24344. Max. coverage (+): 0. Max coverage (-): 0

Region: NODE\_315754\_length\_34971\_cov\_28.695518 24345-24357. Max. coverage (+): 0.11. Max coverage (-): 0

Region: NODE\_315754\_length\_34971\_cov\_28.695518 24358-24371. Max. coverage (+): 0.07. Max coverage (-): 0.04

Region: NODE\_315754\_length\_34971\_cov\_28.695518 24372-24384. Max. coverage (+): 0.01. Max coverage (-): 0.01

Region: NODE\_315754\_length\_34971\_cov\_28.695518 24385-24398. Max. coverage (+): 0.01. Max coverage (-): 0.02

Region: NODE\_315754\_length\_34971\_cov\_28.695518 24399-24411. Max. coverage (+): 0.02. Max coverage (-): 0

Region: NODE\_315754\_length\_34971\_cov\_28.695518 24412-24425. Max. coverage (+): 0.13. Max coverage (-): 0

Region: NODE\_315754\_length\_34971\_cov\_28.695518 24426-24439. Max. coverage (+): 0. Max coverage (-): 0.11

Region: NODE\_315754\_length\_34971\_cov\_28.695518 24440-24452. Max. coverage (+): 0.02. Max coverage (-): 0.08

Region: NODE\_315754\_length\_34971\_cov\_28.695518 24453-24466. Max. coverage (+): 0.02. Max coverage (-): 0

Region: NODE\_315754\_length\_34971\_cov\_28.695518 24467-24479. Max. coverage (+): 0. Max coverage (-): 0

Region: NODE\_315754\_length\_34971\_cov\_28.695518 24480-24493. Max. coverage (+): 0. Max coverage (-): 0

Region: NODE\_315754\_length\_34971\_cov\_28.695518 24494-24506. Max. coverage (+): 0.04. Max coverage (-): 0

Region: NODE\_315754\_length\_34971\_cov\_28.695518 24507-24520. Max. coverage (+): 0. Max coverage (-): 0

Region: NODE\_315754\_length\_34971\_cov\_28.695518 24521-24534. Max. coverage (+): 0.08. Max coverage (-): 0

Region: NODE\_315754\_length\_34971\_cov\_28.695518 24535-24547. Max. coverage (+): 0.08. Max coverage (-): 0

Region: NODE\_315754\_length\_34971\_cov\_28.695518 24548-24561. Max. coverage (+): 0. Max coverage (-): 0

Region: NODE\_315754\_length\_34971\_cov\_28.695518 24562-24574. Max. coverage (+): 0. Max coverage (-): 0

Region: NODE\_315754\_length\_34971\_cov\_28.695518 24575-24588. Max. coverage (+): 0. Max coverage (-): 0

Region: NODE\_315754\_length\_34971\_cov\_28.695518 24589-24601. Max. coverage (+): 0. Max coverage (-): 0.04

Region: NODE\_315754\_length\_34971\_cov\_28.695518 24602-24615. Max. coverage (+): 0.08. Max coverage (-): 0

Region: NODE\_315754\_length\_34971\_cov\_28.695518 24616-24629. Max. coverage (+): 0.28. Max coverage (-): 0

Region: NODE\_315754\_length\_34971\_cov\_28.695518 24630-24642. Max. coverage (+): 0. Max coverage (-): 0

Region: NODE\_315754\_length\_34971\_cov\_28.695518 24643-24656. Max. coverage (+): 0.48. Max coverage (-): 0

Region: NODE\_315754\_length\_34971\_cov\_28.695518 24657-24669. Max. coverage (+): 0.32. Max coverage (-): 0

Region: NODE\_315754\_length\_34971\_cov\_28.695518 24670-24683. Max. coverage (+): 0.08. Max coverage (-): 0

Region: NODE\_315754\_length\_34971\_cov\_28.695518 24684-24696. Max. coverage (+): 0.44. Max coverage (-): 0

Region: NODE\_315754\_length\_34971\_cov\_28.695518 24697-24710. Max. coverage (+): 0.44. Max coverage (-): 0

Region: NODE\_315754\_length\_34971\_cov\_28.695518 24711-24724. Max. coverage (+): 0. Max coverage (-): 0

Region: NODE\_315754\_length\_34971\_cov\_28.695518 24725-24737. Max. coverage (+): 0.04. Max coverage (-): 0

Region: NODE\_315754\_length\_34971\_cov\_28.695518 24738-24751. Max. coverage (+): 0.24. Max coverage (-): 0

Region: NODE\_315754\_length\_34971\_cov\_28.695518 24752-24764. Max. coverage (+): 0.36. Max coverage (-): 0

Region: NODE\_315754\_length\_34971\_cov\_28.695518 24765-24778. Max. coverage (+): 0. Max coverage (-): 0

Region: NODE\_315754\_length\_34971\_cov\_28.695518 24779-24791. Max. coverage (+): 0. Max coverage (-): 0

Region: NODE\_315754\_length\_34971\_cov\_28.695518 24792-24805. Max. coverage (+): 0.04. Max coverage (-): 0.08

Region: NODE\_315754\_length\_34971\_cov\_28.695518 24806-24819. Max. coverage (+): 0.36. Max coverage (-): 0.04

Region: NODE\_315754\_length\_34971\_cov\_28.695518 24820-24832. Max. coverage (+): 0. Max coverage (-): 0

Region: NODE\_315754\_length\_34971\_cov\_28.695518 24833-24846. Max. coverage (+): 0.61. Max coverage (-): 0

Region: NODE\_315754\_length\_34971\_cov\_28.695518 24847-24859. Max. coverage (+): 0.04. Max coverage (-): 0

Region: NODE\_315754\_length\_34971\_cov\_28.695518 24860-24873. Max. coverage (+): 0.32. Max coverage (-): 0

Region: NODE\_315754\_length\_34971\_cov\_28.695518 24874-24886. Max. coverage (+): 0.08. Max coverage (-): 0.08

Region: NODE\_315754\_length\_34971\_cov\_28.695518 24887-24900. Max. coverage (+): 0.16. Max coverage (-): 0.08

Region: NODE\_315754\_length\_34971\_cov\_28.695518 24901-24914. Max. coverage (+): 0. Max coverage (-): 0.08

Region: NODE\_315754\_length\_34971\_cov\_28.695518 24915-24927. Max. coverage (+): 0. Max coverage (-): 0

Region: NODE\_315754\_length\_34971\_cov\_28.695518 24928-24941. Max. coverage (+): 0.65. Max coverage (-): 0

Region: NODE\_315754\_length\_34971\_cov\_28.695518 24942-24954. Max. coverage (+): 0. Max coverage (-): 0

Region: NODE\_315754\_length\_34971\_cov\_28.695518 24955-24968. Max. coverage (+): 0.04. Max coverage (-): 0

Region: NODE\_315754\_length\_34971\_cov\_28.695518 24969-24982. Max. coverage (+): 0. Max coverage (-): 0.04

Region: NODE\_315754\_length\_34971\_cov\_28.695518 24983-24995. Max. coverage (+): 0.89. Max coverage (-): 0

Region: NODE\_315754\_length\_34971\_cov\_28.695518 24996-25009. Max. coverage (+): 0.93. Max coverage (-): 0.08

Region: NODE\_315754\_length\_34971\_cov\_28.695518 25010-25022. Max. coverage (+): 0.04. Max coverage (-): 0

Region: NODE\_315754\_length\_34971\_cov\_28.695518 25023-25036. Max. coverage (+): 0.36. Max coverage (-): 0

Region: NODE\_315754\_length\_34971\_cov\_28.695518 25037-25049. Max. coverage (+): 0. Max coverage (-): 0.4

Region: NODE\_315754\_length\_34971\_cov\_28.695518 25050-25063. Max. coverage (+): 0.89. Max coverage (-): 0.36

Region: NODE\_315754\_length\_34971\_cov\_28.695518 25064-25077. Max. coverage (+): 0.12. Max coverage (-): 0.04

Region: NODE\_315754\_length\_34971\_cov\_28.695518 25078-25090. Max. coverage (+): 0. Max coverage (-): 0.12

Region: NODE\_315754\_length\_34971\_cov\_28.695518 25091-25104. Max. coverage (+): 0.04. Max coverage (-): 0.12

Region: NODE\_315754\_length\_34971\_cov\_28.695518 25105-25117. Max. coverage (+): 1.9. Max coverage (-): 0

Region: NODE\_315754\_length\_34971\_cov\_28.695518 25118-25131. Max. coverage (+): 0. Max coverage (-): 0.08

Region: NODE\_315754\_length\_34971\_cov\_28.695518 25132-25144. Max. coverage (+): 0. Max coverage (-): 0

Region: NODE\_315754\_length\_34971\_cov\_28.695518 25145-25158. Max. coverage (+): 0.04. Max coverage (-): 0

Region: NODE\_315754\_length\_34971\_cov\_28.695518 25159-25172. Max. coverage (+): 0.04. Max coverage (-): 0

Region: NODE\_315754\_length\_34971\_cov\_28.695518 25173-25185. Max. coverage (+): 1.17. Max coverage (-): 0

Region: NODE\_315754\_length\_34971\_cov\_28.695518 25186-25199. Max. coverage (+): 0.04. Max coverage (-): 0.08

Region: NODE\_315754\_length\_34971\_cov\_28.695518 25200-25212. Max. coverage (+): 0. Max coverage (-): 0

Region: NODE\_315754\_length\_34971\_cov\_28.695518 25213-25226. Max. coverage (+): 0.04. Max coverage (-): 0.04

Region: NODE\_315754\_length\_34971\_cov\_28.695518 25227-25239. Max. coverage (+): 0.08. Max coverage (-): 0.04

Region: NODE\_315754\_length\_34971\_cov\_28.695518 25240-25253. Max. coverage (+): 1.37. Max coverage (-): 0

Region: NODE\_315754\_length\_34971\_cov\_28.695518 25254-25267. Max. coverage (+): 0.32. Max coverage (-): 0

Region: NODE\_315754\_length\_34971\_cov\_28.695518 25268-25280. Max. coverage (+): 0.32. Max coverage (-): 0

Region: NODE\_315754\_length\_34971\_cov\_28.695518 25281-25294. Max. coverage (+): 1.25. Max coverage (-): 0

Region: NODE\_315754\_length\_34971\_cov\_28.695518 25295-25307. Max. coverage (+): 1.13. Max coverage (-): 0

Region: NODE\_315754\_length\_34971\_cov\_28.695518 25308-25321. Max. coverage (+): 0.48. Max coverage (-): 0

Region: NODE\_315754\_length\_34971\_cov\_28.695518 25322-25334. Max. coverage (+): 0.08. Max coverage (-): 0

Region: NODE\_315754\_length\_34971\_cov\_28.695518 25335-25348. Max. coverage (+): 0.04. Max coverage (-): 0

Region: NODE\_315754\_length\_34971\_cov\_28.695518 25349-25362. Max. coverage (+): 0. Max coverage (-): 0

Region: NODE\_315754\_length\_34971\_cov\_28.695518 25363-25375. Max. coverage (+): 0.04. Max coverage (-): 1.41

Region: NODE\_315754\_length\_34971\_cov\_28.695518 25376-25389. Max. coverage (+): 0.44. Max coverage (-): 1.13

Region: NODE\_315754\_length\_34971\_cov\_28.695518 25390-25402. Max. coverage (+): 0.73. Max coverage (-): 0

Region: NODE\_315754\_length\_34971\_cov\_28.695518 25403-25416. Max. coverage (+): 0.04. Max coverage (-): 0

Region: NODE\_315754\_length\_34971\_cov\_28.695518 25417-25429. Max. coverage (+): 0.2. Max coverage (-): 0

Region: NODE\_315754\_length\_34971\_cov\_28.695518 25430-25443. Max. coverage (+): 50.96. Max coverage (-): 0

Region: NODE\_315754\_length\_34971\_cov\_28.695518 25444-25457. Max. coverage (+): 0.04. Max coverage (-): 0

Region: NODE\_315754\_length\_34971\_cov\_28.695518 25458-25470. Max. coverage (+): 0.04. Max coverage (-): 0

Region: NODE\_315754\_length\_34971\_cov\_28.695518 25471-25484. Max. coverage (+): 2.3. Max coverage (-): 0

Region: NODE\_315754\_length\_34971\_cov\_28.695518 25485-25497. Max. coverage (+): 0.44. Max coverage (-): 0.04

Region: NODE\_315754\_length\_34971\_cov\_28.695518 25498-25511. Max. coverage (+): 0.81. Max coverage (-): 0.04

Region: NODE\_315754\_length\_34971\_cov\_28.695518 25512-25524. Max. coverage (+): 1.33. Max coverage (-): 0

Region: NODE\_315754\_length\_34971\_cov\_28.695518 25525-25538. Max. coverage (+): 2.5. Max coverage (-): 0.16

Region: NODE\_315754\_length\_34971\_cov\_28.695518 25539-25552. Max. coverage (+): 5.57. Max coverage (-): 0.08

Region: NODE\_315754\_length\_34971\_cov\_28.695518 25553-25565. Max. coverage (+): 5.65. Max coverage (-): 0

Region: NODE\_315754\_length\_34971\_cov\_28.695518 25566-25579. Max. coverage (+): 0. Max coverage (-): 0.04

Region: NODE\_315754\_length\_34971\_cov\_28.695518 25580-25592. Max. coverage (+): 0.08. Max coverage (-): 0.08

Region: NODE\_315754\_length\_34971\_cov\_28.695518 25593-25606. Max. coverage (+): 4.36. Max coverage (-): 0.04

Region: NODE\_315754\_length\_34971\_cov\_28.695518 25607-25619. Max. coverage (+): 0.24. Max coverage (-): 0.04

Region: NODE\_315754\_length\_34971\_cov\_28.695518 25620-25633. Max. coverage (+): 1.98. Max coverage (-): 0.04

Region: NODE\_315754\_length\_34971\_cov\_28.695518 25634-25647. Max. coverage (+): 0.12. Max coverage (-): 0.04

Region: NODE\_315754\_length\_34971\_cov\_28.695518 25648-25660. Max. coverage (+): 0.2. Max coverage (-): 0

Region: NODE\_315754\_length\_34971\_cov\_28.695518 25661-25674. Max. coverage (+): 0.16. Max coverage (-): 0

Region: NODE\_315754\_length\_34971\_cov\_28.695518 25675-25687. Max. coverage (+): 0.08. Max coverage (-): 0

Region: NODE\_315754\_length\_34971\_cov\_28.695518 25688-25701. Max. coverage (+): 0.12. Max coverage (-): 0.65

Region: NODE\_315754\_length\_34971\_cov\_28.695518 25702-25714. Max. coverage (+): 0.69. Max coverage (-): 0

Region: NODE\_315754\_length\_34971\_cov\_28.695518 25715-25728. Max. coverage (+): 0.2. Max coverage (-): 0.04

Region: NODE\_315754\_length\_34971\_cov\_28.695518 25729-25742. Max. coverage (+): 0.12. Max coverage (-): 1.21

Region: NODE\_315754\_length\_34971\_cov\_28.695518 25743-25755. Max. coverage (+): 0.2. Max coverage (-): 0.16

Region: NODE\_315754\_length\_34971\_cov\_28.695518 25756-25769. Max. coverage (+): 0.12. Max coverage (-): 0

Region: NODE\_315754\_length\_34971\_cov\_28.695518 25770-25782. Max. coverage (+): 0. Max coverage (-): 0.12

Region: NODE\_315754\_length\_34971\_cov\_28.695518 25783-25796. Max. coverage (+): 0.08. Max coverage (-): 0.12

Region: NODE\_315754\_length\_34971\_cov\_28.695518 25797-25809. Max. coverage (+): 0.12. Max coverage (-): 0

Region: NODE\_315754\_length\_34971\_cov\_28.695518 25810-25823. Max. coverage (+): 0.16. Max coverage (-): 0.08

Region: NODE\_315754\_length\_34971\_cov\_28.695518 25824-25837. Max. coverage (+): 0. Max coverage (-): 0.08

Region: NODE\_315754\_length\_34971\_cov\_28.695518 25838-25850. Max. coverage (+): 0.24. Max coverage (-): 0

Region: NODE\_315754\_length\_34971\_cov\_28.695518 25851-25864. Max. coverage (+): 0. Max coverage (-): 0.48

Region: NODE\_315754\_length\_34971\_cov\_28.695518 25865-25877. Max. coverage (+): 0.16. Max coverage (-): 0.28

Region: NODE\_315754\_length\_34971\_cov\_28.695518 25878-25891. Max. coverage (+): 0.12. Max coverage (-): 0

Region: NODE\_315754\_length\_34971\_cov\_28.695518 25892-25904. Max. coverage (+): 0.16. Max coverage (-): 0.04

Region: NODE\_315754\_length\_34971\_cov\_28.695518 25905-25918. Max. coverage (+): 2.18. Max coverage (-): 0.04

Region: NODE\_315754\_length\_34971\_cov\_28.695518 25919-25932. Max. coverage (+): 1.13. Max coverage (-): 0

Region: NODE\_315754\_length\_34971\_cov\_28.695518 25933-25945. Max. coverage (+): 0.77. Max coverage (-): 0

Region: NODE\_315754\_length\_34971\_cov\_28.695518 25946-25959. Max. coverage (+): 0. Max coverage (-): 0

Region: NODE\_315754\_length\_34971\_cov\_28.695518 25960-25972. Max. coverage (+): 0. Max coverage (-): 0.04

Region: NODE\_315754\_length\_34971\_cov\_28.695518 25973-25986. Max. coverage (+): 0.28. Max coverage (-): 0.32

Region: NODE\_315754\_length\_34971\_cov\_28.695518 25987-25999. Max. coverage (+): 4.97. Max coverage (-): 0

Region: NODE\_315754\_length\_34971\_cov\_28.695518 26000-26013. Max. coverage (+): 0.12. Max coverage (-): 0

Region: NODE\_315754\_length\_34971\_cov\_28.695518 26014-26027. Max. coverage (+): 0.12. Max coverage (-): 0

Region: NODE\_315754\_length\_34971\_cov\_28.695518 26028-26040. Max. coverage (+): 0. Max coverage (-): 0

Region: NODE\_315754\_length\_34971\_cov\_28.695518 26041-26054. Max. coverage (+): 0. Max coverage (-): 0.04

Region: NODE\_315754\_length\_34971\_cov\_28.695518 26055-26067. Max. coverage (+): 0.24. Max coverage (-): 0.04

Region: NODE\_315754\_length\_34971\_cov\_28.695518 26068-26081. Max. coverage (+): 0.36. Max coverage (-): 0.04

Region: NODE\_315754\_length\_34971\_cov\_28.695518 26082-26094. Max. coverage (+): 0.08. Max coverage (-): 0

Region: NODE\_315754\_length\_34971\_cov\_28.695518 26095-26108. Max. coverage (+): 0.2. Max coverage (-): 0

Region: NODE\_315754\_length\_34971\_cov\_28.695518 26109-26122. Max. coverage (+): 0.04. Max coverage (-): 0

Region: NODE\_315754\_length\_34971\_cov\_28.695518 26123-26135. Max. coverage (+): 0.04. Max coverage (-): 0

Region: NODE\_315754\_length\_34971\_cov\_28.695518 26136-26149. Max. coverage (+): 0.16. Max coverage (-): 0

Region: NODE\_315754\_length\_34971\_cov\_28.695518 26150-26162. Max. coverage (+): 0.08. Max coverage (-): 0.04

Region: NODE\_315754\_length\_34971\_cov\_28.695518 26163-26176. Max. coverage (+): 0.04. Max coverage (-): 0.04

Region: NODE\_315754\_length\_34971\_cov\_28.695518 26177-26189. Max. coverage (+): 0. Max coverage (-): 0

Region: NODE\_315754\_length\_34971\_cov\_28.695518 26190-26203. Max. coverage (+): 0. Max coverage (-): 0

Region: NODE\_315754\_length\_34971\_cov\_28.695518 26204-26217. Max. coverage (+): 1.57. Max coverage (-): 0

Region: NODE\_315754\_length\_34971\_cov\_28.695518 26218-26230. Max. coverage (+): 0.44. Max coverage (-): 0.04

Region: NODE\_315754\_length\_34971\_cov\_28.695518 26231-26244. Max. coverage (+): 0.12. Max coverage (-): 0.04

Region: NODE\_315754\_length\_34971\_cov\_28.695518 26245-26257. Max. coverage (+): 0.28. Max coverage (-): 0

Region: NODE\_315754\_length\_34971\_cov\_28.695518 26258-26271. Max. coverage (+): 0.24. Max coverage (-): 0

Region: NODE\_315754\_length\_34971\_cov\_28.695518 26272-26284. Max. coverage (+): 1.57. Max coverage (-): 0

Region: NODE\_315754\_length\_34971\_cov\_28.695518 26285-26298. Max. coverage (+): 0.97. Max coverage (-): 0.2

Region: NODE\_315754\_length\_34971\_cov\_28.695518 26299-26312. Max. coverage (+): 1.17. Max coverage (-): 0.04

Region: NODE\_315754\_length\_34971\_cov\_28.695518 26313-26325. Max. coverage (+): 0.32. Max coverage (-): 0

Region: NODE\_315754\_length\_34971\_cov\_28.695518 26326-26339. Max. coverage (+): 0.28. Max coverage (-): 0

Region: NODE\_315754\_length\_34971\_cov\_28.695518 26340-26352. Max. coverage (+): 1.78. Max coverage (-): 0.04

Region: NODE\_315754\_length\_34971\_cov\_28.695518 26353-26366. Max. coverage (+): 0.44. Max coverage (-): 0.04

Region: NODE\_315754\_length\_34971\_cov\_28.695518 26367-26379. Max. coverage (+): 0.04. Max coverage (-): 0

Region: NODE\_315754\_length\_34971\_cov\_28.695518 26380-26393. Max. coverage (+): 0. Max coverage (-): 0.12

Region: NODE\_315754\_length\_34971\_cov\_28.695518 26394-26407. Max. coverage (+): 2.71. Max coverage (-): 0.08

Region: NODE\_315754\_length\_34971\_cov\_28.695518 26408-26420. Max. coverage (+): 0.04. Max coverage (-): 0.08

Region: NODE\_315754\_length\_34971\_cov\_28.695518 26421-26434. Max. coverage (+): 0.04. Max coverage (-): 0.04

Region: NODE\_315754\_length\_34971\_cov\_28.695518 26435-26447. Max. coverage (+): 0.08. Max coverage (-): 0.04

Region: NODE\_315754\_length\_34971\_cov\_28.695518 26448-26461. Max. coverage (+): 0.2. Max coverage (-): 0.12

Region: NODE\_315754\_length\_34971\_cov\_28.695518 26462-26474. Max. coverage (+): 0.16. Max coverage (-): 0

Region: NODE\_315754\_length\_34971\_cov\_28.695518 26475-26488. Max. coverage (+): 0.69. Max coverage (-): 0

Region: NODE\_315754\_length\_34971\_cov\_28.695518 26489-26502. Max. coverage (+): 0.73. Max coverage (-): 0

Region: NODE\_315754\_length\_34971\_cov\_28.695518 26503-26515. Max. coverage (+): 0.04. Max coverage (-): 0

Region: NODE\_315754\_length\_34971\_cov\_28.695518 26516-26529. Max. coverage (+): 2.46. Max coverage (-): 0

Region: NODE\_315754\_length\_34971\_cov\_28.695518 26530-26542. Max. coverage (+): 0.12. Max coverage (-): 0

Region: NODE\_315754\_length\_34971\_cov\_28.695518 26543-26556. Max. coverage (+): 0.04. Max coverage (-): 0.12

Region: NODE\_315754\_length\_34971\_cov\_28.695518 26557-26569. Max. coverage (+): 0.2. Max coverage (-): 0

Region: NODE\_315754\_length\_34971\_cov\_28.695518 26570-26583. Max. coverage (+): 1.37. Max coverage (-): 0

Region: NODE\_315754\_length\_34971\_cov\_28.695518 26584-26597. Max. coverage (+): 0.2. Max coverage (-): 0

Region: NODE\_315754\_length\_34971\_cov\_28.695518 26598-26610. Max. coverage (+): 0.12. Max coverage (-): 0.08

Region: NODE\_315754\_length\_34971\_cov\_28.695518 26611-26624. Max. coverage (+): 0.44. Max coverage (-): 0

Region: NODE\_315754\_length\_34971\_cov\_28.695518 26625-26637. Max. coverage (+): 0.28. Max coverage (-): 0.08

Region: NODE\_315754\_length\_34971\_cov\_28.695518 26638-26651. Max. coverage (+): 0.24. Max coverage (-): 0.08

Region: NODE\_315754\_length\_34971\_cov\_28.695518 26652-26664. Max. coverage (+): 0.28. Max coverage (-): 0

Region: NODE\_315754\_length\_34971\_cov\_28.695518 26665-26678. Max. coverage (+): 0.04. Max coverage (-): 0

Region: NODE\_315754\_length\_34971\_cov\_28.695518 26679-26692. Max. coverage (+): 0. Max coverage (-): 0

Region: NODE\_315754\_length\_34971\_cov\_28.695518 26693-26705. Max. coverage (+): 0.04. Max coverage (-): 0

Region: NODE\_315754\_length\_34971\_cov\_28.695518 26706-26719. Max. coverage (+): 0.04. Max coverage (-): 0

Region: NODE\_315754\_length\_34971\_cov\_28.695518 26720-26732. Max. coverage (+): 0. Max coverage (-): 0

Region: NODE\_315754\_length\_34971\_cov\_28.695518 26733-26746. Max. coverage (+): 0. Max coverage (-): 0

Region: NODE\_315754\_length\_34971\_cov\_28.695518 26747-26759. Max. coverage (+): 0. Max coverage (-): 0

Region: NODE\_315754\_length\_34971\_cov\_28.695518 26760-26773. Max. coverage (+): 0. Max coverage (-): 0

Region: NODE\_315754\_length\_34971\_cov\_28.695518 26774-26787. Max. coverage (+): 0.12. Max coverage (-): 0.32

Region: NODE\_315754\_length\_34971\_cov\_28.695518 26788-26800. Max. coverage (+): 0.08. Max coverage (-): 0.24

Region: NODE\_315754\_length\_34971\_cov\_28.695518 26801-26814. Max. coverage (+): 0.24. Max coverage (-): 0.12

Region: NODE\_315754\_length\_34971\_cov\_28.695518 26815-26827. Max. coverage (+): 0.16. Max coverage (-): 0

Region: NODE\_315754\_length\_34971\_cov\_28.695518 26828-26841. Max. coverage (+): 0.16. Max coverage (-): 0.04

Region: NODE\_315754\_length\_34971\_cov\_28.695518 26842-26854. Max. coverage (+): 0.36. Max coverage (-): 0.04

Region: NODE\_315754\_length\_34971\_cov\_28.695518 26855-26868. Max. coverage (+): 0.08. Max coverage (-): 0

Region: NODE\_315754\_length\_34971\_cov\_28.695518 26869-26882. Max. coverage (+): 0.32. Max coverage (-): 0.04

Region: NODE\_315754\_length\_34971\_cov\_28.695518 26883-26895. Max. coverage (+): 0.12. Max coverage (-): 0.28

Region: NODE\_315754\_length\_34971\_cov\_28.695518 26896-26909. Max. coverage (+): 0.16. Max coverage (-): 0.52

Region: NODE\_315754\_length\_34971\_cov\_28.695518 26910-26922. Max. coverage (+): 0.28. Max coverage (-): 0

Region: NODE\_315754\_length\_34971\_cov\_28.695518 26923-26936. Max. coverage (+): 0.28. Max coverage (-): 0.04

Region: NODE\_315754\_length\_34971\_cov\_28.695518 26937-26949. Max. coverage (+): 0. Max coverage (-): 0.04

Region: NODE\_315754\_length\_34971\_cov\_28.695518 26950-26963. Max. coverage (+): 0.28. Max coverage (-): 0.04

Region: NODE\_315754\_length\_34971\_cov\_28.695518 26964-26977. Max. coverage (+): 0. Max coverage (-): 0

Region: NODE\_315754\_length\_34971\_cov\_28.695518 26978-26990. Max. coverage (+): 0. Max coverage (-): 0.04

Region: NODE\_315754\_length\_34971\_cov\_28.695518 26991-27004. Max. coverage (+): 0.16. Max coverage (-): 0

Region: NODE\_315754\_length\_34971\_cov\_28.695518 27005-27017. Max. coverage (+): 0.04. Max coverage (-): 0

Region: NODE\_315754\_length\_34971\_cov\_28.695518 27018-27031. Max. coverage (+): 0.04. Max coverage (-): 0

Region: NODE\_315754\_length\_34971\_cov\_28.695518 27032-27044. Max. coverage (+): 0.4. Max coverage (-): 0

Region: NODE\_315754\_length\_34971\_cov\_28.695518 27045-27058. Max. coverage (+): 0.36. Max coverage (-): 0

Region: NODE\_315754\_length\_34971\_cov\_28.695518 27059-27072. Max. coverage (+): 0.36. Max coverage (-): 0

Region: NODE\_315754\_length\_34971\_cov\_28.695518 27073-27085. Max. coverage (+): 0.28. Max coverage (-): 0

Region: NODE\_315754\_length\_34971\_cov\_28.695518 27086-27099. Max. coverage (+): 0.08. Max coverage (-): 0.32

Region: NODE\_315754\_length\_34971\_cov\_28.695518 27100-27112. Max. coverage (+): 0.36. Max coverage (-): 0.08

Region: NODE\_315754\_length\_34971\_cov\_28.695518 27113-27126. Max. coverage (+): 0.36. Max coverage (-): 0.24

Region: NODE\_315754\_length\_34971\_cov\_28.695518 27127-27139. Max. coverage (+): 0.2. Max coverage (-): 0.32

Region: NODE\_315754\_length\_34971\_cov\_28.695518 27140-27153. Max. coverage (+): 0.61. Max coverage (-): 0.04

Region: NODE\_315754\_length\_34971\_cov\_28.695518 27154-27167. Max. coverage (+): 0.04. Max coverage (-): 0.08

Region: NODE\_315754\_length\_34971\_cov\_28.695518 27168-27180. Max. coverage (+): 0.16. Max coverage (-): 0.04

Region: NODE\_315754\_length\_34971\_cov\_28.695518 27181-27194. Max. coverage (+): 0.16. Max coverage (-): 0

Region: NODE\_315754\_length\_34971\_cov\_28.695518 27195-27207. Max. coverage (+): 0.04. Max coverage (-): 0

Region: NODE\_315754\_length\_34971\_cov\_28.695518 27208-27221. Max. coverage (+): 0.2. Max coverage (-): 0

Region: NODE\_315754\_length\_34971\_cov\_28.695518 27222-27234. Max. coverage (+): 0.16. Max coverage (-): 0

Region: NODE\_315754\_length\_34971\_cov\_28.695518 27235-27248. Max. coverage (+): 0.12. Max coverage (-): 0

Region: NODE\_315754\_length\_34971\_cov\_28.695518 27249-27262. Max. coverage (+): 0.08. Max coverage (-): 0.04

Region: NODE\_315754\_length\_34971\_cov\_28.695518 27263-27275. Max. coverage (+): 0.12. Max coverage (-): 0

Region: NODE\_315754\_length\_34971\_cov\_28.695518 27276-27289. Max. coverage (+): 0.12. Max coverage (-): 0

Region: NODE\_315754\_length\_34971\_cov\_28.695518 27290-27302. Max. coverage (+): 0.04. Max coverage (-): 0

Region: NODE\_315754\_length\_34971\_cov\_28.695518 27303-27316. Max. coverage (+): 0. Max coverage (-): 0

Region: NODE\_315754\_length\_34971\_cov\_28.695518 27317-27329. Max. coverage (+): 0. Max coverage (-): 0.04

Region: NODE\_315754\_length\_34971\_cov\_28.695518 27330-27343. Max. coverage (+): 0. Max coverage (-): 0.04

Region: NODE\_315754\_length\_34971\_cov\_28.695518 27344-27357. Max. coverage (+): 0. Max coverage (-): 0

Region: NODE\_315754\_length\_34971\_cov\_28.695518 27358-27370. Max. coverage (+): 0.08. Max coverage (-): 0

Region: NODE\_315754\_length\_34971\_cov\_28.695518 27371-27384. Max. coverage (+): 0.08. Max coverage (-): 0

Region: NODE\_315754\_length\_34971\_cov\_28.695518 27385-27397. Max. coverage (+): 0.04. Max coverage (-): 0.04

Region: NODE\_315754\_length\_34971\_cov\_28.695518 27398-27411. Max. coverage (+): 6.3. Max coverage (-): 0

Region: NODE\_315754\_length\_34971\_cov\_28.695518 27412-27424. Max. coverage (+): 0.08. Max coverage (-): 0.08

Region: NODE\_315754\_length\_34971\_cov\_28.695518 27425-27438. Max. coverage (+): 0.04. Max coverage (-): 0.2

Region: NODE\_315754\_length\_34971\_cov\_28.695518 27439-27452. Max. coverage (+): 0.12. Max coverage (-): 0.04

Region: NODE\_315754\_length\_34971\_cov\_28.695518 27453-27465. Max. coverage (+): 0. Max coverage (-): 0

Region: NODE\_315754\_length\_34971\_cov\_28.695518 27466-27479. Max. coverage (+): 0.04. Max coverage (-): 0

Region: NODE\_315754\_length\_34971\_cov\_28.695518 27480-27492. Max. coverage (+): 0.04. Max coverage (-): 0

Region: NODE\_315754\_length\_34971\_cov\_28.695518 27493-27506. Max. coverage (+): 0. Max coverage (-): 0

Region: NODE\_315754\_length\_34971\_cov\_28.695518 27507-27519. Max. coverage (+): 0.16. Max coverage (-): 0

Region: NODE\_315754\_length\_34971\_cov\_28.695518 27520-27533. Max. coverage (+): 0.16. Max coverage (-): 0

Region: NODE\_315754\_length\_34971\_cov\_28.695518 27534-27547. Max. coverage (+): 0.08. Max coverage (-): 0

Region: NODE\_315754\_length\_34971\_cov\_28.695518 27548-27560. Max. coverage (+): 0. Max coverage (-): 0

Region: NODE\_315754\_length\_34971\_cov\_28.695518 27561-27574. Max. coverage (+): 0. Max coverage (-): 0

Region: NODE\_315754\_length\_34971\_cov\_28.695518 27575-27587. Max. coverage (+): 0. Max coverage (-): 0.16

Region: NODE\_315754\_length\_34971\_cov\_28.695518 27588-27601. Max. coverage (+): 0.12. Max coverage (-): 0

Region: NODE\_315754\_length\_34971\_cov\_28.695518 27602-27614. Max. coverage (+): 0. Max coverage (-): 0.04

Region: NODE\_315754\_length\_34971\_cov\_28.695518 27615-27628. Max. coverage (+): 0. Max coverage (-): 0

Region: NODE\_315754\_length\_34971\_cov\_28.695518 27629-27642. Max. coverage (+): 0. Max coverage (-): 0

Region: NODE\_315754\_length\_34971\_cov\_28.695518 27643-27655. Max. coverage (+): 0. Max coverage (-): 0

Region: NODE\_315754\_length\_34971\_cov\_28.695518 27656-27669. Max. coverage (+): 0.04. Max coverage (-): 0

Region: NODE\_315754\_length\_34971\_cov\_28.695518 27670-27682. Max. coverage (+): 0.04. Max coverage (-): 0

Region: NODE\_315754\_length\_34971\_cov\_28.695518 27683-27696. Max. coverage (+): 0. Max coverage (-): 0

Region: NODE\_315754\_length\_34971\_cov\_28.695518 27697-27709. Max. coverage (+): 0.12. Max coverage (-): 0

Region: NODE\_315754\_length\_34971\_cov\_28.695518 27710-27723. Max. coverage (+): 0. Max coverage (-): 0

Region: NODE\_315754\_length\_34971\_cov\_28.695518 27724-27737. Max. coverage (+): 0.08. Max coverage (-): 0

Region: NODE\_315754\_length\_34971\_cov\_28.695518 27738-27750. Max. coverage (+): 0.04. Max coverage (-): 0.04

Region: NODE\_315754\_length\_34971\_cov\_28.695518 27751-27764. Max. coverage (+): 0.04. Max coverage (-): 0.04

Region: NODE\_315754\_length\_34971\_cov\_28.695518 27765-27777. Max. coverage (+): 0.04. Max coverage (-): 0

Region: NODE\_315754\_length\_34971\_cov\_28.695518 27778-27791. Max. coverage (+): 0.04. Max coverage (-): 0

Region: NODE\_315754\_length\_34971\_cov\_28.695518 27792-27804. Max. coverage (+): 0. Max coverage (-): 0.2

Region: NODE\_315754\_length\_34971\_cov\_28.695518 27805-27818. Max. coverage (+): 0.12. Max coverage (-): 0

Region: NODE\_315754\_length\_34971\_cov\_28.695518 27819-27832. Max. coverage (+): 0.16. Max coverage (-): 0

Region: NODE\_315754\_length\_34971\_cov\_28.695518 27833-27845. Max. coverage (+): 0.04. Max coverage (-): 0

Region: NODE\_315754\_length\_34971\_cov\_28.695518 27846-27859. Max. coverage (+): 0.08. Max coverage (-): 0

Region: NODE\_315754\_length\_34971\_cov\_28.695518 27860-27872. Max. coverage (+): 0.08. Max coverage (-): 0

Region: NODE\_315754\_length\_34971\_cov\_28.695518 27873-27886. Max. coverage (+): 0.57. Max coverage (-): 0

Region: NODE\_315754\_length\_34971\_cov\_28.695518 27887-27899. Max. coverage (+): 0.12. Max coverage (-): 0

Region: NODE\_315754\_length\_34971\_cov\_28.695518 27900-27913. Max. coverage (+): 0.2. Max coverage (-): 0.04

Region: NODE\_315754\_length\_34971\_cov\_28.695518 27914-27927. Max. coverage (+): 0. Max coverage (-): 0

Region: NODE\_315754\_length\_34971\_cov\_28.695518 27928-27940. Max. coverage (+): 0.04. Max coverage (-): 0

Region: NODE\_315754\_length\_34971\_cov\_28.695518 27941-27954. Max. coverage (+): 0.04. Max coverage (-): 0.04

Region: NODE\_315754\_length\_34971\_cov\_28.695518 27955-27967. Max. coverage (+): 0.04. Max coverage (-): 0

Region: NODE\_315754\_length\_34971\_cov\_28.695518 27968-27981. Max. coverage (+): 0. Max coverage (-): 0

Region: NODE\_315754\_length\_34971\_cov\_28.695518 27982-27994. Max. coverage (+): 0. Max coverage (-): 0

Region: NODE\_315754\_length\_34971\_cov\_28.695518 27995-28008. Max. coverage (+): 0.04. Max coverage (-): 0

Region: NODE\_315754\_length\_34971\_cov\_28.695518 28009-28022. Max. coverage (+): 0.04. Max coverage (-): 0

Region: NODE\_315754\_length\_34971\_cov\_28.695518 28023-28035. Max. coverage (+): 0.04. Max coverage (-): 0

Region: NODE\_315754\_length\_34971\_cov\_28.695518 28036-28049. Max. coverage (+): 0.04. Max coverage (-): 0.04

Region: NODE\_315754\_length\_34971\_cov\_28.695518 28050-28062. Max. coverage (+): 0.12. Max coverage (-): 0.04

Region: NODE\_315754\_length\_34971\_cov\_28.695518 28063-28076. Max. coverage (+): 0.08. Max coverage (-): 0

Region: NODE\_315754\_length\_34971\_cov\_28.695518 28077-28089. Max. coverage (+): 0.08. Max coverage (-): 0

Region: NODE\_315754\_length\_34971\_cov\_28.695518 28090-28103. Max. coverage (+): 0.04. Max coverage (-): 0

Region: NODE\_315754\_length\_34971\_cov\_28.695518 28104-28117. Max. coverage (+): 0. Max coverage (-): 0

Region: NODE\_315754\_length\_34971\_cov\_28.695518 28118-28130. Max. coverage (+): 0. Max coverage (-): 0

Region: NODE\_315754\_length\_34971\_cov\_28.695518 28131-28144. Max. coverage (+): 0.08. Max coverage (-): 0.04

Region: NODE\_315754\_length\_34971\_cov\_28.695518 28145-28157. Max. coverage (+): 0.08. Max coverage (-): 0

Region: NODE\_315754\_length\_34971\_cov\_28.695518 28158-28171. Max. coverage (+): 0.04. Max coverage (-): 0.08

Region: NODE\_315754\_length\_34971\_cov\_28.695518 28172-28184. Max. coverage (+): 0.08. Max coverage (-): 0

Region: NODE\_315754\_length\_34971\_cov\_28.695518 28185-28198. Max. coverage (+): 0. Max coverage (-): 0

Region: NODE\_315754\_length\_34971\_cov\_28.695518 28199-28212. Max. coverage (+): 0.08. Max coverage (-): 0.08

Region: NODE\_315754\_length\_34971\_cov\_28.695518 28213-28225. Max. coverage (+): 0. Max coverage (-): 0

Region: NODE\_315754\_length\_34971\_cov\_28.695518 28226-28239. Max. coverage (+): 0.04. Max coverage (-): 0

Region: NODE\_315754\_length\_34971\_cov\_28.695518 28240-28252. Max. coverage (+): 0.04. Max coverage (-): 0

Region: NODE\_315754\_length\_34971\_cov\_28.695518 28253-28266. Max. coverage (+): 0.04. Max coverage (-): 0

Region: NODE\_315754\_length\_34971\_cov\_28.695518 28267-28279. Max. coverage (+): 0. Max coverage (-): 0

Region: NODE\_315754\_length\_34971\_cov\_28.695518 28280-28293. Max. coverage (+): 0. Max coverage (-): 0

Region: NODE\_315754\_length\_34971\_cov\_28.695518 28294-28307. Max. coverage (+): 0.16. Max coverage (-): 0

Region: NODE\_315754\_length\_34971\_cov\_28.695518 28308-28320. Max. coverage (+): 0.16. Max coverage (-): 0

Region: NODE\_315754\_length\_34971\_cov\_28.695518 28321-28334. Max. coverage (+): 0. Max coverage (-): 0.04

Region: NODE\_315754\_length\_34971\_cov\_28.695518 28335-28347. Max. coverage (+): 0.04. Max coverage (-): 0

Region: NODE\_315754\_length\_34971\_cov\_28.695518 28348-28361. Max. coverage (+): 0.04. Max coverage (-): 0

Region: NODE\_315754\_length\_34971\_cov\_28.695518 28362-28375. Max. coverage (+): 0.04. Max coverage (-): 0

Region: NODE\_315754\_length\_34971\_cov\_28.695518 28376-28388. Max. coverage (+): 0.04. Max coverage (-): 0

Region: NODE\_315754\_length\_34971\_cov\_28.695518 28389-28402. Max. coverage (+): 0.08. Max coverage (-): 0

Region: NODE\_315754\_length\_34971\_cov\_28.695518 28403-28415. Max. coverage (+): 0. Max coverage (-): 0

Region: NODE\_315754\_length\_34971\_cov\_28.695518 28416-28429. Max. coverage (+): 0. Max coverage (-): 0

Region: NODE\_315754\_length\_34971\_cov\_28.695518 28430-28442. Max. coverage (+): 0. Max coverage (-): 0

Region: NODE\_315754\_length\_34971\_cov\_28.695518 28443-28456. Max. coverage (+): 0.12. Max coverage (-): 0

Region: NODE\_315754\_length\_34971\_cov\_28.695518 28457-28470. Max. coverage (+): 0.12. Max coverage (-): 0

Region: NODE\_315754\_length\_34971\_cov\_28.695518 28471-28483. Max. coverage (+): 0. Max coverage (-): 0

Region: NODE\_315754\_length\_34971\_cov\_28.695518 28484-28497. Max. coverage (+): 0. Max coverage (-): 0

Region: NODE\_315754\_length\_34971\_cov\_28.695518 28498-28510. Max. coverage (+): 0. Max coverage (-): 0.04

Region: NODE\_315754\_length\_34971\_cov\_28.695518 28511-28524. Max. coverage (+): 0.61. Max coverage (-): 0.04

Region: NODE\_315754\_length\_34971\_cov\_28.695518 28525-28537. Max. coverage (+): 0.08. Max coverage (-): 0

Region: NODE\_315754\_length\_34971\_cov\_28.695518 28538-28551. Max. coverage (+): 0.04. Max coverage (-): 0

Region: NODE\_315754\_length\_34971\_cov\_28.695518 28552-28565. Max. coverage (+): 0.08. Max coverage (-): 0

Region: NODE\_315754\_length\_34971\_cov\_28.695518 28566-28578. Max. coverage (+): 0.2. Max coverage (-): 0

Region: NODE\_315754\_length\_34971\_cov\_28.695518 28579-28592. Max. coverage (+): 0.2. Max coverage (-): 0

Region: NODE\_315754\_length\_34971\_cov\_28.695518 28593-28605. Max. coverage (+): 0. Max coverage (-): 0

Region: NODE\_315754\_length\_34971\_cov\_28.695518 28606-28619. Max. coverage (+): 0.08. Max coverage (-): 0

Region: NODE\_315754\_length\_34971\_cov\_28.695518 28620-28632. Max. coverage (+): 0.04. Max coverage (-): 0.08

Region: NODE\_315754\_length\_34971\_cov\_28.695518 28633-28646. Max. coverage (+): 0. Max coverage (-): 0.12

Region: NODE\_315754\_length\_34971\_cov\_28.695518 28647-28660. Max. coverage (+): 0.08. Max coverage (-): 0

Region: NODE\_315754\_length\_34971\_cov\_28.695518 28661-28673. Max. coverage (+): 0.08. Max coverage (-): 0

Region: NODE\_315754\_length\_34971\_cov\_28.695518 28674-28687. Max. coverage (+): 0.2. Max coverage (-): 0

Region: NODE\_315754\_length\_34971\_cov\_28.695518 28688-28700. Max. coverage (+): 0.04. Max coverage (-): 0.08

Region: NODE\_315754\_length\_34971\_cov\_28.695518 28701-28714. Max. coverage (+): 0.04. Max coverage (-): 0.08

Region: NODE\_315754\_length\_34971\_cov\_28.695518 28715-28727. Max. coverage (+): 0.08. Max coverage (-): 0

Region: NODE\_315754\_length\_34971\_cov\_28.695518 28728-28741. Max. coverage (+): 0.08. Max coverage (-): 0.04

Region: NODE\_315754\_length\_34971\_cov\_28.695518 28742-28755. Max. coverage (+): 0. Max coverage (-): 0.04

Region: NODE\_315754\_length\_34971\_cov\_28.695518 28756-28768. Max. coverage (+): 0.24. Max coverage (-): 0

Region: NODE\_315754\_length\_34971\_cov\_28.695518 28769-28782. Max. coverage (+): 0.04. Max coverage (-): 0

Region: NODE\_315754\_length\_34971\_cov\_28.695518 28783-28795. Max. coverage (+): 0.04. Max coverage (-): 0

Region: NODE\_315754\_length\_34971\_cov\_28.695518 28796-28809. Max. coverage (+): 0. Max coverage (-): 0

Region: NODE\_315754\_length\_34971\_cov\_28.695518 28810-28822. Max. coverage (+): 0. Max coverage (-): 0.16

Region: NODE\_315754\_length\_34971\_cov\_28.695518 28823-28836. Max. coverage (+): 0. Max coverage (-): 0.12

Region: NODE\_315754\_length\_34971\_cov\_28.695518 28837-28850. Max. coverage (+): 0. Max coverage (-): 0

Region: NODE\_315754\_length\_34971\_cov\_28.695518 28851-28863. Max. coverage (+): 0. Max coverage (-): 0

Region: NODE\_315754\_length\_34971\_cov\_28.695518 28864-28877. Max. coverage (+): 0.04. Max coverage (-): 0

Region: NODE\_315754\_length\_34971\_cov\_28.695518 28878-28890. Max. coverage (+): 0.04. Max coverage (-): 0.04

Region: NODE\_315754\_length\_34971\_cov\_28.695518 28891-28904. Max. coverage (+): 0. Max coverage (-): 0

Region: NODE\_315754\_length\_34971\_cov\_28.695518 28905-28917. Max. coverage (+): 0. Max coverage (-): 0.08

Region: NODE\_315754\_length\_34971\_cov\_28.695518 28918-28931. Max. coverage (+): 0.32. Max coverage (-): 0

Region: NODE\_315754\_length\_34971\_cov\_28.695518 28932-28945. Max. coverage (+): 0. Max coverage (-): 0.04

Region: NODE\_315754\_length\_34971\_cov\_28.695518 28946-28958. Max. coverage (+): 0. Max coverage (-): 0

Region: NODE\_315754\_length\_34971\_cov\_28.695518 28959-28972. Max. coverage (+): 0.04. Max coverage (-): 0

Region: NODE\_315754\_length\_34971\_cov\_28.695518 28973-28985. Max. coverage (+): 0. Max coverage (-): 0.08

Region: NODE\_315754\_length\_34971\_cov\_28.695518 28986-28999. Max. coverage (+): 0.12. Max coverage (-): 0

Region: NODE\_315754\_length\_34971\_cov\_28.695518 29000-29012. Max. coverage (+): 0.04. Max coverage (-): 0

Region: NODE\_315754\_length\_34971\_cov\_28.695518 29013-29026. Max. coverage (+): 0.04. Max coverage (-): 0

Region: NODE\_315754\_length\_34971\_cov\_28.695518 29027-29040. Max. coverage (+): 0.97. Max coverage (-): 0.04

Region: NODE\_315754\_length\_34971\_cov\_28.695518 29041-29053. Max. coverage (+): 0.89. Max coverage (-): 0.12

Region: NODE\_315754\_length\_34971\_cov\_28.695518 29054-29067. Max. coverage (+): 0.32. Max coverage (-): 0

Region: NODE\_315754\_length\_34971\_cov\_28.695518 29068-29080. Max. coverage (+): 0. Max coverage (-): 0

Region: NODE\_315754\_length\_34971\_cov\_28.695518 29081-29094. Max. coverage (+): 0.08. Max coverage (-): 0.04

Region: NODE\_315754\_length\_34971\_cov\_28.695518 29095-29107. Max. coverage (+): 0.08. Max coverage (-): 0.04

Region: NODE\_315754\_length\_34971\_cov\_28.695518 29108-29121. Max. coverage (+): 0. Max coverage (-): 0

Region: NODE\_315754\_length\_34971\_cov\_28.695518 29122-29135. Max. coverage (+): 0.24. Max coverage (-): 0

Region: NODE\_315754\_length\_34971\_cov\_28.695518 29136-29148. Max. coverage (+): 0. Max coverage (-): 0

Region: NODE\_315754\_length\_34971\_cov\_28.695518 29149-29162. Max. coverage (+): 0. Max coverage (-): 0.24

Region: NODE\_315754\_length\_34971\_cov\_28.695518 29163-29175. Max. coverage (+): 0.08. Max coverage (-): 0

Region: NODE\_315754\_length\_34971\_cov\_28.695518 29176-29189. Max. coverage (+): 0. Max coverage (-): 0

Region: NODE\_315754\_length\_34971\_cov\_28.695518 29190-29202. Max. coverage (+): 0.2. Max coverage (-): 0

Region: NODE\_315754\_length\_34971\_cov\_28.695518 29203-29216. Max. coverage (+): 0.57. Max coverage (-): 0

Region: NODE\_315754\_length\_34971\_cov\_28.695518 29217-29230. Max. coverage (+): 0.04. Max coverage (-): 0

Region: NODE\_315754\_length\_34971\_cov\_28.695518 29231-29243. Max. coverage (+): 0.04. Max coverage (-): 0

Region: NODE\_315754\_length\_34971\_cov\_28.695518 29244-29257. Max. coverage (+): 0.12. Max coverage (-): 0

Region: NODE\_315754\_length\_34971\_cov\_28.695518 29258-29270. Max. coverage (+): 0. Max coverage (-): 0

Region: NODE\_315754\_length\_34971\_cov\_28.695518 29271-29284. Max. coverage (+): 0.2. Max coverage (-): 0

Region: NODE\_315754\_length\_34971\_cov\_28.695518 29285-29297. Max. coverage (+): 0.12. Max coverage (-): 0

Region: NODE\_315754\_length\_34971\_cov\_28.695518 29298-29311. Max. coverage (+): 0.04. Max coverage (-): 0

Region: NODE\_315754\_length\_34971\_cov\_28.695518 29312-29325. Max. coverage (+): 0.04. Max coverage (-): 0

Region: NODE\_315754\_length\_34971\_cov\_28.695518 29326-29338. Max. coverage (+): 0.04. Max coverage (-): 0

Region: NODE\_315754\_length\_34971\_cov\_28.695518 29339-29352. Max. coverage (+): 0. Max coverage (-): 0

Region: NODE\_315754\_length\_34971\_cov\_28.695518 29353-29365. Max. coverage (+): 0.32. Max coverage (-): 0

Region: NODE\_315754\_length\_34971\_cov\_28.695518 29366-29379. Max. coverage (+): 0. Max coverage (-): 0

Region: NODE\_315754\_length\_34971\_cov\_28.695518 29380-29392. Max. coverage (+): 0. Max coverage (-): 0

Region: NODE\_315754\_length\_34971\_cov\_28.695518 29393-29406. Max. coverage (+): 0.16. Max coverage (-): 0

Region: NODE\_315754\_length\_34971\_cov\_28.695518 29407-29420. Max. coverage (+): 0.93. Max coverage (-): 0

Region: NODE\_315754\_length\_34971\_cov\_28.695518 29421-29433. Max. coverage (+): 0. Max coverage (-): 0

Region: NODE\_315754\_length\_34971\_cov\_28.695518 29434-29447. Max. coverage (+): 0. Max coverage (-): 0

Region: NODE\_315754\_length\_34971\_cov\_28.695518 29448-29460. Max. coverage (+): 0.2. Max coverage (-): 0

Region: NODE\_315754\_length\_34971\_cov\_28.695518 29461-29474. Max. coverage (+): 0. Max coverage (-): 0

Region: NODE\_315754\_length\_34971\_cov\_28.695518 29475-29487. Max. coverage (+): 0.04. Max coverage (-): 0

Region: NODE\_315754\_length\_34971\_cov\_28.695518 29488-29501. Max. coverage (+): 0.04. Max coverage (-): 0

Region: NODE\_315754\_length\_34971\_cov\_28.695518 29502-29515. Max. coverage (+): 0. Max coverage (-): 0

Region: NODE\_315754\_length\_34971\_cov\_28.695518 29516-29528. Max. coverage (+): 0. Max coverage (-): 0

Region: NODE\_315754\_length\_34971\_cov\_28.695518 29529-29542. Max. coverage (+): 0.04. Max coverage (-): 0

Region: NODE\_315754\_length\_34971\_cov\_28.695518 29543-29555. Max. coverage (+): 0. Max coverage (-): 0

Region: NODE\_315754\_length\_34971\_cov\_28.695518 29556-29569. Max. coverage (+): 0.08. Max coverage (-): 0

Region: NODE\_315754\_length\_34971\_cov\_28.695518 29570-29582. Max. coverage (+): 0. Max coverage (-): 0

Region: NODE\_315754\_length\_34971\_cov\_28.695518 29583-29596. Max. coverage (+): 0.04. Max coverage (-): 0

Region: NODE\_315754\_length\_34971\_cov\_28.695518 29597-29610. Max. coverage (+): 0.04. Max coverage (-): 0

Region: NODE\_315754\_length\_34971\_cov\_28.695518 29611-29623. Max. coverage (+): 0.04. Max coverage (-): 0

Region: NODE\_315754\_length\_34971\_cov\_28.695518 29624-29637. Max. coverage (+): 0.24. Max coverage (-): 0

Region: NODE\_315754\_length\_34971\_cov\_28.695518 29638-29650. Max. coverage (+): 0.12. Max coverage (-): 0

Region: NODE\_315754\_length\_34971\_cov\_28.695518 29651-29664. Max. coverage (+): 0. Max coverage (-): 0

Region: NODE\_315754\_length\_34971\_cov\_28.695518 29665-29677. Max. coverage (+): 0.28. Max coverage (-): 0

Region: NODE\_315754\_length\_34971\_cov\_28.695518 29678-29691. Max. coverage (+): 0.12. Max coverage (-): 0

Region: NODE\_315754\_length\_34971\_cov\_28.695518 29692-29705. Max. coverage (+): 0.08. Max coverage (-): 0

Region: NODE\_315754\_length\_34971\_cov\_28.695518 29706-29718. Max. coverage (+): 0. Max coverage (-): 0

Region: NODE\_315754\_length\_34971\_cov\_28.695518 29719-29732. Max. coverage (+): 0.48. Max coverage (-): 0

Region: NODE\_315754\_length\_34971\_cov\_28.695518 29733-29745. Max. coverage (+): 0.44. Max coverage (-): 0

Region: NODE\_315754\_length\_34971\_cov\_28.695518 29746-29759. Max. coverage (+): 0.08. Max coverage (-): 0

Region: NODE\_315754\_length\_34971\_cov\_28.695518 29760-29772. Max. coverage (+): 0. Max coverage (-): 0

Region: NODE\_315754\_length\_34971\_cov\_28.695518 29773-29786. Max. coverage (+): 0.04. Max coverage (-): 0.04

Region: NODE\_315754\_length\_34971\_cov\_28.695518 29787-29800. Max. coverage (+): 0.04. Max coverage (-): 0.12

Region: NODE\_315754\_length\_34971\_cov\_28.695518 29801-29813. Max. coverage (+): 0.04. Max coverage (-): 0.08

Region: NODE\_315754\_length\_34971\_cov\_28.695518 29814-29827. Max. coverage (+): 0. Max coverage (-): 0

Region: NODE\_315754\_length\_34971\_cov\_28.695518 29828-29840. Max. coverage (+): 0.12. Max coverage (-): 0

Region: NODE\_315754\_length\_34971\_cov\_28.695518 29841-29854. Max. coverage (+): 0.12. Max coverage (-): 0.08

Region: NODE\_315754\_length\_34971\_cov\_28.695518 29855-29867. Max. coverage (+): 0.08. Max coverage (-): 0

Region: NODE\_315754\_length\_34971\_cov\_28.695518 29868-29881. Max. coverage (+): 0. Max coverage (-): 0

Region: NODE\_315754\_length\_34971\_cov\_28.695518 29882-29895. Max. coverage (+): 0.04. Max coverage (-): 0.04

Region: NODE\_315754\_length\_34971\_cov\_28.695518 29896-29908. Max. coverage (+): 0.12. Max coverage (-): 0

Region: NODE\_315754\_length\_34971\_cov\_28.695518 29909-29922. Max. coverage (+): 0. Max coverage (-): 0.04

Region: NODE\_315754\_length\_34971\_cov\_28.695518 29923-29935. Max. coverage (+): 0. Max coverage (-): 0

Region: NODE\_315754\_length\_34971\_cov\_28.695518 29936-29949. Max. coverage (+): 0.08. Max coverage (-): 0

Region: NODE\_315754\_length\_34971\_cov\_28.695518 29950-29962. Max. coverage (+): 0. Max coverage (-): 0

Region: NODE\_315754\_length\_34971\_cov\_28.695518 29963-29976. Max. coverage (+): 0.12. Max coverage (-): 0

Region: NODE\_315754\_length\_34971\_cov\_28.695518 29977-29990. Max. coverage (+): 0. Max coverage (-): 0

Region: NODE\_315754\_length\_34971\_cov\_28.695518 29991-30003. Max. coverage (+): 0.04. Max coverage (-): 0.08

Region: NODE\_315754\_length\_34971\_cov\_28.695518 30004-30017. Max. coverage (+): 0.04. Max coverage (-): 0

Region: NODE\_315754\_length\_34971\_cov\_28.695518 30018-. Max. coverage (+): 0. Max coverage (-): 0

RepeatMasker Color Code

**+**

100-98% Identity

<98-95% Identity

<95-90% Identity

<90-85% Identity

<85-80% Identity

<80-75% Identity

<75-70% Identity

<70% Identity

**-**

Gene Set Color Code

**+**

Gene

Pseudogene

Other

**-**

Topology/Coverage Color Code

Coverage Plus Strand

Coverage Minus Strand

Mainstrand: Plus

Mainstrand: Minus

Complementary Strand

Flanking Region  
(if option -flank >0)

Gene Set Annotation  
  
RepeatMasker Annotation  

**1. AlRepB-923**: 23263-23478 (-), Divergence to consensus: 8%  
**2. AlRepB-923**: 23566-24549 (-), Divergence to consensus: 8.5%  
**3. Tc1-17a\_Xen**: 27075-27180 (-), Divergence to consensus: 36.2%  
**4. GA-rich**: 27649-27688 (+), Divergence to consensus: 17.1%  
**5. AlRepB-26**: 28410-28514 (+), Divergence to consensus: 36.4%  
**6. AlRepB-26**: 28527-28775 (-), Divergence to consensus: 41.5%  
**7. AlRepC-693**: 28657-28934 (-), Divergence to consensus: 37.9%  
**8. AlRepD-1165**: 29044-29132 (+), Divergence to consensus: 19.5%

  
Transcription Factor Binding Sites  

**RHOXF1** (Sequence: GGATCA (-): 24091)  
**RHOXF1** (Sequence: GGCTTA (-): 24364)  
**RHOXF1** (Sequence: AGATTA (-): 24874)  
**RHOXF1** (Sequence: AGCTTA (-): 25049)  
**RHOXF1** (Sequence: GGATTA (-): 25616)  
**RHOXF1** (Sequence: AGATTA (-): 25625)  
**RHOXF1** (Sequence: AGATTA (-): 25834)  
**RHOXF1** (Sequence: AGATTA (-): 25902)  
**RHOXF1** (Sequence: GGATTA (-): 26101)  
**RHOXF1** (Sequence: GGATTA (-): 26278)  
**RHOXF1** (Sequence: AGATCA (-): 26642)  
**RHOXF1** (Sequence: GGATCA (-): 26664)  
**RHOXF1** (Sequence: GGCTCA (-): 27102)  
**RHOXF1** (Sequence: AGCTTA (-): 27786)  
**RHOXF1** (Sequence: AGCTCA (-): 27893)  
**RHOXF1** (Sequence: AGCTCA (-): 28091)  
**RHOXF1** (Sequence: AGATTA (-): 29276)  
**RHOXF1** (Sequence: GGATTA (-): 29372)  
**RHOXF1** (Sequence: GGATTA (-): 29856)  
**RHOXF1** (Sequence: TAATCT (+): 23436)  
**RHOXF1** (Sequence: TAATCT (+): 23844)  
**RHOXF1** (Sequence: TGATCT (+): 24742)  
**RHOXF1** (Sequence: TAATCC (+): 25565)  
**RHOXF1** (Sequence: TAAGCT (+): 26224)  
**RHOXF1** (Sequence: TGATCT (+): 26671)  
**RHOXF1** (Sequence: TAATCT (+): 26787)  
**RHOXF1** (Sequence: TAAGCT (+): 27025)  
**RHOXF1** (Sequence: TAAGCC (+): 27256)  
**RHOXF1** (Sequence: TGATCT (+): 27632)  
**RHOXF1** (Sequence: TGAGCT (+): 27827)  
**RHOXF1** (Sequence: TGAGCT (+): 28089)  
**RHOXF1** (Sequence: TGAGCT (+): 28163)  
**RHOXF1** (Sequence: TGAGCC (+): 28264)  
**RHOXF1** (Sequence: TGAGCC (+): 29050)  
**RHOXF1** (Sequence: TAATCT (+): 29790)  
**Lhx8** (Sequence: TTAATTAA (-): 26057)  
**Lhx8** (Sequence: TTAATTAG (-): 26605)  
**Gata4** (Sequence: GTTATCT (+): 26855)  
**FOXO3\_hsa** (Sequence: GTAAACAA (+): 23413)  
**FOXO3\_hsa** (Sequence: GTAAACAT (+): 24027)  
**SOX9** (Sequence: AACAATAG (-): 29351)  
**FOXP1** (Sequence: GTAAACA (+): 23413)  
**FOXP1** (Sequence: GTAAACA (+): 24027)  
**FOXP1** (Sequence: GTAAACA (+): 24033)  
**FOXO3\_mmu** (Sequence: TGTTTACA (-): 23793)  
**FOXO3\_mmu** (Sequence: TGTTTTCA (-): 27215)  
**FOXO3\_mmu** (Sequence: TGTTTTGA (-): 28798)  
**Sox5** (Sequence: ATTGTT (+): 23791)  
**Sox5** (Sequence: ATTGTT (+): 24916)  
**Sox5** (Sequence: ATTGTT (+): 28031)  
**Sox5** (Sequence: ATTGTT (+): 28990)  
**Sox5** (Sequence: ATTGTT (+): 29176)  
**FIGLA** (Sequence: TACAGCTGGA (-): 27985)  
**SOX9** (Sequence: TTATTGTT (+): 23789)  
**SOX9** (Sequence: CCATTGTT (+): 28029)  
**SOX9** (Sequence: TCATTGTT (+): 28988)  
**FOXO3\_mmu** (Sequence: GGTAAACA (+): 24026)  
**Nobox** (Sequence: AGCAATTA (-): 29251)  
**FOXO1** (Sequence: ATAAACAGC (-): 24033)  
**FOXO1** (Sequence: AAAAACAAG (-): 27957)  
**FOXO3\_hsa** (Sequence: TTGTTTAC (-): 23792)  
**FOXP1** (Sequence: TGTTTAC (-): 23793)  
**Nobox** (Sequence: TAATTAGC (+): 26606)  
**POU2F1** (Sequence: ATTAAAATA (-): 26301)  
**Rhox11** (Sequence: AAAACACCA (-): 24775)  
**Sox5** (Sequence: AACAAT (-): 23416)  
**Sox5** (Sequence: AACAAT (-): 24852)  
**Sox5** (Sequence: AACAAT (-): 29351)  
**POU2F1** (Sequence: TATGTAAAT (+): 26127)  
**POU5F1** (Sequence: ATGCAAA (+): 26948)
