# Supplementary material for: First characterization of PIWI-interacting RNA clusters in a cichlid fish with a B chromosome
Source: BMC Biol. 2022 Sep 21;20:204. doi: 10.1186/s12915-022-01403-2 (PMC9490952; doi:10.1186/s12915-022-01403-2)
Supplement: Supplementary file 1 — Additional file 1. Zipped folder with fasta and interactive html piRNA cluster information for the A. latifasciata genome. The nomenclature is as follows: number-pirna-cluster_sex_B-presence (f, female; m, male; 0b, without B chromosome; 1b, with B chromosome). [file 12915_2022_1403_MOESM1_ESM.zip › 123_m0b.html]

piRNA cluster 123\_m0b 59


Predicted piRNA cluster no. 123\_m0b
  

Show proTRAC run info
Hide proTRAC run info

/\  
                \_\_\_\_\_\_\_\_\_\_\_\_\_\_\_\_\_\_\_\_\_\_\_/\\_\_\_ /  \\_\_\_\_\_\_\_  
               I                      /  \  /    \      I  
               I     pro             /    \/      \     I  
               I        TRAC        /               \   I  
               I   \_\_\_\_\_\_\_\_\_\_\_\_\_\_\_\_/\_\_\_\_\_\_\_\_\_\_\_\_\_\_\_\_\_\\_ I  
               I   \              /                     I  
               I    \            /                      I  
               I     \  /\      /       V.2.4.2         I  
               I      \/  \    /                        I  
               I\_\_\_\_\_\_\_\_\_\_\_\  /\_\_\_\_\_\_\_\_\_\_\_\_\_\_\_\_\_\_\_\_\_\_\_\_\_I  
                            \/  
  
  
================================= proTRAC ====================================  
VERSION: .......... 2.4.2  
LAST MODIFIED: .... 11. May 2018  
  
Please cite:  
Rosenkranz D, Zischler H. proTRAC - a software for probabilistic piRNA cluster  
detection, visualization and analysis. 2012. BMC Bioinformatics 13:5.  
  
  
Contact:  
David Rosenkranz  
Institute of Organismic and Molecular Evolutionary Biology  
Dept. Anthropology, small RNA group  
Johannes Gutenberg University Mainz  
email: rosenkranz@uni-mainz.de  
  
You can find the latest proTRAC version at:  
http://sourceforge.net/projects/protrac/files  
http://www.smallRNAgroup-mainz.de/software  
==============================================================================  
  
PARAMETERS:  
Map file: ...............piwi-machos-0B.fa-collapse.map  
Genome file: ............../../../0B\_ala\_genome.fa  
RepeatMasker annotation: Alatifasciata-all0B-maryan-v2.fa\_corrected.out  
GeneSet:................./guest-storage/Data/annotation/Alatifasciata\_all0B\_maryan-v2\_out2017.gff  
  
Significant (p<=0.01) hit density will be calculated based  
on observed hit distribution.  
  
Sliding window size: ........................................ 5000 bp  
Sliding window increament: .................................. 1000 bp  
Normalize each hit by number of genomic hits: ............... yes  
Normalize each hit by number of sequence reads: ............. yes  
Normalize values (-> per million mapped reads): ............. yes  
Min. fraction of hits with 1T(U) or 10A: .................... 0.75  
Alternatively: Min. fraction of hits with 1T(U) and 10A: .... 0.5  
Min. fraction of hits with typical piRNA length: ............ 0.75  
Typical piRNA length: ....................................... 24-32 nt  
Min. size of a piRNA cluster: ............................... 1000 bp.  
Min. number of hits (absolute): ............................. 0  
Min. number of hits (normalized): ........................... 0  
Min. fraction of hits on the mainstrand: .................... 0.75  
Top fraction of mapped sequences (in terms of read counts): . 1%  
Top fraction accounts for max. n% of sequence reads: ........ 90%  
Min. fraction of hits on each arm of a bidirectional cluster: 0.05  
Output html file for each cluster: .......................... yes  
Output a summary table: ..................................... yes  
Output a FASTA file for each cluster (piRNA sequences): ..... yes  
Output a FASTA file comprising cluster sequences: ........... yes  
Output a GTF file for predicted piRNA clusters: ..............yes  
Search DNA motifs in clusters: .............................. yes  
Output flanking sequences: +/- .............................. 0 bp  
Output ~.pTi file: .......................................... no  
==============================================================================  
  
  
Genome size (without gaps): ............ 758543724 bp  
Gaps (N/X/-): .......................... 417479 bp  
Mapped reads: .......................... 24765598  
Non-identical sequences: ............... 6158275  
Genomic hits: .......................... 53103584  
Significant densitiy of mapped reads: .. 763.098963422187 reads/kb

Show proTRAC cluster info
Hide proTRAC cluster info

|  |  |
| --- | --- |
| Location | NODE\_316089\_length\_2387\_cov\_23.258902 |
| Coordinates | 3-2450 |
| Size [bp] | 2448 |
| Sequence hit loci | 3881 |
| Mapped reads (normalized) | 21313.1 |
| Mapped reads (normalized) per kb | 8706.3 |
| Normalized reads with 1T (1U) | 80.7% |
| Normalized reads with 10A | 40.8% |
| Normalized reads with length 24-32 nt | 99.2% |
| Normalized reads on the main strand(s) | 95.4% |
| Predicted directionality | mono:plus |

100%

0%

1T (1U)  
reads

10A reads

24-32 nt  
reads

reads on mainstrand

**Either the amount of reads with 1T (1U) OR 10A has to exceed 75% (set with option: -1Tor10A)  
Alternatively the amount of reads with 1T (1U) AND 10A has to exceed 50% (set with option: -1Tand10A)  
Minimum amount of reads with preferred size is 75% (set with option: -pisize)  
Minimum amount of reads on the main strand(s) is 75% (set with option: -clstrand)**

Show read coverage
Hide read coverage

WHAT DO I SEE HERE?  
This chart shows the location of mapped sequence reads within a predicted piRNA cluster. The color refers to the number of genomic hits produced by the sequence read in question. A dark red bar indicates that this sequence read produces many other hits elsewhere in the genome. Many adjacent red or yellow bars can indicate the presence of a multi-copy element such as transposons or rRNA genes. A dark green bar indicates that this sequence read maps uniquely to this locus.

1 hit

2-5 hits

6-10 hits

11-20 hits

21-50 hits

51-100 hits

> 100 hits

NODE\_316089\_length\_2387\_cov\_23.258902

3

2450

Gene Set

RepeatMasker

Mapped  
Reads

117.36

plus strand

minus strand

117.36

Region: NODE\_316089\_length\_2387\_cov\_23.258902 30019-5. Max. coverage (+): 0. Max coverage (-): 0

Region: NODE\_316089\_length\_2387\_cov\_23.258902 6-10. Max. coverage (+): 0. Max coverage (-): 0

Region: NODE\_316089\_length\_2387\_cov\_23.258902 11-15. Max. coverage (+): 0. Max coverage (-): 0

Region: NODE\_316089\_length\_2387\_cov\_23.258902 16-20. Max. coverage (+): 0. Max coverage (-): 0

Region: NODE\_316089\_length\_2387\_cov\_23.258902 21-25. Max. coverage (+): 0. Max coverage (-): 0

Region: NODE\_316089\_length\_2387\_cov\_23.258902 26-29. Max. coverage (+): 0. Max coverage (-): 0

Region: NODE\_316089\_length\_2387\_cov\_23.258902 30-34. Max. coverage (+): 0. Max coverage (-): 0

Region: NODE\_316089\_length\_2387\_cov\_23.258902 35-39. Max. coverage (+): 0.2. Max coverage (-): 0

Region: NODE\_316089\_length\_2387\_cov\_23.258902 40-44. Max. coverage (+): 34.41. Max coverage (-): 0

Region: NODE\_316089\_length\_2387\_cov\_23.258902 45-49. Max. coverage (+): 0. Max coverage (-): 0

Region: NODE\_316089\_length\_2387\_cov\_23.258902 50-54. Max. coverage (+): 0. Max coverage (-): 0

Region: NODE\_316089\_length\_2387\_cov\_23.258902 55-59. Max. coverage (+): 0.04. Max coverage (-): 0.4

Region: NODE\_316089\_length\_2387\_cov\_23.258902 60-64. Max. coverage (+): 0.2. Max coverage (-): 0.2

Region: NODE\_316089\_length\_2387\_cov\_23.258902 65-69. Max. coverage (+): 0.04. Max coverage (-): 0.04

Region: NODE\_316089\_length\_2387\_cov\_23.258902 70-73. Max. coverage (+): 0.04. Max coverage (-): 0.04

Region: NODE\_316089\_length\_2387\_cov\_23.258902 74-78. Max. coverage (+): 0.2. Max coverage (-): 0

Region: NODE\_316089\_length\_2387\_cov\_23.258902 79-83. Max. coverage (+): 0. Max coverage (-): 0

Region: NODE\_316089\_length\_2387\_cov\_23.258902 84-88. Max. coverage (+): 0.36. Max coverage (-): 0

Region: NODE\_316089\_length\_2387\_cov\_23.258902 89-93. Max. coverage (+): 0.12. Max coverage (-): 0

Region: NODE\_316089\_length\_2387\_cov\_23.258902 94-98. Max. coverage (+): 0.04. Max coverage (-): 0

Region: NODE\_316089\_length\_2387\_cov\_23.258902 99-103. Max. coverage (+): 0.08. Max coverage (-): 0

Region: NODE\_316089\_length\_2387\_cov\_23.258902 104-108. Max. coverage (+): 0. Max coverage (-): 0

Region: NODE\_316089\_length\_2387\_cov\_23.258902 109-113. Max. coverage (+): 0. Max coverage (-): 0.12

Region: NODE\_316089\_length\_2387\_cov\_23.258902 114-118. Max. coverage (+): 0.04. Max coverage (-): 0.16

Region: NODE\_316089\_length\_2387\_cov\_23.258902 119-122. Max. coverage (+): 0.04. Max coverage (-): 0

Region: NODE\_316089\_length\_2387\_cov\_23.258902 123-127. Max. coverage (+): 0. Max coverage (-): 0.04

Region: NODE\_316089\_length\_2387\_cov\_23.258902 128-132. Max. coverage (+): 0. Max coverage (-): 0

Region: NODE\_316089\_length\_2387\_cov\_23.258902 133-137. Max. coverage (+): 0.04. Max coverage (-): 0

Region: NODE\_316089\_length\_2387\_cov\_23.258902 138-142. Max. coverage (+): 0.97. Max coverage (-): 0.08

Region: NODE\_316089\_length\_2387\_cov\_23.258902 143-147. Max. coverage (+): 1.53. Max coverage (-): 0.04

Region: NODE\_316089\_length\_2387\_cov\_23.258902 148-152. Max. coverage (+): 1.49. Max coverage (-): 0.04

Region: NODE\_316089\_length\_2387\_cov\_23.258902 153-157. Max. coverage (+): 0.04. Max coverage (-): 0.04

Region: NODE\_316089\_length\_2387\_cov\_23.258902 158-162. Max. coverage (+): 0.08. Max coverage (-): 0

Region: NODE\_316089\_length\_2387\_cov\_23.258902 163-167. Max. coverage (+): 0.04. Max coverage (-): 0

Region: NODE\_316089\_length\_2387\_cov\_23.258902 168-171. Max. coverage (+): 0.36. Max coverage (-): 0.04

Region: NODE\_316089\_length\_2387\_cov\_23.258902 172-176. Max. coverage (+): 0.08. Max coverage (-): 0.12

Region: NODE\_316089\_length\_2387\_cov\_23.258902 177-181. Max. coverage (+): 0. Max coverage (-): 0

Region: NODE\_316089\_length\_2387\_cov\_23.258902 182-186. Max. coverage (+): 0. Max coverage (-): 0

Region: NODE\_316089\_length\_2387\_cov\_23.258902 187-191. Max. coverage (+): 0.65. Max coverage (-): 0

Region: NODE\_316089\_length\_2387\_cov\_23.258902 192-196. Max. coverage (+): 0.4. Max coverage (-): 0

Region: NODE\_316089\_length\_2387\_cov\_23.258902 197-201. Max. coverage (+): 0.04. Max coverage (-): 0

Region: NODE\_316089\_length\_2387\_cov\_23.258902 202-206. Max. coverage (+): 0.08. Max coverage (-): 0.2

Region: NODE\_316089\_length\_2387\_cov\_23.258902 207-211. Max. coverage (+): 0. Max coverage (-): 0.2

Region: NODE\_316089\_length\_2387\_cov\_23.258902 212-215. Max. coverage (+): 0.08. Max coverage (-): 0

Region: NODE\_316089\_length\_2387\_cov\_23.258902 216-220. Max. coverage (+): 1.01. Max coverage (-): 0

Region: NODE\_316089\_length\_2387\_cov\_23.258902 221-225. Max. coverage (+): 0.97. Max coverage (-): 0.04

Region: NODE\_316089\_length\_2387\_cov\_23.258902 226-230. Max. coverage (+): 0.85. Max coverage (-): 0

Region: NODE\_316089\_length\_2387\_cov\_23.258902 231-235. Max. coverage (+): 0.16. Max coverage (-): 0

Region: NODE\_316089\_length\_2387\_cov\_23.258902 236-240. Max. coverage (+): 0. Max coverage (-): 0

Region: NODE\_316089\_length\_2387\_cov\_23.258902 241-245. Max. coverage (+): 0.04. Max coverage (-): 0

Region: NODE\_316089\_length\_2387\_cov\_23.258902 246-250. Max. coverage (+): 0. Max coverage (-): 0

Region: NODE\_316089\_length\_2387\_cov\_23.258902 251-255. Max. coverage (+): 0. Max coverage (-): 0

Region: NODE\_316089\_length\_2387\_cov\_23.258902 256-260. Max. coverage (+): 0.04. Max coverage (-): 0

Region: NODE\_316089\_length\_2387\_cov\_23.258902 261-264. Max. coverage (+): 0.08. Max coverage (-): 0.08

Region: NODE\_316089\_length\_2387\_cov\_23.258902 265-269. Max. coverage (+): 0.08. Max coverage (-): 0.2

Region: NODE\_316089\_length\_2387\_cov\_23.258902 270-274. Max. coverage (+): 1.45. Max coverage (-): 0

Region: NODE\_316089\_length\_2387\_cov\_23.258902 275-279. Max. coverage (+): 1.09. Max coverage (-): 0

Region: NODE\_316089\_length\_2387\_cov\_23.258902 280-284. Max. coverage (+): 0.97. Max coverage (-): 0

Region: NODE\_316089\_length\_2387\_cov\_23.258902 285-289. Max. coverage (+): 0.08. Max coverage (-): 0

Region: NODE\_316089\_length\_2387\_cov\_23.258902 290-294. Max. coverage (+): 0. Max coverage (-): 0

Region: NODE\_316089\_length\_2387\_cov\_23.258902 295-299. Max. coverage (+): 0.04. Max coverage (-): 0.03

Region: NODE\_316089\_length\_2387\_cov\_23.258902 300-304. Max. coverage (+): 0.04. Max coverage (-): 0.08

Region: NODE\_316089\_length\_2387\_cov\_23.258902 305-308. Max. coverage (+): 0. Max coverage (-): 0

Region: NODE\_316089\_length\_2387\_cov\_23.258902 309-313. Max. coverage (+): 0. Max coverage (-): 0

Region: NODE\_316089\_length\_2387\_cov\_23.258902 314-318. Max. coverage (+): 0.02. Max coverage (-): 0

Region: NODE\_316089\_length\_2387\_cov\_23.258902 319-323. Max. coverage (+): 0.02. Max coverage (-): 0

Region: NODE\_316089\_length\_2387\_cov\_23.258902 324-328. Max. coverage (+): 0.08. Max coverage (-): 0

Region: NODE\_316089\_length\_2387\_cov\_23.258902 329-333. Max. coverage (+): 0.08. Max coverage (-): 0

Region: NODE\_316089\_length\_2387\_cov\_23.258902 334-338. Max. coverage (+): 0. Max coverage (-): 0.04

Region: NODE\_316089\_length\_2387\_cov\_23.258902 339-343. Max. coverage (+): 0.48. Max coverage (-): 0.04

Region: NODE\_316089\_length\_2387\_cov\_23.258902 344-348. Max. coverage (+): 0.2. Max coverage (-): 0.12

Region: NODE\_316089\_length\_2387\_cov\_23.258902 349-353. Max. coverage (+): 0. Max coverage (-): 0.04

Region: NODE\_316089\_length\_2387\_cov\_23.258902 354-357. Max. coverage (+): 0. Max coverage (-): 0.44

Region: NODE\_316089\_length\_2387\_cov\_23.258902 358-362. Max. coverage (+): 0.04. Max coverage (-): 0.32

Region: NODE\_316089\_length\_2387\_cov\_23.258902 363-367. Max. coverage (+): 0.2. Max coverage (-): 0.04

Region: NODE\_316089\_length\_2387\_cov\_23.258902 368-372. Max. coverage (+): 0.24. Max coverage (-): 0.2

Region: NODE\_316089\_length\_2387\_cov\_23.258902 373-377. Max. coverage (+): 0.77. Max coverage (-): 0

Region: NODE\_316089\_length\_2387\_cov\_23.258902 378-382. Max. coverage (+): 0.28. Max coverage (-): 0.16

Region: NODE\_316089\_length\_2387\_cov\_23.258902 383-387. Max. coverage (+): 0.16. Max coverage (-): 0.12

Region: NODE\_316089\_length\_2387\_cov\_23.258902 388-392. Max. coverage (+): 0.57. Max coverage (-): 0.08

Region: NODE\_316089\_length\_2387\_cov\_23.258902 393-397. Max. coverage (+): 1.29. Max coverage (-): 0.08

Region: NODE\_316089\_length\_2387\_cov\_23.258902 398-402. Max. coverage (+): 1.66. Max coverage (-): 0

Region: NODE\_316089\_length\_2387\_cov\_23.258902 403-406. Max. coverage (+): 2.62. Max coverage (-): 0

Region: NODE\_316089\_length\_2387\_cov\_23.258902 407-411. Max. coverage (+): 0.16. Max coverage (-): 0.04

Region: NODE\_316089\_length\_2387\_cov\_23.258902 412-416. Max. coverage (+): 0.2. Max coverage (-): 0.04

Region: NODE\_316089\_length\_2387\_cov\_23.258902 417-421. Max. coverage (+): 0.08. Max coverage (-): 0

Region: NODE\_316089\_length\_2387\_cov\_23.258902 422-426. Max. coverage (+): 0.08. Max coverage (-): 0

Region: NODE\_316089\_length\_2387\_cov\_23.258902 427-431. Max. coverage (+): 0.12. Max coverage (-): 0.08

Region: NODE\_316089\_length\_2387\_cov\_23.258902 432-436. Max. coverage (+): 0. Max coverage (-): 0.08

Region: NODE\_316089\_length\_2387\_cov\_23.258902 437-441. Max. coverage (+): 0.04. Max coverage (-): 0.24

Region: NODE\_316089\_length\_2387\_cov\_23.258902 442-446. Max. coverage (+): 0.08. Max coverage (-): 0.02

Region: NODE\_316089\_length\_2387\_cov\_23.258902 447-450. Max. coverage (+): 0.32. Max coverage (-): 0.06

Region: NODE\_316089\_length\_2387\_cov\_23.258902 451-455. Max. coverage (+): 0.65. Max coverage (-): 0.14

Region: NODE\_316089\_length\_2387\_cov\_23.258902 456-460. Max. coverage (+): 0.4. Max coverage (-): 0.16

Region: NODE\_316089\_length\_2387\_cov\_23.258902 461-465. Max. coverage (+): 4.14. Max coverage (-): 0.02

Region: NODE\_316089\_length\_2387\_cov\_23.258902 466-470. Max. coverage (+): 12.15. Max coverage (-): 0

Region: NODE\_316089\_length\_2387\_cov\_23.258902 471-475. Max. coverage (+): 12.19. Max coverage (-): 0.08

Region: NODE\_316089\_length\_2387\_cov\_23.258902 476-480. Max. coverage (+): 0.73. Max coverage (-): 0.08

Region: NODE\_316089\_length\_2387\_cov\_23.258902 481-485. Max. coverage (+): 0.24. Max coverage (-): 0

Region: NODE\_316089\_length\_2387\_cov\_23.258902 486-490. Max. coverage (+): 0.12. Max coverage (-): 0

Region: NODE\_316089\_length\_2387\_cov\_23.258902 491-495. Max. coverage (+): 0.28. Max coverage (-): 0.08

Region: NODE\_316089\_length\_2387\_cov\_23.258902 496-499. Max. coverage (+): 0. Max coverage (-): 0.16

Region: NODE\_316089\_length\_2387\_cov\_23.258902 500-504. Max. coverage (+): 0.08. Max coverage (-): 0.16

Region: NODE\_316089\_length\_2387\_cov\_23.258902 505-509. Max. coverage (+): 1.05. Max coverage (-): 0.16

Region: NODE\_316089\_length\_2387\_cov\_23.258902 510-514. Max. coverage (+): 1.29. Max coverage (-): 0

Region: NODE\_316089\_length\_2387\_cov\_23.258902 515-519. Max. coverage (+): 0.4. Max coverage (-): 0

Region: NODE\_316089\_length\_2387\_cov\_23.258902 520-524. Max. coverage (+): 0.81. Max coverage (-): 0

Region: NODE\_316089\_length\_2387\_cov\_23.258902 525-529. Max. coverage (+): 0.08. Max coverage (-): 0.06

Region: NODE\_316089\_length\_2387\_cov\_23.258902 530-534. Max. coverage (+): 0.57. Max coverage (-): 0.02

Region: NODE\_316089\_length\_2387\_cov\_23.258902 535-539. Max. coverage (+): 1.7. Max coverage (-): 0.08

Region: NODE\_316089\_length\_2387\_cov\_23.258902 540-544. Max. coverage (+): 0.04. Max coverage (-): 0.18

Region: NODE\_316089\_length\_2387\_cov\_23.258902 545-548. Max. coverage (+): 0.08. Max coverage (-): 0.1

Region: NODE\_316089\_length\_2387\_cov\_23.258902 549-553. Max. coverage (+): 2.14. Max coverage (-): 0.1

Region: NODE\_316089\_length\_2387\_cov\_23.258902 554-558. Max. coverage (+): 29.15. Max coverage (-): 0

Region: NODE\_316089\_length\_2387\_cov\_23.258902 559-563. Max. coverage (+): 29.76. Max coverage (-): 0.04

Region: NODE\_316089\_length\_2387\_cov\_23.258902 564-568. Max. coverage (+): 7.47. Max coverage (-): 0.04

Region: NODE\_316089\_length\_2387\_cov\_23.258902 569-573. Max. coverage (+): 4.76. Max coverage (-): 0

Region: NODE\_316089\_length\_2387\_cov\_23.258902 574-578. Max. coverage (+): 0.04. Max coverage (-): 0.12

Region: NODE\_316089\_length\_2387\_cov\_23.258902 579-583. Max. coverage (+): 0.02. Max coverage (-): 1.59

Region: NODE\_316089\_length\_2387\_cov\_23.258902 584-588. Max. coverage (+): 0. Max coverage (-): 0.93

Region: NODE\_316089\_length\_2387\_cov\_23.258902 589-592. Max. coverage (+): 0.32. Max coverage (-): 1.49

Region: NODE\_316089\_length\_2387\_cov\_23.258902 593-597. Max. coverage (+): 0.69. Max coverage (-): 0.44

Region: NODE\_316089\_length\_2387\_cov\_23.258902 598-602. Max. coverage (+): 1.53. Max coverage (-): 0.12

Region: NODE\_316089\_length\_2387\_cov\_23.258902 603-607. Max. coverage (+): 0.52. Max coverage (-): 0

Region: NODE\_316089\_length\_2387\_cov\_23.258902 608-612. Max. coverage (+): 0.12. Max coverage (-): 0

Region: NODE\_316089\_length\_2387\_cov\_23.258902 613-617. Max. coverage (+): 0.04. Max coverage (-): 0.08

Region: NODE\_316089\_length\_2387\_cov\_23.258902 618-622. Max. coverage (+): 0.04. Max coverage (-): 0

Region: NODE\_316089\_length\_2387\_cov\_23.258902 623-627. Max. coverage (+): 0.36. Max coverage (-): 0

Region: NODE\_316089\_length\_2387\_cov\_23.258902 628-632. Max. coverage (+): 0.57. Max coverage (-): 0.08

Region: NODE\_316089\_length\_2387\_cov\_23.258902 633-637. Max. coverage (+): 0.67. Max coverage (-): 0

Region: NODE\_316089\_length\_2387\_cov\_23.258902 638-641. Max. coverage (+): 0.99. Max coverage (-): 0

Region: NODE\_316089\_length\_2387\_cov\_23.258902 642-646. Max. coverage (+): 3.12. Max coverage (-): 0

Region: NODE\_316089\_length\_2387\_cov\_23.258902 647-651. Max. coverage (+): 0.79. Max coverage (-): 0

Region: NODE\_316089\_length\_2387\_cov\_23.258902 652-656. Max. coverage (+): 0.22. Max coverage (-): 0.02

Region: NODE\_316089\_length\_2387\_cov\_23.258902 657-661. Max. coverage (+): 0.04. Max coverage (-): 0

Region: NODE\_316089\_length\_2387\_cov\_23.258902 662-666. Max. coverage (+): 0. Max coverage (-): 0

Region: NODE\_316089\_length\_2387\_cov\_23.258902 667-671. Max. coverage (+): 0.1. Max coverage (-): 0.06

Region: NODE\_316089\_length\_2387\_cov\_23.258902 672-676. Max. coverage (+): 0.08. Max coverage (-): 0.14

Region: NODE\_316089\_length\_2387\_cov\_23.258902 677-681. Max. coverage (+): 0.06. Max coverage (-): 0.16

Region: NODE\_316089\_length\_2387\_cov\_23.258902 682-685. Max. coverage (+): 0.2. Max coverage (-): 0

Region: NODE\_316089\_length\_2387\_cov\_23.258902 686-690. Max. coverage (+): 0.32. Max coverage (-): 0

Region: NODE\_316089\_length\_2387\_cov\_23.258902 691-695. Max. coverage (+): 3.15. Max coverage (-): 0.04

Region: NODE\_316089\_length\_2387\_cov\_23.258902 696-700. Max. coverage (+): 2.83. Max coverage (-): 0

Region: NODE\_316089\_length\_2387\_cov\_23.258902 701-705. Max. coverage (+): 0.89. Max coverage (-): 0

Region: NODE\_316089\_length\_2387\_cov\_23.258902 706-710. Max. coverage (+): 1.13. Max coverage (-): 0.08

Region: NODE\_316089\_length\_2387\_cov\_23.258902 711-715. Max. coverage (+): 0.36. Max coverage (-): 0.2

Region: NODE\_316089\_length\_2387\_cov\_23.258902 716-720. Max. coverage (+): 0.08. Max coverage (-): 0.04

Region: NODE\_316089\_length\_2387\_cov\_23.258902 721-725. Max. coverage (+): 1.35. Max coverage (-): 0

Region: NODE\_316089\_length\_2387\_cov\_23.258902 726-730. Max. coverage (+): 117.36. Max coverage (-): 0

Region: NODE\_316089\_length\_2387\_cov\_23.258902 731-734. Max. coverage (+): 3.8. Max coverage (-): 0

Region: NODE\_316089\_length\_2387\_cov\_23.258902 735-739. Max. coverage (+): 1.37. Max coverage (-): 0.08

Region: NODE\_316089\_length\_2387\_cov\_23.258902 740-744. Max. coverage (+): 0.04. Max coverage (-): 0.32

Region: NODE\_316089\_length\_2387\_cov\_23.258902 745-749. Max. coverage (+): 0.16. Max coverage (-): 0.36

Region: NODE\_316089\_length\_2387\_cov\_23.258902 750-754. Max. coverage (+): 2.38. Max coverage (-): 0.24

Region: NODE\_316089\_length\_2387\_cov\_23.258902 755-759. Max. coverage (+): 8.26. Max coverage (-): 0.04

Region: NODE\_316089\_length\_2387\_cov\_23.258902 760-764. Max. coverage (+): 4.87. Max coverage (-): 0.02

Region: NODE\_316089\_length\_2387\_cov\_23.258902 765-769. Max. coverage (+): 49.38. Max coverage (-): 0.02

Region: NODE\_316089\_length\_2387\_cov\_23.258902 770-774. Max. coverage (+): 0.26. Max coverage (-): 0.08

Region: NODE\_316089\_length\_2387\_cov\_23.258902 775-779. Max. coverage (+): 1.78. Max coverage (-): 0.12

Region: NODE\_316089\_length\_2387\_cov\_23.258902 780-783. Max. coverage (+): 0.04. Max coverage (-): 0.04

Region: NODE\_316089\_length\_2387\_cov\_23.258902 784-788. Max. coverage (+): 0. Max coverage (-): 0.04

Region: NODE\_316089\_length\_2387\_cov\_23.258902 789-793. Max. coverage (+): 0.12. Max coverage (-): 0

Region: NODE\_316089\_length\_2387\_cov\_23.258902 794-798. Max. coverage (+): 2.46. Max coverage (-): 0

Region: NODE\_316089\_length\_2387\_cov\_23.258902 799-803. Max. coverage (+): 1.01. Max coverage (-): 0

Region: NODE\_316089\_length\_2387\_cov\_23.258902 804-808. Max. coverage (+): 0. Max coverage (-): 0.02

Region: NODE\_316089\_length\_2387\_cov\_23.258902 809-813. Max. coverage (+): 0.3. Max coverage (-): 0.26

Region: NODE\_316089\_length\_2387\_cov\_23.258902 814-818. Max. coverage (+): 0.28. Max coverage (-): 0.22

Region: NODE\_316089\_length\_2387\_cov\_23.258902 819-823. Max. coverage (+): 0.16. Max coverage (-): 0.44

Region: NODE\_316089\_length\_2387\_cov\_23.258902 824-827. Max. coverage (+): 5.03. Max coverage (-): 0.28

Region: NODE\_316089\_length\_2387\_cov\_23.258902 828-832. Max. coverage (+): 1.41. Max coverage (-): 0.02

Region: NODE\_316089\_length\_2387\_cov\_23.258902 833-837. Max. coverage (+): 1.01. Max coverage (-): 0.02

Region: NODE\_316089\_length\_2387\_cov\_23.258902 838-842. Max. coverage (+): 2.02. Max coverage (-): 0.04

Region: NODE\_316089\_length\_2387\_cov\_23.258902 843-847. Max. coverage (+): 0.16. Max coverage (-): 0.04

Region: NODE\_316089\_length\_2387\_cov\_23.258902 848-852. Max. coverage (+): 0.16. Max coverage (-): 0

Region: NODE\_316089\_length\_2387\_cov\_23.258902 853-857. Max. coverage (+): 0. Max coverage (-): 0

Region: NODE\_316089\_length\_2387\_cov\_23.258902 858-862. Max. coverage (+): 0.04. Max coverage (-): 0

Region: NODE\_316089\_length\_2387\_cov\_23.258902 863-867. Max. coverage (+): 0.81. Max coverage (-): 0.12

Region: NODE\_316089\_length\_2387\_cov\_23.258902 868-872. Max. coverage (+): 2.06. Max coverage (-): 0.16

Region: NODE\_316089\_length\_2387\_cov\_23.258902 873-876. Max. coverage (+): 1.98. Max coverage (-): 0.08

Region: NODE\_316089\_length\_2387\_cov\_23.258902 877-881. Max. coverage (+): 1.41. Max coverage (-): 0.04

Region: NODE\_316089\_length\_2387\_cov\_23.258902 882-886. Max. coverage (+): 6.99. Max coverage (-): 0.04

Region: NODE\_316089\_length\_2387\_cov\_23.258902 887-891. Max. coverage (+): 0.42. Max coverage (-): 0.12

Region: NODE\_316089\_length\_2387\_cov\_23.258902 892-896. Max. coverage (+): 0.36. Max coverage (-): 0.4

Region: NODE\_316089\_length\_2387\_cov\_23.258902 897-901. Max. coverage (+): 0.44. Max coverage (-): 0.08

Region: NODE\_316089\_length\_2387\_cov\_23.258902 902-906. Max. coverage (+): 0.48. Max coverage (-): 0.12

Region: NODE\_316089\_length\_2387\_cov\_23.258902 907-911. Max. coverage (+): 11.91. Max coverage (-): 0.08

Region: NODE\_316089\_length\_2387\_cov\_23.258902 912-916. Max. coverage (+): 6.3. Max coverage (-): 0.24

Region: NODE\_316089\_length\_2387\_cov\_23.258902 917-920. Max. coverage (+): 0. Max coverage (-): 0.04

Region: NODE\_316089\_length\_2387\_cov\_23.258902 921-925. Max. coverage (+): 2.34. Max coverage (-): 0.02

Region: NODE\_316089\_length\_2387\_cov\_23.258902 926-930. Max. coverage (+): 4.89. Max coverage (-): 0.1

Region: NODE\_316089\_length\_2387\_cov\_23.258902 931-935. Max. coverage (+): 5.85. Max coverage (-): 0.04

Region: NODE\_316089\_length\_2387\_cov\_23.258902 936-940. Max. coverage (+): 18.86. Max coverage (-): 0

Region: NODE\_316089\_length\_2387\_cov\_23.258902 941-945. Max. coverage (+): 25.92. Max coverage (-): 0

Region: NODE\_316089\_length\_2387\_cov\_23.258902 946-950. Max. coverage (+): 1.49. Max coverage (-): 0.28

Region: NODE\_316089\_length\_2387\_cov\_23.258902 951-955. Max. coverage (+): 1.78. Max coverage (-): 0.28

Region: NODE\_316089\_length\_2387\_cov\_23.258902 956-960. Max. coverage (+): 0.77. Max coverage (-): 0

Region: NODE\_316089\_length\_2387\_cov\_23.258902 961-965. Max. coverage (+): 0.17. Max coverage (-): 0.01

Region: NODE\_316089\_length\_2387\_cov\_23.258902 966-969. Max. coverage (+): 1.74. Max coverage (-): 0

Region: NODE\_316089\_length\_2387\_cov\_23.258902 970-974. Max. coverage (+): 6.12. Max coverage (-): 0.16

Region: NODE\_316089\_length\_2387\_cov\_23.258902 975-979. Max. coverage (+): 0.69. Max coverage (-): 0.77

Region: NODE\_316089\_length\_2387\_cov\_23.258902 980-984. Max. coverage (+): 0.08. Max coverage (-): 0.57

Region: NODE\_316089\_length\_2387\_cov\_23.258902 985-989. Max. coverage (+): 0.61. Max coverage (-): 0.16

Region: NODE\_316089\_length\_2387\_cov\_23.258902 990-994. Max. coverage (+): 1.29. Max coverage (-): 0

Region: NODE\_316089\_length\_2387\_cov\_23.258902 995-999. Max. coverage (+): 0.24. Max coverage (-): 0

Region: NODE\_316089\_length\_2387\_cov\_23.258902 1000-1004. Max. coverage (+): 0.16. Max coverage (-): 0.04

Region: NODE\_316089\_length\_2387\_cov\_23.258902 1005-1009. Max. coverage (+): 0.2. Max coverage (-): 0.04

Region: NODE\_316089\_length\_2387\_cov\_23.258902 1010-1014. Max. coverage (+): 0.85. Max coverage (-): 0.2

Region: NODE\_316089\_length\_2387\_cov\_23.258902 1015-1018. Max. coverage (+): 0.85. Max coverage (-): 0.08

Region: NODE\_316089\_length\_2387\_cov\_23.258902 1019-1023. Max. coverage (+): 0.61. Max coverage (-): 0.08

Region: NODE\_316089\_length\_2387\_cov\_23.258902 1024-1028. Max. coverage (+): 1.37. Max coverage (-): 0.04

Region: NODE\_316089\_length\_2387\_cov\_23.258902 1029-1033. Max. coverage (+): 0.12. Max coverage (-): 0.08

Region: NODE\_316089\_length\_2387\_cov\_23.258902 1034-1038. Max. coverage (+): 0.73. Max coverage (-): 0.69

Region: NODE\_316089\_length\_2387\_cov\_23.258902 1039-1043. Max. coverage (+): 0.04. Max coverage (-): 0.69

Region: NODE\_316089\_length\_2387\_cov\_23.258902 1044-1048. Max. coverage (+): 0.24. Max coverage (-): 0

Region: NODE\_316089\_length\_2387\_cov\_23.258902 1049-1053. Max. coverage (+): 0.57. Max coverage (-): 0

Region: NODE\_316089\_length\_2387\_cov\_23.258902 1054-1058. Max. coverage (+): 0.48. Max coverage (-): 0

Region: NODE\_316089\_length\_2387\_cov\_23.258902 1059-1062. Max. coverage (+): 0.52. Max coverage (-): 0.04

Region: NODE\_316089\_length\_2387\_cov\_23.258902 1063-1067. Max. coverage (+): 0.24. Max coverage (-): 0

Region: NODE\_316089\_length\_2387\_cov\_23.258902 1068-1072. Max. coverage (+): 1.62. Max coverage (-): 0

Region: NODE\_316089\_length\_2387\_cov\_23.258902 1073-1077. Max. coverage (+): 0.08. Max coverage (-): 0

Region: NODE\_316089\_length\_2387\_cov\_23.258902 1078-1082. Max. coverage (+): 0.08. Max coverage (-): 0

Region: NODE\_316089\_length\_2387\_cov\_23.258902 1083-1087. Max. coverage (+): 0.28. Max coverage (-): 0.81

Region: NODE\_316089\_length\_2387\_cov\_23.258902 1088-1092. Max. coverage (+): 0.32. Max coverage (-): 1.09

Region: NODE\_316089\_length\_2387\_cov\_23.258902 1093-1097. Max. coverage (+): 0. Max coverage (-): 0.18

Region: NODE\_316089\_length\_2387\_cov\_23.258902 1098-1102. Max. coverage (+): 0.22. Max coverage (-): 0.02

Region: NODE\_316089\_length\_2387\_cov\_23.258902 1103-1107. Max. coverage (+): 13.43. Max coverage (-): 0.02

Region: NODE\_316089\_length\_2387\_cov\_23.258902 1108-1111. Max. coverage (+): 3.04. Max coverage (-): 0

Region: NODE\_316089\_length\_2387\_cov\_23.258902 1112-1116. Max. coverage (+): 0.14. Max coverage (-): 0

Region: NODE\_316089\_length\_2387\_cov\_23.258902 1117-1121. Max. coverage (+): 0.03. Max coverage (-): 0

Region: NODE\_316089\_length\_2387\_cov\_23.258902 1122-1126. Max. coverage (+): 0.11. Max coverage (-): 0

Region: NODE\_316089\_length\_2387\_cov\_23.258902 1127-1131. Max. coverage (+): 0. Max coverage (-): 0.06

Region: NODE\_316089\_length\_2387\_cov\_23.258902 1132-1136. Max. coverage (+): 0.08. Max coverage (-): 0.44

Region: NODE\_316089\_length\_2387\_cov\_23.258902 1137-1141. Max. coverage (+): 0.4. Max coverage (-): 0

Region: NODE\_316089\_length\_2387\_cov\_23.258902 1142-1146. Max. coverage (+): 0.48. Max coverage (-): 0

Region: NODE\_316089\_length\_2387\_cov\_23.258902 1147-1151. Max. coverage (+): 17.28. Max coverage (-): 0

Region: NODE\_316089\_length\_2387\_cov\_23.258902 1152-1156. Max. coverage (+): 0.04. Max coverage (-): 0

Region: NODE\_316089\_length\_2387\_cov\_23.258902 1157-1160. Max. coverage (+): 0.04. Max coverage (-): 0

Region: NODE\_316089\_length\_2387\_cov\_23.258902 1161-1165. Max. coverage (+): 0. Max coverage (-): 0.08

Region: NODE\_316089\_length\_2387\_cov\_23.258902 1166-1170. Max. coverage (+): 0.07. Max coverage (-): 0.04

Region: NODE\_316089\_length\_2387\_cov\_23.258902 1171-1175. Max. coverage (+): 0.08. Max coverage (-): 0

Region: NODE\_316089\_length\_2387\_cov\_23.258902 1176-1180. Max. coverage (+): 0.4. Max coverage (-): 0.03

Region: NODE\_316089\_length\_2387\_cov\_23.258902 1181-1185. Max. coverage (+): 0.05. Max coverage (-): 0.16

Region: NODE\_316089\_length\_2387\_cov\_23.258902 1186-1190. Max. coverage (+): 0.02. Max coverage (-): 0.12

Region: NODE\_316089\_length\_2387\_cov\_23.258902 1191-1195. Max. coverage (+): 0. Max coverage (-): 0.12

Region: NODE\_316089\_length\_2387\_cov\_23.258902 1196-1200. Max. coverage (+): 0.2. Max coverage (-): 0.12

Region: NODE\_316089\_length\_2387\_cov\_23.258902 1201-1204. Max. coverage (+): 1.68. Max coverage (-): 0

Region: NODE\_316089\_length\_2387\_cov\_23.258902 1205-1209. Max. coverage (+): 1.23. Max coverage (-): 0

Region: NODE\_316089\_length\_2387\_cov\_23.258902 1210-1214. Max. coverage (+): 1.21. Max coverage (-): 0

Region: NODE\_316089\_length\_2387\_cov\_23.258902 1215-1219. Max. coverage (+): 0. Max coverage (-): 0.32

Region: NODE\_316089\_length\_2387\_cov\_23.258902 1220-1224. Max. coverage (+): 0.02. Max coverage (-): 0.46

Region: NODE\_316089\_length\_2387\_cov\_23.258902 1225-1229. Max. coverage (+): 0.34. Max coverage (-): 0.2

Region: NODE\_316089\_length\_2387\_cov\_23.258902 1230-1234. Max. coverage (+): 1.19. Max coverage (-): 0.02

Region: NODE\_316089\_length\_2387\_cov\_23.258902 1235-1239. Max. coverage (+): 20.79. Max coverage (-): 0

Region: NODE\_316089\_length\_2387\_cov\_23.258902 1240-1244. Max. coverage (+): 2.22. Max coverage (-): 0

Region: NODE\_316089\_length\_2387\_cov\_23.258902 1245-1249. Max. coverage (+): 1.09. Max coverage (-): 0

Region: NODE\_316089\_length\_2387\_cov\_23.258902 1250-1253. Max. coverage (+): 0.08. Max coverage (-): 0.04

Region: NODE\_316089\_length\_2387\_cov\_23.258902 1254-1258. Max. coverage (+): 0.32. Max coverage (-): 0.04

Region: NODE\_316089\_length\_2387\_cov\_23.258902 1259-1263. Max. coverage (+): 0.2. Max coverage (-): 0.12

Region: NODE\_316089\_length\_2387\_cov\_23.258902 1264-1268. Max. coverage (+): 0. Max coverage (-): 0.24

Region: NODE\_316089\_length\_2387\_cov\_23.258902 1269-1273. Max. coverage (+): 0. Max coverage (-): 0.4

Region: NODE\_316089\_length\_2387\_cov\_23.258902 1274-1278. Max. coverage (+): 0.04. Max coverage (-): 0.24

Region: NODE\_316089\_length\_2387\_cov\_23.258902 1279-1283. Max. coverage (+): 0.04. Max coverage (-): 0.16

Region: NODE\_316089\_length\_2387\_cov\_23.258902 1284-1288. Max. coverage (+): 4. Max coverage (-): 0

Region: NODE\_316089\_length\_2387\_cov\_23.258902 1289-1293. Max. coverage (+): 11.43. Max coverage (-): 0

Region: NODE\_316089\_length\_2387\_cov\_23.258902 1294-1297. Max. coverage (+): 11.47. Max coverage (-): 0

Region: NODE\_316089\_length\_2387\_cov\_23.258902 1298-1302. Max. coverage (+): 0.02. Max coverage (-): 0

Region: NODE\_316089\_length\_2387\_cov\_23.258902 1303-1307. Max. coverage (+): 0.26. Max coverage (-): 0

Region: NODE\_316089\_length\_2387\_cov\_23.258902 1308-1312. Max. coverage (+): 0.16. Max coverage (-): 0

Region: NODE\_316089\_length\_2387\_cov\_23.258902 1313-1317. Max. coverage (+): 0.65. Max coverage (-): 0

Region: NODE\_316089\_length\_2387\_cov\_23.258902 1318-1322. Max. coverage (+): 7.75. Max coverage (-): 0

Region: NODE\_316089\_length\_2387\_cov\_23.258902 1323-1327. Max. coverage (+): 7.91. Max coverage (-): 0

Region: NODE\_316089\_length\_2387\_cov\_23.258902 1328-1332. Max. coverage (+): 0.04. Max coverage (-): 0.04

Region: NODE\_316089\_length\_2387\_cov\_23.258902 1333-1337. Max. coverage (+): 3.51. Max coverage (-): 0

Region: NODE\_316089\_length\_2387\_cov\_23.258902 1338-1342. Max. coverage (+): 5.33. Max coverage (-): 0.04

Region: NODE\_316089\_length\_2387\_cov\_23.258902 1343-1346. Max. coverage (+): 2.95. Max coverage (-): 0.04

Region: NODE\_316089\_length\_2387\_cov\_23.258902 1347-1351. Max. coverage (+): 2.66. Max coverage (-): 0

Region: NODE\_316089\_length\_2387\_cov\_23.258902 1352-1356. Max. coverage (+): 8.92. Max coverage (-): 0.12

Region: NODE\_316089\_length\_2387\_cov\_23.258902 1357-1361. Max. coverage (+): 10.05. Max coverage (-): 0.24

Region: NODE\_316089\_length\_2387\_cov\_23.258902 1362-1366. Max. coverage (+): 0.52. Max coverage (-): 0.24

Region: NODE\_316089\_length\_2387\_cov\_23.258902 1367-1371. Max. coverage (+): 0.28. Max coverage (-): 0.16

Region: NODE\_316089\_length\_2387\_cov\_23.258902 1372-1376. Max. coverage (+): 0.52. Max coverage (-): 0.08

Region: NODE\_316089\_length\_2387\_cov\_23.258902 1377-1381. Max. coverage (+): 0.4. Max coverage (-): 0

Region: NODE\_316089\_length\_2387\_cov\_23.258902 1382-1386. Max. coverage (+): 1.86. Max coverage (-): 0

Region: NODE\_316089\_length\_2387\_cov\_23.258902 1387-1391. Max. coverage (+): 1.94. Max coverage (-): 0

Region: NODE\_316089\_length\_2387\_cov\_23.258902 1392-1395. Max. coverage (+): 0.65. Max coverage (-): 0.16

Region: NODE\_316089\_length\_2387\_cov\_23.258902 1396-1400. Max. coverage (+): 0. Max coverage (-): 0.32

Region: NODE\_316089\_length\_2387\_cov\_23.258902 1401-1405. Max. coverage (+): 0. Max coverage (-): 0.16

Region: NODE\_316089\_length\_2387\_cov\_23.258902 1406-1410. Max. coverage (+): 0.77. Max coverage (-): 0.04

Region: NODE\_316089\_length\_2387\_cov\_23.258902 1411-1415. Max. coverage (+): 13.65. Max coverage (-): 0.02

Region: NODE\_316089\_length\_2387\_cov\_23.258902 1416-1420. Max. coverage (+): 1.18. Max coverage (-): 0

Region: NODE\_316089\_length\_2387\_cov\_23.258902 1421-1425. Max. coverage (+): 0. Max coverage (-): 0.04

Region: NODE\_316089\_length\_2387\_cov\_23.258902 1426-1430. Max. coverage (+): 0.04. Max coverage (-): 0

Region: NODE\_316089\_length\_2387\_cov\_23.258902 1431-1435. Max. coverage (+): 0.04. Max coverage (-): 0.04

Region: NODE\_316089\_length\_2387\_cov\_23.258902 1436-1439. Max. coverage (+): 0.4. Max coverage (-): 0.04

Region: NODE\_316089\_length\_2387\_cov\_23.258902 1440-1444. Max. coverage (+): 0.16. Max coverage (-): 0

Region: NODE\_316089\_length\_2387\_cov\_23.258902 1445-1449. Max. coverage (+): 0.24. Max coverage (-): 0.08

Region: NODE\_316089\_length\_2387\_cov\_23.258902 1450-1454. Max. coverage (+): 0.28. Max coverage (-): 0.08

Region: NODE\_316089\_length\_2387\_cov\_23.258902 1455-1459. Max. coverage (+): 0.08. Max coverage (-): 0

Region: NODE\_316089\_length\_2387\_cov\_23.258902 1460-1464. Max. coverage (+): 0. Max coverage (-): 1.43

Region: NODE\_316089\_length\_2387\_cov\_23.258902 1465-1469. Max. coverage (+): 0.04. Max coverage (-): 3.74

Region: NODE\_316089\_length\_2387\_cov\_23.258902 1470-1474. Max. coverage (+): 0.12. Max coverage (-): 0.46

Region: NODE\_316089\_length\_2387\_cov\_23.258902 1475-1479. Max. coverage (+): 1.41. Max coverage (-): 0.16

Region: NODE\_316089\_length\_2387\_cov\_23.258902 1480-1484. Max. coverage (+): 1.41. Max coverage (-): 0.2

Region: NODE\_316089\_length\_2387\_cov\_23.258902 1485-1488. Max. coverage (+): 0.77. Max coverage (-): 0

Region: NODE\_316089\_length\_2387\_cov\_23.258902 1489-1493. Max. coverage (+): 0.26. Max coverage (-): 0.06

Region: NODE\_316089\_length\_2387\_cov\_23.258902 1494-1498. Max. coverage (+): 2. Max coverage (-): 0.02

Region: NODE\_316089\_length\_2387\_cov\_23.258902 1499-1503. Max. coverage (+): 2.1. Max coverage (-): 0.2

Region: NODE\_316089\_length\_2387\_cov\_23.258902 1504-1508. Max. coverage (+): 0.12. Max coverage (-): 0.2

Region: NODE\_316089\_length\_2387\_cov\_23.258902 1509-1513. Max. coverage (+): 9.65. Max coverage (-): 0

Region: NODE\_316089\_length\_2387\_cov\_23.258902 1514-1518. Max. coverage (+): 10.98. Max coverage (-): 0

Region: NODE\_316089\_length\_2387\_cov\_23.258902 1519-1523. Max. coverage (+): 51.28. Max coverage (-): 0.08

Region: NODE\_316089\_length\_2387\_cov\_23.258902 1524-1528. Max. coverage (+): 1.29. Max coverage (-): 0.08

Region: NODE\_316089\_length\_2387\_cov\_23.258902 1529-1532. Max. coverage (+): 0.69. Max coverage (-): 0

Region: NODE\_316089\_length\_2387\_cov\_23.258902 1533-1537. Max. coverage (+): 0.97. Max coverage (-): 0.08

Region: NODE\_316089\_length\_2387\_cov\_23.258902 1538-1542. Max. coverage (+): 0.28. Max coverage (-): 0.16

Region: NODE\_316089\_length\_2387\_cov\_23.258902 1543-1547. Max. coverage (+): 1.01. Max coverage (-): 0.16

Region: NODE\_316089\_length\_2387\_cov\_23.258902 1548-1552. Max. coverage (+): 1.01. Max coverage (-): 0.16

Region: NODE\_316089\_length\_2387\_cov\_23.258902 1553-1557. Max. coverage (+): 0.32. Max coverage (-): 0.12

Region: NODE\_316089\_length\_2387\_cov\_23.258902 1558-1562. Max. coverage (+): 0.63. Max coverage (-): 0.07

Region: NODE\_316089\_length\_2387\_cov\_23.258902 1563-1567. Max. coverage (+): 0.34. Max coverage (-): 0.05

Region: NODE\_316089\_length\_2387\_cov\_23.258902 1568-1572. Max. coverage (+): 1.03. Max coverage (-): 0.03

Region: NODE\_316089\_length\_2387\_cov\_23.258902 1573-1577. Max. coverage (+): 0.1. Max coverage (-): 0.02

Region: NODE\_316089\_length\_2387\_cov\_23.258902 1578-1581. Max. coverage (+): 0.1. Max coverage (-): 0

Region: NODE\_316089\_length\_2387\_cov\_23.258902 1582-1586. Max. coverage (+): 0.73. Max coverage (-): 0.02

Region: NODE\_316089\_length\_2387\_cov\_23.258902 1587-1591. Max. coverage (+): 0.63. Max coverage (-): 0.16

Region: NODE\_316089\_length\_2387\_cov\_23.258902 1592-1596. Max. coverage (+): 6.06. Max coverage (-): 0.4

Region: NODE\_316089\_length\_2387\_cov\_23.258902 1597-1601. Max. coverage (+): 22.57. Max coverage (-): 1.13

Region: NODE\_316089\_length\_2387\_cov\_23.258902 1602-1606. Max. coverage (+): 2.62. Max coverage (-): 0.97

Region: NODE\_316089\_length\_2387\_cov\_23.258902 1607-1611. Max. coverage (+): 2.71. Max coverage (-): 0.04

Region: NODE\_316089\_length\_2387\_cov\_23.258902 1612-1616. Max. coverage (+): 0.28. Max coverage (-): 0.28

Region: NODE\_316089\_length\_2387\_cov\_23.258902 1617-1621. Max. coverage (+): 0.08. Max coverage (-): 0.16

Region: NODE\_316089\_length\_2387\_cov\_23.258902 1622-1626. Max. coverage (+): 0.1. Max coverage (-): 0.08

Region: NODE\_316089\_length\_2387\_cov\_23.258902 1627-1630. Max. coverage (+): 0.16. Max coverage (-): 0.08

Region: NODE\_316089\_length\_2387\_cov\_23.258902 1631-1635. Max. coverage (+): 0.2. Max coverage (-): 0.16

Region: NODE\_316089\_length\_2387\_cov\_23.258902 1636-1640. Max. coverage (+): 0.04. Max coverage (-): 0.16

Region: NODE\_316089\_length\_2387\_cov\_23.258902 1641-1645. Max. coverage (+): 0.04. Max coverage (-): 0

Region: NODE\_316089\_length\_2387\_cov\_23.258902 1646-1650. Max. coverage (+): 0.69. Max coverage (-): 0

Region: NODE\_316089\_length\_2387\_cov\_23.258902 1651-1655. Max. coverage (+): 1.66. Max coverage (-): 0.16

Region: NODE\_316089\_length\_2387\_cov\_23.258902 1656-1660. Max. coverage (+): 0.52. Max coverage (-): 1.17

Region: NODE\_316089\_length\_2387\_cov\_23.258902 1661-1665. Max. coverage (+): 0.5. Max coverage (-): 0.28

Region: NODE\_316089\_length\_2387\_cov\_23.258902 1666-1670. Max. coverage (+): 0.36. Max coverage (-): 0.32

Region: NODE\_316089\_length\_2387\_cov\_23.258902 1671-1674. Max. coverage (+): 0.24. Max coverage (-): 0.12

Region: NODE\_316089\_length\_2387\_cov\_23.258902 1675-1679. Max. coverage (+): 1.7. Max coverage (-): 0

Region: NODE\_316089\_length\_2387\_cov\_23.258902 1680-1684. Max. coverage (+): 1.62. Max coverage (-): 0

Region: NODE\_316089\_length\_2387\_cov\_23.258902 1685-1689. Max. coverage (+): 1.33. Max coverage (-): 0.2

Region: NODE\_316089\_length\_2387\_cov\_23.258902 1690-1694. Max. coverage (+): 0.69. Max coverage (-): 0.2

Region: NODE\_316089\_length\_2387\_cov\_23.258902 1695-1699. Max. coverage (+): 0.16. Max coverage (-): 0.04

Region: NODE\_316089\_length\_2387\_cov\_23.258902 1700-1704. Max. coverage (+): 0.28. Max coverage (-): 0.36

Region: NODE\_316089\_length\_2387\_cov\_23.258902 1705-1709. Max. coverage (+): 0.2. Max coverage (-): 0.32

Region: NODE\_316089\_length\_2387\_cov\_23.258902 1710-1714. Max. coverage (+): 0.08. Max coverage (-): 0.12

Region: NODE\_316089\_length\_2387\_cov\_23.258902 1715-1719. Max. coverage (+): 0.2. Max coverage (-): 0.12

Region: NODE\_316089\_length\_2387\_cov\_23.258902 1720-1723. Max. coverage (+): 0.85. Max coverage (-): 0.08

Region: NODE\_316089\_length\_2387\_cov\_23.258902 1724-1728. Max. coverage (+): 1.9. Max coverage (-): 0.04

Region: NODE\_316089\_length\_2387\_cov\_23.258902 1729-1733. Max. coverage (+): 3.55. Max coverage (-): 0.12

Region: NODE\_316089\_length\_2387\_cov\_23.258902 1734-1738. Max. coverage (+): 2.83. Max coverage (-): 0

Region: NODE\_316089\_length\_2387\_cov\_23.258902 1739-1743. Max. coverage (+): 3.51. Max coverage (-): 0.04

Region: NODE\_316089\_length\_2387\_cov\_23.258902 1744-1748. Max. coverage (+): 0.81. Max coverage (-): 0.04

Region: NODE\_316089\_length\_2387\_cov\_23.258902 1749-1753. Max. coverage (+): 0.16. Max coverage (-): 0.32

Region: NODE\_316089\_length\_2387\_cov\_23.258902 1754-1758. Max. coverage (+): 0.2. Max coverage (-): 0.28

Region: NODE\_316089\_length\_2387\_cov\_23.258902 1759-1763. Max. coverage (+): 0.2. Max coverage (-): 0.12

Region: NODE\_316089\_length\_2387\_cov\_23.258902 1764-1768. Max. coverage (+): 0.44. Max coverage (-): 0

Region: NODE\_316089\_length\_2387\_cov\_23.258902 1769-1772. Max. coverage (+): 11.71. Max coverage (-): 0

Region: NODE\_316089\_length\_2387\_cov\_23.258902 1773-1777. Max. coverage (+): 2.87. Max coverage (-): 0.04

Region: NODE\_316089\_length\_2387\_cov\_23.258902 1778-1782. Max. coverage (+): 0.61. Max coverage (-): 0.04

Region: NODE\_316089\_length\_2387\_cov\_23.258902 1783-1787. Max. coverage (+): 0.36. Max coverage (-): 0.08

Region: NODE\_316089\_length\_2387\_cov\_23.258902 1788-1792. Max. coverage (+): 0.93. Max coverage (-): 0

Region: NODE\_316089\_length\_2387\_cov\_23.258902 1793-1797. Max. coverage (+): 0.04. Max coverage (-): 0

Region: NODE\_316089\_length\_2387\_cov\_23.258902 1798-1802. Max. coverage (+): 0.24. Max coverage (-): 0

Region: NODE\_316089\_length\_2387\_cov\_23.258902 1803-1807. Max. coverage (+): 0.52. Max coverage (-): 0

Region: NODE\_316089\_length\_2387\_cov\_23.258902 1808-1812. Max. coverage (+): 0.57. Max coverage (-): 0

Region: NODE\_316089\_length\_2387\_cov\_23.258902 1813-1816. Max. coverage (+): 0.52. Max coverage (-): 0

Region: NODE\_316089\_length\_2387\_cov\_23.258902 1817-1821. Max. coverage (+): 1.33. Max coverage (-): 0.08

Region: NODE\_316089\_length\_2387\_cov\_23.258902 1822-1826. Max. coverage (+): 0.93. Max coverage (-): 0.08

Region: NODE\_316089\_length\_2387\_cov\_23.258902 1827-1831. Max. coverage (+): 0.44. Max coverage (-): 0.12

Region: NODE\_316089\_length\_2387\_cov\_23.258902 1832-1836. Max. coverage (+): 0.81. Max coverage (-): 0.16

Region: NODE\_316089\_length\_2387\_cov\_23.258902 1837-1841. Max. coverage (+): 0.52. Max coverage (-): 0

Region: NODE\_316089\_length\_2387\_cov\_23.258902 1842-1846. Max. coverage (+): 0.24. Max coverage (-): 0

Region: NODE\_316089\_length\_2387\_cov\_23.258902 1847-1851. Max. coverage (+): 1.01. Max coverage (-): 0.04

Region: NODE\_316089\_length\_2387\_cov\_23.258902 1852-1856. Max. coverage (+): 1.21. Max coverage (-): 0.04

Region: NODE\_316089\_length\_2387\_cov\_23.258902 1857-1861. Max. coverage (+): 0.16. Max coverage (-): 0

Region: NODE\_316089\_length\_2387\_cov\_23.258902 1862-1865. Max. coverage (+): 0.16. Max coverage (-): 0

Region: NODE\_316089\_length\_2387\_cov\_23.258902 1866-1870. Max. coverage (+): 0.12. Max coverage (-): 0.04

Region: NODE\_316089\_length\_2387\_cov\_23.258902 1871-1875. Max. coverage (+): 5.53. Max coverage (-): 0.04

Region: NODE\_316089\_length\_2387\_cov\_23.258902 1876-1880. Max. coverage (+): 5.65. Max coverage (-): 0.08

Region: NODE\_316089\_length\_2387\_cov\_23.258902 1881-1885. Max. coverage (+): 0.08. Max coverage (-): 0

Region: NODE\_316089\_length\_2387\_cov\_23.258902 1886-1890. Max. coverage (+): 0.08. Max coverage (-): 0.12

Region: NODE\_316089\_length\_2387\_cov\_23.258902 1891-1895. Max. coverage (+): 0.08. Max coverage (-): 0.28

Region: NODE\_316089\_length\_2387\_cov\_23.258902 1896-1900. Max. coverage (+): 0. Max coverage (-): 0.08

Region: NODE\_316089\_length\_2387\_cov\_23.258902 1901-1905. Max. coverage (+): 0. Max coverage (-): 0.04

Region: NODE\_316089\_length\_2387\_cov\_23.258902 1906-1909. Max. coverage (+): 0.2. Max coverage (-): 0.04

Region: NODE\_316089\_length\_2387\_cov\_23.258902 1910-1914. Max. coverage (+): 0.32. Max coverage (-): 0

Region: NODE\_316089\_length\_2387\_cov\_23.258902 1915-1919. Max. coverage (+): 0.04. Max coverage (-): 0.08

Region: NODE\_316089\_length\_2387\_cov\_23.258902 1920-1924. Max. coverage (+): 0.32. Max coverage (-): 0.08

Region: NODE\_316089\_length\_2387\_cov\_23.258902 1925-1929. Max. coverage (+): 0.24. Max coverage (-): 0

Region: NODE\_316089\_length\_2387\_cov\_23.258902 1930-1934. Max. coverage (+): 20.39. Max coverage (-): 0.16

Region: NODE\_316089\_length\_2387\_cov\_23.258902 1935-1939. Max. coverage (+): 20.31. Max coverage (-): 0.36

Region: NODE\_316089\_length\_2387\_cov\_23.258902 1940-1944. Max. coverage (+): 20.47. Max coverage (-): 0.28

Region: NODE\_316089\_length\_2387\_cov\_23.258902 1945-1949. Max. coverage (+): 21.49. Max coverage (-): 0.1

Region: NODE\_316089\_length\_2387\_cov\_23.258902 1950-1954. Max. coverage (+): 0.16. Max coverage (-): 0.04

Region: NODE\_316089\_length\_2387\_cov\_23.258902 1955-1958. Max. coverage (+): 0.04. Max coverage (-): 0.04

Region: NODE\_316089\_length\_2387\_cov\_23.258902 1959-1963. Max. coverage (+): 0.12. Max coverage (-): 0

Region: NODE\_316089\_length\_2387\_cov\_23.258902 1964-1968. Max. coverage (+): 0.12. Max coverage (-): 0

Region: NODE\_316089\_length\_2387\_cov\_23.258902 1969-1973. Max. coverage (+): 0. Max coverage (-): 0.14

Region: NODE\_316089\_length\_2387\_cov\_23.258902 1974-1978. Max. coverage (+): 1.57. Max coverage (-): 0.12

Region: NODE\_316089\_length\_2387\_cov\_23.258902 1979-1983. Max. coverage (+): 1.25. Max coverage (-): 0.08

Region: NODE\_316089\_length\_2387\_cov\_23.258902 1984-1988. Max. coverage (+): 1.21. Max coverage (-): 0

Region: NODE\_316089\_length\_2387\_cov\_23.258902 1989-1993. Max. coverage (+): 1.7. Max coverage (-): 0.08

Region: NODE\_316089\_length\_2387\_cov\_23.258902 1994-1998. Max. coverage (+): 1.9. Max coverage (-): 0.04

Region: NODE\_316089\_length\_2387\_cov\_23.258902 1999-2003. Max. coverage (+): 0.32. Max coverage (-): 0

Region: NODE\_316089\_length\_2387\_cov\_23.258902 2004-2007. Max. coverage (+): 0.16. Max coverage (-): 0

Region: NODE\_316089\_length\_2387\_cov\_23.258902 2008-2012. Max. coverage (+): 0.61. Max coverage (-): 0

Region: NODE\_316089\_length\_2387\_cov\_23.258902 2013-2017. Max. coverage (+): 0. Max coverage (-): 0

Region: NODE\_316089\_length\_2387\_cov\_23.258902 2018-2022. Max. coverage (+): 0. Max coverage (-): 0

Region: NODE\_316089\_length\_2387\_cov\_23.258902 2023-2027. Max. coverage (+): 0.73. Max coverage (-): 0.32

Region: NODE\_316089\_length\_2387\_cov\_23.258902 2028-2032. Max. coverage (+): 0.81. Max coverage (-): 0.24

Region: NODE\_316089\_length\_2387\_cov\_23.258902 2033-2037. Max. coverage (+): 0.44. Max coverage (-): 0.16

Region: NODE\_316089\_length\_2387\_cov\_23.258902 2038-2042. Max. coverage (+): 0.65. Max coverage (-): 0.12

Region: NODE\_316089\_length\_2387\_cov\_23.258902 2043-2047. Max. coverage (+): 0.81. Max coverage (-): 0.04

Region: NODE\_316089\_length\_2387\_cov\_23.258902 2048-2051. Max. coverage (+): 0.12. Max coverage (-): 0

Region: NODE\_316089\_length\_2387\_cov\_23.258902 2052-2056. Max. coverage (+): 1.17. Max coverage (-): 0

Region: NODE\_316089\_length\_2387\_cov\_23.258902 2057-2061. Max. coverage (+): 1.29. Max coverage (-): 0

Region: NODE\_316089\_length\_2387\_cov\_23.258902 2062-2066. Max. coverage (+): 0.85. Max coverage (-): 0

Region: NODE\_316089\_length\_2387\_cov\_23.258902 2067-2071. Max. coverage (+): 0.44. Max coverage (-): 0

Region: NODE\_316089\_length\_2387\_cov\_23.258902 2072-2076. Max. coverage (+): 1.21. Max coverage (-): 0.04

Region: NODE\_316089\_length\_2387\_cov\_23.258902 2077-2081. Max. coverage (+): 1.09. Max coverage (-): 0.04

Region: NODE\_316089\_length\_2387\_cov\_23.258902 2082-2086. Max. coverage (+): 4.9. Max coverage (-): 0

Region: NODE\_316089\_length\_2387\_cov\_23.258902 2087-2091. Max. coverage (+): 4.35. Max coverage (-): 0

Region: NODE\_316089\_length\_2387\_cov\_23.258902 2092-2096. Max. coverage (+): 0.01. Max coverage (-): 0.02

Region: NODE\_316089\_length\_2387\_cov\_23.258902 2097-2100. Max. coverage (+): 0.16. Max coverage (-): 0.2

Region: NODE\_316089\_length\_2387\_cov\_23.258902 2101-2105. Max. coverage (+): 1.37. Max coverage (-): 0.24

Region: NODE\_316089\_length\_2387\_cov\_23.258902 2106-2110. Max. coverage (+): 3.88. Max coverage (-): 0.04

Region: NODE\_316089\_length\_2387\_cov\_23.258902 2111-2115. Max. coverage (+): 3.71. Max coverage (-): 0.04

Region: NODE\_316089\_length\_2387\_cov\_23.258902 2116-2120. Max. coverage (+): 2.42. Max coverage (-): 0.04

Region: NODE\_316089\_length\_2387\_cov\_23.258902 2121-2125. Max. coverage (+): 1.06. Max coverage (-): 0.04

Region: NODE\_316089\_length\_2387\_cov\_23.258902 2126-2130. Max. coverage (+): 0. Max coverage (-): 0

Region: NODE\_316089\_length\_2387\_cov\_23.258902 2131-2135. Max. coverage (+): 0. Max coverage (-): 0

Region: NODE\_316089\_length\_2387\_cov\_23.258902 2136-2140. Max. coverage (+): 0.52. Max coverage (-): 0

Region: NODE\_316089\_length\_2387\_cov\_23.258902 2141-2144. Max. coverage (+): 1.82. Max coverage (-): 0

Region: NODE\_316089\_length\_2387\_cov\_23.258902 2145-2149. Max. coverage (+): 1.82. Max coverage (-): 0

Region: NODE\_316089\_length\_2387\_cov\_23.258902 2150-2154. Max. coverage (+): 0.52. Max coverage (-): 0

Region: NODE\_316089\_length\_2387\_cov\_23.258902 2155-2159. Max. coverage (+): 0.04. Max coverage (-): 0

Region: NODE\_316089\_length\_2387\_cov\_23.258902 2160-2164. Max. coverage (+): 0. Max coverage (-): 0.2

Region: NODE\_316089\_length\_2387\_cov\_23.258902 2165-2169. Max. coverage (+): 0.16. Max coverage (-): 0.32

Region: NODE\_316089\_length\_2387\_cov\_23.258902 2170-2174. Max. coverage (+): 4. Max coverage (-): 0

Region: NODE\_316089\_length\_2387\_cov\_23.258902 2175-2179. Max. coverage (+): 5.29. Max coverage (-): 0

Region: NODE\_316089\_length\_2387\_cov\_23.258902 2180-2184. Max. coverage (+): 2.3. Max coverage (-): 0

Region: NODE\_316089\_length\_2387\_cov\_23.258902 2185-2189. Max. coverage (+): 1.33. Max coverage (-): 0

Region: NODE\_316089\_length\_2387\_cov\_23.258902 2190-2193. Max. coverage (+): 0.04. Max coverage (-): 0.02

Region: NODE\_316089\_length\_2387\_cov\_23.258902 2194-2198. Max. coverage (+): 0.06. Max coverage (-): 0.12

Region: NODE\_316089\_length\_2387\_cov\_23.258902 2199-2203. Max. coverage (+): 0. Max coverage (-): 0.1

Region: NODE\_316089\_length\_2387\_cov\_23.258902 2204-2208. Max. coverage (+): 0.03. Max coverage (-): 0.01

Region: NODE\_316089\_length\_2387\_cov\_23.258902 2209-2213. Max. coverage (+): 1.72. Max coverage (-): 0.01

Region: NODE\_316089\_length\_2387\_cov\_23.258902 2214-2218. Max. coverage (+): 0.4. Max coverage (-): 0

Region: NODE\_316089\_length\_2387\_cov\_23.258902 2219-2223. Max. coverage (+): 0.41. Max coverage (-): 0

Region: NODE\_316089\_length\_2387\_cov\_23.258902 2224-2228. Max. coverage (+): 0. Max coverage (-): 0

Region: NODE\_316089\_length\_2387\_cov\_23.258902 2229-2233. Max. coverage (+): 0.01. Max coverage (-): 0.01

Region: NODE\_316089\_length\_2387\_cov\_23.258902 2234-2238. Max. coverage (+): 0. Max coverage (-): 0.01

Region: NODE\_316089\_length\_2387\_cov\_23.258902 2239-2242. Max. coverage (+): 0.12. Max coverage (-): 0

Region: NODE\_316089\_length\_2387\_cov\_23.258902 2243-2247. Max. coverage (+): 0.2. Max coverage (-): 0

Region: NODE\_316089\_length\_2387\_cov\_23.258902 2248-2252. Max. coverage (+): 0.1. Max coverage (-): 0

Region: NODE\_316089\_length\_2387\_cov\_23.258902 2253-2257. Max. coverage (+): 0.02. Max coverage (-): 0

Region: NODE\_316089\_length\_2387\_cov\_23.258902 2258-2262. Max. coverage (+): 0.16. Max coverage (-): 0.12

Region: NODE\_316089\_length\_2387\_cov\_23.258902 2263-2267. Max. coverage (+): 0.02. Max coverage (-): 0.16

Region: NODE\_316089\_length\_2387\_cov\_23.258902 2268-2272. Max. coverage (+): 0.04. Max coverage (-): 0.2

Region: NODE\_316089\_length\_2387\_cov\_23.258902 2273-2277. Max. coverage (+): 0.06. Max coverage (-): 0.1

Region: NODE\_316089\_length\_2387\_cov\_23.258902 2278-2282. Max. coverage (+): 0.3. Max coverage (-): 0.01

Region: NODE\_316089\_length\_2387\_cov\_23.258902 2283-2286. Max. coverage (+): 0.1. Max coverage (-): 0

Region: NODE\_316089\_length\_2387\_cov\_23.258902 2287-2291. Max. coverage (+): 0.02. Max coverage (-): 0

Region: NODE\_316089\_length\_2387\_cov\_23.258902 2292-2296. Max. coverage (+): 0. Max coverage (-): 0.01

Region: NODE\_316089\_length\_2387\_cov\_23.258902 2297-2301. Max. coverage (+): 0. Max coverage (-): 0

Region: NODE\_316089\_length\_2387\_cov\_23.258902 2302-2306. Max. coverage (+): 0.06. Max coverage (-): 0

Region: NODE\_316089\_length\_2387\_cov\_23.258902 2307-2311. Max. coverage (+): 0.17. Max coverage (-): 0.5

Region: NODE\_316089\_length\_2387\_cov\_23.258902 2312-2316. Max. coverage (+): 1.62. Max coverage (-): 0.55

Region: NODE\_316089\_length\_2387\_cov\_23.258902 2317-2321. Max. coverage (+): 2.14. Max coverage (-): 0.06

Region: NODE\_316089\_length\_2387\_cov\_23.258902 2322-2326. Max. coverage (+): 0.02. Max coverage (-): 0

Region: NODE\_316089\_length\_2387\_cov\_23.258902 2327-2331. Max. coverage (+): 3.27. Max coverage (-): 0

Region: NODE\_316089\_length\_2387\_cov\_23.258902 2332-2335. Max. coverage (+): 0.4. Max coverage (-): 0

Region: NODE\_316089\_length\_2387\_cov\_23.258902 2336-2340. Max. coverage (+): 0.2. Max coverage (-): 0.16

Region: NODE\_316089\_length\_2387\_cov\_23.258902 2341-2345. Max. coverage (+): 0.2. Max coverage (-): 0.32

Region: NODE\_316089\_length\_2387\_cov\_23.258902 2346-2350. Max. coverage (+): 0.16. Max coverage (-): 0

Region: NODE\_316089\_length\_2387\_cov\_23.258902 2351-2355. Max. coverage (+): 0.24. Max coverage (-): 0.12

Region: NODE\_316089\_length\_2387\_cov\_23.258902 2356-2360. Max. coverage (+): 2.1. Max coverage (-): 0.16

Region: NODE\_316089\_length\_2387\_cov\_23.258902 2361-2365. Max. coverage (+): 2.1. Max coverage (-): 0

Region: NODE\_316089\_length\_2387\_cov\_23.258902 2366-2370. Max. coverage (+): 0.26. Max coverage (-): 0.04

Region: NODE\_316089\_length\_2387\_cov\_23.258902 2371-2375. Max. coverage (+): 1.59. Max coverage (-): 0.1

Region: NODE\_316089\_length\_2387\_cov\_23.258902 2376-2380. Max. coverage (+): 1.41. Max coverage (-): 0

Region: NODE\_316089\_length\_2387\_cov\_23.258902 2381-2384. Max. coverage (+): 0.69. Max coverage (-): 0

Region: NODE\_316089\_length\_2387\_cov\_23.258902 2385-2389. Max. coverage (+): 4.16. Max coverage (-): 0

Region: NODE\_316089\_length\_2387\_cov\_23.258902 2390-2394. Max. coverage (+): 4.36. Max coverage (-): 0

Region: NODE\_316089\_length\_2387\_cov\_23.258902 2395-2399. Max. coverage (+): 0.4. Max coverage (-): 0.12

Region: NODE\_316089\_length\_2387\_cov\_23.258902 2400-2404. Max. coverage (+): 0.09. Max coverage (-): 0.05

Region: NODE\_316089\_length\_2387\_cov\_23.258902 2405-2409. Max. coverage (+): 0.03. Max coverage (-): 0.01

Region: NODE\_316089\_length\_2387\_cov\_23.258902 2410-2414. Max. coverage (+): 0.6. Max coverage (-): 0

Region: NODE\_316089\_length\_2387\_cov\_23.258902 2415-2419. Max. coverage (+): 0.07. Max coverage (-): 0.04

Region: NODE\_316089\_length\_2387\_cov\_23.258902 2420-2424. Max. coverage (+): 0.11. Max coverage (-): 0.05

Region: NODE\_316089\_length\_2387\_cov\_23.258902 2425-2428. Max. coverage (+): 0.08. Max coverage (-): 0.02

Region: NODE\_316089\_length\_2387\_cov\_23.258902 2429-2433. Max. coverage (+): 0.01. Max coverage (-): 0

Region: NODE\_316089\_length\_2387\_cov\_23.258902 2434-2438. Max. coverage (+): 0. Max coverage (-): 0

Region: NODE\_316089\_length\_2387\_cov\_23.258902 2439-2443. Max. coverage (+): 0. Max coverage (-): 0

Region: NODE\_316089\_length\_2387\_cov\_23.258902 2444-2448. Max. coverage (+): 0. Max coverage (-): 0

Region: NODE\_316089\_length\_2387\_cov\_23.258902 2449-. Max. coverage (+): 0. Max coverage (-): 0

RepeatMasker Color Code

**+**

100-98% Identity

<98-95% Identity

<95-90% Identity

<90-85% Identity

<85-80% Identity

<80-75% Identity

<75-70% Identity

<70% Identity

**-**

Gene Set Color Code

**+**

Gene

Pseudogene

Other

**-**

Topology/Coverage Color Code

Coverage Plus Strand

Coverage Minus Strand

Mainstrand: Plus

Mainstrand: Minus

Complementary Strand

Flanking Region  
(if option -flank >0)

Gene Set Annotation  
  
RepeatMasker Annotation  

**1. AlRepD-2135**: 1-61 (-), Divergence to consensus: 0%  
**2. AlRepE-3612**: 179-267 (-), Divergence to consensus: 13.5%  
**3. Tc1-2\_PM**: 180-462 (+), Divergence to consensus: 35.8%  
**4. Tc1-13\_Xt**: 484-547 (+), Divergence to consensus: 25%  
**5. Tc1-2\_PM**: 553-628 (+), Divergence to consensus: 34.2%  
**6. L1-52\_DR**: 995-1242 (+), Divergence to consensus: 41.5%  
**7. L1-15c\_Lch**: 1205-1326 (+), Divergence to consensus: 36.1%  
**8. AlRepD-2046**: 1662-1704 (-), Divergence to consensus: 16.2%  
**9. AlRepE-7493**: 1756-2073 (+), Divergence to consensus: 23.5%

  
Transcription Factor Binding Sites  

**RHOXF1** (Sequence: AGATCA (-): 221)  
**RHOXF1** (Sequence: AGATCA (-): 542)  
**RHOXF1** (Sequence: AGCTTA (-): 1425)  
**RHOXF1** (Sequence: AGATCA (-): 1995)  
**RHOXF1** (Sequence: AGCTCA (-): 2334)  
**RHOXF1** (Sequence: GGATTA (-): 2383)  
**RHOXF1** (Sequence: AGATCA (-): 2444)  
**RHOXF1** (Sequence: TAAGCT (+): 925)  
**RHOXF1** (Sequence: TAAGCC (+): 1141)  
**RHOXF1** (Sequence: TAAGCC (+): 1201)  
**RHOXF1** (Sequence: TGAGCT (+): 1344)  
**RHOXF1** (Sequence: TGAGCT (+): 1621)  
**RHOXF1** (Sequence: TGATCT (+): 1744)  
**RHOXF1** (Sequence: TGATCT (+): 2303)  
**FOXO3\_hsa** (Sequence: GTAAACAT (+): 2011)  
**FOXP1** (Sequence: GTAAACA (+): 2011)  
**Sox5** (Sequence: ATTGTT (+): 1109)  
**Sox5** (Sequence: ATTGTT (+): 2217)  
**Nobox** (Sequence: TAATTGCT (+): 2288)  
**Rhox11** (Sequence: ATTACACCA (-): 244)  
**Sox5** (Sequence: AACAAT (-): 458)  
**Sox5** (Sequence: AACAAT (-): 699)
