# Supplementary material for: First characterization of PIWI-interacting RNA clusters in a cichlid fish with a B chromosome
Source: BMC Biol. 2022 Sep 21;20:204. doi: 10.1186/s12915-022-01403-2 (PMC9490952; doi:10.1186/s12915-022-01403-2)
Supplement: Supplementary file 1 — Additional file 1. Zipped folder with fasta and interactive html piRNA cluster information for the A. latifasciata genome. The nomenclature is as follows: number-pirna-cluster_sex_B-presence (f, female; m, male; 0b, without B chromosome; 1b, with B chromosome). [file 12915_2022_1403_MOESM1_ESM.zip › 123_m1b.html]

piRNA cluster 123\_m1b 71


Predicted piRNA cluster no. 123\_m1b
  

Show proTRAC run info
Hide proTRAC run info

/\  
                \_\_\_\_\_\_\_\_\_\_\_\_\_\_\_\_\_\_\_\_\_\_\_/\\_\_\_ /  \\_\_\_\_\_\_\_  
               I                      /  \  /    \      I  
               I     pro             /    \/      \     I  
               I        TRAC        /               \   I  
               I   \_\_\_\_\_\_\_\_\_\_\_\_\_\_\_\_/\_\_\_\_\_\_\_\_\_\_\_\_\_\_\_\_\_\\_ I  
               I   \              /                     I  
               I    \            /                      I  
               I     \  /\      /       V.2.4.2         I  
               I      \/  \    /                        I  
               I\_\_\_\_\_\_\_\_\_\_\_\  /\_\_\_\_\_\_\_\_\_\_\_\_\_\_\_\_\_\_\_\_\_\_\_\_\_I  
                            \/  
  
  
================================= proTRAC ====================================  
VERSION: .......... 2.4.2  
LAST MODIFIED: .... 11. May 2018  
  
Please cite:  
Rosenkranz D, Zischler H. proTRAC - a software for probabilistic piRNA cluster  
detection, visualization and analysis. 2012. BMC Bioinformatics 13:5.  
  
  
Contact:  
David Rosenkranz  
Institute of Organismic and Molecular Evolutionary Biology  
Dept. Anthropology, small RNA group  
Johannes Gutenberg University Mainz  
email: rosenkranz@uni-mainz.de  
  
You can find the latest proTRAC version at:  
http://sourceforge.net/projects/protrac/files  
http://www.smallRNAgroup-mainz.de/software  
==============================================================================  
  
PARAMETERS:  
Map file: ...............piwi-machos-1B.fa-collapse.map  
Genome file: ............../../../0B\_ala\_genome.fa  
RepeatMasker annotation: Alatifasciata-all0B-maryan-v2.fa\_corrected.out  
GeneSet:................./guest-storage/Data/annotation/Alatifasciata\_all0B\_maryan-v2\_out2017.gff  
  
Significant (p<=0.01) hit density will be calculated based  
on observed hit distribution.  
  
Sliding window size: ........................................ 5000 bp  
Sliding window increament: .................................. 1000 bp  
Normalize each hit by number of genomic hits: ............... yes  
Normalize each hit by number of sequence reads: ............. yes  
Normalize values (-> per million mapped reads): ............. yes  
Min. fraction of hits with 1T(U) or 10A: .................... 0.75  
Alternatively: Min. fraction of hits with 1T(U) and 10A: .... 0.5  
Min. fraction of hits with typical piRNA length: ............ 0.75  
Typical piRNA length: ....................................... 24-32 nt  
Min. size of a piRNA cluster: ............................... 1000 bp.  
Min. number of hits (absolute): ............................. 0  
Min. number of hits (normalized): ........................... 0  
Min. fraction of hits on the mainstrand: .................... 0.75  
Top fraction of mapped sequences (in terms of read counts): . 1%  
Top fraction accounts for max. n% of sequence reads: ........ 90%  
Min. fraction of hits on each arm of a bidirectional cluster: 0.05  
Output html file for each cluster: .......................... yes  
Output a summary table: ..................................... yes  
Output a FASTA file for each cluster (piRNA sequences): ..... yes  
Output a FASTA file comprising cluster sequences: ........... yes  
Output a GTF file for predicted piRNA clusters: ..............yes  
Search DNA motifs in clusters: .............................. yes  
Output flanking sequences: +/- .............................. 0 bp  
Output ~.pTi file: .......................................... no  
==============================================================================  
  
  
Genome size (without gaps): ............ 758543724 bp  
Gaps (N/X/-): .......................... 417479 bp  
Mapped reads: .......................... 26973943  
Non-identical sequences: ............... 6209225  
Genomic hits: .......................... 48438990  
Significant densitiy of mapped reads: .. 821.144211136946 reads/kb

Show proTRAC cluster info
Hide proTRAC cluster info

|  |  |
| --- | --- |
| Location | NODE\_316089\_length\_2387\_cov\_23.258902 |
| Coordinates | 1-2451 |
| Size [bp] | 2451 |
| Sequence hit loci | 4408 |
| Mapped reads (normalized) | 26496.3 |
| Mapped reads (normalized) per kb | 10810.4 |
| Normalized reads with 1T (1U) | 83.5% |
| Normalized reads with 10A | 36.1% |
| Normalized reads with length 24-32 nt | 99.1% |
| Normalized reads on the main strand(s) | 95.7% |
| Predicted directionality | mono:plus |

100%

0%

1T (1U)  
reads

10A reads

24-32 nt  
reads

reads on mainstrand

**Either the amount of reads with 1T (1U) OR 10A has to exceed 75% (set with option: -1Tor10A)  
Alternatively the amount of reads with 1T (1U) AND 10A has to exceed 50% (set with option: -1Tand10A)  
Minimum amount of reads with preferred size is 75% (set with option: -pisize)  
Minimum amount of reads on the main strand(s) is 75% (set with option: -clstrand)**

Show read coverage
Hide read coverage

WHAT DO I SEE HERE?  
This chart shows the location of mapped sequence reads within a predicted piRNA cluster. The color refers to the number of genomic hits produced by the sequence read in question. A dark red bar indicates that this sequence read produces many other hits elsewhere in the genome. Many adjacent red or yellow bars can indicate the presence of a multi-copy element such as transposons or rRNA genes. A dark green bar indicates that this sequence read maps uniquely to this locus.

1 hit

2-5 hits

6-10 hits

11-20 hits

21-50 hits

51-100 hits

> 100 hits

NODE\_316089\_length\_2387\_cov\_23.258902

1

2451

Gene Set

RepeatMasker

Mapped  
Reads

171.67

plus strand

minus strand

171.67

Region: NODE\_316089\_length\_2387\_cov\_23.258902 64825-3. Max. coverage (+): 0. Max coverage (-): 0

Region: NODE\_316089\_length\_2387\_cov\_23.258902 4-8. Max. coverage (+): 0. Max coverage (-): 0

Region: NODE\_316089\_length\_2387\_cov\_23.258902 9-13. Max. coverage (+): 0. Max coverage (-): 0

Region: NODE\_316089\_length\_2387\_cov\_23.258902 14-18. Max. coverage (+): 0. Max coverage (-): 0

Region: NODE\_316089\_length\_2387\_cov\_23.258902 19-23. Max. coverage (+): 0. Max coverage (-): 0

Region: NODE\_316089\_length\_2387\_cov\_23.258902 24-27. Max. coverage (+): 0. Max coverage (-): 0

Region: NODE\_316089\_length\_2387\_cov\_23.258902 28-32. Max. coverage (+): 0. Max coverage (-): 0

Region: NODE\_316089\_length\_2387\_cov\_23.258902 33-37. Max. coverage (+): 0.07. Max coverage (-): 0

Region: NODE\_316089\_length\_2387\_cov\_23.258902 38-42. Max. coverage (+): 27.95. Max coverage (-): 0

Region: NODE\_316089\_length\_2387\_cov\_23.258902 43-47. Max. coverage (+): 0.04. Max coverage (-): 0

Region: NODE\_316089\_length\_2387\_cov\_23.258902 48-52. Max. coverage (+): 0. Max coverage (-): 0

Region: NODE\_316089\_length\_2387\_cov\_23.258902 53-57. Max. coverage (+): 0. Max coverage (-): 0.67

Region: NODE\_316089\_length\_2387\_cov\_23.258902 58-62. Max. coverage (+): 0. Max coverage (-): 0.56

Region: NODE\_316089\_length\_2387\_cov\_23.258902 63-67. Max. coverage (+): 0.07. Max coverage (-): 0.04

Region: NODE\_316089\_length\_2387\_cov\_23.258902 68-72. Max. coverage (+): 0. Max coverage (-): 0

Region: NODE\_316089\_length\_2387\_cov\_23.258902 73-76. Max. coverage (+): 0.26. Max coverage (-): 0

Region: NODE\_316089\_length\_2387\_cov\_23.258902 77-81. Max. coverage (+): 0.26. Max coverage (-): 0.04

Region: NODE\_316089\_length\_2387\_cov\_23.258902 82-86. Max. coverage (+): 0.15. Max coverage (-): 0.04

Region: NODE\_316089\_length\_2387\_cov\_23.258902 87-91. Max. coverage (+): 0.19. Max coverage (-): 0

Region: NODE\_316089\_length\_2387\_cov\_23.258902 92-96. Max. coverage (+): 0. Max coverage (-): 0

Region: NODE\_316089\_length\_2387\_cov\_23.258902 97-101. Max. coverage (+): 0. Max coverage (-): 0

Region: NODE\_316089\_length\_2387\_cov\_23.258902 102-106. Max. coverage (+): 0. Max coverage (-): 0

Region: NODE\_316089\_length\_2387\_cov\_23.258902 107-111. Max. coverage (+): 0.04. Max coverage (-): 0

Region: NODE\_316089\_length\_2387\_cov\_23.258902 112-116. Max. coverage (+): 0.04. Max coverage (-): 0.04

Region: NODE\_316089\_length\_2387\_cov\_23.258902 117-121. Max. coverage (+): 0.07. Max coverage (-): 0

Region: NODE\_316089\_length\_2387\_cov\_23.258902 122-126. Max. coverage (+): 0.04. Max coverage (-): 0

Region: NODE\_316089\_length\_2387\_cov\_23.258902 127-130. Max. coverage (+): 0.07. Max coverage (-): 0

Region: NODE\_316089\_length\_2387\_cov\_23.258902 131-135. Max. coverage (+): 0.04. Max coverage (-): 0

Region: NODE\_316089\_length\_2387\_cov\_23.258902 136-140. Max. coverage (+): 0.52. Max coverage (-): 0

Region: NODE\_316089\_length\_2387\_cov\_23.258902 141-145. Max. coverage (+): 0.93. Max coverage (-): 0.04

Region: NODE\_316089\_length\_2387\_cov\_23.258902 146-150. Max. coverage (+): 1.48. Max coverage (-): 0.04

Region: NODE\_316089\_length\_2387\_cov\_23.258902 151-155. Max. coverage (+): 1.45. Max coverage (-): 0

Region: NODE\_316089\_length\_2387\_cov\_23.258902 156-160. Max. coverage (+): 0.04. Max coverage (-): 0

Region: NODE\_316089\_length\_2387\_cov\_23.258902 161-165. Max. coverage (+): 0.07. Max coverage (-): 0.04

Region: NODE\_316089\_length\_2387\_cov\_23.258902 166-170. Max. coverage (+): 0.04. Max coverage (-): 0

Region: NODE\_316089\_length\_2387\_cov\_23.258902 171-175. Max. coverage (+): 0.07. Max coverage (-): 0.19

Region: NODE\_316089\_length\_2387\_cov\_23.258902 176-179. Max. coverage (+): 0. Max coverage (-): 0.11

Region: NODE\_316089\_length\_2387\_cov\_23.258902 180-184. Max. coverage (+): 0. Max coverage (-): 0

Region: NODE\_316089\_length\_2387\_cov\_23.258902 185-189. Max. coverage (+): 0.15. Max coverage (-): 0.04

Region: NODE\_316089\_length\_2387\_cov\_23.258902 190-194. Max. coverage (+): 0.85. Max coverage (-): 0.04

Region: NODE\_316089\_length\_2387\_cov\_23.258902 195-199. Max. coverage (+): 0. Max coverage (-): 0

Region: NODE\_316089\_length\_2387\_cov\_23.258902 200-204. Max. coverage (+): 0.04. Max coverage (-): 0

Region: NODE\_316089\_length\_2387\_cov\_23.258902 205-209. Max. coverage (+): 0.04. Max coverage (-): 0.04

Region: NODE\_316089\_length\_2387\_cov\_23.258902 210-214. Max. coverage (+): 0.19. Max coverage (-): 0

Region: NODE\_316089\_length\_2387\_cov\_23.258902 215-219. Max. coverage (+): 1.19. Max coverage (-): 0.04

Region: NODE\_316089\_length\_2387\_cov\_23.258902 220-224. Max. coverage (+): 1.33. Max coverage (-): 0.11

Region: NODE\_316089\_length\_2387\_cov\_23.258902 225-228. Max. coverage (+): 0.74. Max coverage (-): 0.04

Region: NODE\_316089\_length\_2387\_cov\_23.258902 229-233. Max. coverage (+): 0.07. Max coverage (-): 0.04

Region: NODE\_316089\_length\_2387\_cov\_23.258902 234-238. Max. coverage (+): 0. Max coverage (-): 0

Region: NODE\_316089\_length\_2387\_cov\_23.258902 239-243. Max. coverage (+): 0.04. Max coverage (-): 0

Region: NODE\_316089\_length\_2387\_cov\_23.258902 244-248. Max. coverage (+): 0.11. Max coverage (-): 0

Region: NODE\_316089\_length\_2387\_cov\_23.258902 249-253. Max. coverage (+): 0.11. Max coverage (-): 0.04

Region: NODE\_316089\_length\_2387\_cov\_23.258902 254-258. Max. coverage (+): 0. Max coverage (-): 0

Region: NODE\_316089\_length\_2387\_cov\_23.258902 259-263. Max. coverage (+): 0.07. Max coverage (-): 0.04

Region: NODE\_316089\_length\_2387\_cov\_23.258902 264-268. Max. coverage (+): 0.07. Max coverage (-): 0.15

Region: NODE\_316089\_length\_2387\_cov\_23.258902 269-273. Max. coverage (+): 0.7. Max coverage (-): 0.07

Region: NODE\_316089\_length\_2387\_cov\_23.258902 274-277. Max. coverage (+): 1.67. Max coverage (-): 0

Region: NODE\_316089\_length\_2387\_cov\_23.258902 278-282. Max. coverage (+): 0.93. Max coverage (-): 0.04

Region: NODE\_316089\_length\_2387\_cov\_23.258902 283-287. Max. coverage (+): 0.48. Max coverage (-): 0

Region: NODE\_316089\_length\_2387\_cov\_23.258902 288-292. Max. coverage (+): 0. Max coverage (-): 0

Region: NODE\_316089\_length\_2387\_cov\_23.258902 293-297. Max. coverage (+): 0. Max coverage (-): 0.01

Region: NODE\_316089\_length\_2387\_cov\_23.258902 298-302. Max. coverage (+): 0.07. Max coverage (-): 0.03

Region: NODE\_316089\_length\_2387\_cov\_23.258902 303-307. Max. coverage (+): 0. Max coverage (-): 0

Region: NODE\_316089\_length\_2387\_cov\_23.258902 308-312. Max. coverage (+): 0. Max coverage (-): 0.02

Region: NODE\_316089\_length\_2387\_cov\_23.258902 313-317. Max. coverage (+): 0. Max coverage (-): 0

Region: NODE\_316089\_length\_2387\_cov\_23.258902 318-322. Max. coverage (+): 0.07. Max coverage (-): 0

Region: NODE\_316089\_length\_2387\_cov\_23.258902 323-326. Max. coverage (+): 0. Max coverage (-): 0

Region: NODE\_316089\_length\_2387\_cov\_23.258902 327-331. Max. coverage (+): 0.3. Max coverage (-): 0

Region: NODE\_316089\_length\_2387\_cov\_23.258902 332-336. Max. coverage (+): 0.19. Max coverage (-): 0

Region: NODE\_316089\_length\_2387\_cov\_23.258902 337-341. Max. coverage (+): 0.82. Max coverage (-): 0

Region: NODE\_316089\_length\_2387\_cov\_23.258902 342-346. Max. coverage (+): 0.85. Max coverage (-): 0.07

Region: NODE\_316089\_length\_2387\_cov\_23.258902 347-351. Max. coverage (+): 0. Max coverage (-): 0.04

Region: NODE\_316089\_length\_2387\_cov\_23.258902 352-356. Max. coverage (+): 0. Max coverage (-): 0.63

Region: NODE\_316089\_length\_2387\_cov\_23.258902 357-361. Max. coverage (+): 0. Max coverage (-): 0.63

Region: NODE\_316089\_length\_2387\_cov\_23.258902 362-366. Max. coverage (+): 0.19. Max coverage (-): 0.04

Region: NODE\_316089\_length\_2387\_cov\_23.258902 367-371. Max. coverage (+): 0.22. Max coverage (-): 0.22

Region: NODE\_316089\_length\_2387\_cov\_23.258902 372-376. Max. coverage (+): 0.74. Max coverage (-): 0.04

Region: NODE\_316089\_length\_2387\_cov\_23.258902 377-380. Max. coverage (+): 0.74. Max coverage (-): 0.15

Region: NODE\_316089\_length\_2387\_cov\_23.258902 381-385. Max. coverage (+): 0.15. Max coverage (-): 0.19

Region: NODE\_316089\_length\_2387\_cov\_23.258902 386-390. Max. coverage (+): 0.52. Max coverage (-): 0.11

Region: NODE\_316089\_length\_2387\_cov\_23.258902 391-395. Max. coverage (+): 2.34. Max coverage (-): 0.04

Region: NODE\_316089\_length\_2387\_cov\_23.258902 396-400. Max. coverage (+): 2.37. Max coverage (-): 0

Region: NODE\_316089\_length\_2387\_cov\_23.258902 401-405. Max. coverage (+): 2.19. Max coverage (-): 0

Region: NODE\_316089\_length\_2387\_cov\_23.258902 406-410. Max. coverage (+): 0.26. Max coverage (-): 0

Region: NODE\_316089\_length\_2387\_cov\_23.258902 411-415. Max. coverage (+): 0.26. Max coverage (-): 0.07

Region: NODE\_316089\_length\_2387\_cov\_23.258902 416-420. Max. coverage (+): 0.3. Max coverage (-): 0

Region: NODE\_316089\_length\_2387\_cov\_23.258902 421-425. Max. coverage (+): 0. Max coverage (-): 0.04

Region: NODE\_316089\_length\_2387\_cov\_23.258902 426-429. Max. coverage (+): 0.04. Max coverage (-): 0

Region: NODE\_316089\_length\_2387\_cov\_23.258902 430-434. Max. coverage (+): 0.07. Max coverage (-): 0.04

Region: NODE\_316089\_length\_2387\_cov\_23.258902 435-439. Max. coverage (+): 0. Max coverage (-): 0.59

Region: NODE\_316089\_length\_2387\_cov\_23.258902 440-444. Max. coverage (+): 0.17. Max coverage (-): 0.02

Region: NODE\_316089\_length\_2387\_cov\_23.258902 445-449. Max. coverage (+): 0.44. Max coverage (-): 0.06

Region: NODE\_316089\_length\_2387\_cov\_23.258902 450-454. Max. coverage (+): 1.71. Max coverage (-): 0.09

Region: NODE\_316089\_length\_2387\_cov\_23.258902 455-459. Max. coverage (+): 1.41. Max coverage (-): 0.11

Region: NODE\_316089\_length\_2387\_cov\_23.258902 460-464. Max. coverage (+): 3.02. Max coverage (-): 0.09

Region: NODE\_316089\_length\_2387\_cov\_23.258902 465-469. Max. coverage (+): 3.95. Max coverage (-): 0

Region: NODE\_316089\_length\_2387\_cov\_23.258902 470-474. Max. coverage (+): 9.19. Max coverage (-): 0.04

Region: NODE\_316089\_length\_2387\_cov\_23.258902 475-478. Max. coverage (+): 1.04. Max coverage (-): 0.04

Region: NODE\_316089\_length\_2387\_cov\_23.258902 479-483. Max. coverage (+): 0.15. Max coverage (-): 0.04

Region: NODE\_316089\_length\_2387\_cov\_23.258902 484-488. Max. coverage (+): 0.3. Max coverage (-): 0

Region: NODE\_316089\_length\_2387\_cov\_23.258902 489-493. Max. coverage (+): 0.35. Max coverage (-): 0

Region: NODE\_316089\_length\_2387\_cov\_23.258902 494-498. Max. coverage (+): 0.19. Max coverage (-): 0.04

Region: NODE\_316089\_length\_2387\_cov\_23.258902 499-503. Max. coverage (+): 0.11. Max coverage (-): 0.04

Region: NODE\_316089\_length\_2387\_cov\_23.258902 504-508. Max. coverage (+): 0.67. Max coverage (-): 0.19

Region: NODE\_316089\_length\_2387\_cov\_23.258902 509-513. Max. coverage (+): 1.96. Max coverage (-): 0.11

Region: NODE\_316089\_length\_2387\_cov\_23.258902 514-518. Max. coverage (+): 0.56. Max coverage (-): 0.15

Region: NODE\_316089\_length\_2387\_cov\_23.258902 519-523. Max. coverage (+): 0.67. Max coverage (-): 0.07

Region: NODE\_316089\_length\_2387\_cov\_23.258902 524-527. Max. coverage (+): 0.11. Max coverage (-): 0

Region: NODE\_316089\_length\_2387\_cov\_23.258902 528-532. Max. coverage (+): 0.13. Max coverage (-): 0.02

Region: NODE\_316089\_length\_2387\_cov\_23.258902 533-537. Max. coverage (+): 1.45. Max coverage (-): 0.04

Region: NODE\_316089\_length\_2387\_cov\_23.258902 538-542. Max. coverage (+): 0.95. Max coverage (-): 0.13

Region: NODE\_316089\_length\_2387\_cov\_23.258902 543-547. Max. coverage (+): 0.19. Max coverage (-): 0.15

Region: NODE\_316089\_length\_2387\_cov\_23.258902 548-552. Max. coverage (+): 0.04. Max coverage (-): 0.15

Region: NODE\_316089\_length\_2387\_cov\_23.258902 553-557. Max. coverage (+): 1.48. Max coverage (-): 0.04

Region: NODE\_316089\_length\_2387\_cov\_23.258902 558-562. Max. coverage (+): 36.18. Max coverage (-): 0.04

Region: NODE\_316089\_length\_2387\_cov\_23.258902 563-567. Max. coverage (+): 6.34. Max coverage (-): 0.04

Region: NODE\_316089\_length\_2387\_cov\_23.258902 568-572. Max. coverage (+): 5.19. Max coverage (-): 0.07

Region: NODE\_316089\_length\_2387\_cov\_23.258902 573-576. Max. coverage (+): 0.04. Max coverage (-): 0.04

Region: NODE\_316089\_length\_2387\_cov\_23.258902 577-581. Max. coverage (+): 0.04. Max coverage (-): 1.26

Region: NODE\_316089\_length\_2387\_cov\_23.258902 582-586. Max. coverage (+): 0. Max coverage (-): 2

Region: NODE\_316089\_length\_2387\_cov\_23.258902 587-591. Max. coverage (+): 0.44. Max coverage (-): 1.69

Region: NODE\_316089\_length\_2387\_cov\_23.258902 592-596. Max. coverage (+): 1.63. Max coverage (-): 0.59

Region: NODE\_316089\_length\_2387\_cov\_23.258902 597-601. Max. coverage (+): 1.78. Max coverage (-): 0.59

Region: NODE\_316089\_length\_2387\_cov\_23.258902 602-606. Max. coverage (+): 0.67. Max coverage (-): 0.07

Region: NODE\_316089\_length\_2387\_cov\_23.258902 607-611. Max. coverage (+): 0.07. Max coverage (-): 0.04

Region: NODE\_316089\_length\_2387\_cov\_23.258902 612-616. Max. coverage (+): 0.04. Max coverage (-): 0.11

Region: NODE\_316089\_length\_2387\_cov\_23.258902 617-621. Max. coverage (+): 0.15. Max coverage (-): 0.07

Region: NODE\_316089\_length\_2387\_cov\_23.258902 622-626. Max. coverage (+): 0.11. Max coverage (-): 0.11

Region: NODE\_316089\_length\_2387\_cov\_23.258902 627-630. Max. coverage (+): 0.22. Max coverage (-): 0.33

Region: NODE\_316089\_length\_2387\_cov\_23.258902 631-635. Max. coverage (+): 0.74. Max coverage (-): 0.07

Region: NODE\_316089\_length\_2387\_cov\_23.258902 636-640. Max. coverage (+): 0.8. Max coverage (-): 0

Region: NODE\_316089\_length\_2387\_cov\_23.258902 641-645. Max. coverage (+): 3.95. Max coverage (-): 0

Region: NODE\_316089\_length\_2387\_cov\_23.258902 646-650. Max. coverage (+): 0.95. Max coverage (-): 0

Region: NODE\_316089\_length\_2387\_cov\_23.258902 651-655. Max. coverage (+): 0.32. Max coverage (-): 0.04

Region: NODE\_316089\_length\_2387\_cov\_23.258902 656-660. Max. coverage (+): 0.09. Max coverage (-): 0

Region: NODE\_316089\_length\_2387\_cov\_23.258902 661-665. Max. coverage (+): 0. Max coverage (-): 0.07

Region: NODE\_316089\_length\_2387\_cov\_23.258902 666-670. Max. coverage (+): 0.05. Max coverage (-): 0.19

Region: NODE\_316089\_length\_2387\_cov\_23.258902 671-675. Max. coverage (+): 0.02. Max coverage (-): 0.13

Region: NODE\_316089\_length\_2387\_cov\_23.258902 676-679. Max. coverage (+): 0.02. Max coverage (-): 0.15

Region: NODE\_316089\_length\_2387\_cov\_23.258902 680-684. Max. coverage (+): 0.41. Max coverage (-): 0.15

Region: NODE\_316089\_length\_2387\_cov\_23.258902 685-689. Max. coverage (+): 0.41. Max coverage (-): 0

Region: NODE\_316089\_length\_2387\_cov\_23.258902 690-694. Max. coverage (+): 3.34. Max coverage (-): 0

Region: NODE\_316089\_length\_2387\_cov\_23.258902 695-699. Max. coverage (+): 3.45. Max coverage (-): 0

Region: NODE\_316089\_length\_2387\_cov\_23.258902 700-704. Max. coverage (+): 0.19. Max coverage (-): 0

Region: NODE\_316089\_length\_2387\_cov\_23.258902 705-709. Max. coverage (+): 1.33. Max coverage (-): 0.04

Region: NODE\_316089\_length\_2387\_cov\_23.258902 710-714. Max. coverage (+): 0.37. Max coverage (-): 0.11

Region: NODE\_316089\_length\_2387\_cov\_23.258902 715-719. Max. coverage (+): 0.26. Max coverage (-): 0

Region: NODE\_316089\_length\_2387\_cov\_23.258902 720-724. Max. coverage (+): 0.87. Max coverage (-): 0

Region: NODE\_316089\_length\_2387\_cov\_23.258902 725-728. Max. coverage (+): 171.67. Max coverage (-): 0

Region: NODE\_316089\_length\_2387\_cov\_23.258902 729-733. Max. coverage (+): 49.68. Max coverage (-): 0.04

Region: NODE\_316089\_length\_2387\_cov\_23.258902 734-738. Max. coverage (+): 2.11. Max coverage (-): 0.04

Region: NODE\_316089\_length\_2387\_cov\_23.258902 739-743. Max. coverage (+): 0.26. Max coverage (-): 0.19

Region: NODE\_316089\_length\_2387\_cov\_23.258902 744-748. Max. coverage (+): 0.15. Max coverage (-): 0.22

Region: NODE\_316089\_length\_2387\_cov\_23.258902 749-753. Max. coverage (+): 2.19. Max coverage (-): 0.52

Region: NODE\_316089\_length\_2387\_cov\_23.258902 754-758. Max. coverage (+): 6.3. Max coverage (-): 0.09

Region: NODE\_316089\_length\_2387\_cov\_23.258902 759-763. Max. coverage (+): 5.78. Max coverage (-): 0.09

Region: NODE\_316089\_length\_2387\_cov\_23.258902 764-768. Max. coverage (+): 62.52. Max coverage (-): 0.06

Region: NODE\_316089\_length\_2387\_cov\_23.258902 769-773. Max. coverage (+): 0.09. Max coverage (-): 0

Region: NODE\_316089\_length\_2387\_cov\_23.258902 774-777. Max. coverage (+): 9.9. Max coverage (-): 0.19

Region: NODE\_316089\_length\_2387\_cov\_23.258902 778-782. Max. coverage (+): 9.71. Max coverage (-): 0.19

Region: NODE\_316089\_length\_2387\_cov\_23.258902 783-787. Max. coverage (+): 0.07. Max coverage (-): 0

Region: NODE\_316089\_length\_2387\_cov\_23.258902 788-792. Max. coverage (+): 0.04. Max coverage (-): 0

Region: NODE\_316089\_length\_2387\_cov\_23.258902 793-797. Max. coverage (+): 2.67. Max coverage (-): 0

Region: NODE\_316089\_length\_2387\_cov\_23.258902 798-802. Max. coverage (+): 2.56. Max coverage (-): 0

Region: NODE\_316089\_length\_2387\_cov\_23.258902 803-807. Max. coverage (+): 0. Max coverage (-): 0

Region: NODE\_316089\_length\_2387\_cov\_23.258902 808-812. Max. coverage (+): 0.32. Max coverage (-): 0.06

Region: NODE\_316089\_length\_2387\_cov\_23.258902 813-817. Max. coverage (+): 0.33. Max coverage (-): 0.17

Region: NODE\_316089\_length\_2387\_cov\_23.258902 818-822. Max. coverage (+): 0.41. Max coverage (-): 0.2

Region: NODE\_316089\_length\_2387\_cov\_23.258902 823-826. Max. coverage (+): 4.04. Max coverage (-): 0.2

Region: NODE\_316089\_length\_2387\_cov\_23.258902 827-831. Max. coverage (+): 4. Max coverage (-): 0.11

Region: NODE\_316089\_length\_2387\_cov\_23.258902 832-836. Max. coverage (+): 1.59. Max coverage (-): 0.06

Region: NODE\_316089\_length\_2387\_cov\_23.258902 837-841. Max. coverage (+): 2.22. Max coverage (-): 0.06

Region: NODE\_316089\_length\_2387\_cov\_23.258902 842-846. Max. coverage (+): 0.24. Max coverage (-): 0.04

Region: NODE\_316089\_length\_2387\_cov\_23.258902 847-851. Max. coverage (+): 0.28. Max coverage (-): 0.04

Region: NODE\_316089\_length\_2387\_cov\_23.258902 852-856. Max. coverage (+): 0.24. Max coverage (-): 0

Region: NODE\_316089\_length\_2387\_cov\_23.258902 857-861. Max. coverage (+): 0.07. Max coverage (-): 0

Region: NODE\_316089\_length\_2387\_cov\_23.258902 862-866. Max. coverage (+): 0.37. Max coverage (-): 0.07

Region: NODE\_316089\_length\_2387\_cov\_23.258902 867-871. Max. coverage (+): 1.85. Max coverage (-): 0.07

Region: NODE\_316089\_length\_2387\_cov\_23.258902 872-876. Max. coverage (+): 2.04. Max coverage (-): 0

Region: NODE\_316089\_length\_2387\_cov\_23.258902 877-880. Max. coverage (+): 1.37. Max coverage (-): 0.04

Region: NODE\_316089\_length\_2387\_cov\_23.258902 881-885. Max. coverage (+): 6.97. Max coverage (-): 0.15

Region: NODE\_316089\_length\_2387\_cov\_23.258902 886-890. Max. coverage (+): 0.56. Max coverage (-): 0.11

Region: NODE\_316089\_length\_2387\_cov\_23.258902 891-895. Max. coverage (+): 0.11. Max coverage (-): 0.63

Region: NODE\_316089\_length\_2387\_cov\_23.258902 896-900. Max. coverage (+): 0.52. Max coverage (-): 0.52

Region: NODE\_316089\_length\_2387\_cov\_23.258902 901-905. Max. coverage (+): 0.89. Max coverage (-): 0.19

Region: NODE\_316089\_length\_2387\_cov\_23.258902 906-910. Max. coverage (+): 11.34. Max coverage (-): 0.04

Region: NODE\_316089\_length\_2387\_cov\_23.258902 911-915. Max. coverage (+): 9.08. Max coverage (-): 0.48

Region: NODE\_316089\_length\_2387\_cov\_23.258902 916-920. Max. coverage (+): 0.04. Max coverage (-): 0.04

Region: NODE\_316089\_length\_2387\_cov\_23.258902 921-925. Max. coverage (+): 2.34. Max coverage (-): 0.02

Region: NODE\_316089\_length\_2387\_cov\_23.258902 926-929. Max. coverage (+): 2.06. Max coverage (-): 0.11

Region: NODE\_316089\_length\_2387\_cov\_23.258902 930-934. Max. coverage (+): 7.9. Max coverage (-): 0.67

Region: NODE\_316089\_length\_2387\_cov\_23.258902 935-939. Max. coverage (+): 1.52. Max coverage (-): 0.56

Region: NODE\_316089\_length\_2387\_cov\_23.258902 940-944. Max. coverage (+): 27.58. Max coverage (-): 0.19

Region: NODE\_316089\_length\_2387\_cov\_23.258902 945-949. Max. coverage (+): 0.33. Max coverage (-): 0.04

Region: NODE\_316089\_length\_2387\_cov\_23.258902 950-954. Max. coverage (+): 4.3. Max coverage (-): 0.37

Region: NODE\_316089\_length\_2387\_cov\_23.258902 955-959. Max. coverage (+): 1.96. Max coverage (-): 0.04

Region: NODE\_316089\_length\_2387\_cov\_23.258902 960-964. Max. coverage (+): 0.45. Max coverage (-): 0.02

Region: NODE\_316089\_length\_2387\_cov\_23.258902 965-969. Max. coverage (+): 2.67. Max coverage (-): 0.04

Region: NODE\_316089\_length\_2387\_cov\_23.258902 970-974. Max. coverage (+): 7.06. Max coverage (-): 0.11

Region: NODE\_316089\_length\_2387\_cov\_23.258902 975-978. Max. coverage (+): 0.59. Max coverage (-): 0.67

Region: NODE\_316089\_length\_2387\_cov\_23.258902 979-983. Max. coverage (+): 0.04. Max coverage (-): 0.78

Region: NODE\_316089\_length\_2387\_cov\_23.258902 984-988. Max. coverage (+): 0.82. Max coverage (-): 0.33

Region: NODE\_316089\_length\_2387\_cov\_23.258902 989-993. Max. coverage (+): 2.56. Max coverage (-): 0.11

Region: NODE\_316089\_length\_2387\_cov\_23.258902 994-998. Max. coverage (+): 2.41. Max coverage (-): 0

Region: NODE\_316089\_length\_2387\_cov\_23.258902 999-1003. Max. coverage (+): 0.52. Max coverage (-): 0

Region: NODE\_316089\_length\_2387\_cov\_23.258902 1004-1008. Max. coverage (+): 0.11. Max coverage (-): 0

Region: NODE\_316089\_length\_2387\_cov\_23.258902 1009-1013. Max. coverage (+): 0.67. Max coverage (-): 0.11

Region: NODE\_316089\_length\_2387\_cov\_23.258902 1014-1018. Max. coverage (+): 0.93. Max coverage (-): 0.11

Region: NODE\_316089\_length\_2387\_cov\_23.258902 1019-1023. Max. coverage (+): 0.67. Max coverage (-): 0

Region: NODE\_316089\_length\_2387\_cov\_23.258902 1024-1027. Max. coverage (+): 1.19. Max coverage (-): 0.04

Region: NODE\_316089\_length\_2387\_cov\_23.258902 1028-1032. Max. coverage (+): 1.15. Max coverage (-): 0.07

Region: NODE\_316089\_length\_2387\_cov\_23.258902 1033-1037. Max. coverage (+): 0.3. Max coverage (-): 0.52

Region: NODE\_316089\_length\_2387\_cov\_23.258902 1038-1042. Max. coverage (+): 0.26. Max coverage (-): 0.48

Region: NODE\_316089\_length\_2387\_cov\_23.258902 1043-1047. Max. coverage (+): 0.33. Max coverage (-): 0

Region: NODE\_316089\_length\_2387\_cov\_23.258902 1048-1052. Max. coverage (+): 0.63. Max coverage (-): 0.02

Region: NODE\_316089\_length\_2387\_cov\_23.258902 1053-1057. Max. coverage (+): 0.89. Max coverage (-): 0.02

Region: NODE\_316089\_length\_2387\_cov\_23.258902 1058-1062. Max. coverage (+): 0.89. Max coverage (-): 0

Region: NODE\_316089\_length\_2387\_cov\_23.258902 1063-1067. Max. coverage (+): 0.3. Max coverage (-): 0

Region: NODE\_316089\_length\_2387\_cov\_23.258902 1068-1072. Max. coverage (+): 3.45. Max coverage (-): 0.04

Region: NODE\_316089\_length\_2387\_cov\_23.258902 1073-1076. Max. coverage (+): 0.07. Max coverage (-): 0.04

Region: NODE\_316089\_length\_2387\_cov\_23.258902 1077-1081. Max. coverage (+): 0.15. Max coverage (-): 0.11

Region: NODE\_316089\_length\_2387\_cov\_23.258902 1082-1086. Max. coverage (+): 0.48. Max coverage (-): 0.3

Region: NODE\_316089\_length\_2387\_cov\_23.258902 1087-1091. Max. coverage (+): 0.7. Max coverage (-): 0.33

Region: NODE\_316089\_length\_2387\_cov\_23.258902 1092-1096. Max. coverage (+): 0.07. Max coverage (-): 0.19

Region: NODE\_316089\_length\_2387\_cov\_23.258902 1097-1101. Max. coverage (+): 0.2. Max coverage (-): 0.02

Region: NODE\_316089\_length\_2387\_cov\_23.258902 1102-1106. Max. coverage (+): 19.96. Max coverage (-): 0.02

Region: NODE\_316089\_length\_2387\_cov\_23.258902 1107-1111. Max. coverage (+): 18.71. Max coverage (-): 0

Region: NODE\_316089\_length\_2387\_cov\_23.258902 1112-1116. Max. coverage (+): 0.64. Max coverage (-): 0

Region: NODE\_316089\_length\_2387\_cov\_23.258902 1117-1121. Max. coverage (+): 0.17. Max coverage (-): 0

Region: NODE\_316089\_length\_2387\_cov\_23.258902 1122-1126. Max. coverage (+): 0.14. Max coverage (-): 0.02

Region: NODE\_316089\_length\_2387\_cov\_23.258902 1127-1130. Max. coverage (+): 0.02. Max coverage (-): 0.02

Region: NODE\_316089\_length\_2387\_cov\_23.258902 1131-1135. Max. coverage (+): 0.11. Max coverage (-): 0.52

Region: NODE\_316089\_length\_2387\_cov\_23.258902 1136-1140. Max. coverage (+): 0.13. Max coverage (-): 0.46

Region: NODE\_316089\_length\_2387\_cov\_23.258902 1141-1145. Max. coverage (+): 0.98. Max coverage (-): 0.06

Region: NODE\_316089\_length\_2387\_cov\_23.258902 1146-1150. Max. coverage (+): 19.35. Max coverage (-): 0

Region: NODE\_316089\_length\_2387\_cov\_23.258902 1151-1155. Max. coverage (+): 0.26. Max coverage (-): 0

Region: NODE\_316089\_length\_2387\_cov\_23.258902 1156-1160. Max. coverage (+): 0.04. Max coverage (-): 0

Region: NODE\_316089\_length\_2387\_cov\_23.258902 1161-1165. Max. coverage (+): 0.07. Max coverage (-): 0.07

Region: NODE\_316089\_length\_2387\_cov\_23.258902 1166-1170. Max. coverage (+): 0.1. Max coverage (-): 0

Region: NODE\_316089\_length\_2387\_cov\_23.258902 1171-1175. Max. coverage (+): 0.25. Max coverage (-): 0

Region: NODE\_316089\_length\_2387\_cov\_23.258902 1176-1179. Max. coverage (+): 0.28. Max coverage (-): 0.02

Region: NODE\_316089\_length\_2387\_cov\_23.258902 1180-1184. Max. coverage (+): 0.27. Max coverage (-): 0.06

Region: NODE\_316089\_length\_2387\_cov\_23.258902 1185-1189. Max. coverage (+): 0.07. Max coverage (-): 0.07

Region: NODE\_316089\_length\_2387\_cov\_23.258902 1190-1194. Max. coverage (+): 0.04. Max coverage (-): 0

Region: NODE\_316089\_length\_2387\_cov\_23.258902 1195-1199. Max. coverage (+): 0.17. Max coverage (-): 0.02

Region: NODE\_316089\_length\_2387\_cov\_23.258902 1200-1204. Max. coverage (+): 2.43. Max coverage (-): 0

Region: NODE\_316089\_length\_2387\_cov\_23.258902 1205-1209. Max. coverage (+): 0.52. Max coverage (-): 0

Region: NODE\_316089\_length\_2387\_cov\_23.258902 1210-1214. Max. coverage (+): 0.54. Max coverage (-): 0

Region: NODE\_316089\_length\_2387\_cov\_23.258902 1215-1219. Max. coverage (+): 0. Max coverage (-): 0.35

Region: NODE\_316089\_length\_2387\_cov\_23.258902 1220-1224. Max. coverage (+): 0. Max coverage (-): 0.44

Region: NODE\_316089\_length\_2387\_cov\_23.258902 1225-1228. Max. coverage (+): 0.17. Max coverage (-): 0.07

Region: NODE\_316089\_length\_2387\_cov\_23.258902 1229-1233. Max. coverage (+): 1.95. Max coverage (-): 0.06

Region: NODE\_316089\_length\_2387\_cov\_23.258902 1234-1238. Max. coverage (+): 17.76. Max coverage (-): 0

Region: NODE\_316089\_length\_2387\_cov\_23.258902 1239-1243. Max. coverage (+): 18.05. Max coverage (-): 0

Region: NODE\_316089\_length\_2387\_cov\_23.258902 1244-1248. Max. coverage (+): 2.34. Max coverage (-): 0

Region: NODE\_316089\_length\_2387\_cov\_23.258902 1249-1253. Max. coverage (+): 0.3. Max coverage (-): 0

Region: NODE\_316089\_length\_2387\_cov\_23.258902 1254-1258. Max. coverage (+): 0.63. Max coverage (-): 0.07

Region: NODE\_316089\_length\_2387\_cov\_23.258902 1259-1263. Max. coverage (+): 0.48. Max coverage (-): 0.11

Region: NODE\_316089\_length\_2387\_cov\_23.258902 1264-1268. Max. coverage (+): 0. Max coverage (-): 0.22

Region: NODE\_316089\_length\_2387\_cov\_23.258902 1269-1273. Max. coverage (+): 0. Max coverage (-): 0.67

Region: NODE\_316089\_length\_2387\_cov\_23.258902 1274-1277. Max. coverage (+): 0.04. Max coverage (-): 0.22

Region: NODE\_316089\_length\_2387\_cov\_23.258902 1278-1282. Max. coverage (+): 0.26. Max coverage (-): 0.22

Region: NODE\_316089\_length\_2387\_cov\_23.258902 1283-1287. Max. coverage (+): 7.97. Max coverage (-): 0.07

Region: NODE\_316089\_length\_2387\_cov\_23.258902 1288-1292. Max. coverage (+): 12.09. Max coverage (-): 0

Region: NODE\_316089\_length\_2387\_cov\_23.258902 1293-1297. Max. coverage (+): 12.16. Max coverage (-): 0

Region: NODE\_316089\_length\_2387\_cov\_23.258902 1298-1302. Max. coverage (+): 0.07. Max coverage (-): 0.02

Region: NODE\_316089\_length\_2387\_cov\_23.258902 1303-1307. Max. coverage (+): 0.13. Max coverage (-): 0.04

Region: NODE\_316089\_length\_2387\_cov\_23.258902 1308-1312. Max. coverage (+): 0.3. Max coverage (-): 0

Region: NODE\_316089\_length\_2387\_cov\_23.258902 1313-1317. Max. coverage (+): 1.22. Max coverage (-): 0.04

Region: NODE\_316089\_length\_2387\_cov\_23.258902 1318-1322. Max. coverage (+): 7.64. Max coverage (-): 0.04

Region: NODE\_316089\_length\_2387\_cov\_23.258902 1323-1326. Max. coverage (+): 7.86. Max coverage (-): 0.04

Region: NODE\_316089\_length\_2387\_cov\_23.258902 1327-1331. Max. coverage (+): 0. Max coverage (-): 0.07

Region: NODE\_316089\_length\_2387\_cov\_23.258902 1332-1336. Max. coverage (+): 8.93. Max coverage (-): 0.07

Region: NODE\_316089\_length\_2387\_cov\_23.258902 1337-1341. Max. coverage (+): 12.01. Max coverage (-): 0.07

Region: NODE\_316089\_length\_2387\_cov\_23.258902 1342-1346. Max. coverage (+): 2.74. Max coverage (-): 0.07

Region: NODE\_316089\_length\_2387\_cov\_23.258902 1347-1351. Max. coverage (+): 2.6. Max coverage (-): 0.07

Region: NODE\_316089\_length\_2387\_cov\_23.258902 1352-1356. Max. coverage (+): 6.71. Max coverage (-): 0

Region: NODE\_316089\_length\_2387\_cov\_23.258902 1357-1361. Max. coverage (+): 8.49. Max coverage (-): 0.3

Region: NODE\_316089\_length\_2387\_cov\_23.258902 1362-1366. Max. coverage (+): 1. Max coverage (-): 0.22

Region: NODE\_316089\_length\_2387\_cov\_23.258902 1367-1371. Max. coverage (+): 0.59. Max coverage (-): 0.3

Region: NODE\_316089\_length\_2387\_cov\_23.258902 1372-1376. Max. coverage (+): 0.78. Max coverage (-): 0

Region: NODE\_316089\_length\_2387\_cov\_23.258902 1377-1380. Max. coverage (+): 0.67. Max coverage (-): 0

Region: NODE\_316089\_length\_2387\_cov\_23.258902 1381-1385. Max. coverage (+): 0.85. Max coverage (-): 0

Region: NODE\_316089\_length\_2387\_cov\_23.258902 1386-1390. Max. coverage (+): 4.78. Max coverage (-): 0

Region: NODE\_316089\_length\_2387\_cov\_23.258902 1391-1395. Max. coverage (+): 0.89. Max coverage (-): 0.11

Region: NODE\_316089\_length\_2387\_cov\_23.258902 1396-1400. Max. coverage (+): 0.04. Max coverage (-): 0.41

Region: NODE\_316089\_length\_2387\_cov\_23.258902 1401-1405. Max. coverage (+): 0. Max coverage (-): 0.22

Region: NODE\_316089\_length\_2387\_cov\_23.258902 1406-1410. Max. coverage (+): 0.67. Max coverage (-): 0.02

Region: NODE\_316089\_length\_2387\_cov\_23.258902 1411-1415. Max. coverage (+): 6.58. Max coverage (-): 0.02

Region: NODE\_316089\_length\_2387\_cov\_23.258902 1416-1420. Max. coverage (+): 1.71. Max coverage (-): 0.01

Region: NODE\_316089\_length\_2387\_cov\_23.258902 1421-1425. Max. coverage (+): 0.01. Max coverage (-): 0

Region: NODE\_316089\_length\_2387\_cov\_23.258902 1426-1429. Max. coverage (+): 0.16. Max coverage (-): 0.05

Region: NODE\_316089\_length\_2387\_cov\_23.258902 1430-1434. Max. coverage (+): 0.2. Max coverage (-): 0.04

Region: NODE\_316089\_length\_2387\_cov\_23.258902 1435-1439. Max. coverage (+): 0.44. Max coverage (-): 0.04

Region: NODE\_316089\_length\_2387\_cov\_23.258902 1440-1444. Max. coverage (+): 0.15. Max coverage (-): 0

Region: NODE\_316089\_length\_2387\_cov\_23.258902 1445-1449. Max. coverage (+): 0.93. Max coverage (-): 0.04

Region: NODE\_316089\_length\_2387\_cov\_23.258902 1450-1454. Max. coverage (+): 1.04. Max coverage (-): 0.04

Region: NODE\_316089\_length\_2387\_cov\_23.258902 1455-1459. Max. coverage (+): 0.11. Max coverage (-): 0

Region: NODE\_316089\_length\_2387\_cov\_23.258902 1460-1464. Max. coverage (+): 0. Max coverage (-): 0.98

Region: NODE\_316089\_length\_2387\_cov\_23.258902 1465-1469. Max. coverage (+): 0.07. Max coverage (-): 2.91

Region: NODE\_316089\_length\_2387\_cov\_23.258902 1470-1474. Max. coverage (+): 0.07. Max coverage (-): 0.07

Region: NODE\_316089\_length\_2387\_cov\_23.258902 1475-1478. Max. coverage (+): 0.07. Max coverage (-): 0.04

Region: NODE\_316089\_length\_2387\_cov\_23.258902 1479-1483. Max. coverage (+): 2.04. Max coverage (-): 0.04

Region: NODE\_316089\_length\_2387\_cov\_23.258902 1484-1488. Max. coverage (+): 0.59. Max coverage (-): 0

Region: NODE\_316089\_length\_2387\_cov\_23.258902 1489-1493. Max. coverage (+): 0.35. Max coverage (-): 0.04

Region: NODE\_316089\_length\_2387\_cov\_23.258902 1494-1498. Max. coverage (+): 12.75. Max coverage (-): 0.06

Region: NODE\_316089\_length\_2387\_cov\_23.258902 1499-1503. Max. coverage (+): 12.88. Max coverage (-): 0.13

Region: NODE\_316089\_length\_2387\_cov\_23.258902 1504-1508. Max. coverage (+): 0.15. Max coverage (-): 0.17

Region: NODE\_316089\_length\_2387\_cov\_23.258902 1509-1513. Max. coverage (+): 11.12. Max coverage (-): 0.04

Region: NODE\_316089\_length\_2387\_cov\_23.258902 1514-1518. Max. coverage (+): 13.68. Max coverage (-): 0

Region: NODE\_316089\_length\_2387\_cov\_23.258902 1519-1523. Max. coverage (+): 41.45. Max coverage (-): 0

Region: NODE\_316089\_length\_2387\_cov\_23.258902 1524-1527. Max. coverage (+): 1.33. Max coverage (-): 0

Region: NODE\_316089\_length\_2387\_cov\_23.258902 1528-1532. Max. coverage (+): 1.41. Max coverage (-): 0

Region: NODE\_316089\_length\_2387\_cov\_23.258902 1533-1537. Max. coverage (+): 1.48. Max coverage (-): 0.15

Region: NODE\_316089\_length\_2387\_cov\_23.258902 1538-1542. Max. coverage (+): 0.33. Max coverage (-): 0.22

Region: NODE\_316089\_length\_2387\_cov\_23.258902 1543-1547. Max. coverage (+): 1.15. Max coverage (-): 0.26

Region: NODE\_316089\_length\_2387\_cov\_23.258902 1548-1552. Max. coverage (+): 1.41. Max coverage (-): 0

Region: NODE\_316089\_length\_2387\_cov\_23.258902 1553-1557. Max. coverage (+): 0.25. Max coverage (-): 0.1

Region: NODE\_316089\_length\_2387\_cov\_23.258902 1558-1562. Max. coverage (+): 1.01. Max coverage (-): 0.06

Region: NODE\_316089\_length\_2387\_cov\_23.258902 1563-1567. Max. coverage (+): 0.46. Max coverage (-): 0.09

Region: NODE\_316089\_length\_2387\_cov\_23.258902 1568-1572. Max. coverage (+): 0.96. Max coverage (-): 0.04

Region: NODE\_316089\_length\_2387\_cov\_23.258902 1573-1576. Max. coverage (+): 0.09. Max coverage (-): 0.03

Region: NODE\_316089\_length\_2387\_cov\_23.258902 1577-1581. Max. coverage (+): 0.09. Max coverage (-): 0.02

Region: NODE\_316089\_length\_2387\_cov\_23.258902 1582-1586. Max. coverage (+): 0.65. Max coverage (-): 0

Region: NODE\_316089\_length\_2387\_cov\_23.258902 1587-1591. Max. coverage (+): 0.57. Max coverage (-): 0.19

Region: NODE\_316089\_length\_2387\_cov\_23.258902 1592-1596. Max. coverage (+): 8.42. Max coverage (-): 0.37

Region: NODE\_316089\_length\_2387\_cov\_23.258902 1597-1601. Max. coverage (+): 23.69. Max coverage (-): 1.22

Region: NODE\_316089\_length\_2387\_cov\_23.258902 1602-1606. Max. coverage (+): 4.26. Max coverage (-): 1.15

Region: NODE\_316089\_length\_2387\_cov\_23.258902 1607-1611. Max. coverage (+): 4.34. Max coverage (-): 0.04

Region: NODE\_316089\_length\_2387\_cov\_23.258902 1612-1616. Max. coverage (+): 0.63. Max coverage (-): 0.33

Region: NODE\_316089\_length\_2387\_cov\_23.258902 1617-1621. Max. coverage (+): 0.48. Max coverage (-): 0.3

Region: NODE\_316089\_length\_2387\_cov\_23.258902 1622-1626. Max. coverage (+): 0.04. Max coverage (-): 0.24

Region: NODE\_316089\_length\_2387\_cov\_23.258902 1627-1630. Max. coverage (+): 0.17. Max coverage (-): 0.22

Region: NODE\_316089\_length\_2387\_cov\_23.258902 1631-1635. Max. coverage (+): 0.15. Max coverage (-): 0.07

Region: NODE\_316089\_length\_2387\_cov\_23.258902 1636-1640. Max. coverage (+): 0. Max coverage (-): 0.07

Region: NODE\_316089\_length\_2387\_cov\_23.258902 1641-1645. Max. coverage (+): 0. Max coverage (-): 0.04

Region: NODE\_316089\_length\_2387\_cov\_23.258902 1646-1650. Max. coverage (+): 1.15. Max coverage (-): 0

Region: NODE\_316089\_length\_2387\_cov\_23.258902 1651-1655. Max. coverage (+): 3.45. Max coverage (-): 0.3

Region: NODE\_316089\_length\_2387\_cov\_23.258902 1656-1660. Max. coverage (+): 0.89. Max coverage (-): 1.22

Region: NODE\_316089\_length\_2387\_cov\_23.258902 1661-1665. Max. coverage (+): 0.59. Max coverage (-): 0.22

Region: NODE\_316089\_length\_2387\_cov\_23.258902 1666-1670. Max. coverage (+): 0.15. Max coverage (-): 0.3

Region: NODE\_316089\_length\_2387\_cov\_23.258902 1671-1675. Max. coverage (+): 0.22. Max coverage (-): 0.26

Region: NODE\_316089\_length\_2387\_cov\_23.258902 1676-1679. Max. coverage (+): 0.89. Max coverage (-): 0.04

Region: NODE\_316089\_length\_2387\_cov\_23.258902 1680-1684. Max. coverage (+): 1.04. Max coverage (-): 0.26

Region: NODE\_316089\_length\_2387\_cov\_23.258902 1685-1689. Max. coverage (+): 1.19. Max coverage (-): 0.22

Region: NODE\_316089\_length\_2387\_cov\_23.258902 1690-1694. Max. coverage (+): 0.7. Max coverage (-): 0.15

Region: NODE\_316089\_length\_2387\_cov\_23.258902 1695-1699. Max. coverage (+): 0.26. Max coverage (-): 0

Region: NODE\_316089\_length\_2387\_cov\_23.258902 1700-1704. Max. coverage (+): 0.26. Max coverage (-): 0.22

Region: NODE\_316089\_length\_2387\_cov\_23.258902 1705-1709. Max. coverage (+): 0.22. Max coverage (-): 0.19

Region: NODE\_316089\_length\_2387\_cov\_23.258902 1710-1714. Max. coverage (+): 0. Max coverage (-): 0.07

Region: NODE\_316089\_length\_2387\_cov\_23.258902 1715-1719. Max. coverage (+): 0.26. Max coverage (-): 0.04

Region: NODE\_316089\_length\_2387\_cov\_23.258902 1720-1724. Max. coverage (+): 0.89. Max coverage (-): 0.04

Region: NODE\_316089\_length\_2387\_cov\_23.258902 1725-1728. Max. coverage (+): 2.11. Max coverage (-): 0.26

Region: NODE\_316089\_length\_2387\_cov\_23.258902 1729-1733. Max. coverage (+): 4.52. Max coverage (-): 0.22

Region: NODE\_316089\_length\_2387\_cov\_23.258902 1734-1738. Max. coverage (+): 3.48. Max coverage (-): 0.15

Region: NODE\_316089\_length\_2387\_cov\_23.258902 1739-1743. Max. coverage (+): 4. Max coverage (-): 0.04

Region: NODE\_316089\_length\_2387\_cov\_23.258902 1744-1748. Max. coverage (+): 0.67. Max coverage (-): 0.04

Region: NODE\_316089\_length\_2387\_cov\_23.258902 1749-1753. Max. coverage (+): 0.15. Max coverage (-): 0.22

Region: NODE\_316089\_length\_2387\_cov\_23.258902 1754-1758. Max. coverage (+): 0.15. Max coverage (-): 0.33

Region: NODE\_316089\_length\_2387\_cov\_23.258902 1759-1763. Max. coverage (+): 0.15. Max coverage (-): 0.04

Region: NODE\_316089\_length\_2387\_cov\_23.258902 1764-1768. Max. coverage (+): 0.11. Max coverage (-): 0.04

Region: NODE\_316089\_length\_2387\_cov\_23.258902 1769-1773. Max. coverage (+): 8.97. Max coverage (-): 0

Region: NODE\_316089\_length\_2387\_cov\_23.258902 1774-1777. Max. coverage (+): 1.33. Max coverage (-): 0

Region: NODE\_316089\_length\_2387\_cov\_23.258902 1778-1782. Max. coverage (+): 0.33. Max coverage (-): 0

Region: NODE\_316089\_length\_2387\_cov\_23.258902 1783-1787. Max. coverage (+): 0.56. Max coverage (-): 0.07

Region: NODE\_316089\_length\_2387\_cov\_23.258902 1788-1792. Max. coverage (+): 0.85. Max coverage (-): 0.04

Region: NODE\_316089\_length\_2387\_cov\_23.258902 1793-1797. Max. coverage (+): 0.07. Max coverage (-): 0.04

Region: NODE\_316089\_length\_2387\_cov\_23.258902 1798-1802. Max. coverage (+): 0.15. Max coverage (-): 0.07

Region: NODE\_316089\_length\_2387\_cov\_23.258902 1803-1807. Max. coverage (+): 0.48. Max coverage (-): 0.07

Region: NODE\_316089\_length\_2387\_cov\_23.258902 1808-1812. Max. coverage (+): 0.7. Max coverage (-): 0.04

Region: NODE\_316089\_length\_2387\_cov\_23.258902 1813-1817. Max. coverage (+): 1.56. Max coverage (-): 0.19

Region: NODE\_316089\_length\_2387\_cov\_23.258902 1818-1822. Max. coverage (+): 1.74. Max coverage (-): 0.19

Region: NODE\_316089\_length\_2387\_cov\_23.258902 1823-1826. Max. coverage (+): 0.52. Max coverage (-): 0.11

Region: NODE\_316089\_length\_2387\_cov\_23.258902 1827-1831. Max. coverage (+): 0.59. Max coverage (-): 0.07

Region: NODE\_316089\_length\_2387\_cov\_23.258902 1832-1836. Max. coverage (+): 0.96. Max coverage (-): 0.19

Region: NODE\_316089\_length\_2387\_cov\_23.258902 1837-1841. Max. coverage (+): 0.78. Max coverage (-): 0

Region: NODE\_316089\_length\_2387\_cov\_23.258902 1842-1846. Max. coverage (+): 0.15. Max coverage (-): 0

Region: NODE\_316089\_length\_2387\_cov\_23.258902 1847-1851. Max. coverage (+): 1. Max coverage (-): 0

Region: NODE\_316089\_length\_2387\_cov\_23.258902 1852-1856. Max. coverage (+): 1.19. Max coverage (-): 0.04

Region: NODE\_316089\_length\_2387\_cov\_23.258902 1857-1861. Max. coverage (+): 0.3. Max coverage (-): 0.04

Region: NODE\_316089\_length\_2387\_cov\_23.258902 1862-1866. Max. coverage (+): 0.07. Max coverage (-): 0

Region: NODE\_316089\_length\_2387\_cov\_23.258902 1867-1871. Max. coverage (+): 0.33. Max coverage (-): 0

Region: NODE\_316089\_length\_2387\_cov\_23.258902 1872-1876. Max. coverage (+): 3.52. Max coverage (-): 0

Region: NODE\_316089\_length\_2387\_cov\_23.258902 1877-1880. Max. coverage (+): 0.41. Max coverage (-): 0

Region: NODE\_316089\_length\_2387\_cov\_23.258902 1881-1885. Max. coverage (+): 0.3. Max coverage (-): 0

Region: NODE\_316089\_length\_2387\_cov\_23.258902 1886-1890. Max. coverage (+): 0.22. Max coverage (-): 0.19

Region: NODE\_316089\_length\_2387\_cov\_23.258902 1891-1895. Max. coverage (+): 0.11. Max coverage (-): 0.19

Region: NODE\_316089\_length\_2387\_cov\_23.258902 1896-1900. Max. coverage (+): 0.04. Max coverage (-): 0

Region: NODE\_316089\_length\_2387\_cov\_23.258902 1901-1905. Max. coverage (+): 0. Max coverage (-): 0

Region: NODE\_316089\_length\_2387\_cov\_23.258902 1906-1910. Max. coverage (+): 0.33. Max coverage (-): 0.04

Region: NODE\_316089\_length\_2387\_cov\_23.258902 1911-1915. Max. coverage (+): 0.33. Max coverage (-): 0.07

Region: NODE\_316089\_length\_2387\_cov\_23.258902 1916-1920. Max. coverage (+): 0.11. Max coverage (-): 0.37

Region: NODE\_316089\_length\_2387\_cov\_23.258902 1921-1925. Max. coverage (+): 1.56. Max coverage (-): 0.33

Region: NODE\_316089\_length\_2387\_cov\_23.258902 1926-1929. Max. coverage (+): 0.59. Max coverage (-): 0

Region: NODE\_316089\_length\_2387\_cov\_23.258902 1930-1934. Max. coverage (+): 17.68. Max coverage (-): 0.19

Region: NODE\_316089\_length\_2387\_cov\_23.258902 1935-1939. Max. coverage (+): 17.24. Max coverage (-): 0.59

Region: NODE\_316089\_length\_2387\_cov\_23.258902 1940-1944. Max. coverage (+): 9.86. Max coverage (-): 0.41

Region: NODE\_316089\_length\_2387\_cov\_23.258902 1945-1949. Max. coverage (+): 10.88. Max coverage (-): 0

Region: NODE\_316089\_length\_2387\_cov\_23.258902 1950-1954. Max. coverage (+): 0.06. Max coverage (-): 0

Region: NODE\_316089\_length\_2387\_cov\_23.258902 1955-1959. Max. coverage (+): 0.04. Max coverage (-): 0

Region: NODE\_316089\_length\_2387\_cov\_23.258902 1960-1964. Max. coverage (+): 0.3. Max coverage (-): 0

Region: NODE\_316089\_length\_2387\_cov\_23.258902 1965-1969. Max. coverage (+): 0.07. Max coverage (-): 0

Region: NODE\_316089\_length\_2387\_cov\_23.258902 1970-1974. Max. coverage (+): 0.96. Max coverage (-): 0.07

Region: NODE\_316089\_length\_2387\_cov\_23.258902 1975-1978. Max. coverage (+): 1.41. Max coverage (-): 0.22

Region: NODE\_316089\_length\_2387\_cov\_23.258902 1979-1983. Max. coverage (+): 1.48. Max coverage (-): 0.3

Region: NODE\_316089\_length\_2387\_cov\_23.258902 1984-1988. Max. coverage (+): 1.45. Max coverage (-): 0

Region: NODE\_316089\_length\_2387\_cov\_23.258902 1989-1993. Max. coverage (+): 1.85. Max coverage (-): 0

Region: NODE\_316089\_length\_2387\_cov\_23.258902 1994-1998. Max. coverage (+): 2.08. Max coverage (-): 0.04

Region: NODE\_316089\_length\_2387\_cov\_23.258902 1999-2003. Max. coverage (+): 0.52. Max coverage (-): 0

Region: NODE\_316089\_length\_2387\_cov\_23.258902 2004-2008. Max. coverage (+): 0.15. Max coverage (-): 0

Region: NODE\_316089\_length\_2387\_cov\_23.258902 2009-2013. Max. coverage (+): 0.22. Max coverage (-): 0

Region: NODE\_316089\_length\_2387\_cov\_23.258902 2014-2018. Max. coverage (+): 0. Max coverage (-): 0

Region: NODE\_316089\_length\_2387\_cov\_23.258902 2019-2023. Max. coverage (+): 0. Max coverage (-): 0.52

Region: NODE\_316089\_length\_2387\_cov\_23.258902 2024-2027. Max. coverage (+): 0.41. Max coverage (-): 0.63

Region: NODE\_316089\_length\_2387\_cov\_23.258902 2028-2032. Max. coverage (+): 0.52. Max coverage (-): 0.15

Region: NODE\_316089\_length\_2387\_cov\_23.258902 2033-2037. Max. coverage (+): 1.04. Max coverage (-): 0.11

Region: NODE\_316089\_length\_2387\_cov\_23.258902 2038-2042. Max. coverage (+): 1.52. Max coverage (-): 0.11

Region: NODE\_316089\_length\_2387\_cov\_23.258902 2043-2047. Max. coverage (+): 1.78. Max coverage (-): 0.04

Region: NODE\_316089\_length\_2387\_cov\_23.258902 2048-2052. Max. coverage (+): 0.37. Max coverage (-): 0

Region: NODE\_316089\_length\_2387\_cov\_23.258902 2053-2057. Max. coverage (+): 0.44. Max coverage (-): 0

Region: NODE\_316089\_length\_2387\_cov\_23.258902 2058-2062. Max. coverage (+): 0.63. Max coverage (-): 0

Region: NODE\_316089\_length\_2387\_cov\_23.258902 2063-2067. Max. coverage (+): 0.96. Max coverage (-): 0

Region: NODE\_316089\_length\_2387\_cov\_23.258902 2068-2072. Max. coverage (+): 0.52. Max coverage (-): 0

Region: NODE\_316089\_length\_2387\_cov\_23.258902 2073-2076. Max. coverage (+): 2.71. Max coverage (-): 0

Region: NODE\_316089\_length\_2387\_cov\_23.258902 2077-2081. Max. coverage (+): 3.19. Max coverage (-): 0

Region: NODE\_316089\_length\_2387\_cov\_23.258902 2082-2086. Max. coverage (+): 4.62. Max coverage (-): 0

Region: NODE\_316089\_length\_2387\_cov\_23.258902 2087-2091. Max. coverage (+): 4.01. Max coverage (-): 0

Region: NODE\_316089\_length\_2387\_cov\_23.258902 2092-2096. Max. coverage (+): 0.04. Max coverage (-): 0.02

Region: NODE\_316089\_length\_2387\_cov\_23.258902 2097-2101. Max. coverage (+): 0.26. Max coverage (-): 0.04

Region: NODE\_316089\_length\_2387\_cov\_23.258902 2102-2106. Max. coverage (+): 3.08. Max coverage (-): 0.04

Region: NODE\_316089\_length\_2387\_cov\_23.258902 2107-2111. Max. coverage (+): 5.49. Max coverage (-): 0

Region: NODE\_316089\_length\_2387\_cov\_23.258902 2112-2116. Max. coverage (+): 2.52. Max coverage (-): 0

Region: NODE\_316089\_length\_2387\_cov\_23.258902 2117-2121. Max. coverage (+): 2.3. Max coverage (-): 0

Region: NODE\_316089\_length\_2387\_cov\_23.258902 2122-2126. Max. coverage (+): 0.94. Max coverage (-): 0.05

Region: NODE\_316089\_length\_2387\_cov\_23.258902 2127-2130. Max. coverage (+): 0.05. Max coverage (-): 0.01

Region: NODE\_316089\_length\_2387\_cov\_23.258902 2131-2135. Max. coverage (+): 0.04. Max coverage (-): 0

Region: NODE\_316089\_length\_2387\_cov\_23.258902 2136-2140. Max. coverage (+): 0.5. Max coverage (-): 0

Region: NODE\_316089\_length\_2387\_cov\_23.258902 2141-2145. Max. coverage (+): 2.45. Max coverage (-): 0

Region: NODE\_316089\_length\_2387\_cov\_23.258902 2146-2150. Max. coverage (+): 0.85. Max coverage (-): 0

Region: NODE\_316089\_length\_2387\_cov\_23.258902 2151-2155. Max. coverage (+): 0.33. Max coverage (-): 0

Region: NODE\_316089\_length\_2387\_cov\_23.258902 2156-2160. Max. coverage (+): 0.04. Max coverage (-): 0

Region: NODE\_316089\_length\_2387\_cov\_23.258902 2161-2165. Max. coverage (+): 0. Max coverage (-): 0.22

Region: NODE\_316089\_length\_2387\_cov\_23.258902 2166-2170. Max. coverage (+): 0.15. Max coverage (-): 0.19

Region: NODE\_316089\_length\_2387\_cov\_23.258902 2171-2175. Max. coverage (+): 7.01. Max coverage (-): 0

Region: NODE\_316089\_length\_2387\_cov\_23.258902 2176-2179. Max. coverage (+): 5.38. Max coverage (-): 0

Region: NODE\_316089\_length\_2387\_cov\_23.258902 2180-2184. Max. coverage (+): 2.67. Max coverage (-): 0

Region: NODE\_316089\_length\_2387\_cov\_23.258902 2185-2189. Max. coverage (+): 1.59. Max coverage (-): 0

Region: NODE\_316089\_length\_2387\_cov\_23.258902 2190-2194. Max. coverage (+): 0.11. Max coverage (-): 0.04

Region: NODE\_316089\_length\_2387\_cov\_23.258902 2195-2199. Max. coverage (+): 0.07. Max coverage (-): 0.11

Region: NODE\_316089\_length\_2387\_cov\_23.258902 2200-2204. Max. coverage (+): 0.02. Max coverage (-): 0.07

Region: NODE\_316089\_length\_2387\_cov\_23.258902 2205-2209. Max. coverage (+): 0.05. Max coverage (-): 0.01

Region: NODE\_316089\_length\_2387\_cov\_23.258902 2210-2214. Max. coverage (+): 1.78. Max coverage (-): 0.01

Region: NODE\_316089\_length\_2387\_cov\_23.258902 2215-2219. Max. coverage (+): 0.44. Max coverage (-): 0

Region: NODE\_316089\_length\_2387\_cov\_23.258902 2220-2224. Max. coverage (+): 0.43. Max coverage (-): 0.01

Region: NODE\_316089\_length\_2387\_cov\_23.258902 2225-2228. Max. coverage (+): 0.02. Max coverage (-): 0.01

Region: NODE\_316089\_length\_2387\_cov\_23.258902 2229-2233. Max. coverage (+): 0.01. Max coverage (-): 0.01

Region: NODE\_316089\_length\_2387\_cov\_23.258902 2234-2238. Max. coverage (+): 0.01. Max coverage (-): 0.01

Region: NODE\_316089\_length\_2387\_cov\_23.258902 2239-2243. Max. coverage (+): 0.23. Max coverage (-): 0

Region: NODE\_316089\_length\_2387\_cov\_23.258902 2244-2248. Max. coverage (+): 0.23. Max coverage (-): 0.01

Region: NODE\_316089\_length\_2387\_cov\_23.258902 2249-2253. Max. coverage (+): 0.07. Max coverage (-): 0.04

Region: NODE\_316089\_length\_2387\_cov\_23.258902 2254-2258. Max. coverage (+): 0.02. Max coverage (-): 0.04

Region: NODE\_316089\_length\_2387\_cov\_23.258902 2259-2263. Max. coverage (+): 0.43. Max coverage (-): 0.17

Region: NODE\_316089\_length\_2387\_cov\_23.258902 2264-2268. Max. coverage (+): 0.07. Max coverage (-): 0.24

Region: NODE\_316089\_length\_2387\_cov\_23.258902 2269-2273. Max. coverage (+): 0.09. Max coverage (-): 0.09

Region: NODE\_316089\_length\_2387\_cov\_23.258902 2274-2277. Max. coverage (+): 0.19. Max coverage (-): 0.04

Region: NODE\_316089\_length\_2387\_cov\_23.258902 2278-2282. Max. coverage (+): 0.97. Max coverage (-): 0.01

Region: NODE\_316089\_length\_2387\_cov\_23.258902 2283-2287. Max. coverage (+): 0.18. Max coverage (-): 0

Region: NODE\_316089\_length\_2387\_cov\_23.258902 2288-2292. Max. coverage (+): 0.11. Max coverage (-): 0

Region: NODE\_316089\_length\_2387\_cov\_23.258902 2293-2297. Max. coverage (+): 0.02. Max coverage (-): 0

Region: NODE\_316089\_length\_2387\_cov\_23.258902 2298-2302. Max. coverage (+): 0. Max coverage (-): 0.02

Region: NODE\_316089\_length\_2387\_cov\_23.258902 2303-2307. Max. coverage (+): 0.03. Max coverage (-): 0.19

Region: NODE\_316089\_length\_2387\_cov\_23.258902 2308-2312. Max. coverage (+): 0.28. Max coverage (-): 0.39

Region: NODE\_316089\_length\_2387\_cov\_23.258902 2313-2317. Max. coverage (+): 1.78. Max coverage (-): 0.48

Region: NODE\_316089\_length\_2387\_cov\_23.258902 2318-2322. Max. coverage (+): 1.98. Max coverage (-): 0.02

Region: NODE\_316089\_length\_2387\_cov\_23.258902 2323-2326. Max. coverage (+): 0.13. Max coverage (-): 0

Region: NODE\_316089\_length\_2387\_cov\_23.258902 2327-2331. Max. coverage (+): 3.26. Max coverage (-): 0

Region: NODE\_316089\_length\_2387\_cov\_23.258902 2332-2336. Max. coverage (+): 0.82. Max coverage (-): 0

Region: NODE\_316089\_length\_2387\_cov\_23.258902 2337-2341. Max. coverage (+): 0.15. Max coverage (-): 0.19

Region: NODE\_316089\_length\_2387\_cov\_23.258902 2342-2346. Max. coverage (+): 0.19. Max coverage (-): 0.22

Region: NODE\_316089\_length\_2387\_cov\_23.258902 2347-2351. Max. coverage (+): 0.07. Max coverage (-): 0

Region: NODE\_316089\_length\_2387\_cov\_23.258902 2352-2356. Max. coverage (+): 0.26. Max coverage (-): 0.04

Region: NODE\_316089\_length\_2387\_cov\_23.258902 2357-2361. Max. coverage (+): 2.89. Max coverage (-): 0.04

Region: NODE\_316089\_length\_2387\_cov\_23.258902 2362-2366. Max. coverage (+): 2.63. Max coverage (-): 0.04

Region: NODE\_316089\_length\_2387\_cov\_23.258902 2367-2371. Max. coverage (+): 1.22. Max coverage (-): 0.04

Region: NODE\_316089\_length\_2387\_cov\_23.258902 2372-2376. Max. coverage (+): 1.2. Max coverage (-): 0.06

Region: NODE\_316089\_length\_2387\_cov\_23.258902 2377-2380. Max. coverage (+): 1.28. Max coverage (-): 0.04

Region: NODE\_316089\_length\_2387\_cov\_23.258902 2381-2385. Max. coverage (+): 1.06. Max coverage (-): 0.02

Region: NODE\_316089\_length\_2387\_cov\_23.258902 2386-2390. Max. coverage (+): 5.67. Max coverage (-): 0.04

Region: NODE\_316089\_length\_2387\_cov\_23.258902 2391-2395. Max. coverage (+): 0.67. Max coverage (-): 0.04

Region: NODE\_316089\_length\_2387\_cov\_23.258902 2396-2400. Max. coverage (+): 0.26. Max coverage (-): 0.07

Region: NODE\_316089\_length\_2387\_cov\_23.258902 2401-2405. Max. coverage (+): 0.02. Max coverage (-): 0.01

Region: NODE\_316089\_length\_2387\_cov\_23.258902 2406-2410. Max. coverage (+): 0.13. Max coverage (-): 0

Region: NODE\_316089\_length\_2387\_cov\_23.258902 2411-2415. Max. coverage (+): 0.84. Max coverage (-): 0

Region: NODE\_316089\_length\_2387\_cov\_23.258902 2416-2420. Max. coverage (+): 0.15. Max coverage (-): 0

Region: NODE\_316089\_length\_2387\_cov\_23.258902 2421-2425. Max. coverage (+): 0.04. Max coverage (-): 0.09

Region: NODE\_316089\_length\_2387\_cov\_23.258902 2426-2429. Max. coverage (+): 0.11. Max coverage (-): 0.17

Region: NODE\_316089\_length\_2387\_cov\_23.258902 2430-2434. Max. coverage (+): 0. Max coverage (-): 0

Region: NODE\_316089\_length\_2387\_cov\_23.258902 2435-2439. Max. coverage (+): 0. Max coverage (-): 0

Region: NODE\_316089\_length\_2387\_cov\_23.258902 2440-2444. Max. coverage (+): 0. Max coverage (-): 0

Region: NODE\_316089\_length\_2387\_cov\_23.258902 2445-2449. Max. coverage (+): 0. Max coverage (-): 0

Region: NODE\_316089\_length\_2387\_cov\_23.258902 2450-. Max. coverage (+): 0. Max coverage (-): 0

RepeatMasker Color Code

**+**

100-98% Identity

<98-95% Identity

<95-90% Identity

<90-85% Identity

<85-80% Identity

<80-75% Identity

<75-70% Identity

<70% Identity

**-**

Gene Set Color Code

**+**

Gene

Pseudogene

Other

**-**

Topology/Coverage Color Code

Coverage Plus Strand

Coverage Minus Strand

Mainstrand: Plus

Mainstrand: Minus

Complementary Strand

Flanking Region  
(if option -flank >0)

Gene Set Annotation  
  
RepeatMasker Annotation  

**1. AlRepD-2135**: 1-61 (-), Divergence to consensus: 0%  
**2. AlRepE-3612**: 179-267 (-), Divergence to consensus: 13.5%  
**3. Tc1-2\_PM**: 180-462 (+), Divergence to consensus: 35.8%  
**4. Tc1-13\_Xt**: 484-547 (+), Divergence to consensus: 25%  
**5. Tc1-2\_PM**: 553-628 (+), Divergence to consensus: 34.2%  
**6. L1-52\_DR**: 995-1242 (+), Divergence to consensus: 41.5%  
**7. L1-15c\_Lch**: 1205-1326 (+), Divergence to consensus: 36.1%  
**8. AlRepD-2046**: 1662-1704 (-), Divergence to consensus: 16.2%  
**9. AlRepE-7493**: 1756-2073 (+), Divergence to consensus: 23.5%

  
Transcription Factor Binding Sites  

**RHOXF1** (Sequence: AGATCA (-): 221)  
**RHOXF1** (Sequence: AGATCA (-): 542)  
**RHOXF1** (Sequence: AGCTTA (-): 1425)  
**RHOXF1** (Sequence: AGATCA (-): 1995)  
**RHOXF1** (Sequence: AGCTCA (-): 2334)  
**RHOXF1** (Sequence: GGATTA (-): 2383)  
**RHOXF1** (Sequence: AGATCA (-): 2444)  
**RHOXF1** (Sequence: TAAGCT (+): 925)  
**RHOXF1** (Sequence: TAAGCC (+): 1141)  
**RHOXF1** (Sequence: TAAGCC (+): 1201)  
**RHOXF1** (Sequence: TGAGCT (+): 1344)  
**RHOXF1** (Sequence: TGAGCT (+): 1621)  
**RHOXF1** (Sequence: TGATCT (+): 1744)  
**RHOXF1** (Sequence: TGATCT (+): 2303)  
**FOXO3\_hsa** (Sequence: GTAAACAT (+): 2011)  
**FOXP1** (Sequence: GTAAACA (+): 2011)  
**Sox5** (Sequence: ATTGTT (+): 1109)  
**Sox5** (Sequence: ATTGTT (+): 2217)  
**Nobox** (Sequence: TAATTGCT (+): 2288)  
**Rhox11** (Sequence: ATTACACCA (-): 244)  
**Sox5** (Sequence: AACAAT (-): 458)  
**Sox5** (Sequence: AACAAT (-): 699)
