# Supplementary material for: First characterization of PIWI-interacting RNA clusters in a cichlid fish with a B chromosome
Source: BMC Biol. 2022 Sep 21;20:204. doi: 10.1186/s12915-022-01403-2 (PMC9490952; doi:10.1186/s12915-022-01403-2)
Supplement: Supplementary file 1 — Additional file 1. Zipped folder with fasta and interactive html piRNA cluster information for the A. latifasciata genome. The nomenclature is as follows: number-pirna-cluster_sex_B-presence (f, female; m, male; 0b, without B chromosome; 1b, with B chromosome). [file 12915_2022_1403_MOESM1_ESM.zip › 124_f1b.html]

piRNA cluster 124\_f1b 59


Predicted piRNA cluster no. 124\_f1b
  

Show proTRAC run info
Hide proTRAC run info

/\  
                \_\_\_\_\_\_\_\_\_\_\_\_\_\_\_\_\_\_\_\_\_\_\_/\\_\_\_ /  \\_\_\_\_\_\_\_  
               I                      /  \  /    \      I  
               I     pro             /    \/      \     I  
               I        TRAC        /               \   I  
               I   \_\_\_\_\_\_\_\_\_\_\_\_\_\_\_\_/\_\_\_\_\_\_\_\_\_\_\_\_\_\_\_\_\_\\_ I  
               I   \              /                     I  
               I    \            /                      I  
               I     \  /\      /       V.2.4.2         I  
               I      \/  \    /                        I  
               I\_\_\_\_\_\_\_\_\_\_\_\  /\_\_\_\_\_\_\_\_\_\_\_\_\_\_\_\_\_\_\_\_\_\_\_\_\_I  
                            \/  
  
  
================================= proTRAC ====================================  
VERSION: .......... 2.4.2  
LAST MODIFIED: .... 11. May 2018  
  
Please cite:  
Rosenkranz D, Zischler H. proTRAC - a software for probabilistic piRNA cluster  
detection, visualization and analysis. 2012. BMC Bioinformatics 13:5.  
  
  
Contact:  
David Rosenkranz  
Institute of Organismic and Molecular Evolutionary Biology  
Dept. Anthropology, small RNA group  
Johannes Gutenberg University Mainz  
email: rosenkranz@uni-mainz.de  
  
You can find the latest proTRAC version at:  
http://sourceforge.net/projects/protrac/files  
http://www.smallRNAgroup-mainz.de/software  
==============================================================================  
  
PARAMETERS:  
Map file: ...............piwi-femeas-1B.fa-collapse.map  
Genome file: ............../../../0B\_ala\_genome.fa  
RepeatMasker annotation: Alatifasciata-all0B-maryan-v2.fa\_corrected.out  
GeneSet:................./guest-storage/Data/annotation/Alatifasciata\_all0B\_maryan-v2\_out2017.gff  
  
Significant (p<=0.01) hit density will be calculated based  
on observed hit distribution.  
  
Sliding window size: ........................................ 5000 bp  
Sliding window increament: .................................. 1000 bp  
Normalize each hit by number of genomic hits: ............... yes  
Normalize each hit by number of sequence reads: ............. yes  
Normalize values (-> per million mapped reads): ............. yes  
Min. fraction of hits with 1T(U) or 10A: .................... 0.75  
Alternatively: Min. fraction of hits with 1T(U) and 10A: .... 0.5  
Min. fraction of hits with typical piRNA length: ............ 0.75  
Typical piRNA length: ....................................... 24-32 nt  
Min. size of a piRNA cluster: ............................... 1000 bp.  
Min. number of hits (absolute): ............................. 0  
Min. number of hits (normalized): ........................... 0  
Min. fraction of hits on the mainstrand: .................... 0.75  
Top fraction of mapped sequences (in terms of read counts): . 1%  
Top fraction accounts for max. n% of sequence reads: ........ 90%  
Min. fraction of hits on each arm of a bidirectional cluster: 0.05  
Output html file for each cluster: .......................... yes  
Output a summary table: ..................................... yes  
Output a FASTA file for each cluster (piRNA sequences): ..... yes  
Output a FASTA file comprising cluster sequences: ........... yes  
Output a GTF file for predicted piRNA clusters: ..............yes  
Search DNA motifs in clusters: .............................. yes  
Output flanking sequences: +/- .............................. 0 bp  
Output ~.pTi file: .......................................... no  
==============================================================================  
  
  
Genome size (without gaps): ............ 758543724 bp  
Gaps (N/X/-): .......................... 417479 bp  
Mapped reads: .......................... 10641844  
Non-identical sequences: ............... 2832837  
Genomic hits: .......................... 26056853  
Significant densitiy of mapped reads: .. 368.713530323068 reads/kb

Show proTRAC cluster info
Hide proTRAC cluster info

|  |  |
| --- | --- |
| Location | NODE\_317531\_length\_2809\_cov\_25.625490 |
| Coordinates | 1-2889 |
| Size [bp] | 2889 |
| Sequence hit loci | 1027 |
| Mapped reads (normalized) | 2085.1 |
| Mapped reads (normalized) per kb | 721.8 |
| Normalized reads with 1T (1U) | 75% |
| Normalized reads with 10A | 57.7% |
| Normalized reads with length 24-32 nt | 99.7% |
| Normalized reads on the main strand(s) | 79.8% |
| Predicted directionality | bi:minus-plus (split between 2150 and 2167) |

100%

0%

1T (1U)  
reads

10A reads

24-32 nt  
reads

reads on mainstrand

**Either the amount of reads with 1T (1U) OR 10A has to exceed 75% (set with option: -1Tor10A)  
Alternatively the amount of reads with 1T (1U) AND 10A has to exceed 50% (set with option: -1Tand10A)  
Minimum amount of reads with preferred size is 75% (set with option: -pisize)  
Minimum amount of reads on the main strand(s) is 75% (set with option: -clstrand)**

Show read coverage
Hide read coverage

WHAT DO I SEE HERE?  
This chart shows the location of mapped sequence reads within a predicted piRNA cluster. The color refers to the number of genomic hits produced by the sequence read in question. A dark red bar indicates that this sequence read produces many other hits elsewhere in the genome. Many adjacent red or yellow bars can indicate the presence of a multi-copy element such as transposons or rRNA genes. A dark green bar indicates that this sequence read maps uniquely to this locus.

1 hit

2-5 hits

6-10 hits

11-20 hits

21-50 hits

51-100 hits

> 100 hits

NODE\_317531\_length\_2809\_cov\_25.625490

1

2889

Gene Set

RepeatMasker

Mapped  
Reads

24.9

plus strand

minus strand

24.9

Region: NODE\_317531\_length\_2809\_cov\_25.625490 64835-3. Max. coverage (+): 0. Max coverage (-): 0.28

Region: NODE\_317531\_length\_2809\_cov\_25.625490 4-9. Max. coverage (+): 0. Max coverage (-): 0.47

Region: NODE\_317531\_length\_2809\_cov\_25.625490 10-15. Max. coverage (+): 0. Max coverage (-): 0.47

Region: NODE\_317531\_length\_2809\_cov\_25.625490 16-21. Max. coverage (+): 0.05. Max coverage (-): 0.56

Region: NODE\_317531\_length\_2809\_cov\_25.625490 22-27. Max. coverage (+): 0.09. Max coverage (-): 0.05

Region: NODE\_317531\_length\_2809\_cov\_25.625490 28-32. Max. coverage (+): 1.36. Max coverage (-): 0

Region: NODE\_317531\_length\_2809\_cov\_25.625490 33-38. Max. coverage (+): 0.8. Max coverage (-): 0

Region: NODE\_317531\_length\_2809\_cov\_25.625490 39-44. Max. coverage (+): 0. Max coverage (-): 5.83

Region: NODE\_317531\_length\_2809\_cov\_25.625490 45-50. Max. coverage (+): 0. Max coverage (-): 3.29

Region: NODE\_317531\_length\_2809\_cov\_25.625490 51-55. Max. coverage (+): 0. Max coverage (-): 0.75

Region: NODE\_317531\_length\_2809\_cov\_25.625490 56-61. Max. coverage (+): 0.09. Max coverage (-): 1.03

Region: NODE\_317531\_length\_2809\_cov\_25.625490 62-67. Max. coverage (+): 0.09. Max coverage (-): 0.28

Region: NODE\_317531\_length\_2809\_cov\_25.625490 68-73. Max. coverage (+): 0. Max coverage (-): 0.28

Region: NODE\_317531\_length\_2809\_cov\_25.625490 74-79. Max. coverage (+): 1.13. Max coverage (-): 0.19

Region: NODE\_317531\_length\_2809\_cov\_25.625490 80-84. Max. coverage (+): 0.38. Max coverage (-): 0.09

Region: NODE\_317531\_length\_2809\_cov\_25.625490 85-90. Max. coverage (+): 0. Max coverage (-): 0

Region: NODE\_317531\_length\_2809\_cov\_25.625490 91-96. Max. coverage (+): 0. Max coverage (-): 0.09

Region: NODE\_317531\_length\_2809\_cov\_25.625490 97-102. Max. coverage (+): 0. Max coverage (-): 0

Region: NODE\_317531\_length\_2809\_cov\_25.625490 103-107. Max. coverage (+): 0. Max coverage (-): 0

Region: NODE\_317531\_length\_2809\_cov\_25.625490 108-113. Max. coverage (+): 0. Max coverage (-): 0

Region: NODE\_317531\_length\_2809\_cov\_25.625490 114-119. Max. coverage (+): 0. Max coverage (-): 0

Region: NODE\_317531\_length\_2809\_cov\_25.625490 120-125. Max. coverage (+): 0. Max coverage (-): 0.19

Region: NODE\_317531\_length\_2809\_cov\_25.625490 126-131. Max. coverage (+): 0. Max coverage (-): 0.19

Region: NODE\_317531\_length\_2809\_cov\_25.625490 132-136. Max. coverage (+): 0. Max coverage (-): 0.19

Region: NODE\_317531\_length\_2809\_cov\_25.625490 137-142. Max. coverage (+): 0. Max coverage (-): 0

Region: NODE\_317531\_length\_2809\_cov\_25.625490 143-148. Max. coverage (+): 0.47. Max coverage (-): 0.09

Region: NODE\_317531\_length\_2809\_cov\_25.625490 149-154. Max. coverage (+): 0. Max coverage (-): 0.09

Region: NODE\_317531\_length\_2809\_cov\_25.625490 155-159. Max. coverage (+): 0. Max coverage (-): 0.47

Region: NODE\_317531\_length\_2809\_cov\_25.625490 160-165. Max. coverage (+): 0. Max coverage (-): 0

Region: NODE\_317531\_length\_2809\_cov\_25.625490 166-171. Max. coverage (+): 0. Max coverage (-): 0

Region: NODE\_317531\_length\_2809\_cov\_25.625490 172-177. Max. coverage (+): 0. Max coverage (-): 0

Region: NODE\_317531\_length\_2809\_cov\_25.625490 178-183. Max. coverage (+): 0.19. Max coverage (-): 0.38

Region: NODE\_317531\_length\_2809\_cov\_25.625490 184-188. Max. coverage (+): 0.09. Max coverage (-): 0.28

Region: NODE\_317531\_length\_2809\_cov\_25.625490 189-194. Max. coverage (+): 0.09. Max coverage (-): 0.28

Region: NODE\_317531\_length\_2809\_cov\_25.625490 195-200. Max. coverage (+): 0. Max coverage (-): 0

Region: NODE\_317531\_length\_2809\_cov\_25.625490 201-206. Max. coverage (+): 0.28. Max coverage (-): 0

Region: NODE\_317531\_length\_2809\_cov\_25.625490 207-211. Max. coverage (+): 0.19. Max coverage (-): 0.38

Region: NODE\_317531\_length\_2809\_cov\_25.625490 212-217. Max. coverage (+): 0. Max coverage (-): 3.57

Region: NODE\_317531\_length\_2809\_cov\_25.625490 218-223. Max. coverage (+): 0. Max coverage (-): 17.48

Region: NODE\_317531\_length\_2809\_cov\_25.625490 224-229. Max. coverage (+): 0. Max coverage (-): 0

Region: NODE\_317531\_length\_2809\_cov\_25.625490 230-235. Max. coverage (+): 0.09. Max coverage (-): 0

Region: NODE\_317531\_length\_2809\_cov\_25.625490 236-240. Max. coverage (+): 0.19. Max coverage (-): 0

Region: NODE\_317531\_length\_2809\_cov\_25.625490 241-246. Max. coverage (+): 0. Max coverage (-): 0

Region: NODE\_317531\_length\_2809\_cov\_25.625490 247-252. Max. coverage (+): 0. Max coverage (-): 0

Region: NODE\_317531\_length\_2809\_cov\_25.625490 253-258. Max. coverage (+): 0. Max coverage (-): 0

Region: NODE\_317531\_length\_2809\_cov\_25.625490 259-263. Max. coverage (+): 0. Max coverage (-): 0

Region: NODE\_317531\_length\_2809\_cov\_25.625490 264-269. Max. coverage (+): 0.09. Max coverage (-): 0.38

Region: NODE\_317531\_length\_2809\_cov\_25.625490 270-275. Max. coverage (+): 0. Max coverage (-): 0

Region: NODE\_317531\_length\_2809\_cov\_25.625490 276-281. Max. coverage (+): 0. Max coverage (-): 7.8

Region: NODE\_317531\_length\_2809\_cov\_25.625490 282-287. Max. coverage (+): 0. Max coverage (-): 0.09

Region: NODE\_317531\_length\_2809\_cov\_25.625490 288-292. Max. coverage (+): 0. Max coverage (-): 0

Region: NODE\_317531\_length\_2809\_cov\_25.625490 293-298. Max. coverage (+): 0. Max coverage (-): 0

Region: NODE\_317531\_length\_2809\_cov\_25.625490 299-304. Max. coverage (+): 0. Max coverage (-): 0

Region: NODE\_317531\_length\_2809\_cov\_25.625490 305-310. Max. coverage (+): 0. Max coverage (-): 0

Region: NODE\_317531\_length\_2809\_cov\_25.625490 311-315. Max. coverage (+): 0. Max coverage (-): 0

Region: NODE\_317531\_length\_2809\_cov\_25.625490 316-321. Max. coverage (+): 0. Max coverage (-): 0

Region: NODE\_317531\_length\_2809\_cov\_25.625490 322-327. Max. coverage (+): 0. Max coverage (-): 2.63

Region: NODE\_317531\_length\_2809\_cov\_25.625490 328-333. Max. coverage (+): 0.09. Max coverage (-): 0

Region: NODE\_317531\_length\_2809\_cov\_25.625490 334-339. Max. coverage (+): 0.19. Max coverage (-): 0

Region: NODE\_317531\_length\_2809\_cov\_25.625490 340-344. Max. coverage (+): 0.19. Max coverage (-): 0

Region: NODE\_317531\_length\_2809\_cov\_25.625490 345-350. Max. coverage (+): 0.75. Max coverage (-): 0

Region: NODE\_317531\_length\_2809\_cov\_25.625490 351-356. Max. coverage (+): 0.19. Max coverage (-): 0

Region: NODE\_317531\_length\_2809\_cov\_25.625490 357-362. Max. coverage (+): 0. Max coverage (-): 0

Region: NODE\_317531\_length\_2809\_cov\_25.625490 363-367. Max. coverage (+): 0.09. Max coverage (-): 0.28

Region: NODE\_317531\_length\_2809\_cov\_25.625490 368-373. Max. coverage (+): 0.19. Max coverage (-): 0.28

Region: NODE\_317531\_length\_2809\_cov\_25.625490 374-379. Max. coverage (+): 0. Max coverage (-): 0.28

Region: NODE\_317531\_length\_2809\_cov\_25.625490 380-385. Max. coverage (+): 0.09. Max coverage (-): 0.47

Region: NODE\_317531\_length\_2809\_cov\_25.625490 386-391. Max. coverage (+): 0.09. Max coverage (-): 0.09

Region: NODE\_317531\_length\_2809\_cov\_25.625490 392-396. Max. coverage (+): 0.09. Max coverage (-): 0.19

Region: NODE\_317531\_length\_2809\_cov\_25.625490 397-402. Max. coverage (+): 0. Max coverage (-): 0.19

Region: NODE\_317531\_length\_2809\_cov\_25.625490 403-408. Max. coverage (+): 0. Max coverage (-): 0.19

Region: NODE\_317531\_length\_2809\_cov\_25.625490 409-414. Max. coverage (+): 0. Max coverage (-): 0.09

Region: NODE\_317531\_length\_2809\_cov\_25.625490 415-419. Max. coverage (+): 0. Max coverage (-): 0.28

Region: NODE\_317531\_length\_2809\_cov\_25.625490 420-425. Max. coverage (+): 0.28. Max coverage (-): 0.09

Region: NODE\_317531\_length\_2809\_cov\_25.625490 426-431. Max. coverage (+): 0.28. Max coverage (-): 0.09

Region: NODE\_317531\_length\_2809\_cov\_25.625490 432-437. Max. coverage (+): 0.09. Max coverage (-): 0.66

Region: NODE\_317531\_length\_2809\_cov\_25.625490 438-443. Max. coverage (+): 0. Max coverage (-): 0.28

Region: NODE\_317531\_length\_2809\_cov\_25.625490 444-448. Max. coverage (+): 0. Max coverage (-): 0

Region: NODE\_317531\_length\_2809\_cov\_25.625490 449-454. Max. coverage (+): 0. Max coverage (-): 0.09

Region: NODE\_317531\_length\_2809\_cov\_25.625490 455-460. Max. coverage (+): 0. Max coverage (-): 0

Region: NODE\_317531\_length\_2809\_cov\_25.625490 461-466. Max. coverage (+): 0.09. Max coverage (-): 0

Region: NODE\_317531\_length\_2809\_cov\_25.625490 467-471. Max. coverage (+): 0.09. Max coverage (-): 0.28

Region: NODE\_317531\_length\_2809\_cov\_25.625490 472-477. Max. coverage (+): 0. Max coverage (-): 0.28

Region: NODE\_317531\_length\_2809\_cov\_25.625490 478-483. Max. coverage (+): 0. Max coverage (-): 3.1

Region: NODE\_317531\_length\_2809\_cov\_25.625490 484-489. Max. coverage (+): 0.19. Max coverage (-): 0.75

Region: NODE\_317531\_length\_2809\_cov\_25.625490 490-495. Max. coverage (+): 0.09. Max coverage (-): 0.09

Region: NODE\_317531\_length\_2809\_cov\_25.625490 496-500. Max. coverage (+): 0.19. Max coverage (-): 0.19

Region: NODE\_317531\_length\_2809\_cov\_25.625490 501-506. Max. coverage (+): 0. Max coverage (-): 0.28

Region: NODE\_317531\_length\_2809\_cov\_25.625490 507-512. Max. coverage (+): 0. Max coverage (-): 0.09

Region: NODE\_317531\_length\_2809\_cov\_25.625490 513-518. Max. coverage (+): 0.09. Max coverage (-): 0.09

Region: NODE\_317531\_length\_2809\_cov\_25.625490 519-523. Max. coverage (+): 0.19. Max coverage (-): 0.09

Region: NODE\_317531\_length\_2809\_cov\_25.625490 524-529. Max. coverage (+): 0.19. Max coverage (-): 0.47

Region: NODE\_317531\_length\_2809\_cov\_25.625490 530-535. Max. coverage (+): 0. Max coverage (-): 0.47

Region: NODE\_317531\_length\_2809\_cov\_25.625490 536-541. Max. coverage (+): 0. Max coverage (-): 0.09

Region: NODE\_317531\_length\_2809\_cov\_25.625490 542-547. Max. coverage (+): 0. Max coverage (-): 0

Region: NODE\_317531\_length\_2809\_cov\_25.625490 548-552. Max. coverage (+): 0. Max coverage (-): 0.56

Region: NODE\_317531\_length\_2809\_cov\_25.625490 553-558. Max. coverage (+): 0. Max coverage (-): 0.56

Region: NODE\_317531\_length\_2809\_cov\_25.625490 559-564. Max. coverage (+): 0. Max coverage (-): 0.09

Region: NODE\_317531\_length\_2809\_cov\_25.625490 565-570. Max. coverage (+): 0. Max coverage (-): 0

Region: NODE\_317531\_length\_2809\_cov\_25.625490 571-575. Max. coverage (+): 0. Max coverage (-): 0.09

Region: NODE\_317531\_length\_2809\_cov\_25.625490 576-581. Max. coverage (+): 0. Max coverage (-): 0

Region: NODE\_317531\_length\_2809\_cov\_25.625490 582-587. Max. coverage (+): 0. Max coverage (-): 0

Region: NODE\_317531\_length\_2809\_cov\_25.625490 588-593. Max. coverage (+): 0. Max coverage (-): 0

Region: NODE\_317531\_length\_2809\_cov\_25.625490 594-599. Max. coverage (+): 0. Max coverage (-): 0.09

Region: NODE\_317531\_length\_2809\_cov\_25.625490 600-604. Max. coverage (+): 0.09. Max coverage (-): 0

Region: NODE\_317531\_length\_2809\_cov\_25.625490 605-610. Max. coverage (+): 0. Max coverage (-): 0

Region: NODE\_317531\_length\_2809\_cov\_25.625490 611-616. Max. coverage (+): 0. Max coverage (-): 0.66

Region: NODE\_317531\_length\_2809\_cov\_25.625490 617-622. Max. coverage (+): 0. Max coverage (-): 24.9

Region: NODE\_317531\_length\_2809\_cov\_25.625490 623-627. Max. coverage (+): 0. Max coverage (-): 0

Region: NODE\_317531\_length\_2809\_cov\_25.625490 628-633. Max. coverage (+): 0. Max coverage (-): 0

Region: NODE\_317531\_length\_2809\_cov\_25.625490 634-639. Max. coverage (+): 0. Max coverage (-): 0

Region: NODE\_317531\_length\_2809\_cov\_25.625490 640-645. Max. coverage (+): 0. Max coverage (-): 0

Region: NODE\_317531\_length\_2809\_cov\_25.625490 646-651. Max. coverage (+): 0. Max coverage (-): 0

Region: NODE\_317531\_length\_2809\_cov\_25.625490 652-656. Max. coverage (+): 0. Max coverage (-): 0.94

Region: NODE\_317531\_length\_2809\_cov\_25.625490 657-662. Max. coverage (+): 0. Max coverage (-): 0.09

Region: NODE\_317531\_length\_2809\_cov\_25.625490 663-668. Max. coverage (+): 0. Max coverage (-): 0

Region: NODE\_317531\_length\_2809\_cov\_25.625490 669-674. Max. coverage (+): 0.09. Max coverage (-): 0

Region: NODE\_317531\_length\_2809\_cov\_25.625490 675-679. Max. coverage (+): 0.09. Max coverage (-): 0.38

Region: NODE\_317531\_length\_2809\_cov\_25.625490 680-685. Max. coverage (+): 0. Max coverage (-): 0.28

Region: NODE\_317531\_length\_2809\_cov\_25.625490 686-691. Max. coverage (+): 0.09. Max coverage (-): 2.26

Region: NODE\_317531\_length\_2809\_cov\_25.625490 692-697. Max. coverage (+): 0.09. Max coverage (-): 6.86

Region: NODE\_317531\_length\_2809\_cov\_25.625490 698-703. Max. coverage (+): 0. Max coverage (-): 6.58

Region: NODE\_317531\_length\_2809\_cov\_25.625490 704-708. Max. coverage (+): 0. Max coverage (-): 0

Region: NODE\_317531\_length\_2809\_cov\_25.625490 709-714. Max. coverage (+): 0.94. Max coverage (-): 1.69

Region: NODE\_317531\_length\_2809\_cov\_25.625490 715-720. Max. coverage (+): 0. Max coverage (-): 0.47

Region: NODE\_317531\_length\_2809\_cov\_25.625490 721-726. Max. coverage (+): 0. Max coverage (-): 5.92

Region: NODE\_317531\_length\_2809\_cov\_25.625490 727-731. Max. coverage (+): 0. Max coverage (-): 0.38

Region: NODE\_317531\_length\_2809\_cov\_25.625490 732-737. Max. coverage (+): 0. Max coverage (-): 0

Region: NODE\_317531\_length\_2809\_cov\_25.625490 738-743. Max. coverage (+): 0. Max coverage (-): 0

Region: NODE\_317531\_length\_2809\_cov\_25.625490 744-749. Max. coverage (+): 0. Max coverage (-): 0

Region: NODE\_317531\_length\_2809\_cov\_25.625490 750-755. Max. coverage (+): 0. Max coverage (-): 0.94

Region: NODE\_317531\_length\_2809\_cov\_25.625490 756-760. Max. coverage (+): 0. Max coverage (-): 1.03

Region: NODE\_317531\_length\_2809\_cov\_25.625490 761-766. Max. coverage (+): 0. Max coverage (-): 1.13

Region: NODE\_317531\_length\_2809\_cov\_25.625490 767-772. Max. coverage (+): 0. Max coverage (-): 0

Region: NODE\_317531\_length\_2809\_cov\_25.625490 773-778. Max. coverage (+): 0. Max coverage (-): 0.09

Region: NODE\_317531\_length\_2809\_cov\_25.625490 779-783. Max. coverage (+): 0. Max coverage (-): 0.09

Region: NODE\_317531\_length\_2809\_cov\_25.625490 784-789. Max. coverage (+): 0. Max coverage (-): 0.23

Region: NODE\_317531\_length\_2809\_cov\_25.625490 790-795. Max. coverage (+): 0.06. Max coverage (-): 1.67

Region: NODE\_317531\_length\_2809\_cov\_25.625490 796-801. Max. coverage (+): 0. Max coverage (-): 0.43

Region: NODE\_317531\_length\_2809\_cov\_25.625490 802-807. Max. coverage (+): 0.09. Max coverage (-): 0.09

Region: NODE\_317531\_length\_2809\_cov\_25.625490 808-812. Max. coverage (+): 0.19. Max coverage (-): 0.09

Region: NODE\_317531\_length\_2809\_cov\_25.625490 813-818. Max. coverage (+): 0. Max coverage (-): 0.09

Region: NODE\_317531\_length\_2809\_cov\_25.625490 819-824. Max. coverage (+): 0. Max coverage (-): 0.09

Region: NODE\_317531\_length\_2809\_cov\_25.625490 825-830. Max. coverage (+): 0. Max coverage (-): 0

Region: NODE\_317531\_length\_2809\_cov\_25.625490 831-835. Max. coverage (+): 0. Max coverage (-): 0

Region: NODE\_317531\_length\_2809\_cov\_25.625490 836-841. Max. coverage (+): 0.38. Max coverage (-): 0.19

Region: NODE\_317531\_length\_2809\_cov\_25.625490 842-847. Max. coverage (+): 0.09. Max coverage (-): 0

Region: NODE\_317531\_length\_2809\_cov\_25.625490 848-853. Max. coverage (+): 0. Max coverage (-): 0

Region: NODE\_317531\_length\_2809\_cov\_25.625490 854-859. Max. coverage (+): 0.09. Max coverage (-): 0

Region: NODE\_317531\_length\_2809\_cov\_25.625490 860-864. Max. coverage (+): 0. Max coverage (-): 0

Region: NODE\_317531\_length\_2809\_cov\_25.625490 865-870. Max. coverage (+): 0. Max coverage (-): 0

Region: NODE\_317531\_length\_2809\_cov\_25.625490 871-876. Max. coverage (+): 0. Max coverage (-): 0

Region: NODE\_317531\_length\_2809\_cov\_25.625490 877-882. Max. coverage (+): 0. Max coverage (-): 0

Region: NODE\_317531\_length\_2809\_cov\_25.625490 883-887. Max. coverage (+): 0. Max coverage (-): 0

Region: NODE\_317531\_length\_2809\_cov\_25.625490 888-893. Max. coverage (+): 0. Max coverage (-): 0

Region: NODE\_317531\_length\_2809\_cov\_25.625490 894-899. Max. coverage (+): 0. Max coverage (-): 0

Region: NODE\_317531\_length\_2809\_cov\_25.625490 900-905. Max. coverage (+): 0. Max coverage (-): 0

Region: NODE\_317531\_length\_2809\_cov\_25.625490 906-911. Max. coverage (+): 0. Max coverage (-): 0

Region: NODE\_317531\_length\_2809\_cov\_25.625490 912-916. Max. coverage (+): 0. Max coverage (-): 0

Region: NODE\_317531\_length\_2809\_cov\_25.625490 917-922. Max. coverage (+): 0. Max coverage (-): 0

Region: NODE\_317531\_length\_2809\_cov\_25.625490 923-928. Max. coverage (+): 0. Max coverage (-): 0

Region: NODE\_317531\_length\_2809\_cov\_25.625490 929-934. Max. coverage (+): 0. Max coverage (-): 0

Region: NODE\_317531\_length\_2809\_cov\_25.625490 935-939. Max. coverage (+): 0. Max coverage (-): 0

Region: NODE\_317531\_length\_2809\_cov\_25.625490 940-945. Max. coverage (+): 0. Max coverage (-): 0

Region: NODE\_317531\_length\_2809\_cov\_25.625490 946-951. Max. coverage (+): 0. Max coverage (-): 0

Region: NODE\_317531\_length\_2809\_cov\_25.625490 952-957. Max. coverage (+): 0. Max coverage (-): 0.09

Region: NODE\_317531\_length\_2809\_cov\_25.625490 958-963. Max. coverage (+): 0. Max coverage (-): 0

Region: NODE\_317531\_length\_2809\_cov\_25.625490 964-968. Max. coverage (+): 0. Max coverage (-): 0

Region: NODE\_317531\_length\_2809\_cov\_25.625490 969-974. Max. coverage (+): 0. Max coverage (-): 0.85

Region: NODE\_317531\_length\_2809\_cov\_25.625490 975-980. Max. coverage (+): 0. Max coverage (-): 1.03

Region: NODE\_317531\_length\_2809\_cov\_25.625490 981-986. Max. coverage (+): 0. Max coverage (-): 0.19

Region: NODE\_317531\_length\_2809\_cov\_25.625490 987-991. Max. coverage (+): 0.01. Max coverage (-): 0

Region: NODE\_317531\_length\_2809\_cov\_25.625490 992-997. Max. coverage (+): 0.02. Max coverage (-): 0

Region: NODE\_317531\_length\_2809\_cov\_25.625490 998-1003. Max. coverage (+): 0. Max coverage (-): 0

Region: NODE\_317531\_length\_2809\_cov\_25.625490 1004-1009. Max. coverage (+): 0. Max coverage (-): 0.03

Region: NODE\_317531\_length\_2809\_cov\_25.625490 1010-1015. Max. coverage (+): 0. Max coverage (-): 0.18

Region: NODE\_317531\_length\_2809\_cov\_25.625490 1016-1020. Max. coverage (+): 0. Max coverage (-): 0.17

Region: NODE\_317531\_length\_2809\_cov\_25.625490 1021-1026. Max. coverage (+): 0. Max coverage (-): 0

Region: NODE\_317531\_length\_2809\_cov\_25.625490 1027-1032. Max. coverage (+): 0.04. Max coverage (-): 0

Region: NODE\_317531\_length\_2809\_cov\_25.625490 1033-1038. Max. coverage (+): 0.04. Max coverage (-): 0

Region: NODE\_317531\_length\_2809\_cov\_25.625490 1039-1043. Max. coverage (+): 0. Max coverage (-): 0.04

Region: NODE\_317531\_length\_2809\_cov\_25.625490 1044-1049. Max. coverage (+): 0. Max coverage (-): 0.09

Region: NODE\_317531\_length\_2809\_cov\_25.625490 1050-1055. Max. coverage (+): 0. Max coverage (-): 0.02

Region: NODE\_317531\_length\_2809\_cov\_25.625490 1056-1061. Max. coverage (+): 0. Max coverage (-): 0.12

Region: NODE\_317531\_length\_2809\_cov\_25.625490 1062-1067. Max. coverage (+): 0. Max coverage (-): 0

Region: NODE\_317531\_length\_2809\_cov\_25.625490 1068-1072. Max. coverage (+): 0.06. Max coverage (-): 0.44

Region: NODE\_317531\_length\_2809\_cov\_25.625490 1073-1078. Max. coverage (+): 0.06. Max coverage (-): 0.39

Region: NODE\_317531\_length\_2809\_cov\_25.625490 1079-1084. Max. coverage (+): 0. Max coverage (-): 0

Region: NODE\_317531\_length\_2809\_cov\_25.625490 1085-1090. Max. coverage (+): 0. Max coverage (-): 0

Region: NODE\_317531\_length\_2809\_cov\_25.625490 1091-1095. Max. coverage (+): 0. Max coverage (-): 0

Region: NODE\_317531\_length\_2809\_cov\_25.625490 1096-1101. Max. coverage (+): 0. Max coverage (-): 0.05

Region: NODE\_317531\_length\_2809\_cov\_25.625490 1102-1107. Max. coverage (+): 0. Max coverage (-): 0

Region: NODE\_317531\_length\_2809\_cov\_25.625490 1108-1113. Max. coverage (+): 0. Max coverage (-): 0

Region: NODE\_317531\_length\_2809\_cov\_25.625490 1114-1119. Max. coverage (+): 0.01. Max coverage (-): 0

Region: NODE\_317531\_length\_2809\_cov\_25.625490 1120-1124. Max. coverage (+): 0. Max coverage (-): 0

Region: NODE\_317531\_length\_2809\_cov\_25.625490 1125-1130. Max. coverage (+): 0. Max coverage (-): 0

Region: NODE\_317531\_length\_2809\_cov\_25.625490 1131-1136. Max. coverage (+): 0. Max coverage (-): 0

Region: NODE\_317531\_length\_2809\_cov\_25.625490 1137-1142. Max. coverage (+): 0. Max coverage (-): 0

Region: NODE\_317531\_length\_2809\_cov\_25.625490 1143-1147. Max. coverage (+): 0. Max coverage (-): 0

Region: NODE\_317531\_length\_2809\_cov\_25.625490 1148-1153. Max. coverage (+): 0. Max coverage (-): 0

Region: NODE\_317531\_length\_2809\_cov\_25.625490 1154-1159. Max. coverage (+): 0. Max coverage (-): 0

Region: NODE\_317531\_length\_2809\_cov\_25.625490 1160-1165. Max. coverage (+): 0. Max coverage (-): 0

Region: NODE\_317531\_length\_2809\_cov\_25.625490 1166-1171. Max. coverage (+): 0. Max coverage (-): 0

Region: NODE\_317531\_length\_2809\_cov\_25.625490 1172-1176. Max. coverage (+): 0. Max coverage (-): 0

Region: NODE\_317531\_length\_2809\_cov\_25.625490 1177-1182. Max. coverage (+): 0. Max coverage (-): 0

Region: NODE\_317531\_length\_2809\_cov\_25.625490 1183-1188. Max. coverage (+): 0.01. Max coverage (-): 0

Region: NODE\_317531\_length\_2809\_cov\_25.625490 1189-1194. Max. coverage (+): 0.01. Max coverage (-): 0

Region: NODE\_317531\_length\_2809\_cov\_25.625490 1195-1199. Max. coverage (+): 0. Max coverage (-): 0

Region: NODE\_317531\_length\_2809\_cov\_25.625490 1200-1205. Max. coverage (+): 0. Max coverage (-): 0

Region: NODE\_317531\_length\_2809\_cov\_25.625490 1206-1211. Max. coverage (+): 0. Max coverage (-): 0

Region: NODE\_317531\_length\_2809\_cov\_25.625490 1212-1217. Max. coverage (+): 0. Max coverage (-): 0

Region: NODE\_317531\_length\_2809\_cov\_25.625490 1218-1223. Max. coverage (+): 0. Max coverage (-): 0

Region: NODE\_317531\_length\_2809\_cov\_25.625490 1224-1228. Max. coverage (+): 0. Max coverage (-): 0

Region: NODE\_317531\_length\_2809\_cov\_25.625490 1229-1234. Max. coverage (+): 0. Max coverage (-): 0

Region: NODE\_317531\_length\_2809\_cov\_25.625490 1235-1240. Max. coverage (+): 0. Max coverage (-): 0

Region: NODE\_317531\_length\_2809\_cov\_25.625490 1241-1246. Max. coverage (+): 0. Max coverage (-): 0

Region: NODE\_317531\_length\_2809\_cov\_25.625490 1247-1251. Max. coverage (+): 0. Max coverage (-): 0

Region: NODE\_317531\_length\_2809\_cov\_25.625490 1252-1257. Max. coverage (+): 0. Max coverage (-): 0

Region: NODE\_317531\_length\_2809\_cov\_25.625490 1258-1263. Max. coverage (+): 0. Max coverage (-): 0

Region: NODE\_317531\_length\_2809\_cov\_25.625490 1264-1269. Max. coverage (+): 0. Max coverage (-): 0

Region: NODE\_317531\_length\_2809\_cov\_25.625490 1270-1275. Max. coverage (+): 0. Max coverage (-): 0

Region: NODE\_317531\_length\_2809\_cov\_25.625490 1276-1280. Max. coverage (+): 0. Max coverage (-): 0

Region: NODE\_317531\_length\_2809\_cov\_25.625490 1281-1286. Max. coverage (+): 0. Max coverage (-): 0

Region: NODE\_317531\_length\_2809\_cov\_25.625490 1287-1292. Max. coverage (+): 0. Max coverage (-): 0

Region: NODE\_317531\_length\_2809\_cov\_25.625490 1293-1298. Max. coverage (+): 0. Max coverage (-): 0

Region: NODE\_317531\_length\_2809\_cov\_25.625490 1299-1303. Max. coverage (+): 0. Max coverage (-): 0

Region: NODE\_317531\_length\_2809\_cov\_25.625490 1304-1309. Max. coverage (+): 0. Max coverage (-): 0

Region: NODE\_317531\_length\_2809\_cov\_25.625490 1310-1315. Max. coverage (+): 0. Max coverage (-): 0

Region: NODE\_317531\_length\_2809\_cov\_25.625490 1316-1321. Max. coverage (+): 0. Max coverage (-): 0

Region: NODE\_317531\_length\_2809\_cov\_25.625490 1322-1327. Max. coverage (+): 0. Max coverage (-): 0

Region: NODE\_317531\_length\_2809\_cov\_25.625490 1328-1332. Max. coverage (+): 0. Max coverage (-): 0

Region: NODE\_317531\_length\_2809\_cov\_25.625490 1333-1338. Max. coverage (+): 0. Max coverage (-): 0

Region: NODE\_317531\_length\_2809\_cov\_25.625490 1339-1344. Max. coverage (+): 0. Max coverage (-): 0

Region: NODE\_317531\_length\_2809\_cov\_25.625490 1345-1350. Max. coverage (+): 0. Max coverage (-): 0

Region: NODE\_317531\_length\_2809\_cov\_25.625490 1351-1355. Max. coverage (+): 0. Max coverage (-): 0

Region: NODE\_317531\_length\_2809\_cov\_25.625490 1356-1361. Max. coverage (+): 0. Max coverage (-): 0

Region: NODE\_317531\_length\_2809\_cov\_25.625490 1362-1367. Max. coverage (+): 0. Max coverage (-): 0

Region: NODE\_317531\_length\_2809\_cov\_25.625490 1368-1373. Max. coverage (+): 0. Max coverage (-): 0

Region: NODE\_317531\_length\_2809\_cov\_25.625490 1374-1379. Max. coverage (+): 0. Max coverage (-): 0

Region: NODE\_317531\_length\_2809\_cov\_25.625490 1380-1384. Max. coverage (+): 0. Max coverage (-): 0

Region: NODE\_317531\_length\_2809\_cov\_25.625490 1385-1390. Max. coverage (+): 0. Max coverage (-): 0.04

Region: NODE\_317531\_length\_2809\_cov\_25.625490 1391-1396. Max. coverage (+): 0. Max coverage (-): 0.05

Region: NODE\_317531\_length\_2809\_cov\_25.625490 1397-1402. Max. coverage (+): 0. Max coverage (-): 0

Region: NODE\_317531\_length\_2809\_cov\_25.625490 1403-1407. Max. coverage (+): 0. Max coverage (-): 0

Region: NODE\_317531\_length\_2809\_cov\_25.625490 1408-1413. Max. coverage (+): 0.07. Max coverage (-): 0

Region: NODE\_317531\_length\_2809\_cov\_25.625490 1414-1419. Max. coverage (+): 0.03. Max coverage (-): 0

Region: NODE\_317531\_length\_2809\_cov\_25.625490 1420-1425. Max. coverage (+): 0. Max coverage (-): 0

Region: NODE\_317531\_length\_2809\_cov\_25.625490 1426-1431. Max. coverage (+): 0. Max coverage (-): 0

Region: NODE\_317531\_length\_2809\_cov\_25.625490 1432-1436. Max. coverage (+): 0. Max coverage (-): 0.01

Region: NODE\_317531\_length\_2809\_cov\_25.625490 1437-1442. Max. coverage (+): 0.09. Max coverage (-): 0.01

Region: NODE\_317531\_length\_2809\_cov\_25.625490 1443-1448. Max. coverage (+): 0.09. Max coverage (-): 0

Region: NODE\_317531\_length\_2809\_cov\_25.625490 1449-1454. Max. coverage (+): 0.09. Max coverage (-): 0

Region: NODE\_317531\_length\_2809\_cov\_25.625490 1455-1459. Max. coverage (+): 0.09. Max coverage (-): 0

Region: NODE\_317531\_length\_2809\_cov\_25.625490 1460-1465. Max. coverage (+): 0. Max coverage (-): 0.11

Region: NODE\_317531\_length\_2809\_cov\_25.625490 1466-1471. Max. coverage (+): 0. Max coverage (-): 0.09

Region: NODE\_317531\_length\_2809\_cov\_25.625490 1472-1477. Max. coverage (+): 0. Max coverage (-): 0

Region: NODE\_317531\_length\_2809\_cov\_25.625490 1478-1483. Max. coverage (+): 0. Max coverage (-): 0

Region: NODE\_317531\_length\_2809\_cov\_25.625490 1484-1488. Max. coverage (+): 0. Max coverage (-): 0

Region: NODE\_317531\_length\_2809\_cov\_25.625490 1489-1494. Max. coverage (+): 0.09. Max coverage (-): 0

Region: NODE\_317531\_length\_2809\_cov\_25.625490 1495-1500. Max. coverage (+): 0. Max coverage (-): 0

Region: NODE\_317531\_length\_2809\_cov\_25.625490 1501-1506. Max. coverage (+): 0. Max coverage (-): 0.09

Region: NODE\_317531\_length\_2809\_cov\_25.625490 1507-1511. Max. coverage (+): 0. Max coverage (-): 0.09

Region: NODE\_317531\_length\_2809\_cov\_25.625490 1512-1517. Max. coverage (+): 0. Max coverage (-): 0

Region: NODE\_317531\_length\_2809\_cov\_25.625490 1518-1523. Max. coverage (+): 0. Max coverage (-): 0

Region: NODE\_317531\_length\_2809\_cov\_25.625490 1524-1529. Max. coverage (+): 0. Max coverage (-): 0

Region: NODE\_317531\_length\_2809\_cov\_25.625490 1530-1535. Max. coverage (+): 0. Max coverage (-): 0

Region: NODE\_317531\_length\_2809\_cov\_25.625490 1536-1540. Max. coverage (+): 0. Max coverage (-): 0

Region: NODE\_317531\_length\_2809\_cov\_25.625490 1541-1546. Max. coverage (+): 0. Max coverage (-): 0

Region: NODE\_317531\_length\_2809\_cov\_25.625490 1547-1552. Max. coverage (+): 0. Max coverage (-): 0

Region: NODE\_317531\_length\_2809\_cov\_25.625490 1553-1558. Max. coverage (+): 0. Max coverage (-): 0

Region: NODE\_317531\_length\_2809\_cov\_25.625490 1559-1563. Max. coverage (+): 0. Max coverage (-): 0

Region: NODE\_317531\_length\_2809\_cov\_25.625490 1564-1569. Max. coverage (+): 0. Max coverage (-): 0

Region: NODE\_317531\_length\_2809\_cov\_25.625490 1570-1575. Max. coverage (+): 0. Max coverage (-): 0

Region: NODE\_317531\_length\_2809\_cov\_25.625490 1576-1581. Max. coverage (+): 0. Max coverage (-): 0

Region: NODE\_317531\_length\_2809\_cov\_25.625490 1582-1587. Max. coverage (+): 0. Max coverage (-): 0

Region: NODE\_317531\_length\_2809\_cov\_25.625490 1588-1592. Max. coverage (+): 0. Max coverage (-): 0

Region: NODE\_317531\_length\_2809\_cov\_25.625490 1593-1598. Max. coverage (+): 0. Max coverage (-): 0.09

Region: NODE\_317531\_length\_2809\_cov\_25.625490 1599-1604. Max. coverage (+): 0.09. Max coverage (-): 0.09

Region: NODE\_317531\_length\_2809\_cov\_25.625490 1605-1610. Max. coverage (+): 0. Max coverage (-): 0.09

Region: NODE\_317531\_length\_2809\_cov\_25.625490 1611-1615. Max. coverage (+): 0. Max coverage (-): 0

Region: NODE\_317531\_length\_2809\_cov\_25.625490 1616-1621. Max. coverage (+): 0. Max coverage (-): 0

Region: NODE\_317531\_length\_2809\_cov\_25.625490 1622-1627. Max. coverage (+): 0. Max coverage (-): 0

Region: NODE\_317531\_length\_2809\_cov\_25.625490 1628-1633. Max. coverage (+): 0. Max coverage (-): 0

Region: NODE\_317531\_length\_2809\_cov\_25.625490 1634-1639. Max. coverage (+): 0. Max coverage (-): 0

Region: NODE\_317531\_length\_2809\_cov\_25.625490 1640-1644. Max. coverage (+): 0. Max coverage (-): 0

Region: NODE\_317531\_length\_2809\_cov\_25.625490 1645-1650. Max. coverage (+): 0.19. Max coverage (-): 0

Region: NODE\_317531\_length\_2809\_cov\_25.625490 1651-1656. Max. coverage (+): 0.19. Max coverage (-): 0

Region: NODE\_317531\_length\_2809\_cov\_25.625490 1657-1662. Max. coverage (+): 0. Max coverage (-): 0

Region: NODE\_317531\_length\_2809\_cov\_25.625490 1663-1667. Max. coverage (+): 0. Max coverage (-): 0

Region: NODE\_317531\_length\_2809\_cov\_25.625490 1668-1673. Max. coverage (+): 0. Max coverage (-): 0

Region: NODE\_317531\_length\_2809\_cov\_25.625490 1674-1679. Max. coverage (+): 0. Max coverage (-): 0

Region: NODE\_317531\_length\_2809\_cov\_25.625490 1680-1685. Max. coverage (+): 0. Max coverage (-): 0

Region: NODE\_317531\_length\_2809\_cov\_25.625490 1686-1691. Max. coverage (+): 0. Max coverage (-): 0

Region: NODE\_317531\_length\_2809\_cov\_25.625490 1692-1696. Max. coverage (+): 0. Max coverage (-): 0

Region: NODE\_317531\_length\_2809\_cov\_25.625490 1697-1702. Max. coverage (+): 0. Max coverage (-): 0.19

Region: NODE\_317531\_length\_2809\_cov\_25.625490 1703-1708. Max. coverage (+): 0.09. Max coverage (-): 0.19

Region: NODE\_317531\_length\_2809\_cov\_25.625490 1709-1714. Max. coverage (+): 0. Max coverage (-): 0.09

Region: NODE\_317531\_length\_2809\_cov\_25.625490 1715-1719. Max. coverage (+): 0. Max coverage (-): 0.09

Region: NODE\_317531\_length\_2809\_cov\_25.625490 1720-1725. Max. coverage (+): 0. Max coverage (-): 0.09

Region: NODE\_317531\_length\_2809\_cov\_25.625490 1726-1731. Max. coverage (+): 0.28. Max coverage (-): 0

Region: NODE\_317531\_length\_2809\_cov\_25.625490 1732-1737. Max. coverage (+): 0.09. Max coverage (-): 0

Region: NODE\_317531\_length\_2809\_cov\_25.625490 1738-1743. Max. coverage (+): 0. Max coverage (-): 0.19

Region: NODE\_317531\_length\_2809\_cov\_25.625490 1744-1748. Max. coverage (+): 0. Max coverage (-): 0

Region: NODE\_317531\_length\_2809\_cov\_25.625490 1749-1754. Max. coverage (+): 0. Max coverage (-): 0

Region: NODE\_317531\_length\_2809\_cov\_25.625490 1755-1760. Max. coverage (+): 0.19. Max coverage (-): 0

Region: NODE\_317531\_length\_2809\_cov\_25.625490 1761-1766. Max. coverage (+): 0.19. Max coverage (-): 0.09

Region: NODE\_317531\_length\_2809\_cov\_25.625490 1767-1771. Max. coverage (+): 0.09. Max coverage (-): 0.09

Region: NODE\_317531\_length\_2809\_cov\_25.625490 1772-1777. Max. coverage (+): 0. Max coverage (-): 0.09

Region: NODE\_317531\_length\_2809\_cov\_25.625490 1778-1783. Max. coverage (+): 0. Max coverage (-): 0

Region: NODE\_317531\_length\_2809\_cov\_25.625490 1784-1789. Max. coverage (+): 0.38. Max coverage (-): 0

Region: NODE\_317531\_length\_2809\_cov\_25.625490 1790-1795. Max. coverage (+): 0.38. Max coverage (-): 0

Region: NODE\_317531\_length\_2809\_cov\_25.625490 1796-1800. Max. coverage (+): 0. Max coverage (-): 0.28

Region: NODE\_317531\_length\_2809\_cov\_25.625490 1801-1806. Max. coverage (+): 0. Max coverage (-): 0.38

Region: NODE\_317531\_length\_2809\_cov\_25.625490 1807-1812. Max. coverage (+): 0. Max coverage (-): 0

Region: NODE\_317531\_length\_2809\_cov\_25.625490 1813-1818. Max. coverage (+): 0. Max coverage (-): 0

Region: NODE\_317531\_length\_2809\_cov\_25.625490 1819-1823. Max. coverage (+): 0. Max coverage (-): 0

Region: NODE\_317531\_length\_2809\_cov\_25.625490 1824-1829. Max. coverage (+): 0. Max coverage (-): 0.09

Region: NODE\_317531\_length\_2809\_cov\_25.625490 1830-1835. Max. coverage (+): 0. Max coverage (-): 0

Region: NODE\_317531\_length\_2809\_cov\_25.625490 1836-1841. Max. coverage (+): 0.09. Max coverage (-): 0

Region: NODE\_317531\_length\_2809\_cov\_25.625490 1842-1847. Max. coverage (+): 0.19. Max coverage (-): 0.09

Region: NODE\_317531\_length\_2809\_cov\_25.625490 1848-1852. Max. coverage (+): 0.09. Max coverage (-): 0.09

Region: NODE\_317531\_length\_2809\_cov\_25.625490 1853-1858. Max. coverage (+): 0. Max coverage (-): 0

Region: NODE\_317531\_length\_2809\_cov\_25.625490 1859-1864. Max. coverage (+): 0. Max coverage (-): 0

Region: NODE\_317531\_length\_2809\_cov\_25.625490 1865-1870. Max. coverage (+): 0.28. Max coverage (-): 0.09

Region: NODE\_317531\_length\_2809\_cov\_25.625490 1871-1875. Max. coverage (+): 0.28. Max coverage (-): 0

Region: NODE\_317531\_length\_2809\_cov\_25.625490 1876-1881. Max. coverage (+): 0. Max coverage (-): 0

Region: NODE\_317531\_length\_2809\_cov\_25.625490 1882-1887. Max. coverage (+): 0.09. Max coverage (-): 0

Region: NODE\_317531\_length\_2809\_cov\_25.625490 1888-1893. Max. coverage (+): 0. Max coverage (-): 0

Region: NODE\_317531\_length\_2809\_cov\_25.625490 1894-1899. Max. coverage (+): 0.09. Max coverage (-): 0.09

Region: NODE\_317531\_length\_2809\_cov\_25.625490 1900-1904. Max. coverage (+): 0.66. Max coverage (-): 0

Region: NODE\_317531\_length\_2809\_cov\_25.625490 1905-1910. Max. coverage (+): 0.56. Max coverage (-): 0

Region: NODE\_317531\_length\_2809\_cov\_25.625490 1911-1916. Max. coverage (+): 0.28. Max coverage (-): 0.19

Region: NODE\_317531\_length\_2809\_cov\_25.625490 1917-1922. Max. coverage (+): 0.09. Max coverage (-): 0

Region: NODE\_317531\_length\_2809\_cov\_25.625490 1923-1927. Max. coverage (+): 0. Max coverage (-): 0

Region: NODE\_317531\_length\_2809\_cov\_25.625490 1928-1933. Max. coverage (+): 0.19. Max coverage (-): 0

Region: NODE\_317531\_length\_2809\_cov\_25.625490 1934-1939. Max. coverage (+): 0.28. Max coverage (-): 0

Region: NODE\_317531\_length\_2809\_cov\_25.625490 1940-1945. Max. coverage (+): 0.28. Max coverage (-): 0

Region: NODE\_317531\_length\_2809\_cov\_25.625490 1946-1951. Max. coverage (+): 0.28. Max coverage (-): 0

Region: NODE\_317531\_length\_2809\_cov\_25.625490 1952-1956. Max. coverage (+): 0.56. Max coverage (-): 0

Region: NODE\_317531\_length\_2809\_cov\_25.625490 1957-1962. Max. coverage (+): 0.09. Max coverage (-): 0.09

Region: NODE\_317531\_length\_2809\_cov\_25.625490 1963-1968. Max. coverage (+): 0. Max coverage (-): 0.09

Region: NODE\_317531\_length\_2809\_cov\_25.625490 1969-1974. Max. coverage (+): 0. Max coverage (-): 0.19

Region: NODE\_317531\_length\_2809\_cov\_25.625490 1975-1979. Max. coverage (+): 0. Max coverage (-): 0.19

Region: NODE\_317531\_length\_2809\_cov\_25.625490 1980-1985. Max. coverage (+): 0. Max coverage (-): 0.19

Region: NODE\_317531\_length\_2809\_cov\_25.625490 1986-1991. Max. coverage (+): 0.47. Max coverage (-): 0

Region: NODE\_317531\_length\_2809\_cov\_25.625490 1992-1997. Max. coverage (+): 14.28. Max coverage (-): 0

Region: NODE\_317531\_length\_2809\_cov\_25.625490 1998-2003. Max. coverage (+): 0. Max coverage (-): 0

Region: NODE\_317531\_length\_2809\_cov\_25.625490 2004-2008. Max. coverage (+): 0. Max coverage (-): 0

Region: NODE\_317531\_length\_2809\_cov\_25.625490 2009-2014. Max. coverage (+): 0. Max coverage (-): 0.85

Region: NODE\_317531\_length\_2809\_cov\_25.625490 2015-2020. Max. coverage (+): 0. Max coverage (-): 0.94

Region: NODE\_317531\_length\_2809\_cov\_25.625490 2021-2026. Max. coverage (+): 0.09. Max coverage (-): 0

Region: NODE\_317531\_length\_2809\_cov\_25.625490 2027-2031. Max. coverage (+): 0.09. Max coverage (-): 0

Region: NODE\_317531\_length\_2809\_cov\_25.625490 2032-2037. Max. coverage (+): 0. Max coverage (-): 0

Region: NODE\_317531\_length\_2809\_cov\_25.625490 2038-2043. Max. coverage (+): 2.26. Max coverage (-): 0

Region: NODE\_317531\_length\_2809\_cov\_25.625490 2044-2049. Max. coverage (+): 2.26. Max coverage (-): 0

Region: NODE\_317531\_length\_2809\_cov\_25.625490 2050-2055. Max. coverage (+): 0. Max coverage (-): 0

Region: NODE\_317531\_length\_2809\_cov\_25.625490 2056-2060. Max. coverage (+): 0. Max coverage (-): 0

Region: NODE\_317531\_length\_2809\_cov\_25.625490 2061-2066. Max. coverage (+): 0. Max coverage (-): 0

Region: NODE\_317531\_length\_2809\_cov\_25.625490 2067-2072. Max. coverage (+): 0. Max coverage (-): 0

Region: NODE\_317531\_length\_2809\_cov\_25.625490 2073-2078. Max. coverage (+): 0.09. Max coverage (-): 0

Region: NODE\_317531\_length\_2809\_cov\_25.625490 2079-2083. Max. coverage (+): 0. Max coverage (-): 0

Region: NODE\_317531\_length\_2809\_cov\_25.625490 2084-2089. Max. coverage (+): 0. Max coverage (-): 0

Region: NODE\_317531\_length\_2809\_cov\_25.625490 2090-2095. Max. coverage (+): 0. Max coverage (-): 0

Region: NODE\_317531\_length\_2809\_cov\_25.625490 2096-2101. Max. coverage (+): 0.09. Max coverage (-): 0

Region: NODE\_317531\_length\_2809\_cov\_25.625490 2102-2107. Max. coverage (+): 0. Max coverage (-): 0.09

Region: NODE\_317531\_length\_2809\_cov\_25.625490 2108-2112. Max. coverage (+): 0. Max coverage (-): 0.19

Region: NODE\_317531\_length\_2809\_cov\_25.625490 2113-2118. Max. coverage (+): 0. Max coverage (-): 0

Region: NODE\_317531\_length\_2809\_cov\_25.625490 2119-2124. Max. coverage (+): 1.41. Max coverage (-): 0

Region: NODE\_317531\_length\_2809\_cov\_25.625490 2125-2130. Max. coverage (+): 1.32. Max coverage (-): 0

Region: NODE\_317531\_length\_2809\_cov\_25.625490 2131-2135. Max. coverage (+): 0. Max coverage (-): 0

Region: NODE\_317531\_length\_2809\_cov\_25.625490 2136-2141. Max. coverage (+): 0. Max coverage (-): 0

Region: NODE\_317531\_length\_2809\_cov\_25.625490 2142-2147. Max. coverage (+): 0. Max coverage (-): 0.66

Region: NODE\_317531\_length\_2809\_cov\_25.625490 2148-2153. Max. coverage (+): 0. Max coverage (-): 10.52

Region: NODE\_317531\_length\_2809\_cov\_25.625490 2154-2159. Max. coverage (+): 0. Max coverage (-): 0

Region: NODE\_317531\_length\_2809\_cov\_25.625490 2160-2164. Max. coverage (+): 0. Max coverage (-): 0

Region: NODE\_317531\_length\_2809\_cov\_25.625490 2165-2170. Max. coverage (+): 2.82. Max coverage (-): 0

Region: NODE\_317531\_length\_2809\_cov\_25.625490 2171-2176. Max. coverage (+): 0. Max coverage (-): 0

Region: NODE\_317531\_length\_2809\_cov\_25.625490 2177-2182. Max. coverage (+): 0. Max coverage (-): 0

Region: NODE\_317531\_length\_2809\_cov\_25.625490 2183-2187. Max. coverage (+): 0. Max coverage (-): 0

Region: NODE\_317531\_length\_2809\_cov\_25.625490 2188-2193. Max. coverage (+): 0. Max coverage (-): 0

Region: NODE\_317531\_length\_2809\_cov\_25.625490 2194-2199. Max. coverage (+): 0. Max coverage (-): 0.09

Region: NODE\_317531\_length\_2809\_cov\_25.625490 2200-2205. Max. coverage (+): 0. Max coverage (-): 0

Region: NODE\_317531\_length\_2809\_cov\_25.625490 2206-2211. Max. coverage (+): 0. Max coverage (-): 0.09

Region: NODE\_317531\_length\_2809\_cov\_25.625490 2212-2216. Max. coverage (+): 0. Max coverage (-): 0.19

Region: NODE\_317531\_length\_2809\_cov\_25.625490 2217-2222. Max. coverage (+): 0. Max coverage (-): 0

Region: NODE\_317531\_length\_2809\_cov\_25.625490 2223-2228. Max. coverage (+): 0. Max coverage (-): 0

Region: NODE\_317531\_length\_2809\_cov\_25.625490 2229-2234. Max. coverage (+): 0.09. Max coverage (-): 0

Region: NODE\_317531\_length\_2809\_cov\_25.625490 2235-2239. Max. coverage (+): 0. Max coverage (-): 0

Region: NODE\_317531\_length\_2809\_cov\_25.625490 2240-2245. Max. coverage (+): 0. Max coverage (-): 0

Region: NODE\_317531\_length\_2809\_cov\_25.625490 2246-2251. Max. coverage (+): 0.75. Max coverage (-): 0.09

Region: NODE\_317531\_length\_2809\_cov\_25.625490 2252-2257. Max. coverage (+): 0.75. Max coverage (-): 0

Region: NODE\_317531\_length\_2809\_cov\_25.625490 2258-2263. Max. coverage (+): 0. Max coverage (-): 0

Region: NODE\_317531\_length\_2809\_cov\_25.625490 2264-2268. Max. coverage (+): 0. Max coverage (-): 0

Region: NODE\_317531\_length\_2809\_cov\_25.625490 2269-2274. Max. coverage (+): 0.38. Max coverage (-): 0

Region: NODE\_317531\_length\_2809\_cov\_25.625490 2275-2280. Max. coverage (+): 0.85. Max coverage (-): 0

Region: NODE\_317531\_length\_2809\_cov\_25.625490 2281-2286. Max. coverage (+): 0. Max coverage (-): 0

Region: NODE\_317531\_length\_2809\_cov\_25.625490 2287-2291. Max. coverage (+): 0. Max coverage (-): 0

Region: NODE\_317531\_length\_2809\_cov\_25.625490 2292-2297. Max. coverage (+): 0. Max coverage (-): 0.09

Region: NODE\_317531\_length\_2809\_cov\_25.625490 2298-2303. Max. coverage (+): 0. Max coverage (-): 0

Region: NODE\_317531\_length\_2809\_cov\_25.625490 2304-2309. Max. coverage (+): 0. Max coverage (-): 0.09

Region: NODE\_317531\_length\_2809\_cov\_25.625490 2310-2315. Max. coverage (+): 0.28. Max coverage (-): 0.09

Region: NODE\_317531\_length\_2809\_cov\_25.625490 2316-2320. Max. coverage (+): 0.09. Max coverage (-): 0

Region: NODE\_317531\_length\_2809\_cov\_25.625490 2321-2326. Max. coverage (+): 0.66. Max coverage (-): 0.19

Region: NODE\_317531\_length\_2809\_cov\_25.625490 2327-2332. Max. coverage (+): 0.47. Max coverage (-): 0.09

Region: NODE\_317531\_length\_2809\_cov\_25.625490 2333-2338. Max. coverage (+): 0.09. Max coverage (-): 0

Region: NODE\_317531\_length\_2809\_cov\_25.625490 2339-2343. Max. coverage (+): 0. Max coverage (-): 0

Region: NODE\_317531\_length\_2809\_cov\_25.625490 2344-2349. Max. coverage (+): 0. Max coverage (-): 0

Region: NODE\_317531\_length\_2809\_cov\_25.625490 2350-2355. Max. coverage (+): 0.09. Max coverage (-): 0

Region: NODE\_317531\_length\_2809\_cov\_25.625490 2356-2361. Max. coverage (+): 0.66. Max coverage (-): 0

Region: NODE\_317531\_length\_2809\_cov\_25.625490 2362-2367. Max. coverage (+): 0. Max coverage (-): 0

Region: NODE\_317531\_length\_2809\_cov\_25.625490 2368-2372. Max. coverage (+): 0. Max coverage (-): 0

Region: NODE\_317531\_length\_2809\_cov\_25.625490 2373-2378. Max. coverage (+): 0. Max coverage (-): 0

Region: NODE\_317531\_length\_2809\_cov\_25.625490 2379-2384. Max. coverage (+): 0. Max coverage (-): 0

Region: NODE\_317531\_length\_2809\_cov\_25.625490 2385-2390. Max. coverage (+): 0. Max coverage (-): 0.04

Region: NODE\_317531\_length\_2809\_cov\_25.625490 2391-2395. Max. coverage (+): 0.09. Max coverage (-): 0

Region: NODE\_317531\_length\_2809\_cov\_25.625490 2396-2401. Max. coverage (+): 0.75. Max coverage (-): 0

Region: NODE\_317531\_length\_2809\_cov\_25.625490 2402-2407. Max. coverage (+): 0.38. Max coverage (-): 0.09

Region: NODE\_317531\_length\_2809\_cov\_25.625490 2408-2413. Max. coverage (+): 0.38. Max coverage (-): 0.28

Region: NODE\_317531\_length\_2809\_cov\_25.625490 2414-2419. Max. coverage (+): 0. Max coverage (-): 0.09

Region: NODE\_317531\_length\_2809\_cov\_25.625490 2420-2424. Max. coverage (+): 0. Max coverage (-): 0

Region: NODE\_317531\_length\_2809\_cov\_25.625490 2425-2430. Max. coverage (+): 0.38. Max coverage (-): 0

Region: NODE\_317531\_length\_2809\_cov\_25.625490 2431-2436. Max. coverage (+): 0.47. Max coverage (-): 0

Region: NODE\_317531\_length\_2809\_cov\_25.625490 2437-2442. Max. coverage (+): 1.03. Max coverage (-): 0.09

Region: NODE\_317531\_length\_2809\_cov\_25.625490 2443-2447. Max. coverage (+): 0.19. Max coverage (-): 0.09

Region: NODE\_317531\_length\_2809\_cov\_25.625490 2448-2453. Max. coverage (+): 0. Max coverage (-): 0

Region: NODE\_317531\_length\_2809\_cov\_25.625490 2454-2459. Max. coverage (+): 0. Max coverage (-): 0.09

Region: NODE\_317531\_length\_2809\_cov\_25.625490 2460-2465. Max. coverage (+): 0. Max coverage (-): 0.09

Region: NODE\_317531\_length\_2809\_cov\_25.625490 2466-2471. Max. coverage (+): 0.94. Max coverage (-): 0.09

Region: NODE\_317531\_length\_2809\_cov\_25.625490 2472-2476. Max. coverage (+): 0.56. Max coverage (-): 0.09

Region: NODE\_317531\_length\_2809\_cov\_25.625490 2477-2482. Max. coverage (+): 0.28. Max coverage (-): 0

Region: NODE\_317531\_length\_2809\_cov\_25.625490 2483-2488. Max. coverage (+): 0. Max coverage (-): 0.19

Region: NODE\_317531\_length\_2809\_cov\_25.625490 2489-2494. Max. coverage (+): 0.38. Max coverage (-): 0.19

Region: NODE\_317531\_length\_2809\_cov\_25.625490 2495-2499. Max. coverage (+): 0.47. Max coverage (-): 0

Region: NODE\_317531\_length\_2809\_cov\_25.625490 2500-2505. Max. coverage (+): 0.28. Max coverage (-): 0.19

Region: NODE\_317531\_length\_2809\_cov\_25.625490 2506-2511. Max. coverage (+): 0. Max coverage (-): 0

Region: NODE\_317531\_length\_2809\_cov\_25.625490 2512-2517. Max. coverage (+): 0. Max coverage (-): 0

Region: NODE\_317531\_length\_2809\_cov\_25.625490 2518-2523. Max. coverage (+): 0.56. Max coverage (-): 0

Region: NODE\_317531\_length\_2809\_cov\_25.625490 2524-2528. Max. coverage (+): 0.47. Max coverage (-): 0

Region: NODE\_317531\_length\_2809\_cov\_25.625490 2529-2534. Max. coverage (+): 0.09. Max coverage (-): 0

Region: NODE\_317531\_length\_2809\_cov\_25.625490 2535-2540. Max. coverage (+): 0.09. Max coverage (-): 0.75

Region: NODE\_317531\_length\_2809\_cov\_25.625490 2541-2546. Max. coverage (+): 0.19. Max coverage (-): 0.75

Region: NODE\_317531\_length\_2809\_cov\_25.625490 2547-2551. Max. coverage (+): 0.19. Max coverage (-): 0

Region: NODE\_317531\_length\_2809\_cov\_25.625490 2552-2557. Max. coverage (+): 5.64. Max coverage (-): 0

Region: NODE\_317531\_length\_2809\_cov\_25.625490 2558-2563. Max. coverage (+): 4.89. Max coverage (-): 0

Region: NODE\_317531\_length\_2809\_cov\_25.625490 2564-2569. Max. coverage (+): 0. Max coverage (-): 0

Region: NODE\_317531\_length\_2809\_cov\_25.625490 2570-2575. Max. coverage (+): 0.09. Max coverage (-): 0

Region: NODE\_317531\_length\_2809\_cov\_25.625490 2576-2580. Max. coverage (+): 0.09. Max coverage (-): 0

Region: NODE\_317531\_length\_2809\_cov\_25.625490 2581-2586. Max. coverage (+): 0.09. Max coverage (-): 0.09

Region: NODE\_317531\_length\_2809\_cov\_25.625490 2587-2592. Max. coverage (+): 0. Max coverage (-): 0

Region: NODE\_317531\_length\_2809\_cov\_25.625490 2593-2598. Max. coverage (+): 0. Max coverage (-): 0

Region: NODE\_317531\_length\_2809\_cov\_25.625490 2599-2603. Max. coverage (+): 0.28. Max coverage (-): 0

Region: NODE\_317531\_length\_2809\_cov\_25.625490 2604-2609. Max. coverage (+): 0. Max coverage (-): 0

Region: NODE\_317531\_length\_2809\_cov\_25.625490 2610-2615. Max. coverage (+): 0. Max coverage (-): 0.19

Region: NODE\_317531\_length\_2809\_cov\_25.625490 2616-2621. Max. coverage (+): 0. Max coverage (-): 0

Region: NODE\_317531\_length\_2809\_cov\_25.625490 2622-2627. Max. coverage (+): 0.09. Max coverage (-): 0

Region: NODE\_317531\_length\_2809\_cov\_25.625490 2628-2632. Max. coverage (+): 0.09. Max coverage (-): 0

Region: NODE\_317531\_length\_2809\_cov\_25.625490 2633-2638. Max. coverage (+): 0. Max coverage (-): 0

Region: NODE\_317531\_length\_2809\_cov\_25.625490 2639-2644. Max. coverage (+): 0. Max coverage (-): 0

Region: NODE\_317531\_length\_2809\_cov\_25.625490 2645-2650. Max. coverage (+): 0. Max coverage (-): 0

Region: NODE\_317531\_length\_2809\_cov\_25.625490 2651-2655. Max. coverage (+): 0. Max coverage (-): 0

Region: NODE\_317531\_length\_2809\_cov\_25.625490 2656-2661. Max. coverage (+): 0.09. Max coverage (-): 0

Region: NODE\_317531\_length\_2809\_cov\_25.625490 2662-2667. Max. coverage (+): 0. Max coverage (-): 0

Region: NODE\_317531\_length\_2809\_cov\_25.625490 2668-2673. Max. coverage (+): 0. Max coverage (-): 0

Region: NODE\_317531\_length\_2809\_cov\_25.625490 2674-2679. Max. coverage (+): 1.32. Max coverage (-): 0

Region: NODE\_317531\_length\_2809\_cov\_25.625490 2680-2684. Max. coverage (+): 0.66. Max coverage (-): 0.09

Region: NODE\_317531\_length\_2809\_cov\_25.625490 2685-2690. Max. coverage (+): 0. Max coverage (-): 0.09

Region: NODE\_317531\_length\_2809\_cov\_25.625490 2691-2696. Max. coverage (+): 0. Max coverage (-): 0

Region: NODE\_317531\_length\_2809\_cov\_25.625490 2697-2702. Max. coverage (+): 0. Max coverage (-): 0.09

Region: NODE\_317531\_length\_2809\_cov\_25.625490 2703-2707. Max. coverage (+): 0. Max coverage (-): 0.09

Region: NODE\_317531\_length\_2809\_cov\_25.625490 2708-2713. Max. coverage (+): 0. Max coverage (-): 0

Region: NODE\_317531\_length\_2809\_cov\_25.625490 2714-2719. Max. coverage (+): 0.75. Max coverage (-): 0.09

Region: NODE\_317531\_length\_2809\_cov\_25.625490 2720-2725. Max. coverage (+): 0. Max coverage (-): 0

Region: NODE\_317531\_length\_2809\_cov\_25.625490 2726-2731. Max. coverage (+): 0. Max coverage (-): 0

Region: NODE\_317531\_length\_2809\_cov\_25.625490 2732-2736. Max. coverage (+): 0. Max coverage (-): 0

Region: NODE\_317531\_length\_2809\_cov\_25.625490 2737-2742. Max. coverage (+): 0. Max coverage (-): 0

Region: NODE\_317531\_length\_2809\_cov\_25.625490 2743-2748. Max. coverage (+): 0. Max coverage (-): 0

Region: NODE\_317531\_length\_2809\_cov\_25.625490 2749-2754. Max. coverage (+): 0.19. Max coverage (-): 0

Region: NODE\_317531\_length\_2809\_cov\_25.625490 2755-2759. Max. coverage (+): 0.19. Max coverage (-): 0

Region: NODE\_317531\_length\_2809\_cov\_25.625490 2760-2765. Max. coverage (+): 1.32. Max coverage (-): 0.09

Region: NODE\_317531\_length\_2809\_cov\_25.625490 2766-2771. Max. coverage (+): 1.32. Max coverage (-): 0

Region: NODE\_317531\_length\_2809\_cov\_25.625490 2772-2777. Max. coverage (+): 0. Max coverage (-): 0

Region: NODE\_317531\_length\_2809\_cov\_25.625490 2778-2783. Max. coverage (+): 1.69. Max coverage (-): 0

Region: NODE\_317531\_length\_2809\_cov\_25.625490 2784-2788. Max. coverage (+): 1.69. Max coverage (-): 0.09

Region: NODE\_317531\_length\_2809\_cov\_25.625490 2789-2794. Max. coverage (+): 0.19. Max coverage (-): 0.09

Region: NODE\_317531\_length\_2809\_cov\_25.625490 2795-2800. Max. coverage (+): 0.38. Max coverage (-): 0.09

Region: NODE\_317531\_length\_2809\_cov\_25.625490 2801-2806. Max. coverage (+): 0.09. Max coverage (-): 0.19

Region: NODE\_317531\_length\_2809\_cov\_25.625490 2807-2811. Max. coverage (+): 0.09. Max coverage (-): 0

Region: NODE\_317531\_length\_2809\_cov\_25.625490 2812-2817. Max. coverage (+): 0.09. Max coverage (-): 0

Region: NODE\_317531\_length\_2809\_cov\_25.625490 2818-2823. Max. coverage (+): 0.47. Max coverage (-): 0

Region: NODE\_317531\_length\_2809\_cov\_25.625490 2824-2829. Max. coverage (+): 0.09. Max coverage (-): 0

Region: NODE\_317531\_length\_2809\_cov\_25.625490 2830-2835. Max. coverage (+): 0.09. Max coverage (-): 0.28

Region: NODE\_317531\_length\_2809\_cov\_25.625490 2836-2840. Max. coverage (+): 0. Max coverage (-): 0.19

Region: NODE\_317531\_length\_2809\_cov\_25.625490 2841-2846. Max. coverage (+): 0.75. Max coverage (-): 0

Region: NODE\_317531\_length\_2809\_cov\_25.625490 2847-2852. Max. coverage (+): 0.66. Max coverage (-): 0

Region: NODE\_317531\_length\_2809\_cov\_25.625490 2853-2858. Max. coverage (+): 0.56. Max coverage (-): 0

Region: NODE\_317531\_length\_2809\_cov\_25.625490 2859-2863. Max. coverage (+): 3.19. Max coverage (-): 0

Region: NODE\_317531\_length\_2809\_cov\_25.625490 2864-2869. Max. coverage (+): 0. Max coverage (-): 0

Region: NODE\_317531\_length\_2809\_cov\_25.625490 2870-2875. Max. coverage (+): 0. Max coverage (-): 0

Region: NODE\_317531\_length\_2809\_cov\_25.625490 2876-2881. Max. coverage (+): 0. Max coverage (-): 0

Region: NODE\_317531\_length\_2809\_cov\_25.625490 2882-2887. Max. coverage (+): 0. Max coverage (-): 0

Region: NODE\_317531\_length\_2809\_cov\_25.625490 2888-. Max. coverage (+): 0. Max coverage (-): 0

RepeatMasker Color Code

**+**

100-98% Identity

<98-95% Identity

<95-90% Identity

<90-85% Identity

<85-80% Identity

<80-75% Identity

<75-70% Identity

<70% Identity

**-**

Gene Set Color Code

**+**

Gene

Pseudogene

Other

**-**

Topology/Coverage Color Code

Coverage Plus Strand

Coverage Minus Strand

Mainstrand: Plus

Mainstrand: Minus

Complementary Strand

Flanking Region  
(if option -flank >0)

Gene Set Annotation  
  
RepeatMasker Annotation  

**1. SINE\_AFC**: 993-1170 (+), Divergence to consensus: 7.9%  
**2. AlRepA-24**: 1182-1251 (+), Divergence to consensus: 5.7%  
**3. AlRepA-24**: 1251-1314 (+), Divergence to consensus: 3.1%  
**4. AlRepD-5194**: 1317-1481 (+), Divergence to consensus: 23%  
**5. AlRepD-2811**: 1699-1881 (-), Divergence to consensus: 31.7%  
**6. AlRepC-800**: 2013-2427 (-), Divergence to consensus: 37.6%  
**7. (A)n**: 2878-2898 (+), Divergence to consensus: 0%

  
Transcription Factor Binding Sites  

**RHOXF1** (Sequence: GGCTCA (-): 1006)  
**RHOXF1** (Sequence: AGATTA (-): 1583)  
**RHOXF1** (Sequence: GGCTCA (-): 1965)  
**RHOXF1** (Sequence: AGCTCA (-): 2138)  
**RHOXF1** (Sequence: AGATTA (-): 2337)  
**RHOXF1** (Sequence: GGATTA (-): 2779)  
**RHOXF1** (Sequence: TAATCC (+): 2429)  
**Lhx8** (Sequence: TTAATTAA (-): 290)  
**Lhx8** (Sequence: CTAATTAA (-): 304)  
**FOXO3\_hsa** (Sequence: GTAAACAT (+): 2636)  
**FOXP1** (Sequence: GTAAACA (+): 2636)  
**FOXO1** (Sequence: CCTGTTTTC (+): 2375)  
**FOXO3\_mmu** (Sequence: TGTTTTCC (-): 2377)  
**Sox5** (Sequence: ATTGTT (+): 1615)  
**FOXO3\_mmu** (Sequence: TCAAAACA (+): 543)  
**FOXO3\_mmu** (Sequence: TGTAAACA (+): 2635)  
**FOXO3\_mmu** (Sequence: TCAAAACA (+): 2825)  
**Nobox** (Sequence: ACTAATTA (-): 303)  
**Nobox** (Sequence: AGTAATTA (-): 2547)  
**POU2F1** (Sequence: ATTTAAATA (-): 2320)  
**POU2F1** (Sequence: TATTTAAAT (+): 2319)
