# Supplementary material for: First characterization of PIWI-interacting RNA clusters in a cichlid fish with a B chromosome
Source: BMC Biol. 2022 Sep 21;20:204. doi: 10.1186/s12915-022-01403-2 (PMC9490952; doi:10.1186/s12915-022-01403-2)
Supplement: Supplementary file 1 — Additional file 1. Zipped folder with fasta and interactive html piRNA cluster information for the A. latifasciata genome. The nomenclature is as follows: number-pirna-cluster_sex_B-presence (f, female; m, male; 0b, without B chromosome; 1b, with B chromosome). [file 12915_2022_1403_MOESM1_ESM.zip › 125_m1b.html]

piRNA cluster 125\_m1b 72


Predicted piRNA cluster no. 125\_m1b
  

Show proTRAC run info
Hide proTRAC run info

/\  
                \_\_\_\_\_\_\_\_\_\_\_\_\_\_\_\_\_\_\_\_\_\_\_/\\_\_\_ /  \\_\_\_\_\_\_\_  
               I                      /  \  /    \      I  
               I     pro             /    \/      \     I  
               I        TRAC        /               \   I  
               I   \_\_\_\_\_\_\_\_\_\_\_\_\_\_\_\_/\_\_\_\_\_\_\_\_\_\_\_\_\_\_\_\_\_\\_ I  
               I   \              /                     I  
               I    \            /                      I  
               I     \  /\      /       V.2.4.2         I  
               I      \/  \    /                        I  
               I\_\_\_\_\_\_\_\_\_\_\_\  /\_\_\_\_\_\_\_\_\_\_\_\_\_\_\_\_\_\_\_\_\_\_\_\_\_I  
                            \/  
  
  
================================= proTRAC ====================================  
VERSION: .......... 2.4.2  
LAST MODIFIED: .... 11. May 2018  
  
Please cite:  
Rosenkranz D, Zischler H. proTRAC - a software for probabilistic piRNA cluster  
detection, visualization and analysis. 2012. BMC Bioinformatics 13:5.  
  
  
Contact:  
David Rosenkranz  
Institute of Organismic and Molecular Evolutionary Biology  
Dept. Anthropology, small RNA group  
Johannes Gutenberg University Mainz  
email: rosenkranz@uni-mainz.de  
  
You can find the latest proTRAC version at:  
http://sourceforge.net/projects/protrac/files  
http://www.smallRNAgroup-mainz.de/software  
==============================================================================  
  
PARAMETERS:  
Map file: ...............piwi-machos-1B.fa-collapse.map  
Genome file: ............../../../0B\_ala\_genome.fa  
RepeatMasker annotation: Alatifasciata-all0B-maryan-v2.fa\_corrected.out  
GeneSet:................./guest-storage/Data/annotation/Alatifasciata\_all0B\_maryan-v2\_out2017.gff  
  
Significant (p<=0.01) hit density will be calculated based  
on observed hit distribution.  
  
Sliding window size: ........................................ 5000 bp  
Sliding window increament: .................................. 1000 bp  
Normalize each hit by number of genomic hits: ............... yes  
Normalize each hit by number of sequence reads: ............. yes  
Normalize values (-> per million mapped reads): ............. yes  
Min. fraction of hits with 1T(U) or 10A: .................... 0.75  
Alternatively: Min. fraction of hits with 1T(U) and 10A: .... 0.5  
Min. fraction of hits with typical piRNA length: ............ 0.75  
Typical piRNA length: ....................................... 24-32 nt  
Min. size of a piRNA cluster: ............................... 1000 bp.  
Min. number of hits (absolute): ............................. 0  
Min. number of hits (normalized): ........................... 0  
Min. fraction of hits on the mainstrand: .................... 0.75  
Top fraction of mapped sequences (in terms of read counts): . 1%  
Top fraction accounts for max. n% of sequence reads: ........ 90%  
Min. fraction of hits on each arm of a bidirectional cluster: 0.05  
Output html file for each cluster: .......................... yes  
Output a summary table: ..................................... yes  
Output a FASTA file for each cluster (piRNA sequences): ..... yes  
Output a FASTA file comprising cluster sequences: ........... yes  
Output a GTF file for predicted piRNA clusters: ..............yes  
Search DNA motifs in clusters: .............................. yes  
Output flanking sequences: +/- .............................. 0 bp  
Output ~.pTi file: .......................................... no  
==============================================================================  
  
  
Genome size (without gaps): ............ 758543724 bp  
Gaps (N/X/-): .......................... 417479 bp  
Mapped reads: .......................... 26973943  
Non-identical sequences: ............... 6209225  
Genomic hits: .......................... 48438990  
Significant densitiy of mapped reads: .. 821.144211136946 reads/kb

Show proTRAC cluster info
Hide proTRAC cluster info

|  |  |
| --- | --- |
| Location | NODE\_318148\_length\_50640\_cov\_30.107899 |
| Coordinates | 1-9027 |
| Size [bp] | 9027 |
| Sequence hit loci | 4549 |
| Mapped reads (normalized) | 19165.6 |
| Mapped reads (normalized) per kb | 2123.1 |
| Normalized reads with 1T (1U) | 78.2% |
| Normalized reads with 10A | 29.6% |
| Normalized reads with length 24-32 nt | 98.8% |
| Normalized reads on the main strand(s) | 93.6% |
| Predicted directionality | mono:plus |

100%

0%

1T (1U)  
reads

10A reads

24-32 nt  
reads

reads on mainstrand

**Either the amount of reads with 1T (1U) OR 10A has to exceed 75% (set with option: -1Tor10A)  
Alternatively the amount of reads with 1T (1U) AND 10A has to exceed 50% (set with option: -1Tand10A)  
Minimum amount of reads with preferred size is 75% (set with option: -pisize)  
Minimum amount of reads on the main strand(s) is 75% (set with option: -clstrand)**

Show read coverage
Hide read coverage

WHAT DO I SEE HERE?  
This chart shows the location of mapped sequence reads within a predicted piRNA cluster. The color refers to the number of genomic hits produced by the sequence read in question. A dark red bar indicates that this sequence read produces many other hits elsewhere in the genome. Many adjacent red or yellow bars can indicate the presence of a multi-copy element such as transposons or rRNA genes. A dark green bar indicates that this sequence read maps uniquely to this locus.

1 hit

2-5 hits

6-10 hits

11-20 hits

21-50 hits

51-100 hits

> 100 hits

NODE\_318148\_length\_50640\_cov\_30.107899

1

9027

Gene Set

RepeatMasker

Mapped  
Reads

166.38

plus strand

minus strand

166.38

Region: NODE\_318148\_length\_50640\_cov\_30.107899 2451-10. Max. coverage (+): 0. Max coverage (-): 0.01

Region: NODE\_318148\_length\_50640\_cov\_30.107899 11-28. Max. coverage (+): 0.03. Max coverage (-): 0.02

Region: NODE\_318148\_length\_50640\_cov\_30.107899 29-46. Max. coverage (+): 0.13. Max coverage (-): 0.04

Region: NODE\_318148\_length\_50640\_cov\_30.107899 47-64. Max. coverage (+): 0.19. Max coverage (-): 0

Region: NODE\_318148\_length\_50640\_cov\_30.107899 65-82. Max. coverage (+): 0.04. Max coverage (-): 0.08

Region: NODE\_318148\_length\_50640\_cov\_30.107899 83-100. Max. coverage (+): 0.11. Max coverage (-): 0

Region: NODE\_318148\_length\_50640\_cov\_30.107899 101-118. Max. coverage (+): 0.06. Max coverage (-): 0

Region: NODE\_318148\_length\_50640\_cov\_30.107899 119-136. Max. coverage (+): 0.12. Max coverage (-): 0.04

Region: NODE\_318148\_length\_50640\_cov\_30.107899 137-154. Max. coverage (+): 0.11. Max coverage (-): 0.03

Region: NODE\_318148\_length\_50640\_cov\_30.107899 155-172. Max. coverage (+): 0.07. Max coverage (-): 0

Region: NODE\_318148\_length\_50640\_cov\_30.107899 173-190. Max. coverage (+): 0.04. Max coverage (-): 0.04

Region: NODE\_318148\_length\_50640\_cov\_30.107899 191-208. Max. coverage (+): 0.32. Max coverage (-): 0.01

Region: NODE\_318148\_length\_50640\_cov\_30.107899 209-226. Max. coverage (+): 0.07. Max coverage (-): 0.01

Region: NODE\_318148\_length\_50640\_cov\_30.107899 227-244. Max. coverage (+): 0.07. Max coverage (-): 0

Region: NODE\_318148\_length\_50640\_cov\_30.107899 245-262. Max. coverage (+): 0.04. Max coverage (-): 0

Region: NODE\_318148\_length\_50640\_cov\_30.107899 263-280. Max. coverage (+): 0. Max coverage (-): 0

Region: NODE\_318148\_length\_50640\_cov\_30.107899 281-298. Max. coverage (+): 0.11. Max coverage (-): 0.04

Region: NODE\_318148\_length\_50640\_cov\_30.107899 299-316. Max. coverage (+): 0.12. Max coverage (-): 0.16

Region: NODE\_318148\_length\_50640\_cov\_30.107899 317-334. Max. coverage (+): 0.01. Max coverage (-): 0.07

Region: NODE\_318148\_length\_50640\_cov\_30.107899 335-353. Max. coverage (+): 0.76. Max coverage (-): 0.01

Region: NODE\_318148\_length\_50640\_cov\_30.107899 354-371. Max. coverage (+): 0.26. Max coverage (-): 0

Region: NODE\_318148\_length\_50640\_cov\_30.107899 372-389. Max. coverage (+): 0.22. Max coverage (-): 0

Region: NODE\_318148\_length\_50640\_cov\_30.107899 390-407. Max. coverage (+): 0.07. Max coverage (-): 0.04

Region: NODE\_318148\_length\_50640\_cov\_30.107899 408-425. Max. coverage (+): 0.07. Max coverage (-): 0.04

Region: NODE\_318148\_length\_50640\_cov\_30.107899 426-443. Max. coverage (+): 0.19. Max coverage (-): 0.07

Region: NODE\_318148\_length\_50640\_cov\_30.107899 444-461. Max. coverage (+): 0.3. Max coverage (-): 0.04

Region: NODE\_318148\_length\_50640\_cov\_30.107899 462-479. Max. coverage (+): 0.63. Max coverage (-): 0.07

Region: NODE\_318148\_length\_50640\_cov\_30.107899 480-497. Max. coverage (+): 0. Max coverage (-): 0

Region: NODE\_318148\_length\_50640\_cov\_30.107899 498-515. Max. coverage (+): 0.07. Max coverage (-): 0.19

Region: NODE\_318148\_length\_50640\_cov\_30.107899 516-533. Max. coverage (+): 1.48. Max coverage (-): 0.19

Region: NODE\_318148\_length\_50640\_cov\_30.107899 534-551. Max. coverage (+): 0.04. Max coverage (-): 0

Region: NODE\_318148\_length\_50640\_cov\_30.107899 552-569. Max. coverage (+): 0.19. Max coverage (-): 0.07

Region: NODE\_318148\_length\_50640\_cov\_30.107899 570-587. Max. coverage (+): 0.04. Max coverage (-): 0

Region: NODE\_318148\_length\_50640\_cov\_30.107899 588-605. Max. coverage (+): 0.04. Max coverage (-): 0.04

Region: NODE\_318148\_length\_50640\_cov\_30.107899 606-623. Max. coverage (+): 6.9. Max coverage (-): 0.11

Region: NODE\_318148\_length\_50640\_cov\_30.107899 624-641. Max. coverage (+): 0.07. Max coverage (-): 0.07

Region: NODE\_318148\_length\_50640\_cov\_30.107899 642-659. Max. coverage (+): 0.15. Max coverage (-): 0.41

Region: NODE\_318148\_length\_50640\_cov\_30.107899 660-678. Max. coverage (+): 1.96. Max coverage (-): 0

Region: NODE\_318148\_length\_50640\_cov\_30.107899 679-696. Max. coverage (+): 0.37. Max coverage (-): 0.04

Region: NODE\_318148\_length\_50640\_cov\_30.107899 697-714. Max. coverage (+): 3.97. Max coverage (-): 0.22

Region: NODE\_318148\_length\_50640\_cov\_30.107899 715-732. Max. coverage (+): 11.31. Max coverage (-): 0

Region: NODE\_318148\_length\_50640\_cov\_30.107899 733-750. Max. coverage (+): 1.08. Max coverage (-): 0.11

Region: NODE\_318148\_length\_50640\_cov\_30.107899 751-768. Max. coverage (+): 1.19. Max coverage (-): 0.11

Region: NODE\_318148\_length\_50640\_cov\_30.107899 769-786. Max. coverage (+): 1.19. Max coverage (-): 0.22

Region: NODE\_318148\_length\_50640\_cov\_30.107899 787-804. Max. coverage (+): 1.19. Max coverage (-): 0.04

Region: NODE\_318148\_length\_50640\_cov\_30.107899 805-822. Max. coverage (+): 0.56. Max coverage (-): 0

Region: NODE\_318148\_length\_50640\_cov\_30.107899 823-840. Max. coverage (+): 1.41. Max coverage (-): 0.19

Region: NODE\_318148\_length\_50640\_cov\_30.107899 841-858. Max. coverage (+): 0.7. Max coverage (-): 0

Region: NODE\_318148\_length\_50640\_cov\_30.107899 859-876. Max. coverage (+): 0.22. Max coverage (-): 0.63

Region: NODE\_318148\_length\_50640\_cov\_30.107899 877-894. Max. coverage (+): 0.22. Max coverage (-): 0.15

Region: NODE\_318148\_length\_50640\_cov\_30.107899 895-912. Max. coverage (+): 0.3. Max coverage (-): 0.04

Region: NODE\_318148\_length\_50640\_cov\_30.107899 913-930. Max. coverage (+): 1.96. Max coverage (-): 0.04

Region: NODE\_318148\_length\_50640\_cov\_30.107899 931-948. Max. coverage (+): 0.37. Max coverage (-): 0.04

Region: NODE\_318148\_length\_50640\_cov\_30.107899 949-966. Max. coverage (+): 0.26. Max coverage (-): 0.41

Region: NODE\_318148\_length\_50640\_cov\_30.107899 967-984. Max. coverage (+): 0.33. Max coverage (-): 0.11

Region: NODE\_318148\_length\_50640\_cov\_30.107899 985-1002. Max. coverage (+): 0.93. Max coverage (-): 0.04

Region: NODE\_318148\_length\_50640\_cov\_30.107899 1003-1021. Max. coverage (+): 0.52. Max coverage (-): 0.26

Region: NODE\_318148\_length\_50640\_cov\_30.107899 1022-1039. Max. coverage (+): 9.86. Max coverage (-): 0.37

Region: NODE\_318148\_length\_50640\_cov\_30.107899 1040-1057. Max. coverage (+): 9.79. Max coverage (-): 0.04

Region: NODE\_318148\_length\_50640\_cov\_30.107899 1058-1075. Max. coverage (+): 0.33. Max coverage (-): 0.07

Region: NODE\_318148\_length\_50640\_cov\_30.107899 1076-1093. Max. coverage (+): 1.89. Max coverage (-): 0.04

Region: NODE\_318148\_length\_50640\_cov\_30.107899 1094-1111. Max. coverage (+): 0.19. Max coverage (-): 0.22

Region: NODE\_318148\_length\_50640\_cov\_30.107899 1112-1129. Max. coverage (+): 0.44. Max coverage (-): 0.22

Region: NODE\_318148\_length\_50640\_cov\_30.107899 1130-1147. Max. coverage (+): 0.33. Max coverage (-): 0.04

Region: NODE\_318148\_length\_50640\_cov\_30.107899 1148-1165. Max. coverage (+): 0.04. Max coverage (-): 0.07

Region: NODE\_318148\_length\_50640\_cov\_30.107899 1166-1183. Max. coverage (+): 166.38. Max coverage (-): 0

Region: NODE\_318148\_length\_50640\_cov\_30.107899 1184-1201. Max. coverage (+): 0. Max coverage (-): 0

Region: NODE\_318148\_length\_50640\_cov\_30.107899 1202-1219. Max. coverage (+): 0.44. Max coverage (-): 0.11

Region: NODE\_318148\_length\_50640\_cov\_30.107899 1220-1237. Max. coverage (+): 0.7. Max coverage (-): 0.04

Region: NODE\_318148\_length\_50640\_cov\_30.107899 1238-1255. Max. coverage (+): 0.3. Max coverage (-): 0.07

Region: NODE\_318148\_length\_50640\_cov\_30.107899 1256-1273. Max. coverage (+): 3.23. Max coverage (-): 0.07

Region: NODE\_318148\_length\_50640\_cov\_30.107899 1274-1291. Max. coverage (+): 0.04. Max coverage (-): 0.3

Region: NODE\_318148\_length\_50640\_cov\_30.107899 1292-1309. Max. coverage (+): 3.56. Max coverage (-): 0

Region: NODE\_318148\_length\_50640\_cov\_30.107899 1310-1327. Max. coverage (+): 0.04. Max coverage (-): 0.04

Region: NODE\_318148\_length\_50640\_cov\_30.107899 1328-1346. Max. coverage (+): 20.28. Max coverage (-): 0

Region: NODE\_318148\_length\_50640\_cov\_30.107899 1347-1364. Max. coverage (+): 7.71. Max coverage (-): 0

Region: NODE\_318148\_length\_50640\_cov\_30.107899 1365-1382. Max. coverage (+): 0.56. Max coverage (-): 0.04

Region: NODE\_318148\_length\_50640\_cov\_30.107899 1383-1400. Max. coverage (+): 0.41. Max coverage (-): 0

Region: NODE\_318148\_length\_50640\_cov\_30.107899 1401-1418. Max. coverage (+): 0.3. Max coverage (-): 0.04

Region: NODE\_318148\_length\_50640\_cov\_30.107899 1419-1436. Max. coverage (+): 1.08. Max coverage (-): 0

Region: NODE\_318148\_length\_50640\_cov\_30.107899 1437-1454. Max. coverage (+): 0.11. Max coverage (-): 0

Region: NODE\_318148\_length\_50640\_cov\_30.107899 1455-1472. Max. coverage (+): 0.04. Max coverage (-): 0.07

Region: NODE\_318148\_length\_50640\_cov\_30.107899 1473-1490. Max. coverage (+): 0.26. Max coverage (-): 0

Region: NODE\_318148\_length\_50640\_cov\_30.107899 1491-1508. Max. coverage (+): 0.07. Max coverage (-): 0

Region: NODE\_318148\_length\_50640\_cov\_30.107899 1509-1526. Max. coverage (+): 2.08. Max coverage (-): 0

Region: NODE\_318148\_length\_50640\_cov\_30.107899 1527-1544. Max. coverage (+): 0.04. Max coverage (-): 0.11

Region: NODE\_318148\_length\_50640\_cov\_30.107899 1545-1562. Max. coverage (+): 2.41. Max coverage (-): 0

Region: NODE\_318148\_length\_50640\_cov\_30.107899 1563-1580. Max. coverage (+): 2.82. Max coverage (-): 1.74

Region: NODE\_318148\_length\_50640\_cov\_30.107899 1581-1598. Max. coverage (+): 14.75. Max coverage (-): 1.56

Region: NODE\_318148\_length\_50640\_cov\_30.107899 1599-1616. Max. coverage (+): 0.33. Max coverage (-): 0

Region: NODE\_318148\_length\_50640\_cov\_30.107899 1617-1634. Max. coverage (+): 1.26. Max coverage (-): 0.04

Region: NODE\_318148\_length\_50640\_cov\_30.107899 1635-1652. Max. coverage (+): 10.79. Max coverage (-): 0.11

Region: NODE\_318148\_length\_50640\_cov\_30.107899 1653-1670. Max. coverage (+): 4.78. Max coverage (-): 0

Region: NODE\_318148\_length\_50640\_cov\_30.107899 1671-1689. Max. coverage (+): 0.04. Max coverage (-): 0.04

Region: NODE\_318148\_length\_50640\_cov\_30.107899 1690-1707. Max. coverage (+): 0.33. Max coverage (-): 0

Region: NODE\_318148\_length\_50640\_cov\_30.107899 1708-1725. Max. coverage (+): 0. Max coverage (-): 0.15

Region: NODE\_318148\_length\_50640\_cov\_30.107899 1726-1743. Max. coverage (+): 4.67. Max coverage (-): 0.11

Region: NODE\_318148\_length\_50640\_cov\_30.107899 1744-1761. Max. coverage (+): 3.11. Max coverage (-): 0

Region: NODE\_318148\_length\_50640\_cov\_30.107899 1762-1779. Max. coverage (+): 0.33. Max coverage (-): 0

Region: NODE\_318148\_length\_50640\_cov\_30.107899 1780-1797. Max. coverage (+): 0.85. Max coverage (-): 0

Region: NODE\_318148\_length\_50640\_cov\_30.107899 1798-1815. Max. coverage (+): 0.3. Max coverage (-): 0

Region: NODE\_318148\_length\_50640\_cov\_30.107899 1816-1833. Max. coverage (+): 0.19. Max coverage (-): 0

Region: NODE\_318148\_length\_50640\_cov\_30.107899 1834-1851. Max. coverage (+): 0.04. Max coverage (-): 0

Region: NODE\_318148\_length\_50640\_cov\_30.107899 1852-1869. Max. coverage (+): 0.3. Max coverage (-): 0

Region: NODE\_318148\_length\_50640\_cov\_30.107899 1870-1887. Max. coverage (+): 0. Max coverage (-): 0

Region: NODE\_318148\_length\_50640\_cov\_30.107899 1888-1905. Max. coverage (+): 0.04. Max coverage (-): 0

Region: NODE\_318148\_length\_50640\_cov\_30.107899 1906-1923. Max. coverage (+): 0.07. Max coverage (-): 0.04

Region: NODE\_318148\_length\_50640\_cov\_30.107899 1924-1941. Max. coverage (+): 0.26. Max coverage (-): 0

Region: NODE\_318148\_length\_50640\_cov\_30.107899 1942-1959. Max. coverage (+): 1.22. Max coverage (-): 0.11

Region: NODE\_318148\_length\_50640\_cov\_30.107899 1960-1977. Max. coverage (+): 0.82. Max coverage (-): 0.11

Region: NODE\_318148\_length\_50640\_cov\_30.107899 1978-1995. Max. coverage (+): 0.56. Max coverage (-): 0

Region: NODE\_318148\_length\_50640\_cov\_30.107899 1996-2014. Max. coverage (+): 0.52. Max coverage (-): 1.15

Region: NODE\_318148\_length\_50640\_cov\_30.107899 2015-2032. Max. coverage (+): 0.82. Max coverage (-): 1.15

Region: NODE\_318148\_length\_50640\_cov\_30.107899 2033-2050. Max. coverage (+): 0.07. Max coverage (-): 0.04

Region: NODE\_318148\_length\_50640\_cov\_30.107899 2051-2068. Max. coverage (+): 2.08. Max coverage (-): 0.04

Region: NODE\_318148\_length\_50640\_cov\_30.107899 2069-2086. Max. coverage (+): 0.04. Max coverage (-): 1.45

Region: NODE\_318148\_length\_50640\_cov\_30.107899 2087-2104. Max. coverage (+): 2.19. Max coverage (-): 0

Region: NODE\_318148\_length\_50640\_cov\_30.107899 2105-2122. Max. coverage (+): 0.04. Max coverage (-): 0.19

Region: NODE\_318148\_length\_50640\_cov\_30.107899 2123-2140. Max. coverage (+): 0.3. Max coverage (-): 0.48

Region: NODE\_318148\_length\_50640\_cov\_30.107899 2141-2158. Max. coverage (+): 0.41. Max coverage (-): 0.07

Region: NODE\_318148\_length\_50640\_cov\_30.107899 2159-2176. Max. coverage (+): 0.44. Max coverage (-): 0

Region: NODE\_318148\_length\_50640\_cov\_30.107899 2177-2194. Max. coverage (+): 0. Max coverage (-): 0.07

Region: NODE\_318148\_length\_50640\_cov\_30.107899 2195-2212. Max. coverage (+): 1.52. Max coverage (-): 0.07

Region: NODE\_318148\_length\_50640\_cov\_30.107899 2213-2230. Max. coverage (+): 0.26. Max coverage (-): 0.04

Region: NODE\_318148\_length\_50640\_cov\_30.107899 2231-2248. Max. coverage (+): 0.44. Max coverage (-): 0

Region: NODE\_318148\_length\_50640\_cov\_30.107899 2249-2266. Max. coverage (+): 1.3. Max coverage (-): 0.19

Region: NODE\_318148\_length\_50640\_cov\_30.107899 2267-2284. Max. coverage (+): 2.41. Max coverage (-): 0.22

Region: NODE\_318148\_length\_50640\_cov\_30.107899 2285-2302. Max. coverage (+): 2.26. Max coverage (-): 0.04

Region: NODE\_318148\_length\_50640\_cov\_30.107899 2303-2320. Max. coverage (+): 0.52. Max coverage (-): 0.26

Region: NODE\_318148\_length\_50640\_cov\_30.107899 2321-2338. Max. coverage (+): 12.64. Max coverage (-): 0.3

Region: NODE\_318148\_length\_50640\_cov\_30.107899 2339-2357. Max. coverage (+): 10.6. Max coverage (-): 0

Region: NODE\_318148\_length\_50640\_cov\_30.107899 2358-2375. Max. coverage (+): 0.07. Max coverage (-): 0.19

Region: NODE\_318148\_length\_50640\_cov\_30.107899 2376-2393. Max. coverage (+): 4.12. Max coverage (-): 0.04

Region: NODE\_318148\_length\_50640\_cov\_30.107899 2394-2411. Max. coverage (+): 2.04. Max coverage (-): 0.07

Region: NODE\_318148\_length\_50640\_cov\_30.107899 2412-2429. Max. coverage (+): 2.71. Max coverage (-): 0.11

Region: NODE\_318148\_length\_50640\_cov\_30.107899 2430-2447. Max. coverage (+): 0.48. Max coverage (-): 0.04

Region: NODE\_318148\_length\_50640\_cov\_30.107899 2448-2465. Max. coverage (+): 0.78. Max coverage (-): 0

Region: NODE\_318148\_length\_50640\_cov\_30.107899 2466-2483. Max. coverage (+): 0.07. Max coverage (-): 0.04

Region: NODE\_318148\_length\_50640\_cov\_30.107899 2484-2501. Max. coverage (+): 0.07. Max coverage (-): 0.07

Region: NODE\_318148\_length\_50640\_cov\_30.107899 2502-2519. Max. coverage (+): 0.22. Max coverage (-): 0.7

Region: NODE\_318148\_length\_50640\_cov\_30.107899 2520-2537. Max. coverage (+): 3.23. Max coverage (-): 0.59

Region: NODE\_318148\_length\_50640\_cov\_30.107899 2538-2555. Max. coverage (+): 2.15. Max coverage (-): 0.07

Region: NODE\_318148\_length\_50640\_cov\_30.107899 2556-2573. Max. coverage (+): 2.89. Max coverage (-): 0.07

Region: NODE\_318148\_length\_50640\_cov\_30.107899 2574-2591. Max. coverage (+): 1.52. Max coverage (-): 0.22

Region: NODE\_318148\_length\_50640\_cov\_30.107899 2592-2609. Max. coverage (+): 1.08. Max coverage (-): 0.41

Region: NODE\_318148\_length\_50640\_cov\_30.107899 2610-2627. Max. coverage (+): 1.56. Max coverage (-): 0.07

Region: NODE\_318148\_length\_50640\_cov\_30.107899 2628-2645. Max. coverage (+): 0.63. Max coverage (-): 0.07

Region: NODE\_318148\_length\_50640\_cov\_30.107899 2646-2663. Max. coverage (+): 0.22. Max coverage (-): 0.04

Region: NODE\_318148\_length\_50640\_cov\_30.107899 2664-2682. Max. coverage (+): 9.56. Max coverage (-): 0.07

Region: NODE\_318148\_length\_50640\_cov\_30.107899 2683-2700. Max. coverage (+): 6.78. Max coverage (-): 0.07

Region: NODE\_318148\_length\_50640\_cov\_30.107899 2701-2718. Max. coverage (+): 0.07. Max coverage (-): 0

Region: NODE\_318148\_length\_50640\_cov\_30.107899 2719-2736. Max. coverage (+): 3.37. Max coverage (-): 0.04

Region: NODE\_318148\_length\_50640\_cov\_30.107899 2737-2754. Max. coverage (+): 0.44. Max coverage (-): 0

Region: NODE\_318148\_length\_50640\_cov\_30.107899 2755-2772. Max. coverage (+): 1.22. Max coverage (-): 0.07

Region: NODE\_318148\_length\_50640\_cov\_30.107899 2773-2790. Max. coverage (+): 0.33. Max coverage (-): 0.15

Region: NODE\_318148\_length\_50640\_cov\_30.107899 2791-2808. Max. coverage (+): 0.48. Max coverage (-): 0.11

Region: NODE\_318148\_length\_50640\_cov\_30.107899 2809-2826. Max. coverage (+): 0.11. Max coverage (-): 2.74

Region: NODE\_318148\_length\_50640\_cov\_30.107899 2827-2844. Max. coverage (+): 1.52. Max coverage (-): 0.82

Region: NODE\_318148\_length\_50640\_cov\_30.107899 2845-2862. Max. coverage (+): 0.15. Max coverage (-): 0.07

Region: NODE\_318148\_length\_50640\_cov\_30.107899 2863-2880. Max. coverage (+): 8.86. Max coverage (-): 0

Region: NODE\_318148\_length\_50640\_cov\_30.107899 2881-2898. Max. coverage (+): 8.93. Max coverage (-): 0.07

Region: NODE\_318148\_length\_50640\_cov\_30.107899 2899-2916. Max. coverage (+): 10.45. Max coverage (-): 0.11

Region: NODE\_318148\_length\_50640\_cov\_30.107899 2917-2934. Max. coverage (+): 0.07. Max coverage (-): 0.04

Region: NODE\_318148\_length\_50640\_cov\_30.107899 2935-2952. Max. coverage (+): 0.07. Max coverage (-): 0

Region: NODE\_318148\_length\_50640\_cov\_30.107899 2953-2970. Max. coverage (+): 2.37. Max coverage (-): 0

Region: NODE\_318148\_length\_50640\_cov\_30.107899 2971-2988. Max. coverage (+): 1.85. Max coverage (-): 0.04

Region: NODE\_318148\_length\_50640\_cov\_30.107899 2989-3006. Max. coverage (+): 0.22. Max coverage (-): 0

Region: NODE\_318148\_length\_50640\_cov\_30.107899 3007-3025. Max. coverage (+): 0. Max coverage (-): 0

Region: NODE\_318148\_length\_50640\_cov\_30.107899 3026-3043. Max. coverage (+): 0. Max coverage (-): 0

Region: NODE\_318148\_length\_50640\_cov\_30.107899 3044-3061. Max. coverage (+): 0. Max coverage (-): 0

Region: NODE\_318148\_length\_50640\_cov\_30.107899 3062-3079. Max. coverage (+): 0. Max coverage (-): 0.04

Region: NODE\_318148\_length\_50640\_cov\_30.107899 3080-3097. Max. coverage (+): 0.07. Max coverage (-): 0.04

Region: NODE\_318148\_length\_50640\_cov\_30.107899 3098-3115. Max. coverage (+): 0.07. Max coverage (-): 0

Region: NODE\_318148\_length\_50640\_cov\_30.107899 3116-3133. Max. coverage (+): 0.15. Max coverage (-): 0.04

Region: NODE\_318148\_length\_50640\_cov\_30.107899 3134-3151. Max. coverage (+): 0.11. Max coverage (-): 0.04

Region: NODE\_318148\_length\_50640\_cov\_30.107899 3152-3169. Max. coverage (+): 0. Max coverage (-): 0

Region: NODE\_318148\_length\_50640\_cov\_30.107899 3170-3187. Max. coverage (+): 0. Max coverage (-): 0

Region: NODE\_318148\_length\_50640\_cov\_30.107899 3188-3205. Max. coverage (+): 0.11. Max coverage (-): 0

Region: NODE\_318148\_length\_50640\_cov\_30.107899 3206-3223. Max. coverage (+): 0.04. Max coverage (-): 0

Region: NODE\_318148\_length\_50640\_cov\_30.107899 3224-3241. Max. coverage (+): 0.22. Max coverage (-): 0

Region: NODE\_318148\_length\_50640\_cov\_30.107899 3242-3259. Max. coverage (+): 0.04. Max coverage (-): 0

Region: NODE\_318148\_length\_50640\_cov\_30.107899 3260-3277. Max. coverage (+): 0.04. Max coverage (-): 0

Region: NODE\_318148\_length\_50640\_cov\_30.107899 3278-3295. Max. coverage (+): 0.07. Max coverage (-): 0

Region: NODE\_318148\_length\_50640\_cov\_30.107899 3296-3313. Max. coverage (+): 0. Max coverage (-): 0

Region: NODE\_318148\_length\_50640\_cov\_30.107899 3314-3331. Max. coverage (+): 0. Max coverage (-): 0

Region: NODE\_318148\_length\_50640\_cov\_30.107899 3332-3350. Max. coverage (+): 0.04. Max coverage (-): 0

Region: NODE\_318148\_length\_50640\_cov\_30.107899 3351-3368. Max. coverage (+): 0.11. Max coverage (-): 0

Region: NODE\_318148\_length\_50640\_cov\_30.107899 3369-3386. Max. coverage (+): 0.07. Max coverage (-): 0

Region: NODE\_318148\_length\_50640\_cov\_30.107899 3387-3404. Max. coverage (+): 0.07. Max coverage (-): 0

Region: NODE\_318148\_length\_50640\_cov\_30.107899 3405-3422. Max. coverage (+): 0.07. Max coverage (-): 0

Region: NODE\_318148\_length\_50640\_cov\_30.107899 3423-3440. Max. coverage (+): 0.74. Max coverage (-): 0

Region: NODE\_318148\_length\_50640\_cov\_30.107899 3441-3458. Max. coverage (+): 0.04. Max coverage (-): 0

Region: NODE\_318148\_length\_50640\_cov\_30.107899 3459-3476. Max. coverage (+): 0.11. Max coverage (-): 0

Region: NODE\_318148\_length\_50640\_cov\_30.107899 3477-3494. Max. coverage (+): 0.11. Max coverage (-): 0

Region: NODE\_318148\_length\_50640\_cov\_30.107899 3495-3512. Max. coverage (+): 0.07. Max coverage (-): 0

Region: NODE\_318148\_length\_50640\_cov\_30.107899 3513-3530. Max. coverage (+): 0.04. Max coverage (-): 0

Region: NODE\_318148\_length\_50640\_cov\_30.107899 3531-3548. Max. coverage (+): 0.82. Max coverage (-): 0

Region: NODE\_318148\_length\_50640\_cov\_30.107899 3549-3566. Max. coverage (+): 0.78. Max coverage (-): 0

Region: NODE\_318148\_length\_50640\_cov\_30.107899 3567-3584. Max. coverage (+): 0.19. Max coverage (-): 0

Region: NODE\_318148\_length\_50640\_cov\_30.107899 3585-3602. Max. coverage (+): 0.19. Max coverage (-): 0

Region: NODE\_318148\_length\_50640\_cov\_30.107899 3603-3620. Max. coverage (+): 0.15. Max coverage (-): 0

Region: NODE\_318148\_length\_50640\_cov\_30.107899 3621-3638. Max. coverage (+): 0. Max coverage (-): 0

Region: NODE\_318148\_length\_50640\_cov\_30.107899 3639-3656. Max. coverage (+): 0. Max coverage (-): 0.04

Region: NODE\_318148\_length\_50640\_cov\_30.107899 3657-3674. Max. coverage (+): 0.22. Max coverage (-): 0.04

Region: NODE\_318148\_length\_50640\_cov\_30.107899 3675-3693. Max. coverage (+): 0.15. Max coverage (-): 0.04

Region: NODE\_318148\_length\_50640\_cov\_30.107899 3694-3711. Max. coverage (+): 0.37. Max coverage (-): 0

Region: NODE\_318148\_length\_50640\_cov\_30.107899 3712-3729. Max. coverage (+): 0.04. Max coverage (-): 0.07

Region: NODE\_318148\_length\_50640\_cov\_30.107899 3730-3747. Max. coverage (+): 1.48. Max coverage (-): 0.11

Region: NODE\_318148\_length\_50640\_cov\_30.107899 3748-3765. Max. coverage (+): 0.2. Max coverage (-): 0.07

Region: NODE\_318148\_length\_50640\_cov\_30.107899 3766-3783. Max. coverage (+): 0.8. Max coverage (-): 0.07

Region: NODE\_318148\_length\_50640\_cov\_30.107899 3784-3801. Max. coverage (+): 0.19. Max coverage (-): 0.82

Region: NODE\_318148\_length\_50640\_cov\_30.107899 3802-3819. Max. coverage (+): 15.16. Max coverage (-): 0.63

Region: NODE\_318148\_length\_50640\_cov\_30.107899 3820-3837. Max. coverage (+): 0.26. Max coverage (-): 0.07

Region: NODE\_318148\_length\_50640\_cov\_30.107899 3838-3855. Max. coverage (+): 0.85. Max coverage (-): 1.63

Region: NODE\_318148\_length\_50640\_cov\_30.107899 3856-3873. Max. coverage (+): 0.41. Max coverage (-): 0.04

Region: NODE\_318148\_length\_50640\_cov\_30.107899 3874-3891. Max. coverage (+): 0.04. Max coverage (-): 0

Region: NODE\_318148\_length\_50640\_cov\_30.107899 3892-3909. Max. coverage (+): 1.3. Max coverage (-): 0

Region: NODE\_318148\_length\_50640\_cov\_30.107899 3910-3927. Max. coverage (+): 0.04. Max coverage (-): 0

Region: NODE\_318148\_length\_50640\_cov\_30.107899 3928-3945. Max. coverage (+): 0.19. Max coverage (-): 0

Region: NODE\_318148\_length\_50640\_cov\_30.107899 3946-3963. Max. coverage (+): 0. Max coverage (-): 0.11

Region: NODE\_318148\_length\_50640\_cov\_30.107899 3964-3981. Max. coverage (+): 0.11. Max coverage (-): 0.11

Region: NODE\_318148\_length\_50640\_cov\_30.107899 3982-3999. Max. coverage (+): 1.04. Max coverage (-): 0

Region: NODE\_318148\_length\_50640\_cov\_30.107899 4000-4018. Max. coverage (+): 0.07. Max coverage (-): 0.07

Region: NODE\_318148\_length\_50640\_cov\_30.107899 4019-4036. Max. coverage (+): 0.59. Max coverage (-): 0

Region: NODE\_318148\_length\_50640\_cov\_30.107899 4037-4054. Max. coverage (+): 0.67. Max coverage (-): 0

Region: NODE\_318148\_length\_50640\_cov\_30.107899 4055-4072. Max. coverage (+): 15.46. Max coverage (-): 0

Region: NODE\_318148\_length\_50640\_cov\_30.107899 4073-4090. Max. coverage (+): 0.22. Max coverage (-): 0.15

Region: NODE\_318148\_length\_50640\_cov\_30.107899 4091-4108. Max. coverage (+): 1. Max coverage (-): 0

Region: NODE\_318148\_length\_50640\_cov\_30.107899 4109-4126. Max. coverage (+): 0.07. Max coverage (-): 0.04

Region: NODE\_318148\_length\_50640\_cov\_30.107899 4127-4144. Max. coverage (+): 3.6. Max coverage (-): 0

Region: NODE\_318148\_length\_50640\_cov\_30.107899 4145-4162. Max. coverage (+): 0.19. Max coverage (-): 0.04

Region: NODE\_318148\_length\_50640\_cov\_30.107899 4163-4180. Max. coverage (+): 0.33. Max coverage (-): 0

Region: NODE\_318148\_length\_50640\_cov\_30.107899 4181-4198. Max. coverage (+): 0.22. Max coverage (-): 0.26

Region: NODE\_318148\_length\_50640\_cov\_30.107899 4199-4216. Max. coverage (+): 1.48. Max coverage (-): 0.26

Region: NODE\_318148\_length\_50640\_cov\_30.107899 4217-4234. Max. coverage (+): 2.26. Max coverage (-): 0

Region: NODE\_318148\_length\_50640\_cov\_30.107899 4235-4252. Max. coverage (+): 0.19. Max coverage (-): 0

Region: NODE\_318148\_length\_50640\_cov\_30.107899 4253-4270. Max. coverage (+): 0.3. Max coverage (-): 0.07

Region: NODE\_318148\_length\_50640\_cov\_30.107899 4271-4288. Max. coverage (+): 0.56. Max coverage (-): 0.04

Region: NODE\_318148\_length\_50640\_cov\_30.107899 4289-4306. Max. coverage (+): 0.26. Max coverage (-): 0

Region: NODE\_318148\_length\_50640\_cov\_30.107899 4307-4324. Max. coverage (+): 0.04. Max coverage (-): 0.15

Region: NODE\_318148\_length\_50640\_cov\_30.107899 4325-4342. Max. coverage (+): 2.15. Max coverage (-): 0.26

Region: NODE\_318148\_length\_50640\_cov\_30.107899 4343-4361. Max. coverage (+): 2.19. Max coverage (-): 0

Region: NODE\_318148\_length\_50640\_cov\_30.107899 4362-4379. Max. coverage (+): 0.26. Max coverage (-): 0.04

Region: NODE\_318148\_length\_50640\_cov\_30.107899 4380-4397. Max. coverage (+): 0.41. Max coverage (-): 0.04

Region: NODE\_318148\_length\_50640\_cov\_30.107899 4398-4415. Max. coverage (+): 0.26. Max coverage (-): 0.07

Region: NODE\_318148\_length\_50640\_cov\_30.107899 4416-4433. Max. coverage (+): 0.22. Max coverage (-): 0.07

Region: NODE\_318148\_length\_50640\_cov\_30.107899 4434-4451. Max. coverage (+): 0. Max coverage (-): 0

Region: NODE\_318148\_length\_50640\_cov\_30.107899 4452-4469. Max. coverage (+): 0.41. Max coverage (-): 0

Region: NODE\_318148\_length\_50640\_cov\_30.107899 4470-4487. Max. coverage (+): 0.15. Max coverage (-): 0

Region: NODE\_318148\_length\_50640\_cov\_30.107899 4488-4505. Max. coverage (+): 0.48. Max coverage (-): 0

Region: NODE\_318148\_length\_50640\_cov\_30.107899 4506-4523. Max. coverage (+): 0.15. Max coverage (-): 0

Region: NODE\_318148\_length\_50640\_cov\_30.107899 4524-4541. Max. coverage (+): 3.11. Max coverage (-): 0

Region: NODE\_318148\_length\_50640\_cov\_30.107899 4542-4559. Max. coverage (+): 0.96. Max coverage (-): 0.26

Region: NODE\_318148\_length\_50640\_cov\_30.107899 4560-4577. Max. coverage (+): 0. Max coverage (-): 0.04

Region: NODE\_318148\_length\_50640\_cov\_30.107899 4578-4595. Max. coverage (+): 0.07. Max coverage (-): 0.04

Region: NODE\_318148\_length\_50640\_cov\_30.107899 4596-4613. Max. coverage (+): 0.07. Max coverage (-): 0

Region: NODE\_318148\_length\_50640\_cov\_30.107899 4614-4631. Max. coverage (+): 0.19. Max coverage (-): 0

Region: NODE\_318148\_length\_50640\_cov\_30.107899 4632-4649. Max. coverage (+): 0.07. Max coverage (-): 0.19

Region: NODE\_318148\_length\_50640\_cov\_30.107899 4650-4667. Max. coverage (+): 0.04. Max coverage (-): 0.19

Region: NODE\_318148\_length\_50640\_cov\_30.107899 4668-4686. Max. coverage (+): 1.56. Max coverage (-): 0.19

Region: NODE\_318148\_length\_50640\_cov\_30.107899 4687-4704. Max. coverage (+): 1.11. Max coverage (-): 0

Region: NODE\_318148\_length\_50640\_cov\_30.107899 4705-4722. Max. coverage (+): 0.26. Max coverage (-): 0

Region: NODE\_318148\_length\_50640\_cov\_30.107899 4723-4740. Max. coverage (+): 0.26. Max coverage (-): 0.33

Region: NODE\_318148\_length\_50640\_cov\_30.107899 4741-4758. Max. coverage (+): 3.67. Max coverage (-): 0

Region: NODE\_318148\_length\_50640\_cov\_30.107899 4759-4776. Max. coverage (+): 0.15. Max coverage (-): 0

Region: NODE\_318148\_length\_50640\_cov\_30.107899 4777-4794. Max. coverage (+): 0.11. Max coverage (-): 0.07

Region: NODE\_318148\_length\_50640\_cov\_30.107899 4795-4812. Max. coverage (+): 0.19. Max coverage (-): 0

Region: NODE\_318148\_length\_50640\_cov\_30.107899 4813-4830. Max. coverage (+): 0.15. Max coverage (-): 0.04

Region: NODE\_318148\_length\_50640\_cov\_30.107899 4831-4848. Max. coverage (+): 1.11. Max coverage (-): 0.04

Region: NODE\_318148\_length\_50640\_cov\_30.107899 4849-4866. Max. coverage (+): 2.48. Max coverage (-): 0

Region: NODE\_318148\_length\_50640\_cov\_30.107899 4867-4884. Max. coverage (+): 0. Max coverage (-): 0

Region: NODE\_318148\_length\_50640\_cov\_30.107899 4885-4902. Max. coverage (+): 0.19. Max coverage (-): 0.59

Region: NODE\_318148\_length\_50640\_cov\_30.107899 4903-4920. Max. coverage (+): 0.33. Max coverage (-): 0.59

Region: NODE\_318148\_length\_50640\_cov\_30.107899 4921-4938. Max. coverage (+): 0.15. Max coverage (-): 0.15

Region: NODE\_318148\_length\_50640\_cov\_30.107899 4939-4956. Max. coverage (+): 0.07. Max coverage (-): 0.11

Region: NODE\_318148\_length\_50640\_cov\_30.107899 4957-4974. Max. coverage (+): 0.3. Max coverage (-): 0.04

Region: NODE\_318148\_length\_50640\_cov\_30.107899 4975-4992. Max. coverage (+): 0.26. Max coverage (-): 0.04

Region: NODE\_318148\_length\_50640\_cov\_30.107899 4993-5010. Max. coverage (+): 1.33. Max coverage (-): 0

Region: NODE\_318148\_length\_50640\_cov\_30.107899 5011-5029. Max. coverage (+): 0.15. Max coverage (-): 0.04

Region: NODE\_318148\_length\_50640\_cov\_30.107899 5030-5047. Max. coverage (+): 0.85. Max coverage (-): 0.07

Region: NODE\_318148\_length\_50640\_cov\_30.107899 5048-5065. Max. coverage (+): 1.26. Max coverage (-): 0

Region: NODE\_318148\_length\_50640\_cov\_30.107899 5066-5083. Max. coverage (+): 1.3. Max coverage (-): 0.04

Region: NODE\_318148\_length\_50640\_cov\_30.107899 5084-5101. Max. coverage (+): 0.41. Max coverage (-): 2.19

Region: NODE\_318148\_length\_50640\_cov\_30.107899 5102-5119. Max. coverage (+): 0.26. Max coverage (-): 0

Region: NODE\_318148\_length\_50640\_cov\_30.107899 5120-5137. Max. coverage (+): 0. Max coverage (-): 0

Region: NODE\_318148\_length\_50640\_cov\_30.107899 5138-5155. Max. coverage (+): 0.33. Max coverage (-): 0.11

Region: NODE\_318148\_length\_50640\_cov\_30.107899 5156-5173. Max. coverage (+): 0.7. Max coverage (-): 0

Region: NODE\_318148\_length\_50640\_cov\_30.107899 5174-5191. Max. coverage (+): 0.07. Max coverage (-): 0

Region: NODE\_318148\_length\_50640\_cov\_30.107899 5192-5209. Max. coverage (+): 0.22. Max coverage (-): 0

Region: NODE\_318148\_length\_50640\_cov\_30.107899 5210-5227. Max. coverage (+): 1.45. Max coverage (-): 0.04

Region: NODE\_318148\_length\_50640\_cov\_30.107899 5228-5245. Max. coverage (+): 0. Max coverage (-): 0.26

Region: NODE\_318148\_length\_50640\_cov\_30.107899 5246-5263. Max. coverage (+): 0.07. Max coverage (-): 0.3

Region: NODE\_318148\_length\_50640\_cov\_30.107899 5264-5281. Max. coverage (+): 0.04. Max coverage (-): 0

Region: NODE\_318148\_length\_50640\_cov\_30.107899 5282-5299. Max. coverage (+): 0.04. Max coverage (-): 0

Region: NODE\_318148\_length\_50640\_cov\_30.107899 5300-5317. Max. coverage (+): 0.04. Max coverage (-): 0

Region: NODE\_318148\_length\_50640\_cov\_30.107899 5318-5335. Max. coverage (+): 14.61. Max coverage (-): 0.04

Region: NODE\_318148\_length\_50640\_cov\_30.107899 5336-5354. Max. coverage (+): 0.11. Max coverage (-): 0.07

Region: NODE\_318148\_length\_50640\_cov\_30.107899 5355-5372. Max. coverage (+): 1.22. Max coverage (-): 0.04

Region: NODE\_318148\_length\_50640\_cov\_30.107899 5373-5390. Max. coverage (+): 0.41. Max coverage (-): 0

Region: NODE\_318148\_length\_50640\_cov\_30.107899 5391-5408. Max. coverage (+): 0. Max coverage (-): 0

Region: NODE\_318148\_length\_50640\_cov\_30.107899 5409-5426. Max. coverage (+): 0.15. Max coverage (-): 0

Region: NODE\_318148\_length\_50640\_cov\_30.107899 5427-5444. Max. coverage (+): 0.67. Max coverage (-): 0.04

Region: NODE\_318148\_length\_50640\_cov\_30.107899 5445-5462. Max. coverage (+): 0.3. Max coverage (-): 0.04

Region: NODE\_318148\_length\_50640\_cov\_30.107899 5463-5480. Max. coverage (+): 0.07. Max coverage (-): 0.04

Region: NODE\_318148\_length\_50640\_cov\_30.107899 5481-5498. Max. coverage (+): 0. Max coverage (-): 0

Region: NODE\_318148\_length\_50640\_cov\_30.107899 5499-5516. Max. coverage (+): 0.07. Max coverage (-): 0

Region: NODE\_318148\_length\_50640\_cov\_30.107899 5517-5534. Max. coverage (+): 0.04. Max coverage (-): 0

Region: NODE\_318148\_length\_50640\_cov\_30.107899 5535-5552. Max. coverage (+): 0.3. Max coverage (-): 0

Region: NODE\_318148\_length\_50640\_cov\_30.107899 5553-5570. Max. coverage (+): 0. Max coverage (-): 0.07

Region: NODE\_318148\_length\_50640\_cov\_30.107899 5571-5588. Max. coverage (+): 0.15. Max coverage (-): 0

Region: NODE\_318148\_length\_50640\_cov\_30.107899 5589-5606. Max. coverage (+): 0.04. Max coverage (-): 0

Region: NODE\_318148\_length\_50640\_cov\_30.107899 5607-5624. Max. coverage (+): 0. Max coverage (-): 0.11

Region: NODE\_318148\_length\_50640\_cov\_30.107899 5625-5642. Max. coverage (+): 0.07. Max coverage (-): 0

Region: NODE\_318148\_length\_50640\_cov\_30.107899 5643-5660. Max. coverage (+): 0. Max coverage (-): 0

Region: NODE\_318148\_length\_50640\_cov\_30.107899 5661-5678. Max. coverage (+): 0.07. Max coverage (-): 0.07

Region: NODE\_318148\_length\_50640\_cov\_30.107899 5679-5697. Max. coverage (+): 0.04. Max coverage (-): 0.04

Region: NODE\_318148\_length\_50640\_cov\_30.107899 5698-5715. Max. coverage (+): 0.15. Max coverage (-): 0.52

Region: NODE\_318148\_length\_50640\_cov\_30.107899 5716-5733. Max. coverage (+): 0.52. Max coverage (-): 0

Region: NODE\_318148\_length\_50640\_cov\_30.107899 5734-5751. Max. coverage (+): 0.3. Max coverage (-): 0.11

Region: NODE\_318148\_length\_50640\_cov\_30.107899 5752-5769. Max. coverage (+): 2.52. Max coverage (-): 0

Region: NODE\_318148\_length\_50640\_cov\_30.107899 5770-5787. Max. coverage (+): 0. Max coverage (-): 0

Region: NODE\_318148\_length\_50640\_cov\_30.107899 5788-5805. Max. coverage (+): 0.07. Max coverage (-): 0

Region: NODE\_318148\_length\_50640\_cov\_30.107899 5806-5823. Max. coverage (+): 0.15. Max coverage (-): 0

Region: NODE\_318148\_length\_50640\_cov\_30.107899 5824-5841. Max. coverage (+): 1.41. Max coverage (-): 0.04

Region: NODE\_318148\_length\_50640\_cov\_30.107899 5842-5859. Max. coverage (+): 0. Max coverage (-): 0.04

Region: NODE\_318148\_length\_50640\_cov\_30.107899 5860-5877. Max. coverage (+): 0.26. Max coverage (-): 0

Region: NODE\_318148\_length\_50640\_cov\_30.107899 5878-5895. Max. coverage (+): 0.11. Max coverage (-): 0

Region: NODE\_318148\_length\_50640\_cov\_30.107899 5896-5913. Max. coverage (+): 0.19. Max coverage (-): 0.04

Region: NODE\_318148\_length\_50640\_cov\_30.107899 5914-5931. Max. coverage (+): 0.63. Max coverage (-): 0.04

Region: NODE\_318148\_length\_50640\_cov\_30.107899 5932-5949. Max. coverage (+): 0.04. Max coverage (-): 0.07

Region: NODE\_318148\_length\_50640\_cov\_30.107899 5950-5967. Max. coverage (+): 0. Max coverage (-): 0.04

Region: NODE\_318148\_length\_50640\_cov\_30.107899 5968-5985. Max. coverage (+): 0.04. Max coverage (-): 0.04

Region: NODE\_318148\_length\_50640\_cov\_30.107899 5986-6003. Max. coverage (+): 0. Max coverage (-): 0

Region: NODE\_318148\_length\_50640\_cov\_30.107899 6004-6022. Max. coverage (+): 0.11. Max coverage (-): 0

Region: NODE\_318148\_length\_50640\_cov\_30.107899 6023-6040. Max. coverage (+): 0. Max coverage (-): 0.07

Region: NODE\_318148\_length\_50640\_cov\_30.107899 6041-6058. Max. coverage (+): 1.26. Max coverage (-): 0.15

Region: NODE\_318148\_length\_50640\_cov\_30.107899 6059-6076. Max. coverage (+): 0.15. Max coverage (-): 0.59

Region: NODE\_318148\_length\_50640\_cov\_30.107899 6077-6094. Max. coverage (+): 0.11. Max coverage (-): 0

Region: NODE\_318148\_length\_50640\_cov\_30.107899 6095-6112. Max. coverage (+): 0.89. Max coverage (-): 0

Region: NODE\_318148\_length\_50640\_cov\_30.107899 6113-6130. Max. coverage (+): 0.96. Max coverage (-): 0.37

Region: NODE\_318148\_length\_50640\_cov\_30.107899 6131-6148. Max. coverage (+): 1.33. Max coverage (-): 0.04

Region: NODE\_318148\_length\_50640\_cov\_30.107899 6149-6166. Max. coverage (+): 1.59. Max coverage (-): 0

Region: NODE\_318148\_length\_50640\_cov\_30.107899 6167-6184. Max. coverage (+): 0.48. Max coverage (-): 0.19

Region: NODE\_318148\_length\_50640\_cov\_30.107899 6185-6202. Max. coverage (+): 0.22. Max coverage (-): 0

Region: NODE\_318148\_length\_50640\_cov\_30.107899 6203-6220. Max. coverage (+): 0.82. Max coverage (-): 0.07

Region: NODE\_318148\_length\_50640\_cov\_30.107899 6221-6238. Max. coverage (+): 0.22. Max coverage (-): 0.11

Region: NODE\_318148\_length\_50640\_cov\_30.107899 6239-6256. Max. coverage (+): 0.15. Max coverage (-): 0.04

Region: NODE\_318148\_length\_50640\_cov\_30.107899 6257-6274. Max. coverage (+): 0.15. Max coverage (-): 0.04

Region: NODE\_318148\_length\_50640\_cov\_30.107899 6275-6292. Max. coverage (+): 0.41. Max coverage (-): 0.04

Region: NODE\_318148\_length\_50640\_cov\_30.107899 6293-6310. Max. coverage (+): 0.22. Max coverage (-): 0

Region: NODE\_318148\_length\_50640\_cov\_30.107899 6311-6328. Max. coverage (+): 0.48. Max coverage (-): 0

Region: NODE\_318148\_length\_50640\_cov\_30.107899 6329-6346. Max. coverage (+): 0.07. Max coverage (-): 0.04

Region: NODE\_318148\_length\_50640\_cov\_30.107899 6347-6365. Max. coverage (+): 0.19. Max coverage (-): 0

Region: NODE\_318148\_length\_50640\_cov\_30.107899 6366-6383. Max. coverage (+): 0.74. Max coverage (-): 0.04

Region: NODE\_318148\_length\_50640\_cov\_30.107899 6384-6401. Max. coverage (+): 0.22. Max coverage (-): 0

Region: NODE\_318148\_length\_50640\_cov\_30.107899 6402-6419. Max. coverage (+): 0.11. Max coverage (-): 0.04

Region: NODE\_318148\_length\_50640\_cov\_30.107899 6420-6437. Max. coverage (+): 0.04. Max coverage (-): 0.04

Region: NODE\_318148\_length\_50640\_cov\_30.107899 6438-6455. Max. coverage (+): 0.56. Max coverage (-): 0

Region: NODE\_318148\_length\_50640\_cov\_30.107899 6456-6473. Max. coverage (+): 0. Max coverage (-): 0.59

Region: NODE\_318148\_length\_50640\_cov\_30.107899 6474-6491. Max. coverage (+): 0. Max coverage (-): 0.52

Region: NODE\_318148\_length\_50640\_cov\_30.107899 6492-6509. Max. coverage (+): 0.04. Max coverage (-): 0

Region: NODE\_318148\_length\_50640\_cov\_30.107899 6510-6527. Max. coverage (+): 2.71. Max coverage (-): 0

Region: NODE\_318148\_length\_50640\_cov\_30.107899 6528-6545. Max. coverage (+): 0.04. Max coverage (-): 0

Region: NODE\_318148\_length\_50640\_cov\_30.107899 6546-6563. Max. coverage (+): 0.19. Max coverage (-): 0.07

Region: NODE\_318148\_length\_50640\_cov\_30.107899 6564-6581. Max. coverage (+): 1.19. Max coverage (-): 0.04

Region: NODE\_318148\_length\_50640\_cov\_30.107899 6582-6599. Max. coverage (+): 0.11. Max coverage (-): 0.19

Region: NODE\_318148\_length\_50640\_cov\_30.107899 6600-6617. Max. coverage (+): 0.44. Max coverage (-): 0.26

Region: NODE\_318148\_length\_50640\_cov\_30.107899 6618-6635. Max. coverage (+): 0.19. Max coverage (-): 0.04

Region: NODE\_318148\_length\_50640\_cov\_30.107899 6636-6653. Max. coverage (+): 0.11. Max coverage (-): 0.04

Region: NODE\_318148\_length\_50640\_cov\_30.107899 6654-6671. Max. coverage (+): 0.22. Max coverage (-): 0.09

Region: NODE\_318148\_length\_50640\_cov\_30.107899 6672-6690. Max. coverage (+): 1.26. Max coverage (-): 0

Region: NODE\_318148\_length\_50640\_cov\_30.107899 6691-6708. Max. coverage (+): 0. Max coverage (-): 0.04

Region: NODE\_318148\_length\_50640\_cov\_30.107899 6709-6726. Max. coverage (+): 4.12. Max coverage (-): 0.02

Region: NODE\_318148\_length\_50640\_cov\_30.107899 6727-6744. Max. coverage (+): 3.47. Max coverage (-): 0.15

Region: NODE\_318148\_length\_50640\_cov\_30.107899 6745-6762. Max. coverage (+): 0.26. Max coverage (-): 0.15

Region: NODE\_318148\_length\_50640\_cov\_30.107899 6763-6780. Max. coverage (+): 0. Max coverage (-): 0

Region: NODE\_318148\_length\_50640\_cov\_30.107899 6781-6798. Max. coverage (+): 0.07. Max coverage (-): 0

Region: NODE\_318148\_length\_50640\_cov\_30.107899 6799-6816. Max. coverage (+): 0.04. Max coverage (-): 0.04

Region: NODE\_318148\_length\_50640\_cov\_30.107899 6817-6834. Max. coverage (+): 0. Max coverage (-): 0

Region: NODE\_318148\_length\_50640\_cov\_30.107899 6835-6852. Max. coverage (+): 0.04. Max coverage (-): 0

Region: NODE\_318148\_length\_50640\_cov\_30.107899 6853-6870. Max. coverage (+): 0.44. Max coverage (-): 0.11

Region: NODE\_318148\_length\_50640\_cov\_30.107899 6871-6888. Max. coverage (+): 0.04. Max coverage (-): 0

Region: NODE\_318148\_length\_50640\_cov\_30.107899 6889-6906. Max. coverage (+): 0.11. Max coverage (-): 0.15

Region: NODE\_318148\_length\_50640\_cov\_30.107899 6907-6924. Max. coverage (+): 0.07. Max coverage (-): 0

Region: NODE\_318148\_length\_50640\_cov\_30.107899 6925-6942. Max. coverage (+): 0. Max coverage (-): 0

Region: NODE\_318148\_length\_50640\_cov\_30.107899 6943-6960. Max. coverage (+): 0. Max coverage (-): 0.04

Region: NODE\_318148\_length\_50640\_cov\_30.107899 6961-6978. Max. coverage (+): 0. Max coverage (-): 0

Region: NODE\_318148\_length\_50640\_cov\_30.107899 6979-6996. Max. coverage (+): 0. Max coverage (-): 0

Region: NODE\_318148\_length\_50640\_cov\_30.107899 6997-7014. Max. coverage (+): 0. Max coverage (-): 0

Region: NODE\_318148\_length\_50640\_cov\_30.107899 7015-7033. Max. coverage (+): 0. Max coverage (-): 0

Region: NODE\_318148\_length\_50640\_cov\_30.107899 7034-7051. Max. coverage (+): 0. Max coverage (-): 0

Region: NODE\_318148\_length\_50640\_cov\_30.107899 7052-7069. Max. coverage (+): 0. Max coverage (-): 0

Region: NODE\_318148\_length\_50640\_cov\_30.107899 7070-7087. Max. coverage (+): 0. Max coverage (-): 0

Region: NODE\_318148\_length\_50640\_cov\_30.107899 7088-7105. Max. coverage (+): 0. Max coverage (-): 0

Region: NODE\_318148\_length\_50640\_cov\_30.107899 7106-7123. Max. coverage (+): 0. Max coverage (-): 0

Region: NODE\_318148\_length\_50640\_cov\_30.107899 7124-7141. Max. coverage (+): 0. Max coverage (-): 0

Region: NODE\_318148\_length\_50640\_cov\_30.107899 7142-7159. Max. coverage (+): 0. Max coverage (-): 0

Region: NODE\_318148\_length\_50640\_cov\_30.107899 7160-7177. Max. coverage (+): 0. Max coverage (-): 0

Region: NODE\_318148\_length\_50640\_cov\_30.107899 7178-7195. Max. coverage (+): 0. Max coverage (-): 0

Region: NODE\_318148\_length\_50640\_cov\_30.107899 7196-7213. Max. coverage (+): 0. Max coverage (-): 0

Region: NODE\_318148\_length\_50640\_cov\_30.107899 7214-7231. Max. coverage (+): 0. Max coverage (-): 0

Region: NODE\_318148\_length\_50640\_cov\_30.107899 7232-7249. Max. coverage (+): 0. Max coverage (-): 0.07

Region: NODE\_318148\_length\_50640\_cov\_30.107899 7250-7267. Max. coverage (+): 0.3. Max coverage (-): 0.37

Region: NODE\_318148\_length\_50640\_cov\_30.107899 7268-7285. Max. coverage (+): 0.26. Max coverage (-): 0.04

Region: NODE\_318148\_length\_50640\_cov\_30.107899 7286-7303. Max. coverage (+): 0.52. Max coverage (-): 0

Region: NODE\_318148\_length\_50640\_cov\_30.107899 7304-7321. Max. coverage (+): 0.48. Max coverage (-): 0.04

Region: NODE\_318148\_length\_50640\_cov\_30.107899 7322-7339. Max. coverage (+): 0.07. Max coverage (-): 0.33

Region: NODE\_318148\_length\_50640\_cov\_30.107899 7340-7358. Max. coverage (+): 1.15. Max coverage (-): 0.22

Region: NODE\_318148\_length\_50640\_cov\_30.107899 7359-7376. Max. coverage (+): 0.07. Max coverage (-): 0.04

Region: NODE\_318148\_length\_50640\_cov\_30.107899 7377-7394. Max. coverage (+): 0.07. Max coverage (-): 0

Region: NODE\_318148\_length\_50640\_cov\_30.107899 7395-7412. Max. coverage (+): 0.04. Max coverage (-): 0

Region: NODE\_318148\_length\_50640\_cov\_30.107899 7413-7430. Max. coverage (+): 0.04. Max coverage (-): 0.04

Region: NODE\_318148\_length\_50640\_cov\_30.107899 7431-7448. Max. coverage (+): 0. Max coverage (-): 0

Region: NODE\_318148\_length\_50640\_cov\_30.107899 7449-7466. Max. coverage (+): 0. Max coverage (-): 0

Region: NODE\_318148\_length\_50640\_cov\_30.107899 7467-7484. Max. coverage (+): 0. Max coverage (-): 0.07

Region: NODE\_318148\_length\_50640\_cov\_30.107899 7485-7502. Max. coverage (+): 0.07. Max coverage (-): 0.04

Region: NODE\_318148\_length\_50640\_cov\_30.107899 7503-7520. Max. coverage (+): 0.04. Max coverage (-): 0

Region: NODE\_318148\_length\_50640\_cov\_30.107899 7521-7538. Max. coverage (+): 0.04. Max coverage (-): 0

Region: NODE\_318148\_length\_50640\_cov\_30.107899 7539-7556. Max. coverage (+): 0. Max coverage (-): 0

Region: NODE\_318148\_length\_50640\_cov\_30.107899 7557-7574. Max. coverage (+): 0.19. Max coverage (-): 0

Region: NODE\_318148\_length\_50640\_cov\_30.107899 7575-7592. Max. coverage (+): 0.3. Max coverage (-): 0

Region: NODE\_318148\_length\_50640\_cov\_30.107899 7593-7610. Max. coverage (+): 1.22. Max coverage (-): 0.04

Region: NODE\_318148\_length\_50640\_cov\_30.107899 7611-7628. Max. coverage (+): 4.34. Max coverage (-): 0

Region: NODE\_318148\_length\_50640\_cov\_30.107899 7629-7646. Max. coverage (+): 0.22. Max coverage (-): 0.11

Region: NODE\_318148\_length\_50640\_cov\_30.107899 7647-7664. Max. coverage (+): 0.33. Max coverage (-): 0.22

Region: NODE\_318148\_length\_50640\_cov\_30.107899 7665-7682. Max. coverage (+): 0.56. Max coverage (-): 0.3

Region: NODE\_318148\_length\_50640\_cov\_30.107899 7683-7701. Max. coverage (+): 0.07. Max coverage (-): 0.15

Region: NODE\_318148\_length\_50640\_cov\_30.107899 7702-7719. Max. coverage (+): 0.04. Max coverage (-): 0

Region: NODE\_318148\_length\_50640\_cov\_30.107899 7720-7737. Max. coverage (+): 0.07. Max coverage (-): 0.11

Region: NODE\_318148\_length\_50640\_cov\_30.107899 7738-7755. Max. coverage (+): 1.11. Max coverage (-): 0.11

Region: NODE\_318148\_length\_50640\_cov\_30.107899 7756-7773. Max. coverage (+): 0.3. Max coverage (-): 0

Region: NODE\_318148\_length\_50640\_cov\_30.107899 7774-7791. Max. coverage (+): 0.04. Max coverage (-): 0.07

Region: NODE\_318148\_length\_50640\_cov\_30.107899 7792-7809. Max. coverage (+): 0.33. Max coverage (-): 0.04

Region: NODE\_318148\_length\_50640\_cov\_30.107899 7810-7827. Max. coverage (+): 0.19. Max coverage (-): 0

Region: NODE\_318148\_length\_50640\_cov\_30.107899 7828-7845. Max. coverage (+): 0.15. Max coverage (-): 0.04

Region: NODE\_318148\_length\_50640\_cov\_30.107899 7846-7863. Max. coverage (+): 0. Max coverage (-): 0.04

Region: NODE\_318148\_length\_50640\_cov\_30.107899 7864-7881. Max. coverage (+): 0.78. Max coverage (-): 0.11

Region: NODE\_318148\_length\_50640\_cov\_30.107899 7882-7899. Max. coverage (+): 0.07. Max coverage (-): 0

Region: NODE\_318148\_length\_50640\_cov\_30.107899 7900-7917. Max. coverage (+): 0.37. Max coverage (-): 0

Region: NODE\_318148\_length\_50640\_cov\_30.107899 7918-7935. Max. coverage (+): 0.37. Max coverage (-): 0.04

Region: NODE\_318148\_length\_50640\_cov\_30.107899 7936-7953. Max. coverage (+): 0.3. Max coverage (-): 0.04

Region: NODE\_318148\_length\_50640\_cov\_30.107899 7954-7971. Max. coverage (+): 0.48. Max coverage (-): 0.15

Region: NODE\_318148\_length\_50640\_cov\_30.107899 7972-7989. Max. coverage (+): 1.19. Max coverage (-): 0

Region: NODE\_318148\_length\_50640\_cov\_30.107899 7990-8007. Max. coverage (+): 0. Max coverage (-): 0.04

Region: NODE\_318148\_length\_50640\_cov\_30.107899 8008-8026. Max. coverage (+): 0.07. Max coverage (-): 0.04

Region: NODE\_318148\_length\_50640\_cov\_30.107899 8027-8044. Max. coverage (+): 0.04. Max coverage (-): 0.07

Region: NODE\_318148\_length\_50640\_cov\_30.107899 8045-8062. Max. coverage (+): 0.11. Max coverage (-): 0.04

Region: NODE\_318148\_length\_50640\_cov\_30.107899 8063-8080. Max. coverage (+): 0. Max coverage (-): 0.04

Region: NODE\_318148\_length\_50640\_cov\_30.107899 8081-8098. Max. coverage (+): 0. Max coverage (-): 0

Region: NODE\_318148\_length\_50640\_cov\_30.107899 8099-8116. Max. coverage (+): 0. Max coverage (-): 0

Region: NODE\_318148\_length\_50640\_cov\_30.107899 8117-8134. Max. coverage (+): 0. Max coverage (-): 0

Region: NODE\_318148\_length\_50640\_cov\_30.107899 8135-8152. Max. coverage (+): 0. Max coverage (-): 0

Region: NODE\_318148\_length\_50640\_cov\_30.107899 8153-8170. Max. coverage (+): 0. Max coverage (-): 0.11

Region: NODE\_318148\_length\_50640\_cov\_30.107899 8171-8188. Max. coverage (+): 0.33. Max coverage (-): 0

Region: NODE\_318148\_length\_50640\_cov\_30.107899 8189-8206. Max. coverage (+): 0.74. Max coverage (-): 0.04

Region: NODE\_318148\_length\_50640\_cov\_30.107899 8207-8224. Max. coverage (+): 1.45. Max coverage (-): 0.04

Region: NODE\_318148\_length\_50640\_cov\_30.107899 8225-8242. Max. coverage (+): 0.37. Max coverage (-): 0

Region: NODE\_318148\_length\_50640\_cov\_30.107899 8243-8260. Max. coverage (+): 0. Max coverage (-): 0.22

Region: NODE\_318148\_length\_50640\_cov\_30.107899 8261-8278. Max. coverage (+): 0.22. Max coverage (-): 0.19

Region: NODE\_318148\_length\_50640\_cov\_30.107899 8279-8296. Max. coverage (+): 1.15. Max coverage (-): 0

Region: NODE\_318148\_length\_50640\_cov\_30.107899 8297-8314. Max. coverage (+): 0.48. Max coverage (-): 0.04

Region: NODE\_318148\_length\_50640\_cov\_30.107899 8315-8332. Max. coverage (+): 0.59. Max coverage (-): 0.07

Region: NODE\_318148\_length\_50640\_cov\_30.107899 8333-8350. Max. coverage (+): 0.07. Max coverage (-): 0

Region: NODE\_318148\_length\_50640\_cov\_30.107899 8351-8369. Max. coverage (+): 0.11. Max coverage (-): 0

Region: NODE\_318148\_length\_50640\_cov\_30.107899 8370-8387. Max. coverage (+): 0.11. Max coverage (-): 0

Region: NODE\_318148\_length\_50640\_cov\_30.107899 8388-8405. Max. coverage (+): 0.07. Max coverage (-): 0.11

Region: NODE\_318148\_length\_50640\_cov\_30.107899 8406-8423. Max. coverage (+): 4.04. Max coverage (-): 0.04

Region: NODE\_318148\_length\_50640\_cov\_30.107899 8424-8441. Max. coverage (+): 2. Max coverage (-): 0

Region: NODE\_318148\_length\_50640\_cov\_30.107899 8442-8459. Max. coverage (+): 0.04. Max coverage (-): 0.04

Region: NODE\_318148\_length\_50640\_cov\_30.107899 8460-8477. Max. coverage (+): 0.04. Max coverage (-): 0

Region: NODE\_318148\_length\_50640\_cov\_30.107899 8478-8495. Max. coverage (+): 0.96. Max coverage (-): 0.04

Region: NODE\_318148\_length\_50640\_cov\_30.107899 8496-8513. Max. coverage (+): 0.48. Max coverage (-): 0.04

Region: NODE\_318148\_length\_50640\_cov\_30.107899 8514-8531. Max. coverage (+): 0.11. Max coverage (-): 0

Region: NODE\_318148\_length\_50640\_cov\_30.107899 8532-8549. Max. coverage (+): 0. Max coverage (-): 0

Region: NODE\_318148\_length\_50640\_cov\_30.107899 8550-8567. Max. coverage (+): 0.11. Max coverage (-): 0.07

Region: NODE\_318148\_length\_50640\_cov\_30.107899 8568-8585. Max. coverage (+): 0.19. Max coverage (-): 0

Region: NODE\_318148\_length\_50640\_cov\_30.107899 8586-8603. Max. coverage (+): 0.19. Max coverage (-): 0.04

Region: NODE\_318148\_length\_50640\_cov\_30.107899 8604-8621. Max. coverage (+): 0.07. Max coverage (-): 0

Region: NODE\_318148\_length\_50640\_cov\_30.107899 8622-8639. Max. coverage (+): 0.04. Max coverage (-): 0

Region: NODE\_318148\_length\_50640\_cov\_30.107899 8640-8657. Max. coverage (+): 1.45. Max coverage (-): 0

Region: NODE\_318148\_length\_50640\_cov\_30.107899 8658-8675. Max. coverage (+): 0. Max coverage (-): 0

Region: NODE\_318148\_length\_50640\_cov\_30.107899 8676-8694. Max. coverage (+): 0.41. Max coverage (-): 0

Region: NODE\_318148\_length\_50640\_cov\_30.107899 8695-8712. Max. coverage (+): 0.15. Max coverage (-): 0

Region: NODE\_318148\_length\_50640\_cov\_30.107899 8713-8730. Max. coverage (+): 0. Max coverage (-): 0.11

Region: NODE\_318148\_length\_50640\_cov\_30.107899 8731-8748. Max. coverage (+): 0.33. Max coverage (-): 0.04

Region: NODE\_318148\_length\_50640\_cov\_30.107899 8749-8766. Max. coverage (+): 0.07. Max coverage (-): 0

Region: NODE\_318148\_length\_50640\_cov\_30.107899 8767-8784. Max. coverage (+): 0. Max coverage (-): 0

Region: NODE\_318148\_length\_50640\_cov\_30.107899 8785-8802. Max. coverage (+): 0. Max coverage (-): 0.04

Region: NODE\_318148\_length\_50640\_cov\_30.107899 8803-8820. Max. coverage (+): 0.67. Max coverage (-): 0

Region: NODE\_318148\_length\_50640\_cov\_30.107899 8821-8838. Max. coverage (+): 0.04. Max coverage (-): 0

Region: NODE\_318148\_length\_50640\_cov\_30.107899 8839-8856. Max. coverage (+): 0.04. Max coverage (-): 0.04

Region: NODE\_318148\_length\_50640\_cov\_30.107899 8857-8874. Max. coverage (+): 0.11. Max coverage (-): 0

Region: NODE\_318148\_length\_50640\_cov\_30.107899 8875-8892. Max. coverage (+): 0.15. Max coverage (-): 0

Region: NODE\_318148\_length\_50640\_cov\_30.107899 8893-8910. Max. coverage (+): 0.07. Max coverage (-): 0

Region: NODE\_318148\_length\_50640\_cov\_30.107899 8911-8928. Max. coverage (+): 0.07. Max coverage (-): 0

Region: NODE\_318148\_length\_50640\_cov\_30.107899 8929-8946. Max. coverage (+): 0.11. Max coverage (-): 0

Region: NODE\_318148\_length\_50640\_cov\_30.107899 8947-8964. Max. coverage (+): 0. Max coverage (-): 0

Region: NODE\_318148\_length\_50640\_cov\_30.107899 8965-8982. Max. coverage (+): 0. Max coverage (-): 0

Region: NODE\_318148\_length\_50640\_cov\_30.107899 8983-9000. Max. coverage (+): 0.07. Max coverage (-): 0

Region: NODE\_318148\_length\_50640\_cov\_30.107899 9001-9018. Max. coverage (+): 0. Max coverage (-): 0

Region: NODE\_318148\_length\_50640\_cov\_30.107899 9019-. Max. coverage (+): 0. Max coverage (-): 0

RepeatMasker Color Code

**+**

100-98% Identity

<98-95% Identity

<95-90% Identity

<90-85% Identity

<85-80% Identity

<80-75% Identity

<75-70% Identity

<70% Identity

**-**

Gene Set Color Code

**+**

Gene

Pseudogene

Other

**-**

Topology/Coverage Color Code

Coverage Plus Strand

Coverage Minus Strand

Mainstrand: Plus

Mainstrand: Minus

Complementary Strand

Flanking Region  
(if option -flank >0)

Gene Set Annotation  
  
RepeatMasker Annotation  

**1. AlRepD-3184**: 183-353 (-), Divergence to consensus: 14%  
**2. AlRepC-373**: 387-551 (-), Divergence to consensus: 46%  
**3. AlRepA-66**: 3020-3107 (-), Divergence to consensus: 26.2%  
**4. AlRepA-66**: 3110-3219 (+), Divergence to consensus: 30.8%  
**5. AlRepD-573**: 3233-3664 (-), Divergence to consensus: 36.3%  
**6. AlRepC-754**: 4990-5050 (-), Divergence to consensus: 21.3%  
**7. AlRepD-1254**: 5062-5339 (-), Divergence to consensus: 40.3%  
**8. AlRepB-103**: 5334-5470 (-), Divergence to consensus: 35.5%  
**9. AlRepD-2221**: 6166-6254 (-), Divergence to consensus: 31.3%  
**10. AlRepC-968**: 6229-6436 (+), Divergence to consensus: 47.1%  
**11. AlRepC-469**: 6432-6503 (+), Divergence to consensus: 27.9%  
**12. (GT)n**: 6823-6853 (+), Divergence to consensus: 0%  
**13. (GT)n**: 7210-7248 (+), Divergence to consensus: 5.3%  
**14. AlRepD-74**: 7764-7838 (-), Divergence to consensus: 30.6%  
**15. AlRepA-118**: 8986-9828 (+), Divergence to consensus: 29.9%

  
Transcription Factor Binding Sites  

**RFX4\_2** (Sequence: CCTAGTTAC (+): 660)  
**RFX4\_2** (Sequence: CTTGGATAC (+): 2972)  
**RFX4\_2** (Sequence: CATGGTTAC (+): 4932)  
**RHOXF1** (Sequence: AGATCA (-): 473)  
**RHOXF1** (Sequence: AGATCA (-): 709)  
**RHOXF1** (Sequence: GGCTCA (-): 1161)  
**RHOXF1** (Sequence: AGCTCA (-): 1193)  
**RHOXF1** (Sequence: GGCTCA (-): 1615)  
**RHOXF1** (Sequence: AGATTA (-): 1749)  
**RHOXF1** (Sequence: AGATTA (-): 3199)  
**RHOXF1** (Sequence: GGCTCA (-): 3703)  
**RHOXF1** (Sequence: GGCTCA (-): 4084)  
**RHOXF1** (Sequence: GGCTTA (-): 4355)  
**RHOXF1** (Sequence: GGATTA (-): 4622)  
**RHOXF1** (Sequence: AGATCA (-): 4742)  
**RHOXF1** (Sequence: AGCTTA (-): 4787)  
**RHOXF1** (Sequence: GGATTA (-): 5007)  
**RHOXF1** (Sequence: GGATTA (-): 5585)  
**RHOXF1** (Sequence: GGCTCA (-): 5596)  
**RHOXF1** (Sequence: AGCTCA (-): 5798)  
**RHOXF1** (Sequence: GGCTCA (-): 5914)  
**RHOXF1** (Sequence: AGATCA (-): 6075)  
**RHOXF1** (Sequence: AGATTA (-): 6092)  
**RHOXF1** (Sequence: GGCTCA (-): 6369)  
**RHOXF1** (Sequence: GGATCA (-): 6569)  
**RHOXF1** (Sequence: AGCTCA (-): 7517)  
**RHOXF1** (Sequence: AGCTCA (-): 7785)  
**RHOXF1** (Sequence: TAATCT (+): 431)  
**RHOXF1** (Sequence: TAATCT (+): 450)  
**RHOXF1** (Sequence: TGAGCC (+): 656)  
**RHOXF1** (Sequence: TGAGCT (+): 1191)  
**RHOXF1** (Sequence: TAATCT (+): 1715)  
**RHOXF1** (Sequence: TAAGCT (+): 2625)  
**RHOXF1** (Sequence: TGATCT (+): 2717)  
**RHOXF1** (Sequence: TAATCC (+): 2806)  
**RHOXF1** (Sequence: TAATCT (+): 3037)  
**RHOXF1** (Sequence: TGAGCT (+): 3098)  
**RHOXF1** (Sequence: TGAGCT (+): 4111)  
**RHOXF1** (Sequence: TAAGCC (+): 4233)  
**RHOXF1** (Sequence: TGAGCC (+): 5426)  
**RHOXF1** (Sequence: TAATCT (+): 6651)  
**RHOXF1** (Sequence: TGAGCT (+): 6747)  
**RHOXF1** (Sequence: TGAGCC (+): 7392)  
**RHOXF1** (Sequence: TGAGCT (+): 7636)  
**RHOXF1** (Sequence: TGATCT (+): 8198)  
**Lhx8** (Sequence: CTAATTAA (-): 990)  
**Lhx8** (Sequence: TTAATTAG (-): 5220)  
**POU5F1** (Sequence: TTTGCAT (-): 3662)  
**FOXO3\_hsa** (Sequence: GTAAACAT (+): 8275)  
**FOXO3\_hsa** (Sequence: GTAAACAA (+): 8296)  
**SOX9** (Sequence: AACAATAG (-): 4635)  
**SOX9** (Sequence: AACAATGA (-): 4844)  
**SOX9** (Sequence: AACAATAA (-): 5576)  
**FOXP1** (Sequence: GTAAACA (+): 286)  
**FOXP1** (Sequence: GTAAACA (+): 1259)  
**FOXP1** (Sequence: GTAAACA (+): 8275)  
**FOXP1** (Sequence: GTAAACA (+): 8296)  
**FOXO1** (Sequence: GCTGTTTAT (+): 3764)  
**FOXO1** (Sequence: GCTGTTTAC (+): 8585)  
**FOXO3\_mmu** (Sequence: TGTTTTCC (-): 1584)  
**FOXO3\_mmu** (Sequence: TGTTTTCA (-): 2380)  
**FOXO3\_mmu** (Sequence: TGTTTACA (-): 5061)  
**FOXO3\_mmu** (Sequence: TGTTTACC (-): 8587)  
**Sox5** (Sequence: ATTGTT (+): 2602)  
**Sox5** (Sequence: ATTGTT (+): 2853)  
**Sox5** (Sequence: ATTGTT (+): 3714)  
**Sox5** (Sequence: ATTGTT (+): 4682)  
**Sox5** (Sequence: ATTGTT (+): 5877)  
**FIGLA** (Sequence: AACAGCTGGA (-): 561)  
**FIGLA** (Sequence: ACCAGCTGTA (-): 2739)  
**FIGLA** (Sequence: ACCAGCTGTA (-): 2798)  
**A-MYB** (Sequence: CCAACTGCCT (-): 259)  
**SOX9** (Sequence: TCATTGTT (+): 2600)  
**FOXO3\_mmu** (Sequence: TGTAAACA (+): 1258)  
**FOXO3\_mmu** (Sequence: TCAAAACA (+): 1312)  
**FOXO3\_mmu** (Sequence: GCAAAACA (+): 3626)  
**FOXO3\_mmu** (Sequence: TCAAAACA (+): 4253)  
**FOXO3\_mmu** (Sequence: TGAAAACA (+): 4418)  
**FOXO3\_mmu** (Sequence: TGAAAACA (+): 5784)  
**FOXO3\_mmu** (Sequence: TCAAAACA (+): 7788)  
**Nobox** (Sequence: ACTAATTA (-): 3013)  
**Nobox** (Sequence: ACTAATTA (-): 3219)  
**Nobox** (Sequence: ACTAATTA (-): 6953)  
**FOXO1** (Sequence: GTAAACAGG (-): 286)  
**FOXO1** (Sequence: GTAAACAAG (-): 8296)  
**FOXO1** (Sequence: AAAAACAGC (-): 8701)  
**FOXO3\_hsa** (Sequence: ATGTTTAC (-): 6310)  
**FOXP1** (Sequence: TGTTTAC (-): 5061)  
**FOXP1** (Sequence: TGTTTAC (-): 6311)  
**FOXP1** (Sequence: TGTTTAC (-): 8587)  
**Nobox** (Sequence: TAATTGCC (+): 2490)  
**Nobox** (Sequence: TAATTACT (+): 5168)  
**Nobox** (Sequence: TAATTAGT (+): 5221)  
**Nobox** (Sequence: TAATTACT (+): 5882)  
**Rhox11** (Sequence: TGGTGTATT (+): 763)  
**Rhox11** (Sequence: TGCTGTTTA (+): 3763)  
**Rhox11** (Sequence: AATACACCA (-): 1298)  
**Rhox11** (Sequence: AATACACCA (-): 1695)  
**Rhox11** (Sequence: AAAACAGCA (-): 6662)  
**Rhox11** (Sequence: AAAACAGCA (-): 8702)  
**Sox5** (Sequence: AACAAT (-): 894)  
**Sox5** (Sequence: AACAAT (-): 1316)  
**Sox5** (Sequence: AACAAT (-): 1445)  
**Sox5** (Sequence: AACAAT (-): 1736)  
**Sox5** (Sequence: AACAAT (-): 1924)  
**Sox5** (Sequence: AACAAT (-): 2251)  
**Sox5** (Sequence: AACAAT (-): 4545)  
**Sox5** (Sequence: AACAAT (-): 4635)  
**Sox5** (Sequence: AACAAT (-): 4844)  
**Sox5** (Sequence: AACAAT (-): 5576)  
**POU5F1** (Sequence: ATGCAAA (+): 889)  
**POU5F1** (Sequence: ATGCAAA (+): 3677)
