# Supplementary material for: First characterization of PIWI-interacting RNA clusters in a cichlid fish with a B chromosome
Source: BMC Biol. 2022 Sep 21;20:204. doi: 10.1186/s12915-022-01403-2 (PMC9490952; doi:10.1186/s12915-022-01403-2)
Supplement: Supplementary file 1 — Additional file 1. Zipped folder with fasta and interactive html piRNA cluster information for the A. latifasciata genome. The nomenclature is as follows: number-pirna-cluster_sex_B-presence (f, female; m, male; 0b, without B chromosome; 1b, with B chromosome). [file 12915_2022_1403_MOESM1_ESM.zip › 126_m1b.html]

piRNA cluster 126\_m1b 73


Predicted piRNA cluster no. 126\_m1b
  

Show proTRAC run info
Hide proTRAC run info

/\  
                \_\_\_\_\_\_\_\_\_\_\_\_\_\_\_\_\_\_\_\_\_\_\_/\\_\_\_ /  \\_\_\_\_\_\_\_  
               I                      /  \  /    \      I  
               I     pro             /    \/      \     I  
               I        TRAC        /               \   I  
               I   \_\_\_\_\_\_\_\_\_\_\_\_\_\_\_\_/\_\_\_\_\_\_\_\_\_\_\_\_\_\_\_\_\_\\_ I  
               I   \              /                     I  
               I    \            /                      I  
               I     \  /\      /       V.2.4.2         I  
               I      \/  \    /                        I  
               I\_\_\_\_\_\_\_\_\_\_\_\  /\_\_\_\_\_\_\_\_\_\_\_\_\_\_\_\_\_\_\_\_\_\_\_\_\_I  
                            \/  
  
  
================================= proTRAC ====================================  
VERSION: .......... 2.4.2  
LAST MODIFIED: .... 11. May 2018  
  
Please cite:  
Rosenkranz D, Zischler H. proTRAC - a software for probabilistic piRNA cluster  
detection, visualization and analysis. 2012. BMC Bioinformatics 13:5.  
  
  
Contact:  
David Rosenkranz  
Institute of Organismic and Molecular Evolutionary Biology  
Dept. Anthropology, small RNA group  
Johannes Gutenberg University Mainz  
email: rosenkranz@uni-mainz.de  
  
You can find the latest proTRAC version at:  
http://sourceforge.net/projects/protrac/files  
http://www.smallRNAgroup-mainz.de/software  
==============================================================================  
  
PARAMETERS:  
Map file: ...............piwi-machos-1B.fa-collapse.map  
Genome file: ............../../../0B\_ala\_genome.fa  
RepeatMasker annotation: Alatifasciata-all0B-maryan-v2.fa\_corrected.out  
GeneSet:................./guest-storage/Data/annotation/Alatifasciata\_all0B\_maryan-v2\_out2017.gff  
  
Significant (p<=0.01) hit density will be calculated based  
on observed hit distribution.  
  
Sliding window size: ........................................ 5000 bp  
Sliding window increament: .................................. 1000 bp  
Normalize each hit by number of genomic hits: ............... yes  
Normalize each hit by number of sequence reads: ............. yes  
Normalize values (-> per million mapped reads): ............. yes  
Min. fraction of hits with 1T(U) or 10A: .................... 0.75  
Alternatively: Min. fraction of hits with 1T(U) and 10A: .... 0.5  
Min. fraction of hits with typical piRNA length: ............ 0.75  
Typical piRNA length: ....................................... 24-32 nt  
Min. size of a piRNA cluster: ............................... 1000 bp.  
Min. number of hits (absolute): ............................. 0  
Min. number of hits (normalized): ........................... 0  
Min. fraction of hits on the mainstrand: .................... 0.75  
Top fraction of mapped sequences (in terms of read counts): . 1%  
Top fraction accounts for max. n% of sequence reads: ........ 90%  
Min. fraction of hits on each arm of a bidirectional cluster: 0.05  
Output html file for each cluster: .......................... yes  
Output a summary table: ..................................... yes  
Output a FASTA file for each cluster (piRNA sequences): ..... yes  
Output a FASTA file comprising cluster sequences: ........... yes  
Output a GTF file for predicted piRNA clusters: ..............yes  
Search DNA motifs in clusters: .............................. yes  
Output flanking sequences: +/- .............................. 0 bp  
Output ~.pTi file: .......................................... no  
==============================================================================  
  
  
Genome size (without gaps): ............ 758543724 bp  
Gaps (N/X/-): .......................... 417479 bp  
Mapped reads: .......................... 26973943  
Non-identical sequences: ............... 6209225  
Genomic hits: .......................... 48438990  
Significant densitiy of mapped reads: .. 821.144211136946 reads/kb

Show proTRAC cluster info
Hide proTRAC cluster info

|  |  |
| --- | --- |
| Location | NODE\_318280\_length\_3803\_cov\_29.618460 |
| Coordinates | 3-3790 |
| Size [bp] | 3788 |
| Sequence hit loci | 2514 |
| Mapped reads (normalized) | 5655 |
| Mapped reads (normalized) per kb | 1492.9 |
| Normalized reads with 1T (1U) | 77.8% |
| Normalized reads with 10A | 44.6% |
| Normalized reads with length 24-32 nt | 98.7% |
| Normalized reads on the main strand(s) | 84.2% |
| Predicted directionality | mono:minus |

100%

0%

1T (1U)  
reads

10A reads

24-32 nt  
reads

reads on mainstrand

**Either the amount of reads with 1T (1U) OR 10A has to exceed 75% (set with option: -1Tor10A)  
Alternatively the amount of reads with 1T (1U) AND 10A has to exceed 50% (set with option: -1Tand10A)  
Minimum amount of reads with preferred size is 75% (set with option: -pisize)  
Minimum amount of reads on the main strand(s) is 75% (set with option: -clstrand)**

Show read coverage
Hide read coverage

WHAT DO I SEE HERE?  
This chart shows the location of mapped sequence reads within a predicted piRNA cluster. The color refers to the number of genomic hits produced by the sequence read in question. A dark red bar indicates that this sequence read produces many other hits elsewhere in the genome. Many adjacent red or yellow bars can indicate the presence of a multi-copy element such as transposons or rRNA genes. A dark green bar indicates that this sequence read maps uniquely to this locus.

1 hit

2-5 hits

6-10 hits

11-20 hits

21-50 hits

51-100 hits

> 100 hits

NODE\_318280\_length\_3803\_cov\_29.618460

3

3790

Gene Set

RepeatMasker

Mapped  
Reads

13.61

plus strand

minus strand

13.61

Region: NODE\_318280\_length\_3803\_cov\_29.618460 9020-6. Max. coverage (+): 0. Max coverage (-): 0

Region: NODE\_318280\_length\_3803\_cov\_29.618460 7-14. Max. coverage (+): 0. Max coverage (-): 0

Region: NODE\_318280\_length\_3803\_cov\_29.618460 15-21. Max. coverage (+): 0. Max coverage (-): 0

Region: NODE\_318280\_length\_3803\_cov\_29.618460 22-29. Max. coverage (+): 0. Max coverage (-): 0

Region: NODE\_318280\_length\_3803\_cov\_29.618460 30-37. Max. coverage (+): 0. Max coverage (-): 0

Region: NODE\_318280\_length\_3803\_cov\_29.618460 38-44. Max. coverage (+): 0. Max coverage (-): 0

Region: NODE\_318280\_length\_3803\_cov\_29.618460 45-52. Max. coverage (+): 0. Max coverage (-): 0

Region: NODE\_318280\_length\_3803\_cov\_29.618460 53-59. Max. coverage (+): 0.04. Max coverage (-): 0.15

Region: NODE\_318280\_length\_3803\_cov\_29.618460 60-67. Max. coverage (+): 0. Max coverage (-): 0.3

Region: NODE\_318280\_length\_3803\_cov\_29.618460 68-74. Max. coverage (+): 0. Max coverage (-): 0.22

Region: NODE\_318280\_length\_3803\_cov\_29.618460 75-82. Max. coverage (+): 0. Max coverage (-): 0.06

Region: NODE\_318280\_length\_3803\_cov\_29.618460 83-90. Max. coverage (+): 0. Max coverage (-): 0

Region: NODE\_318280\_length\_3803\_cov\_29.618460 91-97. Max. coverage (+): 0. Max coverage (-): 0

Region: NODE\_318280\_length\_3803\_cov\_29.618460 98-105. Max. coverage (+): 0. Max coverage (-): 0

Region: NODE\_318280\_length\_3803\_cov\_29.618460 106-112. Max. coverage (+): 0. Max coverage (-): 0.04

Region: NODE\_318280\_length\_3803\_cov\_29.618460 113-120. Max. coverage (+): 0. Max coverage (-): 0

Region: NODE\_318280\_length\_3803\_cov\_29.618460 121-128. Max. coverage (+): 0. Max coverage (-): 0

Region: NODE\_318280\_length\_3803\_cov\_29.618460 129-135. Max. coverage (+): 0. Max coverage (-): 0.07

Region: NODE\_318280\_length\_3803\_cov\_29.618460 136-143. Max. coverage (+): 0. Max coverage (-): 0

Region: NODE\_318280\_length\_3803\_cov\_29.618460 144-150. Max. coverage (+): 0. Max coverage (-): 0.04

Region: NODE\_318280\_length\_3803\_cov\_29.618460 151-158. Max. coverage (+): 0. Max coverage (-): 0.82

Region: NODE\_318280\_length\_3803\_cov\_29.618460 159-165. Max. coverage (+): 0. Max coverage (-): 0.19

Region: NODE\_318280\_length\_3803\_cov\_29.618460 166-173. Max. coverage (+): 0. Max coverage (-): 0.19

Region: NODE\_318280\_length\_3803\_cov\_29.618460 174-181. Max. coverage (+): 0. Max coverage (-): 0.11

Region: NODE\_318280\_length\_3803\_cov\_29.618460 182-188. Max. coverage (+): 0. Max coverage (-): 0.26

Region: NODE\_318280\_length\_3803\_cov\_29.618460 189-196. Max. coverage (+): 0. Max coverage (-): 0.07

Region: NODE\_318280\_length\_3803\_cov\_29.618460 197-203. Max. coverage (+): 0. Max coverage (-): 0.04

Region: NODE\_318280\_length\_3803\_cov\_29.618460 204-211. Max. coverage (+): 0. Max coverage (-): 0

Region: NODE\_318280\_length\_3803\_cov\_29.618460 212-218. Max. coverage (+): 0. Max coverage (-): 0.04

Region: NODE\_318280\_length\_3803\_cov\_29.618460 219-226. Max. coverage (+): 0. Max coverage (-): 0.07

Region: NODE\_318280\_length\_3803\_cov\_29.618460 227-234. Max. coverage (+): 0.04. Max coverage (-): 0.19

Region: NODE\_318280\_length\_3803\_cov\_29.618460 235-241. Max. coverage (+): 0.04. Max coverage (-): 0

Region: NODE\_318280\_length\_3803\_cov\_29.618460 242-249. Max. coverage (+): 0.04. Max coverage (-): 0.04

Region: NODE\_318280\_length\_3803\_cov\_29.618460 250-256. Max. coverage (+): 0. Max coverage (-): 0.04

Region: NODE\_318280\_length\_3803\_cov\_29.618460 257-264. Max. coverage (+): 0. Max coverage (-): 0.04

Region: NODE\_318280\_length\_3803\_cov\_29.618460 265-271. Max. coverage (+): 0.11. Max coverage (-): 0.22

Region: NODE\_318280\_length\_3803\_cov\_29.618460 272-279. Max. coverage (+): 0.04. Max coverage (-): 0.04

Region: NODE\_318280\_length\_3803\_cov\_29.618460 280-287. Max. coverage (+): 0. Max coverage (-): 0.07

Region: NODE\_318280\_length\_3803\_cov\_29.618460 288-294. Max. coverage (+): 0. Max coverage (-): 0.07

Region: NODE\_318280\_length\_3803\_cov\_29.618460 295-302. Max. coverage (+): 0. Max coverage (-): 0

Region: NODE\_318280\_length\_3803\_cov\_29.618460 303-309. Max. coverage (+): 0. Max coverage (-): 0

Region: NODE\_318280\_length\_3803\_cov\_29.618460 310-317. Max. coverage (+): 0. Max coverage (-): 0.04

Region: NODE\_318280\_length\_3803\_cov\_29.618460 318-324. Max. coverage (+): 0. Max coverage (-): 0

Region: NODE\_318280\_length\_3803\_cov\_29.618460 325-332. Max. coverage (+): 0. Max coverage (-): 0

Region: NODE\_318280\_length\_3803\_cov\_29.618460 333-340. Max. coverage (+): 0. Max coverage (-): 0

Region: NODE\_318280\_length\_3803\_cov\_29.618460 341-347. Max. coverage (+): 0.07. Max coverage (-): 0.07

Region: NODE\_318280\_length\_3803\_cov\_29.618460 348-355. Max. coverage (+): 0. Max coverage (-): 0.11

Region: NODE\_318280\_length\_3803\_cov\_29.618460 356-362. Max. coverage (+): 0. Max coverage (-): 0.89

Region: NODE\_318280\_length\_3803\_cov\_29.618460 363-370. Max. coverage (+): 0. Max coverage (-): 0.82

Region: NODE\_318280\_length\_3803\_cov\_29.618460 371-378. Max. coverage (+): 0.02. Max coverage (-): 0.02

Region: NODE\_318280\_length\_3803\_cov\_29.618460 379-385. Max. coverage (+): 0.04. Max coverage (-): 0

Region: NODE\_318280\_length\_3803\_cov\_29.618460 386-393. Max. coverage (+): 0. Max coverage (-): 0

Region: NODE\_318280\_length\_3803\_cov\_29.618460 394-400. Max. coverage (+): 0. Max coverage (-): 0.06

Region: NODE\_318280\_length\_3803\_cov\_29.618460 401-408. Max. coverage (+): 0. Max coverage (-): 0.06

Region: NODE\_318280\_length\_3803\_cov\_29.618460 409-415. Max. coverage (+): 0. Max coverage (-): 0.07

Region: NODE\_318280\_length\_3803\_cov\_29.618460 416-423. Max. coverage (+): 0. Max coverage (-): 0.01

Region: NODE\_318280\_length\_3803\_cov\_29.618460 424-431. Max. coverage (+): 0. Max coverage (-): 0

Region: NODE\_318280\_length\_3803\_cov\_29.618460 432-438. Max. coverage (+): 0. Max coverage (-): 0.09

Region: NODE\_318280\_length\_3803\_cov\_29.618460 439-446. Max. coverage (+): 0.01. Max coverage (-): 0

Region: NODE\_318280\_length\_3803\_cov\_29.618460 447-453. Max. coverage (+): 0.01. Max coverage (-): 0.12

Region: NODE\_318280\_length\_3803\_cov\_29.618460 454-461. Max. coverage (+): 0.01. Max coverage (-): 0.09

Region: NODE\_318280\_length\_3803\_cov\_29.618460 462-468. Max. coverage (+): 0. Max coverage (-): 0.02

Region: NODE\_318280\_length\_3803\_cov\_29.618460 469-476. Max. coverage (+): 0. Max coverage (-): 0.33

Region: NODE\_318280\_length\_3803\_cov\_29.618460 477-484. Max. coverage (+): 0. Max coverage (-): 0.28

Region: NODE\_318280\_length\_3803\_cov\_29.618460 485-491. Max. coverage (+): 0. Max coverage (-): 0.56

Region: NODE\_318280\_length\_3803\_cov\_29.618460 492-499. Max. coverage (+): 0.52. Max coverage (-): 0.26

Region: NODE\_318280\_length\_3803\_cov\_29.618460 500-506. Max. coverage (+): 0.52. Max coverage (-): 0.26

Region: NODE\_318280\_length\_3803\_cov\_29.618460 507-514. Max. coverage (+): 0.19. Max coverage (-): 0.11

Region: NODE\_318280\_length\_3803\_cov\_29.618460 515-521. Max. coverage (+): 0. Max coverage (-): 0.2

Region: NODE\_318280\_length\_3803\_cov\_29.618460 522-529. Max. coverage (+): 0.02. Max coverage (-): 0

Region: NODE\_318280\_length\_3803\_cov\_29.618460 530-537. Max. coverage (+): 0. Max coverage (-): 0.93

Region: NODE\_318280\_length\_3803\_cov\_29.618460 538-544. Max. coverage (+): 0. Max coverage (-): 1.06

Region: NODE\_318280\_length\_3803\_cov\_29.618460 545-552. Max. coverage (+): 0.11. Max coverage (-): 0.11

Region: NODE\_318280\_length\_3803\_cov\_29.618460 553-559. Max. coverage (+): 0.11. Max coverage (-): 0

Region: NODE\_318280\_length\_3803\_cov\_29.618460 560-567. Max. coverage (+): 0. Max coverage (-): 0

Region: NODE\_318280\_length\_3803\_cov\_29.618460 568-574. Max. coverage (+): 0. Max coverage (-): 10.6

Region: NODE\_318280\_length\_3803\_cov\_29.618460 575-582. Max. coverage (+): 0. Max coverage (-): 0.59

Region: NODE\_318280\_length\_3803\_cov\_29.618460 583-590. Max. coverage (+): 0. Max coverage (-): 0.3

Region: NODE\_318280\_length\_3803\_cov\_29.618460 591-597. Max. coverage (+): 0. Max coverage (-): 0.07

Region: NODE\_318280\_length\_3803\_cov\_29.618460 598-605. Max. coverage (+): 0.11. Max coverage (-): 0.11

Region: NODE\_318280\_length\_3803\_cov\_29.618460 606-612. Max. coverage (+): 0. Max coverage (-): 0.07

Region: NODE\_318280\_length\_3803\_cov\_29.618460 613-620. Max. coverage (+): 0. Max coverage (-): 1.85

Region: NODE\_318280\_length\_3803\_cov\_29.618460 621-628. Max. coverage (+): 0.02. Max coverage (-): 2.15

Region: NODE\_318280\_length\_3803\_cov\_29.618460 629-635. Max. coverage (+): 0.02. Max coverage (-): 0.15

Region: NODE\_318280\_length\_3803\_cov\_29.618460 636-643. Max. coverage (+): 0.04. Max coverage (-): 0.56

Region: NODE\_318280\_length\_3803\_cov\_29.618460 644-650. Max. coverage (+): 0.02. Max coverage (-): 0.04

Region: NODE\_318280\_length\_3803\_cov\_29.618460 651-658. Max. coverage (+): 0.44. Max coverage (-): 0.02

Region: NODE\_318280\_length\_3803\_cov\_29.618460 659-665. Max. coverage (+): 0.44. Max coverage (-): 0.07

Region: NODE\_318280\_length\_3803\_cov\_29.618460 666-673. Max. coverage (+): 0. Max coverage (-): 1.26

Region: NODE\_318280\_length\_3803\_cov\_29.618460 674-681. Max. coverage (+): 0.04. Max coverage (-): 1.08

Region: NODE\_318280\_length\_3803\_cov\_29.618460 682-688. Max. coverage (+): 0.02. Max coverage (-): 1.09

Region: NODE\_318280\_length\_3803\_cov\_29.618460 689-696. Max. coverage (+): 0.04. Max coverage (-): 0.06

Region: NODE\_318280\_length\_3803\_cov\_29.618460 697-703. Max. coverage (+): 0.07. Max coverage (-): 0.09

Region: NODE\_318280\_length\_3803\_cov\_29.618460 704-711. Max. coverage (+): 0.15. Max coverage (-): 1

Region: NODE\_318280\_length\_3803\_cov\_29.618460 712-718. Max. coverage (+): 0. Max coverage (-): 0.78

Region: NODE\_318280\_length\_3803\_cov\_29.618460 719-726. Max. coverage (+): 0. Max coverage (-): 3.23

Region: NODE\_318280\_length\_3803\_cov\_29.618460 727-734. Max. coverage (+): 0.19. Max coverage (-): 0.07

Region: NODE\_318280\_length\_3803\_cov\_29.618460 735-741. Max. coverage (+): 0. Max coverage (-): 0.04

Region: NODE\_318280\_length\_3803\_cov\_29.618460 742-749. Max. coverage (+): 0. Max coverage (-): 0.26

Region: NODE\_318280\_length\_3803\_cov\_29.618460 750-756. Max. coverage (+): 0. Max coverage (-): 0.11

Region: NODE\_318280\_length\_3803\_cov\_29.618460 757-764. Max. coverage (+): 0.04. Max coverage (-): 0.09

Region: NODE\_318280\_length\_3803\_cov\_29.618460 765-771. Max. coverage (+): 0.04. Max coverage (-): 0.15

Region: NODE\_318280\_length\_3803\_cov\_29.618460 772-779. Max. coverage (+): 0. Max coverage (-): 0.04

Region: NODE\_318280\_length\_3803\_cov\_29.618460 780-787. Max. coverage (+): 0. Max coverage (-): 0

Region: NODE\_318280\_length\_3803\_cov\_29.618460 788-794. Max. coverage (+): 0.04. Max coverage (-): 0.3

Region: NODE\_318280\_length\_3803\_cov\_29.618460 795-802. Max. coverage (+): 0.04. Max coverage (-): 0

Region: NODE\_318280\_length\_3803\_cov\_29.618460 803-809. Max. coverage (+): 0. Max coverage (-): 0.13

Region: NODE\_318280\_length\_3803\_cov\_29.618460 810-817. Max. coverage (+): 0.01. Max coverage (-): 0.22

Region: NODE\_318280\_length\_3803\_cov\_29.618460 818-824. Max. coverage (+): 0. Max coverage (-): 0.06

Region: NODE\_318280\_length\_3803\_cov\_29.618460 825-832. Max. coverage (+): 0.11. Max coverage (-): 0

Region: NODE\_318280\_length\_3803\_cov\_29.618460 833-840. Max. coverage (+): 0. Max coverage (-): 0.44

Region: NODE\_318280\_length\_3803\_cov\_29.618460 841-847. Max. coverage (+): 0. Max coverage (-): 0.78

Region: NODE\_318280\_length\_3803\_cov\_29.618460 848-855. Max. coverage (+): 0. Max coverage (-): 0.93

Region: NODE\_318280\_length\_3803\_cov\_29.618460 856-862. Max. coverage (+): 0. Max coverage (-): 0

Region: NODE\_318280\_length\_3803\_cov\_29.618460 863-870. Max. coverage (+): 0.04. Max coverage (-): 0.06

Region: NODE\_318280\_length\_3803\_cov\_29.618460 871-878. Max. coverage (+): 0. Max coverage (-): 0.06

Region: NODE\_318280\_length\_3803\_cov\_29.618460 879-885. Max. coverage (+): 0. Max coverage (-): 0.33

Region: NODE\_318280\_length\_3803\_cov\_29.618460 886-893. Max. coverage (+): 0. Max coverage (-): 0.37

Region: NODE\_318280\_length\_3803\_cov\_29.618460 894-900. Max. coverage (+): 0.04. Max coverage (-): 0

Region: NODE\_318280\_length\_3803\_cov\_29.618460 901-908. Max. coverage (+): 0. Max coverage (-): 0.15

Region: NODE\_318280\_length\_3803\_cov\_29.618460 909-915. Max. coverage (+): 0. Max coverage (-): 0.54

Region: NODE\_318280\_length\_3803\_cov\_29.618460 916-923. Max. coverage (+): 0. Max coverage (-): 1.64

Region: NODE\_318280\_length\_3803\_cov\_29.618460 924-931. Max. coverage (+): 0. Max coverage (-): 0.04

Region: NODE\_318280\_length\_3803\_cov\_29.618460 932-938. Max. coverage (+): 0. Max coverage (-): 0.48

Region: NODE\_318280\_length\_3803\_cov\_29.618460 939-946. Max. coverage (+): 0. Max coverage (-): 0.07

Region: NODE\_318280\_length\_3803\_cov\_29.618460 947-953. Max. coverage (+): 0.02. Max coverage (-): 0.07

Region: NODE\_318280\_length\_3803\_cov\_29.618460 954-961. Max. coverage (+): 0.02. Max coverage (-): 0

Region: NODE\_318280\_length\_3803\_cov\_29.618460 962-968. Max. coverage (+): 0. Max coverage (-): 0.04

Region: NODE\_318280\_length\_3803\_cov\_29.618460 969-976. Max. coverage (+): 0.04. Max coverage (-): 0.32

Region: NODE\_318280\_length\_3803\_cov\_29.618460 977-984. Max. coverage (+): 0.22. Max coverage (-): 3.56

Region: NODE\_318280\_length\_3803\_cov\_29.618460 985-991. Max. coverage (+): 0.19. Max coverage (-): 2.37

Region: NODE\_318280\_length\_3803\_cov\_29.618460 992-999. Max. coverage (+): 0.26. Max coverage (-): 0

Region: NODE\_318280\_length\_3803\_cov\_29.618460 1000-1006. Max. coverage (+): 0.11. Max coverage (-): 0

Region: NODE\_318280\_length\_3803\_cov\_29.618460 1007-1014. Max. coverage (+): 0. Max coverage (-): 0

Region: NODE\_318280\_length\_3803\_cov\_29.618460 1015-1021. Max. coverage (+): 0. Max coverage (-): 0

Region: NODE\_318280\_length\_3803\_cov\_29.618460 1022-1029. Max. coverage (+): 0. Max coverage (-): 0

Region: NODE\_318280\_length\_3803\_cov\_29.618460 1030-1037. Max. coverage (+): 0. Max coverage (-): 0.15

Region: NODE\_318280\_length\_3803\_cov\_29.618460 1038-1044. Max. coverage (+): 0. Max coverage (-): 0.07

Region: NODE\_318280\_length\_3803\_cov\_29.618460 1045-1052. Max. coverage (+): 0.26. Max coverage (-): 0.04

Region: NODE\_318280\_length\_3803\_cov\_29.618460 1053-1059. Max. coverage (+): 0.26. Max coverage (-): 0.11

Region: NODE\_318280\_length\_3803\_cov\_29.618460 1060-1067. Max. coverage (+): 0. Max coverage (-): 0.93

Region: NODE\_318280\_length\_3803\_cov\_29.618460 1068-1075. Max. coverage (+): 0. Max coverage (-): 1.08

Region: NODE\_318280\_length\_3803\_cov\_29.618460 1076-1082. Max. coverage (+): 0. Max coverage (-): 0

Region: NODE\_318280\_length\_3803\_cov\_29.618460 1083-1090. Max. coverage (+): 0. Max coverage (-): 0.04

Region: NODE\_318280\_length\_3803\_cov\_29.618460 1091-1097. Max. coverage (+): 0. Max coverage (-): 0.37

Region: NODE\_318280\_length\_3803\_cov\_29.618460 1098-1105. Max. coverage (+): 0. Max coverage (-): 0.48

Region: NODE\_318280\_length\_3803\_cov\_29.618460 1106-1112. Max. coverage (+): 0. Max coverage (-): 0

Region: NODE\_318280\_length\_3803\_cov\_29.618460 1113-1120. Max. coverage (+): 0.26. Max coverage (-): 0

Region: NODE\_318280\_length\_3803\_cov\_29.618460 1121-1128. Max. coverage (+): 0.22. Max coverage (-): 0

Region: NODE\_318280\_length\_3803\_cov\_29.618460 1129-1135. Max. coverage (+): 0. Max coverage (-): 0.11

Region: NODE\_318280\_length\_3803\_cov\_29.618460 1136-1143. Max. coverage (+): 0. Max coverage (-): 0.04

Region: NODE\_318280\_length\_3803\_cov\_29.618460 1144-1150. Max. coverage (+): 0.04. Max coverage (-): 0

Region: NODE\_318280\_length\_3803\_cov\_29.618460 1151-1158. Max. coverage (+): 0.07. Max coverage (-): 0.19

Region: NODE\_318280\_length\_3803\_cov\_29.618460 1159-1165. Max. coverage (+): 0. Max coverage (-): 0

Region: NODE\_318280\_length\_3803\_cov\_29.618460 1166-1173. Max. coverage (+): 0.04. Max coverage (-): 0

Region: NODE\_318280\_length\_3803\_cov\_29.618460 1174-1181. Max. coverage (+): 0. Max coverage (-): 0

Region: NODE\_318280\_length\_3803\_cov\_29.618460 1182-1188. Max. coverage (+): 0. Max coverage (-): 0

Region: NODE\_318280\_length\_3803\_cov\_29.618460 1189-1196. Max. coverage (+): 0. Max coverage (-): 0

Region: NODE\_318280\_length\_3803\_cov\_29.618460 1197-1203. Max. coverage (+): 0.52. Max coverage (-): 0.04

Region: NODE\_318280\_length\_3803\_cov\_29.618460 1204-1211. Max. coverage (+): 0.52. Max coverage (-): 0

Region: NODE\_318280\_length\_3803\_cov\_29.618460 1212-1218. Max. coverage (+): 0. Max coverage (-): 0

Region: NODE\_318280\_length\_3803\_cov\_29.618460 1219-1226. Max. coverage (+): 0. Max coverage (-): 0.15

Region: NODE\_318280\_length\_3803\_cov\_29.618460 1227-1234. Max. coverage (+): 0. Max coverage (-): 0.07

Region: NODE\_318280\_length\_3803\_cov\_29.618460 1235-1241. Max. coverage (+): 0. Max coverage (-): 0

Region: NODE\_318280\_length\_3803\_cov\_29.618460 1242-1249. Max. coverage (+): 0. Max coverage (-): 0.15

Region: NODE\_318280\_length\_3803\_cov\_29.618460 1250-1256. Max. coverage (+): 0. Max coverage (-): 0.04

Region: NODE\_318280\_length\_3803\_cov\_29.618460 1257-1264. Max. coverage (+): 0. Max coverage (-): 0.56

Region: NODE\_318280\_length\_3803\_cov\_29.618460 1265-1271. Max. coverage (+): 0. Max coverage (-): 0.56

Region: NODE\_318280\_length\_3803\_cov\_29.618460 1272-1279. Max. coverage (+): 0. Max coverage (-): 0.26

Region: NODE\_318280\_length\_3803\_cov\_29.618460 1280-1287. Max. coverage (+): 0.07. Max coverage (-): 0.11

Region: NODE\_318280\_length\_3803\_cov\_29.618460 1288-1294. Max. coverage (+): 0.04. Max coverage (-): 0.22

Region: NODE\_318280\_length\_3803\_cov\_29.618460 1295-1302. Max. coverage (+): 0.04. Max coverage (-): 0.22

Region: NODE\_318280\_length\_3803\_cov\_29.618460 1303-1309. Max. coverage (+): 0. Max coverage (-): 3.48

Region: NODE\_318280\_length\_3803\_cov\_29.618460 1310-1317. Max. coverage (+): 0. Max coverage (-): 0.3

Region: NODE\_318280\_length\_3803\_cov\_29.618460 1318-1325. Max. coverage (+): 0.11. Max coverage (-): 1

Region: NODE\_318280\_length\_3803\_cov\_29.618460 1326-1332. Max. coverage (+): 0.07. Max coverage (-): 1.45

Region: NODE\_318280\_length\_3803\_cov\_29.618460 1333-1340. Max. coverage (+): 0. Max coverage (-): 0.22

Region: NODE\_318280\_length\_3803\_cov\_29.618460 1341-1347. Max. coverage (+): 0. Max coverage (-): 0

Region: NODE\_318280\_length\_3803\_cov\_29.618460 1348-1355. Max. coverage (+): 0.11. Max coverage (-): 0.04

Region: NODE\_318280\_length\_3803\_cov\_29.618460 1356-1362. Max. coverage (+): 0.11. Max coverage (-): 0.3

Region: NODE\_318280\_length\_3803\_cov\_29.618460 1363-1370. Max. coverage (+): 0.11. Max coverage (-): 4.26

Region: NODE\_318280\_length\_3803\_cov\_29.618460 1371-1378. Max. coverage (+): 0.11. Max coverage (-): 0.3

Region: NODE\_318280\_length\_3803\_cov\_29.618460 1379-1385. Max. coverage (+): 0.26. Max coverage (-): 0.26

Region: NODE\_318280\_length\_3803\_cov\_29.618460 1386-1393. Max. coverage (+): 0.26. Max coverage (-): 1.26

Region: NODE\_318280\_length\_3803\_cov\_29.618460 1394-1400. Max. coverage (+): 0.04. Max coverage (-): 10.75

Region: NODE\_318280\_length\_3803\_cov\_29.618460 1401-1408. Max. coverage (+): 0. Max coverage (-): 1.3

Region: NODE\_318280\_length\_3803\_cov\_29.618460 1409-1415. Max. coverage (+): 0.07. Max coverage (-): 0.3

Region: NODE\_318280\_length\_3803\_cov\_29.618460 1416-1423. Max. coverage (+): 0. Max coverage (-): 0.26

Region: NODE\_318280\_length\_3803\_cov\_29.618460 1424-1431. Max. coverage (+): 0.11. Max coverage (-): 0.07

Region: NODE\_318280\_length\_3803\_cov\_29.618460 1432-1438. Max. coverage (+): 0. Max coverage (-): 0.52

Region: NODE\_318280\_length\_3803\_cov\_29.618460 1439-1446. Max. coverage (+): 0.07. Max coverage (-): 0.96

Region: NODE\_318280\_length\_3803\_cov\_29.618460 1447-1453. Max. coverage (+): 0.11. Max coverage (-): 0.7

Region: NODE\_318280\_length\_3803\_cov\_29.618460 1454-1461. Max. coverage (+): 0.26. Max coverage (-): 0.11

Region: NODE\_318280\_length\_3803\_cov\_29.618460 1462-1468. Max. coverage (+): 0.04. Max coverage (-): 13.61

Region: NODE\_318280\_length\_3803\_cov\_29.618460 1469-1476. Max. coverage (+): 0. Max coverage (-): 13.46

Region: NODE\_318280\_length\_3803\_cov\_29.618460 1477-1484. Max. coverage (+): 0.04. Max coverage (-): 0.07

Region: NODE\_318280\_length\_3803\_cov\_29.618460 1485-1491. Max. coverage (+): 0.04. Max coverage (-): 0.11

Region: NODE\_318280\_length\_3803\_cov\_29.618460 1492-1499. Max. coverage (+): 0.04. Max coverage (-): 0.3

Region: NODE\_318280\_length\_3803\_cov\_29.618460 1500-1506. Max. coverage (+): 0. Max coverage (-): 0.26

Region: NODE\_318280\_length\_3803\_cov\_29.618460 1507-1514. Max. coverage (+): 0.04. Max coverage (-): 0.19

Region: NODE\_318280\_length\_3803\_cov\_29.618460 1515-1521. Max. coverage (+): 0.7. Max coverage (-): 0

Region: NODE\_318280\_length\_3803\_cov\_29.618460 1522-1529. Max. coverage (+): 1.04. Max coverage (-): 0.07

Region: NODE\_318280\_length\_3803\_cov\_29.618460 1530-1537. Max. coverage (+): 0.78. Max coverage (-): 0.07

Region: NODE\_318280\_length\_3803\_cov\_29.618460 1538-1544. Max. coverage (+): 0.04. Max coverage (-): 0

Region: NODE\_318280\_length\_3803\_cov\_29.618460 1545-1552. Max. coverage (+): 0. Max coverage (-): 0

Region: NODE\_318280\_length\_3803\_cov\_29.618460 1553-1559. Max. coverage (+): 0.04. Max coverage (-): 0.04

Region: NODE\_318280\_length\_3803\_cov\_29.618460 1560-1567. Max. coverage (+): 0.04. Max coverage (-): 0

Region: NODE\_318280\_length\_3803\_cov\_29.618460 1568-1575. Max. coverage (+): 0.04. Max coverage (-): 0.04

Region: NODE\_318280\_length\_3803\_cov\_29.618460 1576-1582. Max. coverage (+): 0.04. Max coverage (-): 0.04

Region: NODE\_318280\_length\_3803\_cov\_29.618460 1583-1590. Max. coverage (+): 0.07. Max coverage (-): 0.07

Region: NODE\_318280\_length\_3803\_cov\_29.618460 1591-1597. Max. coverage (+): 0. Max coverage (-): 0.26

Region: NODE\_318280\_length\_3803\_cov\_29.618460 1598-1605. Max. coverage (+): 0.04. Max coverage (-): 0.37

Region: NODE\_318280\_length\_3803\_cov\_29.618460 1606-1612. Max. coverage (+): 0.04. Max coverage (-): 0.59

Region: NODE\_318280\_length\_3803\_cov\_29.618460 1613-1620. Max. coverage (+): 0.04. Max coverage (-): 0.26

Region: NODE\_318280\_length\_3803\_cov\_29.618460 1621-1628. Max. coverage (+): 0.04. Max coverage (-): 0.19

Region: NODE\_318280\_length\_3803\_cov\_29.618460 1629-1635. Max. coverage (+): 0. Max coverage (-): 0.11

Region: NODE\_318280\_length\_3803\_cov\_29.618460 1636-1643. Max. coverage (+): 0. Max coverage (-): 0.22

Region: NODE\_318280\_length\_3803\_cov\_29.618460 1644-1650. Max. coverage (+): 0.04. Max coverage (-): 0.15

Region: NODE\_318280\_length\_3803\_cov\_29.618460 1651-1658. Max. coverage (+): 0.07. Max coverage (-): 0.11

Region: NODE\_318280\_length\_3803\_cov\_29.618460 1659-1665. Max. coverage (+): 1.19. Max coverage (-): 0.04

Region: NODE\_318280\_length\_3803\_cov\_29.618460 1666-1673. Max. coverage (+): 0.04. Max coverage (-): 0.04

Region: NODE\_318280\_length\_3803\_cov\_29.618460 1674-1681. Max. coverage (+): 0.07. Max coverage (-): 0.07

Region: NODE\_318280\_length\_3803\_cov\_29.618460 1682-1688. Max. coverage (+): 0. Max coverage (-): 0

Region: NODE\_318280\_length\_3803\_cov\_29.618460 1689-1696. Max. coverage (+): 0. Max coverage (-): 0.56

Region: NODE\_318280\_length\_3803\_cov\_29.618460 1697-1703. Max. coverage (+): 0. Max coverage (-): 0.85

Region: NODE\_318280\_length\_3803\_cov\_29.618460 1704-1711. Max. coverage (+): 0.52. Max coverage (-): 0.11

Region: NODE\_318280\_length\_3803\_cov\_29.618460 1712-1718. Max. coverage (+): 0.52. Max coverage (-): 0.07

Region: NODE\_318280\_length\_3803\_cov\_29.618460 1719-1726. Max. coverage (+): 0. Max coverage (-): 0.07

Region: NODE\_318280\_length\_3803\_cov\_29.618460 1727-1734. Max. coverage (+): 0. Max coverage (-): 3.11

Region: NODE\_318280\_length\_3803\_cov\_29.618460 1735-1741. Max. coverage (+): 0. Max coverage (-): 0.56

Region: NODE\_318280\_length\_3803\_cov\_29.618460 1742-1749. Max. coverage (+): 0.33. Max coverage (-): 0.04

Region: NODE\_318280\_length\_3803\_cov\_29.618460 1750-1756. Max. coverage (+): 0.33. Max coverage (-): 0.04

Region: NODE\_318280\_length\_3803\_cov\_29.618460 1757-1764. Max. coverage (+): 0. Max coverage (-): 0.15

Region: NODE\_318280\_length\_3803\_cov\_29.618460 1765-1771. Max. coverage (+): 0. Max coverage (-): 0.89

Region: NODE\_318280\_length\_3803\_cov\_29.618460 1772-1779. Max. coverage (+): 0.04. Max coverage (-): 0.3

Region: NODE\_318280\_length\_3803\_cov\_29.618460 1780-1787. Max. coverage (+): 0.07. Max coverage (-): 0.04

Region: NODE\_318280\_length\_3803\_cov\_29.618460 1788-1794. Max. coverage (+): 0.11. Max coverage (-): 0.07

Region: NODE\_318280\_length\_3803\_cov\_29.618460 1795-1802. Max. coverage (+): 0. Max coverage (-): 0.04

Region: NODE\_318280\_length\_3803\_cov\_29.618460 1803-1809. Max. coverage (+): 0. Max coverage (-): 1.08

Region: NODE\_318280\_length\_3803\_cov\_29.618460 1810-1817. Max. coverage (+): 0. Max coverage (-): 0

Region: NODE\_318280\_length\_3803\_cov\_29.618460 1818-1825. Max. coverage (+): 0. Max coverage (-): 0.04

Region: NODE\_318280\_length\_3803\_cov\_29.618460 1826-1832. Max. coverage (+): 0. Max coverage (-): 0.37

Region: NODE\_318280\_length\_3803\_cov\_29.618460 1833-1840. Max. coverage (+): 0. Max coverage (-): 0.15

Region: NODE\_318280\_length\_3803\_cov\_29.618460 1841-1847. Max. coverage (+): 0. Max coverage (-): 0.11

Region: NODE\_318280\_length\_3803\_cov\_29.618460 1848-1855. Max. coverage (+): 0.04. Max coverage (-): 0.07

Region: NODE\_318280\_length\_3803\_cov\_29.618460 1856-1862. Max. coverage (+): 0. Max coverage (-): 0.11

Region: NODE\_318280\_length\_3803\_cov\_29.618460 1863-1870. Max. coverage (+): 0. Max coverage (-): 1.3

Region: NODE\_318280\_length\_3803\_cov\_29.618460 1871-1878. Max. coverage (+): 0. Max coverage (-): 0.11

Region: NODE\_318280\_length\_3803\_cov\_29.618460 1879-1885. Max. coverage (+): 0. Max coverage (-): 0

Region: NODE\_318280\_length\_3803\_cov\_29.618460 1886-1893. Max. coverage (+): 0. Max coverage (-): 0.19

Region: NODE\_318280\_length\_3803\_cov\_29.618460 1894-1900. Max. coverage (+): 0. Max coverage (-): 0.52

Region: NODE\_318280\_length\_3803\_cov\_29.618460 1901-1908. Max. coverage (+): 0. Max coverage (-): 0

Region: NODE\_318280\_length\_3803\_cov\_29.618460 1909-1915. Max. coverage (+): 0.11. Max coverage (-): 0.04

Region: NODE\_318280\_length\_3803\_cov\_29.618460 1916-1923. Max. coverage (+): 0.11. Max coverage (-): 0

Region: NODE\_318280\_length\_3803\_cov\_29.618460 1924-1931. Max. coverage (+): 0. Max coverage (-): 0.11

Region: NODE\_318280\_length\_3803\_cov\_29.618460 1932-1938. Max. coverage (+): 0.04. Max coverage (-): 0.15

Region: NODE\_318280\_length\_3803\_cov\_29.618460 1939-1946. Max. coverage (+): 0.04. Max coverage (-): 0.04

Region: NODE\_318280\_length\_3803\_cov\_29.618460 1947-1953. Max. coverage (+): 0.04. Max coverage (-): 0.07

Region: NODE\_318280\_length\_3803\_cov\_29.618460 1954-1961. Max. coverage (+): 0. Max coverage (-): 0.19

Region: NODE\_318280\_length\_3803\_cov\_29.618460 1962-1968. Max. coverage (+): 0. Max coverage (-): 0.04

Region: NODE\_318280\_length\_3803\_cov\_29.618460 1969-1976. Max. coverage (+): 0. Max coverage (-): 0.07

Region: NODE\_318280\_length\_3803\_cov\_29.618460 1977-1984. Max. coverage (+): 0. Max coverage (-): 0.07

Region: NODE\_318280\_length\_3803\_cov\_29.618460 1985-1991. Max. coverage (+): 0. Max coverage (-): 0.04

Region: NODE\_318280\_length\_3803\_cov\_29.618460 1992-1999. Max. coverage (+): 0. Max coverage (-): 0.04

Region: NODE\_318280\_length\_3803\_cov\_29.618460 2000-2006. Max. coverage (+): 0. Max coverage (-): 0.3

Region: NODE\_318280\_length\_3803\_cov\_29.618460 2007-2014. Max. coverage (+): 0. Max coverage (-): 0.3

Region: NODE\_318280\_length\_3803\_cov\_29.618460 2015-2022. Max. coverage (+): 0.07. Max coverage (-): 0.11

Region: NODE\_318280\_length\_3803\_cov\_29.618460 2023-2029. Max. coverage (+): 0.04. Max coverage (-): 0.07

Region: NODE\_318280\_length\_3803\_cov\_29.618460 2030-2037. Max. coverage (+): 0.01. Max coverage (-): 0.01

Region: NODE\_318280\_length\_3803\_cov\_29.618460 2038-2044. Max. coverage (+): 0.01. Max coverage (-): 0

Region: NODE\_318280\_length\_3803\_cov\_29.618460 2045-2052. Max. coverage (+): 0. Max coverage (-): 0

Region: NODE\_318280\_length\_3803\_cov\_29.618460 2053-2059. Max. coverage (+): 0. Max coverage (-): 0.03

Region: NODE\_318280\_length\_3803\_cov\_29.618460 2060-2067. Max. coverage (+): 0. Max coverage (-): 0.05

Region: NODE\_318280\_length\_3803\_cov\_29.618460 2068-2075. Max. coverage (+): 0. Max coverage (-): 0.01

Region: NODE\_318280\_length\_3803\_cov\_29.618460 2076-2082. Max. coverage (+): 0.05. Max coverage (-): 0.01

Region: NODE\_318280\_length\_3803\_cov\_29.618460 2083-2090. Max. coverage (+): 0.03. Max coverage (-): 0

Region: NODE\_318280\_length\_3803\_cov\_29.618460 2091-2097. Max. coverage (+): 0.01. Max coverage (-): 0.01

Region: NODE\_318280\_length\_3803\_cov\_29.618460 2098-2105. Max. coverage (+): 0.01. Max coverage (-): 0.21

Region: NODE\_318280\_length\_3803\_cov\_29.618460 2106-2112. Max. coverage (+): 0. Max coverage (-): 0.34

Region: NODE\_318280\_length\_3803\_cov\_29.618460 2113-2120. Max. coverage (+): 0.02. Max coverage (-): 0.05

Region: NODE\_318280\_length\_3803\_cov\_29.618460 2121-2128. Max. coverage (+): 0. Max coverage (-): 0

Region: NODE\_318280\_length\_3803\_cov\_29.618460 2129-2135. Max. coverage (+): 0. Max coverage (-): 0

Region: NODE\_318280\_length\_3803\_cov\_29.618460 2136-2143. Max. coverage (+): 0. Max coverage (-): 0

Region: NODE\_318280\_length\_3803\_cov\_29.618460 2144-2150. Max. coverage (+): 0. Max coverage (-): 0

Region: NODE\_318280\_length\_3803\_cov\_29.618460 2151-2158. Max. coverage (+): 0. Max coverage (-): 3.26

Region: NODE\_318280\_length\_3803\_cov\_29.618460 2159-2165. Max. coverage (+): 0. Max coverage (-): 12.38

Region: NODE\_318280\_length\_3803\_cov\_29.618460 2166-2173. Max. coverage (+): 0.01. Max coverage (-): 5.49

Region: NODE\_318280\_length\_3803\_cov\_29.618460 2174-2181. Max. coverage (+): 0.12. Max coverage (-): 0.01

Region: NODE\_318280\_length\_3803\_cov\_29.618460 2182-2188. Max. coverage (+): 0.12. Max coverage (-): 0.02

Region: NODE\_318280\_length\_3803\_cov\_29.618460 2189-2196. Max. coverage (+): 0.01. Max coverage (-): 0.07

Region: NODE\_318280\_length\_3803\_cov\_29.618460 2197-2203. Max. coverage (+): 0.01. Max coverage (-): 0.02

Region: NODE\_318280\_length\_3803\_cov\_29.618460 2204-2211. Max. coverage (+): 0. Max coverage (-): 0

Region: NODE\_318280\_length\_3803\_cov\_29.618460 2212-2218. Max. coverage (+): 0. Max coverage (-): 0.04

Region: NODE\_318280\_length\_3803\_cov\_29.618460 2219-2226. Max. coverage (+): 0. Max coverage (-): 0.26

Region: NODE\_318280\_length\_3803\_cov\_29.618460 2227-2234. Max. coverage (+): 0.04. Max coverage (-): 0.19

Region: NODE\_318280\_length\_3803\_cov\_29.618460 2235-2241. Max. coverage (+): 0.07. Max coverage (-): 0.01

Region: NODE\_318280\_length\_3803\_cov\_29.618460 2242-2249. Max. coverage (+): 0.01. Max coverage (-): 0.01

Region: NODE\_318280\_length\_3803\_cov\_29.618460 2250-2256. Max. coverage (+): 0. Max coverage (-): 0.07

Region: NODE\_318280\_length\_3803\_cov\_29.618460 2257-2264. Max. coverage (+): 0.04. Max coverage (-): 0.11

Region: NODE\_318280\_length\_3803\_cov\_29.618460 2265-2272. Max. coverage (+): 0.07. Max coverage (-): 0.07

Region: NODE\_318280\_length\_3803\_cov\_29.618460 2273-2279. Max. coverage (+): 0.04. Max coverage (-): 0.15

Region: NODE\_318280\_length\_3803\_cov\_29.618460 2280-2287. Max. coverage (+): 0.04. Max coverage (-): 0.3

Region: NODE\_318280\_length\_3803\_cov\_29.618460 2288-2294. Max. coverage (+): 0.3. Max coverage (-): 0.19

Region: NODE\_318280\_length\_3803\_cov\_29.618460 2295-2302. Max. coverage (+): 0.11. Max coverage (-): 0

Region: NODE\_318280\_length\_3803\_cov\_29.618460 2303-2309. Max. coverage (+): 0.07. Max coverage (-): 0.12

Region: NODE\_318280\_length\_3803\_cov\_29.618460 2310-2317. Max. coverage (+): 0.02. Max coverage (-): 0.33

Region: NODE\_318280\_length\_3803\_cov\_29.618460 2318-2325. Max. coverage (+): 0.04. Max coverage (-): 0.1

Region: NODE\_318280\_length\_3803\_cov\_29.618460 2326-2332. Max. coverage (+): 0.05. Max coverage (-): 0.04

Region: NODE\_318280\_length\_3803\_cov\_29.618460 2333-2340. Max. coverage (+): 0.02. Max coverage (-): 0.02

Region: NODE\_318280\_length\_3803\_cov\_29.618460 2341-2347. Max. coverage (+): 0.04. Max coverage (-): 0.34

Region: NODE\_318280\_length\_3803\_cov\_29.618460 2348-2355. Max. coverage (+): 0.03. Max coverage (-): 0.23

Region: NODE\_318280\_length\_3803\_cov\_29.618460 2356-2362. Max. coverage (+): 0.02. Max coverage (-): 0.03

Region: NODE\_318280\_length\_3803\_cov\_29.618460 2363-2370. Max. coverage (+): 0.03. Max coverage (-): 0.04

Region: NODE\_318280\_length\_3803\_cov\_29.618460 2371-2378. Max. coverage (+): 0.24. Max coverage (-): 0

Region: NODE\_318280\_length\_3803\_cov\_29.618460 2379-2385. Max. coverage (+): 0.01. Max coverage (-): 0.01

Region: NODE\_318280\_length\_3803\_cov\_29.618460 2386-2393. Max. coverage (+): 0. Max coverage (-): 0.04

Region: NODE\_318280\_length\_3803\_cov\_29.618460 2394-2400. Max. coverage (+): 0. Max coverage (-): 0.04

Region: NODE\_318280\_length\_3803\_cov\_29.618460 2401-2408. Max. coverage (+): 0.01. Max coverage (-): 0

Region: NODE\_318280\_length\_3803\_cov\_29.618460 2409-2415. Max. coverage (+): 0.04. Max coverage (-): 0.01

Region: NODE\_318280\_length\_3803\_cov\_29.618460 2416-2423. Max. coverage (+): 0.01. Max coverage (-): 0.01

Region: NODE\_318280\_length\_3803\_cov\_29.618460 2424-2431. Max. coverage (+): 0. Max coverage (-): 0.01

Region: NODE\_318280\_length\_3803\_cov\_29.618460 2432-2438. Max. coverage (+): 0. Max coverage (-): 0

Region: NODE\_318280\_length\_3803\_cov\_29.618460 2439-2446. Max. coverage (+): 0. Max coverage (-): 0.04

Region: NODE\_318280\_length\_3803\_cov\_29.618460 2447-2453. Max. coverage (+): 0. Max coverage (-): 0.04

Region: NODE\_318280\_length\_3803\_cov\_29.618460 2454-2461. Max. coverage (+): 0. Max coverage (-): 0.03

Region: NODE\_318280\_length\_3803\_cov\_29.618460 2462-2468. Max. coverage (+): 0. Max coverage (-): 0.07

Region: NODE\_318280\_length\_3803\_cov\_29.618460 2469-2476. Max. coverage (+): 0. Max coverage (-): 0.03

Region: NODE\_318280\_length\_3803\_cov\_29.618460 2477-2484. Max. coverage (+): 0.01. Max coverage (-): 0.06

Region: NODE\_318280\_length\_3803\_cov\_29.618460 2485-2491. Max. coverage (+): 0.01. Max coverage (-): 0.01

Region: NODE\_318280\_length\_3803\_cov\_29.618460 2492-2499. Max. coverage (+): 0. Max coverage (-): 0.05

Region: NODE\_318280\_length\_3803\_cov\_29.618460 2500-2506. Max. coverage (+): 0. Max coverage (-): 0.03

Region: NODE\_318280\_length\_3803\_cov\_29.618460 2507-2514. Max. coverage (+): 0.07. Max coverage (-): 0

Region: NODE\_318280\_length\_3803\_cov\_29.618460 2515-2522. Max. coverage (+): 0.07. Max coverage (-): 0

Region: NODE\_318280\_length\_3803\_cov\_29.618460 2523-2529. Max. coverage (+): 0. Max coverage (-): 0.33

Region: NODE\_318280\_length\_3803\_cov\_29.618460 2530-2537. Max. coverage (+): 0. Max coverage (-): 0.37

Region: NODE\_318280\_length\_3803\_cov\_29.618460 2538-2544. Max. coverage (+): 0.1. Max coverage (-): 0.08

Region: NODE\_318280\_length\_3803\_cov\_29.618460 2545-2552. Max. coverage (+): 1.08. Max coverage (-): 0.41

Region: NODE\_318280\_length\_3803\_cov\_29.618460 2553-2559. Max. coverage (+): 0.74. Max coverage (-): 0

Region: NODE\_318280\_length\_3803\_cov\_29.618460 2560-2567. Max. coverage (+): 0. Max coverage (-): 0

Region: NODE\_318280\_length\_3803\_cov\_29.618460 2568-2575. Max. coverage (+): 0. Max coverage (-): 0.22

Region: NODE\_318280\_length\_3803\_cov\_29.618460 2576-2582. Max. coverage (+): 0. Max coverage (-): 0.22

Region: NODE\_318280\_length\_3803\_cov\_29.618460 2583-2590. Max. coverage (+): 0.33. Max coverage (-): 0.19

Region: NODE\_318280\_length\_3803\_cov\_29.618460 2591-2597. Max. coverage (+): 0.3. Max coverage (-): 0.07

Region: NODE\_318280\_length\_3803\_cov\_29.618460 2598-2605. Max. coverage (+): 1.61. Max coverage (-): 0

Region: NODE\_318280\_length\_3803\_cov\_29.618460 2606-2612. Max. coverage (+): 0. Max coverage (-): 0.07

Region: NODE\_318280\_length\_3803\_cov\_29.618460 2613-2620. Max. coverage (+): 0.04. Max coverage (-): 0.11

Region: NODE\_318280\_length\_3803\_cov\_29.618460 2621-2628. Max. coverage (+): 0.04. Max coverage (-): 0.04

Region: NODE\_318280\_length\_3803\_cov\_29.618460 2629-2635. Max. coverage (+): 0.15. Max coverage (-): 0.07

Region: NODE\_318280\_length\_3803\_cov\_29.618460 2636-2643. Max. coverage (+): 0.04. Max coverage (-): 0.04

Region: NODE\_318280\_length\_3803\_cov\_29.618460 2644-2650. Max. coverage (+): 0.04. Max coverage (-): 0.67

Region: NODE\_318280\_length\_3803\_cov\_29.618460 2651-2658. Max. coverage (+): 0.04. Max coverage (-): 2.52

Region: NODE\_318280\_length\_3803\_cov\_29.618460 2659-2665. Max. coverage (+): 0.11. Max coverage (-): 0.63

Region: NODE\_318280\_length\_3803\_cov\_29.618460 2666-2673. Max. coverage (+): 0.22. Max coverage (-): 0.11

Region: NODE\_318280\_length\_3803\_cov\_29.618460 2674-2681. Max. coverage (+): 0. Max coverage (-): 0.19

Region: NODE\_318280\_length\_3803\_cov\_29.618460 2682-2688. Max. coverage (+): 0. Max coverage (-): 1.85

Region: NODE\_318280\_length\_3803\_cov\_29.618460 2689-2696. Max. coverage (+): 0.04. Max coverage (-): 1.85

Region: NODE\_318280\_length\_3803\_cov\_29.618460 2697-2703. Max. coverage (+): 0. Max coverage (-): 0.11

Region: NODE\_318280\_length\_3803\_cov\_29.618460 2704-2711. Max. coverage (+): 0. Max coverage (-): 0

Region: NODE\_318280\_length\_3803\_cov\_29.618460 2712-2718. Max. coverage (+): 0. Max coverage (-): 0

Region: NODE\_318280\_length\_3803\_cov\_29.618460 2719-2726. Max. coverage (+): 0.15. Max coverage (-): 0.19

Region: NODE\_318280\_length\_3803\_cov\_29.618460 2727-2734. Max. coverage (+): 0.11. Max coverage (-): 0.22

Region: NODE\_318280\_length\_3803\_cov\_29.618460 2735-2741. Max. coverage (+): 0.82. Max coverage (-): 0.19

Region: NODE\_318280\_length\_3803\_cov\_29.618460 2742-2749. Max. coverage (+): 0.74. Max coverage (-): 0.07

Region: NODE\_318280\_length\_3803\_cov\_29.618460 2750-2756. Max. coverage (+): 0. Max coverage (-): 0.41

Region: NODE\_318280\_length\_3803\_cov\_29.618460 2757-2764. Max. coverage (+): 0. Max coverage (-): 0.41

Region: NODE\_318280\_length\_3803\_cov\_29.618460 2765-2772. Max. coverage (+): 0.04. Max coverage (-): 0.04

Region: NODE\_318280\_length\_3803\_cov\_29.618460 2773-2779. Max. coverage (+): 0.07. Max coverage (-): 0.07

Region: NODE\_318280\_length\_3803\_cov\_29.618460 2780-2787. Max. coverage (+): 0. Max coverage (-): 0.11

Region: NODE\_318280\_length\_3803\_cov\_29.618460 2788-2794. Max. coverage (+): 0.04. Max coverage (-): 0.15

Region: NODE\_318280\_length\_3803\_cov\_29.618460 2795-2802. Max. coverage (+): 0. Max coverage (-): 0.78

Region: NODE\_318280\_length\_3803\_cov\_29.618460 2803-2809. Max. coverage (+): 0.04. Max coverage (-): 0.15

Region: NODE\_318280\_length\_3803\_cov\_29.618460 2810-2817. Max. coverage (+): 1.74. Max coverage (-): 0.04

Region: NODE\_318280\_length\_3803\_cov\_29.618460 2818-2825. Max. coverage (+): 1.04. Max coverage (-): 0

Region: NODE\_318280\_length\_3803\_cov\_29.618460 2826-2832. Max. coverage (+): 0. Max coverage (-): 2.34

Region: NODE\_318280\_length\_3803\_cov\_29.618460 2833-2840. Max. coverage (+): 0. Max coverage (-): 0.82

Region: NODE\_318280\_length\_3803\_cov\_29.618460 2841-2847. Max. coverage (+): 0.04. Max coverage (-): 0

Region: NODE\_318280\_length\_3803\_cov\_29.618460 2848-2855. Max. coverage (+): 0. Max coverage (-): 0

Region: NODE\_318280\_length\_3803\_cov\_29.618460 2856-2862. Max. coverage (+): 0. Max coverage (-): 0.07

Region: NODE\_318280\_length\_3803\_cov\_29.618460 2863-2870. Max. coverage (+): 0. Max coverage (-): 0.19

Region: NODE\_318280\_length\_3803\_cov\_29.618460 2871-2878. Max. coverage (+): 0. Max coverage (-): 0.04

Region: NODE\_318280\_length\_3803\_cov\_29.618460 2879-2885. Max. coverage (+): 0. Max coverage (-): 0

Region: NODE\_318280\_length\_3803\_cov\_29.618460 2886-2893. Max. coverage (+): 0. Max coverage (-): 0

Region: NODE\_318280\_length\_3803\_cov\_29.618460 2894-2900. Max. coverage (+): 0. Max coverage (-): 0.01

Region: NODE\_318280\_length\_3803\_cov\_29.618460 2901-2908. Max. coverage (+): 0.01. Max coverage (-): 0.01

Region: NODE\_318280\_length\_3803\_cov\_29.618460 2909-2915. Max. coverage (+): 0. Max coverage (-): 0

Region: NODE\_318280\_length\_3803\_cov\_29.618460 2916-2923. Max. coverage (+): 0.01. Max coverage (-): 0

Region: NODE\_318280\_length\_3803\_cov\_29.618460 2924-2931. Max. coverage (+): 0. Max coverage (-): 0.02

Region: NODE\_318280\_length\_3803\_cov\_29.618460 2932-2938. Max. coverage (+): 0. Max coverage (-): 0

Region: NODE\_318280\_length\_3803\_cov\_29.618460 2939-2946. Max. coverage (+): 0.19. Max coverage (-): 0

Region: NODE\_318280\_length\_3803\_cov\_29.618460 2947-2953. Max. coverage (+): 0.09. Max coverage (-): 0.09

Region: NODE\_318280\_length\_3803\_cov\_29.618460 2954-2961. Max. coverage (+): 0.02. Max coverage (-): 2.25

Region: NODE\_318280\_length\_3803\_cov\_29.618460 2962-2969. Max. coverage (+): 0.06. Max coverage (-): 2.25

Region: NODE\_318280\_length\_3803\_cov\_29.618460 2970-2976. Max. coverage (+): 0. Max coverage (-): 0

Region: NODE\_318280\_length\_3803\_cov\_29.618460 2977-2984. Max. coverage (+): 0. Max coverage (-): 0

Region: NODE\_318280\_length\_3803\_cov\_29.618460 2985-2991. Max. coverage (+): 0. Max coverage (-): 0.05

Region: NODE\_318280\_length\_3803\_cov\_29.618460 2992-2999. Max. coverage (+): 0.06. Max coverage (-): 0.28

Region: NODE\_318280\_length\_3803\_cov\_29.618460 3000-3006. Max. coverage (+): 0.07. Max coverage (-): 0.04

Region: NODE\_318280\_length\_3803\_cov\_29.618460 3007-3014. Max. coverage (+): 0.11. Max coverage (-): 0.11

Region: NODE\_318280\_length\_3803\_cov\_29.618460 3015-3022. Max. coverage (+): 0.07. Max coverage (-): 0.11

Region: NODE\_318280\_length\_3803\_cov\_29.618460 3023-3029. Max. coverage (+): 0. Max coverage (-): 0

Region: NODE\_318280\_length\_3803\_cov\_29.618460 3030-3037. Max. coverage (+): 0. Max coverage (-): 0.44

Region: NODE\_318280\_length\_3803\_cov\_29.618460 3038-3044. Max. coverage (+): 0. Max coverage (-): 1.33

Region: NODE\_318280\_length\_3803\_cov\_29.618460 3045-3052. Max. coverage (+): 0. Max coverage (-): 0.07

Region: NODE\_318280\_length\_3803\_cov\_29.618460 3053-3059. Max. coverage (+): 0. Max coverage (-): 0.07

Region: NODE\_318280\_length\_3803\_cov\_29.618460 3060-3067. Max. coverage (+): 0.07. Max coverage (-): 0.15

Region: NODE\_318280\_length\_3803\_cov\_29.618460 3068-3075. Max. coverage (+): 0.11. Max coverage (-): 0.15

Region: NODE\_318280\_length\_3803\_cov\_29.618460 3076-3082. Max. coverage (+): 0.11. Max coverage (-): 0.85

Region: NODE\_318280\_length\_3803\_cov\_29.618460 3083-3090. Max. coverage (+): 0.11. Max coverage (-): 0.83

Region: NODE\_318280\_length\_3803\_cov\_29.618460 3091-3097. Max. coverage (+): 0.04. Max coverage (-): 0.48

Region: NODE\_318280\_length\_3803\_cov\_29.618460 3098-3105. Max. coverage (+): 0. Max coverage (-): 0.67

Region: NODE\_318280\_length\_3803\_cov\_29.618460 3106-3112. Max. coverage (+): 0. Max coverage (-): 0.11

Region: NODE\_318280\_length\_3803\_cov\_29.618460 3113-3120. Max. coverage (+): 0.04. Max coverage (-): 0.05

Region: NODE\_318280\_length\_3803\_cov\_29.618460 3121-3128. Max. coverage (+): 1.71. Max coverage (-): 0.11

Region: NODE\_318280\_length\_3803\_cov\_29.618460 3129-3135. Max. coverage (+): 1.89. Max coverage (-): 0.11

Region: NODE\_318280\_length\_3803\_cov\_29.618460 3136-3143. Max. coverage (+): 0.11. Max coverage (-): 0

Region: NODE\_318280\_length\_3803\_cov\_29.618460 3144-3150. Max. coverage (+): 0. Max coverage (-): 0.04

Region: NODE\_318280\_length\_3803\_cov\_29.618460 3151-3158. Max. coverage (+): 0. Max coverage (-): 1.08

Region: NODE\_318280\_length\_3803\_cov\_29.618460 3159-3165. Max. coverage (+): 0. Max coverage (-): 3.67

Region: NODE\_318280\_length\_3803\_cov\_29.618460 3166-3173. Max. coverage (+): 0. Max coverage (-): 0.19

Region: NODE\_318280\_length\_3803\_cov\_29.618460 3174-3181. Max. coverage (+): 0.04. Max coverage (-): 0.26

Region: NODE\_318280\_length\_3803\_cov\_29.618460 3182-3188. Max. coverage (+): 0.04. Max coverage (-): 0.19

Region: NODE\_318280\_length\_3803\_cov\_29.618460 3189-3196. Max. coverage (+): 0. Max coverage (-): 0.04

Region: NODE\_318280\_length\_3803\_cov\_29.618460 3197-3203. Max. coverage (+): 0. Max coverage (-): 0.04

Region: NODE\_318280\_length\_3803\_cov\_29.618460 3204-3211. Max. coverage (+): 0. Max coverage (-): 0.22

Region: NODE\_318280\_length\_3803\_cov\_29.618460 3212-3219. Max. coverage (+): 0. Max coverage (-): 0.04

Region: NODE\_318280\_length\_3803\_cov\_29.618460 3220-3226. Max. coverage (+): 0.37. Max coverage (-): 0.19

Region: NODE\_318280\_length\_3803\_cov\_29.618460 3227-3234. Max. coverage (+): 0.37. Max coverage (-): 0.26

Region: NODE\_318280\_length\_3803\_cov\_29.618460 3235-3241. Max. coverage (+): 0.07. Max coverage (-): 1.67

Region: NODE\_318280\_length\_3803\_cov\_29.618460 3242-3249. Max. coverage (+): 0.07. Max coverage (-): 0.44

Region: NODE\_318280\_length\_3803\_cov\_29.618460 3250-3256. Max. coverage (+): 0.15. Max coverage (-): 0.04

Region: NODE\_318280\_length\_3803\_cov\_29.618460 3257-3264. Max. coverage (+): 0.07. Max coverage (-): 0.15

Region: NODE\_318280\_length\_3803\_cov\_29.618460 3265-3272. Max. coverage (+): 0.04. Max coverage (-): 0.63

Region: NODE\_318280\_length\_3803\_cov\_29.618460 3273-3279. Max. coverage (+): 0.26. Max coverage (-): 0.67

Region: NODE\_318280\_length\_3803\_cov\_29.618460 3280-3287. Max. coverage (+): 0.19. Max coverage (-): 0.07

Region: NODE\_318280\_length\_3803\_cov\_29.618460 3288-3294. Max. coverage (+): 0.15. Max coverage (-): 0.11

Region: NODE\_318280\_length\_3803\_cov\_29.618460 3295-3302. Max. coverage (+): 0.04. Max coverage (-): 0.19

Region: NODE\_318280\_length\_3803\_cov\_29.618460 3303-3309. Max. coverage (+): 0.07. Max coverage (-): 0.15

Region: NODE\_318280\_length\_3803\_cov\_29.618460 3310-3317. Max. coverage (+): 2.04. Max coverage (-): 0

Region: NODE\_318280\_length\_3803\_cov\_29.618460 3318-3325. Max. coverage (+): 1.89. Max coverage (-): 0.07

Region: NODE\_318280\_length\_3803\_cov\_29.618460 3326-3332. Max. coverage (+): 0.04. Max coverage (-): 0.3

Region: NODE\_318280\_length\_3803\_cov\_29.618460 3333-3340. Max. coverage (+): 0. Max coverage (-): 0.22

Region: NODE\_318280\_length\_3803\_cov\_29.618460 3341-3347. Max. coverage (+): 0.07. Max coverage (-): 0.41

Region: NODE\_318280\_length\_3803\_cov\_29.618460 3348-3355. Max. coverage (+): 0.07. Max coverage (-): 0.04

Region: NODE\_318280\_length\_3803\_cov\_29.618460 3356-3362. Max. coverage (+): 0.04. Max coverage (-): 0.11

Region: NODE\_318280\_length\_3803\_cov\_29.618460 3363-3370. Max. coverage (+): 0.04. Max coverage (-): 0.04

Region: NODE\_318280\_length\_3803\_cov\_29.618460 3371-3378. Max. coverage (+): 0. Max coverage (-): 4.49

Region: NODE\_318280\_length\_3803\_cov\_29.618460 3379-3385. Max. coverage (+): 0.04. Max coverage (-): 4.52

Region: NODE\_318280\_length\_3803\_cov\_29.618460 3386-3393. Max. coverage (+): 0.04. Max coverage (-): 0.67

Region: NODE\_318280\_length\_3803\_cov\_29.618460 3394-3400. Max. coverage (+): 0. Max coverage (-): 0.11

Region: NODE\_318280\_length\_3803\_cov\_29.618460 3401-3408. Max. coverage (+): 0. Max coverage (-): 0.07

Region: NODE\_318280\_length\_3803\_cov\_29.618460 3409-3415. Max. coverage (+): 0. Max coverage (-): 0.22

Region: NODE\_318280\_length\_3803\_cov\_29.618460 3416-3423. Max. coverage (+): 0.07. Max coverage (-): 0.15

Region: NODE\_318280\_length\_3803\_cov\_29.618460 3424-3431. Max. coverage (+): 0.11. Max coverage (-): 0

Region: NODE\_318280\_length\_3803\_cov\_29.618460 3432-3438. Max. coverage (+): 0.59. Max coverage (-): 0.19

Region: NODE\_318280\_length\_3803\_cov\_29.618460 3439-3446. Max. coverage (+): 0.59. Max coverage (-): 0.04

Region: NODE\_318280\_length\_3803\_cov\_29.618460 3447-3453. Max. coverage (+): 0.07. Max coverage (-): 0

Region: NODE\_318280\_length\_3803\_cov\_29.618460 3454-3461. Max. coverage (+): 0.74. Max coverage (-): 0

Region: NODE\_318280\_length\_3803\_cov\_29.618460 3462-3469. Max. coverage (+): 0.07. Max coverage (-): 0.04

Region: NODE\_318280\_length\_3803\_cov\_29.618460 3470-3476. Max. coverage (+): 0.22. Max coverage (-): 0.15

Region: NODE\_318280\_length\_3803\_cov\_29.618460 3477-3484. Max. coverage (+): 0.15. Max coverage (-): 0.37

Region: NODE\_318280\_length\_3803\_cov\_29.618460 3485-3491. Max. coverage (+): 0.07. Max coverage (-): 0.41

Region: NODE\_318280\_length\_3803\_cov\_29.618460 3492-3499. Max. coverage (+): 0.07. Max coverage (-): 0.26

Region: NODE\_318280\_length\_3803\_cov\_29.618460 3500-3506. Max. coverage (+): 0.07. Max coverage (-): 0.33

Region: NODE\_318280\_length\_3803\_cov\_29.618460 3507-3514. Max. coverage (+): 0. Max coverage (-): 0.11

Region: NODE\_318280\_length\_3803\_cov\_29.618460 3515-3522. Max. coverage (+): 0. Max coverage (-): 0.04

Region: NODE\_318280\_length\_3803\_cov\_29.618460 3523-3529. Max. coverage (+): 0. Max coverage (-): 0.04

Region: NODE\_318280\_length\_3803\_cov\_29.618460 3530-3537. Max. coverage (+): 0. Max coverage (-): 0.07

Region: NODE\_318280\_length\_3803\_cov\_29.618460 3538-3544. Max. coverage (+): 0. Max coverage (-): 0.04

Region: NODE\_318280\_length\_3803\_cov\_29.618460 3545-3552. Max. coverage (+): 0. Max coverage (-): 0.04

Region: NODE\_318280\_length\_3803\_cov\_29.618460 3553-3559. Max. coverage (+): 0. Max coverage (-): 0.04

Region: NODE\_318280\_length\_3803\_cov\_29.618460 3560-3567. Max. coverage (+): 0. Max coverage (-): 0.04

Region: NODE\_318280\_length\_3803\_cov\_29.618460 3568-3575. Max. coverage (+): 0. Max coverage (-): 0

Region: NODE\_318280\_length\_3803\_cov\_29.618460 3576-3582. Max. coverage (+): 0. Max coverage (-): 0

Region: NODE\_318280\_length\_3803\_cov\_29.618460 3583-3590. Max. coverage (+): 0. Max coverage (-): 0

Region: NODE\_318280\_length\_3803\_cov\_29.618460 3591-3597. Max. coverage (+): 0. Max coverage (-): 0.15

Region: NODE\_318280\_length\_3803\_cov\_29.618460 3598-3605. Max. coverage (+): 0. Max coverage (-): 0.22

Region: NODE\_318280\_length\_3803\_cov\_29.618460 3606-3612. Max. coverage (+): 0.11. Max coverage (-): 0.04

Region: NODE\_318280\_length\_3803\_cov\_29.618460 3613-3620. Max. coverage (+): 0.04. Max coverage (-): 0

Region: NODE\_318280\_length\_3803\_cov\_29.618460 3621-3628. Max. coverage (+): 0. Max coverage (-): 0.04

Region: NODE\_318280\_length\_3803\_cov\_29.618460 3629-3635. Max. coverage (+): 0. Max coverage (-): 0

Region: NODE\_318280\_length\_3803\_cov\_29.618460 3636-3643. Max. coverage (+): 0. Max coverage (-): 0.04

Region: NODE\_318280\_length\_3803\_cov\_29.618460 3644-3650. Max. coverage (+): 0. Max coverage (-): 0

Region: NODE\_318280\_length\_3803\_cov\_29.618460 3651-3658. Max. coverage (+): 0. Max coverage (-): 0

Region: NODE\_318280\_length\_3803\_cov\_29.618460 3659-3665. Max. coverage (+): 0. Max coverage (-): 0.01

Region: NODE\_318280\_length\_3803\_cov\_29.618460 3666-3673. Max. coverage (+): 0. Max coverage (-): 0.01

Region: NODE\_318280\_length\_3803\_cov\_29.618460 3674-3681. Max. coverage (+): 0. Max coverage (-): 0

Region: NODE\_318280\_length\_3803\_cov\_29.618460 3682-3688. Max. coverage (+): 0. Max coverage (-): 0

Region: NODE\_318280\_length\_3803\_cov\_29.618460 3689-3696. Max. coverage (+): 0.04. Max coverage (-): 0

Region: NODE\_318280\_length\_3803\_cov\_29.618460 3697-3703. Max. coverage (+): 0.04. Max coverage (-): 0

Region: NODE\_318280\_length\_3803\_cov\_29.618460 3704-3711. Max. coverage (+): 0. Max coverage (-): 0

Region: NODE\_318280\_length\_3803\_cov\_29.618460 3712-3719. Max. coverage (+): 0. Max coverage (-): 0

Region: NODE\_318280\_length\_3803\_cov\_29.618460 3720-3726. Max. coverage (+): 0. Max coverage (-): 0

Region: NODE\_318280\_length\_3803\_cov\_29.618460 3727-3734. Max. coverage (+): 0. Max coverage (-): 0

Region: NODE\_318280\_length\_3803\_cov\_29.618460 3735-3741. Max. coverage (+): 0. Max coverage (-): 0.01

Region: NODE\_318280\_length\_3803\_cov\_29.618460 3742-3749. Max. coverage (+): 0. Max coverage (-): 0.01

Region: NODE\_318280\_length\_3803\_cov\_29.618460 3750-3756. Max. coverage (+): 0. Max coverage (-): 0

Region: NODE\_318280\_length\_3803\_cov\_29.618460 3757-3764. Max. coverage (+): 0.01. Max coverage (-): 0

Region: NODE\_318280\_length\_3803\_cov\_29.618460 3765-3772. Max. coverage (+): 0. Max coverage (-): 0

Region: NODE\_318280\_length\_3803\_cov\_29.618460 3773-3779. Max. coverage (+): 0. Max coverage (-): 0

Region: NODE\_318280\_length\_3803\_cov\_29.618460 3780-3787. Max. coverage (+): 0. Max coverage (-): 0

Region: NODE\_318280\_length\_3803\_cov\_29.618460 3788-. Max. coverage (+): 0. Max coverage (-): 0

RepeatMasker Color Code

**+**

100-98% Identity

<98-95% Identity

<95-90% Identity

<90-85% Identity

<85-80% Identity

<80-75% Identity

<75-70% Identity

<70% Identity

**-**

Gene Set Color Code

**+**

Gene

Pseudogene

Other

**-**

Topology/Coverage Color Code

Coverage Plus Strand

Coverage Minus Strand

Mainstrand: Plus

Mainstrand: Minus

Complementary Strand

Flanking Region  
(if option -flank >0)

Gene Set Annotation  
  
RepeatMasker Annotation  

**1. Tc1-2\_FR**: 4-59 (-), Divergence to consensus: 1.8%  
**2. AlRepA-66**: 168-240 (-), Divergence to consensus: 23.7%  
**3. AlRepA-66**: 210-307 (+), Divergence to consensus: 44.8%  
**4. hAT-14\_DR**: 435-750 (+), Divergence to consensus: 36.6%  
**5. Tc1-5\_Xt**: 754-1223 (-), Divergence to consensus: 24.8%  
**6. AlRepC-312**: 1224-1277 (-), Divergence to consensus: 28%  
**7. CryptonV-2\_DR**: 1621-1980 (+), Divergence to consensus: 43.3%  
**8. AlRepD-1421**: 2014-2563 (-), Divergence to consensus: 15.9%  
**9. AlRepC-3303**: 2881-3139 (+), Divergence to consensus: 61%  
**10. AlRepC-1114**: 3620-3662 (-), Divergence to consensus: 11.6%  
**11. AlRepB-356**: 3659-3792 (+), Divergence to consensus: 4.5%

  
Transcription Factor Binding Sites  

**RHOXF1** (Sequence: AGATTA (-): 124)  
**RHOXF1** (Sequence: GGCTTA (-): 537)  
**RHOXF1** (Sequence: AGCTTA (-): 553)  
**RHOXF1** (Sequence: AGATCA (-): 1477)  
**RHOXF1** (Sequence: AGCTCA (-): 2212)  
**RHOXF1** (Sequence: GGATCA (-): 2325)  
**RHOXF1** (Sequence: GGATCA (-): 2426)  
**RHOXF1** (Sequence: AGATCA (-): 2677)  
**RHOXF1** (Sequence: AGCTTA (-): 3632)  
**RHOXF1** (Sequence: TAATCT (+): 178)  
**RHOXF1** (Sequence: TGAGCT (+): 1376)  
**RHOXF1** (Sequence: TAAGCC (+): 1908)  
**RHOXF1** (Sequence: TGATCC (+): 2180)  
**RHOXF1** (Sequence: TGAGCC (+): 2439)  
**RHOXF1** (Sequence: TAATCC (+): 3617)  
**Lhx8** (Sequence: CTAATTAA (-): 1903)  
**FOXO3\_hsa** (Sequence: GTAAACAT (+): 798)  
**FOXP1** (Sequence: GTAAACA (+): 798)  
**FOXO1** (Sequence: GTTGTTTTT (+): 3724)  
**FOXO3\_mmu** (Sequence: TGTTTAGA (-): 1088)  
**Sox5** (Sequence: ATTGTT (+): 2927)  
**FOXO3\_mmu** (Sequence: TGTAAACA (+): 797)  
**FOXO3\_mmu** (Sequence: GCTAAACA (+): 1598)  
**FOXO3\_mmu** (Sequence: TCAAAACA (+): 1632)  
**FOXO1** (Sequence: AAAAACAAG (-): 656)  
**FOXO1** (Sequence: AAAAACAGG (-): 3007)  
**FOXO1** (Sequence: ATAAACAGG (-): 3198)  
**FOXO1** (Sequence: AAAAACAAG (-): 3412)  
**Nobox** (Sequence: TAATTGCT (+): 128)  
**Nobox** (Sequence: TAATTGCT (+): 1043)  
**Rhox11** (Sequence: ATAACACCA (-): 3637)  
**Gata4** (Sequence: AGATAAC (-): 2128)  
**Sox5** (Sequence: AACAAT (-): 141)  
**Sox5** (Sequence: AACAAT (-): 1007)  
**Sox5** (Sequence: AACAAT (-): 3175)  
**POU5F1** (Sequence: ATGCAAA (+): 1991)
