# Supplementary material for: First characterization of PIWI-interacting RNA clusters in a cichlid fish with a B chromosome
Source: BMC Biol. 2022 Sep 21;20:204. doi: 10.1186/s12915-022-01403-2 (PMC9490952; doi:10.1186/s12915-022-01403-2)
Supplement: Supplementary file 1 — Additional file 1. Zipped folder with fasta and interactive html piRNA cluster information for the A. latifasciata genome. The nomenclature is as follows: number-pirna-cluster_sex_B-presence (f, female; m, male; 0b, without B chromosome; 1b, with B chromosome). [file 12915_2022_1403_MOESM1_ESM.zip › 127_m0b.html]

piRNA cluster 127\_m0b 60


Predicted piRNA cluster no. 127\_m0b
  

Show proTRAC run info
Hide proTRAC run info

/\  
                \_\_\_\_\_\_\_\_\_\_\_\_\_\_\_\_\_\_\_\_\_\_\_/\\_\_\_ /  \\_\_\_\_\_\_\_  
               I                      /  \  /    \      I  
               I     pro             /    \/      \     I  
               I        TRAC        /               \   I  
               I   \_\_\_\_\_\_\_\_\_\_\_\_\_\_\_\_/\_\_\_\_\_\_\_\_\_\_\_\_\_\_\_\_\_\\_ I  
               I   \              /                     I  
               I    \            /                      I  
               I     \  /\      /       V.2.4.2         I  
               I      \/  \    /                        I  
               I\_\_\_\_\_\_\_\_\_\_\_\  /\_\_\_\_\_\_\_\_\_\_\_\_\_\_\_\_\_\_\_\_\_\_\_\_\_I  
                            \/  
  
  
================================= proTRAC ====================================  
VERSION: .......... 2.4.2  
LAST MODIFIED: .... 11. May 2018  
  
Please cite:  
Rosenkranz D, Zischler H. proTRAC - a software for probabilistic piRNA cluster  
detection, visualization and analysis. 2012. BMC Bioinformatics 13:5.  
  
  
Contact:  
David Rosenkranz  
Institute of Organismic and Molecular Evolutionary Biology  
Dept. Anthropology, small RNA group  
Johannes Gutenberg University Mainz  
email: rosenkranz@uni-mainz.de  
  
You can find the latest proTRAC version at:  
http://sourceforge.net/projects/protrac/files  
http://www.smallRNAgroup-mainz.de/software  
==============================================================================  
  
PARAMETERS:  
Map file: ...............piwi-machos-0B.fa-collapse.map  
Genome file: ............../../../0B\_ala\_genome.fa  
RepeatMasker annotation: Alatifasciata-all0B-maryan-v2.fa\_corrected.out  
GeneSet:................./guest-storage/Data/annotation/Alatifasciata\_all0B\_maryan-v2\_out2017.gff  
  
Significant (p<=0.01) hit density will be calculated based  
on observed hit distribution.  
  
Sliding window size: ........................................ 5000 bp  
Sliding window increament: .................................. 1000 bp  
Normalize each hit by number of genomic hits: ............... yes  
Normalize each hit by number of sequence reads: ............. yes  
Normalize values (-> per million mapped reads): ............. yes  
Min. fraction of hits with 1T(U) or 10A: .................... 0.75  
Alternatively: Min. fraction of hits with 1T(U) and 10A: .... 0.5  
Min. fraction of hits with typical piRNA length: ............ 0.75  
Typical piRNA length: ....................................... 24-32 nt  
Min. size of a piRNA cluster: ............................... 1000 bp.  
Min. number of hits (absolute): ............................. 0  
Min. number of hits (normalized): ........................... 0  
Min. fraction of hits on the mainstrand: .................... 0.75  
Top fraction of mapped sequences (in terms of read counts): . 1%  
Top fraction accounts for max. n% of sequence reads: ........ 90%  
Min. fraction of hits on each arm of a bidirectional cluster: 0.05  
Output html file for each cluster: .......................... yes  
Output a summary table: ..................................... yes  
Output a FASTA file for each cluster (piRNA sequences): ..... yes  
Output a FASTA file comprising cluster sequences: ........... yes  
Output a GTF file for predicted piRNA clusters: ..............yes  
Search DNA motifs in clusters: .............................. yes  
Output flanking sequences: +/- .............................. 0 bp  
Output ~.pTi file: .......................................... no  
==============================================================================  
  
  
Genome size (without gaps): ............ 758543724 bp  
Gaps (N/X/-): .......................... 417479 bp  
Mapped reads: .......................... 24765598  
Non-identical sequences: ............... 6158275  
Genomic hits: .......................... 53103584  
Significant densitiy of mapped reads: .. 763.098963422187 reads/kb

Show proTRAC cluster info
Hide proTRAC cluster info

|  |  |
| --- | --- |
| Location | NODE\_319315\_length\_10503\_cov\_30.863943 |
| Coordinates | 2002-10614 |
| Size [bp] | 8613 |
| Sequence hit loci | 5504 |
| Mapped reads (normalized) | 20147.3 |
| Mapped reads (normalized) per kb | 2339.2 |
| Normalized reads with 1T (1U) | 78.8% |
| Normalized reads with 10A | 46% |
| Normalized reads with length 24-32 nt | 99.1% |
| Normalized reads on the main strand(s) | 90.2% |
| Predicted directionality | mono:plus |

100%

0%

1T (1U)  
reads

10A reads

24-32 nt  
reads

reads on mainstrand

**Either the amount of reads with 1T (1U) OR 10A has to exceed 75% (set with option: -1Tor10A)  
Alternatively the amount of reads with 1T (1U) AND 10A has to exceed 50% (set with option: -1Tand10A)  
Minimum amount of reads with preferred size is 75% (set with option: -pisize)  
Minimum amount of reads on the main strand(s) is 75% (set with option: -clstrand)**

Show read coverage
Hide read coverage

WHAT DO I SEE HERE?  
This chart shows the location of mapped sequence reads within a predicted piRNA cluster. The color refers to the number of genomic hits produced by the sequence read in question. A dark red bar indicates that this sequence read produces many other hits elsewhere in the genome. Many adjacent red or yellow bars can indicate the presence of a multi-copy element such as transposons or rRNA genes. A dark green bar indicates that this sequence read maps uniquely to this locus.

1 hit

2-5 hits

6-10 hits

11-20 hits

21-50 hits

51-100 hits

> 100 hits

NODE\_319315\_length\_10503\_cov\_30.863943

2002

10614

Gene Set

RepeatMasker

Mapped  
Reads

73.97

plus strand

minus strand

73.97

Region: NODE\_319315\_length\_10503\_cov\_30.863943 2450-2010. Max. coverage (+): 0.12. Max coverage (-): 0.04

Region: NODE\_319315\_length\_10503\_cov\_30.863943 2011-2027. Max. coverage (+): 0.04. Max coverage (-): 0

Region: NODE\_319315\_length\_10503\_cov\_30.863943 2028-2045. Max. coverage (+): 0. Max coverage (-): 0

Region: NODE\_319315\_length\_10503\_cov\_30.863943 2046-2062. Max. coverage (+): 0.04. Max coverage (-): 0.04

Region: NODE\_319315\_length\_10503\_cov\_30.863943 2063-2079. Max. coverage (+): 0.04. Max coverage (-): 0.16

Region: NODE\_319315\_length\_10503\_cov\_30.863943 2080-2096. Max. coverage (+): 0. Max coverage (-): 0

Region: NODE\_319315\_length\_10503\_cov\_30.863943 2097-2113. Max. coverage (+): 0.16. Max coverage (-): 0

Region: NODE\_319315\_length\_10503\_cov\_30.863943 2114-2131. Max. coverage (+): 0. Max coverage (-): 0

Region: NODE\_319315\_length\_10503\_cov\_30.863943 2132-2148. Max. coverage (+): 0.08. Max coverage (-): 0.04

Region: NODE\_319315\_length\_10503\_cov\_30.863943 2149-2165. Max. coverage (+): 0.36. Max coverage (-): 0

Region: NODE\_319315\_length\_10503\_cov\_30.863943 2166-2182. Max. coverage (+): 0. Max coverage (-): 0.2

Region: NODE\_319315\_length\_10503\_cov\_30.863943 2183-2200. Max. coverage (+): 0. Max coverage (-): 0.16

Region: NODE\_319315\_length\_10503\_cov\_30.863943 2201-2217. Max. coverage (+): 0. Max coverage (-): 0

Region: NODE\_319315\_length\_10503\_cov\_30.863943 2218-2234. Max. coverage (+): 0. Max coverage (-): 0

Region: NODE\_319315\_length\_10503\_cov\_30.863943 2235-2251. Max. coverage (+): 0.04. Max coverage (-): 0

Region: NODE\_319315\_length\_10503\_cov\_30.863943 2252-2269. Max. coverage (+): 0.12. Max coverage (-): 0.36

Region: NODE\_319315\_length\_10503\_cov\_30.863943 2270-2286. Max. coverage (+): 0.04. Max coverage (-): 0.36

Region: NODE\_319315\_length\_10503\_cov\_30.863943 2287-2303. Max. coverage (+): 0.08. Max coverage (-): 0.32

Region: NODE\_319315\_length\_10503\_cov\_30.863943 2304-2320. Max. coverage (+): 0.04. Max coverage (-): 0.16

Region: NODE\_319315\_length\_10503\_cov\_30.863943 2321-2337. Max. coverage (+): 0. Max coverage (-): 0.44

Region: NODE\_319315\_length\_10503\_cov\_30.863943 2338-2355. Max. coverage (+): 0.12. Max coverage (-): 0.4

Region: NODE\_319315\_length\_10503\_cov\_30.863943 2356-2372. Max. coverage (+): 0.2. Max coverage (-): 0

Region: NODE\_319315\_length\_10503\_cov\_30.863943 2373-2389. Max. coverage (+): 0.04. Max coverage (-): 0.65

Region: NODE\_319315\_length\_10503\_cov\_30.863943 2390-2406. Max. coverage (+): 1.66. Max coverage (-): 0.04

Region: NODE\_319315\_length\_10503\_cov\_30.863943 2407-2424. Max. coverage (+): 0.28. Max coverage (-): 1.05

Region: NODE\_319315\_length\_10503\_cov\_30.863943 2425-2441. Max. coverage (+): 0.08. Max coverage (-): 1.13

Region: NODE\_319315\_length\_10503\_cov\_30.863943 2442-2458. Max. coverage (+): 0.12. Max coverage (-): 0.36

Region: NODE\_319315\_length\_10503\_cov\_30.863943 2459-2475. Max. coverage (+): 0.73. Max coverage (-): 0.16

Region: NODE\_319315\_length\_10503\_cov\_30.863943 2476-2492. Max. coverage (+): 0.61. Max coverage (-): 0.08

Region: NODE\_319315\_length\_10503\_cov\_30.863943 2493-2510. Max. coverage (+): 0.12. Max coverage (-): 0.08

Region: NODE\_319315\_length\_10503\_cov\_30.863943 2511-2527. Max. coverage (+): 0.12. Max coverage (-): 0.28

Region: NODE\_319315\_length\_10503\_cov\_30.863943 2528-2544. Max. coverage (+): 0. Max coverage (-): 0

Region: NODE\_319315\_length\_10503\_cov\_30.863943 2545-2561. Max. coverage (+): 0.16. Max coverage (-): 0.04

Region: NODE\_319315\_length\_10503\_cov\_30.863943 2562-2579. Max. coverage (+): 0.12. Max coverage (-): 0.08

Region: NODE\_319315\_length\_10503\_cov\_30.863943 2580-2596. Max. coverage (+): 0. Max coverage (-): 0.24

Region: NODE\_319315\_length\_10503\_cov\_30.863943 2597-2613. Max. coverage (+): 0. Max coverage (-): 0.12

Region: NODE\_319315\_length\_10503\_cov\_30.863943 2614-2630. Max. coverage (+): 0. Max coverage (-): 0.08

Region: NODE\_319315\_length\_10503\_cov\_30.863943 2631-2647. Max. coverage (+): 0. Max coverage (-): 0.16

Region: NODE\_319315\_length\_10503\_cov\_30.863943 2648-2665. Max. coverage (+): 0.08. Max coverage (-): 0.16

Region: NODE\_319315\_length\_10503\_cov\_30.863943 2666-2682. Max. coverage (+): 0. Max coverage (-): 0.04

Region: NODE\_319315\_length\_10503\_cov\_30.863943 2683-2699. Max. coverage (+): 0.08. Max coverage (-): 0.16

Region: NODE\_319315\_length\_10503\_cov\_30.863943 2700-2716. Max. coverage (+): 0.36. Max coverage (-): 0.36

Region: NODE\_319315\_length\_10503\_cov\_30.863943 2717-2734. Max. coverage (+): 0.08. Max coverage (-): 0.08

Region: NODE\_319315\_length\_10503\_cov\_30.863943 2735-2751. Max. coverage (+): 0.08. Max coverage (-): 0.08

Region: NODE\_319315\_length\_10503\_cov\_30.863943 2752-2768. Max. coverage (+): 0.28. Max coverage (-): 0.04

Region: NODE\_319315\_length\_10503\_cov\_30.863943 2769-2785. Max. coverage (+): 0. Max coverage (-): 0.2

Region: NODE\_319315\_length\_10503\_cov\_30.863943 2786-2803. Max. coverage (+): 0.16. Max coverage (-): 0.08

Region: NODE\_319315\_length\_10503\_cov\_30.863943 2804-2820. Max. coverage (+): 0.2. Max coverage (-): 0.36

Region: NODE\_319315\_length\_10503\_cov\_30.863943 2821-2837. Max. coverage (+): 0.24. Max coverage (-): 0.44

Region: NODE\_319315\_length\_10503\_cov\_30.863943 2838-2854. Max. coverage (+): 1.13. Max coverage (-): 0

Region: NODE\_319315\_length\_10503\_cov\_30.863943 2855-2871. Max. coverage (+): 0.2. Max coverage (-): 0.04

Region: NODE\_319315\_length\_10503\_cov\_30.863943 2872-2889. Max. coverage (+): 0.08. Max coverage (-): 0.04

Region: NODE\_319315\_length\_10503\_cov\_30.863943 2890-2906. Max. coverage (+): 0.12. Max coverage (-): 0.04

Region: NODE\_319315\_length\_10503\_cov\_30.863943 2907-2923. Max. coverage (+): 0.04. Max coverage (-): 0

Region: NODE\_319315\_length\_10503\_cov\_30.863943 2924-2940. Max. coverage (+): 0.04. Max coverage (-): 0

Region: NODE\_319315\_length\_10503\_cov\_30.863943 2941-2958. Max. coverage (+): 0.24. Max coverage (-): 0.04

Region: NODE\_319315\_length\_10503\_cov\_30.863943 2959-2975. Max. coverage (+): 0.2. Max coverage (-): 0

Region: NODE\_319315\_length\_10503\_cov\_30.863943 2976-2992. Max. coverage (+): 0.16. Max coverage (-): 0.04

Region: NODE\_319315\_length\_10503\_cov\_30.863943 2993-3009. Max. coverage (+): 0.12. Max coverage (-): 0

Region: NODE\_319315\_length\_10503\_cov\_30.863943 3010-3026. Max. coverage (+): 0.04. Max coverage (-): 0.12

Region: NODE\_319315\_length\_10503\_cov\_30.863943 3027-3044. Max. coverage (+): 0.85. Max coverage (-): 0

Region: NODE\_319315\_length\_10503\_cov\_30.863943 3045-3061. Max. coverage (+): 0.04. Max coverage (-): 0

Region: NODE\_319315\_length\_10503\_cov\_30.863943 3062-3078. Max. coverage (+): 0.04. Max coverage (-): 0.24

Region: NODE\_319315\_length\_10503\_cov\_30.863943 3079-3095. Max. coverage (+): 0.73. Max coverage (-): 0.12

Region: NODE\_319315\_length\_10503\_cov\_30.863943 3096-3113. Max. coverage (+): 0.24. Max coverage (-): 0.12

Region: NODE\_319315\_length\_10503\_cov\_30.863943 3114-3130. Max. coverage (+): 0.44. Max coverage (-): 0.12

Region: NODE\_319315\_length\_10503\_cov\_30.863943 3131-3147. Max. coverage (+): 0.16. Max coverage (-): 0.08

Region: NODE\_319315\_length\_10503\_cov\_30.863943 3148-3164. Max. coverage (+): 0.69. Max coverage (-): 0

Region: NODE\_319315\_length\_10503\_cov\_30.863943 3165-3181. Max. coverage (+): 0.12. Max coverage (-): 0

Region: NODE\_319315\_length\_10503\_cov\_30.863943 3182-3199. Max. coverage (+): 0.2. Max coverage (-): 0.04

Region: NODE\_319315\_length\_10503\_cov\_30.863943 3200-3216. Max. coverage (+): 0.2. Max coverage (-): 0.52

Region: NODE\_319315\_length\_10503\_cov\_30.863943 3217-3233. Max. coverage (+): 0.97. Max coverage (-): 0.16

Region: NODE\_319315\_length\_10503\_cov\_30.863943 3234-3250. Max. coverage (+): 0.4. Max coverage (-): 0.12

Region: NODE\_319315\_length\_10503\_cov\_30.863943 3251-3268. Max. coverage (+): 0.32. Max coverage (-): 0.04

Region: NODE\_319315\_length\_10503\_cov\_30.863943 3269-3285. Max. coverage (+): 0.04. Max coverage (-): 0.08

Region: NODE\_319315\_length\_10503\_cov\_30.863943 3286-3302. Max. coverage (+): 0.04. Max coverage (-): 0.2

Region: NODE\_319315\_length\_10503\_cov\_30.863943 3303-3319. Max. coverage (+): 0.2. Max coverage (-): 0.08

Region: NODE\_319315\_length\_10503\_cov\_30.863943 3320-3337. Max. coverage (+): 0.04. Max coverage (-): 0.12

Region: NODE\_319315\_length\_10503\_cov\_30.863943 3338-3354. Max. coverage (+): 0.77. Max coverage (-): 0.08

Region: NODE\_319315\_length\_10503\_cov\_30.863943 3355-3371. Max. coverage (+): 0.32. Max coverage (-): 0.12

Region: NODE\_319315\_length\_10503\_cov\_30.863943 3372-3388. Max. coverage (+): 0.24. Max coverage (-): 0

Region: NODE\_319315\_length\_10503\_cov\_30.863943 3389-3405. Max. coverage (+): 1.49. Max coverage (-): 0.08

Region: NODE\_319315\_length\_10503\_cov\_30.863943 3406-3423. Max. coverage (+): 0.04. Max coverage (-): 0.28

Region: NODE\_319315\_length\_10503\_cov\_30.863943 3424-3440. Max. coverage (+): 0.08. Max coverage (-): 0.12

Region: NODE\_319315\_length\_10503\_cov\_30.863943 3441-3457. Max. coverage (+): 0. Max coverage (-): 0.04

Region: NODE\_319315\_length\_10503\_cov\_30.863943 3458-3474. Max. coverage (+): 0.08. Max coverage (-): 0.04

Region: NODE\_319315\_length\_10503\_cov\_30.863943 3475-3492. Max. coverage (+): 0.12. Max coverage (-): 0.12

Region: NODE\_319315\_length\_10503\_cov\_30.863943 3493-3509. Max. coverage (+): 0. Max coverage (-): 0.12

Region: NODE\_319315\_length\_10503\_cov\_30.863943 3510-3526. Max. coverage (+): 0.28. Max coverage (-): 0.04

Region: NODE\_319315\_length\_10503\_cov\_30.863943 3527-3543. Max. coverage (+): 0.73. Max coverage (-): 0

Region: NODE\_319315\_length\_10503\_cov\_30.863943 3544-3560. Max. coverage (+): 0. Max coverage (-): 0.16

Region: NODE\_319315\_length\_10503\_cov\_30.863943 3561-3578. Max. coverage (+): 1.33. Max coverage (-): 0.16

Region: NODE\_319315\_length\_10503\_cov\_30.863943 3579-3595. Max. coverage (+): 0.4. Max coverage (-): 0

Region: NODE\_319315\_length\_10503\_cov\_30.863943 3596-3612. Max. coverage (+): 0.12. Max coverage (-): 0.04

Region: NODE\_319315\_length\_10503\_cov\_30.863943 3613-3629. Max. coverage (+): 0.12. Max coverage (-): 0.12

Region: NODE\_319315\_length\_10503\_cov\_30.863943 3630-3647. Max. coverage (+): 1.33. Max coverage (-): 0.12

Region: NODE\_319315\_length\_10503\_cov\_30.863943 3648-3664. Max. coverage (+): 0.2. Max coverage (-): 0.04

Region: NODE\_319315\_length\_10503\_cov\_30.863943 3665-3681. Max. coverage (+): 0. Max coverage (-): 0

Region: NODE\_319315\_length\_10503\_cov\_30.863943 3682-3698. Max. coverage (+): 0.12. Max coverage (-): 0.08

Region: NODE\_319315\_length\_10503\_cov\_30.863943 3699-3715. Max. coverage (+): 0.57. Max coverage (-): 0.08

Region: NODE\_319315\_length\_10503\_cov\_30.863943 3716-3733. Max. coverage (+): 0.61. Max coverage (-): 0

Region: NODE\_319315\_length\_10503\_cov\_30.863943 3734-3750. Max. coverage (+): 0.16. Max coverage (-): 0

Region: NODE\_319315\_length\_10503\_cov\_30.863943 3751-3767. Max. coverage (+): 0.28. Max coverage (-): 0

Region: NODE\_319315\_length\_10503\_cov\_30.863943 3768-3784. Max. coverage (+): 0. Max coverage (-): 0

Region: NODE\_319315\_length\_10503\_cov\_30.863943 3785-3802. Max. coverage (+): 0. Max coverage (-): 0

Region: NODE\_319315\_length\_10503\_cov\_30.863943 3803-3819. Max. coverage (+): 0.16. Max coverage (-): 0

Region: NODE\_319315\_length\_10503\_cov\_30.863943 3820-3836. Max. coverage (+): 0.12. Max coverage (-): 0

Region: NODE\_319315\_length\_10503\_cov\_30.863943 3837-3853. Max. coverage (+): 0.16. Max coverage (-): 0

Region: NODE\_319315\_length\_10503\_cov\_30.863943 3854-3871. Max. coverage (+): 0.2. Max coverage (-): 0.04

Region: NODE\_319315\_length\_10503\_cov\_30.863943 3872-3888. Max. coverage (+): 0.04. Max coverage (-): 0

Region: NODE\_319315\_length\_10503\_cov\_30.863943 3889-3905. Max. coverage (+): 0.08. Max coverage (-): 0

Region: NODE\_319315\_length\_10503\_cov\_30.863943 3906-3922. Max. coverage (+): 0.08. Max coverage (-): 0

Region: NODE\_319315\_length\_10503\_cov\_30.863943 3923-3939. Max. coverage (+): 0.08. Max coverage (-): 0

Region: NODE\_319315\_length\_10503\_cov\_30.863943 3940-3957. Max. coverage (+): 0.12. Max coverage (-): 0.2

Region: NODE\_319315\_length\_10503\_cov\_30.863943 3958-3974. Max. coverage (+): 2.14. Max coverage (-): 0

Region: NODE\_319315\_length\_10503\_cov\_30.863943 3975-3991. Max. coverage (+): 0.77. Max coverage (-): 0.85

Region: NODE\_319315\_length\_10503\_cov\_30.863943 3992-4008. Max. coverage (+): 0.44. Max coverage (-): 0

Region: NODE\_319315\_length\_10503\_cov\_30.863943 4009-4026. Max. coverage (+): 0.12. Max coverage (-): 0.32

Region: NODE\_319315\_length\_10503\_cov\_30.863943 4027-4043. Max. coverage (+): 0.12. Max coverage (-): 0.32

Region: NODE\_319315\_length\_10503\_cov\_30.863943 4044-4060. Max. coverage (+): 0.12. Max coverage (-): 0.04

Region: NODE\_319315\_length\_10503\_cov\_30.863943 4061-4077. Max. coverage (+): 0.52. Max coverage (-): 0.04

Region: NODE\_319315\_length\_10503\_cov\_30.863943 4078-4094. Max. coverage (+): 0.28. Max coverage (-): 0.08

Region: NODE\_319315\_length\_10503\_cov\_30.863943 4095-4112. Max. coverage (+): 0.2. Max coverage (-): 0.08

Region: NODE\_319315\_length\_10503\_cov\_30.863943 4113-4129. Max. coverage (+): 0.08. Max coverage (-): 0.04

Region: NODE\_319315\_length\_10503\_cov\_30.863943 4130-4146. Max. coverage (+): 0.2. Max coverage (-): 0.04

Region: NODE\_319315\_length\_10503\_cov\_30.863943 4147-4163. Max. coverage (+): 0.2. Max coverage (-): 0.08

Region: NODE\_319315\_length\_10503\_cov\_30.863943 4164-4181. Max. coverage (+): 0.08. Max coverage (-): 0.12

Region: NODE\_319315\_length\_10503\_cov\_30.863943 4182-4198. Max. coverage (+): 0.32. Max coverage (-): 0.81

Region: NODE\_319315\_length\_10503\_cov\_30.863943 4199-4215. Max. coverage (+): 0.08. Max coverage (-): 0

Region: NODE\_319315\_length\_10503\_cov\_30.863943 4216-4232. Max. coverage (+): 0.77. Max coverage (-): 0

Region: NODE\_319315\_length\_10503\_cov\_30.863943 4233-4249. Max. coverage (+): 0.16. Max coverage (-): 0.08

Region: NODE\_319315\_length\_10503\_cov\_30.863943 4250-4267. Max. coverage (+): 0.04. Max coverage (-): 0.81

Region: NODE\_319315\_length\_10503\_cov\_30.863943 4268-4284. Max. coverage (+): 0.04. Max coverage (-): 0

Region: NODE\_319315\_length\_10503\_cov\_30.863943 4285-4301. Max. coverage (+): 0.12. Max coverage (-): 0.08

Region: NODE\_319315\_length\_10503\_cov\_30.863943 4302-4318. Max. coverage (+): 0.08. Max coverage (-): 0.04

Region: NODE\_319315\_length\_10503\_cov\_30.863943 4319-4336. Max. coverage (+): 0.57. Max coverage (-): 0.04

Region: NODE\_319315\_length\_10503\_cov\_30.863943 4337-4353. Max. coverage (+): 0.36. Max coverage (-): 0.04

Region: NODE\_319315\_length\_10503\_cov\_30.863943 4354-4370. Max. coverage (+): 0.12. Max coverage (-): 0.04

Region: NODE\_319315\_length\_10503\_cov\_30.863943 4371-4387. Max. coverage (+): 0.12. Max coverage (-): 0

Region: NODE\_319315\_length\_10503\_cov\_30.863943 4388-4405. Max. coverage (+): 0.12. Max coverage (-): 0.08

Region: NODE\_319315\_length\_10503\_cov\_30.863943 4406-4422. Max. coverage (+): 1.29. Max coverage (-): 0

Region: NODE\_319315\_length\_10503\_cov\_30.863943 4423-4439. Max. coverage (+): 0.08. Max coverage (-): 0

Region: NODE\_319315\_length\_10503\_cov\_30.863943 4440-4456. Max. coverage (+): 3.51. Max coverage (-): 0.48

Region: NODE\_319315\_length\_10503\_cov\_30.863943 4457-4473. Max. coverage (+): 10.54. Max coverage (-): 0.08

Region: NODE\_319315\_length\_10503\_cov\_30.863943 4474-4491. Max. coverage (+): 0.08. Max coverage (-): 0.08

Region: NODE\_319315\_length\_10503\_cov\_30.863943 4492-4508. Max. coverage (+): 0.08. Max coverage (-): 0.32

Region: NODE\_319315\_length\_10503\_cov\_30.863943 4509-4525. Max. coverage (+): 0.93. Max coverage (-): 0.08

Region: NODE\_319315\_length\_10503\_cov\_30.863943 4526-4542. Max. coverage (+): 0.44. Max coverage (-): 0.12

Region: NODE\_319315\_length\_10503\_cov\_30.863943 4543-4560. Max. coverage (+): 1.45. Max coverage (-): 0.16

Region: NODE\_319315\_length\_10503\_cov\_30.863943 4561-4577. Max. coverage (+): 3.55. Max coverage (-): 0.12

Region: NODE\_319315\_length\_10503\_cov\_30.863943 4578-4594. Max. coverage (+): 0.73. Max coverage (-): 0.08

Region: NODE\_319315\_length\_10503\_cov\_30.863943 4595-4611. Max. coverage (+): 2.1. Max coverage (-): 0.08

Region: NODE\_319315\_length\_10503\_cov\_30.863943 4612-4628. Max. coverage (+): 0.4. Max coverage (-): 0.08

Region: NODE\_319315\_length\_10503\_cov\_30.863943 4629-4646. Max. coverage (+): 3.15. Max coverage (-): 0.04

Region: NODE\_319315\_length\_10503\_cov\_30.863943 4647-4663. Max. coverage (+): 0.32. Max coverage (-): 0.08

Region: NODE\_319315\_length\_10503\_cov\_30.863943 4664-4680. Max. coverage (+): 1.37. Max coverage (-): 0.12

Region: NODE\_319315\_length\_10503\_cov\_30.863943 4681-4697. Max. coverage (+): 0.08. Max coverage (-): 0.08

Region: NODE\_319315\_length\_10503\_cov\_30.863943 4698-4715. Max. coverage (+): 0.16. Max coverage (-): 0.12

Region: NODE\_319315\_length\_10503\_cov\_30.863943 4716-4732. Max. coverage (+): 0.16. Max coverage (-): 0.04

Region: NODE\_319315\_length\_10503\_cov\_30.863943 4733-4749. Max. coverage (+): 0.65. Max coverage (-): 0.04

Region: NODE\_319315\_length\_10503\_cov\_30.863943 4750-4766. Max. coverage (+): 0.08. Max coverage (-): 0.04

Region: NODE\_319315\_length\_10503\_cov\_30.863943 4767-4783. Max. coverage (+): 0.77. Max coverage (-): 0

Region: NODE\_319315\_length\_10503\_cov\_30.863943 4784-4801. Max. coverage (+): 0.16. Max coverage (-): 0

Region: NODE\_319315\_length\_10503\_cov\_30.863943 4802-4818. Max. coverage (+): 0.12. Max coverage (-): 0

Region: NODE\_319315\_length\_10503\_cov\_30.863943 4819-4835. Max. coverage (+): 0.69. Max coverage (-): 0

Region: NODE\_319315\_length\_10503\_cov\_30.863943 4836-4852. Max. coverage (+): 0.04. Max coverage (-): 0.08

Region: NODE\_319315\_length\_10503\_cov\_30.863943 4853-4870. Max. coverage (+): 0. Max coverage (-): 0

Region: NODE\_319315\_length\_10503\_cov\_30.863943 4871-4887. Max. coverage (+): 0. Max coverage (-): 0.16

Region: NODE\_319315\_length\_10503\_cov\_30.863943 4888-4904. Max. coverage (+): 0.16. Max coverage (-): 0.2

Region: NODE\_319315\_length\_10503\_cov\_30.863943 4905-4921. Max. coverage (+): 0.16. Max coverage (-): 0.16

Region: NODE\_319315\_length\_10503\_cov\_30.863943 4922-4939. Max. coverage (+): 0.04. Max coverage (-): 0.04

Region: NODE\_319315\_length\_10503\_cov\_30.863943 4940-4956. Max. coverage (+): 0.08. Max coverage (-): 0

Region: NODE\_319315\_length\_10503\_cov\_30.863943 4957-4973. Max. coverage (+): 0.12. Max coverage (-): 0.04

Region: NODE\_319315\_length\_10503\_cov\_30.863943 4974-4990. Max. coverage (+): 0. Max coverage (-): 0.24

Region: NODE\_319315\_length\_10503\_cov\_30.863943 4991-5007. Max. coverage (+): 0.36. Max coverage (-): 0.12

Region: NODE\_319315\_length\_10503\_cov\_30.863943 5008-5025. Max. coverage (+): 0. Max coverage (-): 0.12

Region: NODE\_319315\_length\_10503\_cov\_30.863943 5026-5042. Max. coverage (+): 0.04. Max coverage (-): 0.08

Region: NODE\_319315\_length\_10503\_cov\_30.863943 5043-5059. Max. coverage (+): 1.82. Max coverage (-): 0

Region: NODE\_319315\_length\_10503\_cov\_30.863943 5060-5076. Max. coverage (+): 0.04. Max coverage (-): 0

Region: NODE\_319315\_length\_10503\_cov\_30.863943 5077-5094. Max. coverage (+): 0. Max coverage (-): 0.04

Region: NODE\_319315\_length\_10503\_cov\_30.863943 5095-5111. Max. coverage (+): 0.12. Max coverage (-): 0.04

Region: NODE\_319315\_length\_10503\_cov\_30.863943 5112-5128. Max. coverage (+): 0.08. Max coverage (-): 0

Region: NODE\_319315\_length\_10503\_cov\_30.863943 5129-5145. Max. coverage (+): 0. Max coverage (-): 0.03

Region: NODE\_319315\_length\_10503\_cov\_30.863943 5146-5162. Max. coverage (+): 0.01. Max coverage (-): 0

Region: NODE\_319315\_length\_10503\_cov\_30.863943 5163-5180. Max. coverage (+): 0. Max coverage (-): 0

Region: NODE\_319315\_length\_10503\_cov\_30.863943 5181-5197. Max. coverage (+): 0. Max coverage (-): 0

Region: NODE\_319315\_length\_10503\_cov\_30.863943 5198-5214. Max. coverage (+): 0. Max coverage (-): 0

Region: NODE\_319315\_length\_10503\_cov\_30.863943 5215-5231. Max. coverage (+): 0. Max coverage (-): 0

Region: NODE\_319315\_length\_10503\_cov\_30.863943 5232-5249. Max. coverage (+): 0.08. Max coverage (-): 0

Region: NODE\_319315\_length\_10503\_cov\_30.863943 5250-5266. Max. coverage (+): 0.04. Max coverage (-): 0

Region: NODE\_319315\_length\_10503\_cov\_30.863943 5267-5283. Max. coverage (+): 0. Max coverage (-): 0

Region: NODE\_319315\_length\_10503\_cov\_30.863943 5284-5300. Max. coverage (+): 0. Max coverage (-): 0

Region: NODE\_319315\_length\_10503\_cov\_30.863943 5301-5318. Max. coverage (+): 0.08. Max coverage (-): 0

Region: NODE\_319315\_length\_10503\_cov\_30.863943 5319-5335. Max. coverage (+): 0.08. Max coverage (-): 0

Region: NODE\_319315\_length\_10503\_cov\_30.863943 5336-5352. Max. coverage (+): 0. Max coverage (-): 0

Region: NODE\_319315\_length\_10503\_cov\_30.863943 5353-5369. Max. coverage (+): 0.01. Max coverage (-): 0

Region: NODE\_319315\_length\_10503\_cov\_30.863943 5370-5386. Max. coverage (+): 0.01. Max coverage (-): 0

Region: NODE\_319315\_length\_10503\_cov\_30.863943 5387-5404. Max. coverage (+): 0. Max coverage (-): 0

Region: NODE\_319315\_length\_10503\_cov\_30.863943 5405-5421. Max. coverage (+): 0. Max coverage (-): 0

Region: NODE\_319315\_length\_10503\_cov\_30.863943 5422-5438. Max. coverage (+): 0.01. Max coverage (-): 0

Region: NODE\_319315\_length\_10503\_cov\_30.863943 5439-5455. Max. coverage (+): 0. Max coverage (-): 0

Region: NODE\_319315\_length\_10503\_cov\_30.863943 5456-5473. Max. coverage (+): 0. Max coverage (-): 0

Region: NODE\_319315\_length\_10503\_cov\_30.863943 5474-5490. Max. coverage (+): 0.01. Max coverage (-): 0

Region: NODE\_319315\_length\_10503\_cov\_30.863943 5491-5507. Max. coverage (+): 0.2. Max coverage (-): 0

Region: NODE\_319315\_length\_10503\_cov\_30.863943 5508-5524. Max. coverage (+): 0.08. Max coverage (-): 0.04

Region: NODE\_319315\_length\_10503\_cov\_30.863943 5525-5541. Max. coverage (+): 0.04. Max coverage (-): 0.04

Region: NODE\_319315\_length\_10503\_cov\_30.863943 5542-5559. Max. coverage (+): 0.1. Max coverage (-): 0.04

Region: NODE\_319315\_length\_10503\_cov\_30.863943 5560-5576. Max. coverage (+): 0.04. Max coverage (-): 0

Region: NODE\_319315\_length\_10503\_cov\_30.863943 5577-5593. Max. coverage (+): 0. Max coverage (-): 0

Region: NODE\_319315\_length\_10503\_cov\_30.863943 5594-5610. Max. coverage (+): 0. Max coverage (-): 0

Region: NODE\_319315\_length\_10503\_cov\_30.863943 5611-5628. Max. coverage (+): 0.12. Max coverage (-): 0.04

Region: NODE\_319315\_length\_10503\_cov\_30.863943 5629-5645. Max. coverage (+): 0.08. Max coverage (-): 0.04

Region: NODE\_319315\_length\_10503\_cov\_30.863943 5646-5662. Max. coverage (+): 0.24. Max coverage (-): 0.04

Region: NODE\_319315\_length\_10503\_cov\_30.863943 5663-5679. Max. coverage (+): 0.04. Max coverage (-): 0

Region: NODE\_319315\_length\_10503\_cov\_30.863943 5680-5696. Max. coverage (+): 0.52. Max coverage (-): 0

Region: NODE\_319315\_length\_10503\_cov\_30.863943 5697-5714. Max. coverage (+): 0.32. Max coverage (-): 0.08

Region: NODE\_319315\_length\_10503\_cov\_30.863943 5715-5731. Max. coverage (+): 0. Max coverage (-): 0.04

Region: NODE\_319315\_length\_10503\_cov\_30.863943 5732-5748. Max. coverage (+): 0. Max coverage (-): 0

Region: NODE\_319315\_length\_10503\_cov\_30.863943 5749-5765. Max. coverage (+): 0.05. Max coverage (-): 0.12

Region: NODE\_319315\_length\_10503\_cov\_30.863943 5766-5783. Max. coverage (+): 0. Max coverage (-): 0

Region: NODE\_319315\_length\_10503\_cov\_30.863943 5784-5800. Max. coverage (+): 0.12. Max coverage (-): 0

Region: NODE\_319315\_length\_10503\_cov\_30.863943 5801-5817. Max. coverage (+): 0. Max coverage (-): 0.04

Region: NODE\_319315\_length\_10503\_cov\_30.863943 5818-5834. Max. coverage (+): 0. Max coverage (-): 0

Region: NODE\_319315\_length\_10503\_cov\_30.863943 5835-5852. Max. coverage (+): 0. Max coverage (-): 0

Region: NODE\_319315\_length\_10503\_cov\_30.863943 5853-5869. Max. coverage (+): 0. Max coverage (-): 0

Region: NODE\_319315\_length\_10503\_cov\_30.863943 5870-5886. Max. coverage (+): 0.04. Max coverage (-): 0

Region: NODE\_319315\_length\_10503\_cov\_30.863943 5887-5903. Max. coverage (+): 0.04. Max coverage (-): 0

Region: NODE\_319315\_length\_10503\_cov\_30.863943 5904-5920. Max. coverage (+): 0.4. Max coverage (-): 0

Region: NODE\_319315\_length\_10503\_cov\_30.863943 5921-5938. Max. coverage (+): 0.04. Max coverage (-): 0

Region: NODE\_319315\_length\_10503\_cov\_30.863943 5939-5955. Max. coverage (+): 0.08. Max coverage (-): 0

Region: NODE\_319315\_length\_10503\_cov\_30.863943 5956-5972. Max. coverage (+): 0.16. Max coverage (-): 0

Region: NODE\_319315\_length\_10503\_cov\_30.863943 5973-5989. Max. coverage (+): 0.12. Max coverage (-): 0.32

Region: NODE\_319315\_length\_10503\_cov\_30.863943 5990-6007. Max. coverage (+): 0. Max coverage (-): 0

Region: NODE\_319315\_length\_10503\_cov\_30.863943 6008-6024. Max. coverage (+): 0.08. Max coverage (-): 0.04

Region: NODE\_319315\_length\_10503\_cov\_30.863943 6025-6041. Max. coverage (+): 0.12. Max coverage (-): 0.04

Region: NODE\_319315\_length\_10503\_cov\_30.863943 6042-6058. Max. coverage (+): 0.28. Max coverage (-): 0.08

Region: NODE\_319315\_length\_10503\_cov\_30.863943 6059-6075. Max. coverage (+): 0. Max coverage (-): 0.04

Region: NODE\_319315\_length\_10503\_cov\_30.863943 6076-6093. Max. coverage (+): 0. Max coverage (-): 0

Region: NODE\_319315\_length\_10503\_cov\_30.863943 6094-6110. Max. coverage (+): 0.04. Max coverage (-): 0

Region: NODE\_319315\_length\_10503\_cov\_30.863943 6111-6127. Max. coverage (+): 1.33. Max coverage (-): 0

Region: NODE\_319315\_length\_10503\_cov\_30.863943 6128-6144. Max. coverage (+): 0.16. Max coverage (-): 0.12

Region: NODE\_319315\_length\_10503\_cov\_30.863943 6145-6162. Max. coverage (+): 0.2. Max coverage (-): 0.04

Region: NODE\_319315\_length\_10503\_cov\_30.863943 6163-6179. Max. coverage (+): 0.24. Max coverage (-): 0.04

Region: NODE\_319315\_length\_10503\_cov\_30.863943 6180-6196. Max. coverage (+): 0.08. Max coverage (-): 0.04

Region: NODE\_319315\_length\_10503\_cov\_30.863943 6197-6213. Max. coverage (+): 0.12. Max coverage (-): 0

Region: NODE\_319315\_length\_10503\_cov\_30.863943 6214-6230. Max. coverage (+): 0.12. Max coverage (-): 0.04

Region: NODE\_319315\_length\_10503\_cov\_30.863943 6231-6248. Max. coverage (+): 0.61. Max coverage (-): 0.04

Region: NODE\_319315\_length\_10503\_cov\_30.863943 6249-6265. Max. coverage (+): 2.91. Max coverage (-): 1.17

Region: NODE\_319315\_length\_10503\_cov\_30.863943 6266-6282. Max. coverage (+): 2.83. Max coverage (-): 0.32

Region: NODE\_319315\_length\_10503\_cov\_30.863943 6283-6299. Max. coverage (+): 1.7. Max coverage (-): 0.36

Region: NODE\_319315\_length\_10503\_cov\_30.863943 6300-6317. Max. coverage (+): 0.12. Max coverage (-): 0.16

Region: NODE\_319315\_length\_10503\_cov\_30.863943 6318-6334. Max. coverage (+): 0.44. Max coverage (-): 0.4

Region: NODE\_319315\_length\_10503\_cov\_30.863943 6335-6351. Max. coverage (+): 0.93. Max coverage (-): 0.04

Region: NODE\_319315\_length\_10503\_cov\_30.863943 6352-6368. Max. coverage (+): 1.62. Max coverage (-): 0.12

Region: NODE\_319315\_length\_10503\_cov\_30.863943 6369-6386. Max. coverage (+): 2.99. Max coverage (-): 0.08

Region: NODE\_319315\_length\_10503\_cov\_30.863943 6387-6403. Max. coverage (+): 0.24. Max coverage (-): 0

Region: NODE\_319315\_length\_10503\_cov\_30.863943 6404-6420. Max. coverage (+): 10.34. Max coverage (-): 0

Region: NODE\_319315\_length\_10503\_cov\_30.863943 6421-6437. Max. coverage (+): 0.4. Max coverage (-): 0

Region: NODE\_319315\_length\_10503\_cov\_30.863943 6438-6454. Max. coverage (+): 1.21. Max coverage (-): 0.08

Region: NODE\_319315\_length\_10503\_cov\_30.863943 6455-6472. Max. coverage (+): 0.73. Max coverage (-): 0.04

Region: NODE\_319315\_length\_10503\_cov\_30.863943 6473-6489. Max. coverage (+): 3.63. Max coverage (-): 0.36

Region: NODE\_319315\_length\_10503\_cov\_30.863943 6490-6506. Max. coverage (+): 3.96. Max coverage (-): 0.04

Region: NODE\_319315\_length\_10503\_cov\_30.863943 6507-6523. Max. coverage (+): 0.44. Max coverage (-): 0.32

Region: NODE\_319315\_length\_10503\_cov\_30.863943 6524-6541. Max. coverage (+): 25.48. Max coverage (-): 0.08

Region: NODE\_319315\_length\_10503\_cov\_30.863943 6542-6558. Max. coverage (+): 0.24. Max coverage (-): 0.48

Region: NODE\_319315\_length\_10503\_cov\_30.863943 6559-6575. Max. coverage (+): 0.24. Max coverage (-): 0.04

Region: NODE\_319315\_length\_10503\_cov\_30.863943 6576-6592. Max. coverage (+): 0.08. Max coverage (-): 0.2

Region: NODE\_319315\_length\_10503\_cov\_30.863943 6593-6609. Max. coverage (+): 10.38. Max coverage (-): 0

Region: NODE\_319315\_length\_10503\_cov\_30.863943 6610-6627. Max. coverage (+): 0.04. Max coverage (-): 0.16

Region: NODE\_319315\_length\_10503\_cov\_30.863943 6628-6644. Max. coverage (+): 1.37. Max coverage (-): 0.48

Region: NODE\_319315\_length\_10503\_cov\_30.863943 6645-6661. Max. coverage (+): 6.22. Max coverage (-): 0.04

Region: NODE\_319315\_length\_10503\_cov\_30.863943 6662-6678. Max. coverage (+): 0.12. Max coverage (-): 0.04

Region: NODE\_319315\_length\_10503\_cov\_30.863943 6679-6696. Max. coverage (+): 5.94. Max coverage (-): 0.28

Region: NODE\_319315\_length\_10503\_cov\_30.863943 6697-6713. Max. coverage (+): 6.26. Max coverage (-): 0

Region: NODE\_319315\_length\_10503\_cov\_30.863943 6714-6730. Max. coverage (+): 0.4. Max coverage (-): 0.89

Region: NODE\_319315\_length\_10503\_cov\_30.863943 6731-6747. Max. coverage (+): 3.23. Max coverage (-): 0.08

Region: NODE\_319315\_length\_10503\_cov\_30.863943 6748-6764. Max. coverage (+): 0.65. Max coverage (-): 0.85

Region: NODE\_319315\_length\_10503\_cov\_30.863943 6765-6782. Max. coverage (+): 73.97. Max coverage (-): 0.24

Region: NODE\_319315\_length\_10503\_cov\_30.863943 6783-6799. Max. coverage (+): 1.33. Max coverage (-): 0.04

Region: NODE\_319315\_length\_10503\_cov\_30.863943 6800-6816. Max. coverage (+): 1.09. Max coverage (-): 0.04

Region: NODE\_319315\_length\_10503\_cov\_30.863943 6817-6833. Max. coverage (+): 0.24. Max coverage (-): 0.89

Region: NODE\_319315\_length\_10503\_cov\_30.863943 6834-6851. Max. coverage (+): 2.46. Max coverage (-): 0.57

Region: NODE\_319315\_length\_10503\_cov\_30.863943 6852-6868. Max. coverage (+): 0.36. Max coverage (-): 0.04

Region: NODE\_319315\_length\_10503\_cov\_30.863943 6869-6885. Max. coverage (+): 2.14. Max coverage (-): 0.12

Region: NODE\_319315\_length\_10503\_cov\_30.863943 6886-6902. Max. coverage (+): 1.45. Max coverage (-): 0.08

Region: NODE\_319315\_length\_10503\_cov\_30.863943 6903-6920. Max. coverage (+): 0.93. Max coverage (-): 0.04

Region: NODE\_319315\_length\_10503\_cov\_30.863943 6921-6937. Max. coverage (+): 5.05. Max coverage (-): 0.32

Region: NODE\_319315\_length\_10503\_cov\_30.863943 6938-6954. Max. coverage (+): 0.61. Max coverage (-): 0.04

Region: NODE\_319315\_length\_10503\_cov\_30.863943 6955-6971. Max. coverage (+): 0. Max coverage (-): 0.08

Region: NODE\_319315\_length\_10503\_cov\_30.863943 6972-6988. Max. coverage (+): 10.54. Max coverage (-): 1.25

Region: NODE\_319315\_length\_10503\_cov\_30.863943 6989-7006. Max. coverage (+): 10.5. Max coverage (-): 3.31

Region: NODE\_319315\_length\_10503\_cov\_30.863943 7007-7023. Max. coverage (+): 3.27. Max coverage (-): 3.39

Region: NODE\_319315\_length\_10503\_cov\_30.863943 7024-7040. Max. coverage (+): 2.91. Max coverage (-): 0.04

Region: NODE\_319315\_length\_10503\_cov\_30.863943 7041-7057. Max. coverage (+): 0.61. Max coverage (-): 0.16

Region: NODE\_319315\_length\_10503\_cov\_30.863943 7058-7075. Max. coverage (+): 1.17. Max coverage (-): 0

Region: NODE\_319315\_length\_10503\_cov\_30.863943 7076-7092. Max. coverage (+): 0.16. Max coverage (-): 0.04

Region: NODE\_319315\_length\_10503\_cov\_30.863943 7093-7109. Max. coverage (+): 0.2. Max coverage (-): 0.28

Region: NODE\_319315\_length\_10503\_cov\_30.863943 7110-7126. Max. coverage (+): 0.32. Max coverage (-): 0

Region: NODE\_319315\_length\_10503\_cov\_30.863943 7127-7143. Max. coverage (+): 1.33. Max coverage (-): 0.08

Region: NODE\_319315\_length\_10503\_cov\_30.863943 7144-7161. Max. coverage (+): 0.12. Max coverage (-): 0.69

Region: NODE\_319315\_length\_10503\_cov\_30.863943 7162-7178. Max. coverage (+): 1.74. Max coverage (-): 0.04

Region: NODE\_319315\_length\_10503\_cov\_30.863943 7179-7195. Max. coverage (+): 1.82. Max coverage (-): 0.04

Region: NODE\_319315\_length\_10503\_cov\_30.863943 7196-7212. Max. coverage (+): 1.41. Max coverage (-): 0.24

Region: NODE\_319315\_length\_10503\_cov\_30.863943 7213-7230. Max. coverage (+): 1.41. Max coverage (-): 0.57

Region: NODE\_319315\_length\_10503\_cov\_30.863943 7231-7247. Max. coverage (+): 1.7. Max coverage (-): 0.12

Region: NODE\_319315\_length\_10503\_cov\_30.863943 7248-7264. Max. coverage (+): 0.48. Max coverage (-): 0.04

Region: NODE\_319315\_length\_10503\_cov\_30.863943 7265-7281. Max. coverage (+): 0.24. Max coverage (-): 0.04

Region: NODE\_319315\_length\_10503\_cov\_30.863943 7282-7298. Max. coverage (+): 3.27. Max coverage (-): 0.04

Region: NODE\_319315\_length\_10503\_cov\_30.863943 7299-7316. Max. coverage (+): 3.27. Max coverage (-): 0.04

Region: NODE\_319315\_length\_10503\_cov\_30.863943 7317-7333. Max. coverage (+): 0.65. Max coverage (-): 0.48

Region: NODE\_319315\_length\_10503\_cov\_30.863943 7334-7350. Max. coverage (+): 0.24. Max coverage (-): 0

Region: NODE\_319315\_length\_10503\_cov\_30.863943 7351-7367. Max. coverage (+): 0.16. Max coverage (-): 0.12

Region: NODE\_319315\_length\_10503\_cov\_30.863943 7368-7385. Max. coverage (+): 1.09. Max coverage (-): 0.04

Region: NODE\_319315\_length\_10503\_cov\_30.863943 7386-7402. Max. coverage (+): 0.04. Max coverage (-): 0.28

Region: NODE\_319315\_length\_10503\_cov\_30.863943 7403-7419. Max. coverage (+): 3.59. Max coverage (-): 0.16

Region: NODE\_319315\_length\_10503\_cov\_30.863943 7420-7436. Max. coverage (+): 1.86. Max coverage (-): 0

Region: NODE\_319315\_length\_10503\_cov\_30.863943 7437-7454. Max. coverage (+): 1.57. Max coverage (-): 0

Region: NODE\_319315\_length\_10503\_cov\_30.863943 7455-7471. Max. coverage (+): 0. Max coverage (-): 0

Region: NODE\_319315\_length\_10503\_cov\_30.863943 7472-7488. Max. coverage (+): 4. Max coverage (-): 0.04

Region: NODE\_319315\_length\_10503\_cov\_30.863943 7489-7505. Max. coverage (+): 4.24. Max coverage (-): 0.52

Region: NODE\_319315\_length\_10503\_cov\_30.863943 7506-7522. Max. coverage (+): 25.68. Max coverage (-): 0.48

Region: NODE\_319315\_length\_10503\_cov\_30.863943 7523-7540. Max. coverage (+): 0.61. Max coverage (-): 0

Region: NODE\_319315\_length\_10503\_cov\_30.863943 7541-7557. Max. coverage (+): 2.42. Max coverage (-): 0

Region: NODE\_319315\_length\_10503\_cov\_30.863943 7558-7574. Max. coverage (+): 14.09. Max coverage (-): 0.08

Region: NODE\_319315\_length\_10503\_cov\_30.863943 7575-7591. Max. coverage (+): 1.49. Max coverage (-): 0

Region: NODE\_319315\_length\_10503\_cov\_30.863943 7592-7609. Max. coverage (+): 2.91. Max coverage (-): 0.85

Region: NODE\_319315\_length\_10503\_cov\_30.863943 7610-7626. Max. coverage (+): 6.38. Max coverage (-): 0.04

Region: NODE\_319315\_length\_10503\_cov\_30.863943 7627-7643. Max. coverage (+): 0.48. Max coverage (-): 0.12

Region: NODE\_319315\_length\_10503\_cov\_30.863943 7644-7660. Max. coverage (+): 0.36. Max coverage (-): 0.2

Region: NODE\_319315\_length\_10503\_cov\_30.863943 7661-7677. Max. coverage (+): 0.32. Max coverage (-): 0.08

Region: NODE\_319315\_length\_10503\_cov\_30.863943 7678-7695. Max. coverage (+): 2.91. Max coverage (-): 0.12

Region: NODE\_319315\_length\_10503\_cov\_30.863943 7696-7712. Max. coverage (+): 1.37. Max coverage (-): 0

Region: NODE\_319315\_length\_10503\_cov\_30.863943 7713-7729. Max. coverage (+): 1.29. Max coverage (-): 0.08

Region: NODE\_319315\_length\_10503\_cov\_30.863943 7730-7746. Max. coverage (+): 0.28. Max coverage (-): 0.04

Region: NODE\_319315\_length\_10503\_cov\_30.863943 7747-7764. Max. coverage (+): 0.44. Max coverage (-): 0.04

Region: NODE\_319315\_length\_10503\_cov\_30.863943 7765-7781. Max. coverage (+): 1.74. Max coverage (-): 0.32

Region: NODE\_319315\_length\_10503\_cov\_30.863943 7782-7798. Max. coverage (+): 0.24. Max coverage (-): 0

Region: NODE\_319315\_length\_10503\_cov\_30.863943 7799-7815. Max. coverage (+): 0.16. Max coverage (-): 0.2

Region: NODE\_319315\_length\_10503\_cov\_30.863943 7816-7833. Max. coverage (+): 0.93. Max coverage (-): 0.12

Region: NODE\_319315\_length\_10503\_cov\_30.863943 7834-7850. Max. coverage (+): 0.89. Max coverage (-): 0.16

Region: NODE\_319315\_length\_10503\_cov\_30.863943 7851-7867. Max. coverage (+): 0.16. Max coverage (-): 0.36

Region: NODE\_319315\_length\_10503\_cov\_30.863943 7868-7884. Max. coverage (+): 1.94. Max coverage (-): 0.04

Region: NODE\_319315\_length\_10503\_cov\_30.863943 7885-7901. Max. coverage (+): 0.16. Max coverage (-): 0

Region: NODE\_319315\_length\_10503\_cov\_30.863943 7902-7919. Max. coverage (+): 6.82. Max coverage (-): 0.04

Region: NODE\_319315\_length\_10503\_cov\_30.863943 7920-7936. Max. coverage (+): 0.73. Max coverage (-): 0.08

Region: NODE\_319315\_length\_10503\_cov\_30.863943 7937-7953. Max. coverage (+): 1.05. Max coverage (-): 0

Region: NODE\_319315\_length\_10503\_cov\_30.863943 7954-7970. Max. coverage (+): 1.13. Max coverage (-): 0.52

Region: NODE\_319315\_length\_10503\_cov\_30.863943 7971-7988. Max. coverage (+): 5.94. Max coverage (-): 0.32

Region: NODE\_319315\_length\_10503\_cov\_30.863943 7989-8005. Max. coverage (+): 0.28. Max coverage (-): 0.04

Region: NODE\_319315\_length\_10503\_cov\_30.863943 8006-8022. Max. coverage (+): 0.04. Max coverage (-): 0.08

Region: NODE\_319315\_length\_10503\_cov\_30.863943 8023-8039. Max. coverage (+): 9.29. Max coverage (-): 0.12

Region: NODE\_319315\_length\_10503\_cov\_30.863943 8040-8056. Max. coverage (+): 1.62. Max coverage (-): 0

Region: NODE\_319315\_length\_10503\_cov\_30.863943 8057-8074. Max. coverage (+): 0.36. Max coverage (-): 1.82

Region: NODE\_319315\_length\_10503\_cov\_30.863943 8075-8091. Max. coverage (+): 6.46. Max coverage (-): 0.08

Region: NODE\_319315\_length\_10503\_cov\_30.863943 8092-8108. Max. coverage (+): 0.04. Max coverage (-): 0.08

Region: NODE\_319315\_length\_10503\_cov\_30.863943 8109-8125. Max. coverage (+): 0.52. Max coverage (-): 0.08

Region: NODE\_319315\_length\_10503\_cov\_30.863943 8126-8143. Max. coverage (+): 0.08. Max coverage (-): 0.16

Region: NODE\_319315\_length\_10503\_cov\_30.863943 8144-8160. Max. coverage (+): 0.48. Max coverage (-): 0.16

Region: NODE\_319315\_length\_10503\_cov\_30.863943 8161-8177. Max. coverage (+): 0.12. Max coverage (-): 0.04

Region: NODE\_319315\_length\_10503\_cov\_30.863943 8178-8194. Max. coverage (+): 0.89. Max coverage (-): 0.08

Region: NODE\_319315\_length\_10503\_cov\_30.863943 8195-8211. Max. coverage (+): 6.54. Max coverage (-): 0

Region: NODE\_319315\_length\_10503\_cov\_30.863943 8212-8229. Max. coverage (+): 0. Max coverage (-): 0.16

Region: NODE\_319315\_length\_10503\_cov\_30.863943 8230-8246. Max. coverage (+): 20.43. Max coverage (-): 0.04

Region: NODE\_319315\_length\_10503\_cov\_30.863943 8247-8263. Max. coverage (+): 0.12. Max coverage (-): 0.04

Region: NODE\_319315\_length\_10503\_cov\_30.863943 8264-8280. Max. coverage (+): 0.52. Max coverage (-): 0.04

Region: NODE\_319315\_length\_10503\_cov\_30.863943 8281-8298. Max. coverage (+): 0.52. Max coverage (-): 0.04

Region: NODE\_319315\_length\_10503\_cov\_30.863943 8299-8315. Max. coverage (+): 1.25. Max coverage (-): 0.04

Region: NODE\_319315\_length\_10503\_cov\_30.863943 8316-8332. Max. coverage (+): 0.57. Max coverage (-): 0.04

Region: NODE\_319315\_length\_10503\_cov\_30.863943 8333-8349. Max. coverage (+): 2.22. Max coverage (-): 0

Region: NODE\_319315\_length\_10503\_cov\_30.863943 8350-8367. Max. coverage (+): 0.08. Max coverage (-): 0.24

Region: NODE\_319315\_length\_10503\_cov\_30.863943 8368-8384. Max. coverage (+): 2.02. Max coverage (-): 0.2

Region: NODE\_319315\_length\_10503\_cov\_30.863943 8385-8401. Max. coverage (+): 2.18. Max coverage (-): 0.04

Region: NODE\_319315\_length\_10503\_cov\_30.863943 8402-8418. Max. coverage (+): 0.16. Max coverage (-): 0.04

Region: NODE\_319315\_length\_10503\_cov\_30.863943 8419-8435. Max. coverage (+): 0.28. Max coverage (-): 0

Region: NODE\_319315\_length\_10503\_cov\_30.863943 8436-8453. Max. coverage (+): 0.32. Max coverage (-): 0.04

Region: NODE\_319315\_length\_10503\_cov\_30.863943 8454-8470. Max. coverage (+): 1.13. Max coverage (-): 0

Region: NODE\_319315\_length\_10503\_cov\_30.863943 8471-8487. Max. coverage (+): 8.44. Max coverage (-): 0

Region: NODE\_319315\_length\_10503\_cov\_30.863943 8488-8504. Max. coverage (+): 1.37. Max coverage (-): 0.04

Region: NODE\_319315\_length\_10503\_cov\_30.863943 8505-8522. Max. coverage (+): 0.44. Max coverage (-): 0

Region: NODE\_319315\_length\_10503\_cov\_30.863943 8523-8539. Max. coverage (+): 2.71. Max coverage (-): 0

Region: NODE\_319315\_length\_10503\_cov\_30.863943 8540-8556. Max. coverage (+): 2.95. Max coverage (-): 0.04

Region: NODE\_319315\_length\_10503\_cov\_30.863943 8557-8573. Max. coverage (+): 0.44. Max coverage (-): 0.12

Region: NODE\_319315\_length\_10503\_cov\_30.863943 8574-8590. Max. coverage (+): 0.77. Max coverage (-): 0

Region: NODE\_319315\_length\_10503\_cov\_30.863943 8591-8608. Max. coverage (+): 0.2. Max coverage (-): 0

Region: NODE\_319315\_length\_10503\_cov\_30.863943 8609-8625. Max. coverage (+): 1.66. Max coverage (-): 0.04

Region: NODE\_319315\_length\_10503\_cov\_30.863943 8626-8642. Max. coverage (+): 2.06. Max coverage (-): 0.04

Region: NODE\_319315\_length\_10503\_cov\_30.863943 8643-8659. Max. coverage (+): 0.44. Max coverage (-): 0.04

Region: NODE\_319315\_length\_10503\_cov\_30.863943 8660-8677. Max. coverage (+): 0.69. Max coverage (-): 0.04

Region: NODE\_319315\_length\_10503\_cov\_30.863943 8678-8694. Max. coverage (+): 2.18. Max coverage (-): 0.08

Region: NODE\_319315\_length\_10503\_cov\_30.863943 8695-8711. Max. coverage (+): 1.05. Max coverage (-): 0.08

Region: NODE\_319315\_length\_10503\_cov\_30.863943 8712-8728. Max. coverage (+): 1.86. Max coverage (-): 0.12

Region: NODE\_319315\_length\_10503\_cov\_30.863943 8729-8745. Max. coverage (+): 11.87. Max coverage (-): 0.08

Region: NODE\_319315\_length\_10503\_cov\_30.863943 8746-8763. Max. coverage (+): 10.3. Max coverage (-): 1.21

Region: NODE\_319315\_length\_10503\_cov\_30.863943 8764-8780. Max. coverage (+): 10.82. Max coverage (-): 0

Region: NODE\_319315\_length\_10503\_cov\_30.863943 8781-8797. Max. coverage (+): 0.52. Max coverage (-): 0

Region: NODE\_319315\_length\_10503\_cov\_30.863943 8798-8814. Max. coverage (+): 6.46. Max coverage (-): 0.28

Region: NODE\_319315\_length\_10503\_cov\_30.863943 8815-8832. Max. coverage (+): 0.52. Max coverage (-): 0.32

Region: NODE\_319315\_length\_10503\_cov\_30.863943 8833-8849. Max. coverage (+): 0.28. Max coverage (-): 0

Region: NODE\_319315\_length\_10503\_cov\_30.863943 8850-8866. Max. coverage (+): 1.29. Max coverage (-): 2.3

Region: NODE\_319315\_length\_10503\_cov\_30.863943 8867-8883. Max. coverage (+): 2.58. Max coverage (-): 0.32

Region: NODE\_319315\_length\_10503\_cov\_30.863943 8884-8901. Max. coverage (+): 0.4. Max coverage (-): 2.14

Region: NODE\_319315\_length\_10503\_cov\_30.863943 8902-8918. Max. coverage (+): 0.4. Max coverage (-): 4.2

Region: NODE\_319315\_length\_10503\_cov\_30.863943 8919-8935. Max. coverage (+): 0.73. Max coverage (-): 0.04

Region: NODE\_319315\_length\_10503\_cov\_30.863943 8936-8952. Max. coverage (+): 0.36. Max coverage (-): 0.44

Region: NODE\_319315\_length\_10503\_cov\_30.863943 8953-8969. Max. coverage (+): 0.32. Max coverage (-): 0

Region: NODE\_319315\_length\_10503\_cov\_30.863943 8970-8987. Max. coverage (+): 0.08. Max coverage (-): 0.04

Region: NODE\_319315\_length\_10503\_cov\_30.863943 8988-9004. Max. coverage (+): 0.4. Max coverage (-): 0.08

Region: NODE\_319315\_length\_10503\_cov\_30.863943 9005-9021. Max. coverage (+): 0.12. Max coverage (-): 0.16

Region: NODE\_319315\_length\_10503\_cov\_30.863943 9022-9038. Max. coverage (+): 0.93. Max coverage (-): 0.08

Region: NODE\_319315\_length\_10503\_cov\_30.863943 9039-9056. Max. coverage (+): 0.36. Max coverage (-): 0

Region: NODE\_319315\_length\_10503\_cov\_30.863943 9057-9073. Max. coverage (+): 1.13. Max coverage (-): 0.12

Region: NODE\_319315\_length\_10503\_cov\_30.863943 9074-9090. Max. coverage (+): 0.48. Max coverage (-): 0.16

Region: NODE\_319315\_length\_10503\_cov\_30.863943 9091-9107. Max. coverage (+): 2.79. Max coverage (-): 0.28

Region: NODE\_319315\_length\_10503\_cov\_30.863943 9108-9124. Max. coverage (+): 2.95. Max coverage (-): 0.04

Region: NODE\_319315\_length\_10503\_cov\_30.863943 9125-9142. Max. coverage (+): 0.24. Max coverage (-): 0.4

Region: NODE\_319315\_length\_10503\_cov\_30.863943 9143-9159. Max. coverage (+): 3.51. Max coverage (-): 0.24

Region: NODE\_319315\_length\_10503\_cov\_30.863943 9160-9176. Max. coverage (+): 0.08. Max coverage (-): 0

Region: NODE\_319315\_length\_10503\_cov\_30.863943 9177-9193. Max. coverage (+): 0.65. Max coverage (-): 0.08

Region: NODE\_319315\_length\_10503\_cov\_30.863943 9194-9211. Max. coverage (+): 0.48. Max coverage (-): 0.08

Region: NODE\_319315\_length\_10503\_cov\_30.863943 9212-9228. Max. coverage (+): 0.4. Max coverage (-): 0

Region: NODE\_319315\_length\_10503\_cov\_30.863943 9229-9245. Max. coverage (+): 0.12. Max coverage (-): 0.16

Region: NODE\_319315\_length\_10503\_cov\_30.863943 9246-9262. Max. coverage (+): 7.31. Max coverage (-): 0.2

Region: NODE\_319315\_length\_10503\_cov\_30.863943 9263-9279. Max. coverage (+): 0.12. Max coverage (-): 0.16

Region: NODE\_319315\_length\_10503\_cov\_30.863943 9280-9297. Max. coverage (+): 0.04. Max coverage (-): 0.04

Region: NODE\_319315\_length\_10503\_cov\_30.863943 9298-9314. Max. coverage (+): 0.08. Max coverage (-): 0.04

Region: NODE\_319315\_length\_10503\_cov\_30.863943 9315-9331. Max. coverage (+): 0.04. Max coverage (-): 0

Region: NODE\_319315\_length\_10503\_cov\_30.863943 9332-9348. Max. coverage (+): 0.2. Max coverage (-): 0.04

Region: NODE\_319315\_length\_10503\_cov\_30.863943 9349-9366. Max. coverage (+): 0.44. Max coverage (-): 0.12

Region: NODE\_319315\_length\_10503\_cov\_30.863943 9367-9383. Max. coverage (+): 0.28. Max coverage (-): 0.04

Region: NODE\_319315\_length\_10503\_cov\_30.863943 9384-9400. Max. coverage (+): 1.86. Max coverage (-): 0.12

Region: NODE\_319315\_length\_10503\_cov\_30.863943 9401-9417. Max. coverage (+): 0.61. Max coverage (-): 0.04

Region: NODE\_319315\_length\_10503\_cov\_30.863943 9418-9435. Max. coverage (+): 0.32. Max coverage (-): 0

Region: NODE\_319315\_length\_10503\_cov\_30.863943 9436-9452. Max. coverage (+): 0.28. Max coverage (-): 0.04

Region: NODE\_319315\_length\_10503\_cov\_30.863943 9453-9469. Max. coverage (+): 1.98. Max coverage (-): 0.04

Region: NODE\_319315\_length\_10503\_cov\_30.863943 9470-9486. Max. coverage (+): 0.01. Max coverage (-): 0

Region: NODE\_319315\_length\_10503\_cov\_30.863943 9487-9503. Max. coverage (+): 0.27. Max coverage (-): 0.04

Region: NODE\_319315\_length\_10503\_cov\_30.863943 9504-9521. Max. coverage (+): 1.57. Max coverage (-): 0.28

Region: NODE\_319315\_length\_10503\_cov\_30.863943 9522-9538. Max. coverage (+): 1.09. Max coverage (-): 0

Region: NODE\_319315\_length\_10503\_cov\_30.863943 9539-9555. Max. coverage (+): 0.08. Max coverage (-): 0

Region: NODE\_319315\_length\_10503\_cov\_30.863943 9556-9572. Max. coverage (+): 0.81. Max coverage (-): 0.04

Region: NODE\_319315\_length\_10503\_cov\_30.863943 9573-9590. Max. coverage (+): 0.24. Max coverage (-): 0.32

Region: NODE\_319315\_length\_10503\_cov\_30.863943 9591-9607. Max. coverage (+): 2.71. Max coverage (-): 0.44

Region: NODE\_319315\_length\_10503\_cov\_30.863943 9608-9624. Max. coverage (+): 0.28. Max coverage (-): 0.04

Region: NODE\_319315\_length\_10503\_cov\_30.863943 9625-9641. Max. coverage (+): 0.16. Max coverage (-): 0.04

Region: NODE\_319315\_length\_10503\_cov\_30.863943 9642-9658. Max. coverage (+): 3.43. Max coverage (-): 0.12

Region: NODE\_319315\_length\_10503\_cov\_30.863943 9659-9676. Max. coverage (+): 3.92. Max coverage (-): 0.28

Region: NODE\_319315\_length\_10503\_cov\_30.863943 9677-9693. Max. coverage (+): 0.61. Max coverage (-): 0

Region: NODE\_319315\_length\_10503\_cov\_30.863943 9694-9710. Max. coverage (+): 0.16. Max coverage (-): 0.04

Region: NODE\_319315\_length\_10503\_cov\_30.863943 9711-9727. Max. coverage (+): 0.12. Max coverage (-): 0

Region: NODE\_319315\_length\_10503\_cov\_30.863943 9728-9745. Max. coverage (+): 0.08. Max coverage (-): 0

Region: NODE\_319315\_length\_10503\_cov\_30.863943 9746-9762. Max. coverage (+): 0.12. Max coverage (-): 0

Region: NODE\_319315\_length\_10503\_cov\_30.863943 9763-9779. Max. coverage (+): 10.66. Max coverage (-): 0

Region: NODE\_319315\_length\_10503\_cov\_30.863943 9780-9796. Max. coverage (+): 0.73. Max coverage (-): 0.04

Region: NODE\_319315\_length\_10503\_cov\_30.863943 9797-9813. Max. coverage (+): 0.97. Max coverage (-): 0.08

Region: NODE\_319315\_length\_10503\_cov\_30.863943 9814-9831. Max. coverage (+): 5.41. Max coverage (-): 0

Region: NODE\_319315\_length\_10503\_cov\_30.863943 9832-9848. Max. coverage (+): 5.45. Max coverage (-): 0

Region: NODE\_319315\_length\_10503\_cov\_30.863943 9849-9865. Max. coverage (+): 0.08. Max coverage (-): 0.69

Region: NODE\_319315\_length\_10503\_cov\_30.863943 9866-9882. Max. coverage (+): 0.89. Max coverage (-): 0.52

Region: NODE\_319315\_length\_10503\_cov\_30.863943 9883-9900. Max. coverage (+): 1.21. Max coverage (-): 0.04

Region: NODE\_319315\_length\_10503\_cov\_30.863943 9901-9917. Max. coverage (+): 0.32. Max coverage (-): 0

Region: NODE\_319315\_length\_10503\_cov\_30.863943 9918-9934. Max. coverage (+): 17.16. Max coverage (-): 0

Region: NODE\_319315\_length\_10503\_cov\_30.863943 9935-9951. Max. coverage (+): 18.7. Max coverage (-): 0

Region: NODE\_319315\_length\_10503\_cov\_30.863943 9952-9969. Max. coverage (+): 0.16. Max coverage (-): 0.81

Region: NODE\_319315\_length\_10503\_cov\_30.863943 9970-9986. Max. coverage (+): 8.2. Max coverage (-): 0.57

Region: NODE\_319315\_length\_10503\_cov\_30.863943 9987-10003. Max. coverage (+): 1.98. Max coverage (-): 0.2

Region: NODE\_319315\_length\_10503\_cov\_30.863943 10004-10020. Max. coverage (+): 0.48. Max coverage (-): 0.04

Region: NODE\_319315\_length\_10503\_cov\_30.863943 10021-10037. Max. coverage (+): 0.61. Max coverage (-): 0.24

Region: NODE\_319315\_length\_10503\_cov\_30.863943 10038-10055. Max. coverage (+): 11.87. Max coverage (-): 0

Region: NODE\_319315\_length\_10503\_cov\_30.863943 10056-10072. Max. coverage (+): 1.49. Max coverage (-): 0.04

Region: NODE\_319315\_length\_10503\_cov\_30.863943 10073-10089. Max. coverage (+): 0.48. Max coverage (-): 0.16

Region: NODE\_319315\_length\_10503\_cov\_30.863943 10090-10106. Max. coverage (+): 1.05. Max coverage (-): 0

Region: NODE\_319315\_length\_10503\_cov\_30.863943 10107-10124. Max. coverage (+): 0.81. Max coverage (-): 0

Region: NODE\_319315\_length\_10503\_cov\_30.863943 10125-10141. Max. coverage (+): 0. Max coverage (-): 0.44

Region: NODE\_319315\_length\_10503\_cov\_30.863943 10142-10158. Max. coverage (+): 0.32. Max coverage (-): 0.08

Region: NODE\_319315\_length\_10503\_cov\_30.863943 10159-10175. Max. coverage (+): 0.2. Max coverage (-): 0.16

Region: NODE\_319315\_length\_10503\_cov\_30.863943 10176-10192. Max. coverage (+): 0.36. Max coverage (-): 0.04

Region: NODE\_319315\_length\_10503\_cov\_30.863943 10193-10210. Max. coverage (+): 9.37. Max coverage (-): 0.08

Region: NODE\_319315\_length\_10503\_cov\_30.863943 10211-10227. Max. coverage (+): 0.16. Max coverage (-): 0.08

Region: NODE\_319315\_length\_10503\_cov\_30.863943 10228-10244. Max. coverage (+): 0.08. Max coverage (-): 0

Region: NODE\_319315\_length\_10503\_cov\_30.863943 10245-10261. Max. coverage (+): 0.08. Max coverage (-): 0.04

Region: NODE\_319315\_length\_10503\_cov\_30.863943 10262-10279. Max. coverage (+): 0.32. Max coverage (-): 0.04

Region: NODE\_319315\_length\_10503\_cov\_30.863943 10280-10296. Max. coverage (+): 1.21. Max coverage (-): 0

Region: NODE\_319315\_length\_10503\_cov\_30.863943 10297-10313. Max. coverage (+): 0.24. Max coverage (-): 0.04

Region: NODE\_319315\_length\_10503\_cov\_30.863943 10314-10330. Max. coverage (+): 0.32. Max coverage (-): 0.2

Region: NODE\_319315\_length\_10503\_cov\_30.863943 10331-10347. Max. coverage (+): 1.05. Max coverage (-): 0

Region: NODE\_319315\_length\_10503\_cov\_30.863943 10348-10365. Max. coverage (+): 0.4. Max coverage (-): 0.28

Region: NODE\_319315\_length\_10503\_cov\_30.863943 10366-10382. Max. coverage (+): 0.57. Max coverage (-): 0

Region: NODE\_319315\_length\_10503\_cov\_30.863943 10383-10399. Max. coverage (+): 0.28. Max coverage (-): 0

Region: NODE\_319315\_length\_10503\_cov\_30.863943 10400-10416. Max. coverage (+): 0.48. Max coverage (-): 0

Region: NODE\_319315\_length\_10503\_cov\_30.863943 10417-10434. Max. coverage (+): 0.24. Max coverage (-): 0.04

Region: NODE\_319315\_length\_10503\_cov\_30.863943 10435-10451. Max. coverage (+): 0.04. Max coverage (-): 0.04

Region: NODE\_319315\_length\_10503\_cov\_30.863943 10452-10468. Max. coverage (+): 0.04. Max coverage (-): 0

Region: NODE\_319315\_length\_10503\_cov\_30.863943 10469-10485. Max. coverage (+): 0.08. Max coverage (-): 0.04

Region: NODE\_319315\_length\_10503\_cov\_30.863943 10486-10503. Max. coverage (+): 0. Max coverage (-): 0.04

Region: NODE\_319315\_length\_10503\_cov\_30.863943 10504-10520. Max. coverage (+): 0.36. Max coverage (-): 0.65

Region: NODE\_319315\_length\_10503\_cov\_30.863943 10521-10537. Max. coverage (+): 1.66. Max coverage (-): 0.04

Region: NODE\_319315\_length\_10503\_cov\_30.863943 10538-10554. Max. coverage (+): 0.26. Max coverage (-): 0.02

Region: NODE\_319315\_length\_10503\_cov\_30.863943 10555-10571. Max. coverage (+): 0. Max coverage (-): 0.02

Region: NODE\_319315\_length\_10503\_cov\_30.863943 10572-10589. Max. coverage (+): 0.01. Max coverage (-): 0

Region: NODE\_319315\_length\_10503\_cov\_30.863943 10590-10606. Max. coverage (+): 0.01. Max coverage (-): 0

Region: NODE\_319315\_length\_10503\_cov\_30.863943 10607-. Max. coverage (+): 0. Max coverage (-): 0

RepeatMasker Color Code

**+**

100-98% Identity

<98-95% Identity

<95-90% Identity

<90-85% Identity

<85-80% Identity

<80-75% Identity

<75-70% Identity

<70% Identity

**-**

Gene Set Color Code

**+**

Gene

Pseudogene

Other

**-**

Topology/Coverage Color Code

Coverage Plus Strand

Coverage Minus Strand

Mainstrand: Plus

Mainstrand: Minus

Complementary Strand

Flanking Region  
(if option -flank >0)

Gene Set Annotation  
  
RepeatMasker Annotation  

**1. AlRepC-169**: 1708-2008 (-), Divergence to consensus: 14.6%  
**2. A-rich**: 2920-2972 (+), Divergence to consensus: 30.5%  
**3. AlRepD-870**: 3695-3740 (+), Divergence to consensus: 19.6%  
**4. AlRepA-115**: 4796-4889 (+), Divergence to consensus: 24.5%  
**5. AlRepB-784**: 5136-5811 (+), Divergence to consensus: 11.7%  
**6. AlRepB-250**: 7149-7247 (-), Divergence to consensus: 27.3%  
**7. AlRepB-250**: 7245-7297 (-), Divergence to consensus: 28.3%  
**8. (GGA)n**: 8132-8148 (+), Divergence to consensus: 0%  
**9. AlRepC-1837**: 8432-8588 (-), Divergence to consensus: 38.9%  
**10. AlRepA-93**: 8616-8692 (-), Divergence to consensus: 35.1%  
**11. AlRepC-693**: 8972-9034 (-), Divergence to consensus: 22.6%  
**12. AlRepD-886**: 9132-9258 (-), Divergence to consensus: 30.4%  
**13. RTE-2\_AFC**: 9394-9546 (+), Divergence to consensus: 32.2%  
**14. AlRepC-234**: 10540-10621 (+), Divergence to consensus: 8.5%

  
Transcription Factor Binding Sites  

**RHOXF1** (Sequence: AGATCA (-): 2174)  
**RHOXF1** (Sequence: AGATTA (-): 3037)  
**RHOXF1** (Sequence: AGCTTA (-): 3217)  
**RHOXF1** (Sequence: AGATTA (-): 3526)  
**RHOXF1** (Sequence: AGATTA (-): 3961)  
**RHOXF1** (Sequence: AGCTCA (-): 4024)  
**RHOXF1** (Sequence: AGCTCA (-): 4187)  
**RHOXF1** (Sequence: AGCTTA (-): 4876)  
**RHOXF1** (Sequence: AGCTCA (-): 5440)  
**RHOXF1** (Sequence: GGCTCA (-): 5979)  
**RHOXF1** (Sequence: AGATTA (-): 6597)  
**RHOXF1** (Sequence: GGATCA (-): 7381)  
**RHOXF1** (Sequence: GGCTCA (-): 8123)  
**RHOXF1** (Sequence: AGATTA (-): 9405)  
**RHOXF1** (Sequence: AGCTCA (-): 9671)  
**RHOXF1** (Sequence: AGCTTA (-): 10068)  
**RHOXF1** (Sequence: GGCTTA (-): 10224)  
**RHOXF1** (Sequence: AGCTTA (-): 10356)  
**RHOXF1** (Sequence: TAAGCC (+): 2394)  
**RHOXF1** (Sequence: TGATCC (+): 2955)  
**RHOXF1** (Sequence: TAATCC (+): 4337)  
**RHOXF1** (Sequence: TAAGCT (+): 5199)  
**RHOXF1** (Sequence: TAATCT (+): 6162)  
**RHOXF1** (Sequence: TGAGCT (+): 6240)  
**RHOXF1** (Sequence: TAATCT (+): 6242)  
**RHOXF1** (Sequence: TAATCT (+): 6527)  
**RHOXF1** (Sequence: TGATCT (+): 7510)  
**RHOXF1** (Sequence: TGAGCT (+): 8295)  
**RHOXF1** (Sequence: TGATCT (+): 8733)  
**RHOXF1** (Sequence: TGATCT (+): 9648)  
**RHOXF1** (Sequence: TAATCT (+): 9696)  
**RHOXF1** (Sequence: TGATCT (+): 10196)  
**Gata4** (Sequence: CTTATCT (+): 10226)  
**POU5F1** (Sequence: TTTGCAT (-): 5900)  
**POU5F1** (Sequence: TTTGCAT (-): 7915)  
**POU5F1** (Sequence: TTTGCAT (-): 9288)  
**RFX4\_1** (Sequence: GTTGCTAGG (-): 8885)  
**FOXO3\_hsa** (Sequence: GTAAACAA (+): 5864)  
**SOX9** (Sequence: AACAATGG (-): 5379)  
**SOX9** (Sequence: AACAATGA (-): 9308)  
**FOXP1** (Sequence: GTAAACA (+): 4655)  
**FOXP1** (Sequence: GTAAACA (+): 5864)  
**FOXO1** (Sequence: CTTGTTTAT (+): 4999)  
**FOXO1** (Sequence: CTTGTTTAT (+): 5406)  
**FOXO1** (Sequence: CCTGTTTAT (+): 8835)  
**FOXO1** (Sequence: GTTGTTTTC (+): 9684)  
**FOXO1** (Sequence: GCTGTTTTT (+): 10414)  
**FOXO3\_mmu** (Sequence: TGTTTAGA (-): 2169)  
**FOXO3\_mmu** (Sequence: TGTTTTCC (-): 4071)  
**FOXO3\_mmu** (Sequence: TGTTTAGA (-): 7131)  
**Sox5** (Sequence: ATTGTT (+): 2588)  
**Sox5** (Sequence: ATTGTT (+): 5117)  
**Sox5** (Sequence: ATTGTT (+): 5187)  
**Sox5** (Sequence: ATTGTT (+): 5249)  
**Sox5** (Sequence: ATTGTT (+): 7307)  
**Sox5** (Sequence: ATTGTT (+): 7486)  
**Sox5** (Sequence: ATTGTT (+): 9411)  
**Mybl1\_1** (Sequence: TAACGGTT (-): 2402)  
**SOX9** (Sequence: CTATTGTT (+): 2586)  
**SOX9** (Sequence: CTATTGTT (+): 5185)  
**SOX9** (Sequence: TTATTGTT (+): 7305)  
**FOXO3\_mmu** (Sequence: TGTAAACA (+): 4654)  
**FOXO3\_mmu** (Sequence: GGAAAACA (+): 8390)  
**Nobox** (Sequence: GCTAATTA (-): 3362)  
**Nobox** (Sequence: AGTAATTA (-): 4930)  
**FOXO1** (Sequence: GAAAACAAG (-): 8403)  
**FOXO3\_hsa** (Sequence: ATGTTTAC (-): 9771)  
**FOXP1** (Sequence: TGTTTAC (-): 5236)  
**FOXP1** (Sequence: TGTTTAC (-): 9772)  
**Nobox** (Sequence: TAATTACT (+): 3364)  
**Nobox** (Sequence: TAATTGCT (+): 6532)  
**Nobox** (Sequence: TAATTAGC (+): 9335)  
**POU2F1** (Sequence: ATTAACATA (-): 3125)  
**Rhox11** (Sequence: CGCTGTAAA (+): 8057)  
**Rhox11** (Sequence: CGGTGTAAT (+): 9957)  
**Rhox11** (Sequence: TTTACAGCA (-): 3564)  
**Sox5** (Sequence: AACAAT (-): 2527)  
**Sox5** (Sequence: AACAAT (-): 4599)  
**Sox5** (Sequence: AACAAT (-): 5379)  
**Sox5** (Sequence: AACAAT (-): 8286)  
**Sox5** (Sequence: AACAAT (-): 8449)  
**Sox5** (Sequence: AACAAT (-): 9308)  
**POU5F1** (Sequence: ATGCAAA (+): 2674)
