# Supplementary material for: First characterization of PIWI-interacting RNA clusters in a cichlid fish with a B chromosome
Source: BMC Biol. 2022 Sep 21;20:204. doi: 10.1186/s12915-022-01403-2 (PMC9490952; doi:10.1186/s12915-022-01403-2)
Supplement: Supplementary file 1 — Additional file 1. Zipped folder with fasta and interactive html piRNA cluster information for the A. latifasciata genome. The nomenclature is as follows: number-pirna-cluster_sex_B-presence (f, female; m, male; 0b, without B chromosome; 1b, with B chromosome). [file 12915_2022_1403_MOESM1_ESM.zip › 127_m1b.html]

piRNA cluster 127\_m1b 74


Predicted piRNA cluster no. 127\_m1b
  

Show proTRAC run info
Hide proTRAC run info

/\  
                \_\_\_\_\_\_\_\_\_\_\_\_\_\_\_\_\_\_\_\_\_\_\_/\\_\_\_ /  \\_\_\_\_\_\_\_  
               I                      /  \  /    \      I  
               I     pro             /    \/      \     I  
               I        TRAC        /               \   I  
               I   \_\_\_\_\_\_\_\_\_\_\_\_\_\_\_\_/\_\_\_\_\_\_\_\_\_\_\_\_\_\_\_\_\_\\_ I  
               I   \              /                     I  
               I    \            /                      I  
               I     \  /\      /       V.2.4.2         I  
               I      \/  \    /                        I  
               I\_\_\_\_\_\_\_\_\_\_\_\  /\_\_\_\_\_\_\_\_\_\_\_\_\_\_\_\_\_\_\_\_\_\_\_\_\_I  
                            \/  
  
  
================================= proTRAC ====================================  
VERSION: .......... 2.4.2  
LAST MODIFIED: .... 11. May 2018  
  
Please cite:  
Rosenkranz D, Zischler H. proTRAC - a software for probabilistic piRNA cluster  
detection, visualization and analysis. 2012. BMC Bioinformatics 13:5.  
  
  
Contact:  
David Rosenkranz  
Institute of Organismic and Molecular Evolutionary Biology  
Dept. Anthropology, small RNA group  
Johannes Gutenberg University Mainz  
email: rosenkranz@uni-mainz.de  
  
You can find the latest proTRAC version at:  
http://sourceforge.net/projects/protrac/files  
http://www.smallRNAgroup-mainz.de/software  
==============================================================================  
  
PARAMETERS:  
Map file: ...............piwi-machos-1B.fa-collapse.map  
Genome file: ............../../../0B\_ala\_genome.fa  
RepeatMasker annotation: Alatifasciata-all0B-maryan-v2.fa\_corrected.out  
GeneSet:................./guest-storage/Data/annotation/Alatifasciata\_all0B\_maryan-v2\_out2017.gff  
  
Significant (p<=0.01) hit density will be calculated based  
on observed hit distribution.  
  
Sliding window size: ........................................ 5000 bp  
Sliding window increament: .................................. 1000 bp  
Normalize each hit by number of genomic hits: ............... yes  
Normalize each hit by number of sequence reads: ............. yes  
Normalize values (-> per million mapped reads): ............. yes  
Min. fraction of hits with 1T(U) or 10A: .................... 0.75  
Alternatively: Min. fraction of hits with 1T(U) and 10A: .... 0.5  
Min. fraction of hits with typical piRNA length: ............ 0.75  
Typical piRNA length: ....................................... 24-32 nt  
Min. size of a piRNA cluster: ............................... 1000 bp.  
Min. number of hits (absolute): ............................. 0  
Min. number of hits (normalized): ........................... 0  
Min. fraction of hits on the mainstrand: .................... 0.75  
Top fraction of mapped sequences (in terms of read counts): . 1%  
Top fraction accounts for max. n% of sequence reads: ........ 90%  
Min. fraction of hits on each arm of a bidirectional cluster: 0.05  
Output html file for each cluster: .......................... yes  
Output a summary table: ..................................... yes  
Output a FASTA file for each cluster (piRNA sequences): ..... yes  
Output a FASTA file comprising cluster sequences: ........... yes  
Output a GTF file for predicted piRNA clusters: ..............yes  
Search DNA motifs in clusters: .............................. yes  
Output flanking sequences: +/- .............................. 0 bp  
Output ~.pTi file: .......................................... no  
==============================================================================  
  
  
Genome size (without gaps): ............ 758543724 bp  
Gaps (N/X/-): .......................... 417479 bp  
Mapped reads: .......................... 26973943  
Non-identical sequences: ............... 6209225  
Genomic hits: .......................... 48438990  
Significant densitiy of mapped reads: .. 821.144211136946 reads/kb

Show proTRAC cluster info
Hide proTRAC cluster info

|  |  |
| --- | --- |
| Location | NODE\_319315\_length\_10503\_cov\_30.863943 |
| Coordinates | 7-10569 |
| Size [bp] | 10563 |
| Sequence hit loci | 7216 |
| Mapped reads (normalized) | 22942.6 |
| Mapped reads (normalized) per kb | 2172 |
| Normalized reads with 1T (1U) | 77.9% |
| Normalized reads with 10A | 45.1% |
| Normalized reads with length 24-32 nt | 98.7% |
| Normalized reads on the main strand(s) | 89.5% |
| Predicted directionality | bi:minus-plus (split between 2398 and 2399) |

100%

0%

1T (1U)  
reads

10A reads

24-32 nt  
reads

reads on mainstrand

**Either the amount of reads with 1T (1U) OR 10A has to exceed 75% (set with option: -1Tor10A)  
Alternatively the amount of reads with 1T (1U) AND 10A has to exceed 50% (set with option: -1Tand10A)  
Minimum amount of reads with preferred size is 75% (set with option: -pisize)  
Minimum amount of reads on the main strand(s) is 75% (set with option: -clstrand)**

Show read coverage
Hide read coverage

WHAT DO I SEE HERE?  
This chart shows the location of mapped sequence reads within a predicted piRNA cluster. The color refers to the number of genomic hits produced by the sequence read in question. A dark red bar indicates that this sequence read produces many other hits elsewhere in the genome. Many adjacent red or yellow bars can indicate the presence of a multi-copy element such as transposons or rRNA genes. A dark green bar indicates that this sequence read maps uniquely to this locus.

1 hit

2-5 hits

6-10 hits

11-20 hits

21-50 hits

51-100 hits

> 100 hits

NODE\_319315\_length\_10503\_cov\_30.863943

7

10569

Gene Set

RepeatMasker

Mapped  
Reads

30.7

plus strand

minus strand

30.7

Region: NODE\_319315\_length\_10503\_cov\_30.863943 3789-17. Max. coverage (+): 0.04. Max coverage (-): 0.19

Region: NODE\_319315\_length\_10503\_cov\_30.863943 18-38. Max. coverage (+): 0.11. Max coverage (-): 1.93

Region: NODE\_319315\_length\_10503\_cov\_30.863943 39-59. Max. coverage (+): 0.04. Max coverage (-): 0.93

Region: NODE\_319315\_length\_10503\_cov\_30.863943 60-80. Max. coverage (+): 0.07. Max coverage (-): 1.04

Region: NODE\_319315\_length\_10503\_cov\_30.863943 81-102. Max. coverage (+): 0.07. Max coverage (-): 0.19

Region: NODE\_319315\_length\_10503\_cov\_30.863943 103-123. Max. coverage (+): 0.07. Max coverage (-): 3.89

Region: NODE\_319315\_length\_10503\_cov\_30.863943 124-144. Max. coverage (+): 0.93. Max coverage (-): 3.3

Region: NODE\_319315\_length\_10503\_cov\_30.863943 145-165. Max. coverage (+): 0.07. Max coverage (-): 0.26

Region: NODE\_319315\_length\_10503\_cov\_30.863943 166-186. Max. coverage (+): 0.04. Max coverage (-): 2.93

Region: NODE\_319315\_length\_10503\_cov\_30.863943 187-207. Max. coverage (+): 0.07. Max coverage (-): 2.82

Region: NODE\_319315\_length\_10503\_cov\_30.863943 208-228. Max. coverage (+): 0.19. Max coverage (-): 0.41

Region: NODE\_319315\_length\_10503\_cov\_30.863943 229-249. Max. coverage (+): 0. Max coverage (-): 1.52

Region: NODE\_319315\_length\_10503\_cov\_30.863943 250-271. Max. coverage (+): 0.37. Max coverage (-): 0.3

Region: NODE\_319315\_length\_10503\_cov\_30.863943 272-292. Max. coverage (+): 0.07. Max coverage (-): 2.63

Region: NODE\_319315\_length\_10503\_cov\_30.863943 293-313. Max. coverage (+): 0.59. Max coverage (-): 1.48

Region: NODE\_319315\_length\_10503\_cov\_30.863943 314-334. Max. coverage (+): 0.11. Max coverage (-): 0.63

Region: NODE\_319315\_length\_10503\_cov\_30.863943 335-355. Max. coverage (+): 0.04. Max coverage (-): 0.22

Region: NODE\_319315\_length\_10503\_cov\_30.863943 356-376. Max. coverage (+): 0.11. Max coverage (-): 0.22

Region: NODE\_319315\_length\_10503\_cov\_30.863943 377-397. Max. coverage (+): 1.08. Max coverage (-): 6.27

Region: NODE\_319315\_length\_10503\_cov\_30.863943 398-418. Max. coverage (+): 0.93. Max coverage (-): 0.41

Region: NODE\_319315\_length\_10503\_cov\_30.863943 419-440. Max. coverage (+): 0.3. Max coverage (-): 1

Region: NODE\_319315\_length\_10503\_cov\_30.863943 441-461. Max. coverage (+): 0.04. Max coverage (-): 0.7

Region: NODE\_319315\_length\_10503\_cov\_30.863943 462-482. Max. coverage (+): 0. Max coverage (-): 0.44

Region: NODE\_319315\_length\_10503\_cov\_30.863943 483-503. Max. coverage (+): 0.07. Max coverage (-): 6.67

Region: NODE\_319315\_length\_10503\_cov\_30.863943 504-524. Max. coverage (+): 0. Max coverage (-): 0.15

Region: NODE\_319315\_length\_10503\_cov\_30.863943 525-545. Max. coverage (+): 0.41. Max coverage (-): 9.31

Region: NODE\_319315\_length\_10503\_cov\_30.863943 546-566. Max. coverage (+): 0. Max coverage (-): 7.19

Region: NODE\_319315\_length\_10503\_cov\_30.863943 567-587. Max. coverage (+): 0.04. Max coverage (-): 6.23

Region: NODE\_319315\_length\_10503\_cov\_30.863943 588-609. Max. coverage (+): 0.07. Max coverage (-): 3.56

Region: NODE\_319315\_length\_10503\_cov\_30.863943 610-630. Max. coverage (+): 0.07. Max coverage (-): 0.07

Region: NODE\_319315\_length\_10503\_cov\_30.863943 631-651. Max. coverage (+): 0.04. Max coverage (-): 0.59

Region: NODE\_319315\_length\_10503\_cov\_30.863943 652-672. Max. coverage (+): 0.74. Max coverage (-): 3.97

Region: NODE\_319315\_length\_10503\_cov\_30.863943 673-693. Max. coverage (+): 0.04. Max coverage (-): 1.04

Region: NODE\_319315\_length\_10503\_cov\_30.863943 694-714. Max. coverage (+): 0.04. Max coverage (-): 0.82

Region: NODE\_319315\_length\_10503\_cov\_30.863943 715-735. Max. coverage (+): 0.07. Max coverage (-): 0.41

Region: NODE\_319315\_length\_10503\_cov\_30.863943 736-756. Max. coverage (+): 0.19. Max coverage (-): 0

Region: NODE\_319315\_length\_10503\_cov\_30.863943 757-778. Max. coverage (+): 0.26. Max coverage (-): 1.74

Region: NODE\_319315\_length\_10503\_cov\_30.863943 779-799. Max. coverage (+): 1.56. Max coverage (-): 1.78

Region: NODE\_319315\_length\_10503\_cov\_30.863943 800-820. Max. coverage (+): 0.33. Max coverage (-): 1.93

Region: NODE\_319315\_length\_10503\_cov\_30.863943 821-841. Max. coverage (+): 0.33. Max coverage (-): 0.33

Region: NODE\_319315\_length\_10503\_cov\_30.863943 842-862. Max. coverage (+): 0.26. Max coverage (-): 0.56

Region: NODE\_319315\_length\_10503\_cov\_30.863943 863-883. Max. coverage (+): 0.07. Max coverage (-): 0.59

Region: NODE\_319315\_length\_10503\_cov\_30.863943 884-904. Max. coverage (+): 0.11. Max coverage (-): 3.23

Region: NODE\_319315\_length\_10503\_cov\_30.863943 905-925. Max. coverage (+): 0.7. Max coverage (-): 6.38

Region: NODE\_319315\_length\_10503\_cov\_30.863943 926-947. Max. coverage (+): 0.33. Max coverage (-): 0.7

Region: NODE\_319315\_length\_10503\_cov\_30.863943 948-968. Max. coverage (+): 0. Max coverage (-): 0.3

Region: NODE\_319315\_length\_10503\_cov\_30.863943 969-989. Max. coverage (+): 0. Max coverage (-): 0.15

Region: NODE\_319315\_length\_10503\_cov\_30.863943 990-1010. Max. coverage (+): 0.04. Max coverage (-): 0.07

Region: NODE\_319315\_length\_10503\_cov\_30.863943 1011-1031. Max. coverage (+): 0.11. Max coverage (-): 0.26

Region: NODE\_319315\_length\_10503\_cov\_30.863943 1032-1052. Max. coverage (+): 0.22. Max coverage (-): 0.04

Region: NODE\_319315\_length\_10503\_cov\_30.863943 1053-1073. Max. coverage (+): 0.04. Max coverage (-): 1.48

Region: NODE\_319315\_length\_10503\_cov\_30.863943 1074-1094. Max. coverage (+): 0.07. Max coverage (-): 0.22

Region: NODE\_319315\_length\_10503\_cov\_30.863943 1095-1116. Max. coverage (+): 0.07. Max coverage (-): 0.04

Region: NODE\_319315\_length\_10503\_cov\_30.863943 1117-1137. Max. coverage (+): 0. Max coverage (-): 0.19

Region: NODE\_319315\_length\_10503\_cov\_30.863943 1138-1158. Max. coverage (+): 1.15. Max coverage (-): 0.04

Region: NODE\_319315\_length\_10503\_cov\_30.863943 1159-1179. Max. coverage (+): 0.04. Max coverage (-): 0.04

Region: NODE\_319315\_length\_10503\_cov\_30.863943 1180-1200. Max. coverage (+): 0.22. Max coverage (-): 0.19

Region: NODE\_319315\_length\_10503\_cov\_30.863943 1201-1221. Max. coverage (+): 0. Max coverage (-): 0.09

Region: NODE\_319315\_length\_10503\_cov\_30.863943 1222-1242. Max. coverage (+): 0.01. Max coverage (-): 0

Region: NODE\_319315\_length\_10503\_cov\_30.863943 1243-1263. Max. coverage (+): 0. Max coverage (-): 0

Region: NODE\_319315\_length\_10503\_cov\_30.863943 1264-1285. Max. coverage (+): 0. Max coverage (-): 0

Region: NODE\_319315\_length\_10503\_cov\_30.863943 1286-1306. Max. coverage (+): 0. Max coverage (-): 0.11

Region: NODE\_319315\_length\_10503\_cov\_30.863943 1307-1327. Max. coverage (+): 0. Max coverage (-): 0.19

Region: NODE\_319315\_length\_10503\_cov\_30.863943 1328-1348. Max. coverage (+): 0. Max coverage (-): 0

Region: NODE\_319315\_length\_10503\_cov\_30.863943 1349-1369. Max. coverage (+): 0. Max coverage (-): 0.02

Region: NODE\_319315\_length\_10503\_cov\_30.863943 1370-1390. Max. coverage (+): 0. Max coverage (-): 0.02

Region: NODE\_319315\_length\_10503\_cov\_30.863943 1391-1411. Max. coverage (+): 0. Max coverage (-): 0

Region: NODE\_319315\_length\_10503\_cov\_30.863943 1412-1433. Max. coverage (+): 0.01. Max coverage (-): 0.04

Region: NODE\_319315\_length\_10503\_cov\_30.863943 1434-1454. Max. coverage (+): 0. Max coverage (-): 0

Region: NODE\_319315\_length\_10503\_cov\_30.863943 1455-1475. Max. coverage (+): 0. Max coverage (-): 0

Region: NODE\_319315\_length\_10503\_cov\_30.863943 1476-1496. Max. coverage (+): 0. Max coverage (-): 0

Region: NODE\_319315\_length\_10503\_cov\_30.863943 1497-1517. Max. coverage (+): 0. Max coverage (-): 0.04

Region: NODE\_319315\_length\_10503\_cov\_30.863943 1518-1538. Max. coverage (+): 0. Max coverage (-): 0.02

Region: NODE\_319315\_length\_10503\_cov\_30.863943 1539-1559. Max. coverage (+): 0. Max coverage (-): 0

Region: NODE\_319315\_length\_10503\_cov\_30.863943 1560-1580. Max. coverage (+): 0.01. Max coverage (-): 0

Region: NODE\_319315\_length\_10503\_cov\_30.863943 1581-1602. Max. coverage (+): 0. Max coverage (-): 0.07

Region: NODE\_319315\_length\_10503\_cov\_30.863943 1603-1623. Max. coverage (+): 0.04. Max coverage (-): 0.06

Region: NODE\_319315\_length\_10503\_cov\_30.863943 1624-1644. Max. coverage (+): 0.04. Max coverage (-): 0.04

Region: NODE\_319315\_length\_10503\_cov\_30.863943 1645-1665. Max. coverage (+): 0.04. Max coverage (-): 0.22

Region: NODE\_319315\_length\_10503\_cov\_30.863943 1666-1686. Max. coverage (+): 0.02. Max coverage (-): 0.04

Region: NODE\_319315\_length\_10503\_cov\_30.863943 1687-1707. Max. coverage (+): 0. Max coverage (-): 0

Region: NODE\_319315\_length\_10503\_cov\_30.863943 1708-1728. Max. coverage (+): 0. Max coverage (-): 0

Region: NODE\_319315\_length\_10503\_cov\_30.863943 1729-1749. Max. coverage (+): 0.01. Max coverage (-): 0

Region: NODE\_319315\_length\_10503\_cov\_30.863943 1750-1771. Max. coverage (+): 0. Max coverage (-): 0.05

Region: NODE\_319315\_length\_10503\_cov\_30.863943 1772-1792. Max. coverage (+): 0.01. Max coverage (-): 0.04

Region: NODE\_319315\_length\_10503\_cov\_30.863943 1793-1813. Max. coverage (+): 0. Max coverage (-): 0

Region: NODE\_319315\_length\_10503\_cov\_30.863943 1814-1834. Max. coverage (+): 0.01. Max coverage (-): 0

Region: NODE\_319315\_length\_10503\_cov\_30.863943 1835-1855. Max. coverage (+): 0. Max coverage (-): 0

Region: NODE\_319315\_length\_10503\_cov\_30.863943 1856-1876. Max. coverage (+): 0.07. Max coverage (-): 0.07

Region: NODE\_319315\_length\_10503\_cov\_30.863943 1877-1897. Max. coverage (+): 0.22. Max coverage (-): 0

Region: NODE\_319315\_length\_10503\_cov\_30.863943 1898-1918. Max. coverage (+): 0. Max coverage (-): 0.04

Region: NODE\_319315\_length\_10503\_cov\_30.863943 1919-1940. Max. coverage (+): 0.04. Max coverage (-): 0.04

Region: NODE\_319315\_length\_10503\_cov\_30.863943 1941-1961. Max. coverage (+): 0.04. Max coverage (-): 0.61

Region: NODE\_319315\_length\_10503\_cov\_30.863943 1962-1982. Max. coverage (+): 0.06. Max coverage (-): 0.01

Region: NODE\_319315\_length\_10503\_cov\_30.863943 1983-2003. Max. coverage (+): 0.15. Max coverage (-): 0.01

Region: NODE\_319315\_length\_10503\_cov\_30.863943 2004-2024. Max. coverage (+): 0.3. Max coverage (-): 0.11

Region: NODE\_319315\_length\_10503\_cov\_30.863943 2025-2045. Max. coverage (+): 0.07. Max coverage (-): 0.04

Region: NODE\_319315\_length\_10503\_cov\_30.863943 2046-2066. Max. coverage (+): 0.04. Max coverage (-): 0

Region: NODE\_319315\_length\_10503\_cov\_30.863943 2067-2087. Max. coverage (+): 0.04. Max coverage (-): 0.26

Region: NODE\_319315\_length\_10503\_cov\_30.863943 2088-2109. Max. coverage (+): 0.22. Max coverage (-): 0.04

Region: NODE\_319315\_length\_10503\_cov\_30.863943 2110-2130. Max. coverage (+): 0.04. Max coverage (-): 0.15

Region: NODE\_319315\_length\_10503\_cov\_30.863943 2131-2151. Max. coverage (+): 0.11. Max coverage (-): 0.11

Region: NODE\_319315\_length\_10503\_cov\_30.863943 2152-2172. Max. coverage (+): 0.63. Max coverage (-): 0.07

Region: NODE\_319315\_length\_10503\_cov\_30.863943 2173-2193. Max. coverage (+): 0.04. Max coverage (-): 0.48

Region: NODE\_319315\_length\_10503\_cov\_30.863943 2194-2214. Max. coverage (+): 0.04. Max coverage (-): 0

Region: NODE\_319315\_length\_10503\_cov\_30.863943 2215-2235. Max. coverage (+): 0. Max coverage (-): 0

Region: NODE\_319315\_length\_10503\_cov\_30.863943 2236-2256. Max. coverage (+): 0.15. Max coverage (-): 0.04

Region: NODE\_319315\_length\_10503\_cov\_30.863943 2257-2278. Max. coverage (+): 0.07. Max coverage (-): 0.59

Region: NODE\_319315\_length\_10503\_cov\_30.863943 2279-2299. Max. coverage (+): 0.04. Max coverage (-): 0.7

Region: NODE\_319315\_length\_10503\_cov\_30.863943 2300-2320. Max. coverage (+): 0.07. Max coverage (-): 0.89

Region: NODE\_319315\_length\_10503\_cov\_30.863943 2321-2341. Max. coverage (+): 0. Max coverage (-): 0.7

Region: NODE\_319315\_length\_10503\_cov\_30.863943 2342-2362. Max. coverage (+): 0.04. Max coverage (-): 0.37

Region: NODE\_319315\_length\_10503\_cov\_30.863943 2363-2383. Max. coverage (+): 0.19. Max coverage (-): 0

Region: NODE\_319315\_length\_10503\_cov\_30.863943 2384-2404. Max. coverage (+): 4.37. Max coverage (-): 0.48

Region: NODE\_319315\_length\_10503\_cov\_30.863943 2405-2425. Max. coverage (+): 4.23. Max coverage (-): 1.89

Region: NODE\_319315\_length\_10503\_cov\_30.863943 2426-2447. Max. coverage (+): 0.11. Max coverage (-): 0.96

Region: NODE\_319315\_length\_10503\_cov\_30.863943 2448-2468. Max. coverage (+): 0.11. Max coverage (-): 0.22

Region: NODE\_319315\_length\_10503\_cov\_30.863943 2469-2489. Max. coverage (+): 1. Max coverage (-): 0.15

Region: NODE\_319315\_length\_10503\_cov\_30.863943 2490-2510. Max. coverage (+): 0.04. Max coverage (-): 0.11

Region: NODE\_319315\_length\_10503\_cov\_30.863943 2511-2531. Max. coverage (+): 0.15. Max coverage (-): 0.19

Region: NODE\_319315\_length\_10503\_cov\_30.863943 2532-2552. Max. coverage (+): 0.07. Max coverage (-): 0.07

Region: NODE\_319315\_length\_10503\_cov\_30.863943 2553-2573. Max. coverage (+): 0.22. Max coverage (-): 0.15

Region: NODE\_319315\_length\_10503\_cov\_30.863943 2574-2594. Max. coverage (+): 0. Max coverage (-): 0.11

Region: NODE\_319315\_length\_10503\_cov\_30.863943 2595-2616. Max. coverage (+): 0. Max coverage (-): 0.15

Region: NODE\_319315\_length\_10503\_cov\_30.863943 2617-2637. Max. coverage (+): 0. Max coverage (-): 0.07

Region: NODE\_319315\_length\_10503\_cov\_30.863943 2638-2658. Max. coverage (+): 0. Max coverage (-): 0.19

Region: NODE\_319315\_length\_10503\_cov\_30.863943 2659-2679. Max. coverage (+): 0.04. Max coverage (-): 0.11

Region: NODE\_319315\_length\_10503\_cov\_30.863943 2680-2700. Max. coverage (+): 0.11. Max coverage (-): 0.33

Region: NODE\_319315\_length\_10503\_cov\_30.863943 2701-2721. Max. coverage (+): 0.59. Max coverage (-): 1.04

Region: NODE\_319315\_length\_10503\_cov\_30.863943 2722-2742. Max. coverage (+): 0.19. Max coverage (-): 0.37

Region: NODE\_319315\_length\_10503\_cov\_30.863943 2743-2763. Max. coverage (+): 0.48. Max coverage (-): 0.07

Region: NODE\_319315\_length\_10503\_cov\_30.863943 2764-2785. Max. coverage (+): 0. Max coverage (-): 0.85

Region: NODE\_319315\_length\_10503\_cov\_30.863943 2786-2806. Max. coverage (+): 0.37. Max coverage (-): 0.15

Region: NODE\_319315\_length\_10503\_cov\_30.863943 2807-2827. Max. coverage (+): 0.04. Max coverage (-): 0.59

Region: NODE\_319315\_length\_10503\_cov\_30.863943 2828-2848. Max. coverage (+): 1.15. Max coverage (-): 0.15

Region: NODE\_319315\_length\_10503\_cov\_30.863943 2849-2869. Max. coverage (+): 2.3. Max coverage (-): 0.04

Region: NODE\_319315\_length\_10503\_cov\_30.863943 2870-2890. Max. coverage (+): 0.04. Max coverage (-): 0.22

Region: NODE\_319315\_length\_10503\_cov\_30.863943 2891-2911. Max. coverage (+): 0.11. Max coverage (-): 0.04

Region: NODE\_319315\_length\_10503\_cov\_30.863943 2912-2932. Max. coverage (+): 0. Max coverage (-): 0

Region: NODE\_319315\_length\_10503\_cov\_30.863943 2933-2954. Max. coverage (+): 0.3. Max coverage (-): 0

Region: NODE\_319315\_length\_10503\_cov\_30.863943 2955-2975. Max. coverage (+): 0.33. Max coverage (-): 0

Region: NODE\_319315\_length\_10503\_cov\_30.863943 2976-2996. Max. coverage (+): 0.11. Max coverage (-): 0

Region: NODE\_319315\_length\_10503\_cov\_30.863943 2997-3017. Max. coverage (+): 0.04. Max coverage (-): 0.07

Region: NODE\_319315\_length\_10503\_cov\_30.863943 3018-3038. Max. coverage (+): 0.63. Max coverage (-): 0.15

Region: NODE\_319315\_length\_10503\_cov\_30.863943 3039-3059. Max. coverage (+): 0.96. Max coverage (-): 0.04

Region: NODE\_319315\_length\_10503\_cov\_30.863943 3060-3080. Max. coverage (+): 0.07. Max coverage (-): 0.33

Region: NODE\_319315\_length\_10503\_cov\_30.863943 3081-3101. Max. coverage (+): 0.78. Max coverage (-): 0.11

Region: NODE\_319315\_length\_10503\_cov\_30.863943 3102-3123. Max. coverage (+): 0.74. Max coverage (-): 0.26

Region: NODE\_319315\_length\_10503\_cov\_30.863943 3124-3144. Max. coverage (+): 0.3. Max coverage (-): 0.11

Region: NODE\_319315\_length\_10503\_cov\_30.863943 3145-3165. Max. coverage (+): 1.11. Max coverage (-): 0.04

Region: NODE\_319315\_length\_10503\_cov\_30.863943 3166-3186. Max. coverage (+): 0.04. Max coverage (-): 0.07

Region: NODE\_319315\_length\_10503\_cov\_30.863943 3187-3207. Max. coverage (+): 0.37. Max coverage (-): 0.26

Region: NODE\_319315\_length\_10503\_cov\_30.863943 3208-3228. Max. coverage (+): 1.71. Max coverage (-): 0.82

Region: NODE\_319315\_length\_10503\_cov\_30.863943 3229-3249. Max. coverage (+): 1. Max coverage (-): 0.22

Region: NODE\_319315\_length\_10503\_cov\_30.863943 3250-3270. Max. coverage (+): 1. Max coverage (-): 0.04

Region: NODE\_319315\_length\_10503\_cov\_30.863943 3271-3292. Max. coverage (+): 0.04. Max coverage (-): 0.11

Region: NODE\_319315\_length\_10503\_cov\_30.863943 3293-3313. Max. coverage (+): 0.19. Max coverage (-): 0.37

Region: NODE\_319315\_length\_10503\_cov\_30.863943 3314-3334. Max. coverage (+): 0.19. Max coverage (-): 0.19

Region: NODE\_319315\_length\_10503\_cov\_30.863943 3335-3355. Max. coverage (+): 2.48. Max coverage (-): 0.11

Region: NODE\_319315\_length\_10503\_cov\_30.863943 3356-3376. Max. coverage (+): 0.41. Max coverage (-): 0.15

Region: NODE\_319315\_length\_10503\_cov\_30.863943 3377-3397. Max. coverage (+): 0.85. Max coverage (-): 0.07

Region: NODE\_319315\_length\_10503\_cov\_30.863943 3398-3418. Max. coverage (+): 1.48. Max coverage (-): 0.48

Region: NODE\_319315\_length\_10503\_cov\_30.863943 3419-3439. Max. coverage (+): 0.22. Max coverage (-): 0.82

Region: NODE\_319315\_length\_10503\_cov\_30.863943 3440-3461. Max. coverage (+): 0.04. Max coverage (-): 0.04

Region: NODE\_319315\_length\_10503\_cov\_30.863943 3462-3482. Max. coverage (+): 0.67. Max coverage (-): 0.04

Region: NODE\_319315\_length\_10503\_cov\_30.863943 3483-3503. Max. coverage (+): 0.04. Max coverage (-): 0.37

Region: NODE\_319315\_length\_10503\_cov\_30.863943 3504-3524. Max. coverage (+): 3.19. Max coverage (-): 0.89

Region: NODE\_319315\_length\_10503\_cov\_30.863943 3525-3545. Max. coverage (+): 0.41. Max coverage (-): 0.04

Region: NODE\_319315\_length\_10503\_cov\_30.863943 3546-3566. Max. coverage (+): 0.63. Max coverage (-): 0.11

Region: NODE\_319315\_length\_10503\_cov\_30.863943 3567-3587. Max. coverage (+): 0.78. Max coverage (-): 0.07

Region: NODE\_319315\_length\_10503\_cov\_30.863943 3588-3608. Max. coverage (+): 0.07. Max coverage (-): 0.04

Region: NODE\_319315\_length\_10503\_cov\_30.863943 3609-3630. Max. coverage (+): 0.07. Max coverage (-): 0.19

Region: NODE\_319315\_length\_10503\_cov\_30.863943 3631-3651. Max. coverage (+): 3.04. Max coverage (-): 0.11

Region: NODE\_319315\_length\_10503\_cov\_30.863943 3652-3672. Max. coverage (+): 0.3. Max coverage (-): 0.04

Region: NODE\_319315\_length\_10503\_cov\_30.863943 3673-3693. Max. coverage (+): 0.3. Max coverage (-): 0.04

Region: NODE\_319315\_length\_10503\_cov\_30.863943 3694-3714. Max. coverage (+): 0.93. Max coverage (-): 0.04

Region: NODE\_319315\_length\_10503\_cov\_30.863943 3715-3735. Max. coverage (+): 0.82. Max coverage (-): 0

Region: NODE\_319315\_length\_10503\_cov\_30.863943 3736-3756. Max. coverage (+): 0.22. Max coverage (-): 0.07

Region: NODE\_319315\_length\_10503\_cov\_30.863943 3757-3777. Max. coverage (+): 0.19. Max coverage (-): 0

Region: NODE\_319315\_length\_10503\_cov\_30.863943 3778-3799. Max. coverage (+): 0.11. Max coverage (-): 0.04

Region: NODE\_319315\_length\_10503\_cov\_30.863943 3800-3820. Max. coverage (+): 0.15. Max coverage (-): 0.04

Region: NODE\_319315\_length\_10503\_cov\_30.863943 3821-3841. Max. coverage (+): 0.33. Max coverage (-): 0.04

Region: NODE\_319315\_length\_10503\_cov\_30.863943 3842-3862. Max. coverage (+): 0.85. Max coverage (-): 0.04

Region: NODE\_319315\_length\_10503\_cov\_30.863943 3863-3883. Max. coverage (+): 0.11. Max coverage (-): 0.15

Region: NODE\_319315\_length\_10503\_cov\_30.863943 3884-3904. Max. coverage (+): 0. Max coverage (-): 0.15

Region: NODE\_319315\_length\_10503\_cov\_30.863943 3905-3925. Max. coverage (+): 0.15. Max coverage (-): 0.15

Region: NODE\_319315\_length\_10503\_cov\_30.863943 3926-3946. Max. coverage (+): 0. Max coverage (-): 0

Region: NODE\_319315\_length\_10503\_cov\_30.863943 3947-3968. Max. coverage (+): 0.59. Max coverage (-): 0.41

Region: NODE\_319315\_length\_10503\_cov\_30.863943 3969-3989. Max. coverage (+): 3.52. Max coverage (-): 0.78

Region: NODE\_319315\_length\_10503\_cov\_30.863943 3990-4010. Max. coverage (+): 0.67. Max coverage (-): 0

Region: NODE\_319315\_length\_10503\_cov\_30.863943 4011-4031. Max. coverage (+): 0.26. Max coverage (-): 0.22

Region: NODE\_319315\_length\_10503\_cov\_30.863943 4032-4052. Max. coverage (+): 0.11. Max coverage (-): 0.04

Region: NODE\_319315\_length\_10503\_cov\_30.863943 4053-4073. Max. coverage (+): 1.15. Max coverage (-): 0.04

Region: NODE\_319315\_length\_10503\_cov\_30.863943 4074-4094. Max. coverage (+): 0.59. Max coverage (-): 0.22

Region: NODE\_319315\_length\_10503\_cov\_30.863943 4095-4116. Max. coverage (+): 0.22. Max coverage (-): 0.11

Region: NODE\_319315\_length\_10503\_cov\_30.863943 4117-4137. Max. coverage (+): 0.04. Max coverage (-): 0.07

Region: NODE\_319315\_length\_10503\_cov\_30.863943 4138-4158. Max. coverage (+): 1. Max coverage (-): 0

Region: NODE\_319315\_length\_10503\_cov\_30.863943 4159-4179. Max. coverage (+): 0.67. Max coverage (-): 0.04

Region: NODE\_319315\_length\_10503\_cov\_30.863943 4180-4200. Max. coverage (+): 0.26. Max coverage (-): 0.85

Region: NODE\_319315\_length\_10503\_cov\_30.863943 4201-4221. Max. coverage (+): 0.93. Max coverage (-): 0.04

Region: NODE\_319315\_length\_10503\_cov\_30.863943 4222-4242. Max. coverage (+): 1.22. Max coverage (-): 0

Region: NODE\_319315\_length\_10503\_cov\_30.863943 4243-4263. Max. coverage (+): 0.04. Max coverage (-): 1.26

Region: NODE\_319315\_length\_10503\_cov\_30.863943 4264-4285. Max. coverage (+): 0.07. Max coverage (-): 0.04

Region: NODE\_319315\_length\_10503\_cov\_30.863943 4286-4306. Max. coverage (+): 0.07. Max coverage (-): 0.22

Region: NODE\_319315\_length\_10503\_cov\_30.863943 4307-4327. Max. coverage (+): 0.07. Max coverage (-): 0.07

Region: NODE\_319315\_length\_10503\_cov\_30.863943 4328-4348. Max. coverage (+): 1.41. Max coverage (-): 0

Region: NODE\_319315\_length\_10503\_cov\_30.863943 4349-4369. Max. coverage (+): 0.07. Max coverage (-): 0

Region: NODE\_319315\_length\_10503\_cov\_30.863943 4370-4390. Max. coverage (+): 0.11. Max coverage (-): 0

Region: NODE\_319315\_length\_10503\_cov\_30.863943 4391-4411. Max. coverage (+): 0.7. Max coverage (-): 0.04

Region: NODE\_319315\_length\_10503\_cov\_30.863943 4412-4432. Max. coverage (+): 1.78. Max coverage (-): 0

Region: NODE\_319315\_length\_10503\_cov\_30.863943 4433-4454. Max. coverage (+): 3.82. Max coverage (-): 0.41

Region: NODE\_319315\_length\_10503\_cov\_30.863943 4455-4475. Max. coverage (+): 11.64. Max coverage (-): 0.11

Region: NODE\_319315\_length\_10503\_cov\_30.863943 4476-4496. Max. coverage (+): 0.26. Max coverage (-): 0.19

Region: NODE\_319315\_length\_10503\_cov\_30.863943 4497-4517. Max. coverage (+): 1.04. Max coverage (-): 0.15

Region: NODE\_319315\_length\_10503\_cov\_30.863943 4518-4538. Max. coverage (+): 1.04. Max coverage (-): 0.07

Region: NODE\_319315\_length\_10503\_cov\_30.863943 4539-4559. Max. coverage (+): 0.67. Max coverage (-): 0.44

Region: NODE\_319315\_length\_10503\_cov\_30.863943 4560-4580. Max. coverage (+): 4. Max coverage (-): 0.07

Region: NODE\_319315\_length\_10503\_cov\_30.863943 4581-4601. Max. coverage (+): 1.22. Max coverage (-): 0.04

Region: NODE\_319315\_length\_10503\_cov\_30.863943 4602-4623. Max. coverage (+): 2.45. Max coverage (-): 0.07

Region: NODE\_319315\_length\_10503\_cov\_30.863943 4624-4644. Max. coverage (+): 2.22. Max coverage (-): 0.19

Region: NODE\_319315\_length\_10503\_cov\_30.863943 4645-4665. Max. coverage (+): 0.89. Max coverage (-): 0.11

Region: NODE\_319315\_length\_10503\_cov\_30.863943 4666-4686. Max. coverage (+): 1.63. Max coverage (-): 0.19

Region: NODE\_319315\_length\_10503\_cov\_30.863943 4687-4707. Max. coverage (+): 0.07. Max coverage (-): 0.07

Region: NODE\_319315\_length\_10503\_cov\_30.863943 4708-4728. Max. coverage (+): 0.26. Max coverage (-): 0.15

Region: NODE\_319315\_length\_10503\_cov\_30.863943 4729-4749. Max. coverage (+): 1.48. Max coverage (-): 0.07

Region: NODE\_319315\_length\_10503\_cov\_30.863943 4750-4770. Max. coverage (+): 0.19. Max coverage (-): 0.07

Region: NODE\_319315\_length\_10503\_cov\_30.863943 4771-4792. Max. coverage (+): 1.26. Max coverage (-): 0.07

Region: NODE\_319315\_length\_10503\_cov\_30.863943 4793-4813. Max. coverage (+): 0.22. Max coverage (-): 0

Region: NODE\_319315\_length\_10503\_cov\_30.863943 4814-4834. Max. coverage (+): 0.78. Max coverage (-): 0.04

Region: NODE\_319315\_length\_10503\_cov\_30.863943 4835-4855. Max. coverage (+): 0.78. Max coverage (-): 0

Region: NODE\_319315\_length\_10503\_cov\_30.863943 4856-4876. Max. coverage (+): 0.07. Max coverage (-): 0

Region: NODE\_319315\_length\_10503\_cov\_30.863943 4877-4897. Max. coverage (+): 0.07. Max coverage (-): 0.11

Region: NODE\_319315\_length\_10503\_cov\_30.863943 4898-4918. Max. coverage (+): 0.04. Max coverage (-): 0.11

Region: NODE\_319315\_length\_10503\_cov\_30.863943 4919-4939. Max. coverage (+): 0.04. Max coverage (-): 0.04

Region: NODE\_319315\_length\_10503\_cov\_30.863943 4940-4961. Max. coverage (+): 0.11. Max coverage (-): 0.04

Region: NODE\_319315\_length\_10503\_cov\_30.863943 4962-4982. Max. coverage (+): 0.19. Max coverage (-): 0.33

Region: NODE\_319315\_length\_10503\_cov\_30.863943 4983-5003. Max. coverage (+): 0.33. Max coverage (-): 0.33

Region: NODE\_319315\_length\_10503\_cov\_30.863943 5004-5024. Max. coverage (+): 0.07. Max coverage (-): 0.07

Region: NODE\_319315\_length\_10503\_cov\_30.863943 5025-5045. Max. coverage (+): 0.11. Max coverage (-): 0.11

Region: NODE\_319315\_length\_10503\_cov\_30.863943 5046-5066. Max. coverage (+): 2.52. Max coverage (-): 0

Region: NODE\_319315\_length\_10503\_cov\_30.863943 5067-5087. Max. coverage (+): 0. Max coverage (-): 0.07

Region: NODE\_319315\_length\_10503\_cov\_30.863943 5088-5108. Max. coverage (+): 0.15. Max coverage (-): 0.04

Region: NODE\_319315\_length\_10503\_cov\_30.863943 5109-5130. Max. coverage (+): 0.11. Max coverage (-): 0

Region: NODE\_319315\_length\_10503\_cov\_30.863943 5131-5151. Max. coverage (+): 0.07. Max coverage (-): 0.02

Region: NODE\_319315\_length\_10503\_cov\_30.863943 5152-5172. Max. coverage (+): 0.05. Max coverage (-): 0

Region: NODE\_319315\_length\_10503\_cov\_30.863943 5173-5193. Max. coverage (+): 0.02. Max coverage (-): 0

Region: NODE\_319315\_length\_10503\_cov\_30.863943 5194-5214. Max. coverage (+): 0. Max coverage (-): 0

Region: NODE\_319315\_length\_10503\_cov\_30.863943 5215-5235. Max. coverage (+): 0. Max coverage (-): 0

Region: NODE\_319315\_length\_10503\_cov\_30.863943 5236-5256. Max. coverage (+): 0.07. Max coverage (-): 0.04

Region: NODE\_319315\_length\_10503\_cov\_30.863943 5257-5277. Max. coverage (+): 0.07. Max coverage (-): 0

Region: NODE\_319315\_length\_10503\_cov\_30.863943 5278-5299. Max. coverage (+): 0. Max coverage (-): 0

Region: NODE\_319315\_length\_10503\_cov\_30.863943 5300-5320. Max. coverage (+): 0.04. Max coverage (-): 0

Region: NODE\_319315\_length\_10503\_cov\_30.863943 5321-5341. Max. coverage (+): 0.11. Max coverage (-): 0

Region: NODE\_319315\_length\_10503\_cov\_30.863943 5342-5362. Max. coverage (+): 0.04. Max coverage (-): 0

Region: NODE\_319315\_length\_10503\_cov\_30.863943 5363-5383. Max. coverage (+): 0.01. Max coverage (-): 0

Region: NODE\_319315\_length\_10503\_cov\_30.863943 5384-5404. Max. coverage (+): 0. Max coverage (-): 0

Region: NODE\_319315\_length\_10503\_cov\_30.863943 5405-5425. Max. coverage (+): 0. Max coverage (-): 0

Region: NODE\_319315\_length\_10503\_cov\_30.863943 5426-5446. Max. coverage (+): 0.01. Max coverage (-): 0

Region: NODE\_319315\_length\_10503\_cov\_30.863943 5447-5468. Max. coverage (+): 0. Max coverage (-): 0.04

Region: NODE\_319315\_length\_10503\_cov\_30.863943 5469-5489. Max. coverage (+): 0. Max coverage (-): 0.04

Region: NODE\_319315\_length\_10503\_cov\_30.863943 5490-5510. Max. coverage (+): 0.22. Max coverage (-): 0.02

Region: NODE\_319315\_length\_10503\_cov\_30.863943 5511-5531. Max. coverage (+): 0.19. Max coverage (-): 0.02

Region: NODE\_319315\_length\_10503\_cov\_30.863943 5532-5552. Max. coverage (+): 0.04. Max coverage (-): 0.11

Region: NODE\_319315\_length\_10503\_cov\_30.863943 5553-5573. Max. coverage (+): 0.04. Max coverage (-): 0

Region: NODE\_319315\_length\_10503\_cov\_30.863943 5574-5594. Max. coverage (+): 0.01. Max coverage (-): 0

Region: NODE\_319315\_length\_10503\_cov\_30.863943 5595-5615. Max. coverage (+): 0. Max coverage (-): 0

Region: NODE\_319315\_length\_10503\_cov\_30.863943 5616-5637. Max. coverage (+): 0.04. Max coverage (-): 0

Region: NODE\_319315\_length\_10503\_cov\_30.863943 5638-5658. Max. coverage (+): 0.16. Max coverage (-): 0.07

Region: NODE\_319315\_length\_10503\_cov\_30.863943 5659-5679. Max. coverage (+): 0. Max coverage (-): 0

Region: NODE\_319315\_length\_10503\_cov\_30.863943 5680-5700. Max. coverage (+): 0.26. Max coverage (-): 0.07

Region: NODE\_319315\_length\_10503\_cov\_30.863943 5701-5721. Max. coverage (+): 0.04. Max coverage (-): 0.07

Region: NODE\_319315\_length\_10503\_cov\_30.863943 5722-5742. Max. coverage (+): 0.04. Max coverage (-): 0

Region: NODE\_319315\_length\_10503\_cov\_30.863943 5743-5763. Max. coverage (+): 0. Max coverage (-): 0.04

Region: NODE\_319315\_length\_10503\_cov\_30.863943 5764-5784. Max. coverage (+): 0.04. Max coverage (-): 0.04

Region: NODE\_319315\_length\_10503\_cov\_30.863943 5785-5806. Max. coverage (+): 0.04. Max coverage (-): 0

Region: NODE\_319315\_length\_10503\_cov\_30.863943 5807-5827. Max. coverage (+): 0. Max coverage (-): 0

Region: NODE\_319315\_length\_10503\_cov\_30.863943 5828-5848. Max. coverage (+): 0. Max coverage (-): 0

Region: NODE\_319315\_length\_10503\_cov\_30.863943 5849-5869. Max. coverage (+): 0. Max coverage (-): 0

Region: NODE\_319315\_length\_10503\_cov\_30.863943 5870-5890. Max. coverage (+): 0. Max coverage (-): 0

Region: NODE\_319315\_length\_10503\_cov\_30.863943 5891-5911. Max. coverage (+): 0.19. Max coverage (-): 0

Region: NODE\_319315\_length\_10503\_cov\_30.863943 5912-5932. Max. coverage (+): 0.26. Max coverage (-): 0

Region: NODE\_319315\_length\_10503\_cov\_30.863943 5933-5953. Max. coverage (+): 0.04. Max coverage (-): 0

Region: NODE\_319315\_length\_10503\_cov\_30.863943 5954-5975. Max. coverage (+): 0.04. Max coverage (-): 0.04

Region: NODE\_319315\_length\_10503\_cov\_30.863943 5976-5996. Max. coverage (+): 0.04. Max coverage (-): 0.04

Region: NODE\_319315\_length\_10503\_cov\_30.863943 5997-6017. Max. coverage (+): 0. Max coverage (-): 0.04

Region: NODE\_319315\_length\_10503\_cov\_30.863943 6018-6038. Max. coverage (+): 0.07. Max coverage (-): 0.04

Region: NODE\_319315\_length\_10503\_cov\_30.863943 6039-6059. Max. coverage (+): 0.04. Max coverage (-): 0

Region: NODE\_319315\_length\_10503\_cov\_30.863943 6060-6080. Max. coverage (+): 0. Max coverage (-): 0

Region: NODE\_319315\_length\_10503\_cov\_30.863943 6081-6101. Max. coverage (+): 0. Max coverage (-): 0

Region: NODE\_319315\_length\_10503\_cov\_30.863943 6102-6122. Max. coverage (+): 0.33. Max coverage (-): 0

Region: NODE\_319315\_length\_10503\_cov\_30.863943 6123-6144. Max. coverage (+): 0.04. Max coverage (-): 0

Region: NODE\_319315\_length\_10503\_cov\_30.863943 6145-6165. Max. coverage (+): 0.07. Max coverage (-): 0

Region: NODE\_319315\_length\_10503\_cov\_30.863943 6166-6186. Max. coverage (+): 0.15. Max coverage (-): 0

Region: NODE\_319315\_length\_10503\_cov\_30.863943 6187-6207. Max. coverage (+): 0.07. Max coverage (-): 0

Region: NODE\_319315\_length\_10503\_cov\_30.863943 6208-6228. Max. coverage (+): 0.07. Max coverage (-): 0.04

Region: NODE\_319315\_length\_10503\_cov\_30.863943 6229-6249. Max. coverage (+): 0.41. Max coverage (-): 0.07

Region: NODE\_319315\_length\_10503\_cov\_30.863943 6250-6270. Max. coverage (+): 2.41. Max coverage (-): 0.48

Region: NODE\_319315\_length\_10503\_cov\_30.863943 6271-6291. Max. coverage (+): 1. Max coverage (-): 0.11

Region: NODE\_319315\_length\_10503\_cov\_30.863943 6292-6313. Max. coverage (+): 0. Max coverage (-): 0.11

Region: NODE\_319315\_length\_10503\_cov\_30.863943 6314-6334. Max. coverage (+): 0.19. Max coverage (-): 0.3

Region: NODE\_319315\_length\_10503\_cov\_30.863943 6335-6355. Max. coverage (+): 0.67. Max coverage (-): 0

Region: NODE\_319315\_length\_10503\_cov\_30.863943 6356-6376. Max. coverage (+): 1.3. Max coverage (-): 0.11

Region: NODE\_319315\_length\_10503\_cov\_30.863943 6377-6397. Max. coverage (+): 0.26. Max coverage (-): 0

Region: NODE\_319315\_length\_10503\_cov\_30.863943 6398-6418. Max. coverage (+): 2.74. Max coverage (-): 0

Region: NODE\_319315\_length\_10503\_cov\_30.863943 6419-6439. Max. coverage (+): 0.52. Max coverage (-): 0

Region: NODE\_319315\_length\_10503\_cov\_30.863943 6440-6460. Max. coverage (+): 0.56. Max coverage (-): 0.04

Region: NODE\_319315\_length\_10503\_cov\_30.863943 6461-6482. Max. coverage (+): 1.59. Max coverage (-): 0.11

Region: NODE\_319315\_length\_10503\_cov\_30.863943 6483-6503. Max. coverage (+): 1.63. Max coverage (-): 0.04

Region: NODE\_319315\_length\_10503\_cov\_30.863943 6504-6524. Max. coverage (+): 0.67. Max coverage (-): 0.11

Region: NODE\_319315\_length\_10503\_cov\_30.863943 6525-6545. Max. coverage (+): 12.83. Max coverage (-): 0.11

Region: NODE\_319315\_length\_10503\_cov\_30.863943 6546-6566. Max. coverage (+): 0.04. Max coverage (-): 0.11

Region: NODE\_319315\_length\_10503\_cov\_30.863943 6567-6587. Max. coverage (+): 0.15. Max coverage (-): 0.19

Region: NODE\_319315\_length\_10503\_cov\_30.863943 6588-6608. Max. coverage (+): 2.11. Max coverage (-): 0.15

Region: NODE\_319315\_length\_10503\_cov\_30.863943 6609-6630. Max. coverage (+): 0. Max coverage (-): 0.07

Region: NODE\_319315\_length\_10503\_cov\_30.863943 6631-6651. Max. coverage (+): 1.3. Max coverage (-): 0

Region: NODE\_319315\_length\_10503\_cov\_30.863943 6652-6672. Max. coverage (+): 0.07. Max coverage (-): 0

Region: NODE\_319315\_length\_10503\_cov\_30.863943 6673-6693. Max. coverage (+): 0.07. Max coverage (-): 0.07

Region: NODE\_319315\_length\_10503\_cov\_30.863943 6694-6714. Max. coverage (+): 2.78. Max coverage (-): 0

Region: NODE\_319315\_length\_10503\_cov\_30.863943 6715-6735. Max. coverage (+): 0.22. Max coverage (-): 0.22

Region: NODE\_319315\_length\_10503\_cov\_30.863943 6736-6756. Max. coverage (+): 1.59. Max coverage (-): 0.04

Region: NODE\_319315\_length\_10503\_cov\_30.863943 6757-6777. Max. coverage (+): 0.33. Max coverage (-): 0.11

Region: NODE\_319315\_length\_10503\_cov\_30.863943 6778-6799. Max. coverage (+): 30.7. Max coverage (-): 0.04

Region: NODE\_319315\_length\_10503\_cov\_30.863943 6800-6820. Max. coverage (+): 0.52. Max coverage (-): 0.04

Region: NODE\_319315\_length\_10503\_cov\_30.863943 6821-6841. Max. coverage (+): 0.7. Max coverage (-): 0.48

Region: NODE\_319315\_length\_10503\_cov\_30.863943 6842-6862. Max. coverage (+): 2. Max coverage (-): 0.04

Region: NODE\_319315\_length\_10503\_cov\_30.863943 6863-6883. Max. coverage (+): 1. Max coverage (-): 0.11

Region: NODE\_319315\_length\_10503\_cov\_30.863943 6884-6904. Max. coverage (+): 0.93. Max coverage (-): 0

Region: NODE\_319315\_length\_10503\_cov\_30.863943 6905-6925. Max. coverage (+): 0.96. Max coverage (-): 0.15

Region: NODE\_319315\_length\_10503\_cov\_30.863943 6926-6946. Max. coverage (+): 3. Max coverage (-): 0.19

Region: NODE\_319315\_length\_10503\_cov\_30.863943 6947-6968. Max. coverage (+): 0.33. Max coverage (-): 0.07

Region: NODE\_319315\_length\_10503\_cov\_30.863943 6969-6989. Max. coverage (+): 3.86. Max coverage (-): 0.3

Region: NODE\_319315\_length\_10503\_cov\_30.863943 6990-7010. Max. coverage (+): 3.6. Max coverage (-): 2.11

Region: NODE\_319315\_length\_10503\_cov\_30.863943 7011-7031. Max. coverage (+): 1.63. Max coverage (-): 0.15

Region: NODE\_319315\_length\_10503\_cov\_30.863943 7032-7052. Max. coverage (+): 0.48. Max coverage (-): 0.07

Region: NODE\_319315\_length\_10503\_cov\_30.863943 7053-7073. Max. coverage (+): 0.82. Max coverage (-): 0.07

Region: NODE\_319315\_length\_10503\_cov\_30.863943 7074-7094. Max. coverage (+): 0.15. Max coverage (-): 0.04

Region: NODE\_319315\_length\_10503\_cov\_30.863943 7095-7115. Max. coverage (+): 0.07. Max coverage (-): 0.11

Region: NODE\_319315\_length\_10503\_cov\_30.863943 7116-7137. Max. coverage (+): 1.19. Max coverage (-): 0.04

Region: NODE\_319315\_length\_10503\_cov\_30.863943 7138-7158. Max. coverage (+): 0.59. Max coverage (-): 0.41

Region: NODE\_319315\_length\_10503\_cov\_30.863943 7159-7179. Max. coverage (+): 1.26. Max coverage (-): 0

Region: NODE\_319315\_length\_10503\_cov\_30.863943 7180-7200. Max. coverage (+): 1.11. Max coverage (-): 0.19

Region: NODE\_319315\_length\_10503\_cov\_30.863943 7201-7221. Max. coverage (+): 2.11. Max coverage (-): 0.33

Region: NODE\_319315\_length\_10503\_cov\_30.863943 7222-7242. Max. coverage (+): 0.89. Max coverage (-): 0.07

Region: NODE\_319315\_length\_10503\_cov\_30.863943 7243-7263. Max. coverage (+): 0.59. Max coverage (-): 0

Region: NODE\_319315\_length\_10503\_cov\_30.863943 7264-7284. Max. coverage (+): 0.3. Max coverage (-): 0.04

Region: NODE\_319315\_length\_10503\_cov\_30.863943 7285-7306. Max. coverage (+): 2.34. Max coverage (-): 0.04

Region: NODE\_319315\_length\_10503\_cov\_30.863943 7307-7327. Max. coverage (+): 0.48. Max coverage (-): 0.19

Region: NODE\_319315\_length\_10503\_cov\_30.863943 7328-7348. Max. coverage (+): 0.74. Max coverage (-): 0.15

Region: NODE\_319315\_length\_10503\_cov\_30.863943 7349-7369. Max. coverage (+): 0.67. Max coverage (-): 0.04

Region: NODE\_319315\_length\_10503\_cov\_30.863943 7370-7390. Max. coverage (+): 0.82. Max coverage (-): 0

Region: NODE\_319315\_length\_10503\_cov\_30.863943 7391-7411. Max. coverage (+): 0.52. Max coverage (-): 0.15

Region: NODE\_319315\_length\_10503\_cov\_30.863943 7412-7432. Max. coverage (+): 2.34. Max coverage (-): 0.07

Region: NODE\_319315\_length\_10503\_cov\_30.863943 7433-7453. Max. coverage (+): 1.63. Max coverage (-): 0.04

Region: NODE\_319315\_length\_10503\_cov\_30.863943 7454-7475. Max. coverage (+): 1.63. Max coverage (-): 0.04

Region: NODE\_319315\_length\_10503\_cov\_30.863943 7476-7496. Max. coverage (+): 4.37. Max coverage (-): 0.19

Region: NODE\_319315\_length\_10503\_cov\_30.863943 7497-7517. Max. coverage (+): 0.67. Max coverage (-): 0.26

Region: NODE\_319315\_length\_10503\_cov\_30.863943 7518-7538. Max. coverage (+): 26.06. Max coverage (-): 0

Region: NODE\_319315\_length\_10503\_cov\_30.863943 7539-7559. Max. coverage (+): 2.67. Max coverage (-): 0.07

Region: NODE\_319315\_length\_10503\_cov\_30.863943 7560-7580. Max. coverage (+): 9.86. Max coverage (-): 0.04

Region: NODE\_319315\_length\_10503\_cov\_30.863943 7581-7601. Max. coverage (+): 2.41. Max coverage (-): 0.07

Region: NODE\_319315\_length\_10503\_cov\_30.863943 7602-7622. Max. coverage (+): 5.71. Max coverage (-): 1

Region: NODE\_319315\_length\_10503\_cov\_30.863943 7623-7644. Max. coverage (+): 3.97. Max coverage (-): 0.11

Region: NODE\_319315\_length\_10503\_cov\_30.863943 7645-7665. Max. coverage (+): 0.22. Max coverage (-): 0.04

Region: NODE\_319315\_length\_10503\_cov\_30.863943 7666-7686. Max. coverage (+): 2.37. Max coverage (-): 0

Region: NODE\_319315\_length\_10503\_cov\_30.863943 7687-7707. Max. coverage (+): 1.41. Max coverage (-): 0.07

Region: NODE\_319315\_length\_10503\_cov\_30.863943 7708-7728. Max. coverage (+): 0.82. Max coverage (-): 0.04

Region: NODE\_319315\_length\_10503\_cov\_30.863943 7729-7749. Max. coverage (+): 0.41. Max coverage (-): 0.04

Region: NODE\_319315\_length\_10503\_cov\_30.863943 7750-7770. Max. coverage (+): 0.48. Max coverage (-): 0.11

Region: NODE\_319315\_length\_10503\_cov\_30.863943 7771-7791. Max. coverage (+): 0.96. Max coverage (-): 0.37

Region: NODE\_319315\_length\_10503\_cov\_30.863943 7792-7813. Max. coverage (+): 0.22. Max coverage (-): 0.07

Region: NODE\_319315\_length\_10503\_cov\_30.863943 7814-7834. Max. coverage (+): 0.7. Max coverage (-): 0.04

Region: NODE\_319315\_length\_10503\_cov\_30.863943 7835-7855. Max. coverage (+): 0.89. Max coverage (-): 0.15

Region: NODE\_319315\_length\_10503\_cov\_30.863943 7856-7876. Max. coverage (+): 1.08. Max coverage (-): 0.19

Region: NODE\_319315\_length\_10503\_cov\_30.863943 7877-7897. Max. coverage (+): 0.11. Max coverage (-): 0

Region: NODE\_319315\_length\_10503\_cov\_30.863943 7898-7918. Max. coverage (+): 4.45. Max coverage (-): 0.04

Region: NODE\_319315\_length\_10503\_cov\_30.863943 7919-7939. Max. coverage (+): 0.93. Max coverage (-): 0.07

Region: NODE\_319315\_length\_10503\_cov\_30.863943 7940-7960. Max. coverage (+): 1.96. Max coverage (-): 0

Region: NODE\_319315\_length\_10503\_cov\_30.863943 7961-7982. Max. coverage (+): 0.11. Max coverage (-): 0.26

Region: NODE\_319315\_length\_10503\_cov\_30.863943 7983-8003. Max. coverage (+): 3.34. Max coverage (-): 0.04

Region: NODE\_319315\_length\_10503\_cov\_30.863943 8004-8024. Max. coverage (+): 0.07. Max coverage (-): 0.11

Region: NODE\_319315\_length\_10503\_cov\_30.863943 8025-8045. Max. coverage (+): 3.93. Max coverage (-): 0.07

Region: NODE\_319315\_length\_10503\_cov\_30.863943 8046-8066. Max. coverage (+): 0.67. Max coverage (-): 0.78

Region: NODE\_319315\_length\_10503\_cov\_30.863943 8067-8087. Max. coverage (+): 5.75. Max coverage (-): 0.7

Region: NODE\_319315\_length\_10503\_cov\_30.863943 8088-8108. Max. coverage (+): 0.44. Max coverage (-): 0.11

Region: NODE\_319315\_length\_10503\_cov\_30.863943 8109-8129. Max. coverage (+): 0.82. Max coverage (-): 0.15

Region: NODE\_319315\_length\_10503\_cov\_30.863943 8130-8151. Max. coverage (+): 0.04. Max coverage (-): 0.11

Region: NODE\_319315\_length\_10503\_cov\_30.863943 8152-8172. Max. coverage (+): 0.19. Max coverage (-): 0

Region: NODE\_319315\_length\_10503\_cov\_30.863943 8173-8193. Max. coverage (+): 0.41. Max coverage (-): 0.11

Region: NODE\_319315\_length\_10503\_cov\_30.863943 8194-8214. Max. coverage (+): 4.34. Max coverage (-): 0.04

Region: NODE\_319315\_length\_10503\_cov\_30.863943 8215-8235. Max. coverage (+): 0.74. Max coverage (-): 0.04

Region: NODE\_319315\_length\_10503\_cov\_30.863943 8236-8256. Max. coverage (+): 19.35. Max coverage (-): 0.04

Region: NODE\_319315\_length\_10503\_cov\_30.863943 8257-8277. Max. coverage (+): 0.07. Max coverage (-): 0

Region: NODE\_319315\_length\_10503\_cov\_30.863943 8278-8298. Max. coverage (+): 0.41. Max coverage (-): 0

Region: NODE\_319315\_length\_10503\_cov\_30.863943 8299-8320. Max. coverage (+): 0.93. Max coverage (-): 0

Region: NODE\_319315\_length\_10503\_cov\_30.863943 8321-8341. Max. coverage (+): 1.82. Max coverage (-): 0

Region: NODE\_319315\_length\_10503\_cov\_30.863943 8342-8362. Max. coverage (+): 0.85. Max coverage (-): 0.04

Region: NODE\_319315\_length\_10503\_cov\_30.863943 8363-8383. Max. coverage (+): 1.74. Max coverage (-): 0.07

Region: NODE\_319315\_length\_10503\_cov\_30.863943 8384-8404. Max. coverage (+): 2.37. Max coverage (-): 0.04

Region: NODE\_319315\_length\_10503\_cov\_30.863943 8405-8425. Max. coverage (+): 0.19. Max coverage (-): 0.04

Region: NODE\_319315\_length\_10503\_cov\_30.863943 8426-8446. Max. coverage (+): 0.26. Max coverage (-): 0

Region: NODE\_319315\_length\_10503\_cov\_30.863943 8447-8467. Max. coverage (+): 0.93. Max coverage (-): 0

Region: NODE\_319315\_length\_10503\_cov\_30.863943 8468-8489. Max. coverage (+): 7.56. Max coverage (-): 0.11

Region: NODE\_319315\_length\_10503\_cov\_30.863943 8490-8510. Max. coverage (+): 0.22. Max coverage (-): 0.11

Region: NODE\_319315\_length\_10503\_cov\_30.863943 8511-8531. Max. coverage (+): 0.33. Max coverage (-): 0.11

Region: NODE\_319315\_length\_10503\_cov\_30.863943 8532-8552. Max. coverage (+): 3.15. Max coverage (-): 0.04

Region: NODE\_319315\_length\_10503\_cov\_30.863943 8553-8573. Max. coverage (+): 0.26. Max coverage (-): 0.07

Region: NODE\_319315\_length\_10503\_cov\_30.863943 8574-8594. Max. coverage (+): 1.45. Max coverage (-): 0

Region: NODE\_319315\_length\_10503\_cov\_30.863943 8595-8615. Max. coverage (+): 0.19. Max coverage (-): 0.04

Region: NODE\_319315\_length\_10503\_cov\_30.863943 8616-8636. Max. coverage (+): 4.12. Max coverage (-): 0.04

Region: NODE\_319315\_length\_10503\_cov\_30.863943 8637-8658. Max. coverage (+): 0.33. Max coverage (-): 0.04

Region: NODE\_319315\_length\_10503\_cov\_30.863943 8659-8679. Max. coverage (+): 0.67. Max coverage (-): 0.04

Region: NODE\_319315\_length\_10503\_cov\_30.863943 8680-8700. Max. coverage (+): 2.15. Max coverage (-): 0

Region: NODE\_319315\_length\_10503\_cov\_30.863943 8701-8721. Max. coverage (+): 1.19. Max coverage (-): 0.07

Region: NODE\_319315\_length\_10503\_cov\_30.863943 8722-8742. Max. coverage (+): 8.56. Max coverage (-): 0.07

Region: NODE\_319315\_length\_10503\_cov\_30.863943 8743-8763. Max. coverage (+): 13.31. Max coverage (-): 1.26

Region: NODE\_319315\_length\_10503\_cov\_30.863943 8764-8784. Max. coverage (+): 14.46. Max coverage (-): 0.04

Region: NODE\_319315\_length\_10503\_cov\_30.863943 8785-8805. Max. coverage (+): 1.15. Max coverage (-): 0.04

Region: NODE\_319315\_length\_10503\_cov\_30.863943 8806-8827. Max. coverage (+): 4.86. Max coverage (-): 0.37

Region: NODE\_319315\_length\_10503\_cov\_30.863943 8828-8848. Max. coverage (+): 1. Max coverage (-): 0

Region: NODE\_319315\_length\_10503\_cov\_30.863943 8849-8869. Max. coverage (+): 0.74. Max coverage (-): 2.45

Region: NODE\_319315\_length\_10503\_cov\_30.863943 8870-8890. Max. coverage (+): 3. Max coverage (-): 0.3

Region: NODE\_319315\_length\_10503\_cov\_30.863943 8891-8911. Max. coverage (+): 0.19. Max coverage (-): 3.26

Region: NODE\_319315\_length\_10503\_cov\_30.863943 8912-8932. Max. coverage (+): 2.85. Max coverage (-): 0.07

Region: NODE\_319315\_length\_10503\_cov\_30.863943 8933-8953. Max. coverage (+): 0.48. Max coverage (-): 0.37

Region: NODE\_319315\_length\_10503\_cov\_30.863943 8954-8974. Max. coverage (+): 0.33. Max coverage (-): 0.04

Region: NODE\_319315\_length\_10503\_cov\_30.863943 8975-8996. Max. coverage (+): 0.19. Max coverage (-): 0.04

Region: NODE\_319315\_length\_10503\_cov\_30.863943 8997-9017. Max. coverage (+): 0.37. Max coverage (-): 0.04

Region: NODE\_319315\_length\_10503\_cov\_30.863943 9018-9038. Max. coverage (+): 0.89. Max coverage (-): 0.04

Region: NODE\_319315\_length\_10503\_cov\_30.863943 9039-9059. Max. coverage (+): 0.44. Max coverage (-): 0.04

Region: NODE\_319315\_length\_10503\_cov\_30.863943 9060-9080. Max. coverage (+): 0.74. Max coverage (-): 0.19

Region: NODE\_319315\_length\_10503\_cov\_30.863943 9081-9101. Max. coverage (+): 0.93. Max coverage (-): 0.19

Region: NODE\_319315\_length\_10503\_cov\_30.863943 9102-9122. Max. coverage (+): 1.74. Max coverage (-): 0.04

Region: NODE\_319315\_length\_10503\_cov\_30.863943 9123-9143. Max. coverage (+): 0.15. Max coverage (-): 0.33

Region: NODE\_319315\_length\_10503\_cov\_30.863943 9144-9165. Max. coverage (+): 2. Max coverage (-): 0.11

Region: NODE\_319315\_length\_10503\_cov\_30.863943 9166-9186. Max. coverage (+): 0.78. Max coverage (-): 0.04

Region: NODE\_319315\_length\_10503\_cov\_30.863943 9187-9207. Max. coverage (+): 0.44. Max coverage (-): 0.15

Region: NODE\_319315\_length\_10503\_cov\_30.863943 9208-9228. Max. coverage (+): 0.67. Max coverage (-): 0.04

Region: NODE\_319315\_length\_10503\_cov\_30.863943 9229-9249. Max. coverage (+): 1.22. Max coverage (-): 0.19

Region: NODE\_319315\_length\_10503\_cov\_30.863943 9250-9270. Max. coverage (+): 11.46. Max coverage (-): 0.19

Region: NODE\_319315\_length\_10503\_cov\_30.863943 9271-9291. Max. coverage (+): 0.15. Max coverage (-): 0

Region: NODE\_319315\_length\_10503\_cov\_30.863943 9292-9313. Max. coverage (+): 0.04. Max coverage (-): 0.04

Region: NODE\_319315\_length\_10503\_cov\_30.863943 9314-9334. Max. coverage (+): 0. Max coverage (-): 0

Region: NODE\_319315\_length\_10503\_cov\_30.863943 9335-9355. Max. coverage (+): 0.3. Max coverage (-): 0.15

Region: NODE\_319315\_length\_10503\_cov\_30.863943 9356-9376. Max. coverage (+): 0.15. Max coverage (-): 0.11

Region: NODE\_319315\_length\_10503\_cov\_30.863943 9377-9397. Max. coverage (+): 1.63. Max coverage (-): 0.22

Region: NODE\_319315\_length\_10503\_cov\_30.863943 9398-9418. Max. coverage (+): 1.89. Max coverage (-): 0.04

Region: NODE\_319315\_length\_10503\_cov\_30.863943 9419-9439. Max. coverage (+): 0.48. Max coverage (-): 0.04

Region: NODE\_319315\_length\_10503\_cov\_30.863943 9440-9460. Max. coverage (+): 1.45. Max coverage (-): 0.07

Region: NODE\_319315\_length\_10503\_cov\_30.863943 9461-9482. Max. coverage (+): 3.04. Max coverage (-): 0.01

Region: NODE\_319315\_length\_10503\_cov\_30.863943 9483-9503. Max. coverage (+): 0.19. Max coverage (-): 0.04

Region: NODE\_319315\_length\_10503\_cov\_30.863943 9504-9524. Max. coverage (+): 0.52. Max coverage (-): 0.15

Region: NODE\_319315\_length\_10503\_cov\_30.863943 9525-9545. Max. coverage (+): 0.96. Max coverage (-): 0

Region: NODE\_319315\_length\_10503\_cov\_30.863943 9546-9566. Max. coverage (+): 0.41. Max coverage (-): 0.11

Region: NODE\_319315\_length\_10503\_cov\_30.863943 9567-9587. Max. coverage (+): 0.22. Max coverage (-): 0.04

Region: NODE\_319315\_length\_10503\_cov\_30.863943 9588-9608. Max. coverage (+): 3.67. Max coverage (-): 0.26

Region: NODE\_319315\_length\_10503\_cov\_30.863943 9609-9629. Max. coverage (+): 0.3. Max coverage (-): 0.04

Region: NODE\_319315\_length\_10503\_cov\_30.863943 9630-9651. Max. coverage (+): 0.3. Max coverage (-): 0.07

Region: NODE\_319315\_length\_10503\_cov\_30.863943 9652-9672. Max. coverage (+): 1.19. Max coverage (-): 0.15

Region: NODE\_319315\_length\_10503\_cov\_30.863943 9673-9693. Max. coverage (+): 0.26. Max coverage (-): 0.04

Region: NODE\_319315\_length\_10503\_cov\_30.863943 9694-9714. Max. coverage (+): 0.22. Max coverage (-): 0

Region: NODE\_319315\_length\_10503\_cov\_30.863943 9715-9735. Max. coverage (+): 0.07. Max coverage (-): 0

Region: NODE\_319315\_length\_10503\_cov\_30.863943 9736-9756. Max. coverage (+): 0.11. Max coverage (-): 0

Region: NODE\_319315\_length\_10503\_cov\_30.863943 9757-9777. Max. coverage (+): 10.97. Max coverage (-): 0

Region: NODE\_319315\_length\_10503\_cov\_30.863943 9778-9798. Max. coverage (+): 1.89. Max coverage (-): 0.04

Region: NODE\_319315\_length\_10503\_cov\_30.863943 9799-9820. Max. coverage (+): 0.63. Max coverage (-): 0.04

Region: NODE\_319315\_length\_10503\_cov\_30.863943 9821-9841. Max. coverage (+): 7.34. Max coverage (-): 0

Region: NODE\_319315\_length\_10503\_cov\_30.863943 9842-9862. Max. coverage (+): 0.59. Max coverage (-): 0.56

Region: NODE\_319315\_length\_10503\_cov\_30.863943 9863-9883. Max. coverage (+): 2.11. Max coverage (-): 0.7

Region: NODE\_319315\_length\_10503\_cov\_30.863943 9884-9904. Max. coverage (+): 0.82. Max coverage (-): 0.11

Region: NODE\_319315\_length\_10503\_cov\_30.863943 9905-9925. Max. coverage (+): 0.37. Max coverage (-): 0.04

Region: NODE\_319315\_length\_10503\_cov\_30.863943 9926-9946. Max. coverage (+): 15.57. Max coverage (-): 0

Region: NODE\_319315\_length\_10503\_cov\_30.863943 9947-9967. Max. coverage (+): 0.15. Max coverage (-): 0.44

Region: NODE\_319315\_length\_10503\_cov\_30.863943 9968-9989. Max. coverage (+): 3.97. Max coverage (-): 0.41

Region: NODE\_319315\_length\_10503\_cov\_30.863943 9990-10010. Max. coverage (+): 0.96. Max coverage (-): 0.04

Region: NODE\_319315\_length\_10503\_cov\_30.863943 10011-10031. Max. coverage (+): 0.56. Max coverage (-): 0.15

Region: NODE\_319315\_length\_10503\_cov\_30.863943 10032-10052. Max. coverage (+): 9.19. Max coverage (-): 0.15

Region: NODE\_319315\_length\_10503\_cov\_30.863943 10053-10073. Max. coverage (+): 2.3. Max coverage (-): 0

Region: NODE\_319315\_length\_10503\_cov\_30.863943 10074-10094. Max. coverage (+): 0.78. Max coverage (-): 0.07

Region: NODE\_319315\_length\_10503\_cov\_30.863943 10095-10115. Max. coverage (+): 0.89. Max coverage (-): 0

Region: NODE\_319315\_length\_10503\_cov\_30.863943 10116-10136. Max. coverage (+): 0.15. Max coverage (-): 0.3

Region: NODE\_319315\_length\_10503\_cov\_30.863943 10137-10158. Max. coverage (+): 0.52. Max coverage (-): 0.26

Region: NODE\_319315\_length\_10503\_cov\_30.863943 10159-10179. Max. coverage (+): 0.33. Max coverage (-): 0.19

Region: NODE\_319315\_length\_10503\_cov\_30.863943 10180-10200. Max. coverage (+): 10.12. Max coverage (-): 0.3

Region: NODE\_319315\_length\_10503\_cov\_30.863943 10201-10221. Max. coverage (+): 0.96. Max coverage (-): 0.11

Region: NODE\_319315\_length\_10503\_cov\_30.863943 10222-10242. Max. coverage (+): 0.3. Max coverage (-): 0.04

Region: NODE\_319315\_length\_10503\_cov\_30.863943 10243-10263. Max. coverage (+): 0.15. Max coverage (-): 0.07

Region: NODE\_319315\_length\_10503\_cov\_30.863943 10264-10284. Max. coverage (+): 0.96. Max coverage (-): 0.19

Region: NODE\_319315\_length\_10503\_cov\_30.863943 10285-10305. Max. coverage (+): 0.85. Max coverage (-): 0

Region: NODE\_319315\_length\_10503\_cov\_30.863943 10306-10327. Max. coverage (+): 0.26. Max coverage (-): 0.07

Region: NODE\_319315\_length\_10503\_cov\_30.863943 10328-10348. Max. coverage (+): 0.93. Max coverage (-): 0.04

Region: NODE\_319315\_length\_10503\_cov\_30.863943 10349-10369. Max. coverage (+): 0.33. Max coverage (-): 0.11

Region: NODE\_319315\_length\_10503\_cov\_30.863943 10370-10390. Max. coverage (+): 0.15. Max coverage (-): 0.04

Region: NODE\_319315\_length\_10503\_cov\_30.863943 10391-10411. Max. coverage (+): 0.3. Max coverage (-): 0.04

Region: NODE\_319315\_length\_10503\_cov\_30.863943 10412-10432. Max. coverage (+): 0.22. Max coverage (-): 0.04

Region: NODE\_319315\_length\_10503\_cov\_30.863943 10433-10453. Max. coverage (+): 0.07. Max coverage (-): 0.04

Region: NODE\_319315\_length\_10503\_cov\_30.863943 10454-10474. Max. coverage (+): 0.04. Max coverage (-): 0.04

Region: NODE\_319315\_length\_10503\_cov\_30.863943 10475-10496. Max. coverage (+): 0. Max coverage (-): 0

Region: NODE\_319315\_length\_10503\_cov\_30.863943 10497-10517. Max. coverage (+): 0. Max coverage (-): 0.26

Region: NODE\_319315\_length\_10503\_cov\_30.863943 10518-10538. Max. coverage (+): 0.85. Max coverage (-): 0.26

Region: NODE\_319315\_length\_10503\_cov\_30.863943 10539-10559. Max. coverage (+): 0.19. Max coverage (-): 0

Region: NODE\_319315\_length\_10503\_cov\_30.863943 10560-. Max. coverage (+): 0. Max coverage (-): 0

RepeatMasker Color Code

**+**

100-98% Identity

<98-95% Identity

<95-90% Identity

<90-85% Identity

<85-80% Identity

<80-75% Identity

<75-70% Identity

<70% Identity

**-**

Gene Set Color Code

**+**

Gene

Pseudogene

Other

**-**

Topology/Coverage Color Code

Coverage Plus Strand

Coverage Minus Strand

Mainstrand: Plus

Mainstrand: Minus

Complementary Strand

Flanking Region  
(if option -flank >0)

Gene Set Annotation  
  
RepeatMasker Annotation  

**1. AlRepB-392**: 9-232 (-), Divergence to consensus: 27.6%  
**2. AlRepD-2195**: 551-653 (+), Divergence to consensus: 34.1%  
**3. AlRepD-148**: 851-1034 (+), Divergence to consensus: 42.7%  
**4. AlRepC-488**: 1107-1159 (+), Divergence to consensus: 20.7%  
**5. AlRepC-169**: 1166-1527 (-), Divergence to consensus: 16.1%  
**6. EnSpm-N17\_DR**: 1510-1602 (+), Divergence to consensus: 32.5%  
**7. SINE\_AFC**: 1529-1666 (+), Divergence to consensus: 10.1%  
**8. SINE\_AFC**: 1665-1720 (+), Divergence to consensus: 17.9%  
**9. AlRepC-169**: 1708-2008 (-), Divergence to consensus: 14.6%  
**10. A-rich**: 2920-2972 (+), Divergence to consensus: 30.5%  
**11. AlRepD-870**: 3695-3740 (+), Divergence to consensus: 19.6%  
**12. AlRepA-115**: 4796-4889 (+), Divergence to consensus: 24.5%  
**13. AlRepB-784**: 5136-5811 (+), Divergence to consensus: 11.7%  
**14. AlRepB-250**: 7149-7247 (-), Divergence to consensus: 27.3%  
**15. AlRepB-250**: 7245-7297 (-), Divergence to consensus: 28.3%  
**16. (GGA)n**: 8132-8148 (+), Divergence to consensus: 0%  
**17. AlRepC-1837**: 8432-8588 (-), Divergence to consensus: 38.9%  
**18. AlRepA-93**: 8616-8692 (-), Divergence to consensus: 35.1%  
**19. AlRepC-693**: 8972-9034 (-), Divergence to consensus: 22.6%  
**20. AlRepD-886**: 9132-9258 (-), Divergence to consensus: 30.4%  
**21. RTE-2\_AFC**: 9394-9546 (+), Divergence to consensus: 32.2%  
**22. AlRepC-234**: 10540-10621 (+), Divergence to consensus: 8.5%

  
Transcription Factor Binding Sites  

**RHOXF1** (Sequence: GGCTTA (-): 60)  
**RHOXF1** (Sequence: AGATTA (-): 328)  
**RHOXF1** (Sequence: GGCTCA (-): 549)  
**RHOXF1** (Sequence: GGCTCA (-): 1539)  
**RHOXF1** (Sequence: AGATCA (-): 2174)  
**RHOXF1** (Sequence: AGATTA (-): 3037)  
**RHOXF1** (Sequence: AGCTTA (-): 3217)  
**RHOXF1** (Sequence: AGATTA (-): 3526)  
**RHOXF1** (Sequence: AGATTA (-): 3961)  
**RHOXF1** (Sequence: AGCTCA (-): 4024)  
**RHOXF1** (Sequence: AGCTCA (-): 4187)  
**RHOXF1** (Sequence: AGCTTA (-): 4876)  
**RHOXF1** (Sequence: AGCTCA (-): 5440)  
**RHOXF1** (Sequence: GGCTCA (-): 5979)  
**RHOXF1** (Sequence: AGATTA (-): 6597)  
**RHOXF1** (Sequence: GGATCA (-): 7381)  
**RHOXF1** (Sequence: GGCTCA (-): 8123)  
**RHOXF1** (Sequence: AGATTA (-): 9405)  
**RHOXF1** (Sequence: AGCTCA (-): 9671)  
**RHOXF1** (Sequence: AGCTTA (-): 10068)  
**RHOXF1** (Sequence: GGCTTA (-): 10224)  
**RHOXF1** (Sequence: AGCTTA (-): 10356)  
**RHOXF1** (Sequence: TGAGCC (+): 117)  
**RHOXF1** (Sequence: TAAGCT (+): 180)  
**RHOXF1** (Sequence: TGATCC (+): 231)  
**RHOXF1** (Sequence: TAAGCT (+): 709)  
**RHOXF1** (Sequence: TAATCT (+): 718)  
**RHOXF1** (Sequence: TGATCC (+): 958)  
**RHOXF1** (Sequence: TAAGCC (+): 2394)  
**RHOXF1** (Sequence: TGATCC (+): 2955)  
**RHOXF1** (Sequence: TAATCC (+): 4337)  
**RHOXF1** (Sequence: TAAGCT (+): 5199)  
**RHOXF1** (Sequence: TAATCT (+): 6162)  
**RHOXF1** (Sequence: TGAGCT (+): 6240)  
**RHOXF1** (Sequence: TAATCT (+): 6242)  
**RHOXF1** (Sequence: TAATCT (+): 6527)  
**RHOXF1** (Sequence: TGATCT (+): 7510)  
**RHOXF1** (Sequence: TGAGCT (+): 8295)  
**RHOXF1** (Sequence: TGATCT (+): 8733)  
**RHOXF1** (Sequence: TGATCT (+): 9648)  
**RHOXF1** (Sequence: TAATCT (+): 9696)  
**RHOXF1** (Sequence: TGATCT (+): 10196)  
**Gata4** (Sequence: CTTATCT (+): 304)  
**Gata4** (Sequence: CTTATCT (+): 10226)  
**POU5F1** (Sequence: TTTGCAT (-): 5900)  
**POU5F1** (Sequence: TTTGCAT (-): 7915)  
**POU5F1** (Sequence: TTTGCAT (-): 9288)  
**RFX4\_1** (Sequence: GTTGCTAGG (-): 8885)  
**FOXO3\_hsa** (Sequence: GTAAACAA (+): 5864)  
**SOX9** (Sequence: AACAATAA (-): 580)  
**SOX9** (Sequence: AACAATGG (-): 5379)  
**SOX9** (Sequence: AACAATGA (-): 9308)  
**FOXP1** (Sequence: GTAAACA (+): 4655)  
**FOXP1** (Sequence: GTAAACA (+): 5864)  
**FOXO1** (Sequence: CTTGTTTAT (+): 1795)  
**FOXO1** (Sequence: CTTGTTTAT (+): 4999)  
**FOXO1** (Sequence: CTTGTTTAT (+): 5406)  
**FOXO1** (Sequence: CCTGTTTAT (+): 8835)  
**FOXO1** (Sequence: GTTGTTTTC (+): 9684)  
**FOXO1** (Sequence: GCTGTTTTT (+): 10414)  
**FOXO3\_mmu** (Sequence: TGTTTACA (-): 597)  
**FOXO3\_mmu** (Sequence: TGTTTAGA (-): 2169)  
**FOXO3\_mmu** (Sequence: TGTTTTCC (-): 4071)  
**FOXO3\_mmu** (Sequence: TGTTTAGA (-): 7131)  
**Sox5** (Sequence: ATTGTT (+): 1307)  
**Sox5** (Sequence: ATTGTT (+): 1349)  
**Sox5** (Sequence: ATTGTT (+): 1518)  
**Sox5** (Sequence: ATTGTT (+): 1814)  
**Sox5** (Sequence: ATTGTT (+): 1832)  
**Sox5** (Sequence: ATTGTT (+): 2588)  
**Sox5** (Sequence: ATTGTT (+): 5117)  
**Sox5** (Sequence: ATTGTT (+): 5187)  
**Sox5** (Sequence: ATTGTT (+): 5249)  
**Sox5** (Sequence: ATTGTT (+): 7307)  
**Sox5** (Sequence: ATTGTT (+): 7486)  
**Sox5** (Sequence: ATTGTT (+): 9411)  
**Mybl1\_1** (Sequence: TAACGGTT (-): 2402)  
**SOX9** (Sequence: TTATTGTT (+): 1516)  
**SOX9** (Sequence: TCATTGTT (+): 1812)  
**SOX9** (Sequence: CTATTGTT (+): 2586)  
**SOX9** (Sequence: CTATTGTT (+): 5185)  
**SOX9** (Sequence: TTATTGTT (+): 7305)  
**FOXO3\_mmu** (Sequence: TGTAAACA (+): 4654)  
**FOXO3\_mmu** (Sequence: GGAAAACA (+): 8390)  
**Nobox** (Sequence: GCTAATTA (-): 3362)  
**Nobox** (Sequence: AGTAATTA (-): 4930)  
**FOXO1** (Sequence: GAAAACAAG (-): 8403)  
**FOXO3\_hsa** (Sequence: TTGTTTAC (-): 596)  
**FOXO3\_hsa** (Sequence: ATGTTTAC (-): 897)  
**FOXO3\_hsa** (Sequence: ATGTTTAC (-): 9771)  
**FOXP1** (Sequence: TGTTTAC (-): 597)  
**FOXP1** (Sequence: TGTTTAC (-): 898)  
**FOXP1** (Sequence: TGTTTAC (-): 5236)  
**FOXP1** (Sequence: TGTTTAC (-): 9772)  
**Nobox** (Sequence: TAATTAGT (+): 454)  
**Nobox** (Sequence: TAATTACT (+): 3364)  
**Nobox** (Sequence: TAATTGCT (+): 6532)  
**Nobox** (Sequence: TAATTAGC (+): 9335)  
**POU2F1** (Sequence: ATTAACATA (-): 3125)  
**Rhox11** (Sequence: CGCTGTAAA (+): 8057)  
**Rhox11** (Sequence: CGGTGTAAT (+): 9957)  
**Rhox11** (Sequence: TTTACAGCA (-): 3564)  
**Sox5** (Sequence: AACAAT (-): 580)  
**Sox5** (Sequence: AACAAT (-): 2527)  
**Sox5** (Sequence: AACAAT (-): 4599)  
**Sox5** (Sequence: AACAAT (-): 5379)  
**Sox5** (Sequence: AACAAT (-): 8286)  
**Sox5** (Sequence: AACAAT (-): 8449)  
**Sox5** (Sequence: AACAAT (-): 9308)  
**POU5F1** (Sequence: ATGCAAA (+): 2674)
