# Supplementary material for: First characterization of PIWI-interacting RNA clusters in a cichlid fish with a B chromosome
Source: BMC Biol. 2022 Sep 21;20:204. doi: 10.1186/s12915-022-01403-2 (PMC9490952; doi:10.1186/s12915-022-01403-2)
Supplement: Supplementary file 1 — Additional file 1. Zipped folder with fasta and interactive html piRNA cluster information for the A. latifasciata genome. The nomenclature is as follows: number-pirna-cluster_sex_B-presence (f, female; m, male; 0b, without B chromosome; 1b, with B chromosome). [file 12915_2022_1403_MOESM1_ESM.zip › 128_m0b.html]

piRNA cluster 128\_m0b 61


Predicted piRNA cluster no. 128\_m0b
  

Show proTRAC run info
Hide proTRAC run info

/\  
                \_\_\_\_\_\_\_\_\_\_\_\_\_\_\_\_\_\_\_\_\_\_\_/\\_\_\_ /  \\_\_\_\_\_\_\_  
               I                      /  \  /    \      I  
               I     pro             /    \/      \     I  
               I        TRAC        /               \   I  
               I   \_\_\_\_\_\_\_\_\_\_\_\_\_\_\_\_/\_\_\_\_\_\_\_\_\_\_\_\_\_\_\_\_\_\\_ I  
               I   \              /                     I  
               I    \            /                      I  
               I     \  /\      /       V.2.4.2         I  
               I      \/  \    /                        I  
               I\_\_\_\_\_\_\_\_\_\_\_\  /\_\_\_\_\_\_\_\_\_\_\_\_\_\_\_\_\_\_\_\_\_\_\_\_\_I  
                            \/  
  
  
================================= proTRAC ====================================  
VERSION: .......... 2.4.2  
LAST MODIFIED: .... 11. May 2018  
  
Please cite:  
Rosenkranz D, Zischler H. proTRAC - a software for probabilistic piRNA cluster  
detection, visualization and analysis. 2012. BMC Bioinformatics 13:5.  
  
  
Contact:  
David Rosenkranz  
Institute of Organismic and Molecular Evolutionary Biology  
Dept. Anthropology, small RNA group  
Johannes Gutenberg University Mainz  
email: rosenkranz@uni-mainz.de  
  
You can find the latest proTRAC version at:  
http://sourceforge.net/projects/protrac/files  
http://www.smallRNAgroup-mainz.de/software  
==============================================================================  
  
PARAMETERS:  
Map file: ...............piwi-machos-0B.fa-collapse.map  
Genome file: ............../../../0B\_ala\_genome.fa  
RepeatMasker annotation: Alatifasciata-all0B-maryan-v2.fa\_corrected.out  
GeneSet:................./guest-storage/Data/annotation/Alatifasciata\_all0B\_maryan-v2\_out2017.gff  
  
Significant (p<=0.01) hit density will be calculated based  
on observed hit distribution.  
  
Sliding window size: ........................................ 5000 bp  
Sliding window increament: .................................. 1000 bp  
Normalize each hit by number of genomic hits: ............... yes  
Normalize each hit by number of sequence reads: ............. yes  
Normalize values (-> per million mapped reads): ............. yes  
Min. fraction of hits with 1T(U) or 10A: .................... 0.75  
Alternatively: Min. fraction of hits with 1T(U) and 10A: .... 0.5  
Min. fraction of hits with typical piRNA length: ............ 0.75  
Typical piRNA length: ....................................... 24-32 nt  
Min. size of a piRNA cluster: ............................... 1000 bp.  
Min. number of hits (absolute): ............................. 0  
Min. number of hits (normalized): ........................... 0  
Min. fraction of hits on the mainstrand: .................... 0.75  
Top fraction of mapped sequences (in terms of read counts): . 1%  
Top fraction accounts for max. n% of sequence reads: ........ 90%  
Min. fraction of hits on each arm of a bidirectional cluster: 0.05  
Output html file for each cluster: .......................... yes  
Output a summary table: ..................................... yes  
Output a FASTA file for each cluster (piRNA sequences): ..... yes  
Output a FASTA file comprising cluster sequences: ........... yes  
Output a GTF file for predicted piRNA clusters: ..............yes  
Search DNA motifs in clusters: .............................. yes  
Output flanking sequences: +/- .............................. 0 bp  
Output ~.pTi file: .......................................... no  
==============================================================================  
  
  
Genome size (without gaps): ............ 758543724 bp  
Gaps (N/X/-): .......................... 417479 bp  
Mapped reads: .......................... 24765598  
Non-identical sequences: ............... 6158275  
Genomic hits: .......................... 53103584  
Significant densitiy of mapped reads: .. 763.098963422187 reads/kb

Show proTRAC cluster info
Hide proTRAC cluster info

|  |  |
| --- | --- |
| Location | NODE\_324426\_length\_69311\_cov\_30.906147 |
| Coordinates | 61462-65823 |
| Size [bp] | 4362 |
| Sequence hit loci | 1383 |
| Mapped reads (normalized) | 4172.9 |
| Mapped reads (normalized) per kb | 956.7 |
| Normalized reads with 1T (1U) | 80.3% |
| Normalized reads with 10A | 44.2% |
| Normalized reads with length 24-32 nt | 98.2% |
| Normalized reads on the main strand(s) | 93.1% |
| Predicted directionality | mono:plus |

100%

0%

1T (1U)  
reads

10A reads

24-32 nt  
reads

reads on mainstrand

**Either the amount of reads with 1T (1U) OR 10A has to exceed 75% (set with option: -1Tor10A)  
Alternatively the amount of reads with 1T (1U) AND 10A has to exceed 50% (set with option: -1Tand10A)  
Minimum amount of reads with preferred size is 75% (set with option: -pisize)  
Minimum amount of reads on the main strand(s) is 75% (set with option: -clstrand)**

Show read coverage
Hide read coverage

WHAT DO I SEE HERE?  
This chart shows the location of mapped sequence reads within a predicted piRNA cluster. The color refers to the number of genomic hits produced by the sequence read in question. A dark red bar indicates that this sequence read produces many other hits elsewhere in the genome. Many adjacent red or yellow bars can indicate the presence of a multi-copy element such as transposons or rRNA genes. A dark green bar indicates that this sequence read maps uniquely to this locus.

1 hit

2-5 hits

6-10 hits

11-20 hits

21-50 hits

51-100 hits

> 100 hits

NODE\_324426\_length\_69311\_cov\_30.906147

61462

65823

Gene Set

RepeatMasker

Mapped  
Reads

15.26

plus strand

minus strand

15.26

Region: NODE\_324426\_length\_69311\_cov\_30.906147 10608-61466. Max. coverage (+): 0. Max coverage (-): 0

Region: NODE\_324426\_length\_69311\_cov\_30.906147 61467-61475. Max. coverage (+): 0. Max coverage (-): 0.01

Region: NODE\_324426\_length\_69311\_cov\_30.906147 61476-61483. Max. coverage (+): 0. Max coverage (-): 0

Region: NODE\_324426\_length\_69311\_cov\_30.906147 61484-61492. Max. coverage (+): 0. Max coverage (-): 0

Region: NODE\_324426\_length\_69311\_cov\_30.906147 61493-61501. Max. coverage (+): 0. Max coverage (-): 0

Region: NODE\_324426\_length\_69311\_cov\_30.906147 61502-61509. Max. coverage (+): 0. Max coverage (-): 0.01

Region: NODE\_324426\_length\_69311\_cov\_30.906147 61510-61518. Max. coverage (+): 0. Max coverage (-): 0.02

Region: NODE\_324426\_length\_69311\_cov\_30.906147 61519-61527. Max. coverage (+): 0. Max coverage (-): 0.01

Region: NODE\_324426\_length\_69311\_cov\_30.906147 61528-61536. Max. coverage (+): 0. Max coverage (-): 0

Region: NODE\_324426\_length\_69311\_cov\_30.906147 61537-61544. Max. coverage (+): 0. Max coverage (-): 0

Region: NODE\_324426\_length\_69311\_cov\_30.906147 61545-61553. Max. coverage (+): 0.09. Max coverage (-): 0

Region: NODE\_324426\_length\_69311\_cov\_30.906147 61554-61562. Max. coverage (+): 0.01. Max coverage (-): 0

Region: NODE\_324426\_length\_69311\_cov\_30.906147 61563-61571. Max. coverage (+): 0. Max coverage (-): 0

Region: NODE\_324426\_length\_69311\_cov\_30.906147 61572-61579. Max. coverage (+): 1.41. Max coverage (-): 0

Region: NODE\_324426\_length\_69311\_cov\_30.906147 61580-61588. Max. coverage (+): 1.53. Max coverage (-): 0

Region: NODE\_324426\_length\_69311\_cov\_30.906147 61589-61597. Max. coverage (+): 0.04. Max coverage (-): 0.28

Region: NODE\_324426\_length\_69311\_cov\_30.906147 61598-61605. Max. coverage (+): 0. Max coverage (-): 0.04

Region: NODE\_324426\_length\_69311\_cov\_30.906147 61606-61614. Max. coverage (+): 0.22. Max coverage (-): 0

Region: NODE\_324426\_length\_69311\_cov\_30.906147 61615-61623. Max. coverage (+): 0. Max coverage (-): 0.04

Region: NODE\_324426\_length\_69311\_cov\_30.906147 61624-61632. Max. coverage (+): 0. Max coverage (-): 0.04

Region: NODE\_324426\_length\_69311\_cov\_30.906147 61633-61640. Max. coverage (+): 0. Max coverage (-): 0

Region: NODE\_324426\_length\_69311\_cov\_30.906147 61641-61649. Max. coverage (+): 0.04. Max coverage (-): 0

Region: NODE\_324426\_length\_69311\_cov\_30.906147 61650-61658. Max. coverage (+): 0. Max coverage (-): 0.04

Region: NODE\_324426\_length\_69311\_cov\_30.906147 61659-61667. Max. coverage (+): 0. Max coverage (-): 0

Region: NODE\_324426\_length\_69311\_cov\_30.906147 61668-61675. Max. coverage (+): 0. Max coverage (-): 0

Region: NODE\_324426\_length\_69311\_cov\_30.906147 61676-61684. Max. coverage (+): 0. Max coverage (-): 0

Region: NODE\_324426\_length\_69311\_cov\_30.906147 61685-61693. Max. coverage (+): 0. Max coverage (-): 0

Region: NODE\_324426\_length\_69311\_cov\_30.906147 61694-61701. Max. coverage (+): 0.04. Max coverage (-): 0.08

Region: NODE\_324426\_length\_69311\_cov\_30.906147 61702-61710. Max. coverage (+): 0.12. Max coverage (-): 0.08

Region: NODE\_324426\_length\_69311\_cov\_30.906147 61711-61719. Max. coverage (+): 0.24. Max coverage (-): 0

Region: NODE\_324426\_length\_69311\_cov\_30.906147 61720-61728. Max. coverage (+): 0.16. Max coverage (-): 0.12

Region: NODE\_324426\_length\_69311\_cov\_30.906147 61729-61736. Max. coverage (+): 0. Max coverage (-): 0.12

Region: NODE\_324426\_length\_69311\_cov\_30.906147 61737-61745. Max. coverage (+): 0.2. Max coverage (-): 0

Region: NODE\_324426\_length\_69311\_cov\_30.906147 61746-61754. Max. coverage (+): 0. Max coverage (-): 0

Region: NODE\_324426\_length\_69311\_cov\_30.906147 61755-61762. Max. coverage (+): 0.08. Max coverage (-): 0

Region: NODE\_324426\_length\_69311\_cov\_30.906147 61763-61771. Max. coverage (+): 0.24. Max coverage (-): 0.08

Region: NODE\_324426\_length\_69311\_cov\_30.906147 61772-61780. Max. coverage (+): 0.65. Max coverage (-): 0.16

Region: NODE\_324426\_length\_69311\_cov\_30.906147 61781-61789. Max. coverage (+): 0.44. Max coverage (-): 0

Region: NODE\_324426\_length\_69311\_cov\_30.906147 61790-61797. Max. coverage (+): 1.27. Max coverage (-): 0.08

Region: NODE\_324426\_length\_69311\_cov\_30.906147 61798-61806. Max. coverage (+): 0. Max coverage (-): 0.04

Region: NODE\_324426\_length\_69311\_cov\_30.906147 61807-61815. Max. coverage (+): 2.42. Max coverage (-): 0

Region: NODE\_324426\_length\_69311\_cov\_30.906147 61816-61824. Max. coverage (+): 1.13. Max coverage (-): 0

Region: NODE\_324426\_length\_69311\_cov\_30.906147 61825-61832. Max. coverage (+): 0.12. Max coverage (-): 0.2

Region: NODE\_324426\_length\_69311\_cov\_30.906147 61833-61841. Max. coverage (+): 0. Max coverage (-): 0.2

Region: NODE\_324426\_length\_69311\_cov\_30.906147 61842-61850. Max. coverage (+): 11.51. Max coverage (-): 0

Region: NODE\_324426\_length\_69311\_cov\_30.906147 61851-61858. Max. coverage (+): 11.39. Max coverage (-): 0

Region: NODE\_324426\_length\_69311\_cov\_30.906147 61859-61867. Max. coverage (+): 0.2. Max coverage (-): 0

Region: NODE\_324426\_length\_69311\_cov\_30.906147 61868-61876. Max. coverage (+): 0. Max coverage (-): 2.06

Region: NODE\_324426\_length\_69311\_cov\_30.906147 61877-61885. Max. coverage (+): 0. Max coverage (-): 1.8

Region: NODE\_324426\_length\_69311\_cov\_30.906147 61886-61893. Max. coverage (+): 2.62. Max coverage (-): 0

Region: NODE\_324426\_length\_69311\_cov\_30.906147 61894-61902. Max. coverage (+): 0.28. Max coverage (-): 0

Region: NODE\_324426\_length\_69311\_cov\_30.906147 61903-61911. Max. coverage (+): 0.08. Max coverage (-): 0

Region: NODE\_324426\_length\_69311\_cov\_30.906147 61912-61920. Max. coverage (+): 0.12. Max coverage (-): 0.08

Region: NODE\_324426\_length\_69311\_cov\_30.906147 61921-61928. Max. coverage (+): 0.2. Max coverage (-): 0

Region: NODE\_324426\_length\_69311\_cov\_30.906147 61929-61937. Max. coverage (+): 0.24. Max coverage (-): 0

Region: NODE\_324426\_length\_69311\_cov\_30.906147 61938-61946. Max. coverage (+): 3.35. Max coverage (-): 0.08

Region: NODE\_324426\_length\_69311\_cov\_30.906147 61947-61954. Max. coverage (+): 3.47. Max coverage (-): 0.04

Region: NODE\_324426\_length\_69311\_cov\_30.906147 61955-61963. Max. coverage (+): 2.18. Max coverage (-): 0

Region: NODE\_324426\_length\_69311\_cov\_30.906147 61964-61972. Max. coverage (+): 0.24. Max coverage (-): 0

Region: NODE\_324426\_length\_69311\_cov\_30.906147 61973-61981. Max. coverage (+): 0.3. Max coverage (-): 0

Region: NODE\_324426\_length\_69311\_cov\_30.906147 61982-61989. Max. coverage (+): 0.05. Max coverage (-): 0.01

Region: NODE\_324426\_length\_69311\_cov\_30.906147 61990-61998. Max. coverage (+): 0.08. Max coverage (-): 0.04

Region: NODE\_324426\_length\_69311\_cov\_30.906147 61999-62007. Max. coverage (+): 0.2. Max coverage (-): 0

Region: NODE\_324426\_length\_69311\_cov\_30.906147 62008-62015. Max. coverage (+): 0.12. Max coverage (-): 0

Region: NODE\_324426\_length\_69311\_cov\_30.906147 62016-62024. Max. coverage (+): 2.46. Max coverage (-): 0.04

Region: NODE\_324426\_length\_69311\_cov\_30.906147 62025-62033. Max. coverage (+): 0.48. Max coverage (-): 0.16

Region: NODE\_324426\_length\_69311\_cov\_30.906147 62034-62042. Max. coverage (+): 0.04. Max coverage (-): 0.08

Region: NODE\_324426\_length\_69311\_cov\_30.906147 62043-62050. Max. coverage (+): 0.24. Max coverage (-): 0.02

Region: NODE\_324426\_length\_69311\_cov\_30.906147 62051-62059. Max. coverage (+): 0.34. Max coverage (-): 0.03

Region: NODE\_324426\_length\_69311\_cov\_30.906147 62060-62068. Max. coverage (+): 0.23. Max coverage (-): 0.01

Region: NODE\_324426\_length\_69311\_cov\_30.906147 62069-62077. Max. coverage (+): 0.23. Max coverage (-): 0

Region: NODE\_324426\_length\_69311\_cov\_30.906147 62078-62085. Max. coverage (+): 0.03. Max coverage (-): 0.03

Region: NODE\_324426\_length\_69311\_cov\_30.906147 62086-62094. Max. coverage (+): 0. Max coverage (-): 0.03

Region: NODE\_324426\_length\_69311\_cov\_30.906147 62095-62103. Max. coverage (+): 0.32. Max coverage (-): 0.04

Region: NODE\_324426\_length\_69311\_cov\_30.906147 62104-62111. Max. coverage (+): 0.24. Max coverage (-): 0

Region: NODE\_324426\_length\_69311\_cov\_30.906147 62112-62120. Max. coverage (+): 0. Max coverage (-): 0.07

Region: NODE\_324426\_length\_69311\_cov\_30.906147 62121-62129. Max. coverage (+): 1.25. Max coverage (-): 0

Region: NODE\_324426\_length\_69311\_cov\_30.906147 62130-62138. Max. coverage (+): 1.25. Max coverage (-): 0

Region: NODE\_324426\_length\_69311\_cov\_30.906147 62139-62146. Max. coverage (+): 0.24. Max coverage (-): 0

Region: NODE\_324426\_length\_69311\_cov\_30.906147 62147-62155. Max. coverage (+): 0.04. Max coverage (-): 0

Region: NODE\_324426\_length\_69311\_cov\_30.906147 62156-62164. Max. coverage (+): 0.57. Max coverage (-): 0

Region: NODE\_324426\_length\_69311\_cov\_30.906147 62165-62173. Max. coverage (+): 0.16. Max coverage (-): 0.16

Region: NODE\_324426\_length\_69311\_cov\_30.906147 62174-62181. Max. coverage (+): 0.61. Max coverage (-): 0.08

Region: NODE\_324426\_length\_69311\_cov\_30.906147 62182-62190. Max. coverage (+): 0.67. Max coverage (-): 0

Region: NODE\_324426\_length\_69311\_cov\_30.906147 62191-62199. Max. coverage (+): 0.95. Max coverage (-): 0.08

Region: NODE\_324426\_length\_69311\_cov\_30.906147 62200-62207. Max. coverage (+): 0. Max coverage (-): 0.32

Region: NODE\_324426\_length\_69311\_cov\_30.906147 62208-62216. Max. coverage (+): 1.82. Max coverage (-): 0.04

Region: NODE\_324426\_length\_69311\_cov\_30.906147 62217-62225. Max. coverage (+): 2.66. Max coverage (-): 0

Region: NODE\_324426\_length\_69311\_cov\_30.906147 62226-62234. Max. coverage (+): 0.04. Max coverage (-): 0.08

Region: NODE\_324426\_length\_69311\_cov\_30.906147 62235-62242. Max. coverage (+): 0. Max coverage (-): 0.08

Region: NODE\_324426\_length\_69311\_cov\_30.906147 62243-62251. Max. coverage (+): 3.23. Max coverage (-): 0.04

Region: NODE\_324426\_length\_69311\_cov\_30.906147 62252-62260. Max. coverage (+): 4.24. Max coverage (-): 0

Region: NODE\_324426\_length\_69311\_cov\_30.906147 62261-62268. Max. coverage (+): 0.04. Max coverage (-): 0.04

Region: NODE\_324426\_length\_69311\_cov\_30.906147 62269-62277. Max. coverage (+): 0.16. Max coverage (-): 0

Region: NODE\_324426\_length\_69311\_cov\_30.906147 62278-62286. Max. coverage (+): 4.36. Max coverage (-): 0

Region: NODE\_324426\_length\_69311\_cov\_30.906147 62287-62295. Max. coverage (+): 0.36. Max coverage (-): 0

Region: NODE\_324426\_length\_69311\_cov\_30.906147 62296-62303. Max. coverage (+): 0.36. Max coverage (-): 0

Region: NODE\_324426\_length\_69311\_cov\_30.906147 62304-62312. Max. coverage (+): 0.08. Max coverage (-): 0

Region: NODE\_324426\_length\_69311\_cov\_30.906147 62313-62321. Max. coverage (+): 0.04. Max coverage (-): 0

Region: NODE\_324426\_length\_69311\_cov\_30.906147 62322-62330. Max. coverage (+): 0.4. Max coverage (-): 0

Region: NODE\_324426\_length\_69311\_cov\_30.906147 62331-62338. Max. coverage (+): 9.61. Max coverage (-): 0.12

Region: NODE\_324426\_length\_69311\_cov\_30.906147 62339-62347. Max. coverage (+): 0.32. Max coverage (-): 0.08

Region: NODE\_324426\_length\_69311\_cov\_30.906147 62348-62356. Max. coverage (+): 1.1. Max coverage (-): 0.01

Region: NODE\_324426\_length\_69311\_cov\_30.906147 62357-62364. Max. coverage (+): 0.97. Max coverage (-): 0

Region: NODE\_324426\_length\_69311\_cov\_30.906147 62365-62373. Max. coverage (+): 3.27. Max coverage (-): 0

Region: NODE\_324426\_length\_69311\_cov\_30.906147 62374-62382. Max. coverage (+): 0.08. Max coverage (-): 0.04

Region: NODE\_324426\_length\_69311\_cov\_30.906147 62383-62391. Max. coverage (+): 0.2. Max coverage (-): 0

Region: NODE\_324426\_length\_69311\_cov\_30.906147 62392-62399. Max. coverage (+): 0.85. Max coverage (-): 0.04

Region: NODE\_324426\_length\_69311\_cov\_30.906147 62400-62408. Max. coverage (+): 3.67. Max coverage (-): 0.04

Region: NODE\_324426\_length\_69311\_cov\_30.906147 62409-62417. Max. coverage (+): 3.11. Max coverage (-): 0.04

Region: NODE\_324426\_length\_69311\_cov\_30.906147 62418-62426. Max. coverage (+): 2.18. Max coverage (-): 0

Region: NODE\_324426\_length\_69311\_cov\_30.906147 62427-62434. Max. coverage (+): 2.02. Max coverage (-): 0

Region: NODE\_324426\_length\_69311\_cov\_30.906147 62435-62443. Max. coverage (+): 0.04. Max coverage (-): 0.44

Region: NODE\_324426\_length\_69311\_cov\_30.906147 62444-62452. Max. coverage (+): 0. Max coverage (-): 0.44

Region: NODE\_324426\_length\_69311\_cov\_30.906147 62453-62460. Max. coverage (+): 3.31. Max coverage (-): 0

Region: NODE\_324426\_length\_69311\_cov\_30.906147 62461-62469. Max. coverage (+): 3.31. Max coverage (-): 0.08

Region: NODE\_324426\_length\_69311\_cov\_30.906147 62470-62478. Max. coverage (+): 0.04. Max coverage (-): 0.08

Region: NODE\_324426\_length\_69311\_cov\_30.906147 62479-62487. Max. coverage (+): 0.04. Max coverage (-): 0.12

Region: NODE\_324426\_length\_69311\_cov\_30.906147 62488-62495. Max. coverage (+): 1.17. Max coverage (-): 0.08

Region: NODE\_324426\_length\_69311\_cov\_30.906147 62496-62504. Max. coverage (+): 0.65. Max coverage (-): 0.52

Region: NODE\_324426\_length\_69311\_cov\_30.906147 62505-62513. Max. coverage (+): 0.32. Max coverage (-): 0

Region: NODE\_324426\_length\_69311\_cov\_30.906147 62514-62521. Max. coverage (+): 0.2. Max coverage (-): 0

Region: NODE\_324426\_length\_69311\_cov\_30.906147 62522-62530. Max. coverage (+): 0. Max coverage (-): 0

Region: NODE\_324426\_length\_69311\_cov\_30.906147 62531-62539. Max. coverage (+): 0. Max coverage (-): 0

Region: NODE\_324426\_length\_69311\_cov\_30.906147 62540-62548. Max. coverage (+): 0.81. Max coverage (-): 0

Region: NODE\_324426\_length\_69311\_cov\_30.906147 62549-62556. Max. coverage (+): 0.4. Max coverage (-): 0

Region: NODE\_324426\_length\_69311\_cov\_30.906147 62557-62565. Max. coverage (+): 0.16. Max coverage (-): 0.04

Region: NODE\_324426\_length\_69311\_cov\_30.906147 62566-62574. Max. coverage (+): 0.3. Max coverage (-): 0.18

Region: NODE\_324426\_length\_69311\_cov\_30.906147 62575-62583. Max. coverage (+): 0.16. Max coverage (-): 0.16

Region: NODE\_324426\_length\_69311\_cov\_30.906147 62584-62591. Max. coverage (+): 0.52. Max coverage (-): 0

Region: NODE\_324426\_length\_69311\_cov\_30.906147 62592-62600. Max. coverage (+): 0. Max coverage (-): 0.12

Region: NODE\_324426\_length\_69311\_cov\_30.906147 62601-62609. Max. coverage (+): 0. Max coverage (-): 0.04

Region: NODE\_324426\_length\_69311\_cov\_30.906147 62610-62617. Max. coverage (+): 0.2. Max coverage (-): 0.04

Region: NODE\_324426\_length\_69311\_cov\_30.906147 62618-62626. Max. coverage (+): 0.2. Max coverage (-): 0.12

Region: NODE\_324426\_length\_69311\_cov\_30.906147 62627-62635. Max. coverage (+): 10.6. Max coverage (-): 0

Region: NODE\_324426\_length\_69311\_cov\_30.906147 62636-62644. Max. coverage (+): 10.46. Max coverage (-): 0.06

Region: NODE\_324426\_length\_69311\_cov\_30.906147 62645-62652. Max. coverage (+): 0.3. Max coverage (-): 0.06

Region: NODE\_324426\_length\_69311\_cov\_30.906147 62653-62661. Max. coverage (+): 0.24. Max coverage (-): 0.02

Region: NODE\_324426\_length\_69311\_cov\_30.906147 62662-62670. Max. coverage (+): 1.53. Max coverage (-): 0

Region: NODE\_324426\_length\_69311\_cov\_30.906147 62671-62678. Max. coverage (+): 0.04. Max coverage (-): 0

Region: NODE\_324426\_length\_69311\_cov\_30.906147 62679-62687. Max. coverage (+): 0.04. Max coverage (-): 0

Region: NODE\_324426\_length\_69311\_cov\_30.906147 62688-62696. Max. coverage (+): 0.57. Max coverage (-): 0

Region: NODE\_324426\_length\_69311\_cov\_30.906147 62697-62705. Max. coverage (+): 0.65. Max coverage (-): 0

Region: NODE\_324426\_length\_69311\_cov\_30.906147 62706-62713. Max. coverage (+): 0.93. Max coverage (-): 0

Region: NODE\_324426\_length\_69311\_cov\_30.906147 62714-62722. Max. coverage (+): 0.04. Max coverage (-): 0

Region: NODE\_324426\_length\_69311\_cov\_30.906147 62723-62731. Max. coverage (+): 0.12. Max coverage (-): 0

Region: NODE\_324426\_length\_69311\_cov\_30.906147 62732-62740. Max. coverage (+): 0.12. Max coverage (-): 0

Region: NODE\_324426\_length\_69311\_cov\_30.906147 62741-62748. Max. coverage (+): 0.08. Max coverage (-): 0

Region: NODE\_324426\_length\_69311\_cov\_30.906147 62749-62757. Max. coverage (+): 0.04. Max coverage (-): 0.08

Region: NODE\_324426\_length\_69311\_cov\_30.906147 62758-62766. Max. coverage (+): 0.04. Max coverage (-): 0.08

Region: NODE\_324426\_length\_69311\_cov\_30.906147 62767-62774. Max. coverage (+): 0.12. Max coverage (-): 0.04

Region: NODE\_324426\_length\_69311\_cov\_30.906147 62775-62783. Max. coverage (+): 1.53. Max coverage (-): 0

Region: NODE\_324426\_length\_69311\_cov\_30.906147 62784-62792. Max. coverage (+): 0.24. Max coverage (-): 0

Region: NODE\_324426\_length\_69311\_cov\_30.906147 62793-62801. Max. coverage (+): 0. Max coverage (-): 0

Region: NODE\_324426\_length\_69311\_cov\_30.906147 62802-62809. Max. coverage (+): 4.36. Max coverage (-): 0

Region: NODE\_324426\_length\_69311\_cov\_30.906147 62810-62818. Max. coverage (+): 8.8. Max coverage (-): 0

Region: NODE\_324426\_length\_69311\_cov\_30.906147 62819-62827. Max. coverage (+): 0.36. Max coverage (-): 0.04

Region: NODE\_324426\_length\_69311\_cov\_30.906147 62828-62836. Max. coverage (+): 0.2. Max coverage (-): 0

Region: NODE\_324426\_length\_69311\_cov\_30.906147 62837-62844. Max. coverage (+): 1.07. Max coverage (-): 0

Region: NODE\_324426\_length\_69311\_cov\_30.906147 62845-62853. Max. coverage (+): 0.55. Max coverage (-): 0.04

Region: NODE\_324426\_length\_69311\_cov\_30.906147 62854-62862. Max. coverage (+): 0.57. Max coverage (-): 0.02

Region: NODE\_324426\_length\_69311\_cov\_30.906147 62863-62870. Max. coverage (+): 0.48. Max coverage (-): 0

Region: NODE\_324426\_length\_69311\_cov\_30.906147 62871-62879. Max. coverage (+): 0.12. Max coverage (-): 0.24

Region: NODE\_324426\_length\_69311\_cov\_30.906147 62880-62888. Max. coverage (+): 0.16. Max coverage (-): 0.16

Region: NODE\_324426\_length\_69311\_cov\_30.906147 62889-62897. Max. coverage (+): 0.44. Max coverage (-): 1.25

Region: NODE\_324426\_length\_69311\_cov\_30.906147 62898-62905. Max. coverage (+): 0.44. Max coverage (-): 0.12

Region: NODE\_324426\_length\_69311\_cov\_30.906147 62906-62914. Max. coverage (+): 0.04. Max coverage (-): 0

Region: NODE\_324426\_length\_69311\_cov\_30.906147 62915-62923. Max. coverage (+): 0.24. Max coverage (-): 0

Region: NODE\_324426\_length\_69311\_cov\_30.906147 62924-62931. Max. coverage (+): 1.17. Max coverage (-): 0

Region: NODE\_324426\_length\_69311\_cov\_30.906147 62932-62940. Max. coverage (+): 0.04. Max coverage (-): 0

Region: NODE\_324426\_length\_69311\_cov\_30.906147 62941-62949. Max. coverage (+): 0.69. Max coverage (-): 0.08

Region: NODE\_324426\_length\_69311\_cov\_30.906147 62950-62958. Max. coverage (+): 0.08. Max coverage (-): 0

Region: NODE\_324426\_length\_69311\_cov\_30.906147 62959-62966. Max. coverage (+): 0.16. Max coverage (-): 0.12

Region: NODE\_324426\_length\_69311\_cov\_30.906147 62967-62975. Max. coverage (+): 1.01. Max coverage (-): 0.12

Region: NODE\_324426\_length\_69311\_cov\_30.906147 62976-62984. Max. coverage (+): 1.17. Max coverage (-): 0

Region: NODE\_324426\_length\_69311\_cov\_30.906147 62985-62993. Max. coverage (+): 0.04. Max coverage (-): 0.02

Region: NODE\_324426\_length\_69311\_cov\_30.906147 62994-63001. Max. coverage (+): 0.04. Max coverage (-): 0

Region: NODE\_324426\_length\_69311\_cov\_30.906147 63002-63010. Max. coverage (+): 1.66. Max coverage (-): 0

Region: NODE\_324426\_length\_69311\_cov\_30.906147 63011-63019. Max. coverage (+): 0.4. Max coverage (-): 0

Region: NODE\_324426\_length\_69311\_cov\_30.906147 63020-63027. Max. coverage (+): 0.08. Max coverage (-): 0

Region: NODE\_324426\_length\_69311\_cov\_30.906147 63028-63036. Max. coverage (+): 0.12. Max coverage (-): 0.24

Region: NODE\_324426\_length\_69311\_cov\_30.906147 63037-63045. Max. coverage (+): 0.22. Max coverage (-): 0

Region: NODE\_324426\_length\_69311\_cov\_30.906147 63046-63054. Max. coverage (+): 0.44. Max coverage (-): 0

Region: NODE\_324426\_length\_69311\_cov\_30.906147 63055-63062. Max. coverage (+): 0.12. Max coverage (-): 0.04

Region: NODE\_324426\_length\_69311\_cov\_30.906147 63063-63071. Max. coverage (+): 0.04. Max coverage (-): 0.12

Region: NODE\_324426\_length\_69311\_cov\_30.906147 63072-63080. Max. coverage (+): 0.36. Max coverage (-): 0

Region: NODE\_324426\_length\_69311\_cov\_30.906147 63081-63089. Max. coverage (+): 0.36. Max coverage (-): 0.08

Region: NODE\_324426\_length\_69311\_cov\_30.906147 63090-63097. Max. coverage (+): 0.04. Max coverage (-): 0

Region: NODE\_324426\_length\_69311\_cov\_30.906147 63098-63106. Max. coverage (+): 0.08. Max coverage (-): 0

Region: NODE\_324426\_length\_69311\_cov\_30.906147 63107-63115. Max. coverage (+): 0.08. Max coverage (-): 0

Region: NODE\_324426\_length\_69311\_cov\_30.906147 63116-63123. Max. coverage (+): 0. Max coverage (-): 0

Region: NODE\_324426\_length\_69311\_cov\_30.906147 63124-63132. Max. coverage (+): 0. Max coverage (-): 0.12

Region: NODE\_324426\_length\_69311\_cov\_30.906147 63133-63141. Max. coverage (+): 0.08. Max coverage (-): 0.12

Region: NODE\_324426\_length\_69311\_cov\_30.906147 63142-63150. Max. coverage (+): 0.08. Max coverage (-): 0.04

Region: NODE\_324426\_length\_69311\_cov\_30.906147 63151-63158. Max. coverage (+): 0. Max coverage (-): 0

Region: NODE\_324426\_length\_69311\_cov\_30.906147 63159-63167. Max. coverage (+): 0. Max coverage (-): 0.04

Region: NODE\_324426\_length\_69311\_cov\_30.906147 63168-63176. Max. coverage (+): 0.24. Max coverage (-): 0.04

Region: NODE\_324426\_length\_69311\_cov\_30.906147 63177-63184. Max. coverage (+): 0.24. Max coverage (-): 0

Region: NODE\_324426\_length\_69311\_cov\_30.906147 63185-63193. Max. coverage (+): 0.24. Max coverage (-): 0

Region: NODE\_324426\_length\_69311\_cov\_30.906147 63194-63202. Max. coverage (+): 0.36. Max coverage (-): 0

Region: NODE\_324426\_length\_69311\_cov\_30.906147 63203-63211. Max. coverage (+): 1.33. Max coverage (-): 0

Region: NODE\_324426\_length\_69311\_cov\_30.906147 63212-63219. Max. coverage (+): 0.16. Max coverage (-): 0.08

Region: NODE\_324426\_length\_69311\_cov\_30.906147 63220-63228. Max. coverage (+): 0.08. Max coverage (-): 0.04

Region: NODE\_324426\_length\_69311\_cov\_30.906147 63229-63237. Max. coverage (+): 14.98. Max coverage (-): 0

Region: NODE\_324426\_length\_69311\_cov\_30.906147 63238-63246. Max. coverage (+): 15.26. Max coverage (-): 0

Region: NODE\_324426\_length\_69311\_cov\_30.906147 63247-63254. Max. coverage (+): 0.08. Max coverage (-): 0.04

Region: NODE\_324426\_length\_69311\_cov\_30.906147 63255-63263. Max. coverage (+): 0.69. Max coverage (-): 0.04

Region: NODE\_324426\_length\_69311\_cov\_30.906147 63264-63272. Max. coverage (+): 0.57. Max coverage (-): 0.61

Region: NODE\_324426\_length\_69311\_cov\_30.906147 63273-63280. Max. coverage (+): 0.12. Max coverage (-): 0.61

Region: NODE\_324426\_length\_69311\_cov\_30.906147 63281-63289. Max. coverage (+): 1.29. Max coverage (-): 0.04

Region: NODE\_324426\_length\_69311\_cov\_30.906147 63290-63298. Max. coverage (+): 1.17. Max coverage (-): 0

Region: NODE\_324426\_length\_69311\_cov\_30.906147 63299-63307. Max. coverage (+): 0.04. Max coverage (-): 0.12

Region: NODE\_324426\_length\_69311\_cov\_30.906147 63308-63315. Max. coverage (+): 0.04. Max coverage (-): 0.61

Region: NODE\_324426\_length\_69311\_cov\_30.906147 63316-63324. Max. coverage (+): 1.98. Max coverage (-): 0.02

Region: NODE\_324426\_length\_69311\_cov\_30.906147 63325-63333. Max. coverage (+): 1.74. Max coverage (-): 0.02

Region: NODE\_324426\_length\_69311\_cov\_30.906147 63334-63342. Max. coverage (+): 0.24. Max coverage (-): 0

Region: NODE\_324426\_length\_69311\_cov\_30.906147 63343-63350. Max. coverage (+): 0.16. Max coverage (-): 0.04

Region: NODE\_324426\_length\_69311\_cov\_30.906147 63351-63359. Max. coverage (+): 0.04. Max coverage (-): 0.04

Region: NODE\_324426\_length\_69311\_cov\_30.906147 63360-63368. Max. coverage (+): 0. Max coverage (-): 0.04

Region: NODE\_324426\_length\_69311\_cov\_30.906147 63369-63376. Max. coverage (+): 0. Max coverage (-): 0

Region: NODE\_324426\_length\_69311\_cov\_30.906147 63377-63385. Max. coverage (+): 0.2. Max coverage (-): 0

Region: NODE\_324426\_length\_69311\_cov\_30.906147 63386-63394. Max. coverage (+): 0.89. Max coverage (-): 0

Region: NODE\_324426\_length\_69311\_cov\_30.906147 63395-63403. Max. coverage (+): 1.45. Max coverage (-): 0

Region: NODE\_324426\_length\_69311\_cov\_30.906147 63404-63411. Max. coverage (+): 0. Max coverage (-): 0

Region: NODE\_324426\_length\_69311\_cov\_30.906147 63412-63420. Max. coverage (+): 0. Max coverage (-): 0

Region: NODE\_324426\_length\_69311\_cov\_30.906147 63421-63429. Max. coverage (+): 0. Max coverage (-): 0

Region: NODE\_324426\_length\_69311\_cov\_30.906147 63430-63437. Max. coverage (+): 0. Max coverage (-): 0

Region: NODE\_324426\_length\_69311\_cov\_30.906147 63438-63446. Max. coverage (+): 0. Max coverage (-): 0

Region: NODE\_324426\_length\_69311\_cov\_30.906147 63447-63455. Max. coverage (+): 0. Max coverage (-): 0

Region: NODE\_324426\_length\_69311\_cov\_30.906147 63456-63464. Max. coverage (+): 1.86. Max coverage (-): 0.04

Region: NODE\_324426\_length\_69311\_cov\_30.906147 63465-63472. Max. coverage (+): 1.7. Max coverage (-): 0.04

Region: NODE\_324426\_length\_69311\_cov\_30.906147 63473-63481. Max. coverage (+): 0.36. Max coverage (-): 0

Region: NODE\_324426\_length\_69311\_cov\_30.906147 63482-63490. Max. coverage (+): 0.12. Max coverage (-): 0

Region: NODE\_324426\_length\_69311\_cov\_30.906147 63491-63499. Max. coverage (+): 0.08. Max coverage (-): 0

Region: NODE\_324426\_length\_69311\_cov\_30.906147 63500-63507. Max. coverage (+): 0.08. Max coverage (-): 0

Region: NODE\_324426\_length\_69311\_cov\_30.906147 63508-63516. Max. coverage (+): 0.12. Max coverage (-): 0

Region: NODE\_324426\_length\_69311\_cov\_30.906147 63517-63525. Max. coverage (+): 0.12. Max coverage (-): 0

Region: NODE\_324426\_length\_69311\_cov\_30.906147 63526-63533. Max. coverage (+): 0.12. Max coverage (-): 0

Region: NODE\_324426\_length\_69311\_cov\_30.906147 63534-63542. Max. coverage (+): 0.01. Max coverage (-): 0

Region: NODE\_324426\_length\_69311\_cov\_30.906147 63543-63551. Max. coverage (+): 0.01. Max coverage (-): 0

Region: NODE\_324426\_length\_69311\_cov\_30.906147 63552-63560. Max. coverage (+): 0.12. Max coverage (-): 0.04

Region: NODE\_324426\_length\_69311\_cov\_30.906147 63561-63568. Max. coverage (+): 0.12. Max coverage (-): 0

Region: NODE\_324426\_length\_69311\_cov\_30.906147 63569-63577. Max. coverage (+): 0.04. Max coverage (-): 0

Region: NODE\_324426\_length\_69311\_cov\_30.906147 63578-63586. Max. coverage (+): 0.04. Max coverage (-): 0.04

Region: NODE\_324426\_length\_69311\_cov\_30.906147 63587-63595. Max. coverage (+): 0.01. Max coverage (-): 0

Region: NODE\_324426\_length\_69311\_cov\_30.906147 63596-63603. Max. coverage (+): 0.02. Max coverage (-): 0.01

Region: NODE\_324426\_length\_69311\_cov\_30.906147 63604-63612. Max. coverage (+): 0.81. Max coverage (-): 0

Region: NODE\_324426\_length\_69311\_cov\_30.906147 63613-63621. Max. coverage (+): 0.81. Max coverage (-): 0

Region: NODE\_324426\_length\_69311\_cov\_30.906147 63622-63629. Max. coverage (+): 0.2. Max coverage (-): 0

Region: NODE\_324426\_length\_69311\_cov\_30.906147 63630-63638. Max. coverage (+): 0.16. Max coverage (-): 0

Region: NODE\_324426\_length\_69311\_cov\_30.906147 63639-63647. Max. coverage (+): 0. Max coverage (-): 0

Region: NODE\_324426\_length\_69311\_cov\_30.906147 63648-63656. Max. coverage (+): 0. Max coverage (-): 0

Region: NODE\_324426\_length\_69311\_cov\_30.906147 63657-63664. Max. coverage (+): 0. Max coverage (-): 0

Region: NODE\_324426\_length\_69311\_cov\_30.906147 63665-63673. Max. coverage (+): 0. Max coverage (-): 0

Region: NODE\_324426\_length\_69311\_cov\_30.906147 63674-63682. Max. coverage (+): 0. Max coverage (-): 0

Region: NODE\_324426\_length\_69311\_cov\_30.906147 63683-63690. Max. coverage (+): 0. Max coverage (-): 0

Region: NODE\_324426\_length\_69311\_cov\_30.906147 63691-63699. Max. coverage (+): 0. Max coverage (-): 0

Region: NODE\_324426\_length\_69311\_cov\_30.906147 63700-63708. Max. coverage (+): 0. Max coverage (-): 0

Region: NODE\_324426\_length\_69311\_cov\_30.906147 63709-63717. Max. coverage (+): 0. Max coverage (-): 0

Region: NODE\_324426\_length\_69311\_cov\_30.906147 63718-63725. Max. coverage (+): 0. Max coverage (-): 0

Region: NODE\_324426\_length\_69311\_cov\_30.906147 63726-63734. Max. coverage (+): 0. Max coverage (-): 0

Region: NODE\_324426\_length\_69311\_cov\_30.906147 63735-63743. Max. coverage (+): 0.04. Max coverage (-): 0

Region: NODE\_324426\_length\_69311\_cov\_30.906147 63744-63752. Max. coverage (+): 0.04. Max coverage (-): 0

Region: NODE\_324426\_length\_69311\_cov\_30.906147 63753-63760. Max. coverage (+): 0.04. Max coverage (-): 0

Region: NODE\_324426\_length\_69311\_cov\_30.906147 63761-63769. Max. coverage (+): 0.08. Max coverage (-): 0

Region: NODE\_324426\_length\_69311\_cov\_30.906147 63770-63778. Max. coverage (+): 0.08. Max coverage (-): 0

Region: NODE\_324426\_length\_69311\_cov\_30.906147 63779-63786. Max. coverage (+): 0. Max coverage (-): 0

Region: NODE\_324426\_length\_69311\_cov\_30.906147 63787-63795. Max. coverage (+): 0.04. Max coverage (-): 0

Region: NODE\_324426\_length\_69311\_cov\_30.906147 63796-63804. Max. coverage (+): 0.16. Max coverage (-): 0

Region: NODE\_324426\_length\_69311\_cov\_30.906147 63805-63813. Max. coverage (+): 0.04. Max coverage (-): 0

Region: NODE\_324426\_length\_69311\_cov\_30.906147 63814-63821. Max. coverage (+): 0. Max coverage (-): 0

Region: NODE\_324426\_length\_69311\_cov\_30.906147 63822-63830. Max. coverage (+): 0. Max coverage (-): 0

Region: NODE\_324426\_length\_69311\_cov\_30.906147 63831-63839. Max. coverage (+): 0. Max coverage (-): 0

Region: NODE\_324426\_length\_69311\_cov\_30.906147 63840-63848. Max. coverage (+): 0. Max coverage (-): 0

Region: NODE\_324426\_length\_69311\_cov\_30.906147 63849-63856. Max. coverage (+): 0.2. Max coverage (-): 0

Region: NODE\_324426\_length\_69311\_cov\_30.906147 63857-63865. Max. coverage (+): 0.2. Max coverage (-): 0

Region: NODE\_324426\_length\_69311\_cov\_30.906147 63866-63874. Max. coverage (+): 0.04. Max coverage (-): 0

Region: NODE\_324426\_length\_69311\_cov\_30.906147 63875-63882. Max. coverage (+): 0.04. Max coverage (-): 0

Region: NODE\_324426\_length\_69311\_cov\_30.906147 63883-63891. Max. coverage (+): 0. Max coverage (-): 0

Region: NODE\_324426\_length\_69311\_cov\_30.906147 63892-63900. Max. coverage (+): 0. Max coverage (-): 0

Region: NODE\_324426\_length\_69311\_cov\_30.906147 63901-63909. Max. coverage (+): 0. Max coverage (-): 0

Region: NODE\_324426\_length\_69311\_cov\_30.906147 63910-63917. Max. coverage (+): 0.12. Max coverage (-): 0

Region: NODE\_324426\_length\_69311\_cov\_30.906147 63918-63926. Max. coverage (+): 0. Max coverage (-): 0

Region: NODE\_324426\_length\_69311\_cov\_30.906147 63927-63935. Max. coverage (+): 0. Max coverage (-): 0

Region: NODE\_324426\_length\_69311\_cov\_30.906147 63936-63943. Max. coverage (+): 0. Max coverage (-): 0.12

Region: NODE\_324426\_length\_69311\_cov\_30.906147 63944-63952. Max. coverage (+): 0. Max coverage (-): 0.12

Region: NODE\_324426\_length\_69311\_cov\_30.906147 63953-63961. Max. coverage (+): 0. Max coverage (-): 0

Region: NODE\_324426\_length\_69311\_cov\_30.906147 63962-63970. Max. coverage (+): 0. Max coverage (-): 0

Region: NODE\_324426\_length\_69311\_cov\_30.906147 63971-63978. Max. coverage (+): 0. Max coverage (-): 0

Region: NODE\_324426\_length\_69311\_cov\_30.906147 63979-63987. Max. coverage (+): 0. Max coverage (-): 0.04

Region: NODE\_324426\_length\_69311\_cov\_30.906147 63988-63996. Max. coverage (+): 0. Max coverage (-): 0.04

Region: NODE\_324426\_length\_69311\_cov\_30.906147 63997-64005. Max. coverage (+): 0. Max coverage (-): 0

Region: NODE\_324426\_length\_69311\_cov\_30.906147 64006-64013. Max. coverage (+): 0. Max coverage (-): 0

Region: NODE\_324426\_length\_69311\_cov\_30.906147 64014-64022. Max. coverage (+): 0. Max coverage (-): 0

Region: NODE\_324426\_length\_69311\_cov\_30.906147 64023-64031. Max. coverage (+): 0. Max coverage (-): 0

Region: NODE\_324426\_length\_69311\_cov\_30.906147 64032-64039. Max. coverage (+): 0. Max coverage (-): 0

Region: NODE\_324426\_length\_69311\_cov\_30.906147 64040-64048. Max. coverage (+): 0. Max coverage (-): 0

Region: NODE\_324426\_length\_69311\_cov\_30.906147 64049-64057. Max. coverage (+): 0. Max coverage (-): 0

Region: NODE\_324426\_length\_69311\_cov\_30.906147 64058-64066. Max. coverage (+): 0. Max coverage (-): 0

Region: NODE\_324426\_length\_69311\_cov\_30.906147 64067-64074. Max. coverage (+): 0. Max coverage (-): 0

Region: NODE\_324426\_length\_69311\_cov\_30.906147 64075-64083. Max. coverage (+): 0.08. Max coverage (-): 0

Region: NODE\_324426\_length\_69311\_cov\_30.906147 64084-64092. Max. coverage (+): 0. Max coverage (-): 0

Region: NODE\_324426\_length\_69311\_cov\_30.906147 64093-64101. Max. coverage (+): 0. Max coverage (-): 0

Region: NODE\_324426\_length\_69311\_cov\_30.906147 64102-64109. Max. coverage (+): 0. Max coverage (-): 0

Region: NODE\_324426\_length\_69311\_cov\_30.906147 64110-64118. Max. coverage (+): 0. Max coverage (-): 0.04

Region: NODE\_324426\_length\_69311\_cov\_30.906147 64119-64127. Max. coverage (+): 0. Max coverage (-): 0.04

Region: NODE\_324426\_length\_69311\_cov\_30.906147 64128-64135. Max. coverage (+): 0. Max coverage (-): 0

Region: NODE\_324426\_length\_69311\_cov\_30.906147 64136-64144. Max. coverage (+): 0. Max coverage (-): 0

Region: NODE\_324426\_length\_69311\_cov\_30.906147 64145-64153. Max. coverage (+): 0. Max coverage (-): 0

Region: NODE\_324426\_length\_69311\_cov\_30.906147 64154-64162. Max. coverage (+): 0.08. Max coverage (-): 0

Region: NODE\_324426\_length\_69311\_cov\_30.906147 64163-64170. Max. coverage (+): 0. Max coverage (-): 0

Region: NODE\_324426\_length\_69311\_cov\_30.906147 64171-64179. Max. coverage (+): 0. Max coverage (-): 0

Region: NODE\_324426\_length\_69311\_cov\_30.906147 64180-64188. Max. coverage (+): 0. Max coverage (-): 0

Region: NODE\_324426\_length\_69311\_cov\_30.906147 64189-64196. Max. coverage (+): 0. Max coverage (-): 0

Region: NODE\_324426\_length\_69311\_cov\_30.906147 64197-64205. Max. coverage (+): 0. Max coverage (-): 0

Region: NODE\_324426\_length\_69311\_cov\_30.906147 64206-64214. Max. coverage (+): 0. Max coverage (-): 0

Region: NODE\_324426\_length\_69311\_cov\_30.906147 64215-64223. Max. coverage (+): 0. Max coverage (-): 0

Region: NODE\_324426\_length\_69311\_cov\_30.906147 64224-64231. Max. coverage (+): 0.08. Max coverage (-): 0

Region: NODE\_324426\_length\_69311\_cov\_30.906147 64232-64240. Max. coverage (+): 0.08. Max coverage (-): 0

Region: NODE\_324426\_length\_69311\_cov\_30.906147 64241-64249. Max. coverage (+): 0. Max coverage (-): 0

Region: NODE\_324426\_length\_69311\_cov\_30.906147 64250-64258. Max. coverage (+): 0. Max coverage (-): 0

Region: NODE\_324426\_length\_69311\_cov\_30.906147 64259-64266. Max. coverage (+): 0. Max coverage (-): 0.04

Region: NODE\_324426\_length\_69311\_cov\_30.906147 64267-64275. Max. coverage (+): 0. Max coverage (-): 0

Region: NODE\_324426\_length\_69311\_cov\_30.906147 64276-64284. Max. coverage (+): 0. Max coverage (-): 0

Region: NODE\_324426\_length\_69311\_cov\_30.906147 64285-64292. Max. coverage (+): 0. Max coverage (-): 0

Region: NODE\_324426\_length\_69311\_cov\_30.906147 64293-64301. Max. coverage (+): 0. Max coverage (-): 0

Region: NODE\_324426\_length\_69311\_cov\_30.906147 64302-64310. Max. coverage (+): 0. Max coverage (-): 0

Region: NODE\_324426\_length\_69311\_cov\_30.906147 64311-64319. Max. coverage (+): 0. Max coverage (-): 0.01

Region: NODE\_324426\_length\_69311\_cov\_30.906147 64320-64327. Max. coverage (+): 0. Max coverage (-): 0

Region: NODE\_324426\_length\_69311\_cov\_30.906147 64328-64336. Max. coverage (+): 0. Max coverage (-): 0

Region: NODE\_324426\_length\_69311\_cov\_30.906147 64337-64345. Max. coverage (+): 0. Max coverage (-): 0

Region: NODE\_324426\_length\_69311\_cov\_30.906147 64346-64354. Max. coverage (+): 0. Max coverage (-): 0

Region: NODE\_324426\_length\_69311\_cov\_30.906147 64355-64362. Max. coverage (+): 0. Max coverage (-): 0

Region: NODE\_324426\_length\_69311\_cov\_30.906147 64363-64371. Max. coverage (+): 0. Max coverage (-): 0

Region: NODE\_324426\_length\_69311\_cov\_30.906147 64372-64380. Max. coverage (+): 0. Max coverage (-): 0

Region: NODE\_324426\_length\_69311\_cov\_30.906147 64381-64388. Max. coverage (+): 0. Max coverage (-): 0

Region: NODE\_324426\_length\_69311\_cov\_30.906147 64389-64397. Max. coverage (+): 0. Max coverage (-): 0.04

Region: NODE\_324426\_length\_69311\_cov\_30.906147 64398-64406. Max. coverage (+): 0. Max coverage (-): 0

Region: NODE\_324426\_length\_69311\_cov\_30.906147 64407-64415. Max. coverage (+): 0. Max coverage (-): 0

Region: NODE\_324426\_length\_69311\_cov\_30.906147 64416-64423. Max. coverage (+): 0. Max coverage (-): 0

Region: NODE\_324426\_length\_69311\_cov\_30.906147 64424-64432. Max. coverage (+): 0. Max coverage (-): 0

Region: NODE\_324426\_length\_69311\_cov\_30.906147 64433-64441. Max. coverage (+): 0. Max coverage (-): 0

Region: NODE\_324426\_length\_69311\_cov\_30.906147 64442-64449. Max. coverage (+): 0. Max coverage (-): 0

Region: NODE\_324426\_length\_69311\_cov\_30.906147 64450-64458. Max. coverage (+): 0. Max coverage (-): 0

Region: NODE\_324426\_length\_69311\_cov\_30.906147 64459-64467. Max. coverage (+): 0. Max coverage (-): 0

Region: NODE\_324426\_length\_69311\_cov\_30.906147 64468-64476. Max. coverage (+): 0. Max coverage (-): 0

Region: NODE\_324426\_length\_69311\_cov\_30.906147 64477-64484. Max. coverage (+): 0. Max coverage (-): 0

Region: NODE\_324426\_length\_69311\_cov\_30.906147 64485-64493. Max. coverage (+): 0. Max coverage (-): 0

Region: NODE\_324426\_length\_69311\_cov\_30.906147 64494-64502. Max. coverage (+): 0. Max coverage (-): 0

Region: NODE\_324426\_length\_69311\_cov\_30.906147 64503-64511. Max. coverage (+): 0. Max coverage (-): 0

Region: NODE\_324426\_length\_69311\_cov\_30.906147 64512-64519. Max. coverage (+): 0. Max coverage (-): 0

Region: NODE\_324426\_length\_69311\_cov\_30.906147 64520-64528. Max. coverage (+): 0. Max coverage (-): 0

Region: NODE\_324426\_length\_69311\_cov\_30.906147 64529-64537. Max. coverage (+): 0. Max coverage (-): 0

Region: NODE\_324426\_length\_69311\_cov\_30.906147 64538-64545. Max. coverage (+): 0. Max coverage (-): 0

Region: NODE\_324426\_length\_69311\_cov\_30.906147 64546-64554. Max. coverage (+): 0. Max coverage (-): 0

Region: NODE\_324426\_length\_69311\_cov\_30.906147 64555-64563. Max. coverage (+): 0.04. Max coverage (-): 0

Region: NODE\_324426\_length\_69311\_cov\_30.906147 64564-64572. Max. coverage (+): 0. Max coverage (-): 0

Region: NODE\_324426\_length\_69311\_cov\_30.906147 64573-64580. Max. coverage (+): 0. Max coverage (-): 0

Region: NODE\_324426\_length\_69311\_cov\_30.906147 64581-64589. Max. coverage (+): 0. Max coverage (-): 0

Region: NODE\_324426\_length\_69311\_cov\_30.906147 64590-64598. Max. coverage (+): 0. Max coverage (-): 0

Region: NODE\_324426\_length\_69311\_cov\_30.906147 64599-64607. Max. coverage (+): 0. Max coverage (-): 0

Region: NODE\_324426\_length\_69311\_cov\_30.906147 64608-64615. Max. coverage (+): 0. Max coverage (-): 0

Region: NODE\_324426\_length\_69311\_cov\_30.906147 64616-64624. Max. coverage (+): 0. Max coverage (-): 0

Region: NODE\_324426\_length\_69311\_cov\_30.906147 64625-64633. Max. coverage (+): 0. Max coverage (-): 0

Region: NODE\_324426\_length\_69311\_cov\_30.906147 64634-64641. Max. coverage (+): 0. Max coverage (-): 0

Region: NODE\_324426\_length\_69311\_cov\_30.906147 64642-64650. Max. coverage (+): 0. Max coverage (-): 0

Region: NODE\_324426\_length\_69311\_cov\_30.906147 64651-64659. Max. coverage (+): 0. Max coverage (-): 0

Region: NODE\_324426\_length\_69311\_cov\_30.906147 64660-64668. Max. coverage (+): 0.04. Max coverage (-): 0

Region: NODE\_324426\_length\_69311\_cov\_30.906147 64669-64676. Max. coverage (+): 0.12. Max coverage (-): 0

Region: NODE\_324426\_length\_69311\_cov\_30.906147 64677-64685. Max. coverage (+): 0. Max coverage (-): 0

Region: NODE\_324426\_length\_69311\_cov\_30.906147 64686-64694. Max. coverage (+): 0. Max coverage (-): 0

Region: NODE\_324426\_length\_69311\_cov\_30.906147 64695-64702. Max. coverage (+): 0. Max coverage (-): 0

Region: NODE\_324426\_length\_69311\_cov\_30.906147 64703-64711. Max. coverage (+): 0. Max coverage (-): 0

Region: NODE\_324426\_length\_69311\_cov\_30.906147 64712-64720. Max. coverage (+): 0. Max coverage (-): 0

Region: NODE\_324426\_length\_69311\_cov\_30.906147 64721-64729. Max. coverage (+): 0. Max coverage (-): 0

Region: NODE\_324426\_length\_69311\_cov\_30.906147 64730-64737. Max. coverage (+): 0. Max coverage (-): 0

Region: NODE\_324426\_length\_69311\_cov\_30.906147 64738-64746. Max. coverage (+): 0. Max coverage (-): 0

Region: NODE\_324426\_length\_69311\_cov\_30.906147 64747-64755. Max. coverage (+): 0. Max coverage (-): 0

Region: NODE\_324426\_length\_69311\_cov\_30.906147 64756-64764. Max. coverage (+): 0. Max coverage (-): 0

Region: NODE\_324426\_length\_69311\_cov\_30.906147 64765-64772. Max. coverage (+): 0. Max coverage (-): 0

Region: NODE\_324426\_length\_69311\_cov\_30.906147 64773-64781. Max. coverage (+): 0. Max coverage (-): 0

Region: NODE\_324426\_length\_69311\_cov\_30.906147 64782-64790. Max. coverage (+): 0. Max coverage (-): 0

Region: NODE\_324426\_length\_69311\_cov\_30.906147 64791-64798. Max. coverage (+): 0. Max coverage (-): 0

Region: NODE\_324426\_length\_69311\_cov\_30.906147 64799-64807. Max. coverage (+): 0. Max coverage (-): 0

Region: NODE\_324426\_length\_69311\_cov\_30.906147 64808-64816. Max. coverage (+): 0. Max coverage (-): 0

Region: NODE\_324426\_length\_69311\_cov\_30.906147 64817-64825. Max. coverage (+): 0. Max coverage (-): 0

Region: NODE\_324426\_length\_69311\_cov\_30.906147 64826-64833. Max. coverage (+): 0. Max coverage (-): 0

Region: NODE\_324426\_length\_69311\_cov\_30.906147 64834-64842. Max. coverage (+): 0. Max coverage (-): 0

Region: NODE\_324426\_length\_69311\_cov\_30.906147 64843-64851. Max. coverage (+): 0. Max coverage (-): 0

Region: NODE\_324426\_length\_69311\_cov\_30.906147 64852-64859. Max. coverage (+): 0. Max coverage (-): 0

Region: NODE\_324426\_length\_69311\_cov\_30.906147 64860-64868. Max. coverage (+): 0. Max coverage (-): 0

Region: NODE\_324426\_length\_69311\_cov\_30.906147 64869-64877. Max. coverage (+): 0. Max coverage (-): 0

Region: NODE\_324426\_length\_69311\_cov\_30.906147 64878-64886. Max. coverage (+): 0. Max coverage (-): 0

Region: NODE\_324426\_length\_69311\_cov\_30.906147 64887-64894. Max. coverage (+): 0. Max coverage (-): 0

Region: NODE\_324426\_length\_69311\_cov\_30.906147 64895-64903. Max. coverage (+): 0. Max coverage (-): 0

Region: NODE\_324426\_length\_69311\_cov\_30.906147 64904-64912. Max. coverage (+): 0. Max coverage (-): 0

Region: NODE\_324426\_length\_69311\_cov\_30.906147 64913-64921. Max. coverage (+): 0. Max coverage (-): 0

Region: NODE\_324426\_length\_69311\_cov\_30.906147 64922-64929. Max. coverage (+): 0. Max coverage (-): 0

Region: NODE\_324426\_length\_69311\_cov\_30.906147 64930-64938. Max. coverage (+): 0. Max coverage (-): 0

Region: NODE\_324426\_length\_69311\_cov\_30.906147 64939-64947. Max. coverage (+): 0. Max coverage (-): 0

Region: NODE\_324426\_length\_69311\_cov\_30.906147 64948-64955. Max. coverage (+): 0. Max coverage (-): 0

Region: NODE\_324426\_length\_69311\_cov\_30.906147 64956-64964. Max. coverage (+): 0. Max coverage (-): 0

Region: NODE\_324426\_length\_69311\_cov\_30.906147 64965-64973. Max. coverage (+): 0. Max coverage (-): 0

Region: NODE\_324426\_length\_69311\_cov\_30.906147 64974-64982. Max. coverage (+): 0. Max coverage (-): 0

Region: NODE\_324426\_length\_69311\_cov\_30.906147 64983-64990. Max. coverage (+): 0. Max coverage (-): 0

Region: NODE\_324426\_length\_69311\_cov\_30.906147 64991-64999. Max. coverage (+): 0. Max coverage (-): 0

Region: NODE\_324426\_length\_69311\_cov\_30.906147 65000-65008. Max. coverage (+): 0. Max coverage (-): 0

Region: NODE\_324426\_length\_69311\_cov\_30.906147 65009-65017. Max. coverage (+): 0. Max coverage (-): 0

Region: NODE\_324426\_length\_69311\_cov\_30.906147 65018-65025. Max. coverage (+): 0. Max coverage (-): 0

Region: NODE\_324426\_length\_69311\_cov\_30.906147 65026-65034. Max. coverage (+): 0. Max coverage (-): 0

Region: NODE\_324426\_length\_69311\_cov\_30.906147 65035-65043. Max. coverage (+): 0. Max coverage (-): 0

Region: NODE\_324426\_length\_69311\_cov\_30.906147 65044-65051. Max. coverage (+): 0. Max coverage (-): 0

Region: NODE\_324426\_length\_69311\_cov\_30.906147 65052-65060. Max. coverage (+): 0. Max coverage (-): 0

Region: NODE\_324426\_length\_69311\_cov\_30.906147 65061-65069. Max. coverage (+): 0. Max coverage (-): 0

Region: NODE\_324426\_length\_69311\_cov\_30.906147 65070-65078. Max. coverage (+): 0. Max coverage (-): 0

Region: NODE\_324426\_length\_69311\_cov\_30.906147 65079-65086. Max. coverage (+): 0. Max coverage (-): 0

Region: NODE\_324426\_length\_69311\_cov\_30.906147 65087-65095. Max. coverage (+): 0. Max coverage (-): 0

Region: NODE\_324426\_length\_69311\_cov\_30.906147 65096-65104. Max. coverage (+): 0. Max coverage (-): 0

Region: NODE\_324426\_length\_69311\_cov\_30.906147 65105-65112. Max. coverage (+): 0. Max coverage (-): 0

Region: NODE\_324426\_length\_69311\_cov\_30.906147 65113-65121. Max. coverage (+): 0. Max coverage (-): 0

Region: NODE\_324426\_length\_69311\_cov\_30.906147 65122-65130. Max. coverage (+): 0. Max coverage (-): 0

Region: NODE\_324426\_length\_69311\_cov\_30.906147 65131-65139. Max. coverage (+): 0. Max coverage (-): 0

Region: NODE\_324426\_length\_69311\_cov\_30.906147 65140-65147. Max. coverage (+): 0. Max coverage (-): 0

Region: NODE\_324426\_length\_69311\_cov\_30.906147 65148-65156. Max. coverage (+): 0. Max coverage (-): 0

Region: NODE\_324426\_length\_69311\_cov\_30.906147 65157-65165. Max. coverage (+): 0. Max coverage (-): 0

Region: NODE\_324426\_length\_69311\_cov\_30.906147 65166-65174. Max. coverage (+): 0. Max coverage (-): 0

Region: NODE\_324426\_length\_69311\_cov\_30.906147 65175-65182. Max. coverage (+): 0. Max coverage (-): 0

Region: NODE\_324426\_length\_69311\_cov\_30.906147 65183-65191. Max. coverage (+): 0. Max coverage (-): 0

Region: NODE\_324426\_length\_69311\_cov\_30.906147 65192-65200. Max. coverage (+): 0. Max coverage (-): 0

Region: NODE\_324426\_length\_69311\_cov\_30.906147 65201-65208. Max. coverage (+): 0. Max coverage (-): 0

Region: NODE\_324426\_length\_69311\_cov\_30.906147 65209-65217. Max. coverage (+): 0. Max coverage (-): 0

Region: NODE\_324426\_length\_69311\_cov\_30.906147 65218-65226. Max. coverage (+): 0. Max coverage (-): 0

Region: NODE\_324426\_length\_69311\_cov\_30.906147 65227-65235. Max. coverage (+): 0. Max coverage (-): 0

Region: NODE\_324426\_length\_69311\_cov\_30.906147 65236-65243. Max. coverage (+): 0. Max coverage (-): 0

Region: NODE\_324426\_length\_69311\_cov\_30.906147 65244-65252. Max. coverage (+): 0. Max coverage (-): 0

Region: NODE\_324426\_length\_69311\_cov\_30.906147 65253-65261. Max. coverage (+): 0. Max coverage (-): 0

Region: NODE\_324426\_length\_69311\_cov\_30.906147 65262-65270. Max. coverage (+): 0. Max coverage (-): 0

Region: NODE\_324426\_length\_69311\_cov\_30.906147 65271-65278. Max. coverage (+): 0. Max coverage (-): 0

Region: NODE\_324426\_length\_69311\_cov\_30.906147 65279-65287. Max. coverage (+): 0. Max coverage (-): 0

Region: NODE\_324426\_length\_69311\_cov\_30.906147 65288-65296. Max. coverage (+): 0. Max coverage (-): 0

Region: NODE\_324426\_length\_69311\_cov\_30.906147 65297-65304. Max. coverage (+): 0. Max coverage (-): 0

Region: NODE\_324426\_length\_69311\_cov\_30.906147 65305-65313. Max. coverage (+): 0. Max coverage (-): 0

Region: NODE\_324426\_length\_69311\_cov\_30.906147 65314-65322. Max. coverage (+): 0. Max coverage (-): 0

Region: NODE\_324426\_length\_69311\_cov\_30.906147 65323-65331. Max. coverage (+): 0. Max coverage (-): 0

Region: NODE\_324426\_length\_69311\_cov\_30.906147 65332-65339. Max. coverage (+): 0. Max coverage (-): 0

Region: NODE\_324426\_length\_69311\_cov\_30.906147 65340-65348. Max. coverage (+): 0. Max coverage (-): 0

Region: NODE\_324426\_length\_69311\_cov\_30.906147 65349-65357. Max. coverage (+): 0. Max coverage (-): 0

Region: NODE\_324426\_length\_69311\_cov\_30.906147 65358-65365. Max. coverage (+): 0. Max coverage (-): 0

Region: NODE\_324426\_length\_69311\_cov\_30.906147 65366-65374. Max. coverage (+): 0. Max coverage (-): 0

Region: NODE\_324426\_length\_69311\_cov\_30.906147 65375-65383. Max. coverage (+): 0. Max coverage (-): 0

Region: NODE\_324426\_length\_69311\_cov\_30.906147 65384-65392. Max. coverage (+): 0. Max coverage (-): 0

Region: NODE\_324426\_length\_69311\_cov\_30.906147 65393-65400. Max. coverage (+): 0. Max coverage (-): 0

Region: NODE\_324426\_length\_69311\_cov\_30.906147 65401-65409. Max. coverage (+): 0. Max coverage (-): 0

Region: NODE\_324426\_length\_69311\_cov\_30.906147 65410-65418. Max. coverage (+): 0. Max coverage (-): 0

Region: NODE\_324426\_length\_69311\_cov\_30.906147 65419-65427. Max. coverage (+): 0. Max coverage (-): 0

Region: NODE\_324426\_length\_69311\_cov\_30.906147 65428-65435. Max. coverage (+): 0. Max coverage (-): 0

Region: NODE\_324426\_length\_69311\_cov\_30.906147 65436-65444. Max. coverage (+): 0. Max coverage (-): 0

Region: NODE\_324426\_length\_69311\_cov\_30.906147 65445-65453. Max. coverage (+): 0. Max coverage (-): 0

Region: NODE\_324426\_length\_69311\_cov\_30.906147 65454-65461. Max. coverage (+): 0. Max coverage (-): 0

Region: NODE\_324426\_length\_69311\_cov\_30.906147 65462-65470. Max. coverage (+): 0. Max coverage (-): 0

Region: NODE\_324426\_length\_69311\_cov\_30.906147 65471-65479. Max. coverage (+): 0. Max coverage (-): 0

Region: NODE\_324426\_length\_69311\_cov\_30.906147 65480-65488. Max. coverage (+): 0. Max coverage (-): 0

Region: NODE\_324426\_length\_69311\_cov\_30.906147 65489-65496. Max. coverage (+): 0. Max coverage (-): 0

Region: NODE\_324426\_length\_69311\_cov\_30.906147 65497-65505. Max. coverage (+): 0. Max coverage (-): 0

Region: NODE\_324426\_length\_69311\_cov\_30.906147 65506-65514. Max. coverage (+): 0. Max coverage (-): 0

Region: NODE\_324426\_length\_69311\_cov\_30.906147 65515-65523. Max. coverage (+): 0. Max coverage (-): 0

Region: NODE\_324426\_length\_69311\_cov\_30.906147 65524-65531. Max. coverage (+): 0. Max coverage (-): 0

Region: NODE\_324426\_length\_69311\_cov\_30.906147 65532-65540. Max. coverage (+): 0. Max coverage (-): 0

Region: NODE\_324426\_length\_69311\_cov\_30.906147 65541-65549. Max. coverage (+): 0. Max coverage (-): 0

Region: NODE\_324426\_length\_69311\_cov\_30.906147 65550-65557. Max. coverage (+): 0. Max coverage (-): 0

Region: NODE\_324426\_length\_69311\_cov\_30.906147 65558-65566. Max. coverage (+): 0. Max coverage (-): 0

Region: NODE\_324426\_length\_69311\_cov\_30.906147 65567-65575. Max. coverage (+): 0. Max coverage (-): 0

Region: NODE\_324426\_length\_69311\_cov\_30.906147 65576-65584. Max. coverage (+): 0. Max coverage (-): 0

Region: NODE\_324426\_length\_69311\_cov\_30.906147 65585-65592. Max. coverage (+): 0. Max coverage (-): 0

Region: NODE\_324426\_length\_69311\_cov\_30.906147 65593-65601. Max. coverage (+): 0. Max coverage (-): 0

Region: NODE\_324426\_length\_69311\_cov\_30.906147 65602-65610. Max. coverage (+): 0. Max coverage (-): 0

Region: NODE\_324426\_length\_69311\_cov\_30.906147 65611-65618. Max. coverage (+): 0. Max coverage (-): 0

Region: NODE\_324426\_length\_69311\_cov\_30.906147 65619-65627. Max. coverage (+): 0. Max coverage (-): 0

Region: NODE\_324426\_length\_69311\_cov\_30.906147 65628-65636. Max. coverage (+): 0. Max coverage (-): 0

Region: NODE\_324426\_length\_69311\_cov\_30.906147 65637-65645. Max. coverage (+): 0. Max coverage (-): 0

Region: NODE\_324426\_length\_69311\_cov\_30.906147 65646-65653. Max. coverage (+): 0. Max coverage (-): 0

Region: NODE\_324426\_length\_69311\_cov\_30.906147 65654-65662. Max. coverage (+): 0.04. Max coverage (-): 0

Region: NODE\_324426\_length\_69311\_cov\_30.906147 65663-65671. Max. coverage (+): 0. Max coverage (-): 0

Region: NODE\_324426\_length\_69311\_cov\_30.906147 65672-65680. Max. coverage (+): 0. Max coverage (-): 0

Region: NODE\_324426\_length\_69311\_cov\_30.906147 65681-65688. Max. coverage (+): 0. Max coverage (-): 0

Region: NODE\_324426\_length\_69311\_cov\_30.906147 65689-65697. Max. coverage (+): 0. Max coverage (-): 0

Region: NODE\_324426\_length\_69311\_cov\_30.906147 65698-65706. Max. coverage (+): 0. Max coverage (-): 0

Region: NODE\_324426\_length\_69311\_cov\_30.906147 65707-65714. Max. coverage (+): 0. Max coverage (-): 0

Region: NODE\_324426\_length\_69311\_cov\_30.906147 65715-65723. Max. coverage (+): 0. Max coverage (-): 0

Region: NODE\_324426\_length\_69311\_cov\_30.906147 65724-65732. Max. coverage (+): 0. Max coverage (-): 0

Region: NODE\_324426\_length\_69311\_cov\_30.906147 65733-65741. Max. coverage (+): 0. Max coverage (-): 0

Region: NODE\_324426\_length\_69311\_cov\_30.906147 65742-65749. Max. coverage (+): 0. Max coverage (-): 0

Region: NODE\_324426\_length\_69311\_cov\_30.906147 65750-65758. Max. coverage (+): 0. Max coverage (-): 0

Region: NODE\_324426\_length\_69311\_cov\_30.906147 65759-65767. Max. coverage (+): 0. Max coverage (-): 0

Region: NODE\_324426\_length\_69311\_cov\_30.906147 65768-65776. Max. coverage (+): 0. Max coverage (-): 0

Region: NODE\_324426\_length\_69311\_cov\_30.906147 65777-65784. Max. coverage (+): 0. Max coverage (-): 0

Region: NODE\_324426\_length\_69311\_cov\_30.906147 65785-65793. Max. coverage (+): 0. Max coverage (-): 0

Region: NODE\_324426\_length\_69311\_cov\_30.906147 65794-65802. Max. coverage (+): 0.04. Max coverage (-): 0

Region: NODE\_324426\_length\_69311\_cov\_30.906147 65803-65810. Max. coverage (+): 0.04. Max coverage (-): 0

Region: NODE\_324426\_length\_69311\_cov\_30.906147 65811-65819. Max. coverage (+): 0. Max coverage (-): 0

Region: NODE\_324426\_length\_69311\_cov\_30.906147 65820-. Max. coverage (+): 0. Max coverage (-): 0

RepeatMasker Color Code

**+**

100-98% Identity

<98-95% Identity

<95-90% Identity

<90-85% Identity

<85-80% Identity

<80-75% Identity

<75-70% Identity

<70% Identity

**-**

Gene Set Color Code

**+**

Gene

Pseudogene

Other

**-**

Topology/Coverage Color Code

Coverage Plus Strand

Coverage Minus Strand

Mainstrand: Plus

Mainstrand: Minus

Complementary Strand

Flanking Region  
(if option -flank >0)

Gene Set Annotation  
  
RepeatMasker Annotation  

**1. (ATCAA)n**: 61448-61476 (+), Divergence to consensus: 0%  
**2. REX1-1\_AFC**: 61477-61546 (-), Divergence to consensus: 5.7%  
**3. P-1\_DR**: 61548-63141 (-), Divergence to consensus: 22%  
**4. P-1\_DR**: 63214-63407 (-), Divergence to consensus: 33.2%  
**5. P-1\_DR**: 63458-63535 (-), Divergence to consensus: 22.1%  
**6. AlRepB-180**: 63522-63955 (+), Divergence to consensus: 38.2%  
**7. AlRepA-93**: 64061-64462 (+), Divergence to consensus: 25.9%  
**8. AlRepB-269**: 64643-64717 (+), Divergence to consensus: 12%  
**9. AlRepC-14**: 64725-64916 (-), Divergence to consensus: 22.3%  
**10. TguLTRK7a**: 65223-65273 (+), Divergence to consensus: 25.7%

  
Transcription Factor Binding Sites  

**RHOXF1** (Sequence: GGCTTA (-): 61824)  
**RHOXF1** (Sequence: GGATCA (-): 62162)  
**RHOXF1** (Sequence: GGATCA (-): 62638)  
**RHOXF1** (Sequence: AGATCA (-): 62870)  
**RHOXF1** (Sequence: AGATCA (-): 64132)  
**RHOXF1** (Sequence: TGAGCC (+): 62032)  
**RHOXF1** (Sequence: TAATCT (+): 62078)  
**RHOXF1** (Sequence: TGATCC (+): 62355)  
**RHOXF1** (Sequence: TAATCC (+): 62631)  
**RHOXF1** (Sequence: TGATCC (+): 63046)  
**RHOXF1** (Sequence: TGAGCC (+): 63264)  
**RHOXF1** (Sequence: TGATCT (+): 64050)  
**RHOXF1** (Sequence: TGAGCC (+): 64320)  
**RHOXF1** (Sequence: TGAGCT (+): 64692)  
**RHOXF1** (Sequence: TAATCC (+): 65394)  
**Lhx8** (Sequence: TTAATTAG (-): 61827)  
**Gata4** (Sequence: CTTATCT (+): 63946)  
**POU5F1** (Sequence: TTTGCAT (-): 63193)  
**POU5F1** (Sequence: TTTGCAT (-): 63822)  
**RFX4\_2** (Sequence: GTAACTATG (-): 63096)  
**SOX9** (Sequence: AACAATGA (-): 62301)  
**Sox5** (Sequence: ATTGTT (+): 63556)  
**Sox5** (Sequence: ATTGTT (+): 64409)  
**Sox5** (Sequence: ATTGTT (+): 64951)  
**Mybl1\_1** (Sequence: TAACGGTT (-): 64396)  
**FIGLA** (Sequence: AACAGCTGTT (-): 61861)  
**FOXO3\_mmu** (Sequence: TCAAAACA (+): 64597)  
**FOXO3\_mmu** (Sequence: TCAAAACA (+): 65526)  
**Nobox** (Sequence: GGTAATTA (-): 63566)  
**Nobox** (Sequence: AGTAATTA (-): 63651)  
**Nobox** (Sequence: TAATTGGC (+): 63160)  
**Rhox11** (Sequence: TGCTGTAAT (+): 63155)  
**Sox5** (Sequence: AACAAT (-): 62301)  
**Sox5** (Sequence: AACAAT (-): 63662)  
**Sox5** (Sequence: AACAAT (-): 65530)  
**POU2F1** (Sequence: TATTTTAAT (+): 63952)  
**POU5F1** (Sequence: ATGCAAA (+): 62117)
