# Supplementary material for: First characterization of PIWI-interacting RNA clusters in a cichlid fish with a B chromosome
Source: BMC Biol. 2022 Sep 21;20:204. doi: 10.1186/s12915-022-01403-2 (PMC9490952; doi:10.1186/s12915-022-01403-2)
Supplement: Supplementary file 1 — Additional file 1. Zipped folder with fasta and interactive html piRNA cluster information for the A. latifasciata genome. The nomenclature is as follows: number-pirna-cluster_sex_B-presence (f, female; m, male; 0b, without B chromosome; 1b, with B chromosome). [file 12915_2022_1403_MOESM1_ESM.zip › 128_m1b.html]

piRNA cluster 128\_m1b 75


Predicted piRNA cluster no. 128\_m1b
  

Show proTRAC run info
Hide proTRAC run info

/\  
                \_\_\_\_\_\_\_\_\_\_\_\_\_\_\_\_\_\_\_\_\_\_\_/\\_\_\_ /  \\_\_\_\_\_\_\_  
               I                      /  \  /    \      I  
               I     pro             /    \/      \     I  
               I        TRAC        /               \   I  
               I   \_\_\_\_\_\_\_\_\_\_\_\_\_\_\_\_/\_\_\_\_\_\_\_\_\_\_\_\_\_\_\_\_\_\\_ I  
               I   \              /                     I  
               I    \            /                      I  
               I     \  /\      /       V.2.4.2         I  
               I      \/  \    /                        I  
               I\_\_\_\_\_\_\_\_\_\_\_\  /\_\_\_\_\_\_\_\_\_\_\_\_\_\_\_\_\_\_\_\_\_\_\_\_\_I  
                            \/  
  
  
================================= proTRAC ====================================  
VERSION: .......... 2.4.2  
LAST MODIFIED: .... 11. May 2018  
  
Please cite:  
Rosenkranz D, Zischler H. proTRAC - a software for probabilistic piRNA cluster  
detection, visualization and analysis. 2012. BMC Bioinformatics 13:5.  
  
  
Contact:  
David Rosenkranz  
Institute of Organismic and Molecular Evolutionary Biology  
Dept. Anthropology, small RNA group  
Johannes Gutenberg University Mainz  
email: rosenkranz@uni-mainz.de  
  
You can find the latest proTRAC version at:  
http://sourceforge.net/projects/protrac/files  
http://www.smallRNAgroup-mainz.de/software  
==============================================================================  
  
PARAMETERS:  
Map file: ...............piwi-machos-1B.fa-collapse.map  
Genome file: ............../../../0B\_ala\_genome.fa  
RepeatMasker annotation: Alatifasciata-all0B-maryan-v2.fa\_corrected.out  
GeneSet:................./guest-storage/Data/annotation/Alatifasciata\_all0B\_maryan-v2\_out2017.gff  
  
Significant (p<=0.01) hit density will be calculated based  
on observed hit distribution.  
  
Sliding window size: ........................................ 5000 bp  
Sliding window increament: .................................. 1000 bp  
Normalize each hit by number of genomic hits: ............... yes  
Normalize each hit by number of sequence reads: ............. yes  
Normalize values (-> per million mapped reads): ............. yes  
Min. fraction of hits with 1T(U) or 10A: .................... 0.75  
Alternatively: Min. fraction of hits with 1T(U) and 10A: .... 0.5  
Min. fraction of hits with typical piRNA length: ............ 0.75  
Typical piRNA length: ....................................... 24-32 nt  
Min. size of a piRNA cluster: ............................... 1000 bp.  
Min. number of hits (absolute): ............................. 0  
Min. number of hits (normalized): ........................... 0  
Min. fraction of hits on the mainstrand: .................... 0.75  
Top fraction of mapped sequences (in terms of read counts): . 1%  
Top fraction accounts for max. n% of sequence reads: ........ 90%  
Min. fraction of hits on each arm of a bidirectional cluster: 0.05  
Output html file for each cluster: .......................... yes  
Output a summary table: ..................................... yes  
Output a FASTA file for each cluster (piRNA sequences): ..... yes  
Output a FASTA file comprising cluster sequences: ........... yes  
Output a GTF file for predicted piRNA clusters: ..............yes  
Search DNA motifs in clusters: .............................. yes  
Output flanking sequences: +/- .............................. 0 bp  
Output ~.pTi file: .......................................... no  
==============================================================================  
  
  
Genome size (without gaps): ............ 758543724 bp  
Gaps (N/X/-): .......................... 417479 bp  
Mapped reads: .......................... 26973943  
Non-identical sequences: ............... 6209225  
Genomic hits: .......................... 48438990  
Significant densitiy of mapped reads: .. 821.144211136946 reads/kb

Show proTRAC cluster info
Hide proTRAC cluster info

|  |  |
| --- | --- |
| Location | NODE\_324426\_length\_69311\_cov\_30.906147 |
| Coordinates | 61452-66925 |
| Size [bp] | 5474 |
| Sequence hit loci | 2456 |
| Mapped reads (normalized) | 12557.2 |
| Mapped reads (normalized) per kb | 2294 |
| Normalized reads with 1T (1U) | 83.7% |
| Normalized reads with 10A | 39.4% |
| Normalized reads with length 24-32 nt | 97.8% |
| Normalized reads on the main strand(s) | 95.6% |
| Predicted directionality | mono:plus |

100%

0%

1T (1U)  
reads

10A reads

24-32 nt  
reads

reads on mainstrand

**Either the amount of reads with 1T (1U) OR 10A has to exceed 75% (set with option: -1Tor10A)  
Alternatively the amount of reads with 1T (1U) AND 10A has to exceed 50% (set with option: -1Tand10A)  
Minimum amount of reads with preferred size is 75% (set with option: -pisize)  
Minimum amount of reads on the main strand(s) is 75% (set with option: -clstrand)**

Show read coverage
Hide read coverage

WHAT DO I SEE HERE?  
This chart shows the location of mapped sequence reads within a predicted piRNA cluster. The color refers to the number of genomic hits produced by the sequence read in question. A dark red bar indicates that this sequence read produces many other hits elsewhere in the genome. Many adjacent red or yellow bars can indicate the presence of a multi-copy element such as transposons or rRNA genes. A dark green bar indicates that this sequence read maps uniquely to this locus.

1 hit

2-5 hits

6-10 hits

11-20 hits

21-50 hits

51-100 hits

> 100 hits

NODE\_324426\_length\_69311\_cov\_30.906147

61452

66925

Gene Set

RepeatMasker

Mapped  
Reads

61.87

plus strand

minus strand

61.87

Region: NODE\_324426\_length\_69311\_cov\_30.906147 10561-61457. Max. coverage (+): 0. Max coverage (-): 0

Region: NODE\_324426\_length\_69311\_cov\_30.906147 61458-61468. Max. coverage (+): 0. Max coverage (-): 0.01

Region: NODE\_324426\_length\_69311\_cov\_30.906147 61469-61479. Max. coverage (+): 0. Max coverage (-): 0.01

Region: NODE\_324426\_length\_69311\_cov\_30.906147 61480-61490. Max. coverage (+): 0. Max coverage (-): 0

Region: NODE\_324426\_length\_69311\_cov\_30.906147 61491-61501. Max. coverage (+): 0. Max coverage (-): 0

Region: NODE\_324426\_length\_69311\_cov\_30.906147 61502-61512. Max. coverage (+): 0. Max coverage (-): 0.01

Region: NODE\_324426\_length\_69311\_cov\_30.906147 61513-61523. Max. coverage (+): 0. Max coverage (-): 0.01

Region: NODE\_324426\_length\_69311\_cov\_30.906147 61524-61534. Max. coverage (+): 0. Max coverage (-): 0

Region: NODE\_324426\_length\_69311\_cov\_30.906147 61535-61545. Max. coverage (+): 0. Max coverage (-): 0

Region: NODE\_324426\_length\_69311\_cov\_30.906147 61546-61556. Max. coverage (+): 0.46. Max coverage (-): 0

Region: NODE\_324426\_length\_69311\_cov\_30.906147 61557-61566. Max. coverage (+): 0.04. Max coverage (-): 0

Region: NODE\_324426\_length\_69311\_cov\_30.906147 61567-61577. Max. coverage (+): 2.19. Max coverage (-): 0

Region: NODE\_324426\_length\_69311\_cov\_30.906147 61578-61588. Max. coverage (+): 3.82. Max coverage (-): 0

Region: NODE\_324426\_length\_69311\_cov\_30.906147 61589-61599. Max. coverage (+): 0.15. Max coverage (-): 0.32

Region: NODE\_324426\_length\_69311\_cov\_30.906147 61600-61610. Max. coverage (+): 0.45. Max coverage (-): 0.04

Region: NODE\_324426\_length\_69311\_cov\_30.906147 61611-61621. Max. coverage (+): 0.42. Max coverage (-): 0.11

Region: NODE\_324426\_length\_69311\_cov\_30.906147 61622-61632. Max. coverage (+): 0.04. Max coverage (-): 0.11

Region: NODE\_324426\_length\_69311\_cov\_30.906147 61633-61643. Max. coverage (+): 0.11. Max coverage (-): 0

Region: NODE\_324426\_length\_69311\_cov\_30.906147 61644-61654. Max. coverage (+): 0. Max coverage (-): 0.07

Region: NODE\_324426\_length\_69311\_cov\_30.906147 61655-61665. Max. coverage (+): 0. Max coverage (-): 0

Region: NODE\_324426\_length\_69311\_cov\_30.906147 61666-61676. Max. coverage (+): 0. Max coverage (-): 0

Region: NODE\_324426\_length\_69311\_cov\_30.906147 61677-61687. Max. coverage (+): 0. Max coverage (-): 0.15

Region: NODE\_324426\_length\_69311\_cov\_30.906147 61688-61698. Max. coverage (+): 0. Max coverage (-): 0.15

Region: NODE\_324426\_length\_69311\_cov\_30.906147 61699-61709. Max. coverage (+): 0.26. Max coverage (-): 0

Region: NODE\_324426\_length\_69311\_cov\_30.906147 61710-61720. Max. coverage (+): 1. Max coverage (-): 0

Region: NODE\_324426\_length\_69311\_cov\_30.906147 61721-61731. Max. coverage (+): 0.19. Max coverage (-): 0.04

Region: NODE\_324426\_length\_69311\_cov\_30.906147 61732-61742. Max. coverage (+): 0.99. Max coverage (-): 0

Region: NODE\_324426\_length\_69311\_cov\_30.906147 61743-61753. Max. coverage (+): 1.03. Max coverage (-): 0

Region: NODE\_324426\_length\_69311\_cov\_30.906147 61754-61764. Max. coverage (+): 0.15. Max coverage (-): 0

Region: NODE\_324426\_length\_69311\_cov\_30.906147 61765-61774. Max. coverage (+): 0.93. Max coverage (-): 0.3

Region: NODE\_324426\_length\_69311\_cov\_30.906147 61775-61785. Max. coverage (+): 1.11. Max coverage (-): 0.3

Region: NODE\_324426\_length\_69311\_cov\_30.906147 61786-61796. Max. coverage (+): 6.34. Max coverage (-): 0.07

Region: NODE\_324426\_length\_69311\_cov\_30.906147 61797-61807. Max. coverage (+): 0.11. Max coverage (-): 0.07

Region: NODE\_324426\_length\_69311\_cov\_30.906147 61808-61818. Max. coverage (+): 3.97. Max coverage (-): 0.02

Region: NODE\_324426\_length\_69311\_cov\_30.906147 61819-61829. Max. coverage (+): 0.56. Max coverage (-): 0.52

Region: NODE\_324426\_length\_69311\_cov\_30.906147 61830-61840. Max. coverage (+): 0.04. Max coverage (-): 0.44

Region: NODE\_324426\_length\_69311\_cov\_30.906147 61841-61851. Max. coverage (+): 28.29. Max coverage (-): 0

Region: NODE\_324426\_length\_69311\_cov\_30.906147 61852-61862. Max. coverage (+): 13.46. Max coverage (-): 0

Region: NODE\_324426\_length\_69311\_cov\_30.906147 61863-61873. Max. coverage (+): 0.19. Max coverage (-): 3.19

Region: NODE\_324426\_length\_69311\_cov\_30.906147 61874-61884. Max. coverage (+): 0.04. Max coverage (-): 3.21

Region: NODE\_324426\_length\_69311\_cov\_30.906147 61885-61895. Max. coverage (+): 4.04. Max coverage (-): 0.04

Region: NODE\_324426\_length\_69311\_cov\_30.906147 61896-61906. Max. coverage (+): 0.11. Max coverage (-): 0.11

Region: NODE\_324426\_length\_69311\_cov\_30.906147 61907-61917. Max. coverage (+): 0.22. Max coverage (-): 0.33

Region: NODE\_324426\_length\_69311\_cov\_30.906147 61918-61928. Max. coverage (+): 0.48. Max coverage (-): 0.15

Region: NODE\_324426\_length\_69311\_cov\_30.906147 61929-61939. Max. coverage (+): 0.89. Max coverage (-): 0.04

Region: NODE\_324426\_length\_69311\_cov\_30.906147 61940-61950. Max. coverage (+): 8.93. Max coverage (-): 0.07

Region: NODE\_324426\_length\_69311\_cov\_30.906147 61951-61961. Max. coverage (+): 3.37. Max coverage (-): 0.04

Region: NODE\_324426\_length\_69311\_cov\_30.906147 61962-61972. Max. coverage (+): 3.37. Max coverage (-): 0

Region: NODE\_324426\_length\_69311\_cov\_30.906147 61973-61982. Max. coverage (+): 1.35. Max coverage (-): 0.01

Region: NODE\_324426\_length\_69311\_cov\_30.906147 61983-61993. Max. coverage (+): 0.22. Max coverage (-): 0.04

Region: NODE\_324426\_length\_69311\_cov\_30.906147 61994-62004. Max. coverage (+): 0.44. Max coverage (-): 0.04

Region: NODE\_324426\_length\_69311\_cov\_30.906147 62005-62015. Max. coverage (+): 0.59. Max coverage (-): 0.04

Region: NODE\_324426\_length\_69311\_cov\_30.906147 62016-62026. Max. coverage (+): 7.16. Max coverage (-): 0.11

Region: NODE\_324426\_length\_69311\_cov\_30.906147 62027-62037. Max. coverage (+): 0.07. Max coverage (-): 0.07

Region: NODE\_324426\_length\_69311\_cov\_30.906147 62038-62048. Max. coverage (+): 0.04. Max coverage (-): 0.02

Region: NODE\_324426\_length\_69311\_cov\_30.906147 62049-62059. Max. coverage (+): 0.41. Max coverage (-): 0.11

Region: NODE\_324426\_length\_69311\_cov\_30.906147 62060-62070. Max. coverage (+): 0.44. Max coverage (-): 0.06

Region: NODE\_324426\_length\_69311\_cov\_30.906147 62071-62081. Max. coverage (+): 0.48. Max coverage (-): 0.02

Region: NODE\_324426\_length\_69311\_cov\_30.906147 62082-62092. Max. coverage (+): 0.06. Max coverage (-): 0.02

Region: NODE\_324426\_length\_69311\_cov\_30.906147 62093-62103. Max. coverage (+): 0.59. Max coverage (-): 0.04

Region: NODE\_324426\_length\_69311\_cov\_30.906147 62104-62114. Max. coverage (+): 0.56. Max coverage (-): 0.07

Region: NODE\_324426\_length\_69311\_cov\_30.906147 62115-62125. Max. coverage (+): 0.11. Max coverage (-): 0.09

Region: NODE\_324426\_length\_69311\_cov\_30.906147 62126-62136. Max. coverage (+): 3.34. Max coverage (-): 0

Region: NODE\_324426\_length\_69311\_cov\_30.906147 62137-62147. Max. coverage (+): 0.15. Max coverage (-): 0

Region: NODE\_324426\_length\_69311\_cov\_30.906147 62148-62158. Max. coverage (+): 1.71. Max coverage (-): 0.04

Region: NODE\_324426\_length\_69311\_cov\_30.906147 62159-62169. Max. coverage (+): 1.71. Max coverage (-): 0

Region: NODE\_324426\_length\_69311\_cov\_30.906147 62170-62180. Max. coverage (+): 1.48. Max coverage (-): 0.04

Region: NODE\_324426\_length\_69311\_cov\_30.906147 62181-62190. Max. coverage (+): 1.63. Max coverage (-): 0

Region: NODE\_324426\_length\_69311\_cov\_30.906147 62191-62201. Max. coverage (+): 1.24. Max coverage (-): 0.19

Region: NODE\_324426\_length\_69311\_cov\_30.906147 62202-62212. Max. coverage (+): 0.11. Max coverage (-): 0.59

Region: NODE\_324426\_length\_69311\_cov\_30.906147 62213-62223. Max. coverage (+): 20.32. Max coverage (-): 0.04

Region: NODE\_324426\_length\_69311\_cov\_30.906147 62224-62234. Max. coverage (+): 0.3. Max coverage (-): 0.15

Region: NODE\_324426\_length\_69311\_cov\_30.906147 62235-62245. Max. coverage (+): 0.33. Max coverage (-): 0.56

Region: NODE\_324426\_length\_69311\_cov\_30.906147 62246-62256. Max. coverage (+): 25.21. Max coverage (-): 0.04

Region: NODE\_324426\_length\_69311\_cov\_30.906147 62257-62267. Max. coverage (+): 2.19. Max coverage (-): 0.11

Region: NODE\_324426\_length\_69311\_cov\_30.906147 62268-62278. Max. coverage (+): 14.5. Max coverage (-): 0.04

Region: NODE\_324426\_length\_69311\_cov\_30.906147 62279-62289. Max. coverage (+): 15.83. Max coverage (-): 0

Region: NODE\_324426\_length\_69311\_cov\_30.906147 62290-62300. Max. coverage (+): 0.7. Max coverage (-): 0

Region: NODE\_324426\_length\_69311\_cov\_30.906147 62301-62311. Max. coverage (+): 0.22. Max coverage (-): 0

Region: NODE\_324426\_length\_69311\_cov\_30.906147 62312-62322. Max. coverage (+): 0.22. Max coverage (-): 0.15

Region: NODE\_324426\_length\_69311\_cov\_30.906147 62323-62333. Max. coverage (+): 26.06. Max coverage (-): 0.04

Region: NODE\_324426\_length\_69311\_cov\_30.906147 62334-62344. Max. coverage (+): 26.14. Max coverage (-): 0.19

Region: NODE\_324426\_length\_69311\_cov\_30.906147 62345-62355. Max. coverage (+): 4.02. Max coverage (-): 0.04

Region: NODE\_324426\_length\_69311\_cov\_30.906147 62356-62366. Max. coverage (+): 8.75. Max coverage (-): 0

Region: NODE\_324426\_length\_69311\_cov\_30.906147 62367-62377. Max. coverage (+): 8.12. Max coverage (-): 0.04

Region: NODE\_324426\_length\_69311\_cov\_30.906147 62378-62388. Max. coverage (+): 0.56. Max coverage (-): 0.07

Region: NODE\_324426\_length\_69311\_cov\_30.906147 62389-62399. Max. coverage (+): 2.15. Max coverage (-): 0.04

Region: NODE\_324426\_length\_69311\_cov\_30.906147 62400-62409. Max. coverage (+): 6.82. Max coverage (-): 0.04

Region: NODE\_324426\_length\_69311\_cov\_30.906147 62410-62420. Max. coverage (+): 2.48. Max coverage (-): 0.04

Region: NODE\_324426\_length\_69311\_cov\_30.906147 62421-62431. Max. coverage (+): 5.26. Max coverage (-): 0

Region: NODE\_324426\_length\_69311\_cov\_30.906147 62432-62442. Max. coverage (+): 0.48. Max coverage (-): 0.15

Region: NODE\_324426\_length\_69311\_cov\_30.906147 62443-62453. Max. coverage (+): 0. Max coverage (-): 0.19

Region: NODE\_324426\_length\_69311\_cov\_30.906147 62454-62464. Max. coverage (+): 5.08. Max coverage (-): 0.04

Region: NODE\_324426\_length\_69311\_cov\_30.906147 62465-62475. Max. coverage (+): 0.26. Max coverage (-): 0.04

Region: NODE\_324426\_length\_69311\_cov\_30.906147 62476-62486. Max. coverage (+): 0.26. Max coverage (-): 0.07

Region: NODE\_324426\_length\_69311\_cov\_30.906147 62487-62497. Max. coverage (+): 2.19. Max coverage (-): 0.93

Region: NODE\_324426\_length\_69311\_cov\_30.906147 62498-62508. Max. coverage (+): 0.22. Max coverage (-): 1.04

Region: NODE\_324426\_length\_69311\_cov\_30.906147 62509-62519. Max. coverage (+): 0.93. Max coverage (-): 0

Region: NODE\_324426\_length\_69311\_cov\_30.906147 62520-62530. Max. coverage (+): 0. Max coverage (-): 0

Region: NODE\_324426\_length\_69311\_cov\_30.906147 62531-62541. Max. coverage (+): 0.37. Max coverage (-): 0.04

Region: NODE\_324426\_length\_69311\_cov\_30.906147 62542-62552. Max. coverage (+): 2. Max coverage (-): 0

Region: NODE\_324426\_length\_69311\_cov\_30.906147 62553-62563. Max. coverage (+): 0.89. Max coverage (-): 0.11

Region: NODE\_324426\_length\_69311\_cov\_30.906147 62564-62574. Max. coverage (+): 0.61. Max coverage (-): 0.11

Region: NODE\_324426\_length\_69311\_cov\_30.906147 62575-62585. Max. coverage (+): 0.59. Max coverage (-): 0.06

Region: NODE\_324426\_length\_69311\_cov\_30.906147 62586-62596. Max. coverage (+): 1.04. Max coverage (-): 0.04

Region: NODE\_324426\_length\_69311\_cov\_30.906147 62597-62607. Max. coverage (+): 0.07. Max coverage (-): 0.07

Region: NODE\_324426\_length\_69311\_cov\_30.906147 62608-62617. Max. coverage (+): 0.48. Max coverage (-): 0.07

Region: NODE\_324426\_length\_69311\_cov\_30.906147 62618-62628. Max. coverage (+): 0.41. Max coverage (-): 0.11

Region: NODE\_324426\_length\_69311\_cov\_30.906147 62629-62639. Max. coverage (+): 29.08. Max coverage (-): 0.04

Region: NODE\_324426\_length\_69311\_cov\_30.906147 62640-62650. Max. coverage (+): 1.35. Max coverage (-): 0.11

Region: NODE\_324426\_length\_69311\_cov\_30.906147 62651-62661. Max. coverage (+): 0.13. Max coverage (-): 0.11

Region: NODE\_324426\_length\_69311\_cov\_30.906147 62662-62672. Max. coverage (+): 4.34. Max coverage (-): 0.11

Region: NODE\_324426\_length\_69311\_cov\_30.906147 62673-62683. Max. coverage (+): 0.07. Max coverage (-): 0

Region: NODE\_324426\_length\_69311\_cov\_30.906147 62684-62694. Max. coverage (+): 0.07. Max coverage (-): 0

Region: NODE\_324426\_length\_69311\_cov\_30.906147 62695-62705. Max. coverage (+): 1.78. Max coverage (-): 0

Region: NODE\_324426\_length\_69311\_cov\_30.906147 62706-62716. Max. coverage (+): 4.56. Max coverage (-): 0.04

Region: NODE\_324426\_length\_69311\_cov\_30.906147 62717-62727. Max. coverage (+): 0.22. Max coverage (-): 0

Region: NODE\_324426\_length\_69311\_cov\_30.906147 62728-62738. Max. coverage (+): 0.48. Max coverage (-): 0

Region: NODE\_324426\_length\_69311\_cov\_30.906147 62739-62749. Max. coverage (+): 0.19. Max coverage (-): 0

Region: NODE\_324426\_length\_69311\_cov\_30.906147 62750-62760. Max. coverage (+): 0.15. Max coverage (-): 0.11

Region: NODE\_324426\_length\_69311\_cov\_30.906147 62761-62771. Max. coverage (+): 0.07. Max coverage (-): 0.11

Region: NODE\_324426\_length\_69311\_cov\_30.906147 62772-62782. Max. coverage (+): 2.15. Max coverage (-): 0

Region: NODE\_324426\_length\_69311\_cov\_30.906147 62783-62793. Max. coverage (+): 2.15. Max coverage (-): 0

Region: NODE\_324426\_length\_69311\_cov\_30.906147 62794-62804. Max. coverage (+): 0.19. Max coverage (-): 0

Region: NODE\_324426\_length\_69311\_cov\_30.906147 62805-62815. Max. coverage (+): 20.17. Max coverage (-): 0

Region: NODE\_324426\_length\_69311\_cov\_30.906147 62816-62825. Max. coverage (+): 1.45. Max coverage (-): 0.04

Region: NODE\_324426\_length\_69311\_cov\_30.906147 62826-62836. Max. coverage (+): 0.63. Max coverage (-): 0.04

Region: NODE\_324426\_length\_69311\_cov\_30.906147 62837-62847. Max. coverage (+): 2.32. Max coverage (-): 0

Region: NODE\_324426\_length\_69311\_cov\_30.906147 62848-62858. Max. coverage (+): 0.19. Max coverage (-): 0.02

Region: NODE\_324426\_length\_69311\_cov\_30.906147 62859-62869. Max. coverage (+): 1.3. Max coverage (-): 0

Region: NODE\_324426\_length\_69311\_cov\_30.906147 62870-62880. Max. coverage (+): 0.15. Max coverage (-): 0.7

Region: NODE\_324426\_length\_69311\_cov\_30.906147 62881-62891. Max. coverage (+): 0.15. Max coverage (-): 2.04

Region: NODE\_324426\_length\_69311\_cov\_30.906147 62892-62902. Max. coverage (+): 0.96. Max coverage (-): 2.11

Region: NODE\_324426\_length\_69311\_cov\_30.906147 62903-62913. Max. coverage (+): 0.59. Max coverage (-): 0

Region: NODE\_324426\_length\_69311\_cov\_30.906147 62914-62924. Max. coverage (+): 0.74. Max coverage (-): 0.04

Region: NODE\_324426\_length\_69311\_cov\_30.906147 62925-62935. Max. coverage (+): 2.34. Max coverage (-): 0.04

Region: NODE\_324426\_length\_69311\_cov\_30.906147 62936-62946. Max. coverage (+): 3.11. Max coverage (-): 0.04

Region: NODE\_324426\_length\_69311\_cov\_30.906147 62947-62957. Max. coverage (+): 3.11. Max coverage (-): 0.04

Region: NODE\_324426\_length\_69311\_cov\_30.906147 62958-62968. Max. coverage (+): 0.33. Max coverage (-): 0.04

Region: NODE\_324426\_length\_69311\_cov\_30.906147 62969-62979. Max. coverage (+): 3.04. Max coverage (-): 0

Region: NODE\_324426\_length\_69311\_cov\_30.906147 62980-62990. Max. coverage (+): 1.89. Max coverage (-): 0.02

Region: NODE\_324426\_length\_69311\_cov\_30.906147 62991-63001. Max. coverage (+): 0.22. Max coverage (-): 0

Region: NODE\_324426\_length\_69311\_cov\_30.906147 63002-63012. Max. coverage (+): 4.71. Max coverage (-): 0

Region: NODE\_324426\_length\_69311\_cov\_30.906147 63013-63023. Max. coverage (+): 0.56. Max coverage (-): 0

Region: NODE\_324426\_length\_69311\_cov\_30.906147 63024-63033. Max. coverage (+): 0.15. Max coverage (-): 0.33

Region: NODE\_324426\_length\_69311\_cov\_30.906147 63034-63044. Max. coverage (+): 0.82. Max coverage (-): 0.48

Region: NODE\_324426\_length\_69311\_cov\_30.906147 63045-63055. Max. coverage (+): 0.82. Max coverage (-): 0.22

Region: NODE\_324426\_length\_69311\_cov\_30.906147 63056-63066. Max. coverage (+): 0.22. Max coverage (-): 0.04

Region: NODE\_324426\_length\_69311\_cov\_30.906147 63067-63077. Max. coverage (+): 0.04. Max coverage (-): 0.04

Region: NODE\_324426\_length\_69311\_cov\_30.906147 63078-63088. Max. coverage (+): 1.11. Max coverage (-): 0.26

Region: NODE\_324426\_length\_69311\_cov\_30.906147 63089-63099. Max. coverage (+): 0.63. Max coverage (-): 0.11

Region: NODE\_324426\_length\_69311\_cov\_30.906147 63100-63110. Max. coverage (+): 0.56. Max coverage (-): 0

Region: NODE\_324426\_length\_69311\_cov\_30.906147 63111-63121. Max. coverage (+): 0.04. Max coverage (-): 0

Region: NODE\_324426\_length\_69311\_cov\_30.906147 63122-63132. Max. coverage (+): 0.07. Max coverage (-): 0.19

Region: NODE\_324426\_length\_69311\_cov\_30.906147 63133-63143. Max. coverage (+): 0.67. Max coverage (-): 0.19

Region: NODE\_324426\_length\_69311\_cov\_30.906147 63144-63154. Max. coverage (+): 0.22. Max coverage (-): 0

Region: NODE\_324426\_length\_69311\_cov\_30.906147 63155-63165. Max. coverage (+): 0.04. Max coverage (-): 0.07

Region: NODE\_324426\_length\_69311\_cov\_30.906147 63166-63176. Max. coverage (+): 0.26. Max coverage (-): 0.07

Region: NODE\_324426\_length\_69311\_cov\_30.906147 63177-63187. Max. coverage (+): 0.3. Max coverage (-): 0.07

Region: NODE\_324426\_length\_69311\_cov\_30.906147 63188-63198. Max. coverage (+): 1.48. Max coverage (-): 0

Region: NODE\_324426\_length\_69311\_cov\_30.906147 63199-63209. Max. coverage (+): 2.08. Max coverage (-): 0

Region: NODE\_324426\_length\_69311\_cov\_30.906147 63210-63220. Max. coverage (+): 0.67. Max coverage (-): 0.04

Region: NODE\_324426\_length\_69311\_cov\_30.906147 63221-63231. Max. coverage (+): 0.07. Max coverage (-): 0.04

Region: NODE\_324426\_length\_69311\_cov\_30.906147 63232-63241. Max. coverage (+): 61.87. Max coverage (-): 0

Region: NODE\_324426\_length\_69311\_cov\_30.906147 63242-63252. Max. coverage (+): 1.82. Max coverage (-): 0.11

Region: NODE\_324426\_length\_69311\_cov\_30.906147 63253-63263. Max. coverage (+): 0.37. Max coverage (-): 0

Region: NODE\_324426\_length\_69311\_cov\_30.906147 63264-63274. Max. coverage (+): 0.44. Max coverage (-): 0.85

Region: NODE\_324426\_length\_69311\_cov\_30.906147 63275-63285. Max. coverage (+): 1.08. Max coverage (-): 0.19

Region: NODE\_324426\_length\_69311\_cov\_30.906147 63286-63296. Max. coverage (+): 3.67. Max coverage (-): 0.07

Region: NODE\_324426\_length\_69311\_cov\_30.906147 63297-63307. Max. coverage (+): 0. Max coverage (-): 0.15

Region: NODE\_324426\_length\_69311\_cov\_30.906147 63308-63318. Max. coverage (+): 0.15. Max coverage (-): 1.59

Region: NODE\_324426\_length\_69311\_cov\_30.906147 63319-63329. Max. coverage (+): 3.76. Max coverage (-): 0

Region: NODE\_324426\_length\_69311\_cov\_30.906147 63330-63340. Max. coverage (+): 0.15. Max coverage (-): 0.04

Region: NODE\_324426\_length\_69311\_cov\_30.906147 63341-63351. Max. coverage (+): 0.11. Max coverage (-): 0.04

Region: NODE\_324426\_length\_69311\_cov\_30.906147 63352-63362. Max. coverage (+): 0.15. Max coverage (-): 0

Region: NODE\_324426\_length\_69311\_cov\_30.906147 63363-63373. Max. coverage (+): 0.15. Max coverage (-): 0.11

Region: NODE\_324426\_length\_69311\_cov\_30.906147 63374-63384. Max. coverage (+): 0.22. Max coverage (-): 0

Region: NODE\_324426\_length\_69311\_cov\_30.906147 63385-63395. Max. coverage (+): 3.19. Max coverage (-): 0.04

Region: NODE\_324426\_length\_69311\_cov\_30.906147 63396-63406. Max. coverage (+): 5. Max coverage (-): 0

Region: NODE\_324426\_length\_69311\_cov\_30.906147 63407-63417. Max. coverage (+): 0. Max coverage (-): 0

Region: NODE\_324426\_length\_69311\_cov\_30.906147 63418-63428. Max. coverage (+): 0. Max coverage (-): 0

Region: NODE\_324426\_length\_69311\_cov\_30.906147 63429-63439. Max. coverage (+): 0. Max coverage (-): 0

Region: NODE\_324426\_length\_69311\_cov\_30.906147 63440-63450. Max. coverage (+): 0.04. Max coverage (-): 0

Region: NODE\_324426\_length\_69311\_cov\_30.906147 63451-63460. Max. coverage (+): 0.59. Max coverage (-): 0.04

Region: NODE\_324426\_length\_69311\_cov\_30.906147 63461-63471. Max. coverage (+): 8.79. Max coverage (-): 0.04

Region: NODE\_324426\_length\_69311\_cov\_30.906147 63472-63482. Max. coverage (+): 1.82. Max coverage (-): 0

Region: NODE\_324426\_length\_69311\_cov\_30.906147 63483-63493. Max. coverage (+): 0.33. Max coverage (-): 0

Region: NODE\_324426\_length\_69311\_cov\_30.906147 63494-63504. Max. coverage (+): 0.41. Max coverage (-): 0.04

Region: NODE\_324426\_length\_69311\_cov\_30.906147 63505-63515. Max. coverage (+): 0.26. Max coverage (-): 0.07

Region: NODE\_324426\_length\_69311\_cov\_30.906147 63516-63526. Max. coverage (+): 0.74. Max coverage (-): 0.04

Region: NODE\_324426\_length\_69311\_cov\_30.906147 63527-63537. Max. coverage (+): 0.22. Max coverage (-): 0.04

Region: NODE\_324426\_length\_69311\_cov\_30.906147 63538-63548. Max. coverage (+): 0.01. Max coverage (-): 0

Region: NODE\_324426\_length\_69311\_cov\_30.906147 63549-63559. Max. coverage (+): 0.12. Max coverage (-): 0.04

Region: NODE\_324426\_length\_69311\_cov\_30.906147 63560-63570. Max. coverage (+): 0.52. Max coverage (-): 0

Region: NODE\_324426\_length\_69311\_cov\_30.906147 63571-63581. Max. coverage (+): 0. Max coverage (-): 0.04

Region: NODE\_324426\_length\_69311\_cov\_30.906147 63582-63592. Max. coverage (+): 0. Max coverage (-): 0.04

Region: NODE\_324426\_length\_69311\_cov\_30.906147 63593-63603. Max. coverage (+): 0.07. Max coverage (-): 0

Region: NODE\_324426\_length\_69311\_cov\_30.906147 63604-63614. Max. coverage (+): 0.93. Max coverage (-): 0

Region: NODE\_324426\_length\_69311\_cov\_30.906147 63615-63625. Max. coverage (+): 0.56. Max coverage (-): 0

Region: NODE\_324426\_length\_69311\_cov\_30.906147 63626-63636. Max. coverage (+): 0.56. Max coverage (-): 0.04

Region: NODE\_324426\_length\_69311\_cov\_30.906147 63637-63647. Max. coverage (+): 0.11. Max coverage (-): 0

Region: NODE\_324426\_length\_69311\_cov\_30.906147 63648-63658. Max. coverage (+): 0.07. Max coverage (-): 0

Region: NODE\_324426\_length\_69311\_cov\_30.906147 63659-63668. Max. coverage (+): 0. Max coverage (-): 0

Region: NODE\_324426\_length\_69311\_cov\_30.906147 63669-63679. Max. coverage (+): 0. Max coverage (-): 0

Region: NODE\_324426\_length\_69311\_cov\_30.906147 63680-63690. Max. coverage (+): 0. Max coverage (-): 0

Region: NODE\_324426\_length\_69311\_cov\_30.906147 63691-63701. Max. coverage (+): 0.07. Max coverage (-): 0

Region: NODE\_324426\_length\_69311\_cov\_30.906147 63702-63712. Max. coverage (+): 0.07. Max coverage (-): 0

Region: NODE\_324426\_length\_69311\_cov\_30.906147 63713-63723. Max. coverage (+): 0. Max coverage (-): 0

Region: NODE\_324426\_length\_69311\_cov\_30.906147 63724-63734. Max. coverage (+): 0.12. Max coverage (-): 0

Region: NODE\_324426\_length\_69311\_cov\_30.906147 63735-63745. Max. coverage (+): 0.26. Max coverage (-): 0.04

Region: NODE\_324426\_length\_69311\_cov\_30.906147 63746-63756. Max. coverage (+): 0.22. Max coverage (-): 0.04

Region: NODE\_324426\_length\_69311\_cov\_30.906147 63757-63767. Max. coverage (+): 0. Max coverage (-): 0

Region: NODE\_324426\_length\_69311\_cov\_30.906147 63768-63778. Max. coverage (+): 0.04. Max coverage (-): 0

Region: NODE\_324426\_length\_69311\_cov\_30.906147 63779-63789. Max. coverage (+): 0. Max coverage (-): 0

Region: NODE\_324426\_length\_69311\_cov\_30.906147 63790-63800. Max. coverage (+): 0.26. Max coverage (-): 0

Region: NODE\_324426\_length\_69311\_cov\_30.906147 63801-63811. Max. coverage (+): 0.26. Max coverage (-): 0

Region: NODE\_324426\_length\_69311\_cov\_30.906147 63812-63822. Max. coverage (+): 0.01. Max coverage (-): 0

Region: NODE\_324426\_length\_69311\_cov\_30.906147 63823-63833. Max. coverage (+): 0.01. Max coverage (-): 0

Region: NODE\_324426\_length\_69311\_cov\_30.906147 63834-63844. Max. coverage (+): 0.11. Max coverage (-): 0

Region: NODE\_324426\_length\_69311\_cov\_30.906147 63845-63855. Max. coverage (+): 0.41. Max coverage (-): 0

Region: NODE\_324426\_length\_69311\_cov\_30.906147 63856-63866. Max. coverage (+): 0.44. Max coverage (-): 0

Region: NODE\_324426\_length\_69311\_cov\_30.906147 63867-63876. Max. coverage (+): 0.07. Max coverage (-): 0

Region: NODE\_324426\_length\_69311\_cov\_30.906147 63877-63887. Max. coverage (+): 0.07. Max coverage (-): 0.04

Region: NODE\_324426\_length\_69311\_cov\_30.906147 63888-63898. Max. coverage (+): 0.11. Max coverage (-): 0

Region: NODE\_324426\_length\_69311\_cov\_30.906147 63899-63909. Max. coverage (+): 0.04. Max coverage (-): 0

Region: NODE\_324426\_length\_69311\_cov\_30.906147 63910-63920. Max. coverage (+): 0.07. Max coverage (-): 0

Region: NODE\_324426\_length\_69311\_cov\_30.906147 63921-63931. Max. coverage (+): 0.19. Max coverage (-): 0

Region: NODE\_324426\_length\_69311\_cov\_30.906147 63932-63942. Max. coverage (+): 0. Max coverage (-): 0.15

Region: NODE\_324426\_length\_69311\_cov\_30.906147 63943-63953. Max. coverage (+): 0. Max coverage (-): 0.15

Region: NODE\_324426\_length\_69311\_cov\_30.906147 63954-63964. Max. coverage (+): 0. Max coverage (-): 0

Region: NODE\_324426\_length\_69311\_cov\_30.906147 63965-63975. Max. coverage (+): 0. Max coverage (-): 0

Region: NODE\_324426\_length\_69311\_cov\_30.906147 63976-63986. Max. coverage (+): 0. Max coverage (-): 0

Region: NODE\_324426\_length\_69311\_cov\_30.906147 63987-63997. Max. coverage (+): 0. Max coverage (-): 0

Region: NODE\_324426\_length\_69311\_cov\_30.906147 63998-64008. Max. coverage (+): 0.04. Max coverage (-): 0

Region: NODE\_324426\_length\_69311\_cov\_30.906147 64009-64019. Max. coverage (+): 0.04. Max coverage (-): 0

Region: NODE\_324426\_length\_69311\_cov\_30.906147 64020-64030. Max. coverage (+): 0. Max coverage (-): 0

Region: NODE\_324426\_length\_69311\_cov\_30.906147 64031-64041. Max. coverage (+): 0.07. Max coverage (-): 0

Region: NODE\_324426\_length\_69311\_cov\_30.906147 64042-64052. Max. coverage (+): 0. Max coverage (-): 0

Region: NODE\_324426\_length\_69311\_cov\_30.906147 64053-64063. Max. coverage (+): 0.04. Max coverage (-): 0

Region: NODE\_324426\_length\_69311\_cov\_30.906147 64064-64074. Max. coverage (+): 0.04. Max coverage (-): 0

Region: NODE\_324426\_length\_69311\_cov\_30.906147 64075-64084. Max. coverage (+): 0.07. Max coverage (-): 0

Region: NODE\_324426\_length\_69311\_cov\_30.906147 64085-64095. Max. coverage (+): 0. Max coverage (-): 0

Region: NODE\_324426\_length\_69311\_cov\_30.906147 64096-64106. Max. coverage (+): 0. Max coverage (-): 0

Region: NODE\_324426\_length\_69311\_cov\_30.906147 64107-64117. Max. coverage (+): 0. Max coverage (-): 0

Region: NODE\_324426\_length\_69311\_cov\_30.906147 64118-64128. Max. coverage (+): 0.04. Max coverage (-): 0.04

Region: NODE\_324426\_length\_69311\_cov\_30.906147 64129-64139. Max. coverage (+): 0. Max coverage (-): 0

Region: NODE\_324426\_length\_69311\_cov\_30.906147 64140-64150. Max. coverage (+): 0. Max coverage (-): 0

Region: NODE\_324426\_length\_69311\_cov\_30.906147 64151-64161. Max. coverage (+): 0. Max coverage (-): 0

Region: NODE\_324426\_length\_69311\_cov\_30.906147 64162-64172. Max. coverage (+): 0. Max coverage (-): 0

Region: NODE\_324426\_length\_69311\_cov\_30.906147 64173-64183. Max. coverage (+): 0. Max coverage (-): 0

Region: NODE\_324426\_length\_69311\_cov\_30.906147 64184-64194. Max. coverage (+): 0. Max coverage (-): 0

Region: NODE\_324426\_length\_69311\_cov\_30.906147 64195-64205. Max. coverage (+): 0. Max coverage (-): 0

Region: NODE\_324426\_length\_69311\_cov\_30.906147 64206-64216. Max. coverage (+): 0.04. Max coverage (-): 0

Region: NODE\_324426\_length\_69311\_cov\_30.906147 64217-64227. Max. coverage (+): 0.04. Max coverage (-): 0

Region: NODE\_324426\_length\_69311\_cov\_30.906147 64228-64238. Max. coverage (+): 0. Max coverage (-): 0

Region: NODE\_324426\_length\_69311\_cov\_30.906147 64239-64249. Max. coverage (+): 0. Max coverage (-): 0

Region: NODE\_324426\_length\_69311\_cov\_30.906147 64250-64260. Max. coverage (+): 0. Max coverage (-): 0

Region: NODE\_324426\_length\_69311\_cov\_30.906147 64261-64271. Max. coverage (+): 0. Max coverage (-): 0

Region: NODE\_324426\_length\_69311\_cov\_30.906147 64272-64282. Max. coverage (+): 0.04. Max coverage (-): 0

Region: NODE\_324426\_length\_69311\_cov\_30.906147 64283-64293. Max. coverage (+): 0.04. Max coverage (-): 0

Region: NODE\_324426\_length\_69311\_cov\_30.906147 64294-64303. Max. coverage (+): 0.04. Max coverage (-): 0

Region: NODE\_324426\_length\_69311\_cov\_30.906147 64304-64314. Max. coverage (+): 0. Max coverage (-): 0

Region: NODE\_324426\_length\_69311\_cov\_30.906147 64315-64325. Max. coverage (+): 0. Max coverage (-): 0

Region: NODE\_324426\_length\_69311\_cov\_30.906147 64326-64336. Max. coverage (+): 0. Max coverage (-): 0

Region: NODE\_324426\_length\_69311\_cov\_30.906147 64337-64347. Max. coverage (+): 0.07. Max coverage (-): 0

Region: NODE\_324426\_length\_69311\_cov\_30.906147 64348-64358. Max. coverage (+): 0.07. Max coverage (-): 0

Region: NODE\_324426\_length\_69311\_cov\_30.906147 64359-64369. Max. coverage (+): 0. Max coverage (-): 0

Region: NODE\_324426\_length\_69311\_cov\_30.906147 64370-64380. Max. coverage (+): 0. Max coverage (-): 0

Region: NODE\_324426\_length\_69311\_cov\_30.906147 64381-64391. Max. coverage (+): 0. Max coverage (-): 0

Region: NODE\_324426\_length\_69311\_cov\_30.906147 64392-64402. Max. coverage (+): 0. Max coverage (-): 0.07

Region: NODE\_324426\_length\_69311\_cov\_30.906147 64403-64413. Max. coverage (+): 0. Max coverage (-): 0.04

Region: NODE\_324426\_length\_69311\_cov\_30.906147 64414-64424. Max. coverage (+): 0. Max coverage (-): 0

Region: NODE\_324426\_length\_69311\_cov\_30.906147 64425-64435. Max. coverage (+): 0. Max coverage (-): 0

Region: NODE\_324426\_length\_69311\_cov\_30.906147 64436-64446. Max. coverage (+): 0. Max coverage (-): 0

Region: NODE\_324426\_length\_69311\_cov\_30.906147 64447-64457. Max. coverage (+): 0.04. Max coverage (-): 0

Region: NODE\_324426\_length\_69311\_cov\_30.906147 64458-64468. Max. coverage (+): 0.04. Max coverage (-): 0

Region: NODE\_324426\_length\_69311\_cov\_30.906147 64469-64479. Max. coverage (+): 0. Max coverage (-): 0

Region: NODE\_324426\_length\_69311\_cov\_30.906147 64480-64490. Max. coverage (+): 0. Max coverage (-): 0

Region: NODE\_324426\_length\_69311\_cov\_30.906147 64491-64501. Max. coverage (+): 0. Max coverage (-): 0

Region: NODE\_324426\_length\_69311\_cov\_30.906147 64502-64511. Max. coverage (+): 0. Max coverage (-): 0

Region: NODE\_324426\_length\_69311\_cov\_30.906147 64512-64522. Max. coverage (+): 0. Max coverage (-): 0

Region: NODE\_324426\_length\_69311\_cov\_30.906147 64523-64533. Max. coverage (+): 0. Max coverage (-): 0

Region: NODE\_324426\_length\_69311\_cov\_30.906147 64534-64544. Max. coverage (+): 0. Max coverage (-): 0

Region: NODE\_324426\_length\_69311\_cov\_30.906147 64545-64555. Max. coverage (+): 0. Max coverage (-): 0

Region: NODE\_324426\_length\_69311\_cov\_30.906147 64556-64566. Max. coverage (+): 0. Max coverage (-): 0

Region: NODE\_324426\_length\_69311\_cov\_30.906147 64567-64577. Max. coverage (+): 0. Max coverage (-): 0

Region: NODE\_324426\_length\_69311\_cov\_30.906147 64578-64588. Max. coverage (+): 0. Max coverage (-): 0

Region: NODE\_324426\_length\_69311\_cov\_30.906147 64589-64599. Max. coverage (+): 0. Max coverage (-): 0

Region: NODE\_324426\_length\_69311\_cov\_30.906147 64600-64610. Max. coverage (+): 0. Max coverage (-): 0

Region: NODE\_324426\_length\_69311\_cov\_30.906147 64611-64621. Max. coverage (+): 0. Max coverage (-): 0

Region: NODE\_324426\_length\_69311\_cov\_30.906147 64622-64632. Max. coverage (+): 0. Max coverage (-): 0

Region: NODE\_324426\_length\_69311\_cov\_30.906147 64633-64643. Max. coverage (+): 0. Max coverage (-): 0

Region: NODE\_324426\_length\_69311\_cov\_30.906147 64644-64654. Max. coverage (+): 0. Max coverage (-): 0

Region: NODE\_324426\_length\_69311\_cov\_30.906147 64655-64665. Max. coverage (+): 0. Max coverage (-): 0

Region: NODE\_324426\_length\_69311\_cov\_30.906147 64666-64676. Max. coverage (+): 0. Max coverage (-): 0

Region: NODE\_324426\_length\_69311\_cov\_30.906147 64677-64687. Max. coverage (+): 0. Max coverage (-): 0

Region: NODE\_324426\_length\_69311\_cov\_30.906147 64688-64698. Max. coverage (+): 0. Max coverage (-): 0

Region: NODE\_324426\_length\_69311\_cov\_30.906147 64699-64709. Max. coverage (+): 0. Max coverage (-): 0

Region: NODE\_324426\_length\_69311\_cov\_30.906147 64710-64719. Max. coverage (+): 0. Max coverage (-): 0

Region: NODE\_324426\_length\_69311\_cov\_30.906147 64720-64730. Max. coverage (+): 0. Max coverage (-): 0

Region: NODE\_324426\_length\_69311\_cov\_30.906147 64731-64741. Max. coverage (+): 0. Max coverage (-): 0

Region: NODE\_324426\_length\_69311\_cov\_30.906147 64742-64752. Max. coverage (+): 0. Max coverage (-): 0

Region: NODE\_324426\_length\_69311\_cov\_30.906147 64753-64763. Max. coverage (+): 0. Max coverage (-): 0

Region: NODE\_324426\_length\_69311\_cov\_30.906147 64764-64774. Max. coverage (+): 0. Max coverage (-): 0

Region: NODE\_324426\_length\_69311\_cov\_30.906147 64775-64785. Max. coverage (+): 0. Max coverage (-): 0

Region: NODE\_324426\_length\_69311\_cov\_30.906147 64786-64796. Max. coverage (+): 0. Max coverage (-): 0

Region: NODE\_324426\_length\_69311\_cov\_30.906147 64797-64807. Max. coverage (+): 0. Max coverage (-): 0

Region: NODE\_324426\_length\_69311\_cov\_30.906147 64808-64818. Max. coverage (+): 0. Max coverage (-): 0

Region: NODE\_324426\_length\_69311\_cov\_30.906147 64819-64829. Max. coverage (+): 0. Max coverage (-): 0

Region: NODE\_324426\_length\_69311\_cov\_30.906147 64830-64840. Max. coverage (+): 0. Max coverage (-): 0

Region: NODE\_324426\_length\_69311\_cov\_30.906147 64841-64851. Max. coverage (+): 0. Max coverage (-): 0

Region: NODE\_324426\_length\_69311\_cov\_30.906147 64852-64862. Max. coverage (+): 0. Max coverage (-): 0

Region: NODE\_324426\_length\_69311\_cov\_30.906147 64863-64873. Max. coverage (+): 0. Max coverage (-): 0

Region: NODE\_324426\_length\_69311\_cov\_30.906147 64874-64884. Max. coverage (+): 0. Max coverage (-): 0

Region: NODE\_324426\_length\_69311\_cov\_30.906147 64885-64895. Max. coverage (+): 0. Max coverage (-): 0

Region: NODE\_324426\_length\_69311\_cov\_30.906147 64896-64906. Max. coverage (+): 0.04. Max coverage (-): 0

Region: NODE\_324426\_length\_69311\_cov\_30.906147 64907-64917. Max. coverage (+): 0. Max coverage (-): 0

Region: NODE\_324426\_length\_69311\_cov\_30.906147 64918-64927. Max. coverage (+): 0. Max coverage (-): 0.04

Region: NODE\_324426\_length\_69311\_cov\_30.906147 64928-64938. Max. coverage (+): 0. Max coverage (-): 0

Region: NODE\_324426\_length\_69311\_cov\_30.906147 64939-64949. Max. coverage (+): 0. Max coverage (-): 0

Region: NODE\_324426\_length\_69311\_cov\_30.906147 64950-64960. Max. coverage (+): 0. Max coverage (-): 0

Region: NODE\_324426\_length\_69311\_cov\_30.906147 64961-64971. Max. coverage (+): 0. Max coverage (-): 0

Region: NODE\_324426\_length\_69311\_cov\_30.906147 64972-64982. Max. coverage (+): 0. Max coverage (-): 0

Region: NODE\_324426\_length\_69311\_cov\_30.906147 64983-64993. Max. coverage (+): 0. Max coverage (-): 0

Region: NODE\_324426\_length\_69311\_cov\_30.906147 64994-65004. Max. coverage (+): 0. Max coverage (-): 0

Region: NODE\_324426\_length\_69311\_cov\_30.906147 65005-65015. Max. coverage (+): 0. Max coverage (-): 0

Region: NODE\_324426\_length\_69311\_cov\_30.906147 65016-65026. Max. coverage (+): 0. Max coverage (-): 0

Region: NODE\_324426\_length\_69311\_cov\_30.906147 65027-65037. Max. coverage (+): 0. Max coverage (-): 0

Region: NODE\_324426\_length\_69311\_cov\_30.906147 65038-65048. Max. coverage (+): 0. Max coverage (-): 0

Region: NODE\_324426\_length\_69311\_cov\_30.906147 65049-65059. Max. coverage (+): 0. Max coverage (-): 0

Region: NODE\_324426\_length\_69311\_cov\_30.906147 65060-65070. Max. coverage (+): 0.04. Max coverage (-): 0

Region: NODE\_324426\_length\_69311\_cov\_30.906147 65071-65081. Max. coverage (+): 0.04. Max coverage (-): 0

Region: NODE\_324426\_length\_69311\_cov\_30.906147 65082-65092. Max. coverage (+): 0. Max coverage (-): 0

Region: NODE\_324426\_length\_69311\_cov\_30.906147 65093-65103. Max. coverage (+): 0. Max coverage (-): 0

Region: NODE\_324426\_length\_69311\_cov\_30.906147 65104-65114. Max. coverage (+): 0. Max coverage (-): 0

Region: NODE\_324426\_length\_69311\_cov\_30.906147 65115-65125. Max. coverage (+): 0. Max coverage (-): 0.04

Region: NODE\_324426\_length\_69311\_cov\_30.906147 65126-65136. Max. coverage (+): 0.04. Max coverage (-): 0

Region: NODE\_324426\_length\_69311\_cov\_30.906147 65137-65146. Max. coverage (+): 0.04. Max coverage (-): 0

Region: NODE\_324426\_length\_69311\_cov\_30.906147 65147-65157. Max. coverage (+): 0. Max coverage (-): 0

Region: NODE\_324426\_length\_69311\_cov\_30.906147 65158-65168. Max. coverage (+): 0. Max coverage (-): 0

Region: NODE\_324426\_length\_69311\_cov\_30.906147 65169-65179. Max. coverage (+): 0. Max coverage (-): 0

Region: NODE\_324426\_length\_69311\_cov\_30.906147 65180-65190. Max. coverage (+): 0. Max coverage (-): 0

Region: NODE\_324426\_length\_69311\_cov\_30.906147 65191-65201. Max. coverage (+): 0. Max coverage (-): 0

Region: NODE\_324426\_length\_69311\_cov\_30.906147 65202-65212. Max. coverage (+): 0. Max coverage (-): 0

Region: NODE\_324426\_length\_69311\_cov\_30.906147 65213-65223. Max. coverage (+): 0. Max coverage (-): 0

Region: NODE\_324426\_length\_69311\_cov\_30.906147 65224-65234. Max. coverage (+): 0. Max coverage (-): 0

Region: NODE\_324426\_length\_69311\_cov\_30.906147 65235-65245. Max. coverage (+): 0. Max coverage (-): 0

Region: NODE\_324426\_length\_69311\_cov\_30.906147 65246-65256. Max. coverage (+): 0. Max coverage (-): 0

Region: NODE\_324426\_length\_69311\_cov\_30.906147 65257-65267. Max. coverage (+): 0. Max coverage (-): 0

Region: NODE\_324426\_length\_69311\_cov\_30.906147 65268-65278. Max. coverage (+): 0. Max coverage (-): 0

Region: NODE\_324426\_length\_69311\_cov\_30.906147 65279-65289. Max. coverage (+): 0. Max coverage (-): 0

Region: NODE\_324426\_length\_69311\_cov\_30.906147 65290-65300. Max. coverage (+): 0. Max coverage (-): 0

Region: NODE\_324426\_length\_69311\_cov\_30.906147 65301-65311. Max. coverage (+): 0. Max coverage (-): 0

Region: NODE\_324426\_length\_69311\_cov\_30.906147 65312-65322. Max. coverage (+): 0. Max coverage (-): 0

Region: NODE\_324426\_length\_69311\_cov\_30.906147 65323-65333. Max. coverage (+): 0. Max coverage (-): 0

Region: NODE\_324426\_length\_69311\_cov\_30.906147 65334-65344. Max. coverage (+): 0. Max coverage (-): 0

Region: NODE\_324426\_length\_69311\_cov\_30.906147 65345-65354. Max. coverage (+): 0. Max coverage (-): 0

Region: NODE\_324426\_length\_69311\_cov\_30.906147 65355-65365. Max. coverage (+): 0. Max coverage (-): 0

Region: NODE\_324426\_length\_69311\_cov\_30.906147 65366-65376. Max. coverage (+): 0. Max coverage (-): 0

Region: NODE\_324426\_length\_69311\_cov\_30.906147 65377-65387. Max. coverage (+): 0. Max coverage (-): 0

Region: NODE\_324426\_length\_69311\_cov\_30.906147 65388-65398. Max. coverage (+): 0. Max coverage (-): 0

Region: NODE\_324426\_length\_69311\_cov\_30.906147 65399-65409. Max. coverage (+): 0. Max coverage (-): 0

Region: NODE\_324426\_length\_69311\_cov\_30.906147 65410-65420. Max. coverage (+): 0. Max coverage (-): 0

Region: NODE\_324426\_length\_69311\_cov\_30.906147 65421-65431. Max. coverage (+): 0. Max coverage (-): 0

Region: NODE\_324426\_length\_69311\_cov\_30.906147 65432-65442. Max. coverage (+): 0.04. Max coverage (-): 0

Region: NODE\_324426\_length\_69311\_cov\_30.906147 65443-65453. Max. coverage (+): 0. Max coverage (-): 0

Region: NODE\_324426\_length\_69311\_cov\_30.906147 65454-65464. Max. coverage (+): 0. Max coverage (-): 0

Region: NODE\_324426\_length\_69311\_cov\_30.906147 65465-65475. Max. coverage (+): 0. Max coverage (-): 0

Region: NODE\_324426\_length\_69311\_cov\_30.906147 65476-65486. Max. coverage (+): 0. Max coverage (-): 0

Region: NODE\_324426\_length\_69311\_cov\_30.906147 65487-65497. Max. coverage (+): 0. Max coverage (-): 0

Region: NODE\_324426\_length\_69311\_cov\_30.906147 65498-65508. Max. coverage (+): 0. Max coverage (-): 0

Region: NODE\_324426\_length\_69311\_cov\_30.906147 65509-65519. Max. coverage (+): 0. Max coverage (-): 0

Region: NODE\_324426\_length\_69311\_cov\_30.906147 65520-65530. Max. coverage (+): 0. Max coverage (-): 0

Region: NODE\_324426\_length\_69311\_cov\_30.906147 65531-65541. Max. coverage (+): 0. Max coverage (-): 0

Region: NODE\_324426\_length\_69311\_cov\_30.906147 65542-65552. Max. coverage (+): 0. Max coverage (-): 0

Region: NODE\_324426\_length\_69311\_cov\_30.906147 65553-65562. Max. coverage (+): 0. Max coverage (-): 0

Region: NODE\_324426\_length\_69311\_cov\_30.906147 65563-65573. Max. coverage (+): 0. Max coverage (-): 0

Region: NODE\_324426\_length\_69311\_cov\_30.906147 65574-65584. Max. coverage (+): 0. Max coverage (-): 0

Region: NODE\_324426\_length\_69311\_cov\_30.906147 65585-65595. Max. coverage (+): 0. Max coverage (-): 0

Region: NODE\_324426\_length\_69311\_cov\_30.906147 65596-65606. Max. coverage (+): 0. Max coverage (-): 0

Region: NODE\_324426\_length\_69311\_cov\_30.906147 65607-65617. Max. coverage (+): 0. Max coverage (-): 0

Region: NODE\_324426\_length\_69311\_cov\_30.906147 65618-65628. Max. coverage (+): 0. Max coverage (-): 0

Region: NODE\_324426\_length\_69311\_cov\_30.906147 65629-65639. Max. coverage (+): 0. Max coverage (-): 0

Region: NODE\_324426\_length\_69311\_cov\_30.906147 65640-65650. Max. coverage (+): 0. Max coverage (-): 0

Region: NODE\_324426\_length\_69311\_cov\_30.906147 65651-65661. Max. coverage (+): 0. Max coverage (-): 0

Region: NODE\_324426\_length\_69311\_cov\_30.906147 65662-65672. Max. coverage (+): 0. Max coverage (-): 0

Region: NODE\_324426\_length\_69311\_cov\_30.906147 65673-65683. Max. coverage (+): 0. Max coverage (-): 0

Region: NODE\_324426\_length\_69311\_cov\_30.906147 65684-65694. Max. coverage (+): 0. Max coverage (-): 0

Region: NODE\_324426\_length\_69311\_cov\_30.906147 65695-65705. Max. coverage (+): 0. Max coverage (-): 0

Region: NODE\_324426\_length\_69311\_cov\_30.906147 65706-65716. Max. coverage (+): 0. Max coverage (-): 0

Region: NODE\_324426\_length\_69311\_cov\_30.906147 65717-65727. Max. coverage (+): 0. Max coverage (-): 0

Region: NODE\_324426\_length\_69311\_cov\_30.906147 65728-65738. Max. coverage (+): 0. Max coverage (-): 0

Region: NODE\_324426\_length\_69311\_cov\_30.906147 65739-65749. Max. coverage (+): 0. Max coverage (-): 0

Region: NODE\_324426\_length\_69311\_cov\_30.906147 65750-65760. Max. coverage (+): 0. Max coverage (-): 0

Region: NODE\_324426\_length\_69311\_cov\_30.906147 65761-65770. Max. coverage (+): 0. Max coverage (-): 0

Region: NODE\_324426\_length\_69311\_cov\_30.906147 65771-65781. Max. coverage (+): 0. Max coverage (-): 0

Region: NODE\_324426\_length\_69311\_cov\_30.906147 65782-65792. Max. coverage (+): 0. Max coverage (-): 0

Region: NODE\_324426\_length\_69311\_cov\_30.906147 65793-65803. Max. coverage (+): 0. Max coverage (-): 0

Region: NODE\_324426\_length\_69311\_cov\_30.906147 65804-65814. Max. coverage (+): 0. Max coverage (-): 0

Region: NODE\_324426\_length\_69311\_cov\_30.906147 65815-65825. Max. coverage (+): 0. Max coverage (-): 0

Region: NODE\_324426\_length\_69311\_cov\_30.906147 65826-65836. Max. coverage (+): 0. Max coverage (-): 0

Region: NODE\_324426\_length\_69311\_cov\_30.906147 65837-65847. Max. coverage (+): 0. Max coverage (-): 0

Region: NODE\_324426\_length\_69311\_cov\_30.906147 65848-65858. Max. coverage (+): 0. Max coverage (-): 0

Region: NODE\_324426\_length\_69311\_cov\_30.906147 65859-65869. Max. coverage (+): 0. Max coverage (-): 0

Region: NODE\_324426\_length\_69311\_cov\_30.906147 65870-65880. Max. coverage (+): 0. Max coverage (-): 0

Region: NODE\_324426\_length\_69311\_cov\_30.906147 65881-65891. Max. coverage (+): 0. Max coverage (-): 0

Region: NODE\_324426\_length\_69311\_cov\_30.906147 65892-65902. Max. coverage (+): 0. Max coverage (-): 0

Region: NODE\_324426\_length\_69311\_cov\_30.906147 65903-65913. Max. coverage (+): 0. Max coverage (-): 0

Region: NODE\_324426\_length\_69311\_cov\_30.906147 65914-65924. Max. coverage (+): 0. Max coverage (-): 0

Region: NODE\_324426\_length\_69311\_cov\_30.906147 65925-65935. Max. coverage (+): 0. Max coverage (-): 0

Region: NODE\_324426\_length\_69311\_cov\_30.906147 65936-65946. Max. coverage (+): 0. Max coverage (-): 0

Region: NODE\_324426\_length\_69311\_cov\_30.906147 65947-65957. Max. coverage (+): 0. Max coverage (-): 0

Region: NODE\_324426\_length\_69311\_cov\_30.906147 65958-65968. Max. coverage (+): 0. Max coverage (-): 0

Region: NODE\_324426\_length\_69311\_cov\_30.906147 65969-65978. Max. coverage (+): 0. Max coverage (-): 0

Region: NODE\_324426\_length\_69311\_cov\_30.906147 65979-65989. Max. coverage (+): 0. Max coverage (-): 0

Region: NODE\_324426\_length\_69311\_cov\_30.906147 65990-66000. Max. coverage (+): 0. Max coverage (-): 0

Region: NODE\_324426\_length\_69311\_cov\_30.906147 66001-66011. Max. coverage (+): 0. Max coverage (-): 0

Region: NODE\_324426\_length\_69311\_cov\_30.906147 66012-66022. Max. coverage (+): 0. Max coverage (-): 0

Region: NODE\_324426\_length\_69311\_cov\_30.906147 66023-66033. Max. coverage (+): 0. Max coverage (-): 0

Region: NODE\_324426\_length\_69311\_cov\_30.906147 66034-66044. Max. coverage (+): 0. Max coverage (-): 0

Region: NODE\_324426\_length\_69311\_cov\_30.906147 66045-66055. Max. coverage (+): 0. Max coverage (-): 0

Region: NODE\_324426\_length\_69311\_cov\_30.906147 66056-66066. Max. coverage (+): 0. Max coverage (-): 0

Region: NODE\_324426\_length\_69311\_cov\_30.906147 66067-66077. Max. coverage (+): 0. Max coverage (-): 0

Region: NODE\_324426\_length\_69311\_cov\_30.906147 66078-66088. Max. coverage (+): 0. Max coverage (-): 0

Region: NODE\_324426\_length\_69311\_cov\_30.906147 66089-66099. Max. coverage (+): 0. Max coverage (-): 0

Region: NODE\_324426\_length\_69311\_cov\_30.906147 66100-66110. Max. coverage (+): 0. Max coverage (-): 0

Region: NODE\_324426\_length\_69311\_cov\_30.906147 66111-66121. Max. coverage (+): 0. Max coverage (-): 0

Region: NODE\_324426\_length\_69311\_cov\_30.906147 66122-66132. Max. coverage (+): 0. Max coverage (-): 0

Region: NODE\_324426\_length\_69311\_cov\_30.906147 66133-66143. Max. coverage (+): 0.04. Max coverage (-): 0

Region: NODE\_324426\_length\_69311\_cov\_30.906147 66144-66154. Max. coverage (+): 0. Max coverage (-): 0

Region: NODE\_324426\_length\_69311\_cov\_30.906147 66155-66165. Max. coverage (+): 0. Max coverage (-): 0

Region: NODE\_324426\_length\_69311\_cov\_30.906147 66166-66176. Max. coverage (+): 0. Max coverage (-): 0

Region: NODE\_324426\_length\_69311\_cov\_30.906147 66177-66187. Max. coverage (+): 0. Max coverage (-): 0

Region: NODE\_324426\_length\_69311\_cov\_30.906147 66188-66197. Max. coverage (+): 0. Max coverage (-): 0

Region: NODE\_324426\_length\_69311\_cov\_30.906147 66198-66208. Max. coverage (+): 0. Max coverage (-): 0

Region: NODE\_324426\_length\_69311\_cov\_30.906147 66209-66219. Max. coverage (+): 0. Max coverage (-): 0

Region: NODE\_324426\_length\_69311\_cov\_30.906147 66220-66230. Max. coverage (+): 0. Max coverage (-): 0

Region: NODE\_324426\_length\_69311\_cov\_30.906147 66231-66241. Max. coverage (+): 0. Max coverage (-): 0

Region: NODE\_324426\_length\_69311\_cov\_30.906147 66242-66252. Max. coverage (+): 0. Max coverage (-): 0

Region: NODE\_324426\_length\_69311\_cov\_30.906147 66253-66263. Max. coverage (+): 0. Max coverage (-): 0

Region: NODE\_324426\_length\_69311\_cov\_30.906147 66264-66274. Max. coverage (+): 0. Max coverage (-): 0

Region: NODE\_324426\_length\_69311\_cov\_30.906147 66275-66285. Max. coverage (+): 0. Max coverage (-): 0

Region: NODE\_324426\_length\_69311\_cov\_30.906147 66286-66296. Max. coverage (+): 0. Max coverage (-): 0

Region: NODE\_324426\_length\_69311\_cov\_30.906147 66297-66307. Max. coverage (+): 0. Max coverage (-): 0

Region: NODE\_324426\_length\_69311\_cov\_30.906147 66308-66318. Max. coverage (+): 0. Max coverage (-): 0

Region: NODE\_324426\_length\_69311\_cov\_30.906147 66319-66329. Max. coverage (+): 0. Max coverage (-): 0

Region: NODE\_324426\_length\_69311\_cov\_30.906147 66330-66340. Max. coverage (+): 0. Max coverage (-): 0

Region: NODE\_324426\_length\_69311\_cov\_30.906147 66341-66351. Max. coverage (+): 0. Max coverage (-): 0

Region: NODE\_324426\_length\_69311\_cov\_30.906147 66352-66362. Max. coverage (+): 0. Max coverage (-): 0

Region: NODE\_324426\_length\_69311\_cov\_30.906147 66363-66373. Max. coverage (+): 0. Max coverage (-): 0

Region: NODE\_324426\_length\_69311\_cov\_30.906147 66374-66384. Max. coverage (+): 0. Max coverage (-): 0

Region: NODE\_324426\_length\_69311\_cov\_30.906147 66385-66395. Max. coverage (+): 0. Max coverage (-): 0

Region: NODE\_324426\_length\_69311\_cov\_30.906147 66396-66405. Max. coverage (+): 0. Max coverage (-): 0.04

Region: NODE\_324426\_length\_69311\_cov\_30.906147 66406-66416. Max. coverage (+): 0. Max coverage (-): 0

Region: NODE\_324426\_length\_69311\_cov\_30.906147 66417-66427. Max. coverage (+): 0. Max coverage (-): 0

Region: NODE\_324426\_length\_69311\_cov\_30.906147 66428-66438. Max. coverage (+): 0. Max coverage (-): 0

Region: NODE\_324426\_length\_69311\_cov\_30.906147 66439-66449. Max. coverage (+): 0. Max coverage (-): 0

Region: NODE\_324426\_length\_69311\_cov\_30.906147 66450-66460. Max. coverage (+): 0. Max coverage (-): 0

Region: NODE\_324426\_length\_69311\_cov\_30.906147 66461-66471. Max. coverage (+): 0. Max coverage (-): 0

Region: NODE\_324426\_length\_69311\_cov\_30.906147 66472-66482. Max. coverage (+): 0. Max coverage (-): 0

Region: NODE\_324426\_length\_69311\_cov\_30.906147 66483-66493. Max. coverage (+): 0. Max coverage (-): 0

Region: NODE\_324426\_length\_69311\_cov\_30.906147 66494-66504. Max. coverage (+): 0. Max coverage (-): 0

Region: NODE\_324426\_length\_69311\_cov\_30.906147 66505-66515. Max. coverage (+): 0. Max coverage (-): 0

Region: NODE\_324426\_length\_69311\_cov\_30.906147 66516-66526. Max. coverage (+): 0.04. Max coverage (-): 0

Region: NODE\_324426\_length\_69311\_cov\_30.906147 66527-66537. Max. coverage (+): 0. Max coverage (-): 0

Region: NODE\_324426\_length\_69311\_cov\_30.906147 66538-66548. Max. coverage (+): 0. Max coverage (-): 0

Region: NODE\_324426\_length\_69311\_cov\_30.906147 66549-66559. Max. coverage (+): 0. Max coverage (-): 0

Region: NODE\_324426\_length\_69311\_cov\_30.906147 66560-66570. Max. coverage (+): 0. Max coverage (-): 0

Region: NODE\_324426\_length\_69311\_cov\_30.906147 66571-66581. Max. coverage (+): 0. Max coverage (-): 0

Region: NODE\_324426\_length\_69311\_cov\_30.906147 66582-66592. Max. coverage (+): 0. Max coverage (-): 0

Region: NODE\_324426\_length\_69311\_cov\_30.906147 66593-66603. Max. coverage (+): 0. Max coverage (-): 0

Region: NODE\_324426\_length\_69311\_cov\_30.906147 66604-66613. Max. coverage (+): 0. Max coverage (-): 0

Region: NODE\_324426\_length\_69311\_cov\_30.906147 66614-66624. Max. coverage (+): 0. Max coverage (-): 0

Region: NODE\_324426\_length\_69311\_cov\_30.906147 66625-66635. Max. coverage (+): 0. Max coverage (-): 0

Region: NODE\_324426\_length\_69311\_cov\_30.906147 66636-66646. Max. coverage (+): 0. Max coverage (-): 0

Region: NODE\_324426\_length\_69311\_cov\_30.906147 66647-66657. Max. coverage (+): 0. Max coverage (-): 0

Region: NODE\_324426\_length\_69311\_cov\_30.906147 66658-66668. Max. coverage (+): 0. Max coverage (-): 0

Region: NODE\_324426\_length\_69311\_cov\_30.906147 66669-66679. Max. coverage (+): 0. Max coverage (-): 0

Region: NODE\_324426\_length\_69311\_cov\_30.906147 66680-66690. Max. coverage (+): 0. Max coverage (-): 0

Region: NODE\_324426\_length\_69311\_cov\_30.906147 66691-66701. Max. coverage (+): 0. Max coverage (-): 0

Region: NODE\_324426\_length\_69311\_cov\_30.906147 66702-66712. Max. coverage (+): 0. Max coverage (-): 0

Region: NODE\_324426\_length\_69311\_cov\_30.906147 66713-66723. Max. coverage (+): 0. Max coverage (-): 0

Region: NODE\_324426\_length\_69311\_cov\_30.906147 66724-66734. Max. coverage (+): 0. Max coverage (-): 0

Region: NODE\_324426\_length\_69311\_cov\_30.906147 66735-66745. Max. coverage (+): 0. Max coverage (-): 0

Region: NODE\_324426\_length\_69311\_cov\_30.906147 66746-66756. Max. coverage (+): 0. Max coverage (-): 0

Region: NODE\_324426\_length\_69311\_cov\_30.906147 66757-66767. Max. coverage (+): 0. Max coverage (-): 0

Region: NODE\_324426\_length\_69311\_cov\_30.906147 66768-66778. Max. coverage (+): 0. Max coverage (-): 0

Region: NODE\_324426\_length\_69311\_cov\_30.906147 66779-66789. Max. coverage (+): 0. Max coverage (-): 0

Region: NODE\_324426\_length\_69311\_cov\_30.906147 66790-66800. Max. coverage (+): 0. Max coverage (-): 0

Region: NODE\_324426\_length\_69311\_cov\_30.906147 66801-66811. Max. coverage (+): 0. Max coverage (-): 0

Region: NODE\_324426\_length\_69311\_cov\_30.906147 66812-66821. Max. coverage (+): 0. Max coverage (-): 0

Region: NODE\_324426\_length\_69311\_cov\_30.906147 66822-66832. Max. coverage (+): 0. Max coverage (-): 0

Region: NODE\_324426\_length\_69311\_cov\_30.906147 66833-66843. Max. coverage (+): 0. Max coverage (-): 0

Region: NODE\_324426\_length\_69311\_cov\_30.906147 66844-66854. Max. coverage (+): 0. Max coverage (-): 0

Region: NODE\_324426\_length\_69311\_cov\_30.906147 66855-66865. Max. coverage (+): 0. Max coverage (-): 0

Region: NODE\_324426\_length\_69311\_cov\_30.906147 66866-66876. Max. coverage (+): 0. Max coverage (-): 0

Region: NODE\_324426\_length\_69311\_cov\_30.906147 66877-66887. Max. coverage (+): 0.01. Max coverage (-): 0

Region: NODE\_324426\_length\_69311\_cov\_30.906147 66888-66898. Max. coverage (+): 0. Max coverage (-): 0.04

Region: NODE\_324426\_length\_69311\_cov\_30.906147 66899-66909. Max. coverage (+): 0. Max coverage (-): 0.04

Region: NODE\_324426\_length\_69311\_cov\_30.906147 66910-66920. Max. coverage (+): 0. Max coverage (-): 0

Region: NODE\_324426\_length\_69311\_cov\_30.906147 66921-. Max. coverage (+): 0. Max coverage (-): 0

RepeatMasker Color Code

**+**

100-98% Identity

<98-95% Identity

<95-90% Identity

<90-85% Identity

<85-80% Identity

<80-75% Identity

<75-70% Identity

<70% Identity

**-**

Gene Set Color Code

**+**

Gene

Pseudogene

Other

**-**

Topology/Coverage Color Code

Coverage Plus Strand

Coverage Minus Strand

Mainstrand: Plus

Mainstrand: Minus

Complementary Strand

Flanking Region  
(if option -flank >0)

Gene Set Annotation  
  
RepeatMasker Annotation  

**1. (ATCAA)n**: 61448-61476 (+), Divergence to consensus: 0%  
**2. REX1-1\_AFC**: 61477-61546 (-), Divergence to consensus: 5.7%  
**3. P-1\_DR**: 61548-63141 (-), Divergence to consensus: 22%  
**4. P-1\_DR**: 63214-63407 (-), Divergence to consensus: 33.2%  
**5. P-1\_DR**: 63458-63535 (-), Divergence to consensus: 22.1%  
**6. AlRepB-180**: 63522-63955 (+), Divergence to consensus: 38.2%  
**7. AlRepA-93**: 64061-64462 (+), Divergence to consensus: 25.9%  
**8. AlRepB-269**: 64643-64717 (+), Divergence to consensus: 12%  
**9. AlRepC-14**: 64725-64916 (-), Divergence to consensus: 22.3%  
**10. TguLTRK7a**: 65223-65273 (+), Divergence to consensus: 25.7%  
**11. AlRepA-297**: 66506-66524 (+), Divergence to consensus: 38.9%  
**12. AlRepD-175**: 66525-66624 (-), Divergence to consensus: 26.6%  
**13. AlRepA-297**: 66625-66799 (+), Divergence to consensus: 38.9%  
**14. AlRepE-1026**: 66651-66802 (-), Divergence to consensus: 27%  
**15. AlRepD-775**: 66803-66865 (+), Divergence to consensus: 22.6%  
**16. AlRepC-2883**: 66856-66920 (-), Divergence to consensus: 4.6%

  
Transcription Factor Binding Sites  

**RHOXF1** (Sequence: GGCTTA (-): 61824)  
**RHOXF1** (Sequence: GGATCA (-): 62162)  
**RHOXF1** (Sequence: GGATCA (-): 62638)  
**RHOXF1** (Sequence: AGATCA (-): 62870)  
**RHOXF1** (Sequence: AGATCA (-): 64132)  
**RHOXF1** (Sequence: GGCTTA (-): 65918)  
**RHOXF1** (Sequence: AGATCA (-): 66345)  
**RHOXF1** (Sequence: TGAGCC (+): 62032)  
**RHOXF1** (Sequence: TAATCT (+): 62078)  
**RHOXF1** (Sequence: TGATCC (+): 62355)  
**RHOXF1** (Sequence: TAATCC (+): 62631)  
**RHOXF1** (Sequence: TGATCC (+): 63046)  
**RHOXF1** (Sequence: TGAGCC (+): 63264)  
**RHOXF1** (Sequence: TGATCT (+): 64050)  
**RHOXF1** (Sequence: TGAGCC (+): 64320)  
**RHOXF1** (Sequence: TGAGCT (+): 64692)  
**RHOXF1** (Sequence: TAATCC (+): 65394)  
**Lhx8** (Sequence: TTAATTAG (-): 61827)  
**Gata4** (Sequence: CTTATCT (+): 63946)  
**POU5F1** (Sequence: TTTGCAT (-): 63193)  
**POU5F1** (Sequence: TTTGCAT (-): 63822)  
**RFX4\_2** (Sequence: GTAACTATG (-): 63096)  
**SOX9** (Sequence: AACAATGA (-): 62301)  
**SOX9** (Sequence: AACAATAA (-): 66193)  
**Sox5** (Sequence: ATTGTT (+): 63556)  
**Sox5** (Sequence: ATTGTT (+): 64409)  
**Sox5** (Sequence: ATTGTT (+): 64951)  
**Sox5** (Sequence: ATTGTT (+): 65991)  
**Sox5** (Sequence: ATTGTT (+): 66555)  
**Mybl1\_1** (Sequence: TAACGGTT (-): 64396)  
**FIGLA** (Sequence: AACAGCTGTT (-): 61861)  
**SOX9** (Sequence: TCATTGTT (+): 66553)  
**FOXO3\_mmu** (Sequence: TCAAAACA (+): 64597)  
**FOXO3\_mmu** (Sequence: TCAAAACA (+): 65526)  
**Nobox** (Sequence: GGTAATTA (-): 63566)  
**Nobox** (Sequence: AGTAATTA (-): 63651)  
**FOXO1** (Sequence: AAAAACAAG (-): 65874)  
**FOXO1** (Sequence: AAAAACAAC (-): 65924)  
**Nobox** (Sequence: TAATTGGC (+): 63160)  
**Rhox11** (Sequence: TGCTGTAAT (+): 63155)  
**Rhox11** (Sequence: CGGTGTTTT (+): 66521)  
**Sox5** (Sequence: AACAAT (-): 62301)  
**Sox5** (Sequence: AACAAT (-): 63662)  
**Sox5** (Sequence: AACAAT (-): 65530)  
**Sox5** (Sequence: AACAAT (-): 65982)  
**Sox5** (Sequence: AACAAT (-): 66142)  
**Sox5** (Sequence: AACAAT (-): 66193)  
**POU2F1** (Sequence: TATTTTAAT (+): 63952)  
**POU5F1** (Sequence: ATGCAAA (+): 62117)
