# Supplementary material for: First characterization of PIWI-interacting RNA clusters in a cichlid fish with a B chromosome
Source: BMC Biol. 2022 Sep 21;20:204. doi: 10.1186/s12915-022-01403-2 (PMC9490952; doi:10.1186/s12915-022-01403-2)
Supplement: Supplementary file 1 — Additional file 1. Zipped folder with fasta and interactive html piRNA cluster information for the A. latifasciata genome. The nomenclature is as follows: number-pirna-cluster_sex_B-presence (f, female; m, male; 0b, without B chromosome; 1b, with B chromosome). [file 12915_2022_1403_MOESM1_ESM.zip › 129_f0b.html]

piRNA cluster 129\_f0b 63


Predicted piRNA cluster no. 129\_f0b
  

Show proTRAC run info
Hide proTRAC run info

/\  
                \_\_\_\_\_\_\_\_\_\_\_\_\_\_\_\_\_\_\_\_\_\_\_/\\_\_\_ /  \\_\_\_\_\_\_\_  
               I                      /  \  /    \      I  
               I     pro             /    \/      \     I  
               I        TRAC        /               \   I  
               I   \_\_\_\_\_\_\_\_\_\_\_\_\_\_\_\_/\_\_\_\_\_\_\_\_\_\_\_\_\_\_\_\_\_\\_ I  
               I   \              /                     I  
               I    \            /                      I  
               I     \  /\      /       V.2.4.2         I  
               I      \/  \    /                        I  
               I\_\_\_\_\_\_\_\_\_\_\_\  /\_\_\_\_\_\_\_\_\_\_\_\_\_\_\_\_\_\_\_\_\_\_\_\_\_I  
                            \/  
  
  
================================= proTRAC ====================================  
VERSION: .......... 2.4.2  
LAST MODIFIED: .... 11. May 2018  
  
Please cite:  
Rosenkranz D, Zischler H. proTRAC - a software for probabilistic piRNA cluster  
detection, visualization and analysis. 2012. BMC Bioinformatics 13:5.  
  
  
Contact:  
David Rosenkranz  
Institute of Organismic and Molecular Evolutionary Biology  
Dept. Anthropology, small RNA group  
Johannes Gutenberg University Mainz  
email: rosenkranz@uni-mainz.de  
  
You can find the latest proTRAC version at:  
http://sourceforge.net/projects/protrac/files  
http://www.smallRNAgroup-mainz.de/software  
==============================================================================  
  
PARAMETERS:  
Map file: ...............piwi-femeas-0B.fa-collapse.map  
Genome file: ............../../../0B\_ala\_genome.fa  
RepeatMasker annotation: Alatifasciata-all0B-maryan-v2.fa\_corrected.out  
GeneSet:................./guest-storage/Data/annotation/Alatifasciata\_all0B\_maryan-v2\_out2017.gff  
  
Significant (p<=0.01) hit density will be calculated based  
on observed hit distribution.  
  
Sliding window size: ........................................ 5000 bp  
Sliding window increament: .................................. 1000 bp  
Normalize each hit by number of genomic hits: ............... yes  
Normalize each hit by number of sequence reads: ............. yes  
Normalize values (-> per million mapped reads): ............. yes  
Min. fraction of hits with 1T(U) or 10A: .................... 0.75  
Alternatively: Min. fraction of hits with 1T(U) and 10A: .... 0.5  
Min. fraction of hits with typical piRNA length: ............ 0.75  
Typical piRNA length: ....................................... 24-32 nt  
Min. size of a piRNA cluster: ............................... 1000 bp.  
Min. number of hits (absolute): ............................. 0  
Min. number of hits (normalized): ........................... 0  
Min. fraction of hits on the mainstrand: .................... 0.75  
Top fraction of mapped sequences (in terms of read counts): . 1%  
Top fraction accounts for max. n% of sequence reads: ........ 90%  
Min. fraction of hits on each arm of a bidirectional cluster: 0.05  
Output html file for each cluster: .......................... yes  
Output a summary table: ..................................... yes  
Output a FASTA file for each cluster (piRNA sequences): ..... yes  
Output a FASTA file comprising cluster sequences: ........... yes  
Output a GTF file for predicted piRNA clusters: ..............yes  
Search DNA motifs in clusters: .............................. yes  
Output flanking sequences: +/- .............................. 0 bp  
Output ~.pTi file: .......................................... no  
==============================================================================  
  
  
Genome size (without gaps): ............ 758543724 bp  
Gaps (N/X/-): .......................... 417479 bp  
Mapped reads: .......................... 13052187  
Non-identical sequences: ............... 3338911  
Genomic hits: .......................... 28737726  
Significant densitiy of mapped reads: .. 470.083249848448 reads/kb

Show proTRAC cluster info
Hide proTRAC cluster info

|  |  |
| --- | --- |
| Location | NODE\_328441\_length\_53725\_cov\_29.545444 |
| Coordinates | 50135-53964 |
| Size [bp] | 3830 |
| Sequence hit loci | 2372 |
| Mapped reads (normalized) | 4474.3 |
| Mapped reads (normalized) per kb | 1168.2 |
| Normalized reads with 1T (1U) | 75.7% |
| Normalized reads with 10A | 39.9% |
| Normalized reads with length 24-32 nt | 99.3% |
| Normalized reads on the main strand(s) | 86.1% |
| Predicted directionality | mono:plus |

100%

0%

1T (1U)  
reads

10A reads

24-32 nt  
reads

reads on mainstrand

**Either the amount of reads with 1T (1U) OR 10A has to exceed 75% (set with option: -1Tor10A)  
Alternatively the amount of reads with 1T (1U) AND 10A has to exceed 50% (set with option: -1Tand10A)  
Minimum amount of reads with preferred size is 75% (set with option: -pisize)  
Minimum amount of reads on the main strand(s) is 75% (set with option: -clstrand)**

Show read coverage
Hide read coverage

WHAT DO I SEE HERE?  
This chart shows the location of mapped sequence reads within a predicted piRNA cluster. The color refers to the number of genomic hits produced by the sequence read in question. A dark red bar indicates that this sequence read produces many other hits elsewhere in the genome. Many adjacent red or yellow bars can indicate the presence of a multi-copy element such as transposons or rRNA genes. A dark green bar indicates that this sequence read maps uniquely to this locus.

1 hit

2-5 hits

6-10 hits

11-20 hits

21-50 hits

51-100 hits

> 100 hits

NODE\_328441\_length\_53725\_cov\_29.545444

50135

53964

Gene Set

RepeatMasker

Mapped  
Reads

49.95

plus strand

minus strand

49.95

Region: NODE\_328441\_length\_53725\_cov\_29.545444 66261-50138. Max. coverage (+): 0.01. Max coverage (-): 0.01

Region: NODE\_328441\_length\_53725\_cov\_29.545444 50139-50146. Max. coverage (+): 0.01. Max coverage (-): 0.02

Region: NODE\_328441\_length\_53725\_cov\_29.545444 50147-50154. Max. coverage (+): 0.01. Max coverage (-): 0

Region: NODE\_328441\_length\_53725\_cov\_29.545444 50155-50161. Max. coverage (+): 0.01. Max coverage (-): 0

Region: NODE\_328441\_length\_53725\_cov\_29.545444 50162-50169. Max. coverage (+): 0. Max coverage (-): 0

Region: NODE\_328441\_length\_53725\_cov\_29.545444 50170-50177. Max. coverage (+): 0. Max coverage (-): 0

Region: NODE\_328441\_length\_53725\_cov\_29.545444 50178-50184. Max. coverage (+): 0. Max coverage (-): 0

Region: NODE\_328441\_length\_53725\_cov\_29.545444 50185-50192. Max. coverage (+): 0. Max coverage (-): 0

Region: NODE\_328441\_length\_53725\_cov\_29.545444 50193-50200. Max. coverage (+): 0. Max coverage (-): 0

Region: NODE\_328441\_length\_53725\_cov\_29.545444 50201-50207. Max. coverage (+): 0. Max coverage (-): 0

Region: NODE\_328441\_length\_53725\_cov\_29.545444 50208-50215. Max. coverage (+): 0. Max coverage (-): 0.03

Region: NODE\_328441\_length\_53725\_cov\_29.545444 50216-50223. Max. coverage (+): 0. Max coverage (-): 0.03

Region: NODE\_328441\_length\_53725\_cov\_29.545444 50224-50230. Max. coverage (+): 0. Max coverage (-): 0

Region: NODE\_328441\_length\_53725\_cov\_29.545444 50231-50238. Max. coverage (+): 0. Max coverage (-): 0

Region: NODE\_328441\_length\_53725\_cov\_29.545444 50239-50246. Max. coverage (+): 0. Max coverage (-): 0

Region: NODE\_328441\_length\_53725\_cov\_29.545444 50247-50253. Max. coverage (+): 0. Max coverage (-): 0

Region: NODE\_328441\_length\_53725\_cov\_29.545444 50254-50261. Max. coverage (+): 0. Max coverage (-): 0

Region: NODE\_328441\_length\_53725\_cov\_29.545444 50262-50269. Max. coverage (+): 0. Max coverage (-): 0.11

Region: NODE\_328441\_length\_53725\_cov\_29.545444 50270-50276. Max. coverage (+): 0. Max coverage (-): 0.08

Region: NODE\_328441\_length\_53725\_cov\_29.545444 50277-50284. Max. coverage (+): 0.02. Max coverage (-): 0.08

Region: NODE\_328441\_length\_53725\_cov\_29.545444 50285-50292. Max. coverage (+): 0.03. Max coverage (-): 0

Region: NODE\_328441\_length\_53725\_cov\_29.545444 50293-50299. Max. coverage (+): 0. Max coverage (-): 0

Region: NODE\_328441\_length\_53725\_cov\_29.545444 50300-50307. Max. coverage (+): 0. Max coverage (-): 0

Region: NODE\_328441\_length\_53725\_cov\_29.545444 50308-50315. Max. coverage (+): 0. Max coverage (-): 0.03

Region: NODE\_328441\_length\_53725\_cov\_29.545444 50316-50322. Max. coverage (+): 0. Max coverage (-): 0.03

Region: NODE\_328441\_length\_53725\_cov\_29.545444 50323-50330. Max. coverage (+): 0. Max coverage (-): 0

Region: NODE\_328441\_length\_53725\_cov\_29.545444 50331-50337. Max. coverage (+): 0. Max coverage (-): 0

Region: NODE\_328441\_length\_53725\_cov\_29.545444 50338-50345. Max. coverage (+): 0. Max coverage (-): 0

Region: NODE\_328441\_length\_53725\_cov\_29.545444 50346-50353. Max. coverage (+): 0. Max coverage (-): 0

Region: NODE\_328441\_length\_53725\_cov\_29.545444 50354-50360. Max. coverage (+): 0. Max coverage (-): 0

Region: NODE\_328441\_length\_53725\_cov\_29.545444 50361-50368. Max. coverage (+): 0. Max coverage (-): 0

Region: NODE\_328441\_length\_53725\_cov\_29.545444 50369-50376. Max. coverage (+): 0. Max coverage (-): 0

Region: NODE\_328441\_length\_53725\_cov\_29.545444 50377-50383. Max. coverage (+): 0. Max coverage (-): 0.03

Region: NODE\_328441\_length\_53725\_cov\_29.545444 50384-50391. Max. coverage (+): 0. Max coverage (-): 0.19

Region: NODE\_328441\_length\_53725\_cov\_29.545444 50392-50399. Max. coverage (+): 0. Max coverage (-): 0

Region: NODE\_328441\_length\_53725\_cov\_29.545444 50400-50406. Max. coverage (+): 0. Max coverage (-): 0

Region: NODE\_328441\_length\_53725\_cov\_29.545444 50407-50414. Max. coverage (+): 0. Max coverage (-): 0

Region: NODE\_328441\_length\_53725\_cov\_29.545444 50415-50422. Max. coverage (+): 0. Max coverage (-): 0

Region: NODE\_328441\_length\_53725\_cov\_29.545444 50423-50429. Max. coverage (+): 0. Max coverage (-): 0

Region: NODE\_328441\_length\_53725\_cov\_29.545444 50430-50437. Max. coverage (+): 0. Max coverage (-): 0

Region: NODE\_328441\_length\_53725\_cov\_29.545444 50438-50445. Max. coverage (+): 0. Max coverage (-): 0

Region: NODE\_328441\_length\_53725\_cov\_29.545444 50446-50452. Max. coverage (+): 0. Max coverage (-): 0.02

Region: NODE\_328441\_length\_53725\_cov\_29.545444 50453-50460. Max. coverage (+): 0. Max coverage (-): 0.02

Region: NODE\_328441\_length\_53725\_cov\_29.545444 50461-50468. Max. coverage (+): 0. Max coverage (-): 0

Region: NODE\_328441\_length\_53725\_cov\_29.545444 50469-50475. Max. coverage (+): 0. Max coverage (-): 0

Region: NODE\_328441\_length\_53725\_cov\_29.545444 50476-50483. Max. coverage (+): 0. Max coverage (-): 0

Region: NODE\_328441\_length\_53725\_cov\_29.545444 50484-50491. Max. coverage (+): 0. Max coverage (-): 0

Region: NODE\_328441\_length\_53725\_cov\_29.545444 50492-50498. Max. coverage (+): 0. Max coverage (-): 0

Region: NODE\_328441\_length\_53725\_cov\_29.545444 50499-50506. Max. coverage (+): 0. Max coverage (-): 0

Region: NODE\_328441\_length\_53725\_cov\_29.545444 50507-50514. Max. coverage (+): 0. Max coverage (-): 0

Region: NODE\_328441\_length\_53725\_cov\_29.545444 50515-50521. Max. coverage (+): 0. Max coverage (-): 0

Region: NODE\_328441\_length\_53725\_cov\_29.545444 50522-50529. Max. coverage (+): 0. Max coverage (-): 0

Region: NODE\_328441\_length\_53725\_cov\_29.545444 50530-50537. Max. coverage (+): 0. Max coverage (-): 0

Region: NODE\_328441\_length\_53725\_cov\_29.545444 50538-50544. Max. coverage (+): 0. Max coverage (-): 0

Region: NODE\_328441\_length\_53725\_cov\_29.545444 50545-50552. Max. coverage (+): 0.69. Max coverage (-): 0

Region: NODE\_328441\_length\_53725\_cov\_29.545444 50553-50560. Max. coverage (+): 0.69. Max coverage (-): 0

Region: NODE\_328441\_length\_53725\_cov\_29.545444 50561-50567. Max. coverage (+): 0. Max coverage (-): 0.19

Region: NODE\_328441\_length\_53725\_cov\_29.545444 50568-50575. Max. coverage (+): 0. Max coverage (-): 0

Region: NODE\_328441\_length\_53725\_cov\_29.545444 50576-50583. Max. coverage (+): 0. Max coverage (-): 0

Region: NODE\_328441\_length\_53725\_cov\_29.545444 50584-50590. Max. coverage (+): 0. Max coverage (-): 0

Region: NODE\_328441\_length\_53725\_cov\_29.545444 50591-50598. Max. coverage (+): 0. Max coverage (-): 0

Region: NODE\_328441\_length\_53725\_cov\_29.545444 50599-50606. Max. coverage (+): 0.54. Max coverage (-): 0

Region: NODE\_328441\_length\_53725\_cov\_29.545444 50607-50613. Max. coverage (+): 0.18. Max coverage (-): 0

Region: NODE\_328441\_length\_53725\_cov\_29.545444 50614-50621. Max. coverage (+): 0.04. Max coverage (-): 0

Region: NODE\_328441\_length\_53725\_cov\_29.545444 50622-50629. Max. coverage (+): 0. Max coverage (-): 0.11

Region: NODE\_328441\_length\_53725\_cov\_29.545444 50630-50636. Max. coverage (+): 0. Max coverage (-): 0.11

Region: NODE\_328441\_length\_53725\_cov\_29.545444 50637-50644. Max. coverage (+): 0. Max coverage (-): 0

Region: NODE\_328441\_length\_53725\_cov\_29.545444 50645-50652. Max. coverage (+): 0. Max coverage (-): 0

Region: NODE\_328441\_length\_53725\_cov\_29.545444 50653-50659. Max. coverage (+): 0. Max coverage (-): 0.08

Region: NODE\_328441\_length\_53725\_cov\_29.545444 50660-50667. Max. coverage (+): 0.04. Max coverage (-): 0.11

Region: NODE\_328441\_length\_53725\_cov\_29.545444 50668-50675. Max. coverage (+): 0.04. Max coverage (-): 0.1

Region: NODE\_328441\_length\_53725\_cov\_29.545444 50676-50682. Max. coverage (+): 0.34. Max coverage (-): 0.02

Region: NODE\_328441\_length\_53725\_cov\_29.545444 50683-50690. Max. coverage (+): 0.03. Max coverage (-): 0

Region: NODE\_328441\_length\_53725\_cov\_29.545444 50691-50698. Max. coverage (+): 1.01. Max coverage (-): 0

Region: NODE\_328441\_length\_53725\_cov\_29.545444 50699-50705. Max. coverage (+): 0. Max coverage (-): 0.08

Region: NODE\_328441\_length\_53725\_cov\_29.545444 50706-50713. Max. coverage (+): 0.19. Max coverage (-): 0.08

Region: NODE\_328441\_length\_53725\_cov\_29.545444 50714-50720. Max. coverage (+): 0.19. Max coverage (-): 0.23

Region: NODE\_328441\_length\_53725\_cov\_29.545444 50721-50728. Max. coverage (+): 0.04. Max coverage (-): 1.53

Region: NODE\_328441\_length\_53725\_cov\_29.545444 50729-50736. Max. coverage (+): 0. Max coverage (-): 0.11

Region: NODE\_328441\_length\_53725\_cov\_29.545444 50737-50743. Max. coverage (+): 0. Max coverage (-): 0.08

Region: NODE\_328441\_length\_53725\_cov\_29.545444 50744-50751. Max. coverage (+): 0. Max coverage (-): 0.15

Region: NODE\_328441\_length\_53725\_cov\_29.545444 50752-50759. Max. coverage (+): 0.31. Max coverage (-): 0.15

Region: NODE\_328441\_length\_53725\_cov\_29.545444 50760-50766. Max. coverage (+): 0.31. Max coverage (-): 0

Region: NODE\_328441\_length\_53725\_cov\_29.545444 50767-50774. Max. coverage (+): 0. Max coverage (-): 0.11

Region: NODE\_328441\_length\_53725\_cov\_29.545444 50775-50782. Max. coverage (+): 1.07. Max coverage (-): 0.11

Region: NODE\_328441\_length\_53725\_cov\_29.545444 50783-50789. Max. coverage (+): 2.03. Max coverage (-): 0

Region: NODE\_328441\_length\_53725\_cov\_29.545444 50790-50797. Max. coverage (+): 7.89. Max coverage (-): 0

Region: NODE\_328441\_length\_53725\_cov\_29.545444 50798-50805. Max. coverage (+): 0. Max coverage (-): 0

Region: NODE\_328441\_length\_53725\_cov\_29.545444 50806-50812. Max. coverage (+): 0.04. Max coverage (-): 0.11

Region: NODE\_328441\_length\_53725\_cov\_29.545444 50813-50820. Max. coverage (+): 0.88. Max coverage (-): 0.11

Region: NODE\_328441\_length\_53725\_cov\_29.545444 50821-50828. Max. coverage (+): 0.1. Max coverage (-): 0

Region: NODE\_328441\_length\_53725\_cov\_29.545444 50829-50835. Max. coverage (+): 0. Max coverage (-): 0

Region: NODE\_328441\_length\_53725\_cov\_29.545444 50836-50843. Max. coverage (+): 0. Max coverage (-): 0

Region: NODE\_328441\_length\_53725\_cov\_29.545444 50844-50851. Max. coverage (+): 0. Max coverage (-): 0

Region: NODE\_328441\_length\_53725\_cov\_29.545444 50852-50858. Max. coverage (+): 0. Max coverage (-): 0

Region: NODE\_328441\_length\_53725\_cov\_29.545444 50859-50866. Max. coverage (+): 0. Max coverage (-): 0

Region: NODE\_328441\_length\_53725\_cov\_29.545444 50867-50874. Max. coverage (+): 0. Max coverage (-): 0

Region: NODE\_328441\_length\_53725\_cov\_29.545444 50875-50881. Max. coverage (+): 0. Max coverage (-): 0

Region: NODE\_328441\_length\_53725\_cov\_29.545444 50882-50889. Max. coverage (+): 0.08. Max coverage (-): 0

Region: NODE\_328441\_length\_53725\_cov\_29.545444 50890-50897. Max. coverage (+): 0.08. Max coverage (-): 0

Region: NODE\_328441\_length\_53725\_cov\_29.545444 50898-50904. Max. coverage (+): 0. Max coverage (-): 0

Region: NODE\_328441\_length\_53725\_cov\_29.545444 50905-50912. Max. coverage (+): 0. Max coverage (-): 0

Region: NODE\_328441\_length\_53725\_cov\_29.545444 50913-50920. Max. coverage (+): 0. Max coverage (-): 0

Region: NODE\_328441\_length\_53725\_cov\_29.545444 50921-50927. Max. coverage (+): 0. Max coverage (-): 0

Region: NODE\_328441\_length\_53725\_cov\_29.545444 50928-50935. Max. coverage (+): 0. Max coverage (-): 0.08

Region: NODE\_328441\_length\_53725\_cov\_29.545444 50936-50943. Max. coverage (+): 0. Max coverage (-): 0.18

Region: NODE\_328441\_length\_53725\_cov\_29.545444 50944-50950. Max. coverage (+): 0. Max coverage (-): 0.01

Region: NODE\_328441\_length\_53725\_cov\_29.545444 50951-50958. Max. coverage (+): 23.82. Max coverage (-): 0.01

Region: NODE\_328441\_length\_53725\_cov\_29.545444 50959-50966. Max. coverage (+): 24.7. Max coverage (-): 0.01

Region: NODE\_328441\_length\_53725\_cov\_29.545444 50967-50973. Max. coverage (+): 0.2. Max coverage (-): 0.03

Region: NODE\_328441\_length\_53725\_cov\_29.545444 50974-50981. Max. coverage (+): 0.03. Max coverage (-): 0.02

Region: NODE\_328441\_length\_53725\_cov\_29.545444 50982-50989. Max. coverage (+): 0. Max coverage (-): 0

Region: NODE\_328441\_length\_53725\_cov\_29.545444 50990-50996. Max. coverage (+): 0. Max coverage (-): 0

Region: NODE\_328441\_length\_53725\_cov\_29.545444 50997-51004. Max. coverage (+): 0.21. Max coverage (-): 0

Region: NODE\_328441\_length\_53725\_cov\_29.545444 51005-51012. Max. coverage (+): 0.18. Max coverage (-): 0

Region: NODE\_328441\_length\_53725\_cov\_29.545444 51013-51019. Max. coverage (+): 0.02. Max coverage (-): 0

Region: NODE\_328441\_length\_53725\_cov\_29.545444 51020-51027. Max. coverage (+): 0.08. Max coverage (-): 0.02

Region: NODE\_328441\_length\_53725\_cov\_29.545444 51028-51035. Max. coverage (+): 5.23. Max coverage (-): 0.02

Region: NODE\_328441\_length\_53725\_cov\_29.545444 51036-51042. Max. coverage (+): 0.18. Max coverage (-): 0

Region: NODE\_328441\_length\_53725\_cov\_29.545444 51043-51050. Max. coverage (+): 0.1. Max coverage (-): 0

Region: NODE\_328441\_length\_53725\_cov\_29.545444 51051-51058. Max. coverage (+): 1.18. Max coverage (-): 0.01

Region: NODE\_328441\_length\_53725\_cov\_29.545444 51059-51065. Max. coverage (+): 0.03. Max coverage (-): 0

Region: NODE\_328441\_length\_53725\_cov\_29.545444 51066-51073. Max. coverage (+): 0.03. Max coverage (-): 0

Region: NODE\_328441\_length\_53725\_cov\_29.545444 51074-51081. Max. coverage (+): 0. Max coverage (-): 0.2

Region: NODE\_328441\_length\_53725\_cov\_29.545444 51082-51088. Max. coverage (+): 0.04. Max coverage (-): 0.03

Region: NODE\_328441\_length\_53725\_cov\_29.545444 51089-51096. Max. coverage (+): 0.04. Max coverage (-): 0

Region: NODE\_328441\_length\_53725\_cov\_29.545444 51097-51103. Max. coverage (+): 0. Max coverage (-): 0

Region: NODE\_328441\_length\_53725\_cov\_29.545444 51104-51111. Max. coverage (+): 0. Max coverage (-): 0

Region: NODE\_328441\_length\_53725\_cov\_29.545444 51112-51119. Max. coverage (+): 0. Max coverage (-): 0

Region: NODE\_328441\_length\_53725\_cov\_29.545444 51120-51126. Max. coverage (+): 0. Max coverage (-): 0

Region: NODE\_328441\_length\_53725\_cov\_29.545444 51127-51134. Max. coverage (+): 0. Max coverage (-): 0

Region: NODE\_328441\_length\_53725\_cov\_29.545444 51135-51142. Max. coverage (+): 0.11. Max coverage (-): 0

Region: NODE\_328441\_length\_53725\_cov\_29.545444 51143-51149. Max. coverage (+): 1.96. Max coverage (-): 0.02

Region: NODE\_328441\_length\_53725\_cov\_29.545444 51150-51157. Max. coverage (+): 6.15. Max coverage (-): 0.08

Region: NODE\_328441\_length\_53725\_cov\_29.545444 51158-51165. Max. coverage (+): 21.98. Max coverage (-): 0.23

Region: NODE\_328441\_length\_53725\_cov\_29.545444 51166-51172. Max. coverage (+): 0.61. Max coverage (-): 0.23

Region: NODE\_328441\_length\_53725\_cov\_29.545444 51173-51180. Max. coverage (+): 2.07. Max coverage (-): 0.23

Region: NODE\_328441\_length\_53725\_cov\_29.545444 51181-51188. Max. coverage (+): 2.45. Max coverage (-): 0

Region: NODE\_328441\_length\_53725\_cov\_29.545444 51189-51195. Max. coverage (+): 2.65. Max coverage (-): 0.03

Region: NODE\_328441\_length\_53725\_cov\_29.545444 51196-51203. Max. coverage (+): 0.7. Max coverage (-): 0.28

Region: NODE\_328441\_length\_53725\_cov\_29.545444 51204-51211. Max. coverage (+): 0.01. Max coverage (-): 2.6

Region: NODE\_328441\_length\_53725\_cov\_29.545444 51212-51218. Max. coverage (+): 0.11. Max coverage (-): 0.34

Region: NODE\_328441\_length\_53725\_cov\_29.545444 51219-51226. Max. coverage (+): 3.68. Max coverage (-): 0

Region: NODE\_328441\_length\_53725\_cov\_29.545444 51227-51234. Max. coverage (+): 3.27. Max coverage (-): 0.03

Region: NODE\_328441\_length\_53725\_cov\_29.545444 51235-51241. Max. coverage (+): 0.2. Max coverage (-): 0

Region: NODE\_328441\_length\_53725\_cov\_29.545444 51242-51249. Max. coverage (+): 0. Max coverage (-): 0.03

Region: NODE\_328441\_length\_53725\_cov\_29.545444 51250-51257. Max. coverage (+): 0.02. Max coverage (-): 0.11

Region: NODE\_328441\_length\_53725\_cov\_29.545444 51258-51264. Max. coverage (+): 0.04. Max coverage (-): 0.09

Region: NODE\_328441\_length\_53725\_cov\_29.545444 51265-51272. Max. coverage (+): 0.11. Max coverage (-): 0

Region: NODE\_328441\_length\_53725\_cov\_29.545444 51273-51280. Max. coverage (+): 1.16. Max coverage (-): 0

Region: NODE\_328441\_length\_53725\_cov\_29.545444 51281-51287. Max. coverage (+): 0.21. Max coverage (-): 0

Region: NODE\_328441\_length\_53725\_cov\_29.545444 51288-51295. Max. coverage (+): 0.04. Max coverage (-): 0.04

Region: NODE\_328441\_length\_53725\_cov\_29.545444 51296-51303. Max. coverage (+): 0. Max coverage (-): 0.19

Region: NODE\_328441\_length\_53725\_cov\_29.545444 51304-51310. Max. coverage (+): 0.52. Max coverage (-): 0.04

Region: NODE\_328441\_length\_53725\_cov\_29.545444 51311-51318. Max. coverage (+): 0.69. Max coverage (-): 0.02

Region: NODE\_328441\_length\_53725\_cov\_29.545444 51319-51326. Max. coverage (+): 0.04. Max coverage (-): 0.04

Region: NODE\_328441\_length\_53725\_cov\_29.545444 51327-51333. Max. coverage (+): 0.04. Max coverage (-): 0

Region: NODE\_328441\_length\_53725\_cov\_29.545444 51334-51341. Max. coverage (+): 32.42. Max coverage (-): 0

Region: NODE\_328441\_length\_53725\_cov\_29.545444 51342-51349. Max. coverage (+): 32.7. Max coverage (-): 0

Region: NODE\_328441\_length\_53725\_cov\_29.545444 51350-51356. Max. coverage (+): 0.18. Max coverage (-): 0

Region: NODE\_328441\_length\_53725\_cov\_29.545444 51357-51364. Max. coverage (+): 0.04. Max coverage (-): 0

Region: NODE\_328441\_length\_53725\_cov\_29.545444 51365-51372. Max. coverage (+): 0.11. Max coverage (-): 0

Region: NODE\_328441\_length\_53725\_cov\_29.545444 51373-51379. Max. coverage (+): 0.15. Max coverage (-): 0.15

Region: NODE\_328441\_length\_53725\_cov\_29.545444 51380-51387. Max. coverage (+): 0.11. Max coverage (-): 0.15

Region: NODE\_328441\_length\_53725\_cov\_29.545444 51388-51395. Max. coverage (+): 0.1. Max coverage (-): 0

Region: NODE\_328441\_length\_53725\_cov\_29.545444 51396-51402. Max. coverage (+): 0.03. Max coverage (-): 0

Region: NODE\_328441\_length\_53725\_cov\_29.545444 51403-51410. Max. coverage (+): 0.15. Max coverage (-): 0.08

Region: NODE\_328441\_length\_53725\_cov\_29.545444 51411-51418. Max. coverage (+): 0. Max coverage (-): 0.31

Region: NODE\_328441\_length\_53725\_cov\_29.545444 51419-51425. Max. coverage (+): 0. Max coverage (-): 0.23

Region: NODE\_328441\_length\_53725\_cov\_29.545444 51426-51433. Max. coverage (+): 5.67. Max coverage (-): 0

Region: NODE\_328441\_length\_53725\_cov\_29.545444 51434-51441. Max. coverage (+): 1.46. Max coverage (-): 0.15

Region: NODE\_328441\_length\_53725\_cov\_29.545444 51442-51448. Max. coverage (+): 0. Max coverage (-): 0.27

Region: NODE\_328441\_length\_53725\_cov\_29.545444 51449-51456. Max. coverage (+): 0.06. Max coverage (-): 0.31

Region: NODE\_328441\_length\_53725\_cov\_29.545444 51457-51464. Max. coverage (+): 0.11. Max coverage (-): 0.06

Region: NODE\_328441\_length\_53725\_cov\_29.545444 51465-51471. Max. coverage (+): 0.11. Max coverage (-): 0.29

Region: NODE\_328441\_length\_53725\_cov\_29.545444 51472-51479. Max. coverage (+): 0. Max coverage (-): 0.25

Region: NODE\_328441\_length\_53725\_cov\_29.545444 51480-51486. Max. coverage (+): 0. Max coverage (-): 0

Region: NODE\_328441\_length\_53725\_cov\_29.545444 51487-51494. Max. coverage (+): 0. Max coverage (-): 0

Region: NODE\_328441\_length\_53725\_cov\_29.545444 51495-51502. Max. coverage (+): 0. Max coverage (-): 0

Region: NODE\_328441\_length\_53725\_cov\_29.545444 51503-51509. Max. coverage (+): 0.14. Max coverage (-): 0

Region: NODE\_328441\_length\_53725\_cov\_29.545444 51510-51517. Max. coverage (+): 0.1. Max coverage (-): 0

Region: NODE\_328441\_length\_53725\_cov\_29.545444 51518-51525. Max. coverage (+): 0. Max coverage (-): 0

Region: NODE\_328441\_length\_53725\_cov\_29.545444 51526-51532. Max. coverage (+): 0. Max coverage (-): 0.04

Region: NODE\_328441\_length\_53725\_cov\_29.545444 51533-51540. Max. coverage (+): 0. Max coverage (-): 0.04

Region: NODE\_328441\_length\_53725\_cov\_29.545444 51541-51548. Max. coverage (+): 0. Max coverage (-): 0

Region: NODE\_328441\_length\_53725\_cov\_29.545444 51549-51555. Max. coverage (+): 0. Max coverage (-): 0

Region: NODE\_328441\_length\_53725\_cov\_29.545444 51556-51563. Max. coverage (+): 0. Max coverage (-): 0

Region: NODE\_328441\_length\_53725\_cov\_29.545444 51564-51571. Max. coverage (+): 0.05. Max coverage (-): 0

Region: NODE\_328441\_length\_53725\_cov\_29.545444 51572-51578. Max. coverage (+): 0.08. Max coverage (-): 0

Region: NODE\_328441\_length\_53725\_cov\_29.545444 51579-51586. Max. coverage (+): 0.04. Max coverage (-): 0

Region: NODE\_328441\_length\_53725\_cov\_29.545444 51587-51594. Max. coverage (+): 0. Max coverage (-): 0

Region: NODE\_328441\_length\_53725\_cov\_29.545444 51595-51601. Max. coverage (+): 0. Max coverage (-): 0

Region: NODE\_328441\_length\_53725\_cov\_29.545444 51602-51609. Max. coverage (+): 0.08. Max coverage (-): 0

Region: NODE\_328441\_length\_53725\_cov\_29.545444 51610-51617. Max. coverage (+): 0. Max coverage (-): 0

Region: NODE\_328441\_length\_53725\_cov\_29.545444 51618-51624. Max. coverage (+): 0. Max coverage (-): 0

Region: NODE\_328441\_length\_53725\_cov\_29.545444 51625-51632. Max. coverage (+): 0. Max coverage (-): 0

Region: NODE\_328441\_length\_53725\_cov\_29.545444 51633-51640. Max. coverage (+): 0. Max coverage (-): 0

Region: NODE\_328441\_length\_53725\_cov\_29.545444 51641-51647. Max. coverage (+): 0.11. Max coverage (-): 0

Region: NODE\_328441\_length\_53725\_cov\_29.545444 51648-51655. Max. coverage (+): 0.2. Max coverage (-): 0

Region: NODE\_328441\_length\_53725\_cov\_29.545444 51656-51663. Max. coverage (+): 0.21. Max coverage (-): 0.06

Region: NODE\_328441\_length\_53725\_cov\_29.545444 51664-51670. Max. coverage (+): 0. Max coverage (-): 0

Region: NODE\_328441\_length\_53725\_cov\_29.545444 51671-51678. Max. coverage (+): 0. Max coverage (-): 0

Region: NODE\_328441\_length\_53725\_cov\_29.545444 51679-51686. Max. coverage (+): 0. Max coverage (-): 0

Region: NODE\_328441\_length\_53725\_cov\_29.545444 51687-51693. Max. coverage (+): 0. Max coverage (-): 0

Region: NODE\_328441\_length\_53725\_cov\_29.545444 51694-51701. Max. coverage (+): 0.13. Max coverage (-): 0

Region: NODE\_328441\_length\_53725\_cov\_29.545444 51702-51709. Max. coverage (+): 1.05. Max coverage (-): 0

Region: NODE\_328441\_length\_53725\_cov\_29.545444 51710-51716. Max. coverage (+): 0.02. Max coverage (-): 0

Region: NODE\_328441\_length\_53725\_cov\_29.545444 51717-51724. Max. coverage (+): 0. Max coverage (-): 0.08

Region: NODE\_328441\_length\_53725\_cov\_29.545444 51725-51732. Max. coverage (+): 0. Max coverage (-): 0

Region: NODE\_328441\_length\_53725\_cov\_29.545444 51733-51739. Max. coverage (+): 0.08. Max coverage (-): 0

Region: NODE\_328441\_length\_53725\_cov\_29.545444 51740-51747. Max. coverage (+): 0.1. Max coverage (-): 0

Region: NODE\_328441\_length\_53725\_cov\_29.545444 51748-51755. Max. coverage (+): 4.71. Max coverage (-): 0

Region: NODE\_328441\_length\_53725\_cov\_29.545444 51756-51762. Max. coverage (+): 4.74. Max coverage (-): 0.09

Region: NODE\_328441\_length\_53725\_cov\_29.545444 51763-51770. Max. coverage (+): 0.46. Max coverage (-): 0.09

Region: NODE\_328441\_length\_53725\_cov\_29.545444 51771-51778. Max. coverage (+): 0.02. Max coverage (-): 0.1

Region: NODE\_328441\_length\_53725\_cov\_29.545444 51779-51785. Max. coverage (+): 0.04. Max coverage (-): 0.1

Region: NODE\_328441\_length\_53725\_cov\_29.545444 51786-51793. Max. coverage (+): 0.49. Max coverage (-): 0.04

Region: NODE\_328441\_length\_53725\_cov\_29.545444 51794-51801. Max. coverage (+): 1.7. Max coverage (-): 0.01

Region: NODE\_328441\_length\_53725\_cov\_29.545444 51802-51808. Max. coverage (+): 0. Max coverage (-): 0.31

Region: NODE\_328441\_length\_53725\_cov\_29.545444 51809-51816. Max. coverage (+): 0. Max coverage (-): 0.31

Region: NODE\_328441\_length\_53725\_cov\_29.545444 51817-51824. Max. coverage (+): 0. Max coverage (-): 0.03

Region: NODE\_328441\_length\_53725\_cov\_29.545444 51825-51831. Max. coverage (+): 0.1. Max coverage (-): 0

Region: NODE\_328441\_length\_53725\_cov\_29.545444 51832-51839. Max. coverage (+): 0.08. Max coverage (-): 0.1

Region: NODE\_328441\_length\_53725\_cov\_29.545444 51840-51847. Max. coverage (+): 0. Max coverage (-): 0.15

Region: NODE\_328441\_length\_53725\_cov\_29.545444 51848-51854. Max. coverage (+): 0. Max coverage (-): 0

Region: NODE\_328441\_length\_53725\_cov\_29.545444 51855-51862. Max. coverage (+): 3.22. Max coverage (-): 0

Region: NODE\_328441\_length\_53725\_cov\_29.545444 51863-51869. Max. coverage (+): 0.94. Max coverage (-): 0

Region: NODE\_328441\_length\_53725\_cov\_29.545444 51870-51877. Max. coverage (+): 0. Max coverage (-): 0

Region: NODE\_328441\_length\_53725\_cov\_29.545444 51878-51885. Max. coverage (+): 0. Max coverage (-): 0

Region: NODE\_328441\_length\_53725\_cov\_29.545444 51886-51892. Max. coverage (+): 0.23. Max coverage (-): 0.03

Region: NODE\_328441\_length\_53725\_cov\_29.545444 51893-51900. Max. coverage (+): 0.2. Max coverage (-): 0

Region: NODE\_328441\_length\_53725\_cov\_29.545444 51901-51908. Max. coverage (+): 0.74. Max coverage (-): 0.02

Region: NODE\_328441\_length\_53725\_cov\_29.545444 51909-51915. Max. coverage (+): 0.28. Max coverage (-): 0

Region: NODE\_328441\_length\_53725\_cov\_29.545444 51916-51923. Max. coverage (+): 0.23. Max coverage (-): 0

Region: NODE\_328441\_length\_53725\_cov\_29.545444 51924-51931. Max. coverage (+): 0.23. Max coverage (-): 0

Region: NODE\_328441\_length\_53725\_cov\_29.545444 51932-51938. Max. coverage (+): 3.73. Max coverage (-): 0.05

Region: NODE\_328441\_length\_53725\_cov\_29.545444 51939-51946. Max. coverage (+): 3.73. Max coverage (-): 0.33

Region: NODE\_328441\_length\_53725\_cov\_29.545444 51947-51954. Max. coverage (+): 0. Max coverage (-): 0.33

Region: NODE\_328441\_length\_53725\_cov\_29.545444 51955-51961. Max. coverage (+): 0. Max coverage (-): 0.08

Region: NODE\_328441\_length\_53725\_cov\_29.545444 51962-51969. Max. coverage (+): 0. Max coverage (-): 0

Region: NODE\_328441\_length\_53725\_cov\_29.545444 51970-51977. Max. coverage (+): 0. Max coverage (-): 0

Region: NODE\_328441\_length\_53725\_cov\_29.545444 51978-51984. Max. coverage (+): 0. Max coverage (-): 0.2

Region: NODE\_328441\_length\_53725\_cov\_29.545444 51985-51992. Max. coverage (+): 0.35. Max coverage (-): 0.15

Region: NODE\_328441\_length\_53725\_cov\_29.545444 51993-52000. Max. coverage (+): 0.39. Max coverage (-): 0.07

Region: NODE\_328441\_length\_53725\_cov\_29.545444 52001-52007. Max. coverage (+): 0.1. Max coverage (-): 0

Region: NODE\_328441\_length\_53725\_cov\_29.545444 52008-52015. Max. coverage (+): 0. Max coverage (-): 0

Region: NODE\_328441\_length\_53725\_cov\_29.545444 52016-52023. Max. coverage (+): 0. Max coverage (-): 0

Region: NODE\_328441\_length\_53725\_cov\_29.545444 52024-52030. Max. coverage (+): 0.03. Max coverage (-): 0

Region: NODE\_328441\_length\_53725\_cov\_29.545444 52031-52038. Max. coverage (+): 0.77. Max coverage (-): 0.12

Region: NODE\_328441\_length\_53725\_cov\_29.545444 52039-52046. Max. coverage (+): 0.35. Max coverage (-): 0

Region: NODE\_328441\_length\_53725\_cov\_29.545444 52047-52053. Max. coverage (+): 0.4. Max coverage (-): 0.04

Region: NODE\_328441\_length\_53725\_cov\_29.545444 52054-52061. Max. coverage (+): 0.15. Max coverage (-): 0.06

Region: NODE\_328441\_length\_53725\_cov\_29.545444 52062-52069. Max. coverage (+): 0.25. Max coverage (-): 0.04

Region: NODE\_328441\_length\_53725\_cov\_29.545444 52070-52076. Max. coverage (+): 0.23. Max coverage (-): 0

Region: NODE\_328441\_length\_53725\_cov\_29.545444 52077-52084. Max. coverage (+): 3.62. Max coverage (-): 0.04

Region: NODE\_328441\_length\_53725\_cov\_29.545444 52085-52092. Max. coverage (+): 0.06. Max coverage (-): 0.42

Region: NODE\_328441\_length\_53725\_cov\_29.545444 52093-52099. Max. coverage (+): 0.03. Max coverage (-): 0.58

Region: NODE\_328441\_length\_53725\_cov\_29.545444 52100-52107. Max. coverage (+): 0.51. Max coverage (-): 0

Region: NODE\_328441\_length\_53725\_cov\_29.545444 52108-52115. Max. coverage (+): 0.43. Max coverage (-): 0

Region: NODE\_328441\_length\_53725\_cov\_29.545444 52116-52122. Max. coverage (+): 0.08. Max coverage (-): 0.1

Region: NODE\_328441\_length\_53725\_cov\_29.545444 52123-52130. Max. coverage (+): 0.05. Max coverage (-): 0.51

Region: NODE\_328441\_length\_53725\_cov\_29.545444 52131-52138. Max. coverage (+): 7.84. Max coverage (-): 0.28

Region: NODE\_328441\_length\_53725\_cov\_29.545444 52139-52145. Max. coverage (+): 7.74. Max coverage (-): 0

Region: NODE\_328441\_length\_53725\_cov\_29.545444 52146-52153. Max. coverage (+): 0.06. Max coverage (-): 0.02

Region: NODE\_328441\_length\_53725\_cov\_29.545444 52154-52161. Max. coverage (+): 0. Max coverage (-): 1.93

Region: NODE\_328441\_length\_53725\_cov\_29.545444 52162-52168. Max. coverage (+): 0.12. Max coverage (-): 2.05

Region: NODE\_328441\_length\_53725\_cov\_29.545444 52169-52176. Max. coverage (+): 0.21. Max coverage (-): 0.05

Region: NODE\_328441\_length\_53725\_cov\_29.545444 52177-52184. Max. coverage (+): 0.2. Max coverage (-): 0.04

Region: NODE\_328441\_length\_53725\_cov\_29.545444 52185-52191. Max. coverage (+): 0.12. Max coverage (-): 0.2

Region: NODE\_328441\_length\_53725\_cov\_29.545444 52192-52199. Max. coverage (+): 0. Max coverage (-): 0.01

Region: NODE\_328441\_length\_53725\_cov\_29.545444 52200-52207. Max. coverage (+): 0. Max coverage (-): 0

Region: NODE\_328441\_length\_53725\_cov\_29.545444 52208-52214. Max. coverage (+): 0. Max coverage (-): 0

Region: NODE\_328441\_length\_53725\_cov\_29.545444 52215-52222. Max. coverage (+): 0.1. Max coverage (-): 0.02

Region: NODE\_328441\_length\_53725\_cov\_29.545444 52223-52230. Max. coverage (+): 0.35. Max coverage (-): 0.29

Region: NODE\_328441\_length\_53725\_cov\_29.545444 52231-52237. Max. coverage (+): 0.12. Max coverage (-): 0.11

Region: NODE\_328441\_length\_53725\_cov\_29.545444 52238-52245. Max. coverage (+): 0. Max coverage (-): 0.08

Region: NODE\_328441\_length\_53725\_cov\_29.545444 52246-52252. Max. coverage (+): 0. Max coverage (-): 0

Region: NODE\_328441\_length\_53725\_cov\_29.545444 52253-52260. Max. coverage (+): 0. Max coverage (-): 0

Region: NODE\_328441\_length\_53725\_cov\_29.545444 52261-52268. Max. coverage (+): 0.38. Max coverage (-): 0.03

Region: NODE\_328441\_length\_53725\_cov\_29.545444 52269-52275. Max. coverage (+): 0.05. Max coverage (-): 0

Region: NODE\_328441\_length\_53725\_cov\_29.545444 52276-52283. Max. coverage (+): 0.17. Max coverage (-): 0

Region: NODE\_328441\_length\_53725\_cov\_29.545444 52284-52291. Max. coverage (+): 0. Max coverage (-): 0

Region: NODE\_328441\_length\_53725\_cov\_29.545444 52292-52298. Max. coverage (+): 0. Max coverage (-): 0.12

Region: NODE\_328441\_length\_53725\_cov\_29.545444 52299-52306. Max. coverage (+): 0.14. Max coverage (-): 0.18

Region: NODE\_328441\_length\_53725\_cov\_29.545444 52307-52314. Max. coverage (+): 0.7. Max coverage (-): 0.16

Region: NODE\_328441\_length\_53725\_cov\_29.545444 52315-52321. Max. coverage (+): 0.63. Max coverage (-): 0.16

Region: NODE\_328441\_length\_53725\_cov\_29.545444 52322-52329. Max. coverage (+): 0.1. Max coverage (-): 0.04

Region: NODE\_328441\_length\_53725\_cov\_29.545444 52330-52337. Max. coverage (+): 0.27. Max coverage (-): 0.06

Region: NODE\_328441\_length\_53725\_cov\_29.545444 52338-52344. Max. coverage (+): 1.03. Max coverage (-): 0.04

Region: NODE\_328441\_length\_53725\_cov\_29.545444 52345-52352. Max. coverage (+): 0.77. Max coverage (-): 0.17

Region: NODE\_328441\_length\_53725\_cov\_29.545444 52353-52360. Max. coverage (+): 0.15. Max coverage (-): 0.19

Region: NODE\_328441\_length\_53725\_cov\_29.545444 52361-52367. Max. coverage (+): 0.1. Max coverage (-): 0

Region: NODE\_328441\_length\_53725\_cov\_29.545444 52368-52375. Max. coverage (+): 1.43. Max coverage (-): 0.04

Region: NODE\_328441\_length\_53725\_cov\_29.545444 52376-52383. Max. coverage (+): 0.1. Max coverage (-): 0.03

Region: NODE\_328441\_length\_53725\_cov\_29.545444 52384-52390. Max. coverage (+): 0.02. Max coverage (-): 0

Region: NODE\_328441\_length\_53725\_cov\_29.545444 52391-52398. Max. coverage (+): 0.03. Max coverage (-): 0

Region: NODE\_328441\_length\_53725\_cov\_29.545444 52399-52406. Max. coverage (+): 0.03. Max coverage (-): 0.05

Region: NODE\_328441\_length\_53725\_cov\_29.545444 52407-52413. Max. coverage (+): 0.36. Max coverage (-): 0.02

Region: NODE\_328441\_length\_53725\_cov\_29.545444 52414-52421. Max. coverage (+): 0.48. Max coverage (-): 0

Region: NODE\_328441\_length\_53725\_cov\_29.545444 52422-52429. Max. coverage (+): 0.06. Max coverage (-): 0

Region: NODE\_328441\_length\_53725\_cov\_29.545444 52430-52436. Max. coverage (+): 0. Max coverage (-): 0

Region: NODE\_328441\_length\_53725\_cov\_29.545444 52437-52444. Max. coverage (+): 0. Max coverage (-): 0

Region: NODE\_328441\_length\_53725\_cov\_29.545444 52445-52452. Max. coverage (+): 0. Max coverage (-): 0

Region: NODE\_328441\_length\_53725\_cov\_29.545444 52453-52459. Max. coverage (+): 0. Max coverage (-): 0

Region: NODE\_328441\_length\_53725\_cov\_29.545444 52460-52467. Max. coverage (+): 0. Max coverage (-): 0

Region: NODE\_328441\_length\_53725\_cov\_29.545444 52468-52475. Max. coverage (+): 0. Max coverage (-): 0

Region: NODE\_328441\_length\_53725\_cov\_29.545444 52476-52482. Max. coverage (+): 0. Max coverage (-): 0

Region: NODE\_328441\_length\_53725\_cov\_29.545444 52483-52490. Max. coverage (+): 0. Max coverage (-): 0

Region: NODE\_328441\_length\_53725\_cov\_29.545444 52491-52498. Max. coverage (+): 0. Max coverage (-): 0

Region: NODE\_328441\_length\_53725\_cov\_29.545444 52499-52505. Max. coverage (+): 0. Max coverage (-): 0

Region: NODE\_328441\_length\_53725\_cov\_29.545444 52506-52513. Max. coverage (+): 0. Max coverage (-): 0

Region: NODE\_328441\_length\_53725\_cov\_29.545444 52514-52521. Max. coverage (+): 0. Max coverage (-): 0

Region: NODE\_328441\_length\_53725\_cov\_29.545444 52522-52528. Max. coverage (+): 0. Max coverage (-): 0

Region: NODE\_328441\_length\_53725\_cov\_29.545444 52529-52536. Max. coverage (+): 0. Max coverage (-): 0

Region: NODE\_328441\_length\_53725\_cov\_29.545444 52537-52544. Max. coverage (+): 0. Max coverage (-): 0

Region: NODE\_328441\_length\_53725\_cov\_29.545444 52545-52551. Max. coverage (+): 0. Max coverage (-): 0

Region: NODE\_328441\_length\_53725\_cov\_29.545444 52552-52559. Max. coverage (+): 0. Max coverage (-): 0

Region: NODE\_328441\_length\_53725\_cov\_29.545444 52560-52567. Max. coverage (+): 1.98. Max coverage (-): 0

Region: NODE\_328441\_length\_53725\_cov\_29.545444 52568-52574. Max. coverage (+): 0.25. Max coverage (-): 0

Region: NODE\_328441\_length\_53725\_cov\_29.545444 52575-52582. Max. coverage (+): 0.05. Max coverage (-): 0

Region: NODE\_328441\_length\_53725\_cov\_29.545444 52583-52590. Max. coverage (+): 0.13. Max coverage (-): 0

Region: NODE\_328441\_length\_53725\_cov\_29.545444 52591-52597. Max. coverage (+): 2.49. Max coverage (-): 0

Region: NODE\_328441\_length\_53725\_cov\_29.545444 52598-52605. Max. coverage (+): 1.08. Max coverage (-): 0.28

Region: NODE\_328441\_length\_53725\_cov\_29.545444 52606-52613. Max. coverage (+): 0. Max coverage (-): 4.72

Region: NODE\_328441\_length\_53725\_cov\_29.545444 52614-52620. Max. coverage (+): 0. Max coverage (-): 0.03

Region: NODE\_328441\_length\_53725\_cov\_29.545444 52621-52628. Max. coverage (+): 4.29. Max coverage (-): 0

Region: NODE\_328441\_length\_53725\_cov\_29.545444 52629-52635. Max. coverage (+): 3.87. Max coverage (-): 0

Region: NODE\_328441\_length\_53725\_cov\_29.545444 52636-52643. Max. coverage (+): 0. Max coverage (-): 0

Region: NODE\_328441\_length\_53725\_cov\_29.545444 52644-52651. Max. coverage (+): 0. Max coverage (-): 0.08

Region: NODE\_328441\_length\_53725\_cov\_29.545444 52652-52658. Max. coverage (+): 0.08. Max coverage (-): 0.04

Region: NODE\_328441\_length\_53725\_cov\_29.545444 52659-52666. Max. coverage (+): 0.54. Max coverage (-): 0

Region: NODE\_328441\_length\_53725\_cov\_29.545444 52667-52674. Max. coverage (+): 0. Max coverage (-): 0.08

Region: NODE\_328441\_length\_53725\_cov\_29.545444 52675-52681. Max. coverage (+): 0. Max coverage (-): 0

Region: NODE\_328441\_length\_53725\_cov\_29.545444 52682-52689. Max. coverage (+): 0.5. Max coverage (-): 0

Region: NODE\_328441\_length\_53725\_cov\_29.545444 52690-52697. Max. coverage (+): 0.5. Max coverage (-): 0

Region: NODE\_328441\_length\_53725\_cov\_29.545444 52698-52704. Max. coverage (+): 0. Max coverage (-): 0

Region: NODE\_328441\_length\_53725\_cov\_29.545444 52705-52712. Max. coverage (+): 0. Max coverage (-): 0

Region: NODE\_328441\_length\_53725\_cov\_29.545444 52713-52720. Max. coverage (+): 0.3. Max coverage (-): 0

Region: NODE\_328441\_length\_53725\_cov\_29.545444 52721-52727. Max. coverage (+): 0.3. Max coverage (-): 0

Region: NODE\_328441\_length\_53725\_cov\_29.545444 52728-52735. Max. coverage (+): 0.09. Max coverage (-): 0.03

Region: NODE\_328441\_length\_53725\_cov\_29.545444 52736-52743. Max. coverage (+): 0. Max coverage (-): 0.03

Region: NODE\_328441\_length\_53725\_cov\_29.545444 52744-52750. Max. coverage (+): 0. Max coverage (-): 0

Region: NODE\_328441\_length\_53725\_cov\_29.545444 52751-52758. Max. coverage (+): 0. Max coverage (-): 0

Region: NODE\_328441\_length\_53725\_cov\_29.545444 52759-52766. Max. coverage (+): 0. Max coverage (-): 0

Region: NODE\_328441\_length\_53725\_cov\_29.545444 52767-52773. Max. coverage (+): 0. Max coverage (-): 0

Region: NODE\_328441\_length\_53725\_cov\_29.545444 52774-52781. Max. coverage (+): 0. Max coverage (-): 0

Region: NODE\_328441\_length\_53725\_cov\_29.545444 52782-52789. Max. coverage (+): 0. Max coverage (-): 0

Region: NODE\_328441\_length\_53725\_cov\_29.545444 52790-52796. Max. coverage (+): 0. Max coverage (-): 0

Region: NODE\_328441\_length\_53725\_cov\_29.545444 52797-52804. Max. coverage (+): 0. Max coverage (-): 0

Region: NODE\_328441\_length\_53725\_cov\_29.545444 52805-52812. Max. coverage (+): 0. Max coverage (-): 0

Region: NODE\_328441\_length\_53725\_cov\_29.545444 52813-52819. Max. coverage (+): 0. Max coverage (-): 0.04

Region: NODE\_328441\_length\_53725\_cov\_29.545444 52820-52827. Max. coverage (+): 0.03. Max coverage (-): 0

Region: NODE\_328441\_length\_53725\_cov\_29.545444 52828-52835. Max. coverage (+): 0. Max coverage (-): 0

Region: NODE\_328441\_length\_53725\_cov\_29.545444 52836-52842. Max. coverage (+): 0. Max coverage (-): 0

Region: NODE\_328441\_length\_53725\_cov\_29.545444 52843-52850. Max. coverage (+): 0. Max coverage (-): 0

Region: NODE\_328441\_length\_53725\_cov\_29.545444 52851-52858. Max. coverage (+): 0. Max coverage (-): 0

Region: NODE\_328441\_length\_53725\_cov\_29.545444 52859-52865. Max. coverage (+): 0. Max coverage (-): 0

Region: NODE\_328441\_length\_53725\_cov\_29.545444 52866-52873. Max. coverage (+): 0. Max coverage (-): 0.04

Region: NODE\_328441\_length\_53725\_cov\_29.545444 52874-52881. Max. coverage (+): 0. Max coverage (-): 0.04

Region: NODE\_328441\_length\_53725\_cov\_29.545444 52882-52888. Max. coverage (+): 2.38. Max coverage (-): 0

Region: NODE\_328441\_length\_53725\_cov\_29.545444 52889-52896. Max. coverage (+): 2.83. Max coverage (-): 0

Region: NODE\_328441\_length\_53725\_cov\_29.545444 52897-52904. Max. coverage (+): 0.31. Max coverage (-): 0

Region: NODE\_328441\_length\_53725\_cov\_29.545444 52905-52911. Max. coverage (+): 0.08. Max coverage (-): 0.04

Region: NODE\_328441\_length\_53725\_cov\_29.545444 52912-52919. Max. coverage (+): 0. Max coverage (-): 0

Region: NODE\_328441\_length\_53725\_cov\_29.545444 52920-52927. Max. coverage (+): 0. Max coverage (-): 0

Region: NODE\_328441\_length\_53725\_cov\_29.545444 52928-52934. Max. coverage (+): 0. Max coverage (-): 0

Region: NODE\_328441\_length\_53725\_cov\_29.545444 52935-52942. Max. coverage (+): 0. Max coverage (-): 0

Region: NODE\_328441\_length\_53725\_cov\_29.545444 52943-52950. Max. coverage (+): 0. Max coverage (-): 0

Region: NODE\_328441\_length\_53725\_cov\_29.545444 52951-52957. Max. coverage (+): 0. Max coverage (-): 0

Region: NODE\_328441\_length\_53725\_cov\_29.545444 52958-52965. Max. coverage (+): 0. Max coverage (-): 0

Region: NODE\_328441\_length\_53725\_cov\_29.545444 52966-52973. Max. coverage (+): 0.33. Max coverage (-): 2.38

Region: NODE\_328441\_length\_53725\_cov\_29.545444 52974-52980. Max. coverage (+): 0. Max coverage (-): 3.56

Region: NODE\_328441\_length\_53725\_cov\_29.545444 52981-52988. Max. coverage (+): 0.08. Max coverage (-): 0.23

Region: NODE\_328441\_length\_53725\_cov\_29.545444 52989-52996. Max. coverage (+): 0. Max coverage (-): 0

Region: NODE\_328441\_length\_53725\_cov\_29.545444 52997-53003. Max. coverage (+): 0. Max coverage (-): 0

Region: NODE\_328441\_length\_53725\_cov\_29.545444 53004-53011. Max. coverage (+): 0. Max coverage (-): 0.08

Region: NODE\_328441\_length\_53725\_cov\_29.545444 53012-53018. Max. coverage (+): 0. Max coverage (-): 0

Region: NODE\_328441\_length\_53725\_cov\_29.545444 53019-53026. Max. coverage (+): 0. Max coverage (-): 0

Region: NODE\_328441\_length\_53725\_cov\_29.545444 53027-53034. Max. coverage (+): 0.08. Max coverage (-): 0

Region: NODE\_328441\_length\_53725\_cov\_29.545444 53035-53041. Max. coverage (+): 0.08. Max coverage (-): 0

Region: NODE\_328441\_length\_53725\_cov\_29.545444 53042-53049. Max. coverage (+): 0. Max coverage (-): 0

Region: NODE\_328441\_length\_53725\_cov\_29.545444 53050-53057. Max. coverage (+): 0. Max coverage (-): 0

Region: NODE\_328441\_length\_53725\_cov\_29.545444 53058-53064. Max. coverage (+): 0. Max coverage (-): 0

Region: NODE\_328441\_length\_53725\_cov\_29.545444 53065-53072. Max. coverage (+): 0. Max coverage (-): 0

Region: NODE\_328441\_length\_53725\_cov\_29.545444 53073-53080. Max. coverage (+): 0. Max coverage (-): 0

Region: NODE\_328441\_length\_53725\_cov\_29.545444 53081-53087. Max. coverage (+): 0. Max coverage (-): 0

Region: NODE\_328441\_length\_53725\_cov\_29.545444 53088-53095. Max. coverage (+): 0. Max coverage (-): 0.03

Region: NODE\_328441\_length\_53725\_cov\_29.545444 53096-53103. Max. coverage (+): 0. Max coverage (-): 0

Region: NODE\_328441\_length\_53725\_cov\_29.545444 53104-53110. Max. coverage (+): 0. Max coverage (-): 0

Region: NODE\_328441\_length\_53725\_cov\_29.545444 53111-53118. Max. coverage (+): 0. Max coverage (-): 0

Region: NODE\_328441\_length\_53725\_cov\_29.545444 53119-53126. Max. coverage (+): 0. Max coverage (-): 0.08

Region: NODE\_328441\_length\_53725\_cov\_29.545444 53127-53133. Max. coverage (+): 0. Max coverage (-): 0.08

Region: NODE\_328441\_length\_53725\_cov\_29.545444 53134-53141. Max. coverage (+): 0. Max coverage (-): 0

Region: NODE\_328441\_length\_53725\_cov\_29.545444 53142-53149. Max. coverage (+): 0.15. Max coverage (-): 0

Region: NODE\_328441\_length\_53725\_cov\_29.545444 53150-53156. Max. coverage (+): 0.15. Max coverage (-): 0

Region: NODE\_328441\_length\_53725\_cov\_29.545444 53157-53164. Max. coverage (+): 0. Max coverage (-): 0

Region: NODE\_328441\_length\_53725\_cov\_29.545444 53165-53172. Max. coverage (+): 0.34. Max coverage (-): 0.13

Region: NODE\_328441\_length\_53725\_cov\_29.545444 53173-53179. Max. coverage (+): 4.9. Max coverage (-): 0

Region: NODE\_328441\_length\_53725\_cov\_29.545444 53180-53187. Max. coverage (+): 5.02. Max coverage (-): 0

Region: NODE\_328441\_length\_53725\_cov\_29.545444 53188-53195. Max. coverage (+): 2.09. Max coverage (-): 0

Region: NODE\_328441\_length\_53725\_cov\_29.545444 53196-53202. Max. coverage (+): 0.08. Max coverage (-): 0.03

Region: NODE\_328441\_length\_53725\_cov\_29.545444 53203-53210. Max. coverage (+): 0. Max coverage (-): 0.03

Region: NODE\_328441\_length\_53725\_cov\_29.545444 53211-53218. Max. coverage (+): 0.03. Max coverage (-): 0

Region: NODE\_328441\_length\_53725\_cov\_29.545444 53219-53225. Max. coverage (+): 1.44. Max coverage (-): 0.02

Region: NODE\_328441\_length\_53725\_cov\_29.545444 53226-53233. Max. coverage (+): 3.97. Max coverage (-): 0.13

Region: NODE\_328441\_length\_53725\_cov\_29.545444 53234-53241. Max. coverage (+): 3.63. Max coverage (-): 0.05

Region: NODE\_328441\_length\_53725\_cov\_29.545444 53242-53248. Max. coverage (+): 0.03. Max coverage (-): 0.05

Region: NODE\_328441\_length\_53725\_cov\_29.545444 53249-53256. Max. coverage (+): 0.03. Max coverage (-): 0

Region: NODE\_328441\_length\_53725\_cov\_29.545444 53257-53264. Max. coverage (+): 0.04. Max coverage (-): 0.06

Region: NODE\_328441\_length\_53725\_cov\_29.545444 53265-53271. Max. coverage (+): 0.04. Max coverage (-): 0.15

Region: NODE\_328441\_length\_53725\_cov\_29.545444 53272-53279. Max. coverage (+): 2.09. Max coverage (-): 0.1

Region: NODE\_328441\_length\_53725\_cov\_29.545444 53280-53287. Max. coverage (+): 0.74. Max coverage (-): 0

Region: NODE\_328441\_length\_53725\_cov\_29.545444 53288-53294. Max. coverage (+): 0.36. Max coverage (-): 0

Region: NODE\_328441\_length\_53725\_cov\_29.545444 53295-53302. Max. coverage (+): 0. Max coverage (-): 0

Region: NODE\_328441\_length\_53725\_cov\_29.545444 53303-53310. Max. coverage (+): 0. Max coverage (-): 0

Region: NODE\_328441\_length\_53725\_cov\_29.545444 53311-53317. Max. coverage (+): 0.04. Max coverage (-): 0

Region: NODE\_328441\_length\_53725\_cov\_29.545444 53318-53325. Max. coverage (+): 0.42. Max coverage (-): 0.04

Region: NODE\_328441\_length\_53725\_cov\_29.545444 53326-53333. Max. coverage (+): 0.34. Max coverage (-): 0.04

Region: NODE\_328441\_length\_53725\_cov\_29.545444 53334-53340. Max. coverage (+): 0. Max coverage (-): 0.08

Region: NODE\_328441\_length\_53725\_cov\_29.545444 53341-53348. Max. coverage (+): 0. Max coverage (-): 0

Region: NODE\_328441\_length\_53725\_cov\_29.545444 53349-53356. Max. coverage (+): 1.14. Max coverage (-): 0

Region: NODE\_328441\_length\_53725\_cov\_29.545444 53357-53363. Max. coverage (+): 1.21. Max coverage (-): 0.04

Region: NODE\_328441\_length\_53725\_cov\_29.545444 53364-53371. Max. coverage (+): 0.04. Max coverage (-): 0.38

Region: NODE\_328441\_length\_53725\_cov\_29.545444 53372-53379. Max. coverage (+): 0. Max coverage (-): 1.15

Region: NODE\_328441\_length\_53725\_cov\_29.545444 53380-53386. Max. coverage (+): 0.8. Max coverage (-): 0.46

Region: NODE\_328441\_length\_53725\_cov\_29.545444 53387-53394. Max. coverage (+): 5.16. Max coverage (-): 0

Region: NODE\_328441\_length\_53725\_cov\_29.545444 53395-53401. Max. coverage (+): 3.54. Max coverage (-): 0.04

Region: NODE\_328441\_length\_53725\_cov\_29.545444 53402-53409. Max. coverage (+): 1.72. Max coverage (-): 0.08

Region: NODE\_328441\_length\_53725\_cov\_29.545444 53410-53417. Max. coverage (+): 0.04. Max coverage (-): 0.19

Region: NODE\_328441\_length\_53725\_cov\_29.545444 53418-53424. Max. coverage (+): 0.46. Max coverage (-): 0.31

Region: NODE\_328441\_length\_53725\_cov\_29.545444 53425-53432. Max. coverage (+): 0.42. Max coverage (-): 1.07

Region: NODE\_328441\_length\_53725\_cov\_29.545444 53433-53440. Max. coverage (+): 0.27. Max coverage (-): 0.08

Region: NODE\_328441\_length\_53725\_cov\_29.545444 53441-53447. Max. coverage (+): 49.95. Max coverage (-): 0

Region: NODE\_328441\_length\_53725\_cov\_29.545444 53448-53455. Max. coverage (+): 49.95. Max coverage (-): 0

Region: NODE\_328441\_length\_53725\_cov\_29.545444 53456-53463. Max. coverage (+): 0.38. Max coverage (-): 0

Region: NODE\_328441\_length\_53725\_cov\_29.545444 53464-53470. Max. coverage (+): 2.22. Max coverage (-): 0.02

Region: NODE\_328441\_length\_53725\_cov\_29.545444 53471-53478. Max. coverage (+): 0.33. Max coverage (-): 0.02

Region: NODE\_328441\_length\_53725\_cov\_29.545444 53479-53486. Max. coverage (+): 0.33. Max coverage (-): 0.17

Region: NODE\_328441\_length\_53725\_cov\_29.545444 53487-53493. Max. coverage (+): 0.06. Max coverage (-): 0.15

Region: NODE\_328441\_length\_53725\_cov\_29.545444 53494-53501. Max. coverage (+): 0.1. Max coverage (-): 0.03

Region: NODE\_328441\_length\_53725\_cov\_29.545444 53502-53509. Max. coverage (+): 3.63. Max coverage (-): 0.03

Region: NODE\_328441\_length\_53725\_cov\_29.545444 53510-53516. Max. coverage (+): 0.36. Max coverage (-): 0.31

Region: NODE\_328441\_length\_53725\_cov\_29.545444 53517-53524. Max. coverage (+): 0.1. Max coverage (-): 0.15

Region: NODE\_328441\_length\_53725\_cov\_29.545444 53525-53532. Max. coverage (+): 0.92. Max coverage (-): 0.13

Region: NODE\_328441\_length\_53725\_cov\_29.545444 53533-53539. Max. coverage (+): 0.3. Max coverage (-): 0.31

Region: NODE\_328441\_length\_53725\_cov\_29.545444 53540-53547. Max. coverage (+): 0.57. Max coverage (-): 0.66

Region: NODE\_328441\_length\_53725\_cov\_29.545444 53548-53555. Max. coverage (+): 1.12. Max coverage (-): 0.59

Region: NODE\_328441\_length\_53725\_cov\_29.545444 53556-53562. Max. coverage (+): 1.17. Max coverage (-): 0

Region: NODE\_328441\_length\_53725\_cov\_29.545444 53563-53570. Max. coverage (+): 0.46. Max coverage (-): 0.11

Region: NODE\_328441\_length\_53725\_cov\_29.545444 53571-53578. Max. coverage (+): 0.11. Max coverage (-): 9.46

Region: NODE\_328441\_length\_53725\_cov\_29.545444 53579-53585. Max. coverage (+): 0.23. Max coverage (-): 4.44

Region: NODE\_328441\_length\_53725\_cov\_29.545444 53586-53593. Max. coverage (+): 6.9. Max coverage (-): 0.04

Region: NODE\_328441\_length\_53725\_cov\_29.545444 53594-53601. Max. coverage (+): 6.93. Max coverage (-): 0

Region: NODE\_328441\_length\_53725\_cov\_29.545444 53602-53608. Max. coverage (+): 0.31. Max coverage (-): 0

Region: NODE\_328441\_length\_53725\_cov\_29.545444 53609-53616. Max. coverage (+): 0.31. Max coverage (-): 0.04

Region: NODE\_328441\_length\_53725\_cov\_29.545444 53617-53624. Max. coverage (+): 0.11. Max coverage (-): 0.04

Region: NODE\_328441\_length\_53725\_cov\_29.545444 53625-53631. Max. coverage (+): 0.27. Max coverage (-): 0.04

Region: NODE\_328441\_length\_53725\_cov\_29.545444 53632-53639. Max. coverage (+): 0.02. Max coverage (-): 0

Region: NODE\_328441\_length\_53725\_cov\_29.545444 53640-53647. Max. coverage (+): 0. Max coverage (-): 0

Region: NODE\_328441\_length\_53725\_cov\_29.545444 53648-53654. Max. coverage (+): 0. Max coverage (-): 0

Region: NODE\_328441\_length\_53725\_cov\_29.545444 53655-53662. Max. coverage (+): 0. Max coverage (-): 0

Region: NODE\_328441\_length\_53725\_cov\_29.545444 53663-53670. Max. coverage (+): 0.38. Max coverage (-): 0

Region: NODE\_328441\_length\_53725\_cov\_29.545444 53671-53677. Max. coverage (+): 0. Max coverage (-): 0.15

Region: NODE\_328441\_length\_53725\_cov\_29.545444 53678-53685. Max. coverage (+): 0.23. Max coverage (-): 0.15

Region: NODE\_328441\_length\_53725\_cov\_29.545444 53686-53693. Max. coverage (+): 0.31. Max coverage (-): 0.15

Region: NODE\_328441\_length\_53725\_cov\_29.545444 53694-53700. Max. coverage (+): 0.08. Max coverage (-): 0.08

Region: NODE\_328441\_length\_53725\_cov\_29.545444 53701-53708. Max. coverage (+): 0.11. Max coverage (-): 0.08

Region: NODE\_328441\_length\_53725\_cov\_29.545444 53709-53716. Max. coverage (+): 0.31. Max coverage (-): 0.59

Region: NODE\_328441\_length\_53725\_cov\_29.545444 53717-53723. Max. coverage (+): 0.14. Max coverage (-): 0.59

Region: NODE\_328441\_length\_53725\_cov\_29.545444 53724-53731. Max. coverage (+): 0.7. Max coverage (-): 0.15

Region: NODE\_328441\_length\_53725\_cov\_29.545444 53732-53739. Max. coverage (+): 0.81. Max coverage (-): 0

Region: NODE\_328441\_length\_53725\_cov\_29.545444 53740-53746. Max. coverage (+): 0.37. Max coverage (-): 0.02

Region: NODE\_328441\_length\_53725\_cov\_29.545444 53747-53754. Max. coverage (+): 0.02. Max coverage (-): 0.03

Region: NODE\_328441\_length\_53725\_cov\_29.545444 53755-53762. Max. coverage (+): 0.99. Max coverage (-): 0

Region: NODE\_328441\_length\_53725\_cov\_29.545444 53763-53769. Max. coverage (+): 1.07. Max coverage (-): 0

Region: NODE\_328441\_length\_53725\_cov\_29.545444 53770-53777. Max. coverage (+): 0. Max coverage (-): 0.03

Region: NODE\_328441\_length\_53725\_cov\_29.545444 53778-53784. Max. coverage (+): 0. Max coverage (-): 0

Region: NODE\_328441\_length\_53725\_cov\_29.545444 53785-53792. Max. coverage (+): 0.49. Max coverage (-): 0

Region: NODE\_328441\_length\_53725\_cov\_29.545444 53793-53800. Max. coverage (+): 0.48. Max coverage (-): 0.09

Region: NODE\_328441\_length\_53725\_cov\_29.545444 53801-53807. Max. coverage (+): 0.06. Max coverage (-): 0.11

Region: NODE\_328441\_length\_53725\_cov\_29.545444 53808-53815. Max. coverage (+): 0.03. Max coverage (-): 0.05

Region: NODE\_328441\_length\_53725\_cov\_29.545444 53816-53823. Max. coverage (+): 0. Max coverage (-): 0

Region: NODE\_328441\_length\_53725\_cov\_29.545444 53824-53830. Max. coverage (+): 0. Max coverage (-): 0

Region: NODE\_328441\_length\_53725\_cov\_29.545444 53831-53838. Max. coverage (+): 0.15. Max coverage (-): 0

Region: NODE\_328441\_length\_53725\_cov\_29.545444 53839-53846. Max. coverage (+): 0.15. Max coverage (-): 0

Region: NODE\_328441\_length\_53725\_cov\_29.545444 53847-53853. Max. coverage (+): 0.05. Max coverage (-): 0

Region: NODE\_328441\_length\_53725\_cov\_29.545444 53854-53861. Max. coverage (+): 0. Max coverage (-): 0

Region: NODE\_328441\_length\_53725\_cov\_29.545444 53862-53869. Max. coverage (+): 0.08. Max coverage (-): 0.18

Region: NODE\_328441\_length\_53725\_cov\_29.545444 53870-53876. Max. coverage (+): 0.08. Max coverage (-): 0.36

Region: NODE\_328441\_length\_53725\_cov\_29.545444 53877-53884. Max. coverage (+): 0. Max coverage (-): 0.15

Region: NODE\_328441\_length\_53725\_cov\_29.545444 53885-53892. Max. coverage (+): 0. Max coverage (-): 0

Region: NODE\_328441\_length\_53725\_cov\_29.545444 53893-53899. Max. coverage (+): 0. Max coverage (-): 0

Region: NODE\_328441\_length\_53725\_cov\_29.545444 53900-53907. Max. coverage (+): 0.02. Max coverage (-): 0

Region: NODE\_328441\_length\_53725\_cov\_29.545444 53908-53915. Max. coverage (+): 3.24. Max coverage (-): 0.06

Region: NODE\_328441\_length\_53725\_cov\_29.545444 53916-53922. Max. coverage (+): 2.16. Max coverage (-): 1.67

Region: NODE\_328441\_length\_53725\_cov\_29.545444 53923-53930. Max. coverage (+): 0.04. Max coverage (-): 1.46

Region: NODE\_328441\_length\_53725\_cov\_29.545444 53931-53938. Max. coverage (+): 0.06. Max coverage (-): 0.02

Region: NODE\_328441\_length\_53725\_cov\_29.545444 53939-53945. Max. coverage (+): 0.2. Max coverage (-): 0

Region: NODE\_328441\_length\_53725\_cov\_29.545444 53946-53953. Max. coverage (+): 0. Max coverage (-): 0

Region: NODE\_328441\_length\_53725\_cov\_29.545444 53954-53961. Max. coverage (+): 0. Max coverage (-): 0

Region: NODE\_328441\_length\_53725\_cov\_29.545444 53962-. Max. coverage (+): 0. Max coverage (-): 0

RepeatMasker Color Code

**+**

100-98% Identity

<98-95% Identity

<95-90% Identity

<90-85% Identity

<85-80% Identity

<80-75% Identity

<75-70% Identity

<70% Identity

**-**

Gene Set Color Code

**+**

Gene

Pseudogene

Other

**-**

Topology/Coverage Color Code

Coverage Plus Strand

Coverage Minus Strand

Mainstrand: Plus

Mainstrand: Minus

Complementary Strand

Flanking Region  
(if option -flank >0)

Gene Set Annotation  
  
RepeatMasker Annotation  

**1. (AGAT)n**: 50213-50257 (+), Divergence to consensus: 22.5%  
**2. BEL-7\_GA-I**: 50620-50911 (-), Divergence to consensus: 30.9%  
**3. BEL-7\_GA-I**: 50948-52443 (-), Divergence to consensus: 29.9%  
**4. BEL-7\_GA-I**: 52560-53954 (-), Divergence to consensus: 35.5%

  
Transcription Factor Binding Sites  

**RHOXF1** (Sequence: AGCTCA (-): 50417)  
**RHOXF1** (Sequence: GGCTTA (-): 51657)  
**RHOXF1** (Sequence: GGATCA (-): 51683)  
**RHOXF1** (Sequence: GGATCA (-): 51852)  
**RHOXF1** (Sequence: AGCTTA (-): 53487)  
**RHOXF1** (Sequence: AGATCA (-): 53532)  
**RHOXF1** (Sequence: GGATCA (-): 53590)  
**RHOXF1** (Sequence: TGATCT (+): 53066)  
**RHOXF1** (Sequence: TGAGCT (+): 53485)  
**FOXO3\_mmu** (Sequence: TGTTTTGC (-): 53144)  
**Sox5** (Sequence: ATTGTT (+): 53142)
