# Supplementary material for: First characterization of PIWI-interacting RNA clusters in a cichlid fish with a B chromosome
Source: BMC Biol. 2022 Sep 21;20:204. doi: 10.1186/s12915-022-01403-2 (PMC9490952; doi:10.1186/s12915-022-01403-2)
Supplement: Supplementary file 1 — Additional file 1. Zipped folder with fasta and interactive html piRNA cluster information for the A. latifasciata genome. The nomenclature is as follows: number-pirna-cluster_sex_B-presence (f, female; m, male; 0b, without B chromosome; 1b, with B chromosome). [file 12915_2022_1403_MOESM1_ESM.zip › 12_f0b.html]

piRNA cluster 12\_f0b 27


Predicted piRNA cluster no. 12\_f0b
  

Show proTRAC run info
Hide proTRAC run info

/\  
                \_\_\_\_\_\_\_\_\_\_\_\_\_\_\_\_\_\_\_\_\_\_\_/\\_\_\_ /  \\_\_\_\_\_\_\_  
               I                      /  \  /    \      I  
               I     pro             /    \/      \     I  
               I        TRAC        /               \   I  
               I   \_\_\_\_\_\_\_\_\_\_\_\_\_\_\_\_/\_\_\_\_\_\_\_\_\_\_\_\_\_\_\_\_\_\\_ I  
               I   \              /                     I  
               I    \            /                      I  
               I     \  /\      /       V.2.4.2         I  
               I      \/  \    /                        I  
               I\_\_\_\_\_\_\_\_\_\_\_\  /\_\_\_\_\_\_\_\_\_\_\_\_\_\_\_\_\_\_\_\_\_\_\_\_\_I  
                            \/  
  
  
================================= proTRAC ====================================  
VERSION: .......... 2.4.2  
LAST MODIFIED: .... 11. May 2018  
  
Please cite:  
Rosenkranz D, Zischler H. proTRAC - a software for probabilistic piRNA cluster  
detection, visualization and analysis. 2012. BMC Bioinformatics 13:5.  
  
  
Contact:  
David Rosenkranz  
Institute of Organismic and Molecular Evolutionary Biology  
Dept. Anthropology, small RNA group  
Johannes Gutenberg University Mainz  
email: rosenkranz@uni-mainz.de  
  
You can find the latest proTRAC version at:  
http://sourceforge.net/projects/protrac/files  
http://www.smallRNAgroup-mainz.de/software  
==============================================================================  
  
PARAMETERS:  
Map file: ...............piwi-femeas-0B.fa-collapse.map  
Genome file: ............../../../0B\_ala\_genome.fa  
RepeatMasker annotation: Alatifasciata-all0B-maryan-v2.fa\_corrected.out  
GeneSet:................./guest-storage/Data/annotation/Alatifasciata\_all0B\_maryan-v2\_out2017.gff  
  
Significant (p<=0.01) hit density will be calculated based  
on observed hit distribution.  
  
Sliding window size: ........................................ 5000 bp  
Sliding window increament: .................................. 1000 bp  
Normalize each hit by number of genomic hits: ............... yes  
Normalize each hit by number of sequence reads: ............. yes  
Normalize values (-> per million mapped reads): ............. yes  
Min. fraction of hits with 1T(U) or 10A: .................... 0.75  
Alternatively: Min. fraction of hits with 1T(U) and 10A: .... 0.5  
Min. fraction of hits with typical piRNA length: ............ 0.75  
Typical piRNA length: ....................................... 24-32 nt  
Min. size of a piRNA cluster: ............................... 1000 bp.  
Min. number of hits (absolute): ............................. 0  
Min. number of hits (normalized): ........................... 0  
Min. fraction of hits on the mainstrand: .................... 0.75  
Top fraction of mapped sequences (in terms of read counts): . 1%  
Top fraction accounts for max. n% of sequence reads: ........ 90%  
Min. fraction of hits on each arm of a bidirectional cluster: 0.05  
Output html file for each cluster: .......................... yes  
Output a summary table: ..................................... yes  
Output a FASTA file for each cluster (piRNA sequences): ..... yes  
Output a FASTA file comprising cluster sequences: ........... yes  
Output a GTF file for predicted piRNA clusters: ..............yes  
Search DNA motifs in clusters: .............................. yes  
Output flanking sequences: +/- .............................. 0 bp  
Output ~.pTi file: .......................................... no  
==============================================================================  
  
  
Genome size (without gaps): ............ 758543724 bp  
Gaps (N/X/-): .......................... 417479 bp  
Mapped reads: .......................... 13052187  
Non-identical sequences: ............... 3338911  
Genomic hits: .......................... 28737726  
Significant densitiy of mapped reads: .. 470.083249848448 reads/kb

Show proTRAC cluster info
Hide proTRAC cluster info

|  |  |
| --- | --- |
| Location | NODE\_118139\_length\_1594\_cov\_363.152435 |
| Coordinates | 7-1654 |
| Size [bp] | 1648 |
| Sequence hit loci | 787 |
| Mapped reads (normalized) | 2476.1 |
| Mapped reads (normalized) per kb | 1502.5 |
| Normalized reads with 1T (1U) | 79.2% |
| Normalized reads with 10A | 31.3% |
| Normalized reads with length 24-32 nt | 97.9% |
| Normalized reads on the main strand(s) | 89.9% |
| Predicted directionality | mono:plus |

100%

0%

1T (1U)  
reads

10A reads

24-32 nt  
reads

reads on mainstrand

**Either the amount of reads with 1T (1U) OR 10A has to exceed 75% (set with option: -1Tor10A)  
Alternatively the amount of reads with 1T (1U) AND 10A has to exceed 50% (set with option: -1Tand10A)  
Minimum amount of reads with preferred size is 75% (set with option: -pisize)  
Minimum amount of reads on the main strand(s) is 75% (set with option: -clstrand)**

Show read coverage
Hide read coverage

WHAT DO I SEE HERE?  
This chart shows the location of mapped sequence reads within a predicted piRNA cluster. The color refers to the number of genomic hits produced by the sequence read in question. A dark red bar indicates that this sequence read produces many other hits elsewhere in the genome. Many adjacent red or yellow bars can indicate the presence of a multi-copy element such as transposons or rRNA genes. A dark green bar indicates that this sequence read maps uniquely to this locus.

1 hit

2-5 hits

6-10 hits

11-20 hits

21-50 hits

51-100 hits

> 100 hits

NODE\_118139\_length\_1594\_cov\_363.152435

7

1654

Gene Set

RepeatMasker

Mapped  
Reads

32.41

plus strand

minus strand

32.41

Region: NODE\_118139\_length\_1594\_cov\_363.152435 15775-8. Max. coverage (+): 0.23. Max coverage (-): 0.04

Region: NODE\_118139\_length\_1594\_cov\_363.152435 9-11. Max. coverage (+): 0.19. Max coverage (-): 0

Region: NODE\_118139\_length\_1594\_cov\_363.152435 12-15. Max. coverage (+): 0.08. Max coverage (-): 0.54

Region: NODE\_118139\_length\_1594\_cov\_363.152435 16-18. Max. coverage (+): 0.46. Max coverage (-): 0

Region: NODE\_118139\_length\_1594\_cov\_363.152435 19-21. Max. coverage (+): 0.31. Max coverage (-): 0

Region: NODE\_118139\_length\_1594\_cov\_363.152435 22-25. Max. coverage (+): 0. Max coverage (-): 0.46

Region: NODE\_118139\_length\_1594\_cov\_363.152435 26-28. Max. coverage (+): 0. Max coverage (-): 0.08

Region: NODE\_118139\_length\_1594\_cov\_363.152435 29-31. Max. coverage (+): 0.08. Max coverage (-): 0.08

Region: NODE\_118139\_length\_1594\_cov\_363.152435 32-35. Max. coverage (+): 0.08. Max coverage (-): 0.08

Region: NODE\_118139\_length\_1594\_cov\_363.152435 36-38. Max. coverage (+): 0.08. Max coverage (-): 0

Region: NODE\_118139\_length\_1594\_cov\_363.152435 39-41. Max. coverage (+): 0.08. Max coverage (-): 0

Region: NODE\_118139\_length\_1594\_cov\_363.152435 42-44. Max. coverage (+): 4.52. Max coverage (-): 0

Region: NODE\_118139\_length\_1594\_cov\_363.152435 45-48. Max. coverage (+): 0. Max coverage (-): 0

Region: NODE\_118139\_length\_1594\_cov\_363.152435 49-51. Max. coverage (+): 0. Max coverage (-): 0

Region: NODE\_118139\_length\_1594\_cov\_363.152435 52-54. Max. coverage (+): 0. Max coverage (-): 0

Region: NODE\_118139\_length\_1594\_cov\_363.152435 55-58. Max. coverage (+): 0. Max coverage (-): 0

Region: NODE\_118139\_length\_1594\_cov\_363.152435 59-61. Max. coverage (+): 0. Max coverage (-): 0

Region: NODE\_118139\_length\_1594\_cov\_363.152435 62-64. Max. coverage (+): 0. Max coverage (-): 0

Region: NODE\_118139\_length\_1594\_cov\_363.152435 65-67. Max. coverage (+): 0. Max coverage (-): 0.08

Region: NODE\_118139\_length\_1594\_cov\_363.152435 68-71. Max. coverage (+): 0. Max coverage (-): 0.08

Region: NODE\_118139\_length\_1594\_cov\_363.152435 72-74. Max. coverage (+): 0.15. Max coverage (-): 0.08

Region: NODE\_118139\_length\_1594\_cov\_363.152435 75-77. Max. coverage (+): 0.38. Max coverage (-): 0.08

Region: NODE\_118139\_length\_1594\_cov\_363.152435 78-81. Max. coverage (+): 0. Max coverage (-): 0.08

Region: NODE\_118139\_length\_1594\_cov\_363.152435 82-84. Max. coverage (+): 0.08. Max coverage (-): 0

Region: NODE\_118139\_length\_1594\_cov\_363.152435 85-87. Max. coverage (+): 0.61. Max coverage (-): 0

Region: NODE\_118139\_length\_1594\_cov\_363.152435 88-91. Max. coverage (+): 0.31. Max coverage (-): 0

Region: NODE\_118139\_length\_1594\_cov\_363.152435 92-94. Max. coverage (+): 0. Max coverage (-): 0

Region: NODE\_118139\_length\_1594\_cov\_363.152435 95-97. Max. coverage (+): 0. Max coverage (-): 0

Region: NODE\_118139\_length\_1594\_cov\_363.152435 98-100. Max. coverage (+): 0. Max coverage (-): 0

Region: NODE\_118139\_length\_1594\_cov\_363.152435 101-104. Max. coverage (+): 0. Max coverage (-): 0

Region: NODE\_118139\_length\_1594\_cov\_363.152435 105-107. Max. coverage (+): 0. Max coverage (-): 0

Region: NODE\_118139\_length\_1594\_cov\_363.152435 108-110. Max. coverage (+): 0. Max coverage (-): 0

Region: NODE\_118139\_length\_1594\_cov\_363.152435 111-114. Max. coverage (+): 0. Max coverage (-): 0

Region: NODE\_118139\_length\_1594\_cov\_363.152435 115-117. Max. coverage (+): 0. Max coverage (-): 0

Region: NODE\_118139\_length\_1594\_cov\_363.152435 118-120. Max. coverage (+): 0.08. Max coverage (-): 0

Region: NODE\_118139\_length\_1594\_cov\_363.152435 121-124. Max. coverage (+): 0.15. Max coverage (-): 0

Region: NODE\_118139\_length\_1594\_cov\_363.152435 125-127. Max. coverage (+): 0. Max coverage (-): 0

Region: NODE\_118139\_length\_1594\_cov\_363.152435 128-130. Max. coverage (+): 0.15. Max coverage (-): 0

Region: NODE\_118139\_length\_1594\_cov\_363.152435 131-133. Max. coverage (+): 0.15. Max coverage (-): 0

Region: NODE\_118139\_length\_1594\_cov\_363.152435 134-137. Max. coverage (+): 0.09. Max coverage (-): 0.01

Region: NODE\_118139\_length\_1594\_cov\_363.152435 138-140. Max. coverage (+): 0.01. Max coverage (-): 0

Region: NODE\_118139\_length\_1594\_cov\_363.152435 141-143. Max. coverage (+): 0. Max coverage (-): 0

Region: NODE\_118139\_length\_1594\_cov\_363.152435 144-147. Max. coverage (+): 0.01. Max coverage (-): 0

Region: NODE\_118139\_length\_1594\_cov\_363.152435 148-150. Max. coverage (+): 0.47. Max coverage (-): 0

Region: NODE\_118139\_length\_1594\_cov\_363.152435 151-153. Max. coverage (+): 0.95. Max coverage (-): 0

Region: NODE\_118139\_length\_1594\_cov\_363.152435 154-156. Max. coverage (+): 1.08. Max coverage (-): 0

Region: NODE\_118139\_length\_1594\_cov\_363.152435 157-160. Max. coverage (+): 1.41. Max coverage (-): 0.08

Region: NODE\_118139\_length\_1594\_cov\_363.152435 161-163. Max. coverage (+): 0. Max coverage (-): 0.08

Region: NODE\_118139\_length\_1594\_cov\_363.152435 164-166. Max. coverage (+): 0.08. Max coverage (-): 0

Region: NODE\_118139\_length\_1594\_cov\_363.152435 167-170. Max. coverage (+): 0.08. Max coverage (-): 0.08

Region: NODE\_118139\_length\_1594\_cov\_363.152435 171-173. Max. coverage (+): 0. Max coverage (-): 0

Region: NODE\_118139\_length\_1594\_cov\_363.152435 174-176. Max. coverage (+): 0.15. Max coverage (-): 0.08

Region: NODE\_118139\_length\_1594\_cov\_363.152435 177-180. Max. coverage (+): 1.07. Max coverage (-): 0

Region: NODE\_118139\_length\_1594\_cov\_363.152435 181-183. Max. coverage (+): 0.08. Max coverage (-): 0

Region: NODE\_118139\_length\_1594\_cov\_363.152435 184-186. Max. coverage (+): 0.15. Max coverage (-): 0

Region: NODE\_118139\_length\_1594\_cov\_363.152435 187-189. Max. coverage (+): 0.15. Max coverage (-): 0.08

Region: NODE\_118139\_length\_1594\_cov\_363.152435 190-193. Max. coverage (+): 0. Max coverage (-): 0.08

Region: NODE\_118139\_length\_1594\_cov\_363.152435 194-196. Max. coverage (+): 0.08. Max coverage (-): 0

Region: NODE\_118139\_length\_1594\_cov\_363.152435 197-199. Max. coverage (+): 0.08. Max coverage (-): 0.08

Region: NODE\_118139\_length\_1594\_cov\_363.152435 200-203. Max. coverage (+): 0.31. Max coverage (-): 0.08

Region: NODE\_118139\_length\_1594\_cov\_363.152435 204-206. Max. coverage (+): 0.31. Max coverage (-): 0

Region: NODE\_118139\_length\_1594\_cov\_363.152435 207-209. Max. coverage (+): 6.59. Max coverage (-): 0

Region: NODE\_118139\_length\_1594\_cov\_363.152435 210-212. Max. coverage (+): 8.96. Max coverage (-): 0

Region: NODE\_118139\_length\_1594\_cov\_363.152435 213-216. Max. coverage (+): 0.15. Max coverage (-): 0

Region: NODE\_118139\_length\_1594\_cov\_363.152435 217-219. Max. coverage (+): 0.08. Max coverage (-): 0

Region: NODE\_118139\_length\_1594\_cov\_363.152435 220-222. Max. coverage (+): 0. Max coverage (-): 0

Region: NODE\_118139\_length\_1594\_cov\_363.152435 223-226. Max. coverage (+): 0. Max coverage (-): 0

Region: NODE\_118139\_length\_1594\_cov\_363.152435 227-229. Max. coverage (+): 0. Max coverage (-): 0

Region: NODE\_118139\_length\_1594\_cov\_363.152435 230-232. Max. coverage (+): 0. Max coverage (-): 0.08

Region: NODE\_118139\_length\_1594\_cov\_363.152435 233-236. Max. coverage (+): 0. Max coverage (-): 0.15

Region: NODE\_118139\_length\_1594\_cov\_363.152435 237-239. Max. coverage (+): 0.15. Max coverage (-): 0.15

Region: NODE\_118139\_length\_1594\_cov\_363.152435 240-242. Max. coverage (+): 0.31. Max coverage (-): 0.15

Region: NODE\_118139\_length\_1594\_cov\_363.152435 243-245. Max. coverage (+): 0.84. Max coverage (-): 0.08

Region: NODE\_118139\_length\_1594\_cov\_363.152435 246-249. Max. coverage (+): 0.77. Max coverage (-): 0

Region: NODE\_118139\_length\_1594\_cov\_363.152435 250-252. Max. coverage (+): 0. Max coverage (-): 0

Region: NODE\_118139\_length\_1594\_cov\_363.152435 253-255. Max. coverage (+): 0. Max coverage (-): 0.23

Region: NODE\_118139\_length\_1594\_cov\_363.152435 256-259. Max. coverage (+): 0.69. Max coverage (-): 0.54

Region: NODE\_118139\_length\_1594\_cov\_363.152435 260-262. Max. coverage (+): 0.77. Max coverage (-): 0

Region: NODE\_118139\_length\_1594\_cov\_363.152435 263-265. Max. coverage (+): 0.08. Max coverage (-): 0

Region: NODE\_118139\_length\_1594\_cov\_363.152435 266-269. Max. coverage (+): 0.23. Max coverage (-): 0

Region: NODE\_118139\_length\_1594\_cov\_363.152435 270-272. Max. coverage (+): 2.15. Max coverage (-): 0

Region: NODE\_118139\_length\_1594\_cov\_363.152435 273-275. Max. coverage (+): 2.3. Max coverage (-): 0

Region: NODE\_118139\_length\_1594\_cov\_363.152435 276-278. Max. coverage (+): 0.31. Max coverage (-): 0

Region: NODE\_118139\_length\_1594\_cov\_363.152435 279-282. Max. coverage (+): 0.08. Max coverage (-): 0

Region: NODE\_118139\_length\_1594\_cov\_363.152435 283-285. Max. coverage (+): 0.15. Max coverage (-): 0

Region: NODE\_118139\_length\_1594\_cov\_363.152435 286-288. Max. coverage (+): 2.6. Max coverage (-): 0

Region: NODE\_118139\_length\_1594\_cov\_363.152435 289-292. Max. coverage (+): 2.6. Max coverage (-): 0

Region: NODE\_118139\_length\_1594\_cov\_363.152435 293-295. Max. coverage (+): 0.08. Max coverage (-): 0

Region: NODE\_118139\_length\_1594\_cov\_363.152435 296-298. Max. coverage (+): 0.08. Max coverage (-): 0.15

Region: NODE\_118139\_length\_1594\_cov\_363.152435 299-301. Max. coverage (+): 0.08. Max coverage (-): 0

Region: NODE\_118139\_length\_1594\_cov\_363.152435 302-305. Max. coverage (+): 0. Max coverage (-): 0

Region: NODE\_118139\_length\_1594\_cov\_363.152435 306-308. Max. coverage (+): 0. Max coverage (-): 0

Region: NODE\_118139\_length\_1594\_cov\_363.152435 309-311. Max. coverage (+): 0. Max coverage (-): 0

Region: NODE\_118139\_length\_1594\_cov\_363.152435 312-315. Max. coverage (+): 0. Max coverage (-): 0.08

Region: NODE\_118139\_length\_1594\_cov\_363.152435 316-318. Max. coverage (+): 0. Max coverage (-): 0.08

Region: NODE\_118139\_length\_1594\_cov\_363.152435 319-321. Max. coverage (+): 0. Max coverage (-): 0.08

Region: NODE\_118139\_length\_1594\_cov\_363.152435 322-325. Max. coverage (+): 0. Max coverage (-): 0.23

Region: NODE\_118139\_length\_1594\_cov\_363.152435 326-328. Max. coverage (+): 0.08. Max coverage (-): 0.08

Region: NODE\_118139\_length\_1594\_cov\_363.152435 329-331. Max. coverage (+): 0.08. Max coverage (-): 0

Region: NODE\_118139\_length\_1594\_cov\_363.152435 332-334. Max. coverage (+): 0.15. Max coverage (-): 0

Region: NODE\_118139\_length\_1594\_cov\_363.152435 335-338. Max. coverage (+): 1. Max coverage (-): 0

Region: NODE\_118139\_length\_1594\_cov\_363.152435 339-341. Max. coverage (+): 1.15. Max coverage (-): 0

Region: NODE\_118139\_length\_1594\_cov\_363.152435 342-344. Max. coverage (+): 0.84. Max coverage (-): 0

Region: NODE\_118139\_length\_1594\_cov\_363.152435 345-348. Max. coverage (+): 0.08. Max coverage (-): 0

Region: NODE\_118139\_length\_1594\_cov\_363.152435 349-351. Max. coverage (+): 0. Max coverage (-): 0.08

Region: NODE\_118139\_length\_1594\_cov\_363.152435 352-354. Max. coverage (+): 0.08. Max coverage (-): 0.23

Region: NODE\_118139\_length\_1594\_cov\_363.152435 355-358. Max. coverage (+): 0.08. Max coverage (-): 0.15

Region: NODE\_118139\_length\_1594\_cov\_363.152435 359-361. Max. coverage (+): 0. Max coverage (-): 0

Region: NODE\_118139\_length\_1594\_cov\_363.152435 362-364. Max. coverage (+): 0. Max coverage (-): 0

Region: NODE\_118139\_length\_1594\_cov\_363.152435 365-367. Max. coverage (+): 0. Max coverage (-): 0

Region: NODE\_118139\_length\_1594\_cov\_363.152435 368-371. Max. coverage (+): 0.15. Max coverage (-): 0

Region: NODE\_118139\_length\_1594\_cov\_363.152435 372-374. Max. coverage (+): 6.67. Max coverage (-): 0

Region: NODE\_118139\_length\_1594\_cov\_363.152435 375-377. Max. coverage (+): 6.28. Max coverage (-): 0

Region: NODE\_118139\_length\_1594\_cov\_363.152435 378-381. Max. coverage (+): 0.08. Max coverage (-): 0

Region: NODE\_118139\_length\_1594\_cov\_363.152435 382-384. Max. coverage (+): 0. Max coverage (-): 0

Region: NODE\_118139\_length\_1594\_cov\_363.152435 385-387. Max. coverage (+): 0. Max coverage (-): 0.08

Region: NODE\_118139\_length\_1594\_cov\_363.152435 388-390. Max. coverage (+): 0. Max coverage (-): 0.08

Region: NODE\_118139\_length\_1594\_cov\_363.152435 391-394. Max. coverage (+): 0. Max coverage (-): 0

Region: NODE\_118139\_length\_1594\_cov\_363.152435 395-397. Max. coverage (+): 0. Max coverage (-): 0

Region: NODE\_118139\_length\_1594\_cov\_363.152435 398-400. Max. coverage (+): 0. Max coverage (-): 0

Region: NODE\_118139\_length\_1594\_cov\_363.152435 401-404. Max. coverage (+): 0.15. Max coverage (-): 0.08

Region: NODE\_118139\_length\_1594\_cov\_363.152435 405-407. Max. coverage (+): 0.23. Max coverage (-): 0.08

Region: NODE\_118139\_length\_1594\_cov\_363.152435 408-410. Max. coverage (+): 0. Max coverage (-): 0

Region: NODE\_118139\_length\_1594\_cov\_363.152435 411-414. Max. coverage (+): 0. Max coverage (-): 0

Region: NODE\_118139\_length\_1594\_cov\_363.152435 415-417. Max. coverage (+): 0. Max coverage (-): 0.15

Region: NODE\_118139\_length\_1594\_cov\_363.152435 418-420. Max. coverage (+): 0.08. Max coverage (-): 0.15

Region: NODE\_118139\_length\_1594\_cov\_363.152435 421-423. Max. coverage (+): 0.08. Max coverage (-): 0

Region: NODE\_118139\_length\_1594\_cov\_363.152435 424-427. Max. coverage (+): 0. Max coverage (-): 0

Region: NODE\_118139\_length\_1594\_cov\_363.152435 428-430. Max. coverage (+): 0.92. Max coverage (-): 0

Region: NODE\_118139\_length\_1594\_cov\_363.152435 431-433. Max. coverage (+): 1.3. Max coverage (-): 0

Region: NODE\_118139\_length\_1594\_cov\_363.152435 434-437. Max. coverage (+): 1.07. Max coverage (-): 0

Region: NODE\_118139\_length\_1594\_cov\_363.152435 438-440. Max. coverage (+): 0.15. Max coverage (-): 0

Region: NODE\_118139\_length\_1594\_cov\_363.152435 441-443. Max. coverage (+): 0.08. Max coverage (-): 0.23

Region: NODE\_118139\_length\_1594\_cov\_363.152435 444-447. Max. coverage (+): 0.08. Max coverage (-): 0.54

Region: NODE\_118139\_length\_1594\_cov\_363.152435 448-450. Max. coverage (+): 0.01. Max coverage (-): 0.09

Region: NODE\_118139\_length\_1594\_cov\_363.152435 451-453. Max. coverage (+): 0.01. Max coverage (-): 0.1

Region: NODE\_118139\_length\_1594\_cov\_363.152435 454-456. Max. coverage (+): 0.01. Max coverage (-): 0.01

Region: NODE\_118139\_length\_1594\_cov\_363.152435 457-460. Max. coverage (+): 3.83. Max coverage (-): 0.01

Region: NODE\_118139\_length\_1594\_cov\_363.152435 461-463. Max. coverage (+): 3.83. Max coverage (-): 0

Region: NODE\_118139\_length\_1594\_cov\_363.152435 464-466. Max. coverage (+): 5.36. Max coverage (-): 0

Region: NODE\_118139\_length\_1594\_cov\_363.152435 467-470. Max. coverage (+): 0.69. Max coverage (-): 0.08

Region: NODE\_118139\_length\_1594\_cov\_363.152435 471-473. Max. coverage (+): 1.92. Max coverage (-): 0

Region: NODE\_118139\_length\_1594\_cov\_363.152435 474-476. Max. coverage (+): 1.15. Max coverage (-): 0

Region: NODE\_118139\_length\_1594\_cov\_363.152435 477-479. Max. coverage (+): 0. Max coverage (-): 0.08

Region: NODE\_118139\_length\_1594\_cov\_363.152435 480-483. Max. coverage (+): 0. Max coverage (-): 0.08

Region: NODE\_118139\_length\_1594\_cov\_363.152435 484-486. Max. coverage (+): 0. Max coverage (-): 0

Region: NODE\_118139\_length\_1594\_cov\_363.152435 487-489. Max. coverage (+): 0. Max coverage (-): 0

Region: NODE\_118139\_length\_1594\_cov\_363.152435 490-493. Max. coverage (+): 0.23. Max coverage (-): 0

Region: NODE\_118139\_length\_1594\_cov\_363.152435 494-496. Max. coverage (+): 0. Max coverage (-): 0

Region: NODE\_118139\_length\_1594\_cov\_363.152435 497-499. Max. coverage (+): 0. Max coverage (-): 0

Region: NODE\_118139\_length\_1594\_cov\_363.152435 500-503. Max. coverage (+): 0.08. Max coverage (-): 0

Region: NODE\_118139\_length\_1594\_cov\_363.152435 504-506. Max. coverage (+): 0. Max coverage (-): 0

Region: NODE\_118139\_length\_1594\_cov\_363.152435 507-509. Max. coverage (+): 0. Max coverage (-): 0

Region: NODE\_118139\_length\_1594\_cov\_363.152435 510-512. Max. coverage (+): 0. Max coverage (-): 0

Region: NODE\_118139\_length\_1594\_cov\_363.152435 513-516. Max. coverage (+): 0.08. Max coverage (-): 0

Region: NODE\_118139\_length\_1594\_cov\_363.152435 517-519. Max. coverage (+): 0.08. Max coverage (-): 0

Region: NODE\_118139\_length\_1594\_cov\_363.152435 520-522. Max. coverage (+): 1.69. Max coverage (-): 0

Region: NODE\_118139\_length\_1594\_cov\_363.152435 523-526. Max. coverage (+): 6.97. Max coverage (-): 0

Region: NODE\_118139\_length\_1594\_cov\_363.152435 527-529. Max. coverage (+): 7.05. Max coverage (-): 0

Region: NODE\_118139\_length\_1594\_cov\_363.152435 530-532. Max. coverage (+): 0. Max coverage (-): 0

Region: NODE\_118139\_length\_1594\_cov\_363.152435 533-536. Max. coverage (+): 0.08. Max coverage (-): 0

Region: NODE\_118139\_length\_1594\_cov\_363.152435 537-539. Max. coverage (+): 0.08. Max coverage (-): 0

Region: NODE\_118139\_length\_1594\_cov\_363.152435 540-542. Max. coverage (+): 0. Max coverage (-): 0

Region: NODE\_118139\_length\_1594\_cov\_363.152435 543-545. Max. coverage (+): 0. Max coverage (-): 0.08

Region: NODE\_118139\_length\_1594\_cov\_363.152435 546-549. Max. coverage (+): 0. Max coverage (-): 0

Region: NODE\_118139\_length\_1594\_cov\_363.152435 550-552. Max. coverage (+): 0. Max coverage (-): 0

Region: NODE\_118139\_length\_1594\_cov\_363.152435 553-555. Max. coverage (+): 0. Max coverage (-): 0

Region: NODE\_118139\_length\_1594\_cov\_363.152435 556-559. Max. coverage (+): 1.76. Max coverage (-): 0.08

Region: NODE\_118139\_length\_1594\_cov\_363.152435 560-562. Max. coverage (+): 2.07. Max coverage (-): 0.08

Region: NODE\_118139\_length\_1594\_cov\_363.152435 563-565. Max. coverage (+): 0.08. Max coverage (-): 0.15

Region: NODE\_118139\_length\_1594\_cov\_363.152435 566-568. Max. coverage (+): 0. Max coverage (-): 1

Region: NODE\_118139\_length\_1594\_cov\_363.152435 569-572. Max. coverage (+): 0. Max coverage (-): 1

Region: NODE\_118139\_length\_1594\_cov\_363.152435 573-575. Max. coverage (+): 0. Max coverage (-): 0

Region: NODE\_118139\_length\_1594\_cov\_363.152435 576-578. Max. coverage (+): 0.08. Max coverage (-): 0

Region: NODE\_118139\_length\_1594\_cov\_363.152435 579-582. Max. coverage (+): 0.46. Max coverage (-): 0

Region: NODE\_118139\_length\_1594\_cov\_363.152435 583-585. Max. coverage (+): 0. Max coverage (-): 0

Region: NODE\_118139\_length\_1594\_cov\_363.152435 586-588. Max. coverage (+): 0.31. Max coverage (-): 0

Region: NODE\_118139\_length\_1594\_cov\_363.152435 589-592. Max. coverage (+): 0.15. Max coverage (-): 0

Region: NODE\_118139\_length\_1594\_cov\_363.152435 593-595. Max. coverage (+): 0.15. Max coverage (-): 0

Region: NODE\_118139\_length\_1594\_cov\_363.152435 596-598. Max. coverage (+): 0.08. Max coverage (-): 0

Region: NODE\_118139\_length\_1594\_cov\_363.152435 599-601. Max. coverage (+): 0. Max coverage (-): 0

Region: NODE\_118139\_length\_1594\_cov\_363.152435 602-605. Max. coverage (+): 0. Max coverage (-): 0.23

Region: NODE\_118139\_length\_1594\_cov\_363.152435 606-608. Max. coverage (+): 0. Max coverage (-): 0.08

Region: NODE\_118139\_length\_1594\_cov\_363.152435 609-611. Max. coverage (+): 0.15. Max coverage (-): 0.08

Region: NODE\_118139\_length\_1594\_cov\_363.152435 612-615. Max. coverage (+): 0. Max coverage (-): 0.08

Region: NODE\_118139\_length\_1594\_cov\_363.152435 616-618. Max. coverage (+): 0.08. Max coverage (-): 0

Region: NODE\_118139\_length\_1594\_cov\_363.152435 619-621. Max. coverage (+): 0.08. Max coverage (-): 0

Region: NODE\_118139\_length\_1594\_cov\_363.152435 622-624. Max. coverage (+): 0.08. Max coverage (-): 0.08

Region: NODE\_118139\_length\_1594\_cov\_363.152435 625-628. Max. coverage (+): 0.08. Max coverage (-): 0

Region: NODE\_118139\_length\_1594\_cov\_363.152435 629-631. Max. coverage (+): 0. Max coverage (-): 0

Region: NODE\_118139\_length\_1594\_cov\_363.152435 632-634. Max. coverage (+): 0. Max coverage (-): 0

Region: NODE\_118139\_length\_1594\_cov\_363.152435 635-638. Max. coverage (+): 0. Max coverage (-): 0

Region: NODE\_118139\_length\_1594\_cov\_363.152435 639-641. Max. coverage (+): 0. Max coverage (-): 0

Region: NODE\_118139\_length\_1594\_cov\_363.152435 642-644. Max. coverage (+): 0.08. Max coverage (-): 0.15

Region: NODE\_118139\_length\_1594\_cov\_363.152435 645-648. Max. coverage (+): 0.08. Max coverage (-): 0.15

Region: NODE\_118139\_length\_1594\_cov\_363.152435 649-651. Max. coverage (+): 0. Max coverage (-): 0

Region: NODE\_118139\_length\_1594\_cov\_363.152435 652-654. Max. coverage (+): 0. Max coverage (-): 0

Region: NODE\_118139\_length\_1594\_cov\_363.152435 655-657. Max. coverage (+): 0. Max coverage (-): 0

Region: NODE\_118139\_length\_1594\_cov\_363.152435 658-661. Max. coverage (+): 0.92. Max coverage (-): 0

Region: NODE\_118139\_length\_1594\_cov\_363.152435 662-664. Max. coverage (+): 1. Max coverage (-): 0.08

Region: NODE\_118139\_length\_1594\_cov\_363.152435 665-667. Max. coverage (+): 0.54. Max coverage (-): 0.08

Region: NODE\_118139\_length\_1594\_cov\_363.152435 668-671. Max. coverage (+): 0.38. Max coverage (-): 0.08

Region: NODE\_118139\_length\_1594\_cov\_363.152435 672-674. Max. coverage (+): 0.08. Max coverage (-): 0.08

Region: NODE\_118139\_length\_1594\_cov\_363.152435 675-677. Max. coverage (+): 0.08. Max coverage (-): 0

Region: NODE\_118139\_length\_1594\_cov\_363.152435 678-681. Max. coverage (+): 0. Max coverage (-): 0

Region: NODE\_118139\_length\_1594\_cov\_363.152435 682-684. Max. coverage (+): 0. Max coverage (-): 0

Region: NODE\_118139\_length\_1594\_cov\_363.152435 685-687. Max. coverage (+): 0.08. Max coverage (-): 0

Region: NODE\_118139\_length\_1594\_cov\_363.152435 688-690. Max. coverage (+): 0.08. Max coverage (-): 0

Region: NODE\_118139\_length\_1594\_cov\_363.152435 691-694. Max. coverage (+): 0. Max coverage (-): 0

Region: NODE\_118139\_length\_1594\_cov\_363.152435 695-697. Max. coverage (+): 0.15. Max coverage (-): 0

Region: NODE\_118139\_length\_1594\_cov\_363.152435 698-700. Max. coverage (+): 0.15. Max coverage (-): 0

Region: NODE\_118139\_length\_1594\_cov\_363.152435 701-704. Max. coverage (+): 0. Max coverage (-): 0

Region: NODE\_118139\_length\_1594\_cov\_363.152435 705-707. Max. coverage (+): 0. Max coverage (-): 0

Region: NODE\_118139\_length\_1594\_cov\_363.152435 708-710. Max. coverage (+): 0. Max coverage (-): 0

Region: NODE\_118139\_length\_1594\_cov\_363.152435 711-713. Max. coverage (+): 0. Max coverage (-): 0

Region: NODE\_118139\_length\_1594\_cov\_363.152435 714-717. Max. coverage (+): 0.15. Max coverage (-): 0

Region: NODE\_118139\_length\_1594\_cov\_363.152435 718-720. Max. coverage (+): 0.23. Max coverage (-): 0

Region: NODE\_118139\_length\_1594\_cov\_363.152435 721-723. Max. coverage (+): 3.83. Max coverage (-): 0

Region: NODE\_118139\_length\_1594\_cov\_363.152435 724-727. Max. coverage (+): 32.41. Max coverage (-): 0

Region: NODE\_118139\_length\_1594\_cov\_363.152435 728-730. Max. coverage (+): 30.03. Max coverage (-): 0

Region: NODE\_118139\_length\_1594\_cov\_363.152435 731-733. Max. coverage (+): 0.46. Max coverage (-): 0

Region: NODE\_118139\_length\_1594\_cov\_363.152435 734-737. Max. coverage (+): 0.08. Max coverage (-): 0

Region: NODE\_118139\_length\_1594\_cov\_363.152435 738-740. Max. coverage (+): 0. Max coverage (-): 0

Region: NODE\_118139\_length\_1594\_cov\_363.152435 741-743. Max. coverage (+): 0. Max coverage (-): 0.08

Region: NODE\_118139\_length\_1594\_cov\_363.152435 744-746. Max. coverage (+): 0. Max coverage (-): 0

Region: NODE\_118139\_length\_1594\_cov\_363.152435 747-750. Max. coverage (+): 0. Max coverage (-): 0

Region: NODE\_118139\_length\_1594\_cov\_363.152435 751-753. Max. coverage (+): 0. Max coverage (-): 0

Region: NODE\_118139\_length\_1594\_cov\_363.152435 754-756. Max. coverage (+): 0. Max coverage (-): 0

Region: NODE\_118139\_length\_1594\_cov\_363.152435 757-760. Max. coverage (+): 0.01. Max coverage (-): 0

Region: NODE\_118139\_length\_1594\_cov\_363.152435 761-763. Max. coverage (+): 0.69. Max coverage (-): 0.08

Region: NODE\_118139\_length\_1594\_cov\_363.152435 764-766. Max. coverage (+): 0.69. Max coverage (-): 0

Region: NODE\_118139\_length\_1594\_cov\_363.152435 767-770. Max. coverage (+): 3.29. Max coverage (-): 0

Region: NODE\_118139\_length\_1594\_cov\_363.152435 771-773. Max. coverage (+): 0. Max coverage (-): 0

Region: NODE\_118139\_length\_1594\_cov\_363.152435 774-776. Max. coverage (+): 0. Max coverage (-): 0.08

Region: NODE\_118139\_length\_1594\_cov\_363.152435 777-779. Max. coverage (+): 0. Max coverage (-): 0.08

Region: NODE\_118139\_length\_1594\_cov\_363.152435 780-783. Max. coverage (+): 0. Max coverage (-): 0

Region: NODE\_118139\_length\_1594\_cov\_363.152435 784-786. Max. coverage (+): 0. Max coverage (-): 0

Region: NODE\_118139\_length\_1594\_cov\_363.152435 787-789. Max. coverage (+): 0.08. Max coverage (-): 0.08

Region: NODE\_118139\_length\_1594\_cov\_363.152435 790-793. Max. coverage (+): 0.08. Max coverage (-): 0.08

Region: NODE\_118139\_length\_1594\_cov\_363.152435 794-796. Max. coverage (+): 1.15. Max coverage (-): 0

Region: NODE\_118139\_length\_1594\_cov\_363.152435 797-799. Max. coverage (+): 1.15. Max coverage (-): 0

Region: NODE\_118139\_length\_1594\_cov\_363.152435 800-802. Max. coverage (+): 0. Max coverage (-): 0

Region: NODE\_118139\_length\_1594\_cov\_363.152435 803-806. Max. coverage (+): 0. Max coverage (-): 0.08

Region: NODE\_118139\_length\_1594\_cov\_363.152435 807-809. Max. coverage (+): 0. Max coverage (-): 0.15

Region: NODE\_118139\_length\_1594\_cov\_363.152435 810-812. Max. coverage (+): 0. Max coverage (-): 0

Region: NODE\_118139\_length\_1594\_cov\_363.152435 813-816. Max. coverage (+): 0.06. Max coverage (-): 0

Region: NODE\_118139\_length\_1594\_cov\_363.152435 817-819. Max. coverage (+): 0. Max coverage (-): 0

Region: NODE\_118139\_length\_1594\_cov\_363.152435 820-822. Max. coverage (+): 0. Max coverage (-): 0

Region: NODE\_118139\_length\_1594\_cov\_363.152435 823-826. Max. coverage (+): 1.76. Max coverage (-): 0

Region: NODE\_118139\_length\_1594\_cov\_363.152435 827-829. Max. coverage (+): 1.76. Max coverage (-): 0

Region: NODE\_118139\_length\_1594\_cov\_363.152435 830-832. Max. coverage (+): 0. Max coverage (-): 0

Region: NODE\_118139\_length\_1594\_cov\_363.152435 833-835. Max. coverage (+): 0. Max coverage (-): 0

Region: NODE\_118139\_length\_1594\_cov\_363.152435 836-839. Max. coverage (+): 0. Max coverage (-): 0.08

Region: NODE\_118139\_length\_1594\_cov\_363.152435 840-842. Max. coverage (+): 0. Max coverage (-): 0

Region: NODE\_118139\_length\_1594\_cov\_363.152435 843-845. Max. coverage (+): 0. Max coverage (-): 0

Region: NODE\_118139\_length\_1594\_cov\_363.152435 846-849. Max. coverage (+): 0. Max coverage (-): 0

Region: NODE\_118139\_length\_1594\_cov\_363.152435 850-852. Max. coverage (+): 0.01. Max coverage (-): 0

Region: NODE\_118139\_length\_1594\_cov\_363.152435 853-855. Max. coverage (+): 0.01. Max coverage (-): 0.05

Region: NODE\_118139\_length\_1594\_cov\_363.152435 856-859. Max. coverage (+): 0.03. Max coverage (-): 0.04

Region: NODE\_118139\_length\_1594\_cov\_363.152435 860-862. Max. coverage (+): 0.03. Max coverage (-): 0.03

Region: NODE\_118139\_length\_1594\_cov\_363.152435 863-865. Max. coverage (+): 0. Max coverage (-): 0

Region: NODE\_118139\_length\_1594\_cov\_363.152435 866-868. Max. coverage (+): 0. Max coverage (-): 0.07

Region: NODE\_118139\_length\_1594\_cov\_363.152435 869-872. Max. coverage (+): 0. Max coverage (-): 0.03

Region: NODE\_118139\_length\_1594\_cov\_363.152435 873-875. Max. coverage (+): 0.72. Max coverage (-): 0

Region: NODE\_118139\_length\_1594\_cov\_363.152435 876-878. Max. coverage (+): 0.72. Max coverage (-): 0

Region: NODE\_118139\_length\_1594\_cov\_363.152435 879-882. Max. coverage (+): 0.31. Max coverage (-): 0

Region: NODE\_118139\_length\_1594\_cov\_363.152435 883-885. Max. coverage (+): 0.15. Max coverage (-): 0

Region: NODE\_118139\_length\_1594\_cov\_363.152435 886-888. Max. coverage (+): 0. Max coverage (-): 0

Region: NODE\_118139\_length\_1594\_cov\_363.152435 889-891. Max. coverage (+): 0. Max coverage (-): 2.91

Region: NODE\_118139\_length\_1594\_cov\_363.152435 892-895. Max. coverage (+): 0. Max coverage (-): 4.21

Region: NODE\_118139\_length\_1594\_cov\_363.152435 896-898. Max. coverage (+): 0.31. Max coverage (-): 0.54

Region: NODE\_118139\_length\_1594\_cov\_363.152435 899-901. Max. coverage (+): 0.31. Max coverage (-): 0.08

Region: NODE\_118139\_length\_1594\_cov\_363.152435 902-905. Max. coverage (+): 0. Max coverage (-): 0.83

Region: NODE\_118139\_length\_1594\_cov\_363.152435 906-908. Max. coverage (+): 0. Max coverage (-): 0.18

Region: NODE\_118139\_length\_1594\_cov\_363.152435 909-911. Max. coverage (+): 0.03. Max coverage (-): 0

Region: NODE\_118139\_length\_1594\_cov\_363.152435 912-915. Max. coverage (+): 0.02. Max coverage (-): 0

Region: NODE\_118139\_length\_1594\_cov\_363.152435 916-918. Max. coverage (+): 0. Max coverage (-): 0

Region: NODE\_118139\_length\_1594\_cov\_363.152435 919-921. Max. coverage (+): 0. Max coverage (-): 0

Region: NODE\_118139\_length\_1594\_cov\_363.152435 922-924. Max. coverage (+): 4.08. Max coverage (-): 0

Region: NODE\_118139\_length\_1594\_cov\_363.152435 925-928. Max. coverage (+): 3.85. Max coverage (-): 0

Region: NODE\_118139\_length\_1594\_cov\_363.152435 929-931. Max. coverage (+): 0.08. Max coverage (-): 0

Region: NODE\_118139\_length\_1594\_cov\_363.152435 932-934. Max. coverage (+): 0.15. Max coverage (-): 0

Region: NODE\_118139\_length\_1594\_cov\_363.152435 935-938. Max. coverage (+): 0. Max coverage (-): 0

Region: NODE\_118139\_length\_1594\_cov\_363.152435 939-941. Max. coverage (+): 0. Max coverage (-): 0

Region: NODE\_118139\_length\_1594\_cov\_363.152435 942-944. Max. coverage (+): 0. Max coverage (-): 0

Region: NODE\_118139\_length\_1594\_cov\_363.152435 945-948. Max. coverage (+): 0. Max coverage (-): 0

Region: NODE\_118139\_length\_1594\_cov\_363.152435 949-951. Max. coverage (+): 0. Max coverage (-): 0.08

Region: NODE\_118139\_length\_1594\_cov\_363.152435 952-954. Max. coverage (+): 0. Max coverage (-): 0.08

Region: NODE\_118139\_length\_1594\_cov\_363.152435 955-957. Max. coverage (+): 0. Max coverage (-): 0

Region: NODE\_118139\_length\_1594\_cov\_363.152435 958-961. Max. coverage (+): 0. Max coverage (-): 0

Region: NODE\_118139\_length\_1594\_cov\_363.152435 962-964. Max. coverage (+): 0. Max coverage (-): 0

Region: NODE\_118139\_length\_1594\_cov\_363.152435 965-967. Max. coverage (+): 0. Max coverage (-): 0.61

Region: NODE\_118139\_length\_1594\_cov\_363.152435 968-971. Max. coverage (+): 0. Max coverage (-): 0.61

Region: NODE\_118139\_length\_1594\_cov\_363.152435 972-974. Max. coverage (+): 0.08. Max coverage (-): 0.08

Region: NODE\_118139\_length\_1594\_cov\_363.152435 975-977. Max. coverage (+): 0.08. Max coverage (-): 0

Region: NODE\_118139\_length\_1594\_cov\_363.152435 978-980. Max. coverage (+): 0.08. Max coverage (-): 0.08

Region: NODE\_118139\_length\_1594\_cov\_363.152435 981-984. Max. coverage (+): 0. Max coverage (-): 0

Region: NODE\_118139\_length\_1594\_cov\_363.152435 985-987. Max. coverage (+): 0. Max coverage (-): 0.08

Region: NODE\_118139\_length\_1594\_cov\_363.152435 988-990. Max. coverage (+): 0. Max coverage (-): 0.08

Region: NODE\_118139\_length\_1594\_cov\_363.152435 991-994. Max. coverage (+): 0. Max coverage (-): 0.08

Region: NODE\_118139\_length\_1594\_cov\_363.152435 995-997. Max. coverage (+): 0.08. Max coverage (-): 0

Region: NODE\_118139\_length\_1594\_cov\_363.152435 998-1000. Max. coverage (+): 0.15. Max coverage (-): 0.08

Region: NODE\_118139\_length\_1594\_cov\_363.152435 1001-1004. Max. coverage (+): 0.92. Max coverage (-): 0

Region: NODE\_118139\_length\_1594\_cov\_363.152435 1005-1007. Max. coverage (+): 0.54. Max coverage (-): 0

Region: NODE\_118139\_length\_1594\_cov\_363.152435 1008-1010. Max. coverage (+): 0.54. Max coverage (-): 0

Region: NODE\_118139\_length\_1594\_cov\_363.152435 1011-1013. Max. coverage (+): 0.92. Max coverage (-): 0

Region: NODE\_118139\_length\_1594\_cov\_363.152435 1014-1017. Max. coverage (+): 3.91. Max coverage (-): 0

Region: NODE\_118139\_length\_1594\_cov\_363.152435 1018-1020. Max. coverage (+): 3.91. Max coverage (-): 0.08

Region: NODE\_118139\_length\_1594\_cov\_363.152435 1021-1023. Max. coverage (+): 0.15. Max coverage (-): 0.08

Region: NODE\_118139\_length\_1594\_cov\_363.152435 1024-1027. Max. coverage (+): 0.15. Max coverage (-): 0

Region: NODE\_118139\_length\_1594\_cov\_363.152435 1028-1030. Max. coverage (+): 0. Max coverage (-): 0.38

Region: NODE\_118139\_length\_1594\_cov\_363.152435 1031-1033. Max. coverage (+): 0. Max coverage (-): 0.46

Region: NODE\_118139\_length\_1594\_cov\_363.152435 1034-1036. Max. coverage (+): 1.3. Max coverage (-): 0.08

Region: NODE\_118139\_length\_1594\_cov\_363.152435 1037-1040. Max. coverage (+): 2.3. Max coverage (-): 0.15

Region: NODE\_118139\_length\_1594\_cov\_363.152435 1041-1043. Max. coverage (+): 0.08. Max coverage (-): 0.15

Region: NODE\_118139\_length\_1594\_cov\_363.152435 1044-1046. Max. coverage (+): 0.08. Max coverage (-): 0

Region: NODE\_118139\_length\_1594\_cov\_363.152435 1047-1050. Max. coverage (+): 0.23. Max coverage (-): 0

Region: NODE\_118139\_length\_1594\_cov\_363.152435 1051-1053. Max. coverage (+): 0.23. Max coverage (-): 0

Region: NODE\_118139\_length\_1594\_cov\_363.152435 1054-1056. Max. coverage (+): 0.38. Max coverage (-): 0

Region: NODE\_118139\_length\_1594\_cov\_363.152435 1057-1060. Max. coverage (+): 0.38. Max coverage (-): 0

Region: NODE\_118139\_length\_1594\_cov\_363.152435 1061-1063. Max. coverage (+): 0. Max coverage (-): 0

Region: NODE\_118139\_length\_1594\_cov\_363.152435 1064-1066. Max. coverage (+): 0. Max coverage (-): 0

Region: NODE\_118139\_length\_1594\_cov\_363.152435 1067-1069. Max. coverage (+): 0. Max coverage (-): 0.08

Region: NODE\_118139\_length\_1594\_cov\_363.152435 1070-1073. Max. coverage (+): 0. Max coverage (-): 0.08

Region: NODE\_118139\_length\_1594\_cov\_363.152435 1074-1076. Max. coverage (+): 0. Max coverage (-): 0

Region: NODE\_118139\_length\_1594\_cov\_363.152435 1077-1079. Max. coverage (+): 0. Max coverage (-): 0

Region: NODE\_118139\_length\_1594\_cov\_363.152435 1080-1083. Max. coverage (+): 0.23. Max coverage (-): 0

Region: NODE\_118139\_length\_1594\_cov\_363.152435 1084-1086. Max. coverage (+): 0.31. Max coverage (-): 0

Region: NODE\_118139\_length\_1594\_cov\_363.152435 1087-1089. Max. coverage (+): 0.08. Max coverage (-): 0

Region: NODE\_118139\_length\_1594\_cov\_363.152435 1090-1093. Max. coverage (+): 0. Max coverage (-): 0

Region: NODE\_118139\_length\_1594\_cov\_363.152435 1094-1096. Max. coverage (+): 0. Max coverage (-): 0

Region: NODE\_118139\_length\_1594\_cov\_363.152435 1097-1099. Max. coverage (+): 0. Max coverage (-): 0.15

Region: NODE\_118139\_length\_1594\_cov\_363.152435 1100-1102. Max. coverage (+): 0.08. Max coverage (-): 0.15

Region: NODE\_118139\_length\_1594\_cov\_363.152435 1103-1106. Max. coverage (+): 0.08. Max coverage (-): 0.03

Region: NODE\_118139\_length\_1594\_cov\_363.152435 1107-1109. Max. coverage (+): 0. Max coverage (-): 0

Region: NODE\_118139\_length\_1594\_cov\_363.152435 1110-1112. Max. coverage (+): 0. Max coverage (-): 0

Region: NODE\_118139\_length\_1594\_cov\_363.152435 1113-1116. Max. coverage (+): 0. Max coverage (-): 0

Region: NODE\_118139\_length\_1594\_cov\_363.152435 1117-1119. Max. coverage (+): 1. Max coverage (-): 0

Region: NODE\_118139\_length\_1594\_cov\_363.152435 1120-1122. Max. coverage (+): 1.46. Max coverage (-): 0

Region: NODE\_118139\_length\_1594\_cov\_363.152435 1123-1125. Max. coverage (+): 0.61. Max coverage (-): 0

Region: NODE\_118139\_length\_1594\_cov\_363.152435 1126-1129. Max. coverage (+): 0. Max coverage (-): 0

Region: NODE\_118139\_length\_1594\_cov\_363.152435 1130-1132. Max. coverage (+): 0. Max coverage (-): 0.08

Region: NODE\_118139\_length\_1594\_cov\_363.152435 1133-1135. Max. coverage (+): 0. Max coverage (-): 0.46

Region: NODE\_118139\_length\_1594\_cov\_363.152435 1136-1139. Max. coverage (+): 0. Max coverage (-): 0.54

Region: NODE\_118139\_length\_1594\_cov\_363.152435 1140-1142. Max. coverage (+): 0. Max coverage (-): 0.23

Region: NODE\_118139\_length\_1594\_cov\_363.152435 1143-1145. Max. coverage (+): 0. Max coverage (-): 0.23

Region: NODE\_118139\_length\_1594\_cov\_363.152435 1146-1149. Max. coverage (+): 0. Max coverage (-): 0

Region: NODE\_118139\_length\_1594\_cov\_363.152435 1150-1152. Max. coverage (+): 0.54. Max coverage (-): 0

Region: NODE\_118139\_length\_1594\_cov\_363.152435 1153-1155. Max. coverage (+): 3.06. Max coverage (-): 0

Region: NODE\_118139\_length\_1594\_cov\_363.152435 1156-1158. Max. coverage (+): 3.37. Max coverage (-): 0

Region: NODE\_118139\_length\_1594\_cov\_363.152435 1159-1162. Max. coverage (+): 0.46. Max coverage (-): 0

Region: NODE\_118139\_length\_1594\_cov\_363.152435 1163-1165. Max. coverage (+): 0.08. Max coverage (-): 0.04

Region: NODE\_118139\_length\_1594\_cov\_363.152435 1166-1168. Max. coverage (+): 0. Max coverage (-): 0.04

Region: NODE\_118139\_length\_1594\_cov\_363.152435 1169-1172. Max. coverage (+): 0. Max coverage (-): 0

Region: NODE\_118139\_length\_1594\_cov\_363.152435 1173-1175. Max. coverage (+): 0. Max coverage (-): 0

Region: NODE\_118139\_length\_1594\_cov\_363.152435 1176-1178. Max. coverage (+): 0. Max coverage (-): 0

Region: NODE\_118139\_length\_1594\_cov\_363.152435 1179-1182. Max. coverage (+): 0. Max coverage (-): 0

Region: NODE\_118139\_length\_1594\_cov\_363.152435 1183-1185. Max. coverage (+): 0. Max coverage (-): 0

Region: NODE\_118139\_length\_1594\_cov\_363.152435 1186-1188. Max. coverage (+): 0. Max coverage (-): 0

Region: NODE\_118139\_length\_1594\_cov\_363.152435 1189-1191. Max. coverage (+): 0. Max coverage (-): 0

Region: NODE\_118139\_length\_1594\_cov\_363.152435 1192-1195. Max. coverage (+): 0. Max coverage (-): 0.38

Region: NODE\_118139\_length\_1594\_cov\_363.152435 1196-1198. Max. coverage (+): 0.08. Max coverage (-): 0.38

Region: NODE\_118139\_length\_1594\_cov\_363.152435 1199-1201. Max. coverage (+): 0.08. Max coverage (-): 0

Region: NODE\_118139\_length\_1594\_cov\_363.152435 1202-1205. Max. coverage (+): 0.08. Max coverage (-): 0

Region: NODE\_118139\_length\_1594\_cov\_363.152435 1206-1208. Max. coverage (+): 7.13. Max coverage (-): 0.08

Region: NODE\_118139\_length\_1594\_cov\_363.152435 1209-1211. Max. coverage (+): 11.57. Max coverage (-): 0.23

Region: NODE\_118139\_length\_1594\_cov\_363.152435 1212-1214. Max. coverage (+): 4.67. Max coverage (-): 0.15

Region: NODE\_118139\_length\_1594\_cov\_363.152435 1215-1218. Max. coverage (+): 0. Max coverage (-): 0

Region: NODE\_118139\_length\_1594\_cov\_363.152435 1219-1221. Max. coverage (+): 3.22. Max coverage (-): 0

Region: NODE\_118139\_length\_1594\_cov\_363.152435 1222-1224. Max. coverage (+): 3.22. Max coverage (-): 0

Region: NODE\_118139\_length\_1594\_cov\_363.152435 1225-1228. Max. coverage (+): 0.31. Max coverage (-): 0

Region: NODE\_118139\_length\_1594\_cov\_363.152435 1229-1231. Max. coverage (+): 0.38. Max coverage (-): 0

Region: NODE\_118139\_length\_1594\_cov\_363.152435 1232-1234. Max. coverage (+): 0.08. Max coverage (-): 0

Region: NODE\_118139\_length\_1594\_cov\_363.152435 1235-1238. Max. coverage (+): 0. Max coverage (-): 0

Region: NODE\_118139\_length\_1594\_cov\_363.152435 1239-1241. Max. coverage (+): 0. Max coverage (-): 0.08

Region: NODE\_118139\_length\_1594\_cov\_363.152435 1242-1244. Max. coverage (+): 0. Max coverage (-): 0.08

Region: NODE\_118139\_length\_1594\_cov\_363.152435 1245-1247. Max. coverage (+): 0. Max coverage (-): 0

Region: NODE\_118139\_length\_1594\_cov\_363.152435 1248-1251. Max. coverage (+): 0.08. Max coverage (-): 0

Region: NODE\_118139\_length\_1594\_cov\_363.152435 1252-1254. Max. coverage (+): 0.31. Max coverage (-): 0

Region: NODE\_118139\_length\_1594\_cov\_363.152435 1255-1257. Max. coverage (+): 0.38. Max coverage (-): 0

Region: NODE\_118139\_length\_1594\_cov\_363.152435 1258-1261. Max. coverage (+): 0.08. Max coverage (-): 0

Region: NODE\_118139\_length\_1594\_cov\_363.152435 1262-1264. Max. coverage (+): 0.15. Max coverage (-): 0

Region: NODE\_118139\_length\_1594\_cov\_363.152435 1265-1267. Max. coverage (+): 0.61. Max coverage (-): 0

Region: NODE\_118139\_length\_1594\_cov\_363.152435 1268-1271. Max. coverage (+): 1.23. Max coverage (-): 0

Region: NODE\_118139\_length\_1594\_cov\_363.152435 1272-1274. Max. coverage (+): 0.46. Max coverage (-): 0

Region: NODE\_118139\_length\_1594\_cov\_363.152435 1275-1277. Max. coverage (+): 0. Max coverage (-): 0

Region: NODE\_118139\_length\_1594\_cov\_363.152435 1278-1280. Max. coverage (+): 0. Max coverage (-): 0

Region: NODE\_118139\_length\_1594\_cov\_363.152435 1281-1284. Max. coverage (+): 0. Max coverage (-): 0.15

Region: NODE\_118139\_length\_1594\_cov\_363.152435 1285-1287. Max. coverage (+): 0.08. Max coverage (-): 0.23

Region: NODE\_118139\_length\_1594\_cov\_363.152435 1288-1290. Max. coverage (+): 0.1. Max coverage (-): 0.1

Region: NODE\_118139\_length\_1594\_cov\_363.152435 1291-1294. Max. coverage (+): 0.06. Max coverage (-): 0

Region: NODE\_118139\_length\_1594\_cov\_363.152435 1295-1297. Max. coverage (+): 0.02. Max coverage (-): 0

Region: NODE\_118139\_length\_1594\_cov\_363.152435 1298-1300. Max. coverage (+): 4.23. Max coverage (-): 0

Region: NODE\_118139\_length\_1594\_cov\_363.152435 1301-1303. Max. coverage (+): 4.21. Max coverage (-): 0

Region: NODE\_118139\_length\_1594\_cov\_363.152435 1304-1307. Max. coverage (+): 0.15. Max coverage (-): 0

Region: NODE\_118139\_length\_1594\_cov\_363.152435 1308-1310. Max. coverage (+): 0.08. Max coverage (-): 0

Region: NODE\_118139\_length\_1594\_cov\_363.152435 1311-1313. Max. coverage (+): 0.08. Max coverage (-): 0

Region: NODE\_118139\_length\_1594\_cov\_363.152435 1314-1317. Max. coverage (+): 0. Max coverage (-): 0

Region: NODE\_118139\_length\_1594\_cov\_363.152435 1318-1320. Max. coverage (+): 0. Max coverage (-): 0

Region: NODE\_118139\_length\_1594\_cov\_363.152435 1321-1323. Max. coverage (+): 0. Max coverage (-): 0

Region: NODE\_118139\_length\_1594\_cov\_363.152435 1324-1327. Max. coverage (+): 0. Max coverage (-): 0

Region: NODE\_118139\_length\_1594\_cov\_363.152435 1328-1330. Max. coverage (+): 0. Max coverage (-): 0

Region: NODE\_118139\_length\_1594\_cov\_363.152435 1331-1333. Max. coverage (+): 0.08. Max coverage (-): 0

Region: NODE\_118139\_length\_1594\_cov\_363.152435 1334-1336. Max. coverage (+): 0.54. Max coverage (-): 0

Region: NODE\_118139\_length\_1594\_cov\_363.152435 1337-1340. Max. coverage (+): 0.46. Max coverage (-): 0

Region: NODE\_118139\_length\_1594\_cov\_363.152435 1341-1343. Max. coverage (+): 0. Max coverage (-): 0

Region: NODE\_118139\_length\_1594\_cov\_363.152435 1344-1346. Max. coverage (+): 0. Max coverage (-): 0

Region: NODE\_118139\_length\_1594\_cov\_363.152435 1347-1350. Max. coverage (+): 0. Max coverage (-): 0

Region: NODE\_118139\_length\_1594\_cov\_363.152435 1351-1353. Max. coverage (+): 0. Max coverage (-): 0.31

Region: NODE\_118139\_length\_1594\_cov\_363.152435 1354-1356. Max. coverage (+): 0. Max coverage (-): 0.31

Region: NODE\_118139\_length\_1594\_cov\_363.152435 1357-1360. Max. coverage (+): 0.08. Max coverage (-): 0

Region: NODE\_118139\_length\_1594\_cov\_363.152435 1361-1363. Max. coverage (+): 0.08. Max coverage (-): 0

Region: NODE\_118139\_length\_1594\_cov\_363.152435 1364-1366. Max. coverage (+): 0. Max coverage (-): 0.08

Region: NODE\_118139\_length\_1594\_cov\_363.152435 1367-1369. Max. coverage (+): 0. Max coverage (-): 0.08

Region: NODE\_118139\_length\_1594\_cov\_363.152435 1370-1373. Max. coverage (+): 0.38. Max coverage (-): 0

Region: NODE\_118139\_length\_1594\_cov\_363.152435 1374-1376. Max. coverage (+): 0. Max coverage (-): 0

Region: NODE\_118139\_length\_1594\_cov\_363.152435 1377-1379. Max. coverage (+): 0. Max coverage (-): 0

Region: NODE\_118139\_length\_1594\_cov\_363.152435 1380-1383. Max. coverage (+): 0. Max coverage (-): 0

Region: NODE\_118139\_length\_1594\_cov\_363.152435 1384-1386. Max. coverage (+): 0. Max coverage (-): 0

Region: NODE\_118139\_length\_1594\_cov\_363.152435 1387-1389. Max. coverage (+): 0. Max coverage (-): 0

Region: NODE\_118139\_length\_1594\_cov\_363.152435 1390-1392. Max. coverage (+): 0. Max coverage (-): 0.08

Region: NODE\_118139\_length\_1594\_cov\_363.152435 1393-1396. Max. coverage (+): 0.08. Max coverage (-): 0.08

Region: NODE\_118139\_length\_1594\_cov\_363.152435 1397-1399. Max. coverage (+): 0.08. Max coverage (-): 0

Region: NODE\_118139\_length\_1594\_cov\_363.152435 1400-1402. Max. coverage (+): 0. Max coverage (-): 0

Region: NODE\_118139\_length\_1594\_cov\_363.152435 1403-1406. Max. coverage (+): 0.08. Max coverage (-): 0

Region: NODE\_118139\_length\_1594\_cov\_363.152435 1407-1409. Max. coverage (+): 0.08. Max coverage (-): 0

Region: NODE\_118139\_length\_1594\_cov\_363.152435 1410-1412. Max. coverage (+): 0.15. Max coverage (-): 0.15

Region: NODE\_118139\_length\_1594\_cov\_363.152435 1413-1416. Max. coverage (+): 0.23. Max coverage (-): 0.15

Region: NODE\_118139\_length\_1594\_cov\_363.152435 1417-1419. Max. coverage (+): 0. Max coverage (-): 0

Region: NODE\_118139\_length\_1594\_cov\_363.152435 1420-1422. Max. coverage (+): 0. Max coverage (-): 0

Region: NODE\_118139\_length\_1594\_cov\_363.152435 1423-1425. Max. coverage (+): 0. Max coverage (-): 0

Region: NODE\_118139\_length\_1594\_cov\_363.152435 1426-1429. Max. coverage (+): 0. Max coverage (-): 0

Region: NODE\_118139\_length\_1594\_cov\_363.152435 1430-1432. Max. coverage (+): 0. Max coverage (-): 0

Region: NODE\_118139\_length\_1594\_cov\_363.152435 1433-1435. Max. coverage (+): 0. Max coverage (-): 0

Region: NODE\_118139\_length\_1594\_cov\_363.152435 1436-1439. Max. coverage (+): 0. Max coverage (-): 0

Region: NODE\_118139\_length\_1594\_cov\_363.152435 1440-1442. Max. coverage (+): 0. Max coverage (-): 0

Region: NODE\_118139\_length\_1594\_cov\_363.152435 1443-1445. Max. coverage (+): 0. Max coverage (-): 0.31

Region: NODE\_118139\_length\_1594\_cov\_363.152435 1446-1448. Max. coverage (+): 0. Max coverage (-): 0.31

Region: NODE\_118139\_length\_1594\_cov\_363.152435 1449-1452. Max. coverage (+): 0. Max coverage (-): 0

Region: NODE\_118139\_length\_1594\_cov\_363.152435 1453-1455. Max. coverage (+): 0. Max coverage (-): 0

Region: NODE\_118139\_length\_1594\_cov\_363.152435 1456-1458. Max. coverage (+): 1. Max coverage (-): 0

Region: NODE\_118139\_length\_1594\_cov\_363.152435 1459-1462. Max. coverage (+): 1.03. Max coverage (-): 0

Region: NODE\_118139\_length\_1594\_cov\_363.152435 1463-1465. Max. coverage (+): 0.27. Max coverage (-): 0

Region: NODE\_118139\_length\_1594\_cov\_363.152435 1466-1468. Max. coverage (+): 0.23. Max coverage (-): 0

Region: NODE\_118139\_length\_1594\_cov\_363.152435 1469-1472. Max. coverage (+): 0. Max coverage (-): 0

Region: NODE\_118139\_length\_1594\_cov\_363.152435 1473-1475. Max. coverage (+): 0. Max coverage (-): 0

Region: NODE\_118139\_length\_1594\_cov\_363.152435 1476-1478. Max. coverage (+): 0.08. Max coverage (-): 0

Region: NODE\_118139\_length\_1594\_cov\_363.152435 1479-1481. Max. coverage (+): 0.08. Max coverage (-): 0

Region: NODE\_118139\_length\_1594\_cov\_363.152435 1482-1485. Max. coverage (+): 0. Max coverage (-): 0

Region: NODE\_118139\_length\_1594\_cov\_363.152435 1486-1488. Max. coverage (+): 0. Max coverage (-): 0

Region: NODE\_118139\_length\_1594\_cov\_363.152435 1489-1491. Max. coverage (+): 0. Max coverage (-): 0

Region: NODE\_118139\_length\_1594\_cov\_363.152435 1492-1495. Max. coverage (+): 0. Max coverage (-): 0

Region: NODE\_118139\_length\_1594\_cov\_363.152435 1496-1498. Max. coverage (+): 0. Max coverage (-): 0

Region: NODE\_118139\_length\_1594\_cov\_363.152435 1499-1501. Max. coverage (+): 0. Max coverage (-): 0

Region: NODE\_118139\_length\_1594\_cov\_363.152435 1502-1505. Max. coverage (+): 0.01. Max coverage (-): 0

Region: NODE\_118139\_length\_1594\_cov\_363.152435 1506-1508. Max. coverage (+): 0.1. Max coverage (-): 0

Region: NODE\_118139\_length\_1594\_cov\_363.152435 1509-1511. Max. coverage (+): 0.09. Max coverage (-): 0

Region: NODE\_118139\_length\_1594\_cov\_363.152435 1512-1514. Max. coverage (+): 0. Max coverage (-): 0

Region: NODE\_118139\_length\_1594\_cov\_363.152435 1515-1518. Max. coverage (+): 0. Max coverage (-): 0

Region: NODE\_118139\_length\_1594\_cov\_363.152435 1519-1521. Max. coverage (+): 0. Max coverage (-): 0

Region: NODE\_118139\_length\_1594\_cov\_363.152435 1522-1524. Max. coverage (+): 0. Max coverage (-): 0

Region: NODE\_118139\_length\_1594\_cov\_363.152435 1525-1528. Max. coverage (+): 0. Max coverage (-): 0

Region: NODE\_118139\_length\_1594\_cov\_363.152435 1529-1531. Max. coverage (+): 0. Max coverage (-): 0

Region: NODE\_118139\_length\_1594\_cov\_363.152435 1532-1534. Max. coverage (+): 0. Max coverage (-): 0

Region: NODE\_118139\_length\_1594\_cov\_363.152435 1535-1537. Max. coverage (+): 0.08. Max coverage (-): 0

Region: NODE\_118139\_length\_1594\_cov\_363.152435 1538-1541. Max. coverage (+): 0.08. Max coverage (-): 0

Region: NODE\_118139\_length\_1594\_cov\_363.152435 1542-1544. Max. coverage (+): 0. Max coverage (-): 0

Region: NODE\_118139\_length\_1594\_cov\_363.152435 1545-1547. Max. coverage (+): 0. Max coverage (-): 0

Region: NODE\_118139\_length\_1594\_cov\_363.152435 1548-1551. Max. coverage (+): 0.31. Max coverage (-): 0

Region: NODE\_118139\_length\_1594\_cov\_363.152435 1552-1554. Max. coverage (+): 0.31. Max coverage (-): 0.23

Region: NODE\_118139\_length\_1594\_cov\_363.152435 1555-1557. Max. coverage (+): 0.08. Max coverage (-): 0.23

Region: NODE\_118139\_length\_1594\_cov\_363.152435 1558-1561. Max. coverage (+): 0.61. Max coverage (-): 0

Region: NODE\_118139\_length\_1594\_cov\_363.152435 1562-1564. Max. coverage (+): 0.61. Max coverage (-): 0

Region: NODE\_118139\_length\_1594\_cov\_363.152435 1565-1567. Max. coverage (+): 0. Max coverage (-): 0.08

Region: NODE\_118139\_length\_1594\_cov\_363.152435 1568-1570. Max. coverage (+): 0. Max coverage (-): 0.08

Region: NODE\_118139\_length\_1594\_cov\_363.152435 1571-1574. Max. coverage (+): 0.69. Max coverage (-): 0

Region: NODE\_118139\_length\_1594\_cov\_363.152435 1575-1577. Max. coverage (+): 0.77. Max coverage (-): 0.08

Region: NODE\_118139\_length\_1594\_cov\_363.152435 1578-1580. Max. coverage (+): 0.08. Max coverage (-): 0.15

Region: NODE\_118139\_length\_1594\_cov\_363.152435 1581-1584. Max. coverage (+): 0.08. Max coverage (-): 0.08

Region: NODE\_118139\_length\_1594\_cov\_363.152435 1585-1587. Max. coverage (+): 0. Max coverage (-): 0

Region: NODE\_118139\_length\_1594\_cov\_363.152435 1588-1590. Max. coverage (+): 0. Max coverage (-): 0

Region: NODE\_118139\_length\_1594\_cov\_363.152435 1591-1594. Max. coverage (+): 0. Max coverage (-): 0.08

Region: NODE\_118139\_length\_1594\_cov\_363.152435 1595-1597. Max. coverage (+): 0. Max coverage (-): 0.08

Region: NODE\_118139\_length\_1594\_cov\_363.152435 1598-1600. Max. coverage (+): 0. Max coverage (-): 0

Region: NODE\_118139\_length\_1594\_cov\_363.152435 1601-1603. Max. coverage (+): 0. Max coverage (-): 0

Region: NODE\_118139\_length\_1594\_cov\_363.152435 1604-1607. Max. coverage (+): 0.09. Max coverage (-): 0

Region: NODE\_118139\_length\_1594\_cov\_363.152435 1608-1610. Max. coverage (+): 2.52. Max coverage (-): 0

Region: NODE\_118139\_length\_1594\_cov\_363.152435 1611-1613. Max. coverage (+): 2.52. Max coverage (-): 0

Region: NODE\_118139\_length\_1594\_cov\_363.152435 1614-1617. Max. coverage (+): 0.01. Max coverage (-): 0

Region: NODE\_118139\_length\_1594\_cov\_363.152435 1618-1620. Max. coverage (+): 0. Max coverage (-): 0

Region: NODE\_118139\_length\_1594\_cov\_363.152435 1621-1623. Max. coverage (+): 0. Max coverage (-): 0.01

Region: NODE\_118139\_length\_1594\_cov\_363.152435 1624-1626. Max. coverage (+): 0.01. Max coverage (-): 0.03

Region: NODE\_118139\_length\_1594\_cov\_363.152435 1627-1630. Max. coverage (+): 0.01. Max coverage (-): 0.03

Region: NODE\_118139\_length\_1594\_cov\_363.152435 1631-1633. Max. coverage (+): 0. Max coverage (-): 0

Region: NODE\_118139\_length\_1594\_cov\_363.152435 1634-1636. Max. coverage (+): 0. Max coverage (-): 0

Region: NODE\_118139\_length\_1594\_cov\_363.152435 1637-1640. Max. coverage (+): 0. Max coverage (-): 0

Region: NODE\_118139\_length\_1594\_cov\_363.152435 1641-1643. Max. coverage (+): 0. Max coverage (-): 0

Region: NODE\_118139\_length\_1594\_cov\_363.152435 1644-1646. Max. coverage (+): 0. Max coverage (-): 0

Region: NODE\_118139\_length\_1594\_cov\_363.152435 1647-1650. Max. coverage (+): 0. Max coverage (-): 0

Region: NODE\_118139\_length\_1594\_cov\_363.152435 1651-1653. Max. coverage (+): 0. Max coverage (-): 0

Region: NODE\_118139\_length\_1594\_cov\_363.152435 1654-. Max. coverage (+): 0. Max coverage (-): 0

RepeatMasker Color Code

**+**

100-98% Identity

<98-95% Identity

<95-90% Identity

<90-85% Identity

<85-80% Identity

<80-75% Identity

<75-70% Identity

<70% Identity

**-**

Gene Set Color Code

**+**

Gene

Pseudogene

Other

**-**

Topology/Coverage Color Code

Coverage Plus Strand

Coverage Minus Strand

Mainstrand: Plus

Mainstrand: Minus

Complementary Strand

Flanking Region  
(if option -flank >0)

Gene Set Annotation  
  
RepeatMasker Annotation  

**1. L1-24\_DR**: 81-1650 (+), Divergence to consensus: 41.2%

  
Transcription Factor Binding Sites  

**RHOXF1** (Sequence: AGCTTA (-): 13)  
**RHOXF1** (Sequence: AGATCA (-): 256)  
**RHOXF1** (Sequence: GGATCA (-): 350)  
**RHOXF1** (Sequence: GGATCA (-): 386)  
**RHOXF1** (Sequence: AGCTTA (-): 876)  
**RHOXF1** (Sequence: GGCTCA (-): 1109)  
**RHOXF1** (Sequence: GGATCA (-): 1329)  
**RHOXF1** (Sequence: AGCTTA (-): 1345)  
**RHOXF1** (Sequence: AGATCA (-): 1372)  
**RHOXF1** (Sequence: AGCTCA (-): 1497)  
**RHOXF1** (Sequence: TGAGCT (+): 874)  
**RHOXF1** (Sequence: TGAGCT (+): 1037)  
**RHOXF1** (Sequence: TGAGCC (+): 1072)  
**RHOXF1** (Sequence: TGAGCC (+): 1100)  
**RHOXF1** (Sequence: TGAGCT (+): 1343)  
**FOXO3\_mmu** (Sequence: TGTTTTGA (-): 929)  
**Nobox** (Sequence: AGCAATTA (-): 37)  
**Nobox** (Sequence: ACCAATTA (-): 440)  
**FOXO1** (Sequence: GAAAACAAC (-): 112)  
**Rhox11** (Sequence: TGCTGTTTT (+): 926)  
**POU5F1** (Sequence: ATGCAAA (+): 231)
