# Supplementary material for: First characterization of PIWI-interacting RNA clusters in a cichlid fish with a B chromosome
Source: BMC Biol. 2022 Sep 21;20:204. doi: 10.1186/s12915-022-01403-2 (PMC9490952; doi:10.1186/s12915-022-01403-2)
Supplement: Supplementary file 1 — Additional file 1. Zipped folder with fasta and interactive html piRNA cluster information for the A. latifasciata genome. The nomenclature is as follows: number-pirna-cluster_sex_B-presence (f, female; m, male; 0b, without B chromosome; 1b, with B chromosome). [file 12915_2022_1403_MOESM1_ESM.zip › 130_m1b.html]

piRNA cluster 130\_m1b 76


Predicted piRNA cluster no. 130\_m1b
  

Show proTRAC run info
Hide proTRAC run info

/\  
                \_\_\_\_\_\_\_\_\_\_\_\_\_\_\_\_\_\_\_\_\_\_\_/\\_\_\_ /  \\_\_\_\_\_\_\_  
               I                      /  \  /    \      I  
               I     pro             /    \/      \     I  
               I        TRAC        /               \   I  
               I   \_\_\_\_\_\_\_\_\_\_\_\_\_\_\_\_/\_\_\_\_\_\_\_\_\_\_\_\_\_\_\_\_\_\\_ I  
               I   \              /                     I  
               I    \            /                      I  
               I     \  /\      /       V.2.4.2         I  
               I      \/  \    /                        I  
               I\_\_\_\_\_\_\_\_\_\_\_\  /\_\_\_\_\_\_\_\_\_\_\_\_\_\_\_\_\_\_\_\_\_\_\_\_\_I  
                            \/  
  
  
================================= proTRAC ====================================  
VERSION: .......... 2.4.2  
LAST MODIFIED: .... 11. May 2018  
  
Please cite:  
Rosenkranz D, Zischler H. proTRAC - a software for probabilistic piRNA cluster  
detection, visualization and analysis. 2012. BMC Bioinformatics 13:5.  
  
  
Contact:  
David Rosenkranz  
Institute of Organismic and Molecular Evolutionary Biology  
Dept. Anthropology, small RNA group  
Johannes Gutenberg University Mainz  
email: rosenkranz@uni-mainz.de  
  
You can find the latest proTRAC version at:  
http://sourceforge.net/projects/protrac/files  
http://www.smallRNAgroup-mainz.de/software  
==============================================================================  
  
PARAMETERS:  
Map file: ...............piwi-machos-1B.fa-collapse.map  
Genome file: ............../../../0B\_ala\_genome.fa  
RepeatMasker annotation: Alatifasciata-all0B-maryan-v2.fa\_corrected.out  
GeneSet:................./guest-storage/Data/annotation/Alatifasciata\_all0B\_maryan-v2\_out2017.gff  
  
Significant (p<=0.01) hit density will be calculated based  
on observed hit distribution.  
  
Sliding window size: ........................................ 5000 bp  
Sliding window increament: .................................. 1000 bp  
Normalize each hit by number of genomic hits: ............... yes  
Normalize each hit by number of sequence reads: ............. yes  
Normalize values (-> per million mapped reads): ............. yes  
Min. fraction of hits with 1T(U) or 10A: .................... 0.75  
Alternatively: Min. fraction of hits with 1T(U) and 10A: .... 0.5  
Min. fraction of hits with typical piRNA length: ............ 0.75  
Typical piRNA length: ....................................... 24-32 nt  
Min. size of a piRNA cluster: ............................... 1000 bp.  
Min. number of hits (absolute): ............................. 0  
Min. number of hits (normalized): ........................... 0  
Min. fraction of hits on the mainstrand: .................... 0.75  
Top fraction of mapped sequences (in terms of read counts): . 1%  
Top fraction accounts for max. n% of sequence reads: ........ 90%  
Min. fraction of hits on each arm of a bidirectional cluster: 0.05  
Output html file for each cluster: .......................... yes  
Output a summary table: ..................................... yes  
Output a FASTA file for each cluster (piRNA sequences): ..... yes  
Output a FASTA file comprising cluster sequences: ........... yes  
Output a GTF file for predicted piRNA clusters: ..............yes  
Search DNA motifs in clusters: .............................. yes  
Output flanking sequences: +/- .............................. 0 bp  
Output ~.pTi file: .......................................... no  
==============================================================================  
  
  
Genome size (without gaps): ............ 758543724 bp  
Gaps (N/X/-): .......................... 417479 bp  
Mapped reads: .......................... 26973943  
Non-identical sequences: ............... 6209225  
Genomic hits: .......................... 48438990  
Significant densitiy of mapped reads: .. 821.144211136946 reads/kb

Show proTRAC cluster info
Hide proTRAC cluster info

|  |  |
| --- | --- |
| Location | NODE\_329218\_length\_1765\_cov\_29.614164 |
| Coordinates | 1-1824 |
| Size [bp] | 1824 |
| Sequence hit loci | 3893 |
| Mapped reads (normalized) | 28498 |
| Mapped reads (normalized) per kb | 15623.9 |
| Normalized reads with 1T (1U) | 87.8% |
| Normalized reads with 10A | 27.4% |
| Normalized reads with length 24-32 nt | 98.9% |
| Normalized reads on the main strand(s) | 95.2% |
| Predicted directionality | mono:plus |

100%

0%

1T (1U)  
reads

10A reads

24-32 nt  
reads

reads on mainstrand

**Either the amount of reads with 1T (1U) OR 10A has to exceed 75% (set with option: -1Tor10A)  
Alternatively the amount of reads with 1T (1U) AND 10A has to exceed 50% (set with option: -1Tand10A)  
Minimum amount of reads with preferred size is 75% (set with option: -pisize)  
Minimum amount of reads on the main strand(s) is 75% (set with option: -clstrand)**

Show read coverage
Hide read coverage

WHAT DO I SEE HERE?  
This chart shows the location of mapped sequence reads within a predicted piRNA cluster. The color refers to the number of genomic hits produced by the sequence read in question. A dark red bar indicates that this sequence read produces many other hits elsewhere in the genome. Many adjacent red or yellow bars can indicate the presence of a multi-copy element such as transposons or rRNA genes. A dark green bar indicates that this sequence read maps uniquely to this locus.

1 hit

2-5 hits

6-10 hits

11-20 hits

21-50 hits

51-100 hits

> 100 hits

NODE\_329218\_length\_1765\_cov\_29.614164

1

1824

Gene Set

RepeatMasker

Mapped  
Reads

122.3

plus strand

minus strand

122.3

Region: NODE\_329218\_length\_1765\_cov\_29.614164 66922-2. Max. coverage (+): 0.26. Max coverage (-): 0

Region: NODE\_329218\_length\_1765\_cov\_29.614164 3-6. Max. coverage (+): 0.03. Max coverage (-): 0

Region: NODE\_329218\_length\_1765\_cov\_29.614164 7-10. Max. coverage (+): 0.01. Max coverage (-): 0

Region: NODE\_329218\_length\_1765\_cov\_29.614164 11-13. Max. coverage (+): 0.07. Max coverage (-): 0

Region: NODE\_329218\_length\_1765\_cov\_29.614164 14-17. Max. coverage (+): 0.07. Max coverage (-): 0

Region: NODE\_329218\_length\_1765\_cov\_29.614164 18-21. Max. coverage (+): 0.03. Max coverage (-): 0

Region: NODE\_329218\_length\_1765\_cov\_29.614164 22-24. Max. coverage (+): 0. Max coverage (-): 0.01

Region: NODE\_329218\_length\_1765\_cov\_29.614164 25-28. Max. coverage (+): 0. Max coverage (-): 0.02

Region: NODE\_329218\_length\_1765\_cov\_29.614164 29-32. Max. coverage (+): 0. Max coverage (-): 0

Region: NODE\_329218\_length\_1765\_cov\_29.614164 33-35. Max. coverage (+): 0. Max coverage (-): 0

Region: NODE\_329218\_length\_1765\_cov\_29.614164 36-39. Max. coverage (+): 0. Max coverage (-): 0

Region: NODE\_329218\_length\_1765\_cov\_29.614164 40-42. Max. coverage (+): 0. Max coverage (-): 0

Region: NODE\_329218\_length\_1765\_cov\_29.614164 43-46. Max. coverage (+): 0.07. Max coverage (-): 0

Region: NODE\_329218\_length\_1765\_cov\_29.614164 47-50. Max. coverage (+): 0.11. Max coverage (-): 0

Region: NODE\_329218\_length\_1765\_cov\_29.614164 51-53. Max. coverage (+): 0.07. Max coverage (-): 0

Region: NODE\_329218\_length\_1765\_cov\_29.614164 54-57. Max. coverage (+): 0. Max coverage (-): 0

Region: NODE\_329218\_length\_1765\_cov\_29.614164 58-61. Max. coverage (+): 0. Max coverage (-): 0

Region: NODE\_329218\_length\_1765\_cov\_29.614164 62-64. Max. coverage (+): 0. Max coverage (-): 0

Region: NODE\_329218\_length\_1765\_cov\_29.614164 65-68. Max. coverage (+): 0. Max coverage (-): 0.09

Region: NODE\_329218\_length\_1765\_cov\_29.614164 69-72. Max. coverage (+): 0. Max coverage (-): 0.02

Region: NODE\_329218\_length\_1765\_cov\_29.614164 73-75. Max. coverage (+): 0.02. Max coverage (-): 0.03

Region: NODE\_329218\_length\_1765\_cov\_29.614164 76-79. Max. coverage (+): 0.04. Max coverage (-): 0.02

Region: NODE\_329218\_length\_1765\_cov\_29.614164 80-83. Max. coverage (+): 2.92. Max coverage (-): 0

Region: NODE\_329218\_length\_1765\_cov\_29.614164 84-86. Max. coverage (+): 4.36. Max coverage (-): 0

Region: NODE\_329218\_length\_1765\_cov\_29.614164 87-90. Max. coverage (+): 0.02. Max coverage (-): 0

Region: NODE\_329218\_length\_1765\_cov\_29.614164 91-94. Max. coverage (+): 0.02. Max coverage (-): 0

Region: NODE\_329218\_length\_1765\_cov\_29.614164 95-97. Max. coverage (+): 0. Max coverage (-): 0

Region: NODE\_329218\_length\_1765\_cov\_29.614164 98-101. Max. coverage (+): 0. Max coverage (-): 0

Region: NODE\_329218\_length\_1765\_cov\_29.614164 102-104. Max. coverage (+): 0. Max coverage (-): 0

Region: NODE\_329218\_length\_1765\_cov\_29.614164 105-108. Max. coverage (+): 0. Max coverage (-): 0

Region: NODE\_329218\_length\_1765\_cov\_29.614164 109-112. Max. coverage (+): 0.01. Max coverage (-): 0

Region: NODE\_329218\_length\_1765\_cov\_29.614164 113-115. Max. coverage (+): 0.02. Max coverage (-): 0.01

Region: NODE\_329218\_length\_1765\_cov\_29.614164 116-119. Max. coverage (+): 0.01. Max coverage (-): 0

Region: NODE\_329218\_length\_1765\_cov\_29.614164 120-123. Max. coverage (+): 0. Max coverage (-): 0

Region: NODE\_329218\_length\_1765\_cov\_29.614164 124-126. Max. coverage (+): 0. Max coverage (-): 0

Region: NODE\_329218\_length\_1765\_cov\_29.614164 127-130. Max. coverage (+): 0.02. Max coverage (-): 0.01

Region: NODE\_329218\_length\_1765\_cov\_29.614164 131-134. Max. coverage (+): 0.16. Max coverage (-): 0.01

Region: NODE\_329218\_length\_1765\_cov\_29.614164 135-137. Max. coverage (+): 2.13. Max coverage (-): 0

Region: NODE\_329218\_length\_1765\_cov\_29.614164 138-141. Max. coverage (+): 2.06. Max coverage (-): 0.01

Region: NODE\_329218\_length\_1765\_cov\_29.614164 142-145. Max. coverage (+): 0.98. Max coverage (-): 0.01

Region: NODE\_329218\_length\_1765\_cov\_29.614164 146-148. Max. coverage (+): 1.13. Max coverage (-): 0.01

Region: NODE\_329218\_length\_1765\_cov\_29.614164 149-152. Max. coverage (+): 1.2. Max coverage (-): 0.02

Region: NODE\_329218\_length\_1765\_cov\_29.614164 153-156. Max. coverage (+): 0.2. Max coverage (-): 0.08

Region: NODE\_329218\_length\_1765\_cov\_29.614164 157-159. Max. coverage (+): 0.01. Max coverage (-): 0.07

Region: NODE\_329218\_length\_1765\_cov\_29.614164 160-163. Max. coverage (+): 0.05. Max coverage (-): 0.1

Region: NODE\_329218\_length\_1765\_cov\_29.614164 164-166. Max. coverage (+): 0.03. Max coverage (-): 0.07

Region: NODE\_329218\_length\_1765\_cov\_29.614164 167-170. Max. coverage (+): 0.29. Max coverage (-): 0.14

Region: NODE\_329218\_length\_1765\_cov\_29.614164 171-174. Max. coverage (+): 1. Max coverage (-): 0.13

Region: NODE\_329218\_length\_1765\_cov\_29.614164 175-177. Max. coverage (+): 0.87. Max coverage (-): 0.01

Region: NODE\_329218\_length\_1765\_cov\_29.614164 178-181. Max. coverage (+): 1.36. Max coverage (-): 0.02

Region: NODE\_329218\_length\_1765\_cov\_29.614164 182-185. Max. coverage (+): 0.2. Max coverage (-): 0.02

Region: NODE\_329218\_length\_1765\_cov\_29.614164 186-188. Max. coverage (+): 0.07. Max coverage (-): 0

Region: NODE\_329218\_length\_1765\_cov\_29.614164 189-192. Max. coverage (+): 0.07. Max coverage (-): 0.01

Region: NODE\_329218\_length\_1765\_cov\_29.614164 193-196. Max. coverage (+): 0.05. Max coverage (-): 0.01

Region: NODE\_329218\_length\_1765\_cov\_29.614164 197-199. Max. coverage (+): 0. Max coverage (-): 0.01

Region: NODE\_329218\_length\_1765\_cov\_29.614164 200-203. Max. coverage (+): 0.02. Max coverage (-): 0

Region: NODE\_329218\_length\_1765\_cov\_29.614164 204-207. Max. coverage (+): 0.02. Max coverage (-): 0

Region: NODE\_329218\_length\_1765\_cov\_29.614164 208-210. Max. coverage (+): 0.03. Max coverage (-): 0.02

Region: NODE\_329218\_length\_1765\_cov\_29.614164 211-214. Max. coverage (+): 0.06. Max coverage (-): 0.02

Region: NODE\_329218\_length\_1765\_cov\_29.614164 215-218. Max. coverage (+): 0.43. Max coverage (-): 0.01

Region: NODE\_329218\_length\_1765\_cov\_29.614164 219-221. Max. coverage (+): 0.14. Max coverage (-): 0.01

Region: NODE\_329218\_length\_1765\_cov\_29.614164 222-225. Max. coverage (+): 0.09. Max coverage (-): 0

Region: NODE\_329218\_length\_1765\_cov\_29.614164 226-228. Max. coverage (+): 0.18. Max coverage (-): 0

Region: NODE\_329218\_length\_1765\_cov\_29.614164 229-232. Max. coverage (+): 0.07. Max coverage (-): 0

Region: NODE\_329218\_length\_1765\_cov\_29.614164 233-236. Max. coverage (+): 0.88. Max coverage (-): 0

Region: NODE\_329218\_length\_1765\_cov\_29.614164 237-239. Max. coverage (+): 0.1. Max coverage (-): 0

Region: NODE\_329218\_length\_1765\_cov\_29.614164 240-243. Max. coverage (+): 0.07. Max coverage (-): 0

Region: NODE\_329218\_length\_1765\_cov\_29.614164 244-247. Max. coverage (+): 0.12. Max coverage (-): 0.01

Region: NODE\_329218\_length\_1765\_cov\_29.614164 248-250. Max. coverage (+): 0.13. Max coverage (-): 0.09

Region: NODE\_329218\_length\_1765\_cov\_29.614164 251-254. Max. coverage (+): 0.06. Max coverage (-): 0.09

Region: NODE\_329218\_length\_1765\_cov\_29.614164 255-258. Max. coverage (+): 0.46. Max coverage (-): 0.02

Region: NODE\_329218\_length\_1765\_cov\_29.614164 259-261. Max. coverage (+): 0.08. Max coverage (-): 0

Region: NODE\_329218\_length\_1765\_cov\_29.614164 262-265. Max. coverage (+): 0.04. Max coverage (-): 0

Region: NODE\_329218\_length\_1765\_cov\_29.614164 266-269. Max. coverage (+): 0.89. Max coverage (-): 0

Region: NODE\_329218\_length\_1765\_cov\_29.614164 270-272. Max. coverage (+): 0.95. Max coverage (-): 0

Region: NODE\_329218\_length\_1765\_cov\_29.614164 273-276. Max. coverage (+): 0.08. Max coverage (-): 0

Region: NODE\_329218\_length\_1765\_cov\_29.614164 277-280. Max. coverage (+): 0.01. Max coverage (-): 0

Region: NODE\_329218\_length\_1765\_cov\_29.614164 281-283. Max. coverage (+): 0. Max coverage (-): 0

Region: NODE\_329218\_length\_1765\_cov\_29.614164 284-287. Max. coverage (+): 0. Max coverage (-): 0

Region: NODE\_329218\_length\_1765\_cov\_29.614164 288-291. Max. coverage (+): 0. Max coverage (-): 0

Region: NODE\_329218\_length\_1765\_cov\_29.614164 292-294. Max. coverage (+): 0. Max coverage (-): 0.01

Region: NODE\_329218\_length\_1765\_cov\_29.614164 295-298. Max. coverage (+): 0. Max coverage (-): 0

Region: NODE\_329218\_length\_1765\_cov\_29.614164 299-301. Max. coverage (+): 0. Max coverage (-): 0

Region: NODE\_329218\_length\_1765\_cov\_29.614164 302-305. Max. coverage (+): 0. Max coverage (-): 0

Region: NODE\_329218\_length\_1765\_cov\_29.614164 306-309. Max. coverage (+): 0. Max coverage (-): 0.01

Region: NODE\_329218\_length\_1765\_cov\_29.614164 310-312. Max. coverage (+): 0. Max coverage (-): 0

Region: NODE\_329218\_length\_1765\_cov\_29.614164 313-316. Max. coverage (+): 0.01. Max coverage (-): 0

Region: NODE\_329218\_length\_1765\_cov\_29.614164 317-320. Max. coverage (+): 0.57. Max coverage (-): 0

Region: NODE\_329218\_length\_1765\_cov\_29.614164 321-323. Max. coverage (+): 0.14. Max coverage (-): 0

Region: NODE\_329218\_length\_1765\_cov\_29.614164 324-327. Max. coverage (+): 0.08. Max coverage (-): 0

Region: NODE\_329218\_length\_1765\_cov\_29.614164 328-331. Max. coverage (+): 0.54. Max coverage (-): 0.02

Region: NODE\_329218\_length\_1765\_cov\_29.614164 332-334. Max. coverage (+): 0.24. Max coverage (-): 0.03

Region: NODE\_329218\_length\_1765\_cov\_29.614164 335-338. Max. coverage (+): 0.05. Max coverage (-): 0

Region: NODE\_329218\_length\_1765\_cov\_29.614164 339-342. Max. coverage (+): 0.25. Max coverage (-): 0

Region: NODE\_329218\_length\_1765\_cov\_29.614164 343-345. Max. coverage (+): 0.18. Max coverage (-): 0

Region: NODE\_329218\_length\_1765\_cov\_29.614164 346-349. Max. coverage (+): 1.02. Max coverage (-): 0.01

Region: NODE\_329218\_length\_1765\_cov\_29.614164 350-353. Max. coverage (+): 0.43. Max coverage (-): 0.04

Region: NODE\_329218\_length\_1765\_cov\_29.614164 354-356. Max. coverage (+): 0.01. Max coverage (-): 0.04

Region: NODE\_329218\_length\_1765\_cov\_29.614164 357-360. Max. coverage (+): 0.01. Max coverage (-): 0

Region: NODE\_329218\_length\_1765\_cov\_29.614164 361-363. Max. coverage (+): 0.01. Max coverage (-): 0

Region: NODE\_329218\_length\_1765\_cov\_29.614164 364-367. Max. coverage (+): 0.7. Max coverage (-): 0

Region: NODE\_329218\_length\_1765\_cov\_29.614164 368-371. Max. coverage (+): 0.92. Max coverage (-): 0.03

Region: NODE\_329218\_length\_1765\_cov\_29.614164 372-374. Max. coverage (+): 0.51. Max coverage (-): 0.03

Region: NODE\_329218\_length\_1765\_cov\_29.614164 375-378. Max. coverage (+): 0.17. Max coverage (-): 0

Region: NODE\_329218\_length\_1765\_cov\_29.614164 379-382. Max. coverage (+): 0.13. Max coverage (-): 0.01

Region: NODE\_329218\_length\_1765\_cov\_29.614164 383-385. Max. coverage (+): 0.17. Max coverage (-): 0

Region: NODE\_329218\_length\_1765\_cov\_29.614164 386-389. Max. coverage (+): 0.4. Max coverage (-): 0

Region: NODE\_329218\_length\_1765\_cov\_29.614164 390-393. Max. coverage (+): 1.7. Max coverage (-): 0

Region: NODE\_329218\_length\_1765\_cov\_29.614164 394-396. Max. coverage (+): 0.44. Max coverage (-): 0.01

Region: NODE\_329218\_length\_1765\_cov\_29.614164 397-400. Max. coverage (+): 0.14. Max coverage (-): 0.01

Region: NODE\_329218\_length\_1765\_cov\_29.614164 401-404. Max. coverage (+): 0.01. Max coverage (-): 0.03

Region: NODE\_329218\_length\_1765\_cov\_29.614164 405-407. Max. coverage (+): 0.01. Max coverage (-): 0.06

Region: NODE\_329218\_length\_1765\_cov\_29.614164 408-411. Max. coverage (+): 0.01. Max coverage (-): 0.05

Region: NODE\_329218\_length\_1765\_cov\_29.614164 412-415. Max. coverage (+): 0. Max coverage (-): 0

Region: NODE\_329218\_length\_1765\_cov\_29.614164 416-418. Max. coverage (+): 0. Max coverage (-): 0

Region: NODE\_329218\_length\_1765\_cov\_29.614164 419-422. Max. coverage (+): 0. Max coverage (-): 0

Region: NODE\_329218\_length\_1765\_cov\_29.614164 423-425. Max. coverage (+): 0. Max coverage (-): 0

Region: NODE\_329218\_length\_1765\_cov\_29.614164 426-429. Max. coverage (+): 0. Max coverage (-): 0

Region: NODE\_329218\_length\_1765\_cov\_29.614164 430-433. Max. coverage (+): 0. Max coverage (-): 0

Region: NODE\_329218\_length\_1765\_cov\_29.614164 434-436. Max. coverage (+): 0.01. Max coverage (-): 0.03

Region: NODE\_329218\_length\_1765\_cov\_29.614164 437-440. Max. coverage (+): 0.02. Max coverage (-): 0.04

Region: NODE\_329218\_length\_1765\_cov\_29.614164 441-444. Max. coverage (+): 0.02. Max coverage (-): 0

Region: NODE\_329218\_length\_1765\_cov\_29.614164 445-447. Max. coverage (+): 0.01. Max coverage (-): 0

Region: NODE\_329218\_length\_1765\_cov\_29.614164 448-451. Max. coverage (+): 0.09. Max coverage (-): 0

Region: NODE\_329218\_length\_1765\_cov\_29.614164 452-455. Max. coverage (+): 0.21. Max coverage (-): 0

Region: NODE\_329218\_length\_1765\_cov\_29.614164 456-458. Max. coverage (+): 0.01. Max coverage (-): 0

Region: NODE\_329218\_length\_1765\_cov\_29.614164 459-462. Max. coverage (+): 0. Max coverage (-): 0

Region: NODE\_329218\_length\_1765\_cov\_29.614164 463-466. Max. coverage (+): 0.02. Max coverage (-): 0

Region: NODE\_329218\_length\_1765\_cov\_29.614164 467-469. Max. coverage (+): 0.21. Max coverage (-): 0

Region: NODE\_329218\_length\_1765\_cov\_29.614164 470-473. Max. coverage (+): 0.37. Max coverage (-): 0.06

Region: NODE\_329218\_length\_1765\_cov\_29.614164 474-477. Max. coverage (+): 0.35. Max coverage (-): 0.26

Region: NODE\_329218\_length\_1765\_cov\_29.614164 478-480. Max. coverage (+): 0.01. Max coverage (-): 0.06

Region: NODE\_329218\_length\_1765\_cov\_29.614164 481-484. Max. coverage (+): 0.59. Max coverage (-): 0.07

Region: NODE\_329218\_length\_1765\_cov\_29.614164 485-488. Max. coverage (+): 95.57. Max coverage (-): 0.85

Region: NODE\_329218\_length\_1765\_cov\_29.614164 489-491. Max. coverage (+): 0.19. Max coverage (-): 0.85

Region: NODE\_329218\_length\_1765\_cov\_29.614164 492-495. Max. coverage (+): 0.33. Max coverage (-): 1.89

Region: NODE\_329218\_length\_1765\_cov\_29.614164 496-498. Max. coverage (+): 0.04. Max coverage (-): 0.41

Region: NODE\_329218\_length\_1765\_cov\_29.614164 499-502. Max. coverage (+): 0.44. Max coverage (-): 0.85

Region: NODE\_329218\_length\_1765\_cov\_29.614164 503-506. Max. coverage (+): 48.86. Max coverage (-): 0.33

Region: NODE\_329218\_length\_1765\_cov\_29.614164 507-509. Max. coverage (+): 1.11. Max coverage (-): 0.07

Region: NODE\_329218\_length\_1765\_cov\_29.614164 510-513. Max. coverage (+): 0.78. Max coverage (-): 0.07

Region: NODE\_329218\_length\_1765\_cov\_29.614164 514-517. Max. coverage (+): 0.7. Max coverage (-): 0

Region: NODE\_329218\_length\_1765\_cov\_29.614164 518-520. Max. coverage (+): 0.52. Max coverage (-): 0

Region: NODE\_329218\_length\_1765\_cov\_29.614164 521-524. Max. coverage (+): 0. Max coverage (-): 0

Region: NODE\_329218\_length\_1765\_cov\_29.614164 525-528. Max. coverage (+): 0.85. Max coverage (-): 0

Region: NODE\_329218\_length\_1765\_cov\_29.614164 529-531. Max. coverage (+): 1.19. Max coverage (-): 0

Region: NODE\_329218\_length\_1765\_cov\_29.614164 532-535. Max. coverage (+): 0.59. Max coverage (-): 0.04

Region: NODE\_329218\_length\_1765\_cov\_29.614164 536-539. Max. coverage (+): 0.33. Max coverage (-): 1.82

Region: NODE\_329218\_length\_1765\_cov\_29.614164 540-542. Max. coverage (+): 0.11. Max coverage (-): 1.82

Region: NODE\_329218\_length\_1765\_cov\_29.614164 543-546. Max. coverage (+): 0.07. Max coverage (-): 1.41

Region: NODE\_329218\_length\_1765\_cov\_29.614164 547-550. Max. coverage (+): 0.89. Max coverage (-): 0.37

Region: NODE\_329218\_length\_1765\_cov\_29.614164 551-553. Max. coverage (+): 0.96. Max coverage (-): 0.19

Region: NODE\_329218\_length\_1765\_cov\_29.614164 554-557. Max. coverage (+): 10.08. Max coverage (-): 0.15

Region: NODE\_329218\_length\_1765\_cov\_29.614164 558-560. Max. coverage (+): 13.46. Max coverage (-): 0.11

Region: NODE\_329218\_length\_1765\_cov\_29.614164 561-564. Max. coverage (+): 4.41. Max coverage (-): 0.07

Region: NODE\_329218\_length\_1765\_cov\_29.614164 565-568. Max. coverage (+): 1.67. Max coverage (-): 0.07

Region: NODE\_329218\_length\_1765\_cov\_29.614164 569-571. Max. coverage (+): 0. Max coverage (-): 0.11

Region: NODE\_329218\_length\_1765\_cov\_29.614164 572-575. Max. coverage (+): 0.11. Max coverage (-): 0.07

Region: NODE\_329218\_length\_1765\_cov\_29.614164 576-579. Max. coverage (+): 0.07. Max coverage (-): 0.19

Region: NODE\_329218\_length\_1765\_cov\_29.614164 580-582. Max. coverage (+): 0.89. Max coverage (-): 0.15

Region: NODE\_329218\_length\_1765\_cov\_29.614164 583-586. Max. coverage (+): 3.08. Max coverage (-): 0.11

Region: NODE\_329218\_length\_1765\_cov\_29.614164 587-590. Max. coverage (+): 0.82. Max coverage (-): 0.04

Region: NODE\_329218\_length\_1765\_cov\_29.614164 591-593. Max. coverage (+): 0.3. Max coverage (-): 0.04

Region: NODE\_329218\_length\_1765\_cov\_29.614164 594-597. Max. coverage (+): 3.71. Max coverage (-): 0.04

Region: NODE\_329218\_length\_1765\_cov\_29.614164 598-601. Max. coverage (+): 0.3. Max coverage (-): 0.15

Region: NODE\_329218\_length\_1765\_cov\_29.614164 602-604. Max. coverage (+): 0.07. Max coverage (-): 0.11

Region: NODE\_329218\_length\_1765\_cov\_29.614164 605-608. Max. coverage (+): 0.04. Max coverage (-): 0.19

Region: NODE\_329218\_length\_1765\_cov\_29.614164 609-612. Max. coverage (+): 0.04. Max coverage (-): 0.26

Region: NODE\_329218\_length\_1765\_cov\_29.614164 613-615. Max. coverage (+): 0.04. Max coverage (-): 0

Region: NODE\_329218\_length\_1765\_cov\_29.614164 616-619. Max. coverage (+): 0.74. Max coverage (-): 0.48

Region: NODE\_329218\_length\_1765\_cov\_29.614164 620-622. Max. coverage (+): 0.22. Max coverage (-): 0.04

Region: NODE\_329218\_length\_1765\_cov\_29.614164 623-626. Max. coverage (+): 122.3. Max coverage (-): 0

Region: NODE\_329218\_length\_1765\_cov\_29.614164 627-630. Max. coverage (+): 5.56. Max coverage (-): 0.22

Region: NODE\_329218\_length\_1765\_cov\_29.614164 631-633. Max. coverage (+): 37.93. Max coverage (-): 0.26

Region: NODE\_329218\_length\_1765\_cov\_29.614164 634-637. Max. coverage (+): 38.18. Max coverage (-): 0.19

Region: NODE\_329218\_length\_1765\_cov\_29.614164 638-641. Max. coverage (+): 0.26. Max coverage (-): 0.15

Region: NODE\_329218\_length\_1765\_cov\_29.614164 642-644. Max. coverage (+): 0.3. Max coverage (-): 0.37

Region: NODE\_329218\_length\_1765\_cov\_29.614164 645-648. Max. coverage (+): 0.56. Max coverage (-): 0.93

Region: NODE\_329218\_length\_1765\_cov\_29.614164 649-652. Max. coverage (+): 0.93. Max coverage (-): 0.26

Region: NODE\_329218\_length\_1765\_cov\_29.614164 653-655. Max. coverage (+): 0.59. Max coverage (-): 0.22

Region: NODE\_329218\_length\_1765\_cov\_29.614164 656-659. Max. coverage (+): 1.11. Max coverage (-): 0.48

Region: NODE\_329218\_length\_1765\_cov\_29.614164 660-663. Max. coverage (+): 1.11. Max coverage (-): 8.53

Region: NODE\_329218\_length\_1765\_cov\_29.614164 664-666. Max. coverage (+): 0.19. Max coverage (-): 8.34

Region: NODE\_329218\_length\_1765\_cov\_29.614164 667-670. Max. coverage (+): 0.44. Max coverage (-): 0.3

Region: NODE\_329218\_length\_1765\_cov\_29.614164 671-674. Max. coverage (+): 1.22. Max coverage (-): 0.15

Region: NODE\_329218\_length\_1765\_cov\_29.614164 675-677. Max. coverage (+): 9.9. Max coverage (-): 0

Region: NODE\_329218\_length\_1765\_cov\_29.614164 678-681. Max. coverage (+): 10.45. Max coverage (-): 0.52

Region: NODE\_329218\_length\_1765\_cov\_29.614164 682-684. Max. coverage (+): 1.85. Max coverage (-): 0.59

Region: NODE\_329218\_length\_1765\_cov\_29.614164 685-688. Max. coverage (+): 2.04. Max coverage (-): 0.07

Region: NODE\_329218\_length\_1765\_cov\_29.614164 689-692. Max. coverage (+): 0.74. Max coverage (-): 0.07

Region: NODE\_329218\_length\_1765\_cov\_29.614164 693-695. Max. coverage (+): 0.7. Max coverage (-): 0.04

Region: NODE\_329218\_length\_1765\_cov\_29.614164 696-699. Max. coverage (+): 1.59. Max coverage (-): 0

Region: NODE\_329218\_length\_1765\_cov\_29.614164 700-703. Max. coverage (+): 0.04. Max coverage (-): 0

Region: NODE\_329218\_length\_1765\_cov\_29.614164 704-706. Max. coverage (+): 0.78. Max coverage (-): 0

Region: NODE\_329218\_length\_1765\_cov\_29.614164 707-710. Max. coverage (+): 0. Max coverage (-): 0

Region: NODE\_329218\_length\_1765\_cov\_29.614164 711-714. Max. coverage (+): 0. Max coverage (-): 0

Region: NODE\_329218\_length\_1765\_cov\_29.614164 715-717. Max. coverage (+): 0. Max coverage (-): 0

Region: NODE\_329218\_length\_1765\_cov\_29.614164 718-721. Max. coverage (+): 3.74. Max coverage (-): 0.15

Region: NODE\_329218\_length\_1765\_cov\_29.614164 722-725. Max. coverage (+): 6.19. Max coverage (-): 0.07

Region: NODE\_329218\_length\_1765\_cov\_29.614164 726-728. Max. coverage (+): 11.2. Max coverage (-): 0

Region: NODE\_329218\_length\_1765\_cov\_29.614164 729-732. Max. coverage (+): 0.11. Max coverage (-): 0

Region: NODE\_329218\_length\_1765\_cov\_29.614164 733-736. Max. coverage (+): 0.22. Max coverage (-): 0

Region: NODE\_329218\_length\_1765\_cov\_29.614164 737-739. Max. coverage (+): 1.67. Max coverage (-): 0

Region: NODE\_329218\_length\_1765\_cov\_29.614164 740-743. Max. coverage (+): 0.59. Max coverage (-): 0.07

Region: NODE\_329218\_length\_1765\_cov\_29.614164 744-747. Max. coverage (+): 2.97. Max coverage (-): 0.07

Region: NODE\_329218\_length\_1765\_cov\_29.614164 748-750. Max. coverage (+): 4.49. Max coverage (-): 1.08

Region: NODE\_329218\_length\_1765\_cov\_29.614164 751-754. Max. coverage (+): 5.67. Max coverage (-): 0.11

Region: NODE\_329218\_length\_1765\_cov\_29.614164 755-757. Max. coverage (+): 3.93. Max coverage (-): 0.33

Region: NODE\_329218\_length\_1765\_cov\_29.614164 758-761. Max. coverage (+): 1.15. Max coverage (-): 0.07

Region: NODE\_329218\_length\_1765\_cov\_29.614164 762-765. Max. coverage (+): 0.33. Max coverage (-): 0.26

Region: NODE\_329218\_length\_1765\_cov\_29.614164 766-768. Max. coverage (+): 0.11. Max coverage (-): 1

Region: NODE\_329218\_length\_1765\_cov\_29.614164 769-772. Max. coverage (+): 1.04. Max coverage (-): 0.41

Region: NODE\_329218\_length\_1765\_cov\_29.614164 773-776. Max. coverage (+): 0.82. Max coverage (-): 0.56

Region: NODE\_329218\_length\_1765\_cov\_29.614164 777-779. Max. coverage (+): 0.3. Max coverage (-): 0.15

Region: NODE\_329218\_length\_1765\_cov\_29.614164 780-783. Max. coverage (+): 1.04. Max coverage (-): 0.07

Region: NODE\_329218\_length\_1765\_cov\_29.614164 784-787. Max. coverage (+): 1.26. Max coverage (-): 0.63

Region: NODE\_329218\_length\_1765\_cov\_29.614164 788-790. Max. coverage (+): 2.22. Max coverage (-): 0.07

Region: NODE\_329218\_length\_1765\_cov\_29.614164 791-794. Max. coverage (+): 3.86. Max coverage (-): 0.15

Region: NODE\_329218\_length\_1765\_cov\_29.614164 795-798. Max. coverage (+): 1.22. Max coverage (-): 0.07

Region: NODE\_329218\_length\_1765\_cov\_29.614164 799-801. Max. coverage (+): 11.72. Max coverage (-): 0.04

Region: NODE\_329218\_length\_1765\_cov\_29.614164 802-805. Max. coverage (+): 0.04. Max coverage (-): 0.04

Region: NODE\_329218\_length\_1765\_cov\_29.614164 806-809. Max. coverage (+): 0.67. Max coverage (-): 0

Region: NODE\_329218\_length\_1765\_cov\_29.614164 810-812. Max. coverage (+): 0.7. Max coverage (-): 0

Region: NODE\_329218\_length\_1765\_cov\_29.614164 813-816. Max. coverage (+): 0.26. Max coverage (-): 0.04

Region: NODE\_329218\_length\_1765\_cov\_29.614164 817-819. Max. coverage (+): 0.26. Max coverage (-): 0.04

Region: NODE\_329218\_length\_1765\_cov\_29.614164 820-823. Max. coverage (+): 0.07. Max coverage (-): 0

Region: NODE\_329218\_length\_1765\_cov\_29.614164 824-827. Max. coverage (+): 0. Max coverage (-): 0

Region: NODE\_329218\_length\_1765\_cov\_29.614164 828-830. Max. coverage (+): 0. Max coverage (-): 0

Region: NODE\_329218\_length\_1765\_cov\_29.614164 831-834. Max. coverage (+): 0. Max coverage (-): 0.93

Region: NODE\_329218\_length\_1765\_cov\_29.614164 835-838. Max. coverage (+): 0. Max coverage (-): 0.96

Region: NODE\_329218\_length\_1765\_cov\_29.614164 839-841. Max. coverage (+): 0.07. Max coverage (-): 0.07

Region: NODE\_329218\_length\_1765\_cov\_29.614164 842-845. Max. coverage (+): 0.11. Max coverage (-): 0

Region: NODE\_329218\_length\_1765\_cov\_29.614164 846-849. Max. coverage (+): 0. Max coverage (-): 0

Region: NODE\_329218\_length\_1765\_cov\_29.614164 850-852. Max. coverage (+): 0.56. Max coverage (-): 0

Region: NODE\_329218\_length\_1765\_cov\_29.614164 853-856. Max. coverage (+): 0.74. Max coverage (-): 0

Region: NODE\_329218\_length\_1765\_cov\_29.614164 857-860. Max. coverage (+): 0.04. Max coverage (-): 0

Region: NODE\_329218\_length\_1765\_cov\_29.614164 861-863. Max. coverage (+): 0. Max coverage (-): 0

Region: NODE\_329218\_length\_1765\_cov\_29.614164 864-867. Max. coverage (+): 0. Max coverage (-): 0.15

Region: NODE\_329218\_length\_1765\_cov\_29.614164 868-871. Max. coverage (+): 0. Max coverage (-): 0.15

Region: NODE\_329218\_length\_1765\_cov\_29.614164 872-874. Max. coverage (+): 0.04. Max coverage (-): 0

Region: NODE\_329218\_length\_1765\_cov\_29.614164 875-878. Max. coverage (+): 0.19. Max coverage (-): 0.07

Region: NODE\_329218\_length\_1765\_cov\_29.614164 879-881. Max. coverage (+): 0.44. Max coverage (-): 0.04

Region: NODE\_329218\_length\_1765\_cov\_29.614164 882-885. Max. coverage (+): 15.79. Max coverage (-): 0.11

Region: NODE\_329218\_length\_1765\_cov\_29.614164 886-889. Max. coverage (+): 2.37. Max coverage (-): 0.11

Region: NODE\_329218\_length\_1765\_cov\_29.614164 890-892. Max. coverage (+): 21.65. Max coverage (-): 0.07

Region: NODE\_329218\_length\_1765\_cov\_29.614164 893-896. Max. coverage (+): 5.6. Max coverage (-): 0

Region: NODE\_329218\_length\_1765\_cov\_29.614164 897-900. Max. coverage (+): 2.08. Max coverage (-): 0

Region: NODE\_329218\_length\_1765\_cov\_29.614164 901-903. Max. coverage (+): 1.89. Max coverage (-): 0.04

Region: NODE\_329218\_length\_1765\_cov\_29.614164 904-907. Max. coverage (+): 0.37. Max coverage (-): 0.04

Region: NODE\_329218\_length\_1765\_cov\_29.614164 908-911. Max. coverage (+): 0.11. Max coverage (-): 0.04

Region: NODE\_329218\_length\_1765\_cov\_29.614164 912-914. Max. coverage (+): 0.13. Max coverage (-): 0

Region: NODE\_329218\_length\_1765\_cov\_29.614164 915-918. Max. coverage (+): 0.07. Max coverage (-): 0

Region: NODE\_329218\_length\_1765\_cov\_29.614164 919-922. Max. coverage (+): 0.1. Max coverage (-): 0

Region: NODE\_329218\_length\_1765\_cov\_29.614164 923-925. Max. coverage (+): 2.04. Max coverage (-): 0

Region: NODE\_329218\_length\_1765\_cov\_29.614164 926-929. Max. coverage (+): 1.75. Max coverage (-): 0.31

Region: NODE\_329218\_length\_1765\_cov\_29.614164 930-933. Max. coverage (+): 0. Max coverage (-): 0.09

Region: NODE\_329218\_length\_1765\_cov\_29.614164 934-936. Max. coverage (+): 0. Max coverage (-): 0

Region: NODE\_329218\_length\_1765\_cov\_29.614164 937-940. Max. coverage (+): 0. Max coverage (-): 0.01

Region: NODE\_329218\_length\_1765\_cov\_29.614164 941-944. Max. coverage (+): 0.11. Max coverage (-): 0

Region: NODE\_329218\_length\_1765\_cov\_29.614164 945-947. Max. coverage (+): 0.09. Max coverage (-): 0

Region: NODE\_329218\_length\_1765\_cov\_29.614164 948-951. Max. coverage (+): 0.02. Max coverage (-): 0

Region: NODE\_329218\_length\_1765\_cov\_29.614164 952-954. Max. coverage (+): 0.01. Max coverage (-): 0

Region: NODE\_329218\_length\_1765\_cov\_29.614164 955-958. Max. coverage (+): 0. Max coverage (-): 0

Region: NODE\_329218\_length\_1765\_cov\_29.614164 959-962. Max. coverage (+): 0. Max coverage (-): 0

Region: NODE\_329218\_length\_1765\_cov\_29.614164 963-965. Max. coverage (+): 0. Max coverage (-): 0

Region: NODE\_329218\_length\_1765\_cov\_29.614164 966-969. Max. coverage (+): 0. Max coverage (-): 0.04

Region: NODE\_329218\_length\_1765\_cov\_29.614164 970-973. Max. coverage (+): 0.7. Max coverage (-): 0.04

Region: NODE\_329218\_length\_1765\_cov\_29.614164 974-976. Max. coverage (+): 3.71. Max coverage (-): 0.04

Region: NODE\_329218\_length\_1765\_cov\_29.614164 977-980. Max. coverage (+): 3.56. Max coverage (-): 0.07

Region: NODE\_329218\_length\_1765\_cov\_29.614164 981-984. Max. coverage (+): 0.56. Max coverage (-): 0.11

Region: NODE\_329218\_length\_1765\_cov\_29.614164 985-987. Max. coverage (+): 0.87. Max coverage (-): 0.07

Region: NODE\_329218\_length\_1765\_cov\_29.614164 988-991. Max. coverage (+): 15.68. Max coverage (-): 0

Region: NODE\_329218\_length\_1765\_cov\_29.614164 992-995. Max. coverage (+): 0.82. Max coverage (-): 0.11

Region: NODE\_329218\_length\_1765\_cov\_29.614164 996-998. Max. coverage (+): 1.22. Max coverage (-): 0

Region: NODE\_329218\_length\_1765\_cov\_29.614164 999-1002. Max. coverage (+): 1.28. Max coverage (-): 0

Region: NODE\_329218\_length\_1765\_cov\_29.614164 1003-1006. Max. coverage (+): 2.61. Max coverage (-): 0.06

Region: NODE\_329218\_length\_1765\_cov\_29.614164 1007-1009. Max. coverage (+): 3.11. Max coverage (-): 0.04

Region: NODE\_329218\_length\_1765\_cov\_29.614164 1010-1013. Max. coverage (+): 3.3. Max coverage (-): 0.19

Region: NODE\_329218\_length\_1765\_cov\_29.614164 1014-1016. Max. coverage (+): 0.52. Max coverage (-): 0.39

Region: NODE\_329218\_length\_1765\_cov\_29.614164 1017-1020. Max. coverage (+): 0.17. Max coverage (-): 0.48

Region: NODE\_329218\_length\_1765\_cov\_29.614164 1021-1024. Max. coverage (+): 5.62. Max coverage (-): 0.83

Region: NODE\_329218\_length\_1765\_cov\_29.614164 1025-1027. Max. coverage (+): 21.37. Max coverage (-): 0.5

Region: NODE\_329218\_length\_1765\_cov\_29.614164 1028-1031. Max. coverage (+): 17.83. Max coverage (-): 0.07

Region: NODE\_329218\_length\_1765\_cov\_29.614164 1032-1035. Max. coverage (+): 3.19. Max coverage (-): 0.06

Region: NODE\_329218\_length\_1765\_cov\_29.614164 1036-1038. Max. coverage (+): 3.23. Max coverage (-): 0.02

Region: NODE\_329218\_length\_1765\_cov\_29.614164 1039-1042. Max. coverage (+): 3.39. Max coverage (-): 0.02

Region: NODE\_329218\_length\_1765\_cov\_29.614164 1043-1046. Max. coverage (+): 4.62. Max coverage (-): 0.04

Region: NODE\_329218\_length\_1765\_cov\_29.614164 1047-1049. Max. coverage (+): 1.76. Max coverage (-): 0.11

Region: NODE\_329218\_length\_1765\_cov\_29.614164 1050-1053. Max. coverage (+): 1.63. Max coverage (-): 0.09

Region: NODE\_329218\_length\_1765\_cov\_29.614164 1054-1057. Max. coverage (+): 0.06. Max coverage (-): 0.43

Region: NODE\_329218\_length\_1765\_cov\_29.614164 1058-1060. Max. coverage (+): 0.06. Max coverage (-): 0.39

Region: NODE\_329218\_length\_1765\_cov\_29.614164 1061-1064. Max. coverage (+): 0.59. Max coverage (-): 0.78

Region: NODE\_329218\_length\_1765\_cov\_29.614164 1065-1068. Max. coverage (+): 1.48. Max coverage (-): 3.89

Region: NODE\_329218\_length\_1765\_cov\_29.614164 1069-1071. Max. coverage (+): 0.07. Max coverage (-): 3.78

Region: NODE\_329218\_length\_1765\_cov\_29.614164 1072-1075. Max. coverage (+): 3.37. Max coverage (-): 0.37

Region: NODE\_329218\_length\_1765\_cov\_29.614164 1076-1078. Max. coverage (+): 3.86. Max coverage (-): 0.37

Region: NODE\_329218\_length\_1765\_cov\_29.614164 1079-1082. Max. coverage (+): 3.63. Max coverage (-): 0.48

Region: NODE\_329218\_length\_1765\_cov\_29.614164 1083-1086. Max. coverage (+): 61.17. Max coverage (-): 0.33

Region: NODE\_329218\_length\_1765\_cov\_29.614164 1087-1089. Max. coverage (+): 1.24. Max coverage (-): 0.24

Region: NODE\_329218\_length\_1765\_cov\_29.614164 1090-1093. Max. coverage (+): 1.09. Max coverage (-): 0.09

Region: NODE\_329218\_length\_1765\_cov\_29.614164 1094-1097. Max. coverage (+): 1.04. Max coverage (-): 0.07

Region: NODE\_329218\_length\_1765\_cov\_29.614164 1098-1100. Max. coverage (+): 0.07. Max coverage (-): 0.04

Region: NODE\_329218\_length\_1765\_cov\_29.614164 1101-1104. Max. coverage (+): 0.11. Max coverage (-): 0.02

Region: NODE\_329218\_length\_1765\_cov\_29.614164 1105-1108. Max. coverage (+): 0.04. Max coverage (-): 0.06

Region: NODE\_329218\_length\_1765\_cov\_29.614164 1109-1111. Max. coverage (+): 0.33. Max coverage (-): 0.06

Region: NODE\_329218\_length\_1765\_cov\_29.614164 1112-1115. Max. coverage (+): 1.63. Max coverage (-): 0.02

Region: NODE\_329218\_length\_1765\_cov\_29.614164 1116-1119. Max. coverage (+): 0.93. Max coverage (-): 0

Region: NODE\_329218\_length\_1765\_cov\_29.614164 1120-1122. Max. coverage (+): 0.04. Max coverage (-): 0

Region: NODE\_329218\_length\_1765\_cov\_29.614164 1123-1126. Max. coverage (+): 0.15. Max coverage (-): 0

Region: NODE\_329218\_length\_1765\_cov\_29.614164 1127-1130. Max. coverage (+): 0.19. Max coverage (-): 0

Region: NODE\_329218\_length\_1765\_cov\_29.614164 1131-1133. Max. coverage (+): 0.19. Max coverage (-): 0

Region: NODE\_329218\_length\_1765\_cov\_29.614164 1134-1137. Max. coverage (+): 0. Max coverage (-): 0

Region: NODE\_329218\_length\_1765\_cov\_29.614164 1138-1140. Max. coverage (+): 0.07. Max coverage (-): 0

Region: NODE\_329218\_length\_1765\_cov\_29.614164 1141-1144. Max. coverage (+): 0.3. Max coverage (-): 0

Region: NODE\_329218\_length\_1765\_cov\_29.614164 1145-1148. Max. coverage (+): 0.93. Max coverage (-): 0

Region: NODE\_329218\_length\_1765\_cov\_29.614164 1149-1151. Max. coverage (+): 0.44. Max coverage (-): 0.33

Region: NODE\_329218\_length\_1765\_cov\_29.614164 1152-1155. Max. coverage (+): 0.15. Max coverage (-): 0.33

Region: NODE\_329218\_length\_1765\_cov\_29.614164 1156-1159. Max. coverage (+): 2.6. Max coverage (-): 0.33

Region: NODE\_329218\_length\_1765\_cov\_29.614164 1160-1162. Max. coverage (+): 4.08. Max coverage (-): 0.07

Region: NODE\_329218\_length\_1765\_cov\_29.614164 1163-1166. Max. coverage (+): 3.34. Max coverage (-): 0.41

Region: NODE\_329218\_length\_1765\_cov\_29.614164 1167-1170. Max. coverage (+): 3.74. Max coverage (-): 0.41

Region: NODE\_329218\_length\_1765\_cov\_29.614164 1171-1173. Max. coverage (+): 0.82. Max coverage (-): 0.04

Region: NODE\_329218\_length\_1765\_cov\_29.614164 1174-1177. Max. coverage (+): 0.04. Max coverage (-): 0.04

Region: NODE\_329218\_length\_1765\_cov\_29.614164 1178-1181. Max. coverage (+): 36.44. Max coverage (-): 0.07

Region: NODE\_329218\_length\_1765\_cov\_29.614164 1182-1184. Max. coverage (+): 39.52. Max coverage (-): 0

Region: NODE\_329218\_length\_1765\_cov\_29.614164 1185-1188. Max. coverage (+): 3.45. Max coverage (-): 0

Region: NODE\_329218\_length\_1765\_cov\_29.614164 1189-1192. Max. coverage (+): 0.52. Max coverage (-): 0

Region: NODE\_329218\_length\_1765\_cov\_29.614164 1193-1195. Max. coverage (+): 0.07. Max coverage (-): 0

Region: NODE\_329218\_length\_1765\_cov\_29.614164 1196-1199. Max. coverage (+): 0.04. Max coverage (-): 0

Region: NODE\_329218\_length\_1765\_cov\_29.614164 1200-1203. Max. coverage (+): 0.26. Max coverage (-): 0

Region: NODE\_329218\_length\_1765\_cov\_29.614164 1204-1206. Max. coverage (+): 0.19. Max coverage (-): 0

Region: NODE\_329218\_length\_1765\_cov\_29.614164 1207-1210. Max. coverage (+): 0.04. Max coverage (-): 0.19

Region: NODE\_329218\_length\_1765\_cov\_29.614164 1211-1213. Max. coverage (+): 0.04. Max coverage (-): 0.19

Region: NODE\_329218\_length\_1765\_cov\_29.614164 1214-1217. Max. coverage (+): 0.07. Max coverage (-): 0

Region: NODE\_329218\_length\_1765\_cov\_29.614164 1218-1221. Max. coverage (+): 0.07. Max coverage (-): 0

Region: NODE\_329218\_length\_1765\_cov\_29.614164 1222-1224. Max. coverage (+): 0. Max coverage (-): 0

Region: NODE\_329218\_length\_1765\_cov\_29.614164 1225-1228. Max. coverage (+): 0.52. Max coverage (-): 0.04

Region: NODE\_329218\_length\_1765\_cov\_29.614164 1229-1232. Max. coverage (+): 0.65. Max coverage (-): 0

Region: NODE\_329218\_length\_1765\_cov\_29.614164 1233-1235. Max. coverage (+): 0.65. Max coverage (-): 0

Region: NODE\_329218\_length\_1765\_cov\_29.614164 1236-1239. Max. coverage (+): 0. Max coverage (-): 0

Region: NODE\_329218\_length\_1765\_cov\_29.614164 1240-1243. Max. coverage (+): 0.04. Max coverage (-): 0

Region: NODE\_329218\_length\_1765\_cov\_29.614164 1244-1246. Max. coverage (+): 0.06. Max coverage (-): 0.04

Region: NODE\_329218\_length\_1765\_cov\_29.614164 1247-1250. Max. coverage (+): 0.06. Max coverage (-): 0.11

Region: NODE\_329218\_length\_1765\_cov\_29.614164 1251-1254. Max. coverage (+): 0.44. Max coverage (-): 0.13

Region: NODE\_329218\_length\_1765\_cov\_29.614164 1255-1257. Max. coverage (+): 0.74. Max coverage (-): 0.07

Region: NODE\_329218\_length\_1765\_cov\_29.614164 1258-1261. Max. coverage (+): 5.52. Max coverage (-): 0.07

Region: NODE\_329218\_length\_1765\_cov\_29.614164 1262-1265. Max. coverage (+): 4.75. Max coverage (-): 0.22

Region: NODE\_329218\_length\_1765\_cov\_29.614164 1266-1268. Max. coverage (+): 0.19. Max coverage (-): 0.22

Region: NODE\_329218\_length\_1765\_cov\_29.614164 1269-1272. Max. coverage (+): 0.07. Max coverage (-): 0.11

Region: NODE\_329218\_length\_1765\_cov\_29.614164 1273-1275. Max. coverage (+): 1.87. Max coverage (-): 0.15

Region: NODE\_329218\_length\_1765\_cov\_29.614164 1276-1279. Max. coverage (+): 4.62. Max coverage (-): 0.04

Region: NODE\_329218\_length\_1765\_cov\_29.614164 1280-1283. Max. coverage (+): 2.47. Max coverage (-): 0

Region: NODE\_329218\_length\_1765\_cov\_29.614164 1284-1286. Max. coverage (+): 2.21. Max coverage (-): 0

Region: NODE\_329218\_length\_1765\_cov\_29.614164 1287-1290. Max. coverage (+): 0. Max coverage (-): 0

Region: NODE\_329218\_length\_1765\_cov\_29.614164 1291-1294. Max. coverage (+): 0. Max coverage (-): 0

Region: NODE\_329218\_length\_1765\_cov\_29.614164 1295-1297. Max. coverage (+): 0. Max coverage (-): 0

Region: NODE\_329218\_length\_1765\_cov\_29.614164 1298-1301. Max. coverage (+): 0. Max coverage (-): 0

Region: NODE\_329218\_length\_1765\_cov\_29.614164 1302-1305. Max. coverage (+): 0. Max coverage (-): 0

Region: NODE\_329218\_length\_1765\_cov\_29.614164 1306-1308. Max. coverage (+): 0. Max coverage (-): 0

Region: NODE\_329218\_length\_1765\_cov\_29.614164 1309-1312. Max. coverage (+): 0. Max coverage (-): 0

Region: NODE\_329218\_length\_1765\_cov\_29.614164 1313-1316. Max. coverage (+): 0.04. Max coverage (-): 0

Region: NODE\_329218\_length\_1765\_cov\_29.614164 1317-1319. Max. coverage (+): 0.07. Max coverage (-): 0

Region: NODE\_329218\_length\_1765\_cov\_29.614164 1320-1323. Max. coverage (+): 0.04. Max coverage (-): 0

Region: NODE\_329218\_length\_1765\_cov\_29.614164 1324-1327. Max. coverage (+): 0.07. Max coverage (-): 0

Region: NODE\_329218\_length\_1765\_cov\_29.614164 1328-1330. Max. coverage (+): 0.07. Max coverage (-): 0

Region: NODE\_329218\_length\_1765\_cov\_29.614164 1331-1334. Max. coverage (+): 0. Max coverage (-): 0.07

Region: NODE\_329218\_length\_1765\_cov\_29.614164 1335-1337. Max. coverage (+): 0. Max coverage (-): 0.07

Region: NODE\_329218\_length\_1765\_cov\_29.614164 1338-1341. Max. coverage (+): 0.07. Max coverage (-): 0.04

Region: NODE\_329218\_length\_1765\_cov\_29.614164 1342-1345. Max. coverage (+): 0.09. Max coverage (-): 0.04

Region: NODE\_329218\_length\_1765\_cov\_29.614164 1346-1348. Max. coverage (+): 0.19. Max coverage (-): 0.04

Region: NODE\_329218\_length\_1765\_cov\_29.614164 1349-1352. Max. coverage (+): 54.05. Max coverage (-): 0

Region: NODE\_329218\_length\_1765\_cov\_29.614164 1353-1356. Max. coverage (+): 53.44. Max coverage (-): 0

Region: NODE\_329218\_length\_1765\_cov\_29.614164 1357-1359. Max. coverage (+): 0.98. Max coverage (-): 0

Region: NODE\_329218\_length\_1765\_cov\_29.614164 1360-1363. Max. coverage (+): 0.85. Max coverage (-): 0

Region: NODE\_329218\_length\_1765\_cov\_29.614164 1364-1367. Max. coverage (+): 0.02. Max coverage (-): 0

Region: NODE\_329218\_length\_1765\_cov\_29.614164 1368-1370. Max. coverage (+): 0. Max coverage (-): 0

Region: NODE\_329218\_length\_1765\_cov\_29.614164 1371-1374. Max. coverage (+): 0.13. Max coverage (-): 0.11

Region: NODE\_329218\_length\_1765\_cov\_29.614164 1375-1378. Max. coverage (+): 0.04. Max coverage (-): 0.09

Region: NODE\_329218\_length\_1765\_cov\_29.614164 1379-1381. Max. coverage (+): 0. Max coverage (-): 0.02

Region: NODE\_329218\_length\_1765\_cov\_29.614164 1382-1385. Max. coverage (+): 0. Max coverage (-): 0

Region: NODE\_329218\_length\_1765\_cov\_29.614164 1386-1389. Max. coverage (+): 0. Max coverage (-): 0

Region: NODE\_329218\_length\_1765\_cov\_29.614164 1390-1392. Max. coverage (+): 0.7. Max coverage (-): 0

Region: NODE\_329218\_length\_1765\_cov\_29.614164 1393-1396. Max. coverage (+): 0.7. Max coverage (-): 0

Region: NODE\_329218\_length\_1765\_cov\_29.614164 1397-1400. Max. coverage (+): 0. Max coverage (-): 0.04

Region: NODE\_329218\_length\_1765\_cov\_29.614164 1401-1403. Max. coverage (+): 0.04. Max coverage (-): 0.07

Region: NODE\_329218\_length\_1765\_cov\_29.614164 1404-1407. Max. coverage (+): 0.04. Max coverage (-): 0.19

Region: NODE\_329218\_length\_1765\_cov\_29.614164 1408-1410. Max. coverage (+): 0. Max coverage (-): 0.26

Region: NODE\_329218\_length\_1765\_cov\_29.614164 1411-1414. Max. coverage (+): 0. Max coverage (-): 0.15

Region: NODE\_329218\_length\_1765\_cov\_29.614164 1415-1418. Max. coverage (+): 0.07. Max coverage (-): 0.04

Region: NODE\_329218\_length\_1765\_cov\_29.614164 1419-1421. Max. coverage (+): 0.07. Max coverage (-): 0.04

Region: NODE\_329218\_length\_1765\_cov\_29.614164 1422-1425. Max. coverage (+): 0.07. Max coverage (-): 0.11

Region: NODE\_329218\_length\_1765\_cov\_29.614164 1426-1429. Max. coverage (+): 0.07. Max coverage (-): 0.11

Region: NODE\_329218\_length\_1765\_cov\_29.614164 1430-1432. Max. coverage (+): 0.07. Max coverage (-): 0.48

Region: NODE\_329218\_length\_1765\_cov\_29.614164 1433-1436. Max. coverage (+): 0. Max coverage (-): 2.02

Region: NODE\_329218\_length\_1765\_cov\_29.614164 1437-1440. Max. coverage (+): 0. Max coverage (-): 1.65

Region: NODE\_329218\_length\_1765\_cov\_29.614164 1441-1443. Max. coverage (+): 0. Max coverage (-): 0.04

Region: NODE\_329218\_length\_1765\_cov\_29.614164 1444-1447. Max. coverage (+): 0.82. Max coverage (-): 0.02

Region: NODE\_329218\_length\_1765\_cov\_29.614164 1448-1451. Max. coverage (+): 74.65. Max coverage (-): 0.04

Region: NODE\_329218\_length\_1765\_cov\_29.614164 1452-1454. Max. coverage (+): 81.75. Max coverage (-): 0.04

Region: NODE\_329218\_length\_1765\_cov\_29.614164 1455-1458. Max. coverage (+): 15.35. Max coverage (-): 0

Region: NODE\_329218\_length\_1765\_cov\_29.614164 1459-1462. Max. coverage (+): 1.26. Max coverage (-): 0

Region: NODE\_329218\_length\_1765\_cov\_29.614164 1463-1465. Max. coverage (+): 0.07. Max coverage (-): 0.04

Region: NODE\_329218\_length\_1765\_cov\_29.614164 1466-1469. Max. coverage (+): 0. Max coverage (-): 0.04

Region: NODE\_329218\_length\_1765\_cov\_29.614164 1470-1472. Max. coverage (+): 0.02. Max coverage (-): 0

Region: NODE\_329218\_length\_1765\_cov\_29.614164 1473-1476. Max. coverage (+): 0.41. Max coverage (-): 0

Region: NODE\_329218\_length\_1765\_cov\_29.614164 1477-1480. Max. coverage (+): 0.3. Max coverage (-): 0

Region: NODE\_329218\_length\_1765\_cov\_29.614164 1481-1483. Max. coverage (+): 1.26. Max coverage (-): 0.15

Region: NODE\_329218\_length\_1765\_cov\_29.614164 1484-1487. Max. coverage (+): 2.82. Max coverage (-): 0.67

Region: NODE\_329218\_length\_1765\_cov\_29.614164 1488-1491. Max. coverage (+): 0.37. Max coverage (-): 0.74

Region: NODE\_329218\_length\_1765\_cov\_29.614164 1492-1494. Max. coverage (+): 0.11. Max coverage (-): 0.04

Region: NODE\_329218\_length\_1765\_cov\_29.614164 1495-1498. Max. coverage (+): 0.22. Max coverage (-): 0.04

Region: NODE\_329218\_length\_1765\_cov\_29.614164 1499-1502. Max. coverage (+): 0.63. Max coverage (-): 0

Region: NODE\_329218\_length\_1765\_cov\_29.614164 1503-1505. Max. coverage (+): 0.68. Max coverage (-): 0

Region: NODE\_329218\_length\_1765\_cov\_29.614164 1506-1509. Max. coverage (+): 0.1. Max coverage (-): 0

Region: NODE\_329218\_length\_1765\_cov\_29.614164 1510-1513. Max. coverage (+): 0.01. Max coverage (-): 0

Region: NODE\_329218\_length\_1765\_cov\_29.614164 1514-1516. Max. coverage (+): 0. Max coverage (-): 0.01

Region: NODE\_329218\_length\_1765\_cov\_29.614164 1517-1520. Max. coverage (+): 0. Max coverage (-): 0.01

Region: NODE\_329218\_length\_1765\_cov\_29.614164 1521-1524. Max. coverage (+): 0. Max coverage (-): 0.04

Region: NODE\_329218\_length\_1765\_cov\_29.614164 1525-1527. Max. coverage (+): 0.11. Max coverage (-): 0.32

Region: NODE\_329218\_length\_1765\_cov\_29.614164 1528-1531. Max. coverage (+): 0.11. Max coverage (-): 0.36

Region: NODE\_329218\_length\_1765\_cov\_29.614164 1532-1534. Max. coverage (+): 23.1. Max coverage (-): 0

Region: NODE\_329218\_length\_1765\_cov\_29.614164 1535-1538. Max. coverage (+): 23.13. Max coverage (-): 0

Region: NODE\_329218\_length\_1765\_cov\_29.614164 1539-1542. Max. coverage (+): 1.63. Max coverage (-): 0

Region: NODE\_329218\_length\_1765\_cov\_29.614164 1543-1545. Max. coverage (+): 4.41. Max coverage (-): 0

Region: NODE\_329218\_length\_1765\_cov\_29.614164 1546-1549. Max. coverage (+): 5.19. Max coverage (-): 0.04

Region: NODE\_329218\_length\_1765\_cov\_29.614164 1550-1553. Max. coverage (+): 0.22. Max coverage (-): 0.01

Region: NODE\_329218\_length\_1765\_cov\_29.614164 1554-1556. Max. coverage (+): 0.1. Max coverage (-): 0.01

Region: NODE\_329218\_length\_1765\_cov\_29.614164 1557-1560. Max. coverage (+): 0.31. Max coverage (-): 0.01

Region: NODE\_329218\_length\_1765\_cov\_29.614164 1561-1564. Max. coverage (+): 0.17. Max coverage (-): 0.53

Region: NODE\_329218\_length\_1765\_cov\_29.614164 1565-1567. Max. coverage (+): 2.57. Max coverage (-): 0.53

Region: NODE\_329218\_length\_1765\_cov\_29.614164 1568-1571. Max. coverage (+): 2.78. Max coverage (-): 0.14

Region: NODE\_329218\_length\_1765\_cov\_29.614164 1572-1575. Max. coverage (+): 1.12. Max coverage (-): 0.07

Region: NODE\_329218\_length\_1765\_cov\_29.614164 1576-1578. Max. coverage (+): 1.32. Max coverage (-): 0.04

Region: NODE\_329218\_length\_1765\_cov\_29.614164 1579-1582. Max. coverage (+): 6.01. Max coverage (-): 0.69

Region: NODE\_329218\_length\_1765\_cov\_29.614164 1583-1586. Max. coverage (+): 6.07. Max coverage (-): 0.79

Region: NODE\_329218\_length\_1765\_cov\_29.614164 1587-1589. Max. coverage (+): 0.47. Max coverage (-): 0.01

Region: NODE\_329218\_length\_1765\_cov\_29.614164 1590-1593. Max. coverage (+): 0.04. Max coverage (-): 0.01

Region: NODE\_329218\_length\_1765\_cov\_29.614164 1594-1596. Max. coverage (+): 1.93. Max coverage (-): 0.01

Region: NODE\_329218\_length\_1765\_cov\_29.614164 1597-1600. Max. coverage (+): 35.56. Max coverage (-): 0

Region: NODE\_329218\_length\_1765\_cov\_29.614164 1601-1604. Max. coverage (+): 3.45. Max coverage (-): 0

Region: NODE\_329218\_length\_1765\_cov\_29.614164 1605-1607. Max. coverage (+): 0.7. Max coverage (-): 0.07

Region: NODE\_329218\_length\_1765\_cov\_29.614164 1608-1611. Max. coverage (+): 0.63. Max coverage (-): 0.11

Region: NODE\_329218\_length\_1765\_cov\_29.614164 1612-1615. Max. coverage (+): 0.67. Max coverage (-): 0

Region: NODE\_329218\_length\_1765\_cov\_29.614164 1616-1618. Max. coverage (+): 0.04. Max coverage (-): 0

Region: NODE\_329218\_length\_1765\_cov\_29.614164 1619-1622. Max. coverage (+): 0.04. Max coverage (-): 0

Region: NODE\_329218\_length\_1765\_cov\_29.614164 1623-1626. Max. coverage (+): 0.06. Max coverage (-): 0

Region: NODE\_329218\_length\_1765\_cov\_29.614164 1627-1629. Max. coverage (+): 0.19. Max coverage (-): 0

Region: NODE\_329218\_length\_1765\_cov\_29.614164 1630-1633. Max. coverage (+): 0.41. Max coverage (-): 0

Region: NODE\_329218\_length\_1765\_cov\_29.614164 1634-1637. Max. coverage (+): 11.81. Max coverage (-): 0.06

Region: NODE\_329218\_length\_1765\_cov\_29.614164 1638-1640. Max. coverage (+): 11.66. Max coverage (-): 0.04

Region: NODE\_329218\_length\_1765\_cov\_29.614164 1641-1644. Max. coverage (+): 9.81. Max coverage (-): 0

Region: NODE\_329218\_length\_1765\_cov\_29.614164 1645-1648. Max. coverage (+): 1.09. Max coverage (-): 0

Region: NODE\_329218\_length\_1765\_cov\_29.614164 1649-1651. Max. coverage (+): 0.02. Max coverage (-): 0

Region: NODE\_329218\_length\_1765\_cov\_29.614164 1652-1655. Max. coverage (+): 0.02. Max coverage (-): 0.09

Region: NODE\_329218\_length\_1765\_cov\_29.614164 1656-1659. Max. coverage (+): 0. Max coverage (-): 0.19

Region: NODE\_329218\_length\_1765\_cov\_29.614164 1660-1662. Max. coverage (+): 0. Max coverage (-): 0.19

Region: NODE\_329218\_length\_1765\_cov\_29.614164 1663-1666. Max. coverage (+): 0.15. Max coverage (-): 0.02

Region: NODE\_329218\_length\_1765\_cov\_29.614164 1667-1669. Max. coverage (+): 0.15. Max coverage (-): 0

Region: NODE\_329218\_length\_1765\_cov\_29.614164 1670-1673. Max. coverage (+): 0. Max coverage (-): 0.04

Region: NODE\_329218\_length\_1765\_cov\_29.614164 1674-1677. Max. coverage (+): 0.04. Max coverage (-): 0.04

Region: NODE\_329218\_length\_1765\_cov\_29.614164 1678-1680. Max. coverage (+): 0.11. Max coverage (-): 0.02

Region: NODE\_329218\_length\_1765\_cov\_29.614164 1681-1684. Max. coverage (+): 0.32. Max coverage (-): 0.01

Region: NODE\_329218\_length\_1765\_cov\_29.614164 1685-1688. Max. coverage (+): 0.05. Max coverage (-): 0.02

Region: NODE\_329218\_length\_1765\_cov\_29.614164 1689-1691. Max. coverage (+): 0.06. Max coverage (-): 0.04

Region: NODE\_329218\_length\_1765\_cov\_29.614164 1692-1695. Max. coverage (+): 0.43. Max coverage (-): 0.02

Region: NODE\_329218\_length\_1765\_cov\_29.614164 1696-1699. Max. coverage (+): 2.71. Max coverage (-): 0

Region: NODE\_329218\_length\_1765\_cov\_29.614164 1700-1702. Max. coverage (+): 5.32. Max coverage (-): 0

Region: NODE\_329218\_length\_1765\_cov\_29.614164 1703-1706. Max. coverage (+): 3.93. Max coverage (-): 0.26

Region: NODE\_329218\_length\_1765\_cov\_29.614164 1707-1710. Max. coverage (+): 3.37. Max coverage (-): 0.26

Region: NODE\_329218\_length\_1765\_cov\_29.614164 1711-1713. Max. coverage (+): 1.89. Max coverage (-): 0.3

Region: NODE\_329218\_length\_1765\_cov\_29.614164 1714-1717. Max. coverage (+): 0.19. Max coverage (-): 0.19

Region: NODE\_329218\_length\_1765\_cov\_29.614164 1718-1721. Max. coverage (+): 0.96. Max coverage (-): 0.04

Region: NODE\_329218\_length\_1765\_cov\_29.614164 1722-1724. Max. coverage (+): 0.74. Max coverage (-): 0

Region: NODE\_329218\_length\_1765\_cov\_29.614164 1725-1728. Max. coverage (+): 0.74. Max coverage (-): 0

Region: NODE\_329218\_length\_1765\_cov\_29.614164 1729-1731. Max. coverage (+): 2.48. Max coverage (-): 0

Region: NODE\_329218\_length\_1765\_cov\_29.614164 1732-1735. Max. coverage (+): 2.52. Max coverage (-): 0.07

Region: NODE\_329218\_length\_1765\_cov\_29.614164 1736-1739. Max. coverage (+): 1.85. Max coverage (-): 0.04

Region: NODE\_329218\_length\_1765\_cov\_29.614164 1740-1742. Max. coverage (+): 0. Max coverage (-): 0

Region: NODE\_329218\_length\_1765\_cov\_29.614164 1743-1746. Max. coverage (+): 0.04. Max coverage (-): 0.04

Region: NODE\_329218\_length\_1765\_cov\_29.614164 1747-1750. Max. coverage (+): 0.26. Max coverage (-): 0.04

Region: NODE\_329218\_length\_1765\_cov\_29.614164 1751-1753. Max. coverage (+): 0.07. Max coverage (-): 0

Region: NODE\_329218\_length\_1765\_cov\_29.614164 1754-1757. Max. coverage (+): 2.45. Max coverage (-): 0

Region: NODE\_329218\_length\_1765\_cov\_29.614164 1758-1761. Max. coverage (+): 6.45. Max coverage (-): 0

Region: NODE\_329218\_length\_1765\_cov\_29.614164 1762-1764. Max. coverage (+): 2.01. Max coverage (-): 0.01

Region: NODE\_329218\_length\_1765\_cov\_29.614164 1765-1768. Max. coverage (+): 1. Max coverage (-): 0.01

Region: NODE\_329218\_length\_1765\_cov\_29.614164 1769-1772. Max. coverage (+): 0.34. Max coverage (-): 0

Region: NODE\_329218\_length\_1765\_cov\_29.614164 1773-1775. Max. coverage (+): 0. Max coverage (-): 0

Region: NODE\_329218\_length\_1765\_cov\_29.614164 1776-1779. Max. coverage (+): 0.06. Max coverage (-): 0.02

Region: NODE\_329218\_length\_1765\_cov\_29.614164 1780-1783. Max. coverage (+): 0.08. Max coverage (-): 0.03

Region: NODE\_329218\_length\_1765\_cov\_29.614164 1784-1786. Max. coverage (+): 0.07. Max coverage (-): 0.01

Region: NODE\_329218\_length\_1765\_cov\_29.614164 1787-1790. Max. coverage (+): 0.81. Max coverage (-): 0.02

Region: NODE\_329218\_length\_1765\_cov\_29.614164 1791-1793. Max. coverage (+): 1.23. Max coverage (-): 0.06

Region: NODE\_329218\_length\_1765\_cov\_29.614164 1794-1797. Max. coverage (+): 1.83. Max coverage (-): 0.07

Region: NODE\_329218\_length\_1765\_cov\_29.614164 1798-1801. Max. coverage (+): 1.37. Max coverage (-): 0.08

Region: NODE\_329218\_length\_1765\_cov\_29.614164 1802-1804. Max. coverage (+): 0.04. Max coverage (-): 0.01

Region: NODE\_329218\_length\_1765\_cov\_29.614164 1805-1808. Max. coverage (+): 0. Max coverage (-): 0

Region: NODE\_329218\_length\_1765\_cov\_29.614164 1809-1812. Max. coverage (+): 0. Max coverage (-): 0

Region: NODE\_329218\_length\_1765\_cov\_29.614164 1813-1815. Max. coverage (+): 0. Max coverage (-): 0

Region: NODE\_329218\_length\_1765\_cov\_29.614164 1816-1819. Max. coverage (+): 0. Max coverage (-): 0

Region: NODE\_329218\_length\_1765\_cov\_29.614164 1820-1823. Max. coverage (+): 0. Max coverage (-): 0

Region: NODE\_329218\_length\_1765\_cov\_29.614164 1824-. Max. coverage (+): 0. Max coverage (-): 0

RepeatMasker Color Code

**+**

100-98% Identity

<98-95% Identity

<95-90% Identity

<90-85% Identity

<85-80% Identity

<80-75% Identity

<75-70% Identity

<70% Identity

**-**

Gene Set Color Code

**+**

Gene

Pseudogene

Other

**-**

Topology/Coverage Color Code

Coverage Plus Strand

Coverage Minus Strand

Mainstrand: Plus

Mainstrand: Minus

Complementary Strand

Flanking Region  
(if option -flank >0)

Gene Set Annotation  
  
RepeatMasker Annotation  

**1. AlRepD-1346**: 1-506 (-), Divergence to consensus: 25.6%  
**2. (ATAA)n**: 909-951 (+), Divergence to consensus: 23.9%  
**3. Tc1-2\_PM**: 1060-1148 (-), Divergence to consensus: 20.6%  
**4. (AATT)n**: 1291-1334 (+), Divergence to consensus: 25.7%

  
Transcription Factor Binding Sites  

**RHOXF1** (Sequence: AGCTTA (-): 1660)  
**RHOXF1** (Sequence: AGCTTA (-): 1760)  
**RHOXF1** (Sequence: TGAGCC (+): 208)  
**Lhx8** (Sequence: TTAATTAA (-): 382)  
**POU5F1** (Sequence: TTTGCAT (-): 737)  
**FOXP1** (Sequence: GTAAACA (+): 891)  
**FOXP1** (Sequence: GTAAACA (+): 1452)  
**Sox5** (Sequence: ATTGTT (+): 637)  
**Sox5** (Sequence: ATTGTT (+): 1635)  
**FOXO3\_mmu** (Sequence: TGTAAACA (+): 890)  
**FOXO3\_mmu** (Sequence: TGTAAACA (+): 1451)  
**FOXO1** (Sequence: ATAAACAAG (-): 920)  
**FOXO1** (Sequence: ATAAACAAG (-): 1479)  
**Nobox** (Sequence: TAATTACT (+): 1295)  
**Nobox** (Sequence: TAATTACT (+): 1322)  
**POU2F1** (Sequence: ATTTAAATA (-): 32)
