# Supplementary material for: First characterization of PIWI-interacting RNA clusters in a cichlid fish with a B chromosome
Source: BMC Biol. 2022 Sep 21;20:204. doi: 10.1186/s12915-022-01403-2 (PMC9490952; doi:10.1186/s12915-022-01403-2)
Supplement: Supplementary file 1 — Additional file 1. Zipped folder with fasta and interactive html piRNA cluster information for the A. latifasciata genome. The nomenclature is as follows: number-pirna-cluster_sex_B-presence (f, female; m, male; 0b, without B chromosome; 1b, with B chromosome). [file 12915_2022_1403_MOESM1_ESM.zip › 131_m1b.html]

piRNA cluster 131\_m1b 77


Predicted piRNA cluster no. 131\_m1b
  

Show proTRAC run info
Hide proTRAC run info

/\  
                \_\_\_\_\_\_\_\_\_\_\_\_\_\_\_\_\_\_\_\_\_\_\_/\\_\_\_ /  \\_\_\_\_\_\_\_  
               I                      /  \  /    \      I  
               I     pro             /    \/      \     I  
               I        TRAC        /               \   I  
               I   \_\_\_\_\_\_\_\_\_\_\_\_\_\_\_\_/\_\_\_\_\_\_\_\_\_\_\_\_\_\_\_\_\_\\_ I  
               I   \              /                     I  
               I    \            /                      I  
               I     \  /\      /       V.2.4.2         I  
               I      \/  \    /                        I  
               I\_\_\_\_\_\_\_\_\_\_\_\  /\_\_\_\_\_\_\_\_\_\_\_\_\_\_\_\_\_\_\_\_\_\_\_\_\_I  
                            \/  
  
  
================================= proTRAC ====================================  
VERSION: .......... 2.4.2  
LAST MODIFIED: .... 11. May 2018  
  
Please cite:  
Rosenkranz D, Zischler H. proTRAC - a software for probabilistic piRNA cluster  
detection, visualization and analysis. 2012. BMC Bioinformatics 13:5.  
  
  
Contact:  
David Rosenkranz  
Institute of Organismic and Molecular Evolutionary Biology  
Dept. Anthropology, small RNA group  
Johannes Gutenberg University Mainz  
email: rosenkranz@uni-mainz.de  
  
You can find the latest proTRAC version at:  
http://sourceforge.net/projects/protrac/files  
http://www.smallRNAgroup-mainz.de/software  
==============================================================================  
  
PARAMETERS:  
Map file: ...............piwi-machos-1B.fa-collapse.map  
Genome file: ............../../../0B\_ala\_genome.fa  
RepeatMasker annotation: Alatifasciata-all0B-maryan-v2.fa\_corrected.out  
GeneSet:................./guest-storage/Data/annotation/Alatifasciata\_all0B\_maryan-v2\_out2017.gff  
  
Significant (p<=0.01) hit density will be calculated based  
on observed hit distribution.  
  
Sliding window size: ........................................ 5000 bp  
Sliding window increament: .................................. 1000 bp  
Normalize each hit by number of genomic hits: ............... yes  
Normalize each hit by number of sequence reads: ............. yes  
Normalize values (-> per million mapped reads): ............. yes  
Min. fraction of hits with 1T(U) or 10A: .................... 0.75  
Alternatively: Min. fraction of hits with 1T(U) and 10A: .... 0.5  
Min. fraction of hits with typical piRNA length: ............ 0.75  
Typical piRNA length: ....................................... 24-32 nt  
Min. size of a piRNA cluster: ............................... 1000 bp.  
Min. number of hits (absolute): ............................. 0  
Min. number of hits (normalized): ........................... 0  
Min. fraction of hits on the mainstrand: .................... 0.75  
Top fraction of mapped sequences (in terms of read counts): . 1%  
Top fraction accounts for max. n% of sequence reads: ........ 90%  
Min. fraction of hits on each arm of a bidirectional cluster: 0.05  
Output html file for each cluster: .......................... yes  
Output a summary table: ..................................... yes  
Output a FASTA file for each cluster (piRNA sequences): ..... yes  
Output a FASTA file comprising cluster sequences: ........... yes  
Output a GTF file for predicted piRNA clusters: ..............yes  
Search DNA motifs in clusters: .............................. yes  
Output flanking sequences: +/- .............................. 0 bp  
Output ~.pTi file: .......................................... no  
==============================================================================  
  
  
Genome size (without gaps): ............ 758543724 bp  
Gaps (N/X/-): .......................... 417479 bp  
Mapped reads: .......................... 26973943  
Non-identical sequences: ............... 6209225  
Genomic hits: .......................... 48438990  
Significant densitiy of mapped reads: .. 821.144211136946 reads/kb

Show proTRAC cluster info
Hide proTRAC cluster info

|  |  |
| --- | --- |
| Location | NODE\_330285\_length\_10438\_cov\_22.988407 |
| Coordinates | 1-8024 |
| Size [bp] | 8024 |
| Sequence hit loci | 3750 |
| Mapped reads (normalized) | 11742.8 |
| Mapped reads (normalized) per kb | 1463.5 |
| Normalized reads with 1T (1U) | 75.5% |
| Normalized reads with 10A | 45.3% |
| Normalized reads with length 24-32 nt | 98.5% |
| Normalized reads on the main strand(s) | 92.5% |
| Predicted directionality | bi:minus-plus (split between 682 and 682) |

100%

0%

1T (1U)  
reads

10A reads

24-32 nt  
reads

reads on mainstrand

**Either the amount of reads with 1T (1U) OR 10A has to exceed 75% (set with option: -1Tor10A)  
Alternatively the amount of reads with 1T (1U) AND 10A has to exceed 50% (set with option: -1Tand10A)  
Minimum amount of reads with preferred size is 75% (set with option: -pisize)  
Minimum amount of reads on the main strand(s) is 75% (set with option: -clstrand)**

Show read coverage
Hide read coverage

WHAT DO I SEE HERE?  
This chart shows the location of mapped sequence reads within a predicted piRNA cluster. The color refers to the number of genomic hits produced by the sequence read in question. A dark red bar indicates that this sequence read produces many other hits elsewhere in the genome. Many adjacent red or yellow bars can indicate the presence of a multi-copy element such as transposons or rRNA genes. A dark green bar indicates that this sequence read maps uniquely to this locus.

1 hit

2-5 hits

6-10 hits

11-20 hits

21-50 hits

51-100 hits

> 100 hits

NODE\_330285\_length\_10438\_cov\_22.988407

1

8024

Gene Set

RepeatMasker

Mapped  
Reads

23.32

plus strand

minus strand

23.32

Region: NODE\_330285\_length\_10438\_cov\_22.988407 1825-9. Max. coverage (+): 0. Max coverage (-): 0.56

Region: NODE\_330285\_length\_10438\_cov\_22.988407 10-25. Max. coverage (+): 0.26. Max coverage (-): 0

Region: NODE\_330285\_length\_10438\_cov\_22.988407 26-41. Max. coverage (+): 0. Max coverage (-): 0.19

Region: NODE\_330285\_length\_10438\_cov\_22.988407 42-57. Max. coverage (+): 0. Max coverage (-): 0

Region: NODE\_330285\_length\_10438\_cov\_22.988407 58-73. Max. coverage (+): 0. Max coverage (-): 0

Region: NODE\_330285\_length\_10438\_cov\_22.988407 74-89. Max. coverage (+): 0. Max coverage (-): 0.33

Region: NODE\_330285\_length\_10438\_cov\_22.988407 90-105. Max. coverage (+): 0.15. Max coverage (-): 0.07

Region: NODE\_330285\_length\_10438\_cov\_22.988407 106-121. Max. coverage (+): 0. Max coverage (-): 0.07

Region: NODE\_330285\_length\_10438\_cov\_22.988407 122-137. Max. coverage (+): 0. Max coverage (-): 0.07

Region: NODE\_330285\_length\_10438\_cov\_22.988407 138-153. Max. coverage (+): 0.04. Max coverage (-): 0.3

Region: NODE\_330285\_length\_10438\_cov\_22.988407 154-169. Max. coverage (+): 0.04. Max coverage (-): 0.15

Region: NODE\_330285\_length\_10438\_cov\_22.988407 170-185. Max. coverage (+): 0.41. Max coverage (-): 0.04

Region: NODE\_330285\_length\_10438\_cov\_22.988407 186-201. Max. coverage (+): 0. Max coverage (-): 2.71

Region: NODE\_330285\_length\_10438\_cov\_22.988407 202-217. Max. coverage (+): 0. Max coverage (-): 0

Region: NODE\_330285\_length\_10438\_cov\_22.988407 218-233. Max. coverage (+): 0. Max coverage (-): 0.26

Region: NODE\_330285\_length\_10438\_cov\_22.988407 234-249. Max. coverage (+): 0. Max coverage (-): 0.04

Region: NODE\_330285\_length\_10438\_cov\_22.988407 250-265. Max. coverage (+): 0. Max coverage (-): 6.75

Region: NODE\_330285\_length\_10438\_cov\_22.988407 266-281. Max. coverage (+): 0. Max coverage (-): 0.04

Region: NODE\_330285\_length\_10438\_cov\_22.988407 282-297. Max. coverage (+): 0. Max coverage (-): 3.23

Region: NODE\_330285\_length\_10438\_cov\_22.988407 298-313. Max. coverage (+): 0.26. Max coverage (-): 0

Region: NODE\_330285\_length\_10438\_cov\_22.988407 314-329. Max. coverage (+): 0. Max coverage (-): 0.07

Region: NODE\_330285\_length\_10438\_cov\_22.988407 330-346. Max. coverage (+): 0.04. Max coverage (-): 0

Region: NODE\_330285\_length\_10438\_cov\_22.988407 347-362. Max. coverage (+): 0. Max coverage (-): 1.08

Region: NODE\_330285\_length\_10438\_cov\_22.988407 363-378. Max. coverage (+): 0.07. Max coverage (-): 0.04

Region: NODE\_330285\_length\_10438\_cov\_22.988407 379-394. Max. coverage (+): 0.07. Max coverage (-): 0.19

Region: NODE\_330285\_length\_10438\_cov\_22.988407 395-410. Max. coverage (+): 0.15. Max coverage (-): 0

Region: NODE\_330285\_length\_10438\_cov\_22.988407 411-426. Max. coverage (+): 0. Max coverage (-): 0.3

Region: NODE\_330285\_length\_10438\_cov\_22.988407 427-442. Max. coverage (+): 0.11. Max coverage (-): 0.19

Region: NODE\_330285\_length\_10438\_cov\_22.988407 443-458. Max. coverage (+): 0.07. Max coverage (-): 0.04

Region: NODE\_330285\_length\_10438\_cov\_22.988407 459-474. Max. coverage (+): 0. Max coverage (-): 0

Region: NODE\_330285\_length\_10438\_cov\_22.988407 475-490. Max. coverage (+): 0.07. Max coverage (-): 0.04

Region: NODE\_330285\_length\_10438\_cov\_22.988407 491-506. Max. coverage (+): 0.07. Max coverage (-): 0.04

Region: NODE\_330285\_length\_10438\_cov\_22.988407 507-522. Max. coverage (+): 0. Max coverage (-): 0.04

Region: NODE\_330285\_length\_10438\_cov\_22.988407 523-538. Max. coverage (+): 0. Max coverage (-): 0.07

Region: NODE\_330285\_length\_10438\_cov\_22.988407 539-554. Max. coverage (+): 0.07. Max coverage (-): 0.15

Region: NODE\_330285\_length\_10438\_cov\_22.988407 555-570. Max. coverage (+): 0. Max coverage (-): 0.15

Region: NODE\_330285\_length\_10438\_cov\_22.988407 571-586. Max. coverage (+): 0.04. Max coverage (-): 0

Region: NODE\_330285\_length\_10438\_cov\_22.988407 587-602. Max. coverage (+): 0. Max coverage (-): 0

Region: NODE\_330285\_length\_10438\_cov\_22.988407 603-618. Max. coverage (+): 0.22. Max coverage (-): 0

Region: NODE\_330285\_length\_10438\_cov\_22.988407 619-634. Max. coverage (+): 0. Max coverage (-): 0.11

Region: NODE\_330285\_length\_10438\_cov\_22.988407 635-650. Max. coverage (+): 0. Max coverage (-): 0

Region: NODE\_330285\_length\_10438\_cov\_22.988407 651-666. Max. coverage (+): 0. Max coverage (-): 0.07

Region: NODE\_330285\_length\_10438\_cov\_22.988407 667-683. Max. coverage (+): 0.11. Max coverage (-): 0.11

Region: NODE\_330285\_length\_10438\_cov\_22.988407 684-699. Max. coverage (+): 0.15. Max coverage (-): 0.11

Region: NODE\_330285\_length\_10438\_cov\_22.988407 700-715. Max. coverage (+): 0.07. Max coverage (-): 0

Region: NODE\_330285\_length\_10438\_cov\_22.988407 716-731. Max. coverage (+): 0. Max coverage (-): 0.11

Region: NODE\_330285\_length\_10438\_cov\_22.988407 732-747. Max. coverage (+): 0.22. Max coverage (-): 0.04

Region: NODE\_330285\_length\_10438\_cov\_22.988407 748-763. Max. coverage (+): 0.07. Max coverage (-): 0.04

Region: NODE\_330285\_length\_10438\_cov\_22.988407 764-779. Max. coverage (+): 0.33. Max coverage (-): 0.04

Region: NODE\_330285\_length\_10438\_cov\_22.988407 780-795. Max. coverage (+): 0.19. Max coverage (-): 0.07

Region: NODE\_330285\_length\_10438\_cov\_22.988407 796-811. Max. coverage (+): 1.22. Max coverage (-): 0.19

Region: NODE\_330285\_length\_10438\_cov\_22.988407 812-827. Max. coverage (+): 0.33. Max coverage (-): 0

Region: NODE\_330285\_length\_10438\_cov\_22.988407 828-843. Max. coverage (+): 0.11. Max coverage (-): 0.04

Region: NODE\_330285\_length\_10438\_cov\_22.988407 844-859. Max. coverage (+): 0.04. Max coverage (-): 0

Region: NODE\_330285\_length\_10438\_cov\_22.988407 860-875. Max. coverage (+): 0.11. Max coverage (-): 0

Region: NODE\_330285\_length\_10438\_cov\_22.988407 876-891. Max. coverage (+): 0. Max coverage (-): 0.26

Region: NODE\_330285\_length\_10438\_cov\_22.988407 892-907. Max. coverage (+): 2.89. Max coverage (-): 0.07

Region: NODE\_330285\_length\_10438\_cov\_22.988407 908-923. Max. coverage (+): 0.67. Max coverage (-): 0.04

Region: NODE\_330285\_length\_10438\_cov\_22.988407 924-939. Max. coverage (+): 0. Max coverage (-): 0

Region: NODE\_330285\_length\_10438\_cov\_22.988407 940-955. Max. coverage (+): 0.04. Max coverage (-): 0.04

Region: NODE\_330285\_length\_10438\_cov\_22.988407 956-971. Max. coverage (+): 2.63. Max coverage (-): 0.15

Region: NODE\_330285\_length\_10438\_cov\_22.988407 972-987. Max. coverage (+): 4.12. Max coverage (-): 0.04

Region: NODE\_330285\_length\_10438\_cov\_22.988407 988-1003. Max. coverage (+): 2.6. Max coverage (-): 0

Region: NODE\_330285\_length\_10438\_cov\_22.988407 1004-1020. Max. coverage (+): 1.67. Max coverage (-): 0.04

Region: NODE\_330285\_length\_10438\_cov\_22.988407 1021-1036. Max. coverage (+): 1. Max coverage (-): 0.52

Region: NODE\_330285\_length\_10438\_cov\_22.988407 1037-1052. Max. coverage (+): 1.22. Max coverage (-): 0.07

Region: NODE\_330285\_length\_10438\_cov\_22.988407 1053-1068. Max. coverage (+): 0. Max coverage (-): 0.41

Region: NODE\_330285\_length\_10438\_cov\_22.988407 1069-1084. Max. coverage (+): 2.48. Max coverage (-): 0.04

Region: NODE\_330285\_length\_10438\_cov\_22.988407 1085-1100. Max. coverage (+): 0. Max coverage (-): 0

Region: NODE\_330285\_length\_10438\_cov\_22.988407 1101-1116. Max. coverage (+): 0.04. Max coverage (-): 0.07

Region: NODE\_330285\_length\_10438\_cov\_22.988407 1117-1132. Max. coverage (+): 0.22. Max coverage (-): 0

Region: NODE\_330285\_length\_10438\_cov\_22.988407 1133-1148. Max. coverage (+): 0. Max coverage (-): 0.19

Region: NODE\_330285\_length\_10438\_cov\_22.988407 1149-1164. Max. coverage (+): 0.26. Max coverage (-): 0.3

Region: NODE\_330285\_length\_10438\_cov\_22.988407 1165-1180. Max. coverage (+): 2.04. Max coverage (-): 0

Region: NODE\_330285\_length\_10438\_cov\_22.988407 1181-1196. Max. coverage (+): 0.26. Max coverage (-): 0.04

Region: NODE\_330285\_length\_10438\_cov\_22.988407 1197-1212. Max. coverage (+): 0.15. Max coverage (-): 0

Region: NODE\_330285\_length\_10438\_cov\_22.988407 1213-1228. Max. coverage (+): 0. Max coverage (-): 0.07

Region: NODE\_330285\_length\_10438\_cov\_22.988407 1229-1244. Max. coverage (+): 0.89. Max coverage (-): 0.04

Region: NODE\_330285\_length\_10438\_cov\_22.988407 1245-1260. Max. coverage (+): 0.07. Max coverage (-): 0.04

Region: NODE\_330285\_length\_10438\_cov\_22.988407 1261-1276. Max. coverage (+): 0.26. Max coverage (-): 0.19

Region: NODE\_330285\_length\_10438\_cov\_22.988407 1277-1292. Max. coverage (+): 4.56. Max coverage (-): 0.04

Region: NODE\_330285\_length\_10438\_cov\_22.988407 1293-1308. Max. coverage (+): 0.63. Max coverage (-): 0.63

Region: NODE\_330285\_length\_10438\_cov\_22.988407 1309-1324. Max. coverage (+): 0.48. Max coverage (-): 0.37

Region: NODE\_330285\_length\_10438\_cov\_22.988407 1325-1341. Max. coverage (+): 1.08. Max coverage (-): 0

Region: NODE\_330285\_length\_10438\_cov\_22.988407 1342-1357. Max. coverage (+): 4.12. Max coverage (-): 0

Region: NODE\_330285\_length\_10438\_cov\_22.988407 1358-1373. Max. coverage (+): 0.37. Max coverage (-): 0

Region: NODE\_330285\_length\_10438\_cov\_22.988407 1374-1389. Max. coverage (+): 0.33. Max coverage (-): 0.07

Region: NODE\_330285\_length\_10438\_cov\_22.988407 1390-1405. Max. coverage (+): 0.67. Max coverage (-): 0.04

Region: NODE\_330285\_length\_10438\_cov\_22.988407 1406-1421. Max. coverage (+): 1.89. Max coverage (-): 0

Region: NODE\_330285\_length\_10438\_cov\_22.988407 1422-1437. Max. coverage (+): 0.04. Max coverage (-): 0.15

Region: NODE\_330285\_length\_10438\_cov\_22.988407 1438-1453. Max. coverage (+): 5.82. Max coverage (-): 0.15

Region: NODE\_330285\_length\_10438\_cov\_22.988407 1454-1469. Max. coverage (+): 1.11. Max coverage (-): 0.07

Region: NODE\_330285\_length\_10438\_cov\_22.988407 1470-1485. Max. coverage (+): 0.3. Max coverage (-): 1.45

Region: NODE\_330285\_length\_10438\_cov\_22.988407 1486-1501. Max. coverage (+): 2.52. Max coverage (-): 0.15

Region: NODE\_330285\_length\_10438\_cov\_22.988407 1502-1517. Max. coverage (+): 1.19. Max coverage (-): 0

Region: NODE\_330285\_length\_10438\_cov\_22.988407 1518-1533. Max. coverage (+): 0. Max coverage (-): 0

Region: NODE\_330285\_length\_10438\_cov\_22.988407 1534-1549. Max. coverage (+): 1.22. Max coverage (-): 0.04

Region: NODE\_330285\_length\_10438\_cov\_22.988407 1550-1565. Max. coverage (+): 0.07. Max coverage (-): 0.07

Region: NODE\_330285\_length\_10438\_cov\_22.988407 1566-1581. Max. coverage (+): 3.04. Max coverage (-): 0.11

Region: NODE\_330285\_length\_10438\_cov\_22.988407 1582-1597. Max. coverage (+): 0.82. Max coverage (-): 0

Region: NODE\_330285\_length\_10438\_cov\_22.988407 1598-1613. Max. coverage (+): 7.45. Max coverage (-): 0

Region: NODE\_330285\_length\_10438\_cov\_22.988407 1614-1629. Max. coverage (+): 7.79. Max coverage (-): 0.04

Region: NODE\_330285\_length\_10438\_cov\_22.988407 1630-1645. Max. coverage (+): 1.74. Max coverage (-): 0.04

Region: NODE\_330285\_length\_10438\_cov\_22.988407 1646-1661. Max. coverage (+): 1.45. Max coverage (-): 0.19

Region: NODE\_330285\_length\_10438\_cov\_22.988407 1662-1678. Max. coverage (+): 0.07. Max coverage (-): 0.19

Region: NODE\_330285\_length\_10438\_cov\_22.988407 1679-1694. Max. coverage (+): 0.37. Max coverage (-): 0.37

Region: NODE\_330285\_length\_10438\_cov\_22.988407 1695-1710. Max. coverage (+): 0.3. Max coverage (-): 0.15

Region: NODE\_330285\_length\_10438\_cov\_22.988407 1711-1726. Max. coverage (+): 0. Max coverage (-): 0.15

Region: NODE\_330285\_length\_10438\_cov\_22.988407 1727-1742. Max. coverage (+): 3.93. Max coverage (-): 0.04

Region: NODE\_330285\_length\_10438\_cov\_22.988407 1743-1758. Max. coverage (+): 0.3. Max coverage (-): 0.04

Region: NODE\_330285\_length\_10438\_cov\_22.988407 1759-1774. Max. coverage (+): 0.93. Max coverage (-): 0.04

Region: NODE\_330285\_length\_10438\_cov\_22.988407 1775-1790. Max. coverage (+): 0.11. Max coverage (-): 0

Region: NODE\_330285\_length\_10438\_cov\_22.988407 1791-1806. Max. coverage (+): 6.41. Max coverage (-): 0.04

Region: NODE\_330285\_length\_10438\_cov\_22.988407 1807-1822. Max. coverage (+): 6.19. Max coverage (-): 0

Region: NODE\_330285\_length\_10438\_cov\_22.988407 1823-1838. Max. coverage (+): 0.67. Max coverage (-): 0.3

Region: NODE\_330285\_length\_10438\_cov\_22.988407 1839-1854. Max. coverage (+): 3.48. Max coverage (-): 0

Region: NODE\_330285\_length\_10438\_cov\_22.988407 1855-1870. Max. coverage (+): 0.26. Max coverage (-): 0

Region: NODE\_330285\_length\_10438\_cov\_22.988407 1871-1886. Max. coverage (+): 0.37. Max coverage (-): 0

Region: NODE\_330285\_length\_10438\_cov\_22.988407 1887-1902. Max. coverage (+): 0.48. Max coverage (-): 0.04

Region: NODE\_330285\_length\_10438\_cov\_22.988407 1903-1918. Max. coverage (+): 0.33. Max coverage (-): 0.04

Region: NODE\_330285\_length\_10438\_cov\_22.988407 1919-1934. Max. coverage (+): 6.64. Max coverage (-): 0

Region: NODE\_330285\_length\_10438\_cov\_22.988407 1935-1950. Max. coverage (+): 0.67. Max coverage (-): 0

Region: NODE\_330285\_length\_10438\_cov\_22.988407 1951-1966. Max. coverage (+): 0.19. Max coverage (-): 0.07

Region: NODE\_330285\_length\_10438\_cov\_22.988407 1967-1982. Max. coverage (+): 7.08. Max coverage (-): 0

Region: NODE\_330285\_length\_10438\_cov\_22.988407 1983-1998. Max. coverage (+): 0.85. Max coverage (-): 0.04

Region: NODE\_330285\_length\_10438\_cov\_22.988407 1999-2015. Max. coverage (+): 4.41. Max coverage (-): 0.11

Region: NODE\_330285\_length\_10438\_cov\_22.988407 2016-2031. Max. coverage (+): 0.89. Max coverage (-): 0.37

Region: NODE\_330285\_length\_10438\_cov\_22.988407 2032-2047. Max. coverage (+): 1.56. Max coverage (-): 0

Region: NODE\_330285\_length\_10438\_cov\_22.988407 2048-2063. Max. coverage (+): 0.26. Max coverage (-): 0

Region: NODE\_330285\_length\_10438\_cov\_22.988407 2064-2079. Max. coverage (+): 2.08. Max coverage (-): 0.07

Region: NODE\_330285\_length\_10438\_cov\_22.988407 2080-2095. Max. coverage (+): 10.34. Max coverage (-): 0.3

Region: NODE\_330285\_length\_10438\_cov\_22.988407 2096-2111. Max. coverage (+): 0.22. Max coverage (-): 0.04

Region: NODE\_330285\_length\_10438\_cov\_22.988407 2112-2127. Max. coverage (+): 0.41. Max coverage (-): 0.04

Region: NODE\_330285\_length\_10438\_cov\_22.988407 2128-2143. Max. coverage (+): 0. Max coverage (-): 0.04

Region: NODE\_330285\_length\_10438\_cov\_22.988407 2144-2159. Max. coverage (+): 1.3. Max coverage (-): 0

Region: NODE\_330285\_length\_10438\_cov\_22.988407 2160-2175. Max. coverage (+): 0.33. Max coverage (-): 0.11

Region: NODE\_330285\_length\_10438\_cov\_22.988407 2176-2191. Max. coverage (+): 8.6. Max coverage (-): 0.15

Region: NODE\_330285\_length\_10438\_cov\_22.988407 2192-2207. Max. coverage (+): 0.07. Max coverage (-): 0.04

Region: NODE\_330285\_length\_10438\_cov\_22.988407 2208-2223. Max. coverage (+): 0. Max coverage (-): 0

Region: NODE\_330285\_length\_10438\_cov\_22.988407 2224-2239. Max. coverage (+): 0. Max coverage (-): 0.07

Region: NODE\_330285\_length\_10438\_cov\_22.988407 2240-2255. Max. coverage (+): 0.07. Max coverage (-): 0

Region: NODE\_330285\_length\_10438\_cov\_22.988407 2256-2271. Max. coverage (+): 0.11. Max coverage (-): 0.04

Region: NODE\_330285\_length\_10438\_cov\_22.988407 2272-2287. Max. coverage (+): 0.85. Max coverage (-): 0

Region: NODE\_330285\_length\_10438\_cov\_22.988407 2288-2303. Max. coverage (+): 0.11. Max coverage (-): 0.04

Region: NODE\_330285\_length\_10438\_cov\_22.988407 2304-2319. Max. coverage (+): 2.11. Max coverage (-): 0

Region: NODE\_330285\_length\_10438\_cov\_22.988407 2320-2335. Max. coverage (+): 0.3. Max coverage (-): 0

Region: NODE\_330285\_length\_10438\_cov\_22.988407 2336-2352. Max. coverage (+): 0.26. Max coverage (-): 0.04

Region: NODE\_330285\_length\_10438\_cov\_22.988407 2353-2368. Max. coverage (+): 0. Max coverage (-): 0

Region: NODE\_330285\_length\_10438\_cov\_22.988407 2369-2384. Max. coverage (+): 0.04. Max coverage (-): 0.04

Region: NODE\_330285\_length\_10438\_cov\_22.988407 2385-2400. Max. coverage (+): 0.15. Max coverage (-): 0.04

Region: NODE\_330285\_length\_10438\_cov\_22.988407 2401-2416. Max. coverage (+): 0.07. Max coverage (-): 0

Region: NODE\_330285\_length\_10438\_cov\_22.988407 2417-2432. Max. coverage (+): 0.11. Max coverage (-): 0.15

Region: NODE\_330285\_length\_10438\_cov\_22.988407 2433-2448. Max. coverage (+): 1.41. Max coverage (-): 0

Region: NODE\_330285\_length\_10438\_cov\_22.988407 2449-2464. Max. coverage (+): 0.19. Max coverage (-): 0.07

Region: NODE\_330285\_length\_10438\_cov\_22.988407 2465-2480. Max. coverage (+): 0.74. Max coverage (-): 0.48

Region: NODE\_330285\_length\_10438\_cov\_22.988407 2481-2496. Max. coverage (+): 0.26. Max coverage (-): 0.33

Region: NODE\_330285\_length\_10438\_cov\_22.988407 2497-2512. Max. coverage (+): 0.22. Max coverage (-): 0

Region: NODE\_330285\_length\_10438\_cov\_22.988407 2513-2528. Max. coverage (+): 0.3. Max coverage (-): 0.04

Region: NODE\_330285\_length\_10438\_cov\_22.988407 2529-2544. Max. coverage (+): 0.37. Max coverage (-): 0.04

Region: NODE\_330285\_length\_10438\_cov\_22.988407 2545-2560. Max. coverage (+): 0.37. Max coverage (-): 0

Region: NODE\_330285\_length\_10438\_cov\_22.988407 2561-2576. Max. coverage (+): 0.11. Max coverage (-): 0

Region: NODE\_330285\_length\_10438\_cov\_22.988407 2577-2592. Max. coverage (+): 0.93. Max coverage (-): 0

Region: NODE\_330285\_length\_10438\_cov\_22.988407 2593-2608. Max. coverage (+): 0.04. Max coverage (-): 0.26

Region: NODE\_330285\_length\_10438\_cov\_22.988407 2609-2624. Max. coverage (+): 0.67. Max coverage (-): 0.11

Region: NODE\_330285\_length\_10438\_cov\_22.988407 2625-2640. Max. coverage (+): 0.11. Max coverage (-): 0.07

Region: NODE\_330285\_length\_10438\_cov\_22.988407 2641-2656. Max. coverage (+): 0.78. Max coverage (-): 0

Region: NODE\_330285\_length\_10438\_cov\_22.988407 2657-2672. Max. coverage (+): 0.3. Max coverage (-): 0.33

Region: NODE\_330285\_length\_10438\_cov\_22.988407 2673-2689. Max. coverage (+): 0.56. Max coverage (-): 0.07

Region: NODE\_330285\_length\_10438\_cov\_22.988407 2690-2705. Max. coverage (+): 0.22. Max coverage (-): 0.11

Region: NODE\_330285\_length\_10438\_cov\_22.988407 2706-2721. Max. coverage (+): 1.74. Max coverage (-): 0.04

Region: NODE\_330285\_length\_10438\_cov\_22.988407 2722-2737. Max. coverage (+): 0.44. Max coverage (-): 0.11

Region: NODE\_330285\_length\_10438\_cov\_22.988407 2738-2753. Max. coverage (+): 1.19. Max coverage (-): 0.07

Region: NODE\_330285\_length\_10438\_cov\_22.988407 2754-2769. Max. coverage (+): 0.78. Max coverage (-): 0.07

Region: NODE\_330285\_length\_10438\_cov\_22.988407 2770-2785. Max. coverage (+): 1.37. Max coverage (-): 0.07

Region: NODE\_330285\_length\_10438\_cov\_22.988407 2786-2801. Max. coverage (+): 0.19. Max coverage (-): 0.07

Region: NODE\_330285\_length\_10438\_cov\_22.988407 2802-2817. Max. coverage (+): 0.63. Max coverage (-): 0.15

Region: NODE\_330285\_length\_10438\_cov\_22.988407 2818-2833. Max. coverage (+): 0.22. Max coverage (-): 0.07

Region: NODE\_330285\_length\_10438\_cov\_22.988407 2834-2849. Max. coverage (+): 2.78. Max coverage (-): 0.04

Region: NODE\_330285\_length\_10438\_cov\_22.988407 2850-2865. Max. coverage (+): 0.11. Max coverage (-): 0

Region: NODE\_330285\_length\_10438\_cov\_22.988407 2866-2881. Max. coverage (+): 0.11. Max coverage (-): 0

Region: NODE\_330285\_length\_10438\_cov\_22.988407 2882-2897. Max. coverage (+): 15.72. Max coverage (-): 0.04

Region: NODE\_330285\_length\_10438\_cov\_22.988407 2898-2913. Max. coverage (+): 0.07. Max coverage (-): 0.04

Region: NODE\_330285\_length\_10438\_cov\_22.988407 2914-2929. Max. coverage (+): 0. Max coverage (-): 0.04

Region: NODE\_330285\_length\_10438\_cov\_22.988407 2930-2945. Max. coverage (+): 0.07. Max coverage (-): 0

Region: NODE\_330285\_length\_10438\_cov\_22.988407 2946-2961. Max. coverage (+): 0.11. Max coverage (-): 0

Region: NODE\_330285\_length\_10438\_cov\_22.988407 2962-2977. Max. coverage (+): 0. Max coverage (-): 0.04

Region: NODE\_330285\_length\_10438\_cov\_22.988407 2978-2993. Max. coverage (+): 1.08. Max coverage (-): 0

Region: NODE\_330285\_length\_10438\_cov\_22.988407 2994-3009. Max. coverage (+): 1.04. Max coverage (-): 0

Region: NODE\_330285\_length\_10438\_cov\_22.988407 3010-3026. Max. coverage (+): 0.11. Max coverage (-): 0.93

Region: NODE\_330285\_length\_10438\_cov\_22.988407 3027-3042. Max. coverage (+): 0.22. Max coverage (-): 0.44

Region: NODE\_330285\_length\_10438\_cov\_22.988407 3043-3058. Max. coverage (+): 1.15. Max coverage (-): 0

Region: NODE\_330285\_length\_10438\_cov\_22.988407 3059-3074. Max. coverage (+): 0. Max coverage (-): 0.07

Region: NODE\_330285\_length\_10438\_cov\_22.988407 3075-3090. Max. coverage (+): 0.15. Max coverage (-): 0.04

Region: NODE\_330285\_length\_10438\_cov\_22.988407 3091-3106. Max. coverage (+): 0. Max coverage (-): 0

Region: NODE\_330285\_length\_10438\_cov\_22.988407 3107-3122. Max. coverage (+): 1.41. Max coverage (-): 0.11

Region: NODE\_330285\_length\_10438\_cov\_22.988407 3123-3138. Max. coverage (+): 3. Max coverage (-): 0.11

Region: NODE\_330285\_length\_10438\_cov\_22.988407 3139-3154. Max. coverage (+): 5.41. Max coverage (-): 0.04

Region: NODE\_330285\_length\_10438\_cov\_22.988407 3155-3170. Max. coverage (+): 0.07. Max coverage (-): 0.04

Region: NODE\_330285\_length\_10438\_cov\_22.988407 3171-3186. Max. coverage (+): 0.44. Max coverage (-): 0.04

Region: NODE\_330285\_length\_10438\_cov\_22.988407 3187-3202. Max. coverage (+): 23.32. Max coverage (-): 0.07

Region: NODE\_330285\_length\_10438\_cov\_22.988407 3203-3218. Max. coverage (+): 0.15. Max coverage (-): 0.11

Region: NODE\_330285\_length\_10438\_cov\_22.988407 3219-3234. Max. coverage (+): 1.19. Max coverage (-): 0.63

Region: NODE\_330285\_length\_10438\_cov\_22.988407 3235-3250. Max. coverage (+): 1.52. Max coverage (-): 0.04

Region: NODE\_330285\_length\_10438\_cov\_22.988407 3251-3266. Max. coverage (+): 0.15. Max coverage (-): 0.22

Region: NODE\_330285\_length\_10438\_cov\_22.988407 3267-3282. Max. coverage (+): 0.07. Max coverage (-): 0.04

Region: NODE\_330285\_length\_10438\_cov\_22.988407 3283-3298. Max. coverage (+): 1.52. Max coverage (-): 0.04

Region: NODE\_330285\_length\_10438\_cov\_22.988407 3299-3314. Max. coverage (+): 1.52. Max coverage (-): 0

Region: NODE\_330285\_length\_10438\_cov\_22.988407 3315-3330. Max. coverage (+): 0. Max coverage (-): 0

Region: NODE\_330285\_length\_10438\_cov\_22.988407 3331-3347. Max. coverage (+): 0. Max coverage (-): 0

Region: NODE\_330285\_length\_10438\_cov\_22.988407 3348-3363. Max. coverage (+): 0. Max coverage (-): 0

Region: NODE\_330285\_length\_10438\_cov\_22.988407 3364-3379. Max. coverage (+): 0. Max coverage (-): 0

Region: NODE\_330285\_length\_10438\_cov\_22.988407 3380-3395. Max. coverage (+): 0. Max coverage (-): 0

Region: NODE\_330285\_length\_10438\_cov\_22.988407 3396-3411. Max. coverage (+): 0. Max coverage (-): 0

Region: NODE\_330285\_length\_10438\_cov\_22.988407 3412-3427. Max. coverage (+): 0. Max coverage (-): 0

Region: NODE\_330285\_length\_10438\_cov\_22.988407 3428-3443. Max. coverage (+): 0. Max coverage (-): 0

Region: NODE\_330285\_length\_10438\_cov\_22.988407 3444-3459. Max. coverage (+): 0. Max coverage (-): 0

Region: NODE\_330285\_length\_10438\_cov\_22.988407 3460-3475. Max. coverage (+): 0. Max coverage (-): 0

Region: NODE\_330285\_length\_10438\_cov\_22.988407 3476-3491. Max. coverage (+): 0. Max coverage (-): 0

Region: NODE\_330285\_length\_10438\_cov\_22.988407 3492-3507. Max. coverage (+): 0.04. Max coverage (-): 0.19

Region: NODE\_330285\_length\_10438\_cov\_22.988407 3508-3523. Max. coverage (+): 0.15. Max coverage (-): 0.19

Region: NODE\_330285\_length\_10438\_cov\_22.988407 3524-3539. Max. coverage (+): 1.26. Max coverage (-): 0.04

Region: NODE\_330285\_length\_10438\_cov\_22.988407 3540-3555. Max. coverage (+): 0.07. Max coverage (-): 0

Region: NODE\_330285\_length\_10438\_cov\_22.988407 3556-3571. Max. coverage (+): 0.56. Max coverage (-): 0

Region: NODE\_330285\_length\_10438\_cov\_22.988407 3572-3587. Max. coverage (+): 0.63. Max coverage (-): 0.04

Region: NODE\_330285\_length\_10438\_cov\_22.988407 3588-3603. Max. coverage (+): 0. Max coverage (-): 0

Region: NODE\_330285\_length\_10438\_cov\_22.988407 3604-3619. Max. coverage (+): 0.07. Max coverage (-): 0.04

Region: NODE\_330285\_length\_10438\_cov\_22.988407 3620-3635. Max. coverage (+): 8.08. Max coverage (-): 0

Region: NODE\_330285\_length\_10438\_cov\_22.988407 3636-3651. Max. coverage (+): 0. Max coverage (-): 0.04

Region: NODE\_330285\_length\_10438\_cov\_22.988407 3652-3667. Max. coverage (+): 0.74. Max coverage (-): 0

Region: NODE\_330285\_length\_10438\_cov\_22.988407 3668-3684. Max. coverage (+): 1.85. Max coverage (-): 0.15

Region: NODE\_330285\_length\_10438\_cov\_22.988407 3685-3700. Max. coverage (+): 0.11. Max coverage (-): 0.3

Region: NODE\_330285\_length\_10438\_cov\_22.988407 3701-3716. Max. coverage (+): 2.22. Max coverage (-): 0.11

Region: NODE\_330285\_length\_10438\_cov\_22.988407 3717-3732. Max. coverage (+): 2.22. Max coverage (-): 0.15

Region: NODE\_330285\_length\_10438\_cov\_22.988407 3733-3748. Max. coverage (+): 8.56. Max coverage (-): 0.26

Region: NODE\_330285\_length\_10438\_cov\_22.988407 3749-3764. Max. coverage (+): 8.45. Max coverage (-): 0

Region: NODE\_330285\_length\_10438\_cov\_22.988407 3765-3780. Max. coverage (+): 0. Max coverage (-): 0.04

Region: NODE\_330285\_length\_10438\_cov\_22.988407 3781-3796. Max. coverage (+): 0.11. Max coverage (-): 0.07

Region: NODE\_330285\_length\_10438\_cov\_22.988407 3797-3812. Max. coverage (+): 0.22. Max coverage (-): 0.04

Region: NODE\_330285\_length\_10438\_cov\_22.988407 3813-3828. Max. coverage (+): 0.22. Max coverage (-): 0.07

Region: NODE\_330285\_length\_10438\_cov\_22.988407 3829-3844. Max. coverage (+): 0.11. Max coverage (-): 0.07

Region: NODE\_330285\_length\_10438\_cov\_22.988407 3845-3860. Max. coverage (+): 0.04. Max coverage (-): 0.22

Region: NODE\_330285\_length\_10438\_cov\_22.988407 3861-3876. Max. coverage (+): 0.93. Max coverage (-): 0.04

Region: NODE\_330285\_length\_10438\_cov\_22.988407 3877-3892. Max. coverage (+): 0.15. Max coverage (-): 0.04

Region: NODE\_330285\_length\_10438\_cov\_22.988407 3893-3908. Max. coverage (+): 0.04. Max coverage (-): 0

Region: NODE\_330285\_length\_10438\_cov\_22.988407 3909-3924. Max. coverage (+): 0.74. Max coverage (-): 0

Region: NODE\_330285\_length\_10438\_cov\_22.988407 3925-3940. Max. coverage (+): 0.04. Max coverage (-): 0.04

Region: NODE\_330285\_length\_10438\_cov\_22.988407 3941-3956. Max. coverage (+): 0.07. Max coverage (-): 0

Region: NODE\_330285\_length\_10438\_cov\_22.988407 3957-3972. Max. coverage (+): 0.11. Max coverage (-): 0

Region: NODE\_330285\_length\_10438\_cov\_22.988407 3973-3988. Max. coverage (+): 0.22. Max coverage (-): 0.04

Region: NODE\_330285\_length\_10438\_cov\_22.988407 3989-4004. Max. coverage (+): 0.74. Max coverage (-): 0.04

Region: NODE\_330285\_length\_10438\_cov\_22.988407 4005-4021. Max. coverage (+): 0.41. Max coverage (-): 0.04

Region: NODE\_330285\_length\_10438\_cov\_22.988407 4022-4037. Max. coverage (+): 0.11. Max coverage (-): 0.04

Region: NODE\_330285\_length\_10438\_cov\_22.988407 4038-4053. Max. coverage (+): 0. Max coverage (-): 0.07

Region: NODE\_330285\_length\_10438\_cov\_22.988407 4054-4069. Max. coverage (+): 1. Max coverage (-): 0.04

Region: NODE\_330285\_length\_10438\_cov\_22.988407 4070-4085. Max. coverage (+): 1.08. Max coverage (-): 0

Region: NODE\_330285\_length\_10438\_cov\_22.988407 4086-4101. Max. coverage (+): 0.3. Max coverage (-): 0.07

Region: NODE\_330285\_length\_10438\_cov\_22.988407 4102-4117. Max. coverage (+): 3.34. Max coverage (-): 0.15

Region: NODE\_330285\_length\_10438\_cov\_22.988407 4118-4133. Max. coverage (+): 0.04. Max coverage (-): 0.11

Region: NODE\_330285\_length\_10438\_cov\_22.988407 4134-4149. Max. coverage (+): 1.41. Max coverage (-): 0.11

Region: NODE\_330285\_length\_10438\_cov\_22.988407 4150-4165. Max. coverage (+): 0.7. Max coverage (-): 0.22

Region: NODE\_330285\_length\_10438\_cov\_22.988407 4166-4181. Max. coverage (+): 3.52. Max coverage (-): 0.04

Region: NODE\_330285\_length\_10438\_cov\_22.988407 4182-4197. Max. coverage (+): 0.07. Max coverage (-): 0

Region: NODE\_330285\_length\_10438\_cov\_22.988407 4198-4213. Max. coverage (+): 6.3. Max coverage (-): 0

Region: NODE\_330285\_length\_10438\_cov\_22.988407 4214-4229. Max. coverage (+): 13.94. Max coverage (-): 0

Region: NODE\_330285\_length\_10438\_cov\_22.988407 4230-4245. Max. coverage (+): 0.07. Max coverage (-): 0.07

Region: NODE\_330285\_length\_10438\_cov\_22.988407 4246-4261. Max. coverage (+): 0.07. Max coverage (-): 0

Region: NODE\_330285\_length\_10438\_cov\_22.988407 4262-4277. Max. coverage (+): 0.04. Max coverage (-): 0.04

Region: NODE\_330285\_length\_10438\_cov\_22.988407 4278-4293. Max. coverage (+): 0.07. Max coverage (-): 0

Region: NODE\_330285\_length\_10438\_cov\_22.988407 4294-4309. Max. coverage (+): 0.19. Max coverage (-): 0.26

Region: NODE\_330285\_length\_10438\_cov\_22.988407 4310-4325. Max. coverage (+): 0.15. Max coverage (-): 0.26

Region: NODE\_330285\_length\_10438\_cov\_22.988407 4326-4341. Max. coverage (+): 0.41. Max coverage (-): 0.33

Region: NODE\_330285\_length\_10438\_cov\_22.988407 4342-4358. Max. coverage (+): 4.37. Max coverage (-): 0.04

Region: NODE\_330285\_length\_10438\_cov\_22.988407 4359-4374. Max. coverage (+): 3.19. Max coverage (-): 0.67

Region: NODE\_330285\_length\_10438\_cov\_22.988407 4375-4390. Max. coverage (+): 0.78. Max coverage (-): 0

Region: NODE\_330285\_length\_10438\_cov\_22.988407 4391-4406. Max. coverage (+): 0. Max coverage (-): 0

Region: NODE\_330285\_length\_10438\_cov\_22.988407 4407-4422. Max. coverage (+): 0.19. Max coverage (-): 0.33

Region: NODE\_330285\_length\_10438\_cov\_22.988407 4423-4438. Max. coverage (+): 4.12. Max coverage (-): 0.04

Region: NODE\_330285\_length\_10438\_cov\_22.988407 4439-4454. Max. coverage (+): 0.85. Max coverage (-): 0

Region: NODE\_330285\_length\_10438\_cov\_22.988407 4455-4470. Max. coverage (+): 0.37. Max coverage (-): 0

Region: NODE\_330285\_length\_10438\_cov\_22.988407 4471-4486. Max. coverage (+): 0.37. Max coverage (-): 0

Region: NODE\_330285\_length\_10438\_cov\_22.988407 4487-4502. Max. coverage (+): 0. Max coverage (-): 0.26

Region: NODE\_330285\_length\_10438\_cov\_22.988407 4503-4518. Max. coverage (+): 1.33. Max coverage (-): 0.04

Region: NODE\_330285\_length\_10438\_cov\_22.988407 4519-4534. Max. coverage (+): 0.3. Max coverage (-): 0.07

Region: NODE\_330285\_length\_10438\_cov\_22.988407 4535-4550. Max. coverage (+): 0.07. Max coverage (-): 0.04

Region: NODE\_330285\_length\_10438\_cov\_22.988407 4551-4566. Max. coverage (+): 0.33. Max coverage (-): 0.19

Region: NODE\_330285\_length\_10438\_cov\_22.988407 4567-4582. Max. coverage (+): 0. Max coverage (-): 0

Region: NODE\_330285\_length\_10438\_cov\_22.988407 4583-4598. Max. coverage (+): 0.22. Max coverage (-): 0

Region: NODE\_330285\_length\_10438\_cov\_22.988407 4599-4614. Max. coverage (+): 0. Max coverage (-): 0.04

Region: NODE\_330285\_length\_10438\_cov\_22.988407 4615-4630. Max. coverage (+): 0.52. Max coverage (-): 0.04

Region: NODE\_330285\_length\_10438\_cov\_22.988407 4631-4646. Max. coverage (+): 0.67. Max coverage (-): 0.15

Region: NODE\_330285\_length\_10438\_cov\_22.988407 4647-4662. Max. coverage (+): 0.48. Max coverage (-): 0.15

Region: NODE\_330285\_length\_10438\_cov\_22.988407 4663-4678. Max. coverage (+): 0.37. Max coverage (-): 0

Region: NODE\_330285\_length\_10438\_cov\_22.988407 4679-4695. Max. coverage (+): 0.78. Max coverage (-): 0.04

Region: NODE\_330285\_length\_10438\_cov\_22.988407 4696-4711. Max. coverage (+): 0.37. Max coverage (-): 0.04

Region: NODE\_330285\_length\_10438\_cov\_22.988407 4712-4727. Max. coverage (+): 1. Max coverage (-): 0.04

Region: NODE\_330285\_length\_10438\_cov\_22.988407 4728-4743. Max. coverage (+): 0.15. Max coverage (-): 0.04

Region: NODE\_330285\_length\_10438\_cov\_22.988407 4744-4759. Max. coverage (+): 0.15. Max coverage (-): 0.07

Region: NODE\_330285\_length\_10438\_cov\_22.988407 4760-4775. Max. coverage (+): 0.78. Max coverage (-): 0.07

Region: NODE\_330285\_length\_10438\_cov\_22.988407 4776-4791. Max. coverage (+): 0.15. Max coverage (-): 0.04

Region: NODE\_330285\_length\_10438\_cov\_22.988407 4792-4807. Max. coverage (+): 0.19. Max coverage (-): 0

Region: NODE\_330285\_length\_10438\_cov\_22.988407 4808-4823. Max. coverage (+): 0.34. Max coverage (-): 0.04

Region: NODE\_330285\_length\_10438\_cov\_22.988407 4824-4839. Max. coverage (+): 1.96. Max coverage (-): 0.07

Region: NODE\_330285\_length\_10438\_cov\_22.988407 4840-4855. Max. coverage (+): 0.56. Max coverage (-): 0.19

Region: NODE\_330285\_length\_10438\_cov\_22.988407 4856-4871. Max. coverage (+): 2.48. Max coverage (-): 0.04

Region: NODE\_330285\_length\_10438\_cov\_22.988407 4872-4887. Max. coverage (+): 1.11. Max coverage (-): 0.04

Region: NODE\_330285\_length\_10438\_cov\_22.988407 4888-4903. Max. coverage (+): 0.48. Max coverage (-): 0.07

Region: NODE\_330285\_length\_10438\_cov\_22.988407 4904-4919. Max. coverage (+): 0.04. Max coverage (-): 0.15

Region: NODE\_330285\_length\_10438\_cov\_22.988407 4920-4935. Max. coverage (+): 0.52. Max coverage (-): 0.04

Region: NODE\_330285\_length\_10438\_cov\_22.988407 4936-4951. Max. coverage (+): 0.07. Max coverage (-): 0.11

Region: NODE\_330285\_length\_10438\_cov\_22.988407 4952-4967. Max. coverage (+): 0. Max coverage (-): 0.07

Region: NODE\_330285\_length\_10438\_cov\_22.988407 4968-4983. Max. coverage (+): 0.07. Max coverage (-): 0

Region: NODE\_330285\_length\_10438\_cov\_22.988407 4984-4999. Max. coverage (+): 0.11. Max coverage (-): 0

Region: NODE\_330285\_length\_10438\_cov\_22.988407 5000-5015. Max. coverage (+): 0.11. Max coverage (-): 0.04

Region: NODE\_330285\_length\_10438\_cov\_22.988407 5016-5032. Max. coverage (+): 0.11. Max coverage (-): 0.04

Region: NODE\_330285\_length\_10438\_cov\_22.988407 5033-5048. Max. coverage (+): 0.22. Max coverage (-): 0.04

Region: NODE\_330285\_length\_10438\_cov\_22.988407 5049-5064. Max. coverage (+): 0.33. Max coverage (-): 0

Region: NODE\_330285\_length\_10438\_cov\_22.988407 5065-5080. Max. coverage (+): 0.11. Max coverage (-): 0

Region: NODE\_330285\_length\_10438\_cov\_22.988407 5081-5096. Max. coverage (+): 0.04. Max coverage (-): 0.04

Region: NODE\_330285\_length\_10438\_cov\_22.988407 5097-5112. Max. coverage (+): 1.59. Max coverage (-): 0.15

Region: NODE\_330285\_length\_10438\_cov\_22.988407 5113-5128. Max. coverage (+): 1.11. Max coverage (-): 0

Region: NODE\_330285\_length\_10438\_cov\_22.988407 5129-5144. Max. coverage (+): 0. Max coverage (-): 0

Region: NODE\_330285\_length\_10438\_cov\_22.988407 5145-5160. Max. coverage (+): 8.56. Max coverage (-): 0

Region: NODE\_330285\_length\_10438\_cov\_22.988407 5161-5176. Max. coverage (+): 8.08. Max coverage (-): 0.19

Region: NODE\_330285\_length\_10438\_cov\_22.988407 5177-5192. Max. coverage (+): 0.07. Max coverage (-): 0.04

Region: NODE\_330285\_length\_10438\_cov\_22.988407 5193-5208. Max. coverage (+): 0.19. Max coverage (-): 0

Region: NODE\_330285\_length\_10438\_cov\_22.988407 5209-5224. Max. coverage (+): 0. Max coverage (-): 0.11

Region: NODE\_330285\_length\_10438\_cov\_22.988407 5225-5240. Max. coverage (+): 0.07. Max coverage (-): 0.07

Region: NODE\_330285\_length\_10438\_cov\_22.988407 5241-5256. Max. coverage (+): 0.04. Max coverage (-): 0

Region: NODE\_330285\_length\_10438\_cov\_22.988407 5257-5272. Max. coverage (+): 0. Max coverage (-): 0

Region: NODE\_330285\_length\_10438\_cov\_22.988407 5273-5288. Max. coverage (+): 0.04. Max coverage (-): 0.15

Region: NODE\_330285\_length\_10438\_cov\_22.988407 5289-5304. Max. coverage (+): 0.04. Max coverage (-): 0

Region: NODE\_330285\_length\_10438\_cov\_22.988407 5305-5320. Max. coverage (+): 0. Max coverage (-): 0

Region: NODE\_330285\_length\_10438\_cov\_22.988407 5321-5336. Max. coverage (+): 0. Max coverage (-): 0

Region: NODE\_330285\_length\_10438\_cov\_22.988407 5337-5353. Max. coverage (+): 0.22. Max coverage (-): 0.04

Region: NODE\_330285\_length\_10438\_cov\_22.988407 5354-5369. Max. coverage (+): 0. Max coverage (-): 0.07

Region: NODE\_330285\_length\_10438\_cov\_22.988407 5370-5385. Max. coverage (+): 0.11. Max coverage (-): 0.07

Region: NODE\_330285\_length\_10438\_cov\_22.988407 5386-5401. Max. coverage (+): 0.19. Max coverage (-): 0

Region: NODE\_330285\_length\_10438\_cov\_22.988407 5402-5417. Max. coverage (+): 0.01. Max coverage (-): 0.01

Region: NODE\_330285\_length\_10438\_cov\_22.988407 5418-5433. Max. coverage (+): 0. Max coverage (-): 0.01

Region: NODE\_330285\_length\_10438\_cov\_22.988407 5434-5449. Max. coverage (+): 0.75. Max coverage (-): 0

Region: NODE\_330285\_length\_10438\_cov\_22.988407 5450-5465. Max. coverage (+): 0.02. Max coverage (-): 0

Region: NODE\_330285\_length\_10438\_cov\_22.988407 5466-5481. Max. coverage (+): 0.01. Max coverage (-): 0

Region: NODE\_330285\_length\_10438\_cov\_22.988407 5482-5497. Max. coverage (+): 0.01. Max coverage (-): 0

Region: NODE\_330285\_length\_10438\_cov\_22.988407 5498-5513. Max. coverage (+): 0. Max coverage (-): 0

Region: NODE\_330285\_length\_10438\_cov\_22.988407 5514-5529. Max. coverage (+): 0. Max coverage (-): 0

Region: NODE\_330285\_length\_10438\_cov\_22.988407 5530-5545. Max. coverage (+): 0.02. Max coverage (-): 0

Region: NODE\_330285\_length\_10438\_cov\_22.988407 5546-5561. Max. coverage (+): 0. Max coverage (-): 0.03

Region: NODE\_330285\_length\_10438\_cov\_22.988407 5562-5577. Max. coverage (+): 0. Max coverage (-): 0

Region: NODE\_330285\_length\_10438\_cov\_22.988407 5578-5593. Max. coverage (+): 0. Max coverage (-): 0

Region: NODE\_330285\_length\_10438\_cov\_22.988407 5594-5609. Max. coverage (+): 0. Max coverage (-): 0

Region: NODE\_330285\_length\_10438\_cov\_22.988407 5610-5625. Max. coverage (+): 0. Max coverage (-): 0

Region: NODE\_330285\_length\_10438\_cov\_22.988407 5626-5641. Max. coverage (+): 0.02. Max coverage (-): 0

Region: NODE\_330285\_length\_10438\_cov\_22.988407 5642-5657. Max. coverage (+): 0. Max coverage (-): 0

Region: NODE\_330285\_length\_10438\_cov\_22.988407 5658-5673. Max. coverage (+): 0.06. Max coverage (-): 0

Region: NODE\_330285\_length\_10438\_cov\_22.988407 5674-5690. Max. coverage (+): 0.06. Max coverage (-): 0.02

Region: NODE\_330285\_length\_10438\_cov\_22.988407 5691-5706. Max. coverage (+): 0. Max coverage (-): 0

Region: NODE\_330285\_length\_10438\_cov\_22.988407 5707-5722. Max. coverage (+): 0. Max coverage (-): 0

Region: NODE\_330285\_length\_10438\_cov\_22.988407 5723-5738. Max. coverage (+): 0. Max coverage (-): 0

Region: NODE\_330285\_length\_10438\_cov\_22.988407 5739-5754. Max. coverage (+): 0. Max coverage (-): 0

Region: NODE\_330285\_length\_10438\_cov\_22.988407 5755-5770. Max. coverage (+): 0. Max coverage (-): 0

Region: NODE\_330285\_length\_10438\_cov\_22.988407 5771-5786. Max. coverage (+): 0. Max coverage (-): 0

Region: NODE\_330285\_length\_10438\_cov\_22.988407 5787-5802. Max. coverage (+): 0. Max coverage (-): 0

Region: NODE\_330285\_length\_10438\_cov\_22.988407 5803-5818. Max. coverage (+): 0. Max coverage (-): 0.03

Region: NODE\_330285\_length\_10438\_cov\_22.988407 5819-5834. Max. coverage (+): 0. Max coverage (-): 0

Region: NODE\_330285\_length\_10438\_cov\_22.988407 5835-5850. Max. coverage (+): 0. Max coverage (-): 0

Region: NODE\_330285\_length\_10438\_cov\_22.988407 5851-5866. Max. coverage (+): 0. Max coverage (-): 0

Region: NODE\_330285\_length\_10438\_cov\_22.988407 5867-5882. Max. coverage (+): 0.02. Max coverage (-): 0

Region: NODE\_330285\_length\_10438\_cov\_22.988407 5883-5898. Max. coverage (+): 0. Max coverage (-): 0

Region: NODE\_330285\_length\_10438\_cov\_22.988407 5899-5914. Max. coverage (+): 0. Max coverage (-): 0

Region: NODE\_330285\_length\_10438\_cov\_22.988407 5915-5930. Max. coverage (+): 0.06. Max coverage (-): 0

Region: NODE\_330285\_length\_10438\_cov\_22.988407 5931-5946. Max. coverage (+): 0.04. Max coverage (-): 0.02

Region: NODE\_330285\_length\_10438\_cov\_22.988407 5947-5962. Max. coverage (+): 0. Max coverage (-): 0

Region: NODE\_330285\_length\_10438\_cov\_22.988407 5963-5978. Max. coverage (+): 0. Max coverage (-): 0

Region: NODE\_330285\_length\_10438\_cov\_22.988407 5979-5994. Max. coverage (+): 0. Max coverage (-): 0

Region: NODE\_330285\_length\_10438\_cov\_22.988407 5995-6010. Max. coverage (+): 0. Max coverage (-): 0

Region: NODE\_330285\_length\_10438\_cov\_22.988407 6011-6027. Max. coverage (+): 0. Max coverage (-): 0

Region: NODE\_330285\_length\_10438\_cov\_22.988407 6028-6043. Max. coverage (+): 0. Max coverage (-): 0

Region: NODE\_330285\_length\_10438\_cov\_22.988407 6044-6059. Max. coverage (+): 0. Max coverage (-): 0

Region: NODE\_330285\_length\_10438\_cov\_22.988407 6060-6075. Max. coverage (+): 0. Max coverage (-): 0

Region: NODE\_330285\_length\_10438\_cov\_22.988407 6076-6091. Max. coverage (+): 0. Max coverage (-): 0

Region: NODE\_330285\_length\_10438\_cov\_22.988407 6092-6107. Max. coverage (+): 0. Max coverage (-): 0

Region: NODE\_330285\_length\_10438\_cov\_22.988407 6108-6123. Max. coverage (+): 0. Max coverage (-): 0

Region: NODE\_330285\_length\_10438\_cov\_22.988407 6124-6139. Max. coverage (+): 0.07. Max coverage (-): 0

Region: NODE\_330285\_length\_10438\_cov\_22.988407 6140-6155. Max. coverage (+): 0.11. Max coverage (-): 0

Region: NODE\_330285\_length\_10438\_cov\_22.988407 6156-6171. Max. coverage (+): 0.04. Max coverage (-): 0.04

Region: NODE\_330285\_length\_10438\_cov\_22.988407 6172-6187. Max. coverage (+): 0.41. Max coverage (-): 0

Region: NODE\_330285\_length\_10438\_cov\_22.988407 6188-6203. Max. coverage (+): 0.07. Max coverage (-): 0

Region: NODE\_330285\_length\_10438\_cov\_22.988407 6204-6219. Max. coverage (+): 0.04. Max coverage (-): 0

Region: NODE\_330285\_length\_10438\_cov\_22.988407 6220-6235. Max. coverage (+): 0.07. Max coverage (-): 0

Region: NODE\_330285\_length\_10438\_cov\_22.988407 6236-6251. Max. coverage (+): 0. Max coverage (-): 0

Region: NODE\_330285\_length\_10438\_cov\_22.988407 6252-6267. Max. coverage (+): 0. Max coverage (-): 0

Region: NODE\_330285\_length\_10438\_cov\_22.988407 6268-6283. Max. coverage (+): 0. Max coverage (-): 0

Region: NODE\_330285\_length\_10438\_cov\_22.988407 6284-6299. Max. coverage (+): 0. Max coverage (-): 0

Region: NODE\_330285\_length\_10438\_cov\_22.988407 6300-6315. Max. coverage (+): 0. Max coverage (-): 0

Region: NODE\_330285\_length\_10438\_cov\_22.988407 6316-6331. Max. coverage (+): 0. Max coverage (-): 0

Region: NODE\_330285\_length\_10438\_cov\_22.988407 6332-6347. Max. coverage (+): 0. Max coverage (-): 0

Region: NODE\_330285\_length\_10438\_cov\_22.988407 6348-6364. Max. coverage (+): 0. Max coverage (-): 0

Region: NODE\_330285\_length\_10438\_cov\_22.988407 6365-6380. Max. coverage (+): 0. Max coverage (-): 0

Region: NODE\_330285\_length\_10438\_cov\_22.988407 6381-6396. Max. coverage (+): 0. Max coverage (-): 0

Region: NODE\_330285\_length\_10438\_cov\_22.988407 6397-6412. Max. coverage (+): 0. Max coverage (-): 0

Region: NODE\_330285\_length\_10438\_cov\_22.988407 6413-6428. Max. coverage (+): 0. Max coverage (-): 0

Region: NODE\_330285\_length\_10438\_cov\_22.988407 6429-6444. Max. coverage (+): 0.01. Max coverage (-): 0

Region: NODE\_330285\_length\_10438\_cov\_22.988407 6445-6460. Max. coverage (+): 0. Max coverage (-): 0

Region: NODE\_330285\_length\_10438\_cov\_22.988407 6461-6476. Max. coverage (+): 0.04. Max coverage (-): 0

Region: NODE\_330285\_length\_10438\_cov\_22.988407 6477-6492. Max. coverage (+): 0. Max coverage (-): 0

Region: NODE\_330285\_length\_10438\_cov\_22.988407 6493-6508. Max. coverage (+): 0. Max coverage (-): 0

Region: NODE\_330285\_length\_10438\_cov\_22.988407 6509-6524. Max. coverage (+): 0.04. Max coverage (-): 0

Region: NODE\_330285\_length\_10438\_cov\_22.988407 6525-6540. Max. coverage (+): 0.04. Max coverage (-): 0.33

Region: NODE\_330285\_length\_10438\_cov\_22.988407 6541-6556. Max. coverage (+): 0.04. Max coverage (-): 0.63

Region: NODE\_330285\_length\_10438\_cov\_22.988407 6557-6572. Max. coverage (+): 0.01. Max coverage (-): 0

Region: NODE\_330285\_length\_10438\_cov\_22.988407 6573-6588. Max. coverage (+): 0. Max coverage (-): 0

Region: NODE\_330285\_length\_10438\_cov\_22.988407 6589-6604. Max. coverage (+): 0.01. Max coverage (-): 0

Region: NODE\_330285\_length\_10438\_cov\_22.988407 6605-6620. Max. coverage (+): 0. Max coverage (-): 0.04

Region: NODE\_330285\_length\_10438\_cov\_22.988407 6621-6636. Max. coverage (+): 0.08. Max coverage (-): 0.04

Region: NODE\_330285\_length\_10438\_cov\_22.988407 6637-6652. Max. coverage (+): 0.01. Max coverage (-): 0

Region: NODE\_330285\_length\_10438\_cov\_22.988407 6653-6668. Max. coverage (+): 0.01. Max coverage (-): 0.02

Region: NODE\_330285\_length\_10438\_cov\_22.988407 6669-6684. Max. coverage (+): 0. Max coverage (-): 0

Region: NODE\_330285\_length\_10438\_cov\_22.988407 6685-6701. Max. coverage (+): 0. Max coverage (-): 0

Region: NODE\_330285\_length\_10438\_cov\_22.988407 6702-6717. Max. coverage (+): 0. Max coverage (-): 0

Region: NODE\_330285\_length\_10438\_cov\_22.988407 6718-6733. Max. coverage (+): 0.04. Max coverage (-): 0

Region: NODE\_330285\_length\_10438\_cov\_22.988407 6734-6749. Max. coverage (+): 0.04. Max coverage (-): 0

Region: NODE\_330285\_length\_10438\_cov\_22.988407 6750-6765. Max. coverage (+): 0.11. Max coverage (-): 0

Region: NODE\_330285\_length\_10438\_cov\_22.988407 6766-6781. Max. coverage (+): 0.04. Max coverage (-): 0

Region: NODE\_330285\_length\_10438\_cov\_22.988407 6782-6797. Max. coverage (+): 0. Max coverage (-): 0

Region: NODE\_330285\_length\_10438\_cov\_22.988407 6798-6813. Max. coverage (+): 0. Max coverage (-): 0

Region: NODE\_330285\_length\_10438\_cov\_22.988407 6814-6829. Max. coverage (+): 1.35. Max coverage (-): 0.02

Region: NODE\_330285\_length\_10438\_cov\_22.988407 6830-6845. Max. coverage (+): 0. Max coverage (-): 0

Region: NODE\_330285\_length\_10438\_cov\_22.988407 6846-6861. Max. coverage (+): 0. Max coverage (-): 0

Region: NODE\_330285\_length\_10438\_cov\_22.988407 6862-6877. Max. coverage (+): 0. Max coverage (-): 0

Region: NODE\_330285\_length\_10438\_cov\_22.988407 6878-6893. Max. coverage (+): 0. Max coverage (-): 0

Region: NODE\_330285\_length\_10438\_cov\_22.988407 6894-6909. Max. coverage (+): 0. Max coverage (-): 0

Region: NODE\_330285\_length\_10438\_cov\_22.988407 6910-6925. Max. coverage (+): 0. Max coverage (-): 0

Region: NODE\_330285\_length\_10438\_cov\_22.988407 6926-6941. Max. coverage (+): 0.02. Max coverage (-): 0.01

Region: NODE\_330285\_length\_10438\_cov\_22.988407 6942-6957. Max. coverage (+): 0.04. Max coverage (-): 0.04

Region: NODE\_330285\_length\_10438\_cov\_22.988407 6958-6973. Max. coverage (+): 0. Max coverage (-): 0

Region: NODE\_330285\_length\_10438\_cov\_22.988407 6974-6989. Max. coverage (+): 0. Max coverage (-): 0

Region: NODE\_330285\_length\_10438\_cov\_22.988407 6990-7005. Max. coverage (+): 0. Max coverage (-): 0

Region: NODE\_330285\_length\_10438\_cov\_22.988407 7006-7021. Max. coverage (+): 0. Max coverage (-): 0.04

Region: NODE\_330285\_length\_10438\_cov\_22.988407 7022-7038. Max. coverage (+): 0.07. Max coverage (-): 0

Region: NODE\_330285\_length\_10438\_cov\_22.988407 7039-7054. Max. coverage (+): 0. Max coverage (-): 0

Region: NODE\_330285\_length\_10438\_cov\_22.988407 7055-7070. Max. coverage (+): 0. Max coverage (-): 0

Region: NODE\_330285\_length\_10438\_cov\_22.988407 7071-7086. Max. coverage (+): 0. Max coverage (-): 0

Region: NODE\_330285\_length\_10438\_cov\_22.988407 7087-7102. Max. coverage (+): 0. Max coverage (-): 0

Region: NODE\_330285\_length\_10438\_cov\_22.988407 7103-7118. Max. coverage (+): 0. Max coverage (-): 0

Region: NODE\_330285\_length\_10438\_cov\_22.988407 7119-7134. Max. coverage (+): 0.04. Max coverage (-): 0

Region: NODE\_330285\_length\_10438\_cov\_22.988407 7135-7150. Max. coverage (+): 0. Max coverage (-): 0

Region: NODE\_330285\_length\_10438\_cov\_22.988407 7151-7166. Max. coverage (+): 0. Max coverage (-): 0

Region: NODE\_330285\_length\_10438\_cov\_22.988407 7167-7182. Max. coverage (+): 0.11. Max coverage (-): 0

Region: NODE\_330285\_length\_10438\_cov\_22.988407 7183-7198. Max. coverage (+): 0.04. Max coverage (-): 0

Region: NODE\_330285\_length\_10438\_cov\_22.988407 7199-7214. Max. coverage (+): 0. Max coverage (-): 0.04

Region: NODE\_330285\_length\_10438\_cov\_22.988407 7215-7230. Max. coverage (+): 0. Max coverage (-): 0

Region: NODE\_330285\_length\_10438\_cov\_22.988407 7231-7246. Max. coverage (+): 0.04. Max coverage (-): 0

Region: NODE\_330285\_length\_10438\_cov\_22.988407 7247-7262. Max. coverage (+): 0.04. Max coverage (-): 0

Region: NODE\_330285\_length\_10438\_cov\_22.988407 7263-7278. Max. coverage (+): 0. Max coverage (-): 0

Region: NODE\_330285\_length\_10438\_cov\_22.988407 7279-7294. Max. coverage (+): 0. Max coverage (-): 0

Region: NODE\_330285\_length\_10438\_cov\_22.988407 7295-7310. Max. coverage (+): 0.07. Max coverage (-): 0

Region: NODE\_330285\_length\_10438\_cov\_22.988407 7311-7326. Max. coverage (+): 0.04. Max coverage (-): 0.22

Region: NODE\_330285\_length\_10438\_cov\_22.988407 7327-7342. Max. coverage (+): 0.11. Max coverage (-): 0.3

Region: NODE\_330285\_length\_10438\_cov\_22.988407 7343-7359. Max. coverage (+): 0.19. Max coverage (-): 0.7

Region: NODE\_330285\_length\_10438\_cov\_22.988407 7360-7375. Max. coverage (+): 0.19. Max coverage (-): 0.04

Region: NODE\_330285\_length\_10438\_cov\_22.988407 7376-7391. Max. coverage (+): 0.04. Max coverage (-): 0.15

Region: NODE\_330285\_length\_10438\_cov\_22.988407 7392-7407. Max. coverage (+): 0.15. Max coverage (-): 0.11

Region: NODE\_330285\_length\_10438\_cov\_22.988407 7408-7423. Max. coverage (+): 0. Max coverage (-): 0

Region: NODE\_330285\_length\_10438\_cov\_22.988407 7424-7439. Max. coverage (+): 0.22. Max coverage (-): 0

Region: NODE\_330285\_length\_10438\_cov\_22.988407 7440-7455. Max. coverage (+): 0. Max coverage (-): 0

Region: NODE\_330285\_length\_10438\_cov\_22.988407 7456-7471. Max. coverage (+): 0. Max coverage (-): 0

Region: NODE\_330285\_length\_10438\_cov\_22.988407 7472-7487. Max. coverage (+): 0.04. Max coverage (-): 0

Region: NODE\_330285\_length\_10438\_cov\_22.988407 7488-7503. Max. coverage (+): 0. Max coverage (-): 0

Region: NODE\_330285\_length\_10438\_cov\_22.988407 7504-7519. Max. coverage (+): 0.04. Max coverage (-): 0

Region: NODE\_330285\_length\_10438\_cov\_22.988407 7520-7535. Max. coverage (+): 0.11. Max coverage (-): 0

Region: NODE\_330285\_length\_10438\_cov\_22.988407 7536-7551. Max. coverage (+): 0.04. Max coverage (-): 0

Region: NODE\_330285\_length\_10438\_cov\_22.988407 7552-7567. Max. coverage (+): 0. Max coverage (-): 0

Region: NODE\_330285\_length\_10438\_cov\_22.988407 7568-7583. Max. coverage (+): 0. Max coverage (-): 0

Region: NODE\_330285\_length\_10438\_cov\_22.988407 7584-7599. Max. coverage (+): 0.04. Max coverage (-): 0

Region: NODE\_330285\_length\_10438\_cov\_22.988407 7600-7615. Max. coverage (+): 0.04. Max coverage (-): 0.04

Region: NODE\_330285\_length\_10438\_cov\_22.988407 7616-7631. Max. coverage (+): 0.41. Max coverage (-): 0

Region: NODE\_330285\_length\_10438\_cov\_22.988407 7632-7647. Max. coverage (+): 0. Max coverage (-): 0

Region: NODE\_330285\_length\_10438\_cov\_22.988407 7648-7663. Max. coverage (+): 0.15. Max coverage (-): 0

Region: NODE\_330285\_length\_10438\_cov\_22.988407 7664-7679. Max. coverage (+): 0.15. Max coverage (-): 0

Region: NODE\_330285\_length\_10438\_cov\_22.988407 7680-7696. Max. coverage (+): 0.11. Max coverage (-): 0

Region: NODE\_330285\_length\_10438\_cov\_22.988407 7697-7712. Max. coverage (+): 0.22. Max coverage (-): 0.04

Region: NODE\_330285\_length\_10438\_cov\_22.988407 7713-7728. Max. coverage (+): 0.15. Max coverage (-): 0

Region: NODE\_330285\_length\_10438\_cov\_22.988407 7729-7744. Max. coverage (+): 0. Max coverage (-): 0.04

Region: NODE\_330285\_length\_10438\_cov\_22.988407 7745-7760. Max. coverage (+): 0.19. Max coverage (-): 0

Region: NODE\_330285\_length\_10438\_cov\_22.988407 7761-7776. Max. coverage (+): 0.11. Max coverage (-): 0.04

Region: NODE\_330285\_length\_10438\_cov\_22.988407 7777-7792. Max. coverage (+): 0.15. Max coverage (-): 0

Region: NODE\_330285\_length\_10438\_cov\_22.988407 7793-7808. Max. coverage (+): 1.33. Max coverage (-): 0.11

Region: NODE\_330285\_length\_10438\_cov\_22.988407 7809-7824. Max. coverage (+): 0.48. Max coverage (-): 0.11

Region: NODE\_330285\_length\_10438\_cov\_22.988407 7825-7840. Max. coverage (+): 0.48. Max coverage (-): 0

Region: NODE\_330285\_length\_10438\_cov\_22.988407 7841-7856. Max. coverage (+): 0.19. Max coverage (-): 0.04

Region: NODE\_330285\_length\_10438\_cov\_22.988407 7857-7872. Max. coverage (+): 0.19. Max coverage (-): 0.07

Region: NODE\_330285\_length\_10438\_cov\_22.988407 7873-7888. Max. coverage (+): 0.19. Max coverage (-): 0.04

Region: NODE\_330285\_length\_10438\_cov\_22.988407 7889-7904. Max. coverage (+): 0.15. Max coverage (-): 0

Region: NODE\_330285\_length\_10438\_cov\_22.988407 7905-7920. Max. coverage (+): 0.3. Max coverage (-): 0

Region: NODE\_330285\_length\_10438\_cov\_22.988407 7921-7936. Max. coverage (+): 0.15. Max coverage (-): 0.41

Region: NODE\_330285\_length\_10438\_cov\_22.988407 7937-7952. Max. coverage (+): 1.08. Max coverage (-): 0

Region: NODE\_330285\_length\_10438\_cov\_22.988407 7953-7968. Max. coverage (+): 0.7. Max coverage (-): 0

Region: NODE\_330285\_length\_10438\_cov\_22.988407 7969-7984. Max. coverage (+): 0.07. Max coverage (-): 0

Region: NODE\_330285\_length\_10438\_cov\_22.988407 7985-8000. Max. coverage (+): 0.04. Max coverage (-): 0

Region: NODE\_330285\_length\_10438\_cov\_22.988407 8001-8016. Max. coverage (+): 0.07. Max coverage (-): 0

Region: NODE\_330285\_length\_10438\_cov\_22.988407 8017-. Max. coverage (+): 0. Max coverage (-): 0

RepeatMasker Color Code

**+**

100-98% Identity

<98-95% Identity

<95-90% Identity

<90-85% Identity

<85-80% Identity

<80-75% Identity

<75-70% Identity

<70% Identity

**-**

Gene Set Color Code

**+**

Gene

Pseudogene

Other

**-**

Topology/Coverage Color Code

Coverage Plus Strand

Coverage Minus Strand

Mainstrand: Plus

Mainstrand: Minus

Complementary Strand

Flanking Region  
(if option -flank >0)

Gene Set Annotation  
  
RepeatMasker Annotation  

**1. (GCA)n**: 935-969 (+), Divergence to consensus: 14.9%  
**2. (TTATA)n**: 2920-2951 (+), Divergence to consensus: 12.9%  
**3. hAT-6\_GA**: 4666-4697 (-), Divergence to consensus: 12.5%  
**4. AlRepA-4**: 4692-4878 (-), Divergence to consensus: 27.7%  
**5. AlRepA-4**: 4948-5159 (-), Divergence to consensus: 38.8%  
**6. (CA)n**: 5330-5348 (+), Divergence to consensus: 0%  
**7. AlRepC-1433**: 5387-5532 (-), Divergence to consensus: 3.5%  
**8. AlRepC-743**: 5531-5747 (-), Divergence to consensus: 20.3%  
**9. AlRepC-1433**: 5749-5803 (-), Divergence to consensus: 7.3%  
**10. AlRepC-743**: 5805-6002 (-), Divergence to consensus: 21.8%  
**11. AlRepC-1433**: 5993-6254 (-), Divergence to consensus: 8.4%  
**12. AlRepB-392**: 6256-6315 (-), Divergence to consensus: 1.7%  
**13. AlRepB-392**: 6418-6541 (-), Divergence to consensus: 11.3%  
**14. AlRepB-392**: 6556-6943 (-), Divergence to consensus: 14.6%  
**15. Chap3a\_Cis**: 6915-6959 (-), Divergence to consensus: 13.3%  
**16. AlRepD-688**: 7624-7691 (+), Divergence to consensus: 22.1%  
**17. AlRepE-2751**: 7698-7853 (-), Divergence to consensus: 45.2%

  
Transcription Factor Binding Sites  

**RHOXF1** (Sequence: AGCTCA (-): 163)  
**RHOXF1** (Sequence: GGATTA (-): 314)  
**RHOXF1** (Sequence: GGCTCA (-): 447)  
**RHOXF1** (Sequence: AGATTA (-): 1982)  
**RHOXF1** (Sequence: GGCTTA (-): 4160)  
**RHOXF1** (Sequence: AGATCA (-): 4662)  
**RHOXF1** (Sequence: GGCTCA (-): 5277)  
**RHOXF1** (Sequence: AGATCA (-): 5641)  
**RHOXF1** (Sequence: AGATCA (-): 5679)  
**RHOXF1** (Sequence: AGATCA (-): 5894)  
**RHOXF1** (Sequence: AGATCA (-): 5932)  
**RHOXF1** (Sequence: TAATCC (+): 372)  
**RHOXF1** (Sequence: TAAGCT (+): 2136)  
**RHOXF1** (Sequence: TAATCT (+): 2982)  
**RHOXF1** (Sequence: TGAGCC (+): 3165)  
**RHOXF1** (Sequence: TGATCT (+): 3257)  
**RHOXF1** (Sequence: TAATCC (+): 3623)  
**RHOXF1** (Sequence: TGAGCT (+): 3679)  
**RHOXF1** (Sequence: TGAGCT (+): 4022)  
**RHOXF1** (Sequence: TGAGCT (+): 4620)  
**RHOXF1** (Sequence: TAAGCT (+): 4882)  
**RHOXF1** (Sequence: TGATCC (+): 6125)  
**RHOXF1** (Sequence: TGAGCC (+): 6149)  
**RHOXF1** (Sequence: TAAGCT (+): 6589)  
**RHOXF1** (Sequence: TAATCT (+): 6818)  
**RHOXF1** (Sequence: TGATCT (+): 7256)  
**RHOXF1** (Sequence: TGAGCT (+): 7845)  
**POU5F1** (Sequence: TTTGCAT (-): 4341)  
**RFX4\_2** (Sequence: GTATCCAAG (-): 1619)  
**RFX4\_2** (Sequence: GTATCCAAG (-): 2468)  
**RFX4\_1** (Sequence: GTTGCCATG (-): 1489)  
**FOXO1** (Sequence: CCTGTTTTC (+): 251)  
**FOXO3\_mmu** (Sequence: TGTTTAGC (-): 33)  
**FOXO3\_mmu** (Sequence: TGTTTACA (-): 92)  
**FOXO3\_mmu** (Sequence: TGTTTTCC (-): 253)  
**FOXO3\_mmu** (Sequence: TGTTTTGA (-): 2059)  
**Sox5** (Sequence: ATTGTT (+): 31)  
**Sox5** (Sequence: ATTGTT (+): 1567)  
**Sox5** (Sequence: ATTGTT (+): 5492)  
**FIGLA** (Sequence: ACCACCTGGA (-): 6907)  
**FOXO3\_mmu** (Sequence: TGAAAACA (+): 5020)  
**Nobox** (Sequence: GCTAATTA (-): 6879)  
**FOXO1** (Sequence: AAAAACAAG (-): 7519)  
**FOXP1** (Sequence: TGTTTAC (-): 92)  
**Nobox** (Sequence: TAATTACT (+): 2666)  
**Nobox** (Sequence: TAATTACT (+): 3995)  
**Nobox** (Sequence: TAATTGCT (+): 4965)  
**Nobox** (Sequence: TAATTGGC (+): 6677)  
**Nobox** (Sequence: TAATTACT (+): 6881)  
**POU2F1** (Sequence: ATTTAAATA (-): 624)  
**POU2F1** (Sequence: ATTAAAATA (-): 5596)  
**POU2F1** (Sequence: ATTAAAATA (-): 5849)  
**Rhox11** (Sequence: TGGTGTATA (+): 4986)  
**Rhox11** (Sequence: TATACAGCA (-): 6969)  
**Gata4** (Sequence: AGATAAC (-): 5476)  
**Sox5** (Sequence: AACAAT (-): 2189)  
**Sox5** (Sequence: AACAAT (-): 5605)  
**Sox5** (Sequence: AACAAT (-): 5610)  
**Sox5** (Sequence: AACAAT (-): 5858)  
**Sox5** (Sequence: AACAAT (-): 5863)
