# Supplementary material for: First characterization of PIWI-interacting RNA clusters in a cichlid fish with a B chromosome
Source: BMC Biol. 2022 Sep 21;20:204. doi: 10.1186/s12915-022-01403-2 (PMC9490952; doi:10.1186/s12915-022-01403-2)
Supplement: Supplementary file 1 — Additional file 1. Zipped folder with fasta and interactive html piRNA cluster information for the A. latifasciata genome. The nomenclature is as follows: number-pirna-cluster_sex_B-presence (f, female; m, male; 0b, without B chromosome; 1b, with B chromosome). [file 12915_2022_1403_MOESM1_ESM.zip › 132_m0b.html]

piRNA cluster 132\_m0b 63


Predicted piRNA cluster no. 132\_m0b
  

Show proTRAC run info
Hide proTRAC run info

/\  
                \_\_\_\_\_\_\_\_\_\_\_\_\_\_\_\_\_\_\_\_\_\_\_/\\_\_\_ /  \\_\_\_\_\_\_\_  
               I                      /  \  /    \      I  
               I     pro             /    \/      \     I  
               I        TRAC        /               \   I  
               I   \_\_\_\_\_\_\_\_\_\_\_\_\_\_\_\_/\_\_\_\_\_\_\_\_\_\_\_\_\_\_\_\_\_\\_ I  
               I   \              /                     I  
               I    \            /                      I  
               I     \  /\      /       V.2.4.2         I  
               I      \/  \    /                        I  
               I\_\_\_\_\_\_\_\_\_\_\_\  /\_\_\_\_\_\_\_\_\_\_\_\_\_\_\_\_\_\_\_\_\_\_\_\_\_I  
                            \/  
  
  
================================= proTRAC ====================================  
VERSION: .......... 2.4.2  
LAST MODIFIED: .... 11. May 2018  
  
Please cite:  
Rosenkranz D, Zischler H. proTRAC - a software for probabilistic piRNA cluster  
detection, visualization and analysis. 2012. BMC Bioinformatics 13:5.  
  
  
Contact:  
David Rosenkranz  
Institute of Organismic and Molecular Evolutionary Biology  
Dept. Anthropology, small RNA group  
Johannes Gutenberg University Mainz  
email: rosenkranz@uni-mainz.de  
  
You can find the latest proTRAC version at:  
http://sourceforge.net/projects/protrac/files  
http://www.smallRNAgroup-mainz.de/software  
==============================================================================  
  
PARAMETERS:  
Map file: ...............piwi-machos-0B.fa-collapse.map  
Genome file: ............../../../0B\_ala\_genome.fa  
RepeatMasker annotation: Alatifasciata-all0B-maryan-v2.fa\_corrected.out  
GeneSet:................./guest-storage/Data/annotation/Alatifasciata\_all0B\_maryan-v2\_out2017.gff  
  
Significant (p<=0.01) hit density will be calculated based  
on observed hit distribution.  
  
Sliding window size: ........................................ 5000 bp  
Sliding window increament: .................................. 1000 bp  
Normalize each hit by number of genomic hits: ............... yes  
Normalize each hit by number of sequence reads: ............. yes  
Normalize values (-> per million mapped reads): ............. yes  
Min. fraction of hits with 1T(U) or 10A: .................... 0.75  
Alternatively: Min. fraction of hits with 1T(U) and 10A: .... 0.5  
Min. fraction of hits with typical piRNA length: ............ 0.75  
Typical piRNA length: ....................................... 24-32 nt  
Min. size of a piRNA cluster: ............................... 1000 bp.  
Min. number of hits (absolute): ............................. 0  
Min. number of hits (normalized): ........................... 0  
Min. fraction of hits on the mainstrand: .................... 0.75  
Top fraction of mapped sequences (in terms of read counts): . 1%  
Top fraction accounts for max. n% of sequence reads: ........ 90%  
Min. fraction of hits on each arm of a bidirectional cluster: 0.05  
Output html file for each cluster: .......................... yes  
Output a summary table: ..................................... yes  
Output a FASTA file for each cluster (piRNA sequences): ..... yes  
Output a FASTA file comprising cluster sequences: ........... yes  
Output a GTF file for predicted piRNA clusters: ..............yes  
Search DNA motifs in clusters: .............................. yes  
Output flanking sequences: +/- .............................. 0 bp  
Output ~.pTi file: .......................................... no  
==============================================================================  
  
  
Genome size (without gaps): ............ 758543724 bp  
Gaps (N/X/-): .......................... 417479 bp  
Mapped reads: .......................... 24765598  
Non-identical sequences: ............... 6158275  
Genomic hits: .......................... 53103584  
Significant densitiy of mapped reads: .. 763.098963422187 reads/kb

Show proTRAC cluster info
Hide proTRAC cluster info

|  |  |
| --- | --- |
| Location | NODE\_331560\_length\_1271\_cov\_18.383949 |
| Coordinates | 1-1326 |
| Size [bp] | 1326 |
| Sequence hit loci | 1356 |
| Mapped reads (normalized) | 4104.6 |
| Mapped reads (normalized) per kb | 3095.4 |
| Normalized reads with 1T (1U) | 80.3% |
| Normalized reads with 10A | 24.4% |
| Normalized reads with length 24-32 nt | 98.8% |
| Normalized reads on the main strand(s) | 90.6% |
| Predicted directionality | mono:minus |

100%

0%

1T (1U)  
reads

10A reads

24-32 nt  
reads

reads on mainstrand

**Either the amount of reads with 1T (1U) OR 10A has to exceed 75% (set with option: -1Tor10A)  
Alternatively the amount of reads with 1T (1U) AND 10A has to exceed 50% (set with option: -1Tand10A)  
Minimum amount of reads with preferred size is 75% (set with option: -pisize)  
Minimum amount of reads on the main strand(s) is 75% (set with option: -clstrand)**

Show read coverage
Hide read coverage

WHAT DO I SEE HERE?  
This chart shows the location of mapped sequence reads within a predicted piRNA cluster. The color refers to the number of genomic hits produced by the sequence read in question. A dark red bar indicates that this sequence read produces many other hits elsewhere in the genome. Many adjacent red or yellow bars can indicate the presence of a multi-copy element such as transposons or rRNA genes. A dark green bar indicates that this sequence read maps uniquely to this locus.

1 hit

2-5 hits

6-10 hits

11-20 hits

21-50 hits

51-100 hits

> 100 hits

NODE\_331560\_length\_1271\_cov\_18.383949

1

1326

Gene Set

RepeatMasker

Mapped  
Reads

20.75

plus strand

minus strand

20.75

Region: NODE\_331560\_length\_1271\_cov\_18.383949 1825-2. Max. coverage (+): 0. Max coverage (-): 0

Region: NODE\_331560\_length\_1271\_cov\_18.383949 3-4. Max. coverage (+): 0. Max coverage (-): 0

Region: NODE\_331560\_length\_1271\_cov\_18.383949 5-7. Max. coverage (+): 0. Max coverage (-): 0

Region: NODE\_331560\_length\_1271\_cov\_18.383949 8-10. Max. coverage (+): 0. Max coverage (-): 0

Region: NODE\_331560\_length\_1271\_cov\_18.383949 11-12. Max. coverage (+): 0. Max coverage (-): 0

Region: NODE\_331560\_length\_1271\_cov\_18.383949 13-15. Max. coverage (+): 0. Max coverage (-): 0

Region: NODE\_331560\_length\_1271\_cov\_18.383949 16-18. Max. coverage (+): 0. Max coverage (-): 0

Region: NODE\_331560\_length\_1271\_cov\_18.383949 19-20. Max. coverage (+): 0. Max coverage (-): 0

Region: NODE\_331560\_length\_1271\_cov\_18.383949 21-23. Max. coverage (+): 0. Max coverage (-): 0

Region: NODE\_331560\_length\_1271\_cov\_18.383949 24-26. Max. coverage (+): 0. Max coverage (-): 0

Region: NODE\_331560\_length\_1271\_cov\_18.383949 27-28. Max. coverage (+): 0. Max coverage (-): 0

Region: NODE\_331560\_length\_1271\_cov\_18.383949 29-31. Max. coverage (+): 0. Max coverage (-): 0

Region: NODE\_331560\_length\_1271\_cov\_18.383949 32-34. Max. coverage (+): 0. Max coverage (-): 0

Region: NODE\_331560\_length\_1271\_cov\_18.383949 35-36. Max. coverage (+): 0. Max coverage (-): 0

Region: NODE\_331560\_length\_1271\_cov\_18.383949 37-39. Max. coverage (+): 0. Max coverage (-): 0.04

Region: NODE\_331560\_length\_1271\_cov\_18.383949 40-42. Max. coverage (+): 0.12. Max coverage (-): 0.04

Region: NODE\_331560\_length\_1271\_cov\_18.383949 43-44. Max. coverage (+): 0.08. Max coverage (-): 0

Region: NODE\_331560\_length\_1271\_cov\_18.383949 45-47. Max. coverage (+): 0. Max coverage (-): 0

Region: NODE\_331560\_length\_1271\_cov\_18.383949 48-50. Max. coverage (+): 0.12. Max coverage (-): 0.08

Region: NODE\_331560\_length\_1271\_cov\_18.383949 51-52. Max. coverage (+): 0. Max coverage (-): 0.08

Region: NODE\_331560\_length\_1271\_cov\_18.383949 53-55. Max. coverage (+): 0. Max coverage (-): 0.08

Region: NODE\_331560\_length\_1271\_cov\_18.383949 56-58. Max. coverage (+): 0. Max coverage (-): 0.04

Region: NODE\_331560\_length\_1271\_cov\_18.383949 59-60. Max. coverage (+): 0.04. Max coverage (-): 0.04

Region: NODE\_331560\_length\_1271\_cov\_18.383949 61-63. Max. coverage (+): 0. Max coverage (-): 0

Region: NODE\_331560\_length\_1271\_cov\_18.383949 64-65. Max. coverage (+): 0. Max coverage (-): 0

Region: NODE\_331560\_length\_1271\_cov\_18.383949 66-68. Max. coverage (+): 0. Max coverage (-): 0

Region: NODE\_331560\_length\_1271\_cov\_18.383949 69-71. Max. coverage (+): 0. Max coverage (-): 0

Region: NODE\_331560\_length\_1271\_cov\_18.383949 72-73. Max. coverage (+): 0. Max coverage (-): 0

Region: NODE\_331560\_length\_1271\_cov\_18.383949 74-76. Max. coverage (+): 0. Max coverage (-): 0

Region: NODE\_331560\_length\_1271\_cov\_18.383949 77-79. Max. coverage (+): 0. Max coverage (-): 0.08

Region: NODE\_331560\_length\_1271\_cov\_18.383949 80-81. Max. coverage (+): 0. Max coverage (-): 0.4

Region: NODE\_331560\_length\_1271\_cov\_18.383949 82-84. Max. coverage (+): 0. Max coverage (-): 0.53

Region: NODE\_331560\_length\_1271\_cov\_18.383949 85-87. Max. coverage (+): 0. Max coverage (-): 0

Region: NODE\_331560\_length\_1271\_cov\_18.383949 88-89. Max. coverage (+): 0. Max coverage (-): 0.12

Region: NODE\_331560\_length\_1271\_cov\_18.383949 90-92. Max. coverage (+): 0.04. Max coverage (-): 0.08

Region: NODE\_331560\_length\_1271\_cov\_18.383949 93-95. Max. coverage (+): 0. Max coverage (-): 0

Region: NODE\_331560\_length\_1271\_cov\_18.383949 96-97. Max. coverage (+): 0. Max coverage (-): 0

Region: NODE\_331560\_length\_1271\_cov\_18.383949 98-100. Max. coverage (+): 0.12. Max coverage (-): 0.16

Region: NODE\_331560\_length\_1271\_cov\_18.383949 101-103. Max. coverage (+): 0.57. Max coverage (-): 0.32

Region: NODE\_331560\_length\_1271\_cov\_18.383949 104-105. Max. coverage (+): 0. Max coverage (-): 0.04

Region: NODE\_331560\_length\_1271\_cov\_18.383949 106-108. Max. coverage (+): 0. Max coverage (-): 0

Region: NODE\_331560\_length\_1271\_cov\_18.383949 109-111. Max. coverage (+): 0. Max coverage (-): 0

Region: NODE\_331560\_length\_1271\_cov\_18.383949 112-113. Max. coverage (+): 0. Max coverage (-): 0

Region: NODE\_331560\_length\_1271\_cov\_18.383949 114-116. Max. coverage (+): 0. Max coverage (-): 0.16

Region: NODE\_331560\_length\_1271\_cov\_18.383949 117-119. Max. coverage (+): 0. Max coverage (-): 0.16

Region: NODE\_331560\_length\_1271\_cov\_18.383949 120-121. Max. coverage (+): 0. Max coverage (-): 0.04

Region: NODE\_331560\_length\_1271\_cov\_18.383949 122-124. Max. coverage (+): 0. Max coverage (-): 0.04

Region: NODE\_331560\_length\_1271\_cov\_18.383949 125-126. Max. coverage (+): 0. Max coverage (-): 0.04

Region: NODE\_331560\_length\_1271\_cov\_18.383949 127-129. Max. coverage (+): 0. Max coverage (-): 0

Region: NODE\_331560\_length\_1271\_cov\_18.383949 130-132. Max. coverage (+): 0. Max coverage (-): 0

Region: NODE\_331560\_length\_1271\_cov\_18.383949 133-134. Max. coverage (+): 0. Max coverage (-): 0

Region: NODE\_331560\_length\_1271\_cov\_18.383949 135-137. Max. coverage (+): 0. Max coverage (-): 0

Region: NODE\_331560\_length\_1271\_cov\_18.383949 138-140. Max. coverage (+): 0. Max coverage (-): 0

Region: NODE\_331560\_length\_1271\_cov\_18.383949 141-142. Max. coverage (+): 0. Max coverage (-): 0

Region: NODE\_331560\_length\_1271\_cov\_18.383949 143-145. Max. coverage (+): 0. Max coverage (-): 0

Region: NODE\_331560\_length\_1271\_cov\_18.383949 146-148. Max. coverage (+): 0. Max coverage (-): 0

Region: NODE\_331560\_length\_1271\_cov\_18.383949 149-150. Max. coverage (+): 0. Max coverage (-): 0

Region: NODE\_331560\_length\_1271\_cov\_18.383949 151-153. Max. coverage (+): 0. Max coverage (-): 0

Region: NODE\_331560\_length\_1271\_cov\_18.383949 154-156. Max. coverage (+): 0. Max coverage (-): 0

Region: NODE\_331560\_length\_1271\_cov\_18.383949 157-158. Max. coverage (+): 0. Max coverage (-): 0

Region: NODE\_331560\_length\_1271\_cov\_18.383949 159-161. Max. coverage (+): 0. Max coverage (-): 0

Region: NODE\_331560\_length\_1271\_cov\_18.383949 162-164. Max. coverage (+): 0. Max coverage (-): 0

Region: NODE\_331560\_length\_1271\_cov\_18.383949 165-166. Max. coverage (+): 0. Max coverage (-): 0.08

Region: NODE\_331560\_length\_1271\_cov\_18.383949 167-169. Max. coverage (+): 0. Max coverage (-): 0.48

Region: NODE\_331560\_length\_1271\_cov\_18.383949 170-172. Max. coverage (+): 0. Max coverage (-): 0.44

Region: NODE\_331560\_length\_1271\_cov\_18.383949 173-174. Max. coverage (+): 0. Max coverage (-): 0.12

Region: NODE\_331560\_length\_1271\_cov\_18.383949 175-177. Max. coverage (+): 0.08. Max coverage (-): 0.12

Region: NODE\_331560\_length\_1271\_cov\_18.383949 178-180. Max. coverage (+): 0.08. Max coverage (-): 0

Region: NODE\_331560\_length\_1271\_cov\_18.383949 181-182. Max. coverage (+): 0. Max coverage (-): 0

Region: NODE\_331560\_length\_1271\_cov\_18.383949 183-185. Max. coverage (+): 0. Max coverage (-): 0

Region: NODE\_331560\_length\_1271\_cov\_18.383949 186-187. Max. coverage (+): 0. Max coverage (-): 0.04

Region: NODE\_331560\_length\_1271\_cov\_18.383949 188-190. Max. coverage (+): 0. Max coverage (-): 0.89

Region: NODE\_331560\_length\_1271\_cov\_18.383949 191-193. Max. coverage (+): 0. Max coverage (-): 0.08

Region: NODE\_331560\_length\_1271\_cov\_18.383949 194-195. Max. coverage (+): 0. Max coverage (-): 0.08

Region: NODE\_331560\_length\_1271\_cov\_18.383949 196-198. Max. coverage (+): 0. Max coverage (-): 0.4

Region: NODE\_331560\_length\_1271\_cov\_18.383949 199-201. Max. coverage (+): 0. Max coverage (-): 6.3

Region: NODE\_331560\_length\_1271\_cov\_18.383949 202-203. Max. coverage (+): 0. Max coverage (-): 6.58

Region: NODE\_331560\_length\_1271\_cov\_18.383949 204-206. Max. coverage (+): 0. Max coverage (-): 0.57

Region: NODE\_331560\_length\_1271\_cov\_18.383949 207-209. Max. coverage (+): 0.08. Max coverage (-): 0.24

Region: NODE\_331560\_length\_1271\_cov\_18.383949 210-211. Max. coverage (+): 0.2. Max coverage (-): 0.97

Region: NODE\_331560\_length\_1271\_cov\_18.383949 212-214. Max. coverage (+): 0.16. Max coverage (-): 1.01

Region: NODE\_331560\_length\_1271\_cov\_18.383949 215-217. Max. coverage (+): 1.33. Max coverage (-): 0.24

Region: NODE\_331560\_length\_1271\_cov\_18.383949 218-219. Max. coverage (+): 1.33. Max coverage (-): 0.08

Region: NODE\_331560\_length\_1271\_cov\_18.383949 220-222. Max. coverage (+): 0. Max coverage (-): 0.12

Region: NODE\_331560\_length\_1271\_cov\_18.383949 223-225. Max. coverage (+): 0.16. Max coverage (-): 0.28

Region: NODE\_331560\_length\_1271\_cov\_18.383949 226-227. Max. coverage (+): 0.08. Max coverage (-): 0.12

Region: NODE\_331560\_length\_1271\_cov\_18.383949 228-230. Max. coverage (+): 0.04. Max coverage (-): 0.12

Region: NODE\_331560\_length\_1271\_cov\_18.383949 231-233. Max. coverage (+): 0.04. Max coverage (-): 0.44

Region: NODE\_331560\_length\_1271\_cov\_18.383949 234-235. Max. coverage (+): 0.08. Max coverage (-): 0.4

Region: NODE\_331560\_length\_1271\_cov\_18.383949 236-238. Max. coverage (+): 0.08. Max coverage (-): 0.24

Region: NODE\_331560\_length\_1271\_cov\_18.383949 239-241. Max. coverage (+): 0. Max coverage (-): 0.28

Region: NODE\_331560\_length\_1271\_cov\_18.383949 242-243. Max. coverage (+): 0. Max coverage (-): 0.12

Region: NODE\_331560\_length\_1271\_cov\_18.383949 244-246. Max. coverage (+): 0.08. Max coverage (-): 0.32

Region: NODE\_331560\_length\_1271\_cov\_18.383949 247-248. Max. coverage (+): 0.04. Max coverage (-): 0.16

Region: NODE\_331560\_length\_1271\_cov\_18.383949 249-251. Max. coverage (+): 0.08. Max coverage (-): 0.24

Region: NODE\_331560\_length\_1271\_cov\_18.383949 252-254. Max. coverage (+): 0.04. Max coverage (-): 0.44

Region: NODE\_331560\_length\_1271\_cov\_18.383949 255-256. Max. coverage (+): 0.04. Max coverage (-): 0.73

Region: NODE\_331560\_length\_1271\_cov\_18.383949 257-259. Max. coverage (+): 0. Max coverage (-): 1.05

Region: NODE\_331560\_length\_1271\_cov\_18.383949 260-262. Max. coverage (+): 0. Max coverage (-): 0.12

Region: NODE\_331560\_length\_1271\_cov\_18.383949 263-264. Max. coverage (+): 0. Max coverage (-): 0.04

Region: NODE\_331560\_length\_1271\_cov\_18.383949 265-267. Max. coverage (+): 0.12. Max coverage (-): 0.04

Region: NODE\_331560\_length\_1271\_cov\_18.383949 268-270. Max. coverage (+): 0.2. Max coverage (-): 0.12

Region: NODE\_331560\_length\_1271\_cov\_18.383949 271-272. Max. coverage (+): 0.24. Max coverage (-): 0.12

Region: NODE\_331560\_length\_1271\_cov\_18.383949 273-275. Max. coverage (+): 0.08. Max coverage (-): 0

Region: NODE\_331560\_length\_1271\_cov\_18.383949 276-278. Max. coverage (+): 0.08. Max coverage (-): 0

Region: NODE\_331560\_length\_1271\_cov\_18.383949 279-280. Max. coverage (+): 0. Max coverage (-): 0

Region: NODE\_331560\_length\_1271\_cov\_18.383949 281-283. Max. coverage (+): 0. Max coverage (-): 0

Region: NODE\_331560\_length\_1271\_cov\_18.383949 284-286. Max. coverage (+): 0. Max coverage (-): 0.04

Region: NODE\_331560\_length\_1271\_cov\_18.383949 287-288. Max. coverage (+): 0. Max coverage (-): 0.4

Region: NODE\_331560\_length\_1271\_cov\_18.383949 289-291. Max. coverage (+): 0. Max coverage (-): 1.29

Region: NODE\_331560\_length\_1271\_cov\_18.383949 292-294. Max. coverage (+): 0. Max coverage (-): 1.01

Region: NODE\_331560\_length\_1271\_cov\_18.383949 295-296. Max. coverage (+): 0. Max coverage (-): 0.85

Region: NODE\_331560\_length\_1271\_cov\_18.383949 297-299. Max. coverage (+): 0. Max coverage (-): 0.44

Region: NODE\_331560\_length\_1271\_cov\_18.383949 300-302. Max. coverage (+): 0. Max coverage (-): 0.2

Region: NODE\_331560\_length\_1271\_cov\_18.383949 303-304. Max. coverage (+): 0. Max coverage (-): 0.24

Region: NODE\_331560\_length\_1271\_cov\_18.383949 305-307. Max. coverage (+): 0. Max coverage (-): 0.12

Region: NODE\_331560\_length\_1271\_cov\_18.383949 308-309. Max. coverage (+): 0.2. Max coverage (-): 1.21

Region: NODE\_331560\_length\_1271\_cov\_18.383949 310-312. Max. coverage (+): 0.2. Max coverage (-): 1.37

Region: NODE\_331560\_length\_1271\_cov\_18.383949 313-315. Max. coverage (+): 0. Max coverage (-): 0.2

Region: NODE\_331560\_length\_1271\_cov\_18.383949 316-317. Max. coverage (+): 0. Max coverage (-): 0.04

Region: NODE\_331560\_length\_1271\_cov\_18.383949 318-320. Max. coverage (+): 0. Max coverage (-): 0.04

Region: NODE\_331560\_length\_1271\_cov\_18.383949 321-323. Max. coverage (+): 0. Max coverage (-): 0.04

Region: NODE\_331560\_length\_1271\_cov\_18.383949 324-325. Max. coverage (+): 0. Max coverage (-): 0.32

Region: NODE\_331560\_length\_1271\_cov\_18.383949 326-328. Max. coverage (+): 0. Max coverage (-): 2.06

Region: NODE\_331560\_length\_1271\_cov\_18.383949 329-331. Max. coverage (+): 0. Max coverage (-): 0.81

Region: NODE\_331560\_length\_1271\_cov\_18.383949 332-333. Max. coverage (+): 0. Max coverage (-): 0.2

Region: NODE\_331560\_length\_1271\_cov\_18.383949 334-336. Max. coverage (+): 0. Max coverage (-): 0.12

Region: NODE\_331560\_length\_1271\_cov\_18.383949 337-339. Max. coverage (+): 0. Max coverage (-): 0.12

Region: NODE\_331560\_length\_1271\_cov\_18.383949 340-341. Max. coverage (+): 0.04. Max coverage (-): 0.28

Region: NODE\_331560\_length\_1271\_cov\_18.383949 342-344. Max. coverage (+): 0.04. Max coverage (-): 1.05

Region: NODE\_331560\_length\_1271\_cov\_18.383949 345-347. Max. coverage (+): 0. Max coverage (-): 0.69

Region: NODE\_331560\_length\_1271\_cov\_18.383949 348-349. Max. coverage (+): 0. Max coverage (-): 0.08

Region: NODE\_331560\_length\_1271\_cov\_18.383949 350-352. Max. coverage (+): 0. Max coverage (-): 0.28

Region: NODE\_331560\_length\_1271\_cov\_18.383949 353-355. Max. coverage (+): 0. Max coverage (-): 0.4

Region: NODE\_331560\_length\_1271\_cov\_18.383949 356-357. Max. coverage (+): 0.08. Max coverage (-): 0.32

Region: NODE\_331560\_length\_1271\_cov\_18.383949 358-360. Max. coverage (+): 0.12. Max coverage (-): 0.44

Region: NODE\_331560\_length\_1271\_cov\_18.383949 361-362. Max. coverage (+): 0.04. Max coverage (-): 0.44

Region: NODE\_331560\_length\_1271\_cov\_18.383949 363-365. Max. coverage (+): 0. Max coverage (-): 0.04

Region: NODE\_331560\_length\_1271\_cov\_18.383949 366-368. Max. coverage (+): 0. Max coverage (-): 0.24

Region: NODE\_331560\_length\_1271\_cov\_18.383949 369-370. Max. coverage (+): 0. Max coverage (-): 0.24

Region: NODE\_331560\_length\_1271\_cov\_18.383949 371-373. Max. coverage (+): 0.04. Max coverage (-): 3.11

Region: NODE\_331560\_length\_1271\_cov\_18.383949 374-376. Max. coverage (+): 0. Max coverage (-): 3.63

Region: NODE\_331560\_length\_1271\_cov\_18.383949 377-378. Max. coverage (+): 0. Max coverage (-): 3.15

Region: NODE\_331560\_length\_1271\_cov\_18.383949 379-381. Max. coverage (+): 0. Max coverage (-): 1.57

Region: NODE\_331560\_length\_1271\_cov\_18.383949 382-384. Max. coverage (+): 0. Max coverage (-): 0.93

Region: NODE\_331560\_length\_1271\_cov\_18.383949 385-386. Max. coverage (+): 0. Max coverage (-): 1.01

Region: NODE\_331560\_length\_1271\_cov\_18.383949 387-389. Max. coverage (+): 0.28. Max coverage (-): 1.41

Region: NODE\_331560\_length\_1271\_cov\_18.383949 390-392. Max. coverage (+): 0.28. Max coverage (-): 0.24

Region: NODE\_331560\_length\_1271\_cov\_18.383949 393-394. Max. coverage (+): 0.2. Max coverage (-): 0

Region: NODE\_331560\_length\_1271\_cov\_18.383949 395-397. Max. coverage (+): 0.48. Max coverage (-): 0.12

Region: NODE\_331560\_length\_1271\_cov\_18.383949 398-400. Max. coverage (+): 0.16. Max coverage (-): 0.28

Region: NODE\_331560\_length\_1271\_cov\_18.383949 401-402. Max. coverage (+): 0.36. Max coverage (-): 0.08

Region: NODE\_331560\_length\_1271\_cov\_18.383949 403-405. Max. coverage (+): 0.57. Max coverage (-): 0.16

Region: NODE\_331560\_length\_1271\_cov\_18.383949 406-408. Max. coverage (+): 0.65. Max coverage (-): 0.16

Region: NODE\_331560\_length\_1271\_cov\_18.383949 409-410. Max. coverage (+): 0.24. Max coverage (-): 0.12

Region: NODE\_331560\_length\_1271\_cov\_18.383949 411-413. Max. coverage (+): 0.36. Max coverage (-): 0.2

Region: NODE\_331560\_length\_1271\_cov\_18.383949 414-416. Max. coverage (+): 0.2. Max coverage (-): 0.57

Region: NODE\_331560\_length\_1271\_cov\_18.383949 417-418. Max. coverage (+): 0. Max coverage (-): 0.73

Region: NODE\_331560\_length\_1271\_cov\_18.383949 419-421. Max. coverage (+): 0. Max coverage (-): 1.09

Region: NODE\_331560\_length\_1271\_cov\_18.383949 422-423. Max. coverage (+): 0. Max coverage (-): 4.4

Region: NODE\_331560\_length\_1271\_cov\_18.383949 424-426. Max. coverage (+): 0. Max coverage (-): 3.39

Region: NODE\_331560\_length\_1271\_cov\_18.383949 427-429. Max. coverage (+): 0. Max coverage (-): 0.81

Region: NODE\_331560\_length\_1271\_cov\_18.383949 430-431. Max. coverage (+): 0. Max coverage (-): 1.09

Region: NODE\_331560\_length\_1271\_cov\_18.383949 432-434. Max. coverage (+): 0.04. Max coverage (-): 0.48

Region: NODE\_331560\_length\_1271\_cov\_18.383949 435-437. Max. coverage (+): 0.36. Max coverage (-): 0.57

Region: NODE\_331560\_length\_1271\_cov\_18.383949 438-439. Max. coverage (+): 0.36. Max coverage (-): 0.24

Region: NODE\_331560\_length\_1271\_cov\_18.383949 440-442. Max. coverage (+): 0. Max coverage (-): 0.24

Region: NODE\_331560\_length\_1271\_cov\_18.383949 443-445. Max. coverage (+): 0.08. Max coverage (-): 0.2

Region: NODE\_331560\_length\_1271\_cov\_18.383949 446-447. Max. coverage (+): 0.08. Max coverage (-): 0.08

Region: NODE\_331560\_length\_1271\_cov\_18.383949 448-450. Max. coverage (+): 0.12. Max coverage (-): 0.24

Region: NODE\_331560\_length\_1271\_cov\_18.383949 451-453. Max. coverage (+): 0.08. Max coverage (-): 0.52

Region: NODE\_331560\_length\_1271\_cov\_18.383949 454-455. Max. coverage (+): 0. Max coverage (-): 0.61

Region: NODE\_331560\_length\_1271\_cov\_18.383949 456-458. Max. coverage (+): 0. Max coverage (-): 0.81

Region: NODE\_331560\_length\_1271\_cov\_18.383949 459-461. Max. coverage (+): 0. Max coverage (-): 2.62

Region: NODE\_331560\_length\_1271\_cov\_18.383949 462-463. Max. coverage (+): 0. Max coverage (-): 0.85

Region: NODE\_331560\_length\_1271\_cov\_18.383949 464-466. Max. coverage (+): 0. Max coverage (-): 0.48

Region: NODE\_331560\_length\_1271\_cov\_18.383949 467-469. Max. coverage (+): 0. Max coverage (-): 0.2

Region: NODE\_331560\_length\_1271\_cov\_18.383949 470-471. Max. coverage (+): 0.04. Max coverage (-): 0.24

Region: NODE\_331560\_length\_1271\_cov\_18.383949 472-474. Max. coverage (+): 0.04. Max coverage (-): 0.12

Region: NODE\_331560\_length\_1271\_cov\_18.383949 475-477. Max. coverage (+): 0. Max coverage (-): 0.73

Region: NODE\_331560\_length\_1271\_cov\_18.383949 478-479. Max. coverage (+): 0. Max coverage (-): 0.65

Region: NODE\_331560\_length\_1271\_cov\_18.383949 480-482. Max. coverage (+): 0. Max coverage (-): 0.2

Region: NODE\_331560\_length\_1271\_cov\_18.383949 483-484. Max. coverage (+): 0. Max coverage (-): 0.12

Region: NODE\_331560\_length\_1271\_cov\_18.383949 485-487. Max. coverage (+): 0.08. Max coverage (-): 0.44

Region: NODE\_331560\_length\_1271\_cov\_18.383949 488-490. Max. coverage (+): 0.12. Max coverage (-): 0.44

Region: NODE\_331560\_length\_1271\_cov\_18.383949 491-492. Max. coverage (+): 0. Max coverage (-): 0.16

Region: NODE\_331560\_length\_1271\_cov\_18.383949 493-495. Max. coverage (+): 0. Max coverage (-): 0.2

Region: NODE\_331560\_length\_1271\_cov\_18.383949 496-498. Max. coverage (+): 0. Max coverage (-): 10.09

Region: NODE\_331560\_length\_1271\_cov\_18.383949 499-500. Max. coverage (+): 0. Max coverage (-): 10.46

Region: NODE\_331560\_length\_1271\_cov\_18.383949 501-503. Max. coverage (+): 0. Max coverage (-): 2.34

Region: NODE\_331560\_length\_1271\_cov\_18.383949 504-506. Max. coverage (+): 0. Max coverage (-): 2.26

Region: NODE\_331560\_length\_1271\_cov\_18.383949 507-508. Max. coverage (+): 0. Max coverage (-): 0.36

Region: NODE\_331560\_length\_1271\_cov\_18.383949 509-511. Max. coverage (+): 0. Max coverage (-): 0.04

Region: NODE\_331560\_length\_1271\_cov\_18.383949 512-514. Max. coverage (+): 0. Max coverage (-): 0.12

Region: NODE\_331560\_length\_1271\_cov\_18.383949 515-516. Max. coverage (+): 0. Max coverage (-): 0.12

Region: NODE\_331560\_length\_1271\_cov\_18.383949 517-519. Max. coverage (+): 0.16. Max coverage (-): 0.08

Region: NODE\_331560\_length\_1271\_cov\_18.383949 520-522. Max. coverage (+): 0.12. Max coverage (-): 0

Region: NODE\_331560\_length\_1271\_cov\_18.383949 523-524. Max. coverage (+): 0.04. Max coverage (-): 0.04

Region: NODE\_331560\_length\_1271\_cov\_18.383949 525-527. Max. coverage (+): 0. Max coverage (-): 0.08

Region: NODE\_331560\_length\_1271\_cov\_18.383949 528-530. Max. coverage (+): 0. Max coverage (-): 0.12

Region: NODE\_331560\_length\_1271\_cov\_18.383949 531-532. Max. coverage (+): 0. Max coverage (-): 0.28

Region: NODE\_331560\_length\_1271\_cov\_18.383949 533-535. Max. coverage (+): 0. Max coverage (-): 0.28

Region: NODE\_331560\_length\_1271\_cov\_18.383949 536-538. Max. coverage (+): 0. Max coverage (-): 0.04

Region: NODE\_331560\_length\_1271\_cov\_18.383949 539-540. Max. coverage (+): 0. Max coverage (-): 0

Region: NODE\_331560\_length\_1271\_cov\_18.383949 541-543. Max. coverage (+): 0. Max coverage (-): 0

Region: NODE\_331560\_length\_1271\_cov\_18.383949 544-545. Max. coverage (+): 0. Max coverage (-): 0.24

Region: NODE\_331560\_length\_1271\_cov\_18.383949 546-548. Max. coverage (+): 0. Max coverage (-): 0.32

Region: NODE\_331560\_length\_1271\_cov\_18.383949 549-551. Max. coverage (+): 0.36. Max coverage (-): 0.32

Region: NODE\_331560\_length\_1271\_cov\_18.383949 552-553. Max. coverage (+): 0.77. Max coverage (-): 0.2

Region: NODE\_331560\_length\_1271\_cov\_18.383949 554-556. Max. coverage (+): 0.89. Max coverage (-): 0.2

Region: NODE\_331560\_length\_1271\_cov\_18.383949 557-559. Max. coverage (+): 0.12. Max coverage (-): 0.16

Region: NODE\_331560\_length\_1271\_cov\_18.383949 560-561. Max. coverage (+): 0.12. Max coverage (-): 0.2

Region: NODE\_331560\_length\_1271\_cov\_18.383949 562-564. Max. coverage (+): 0.28. Max coverage (-): 0.24

Region: NODE\_331560\_length\_1271\_cov\_18.383949 565-567. Max. coverage (+): 0.77. Max coverage (-): 0.04

Region: NODE\_331560\_length\_1271\_cov\_18.383949 568-569. Max. coverage (+): 0.73. Max coverage (-): 0.04

Region: NODE\_331560\_length\_1271\_cov\_18.383949 570-572. Max. coverage (+): 0. Max coverage (-): 0

Region: NODE\_331560\_length\_1271\_cov\_18.383949 573-575. Max. coverage (+): 0. Max coverage (-): 0

Region: NODE\_331560\_length\_1271\_cov\_18.383949 576-577. Max. coverage (+): 0. Max coverage (-): 0

Region: NODE\_331560\_length\_1271\_cov\_18.383949 578-580. Max. coverage (+): 0. Max coverage (-): 0

Region: NODE\_331560\_length\_1271\_cov\_18.383949 581-583. Max. coverage (+): 0. Max coverage (-): 0

Region: NODE\_331560\_length\_1271\_cov\_18.383949 584-585. Max. coverage (+): 0. Max coverage (-): 0.16

Region: NODE\_331560\_length\_1271\_cov\_18.383949 586-588. Max. coverage (+): 0. Max coverage (-): 0.2

Region: NODE\_331560\_length\_1271\_cov\_18.383949 589-591. Max. coverage (+): 0. Max coverage (-): 0.04

Region: NODE\_331560\_length\_1271\_cov\_18.383949 592-593. Max. coverage (+): 0. Max coverage (-): 0

Region: NODE\_331560\_length\_1271\_cov\_18.383949 594-596. Max. coverage (+): 0. Max coverage (-): 0.28

Region: NODE\_331560\_length\_1271\_cov\_18.383949 597-599. Max. coverage (+): 0. Max coverage (-): 0.32

Region: NODE\_331560\_length\_1271\_cov\_18.383949 600-601. Max. coverage (+): 0. Max coverage (-): 0.08

Region: NODE\_331560\_length\_1271\_cov\_18.383949 602-604. Max. coverage (+): 0. Max coverage (-): 0.16

Region: NODE\_331560\_length\_1271\_cov\_18.383949 605-606. Max. coverage (+): 0. Max coverage (-): 0

Region: NODE\_331560\_length\_1271\_cov\_18.383949 607-609. Max. coverage (+): 0. Max coverage (-): 0

Region: NODE\_331560\_length\_1271\_cov\_18.383949 610-612. Max. coverage (+): 0. Max coverage (-): 0.08

Region: NODE\_331560\_length\_1271\_cov\_18.383949 613-614. Max. coverage (+): 0. Max coverage (-): 0

Region: NODE\_331560\_length\_1271\_cov\_18.383949 615-617. Max. coverage (+): 0. Max coverage (-): 0

Region: NODE\_331560\_length\_1271\_cov\_18.383949 618-620. Max. coverage (+): 0. Max coverage (-): 0.44

Region: NODE\_331560\_length\_1271\_cov\_18.383949 621-622. Max. coverage (+): 0. Max coverage (-): 0.4

Region: NODE\_331560\_length\_1271\_cov\_18.383949 623-625. Max. coverage (+): 0. Max coverage (-): 0

Region: NODE\_331560\_length\_1271\_cov\_18.383949 626-628. Max. coverage (+): 0. Max coverage (-): 0

Region: NODE\_331560\_length\_1271\_cov\_18.383949 629-630. Max. coverage (+): 0. Max coverage (-): 0

Region: NODE\_331560\_length\_1271\_cov\_18.383949 631-633. Max. coverage (+): 0.04. Max coverage (-): 0.12

Region: NODE\_331560\_length\_1271\_cov\_18.383949 634-636. Max. coverage (+): 0.04. Max coverage (-): 0.12

Region: NODE\_331560\_length\_1271\_cov\_18.383949 637-638. Max. coverage (+): 0. Max coverage (-): 0

Region: NODE\_331560\_length\_1271\_cov\_18.383949 639-641. Max. coverage (+): 0. Max coverage (-): 0

Region: NODE\_331560\_length\_1271\_cov\_18.383949 642-644. Max. coverage (+): 0. Max coverage (-): 0

Region: NODE\_331560\_length\_1271\_cov\_18.383949 645-646. Max. coverage (+): 0. Max coverage (-): 0

Region: NODE\_331560\_length\_1271\_cov\_18.383949 647-649. Max. coverage (+): 0. Max coverage (-): 0

Region: NODE\_331560\_length\_1271\_cov\_18.383949 650-652. Max. coverage (+): 0. Max coverage (-): 0

Region: NODE\_331560\_length\_1271\_cov\_18.383949 653-654. Max. coverage (+): 0. Max coverage (-): 0

Region: NODE\_331560\_length\_1271\_cov\_18.383949 655-657. Max. coverage (+): 0. Max coverage (-): 0

Region: NODE\_331560\_length\_1271\_cov\_18.383949 658-660. Max. coverage (+): 0. Max coverage (-): 0

Region: NODE\_331560\_length\_1271\_cov\_18.383949 661-662. Max. coverage (+): 0. Max coverage (-): 0

Region: NODE\_331560\_length\_1271\_cov\_18.383949 663-665. Max. coverage (+): 0. Max coverage (-): 0

Region: NODE\_331560\_length\_1271\_cov\_18.383949 666-667. Max. coverage (+): 0. Max coverage (-): 0

Region: NODE\_331560\_length\_1271\_cov\_18.383949 668-670. Max. coverage (+): 0.04. Max coverage (-): 0

Region: NODE\_331560\_length\_1271\_cov\_18.383949 671-673. Max. coverage (+): 0.04. Max coverage (-): 0

Region: NODE\_331560\_length\_1271\_cov\_18.383949 674-675. Max. coverage (+): 0. Max coverage (-): 0

Region: NODE\_331560\_length\_1271\_cov\_18.383949 676-678. Max. coverage (+): 0. Max coverage (-): 0

Region: NODE\_331560\_length\_1271\_cov\_18.383949 679-681. Max. coverage (+): 0. Max coverage (-): 0

Region: NODE\_331560\_length\_1271\_cov\_18.383949 682-683. Max. coverage (+): 0. Max coverage (-): 0

Region: NODE\_331560\_length\_1271\_cov\_18.383949 684-686. Max. coverage (+): 0. Max coverage (-): 0

Region: NODE\_331560\_length\_1271\_cov\_18.383949 687-689. Max. coverage (+): 0. Max coverage (-): 0

Region: NODE\_331560\_length\_1271\_cov\_18.383949 690-691. Max. coverage (+): 0. Max coverage (-): 0

Region: NODE\_331560\_length\_1271\_cov\_18.383949 692-694. Max. coverage (+): 0. Max coverage (-): 0

Region: NODE\_331560\_length\_1271\_cov\_18.383949 695-697. Max. coverage (+): 0. Max coverage (-): 0

Region: NODE\_331560\_length\_1271\_cov\_18.383949 698-699. Max. coverage (+): 0. Max coverage (-): 0

Region: NODE\_331560\_length\_1271\_cov\_18.383949 700-702. Max. coverage (+): 0. Max coverage (-): 0.04

Region: NODE\_331560\_length\_1271\_cov\_18.383949 703-705. Max. coverage (+): 0. Max coverage (-): 0.12

Region: NODE\_331560\_length\_1271\_cov\_18.383949 706-707. Max. coverage (+): 0. Max coverage (-): 0.16

Region: NODE\_331560\_length\_1271\_cov\_18.383949 708-710. Max. coverage (+): 0. Max coverage (-): 0.12

Region: NODE\_331560\_length\_1271\_cov\_18.383949 711-713. Max. coverage (+): 0. Max coverage (-): 0.04

Region: NODE\_331560\_length\_1271\_cov\_18.383949 714-715. Max. coverage (+): 0. Max coverage (-): 0.12

Region: NODE\_331560\_length\_1271\_cov\_18.383949 716-718. Max. coverage (+): 0. Max coverage (-): 0.12

Region: NODE\_331560\_length\_1271\_cov\_18.383949 719-721. Max. coverage (+): 0. Max coverage (-): 0

Region: NODE\_331560\_length\_1271\_cov\_18.383949 722-723. Max. coverage (+): 0. Max coverage (-): 0

Region: NODE\_331560\_length\_1271\_cov\_18.383949 724-726. Max. coverage (+): 0. Max coverage (-): 0.32

Region: NODE\_331560\_length\_1271\_cov\_18.383949 727-728. Max. coverage (+): 0. Max coverage (-): 0.32

Region: NODE\_331560\_length\_1271\_cov\_18.383949 729-731. Max. coverage (+): 0.04. Max coverage (-): 0.12

Region: NODE\_331560\_length\_1271\_cov\_18.383949 732-734. Max. coverage (+): 0. Max coverage (-): 0

Region: NODE\_331560\_length\_1271\_cov\_18.383949 735-736. Max. coverage (+): 0. Max coverage (-): 0

Region: NODE\_331560\_length\_1271\_cov\_18.383949 737-739. Max. coverage (+): 0. Max coverage (-): 0

Region: NODE\_331560\_length\_1271\_cov\_18.383949 740-742. Max. coverage (+): 0.04. Max coverage (-): 0

Region: NODE\_331560\_length\_1271\_cov\_18.383949 743-744. Max. coverage (+): 0.12. Max coverage (-): 0.04

Region: NODE\_331560\_length\_1271\_cov\_18.383949 745-747. Max. coverage (+): 0.12. Max coverage (-): 0.08

Region: NODE\_331560\_length\_1271\_cov\_18.383949 748-750. Max. coverage (+): 0.04. Max coverage (-): 0.2

Region: NODE\_331560\_length\_1271\_cov\_18.383949 751-752. Max. coverage (+): 0. Max coverage (-): 0.04

Region: NODE\_331560\_length\_1271\_cov\_18.383949 753-755. Max. coverage (+): 0. Max coverage (-): 0.08

Region: NODE\_331560\_length\_1271\_cov\_18.383949 756-758. Max. coverage (+): 0. Max coverage (-): 0

Region: NODE\_331560\_length\_1271\_cov\_18.383949 759-760. Max. coverage (+): 0. Max coverage (-): 0

Region: NODE\_331560\_length\_1271\_cov\_18.383949 761-763. Max. coverage (+): 0. Max coverage (-): 0

Region: NODE\_331560\_length\_1271\_cov\_18.383949 764-766. Max. coverage (+): 0. Max coverage (-): 0

Region: NODE\_331560\_length\_1271\_cov\_18.383949 767-768. Max. coverage (+): 0. Max coverage (-): 0

Region: NODE\_331560\_length\_1271\_cov\_18.383949 769-771. Max. coverage (+): 0. Max coverage (-): 0

Region: NODE\_331560\_length\_1271\_cov\_18.383949 772-774. Max. coverage (+): 0. Max coverage (-): 7.51

Region: NODE\_331560\_length\_1271\_cov\_18.383949 775-776. Max. coverage (+): 0.04. Max coverage (-): 2.02

Region: NODE\_331560\_length\_1271\_cov\_18.383949 777-779. Max. coverage (+): 0.36. Max coverage (-): 1.98

Region: NODE\_331560\_length\_1271\_cov\_18.383949 780-782. Max. coverage (+): 0.69. Max coverage (-): 1.57

Region: NODE\_331560\_length\_1271\_cov\_18.383949 783-784. Max. coverage (+): 0.08. Max coverage (-): 1.25

Region: NODE\_331560\_length\_1271\_cov\_18.383949 785-787. Max. coverage (+): 0.08. Max coverage (-): 2.91

Region: NODE\_331560\_length\_1271\_cov\_18.383949 788-789. Max. coverage (+): 0. Max coverage (-): 0.2

Region: NODE\_331560\_length\_1271\_cov\_18.383949 790-792. Max. coverage (+): 0.04. Max coverage (-): 0.12

Region: NODE\_331560\_length\_1271\_cov\_18.383949 793-795. Max. coverage (+): 0. Max coverage (-): 0.04

Region: NODE\_331560\_length\_1271\_cov\_18.383949 796-797. Max. coverage (+): 0. Max coverage (-): 0

Region: NODE\_331560\_length\_1271\_cov\_18.383949 798-800. Max. coverage (+): 0. Max coverage (-): 0.04

Region: NODE\_331560\_length\_1271\_cov\_18.383949 801-803. Max. coverage (+): 0. Max coverage (-): 1.09

Region: NODE\_331560\_length\_1271\_cov\_18.383949 804-805. Max. coverage (+): 0. Max coverage (-): 1.21

Region: NODE\_331560\_length\_1271\_cov\_18.383949 806-808. Max. coverage (+): 0. Max coverage (-): 20.71

Region: NODE\_331560\_length\_1271\_cov\_18.383949 809-811. Max. coverage (+): 0. Max coverage (-): 20.75

Region: NODE\_331560\_length\_1271\_cov\_18.383949 812-813. Max. coverage (+): 0. Max coverage (-): 6.02

Region: NODE\_331560\_length\_1271\_cov\_18.383949 814-816. Max. coverage (+): 0. Max coverage (-): 6.02

Region: NODE\_331560\_length\_1271\_cov\_18.383949 817-819. Max. coverage (+): 0.04. Max coverage (-): 0.28

Region: NODE\_331560\_length\_1271\_cov\_18.383949 820-821. Max. coverage (+): 0.04. Max coverage (-): 0.32

Region: NODE\_331560\_length\_1271\_cov\_18.383949 822-824. Max. coverage (+): 0.04. Max coverage (-): 0.36

Region: NODE\_331560\_length\_1271\_cov\_18.383949 825-827. Max. coverage (+): 0.04. Max coverage (-): 0.65

Region: NODE\_331560\_length\_1271\_cov\_18.383949 828-829. Max. coverage (+): 0.04. Max coverage (-): 0.65

Region: NODE\_331560\_length\_1271\_cov\_18.383949 830-832. Max. coverage (+): 0. Max coverage (-): 1.17

Region: NODE\_331560\_length\_1271\_cov\_18.383949 833-835. Max. coverage (+): 0. Max coverage (-): 0.12

Region: NODE\_331560\_length\_1271\_cov\_18.383949 836-837. Max. coverage (+): 0. Max coverage (-): 0

Region: NODE\_331560\_length\_1271\_cov\_18.383949 838-840. Max. coverage (+): 0. Max coverage (-): 0.02

Region: NODE\_331560\_length\_1271\_cov\_18.383949 841-843. Max. coverage (+): 0.06. Max coverage (-): 0

Region: NODE\_331560\_length\_1271\_cov\_18.383949 844-845. Max. coverage (+): 0.06. Max coverage (-): 0

Region: NODE\_331560\_length\_1271\_cov\_18.383949 846-848. Max. coverage (+): 0.02. Max coverage (-): 0

Region: NODE\_331560\_length\_1271\_cov\_18.383949 849-850. Max. coverage (+): 0. Max coverage (-): 0

Region: NODE\_331560\_length\_1271\_cov\_18.383949 851-853. Max. coverage (+): 0. Max coverage (-): 0.08

Region: NODE\_331560\_length\_1271\_cov\_18.383949 854-856. Max. coverage (+): 0. Max coverage (-): 0.16

Region: NODE\_331560\_length\_1271\_cov\_18.383949 857-858. Max. coverage (+): 0. Max coverage (-): 0.16

Region: NODE\_331560\_length\_1271\_cov\_18.383949 859-861. Max. coverage (+): 0. Max coverage (-): 0.48

Region: NODE\_331560\_length\_1271\_cov\_18.383949 862-864. Max. coverage (+): 0. Max coverage (-): 2.54

Region: NODE\_331560\_length\_1271\_cov\_18.383949 865-866. Max. coverage (+): 0. Max coverage (-): 2.87

Region: NODE\_331560\_length\_1271\_cov\_18.383949 867-869. Max. coverage (+): 0. Max coverage (-): 0.61

Region: NODE\_331560\_length\_1271\_cov\_18.383949 870-872. Max. coverage (+): 0. Max coverage (-): 0.04

Region: NODE\_331560\_length\_1271\_cov\_18.383949 873-874. Max. coverage (+): 0. Max coverage (-): 0.04

Region: NODE\_331560\_length\_1271\_cov\_18.383949 875-877. Max. coverage (+): 0. Max coverage (-): 1.21

Region: NODE\_331560\_length\_1271\_cov\_18.383949 878-880. Max. coverage (+): 0.08. Max coverage (-): 0

Region: NODE\_331560\_length\_1271\_cov\_18.383949 881-882. Max. coverage (+): 0.1. Max coverage (-): 0.14

Region: NODE\_331560\_length\_1271\_cov\_18.383949 883-885. Max. coverage (+): 0.02. Max coverage (-): 0.14

Region: NODE\_331560\_length\_1271\_cov\_18.383949 886-888. Max. coverage (+): 0.02. Max coverage (-): 0.14

Region: NODE\_331560\_length\_1271\_cov\_18.383949 889-890. Max. coverage (+): 0. Max coverage (-): 0.04

Region: NODE\_331560\_length\_1271\_cov\_18.383949 891-893. Max. coverage (+): 0.02. Max coverage (-): 0.12

Region: NODE\_331560\_length\_1271\_cov\_18.383949 894-896. Max. coverage (+): 0.02. Max coverage (-): 0.2

Region: NODE\_331560\_length\_1271\_cov\_18.383949 897-898. Max. coverage (+): 0. Max coverage (-): 0.42

Region: NODE\_331560\_length\_1271\_cov\_18.383949 899-901. Max. coverage (+): 0. Max coverage (-): 0.65

Region: NODE\_331560\_length\_1271\_cov\_18.383949 902-904. Max. coverage (+): 0. Max coverage (-): 1.09

Region: NODE\_331560\_length\_1271\_cov\_18.383949 905-906. Max. coverage (+): 0. Max coverage (-): 0.48

Region: NODE\_331560\_length\_1271\_cov\_18.383949 907-909. Max. coverage (+): 0. Max coverage (-): 0.04

Region: NODE\_331560\_length\_1271\_cov\_18.383949 910-911. Max. coverage (+): 0. Max coverage (-): 0

Region: NODE\_331560\_length\_1271\_cov\_18.383949 912-914. Max. coverage (+): 0.04. Max coverage (-): 0

Region: NODE\_331560\_length\_1271\_cov\_18.383949 915-917. Max. coverage (+): 0.04. Max coverage (-): 0

Region: NODE\_331560\_length\_1271\_cov\_18.383949 918-919. Max. coverage (+): 0. Max coverage (-): 0

Region: NODE\_331560\_length\_1271\_cov\_18.383949 920-922. Max. coverage (+): 0.08. Max coverage (-): 0.04

Region: NODE\_331560\_length\_1271\_cov\_18.383949 923-925. Max. coverage (+): 0. Max coverage (-): 0.04

Region: NODE\_331560\_length\_1271\_cov\_18.383949 926-927. Max. coverage (+): 0. Max coverage (-): 0.04

Region: NODE\_331560\_length\_1271\_cov\_18.383949 928-930. Max. coverage (+): 0. Max coverage (-): 0.04

Region: NODE\_331560\_length\_1271\_cov\_18.383949 931-933. Max. coverage (+): 0. Max coverage (-): 0.12

Region: NODE\_331560\_length\_1271\_cov\_18.383949 934-935. Max. coverage (+): 0. Max coverage (-): 0.12

Region: NODE\_331560\_length\_1271\_cov\_18.383949 936-938. Max. coverage (+): 0. Max coverage (-): 0

Region: NODE\_331560\_length\_1271\_cov\_18.383949 939-941. Max. coverage (+): 0.04. Max coverage (-): 0

Region: NODE\_331560\_length\_1271\_cov\_18.383949 942-943. Max. coverage (+): 0.2. Max coverage (-): 0

Region: NODE\_331560\_length\_1271\_cov\_18.383949 944-946. Max. coverage (+): 0.16. Max coverage (-): 0

Region: NODE\_331560\_length\_1271\_cov\_18.383949 947-949. Max. coverage (+): 0.12. Max coverage (-): 0.28

Region: NODE\_331560\_length\_1271\_cov\_18.383949 950-951. Max. coverage (+): 0.08. Max coverage (-): 0.61

Region: NODE\_331560\_length\_1271\_cov\_18.383949 952-954. Max. coverage (+): 0.36. Max coverage (-): 0.48

Region: NODE\_331560\_length\_1271\_cov\_18.383949 955-957. Max. coverage (+): 0.28. Max coverage (-): 0.24

Region: NODE\_331560\_length\_1271\_cov\_18.383949 958-959. Max. coverage (+): 0.04. Max coverage (-): 0.12

Region: NODE\_331560\_length\_1271\_cov\_18.383949 960-962. Max. coverage (+): 0.04. Max coverage (-): 0.16

Region: NODE\_331560\_length\_1271\_cov\_18.383949 963-965. Max. coverage (+): 0.1. Max coverage (-): 0.12

Region: NODE\_331560\_length\_1271\_cov\_18.383949 966-967. Max. coverage (+): 0.08. Max coverage (-): 0.02

Region: NODE\_331560\_length\_1271\_cov\_18.383949 968-970. Max. coverage (+): 0. Max coverage (-): 0.61

Region: NODE\_331560\_length\_1271\_cov\_18.383949 971-972. Max. coverage (+): 0. Max coverage (-): 0.2

Region: NODE\_331560\_length\_1271\_cov\_18.383949 973-975. Max. coverage (+): 0. Max coverage (-): 0.04

Region: NODE\_331560\_length\_1271\_cov\_18.383949 976-978. Max. coverage (+): 0. Max coverage (-): 0.06

Region: NODE\_331560\_length\_1271\_cov\_18.383949 979-980. Max. coverage (+): 0. Max coverage (-): 0.02

Region: NODE\_331560\_length\_1271\_cov\_18.383949 981-983. Max. coverage (+): 0. Max coverage (-): 0

Region: NODE\_331560\_length\_1271\_cov\_18.383949 984-986. Max. coverage (+): 0. Max coverage (-): 0

Region: NODE\_331560\_length\_1271\_cov\_18.383949 987-988. Max. coverage (+): 0. Max coverage (-): 0

Region: NODE\_331560\_length\_1271\_cov\_18.383949 989-991. Max. coverage (+): 0. Max coverage (-): 0.04

Region: NODE\_331560\_length\_1271\_cov\_18.383949 992-994. Max. coverage (+): 0. Max coverage (-): 0.04

Region: NODE\_331560\_length\_1271\_cov\_18.383949 995-996. Max. coverage (+): 0. Max coverage (-): 0.12

Region: NODE\_331560\_length\_1271\_cov\_18.383949 997-999. Max. coverage (+): 0. Max coverage (-): 0.08

Region: NODE\_331560\_length\_1271\_cov\_18.383949 1000-1002. Max. coverage (+): 0. Max coverage (-): 0.85

Region: NODE\_331560\_length\_1271\_cov\_18.383949 1003-1004. Max. coverage (+): 0. Max coverage (-): 0.85

Region: NODE\_331560\_length\_1271\_cov\_18.383949 1005-1007. Max. coverage (+): 0. Max coverage (-): 0.08

Region: NODE\_331560\_length\_1271\_cov\_18.383949 1008-1010. Max. coverage (+): 0. Max coverage (-): 0.24

Region: NODE\_331560\_length\_1271\_cov\_18.383949 1011-1012. Max. coverage (+): 0. Max coverage (-): 0.77

Region: NODE\_331560\_length\_1271\_cov\_18.383949 1013-1015. Max. coverage (+): 0.04. Max coverage (-): 1.57

Region: NODE\_331560\_length\_1271\_cov\_18.383949 1016-1018. Max. coverage (+): 0.16. Max coverage (-): 1.25

Region: NODE\_331560\_length\_1271\_cov\_18.383949 1019-1020. Max. coverage (+): 0.12. Max coverage (-): 0.24

Region: NODE\_331560\_length\_1271\_cov\_18.383949 1021-1023. Max. coverage (+): 0. Max coverage (-): 0

Region: NODE\_331560\_length\_1271\_cov\_18.383949 1024-1025. Max. coverage (+): 0. Max coverage (-): 0.08

Region: NODE\_331560\_length\_1271\_cov\_18.383949 1026-1028. Max. coverage (+): 0. Max coverage (-): 0.24

Region: NODE\_331560\_length\_1271\_cov\_18.383949 1029-1031. Max. coverage (+): 0. Max coverage (-): 0.24

Region: NODE\_331560\_length\_1271\_cov\_18.383949 1032-1033. Max. coverage (+): 0. Max coverage (-): 0.08

Region: NODE\_331560\_length\_1271\_cov\_18.383949 1034-1036. Max. coverage (+): 0. Max coverage (-): 0

Region: NODE\_331560\_length\_1271\_cov\_18.383949 1037-1039. Max. coverage (+): 0. Max coverage (-): 0

Region: NODE\_331560\_length\_1271\_cov\_18.383949 1040-1041. Max. coverage (+): 0. Max coverage (-): 0

Region: NODE\_331560\_length\_1271\_cov\_18.383949 1042-1044. Max. coverage (+): 0. Max coverage (-): 1.86

Region: NODE\_331560\_length\_1271\_cov\_18.383949 1045-1047. Max. coverage (+): 0. Max coverage (-): 1.86

Region: NODE\_331560\_length\_1271\_cov\_18.383949 1048-1049. Max. coverage (+): 0. Max coverage (-): 0.04

Region: NODE\_331560\_length\_1271\_cov\_18.383949 1050-1052. Max. coverage (+): 0. Max coverage (-): 0.12

Region: NODE\_331560\_length\_1271\_cov\_18.383949 1053-1055. Max. coverage (+): 0. Max coverage (-): 0.12

Region: NODE\_331560\_length\_1271\_cov\_18.383949 1056-1057. Max. coverage (+): 0. Max coverage (-): 0.16

Region: NODE\_331560\_length\_1271\_cov\_18.383949 1058-1060. Max. coverage (+): 0. Max coverage (-): 0.28

Region: NODE\_331560\_length\_1271\_cov\_18.383949 1061-1063. Max. coverage (+): 0.04. Max coverage (-): 0.36

Region: NODE\_331560\_length\_1271\_cov\_18.383949 1064-1065. Max. coverage (+): 0.28. Max coverage (-): 1.29

Region: NODE\_331560\_length\_1271\_cov\_18.383949 1066-1068. Max. coverage (+): 0.28. Max coverage (-): 1.31

Region: NODE\_331560\_length\_1271\_cov\_18.383949 1069-1071. Max. coverage (+): 0.08. Max coverage (-): 0.32

Region: NODE\_331560\_length\_1271\_cov\_18.383949 1072-1073. Max. coverage (+): 0.16. Max coverage (-): 0.32

Region: NODE\_331560\_length\_1271\_cov\_18.383949 1074-1076. Max. coverage (+): 0.14. Max coverage (-): 0.5

Region: NODE\_331560\_length\_1271\_cov\_18.383949 1077-1079. Max. coverage (+): 0.12. Max coverage (-): 1.03

Region: NODE\_331560\_length\_1271\_cov\_18.383949 1080-1081. Max. coverage (+): 0.2. Max coverage (-): 1.05

Region: NODE\_331560\_length\_1271\_cov\_18.383949 1082-1084. Max. coverage (+): 0.08. Max coverage (-): 0.52

Region: NODE\_331560\_length\_1271\_cov\_18.383949 1085-1086. Max. coverage (+): 0. Max coverage (-): 0.4

Region: NODE\_331560\_length\_1271\_cov\_18.383949 1087-1089. Max. coverage (+): 0. Max coverage (-): 0.2

Region: NODE\_331560\_length\_1271\_cov\_18.383949 1090-1092. Max. coverage (+): 0. Max coverage (-): 0.44

Region: NODE\_331560\_length\_1271\_cov\_18.383949 1093-1094. Max. coverage (+): 0. Max coverage (-): 7.91

Region: NODE\_331560\_length\_1271\_cov\_18.383949 1095-1097. Max. coverage (+): 0. Max coverage (-): 7.91

Region: NODE\_331560\_length\_1271\_cov\_18.383949 1098-1100. Max. coverage (+): 0. Max coverage (-): 1.05

Region: NODE\_331560\_length\_1271\_cov\_18.383949 1101-1102. Max. coverage (+): 0. Max coverage (-): 1.05

Region: NODE\_331560\_length\_1271\_cov\_18.383949 1103-1105. Max. coverage (+): 0. Max coverage (-): 0.52

Region: NODE\_331560\_length\_1271\_cov\_18.383949 1106-1108. Max. coverage (+): 0. Max coverage (-): 0.61

Region: NODE\_331560\_length\_1271\_cov\_18.383949 1109-1110. Max. coverage (+): 0.04. Max coverage (-): 0.12

Region: NODE\_331560\_length\_1271\_cov\_18.383949 1111-1113. Max. coverage (+): 0.36. Max coverage (-): 0.2

Region: NODE\_331560\_length\_1271\_cov\_18.383949 1114-1116. Max. coverage (+): 0.4. Max coverage (-): 0.28

Region: NODE\_331560\_length\_1271\_cov\_18.383949 1117-1118. Max. coverage (+): 0.04. Max coverage (-): 0.12

Region: NODE\_331560\_length\_1271\_cov\_18.383949 1119-1121. Max. coverage (+): 0. Max coverage (-): 0.04

Region: NODE\_331560\_length\_1271\_cov\_18.383949 1122-1124. Max. coverage (+): 0.04. Max coverage (-): 0.16

Region: NODE\_331560\_length\_1271\_cov\_18.383949 1125-1126. Max. coverage (+): 0.08. Max coverage (-): 0.16

Region: NODE\_331560\_length\_1271\_cov\_18.383949 1127-1129. Max. coverage (+): 0.12. Max coverage (-): 0.08

Region: NODE\_331560\_length\_1271\_cov\_18.383949 1130-1132. Max. coverage (+): 0.08. Max coverage (-): 0.08

Region: NODE\_331560\_length\_1271\_cov\_18.383949 1133-1134. Max. coverage (+): 0. Max coverage (-): 0

Region: NODE\_331560\_length\_1271\_cov\_18.383949 1135-1137. Max. coverage (+): 0. Max coverage (-): 0

Region: NODE\_331560\_length\_1271\_cov\_18.383949 1138-1140. Max. coverage (+): 0. Max coverage (-): 0

Region: NODE\_331560\_length\_1271\_cov\_18.383949 1141-1142. Max. coverage (+): 0. Max coverage (-): 0

Region: NODE\_331560\_length\_1271\_cov\_18.383949 1143-1145. Max. coverage (+): 0. Max coverage (-): 0

Region: NODE\_331560\_length\_1271\_cov\_18.383949 1146-1147. Max. coverage (+): 0.04. Max coverage (-): 0

Region: NODE\_331560\_length\_1271\_cov\_18.383949 1148-1150. Max. coverage (+): 0.04. Max coverage (-): 0.2

Region: NODE\_331560\_length\_1271\_cov\_18.383949 1151-1153. Max. coverage (+): 0.04. Max coverage (-): 0.2

Region: NODE\_331560\_length\_1271\_cov\_18.383949 1154-1155. Max. coverage (+): 0.12. Max coverage (-): 0.16

Region: NODE\_331560\_length\_1271\_cov\_18.383949 1156-1158. Max. coverage (+): 0.12. Max coverage (-): 0.24

Region: NODE\_331560\_length\_1271\_cov\_18.383949 1159-1161. Max. coverage (+): 0.08. Max coverage (-): 0.08

Region: NODE\_331560\_length\_1271\_cov\_18.383949 1162-1163. Max. coverage (+): 0.04. Max coverage (-): 0.04

Region: NODE\_331560\_length\_1271\_cov\_18.383949 1164-1166. Max. coverage (+): 0. Max coverage (-): 0.08

Region: NODE\_331560\_length\_1271\_cov\_18.383949 1167-1169. Max. coverage (+): 0. Max coverage (-): 0.04

Region: NODE\_331560\_length\_1271\_cov\_18.383949 1170-1171. Max. coverage (+): 0. Max coverage (-): 0

Region: NODE\_331560\_length\_1271\_cov\_18.383949 1172-1174. Max. coverage (+): 0. Max coverage (-): 0.77

Region: NODE\_331560\_length\_1271\_cov\_18.383949 1175-1177. Max. coverage (+): 0.08. Max coverage (-): 0.77

Region: NODE\_331560\_length\_1271\_cov\_18.383949 1178-1179. Max. coverage (+): 0.16. Max coverage (-): 0.28

Region: NODE\_331560\_length\_1271\_cov\_18.383949 1180-1182. Max. coverage (+): 0.08. Max coverage (-): 0.12

Region: NODE\_331560\_length\_1271\_cov\_18.383949 1183-1185. Max. coverage (+): 0.08. Max coverage (-): 0.32

Region: NODE\_331560\_length\_1271\_cov\_18.383949 1186-1187. Max. coverage (+): 0.12. Max coverage (-): 0.32

Region: NODE\_331560\_length\_1271\_cov\_18.383949 1188-1190. Max. coverage (+): 0.08. Max coverage (-): 0.28

Region: NODE\_331560\_length\_1271\_cov\_18.383949 1191-1193. Max. coverage (+): 0.04. Max coverage (-): 0.24

Region: NODE\_331560\_length\_1271\_cov\_18.383949 1194-1195. Max. coverage (+): 0. Max coverage (-): 0

Region: NODE\_331560\_length\_1271\_cov\_18.383949 1196-1198. Max. coverage (+): 0. Max coverage (-): 0

Region: NODE\_331560\_length\_1271\_cov\_18.383949 1199-1201. Max. coverage (+): 0. Max coverage (-): 0

Region: NODE\_331560\_length\_1271\_cov\_18.383949 1202-1203. Max. coverage (+): 0.04. Max coverage (-): 0

Region: NODE\_331560\_length\_1271\_cov\_18.383949 1204-1206. Max. coverage (+): 0.04. Max coverage (-): 0

Region: NODE\_331560\_length\_1271\_cov\_18.383949 1207-1208. Max. coverage (+): 0.04. Max coverage (-): 0.04

Region: NODE\_331560\_length\_1271\_cov\_18.383949 1209-1211. Max. coverage (+): 0.04. Max coverage (-): 0.12

Region: NODE\_331560\_length\_1271\_cov\_18.383949 1212-1214. Max. coverage (+): 0. Max coverage (-): 0.12

Region: NODE\_331560\_length\_1271\_cov\_18.383949 1215-1216. Max. coverage (+): 0. Max coverage (-): 0.08

Region: NODE\_331560\_length\_1271\_cov\_18.383949 1217-1219. Max. coverage (+): 0.12. Max coverage (-): 0.04

Region: NODE\_331560\_length\_1271\_cov\_18.383949 1220-1222. Max. coverage (+): 0.24. Max coverage (-): 0.04

Region: NODE\_331560\_length\_1271\_cov\_18.383949 1223-1224. Max. coverage (+): 0.2. Max coverage (-): 0.16

Region: NODE\_331560\_length\_1271\_cov\_18.383949 1225-1227. Max. coverage (+): 0.16. Max coverage (-): 0.24

Region: NODE\_331560\_length\_1271\_cov\_18.383949 1228-1230. Max. coverage (+): 0. Max coverage (-): 0.16

Region: NODE\_331560\_length\_1271\_cov\_18.383949 1231-1232. Max. coverage (+): 0. Max coverage (-): 0.08

Region: NODE\_331560\_length\_1271\_cov\_18.383949 1233-1235. Max. coverage (+): 0.04. Max coverage (-): 1.09

Region: NODE\_331560\_length\_1271\_cov\_18.383949 1236-1238. Max. coverage (+): 0.08. Max coverage (-): 1.09

Region: NODE\_331560\_length\_1271\_cov\_18.383949 1239-1240. Max. coverage (+): 0.08. Max coverage (-): 0.32

Region: NODE\_331560\_length\_1271\_cov\_18.383949 1241-1243. Max. coverage (+): 0.04. Max coverage (-): 0.28

Region: NODE\_331560\_length\_1271\_cov\_18.383949 1244-1246. Max. coverage (+): 0. Max coverage (-): 0

Region: NODE\_331560\_length\_1271\_cov\_18.383949 1247-1248. Max. coverage (+): 0. Max coverage (-): 0

Region: NODE\_331560\_length\_1271\_cov\_18.383949 1249-1251. Max. coverage (+): 0. Max coverage (-): 0

Region: NODE\_331560\_length\_1271\_cov\_18.383949 1252-1254. Max. coverage (+): 0.04. Max coverage (-): 0

Region: NODE\_331560\_length\_1271\_cov\_18.383949 1255-1256. Max. coverage (+): 0.04. Max coverage (-): 0

Region: NODE\_331560\_length\_1271\_cov\_18.383949 1257-1259. Max. coverage (+): 0.04. Max coverage (-): 0

Region: NODE\_331560\_length\_1271\_cov\_18.383949 1260-1262. Max. coverage (+): 0. Max coverage (-): 0

Region: NODE\_331560\_length\_1271\_cov\_18.383949 1263-1264. Max. coverage (+): 0. Max coverage (-): 0.04

Region: NODE\_331560\_length\_1271\_cov\_18.383949 1265-1267. Max. coverage (+): 0. Max coverage (-): 0.13

Region: NODE\_331560\_length\_1271\_cov\_18.383949 1268-1269. Max. coverage (+): 0. Max coverage (-): 0.1

Region: NODE\_331560\_length\_1271\_cov\_18.383949 1270-1272. Max. coverage (+): 0. Max coverage (-): 0.02

Region: NODE\_331560\_length\_1271\_cov\_18.383949 1273-1275. Max. coverage (+): 0. Max coverage (-): 0

Region: NODE\_331560\_length\_1271\_cov\_18.383949 1276-1277. Max. coverage (+): 0. Max coverage (-): 0

Region: NODE\_331560\_length\_1271\_cov\_18.383949 1278-1280. Max. coverage (+): 0. Max coverage (-): 0

Region: NODE\_331560\_length\_1271\_cov\_18.383949 1281-1283. Max. coverage (+): 0. Max coverage (-): 0

Region: NODE\_331560\_length\_1271\_cov\_18.383949 1284-1285. Max. coverage (+): 0.02. Max coverage (-): 0

Region: NODE\_331560\_length\_1271\_cov\_18.383949 1286-1288. Max. coverage (+): 0.02. Max coverage (-): 0

Region: NODE\_331560\_length\_1271\_cov\_18.383949 1289-1291. Max. coverage (+): 0. Max coverage (-): 0

Region: NODE\_331560\_length\_1271\_cov\_18.383949 1292-1293. Max. coverage (+): 0. Max coverage (-): 0

Region: NODE\_331560\_length\_1271\_cov\_18.383949 1294-1296. Max. coverage (+): 0. Max coverage (-): 0.01

Region: NODE\_331560\_length\_1271\_cov\_18.383949 1297-1299. Max. coverage (+): 0.01. Max coverage (-): 0.01

Region: NODE\_331560\_length\_1271\_cov\_18.383949 1300-1301. Max. coverage (+): 0.01. Max coverage (-): 0

Region: NODE\_331560\_length\_1271\_cov\_18.383949 1302-1304. Max. coverage (+): 0.01. Max coverage (-): 0

Region: NODE\_331560\_length\_1271\_cov\_18.383949 1305-1307. Max. coverage (+): 0. Max coverage (-): 0

Region: NODE\_331560\_length\_1271\_cov\_18.383949 1308-1309. Max. coverage (+): 0. Max coverage (-): 0

Region: NODE\_331560\_length\_1271\_cov\_18.383949 1310-1312. Max. coverage (+): 0. Max coverage (-): 0

Region: NODE\_331560\_length\_1271\_cov\_18.383949 1313-1315. Max. coverage (+): 0. Max coverage (-): 0

Region: NODE\_331560\_length\_1271\_cov\_18.383949 1316-1317. Max. coverage (+): 0. Max coverage (-): 0

Region: NODE\_331560\_length\_1271\_cov\_18.383949 1318-1320. Max. coverage (+): 0. Max coverage (-): 0

Region: NODE\_331560\_length\_1271\_cov\_18.383949 1321-1323. Max. coverage (+): 0. Max coverage (-): 0

Region: NODE\_331560\_length\_1271\_cov\_18.383949 1324-1325. Max. coverage (+): 0. Max coverage (-): 0

Region: NODE\_331560\_length\_1271\_cov\_18.383949 1326-. Max. coverage (+): 0. Max coverage (-): 0

RepeatMasker Color Code

**+**

100-98% Identity

<98-95% Identity

<95-90% Identity

<90-85% Identity

<85-80% Identity

<80-75% Identity

<75-70% Identity

<70% Identity

**-**

Gene Set Color Code

**+**

Gene

Pseudogene

Other

**-**

Topology/Coverage Color Code

Coverage Plus Strand

Coverage Minus Strand

Mainstrand: Plus

Mainstrand: Minus

Complementary Strand

Flanking Region  
(if option -flank >0)

Gene Set Annotation  
  
RepeatMasker Annotation  

**1. AlRepB-127**: 1-64 (-), Divergence to consensus: 3.2%  
**2. AlRepC-959**: 1272-1335 (-), Divergence to consensus: 1.6%

  
Transcription Factor Binding Sites  

**RHOXF1** (Sequence: AGCTTA (-): 461)  
**RHOXF1** (Sequence: GGCTTA (-): 1315)  
**RHOXF1** (Sequence: TGAGCC (+): 507)  
**RHOXF1** (Sequence: TAATCT (+): 922)  
**RHOXF1** (Sequence: TGAGCT (+): 1087)  
**RHOXF1** (Sequence: TGAGCT (+): 1170)  
**POU5F1** (Sequence: TTTGCAT (-): 1286)  
**Nobox** (Sequence: AGCAATTA (-): 777)  
**Rhox11** (Sequence: TTTACAGCA (-): 1101)  
**Sox5** (Sequence: AACAAT (-): 851)  
**Sox5** (Sequence: AACAAT (-): 937)  
**POU2F1** (Sequence: TATTTAAAT (+): 681)  
**POU2F1** (Sequence: TATTCTAAT (+): 1051)
