# Supplementary material for: First characterization of PIWI-interacting RNA clusters in a cichlid fish with a B chromosome
Source: BMC Biol. 2022 Sep 21;20:204. doi: 10.1186/s12915-022-01403-2 (PMC9490952; doi:10.1186/s12915-022-01403-2)
Supplement: Supplementary file 1 — Additional file 1. Zipped folder with fasta and interactive html piRNA cluster information for the A. latifasciata genome. The nomenclature is as follows: number-pirna-cluster_sex_B-presence (f, female; m, male; 0b, without B chromosome; 1b, with B chromosome). [file 12915_2022_1403_MOESM1_ESM.zip › 132_m1b.html]

piRNA cluster 132\_m1b 78


Predicted piRNA cluster no. 132\_m1b
  

Show proTRAC run info
Hide proTRAC run info

/\  
                \_\_\_\_\_\_\_\_\_\_\_\_\_\_\_\_\_\_\_\_\_\_\_/\\_\_\_ /  \\_\_\_\_\_\_\_  
               I                      /  \  /    \      I  
               I     pro             /    \/      \     I  
               I        TRAC        /               \   I  
               I   \_\_\_\_\_\_\_\_\_\_\_\_\_\_\_\_/\_\_\_\_\_\_\_\_\_\_\_\_\_\_\_\_\_\\_ I  
               I   \              /                     I  
               I    \            /                      I  
               I     \  /\      /       V.2.4.2         I  
               I      \/  \    /                        I  
               I\_\_\_\_\_\_\_\_\_\_\_\  /\_\_\_\_\_\_\_\_\_\_\_\_\_\_\_\_\_\_\_\_\_\_\_\_\_I  
                            \/  
  
  
================================= proTRAC ====================================  
VERSION: .......... 2.4.2  
LAST MODIFIED: .... 11. May 2018  
  
Please cite:  
Rosenkranz D, Zischler H. proTRAC - a software for probabilistic piRNA cluster  
detection, visualization and analysis. 2012. BMC Bioinformatics 13:5.  
  
  
Contact:  
David Rosenkranz  
Institute of Organismic and Molecular Evolutionary Biology  
Dept. Anthropology, small RNA group  
Johannes Gutenberg University Mainz  
email: rosenkranz@uni-mainz.de  
  
You can find the latest proTRAC version at:  
http://sourceforge.net/projects/protrac/files  
http://www.smallRNAgroup-mainz.de/software  
==============================================================================  
  
PARAMETERS:  
Map file: ...............piwi-machos-1B.fa-collapse.map  
Genome file: ............../../../0B\_ala\_genome.fa  
RepeatMasker annotation: Alatifasciata-all0B-maryan-v2.fa\_corrected.out  
GeneSet:................./guest-storage/Data/annotation/Alatifasciata\_all0B\_maryan-v2\_out2017.gff  
  
Significant (p<=0.01) hit density will be calculated based  
on observed hit distribution.  
  
Sliding window size: ........................................ 5000 bp  
Sliding window increament: .................................. 1000 bp  
Normalize each hit by number of genomic hits: ............... yes  
Normalize each hit by number of sequence reads: ............. yes  
Normalize values (-> per million mapped reads): ............. yes  
Min. fraction of hits with 1T(U) or 10A: .................... 0.75  
Alternatively: Min. fraction of hits with 1T(U) and 10A: .... 0.5  
Min. fraction of hits with typical piRNA length: ............ 0.75  
Typical piRNA length: ....................................... 24-32 nt  
Min. size of a piRNA cluster: ............................... 1000 bp.  
Min. number of hits (absolute): ............................. 0  
Min. number of hits (normalized): ........................... 0  
Min. fraction of hits on the mainstrand: .................... 0.75  
Top fraction of mapped sequences (in terms of read counts): . 1%  
Top fraction accounts for max. n% of sequence reads: ........ 90%  
Min. fraction of hits on each arm of a bidirectional cluster: 0.05  
Output html file for each cluster: .......................... yes  
Output a summary table: ..................................... yes  
Output a FASTA file for each cluster (piRNA sequences): ..... yes  
Output a FASTA file comprising cluster sequences: ........... yes  
Output a GTF file for predicted piRNA clusters: ..............yes  
Search DNA motifs in clusters: .............................. yes  
Output flanking sequences: +/- .............................. 0 bp  
Output ~.pTi file: .......................................... no  
==============================================================================  
  
  
Genome size (without gaps): ............ 758543724 bp  
Gaps (N/X/-): .......................... 417479 bp  
Mapped reads: .......................... 26973943  
Non-identical sequences: ............... 6209225  
Genomic hits: .......................... 48438990  
Significant densitiy of mapped reads: .. 821.144211136946 reads/kb

Show proTRAC cluster info
Hide proTRAC cluster info

|  |  |
| --- | --- |
| Location | NODE\_331560\_length\_1271\_cov\_18.383949 |
| Coordinates | 4-1335 |
| Size [bp] | 1332 |
| Sequence hit loci | 1299 |
| Mapped reads (normalized) | 5693.1 |
| Mapped reads (normalized) per kb | 4274.1 |
| Normalized reads with 1T (1U) | 87.2% |
| Normalized reads with 10A | 16.6% |
| Normalized reads with length 24-32 nt | 99.2% |
| Normalized reads on the main strand(s) | 94.9% |
| Predicted directionality | mono:minus |

100%

0%

1T (1U)  
reads

10A reads

24-32 nt  
reads

reads on mainstrand

**Either the amount of reads with 1T (1U) OR 10A has to exceed 75% (set with option: -1Tor10A)  
Alternatively the amount of reads with 1T (1U) AND 10A has to exceed 50% (set with option: -1Tand10A)  
Minimum amount of reads with preferred size is 75% (set with option: -pisize)  
Minimum amount of reads on the main strand(s) is 75% (set with option: -clstrand)**

Show read coverage
Hide read coverage

WHAT DO I SEE HERE?  
This chart shows the location of mapped sequence reads within a predicted piRNA cluster. The color refers to the number of genomic hits produced by the sequence read in question. A dark red bar indicates that this sequence read produces many other hits elsewhere in the genome. Many adjacent red or yellow bars can indicate the presence of a multi-copy element such as transposons or rRNA genes. A dark green bar indicates that this sequence read maps uniquely to this locus.

1 hit

2-5 hits

6-10 hits

11-20 hits

21-50 hits

51-100 hits

> 100 hits

NODE\_331560\_length\_1271\_cov\_18.383949

4

1335

Gene Set

RepeatMasker

Mapped  
Reads

64.88

plus strand

minus strand

64.88

Region: NODE\_331560\_length\_1271\_cov\_18.383949 8018-5. Max. coverage (+): 0. Max coverage (-): 0

Region: NODE\_331560\_length\_1271\_cov\_18.383949 6-7. Max. coverage (+): 0. Max coverage (-): 0

Region: NODE\_331560\_length\_1271\_cov\_18.383949 8-10. Max. coverage (+): 0. Max coverage (-): 0

Region: NODE\_331560\_length\_1271\_cov\_18.383949 11-13. Max. coverage (+): 0. Max coverage (-): 0

Region: NODE\_331560\_length\_1271\_cov\_18.383949 14-15. Max. coverage (+): 0. Max coverage (-): 0

Region: NODE\_331560\_length\_1271\_cov\_18.383949 16-18. Max. coverage (+): 0. Max coverage (-): 0

Region: NODE\_331560\_length\_1271\_cov\_18.383949 19-21. Max. coverage (+): 0. Max coverage (-): 0

Region: NODE\_331560\_length\_1271\_cov\_18.383949 22-23. Max. coverage (+): 0. Max coverage (-): 0

Region: NODE\_331560\_length\_1271\_cov\_18.383949 24-26. Max. coverage (+): 0. Max coverage (-): 0

Region: NODE\_331560\_length\_1271\_cov\_18.383949 27-29. Max. coverage (+): 0. Max coverage (-): 0

Region: NODE\_331560\_length\_1271\_cov\_18.383949 30-31. Max. coverage (+): 0. Max coverage (-): 0

Region: NODE\_331560\_length\_1271\_cov\_18.383949 32-34. Max. coverage (+): 0. Max coverage (-): 0

Region: NODE\_331560\_length\_1271\_cov\_18.383949 35-37. Max. coverage (+): 0. Max coverage (-): 0

Region: NODE\_331560\_length\_1271\_cov\_18.383949 38-39. Max. coverage (+): 0. Max coverage (-): 0.01

Region: NODE\_331560\_length\_1271\_cov\_18.383949 40-42. Max. coverage (+): 0. Max coverage (-): 0.04

Region: NODE\_331560\_length\_1271\_cov\_18.383949 43-45. Max. coverage (+): 0. Max coverage (-): 0.04

Region: NODE\_331560\_length\_1271\_cov\_18.383949 46-47. Max. coverage (+): 0. Max coverage (-): 0

Region: NODE\_331560\_length\_1271\_cov\_18.383949 48-50. Max. coverage (+): 0.15. Max coverage (-): 0.04

Region: NODE\_331560\_length\_1271\_cov\_18.383949 51-53. Max. coverage (+): 0.04. Max coverage (-): 0.04

Region: NODE\_331560\_length\_1271\_cov\_18.383949 54-55. Max. coverage (+): 0. Max coverage (-): 0

Region: NODE\_331560\_length\_1271\_cov\_18.383949 56-58. Max. coverage (+): 0. Max coverage (-): 0

Region: NODE\_331560\_length\_1271\_cov\_18.383949 59-61. Max. coverage (+): 0. Max coverage (-): 0

Region: NODE\_331560\_length\_1271\_cov\_18.383949 62-63. Max. coverage (+): 0. Max coverage (-): 0

Region: NODE\_331560\_length\_1271\_cov\_18.383949 64-66. Max. coverage (+): 0. Max coverage (-): 0

Region: NODE\_331560\_length\_1271\_cov\_18.383949 67-69. Max. coverage (+): 0. Max coverage (-): 0

Region: NODE\_331560\_length\_1271\_cov\_18.383949 70-71. Max. coverage (+): 0. Max coverage (-): 0.04

Region: NODE\_331560\_length\_1271\_cov\_18.383949 72-74. Max. coverage (+): 0. Max coverage (-): 0.04

Region: NODE\_331560\_length\_1271\_cov\_18.383949 75-77. Max. coverage (+): 0. Max coverage (-): 0.04

Region: NODE\_331560\_length\_1271\_cov\_18.383949 78-79. Max. coverage (+): 0.07. Max coverage (-): 0

Region: NODE\_331560\_length\_1271\_cov\_18.383949 80-82. Max. coverage (+): 0.04. Max coverage (-): 0.04

Region: NODE\_331560\_length\_1271\_cov\_18.383949 83-85. Max. coverage (+): 0. Max coverage (-): 0.04

Region: NODE\_331560\_length\_1271\_cov\_18.383949 86-87. Max. coverage (+): 0.04. Max coverage (-): 0

Region: NODE\_331560\_length\_1271\_cov\_18.383949 88-90. Max. coverage (+): 0.04. Max coverage (-): 0

Region: NODE\_331560\_length\_1271\_cov\_18.383949 91-93. Max. coverage (+): 0. Max coverage (-): 0

Region: NODE\_331560\_length\_1271\_cov\_18.383949 94-95. Max. coverage (+): 0. Max coverage (-): 0

Region: NODE\_331560\_length\_1271\_cov\_18.383949 96-98. Max. coverage (+): 0.04. Max coverage (-): 0

Region: NODE\_331560\_length\_1271\_cov\_18.383949 99-101. Max. coverage (+): 0.22. Max coverage (-): 0.33

Region: NODE\_331560\_length\_1271\_cov\_18.383949 102-103. Max. coverage (+): 0.26. Max coverage (-): 0.33

Region: NODE\_331560\_length\_1271\_cov\_18.383949 104-106. Max. coverage (+): 0.04. Max coverage (-): 0.04

Region: NODE\_331560\_length\_1271\_cov\_18.383949 107-109. Max. coverage (+): 0. Max coverage (-): 0

Region: NODE\_331560\_length\_1271\_cov\_18.383949 110-111. Max. coverage (+): 0. Max coverage (-): 0.04

Region: NODE\_331560\_length\_1271\_cov\_18.383949 112-114. Max. coverage (+): 0. Max coverage (-): 0.04

Region: NODE\_331560\_length\_1271\_cov\_18.383949 115-117. Max. coverage (+): 0. Max coverage (-): 0

Region: NODE\_331560\_length\_1271\_cov\_18.383949 118-119. Max. coverage (+): 0. Max coverage (-): 0

Region: NODE\_331560\_length\_1271\_cov\_18.383949 120-122. Max. coverage (+): 0. Max coverage (-): 0.04

Region: NODE\_331560\_length\_1271\_cov\_18.383949 123-125. Max. coverage (+): 0. Max coverage (-): 0

Region: NODE\_331560\_length\_1271\_cov\_18.383949 126-127. Max. coverage (+): 0. Max coverage (-): 0.04

Region: NODE\_331560\_length\_1271\_cov\_18.383949 128-130. Max. coverage (+): 0. Max coverage (-): 0.11

Region: NODE\_331560\_length\_1271\_cov\_18.383949 131-133. Max. coverage (+): 0. Max coverage (-): 0.11

Region: NODE\_331560\_length\_1271\_cov\_18.383949 134-135. Max. coverage (+): 0. Max coverage (-): 0

Region: NODE\_331560\_length\_1271\_cov\_18.383949 136-138. Max. coverage (+): 0. Max coverage (-): 0

Region: NODE\_331560\_length\_1271\_cov\_18.383949 139-141. Max. coverage (+): 0. Max coverage (-): 0.04

Region: NODE\_331560\_length\_1271\_cov\_18.383949 142-143. Max. coverage (+): 0. Max coverage (-): 0

Region: NODE\_331560\_length\_1271\_cov\_18.383949 144-146. Max. coverage (+): 0. Max coverage (-): 0

Region: NODE\_331560\_length\_1271\_cov\_18.383949 147-149. Max. coverage (+): 0. Max coverage (-): 0

Region: NODE\_331560\_length\_1271\_cov\_18.383949 150-151. Max. coverage (+): 0. Max coverage (-): 0

Region: NODE\_331560\_length\_1271\_cov\_18.383949 152-154. Max. coverage (+): 0. Max coverage (-): 0

Region: NODE\_331560\_length\_1271\_cov\_18.383949 155-157. Max. coverage (+): 0. Max coverage (-): 0

Region: NODE\_331560\_length\_1271\_cov\_18.383949 158-159. Max. coverage (+): 0. Max coverage (-): 0

Region: NODE\_331560\_length\_1271\_cov\_18.383949 160-162. Max. coverage (+): 0. Max coverage (-): 0

Region: NODE\_331560\_length\_1271\_cov\_18.383949 163-165. Max. coverage (+): 0. Max coverage (-): 0.22

Region: NODE\_331560\_length\_1271\_cov\_18.383949 166-167. Max. coverage (+): 0. Max coverage (-): 0.19

Region: NODE\_331560\_length\_1271\_cov\_18.383949 168-170. Max. coverage (+): 0. Max coverage (-): 0.33

Region: NODE\_331560\_length\_1271\_cov\_18.383949 171-173. Max. coverage (+): 0. Max coverage (-): 0.15

Region: NODE\_331560\_length\_1271\_cov\_18.383949 174-175. Max. coverage (+): 0. Max coverage (-): 0.04

Region: NODE\_331560\_length\_1271\_cov\_18.383949 176-178. Max. coverage (+): 0. Max coverage (-): 0.04

Region: NODE\_331560\_length\_1271\_cov\_18.383949 179-181. Max. coverage (+): 0. Max coverage (-): 0

Region: NODE\_331560\_length\_1271\_cov\_18.383949 182-183. Max. coverage (+): 0. Max coverage (-): 0

Region: NODE\_331560\_length\_1271\_cov\_18.383949 184-186. Max. coverage (+): 0. Max coverage (-): 0

Region: NODE\_331560\_length\_1271\_cov\_18.383949 187-189. Max. coverage (+): 0. Max coverage (-): 0.89

Region: NODE\_331560\_length\_1271\_cov\_18.383949 190-191. Max. coverage (+): 0. Max coverage (-): 0.89

Region: NODE\_331560\_length\_1271\_cov\_18.383949 192-194. Max. coverage (+): 0. Max coverage (-): 0.11

Region: NODE\_331560\_length\_1271\_cov\_18.383949 195-197. Max. coverage (+): 0. Max coverage (-): 0.59

Region: NODE\_331560\_length\_1271\_cov\_18.383949 198-199. Max. coverage (+): 0. Max coverage (-): 0.7

Region: NODE\_331560\_length\_1271\_cov\_18.383949 200-202. Max. coverage (+): 0. Max coverage (-): 5.97

Region: NODE\_331560\_length\_1271\_cov\_18.383949 203-205. Max. coverage (+): 0. Max coverage (-): 1.74

Region: NODE\_331560\_length\_1271\_cov\_18.383949 206-207. Max. coverage (+): 0. Max coverage (-): 0.19

Region: NODE\_331560\_length\_1271\_cov\_18.383949 208-210. Max. coverage (+): 0.04. Max coverage (-): 0.15

Region: NODE\_331560\_length\_1271\_cov\_18.383949 211-213. Max. coverage (+): 0.04. Max coverage (-): 0.44

Region: NODE\_331560\_length\_1271\_cov\_18.383949 214-215. Max. coverage (+): 0. Max coverage (-): 0.07

Region: NODE\_331560\_length\_1271\_cov\_18.383949 216-218. Max. coverage (+): 1.11. Max coverage (-): 0.07

Region: NODE\_331560\_length\_1271\_cov\_18.383949 219-221. Max. coverage (+): 1.11. Max coverage (-): 0.11

Region: NODE\_331560\_length\_1271\_cov\_18.383949 222-223. Max. coverage (+): 0.11. Max coverage (-): 0.07

Region: NODE\_331560\_length\_1271\_cov\_18.383949 224-226. Max. coverage (+): 0.15. Max coverage (-): 0.07

Region: NODE\_331560\_length\_1271\_cov\_18.383949 227-229. Max. coverage (+): 0.07. Max coverage (-): 0.07

Region: NODE\_331560\_length\_1271\_cov\_18.383949 230-231. Max. coverage (+): 0.11. Max coverage (-): 0.04

Region: NODE\_331560\_length\_1271\_cov\_18.383949 232-234. Max. coverage (+): 0.04. Max coverage (-): 0.19

Region: NODE\_331560\_length\_1271\_cov\_18.383949 235-237. Max. coverage (+): 0. Max coverage (-): 0.19

Region: NODE\_331560\_length\_1271\_cov\_18.383949 238-239. Max. coverage (+): 0. Max coverage (-): 0.26

Region: NODE\_331560\_length\_1271\_cov\_18.383949 240-242. Max. coverage (+): 0. Max coverage (-): 0.3

Region: NODE\_331560\_length\_1271\_cov\_18.383949 243-245. Max. coverage (+): 0. Max coverage (-): 0.26

Region: NODE\_331560\_length\_1271\_cov\_18.383949 246-247. Max. coverage (+): 0. Max coverage (-): 0.33

Region: NODE\_331560\_length\_1271\_cov\_18.383949 248-250. Max. coverage (+): 0.07. Max coverage (-): 0.3

Region: NODE\_331560\_length\_1271\_cov\_18.383949 251-253. Max. coverage (+): 0.07. Max coverage (-): 0.22

Region: NODE\_331560\_length\_1271\_cov\_18.383949 254-255. Max. coverage (+): 0. Max coverage (-): 0.19

Region: NODE\_331560\_length\_1271\_cov\_18.383949 256-258. Max. coverage (+): 0.07. Max coverage (-): 0.67

Region: NODE\_331560\_length\_1271\_cov\_18.383949 259-261. Max. coverage (+): 0.07. Max coverage (-): 0.26

Region: NODE\_331560\_length\_1271\_cov\_18.383949 262-263. Max. coverage (+): 0.07. Max coverage (-): 0

Region: NODE\_331560\_length\_1271\_cov\_18.383949 264-266. Max. coverage (+): 0.07. Max coverage (-): 0.07

Region: NODE\_331560\_length\_1271\_cov\_18.383949 267-269. Max. coverage (+): 0.15. Max coverage (-): 0.11

Region: NODE\_331560\_length\_1271\_cov\_18.383949 270-271. Max. coverage (+): 0.15. Max coverage (-): 0.04

Region: NODE\_331560\_length\_1271\_cov\_18.383949 272-274. Max. coverage (+): 0.04. Max coverage (-): 0

Region: NODE\_331560\_length\_1271\_cov\_18.383949 275-277. Max. coverage (+): 0. Max coverage (-): 0

Region: NODE\_331560\_length\_1271\_cov\_18.383949 278-279. Max. coverage (+): 0. Max coverage (-): 0.04

Region: NODE\_331560\_length\_1271\_cov\_18.383949 280-282. Max. coverage (+): 0. Max coverage (-): 0.04

Region: NODE\_331560\_length\_1271\_cov\_18.383949 283-285. Max. coverage (+): 0. Max coverage (-): 0

Region: NODE\_331560\_length\_1271\_cov\_18.383949 286-287. Max. coverage (+): 0. Max coverage (-): 0.07

Region: NODE\_331560\_length\_1271\_cov\_18.383949 288-290. Max. coverage (+): 0.04. Max coverage (-): 0.82

Region: NODE\_331560\_length\_1271\_cov\_18.383949 291-293. Max. coverage (+): 0.04. Max coverage (-): 0.82

Region: NODE\_331560\_length\_1271\_cov\_18.383949 294-295. Max. coverage (+): 0. Max coverage (-): 0.67

Region: NODE\_331560\_length\_1271\_cov\_18.383949 296-298. Max. coverage (+): 0. Max coverage (-): 0.44

Region: NODE\_331560\_length\_1271\_cov\_18.383949 299-301. Max. coverage (+): 0. Max coverage (-): 0.11

Region: NODE\_331560\_length\_1271\_cov\_18.383949 302-303. Max. coverage (+): 0. Max coverage (-): 0.11

Region: NODE\_331560\_length\_1271\_cov\_18.383949 304-306. Max. coverage (+): 0. Max coverage (-): 0.07

Region: NODE\_331560\_length\_1271\_cov\_18.383949 307-309. Max. coverage (+): 0.07. Max coverage (-): 0.7

Region: NODE\_331560\_length\_1271\_cov\_18.383949 310-311. Max. coverage (+): 0.07. Max coverage (-): 0.85

Region: NODE\_331560\_length\_1271\_cov\_18.383949 312-314. Max. coverage (+): 0.04. Max coverage (-): 0.22

Region: NODE\_331560\_length\_1271\_cov\_18.383949 315-317. Max. coverage (+): 0. Max coverage (-): 0.07

Region: NODE\_331560\_length\_1271\_cov\_18.383949 318-319. Max. coverage (+): 0. Max coverage (-): 0

Region: NODE\_331560\_length\_1271\_cov\_18.383949 320-322. Max. coverage (+): 0. Max coverage (-): 0.07

Region: NODE\_331560\_length\_1271\_cov\_18.383949 323-325. Max. coverage (+): 0. Max coverage (-): 0.37

Region: NODE\_331560\_length\_1271\_cov\_18.383949 326-327. Max. coverage (+): 0. Max coverage (-): 1.71

Region: NODE\_331560\_length\_1271\_cov\_18.383949 328-330. Max. coverage (+): 0. Max coverage (-): 1.33

Region: NODE\_331560\_length\_1271\_cov\_18.383949 331-333. Max. coverage (+): 0.04. Max coverage (-): 0.63

Region: NODE\_331560\_length\_1271\_cov\_18.383949 334-335. Max. coverage (+): 0.04. Max coverage (-): 0.15

Region: NODE\_331560\_length\_1271\_cov\_18.383949 336-338. Max. coverage (+): 0. Max coverage (-): 0.04

Region: NODE\_331560\_length\_1271\_cov\_18.383949 339-340. Max. coverage (+): 0. Max coverage (-): 0.04

Region: NODE\_331560\_length\_1271\_cov\_18.383949 341-343. Max. coverage (+): 0. Max coverage (-): 0.82

Region: NODE\_331560\_length\_1271\_cov\_18.383949 344-346. Max. coverage (+): 0. Max coverage (-): 0.56

Region: NODE\_331560\_length\_1271\_cov\_18.383949 347-348. Max. coverage (+): 0. Max coverage (-): 0

Region: NODE\_331560\_length\_1271\_cov\_18.383949 349-351. Max. coverage (+): 0. Max coverage (-): 0.19

Region: NODE\_331560\_length\_1271\_cov\_18.383949 352-354. Max. coverage (+): 0. Max coverage (-): 0.67

Region: NODE\_331560\_length\_1271\_cov\_18.383949 355-356. Max. coverage (+): 0. Max coverage (-): 0.33

Region: NODE\_331560\_length\_1271\_cov\_18.383949 357-359. Max. coverage (+): 0. Max coverage (-): 0.15

Region: NODE\_331560\_length\_1271\_cov\_18.383949 360-362. Max. coverage (+): 0.04. Max coverage (-): 0.26

Region: NODE\_331560\_length\_1271\_cov\_18.383949 363-364. Max. coverage (+): 0. Max coverage (-): 0

Region: NODE\_331560\_length\_1271\_cov\_18.383949 365-367. Max. coverage (+): 0. Max coverage (-): 0.37

Region: NODE\_331560\_length\_1271\_cov\_18.383949 368-370. Max. coverage (+): 0.07. Max coverage (-): 0.41

Region: NODE\_331560\_length\_1271\_cov\_18.383949 371-372. Max. coverage (+): 0.07. Max coverage (-): 0.52

Region: NODE\_331560\_length\_1271\_cov\_18.383949 373-375. Max. coverage (+): 0.07. Max coverage (-): 2.52

Region: NODE\_331560\_length\_1271\_cov\_18.383949 376-378. Max. coverage (+): 0. Max coverage (-): 3.3

Region: NODE\_331560\_length\_1271\_cov\_18.383949 379-380. Max. coverage (+): 0. Max coverage (-): 1.71

Region: NODE\_331560\_length\_1271\_cov\_18.383949 381-383. Max. coverage (+): 0. Max coverage (-): 1.08

Region: NODE\_331560\_length\_1271\_cov\_18.383949 384-386. Max. coverage (+): 0.04. Max coverage (-): 1.19

Region: NODE\_331560\_length\_1271\_cov\_18.383949 387-388. Max. coverage (+): 0.04. Max coverage (-): 1.74

Region: NODE\_331560\_length\_1271\_cov\_18.383949 389-391. Max. coverage (+): 0.07. Max coverage (-): 0.67

Region: NODE\_331560\_length\_1271\_cov\_18.383949 392-394. Max. coverage (+): 0.11. Max coverage (-): 0.07

Region: NODE\_331560\_length\_1271\_cov\_18.383949 395-396. Max. coverage (+): 0.11. Max coverage (-): 0.11

Region: NODE\_331560\_length\_1271\_cov\_18.383949 397-399. Max. coverage (+): 0.11. Max coverage (-): 0.04

Region: NODE\_331560\_length\_1271\_cov\_18.383949 400-402. Max. coverage (+): 0.15. Max coverage (-): 0.26

Region: NODE\_331560\_length\_1271\_cov\_18.383949 403-404. Max. coverage (+): 0.15. Max coverage (-): 0.44

Region: NODE\_331560\_length\_1271\_cov\_18.383949 405-407. Max. coverage (+): 0.19. Max coverage (-): 0.19

Region: NODE\_331560\_length\_1271\_cov\_18.383949 408-410. Max. coverage (+): 0.15. Max coverage (-): 0.11

Region: NODE\_331560\_length\_1271\_cov\_18.383949 411-412. Max. coverage (+): 0.22. Max coverage (-): 0.22

Region: NODE\_331560\_length\_1271\_cov\_18.383949 413-415. Max. coverage (+): 0.19. Max coverage (-): 0.22

Region: NODE\_331560\_length\_1271\_cov\_18.383949 416-418. Max. coverage (+): 0. Max coverage (-): 0.56

Region: NODE\_331560\_length\_1271\_cov\_18.383949 419-420. Max. coverage (+): 0. Max coverage (-): 0.56

Region: NODE\_331560\_length\_1271\_cov\_18.383949 421-423. Max. coverage (+): 0.04. Max coverage (-): 2.63

Region: NODE\_331560\_length\_1271\_cov\_18.383949 424-426. Max. coverage (+): 0.04. Max coverage (-): 2.34

Region: NODE\_331560\_length\_1271\_cov\_18.383949 427-428. Max. coverage (+): 0. Max coverage (-): 0.07

Region: NODE\_331560\_length\_1271\_cov\_18.383949 429-431. Max. coverage (+): 0.04. Max coverage (-): 0.37

Region: NODE\_331560\_length\_1271\_cov\_18.383949 432-434. Max. coverage (+): 0.04. Max coverage (-): 0.19

Region: NODE\_331560\_length\_1271\_cov\_18.383949 435-436. Max. coverage (+): 0.19. Max coverage (-): 0.19

Region: NODE\_331560\_length\_1271\_cov\_18.383949 437-439. Max. coverage (+): 0.15. Max coverage (-): 0.26

Region: NODE\_331560\_length\_1271\_cov\_18.383949 440-442. Max. coverage (+): 0. Max coverage (-): 0.3

Region: NODE\_331560\_length\_1271\_cov\_18.383949 443-444. Max. coverage (+): 0. Max coverage (-): 0.07

Region: NODE\_331560\_length\_1271\_cov\_18.383949 445-447. Max. coverage (+): 0. Max coverage (-): 0.15

Region: NODE\_331560\_length\_1271\_cov\_18.383949 448-450. Max. coverage (+): 0.04. Max coverage (-): 0.22

Region: NODE\_331560\_length\_1271\_cov\_18.383949 451-452. Max. coverage (+): 0.04. Max coverage (-): 0.04

Region: NODE\_331560\_length\_1271\_cov\_18.383949 453-455. Max. coverage (+): 0. Max coverage (-): 0.3

Region: NODE\_331560\_length\_1271\_cov\_18.383949 456-458. Max. coverage (+): 0. Max coverage (-): 0.89

Region: NODE\_331560\_length\_1271\_cov\_18.383949 459-460. Max. coverage (+): 0. Max coverage (-): 3.6

Region: NODE\_331560\_length\_1271\_cov\_18.383949 461-463. Max. coverage (+): 0. Max coverage (-): 3.11

Region: NODE\_331560\_length\_1271\_cov\_18.383949 464-466. Max. coverage (+): 0. Max coverage (-): 0.37

Region: NODE\_331560\_length\_1271\_cov\_18.383949 467-468. Max. coverage (+): 0. Max coverage (-): 0

Region: NODE\_331560\_length\_1271\_cov\_18.383949 469-471. Max. coverage (+): 0.04. Max coverage (-): 0.11

Region: NODE\_331560\_length\_1271\_cov\_18.383949 472-474. Max. coverage (+): 0.04. Max coverage (-): 0.04

Region: NODE\_331560\_length\_1271\_cov\_18.383949 475-476. Max. coverage (+): 0. Max coverage (-): 0.41

Region: NODE\_331560\_length\_1271\_cov\_18.383949 477-479. Max. coverage (+): 0. Max coverage (-): 0.44

Region: NODE\_331560\_length\_1271\_cov\_18.383949 480-482. Max. coverage (+): 0. Max coverage (-): 0.15

Region: NODE\_331560\_length\_1271\_cov\_18.383949 483-484. Max. coverage (+): 0. Max coverage (-): 0.07

Region: NODE\_331560\_length\_1271\_cov\_18.383949 485-487. Max. coverage (+): 0.07. Max coverage (-): 0.07

Region: NODE\_331560\_length\_1271\_cov\_18.383949 488-490. Max. coverage (+): 0.07. Max coverage (-): 0.07

Region: NODE\_331560\_length\_1271\_cov\_18.383949 491-492. Max. coverage (+): 0. Max coverage (-): 0.37

Region: NODE\_331560\_length\_1271\_cov\_18.383949 493-495. Max. coverage (+): 0. Max coverage (-): 0.3

Region: NODE\_331560\_length\_1271\_cov\_18.383949 496-498. Max. coverage (+): 0. Max coverage (-): 10.97

Region: NODE\_331560\_length\_1271\_cov\_18.383949 499-500. Max. coverage (+): 0. Max coverage (-): 11.16

Region: NODE\_331560\_length\_1271\_cov\_18.383949 501-503. Max. coverage (+): 0. Max coverage (-): 1.11

Region: NODE\_331560\_length\_1271\_cov\_18.383949 504-506. Max. coverage (+): 0. Max coverage (-): 1.04

Region: NODE\_331560\_length\_1271\_cov\_18.383949 507-508. Max. coverage (+): 0. Max coverage (-): 0.11

Region: NODE\_331560\_length\_1271\_cov\_18.383949 509-511. Max. coverage (+): 0. Max coverage (-): 0

Region: NODE\_331560\_length\_1271\_cov\_18.383949 512-514. Max. coverage (+): 0. Max coverage (-): 0.04

Region: NODE\_331560\_length\_1271\_cov\_18.383949 515-516. Max. coverage (+): 0. Max coverage (-): 0.11

Region: NODE\_331560\_length\_1271\_cov\_18.383949 517-519. Max. coverage (+): 0.07. Max coverage (-): 0.11

Region: NODE\_331560\_length\_1271\_cov\_18.383949 520-522. Max. coverage (+): 0.04. Max coverage (-): 0.04

Region: NODE\_331560\_length\_1271\_cov\_18.383949 523-524. Max. coverage (+): 0. Max coverage (-): 0.04

Region: NODE\_331560\_length\_1271\_cov\_18.383949 525-527. Max. coverage (+): 0. Max coverage (-): 0.15

Region: NODE\_331560\_length\_1271\_cov\_18.383949 528-530. Max. coverage (+): 0. Max coverage (-): 0.19

Region: NODE\_331560\_length\_1271\_cov\_18.383949 531-532. Max. coverage (+): 0. Max coverage (-): 0.37

Region: NODE\_331560\_length\_1271\_cov\_18.383949 533-535. Max. coverage (+): 0. Max coverage (-): 0.37

Region: NODE\_331560\_length\_1271\_cov\_18.383949 536-538. Max. coverage (+): 0. Max coverage (-): 0

Region: NODE\_331560\_length\_1271\_cov\_18.383949 539-540. Max. coverage (+): 0. Max coverage (-): 0

Region: NODE\_331560\_length\_1271\_cov\_18.383949 541-543. Max. coverage (+): 0. Max coverage (-): 0

Region: NODE\_331560\_length\_1271\_cov\_18.383949 544-546. Max. coverage (+): 0. Max coverage (-): 0.44

Region: NODE\_331560\_length\_1271\_cov\_18.383949 547-548. Max. coverage (+): 0. Max coverage (-): 0.15

Region: NODE\_331560\_length\_1271\_cov\_18.383949 549-551. Max. coverage (+): 0.07. Max coverage (-): 0.22

Region: NODE\_331560\_length\_1271\_cov\_18.383949 552-554. Max. coverage (+): 0.48. Max coverage (-): 0.26

Region: NODE\_331560\_length\_1271\_cov\_18.383949 555-556. Max. coverage (+): 0.33. Max coverage (-): 0.04

Region: NODE\_331560\_length\_1271\_cov\_18.383949 557-559. Max. coverage (+): 0.07. Max coverage (-): 0.15

Region: NODE\_331560\_length\_1271\_cov\_18.383949 560-562. Max. coverage (+): 0.04. Max coverage (-): 0.04

Region: NODE\_331560\_length\_1271\_cov\_18.383949 563-564. Max. coverage (+): 0.11. Max coverage (-): 0.04

Region: NODE\_331560\_length\_1271\_cov\_18.383949 565-567. Max. coverage (+): 1. Max coverage (-): 0.04

Region: NODE\_331560\_length\_1271\_cov\_18.383949 568-570. Max. coverage (+): 1.04. Max coverage (-): 0

Region: NODE\_331560\_length\_1271\_cov\_18.383949 571-572. Max. coverage (+): 0.07. Max coverage (-): 0

Region: NODE\_331560\_length\_1271\_cov\_18.383949 573-575. Max. coverage (+): 0.04. Max coverage (-): 0

Region: NODE\_331560\_length\_1271\_cov\_18.383949 576-578. Max. coverage (+): 0. Max coverage (-): 0

Region: NODE\_331560\_length\_1271\_cov\_18.383949 579-580. Max. coverage (+): 0. Max coverage (-): 0

Region: NODE\_331560\_length\_1271\_cov\_18.383949 581-583. Max. coverage (+): 0. Max coverage (-): 0

Region: NODE\_331560\_length\_1271\_cov\_18.383949 584-586. Max. coverage (+): 0. Max coverage (-): 0.22

Region: NODE\_331560\_length\_1271\_cov\_18.383949 587-588. Max. coverage (+): 0. Max coverage (-): 0.04

Region: NODE\_331560\_length\_1271\_cov\_18.383949 589-591. Max. coverage (+): 0. Max coverage (-): 0

Region: NODE\_331560\_length\_1271\_cov\_18.383949 592-594. Max. coverage (+): 0. Max coverage (-): 0.04

Region: NODE\_331560\_length\_1271\_cov\_18.383949 595-596. Max. coverage (+): 0. Max coverage (-): 0.07

Region: NODE\_331560\_length\_1271\_cov\_18.383949 597-599. Max. coverage (+): 0. Max coverage (-): 0.15

Region: NODE\_331560\_length\_1271\_cov\_18.383949 600-602. Max. coverage (+): 0. Max coverage (-): 0.15

Region: NODE\_331560\_length\_1271\_cov\_18.383949 603-604. Max. coverage (+): 0. Max coverage (-): 0.07

Region: NODE\_331560\_length\_1271\_cov\_18.383949 605-607. Max. coverage (+): 0. Max coverage (-): 0

Region: NODE\_331560\_length\_1271\_cov\_18.383949 608-610. Max. coverage (+): 0. Max coverage (-): 0.15

Region: NODE\_331560\_length\_1271\_cov\_18.383949 611-612. Max. coverage (+): 0. Max coverage (-): 0.15

Region: NODE\_331560\_length\_1271\_cov\_18.383949 613-615. Max. coverage (+): 0. Max coverage (-): 0.04

Region: NODE\_331560\_length\_1271\_cov\_18.383949 616-618. Max. coverage (+): 0. Max coverage (-): 0.04

Region: NODE\_331560\_length\_1271\_cov\_18.383949 619-620. Max. coverage (+): 0. Max coverage (-): 0.37

Region: NODE\_331560\_length\_1271\_cov\_18.383949 621-623. Max. coverage (+): 0. Max coverage (-): 0.37

Region: NODE\_331560\_length\_1271\_cov\_18.383949 624-626. Max. coverage (+): 0. Max coverage (-): 0.07

Region: NODE\_331560\_length\_1271\_cov\_18.383949 627-628. Max. coverage (+): 0. Max coverage (-): 0

Region: NODE\_331560\_length\_1271\_cov\_18.383949 629-631. Max. coverage (+): 0. Max coverage (-): 0

Region: NODE\_331560\_length\_1271\_cov\_18.383949 632-634. Max. coverage (+): 0. Max coverage (-): 0.11

Region: NODE\_331560\_length\_1271\_cov\_18.383949 635-636. Max. coverage (+): 0. Max coverage (-): 0.04

Region: NODE\_331560\_length\_1271\_cov\_18.383949 637-639. Max. coverage (+): 0. Max coverage (-): 0.04

Region: NODE\_331560\_length\_1271\_cov\_18.383949 640-642. Max. coverage (+): 0. Max coverage (-): 0

Region: NODE\_331560\_length\_1271\_cov\_18.383949 643-644. Max. coverage (+): 0. Max coverage (-): 0

Region: NODE\_331560\_length\_1271\_cov\_18.383949 645-647. Max. coverage (+): 0. Max coverage (-): 0

Region: NODE\_331560\_length\_1271\_cov\_18.383949 648-650. Max. coverage (+): 0. Max coverage (-): 0

Region: NODE\_331560\_length\_1271\_cov\_18.383949 651-652. Max. coverage (+): 0. Max coverage (-): 0

Region: NODE\_331560\_length\_1271\_cov\_18.383949 653-655. Max. coverage (+): 0. Max coverage (-): 0

Region: NODE\_331560\_length\_1271\_cov\_18.383949 656-658. Max. coverage (+): 0. Max coverage (-): 0

Region: NODE\_331560\_length\_1271\_cov\_18.383949 659-660. Max. coverage (+): 0. Max coverage (-): 0

Region: NODE\_331560\_length\_1271\_cov\_18.383949 661-663. Max. coverage (+): 0. Max coverage (-): 0

Region: NODE\_331560\_length\_1271\_cov\_18.383949 664-666. Max. coverage (+): 0. Max coverage (-): 0

Region: NODE\_331560\_length\_1271\_cov\_18.383949 667-668. Max. coverage (+): 0. Max coverage (-): 0

Region: NODE\_331560\_length\_1271\_cov\_18.383949 669-671. Max. coverage (+): 0.04. Max coverage (-): 0

Region: NODE\_331560\_length\_1271\_cov\_18.383949 672-673. Max. coverage (+): 0.04. Max coverage (-): 0

Region: NODE\_331560\_length\_1271\_cov\_18.383949 674-676. Max. coverage (+): 0. Max coverage (-): 0.04

Region: NODE\_331560\_length\_1271\_cov\_18.383949 677-679. Max. coverage (+): 0. Max coverage (-): 0.04

Region: NODE\_331560\_length\_1271\_cov\_18.383949 680-681. Max. coverage (+): 0. Max coverage (-): 0

Region: NODE\_331560\_length\_1271\_cov\_18.383949 682-684. Max. coverage (+): 0. Max coverage (-): 0

Region: NODE\_331560\_length\_1271\_cov\_18.383949 685-687. Max. coverage (+): 0. Max coverage (-): 0

Region: NODE\_331560\_length\_1271\_cov\_18.383949 688-689. Max. coverage (+): 0. Max coverage (-): 0

Region: NODE\_331560\_length\_1271\_cov\_18.383949 690-692. Max. coverage (+): 0. Max coverage (-): 0

Region: NODE\_331560\_length\_1271\_cov\_18.383949 693-695. Max. coverage (+): 0. Max coverage (-): 0

Region: NODE\_331560\_length\_1271\_cov\_18.383949 696-697. Max. coverage (+): 0. Max coverage (-): 0

Region: NODE\_331560\_length\_1271\_cov\_18.383949 698-700. Max. coverage (+): 0. Max coverage (-): 0

Region: NODE\_331560\_length\_1271\_cov\_18.383949 701-703. Max. coverage (+): 0.04. Max coverage (-): 0.04

Region: NODE\_331560\_length\_1271\_cov\_18.383949 704-705. Max. coverage (+): 0.04. Max coverage (-): 0

Region: NODE\_331560\_length\_1271\_cov\_18.383949 706-708. Max. coverage (+): 0. Max coverage (-): 0.48

Region: NODE\_331560\_length\_1271\_cov\_18.383949 709-711. Max. coverage (+): 0. Max coverage (-): 0.44

Region: NODE\_331560\_length\_1271\_cov\_18.383949 712-713. Max. coverage (+): 0. Max coverage (-): 0.04

Region: NODE\_331560\_length\_1271\_cov\_18.383949 714-716. Max. coverage (+): 0. Max coverage (-): 0.22

Region: NODE\_331560\_length\_1271\_cov\_18.383949 717-719. Max. coverage (+): 0. Max coverage (-): 0

Region: NODE\_331560\_length\_1271\_cov\_18.383949 720-721. Max. coverage (+): 0. Max coverage (-): 0

Region: NODE\_331560\_length\_1271\_cov\_18.383949 722-724. Max. coverage (+): 0. Max coverage (-): 0.11

Region: NODE\_331560\_length\_1271\_cov\_18.383949 725-727. Max. coverage (+): 0. Max coverage (-): 0.19

Region: NODE\_331560\_length\_1271\_cov\_18.383949 728-729. Max. coverage (+): 0. Max coverage (-): 0.19

Region: NODE\_331560\_length\_1271\_cov\_18.383949 730-732. Max. coverage (+): 0. Max coverage (-): 0

Region: NODE\_331560\_length\_1271\_cov\_18.383949 733-735. Max. coverage (+): 0. Max coverage (-): 0.04

Region: NODE\_331560\_length\_1271\_cov\_18.383949 736-737. Max. coverage (+): 0. Max coverage (-): 0.04

Region: NODE\_331560\_length\_1271\_cov\_18.383949 738-740. Max. coverage (+): 0.04. Max coverage (-): 0

Region: NODE\_331560\_length\_1271\_cov\_18.383949 741-743. Max. coverage (+): 0.04. Max coverage (-): 0

Region: NODE\_331560\_length\_1271\_cov\_18.383949 744-745. Max. coverage (+): 0.22. Max coverage (-): 0

Region: NODE\_331560\_length\_1271\_cov\_18.383949 746-748. Max. coverage (+): 0.26. Max coverage (-): 0.04

Region: NODE\_331560\_length\_1271\_cov\_18.383949 749-751. Max. coverage (+): 0. Max coverage (-): 0.04

Region: NODE\_331560\_length\_1271\_cov\_18.383949 752-753. Max. coverage (+): 0. Max coverage (-): 0.11

Region: NODE\_331560\_length\_1271\_cov\_18.383949 754-756. Max. coverage (+): 0. Max coverage (-): 0.15

Region: NODE\_331560\_length\_1271\_cov\_18.383949 757-759. Max. coverage (+): 0. Max coverage (-): 0

Region: NODE\_331560\_length\_1271\_cov\_18.383949 760-761. Max. coverage (+): 0. Max coverage (-): 0

Region: NODE\_331560\_length\_1271\_cov\_18.383949 762-764. Max. coverage (+): 0. Max coverage (-): 0

Region: NODE\_331560\_length\_1271\_cov\_18.383949 765-767. Max. coverage (+): 0. Max coverage (-): 0

Region: NODE\_331560\_length\_1271\_cov\_18.383949 768-769. Max. coverage (+): 0. Max coverage (-): 0

Region: NODE\_331560\_length\_1271\_cov\_18.383949 770-772. Max. coverage (+): 0.04. Max coverage (-): 9.86

Region: NODE\_331560\_length\_1271\_cov\_18.383949 773-775. Max. coverage (+): 0.04. Max coverage (-): 10.79

Region: NODE\_331560\_length\_1271\_cov\_18.383949 776-777. Max. coverage (+): 0.04. Max coverage (-): 2.37

Region: NODE\_331560\_length\_1271\_cov\_18.383949 778-780. Max. coverage (+): 0.37. Max coverage (-): 1.52

Region: NODE\_331560\_length\_1271\_cov\_18.383949 781-783. Max. coverage (+): 0.22. Max coverage (-): 1.48

Region: NODE\_331560\_length\_1271\_cov\_18.383949 784-785. Max. coverage (+): 0.07. Max coverage (-): 2.26

Region: NODE\_331560\_length\_1271\_cov\_18.383949 786-788. Max. coverage (+): 0.07. Max coverage (-): 2.56

Region: NODE\_331560\_length\_1271\_cov\_18.383949 789-791. Max. coverage (+): 0. Max coverage (-): 0.37

Region: NODE\_331560\_length\_1271\_cov\_18.383949 792-793. Max. coverage (+): 0. Max coverage (-): 0

Region: NODE\_331560\_length\_1271\_cov\_18.383949 794-796. Max. coverage (+): 0. Max coverage (-): 0

Region: NODE\_331560\_length\_1271\_cov\_18.383949 797-799. Max. coverage (+): 0. Max coverage (-): 0.07

Region: NODE\_331560\_length\_1271\_cov\_18.383949 800-801. Max. coverage (+): 0. Max coverage (-): 0.44

Region: NODE\_331560\_length\_1271\_cov\_18.383949 802-804. Max. coverage (+): 0.04. Max coverage (-): 1.3

Region: NODE\_331560\_length\_1271\_cov\_18.383949 805-807. Max. coverage (+): 0. Max coverage (-): 1.3

Region: NODE\_331560\_length\_1271\_cov\_18.383949 808-809. Max. coverage (+): 0. Max coverage (-): 64.88

Region: NODE\_331560\_length\_1271\_cov\_18.383949 810-812. Max. coverage (+): 0. Max coverage (-): 64.88

Region: NODE\_331560\_length\_1271\_cov\_18.383949 813-815. Max. coverage (+): 0. Max coverage (-): 9.16

Region: NODE\_331560\_length\_1271\_cov\_18.383949 816-817. Max. coverage (+): 0. Max coverage (-): 0.41

Region: NODE\_331560\_length\_1271\_cov\_18.383949 818-820. Max. coverage (+): 0. Max coverage (-): 0.48

Region: NODE\_331560\_length\_1271\_cov\_18.383949 821-823. Max. coverage (+): 0. Max coverage (-): 0.59

Region: NODE\_331560\_length\_1271\_cov\_18.383949 824-825. Max. coverage (+): 0. Max coverage (-): 0.22

Region: NODE\_331560\_length\_1271\_cov\_18.383949 826-828. Max. coverage (+): 0.07. Max coverage (-): 0.41

Region: NODE\_331560\_length\_1271\_cov\_18.383949 829-831. Max. coverage (+): 0.07. Max coverage (-): 1.33

Region: NODE\_331560\_length\_1271\_cov\_18.383949 832-833. Max. coverage (+): 0.04. Max coverage (-): 1.3

Region: NODE\_331560\_length\_1271\_cov\_18.383949 834-836. Max. coverage (+): 0.04. Max coverage (-): 0

Region: NODE\_331560\_length\_1271\_cov\_18.383949 837-839. Max. coverage (+): 0. Max coverage (-): 0.04

Region: NODE\_331560\_length\_1271\_cov\_18.383949 840-841. Max. coverage (+): 0.04. Max coverage (-): 0

Region: NODE\_331560\_length\_1271\_cov\_18.383949 842-844. Max. coverage (+): 0.09. Max coverage (-): 0.02

Region: NODE\_331560\_length\_1271\_cov\_18.383949 845-847. Max. coverage (+): 0.02. Max coverage (-): 0

Region: NODE\_331560\_length\_1271\_cov\_18.383949 848-849. Max. coverage (+): 0.02. Max coverage (-): 0

Region: NODE\_331560\_length\_1271\_cov\_18.383949 850-852. Max. coverage (+): 0. Max coverage (-): 0.07

Region: NODE\_331560\_length\_1271\_cov\_18.383949 853-855. Max. coverage (+): 0. Max coverage (-): 0.11

Region: NODE\_331560\_length\_1271\_cov\_18.383949 856-857. Max. coverage (+): 0. Max coverage (-): 0.3

Region: NODE\_331560\_length\_1271\_cov\_18.383949 858-860. Max. coverage (+): 0. Max coverage (-): 0.56

Region: NODE\_331560\_length\_1271\_cov\_18.383949 861-863. Max. coverage (+): 0. Max coverage (-): 1.04

Region: NODE\_331560\_length\_1271\_cov\_18.383949 864-865. Max. coverage (+): 0. Max coverage (-): 4.75

Region: NODE\_331560\_length\_1271\_cov\_18.383949 866-868. Max. coverage (+): 0. Max coverage (-): 5.38

Region: NODE\_331560\_length\_1271\_cov\_18.383949 869-871. Max. coverage (+): 0. Max coverage (-): 0.15

Region: NODE\_331560\_length\_1271\_cov\_18.383949 872-873. Max. coverage (+): 0. Max coverage (-): 0.11

Region: NODE\_331560\_length\_1271\_cov\_18.383949 874-876. Max. coverage (+): 0.07. Max coverage (-): 0.56

Region: NODE\_331560\_length\_1271\_cov\_18.383949 877-879. Max. coverage (+): 0.07. Max coverage (-): 0.44

Region: NODE\_331560\_length\_1271\_cov\_18.383949 880-881. Max. coverage (+): 0.09. Max coverage (-): 0.04

Region: NODE\_331560\_length\_1271\_cov\_18.383949 882-884. Max. coverage (+): 0.09. Max coverage (-): 0.04

Region: NODE\_331560\_length\_1271\_cov\_18.383949 885-887. Max. coverage (+): 0.06. Max coverage (-): 0.07

Region: NODE\_331560\_length\_1271\_cov\_18.383949 888-889. Max. coverage (+): 0. Max coverage (-): 0.06

Region: NODE\_331560\_length\_1271\_cov\_18.383949 890-892. Max. coverage (+): 0. Max coverage (-): 0.06

Region: NODE\_331560\_length\_1271\_cov\_18.383949 893-895. Max. coverage (+): 0. Max coverage (-): 0.24

Region: NODE\_331560\_length\_1271\_cov\_18.383949 896-897. Max. coverage (+): 0. Max coverage (-): 0.95

Region: NODE\_331560\_length\_1271\_cov\_18.383949 898-900. Max. coverage (+): 0. Max coverage (-): 1.22

Region: NODE\_331560\_length\_1271\_cov\_18.383949 901-903. Max. coverage (+): 0. Max coverage (-): 0.52

Region: NODE\_331560\_length\_1271\_cov\_18.383949 904-905. Max. coverage (+): 0. Max coverage (-): 0.17

Region: NODE\_331560\_length\_1271\_cov\_18.383949 906-908. Max. coverage (+): 0. Max coverage (-): 0.04

Region: NODE\_331560\_length\_1271\_cov\_18.383949 909-911. Max. coverage (+): 0. Max coverage (-): 0.04

Region: NODE\_331560\_length\_1271\_cov\_18.383949 912-913. Max. coverage (+): 0. Max coverage (-): 0.04

Region: NODE\_331560\_length\_1271\_cov\_18.383949 914-916. Max. coverage (+): 0.02. Max coverage (-): 0

Region: NODE\_331560\_length\_1271\_cov\_18.383949 917-919. Max. coverage (+): 0.02. Max coverage (-): 0

Region: NODE\_331560\_length\_1271\_cov\_18.383949 920-921. Max. coverage (+): 0. Max coverage (-): 0.04

Region: NODE\_331560\_length\_1271\_cov\_18.383949 922-924. Max. coverage (+): 0. Max coverage (-): 0.04

Region: NODE\_331560\_length\_1271\_cov\_18.383949 925-927. Max. coverage (+): 0. Max coverage (-): 0.15

Region: NODE\_331560\_length\_1271\_cov\_18.383949 928-929. Max. coverage (+): 0. Max coverage (-): 0

Region: NODE\_331560\_length\_1271\_cov\_18.383949 930-932. Max. coverage (+): 0. Max coverage (-): 0

Region: NODE\_331560\_length\_1271\_cov\_18.383949 933-935. Max. coverage (+): 0. Max coverage (-): 0.11

Region: NODE\_331560\_length\_1271\_cov\_18.383949 936-937. Max. coverage (+): 0. Max coverage (-): 0

Region: NODE\_331560\_length\_1271\_cov\_18.383949 938-940. Max. coverage (+): 0.04. Max coverage (-): 0.04

Region: NODE\_331560\_length\_1271\_cov\_18.383949 941-943. Max. coverage (+): 0.04. Max coverage (-): 0.07

Region: NODE\_331560\_length\_1271\_cov\_18.383949 944-945. Max. coverage (+): 0.07. Max coverage (-): 0.11

Region: NODE\_331560\_length\_1271\_cov\_18.383949 946-948. Max. coverage (+): 0.19. Max coverage (-): 0.48

Region: NODE\_331560\_length\_1271\_cov\_18.383949 949-951. Max. coverage (+): 0.11. Max coverage (-): 1.45

Region: NODE\_331560\_length\_1271\_cov\_18.383949 952-953. Max. coverage (+): 0.22. Max coverage (-): 1.33

Region: NODE\_331560\_length\_1271\_cov\_18.383949 954-956. Max. coverage (+): 0.22. Max coverage (-): 0.52

Region: NODE\_331560\_length\_1271\_cov\_18.383949 957-959. Max. coverage (+): 0. Max coverage (-): 0.24

Region: NODE\_331560\_length\_1271\_cov\_18.383949 960-961. Max. coverage (+): 0. Max coverage (-): 0.32

Region: NODE\_331560\_length\_1271\_cov\_18.383949 962-964. Max. coverage (+): 0.04. Max coverage (-): 0.26

Region: NODE\_331560\_length\_1271\_cov\_18.383949 965-967. Max. coverage (+): 0.09. Max coverage (-): 0.17

Region: NODE\_331560\_length\_1271\_cov\_18.383949 968-969. Max. coverage (+): 0.04. Max coverage (-): 1.35

Region: NODE\_331560\_length\_1271\_cov\_18.383949 970-972. Max. coverage (+): 0.02. Max coverage (-): 1.22

Region: NODE\_331560\_length\_1271\_cov\_18.383949 973-975. Max. coverage (+): 0. Max coverage (-): 0.02

Region: NODE\_331560\_length\_1271\_cov\_18.383949 976-977. Max. coverage (+): 0. Max coverage (-): 0.04

Region: NODE\_331560\_length\_1271\_cov\_18.383949 978-980. Max. coverage (+): 0. Max coverage (-): 0.06

Region: NODE\_331560\_length\_1271\_cov\_18.383949 981-983. Max. coverage (+): 0. Max coverage (-): 0

Region: NODE\_331560\_length\_1271\_cov\_18.383949 984-985. Max. coverage (+): 0. Max coverage (-): 0

Region: NODE\_331560\_length\_1271\_cov\_18.383949 986-988. Max. coverage (+): 0. Max coverage (-): 0

Region: NODE\_331560\_length\_1271\_cov\_18.383949 989-991. Max. coverage (+): 0. Max coverage (-): 0.04

Region: NODE\_331560\_length\_1271\_cov\_18.383949 992-993. Max. coverage (+): 0. Max coverage (-): 0.04

Region: NODE\_331560\_length\_1271\_cov\_18.383949 994-996. Max. coverage (+): 0. Max coverage (-): 0.04

Region: NODE\_331560\_length\_1271\_cov\_18.383949 997-999. Max. coverage (+): 0. Max coverage (-): 0.04

Region: NODE\_331560\_length\_1271\_cov\_18.383949 1000-1001. Max. coverage (+): 0. Max coverage (-): 1.33

Region: NODE\_331560\_length\_1271\_cov\_18.383949 1002-1004. Max. coverage (+): 0. Max coverage (-): 1.45

Region: NODE\_331560\_length\_1271\_cov\_18.383949 1005-1006. Max. coverage (+): 0. Max coverage (-): 0.15

Region: NODE\_331560\_length\_1271\_cov\_18.383949 1007-1009. Max. coverage (+): 0. Max coverage (-): 0

Region: NODE\_331560\_length\_1271\_cov\_18.383949 1010-1012. Max. coverage (+): 0. Max coverage (-): 0.33

Region: NODE\_331560\_length\_1271\_cov\_18.383949 1013-1014. Max. coverage (+): 0. Max coverage (-): 0.52

Region: NODE\_331560\_length\_1271\_cov\_18.383949 1015-1017. Max. coverage (+): 0.11. Max coverage (-): 0.26

Region: NODE\_331560\_length\_1271\_cov\_18.383949 1018-1020. Max. coverage (+): 0.11. Max coverage (-): 0.07

Region: NODE\_331560\_length\_1271\_cov\_18.383949 1021-1022. Max. coverage (+): 0. Max coverage (-): 0.04

Region: NODE\_331560\_length\_1271\_cov\_18.383949 1023-1025. Max. coverage (+): 0.04. Max coverage (-): 0.04

Region: NODE\_331560\_length\_1271\_cov\_18.383949 1026-1028. Max. coverage (+): 0.04. Max coverage (-): 0.44

Region: NODE\_331560\_length\_1271\_cov\_18.383949 1029-1030. Max. coverage (+): 0. Max coverage (-): 0.52

Region: NODE\_331560\_length\_1271\_cov\_18.383949 1031-1033. Max. coverage (+): 0. Max coverage (-): 0.19

Region: NODE\_331560\_length\_1271\_cov\_18.383949 1034-1036. Max. coverage (+): 0. Max coverage (-): 0.11

Region: NODE\_331560\_length\_1271\_cov\_18.383949 1037-1038. Max. coverage (+): 0. Max coverage (-): 0

Region: NODE\_331560\_length\_1271\_cov\_18.383949 1039-1041. Max. coverage (+): 0. Max coverage (-): 0

Region: NODE\_331560\_length\_1271\_cov\_18.383949 1042-1044. Max. coverage (+): 0. Max coverage (-): 1.59

Region: NODE\_331560\_length\_1271\_cov\_18.383949 1045-1046. Max. coverage (+): 0. Max coverage (-): 1.63

Region: NODE\_331560\_length\_1271\_cov\_18.383949 1047-1049. Max. coverage (+): 0. Max coverage (-): 0.19

Region: NODE\_331560\_length\_1271\_cov\_18.383949 1050-1052. Max. coverage (+): 0. Max coverage (-): 0.26

Region: NODE\_331560\_length\_1271\_cov\_18.383949 1053-1054. Max. coverage (+): 0. Max coverage (-): 0.26

Region: NODE\_331560\_length\_1271\_cov\_18.383949 1055-1057. Max. coverage (+): 0. Max coverage (-): 0.11

Region: NODE\_331560\_length\_1271\_cov\_18.383949 1058-1060. Max. coverage (+): 0. Max coverage (-): 0.11

Region: NODE\_331560\_length\_1271\_cov\_18.383949 1061-1062. Max. coverage (+): 0. Max coverage (-): 0.15

Region: NODE\_331560\_length\_1271\_cov\_18.383949 1063-1065. Max. coverage (+): 0.15. Max coverage (-): 0.85

Region: NODE\_331560\_length\_1271\_cov\_18.383949 1066-1068. Max. coverage (+): 0.19. Max coverage (-): 0.96

Region: NODE\_331560\_length\_1271\_cov\_18.383949 1069-1070. Max. coverage (+): 0.06. Max coverage (-): 0.33

Region: NODE\_331560\_length\_1271\_cov\_18.383949 1071-1073. Max. coverage (+): 0.26. Max coverage (-): 0.17

Region: NODE\_331560\_length\_1271\_cov\_18.383949 1074-1076. Max. coverage (+): 0.32. Max coverage (-): 0.24

Region: NODE\_331560\_length\_1271\_cov\_18.383949 1077-1078. Max. coverage (+): 0.11. Max coverage (-): 0.65

Region: NODE\_331560\_length\_1271\_cov\_18.383949 1079-1081. Max. coverage (+): 0.02. Max coverage (-): 0.59

Region: NODE\_331560\_length\_1271\_cov\_18.383949 1082-1084. Max. coverage (+): 0. Max coverage (-): 0.41

Region: NODE\_331560\_length\_1271\_cov\_18.383949 1085-1086. Max. coverage (+): 0. Max coverage (-): 0.41

Region: NODE\_331560\_length\_1271\_cov\_18.383949 1087-1089. Max. coverage (+): 0. Max coverage (-): 0.26

Region: NODE\_331560\_length\_1271\_cov\_18.383949 1090-1092. Max. coverage (+): 0.04. Max coverage (-): 0.63

Region: NODE\_331560\_length\_1271\_cov\_18.383949 1093-1094. Max. coverage (+): 0.04. Max coverage (-): 12.46

Region: NODE\_331560\_length\_1271\_cov\_18.383949 1095-1097. Max. coverage (+): 0. Max coverage (-): 12.49

Region: NODE\_331560\_length\_1271\_cov\_18.383949 1098-1100. Max. coverage (+): 0. Max coverage (-): 1.63

Region: NODE\_331560\_length\_1271\_cov\_18.383949 1101-1102. Max. coverage (+): 0. Max coverage (-): 1.67

Region: NODE\_331560\_length\_1271\_cov\_18.383949 1103-1105. Max. coverage (+): 0. Max coverage (-): 0.7

Region: NODE\_331560\_length\_1271\_cov\_18.383949 1106-1108. Max. coverage (+): 0. Max coverage (-): 0.7

Region: NODE\_331560\_length\_1271\_cov\_18.383949 1109-1110. Max. coverage (+): 0. Max coverage (-): 0.11

Region: NODE\_331560\_length\_1271\_cov\_18.383949 1111-1113. Max. coverage (+): 0.37. Max coverage (-): 0.33

Region: NODE\_331560\_length\_1271\_cov\_18.383949 1114-1116. Max. coverage (+): 0.37. Max coverage (-): 0.41

Region: NODE\_331560\_length\_1271\_cov\_18.383949 1117-1118. Max. coverage (+): 0. Max coverage (-): 0.11

Region: NODE\_331560\_length\_1271\_cov\_18.383949 1119-1121. Max. coverage (+): 0.04. Max coverage (-): 0.07

Region: NODE\_331560\_length\_1271\_cov\_18.383949 1122-1124. Max. coverage (+): 0.15. Max coverage (-): 0.3

Region: NODE\_331560\_length\_1271\_cov\_18.383949 1125-1126. Max. coverage (+): 0.15. Max coverage (-): 0.3

Region: NODE\_331560\_length\_1271\_cov\_18.383949 1127-1129. Max. coverage (+): 0.07. Max coverage (-): 0.11

Region: NODE\_331560\_length\_1271\_cov\_18.383949 1130-1132. Max. coverage (+): 0.07. Max coverage (-): 0.11

Region: NODE\_331560\_length\_1271\_cov\_18.383949 1133-1134. Max. coverage (+): 0. Max coverage (-): 0

Region: NODE\_331560\_length\_1271\_cov\_18.383949 1135-1137. Max. coverage (+): 0. Max coverage (-): 0

Region: NODE\_331560\_length\_1271\_cov\_18.383949 1138-1140. Max. coverage (+): 0. Max coverage (-): 0

Region: NODE\_331560\_length\_1271\_cov\_18.383949 1141-1142. Max. coverage (+): 0. Max coverage (-): 0.04

Region: NODE\_331560\_length\_1271\_cov\_18.383949 1143-1145. Max. coverage (+): 0. Max coverage (-): 0.04

Region: NODE\_331560\_length\_1271\_cov\_18.383949 1146-1148. Max. coverage (+): 0. Max coverage (-): 0

Region: NODE\_331560\_length\_1271\_cov\_18.383949 1149-1150. Max. coverage (+): 0. Max coverage (-): 0.04

Region: NODE\_331560\_length\_1271\_cov\_18.383949 1151-1153. Max. coverage (+): 0. Max coverage (-): 0.04

Region: NODE\_331560\_length\_1271\_cov\_18.383949 1154-1156. Max. coverage (+): 0.07. Max coverage (-): 0.11

Region: NODE\_331560\_length\_1271\_cov\_18.383949 1157-1158. Max. coverage (+): 0.11. Max coverage (-): 0.11

Region: NODE\_331560\_length\_1271\_cov\_18.383949 1159-1161. Max. coverage (+): 0.07. Max coverage (-): 0.07

Region: NODE\_331560\_length\_1271\_cov\_18.383949 1162-1164. Max. coverage (+): 0.04. Max coverage (-): 0.04

Region: NODE\_331560\_length\_1271\_cov\_18.383949 1165-1166. Max. coverage (+): 0. Max coverage (-): 0.04

Region: NODE\_331560\_length\_1271\_cov\_18.383949 1167-1169. Max. coverage (+): 0. Max coverage (-): 0.04

Region: NODE\_331560\_length\_1271\_cov\_18.383949 1170-1172. Max. coverage (+): 0. Max coverage (-): 0.48

Region: NODE\_331560\_length\_1271\_cov\_18.383949 1173-1174. Max. coverage (+): 0. Max coverage (-): 0.93

Region: NODE\_331560\_length\_1271\_cov\_18.383949 1175-1177. Max. coverage (+): 0.04. Max coverage (-): 0.93

Region: NODE\_331560\_length\_1271\_cov\_18.383949 1178-1180. Max. coverage (+): 0.07. Max coverage (-): 0.15

Region: NODE\_331560\_length\_1271\_cov\_18.383949 1181-1182. Max. coverage (+): 0.04. Max coverage (-): 0.11

Region: NODE\_331560\_length\_1271\_cov\_18.383949 1183-1185. Max. coverage (+): 0. Max coverage (-): 0.15

Region: NODE\_331560\_length\_1271\_cov\_18.383949 1186-1188. Max. coverage (+): 0. Max coverage (-): 0.11

Region: NODE\_331560\_length\_1271\_cov\_18.383949 1189-1190. Max. coverage (+): 0.11. Max coverage (-): 0.37

Region: NODE\_331560\_length\_1271\_cov\_18.383949 1191-1193. Max. coverage (+): 0.19. Max coverage (-): 0.3

Region: NODE\_331560\_length\_1271\_cov\_18.383949 1194-1196. Max. coverage (+): 0.11. Max coverage (-): 0.04

Region: NODE\_331560\_length\_1271\_cov\_18.383949 1197-1198. Max. coverage (+): 0.04. Max coverage (-): 0

Region: NODE\_331560\_length\_1271\_cov\_18.383949 1199-1201. Max. coverage (+): 0. Max coverage (-): 0.11

Region: NODE\_331560\_length\_1271\_cov\_18.383949 1202-1204. Max. coverage (+): 0. Max coverage (-): 0.11

Region: NODE\_331560\_length\_1271\_cov\_18.383949 1205-1206. Max. coverage (+): 0.04. Max coverage (-): 0

Region: NODE\_331560\_length\_1271\_cov\_18.383949 1207-1209. Max. coverage (+): 0.04. Max coverage (-): 0

Region: NODE\_331560\_length\_1271\_cov\_18.383949 1210-1212. Max. coverage (+): 0. Max coverage (-): 0

Region: NODE\_331560\_length\_1271\_cov\_18.383949 1213-1214. Max. coverage (+): 0. Max coverage (-): 0.07

Region: NODE\_331560\_length\_1271\_cov\_18.383949 1215-1217. Max. coverage (+): 0.07. Max coverage (-): 0.07

Region: NODE\_331560\_length\_1271\_cov\_18.383949 1218-1220. Max. coverage (+): 0.19. Max coverage (-): 0.15

Region: NODE\_331560\_length\_1271\_cov\_18.383949 1221-1222. Max. coverage (+): 0.11. Max coverage (-): 0.15

Region: NODE\_331560\_length\_1271\_cov\_18.383949 1223-1225. Max. coverage (+): 0.11. Max coverage (-): 0.11

Region: NODE\_331560\_length\_1271\_cov\_18.383949 1226-1228. Max. coverage (+): 0. Max coverage (-): 0.15

Region: NODE\_331560\_length\_1271\_cov\_18.383949 1229-1230. Max. coverage (+): 0. Max coverage (-): 0.3

Region: NODE\_331560\_length\_1271\_cov\_18.383949 1231-1233. Max. coverage (+): 0.04. Max coverage (-): 0.44

Region: NODE\_331560\_length\_1271\_cov\_18.383949 1234-1236. Max. coverage (+): 0.04. Max coverage (-): 1.41

Region: NODE\_331560\_length\_1271\_cov\_18.383949 1237-1238. Max. coverage (+): 0. Max coverage (-): 1.45

Region: NODE\_331560\_length\_1271\_cov\_18.383949 1239-1241. Max. coverage (+): 0. Max coverage (-): 0.41

Region: NODE\_331560\_length\_1271\_cov\_18.383949 1242-1244. Max. coverage (+): 0. Max coverage (-): 0.22

Region: NODE\_331560\_length\_1271\_cov\_18.383949 1245-1246. Max. coverage (+): 0. Max coverage (-): 0

Region: NODE\_331560\_length\_1271\_cov\_18.383949 1247-1249. Max. coverage (+): 0.04. Max coverage (-): 0

Region: NODE\_331560\_length\_1271\_cov\_18.383949 1250-1252. Max. coverage (+): 0.07. Max coverage (-): 0

Region: NODE\_331560\_length\_1271\_cov\_18.383949 1253-1254. Max. coverage (+): 0.04. Max coverage (-): 0

Region: NODE\_331560\_length\_1271\_cov\_18.383949 1255-1257. Max. coverage (+): 0.04. Max coverage (-): 0.07

Region: NODE\_331560\_length\_1271\_cov\_18.383949 1258-1260. Max. coverage (+): 0. Max coverage (-): 0.07

Region: NODE\_331560\_length\_1271\_cov\_18.383949 1261-1262. Max. coverage (+): 0. Max coverage (-): 0

Region: NODE\_331560\_length\_1271\_cov\_18.383949 1263-1265. Max. coverage (+): 0. Max coverage (-): 0.07

Region: NODE\_331560\_length\_1271\_cov\_18.383949 1266-1268. Max. coverage (+): 0. Max coverage (-): 0.07

Region: NODE\_331560\_length\_1271\_cov\_18.383949 1269-1270. Max. coverage (+): 0. Max coverage (-): 0.01

Region: NODE\_331560\_length\_1271\_cov\_18.383949 1271-1273. Max. coverage (+): 0. Max coverage (-): 0.01

Region: NODE\_331560\_length\_1271\_cov\_18.383949 1274-1276. Max. coverage (+): 0. Max coverage (-): 0

Region: NODE\_331560\_length\_1271\_cov\_18.383949 1277-1278. Max. coverage (+): 0. Max coverage (-): 0

Region: NODE\_331560\_length\_1271\_cov\_18.383949 1279-1281. Max. coverage (+): 0. Max coverage (-): 0

Region: NODE\_331560\_length\_1271\_cov\_18.383949 1282-1284. Max. coverage (+): 0. Max coverage (-): 0

Region: NODE\_331560\_length\_1271\_cov\_18.383949 1285-1286. Max. coverage (+): 0.02. Max coverage (-): 0

Region: NODE\_331560\_length\_1271\_cov\_18.383949 1287-1289. Max. coverage (+): 0.02. Max coverage (-): 0

Region: NODE\_331560\_length\_1271\_cov\_18.383949 1290-1292. Max. coverage (+): 0. Max coverage (-): 0

Region: NODE\_331560\_length\_1271\_cov\_18.383949 1293-1294. Max. coverage (+): 0. Max coverage (-): 0

Region: NODE\_331560\_length\_1271\_cov\_18.383949 1295-1297. Max. coverage (+): 0. Max coverage (-): 0

Region: NODE\_331560\_length\_1271\_cov\_18.383949 1298-1300. Max. coverage (+): 0. Max coverage (-): 0

Region: NODE\_331560\_length\_1271\_cov\_18.383949 1301-1302. Max. coverage (+): 0. Max coverage (-): 0

Region: NODE\_331560\_length\_1271\_cov\_18.383949 1303-1305. Max. coverage (+): 0. Max coverage (-): 0

Region: NODE\_331560\_length\_1271\_cov\_18.383949 1306-1308. Max. coverage (+): 0. Max coverage (-): 0.04

Region: NODE\_331560\_length\_1271\_cov\_18.383949 1309-1310. Max. coverage (+): 0. Max coverage (-): 0.06

Region: NODE\_331560\_length\_1271\_cov\_18.383949 1311-1313. Max. coverage (+): 0. Max coverage (-): 0.06

Region: NODE\_331560\_length\_1271\_cov\_18.383949 1314-1316. Max. coverage (+): 0. Max coverage (-): 0

Region: NODE\_331560\_length\_1271\_cov\_18.383949 1317-1318. Max. coverage (+): 0. Max coverage (-): 0

Region: NODE\_331560\_length\_1271\_cov\_18.383949 1319-1321. Max. coverage (+): 0. Max coverage (-): 0

Region: NODE\_331560\_length\_1271\_cov\_18.383949 1322-1324. Max. coverage (+): 0. Max coverage (-): 0

Region: NODE\_331560\_length\_1271\_cov\_18.383949 1325-1326. Max. coverage (+): 0. Max coverage (-): 0

Region: NODE\_331560\_length\_1271\_cov\_18.383949 1327-1329. Max. coverage (+): 0. Max coverage (-): 0

Region: NODE\_331560\_length\_1271\_cov\_18.383949 1330-1332. Max. coverage (+): 0. Max coverage (-): 0

Region: NODE\_331560\_length\_1271\_cov\_18.383949 1333-1334. Max. coverage (+): 0. Max coverage (-): 0

Region: NODE\_331560\_length\_1271\_cov\_18.383949 1335-. Max. coverage (+): 0. Max coverage (-): 0

RepeatMasker Color Code

**+**

100-98% Identity

<98-95% Identity

<95-90% Identity

<90-85% Identity

<85-80% Identity

<80-75% Identity

<75-70% Identity

<70% Identity

**-**

Gene Set Color Code

**+**

Gene

Pseudogene

Other

**-**

Topology/Coverage Color Code

Coverage Plus Strand

Coverage Minus Strand

Mainstrand: Plus

Mainstrand: Minus

Complementary Strand

Flanking Region  
(if option -flank >0)

Gene Set Annotation  
  
RepeatMasker Annotation  

**1. AlRepB-127**: 1-64 (-), Divergence to consensus: 3.2%  
**2. AlRepC-959**: 1272-1335 (-), Divergence to consensus: 1.6%

  
Transcription Factor Binding Sites  

**RHOXF1** (Sequence: AGCTTA (-): 461)  
**RHOXF1** (Sequence: GGCTTA (-): 1315)  
**RHOXF1** (Sequence: TGAGCC (+): 507)  
**RHOXF1** (Sequence: TAATCT (+): 922)  
**RHOXF1** (Sequence: TGAGCT (+): 1087)  
**RHOXF1** (Sequence: TGAGCT (+): 1170)  
**POU5F1** (Sequence: TTTGCAT (-): 1286)  
**Nobox** (Sequence: AGCAATTA (-): 777)  
**Rhox11** (Sequence: TTTACAGCA (-): 1101)  
**Sox5** (Sequence: AACAAT (-): 851)  
**Sox5** (Sequence: AACAAT (-): 937)  
**POU2F1** (Sequence: TATTTAAAT (+): 681)  
**POU2F1** (Sequence: TATTCTAAT (+): 1051)
