# Supplementary material for: First characterization of PIWI-interacting RNA clusters in a cichlid fish with a B chromosome
Source: BMC Biol. 2022 Sep 21;20:204. doi: 10.1186/s12915-022-01403-2 (PMC9490952; doi:10.1186/s12915-022-01403-2)
Supplement: Supplementary file 1 — Additional file 1. Zipped folder with fasta and interactive html piRNA cluster information for the A. latifasciata genome. The nomenclature is as follows: number-pirna-cluster_sex_B-presence (f, female; m, male; 0b, without B chromosome; 1b, with B chromosome). [file 12915_2022_1403_MOESM1_ESM.zip › 133_f0b.html]

piRNA cluster 133\_f0b 13


Predicted piRNA cluster no. 133\_f0b
  

Show proTRAC run info
Hide proTRAC run info

/\  
                \_\_\_\_\_\_\_\_\_\_\_\_\_\_\_\_\_\_\_\_\_\_\_/\\_\_\_ /  \\_\_\_\_\_\_\_  
               I                      /  \  /    \      I  
               I     pro             /    \/      \     I  
               I        TRAC        /               \   I  
               I   \_\_\_\_\_\_\_\_\_\_\_\_\_\_\_\_/\_\_\_\_\_\_\_\_\_\_\_\_\_\_\_\_\_\\_ I  
               I   \              /                     I  
               I    \            /                      I  
               I     \  /\      /       V.2.4.2         I  
               I      \/  \    /                        I  
               I\_\_\_\_\_\_\_\_\_\_\_\  /\_\_\_\_\_\_\_\_\_\_\_\_\_\_\_\_\_\_\_\_\_\_\_\_\_I  
                            \/  
  
  
================================= proTRAC ====================================  
VERSION: .......... 2.4.2  
LAST MODIFIED: .... 11. May 2018  
  
Please cite:  
Rosenkranz D, Zischler H. proTRAC - a software for probabilistic piRNA cluster  
detection, visualization and analysis. 2012. BMC Bioinformatics 13:5.  
  
  
Contact:  
David Rosenkranz  
Institute of Organismic and Molecular Evolutionary Biology  
Dept. Anthropology, small RNA group  
Johannes Gutenberg University Mainz  
email: rosenkranz@uni-mainz.de  
  
You can find the latest proTRAC version at:  
http://sourceforge.net/projects/protrac/files  
http://www.smallRNAgroup-mainz.de/software  
==============================================================================  
  
PARAMETERS:  
Map file: ...............piwi-femeas-0B.fa-collapse.map  
Genome file: ............../../../0B\_ala\_genome.fa  
RepeatMasker annotation: Alatifasciata-all0B-maryan-v2.fa\_corrected.out  
GeneSet:................./guest-storage/Data/annotation/Alatifasciata\_all0B\_maryan-v2\_out2017.gff  
  
Significant (p<=0.01) hit density will be calculated based  
on observed hit distribution.  
  
Sliding window size: ........................................ 5000 bp  
Sliding window increament: .................................. 1000 bp  
Normalize each hit by number of genomic hits: ............... yes  
Normalize each hit by number of sequence reads: ............. yes  
Normalize values (-> per million mapped reads): ............. yes  
Min. fraction of hits with 1T(U) or 10A: .................... 0.75  
Alternatively: Min. fraction of hits with 1T(U) and 10A: .... 0.5  
Min. fraction of hits with typical piRNA length: ............ 0.75  
Typical piRNA length: ....................................... 24-32 nt  
Min. size of a piRNA cluster: ............................... 1000 bp.  
Min. number of hits (absolute): ............................. 0  
Min. number of hits (normalized): ........................... 0  
Min. fraction of hits on the mainstrand: .................... 0.75  
Top fraction of mapped sequences (in terms of read counts): . 1%  
Top fraction accounts for max. n% of sequence reads: ........ 90%  
Min. fraction of hits on each arm of a bidirectional cluster: 0.05  
Output html file for each cluster: .......................... yes  
Output a summary table: ..................................... yes  
Output a FASTA file for each cluster (piRNA sequences): ..... yes  
Output a FASTA file comprising cluster sequences: ........... yes  
Output a GTF file for predicted piRNA clusters: ..............yes  
Search DNA motifs in clusters: .............................. yes  
Output flanking sequences: +/- .............................. 0 bp  
Output ~.pTi file: .......................................... no  
==============================================================================  
  
  
Genome size (without gaps): ............ 758543724 bp  
Gaps (N/X/-): .......................... 417479 bp  
Mapped reads: .......................... 13052187  
Non-identical sequences: ............... 3338911  
Genomic hits: .......................... 28737726  
Significant densitiy of mapped reads: .. 470.083249848448 reads/kb

Show proTRAC cluster info
Hide proTRAC cluster info

|  |  |
| --- | --- |
| Location | NODE\_34239\_length\_42430\_cov\_28.169903 |
| Coordinates | 37398-41999 |
| Size [bp] | 4602 |
| Sequence hit loci | 122 |
| Mapped reads (normalized) | 25345.5 |
| Mapped reads (normalized) per kb | 5507.5 |
| Normalized reads with 1T (1U) | 99.4% |
| Normalized reads with 10A | 0% |
| Normalized reads with length 24-32 nt | 100% |
| Normalized reads on the main strand(s) | 100% |
| Predicted directionality | mono:plus |

100%

0%

1T (1U)  
reads

10A reads

24-32 nt  
reads

reads on mainstrand

**Either the amount of reads with 1T (1U) OR 10A has to exceed 75% (set with option: -1Tor10A)  
Alternatively the amount of reads with 1T (1U) AND 10A has to exceed 50% (set with option: -1Tand10A)  
Minimum amount of reads with preferred size is 75% (set with option: -pisize)  
Minimum amount of reads on the main strand(s) is 75% (set with option: -clstrand)**

Show read coverage
Hide read coverage

WHAT DO I SEE HERE?  
This chart shows the location of mapped sequence reads within a predicted piRNA cluster. The color refers to the number of genomic hits produced by the sequence read in question. A dark red bar indicates that this sequence read produces many other hits elsewhere in the genome. Many adjacent red or yellow bars can indicate the presence of a multi-copy element such as transposons or rRNA genes. A dark green bar indicates that this sequence read maps uniquely to this locus.

1 hit

2-5 hits

6-10 hits

11-20 hits

21-50 hits

51-100 hits

> 100 hits

NODE\_34239\_length\_42430\_cov\_28.169903

37398

41999

Gene Set

RepeatMasker

Mapped  
Reads

981.29

plus strand

minus strand

981.29

Region: NODE\_34239\_length\_42430\_cov\_28.169903 1805-37402. Max. coverage (+): 0.08. Max coverage (-): 0

Region: NODE\_34239\_length\_42430\_cov\_28.169903 37403-37411. Max. coverage (+): 0. Max coverage (-): 0

Region: NODE\_34239\_length\_42430\_cov\_28.169903 37412-37421. Max. coverage (+): 0.38. Max coverage (-): 0

Region: NODE\_34239\_length\_42430\_cov\_28.169903 37422-37430. Max. coverage (+): 981.29. Max coverage (-): 0

Region: NODE\_34239\_length\_42430\_cov\_28.169903 37431-37439. Max. coverage (+): 0. Max coverage (-): 0

Region: NODE\_34239\_length\_42430\_cov\_28.169903 37440-37448. Max. coverage (+): 0. Max coverage (-): 0

Region: NODE\_34239\_length\_42430\_cov\_28.169903 37449-37457. Max. coverage (+): 0. Max coverage (-): 0

Region: NODE\_34239\_length\_42430\_cov\_28.169903 37458-37467. Max. coverage (+): 5.13. Max coverage (-): 0

Region: NODE\_34239\_length\_42430\_cov\_28.169903 37468-37476. Max. coverage (+): 5.13. Max coverage (-): 0

Region: NODE\_34239\_length\_42430\_cov\_28.169903 37477-37485. Max. coverage (+): 0. Max coverage (-): 0

Region: NODE\_34239\_length\_42430\_cov\_28.169903 37486-37494. Max. coverage (+): 0. Max coverage (-): 0

Region: NODE\_34239\_length\_42430\_cov\_28.169903 37495-37503. Max. coverage (+): 0. Max coverage (-): 0

Region: NODE\_34239\_length\_42430\_cov\_28.169903 37504-37513. Max. coverage (+): 0. Max coverage (-): 0

Region: NODE\_34239\_length\_42430\_cov\_28.169903 37514-37522. Max. coverage (+): 0. Max coverage (-): 0

Region: NODE\_34239\_length\_42430\_cov\_28.169903 37523-37531. Max. coverage (+): 0. Max coverage (-): 0

Region: NODE\_34239\_length\_42430\_cov\_28.169903 37532-37540. Max. coverage (+): 0. Max coverage (-): 0

Region: NODE\_34239\_length\_42430\_cov\_28.169903 37541-37549. Max. coverage (+): 0. Max coverage (-): 0

Region: NODE\_34239\_length\_42430\_cov\_28.169903 37550-37559. Max. coverage (+): 0. Max coverage (-): 0

Region: NODE\_34239\_length\_42430\_cov\_28.169903 37560-37568. Max. coverage (+): 0. Max coverage (-): 0

Region: NODE\_34239\_length\_42430\_cov\_28.169903 37569-37577. Max. coverage (+): 0. Max coverage (-): 0

Region: NODE\_34239\_length\_42430\_cov\_28.169903 37578-37586. Max. coverage (+): 0. Max coverage (-): 0

Region: NODE\_34239\_length\_42430\_cov\_28.169903 37587-37595. Max. coverage (+): 0. Max coverage (-): 0

Region: NODE\_34239\_length\_42430\_cov\_28.169903 37596-37605. Max. coverage (+): 0. Max coverage (-): 0

Region: NODE\_34239\_length\_42430\_cov\_28.169903 37606-37614. Max. coverage (+): 0. Max coverage (-): 0

Region: NODE\_34239\_length\_42430\_cov\_28.169903 37615-37623. Max. coverage (+): 0. Max coverage (-): 0

Region: NODE\_34239\_length\_42430\_cov\_28.169903 37624-37632. Max. coverage (+): 0. Max coverage (-): 0

Region: NODE\_34239\_length\_42430\_cov\_28.169903 37633-37641. Max. coverage (+): 0. Max coverage (-): 0

Region: NODE\_34239\_length\_42430\_cov\_28.169903 37642-37651. Max. coverage (+): 0. Max coverage (-): 0

Region: NODE\_34239\_length\_42430\_cov\_28.169903 37652-37660. Max. coverage (+): 0. Max coverage (-): 0

Region: NODE\_34239\_length\_42430\_cov\_28.169903 37661-37669. Max. coverage (+): 0. Max coverage (-): 0

Region: NODE\_34239\_length\_42430\_cov\_28.169903 37670-37678. Max. coverage (+): 0. Max coverage (-): 0

Region: NODE\_34239\_length\_42430\_cov\_28.169903 37679-37687. Max. coverage (+): 0. Max coverage (-): 0

Region: NODE\_34239\_length\_42430\_cov\_28.169903 37688-37697. Max. coverage (+): 0. Max coverage (-): 0

Region: NODE\_34239\_length\_42430\_cov\_28.169903 37698-37706. Max. coverage (+): 0. Max coverage (-): 0

Region: NODE\_34239\_length\_42430\_cov\_28.169903 37707-37715. Max. coverage (+): 0. Max coverage (-): 0

Region: NODE\_34239\_length\_42430\_cov\_28.169903 37716-37724. Max. coverage (+): 0. Max coverage (-): 0

Region: NODE\_34239\_length\_42430\_cov\_28.169903 37725-37733. Max. coverage (+): 0. Max coverage (-): 0

Region: NODE\_34239\_length\_42430\_cov\_28.169903 37734-37743. Max. coverage (+): 0. Max coverage (-): 0

Region: NODE\_34239\_length\_42430\_cov\_28.169903 37744-37752. Max. coverage (+): 0. Max coverage (-): 0

Region: NODE\_34239\_length\_42430\_cov\_28.169903 37753-37761. Max. coverage (+): 954.28. Max coverage (-): 0

Region: NODE\_34239\_length\_42430\_cov\_28.169903 37762-37770. Max. coverage (+): 0.08. Max coverage (-): 0

Region: NODE\_34239\_length\_42430\_cov\_28.169903 37771-37779. Max. coverage (+): 0. Max coverage (-): 0

Region: NODE\_34239\_length\_42430\_cov\_28.169903 37780-37789. Max. coverage (+): 0. Max coverage (-): 0

Region: NODE\_34239\_length\_42430\_cov\_28.169903 37790-37798. Max. coverage (+): 0. Max coverage (-): 0

Region: NODE\_34239\_length\_42430\_cov\_28.169903 37799-37807. Max. coverage (+): 0. Max coverage (-): 0

Region: NODE\_34239\_length\_42430\_cov\_28.169903 37808-37816. Max. coverage (+): 0. Max coverage (-): 0

Region: NODE\_34239\_length\_42430\_cov\_28.169903 37817-37825. Max. coverage (+): 0. Max coverage (-): 0

Region: NODE\_34239\_length\_42430\_cov\_28.169903 37826-37835. Max. coverage (+): 0. Max coverage (-): 0

Region: NODE\_34239\_length\_42430\_cov\_28.169903 37836-37844. Max. coverage (+): 0. Max coverage (-): 0

Region: NODE\_34239\_length\_42430\_cov\_28.169903 37845-37853. Max. coverage (+): 0. Max coverage (-): 0

Region: NODE\_34239\_length\_42430\_cov\_28.169903 37854-37862. Max. coverage (+): 0. Max coverage (-): 0

Region: NODE\_34239\_length\_42430\_cov\_28.169903 37863-37872. Max. coverage (+): 0. Max coverage (-): 0

Region: NODE\_34239\_length\_42430\_cov\_28.169903 37873-37881. Max. coverage (+): 0. Max coverage (-): 0

Region: NODE\_34239\_length\_42430\_cov\_28.169903 37882-37890. Max. coverage (+): 0. Max coverage (-): 0

Region: NODE\_34239\_length\_42430\_cov\_28.169903 37891-37899. Max. coverage (+): 0. Max coverage (-): 0

Region: NODE\_34239\_length\_42430\_cov\_28.169903 37900-37908. Max. coverage (+): 0. Max coverage (-): 0

Region: NODE\_34239\_length\_42430\_cov\_28.169903 37909-37918. Max. coverage (+): 0. Max coverage (-): 0

Region: NODE\_34239\_length\_42430\_cov\_28.169903 37919-37927. Max. coverage (+): 0. Max coverage (-): 0

Region: NODE\_34239\_length\_42430\_cov\_28.169903 37928-37936. Max. coverage (+): 0. Max coverage (-): 0

Region: NODE\_34239\_length\_42430\_cov\_28.169903 37937-37945. Max. coverage (+): 0. Max coverage (-): 0

Region: NODE\_34239\_length\_42430\_cov\_28.169903 37946-37954. Max. coverage (+): 0. Max coverage (-): 0

Region: NODE\_34239\_length\_42430\_cov\_28.169903 37955-37964. Max. coverage (+): 0. Max coverage (-): 0

Region: NODE\_34239\_length\_42430\_cov\_28.169903 37965-37973. Max. coverage (+): 0. Max coverage (-): 0

Region: NODE\_34239\_length\_42430\_cov\_28.169903 37974-37982. Max. coverage (+): 0. Max coverage (-): 0

Region: NODE\_34239\_length\_42430\_cov\_28.169903 37983-37991. Max. coverage (+): 0. Max coverage (-): 0

Region: NODE\_34239\_length\_42430\_cov\_28.169903 37992-38000. Max. coverage (+): 0. Max coverage (-): 0

Region: NODE\_34239\_length\_42430\_cov\_28.169903 38001-38010. Max. coverage (+): 0. Max coverage (-): 0

Region: NODE\_34239\_length\_42430\_cov\_28.169903 38011-38019. Max. coverage (+): 0. Max coverage (-): 0

Region: NODE\_34239\_length\_42430\_cov\_28.169903 38020-38028. Max. coverage (+): 0. Max coverage (-): 0

Region: NODE\_34239\_length\_42430\_cov\_28.169903 38029-38037. Max. coverage (+): 0. Max coverage (-): 0

Region: NODE\_34239\_length\_42430\_cov\_28.169903 38038-38046. Max. coverage (+): 0. Max coverage (-): 0

Region: NODE\_34239\_length\_42430\_cov\_28.169903 38047-38056. Max. coverage (+): 0. Max coverage (-): 0

Region: NODE\_34239\_length\_42430\_cov\_28.169903 38057-38065. Max. coverage (+): 0. Max coverage (-): 0

Region: NODE\_34239\_length\_42430\_cov\_28.169903 38066-38074. Max. coverage (+): 0. Max coverage (-): 0

Region: NODE\_34239\_length\_42430\_cov\_28.169903 38075-38083. Max. coverage (+): 0. Max coverage (-): 0

Region: NODE\_34239\_length\_42430\_cov\_28.169903 38084-38092. Max. coverage (+): 0. Max coverage (-): 0

Region: NODE\_34239\_length\_42430\_cov\_28.169903 38093-38102. Max. coverage (+): 0. Max coverage (-): 0

Region: NODE\_34239\_length\_42430\_cov\_28.169903 38103-38111. Max. coverage (+): 0. Max coverage (-): 0

Region: NODE\_34239\_length\_42430\_cov\_28.169903 38112-38120. Max. coverage (+): 0. Max coverage (-): 0

Region: NODE\_34239\_length\_42430\_cov\_28.169903 38121-38129. Max. coverage (+): 0. Max coverage (-): 0

Region: NODE\_34239\_length\_42430\_cov\_28.169903 38130-38138. Max. coverage (+): 0. Max coverage (-): 0

Region: NODE\_34239\_length\_42430\_cov\_28.169903 38139-38148. Max. coverage (+): 0. Max coverage (-): 0

Region: NODE\_34239\_length\_42430\_cov\_28.169903 38149-38157. Max. coverage (+): 0. Max coverage (-): 0

Region: NODE\_34239\_length\_42430\_cov\_28.169903 38158-38166. Max. coverage (+): 0. Max coverage (-): 0

Region: NODE\_34239\_length\_42430\_cov\_28.169903 38167-38175. Max. coverage (+): 0. Max coverage (-): 0

Region: NODE\_34239\_length\_42430\_cov\_28.169903 38176-38184. Max. coverage (+): 0. Max coverage (-): 0

Region: NODE\_34239\_length\_42430\_cov\_28.169903 38185-38194. Max. coverage (+): 0. Max coverage (-): 0

Region: NODE\_34239\_length\_42430\_cov\_28.169903 38195-38203. Max. coverage (+): 0. Max coverage (-): 0

Region: NODE\_34239\_length\_42430\_cov\_28.169903 38204-38212. Max. coverage (+): 0. Max coverage (-): 0

Region: NODE\_34239\_length\_42430\_cov\_28.169903 38213-38221. Max. coverage (+): 0. Max coverage (-): 0

Region: NODE\_34239\_length\_42430\_cov\_28.169903 38222-38230. Max. coverage (+): 0. Max coverage (-): 0

Region: NODE\_34239\_length\_42430\_cov\_28.169903 38231-38240. Max. coverage (+): 0. Max coverage (-): 0

Region: NODE\_34239\_length\_42430\_cov\_28.169903 38241-38249. Max. coverage (+): 0. Max coverage (-): 0

Region: NODE\_34239\_length\_42430\_cov\_28.169903 38250-38258. Max. coverage (+): 0. Max coverage (-): 0

Region: NODE\_34239\_length\_42430\_cov\_28.169903 38259-38267. Max. coverage (+): 0. Max coverage (-): 0

Region: NODE\_34239\_length\_42430\_cov\_28.169903 38268-38276. Max. coverage (+): 0. Max coverage (-): 0

Region: NODE\_34239\_length\_42430\_cov\_28.169903 38277-38286. Max. coverage (+): 0. Max coverage (-): 0

Region: NODE\_34239\_length\_42430\_cov\_28.169903 38287-38295. Max. coverage (+): 0. Max coverage (-): 0

Region: NODE\_34239\_length\_42430\_cov\_28.169903 38296-38304. Max. coverage (+): 0. Max coverage (-): 0

Region: NODE\_34239\_length\_42430\_cov\_28.169903 38305-38313. Max. coverage (+): 0. Max coverage (-): 0

Region: NODE\_34239\_length\_42430\_cov\_28.169903 38314-38323. Max. coverage (+): 0.15. Max coverage (-): 0

Region: NODE\_34239\_length\_42430\_cov\_28.169903 38324-38332. Max. coverage (+): 0.08. Max coverage (-): 0

Region: NODE\_34239\_length\_42430\_cov\_28.169903 38333-38341. Max. coverage (+): 0. Max coverage (-): 0

Region: NODE\_34239\_length\_42430\_cov\_28.169903 38342-38350. Max. coverage (+): 0. Max coverage (-): 0

Region: NODE\_34239\_length\_42430\_cov\_28.169903 38351-38359. Max. coverage (+): 0. Max coverage (-): 0

Region: NODE\_34239\_length\_42430\_cov\_28.169903 38360-38369. Max. coverage (+): 0. Max coverage (-): 0

Region: NODE\_34239\_length\_42430\_cov\_28.169903 38370-38378. Max. coverage (+): 0. Max coverage (-): 0

Region: NODE\_34239\_length\_42430\_cov\_28.169903 38379-38387. Max. coverage (+): 0. Max coverage (-): 0

Region: NODE\_34239\_length\_42430\_cov\_28.169903 38388-38396. Max. coverage (+): 0. Max coverage (-): 0

Region: NODE\_34239\_length\_42430\_cov\_28.169903 38397-38405. Max. coverage (+): 0. Max coverage (-): 0

Region: NODE\_34239\_length\_42430\_cov\_28.169903 38406-38415. Max. coverage (+): 0. Max coverage (-): 0

Region: NODE\_34239\_length\_42430\_cov\_28.169903 38416-38424. Max. coverage (+): 0. Max coverage (-): 0

Region: NODE\_34239\_length\_42430\_cov\_28.169903 38425-38433. Max. coverage (+): 0. Max coverage (-): 0

Region: NODE\_34239\_length\_42430\_cov\_28.169903 38434-38442. Max. coverage (+): 0. Max coverage (-): 0

Region: NODE\_34239\_length\_42430\_cov\_28.169903 38443-38451. Max. coverage (+): 0. Max coverage (-): 0

Region: NODE\_34239\_length\_42430\_cov\_28.169903 38452-38461. Max. coverage (+): 0. Max coverage (-): 0

Region: NODE\_34239\_length\_42430\_cov\_28.169903 38462-38470. Max. coverage (+): 0. Max coverage (-): 0

Region: NODE\_34239\_length\_42430\_cov\_28.169903 38471-38479. Max. coverage (+): 0. Max coverage (-): 0

Region: NODE\_34239\_length\_42430\_cov\_28.169903 38480-38488. Max. coverage (+): 0. Max coverage (-): 0

Region: NODE\_34239\_length\_42430\_cov\_28.169903 38489-38497. Max. coverage (+): 0. Max coverage (-): 0

Region: NODE\_34239\_length\_42430\_cov\_28.169903 38498-38507. Max. coverage (+): 0. Max coverage (-): 0

Region: NODE\_34239\_length\_42430\_cov\_28.169903 38508-38516. Max. coverage (+): 0. Max coverage (-): 0

Region: NODE\_34239\_length\_42430\_cov\_28.169903 38517-38525. Max. coverage (+): 0. Max coverage (-): 0

Region: NODE\_34239\_length\_42430\_cov\_28.169903 38526-38534. Max. coverage (+): 0. Max coverage (-): 0

Region: NODE\_34239\_length\_42430\_cov\_28.169903 38535-38543. Max. coverage (+): 0. Max coverage (-): 0

Region: NODE\_34239\_length\_42430\_cov\_28.169903 38544-38553. Max. coverage (+): 0. Max coverage (-): 0

Region: NODE\_34239\_length\_42430\_cov\_28.169903 38554-38562. Max. coverage (+): 0. Max coverage (-): 0

Region: NODE\_34239\_length\_42430\_cov\_28.169903 38563-38571. Max. coverage (+): 0. Max coverage (-): 0

Region: NODE\_34239\_length\_42430\_cov\_28.169903 38572-38580. Max. coverage (+): 0. Max coverage (-): 0

Region: NODE\_34239\_length\_42430\_cov\_28.169903 38581-38589. Max. coverage (+): 0. Max coverage (-): 0

Region: NODE\_34239\_length\_42430\_cov\_28.169903 38590-38599. Max. coverage (+): 0. Max coverage (-): 0

Region: NODE\_34239\_length\_42430\_cov\_28.169903 38600-38608. Max. coverage (+): 0. Max coverage (-): 0

Region: NODE\_34239\_length\_42430\_cov\_28.169903 38609-38617. Max. coverage (+): 0. Max coverage (-): 0

Region: NODE\_34239\_length\_42430\_cov\_28.169903 38618-38626. Max. coverage (+): 0. Max coverage (-): 0

Region: NODE\_34239\_length\_42430\_cov\_28.169903 38627-38635. Max. coverage (+): 0. Max coverage (-): 0

Region: NODE\_34239\_length\_42430\_cov\_28.169903 38636-38645. Max. coverage (+): 0. Max coverage (-): 0

Region: NODE\_34239\_length\_42430\_cov\_28.169903 38646-38654. Max. coverage (+): 0. Max coverage (-): 0

Region: NODE\_34239\_length\_42430\_cov\_28.169903 38655-38663. Max. coverage (+): 0. Max coverage (-): 0

Region: NODE\_34239\_length\_42430\_cov\_28.169903 38664-38672. Max. coverage (+): 0. Max coverage (-): 0

Region: NODE\_34239\_length\_42430\_cov\_28.169903 38673-38681. Max. coverage (+): 0. Max coverage (-): 0

Region: NODE\_34239\_length\_42430\_cov\_28.169903 38682-38691. Max. coverage (+): 0. Max coverage (-): 0

Region: NODE\_34239\_length\_42430\_cov\_28.169903 38692-38700. Max. coverage (+): 0. Max coverage (-): 0

Region: NODE\_34239\_length\_42430\_cov\_28.169903 38701-38709. Max. coverage (+): 0. Max coverage (-): 0

Region: NODE\_34239\_length\_42430\_cov\_28.169903 38710-38718. Max. coverage (+): 0. Max coverage (-): 0

Region: NODE\_34239\_length\_42430\_cov\_28.169903 38719-38727. Max. coverage (+): 0.08. Max coverage (-): 0

Region: NODE\_34239\_length\_42430\_cov\_28.169903 38728-38737. Max. coverage (+): 0. Max coverage (-): 0

Region: NODE\_34239\_length\_42430\_cov\_28.169903 38738-38746. Max. coverage (+): 0. Max coverage (-): 0

Region: NODE\_34239\_length\_42430\_cov\_28.169903 38747-38755. Max. coverage (+): 0. Max coverage (-): 0

Region: NODE\_34239\_length\_42430\_cov\_28.169903 38756-38764. Max. coverage (+): 0. Max coverage (-): 0

Region: NODE\_34239\_length\_42430\_cov\_28.169903 38765-38773. Max. coverage (+): 0. Max coverage (-): 0

Region: NODE\_34239\_length\_42430\_cov\_28.169903 38774-38783. Max. coverage (+): 0. Max coverage (-): 0

Region: NODE\_34239\_length\_42430\_cov\_28.169903 38784-38792. Max. coverage (+): 0. Max coverage (-): 0

Region: NODE\_34239\_length\_42430\_cov\_28.169903 38793-38801. Max. coverage (+): 0. Max coverage (-): 0

Region: NODE\_34239\_length\_42430\_cov\_28.169903 38802-38810. Max. coverage (+): 0. Max coverage (-): 0

Region: NODE\_34239\_length\_42430\_cov\_28.169903 38811-38820. Max. coverage (+): 0. Max coverage (-): 0

Region: NODE\_34239\_length\_42430\_cov\_28.169903 38821-38829. Max. coverage (+): 0. Max coverage (-): 0

Region: NODE\_34239\_length\_42430\_cov\_28.169903 38830-38838. Max. coverage (+): 0. Max coverage (-): 0

Region: NODE\_34239\_length\_42430\_cov\_28.169903 38839-38847. Max. coverage (+): 0. Max coverage (-): 0

Region: NODE\_34239\_length\_42430\_cov\_28.169903 38848-38856. Max. coverage (+): 0. Max coverage (-): 0

Region: NODE\_34239\_length\_42430\_cov\_28.169903 38857-38866. Max. coverage (+): 0. Max coverage (-): 0

Region: NODE\_34239\_length\_42430\_cov\_28.169903 38867-38875. Max. coverage (+): 0. Max coverage (-): 0

Region: NODE\_34239\_length\_42430\_cov\_28.169903 38876-38884. Max. coverage (+): 0. Max coverage (-): 0

Region: NODE\_34239\_length\_42430\_cov\_28.169903 38885-38893. Max. coverage (+): 0. Max coverage (-): 0

Region: NODE\_34239\_length\_42430\_cov\_28.169903 38894-38902. Max. coverage (+): 0. Max coverage (-): 0

Region: NODE\_34239\_length\_42430\_cov\_28.169903 38903-38912. Max. coverage (+): 0. Max coverage (-): 0

Region: NODE\_34239\_length\_42430\_cov\_28.169903 38913-38921. Max. coverage (+): 0. Max coverage (-): 0

Region: NODE\_34239\_length\_42430\_cov\_28.169903 38922-38930. Max. coverage (+): 0. Max coverage (-): 0

Region: NODE\_34239\_length\_42430\_cov\_28.169903 38931-38939. Max. coverage (+): 0. Max coverage (-): 0

Region: NODE\_34239\_length\_42430\_cov\_28.169903 38940-38948. Max. coverage (+): 0. Max coverage (-): 0

Region: NODE\_34239\_length\_42430\_cov\_28.169903 38949-38958. Max. coverage (+): 0. Max coverage (-): 0

Region: NODE\_34239\_length\_42430\_cov\_28.169903 38959-38967. Max. coverage (+): 0. Max coverage (-): 0

Region: NODE\_34239\_length\_42430\_cov\_28.169903 38968-38976. Max. coverage (+): 0. Max coverage (-): 0

Region: NODE\_34239\_length\_42430\_cov\_28.169903 38977-38985. Max. coverage (+): 0. Max coverage (-): 0

Region: NODE\_34239\_length\_42430\_cov\_28.169903 38986-38994. Max. coverage (+): 0. Max coverage (-): 0

Region: NODE\_34239\_length\_42430\_cov\_28.169903 38995-39004. Max. coverage (+): 0. Max coverage (-): 0

Region: NODE\_34239\_length\_42430\_cov\_28.169903 39005-39013. Max. coverage (+): 0. Max coverage (-): 0

Region: NODE\_34239\_length\_42430\_cov\_28.169903 39014-39022. Max. coverage (+): 0. Max coverage (-): 0

Region: NODE\_34239\_length\_42430\_cov\_28.169903 39023-39031. Max. coverage (+): 0. Max coverage (-): 0

Region: NODE\_34239\_length\_42430\_cov\_28.169903 39032-39040. Max. coverage (+): 0. Max coverage (-): 0

Region: NODE\_34239\_length\_42430\_cov\_28.169903 39041-39050. Max. coverage (+): 0. Max coverage (-): 0

Region: NODE\_34239\_length\_42430\_cov\_28.169903 39051-39059. Max. coverage (+): 0. Max coverage (-): 0

Region: NODE\_34239\_length\_42430\_cov\_28.169903 39060-39068. Max. coverage (+): 0. Max coverage (-): 0

Region: NODE\_34239\_length\_42430\_cov\_28.169903 39069-39077. Max. coverage (+): 0. Max coverage (-): 0

Region: NODE\_34239\_length\_42430\_cov\_28.169903 39078-39086. Max. coverage (+): 0. Max coverage (-): 0

Region: NODE\_34239\_length\_42430\_cov\_28.169903 39087-39096. Max. coverage (+): 0. Max coverage (-): 0

Region: NODE\_34239\_length\_42430\_cov\_28.169903 39097-39105. Max. coverage (+): 0. Max coverage (-): 0

Region: NODE\_34239\_length\_42430\_cov\_28.169903 39106-39114. Max. coverage (+): 0. Max coverage (-): 0

Region: NODE\_34239\_length\_42430\_cov\_28.169903 39115-39123. Max. coverage (+): 0. Max coverage (-): 0

Region: NODE\_34239\_length\_42430\_cov\_28.169903 39124-39132. Max. coverage (+): 0. Max coverage (-): 0

Region: NODE\_34239\_length\_42430\_cov\_28.169903 39133-39142. Max. coverage (+): 0. Max coverage (-): 0

Region: NODE\_34239\_length\_42430\_cov\_28.169903 39143-39151. Max. coverage (+): 0. Max coverage (-): 0

Region: NODE\_34239\_length\_42430\_cov\_28.169903 39152-39160. Max. coverage (+): 0. Max coverage (-): 0

Region: NODE\_34239\_length\_42430\_cov\_28.169903 39161-39169. Max. coverage (+): 0. Max coverage (-): 0

Region: NODE\_34239\_length\_42430\_cov\_28.169903 39170-39178. Max. coverage (+): 0. Max coverage (-): 0

Region: NODE\_34239\_length\_42430\_cov\_28.169903 39179-39188. Max. coverage (+): 0. Max coverage (-): 0

Region: NODE\_34239\_length\_42430\_cov\_28.169903 39189-39197. Max. coverage (+): 0. Max coverage (-): 0

Region: NODE\_34239\_length\_42430\_cov\_28.169903 39198-39206. Max. coverage (+): 0. Max coverage (-): 0

Region: NODE\_34239\_length\_42430\_cov\_28.169903 39207-39215. Max. coverage (+): 0. Max coverage (-): 0

Region: NODE\_34239\_length\_42430\_cov\_28.169903 39216-39224. Max. coverage (+): 0. Max coverage (-): 0

Region: NODE\_34239\_length\_42430\_cov\_28.169903 39225-39234. Max. coverage (+): 0. Max coverage (-): 0

Region: NODE\_34239\_length\_42430\_cov\_28.169903 39235-39243. Max. coverage (+): 0. Max coverage (-): 0

Region: NODE\_34239\_length\_42430\_cov\_28.169903 39244-39252. Max. coverage (+): 0. Max coverage (-): 0

Region: NODE\_34239\_length\_42430\_cov\_28.169903 39253-39261. Max. coverage (+): 0. Max coverage (-): 0

Region: NODE\_34239\_length\_42430\_cov\_28.169903 39262-39271. Max. coverage (+): 0. Max coverage (-): 0

Region: NODE\_34239\_length\_42430\_cov\_28.169903 39272-39280. Max. coverage (+): 0. Max coverage (-): 0

Region: NODE\_34239\_length\_42430\_cov\_28.169903 39281-39289. Max. coverage (+): 0. Max coverage (-): 0

Region: NODE\_34239\_length\_42430\_cov\_28.169903 39290-39298. Max. coverage (+): 0. Max coverage (-): 0

Region: NODE\_34239\_length\_42430\_cov\_28.169903 39299-39307. Max. coverage (+): 0. Max coverage (-): 0

Region: NODE\_34239\_length\_42430\_cov\_28.169903 39308-39317. Max. coverage (+): 0. Max coverage (-): 0

Region: NODE\_34239\_length\_42430\_cov\_28.169903 39318-39326. Max. coverage (+): 0. Max coverage (-): 0

Region: NODE\_34239\_length\_42430\_cov\_28.169903 39327-39335. Max. coverage (+): 0. Max coverage (-): 0

Region: NODE\_34239\_length\_42430\_cov\_28.169903 39336-39344. Max. coverage (+): 0. Max coverage (-): 0

Region: NODE\_34239\_length\_42430\_cov\_28.169903 39345-39353. Max. coverage (+): 0. Max coverage (-): 0

Region: NODE\_34239\_length\_42430\_cov\_28.169903 39354-39363. Max. coverage (+): 0. Max coverage (-): 0

Region: NODE\_34239\_length\_42430\_cov\_28.169903 39364-39372. Max. coverage (+): 0. Max coverage (-): 0

Region: NODE\_34239\_length\_42430\_cov\_28.169903 39373-39381. Max. coverage (+): 0. Max coverage (-): 0

Region: NODE\_34239\_length\_42430\_cov\_28.169903 39382-39390. Max. coverage (+): 0. Max coverage (-): 0

Region: NODE\_34239\_length\_42430\_cov\_28.169903 39391-39399. Max. coverage (+): 0. Max coverage (-): 0

Region: NODE\_34239\_length\_42430\_cov\_28.169903 39400-39409. Max. coverage (+): 0. Max coverage (-): 0

Region: NODE\_34239\_length\_42430\_cov\_28.169903 39410-39418. Max. coverage (+): 0. Max coverage (-): 0

Region: NODE\_34239\_length\_42430\_cov\_28.169903 39419-39427. Max. coverage (+): 0. Max coverage (-): 0

Region: NODE\_34239\_length\_42430\_cov\_28.169903 39428-39436. Max. coverage (+): 0. Max coverage (-): 0

Region: NODE\_34239\_length\_42430\_cov\_28.169903 39437-39445. Max. coverage (+): 0. Max coverage (-): 0

Region: NODE\_34239\_length\_42430\_cov\_28.169903 39446-39455. Max. coverage (+): 0. Max coverage (-): 0

Region: NODE\_34239\_length\_42430\_cov\_28.169903 39456-39464. Max. coverage (+): 0. Max coverage (-): 0

Region: NODE\_34239\_length\_42430\_cov\_28.169903 39465-39473. Max. coverage (+): 0. Max coverage (-): 0

Region: NODE\_34239\_length\_42430\_cov\_28.169903 39474-39482. Max. coverage (+): 0. Max coverage (-): 0

Region: NODE\_34239\_length\_42430\_cov\_28.169903 39483-39491. Max. coverage (+): 0. Max coverage (-): 0

Region: NODE\_34239\_length\_42430\_cov\_28.169903 39492-39501. Max. coverage (+): 0. Max coverage (-): 0

Region: NODE\_34239\_length\_42430\_cov\_28.169903 39502-39510. Max. coverage (+): 0. Max coverage (-): 0

Region: NODE\_34239\_length\_42430\_cov\_28.169903 39511-39519. Max. coverage (+): 0. Max coverage (-): 0

Region: NODE\_34239\_length\_42430\_cov\_28.169903 39520-39528. Max. coverage (+): 0. Max coverage (-): 0

Region: NODE\_34239\_length\_42430\_cov\_28.169903 39529-39537. Max. coverage (+): 0. Max coverage (-): 0

Region: NODE\_34239\_length\_42430\_cov\_28.169903 39538-39547. Max. coverage (+): 0. Max coverage (-): 0

Region: NODE\_34239\_length\_42430\_cov\_28.169903 39548-39556. Max. coverage (+): 0. Max coverage (-): 0

Region: NODE\_34239\_length\_42430\_cov\_28.169903 39557-39565. Max. coverage (+): 0. Max coverage (-): 0

Region: NODE\_34239\_length\_42430\_cov\_28.169903 39566-39574. Max. coverage (+): 0. Max coverage (-): 0

Region: NODE\_34239\_length\_42430\_cov\_28.169903 39575-39583. Max. coverage (+): 0. Max coverage (-): 0

Region: NODE\_34239\_length\_42430\_cov\_28.169903 39584-39593. Max. coverage (+): 0. Max coverage (-): 0

Region: NODE\_34239\_length\_42430\_cov\_28.169903 39594-39602. Max. coverage (+): 0. Max coverage (-): 0

Region: NODE\_34239\_length\_42430\_cov\_28.169903 39603-39611. Max. coverage (+): 0. Max coverage (-): 0

Region: NODE\_34239\_length\_42430\_cov\_28.169903 39612-39620. Max. coverage (+): 0. Max coverage (-): 0

Region: NODE\_34239\_length\_42430\_cov\_28.169903 39621-39629. Max. coverage (+): 0. Max coverage (-): 0

Region: NODE\_34239\_length\_42430\_cov\_28.169903 39630-39639. Max. coverage (+): 0. Max coverage (-): 0

Region: NODE\_34239\_length\_42430\_cov\_28.169903 39640-39648. Max. coverage (+): 0. Max coverage (-): 0

Region: NODE\_34239\_length\_42430\_cov\_28.169903 39649-39657. Max. coverage (+): 0. Max coverage (-): 0

Region: NODE\_34239\_length\_42430\_cov\_28.169903 39658-39666. Max. coverage (+): 0. Max coverage (-): 0

Region: NODE\_34239\_length\_42430\_cov\_28.169903 39667-39675. Max. coverage (+): 0. Max coverage (-): 0

Region: NODE\_34239\_length\_42430\_cov\_28.169903 39676-39685. Max. coverage (+): 0. Max coverage (-): 0

Region: NODE\_34239\_length\_42430\_cov\_28.169903 39686-39694. Max. coverage (+): 0. Max coverage (-): 0

Region: NODE\_34239\_length\_42430\_cov\_28.169903 39695-39703. Max. coverage (+): 0. Max coverage (-): 0

Region: NODE\_34239\_length\_42430\_cov\_28.169903 39704-39712. Max. coverage (+): 0. Max coverage (-): 0

Region: NODE\_34239\_length\_42430\_cov\_28.169903 39713-39722. Max. coverage (+): 0. Max coverage (-): 0

Region: NODE\_34239\_length\_42430\_cov\_28.169903 39723-39731. Max. coverage (+): 0. Max coverage (-): 0

Region: NODE\_34239\_length\_42430\_cov\_28.169903 39732-39740. Max. coverage (+): 0. Max coverage (-): 0

Region: NODE\_34239\_length\_42430\_cov\_28.169903 39741-39749. Max. coverage (+): 0. Max coverage (-): 0

Region: NODE\_34239\_length\_42430\_cov\_28.169903 39750-39758. Max. coverage (+): 0. Max coverage (-): 0

Region: NODE\_34239\_length\_42430\_cov\_28.169903 39759-39768. Max. coverage (+): 0. Max coverage (-): 0

Region: NODE\_34239\_length\_42430\_cov\_28.169903 39769-39777. Max. coverage (+): 0. Max coverage (-): 0

Region: NODE\_34239\_length\_42430\_cov\_28.169903 39778-39786. Max. coverage (+): 0. Max coverage (-): 0

Region: NODE\_34239\_length\_42430\_cov\_28.169903 39787-39795. Max. coverage (+): 0. Max coverage (-): 0

Region: NODE\_34239\_length\_42430\_cov\_28.169903 39796-39804. Max. coverage (+): 0. Max coverage (-): 0

Region: NODE\_34239\_length\_42430\_cov\_28.169903 39805-39814. Max. coverage (+): 0. Max coverage (-): 0

Region: NODE\_34239\_length\_42430\_cov\_28.169903 39815-39823. Max. coverage (+): 0. Max coverage (-): 0

Region: NODE\_34239\_length\_42430\_cov\_28.169903 39824-39832. Max. coverage (+): 0. Max coverage (-): 0

Region: NODE\_34239\_length\_42430\_cov\_28.169903 39833-39841. Max. coverage (+): 0. Max coverage (-): 0

Region: NODE\_34239\_length\_42430\_cov\_28.169903 39842-39850. Max. coverage (+): 0. Max coverage (-): 0

Region: NODE\_34239\_length\_42430\_cov\_28.169903 39851-39860. Max. coverage (+): 0.08. Max coverage (-): 0

Region: NODE\_34239\_length\_42430\_cov\_28.169903 39861-39869. Max. coverage (+): 0.08. Max coverage (-): 0

Region: NODE\_34239\_length\_42430\_cov\_28.169903 39870-39878. Max. coverage (+): 0. Max coverage (-): 0

Region: NODE\_34239\_length\_42430\_cov\_28.169903 39879-39887. Max. coverage (+): 0. Max coverage (-): 0

Region: NODE\_34239\_length\_42430\_cov\_28.169903 39888-39896. Max. coverage (+): 0. Max coverage (-): 0

Region: NODE\_34239\_length\_42430\_cov\_28.169903 39897-39906. Max. coverage (+): 0. Max coverage (-): 0

Region: NODE\_34239\_length\_42430\_cov\_28.169903 39907-39915. Max. coverage (+): 0. Max coverage (-): 0

Region: NODE\_34239\_length\_42430\_cov\_28.169903 39916-39924. Max. coverage (+): 0. Max coverage (-): 0

Region: NODE\_34239\_length\_42430\_cov\_28.169903 39925-39933. Max. coverage (+): 0. Max coverage (-): 0

Region: NODE\_34239\_length\_42430\_cov\_28.169903 39934-39942. Max. coverage (+): 0. Max coverage (-): 0

Region: NODE\_34239\_length\_42430\_cov\_28.169903 39943-39952. Max. coverage (+): 0. Max coverage (-): 0

Region: NODE\_34239\_length\_42430\_cov\_28.169903 39953-39961. Max. coverage (+): 0. Max coverage (-): 0

Region: NODE\_34239\_length\_42430\_cov\_28.169903 39962-39970. Max. coverage (+): 0. Max coverage (-): 0

Region: NODE\_34239\_length\_42430\_cov\_28.169903 39971-39979. Max. coverage (+): 0. Max coverage (-): 0

Region: NODE\_34239\_length\_42430\_cov\_28.169903 39980-39988. Max. coverage (+): 0. Max coverage (-): 0

Region: NODE\_34239\_length\_42430\_cov\_28.169903 39989-39998. Max. coverage (+): 0. Max coverage (-): 0

Region: NODE\_34239\_length\_42430\_cov\_28.169903 39999-40007. Max. coverage (+): 0. Max coverage (-): 0

Region: NODE\_34239\_length\_42430\_cov\_28.169903 40008-40016. Max. coverage (+): 0. Max coverage (-): 0

Region: NODE\_34239\_length\_42430\_cov\_28.169903 40017-40025. Max. coverage (+): 0. Max coverage (-): 0

Region: NODE\_34239\_length\_42430\_cov\_28.169903 40026-40034. Max. coverage (+): 0. Max coverage (-): 0

Region: NODE\_34239\_length\_42430\_cov\_28.169903 40035-40044. Max. coverage (+): 0. Max coverage (-): 0

Region: NODE\_34239\_length\_42430\_cov\_28.169903 40045-40053. Max. coverage (+): 0. Max coverage (-): 0

Region: NODE\_34239\_length\_42430\_cov\_28.169903 40054-40062. Max. coverage (+): 0. Max coverage (-): 0

Region: NODE\_34239\_length\_42430\_cov\_28.169903 40063-40071. Max. coverage (+): 0. Max coverage (-): 0

Region: NODE\_34239\_length\_42430\_cov\_28.169903 40072-40080. Max. coverage (+): 0. Max coverage (-): 0

Region: NODE\_34239\_length\_42430\_cov\_28.169903 40081-40090. Max. coverage (+): 0. Max coverage (-): 0

Region: NODE\_34239\_length\_42430\_cov\_28.169903 40091-40099. Max. coverage (+): 0. Max coverage (-): 0

Region: NODE\_34239\_length\_42430\_cov\_28.169903 40100-40108. Max. coverage (+): 0. Max coverage (-): 0

Region: NODE\_34239\_length\_42430\_cov\_28.169903 40109-40117. Max. coverage (+): 0. Max coverage (-): 0

Region: NODE\_34239\_length\_42430\_cov\_28.169903 40118-40126. Max. coverage (+): 0. Max coverage (-): 0

Region: NODE\_34239\_length\_42430\_cov\_28.169903 40127-40136. Max. coverage (+): 0. Max coverage (-): 0

Region: NODE\_34239\_length\_42430\_cov\_28.169903 40137-40145. Max. coverage (+): 0. Max coverage (-): 0

Region: NODE\_34239\_length\_42430\_cov\_28.169903 40146-40154. Max. coverage (+): 0. Max coverage (-): 0

Region: NODE\_34239\_length\_42430\_cov\_28.169903 40155-40163. Max. coverage (+): 0. Max coverage (-): 0

Region: NODE\_34239\_length\_42430\_cov\_28.169903 40164-40173. Max. coverage (+): 0. Max coverage (-): 0

Region: NODE\_34239\_length\_42430\_cov\_28.169903 40174-40182. Max. coverage (+): 0. Max coverage (-): 0

Region: NODE\_34239\_length\_42430\_cov\_28.169903 40183-40191. Max. coverage (+): 0. Max coverage (-): 0

Region: NODE\_34239\_length\_42430\_cov\_28.169903 40192-40200. Max. coverage (+): 0. Max coverage (-): 0

Region: NODE\_34239\_length\_42430\_cov\_28.169903 40201-40209. Max. coverage (+): 0. Max coverage (-): 0

Region: NODE\_34239\_length\_42430\_cov\_28.169903 40210-40219. Max. coverage (+): 0. Max coverage (-): 0

Region: NODE\_34239\_length\_42430\_cov\_28.169903 40220-40228. Max. coverage (+): 0. Max coverage (-): 0

Region: NODE\_34239\_length\_42430\_cov\_28.169903 40229-40237. Max. coverage (+): 0. Max coverage (-): 0

Region: NODE\_34239\_length\_42430\_cov\_28.169903 40238-40246. Max. coverage (+): 0. Max coverage (-): 0

Region: NODE\_34239\_length\_42430\_cov\_28.169903 40247-40255. Max. coverage (+): 0. Max coverage (-): 0

Region: NODE\_34239\_length\_42430\_cov\_28.169903 40256-40265. Max. coverage (+): 0.08. Max coverage (-): 0

Region: NODE\_34239\_length\_42430\_cov\_28.169903 40266-40274. Max. coverage (+): 0. Max coverage (-): 0

Region: NODE\_34239\_length\_42430\_cov\_28.169903 40275-40283. Max. coverage (+): 0. Max coverage (-): 0

Region: NODE\_34239\_length\_42430\_cov\_28.169903 40284-40292. Max. coverage (+): 0. Max coverage (-): 0

Region: NODE\_34239\_length\_42430\_cov\_28.169903 40293-40301. Max. coverage (+): 0. Max coverage (-): 0

Region: NODE\_34239\_length\_42430\_cov\_28.169903 40302-40311. Max. coverage (+): 0. Max coverage (-): 0

Region: NODE\_34239\_length\_42430\_cov\_28.169903 40312-40320. Max. coverage (+): 0. Max coverage (-): 0

Region: NODE\_34239\_length\_42430\_cov\_28.169903 40321-40329. Max. coverage (+): 0. Max coverage (-): 0

Region: NODE\_34239\_length\_42430\_cov\_28.169903 40330-40338. Max. coverage (+): 0. Max coverage (-): 0

Region: NODE\_34239\_length\_42430\_cov\_28.169903 40339-40347. Max. coverage (+): 0. Max coverage (-): 0

Region: NODE\_34239\_length\_42430\_cov\_28.169903 40348-40357. Max. coverage (+): 0. Max coverage (-): 0

Region: NODE\_34239\_length\_42430\_cov\_28.169903 40358-40366. Max. coverage (+): 0. Max coverage (-): 0

Region: NODE\_34239\_length\_42430\_cov\_28.169903 40367-40375. Max. coverage (+): 0. Max coverage (-): 0

Region: NODE\_34239\_length\_42430\_cov\_28.169903 40376-40384. Max. coverage (+): 0. Max coverage (-): 0

Region: NODE\_34239\_length\_42430\_cov\_28.169903 40385-40393. Max. coverage (+): 0. Max coverage (-): 0

Region: NODE\_34239\_length\_42430\_cov\_28.169903 40394-40403. Max. coverage (+): 0. Max coverage (-): 0

Region: NODE\_34239\_length\_42430\_cov\_28.169903 40404-40412. Max. coverage (+): 0. Max coverage (-): 0

Region: NODE\_34239\_length\_42430\_cov\_28.169903 40413-40421. Max. coverage (+): 0. Max coverage (-): 0

Region: NODE\_34239\_length\_42430\_cov\_28.169903 40422-40430. Max. coverage (+): 0. Max coverage (-): 0

Region: NODE\_34239\_length\_42430\_cov\_28.169903 40431-40439. Max. coverage (+): 0. Max coverage (-): 0

Region: NODE\_34239\_length\_42430\_cov\_28.169903 40440-40449. Max. coverage (+): 0. Max coverage (-): 0

Region: NODE\_34239\_length\_42430\_cov\_28.169903 40450-40458. Max. coverage (+): 0. Max coverage (-): 0

Region: NODE\_34239\_length\_42430\_cov\_28.169903 40459-40467. Max. coverage (+): 0. Max coverage (-): 0

Region: NODE\_34239\_length\_42430\_cov\_28.169903 40468-40476. Max. coverage (+): 0.08. Max coverage (-): 0

Region: NODE\_34239\_length\_42430\_cov\_28.169903 40477-40485. Max. coverage (+): 0. Max coverage (-): 0

Region: NODE\_34239\_length\_42430\_cov\_28.169903 40486-40495. Max. coverage (+): 0. Max coverage (-): 0

Region: NODE\_34239\_length\_42430\_cov\_28.169903 40496-40504. Max. coverage (+): 0. Max coverage (-): 0

Region: NODE\_34239\_length\_42430\_cov\_28.169903 40505-40513. Max. coverage (+): 0. Max coverage (-): 0

Region: NODE\_34239\_length\_42430\_cov\_28.169903 40514-40522. Max. coverage (+): 0. Max coverage (-): 0

Region: NODE\_34239\_length\_42430\_cov\_28.169903 40523-40531. Max. coverage (+): 0. Max coverage (-): 0

Region: NODE\_34239\_length\_42430\_cov\_28.169903 40532-40541. Max. coverage (+): 0. Max coverage (-): 0

Region: NODE\_34239\_length\_42430\_cov\_28.169903 40542-40550. Max. coverage (+): 0. Max coverage (-): 0

Region: NODE\_34239\_length\_42430\_cov\_28.169903 40551-40559. Max. coverage (+): 0. Max coverage (-): 0

Region: NODE\_34239\_length\_42430\_cov\_28.169903 40560-40568. Max. coverage (+): 0. Max coverage (-): 0

Region: NODE\_34239\_length\_42430\_cov\_28.169903 40569-40577. Max. coverage (+): 0. Max coverage (-): 0

Region: NODE\_34239\_length\_42430\_cov\_28.169903 40578-40587. Max. coverage (+): 0. Max coverage (-): 0

Region: NODE\_34239\_length\_42430\_cov\_28.169903 40588-40596. Max. coverage (+): 0. Max coverage (-): 0

Region: NODE\_34239\_length\_42430\_cov\_28.169903 40597-40605. Max. coverage (+): 0. Max coverage (-): 0

Region: NODE\_34239\_length\_42430\_cov\_28.169903 40606-40614. Max. coverage (+): 0. Max coverage (-): 0

Region: NODE\_34239\_length\_42430\_cov\_28.169903 40615-40624. Max. coverage (+): 0. Max coverage (-): 0

Region: NODE\_34239\_length\_42430\_cov\_28.169903 40625-40633. Max. coverage (+): 0. Max coverage (-): 0

Region: NODE\_34239\_length\_42430\_cov\_28.169903 40634-40642. Max. coverage (+): 0. Max coverage (-): 0

Region: NODE\_34239\_length\_42430\_cov\_28.169903 40643-40651. Max. coverage (+): 0. Max coverage (-): 0

Region: NODE\_34239\_length\_42430\_cov\_28.169903 40652-40660. Max. coverage (+): 0. Max coverage (-): 0

Region: NODE\_34239\_length\_42430\_cov\_28.169903 40661-40670. Max. coverage (+): 0. Max coverage (-): 0

Region: NODE\_34239\_length\_42430\_cov\_28.169903 40671-40679. Max. coverage (+): 0. Max coverage (-): 0

Region: NODE\_34239\_length\_42430\_cov\_28.169903 40680-40688. Max. coverage (+): 0. Max coverage (-): 0

Region: NODE\_34239\_length\_42430\_cov\_28.169903 40689-40697. Max. coverage (+): 0. Max coverage (-): 0

Region: NODE\_34239\_length\_42430\_cov\_28.169903 40698-40706. Max. coverage (+): 0. Max coverage (-): 0

Region: NODE\_34239\_length\_42430\_cov\_28.169903 40707-40716. Max. coverage (+): 0. Max coverage (-): 0

Region: NODE\_34239\_length\_42430\_cov\_28.169903 40717-40725. Max. coverage (+): 0. Max coverage (-): 0

Region: NODE\_34239\_length\_42430\_cov\_28.169903 40726-40734. Max. coverage (+): 0. Max coverage (-): 0

Region: NODE\_34239\_length\_42430\_cov\_28.169903 40735-40743. Max. coverage (+): 0. Max coverage (-): 0

Region: NODE\_34239\_length\_42430\_cov\_28.169903 40744-40752. Max. coverage (+): 0. Max coverage (-): 0

Region: NODE\_34239\_length\_42430\_cov\_28.169903 40753-40762. Max. coverage (+): 0. Max coverage (-): 0

Region: NODE\_34239\_length\_42430\_cov\_28.169903 40763-40771. Max. coverage (+): 0. Max coverage (-): 0

Region: NODE\_34239\_length\_42430\_cov\_28.169903 40772-40780. Max. coverage (+): 0. Max coverage (-): 0

Region: NODE\_34239\_length\_42430\_cov\_28.169903 40781-40789. Max. coverage (+): 0. Max coverage (-): 0

Region: NODE\_34239\_length\_42430\_cov\_28.169903 40790-40798. Max. coverage (+): 0. Max coverage (-): 0

Region: NODE\_34239\_length\_42430\_cov\_28.169903 40799-40808. Max. coverage (+): 0. Max coverage (-): 0

Region: NODE\_34239\_length\_42430\_cov\_28.169903 40809-40817. Max. coverage (+): 0. Max coverage (-): 0

Region: NODE\_34239\_length\_42430\_cov\_28.169903 40818-40826. Max. coverage (+): 0. Max coverage (-): 0

Region: NODE\_34239\_length\_42430\_cov\_28.169903 40827-40835. Max. coverage (+): 0. Max coverage (-): 0

Region: NODE\_34239\_length\_42430\_cov\_28.169903 40836-40844. Max. coverage (+): 0. Max coverage (-): 0

Region: NODE\_34239\_length\_42430\_cov\_28.169903 40845-40854. Max. coverage (+): 0. Max coverage (-): 0

Region: NODE\_34239\_length\_42430\_cov\_28.169903 40855-40863. Max. coverage (+): 0. Max coverage (-): 0

Region: NODE\_34239\_length\_42430\_cov\_28.169903 40864-40872. Max. coverage (+): 0. Max coverage (-): 0

Region: NODE\_34239\_length\_42430\_cov\_28.169903 40873-40881. Max. coverage (+): 0. Max coverage (-): 0

Region: NODE\_34239\_length\_42430\_cov\_28.169903 40882-40890. Max. coverage (+): 0. Max coverage (-): 0

Region: NODE\_34239\_length\_42430\_cov\_28.169903 40891-40900. Max. coverage (+): 0. Max coverage (-): 0

Region: NODE\_34239\_length\_42430\_cov\_28.169903 40901-40909. Max. coverage (+): 0. Max coverage (-): 0

Region: NODE\_34239\_length\_42430\_cov\_28.169903 40910-40918. Max. coverage (+): 0. Max coverage (-): 0

Region: NODE\_34239\_length\_42430\_cov\_28.169903 40919-40927. Max. coverage (+): 0. Max coverage (-): 0

Region: NODE\_34239\_length\_42430\_cov\_28.169903 40928-40936. Max. coverage (+): 0. Max coverage (-): 0

Region: NODE\_34239\_length\_42430\_cov\_28.169903 40937-40946. Max. coverage (+): 0. Max coverage (-): 0

Region: NODE\_34239\_length\_42430\_cov\_28.169903 40947-40955. Max. coverage (+): 0. Max coverage (-): 0

Region: NODE\_34239\_length\_42430\_cov\_28.169903 40956-40964. Max. coverage (+): 0. Max coverage (-): 0

Region: NODE\_34239\_length\_42430\_cov\_28.169903 40965-40973. Max. coverage (+): 0. Max coverage (-): 0

Region: NODE\_34239\_length\_42430\_cov\_28.169903 40974-40982. Max. coverage (+): 0. Max coverage (-): 0

Region: NODE\_34239\_length\_42430\_cov\_28.169903 40983-40992. Max. coverage (+): 0. Max coverage (-): 0

Region: NODE\_34239\_length\_42430\_cov\_28.169903 40993-41001. Max. coverage (+): 0. Max coverage (-): 0

Region: NODE\_34239\_length\_42430\_cov\_28.169903 41002-41010. Max. coverage (+): 0. Max coverage (-): 0

Region: NODE\_34239\_length\_42430\_cov\_28.169903 41011-41019. Max. coverage (+): 0. Max coverage (-): 0

Region: NODE\_34239\_length\_42430\_cov\_28.169903 41020-41028. Max. coverage (+): 0. Max coverage (-): 0

Region: NODE\_34239\_length\_42430\_cov\_28.169903 41029-41038. Max. coverage (+): 0. Max coverage (-): 0

Region: NODE\_34239\_length\_42430\_cov\_28.169903 41039-41047. Max. coverage (+): 0. Max coverage (-): 0

Region: NODE\_34239\_length\_42430\_cov\_28.169903 41048-41056. Max. coverage (+): 0. Max coverage (-): 0

Region: NODE\_34239\_length\_42430\_cov\_28.169903 41057-41065. Max. coverage (+): 0. Max coverage (-): 0

Region: NODE\_34239\_length\_42430\_cov\_28.169903 41066-41074. Max. coverage (+): 0. Max coverage (-): 0

Region: NODE\_34239\_length\_42430\_cov\_28.169903 41075-41084. Max. coverage (+): 0. Max coverage (-): 0

Region: NODE\_34239\_length\_42430\_cov\_28.169903 41085-41093. Max. coverage (+): 0. Max coverage (-): 0

Region: NODE\_34239\_length\_42430\_cov\_28.169903 41094-41102. Max. coverage (+): 0. Max coverage (-): 0

Region: NODE\_34239\_length\_42430\_cov\_28.169903 41103-41111. Max. coverage (+): 0. Max coverage (-): 0

Region: NODE\_34239\_length\_42430\_cov\_28.169903 41112-41121. Max. coverage (+): 0. Max coverage (-): 0

Region: NODE\_34239\_length\_42430\_cov\_28.169903 41122-41130. Max. coverage (+): 0. Max coverage (-): 0

Region: NODE\_34239\_length\_42430\_cov\_28.169903 41131-41139. Max. coverage (+): 0. Max coverage (-): 0

Region: NODE\_34239\_length\_42430\_cov\_28.169903 41140-41148. Max. coverage (+): 0. Max coverage (-): 0

Region: NODE\_34239\_length\_42430\_cov\_28.169903 41149-41157. Max. coverage (+): 0. Max coverage (-): 0

Region: NODE\_34239\_length\_42430\_cov\_28.169903 41158-41167. Max. coverage (+): 0. Max coverage (-): 0

Region: NODE\_34239\_length\_42430\_cov\_28.169903 41168-41176. Max. coverage (+): 0. Max coverage (-): 0

Region: NODE\_34239\_length\_42430\_cov\_28.169903 41177-41185. Max. coverage (+): 0. Max coverage (-): 0

Region: NODE\_34239\_length\_42430\_cov\_28.169903 41186-41194. Max. coverage (+): 0. Max coverage (-): 0

Region: NODE\_34239\_length\_42430\_cov\_28.169903 41195-41203. Max. coverage (+): 0. Max coverage (-): 0

Region: NODE\_34239\_length\_42430\_cov\_28.169903 41204-41213. Max. coverage (+): 0. Max coverage (-): 0

Region: NODE\_34239\_length\_42430\_cov\_28.169903 41214-41222. Max. coverage (+): 0.15. Max coverage (-): 0

Region: NODE\_34239\_length\_42430\_cov\_28.169903 41223-41231. Max. coverage (+): 0. Max coverage (-): 0

Region: NODE\_34239\_length\_42430\_cov\_28.169903 41232-41240. Max. coverage (+): 0. Max coverage (-): 0

Region: NODE\_34239\_length\_42430\_cov\_28.169903 41241-41249. Max. coverage (+): 0. Max coverage (-): 0

Region: NODE\_34239\_length\_42430\_cov\_28.169903 41250-41259. Max. coverage (+): 0. Max coverage (-): 0

Region: NODE\_34239\_length\_42430\_cov\_28.169903 41260-41268. Max. coverage (+): 0.08. Max coverage (-): 0

Region: NODE\_34239\_length\_42430\_cov\_28.169903 41269-41277. Max. coverage (+): 0.08. Max coverage (-): 0

Region: NODE\_34239\_length\_42430\_cov\_28.169903 41278-41286. Max. coverage (+): 0.08. Max coverage (-): 0

Region: NODE\_34239\_length\_42430\_cov\_28.169903 41287-41295. Max. coverage (+): 0.08. Max coverage (-): 0

Region: NODE\_34239\_length\_42430\_cov\_28.169903 41296-41305. Max. coverage (+): 0. Max coverage (-): 0

Region: NODE\_34239\_length\_42430\_cov\_28.169903 41306-41314. Max. coverage (+): 0. Max coverage (-): 0

Region: NODE\_34239\_length\_42430\_cov\_28.169903 41315-41323. Max. coverage (+): 0. Max coverage (-): 0

Region: NODE\_34239\_length\_42430\_cov\_28.169903 41324-41332. Max. coverage (+): 0. Max coverage (-): 0

Region: NODE\_34239\_length\_42430\_cov\_28.169903 41333-41341. Max. coverage (+): 0. Max coverage (-): 0

Region: NODE\_34239\_length\_42430\_cov\_28.169903 41342-41351. Max. coverage (+): 0. Max coverage (-): 0

Region: NODE\_34239\_length\_42430\_cov\_28.169903 41352-41360. Max. coverage (+): 0. Max coverage (-): 0

Region: NODE\_34239\_length\_42430\_cov\_28.169903 41361-41369. Max. coverage (+): 0. Max coverage (-): 0

Region: NODE\_34239\_length\_42430\_cov\_28.169903 41370-41378. Max. coverage (+): 0. Max coverage (-): 0

Region: NODE\_34239\_length\_42430\_cov\_28.169903 41379-41387. Max. coverage (+): 0. Max coverage (-): 0

Region: NODE\_34239\_length\_42430\_cov\_28.169903 41388-41397. Max. coverage (+): 0. Max coverage (-): 0

Region: NODE\_34239\_length\_42430\_cov\_28.169903 41398-41406. Max. coverage (+): 0. Max coverage (-): 0

Region: NODE\_34239\_length\_42430\_cov\_28.169903 41407-41415. Max. coverage (+): 0. Max coverage (-): 0

Region: NODE\_34239\_length\_42430\_cov\_28.169903 41416-41424. Max. coverage (+): 0. Max coverage (-): 0

Region: NODE\_34239\_length\_42430\_cov\_28.169903 41425-41433. Max. coverage (+): 0. Max coverage (-): 0

Region: NODE\_34239\_length\_42430\_cov\_28.169903 41434-41443. Max. coverage (+): 0. Max coverage (-): 0

Region: NODE\_34239\_length\_42430\_cov\_28.169903 41444-41452. Max. coverage (+): 0. Max coverage (-): 0

Region: NODE\_34239\_length\_42430\_cov\_28.169903 41453-41461. Max. coverage (+): 0. Max coverage (-): 0

Region: NODE\_34239\_length\_42430\_cov\_28.169903 41462-41470. Max. coverage (+): 0. Max coverage (-): 0

Region: NODE\_34239\_length\_42430\_cov\_28.169903 41471-41479. Max. coverage (+): 0. Max coverage (-): 0

Region: NODE\_34239\_length\_42430\_cov\_28.169903 41480-41489. Max. coverage (+): 0. Max coverage (-): 0

Region: NODE\_34239\_length\_42430\_cov\_28.169903 41490-41498. Max. coverage (+): 0. Max coverage (-): 0

Region: NODE\_34239\_length\_42430\_cov\_28.169903 41499-41507. Max. coverage (+): 0. Max coverage (-): 0

Region: NODE\_34239\_length\_42430\_cov\_28.169903 41508-41516. Max. coverage (+): 0. Max coverage (-): 0

Region: NODE\_34239\_length\_42430\_cov\_28.169903 41517-41525. Max. coverage (+): 0. Max coverage (-): 0

Region: NODE\_34239\_length\_42430\_cov\_28.169903 41526-41535. Max. coverage (+): 0. Max coverage (-): 0

Region: NODE\_34239\_length\_42430\_cov\_28.169903 41536-41544. Max. coverage (+): 0. Max coverage (-): 0

Region: NODE\_34239\_length\_42430\_cov\_28.169903 41545-41553. Max. coverage (+): 0. Max coverage (-): 0

Region: NODE\_34239\_length\_42430\_cov\_28.169903 41554-41562. Max. coverage (+): 0. Max coverage (-): 0

Region: NODE\_34239\_length\_42430\_cov\_28.169903 41563-41572. Max. coverage (+): 0. Max coverage (-): 0

Region: NODE\_34239\_length\_42430\_cov\_28.169903 41573-41581. Max. coverage (+): 0. Max coverage (-): 0

Region: NODE\_34239\_length\_42430\_cov\_28.169903 41582-41590. Max. coverage (+): 0. Max coverage (-): 0

Region: NODE\_34239\_length\_42430\_cov\_28.169903 41591-41599. Max. coverage (+): 0. Max coverage (-): 0

Region: NODE\_34239\_length\_42430\_cov\_28.169903 41600-41608. Max. coverage (+): 0. Max coverage (-): 0

Region: NODE\_34239\_length\_42430\_cov\_28.169903 41609-41618. Max. coverage (+): 0. Max coverage (-): 0

Region: NODE\_34239\_length\_42430\_cov\_28.169903 41619-41627. Max. coverage (+): 0. Max coverage (-): 0

Region: NODE\_34239\_length\_42430\_cov\_28.169903 41628-41636. Max. coverage (+): 0. Max coverage (-): 0

Region: NODE\_34239\_length\_42430\_cov\_28.169903 41637-41645. Max. coverage (+): 0. Max coverage (-): 0

Region: NODE\_34239\_length\_42430\_cov\_28.169903 41646-41654. Max. coverage (+): 0. Max coverage (-): 0

Region: NODE\_34239\_length\_42430\_cov\_28.169903 41655-41664. Max. coverage (+): 0. Max coverage (-): 0

Region: NODE\_34239\_length\_42430\_cov\_28.169903 41665-41673. Max. coverage (+): 0. Max coverage (-): 0

Region: NODE\_34239\_length\_42430\_cov\_28.169903 41674-41682. Max. coverage (+): 0. Max coverage (-): 0

Region: NODE\_34239\_length\_42430\_cov\_28.169903 41683-41691. Max. coverage (+): 0. Max coverage (-): 0

Region: NODE\_34239\_length\_42430\_cov\_28.169903 41692-41700. Max. coverage (+): 0. Max coverage (-): 0

Region: NODE\_34239\_length\_42430\_cov\_28.169903 41701-41710. Max. coverage (+): 0. Max coverage (-): 0

Region: NODE\_34239\_length\_42430\_cov\_28.169903 41711-41719. Max. coverage (+): 0. Max coverage (-): 0

Region: NODE\_34239\_length\_42430\_cov\_28.169903 41720-41728. Max. coverage (+): 0. Max coverage (-): 0

Region: NODE\_34239\_length\_42430\_cov\_28.169903 41729-41737. Max. coverage (+): 0. Max coverage (-): 0

Region: NODE\_34239\_length\_42430\_cov\_28.169903 41738-41746. Max. coverage (+): 0. Max coverage (-): 0

Region: NODE\_34239\_length\_42430\_cov\_28.169903 41747-41756. Max. coverage (+): 0. Max coverage (-): 0

Region: NODE\_34239\_length\_42430\_cov\_28.169903 41757-41765. Max. coverage (+): 0. Max coverage (-): 0

Region: NODE\_34239\_length\_42430\_cov\_28.169903 41766-41774. Max. coverage (+): 0. Max coverage (-): 0

Region: NODE\_34239\_length\_42430\_cov\_28.169903 41775-41783. Max. coverage (+): 0. Max coverage (-): 0

Region: NODE\_34239\_length\_42430\_cov\_28.169903 41784-41792. Max. coverage (+): 0. Max coverage (-): 0

Region: NODE\_34239\_length\_42430\_cov\_28.169903 41793-41802. Max. coverage (+): 0. Max coverage (-): 0

Region: NODE\_34239\_length\_42430\_cov\_28.169903 41803-41811. Max. coverage (+): 0. Max coverage (-): 0

Region: NODE\_34239\_length\_42430\_cov\_28.169903 41812-41820. Max. coverage (+): 0. Max coverage (-): 0

Region: NODE\_34239\_length\_42430\_cov\_28.169903 41821-41829. Max. coverage (+): 0. Max coverage (-): 0

Region: NODE\_34239\_length\_42430\_cov\_28.169903 41830-41838. Max. coverage (+): 0. Max coverage (-): 0

Region: NODE\_34239\_length\_42430\_cov\_28.169903 41839-41848. Max. coverage (+): 0. Max coverage (-): 0

Region: NODE\_34239\_length\_42430\_cov\_28.169903 41849-41857. Max. coverage (+): 0. Max coverage (-): 0

Region: NODE\_34239\_length\_42430\_cov\_28.169903 41858-41866. Max. coverage (+): 0. Max coverage (-): 0

Region: NODE\_34239\_length\_42430\_cov\_28.169903 41867-41875. Max. coverage (+): 0. Max coverage (-): 0

Region: NODE\_34239\_length\_42430\_cov\_28.169903 41876-41884. Max. coverage (+): 0. Max coverage (-): 0

Region: NODE\_34239\_length\_42430\_cov\_28.169903 41885-41894. Max. coverage (+): 0. Max coverage (-): 0

Region: NODE\_34239\_length\_42430\_cov\_28.169903 41895-41903. Max. coverage (+): 0. Max coverage (-): 0

Region: NODE\_34239\_length\_42430\_cov\_28.169903 41904-41912. Max. coverage (+): 0. Max coverage (-): 0

Region: NODE\_34239\_length\_42430\_cov\_28.169903 41913-41921. Max. coverage (+): 0. Max coverage (-): 0

Region: NODE\_34239\_length\_42430\_cov\_28.169903 41922-41930. Max. coverage (+): 0. Max coverage (-): 0

Region: NODE\_34239\_length\_42430\_cov\_28.169903 41931-41940. Max. coverage (+): 0. Max coverage (-): 0

Region: NODE\_34239\_length\_42430\_cov\_28.169903 41941-41949. Max. coverage (+): 0. Max coverage (-): 0

Region: NODE\_34239\_length\_42430\_cov\_28.169903 41950-41958. Max. coverage (+): 0. Max coverage (-): 0

Region: NODE\_34239\_length\_42430\_cov\_28.169903 41959-41967. Max. coverage (+): 0. Max coverage (-): 0

Region: NODE\_34239\_length\_42430\_cov\_28.169903 41968-41976. Max. coverage (+): 0.08. Max coverage (-): 0

Region: NODE\_34239\_length\_42430\_cov\_28.169903 41977-41986. Max. coverage (+): 0.08. Max coverage (-): 0

Region: NODE\_34239\_length\_42430\_cov\_28.169903 41987-41995. Max. coverage (+): 0. Max coverage (-): 0

Region: NODE\_34239\_length\_42430\_cov\_28.169903 41996-. Max. coverage (+): 0. Max coverage (-): 0

RepeatMasker Color Code

**+**

100-98% Identity

<98-95% Identity

<95-90% Identity

<90-85% Identity

<85-80% Identity

<80-75% Identity

<75-70% Identity

<70% Identity

**-**

Gene Set Color Code

**+**

Gene

Pseudogene

Other

**-**

Topology/Coverage Color Code

Coverage Plus Strand

Coverage Minus Strand

Mainstrand: Plus

Mainstrand: Minus

Complementary Strand

Flanking Region  
(if option -flank >0)

Gene Set Annotation  

**1. unknown (unknownunknown) Tr:unknown**: 38319-40308 (+)  
**2. unknown (unknownunknown) Tr:unknown**: 40735-40893 (+)  
**3. unknown (unknownunknown) Tr:unknown**: 41210-41314 (+)  
**4. unknown (unknownunknown) Tr:unknown**: 41457-41516 (+)  
**5. unknown (unknownunknown) Tr:unknown**: 41873-42493 (+)  
**6. unknown (unknownunknown) Tr:unknown UTR**: 38550-40308 (+)  
**7. unknown (unknownunknown) Tr:unknown UTR**: 40735-40893 (+)  
**8. unknown (unknownunknown) Tr:unknown UTR**: 41210-41314 (+)  
**9. unknown (unknownunknown) Tr:unknown UTR**: 41457-41516 (+)  
**10. unknown (unknownunknown) Tr:unknown UTR**: 41873-42493 (+)

  
RepeatMasker Annotation  

**1. (TCTTT)n**: 40684-40727 (+), Divergence to consensus: 26.4%

  
Transcription Factor Binding Sites  

**RHOXF1** (Sequence: AGCTCA (-): 37937)  
**RHOXF1** (Sequence: AGATTA (-): 38189)  
**RHOXF1** (Sequence: AGATCA (-): 38464)  
**RHOXF1** (Sequence: GGATTA (-): 38781)  
**RHOXF1** (Sequence: GGATTA (-): 39634)  
**RHOXF1** (Sequence: AGATTA (-): 39745)  
**RHOXF1** (Sequence: GGCTCA (-): 40809)  
**RHOXF1** (Sequence: AGCTCA (-): 40818)  
**RHOXF1** (Sequence: AGATCA (-): 40856)  
**RHOXF1** (Sequence: AGCTCA (-): 40859)  
**RHOXF1** (Sequence: AGATCA (-): 41455)  
**RHOXF1** (Sequence: AGATCA (-): 41723)  
**RHOXF1** (Sequence: AGCTCA (-): 41811)  
**RHOXF1** (Sequence: TGATCT (+): 38867)  
**RHOXF1** (Sequence: TGAGCT (+): 39034)  
**RHOXF1** (Sequence: TGAGCT (+): 39347)  
**RHOXF1** (Sequence: TGAGCT (+): 39897)  
**RHOXF1** (Sequence: TGATCC (+): 40450)  
**RHOXF1** (Sequence: TAAGCT (+): 40544)  
**RHOXF1** (Sequence: TGAGCT (+): 41053)  
**RHOXF1** (Sequence: TAATCC (+): 41786)  
**RHOXF1** (Sequence: TGAGCC (+): 41990)  
**FOXO3\_hsa** (Sequence: GTAAACAT (+): 37423)  
**FOXO3\_hsa** (Sequence: GTAAACAT (+): 37758)  
**FOXO3\_hsa** (Sequence: GTAAACAA (+): 40667)  
**SOX9** (Sequence: AACAATGA (-): 41770)  
**FOXP1** (Sequence: GTAAACA (+): 37423)  
**FOXP1** (Sequence: GTAAACA (+): 37758)  
**FOXP1** (Sequence: GTAAACA (+): 40309)  
**FOXP1** (Sequence: GTAAACA (+): 40667)  
**FOXO3\_mmu** (Sequence: TGTTTAGA (-): 39361)  
**FOXO3\_mmu** (Sequence: TGTTTACA (-): 39451)  
**FOXO3\_mmu** (Sequence: TGTTTACA (-): 41384)  
**FOXO3\_mmu** (Sequence: TGTTTTCA (-): 41566)  
**Sox5** (Sequence: ATTGTT (+): 37400)  
**Sox5** (Sequence: ATTGTT (+): 39542)  
**Sox5** (Sequence: ATTGTT (+): 41589)  
**Sox5** (Sequence: ATTGTT (+): 41711)  
**FIGLA** (Sequence: AACACCTGTA (-): 37923)  
**SOX9** (Sequence: CCATTGTT (+): 37398)  
**FOXO3\_mmu** (Sequence: TGTAAACA (+): 37422)  
**FOXO3\_mmu** (Sequence: TGTAAACA (+): 37757)  
**FOXO3\_mmu** (Sequence: GGTAAACA (+): 40308)  
**FOXO1** (Sequence: GTAAACAAC (-): 40667)  
**FOXP1** (Sequence: TGTTTAC (-): 37807)  
**FOXP1** (Sequence: TGTTTAC (-): 39451)  
**FOXP1** (Sequence: TGTTTAC (-): 41384)  
**Rhox11** (Sequence: TGCTGTAAA (+): 37419)  
**Rhox11** (Sequence: CGGTGTTTA (+): 37888)  
**Gata4** (Sequence: AGATAAC (-): 39173)  
**Sox5** (Sequence: AACAAT (-): 39990)  
**Sox5** (Sequence: AACAAT (-): 41770)  
**POU2F1** (Sequence: TATTTAAAT (+): 37720)  
**POU2F1** (Sequence: TATTCAAAT (+): 39592)  
**POU5F1** (Sequence: ATGCAAA (+): 39098)
