# Supplementary material for: First characterization of PIWI-interacting RNA clusters in a cichlid fish with a B chromosome
Source: BMC Biol. 2022 Sep 21;20:204. doi: 10.1186/s12915-022-01403-2 (PMC9490952; doi:10.1186/s12915-022-01403-2)
Supplement: Supplementary file 1 — Additional file 1. Zipped folder with fasta and interactive html piRNA cluster information for the A. latifasciata genome. The nomenclature is as follows: number-pirna-cluster_sex_B-presence (f, female; m, male; 0b, without B chromosome; 1b, with B chromosome). [file 12915_2022_1403_MOESM1_ESM.zip › 133_m1b.html]

piRNA cluster 133\_m1b 12


Predicted piRNA cluster no. 133\_m1b
  

Show proTRAC run info
Hide proTRAC run info

/\  
                \_\_\_\_\_\_\_\_\_\_\_\_\_\_\_\_\_\_\_\_\_\_\_/\\_\_\_ /  \\_\_\_\_\_\_\_  
               I                      /  \  /    \      I  
               I     pro             /    \/      \     I  
               I        TRAC        /               \   I  
               I   \_\_\_\_\_\_\_\_\_\_\_\_\_\_\_\_/\_\_\_\_\_\_\_\_\_\_\_\_\_\_\_\_\_\\_ I  
               I   \              /                     I  
               I    \            /                      I  
               I     \  /\      /       V.2.4.2         I  
               I      \/  \    /                        I  
               I\_\_\_\_\_\_\_\_\_\_\_\  /\_\_\_\_\_\_\_\_\_\_\_\_\_\_\_\_\_\_\_\_\_\_\_\_\_I  
                            \/  
  
  
================================= proTRAC ====================================  
VERSION: .......... 2.4.2  
LAST MODIFIED: .... 11. May 2018  
  
Please cite:  
Rosenkranz D, Zischler H. proTRAC - a software for probabilistic piRNA cluster  
detection, visualization and analysis. 2012. BMC Bioinformatics 13:5.  
  
  
Contact:  
David Rosenkranz  
Institute of Organismic and Molecular Evolutionary Biology  
Dept. Anthropology, small RNA group  
Johannes Gutenberg University Mainz  
email: rosenkranz@uni-mainz.de  
  
You can find the latest proTRAC version at:  
http://sourceforge.net/projects/protrac/files  
http://www.smallRNAgroup-mainz.de/software  
==============================================================================  
  
PARAMETERS:  
Map file: ...............piwi-machos-1B.fa-collapse.map  
Genome file: ............../../../0B\_ala\_genome.fa  
RepeatMasker annotation: Alatifasciata-all0B-maryan-v2.fa\_corrected.out  
GeneSet:................./guest-storage/Data/annotation/Alatifasciata\_all0B\_maryan-v2\_out2017.gff  
  
Significant (p<=0.01) hit density will be calculated based  
on observed hit distribution.  
  
Sliding window size: ........................................ 5000 bp  
Sliding window increament: .................................. 1000 bp  
Normalize each hit by number of genomic hits: ............... yes  
Normalize each hit by number of sequence reads: ............. yes  
Normalize values (-> per million mapped reads): ............. yes  
Min. fraction of hits with 1T(U) or 10A: .................... 0.75  
Alternatively: Min. fraction of hits with 1T(U) and 10A: .... 0.5  
Min. fraction of hits with typical piRNA length: ............ 0.75  
Typical piRNA length: ....................................... 24-32 nt  
Min. size of a piRNA cluster: ............................... 1000 bp.  
Min. number of hits (absolute): ............................. 0  
Min. number of hits (normalized): ........................... 0  
Min. fraction of hits on the mainstrand: .................... 0.75  
Top fraction of mapped sequences (in terms of read counts): . 1%  
Top fraction accounts for max. n% of sequence reads: ........ 90%  
Min. fraction of hits on each arm of a bidirectional cluster: 0.05  
Output html file for each cluster: .......................... yes  
Output a summary table: ..................................... yes  
Output a FASTA file for each cluster (piRNA sequences): ..... yes  
Output a FASTA file comprising cluster sequences: ........... yes  
Output a GTF file for predicted piRNA clusters: ..............yes  
Search DNA motifs in clusters: .............................. yes  
Output flanking sequences: +/- .............................. 0 bp  
Output ~.pTi file: .......................................... no  
==============================================================================  
  
  
Genome size (without gaps): ............ 758543724 bp  
Gaps (N/X/-): .......................... 417479 bp  
Mapped reads: .......................... 26973943  
Non-identical sequences: ............... 6209225  
Genomic hits: .......................... 48438990  
Significant densitiy of mapped reads: .. 821.144211136946 reads/kb

Show proTRAC cluster info
Hide proTRAC cluster info

|  |  |
| --- | --- |
| Location | NODE\_34239\_length\_42430\_cov\_28.169903 |
| Coordinates | 36149-39395 |
| Size [bp] | 3247 |
| Sequence hit loci | 120 |
| Mapped reads (normalized) | 35735.5 |
| Mapped reads (normalized) per kb | 11005.7 |
| Normalized reads with 1T (1U) | 99.4% |
| Normalized reads with 10A | 0% |
| Normalized reads with length 24-32 nt | 100% |
| Normalized reads on the main strand(s) | 100% |
| Predicted directionality | mono:plus |

100%

0%

1T (1U)  
reads

10A reads

24-32 nt  
reads

reads on mainstrand

**Either the amount of reads with 1T (1U) OR 10A has to exceed 75% (set with option: -1Tor10A)  
Alternatively the amount of reads with 1T (1U) AND 10A has to exceed 50% (set with option: -1Tand10A)  
Minimum amount of reads with preferred size is 75% (set with option: -pisize)  
Minimum amount of reads on the main strand(s) is 75% (set with option: -clstrand)**

Show read coverage
Hide read coverage

WHAT DO I SEE HERE?  
This chart shows the location of mapped sequence reads within a predicted piRNA cluster. The color refers to the number of genomic hits produced by the sequence read in question. A dark red bar indicates that this sequence read produces many other hits elsewhere in the genome. Many adjacent red or yellow bars can indicate the presence of a multi-copy element such as transposons or rRNA genes. A dark green bar indicates that this sequence read maps uniquely to this locus.

1 hit

2-5 hits

6-10 hits

11-20 hits

21-50 hits

51-100 hits

> 100 hits

NODE\_34239\_length\_42430\_cov\_28.169903

36149

39395

Gene Set

RepeatMasker

Mapped  
Reads

733.95

plus strand

minus strand

733.95

Region: NODE\_34239\_length\_42430\_cov\_28.169903 1079-36152. Max. coverage (+): 0.04. Max coverage (-): 0

Region: NODE\_34239\_length\_42430\_cov\_28.169903 36153-36158. Max. coverage (+): 0.04. Max coverage (-): 0

Region: NODE\_34239\_length\_42430\_cov\_28.169903 36159-36165. Max. coverage (+): 0. Max coverage (-): 0

Region: NODE\_34239\_length\_42430\_cov\_28.169903 36166-36171. Max. coverage (+): 0. Max coverage (-): 0

Region: NODE\_34239\_length\_42430\_cov\_28.169903 36172-36178. Max. coverage (+): 0. Max coverage (-): 0

Region: NODE\_34239\_length\_42430\_cov\_28.169903 36179-36184. Max. coverage (+): 0. Max coverage (-): 0

Region: NODE\_34239\_length\_42430\_cov\_28.169903 36185-36191. Max. coverage (+): 0. Max coverage (-): 0

Region: NODE\_34239\_length\_42430\_cov\_28.169903 36192-36197. Max. coverage (+): 0. Max coverage (-): 0

Region: NODE\_34239\_length\_42430\_cov\_28.169903 36198-36204. Max. coverage (+): 0. Max coverage (-): 0

Region: NODE\_34239\_length\_42430\_cov\_28.169903 36205-36210. Max. coverage (+): 0. Max coverage (-): 0

Region: NODE\_34239\_length\_42430\_cov\_28.169903 36211-36217. Max. coverage (+): 0. Max coverage (-): 0

Region: NODE\_34239\_length\_42430\_cov\_28.169903 36218-36223. Max. coverage (+): 0. Max coverage (-): 0

Region: NODE\_34239\_length\_42430\_cov\_28.169903 36224-36230. Max. coverage (+): 0. Max coverage (-): 0

Region: NODE\_34239\_length\_42430\_cov\_28.169903 36231-36236. Max. coverage (+): 0. Max coverage (-): 0

Region: NODE\_34239\_length\_42430\_cov\_28.169903 36237-36243. Max. coverage (+): 0. Max coverage (-): 0

Region: NODE\_34239\_length\_42430\_cov\_28.169903 36244-36249. Max. coverage (+): 0. Max coverage (-): 0

Region: NODE\_34239\_length\_42430\_cov\_28.169903 36250-36256. Max. coverage (+): 0. Max coverage (-): 0

Region: NODE\_34239\_length\_42430\_cov\_28.169903 36257-36262. Max. coverage (+): 0. Max coverage (-): 0

Region: NODE\_34239\_length\_42430\_cov\_28.169903 36263-36269. Max. coverage (+): 0. Max coverage (-): 0

Region: NODE\_34239\_length\_42430\_cov\_28.169903 36270-36275. Max. coverage (+): 0. Max coverage (-): 0

Region: NODE\_34239\_length\_42430\_cov\_28.169903 36276-36282. Max. coverage (+): 0. Max coverage (-): 0

Region: NODE\_34239\_length\_42430\_cov\_28.169903 36283-36288. Max. coverage (+): 0. Max coverage (-): 0

Region: NODE\_34239\_length\_42430\_cov\_28.169903 36289-36295. Max. coverage (+): 0. Max coverage (-): 0

Region: NODE\_34239\_length\_42430\_cov\_28.169903 36296-36301. Max. coverage (+): 0. Max coverage (-): 0

Region: NODE\_34239\_length\_42430\_cov\_28.169903 36302-36308. Max. coverage (+): 0. Max coverage (-): 0

Region: NODE\_34239\_length\_42430\_cov\_28.169903 36309-36314. Max. coverage (+): 0. Max coverage (-): 0

Region: NODE\_34239\_length\_42430\_cov\_28.169903 36315-36321. Max. coverage (+): 0. Max coverage (-): 0

Region: NODE\_34239\_length\_42430\_cov\_28.169903 36322-36327. Max. coverage (+): 0. Max coverage (-): 0

Region: NODE\_34239\_length\_42430\_cov\_28.169903 36328-36334. Max. coverage (+): 0. Max coverage (-): 0

Region: NODE\_34239\_length\_42430\_cov\_28.169903 36335-36340. Max. coverage (+): 0. Max coverage (-): 0

Region: NODE\_34239\_length\_42430\_cov\_28.169903 36341-36347. Max. coverage (+): 0. Max coverage (-): 0

Region: NODE\_34239\_length\_42430\_cov\_28.169903 36348-36353. Max. coverage (+): 0. Max coverage (-): 0

Region: NODE\_34239\_length\_42430\_cov\_28.169903 36354-36360. Max. coverage (+): 0. Max coverage (-): 0

Region: NODE\_34239\_length\_42430\_cov\_28.169903 36361-36366. Max. coverage (+): 0. Max coverage (-): 0

Region: NODE\_34239\_length\_42430\_cov\_28.169903 36367-36373. Max. coverage (+): 0. Max coverage (-): 0

Region: NODE\_34239\_length\_42430\_cov\_28.169903 36374-36379. Max. coverage (+): 0. Max coverage (-): 0

Region: NODE\_34239\_length\_42430\_cov\_28.169903 36380-36386. Max. coverage (+): 0. Max coverage (-): 0

Region: NODE\_34239\_length\_42430\_cov\_28.169903 36387-36392. Max. coverage (+): 0. Max coverage (-): 0

Region: NODE\_34239\_length\_42430\_cov\_28.169903 36393-36399. Max. coverage (+): 0. Max coverage (-): 0

Region: NODE\_34239\_length\_42430\_cov\_28.169903 36400-36405. Max. coverage (+): 0. Max coverage (-): 0

Region: NODE\_34239\_length\_42430\_cov\_28.169903 36406-36412. Max. coverage (+): 0. Max coverage (-): 0

Region: NODE\_34239\_length\_42430\_cov\_28.169903 36413-36418. Max. coverage (+): 0. Max coverage (-): 0

Region: NODE\_34239\_length\_42430\_cov\_28.169903 36419-36424. Max. coverage (+): 0. Max coverage (-): 0

Region: NODE\_34239\_length\_42430\_cov\_28.169903 36425-36431. Max. coverage (+): 0. Max coverage (-): 0

Region: NODE\_34239\_length\_42430\_cov\_28.169903 36432-36437. Max. coverage (+): 0. Max coverage (-): 0

Region: NODE\_34239\_length\_42430\_cov\_28.169903 36438-36444. Max. coverage (+): 0. Max coverage (-): 0

Region: NODE\_34239\_length\_42430\_cov\_28.169903 36445-36450. Max. coverage (+): 0. Max coverage (-): 0

Region: NODE\_34239\_length\_42430\_cov\_28.169903 36451-36457. Max. coverage (+): 0. Max coverage (-): 0

Region: NODE\_34239\_length\_42430\_cov\_28.169903 36458-36463. Max. coverage (+): 0. Max coverage (-): 0

Region: NODE\_34239\_length\_42430\_cov\_28.169903 36464-36470. Max. coverage (+): 0. Max coverage (-): 0

Region: NODE\_34239\_length\_42430\_cov\_28.169903 36471-36476. Max. coverage (+): 0. Max coverage (-): 0

Region: NODE\_34239\_length\_42430\_cov\_28.169903 36477-36483. Max. coverage (+): 0. Max coverage (-): 0

Region: NODE\_34239\_length\_42430\_cov\_28.169903 36484-36489. Max. coverage (+): 0. Max coverage (-): 0

Region: NODE\_34239\_length\_42430\_cov\_28.169903 36490-36496. Max. coverage (+): 0. Max coverage (-): 0

Region: NODE\_34239\_length\_42430\_cov\_28.169903 36497-36502. Max. coverage (+): 0. Max coverage (-): 0

Region: NODE\_34239\_length\_42430\_cov\_28.169903 36503-36509. Max. coverage (+): 0. Max coverage (-): 0

Region: NODE\_34239\_length\_42430\_cov\_28.169903 36510-36515. Max. coverage (+): 0. Max coverage (-): 0

Region: NODE\_34239\_length\_42430\_cov\_28.169903 36516-36522. Max. coverage (+): 0. Max coverage (-): 0

Region: NODE\_34239\_length\_42430\_cov\_28.169903 36523-36528. Max. coverage (+): 0. Max coverage (-): 0

Region: NODE\_34239\_length\_42430\_cov\_28.169903 36529-36535. Max. coverage (+): 0. Max coverage (-): 0

Region: NODE\_34239\_length\_42430\_cov\_28.169903 36536-36541. Max. coverage (+): 0. Max coverage (-): 0

Region: NODE\_34239\_length\_42430\_cov\_28.169903 36542-36548. Max. coverage (+): 0. Max coverage (-): 0

Region: NODE\_34239\_length\_42430\_cov\_28.169903 36549-36554. Max. coverage (+): 0. Max coverage (-): 0

Region: NODE\_34239\_length\_42430\_cov\_28.169903 36555-36561. Max. coverage (+): 0. Max coverage (-): 0

Region: NODE\_34239\_length\_42430\_cov\_28.169903 36562-36567. Max. coverage (+): 0. Max coverage (-): 0

Region: NODE\_34239\_length\_42430\_cov\_28.169903 36568-36574. Max. coverage (+): 0. Max coverage (-): 0

Region: NODE\_34239\_length\_42430\_cov\_28.169903 36575-36580. Max. coverage (+): 0. Max coverage (-): 0

Region: NODE\_34239\_length\_42430\_cov\_28.169903 36581-36587. Max. coverage (+): 0. Max coverage (-): 0

Region: NODE\_34239\_length\_42430\_cov\_28.169903 36588-36593. Max. coverage (+): 0. Max coverage (-): 0

Region: NODE\_34239\_length\_42430\_cov\_28.169903 36594-36600. Max. coverage (+): 0. Max coverage (-): 0

Region: NODE\_34239\_length\_42430\_cov\_28.169903 36601-36606. Max. coverage (+): 0. Max coverage (-): 0

Region: NODE\_34239\_length\_42430\_cov\_28.169903 36607-36613. Max. coverage (+): 0. Max coverage (-): 0

Region: NODE\_34239\_length\_42430\_cov\_28.169903 36614-36619. Max. coverage (+): 0. Max coverage (-): 0

Region: NODE\_34239\_length\_42430\_cov\_28.169903 36620-36626. Max. coverage (+): 0. Max coverage (-): 0

Region: NODE\_34239\_length\_42430\_cov\_28.169903 36627-36632. Max. coverage (+): 0. Max coverage (-): 0

Region: NODE\_34239\_length\_42430\_cov\_28.169903 36633-36639. Max. coverage (+): 0. Max coverage (-): 0

Region: NODE\_34239\_length\_42430\_cov\_28.169903 36640-36645. Max. coverage (+): 0. Max coverage (-): 0

Region: NODE\_34239\_length\_42430\_cov\_28.169903 36646-36652. Max. coverage (+): 0. Max coverage (-): 0

Region: NODE\_34239\_length\_42430\_cov\_28.169903 36653-36658. Max. coverage (+): 0. Max coverage (-): 0

Region: NODE\_34239\_length\_42430\_cov\_28.169903 36659-36665. Max. coverage (+): 0. Max coverage (-): 0

Region: NODE\_34239\_length\_42430\_cov\_28.169903 36666-36671. Max. coverage (+): 0. Max coverage (-): 0

Region: NODE\_34239\_length\_42430\_cov\_28.169903 36672-36678. Max. coverage (+): 0. Max coverage (-): 0

Region: NODE\_34239\_length\_42430\_cov\_28.169903 36679-36684. Max. coverage (+): 0. Max coverage (-): 0

Region: NODE\_34239\_length\_42430\_cov\_28.169903 36685-36691. Max. coverage (+): 0. Max coverage (-): 0

Region: NODE\_34239\_length\_42430\_cov\_28.169903 36692-36697. Max. coverage (+): 0. Max coverage (-): 0

Region: NODE\_34239\_length\_42430\_cov\_28.169903 36698-36704. Max. coverage (+): 0. Max coverage (-): 0

Region: NODE\_34239\_length\_42430\_cov\_28.169903 36705-36710. Max. coverage (+): 0. Max coverage (-): 0

Region: NODE\_34239\_length\_42430\_cov\_28.169903 36711-36717. Max. coverage (+): 0. Max coverage (-): 0

Region: NODE\_34239\_length\_42430\_cov\_28.169903 36718-36723. Max. coverage (+): 0. Max coverage (-): 0

Region: NODE\_34239\_length\_42430\_cov\_28.169903 36724-36730. Max. coverage (+): 0. Max coverage (-): 0

Region: NODE\_34239\_length\_42430\_cov\_28.169903 36731-36736. Max. coverage (+): 0. Max coverage (-): 0

Region: NODE\_34239\_length\_42430\_cov\_28.169903 36737-36743. Max. coverage (+): 0. Max coverage (-): 0

Region: NODE\_34239\_length\_42430\_cov\_28.169903 36744-36749. Max. coverage (+): 0. Max coverage (-): 0

Region: NODE\_34239\_length\_42430\_cov\_28.169903 36750-36756. Max. coverage (+): 0. Max coverage (-): 0

Region: NODE\_34239\_length\_42430\_cov\_28.169903 36757-36762. Max. coverage (+): 0. Max coverage (-): 0

Region: NODE\_34239\_length\_42430\_cov\_28.169903 36763-36769. Max. coverage (+): 0. Max coverage (-): 0

Region: NODE\_34239\_length\_42430\_cov\_28.169903 36770-36775. Max. coverage (+): 0. Max coverage (-): 0

Region: NODE\_34239\_length\_42430\_cov\_28.169903 36776-36782. Max. coverage (+): 0. Max coverage (-): 0

Region: NODE\_34239\_length\_42430\_cov\_28.169903 36783-36788. Max. coverage (+): 0. Max coverage (-): 0

Region: NODE\_34239\_length\_42430\_cov\_28.169903 36789-36795. Max. coverage (+): 0. Max coverage (-): 0

Region: NODE\_34239\_length\_42430\_cov\_28.169903 36796-36801. Max. coverage (+): 0. Max coverage (-): 0

Region: NODE\_34239\_length\_42430\_cov\_28.169903 36802-36808. Max. coverage (+): 0. Max coverage (-): 0

Region: NODE\_34239\_length\_42430\_cov\_28.169903 36809-36814. Max. coverage (+): 0. Max coverage (-): 0

Region: NODE\_34239\_length\_42430\_cov\_28.169903 36815-36821. Max. coverage (+): 0. Max coverage (-): 0

Region: NODE\_34239\_length\_42430\_cov\_28.169903 36822-36827. Max. coverage (+): 0. Max coverage (-): 0

Region: NODE\_34239\_length\_42430\_cov\_28.169903 36828-36834. Max. coverage (+): 0. Max coverage (-): 0

Region: NODE\_34239\_length\_42430\_cov\_28.169903 36835-36840. Max. coverage (+): 0. Max coverage (-): 0

Region: NODE\_34239\_length\_42430\_cov\_28.169903 36841-36847. Max. coverage (+): 0. Max coverage (-): 0

Region: NODE\_34239\_length\_42430\_cov\_28.169903 36848-36853. Max. coverage (+): 0. Max coverage (-): 0

Region: NODE\_34239\_length\_42430\_cov\_28.169903 36854-36860. Max. coverage (+): 0. Max coverage (-): 0

Region: NODE\_34239\_length\_42430\_cov\_28.169903 36861-36866. Max. coverage (+): 0. Max coverage (-): 0

Region: NODE\_34239\_length\_42430\_cov\_28.169903 36867-36873. Max. coverage (+): 0. Max coverage (-): 0

Region: NODE\_34239\_length\_42430\_cov\_28.169903 36874-36879. Max. coverage (+): 0. Max coverage (-): 0

Region: NODE\_34239\_length\_42430\_cov\_28.169903 36880-36886. Max. coverage (+): 0. Max coverage (-): 0

Region: NODE\_34239\_length\_42430\_cov\_28.169903 36887-36892. Max. coverage (+): 0. Max coverage (-): 0

Region: NODE\_34239\_length\_42430\_cov\_28.169903 36893-36899. Max. coverage (+): 0. Max coverage (-): 0

Region: NODE\_34239\_length\_42430\_cov\_28.169903 36900-36905. Max. coverage (+): 0. Max coverage (-): 0

Region: NODE\_34239\_length\_42430\_cov\_28.169903 36906-36912. Max. coverage (+): 0. Max coverage (-): 0

Region: NODE\_34239\_length\_42430\_cov\_28.169903 36913-36918. Max. coverage (+): 0. Max coverage (-): 0

Region: NODE\_34239\_length\_42430\_cov\_28.169903 36919-36925. Max. coverage (+): 0. Max coverage (-): 0

Region: NODE\_34239\_length\_42430\_cov\_28.169903 36926-36931. Max. coverage (+): 0. Max coverage (-): 0

Region: NODE\_34239\_length\_42430\_cov\_28.169903 36932-36938. Max. coverage (+): 0. Max coverage (-): 0

Region: NODE\_34239\_length\_42430\_cov\_28.169903 36939-36944. Max. coverage (+): 0. Max coverage (-): 0

Region: NODE\_34239\_length\_42430\_cov\_28.169903 36945-36951. Max. coverage (+): 0. Max coverage (-): 0

Region: NODE\_34239\_length\_42430\_cov\_28.169903 36952-36957. Max. coverage (+): 0. Max coverage (-): 0

Region: NODE\_34239\_length\_42430\_cov\_28.169903 36958-36963. Max. coverage (+): 0. Max coverage (-): 0

Region: NODE\_34239\_length\_42430\_cov\_28.169903 36964-36970. Max. coverage (+): 0. Max coverage (-): 0

Region: NODE\_34239\_length\_42430\_cov\_28.169903 36971-36976. Max. coverage (+): 0. Max coverage (-): 0

Region: NODE\_34239\_length\_42430\_cov\_28.169903 36977-36983. Max. coverage (+): 0. Max coverage (-): 0

Region: NODE\_34239\_length\_42430\_cov\_28.169903 36984-36989. Max. coverage (+): 0. Max coverage (-): 0

Region: NODE\_34239\_length\_42430\_cov\_28.169903 36990-36996. Max. coverage (+): 0. Max coverage (-): 0

Region: NODE\_34239\_length\_42430\_cov\_28.169903 36997-37002. Max. coverage (+): 0. Max coverage (-): 0

Region: NODE\_34239\_length\_42430\_cov\_28.169903 37003-37009. Max. coverage (+): 0. Max coverage (-): 0

Region: NODE\_34239\_length\_42430\_cov\_28.169903 37010-37015. Max. coverage (+): 0. Max coverage (-): 0

Region: NODE\_34239\_length\_42430\_cov\_28.169903 37016-37022. Max. coverage (+): 0. Max coverage (-): 0

Region: NODE\_34239\_length\_42430\_cov\_28.169903 37023-37028. Max. coverage (+): 0. Max coverage (-): 0

Region: NODE\_34239\_length\_42430\_cov\_28.169903 37029-37035. Max. coverage (+): 0. Max coverage (-): 0

Region: NODE\_34239\_length\_42430\_cov\_28.169903 37036-37041. Max. coverage (+): 0. Max coverage (-): 0

Region: NODE\_34239\_length\_42430\_cov\_28.169903 37042-37048. Max. coverage (+): 0. Max coverage (-): 0

Region: NODE\_34239\_length\_42430\_cov\_28.169903 37049-37054. Max. coverage (+): 0. Max coverage (-): 0

Region: NODE\_34239\_length\_42430\_cov\_28.169903 37055-37061. Max. coverage (+): 0. Max coverage (-): 0

Region: NODE\_34239\_length\_42430\_cov\_28.169903 37062-37067. Max. coverage (+): 0. Max coverage (-): 0

Region: NODE\_34239\_length\_42430\_cov\_28.169903 37068-37074. Max. coverage (+): 0. Max coverage (-): 0

Region: NODE\_34239\_length\_42430\_cov\_28.169903 37075-37080. Max. coverage (+): 0. Max coverage (-): 0

Region: NODE\_34239\_length\_42430\_cov\_28.169903 37081-37087. Max. coverage (+): 0. Max coverage (-): 0

Region: NODE\_34239\_length\_42430\_cov\_28.169903 37088-37093. Max. coverage (+): 0. Max coverage (-): 0

Region: NODE\_34239\_length\_42430\_cov\_28.169903 37094-37100. Max. coverage (+): 0. Max coverage (-): 0

Region: NODE\_34239\_length\_42430\_cov\_28.169903 37101-37106. Max. coverage (+): 0. Max coverage (-): 0

Region: NODE\_34239\_length\_42430\_cov\_28.169903 37107-37113. Max. coverage (+): 0. Max coverage (-): 0

Region: NODE\_34239\_length\_42430\_cov\_28.169903 37114-37119. Max. coverage (+): 0. Max coverage (-): 0

Region: NODE\_34239\_length\_42430\_cov\_28.169903 37120-37126. Max. coverage (+): 0. Max coverage (-): 0

Region: NODE\_34239\_length\_42430\_cov\_28.169903 37127-37132. Max. coverage (+): 0. Max coverage (-): 0

Region: NODE\_34239\_length\_42430\_cov\_28.169903 37133-37139. Max. coverage (+): 0. Max coverage (-): 0

Region: NODE\_34239\_length\_42430\_cov\_28.169903 37140-37145. Max. coverage (+): 0. Max coverage (-): 0

Region: NODE\_34239\_length\_42430\_cov\_28.169903 37146-37152. Max. coverage (+): 0. Max coverage (-): 0

Region: NODE\_34239\_length\_42430\_cov\_28.169903 37153-37158. Max. coverage (+): 0. Max coverage (-): 0

Region: NODE\_34239\_length\_42430\_cov\_28.169903 37159-37165. Max. coverage (+): 0. Max coverage (-): 0

Region: NODE\_34239\_length\_42430\_cov\_28.169903 37166-37171. Max. coverage (+): 0. Max coverage (-): 0

Region: NODE\_34239\_length\_42430\_cov\_28.169903 37172-37178. Max. coverage (+): 0. Max coverage (-): 0

Region: NODE\_34239\_length\_42430\_cov\_28.169903 37179-37184. Max. coverage (+): 0. Max coverage (-): 0

Region: NODE\_34239\_length\_42430\_cov\_28.169903 37185-37191. Max. coverage (+): 0. Max coverage (-): 0

Region: NODE\_34239\_length\_42430\_cov\_28.169903 37192-37197. Max. coverage (+): 0. Max coverage (-): 0

Region: NODE\_34239\_length\_42430\_cov\_28.169903 37198-37204. Max. coverage (+): 0. Max coverage (-): 0

Region: NODE\_34239\_length\_42430\_cov\_28.169903 37205-37210. Max. coverage (+): 0. Max coverage (-): 0

Region: NODE\_34239\_length\_42430\_cov\_28.169903 37211-37217. Max. coverage (+): 0. Max coverage (-): 0

Region: NODE\_34239\_length\_42430\_cov\_28.169903 37218-37223. Max. coverage (+): 0. Max coverage (-): 0

Region: NODE\_34239\_length\_42430\_cov\_28.169903 37224-37230. Max. coverage (+): 0. Max coverage (-): 0

Region: NODE\_34239\_length\_42430\_cov\_28.169903 37231-37236. Max. coverage (+): 0. Max coverage (-): 0

Region: NODE\_34239\_length\_42430\_cov\_28.169903 37237-37243. Max. coverage (+): 0. Max coverage (-): 0

Region: NODE\_34239\_length\_42430\_cov\_28.169903 37244-37249. Max. coverage (+): 0. Max coverage (-): 0

Region: NODE\_34239\_length\_42430\_cov\_28.169903 37250-37256. Max. coverage (+): 0. Max coverage (-): 0

Region: NODE\_34239\_length\_42430\_cov\_28.169903 37257-37262. Max. coverage (+): 0. Max coverage (-): 0

Region: NODE\_34239\_length\_42430\_cov\_28.169903 37263-37269. Max. coverage (+): 0. Max coverage (-): 0

Region: NODE\_34239\_length\_42430\_cov\_28.169903 37270-37275. Max. coverage (+): 0. Max coverage (-): 0

Region: NODE\_34239\_length\_42430\_cov\_28.169903 37276-37282. Max. coverage (+): 0. Max coverage (-): 0

Region: NODE\_34239\_length\_42430\_cov\_28.169903 37283-37288. Max. coverage (+): 0. Max coverage (-): 0

Region: NODE\_34239\_length\_42430\_cov\_28.169903 37289-37295. Max. coverage (+): 0. Max coverage (-): 0

Region: NODE\_34239\_length\_42430\_cov\_28.169903 37296-37301. Max. coverage (+): 0. Max coverage (-): 0

Region: NODE\_34239\_length\_42430\_cov\_28.169903 37302-37308. Max. coverage (+): 0. Max coverage (-): 0

Region: NODE\_34239\_length\_42430\_cov\_28.169903 37309-37314. Max. coverage (+): 0. Max coverage (-): 0

Region: NODE\_34239\_length\_42430\_cov\_28.169903 37315-37321. Max. coverage (+): 0. Max coverage (-): 0

Region: NODE\_34239\_length\_42430\_cov\_28.169903 37322-37327. Max. coverage (+): 0. Max coverage (-): 0

Region: NODE\_34239\_length\_42430\_cov\_28.169903 37328-37334. Max. coverage (+): 0. Max coverage (-): 0

Region: NODE\_34239\_length\_42430\_cov\_28.169903 37335-37340. Max. coverage (+): 0. Max coverage (-): 0

Region: NODE\_34239\_length\_42430\_cov\_28.169903 37341-37347. Max. coverage (+): 0. Max coverage (-): 0

Region: NODE\_34239\_length\_42430\_cov\_28.169903 37348-37353. Max. coverage (+): 0. Max coverage (-): 0

Region: NODE\_34239\_length\_42430\_cov\_28.169903 37354-37360. Max. coverage (+): 0. Max coverage (-): 0

Region: NODE\_34239\_length\_42430\_cov\_28.169903 37361-37366. Max. coverage (+): 0. Max coverage (-): 0

Region: NODE\_34239\_length\_42430\_cov\_28.169903 37367-37373. Max. coverage (+): 0. Max coverage (-): 0

Region: NODE\_34239\_length\_42430\_cov\_28.169903 37374-37379. Max. coverage (+): 0. Max coverage (-): 0

Region: NODE\_34239\_length\_42430\_cov\_28.169903 37380-37386. Max. coverage (+): 0. Max coverage (-): 0

Region: NODE\_34239\_length\_42430\_cov\_28.169903 37387-37392. Max. coverage (+): 0. Max coverage (-): 0

Region: NODE\_34239\_length\_42430\_cov\_28.169903 37393-37399. Max. coverage (+): 0. Max coverage (-): 0

Region: NODE\_34239\_length\_42430\_cov\_28.169903 37400-37405. Max. coverage (+): 0. Max coverage (-): 0

Region: NODE\_34239\_length\_42430\_cov\_28.169903 37406-37412. Max. coverage (+): 0. Max coverage (-): 0

Region: NODE\_34239\_length\_42430\_cov\_28.169903 37413-37418. Max. coverage (+): 0. Max coverage (-): 0

Region: NODE\_34239\_length\_42430\_cov\_28.169903 37419-37425. Max. coverage (+): 586.77. Max coverage (-): 0

Region: NODE\_34239\_length\_42430\_cov\_28.169903 37426-37431. Max. coverage (+): 586.51. Max coverage (-): 0

Region: NODE\_34239\_length\_42430\_cov\_28.169903 37432-37438. Max. coverage (+): 0. Max coverage (-): 0

Region: NODE\_34239\_length\_42430\_cov\_28.169903 37439-37444. Max. coverage (+): 0. Max coverage (-): 0

Region: NODE\_34239\_length\_42430\_cov\_28.169903 37445-37451. Max. coverage (+): 0. Max coverage (-): 0

Region: NODE\_34239\_length\_42430\_cov\_28.169903 37452-37457. Max. coverage (+): 0.04. Max coverage (-): 0

Region: NODE\_34239\_length\_42430\_cov\_28.169903 37458-37464. Max. coverage (+): 0.04. Max coverage (-): 0

Region: NODE\_34239\_length\_42430\_cov\_28.169903 37465-37470. Max. coverage (+): 3.87. Max coverage (-): 0

Region: NODE\_34239\_length\_42430\_cov\_28.169903 37471-37477. Max. coverage (+): 0. Max coverage (-): 0

Region: NODE\_34239\_length\_42430\_cov\_28.169903 37478-37483. Max. coverage (+): 0. Max coverage (-): 0

Region: NODE\_34239\_length\_42430\_cov\_28.169903 37484-37490. Max. coverage (+): 0. Max coverage (-): 0

Region: NODE\_34239\_length\_42430\_cov\_28.169903 37491-37496. Max. coverage (+): 0. Max coverage (-): 0

Region: NODE\_34239\_length\_42430\_cov\_28.169903 37497-37502. Max. coverage (+): 0. Max coverage (-): 0

Region: NODE\_34239\_length\_42430\_cov\_28.169903 37503-37509. Max. coverage (+): 0. Max coverage (-): 0

Region: NODE\_34239\_length\_42430\_cov\_28.169903 37510-37515. Max. coverage (+): 0. Max coverage (-): 0

Region: NODE\_34239\_length\_42430\_cov\_28.169903 37516-37522. Max. coverage (+): 0. Max coverage (-): 0

Region: NODE\_34239\_length\_42430\_cov\_28.169903 37523-37528. Max. coverage (+): 0. Max coverage (-): 0

Region: NODE\_34239\_length\_42430\_cov\_28.169903 37529-37535. Max. coverage (+): 0. Max coverage (-): 0

Region: NODE\_34239\_length\_42430\_cov\_28.169903 37536-37541. Max. coverage (+): 0. Max coverage (-): 0

Region: NODE\_34239\_length\_42430\_cov\_28.169903 37542-37548. Max. coverage (+): 0. Max coverage (-): 0

Region: NODE\_34239\_length\_42430\_cov\_28.169903 37549-37554. Max. coverage (+): 0. Max coverage (-): 0

Region: NODE\_34239\_length\_42430\_cov\_28.169903 37555-37561. Max. coverage (+): 0. Max coverage (-): 0

Region: NODE\_34239\_length\_42430\_cov\_28.169903 37562-37567. Max. coverage (+): 0. Max coverage (-): 0

Region: NODE\_34239\_length\_42430\_cov\_28.169903 37568-37574. Max. coverage (+): 0. Max coverage (-): 0

Region: NODE\_34239\_length\_42430\_cov\_28.169903 37575-37580. Max. coverage (+): 0. Max coverage (-): 0

Region: NODE\_34239\_length\_42430\_cov\_28.169903 37581-37587. Max. coverage (+): 0. Max coverage (-): 0

Region: NODE\_34239\_length\_42430\_cov\_28.169903 37588-37593. Max. coverage (+): 0. Max coverage (-): 0

Region: NODE\_34239\_length\_42430\_cov\_28.169903 37594-37600. Max. coverage (+): 0. Max coverage (-): 0

Region: NODE\_34239\_length\_42430\_cov\_28.169903 37601-37606. Max. coverage (+): 0. Max coverage (-): 0

Region: NODE\_34239\_length\_42430\_cov\_28.169903 37607-37613. Max. coverage (+): 0. Max coverage (-): 0

Region: NODE\_34239\_length\_42430\_cov\_28.169903 37614-37619. Max. coverage (+): 0. Max coverage (-): 0

Region: NODE\_34239\_length\_42430\_cov\_28.169903 37620-37626. Max. coverage (+): 0. Max coverage (-): 0

Region: NODE\_34239\_length\_42430\_cov\_28.169903 37627-37632. Max. coverage (+): 0. Max coverage (-): 0

Region: NODE\_34239\_length\_42430\_cov\_28.169903 37633-37639. Max. coverage (+): 0. Max coverage (-): 0

Region: NODE\_34239\_length\_42430\_cov\_28.169903 37640-37645. Max. coverage (+): 0. Max coverage (-): 0

Region: NODE\_34239\_length\_42430\_cov\_28.169903 37646-37652. Max. coverage (+): 0. Max coverage (-): 0

Region: NODE\_34239\_length\_42430\_cov\_28.169903 37653-37658. Max. coverage (+): 0. Max coverage (-): 0

Region: NODE\_34239\_length\_42430\_cov\_28.169903 37659-37665. Max. coverage (+): 0. Max coverage (-): 0

Region: NODE\_34239\_length\_42430\_cov\_28.169903 37666-37671. Max. coverage (+): 0. Max coverage (-): 0

Region: NODE\_34239\_length\_42430\_cov\_28.169903 37672-37678. Max. coverage (+): 0. Max coverage (-): 0

Region: NODE\_34239\_length\_42430\_cov\_28.169903 37679-37684. Max. coverage (+): 0. Max coverage (-): 0

Region: NODE\_34239\_length\_42430\_cov\_28.169903 37685-37691. Max. coverage (+): 0. Max coverage (-): 0

Region: NODE\_34239\_length\_42430\_cov\_28.169903 37692-37697. Max. coverage (+): 0. Max coverage (-): 0

Region: NODE\_34239\_length\_42430\_cov\_28.169903 37698-37704. Max. coverage (+): 0. Max coverage (-): 0

Region: NODE\_34239\_length\_42430\_cov\_28.169903 37705-37710. Max. coverage (+): 0. Max coverage (-): 0

Region: NODE\_34239\_length\_42430\_cov\_28.169903 37711-37717. Max. coverage (+): 0. Max coverage (-): 0

Region: NODE\_34239\_length\_42430\_cov\_28.169903 37718-37723. Max. coverage (+): 0. Max coverage (-): 0

Region: NODE\_34239\_length\_42430\_cov\_28.169903 37724-37730. Max. coverage (+): 0. Max coverage (-): 0

Region: NODE\_34239\_length\_42430\_cov\_28.169903 37731-37736. Max. coverage (+): 0. Max coverage (-): 0

Region: NODE\_34239\_length\_42430\_cov\_28.169903 37737-37743. Max. coverage (+): 0. Max coverage (-): 0

Region: NODE\_34239\_length\_42430\_cov\_28.169903 37744-37749. Max. coverage (+): 0. Max coverage (-): 0

Region: NODE\_34239\_length\_42430\_cov\_28.169903 37750-37756. Max. coverage (+): 0.26. Max coverage (-): 0

Region: NODE\_34239\_length\_42430\_cov\_28.169903 37757-37762. Max. coverage (+): 733.95. Max coverage (-): 0

Region: NODE\_34239\_length\_42430\_cov\_28.169903 37763-37769. Max. coverage (+): 0. Max coverage (-): 0

Region: NODE\_34239\_length\_42430\_cov\_28.169903 37770-37775. Max. coverage (+): 0. Max coverage (-): 0

Region: NODE\_34239\_length\_42430\_cov\_28.169903 37776-37782. Max. coverage (+): 0. Max coverage (-): 0

Region: NODE\_34239\_length\_42430\_cov\_28.169903 37783-37788. Max. coverage (+): 0. Max coverage (-): 0

Region: NODE\_34239\_length\_42430\_cov\_28.169903 37789-37795. Max. coverage (+): 0.04. Max coverage (-): 0

Region: NODE\_34239\_length\_42430\_cov\_28.169903 37796-37801. Max. coverage (+): 0.04. Max coverage (-): 0

Region: NODE\_34239\_length\_42430\_cov\_28.169903 37802-37808. Max. coverage (+): 0. Max coverage (-): 0

Region: NODE\_34239\_length\_42430\_cov\_28.169903 37809-37814. Max. coverage (+): 0. Max coverage (-): 0

Region: NODE\_34239\_length\_42430\_cov\_28.169903 37815-37821. Max. coverage (+): 0. Max coverage (-): 0

Region: NODE\_34239\_length\_42430\_cov\_28.169903 37822-37827. Max. coverage (+): 0. Max coverage (-): 0

Region: NODE\_34239\_length\_42430\_cov\_28.169903 37828-37834. Max. coverage (+): 0. Max coverage (-): 0

Region: NODE\_34239\_length\_42430\_cov\_28.169903 37835-37840. Max. coverage (+): 0. Max coverage (-): 0

Region: NODE\_34239\_length\_42430\_cov\_28.169903 37841-37847. Max. coverage (+): 0. Max coverage (-): 0

Region: NODE\_34239\_length\_42430\_cov\_28.169903 37848-37853. Max. coverage (+): 0. Max coverage (-): 0

Region: NODE\_34239\_length\_42430\_cov\_28.169903 37854-37860. Max. coverage (+): 0. Max coverage (-): 0

Region: NODE\_34239\_length\_42430\_cov\_28.169903 37861-37866. Max. coverage (+): 0. Max coverage (-): 0

Region: NODE\_34239\_length\_42430\_cov\_28.169903 37867-37873. Max. coverage (+): 0. Max coverage (-): 0

Region: NODE\_34239\_length\_42430\_cov\_28.169903 37874-37879. Max. coverage (+): 0. Max coverage (-): 0

Region: NODE\_34239\_length\_42430\_cov\_28.169903 37880-37886. Max. coverage (+): 0. Max coverage (-): 0

Region: NODE\_34239\_length\_42430\_cov\_28.169903 37887-37892. Max. coverage (+): 0. Max coverage (-): 0

Region: NODE\_34239\_length\_42430\_cov\_28.169903 37893-37899. Max. coverage (+): 0. Max coverage (-): 0

Region: NODE\_34239\_length\_42430\_cov\_28.169903 37900-37905. Max. coverage (+): 0. Max coverage (-): 0

Region: NODE\_34239\_length\_42430\_cov\_28.169903 37906-37912. Max. coverage (+): 0. Max coverage (-): 0

Region: NODE\_34239\_length\_42430\_cov\_28.169903 37913-37918. Max. coverage (+): 0. Max coverage (-): 0

Region: NODE\_34239\_length\_42430\_cov\_28.169903 37919-37925. Max. coverage (+): 0. Max coverage (-): 0

Region: NODE\_34239\_length\_42430\_cov\_28.169903 37926-37931. Max. coverage (+): 0. Max coverage (-): 0

Region: NODE\_34239\_length\_42430\_cov\_28.169903 37932-37938. Max. coverage (+): 0. Max coverage (-): 0

Region: NODE\_34239\_length\_42430\_cov\_28.169903 37939-37944. Max. coverage (+): 0. Max coverage (-): 0

Region: NODE\_34239\_length\_42430\_cov\_28.169903 37945-37951. Max. coverage (+): 0. Max coverage (-): 0

Region: NODE\_34239\_length\_42430\_cov\_28.169903 37952-37957. Max. coverage (+): 0. Max coverage (-): 0

Region: NODE\_34239\_length\_42430\_cov\_28.169903 37958-37964. Max. coverage (+): 0. Max coverage (-): 0

Region: NODE\_34239\_length\_42430\_cov\_28.169903 37965-37970. Max. coverage (+): 0. Max coverage (-): 0

Region: NODE\_34239\_length\_42430\_cov\_28.169903 37971-37977. Max. coverage (+): 0. Max coverage (-): 0

Region: NODE\_34239\_length\_42430\_cov\_28.169903 37978-37983. Max. coverage (+): 0. Max coverage (-): 0

Region: NODE\_34239\_length\_42430\_cov\_28.169903 37984-37990. Max. coverage (+): 0. Max coverage (-): 0

Region: NODE\_34239\_length\_42430\_cov\_28.169903 37991-37996. Max. coverage (+): 0. Max coverage (-): 0

Region: NODE\_34239\_length\_42430\_cov\_28.169903 37997-38003. Max. coverage (+): 0. Max coverage (-): 0

Region: NODE\_34239\_length\_42430\_cov\_28.169903 38004-38009. Max. coverage (+): 0. Max coverage (-): 0

Region: NODE\_34239\_length\_42430\_cov\_28.169903 38010-38016. Max. coverage (+): 0. Max coverage (-): 0

Region: NODE\_34239\_length\_42430\_cov\_28.169903 38017-38022. Max. coverage (+): 0. Max coverage (-): 0

Region: NODE\_34239\_length\_42430\_cov\_28.169903 38023-38029. Max. coverage (+): 0. Max coverage (-): 0

Region: NODE\_34239\_length\_42430\_cov\_28.169903 38030-38035. Max. coverage (+): 0. Max coverage (-): 0

Region: NODE\_34239\_length\_42430\_cov\_28.169903 38036-38042. Max. coverage (+): 0. Max coverage (-): 0

Region: NODE\_34239\_length\_42430\_cov\_28.169903 38043-38048. Max. coverage (+): 0. Max coverage (-): 0

Region: NODE\_34239\_length\_42430\_cov\_28.169903 38049-38054. Max. coverage (+): 0. Max coverage (-): 0

Region: NODE\_34239\_length\_42430\_cov\_28.169903 38055-38061. Max. coverage (+): 0. Max coverage (-): 0

Region: NODE\_34239\_length\_42430\_cov\_28.169903 38062-38067. Max. coverage (+): 0. Max coverage (-): 0

Region: NODE\_34239\_length\_42430\_cov\_28.169903 38068-38074. Max. coverage (+): 0. Max coverage (-): 0

Region: NODE\_34239\_length\_42430\_cov\_28.169903 38075-38080. Max. coverage (+): 0. Max coverage (-): 0

Region: NODE\_34239\_length\_42430\_cov\_28.169903 38081-38087. Max. coverage (+): 0. Max coverage (-): 0

Region: NODE\_34239\_length\_42430\_cov\_28.169903 38088-38093. Max. coverage (+): 0. Max coverage (-): 0

Region: NODE\_34239\_length\_42430\_cov\_28.169903 38094-38100. Max. coverage (+): 0. Max coverage (-): 0

Region: NODE\_34239\_length\_42430\_cov\_28.169903 38101-38106. Max. coverage (+): 0. Max coverage (-): 0

Region: NODE\_34239\_length\_42430\_cov\_28.169903 38107-38113. Max. coverage (+): 0. Max coverage (-): 0

Region: NODE\_34239\_length\_42430\_cov\_28.169903 38114-38119. Max. coverage (+): 0. Max coverage (-): 0

Region: NODE\_34239\_length\_42430\_cov\_28.169903 38120-38126. Max. coverage (+): 0. Max coverage (-): 0

Region: NODE\_34239\_length\_42430\_cov\_28.169903 38127-38132. Max. coverage (+): 0. Max coverage (-): 0

Region: NODE\_34239\_length\_42430\_cov\_28.169903 38133-38139. Max. coverage (+): 0. Max coverage (-): 0

Region: NODE\_34239\_length\_42430\_cov\_28.169903 38140-38145. Max. coverage (+): 0. Max coverage (-): 0

Region: NODE\_34239\_length\_42430\_cov\_28.169903 38146-38152. Max. coverage (+): 0. Max coverage (-): 0

Region: NODE\_34239\_length\_42430\_cov\_28.169903 38153-38158. Max. coverage (+): 0. Max coverage (-): 0

Region: NODE\_34239\_length\_42430\_cov\_28.169903 38159-38165. Max. coverage (+): 0. Max coverage (-): 0

Region: NODE\_34239\_length\_42430\_cov\_28.169903 38166-38171. Max. coverage (+): 0. Max coverage (-): 0

Region: NODE\_34239\_length\_42430\_cov\_28.169903 38172-38178. Max. coverage (+): 0. Max coverage (-): 0

Region: NODE\_34239\_length\_42430\_cov\_28.169903 38179-38184. Max. coverage (+): 0. Max coverage (-): 0

Region: NODE\_34239\_length\_42430\_cov\_28.169903 38185-38191. Max. coverage (+): 0. Max coverage (-): 0

Region: NODE\_34239\_length\_42430\_cov\_28.169903 38192-38197. Max. coverage (+): 0. Max coverage (-): 0

Region: NODE\_34239\_length\_42430\_cov\_28.169903 38198-38204. Max. coverage (+): 0. Max coverage (-): 0

Region: NODE\_34239\_length\_42430\_cov\_28.169903 38205-38210. Max. coverage (+): 0. Max coverage (-): 0

Region: NODE\_34239\_length\_42430\_cov\_28.169903 38211-38217. Max. coverage (+): 0. Max coverage (-): 0

Region: NODE\_34239\_length\_42430\_cov\_28.169903 38218-38223. Max. coverage (+): 0. Max coverage (-): 0

Region: NODE\_34239\_length\_42430\_cov\_28.169903 38224-38230. Max. coverage (+): 0. Max coverage (-): 0

Region: NODE\_34239\_length\_42430\_cov\_28.169903 38231-38236. Max. coverage (+): 0. Max coverage (-): 0

Region: NODE\_34239\_length\_42430\_cov\_28.169903 38237-38243. Max. coverage (+): 0. Max coverage (-): 0

Region: NODE\_34239\_length\_42430\_cov\_28.169903 38244-38249. Max. coverage (+): 0. Max coverage (-): 0

Region: NODE\_34239\_length\_42430\_cov\_28.169903 38250-38256. Max. coverage (+): 0. Max coverage (-): 0

Region: NODE\_34239\_length\_42430\_cov\_28.169903 38257-38262. Max. coverage (+): 0. Max coverage (-): 0

Region: NODE\_34239\_length\_42430\_cov\_28.169903 38263-38269. Max. coverage (+): 0. Max coverage (-): 0

Region: NODE\_34239\_length\_42430\_cov\_28.169903 38270-38275. Max. coverage (+): 0. Max coverage (-): 0

Region: NODE\_34239\_length\_42430\_cov\_28.169903 38276-38282. Max. coverage (+): 0. Max coverage (-): 0

Region: NODE\_34239\_length\_42430\_cov\_28.169903 38283-38288. Max. coverage (+): 0. Max coverage (-): 0

Region: NODE\_34239\_length\_42430\_cov\_28.169903 38289-38295. Max. coverage (+): 0. Max coverage (-): 0

Region: NODE\_34239\_length\_42430\_cov\_28.169903 38296-38301. Max. coverage (+): 0. Max coverage (-): 0

Region: NODE\_34239\_length\_42430\_cov\_28.169903 38302-38308. Max. coverage (+): 0. Max coverage (-): 0

Region: NODE\_34239\_length\_42430\_cov\_28.169903 38309-38314. Max. coverage (+): 0. Max coverage (-): 0

Region: NODE\_34239\_length\_42430\_cov\_28.169903 38315-38321. Max. coverage (+): 0. Max coverage (-): 0

Region: NODE\_34239\_length\_42430\_cov\_28.169903 38322-38327. Max. coverage (+): 0. Max coverage (-): 0

Region: NODE\_34239\_length\_42430\_cov\_28.169903 38328-38334. Max. coverage (+): 0. Max coverage (-): 0

Region: NODE\_34239\_length\_42430\_cov\_28.169903 38335-38340. Max. coverage (+): 0. Max coverage (-): 0

Region: NODE\_34239\_length\_42430\_cov\_28.169903 38341-38347. Max. coverage (+): 0. Max coverage (-): 0

Region: NODE\_34239\_length\_42430\_cov\_28.169903 38348-38353. Max. coverage (+): 0. Max coverage (-): 0

Region: NODE\_34239\_length\_42430\_cov\_28.169903 38354-38360. Max. coverage (+): 0. Max coverage (-): 0

Region: NODE\_34239\_length\_42430\_cov\_28.169903 38361-38366. Max. coverage (+): 0.04. Max coverage (-): 0

Region: NODE\_34239\_length\_42430\_cov\_28.169903 38367-38373. Max. coverage (+): 0.04. Max coverage (-): 0

Region: NODE\_34239\_length\_42430\_cov\_28.169903 38374-38379. Max. coverage (+): 0. Max coverage (-): 0

Region: NODE\_34239\_length\_42430\_cov\_28.169903 38380-38386. Max. coverage (+): 0. Max coverage (-): 0

Region: NODE\_34239\_length\_42430\_cov\_28.169903 38387-38392. Max. coverage (+): 0. Max coverage (-): 0

Region: NODE\_34239\_length\_42430\_cov\_28.169903 38393-38399. Max. coverage (+): 0. Max coverage (-): 0

Region: NODE\_34239\_length\_42430\_cov\_28.169903 38400-38405. Max. coverage (+): 0. Max coverage (-): 0

Region: NODE\_34239\_length\_42430\_cov\_28.169903 38406-38412. Max. coverage (+): 0. Max coverage (-): 0

Region: NODE\_34239\_length\_42430\_cov\_28.169903 38413-38418. Max. coverage (+): 0. Max coverage (-): 0

Region: NODE\_34239\_length\_42430\_cov\_28.169903 38419-38425. Max. coverage (+): 0. Max coverage (-): 0

Region: NODE\_34239\_length\_42430\_cov\_28.169903 38426-38431. Max. coverage (+): 0. Max coverage (-): 0

Region: NODE\_34239\_length\_42430\_cov\_28.169903 38432-38438. Max. coverage (+): 0. Max coverage (-): 0

Region: NODE\_34239\_length\_42430\_cov\_28.169903 38439-38444. Max. coverage (+): 0. Max coverage (-): 0

Region: NODE\_34239\_length\_42430\_cov\_28.169903 38445-38451. Max. coverage (+): 0. Max coverage (-): 0

Region: NODE\_34239\_length\_42430\_cov\_28.169903 38452-38457. Max. coverage (+): 0. Max coverage (-): 0

Region: NODE\_34239\_length\_42430\_cov\_28.169903 38458-38464. Max. coverage (+): 0. Max coverage (-): 0

Region: NODE\_34239\_length\_42430\_cov\_28.169903 38465-38470. Max. coverage (+): 0. Max coverage (-): 0

Region: NODE\_34239\_length\_42430\_cov\_28.169903 38471-38477. Max. coverage (+): 0. Max coverage (-): 0

Region: NODE\_34239\_length\_42430\_cov\_28.169903 38478-38483. Max. coverage (+): 0. Max coverage (-): 0

Region: NODE\_34239\_length\_42430\_cov\_28.169903 38484-38490. Max. coverage (+): 0. Max coverage (-): 0

Region: NODE\_34239\_length\_42430\_cov\_28.169903 38491-38496. Max. coverage (+): 0. Max coverage (-): 0

Region: NODE\_34239\_length\_42430\_cov\_28.169903 38497-38503. Max. coverage (+): 0. Max coverage (-): 0

Region: NODE\_34239\_length\_42430\_cov\_28.169903 38504-38509. Max. coverage (+): 0. Max coverage (-): 0

Region: NODE\_34239\_length\_42430\_cov\_28.169903 38510-38516. Max. coverage (+): 0. Max coverage (-): 0

Region: NODE\_34239\_length\_42430\_cov\_28.169903 38517-38522. Max. coverage (+): 0. Max coverage (-): 0

Region: NODE\_34239\_length\_42430\_cov\_28.169903 38523-38529. Max. coverage (+): 0. Max coverage (-): 0

Region: NODE\_34239\_length\_42430\_cov\_28.169903 38530-38535. Max. coverage (+): 0. Max coverage (-): 0

Region: NODE\_34239\_length\_42430\_cov\_28.169903 38536-38542. Max. coverage (+): 0. Max coverage (-): 0

Region: NODE\_34239\_length\_42430\_cov\_28.169903 38543-38548. Max. coverage (+): 0. Max coverage (-): 0

Region: NODE\_34239\_length\_42430\_cov\_28.169903 38549-38555. Max. coverage (+): 0. Max coverage (-): 0

Region: NODE\_34239\_length\_42430\_cov\_28.169903 38556-38561. Max. coverage (+): 0. Max coverage (-): 0

Region: NODE\_34239\_length\_42430\_cov\_28.169903 38562-38568. Max. coverage (+): 0. Max coverage (-): 0

Region: NODE\_34239\_length\_42430\_cov\_28.169903 38569-38574. Max. coverage (+): 0.04. Max coverage (-): 0

Region: NODE\_34239\_length\_42430\_cov\_28.169903 38575-38581. Max. coverage (+): 0. Max coverage (-): 0

Region: NODE\_34239\_length\_42430\_cov\_28.169903 38582-38587. Max. coverage (+): 0. Max coverage (-): 0

Region: NODE\_34239\_length\_42430\_cov\_28.169903 38588-38593. Max. coverage (+): 0. Max coverage (-): 0

Region: NODE\_34239\_length\_42430\_cov\_28.169903 38594-38600. Max. coverage (+): 0. Max coverage (-): 0

Region: NODE\_34239\_length\_42430\_cov\_28.169903 38601-38606. Max. coverage (+): 0. Max coverage (-): 0

Region: NODE\_34239\_length\_42430\_cov\_28.169903 38607-38613. Max. coverage (+): 0. Max coverage (-): 0

Region: NODE\_34239\_length\_42430\_cov\_28.169903 38614-38619. Max. coverage (+): 0. Max coverage (-): 0

Region: NODE\_34239\_length\_42430\_cov\_28.169903 38620-38626. Max. coverage (+): 0. Max coverage (-): 0

Region: NODE\_34239\_length\_42430\_cov\_28.169903 38627-38632. Max. coverage (+): 0. Max coverage (-): 0

Region: NODE\_34239\_length\_42430\_cov\_28.169903 38633-38639. Max. coverage (+): 0. Max coverage (-): 0

Region: NODE\_34239\_length\_42430\_cov\_28.169903 38640-38645. Max. coverage (+): 0. Max coverage (-): 0

Region: NODE\_34239\_length\_42430\_cov\_28.169903 38646-38652. Max. coverage (+): 0. Max coverage (-): 0

Region: NODE\_34239\_length\_42430\_cov\_28.169903 38653-38658. Max. coverage (+): 0. Max coverage (-): 0

Region: NODE\_34239\_length\_42430\_cov\_28.169903 38659-38665. Max. coverage (+): 0. Max coverage (-): 0

Region: NODE\_34239\_length\_42430\_cov\_28.169903 38666-38671. Max. coverage (+): 0. Max coverage (-): 0

Region: NODE\_34239\_length\_42430\_cov\_28.169903 38672-38678. Max. coverage (+): 0. Max coverage (-): 0

Region: NODE\_34239\_length\_42430\_cov\_28.169903 38679-38684. Max. coverage (+): 0. Max coverage (-): 0

Region: NODE\_34239\_length\_42430\_cov\_28.169903 38685-38691. Max. coverage (+): 0. Max coverage (-): 0

Region: NODE\_34239\_length\_42430\_cov\_28.169903 38692-38697. Max. coverage (+): 0. Max coverage (-): 0

Region: NODE\_34239\_length\_42430\_cov\_28.169903 38698-38704. Max. coverage (+): 0. Max coverage (-): 0

Region: NODE\_34239\_length\_42430\_cov\_28.169903 38705-38710. Max. coverage (+): 0. Max coverage (-): 0

Region: NODE\_34239\_length\_42430\_cov\_28.169903 38711-38717. Max. coverage (+): 0. Max coverage (-): 0

Region: NODE\_34239\_length\_42430\_cov\_28.169903 38718-38723. Max. coverage (+): 0. Max coverage (-): 0

Region: NODE\_34239\_length\_42430\_cov\_28.169903 38724-38730. Max. coverage (+): 0. Max coverage (-): 0

Region: NODE\_34239\_length\_42430\_cov\_28.169903 38731-38736. Max. coverage (+): 0. Max coverage (-): 0

Region: NODE\_34239\_length\_42430\_cov\_28.169903 38737-38743. Max. coverage (+): 0. Max coverage (-): 0

Region: NODE\_34239\_length\_42430\_cov\_28.169903 38744-38749. Max. coverage (+): 0. Max coverage (-): 0

Region: NODE\_34239\_length\_42430\_cov\_28.169903 38750-38756. Max. coverage (+): 0. Max coverage (-): 0

Region: NODE\_34239\_length\_42430\_cov\_28.169903 38757-38762. Max. coverage (+): 0. Max coverage (-): 0

Region: NODE\_34239\_length\_42430\_cov\_28.169903 38763-38769. Max. coverage (+): 0. Max coverage (-): 0

Region: NODE\_34239\_length\_42430\_cov\_28.169903 38770-38775. Max. coverage (+): 0. Max coverage (-): 0

Region: NODE\_34239\_length\_42430\_cov\_28.169903 38776-38782. Max. coverage (+): 0. Max coverage (-): 0

Region: NODE\_34239\_length\_42430\_cov\_28.169903 38783-38788. Max. coverage (+): 0. Max coverage (-): 0

Region: NODE\_34239\_length\_42430\_cov\_28.169903 38789-38795. Max. coverage (+): 0. Max coverage (-): 0

Region: NODE\_34239\_length\_42430\_cov\_28.169903 38796-38801. Max. coverage (+): 0. Max coverage (-): 0

Region: NODE\_34239\_length\_42430\_cov\_28.169903 38802-38808. Max. coverage (+): 0. Max coverage (-): 0

Region: NODE\_34239\_length\_42430\_cov\_28.169903 38809-38814. Max. coverage (+): 0. Max coverage (-): 0

Region: NODE\_34239\_length\_42430\_cov\_28.169903 38815-38821. Max. coverage (+): 0. Max coverage (-): 0

Region: NODE\_34239\_length\_42430\_cov\_28.169903 38822-38827. Max. coverage (+): 0. Max coverage (-): 0

Region: NODE\_34239\_length\_42430\_cov\_28.169903 38828-38834. Max. coverage (+): 0. Max coverage (-): 0

Region: NODE\_34239\_length\_42430\_cov\_28.169903 38835-38840. Max. coverage (+): 0. Max coverage (-): 0

Region: NODE\_34239\_length\_42430\_cov\_28.169903 38841-38847. Max. coverage (+): 0. Max coverage (-): 0

Region: NODE\_34239\_length\_42430\_cov\_28.169903 38848-38853. Max. coverage (+): 0. Max coverage (-): 0

Region: NODE\_34239\_length\_42430\_cov\_28.169903 38854-38860. Max. coverage (+): 0. Max coverage (-): 0

Region: NODE\_34239\_length\_42430\_cov\_28.169903 38861-38866. Max. coverage (+): 0. Max coverage (-): 0

Region: NODE\_34239\_length\_42430\_cov\_28.169903 38867-38873. Max. coverage (+): 0. Max coverage (-): 0

Region: NODE\_34239\_length\_42430\_cov\_28.169903 38874-38879. Max. coverage (+): 0. Max coverage (-): 0

Region: NODE\_34239\_length\_42430\_cov\_28.169903 38880-38886. Max. coverage (+): 0. Max coverage (-): 0

Region: NODE\_34239\_length\_42430\_cov\_28.169903 38887-38892. Max. coverage (+): 0. Max coverage (-): 0

Region: NODE\_34239\_length\_42430\_cov\_28.169903 38893-38899. Max. coverage (+): 0. Max coverage (-): 0

Region: NODE\_34239\_length\_42430\_cov\_28.169903 38900-38905. Max. coverage (+): 0. Max coverage (-): 0

Region: NODE\_34239\_length\_42430\_cov\_28.169903 38906-38912. Max. coverage (+): 0. Max coverage (-): 0

Region: NODE\_34239\_length\_42430\_cov\_28.169903 38913-38918. Max. coverage (+): 0. Max coverage (-): 0

Region: NODE\_34239\_length\_42430\_cov\_28.169903 38919-38925. Max. coverage (+): 0. Max coverage (-): 0

Region: NODE\_34239\_length\_42430\_cov\_28.169903 38926-38931. Max. coverage (+): 0. Max coverage (-): 0

Region: NODE\_34239\_length\_42430\_cov\_28.169903 38932-38938. Max. coverage (+): 0. Max coverage (-): 0

Region: NODE\_34239\_length\_42430\_cov\_28.169903 38939-38944. Max. coverage (+): 0. Max coverage (-): 0

Region: NODE\_34239\_length\_42430\_cov\_28.169903 38945-38951. Max. coverage (+): 0. Max coverage (-): 0

Region: NODE\_34239\_length\_42430\_cov\_28.169903 38952-38957. Max. coverage (+): 0. Max coverage (-): 0

Region: NODE\_34239\_length\_42430\_cov\_28.169903 38958-38964. Max. coverage (+): 0. Max coverage (-): 0

Region: NODE\_34239\_length\_42430\_cov\_28.169903 38965-38970. Max. coverage (+): 0. Max coverage (-): 0

Region: NODE\_34239\_length\_42430\_cov\_28.169903 38971-38977. Max. coverage (+): 0. Max coverage (-): 0

Region: NODE\_34239\_length\_42430\_cov\_28.169903 38978-38983. Max. coverage (+): 0. Max coverage (-): 0

Region: NODE\_34239\_length\_42430\_cov\_28.169903 38984-38990. Max. coverage (+): 0. Max coverage (-): 0

Region: NODE\_34239\_length\_42430\_cov\_28.169903 38991-38996. Max. coverage (+): 0. Max coverage (-): 0

Region: NODE\_34239\_length\_42430\_cov\_28.169903 38997-39003. Max. coverage (+): 0. Max coverage (-): 0

Region: NODE\_34239\_length\_42430\_cov\_28.169903 39004-39009. Max. coverage (+): 0. Max coverage (-): 0

Region: NODE\_34239\_length\_42430\_cov\_28.169903 39010-39016. Max. coverage (+): 0. Max coverage (-): 0

Region: NODE\_34239\_length\_42430\_cov\_28.169903 39017-39022. Max. coverage (+): 0. Max coverage (-): 0

Region: NODE\_34239\_length\_42430\_cov\_28.169903 39023-39029. Max. coverage (+): 0. Max coverage (-): 0

Region: NODE\_34239\_length\_42430\_cov\_28.169903 39030-39035. Max. coverage (+): 0. Max coverage (-): 0

Region: NODE\_34239\_length\_42430\_cov\_28.169903 39036-39042. Max. coverage (+): 0. Max coverage (-): 0

Region: NODE\_34239\_length\_42430\_cov\_28.169903 39043-39048. Max. coverage (+): 0. Max coverage (-): 0

Region: NODE\_34239\_length\_42430\_cov\_28.169903 39049-39055. Max. coverage (+): 0. Max coverage (-): 0

Region: NODE\_34239\_length\_42430\_cov\_28.169903 39056-39061. Max. coverage (+): 0. Max coverage (-): 0

Region: NODE\_34239\_length\_42430\_cov\_28.169903 39062-39068. Max. coverage (+): 0. Max coverage (-): 0

Region: NODE\_34239\_length\_42430\_cov\_28.169903 39069-39074. Max. coverage (+): 0. Max coverage (-): 0

Region: NODE\_34239\_length\_42430\_cov\_28.169903 39075-39081. Max. coverage (+): 0. Max coverage (-): 0

Region: NODE\_34239\_length\_42430\_cov\_28.169903 39082-39087. Max. coverage (+): 0. Max coverage (-): 0

Region: NODE\_34239\_length\_42430\_cov\_28.169903 39088-39094. Max. coverage (+): 0. Max coverage (-): 0

Region: NODE\_34239\_length\_42430\_cov\_28.169903 39095-39100. Max. coverage (+): 0. Max coverage (-): 0

Region: NODE\_34239\_length\_42430\_cov\_28.169903 39101-39107. Max. coverage (+): 0. Max coverage (-): 0

Region: NODE\_34239\_length\_42430\_cov\_28.169903 39108-39113. Max. coverage (+): 0. Max coverage (-): 0

Region: NODE\_34239\_length\_42430\_cov\_28.169903 39114-39120. Max. coverage (+): 0. Max coverage (-): 0

Region: NODE\_34239\_length\_42430\_cov\_28.169903 39121-39126. Max. coverage (+): 0. Max coverage (-): 0

Region: NODE\_34239\_length\_42430\_cov\_28.169903 39127-39132. Max. coverage (+): 0. Max coverage (-): 0

Region: NODE\_34239\_length\_42430\_cov\_28.169903 39133-39139. Max. coverage (+): 0. Max coverage (-): 0

Region: NODE\_34239\_length\_42430\_cov\_28.169903 39140-39145. Max. coverage (+): 0. Max coverage (-): 0

Region: NODE\_34239\_length\_42430\_cov\_28.169903 39146-39152. Max. coverage (+): 0. Max coverage (-): 0

Region: NODE\_34239\_length\_42430\_cov\_28.169903 39153-39158. Max. coverage (+): 0. Max coverage (-): 0

Region: NODE\_34239\_length\_42430\_cov\_28.169903 39159-39165. Max. coverage (+): 0. Max coverage (-): 0

Region: NODE\_34239\_length\_42430\_cov\_28.169903 39166-39171. Max. coverage (+): 0. Max coverage (-): 0

Region: NODE\_34239\_length\_42430\_cov\_28.169903 39172-39178. Max. coverage (+): 0. Max coverage (-): 0

Region: NODE\_34239\_length\_42430\_cov\_28.169903 39179-39184. Max. coverage (+): 0. Max coverage (-): 0

Region: NODE\_34239\_length\_42430\_cov\_28.169903 39185-39191. Max. coverage (+): 0. Max coverage (-): 0

Region: NODE\_34239\_length\_42430\_cov\_28.169903 39192-39197. Max. coverage (+): 0. Max coverage (-): 0

Region: NODE\_34239\_length\_42430\_cov\_28.169903 39198-39204. Max. coverage (+): 0. Max coverage (-): 0

Region: NODE\_34239\_length\_42430\_cov\_28.169903 39205-39210. Max. coverage (+): 0. Max coverage (-): 0

Region: NODE\_34239\_length\_42430\_cov\_28.169903 39211-39217. Max. coverage (+): 0. Max coverage (-): 0

Region: NODE\_34239\_length\_42430\_cov\_28.169903 39218-39223. Max. coverage (+): 0. Max coverage (-): 0

Region: NODE\_34239\_length\_42430\_cov\_28.169903 39224-39230. Max. coverage (+): 0. Max coverage (-): 0

Region: NODE\_34239\_length\_42430\_cov\_28.169903 39231-39236. Max. coverage (+): 0. Max coverage (-): 0

Region: NODE\_34239\_length\_42430\_cov\_28.169903 39237-39243. Max. coverage (+): 0. Max coverage (-): 0

Region: NODE\_34239\_length\_42430\_cov\_28.169903 39244-39249. Max. coverage (+): 0. Max coverage (-): 0

Region: NODE\_34239\_length\_42430\_cov\_28.169903 39250-39256. Max. coverage (+): 0. Max coverage (-): 0

Region: NODE\_34239\_length\_42430\_cov\_28.169903 39257-39262. Max. coverage (+): 0. Max coverage (-): 0

Region: NODE\_34239\_length\_42430\_cov\_28.169903 39263-39269. Max. coverage (+): 0. Max coverage (-): 0

Region: NODE\_34239\_length\_42430\_cov\_28.169903 39270-39275. Max. coverage (+): 0. Max coverage (-): 0

Region: NODE\_34239\_length\_42430\_cov\_28.169903 39276-39282. Max. coverage (+): 0. Max coverage (-): 0

Region: NODE\_34239\_length\_42430\_cov\_28.169903 39283-39288. Max. coverage (+): 0. Max coverage (-): 0

Region: NODE\_34239\_length\_42430\_cov\_28.169903 39289-39295. Max. coverage (+): 0. Max coverage (-): 0

Region: NODE\_34239\_length\_42430\_cov\_28.169903 39296-39301. Max. coverage (+): 0. Max coverage (-): 0

Region: NODE\_34239\_length\_42430\_cov\_28.169903 39302-39308. Max. coverage (+): 0. Max coverage (-): 0

Region: NODE\_34239\_length\_42430\_cov\_28.169903 39309-39314. Max. coverage (+): 0. Max coverage (-): 0

Region: NODE\_34239\_length\_42430\_cov\_28.169903 39315-39321. Max. coverage (+): 0. Max coverage (-): 0

Region: NODE\_34239\_length\_42430\_cov\_28.169903 39322-39327. Max. coverage (+): 0. Max coverage (-): 0

Region: NODE\_34239\_length\_42430\_cov\_28.169903 39328-39334. Max. coverage (+): 0. Max coverage (-): 0

Region: NODE\_34239\_length\_42430\_cov\_28.169903 39335-39340. Max. coverage (+): 0. Max coverage (-): 0

Region: NODE\_34239\_length\_42430\_cov\_28.169903 39341-39347. Max. coverage (+): 0. Max coverage (-): 0

Region: NODE\_34239\_length\_42430\_cov\_28.169903 39348-39353. Max. coverage (+): 0. Max coverage (-): 0

Region: NODE\_34239\_length\_42430\_cov\_28.169903 39354-39360. Max. coverage (+): 0. Max coverage (-): 0

Region: NODE\_34239\_length\_42430\_cov\_28.169903 39361-39366. Max. coverage (+): 0.04. Max coverage (-): 0

Region: NODE\_34239\_length\_42430\_cov\_28.169903 39367-39373. Max. coverage (+): 0. Max coverage (-): 0

Region: NODE\_34239\_length\_42430\_cov\_28.169903 39374-39379. Max. coverage (+): 0. Max coverage (-): 0

Region: NODE\_34239\_length\_42430\_cov\_28.169903 39380-39386. Max. coverage (+): 0. Max coverage (-): 0

Region: NODE\_34239\_length\_42430\_cov\_28.169903 39387-39392. Max. coverage (+): 0. Max coverage (-): 0

Region: NODE\_34239\_length\_42430\_cov\_28.169903 39393-. Max. coverage (+): 0. Max coverage (-): 0

RepeatMasker Color Code

**+**

100-98% Identity

<98-95% Identity

<95-90% Identity

<90-85% Identity

<85-80% Identity

<80-75% Identity

<75-70% Identity

<70% Identity

**-**

Gene Set Color Code

**+**

Gene

Pseudogene

Other

**-**

Topology/Coverage Color Code

Coverage Plus Strand

Coverage Minus Strand

Mainstrand: Plus

Mainstrand: Minus

Complementary Strand

Flanking Region  
(if option -flank >0)

Gene Set Annotation  

**1. unknown (unknownunknown) Tr:unknown**: 37180-37275 (+)  
**2. unknown (unknownunknown) Tr:unknown**: 38319-40308 (+)  
**3. unknown (unknownunknown) Tr:unknown UTR**: 38550-40308 (+)

  
RepeatMasker Annotation  
  
Transcription Factor Binding Sites  

**RFX4\_1** (Sequence: CATAGCAAC (+): 36358)  
**RHOXF1** (Sequence: AGATCA (-): 36777)  
**RHOXF1** (Sequence: AGATTA (-): 36874)  
**RHOXF1** (Sequence: AGCTCA (-): 37937)  
**RHOXF1** (Sequence: AGATTA (-): 38189)  
**RHOXF1** (Sequence: AGATCA (-): 38464)  
**RHOXF1** (Sequence: GGATTA (-): 38781)  
**RHOXF1** (Sequence: TGATCC (+): 36320)  
**RHOXF1** (Sequence: TGATCT (+): 36729)  
**RHOXF1** (Sequence: TGAGCT (+): 36946)  
**RHOXF1** (Sequence: TAAGCT (+): 36963)  
**RHOXF1** (Sequence: TGATCT (+): 36965)  
**RHOXF1** (Sequence: TGATCT (+): 38867)  
**RHOXF1** (Sequence: TGAGCT (+): 39034)  
**RHOXF1** (Sequence: TGAGCT (+): 39347)  
**Gata4** (Sequence: CTTATCT (+): 36602)  
**POU5F1** (Sequence: TTTGCAT (-): 36815)  
**FOXO3\_hsa** (Sequence: GTAAACAT (+): 37423)  
**FOXO3\_hsa** (Sequence: GTAAACAT (+): 37758)  
**FOXP1** (Sequence: GTAAACA (+): 37423)  
**FOXP1** (Sequence: GTAAACA (+): 37758)  
**FOXO3\_mmu** (Sequence: TGTTTAGA (-): 39361)  
**Sox5** (Sequence: ATTGTT (+): 37400)  
**FIGLA** (Sequence: AACAGCTGGT (-): 37326)  
**FIGLA** (Sequence: AACACCTGTA (-): 37923)  
**SOX9** (Sequence: CCATTGTT (+): 37398)  
**FOXO3\_mmu** (Sequence: TGTAAACA (+): 37422)  
**FOXO3\_mmu** (Sequence: TGTAAACA (+): 37757)  
**FOXP1** (Sequence: TGTTTAC (-): 37807)  
**Nobox** (Sequence: TAATTGCC (+): 36180)  
**Rhox11** (Sequence: CGGTGTTTT (+): 36838)  
**Rhox11** (Sequence: TGCTGTAAA (+): 37419)  
**Rhox11** (Sequence: CGGTGTTTA (+): 37888)  
**Gata4** (Sequence: AGATAAC (-): 39173)  
**POU2F1** (Sequence: TATTTAAAT (+): 37720)  
**POU5F1** (Sequence: ATGCAAA (+): 39098)
