# Supplementary material for: First characterization of PIWI-interacting RNA clusters in a cichlid fish with a B chromosome
Source: BMC Biol. 2022 Sep 21;20:204. doi: 10.1186/s12915-022-01403-2 (PMC9490952; doi:10.1186/s12915-022-01403-2)
Supplement: Supplementary file 1 — Additional file 1. Zipped folder with fasta and interactive html piRNA cluster information for the A. latifasciata genome. The nomenclature is as follows: number-pirna-cluster_sex_B-presence (f, female; m, male; 0b, without B chromosome; 1b, with B chromosome). [file 12915_2022_1403_MOESM1_ESM.zip › 134_f0b.html]

piRNA cluster 134\_f0b 64


Predicted piRNA cluster no. 134\_f0b
  

Show proTRAC run info
Hide proTRAC run info

/\  
                \_\_\_\_\_\_\_\_\_\_\_\_\_\_\_\_\_\_\_\_\_\_\_/\\_\_\_ /  \\_\_\_\_\_\_\_  
               I                      /  \  /    \      I  
               I     pro             /    \/      \     I  
               I        TRAC        /               \   I  
               I   \_\_\_\_\_\_\_\_\_\_\_\_\_\_\_\_/\_\_\_\_\_\_\_\_\_\_\_\_\_\_\_\_\_\\_ I  
               I   \              /                     I  
               I    \            /                      I  
               I     \  /\      /       V.2.4.2         I  
               I      \/  \    /                        I  
               I\_\_\_\_\_\_\_\_\_\_\_\  /\_\_\_\_\_\_\_\_\_\_\_\_\_\_\_\_\_\_\_\_\_\_\_\_\_I  
                            \/  
  
  
================================= proTRAC ====================================  
VERSION: .......... 2.4.2  
LAST MODIFIED: .... 11. May 2018  
  
Please cite:  
Rosenkranz D, Zischler H. proTRAC - a software for probabilistic piRNA cluster  
detection, visualization and analysis. 2012. BMC Bioinformatics 13:5.  
  
  
Contact:  
David Rosenkranz  
Institute of Organismic and Molecular Evolutionary Biology  
Dept. Anthropology, small RNA group  
Johannes Gutenberg University Mainz  
email: rosenkranz@uni-mainz.de  
  
You can find the latest proTRAC version at:  
http://sourceforge.net/projects/protrac/files  
http://www.smallRNAgroup-mainz.de/software  
==============================================================================  
  
PARAMETERS:  
Map file: ...............piwi-femeas-0B.fa-collapse.map  
Genome file: ............../../../0B\_ala\_genome.fa  
RepeatMasker annotation: Alatifasciata-all0B-maryan-v2.fa\_corrected.out  
GeneSet:................./guest-storage/Data/annotation/Alatifasciata\_all0B\_maryan-v2\_out2017.gff  
  
Significant (p<=0.01) hit density will be calculated based  
on observed hit distribution.  
  
Sliding window size: ........................................ 5000 bp  
Sliding window increament: .................................. 1000 bp  
Normalize each hit by number of genomic hits: ............... yes  
Normalize each hit by number of sequence reads: ............. yes  
Normalize values (-> per million mapped reads): ............. yes  
Min. fraction of hits with 1T(U) or 10A: .................... 0.75  
Alternatively: Min. fraction of hits with 1T(U) and 10A: .... 0.5  
Min. fraction of hits with typical piRNA length: ............ 0.75  
Typical piRNA length: ....................................... 24-32 nt  
Min. size of a piRNA cluster: ............................... 1000 bp.  
Min. number of hits (absolute): ............................. 0  
Min. number of hits (normalized): ........................... 0  
Min. fraction of hits on the mainstrand: .................... 0.75  
Top fraction of mapped sequences (in terms of read counts): . 1%  
Top fraction accounts for max. n% of sequence reads: ........ 90%  
Min. fraction of hits on each arm of a bidirectional cluster: 0.05  
Output html file for each cluster: .......................... yes  
Output a summary table: ..................................... yes  
Output a FASTA file for each cluster (piRNA sequences): ..... yes  
Output a FASTA file comprising cluster sequences: ........... yes  
Output a GTF file for predicted piRNA clusters: ..............yes  
Search DNA motifs in clusters: .............................. yes  
Output flanking sequences: +/- .............................. 0 bp  
Output ~.pTi file: .......................................... no  
==============================================================================  
  
  
Genome size (without gaps): ............ 758543724 bp  
Gaps (N/X/-): .......................... 417479 bp  
Mapped reads: .......................... 13052187  
Non-identical sequences: ............... 3338911  
Genomic hits: .......................... 28737726  
Significant densitiy of mapped reads: .. 470.083249848448 reads/kb

Show proTRAC cluster info
Hide proTRAC cluster info

|  |  |
| --- | --- |
| Location | NODE\_347176\_length\_38458\_cov\_26.791616 |
| Coordinates | 35371-38453 |
| Size [bp] | 3083 |
| Sequence hit loci | 130 |
| Mapped reads (normalized) | 3591.5 |
| Mapped reads (normalized) per kb | 1164.9 |
| Normalized reads with 1T (1U) | 97.2% |
| Normalized reads with 10A | 97% |
| Normalized reads with length 24-32 nt | 99.7% |
| Normalized reads on the main strand(s) | 100% |
| Predicted directionality | mono:minus |

100%

0%

1T (1U)  
reads

10A reads

24-32 nt  
reads

reads on mainstrand

**Either the amount of reads with 1T (1U) OR 10A has to exceed 75% (set with option: -1Tor10A)  
Alternatively the amount of reads with 1T (1U) AND 10A has to exceed 50% (set with option: -1Tand10A)  
Minimum amount of reads with preferred size is 75% (set with option: -pisize)  
Minimum amount of reads on the main strand(s) is 75% (set with option: -clstrand)**

Show read coverage
Hide read coverage

WHAT DO I SEE HERE?  
This chart shows the location of mapped sequence reads within a predicted piRNA cluster. The color refers to the number of genomic hits produced by the sequence read in question. A dark red bar indicates that this sequence read produces many other hits elsewhere in the genome. Many adjacent red or yellow bars can indicate the presence of a multi-copy element such as transposons or rRNA genes. A dark green bar indicates that this sequence read maps uniquely to this locus.

1 hit

2-5 hits

6-10 hits

11-20 hits

21-50 hits

51-100 hits

> 100 hits

NODE\_347176\_length\_38458\_cov\_26.791616

35371

38453

Gene Set

RepeatMasker

Mapped  
Reads

272.56

plus strand

minus strand

272.56

Region: NODE\_347176\_length\_38458\_cov\_26.791616 53963-35374. Max. coverage (+): 0. Max coverage (-): 0.08

Region: NODE\_347176\_length\_38458\_cov\_26.791616 35375-35380. Max. coverage (+): 0. Max coverage (-): 0.08

Region: NODE\_347176\_length\_38458\_cov\_26.791616 35381-35386. Max. coverage (+): 0. Max coverage (-): 0

Region: NODE\_347176\_length\_38458\_cov\_26.791616 35387-35392. Max. coverage (+): 0. Max coverage (-): 0

Region: NODE\_347176\_length\_38458\_cov\_26.791616 35393-35398. Max. coverage (+): 0. Max coverage (-): 0

Region: NODE\_347176\_length\_38458\_cov\_26.791616 35399-35404. Max. coverage (+): 0. Max coverage (-): 0

Region: NODE\_347176\_length\_38458\_cov\_26.791616 35405-35411. Max. coverage (+): 0. Max coverage (-): 0

Region: NODE\_347176\_length\_38458\_cov\_26.791616 35412-35417. Max. coverage (+): 0. Max coverage (-): 0.08

Region: NODE\_347176\_length\_38458\_cov\_26.791616 35418-35423. Max. coverage (+): 0. Max coverage (-): 0.08

Region: NODE\_347176\_length\_38458\_cov\_26.791616 35424-35429. Max. coverage (+): 0. Max coverage (-): 0

Region: NODE\_347176\_length\_38458\_cov\_26.791616 35430-35435. Max. coverage (+): 0. Max coverage (-): 0

Region: NODE\_347176\_length\_38458\_cov\_26.791616 35436-35441. Max. coverage (+): 0. Max coverage (-): 0

Region: NODE\_347176\_length\_38458\_cov\_26.791616 35442-35448. Max. coverage (+): 0. Max coverage (-): 0

Region: NODE\_347176\_length\_38458\_cov\_26.791616 35449-35454. Max. coverage (+): 0. Max coverage (-): 0

Region: NODE\_347176\_length\_38458\_cov\_26.791616 35455-35460. Max. coverage (+): 0. Max coverage (-): 0

Region: NODE\_347176\_length\_38458\_cov\_26.791616 35461-35466. Max. coverage (+): 0. Max coverage (-): 0

Region: NODE\_347176\_length\_38458\_cov\_26.791616 35467-35472. Max. coverage (+): 0. Max coverage (-): 0

Region: NODE\_347176\_length\_38458\_cov\_26.791616 35473-35478. Max. coverage (+): 0. Max coverage (-): 0

Region: NODE\_347176\_length\_38458\_cov\_26.791616 35479-35485. Max. coverage (+): 0. Max coverage (-): 0

Region: NODE\_347176\_length\_38458\_cov\_26.791616 35486-35491. Max. coverage (+): 0. Max coverage (-): 0

Region: NODE\_347176\_length\_38458\_cov\_26.791616 35492-35497. Max. coverage (+): 0. Max coverage (-): 0

Region: NODE\_347176\_length\_38458\_cov\_26.791616 35498-35503. Max. coverage (+): 0. Max coverage (-): 0

Region: NODE\_347176\_length\_38458\_cov\_26.791616 35504-35509. Max. coverage (+): 0. Max coverage (-): 0

Region: NODE\_347176\_length\_38458\_cov\_26.791616 35510-35515. Max. coverage (+): 0. Max coverage (-): 0

Region: NODE\_347176\_length\_38458\_cov\_26.791616 35516-35522. Max. coverage (+): 0. Max coverage (-): 0

Region: NODE\_347176\_length\_38458\_cov\_26.791616 35523-35528. Max. coverage (+): 0. Max coverage (-): 0

Region: NODE\_347176\_length\_38458\_cov\_26.791616 35529-35534. Max. coverage (+): 0. Max coverage (-): 0

Region: NODE\_347176\_length\_38458\_cov\_26.791616 35535-35540. Max. coverage (+): 0. Max coverage (-): 0

Region: NODE\_347176\_length\_38458\_cov\_26.791616 35541-35546. Max. coverage (+): 0. Max coverage (-): 0

Region: NODE\_347176\_length\_38458\_cov\_26.791616 35547-35552. Max. coverage (+): 0. Max coverage (-): 0

Region: NODE\_347176\_length\_38458\_cov\_26.791616 35553-35559. Max. coverage (+): 0. Max coverage (-): 0

Region: NODE\_347176\_length\_38458\_cov\_26.791616 35560-35565. Max. coverage (+): 0. Max coverage (-): 0

Region: NODE\_347176\_length\_38458\_cov\_26.791616 35566-35571. Max. coverage (+): 0. Max coverage (-): 0

Region: NODE\_347176\_length\_38458\_cov\_26.791616 35572-35577. Max. coverage (+): 0. Max coverage (-): 0

Region: NODE\_347176\_length\_38458\_cov\_26.791616 35578-35583. Max. coverage (+): 0. Max coverage (-): 0

Region: NODE\_347176\_length\_38458\_cov\_26.791616 35584-35589. Max. coverage (+): 0. Max coverage (-): 0

Region: NODE\_347176\_length\_38458\_cov\_26.791616 35590-35596. Max. coverage (+): 0. Max coverage (-): 0

Region: NODE\_347176\_length\_38458\_cov\_26.791616 35597-35602. Max. coverage (+): 0. Max coverage (-): 0

Region: NODE\_347176\_length\_38458\_cov\_26.791616 35603-35608. Max. coverage (+): 0. Max coverage (-): 0

Region: NODE\_347176\_length\_38458\_cov\_26.791616 35609-35614. Max. coverage (+): 0. Max coverage (-): 0

Region: NODE\_347176\_length\_38458\_cov\_26.791616 35615-35620. Max. coverage (+): 0. Max coverage (-): 0

Region: NODE\_347176\_length\_38458\_cov\_26.791616 35621-35626. Max. coverage (+): 0. Max coverage (-): 0

Region: NODE\_347176\_length\_38458\_cov\_26.791616 35627-35633. Max. coverage (+): 0. Max coverage (-): 0

Region: NODE\_347176\_length\_38458\_cov\_26.791616 35634-35639. Max. coverage (+): 0. Max coverage (-): 0

Region: NODE\_347176\_length\_38458\_cov\_26.791616 35640-35645. Max. coverage (+): 0. Max coverage (-): 0

Region: NODE\_347176\_length\_38458\_cov\_26.791616 35646-35651. Max. coverage (+): 0. Max coverage (-): 0

Region: NODE\_347176\_length\_38458\_cov\_26.791616 35652-35657. Max. coverage (+): 0. Max coverage (-): 0

Region: NODE\_347176\_length\_38458\_cov\_26.791616 35658-35663. Max. coverage (+): 0. Max coverage (-): 0

Region: NODE\_347176\_length\_38458\_cov\_26.791616 35664-35670. Max. coverage (+): 0. Max coverage (-): 0.08

Region: NODE\_347176\_length\_38458\_cov\_26.791616 35671-35676. Max. coverage (+): 0. Max coverage (-): 0

Region: NODE\_347176\_length\_38458\_cov\_26.791616 35677-35682. Max. coverage (+): 0. Max coverage (-): 0.08

Region: NODE\_347176\_length\_38458\_cov\_26.791616 35683-35688. Max. coverage (+): 0. Max coverage (-): 0.08

Region: NODE\_347176\_length\_38458\_cov\_26.791616 35689-35694. Max. coverage (+): 0. Max coverage (-): 0

Region: NODE\_347176\_length\_38458\_cov\_26.791616 35695-35700. Max. coverage (+): 0. Max coverage (-): 0

Region: NODE\_347176\_length\_38458\_cov\_26.791616 35701-35707. Max. coverage (+): 0. Max coverage (-): 0

Region: NODE\_347176\_length\_38458\_cov\_26.791616 35708-35713. Max. coverage (+): 0. Max coverage (-): 0

Region: NODE\_347176\_length\_38458\_cov\_26.791616 35714-35719. Max. coverage (+): 0. Max coverage (-): 0

Region: NODE\_347176\_length\_38458\_cov\_26.791616 35720-35725. Max. coverage (+): 0. Max coverage (-): 0

Region: NODE\_347176\_length\_38458\_cov\_26.791616 35726-35731. Max. coverage (+): 0. Max coverage (-): 0

Region: NODE\_347176\_length\_38458\_cov\_26.791616 35732-35737. Max. coverage (+): 0. Max coverage (-): 0

Region: NODE\_347176\_length\_38458\_cov\_26.791616 35738-35744. Max. coverage (+): 0. Max coverage (-): 0

Region: NODE\_347176\_length\_38458\_cov\_26.791616 35745-35750. Max. coverage (+): 0. Max coverage (-): 0

Region: NODE\_347176\_length\_38458\_cov\_26.791616 35751-35756. Max. coverage (+): 0. Max coverage (-): 0

Region: NODE\_347176\_length\_38458\_cov\_26.791616 35757-35762. Max. coverage (+): 0. Max coverage (-): 0

Region: NODE\_347176\_length\_38458\_cov\_26.791616 35763-35768. Max. coverage (+): 0. Max coverage (-): 0

Region: NODE\_347176\_length\_38458\_cov\_26.791616 35769-35774. Max. coverage (+): 0. Max coverage (-): 0

Region: NODE\_347176\_length\_38458\_cov\_26.791616 35775-35781. Max. coverage (+): 0. Max coverage (-): 0

Region: NODE\_347176\_length\_38458\_cov\_26.791616 35782-35787. Max. coverage (+): 0. Max coverage (-): 0

Region: NODE\_347176\_length\_38458\_cov\_26.791616 35788-35793. Max. coverage (+): 0. Max coverage (-): 0

Region: NODE\_347176\_length\_38458\_cov\_26.791616 35794-35799. Max. coverage (+): 0. Max coverage (-): 0

Region: NODE\_347176\_length\_38458\_cov\_26.791616 35800-35805. Max. coverage (+): 0. Max coverage (-): 0

Region: NODE\_347176\_length\_38458\_cov\_26.791616 35806-35811. Max. coverage (+): 0. Max coverage (-): 0

Region: NODE\_347176\_length\_38458\_cov\_26.791616 35812-35818. Max. coverage (+): 0. Max coverage (-): 0

Region: NODE\_347176\_length\_38458\_cov\_26.791616 35819-35824. Max. coverage (+): 0. Max coverage (-): 0

Region: NODE\_347176\_length\_38458\_cov\_26.791616 35825-35830. Max. coverage (+): 0. Max coverage (-): 0

Region: NODE\_347176\_length\_38458\_cov\_26.791616 35831-35836. Max. coverage (+): 0. Max coverage (-): 0

Region: NODE\_347176\_length\_38458\_cov\_26.791616 35837-35842. Max. coverage (+): 0. Max coverage (-): 0

Region: NODE\_347176\_length\_38458\_cov\_26.791616 35843-35848. Max. coverage (+): 0. Max coverage (-): 0

Region: NODE\_347176\_length\_38458\_cov\_26.791616 35849-35855. Max. coverage (+): 0. Max coverage (-): 0

Region: NODE\_347176\_length\_38458\_cov\_26.791616 35856-35861. Max. coverage (+): 0. Max coverage (-): 0

Region: NODE\_347176\_length\_38458\_cov\_26.791616 35862-35867. Max. coverage (+): 0. Max coverage (-): 0

Region: NODE\_347176\_length\_38458\_cov\_26.791616 35868-35873. Max. coverage (+): 0. Max coverage (-): 0

Region: NODE\_347176\_length\_38458\_cov\_26.791616 35874-35879. Max. coverage (+): 0. Max coverage (-): 0

Region: NODE\_347176\_length\_38458\_cov\_26.791616 35880-35885. Max. coverage (+): 0. Max coverage (-): 0

Region: NODE\_347176\_length\_38458\_cov\_26.791616 35886-35892. Max. coverage (+): 0. Max coverage (-): 0

Region: NODE\_347176\_length\_38458\_cov\_26.791616 35893-35898. Max. coverage (+): 0. Max coverage (-): 0

Region: NODE\_347176\_length\_38458\_cov\_26.791616 35899-35904. Max. coverage (+): 0. Max coverage (-): 0.08

Region: NODE\_347176\_length\_38458\_cov\_26.791616 35905-35910. Max. coverage (+): 0. Max coverage (-): 0.08

Region: NODE\_347176\_length\_38458\_cov\_26.791616 35911-35916. Max. coverage (+): 0. Max coverage (-): 0

Region: NODE\_347176\_length\_38458\_cov\_26.791616 35917-35922. Max. coverage (+): 0. Max coverage (-): 0

Region: NODE\_347176\_length\_38458\_cov\_26.791616 35923-35929. Max. coverage (+): 0. Max coverage (-): 0

Region: NODE\_347176\_length\_38458\_cov\_26.791616 35930-35935. Max. coverage (+): 0. Max coverage (-): 0

Region: NODE\_347176\_length\_38458\_cov\_26.791616 35936-35941. Max. coverage (+): 0. Max coverage (-): 0

Region: NODE\_347176\_length\_38458\_cov\_26.791616 35942-35947. Max. coverage (+): 0. Max coverage (-): 0

Region: NODE\_347176\_length\_38458\_cov\_26.791616 35948-35953. Max. coverage (+): 0. Max coverage (-): 0

Region: NODE\_347176\_length\_38458\_cov\_26.791616 35954-35959. Max. coverage (+): 0. Max coverage (-): 0

Region: NODE\_347176\_length\_38458\_cov\_26.791616 35960-35966. Max. coverage (+): 0. Max coverage (-): 0

Region: NODE\_347176\_length\_38458\_cov\_26.791616 35967-35972. Max. coverage (+): 0. Max coverage (-): 0

Region: NODE\_347176\_length\_38458\_cov\_26.791616 35973-35978. Max. coverage (+): 0. Max coverage (-): 0

Region: NODE\_347176\_length\_38458\_cov\_26.791616 35979-35984. Max. coverage (+): 0. Max coverage (-): 0

Region: NODE\_347176\_length\_38458\_cov\_26.791616 35985-35990. Max. coverage (+): 0. Max coverage (-): 0

Region: NODE\_347176\_length\_38458\_cov\_26.791616 35991-35996. Max. coverage (+): 0. Max coverage (-): 0

Region: NODE\_347176\_length\_38458\_cov\_26.791616 35997-36003. Max. coverage (+): 0. Max coverage (-): 0

Region: NODE\_347176\_length\_38458\_cov\_26.791616 36004-36009. Max. coverage (+): 0. Max coverage (-): 0

Region: NODE\_347176\_length\_38458\_cov\_26.791616 36010-36015. Max. coverage (+): 0. Max coverage (-): 0

Region: NODE\_347176\_length\_38458\_cov\_26.791616 36016-36021. Max. coverage (+): 0. Max coverage (-): 0

Region: NODE\_347176\_length\_38458\_cov\_26.791616 36022-36027. Max. coverage (+): 0. Max coverage (-): 0

Region: NODE\_347176\_length\_38458\_cov\_26.791616 36028-36033. Max. coverage (+): 0. Max coverage (-): 0

Region: NODE\_347176\_length\_38458\_cov\_26.791616 36034-36040. Max. coverage (+): 0. Max coverage (-): 0

Region: NODE\_347176\_length\_38458\_cov\_26.791616 36041-36046. Max. coverage (+): 0. Max coverage (-): 0

Region: NODE\_347176\_length\_38458\_cov\_26.791616 36047-36052. Max. coverage (+): 0. Max coverage (-): 0

Region: NODE\_347176\_length\_38458\_cov\_26.791616 36053-36058. Max. coverage (+): 0. Max coverage (-): 0

Region: NODE\_347176\_length\_38458\_cov\_26.791616 36059-36064. Max. coverage (+): 0. Max coverage (-): 0

Region: NODE\_347176\_length\_38458\_cov\_26.791616 36065-36070. Max. coverage (+): 0. Max coverage (-): 0

Region: NODE\_347176\_length\_38458\_cov\_26.791616 36071-36077. Max. coverage (+): 0. Max coverage (-): 0

Region: NODE\_347176\_length\_38458\_cov\_26.791616 36078-36083. Max. coverage (+): 0. Max coverage (-): 0

Region: NODE\_347176\_length\_38458\_cov\_26.791616 36084-36089. Max. coverage (+): 0. Max coverage (-): 0.31

Region: NODE\_347176\_length\_38458\_cov\_26.791616 36090-36095. Max. coverage (+): 0. Max coverage (-): 0.31

Region: NODE\_347176\_length\_38458\_cov\_26.791616 36096-36101. Max. coverage (+): 0. Max coverage (-): 0

Region: NODE\_347176\_length\_38458\_cov\_26.791616 36102-36107. Max. coverage (+): 0. Max coverage (-): 0

Region: NODE\_347176\_length\_38458\_cov\_26.791616 36108-36114. Max. coverage (+): 0. Max coverage (-): 0

Region: NODE\_347176\_length\_38458\_cov\_26.791616 36115-36120. Max. coverage (+): 0. Max coverage (-): 0.15

Region: NODE\_347176\_length\_38458\_cov\_26.791616 36121-36126. Max. coverage (+): 0. Max coverage (-): 0.38

Region: NODE\_347176\_length\_38458\_cov\_26.791616 36127-36132. Max. coverage (+): 0. Max coverage (-): 0.08

Region: NODE\_347176\_length\_38458\_cov\_26.791616 36133-36138. Max. coverage (+): 0. Max coverage (-): 0

Region: NODE\_347176\_length\_38458\_cov\_26.791616 36139-36144. Max. coverage (+): 0. Max coverage (-): 0

Region: NODE\_347176\_length\_38458\_cov\_26.791616 36145-36150. Max. coverage (+): 0. Max coverage (-): 0

Region: NODE\_347176\_length\_38458\_cov\_26.791616 36151-36157. Max. coverage (+): 0. Max coverage (-): 14.63

Region: NODE\_347176\_length\_38458\_cov\_26.791616 36158-36163. Max. coverage (+): 0. Max coverage (-): 272.56

Region: NODE\_347176\_length\_38458\_cov\_26.791616 36164-36169. Max. coverage (+): 0. Max coverage (-): 0

Region: NODE\_347176\_length\_38458\_cov\_26.791616 36170-36175. Max. coverage (+): 0. Max coverage (-): 0

Region: NODE\_347176\_length\_38458\_cov\_26.791616 36176-36181. Max. coverage (+): 0. Max coverage (-): 0

Region: NODE\_347176\_length\_38458\_cov\_26.791616 36182-36187. Max. coverage (+): 0. Max coverage (-): 0.23

Region: NODE\_347176\_length\_38458\_cov\_26.791616 36188-36194. Max. coverage (+): 0. Max coverage (-): 0

Region: NODE\_347176\_length\_38458\_cov\_26.791616 36195-36200. Max. coverage (+): 0. Max coverage (-): 0

Region: NODE\_347176\_length\_38458\_cov\_26.791616 36201-36206. Max. coverage (+): 0. Max coverage (-): 0

Region: NODE\_347176\_length\_38458\_cov\_26.791616 36207-36212. Max. coverage (+): 0. Max coverage (-): 0

Region: NODE\_347176\_length\_38458\_cov\_26.791616 36213-36218. Max. coverage (+): 0. Max coverage (-): 0

Region: NODE\_347176\_length\_38458\_cov\_26.791616 36219-36224. Max. coverage (+): 0. Max coverage (-): 0

Region: NODE\_347176\_length\_38458\_cov\_26.791616 36225-36231. Max. coverage (+): 0. Max coverage (-): 0

Region: NODE\_347176\_length\_38458\_cov\_26.791616 36232-36237. Max. coverage (+): 0. Max coverage (-): 0

Region: NODE\_347176\_length\_38458\_cov\_26.791616 36238-36243. Max. coverage (+): 0. Max coverage (-): 0

Region: NODE\_347176\_length\_38458\_cov\_26.791616 36244-36249. Max. coverage (+): 0. Max coverage (-): 0

Region: NODE\_347176\_length\_38458\_cov\_26.791616 36250-36255. Max. coverage (+): 0. Max coverage (-): 0

Region: NODE\_347176\_length\_38458\_cov\_26.791616 36256-36261. Max. coverage (+): 0. Max coverage (-): 0

Region: NODE\_347176\_length\_38458\_cov\_26.791616 36262-36268. Max. coverage (+): 0. Max coverage (-): 0

Region: NODE\_347176\_length\_38458\_cov\_26.791616 36269-36274. Max. coverage (+): 0. Max coverage (-): 0

Region: NODE\_347176\_length\_38458\_cov\_26.791616 36275-36280. Max. coverage (+): 0. Max coverage (-): 0

Region: NODE\_347176\_length\_38458\_cov\_26.791616 36281-36286. Max. coverage (+): 0. Max coverage (-): 0

Region: NODE\_347176\_length\_38458\_cov\_26.791616 36287-36292. Max. coverage (+): 0. Max coverage (-): 0

Region: NODE\_347176\_length\_38458\_cov\_26.791616 36293-36298. Max. coverage (+): 0. Max coverage (-): 0

Region: NODE\_347176\_length\_38458\_cov\_26.791616 36299-36305. Max. coverage (+): 0. Max coverage (-): 0

Region: NODE\_347176\_length\_38458\_cov\_26.791616 36306-36311. Max. coverage (+): 0. Max coverage (-): 0

Region: NODE\_347176\_length\_38458\_cov\_26.791616 36312-36317. Max. coverage (+): 0. Max coverage (-): 0

Region: NODE\_347176\_length\_38458\_cov\_26.791616 36318-36323. Max. coverage (+): 0. Max coverage (-): 0

Region: NODE\_347176\_length\_38458\_cov\_26.791616 36324-36329. Max. coverage (+): 0. Max coverage (-): 0

Region: NODE\_347176\_length\_38458\_cov\_26.791616 36330-36335. Max. coverage (+): 0. Max coverage (-): 0

Region: NODE\_347176\_length\_38458\_cov\_26.791616 36336-36342. Max. coverage (+): 0. Max coverage (-): 0

Region: NODE\_347176\_length\_38458\_cov\_26.791616 36343-36348. Max. coverage (+): 0. Max coverage (-): 0

Region: NODE\_347176\_length\_38458\_cov\_26.791616 36349-36354. Max. coverage (+): 0. Max coverage (-): 0

Region: NODE\_347176\_length\_38458\_cov\_26.791616 36355-36360. Max. coverage (+): 0. Max coverage (-): 0

Region: NODE\_347176\_length\_38458\_cov\_26.791616 36361-36366. Max. coverage (+): 0. Max coverage (-): 0

Region: NODE\_347176\_length\_38458\_cov\_26.791616 36367-36372. Max. coverage (+): 0. Max coverage (-): 0

Region: NODE\_347176\_length\_38458\_cov\_26.791616 36373-36379. Max. coverage (+): 0. Max coverage (-): 0

Region: NODE\_347176\_length\_38458\_cov\_26.791616 36380-36385. Max. coverage (+): 0. Max coverage (-): 0

Region: NODE\_347176\_length\_38458\_cov\_26.791616 36386-36391. Max. coverage (+): 0. Max coverage (-): 0

Region: NODE\_347176\_length\_38458\_cov\_26.791616 36392-36397. Max. coverage (+): 0. Max coverage (-): 0

Region: NODE\_347176\_length\_38458\_cov\_26.791616 36398-36403. Max. coverage (+): 0. Max coverage (-): 0

Region: NODE\_347176\_length\_38458\_cov\_26.791616 36404-36409. Max. coverage (+): 0. Max coverage (-): 0

Region: NODE\_347176\_length\_38458\_cov\_26.791616 36410-36416. Max. coverage (+): 0. Max coverage (-): 0

Region: NODE\_347176\_length\_38458\_cov\_26.791616 36417-36422. Max. coverage (+): 0. Max coverage (-): 0

Region: NODE\_347176\_length\_38458\_cov\_26.791616 36423-36428. Max. coverage (+): 0. Max coverage (-): 0

Region: NODE\_347176\_length\_38458\_cov\_26.791616 36429-36434. Max. coverage (+): 0. Max coverage (-): 0

Region: NODE\_347176\_length\_38458\_cov\_26.791616 36435-36440. Max. coverage (+): 0. Max coverage (-): 0

Region: NODE\_347176\_length\_38458\_cov\_26.791616 36441-36446. Max. coverage (+): 0. Max coverage (-): 0

Region: NODE\_347176\_length\_38458\_cov\_26.791616 36447-36453. Max. coverage (+): 0. Max coverage (-): 0

Region: NODE\_347176\_length\_38458\_cov\_26.791616 36454-36459. Max. coverage (+): 0. Max coverage (-): 0

Region: NODE\_347176\_length\_38458\_cov\_26.791616 36460-36465. Max. coverage (+): 0. Max coverage (-): 0

Region: NODE\_347176\_length\_38458\_cov\_26.791616 36466-36471. Max. coverage (+): 0. Max coverage (-): 0

Region: NODE\_347176\_length\_38458\_cov\_26.791616 36472-36477. Max. coverage (+): 0. Max coverage (-): 0

Region: NODE\_347176\_length\_38458\_cov\_26.791616 36478-36483. Max. coverage (+): 0. Max coverage (-): 0

Region: NODE\_347176\_length\_38458\_cov\_26.791616 36484-36490. Max. coverage (+): 0. Max coverage (-): 0

Region: NODE\_347176\_length\_38458\_cov\_26.791616 36491-36496. Max. coverage (+): 0. Max coverage (-): 0

Region: NODE\_347176\_length\_38458\_cov\_26.791616 36497-36502. Max. coverage (+): 0. Max coverage (-): 0

Region: NODE\_347176\_length\_38458\_cov\_26.791616 36503-36508. Max. coverage (+): 0. Max coverage (-): 0

Region: NODE\_347176\_length\_38458\_cov\_26.791616 36509-36514. Max. coverage (+): 0. Max coverage (-): 0

Region: NODE\_347176\_length\_38458\_cov\_26.791616 36515-36520. Max. coverage (+): 0. Max coverage (-): 0

Region: NODE\_347176\_length\_38458\_cov\_26.791616 36521-36527. Max. coverage (+): 0. Max coverage (-): 0

Region: NODE\_347176\_length\_38458\_cov\_26.791616 36528-36533. Max. coverage (+): 0. Max coverage (-): 0

Region: NODE\_347176\_length\_38458\_cov\_26.791616 36534-36539. Max. coverage (+): 0. Max coverage (-): 0

Region: NODE\_347176\_length\_38458\_cov\_26.791616 36540-36545. Max. coverage (+): 0. Max coverage (-): 0

Region: NODE\_347176\_length\_38458\_cov\_26.791616 36546-36551. Max. coverage (+): 0. Max coverage (-): 0

Region: NODE\_347176\_length\_38458\_cov\_26.791616 36552-36557. Max. coverage (+): 0. Max coverage (-): 0

Region: NODE\_347176\_length\_38458\_cov\_26.791616 36558-36564. Max. coverage (+): 0. Max coverage (-): 0

Region: NODE\_347176\_length\_38458\_cov\_26.791616 36565-36570. Max. coverage (+): 0. Max coverage (-): 0

Region: NODE\_347176\_length\_38458\_cov\_26.791616 36571-36576. Max. coverage (+): 0. Max coverage (-): 0

Region: NODE\_347176\_length\_38458\_cov\_26.791616 36577-36582. Max. coverage (+): 0. Max coverage (-): 0

Region: NODE\_347176\_length\_38458\_cov\_26.791616 36583-36588. Max. coverage (+): 0. Max coverage (-): 0

Region: NODE\_347176\_length\_38458\_cov\_26.791616 36589-36594. Max. coverage (+): 0. Max coverage (-): 0

Region: NODE\_347176\_length\_38458\_cov\_26.791616 36595-36601. Max. coverage (+): 0. Max coverage (-): 0

Region: NODE\_347176\_length\_38458\_cov\_26.791616 36602-36607. Max. coverage (+): 0. Max coverage (-): 0

Region: NODE\_347176\_length\_38458\_cov\_26.791616 36608-36613. Max. coverage (+): 0. Max coverage (-): 0

Region: NODE\_347176\_length\_38458\_cov\_26.791616 36614-36619. Max. coverage (+): 0. Max coverage (-): 0

Region: NODE\_347176\_length\_38458\_cov\_26.791616 36620-36625. Max. coverage (+): 0. Max coverage (-): 0

Region: NODE\_347176\_length\_38458\_cov\_26.791616 36626-36631. Max. coverage (+): 0. Max coverage (-): 0

Region: NODE\_347176\_length\_38458\_cov\_26.791616 36632-36638. Max. coverage (+): 0. Max coverage (-): 0

Region: NODE\_347176\_length\_38458\_cov\_26.791616 36639-36644. Max. coverage (+): 0. Max coverage (-): 0

Region: NODE\_347176\_length\_38458\_cov\_26.791616 36645-36650. Max. coverage (+): 0. Max coverage (-): 0

Region: NODE\_347176\_length\_38458\_cov\_26.791616 36651-36656. Max. coverage (+): 0. Max coverage (-): 0

Region: NODE\_347176\_length\_38458\_cov\_26.791616 36657-36662. Max. coverage (+): 0. Max coverage (-): 0

Region: NODE\_347176\_length\_38458\_cov\_26.791616 36663-36668. Max. coverage (+): 0. Max coverage (-): 0

Region: NODE\_347176\_length\_38458\_cov\_26.791616 36669-36675. Max. coverage (+): 0. Max coverage (-): 0

Region: NODE\_347176\_length\_38458\_cov\_26.791616 36676-36681. Max. coverage (+): 0. Max coverage (-): 0

Region: NODE\_347176\_length\_38458\_cov\_26.791616 36682-36687. Max. coverage (+): 0. Max coverage (-): 0

Region: NODE\_347176\_length\_38458\_cov\_26.791616 36688-36693. Max. coverage (+): 0. Max coverage (-): 0

Region: NODE\_347176\_length\_38458\_cov\_26.791616 36694-36699. Max. coverage (+): 0. Max coverage (-): 0

Region: NODE\_347176\_length\_38458\_cov\_26.791616 36700-36705. Max. coverage (+): 0. Max coverage (-): 0

Region: NODE\_347176\_length\_38458\_cov\_26.791616 36706-36712. Max. coverage (+): 0. Max coverage (-): 0

Region: NODE\_347176\_length\_38458\_cov\_26.791616 36713-36718. Max. coverage (+): 0. Max coverage (-): 0

Region: NODE\_347176\_length\_38458\_cov\_26.791616 36719-36724. Max. coverage (+): 0. Max coverage (-): 0

Region: NODE\_347176\_length\_38458\_cov\_26.791616 36725-36730. Max. coverage (+): 0. Max coverage (-): 0

Region: NODE\_347176\_length\_38458\_cov\_26.791616 36731-36736. Max. coverage (+): 0. Max coverage (-): 0

Region: NODE\_347176\_length\_38458\_cov\_26.791616 36737-36742. Max. coverage (+): 0. Max coverage (-): 0

Region: NODE\_347176\_length\_38458\_cov\_26.791616 36743-36749. Max. coverage (+): 0. Max coverage (-): 0

Region: NODE\_347176\_length\_38458\_cov\_26.791616 36750-36755. Max. coverage (+): 0. Max coverage (-): 0

Region: NODE\_347176\_length\_38458\_cov\_26.791616 36756-36761. Max. coverage (+): 0. Max coverage (-): 0

Region: NODE\_347176\_length\_38458\_cov\_26.791616 36762-36767. Max. coverage (+): 0. Max coverage (-): 0

Region: NODE\_347176\_length\_38458\_cov\_26.791616 36768-36773. Max. coverage (+): 0. Max coverage (-): 0

Region: NODE\_347176\_length\_38458\_cov\_26.791616 36774-36779. Max. coverage (+): 0. Max coverage (-): 0

Region: NODE\_347176\_length\_38458\_cov\_26.791616 36780-36786. Max. coverage (+): 0. Max coverage (-): 0

Region: NODE\_347176\_length\_38458\_cov\_26.791616 36787-36792. Max. coverage (+): 0. Max coverage (-): 0

Region: NODE\_347176\_length\_38458\_cov\_26.791616 36793-36798. Max. coverage (+): 0. Max coverage (-): 0

Region: NODE\_347176\_length\_38458\_cov\_26.791616 36799-36804. Max. coverage (+): 0. Max coverage (-): 0

Region: NODE\_347176\_length\_38458\_cov\_26.791616 36805-36810. Max. coverage (+): 0. Max coverage (-): 0

Region: NODE\_347176\_length\_38458\_cov\_26.791616 36811-36816. Max. coverage (+): 0. Max coverage (-): 0

Region: NODE\_347176\_length\_38458\_cov\_26.791616 36817-36823. Max. coverage (+): 0. Max coverage (-): 0

Region: NODE\_347176\_length\_38458\_cov\_26.791616 36824-36829. Max. coverage (+): 0. Max coverage (-): 0

Region: NODE\_347176\_length\_38458\_cov\_26.791616 36830-36835. Max. coverage (+): 0. Max coverage (-): 0

Region: NODE\_347176\_length\_38458\_cov\_26.791616 36836-36841. Max. coverage (+): 0. Max coverage (-): 0

Region: NODE\_347176\_length\_38458\_cov\_26.791616 36842-36847. Max. coverage (+): 0. Max coverage (-): 0

Region: NODE\_347176\_length\_38458\_cov\_26.791616 36848-36853. Max. coverage (+): 0. Max coverage (-): 0

Region: NODE\_347176\_length\_38458\_cov\_26.791616 36854-36860. Max. coverage (+): 0. Max coverage (-): 0

Region: NODE\_347176\_length\_38458\_cov\_26.791616 36861-36866. Max. coverage (+): 0. Max coverage (-): 0

Region: NODE\_347176\_length\_38458\_cov\_26.791616 36867-36872. Max. coverage (+): 0. Max coverage (-): 0

Region: NODE\_347176\_length\_38458\_cov\_26.791616 36873-36878. Max. coverage (+): 0. Max coverage (-): 0

Region: NODE\_347176\_length\_38458\_cov\_26.791616 36879-36884. Max. coverage (+): 0. Max coverage (-): 0

Region: NODE\_347176\_length\_38458\_cov\_26.791616 36885-36890. Max. coverage (+): 0. Max coverage (-): 0

Region: NODE\_347176\_length\_38458\_cov\_26.791616 36891-36897. Max. coverage (+): 0. Max coverage (-): 0

Region: NODE\_347176\_length\_38458\_cov\_26.791616 36898-36903. Max. coverage (+): 0. Max coverage (-): 0

Region: NODE\_347176\_length\_38458\_cov\_26.791616 36904-36909. Max. coverage (+): 0. Max coverage (-): 0

Region: NODE\_347176\_length\_38458\_cov\_26.791616 36910-36915. Max. coverage (+): 0. Max coverage (-): 0

Region: NODE\_347176\_length\_38458\_cov\_26.791616 36916-36921. Max. coverage (+): 0. Max coverage (-): 0

Region: NODE\_347176\_length\_38458\_cov\_26.791616 36922-36927. Max. coverage (+): 0. Max coverage (-): 0

Region: NODE\_347176\_length\_38458\_cov\_26.791616 36928-36934. Max. coverage (+): 0. Max coverage (-): 0.08

Region: NODE\_347176\_length\_38458\_cov\_26.791616 36935-36940. Max. coverage (+): 0. Max coverage (-): 0

Region: NODE\_347176\_length\_38458\_cov\_26.791616 36941-36946. Max. coverage (+): 0. Max coverage (-): 0

Region: NODE\_347176\_length\_38458\_cov\_26.791616 36947-36952. Max. coverage (+): 0. Max coverage (-): 0

Region: NODE\_347176\_length\_38458\_cov\_26.791616 36953-36958. Max. coverage (+): 0. Max coverage (-): 0

Region: NODE\_347176\_length\_38458\_cov\_26.791616 36959-36964. Max. coverage (+): 0. Max coverage (-): 0

Region: NODE\_347176\_length\_38458\_cov\_26.791616 36965-36971. Max. coverage (+): 0. Max coverage (-): 0

Region: NODE\_347176\_length\_38458\_cov\_26.791616 36972-36977. Max. coverage (+): 0. Max coverage (-): 0

Region: NODE\_347176\_length\_38458\_cov\_26.791616 36978-36983. Max. coverage (+): 0. Max coverage (-): 0

Region: NODE\_347176\_length\_38458\_cov\_26.791616 36984-36989. Max. coverage (+): 0. Max coverage (-): 0

Region: NODE\_347176\_length\_38458\_cov\_26.791616 36990-36995. Max. coverage (+): 0. Max coverage (-): 0

Region: NODE\_347176\_length\_38458\_cov\_26.791616 36996-37001. Max. coverage (+): 0. Max coverage (-): 0

Region: NODE\_347176\_length\_38458\_cov\_26.791616 37002-37008. Max. coverage (+): 0. Max coverage (-): 0

Region: NODE\_347176\_length\_38458\_cov\_26.791616 37009-37014. Max. coverage (+): 0. Max coverage (-): 0

Region: NODE\_347176\_length\_38458\_cov\_26.791616 37015-37020. Max. coverage (+): 0. Max coverage (-): 0

Region: NODE\_347176\_length\_38458\_cov\_26.791616 37021-37026. Max. coverage (+): 0. Max coverage (-): 0

Region: NODE\_347176\_length\_38458\_cov\_26.791616 37027-37032. Max. coverage (+): 0. Max coverage (-): 0

Region: NODE\_347176\_length\_38458\_cov\_26.791616 37033-37038. Max. coverage (+): 0. Max coverage (-): 0

Region: NODE\_347176\_length\_38458\_cov\_26.791616 37039-37045. Max. coverage (+): 0. Max coverage (-): 0

Region: NODE\_347176\_length\_38458\_cov\_26.791616 37046-37051. Max. coverage (+): 0. Max coverage (-): 0

Region: NODE\_347176\_length\_38458\_cov\_26.791616 37052-37057. Max. coverage (+): 0. Max coverage (-): 0

Region: NODE\_347176\_length\_38458\_cov\_26.791616 37058-37063. Max. coverage (+): 0. Max coverage (-): 0

Region: NODE\_347176\_length\_38458\_cov\_26.791616 37064-37069. Max. coverage (+): 0. Max coverage (-): 0

Region: NODE\_347176\_length\_38458\_cov\_26.791616 37070-37075. Max. coverage (+): 0. Max coverage (-): 0

Region: NODE\_347176\_length\_38458\_cov\_26.791616 37076-37082. Max. coverage (+): 0. Max coverage (-): 0

Region: NODE\_347176\_length\_38458\_cov\_26.791616 37083-37088. Max. coverage (+): 0. Max coverage (-): 0

Region: NODE\_347176\_length\_38458\_cov\_26.791616 37089-37094. Max. coverage (+): 0. Max coverage (-): 0

Region: NODE\_347176\_length\_38458\_cov\_26.791616 37095-37100. Max. coverage (+): 0. Max coverage (-): 0

Region: NODE\_347176\_length\_38458\_cov\_26.791616 37101-37106. Max. coverage (+): 0. Max coverage (-): 0

Region: NODE\_347176\_length\_38458\_cov\_26.791616 37107-37112. Max. coverage (+): 0. Max coverage (-): 0

Region: NODE\_347176\_length\_38458\_cov\_26.791616 37113-37119. Max. coverage (+): 0. Max coverage (-): 0

Region: NODE\_347176\_length\_38458\_cov\_26.791616 37120-37125. Max. coverage (+): 0. Max coverage (-): 0

Region: NODE\_347176\_length\_38458\_cov\_26.791616 37126-37131. Max. coverage (+): 0. Max coverage (-): 0

Region: NODE\_347176\_length\_38458\_cov\_26.791616 37132-37137. Max. coverage (+): 0. Max coverage (-): 0

Region: NODE\_347176\_length\_38458\_cov\_26.791616 37138-37143. Max. coverage (+): 0. Max coverage (-): 0

Region: NODE\_347176\_length\_38458\_cov\_26.791616 37144-37149. Max. coverage (+): 0. Max coverage (-): 0

Region: NODE\_347176\_length\_38458\_cov\_26.791616 37150-37156. Max. coverage (+): 0. Max coverage (-): 0.08

Region: NODE\_347176\_length\_38458\_cov\_26.791616 37157-37162. Max. coverage (+): 0. Max coverage (-): 0.08

Region: NODE\_347176\_length\_38458\_cov\_26.791616 37163-37168. Max. coverage (+): 0. Max coverage (-): 0.08

Region: NODE\_347176\_length\_38458\_cov\_26.791616 37169-37174. Max. coverage (+): 0. Max coverage (-): 0.08

Region: NODE\_347176\_length\_38458\_cov\_26.791616 37175-37180. Max. coverage (+): 0. Max coverage (-): 0

Region: NODE\_347176\_length\_38458\_cov\_26.791616 37181-37186. Max. coverage (+): 0. Max coverage (-): 0

Region: NODE\_347176\_length\_38458\_cov\_26.791616 37187-37193. Max. coverage (+): 0. Max coverage (-): 0

Region: NODE\_347176\_length\_38458\_cov\_26.791616 37194-37199. Max. coverage (+): 0. Max coverage (-): 0

Region: NODE\_347176\_length\_38458\_cov\_26.791616 37200-37205. Max. coverage (+): 0. Max coverage (-): 0

Region: NODE\_347176\_length\_38458\_cov\_26.791616 37206-37211. Max. coverage (+): 0. Max coverage (-): 0

Region: NODE\_347176\_length\_38458\_cov\_26.791616 37212-37217. Max. coverage (+): 0. Max coverage (-): 0

Region: NODE\_347176\_length\_38458\_cov\_26.791616 37218-37223. Max. coverage (+): 0. Max coverage (-): 0

Region: NODE\_347176\_length\_38458\_cov\_26.791616 37224-37230. Max. coverage (+): 0. Max coverage (-): 0

Region: NODE\_347176\_length\_38458\_cov\_26.791616 37231-37236. Max. coverage (+): 0. Max coverage (-): 0

Region: NODE\_347176\_length\_38458\_cov\_26.791616 37237-37242. Max. coverage (+): 0. Max coverage (-): 0

Region: NODE\_347176\_length\_38458\_cov\_26.791616 37243-37248. Max. coverage (+): 0. Max coverage (-): 0

Region: NODE\_347176\_length\_38458\_cov\_26.791616 37249-37254. Max. coverage (+): 0. Max coverage (-): 0

Region: NODE\_347176\_length\_38458\_cov\_26.791616 37255-37260. Max. coverage (+): 0. Max coverage (-): 0

Region: NODE\_347176\_length\_38458\_cov\_26.791616 37261-37267. Max. coverage (+): 0. Max coverage (-): 0

Region: NODE\_347176\_length\_38458\_cov\_26.791616 37268-37273. Max. coverage (+): 0. Max coverage (-): 0

Region: NODE\_347176\_length\_38458\_cov\_26.791616 37274-37279. Max. coverage (+): 0. Max coverage (-): 0

Region: NODE\_347176\_length\_38458\_cov\_26.791616 37280-37285. Max. coverage (+): 0. Max coverage (-): 0

Region: NODE\_347176\_length\_38458\_cov\_26.791616 37286-37291. Max. coverage (+): 0. Max coverage (-): 0

Region: NODE\_347176\_length\_38458\_cov\_26.791616 37292-37297. Max. coverage (+): 0. Max coverage (-): 0

Region: NODE\_347176\_length\_38458\_cov\_26.791616 37298-37304. Max. coverage (+): 0. Max coverage (-): 0.08

Region: NODE\_347176\_length\_38458\_cov\_26.791616 37305-37310. Max. coverage (+): 0. Max coverage (-): 0

Region: NODE\_347176\_length\_38458\_cov\_26.791616 37311-37316. Max. coverage (+): 0. Max coverage (-): 0

Region: NODE\_347176\_length\_38458\_cov\_26.791616 37317-37322. Max. coverage (+): 0. Max coverage (-): 0.08

Region: NODE\_347176\_length\_38458\_cov\_26.791616 37323-37328. Max. coverage (+): 0. Max coverage (-): 0.08

Region: NODE\_347176\_length\_38458\_cov\_26.791616 37329-37334. Max. coverage (+): 0. Max coverage (-): 0

Region: NODE\_347176\_length\_38458\_cov\_26.791616 37335-37341. Max. coverage (+): 0. Max coverage (-): 0

Region: NODE\_347176\_length\_38458\_cov\_26.791616 37342-37347. Max. coverage (+): 0. Max coverage (-): 0

Region: NODE\_347176\_length\_38458\_cov\_26.791616 37348-37353. Max. coverage (+): 0. Max coverage (-): 0

Region: NODE\_347176\_length\_38458\_cov\_26.791616 37354-37359. Max. coverage (+): 0. Max coverage (-): 0

Region: NODE\_347176\_length\_38458\_cov\_26.791616 37360-37365. Max. coverage (+): 0. Max coverage (-): 0

Region: NODE\_347176\_length\_38458\_cov\_26.791616 37366-37371. Max. coverage (+): 0. Max coverage (-): 0

Region: NODE\_347176\_length\_38458\_cov\_26.791616 37372-37378. Max. coverage (+): 0. Max coverage (-): 0

Region: NODE\_347176\_length\_38458\_cov\_26.791616 37379-37384. Max. coverage (+): 0. Max coverage (-): 0

Region: NODE\_347176\_length\_38458\_cov\_26.791616 37385-37390. Max. coverage (+): 0. Max coverage (-): 0

Region: NODE\_347176\_length\_38458\_cov\_26.791616 37391-37396. Max. coverage (+): 0. Max coverage (-): 0

Region: NODE\_347176\_length\_38458\_cov\_26.791616 37397-37402. Max. coverage (+): 0. Max coverage (-): 0

Region: NODE\_347176\_length\_38458\_cov\_26.791616 37403-37408. Max. coverage (+): 0. Max coverage (-): 0

Region: NODE\_347176\_length\_38458\_cov\_26.791616 37409-37415. Max. coverage (+): 0. Max coverage (-): 0

Region: NODE\_347176\_length\_38458\_cov\_26.791616 37416-37421. Max. coverage (+): 0. Max coverage (-): 0

Region: NODE\_347176\_length\_38458\_cov\_26.791616 37422-37427. Max. coverage (+): 0. Max coverage (-): 0

Region: NODE\_347176\_length\_38458\_cov\_26.791616 37428-37433. Max. coverage (+): 0. Max coverage (-): 0

Region: NODE\_347176\_length\_38458\_cov\_26.791616 37434-37439. Max. coverage (+): 0. Max coverage (-): 0

Region: NODE\_347176\_length\_38458\_cov\_26.791616 37440-37445. Max. coverage (+): 0. Max coverage (-): 0

Region: NODE\_347176\_length\_38458\_cov\_26.791616 37446-37452. Max. coverage (+): 0. Max coverage (-): 0

Region: NODE\_347176\_length\_38458\_cov\_26.791616 37453-37458. Max. coverage (+): 0. Max coverage (-): 0

Region: NODE\_347176\_length\_38458\_cov\_26.791616 37459-37464. Max. coverage (+): 0. Max coverage (-): 0

Region: NODE\_347176\_length\_38458\_cov\_26.791616 37465-37470. Max. coverage (+): 0. Max coverage (-): 0

Region: NODE\_347176\_length\_38458\_cov\_26.791616 37471-37476. Max. coverage (+): 0. Max coverage (-): 0

Region: NODE\_347176\_length\_38458\_cov\_26.791616 37477-37482. Max. coverage (+): 0. Max coverage (-): 0

Region: NODE\_347176\_length\_38458\_cov\_26.791616 37483-37489. Max. coverage (+): 0. Max coverage (-): 0

Region: NODE\_347176\_length\_38458\_cov\_26.791616 37490-37495. Max. coverage (+): 0. Max coverage (-): 0

Region: NODE\_347176\_length\_38458\_cov\_26.791616 37496-37501. Max. coverage (+): 0. Max coverage (-): 0

Region: NODE\_347176\_length\_38458\_cov\_26.791616 37502-37507. Max. coverage (+): 0. Max coverage (-): 0

Region: NODE\_347176\_length\_38458\_cov\_26.791616 37508-37513. Max. coverage (+): 0. Max coverage (-): 0

Region: NODE\_347176\_length\_38458\_cov\_26.791616 37514-37519. Max. coverage (+): 0. Max coverage (-): 0

Region: NODE\_347176\_length\_38458\_cov\_26.791616 37520-37526. Max. coverage (+): 0. Max coverage (-): 0

Region: NODE\_347176\_length\_38458\_cov\_26.791616 37527-37532. Max. coverage (+): 0. Max coverage (-): 0

Region: NODE\_347176\_length\_38458\_cov\_26.791616 37533-37538. Max. coverage (+): 0. Max coverage (-): 0

Region: NODE\_347176\_length\_38458\_cov\_26.791616 37539-37544. Max. coverage (+): 0. Max coverage (-): 0

Region: NODE\_347176\_length\_38458\_cov\_26.791616 37545-37550. Max. coverage (+): 0. Max coverage (-): 0

Region: NODE\_347176\_length\_38458\_cov\_26.791616 37551-37556. Max. coverage (+): 0. Max coverage (-): 0

Region: NODE\_347176\_length\_38458\_cov\_26.791616 37557-37563. Max. coverage (+): 0. Max coverage (-): 0

Region: NODE\_347176\_length\_38458\_cov\_26.791616 37564-37569. Max. coverage (+): 0. Max coverage (-): 0

Region: NODE\_347176\_length\_38458\_cov\_26.791616 37570-37575. Max. coverage (+): 0. Max coverage (-): 0

Region: NODE\_347176\_length\_38458\_cov\_26.791616 37576-37581. Max. coverage (+): 0. Max coverage (-): 0

Region: NODE\_347176\_length\_38458\_cov\_26.791616 37582-37587. Max. coverage (+): 0. Max coverage (-): 0

Region: NODE\_347176\_length\_38458\_cov\_26.791616 37588-37593. Max. coverage (+): 0. Max coverage (-): 0

Region: NODE\_347176\_length\_38458\_cov\_26.791616 37594-37600. Max. coverage (+): 0. Max coverage (-): 0

Region: NODE\_347176\_length\_38458\_cov\_26.791616 37601-37606. Max. coverage (+): 0. Max coverage (-): 0

Region: NODE\_347176\_length\_38458\_cov\_26.791616 37607-37612. Max. coverage (+): 0. Max coverage (-): 0

Region: NODE\_347176\_length\_38458\_cov\_26.791616 37613-37618. Max. coverage (+): 0. Max coverage (-): 0

Region: NODE\_347176\_length\_38458\_cov\_26.791616 37619-37624. Max. coverage (+): 0. Max coverage (-): 0

Region: NODE\_347176\_length\_38458\_cov\_26.791616 37625-37630. Max. coverage (+): 0. Max coverage (-): 0

Region: NODE\_347176\_length\_38458\_cov\_26.791616 37631-37637. Max. coverage (+): 0. Max coverage (-): 0

Region: NODE\_347176\_length\_38458\_cov\_26.791616 37638-37643. Max. coverage (+): 0. Max coverage (-): 0

Region: NODE\_347176\_length\_38458\_cov\_26.791616 37644-37649. Max. coverage (+): 0. Max coverage (-): 0

Region: NODE\_347176\_length\_38458\_cov\_26.791616 37650-37655. Max. coverage (+): 0. Max coverage (-): 0

Region: NODE\_347176\_length\_38458\_cov\_26.791616 37656-37661. Max. coverage (+): 0. Max coverage (-): 0

Region: NODE\_347176\_length\_38458\_cov\_26.791616 37662-37667. Max. coverage (+): 0. Max coverage (-): 0

Region: NODE\_347176\_length\_38458\_cov\_26.791616 37668-37674. Max. coverage (+): 0. Max coverage (-): 0

Region: NODE\_347176\_length\_38458\_cov\_26.791616 37675-37680. Max. coverage (+): 0. Max coverage (-): 0

Region: NODE\_347176\_length\_38458\_cov\_26.791616 37681-37686. Max. coverage (+): 0. Max coverage (-): 0

Region: NODE\_347176\_length\_38458\_cov\_26.791616 37687-37692. Max. coverage (+): 0. Max coverage (-): 0

Region: NODE\_347176\_length\_38458\_cov\_26.791616 37693-37698. Max. coverage (+): 0. Max coverage (-): 0.08

Region: NODE\_347176\_length\_38458\_cov\_26.791616 37699-37704. Max. coverage (+): 0. Max coverage (-): 0

Region: NODE\_347176\_length\_38458\_cov\_26.791616 37705-37710. Max. coverage (+): 0. Max coverage (-): 0

Region: NODE\_347176\_length\_38458\_cov\_26.791616 37711-37717. Max. coverage (+): 0. Max coverage (-): 0

Region: NODE\_347176\_length\_38458\_cov\_26.791616 37718-37723. Max. coverage (+): 0. Max coverage (-): 0

Region: NODE\_347176\_length\_38458\_cov\_26.791616 37724-37729. Max. coverage (+): 0. Max coverage (-): 0

Region: NODE\_347176\_length\_38458\_cov\_26.791616 37730-37735. Max. coverage (+): 0. Max coverage (-): 0

Region: NODE\_347176\_length\_38458\_cov\_26.791616 37736-37741. Max. coverage (+): 0. Max coverage (-): 0.08

Region: NODE\_347176\_length\_38458\_cov\_26.791616 37742-37747. Max. coverage (+): 0. Max coverage (-): 0

Region: NODE\_347176\_length\_38458\_cov\_26.791616 37748-37754. Max. coverage (+): 0. Max coverage (-): 0

Region: NODE\_347176\_length\_38458\_cov\_26.791616 37755-37760. Max. coverage (+): 0. Max coverage (-): 0

Region: NODE\_347176\_length\_38458\_cov\_26.791616 37761-37766. Max. coverage (+): 0. Max coverage (-): 0

Region: NODE\_347176\_length\_38458\_cov\_26.791616 37767-37772. Max. coverage (+): 0. Max coverage (-): 0

Region: NODE\_347176\_length\_38458\_cov\_26.791616 37773-37778. Max. coverage (+): 0. Max coverage (-): 0

Region: NODE\_347176\_length\_38458\_cov\_26.791616 37779-37784. Max. coverage (+): 0. Max coverage (-): 0

Region: NODE\_347176\_length\_38458\_cov\_26.791616 37785-37791. Max. coverage (+): 0. Max coverage (-): 0

Region: NODE\_347176\_length\_38458\_cov\_26.791616 37792-37797. Max. coverage (+): 0. Max coverage (-): 0

Region: NODE\_347176\_length\_38458\_cov\_26.791616 37798-37803. Max. coverage (+): 0. Max coverage (-): 0

Region: NODE\_347176\_length\_38458\_cov\_26.791616 37804-37809. Max. coverage (+): 0. Max coverage (-): 0

Region: NODE\_347176\_length\_38458\_cov\_26.791616 37810-37815. Max. coverage (+): 0. Max coverage (-): 0

Region: NODE\_347176\_length\_38458\_cov\_26.791616 37816-37821. Max. coverage (+): 0. Max coverage (-): 0

Region: NODE\_347176\_length\_38458\_cov\_26.791616 37822-37828. Max. coverage (+): 0. Max coverage (-): 0

Region: NODE\_347176\_length\_38458\_cov\_26.791616 37829-37834. Max. coverage (+): 0. Max coverage (-): 0

Region: NODE\_347176\_length\_38458\_cov\_26.791616 37835-37840. Max. coverage (+): 0. Max coverage (-): 0

Region: NODE\_347176\_length\_38458\_cov\_26.791616 37841-37846. Max. coverage (+): 0. Max coverage (-): 0

Region: NODE\_347176\_length\_38458\_cov\_26.791616 37847-37852. Max. coverage (+): 0. Max coverage (-): 0

Region: NODE\_347176\_length\_38458\_cov\_26.791616 37853-37858. Max. coverage (+): 0. Max coverage (-): 0

Region: NODE\_347176\_length\_38458\_cov\_26.791616 37859-37865. Max. coverage (+): 0. Max coverage (-): 0

Region: NODE\_347176\_length\_38458\_cov\_26.791616 37866-37871. Max. coverage (+): 0. Max coverage (-): 0

Region: NODE\_347176\_length\_38458\_cov\_26.791616 37872-37877. Max. coverage (+): 0. Max coverage (-): 0

Region: NODE\_347176\_length\_38458\_cov\_26.791616 37878-37883. Max. coverage (+): 0. Max coverage (-): 0

Region: NODE\_347176\_length\_38458\_cov\_26.791616 37884-37889. Max. coverage (+): 0. Max coverage (-): 0

Region: NODE\_347176\_length\_38458\_cov\_26.791616 37890-37895. Max. coverage (+): 0. Max coverage (-): 0

Region: NODE\_347176\_length\_38458\_cov\_26.791616 37896-37902. Max. coverage (+): 0. Max coverage (-): 0

Region: NODE\_347176\_length\_38458\_cov\_26.791616 37903-37908. Max. coverage (+): 0. Max coverage (-): 0

Region: NODE\_347176\_length\_38458\_cov\_26.791616 37909-37914. Max. coverage (+): 0. Max coverage (-): 0

Region: NODE\_347176\_length\_38458\_cov\_26.791616 37915-37920. Max. coverage (+): 0. Max coverage (-): 0

Region: NODE\_347176\_length\_38458\_cov\_26.791616 37921-37926. Max. coverage (+): 0. Max coverage (-): 0

Region: NODE\_347176\_length\_38458\_cov\_26.791616 37927-37932. Max. coverage (+): 0. Max coverage (-): 0

Region: NODE\_347176\_length\_38458\_cov\_26.791616 37933-37939. Max. coverage (+): 0. Max coverage (-): 0

Region: NODE\_347176\_length\_38458\_cov\_26.791616 37940-37945. Max. coverage (+): 0. Max coverage (-): 0.08

Region: NODE\_347176\_length\_38458\_cov\_26.791616 37946-37951. Max. coverage (+): 0. Max coverage (-): 0.08

Region: NODE\_347176\_length\_38458\_cov\_26.791616 37952-37957. Max. coverage (+): 0. Max coverage (-): 0

Region: NODE\_347176\_length\_38458\_cov\_26.791616 37958-37963. Max. coverage (+): 0. Max coverage (-): 0

Region: NODE\_347176\_length\_38458\_cov\_26.791616 37964-37969. Max. coverage (+): 0. Max coverage (-): 0

Region: NODE\_347176\_length\_38458\_cov\_26.791616 37970-37976. Max. coverage (+): 0. Max coverage (-): 0

Region: NODE\_347176\_length\_38458\_cov\_26.791616 37977-37982. Max. coverage (+): 0. Max coverage (-): 0

Region: NODE\_347176\_length\_38458\_cov\_26.791616 37983-37988. Max. coverage (+): 0. Max coverage (-): 0

Region: NODE\_347176\_length\_38458\_cov\_26.791616 37989-37994. Max. coverage (+): 0. Max coverage (-): 0

Region: NODE\_347176\_length\_38458\_cov\_26.791616 37995-38000. Max. coverage (+): 0. Max coverage (-): 0

Region: NODE\_347176\_length\_38458\_cov\_26.791616 38001-38006. Max. coverage (+): 0. Max coverage (-): 0

Region: NODE\_347176\_length\_38458\_cov\_26.791616 38007-38013. Max. coverage (+): 0. Max coverage (-): 0

Region: NODE\_347176\_length\_38458\_cov\_26.791616 38014-38019. Max. coverage (+): 0. Max coverage (-): 0

Region: NODE\_347176\_length\_38458\_cov\_26.791616 38020-38025. Max. coverage (+): 0. Max coverage (-): 0

Region: NODE\_347176\_length\_38458\_cov\_26.791616 38026-38031. Max. coverage (+): 0. Max coverage (-): 0

Region: NODE\_347176\_length\_38458\_cov\_26.791616 38032-38037. Max. coverage (+): 0. Max coverage (-): 0

Region: NODE\_347176\_length\_38458\_cov\_26.791616 38038-38043. Max. coverage (+): 0. Max coverage (-): 0

Region: NODE\_347176\_length\_38458\_cov\_26.791616 38044-38050. Max. coverage (+): 0. Max coverage (-): 0

Region: NODE\_347176\_length\_38458\_cov\_26.791616 38051-38056. Max. coverage (+): 0. Max coverage (-): 0

Region: NODE\_347176\_length\_38458\_cov\_26.791616 38057-38062. Max. coverage (+): 0. Max coverage (-): 0

Region: NODE\_347176\_length\_38458\_cov\_26.791616 38063-38068. Max. coverage (+): 0. Max coverage (-): 0

Region: NODE\_347176\_length\_38458\_cov\_26.791616 38069-38074. Max. coverage (+): 0. Max coverage (-): 0

Region: NODE\_347176\_length\_38458\_cov\_26.791616 38075-38080. Max. coverage (+): 0. Max coverage (-): 0

Region: NODE\_347176\_length\_38458\_cov\_26.791616 38081-38087. Max. coverage (+): 0. Max coverage (-): 0

Region: NODE\_347176\_length\_38458\_cov\_26.791616 38088-38093. Max. coverage (+): 0. Max coverage (-): 0

Region: NODE\_347176\_length\_38458\_cov\_26.791616 38094-38099. Max. coverage (+): 0. Max coverage (-): 0

Region: NODE\_347176\_length\_38458\_cov\_26.791616 38100-38105. Max. coverage (+): 0. Max coverage (-): 0

Region: NODE\_347176\_length\_38458\_cov\_26.791616 38106-38111. Max. coverage (+): 0. Max coverage (-): 0

Region: NODE\_347176\_length\_38458\_cov\_26.791616 38112-38117. Max. coverage (+): 0. Max coverage (-): 0

Region: NODE\_347176\_length\_38458\_cov\_26.791616 38118-38124. Max. coverage (+): 0. Max coverage (-): 0

Region: NODE\_347176\_length\_38458\_cov\_26.791616 38125-38130. Max. coverage (+): 0. Max coverage (-): 0

Region: NODE\_347176\_length\_38458\_cov\_26.791616 38131-38136. Max. coverage (+): 0. Max coverage (-): 0

Region: NODE\_347176\_length\_38458\_cov\_26.791616 38137-38142. Max. coverage (+): 0. Max coverage (-): 0

Region: NODE\_347176\_length\_38458\_cov\_26.791616 38143-38148. Max. coverage (+): 0. Max coverage (-): 0

Region: NODE\_347176\_length\_38458\_cov\_26.791616 38149-38154. Max. coverage (+): 0. Max coverage (-): 0

Region: NODE\_347176\_length\_38458\_cov\_26.791616 38155-38161. Max. coverage (+): 0. Max coverage (-): 0

Region: NODE\_347176\_length\_38458\_cov\_26.791616 38162-38167. Max. coverage (+): 0. Max coverage (-): 0

Region: NODE\_347176\_length\_38458\_cov\_26.791616 38168-38173. Max. coverage (+): 0. Max coverage (-): 0

Region: NODE\_347176\_length\_38458\_cov\_26.791616 38174-38179. Max. coverage (+): 0. Max coverage (-): 0

Region: NODE\_347176\_length\_38458\_cov\_26.791616 38180-38185. Max. coverage (+): 0. Max coverage (-): 0

Region: NODE\_347176\_length\_38458\_cov\_26.791616 38186-38191. Max. coverage (+): 0. Max coverage (-): 0

Region: NODE\_347176\_length\_38458\_cov\_26.791616 38192-38198. Max. coverage (+): 0. Max coverage (-): 0

Region: NODE\_347176\_length\_38458\_cov\_26.791616 38199-38204. Max. coverage (+): 0. Max coverage (-): 0

Region: NODE\_347176\_length\_38458\_cov\_26.791616 38205-38210. Max. coverage (+): 0. Max coverage (-): 0

Region: NODE\_347176\_length\_38458\_cov\_26.791616 38211-38216. Max. coverage (+): 0. Max coverage (-): 0

Region: NODE\_347176\_length\_38458\_cov\_26.791616 38217-38222. Max. coverage (+): 0. Max coverage (-): 0

Region: NODE\_347176\_length\_38458\_cov\_26.791616 38223-38228. Max. coverage (+): 0. Max coverage (-): 0

Region: NODE\_347176\_length\_38458\_cov\_26.791616 38229-38235. Max. coverage (+): 0. Max coverage (-): 0

Region: NODE\_347176\_length\_38458\_cov\_26.791616 38236-38241. Max. coverage (+): 0. Max coverage (-): 0

Region: NODE\_347176\_length\_38458\_cov\_26.791616 38242-38247. Max. coverage (+): 0. Max coverage (-): 0

Region: NODE\_347176\_length\_38458\_cov\_26.791616 38248-38253. Max. coverage (+): 0. Max coverage (-): 0

Region: NODE\_347176\_length\_38458\_cov\_26.791616 38254-38259. Max. coverage (+): 0. Max coverage (-): 0

Region: NODE\_347176\_length\_38458\_cov\_26.791616 38260-38265. Max. coverage (+): 0. Max coverage (-): 0

Region: NODE\_347176\_length\_38458\_cov\_26.791616 38266-38272. Max. coverage (+): 0. Max coverage (-): 0

Region: NODE\_347176\_length\_38458\_cov\_26.791616 38273-38278. Max. coverage (+): 0. Max coverage (-): 0

Region: NODE\_347176\_length\_38458\_cov\_26.791616 38279-38284. Max. coverage (+): 0. Max coverage (-): 0

Region: NODE\_347176\_length\_38458\_cov\_26.791616 38285-38290. Max. coverage (+): 0. Max coverage (-): 0

Region: NODE\_347176\_length\_38458\_cov\_26.791616 38291-38296. Max. coverage (+): 0. Max coverage (-): 0.08

Region: NODE\_347176\_length\_38458\_cov\_26.791616 38297-38302. Max. coverage (+): 0. Max coverage (-): 0.08

Region: NODE\_347176\_length\_38458\_cov\_26.791616 38303-38309. Max. coverage (+): 0. Max coverage (-): 0

Region: NODE\_347176\_length\_38458\_cov\_26.791616 38310-38315. Max. coverage (+): 0. Max coverage (-): 0

Region: NODE\_347176\_length\_38458\_cov\_26.791616 38316-38321. Max. coverage (+): 0. Max coverage (-): 0

Region: NODE\_347176\_length\_38458\_cov\_26.791616 38322-38327. Max. coverage (+): 0. Max coverage (-): 0

Region: NODE\_347176\_length\_38458\_cov\_26.791616 38328-38333. Max. coverage (+): 0. Max coverage (-): 0

Region: NODE\_347176\_length\_38458\_cov\_26.791616 38334-38339. Max. coverage (+): 0. Max coverage (-): 0

Region: NODE\_347176\_length\_38458\_cov\_26.791616 38340-38346. Max. coverage (+): 0. Max coverage (-): 0

Region: NODE\_347176\_length\_38458\_cov\_26.791616 38347-38352. Max. coverage (+): 0. Max coverage (-): 0

Region: NODE\_347176\_length\_38458\_cov\_26.791616 38353-38358. Max. coverage (+): 0. Max coverage (-): 0

Region: NODE\_347176\_length\_38458\_cov\_26.791616 38359-38364. Max. coverage (+): 0. Max coverage (-): 0

Region: NODE\_347176\_length\_38458\_cov\_26.791616 38365-38370. Max. coverage (+): 0. Max coverage (-): 0

Region: NODE\_347176\_length\_38458\_cov\_26.791616 38371-38376. Max. coverage (+): 0. Max coverage (-): 0

Region: NODE\_347176\_length\_38458\_cov\_26.791616 38377-38383. Max. coverage (+): 0. Max coverage (-): 0

Region: NODE\_347176\_length\_38458\_cov\_26.791616 38384-38389. Max. coverage (+): 0. Max coverage (-): 0

Region: NODE\_347176\_length\_38458\_cov\_26.791616 38390-38395. Max. coverage (+): 0. Max coverage (-): 0

Region: NODE\_347176\_length\_38458\_cov\_26.791616 38396-38401. Max. coverage (+): 0. Max coverage (-): 0

Region: NODE\_347176\_length\_38458\_cov\_26.791616 38402-38407. Max. coverage (+): 0. Max coverage (-): 0.08

Region: NODE\_347176\_length\_38458\_cov\_26.791616 38408-38413. Max. coverage (+): 0. Max coverage (-): 0.08

Region: NODE\_347176\_length\_38458\_cov\_26.791616 38414-38420. Max. coverage (+): 0. Max coverage (-): 0

Region: NODE\_347176\_length\_38458\_cov\_26.791616 38421-38426. Max. coverage (+): 0. Max coverage (-): 0.15

Region: NODE\_347176\_length\_38458\_cov\_26.791616 38427-38432. Max. coverage (+): 0. Max coverage (-): 0.08

Region: NODE\_347176\_length\_38458\_cov\_26.791616 38433-38438. Max. coverage (+): 0. Max coverage (-): 0

Region: NODE\_347176\_length\_38458\_cov\_26.791616 38439-38444. Max. coverage (+): 0. Max coverage (-): 0

Region: NODE\_347176\_length\_38458\_cov\_26.791616 38445-38450. Max. coverage (+): 0. Max coverage (-): 0

Region: NODE\_347176\_length\_38458\_cov\_26.791616 38451-. Max. coverage (+): 0. Max coverage (-): 0

RepeatMasker Color Code

**+**

100-98% Identity

<98-95% Identity

<95-90% Identity

<90-85% Identity

<85-80% Identity

<80-75% Identity

<75-70% Identity

<70% Identity

**-**

Gene Set Color Code

**+**

Gene

Pseudogene

Other

**-**

Topology/Coverage Color Code

Coverage Plus Strand

Coverage Minus Strand

Mainstrand: Plus

Mainstrand: Minus

Complementary Strand

Flanking Region  
(if option -flank >0)

Gene Set Annotation  
  
RepeatMasker Annotation  
  
Transcription Factor Binding Sites  

**RHOXF1** (Sequence: AGCTCA (-): 37239)  
**RHOXF1** (Sequence: AGCTTA (-): 37415)  
**RHOXF1** (Sequence: AGCTTA (-): 37648)  
**RHOXF1** (Sequence: GGCTCA (-): 37820)  
**RHOXF1** (Sequence: GGATCA (-): 37849)  
**RHOXF1** (Sequence: GGCTCA (-): 38352)  
**RHOXF1** (Sequence: TAAGCC (+): 35410)  
**RHOXF1** (Sequence: TAAGCT (+): 35781)  
**RHOXF1** (Sequence: TAAGCT (+): 36175)  
**RHOXF1** (Sequence: TAATCC (+): 36883)  
**RHOXF1** (Sequence: TGATCT (+): 37917)  
**RHOXF1** (Sequence: TGAGCC (+): 37989)  
**RHOXF1** (Sequence: TAATCT (+): 38396)  
**SOX9** (Sequence: AACAATGA (-): 36055)  
**SOX9** (Sequence: AACAATGA (-): 36239)  
**SOX9** (Sequence: AACAATAA (-): 37073)  
**Sox5** (Sequence: ATTGTT (+): 37347)  
**Sox5** (Sequence: ATTGTT (+): 38378)  
**SOX9** (Sequence: TCATTGTT (+): 37345)  
**Nobox** (Sequence: GGCAATTA (-): 36598)  
**FOXO1** (Sequence: AAAAACAAG (-): 37764)  
**Nobox** (Sequence: TAATTACC (+): 35558)  
**Rhox11** (Sequence: TAAACACCA (-): 37623)  
**Sox5** (Sequence: AACAAT (-): 36055)  
**Sox5** (Sequence: AACAAT (-): 36239)  
**Sox5** (Sequence: AACAAT (-): 37073)  
**Sox5** (Sequence: AACAAT (-): 37244)  
**POU5F1** (Sequence: ATGCAAA (+): 37751)
