# Supplementary material for: First characterization of PIWI-interacting RNA clusters in a cichlid fish with a B chromosome
Source: BMC Biol. 2022 Sep 21;20:204. doi: 10.1186/s12915-022-01403-2 (PMC9490952; doi:10.1186/s12915-022-01403-2)
Supplement: Supplementary file 1 — Additional file 1. Zipped folder with fasta and interactive html piRNA cluster information for the A. latifasciata genome. The nomenclature is as follows: number-pirna-cluster_sex_B-presence (f, female; m, male; 0b, without B chromosome; 1b, with B chromosome). [file 12915_2022_1403_MOESM1_ESM.zip › 134_f1b.html]

piRNA cluster 134\_f1b 60


Predicted piRNA cluster no. 134\_f1b
  

Show proTRAC run info
Hide proTRAC run info

/\  
                \_\_\_\_\_\_\_\_\_\_\_\_\_\_\_\_\_\_\_\_\_\_\_/\\_\_\_ /  \\_\_\_\_\_\_\_  
               I                      /  \  /    \      I  
               I     pro             /    \/      \     I  
               I        TRAC        /               \   I  
               I   \_\_\_\_\_\_\_\_\_\_\_\_\_\_\_\_/\_\_\_\_\_\_\_\_\_\_\_\_\_\_\_\_\_\\_ I  
               I   \              /                     I  
               I    \            /                      I  
               I     \  /\      /       V.2.4.2         I  
               I      \/  \    /                        I  
               I\_\_\_\_\_\_\_\_\_\_\_\  /\_\_\_\_\_\_\_\_\_\_\_\_\_\_\_\_\_\_\_\_\_\_\_\_\_I  
                            \/  
  
  
================================= proTRAC ====================================  
VERSION: .......... 2.4.2  
LAST MODIFIED: .... 11. May 2018  
  
Please cite:  
Rosenkranz D, Zischler H. proTRAC - a software for probabilistic piRNA cluster  
detection, visualization and analysis. 2012. BMC Bioinformatics 13:5.  
  
  
Contact:  
David Rosenkranz  
Institute of Organismic and Molecular Evolutionary Biology  
Dept. Anthropology, small RNA group  
Johannes Gutenberg University Mainz  
email: rosenkranz@uni-mainz.de  
  
You can find the latest proTRAC version at:  
http://sourceforge.net/projects/protrac/files  
http://www.smallRNAgroup-mainz.de/software  
==============================================================================  
  
PARAMETERS:  
Map file: ...............piwi-femeas-1B.fa-collapse.map  
Genome file: ............../../../0B\_ala\_genome.fa  
RepeatMasker annotation: Alatifasciata-all0B-maryan-v2.fa\_corrected.out  
GeneSet:................./guest-storage/Data/annotation/Alatifasciata\_all0B\_maryan-v2\_out2017.gff  
  
Significant (p<=0.01) hit density will be calculated based  
on observed hit distribution.  
  
Sliding window size: ........................................ 5000 bp  
Sliding window increament: .................................. 1000 bp  
Normalize each hit by number of genomic hits: ............... yes  
Normalize each hit by number of sequence reads: ............. yes  
Normalize values (-> per million mapped reads): ............. yes  
Min. fraction of hits with 1T(U) or 10A: .................... 0.75  
Alternatively: Min. fraction of hits with 1T(U) and 10A: .... 0.5  
Min. fraction of hits with typical piRNA length: ............ 0.75  
Typical piRNA length: ....................................... 24-32 nt  
Min. size of a piRNA cluster: ............................... 1000 bp.  
Min. number of hits (absolute): ............................. 0  
Min. number of hits (normalized): ........................... 0  
Min. fraction of hits on the mainstrand: .................... 0.75  
Top fraction of mapped sequences (in terms of read counts): . 1%  
Top fraction accounts for max. n% of sequence reads: ........ 90%  
Min. fraction of hits on each arm of a bidirectional cluster: 0.05  
Output html file for each cluster: .......................... yes  
Output a summary table: ..................................... yes  
Output a FASTA file for each cluster (piRNA sequences): ..... yes  
Output a FASTA file comprising cluster sequences: ........... yes  
Output a GTF file for predicted piRNA clusters: ..............yes  
Search DNA motifs in clusters: .............................. yes  
Output flanking sequences: +/- .............................. 0 bp  
Output ~.pTi file: .......................................... no  
==============================================================================  
  
  
Genome size (without gaps): ............ 758543724 bp  
Gaps (N/X/-): .......................... 417479 bp  
Mapped reads: .......................... 10641844  
Non-identical sequences: ............... 2832837  
Genomic hits: .......................... 26056853  
Significant densitiy of mapped reads: .. 368.713530323068 reads/kb

Show proTRAC cluster info
Hide proTRAC cluster info

|  |  |
| --- | --- |
| Location | NODE\_347176\_length\_38458\_cov\_26.791616 |
| Coordinates | 35537-38422 |
| Size [bp] | 2886 |
| Sequence hit loci | 127 |
| Mapped reads (normalized) | 3862 |
| Mapped reads (normalized) per kb | 1338.2 |
| Normalized reads with 1T (1U) | 97.3% |
| Normalized reads with 10A | 97.1% |
| Normalized reads with length 24-32 nt | 99.8% |
| Normalized reads on the main strand(s) | 100% |
| Predicted directionality | mono:minus |

100%

0%

1T (1U)  
reads

10A reads

24-32 nt  
reads

reads on mainstrand

**Either the amount of reads with 1T (1U) OR 10A has to exceed 75% (set with option: -1Tor10A)  
Alternatively the amount of reads with 1T (1U) AND 10A has to exceed 50% (set with option: -1Tand10A)  
Minimum amount of reads with preferred size is 75% (set with option: -pisize)  
Minimum amount of reads on the main strand(s) is 75% (set with option: -clstrand)**

Show read coverage
Hide read coverage

WHAT DO I SEE HERE?  
This chart shows the location of mapped sequence reads within a predicted piRNA cluster. The color refers to the number of genomic hits produced by the sequence read in question. A dark red bar indicates that this sequence read produces many other hits elsewhere in the genome. Many adjacent red or yellow bars can indicate the presence of a multi-copy element such as transposons or rRNA genes. A dark green bar indicates that this sequence read maps uniquely to this locus.

1 hit

2-5 hits

6-10 hits

11-20 hits

21-50 hits

51-100 hits

> 100 hits

NODE\_347176\_length\_38458\_cov\_26.791616

35537

38422

Gene Set

RepeatMasker

Mapped  
Reads

360.6

plus strand

minus strand

360.6

Region: NODE\_347176\_length\_38458\_cov\_26.791616 2889-35539. Max. coverage (+): 0. Max coverage (-): 0.09

Region: NODE\_347176\_length\_38458\_cov\_26.791616 35540-35545. Max. coverage (+): 0. Max coverage (-): 0.09

Region: NODE\_347176\_length\_38458\_cov\_26.791616 35546-35551. Max. coverage (+): 0. Max coverage (-): 0

Region: NODE\_347176\_length\_38458\_cov\_26.791616 35552-35557. Max. coverage (+): 0. Max coverage (-): 0

Region: NODE\_347176\_length\_38458\_cov\_26.791616 35558-35562. Max. coverage (+): 0. Max coverage (-): 0

Region: NODE\_347176\_length\_38458\_cov\_26.791616 35563-35568. Max. coverage (+): 0. Max coverage (-): 0

Region: NODE\_347176\_length\_38458\_cov\_26.791616 35569-35574. Max. coverage (+): 0. Max coverage (-): 0

Region: NODE\_347176\_length\_38458\_cov\_26.791616 35575-35580. Max. coverage (+): 0. Max coverage (-): 0

Region: NODE\_347176\_length\_38458\_cov\_26.791616 35581-35586. Max. coverage (+): 0. Max coverage (-): 0

Region: NODE\_347176\_length\_38458\_cov\_26.791616 35587-35591. Max. coverage (+): 0. Max coverage (-): 0

Region: NODE\_347176\_length\_38458\_cov\_26.791616 35592-35597. Max. coverage (+): 0. Max coverage (-): 0

Region: NODE\_347176\_length\_38458\_cov\_26.791616 35598-35603. Max. coverage (+): 0. Max coverage (-): 0

Region: NODE\_347176\_length\_38458\_cov\_26.791616 35604-35609. Max. coverage (+): 0. Max coverage (-): 0

Region: NODE\_347176\_length\_38458\_cov\_26.791616 35610-35614. Max. coverage (+): 0. Max coverage (-): 0

Region: NODE\_347176\_length\_38458\_cov\_26.791616 35615-35620. Max. coverage (+): 0. Max coverage (-): 0

Region: NODE\_347176\_length\_38458\_cov\_26.791616 35621-35626. Max. coverage (+): 0. Max coverage (-): 0

Region: NODE\_347176\_length\_38458\_cov\_26.791616 35627-35632. Max. coverage (+): 0. Max coverage (-): 0

Region: NODE\_347176\_length\_38458\_cov\_26.791616 35633-35638. Max. coverage (+): 0. Max coverage (-): 0

Region: NODE\_347176\_length\_38458\_cov\_26.791616 35639-35643. Max. coverage (+): 0. Max coverage (-): 0

Region: NODE\_347176\_length\_38458\_cov\_26.791616 35644-35649. Max. coverage (+): 0. Max coverage (-): 0

Region: NODE\_347176\_length\_38458\_cov\_26.791616 35650-35655. Max. coverage (+): 0. Max coverage (-): 0.09

Region: NODE\_347176\_length\_38458\_cov\_26.791616 35656-35661. Max. coverage (+): 0. Max coverage (-): 0.09

Region: NODE\_347176\_length\_38458\_cov\_26.791616 35662-35666. Max. coverage (+): 0. Max coverage (-): 0

Region: NODE\_347176\_length\_38458\_cov\_26.791616 35667-35672. Max. coverage (+): 0. Max coverage (-): 0

Region: NODE\_347176\_length\_38458\_cov\_26.791616 35673-35678. Max. coverage (+): 0. Max coverage (-): 0

Region: NODE\_347176\_length\_38458\_cov\_26.791616 35679-35684. Max. coverage (+): 0. Max coverage (-): 0

Region: NODE\_347176\_length\_38458\_cov\_26.791616 35685-35689. Max. coverage (+): 0. Max coverage (-): 0

Region: NODE\_347176\_length\_38458\_cov\_26.791616 35690-35695. Max. coverage (+): 0. Max coverage (-): 0

Region: NODE\_347176\_length\_38458\_cov\_26.791616 35696-35701. Max. coverage (+): 0. Max coverage (-): 0

Region: NODE\_347176\_length\_38458\_cov\_26.791616 35702-35707. Max. coverage (+): 0. Max coverage (-): 0

Region: NODE\_347176\_length\_38458\_cov\_26.791616 35708-35713. Max. coverage (+): 0. Max coverage (-): 0

Region: NODE\_347176\_length\_38458\_cov\_26.791616 35714-35718. Max. coverage (+): 0. Max coverage (-): 0

Region: NODE\_347176\_length\_38458\_cov\_26.791616 35719-35724. Max. coverage (+): 0. Max coverage (-): 0

Region: NODE\_347176\_length\_38458\_cov\_26.791616 35725-35730. Max. coverage (+): 0. Max coverage (-): 0

Region: NODE\_347176\_length\_38458\_cov\_26.791616 35731-35736. Max. coverage (+): 0. Max coverage (-): 0

Region: NODE\_347176\_length\_38458\_cov\_26.791616 35737-35741. Max. coverage (+): 0. Max coverage (-): 0

Region: NODE\_347176\_length\_38458\_cov\_26.791616 35742-35747. Max. coverage (+): 0. Max coverage (-): 0

Region: NODE\_347176\_length\_38458\_cov\_26.791616 35748-35753. Max. coverage (+): 0. Max coverage (-): 0

Region: NODE\_347176\_length\_38458\_cov\_26.791616 35754-35759. Max. coverage (+): 0. Max coverage (-): 0

Region: NODE\_347176\_length\_38458\_cov\_26.791616 35760-35764. Max. coverage (+): 0. Max coverage (-): 0

Region: NODE\_347176\_length\_38458\_cov\_26.791616 35765-35770. Max. coverage (+): 0. Max coverage (-): 0

Region: NODE\_347176\_length\_38458\_cov\_26.791616 35771-35776. Max. coverage (+): 0. Max coverage (-): 0

Region: NODE\_347176\_length\_38458\_cov\_26.791616 35777-35782. Max. coverage (+): 0. Max coverage (-): 0

Region: NODE\_347176\_length\_38458\_cov\_26.791616 35783-35788. Max. coverage (+): 0. Max coverage (-): 0

Region: NODE\_347176\_length\_38458\_cov\_26.791616 35789-35793. Max. coverage (+): 0. Max coverage (-): 0

Region: NODE\_347176\_length\_38458\_cov\_26.791616 35794-35799. Max. coverage (+): 0. Max coverage (-): 0

Region: NODE\_347176\_length\_38458\_cov\_26.791616 35800-35805. Max. coverage (+): 0. Max coverage (-): 0

Region: NODE\_347176\_length\_38458\_cov\_26.791616 35806-35811. Max. coverage (+): 0. Max coverage (-): 0

Region: NODE\_347176\_length\_38458\_cov\_26.791616 35812-35816. Max. coverage (+): 0. Max coverage (-): 0

Region: NODE\_347176\_length\_38458\_cov\_26.791616 35817-35822. Max. coverage (+): 0. Max coverage (-): 0

Region: NODE\_347176\_length\_38458\_cov\_26.791616 35823-35828. Max. coverage (+): 0. Max coverage (-): 0

Region: NODE\_347176\_length\_38458\_cov\_26.791616 35829-35834. Max. coverage (+): 0. Max coverage (-): 0

Region: NODE\_347176\_length\_38458\_cov\_26.791616 35835-35840. Max. coverage (+): 0. Max coverage (-): 0

Region: NODE\_347176\_length\_38458\_cov\_26.791616 35841-35845. Max. coverage (+): 0. Max coverage (-): 0

Region: NODE\_347176\_length\_38458\_cov\_26.791616 35846-35851. Max. coverage (+): 0. Max coverage (-): 0

Region: NODE\_347176\_length\_38458\_cov\_26.791616 35852-35857. Max. coverage (+): 0. Max coverage (-): 0

Region: NODE\_347176\_length\_38458\_cov\_26.791616 35858-35863. Max. coverage (+): 0. Max coverage (-): 0

Region: NODE\_347176\_length\_38458\_cov\_26.791616 35864-35868. Max. coverage (+): 0. Max coverage (-): 0

Region: NODE\_347176\_length\_38458\_cov\_26.791616 35869-35874. Max. coverage (+): 0. Max coverage (-): 0

Region: NODE\_347176\_length\_38458\_cov\_26.791616 35875-35880. Max. coverage (+): 0. Max coverage (-): 0

Region: NODE\_347176\_length\_38458\_cov\_26.791616 35881-35886. Max. coverage (+): 0. Max coverage (-): 0

Region: NODE\_347176\_length\_38458\_cov\_26.791616 35887-35891. Max. coverage (+): 0. Max coverage (-): 0

Region: NODE\_347176\_length\_38458\_cov\_26.791616 35892-35897. Max. coverage (+): 0. Max coverage (-): 0

Region: NODE\_347176\_length\_38458\_cov\_26.791616 35898-35903. Max. coverage (+): 0. Max coverage (-): 0

Region: NODE\_347176\_length\_38458\_cov\_26.791616 35904-35909. Max. coverage (+): 0. Max coverage (-): 0

Region: NODE\_347176\_length\_38458\_cov\_26.791616 35910-35915. Max. coverage (+): 0. Max coverage (-): 0

Region: NODE\_347176\_length\_38458\_cov\_26.791616 35916-35920. Max. coverage (+): 0. Max coverage (-): 0

Region: NODE\_347176\_length\_38458\_cov\_26.791616 35921-35926. Max. coverage (+): 0. Max coverage (-): 0

Region: NODE\_347176\_length\_38458\_cov\_26.791616 35927-35932. Max. coverage (+): 0. Max coverage (-): 0

Region: NODE\_347176\_length\_38458\_cov\_26.791616 35933-35938. Max. coverage (+): 0. Max coverage (-): 0

Region: NODE\_347176\_length\_38458\_cov\_26.791616 35939-35943. Max. coverage (+): 0. Max coverage (-): 0

Region: NODE\_347176\_length\_38458\_cov\_26.791616 35944-35949. Max. coverage (+): 0. Max coverage (-): 0

Region: NODE\_347176\_length\_38458\_cov\_26.791616 35950-35955. Max. coverage (+): 0. Max coverage (-): 0

Region: NODE\_347176\_length\_38458\_cov\_26.791616 35956-35961. Max. coverage (+): 0. Max coverage (-): 0

Region: NODE\_347176\_length\_38458\_cov\_26.791616 35962-35967. Max. coverage (+): 0. Max coverage (-): 0

Region: NODE\_347176\_length\_38458\_cov\_26.791616 35968-35972. Max. coverage (+): 0. Max coverage (-): 0

Region: NODE\_347176\_length\_38458\_cov\_26.791616 35973-35978. Max. coverage (+): 0. Max coverage (-): 0

Region: NODE\_347176\_length\_38458\_cov\_26.791616 35979-35984. Max. coverage (+): 0. Max coverage (-): 0

Region: NODE\_347176\_length\_38458\_cov\_26.791616 35985-35990. Max. coverage (+): 0. Max coverage (-): 0

Region: NODE\_347176\_length\_38458\_cov\_26.791616 35991-35995. Max. coverage (+): 0. Max coverage (-): 0

Region: NODE\_347176\_length\_38458\_cov\_26.791616 35996-36001. Max. coverage (+): 0. Max coverage (-): 0

Region: NODE\_347176\_length\_38458\_cov\_26.791616 36002-36007. Max. coverage (+): 0. Max coverage (-): 0

Region: NODE\_347176\_length\_38458\_cov\_26.791616 36008-36013. Max. coverage (+): 0. Max coverage (-): 0

Region: NODE\_347176\_length\_38458\_cov\_26.791616 36014-36018. Max. coverage (+): 0. Max coverage (-): 0

Region: NODE\_347176\_length\_38458\_cov\_26.791616 36019-36024. Max. coverage (+): 0. Max coverage (-): 0

Region: NODE\_347176\_length\_38458\_cov\_26.791616 36025-36030. Max. coverage (+): 0. Max coverage (-): 0

Region: NODE\_347176\_length\_38458\_cov\_26.791616 36031-36036. Max. coverage (+): 0. Max coverage (-): 0

Region: NODE\_347176\_length\_38458\_cov\_26.791616 36037-36042. Max. coverage (+): 0. Max coverage (-): 0

Region: NODE\_347176\_length\_38458\_cov\_26.791616 36043-36047. Max. coverage (+): 0. Max coverage (-): 0

Region: NODE\_347176\_length\_38458\_cov\_26.791616 36048-36053. Max. coverage (+): 0. Max coverage (-): 0

Region: NODE\_347176\_length\_38458\_cov\_26.791616 36054-36059. Max. coverage (+): 0. Max coverage (-): 0

Region: NODE\_347176\_length\_38458\_cov\_26.791616 36060-36065. Max. coverage (+): 0. Max coverage (-): 0

Region: NODE\_347176\_length\_38458\_cov\_26.791616 36066-36070. Max. coverage (+): 0. Max coverage (-): 0

Region: NODE\_347176\_length\_38458\_cov\_26.791616 36071-36076. Max. coverage (+): 0. Max coverage (-): 0

Region: NODE\_347176\_length\_38458\_cov\_26.791616 36077-36082. Max. coverage (+): 0. Max coverage (-): 0

Region: NODE\_347176\_length\_38458\_cov\_26.791616 36083-36088. Max. coverage (+): 0. Max coverage (-): 0.09

Region: NODE\_347176\_length\_38458\_cov\_26.791616 36089-36093. Max. coverage (+): 0. Max coverage (-): 0.09

Region: NODE\_347176\_length\_38458\_cov\_26.791616 36094-36099. Max. coverage (+): 0. Max coverage (-): 0.47

Region: NODE\_347176\_length\_38458\_cov\_26.791616 36100-36105. Max. coverage (+): 0. Max coverage (-): 0

Region: NODE\_347176\_length\_38458\_cov\_26.791616 36106-36111. Max. coverage (+): 0. Max coverage (-): 0

Region: NODE\_347176\_length\_38458\_cov\_26.791616 36112-36117. Max. coverage (+): 0. Max coverage (-): 0

Region: NODE\_347176\_length\_38458\_cov\_26.791616 36118-36122. Max. coverage (+): 0. Max coverage (-): 0.47

Region: NODE\_347176\_length\_38458\_cov\_26.791616 36123-36128. Max. coverage (+): 0. Max coverage (-): 0.47

Region: NODE\_347176\_length\_38458\_cov\_26.791616 36129-36134. Max. coverage (+): 0. Max coverage (-): 0

Region: NODE\_347176\_length\_38458\_cov\_26.791616 36135-36140. Max. coverage (+): 0. Max coverage (-): 0

Region: NODE\_347176\_length\_38458\_cov\_26.791616 36141-36145. Max. coverage (+): 0. Max coverage (-): 0

Region: NODE\_347176\_length\_38458\_cov\_26.791616 36146-36151. Max. coverage (+): 0. Max coverage (-): 0

Region: NODE\_347176\_length\_38458\_cov\_26.791616 36152-36157. Max. coverage (+): 0. Max coverage (-): 19.31

Region: NODE\_347176\_length\_38458\_cov\_26.791616 36158-36163. Max. coverage (+): 0. Max coverage (-): 360.6

Region: NODE\_347176\_length\_38458\_cov\_26.791616 36164-36169. Max. coverage (+): 0. Max coverage (-): 0

Region: NODE\_347176\_length\_38458\_cov\_26.791616 36170-36174. Max. coverage (+): 0. Max coverage (-): 0

Region: NODE\_347176\_length\_38458\_cov\_26.791616 36175-36180. Max. coverage (+): 0. Max coverage (-): 0

Region: NODE\_347176\_length\_38458\_cov\_26.791616 36181-36186. Max. coverage (+): 0. Max coverage (-): 0

Region: NODE\_347176\_length\_38458\_cov\_26.791616 36187-36192. Max. coverage (+): 0. Max coverage (-): 0

Region: NODE\_347176\_length\_38458\_cov\_26.791616 36193-36197. Max. coverage (+): 0. Max coverage (-): 0

Region: NODE\_347176\_length\_38458\_cov\_26.791616 36198-36203. Max. coverage (+): 0. Max coverage (-): 0

Region: NODE\_347176\_length\_38458\_cov\_26.791616 36204-36209. Max. coverage (+): 0. Max coverage (-): 0

Region: NODE\_347176\_length\_38458\_cov\_26.791616 36210-36215. Max. coverage (+): 0. Max coverage (-): 0

Region: NODE\_347176\_length\_38458\_cov\_26.791616 36216-36220. Max. coverage (+): 0. Max coverage (-): 0

Region: NODE\_347176\_length\_38458\_cov\_26.791616 36221-36226. Max. coverage (+): 0. Max coverage (-): 0

Region: NODE\_347176\_length\_38458\_cov\_26.791616 36227-36232. Max. coverage (+): 0. Max coverage (-): 0

Region: NODE\_347176\_length\_38458\_cov\_26.791616 36233-36238. Max. coverage (+): 0. Max coverage (-): 0

Region: NODE\_347176\_length\_38458\_cov\_26.791616 36239-36244. Max. coverage (+): 0. Max coverage (-): 0

Region: NODE\_347176\_length\_38458\_cov\_26.791616 36245-36249. Max. coverage (+): 0. Max coverage (-): 0

Region: NODE\_347176\_length\_38458\_cov\_26.791616 36250-36255. Max. coverage (+): 0. Max coverage (-): 0

Region: NODE\_347176\_length\_38458\_cov\_26.791616 36256-36261. Max. coverage (+): 0. Max coverage (-): 0

Region: NODE\_347176\_length\_38458\_cov\_26.791616 36262-36267. Max. coverage (+): 0. Max coverage (-): 0

Region: NODE\_347176\_length\_38458\_cov\_26.791616 36268-36272. Max. coverage (+): 0. Max coverage (-): 0

Region: NODE\_347176\_length\_38458\_cov\_26.791616 36273-36278. Max. coverage (+): 0. Max coverage (-): 0

Region: NODE\_347176\_length\_38458\_cov\_26.791616 36279-36284. Max. coverage (+): 0. Max coverage (-): 0

Region: NODE\_347176\_length\_38458\_cov\_26.791616 36285-36290. Max. coverage (+): 0. Max coverage (-): 0

Region: NODE\_347176\_length\_38458\_cov\_26.791616 36291-36296. Max. coverage (+): 0. Max coverage (-): 0

Region: NODE\_347176\_length\_38458\_cov\_26.791616 36297-36301. Max. coverage (+): 0. Max coverage (-): 0

Region: NODE\_347176\_length\_38458\_cov\_26.791616 36302-36307. Max. coverage (+): 0. Max coverage (-): 0

Region: NODE\_347176\_length\_38458\_cov\_26.791616 36308-36313. Max. coverage (+): 0. Max coverage (-): 0

Region: NODE\_347176\_length\_38458\_cov\_26.791616 36314-36319. Max. coverage (+): 0. Max coverage (-): 0

Region: NODE\_347176\_length\_38458\_cov\_26.791616 36320-36324. Max. coverage (+): 0. Max coverage (-): 0

Region: NODE\_347176\_length\_38458\_cov\_26.791616 36325-36330. Max. coverage (+): 0. Max coverage (-): 0

Region: NODE\_347176\_length\_38458\_cov\_26.791616 36331-36336. Max. coverage (+): 0. Max coverage (-): 0

Region: NODE\_347176\_length\_38458\_cov\_26.791616 36337-36342. Max. coverage (+): 0. Max coverage (-): 0

Region: NODE\_347176\_length\_38458\_cov\_26.791616 36343-36347. Max. coverage (+): 0. Max coverage (-): 0

Region: NODE\_347176\_length\_38458\_cov\_26.791616 36348-36353. Max. coverage (+): 0. Max coverage (-): 0

Region: NODE\_347176\_length\_38458\_cov\_26.791616 36354-36359. Max. coverage (+): 0. Max coverage (-): 0

Region: NODE\_347176\_length\_38458\_cov\_26.791616 36360-36365. Max. coverage (+): 0. Max coverage (-): 0

Region: NODE\_347176\_length\_38458\_cov\_26.791616 36366-36371. Max. coverage (+): 0. Max coverage (-): 0

Region: NODE\_347176\_length\_38458\_cov\_26.791616 36372-36376. Max. coverage (+): 0. Max coverage (-): 0

Region: NODE\_347176\_length\_38458\_cov\_26.791616 36377-36382. Max. coverage (+): 0. Max coverage (-): 0

Region: NODE\_347176\_length\_38458\_cov\_26.791616 36383-36388. Max. coverage (+): 0. Max coverage (-): 0

Region: NODE\_347176\_length\_38458\_cov\_26.791616 36389-36394. Max. coverage (+): 0. Max coverage (-): 0

Region: NODE\_347176\_length\_38458\_cov\_26.791616 36395-36399. Max. coverage (+): 0. Max coverage (-): 0

Region: NODE\_347176\_length\_38458\_cov\_26.791616 36400-36405. Max. coverage (+): 0. Max coverage (-): 0

Region: NODE\_347176\_length\_38458\_cov\_26.791616 36406-36411. Max. coverage (+): 0. Max coverage (-): 0

Region: NODE\_347176\_length\_38458\_cov\_26.791616 36412-36417. Max. coverage (+): 0. Max coverage (-): 0

Region: NODE\_347176\_length\_38458\_cov\_26.791616 36418-36423. Max. coverage (+): 0. Max coverage (-): 0

Region: NODE\_347176\_length\_38458\_cov\_26.791616 36424-36428. Max. coverage (+): 0. Max coverage (-): 0

Region: NODE\_347176\_length\_38458\_cov\_26.791616 36429-36434. Max. coverage (+): 0. Max coverage (-): 0

Region: NODE\_347176\_length\_38458\_cov\_26.791616 36435-36440. Max. coverage (+): 0. Max coverage (-): 0

Region: NODE\_347176\_length\_38458\_cov\_26.791616 36441-36446. Max. coverage (+): 0. Max coverage (-): 0

Region: NODE\_347176\_length\_38458\_cov\_26.791616 36447-36451. Max. coverage (+): 0. Max coverage (-): 0

Region: NODE\_347176\_length\_38458\_cov\_26.791616 36452-36457. Max. coverage (+): 0. Max coverage (-): 0

Region: NODE\_347176\_length\_38458\_cov\_26.791616 36458-36463. Max. coverage (+): 0. Max coverage (-): 0

Region: NODE\_347176\_length\_38458\_cov\_26.791616 36464-36469. Max. coverage (+): 0. Max coverage (-): 0

Region: NODE\_347176\_length\_38458\_cov\_26.791616 36470-36474. Max. coverage (+): 0. Max coverage (-): 0

Region: NODE\_347176\_length\_38458\_cov\_26.791616 36475-36480. Max. coverage (+): 0. Max coverage (-): 0

Region: NODE\_347176\_length\_38458\_cov\_26.791616 36481-36486. Max. coverage (+): 0. Max coverage (-): 0

Region: NODE\_347176\_length\_38458\_cov\_26.791616 36487-36492. Max. coverage (+): 0. Max coverage (-): 0

Region: NODE\_347176\_length\_38458\_cov\_26.791616 36493-36498. Max. coverage (+): 0. Max coverage (-): 0

Region: NODE\_347176\_length\_38458\_cov\_26.791616 36499-36503. Max. coverage (+): 0. Max coverage (-): 0

Region: NODE\_347176\_length\_38458\_cov\_26.791616 36504-36509. Max. coverage (+): 0. Max coverage (-): 0

Region: NODE\_347176\_length\_38458\_cov\_26.791616 36510-36515. Max. coverage (+): 0. Max coverage (-): 0

Region: NODE\_347176\_length\_38458\_cov\_26.791616 36516-36521. Max. coverage (+): 0. Max coverage (-): 0

Region: NODE\_347176\_length\_38458\_cov\_26.791616 36522-36526. Max. coverage (+): 0. Max coverage (-): 0

Region: NODE\_347176\_length\_38458\_cov\_26.791616 36527-36532. Max. coverage (+): 0. Max coverage (-): 0

Region: NODE\_347176\_length\_38458\_cov\_26.791616 36533-36538. Max. coverage (+): 0. Max coverage (-): 0

Region: NODE\_347176\_length\_38458\_cov\_26.791616 36539-36544. Max. coverage (+): 0. Max coverage (-): 0

Region: NODE\_347176\_length\_38458\_cov\_26.791616 36545-36549. Max. coverage (+): 0. Max coverage (-): 0

Region: NODE\_347176\_length\_38458\_cov\_26.791616 36550-36555. Max. coverage (+): 0. Max coverage (-): 0

Region: NODE\_347176\_length\_38458\_cov\_26.791616 36556-36561. Max. coverage (+): 0. Max coverage (-): 0

Region: NODE\_347176\_length\_38458\_cov\_26.791616 36562-36567. Max. coverage (+): 0. Max coverage (-): 0

Region: NODE\_347176\_length\_38458\_cov\_26.791616 36568-36573. Max. coverage (+): 0. Max coverage (-): 0

Region: NODE\_347176\_length\_38458\_cov\_26.791616 36574-36578. Max. coverage (+): 0. Max coverage (-): 0

Region: NODE\_347176\_length\_38458\_cov\_26.791616 36579-36584. Max. coverage (+): 0. Max coverage (-): 0

Region: NODE\_347176\_length\_38458\_cov\_26.791616 36585-36590. Max. coverage (+): 0. Max coverage (-): 0

Region: NODE\_347176\_length\_38458\_cov\_26.791616 36591-36596. Max. coverage (+): 0. Max coverage (-): 0

Region: NODE\_347176\_length\_38458\_cov\_26.791616 36597-36601. Max. coverage (+): 0. Max coverage (-): 0

Region: NODE\_347176\_length\_38458\_cov\_26.791616 36602-36607. Max. coverage (+): 0. Max coverage (-): 0

Region: NODE\_347176\_length\_38458\_cov\_26.791616 36608-36613. Max. coverage (+): 0. Max coverage (-): 0

Region: NODE\_347176\_length\_38458\_cov\_26.791616 36614-36619. Max. coverage (+): 0. Max coverage (-): 0

Region: NODE\_347176\_length\_38458\_cov\_26.791616 36620-36625. Max. coverage (+): 0. Max coverage (-): 0

Region: NODE\_347176\_length\_38458\_cov\_26.791616 36626-36630. Max. coverage (+): 0. Max coverage (-): 0

Region: NODE\_347176\_length\_38458\_cov\_26.791616 36631-36636. Max. coverage (+): 0. Max coverage (-): 0

Region: NODE\_347176\_length\_38458\_cov\_26.791616 36637-36642. Max. coverage (+): 0. Max coverage (-): 0

Region: NODE\_347176\_length\_38458\_cov\_26.791616 36643-36648. Max. coverage (+): 0. Max coverage (-): 0

Region: NODE\_347176\_length\_38458\_cov\_26.791616 36649-36653. Max. coverage (+): 0. Max coverage (-): 0

Region: NODE\_347176\_length\_38458\_cov\_26.791616 36654-36659. Max. coverage (+): 0. Max coverage (-): 0

Region: NODE\_347176\_length\_38458\_cov\_26.791616 36660-36665. Max. coverage (+): 0. Max coverage (-): 0.09

Region: NODE\_347176\_length\_38458\_cov\_26.791616 36666-36671. Max. coverage (+): 0. Max coverage (-): 0

Region: NODE\_347176\_length\_38458\_cov\_26.791616 36672-36676. Max. coverage (+): 0. Max coverage (-): 0

Region: NODE\_347176\_length\_38458\_cov\_26.791616 36677-36682. Max. coverage (+): 0. Max coverage (-): 0

Region: NODE\_347176\_length\_38458\_cov\_26.791616 36683-36688. Max. coverage (+): 0. Max coverage (-): 0

Region: NODE\_347176\_length\_38458\_cov\_26.791616 36689-36694. Max. coverage (+): 0. Max coverage (-): 0

Region: NODE\_347176\_length\_38458\_cov\_26.791616 36695-36700. Max. coverage (+): 0. Max coverage (-): 0

Region: NODE\_347176\_length\_38458\_cov\_26.791616 36701-36705. Max. coverage (+): 0. Max coverage (-): 0

Region: NODE\_347176\_length\_38458\_cov\_26.791616 36706-36711. Max. coverage (+): 0. Max coverage (-): 0

Region: NODE\_347176\_length\_38458\_cov\_26.791616 36712-36717. Max. coverage (+): 0. Max coverage (-): 0

Region: NODE\_347176\_length\_38458\_cov\_26.791616 36718-36723. Max. coverage (+): 0. Max coverage (-): 0

Region: NODE\_347176\_length\_38458\_cov\_26.791616 36724-36728. Max. coverage (+): 0. Max coverage (-): 0

Region: NODE\_347176\_length\_38458\_cov\_26.791616 36729-36734. Max. coverage (+): 0. Max coverage (-): 0

Region: NODE\_347176\_length\_38458\_cov\_26.791616 36735-36740. Max. coverage (+): 0. Max coverage (-): 0

Region: NODE\_347176\_length\_38458\_cov\_26.791616 36741-36746. Max. coverage (+): 0. Max coverage (-): 0

Region: NODE\_347176\_length\_38458\_cov\_26.791616 36747-36752. Max. coverage (+): 0. Max coverage (-): 0

Region: NODE\_347176\_length\_38458\_cov\_26.791616 36753-36757. Max. coverage (+): 0. Max coverage (-): 0

Region: NODE\_347176\_length\_38458\_cov\_26.791616 36758-36763. Max. coverage (+): 0. Max coverage (-): 0

Region: NODE\_347176\_length\_38458\_cov\_26.791616 36764-36769. Max. coverage (+): 0. Max coverage (-): 0

Region: NODE\_347176\_length\_38458\_cov\_26.791616 36770-36775. Max. coverage (+): 0. Max coverage (-): 0

Region: NODE\_347176\_length\_38458\_cov\_26.791616 36776-36780. Max. coverage (+): 0. Max coverage (-): 0

Region: NODE\_347176\_length\_38458\_cov\_26.791616 36781-36786. Max. coverage (+): 0. Max coverage (-): 0

Region: NODE\_347176\_length\_38458\_cov\_26.791616 36787-36792. Max. coverage (+): 0. Max coverage (-): 0

Region: NODE\_347176\_length\_38458\_cov\_26.791616 36793-36798. Max. coverage (+): 0. Max coverage (-): 0

Region: NODE\_347176\_length\_38458\_cov\_26.791616 36799-36803. Max. coverage (+): 0. Max coverage (-): 0

Region: NODE\_347176\_length\_38458\_cov\_26.791616 36804-36809. Max. coverage (+): 0. Max coverage (-): 0

Region: NODE\_347176\_length\_38458\_cov\_26.791616 36810-36815. Max. coverage (+): 0. Max coverage (-): 0

Region: NODE\_347176\_length\_38458\_cov\_26.791616 36816-36821. Max. coverage (+): 0. Max coverage (-): 0

Region: NODE\_347176\_length\_38458\_cov\_26.791616 36822-36827. Max. coverage (+): 0. Max coverage (-): 0

Region: NODE\_347176\_length\_38458\_cov\_26.791616 36828-36832. Max. coverage (+): 0. Max coverage (-): 0

Region: NODE\_347176\_length\_38458\_cov\_26.791616 36833-36838. Max. coverage (+): 0. Max coverage (-): 0

Region: NODE\_347176\_length\_38458\_cov\_26.791616 36839-36844. Max. coverage (+): 0. Max coverage (-): 0

Region: NODE\_347176\_length\_38458\_cov\_26.791616 36845-36850. Max. coverage (+): 0. Max coverage (-): 0

Region: NODE\_347176\_length\_38458\_cov\_26.791616 36851-36855. Max. coverage (+): 0. Max coverage (-): 0

Region: NODE\_347176\_length\_38458\_cov\_26.791616 36856-36861. Max. coverage (+): 0. Max coverage (-): 0

Region: NODE\_347176\_length\_38458\_cov\_26.791616 36862-36867. Max. coverage (+): 0. Max coverage (-): 0

Region: NODE\_347176\_length\_38458\_cov\_26.791616 36868-36873. Max. coverage (+): 0. Max coverage (-): 0

Region: NODE\_347176\_length\_38458\_cov\_26.791616 36874-36878. Max. coverage (+): 0. Max coverage (-): 0

Region: NODE\_347176\_length\_38458\_cov\_26.791616 36879-36884. Max. coverage (+): 0. Max coverage (-): 0

Region: NODE\_347176\_length\_38458\_cov\_26.791616 36885-36890. Max. coverage (+): 0. Max coverage (-): 0

Region: NODE\_347176\_length\_38458\_cov\_26.791616 36891-36896. Max. coverage (+): 0. Max coverage (-): 0

Region: NODE\_347176\_length\_38458\_cov\_26.791616 36897-36902. Max. coverage (+): 0. Max coverage (-): 0

Region: NODE\_347176\_length\_38458\_cov\_26.791616 36903-36907. Max. coverage (+): 0. Max coverage (-): 0

Region: NODE\_347176\_length\_38458\_cov\_26.791616 36908-36913. Max. coverage (+): 0. Max coverage (-): 0

Region: NODE\_347176\_length\_38458\_cov\_26.791616 36914-36919. Max. coverage (+): 0. Max coverage (-): 0

Region: NODE\_347176\_length\_38458\_cov\_26.791616 36920-36925. Max. coverage (+): 0. Max coverage (-): 0

Region: NODE\_347176\_length\_38458\_cov\_26.791616 36926-36930. Max. coverage (+): 0. Max coverage (-): 0

Region: NODE\_347176\_length\_38458\_cov\_26.791616 36931-36936. Max. coverage (+): 0. Max coverage (-): 0

Region: NODE\_347176\_length\_38458\_cov\_26.791616 36937-36942. Max. coverage (+): 0. Max coverage (-): 0

Region: NODE\_347176\_length\_38458\_cov\_26.791616 36943-36948. Max. coverage (+): 0. Max coverage (-): 0

Region: NODE\_347176\_length\_38458\_cov\_26.791616 36949-36954. Max. coverage (+): 0. Max coverage (-): 0

Region: NODE\_347176\_length\_38458\_cov\_26.791616 36955-36959. Max. coverage (+): 0. Max coverage (-): 0

Region: NODE\_347176\_length\_38458\_cov\_26.791616 36960-36965. Max. coverage (+): 0. Max coverage (-): 0

Region: NODE\_347176\_length\_38458\_cov\_26.791616 36966-36971. Max. coverage (+): 0. Max coverage (-): 0

Region: NODE\_347176\_length\_38458\_cov\_26.791616 36972-36977. Max. coverage (+): 0. Max coverage (-): 0

Region: NODE\_347176\_length\_38458\_cov\_26.791616 36978-36982. Max. coverage (+): 0. Max coverage (-): 0

Region: NODE\_347176\_length\_38458\_cov\_26.791616 36983-36988. Max. coverage (+): 0. Max coverage (-): 0

Region: NODE\_347176\_length\_38458\_cov\_26.791616 36989-36994. Max. coverage (+): 0. Max coverage (-): 0

Region: NODE\_347176\_length\_38458\_cov\_26.791616 36995-37000. Max. coverage (+): 0. Max coverage (-): 0

Region: NODE\_347176\_length\_38458\_cov\_26.791616 37001-37005. Max. coverage (+): 0. Max coverage (-): 0

Region: NODE\_347176\_length\_38458\_cov\_26.791616 37006-37011. Max. coverage (+): 0. Max coverage (-): 0

Region: NODE\_347176\_length\_38458\_cov\_26.791616 37012-37017. Max. coverage (+): 0. Max coverage (-): 0

Region: NODE\_347176\_length\_38458\_cov\_26.791616 37018-37023. Max. coverage (+): 0. Max coverage (-): 0

Region: NODE\_347176\_length\_38458\_cov\_26.791616 37024-37029. Max. coverage (+): 0. Max coverage (-): 0

Region: NODE\_347176\_length\_38458\_cov\_26.791616 37030-37034. Max. coverage (+): 0. Max coverage (-): 0

Region: NODE\_347176\_length\_38458\_cov\_26.791616 37035-37040. Max. coverage (+): 0. Max coverage (-): 0

Region: NODE\_347176\_length\_38458\_cov\_26.791616 37041-37046. Max. coverage (+): 0. Max coverage (-): 0

Region: NODE\_347176\_length\_38458\_cov\_26.791616 37047-37052. Max. coverage (+): 0. Max coverage (-): 0

Region: NODE\_347176\_length\_38458\_cov\_26.791616 37053-37057. Max. coverage (+): 0. Max coverage (-): 0

Region: NODE\_347176\_length\_38458\_cov\_26.791616 37058-37063. Max. coverage (+): 0. Max coverage (-): 0

Region: NODE\_347176\_length\_38458\_cov\_26.791616 37064-37069. Max. coverage (+): 0. Max coverage (-): 0

Region: NODE\_347176\_length\_38458\_cov\_26.791616 37070-37075. Max. coverage (+): 0. Max coverage (-): 0

Region: NODE\_347176\_length\_38458\_cov\_26.791616 37076-37081. Max. coverage (+): 0. Max coverage (-): 0

Region: NODE\_347176\_length\_38458\_cov\_26.791616 37082-37086. Max. coverage (+): 0. Max coverage (-): 0

Region: NODE\_347176\_length\_38458\_cov\_26.791616 37087-37092. Max. coverage (+): 0. Max coverage (-): 0

Region: NODE\_347176\_length\_38458\_cov\_26.791616 37093-37098. Max. coverage (+): 0. Max coverage (-): 0

Region: NODE\_347176\_length\_38458\_cov\_26.791616 37099-37104. Max. coverage (+): 0. Max coverage (-): 0

Region: NODE\_347176\_length\_38458\_cov\_26.791616 37105-37109. Max. coverage (+): 0. Max coverage (-): 0

Region: NODE\_347176\_length\_38458\_cov\_26.791616 37110-37115. Max. coverage (+): 0. Max coverage (-): 0.09

Region: NODE\_347176\_length\_38458\_cov\_26.791616 37116-37121. Max. coverage (+): 0. Max coverage (-): 0.09

Region: NODE\_347176\_length\_38458\_cov\_26.791616 37122-37127. Max. coverage (+): 0. Max coverage (-): 0

Region: NODE\_347176\_length\_38458\_cov\_26.791616 37128-37132. Max. coverage (+): 0. Max coverage (-): 0

Region: NODE\_347176\_length\_38458\_cov\_26.791616 37133-37138. Max. coverage (+): 0. Max coverage (-): 0

Region: NODE\_347176\_length\_38458\_cov\_26.791616 37139-37144. Max. coverage (+): 0. Max coverage (-): 0

Region: NODE\_347176\_length\_38458\_cov\_26.791616 37145-37150. Max. coverage (+): 0. Max coverage (-): 0

Region: NODE\_347176\_length\_38458\_cov\_26.791616 37151-37156. Max. coverage (+): 0. Max coverage (-): 0.09

Region: NODE\_347176\_length\_38458\_cov\_26.791616 37157-37161. Max. coverage (+): 0. Max coverage (-): 0.09

Region: NODE\_347176\_length\_38458\_cov\_26.791616 37162-37167. Max. coverage (+): 0. Max coverage (-): 0

Region: NODE\_347176\_length\_38458\_cov\_26.791616 37168-37173. Max. coverage (+): 0. Max coverage (-): 0

Region: NODE\_347176\_length\_38458\_cov\_26.791616 37174-37179. Max. coverage (+): 0. Max coverage (-): 0

Region: NODE\_347176\_length\_38458\_cov\_26.791616 37180-37184. Max. coverage (+): 0. Max coverage (-): 0

Region: NODE\_347176\_length\_38458\_cov\_26.791616 37185-37190. Max. coverage (+): 0. Max coverage (-): 0

Region: NODE\_347176\_length\_38458\_cov\_26.791616 37191-37196. Max. coverage (+): 0. Max coverage (-): 0

Region: NODE\_347176\_length\_38458\_cov\_26.791616 37197-37202. Max. coverage (+): 0. Max coverage (-): 0

Region: NODE\_347176\_length\_38458\_cov\_26.791616 37203-37207. Max. coverage (+): 0. Max coverage (-): 0

Region: NODE\_347176\_length\_38458\_cov\_26.791616 37208-37213. Max. coverage (+): 0. Max coverage (-): 0

Region: NODE\_347176\_length\_38458\_cov\_26.791616 37214-37219. Max. coverage (+): 0. Max coverage (-): 0

Region: NODE\_347176\_length\_38458\_cov\_26.791616 37220-37225. Max. coverage (+): 0. Max coverage (-): 0

Region: NODE\_347176\_length\_38458\_cov\_26.791616 37226-37231. Max. coverage (+): 0. Max coverage (-): 0

Region: NODE\_347176\_length\_38458\_cov\_26.791616 37232-37236. Max. coverage (+): 0. Max coverage (-): 0

Region: NODE\_347176\_length\_38458\_cov\_26.791616 37237-37242. Max. coverage (+): 0. Max coverage (-): 0

Region: NODE\_347176\_length\_38458\_cov\_26.791616 37243-37248. Max. coverage (+): 0. Max coverage (-): 0

Region: NODE\_347176\_length\_38458\_cov\_26.791616 37249-37254. Max. coverage (+): 0. Max coverage (-): 0

Region: NODE\_347176\_length\_38458\_cov\_26.791616 37255-37259. Max. coverage (+): 0. Max coverage (-): 0

Region: NODE\_347176\_length\_38458\_cov\_26.791616 37260-37265. Max. coverage (+): 0. Max coverage (-): 0

Region: NODE\_347176\_length\_38458\_cov\_26.791616 37266-37271. Max. coverage (+): 0. Max coverage (-): 0

Region: NODE\_347176\_length\_38458\_cov\_26.791616 37272-37277. Max. coverage (+): 0. Max coverage (-): 0

Region: NODE\_347176\_length\_38458\_cov\_26.791616 37278-37283. Max. coverage (+): 0. Max coverage (-): 0

Region: NODE\_347176\_length\_38458\_cov\_26.791616 37284-37288. Max. coverage (+): 0. Max coverage (-): 0

Region: NODE\_347176\_length\_38458\_cov\_26.791616 37289-37294. Max. coverage (+): 0. Max coverage (-): 0

Region: NODE\_347176\_length\_38458\_cov\_26.791616 37295-37300. Max. coverage (+): 0. Max coverage (-): 0

Region: NODE\_347176\_length\_38458\_cov\_26.791616 37301-37306. Max. coverage (+): 0. Max coverage (-): 0

Region: NODE\_347176\_length\_38458\_cov\_26.791616 37307-37311. Max. coverage (+): 0. Max coverage (-): 0

Region: NODE\_347176\_length\_38458\_cov\_26.791616 37312-37317. Max. coverage (+): 0. Max coverage (-): 0

Region: NODE\_347176\_length\_38458\_cov\_26.791616 37318-37323. Max. coverage (+): 0. Max coverage (-): 0

Region: NODE\_347176\_length\_38458\_cov\_26.791616 37324-37329. Max. coverage (+): 0. Max coverage (-): 0

Region: NODE\_347176\_length\_38458\_cov\_26.791616 37330-37334. Max. coverage (+): 0. Max coverage (-): 0

Region: NODE\_347176\_length\_38458\_cov\_26.791616 37335-37340. Max. coverage (+): 0. Max coverage (-): 0

Region: NODE\_347176\_length\_38458\_cov\_26.791616 37341-37346. Max. coverage (+): 0. Max coverage (-): 0

Region: NODE\_347176\_length\_38458\_cov\_26.791616 37347-37352. Max. coverage (+): 0. Max coverage (-): 0

Region: NODE\_347176\_length\_38458\_cov\_26.791616 37353-37358. Max. coverage (+): 0. Max coverage (-): 0

Region: NODE\_347176\_length\_38458\_cov\_26.791616 37359-37363. Max. coverage (+): 0. Max coverage (-): 0

Region: NODE\_347176\_length\_38458\_cov\_26.791616 37364-37369. Max. coverage (+): 0. Max coverage (-): 0

Region: NODE\_347176\_length\_38458\_cov\_26.791616 37370-37375. Max. coverage (+): 0. Max coverage (-): 0

Region: NODE\_347176\_length\_38458\_cov\_26.791616 37376-37381. Max. coverage (+): 0. Max coverage (-): 0

Region: NODE\_347176\_length\_38458\_cov\_26.791616 37382-37386. Max. coverage (+): 0. Max coverage (-): 0

Region: NODE\_347176\_length\_38458\_cov\_26.791616 37387-37392. Max. coverage (+): 0. Max coverage (-): 0

Region: NODE\_347176\_length\_38458\_cov\_26.791616 37393-37398. Max. coverage (+): 0. Max coverage (-): 0

Region: NODE\_347176\_length\_38458\_cov\_26.791616 37399-37404. Max. coverage (+): 0. Max coverage (-): 0

Region: NODE\_347176\_length\_38458\_cov\_26.791616 37405-37410. Max. coverage (+): 0. Max coverage (-): 0

Region: NODE\_347176\_length\_38458\_cov\_26.791616 37411-37415. Max. coverage (+): 0. Max coverage (-): 0

Region: NODE\_347176\_length\_38458\_cov\_26.791616 37416-37421. Max. coverage (+): 0. Max coverage (-): 0

Region: NODE\_347176\_length\_38458\_cov\_26.791616 37422-37427. Max. coverage (+): 0. Max coverage (-): 0

Region: NODE\_347176\_length\_38458\_cov\_26.791616 37428-37433. Max. coverage (+): 0. Max coverage (-): 0

Region: NODE\_347176\_length\_38458\_cov\_26.791616 37434-37438. Max. coverage (+): 0. Max coverage (-): 0

Region: NODE\_347176\_length\_38458\_cov\_26.791616 37439-37444. Max. coverage (+): 0. Max coverage (-): 0

Region: NODE\_347176\_length\_38458\_cov\_26.791616 37445-37450. Max. coverage (+): 0. Max coverage (-): 0

Region: NODE\_347176\_length\_38458\_cov\_26.791616 37451-37456. Max. coverage (+): 0. Max coverage (-): 0

Region: NODE\_347176\_length\_38458\_cov\_26.791616 37457-37461. Max. coverage (+): 0. Max coverage (-): 0

Region: NODE\_347176\_length\_38458\_cov\_26.791616 37462-37467. Max. coverage (+): 0. Max coverage (-): 0

Region: NODE\_347176\_length\_38458\_cov\_26.791616 37468-37473. Max. coverage (+): 0. Max coverage (-): 0

Region: NODE\_347176\_length\_38458\_cov\_26.791616 37474-37479. Max. coverage (+): 0. Max coverage (-): 0

Region: NODE\_347176\_length\_38458\_cov\_26.791616 37480-37485. Max. coverage (+): 0. Max coverage (-): 0

Region: NODE\_347176\_length\_38458\_cov\_26.791616 37486-37490. Max. coverage (+): 0. Max coverage (-): 0

Region: NODE\_347176\_length\_38458\_cov\_26.791616 37491-37496. Max. coverage (+): 0. Max coverage (-): 0

Region: NODE\_347176\_length\_38458\_cov\_26.791616 37497-37502. Max. coverage (+): 0. Max coverage (-): 0

Region: NODE\_347176\_length\_38458\_cov\_26.791616 37503-37508. Max. coverage (+): 0. Max coverage (-): 0

Region: NODE\_347176\_length\_38458\_cov\_26.791616 37509-37513. Max. coverage (+): 0. Max coverage (-): 0

Region: NODE\_347176\_length\_38458\_cov\_26.791616 37514-37519. Max. coverage (+): 0. Max coverage (-): 0

Region: NODE\_347176\_length\_38458\_cov\_26.791616 37520-37525. Max. coverage (+): 0. Max coverage (-): 0

Region: NODE\_347176\_length\_38458\_cov\_26.791616 37526-37531. Max. coverage (+): 0. Max coverage (-): 0

Region: NODE\_347176\_length\_38458\_cov\_26.791616 37532-37536. Max. coverage (+): 0. Max coverage (-): 0

Region: NODE\_347176\_length\_38458\_cov\_26.791616 37537-37542. Max. coverage (+): 0. Max coverage (-): 0

Region: NODE\_347176\_length\_38458\_cov\_26.791616 37543-37548. Max. coverage (+): 0. Max coverage (-): 0

Region: NODE\_347176\_length\_38458\_cov\_26.791616 37549-37554. Max. coverage (+): 0. Max coverage (-): 0

Region: NODE\_347176\_length\_38458\_cov\_26.791616 37555-37560. Max. coverage (+): 0. Max coverage (-): 0

Region: NODE\_347176\_length\_38458\_cov\_26.791616 37561-37565. Max. coverage (+): 0. Max coverage (-): 0

Region: NODE\_347176\_length\_38458\_cov\_26.791616 37566-37571. Max. coverage (+): 0. Max coverage (-): 0

Region: NODE\_347176\_length\_38458\_cov\_26.791616 37572-37577. Max. coverage (+): 0. Max coverage (-): 0

Region: NODE\_347176\_length\_38458\_cov\_26.791616 37578-37583. Max. coverage (+): 0. Max coverage (-): 0

Region: NODE\_347176\_length\_38458\_cov\_26.791616 37584-37588. Max. coverage (+): 0. Max coverage (-): 0

Region: NODE\_347176\_length\_38458\_cov\_26.791616 37589-37594. Max. coverage (+): 0. Max coverage (-): 0

Region: NODE\_347176\_length\_38458\_cov\_26.791616 37595-37600. Max. coverage (+): 0. Max coverage (-): 0

Region: NODE\_347176\_length\_38458\_cov\_26.791616 37601-37606. Max. coverage (+): 0. Max coverage (-): 0

Region: NODE\_347176\_length\_38458\_cov\_26.791616 37607-37612. Max. coverage (+): 0. Max coverage (-): 0

Region: NODE\_347176\_length\_38458\_cov\_26.791616 37613-37617. Max. coverage (+): 0. Max coverage (-): 0

Region: NODE\_347176\_length\_38458\_cov\_26.791616 37618-37623. Max. coverage (+): 0. Max coverage (-): 0

Region: NODE\_347176\_length\_38458\_cov\_26.791616 37624-37629. Max. coverage (+): 0. Max coverage (-): 0

Region: NODE\_347176\_length\_38458\_cov\_26.791616 37630-37635. Max. coverage (+): 0. Max coverage (-): 0

Region: NODE\_347176\_length\_38458\_cov\_26.791616 37636-37640. Max. coverage (+): 0. Max coverage (-): 0

Region: NODE\_347176\_length\_38458\_cov\_26.791616 37641-37646. Max. coverage (+): 0. Max coverage (-): 0

Region: NODE\_347176\_length\_38458\_cov\_26.791616 37647-37652. Max. coverage (+): 0. Max coverage (-): 0

Region: NODE\_347176\_length\_38458\_cov\_26.791616 37653-37658. Max. coverage (+): 0. Max coverage (-): 0

Region: NODE\_347176\_length\_38458\_cov\_26.791616 37659-37663. Max. coverage (+): 0. Max coverage (-): 0

Region: NODE\_347176\_length\_38458\_cov\_26.791616 37664-37669. Max. coverage (+): 0. Max coverage (-): 0

Region: NODE\_347176\_length\_38458\_cov\_26.791616 37670-37675. Max. coverage (+): 0. Max coverage (-): 0

Region: NODE\_347176\_length\_38458\_cov\_26.791616 37676-37681. Max. coverage (+): 0. Max coverage (-): 0

Region: NODE\_347176\_length\_38458\_cov\_26.791616 37682-37687. Max. coverage (+): 0. Max coverage (-): 0

Region: NODE\_347176\_length\_38458\_cov\_26.791616 37688-37692. Max. coverage (+): 0. Max coverage (-): 0

Region: NODE\_347176\_length\_38458\_cov\_26.791616 37693-37698. Max. coverage (+): 0. Max coverage (-): 0

Region: NODE\_347176\_length\_38458\_cov\_26.791616 37699-37704. Max. coverage (+): 0. Max coverage (-): 0

Region: NODE\_347176\_length\_38458\_cov\_26.791616 37705-37710. Max. coverage (+): 0. Max coverage (-): 0.09

Region: NODE\_347176\_length\_38458\_cov\_26.791616 37711-37715. Max. coverage (+): 0. Max coverage (-): 0.09

Region: NODE\_347176\_length\_38458\_cov\_26.791616 37716-37721. Max. coverage (+): 0. Max coverage (-): 0

Region: NODE\_347176\_length\_38458\_cov\_26.791616 37722-37727. Max. coverage (+): 0. Max coverage (-): 0

Region: NODE\_347176\_length\_38458\_cov\_26.791616 37728-37733. Max. coverage (+): 0. Max coverage (-): 0

Region: NODE\_347176\_length\_38458\_cov\_26.791616 37734-37739. Max. coverage (+): 0. Max coverage (-): 0

Region: NODE\_347176\_length\_38458\_cov\_26.791616 37740-37744. Max. coverage (+): 0. Max coverage (-): 0

Region: NODE\_347176\_length\_38458\_cov\_26.791616 37745-37750. Max. coverage (+): 0. Max coverage (-): 0

Region: NODE\_347176\_length\_38458\_cov\_26.791616 37751-37756. Max. coverage (+): 0. Max coverage (-): 0

Region: NODE\_347176\_length\_38458\_cov\_26.791616 37757-37762. Max. coverage (+): 0. Max coverage (-): 0

Region: NODE\_347176\_length\_38458\_cov\_26.791616 37763-37767. Max. coverage (+): 0. Max coverage (-): 0

Region: NODE\_347176\_length\_38458\_cov\_26.791616 37768-37773. Max. coverage (+): 0. Max coverage (-): 0

Region: NODE\_347176\_length\_38458\_cov\_26.791616 37774-37779. Max. coverage (+): 0. Max coverage (-): 0

Region: NODE\_347176\_length\_38458\_cov\_26.791616 37780-37785. Max. coverage (+): 0. Max coverage (-): 0

Region: NODE\_347176\_length\_38458\_cov\_26.791616 37786-37790. Max. coverage (+): 0. Max coverage (-): 0

Region: NODE\_347176\_length\_38458\_cov\_26.791616 37791-37796. Max. coverage (+): 0. Max coverage (-): 0

Region: NODE\_347176\_length\_38458\_cov\_26.791616 37797-37802. Max. coverage (+): 0. Max coverage (-): 0

Region: NODE\_347176\_length\_38458\_cov\_26.791616 37803-37808. Max. coverage (+): 0. Max coverage (-): 0

Region: NODE\_347176\_length\_38458\_cov\_26.791616 37809-37814. Max. coverage (+): 0. Max coverage (-): 0

Region: NODE\_347176\_length\_38458\_cov\_26.791616 37815-37819. Max. coverage (+): 0. Max coverage (-): 0

Region: NODE\_347176\_length\_38458\_cov\_26.791616 37820-37825. Max. coverage (+): 0. Max coverage (-): 0

Region: NODE\_347176\_length\_38458\_cov\_26.791616 37826-37831. Max. coverage (+): 0. Max coverage (-): 0

Region: NODE\_347176\_length\_38458\_cov\_26.791616 37832-37837. Max. coverage (+): 0. Max coverage (-): 0

Region: NODE\_347176\_length\_38458\_cov\_26.791616 37838-37842. Max. coverage (+): 0. Max coverage (-): 0

Region: NODE\_347176\_length\_38458\_cov\_26.791616 37843-37848. Max. coverage (+): 0. Max coverage (-): 0

Region: NODE\_347176\_length\_38458\_cov\_26.791616 37849-37854. Max. coverage (+): 0. Max coverage (-): 0

Region: NODE\_347176\_length\_38458\_cov\_26.791616 37855-37860. Max. coverage (+): 0. Max coverage (-): 0

Region: NODE\_347176\_length\_38458\_cov\_26.791616 37861-37866. Max. coverage (+): 0. Max coverage (-): 0

Region: NODE\_347176\_length\_38458\_cov\_26.791616 37867-37871. Max. coverage (+): 0. Max coverage (-): 0

Region: NODE\_347176\_length\_38458\_cov\_26.791616 37872-37877. Max. coverage (+): 0. Max coverage (-): 0

Region: NODE\_347176\_length\_38458\_cov\_26.791616 37878-37883. Max. coverage (+): 0. Max coverage (-): 0

Region: NODE\_347176\_length\_38458\_cov\_26.791616 37884-37889. Max. coverage (+): 0. Max coverage (-): 0

Region: NODE\_347176\_length\_38458\_cov\_26.791616 37890-37894. Max. coverage (+): 0. Max coverage (-): 0

Region: NODE\_347176\_length\_38458\_cov\_26.791616 37895-37900. Max. coverage (+): 0. Max coverage (-): 0

Region: NODE\_347176\_length\_38458\_cov\_26.791616 37901-37906. Max. coverage (+): 0. Max coverage (-): 0

Region: NODE\_347176\_length\_38458\_cov\_26.791616 37907-37912. Max. coverage (+): 0. Max coverage (-): 0

Region: NODE\_347176\_length\_38458\_cov\_26.791616 37913-37917. Max. coverage (+): 0. Max coverage (-): 0

Region: NODE\_347176\_length\_38458\_cov\_26.791616 37918-37923. Max. coverage (+): 0. Max coverage (-): 0

Region: NODE\_347176\_length\_38458\_cov\_26.791616 37924-37929. Max. coverage (+): 0. Max coverage (-): 0

Region: NODE\_347176\_length\_38458\_cov\_26.791616 37930-37935. Max. coverage (+): 0. Max coverage (-): 0

Region: NODE\_347176\_length\_38458\_cov\_26.791616 37936-37941. Max. coverage (+): 0. Max coverage (-): 0

Region: NODE\_347176\_length\_38458\_cov\_26.791616 37942-37946. Max. coverage (+): 0. Max coverage (-): 0

Region: NODE\_347176\_length\_38458\_cov\_26.791616 37947-37952. Max. coverage (+): 0. Max coverage (-): 0

Region: NODE\_347176\_length\_38458\_cov\_26.791616 37953-37958. Max. coverage (+): 0. Max coverage (-): 0

Region: NODE\_347176\_length\_38458\_cov\_26.791616 37959-37964. Max. coverage (+): 0. Max coverage (-): 0

Region: NODE\_347176\_length\_38458\_cov\_26.791616 37965-37969. Max. coverage (+): 0. Max coverage (-): 0

Region: NODE\_347176\_length\_38458\_cov\_26.791616 37970-37975. Max. coverage (+): 0. Max coverage (-): 0

Region: NODE\_347176\_length\_38458\_cov\_26.791616 37976-37981. Max. coverage (+): 0. Max coverage (-): 0

Region: NODE\_347176\_length\_38458\_cov\_26.791616 37982-37987. Max. coverage (+): 0. Max coverage (-): 0

Region: NODE\_347176\_length\_38458\_cov\_26.791616 37988-37992. Max. coverage (+): 0. Max coverage (-): 0

Region: NODE\_347176\_length\_38458\_cov\_26.791616 37993-37998. Max. coverage (+): 0. Max coverage (-): 0

Region: NODE\_347176\_length\_38458\_cov\_26.791616 37999-38004. Max. coverage (+): 0. Max coverage (-): 0

Region: NODE\_347176\_length\_38458\_cov\_26.791616 38005-38010. Max. coverage (+): 0. Max coverage (-): 0

Region: NODE\_347176\_length\_38458\_cov\_26.791616 38011-38016. Max. coverage (+): 0. Max coverage (-): 0

Region: NODE\_347176\_length\_38458\_cov\_26.791616 38017-38021. Max. coverage (+): 0. Max coverage (-): 0

Region: NODE\_347176\_length\_38458\_cov\_26.791616 38022-38027. Max. coverage (+): 0. Max coverage (-): 0

Region: NODE\_347176\_length\_38458\_cov\_26.791616 38028-38033. Max. coverage (+): 0. Max coverage (-): 0

Region: NODE\_347176\_length\_38458\_cov\_26.791616 38034-38039. Max. coverage (+): 0. Max coverage (-): 0

Region: NODE\_347176\_length\_38458\_cov\_26.791616 38040-38044. Max. coverage (+): 0. Max coverage (-): 0

Region: NODE\_347176\_length\_38458\_cov\_26.791616 38045-38050. Max. coverage (+): 0. Max coverage (-): 0

Region: NODE\_347176\_length\_38458\_cov\_26.791616 38051-38056. Max. coverage (+): 0. Max coverage (-): 0

Region: NODE\_347176\_length\_38458\_cov\_26.791616 38057-38062. Max. coverage (+): 0. Max coverage (-): 0.09

Region: NODE\_347176\_length\_38458\_cov\_26.791616 38063-38068. Max. coverage (+): 0. Max coverage (-): 0.09

Region: NODE\_347176\_length\_38458\_cov\_26.791616 38069-38073. Max. coverage (+): 0. Max coverage (-): 0

Region: NODE\_347176\_length\_38458\_cov\_26.791616 38074-38079. Max. coverage (+): 0. Max coverage (-): 0

Region: NODE\_347176\_length\_38458\_cov\_26.791616 38080-38085. Max. coverage (+): 0. Max coverage (-): 0

Region: NODE\_347176\_length\_38458\_cov\_26.791616 38086-38091. Max. coverage (+): 0. Max coverage (-): 0

Region: NODE\_347176\_length\_38458\_cov\_26.791616 38092-38096. Max. coverage (+): 0. Max coverage (-): 0

Region: NODE\_347176\_length\_38458\_cov\_26.791616 38097-38102. Max. coverage (+): 0. Max coverage (-): 0

Region: NODE\_347176\_length\_38458\_cov\_26.791616 38103-38108. Max. coverage (+): 0. Max coverage (-): 0

Region: NODE\_347176\_length\_38458\_cov\_26.791616 38109-38114. Max. coverage (+): 0. Max coverage (-): 0

Region: NODE\_347176\_length\_38458\_cov\_26.791616 38115-38119. Max. coverage (+): 0. Max coverage (-): 0

Region: NODE\_347176\_length\_38458\_cov\_26.791616 38120-38125. Max. coverage (+): 0. Max coverage (-): 0

Region: NODE\_347176\_length\_38458\_cov\_26.791616 38126-38131. Max. coverage (+): 0. Max coverage (-): 0

Region: NODE\_347176\_length\_38458\_cov\_26.791616 38132-38137. Max. coverage (+): 0. Max coverage (-): 0

Region: NODE\_347176\_length\_38458\_cov\_26.791616 38138-38143. Max. coverage (+): 0. Max coverage (-): 0

Region: NODE\_347176\_length\_38458\_cov\_26.791616 38144-38148. Max. coverage (+): 0. Max coverage (-): 0

Region: NODE\_347176\_length\_38458\_cov\_26.791616 38149-38154. Max. coverage (+): 0. Max coverage (-): 0

Region: NODE\_347176\_length\_38458\_cov\_26.791616 38155-38160. Max. coverage (+): 0. Max coverage (-): 0

Region: NODE\_347176\_length\_38458\_cov\_26.791616 38161-38166. Max. coverage (+): 0. Max coverage (-): 0

Region: NODE\_347176\_length\_38458\_cov\_26.791616 38167-38171. Max. coverage (+): 0. Max coverage (-): 0

Region: NODE\_347176\_length\_38458\_cov\_26.791616 38172-38177. Max. coverage (+): 0. Max coverage (-): 0

Region: NODE\_347176\_length\_38458\_cov\_26.791616 38178-38183. Max. coverage (+): 0. Max coverage (-): 0

Region: NODE\_347176\_length\_38458\_cov\_26.791616 38184-38189. Max. coverage (+): 0. Max coverage (-): 0

Region: NODE\_347176\_length\_38458\_cov\_26.791616 38190-38195. Max. coverage (+): 0. Max coverage (-): 0

Region: NODE\_347176\_length\_38458\_cov\_26.791616 38196-38200. Max. coverage (+): 0. Max coverage (-): 0

Region: NODE\_347176\_length\_38458\_cov\_26.791616 38201-38206. Max. coverage (+): 0. Max coverage (-): 0

Region: NODE\_347176\_length\_38458\_cov\_26.791616 38207-38212. Max. coverage (+): 0. Max coverage (-): 0

Region: NODE\_347176\_length\_38458\_cov\_26.791616 38213-38218. Max. coverage (+): 0. Max coverage (-): 0

Region: NODE\_347176\_length\_38458\_cov\_26.791616 38219-38223. Max. coverage (+): 0. Max coverage (-): 0

Region: NODE\_347176\_length\_38458\_cov\_26.791616 38224-38229. Max. coverage (+): 0. Max coverage (-): 0

Region: NODE\_347176\_length\_38458\_cov\_26.791616 38230-38235. Max. coverage (+): 0. Max coverage (-): 0

Region: NODE\_347176\_length\_38458\_cov\_26.791616 38236-38241. Max. coverage (+): 0. Max coverage (-): 0

Region: NODE\_347176\_length\_38458\_cov\_26.791616 38242-38246. Max. coverage (+): 0. Max coverage (-): 0

Region: NODE\_347176\_length\_38458\_cov\_26.791616 38247-38252. Max. coverage (+): 0. Max coverage (-): 0

Region: NODE\_347176\_length\_38458\_cov\_26.791616 38253-38258. Max. coverage (+): 0. Max coverage (-): 0

Region: NODE\_347176\_length\_38458\_cov\_26.791616 38259-38264. Max. coverage (+): 0. Max coverage (-): 0

Region: NODE\_347176\_length\_38458\_cov\_26.791616 38265-38270. Max. coverage (+): 0. Max coverage (-): 0

Region: NODE\_347176\_length\_38458\_cov\_26.791616 38271-38275. Max. coverage (+): 0. Max coverage (-): 0

Region: NODE\_347176\_length\_38458\_cov\_26.791616 38276-38281. Max. coverage (+): 0. Max coverage (-): 0

Region: NODE\_347176\_length\_38458\_cov\_26.791616 38282-38287. Max. coverage (+): 0. Max coverage (-): 0

Region: NODE\_347176\_length\_38458\_cov\_26.791616 38288-38293. Max. coverage (+): 0. Max coverage (-): 0

Region: NODE\_347176\_length\_38458\_cov\_26.791616 38294-38298. Max. coverage (+): 0. Max coverage (-): 0

Region: NODE\_347176\_length\_38458\_cov\_26.791616 38299-38304. Max. coverage (+): 0. Max coverage (-): 0

Region: NODE\_347176\_length\_38458\_cov\_26.791616 38305-38310. Max. coverage (+): 0. Max coverage (-): 0

Region: NODE\_347176\_length\_38458\_cov\_26.791616 38311-38316. Max. coverage (+): 0. Max coverage (-): 0

Region: NODE\_347176\_length\_38458\_cov\_26.791616 38317-38321. Max. coverage (+): 0. Max coverage (-): 0

Region: NODE\_347176\_length\_38458\_cov\_26.791616 38322-38327. Max. coverage (+): 0. Max coverage (-): 0

Region: NODE\_347176\_length\_38458\_cov\_26.791616 38328-38333. Max. coverage (+): 0. Max coverage (-): 0

Region: NODE\_347176\_length\_38458\_cov\_26.791616 38334-38339. Max. coverage (+): 0. Max coverage (-): 0

Region: NODE\_347176\_length\_38458\_cov\_26.791616 38340-38345. Max. coverage (+): 0. Max coverage (-): 0

Region: NODE\_347176\_length\_38458\_cov\_26.791616 38346-38350. Max. coverage (+): 0. Max coverage (-): 0

Region: NODE\_347176\_length\_38458\_cov\_26.791616 38351-38356. Max. coverage (+): 0. Max coverage (-): 0

Region: NODE\_347176\_length\_38458\_cov\_26.791616 38357-38362. Max. coverage (+): 0. Max coverage (-): 0

Region: NODE\_347176\_length\_38458\_cov\_26.791616 38363-38368. Max. coverage (+): 0. Max coverage (-): 0

Region: NODE\_347176\_length\_38458\_cov\_26.791616 38369-38373. Max. coverage (+): 0. Max coverage (-): 0

Region: NODE\_347176\_length\_38458\_cov\_26.791616 38374-38379. Max. coverage (+): 0. Max coverage (-): 0

Region: NODE\_347176\_length\_38458\_cov\_26.791616 38380-38385. Max. coverage (+): 0. Max coverage (-): 0

Region: NODE\_347176\_length\_38458\_cov\_26.791616 38386-38391. Max. coverage (+): 0. Max coverage (-): 0.19

Region: NODE\_347176\_length\_38458\_cov\_26.791616 38392-38397. Max. coverage (+): 0. Max coverage (-): 0.19

Region: NODE\_347176\_length\_38458\_cov\_26.791616 38398-38402. Max. coverage (+): 0. Max coverage (-): 0

Region: NODE\_347176\_length\_38458\_cov\_26.791616 38403-38408. Max. coverage (+): 0. Max coverage (-): 0

Region: NODE\_347176\_length\_38458\_cov\_26.791616 38409-38414. Max. coverage (+): 0. Max coverage (-): 0

Region: NODE\_347176\_length\_38458\_cov\_26.791616 38415-38420. Max. coverage (+): 0. Max coverage (-): 0

Region: NODE\_347176\_length\_38458\_cov\_26.791616 38421-. Max. coverage (+): 0. Max coverage (-): 0

RepeatMasker Color Code

**+**

100-98% Identity

<98-95% Identity

<95-90% Identity

<90-85% Identity

<85-80% Identity

<80-75% Identity

<75-70% Identity

<70% Identity

**-**

Gene Set Color Code

**+**

Gene

Pseudogene

Other

**-**

Topology/Coverage Color Code

Coverage Plus Strand

Coverage Minus Strand

Mainstrand: Plus

Mainstrand: Minus

Complementary Strand

Flanking Region  
(if option -flank >0)

Gene Set Annotation  
  
RepeatMasker Annotation  
  
Transcription Factor Binding Sites  

**RHOXF1** (Sequence: AGCTCA (-): 37239)  
**RHOXF1** (Sequence: AGCTTA (-): 37415)  
**RHOXF1** (Sequence: AGCTTA (-): 37648)  
**RHOXF1** (Sequence: GGCTCA (-): 37820)  
**RHOXF1** (Sequence: GGATCA (-): 37849)  
**RHOXF1** (Sequence: GGCTCA (-): 38352)  
**RHOXF1** (Sequence: TAAGCT (+): 35781)  
**RHOXF1** (Sequence: TAAGCT (+): 36175)  
**RHOXF1** (Sequence: TAATCC (+): 36883)  
**RHOXF1** (Sequence: TGATCT (+): 37917)  
**RHOXF1** (Sequence: TGAGCC (+): 37989)  
**RHOXF1** (Sequence: TAATCT (+): 38396)  
**SOX9** (Sequence: AACAATGA (-): 36055)  
**SOX9** (Sequence: AACAATGA (-): 36239)  
**SOX9** (Sequence: AACAATAA (-): 37073)  
**Sox5** (Sequence: ATTGTT (+): 37347)  
**Sox5** (Sequence: ATTGTT (+): 38378)  
**SOX9** (Sequence: TCATTGTT (+): 37345)  
**Nobox** (Sequence: GGCAATTA (-): 36598)  
**FOXO1** (Sequence: AAAAACAAG (-): 37764)  
**Nobox** (Sequence: TAATTACC (+): 35558)  
**Rhox11** (Sequence: TAAACACCA (-): 37623)  
**Sox5** (Sequence: AACAAT (-): 36055)  
**Sox5** (Sequence: AACAAT (-): 36239)  
**Sox5** (Sequence: AACAAT (-): 37073)  
**Sox5** (Sequence: AACAAT (-): 37244)  
**POU5F1** (Sequence: ATGCAAA (+): 37751)
