# Supplementary material for: First characterization of PIWI-interacting RNA clusters in a cichlid fish with a B chromosome
Source: BMC Biol. 2022 Sep 21;20:204. doi: 10.1186/s12915-022-01403-2 (PMC9490952; doi:10.1186/s12915-022-01403-2)
Supplement: Supplementary file 1 — Additional file 1. Zipped folder with fasta and interactive html piRNA cluster information for the A. latifasciata genome. The nomenclature is as follows: number-pirna-cluster_sex_B-presence (f, female; m, male; 0b, without B chromosome; 1b, with B chromosome). [file 12915_2022_1403_MOESM1_ESM.zip › 135_m0b.html]

piRNA cluster 135\_m0b 64


Predicted piRNA cluster no. 135\_m0b
  

Show proTRAC run info
Hide proTRAC run info

/\  
                \_\_\_\_\_\_\_\_\_\_\_\_\_\_\_\_\_\_\_\_\_\_\_/\\_\_\_ /  \\_\_\_\_\_\_\_  
               I                      /  \  /    \      I  
               I     pro             /    \/      \     I  
               I        TRAC        /               \   I  
               I   \_\_\_\_\_\_\_\_\_\_\_\_\_\_\_\_/\_\_\_\_\_\_\_\_\_\_\_\_\_\_\_\_\_\\_ I  
               I   \              /                     I  
               I    \            /                      I  
               I     \  /\      /       V.2.4.2         I  
               I      \/  \    /                        I  
               I\_\_\_\_\_\_\_\_\_\_\_\  /\_\_\_\_\_\_\_\_\_\_\_\_\_\_\_\_\_\_\_\_\_\_\_\_\_I  
                            \/  
  
  
================================= proTRAC ====================================  
VERSION: .......... 2.4.2  
LAST MODIFIED: .... 11. May 2018  
  
Please cite:  
Rosenkranz D, Zischler H. proTRAC - a software for probabilistic piRNA cluster  
detection, visualization and analysis. 2012. BMC Bioinformatics 13:5.  
  
  
Contact:  
David Rosenkranz  
Institute of Organismic and Molecular Evolutionary Biology  
Dept. Anthropology, small RNA group  
Johannes Gutenberg University Mainz  
email: rosenkranz@uni-mainz.de  
  
You can find the latest proTRAC version at:  
http://sourceforge.net/projects/protrac/files  
http://www.smallRNAgroup-mainz.de/software  
==============================================================================  
  
PARAMETERS:  
Map file: ...............piwi-machos-0B.fa-collapse.map  
Genome file: ............../../../0B\_ala\_genome.fa  
RepeatMasker annotation: Alatifasciata-all0B-maryan-v2.fa\_corrected.out  
GeneSet:................./guest-storage/Data/annotation/Alatifasciata\_all0B\_maryan-v2\_out2017.gff  
  
Significant (p<=0.01) hit density will be calculated based  
on observed hit distribution.  
  
Sliding window size: ........................................ 5000 bp  
Sliding window increament: .................................. 1000 bp  
Normalize each hit by number of genomic hits: ............... yes  
Normalize each hit by number of sequence reads: ............. yes  
Normalize values (-> per million mapped reads): ............. yes  
Min. fraction of hits with 1T(U) or 10A: .................... 0.75  
Alternatively: Min. fraction of hits with 1T(U) and 10A: .... 0.5  
Min. fraction of hits with typical piRNA length: ............ 0.75  
Typical piRNA length: ....................................... 24-32 nt  
Min. size of a piRNA cluster: ............................... 1000 bp.  
Min. number of hits (absolute): ............................. 0  
Min. number of hits (normalized): ........................... 0  
Min. fraction of hits on the mainstrand: .................... 0.75  
Top fraction of mapped sequences (in terms of read counts): . 1%  
Top fraction accounts for max. n% of sequence reads: ........ 90%  
Min. fraction of hits on each arm of a bidirectional cluster: 0.05  
Output html file for each cluster: .......................... yes  
Output a summary table: ..................................... yes  
Output a FASTA file for each cluster (piRNA sequences): ..... yes  
Output a FASTA file comprising cluster sequences: ........... yes  
Output a GTF file for predicted piRNA clusters: ..............yes  
Search DNA motifs in clusters: .............................. yes  
Output flanking sequences: +/- .............................. 0 bp  
Output ~.pTi file: .......................................... no  
==============================================================================  
  
  
Genome size (without gaps): ............ 758543724 bp  
Gaps (N/X/-): .......................... 417479 bp  
Mapped reads: .......................... 24765598  
Non-identical sequences: ............... 6158275  
Genomic hits: .......................... 53103584  
Significant densitiy of mapped reads: .. 763.098963422187 reads/kb

Show proTRAC cluster info
Hide proTRAC cluster info

|  |  |
| --- | --- |
| Location | NODE\_347534\_length\_4529\_cov\_29.102673 |
| Coordinates | 1-4654 |
| Size [bp] | 4654 |
| Sequence hit loci | 3877 |
| Mapped reads (normalized) | 9101 |
| Mapped reads (normalized) per kb | 1955.5 |
| Normalized reads with 1T (1U) | 70.4% |
| Normalized reads with 10A | 51.7% |
| Normalized reads with length 24-32 nt | 98.7% |
| Normalized reads on the main strand(s) | 76.6% |
| Predicted directionality | mono:minus |

100%

0%

1T (1U)  
reads

10A reads

24-32 nt  
reads

reads on mainstrand

**Either the amount of reads with 1T (1U) OR 10A has to exceed 75% (set with option: -1Tor10A)  
Alternatively the amount of reads with 1T (1U) AND 10A has to exceed 50% (set with option: -1Tand10A)  
Minimum amount of reads with preferred size is 75% (set with option: -pisize)  
Minimum amount of reads on the main strand(s) is 75% (set with option: -clstrand)**

Show read coverage
Hide read coverage

WHAT DO I SEE HERE?  
This chart shows the location of mapped sequence reads within a predicted piRNA cluster. The color refers to the number of genomic hits produced by the sequence read in question. A dark red bar indicates that this sequence read produces many other hits elsewhere in the genome. Many adjacent red or yellow bars can indicate the presence of a multi-copy element such as transposons or rRNA genes. A dark green bar indicates that this sequence read maps uniquely to this locus.

1 hit

2-5 hits

6-10 hits

11-20 hits

21-50 hits

51-100 hits

> 100 hits

NODE\_347534\_length\_4529\_cov\_29.102673

1

4654

Gene Set

RepeatMasker

Mapped  
Reads

46.68

plus strand

minus strand

46.68

Region: NODE\_347534\_length\_4529\_cov\_29.102673 1327-5. Max. coverage (+): 0.03. Max coverage (-): 0.05

Region: NODE\_347534\_length\_4529\_cov\_29.102673 6-14. Max. coverage (+): 0.13. Max coverage (-): 0.19

Region: NODE\_347534\_length\_4529\_cov\_29.102673 15-24. Max. coverage (+): 0.04. Max coverage (-): 0.32

Region: NODE\_347534\_length\_4529\_cov\_29.102673 25-33. Max. coverage (+): 0.01. Max coverage (-): 0.11

Region: NODE\_347534\_length\_4529\_cov\_29.102673 34-42. Max. coverage (+): 0.08. Max coverage (-): 0.04

Region: NODE\_347534\_length\_4529\_cov\_29.102673 43-52. Max. coverage (+): 0.04. Max coverage (-): 0.16

Region: NODE\_347534\_length\_4529\_cov\_29.102673 53-61. Max. coverage (+): 0. Max coverage (-): 1.86

Region: NODE\_347534\_length\_4529\_cov\_29.102673 62-70. Max. coverage (+): 0.12. Max coverage (-): 0.02

Region: NODE\_347534\_length\_4529\_cov\_29.102673 71-80. Max. coverage (+): 0.12. Max coverage (-): 0.04

Region: NODE\_347534\_length\_4529\_cov\_29.102673 81-89. Max. coverage (+): 0.04. Max coverage (-): 0.08

Region: NODE\_347534\_length\_4529\_cov\_29.102673 90-98. Max. coverage (+): 0. Max coverage (-): 0.12

Region: NODE\_347534\_length\_4529\_cov\_29.102673 99-108. Max. coverage (+): 0.04. Max coverage (-): 0.16

Region: NODE\_347534\_length\_4529\_cov\_29.102673 109-117. Max. coverage (+): 0.1. Max coverage (-): 0.16

Region: NODE\_347534\_length\_4529\_cov\_29.102673 118-126. Max. coverage (+): 0.08. Max coverage (-): 0.04

Region: NODE\_347534\_length\_4529\_cov\_29.102673 127-135. Max. coverage (+): 0. Max coverage (-): 0.24

Region: NODE\_347534\_length\_4529\_cov\_29.102673 136-145. Max. coverage (+): 0. Max coverage (-): 0.28

Region: NODE\_347534\_length\_4529\_cov\_29.102673 146-154. Max. coverage (+): 0.08. Max coverage (-): 0

Region: NODE\_347534\_length\_4529\_cov\_29.102673 155-163. Max. coverage (+): 0.28. Max coverage (-): 0.36

Region: NODE\_347534\_length\_4529\_cov\_29.102673 164-173. Max. coverage (+): 0. Max coverage (-): 0.85

Region: NODE\_347534\_length\_4529\_cov\_29.102673 174-182. Max. coverage (+): 0.35. Max coverage (-): 0.08

Region: NODE\_347534\_length\_4529\_cov\_29.102673 183-191. Max. coverage (+): 0.36. Max coverage (-): 0.04

Region: NODE\_347534\_length\_4529\_cov\_29.102673 192-201. Max. coverage (+): 0.01. Max coverage (-): 0.09

Region: NODE\_347534\_length\_4529\_cov\_29.102673 202-210. Max. coverage (+): 0. Max coverage (-): 0.44

Region: NODE\_347534\_length\_4529\_cov\_29.102673 211-219. Max. coverage (+): 0.04. Max coverage (-): 0.69

Region: NODE\_347534\_length\_4529\_cov\_29.102673 220-229. Max. coverage (+): 0.12. Max coverage (-): 0.61

Region: NODE\_347534\_length\_4529\_cov\_29.102673 230-238. Max. coverage (+): 0.04. Max coverage (-): 0.04

Region: NODE\_347534\_length\_4529\_cov\_29.102673 239-247. Max. coverage (+): 0.04. Max coverage (-): 0.22

Region: NODE\_347534\_length\_4529\_cov\_29.102673 248-256. Max. coverage (+): 0.02. Max coverage (-): 0.2

Region: NODE\_347534\_length\_4529\_cov\_29.102673 257-266. Max. coverage (+): 0.02. Max coverage (-): 0.09

Region: NODE\_347534\_length\_4529\_cov\_29.102673 267-275. Max. coverage (+): 0.02. Max coverage (-): 0.03

Region: NODE\_347534\_length\_4529\_cov\_29.102673 276-284. Max. coverage (+): 0.03. Max coverage (-): 0.04

Region: NODE\_347534\_length\_4529\_cov\_29.102673 285-294. Max. coverage (+): 0.05. Max coverage (-): 0.03

Region: NODE\_347534\_length\_4529\_cov\_29.102673 295-303. Max. coverage (+): 0.07. Max coverage (-): 0.09

Region: NODE\_347534\_length\_4529\_cov\_29.102673 304-312. Max. coverage (+): 0.06. Max coverage (-): 1.17

Region: NODE\_347534\_length\_4529\_cov\_29.102673 313-322. Max. coverage (+): 0. Max coverage (-): 0.03

Region: NODE\_347534\_length\_4529\_cov\_29.102673 323-331. Max. coverage (+): 0. Max coverage (-): 0

Region: NODE\_347534\_length\_4529\_cov\_29.102673 332-340. Max. coverage (+): 0. Max coverage (-): 0

Region: NODE\_347534\_length\_4529\_cov\_29.102673 341-350. Max. coverage (+): 0.02. Max coverage (-): 0.04

Region: NODE\_347534\_length\_4529\_cov\_29.102673 351-359. Max. coverage (+): 0.12. Max coverage (-): 0.08

Region: NODE\_347534\_length\_4529\_cov\_29.102673 360-368. Max. coverage (+): 0. Max coverage (-): 0.44

Region: NODE\_347534\_length\_4529\_cov\_29.102673 369-377. Max. coverage (+): 0.01. Max coverage (-): 0

Region: NODE\_347534\_length\_4529\_cov\_29.102673 378-387. Max. coverage (+): 0. Max coverage (-): 0.02

Region: NODE\_347534\_length\_4529\_cov\_29.102673 388-396. Max. coverage (+): 0.02. Max coverage (-): 0.01

Region: NODE\_347534\_length\_4529\_cov\_29.102673 397-405. Max. coverage (+): 0.08. Max coverage (-): 0.01

Region: NODE\_347534\_length\_4529\_cov\_29.102673 406-415. Max. coverage (+): 1.01. Max coverage (-): 0.12

Region: NODE\_347534\_length\_4529\_cov\_29.102673 416-424. Max. coverage (+): 0.04. Max coverage (-): 0.08

Region: NODE\_347534\_length\_4529\_cov\_29.102673 425-433. Max. coverage (+): 0. Max coverage (-): 0.04

Region: NODE\_347534\_length\_4529\_cov\_29.102673 434-443. Max. coverage (+): 0. Max coverage (-): 0.01

Region: NODE\_347534\_length\_4529\_cov\_29.102673 444-452. Max. coverage (+): 0. Max coverage (-): 0.01

Region: NODE\_347534\_length\_4529\_cov\_29.102673 453-461. Max. coverage (+): 0. Max coverage (-): 0.01

Region: NODE\_347534\_length\_4529\_cov\_29.102673 462-471. Max. coverage (+): 0.01. Max coverage (-): 0.01

Region: NODE\_347534\_length\_4529\_cov\_29.102673 472-480. Max. coverage (+): 0. Max coverage (-): 0

Region: NODE\_347534\_length\_4529\_cov\_29.102673 481-489. Max. coverage (+): 0. Max coverage (-): 0

Region: NODE\_347534\_length\_4529\_cov\_29.102673 490-498. Max. coverage (+): 0. Max coverage (-): 0.02

Region: NODE\_347534\_length\_4529\_cov\_29.102673 499-508. Max. coverage (+): 0. Max coverage (-): 0.08

Region: NODE\_347534\_length\_4529\_cov\_29.102673 509-517. Max. coverage (+): 0. Max coverage (-): 0.04

Region: NODE\_347534\_length\_4529\_cov\_29.102673 518-526. Max. coverage (+): 0. Max coverage (-): 0

Region: NODE\_347534\_length\_4529\_cov\_29.102673 527-536. Max. coverage (+): 0. Max coverage (-): 0

Region: NODE\_347534\_length\_4529\_cov\_29.102673 537-545. Max. coverage (+): 0. Max coverage (-): 0

Region: NODE\_347534\_length\_4529\_cov\_29.102673 546-554. Max. coverage (+): 0. Max coverage (-): 0

Region: NODE\_347534\_length\_4529\_cov\_29.102673 555-564. Max. coverage (+): 0.04. Max coverage (-): 0.04

Region: NODE\_347534\_length\_4529\_cov\_29.102673 565-573. Max. coverage (+): 0.04. Max coverage (-): 2.22

Region: NODE\_347534\_length\_4529\_cov\_29.102673 574-582. Max. coverage (+): 0. Max coverage (-): 13.41

Region: NODE\_347534\_length\_4529\_cov\_29.102673 583-592. Max. coverage (+): 0.03. Max coverage (-): 0.04

Region: NODE\_347534\_length\_4529\_cov\_29.102673 593-601. Max. coverage (+): 0.15. Max coverage (-): 0.08

Region: NODE\_347534\_length\_4529\_cov\_29.102673 602-610. Max. coverage (+): 0.03. Max coverage (-): 0.01

Region: NODE\_347534\_length\_4529\_cov\_29.102673 611-619. Max. coverage (+): 0. Max coverage (-): 0.01

Region: NODE\_347534\_length\_4529\_cov\_29.102673 620-629. Max. coverage (+): 0.06. Max coverage (-): 0.01

Region: NODE\_347534\_length\_4529\_cov\_29.102673 630-638. Max. coverage (+): 0.06. Max coverage (-): 0.02

Region: NODE\_347534\_length\_4529\_cov\_29.102673 639-647. Max. coverage (+): 0.06. Max coverage (-): 0.01

Region: NODE\_347534\_length\_4529\_cov\_29.102673 648-657. Max. coverage (+): 0.08. Max coverage (-): 0.08

Region: NODE\_347534\_length\_4529\_cov\_29.102673 658-666. Max. coverage (+): 0. Max coverage (-): 1.09

Region: NODE\_347534\_length\_4529\_cov\_29.102673 667-675. Max. coverage (+): 0. Max coverage (-): 0.32

Region: NODE\_347534\_length\_4529\_cov\_29.102673 676-685. Max. coverage (+): 0. Max coverage (-): 0.04

Region: NODE\_347534\_length\_4529\_cov\_29.102673 686-694. Max. coverage (+): 0. Max coverage (-): 0

Region: NODE\_347534\_length\_4529\_cov\_29.102673 695-703. Max. coverage (+): 0. Max coverage (-): 0

Region: NODE\_347534\_length\_4529\_cov\_29.102673 704-713. Max. coverage (+): 0. Max coverage (-): 0.08

Region: NODE\_347534\_length\_4529\_cov\_29.102673 714-722. Max. coverage (+): 0. Max coverage (-): 0.16

Region: NODE\_347534\_length\_4529\_cov\_29.102673 723-731. Max. coverage (+): 0. Max coverage (-): 0

Region: NODE\_347534\_length\_4529\_cov\_29.102673 732-740. Max. coverage (+): 0.02. Max coverage (-): 0

Region: NODE\_347534\_length\_4529\_cov\_29.102673 741-750. Max. coverage (+): 0. Max coverage (-): 0.01

Region: NODE\_347534\_length\_4529\_cov\_29.102673 751-759. Max. coverage (+): 0. Max coverage (-): 0

Region: NODE\_347534\_length\_4529\_cov\_29.102673 760-768. Max. coverage (+): 0. Max coverage (-): 0

Region: NODE\_347534\_length\_4529\_cov\_29.102673 769-778. Max. coverage (+): 0. Max coverage (-): 0.02

Region: NODE\_347534\_length\_4529\_cov\_29.102673 779-787. Max. coverage (+): 0. Max coverage (-): 0.02

Region: NODE\_347534\_length\_4529\_cov\_29.102673 788-796. Max. coverage (+): 0. Max coverage (-): 0.01

Region: NODE\_347534\_length\_4529\_cov\_29.102673 797-806. Max. coverage (+): 0. Max coverage (-): 0.01

Region: NODE\_347534\_length\_4529\_cov\_29.102673 807-815. Max. coverage (+): 0.08. Max coverage (-): 0

Region: NODE\_347534\_length\_4529\_cov\_29.102673 816-824. Max. coverage (+): 0.08. Max coverage (-): 0

Region: NODE\_347534\_length\_4529\_cov\_29.102673 825-834. Max. coverage (+): 0. Max coverage (-): 0.31

Region: NODE\_347534\_length\_4529\_cov\_29.102673 835-843. Max. coverage (+): 0. Max coverage (-): 2.44

Region: NODE\_347534\_length\_4529\_cov\_29.102673 844-852. Max. coverage (+): 0.73. Max coverage (-): 0

Region: NODE\_347534\_length\_4529\_cov\_29.102673 853-861. Max. coverage (+): 0.01. Max coverage (-): 0

Region: NODE\_347534\_length\_4529\_cov\_29.102673 862-871. Max. coverage (+): 0. Max coverage (-): 0.08

Region: NODE\_347534\_length\_4529\_cov\_29.102673 872-880. Max. coverage (+): 0.12. Max coverage (-): 0

Region: NODE\_347534\_length\_4529\_cov\_29.102673 881-889. Max. coverage (+): 0.16. Max coverage (-): 0.08

Region: NODE\_347534\_length\_4529\_cov\_29.102673 890-899. Max. coverage (+): 0.12. Max coverage (-): 0.08

Region: NODE\_347534\_length\_4529\_cov\_29.102673 900-908. Max. coverage (+): 0.04. Max coverage (-): 0.08

Region: NODE\_347534\_length\_4529\_cov\_29.102673 909-917. Max. coverage (+): 0. Max coverage (-): 1.74

Region: NODE\_347534\_length\_4529\_cov\_29.102673 918-927. Max. coverage (+): 0. Max coverage (-): 0.65

Region: NODE\_347534\_length\_4529\_cov\_29.102673 928-936. Max. coverage (+): 0. Max coverage (-): 1.17

Region: NODE\_347534\_length\_4529\_cov\_29.102673 937-945. Max. coverage (+): 0. Max coverage (-): 0.04

Region: NODE\_347534\_length\_4529\_cov\_29.102673 946-955. Max. coverage (+): 0.2. Max coverage (-): 0.04

Region: NODE\_347534\_length\_4529\_cov\_29.102673 956-964. Max. coverage (+): 0.12. Max coverage (-): 0

Region: NODE\_347534\_length\_4529\_cov\_29.102673 965-973. Max. coverage (+): 0. Max coverage (-): 0

Region: NODE\_347534\_length\_4529\_cov\_29.102673 974-982. Max. coverage (+): 0. Max coverage (-): 0

Region: NODE\_347534\_length\_4529\_cov\_29.102673 983-992. Max. coverage (+): 0. Max coverage (-): 0

Region: NODE\_347534\_length\_4529\_cov\_29.102673 993-1001. Max. coverage (+): 0. Max coverage (-): 0

Region: NODE\_347534\_length\_4529\_cov\_29.102673 1002-1010. Max. coverage (+): 0. Max coverage (-): 0.04

Region: NODE\_347534\_length\_4529\_cov\_29.102673 1011-1020. Max. coverage (+): 0. Max coverage (-): 0.12

Region: NODE\_347534\_length\_4529\_cov\_29.102673 1021-1029. Max. coverage (+): 0.04. Max coverage (-): 4.85

Region: NODE\_347534\_length\_4529\_cov\_29.102673 1030-1038. Max. coverage (+): 0.12. Max coverage (-): 5.17

Region: NODE\_347534\_length\_4529\_cov\_29.102673 1039-1048. Max. coverage (+): 0.04. Max coverage (-): 0.08

Region: NODE\_347534\_length\_4529\_cov\_29.102673 1049-1057. Max. coverage (+): 0.12. Max coverage (-): 0.08

Region: NODE\_347534\_length\_4529\_cov\_29.102673 1058-1066. Max. coverage (+): 0. Max coverage (-): 0.04

Region: NODE\_347534\_length\_4529\_cov\_29.102673 1067-1076. Max. coverage (+): 0. Max coverage (-): 0.04

Region: NODE\_347534\_length\_4529\_cov\_29.102673 1077-1085. Max. coverage (+): 0. Max coverage (-): 0

Region: NODE\_347534\_length\_4529\_cov\_29.102673 1086-1094. Max. coverage (+): 0. Max coverage (-): 0

Region: NODE\_347534\_length\_4529\_cov\_29.102673 1095-1103. Max. coverage (+): 0. Max coverage (-): 0

Region: NODE\_347534\_length\_4529\_cov\_29.102673 1104-1113. Max. coverage (+): 0. Max coverage (-): 0.32

Region: NODE\_347534\_length\_4529\_cov\_29.102673 1114-1122. Max. coverage (+): 0. Max coverage (-): 0.73

Region: NODE\_347534\_length\_4529\_cov\_29.102673 1123-1131. Max. coverage (+): 0.24. Max coverage (-): 0.4

Region: NODE\_347534\_length\_4529\_cov\_29.102673 1132-1141. Max. coverage (+): 0.04. Max coverage (-): 0.08

Region: NODE\_347534\_length\_4529\_cov\_29.102673 1142-1150. Max. coverage (+): 0. Max coverage (-): 1.53

Region: NODE\_347534\_length\_4529\_cov\_29.102673 1151-1159. Max. coverage (+): 0.48. Max coverage (-): 1.05

Region: NODE\_347534\_length\_4529\_cov\_29.102673 1160-1169. Max. coverage (+): 0.28. Max coverage (-): 0.52

Region: NODE\_347534\_length\_4529\_cov\_29.102673 1170-1178. Max. coverage (+): 1.66. Max coverage (-): 46.68

Region: NODE\_347534\_length\_4529\_cov\_29.102673 1179-1187. Max. coverage (+): 1.09. Max coverage (-): 1.45

Region: NODE\_347534\_length\_4529\_cov\_29.102673 1188-1197. Max. coverage (+): 0.04. Max coverage (-): 1.09

Region: NODE\_347534\_length\_4529\_cov\_29.102673 1198-1206. Max. coverage (+): 0.36. Max coverage (-): 0.04

Region: NODE\_347534\_length\_4529\_cov\_29.102673 1207-1215. Max. coverage (+): 0.65. Max coverage (-): 0.08

Region: NODE\_347534\_length\_4529\_cov\_29.102673 1216-1225. Max. coverage (+): 0. Max coverage (-): 0.04

Region: NODE\_347534\_length\_4529\_cov\_29.102673 1226-1234. Max. coverage (+): 0. Max coverage (-): 0.52

Region: NODE\_347534\_length\_4529\_cov\_29.102673 1235-1243. Max. coverage (+): 0. Max coverage (-): 0.04

Region: NODE\_347534\_length\_4529\_cov\_29.102673 1244-1252. Max. coverage (+): 0.12. Max coverage (-): 0.12

Region: NODE\_347534\_length\_4529\_cov\_29.102673 1253-1262. Max. coverage (+): 0.57. Max coverage (-): 1.25

Region: NODE\_347534\_length\_4529\_cov\_29.102673 1263-1271. Max. coverage (+): 0.44. Max coverage (-): 0.57

Region: NODE\_347534\_length\_4529\_cov\_29.102673 1272-1280. Max. coverage (+): 0.16. Max coverage (-): 0.16

Region: NODE\_347534\_length\_4529\_cov\_29.102673 1281-1290. Max. coverage (+): 0.24. Max coverage (-): 0.09

Region: NODE\_347534\_length\_4529\_cov\_29.102673 1291-1299. Max. coverage (+): 0.04. Max coverage (-): 0

Region: NODE\_347534\_length\_4529\_cov\_29.102673 1300-1308. Max. coverage (+): 0.01. Max coverage (-): 0

Region: NODE\_347534\_length\_4529\_cov\_29.102673 1309-1318. Max. coverage (+): 0. Max coverage (-): 0.08

Region: NODE\_347534\_length\_4529\_cov\_29.102673 1319-1327. Max. coverage (+): 0. Max coverage (-): 0.08

Region: NODE\_347534\_length\_4529\_cov\_29.102673 1328-1336. Max. coverage (+): 0. Max coverage (-): 0.12

Region: NODE\_347534\_length\_4529\_cov\_29.102673 1337-1346. Max. coverage (+): 0. Max coverage (-): 0.12

Region: NODE\_347534\_length\_4529\_cov\_29.102673 1347-1355. Max. coverage (+): 0. Max coverage (-): 0.44

Region: NODE\_347534\_length\_4529\_cov\_29.102673 1356-1364. Max. coverage (+): 0.04. Max coverage (-): 5.45

Region: NODE\_347534\_length\_4529\_cov\_29.102673 1365-1373. Max. coverage (+): 0.04. Max coverage (-): 2.38

Region: NODE\_347534\_length\_4529\_cov\_29.102673 1374-1383. Max. coverage (+): 0.08. Max coverage (-): 4.89

Region: NODE\_347534\_length\_4529\_cov\_29.102673 1384-1392. Max. coverage (+): 0.12. Max coverage (-): 0

Region: NODE\_347534\_length\_4529\_cov\_29.102673 1393-1401. Max. coverage (+): 0.01. Max coverage (-): 0.12

Region: NODE\_347534\_length\_4529\_cov\_29.102673 1402-1411. Max. coverage (+): 0. Max coverage (-): 3.11

Region: NODE\_347534\_length\_4529\_cov\_29.102673 1412-1420. Max. coverage (+): 0.04. Max coverage (-): 0.48

Region: NODE\_347534\_length\_4529\_cov\_29.102673 1421-1429. Max. coverage (+): 0.73. Max coverage (-): 0.48

Region: NODE\_347534\_length\_4529\_cov\_29.102673 1430-1439. Max. coverage (+): 0. Max coverage (-): 0.16

Region: NODE\_347534\_length\_4529\_cov\_29.102673 1440-1448. Max. coverage (+): 0. Max coverage (-): 1.25

Region: NODE\_347534\_length\_4529\_cov\_29.102673 1449-1457. Max. coverage (+): 0.04. Max coverage (-): 10.7

Region: NODE\_347534\_length\_4529\_cov\_29.102673 1458-1467. Max. coverage (+): 0.16. Max coverage (-): 5.29

Region: NODE\_347534\_length\_4529\_cov\_29.102673 1468-1476. Max. coverage (+): 0.16. Max coverage (-): 2.66

Region: NODE\_347534\_length\_4529\_cov\_29.102673 1477-1485. Max. coverage (+): 0.12. Max coverage (-): 0.52

Region: NODE\_347534\_length\_4529\_cov\_29.102673 1486-1494. Max. coverage (+): 0. Max coverage (-): 0.08

Region: NODE\_347534\_length\_4529\_cov\_29.102673 1495-1504. Max. coverage (+): 0. Max coverage (-): 0.04

Region: NODE\_347534\_length\_4529\_cov\_29.102673 1505-1513. Max. coverage (+): 0. Max coverage (-): 0.73

Region: NODE\_347534\_length\_4529\_cov\_29.102673 1514-1522. Max. coverage (+): 0. Max coverage (-): 0.85

Region: NODE\_347534\_length\_4529\_cov\_29.102673 1523-1532. Max. coverage (+): 0. Max coverage (-): 0.04

Region: NODE\_347534\_length\_4529\_cov\_29.102673 1533-1541. Max. coverage (+): 0. Max coverage (-): 0

Region: NODE\_347534\_length\_4529\_cov\_29.102673 1542-1550. Max. coverage (+): 0. Max coverage (-): 0

Region: NODE\_347534\_length\_4529\_cov\_29.102673 1551-1560. Max. coverage (+): 0. Max coverage (-): 0.12

Region: NODE\_347534\_length\_4529\_cov\_29.102673 1561-1569. Max. coverage (+): 0. Max coverage (-): 0.12

Region: NODE\_347534\_length\_4529\_cov\_29.102673 1570-1578. Max. coverage (+): 0. Max coverage (-): 0

Region: NODE\_347534\_length\_4529\_cov\_29.102673 1579-1588. Max. coverage (+): 0.08. Max coverage (-): 0.93

Region: NODE\_347534\_length\_4529\_cov\_29.102673 1589-1597. Max. coverage (+): 0.08. Max coverage (-): 1.66

Region: NODE\_347534\_length\_4529\_cov\_29.102673 1598-1606. Max. coverage (+): 0.08. Max coverage (-): 0.28

Region: NODE\_347534\_length\_4529\_cov\_29.102673 1607-1615. Max. coverage (+): 0.12. Max coverage (-): 0.61

Region: NODE\_347534\_length\_4529\_cov\_29.102673 1616-1625. Max. coverage (+): 0.16. Max coverage (-): 0.57

Region: NODE\_347534\_length\_4529\_cov\_29.102673 1626-1634. Max. coverage (+): 0.12. Max coverage (-): 0.52

Region: NODE\_347534\_length\_4529\_cov\_29.102673 1635-1643. Max. coverage (+): 0.18. Max coverage (-): 0.12

Region: NODE\_347534\_length\_4529\_cov\_29.102673 1644-1653. Max. coverage (+): 0.04. Max coverage (-): 0.12

Region: NODE\_347534\_length\_4529\_cov\_29.102673 1654-1662. Max. coverage (+): 0.28. Max coverage (-): 0

Region: NODE\_347534\_length\_4529\_cov\_29.102673 1663-1671. Max. coverage (+): 0.32. Max coverage (-): 0

Region: NODE\_347534\_length\_4529\_cov\_29.102673 1672-1681. Max. coverage (+): 0. Max coverage (-): 0.08

Region: NODE\_347534\_length\_4529\_cov\_29.102673 1682-1690. Max. coverage (+): 0. Max coverage (-): 0.08

Region: NODE\_347534\_length\_4529\_cov\_29.102673 1691-1699. Max. coverage (+): 0.04. Max coverage (-): 1.78

Region: NODE\_347534\_length\_4529\_cov\_29.102673 1700-1709. Max. coverage (+): 0.04. Max coverage (-): 6.99

Region: NODE\_347534\_length\_4529\_cov\_29.102673 1710-1718. Max. coverage (+): 0.07. Max coverage (-): 6.78

Region: NODE\_347534\_length\_4529\_cov\_29.102673 1719-1727. Max. coverage (+): 0.04. Max coverage (-): 0.04

Region: NODE\_347534\_length\_4529\_cov\_29.102673 1728-1736. Max. coverage (+): 0.04. Max coverage (-): 0.08

Region: NODE\_347534\_length\_4529\_cov\_29.102673 1737-1746. Max. coverage (+): 0. Max coverage (-): 0.12

Region: NODE\_347534\_length\_4529\_cov\_29.102673 1747-1755. Max. coverage (+): 0.08. Max coverage (-): 0.12

Region: NODE\_347534\_length\_4529\_cov\_29.102673 1756-1764. Max. coverage (+): 0.04. Max coverage (-): 0.08

Region: NODE\_347534\_length\_4529\_cov\_29.102673 1765-1774. Max. coverage (+): 0.04. Max coverage (-): 0.16

Region: NODE\_347534\_length\_4529\_cov\_29.102673 1775-1783. Max. coverage (+): 0.04. Max coverage (-): 0

Region: NODE\_347534\_length\_4529\_cov\_29.102673 1784-1792. Max. coverage (+): 0.08. Max coverage (-): 0.16

Region: NODE\_347534\_length\_4529\_cov\_29.102673 1793-1802. Max. coverage (+): 1.09. Max coverage (-): 0.32

Region: NODE\_347534\_length\_4529\_cov\_29.102673 1803-1811. Max. coverage (+): 2.66. Max coverage (-): 0.04

Region: NODE\_347534\_length\_4529\_cov\_29.102673 1812-1820. Max. coverage (+): 0.28. Max coverage (-): 0.36

Region: NODE\_347534\_length\_4529\_cov\_29.102673 1821-1830. Max. coverage (+): 0.04. Max coverage (-): 0.16

Region: NODE\_347534\_length\_4529\_cov\_29.102673 1831-1839. Max. coverage (+): 0. Max coverage (-): 0.08

Region: NODE\_347534\_length\_4529\_cov\_29.102673 1840-1848. Max. coverage (+): 0. Max coverage (-): 0

Region: NODE\_347534\_length\_4529\_cov\_29.102673 1849-1857. Max. coverage (+): 0.02. Max coverage (-): 0

Region: NODE\_347534\_length\_4529\_cov\_29.102673 1858-1867. Max. coverage (+): 0. Max coverage (-): 0

Region: NODE\_347534\_length\_4529\_cov\_29.102673 1868-1876. Max. coverage (+): 0. Max coverage (-): 0.4

Region: NODE\_347534\_length\_4529\_cov\_29.102673 1877-1885. Max. coverage (+): 0. Max coverage (-): 0.36

Region: NODE\_347534\_length\_4529\_cov\_29.102673 1886-1895. Max. coverage (+): 0. Max coverage (-): 0.3

Region: NODE\_347534\_length\_4529\_cov\_29.102673 1896-1904. Max. coverage (+): 0.12. Max coverage (-): 0.08

Region: NODE\_347534\_length\_4529\_cov\_29.102673 1905-1913. Max. coverage (+): 0. Max coverage (-): 6.22

Region: NODE\_347534\_length\_4529\_cov\_29.102673 1914-1923. Max. coverage (+): 0.04. Max coverage (-): 2.22

Region: NODE\_347534\_length\_4529\_cov\_29.102673 1924-1932. Max. coverage (+): 0.04. Max coverage (-): 0.01

Region: NODE\_347534\_length\_4529\_cov\_29.102673 1933-1941. Max. coverage (+): 0. Max coverage (-): 0.02

Region: NODE\_347534\_length\_4529\_cov\_29.102673 1942-1951. Max. coverage (+): 0.04. Max coverage (-): 0.08

Region: NODE\_347534\_length\_4529\_cov\_29.102673 1952-1960. Max. coverage (+): 0.57. Max coverage (-): 0.4

Region: NODE\_347534\_length\_4529\_cov\_29.102673 1961-1969. Max. coverage (+): 0.04. Max coverage (-): 0.4

Region: NODE\_347534\_length\_4529\_cov\_29.102673 1970-1978. Max. coverage (+): 0.04. Max coverage (-): 0.81

Region: NODE\_347534\_length\_4529\_cov\_29.102673 1979-1988. Max. coverage (+): 0.04. Max coverage (-): 0.93

Region: NODE\_347534\_length\_4529\_cov\_29.102673 1989-1997. Max. coverage (+): 0.08. Max coverage (-): 0.28

Region: NODE\_347534\_length\_4529\_cov\_29.102673 1998-2006. Max. coverage (+): 0.04. Max coverage (-): 0.16

Region: NODE\_347534\_length\_4529\_cov\_29.102673 2007-2016. Max. coverage (+): 0.32. Max coverage (-): 0.04

Region: NODE\_347534\_length\_4529\_cov\_29.102673 2017-2025. Max. coverage (+): 0.32. Max coverage (-): 0.24

Region: NODE\_347534\_length\_4529\_cov\_29.102673 2026-2034. Max. coverage (+): 0.12. Max coverage (-): 1.05

Region: NODE\_347534\_length\_4529\_cov\_29.102673 2035-2044. Max. coverage (+): 0.2. Max coverage (-): 0.85

Region: NODE\_347534\_length\_4529\_cov\_29.102673 2045-2053. Max. coverage (+): 0.04. Max coverage (-): 1.25

Region: NODE\_347534\_length\_4529\_cov\_29.102673 2054-2062. Max. coverage (+): 0.12. Max coverage (-): 1.25

Region: NODE\_347534\_length\_4529\_cov\_29.102673 2063-2072. Max. coverage (+): 0.48. Max coverage (-): 0.16

Region: NODE\_347534\_length\_4529\_cov\_29.102673 2073-2081. Max. coverage (+): 0.04. Max coverage (-): 23.18

Region: NODE\_347534\_length\_4529\_cov\_29.102673 2082-2090. Max. coverage (+): 0.04. Max coverage (-): 22.77

Region: NODE\_347534\_length\_4529\_cov\_29.102673 2091-2099. Max. coverage (+): 0.24. Max coverage (-): 1.21

Region: NODE\_347534\_length\_4529\_cov\_29.102673 2100-2109. Max. coverage (+): 1.17. Max coverage (-): 0.12

Region: NODE\_347534\_length\_4529\_cov\_29.102673 2110-2118. Max. coverage (+): 0.28. Max coverage (-): 0.16

Region: NODE\_347534\_length\_4529\_cov\_29.102673 2119-2127. Max. coverage (+): 0.04. Max coverage (-): 0.16

Region: NODE\_347534\_length\_4529\_cov\_29.102673 2128-2137. Max. coverage (+): 0.04. Max coverage (-): 0.32

Region: NODE\_347534\_length\_4529\_cov\_29.102673 2138-2146. Max. coverage (+): 0.48. Max coverage (-): 0.85

Region: NODE\_347534\_length\_4529\_cov\_29.102673 2147-2155. Max. coverage (+): 0.2. Max coverage (-): 0.48

Region: NODE\_347534\_length\_4529\_cov\_29.102673 2156-2165. Max. coverage (+): 0.1. Max coverage (-): 1.27

Region: NODE\_347534\_length\_4529\_cov\_29.102673 2166-2174. Max. coverage (+): 0.2. Max coverage (-): 0.4

Region: NODE\_347534\_length\_4529\_cov\_29.102673 2175-2183. Max. coverage (+): 0.32. Max coverage (-): 0.24

Region: NODE\_347534\_length\_4529\_cov\_29.102673 2184-2193. Max. coverage (+): 0.2. Max coverage (-): 0.19

Region: NODE\_347534\_length\_4529\_cov\_29.102673 2194-2202. Max. coverage (+): 0.04. Max coverage (-): 0.12

Region: NODE\_347534\_length\_4529\_cov\_29.102673 2203-2211. Max. coverage (+): 0.12. Max coverage (-): 0.08

Region: NODE\_347534\_length\_4529\_cov\_29.102673 2212-2220. Max. coverage (+): 0.32. Max coverage (-): 0.08

Region: NODE\_347534\_length\_4529\_cov\_29.102673 2221-2230. Max. coverage (+): 0. Max coverage (-): 4.2

Region: NODE\_347534\_length\_4529\_cov\_29.102673 2231-2239. Max. coverage (+): 0.04. Max coverage (-): 2.99

Region: NODE\_347534\_length\_4529\_cov\_29.102673 2240-2248. Max. coverage (+): 0.24. Max coverage (-): 0

Region: NODE\_347534\_length\_4529\_cov\_29.102673 2249-2258. Max. coverage (+): 0. Max coverage (-): 0

Region: NODE\_347534\_length\_4529\_cov\_29.102673 2259-2267. Max. coverage (+): 0.04. Max coverage (-): 0.04

Region: NODE\_347534\_length\_4529\_cov\_29.102673 2268-2276. Max. coverage (+): 0.16. Max coverage (-): 0.12

Region: NODE\_347534\_length\_4529\_cov\_29.102673 2277-2286. Max. coverage (+): 0.04. Max coverage (-): 0.16

Region: NODE\_347534\_length\_4529\_cov\_29.102673 2287-2295. Max. coverage (+): 0.04. Max coverage (-): 0.16

Region: NODE\_347534\_length\_4529\_cov\_29.102673 2296-2304. Max. coverage (+): 0.32. Max coverage (-): 0.36

Region: NODE\_347534\_length\_4529\_cov\_29.102673 2305-2314. Max. coverage (+): 0.36. Max coverage (-): 5.73

Region: NODE\_347534\_length\_4529\_cov\_29.102673 2315-2323. Max. coverage (+): 0.24. Max coverage (-): 11.18

Region: NODE\_347534\_length\_4529\_cov\_29.102673 2324-2332. Max. coverage (+): 0.16. Max coverage (-): 0.52

Region: NODE\_347534\_length\_4529\_cov\_29.102673 2333-2341. Max. coverage (+): 0. Max coverage (-): 0.36

Region: NODE\_347534\_length\_4529\_cov\_29.102673 2342-2351. Max. coverage (+): 0. Max coverage (-): 0.04

Region: NODE\_347534\_length\_4529\_cov\_29.102673 2352-2360. Max. coverage (+): 0. Max coverage (-): 0.24

Region: NODE\_347534\_length\_4529\_cov\_29.102673 2361-2369. Max. coverage (+): 0.04. Max coverage (-): 0.24

Region: NODE\_347534\_length\_4529\_cov\_29.102673 2370-2379. Max. coverage (+): 0.08. Max coverage (-): 0.04

Region: NODE\_347534\_length\_4529\_cov\_29.102673 2380-2388. Max. coverage (+): 0. Max coverage (-): 0.08

Region: NODE\_347534\_length\_4529\_cov\_29.102673 2389-2397. Max. coverage (+): 0. Max coverage (-): 0

Region: NODE\_347534\_length\_4529\_cov\_29.102673 2398-2407. Max. coverage (+): 0.01. Max coverage (-): 0.09

Region: NODE\_347534\_length\_4529\_cov\_29.102673 2408-2416. Max. coverage (+): 0.03. Max coverage (-): 0.17

Region: NODE\_347534\_length\_4529\_cov\_29.102673 2417-2425. Max. coverage (+): 0.11. Max coverage (-): 0.17

Region: NODE\_347534\_length\_4529\_cov\_29.102673 2426-2435. Max. coverage (+): 0. Max coverage (-): 0.04

Region: NODE\_347534\_length\_4529\_cov\_29.102673 2436-2444. Max. coverage (+): 0.04. Max coverage (-): 0

Region: NODE\_347534\_length\_4529\_cov\_29.102673 2445-2453. Max. coverage (+): 0.04. Max coverage (-): 0

Region: NODE\_347534\_length\_4529\_cov\_29.102673 2454-2462. Max. coverage (+): 0.04. Max coverage (-): 0.04

Region: NODE\_347534\_length\_4529\_cov\_29.102673 2463-2472. Max. coverage (+): 0.12. Max coverage (-): 5.21

Region: NODE\_347534\_length\_4529\_cov\_29.102673 2473-2481. Max. coverage (+): 0.12. Max coverage (-): 0.52

Region: NODE\_347534\_length\_4529\_cov\_29.102673 2482-2490. Max. coverage (+): 0.16. Max coverage (-): 0.36

Region: NODE\_347534\_length\_4529\_cov\_29.102673 2491-2500. Max. coverage (+): 0. Max coverage (-): 0.04

Region: NODE\_347534\_length\_4529\_cov\_29.102673 2501-2509. Max. coverage (+): 0.04. Max coverage (-): 0.08

Region: NODE\_347534\_length\_4529\_cov\_29.102673 2510-2518. Max. coverage (+): 0. Max coverage (-): 0

Region: NODE\_347534\_length\_4529\_cov\_29.102673 2519-2528. Max. coverage (+): 0. Max coverage (-): 0

Region: NODE\_347534\_length\_4529\_cov\_29.102673 2529-2537. Max. coverage (+): 0. Max coverage (-): 0

Region: NODE\_347534\_length\_4529\_cov\_29.102673 2538-2546. Max. coverage (+): 0.73. Max coverage (-): 0

Region: NODE\_347534\_length\_4529\_cov\_29.102673 2547-2556. Max. coverage (+): 0. Max coverage (-): 0

Region: NODE\_347534\_length\_4529\_cov\_29.102673 2557-2565. Max. coverage (+): 0. Max coverage (-): 0.87

Region: NODE\_347534\_length\_4529\_cov\_29.102673 2566-2574. Max. coverage (+): 0.4. Max coverage (-): 1.03

Region: NODE\_347534\_length\_4529\_cov\_29.102673 2575-2583. Max. coverage (+): 1.9. Max coverage (-): 0.22

Region: NODE\_347534\_length\_4529\_cov\_29.102673 2584-2593. Max. coverage (+): 2.18. Max coverage (-): 0.12

Region: NODE\_347534\_length\_4529\_cov\_29.102673 2594-2602. Max. coverage (+): 0.05. Max coverage (-): 0.12

Region: NODE\_347534\_length\_4529\_cov\_29.102673 2603-2611. Max. coverage (+): 0.62. Max coverage (-): 0

Region: NODE\_347534\_length\_4529\_cov\_29.102673 2612-2621. Max. coverage (+): 0. Max coverage (-): 0

Region: NODE\_347534\_length\_4529\_cov\_29.102673 2622-2630. Max. coverage (+): 0. Max coverage (-): 0

Region: NODE\_347534\_length\_4529\_cov\_29.102673 2631-2639. Max. coverage (+): 0. Max coverage (-): 0

Region: NODE\_347534\_length\_4529\_cov\_29.102673 2640-2649. Max. coverage (+): 0. Max coverage (-): 0

Region: NODE\_347534\_length\_4529\_cov\_29.102673 2650-2658. Max. coverage (+): 0. Max coverage (-): 0.08

Region: NODE\_347534\_length\_4529\_cov\_29.102673 2659-2667. Max. coverage (+): 0.12. Max coverage (-): 0.04

Region: NODE\_347534\_length\_4529\_cov\_29.102673 2668-2677. Max. coverage (+): 0.12. Max coverage (-): 0.06

Region: NODE\_347534\_length\_4529\_cov\_29.102673 2678-2686. Max. coverage (+): 0.16. Max coverage (-): 0.04

Region: NODE\_347534\_length\_4529\_cov\_29.102673 2687-2695. Max. coverage (+): 0.04. Max coverage (-): 0.32

Region: NODE\_347534\_length\_4529\_cov\_29.102673 2696-2704. Max. coverage (+): 0. Max coverage (-): 0.36

Region: NODE\_347534\_length\_4529\_cov\_29.102673 2705-2714. Max. coverage (+): 0. Max coverage (-): 0.08

Region: NODE\_347534\_length\_4529\_cov\_29.102673 2715-2723. Max. coverage (+): 0. Max coverage (-): 0

Region: NODE\_347534\_length\_4529\_cov\_29.102673 2724-2732. Max. coverage (+): 0. Max coverage (-): 0.93

Region: NODE\_347534\_length\_4529\_cov\_29.102673 2733-2742. Max. coverage (+): 0. Max coverage (-): 1.29

Region: NODE\_347534\_length\_4529\_cov\_29.102673 2743-2751. Max. coverage (+): 0.17. Max coverage (-): 0.01

Region: NODE\_347534\_length\_4529\_cov\_29.102673 2752-2760. Max. coverage (+): 0.16. Max coverage (-): 0

Region: NODE\_347534\_length\_4529\_cov\_29.102673 2761-2770. Max. coverage (+): 0.03. Max coverage (-): 0.17

Region: NODE\_347534\_length\_4529\_cov\_29.102673 2771-2779. Max. coverage (+): 0.19. Max coverage (-): 0.22

Region: NODE\_347534\_length\_4529\_cov\_29.102673 2780-2788. Max. coverage (+): 0.2. Max coverage (-): 0.17

Region: NODE\_347534\_length\_4529\_cov\_29.102673 2789-2798. Max. coverage (+): 0.04. Max coverage (-): 0

Region: NODE\_347534\_length\_4529\_cov\_29.102673 2799-2807. Max. coverage (+): 0. Max coverage (-): 0

Region: NODE\_347534\_length\_4529\_cov\_29.102673 2808-2816. Max. coverage (+): 0.04. Max coverage (-): 0

Region: NODE\_347534\_length\_4529\_cov\_29.102673 2817-2825. Max. coverage (+): 0. Max coverage (-): 0.16

Region: NODE\_347534\_length\_4529\_cov\_29.102673 2826-2835. Max. coverage (+): 2.38. Max coverage (-): 2.71

Region: NODE\_347534\_length\_4529\_cov\_29.102673 2836-2844. Max. coverage (+): 0.4. Max coverage (-): 0.16

Region: NODE\_347534\_length\_4529\_cov\_29.102673 2845-2853. Max. coverage (+): 0.04. Max coverage (-): 0.04

Region: NODE\_347534\_length\_4529\_cov\_29.102673 2854-2863. Max. coverage (+): 0. Max coverage (-): 0.04

Region: NODE\_347534\_length\_4529\_cov\_29.102673 2864-2872. Max. coverage (+): 0. Max coverage (-): 0

Region: NODE\_347534\_length\_4529\_cov\_29.102673 2873-2881. Max. coverage (+): 0.69. Max coverage (-): 0

Region: NODE\_347534\_length\_4529\_cov\_29.102673 2882-2891. Max. coverage (+): 0.69. Max coverage (-): 0

Region: NODE\_347534\_length\_4529\_cov\_29.102673 2892-2900. Max. coverage (+): 0. Max coverage (-): 0

Region: NODE\_347534\_length\_4529\_cov\_29.102673 2901-2909. Max. coverage (+): 0.04. Max coverage (-): 0

Region: NODE\_347534\_length\_4529\_cov\_29.102673 2910-2919. Max. coverage (+): 0.73. Max coverage (-): 0

Region: NODE\_347534\_length\_4529\_cov\_29.102673 2920-2928. Max. coverage (+): 0. Max coverage (-): 0

Region: NODE\_347534\_length\_4529\_cov\_29.102673 2929-2937. Max. coverage (+): 0.18. Max coverage (-): 0.87

Region: NODE\_347534\_length\_4529\_cov\_29.102673 2938-2946. Max. coverage (+): 0.4. Max coverage (-): 1.03

Region: NODE\_347534\_length\_4529\_cov\_29.102673 2947-2956. Max. coverage (+): 1.9. Max coverage (-): 0.2

Region: NODE\_347534\_length\_4529\_cov\_29.102673 2957-2965. Max. coverage (+): 0.42. Max coverage (-): 0.24

Region: NODE\_347534\_length\_4529\_cov\_29.102673 2966-2974. Max. coverage (+): 0.04. Max coverage (-): 0.24

Region: NODE\_347534\_length\_4529\_cov\_29.102673 2975-2984. Max. coverage (+): 0.08. Max coverage (-): 0.28

Region: NODE\_347534\_length\_4529\_cov\_29.102673 2985-2993. Max. coverage (+): 0.04. Max coverage (-): 0.4

Region: NODE\_347534\_length\_4529\_cov\_29.102673 2994-3002. Max. coverage (+): 0. Max coverage (-): 0

Region: NODE\_347534\_length\_4529\_cov\_29.102673 3003-3012. Max. coverage (+): 0. Max coverage (-): 0

Region: NODE\_347534\_length\_4529\_cov\_29.102673 3013-3021. Max. coverage (+): 0. Max coverage (-): 0

Region: NODE\_347534\_length\_4529\_cov\_29.102673 3022-3030. Max. coverage (+): 0. Max coverage (-): 0

Region: NODE\_347534\_length\_4529\_cov\_29.102673 3031-3040. Max. coverage (+): 0.12. Max coverage (-): 0.04

Region: NODE\_347534\_length\_4529\_cov\_29.102673 3041-3049. Max. coverage (+): 0.12. Max coverage (-): 0.06

Region: NODE\_347534\_length\_4529\_cov\_29.102673 3050-3058. Max. coverage (+): 0.12. Max coverage (-): 0

Region: NODE\_347534\_length\_4529\_cov\_29.102673 3059-3067. Max. coverage (+): 0. Max coverage (-): 0.08

Region: NODE\_347534\_length\_4529\_cov\_29.102673 3068-3077. Max. coverage (+): 0. Max coverage (-): 0.08

Region: NODE\_347534\_length\_4529\_cov\_29.102673 3078-3086. Max. coverage (+): 0. Max coverage (-): 0

Region: NODE\_347534\_length\_4529\_cov\_29.102673 3087-3095. Max. coverage (+): 0. Max coverage (-): 0

Region: NODE\_347534\_length\_4529\_cov\_29.102673 3096-3105. Max. coverage (+): 0. Max coverage (-): 0.25

Region: NODE\_347534\_length\_4529\_cov\_29.102673 3106-3114. Max. coverage (+): 0.01. Max coverage (-): 0

Region: NODE\_347534\_length\_4529\_cov\_29.102673 3115-3123. Max. coverage (+): 1.09. Max coverage (-): 0.04

Region: NODE\_347534\_length\_4529\_cov\_29.102673 3124-3133. Max. coverage (+): 0.28. Max coverage (-): 0.36

Region: NODE\_347534\_length\_4529\_cov\_29.102673 3134-3142. Max. coverage (+): 0. Max coverage (-): 0.32

Region: NODE\_347534\_length\_4529\_cov\_29.102673 3143-3151. Max. coverage (+): 0. Max coverage (-): 0.04

Region: NODE\_347534\_length\_4529\_cov\_29.102673 3152-3161. Max. coverage (+): 0.12. Max coverage (-): 0.28

Region: NODE\_347534\_length\_4529\_cov\_29.102673 3162-3170. Max. coverage (+): 0. Max coverage (-): 0.28

Region: NODE\_347534\_length\_4529\_cov\_29.102673 3171-3179. Max. coverage (+): 0. Max coverage (-): 0

Region: NODE\_347534\_length\_4529\_cov\_29.102673 3180-3188. Max. coverage (+): 0. Max coverage (-): 0

Region: NODE\_347534\_length\_4529\_cov\_29.102673 3189-3198. Max. coverage (+): 0. Max coverage (-): 0

Region: NODE\_347534\_length\_4529\_cov\_29.102673 3199-3207. Max. coverage (+): 0.08. Max coverage (-): 0.04

Region: NODE\_347534\_length\_4529\_cov\_29.102673 3208-3216. Max. coverage (+): 0.12. Max coverage (-): 0.28

Region: NODE\_347534\_length\_4529\_cov\_29.102673 3217-3226. Max. coverage (+): 0. Max coverage (-): 0.12

Region: NODE\_347534\_length\_4529\_cov\_29.102673 3227-3235. Max. coverage (+): 0.04. Max coverage (-): 0.04

Region: NODE\_347534\_length\_4529\_cov\_29.102673 3236-3244. Max. coverage (+): 0. Max coverage (-): 0

Region: NODE\_347534\_length\_4529\_cov\_29.102673 3245-3254. Max. coverage (+): 0. Max coverage (-): 0

Region: NODE\_347534\_length\_4529\_cov\_29.102673 3255-3263. Max. coverage (+): 0. Max coverage (-): 0

Region: NODE\_347534\_length\_4529\_cov\_29.102673 3264-3272. Max. coverage (+): 0. Max coverage (-): 0

Region: NODE\_347534\_length\_4529\_cov\_29.102673 3273-3282. Max. coverage (+): 0.08. Max coverage (-): 0.12

Region: NODE\_347534\_length\_4529\_cov\_29.102673 3283-3291. Max. coverage (+): 0.2. Max coverage (-): 0.08

Region: NODE\_347534\_length\_4529\_cov\_29.102673 3292-3300. Max. coverage (+): 0.24. Max coverage (-): 0.04

Region: NODE\_347534\_length\_4529\_cov\_29.102673 3301-3309. Max. coverage (+): 0.04. Max coverage (-): 0.12

Region: NODE\_347534\_length\_4529\_cov\_29.102673 3310-3319. Max. coverage (+): 0.04. Max coverage (-): 0.04

Region: NODE\_347534\_length\_4529\_cov\_29.102673 3320-3328. Max. coverage (+): 0. Max coverage (-): 0

Region: NODE\_347534\_length\_4529\_cov\_29.102673 3329-3337. Max. coverage (+): 0. Max coverage (-): 0.28

Region: NODE\_347534\_length\_4529\_cov\_29.102673 3338-3347. Max. coverage (+): 0. Max coverage (-): 0.03

Region: NODE\_347534\_length\_4529\_cov\_29.102673 3348-3356. Max. coverage (+): 0.29. Max coverage (-): 0

Region: NODE\_347534\_length\_4529\_cov\_29.102673 3357-3365. Max. coverage (+): 0.04. Max coverage (-): 0.21

Region: NODE\_347534\_length\_4529\_cov\_29.102673 3366-3375. Max. coverage (+): 0.08. Max coverage (-): 0.36

Region: NODE\_347534\_length\_4529\_cov\_29.102673 3376-3384. Max. coverage (+): 0.16. Max coverage (-): 1.74

Region: NODE\_347534\_length\_4529\_cov\_29.102673 3385-3393. Max. coverage (+): 1.13. Max coverage (-): 0.08

Region: NODE\_347534\_length\_4529\_cov\_29.102673 3394-3403. Max. coverage (+): 0.44. Max coverage (-): 0.61

Region: NODE\_347534\_length\_4529\_cov\_29.102673 3404-3412. Max. coverage (+): 0.89. Max coverage (-): 0.61

Region: NODE\_347534\_length\_4529\_cov\_29.102673 3413-3421. Max. coverage (+): 0.16. Max coverage (-): 0.04

Region: NODE\_347534\_length\_4529\_cov\_29.102673 3422-3430. Max. coverage (+): 0.08. Max coverage (-): 0.12

Region: NODE\_347534\_length\_4529\_cov\_29.102673 3431-3440. Max. coverage (+): 0.08. Max coverage (-): 0.44

Region: NODE\_347534\_length\_4529\_cov\_29.102673 3441-3449. Max. coverage (+): 0.04. Max coverage (-): 0.04

Region: NODE\_347534\_length\_4529\_cov\_29.102673 3450-3458. Max. coverage (+): 0.32. Max coverage (-): 0.08

Region: NODE\_347534\_length\_4529\_cov\_29.102673 3459-3468. Max. coverage (+): 0.2. Max coverage (-): 1.01

Region: NODE\_347534\_length\_4529\_cov\_29.102673 3469-3477. Max. coverage (+): 0.04. Max coverage (-): 1.01

Region: NODE\_347534\_length\_4529\_cov\_29.102673 3478-3486. Max. coverage (+): 0.04. Max coverage (-): 0

Region: NODE\_347534\_length\_4529\_cov\_29.102673 3487-3496. Max. coverage (+): 0.04. Max coverage (-): 0.28

Region: NODE\_347534\_length\_4529\_cov\_29.102673 3497-3505. Max. coverage (+): 0.04. Max coverage (-): 0.4

Region: NODE\_347534\_length\_4529\_cov\_29.102673 3506-3514. Max. coverage (+): 0.04. Max coverage (-): 0.08

Region: NODE\_347534\_length\_4529\_cov\_29.102673 3515-3524. Max. coverage (+): 0.12. Max coverage (-): 0.04

Region: NODE\_347534\_length\_4529\_cov\_29.102673 3525-3533. Max. coverage (+): 0.04. Max coverage (-): 0.04

Region: NODE\_347534\_length\_4529\_cov\_29.102673 3534-3542. Max. coverage (+): 0.04. Max coverage (-): 1.01

Region: NODE\_347534\_length\_4529\_cov\_29.102673 3543-3552. Max. coverage (+): 0.04. Max coverage (-): 0.69

Region: NODE\_347534\_length\_4529\_cov\_29.102673 3553-3561. Max. coverage (+): 0.02. Max coverage (-): 0.05

Region: NODE\_347534\_length\_4529\_cov\_29.102673 3562-3570. Max. coverage (+): 0.08. Max coverage (-): 0.01

Region: NODE\_347534\_length\_4529\_cov\_29.102673 3571-3579. Max. coverage (+): 0.32. Max coverage (-): 0.32

Region: NODE\_347534\_length\_4529\_cov\_29.102673 3580-3589. Max. coverage (+): 0.52. Max coverage (-): 0.36

Region: NODE\_347534\_length\_4529\_cov\_29.102673 3590-3598. Max. coverage (+): 0.04. Max coverage (-): 0

Region: NODE\_347534\_length\_4529\_cov\_29.102673 3599-3607. Max. coverage (+): 0.08. Max coverage (-): 0.28

Region: NODE\_347534\_length\_4529\_cov\_29.102673 3608-3617. Max. coverage (+): 0.28. Max coverage (-): 0.28

Region: NODE\_347534\_length\_4529\_cov\_29.102673 3618-3626. Max. coverage (+): 0.28. Max coverage (-): 0.2

Region: NODE\_347534\_length\_4529\_cov\_29.102673 3627-3635. Max. coverage (+): 2.02. Max coverage (-): 0.28

Region: NODE\_347534\_length\_4529\_cov\_29.102673 3636-3645. Max. coverage (+): 0.12. Max coverage (-): 0.04

Region: NODE\_347534\_length\_4529\_cov\_29.102673 3646-3654. Max. coverage (+): 0.02. Max coverage (-): 0

Region: NODE\_347534\_length\_4529\_cov\_29.102673 3655-3663. Max. coverage (+): 0. Max coverage (-): 0.04

Region: NODE\_347534\_length\_4529\_cov\_29.102673 3664-3673. Max. coverage (+): 0.12. Max coverage (-): 0.04

Region: NODE\_347534\_length\_4529\_cov\_29.102673 3674-3682. Max. coverage (+): 0.2. Max coverage (-): 0.04

Region: NODE\_347534\_length\_4529\_cov\_29.102673 3683-3691. Max. coverage (+): 0.2. Max coverage (-): 0.97

Region: NODE\_347534\_length\_4529\_cov\_29.102673 3692-3700. Max. coverage (+): 0.57. Max coverage (-): 0.85

Region: NODE\_347534\_length\_4529\_cov\_29.102673 3701-3710. Max. coverage (+): 9.17. Max coverage (-): 0.08

Region: NODE\_347534\_length\_4529\_cov\_29.102673 3711-3719. Max. coverage (+): 9.04. Max coverage (-): 0.16

Region: NODE\_347534\_length\_4529\_cov\_29.102673 3720-3728. Max. coverage (+): 1.41. Max coverage (-): 0.24

Region: NODE\_347534\_length\_4529\_cov\_29.102673 3729-3738. Max. coverage (+): 0.08. Max coverage (-): 0.08

Region: NODE\_347534\_length\_4529\_cov\_29.102673 3739-3747. Max. coverage (+): 0.36. Max coverage (-): 0.04

Region: NODE\_347534\_length\_4529\_cov\_29.102673 3748-3756. Max. coverage (+): 0.52. Max coverage (-): 0.04

Region: NODE\_347534\_length\_4529\_cov\_29.102673 3757-3766. Max. coverage (+): 0.16. Max coverage (-): 0

Region: NODE\_347534\_length\_4529\_cov\_29.102673 3767-3775. Max. coverage (+): 0.2. Max coverage (-): 0.12

Region: NODE\_347534\_length\_4529\_cov\_29.102673 3776-3784. Max. coverage (+): 0.85. Max coverage (-): 0.52

Region: NODE\_347534\_length\_4529\_cov\_29.102673 3785-3794. Max. coverage (+): 0.73. Max coverage (-): 0.12

Region: NODE\_347534\_length\_4529\_cov\_29.102673 3795-3803. Max. coverage (+): 0.81. Max coverage (-): 0.04

Region: NODE\_347534\_length\_4529\_cov\_29.102673 3804-3812. Max. coverage (+): 0.16. Max coverage (-): 0

Region: NODE\_347534\_length\_4529\_cov\_29.102673 3813-3821. Max. coverage (+): 0. Max coverage (-): 0.69

Region: NODE\_347534\_length\_4529\_cov\_29.102673 3822-3831. Max. coverage (+): 0.16. Max coverage (-): 0.95

Region: NODE\_347534\_length\_4529\_cov\_29.102673 3832-3840. Max. coverage (+): 0.36. Max coverage (-): 0

Region: NODE\_347534\_length\_4529\_cov\_29.102673 3841-3849. Max. coverage (+): 0.04. Max coverage (-): 0

Region: NODE\_347534\_length\_4529\_cov\_29.102673 3850-3859. Max. coverage (+): 1.09. Max coverage (-): 0

Region: NODE\_347534\_length\_4529\_cov\_29.102673 3860-3868. Max. coverage (+): 1.27. Max coverage (-): 0

Region: NODE\_347534\_length\_4529\_cov\_29.102673 3869-3877. Max. coverage (+): 0.12. Max coverage (-): 0

Region: NODE\_347534\_length\_4529\_cov\_29.102673 3878-3887. Max. coverage (+): 0.12. Max coverage (-): 0.08

Region: NODE\_347534\_length\_4529\_cov\_29.102673 3888-3896. Max. coverage (+): 0.48. Max coverage (-): 0.04

Region: NODE\_347534\_length\_4529\_cov\_29.102673 3897-3905. Max. coverage (+): 3.19. Max coverage (-): 0.04

Region: NODE\_347534\_length\_4529\_cov\_29.102673 3906-3915. Max. coverage (+): 1.53. Max coverage (-): 0

Region: NODE\_347534\_length\_4529\_cov\_29.102673 3916-3924. Max. coverage (+): 0.2. Max coverage (-): 0.08

Region: NODE\_347534\_length\_4529\_cov\_29.102673 3925-3933. Max. coverage (+): 0. Max coverage (-): 0

Region: NODE\_347534\_length\_4529\_cov\_29.102673 3934-3942. Max. coverage (+): 0.04. Max coverage (-): 0

Region: NODE\_347534\_length\_4529\_cov\_29.102673 3943-3952. Max. coverage (+): 0.04. Max coverage (-): 0.04

Region: NODE\_347534\_length\_4529\_cov\_29.102673 3953-3961. Max. coverage (+): 0. Max coverage (-): 0.04

Region: NODE\_347534\_length\_4529\_cov\_29.102673 3962-3970. Max. coverage (+): 0. Max coverage (-): 0.28

Region: NODE\_347534\_length\_4529\_cov\_29.102673 3971-3980. Max. coverage (+): 0.16. Max coverage (-): 0.28

Region: NODE\_347534\_length\_4529\_cov\_29.102673 3981-3989. Max. coverage (+): 0.02. Max coverage (-): 0.2

Region: NODE\_347534\_length\_4529\_cov\_29.102673 3990-3998. Max. coverage (+): 0.08. Max coverage (-): 0.79

Region: NODE\_347534\_length\_4529\_cov\_29.102673 3999-4008. Max. coverage (+): 0.2. Max coverage (-): 0.16

Region: NODE\_347534\_length\_4529\_cov\_29.102673 4009-4017. Max. coverage (+): 0.32. Max coverage (-): 0.04

Region: NODE\_347534\_length\_4529\_cov\_29.102673 4018-4026. Max. coverage (+): 0.24. Max coverage (-): 0.16

Region: NODE\_347534\_length\_4529\_cov\_29.102673 4027-4036. Max. coverage (+): 0.04. Max coverage (-): 0

Region: NODE\_347534\_length\_4529\_cov\_29.102673 4037-4045. Max. coverage (+): 0.16. Max coverage (-): 0

Region: NODE\_347534\_length\_4529\_cov\_29.102673 4046-4054. Max. coverage (+): 0.12. Max coverage (-): 0

Region: NODE\_347534\_length\_4529\_cov\_29.102673 4055-4063. Max. coverage (+): 0. Max coverage (-): 0

Region: NODE\_347534\_length\_4529\_cov\_29.102673 4064-4073. Max. coverage (+): 0.24. Max coverage (-): 0.08

Region: NODE\_347534\_length\_4529\_cov\_29.102673 4074-4082. Max. coverage (+): 0.08. Max coverage (-): 0.65

Region: NODE\_347534\_length\_4529\_cov\_29.102673 4083-4091. Max. coverage (+): 0.12. Max coverage (-): 0.73

Region: NODE\_347534\_length\_4529\_cov\_29.102673 4092-4101. Max. coverage (+): 0.24. Max coverage (-): 0.04

Region: NODE\_347534\_length\_4529\_cov\_29.102673 4102-4110. Max. coverage (+): 0.4. Max coverage (-): 0.2

Region: NODE\_347534\_length\_4529\_cov\_29.102673 4111-4119. Max. coverage (+): 0. Max coverage (-): 0.12

Region: NODE\_347534\_length\_4529\_cov\_29.102673 4120-4129. Max. coverage (+): 0.12. Max coverage (-): 0.04

Region: NODE\_347534\_length\_4529\_cov\_29.102673 4130-4138. Max. coverage (+): 0.01. Max coverage (-): 0.01

Region: NODE\_347534\_length\_4529\_cov\_29.102673 4139-4147. Max. coverage (+): 0.25. Max coverage (-): 0.01

Region: NODE\_347534\_length\_4529\_cov\_29.102673 4148-4157. Max. coverage (+): 0. Max coverage (-): 2.86

Region: NODE\_347534\_length\_4529\_cov\_29.102673 4158-4166. Max. coverage (+): 0.04. Max coverage (-): 1.84

Region: NODE\_347534\_length\_4529\_cov\_29.102673 4167-4175. Max. coverage (+): 0.46. Max coverage (-): 0

Region: NODE\_347534\_length\_4529\_cov\_29.102673 4176-4184. Max. coverage (+): 0.03. Max coverage (-): 0.01

Region: NODE\_347534\_length\_4529\_cov\_29.102673 4185-4194. Max. coverage (+): 0. Max coverage (-): 0.01

Region: NODE\_347534\_length\_4529\_cov\_29.102673 4195-4203. Max. coverage (+): 0. Max coverage (-): 0

Region: NODE\_347534\_length\_4529\_cov\_29.102673 4204-4212. Max. coverage (+): 0. Max coverage (-): 0

Region: NODE\_347534\_length\_4529\_cov\_29.102673 4213-4222. Max. coverage (+): 0. Max coverage (-): 0

Region: NODE\_347534\_length\_4529\_cov\_29.102673 4223-4231. Max. coverage (+): 0. Max coverage (-): 0

Region: NODE\_347534\_length\_4529\_cov\_29.102673 4232-4240. Max. coverage (+): 0. Max coverage (-): 0

Region: NODE\_347534\_length\_4529\_cov\_29.102673 4241-4250. Max. coverage (+): 0. Max coverage (-): 0

Region: NODE\_347534\_length\_4529\_cov\_29.102673 4251-4259. Max. coverage (+): 0.16. Max coverage (-): 0

Region: NODE\_347534\_length\_4529\_cov\_29.102673 4260-4268. Max. coverage (+): 0.4. Max coverage (-): 0

Region: NODE\_347534\_length\_4529\_cov\_29.102673 4269-4278. Max. coverage (+): 0.12. Max coverage (-): 0

Region: NODE\_347534\_length\_4529\_cov\_29.102673 4279-4287. Max. coverage (+): 0. Max coverage (-): 0

Region: NODE\_347534\_length\_4529\_cov\_29.102673 4288-4296. Max. coverage (+): 0. Max coverage (-): 0

Region: NODE\_347534\_length\_4529\_cov\_29.102673 4297-4305. Max. coverage (+): 0. Max coverage (-): 0

Region: NODE\_347534\_length\_4529\_cov\_29.102673 4306-4315. Max. coverage (+): 0. Max coverage (-): 0.01

Region: NODE\_347534\_length\_4529\_cov\_29.102673 4316-4324. Max. coverage (+): 0.28. Max coverage (-): 0.01

Region: NODE\_347534\_length\_4529\_cov\_29.102673 4325-4333. Max. coverage (+): 0.28. Max coverage (-): 0

Region: NODE\_347534\_length\_4529\_cov\_29.102673 4334-4343. Max. coverage (+): 0.08. Max coverage (-): 0

Region: NODE\_347534\_length\_4529\_cov\_29.102673 4344-4352. Max. coverage (+): 0. Max coverage (-): 0

Region: NODE\_347534\_length\_4529\_cov\_29.102673 4353-4361. Max. coverage (+): 0.01. Max coverage (-): 0

Region: NODE\_347534\_length\_4529\_cov\_29.102673 4362-4371. Max. coverage (+): 0.03. Max coverage (-): 0.02

Region: NODE\_347534\_length\_4529\_cov\_29.102673 4372-4380. Max. coverage (+): 0.09. Max coverage (-): 0.1

Region: NODE\_347534\_length\_4529\_cov\_29.102673 4381-4389. Max. coverage (+): 0.01. Max coverage (-): 0.09

Region: NODE\_347534\_length\_4529\_cov\_29.102673 4390-4399. Max. coverage (+): 0. Max coverage (-): 0

Region: NODE\_347534\_length\_4529\_cov\_29.102673 4400-4408. Max. coverage (+): 0. Max coverage (-): 0

Region: NODE\_347534\_length\_4529\_cov\_29.102673 4409-4417. Max. coverage (+): 0. Max coverage (-): 0.01

Region: NODE\_347534\_length\_4529\_cov\_29.102673 4418-4426. Max. coverage (+): 0. Max coverage (-): 0.02

Region: NODE\_347534\_length\_4529\_cov\_29.102673 4427-4436. Max. coverage (+): 0.05. Max coverage (-): 0.05

Region: NODE\_347534\_length\_4529\_cov\_29.102673 4437-4445. Max. coverage (+): 0.06. Max coverage (-): 0

Region: NODE\_347534\_length\_4529\_cov\_29.102673 4446-4454. Max. coverage (+): 0. Max coverage (-): 0

Region: NODE\_347534\_length\_4529\_cov\_29.102673 4455-4464. Max. coverage (+): 0. Max coverage (-): 0

Region: NODE\_347534\_length\_4529\_cov\_29.102673 4465-4473. Max. coverage (+): 0. Max coverage (-): 0

Region: NODE\_347534\_length\_4529\_cov\_29.102673 4474-4482. Max. coverage (+): 0. Max coverage (-): 0

Region: NODE\_347534\_length\_4529\_cov\_29.102673 4483-4492. Max. coverage (+): 0. Max coverage (-): 0

Region: NODE\_347534\_length\_4529\_cov\_29.102673 4493-4501. Max. coverage (+): 0. Max coverage (-): 0

Region: NODE\_347534\_length\_4529\_cov\_29.102673 4502-4510. Max. coverage (+): 0. Max coverage (-): 0

Region: NODE\_347534\_length\_4529\_cov\_29.102673 4511-4520. Max. coverage (+): 0. Max coverage (-): 0

Region: NODE\_347534\_length\_4529\_cov\_29.102673 4521-4529. Max. coverage (+): 0. Max coverage (-): 0

Region: NODE\_347534\_length\_4529\_cov\_29.102673 4530-4538. Max. coverage (+): 0. Max coverage (-): 0

Region: NODE\_347534\_length\_4529\_cov\_29.102673 4539-4547. Max. coverage (+): 0. Max coverage (-): 0

Region: NODE\_347534\_length\_4529\_cov\_29.102673 4548-4557. Max. coverage (+): 0. Max coverage (-): 0

Region: NODE\_347534\_length\_4529\_cov\_29.102673 4558-4566. Max. coverage (+): 0. Max coverage (-): 0

Region: NODE\_347534\_length\_4529\_cov\_29.102673 4567-4575. Max. coverage (+): 0. Max coverage (-): 0

Region: NODE\_347534\_length\_4529\_cov\_29.102673 4576-4585. Max. coverage (+): 0. Max coverage (-): 0

Region: NODE\_347534\_length\_4529\_cov\_29.102673 4586-4594. Max. coverage (+): 0. Max coverage (-): 0

Region: NODE\_347534\_length\_4529\_cov\_29.102673 4595-4603. Max. coverage (+): 0. Max coverage (-): 0

Region: NODE\_347534\_length\_4529\_cov\_29.102673 4604-4613. Max. coverage (+): 0. Max coverage (-): 0

Region: NODE\_347534\_length\_4529\_cov\_29.102673 4614-4622. Max. coverage (+): 0.01. Max coverage (-): 0.02

Region: NODE\_347534\_length\_4529\_cov\_29.102673 4623-4631. Max. coverage (+): 0. Max coverage (-): 0

Region: NODE\_347534\_length\_4529\_cov\_29.102673 4632-4641. Max. coverage (+): 0. Max coverage (-): 0

Region: NODE\_347534\_length\_4529\_cov\_29.102673 4642-4650. Max. coverage (+): 0. Max coverage (-): 0

Region: NODE\_347534\_length\_4529\_cov\_29.102673 4651-. Max. coverage (+): 0. Max coverage (-): 0

RepeatMasker Color Code

**+**

100-98% Identity

<98-95% Identity

<95-90% Identity

<90-85% Identity

<85-80% Identity

<80-75% Identity

<75-70% Identity

<70% Identity

**-**

Gene Set Color Code

**+**

Gene

Pseudogene

Other

**-**

Topology/Coverage Color Code

Coverage Plus Strand

Coverage Minus Strand

Mainstrand: Plus

Mainstrand: Minus

Complementary Strand

Flanking Region  
(if option -flank >0)

Gene Set Annotation  
  
RepeatMasker Annotation  

**1. hAT-14\_HM**: 20-217 (-), Divergence to consensus: 38.1%  
**2. AlRepC-733**: 140-236 (+), Divergence to consensus: 27.6%  
**3. DNA-8-18\_DR**: 527-675 (+), Divergence to consensus: 3.4%  
**4. AlRepC-1432**: 677-903 (-), Divergence to consensus: 20.3%  
**5. AlRepB-60**: 904-1002 (+), Divergence to consensus: 36.7%  
**6. AlRepB-59**: 1003-1078 (+), Divergence to consensus: 17.1%  
**7. AlRepB-2**: 1220-1406 (-), Divergence to consensus: 39.8%  
**8. AlRepC-532**: 1418-1486 (-), Divergence to consensus: 17.3%  
**9. A-rich**: 1546-1585 (+), Divergence to consensus: 23.3%  
**10. AlRepC-1020**: 2268-2331 (-), Divergence to consensus: 22%  
**11. AlRepB-420**: 3138-3478 (-), Divergence to consensus: 26.4%  
**12. AlRepD-880**: 3478-3619 (-), Divergence to consensus: 51.1%  
**13. AlRepB-11**: 3817-4106 (+), Divergence to consensus: 39.1%  
**14. AlRepC-1432**: 4126-4406 (+), Divergence to consensus: 12.6%  
**15. AlRepB-356**: 4409-4655 (+), Divergence to consensus: 9.5%

  
Transcription Factor Binding Sites  

**RHOXF1** (Sequence: GGATCA (-): 598)  
**RHOXF1** (Sequence: AGCTTA (-): 2003)  
**RHOXF1** (Sequence: AGCTCA (-): 2228)  
**RHOXF1** (Sequence: GGATTA (-): 3348)  
**RHOXF1** (Sequence: AGATTA (-): 4464)  
**RHOXF1** (Sequence: TAATCC (+): 1114)  
**RHOXF1** (Sequence: TAAGCT (+): 1792)  
**RHOXF1** (Sequence: TGATCC (+): 2626)  
**RHOXF1** (Sequence: TAATCT (+): 3183)  
**RHOXF1** (Sequence: TGATCC (+): 3975)  
**Gata4** (Sequence: CTTATCT (+): 434)  
**POU5F1** (Sequence: TTTGCAT (-): 2643)  
**POU5F1** (Sequence: TTTGCAT (-): 3013)  
**FOXO3\_hsa** (Sequence: GTAAACAA (+): 403)  
**FOXP1** (Sequence: GTAAACA (+): 403)  
**FOXO1** (Sequence: GTTGTTTTC (+): 4600)  
**FOXO3\_mmu** (Sequence: TGTTTACA (-): 2308)  
**FOXO3\_mmu** (Sequence: TGTTTTCA (-): 4547)  
**FOXO3\_mmu** (Sequence: TGTTTTCA (-): 4602)  
**Sox5** (Sequence: ATTGTT (+): 72)  
**Sox5** (Sequence: ATTGTT (+): 2570)  
**Sox5** (Sequence: ATTGTT (+): 2940)  
**FIGLA** (Sequence: AACAGCTGGT (-): 914)  
**FOXO3\_mmu** (Sequence: GGTAAACA (+): 402)  
**FOXO3\_mmu** (Sequence: TGAAAACA (+): 3768)  
**Nobox** (Sequence: GCCAATTA (-): 3644)  
**FOXP1** (Sequence: TGTTTAC (-): 2308)  
**POU2F1** (Sequence: ATTTAAATA (-): 4640)  
**Rhox11** (Sequence: TTTACAGCA (-): 3387)  
**POU2F1** (Sequence: TATTTAAAT (+): 343)  
**POU2F1** (Sequence: TATTCTAAT (+): 3517)  
**POU2F1** (Sequence: TATTTTAAT (+): 4505)  
**POU5F1** (Sequence: ATGCAAA (+): 1397)
