# Supplementary material for: First characterization of PIWI-interacting RNA clusters in a cichlid fish with a B chromosome
Source: BMC Biol. 2022 Sep 21;20:204. doi: 10.1186/s12915-022-01403-2 (PMC9490952; doi:10.1186/s12915-022-01403-2)
Supplement: Supplementary file 1 — Additional file 1. Zipped folder with fasta and interactive html piRNA cluster information for the A. latifasciata genome. The nomenclature is as follows: number-pirna-cluster_sex_B-presence (f, female; m, male; 0b, without B chromosome; 1b, with B chromosome). [file 12915_2022_1403_MOESM1_ESM.zip › 135_m1b.html]

piRNA cluster 135\_m1b 79


Predicted piRNA cluster no. 135\_m1b
  

Show proTRAC run info
Hide proTRAC run info

/\  
                \_\_\_\_\_\_\_\_\_\_\_\_\_\_\_\_\_\_\_\_\_\_\_/\\_\_\_ /  \\_\_\_\_\_\_\_  
               I                      /  \  /    \      I  
               I     pro             /    \/      \     I  
               I        TRAC        /               \   I  
               I   \_\_\_\_\_\_\_\_\_\_\_\_\_\_\_\_/\_\_\_\_\_\_\_\_\_\_\_\_\_\_\_\_\_\\_ I  
               I   \              /                     I  
               I    \            /                      I  
               I     \  /\      /       V.2.4.2         I  
               I      \/  \    /                        I  
               I\_\_\_\_\_\_\_\_\_\_\_\  /\_\_\_\_\_\_\_\_\_\_\_\_\_\_\_\_\_\_\_\_\_\_\_\_\_I  
                            \/  
  
  
================================= proTRAC ====================================  
VERSION: .......... 2.4.2  
LAST MODIFIED: .... 11. May 2018  
  
Please cite:  
Rosenkranz D, Zischler H. proTRAC - a software for probabilistic piRNA cluster  
detection, visualization and analysis. 2012. BMC Bioinformatics 13:5.  
  
  
Contact:  
David Rosenkranz  
Institute of Organismic and Molecular Evolutionary Biology  
Dept. Anthropology, small RNA group  
Johannes Gutenberg University Mainz  
email: rosenkranz@uni-mainz.de  
  
You can find the latest proTRAC version at:  
http://sourceforge.net/projects/protrac/files  
http://www.smallRNAgroup-mainz.de/software  
==============================================================================  
  
PARAMETERS:  
Map file: ...............piwi-machos-1B.fa-collapse.map  
Genome file: ............../../../0B\_ala\_genome.fa  
RepeatMasker annotation: Alatifasciata-all0B-maryan-v2.fa\_corrected.out  
GeneSet:................./guest-storage/Data/annotation/Alatifasciata\_all0B\_maryan-v2\_out2017.gff  
  
Significant (p<=0.01) hit density will be calculated based  
on observed hit distribution.  
  
Sliding window size: ........................................ 5000 bp  
Sliding window increament: .................................. 1000 bp  
Normalize each hit by number of genomic hits: ............... yes  
Normalize each hit by number of sequence reads: ............. yes  
Normalize values (-> per million mapped reads): ............. yes  
Min. fraction of hits with 1T(U) or 10A: .................... 0.75  
Alternatively: Min. fraction of hits with 1T(U) and 10A: .... 0.5  
Min. fraction of hits with typical piRNA length: ............ 0.75  
Typical piRNA length: ....................................... 24-32 nt  
Min. size of a piRNA cluster: ............................... 1000 bp.  
Min. number of hits (absolute): ............................. 0  
Min. number of hits (normalized): ........................... 0  
Min. fraction of hits on the mainstrand: .................... 0.75  
Top fraction of mapped sequences (in terms of read counts): . 1%  
Top fraction accounts for max. n% of sequence reads: ........ 90%  
Min. fraction of hits on each arm of a bidirectional cluster: 0.05  
Output html file for each cluster: .......................... yes  
Output a summary table: ..................................... yes  
Output a FASTA file for each cluster (piRNA sequences): ..... yes  
Output a FASTA file comprising cluster sequences: ........... yes  
Output a GTF file for predicted piRNA clusters: ..............yes  
Search DNA motifs in clusters: .............................. yes  
Output flanking sequences: +/- .............................. 0 bp  
Output ~.pTi file: .......................................... no  
==============================================================================  
  
  
Genome size (without gaps): ............ 758543724 bp  
Gaps (N/X/-): .......................... 417479 bp  
Mapped reads: .......................... 26973943  
Non-identical sequences: ............... 6209225  
Genomic hits: .......................... 48438990  
Significant densitiy of mapped reads: .. 821.144211136946 reads/kb

Show proTRAC cluster info
Hide proTRAC cluster info

|  |  |
| --- | --- |
| Location | NODE\_347534\_length\_4529\_cov\_29.102673 |
| Coordinates | 1-4655 |
| Size [bp] | 4655 |
| Sequence hit loci | 4010 |
| Mapped reads (normalized) | 11091.8 |
| Mapped reads (normalized) per kb | 2382.8 |
| Normalized reads with 1T (1U) | 70.6% |
| Normalized reads with 10A | 56% |
| Normalized reads with length 24-32 nt | 99.3% |
| Normalized reads on the main strand(s) | 83.6% |
| Predicted directionality | bi:minus-plus (split between 3577 and 3579) |

100%

0%

1T (1U)  
reads

10A reads

24-32 nt  
reads

reads on mainstrand

**Either the amount of reads with 1T (1U) OR 10A has to exceed 75% (set with option: -1Tor10A)  
Alternatively the amount of reads with 1T (1U) AND 10A has to exceed 50% (set with option: -1Tand10A)  
Minimum amount of reads with preferred size is 75% (set with option: -pisize)  
Minimum amount of reads on the main strand(s) is 75% (set with option: -clstrand)**

Show read coverage
Hide read coverage

WHAT DO I SEE HERE?  
This chart shows the location of mapped sequence reads within a predicted piRNA cluster. The color refers to the number of genomic hits produced by the sequence read in question. A dark red bar indicates that this sequence read produces many other hits elsewhere in the genome. Many adjacent red or yellow bars can indicate the presence of a multi-copy element such as transposons or rRNA genes. A dark green bar indicates that this sequence read maps uniquely to this locus.

1 hit

2-5 hits

6-10 hits

11-20 hits

21-50 hits

51-100 hits

> 100 hits

NODE\_347534\_length\_4529\_cov\_29.102673

1

4655

Gene Set

RepeatMasker

Mapped  
Reads

54.16

plus strand

minus strand

54.16

Region: NODE\_347534\_length\_4529\_cov\_29.102673 1336-5. Max. coverage (+): 0.05. Max coverage (-): 0.07

Region: NODE\_347534\_length\_4529\_cov\_29.102673 6-14. Max. coverage (+): 0.04. Max coverage (-): 0.23

Region: NODE\_347534\_length\_4529\_cov\_29.102673 15-24. Max. coverage (+): 0.01. Max coverage (-): 0.28

Region: NODE\_347534\_length\_4529\_cov\_29.102673 25-33. Max. coverage (+): 0.01. Max coverage (-): 0.07

Region: NODE\_347534\_length\_4529\_cov\_29.102673 34-42. Max. coverage (+): 0.01. Max coverage (-): 0.07

Region: NODE\_347534\_length\_4529\_cov\_29.102673 43-52. Max. coverage (+): 0. Max coverage (-): 0.11

Region: NODE\_347534\_length\_4529\_cov\_29.102673 53-61. Max. coverage (+): 0. Max coverage (-): 2.63

Region: NODE\_347534\_length\_4529\_cov\_29.102673 62-70. Max. coverage (+): 0.11. Max coverage (-): 0.07

Region: NODE\_347534\_length\_4529\_cov\_29.102673 71-80. Max. coverage (+): 0.17. Max coverage (-): 0.04

Region: NODE\_347534\_length\_4529\_cov\_29.102673 81-89. Max. coverage (+): 0.02. Max coverage (-): 0.04

Region: NODE\_347534\_length\_4529\_cov\_29.102673 90-98. Max. coverage (+): 0. Max coverage (-): 0.19

Region: NODE\_347534\_length\_4529\_cov\_29.102673 99-108. Max. coverage (+): 0. Max coverage (-): 0.19

Region: NODE\_347534\_length\_4529\_cov\_29.102673 109-117. Max. coverage (+): 0.02. Max coverage (-): 0.04

Region: NODE\_347534\_length\_4529\_cov\_29.102673 118-126. Max. coverage (+): 0.07. Max coverage (-): 0.04

Region: NODE\_347534\_length\_4529\_cov\_29.102673 127-135. Max. coverage (+): 0.07. Max coverage (-): 0.33

Region: NODE\_347534\_length\_4529\_cov\_29.102673 136-145. Max. coverage (+): 0.04. Max coverage (-): 0.09

Region: NODE\_347534\_length\_4529\_cov\_29.102673 146-154. Max. coverage (+): 0.07. Max coverage (-): 0.04

Region: NODE\_347534\_length\_4529\_cov\_29.102673 155-163. Max. coverage (+): 0.15. Max coverage (-): 0.33

Region: NODE\_347534\_length\_4529\_cov\_29.102673 164-173. Max. coverage (+): 0.04. Max coverage (-): 0.85

Region: NODE\_347534\_length\_4529\_cov\_29.102673 174-182. Max. coverage (+): 0.3. Max coverage (-): 0.06

Region: NODE\_347534\_length\_4529\_cov\_29.102673 183-191. Max. coverage (+): 0.32. Max coverage (-): 0.06

Region: NODE\_347534\_length\_4529\_cov\_29.102673 192-201. Max. coverage (+): 0.02. Max coverage (-): 0.13

Region: NODE\_347534\_length\_4529\_cov\_29.102673 202-210. Max. coverage (+): 0.01. Max coverage (-): 0.41

Region: NODE\_347534\_length\_4529\_cov\_29.102673 211-219. Max. coverage (+): 0.15. Max coverage (-): 0.41

Region: NODE\_347534\_length\_4529\_cov\_29.102673 220-229. Max. coverage (+): 0.11. Max coverage (-): 0.37

Region: NODE\_347534\_length\_4529\_cov\_29.102673 230-238. Max. coverage (+): 0.07. Max coverage (-): 0.07

Region: NODE\_347534\_length\_4529\_cov\_29.102673 239-247. Max. coverage (+): 0.02. Max coverage (-): 0.11

Region: NODE\_347534\_length\_4529\_cov\_29.102673 248-257. Max. coverage (+): 0. Max coverage (-): 0.06

Region: NODE\_347534\_length\_4529\_cov\_29.102673 258-266. Max. coverage (+): 0.01. Max coverage (-): 0.12

Region: NODE\_347534\_length\_4529\_cov\_29.102673 267-275. Max. coverage (+): 0. Max coverage (-): 0.02

Region: NODE\_347534\_length\_4529\_cov\_29.102673 276-284. Max. coverage (+): 0.06. Max coverage (-): 0.05

Region: NODE\_347534\_length\_4529\_cov\_29.102673 285-294. Max. coverage (+): 0.09. Max coverage (-): 0

Region: NODE\_347534\_length\_4529\_cov\_29.102673 295-303. Max. coverage (+): 0.04. Max coverage (-): 0.17

Region: NODE\_347534\_length\_4529\_cov\_29.102673 304-312. Max. coverage (+): 0.09. Max coverage (-): 1.71

Region: NODE\_347534\_length\_4529\_cov\_29.102673 313-322. Max. coverage (+): 0.02. Max coverage (-): 0.06

Region: NODE\_347534\_length\_4529\_cov\_29.102673 323-331. Max. coverage (+): 0. Max coverage (-): 0

Region: NODE\_347534\_length\_4529\_cov\_29.102673 332-340. Max. coverage (+): 0.01. Max coverage (-): 0

Region: NODE\_347534\_length\_4529\_cov\_29.102673 341-350. Max. coverage (+): 0.02. Max coverage (-): 0.01

Region: NODE\_347534\_length\_4529\_cov\_29.102673 351-359. Max. coverage (+): 0.04. Max coverage (-): 0.07

Region: NODE\_347534\_length\_4529\_cov\_29.102673 360-368. Max. coverage (+): 0.04. Max coverage (-): 0.44

Region: NODE\_347534\_length\_4529\_cov\_29.102673 369-378. Max. coverage (+): 0.04. Max coverage (-): 0

Region: NODE\_347534\_length\_4529\_cov\_29.102673 379-387. Max. coverage (+): 0. Max coverage (-): 0.01

Region: NODE\_347534\_length\_4529\_cov\_29.102673 388-396. Max. coverage (+): 0.01. Max coverage (-): 0.13

Region: NODE\_347534\_length\_4529\_cov\_29.102673 397-405. Max. coverage (+): 0.04. Max coverage (-): 0.02

Region: NODE\_347534\_length\_4529\_cov\_29.102673 406-415. Max. coverage (+): 1.08. Max coverage (-): 0

Region: NODE\_347534\_length\_4529\_cov\_29.102673 416-424. Max. coverage (+): 0. Max coverage (-): 0

Region: NODE\_347534\_length\_4529\_cov\_29.102673 425-433. Max. coverage (+): 0. Max coverage (-): 0

Region: NODE\_347534\_length\_4529\_cov\_29.102673 434-443. Max. coverage (+): 0. Max coverage (-): 0

Region: NODE\_347534\_length\_4529\_cov\_29.102673 444-452. Max. coverage (+): 0. Max coverage (-): 0

Region: NODE\_347534\_length\_4529\_cov\_29.102673 453-461. Max. coverage (+): 0. Max coverage (-): 0.04

Region: NODE\_347534\_length\_4529\_cov\_29.102673 462-471. Max. coverage (+): 0.01. Max coverage (-): 0.01

Region: NODE\_347534\_length\_4529\_cov\_29.102673 472-480. Max. coverage (+): 0.02. Max coverage (-): 0

Region: NODE\_347534\_length\_4529\_cov\_29.102673 481-489. Max. coverage (+): 0.01. Max coverage (-): 0

Region: NODE\_347534\_length\_4529\_cov\_29.102673 490-499. Max. coverage (+): 0. Max coverage (-): 0.06

Region: NODE\_347534\_length\_4529\_cov\_29.102673 500-508. Max. coverage (+): 0. Max coverage (-): 0.05

Region: NODE\_347534\_length\_4529\_cov\_29.102673 509-517. Max. coverage (+): 0. Max coverage (-): 0

Region: NODE\_347534\_length\_4529\_cov\_29.102673 518-527. Max. coverage (+): 0. Max coverage (-): 0

Region: NODE\_347534\_length\_4529\_cov\_29.102673 528-536. Max. coverage (+): 0. Max coverage (-): 0

Region: NODE\_347534\_length\_4529\_cov\_29.102673 537-545. Max. coverage (+): 0. Max coverage (-): 0

Region: NODE\_347534\_length\_4529\_cov\_29.102673 546-554. Max. coverage (+): 0. Max coverage (-): 0

Region: NODE\_347534\_length\_4529\_cov\_29.102673 555-564. Max. coverage (+): 0. Max coverage (-): 0.11

Region: NODE\_347534\_length\_4529\_cov\_29.102673 565-573. Max. coverage (+): 0. Max coverage (-): 2.11

Region: NODE\_347534\_length\_4529\_cov\_29.102673 574-582. Max. coverage (+): 0.04. Max coverage (-): 17.64

Region: NODE\_347534\_length\_4529\_cov\_29.102673 583-592. Max. coverage (+): 0. Max coverage (-): 0.03

Region: NODE\_347534\_length\_4529\_cov\_29.102673 593-601. Max. coverage (+): 0.1. Max coverage (-): 0.05

Region: NODE\_347534\_length\_4529\_cov\_29.102673 602-610. Max. coverage (+): 0.02. Max coverage (-): 0.09

Region: NODE\_347534\_length\_4529\_cov\_29.102673 611-620. Max. coverage (+): 0. Max coverage (-): 0.1

Region: NODE\_347534\_length\_4529\_cov\_29.102673 621-629. Max. coverage (+): 0.05. Max coverage (-): 0.01

Region: NODE\_347534\_length\_4529\_cov\_29.102673 630-638. Max. coverage (+): 0.05. Max coverage (-): 0.01

Region: NODE\_347534\_length\_4529\_cov\_29.102673 639-648. Max. coverage (+): 0.03. Max coverage (-): 0.01

Region: NODE\_347534\_length\_4529\_cov\_29.102673 649-657. Max. coverage (+): 0. Max coverage (-): 0.07

Region: NODE\_347534\_length\_4529\_cov\_29.102673 658-666. Max. coverage (+): 0. Max coverage (-): 0.67

Region: NODE\_347534\_length\_4529\_cov\_29.102673 667-675. Max. coverage (+): 0.04. Max coverage (-): 0.22

Region: NODE\_347534\_length\_4529\_cov\_29.102673 676-685. Max. coverage (+): 0. Max coverage (-): 0

Region: NODE\_347534\_length\_4529\_cov\_29.102673 686-694. Max. coverage (+): 0. Max coverage (-): 0

Region: NODE\_347534\_length\_4529\_cov\_29.102673 695-703. Max. coverage (+): 0. Max coverage (-): 0

Region: NODE\_347534\_length\_4529\_cov\_29.102673 704-713. Max. coverage (+): 0. Max coverage (-): 0.11

Region: NODE\_347534\_length\_4529\_cov\_29.102673 714-722. Max. coverage (+): 0. Max coverage (-): 0.07

Region: NODE\_347534\_length\_4529\_cov\_29.102673 723-731. Max. coverage (+): 0. Max coverage (-): 0

Region: NODE\_347534\_length\_4529\_cov\_29.102673 732-741. Max. coverage (+): 0. Max coverage (-): 0.06

Region: NODE\_347534\_length\_4529\_cov\_29.102673 742-750. Max. coverage (+): 0. Max coverage (-): 0.01

Region: NODE\_347534\_length\_4529\_cov\_29.102673 751-759. Max. coverage (+): 0. Max coverage (-): 0.01

Region: NODE\_347534\_length\_4529\_cov\_29.102673 760-769. Max. coverage (+): 0. Max coverage (-): 0.01

Region: NODE\_347534\_length\_4529\_cov\_29.102673 770-778. Max. coverage (+): 0. Max coverage (-): 0.09

Region: NODE\_347534\_length\_4529\_cov\_29.102673 779-787. Max. coverage (+): 0. Max coverage (-): 0.04

Region: NODE\_347534\_length\_4529\_cov\_29.102673 788-797. Max. coverage (+): 0. Max coverage (-): 0.02

Region: NODE\_347534\_length\_4529\_cov\_29.102673 798-806. Max. coverage (+): 0. Max coverage (-): 0

Region: NODE\_347534\_length\_4529\_cov\_29.102673 807-815. Max. coverage (+): 0. Max coverage (-): 0

Region: NODE\_347534\_length\_4529\_cov\_29.102673 816-824. Max. coverage (+): 0. Max coverage (-): 0

Region: NODE\_347534\_length\_4529\_cov\_29.102673 825-834. Max. coverage (+): 0. Max coverage (-): 0.23

Region: NODE\_347534\_length\_4529\_cov\_29.102673 835-843. Max. coverage (+): 0. Max coverage (-): 2.42

Region: NODE\_347534\_length\_4529\_cov\_29.102673 844-852. Max. coverage (+): 0.42. Max coverage (-): 0

Region: NODE\_347534\_length\_4529\_cov\_29.102673 853-862. Max. coverage (+): 0.01. Max coverage (-): 0

Region: NODE\_347534\_length\_4529\_cov\_29.102673 863-871. Max. coverage (+): 0. Max coverage (-): 0.07

Region: NODE\_347534\_length\_4529\_cov\_29.102673 872-880. Max. coverage (+): 0. Max coverage (-): 0.07

Region: NODE\_347534\_length\_4529\_cov\_29.102673 881-890. Max. coverage (+): 0.11. Max coverage (-): 0.22

Region: NODE\_347534\_length\_4529\_cov\_29.102673 891-899. Max. coverage (+): 0. Max coverage (-): 0.07

Region: NODE\_347534\_length\_4529\_cov\_29.102673 900-908. Max. coverage (+): 0.07. Max coverage (-): 0.22

Region: NODE\_347534\_length\_4529\_cov\_29.102673 909-918. Max. coverage (+): 0. Max coverage (-): 2.34

Region: NODE\_347534\_length\_4529\_cov\_29.102673 919-927. Max. coverage (+): 0. Max coverage (-): 0.56

Region: NODE\_347534\_length\_4529\_cov\_29.102673 928-936. Max. coverage (+): 0. Max coverage (-): 1.3

Region: NODE\_347534\_length\_4529\_cov\_29.102673 937-945. Max. coverage (+): 0. Max coverage (-): 0.04

Region: NODE\_347534\_length\_4529\_cov\_29.102673 946-955. Max. coverage (+): 0.07. Max coverage (-): 0.04

Region: NODE\_347534\_length\_4529\_cov\_29.102673 956-964. Max. coverage (+): 0.04. Max coverage (-): 0.04

Region: NODE\_347534\_length\_4529\_cov\_29.102673 965-973. Max. coverage (+): 0. Max coverage (-): 0.07

Region: NODE\_347534\_length\_4529\_cov\_29.102673 974-983. Max. coverage (+): 0. Max coverage (-): 0

Region: NODE\_347534\_length\_4529\_cov\_29.102673 984-992. Max. coverage (+): 0. Max coverage (-): 0

Region: NODE\_347534\_length\_4529\_cov\_29.102673 993-1001. Max. coverage (+): 0. Max coverage (-): 0

Region: NODE\_347534\_length\_4529\_cov\_29.102673 1002-1011. Max. coverage (+): 0. Max coverage (-): 0.03

Region: NODE\_347534\_length\_4529\_cov\_29.102673 1012-1020. Max. coverage (+): 0. Max coverage (-): 0.17

Region: NODE\_347534\_length\_4529\_cov\_29.102673 1021-1029. Max. coverage (+): 0.04. Max coverage (-): 6.08

Region: NODE\_347534\_length\_4529\_cov\_29.102673 1030-1039. Max. coverage (+): 0.07. Max coverage (-): 6.75

Region: NODE\_347534\_length\_4529\_cov\_29.102673 1040-1048. Max. coverage (+): 0.04. Max coverage (-): 0.19

Region: NODE\_347534\_length\_4529\_cov\_29.102673 1049-1057. Max. coverage (+): 0.15. Max coverage (-): 0.04

Region: NODE\_347534\_length\_4529\_cov\_29.102673 1058-1066. Max. coverage (+): 0. Max coverage (-): 0

Region: NODE\_347534\_length\_4529\_cov\_29.102673 1067-1076. Max. coverage (+): 0. Max coverage (-): 0.04

Region: NODE\_347534\_length\_4529\_cov\_29.102673 1077-1085. Max. coverage (+): 0. Max coverage (-): 0

Region: NODE\_347534\_length\_4529\_cov\_29.102673 1086-1094. Max. coverage (+): 0. Max coverage (-): 0

Region: NODE\_347534\_length\_4529\_cov\_29.102673 1095-1104. Max. coverage (+): 0. Max coverage (-): 0.15

Region: NODE\_347534\_length\_4529\_cov\_29.102673 1105-1113. Max. coverage (+): 0. Max coverage (-): 0.44

Region: NODE\_347534\_length\_4529\_cov\_29.102673 1114-1122. Max. coverage (+): 0. Max coverage (-): 0.85

Region: NODE\_347534\_length\_4529\_cov\_29.102673 1123-1132. Max. coverage (+): 0.22. Max coverage (-): 0.07

Region: NODE\_347534\_length\_4529\_cov\_29.102673 1133-1141. Max. coverage (+): 0. Max coverage (-): 0

Region: NODE\_347534\_length\_4529\_cov\_29.102673 1142-1150. Max. coverage (+): 0. Max coverage (-): 3.15

Region: NODE\_347534\_length\_4529\_cov\_29.102673 1151-1160. Max. coverage (+): 0.3. Max coverage (-): 1.85

Region: NODE\_347534\_length\_4529\_cov\_29.102673 1161-1169. Max. coverage (+): 0.3. Max coverage (-): 0.07

Region: NODE\_347534\_length\_4529\_cov\_29.102673 1170-1178. Max. coverage (+): 1.74. Max coverage (-): 54.16

Region: NODE\_347534\_length\_4529\_cov\_29.102673 1179-1188. Max. coverage (+): 0.7. Max coverage (-): 1.45

Region: NODE\_347534\_length\_4529\_cov\_29.102673 1189-1197. Max. coverage (+): 0.11. Max coverage (-): 0.63

Region: NODE\_347534\_length\_4529\_cov\_29.102673 1198-1206. Max. coverage (+): 1.04. Max coverage (-): 0

Region: NODE\_347534\_length\_4529\_cov\_29.102673 1207-1215. Max. coverage (+): 1.11. Max coverage (-): 0.04

Region: NODE\_347534\_length\_4529\_cov\_29.102673 1216-1225. Max. coverage (+): 0. Max coverage (-): 0.04

Region: NODE\_347534\_length\_4529\_cov\_29.102673 1226-1234. Max. coverage (+): 0.04. Max coverage (-): 0.82

Region: NODE\_347534\_length\_4529\_cov\_29.102673 1235-1243. Max. coverage (+): 0. Max coverage (-): 0.11

Region: NODE\_347534\_length\_4529\_cov\_29.102673 1244-1253. Max. coverage (+): 0.26. Max coverage (-): 0.22

Region: NODE\_347534\_length\_4529\_cov\_29.102673 1254-1262. Max. coverage (+): 0.59. Max coverage (-): 1.96

Region: NODE\_347534\_length\_4529\_cov\_29.102673 1263-1271. Max. coverage (+): 0.59. Max coverage (-): 0.78

Region: NODE\_347534\_length\_4529\_cov\_29.102673 1272-1281. Max. coverage (+): 0.19. Max coverage (-): 0.33

Region: NODE\_347534\_length\_4529\_cov\_29.102673 1282-1290. Max. coverage (+): 0.19. Max coverage (-): 0.07

Region: NODE\_347534\_length\_4529\_cov\_29.102673 1291-1299. Max. coverage (+): 0. Max coverage (-): 0

Region: NODE\_347534\_length\_4529\_cov\_29.102673 1300-1309. Max. coverage (+): 0.02. Max coverage (-): 0

Region: NODE\_347534\_length\_4529\_cov\_29.102673 1310-1318. Max. coverage (+): 0. Max coverage (-): 0.04

Region: NODE\_347534\_length\_4529\_cov\_29.102673 1319-1327. Max. coverage (+): 0. Max coverage (-): 0.19

Region: NODE\_347534\_length\_4529\_cov\_29.102673 1328-1336. Max. coverage (+): 0. Max coverage (-): 0.11

Region: NODE\_347534\_length\_4529\_cov\_29.102673 1337-1346. Max. coverage (+): 0. Max coverage (-): 0.11

Region: NODE\_347534\_length\_4529\_cov\_29.102673 1347-1355. Max. coverage (+): 0. Max coverage (-): 0.85

Region: NODE\_347534\_length\_4529\_cov\_29.102673 1356-1364. Max. coverage (+): 0. Max coverage (-): 8.71

Region: NODE\_347534\_length\_4529\_cov\_29.102673 1365-1374. Max. coverage (+): 0. Max coverage (-): 4.23

Region: NODE\_347534\_length\_4529\_cov\_29.102673 1375-1383. Max. coverage (+): 0.11. Max coverage (-): 6.19

Region: NODE\_347534\_length\_4529\_cov\_29.102673 1384-1392. Max. coverage (+): 0.04. Max coverage (-): 0

Region: NODE\_347534\_length\_4529\_cov\_29.102673 1393-1402. Max. coverage (+): 0. Max coverage (-): 0.19

Region: NODE\_347534\_length\_4529\_cov\_29.102673 1403-1411. Max. coverage (+): 0.04. Max coverage (-): 4.12

Region: NODE\_347534\_length\_4529\_cov\_29.102673 1412-1420. Max. coverage (+): 0.04. Max coverage (-): 0.59

Region: NODE\_347534\_length\_4529\_cov\_29.102673 1421-1430. Max. coverage (+): 0.33. Max coverage (-): 0.52

Region: NODE\_347534\_length\_4529\_cov\_29.102673 1431-1439. Max. coverage (+): 0. Max coverage (-): 0.19

Region: NODE\_347534\_length\_4529\_cov\_29.102673 1440-1448. Max. coverage (+): 0.11. Max coverage (-): 1.3

Region: NODE\_347534\_length\_4529\_cov\_29.102673 1449-1458. Max. coverage (+): 0.07. Max coverage (-): 10.9

Region: NODE\_347534\_length\_4529\_cov\_29.102673 1459-1467. Max. coverage (+): 0.3. Max coverage (-): 3.23

Region: NODE\_347534\_length\_4529\_cov\_29.102673 1468-1476. Max. coverage (+): 0.63. Max coverage (-): 2.52

Region: NODE\_347534\_length\_4529\_cov\_29.102673 1477-1485. Max. coverage (+): 0.48. Max coverage (-): 0.3

Region: NODE\_347534\_length\_4529\_cov\_29.102673 1486-1495. Max. coverage (+): 0. Max coverage (-): 0

Region: NODE\_347534\_length\_4529\_cov\_29.102673 1496-1504. Max. coverage (+): 0. Max coverage (-): 0.52

Region: NODE\_347534\_length\_4529\_cov\_29.102673 1505-1513. Max. coverage (+): 0. Max coverage (-): 1.48

Region: NODE\_347534\_length\_4529\_cov\_29.102673 1514-1523. Max. coverage (+): 0. Max coverage (-): 1.41

Region: NODE\_347534\_length\_4529\_cov\_29.102673 1524-1532. Max. coverage (+): 0. Max coverage (-): 0

Region: NODE\_347534\_length\_4529\_cov\_29.102673 1533-1541. Max. coverage (+): 0. Max coverage (-): 0.04

Region: NODE\_347534\_length\_4529\_cov\_29.102673 1542-1551. Max. coverage (+): 0. Max coverage (-): 0.06

Region: NODE\_347534\_length\_4529\_cov\_29.102673 1552-1560. Max. coverage (+): 0. Max coverage (-): 0.06

Region: NODE\_347534\_length\_4529\_cov\_29.102673 1561-1569. Max. coverage (+): 0. Max coverage (-): 0.11

Region: NODE\_347534\_length\_4529\_cov\_29.102673 1570-1579. Max. coverage (+): 0. Max coverage (-): 0.04

Region: NODE\_347534\_length\_4529\_cov\_29.102673 1580-1588. Max. coverage (+): 0. Max coverage (-): 0.82

Region: NODE\_347534\_length\_4529\_cov\_29.102673 1589-1597. Max. coverage (+): 0.04. Max coverage (-): 1.41

Region: NODE\_347534\_length\_4529\_cov\_29.102673 1598-1606. Max. coverage (+): 0.04. Max coverage (-): 0.41

Region: NODE\_347534\_length\_4529\_cov\_29.102673 1607-1616. Max. coverage (+): 0.04. Max coverage (-): 1

Region: NODE\_347534\_length\_4529\_cov\_29.102673 1617-1625. Max. coverage (+): 0.15. Max coverage (-): 0.59

Region: NODE\_347534\_length\_4529\_cov\_29.102673 1626-1634. Max. coverage (+): 0.04. Max coverage (-): 0.63

Region: NODE\_347534\_length\_4529\_cov\_29.102673 1635-1644. Max. coverage (+): 0.04. Max coverage (-): 0.07

Region: NODE\_347534\_length\_4529\_cov\_29.102673 1645-1653. Max. coverage (+): 0.04. Max coverage (-): 0.15

Region: NODE\_347534\_length\_4529\_cov\_29.102673 1654-1662. Max. coverage (+): 0.96. Max coverage (-): 0.04

Region: NODE\_347534\_length\_4529\_cov\_29.102673 1663-1672. Max. coverage (+): 0.96. Max coverage (-): 0

Region: NODE\_347534\_length\_4529\_cov\_29.102673 1673-1681. Max. coverage (+): 0. Max coverage (-): 0.04

Region: NODE\_347534\_length\_4529\_cov\_29.102673 1682-1690. Max. coverage (+): 0. Max coverage (-): 0.04

Region: NODE\_347534\_length\_4529\_cov\_29.102673 1691-1700. Max. coverage (+): 0.07. Max coverage (-): 1.37

Region: NODE\_347534\_length\_4529\_cov\_29.102673 1701-1709. Max. coverage (+): 0.19. Max coverage (-): 5.86

Region: NODE\_347534\_length\_4529\_cov\_29.102673 1710-1718. Max. coverage (+): 0.04. Max coverage (-): 5.75

Region: NODE\_347534\_length\_4529\_cov\_29.102673 1719-1728. Max. coverage (+): 0.04. Max coverage (-): 0.04

Region: NODE\_347534\_length\_4529\_cov\_29.102673 1729-1737. Max. coverage (+): 0. Max coverage (-): 0.04

Region: NODE\_347534\_length\_4529\_cov\_29.102673 1738-1746. Max. coverage (+): 0. Max coverage (-): 0.11

Region: NODE\_347534\_length\_4529\_cov\_29.102673 1747-1755. Max. coverage (+): 0.07. Max coverage (-): 0.04

Region: NODE\_347534\_length\_4529\_cov\_29.102673 1756-1765. Max. coverage (+): 0.07. Max coverage (-): 0.11

Region: NODE\_347534\_length\_4529\_cov\_29.102673 1766-1774. Max. coverage (+): 0. Max coverage (-): 0.3

Region: NODE\_347534\_length\_4529\_cov\_29.102673 1775-1783. Max. coverage (+): 0. Max coverage (-): 0.11

Region: NODE\_347534\_length\_4529\_cov\_29.102673 1784-1793. Max. coverage (+): 0.04. Max coverage (-): 0

Region: NODE\_347534\_length\_4529\_cov\_29.102673 1794-1802. Max. coverage (+): 2.78. Max coverage (-): 0.48

Region: NODE\_347534\_length\_4529\_cov\_29.102673 1803-1811. Max. coverage (+): 3.15. Max coverage (-): 0.08

Region: NODE\_347534\_length\_4529\_cov\_29.102673 1812-1821. Max. coverage (+): 0.11. Max coverage (-): 0.74

Region: NODE\_347534\_length\_4529\_cov\_29.102673 1822-1830. Max. coverage (+): 0. Max coverage (-): 0.74

Region: NODE\_347534\_length\_4529\_cov\_29.102673 1831-1839. Max. coverage (+): 0. Max coverage (-): 0.04

Region: NODE\_347534\_length\_4529\_cov\_29.102673 1840-1849. Max. coverage (+): 0.04. Max coverage (-): 0.11

Region: NODE\_347534\_length\_4529\_cov\_29.102673 1850-1858. Max. coverage (+): 0.04. Max coverage (-): 0.11

Region: NODE\_347534\_length\_4529\_cov\_29.102673 1859-1867. Max. coverage (+): 0. Max coverage (-): 0

Region: NODE\_347534\_length\_4529\_cov\_29.102673 1868-1876. Max. coverage (+): 0. Max coverage (-): 0.44

Region: NODE\_347534\_length\_4529\_cov\_29.102673 1877-1886. Max. coverage (+): 0.02. Max coverage (-): 0.17

Region: NODE\_347534\_length\_4529\_cov\_29.102673 1887-1895. Max. coverage (+): 0. Max coverage (-): 0.15

Region: NODE\_347534\_length\_4529\_cov\_29.102673 1896-1904. Max. coverage (+): 0. Max coverage (-): 0.16

Region: NODE\_347534\_length\_4529\_cov\_29.102673 1905-1914. Max. coverage (+): 0.07. Max coverage (-): 12.42

Region: NODE\_347534\_length\_4529\_cov\_29.102673 1915-1923. Max. coverage (+): 0.04. Max coverage (-): 3.11

Region: NODE\_347534\_length\_4529\_cov\_29.102673 1924-1932. Max. coverage (+): 0.04. Max coverage (-): 0.04

Region: NODE\_347534\_length\_4529\_cov\_29.102673 1933-1942. Max. coverage (+): 0. Max coverage (-): 0.03

Region: NODE\_347534\_length\_4529\_cov\_29.102673 1943-1951. Max. coverage (+): 0. Max coverage (-): 0

Region: NODE\_347534\_length\_4529\_cov\_29.102673 1952-1960. Max. coverage (+): 0.78. Max coverage (-): 0.37

Region: NODE\_347534\_length\_4529\_cov\_29.102673 1961-1970. Max. coverage (+): 0.15. Max coverage (-): 0.26

Region: NODE\_347534\_length\_4529\_cov\_29.102673 1971-1979. Max. coverage (+): 0. Max coverage (-): 0.63

Region: NODE\_347534\_length\_4529\_cov\_29.102673 1980-1988. Max. coverage (+): 0. Max coverage (-): 0.63

Region: NODE\_347534\_length\_4529\_cov\_29.102673 1989-1997. Max. coverage (+): 0.33. Max coverage (-): 0.07

Region: NODE\_347534\_length\_4529\_cov\_29.102673 1998-2007. Max. coverage (+): 0.04. Max coverage (-): 0.04

Region: NODE\_347534\_length\_4529\_cov\_29.102673 2008-2016. Max. coverage (+): 0.74. Max coverage (-): 0

Region: NODE\_347534\_length\_4529\_cov\_29.102673 2017-2025. Max. coverage (+): 0.67. Max coverage (-): 0.3

Region: NODE\_347534\_length\_4529\_cov\_29.102673 2026-2035. Max. coverage (+): 0.11. Max coverage (-): 1.04

Region: NODE\_347534\_length\_4529\_cov\_29.102673 2036-2044. Max. coverage (+): 0.07. Max coverage (-): 0.7

Region: NODE\_347534\_length\_4529\_cov\_29.102673 2045-2053. Max. coverage (+): 0. Max coverage (-): 1.08

Region: NODE\_347534\_length\_4529\_cov\_29.102673 2054-2063. Max. coverage (+): 0.15. Max coverage (-): 0.74

Region: NODE\_347534\_length\_4529\_cov\_29.102673 2064-2072. Max. coverage (+): 0.85. Max coverage (-): 0.22

Region: NODE\_347534\_length\_4529\_cov\_29.102673 2073-2081. Max. coverage (+): 0. Max coverage (-): 31.36

Region: NODE\_347534\_length\_4529\_cov\_29.102673 2082-2091. Max. coverage (+): 0.07. Max coverage (-): 30.73

Region: NODE\_347534\_length\_4529\_cov\_29.102673 2092-2100. Max. coverage (+): 0.37. Max coverage (-): 1.04

Region: NODE\_347534\_length\_4529\_cov\_29.102673 2101-2109. Max. coverage (+): 0.7. Max coverage (-): 0.26

Region: NODE\_347534\_length\_4529\_cov\_29.102673 2110-2119. Max. coverage (+): 0.3. Max coverage (-): 0.22

Region: NODE\_347534\_length\_4529\_cov\_29.102673 2120-2128. Max. coverage (+): 0. Max coverage (-): 0.22

Region: NODE\_347534\_length\_4529\_cov\_29.102673 2129-2137. Max. coverage (+): 0.04. Max coverage (-): 0.3

Region: NODE\_347534\_length\_4529\_cov\_29.102673 2138-2146. Max. coverage (+): 0.44. Max coverage (-): 0.33

Region: NODE\_347534\_length\_4529\_cov\_29.102673 2147-2156. Max. coverage (+): 0.04. Max coverage (-): 0.3

Region: NODE\_347534\_length\_4529\_cov\_29.102673 2157-2165. Max. coverage (+): 0.04. Max coverage (-): 0.52

Region: NODE\_347534\_length\_4529\_cov\_29.102673 2166-2174. Max. coverage (+): 0.11. Max coverage (-): 0.33

Region: NODE\_347534\_length\_4529\_cov\_29.102673 2175-2184. Max. coverage (+): 0.3. Max coverage (-): 0.07

Region: NODE\_347534\_length\_4529\_cov\_29.102673 2185-2193. Max. coverage (+): 0.15. Max coverage (-): 0.17

Region: NODE\_347534\_length\_4529\_cov\_29.102673 2194-2202. Max. coverage (+): 0.01. Max coverage (-): 0.11

Region: NODE\_347534\_length\_4529\_cov\_29.102673 2203-2212. Max. coverage (+): 0.22. Max coverage (-): 0.04

Region: NODE\_347534\_length\_4529\_cov\_29.102673 2213-2221. Max. coverage (+): 0.3. Max coverage (-): 0.07

Region: NODE\_347534\_length\_4529\_cov\_29.102673 2222-2230. Max. coverage (+): 0. Max coverage (-): 3.71

Region: NODE\_347534\_length\_4529\_cov\_29.102673 2231-2240. Max. coverage (+): 0. Max coverage (-): 2.89

Region: NODE\_347534\_length\_4529\_cov\_29.102673 2241-2249. Max. coverage (+): 0.17. Max coverage (-): 0

Region: NODE\_347534\_length\_4529\_cov\_29.102673 2250-2258. Max. coverage (+): 0.05. Max coverage (-): 0.04

Region: NODE\_347534\_length\_4529\_cov\_29.102673 2259-2267. Max. coverage (+): 0. Max coverage (-): 0.07

Region: NODE\_347534\_length\_4529\_cov\_29.102673 2268-2277. Max. coverage (+): 0.15. Max coverage (-): 0.04

Region: NODE\_347534\_length\_4529\_cov\_29.102673 2278-2286. Max. coverage (+): 0.04. Max coverage (-): 0.07

Region: NODE\_347534\_length\_4529\_cov\_29.102673 2287-2295. Max. coverage (+): 0.07. Max coverage (-): 0.44

Region: NODE\_347534\_length\_4529\_cov\_29.102673 2296-2305. Max. coverage (+): 0.07. Max coverage (-): 0.11

Region: NODE\_347534\_length\_4529\_cov\_29.102673 2306-2314. Max. coverage (+): 0.33. Max coverage (-): 3

Region: NODE\_347534\_length\_4529\_cov\_29.102673 2315-2323. Max. coverage (+): 0.3. Max coverage (-): 6.78

Region: NODE\_347534\_length\_4529\_cov\_29.102673 2324-2333. Max. coverage (+): 0.04. Max coverage (-): 0.52

Region: NODE\_347534\_length\_4529\_cov\_29.102673 2334-2342. Max. coverage (+): 0. Max coverage (-): 0.07

Region: NODE\_347534\_length\_4529\_cov\_29.102673 2343-2351. Max. coverage (+): 0. Max coverage (-): 0.07

Region: NODE\_347534\_length\_4529\_cov\_29.102673 2352-2361. Max. coverage (+): 0.04. Max coverage (-): 0.15

Region: NODE\_347534\_length\_4529\_cov\_29.102673 2362-2370. Max. coverage (+): 0. Max coverage (-): 0.15

Region: NODE\_347534\_length\_4529\_cov\_29.102673 2371-2379. Max. coverage (+): 0. Max coverage (-): 0

Region: NODE\_347534\_length\_4529\_cov\_29.102673 2380-2389. Max. coverage (+): 0. Max coverage (-): 0.11

Region: NODE\_347534\_length\_4529\_cov\_29.102673 2390-2398. Max. coverage (+): 0. Max coverage (-): 0

Region: NODE\_347534\_length\_4529\_cov\_29.102673 2399-2407. Max. coverage (+): 0.07. Max coverage (-): 0.12

Region: NODE\_347534\_length\_4529\_cov\_29.102673 2408-2416. Max. coverage (+): 0.04. Max coverage (-): 0.29

Region: NODE\_347534\_length\_4529\_cov\_29.102673 2417-2426. Max. coverage (+): 0.01. Max coverage (-): 0.15

Region: NODE\_347534\_length\_4529\_cov\_29.102673 2427-2435. Max. coverage (+): 0. Max coverage (-): 0

Region: NODE\_347534\_length\_4529\_cov\_29.102673 2436-2444. Max. coverage (+): 0. Max coverage (-): 0

Region: NODE\_347534\_length\_4529\_cov\_29.102673 2445-2454. Max. coverage (+): 0.07. Max coverage (-): 0

Region: NODE\_347534\_length\_4529\_cov\_29.102673 2455-2463. Max. coverage (+): 0.07. Max coverage (-): 0

Region: NODE\_347534\_length\_4529\_cov\_29.102673 2464-2472. Max. coverage (+): 0.07. Max coverage (-): 2.89

Region: NODE\_347534\_length\_4529\_cov\_29.102673 2473-2482. Max. coverage (+): 0.07. Max coverage (-): 0.44

Region: NODE\_347534\_length\_4529\_cov\_29.102673 2483-2491. Max. coverage (+): 0.07. Max coverage (-): 0.3

Region: NODE\_347534\_length\_4529\_cov\_29.102673 2492-2500. Max. coverage (+): 0.04. Max coverage (-): 0

Region: NODE\_347534\_length\_4529\_cov\_29.102673 2501-2510. Max. coverage (+): 0.07. Max coverage (-): 0

Region: NODE\_347534\_length\_4529\_cov\_29.102673 2511-2519. Max. coverage (+): 0. Max coverage (-): 0

Region: NODE\_347534\_length\_4529\_cov\_29.102673 2520-2528. Max. coverage (+): 0. Max coverage (-): 0.04

Region: NODE\_347534\_length\_4529\_cov\_29.102673 2529-2537. Max. coverage (+): 0. Max coverage (-): 0

Region: NODE\_347534\_length\_4529\_cov\_29.102673 2538-2547. Max. coverage (+): 1.22. Max coverage (-): 0

Region: NODE\_347534\_length\_4529\_cov\_29.102673 2548-2556. Max. coverage (+): 0. Max coverage (-): 0

Region: NODE\_347534\_length\_4529\_cov\_29.102673 2557-2565. Max. coverage (+): 0.06. Max coverage (-): 0.89

Region: NODE\_347534\_length\_4529\_cov\_29.102673 2566-2575. Max. coverage (+): 0.37. Max coverage (-): 1.61

Region: NODE\_347534\_length\_4529\_cov\_29.102673 2576-2584. Max. coverage (+): 2.69. Max coverage (-): 0.09

Region: NODE\_347534\_length\_4529\_cov\_29.102673 2585-2593. Max. coverage (+): 2.72. Max coverage (-): 0.04

Region: NODE\_347534\_length\_4529\_cov\_29.102673 2594-2603. Max. coverage (+): 0.09. Max coverage (-): 0.13

Region: NODE\_347534\_length\_4529\_cov\_29.102673 2604-2612. Max. coverage (+): 0.94. Max coverage (-): 0.19

Region: NODE\_347534\_length\_4529\_cov\_29.102673 2613-2621. Max. coverage (+): 0.07. Max coverage (-): 0.19

Region: NODE\_347534\_length\_4529\_cov\_29.102673 2622-2631. Max. coverage (+): 0. Max coverage (-): 0.04

Region: NODE\_347534\_length\_4529\_cov\_29.102673 2632-2640. Max. coverage (+): 0. Max coverage (-): 0

Region: NODE\_347534\_length\_4529\_cov\_29.102673 2641-2649. Max. coverage (+): 0. Max coverage (-): 0

Region: NODE\_347534\_length\_4529\_cov\_29.102673 2650-2659. Max. coverage (+): 0.04. Max coverage (-): 0.04

Region: NODE\_347534\_length\_4529\_cov\_29.102673 2660-2668. Max. coverage (+): 0.06. Max coverage (-): 0.02

Region: NODE\_347534\_length\_4529\_cov\_29.102673 2669-2677. Max. coverage (+): 0.13. Max coverage (-): 0.06

Region: NODE\_347534\_length\_4529\_cov\_29.102673 2678-2686. Max. coverage (+): 0.13. Max coverage (-): 0.04

Region: NODE\_347534\_length\_4529\_cov\_29.102673 2687-2696. Max. coverage (+): 0.04. Max coverage (-): 0.19

Region: NODE\_347534\_length\_4529\_cov\_29.102673 2697-2705. Max. coverage (+): 0. Max coverage (-): 0.33

Region: NODE\_347534\_length\_4529\_cov\_29.102673 2706-2714. Max. coverage (+): 0. Max coverage (-): 0.07

Region: NODE\_347534\_length\_4529\_cov\_29.102673 2715-2724. Max. coverage (+): 0.02. Max coverage (-): 0

Region: NODE\_347534\_length\_4529\_cov\_29.102673 2725-2733. Max. coverage (+): 0. Max coverage (-): 0.59

Region: NODE\_347534\_length\_4529\_cov\_29.102673 2734-2742. Max. coverage (+): 0. Max coverage (-): 0.85

Region: NODE\_347534\_length\_4529\_cov\_29.102673 2743-2752. Max. coverage (+): 0.17. Max coverage (-): 0

Region: NODE\_347534\_length\_4529\_cov\_29.102673 2753-2761. Max. coverage (+): 0.16. Max coverage (-): 0

Region: NODE\_347534\_length\_4529\_cov\_29.102673 2762-2770. Max. coverage (+): 0.04. Max coverage (-): 0.29

Region: NODE\_347534\_length\_4529\_cov\_29.102673 2771-2780. Max. coverage (+): 0.01. Max coverage (-): 0.28

Region: NODE\_347534\_length\_4529\_cov\_29.102673 2781-2789. Max. coverage (+): 0.04. Max coverage (-): 0.1

Region: NODE\_347534\_length\_4529\_cov\_29.102673 2790-2798. Max. coverage (+): 0.04. Max coverage (-): 0.04

Region: NODE\_347534\_length\_4529\_cov\_29.102673 2799-2807. Max. coverage (+): 0.04. Max coverage (-): 0

Region: NODE\_347534\_length\_4529\_cov\_29.102673 2808-2817. Max. coverage (+): 0.04. Max coverage (-): 0

Region: NODE\_347534\_length\_4529\_cov\_29.102673 2818-2826. Max. coverage (+): 0.07. Max coverage (-): 1.26

Region: NODE\_347534\_length\_4529\_cov\_29.102673 2827-2835. Max. coverage (+): 2.26. Max coverage (-): 2.3

Region: NODE\_347534\_length\_4529\_cov\_29.102673 2836-2845. Max. coverage (+): 0.63. Max coverage (-): 0.26

Region: NODE\_347534\_length\_4529\_cov\_29.102673 2846-2854. Max. coverage (+): 0.19. Max coverage (-): 0.04

Region: NODE\_347534\_length\_4529\_cov\_29.102673 2855-2863. Max. coverage (+): 0. Max coverage (-): 0

Region: NODE\_347534\_length\_4529\_cov\_29.102673 2864-2873. Max. coverage (+): 0.04. Max coverage (-): 0

Region: NODE\_347534\_length\_4529\_cov\_29.102673 2874-2882. Max. coverage (+): 0.52. Max coverage (-): 0.07

Region: NODE\_347534\_length\_4529\_cov\_29.102673 2883-2891. Max. coverage (+): 0.22. Max coverage (-): 0.04

Region: NODE\_347534\_length\_4529\_cov\_29.102673 2892-2901. Max. coverage (+): 0. Max coverage (-): 0

Region: NODE\_347534\_length\_4529\_cov\_29.102673 2902-2910. Max. coverage (+): 1.15. Max coverage (-): 0

Region: NODE\_347534\_length\_4529\_cov\_29.102673 2911-2919. Max. coverage (+): 1.15. Max coverage (-): 0

Region: NODE\_347534\_length\_4529\_cov\_29.102673 2920-2928. Max. coverage (+): 0.02. Max coverage (-): 0.13

Region: NODE\_347534\_length\_4529\_cov\_29.102673 2929-2938. Max. coverage (+): 0.17. Max coverage (-): 0.89

Region: NODE\_347534\_length\_4529\_cov\_29.102673 2939-2947. Max. coverage (+): 2.52. Max coverage (-): 1.61

Region: NODE\_347534\_length\_4529\_cov\_29.102673 2948-2956. Max. coverage (+): 2.69. Max coverage (-): 0.09

Region: NODE\_347534\_length\_4529\_cov\_29.102673 2957-2966. Max. coverage (+): 0.52. Max coverage (-): 0.37

Region: NODE\_347534\_length\_4529\_cov\_29.102673 2967-2975. Max. coverage (+): 0.04. Max coverage (-): 0.37

Region: NODE\_347534\_length\_4529\_cov\_29.102673 2976-2984. Max. coverage (+): 0.15. Max coverage (-): 0.15

Region: NODE\_347534\_length\_4529\_cov\_29.102673 2985-2994. Max. coverage (+): 0.07. Max coverage (-): 0.19

Region: NODE\_347534\_length\_4529\_cov\_29.102673 2995-3003. Max. coverage (+): 0. Max coverage (-): 0

Region: NODE\_347534\_length\_4529\_cov\_29.102673 3004-3012. Max. coverage (+): 0. Max coverage (-): 0

Region: NODE\_347534\_length\_4529\_cov\_29.102673 3013-3022. Max. coverage (+): 0. Max coverage (-): 0

Region: NODE\_347534\_length\_4529\_cov\_29.102673 3023-3031. Max. coverage (+): 0.02. Max coverage (-): 0.02

Region: NODE\_347534\_length\_4529\_cov\_29.102673 3032-3040. Max. coverage (+): 0.06. Max coverage (-): 0.02

Region: NODE\_347534\_length\_4529\_cov\_29.102673 3041-3050. Max. coverage (+): 0.17. Max coverage (-): 0.07

Region: NODE\_347534\_length\_4529\_cov\_29.102673 3051-3059. Max. coverage (+): 0.06. Max coverage (-): 0.04

Region: NODE\_347534\_length\_4529\_cov\_29.102673 3060-3068. Max. coverage (+): 0. Max coverage (-): 0.07

Region: NODE\_347534\_length\_4529\_cov\_29.102673 3069-3077. Max. coverage (+): 0. Max coverage (-): 0

Region: NODE\_347534\_length\_4529\_cov\_29.102673 3078-3087. Max. coverage (+): 0.04. Max coverage (-): 0

Region: NODE\_347534\_length\_4529\_cov\_29.102673 3088-3096. Max. coverage (+): 0.04. Max coverage (-): 0

Region: NODE\_347534\_length\_4529\_cov\_29.102673 3097-3105. Max. coverage (+): 0. Max coverage (-): 0.36

Region: NODE\_347534\_length\_4529\_cov\_29.102673 3106-3115. Max. coverage (+): 0.01. Max coverage (-): 0.04

Region: NODE\_347534\_length\_4529\_cov\_29.102673 3116-3124. Max. coverage (+): 1.53. Max coverage (-): 0.11

Region: NODE\_347534\_length\_4529\_cov\_29.102673 3125-3133. Max. coverage (+): 0.15. Max coverage (-): 0.37

Region: NODE\_347534\_length\_4529\_cov\_29.102673 3134-3143. Max. coverage (+): 0. Max coverage (-): 0.37

Region: NODE\_347534\_length\_4529\_cov\_29.102673 3144-3152. Max. coverage (+): 0. Max coverage (-): 0

Region: NODE\_347534\_length\_4529\_cov\_29.102673 3153-3161. Max. coverage (+): 0.19. Max coverage (-): 0.26

Region: NODE\_347534\_length\_4529\_cov\_29.102673 3162-3171. Max. coverage (+): 0.04. Max coverage (-): 0.26

Region: NODE\_347534\_length\_4529\_cov\_29.102673 3172-3180. Max. coverage (+): 0. Max coverage (-): 0

Region: NODE\_347534\_length\_4529\_cov\_29.102673 3181-3189. Max. coverage (+): 0. Max coverage (-): 0

Region: NODE\_347534\_length\_4529\_cov\_29.102673 3190-3198. Max. coverage (+): 0. Max coverage (-): 0.01

Region: NODE\_347534\_length\_4529\_cov\_29.102673 3199-3208. Max. coverage (+): 0.08. Max coverage (-): 0.19

Region: NODE\_347534\_length\_4529\_cov\_29.102673 3209-3217. Max. coverage (+): 0.19. Max coverage (-): 0.26

Region: NODE\_347534\_length\_4529\_cov\_29.102673 3218-3226. Max. coverage (+): 0. Max coverage (-): 0.11

Region: NODE\_347534\_length\_4529\_cov\_29.102673 3227-3236. Max. coverage (+): 0.04. Max coverage (-): 0

Region: NODE\_347534\_length\_4529\_cov\_29.102673 3237-3245. Max. coverage (+): 0. Max coverage (-): 0

Region: NODE\_347534\_length\_4529\_cov\_29.102673 3246-3254. Max. coverage (+): 0.04. Max coverage (-): 0

Region: NODE\_347534\_length\_4529\_cov\_29.102673 3255-3264. Max. coverage (+): 0. Max coverage (-): 0

Region: NODE\_347534\_length\_4529\_cov\_29.102673 3265-3273. Max. coverage (+): 0. Max coverage (-): 0.04

Region: NODE\_347534\_length\_4529\_cov\_29.102673 3274-3282. Max. coverage (+): 0. Max coverage (-): 0.04

Region: NODE\_347534\_length\_4529\_cov\_29.102673 3283-3292. Max. coverage (+): 0.26. Max coverage (-): 0.07

Region: NODE\_347534\_length\_4529\_cov\_29.102673 3293-3301. Max. coverage (+): 0.3. Max coverage (-): 0.15

Region: NODE\_347534\_length\_4529\_cov\_29.102673 3302-3310. Max. coverage (+): 0.07. Max coverage (-): 0.19

Region: NODE\_347534\_length\_4529\_cov\_29.102673 3311-3320. Max. coverage (+): 0. Max coverage (-): 0

Region: NODE\_347534\_length\_4529\_cov\_29.102673 3321-3329. Max. coverage (+): 0.04. Max coverage (-): 0

Region: NODE\_347534\_length\_4529\_cov\_29.102673 3330-3338. Max. coverage (+): 0. Max coverage (-): 0.32

Region: NODE\_347534\_length\_4529\_cov\_29.102673 3339-3347. Max. coverage (+): 0. Max coverage (-): 0.03

Region: NODE\_347534\_length\_4529\_cov\_29.102673 3348-3357. Max. coverage (+): 0.35. Max coverage (-): 0.06

Region: NODE\_347534\_length\_4529\_cov\_29.102673 3358-3366. Max. coverage (+): 0.06. Max coverage (-): 0.07

Region: NODE\_347534\_length\_4529\_cov\_29.102673 3367-3375. Max. coverage (+): 0.04. Max coverage (-): 0.27

Region: NODE\_347534\_length\_4529\_cov\_29.102673 3376-3385. Max. coverage (+): 0.07. Max coverage (-): 4.17

Region: NODE\_347534\_length\_4529\_cov\_29.102673 3386-3394. Max. coverage (+): 1.08. Max coverage (-): 0.15

Region: NODE\_347534\_length\_4529\_cov\_29.102673 3395-3403. Max. coverage (+): 0.89. Max coverage (-): 0.22

Region: NODE\_347534\_length\_4529\_cov\_29.102673 3404-3413. Max. coverage (+): 1.19. Max coverage (-): 0.22

Region: NODE\_347534\_length\_4529\_cov\_29.102673 3414-3422. Max. coverage (+): 0.11. Max coverage (-): 0.04

Region: NODE\_347534\_length\_4529\_cov\_29.102673 3423-3431. Max. coverage (+): 0.04. Max coverage (-): 0.15

Region: NODE\_347534\_length\_4529\_cov\_29.102673 3432-3441. Max. coverage (+): 0.07. Max coverage (-): 0.22

Region: NODE\_347534\_length\_4529\_cov\_29.102673 3442-3450. Max. coverage (+): 0.56. Max coverage (-): 0.07

Region: NODE\_347534\_length\_4529\_cov\_29.102673 3451-3459. Max. coverage (+): 0.56. Max coverage (-): 0.15

Region: NODE\_347534\_length\_4529\_cov\_29.102673 3460-3468. Max. coverage (+): 0.11. Max coverage (-): 0.89

Region: NODE\_347534\_length\_4529\_cov\_29.102673 3469-3478. Max. coverage (+): 0.07. Max coverage (-): 1.15

Region: NODE\_347534\_length\_4529\_cov\_29.102673 3479-3487. Max. coverage (+): 0.07. Max coverage (-): 0.11

Region: NODE\_347534\_length\_4529\_cov\_29.102673 3488-3496. Max. coverage (+): 0.04. Max coverage (-): 0.26

Region: NODE\_347534\_length\_4529\_cov\_29.102673 3497-3506. Max. coverage (+): 0. Max coverage (-): 0.3

Region: NODE\_347534\_length\_4529\_cov\_29.102673 3507-3515. Max. coverage (+): 0. Max coverage (-): 0.11

Region: NODE\_347534\_length\_4529\_cov\_29.102673 3516-3524. Max. coverage (+): 0.04. Max coverage (-): 0.07

Region: NODE\_347534\_length\_4529\_cov\_29.102673 3525-3534. Max. coverage (+): 0.04. Max coverage (-): 0.07

Region: NODE\_347534\_length\_4529\_cov\_29.102673 3535-3543. Max. coverage (+): 0.04. Max coverage (-): 1.04

Region: NODE\_347534\_length\_4529\_cov\_29.102673 3544-3552. Max. coverage (+): 0.19. Max coverage (-): 0.41

Region: NODE\_347534\_length\_4529\_cov\_29.102673 3553-3562. Max. coverage (+): 0. Max coverage (-): 0.03

Region: NODE\_347534\_length\_4529\_cov\_29.102673 3563-3571. Max. coverage (+): 0. Max coverage (-): 0.04

Region: NODE\_347534\_length\_4529\_cov\_29.102673 3572-3580. Max. coverage (+): 0.22. Max coverage (-): 0.15

Region: NODE\_347534\_length\_4529\_cov\_29.102673 3581-3590. Max. coverage (+): 0.59. Max coverage (-): 0.04

Region: NODE\_347534\_length\_4529\_cov\_29.102673 3591-3599. Max. coverage (+): 0.11. Max coverage (-): 0

Region: NODE\_347534\_length\_4529\_cov\_29.102673 3600-3608. Max. coverage (+): 0.15. Max coverage (-): 0.19

Region: NODE\_347534\_length\_4529\_cov\_29.102673 3609-3617. Max. coverage (+): 0.11. Max coverage (-): 0.22

Region: NODE\_347534\_length\_4529\_cov\_29.102673 3618-3627. Max. coverage (+): 1.96. Max coverage (-): 0.15

Region: NODE\_347534\_length\_4529\_cov\_29.102673 3628-3636. Max. coverage (+): 1.96. Max coverage (-): 0.33

Region: NODE\_347534\_length\_4529\_cov\_29.102673 3637-3645. Max. coverage (+): 0.11. Max coverage (-): 0.22

Region: NODE\_347534\_length\_4529\_cov\_29.102673 3646-3655. Max. coverage (+): 0.02. Max coverage (-): 0.02

Region: NODE\_347534\_length\_4529\_cov\_29.102673 3656-3664. Max. coverage (+): 0. Max coverage (-): 0.02

Region: NODE\_347534\_length\_4529\_cov\_29.102673 3665-3673. Max. coverage (+): 0.07. Max coverage (-): 0.04

Region: NODE\_347534\_length\_4529\_cov\_29.102673 3674-3683. Max. coverage (+): 0.33. Max coverage (-): 0.04

Region: NODE\_347534\_length\_4529\_cov\_29.102673 3684-3692. Max. coverage (+): 0.44. Max coverage (-): 1.22

Region: NODE\_347534\_length\_4529\_cov\_29.102673 3693-3701. Max. coverage (+): 0.7. Max coverage (-): 1.22

Region: NODE\_347534\_length\_4529\_cov\_29.102673 3702-3711. Max. coverage (+): 10.2. Max coverage (-): 0.04

Region: NODE\_347534\_length\_4529\_cov\_29.102673 3712-3720. Max. coverage (+): 0.89. Max coverage (-): 0.15

Region: NODE\_347534\_length\_4529\_cov\_29.102673 3721-3729. Max. coverage (+): 1.74. Max coverage (-): 0.07

Region: NODE\_347534\_length\_4529\_cov\_29.102673 3730-3738. Max. coverage (+): 0.04. Max coverage (-): 0.07

Region: NODE\_347534\_length\_4529\_cov\_29.102673 3739-3748. Max. coverage (+): 0.44. Max coverage (-): 0.11

Region: NODE\_347534\_length\_4529\_cov\_29.102673 3749-3757. Max. coverage (+): 0.74. Max coverage (-): 0.04

Region: NODE\_347534\_length\_4529\_cov\_29.102673 3758-3766. Max. coverage (+): 0.07. Max coverage (-): 0

Region: NODE\_347534\_length\_4529\_cov\_29.102673 3767-3776. Max. coverage (+): 0.07. Max coverage (-): 0.04

Region: NODE\_347534\_length\_4529\_cov\_29.102673 3777-3785. Max. coverage (+): 1.19. Max coverage (-): 0.3

Region: NODE\_347534\_length\_4529\_cov\_29.102673 3786-3794. Max. coverage (+): 0.33. Max coverage (-): 0.22

Region: NODE\_347534\_length\_4529\_cov\_29.102673 3795-3804. Max. coverage (+): 2.67. Max coverage (-): 0.11

Region: NODE\_347534\_length\_4529\_cov\_29.102673 3805-3813. Max. coverage (+): 0. Max coverage (-): 0

Region: NODE\_347534\_length\_4529\_cov\_29.102673 3814-3822. Max. coverage (+): 0. Max coverage (-): 0.82

Region: NODE\_347534\_length\_4529\_cov\_29.102673 3823-3832. Max. coverage (+): 0.15. Max coverage (-): 0.83

Region: NODE\_347534\_length\_4529\_cov\_29.102673 3833-3841. Max. coverage (+): 0.74. Max coverage (-): 0.04

Region: NODE\_347534\_length\_4529\_cov\_29.102673 3842-3850. Max. coverage (+): 0.11. Max coverage (-): 0.04

Region: NODE\_347534\_length\_4529\_cov\_29.102673 3851-3859. Max. coverage (+): 1.33. Max coverage (-): 0

Region: NODE\_347534\_length\_4529\_cov\_29.102673 3860-3869. Max. coverage (+): 1.96. Max coverage (-): 0

Region: NODE\_347534\_length\_4529\_cov\_29.102673 3870-3878. Max. coverage (+): 0.3. Max coverage (-): 0.04

Region: NODE\_347534\_length\_4529\_cov\_29.102673 3879-3887. Max. coverage (+): 0.37. Max coverage (-): 0.15

Region: NODE\_347534\_length\_4529\_cov\_29.102673 3888-3897. Max. coverage (+): 1.15. Max coverage (-): 0.11

Region: NODE\_347534\_length\_4529\_cov\_29.102673 3898-3906. Max. coverage (+): 3.41. Max coverage (-): 0

Region: NODE\_347534\_length\_4529\_cov\_29.102673 3907-3915. Max. coverage (+): 2.48. Max coverage (-): 0.04

Region: NODE\_347534\_length\_4529\_cov\_29.102673 3916-3925. Max. coverage (+): 0.41. Max coverage (-): 0.07

Region: NODE\_347534\_length\_4529\_cov\_29.102673 3926-3934. Max. coverage (+): 0.02. Max coverage (-): 0

Region: NODE\_347534\_length\_4529\_cov\_29.102673 3935-3943. Max. coverage (+): 0. Max coverage (-): 0

Region: NODE\_347534\_length\_4529\_cov\_29.102673 3944-3953. Max. coverage (+): 0.07. Max coverage (-): 0

Region: NODE\_347534\_length\_4529\_cov\_29.102673 3954-3962. Max. coverage (+): 0.04. Max coverage (-): 0

Region: NODE\_347534\_length\_4529\_cov\_29.102673 3963-3971. Max. coverage (+): 0.04. Max coverage (-): 0.19

Region: NODE\_347534\_length\_4529\_cov\_29.102673 3972-3981. Max. coverage (+): 0.22. Max coverage (-): 0.19

Region: NODE\_347534\_length\_4529\_cov\_29.102673 3982-3990. Max. coverage (+): 0.02. Max coverage (-): 0.26

Region: NODE\_347534\_length\_4529\_cov\_29.102673 3991-3999. Max. coverage (+): 0.15. Max coverage (-): 0.22

Region: NODE\_347534\_length\_4529\_cov\_29.102673 4000-4008. Max. coverage (+): 0.19. Max coverage (-): 0.04

Region: NODE\_347534\_length\_4529\_cov\_29.102673 4009-4018. Max. coverage (+): 0.59. Max coverage (-): 0.15

Region: NODE\_347534\_length\_4529\_cov\_29.102673 4019-4027. Max. coverage (+): 0.15. Max coverage (-): 0.15

Region: NODE\_347534\_length\_4529\_cov\_29.102673 4028-4036. Max. coverage (+): 0.41. Max coverage (-): 0.15

Region: NODE\_347534\_length\_4529\_cov\_29.102673 4037-4046. Max. coverage (+): 0.41. Max coverage (-): 0.07

Region: NODE\_347534\_length\_4529\_cov\_29.102673 4047-4055. Max. coverage (+): 0.22. Max coverage (-): 0

Region: NODE\_347534\_length\_4529\_cov\_29.102673 4056-4064. Max. coverage (+): 0.04. Max coverage (-): 0.04

Region: NODE\_347534\_length\_4529\_cov\_29.102673 4065-4074. Max. coverage (+): 0.56. Max coverage (-): 0.04

Region: NODE\_347534\_length\_4529\_cov\_29.102673 4075-4083. Max. coverage (+): 0.26. Max coverage (-): 0.44

Region: NODE\_347534\_length\_4529\_cov\_29.102673 4084-4092. Max. coverage (+): 0.19. Max coverage (-): 0.11

Region: NODE\_347534\_length\_4529\_cov\_29.102673 4093-4102. Max. coverage (+): 0.33. Max coverage (-): 0.04

Region: NODE\_347534\_length\_4529\_cov\_29.102673 4103-4111. Max. coverage (+): 0.33. Max coverage (-): 0.04

Region: NODE\_347534\_length\_4529\_cov\_29.102673 4112-4120. Max. coverage (+): 0.07. Max coverage (-): 0.04

Region: NODE\_347534\_length\_4529\_cov\_29.102673 4121-4129. Max. coverage (+): 0.13. Max coverage (-): 0.04

Region: NODE\_347534\_length\_4529\_cov\_29.102673 4130-4139. Max. coverage (+): 0.03. Max coverage (-): 0

Region: NODE\_347534\_length\_4529\_cov\_29.102673 4140-4148. Max. coverage (+): 0.21. Max coverage (-): 0

Region: NODE\_347534\_length\_4529\_cov\_29.102673 4149-4157. Max. coverage (+): 0. Max coverage (-): 2.22

Region: NODE\_347534\_length\_4529\_cov\_29.102673 4158-4167. Max. coverage (+): 0. Max coverage (-): 1.53

Region: NODE\_347534\_length\_4529\_cov\_29.102673 4168-4176. Max. coverage (+): 0.42. Max coverage (-): 0

Region: NODE\_347534\_length\_4529\_cov\_29.102673 4177-4185. Max. coverage (+): 0.02. Max coverage (-): 0

Region: NODE\_347534\_length\_4529\_cov\_29.102673 4186-4195. Max. coverage (+): 0. Max coverage (-): 0

Region: NODE\_347534\_length\_4529\_cov\_29.102673 4196-4204. Max. coverage (+): 0. Max coverage (-): 0

Region: NODE\_347534\_length\_4529\_cov\_29.102673 4205-4213. Max. coverage (+): 0. Max coverage (-): 0

Region: NODE\_347534\_length\_4529\_cov\_29.102673 4214-4223. Max. coverage (+): 0. Max coverage (-): 0

Region: NODE\_347534\_length\_4529\_cov\_29.102673 4224-4232. Max. coverage (+): 0. Max coverage (-): 0

Region: NODE\_347534\_length\_4529\_cov\_29.102673 4233-4241. Max. coverage (+): 0. Max coverage (-): 0

Region: NODE\_347534\_length\_4529\_cov\_29.102673 4242-4251. Max. coverage (+): 0. Max coverage (-): 0

Region: NODE\_347534\_length\_4529\_cov\_29.102673 4252-4260. Max. coverage (+): 0.19. Max coverage (-): 0

Region: NODE\_347534\_length\_4529\_cov\_29.102673 4261-4269. Max. coverage (+): 0.37. Max coverage (-): 0

Region: NODE\_347534\_length\_4529\_cov\_29.102673 4270-4278. Max. coverage (+): 0.11. Max coverage (-): 0

Region: NODE\_347534\_length\_4529\_cov\_29.102673 4279-4288. Max. coverage (+): 0. Max coverage (-): 0

Region: NODE\_347534\_length\_4529\_cov\_29.102673 4289-4297. Max. coverage (+): 0. Max coverage (-): 0

Region: NODE\_347534\_length\_4529\_cov\_29.102673 4298-4306. Max. coverage (+): 0. Max coverage (-): 0.01

Region: NODE\_347534\_length\_4529\_cov\_29.102673 4307-4316. Max. coverage (+): 0. Max coverage (-): 0.01

Region: NODE\_347534\_length\_4529\_cov\_29.102673 4317-4325. Max. coverage (+): 0.16. Max coverage (-): 0

Region: NODE\_347534\_length\_4529\_cov\_29.102673 4326-4334. Max. coverage (+): 0.11. Max coverage (-): 0

Region: NODE\_347534\_length\_4529\_cov\_29.102673 4335-4344. Max. coverage (+): 0. Max coverage (-): 0

Region: NODE\_347534\_length\_4529\_cov\_29.102673 4345-4353. Max. coverage (+): 0. Max coverage (-): 0

Region: NODE\_347534\_length\_4529\_cov\_29.102673 4354-4362. Max. coverage (+): 0. Max coverage (-): 0

Region: NODE\_347534\_length\_4529\_cov\_29.102673 4363-4372. Max. coverage (+): 0.04. Max coverage (-): 0.01

Region: NODE\_347534\_length\_4529\_cov\_29.102673 4373-4381. Max. coverage (+): 0.05. Max coverage (-): 0.05

Region: NODE\_347534\_length\_4529\_cov\_29.102673 4382-4390. Max. coverage (+): 0. Max coverage (-): 0.03

Region: NODE\_347534\_length\_4529\_cov\_29.102673 4391-4399. Max. coverage (+): 0. Max coverage (-): 0

Region: NODE\_347534\_length\_4529\_cov\_29.102673 4400-4409. Max. coverage (+): 0. Max coverage (-): 0

Region: NODE\_347534\_length\_4529\_cov\_29.102673 4410-4418. Max. coverage (+): 0. Max coverage (-): 0.01

Region: NODE\_347534\_length\_4529\_cov\_29.102673 4419-4427. Max. coverage (+): 0. Max coverage (-): 0.02

Region: NODE\_347534\_length\_4529\_cov\_29.102673 4428-4437. Max. coverage (+): 0.03. Max coverage (-): 0.04

Region: NODE\_347534\_length\_4529\_cov\_29.102673 4438-4446. Max. coverage (+): 0. Max coverage (-): 0

Region: NODE\_347534\_length\_4529\_cov\_29.102673 4447-4455. Max. coverage (+): 0. Max coverage (-): 0

Region: NODE\_347534\_length\_4529\_cov\_29.102673 4456-4465. Max. coverage (+): 0. Max coverage (-): 0

Region: NODE\_347534\_length\_4529\_cov\_29.102673 4466-4474. Max. coverage (+): 0. Max coverage (-): 0

Region: NODE\_347534\_length\_4529\_cov\_29.102673 4475-4483. Max. coverage (+): 0. Max coverage (-): 0

Region: NODE\_347534\_length\_4529\_cov\_29.102673 4484-4493. Max. coverage (+): 0. Max coverage (-): 0

Region: NODE\_347534\_length\_4529\_cov\_29.102673 4494-4502. Max. coverage (+): 0. Max coverage (-): 0

Region: NODE\_347534\_length\_4529\_cov\_29.102673 4503-4511. Max. coverage (+): 0. Max coverage (-): 0

Region: NODE\_347534\_length\_4529\_cov\_29.102673 4512-4521. Max. coverage (+): 0. Max coverage (-): 0

Region: NODE\_347534\_length\_4529\_cov\_29.102673 4522-4530. Max. coverage (+): 0. Max coverage (-): 0

Region: NODE\_347534\_length\_4529\_cov\_29.102673 4531-4539. Max. coverage (+): 0. Max coverage (-): 0

Region: NODE\_347534\_length\_4529\_cov\_29.102673 4540-4548. Max. coverage (+): 0. Max coverage (-): 0

Region: NODE\_347534\_length\_4529\_cov\_29.102673 4549-4558. Max. coverage (+): 0. Max coverage (-): 0

Region: NODE\_347534\_length\_4529\_cov\_29.102673 4559-4567. Max. coverage (+): 0.04. Max coverage (-): 0

Region: NODE\_347534\_length\_4529\_cov\_29.102673 4568-4576. Max. coverage (+): 0.04. Max coverage (-): 0

Region: NODE\_347534\_length\_4529\_cov\_29.102673 4577-4586. Max. coverage (+): 0.02. Max coverage (-): 0

Region: NODE\_347534\_length\_4529\_cov\_29.102673 4587-4595. Max. coverage (+): 0. Max coverage (-): 0

Region: NODE\_347534\_length\_4529\_cov\_29.102673 4596-4604. Max. coverage (+): 0. Max coverage (-): 0

Region: NODE\_347534\_length\_4529\_cov\_29.102673 4605-4614. Max. coverage (+): 0. Max coverage (-): 0

Region: NODE\_347534\_length\_4529\_cov\_29.102673 4615-4623. Max. coverage (+): 0.01. Max coverage (-): 0.01

Region: NODE\_347534\_length\_4529\_cov\_29.102673 4624-4632. Max. coverage (+): 0. Max coverage (-): 0

Region: NODE\_347534\_length\_4529\_cov\_29.102673 4633-4642. Max. coverage (+): 0. Max coverage (-): 0

Region: NODE\_347534\_length\_4529\_cov\_29.102673 4643-4651. Max. coverage (+): 0. Max coverage (-): 0

Region: NODE\_347534\_length\_4529\_cov\_29.102673 4652-. Max. coverage (+): 0. Max coverage (-): 0

RepeatMasker Color Code

**+**

100-98% Identity

<98-95% Identity

<95-90% Identity

<90-85% Identity

<85-80% Identity

<80-75% Identity

<75-70% Identity

<70% Identity

**-**

Gene Set Color Code

**+**

Gene

Pseudogene

Other

**-**

Topology/Coverage Color Code

Coverage Plus Strand

Coverage Minus Strand

Mainstrand: Plus

Mainstrand: Minus

Complementary Strand

Flanking Region  
(if option -flank >0)

Gene Set Annotation  
  
RepeatMasker Annotation  

**1. hAT-14\_HM**: 20-217 (-), Divergence to consensus: 38.1%  
**2. AlRepC-733**: 140-236 (+), Divergence to consensus: 27.6%  
**3. DNA-8-18\_DR**: 527-675 (+), Divergence to consensus: 3.4%  
**4. AlRepC-1432**: 677-903 (-), Divergence to consensus: 20.3%  
**5. AlRepB-60**: 904-1002 (+), Divergence to consensus: 36.7%  
**6. AlRepB-59**: 1003-1078 (+), Divergence to consensus: 17.1%  
**7. AlRepB-2**: 1220-1406 (-), Divergence to consensus: 39.8%  
**8. AlRepC-532**: 1418-1486 (-), Divergence to consensus: 17.3%  
**9. A-rich**: 1546-1585 (+), Divergence to consensus: 23.3%  
**10. AlRepC-1020**: 2268-2331 (-), Divergence to consensus: 22%  
**11. AlRepB-420**: 3138-3478 (-), Divergence to consensus: 26.4%  
**12. AlRepD-880**: 3478-3619 (-), Divergence to consensus: 51.1%  
**13. AlRepB-11**: 3817-4106 (+), Divergence to consensus: 39.1%  
**14. AlRepC-1432**: 4126-4406 (+), Divergence to consensus: 12.6%  
**15. AlRepB-356**: 4409-4655 (+), Divergence to consensus: 9.5%

  
Transcription Factor Binding Sites  

**RHOXF1** (Sequence: GGATCA (-): 598)  
**RHOXF1** (Sequence: AGCTTA (-): 2003)  
**RHOXF1** (Sequence: AGCTCA (-): 2228)  
**RHOXF1** (Sequence: GGATTA (-): 3348)  
**RHOXF1** (Sequence: AGATTA (-): 4464)  
**RHOXF1** (Sequence: TAATCC (+): 1114)  
**RHOXF1** (Sequence: TAAGCT (+): 1792)  
**RHOXF1** (Sequence: TGATCC (+): 2626)  
**RHOXF1** (Sequence: TAATCT (+): 3183)  
**RHOXF1** (Sequence: TGATCC (+): 3975)  
**Gata4** (Sequence: CTTATCT (+): 434)  
**POU5F1** (Sequence: TTTGCAT (-): 2643)  
**POU5F1** (Sequence: TTTGCAT (-): 3013)  
**FOXO3\_hsa** (Sequence: GTAAACAA (+): 403)  
**FOXP1** (Sequence: GTAAACA (+): 403)  
**FOXO1** (Sequence: GTTGTTTTC (+): 4600)  
**FOXO3\_mmu** (Sequence: TGTTTACA (-): 2308)  
**FOXO3\_mmu** (Sequence: TGTTTTCA (-): 4547)  
**FOXO3\_mmu** (Sequence: TGTTTTCA (-): 4602)  
**Sox5** (Sequence: ATTGTT (+): 72)  
**Sox5** (Sequence: ATTGTT (+): 2570)  
**Sox5** (Sequence: ATTGTT (+): 2940)  
**FIGLA** (Sequence: AACAGCTGGT (-): 914)  
**FOXO3\_mmu** (Sequence: GGTAAACA (+): 402)  
**FOXO3\_mmu** (Sequence: TGAAAACA (+): 3768)  
**Nobox** (Sequence: GCCAATTA (-): 3644)  
**FOXP1** (Sequence: TGTTTAC (-): 2308)  
**POU2F1** (Sequence: ATTTAAATA (-): 4640)  
**Rhox11** (Sequence: TTTACAGCA (-): 3387)  
**POU2F1** (Sequence: TATTTAAAT (+): 343)  
**POU2F1** (Sequence: TATTCTAAT (+): 3517)  
**POU2F1** (Sequence: TATTTTAAT (+): 4505)  
**POU5F1** (Sequence: ATGCAAA (+): 1397)
