# Supplementary material for: First characterization of PIWI-interacting RNA clusters in a cichlid fish with a B chromosome
Source: BMC Biol. 2022 Sep 21;20:204. doi: 10.1186/s12915-022-01403-2 (PMC9490952; doi:10.1186/s12915-022-01403-2)
Supplement: Supplementary file 1 — Additional file 1. Zipped folder with fasta and interactive html piRNA cluster information for the A. latifasciata genome. The nomenclature is as follows: number-pirna-cluster_sex_B-presence (f, female; m, male; 0b, without B chromosome; 1b, with B chromosome). [file 12915_2022_1403_MOESM1_ESM.zip › 136_m1b.html]

piRNA cluster 136\_m1b 80


Predicted piRNA cluster no. 136\_m1b
  

Show proTRAC run info
Hide proTRAC run info

/\  
                \_\_\_\_\_\_\_\_\_\_\_\_\_\_\_\_\_\_\_\_\_\_\_/\\_\_\_ /  \\_\_\_\_\_\_\_  
               I                      /  \  /    \      I  
               I     pro             /    \/      \     I  
               I        TRAC        /               \   I  
               I   \_\_\_\_\_\_\_\_\_\_\_\_\_\_\_\_/\_\_\_\_\_\_\_\_\_\_\_\_\_\_\_\_\_\\_ I  
               I   \              /                     I  
               I    \            /                      I  
               I     \  /\      /       V.2.4.2         I  
               I      \/  \    /                        I  
               I\_\_\_\_\_\_\_\_\_\_\_\  /\_\_\_\_\_\_\_\_\_\_\_\_\_\_\_\_\_\_\_\_\_\_\_\_\_I  
                            \/  
  
  
================================= proTRAC ====================================  
VERSION: .......... 2.4.2  
LAST MODIFIED: .... 11. May 2018  
  
Please cite:  
Rosenkranz D, Zischler H. proTRAC - a software for probabilistic piRNA cluster  
detection, visualization and analysis. 2012. BMC Bioinformatics 13:5.  
  
  
Contact:  
David Rosenkranz  
Institute of Organismic and Molecular Evolutionary Biology  
Dept. Anthropology, small RNA group  
Johannes Gutenberg University Mainz  
email: rosenkranz@uni-mainz.de  
  
You can find the latest proTRAC version at:  
http://sourceforge.net/projects/protrac/files  
http://www.smallRNAgroup-mainz.de/software  
==============================================================================  
  
PARAMETERS:  
Map file: ...............piwi-machos-1B.fa-collapse.map  
Genome file: ............../../../0B\_ala\_genome.fa  
RepeatMasker annotation: Alatifasciata-all0B-maryan-v2.fa\_corrected.out  
GeneSet:................./guest-storage/Data/annotation/Alatifasciata\_all0B\_maryan-v2\_out2017.gff  
  
Significant (p<=0.01) hit density will be calculated based  
on observed hit distribution.  
  
Sliding window size: ........................................ 5000 bp  
Sliding window increament: .................................. 1000 bp  
Normalize each hit by number of genomic hits: ............... yes  
Normalize each hit by number of sequence reads: ............. yes  
Normalize values (-> per million mapped reads): ............. yes  
Min. fraction of hits with 1T(U) or 10A: .................... 0.75  
Alternatively: Min. fraction of hits with 1T(U) and 10A: .... 0.5  
Min. fraction of hits with typical piRNA length: ............ 0.75  
Typical piRNA length: ....................................... 24-32 nt  
Min. size of a piRNA cluster: ............................... 1000 bp.  
Min. number of hits (absolute): ............................. 0  
Min. number of hits (normalized): ........................... 0  
Min. fraction of hits on the mainstrand: .................... 0.75  
Top fraction of mapped sequences (in terms of read counts): . 1%  
Top fraction accounts for max. n% of sequence reads: ........ 90%  
Min. fraction of hits on each arm of a bidirectional cluster: 0.05  
Output html file for each cluster: .......................... yes  
Output a summary table: ..................................... yes  
Output a FASTA file for each cluster (piRNA sequences): ..... yes  
Output a FASTA file comprising cluster sequences: ........... yes  
Output a GTF file for predicted piRNA clusters: ..............yes  
Search DNA motifs in clusters: .............................. yes  
Output flanking sequences: +/- .............................. 0 bp  
Output ~.pTi file: .......................................... no  
==============================================================================  
  
  
Genome size (without gaps): ............ 758543724 bp  
Gaps (N/X/-): .......................... 417479 bp  
Mapped reads: .......................... 26973943  
Non-identical sequences: ............... 6209225  
Genomic hits: .......................... 48438990  
Significant densitiy of mapped reads: .. 821.144211136946 reads/kb

Show proTRAC cluster info
Hide proTRAC cluster info

|  |  |
| --- | --- |
| Location | NODE\_353178\_length\_4064\_cov\_15.830709 |
| Coordinates | 16-4125 |
| Size [bp] | 4110 |
| Sequence hit loci | 3375 |
| Mapped reads (normalized) | 7565.4 |
| Mapped reads (normalized) per kb | 1840.7 |
| Normalized reads with 1T (1U) | 74.9% |
| Normalized reads with 10A | 59.3% |
| Normalized reads with length 24-32 nt | 99.2% |
| Normalized reads on the main strand(s) | 93.8% |
| Predicted directionality | mono:minus |

100%

0%

1T (1U)  
reads

10A reads

24-32 nt  
reads

reads on mainstrand

**Either the amount of reads with 1T (1U) OR 10A has to exceed 75% (set with option: -1Tor10A)  
Alternatively the amount of reads with 1T (1U) AND 10A has to exceed 50% (set with option: -1Tand10A)  
Minimum amount of reads with preferred size is 75% (set with option: -pisize)  
Minimum amount of reads on the main strand(s) is 75% (set with option: -clstrand)**

Show read coverage
Hide read coverage

WHAT DO I SEE HERE?  
This chart shows the location of mapped sequence reads within a predicted piRNA cluster. The color refers to the number of genomic hits produced by the sequence read in question. A dark red bar indicates that this sequence read produces many other hits elsewhere in the genome. Many adjacent red or yellow bars can indicate the presence of a multi-copy element such as transposons or rRNA genes. A dark green bar indicates that this sequence read maps uniquely to this locus.

1 hit

2-5 hits

6-10 hits

11-20 hits

21-50 hits

51-100 hits

> 100 hits

NODE\_353178\_length\_4064\_cov\_15.830709

16

4125

Gene Set

RepeatMasker

Mapped  
Reads

38.63

plus strand

minus strand

38.63

Region: NODE\_353178\_length\_4064\_cov\_15.830709 4653-20. Max. coverage (+): 0.01. Max coverage (-): 0.01

Region: NODE\_353178\_length\_4064\_cov\_15.830709 21-28. Max. coverage (+): 0. Max coverage (-): 0.02

Region: NODE\_353178\_length\_4064\_cov\_15.830709 29-36. Max. coverage (+): 0. Max coverage (-): 0.69

Region: NODE\_353178\_length\_4064\_cov\_15.830709 37-44. Max. coverage (+): 0. Max coverage (-): 1.56

Region: NODE\_353178\_length\_4064\_cov\_15.830709 45-52. Max. coverage (+): 0. Max coverage (-): 0.44

Region: NODE\_353178\_length\_4064\_cov\_15.830709 53-61. Max. coverage (+): 0. Max coverage (-): 0.67

Region: NODE\_353178\_length\_4064\_cov\_15.830709 62-69. Max. coverage (+): 0. Max coverage (-): 0.04

Region: NODE\_353178\_length\_4064\_cov\_15.830709 70-77. Max. coverage (+): 0. Max coverage (-): 0

Region: NODE\_353178\_length\_4064\_cov\_15.830709 78-85. Max. coverage (+): 0. Max coverage (-): 0.15

Region: NODE\_353178\_length\_4064\_cov\_15.830709 86-94. Max. coverage (+): 0. Max coverage (-): 0.15

Region: NODE\_353178\_length\_4064\_cov\_15.830709 95-102. Max. coverage (+): 0.11. Max coverage (-): 0

Region: NODE\_353178\_length\_4064\_cov\_15.830709 103-110. Max. coverage (+): 0.07. Max coverage (-): 0.22

Region: NODE\_353178\_length\_4064\_cov\_15.830709 111-118. Max. coverage (+): 0.04. Max coverage (-): 0.04

Region: NODE\_353178\_length\_4064\_cov\_15.830709 119-126. Max. coverage (+): 0.04. Max coverage (-): 0.02

Region: NODE\_353178\_length\_4064\_cov\_15.830709 127-135. Max. coverage (+): 0. Max coverage (-): 0.02

Region: NODE\_353178\_length\_4064\_cov\_15.830709 136-143. Max. coverage (+): 0. Max coverage (-): 0

Region: NODE\_353178\_length\_4064\_cov\_15.830709 144-151. Max. coverage (+): 0.02. Max coverage (-): 0.07

Region: NODE\_353178\_length\_4064\_cov\_15.830709 152-159. Max. coverage (+): 0.04. Max coverage (-): 0.04

Region: NODE\_353178\_length\_4064\_cov\_15.830709 160-168. Max. coverage (+): 0. Max coverage (-): 1.22

Region: NODE\_353178\_length\_4064\_cov\_15.830709 169-176. Max. coverage (+): 0. Max coverage (-): 0.06

Region: NODE\_353178\_length\_4064\_cov\_15.830709 177-184. Max. coverage (+): 0. Max coverage (-): 0.04

Region: NODE\_353178\_length\_4064\_cov\_15.830709 185-192. Max. coverage (+): 0. Max coverage (-): 0.8

Region: NODE\_353178\_length\_4064\_cov\_15.830709 193-200. Max. coverage (+): 0. Max coverage (-): 0.82

Region: NODE\_353178\_length\_4064\_cov\_15.830709 201-209. Max. coverage (+): 0. Max coverage (-): 0.07

Region: NODE\_353178\_length\_4064\_cov\_15.830709 210-217. Max. coverage (+): 0.07. Max coverage (-): 0.04

Region: NODE\_353178\_length\_4064\_cov\_15.830709 218-225. Max. coverage (+): 0.04. Max coverage (-): 0.78

Region: NODE\_353178\_length\_4064\_cov\_15.830709 226-233. Max. coverage (+): 0.02. Max coverage (-): 1.06

Region: NODE\_353178\_length\_4064\_cov\_15.830709 234-242. Max. coverage (+): 0.02. Max coverage (-): 0.46

Region: NODE\_353178\_length\_4064\_cov\_15.830709 243-250. Max. coverage (+): 0. Max coverage (-): 0.13

Region: NODE\_353178\_length\_4064\_cov\_15.830709 251-258. Max. coverage (+): 0. Max coverage (-): 0.04

Region: NODE\_353178\_length\_4064\_cov\_15.830709 259-266. Max. coverage (+): 0. Max coverage (-): 0

Region: NODE\_353178\_length\_4064\_cov\_15.830709 267-274. Max. coverage (+): 0. Max coverage (-): 0.87

Region: NODE\_353178\_length\_4064\_cov\_15.830709 275-283. Max. coverage (+): 0. Max coverage (-): 0.87

Region: NODE\_353178\_length\_4064\_cov\_15.830709 284-291. Max. coverage (+): 0.02. Max coverage (-): 0.19

Region: NODE\_353178\_length\_4064\_cov\_15.830709 292-299. Max. coverage (+): 0.02. Max coverage (-): 0.17

Region: NODE\_353178\_length\_4064\_cov\_15.830709 300-307. Max. coverage (+): 0.06. Max coverage (-): 0.06

Region: NODE\_353178\_length\_4064\_cov\_15.830709 308-316. Max. coverage (+): 0. Max coverage (-): 0.15

Region: NODE\_353178\_length\_4064\_cov\_15.830709 317-324. Max. coverage (+): 0. Max coverage (-): 0

Region: NODE\_353178\_length\_4064\_cov\_15.830709 325-332. Max. coverage (+): 0. Max coverage (-): 0.11

Region: NODE\_353178\_length\_4064\_cov\_15.830709 333-340. Max. coverage (+): 0. Max coverage (-): 0.04

Region: NODE\_353178\_length\_4064\_cov\_15.830709 341-348. Max. coverage (+): 0. Max coverage (-): 0.26

Region: NODE\_353178\_length\_4064\_cov\_15.830709 349-357. Max. coverage (+): 0.02. Max coverage (-): 0.33

Region: NODE\_353178\_length\_4064\_cov\_15.830709 358-365. Max. coverage (+): 0. Max coverage (-): 0.04

Region: NODE\_353178\_length\_4064\_cov\_15.830709 366-373. Max. coverage (+): 0.17. Max coverage (-): 0.76

Region: NODE\_353178\_length\_4064\_cov\_15.830709 374-381. Max. coverage (+): 0.17. Max coverage (-): 0.11

Region: NODE\_353178\_length\_4064\_cov\_15.830709 382-390. Max. coverage (+): 0.02. Max coverage (-): 0

Region: NODE\_353178\_length\_4064\_cov\_15.830709 391-398. Max. coverage (+): 0. Max coverage (-): 0

Region: NODE\_353178\_length\_4064\_cov\_15.830709 399-406. Max. coverage (+): 0. Max coverage (-): 0

Region: NODE\_353178\_length\_4064\_cov\_15.830709 407-414. Max. coverage (+): 0. Max coverage (-): 0.02

Region: NODE\_353178\_length\_4064\_cov\_15.830709 415-422. Max. coverage (+): 0. Max coverage (-): 0.02

Region: NODE\_353178\_length\_4064\_cov\_15.830709 423-431. Max. coverage (+): 0. Max coverage (-): 0.26

Region: NODE\_353178\_length\_4064\_cov\_15.830709 432-439. Max. coverage (+): 0.02. Max coverage (-): 0.2

Region: NODE\_353178\_length\_4064\_cov\_15.830709 440-447. Max. coverage (+): 0.04. Max coverage (-): 0.04

Region: NODE\_353178\_length\_4064\_cov\_15.830709 448-455. Max. coverage (+): 0. Max coverage (-): 0.06

Region: NODE\_353178\_length\_4064\_cov\_15.830709 456-463. Max. coverage (+): 0. Max coverage (-): 0

Region: NODE\_353178\_length\_4064\_cov\_15.830709 464-472. Max. coverage (+): 0. Max coverage (-): 0.02

Region: NODE\_353178\_length\_4064\_cov\_15.830709 473-480. Max. coverage (+): 0.02. Max coverage (-): 0.02

Region: NODE\_353178\_length\_4064\_cov\_15.830709 481-488. Max. coverage (+): 0.02. Max coverage (-): 0

Region: NODE\_353178\_length\_4064\_cov\_15.830709 489-496. Max. coverage (+): 0. Max coverage (-): 0

Region: NODE\_353178\_length\_4064\_cov\_15.830709 497-505. Max. coverage (+): 0. Max coverage (-): 0.07

Region: NODE\_353178\_length\_4064\_cov\_15.830709 506-513. Max. coverage (+): 0. Max coverage (-): 0

Region: NODE\_353178\_length\_4064\_cov\_15.830709 514-521. Max. coverage (+): 0. Max coverage (-): 0.02

Region: NODE\_353178\_length\_4064\_cov\_15.830709 522-529. Max. coverage (+): 0. Max coverage (-): 0.07

Region: NODE\_353178\_length\_4064\_cov\_15.830709 530-537. Max. coverage (+): 0. Max coverage (-): 0.04

Region: NODE\_353178\_length\_4064\_cov\_15.830709 538-546. Max. coverage (+): 0.04. Max coverage (-): 0.44

Region: NODE\_353178\_length\_4064\_cov\_15.830709 547-554. Max. coverage (+): 0. Max coverage (-): 0.04

Region: NODE\_353178\_length\_4064\_cov\_15.830709 555-562. Max. coverage (+): 0. Max coverage (-): 0.06

Region: NODE\_353178\_length\_4064\_cov\_15.830709 563-570. Max. coverage (+): 0. Max coverage (-): 0

Region: NODE\_353178\_length\_4064\_cov\_15.830709 571-579. Max. coverage (+): 0. Max coverage (-): 0.87

Region: NODE\_353178\_length\_4064\_cov\_15.830709 580-587. Max. coverage (+): 0. Max coverage (-): 0.96

Region: NODE\_353178\_length\_4064\_cov\_15.830709 588-595. Max. coverage (+): 0. Max coverage (-): 0.04

Region: NODE\_353178\_length\_4064\_cov\_15.830709 596-603. Max. coverage (+): 0.04. Max coverage (-): 0.06

Region: NODE\_353178\_length\_4064\_cov\_15.830709 604-611. Max. coverage (+): 0. Max coverage (-): 0.46

Region: NODE\_353178\_length\_4064\_cov\_15.830709 612-620. Max. coverage (+): 0. Max coverage (-): 0.07

Region: NODE\_353178\_length\_4064\_cov\_15.830709 621-628. Max. coverage (+): 0. Max coverage (-): 0.02

Region: NODE\_353178\_length\_4064\_cov\_15.830709 629-636. Max. coverage (+): 0. Max coverage (-): 0.02

Region: NODE\_353178\_length\_4064\_cov\_15.830709 637-644. Max. coverage (+): 0.02. Max coverage (-): 0.13

Region: NODE\_353178\_length\_4064\_cov\_15.830709 645-653. Max. coverage (+): 0.02. Max coverage (-): 0.04

Region: NODE\_353178\_length\_4064\_cov\_15.830709 654-661. Max. coverage (+): 0. Max coverage (-): 0.09

Region: NODE\_353178\_length\_4064\_cov\_15.830709 662-669. Max. coverage (+): 0. Max coverage (-): 0

Region: NODE\_353178\_length\_4064\_cov\_15.830709 670-677. Max. coverage (+): 0.02. Max coverage (-): 0.15

Region: NODE\_353178\_length\_4064\_cov\_15.830709 678-685. Max. coverage (+): 0.02. Max coverage (-): 1.11

Region: NODE\_353178\_length\_4064\_cov\_15.830709 686-694. Max. coverage (+): 0. Max coverage (-): 0.59

Region: NODE\_353178\_length\_4064\_cov\_15.830709 695-702. Max. coverage (+): 0. Max coverage (-): 0.04

Region: NODE\_353178\_length\_4064\_cov\_15.830709 703-710. Max. coverage (+): 0. Max coverage (-): 0.02

Region: NODE\_353178\_length\_4064\_cov\_15.830709 711-718. Max. coverage (+): 0. Max coverage (-): 0.02

Region: NODE\_353178\_length\_4064\_cov\_15.830709 719-727. Max. coverage (+): 0. Max coverage (-): 0.17

Region: NODE\_353178\_length\_4064\_cov\_15.830709 728-735. Max. coverage (+): 0. Max coverage (-): 0.89

Region: NODE\_353178\_length\_4064\_cov\_15.830709 736-743. Max. coverage (+): 0. Max coverage (-): 0.17

Region: NODE\_353178\_length\_4064\_cov\_15.830709 744-751. Max. coverage (+): 0. Max coverage (-): 0.59

Region: NODE\_353178\_length\_4064\_cov\_15.830709 752-759. Max. coverage (+): 0. Max coverage (-): 0.11

Region: NODE\_353178\_length\_4064\_cov\_15.830709 760-768. Max. coverage (+): 0. Max coverage (-): 0.04

Region: NODE\_353178\_length\_4064\_cov\_15.830709 769-776. Max. coverage (+): 0.02. Max coverage (-): 0.13

Region: NODE\_353178\_length\_4064\_cov\_15.830709 777-784. Max. coverage (+): 0.02. Max coverage (-): 0.13

Region: NODE\_353178\_length\_4064\_cov\_15.830709 785-792. Max. coverage (+): 0.02. Max coverage (-): 0.24

Region: NODE\_353178\_length\_4064\_cov\_15.830709 793-801. Max. coverage (+): 0.02. Max coverage (-): 0.11

Region: NODE\_353178\_length\_4064\_cov\_15.830709 802-809. Max. coverage (+): 0.07. Max coverage (-): 0.3

Region: NODE\_353178\_length\_4064\_cov\_15.830709 810-817. Max. coverage (+): 0.06. Max coverage (-): 0.02

Region: NODE\_353178\_length\_4064\_cov\_15.830709 818-825. Max. coverage (+): 0.04. Max coverage (-): 0.04

Region: NODE\_353178\_length\_4064\_cov\_15.830709 826-833. Max. coverage (+): 0.04. Max coverage (-): 0.19

Region: NODE\_353178\_length\_4064\_cov\_15.830709 834-842. Max. coverage (+): 0.02. Max coverage (-): 0.09

Region: NODE\_353178\_length\_4064\_cov\_15.830709 843-850. Max. coverage (+): 0. Max coverage (-): 0.07

Region: NODE\_353178\_length\_4064\_cov\_15.830709 851-858. Max. coverage (+): 0. Max coverage (-): 0.02

Region: NODE\_353178\_length\_4064\_cov\_15.830709 859-866. Max. coverage (+): 0. Max coverage (-): 0

Region: NODE\_353178\_length\_4064\_cov\_15.830709 867-874. Max. coverage (+): 0.02. Max coverage (-): 0.48

Region: NODE\_353178\_length\_4064\_cov\_15.830709 875-883. Max. coverage (+): 0. Max coverage (-): 0.15

Region: NODE\_353178\_length\_4064\_cov\_15.830709 884-891. Max. coverage (+): 0.06. Max coverage (-): 0.06

Region: NODE\_353178\_length\_4064\_cov\_15.830709 892-899. Max. coverage (+): 0.13. Max coverage (-): 0.09

Region: NODE\_353178\_length\_4064\_cov\_15.830709 900-907. Max. coverage (+): 0.07. Max coverage (-): 0

Region: NODE\_353178\_length\_4064\_cov\_15.830709 908-916. Max. coverage (+): 0.04. Max coverage (-): 0.07

Region: NODE\_353178\_length\_4064\_cov\_15.830709 917-924. Max. coverage (+): 0. Max coverage (-): 0.11

Region: NODE\_353178\_length\_4064\_cov\_15.830709 925-932. Max. coverage (+): 0.02. Max coverage (-): 0.17

Region: NODE\_353178\_length\_4064\_cov\_15.830709 933-940. Max. coverage (+): 0. Max coverage (-): 0.07

Region: NODE\_353178\_length\_4064\_cov\_15.830709 941-948. Max. coverage (+): 0.17. Max coverage (-): 0

Region: NODE\_353178\_length\_4064\_cov\_15.830709 949-957. Max. coverage (+): 0. Max coverage (-): 0.33

Region: NODE\_353178\_length\_4064\_cov\_15.830709 958-965. Max. coverage (+): 0. Max coverage (-): 0.56

Region: NODE\_353178\_length\_4064\_cov\_15.830709 966-973. Max. coverage (+): 0. Max coverage (-): 0.19

Region: NODE\_353178\_length\_4064\_cov\_15.830709 974-981. Max. coverage (+): 0. Max coverage (-): 0.02

Region: NODE\_353178\_length\_4064\_cov\_15.830709 982-990. Max. coverage (+): 0.02. Max coverage (-): 0.04

Region: NODE\_353178\_length\_4064\_cov\_15.830709 991-998. Max. coverage (+): 0.07. Max coverage (-): 0.15

Region: NODE\_353178\_length\_4064\_cov\_15.830709 999-1006. Max. coverage (+): 0.04. Max coverage (-): 0.44

Region: NODE\_353178\_length\_4064\_cov\_15.830709 1007-1014. Max. coverage (+): 0. Max coverage (-): 5.89

Region: NODE\_353178\_length\_4064\_cov\_15.830709 1015-1022. Max. coverage (+): 0. Max coverage (-): 6.01

Region: NODE\_353178\_length\_4064\_cov\_15.830709 1023-1031. Max. coverage (+): 0.15. Max coverage (-): 0.04

Region: NODE\_353178\_length\_4064\_cov\_15.830709 1032-1039. Max. coverage (+): 0.15. Max coverage (-): 0

Region: NODE\_353178\_length\_4064\_cov\_15.830709 1040-1047. Max. coverage (+): 0. Max coverage (-): 0.11

Region: NODE\_353178\_length\_4064\_cov\_15.830709 1048-1055. Max. coverage (+): 0. Max coverage (-): 0.63

Region: NODE\_353178\_length\_4064\_cov\_15.830709 1056-1064. Max. coverage (+): 0. Max coverage (-): 0.02

Region: NODE\_353178\_length\_4064\_cov\_15.830709 1065-1072. Max. coverage (+): 0.02. Max coverage (-): 0.04

Region: NODE\_353178\_length\_4064\_cov\_15.830709 1073-1080. Max. coverage (+): 0. Max coverage (-): 0.26

Region: NODE\_353178\_length\_4064\_cov\_15.830709 1081-1088. Max. coverage (+): 0.04. Max coverage (-): 0.37

Region: NODE\_353178\_length\_4064\_cov\_15.830709 1089-1096. Max. coverage (+): 0. Max coverage (-): 0.07

Region: NODE\_353178\_length\_4064\_cov\_15.830709 1097-1105. Max. coverage (+): 0. Max coverage (-): 0

Region: NODE\_353178\_length\_4064\_cov\_15.830709 1106-1113. Max. coverage (+): 0. Max coverage (-): 0

Region: NODE\_353178\_length\_4064\_cov\_15.830709 1114-1121. Max. coverage (+): 0. Max coverage (-): 0

Region: NODE\_353178\_length\_4064\_cov\_15.830709 1122-1129. Max. coverage (+): 0. Max coverage (-): 0.04

Region: NODE\_353178\_length\_4064\_cov\_15.830709 1130-1138. Max. coverage (+): 0. Max coverage (-): 1.63

Region: NODE\_353178\_length\_4064\_cov\_15.830709 1139-1146. Max. coverage (+): 0.04. Max coverage (-): 0.07

Region: NODE\_353178\_length\_4064\_cov\_15.830709 1147-1154. Max. coverage (+): 0.07. Max coverage (-): 0.11

Region: NODE\_353178\_length\_4064\_cov\_15.830709 1155-1162. Max. coverage (+): 0.07. Max coverage (-): 0.19

Region: NODE\_353178\_length\_4064\_cov\_15.830709 1163-1170. Max. coverage (+): 0. Max coverage (-): 0.7

Region: NODE\_353178\_length\_4064\_cov\_15.830709 1171-1179. Max. coverage (+): 0.02. Max coverage (-): 0.41

Region: NODE\_353178\_length\_4064\_cov\_15.830709 1180-1187. Max. coverage (+): 0.07. Max coverage (-): 0

Region: NODE\_353178\_length\_4064\_cov\_15.830709 1188-1195. Max. coverage (+): 0.09. Max coverage (-): 0

Region: NODE\_353178\_length\_4064\_cov\_15.830709 1196-1203. Max. coverage (+): 0. Max coverage (-): 0.04

Region: NODE\_353178\_length\_4064\_cov\_15.830709 1204-1212. Max. coverage (+): 0. Max coverage (-): 1.22

Region: NODE\_353178\_length\_4064\_cov\_15.830709 1213-1220. Max. coverage (+): 0. Max coverage (-): 0.19

Region: NODE\_353178\_length\_4064\_cov\_15.830709 1221-1228. Max. coverage (+): 0. Max coverage (-): 0

Region: NODE\_353178\_length\_4064\_cov\_15.830709 1229-1236. Max. coverage (+): 0. Max coverage (-): 0

Region: NODE\_353178\_length\_4064\_cov\_15.830709 1237-1244. Max. coverage (+): 0. Max coverage (-): 1.59

Region: NODE\_353178\_length\_4064\_cov\_15.830709 1245-1253. Max. coverage (+): 0. Max coverage (-): 0.07

Region: NODE\_353178\_length\_4064\_cov\_15.830709 1254-1261. Max. coverage (+): 0.04. Max coverage (-): 0

Region: NODE\_353178\_length\_4064\_cov\_15.830709 1262-1269. Max. coverage (+): 0. Max coverage (-): 0

Region: NODE\_353178\_length\_4064\_cov\_15.830709 1270-1277. Max. coverage (+): 0. Max coverage (-): 0.04

Region: NODE\_353178\_length\_4064\_cov\_15.830709 1278-1285. Max. coverage (+): 0.04. Max coverage (-): 0.11

Region: NODE\_353178\_length\_4064\_cov\_15.830709 1286-1294. Max. coverage (+): 0.07. Max coverage (-): 0.07

Region: NODE\_353178\_length\_4064\_cov\_15.830709 1295-1302. Max. coverage (+): 0.13. Max coverage (-): 0.11

Region: NODE\_353178\_length\_4064\_cov\_15.830709 1303-1310. Max. coverage (+): 0.02. Max coverage (-): 0.22

Region: NODE\_353178\_length\_4064\_cov\_15.830709 1311-1318. Max. coverage (+): 0. Max coverage (-): 0.06

Region: NODE\_353178\_length\_4064\_cov\_15.830709 1319-1327. Max. coverage (+): 0. Max coverage (-): 0

Region: NODE\_353178\_length\_4064\_cov\_15.830709 1328-1335. Max. coverage (+): 0. Max coverage (-): 0

Region: NODE\_353178\_length\_4064\_cov\_15.830709 1336-1343. Max. coverage (+): 0. Max coverage (-): 0

Region: NODE\_353178\_length\_4064\_cov\_15.830709 1344-1351. Max. coverage (+): 0.06. Max coverage (-): 1.08

Region: NODE\_353178\_length\_4064\_cov\_15.830709 1352-1359. Max. coverage (+): 0.06. Max coverage (-): 0.7

Region: NODE\_353178\_length\_4064\_cov\_15.830709 1360-1368. Max. coverage (+): 0. Max coverage (-): 0.06

Region: NODE\_353178\_length\_4064\_cov\_15.830709 1369-1376. Max. coverage (+): 0. Max coverage (-): 0

Region: NODE\_353178\_length\_4064\_cov\_15.830709 1377-1384. Max. coverage (+): 0. Max coverage (-): 1.52

Region: NODE\_353178\_length\_4064\_cov\_15.830709 1385-1392. Max. coverage (+): 0. Max coverage (-): 0.07

Region: NODE\_353178\_length\_4064\_cov\_15.830709 1393-1401. Max. coverage (+): 0. Max coverage (-): 0.15

Region: NODE\_353178\_length\_4064\_cov\_15.830709 1402-1409. Max. coverage (+): 0.09. Max coverage (-): 0.26

Region: NODE\_353178\_length\_4064\_cov\_15.830709 1410-1417. Max. coverage (+): 0.24. Max coverage (-): 0.5

Region: NODE\_353178\_length\_4064\_cov\_15.830709 1418-1425. Max. coverage (+): 0.17. Max coverage (-): 0.35

Region: NODE\_353178\_length\_4064\_cov\_15.830709 1426-1433. Max. coverage (+): 0.07. Max coverage (-): 0.04

Region: NODE\_353178\_length\_4064\_cov\_15.830709 1434-1442. Max. coverage (+): 0.01. Max coverage (-): 0.52

Region: NODE\_353178\_length\_4064\_cov\_15.830709 1443-1450. Max. coverage (+): 0.06. Max coverage (-): 0.47

Region: NODE\_353178\_length\_4064\_cov\_15.830709 1451-1458. Max. coverage (+): 0.05. Max coverage (-): 0.22

Region: NODE\_353178\_length\_4064\_cov\_15.830709 1459-1466. Max. coverage (+): 0. Max coverage (-): 0.11

Region: NODE\_353178\_length\_4064\_cov\_15.830709 1467-1475. Max. coverage (+): 0.04. Max coverage (-): 0.09

Region: NODE\_353178\_length\_4064\_cov\_15.830709 1476-1483. Max. coverage (+): 0. Max coverage (-): 0.09

Region: NODE\_353178\_length\_4064\_cov\_15.830709 1484-1491. Max. coverage (+): 0.01. Max coverage (-): 0.11

Region: NODE\_353178\_length\_4064\_cov\_15.830709 1492-1499. Max. coverage (+): 0. Max coverage (-): 0.14

Region: NODE\_353178\_length\_4064\_cov\_15.830709 1500-1507. Max. coverage (+): 0. Max coverage (-): 0.61

Region: NODE\_353178\_length\_4064\_cov\_15.830709 1508-1516. Max. coverage (+): 0. Max coverage (-): 0.62

Region: NODE\_353178\_length\_4064\_cov\_15.830709 1517-1524. Max. coverage (+): 0.07. Max coverage (-): 0.15

Region: NODE\_353178\_length\_4064\_cov\_15.830709 1525-1532. Max. coverage (+): 0.04. Max coverage (-): 0.23

Region: NODE\_353178\_length\_4064\_cov\_15.830709 1533-1540. Max. coverage (+): 0.02. Max coverage (-): 0.47

Region: NODE\_353178\_length\_4064\_cov\_15.830709 1541-1549. Max. coverage (+): 0.01. Max coverage (-): 0.06

Region: NODE\_353178\_length\_4064\_cov\_15.830709 1550-1557. Max. coverage (+): 0. Max coverage (-): 1.08

Region: NODE\_353178\_length\_4064\_cov\_15.830709 1558-1565. Max. coverage (+): 0. Max coverage (-): 0.59

Region: NODE\_353178\_length\_4064\_cov\_15.830709 1566-1573. Max. coverage (+): 0.04. Max coverage (-): 0.25

Region: NODE\_353178\_length\_4064\_cov\_15.830709 1574-1581. Max. coverage (+): 0.09. Max coverage (-): 0.2

Region: NODE\_353178\_length\_4064\_cov\_15.830709 1582-1590. Max. coverage (+): 0.1. Max coverage (-): 0.43

Region: NODE\_353178\_length\_4064\_cov\_15.830709 1591-1598. Max. coverage (+): 0. Max coverage (-): 0.01

Region: NODE\_353178\_length\_4064\_cov\_15.830709 1599-1606. Max. coverage (+): 0.04. Max coverage (-): 0.04

Region: NODE\_353178\_length\_4064\_cov\_15.830709 1607-1614. Max. coverage (+): 0. Max coverage (-): 0

Region: NODE\_353178\_length\_4064\_cov\_15.830709 1615-1623. Max. coverage (+): 0. Max coverage (-): 0

Region: NODE\_353178\_length\_4064\_cov\_15.830709 1624-1631. Max. coverage (+): 0. Max coverage (-): 0.15

Region: NODE\_353178\_length\_4064\_cov\_15.830709 1632-1639. Max. coverage (+): 0. Max coverage (-): 0.12

Region: NODE\_353178\_length\_4064\_cov\_15.830709 1640-1647. Max. coverage (+): 0.02. Max coverage (-): 0

Region: NODE\_353178\_length\_4064\_cov\_15.830709 1648-1655. Max. coverage (+): 0.04. Max coverage (-): 0

Region: NODE\_353178\_length\_4064\_cov\_15.830709 1656-1664. Max. coverage (+): 0. Max coverage (-): 0

Region: NODE\_353178\_length\_4064\_cov\_15.830709 1665-1672. Max. coverage (+): 0. Max coverage (-): 0

Region: NODE\_353178\_length\_4064\_cov\_15.830709 1673-1680. Max. coverage (+): 0. Max coverage (-): 0

Region: NODE\_353178\_length\_4064\_cov\_15.830709 1681-1688. Max. coverage (+): 0. Max coverage (-): 0

Region: NODE\_353178\_length\_4064\_cov\_15.830709 1689-1696. Max. coverage (+): 0. Max coverage (-): 2.11

Region: NODE\_353178\_length\_4064\_cov\_15.830709 1697-1705. Max. coverage (+): 0. Max coverage (-): 0.13

Region: NODE\_353178\_length\_4064\_cov\_15.830709 1706-1713. Max. coverage (+): 0. Max coverage (-): 0.06

Region: NODE\_353178\_length\_4064\_cov\_15.830709 1714-1721. Max. coverage (+): 0. Max coverage (-): 0.87

Region: NODE\_353178\_length\_4064\_cov\_15.830709 1722-1729. Max. coverage (+): 0. Max coverage (-): 0.22

Region: NODE\_353178\_length\_4064\_cov\_15.830709 1730-1738. Max. coverage (+): 0.11. Max coverage (-): 0.15

Region: NODE\_353178\_length\_4064\_cov\_15.830709 1739-1746. Max. coverage (+): 0.11. Max coverage (-): 0.07

Region: NODE\_353178\_length\_4064\_cov\_15.830709 1747-1754. Max. coverage (+): 0.11. Max coverage (-): 0.07

Region: NODE\_353178\_length\_4064\_cov\_15.830709 1755-1762. Max. coverage (+): 0.11. Max coverage (-): 0.04

Region: NODE\_353178\_length\_4064\_cov\_15.830709 1763-1770. Max. coverage (+): 0. Max coverage (-): 0.48

Region: NODE\_353178\_length\_4064\_cov\_15.830709 1771-1779. Max. coverage (+): 0.02. Max coverage (-): 0.44

Region: NODE\_353178\_length\_4064\_cov\_15.830709 1780-1787. Max. coverage (+): 0. Max coverage (-): 0.11

Region: NODE\_353178\_length\_4064\_cov\_15.830709 1788-1795. Max. coverage (+): 0. Max coverage (-): 0.04

Region: NODE\_353178\_length\_4064\_cov\_15.830709 1796-1803. Max. coverage (+): 0.01. Max coverage (-): 0

Region: NODE\_353178\_length\_4064\_cov\_15.830709 1804-1812. Max. coverage (+): 0.37. Max coverage (-): 0.04

Region: NODE\_353178\_length\_4064\_cov\_15.830709 1813-1820. Max. coverage (+): 0.07. Max coverage (-): 0.56

Region: NODE\_353178\_length\_4064\_cov\_15.830709 1821-1828. Max. coverage (+): 0. Max coverage (-): 2.37

Region: NODE\_353178\_length\_4064\_cov\_15.830709 1829-1836. Max. coverage (+): 0. Max coverage (-): 1.66

Region: NODE\_353178\_length\_4064\_cov\_15.830709 1837-1844. Max. coverage (+): 0. Max coverage (-): 0

Region: NODE\_353178\_length\_4064\_cov\_15.830709 1845-1853. Max. coverage (+): 0. Max coverage (-): 0.07

Region: NODE\_353178\_length\_4064\_cov\_15.830709 1854-1861. Max. coverage (+): 0. Max coverage (-): 1.04

Region: NODE\_353178\_length\_4064\_cov\_15.830709 1862-1869. Max. coverage (+): 0.01. Max coverage (-): 0.31

Region: NODE\_353178\_length\_4064\_cov\_15.830709 1870-1877. Max. coverage (+): 0.04. Max coverage (-): 0.3

Region: NODE\_353178\_length\_4064\_cov\_15.830709 1878-1886. Max. coverage (+): 0.05. Max coverage (-): 0.73

Region: NODE\_353178\_length\_4064\_cov\_15.830709 1887-1894. Max. coverage (+): 0.05. Max coverage (-): 0.83

Region: NODE\_353178\_length\_4064\_cov\_15.830709 1895-1902. Max. coverage (+): 0. Max coverage (-): 0.56

Region: NODE\_353178\_length\_4064\_cov\_15.830709 1903-1910. Max. coverage (+): 0.02. Max coverage (-): 0.04

Region: NODE\_353178\_length\_4064\_cov\_15.830709 1911-1918. Max. coverage (+): 0.02. Max coverage (-): 0.19

Region: NODE\_353178\_length\_4064\_cov\_15.830709 1919-1927. Max. coverage (+): 0.02. Max coverage (-): 0.15

Region: NODE\_353178\_length\_4064\_cov\_15.830709 1928-1935. Max. coverage (+): 0.04. Max coverage (-): 0.04

Region: NODE\_353178\_length\_4064\_cov\_15.830709 1936-1943. Max. coverage (+): 0.07. Max coverage (-): 0.04

Region: NODE\_353178\_length\_4064\_cov\_15.830709 1944-1951. Max. coverage (+): 0.08. Max coverage (-): 0.04

Region: NODE\_353178\_length\_4064\_cov\_15.830709 1952-1960. Max. coverage (+): 0.04. Max coverage (-): 0.09

Region: NODE\_353178\_length\_4064\_cov\_15.830709 1961-1968. Max. coverage (+): 0.01. Max coverage (-): 0.32

Region: NODE\_353178\_length\_4064\_cov\_15.830709 1969-1976. Max. coverage (+): 0.01. Max coverage (-): 0.21

Region: NODE\_353178\_length\_4064\_cov\_15.830709 1977-1984. Max. coverage (+): 0.18. Max coverage (-): 0.11

Region: NODE\_353178\_length\_4064\_cov\_15.830709 1985-1992. Max. coverage (+): 0.39. Max coverage (-): 0.07

Region: NODE\_353178\_length\_4064\_cov\_15.830709 1993-2001. Max. coverage (+): 0.04. Max coverage (-): 2.78

Region: NODE\_353178\_length\_4064\_cov\_15.830709 2002-2009. Max. coverage (+): 0.11. Max coverage (-): 2.89

Region: NODE\_353178\_length\_4064\_cov\_15.830709 2010-2017. Max. coverage (+): 0.19. Max coverage (-): 0.11

Region: NODE\_353178\_length\_4064\_cov\_15.830709 2018-2025. Max. coverage (+): 0.15. Max coverage (-): 35.2

Region: NODE\_353178\_length\_4064\_cov\_15.830709 2026-2034. Max. coverage (+): 0.07. Max coverage (-): 0.59

Region: NODE\_353178\_length\_4064\_cov\_15.830709 2035-2042. Max. coverage (+): 0.19. Max coverage (-): 0.36

Region: NODE\_353178\_length\_4064\_cov\_15.830709 2043-2050. Max. coverage (+): 0.19. Max coverage (-): 0.3

Region: NODE\_353178\_length\_4064\_cov\_15.830709 2051-2058. Max. coverage (+): 0.04. Max coverage (-): 0.3

Region: NODE\_353178\_length\_4064\_cov\_15.830709 2059-2066. Max. coverage (+): 0.07. Max coverage (-): 0.26

Region: NODE\_353178\_length\_4064\_cov\_15.830709 2067-2075. Max. coverage (+): 0.28. Max coverage (-): 0.37

Region: NODE\_353178\_length\_4064\_cov\_15.830709 2076-2083. Max. coverage (+): 0.11. Max coverage (-): 0.68

Region: NODE\_353178\_length\_4064\_cov\_15.830709 2084-2091. Max. coverage (+): 0.02. Max coverage (-): 0.04

Region: NODE\_353178\_length\_4064\_cov\_15.830709 2092-2099. Max. coverage (+): 0. Max coverage (-): 0.02

Region: NODE\_353178\_length\_4064\_cov\_15.830709 2100-2107. Max. coverage (+): 0. Max coverage (-): 0.14

Region: NODE\_353178\_length\_4064\_cov\_15.830709 2108-2116. Max. coverage (+): 0.14. Max coverage (-): 0.05

Region: NODE\_353178\_length\_4064\_cov\_15.830709 2117-2124. Max. coverage (+): 0.08. Max coverage (-): 1.66

Region: NODE\_353178\_length\_4064\_cov\_15.830709 2125-2132. Max. coverage (+): 0. Max coverage (-): 1.62

Region: NODE\_353178\_length\_4064\_cov\_15.830709 2133-2140. Max. coverage (+): 0.01. Max coverage (-): 0.04

Region: NODE\_353178\_length\_4064\_cov\_15.830709 2141-2149. Max. coverage (+): 0.01. Max coverage (-): 0.04

Region: NODE\_353178\_length\_4064\_cov\_15.830709 2150-2157. Max. coverage (+): 0.01. Max coverage (-): 1.84

Region: NODE\_353178\_length\_4064\_cov\_15.830709 2158-2165. Max. coverage (+): 0.01. Max coverage (-): 0.7

Region: NODE\_353178\_length\_4064\_cov\_15.830709 2166-2173. Max. coverage (+): 0.01. Max coverage (-): 0.07

Region: NODE\_353178\_length\_4064\_cov\_15.830709 2174-2181. Max. coverage (+): 0.05. Max coverage (-): 0.14

Region: NODE\_353178\_length\_4064\_cov\_15.830709 2182-2190. Max. coverage (+): 0. Max coverage (-): 0.14

Region: NODE\_353178\_length\_4064\_cov\_15.830709 2191-2198. Max. coverage (+): 0. Max coverage (-): 0.46

Region: NODE\_353178\_length\_4064\_cov\_15.830709 2199-2206. Max. coverage (+): 0.01. Max coverage (-): 0.14

Region: NODE\_353178\_length\_4064\_cov\_15.830709 2207-2214. Max. coverage (+): 0.02. Max coverage (-): 0.07

Region: NODE\_353178\_length\_4064\_cov\_15.830709 2215-2223. Max. coverage (+): 0.07. Max coverage (-): 0.11

Region: NODE\_353178\_length\_4064\_cov\_15.830709 2224-2231. Max. coverage (+): 0.07. Max coverage (-): 1.08

Region: NODE\_353178\_length\_4064\_cov\_15.830709 2232-2239. Max. coverage (+): 0.01. Max coverage (-): 0.11

Region: NODE\_353178\_length\_4064\_cov\_15.830709 2240-2247. Max. coverage (+): 0.01. Max coverage (-): 0.02

Region: NODE\_353178\_length\_4064\_cov\_15.830709 2248-2255. Max. coverage (+): 0.01. Max coverage (-): 0.28

Region: NODE\_353178\_length\_4064\_cov\_15.830709 2256-2264. Max. coverage (+): 0.01. Max coverage (-): 1.88

Region: NODE\_353178\_length\_4064\_cov\_15.830709 2265-2272. Max. coverage (+): 0.05. Max coverage (-): 10.36

Region: NODE\_353178\_length\_4064\_cov\_15.830709 2273-2280. Max. coverage (+): 0.02. Max coverage (-): 0.31

Region: NODE\_353178\_length\_4064\_cov\_15.830709 2281-2288. Max. coverage (+): 0. Max coverage (-): 3.51

Region: NODE\_353178\_length\_4064\_cov\_15.830709 2289-2297. Max. coverage (+): 0. Max coverage (-): 3.63

Region: NODE\_353178\_length\_4064\_cov\_15.830709 2298-2305. Max. coverage (+): 0.01. Max coverage (-): 0.22

Region: NODE\_353178\_length\_4064\_cov\_15.830709 2306-2313. Max. coverage (+): 0.05. Max coverage (-): 1.63

Region: NODE\_353178\_length\_4064\_cov\_15.830709 2314-2321. Max. coverage (+): 0.63. Max coverage (-): 1.52

Region: NODE\_353178\_length\_4064\_cov\_15.830709 2322-2329. Max. coverage (+): 0.67. Max coverage (-): 0

Region: NODE\_353178\_length\_4064\_cov\_15.830709 2330-2338. Max. coverage (+): 0. Max coverage (-): 0.43

Region: NODE\_353178\_length\_4064\_cov\_15.830709 2339-2346. Max. coverage (+): 0. Max coverage (-): 0.41

Region: NODE\_353178\_length\_4064\_cov\_15.830709 2347-2354. Max. coverage (+): 0.78. Max coverage (-): 0.22

Region: NODE\_353178\_length\_4064\_cov\_15.830709 2355-2362. Max. coverage (+): 0.67. Max coverage (-): 0.04

Region: NODE\_353178\_length\_4064\_cov\_15.830709 2363-2371. Max. coverage (+): 0.33. Max coverage (-): 0.04

Region: NODE\_353178\_length\_4064\_cov\_15.830709 2372-2379. Max. coverage (+): 0. Max coverage (-): 3.43

Region: NODE\_353178\_length\_4064\_cov\_15.830709 2380-2387. Max. coverage (+): 0.04. Max coverage (-): 2.76

Region: NODE\_353178\_length\_4064\_cov\_15.830709 2388-2395. Max. coverage (+): 0.19. Max coverage (-): 1.33

Region: NODE\_353178\_length\_4064\_cov\_15.830709 2396-2403. Max. coverage (+): 0.07. Max coverage (-): 2.45

Region: NODE\_353178\_length\_4064\_cov\_15.830709 2404-2412. Max. coverage (+): 0.04. Max coverage (-): 3.52

Region: NODE\_353178\_length\_4064\_cov\_15.830709 2413-2420. Max. coverage (+): 0. Max coverage (-): 0.07

Region: NODE\_353178\_length\_4064\_cov\_15.830709 2421-2428. Max. coverage (+): 0.15. Max coverage (-): 38.63

Region: NODE\_353178\_length\_4064\_cov\_15.830709 2429-2436. Max. coverage (+): 0. Max coverage (-): 13.31

Region: NODE\_353178\_length\_4064\_cov\_15.830709 2437-2445. Max. coverage (+): 0.07. Max coverage (-): 2.67

Region: NODE\_353178\_length\_4064\_cov\_15.830709 2446-2453. Max. coverage (+): 0.07. Max coverage (-): 0.11

Region: NODE\_353178\_length\_4064\_cov\_15.830709 2454-2461. Max. coverage (+): 0. Max coverage (-): 0.52

Region: NODE\_353178\_length\_4064\_cov\_15.830709 2462-2469. Max. coverage (+): 0. Max coverage (-): 0.33

Region: NODE\_353178\_length\_4064\_cov\_15.830709 2470-2477. Max. coverage (+): 0. Max coverage (-): 0.04

Region: NODE\_353178\_length\_4064\_cov\_15.830709 2478-2486. Max. coverage (+): 0.07. Max coverage (-): 1.15

Region: NODE\_353178\_length\_4064\_cov\_15.830709 2487-2494. Max. coverage (+): 0.07. Max coverage (-): 1.37

Region: NODE\_353178\_length\_4064\_cov\_15.830709 2495-2502. Max. coverage (+): 0.04. Max coverage (-): 2.19

Region: NODE\_353178\_length\_4064\_cov\_15.830709 2503-2510. Max. coverage (+): 0. Max coverage (-): 0.3

Region: NODE\_353178\_length\_4064\_cov\_15.830709 2511-2518. Max. coverage (+): 0.67. Max coverage (-): 0.22

Region: NODE\_353178\_length\_4064\_cov\_15.830709 2519-2527. Max. coverage (+): 0.3. Max coverage (-): 0.15

Region: NODE\_353178\_length\_4064\_cov\_15.830709 2528-2535. Max. coverage (+): 0.15. Max coverage (-): 0.74

Region: NODE\_353178\_length\_4064\_cov\_15.830709 2536-2543. Max. coverage (+): 0.07. Max coverage (-): 0.3

Region: NODE\_353178\_length\_4064\_cov\_15.830709 2544-2551. Max. coverage (+): 0.27. Max coverage (-): 0.17

Region: NODE\_353178\_length\_4064\_cov\_15.830709 2552-2560. Max. coverage (+): 0.02. Max coverage (-): 0.41

Region: NODE\_353178\_length\_4064\_cov\_15.830709 2561-2568. Max. coverage (+): 0.01. Max coverage (-): 0.33

Region: NODE\_353178\_length\_4064\_cov\_15.830709 2569-2576. Max. coverage (+): 0. Max coverage (-): 0

Region: NODE\_353178\_length\_4064\_cov\_15.830709 2577-2584. Max. coverage (+): 0.01. Max coverage (-): 0.32

Region: NODE\_353178\_length\_4064\_cov\_15.830709 2585-2592. Max. coverage (+): 0. Max coverage (-): 0.38

Region: NODE\_353178\_length\_4064\_cov\_15.830709 2593-2601. Max. coverage (+): 0.25. Max coverage (-): 0.11

Region: NODE\_353178\_length\_4064\_cov\_15.830709 2602-2609. Max. coverage (+): 0.3. Max coverage (-): 0.06

Region: NODE\_353178\_length\_4064\_cov\_15.830709 2610-2617. Max. coverage (+): 0.02. Max coverage (-): 0.04

Region: NODE\_353178\_length\_4064\_cov\_15.830709 2618-2625. Max. coverage (+): 0. Max coverage (-): 0.27

Region: NODE\_353178\_length\_4064\_cov\_15.830709 2626-2634. Max. coverage (+): 0.01. Max coverage (-): 0.22

Region: NODE\_353178\_length\_4064\_cov\_15.830709 2635-2642. Max. coverage (+): 0. Max coverage (-): 0.01

Region: NODE\_353178\_length\_4064\_cov\_15.830709 2643-2650. Max. coverage (+): 0. Max coverage (-): 0.01

Region: NODE\_353178\_length\_4064\_cov\_15.830709 2651-2658. Max. coverage (+): 0. Max coverage (-): 0.04

Region: NODE\_353178\_length\_4064\_cov\_15.830709 2659-2666. Max. coverage (+): 0. Max coverage (-): 0

Region: NODE\_353178\_length\_4064\_cov\_15.830709 2667-2675. Max. coverage (+): 0. Max coverage (-): 0.02

Region: NODE\_353178\_length\_4064\_cov\_15.830709 2676-2683. Max. coverage (+): 0. Max coverage (-): 0.02

Region: NODE\_353178\_length\_4064\_cov\_15.830709 2684-2691. Max. coverage (+): 0. Max coverage (-): 2.48

Region: NODE\_353178\_length\_4064\_cov\_15.830709 2692-2699. Max. coverage (+): 0. Max coverage (-): 2.45

Region: NODE\_353178\_length\_4064\_cov\_15.830709 2700-2708. Max. coverage (+): 0. Max coverage (-): 0

Region: NODE\_353178\_length\_4064\_cov\_15.830709 2709-2716. Max. coverage (+): 0. Max coverage (-): 0

Region: NODE\_353178\_length\_4064\_cov\_15.830709 2717-2724. Max. coverage (+): 0. Max coverage (-): 0.04

Region: NODE\_353178\_length\_4064\_cov\_15.830709 2725-2732. Max. coverage (+): 0.01. Max coverage (-): 0.09

Region: NODE\_353178\_length\_4064\_cov\_15.830709 2733-2740. Max. coverage (+): 0. Max coverage (-): 1.68

Region: NODE\_353178\_length\_4064\_cov\_15.830709 2741-2749. Max. coverage (+): 0.01. Max coverage (-): 1.68

Region: NODE\_353178\_length\_4064\_cov\_15.830709 2750-2757. Max. coverage (+): 0.01. Max coverage (-): 0.47

Region: NODE\_353178\_length\_4064\_cov\_15.830709 2758-2765. Max. coverage (+): 0. Max coverage (-): 0.02

Region: NODE\_353178\_length\_4064\_cov\_15.830709 2766-2773. Max. coverage (+): 0.38. Max coverage (-): 0.01

Region: NODE\_353178\_length\_4064\_cov\_15.830709 2774-2782. Max. coverage (+): 0.01. Max coverage (-): 0.17

Region: NODE\_353178\_length\_4064\_cov\_15.830709 2783-2790. Max. coverage (+): 0. Max coverage (-): 0.31

Region: NODE\_353178\_length\_4064\_cov\_15.830709 2791-2798. Max. coverage (+): 0.01. Max coverage (-): 0.21

Region: NODE\_353178\_length\_4064\_cov\_15.830709 2799-2806. Max. coverage (+): 0.05. Max coverage (-): 0

Region: NODE\_353178\_length\_4064\_cov\_15.830709 2807-2814. Max. coverage (+): 0.02. Max coverage (-): 0

Region: NODE\_353178\_length\_4064\_cov\_15.830709 2815-2823. Max. coverage (+): 0. Max coverage (-): 1.15

Region: NODE\_353178\_length\_4064\_cov\_15.830709 2824-2831. Max. coverage (+): 0. Max coverage (-): 1.26

Region: NODE\_353178\_length\_4064\_cov\_15.830709 2832-2839. Max. coverage (+): 0.07. Max coverage (-): 0

Region: NODE\_353178\_length\_4064\_cov\_15.830709 2840-2847. Max. coverage (+): 0.26. Max coverage (-): 1.15

Region: NODE\_353178\_length\_4064\_cov\_15.830709 2848-2856. Max. coverage (+): 0.04. Max coverage (-): 0.74

Region: NODE\_353178\_length\_4064\_cov\_15.830709 2857-2864. Max. coverage (+): 0. Max coverage (-): 0.54

Region: NODE\_353178\_length\_4064\_cov\_15.830709 2865-2872. Max. coverage (+): 0. Max coverage (-): 0

Region: NODE\_353178\_length\_4064\_cov\_15.830709 2873-2880. Max. coverage (+): 0. Max coverage (-): 0

Region: NODE\_353178\_length\_4064\_cov\_15.830709 2881-2888. Max. coverage (+): 0. Max coverage (-): 0

Region: NODE\_353178\_length\_4064\_cov\_15.830709 2889-2897. Max. coverage (+): 0. Max coverage (-): 0.04

Region: NODE\_353178\_length\_4064\_cov\_15.830709 2898-2905. Max. coverage (+): 0.02. Max coverage (-): 0.01

Region: NODE\_353178\_length\_4064\_cov\_15.830709 2906-2913. Max. coverage (+): 0.04. Max coverage (-): 0.07

Region: NODE\_353178\_length\_4064\_cov\_15.830709 2914-2921. Max. coverage (+): 0.01. Max coverage (-): 0.11

Region: NODE\_353178\_length\_4064\_cov\_15.830709 2922-2929. Max. coverage (+): 0. Max coverage (-): 0.01

Region: NODE\_353178\_length\_4064\_cov\_15.830709 2930-2938. Max. coverage (+): 0. Max coverage (-): 0

Region: NODE\_353178\_length\_4064\_cov\_15.830709 2939-2946. Max. coverage (+): 0. Max coverage (-): 1.17

Region: NODE\_353178\_length\_4064\_cov\_15.830709 2947-2954. Max. coverage (+): 0. Max coverage (-): 0.78

Region: NODE\_353178\_length\_4064\_cov\_15.830709 2955-2962. Max. coverage (+): 0.02. Max coverage (-): 0.05

Region: NODE\_353178\_length\_4064\_cov\_15.830709 2963-2971. Max. coverage (+): 0.04. Max coverage (-): 0.01

Region: NODE\_353178\_length\_4064\_cov\_15.830709 2972-2979. Max. coverage (+): 0. Max coverage (-): 0.09

Region: NODE\_353178\_length\_4064\_cov\_15.830709 2980-2987. Max. coverage (+): 0. Max coverage (-): 0.06

Region: NODE\_353178\_length\_4064\_cov\_15.830709 2988-2995. Max. coverage (+): 0. Max coverage (-): 0

Region: NODE\_353178\_length\_4064\_cov\_15.830709 2996-3003. Max. coverage (+): 0. Max coverage (-): 0

Region: NODE\_353178\_length\_4064\_cov\_15.830709 3004-3012. Max. coverage (+): 0. Max coverage (-): 0

Region: NODE\_353178\_length\_4064\_cov\_15.830709 3013-3020. Max. coverage (+): 0. Max coverage (-): 0.05

Region: NODE\_353178\_length\_4064\_cov\_15.830709 3021-3028. Max. coverage (+): 0.01. Max coverage (-): 0.14

Region: NODE\_353178\_length\_4064\_cov\_15.830709 3029-3036. Max. coverage (+): 0.01. Max coverage (-): 0.16

Region: NODE\_353178\_length\_4064\_cov\_15.830709 3037-3045. Max. coverage (+): 0.02. Max coverage (-): 0.94

Region: NODE\_353178\_length\_4064\_cov\_15.830709 3046-3053. Max. coverage (+): 0.02. Max coverage (-): 0.05

Region: NODE\_353178\_length\_4064\_cov\_15.830709 3054-3061. Max. coverage (+): 0. Max coverage (-): 0.05

Region: NODE\_353178\_length\_4064\_cov\_15.830709 3062-3069. Max. coverage (+): 0. Max coverage (-): 0.07

Region: NODE\_353178\_length\_4064\_cov\_15.830709 3070-3077. Max. coverage (+): 0.02. Max coverage (-): 0.17

Region: NODE\_353178\_length\_4064\_cov\_15.830709 3078-3086. Max. coverage (+): 0. Max coverage (-): 0.09

Region: NODE\_353178\_length\_4064\_cov\_15.830709 3087-3094. Max. coverage (+): 0.01. Max coverage (-): 0.06

Region: NODE\_353178\_length\_4064\_cov\_15.830709 3095-3102. Max. coverage (+): 0. Max coverage (-): 0.01

Region: NODE\_353178\_length\_4064\_cov\_15.830709 3103-3110. Max. coverage (+): 0. Max coverage (-): 0

Region: NODE\_353178\_length\_4064\_cov\_15.830709 3111-3119. Max. coverage (+): 0. Max coverage (-): 0

Region: NODE\_353178\_length\_4064\_cov\_15.830709 3120-3127. Max. coverage (+): 0. Max coverage (-): 0

Region: NODE\_353178\_length\_4064\_cov\_15.830709 3128-3135. Max. coverage (+): 0. Max coverage (-): 0.26

Region: NODE\_353178\_length\_4064\_cov\_15.830709 3136-3143. Max. coverage (+): 0.02. Max coverage (-): 0.37

Region: NODE\_353178\_length\_4064\_cov\_15.830709 3144-3151. Max. coverage (+): 0.07. Max coverage (-): 0.02

Region: NODE\_353178\_length\_4064\_cov\_15.830709 3152-3160. Max. coverage (+): 0.05. Max coverage (-): 0.23

Region: NODE\_353178\_length\_4064\_cov\_15.830709 3161-3168. Max. coverage (+): 0.06. Max coverage (-): 0.05

Region: NODE\_353178\_length\_4064\_cov\_15.830709 3169-3176. Max. coverage (+): 0.22. Max coverage (-): 0.25

Region: NODE\_353178\_length\_4064\_cov\_15.830709 3177-3184. Max. coverage (+): 0. Max coverage (-): 0.19

Region: NODE\_353178\_length\_4064\_cov\_15.830709 3185-3193. Max. coverage (+): 0. Max coverage (-): 0.77

Region: NODE\_353178\_length\_4064\_cov\_15.830709 3194-3201. Max. coverage (+): 0. Max coverage (-): 0.62

Region: NODE\_353178\_length\_4064\_cov\_15.830709 3202-3209. Max. coverage (+): 0. Max coverage (-): 0.01

Region: NODE\_353178\_length\_4064\_cov\_15.830709 3210-3217. Max. coverage (+): 0. Max coverage (-): 0

Region: NODE\_353178\_length\_4064\_cov\_15.830709 3218-3225. Max. coverage (+): 0. Max coverage (-): 0

Region: NODE\_353178\_length\_4064\_cov\_15.830709 3226-3234. Max. coverage (+): 0. Max coverage (-): 0.07

Region: NODE\_353178\_length\_4064\_cov\_15.830709 3235-3242. Max. coverage (+): 0.52. Max coverage (-): 0.48

Region: NODE\_353178\_length\_4064\_cov\_15.830709 3243-3250. Max. coverage (+): 0.48. Max coverage (-): 0.41

Region: NODE\_353178\_length\_4064\_cov\_15.830709 3251-3258. Max. coverage (+): 0.19. Max coverage (-): 0

Region: NODE\_353178\_length\_4064\_cov\_15.830709 3259-3267. Max. coverage (+): 0.04. Max coverage (-): 0.01

Region: NODE\_353178\_length\_4064\_cov\_15.830709 3268-3275. Max. coverage (+): 0.04. Max coverage (-): 0.11

Region: NODE\_353178\_length\_4064\_cov\_15.830709 3276-3283. Max. coverage (+): 0.01. Max coverage (-): 0.09

Region: NODE\_353178\_length\_4064\_cov\_15.830709 3284-3291. Max. coverage (+): 0.02. Max coverage (-): 0.1

Region: NODE\_353178\_length\_4064\_cov\_15.830709 3292-3299. Max. coverage (+): 0.02. Max coverage (-): 0.06

Region: NODE\_353178\_length\_4064\_cov\_15.830709 3300-3308. Max. coverage (+): 0. Max coverage (-): 0.23

Region: NODE\_353178\_length\_4064\_cov\_15.830709 3309-3316. Max. coverage (+): 0.01. Max coverage (-): 0.26

Region: NODE\_353178\_length\_4064\_cov\_15.830709 3317-3324. Max. coverage (+): 0.01. Max coverage (-): 0.44

Region: NODE\_353178\_length\_4064\_cov\_15.830709 3325-3332. Max. coverage (+): 0.14. Max coverage (-): 0.31

Region: NODE\_353178\_length\_4064\_cov\_15.830709 3333-3340. Max. coverage (+): 0. Max coverage (-): 0.1

Region: NODE\_353178\_length\_4064\_cov\_15.830709 3341-3349. Max. coverage (+): 0. Max coverage (-): 0.31

Region: NODE\_353178\_length\_4064\_cov\_15.830709 3350-3357. Max. coverage (+): 0. Max coverage (-): 0.37

Region: NODE\_353178\_length\_4064\_cov\_15.830709 3358-3365. Max. coverage (+): 0.01. Max coverage (-): 0.06

Region: NODE\_353178\_length\_4064\_cov\_15.830709 3366-3373. Max. coverage (+): 0.01. Max coverage (-): 0.06

Region: NODE\_353178\_length\_4064\_cov\_15.830709 3374-3382. Max. coverage (+): 0. Max coverage (-): 0.09

Region: NODE\_353178\_length\_4064\_cov\_15.830709 3383-3390. Max. coverage (+): 0. Max coverage (-): 0.05

Region: NODE\_353178\_length\_4064\_cov\_15.830709 3391-3398. Max. coverage (+): 0.01. Max coverage (-): 0.11

Region: NODE\_353178\_length\_4064\_cov\_15.830709 3399-3406. Max. coverage (+): 0.01. Max coverage (-): 0.25

Region: NODE\_353178\_length\_4064\_cov\_15.830709 3407-3414. Max. coverage (+): 0.01. Max coverage (-): 0.19

Region: NODE\_353178\_length\_4064\_cov\_15.830709 3415-3423. Max. coverage (+): 0.01. Max coverage (-): 0.17

Region: NODE\_353178\_length\_4064\_cov\_15.830709 3424-3431. Max. coverage (+): 0. Max coverage (-): 0.21

Region: NODE\_353178\_length\_4064\_cov\_15.830709 3432-3439. Max. coverage (+): 0. Max coverage (-): 0.01

Region: NODE\_353178\_length\_4064\_cov\_15.830709 3440-3447. Max. coverage (+): 0. Max coverage (-): 0.23

Region: NODE\_353178\_length\_4064\_cov\_15.830709 3448-3456. Max. coverage (+): 0.01. Max coverage (-): 0.82

Region: NODE\_353178\_length\_4064\_cov\_15.830709 3457-3464. Max. coverage (+): 0.04. Max coverage (-): 0.33

Region: NODE\_353178\_length\_4064\_cov\_15.830709 3465-3472. Max. coverage (+): 0.05. Max coverage (-): 0.05

Region: NODE\_353178\_length\_4064\_cov\_15.830709 3473-3480. Max. coverage (+): 0. Max coverage (-): 0.02

Region: NODE\_353178\_length\_4064\_cov\_15.830709 3481-3488. Max. coverage (+): 0. Max coverage (-): 0.01

Region: NODE\_353178\_length\_4064\_cov\_15.830709 3489-3497. Max. coverage (+): 0. Max coverage (-): 0.2

Region: NODE\_353178\_length\_4064\_cov\_15.830709 3498-3505. Max. coverage (+): 0. Max coverage (-): 0.12

Region: NODE\_353178\_length\_4064\_cov\_15.830709 3506-3513. Max. coverage (+): 0. Max coverage (-): 0.04

Region: NODE\_353178\_length\_4064\_cov\_15.830709 3514-3521. Max. coverage (+): 0. Max coverage (-): 0.19

Region: NODE\_353178\_length\_4064\_cov\_15.830709 3522-3530. Max. coverage (+): 0.15. Max coverage (-): 0.44

Region: NODE\_353178\_length\_4064\_cov\_15.830709 3531-3538. Max. coverage (+): 0.11. Max coverage (-): 0.26

Region: NODE\_353178\_length\_4064\_cov\_15.830709 3539-3546. Max. coverage (+): 0.04. Max coverage (-): 2.97

Region: NODE\_353178\_length\_4064\_cov\_15.830709 3547-3554. Max. coverage (+): 0.04. Max coverage (-): 0.57

Region: NODE\_353178\_length\_4064\_cov\_15.830709 3555-3562. Max. coverage (+): 0.04. Max coverage (-): 0.19

Region: NODE\_353178\_length\_4064\_cov\_15.830709 3563-3571. Max. coverage (+): 0. Max coverage (-): 0.33

Region: NODE\_353178\_length\_4064\_cov\_15.830709 3572-3579. Max. coverage (+): 0. Max coverage (-): 0.22

Region: NODE\_353178\_length\_4064\_cov\_15.830709 3580-3587. Max. coverage (+): 0. Max coverage (-): 0.33

Region: NODE\_353178\_length\_4064\_cov\_15.830709 3588-3595. Max. coverage (+): 0.02. Max coverage (-): 0.54

Region: NODE\_353178\_length\_4064\_cov\_15.830709 3596-3604. Max. coverage (+): 0.02. Max coverage (-): 0.39

Region: NODE\_353178\_length\_4064\_cov\_15.830709 3605-3612. Max. coverage (+): 0. Max coverage (-): 0

Region: NODE\_353178\_length\_4064\_cov\_15.830709 3613-3620. Max. coverage (+): 0. Max coverage (-): 0.04

Region: NODE\_353178\_length\_4064\_cov\_15.830709 3621-3628. Max. coverage (+): 0. Max coverage (-): 0.48

Region: NODE\_353178\_length\_4064\_cov\_15.830709 3629-3636. Max. coverage (+): 0. Max coverage (-): 0.22

Region: NODE\_353178\_length\_4064\_cov\_15.830709 3637-3645. Max. coverage (+): 0.02. Max coverage (-): 0.26

Region: NODE\_353178\_length\_4064\_cov\_15.830709 3646-3653. Max. coverage (+): 0.04. Max coverage (-): 0.17

Region: NODE\_353178\_length\_4064\_cov\_15.830709 3654-3661. Max. coverage (+): 0.02. Max coverage (-): 0.44

Region: NODE\_353178\_length\_4064\_cov\_15.830709 3662-3669. Max. coverage (+): 0.04. Max coverage (-): 0.11

Region: NODE\_353178\_length\_4064\_cov\_15.830709 3670-3678. Max. coverage (+): 0.04. Max coverage (-): 0.07

Region: NODE\_353178\_length\_4064\_cov\_15.830709 3679-3686. Max. coverage (+): 0.02. Max coverage (-): 0.04

Region: NODE\_353178\_length\_4064\_cov\_15.830709 3687-3694. Max. coverage (+): 0.04. Max coverage (-): 0.07

Region: NODE\_353178\_length\_4064\_cov\_15.830709 3695-3702. Max. coverage (+): 0. Max coverage (-): 0.7

Region: NODE\_353178\_length\_4064\_cov\_15.830709 3703-3710. Max. coverage (+): 0. Max coverage (-): 1

Region: NODE\_353178\_length\_4064\_cov\_15.830709 3711-3719. Max. coverage (+): 0. Max coverage (-): 0.15

Region: NODE\_353178\_length\_4064\_cov\_15.830709 3720-3727. Max. coverage (+): 0.02. Max coverage (-): 0

Region: NODE\_353178\_length\_4064\_cov\_15.830709 3728-3735. Max. coverage (+): 0.04. Max coverage (-): 0.02

Region: NODE\_353178\_length\_4064\_cov\_15.830709 3736-3743. Max. coverage (+): 0.02. Max coverage (-): 0.85

Region: NODE\_353178\_length\_4064\_cov\_15.830709 3744-3751. Max. coverage (+): 0.04. Max coverage (-): 0.95

Region: NODE\_353178\_length\_4064\_cov\_15.830709 3752-3760. Max. coverage (+): 0.11. Max coverage (-): 0.15

Region: NODE\_353178\_length\_4064\_cov\_15.830709 3761-3768. Max. coverage (+): 0.19. Max coverage (-): 0.15

Region: NODE\_353178\_length\_4064\_cov\_15.830709 3769-3776. Max. coverage (+): 0.04. Max coverage (-): 0

Region: NODE\_353178\_length\_4064\_cov\_15.830709 3777-3784. Max. coverage (+): 0. Max coverage (-): 0

Region: NODE\_353178\_length\_4064\_cov\_15.830709 3785-3793. Max. coverage (+): 0. Max coverage (-): 0.41

Region: NODE\_353178\_length\_4064\_cov\_15.830709 3794-3801. Max. coverage (+): 0.04. Max coverage (-): 0.33

Region: NODE\_353178\_length\_4064\_cov\_15.830709 3802-3809. Max. coverage (+): 0. Max coverage (-): 0.04

Region: NODE\_353178\_length\_4064\_cov\_15.830709 3810-3817. Max. coverage (+): 0.04. Max coverage (-): 0.01

Region: NODE\_353178\_length\_4064\_cov\_15.830709 3818-3825. Max. coverage (+): 0. Max coverage (-): 0.01

Region: NODE\_353178\_length\_4064\_cov\_15.830709 3826-3834. Max. coverage (+): 0.02. Max coverage (-): 1.11

Region: NODE\_353178\_length\_4064\_cov\_15.830709 3835-3842. Max. coverage (+): 0.12. Max coverage (-): 0.42

Region: NODE\_353178\_length\_4064\_cov\_15.830709 3843-3850. Max. coverage (+): 0.02. Max coverage (-): 0.51

Region: NODE\_353178\_length\_4064\_cov\_15.830709 3851-3858. Max. coverage (+): 0. Max coverage (-): 0.01

Region: NODE\_353178\_length\_4064\_cov\_15.830709 3859-3867. Max. coverage (+): 0. Max coverage (-): 0.01

Region: NODE\_353178\_length\_4064\_cov\_15.830709 3868-3875. Max. coverage (+): 0. Max coverage (-): 0.27

Region: NODE\_353178\_length\_4064\_cov\_15.830709 3876-3883. Max. coverage (+): 0. Max coverage (-): 0.15

Region: NODE\_353178\_length\_4064\_cov\_15.830709 3884-3891. Max. coverage (+): 0. Max coverage (-): 0.06

Region: NODE\_353178\_length\_4064\_cov\_15.830709 3892-3899. Max. coverage (+): 0. Max coverage (-): 0.14

Region: NODE\_353178\_length\_4064\_cov\_15.830709 3900-3908. Max. coverage (+): 0. Max coverage (-): 0.58

Region: NODE\_353178\_length\_4064\_cov\_15.830709 3909-3916. Max. coverage (+): 0.01. Max coverage (-): 0.59

Region: NODE\_353178\_length\_4064\_cov\_15.830709 3917-3924. Max. coverage (+): 0.54. Max coverage (-): 1.57

Region: NODE\_353178\_length\_4064\_cov\_15.830709 3925-3932. Max. coverage (+): 0.56. Max coverage (-): 0.09

Region: NODE\_353178\_length\_4064\_cov\_15.830709 3933-3941. Max. coverage (+): 0.02. Max coverage (-): 0.14

Region: NODE\_353178\_length\_4064\_cov\_15.830709 3942-3949. Max. coverage (+): 0.01. Max coverage (-): 0.04

Region: NODE\_353178\_length\_4064\_cov\_15.830709 3950-3957. Max. coverage (+): 0.01. Max coverage (-): 0.11

Region: NODE\_353178\_length\_4064\_cov\_15.830709 3958-3965. Max. coverage (+): 0.05. Max coverage (-): 0.07

Region: NODE\_353178\_length\_4064\_cov\_15.830709 3966-3973. Max. coverage (+): 0.06. Max coverage (-): 0.02

Region: NODE\_353178\_length\_4064\_cov\_15.830709 3974-3982. Max. coverage (+): 0.04. Max coverage (-): 0.02

Region: NODE\_353178\_length\_4064\_cov\_15.830709 3983-3990. Max. coverage (+): 0. Max coverage (-): 0

Region: NODE\_353178\_length\_4064\_cov\_15.830709 3991-3998. Max. coverage (+): 0. Max coverage (-): 0.15

Region: NODE\_353178\_length\_4064\_cov\_15.830709 3999-4006. Max. coverage (+): 0.04. Max coverage (-): 0.33

Region: NODE\_353178\_length\_4064\_cov\_15.830709 4007-4015. Max. coverage (+): 0.07. Max coverage (-): 1.33

Region: NODE\_353178\_length\_4064\_cov\_15.830709 4016-4023. Max. coverage (+): 0.07. Max coverage (-): 1.37

Region: NODE\_353178\_length\_4064\_cov\_15.830709 4024-4031. Max. coverage (+): 0. Max coverage (-): 0.74

Region: NODE\_353178\_length\_4064\_cov\_15.830709 4032-4039. Max. coverage (+): 0.07. Max coverage (-): 0.04

Region: NODE\_353178\_length\_4064\_cov\_15.830709 4040-4047. Max. coverage (+): 0. Max coverage (-): 0.04

Region: NODE\_353178\_length\_4064\_cov\_15.830709 4048-4056. Max. coverage (+): 0. Max coverage (-): 0.33

Region: NODE\_353178\_length\_4064\_cov\_15.830709 4057-4064. Max. coverage (+): 0. Max coverage (-): 0.4

Region: NODE\_353178\_length\_4064\_cov\_15.830709 4065-4072. Max. coverage (+): 0. Max coverage (-): 0.01

Region: NODE\_353178\_length\_4064\_cov\_15.830709 4073-4080. Max. coverage (+): 0.01. Max coverage (-): 0.01

Region: NODE\_353178\_length\_4064\_cov\_15.830709 4081-4089. Max. coverage (+): 0. Max coverage (-): 0.12

Region: NODE\_353178\_length\_4064\_cov\_15.830709 4090-4097. Max. coverage (+): 0. Max coverage (-): 0.17

Region: NODE\_353178\_length\_4064\_cov\_15.830709 4098-4105. Max. coverage (+): 0. Max coverage (-): 0.27

Region: NODE\_353178\_length\_4064\_cov\_15.830709 4106-4113. Max. coverage (+): 0. Max coverage (-): 0

Region: NODE\_353178\_length\_4064\_cov\_15.830709 4114-4121. Max. coverage (+): 0. Max coverage (-): 0

Region: NODE\_353178\_length\_4064\_cov\_15.830709 4122-. Max. coverage (+): 0. Max coverage (-): 0

RepeatMasker Color Code

**+**

100-98% Identity

<98-95% Identity

<95-90% Identity

<90-85% Identity

<85-80% Identity

<80-75% Identity

<75-70% Identity

<70% Identity

**-**

Gene Set Color Code

**+**

Gene

Pseudogene

Other

**-**

Topology/Coverage Color Code

Coverage Plus Strand

Coverage Minus Strand

Mainstrand: Plus

Mainstrand: Minus

Complementary Strand

Flanking Region  
(if option -flank >0)

Gene Set Annotation  
  
RepeatMasker Annotation  

**1. (TAACTA)n**: 319-357 (+), Divergence to consensus: 20.5%  
**2. (TTTAT)n**: 1097-1132 (+), Divergence to consensus: 17.6%  
**3. (TATT)n**: 1265-1286 (+), Divergence to consensus: 9.8%  
**4. (AGCCAT)n**: 1322-1355 (+), Divergence to consensus: 20.3%  
**5. AlRepD-5449**: 1433-1527 (-), Divergence to consensus: 28.5%  
**6. AlRepD-5953**: 1590-1658 (-), Divergence to consensus: 20.4%  
**7. AlRepD-2347**: 1829-2606 (-), Divergence to consensus: 42.2%  
**8. Rex1\_FurC**: 2682-2763 (-), Divergence to consensus: 25.6%

  
Transcription Factor Binding Sites  

**RHOXF1** (Sequence: AGATCA (-): 452)  
**RHOXF1** (Sequence: AGATTA (-): 514)  
**RHOXF1** (Sequence: AGATTA (-): 1054)  
**RHOXF1** (Sequence: AGCTTA (-): 1246)  
**RHOXF1** (Sequence: GGCTCA (-): 1583)  
**RHOXF1** (Sequence: GGATCA (-): 2242)  
**RHOXF1** (Sequence: AGATTA (-): 2356)  
**RHOXF1** (Sequence: GGATCA (-): 2539)  
**RHOXF1** (Sequence: AGCTTA (-): 2896)  
**RHOXF1** (Sequence: AGATTA (-): 3741)  
**RHOXF1** (Sequence: TAAGCT (+): 1194)  
**RHOXF1** (Sequence: TAATCC (+): 2170)  
**RHOXF1** (Sequence: TAAGCT (+): 2941)  
**RHOXF1** (Sequence: TGAGCT (+): 3260)  
**RHOXF1** (Sequence: TAAGCC (+): 3662)  
**RHOXF1** (Sequence: TGAGCT (+): 3830)  
**Gata4** (Sequence: GTTATCT (+): 3004)  
**Gata4** (Sequence: CTTATCT (+): 3885)  
**POU5F1** (Sequence: TTTGCAT (-): 1627)  
**POU5F1** (Sequence: TTTGCAT (-): 3225)  
**Sox5** (Sequence: ATTGTT (+): 1189)  
**Sox5** (Sequence: ATTGTT (+): 3185)  
**FIGLA** (Sequence: AACAGCTGGT (-): 1467)  
**FIGLA** (Sequence: AACACCTGTA (-): 2491)  
**FOXO3\_mmu** (Sequence: GGAAAACA (+): 1179)  
**FOXO3\_mmu** (Sequence: GCAAAACA (+): 1594)  
**FOXO1** (Sequence: GAAAACAAG (-): 1180)  
**Nobox** (Sequence: TAATTGCT (+): 551)  
**Sox5** (Sequence: AACAAT (-): 1606)  
**Sox5** (Sequence: AACAAT (-): 3234)  
**POU2F1** (Sequence: TATTTAAAT (+): 996)
