# Supplementary material for: First characterization of PIWI-interacting RNA clusters in a cichlid fish with a B chromosome
Source: BMC Biol. 2022 Sep 21;20:204. doi: 10.1186/s12915-022-01403-2 (PMC9490952; doi:10.1186/s12915-022-01403-2)
Supplement: Supplementary file 1 — Additional file 1. Zipped folder with fasta and interactive html piRNA cluster information for the A. latifasciata genome. The nomenclature is as follows: number-pirna-cluster_sex_B-presence (f, female; m, male; 0b, without B chromosome; 1b, with B chromosome). [file 12915_2022_1403_MOESM1_ESM.zip › 137_m0b.html]

piRNA cluster 137\_m0b 65


Predicted piRNA cluster no. 137\_m0b
  

Show proTRAC run info
Hide proTRAC run info

/\  
                \_\_\_\_\_\_\_\_\_\_\_\_\_\_\_\_\_\_\_\_\_\_\_/\\_\_\_ /  \\_\_\_\_\_\_\_  
               I                      /  \  /    \      I  
               I     pro             /    \/      \     I  
               I        TRAC        /               \   I  
               I   \_\_\_\_\_\_\_\_\_\_\_\_\_\_\_\_/\_\_\_\_\_\_\_\_\_\_\_\_\_\_\_\_\_\\_ I  
               I   \              /                     I  
               I    \            /                      I  
               I     \  /\      /       V.2.4.2         I  
               I      \/  \    /                        I  
               I\_\_\_\_\_\_\_\_\_\_\_\  /\_\_\_\_\_\_\_\_\_\_\_\_\_\_\_\_\_\_\_\_\_\_\_\_\_I  
                            \/  
  
  
================================= proTRAC ====================================  
VERSION: .......... 2.4.2  
LAST MODIFIED: .... 11. May 2018  
  
Please cite:  
Rosenkranz D, Zischler H. proTRAC - a software for probabilistic piRNA cluster  
detection, visualization and analysis. 2012. BMC Bioinformatics 13:5.  
  
  
Contact:  
David Rosenkranz  
Institute of Organismic and Molecular Evolutionary Biology  
Dept. Anthropology, small RNA group  
Johannes Gutenberg University Mainz  
email: rosenkranz@uni-mainz.de  
  
You can find the latest proTRAC version at:  
http://sourceforge.net/projects/protrac/files  
http://www.smallRNAgroup-mainz.de/software  
==============================================================================  
  
PARAMETERS:  
Map file: ...............piwi-machos-0B.fa-collapse.map  
Genome file: ............../../../0B\_ala\_genome.fa  
RepeatMasker annotation: Alatifasciata-all0B-maryan-v2.fa\_corrected.out  
GeneSet:................./guest-storage/Data/annotation/Alatifasciata\_all0B\_maryan-v2\_out2017.gff  
  
Significant (p<=0.01) hit density will be calculated based  
on observed hit distribution.  
  
Sliding window size: ........................................ 5000 bp  
Sliding window increament: .................................. 1000 bp  
Normalize each hit by number of genomic hits: ............... yes  
Normalize each hit by number of sequence reads: ............. yes  
Normalize values (-> per million mapped reads): ............. yes  
Min. fraction of hits with 1T(U) or 10A: .................... 0.75  
Alternatively: Min. fraction of hits with 1T(U) and 10A: .... 0.5  
Min. fraction of hits with typical piRNA length: ............ 0.75  
Typical piRNA length: ....................................... 24-32 nt  
Min. size of a piRNA cluster: ............................... 1000 bp.  
Min. number of hits (absolute): ............................. 0  
Min. number of hits (normalized): ........................... 0  
Min. fraction of hits on the mainstrand: .................... 0.75  
Top fraction of mapped sequences (in terms of read counts): . 1%  
Top fraction accounts for max. n% of sequence reads: ........ 90%  
Min. fraction of hits on each arm of a bidirectional cluster: 0.05  
Output html file for each cluster: .......................... yes  
Output a summary table: ..................................... yes  
Output a FASTA file for each cluster (piRNA sequences): ..... yes  
Output a FASTA file comprising cluster sequences: ........... yes  
Output a GTF file for predicted piRNA clusters: ..............yes  
Search DNA motifs in clusters: .............................. yes  
Output flanking sequences: +/- .............................. 0 bp  
Output ~.pTi file: .......................................... no  
==============================================================================  
  
  
Genome size (without gaps): ............ 758543724 bp  
Gaps (N/X/-): .......................... 417479 bp  
Mapped reads: .......................... 24765598  
Non-identical sequences: ............... 6158275  
Genomic hits: .......................... 53103584  
Significant densitiy of mapped reads: .. 763.098963422187 reads/kb

Show proTRAC cluster info
Hide proTRAC cluster info

|  |  |
| --- | --- |
| Location | NODE\_355988\_length\_17263\_cov\_24.156172 |
| Coordinates | 4749-12913 |
| Size [bp] | 8165 |
| Sequence hit loci | 3685 |
| Mapped reads (normalized) | 10445.8 |
| Mapped reads (normalized) per kb | 1279.3 |
| Normalized reads with 1T (1U) | 54.7% |
| Normalized reads with 10A | 50.5% |
| Normalized reads with length 24-32 nt | 99.4% |
| Normalized reads on the main strand(s) | 92.8% |
| Predicted directionality | mono:minus |

100%

0%

1T (1U)  
reads

10A reads

24-32 nt  
reads

reads on mainstrand

**Either the amount of reads with 1T (1U) OR 10A has to exceed 75% (set with option: -1Tor10A)  
Alternatively the amount of reads with 1T (1U) AND 10A has to exceed 50% (set with option: -1Tand10A)  
Minimum amount of reads with preferred size is 75% (set with option: -pisize)  
Minimum amount of reads on the main strand(s) is 75% (set with option: -clstrand)**

Show read coverage
Hide read coverage

WHAT DO I SEE HERE?  
This chart shows the location of mapped sequence reads within a predicted piRNA cluster. The color refers to the number of genomic hits produced by the sequence read in question. A dark red bar indicates that this sequence read produces many other hits elsewhere in the genome. Many adjacent red or yellow bars can indicate the presence of a multi-copy element such as transposons or rRNA genes. A dark green bar indicates that this sequence read maps uniquely to this locus.

1 hit

2-5 hits

6-10 hits

11-20 hits

21-50 hits

51-100 hits

> 100 hits

NODE\_355988\_length\_17263\_cov\_24.156172

4749

12913

Gene Set

RepeatMasker

Mapped  
Reads

24.99

plus strand

minus strand

24.99

Region: NODE\_355988\_length\_17263\_cov\_24.156172 4652-4757. Max. coverage (+): 0. Max coverage (-): 0.17

Region: NODE\_355988\_length\_17263\_cov\_24.156172 4758-4773. Max. coverage (+): 0. Max coverage (-): 0

Region: NODE\_355988\_length\_17263\_cov\_24.156172 4774-4789. Max. coverage (+): 0. Max coverage (-): 0

Region: NODE\_355988\_length\_17263\_cov\_24.156172 4790-4806. Max. coverage (+): 0. Max coverage (-): 0

Region: NODE\_355988\_length\_17263\_cov\_24.156172 4807-4822. Max. coverage (+): 0. Max coverage (-): 0

Region: NODE\_355988\_length\_17263\_cov\_24.156172 4823-4838. Max. coverage (+): 0. Max coverage (-): 0

Region: NODE\_355988\_length\_17263\_cov\_24.156172 4839-4855. Max. coverage (+): 0. Max coverage (-): 0

Region: NODE\_355988\_length\_17263\_cov\_24.156172 4856-4871. Max. coverage (+): 0. Max coverage (-): 0

Region: NODE\_355988\_length\_17263\_cov\_24.156172 4872-4887. Max. coverage (+): 0. Max coverage (-): 0

Region: NODE\_355988\_length\_17263\_cov\_24.156172 4888-4904. Max. coverage (+): 0. Max coverage (-): 0

Region: NODE\_355988\_length\_17263\_cov\_24.156172 4905-4920. Max. coverage (+): 0. Max coverage (-): 0

Region: NODE\_355988\_length\_17263\_cov\_24.156172 4921-4936. Max. coverage (+): 0. Max coverage (-): 0

Region: NODE\_355988\_length\_17263\_cov\_24.156172 4937-4953. Max. coverage (+): 0. Max coverage (-): 0.01

Region: NODE\_355988\_length\_17263\_cov\_24.156172 4954-4969. Max. coverage (+): 0. Max coverage (-): 0

Region: NODE\_355988\_length\_17263\_cov\_24.156172 4970-4985. Max. coverage (+): 0. Max coverage (-): 0.04

Region: NODE\_355988\_length\_17263\_cov\_24.156172 4986-5002. Max. coverage (+): 0.01. Max coverage (-): 0.04

Region: NODE\_355988\_length\_17263\_cov\_24.156172 5003-5018. Max. coverage (+): 0.01. Max coverage (-): 0

Region: NODE\_355988\_length\_17263\_cov\_24.156172 5019-5034. Max. coverage (+): 0. Max coverage (-): 0

Region: NODE\_355988\_length\_17263\_cov\_24.156172 5035-5051. Max. coverage (+): 0. Max coverage (-): 0

Region: NODE\_355988\_length\_17263\_cov\_24.156172 5052-5067. Max. coverage (+): 0. Max coverage (-): 0

Region: NODE\_355988\_length\_17263\_cov\_24.156172 5068-5083. Max. coverage (+): 0.02. Max coverage (-): 0.06

Region: NODE\_355988\_length\_17263\_cov\_24.156172 5084-5100. Max. coverage (+): 0.26. Max coverage (-): 0.2

Region: NODE\_355988\_length\_17263\_cov\_24.156172 5101-5116. Max. coverage (+): 0.18. Max coverage (-): 3.51

Region: NODE\_355988\_length\_17263\_cov\_24.156172 5117-5132. Max. coverage (+): 0.04. Max coverage (-): 2.44

Region: NODE\_355988\_length\_17263\_cov\_24.156172 5133-5149. Max. coverage (+): 0.04. Max coverage (-): 1.88

Region: NODE\_355988\_length\_17263\_cov\_24.156172 5150-5165. Max. coverage (+): 0.06. Max coverage (-): 0.26

Region: NODE\_355988\_length\_17263\_cov\_24.156172 5166-5181. Max. coverage (+): 0.02. Max coverage (-): 0.08

Region: NODE\_355988\_length\_17263\_cov\_24.156172 5182-5198. Max. coverage (+): 0. Max coverage (-): 0.06

Region: NODE\_355988\_length\_17263\_cov\_24.156172 5199-5214. Max. coverage (+): 0. Max coverage (-): 0

Region: NODE\_355988\_length\_17263\_cov\_24.156172 5215-5230. Max. coverage (+): 0. Max coverage (-): 0.1

Region: NODE\_355988\_length\_17263\_cov\_24.156172 5231-5247. Max. coverage (+): 0. Max coverage (-): 0.1

Region: NODE\_355988\_length\_17263\_cov\_24.156172 5248-5263. Max. coverage (+): 0. Max coverage (-): 0

Region: NODE\_355988\_length\_17263\_cov\_24.156172 5264-5279. Max. coverage (+): 0. Max coverage (-): 0

Region: NODE\_355988\_length\_17263\_cov\_24.156172 5280-5296. Max. coverage (+): 0. Max coverage (-): 0

Region: NODE\_355988\_length\_17263\_cov\_24.156172 5297-5312. Max. coverage (+): 0. Max coverage (-): 0

Region: NODE\_355988\_length\_17263\_cov\_24.156172 5313-5328. Max. coverage (+): 0. Max coverage (-): 0

Region: NODE\_355988\_length\_17263\_cov\_24.156172 5329-5345. Max. coverage (+): 0. Max coverage (-): 0

Region: NODE\_355988\_length\_17263\_cov\_24.156172 5346-5361. Max. coverage (+): 0. Max coverage (-): 0

Region: NODE\_355988\_length\_17263\_cov\_24.156172 5362-5377. Max. coverage (+): 0. Max coverage (-): 0

Region: NODE\_355988\_length\_17263\_cov\_24.156172 5378-5394. Max. coverage (+): 0. Max coverage (-): 0

Region: NODE\_355988\_length\_17263\_cov\_24.156172 5395-5410. Max. coverage (+): 0. Max coverage (-): 0

Region: NODE\_355988\_length\_17263\_cov\_24.156172 5411-5426. Max. coverage (+): 0.02. Max coverage (-): 0.75

Region: NODE\_355988\_length\_17263\_cov\_24.156172 5427-5443. Max. coverage (+): 0. Max coverage (-): 0.04

Region: NODE\_355988\_length\_17263\_cov\_24.156172 5444-5459. Max. coverage (+): 0. Max coverage (-): 0.42

Region: NODE\_355988\_length\_17263\_cov\_24.156172 5460-5475. Max. coverage (+): 0.04. Max coverage (-): 2.34

Region: NODE\_355988\_length\_17263\_cov\_24.156172 5476-5492. Max. coverage (+): 0.56. Max coverage (-): 2.45

Region: NODE\_355988\_length\_17263\_cov\_24.156172 5493-5508. Max. coverage (+): 0.02. Max coverage (-): 1.13

Region: NODE\_355988\_length\_17263\_cov\_24.156172 5509-5524. Max. coverage (+): 0.47. Max coverage (-): 0.28

Region: NODE\_355988\_length\_17263\_cov\_24.156172 5525-5541. Max. coverage (+): 0.08. Max coverage (-): 23.97

Region: NODE\_355988\_length\_17263\_cov\_24.156172 5542-5557. Max. coverage (+): 0. Max coverage (-): 24.27

Region: NODE\_355988\_length\_17263\_cov\_24.156172 5558-5573. Max. coverage (+): 0. Max coverage (-): 0

Region: NODE\_355988\_length\_17263\_cov\_24.156172 5574-5589. Max. coverage (+): 0. Max coverage (-): 0

Region: NODE\_355988\_length\_17263\_cov\_24.156172 5590-5606. Max. coverage (+): 0.02. Max coverage (-): 0.06

Region: NODE\_355988\_length\_17263\_cov\_24.156172 5607-5622. Max. coverage (+): 0.04. Max coverage (-): 5.7

Region: NODE\_355988\_length\_17263\_cov\_24.156172 5623-5638. Max. coverage (+): 0.08. Max coverage (-): 3.09

Region: NODE\_355988\_length\_17263\_cov\_24.156172 5639-5655. Max. coverage (+): 0. Max coverage (-): 0.01

Region: NODE\_355988\_length\_17263\_cov\_24.156172 5656-5671. Max. coverage (+): 0. Max coverage (-): 0

Region: NODE\_355988\_length\_17263\_cov\_24.156172 5672-5687. Max. coverage (+): 0. Max coverage (-): 0

Region: NODE\_355988\_length\_17263\_cov\_24.156172 5688-5704. Max. coverage (+): 0. Max coverage (-): 0

Region: NODE\_355988\_length\_17263\_cov\_24.156172 5705-5720. Max. coverage (+): 0.04. Max coverage (-): 0

Region: NODE\_355988\_length\_17263\_cov\_24.156172 5721-5736. Max. coverage (+): 0.01. Max coverage (-): 0

Region: NODE\_355988\_length\_17263\_cov\_24.156172 5737-5753. Max. coverage (+): 0. Max coverage (-): 0.01

Region: NODE\_355988\_length\_17263\_cov\_24.156172 5754-5769. Max. coverage (+): 0. Max coverage (-): 0.02

Region: NODE\_355988\_length\_17263\_cov\_24.156172 5770-5785. Max. coverage (+): 0. Max coverage (-): 0

Region: NODE\_355988\_length\_17263\_cov\_24.156172 5786-5802. Max. coverage (+): 0.01. Max coverage (-): 0

Region: NODE\_355988\_length\_17263\_cov\_24.156172 5803-5818. Max. coverage (+): 0. Max coverage (-): 0.01

Region: NODE\_355988\_length\_17263\_cov\_24.156172 5819-5834. Max. coverage (+): 0. Max coverage (-): 0.01

Region: NODE\_355988\_length\_17263\_cov\_24.156172 5835-5851. Max. coverage (+): 0. Max coverage (-): 0

Region: NODE\_355988\_length\_17263\_cov\_24.156172 5852-5867. Max. coverage (+): 0. Max coverage (-): 0

Region: NODE\_355988\_length\_17263\_cov\_24.156172 5868-5883. Max. coverage (+): 0. Max coverage (-): 0

Region: NODE\_355988\_length\_17263\_cov\_24.156172 5884-5900. Max. coverage (+): 0. Max coverage (-): 0

Region: NODE\_355988\_length\_17263\_cov\_24.156172 5901-5916. Max. coverage (+): 0. Max coverage (-): 0

Region: NODE\_355988\_length\_17263\_cov\_24.156172 5917-5932. Max. coverage (+): 0. Max coverage (-): 0

Region: NODE\_355988\_length\_17263\_cov\_24.156172 5933-5949. Max. coverage (+): 0. Max coverage (-): 0

Region: NODE\_355988\_length\_17263\_cov\_24.156172 5950-5965. Max. coverage (+): 0. Max coverage (-): 0

Region: NODE\_355988\_length\_17263\_cov\_24.156172 5966-5981. Max. coverage (+): 0.04. Max coverage (-): 0.01

Region: NODE\_355988\_length\_17263\_cov\_24.156172 5982-5998. Max. coverage (+): 0.05. Max coverage (-): 0

Region: NODE\_355988\_length\_17263\_cov\_24.156172 5999-6014. Max. coverage (+): 0.01. Max coverage (-): 0

Region: NODE\_355988\_length\_17263\_cov\_24.156172 6015-6030. Max. coverage (+): 0.01. Max coverage (-): 0.4

Region: NODE\_355988\_length\_17263\_cov\_24.156172 6031-6047. Max. coverage (+): 0.05. Max coverage (-): 0.14

Region: NODE\_355988\_length\_17263\_cov\_24.156172 6048-6063. Max. coverage (+): 0.02. Max coverage (-): 0.01

Region: NODE\_355988\_length\_17263\_cov\_24.156172 6064-6079. Max. coverage (+): 0.11. Max coverage (-): 0

Region: NODE\_355988\_length\_17263\_cov\_24.156172 6080-6096. Max. coverage (+): 0.05. Max coverage (-): 0.01

Region: NODE\_355988\_length\_17263\_cov\_24.156172 6097-6112. Max. coverage (+): 0.01. Max coverage (-): 0.06

Region: NODE\_355988\_length\_17263\_cov\_24.156172 6113-6128. Max. coverage (+): 0.05. Max coverage (-): 0.05

Region: NODE\_355988\_length\_17263\_cov\_24.156172 6129-6145. Max. coverage (+): 0.05. Max coverage (-): 0.03

Region: NODE\_355988\_length\_17263\_cov\_24.156172 6146-6161. Max. coverage (+): 0.05. Max coverage (-): 0.05

Region: NODE\_355988\_length\_17263\_cov\_24.156172 6162-6177. Max. coverage (+): 0.03. Max coverage (-): 0.05

Region: NODE\_355988\_length\_17263\_cov\_24.156172 6178-6194. Max. coverage (+): 0. Max coverage (-): 0

Region: NODE\_355988\_length\_17263\_cov\_24.156172 6195-6210. Max. coverage (+): 0. Max coverage (-): 0.09

Region: NODE\_355988\_length\_17263\_cov\_24.156172 6211-6226. Max. coverage (+): 0.05. Max coverage (-): 0.06

Region: NODE\_355988\_length\_17263\_cov\_24.156172 6227-6243. Max. coverage (+): 0.06. Max coverage (-): 0.05

Region: NODE\_355988\_length\_17263\_cov\_24.156172 6244-6259. Max. coverage (+): 0.01. Max coverage (-): 0.17

Region: NODE\_355988\_length\_17263\_cov\_24.156172 6260-6275. Max. coverage (+): 0. Max coverage (-): 0.01

Region: NODE\_355988\_length\_17263\_cov\_24.156172 6276-6292. Max. coverage (+): 0.04. Max coverage (-): 0

Region: NODE\_355988\_length\_17263\_cov\_24.156172 6293-6308. Max. coverage (+): 0.01. Max coverage (-): 0.01

Region: NODE\_355988\_length\_17263\_cov\_24.156172 6309-6324. Max. coverage (+): 0.01. Max coverage (-): 0.02

Region: NODE\_355988\_length\_17263\_cov\_24.156172 6325-6341. Max. coverage (+): 0. Max coverage (-): 0.09

Region: NODE\_355988\_length\_17263\_cov\_24.156172 6342-6357. Max. coverage (+): 0.07. Max coverage (-): 0.01

Region: NODE\_355988\_length\_17263\_cov\_24.156172 6358-6373. Max. coverage (+): 0.12. Max coverage (-): 0.02

Region: NODE\_355988\_length\_17263\_cov\_24.156172 6374-6390. Max. coverage (+): 0.01. Max coverage (-): 0.01

Region: NODE\_355988\_length\_17263\_cov\_24.156172 6391-6406. Max. coverage (+): 0.12. Max coverage (-): 0.01

Region: NODE\_355988\_length\_17263\_cov\_24.156172 6407-6422. Max. coverage (+): 0.12. Max coverage (-): 0.09

Region: NODE\_355988\_length\_17263\_cov\_24.156172 6423-6439. Max. coverage (+): 0. Max coverage (-): 0.13

Region: NODE\_355988\_length\_17263\_cov\_24.156172 6440-6455. Max. coverage (+): 0.02. Max coverage (-): 0.01

Region: NODE\_355988\_length\_17263\_cov\_24.156172 6456-6471. Max. coverage (+): 0. Max coverage (-): 0

Region: NODE\_355988\_length\_17263\_cov\_24.156172 6472-6488. Max. coverage (+): 0.01. Max coverage (-): 0.01

Region: NODE\_355988\_length\_17263\_cov\_24.156172 6489-6504. Max. coverage (+): 0.04. Max coverage (-): 0.07

Region: NODE\_355988\_length\_17263\_cov\_24.156172 6505-6520. Max. coverage (+): 0.04. Max coverage (-): 0

Region: NODE\_355988\_length\_17263\_cov\_24.156172 6521-6537. Max. coverage (+): 0.06. Max coverage (-): 0.03

Region: NODE\_355988\_length\_17263\_cov\_24.156172 6538-6553. Max. coverage (+): 0.03. Max coverage (-): 0.09

Region: NODE\_355988\_length\_17263\_cov\_24.156172 6554-6569. Max. coverage (+): 0.13. Max coverage (-): 0.08

Region: NODE\_355988\_length\_17263\_cov\_24.156172 6570-6586. Max. coverage (+): 0.01. Max coverage (-): 0.11

Region: NODE\_355988\_length\_17263\_cov\_24.156172 6587-6602. Max. coverage (+): 0. Max coverage (-): 0

Region: NODE\_355988\_length\_17263\_cov\_24.156172 6603-6618. Max. coverage (+): 0. Max coverage (-): 0

Region: NODE\_355988\_length\_17263\_cov\_24.156172 6619-6635. Max. coverage (+): 0. Max coverage (-): 0.04

Region: NODE\_355988\_length\_17263\_cov\_24.156172 6636-6651. Max. coverage (+): 0. Max coverage (-): 0.04

Region: NODE\_355988\_length\_17263\_cov\_24.156172 6652-6667. Max. coverage (+): 0. Max coverage (-): 0

Region: NODE\_355988\_length\_17263\_cov\_24.156172 6668-6684. Max. coverage (+): 0.02. Max coverage (-): 0.01

Region: NODE\_355988\_length\_17263\_cov\_24.156172 6685-6700. Max. coverage (+): 0.02. Max coverage (-): 0.02

Region: NODE\_355988\_length\_17263\_cov\_24.156172 6701-6716. Max. coverage (+): 0.03. Max coverage (-): 0.02

Region: NODE\_355988\_length\_17263\_cov\_24.156172 6717-6733. Max. coverage (+): 0.04. Max coverage (-): 0

Region: NODE\_355988\_length\_17263\_cov\_24.156172 6734-6749. Max. coverage (+): 0. Max coverage (-): 0

Region: NODE\_355988\_length\_17263\_cov\_24.156172 6750-6765. Max. coverage (+): 0. Max coverage (-): 0

Region: NODE\_355988\_length\_17263\_cov\_24.156172 6766-6782. Max. coverage (+): 0.04. Max coverage (-): 0.04

Region: NODE\_355988\_length\_17263\_cov\_24.156172 6783-6798. Max. coverage (+): 0.04. Max coverage (-): 0

Region: NODE\_355988\_length\_17263\_cov\_24.156172 6799-6814. Max. coverage (+): 0. Max coverage (-): 0

Region: NODE\_355988\_length\_17263\_cov\_24.156172 6815-6831. Max. coverage (+): 0. Max coverage (-): 0

Region: NODE\_355988\_length\_17263\_cov\_24.156172 6832-6847. Max. coverage (+): 0. Max coverage (-): 0

Region: NODE\_355988\_length\_17263\_cov\_24.156172 6848-6863. Max. coverage (+): 0. Max coverage (-): 0

Region: NODE\_355988\_length\_17263\_cov\_24.156172 6864-6880. Max. coverage (+): 0. Max coverage (-): 0

Region: NODE\_355988\_length\_17263\_cov\_24.156172 6881-6896. Max. coverage (+): 0. Max coverage (-): 0

Region: NODE\_355988\_length\_17263\_cov\_24.156172 6897-6912. Max. coverage (+): 0. Max coverage (-): 0

Region: NODE\_355988\_length\_17263\_cov\_24.156172 6913-6929. Max. coverage (+): 0. Max coverage (-): 0

Region: NODE\_355988\_length\_17263\_cov\_24.156172 6930-6945. Max. coverage (+): 0.08. Max coverage (-): 2.04

Region: NODE\_355988\_length\_17263\_cov\_24.156172 6946-6961. Max. coverage (+): 0.06. Max coverage (-): 1.86

Region: NODE\_355988\_length\_17263\_cov\_24.156172 6962-6978. Max. coverage (+): 1.03. Max coverage (-): 0.4

Region: NODE\_355988\_length\_17263\_cov\_24.156172 6979-6994. Max. coverage (+): 0. Max coverage (-): 0.16

Region: NODE\_355988\_length\_17263\_cov\_24.156172 6995-7010. Max. coverage (+): 0. Max coverage (-): 0.06

Region: NODE\_355988\_length\_17263\_cov\_24.156172 7011-7027. Max. coverage (+): 0.02. Max coverage (-): 0.34

Region: NODE\_355988\_length\_17263\_cov\_24.156172 7028-7043. Max. coverage (+): 0.04. Max coverage (-): 23.42

Region: NODE\_355988\_length\_17263\_cov\_24.156172 7044-7059. Max. coverage (+): 0. Max coverage (-): 0.38

Region: NODE\_355988\_length\_17263\_cov\_24.156172 7060-7076. Max. coverage (+): 0. Max coverage (-): 0

Region: NODE\_355988\_length\_17263\_cov\_24.156172 7077-7092. Max. coverage (+): 0. Max coverage (-): 0

Region: NODE\_355988\_length\_17263\_cov\_24.156172 7093-7108. Max. coverage (+): 0.04. Max coverage (-): 0.08

Region: NODE\_355988\_length\_17263\_cov\_24.156172 7109-7125. Max. coverage (+): 0. Max coverage (-): 0

Region: NODE\_355988\_length\_17263\_cov\_24.156172 7126-7141. Max. coverage (+): 0. Max coverage (-): 1.55

Region: NODE\_355988\_length\_17263\_cov\_24.156172 7142-7157. Max. coverage (+): 0.06. Max coverage (-): 0.69

Region: NODE\_355988\_length\_17263\_cov\_24.156172 7158-7174. Max. coverage (+): 0.08. Max coverage (-): 0.2

Region: NODE\_355988\_length\_17263\_cov\_24.156172 7175-7190. Max. coverage (+): 0.04. Max coverage (-): 0.69

Region: NODE\_355988\_length\_17263\_cov\_24.156172 7191-7206. Max. coverage (+): 0.12. Max coverage (-): 0.85

Region: NODE\_355988\_length\_17263\_cov\_24.156172 7207-7222. Max. coverage (+): 0. Max coverage (-): 0.04

Region: NODE\_355988\_length\_17263\_cov\_24.156172 7223-7239. Max. coverage (+): 0.04. Max coverage (-): 0.14

Region: NODE\_355988\_length\_17263\_cov\_24.156172 7240-7255. Max. coverage (+): 0.04. Max coverage (-): 0.02

Region: NODE\_355988\_length\_17263\_cov\_24.156172 7256-7271. Max. coverage (+): 0. Max coverage (-): 1.13

Region: NODE\_355988\_length\_17263\_cov\_24.156172 7272-7288. Max. coverage (+): 0.08. Max coverage (-): 3.51

Region: NODE\_355988\_length\_17263\_cov\_24.156172 7289-7304. Max. coverage (+): 0.04. Max coverage (-): 0

Region: NODE\_355988\_length\_17263\_cov\_24.156172 7305-7320. Max. coverage (+): 0. Max coverage (-): 0.65

Region: NODE\_355988\_length\_17263\_cov\_24.156172 7321-7337. Max. coverage (+): 0. Max coverage (-): 0.02

Region: NODE\_355988\_length\_17263\_cov\_24.156172 7338-7353. Max. coverage (+): 0. Max coverage (-): 0.04

Region: NODE\_355988\_length\_17263\_cov\_24.156172 7354-7369. Max. coverage (+): 0. Max coverage (-): 0

Region: NODE\_355988\_length\_17263\_cov\_24.156172 7370-7386. Max. coverage (+): 0. Max coverage (-): 0

Region: NODE\_355988\_length\_17263\_cov\_24.156172 7387-7402. Max. coverage (+): 0. Max coverage (-): 0

Region: NODE\_355988\_length\_17263\_cov\_24.156172 7403-7418. Max. coverage (+): 0. Max coverage (-): 0

Region: NODE\_355988\_length\_17263\_cov\_24.156172 7419-7435. Max. coverage (+): 0. Max coverage (-): 0

Region: NODE\_355988\_length\_17263\_cov\_24.156172 7436-7451. Max. coverage (+): 0. Max coverage (-): 0

Region: NODE\_355988\_length\_17263\_cov\_24.156172 7452-7467. Max. coverage (+): 0. Max coverage (-): 0

Region: NODE\_355988\_length\_17263\_cov\_24.156172 7468-7484. Max. coverage (+): 0. Max coverage (-): 0

Region: NODE\_355988\_length\_17263\_cov\_24.156172 7485-7500. Max. coverage (+): 0. Max coverage (-): 0

Region: NODE\_355988\_length\_17263\_cov\_24.156172 7501-7516. Max. coverage (+): 0. Max coverage (-): 0

Region: NODE\_355988\_length\_17263\_cov\_24.156172 7517-7533. Max. coverage (+): 0. Max coverage (-): 0

Region: NODE\_355988\_length\_17263\_cov\_24.156172 7534-7549. Max. coverage (+): 0. Max coverage (-): 0

Region: NODE\_355988\_length\_17263\_cov\_24.156172 7550-7565. Max. coverage (+): 0. Max coverage (-): 0

Region: NODE\_355988\_length\_17263\_cov\_24.156172 7566-7582. Max. coverage (+): 0. Max coverage (-): 0

Region: NODE\_355988\_length\_17263\_cov\_24.156172 7583-7598. Max. coverage (+): 0. Max coverage (-): 0

Region: NODE\_355988\_length\_17263\_cov\_24.156172 7599-7614. Max. coverage (+): 0.04. Max coverage (-): 0

Region: NODE\_355988\_length\_17263\_cov\_24.156172 7615-7631. Max. coverage (+): 0.04. Max coverage (-): 0

Region: NODE\_355988\_length\_17263\_cov\_24.156172 7632-7647. Max. coverage (+): 0. Max coverage (-): 0

Region: NODE\_355988\_length\_17263\_cov\_24.156172 7648-7663. Max. coverage (+): 0. Max coverage (-): 0

Region: NODE\_355988\_length\_17263\_cov\_24.156172 7664-7680. Max. coverage (+): 0. Max coverage (-): 0

Region: NODE\_355988\_length\_17263\_cov\_24.156172 7681-7696. Max. coverage (+): 0. Max coverage (-): 0

Region: NODE\_355988\_length\_17263\_cov\_24.156172 7697-7712. Max. coverage (+): 0. Max coverage (-): 0

Region: NODE\_355988\_length\_17263\_cov\_24.156172 7713-7729. Max. coverage (+): 0. Max coverage (-): 0

Region: NODE\_355988\_length\_17263\_cov\_24.156172 7730-7745. Max. coverage (+): 0. Max coverage (-): 0

Region: NODE\_355988\_length\_17263\_cov\_24.156172 7746-7761. Max. coverage (+): 0. Max coverage (-): 0.04

Region: NODE\_355988\_length\_17263\_cov\_24.156172 7762-7778. Max. coverage (+): 0. Max coverage (-): 0

Region: NODE\_355988\_length\_17263\_cov\_24.156172 7779-7794. Max. coverage (+): 0. Max coverage (-): 0

Region: NODE\_355988\_length\_17263\_cov\_24.156172 7795-7810. Max. coverage (+): 0. Max coverage (-): 0

Region: NODE\_355988\_length\_17263\_cov\_24.156172 7811-7827. Max. coverage (+): 0.04. Max coverage (-): 0

Region: NODE\_355988\_length\_17263\_cov\_24.156172 7828-7843. Max. coverage (+): 0.02. Max coverage (-): 0

Region: NODE\_355988\_length\_17263\_cov\_24.156172 7844-7859. Max. coverage (+): 0. Max coverage (-): 0

Region: NODE\_355988\_length\_17263\_cov\_24.156172 7860-7876. Max. coverage (+): 0.04. Max coverage (-): 0.4

Region: NODE\_355988\_length\_17263\_cov\_24.156172 7877-7892. Max. coverage (+): 0.04. Max coverage (-): 13.59

Region: NODE\_355988\_length\_17263\_cov\_24.156172 7893-7908. Max. coverage (+): 0. Max coverage (-): 0.08

Region: NODE\_355988\_length\_17263\_cov\_24.156172 7909-7925. Max. coverage (+): 0.01. Max coverage (-): 0.03

Region: NODE\_355988\_length\_17263\_cov\_24.156172 7926-7941. Max. coverage (+): 0. Max coverage (-): 0

Region: NODE\_355988\_length\_17263\_cov\_24.156172 7942-7957. Max. coverage (+): 0.24. Max coverage (-): 0.01

Region: NODE\_355988\_length\_17263\_cov\_24.156172 7958-7974. Max. coverage (+): 0.65. Max coverage (-): 0

Region: NODE\_355988\_length\_17263\_cov\_24.156172 7975-7990. Max. coverage (+): 0. Max coverage (-): 0

Region: NODE\_355988\_length\_17263\_cov\_24.156172 7991-8006. Max. coverage (+): 0.04. Max coverage (-): 0

Region: NODE\_355988\_length\_17263\_cov\_24.156172 8007-8023. Max. coverage (+): 0. Max coverage (-): 0

Region: NODE\_355988\_length\_17263\_cov\_24.156172 8024-8039. Max. coverage (+): 0.04. Max coverage (-): 0.12

Region: NODE\_355988\_length\_17263\_cov\_24.156172 8040-8055. Max. coverage (+): 0.08. Max coverage (-): 3.19

Region: NODE\_355988\_length\_17263\_cov\_24.156172 8056-8072. Max. coverage (+): 0.36. Max coverage (-): 0.69

Region: NODE\_355988\_length\_17263\_cov\_24.156172 8073-8088. Max. coverage (+): 0.04. Max coverage (-): 0

Region: NODE\_355988\_length\_17263\_cov\_24.156172 8089-8104. Max. coverage (+): 0. Max coverage (-): 0

Region: NODE\_355988\_length\_17263\_cov\_24.156172 8105-8121. Max. coverage (+): 0. Max coverage (-): 0

Region: NODE\_355988\_length\_17263\_cov\_24.156172 8122-8137. Max. coverage (+): 0.54. Max coverage (-): 0.04

Region: NODE\_355988\_length\_17263\_cov\_24.156172 8138-8153. Max. coverage (+): 0.02. Max coverage (-): 0.81

Region: NODE\_355988\_length\_17263\_cov\_24.156172 8154-8170. Max. coverage (+): 0.03. Max coverage (-): 0.68

Region: NODE\_355988\_length\_17263\_cov\_24.156172 8171-8186. Max. coverage (+): 0. Max coverage (-): 0

Region: NODE\_355988\_length\_17263\_cov\_24.156172 8187-8202. Max. coverage (+): 0. Max coverage (-): 1.45

Region: NODE\_355988\_length\_17263\_cov\_24.156172 8203-8219. Max. coverage (+): 0.52. Max coverage (-): 2.18

Region: NODE\_355988\_length\_17263\_cov\_24.156172 8220-8235. Max. coverage (+): 0.12. Max coverage (-): 5.94

Region: NODE\_355988\_length\_17263\_cov\_24.156172 8236-8251. Max. coverage (+): 0.24. Max coverage (-): 0.44

Region: NODE\_355988\_length\_17263\_cov\_24.156172 8252-8268. Max. coverage (+): 0. Max coverage (-): 8.4

Region: NODE\_355988\_length\_17263\_cov\_24.156172 8269-8284. Max. coverage (+): 0.04. Max coverage (-): 3.59

Region: NODE\_355988\_length\_17263\_cov\_24.156172 8285-8300. Max. coverage (+): 0. Max coverage (-): 21.6

Region: NODE\_355988\_length\_17263\_cov\_24.156172 8301-8317. Max. coverage (+): 0.4. Max coverage (-): 1.09

Region: NODE\_355988\_length\_17263\_cov\_24.156172 8318-8333. Max. coverage (+): 0.32. Max coverage (-): 0.4

Region: NODE\_355988\_length\_17263\_cov\_24.156172 8334-8349. Max. coverage (+): 0.28. Max coverage (-): 2.91

Region: NODE\_355988\_length\_17263\_cov\_24.156172 8350-8366. Max. coverage (+): 0.28. Max coverage (-): 3.71

Region: NODE\_355988\_length\_17263\_cov\_24.156172 8367-8382. Max. coverage (+): 0.32. Max coverage (-): 0.16

Region: NODE\_355988\_length\_17263\_cov\_24.156172 8383-8398. Max. coverage (+): 0. Max coverage (-): 0.08

Region: NODE\_355988\_length\_17263\_cov\_24.156172 8399-8415. Max. coverage (+): 0. Max coverage (-): 0.2

Region: NODE\_355988\_length\_17263\_cov\_24.156172 8416-8431. Max. coverage (+): 8.72. Max coverage (-): 0.16

Region: NODE\_355988\_length\_17263\_cov\_24.156172 8432-8447. Max. coverage (+): 0.08. Max coverage (-): 1.09

Region: NODE\_355988\_length\_17263\_cov\_24.156172 8448-8464. Max. coverage (+): 0.04. Max coverage (-): 2.71

Region: NODE\_355988\_length\_17263\_cov\_24.156172 8465-8480. Max. coverage (+): 0.04. Max coverage (-): 0.65

Region: NODE\_355988\_length\_17263\_cov\_24.156172 8481-8496. Max. coverage (+): 0.04. Max coverage (-): 17.04

Region: NODE\_355988\_length\_17263\_cov\_24.156172 8497-8513. Max. coverage (+): 0. Max coverage (-): 0.08

Region: NODE\_355988\_length\_17263\_cov\_24.156172 8514-8529. Max. coverage (+): 0. Max coverage (-): 24.91

Region: NODE\_355988\_length\_17263\_cov\_24.156172 8530-8545. Max. coverage (+): 0.08. Max coverage (-): 24.99

Region: NODE\_355988\_length\_17263\_cov\_24.156172 8546-8562. Max. coverage (+): 0.36. Max coverage (-): 0.83

Region: NODE\_355988\_length\_17263\_cov\_24.156172 8563-8578. Max. coverage (+): 0.16. Max coverage (-): 5.96

Region: NODE\_355988\_length\_17263\_cov\_24.156172 8579-8594. Max. coverage (+): 0.04. Max coverage (-): 5.71

Region: NODE\_355988\_length\_17263\_cov\_24.156172 8595-8611. Max. coverage (+): 0.57. Max coverage (-): 0

Region: NODE\_355988\_length\_17263\_cov\_24.156172 8612-8627. Max. coverage (+): 0.61. Max coverage (-): 1.51

Region: NODE\_355988\_length\_17263\_cov\_24.156172 8628-8643. Max. coverage (+): 0.09. Max coverage (-): 0.3

Region: NODE\_355988\_length\_17263\_cov\_24.156172 8644-8660. Max. coverage (+): 0.05. Max coverage (-): 2.97

Region: NODE\_355988\_length\_17263\_cov\_24.156172 8661-8676. Max. coverage (+): 0. Max coverage (-): 0.03

Region: NODE\_355988\_length\_17263\_cov\_24.156172 8677-8692. Max. coverage (+): 0. Max coverage (-): 0.08

Region: NODE\_355988\_length\_17263\_cov\_24.156172 8693-8709. Max. coverage (+): 0.04. Max coverage (-): 0.32

Region: NODE\_355988\_length\_17263\_cov\_24.156172 8710-8725. Max. coverage (+): 0.24. Max coverage (-): 0.93

Region: NODE\_355988\_length\_17263\_cov\_24.156172 8726-8741. Max. coverage (+): 0.42. Max coverage (-): 3.7

Region: NODE\_355988\_length\_17263\_cov\_24.156172 8742-8758. Max. coverage (+): 0.03. Max coverage (-): 2.89

Region: NODE\_355988\_length\_17263\_cov\_24.156172 8759-8774. Max. coverage (+): 0.15. Max coverage (-): 2.29

Region: NODE\_355988\_length\_17263\_cov\_24.156172 8775-8790. Max. coverage (+): 0.04. Max coverage (-): 0.05

Region: NODE\_355988\_length\_17263\_cov\_24.156172 8791-8807. Max. coverage (+): 0.01. Max coverage (-): 1.18

Region: NODE\_355988\_length\_17263\_cov\_24.156172 8808-8823. Max. coverage (+): 0.36. Max coverage (-): 1.44

Region: NODE\_355988\_length\_17263\_cov\_24.156172 8824-8839. Max. coverage (+): 0.15. Max coverage (-): 1

Region: NODE\_355988\_length\_17263\_cov\_24.156172 8840-8855. Max. coverage (+): 0.04. Max coverage (-): 0.08

Region: NODE\_355988\_length\_17263\_cov\_24.156172 8856-8872. Max. coverage (+): 0.01. Max coverage (-): 1.6

Region: NODE\_355988\_length\_17263\_cov\_24.156172 8873-8888. Max. coverage (+): 0.12. Max coverage (-): 0.77

Region: NODE\_355988\_length\_17263\_cov\_24.156172 8889-8904. Max. coverage (+): 0.03. Max coverage (-): 0.97

Region: NODE\_355988\_length\_17263\_cov\_24.156172 8905-8921. Max. coverage (+): 0.01. Max coverage (-): 4.71

Region: NODE\_355988\_length\_17263\_cov\_24.156172 8922-8937. Max. coverage (+): 2.26. Max coverage (-): 0.32

Region: NODE\_355988\_length\_17263\_cov\_24.156172 8938-8953. Max. coverage (+): 0.17. Max coverage (-): 7.95

Region: NODE\_355988\_length\_17263\_cov\_24.156172 8954-8970. Max. coverage (+): 0.24. Max coverage (-): 20.3

Region: NODE\_355988\_length\_17263\_cov\_24.156172 8971-8986. Max. coverage (+): 0.07. Max coverage (-): 4.9

Region: NODE\_355988\_length\_17263\_cov\_24.156172 8987-9002. Max. coverage (+): 0.07. Max coverage (-): 0.03

Region: NODE\_355988\_length\_17263\_cov\_24.156172 9003-9019. Max. coverage (+): 0.04. Max coverage (-): 1.92

Region: NODE\_355988\_length\_17263\_cov\_24.156172 9020-9035. Max. coverage (+): 0. Max coverage (-): 0.38

Region: NODE\_355988\_length\_17263\_cov\_24.156172 9036-9051. Max. coverage (+): 0. Max coverage (-): 0.02

Region: NODE\_355988\_length\_17263\_cov\_24.156172 9052-9068. Max. coverage (+): 0.06. Max coverage (-): 0.67

Region: NODE\_355988\_length\_17263\_cov\_24.156172 9069-9084. Max. coverage (+): 0.02. Max coverage (-): 0.26

Region: NODE\_355988\_length\_17263\_cov\_24.156172 9085-9100. Max. coverage (+): 0. Max coverage (-): 0.04

Region: NODE\_355988\_length\_17263\_cov\_24.156172 9101-9117. Max. coverage (+): 0.04. Max coverage (-): 2.53

Region: NODE\_355988\_length\_17263\_cov\_24.156172 9118-9133. Max. coverage (+): 0.01. Max coverage (-): 0.22

Region: NODE\_355988\_length\_17263\_cov\_24.156172 9134-9149. Max. coverage (+): 0.01. Max coverage (-): 0.22

Region: NODE\_355988\_length\_17263\_cov\_24.156172 9150-9166. Max. coverage (+): 0. Max coverage (-): 0.08

Region: NODE\_355988\_length\_17263\_cov\_24.156172 9167-9182. Max. coverage (+): 0. Max coverage (-): 0.24

Region: NODE\_355988\_length\_17263\_cov\_24.156172 9183-9198. Max. coverage (+): 0.34. Max coverage (-): 0.2

Region: NODE\_355988\_length\_17263\_cov\_24.156172 9199-9215. Max. coverage (+): 0.04. Max coverage (-): 2.22

Region: NODE\_355988\_length\_17263\_cov\_24.156172 9216-9231. Max. coverage (+): 0. Max coverage (-): 0.02

Region: NODE\_355988\_length\_17263\_cov\_24.156172 9232-9247. Max. coverage (+): 0.06. Max coverage (-): 0.32

Region: NODE\_355988\_length\_17263\_cov\_24.156172 9248-9264. Max. coverage (+): 0.06. Max coverage (-): 0.12

Region: NODE\_355988\_length\_17263\_cov\_24.156172 9265-9280. Max. coverage (+): 0.06. Max coverage (-): 0

Region: NODE\_355988\_length\_17263\_cov\_24.156172 9281-9296. Max. coverage (+): 0. Max coverage (-): 0

Region: NODE\_355988\_length\_17263\_cov\_24.156172 9297-9313. Max. coverage (+): 0. Max coverage (-): 0

Region: NODE\_355988\_length\_17263\_cov\_24.156172 9314-9329. Max. coverage (+): 0.04. Max coverage (-): 0.08

Region: NODE\_355988\_length\_17263\_cov\_24.156172 9330-9345. Max. coverage (+): 0.48. Max coverage (-): 0.08

Region: NODE\_355988\_length\_17263\_cov\_24.156172 9346-9362. Max. coverage (+): 0. Max coverage (-): 4.04

Region: NODE\_355988\_length\_17263\_cov\_24.156172 9363-9378. Max. coverage (+): 0.02. Max coverage (-): 9.27

Region: NODE\_355988\_length\_17263\_cov\_24.156172 9379-9394. Max. coverage (+): 0.18. Max coverage (-): 0.02

Region: NODE\_355988\_length\_17263\_cov\_24.156172 9395-9411. Max. coverage (+): 0.08. Max coverage (-): 0.12

Region: NODE\_355988\_length\_17263\_cov\_24.156172 9412-9427. Max. coverage (+): 0. Max coverage (-): 0.04

Region: NODE\_355988\_length\_17263\_cov\_24.156172 9428-9443. Max. coverage (+): 0. Max coverage (-): 0

Region: NODE\_355988\_length\_17263\_cov\_24.156172 9444-9460. Max. coverage (+): 0. Max coverage (-): 0

Region: NODE\_355988\_length\_17263\_cov\_24.156172 9461-9476. Max. coverage (+): 0. Max coverage (-): 0

Region: NODE\_355988\_length\_17263\_cov\_24.156172 9477-9492. Max. coverage (+): 0. Max coverage (-): 0

Region: NODE\_355988\_length\_17263\_cov\_24.156172 9493-9509. Max. coverage (+): 0. Max coverage (-): 0

Region: NODE\_355988\_length\_17263\_cov\_24.156172 9510-9525. Max. coverage (+): 0. Max coverage (-): 0

Region: NODE\_355988\_length\_17263\_cov\_24.156172 9526-9541. Max. coverage (+): 0. Max coverage (-): 0

Region: NODE\_355988\_length\_17263\_cov\_24.156172 9542-9558. Max. coverage (+): 0. Max coverage (-): 0.04

Region: NODE\_355988\_length\_17263\_cov\_24.156172 9559-9574. Max. coverage (+): 0. Max coverage (-): 0

Region: NODE\_355988\_length\_17263\_cov\_24.156172 9575-9590. Max. coverage (+): 0. Max coverage (-): 0

Region: NODE\_355988\_length\_17263\_cov\_24.156172 9591-9607. Max. coverage (+): 0. Max coverage (-): 0

Region: NODE\_355988\_length\_17263\_cov\_24.156172 9608-9623. Max. coverage (+): 0. Max coverage (-): 0

Region: NODE\_355988\_length\_17263\_cov\_24.156172 9624-9639. Max. coverage (+): 0. Max coverage (-): 0

Region: NODE\_355988\_length\_17263\_cov\_24.156172 9640-9656. Max. coverage (+): 0. Max coverage (-): 0

Region: NODE\_355988\_length\_17263\_cov\_24.156172 9657-9672. Max. coverage (+): 0. Max coverage (-): 0

Region: NODE\_355988\_length\_17263\_cov\_24.156172 9673-9688. Max. coverage (+): 0. Max coverage (-): 0

Region: NODE\_355988\_length\_17263\_cov\_24.156172 9689-9705. Max. coverage (+): 0. Max coverage (-): 0

Region: NODE\_355988\_length\_17263\_cov\_24.156172 9706-9721. Max. coverage (+): 0. Max coverage (-): 0.16

Region: NODE\_355988\_length\_17263\_cov\_24.156172 9722-9737. Max. coverage (+): 0. Max coverage (-): 0.04

Region: NODE\_355988\_length\_17263\_cov\_24.156172 9738-9754. Max. coverage (+): 0.04. Max coverage (-): 0

Region: NODE\_355988\_length\_17263\_cov\_24.156172 9755-9770. Max. coverage (+): 0. Max coverage (-): 0

Region: NODE\_355988\_length\_17263\_cov\_24.156172 9771-9786. Max. coverage (+): 0. Max coverage (-): 0

Region: NODE\_355988\_length\_17263\_cov\_24.156172 9787-9803. Max. coverage (+): 0. Max coverage (-): 0

Region: NODE\_355988\_length\_17263\_cov\_24.156172 9804-9819. Max. coverage (+): 0. Max coverage (-): 0.08

Region: NODE\_355988\_length\_17263\_cov\_24.156172 9820-9835. Max. coverage (+): 0. Max coverage (-): 0.01

Region: NODE\_355988\_length\_17263\_cov\_24.156172 9836-9852. Max. coverage (+): 0. Max coverage (-): 0

Region: NODE\_355988\_length\_17263\_cov\_24.156172 9853-9868. Max. coverage (+): 0. Max coverage (-): 0

Region: NODE\_355988\_length\_17263\_cov\_24.156172 9869-9884. Max. coverage (+): 0. Max coverage (-): 0

Region: NODE\_355988\_length\_17263\_cov\_24.156172 9885-9901. Max. coverage (+): 0. Max coverage (-): 0

Region: NODE\_355988\_length\_17263\_cov\_24.156172 9902-9917. Max. coverage (+): 0. Max coverage (-): 0

Region: NODE\_355988\_length\_17263\_cov\_24.156172 9918-9933. Max. coverage (+): 0. Max coverage (-): 0

Region: NODE\_355988\_length\_17263\_cov\_24.156172 9934-9950. Max. coverage (+): 0. Max coverage (-): 0

Region: NODE\_355988\_length\_17263\_cov\_24.156172 9951-9966. Max. coverage (+): 0. Max coverage (-): 0

Region: NODE\_355988\_length\_17263\_cov\_24.156172 9967-9982. Max. coverage (+): 0. Max coverage (-): 0

Region: NODE\_355988\_length\_17263\_cov\_24.156172 9983-9999. Max. coverage (+): 0. Max coverage (-): 0

Region: NODE\_355988\_length\_17263\_cov\_24.156172 10000-10015. Max. coverage (+): 0. Max coverage (-): 0

Region: NODE\_355988\_length\_17263\_cov\_24.156172 10016-10031. Max. coverage (+): 0. Max coverage (-): 0.02

Region: NODE\_355988\_length\_17263\_cov\_24.156172 10032-10048. Max. coverage (+): 0.02. Max coverage (-): 0

Region: NODE\_355988\_length\_17263\_cov\_24.156172 10049-10064. Max. coverage (+): 0.04. Max coverage (-): 0

Region: NODE\_355988\_length\_17263\_cov\_24.156172 10065-10080. Max. coverage (+): 0. Max coverage (-): 0

Region: NODE\_355988\_length\_17263\_cov\_24.156172 10081-10097. Max. coverage (+): 0. Max coverage (-): 0.1

Region: NODE\_355988\_length\_17263\_cov\_24.156172 10098-10113. Max. coverage (+): 0.04. Max coverage (-): 0.44

Region: NODE\_355988\_length\_17263\_cov\_24.156172 10114-10129. Max. coverage (+): 0.56. Max coverage (-): 2.46

Region: NODE\_355988\_length\_17263\_cov\_24.156172 10130-10146. Max. coverage (+): 0.02. Max coverage (-): 1.19

Region: NODE\_355988\_length\_17263\_cov\_24.156172 10147-10162. Max. coverage (+): 0.39. Max coverage (-): 0.88

Region: NODE\_355988\_length\_17263\_cov\_24.156172 10163-10178. Max. coverage (+): 0.47. Max coverage (-): 0.03

Region: NODE\_355988\_length\_17263\_cov\_24.156172 10179-10195. Max. coverage (+): 0. Max coverage (-): 24.86

Region: NODE\_355988\_length\_17263\_cov\_24.156172 10196-10211. Max. coverage (+): 0. Max coverage (-): 0

Region: NODE\_355988\_length\_17263\_cov\_24.156172 10212-10227. Max. coverage (+): 0.02. Max coverage (-): 0

Region: NODE\_355988\_length\_17263\_cov\_24.156172 10228-10244. Max. coverage (+): 0.02. Max coverage (-): 0

Region: NODE\_355988\_length\_17263\_cov\_24.156172 10245-10260. Max. coverage (+): 0.02. Max coverage (-): 0

Region: NODE\_355988\_length\_17263\_cov\_24.156172 10261-10276. Max. coverage (+): 0. Max coverage (-): 0

Region: NODE\_355988\_length\_17263\_cov\_24.156172 10277-10293. Max. coverage (+): 0. Max coverage (-): 0.01

Region: NODE\_355988\_length\_17263\_cov\_24.156172 10294-10309. Max. coverage (+): 0.02. Max coverage (-): 0

Region: NODE\_355988\_length\_17263\_cov\_24.156172 10310-10325. Max. coverage (+): 0.01. Max coverage (-): 0

Region: NODE\_355988\_length\_17263\_cov\_24.156172 10326-10342. Max. coverage (+): 0. Max coverage (-): 0

Region: NODE\_355988\_length\_17263\_cov\_24.156172 10343-10358. Max. coverage (+): 0. Max coverage (-): 0

Region: NODE\_355988\_length\_17263\_cov\_24.156172 10359-10374. Max. coverage (+): 0. Max coverage (-): 0.12

Region: NODE\_355988\_length\_17263\_cov\_24.156172 10375-10391. Max. coverage (+): 0. Max coverage (-): 0.04

Region: NODE\_355988\_length\_17263\_cov\_24.156172 10392-10407. Max. coverage (+): 0. Max coverage (-): 0.08

Region: NODE\_355988\_length\_17263\_cov\_24.156172 10408-10423. Max. coverage (+): 0.04. Max coverage (-): 0.08

Region: NODE\_355988\_length\_17263\_cov\_24.156172 10424-10440. Max. coverage (+): 0.16. Max coverage (-): 0.04

Region: NODE\_355988\_length\_17263\_cov\_24.156172 10441-10456. Max. coverage (+): 0.2. Max coverage (-): 0.04

Region: NODE\_355988\_length\_17263\_cov\_24.156172 10457-10472. Max. coverage (+): 0. Max coverage (-): 0.06

Region: NODE\_355988\_length\_17263\_cov\_24.156172 10473-10488. Max. coverage (+): 0. Max coverage (-): 0.14

Region: NODE\_355988\_length\_17263\_cov\_24.156172 10489-10505. Max. coverage (+): 0.08. Max coverage (-): 0

Region: NODE\_355988\_length\_17263\_cov\_24.156172 10506-10521. Max. coverage (+): 0. Max coverage (-): 0

Region: NODE\_355988\_length\_17263\_cov\_24.156172 10522-10537. Max. coverage (+): 0. Max coverage (-): 0.01

Region: NODE\_355988\_length\_17263\_cov\_24.156172 10538-10554. Max. coverage (+): 0. Max coverage (-): 0.04

Region: NODE\_355988\_length\_17263\_cov\_24.156172 10555-10570. Max. coverage (+): 0. Max coverage (-): 0.08

Region: NODE\_355988\_length\_17263\_cov\_24.156172 10571-10586. Max. coverage (+): 0.01. Max coverage (-): 0.08

Region: NODE\_355988\_length\_17263\_cov\_24.156172 10587-10603. Max. coverage (+): 0. Max coverage (-): 0.01

Region: NODE\_355988\_length\_17263\_cov\_24.156172 10604-10619. Max. coverage (+): 0. Max coverage (-): 0.01

Region: NODE\_355988\_length\_17263\_cov\_24.156172 10620-10635. Max. coverage (+): 0. Max coverage (-): 0.07

Region: NODE\_355988\_length\_17263\_cov\_24.156172 10636-10652. Max. coverage (+): 0.02. Max coverage (-): 0.2

Region: NODE\_355988\_length\_17263\_cov\_24.156172 10653-10668. Max. coverage (+): 0.02. Max coverage (-): 0.02

Region: NODE\_355988\_length\_17263\_cov\_24.156172 10669-10684. Max. coverage (+): 0.04. Max coverage (-): 0

Region: NODE\_355988\_length\_17263\_cov\_24.156172 10685-10701. Max. coverage (+): 0. Max coverage (-): 0

Region: NODE\_355988\_length\_17263\_cov\_24.156172 10702-10717. Max. coverage (+): 0. Max coverage (-): 0

Region: NODE\_355988\_length\_17263\_cov\_24.156172 10718-10733. Max. coverage (+): 0. Max coverage (-): 0

Region: NODE\_355988\_length\_17263\_cov\_24.156172 10734-10750. Max. coverage (+): 0. Max coverage (-): 0

Region: NODE\_355988\_length\_17263\_cov\_24.156172 10751-10766. Max. coverage (+): 0. Max coverage (-): 0.12

Region: NODE\_355988\_length\_17263\_cov\_24.156172 10767-10782. Max. coverage (+): 0. Max coverage (-): 0

Region: NODE\_355988\_length\_17263\_cov\_24.156172 10783-10799. Max. coverage (+): 0. Max coverage (-): 0

Region: NODE\_355988\_length\_17263\_cov\_24.156172 10800-10815. Max. coverage (+): 0. Max coverage (-): 0

Region: NODE\_355988\_length\_17263\_cov\_24.156172 10816-10831. Max. coverage (+): 0. Max coverage (-): 0.28

Region: NODE\_355988\_length\_17263\_cov\_24.156172 10832-10848. Max. coverage (+): 0. Max coverage (-): 0.36

Region: NODE\_355988\_length\_17263\_cov\_24.156172 10849-10864. Max. coverage (+): 0.12. Max coverage (-): 0

Region: NODE\_355988\_length\_17263\_cov\_24.156172 10865-10880. Max. coverage (+): 0. Max coverage (-): 0

Region: NODE\_355988\_length\_17263\_cov\_24.156172 10881-10897. Max. coverage (+): 0. Max coverage (-): 0.12

Region: NODE\_355988\_length\_17263\_cov\_24.156172 10898-10913. Max. coverage (+): 0. Max coverage (-): 0

Region: NODE\_355988\_length\_17263\_cov\_24.156172 10914-10929. Max. coverage (+): 0. Max coverage (-): 0

Region: NODE\_355988\_length\_17263\_cov\_24.156172 10930-10946. Max. coverage (+): 0.04. Max coverage (-): 0

Region: NODE\_355988\_length\_17263\_cov\_24.156172 10947-10962. Max. coverage (+): 0.06. Max coverage (-): 0

Region: NODE\_355988\_length\_17263\_cov\_24.156172 10963-10978. Max. coverage (+): 0. Max coverage (-): 0

Region: NODE\_355988\_length\_17263\_cov\_24.156172 10979-10995. Max. coverage (+): 0. Max coverage (-): 0

Region: NODE\_355988\_length\_17263\_cov\_24.156172 10996-11011. Max. coverage (+): 0. Max coverage (-): 0

Region: NODE\_355988\_length\_17263\_cov\_24.156172 11012-11027. Max. coverage (+): 0. Max coverage (-): 0

Region: NODE\_355988\_length\_17263\_cov\_24.156172 11028-11044. Max. coverage (+): 0. Max coverage (-): 0

Region: NODE\_355988\_length\_17263\_cov\_24.156172 11045-11060. Max. coverage (+): 0. Max coverage (-): 0

Region: NODE\_355988\_length\_17263\_cov\_24.156172 11061-11076. Max. coverage (+): 0. Max coverage (-): 0

Region: NODE\_355988\_length\_17263\_cov\_24.156172 11077-11093. Max. coverage (+): 0. Max coverage (-): 0

Region: NODE\_355988\_length\_17263\_cov\_24.156172 11094-11109. Max. coverage (+): 0. Max coverage (-): 0

Region: NODE\_355988\_length\_17263\_cov\_24.156172 11110-11125. Max. coverage (+): 0. Max coverage (-): 0

Region: NODE\_355988\_length\_17263\_cov\_24.156172 11126-11142. Max. coverage (+): 0. Max coverage (-): 0

Region: NODE\_355988\_length\_17263\_cov\_24.156172 11143-11158. Max. coverage (+): 0. Max coverage (-): 0

Region: NODE\_355988\_length\_17263\_cov\_24.156172 11159-11174. Max. coverage (+): 0. Max coverage (-): 0

Region: NODE\_355988\_length\_17263\_cov\_24.156172 11175-11191. Max. coverage (+): 0. Max coverage (-): 0

Region: NODE\_355988\_length\_17263\_cov\_24.156172 11192-11207. Max. coverage (+): 0. Max coverage (-): 0

Region: NODE\_355988\_length\_17263\_cov\_24.156172 11208-11223. Max. coverage (+): 0. Max coverage (-): 0

Region: NODE\_355988\_length\_17263\_cov\_24.156172 11224-11240. Max. coverage (+): 0. Max coverage (-): 0

Region: NODE\_355988\_length\_17263\_cov\_24.156172 11241-11256. Max. coverage (+): 0. Max coverage (-): 0

Region: NODE\_355988\_length\_17263\_cov\_24.156172 11257-11272. Max. coverage (+): 0. Max coverage (-): 0

Region: NODE\_355988\_length\_17263\_cov\_24.156172 11273-11289. Max. coverage (+): 0. Max coverage (-): 0

Region: NODE\_355988\_length\_17263\_cov\_24.156172 11290-11305. Max. coverage (+): 0. Max coverage (-): 0

Region: NODE\_355988\_length\_17263\_cov\_24.156172 11306-11321. Max. coverage (+): 0. Max coverage (-): 0

Region: NODE\_355988\_length\_17263\_cov\_24.156172 11322-11338. Max. coverage (+): 0. Max coverage (-): 0

Region: NODE\_355988\_length\_17263\_cov\_24.156172 11339-11354. Max. coverage (+): 0. Max coverage (-): 0

Region: NODE\_355988\_length\_17263\_cov\_24.156172 11355-11370. Max. coverage (+): 0. Max coverage (-): 0

Region: NODE\_355988\_length\_17263\_cov\_24.156172 11371-11387. Max. coverage (+): 0. Max coverage (-): 0

Region: NODE\_355988\_length\_17263\_cov\_24.156172 11388-11403. Max. coverage (+): 0. Max coverage (-): 0

Region: NODE\_355988\_length\_17263\_cov\_24.156172 11404-11419. Max. coverage (+): 0. Max coverage (-): 0

Region: NODE\_355988\_length\_17263\_cov\_24.156172 11420-11436. Max. coverage (+): 0. Max coverage (-): 0

Region: NODE\_355988\_length\_17263\_cov\_24.156172 11437-11452. Max. coverage (+): 0. Max coverage (-): 0

Region: NODE\_355988\_length\_17263\_cov\_24.156172 11453-11468. Max. coverage (+): 0. Max coverage (-): 0

Region: NODE\_355988\_length\_17263\_cov\_24.156172 11469-11485. Max. coverage (+): 0. Max coverage (-): 0

Region: NODE\_355988\_length\_17263\_cov\_24.156172 11486-11501. Max. coverage (+): 0. Max coverage (-): 0

Region: NODE\_355988\_length\_17263\_cov\_24.156172 11502-11517. Max. coverage (+): 0. Max coverage (-): 0

Region: NODE\_355988\_length\_17263\_cov\_24.156172 11518-11534. Max. coverage (+): 0. Max coverage (-): 0

Region: NODE\_355988\_length\_17263\_cov\_24.156172 11535-11550. Max. coverage (+): 0. Max coverage (-): 0

Region: NODE\_355988\_length\_17263\_cov\_24.156172 11551-11566. Max. coverage (+): 0. Max coverage (-): 0

Region: NODE\_355988\_length\_17263\_cov\_24.156172 11567-11583. Max. coverage (+): 0. Max coverage (-): 0

Region: NODE\_355988\_length\_17263\_cov\_24.156172 11584-11599. Max. coverage (+): 0. Max coverage (-): 0

Region: NODE\_355988\_length\_17263\_cov\_24.156172 11600-11615. Max. coverage (+): 0. Max coverage (-): 0

Region: NODE\_355988\_length\_17263\_cov\_24.156172 11616-11632. Max. coverage (+): 0. Max coverage (-): 0

Region: NODE\_355988\_length\_17263\_cov\_24.156172 11633-11648. Max. coverage (+): 0. Max coverage (-): 0

Region: NODE\_355988\_length\_17263\_cov\_24.156172 11649-11664. Max. coverage (+): 0. Max coverage (-): 0

Region: NODE\_355988\_length\_17263\_cov\_24.156172 11665-11681. Max. coverage (+): 0. Max coverage (-): 0

Region: NODE\_355988\_length\_17263\_cov\_24.156172 11682-11697. Max. coverage (+): 0. Max coverage (-): 0

Region: NODE\_355988\_length\_17263\_cov\_24.156172 11698-11713. Max. coverage (+): 0. Max coverage (-): 0

Region: NODE\_355988\_length\_17263\_cov\_24.156172 11714-11730. Max. coverage (+): 0. Max coverage (-): 0

Region: NODE\_355988\_length\_17263\_cov\_24.156172 11731-11746. Max. coverage (+): 0. Max coverage (-): 0

Region: NODE\_355988\_length\_17263\_cov\_24.156172 11747-11762. Max. coverage (+): 0. Max coverage (-): 0

Region: NODE\_355988\_length\_17263\_cov\_24.156172 11763-11779. Max. coverage (+): 0. Max coverage (-): 0

Region: NODE\_355988\_length\_17263\_cov\_24.156172 11780-11795. Max. coverage (+): 0. Max coverage (-): 0

Region: NODE\_355988\_length\_17263\_cov\_24.156172 11796-11811. Max. coverage (+): 0. Max coverage (-): 0

Region: NODE\_355988\_length\_17263\_cov\_24.156172 11812-11828. Max. coverage (+): 0. Max coverage (-): 0

Region: NODE\_355988\_length\_17263\_cov\_24.156172 11829-11844. Max. coverage (+): 0. Max coverage (-): 0

Region: NODE\_355988\_length\_17263\_cov\_24.156172 11845-11860. Max. coverage (+): 0. Max coverage (-): 0

Region: NODE\_355988\_length\_17263\_cov\_24.156172 11861-11877. Max. coverage (+): 0.05. Max coverage (-): 0

Region: NODE\_355988\_length\_17263\_cov\_24.156172 11878-11893. Max. coverage (+): 0. Max coverage (-): 0

Region: NODE\_355988\_length\_17263\_cov\_24.156172 11894-11909. Max. coverage (+): 0. Max coverage (-): 0

Region: NODE\_355988\_length\_17263\_cov\_24.156172 11910-11926. Max. coverage (+): 0. Max coverage (-): 0

Region: NODE\_355988\_length\_17263\_cov\_24.156172 11927-11942. Max. coverage (+): 0. Max coverage (-): 0

Region: NODE\_355988\_length\_17263\_cov\_24.156172 11943-11958. Max. coverage (+): 0. Max coverage (-): 0

Region: NODE\_355988\_length\_17263\_cov\_24.156172 11959-11975. Max. coverage (+): 0. Max coverage (-): 0

Region: NODE\_355988\_length\_17263\_cov\_24.156172 11976-11991. Max. coverage (+): 0. Max coverage (-): 0

Region: NODE\_355988\_length\_17263\_cov\_24.156172 11992-12007. Max. coverage (+): 0. Max coverage (-): 0

Region: NODE\_355988\_length\_17263\_cov\_24.156172 12008-12024. Max. coverage (+): 0. Max coverage (-): 0

Region: NODE\_355988\_length\_17263\_cov\_24.156172 12025-12040. Max. coverage (+): 0. Max coverage (-): 0

Region: NODE\_355988\_length\_17263\_cov\_24.156172 12041-12056. Max. coverage (+): 0. Max coverage (-): 0

Region: NODE\_355988\_length\_17263\_cov\_24.156172 12057-12073. Max. coverage (+): 0.04. Max coverage (-): 0

Region: NODE\_355988\_length\_17263\_cov\_24.156172 12074-12089. Max. coverage (+): 0. Max coverage (-): 0

Region: NODE\_355988\_length\_17263\_cov\_24.156172 12090-12105. Max. coverage (+): 0. Max coverage (-): 0

Region: NODE\_355988\_length\_17263\_cov\_24.156172 12106-12121. Max. coverage (+): 0. Max coverage (-): 0

Region: NODE\_355988\_length\_17263\_cov\_24.156172 12122-12138. Max. coverage (+): 0. Max coverage (-): 0

Region: NODE\_355988\_length\_17263\_cov\_24.156172 12139-12154. Max. coverage (+): 0. Max coverage (-): 0

Region: NODE\_355988\_length\_17263\_cov\_24.156172 12155-12170. Max. coverage (+): 0. Max coverage (-): 0

Region: NODE\_355988\_length\_17263\_cov\_24.156172 12171-12187. Max. coverage (+): 0. Max coverage (-): 0

Region: NODE\_355988\_length\_17263\_cov\_24.156172 12188-12203. Max. coverage (+): 0. Max coverage (-): 0

Region: NODE\_355988\_length\_17263\_cov\_24.156172 12204-12219. Max. coverage (+): 0. Max coverage (-): 0

Region: NODE\_355988\_length\_17263\_cov\_24.156172 12220-12236. Max. coverage (+): 0. Max coverage (-): 0

Region: NODE\_355988\_length\_17263\_cov\_24.156172 12237-12252. Max. coverage (+): 0. Max coverage (-): 0

Region: NODE\_355988\_length\_17263\_cov\_24.156172 12253-12268. Max. coverage (+): 0. Max coverage (-): 0

Region: NODE\_355988\_length\_17263\_cov\_24.156172 12269-12285. Max. coverage (+): 0. Max coverage (-): 0

Region: NODE\_355988\_length\_17263\_cov\_24.156172 12286-12301. Max. coverage (+): 0. Max coverage (-): 0

Region: NODE\_355988\_length\_17263\_cov\_24.156172 12302-12317. Max. coverage (+): 0. Max coverage (-): 0

Region: NODE\_355988\_length\_17263\_cov\_24.156172 12318-12334. Max. coverage (+): 0. Max coverage (-): 0

Region: NODE\_355988\_length\_17263\_cov\_24.156172 12335-12350. Max. coverage (+): 0. Max coverage (-): 0

Region: NODE\_355988\_length\_17263\_cov\_24.156172 12351-12366. Max. coverage (+): 0. Max coverage (-): 0

Region: NODE\_355988\_length\_17263\_cov\_24.156172 12367-12383. Max. coverage (+): 0. Max coverage (-): 0

Region: NODE\_355988\_length\_17263\_cov\_24.156172 12384-12399. Max. coverage (+): 0. Max coverage (-): 0

Region: NODE\_355988\_length\_17263\_cov\_24.156172 12400-12415. Max. coverage (+): 0. Max coverage (-): 0.04

Region: NODE\_355988\_length\_17263\_cov\_24.156172 12416-12432. Max. coverage (+): 0. Max coverage (-): 0.04

Region: NODE\_355988\_length\_17263\_cov\_24.156172 12433-12448. Max. coverage (+): 0. Max coverage (-): 0

Region: NODE\_355988\_length\_17263\_cov\_24.156172 12449-12464. Max. coverage (+): 0. Max coverage (-): 0

Region: NODE\_355988\_length\_17263\_cov\_24.156172 12465-12481. Max. coverage (+): 0. Max coverage (-): 0

Region: NODE\_355988\_length\_17263\_cov\_24.156172 12482-12497. Max. coverage (+): 0. Max coverage (-): 0

Region: NODE\_355988\_length\_17263\_cov\_24.156172 12498-12513. Max. coverage (+): 0. Max coverage (-): 0

Region: NODE\_355988\_length\_17263\_cov\_24.156172 12514-12530. Max. coverage (+): 0. Max coverage (-): 0

Region: NODE\_355988\_length\_17263\_cov\_24.156172 12531-12546. Max. coverage (+): 0. Max coverage (-): 0

Region: NODE\_355988\_length\_17263\_cov\_24.156172 12547-12562. Max. coverage (+): 0. Max coverage (-): 0

Region: NODE\_355988\_length\_17263\_cov\_24.156172 12563-12579. Max. coverage (+): 0. Max coverage (-): 0

Region: NODE\_355988\_length\_17263\_cov\_24.156172 12580-12595. Max. coverage (+): 0. Max coverage (-): 0

Region: NODE\_355988\_length\_17263\_cov\_24.156172 12596-12611. Max. coverage (+): 0. Max coverage (-): 0

Region: NODE\_355988\_length\_17263\_cov\_24.156172 12612-12628. Max. coverage (+): 0. Max coverage (-): 0

Region: NODE\_355988\_length\_17263\_cov\_24.156172 12629-12644. Max. coverage (+): 0. Max coverage (-): 0

Region: NODE\_355988\_length\_17263\_cov\_24.156172 12645-12660. Max. coverage (+): 0. Max coverage (-): 0

Region: NODE\_355988\_length\_17263\_cov\_24.156172 12661-12677. Max. coverage (+): 0. Max coverage (-): 0

Region: NODE\_355988\_length\_17263\_cov\_24.156172 12678-12693. Max. coverage (+): 0. Max coverage (-): 0

Region: NODE\_355988\_length\_17263\_cov\_24.156172 12694-12709. Max. coverage (+): 0. Max coverage (-): 0

Region: NODE\_355988\_length\_17263\_cov\_24.156172 12710-12726. Max. coverage (+): 0. Max coverage (-): 0

Region: NODE\_355988\_length\_17263\_cov\_24.156172 12727-12742. Max. coverage (+): 0. Max coverage (-): 0

Region: NODE\_355988\_length\_17263\_cov\_24.156172 12743-12758. Max. coverage (+): 0. Max coverage (-): 0

Region: NODE\_355988\_length\_17263\_cov\_24.156172 12759-12775. Max. coverage (+): 0. Max coverage (-): 0

Region: NODE\_355988\_length\_17263\_cov\_24.156172 12776-12791. Max. coverage (+): 0. Max coverage (-): 0

Region: NODE\_355988\_length\_17263\_cov\_24.156172 12792-12807. Max. coverage (+): 0. Max coverage (-): 0

Region: NODE\_355988\_length\_17263\_cov\_24.156172 12808-12824. Max. coverage (+): 0. Max coverage (-): 0

Region: NODE\_355988\_length\_17263\_cov\_24.156172 12825-12840. Max. coverage (+): 0. Max coverage (-): 0

Region: NODE\_355988\_length\_17263\_cov\_24.156172 12841-12856. Max. coverage (+): 0. Max coverage (-): 0

Region: NODE\_355988\_length\_17263\_cov\_24.156172 12857-12873. Max. coverage (+): 0. Max coverage (-): 0

Region: NODE\_355988\_length\_17263\_cov\_24.156172 12874-12889. Max. coverage (+): 0. Max coverage (-): 0.04

Region: NODE\_355988\_length\_17263\_cov\_24.156172 12890-12905. Max. coverage (+): 0. Max coverage (-): 0.04

Region: NODE\_355988\_length\_17263\_cov\_24.156172 12906-. Max. coverage (+): 0. Max coverage (-): 0

RepeatMasker Color Code

**+**

100-98% Identity

<98-95% Identity

<95-90% Identity

<90-85% Identity

<85-80% Identity

<80-75% Identity

<75-70% Identity

<70% Identity

**-**

Gene Set Color Code

**+**

Gene

Pseudogene

Other

**-**

Topology/Coverage Color Code

Coverage Plus Strand

Coverage Minus Strand

Mainstrand: Plus

Mainstrand: Minus

Complementary Strand

Flanking Region  
(if option -flank >0)

Gene Set Annotation  
  
RepeatMasker Annotation  

**1. SINE\_AFC**: 4743-5047 (-), Divergence to consensus: 22.3%  
**2. AlRepE-6108**: 5563-5877 (+), Divergence to consensus: 19%  
**3. AlRepE-6108**: 5962-6769 (+), Divergence to consensus: 29.4%  
**4. AlRepA-485**: 7796-8032 (-), Divergence to consensus: 20.4%  
**5. AlRepC-693**: 8258-8331 (-), Divergence to consensus: 29.9%  
**6. AlRepD-5199**: 8673-8931 (-), Divergence to consensus: 38.8%  
**7. AlRepC-1885**: 8939-9177 (-), Divergence to consensus: 30.4%  
**8. (AAAC)n**: 9478-9506 (+), Divergence to consensus: 14.6%  
**9. AlRepD-1636**: 9895-9969 (+), Divergence to consensus: 26%  
**10. AlRepB-767**: 10216-10359 (+), Divergence to consensus: 25.1%  
**11. AlRepD-1165**: 11820-12012 (+), Divergence to consensus: 28.1%  
**12. REX1-4\_AFC**: 12123-12306 (+), Divergence to consensus: 31.7%  
**13. AlRepD-7900**: 12670-12806 (+), Divergence to consensus: 9.7%  
**14. AlRepB-250**: 12893-12942 (+), Divergence to consensus: 18.3%

  
Transcription Factor Binding Sites  

**RFX4\_2** (Sequence: CTTGGTTAC (+): 8950)  
**RHOXF1** (Sequence: GGCTCA (-): 4985)  
**RHOXF1** (Sequence: GGATTA (-): 5080)  
**RHOXF1** (Sequence: AGCTCA (-): 5111)  
**RHOXF1** (Sequence: AGATTA (-): 5401)  
**RHOXF1** (Sequence: AGATTA (-): 6235)  
**RHOXF1** (Sequence: AGCTCA (-): 6328)  
**RHOXF1** (Sequence: AGATTA (-): 6436)  
**RHOXF1** (Sequence: AGATCA (-): 7290)  
**RHOXF1** (Sequence: AGATTA (-): 7451)  
**RHOXF1** (Sequence: AGCTTA (-): 7878)  
**RHOXF1** (Sequence: GGCTTA (-): 9446)  
**RHOXF1** (Sequence: AGCTTA (-): 9972)  
**RHOXF1** (Sequence: AGATCA (-): 10902)  
**RHOXF1** (Sequence: AGATTA (-): 11429)  
**RHOXF1** (Sequence: GGCTTA (-): 12294)  
**RHOXF1** (Sequence: AGATCA (-): 12482)  
**RHOXF1** (Sequence: GGATCA (-): 12676)  
**RHOXF1** (Sequence: GGCTCA (-): 12802)  
**RHOXF1** (Sequence: TGAGCC (+): 5031)  
**RHOXF1** (Sequence: TAAGCT (+): 5084)  
**RHOXF1** (Sequence: TAATCC (+): 5250)  
**RHOXF1** (Sequence: TAATCT (+): 5624)  
**RHOXF1** (Sequence: TGATCT (+): 5675)  
**RHOXF1** (Sequence: TGATCT (+): 6006)  
**RHOXF1** (Sequence: TAAGCC (+): 6057)  
**RHOXF1** (Sequence: TAATCT (+): 6632)  
**RHOXF1** (Sequence: TAATCT (+): 6697)  
**RHOXF1** (Sequence: TGAGCT (+): 7167)  
**RHOXF1** (Sequence: TGAGCT (+): 7176)  
**RHOXF1** (Sequence: TAAGCT (+): 7582)  
**RHOXF1** (Sequence: TGAGCC (+): 8435)  
**RHOXF1** (Sequence: TGAGCT (+): 10273)  
**RHOXF1** (Sequence: TGATCT (+): 10553)  
**Gata4** (Sequence: CTTATCT (+): 12186)  
**Gata4** (Sequence: CTTATCT (+): 12296)  
**POU5F1** (Sequence: TTTGCAT (-): 12164)  
**RFX4\_1** (Sequence: GTTGCCAGG (-): 8287)  
**SOX9** (Sequence: AACAATGA (-): 5216)  
**SOX9** (Sequence: AACAATGG (-): 8279)  
**FOXO1** (Sequence: CCTGTTTAC (+): 6250)  
**FOXO1** (Sequence: GTTGTTTAC (+): 8755)  
**FOXO1** (Sequence: CCTGTTTTC (+): 10057)  
**FOXO3\_mmu** (Sequence: TGTTTACC (-): 6252)  
**FOXO3\_mmu** (Sequence: TGTTTACA (-): 8757)  
**FOXO3\_mmu** (Sequence: TGTTTTCA (-): 10964)  
**Sox5** (Sequence: ATTGTT (+): 5653)  
**Sox5** (Sequence: ATTGTT (+): 6398)  
**Sox5** (Sequence: ATTGTT (+): 9078)  
**Sox5** (Sequence: ATTGTT (+): 9541)  
**Sox5** (Sequence: ATTGTT (+): 10229)  
**Sox5** (Sequence: ATTGTT (+): 12204)  
**Sox5** (Sequence: ATTGTT (+): 12332)  
**Sox5** (Sequence: ATTGTT (+): 12494)  
**FIGLA** (Sequence: ACCAGGTGGT (-): 10767)  
**FIGLA** (Sequence: AACAGCTGTA (-): 11271)  
**SOX9** (Sequence: TTATTGTT (+): 6396)  
**SOX9** (Sequence: CCATTGTT (+): 12330)  
**SOX9** (Sequence: TTATTGTT (+): 12492)  
**FOXO3\_mmu** (Sequence: TCTAAACA (+): 10895)  
**FOXO3\_mmu** (Sequence: TCAAAACA (+): 11267)  
**FOXO3\_mmu** (Sequence: GCAAAACA (+): 12404)  
**FOXO1** (Sequence: ATAAACAGC (-): 12634)  
**FOXO3\_hsa** (Sequence: TTGTTTAC (-): 8756)  
**FOXP1** (Sequence: TGTTTAC (-): 6252)  
**FOXP1** (Sequence: TGTTTAC (-): 8757)  
**POU2F1** (Sequence: ATTAACATA (-): 5207)  
**POU2F1** (Sequence: ATTTAAATA (-): 10533)  
**Rhox11** (Sequence: TGCTGTAAT (+): 12382)  
**Sox5** (Sequence: AACAAT (-): 5216)  
**Sox5** (Sequence: AACAAT (-): 8279)  
**Sox5** (Sequence: AACAAT (-): 8930)  
**Sox5** (Sequence: AACAAT (-): 12355)  
**POU2F1** (Sequence: TATTTTAAT (+): 6692)
