# Supplementary material for: First characterization of PIWI-interacting RNA clusters in a cichlid fish with a B chromosome
Source: BMC Biol. 2022 Sep 21;20:204. doi: 10.1186/s12915-022-01403-2 (PMC9490952; doi:10.1186/s12915-022-01403-2)
Supplement: Supplementary file 1 — Additional file 1. Zipped folder with fasta and interactive html piRNA cluster information for the A. latifasciata genome. The nomenclature is as follows: number-pirna-cluster_sex_B-presence (f, female; m, male; 0b, without B chromosome; 1b, with B chromosome). [file 12915_2022_1403_MOESM1_ESM.zip › 137_m1b.html]

piRNA cluster 137\_m1b 81


Predicted piRNA cluster no. 137\_m1b
  

Show proTRAC run info
Hide proTRAC run info

/\  
                \_\_\_\_\_\_\_\_\_\_\_\_\_\_\_\_\_\_\_\_\_\_\_/\\_\_\_ /  \\_\_\_\_\_\_\_  
               I                      /  \  /    \      I  
               I     pro             /    \/      \     I  
               I        TRAC        /               \   I  
               I   \_\_\_\_\_\_\_\_\_\_\_\_\_\_\_\_/\_\_\_\_\_\_\_\_\_\_\_\_\_\_\_\_\_\\_ I  
               I   \              /                     I  
               I    \            /                      I  
               I     \  /\      /       V.2.4.2         I  
               I      \/  \    /                        I  
               I\_\_\_\_\_\_\_\_\_\_\_\  /\_\_\_\_\_\_\_\_\_\_\_\_\_\_\_\_\_\_\_\_\_\_\_\_\_I  
                            \/  
  
  
================================= proTRAC ====================================  
VERSION: .......... 2.4.2  
LAST MODIFIED: .... 11. May 2018  
  
Please cite:  
Rosenkranz D, Zischler H. proTRAC - a software for probabilistic piRNA cluster  
detection, visualization and analysis. 2012. BMC Bioinformatics 13:5.  
  
  
Contact:  
David Rosenkranz  
Institute of Organismic and Molecular Evolutionary Biology  
Dept. Anthropology, small RNA group  
Johannes Gutenberg University Mainz  
email: rosenkranz@uni-mainz.de  
  
You can find the latest proTRAC version at:  
http://sourceforge.net/projects/protrac/files  
http://www.smallRNAgroup-mainz.de/software  
==============================================================================  
  
PARAMETERS:  
Map file: ...............piwi-machos-1B.fa-collapse.map  
Genome file: ............../../../0B\_ala\_genome.fa  
RepeatMasker annotation: Alatifasciata-all0B-maryan-v2.fa\_corrected.out  
GeneSet:................./guest-storage/Data/annotation/Alatifasciata\_all0B\_maryan-v2\_out2017.gff  
  
Significant (p<=0.01) hit density will be calculated based  
on observed hit distribution.  
  
Sliding window size: ........................................ 5000 bp  
Sliding window increament: .................................. 1000 bp  
Normalize each hit by number of genomic hits: ............... yes  
Normalize each hit by number of sequence reads: ............. yes  
Normalize values (-> per million mapped reads): ............. yes  
Min. fraction of hits with 1T(U) or 10A: .................... 0.75  
Alternatively: Min. fraction of hits with 1T(U) and 10A: .... 0.5  
Min. fraction of hits with typical piRNA length: ............ 0.75  
Typical piRNA length: ....................................... 24-32 nt  
Min. size of a piRNA cluster: ............................... 1000 bp.  
Min. number of hits (absolute): ............................. 0  
Min. number of hits (normalized): ........................... 0  
Min. fraction of hits on the mainstrand: .................... 0.75  
Top fraction of mapped sequences (in terms of read counts): . 1%  
Top fraction accounts for max. n% of sequence reads: ........ 90%  
Min. fraction of hits on each arm of a bidirectional cluster: 0.05  
Output html file for each cluster: .......................... yes  
Output a summary table: ..................................... yes  
Output a FASTA file for each cluster (piRNA sequences): ..... yes  
Output a FASTA file comprising cluster sequences: ........... yes  
Output a GTF file for predicted piRNA clusters: ..............yes  
Search DNA motifs in clusters: .............................. yes  
Output flanking sequences: +/- .............................. 0 bp  
Output ~.pTi file: .......................................... no  
==============================================================================  
  
  
Genome size (without gaps): ............ 758543724 bp  
Gaps (N/X/-): .......................... 417479 bp  
Mapped reads: .......................... 26973943  
Non-identical sequences: ............... 6209225  
Genomic hits: .......................... 48438990  
Significant densitiy of mapped reads: .. 821.144211136946 reads/kb

Show proTRAC cluster info
Hide proTRAC cluster info

|  |  |
| --- | --- |
| Location | NODE\_355988\_length\_17263\_cov\_24.156172 |
| Coordinates | 4749-12492 |
| Size [bp] | 7744 |
| Sequence hit loci | 3564 |
| Mapped reads (normalized) | 9327.5 |
| Mapped reads (normalized) per kb | 1204.5 |
| Normalized reads with 1T (1U) | 53.2% |
| Normalized reads with 10A | 50.6% |
| Normalized reads with length 24-32 nt | 99% |
| Normalized reads on the main strand(s) | 92.8% |
| Predicted directionality | mono:minus |

100%

0%

1T (1U)  
reads

10A reads

24-32 nt  
reads

reads on mainstrand

**Either the amount of reads with 1T (1U) OR 10A has to exceed 75% (set with option: -1Tor10A)  
Alternatively the amount of reads with 1T (1U) AND 10A has to exceed 50% (set with option: -1Tand10A)  
Minimum amount of reads with preferred size is 75% (set with option: -pisize)  
Minimum amount of reads on the main strand(s) is 75% (set with option: -clstrand)**

Show read coverage
Hide read coverage

WHAT DO I SEE HERE?  
This chart shows the location of mapped sequence reads within a predicted piRNA cluster. The color refers to the number of genomic hits produced by the sequence read in question. A dark red bar indicates that this sequence read produces many other hits elsewhere in the genome. Many adjacent red or yellow bars can indicate the presence of a multi-copy element such as transposons or rRNA genes. A dark green bar indicates that this sequence read maps uniquely to this locus.

1 hit

2-5 hits

6-10 hits

11-20 hits

21-50 hits

51-100 hits

> 100 hits

NODE\_355988\_length\_17263\_cov\_24.156172

4749

12492

Gene Set

RepeatMasker

Mapped  
Reads

26.06

plus strand

minus strand

26.06

Region: NODE\_355988\_length\_17263\_cov\_24.156172 4123-4756. Max. coverage (+): 0.01. Max coverage (-): 0.05

Region: NODE\_355988\_length\_17263\_cov\_24.156172 4757-4772. Max. coverage (+): 0. Max coverage (-): 0

Region: NODE\_355988\_length\_17263\_cov\_24.156172 4773-4787. Max. coverage (+): 0. Max coverage (-): 0

Region: NODE\_355988\_length\_17263\_cov\_24.156172 4788-4803. Max. coverage (+): 0. Max coverage (-): 0

Region: NODE\_355988\_length\_17263\_cov\_24.156172 4804-4818. Max. coverage (+): 0. Max coverage (-): 0

Region: NODE\_355988\_length\_17263\_cov\_24.156172 4819-4834. Max. coverage (+): 0. Max coverage (-): 0

Region: NODE\_355988\_length\_17263\_cov\_24.156172 4835-4849. Max. coverage (+): 0. Max coverage (-): 0

Region: NODE\_355988\_length\_17263\_cov\_24.156172 4850-4865. Max. coverage (+): 0. Max coverage (-): 0

Region: NODE\_355988\_length\_17263\_cov\_24.156172 4866-4880. Max. coverage (+): 0. Max coverage (-): 0

Region: NODE\_355988\_length\_17263\_cov\_24.156172 4881-4896. Max. coverage (+): 0. Max coverage (-): 0

Region: NODE\_355988\_length\_17263\_cov\_24.156172 4897-4911. Max. coverage (+): 0. Max coverage (-): 0

Region: NODE\_355988\_length\_17263\_cov\_24.156172 4912-4927. Max. coverage (+): 0. Max coverage (-): 0

Region: NODE\_355988\_length\_17263\_cov\_24.156172 4928-4942. Max. coverage (+): 0. Max coverage (-): 0

Region: NODE\_355988\_length\_17263\_cov\_24.156172 4943-4958. Max. coverage (+): 0. Max coverage (-): 0

Region: NODE\_355988\_length\_17263\_cov\_24.156172 4959-4973. Max. coverage (+): 0. Max coverage (-): 0

Region: NODE\_355988\_length\_17263\_cov\_24.156172 4974-4989. Max. coverage (+): 0. Max coverage (-): 0.03

Region: NODE\_355988\_length\_17263\_cov\_24.156172 4990-5004. Max. coverage (+): 0.01. Max coverage (-): 0

Region: NODE\_355988\_length\_17263\_cov\_24.156172 5005-5020. Max. coverage (+): 0.01. Max coverage (-): 0

Region: NODE\_355988\_length\_17263\_cov\_24.156172 5021-5035. Max. coverage (+): 0. Max coverage (-): 0

Region: NODE\_355988\_length\_17263\_cov\_24.156172 5036-5051. Max. coverage (+): 0. Max coverage (-): 0

Region: NODE\_355988\_length\_17263\_cov\_24.156172 5052-5066. Max. coverage (+): 0. Max coverage (-): 0

Region: NODE\_355988\_length\_17263\_cov\_24.156172 5067-5081. Max. coverage (+): 0. Max coverage (-): 0.13

Region: NODE\_355988\_length\_17263\_cov\_24.156172 5082-5097. Max. coverage (+): 0.19. Max coverage (-): 0.2

Region: NODE\_355988\_length\_17263\_cov\_24.156172 5098-5112. Max. coverage (+): 0.32. Max coverage (-): 1.52

Region: NODE\_355988\_length\_17263\_cov\_24.156172 5113-5128. Max. coverage (+): 0.06. Max coverage (-): 2.61

Region: NODE\_355988\_length\_17263\_cov\_24.156172 5129-5143. Max. coverage (+): 0.04. Max coverage (-): 0.35

Region: NODE\_355988\_length\_17263\_cov\_24.156172 5144-5159. Max. coverage (+): 0.02. Max coverage (-): 1.19

Region: NODE\_355988\_length\_17263\_cov\_24.156172 5160-5174. Max. coverage (+): 0.06. Max coverage (-): 0.2

Region: NODE\_355988\_length\_17263\_cov\_24.156172 5175-5190. Max. coverage (+): 0.02. Max coverage (-): 0.19

Region: NODE\_355988\_length\_17263\_cov\_24.156172 5191-5205. Max. coverage (+): 0.02. Max coverage (-): 0.06

Region: NODE\_355988\_length\_17263\_cov\_24.156172 5206-5221. Max. coverage (+): 0. Max coverage (-): 0

Region: NODE\_355988\_length\_17263\_cov\_24.156172 5222-5236. Max. coverage (+): 0. Max coverage (-): 0.09

Region: NODE\_355988\_length\_17263\_cov\_24.156172 5237-5252. Max. coverage (+): 0. Max coverage (-): 0

Region: NODE\_355988\_length\_17263\_cov\_24.156172 5253-5267. Max. coverage (+): 0. Max coverage (-): 0

Region: NODE\_355988\_length\_17263\_cov\_24.156172 5268-5283. Max. coverage (+): 0. Max coverage (-): 0

Region: NODE\_355988\_length\_17263\_cov\_24.156172 5284-5298. Max. coverage (+): 0. Max coverage (-): 0

Region: NODE\_355988\_length\_17263\_cov\_24.156172 5299-5314. Max. coverage (+): 0. Max coverage (-): 0

Region: NODE\_355988\_length\_17263\_cov\_24.156172 5315-5329. Max. coverage (+): 0. Max coverage (-): 0.04

Region: NODE\_355988\_length\_17263\_cov\_24.156172 5330-5345. Max. coverage (+): 0. Max coverage (-): 0

Region: NODE\_355988\_length\_17263\_cov\_24.156172 5346-5360. Max. coverage (+): 0. Max coverage (-): 0

Region: NODE\_355988\_length\_17263\_cov\_24.156172 5361-5376. Max. coverage (+): 0. Max coverage (-): 0

Region: NODE\_355988\_length\_17263\_cov\_24.156172 5377-5391. Max. coverage (+): 0. Max coverage (-): 0

Region: NODE\_355988\_length\_17263\_cov\_24.156172 5392-5407. Max. coverage (+): 0. Max coverage (-): 0

Region: NODE\_355988\_length\_17263\_cov\_24.156172 5408-5422. Max. coverage (+): 0. Max coverage (-): 0.7

Region: NODE\_355988\_length\_17263\_cov\_24.156172 5423-5438. Max. coverage (+): 0.04. Max coverage (-): 0.04

Region: NODE\_355988\_length\_17263\_cov\_24.156172 5439-5453. Max. coverage (+): 0. Max coverage (-): 0.06

Region: NODE\_355988\_length\_17263\_cov\_24.156172 5454-5469. Max. coverage (+): 0.06. Max coverage (-): 0.67

Region: NODE\_355988\_length\_17263\_cov\_24.156172 5470-5484. Max. coverage (+): 0.45. Max coverage (-): 2.26

Region: NODE\_355988\_length\_17263\_cov\_24.156172 5485-5500. Max. coverage (+): 0.04. Max coverage (-): 0.5

Region: NODE\_355988\_length\_17263\_cov\_24.156172 5501-5515. Max. coverage (+): 0.07. Max coverage (-): 0.76

Region: NODE\_355988\_length\_17263\_cov\_24.156172 5516-5531. Max. coverage (+): 0.3. Max coverage (-): 0.01

Region: NODE\_355988\_length\_17263\_cov\_24.156172 5532-5546. Max. coverage (+): 0.02. Max coverage (-): 25.51

Region: NODE\_355988\_length\_17263\_cov\_24.156172 5547-5562. Max. coverage (+): 0. Max coverage (-): 0.37

Region: NODE\_355988\_length\_17263\_cov\_24.156172 5563-5577. Max. coverage (+): 0.05. Max coverage (-): 0.01

Region: NODE\_355988\_length\_17263\_cov\_24.156172 5578-5593. Max. coverage (+): 0.02. Max coverage (-): 0.01

Region: NODE\_355988\_length\_17263\_cov\_24.156172 5594-5608. Max. coverage (+): 0. Max coverage (-): 0.15

Region: NODE\_355988\_length\_17263\_cov\_24.156172 5609-5624. Max. coverage (+): 0.06. Max coverage (-): 1.08

Region: NODE\_355988\_length\_17263\_cov\_24.156172 5625-5639. Max. coverage (+): 0.09. Max coverage (-): 0.2

Region: NODE\_355988\_length\_17263\_cov\_24.156172 5640-5655. Max. coverage (+): 0.01. Max coverage (-): 0.02

Region: NODE\_355988\_length\_17263\_cov\_24.156172 5656-5670. Max. coverage (+): 0.02. Max coverage (-): 0

Region: NODE\_355988\_length\_17263\_cov\_24.156172 5671-5686. Max. coverage (+): 0.04. Max coverage (-): 0

Region: NODE\_355988\_length\_17263\_cov\_24.156172 5687-5701. Max. coverage (+): 0. Max coverage (-): 0

Region: NODE\_355988\_length\_17263\_cov\_24.156172 5702-5716. Max. coverage (+): 0.06. Max coverage (-): 0

Region: NODE\_355988\_length\_17263\_cov\_24.156172 5717-5732. Max. coverage (+): 0.02. Max coverage (-): 0

Region: NODE\_355988\_length\_17263\_cov\_24.156172 5733-5747. Max. coverage (+): 0.01. Max coverage (-): 0.01

Region: NODE\_355988\_length\_17263\_cov\_24.156172 5748-5763. Max. coverage (+): 0. Max coverage (-): 0.01

Region: NODE\_355988\_length\_17263\_cov\_24.156172 5764-5778. Max. coverage (+): 0.01. Max coverage (-): 0

Region: NODE\_355988\_length\_17263\_cov\_24.156172 5779-5794. Max. coverage (+): 0. Max coverage (-): 0

Region: NODE\_355988\_length\_17263\_cov\_24.156172 5795-5809. Max. coverage (+): 0.02. Max coverage (-): 0

Region: NODE\_355988\_length\_17263\_cov\_24.156172 5810-5825. Max. coverage (+): 0.01. Max coverage (-): 0.01

Region: NODE\_355988\_length\_17263\_cov\_24.156172 5826-5840. Max. coverage (+): 0.01. Max coverage (-): 0

Region: NODE\_355988\_length\_17263\_cov\_24.156172 5841-5856. Max. coverage (+): 0. Max coverage (-): 0

Region: NODE\_355988\_length\_17263\_cov\_24.156172 5857-5871. Max. coverage (+): 0. Max coverage (-): 0

Region: NODE\_355988\_length\_17263\_cov\_24.156172 5872-5887. Max. coverage (+): 0. Max coverage (-): 0

Region: NODE\_355988\_length\_17263\_cov\_24.156172 5888-5902. Max. coverage (+): 0. Max coverage (-): 0

Region: NODE\_355988\_length\_17263\_cov\_24.156172 5903-5918. Max. coverage (+): 0. Max coverage (-): 0

Region: NODE\_355988\_length\_17263\_cov\_24.156172 5919-5933. Max. coverage (+): 0. Max coverage (-): 0

Region: NODE\_355988\_length\_17263\_cov\_24.156172 5934-5949. Max. coverage (+): 0. Max coverage (-): 0

Region: NODE\_355988\_length\_17263\_cov\_24.156172 5950-5964. Max. coverage (+): 0. Max coverage (-): 0

Region: NODE\_355988\_length\_17263\_cov\_24.156172 5965-5980. Max. coverage (+): 0.01. Max coverage (-): 0.01

Region: NODE\_355988\_length\_17263\_cov\_24.156172 5981-5995. Max. coverage (+): 0.02. Max coverage (-): 0.01

Region: NODE\_355988\_length\_17263\_cov\_24.156172 5996-6011. Max. coverage (+): 0.01. Max coverage (-): 0

Region: NODE\_355988\_length\_17263\_cov\_24.156172 6012-6026. Max. coverage (+): 0.01. Max coverage (-): 0.12

Region: NODE\_355988\_length\_17263\_cov\_24.156172 6027-6042. Max. coverage (+): 0.01. Max coverage (-): 0.12

Region: NODE\_355988\_length\_17263\_cov\_24.156172 6043-6057. Max. coverage (+): 0.01. Max coverage (-): 0

Region: NODE\_355988\_length\_17263\_cov\_24.156172 6058-6073. Max. coverage (+): 0.12. Max coverage (-): 0.05

Region: NODE\_355988\_length\_17263\_cov\_24.156172 6074-6088. Max. coverage (+): 2.58. Max coverage (-): 0.01

Region: NODE\_355988\_length\_17263\_cov\_24.156172 6089-6104. Max. coverage (+): 0.07. Max coverage (-): 0.02

Region: NODE\_355988\_length\_17263\_cov\_24.156172 6105-6119. Max. coverage (+): 0.07. Max coverage (-): 0.03

Region: NODE\_355988\_length\_17263\_cov\_24.156172 6120-6135. Max. coverage (+): 0.01. Max coverage (-): 0.02

Region: NODE\_355988\_length\_17263\_cov\_24.156172 6136-6150. Max. coverage (+): 0.01. Max coverage (-): 0.01

Region: NODE\_355988\_length\_17263\_cov\_24.156172 6151-6166. Max. coverage (+): 0.02. Max coverage (-): 0.01

Region: NODE\_355988\_length\_17263\_cov\_24.156172 6167-6181. Max. coverage (+): 0.02. Max coverage (-): 0.01

Region: NODE\_355988\_length\_17263\_cov\_24.156172 6182-6197. Max. coverage (+): 0.01. Max coverage (-): 0.01

Region: NODE\_355988\_length\_17263\_cov\_24.156172 6198-6212. Max. coverage (+): 0. Max coverage (-): 0.06

Region: NODE\_355988\_length\_17263\_cov\_24.156172 6213-6228. Max. coverage (+): 0.02. Max coverage (-): 0.01

Region: NODE\_355988\_length\_17263\_cov\_24.156172 6229-6243. Max. coverage (+): 0.03. Max coverage (-): 0.01

Region: NODE\_355988\_length\_17263\_cov\_24.156172 6244-6259. Max. coverage (+): 0. Max coverage (-): 0.22

Region: NODE\_355988\_length\_17263\_cov\_24.156172 6260-6274. Max. coverage (+): 0. Max coverage (-): 0.02

Region: NODE\_355988\_length\_17263\_cov\_24.156172 6275-6290. Max. coverage (+): 0.04. Max coverage (-): 0

Region: NODE\_355988\_length\_17263\_cov\_24.156172 6291-6305. Max. coverage (+): 0.04. Max coverage (-): 0

Region: NODE\_355988\_length\_17263\_cov\_24.156172 6306-6321. Max. coverage (+): 0. Max coverage (-): 0.01

Region: NODE\_355988\_length\_17263\_cov\_24.156172 6322-6336. Max. coverage (+): 0. Max coverage (-): 0.06

Region: NODE\_355988\_length\_17263\_cov\_24.156172 6337-6352. Max. coverage (+): 0.08. Max coverage (-): 0.01

Region: NODE\_355988\_length\_17263\_cov\_24.156172 6353-6367. Max. coverage (+): 0.08. Max coverage (-): 0

Region: NODE\_355988\_length\_17263\_cov\_24.156172 6368-6382. Max. coverage (+): 0.03. Max coverage (-): 0

Region: NODE\_355988\_length\_17263\_cov\_24.156172 6383-6398. Max. coverage (+): 0. Max coverage (-): 0.01

Region: NODE\_355988\_length\_17263\_cov\_24.156172 6399-6413. Max. coverage (+): 0.08. Max coverage (-): 0

Region: NODE\_355988\_length\_17263\_cov\_24.156172 6414-6429. Max. coverage (+): 0.01. Max coverage (-): 0.11

Region: NODE\_355988\_length\_17263\_cov\_24.156172 6430-6444. Max. coverage (+): 0. Max coverage (-): 0.01

Region: NODE\_355988\_length\_17263\_cov\_24.156172 6445-6460. Max. coverage (+): 0.01. Max coverage (-): 0

Region: NODE\_355988\_length\_17263\_cov\_24.156172 6461-6475. Max. coverage (+): 0. Max coverage (-): 0

Region: NODE\_355988\_length\_17263\_cov\_24.156172 6476-6491. Max. coverage (+): 0. Max coverage (-): 0.09

Region: NODE\_355988\_length\_17263\_cov\_24.156172 6492-6506. Max. coverage (+): 0.02. Max coverage (-): 0.09

Region: NODE\_355988\_length\_17263\_cov\_24.156172 6507-6522. Max. coverage (+): 0.04. Max coverage (-): 0

Region: NODE\_355988\_length\_17263\_cov\_24.156172 6523-6537. Max. coverage (+): 0.07. Max coverage (-): 0.01

Region: NODE\_355988\_length\_17263\_cov\_24.156172 6538-6553. Max. coverage (+): 0.01. Max coverage (-): 0.09

Region: NODE\_355988\_length\_17263\_cov\_24.156172 6554-6568. Max. coverage (+): 0.1. Max coverage (-): 0.06

Region: NODE\_355988\_length\_17263\_cov\_24.156172 6569-6584. Max. coverage (+): 0. Max coverage (-): 0.1

Region: NODE\_355988\_length\_17263\_cov\_24.156172 6585-6599. Max. coverage (+): 0. Max coverage (-): 0

Region: NODE\_355988\_length\_17263\_cov\_24.156172 6600-6615. Max. coverage (+): 0. Max coverage (-): 0

Region: NODE\_355988\_length\_17263\_cov\_24.156172 6616-6630. Max. coverage (+): 0. Max coverage (-): 0

Region: NODE\_355988\_length\_17263\_cov\_24.156172 6631-6646. Max. coverage (+): 0. Max coverage (-): 0

Region: NODE\_355988\_length\_17263\_cov\_24.156172 6647-6661. Max. coverage (+): 0.01. Max coverage (-): 0

Region: NODE\_355988\_length\_17263\_cov\_24.156172 6662-6677. Max. coverage (+): 0. Max coverage (-): 0.01

Region: NODE\_355988\_length\_17263\_cov\_24.156172 6678-6692. Max. coverage (+): 0.01. Max coverage (-): 0.01

Region: NODE\_355988\_length\_17263\_cov\_24.156172 6693-6708. Max. coverage (+): 0. Max coverage (-): 0.03

Region: NODE\_355988\_length\_17263\_cov\_24.156172 6709-6723. Max. coverage (+): 0.04. Max coverage (-): 0.01

Region: NODE\_355988\_length\_17263\_cov\_24.156172 6724-6739. Max. coverage (+): 0. Max coverage (-): 0

Region: NODE\_355988\_length\_17263\_cov\_24.156172 6740-6754. Max. coverage (+): 0. Max coverage (-): 0

Region: NODE\_355988\_length\_17263\_cov\_24.156172 6755-6770. Max. coverage (+): 0.04. Max coverage (-): 0

Region: NODE\_355988\_length\_17263\_cov\_24.156172 6771-6785. Max. coverage (+): 0. Max coverage (-): 0

Region: NODE\_355988\_length\_17263\_cov\_24.156172 6786-6801. Max. coverage (+): 0. Max coverage (-): 0

Region: NODE\_355988\_length\_17263\_cov\_24.156172 6802-6816. Max. coverage (+): 0. Max coverage (-): 0

Region: NODE\_355988\_length\_17263\_cov\_24.156172 6817-6832. Max. coverage (+): 0. Max coverage (-): 0

Region: NODE\_355988\_length\_17263\_cov\_24.156172 6833-6847. Max. coverage (+): 0. Max coverage (-): 0

Region: NODE\_355988\_length\_17263\_cov\_24.156172 6848-6863. Max. coverage (+): 0.04. Max coverage (-): 0

Region: NODE\_355988\_length\_17263\_cov\_24.156172 6864-6878. Max. coverage (+): 0. Max coverage (-): 0

Region: NODE\_355988\_length\_17263\_cov\_24.156172 6879-6894. Max. coverage (+): 0. Max coverage (-): 0

Region: NODE\_355988\_length\_17263\_cov\_24.156172 6895-6909. Max. coverage (+): 0.04. Max coverage (-): 0

Region: NODE\_355988\_length\_17263\_cov\_24.156172 6910-6925. Max. coverage (+): 0. Max coverage (-): 0.04

Region: NODE\_355988\_length\_17263\_cov\_24.156172 6926-6940. Max. coverage (+): 0.04. Max coverage (-): 0.06

Region: NODE\_355988\_length\_17263\_cov\_24.156172 6941-6956. Max. coverage (+): 0.04. Max coverage (-): 2.09

Region: NODE\_355988\_length\_17263\_cov\_24.156172 6957-6971. Max. coverage (+): 0.8. Max coverage (-): 0.09

Region: NODE\_355988\_length\_17263\_cov\_24.156172 6972-6987. Max. coverage (+): 0.02. Max coverage (-): 0.32

Region: NODE\_355988\_length\_17263\_cov\_24.156172 6988-7002. Max. coverage (+): 0.04. Max coverage (-): 0

Region: NODE\_355988\_length\_17263\_cov\_24.156172 7003-7017. Max. coverage (+): 0. Max coverage (-): 0.33

Region: NODE\_355988\_length\_17263\_cov\_24.156172 7018-7033. Max. coverage (+): 0.04. Max coverage (-): 19.65

Region: NODE\_355988\_length\_17263\_cov\_24.156172 7034-7048. Max. coverage (+): 0.11. Max coverage (-): 2.78

Region: NODE\_355988\_length\_17263\_cov\_24.156172 7049-7064. Max. coverage (+): 0.04. Max coverage (-): 0.22

Region: NODE\_355988\_length\_17263\_cov\_24.156172 7065-7079. Max. coverage (+): 0. Max coverage (-): 0

Region: NODE\_355988\_length\_17263\_cov\_24.156172 7080-7095. Max. coverage (+): 0. Max coverage (-): 0.06

Region: NODE\_355988\_length\_17263\_cov\_24.156172 7096-7110. Max. coverage (+): 0. Max coverage (-): 0.19

Region: NODE\_355988\_length\_17263\_cov\_24.156172 7111-7126. Max. coverage (+): 0. Max coverage (-): 0

Region: NODE\_355988\_length\_17263\_cov\_24.156172 7127-7141. Max. coverage (+): 0. Max coverage (-): 1.58

Region: NODE\_355988\_length\_17263\_cov\_24.156172 7142-7157. Max. coverage (+): 0.07. Max coverage (-): 0.57

Region: NODE\_355988\_length\_17263\_cov\_24.156172 7158-7172. Max. coverage (+): 0.07. Max coverage (-): 0.24

Region: NODE\_355988\_length\_17263\_cov\_24.156172 7173-7188. Max. coverage (+): 0.02. Max coverage (-): 0.5

Region: NODE\_355988\_length\_17263\_cov\_24.156172 7189-7203. Max. coverage (+): 0.07. Max coverage (-): 1

Region: NODE\_355988\_length\_17263\_cov\_24.156172 7204-7219. Max. coverage (+): 0.07. Max coverage (-): 0.04

Region: NODE\_355988\_length\_17263\_cov\_24.156172 7220-7234. Max. coverage (+): 0.02. Max coverage (-): 0.33

Region: NODE\_355988\_length\_17263\_cov\_24.156172 7235-7250. Max. coverage (+): 0.07. Max coverage (-): 0.02

Region: NODE\_355988\_length\_17263\_cov\_24.156172 7251-7265. Max. coverage (+): 0. Max coverage (-): 0.04

Region: NODE\_355988\_length\_17263\_cov\_24.156172 7266-7281. Max. coverage (+): 0.04. Max coverage (-): 3.86

Region: NODE\_355988\_length\_17263\_cov\_24.156172 7282-7296. Max. coverage (+): 0.04. Max coverage (-): 0.02

Region: NODE\_355988\_length\_17263\_cov\_24.156172 7297-7312. Max. coverage (+): 0. Max coverage (-): 0.04

Region: NODE\_355988\_length\_17263\_cov\_24.156172 7313-7327. Max. coverage (+): 0. Max coverage (-): 0.56

Region: NODE\_355988\_length\_17263\_cov\_24.156172 7328-7343. Max. coverage (+): 0. Max coverage (-): 0.09

Region: NODE\_355988\_length\_17263\_cov\_24.156172 7344-7358. Max. coverage (+): 0. Max coverage (-): 0.04

Region: NODE\_355988\_length\_17263\_cov\_24.156172 7359-7374. Max. coverage (+): 0. Max coverage (-): 0

Region: NODE\_355988\_length\_17263\_cov\_24.156172 7375-7389. Max. coverage (+): 0. Max coverage (-): 0

Region: NODE\_355988\_length\_17263\_cov\_24.156172 7390-7405. Max. coverage (+): 0. Max coverage (-): 0

Region: NODE\_355988\_length\_17263\_cov\_24.156172 7406-7420. Max. coverage (+): 0. Max coverage (-): 0

Region: NODE\_355988\_length\_17263\_cov\_24.156172 7421-7436. Max. coverage (+): 0. Max coverage (-): 0

Region: NODE\_355988\_length\_17263\_cov\_24.156172 7437-7451. Max. coverage (+): 0. Max coverage (-): 0

Region: NODE\_355988\_length\_17263\_cov\_24.156172 7452-7467. Max. coverage (+): 0. Max coverage (-): 0

Region: NODE\_355988\_length\_17263\_cov\_24.156172 7468-7482. Max. coverage (+): 0. Max coverage (-): 0

Region: NODE\_355988\_length\_17263\_cov\_24.156172 7483-7498. Max. coverage (+): 0. Max coverage (-): 0

Region: NODE\_355988\_length\_17263\_cov\_24.156172 7499-7513. Max. coverage (+): 0. Max coverage (-): 0

Region: NODE\_355988\_length\_17263\_cov\_24.156172 7514-7529. Max. coverage (+): 0. Max coverage (-): 0

Region: NODE\_355988\_length\_17263\_cov\_24.156172 7530-7544. Max. coverage (+): 0. Max coverage (-): 0

Region: NODE\_355988\_length\_17263\_cov\_24.156172 7545-7560. Max. coverage (+): 0. Max coverage (-): 0

Region: NODE\_355988\_length\_17263\_cov\_24.156172 7561-7575. Max. coverage (+): 0. Max coverage (-): 0

Region: NODE\_355988\_length\_17263\_cov\_24.156172 7576-7591. Max. coverage (+): 0. Max coverage (-): 0

Region: NODE\_355988\_length\_17263\_cov\_24.156172 7592-7606. Max. coverage (+): 0. Max coverage (-): 0

Region: NODE\_355988\_length\_17263\_cov\_24.156172 7607-7622. Max. coverage (+): 0. Max coverage (-): 0

Region: NODE\_355988\_length\_17263\_cov\_24.156172 7623-7637. Max. coverage (+): 0. Max coverage (-): 0

Region: NODE\_355988\_length\_17263\_cov\_24.156172 7638-7652. Max. coverage (+): 0. Max coverage (-): 0

Region: NODE\_355988\_length\_17263\_cov\_24.156172 7653-7668. Max. coverage (+): 0. Max coverage (-): 0

Region: NODE\_355988\_length\_17263\_cov\_24.156172 7669-7683. Max. coverage (+): 0. Max coverage (-): 0

Region: NODE\_355988\_length\_17263\_cov\_24.156172 7684-7699. Max. coverage (+): 0. Max coverage (-): 0

Region: NODE\_355988\_length\_17263\_cov\_24.156172 7700-7714. Max. coverage (+): 0. Max coverage (-): 0

Region: NODE\_355988\_length\_17263\_cov\_24.156172 7715-7730. Max. coverage (+): 0. Max coverage (-): 0

Region: NODE\_355988\_length\_17263\_cov\_24.156172 7731-7745. Max. coverage (+): 0. Max coverage (-): 0

Region: NODE\_355988\_length\_17263\_cov\_24.156172 7746-7761. Max. coverage (+): 0. Max coverage (-): 0

Region: NODE\_355988\_length\_17263\_cov\_24.156172 7762-7776. Max. coverage (+): 0. Max coverage (-): 0

Region: NODE\_355988\_length\_17263\_cov\_24.156172 7777-7792. Max. coverage (+): 0. Max coverage (-): 0

Region: NODE\_355988\_length\_17263\_cov\_24.156172 7793-7807. Max. coverage (+): 0. Max coverage (-): 0

Region: NODE\_355988\_length\_17263\_cov\_24.156172 7808-7823. Max. coverage (+): 0.01. Max coverage (-): 0

Region: NODE\_355988\_length\_17263\_cov\_24.156172 7824-7838. Max. coverage (+): 0.07. Max coverage (-): 0

Region: NODE\_355988\_length\_17263\_cov\_24.156172 7839-7854. Max. coverage (+): 0. Max coverage (-): 0

Region: NODE\_355988\_length\_17263\_cov\_24.156172 7855-7869. Max. coverage (+): 0. Max coverage (-): 0.11

Region: NODE\_355988\_length\_17263\_cov\_24.156172 7870-7885. Max. coverage (+): 0. Max coverage (-): 0.82

Region: NODE\_355988\_length\_17263\_cov\_24.156172 7886-7900. Max. coverage (+): 0.04. Max coverage (-): 4.82

Region: NODE\_355988\_length\_17263\_cov\_24.156172 7901-7916. Max. coverage (+): 0. Max coverage (-): 0.01

Region: NODE\_355988\_length\_17263\_cov\_24.156172 7917-7931. Max. coverage (+): 0.01. Max coverage (-): 0.02

Region: NODE\_355988\_length\_17263\_cov\_24.156172 7932-7947. Max. coverage (+): 0. Max coverage (-): 0

Region: NODE\_355988\_length\_17263\_cov\_24.156172 7948-7962. Max. coverage (+): 0.33. Max coverage (-): 0.04

Region: NODE\_355988\_length\_17263\_cov\_24.156172 7963-7978. Max. coverage (+): 0.26. Max coverage (-): 0

Region: NODE\_355988\_length\_17263\_cov\_24.156172 7979-7993. Max. coverage (+): 0.01. Max coverage (-): 0

Region: NODE\_355988\_length\_17263\_cov\_24.156172 7994-8009. Max. coverage (+): 0.07. Max coverage (-): 0

Region: NODE\_355988\_length\_17263\_cov\_24.156172 8010-8024. Max. coverage (+): 0. Max coverage (-): 0

Region: NODE\_355988\_length\_17263\_cov\_24.156172 8025-8040. Max. coverage (+): 0. Max coverage (-): 0.11

Region: NODE\_355988\_length\_17263\_cov\_24.156172 8041-8055. Max. coverage (+): 0. Max coverage (-): 1.33

Region: NODE\_355988\_length\_17263\_cov\_24.156172 8056-8071. Max. coverage (+): 0.26. Max coverage (-): 0.89

Region: NODE\_355988\_length\_17263\_cov\_24.156172 8072-8086. Max. coverage (+): 0.15. Max coverage (-): 0

Region: NODE\_355988\_length\_17263\_cov\_24.156172 8087-8102. Max. coverage (+): 0. Max coverage (-): 0

Region: NODE\_355988\_length\_17263\_cov\_24.156172 8103-8117. Max. coverage (+): 0. Max coverage (-): 0

Region: NODE\_355988\_length\_17263\_cov\_24.156172 8118-8133. Max. coverage (+): 0.44. Max coverage (-): 0.04

Region: NODE\_355988\_length\_17263\_cov\_24.156172 8134-8148. Max. coverage (+): 0.44. Max coverage (-): 0.7

Region: NODE\_355988\_length\_17263\_cov\_24.156172 8149-8164. Max. coverage (+): 0.04. Max coverage (-): 1.04

Region: NODE\_355988\_length\_17263\_cov\_24.156172 8165-8179. Max. coverage (+): 0. Max coverage (-): 0.01

Region: NODE\_355988\_length\_17263\_cov\_24.156172 8180-8195. Max. coverage (+): 0. Max coverage (-): 0.78

Region: NODE\_355988\_length\_17263\_cov\_24.156172 8196-8210. Max. coverage (+): 0.33. Max coverage (-): 0.19

Region: NODE\_355988\_length\_17263\_cov\_24.156172 8211-8226. Max. coverage (+): 0. Max coverage (-): 2.22

Region: NODE\_355988\_length\_17263\_cov\_24.156172 8227-8241. Max. coverage (+): 0.15. Max coverage (-): 1.08

Region: NODE\_355988\_length\_17263\_cov\_24.156172 8242-8257. Max. coverage (+): 0.07. Max coverage (-): 6.04

Region: NODE\_355988\_length\_17263\_cov\_24.156172 8258-8272. Max. coverage (+): 0.04. Max coverage (-): 2.08

Region: NODE\_355988\_length\_17263\_cov\_24.156172 8273-8288. Max. coverage (+): 0. Max coverage (-): 17.29

Region: NODE\_355988\_length\_17263\_cov\_24.156172 8289-8303. Max. coverage (+): 0.07. Max coverage (-): 14.11

Region: NODE\_355988\_length\_17263\_cov\_24.156172 8304-8318. Max. coverage (+): 0.48. Max coverage (-): 0.67

Region: NODE\_355988\_length\_17263\_cov\_24.156172 8319-8334. Max. coverage (+): 0.26. Max coverage (-): 0.33

Region: NODE\_355988\_length\_17263\_cov\_24.156172 8335-8349. Max. coverage (+): 0.3. Max coverage (-): 3.04

Region: NODE\_355988\_length\_17263\_cov\_24.156172 8350-8365. Max. coverage (+): 0.07. Max coverage (-): 3.11

Region: NODE\_355988\_length\_17263\_cov\_24.156172 8366-8380. Max. coverage (+): 0.07. Max coverage (-): 0.11

Region: NODE\_355988\_length\_17263\_cov\_24.156172 8381-8396. Max. coverage (+): 0.07. Max coverage (-): 0.04

Region: NODE\_355988\_length\_17263\_cov\_24.156172 8397-8411. Max. coverage (+): 0. Max coverage (-): 0.19

Region: NODE\_355988\_length\_17263\_cov\_24.156172 8412-8427. Max. coverage (+): 5.82. Max coverage (-): 0.04

Region: NODE\_355988\_length\_17263\_cov\_24.156172 8428-8442. Max. coverage (+): 0.15. Max coverage (-): 0.59

Region: NODE\_355988\_length\_17263\_cov\_24.156172 8443-8458. Max. coverage (+): 0. Max coverage (-): 2.08

Region: NODE\_355988\_length\_17263\_cov\_24.156172 8459-8473. Max. coverage (+): 0. Max coverage (-): 0.3

Region: NODE\_355988\_length\_17263\_cov\_24.156172 8474-8489. Max. coverage (+): 0. Max coverage (-): 13.09

Region: NODE\_355988\_length\_17263\_cov\_24.156172 8490-8504. Max. coverage (+): 0.07. Max coverage (-): 0.89

Region: NODE\_355988\_length\_17263\_cov\_24.156172 8505-8520. Max. coverage (+): 0. Max coverage (-): 0.04

Region: NODE\_355988\_length\_17263\_cov\_24.156172 8521-8535. Max. coverage (+): 0.04. Max coverage (-): 26.06

Region: NODE\_355988\_length\_17263\_cov\_24.156172 8536-8551. Max. coverage (+): 0.24. Max coverage (-): 0.54

Region: NODE\_355988\_length\_17263\_cov\_24.156172 8552-8566. Max. coverage (+): 0.06. Max coverage (-): 0.2

Region: NODE\_355988\_length\_17263\_cov\_24.156172 8567-8582. Max. coverage (+): 0.11. Max coverage (-): 6.32

Region: NODE\_355988\_length\_17263\_cov\_24.156172 8583-8597. Max. coverage (+): 0. Max coverage (-): 0.13

Region: NODE\_355988\_length\_17263\_cov\_24.156172 8598-8613. Max. coverage (+): 0.33. Max coverage (-): 0.04

Region: NODE\_355988\_length\_17263\_cov\_24.156172 8614-8628. Max. coverage (+): 0.35. Max coverage (-): 0.74

Region: NODE\_355988\_length\_17263\_cov\_24.156172 8629-8644. Max. coverage (+): 0.17. Max coverage (-): 0.22

Region: NODE\_355988\_length\_17263\_cov\_24.156172 8645-8659. Max. coverage (+): 0.19. Max coverage (-): 3.04

Region: NODE\_355988\_length\_17263\_cov\_24.156172 8660-8675. Max. coverage (+): 0. Max coverage (-): 0.04

Region: NODE\_355988\_length\_17263\_cov\_24.156172 8676-8690. Max. coverage (+): 0. Max coverage (-): 0.04

Region: NODE\_355988\_length\_17263\_cov\_24.156172 8691-8706. Max. coverage (+): 0. Max coverage (-): 0.44

Region: NODE\_355988\_length\_17263\_cov\_24.156172 8707-8721. Max. coverage (+): 0.05. Max coverage (-): 0.1

Region: NODE\_355988\_length\_17263\_cov\_24.156172 8722-8737. Max. coverage (+): 0.28. Max coverage (-): 1.71

Region: NODE\_355988\_length\_17263\_cov\_24.156172 8738-8752. Max. coverage (+): 0.02. Max coverage (-): 1.89

Region: NODE\_355988\_length\_17263\_cov\_24.156172 8753-8768. Max. coverage (+): 0.09. Max coverage (-): 2.31

Region: NODE\_355988\_length\_17263\_cov\_24.156172 8769-8783. Max. coverage (+): 0.11. Max coverage (-): 0.09

Region: NODE\_355988\_length\_17263\_cov\_24.156172 8784-8799. Max. coverage (+): 0.05. Max coverage (-): 0.21

Region: NODE\_355988\_length\_17263\_cov\_24.156172 8800-8814. Max. coverage (+): 0.04. Max coverage (-): 0.94

Region: NODE\_355988\_length\_17263\_cov\_24.156172 8815-8830. Max. coverage (+): 0.16. Max coverage (-): 0.89

Region: NODE\_355988\_length\_17263\_cov\_24.156172 8831-8845. Max. coverage (+): 0.01. Max coverage (-): 0.38

Region: NODE\_355988\_length\_17263\_cov\_24.156172 8846-8861. Max. coverage (+): 0.05. Max coverage (-): 0.26

Region: NODE\_355988\_length\_17263\_cov\_24.156172 8862-8876. Max. coverage (+): 0.01. Max coverage (-): 1.17

Region: NODE\_355988\_length\_17263\_cov\_24.156172 8877-8892. Max. coverage (+): 0.07. Max coverage (-): 0.3

Region: NODE\_355988\_length\_17263\_cov\_24.156172 8893-8907. Max. coverage (+): 0. Max coverage (-): 0.79

Region: NODE\_355988\_length\_17263\_cov\_24.156172 8908-8923. Max. coverage (+): 0.02. Max coverage (-): 2.61

Region: NODE\_355988\_length\_17263\_cov\_24.156172 8924-8938. Max. coverage (+): 1.32. Max coverage (-): 0.41

Region: NODE\_355988\_length\_17263\_cov\_24.156172 8939-8953. Max. coverage (+): 0.01. Max coverage (-): 6.61

Region: NODE\_355988\_length\_17263\_cov\_24.156172 8954-8969. Max. coverage (+): 0.19. Max coverage (-): 16.14

Region: NODE\_355988\_length\_17263\_cov\_24.156172 8970-8984. Max. coverage (+): 0.02. Max coverage (-): 8.54

Region: NODE\_355988\_length\_17263\_cov\_24.156172 8985-9000. Max. coverage (+): 0.05. Max coverage (-): 0.02

Region: NODE\_355988\_length\_17263\_cov\_24.156172 9001-9015. Max. coverage (+): 0.04. Max coverage (-): 1.66

Region: NODE\_355988\_length\_17263\_cov\_24.156172 9016-9031. Max. coverage (+): 0.02. Max coverage (-): 1.21

Region: NODE\_355988\_length\_17263\_cov\_24.156172 9032-9046. Max. coverage (+): 0. Max coverage (-): 0

Region: NODE\_355988\_length\_17263\_cov\_24.156172 9047-9062. Max. coverage (+): 0.04. Max coverage (-): 0.04

Region: NODE\_355988\_length\_17263\_cov\_24.156172 9063-9077. Max. coverage (+): 0.02. Max coverage (-): 0.65

Region: NODE\_355988\_length\_17263\_cov\_24.156172 9078-9093. Max. coverage (+): 0. Max coverage (-): 0

Region: NODE\_355988\_length\_17263\_cov\_24.156172 9094-9108. Max. coverage (+): 0. Max coverage (-): 0.98

Region: NODE\_355988\_length\_17263\_cov\_24.156172 9109-9124. Max. coverage (+): 0.01. Max coverage (-): 2.84

Region: NODE\_355988\_length\_17263\_cov\_24.156172 9125-9139. Max. coverage (+): 0.02. Max coverage (-): 0.02

Region: NODE\_355988\_length\_17263\_cov\_24.156172 9140-9155. Max. coverage (+): 0. Max coverage (-): 0.04

Region: NODE\_355988\_length\_17263\_cov\_24.156172 9156-9170. Max. coverage (+): 0.04. Max coverage (-): 0.3

Region: NODE\_355988\_length\_17263\_cov\_24.156172 9171-9186. Max. coverage (+): 0.06. Max coverage (-): 0.48

Region: NODE\_355988\_length\_17263\_cov\_24.156172 9187-9201. Max. coverage (+): 0.04. Max coverage (-): 0.11

Region: NODE\_355988\_length\_17263\_cov\_24.156172 9202-9217. Max. coverage (+): 0. Max coverage (-): 1.3

Region: NODE\_355988\_length\_17263\_cov\_24.156172 9218-9232. Max. coverage (+): 0. Max coverage (-): 0.04

Region: NODE\_355988\_length\_17263\_cov\_24.156172 9233-9248. Max. coverage (+): 0.09. Max coverage (-): 0.19

Region: NODE\_355988\_length\_17263\_cov\_24.156172 9249-9263. Max. coverage (+): 0.04. Max coverage (-): 0.06

Region: NODE\_355988\_length\_17263\_cov\_24.156172 9264-9279. Max. coverage (+): 0.04. Max coverage (-): 0.04

Region: NODE\_355988\_length\_17263\_cov\_24.156172 9280-9294. Max. coverage (+): 0. Max coverage (-): 0

Region: NODE\_355988\_length\_17263\_cov\_24.156172 9295-9310. Max. coverage (+): 0. Max coverage (-): 0

Region: NODE\_355988\_length\_17263\_cov\_24.156172 9311-9325. Max. coverage (+): 0. Max coverage (-): 0

Region: NODE\_355988\_length\_17263\_cov\_24.156172 9326-9341. Max. coverage (+): 0.22. Max coverage (-): 0.15

Region: NODE\_355988\_length\_17263\_cov\_24.156172 9342-9356. Max. coverage (+): 0.26. Max coverage (-): 0.19

Region: NODE\_355988\_length\_17263\_cov\_24.156172 9357-9372. Max. coverage (+): 0. Max coverage (-): 3.67

Region: NODE\_355988\_length\_17263\_cov\_24.156172 9373-9387. Max. coverage (+): 0.02. Max coverage (-): 4.47

Region: NODE\_355988\_length\_17263\_cov\_24.156172 9388-9403. Max. coverage (+): 0.07. Max coverage (-): 0.02

Region: NODE\_355988\_length\_17263\_cov\_24.156172 9404-9418. Max. coverage (+): 0. Max coverage (-): 0.04

Region: NODE\_355988\_length\_17263\_cov\_24.156172 9419-9434. Max. coverage (+): 0. Max coverage (-): 0

Region: NODE\_355988\_length\_17263\_cov\_24.156172 9435-9449. Max. coverage (+): 0. Max coverage (-): 0

Region: NODE\_355988\_length\_17263\_cov\_24.156172 9450-9465. Max. coverage (+): 0. Max coverage (-): 0.04

Region: NODE\_355988\_length\_17263\_cov\_24.156172 9466-9480. Max. coverage (+): 0. Max coverage (-): 0.02

Region: NODE\_355988\_length\_17263\_cov\_24.156172 9481-9496. Max. coverage (+): 0. Max coverage (-): 0.02

Region: NODE\_355988\_length\_17263\_cov\_24.156172 9497-9511. Max. coverage (+): 0. Max coverage (-): 0

Region: NODE\_355988\_length\_17263\_cov\_24.156172 9512-9527. Max. coverage (+): 0. Max coverage (-): 0.04

Region: NODE\_355988\_length\_17263\_cov\_24.156172 9528-9542. Max. coverage (+): 0. Max coverage (-): 0

Region: NODE\_355988\_length\_17263\_cov\_24.156172 9543-9558. Max. coverage (+): 0. Max coverage (-): 0

Region: NODE\_355988\_length\_17263\_cov\_24.156172 9559-9573. Max. coverage (+): 0. Max coverage (-): 0

Region: NODE\_355988\_length\_17263\_cov\_24.156172 9574-9588. Max. coverage (+): 0. Max coverage (-): 0

Region: NODE\_355988\_length\_17263\_cov\_24.156172 9589-9604. Max. coverage (+): 0. Max coverage (-): 0

Region: NODE\_355988\_length\_17263\_cov\_24.156172 9605-9619. Max. coverage (+): 0. Max coverage (-): 0

Region: NODE\_355988\_length\_17263\_cov\_24.156172 9620-9635. Max. coverage (+): 0. Max coverage (-): 0

Region: NODE\_355988\_length\_17263\_cov\_24.156172 9636-9650. Max. coverage (+): 0. Max coverage (-): 0

Region: NODE\_355988\_length\_17263\_cov\_24.156172 9651-9666. Max. coverage (+): 0. Max coverage (-): 0

Region: NODE\_355988\_length\_17263\_cov\_24.156172 9667-9681. Max. coverage (+): 0. Max coverage (-): 0

Region: NODE\_355988\_length\_17263\_cov\_24.156172 9682-9697. Max. coverage (+): 0. Max coverage (-): 0

Region: NODE\_355988\_length\_17263\_cov\_24.156172 9698-9712. Max. coverage (+): 0. Max coverage (-): 0

Region: NODE\_355988\_length\_17263\_cov\_24.156172 9713-9728. Max. coverage (+): 0. Max coverage (-): 0.23

Region: NODE\_355988\_length\_17263\_cov\_24.156172 9729-9743. Max. coverage (+): 0.13. Max coverage (-): 0.03

Region: NODE\_355988\_length\_17263\_cov\_24.156172 9744-9759. Max. coverage (+): 0. Max coverage (-): 0

Region: NODE\_355988\_length\_17263\_cov\_24.156172 9760-9774. Max. coverage (+): 0. Max coverage (-): 0

Region: NODE\_355988\_length\_17263\_cov\_24.156172 9775-9790. Max. coverage (+): 0. Max coverage (-): 0

Region: NODE\_355988\_length\_17263\_cov\_24.156172 9791-9805. Max. coverage (+): 0. Max coverage (-): 0

Region: NODE\_355988\_length\_17263\_cov\_24.156172 9806-9821. Max. coverage (+): 0.02. Max coverage (-): 0.07

Region: NODE\_355988\_length\_17263\_cov\_24.156172 9822-9836. Max. coverage (+): 0. Max coverage (-): 0.01

Region: NODE\_355988\_length\_17263\_cov\_24.156172 9837-9852. Max. coverage (+): 0. Max coverage (-): 0

Region: NODE\_355988\_length\_17263\_cov\_24.156172 9853-9867. Max. coverage (+): 0. Max coverage (-): 0

Region: NODE\_355988\_length\_17263\_cov\_24.156172 9868-9883. Max. coverage (+): 0. Max coverage (-): 0

Region: NODE\_355988\_length\_17263\_cov\_24.156172 9884-9898. Max. coverage (+): 0.04. Max coverage (-): 0

Region: NODE\_355988\_length\_17263\_cov\_24.156172 9899-9914. Max. coverage (+): 0. Max coverage (-): 0

Region: NODE\_355988\_length\_17263\_cov\_24.156172 9915-9929. Max. coverage (+): 0. Max coverage (-): 0

Region: NODE\_355988\_length\_17263\_cov\_24.156172 9930-9945. Max. coverage (+): 0. Max coverage (-): 0

Region: NODE\_355988\_length\_17263\_cov\_24.156172 9946-9960. Max. coverage (+): 0. Max coverage (-): 0

Region: NODE\_355988\_length\_17263\_cov\_24.156172 9961-9976. Max. coverage (+): 0. Max coverage (-): 0

Region: NODE\_355988\_length\_17263\_cov\_24.156172 9977-9991. Max. coverage (+): 0. Max coverage (-): 0

Region: NODE\_355988\_length\_17263\_cov\_24.156172 9992-10007. Max. coverage (+): 0. Max coverage (-): 0

Region: NODE\_355988\_length\_17263\_cov\_24.156172 10008-10022. Max. coverage (+): 0. Max coverage (-): 0

Region: NODE\_355988\_length\_17263\_cov\_24.156172 10023-10038. Max. coverage (+): 0. Max coverage (-): 0

Region: NODE\_355988\_length\_17263\_cov\_24.156172 10039-10053. Max. coverage (+): 0. Max coverage (-): 0

Region: NODE\_355988\_length\_17263\_cov\_24.156172 10054-10069. Max. coverage (+): 0. Max coverage (-): 0

Region: NODE\_355988\_length\_17263\_cov\_24.156172 10070-10084. Max. coverage (+): 0. Max coverage (-): 0.02

Region: NODE\_355988\_length\_17263\_cov\_24.156172 10085-10100. Max. coverage (+): 0. Max coverage (-): 0.69

Region: NODE\_355988\_length\_17263\_cov\_24.156172 10101-10115. Max. coverage (+): 0.07. Max coverage (-): 0.69

Region: NODE\_355988\_length\_17263\_cov\_24.156172 10116-10131. Max. coverage (+): 0.46. Max coverage (-): 2.27

Region: NODE\_355988\_length\_17263\_cov\_24.156172 10132-10146. Max. coverage (+): 0.02. Max coverage (-): 0.91

Region: NODE\_355988\_length\_17263\_cov\_24.156172 10147-10162. Max. coverage (+): 0.26. Max coverage (-): 0.66

Region: NODE\_355988\_length\_17263\_cov\_24.156172 10163-10177. Max. coverage (+): 0.3. Max coverage (-): 0

Region: NODE\_355988\_length\_17263\_cov\_24.156172 10178-10193. Max. coverage (+): 0. Max coverage (-): 25.8

Region: NODE\_355988\_length\_17263\_cov\_24.156172 10194-10208. Max. coverage (+): 0. Max coverage (-): 0

Region: NODE\_355988\_length\_17263\_cov\_24.156172 10209-10224. Max. coverage (+): 0. Max coverage (-): 0

Region: NODE\_355988\_length\_17263\_cov\_24.156172 10225-10239. Max. coverage (+): 0. Max coverage (-): 0

Region: NODE\_355988\_length\_17263\_cov\_24.156172 10240-10254. Max. coverage (+): 0. Max coverage (-): 0

Region: NODE\_355988\_length\_17263\_cov\_24.156172 10255-10270. Max. coverage (+): 0. Max coverage (-): 0

Region: NODE\_355988\_length\_17263\_cov\_24.156172 10271-10285. Max. coverage (+): 0. Max coverage (-): 0

Region: NODE\_355988\_length\_17263\_cov\_24.156172 10286-10301. Max. coverage (+): 0.01. Max coverage (-): 0.01

Region: NODE\_355988\_length\_17263\_cov\_24.156172 10302-10316. Max. coverage (+): 0.02. Max coverage (-): 0

Region: NODE\_355988\_length\_17263\_cov\_24.156172 10317-10332. Max. coverage (+): 0. Max coverage (-): 0

Region: NODE\_355988\_length\_17263\_cov\_24.156172 10333-10347. Max. coverage (+): 0.04. Max coverage (-): 0

Region: NODE\_355988\_length\_17263\_cov\_24.156172 10348-10363. Max. coverage (+): 0. Max coverage (-): 0.11

Region: NODE\_355988\_length\_17263\_cov\_24.156172 10364-10378. Max. coverage (+): 0. Max coverage (-): 0.2

Region: NODE\_355988\_length\_17263\_cov\_24.156172 10379-10394. Max. coverage (+): 0. Max coverage (-): 0.07

Region: NODE\_355988\_length\_17263\_cov\_24.156172 10395-10409. Max. coverage (+): 0. Max coverage (-): 0.15

Region: NODE\_355988\_length\_17263\_cov\_24.156172 10410-10425. Max. coverage (+): 0.07. Max coverage (-): 0.05

Region: NODE\_355988\_length\_17263\_cov\_24.156172 10426-10440. Max. coverage (+): 0.07. Max coverage (-): 0.22

Region: NODE\_355988\_length\_17263\_cov\_24.156172 10441-10456. Max. coverage (+): 0.11. Max coverage (-): 0.19

Region: NODE\_355988\_length\_17263\_cov\_24.156172 10457-10471. Max. coverage (+): 0. Max coverage (-): 0.04

Region: NODE\_355988\_length\_17263\_cov\_24.156172 10472-10487. Max. coverage (+): 0. Max coverage (-): 0.19

Region: NODE\_355988\_length\_17263\_cov\_24.156172 10488-10502. Max. coverage (+): 0.04. Max coverage (-): 0

Region: NODE\_355988\_length\_17263\_cov\_24.156172 10503-10518. Max. coverage (+): 0. Max coverage (-): 0

Region: NODE\_355988\_length\_17263\_cov\_24.156172 10519-10533. Max. coverage (+): 0.05. Max coverage (-): 0.1

Region: NODE\_355988\_length\_17263\_cov\_24.156172 10534-10549. Max. coverage (+): 0. Max coverage (-): 0

Region: NODE\_355988\_length\_17263\_cov\_24.156172 10550-10564. Max. coverage (+): 0. Max coverage (-): 0.01

Region: NODE\_355988\_length\_17263\_cov\_24.156172 10565-10580. Max. coverage (+): 0.01. Max coverage (-): 0.12

Region: NODE\_355988\_length\_17263\_cov\_24.156172 10581-10595. Max. coverage (+): 0.02. Max coverage (-): 0

Region: NODE\_355988\_length\_17263\_cov\_24.156172 10596-10611. Max. coverage (+): 0. Max coverage (-): 0.05

Region: NODE\_355988\_length\_17263\_cov\_24.156172 10612-10626. Max. coverage (+): 0.01. Max coverage (-): 0.01

Region: NODE\_355988\_length\_17263\_cov\_24.156172 10627-10642. Max. coverage (+): 0. Max coverage (-): 0.19

Region: NODE\_355988\_length\_17263\_cov\_24.156172 10643-10657. Max. coverage (+): 0. Max coverage (-): 0.03

Region: NODE\_355988\_length\_17263\_cov\_24.156172 10658-10673. Max. coverage (+): 0.04. Max coverage (-): 0

Region: NODE\_355988\_length\_17263\_cov\_24.156172 10674-10688. Max. coverage (+): 0.07. Max coverage (-): 0

Region: NODE\_355988\_length\_17263\_cov\_24.156172 10689-10704. Max. coverage (+): 0. Max coverage (-): 0

Region: NODE\_355988\_length\_17263\_cov\_24.156172 10705-10719. Max. coverage (+): 0. Max coverage (-): 0.11

Region: NODE\_355988\_length\_17263\_cov\_24.156172 10720-10735. Max. coverage (+): 0. Max coverage (-): 0.07

Region: NODE\_355988\_length\_17263\_cov\_24.156172 10736-10750. Max. coverage (+): 0. Max coverage (-): 0

Region: NODE\_355988\_length\_17263\_cov\_24.156172 10751-10766. Max. coverage (+): 0. Max coverage (-): 0.04

Region: NODE\_355988\_length\_17263\_cov\_24.156172 10767-10781. Max. coverage (+): 0. Max coverage (-): 0

Region: NODE\_355988\_length\_17263\_cov\_24.156172 10782-10797. Max. coverage (+): 0. Max coverage (-): 0

Region: NODE\_355988\_length\_17263\_cov\_24.156172 10798-10812. Max. coverage (+): 0. Max coverage (-): 0

Region: NODE\_355988\_length\_17263\_cov\_24.156172 10813-10828. Max. coverage (+): 0. Max coverage (-): 0

Region: NODE\_355988\_length\_17263\_cov\_24.156172 10829-10843. Max. coverage (+): 0. Max coverage (-): 0.11

Region: NODE\_355988\_length\_17263\_cov\_24.156172 10844-10859. Max. coverage (+): 0.04. Max coverage (-): 0

Region: NODE\_355988\_length\_17263\_cov\_24.156172 10860-10874. Max. coverage (+): 0. Max coverage (-): 0

Region: NODE\_355988\_length\_17263\_cov\_24.156172 10875-10889. Max. coverage (+): 0. Max coverage (-): 0

Region: NODE\_355988\_length\_17263\_cov\_24.156172 10890-10905. Max. coverage (+): 0. Max coverage (-): 0.11

Region: NODE\_355988\_length\_17263\_cov\_24.156172 10906-10920. Max. coverage (+): 0.04. Max coverage (-): 0

Region: NODE\_355988\_length\_17263\_cov\_24.156172 10921-10936. Max. coverage (+): 0. Max coverage (-): 0

Region: NODE\_355988\_length\_17263\_cov\_24.156172 10937-10951. Max. coverage (+): 0.02. Max coverage (-): 0

Region: NODE\_355988\_length\_17263\_cov\_24.156172 10952-10967. Max. coverage (+): 0. Max coverage (-): 0

Region: NODE\_355988\_length\_17263\_cov\_24.156172 10968-10982. Max. coverage (+): 0. Max coverage (-): 0

Region: NODE\_355988\_length\_17263\_cov\_24.156172 10983-10998. Max. coverage (+): 0. Max coverage (-): 0

Region: NODE\_355988\_length\_17263\_cov\_24.156172 10999-11013. Max. coverage (+): 0. Max coverage (-): 0

Region: NODE\_355988\_length\_17263\_cov\_24.156172 11014-11029. Max. coverage (+): 0. Max coverage (-): 0

Region: NODE\_355988\_length\_17263\_cov\_24.156172 11030-11044. Max. coverage (+): 0. Max coverage (-): 0

Region: NODE\_355988\_length\_17263\_cov\_24.156172 11045-11060. Max. coverage (+): 0. Max coverage (-): 0

Region: NODE\_355988\_length\_17263\_cov\_24.156172 11061-11075. Max. coverage (+): 0. Max coverage (-): 0

Region: NODE\_355988\_length\_17263\_cov\_24.156172 11076-11091. Max. coverage (+): 0. Max coverage (-): 0

Region: NODE\_355988\_length\_17263\_cov\_24.156172 11092-11106. Max. coverage (+): 0. Max coverage (-): 0

Region: NODE\_355988\_length\_17263\_cov\_24.156172 11107-11122. Max. coverage (+): 0. Max coverage (-): 0

Region: NODE\_355988\_length\_17263\_cov\_24.156172 11123-11137. Max. coverage (+): 0.04. Max coverage (-): 0

Region: NODE\_355988\_length\_17263\_cov\_24.156172 11138-11153. Max. coverage (+): 0.04. Max coverage (-): 0

Region: NODE\_355988\_length\_17263\_cov\_24.156172 11154-11168. Max. coverage (+): 0. Max coverage (-): 0

Region: NODE\_355988\_length\_17263\_cov\_24.156172 11169-11184. Max. coverage (+): 0. Max coverage (-): 0

Region: NODE\_355988\_length\_17263\_cov\_24.156172 11185-11199. Max. coverage (+): 0. Max coverage (-): 0

Region: NODE\_355988\_length\_17263\_cov\_24.156172 11200-11215. Max. coverage (+): 0. Max coverage (-): 0

Region: NODE\_355988\_length\_17263\_cov\_24.156172 11216-11230. Max. coverage (+): 0. Max coverage (-): 0

Region: NODE\_355988\_length\_17263\_cov\_24.156172 11231-11246. Max. coverage (+): 0. Max coverage (-): 0

Region: NODE\_355988\_length\_17263\_cov\_24.156172 11247-11261. Max. coverage (+): 0. Max coverage (-): 0

Region: NODE\_355988\_length\_17263\_cov\_24.156172 11262-11277. Max. coverage (+): 0.04. Max coverage (-): 0

Region: NODE\_355988\_length\_17263\_cov\_24.156172 11278-11292. Max. coverage (+): 0. Max coverage (-): 0

Region: NODE\_355988\_length\_17263\_cov\_24.156172 11293-11308. Max. coverage (+): 0. Max coverage (-): 0

Region: NODE\_355988\_length\_17263\_cov\_24.156172 11309-11323. Max. coverage (+): 0. Max coverage (-): 0

Region: NODE\_355988\_length\_17263\_cov\_24.156172 11324-11339. Max. coverage (+): 0. Max coverage (-): 0

Region: NODE\_355988\_length\_17263\_cov\_24.156172 11340-11354. Max. coverage (+): 0. Max coverage (-): 0

Region: NODE\_355988\_length\_17263\_cov\_24.156172 11355-11370. Max. coverage (+): 0. Max coverage (-): 0.04

Region: NODE\_355988\_length\_17263\_cov\_24.156172 11371-11385. Max. coverage (+): 0. Max coverage (-): 0

Region: NODE\_355988\_length\_17263\_cov\_24.156172 11386-11401. Max. coverage (+): 0. Max coverage (-): 0

Region: NODE\_355988\_length\_17263\_cov\_24.156172 11402-11416. Max. coverage (+): 0. Max coverage (-): 0

Region: NODE\_355988\_length\_17263\_cov\_24.156172 11417-11432. Max. coverage (+): 0. Max coverage (-): 0

Region: NODE\_355988\_length\_17263\_cov\_24.156172 11433-11447. Max. coverage (+): 0. Max coverage (-): 0

Region: NODE\_355988\_length\_17263\_cov\_24.156172 11448-11463. Max. coverage (+): 0. Max coverage (-): 0

Region: NODE\_355988\_length\_17263\_cov\_24.156172 11464-11478. Max. coverage (+): 0. Max coverage (-): 0

Region: NODE\_355988\_length\_17263\_cov\_24.156172 11479-11494. Max. coverage (+): 0. Max coverage (-): 0

Region: NODE\_355988\_length\_17263\_cov\_24.156172 11495-11509. Max. coverage (+): 0. Max coverage (-): 0

Region: NODE\_355988\_length\_17263\_cov\_24.156172 11510-11524. Max. coverage (+): 0. Max coverage (-): 0

Region: NODE\_355988\_length\_17263\_cov\_24.156172 11525-11540. Max. coverage (+): 0. Max coverage (-): 0

Region: NODE\_355988\_length\_17263\_cov\_24.156172 11541-11555. Max. coverage (+): 0. Max coverage (-): 0

Region: NODE\_355988\_length\_17263\_cov\_24.156172 11556-11571. Max. coverage (+): 0. Max coverage (-): 0

Region: NODE\_355988\_length\_17263\_cov\_24.156172 11572-11586. Max. coverage (+): 0. Max coverage (-): 0

Region: NODE\_355988\_length\_17263\_cov\_24.156172 11587-11602. Max. coverage (+): 0. Max coverage (-): 0

Region: NODE\_355988\_length\_17263\_cov\_24.156172 11603-11617. Max. coverage (+): 0. Max coverage (-): 0

Region: NODE\_355988\_length\_17263\_cov\_24.156172 11618-11633. Max. coverage (+): 0. Max coverage (-): 0

Region: NODE\_355988\_length\_17263\_cov\_24.156172 11634-11648. Max. coverage (+): 0. Max coverage (-): 0

Region: NODE\_355988\_length\_17263\_cov\_24.156172 11649-11664. Max. coverage (+): 0. Max coverage (-): 0

Region: NODE\_355988\_length\_17263\_cov\_24.156172 11665-11679. Max. coverage (+): 0. Max coverage (-): 0

Region: NODE\_355988\_length\_17263\_cov\_24.156172 11680-11695. Max. coverage (+): 0. Max coverage (-): 0

Region: NODE\_355988\_length\_17263\_cov\_24.156172 11696-11710. Max. coverage (+): 0. Max coverage (-): 0

Region: NODE\_355988\_length\_17263\_cov\_24.156172 11711-11726. Max. coverage (+): 0. Max coverage (-): 0

Region: NODE\_355988\_length\_17263\_cov\_24.156172 11727-11741. Max. coverage (+): 0. Max coverage (-): 0

Region: NODE\_355988\_length\_17263\_cov\_24.156172 11742-11757. Max. coverage (+): 0. Max coverage (-): 0

Region: NODE\_355988\_length\_17263\_cov\_24.156172 11758-11772. Max. coverage (+): 0. Max coverage (-): 0

Region: NODE\_355988\_length\_17263\_cov\_24.156172 11773-11788. Max. coverage (+): 0. Max coverage (-): 0

Region: NODE\_355988\_length\_17263\_cov\_24.156172 11789-11803. Max. coverage (+): 0. Max coverage (-): 0

Region: NODE\_355988\_length\_17263\_cov\_24.156172 11804-11819. Max. coverage (+): 0. Max coverage (-): 0

Region: NODE\_355988\_length\_17263\_cov\_24.156172 11820-11834. Max. coverage (+): 0. Max coverage (-): 0

Region: NODE\_355988\_length\_17263\_cov\_24.156172 11835-11850. Max. coverage (+): 0. Max coverage (-): 0

Region: NODE\_355988\_length\_17263\_cov\_24.156172 11851-11865. Max. coverage (+): 0. Max coverage (-): 0

Region: NODE\_355988\_length\_17263\_cov\_24.156172 11866-11881. Max. coverage (+): 0. Max coverage (-): 0

Region: NODE\_355988\_length\_17263\_cov\_24.156172 11882-11896. Max. coverage (+): 0. Max coverage (-): 0

Region: NODE\_355988\_length\_17263\_cov\_24.156172 11897-11912. Max. coverage (+): 0. Max coverage (-): 0

Region: NODE\_355988\_length\_17263\_cov\_24.156172 11913-11927. Max. coverage (+): 0. Max coverage (-): 0

Region: NODE\_355988\_length\_17263\_cov\_24.156172 11928-11943. Max. coverage (+): 0. Max coverage (-): 0

Region: NODE\_355988\_length\_17263\_cov\_24.156172 11944-11958. Max. coverage (+): 0. Max coverage (-): 0

Region: NODE\_355988\_length\_17263\_cov\_24.156172 11959-11974. Max. coverage (+): 0. Max coverage (-): 0

Region: NODE\_355988\_length\_17263\_cov\_24.156172 11975-11989. Max. coverage (+): 0. Max coverage (-): 0

Region: NODE\_355988\_length\_17263\_cov\_24.156172 11990-12005. Max. coverage (+): 0. Max coverage (-): 0

Region: NODE\_355988\_length\_17263\_cov\_24.156172 12006-12020. Max. coverage (+): 0. Max coverage (-): 0

Region: NODE\_355988\_length\_17263\_cov\_24.156172 12021-12036. Max. coverage (+): 0. Max coverage (-): 0

Region: NODE\_355988\_length\_17263\_cov\_24.156172 12037-12051. Max. coverage (+): 0. Max coverage (-): 0

Region: NODE\_355988\_length\_17263\_cov\_24.156172 12052-12067. Max. coverage (+): 0. Max coverage (-): 0

Region: NODE\_355988\_length\_17263\_cov\_24.156172 12068-12082. Max. coverage (+): 0. Max coverage (-): 0

Region: NODE\_355988\_length\_17263\_cov\_24.156172 12083-12098. Max. coverage (+): 0. Max coverage (-): 0

Region: NODE\_355988\_length\_17263\_cov\_24.156172 12099-12113. Max. coverage (+): 0. Max coverage (-): 0

Region: NODE\_355988\_length\_17263\_cov\_24.156172 12114-12129. Max. coverage (+): 0. Max coverage (-): 0

Region: NODE\_355988\_length\_17263\_cov\_24.156172 12130-12144. Max. coverage (+): 0. Max coverage (-): 0

Region: NODE\_355988\_length\_17263\_cov\_24.156172 12145-12160. Max. coverage (+): 0. Max coverage (-): 0

Region: NODE\_355988\_length\_17263\_cov\_24.156172 12161-12175. Max. coverage (+): 0. Max coverage (-): 0

Region: NODE\_355988\_length\_17263\_cov\_24.156172 12176-12190. Max. coverage (+): 0. Max coverage (-): 0

Region: NODE\_355988\_length\_17263\_cov\_24.156172 12191-12206. Max. coverage (+): 0. Max coverage (-): 0

Region: NODE\_355988\_length\_17263\_cov\_24.156172 12207-12221. Max. coverage (+): 0. Max coverage (-): 0

Region: NODE\_355988\_length\_17263\_cov\_24.156172 12222-12237. Max. coverage (+): 0. Max coverage (-): 0

Region: NODE\_355988\_length\_17263\_cov\_24.156172 12238-12252. Max. coverage (+): 0. Max coverage (-): 0

Region: NODE\_355988\_length\_17263\_cov\_24.156172 12253-12268. Max. coverage (+): 0. Max coverage (-): 0

Region: NODE\_355988\_length\_17263\_cov\_24.156172 12269-12283. Max. coverage (+): 0. Max coverage (-): 0

Region: NODE\_355988\_length\_17263\_cov\_24.156172 12284-12299. Max. coverage (+): 0. Max coverage (-): 0

Region: NODE\_355988\_length\_17263\_cov\_24.156172 12300-12314. Max. coverage (+): 0. Max coverage (-): 0

Region: NODE\_355988\_length\_17263\_cov\_24.156172 12315-12330. Max. coverage (+): 0. Max coverage (-): 0

Region: NODE\_355988\_length\_17263\_cov\_24.156172 12331-12345. Max. coverage (+): 0. Max coverage (-): 0

Region: NODE\_355988\_length\_17263\_cov\_24.156172 12346-12361. Max. coverage (+): 0. Max coverage (-): 0

Region: NODE\_355988\_length\_17263\_cov\_24.156172 12362-12376. Max. coverage (+): 0. Max coverage (-): 0

Region: NODE\_355988\_length\_17263\_cov\_24.156172 12377-12392. Max. coverage (+): 0. Max coverage (-): 0

Region: NODE\_355988\_length\_17263\_cov\_24.156172 12393-12407. Max. coverage (+): 0. Max coverage (-): 0

Region: NODE\_355988\_length\_17263\_cov\_24.156172 12408-12423. Max. coverage (+): 0. Max coverage (-): 0

Region: NODE\_355988\_length\_17263\_cov\_24.156172 12424-12438. Max. coverage (+): 0. Max coverage (-): 0

Region: NODE\_355988\_length\_17263\_cov\_24.156172 12439-12454. Max. coverage (+): 0. Max coverage (-): 0

Region: NODE\_355988\_length\_17263\_cov\_24.156172 12455-12469. Max. coverage (+): 0.11. Max coverage (-): 0

Region: NODE\_355988\_length\_17263\_cov\_24.156172 12470-12485. Max. coverage (+): 0. Max coverage (-): 0

Region: NODE\_355988\_length\_17263\_cov\_24.156172 12486-. Max. coverage (+): 0. Max coverage (-): 0

RepeatMasker Color Code

**+**

100-98% Identity

<98-95% Identity

<95-90% Identity

<90-85% Identity

<85-80% Identity

<80-75% Identity

<75-70% Identity

<70% Identity

**-**

Gene Set Color Code

**+**

Gene

Pseudogene

Other

**-**

Topology/Coverage Color Code

Coverage Plus Strand

Coverage Minus Strand

Mainstrand: Plus

Mainstrand: Minus

Complementary Strand

Flanking Region  
(if option -flank >0)

Gene Set Annotation  
  
RepeatMasker Annotation  

**1. SINE\_AFC**: 4743-5047 (-), Divergence to consensus: 22.3%  
**2. AlRepE-6108**: 5563-5877 (+), Divergence to consensus: 19%  
**3. AlRepE-6108**: 5962-6769 (+), Divergence to consensus: 29.4%  
**4. AlRepA-485**: 7796-8032 (-), Divergence to consensus: 20.4%  
**5. AlRepC-693**: 8258-8331 (-), Divergence to consensus: 29.9%  
**6. AlRepD-5199**: 8673-8931 (-), Divergence to consensus: 38.8%  
**7. AlRepC-1885**: 8939-9177 (-), Divergence to consensus: 30.4%  
**8. (AAAC)n**: 9478-9506 (+), Divergence to consensus: 14.6%  
**9. AlRepD-1636**: 9895-9969 (+), Divergence to consensus: 26%  
**10. AlRepB-767**: 10216-10359 (+), Divergence to consensus: 25.1%  
**11. AlRepD-1165**: 11820-12012 (+), Divergence to consensus: 28.1%  
**12. REX1-4\_AFC**: 12123-12306 (+), Divergence to consensus: 31.7%

  
Transcription Factor Binding Sites  

**RFX4\_2** (Sequence: CTTGGTTAC (+): 8950)  
**RHOXF1** (Sequence: GGCTCA (-): 4985)  
**RHOXF1** (Sequence: GGATTA (-): 5080)  
**RHOXF1** (Sequence: AGCTCA (-): 5111)  
**RHOXF1** (Sequence: AGATTA (-): 5401)  
**RHOXF1** (Sequence: AGATTA (-): 6235)  
**RHOXF1** (Sequence: AGCTCA (-): 6328)  
**RHOXF1** (Sequence: AGATTA (-): 6436)  
**RHOXF1** (Sequence: AGATCA (-): 7290)  
**RHOXF1** (Sequence: AGATTA (-): 7451)  
**RHOXF1** (Sequence: AGCTTA (-): 7878)  
**RHOXF1** (Sequence: GGCTTA (-): 9446)  
**RHOXF1** (Sequence: AGCTTA (-): 9972)  
**RHOXF1** (Sequence: AGATCA (-): 10902)  
**RHOXF1** (Sequence: AGATTA (-): 11429)  
**RHOXF1** (Sequence: GGCTTA (-): 12294)  
**RHOXF1** (Sequence: AGATCA (-): 12482)  
**RHOXF1** (Sequence: TGAGCC (+): 5031)  
**RHOXF1** (Sequence: TAAGCT (+): 5084)  
**RHOXF1** (Sequence: TAATCC (+): 5250)  
**RHOXF1** (Sequence: TAATCT (+): 5624)  
**RHOXF1** (Sequence: TGATCT (+): 5675)  
**RHOXF1** (Sequence: TGATCT (+): 6006)  
**RHOXF1** (Sequence: TAAGCC (+): 6057)  
**RHOXF1** (Sequence: TAATCT (+): 6632)  
**RHOXF1** (Sequence: TAATCT (+): 6697)  
**RHOXF1** (Sequence: TGAGCT (+): 7167)  
**RHOXF1** (Sequence: TGAGCT (+): 7176)  
**RHOXF1** (Sequence: TAAGCT (+): 7582)  
**RHOXF1** (Sequence: TGAGCC (+): 8435)  
**RHOXF1** (Sequence: TGAGCT (+): 10273)  
**RHOXF1** (Sequence: TGATCT (+): 10553)  
**Gata4** (Sequence: CTTATCT (+): 12186)  
**Gata4** (Sequence: CTTATCT (+): 12296)  
**POU5F1** (Sequence: TTTGCAT (-): 12164)  
**RFX4\_1** (Sequence: GTTGCCAGG (-): 8287)  
**SOX9** (Sequence: AACAATGA (-): 5216)  
**SOX9** (Sequence: AACAATGG (-): 8279)  
**FOXO1** (Sequence: CCTGTTTAC (+): 6250)  
**FOXO1** (Sequence: GTTGTTTAC (+): 8755)  
**FOXO1** (Sequence: CCTGTTTTC (+): 10057)  
**FOXO3\_mmu** (Sequence: TGTTTACC (-): 6252)  
**FOXO3\_mmu** (Sequence: TGTTTACA (-): 8757)  
**FOXO3\_mmu** (Sequence: TGTTTTCA (-): 10964)  
**Sox5** (Sequence: ATTGTT (+): 5653)  
**Sox5** (Sequence: ATTGTT (+): 6398)  
**Sox5** (Sequence: ATTGTT (+): 9078)  
**Sox5** (Sequence: ATTGTT (+): 9541)  
**Sox5** (Sequence: ATTGTT (+): 10229)  
**Sox5** (Sequence: ATTGTT (+): 12204)  
**Sox5** (Sequence: ATTGTT (+): 12332)  
**FIGLA** (Sequence: ACCAGGTGGT (-): 10767)  
**FIGLA** (Sequence: AACAGCTGTA (-): 11271)  
**SOX9** (Sequence: TTATTGTT (+): 6396)  
**SOX9** (Sequence: CCATTGTT (+): 12330)  
**FOXO3\_mmu** (Sequence: TCTAAACA (+): 10895)  
**FOXO3\_mmu** (Sequence: TCAAAACA (+): 11267)  
**FOXO3\_mmu** (Sequence: GCAAAACA (+): 12404)  
**FOXO3\_hsa** (Sequence: TTGTTTAC (-): 8756)  
**FOXP1** (Sequence: TGTTTAC (-): 6252)  
**FOXP1** (Sequence: TGTTTAC (-): 8757)  
**POU2F1** (Sequence: ATTAACATA (-): 5207)  
**POU2F1** (Sequence: ATTTAAATA (-): 10533)  
**Rhox11** (Sequence: TGCTGTAAT (+): 12382)  
**Sox5** (Sequence: AACAAT (-): 5216)  
**Sox5** (Sequence: AACAAT (-): 8279)  
**Sox5** (Sequence: AACAAT (-): 8930)  
**Sox5** (Sequence: AACAAT (-): 12355)  
**POU2F1** (Sequence: TATTTTAAT (+): 6692)
