# Supplementary material for: First characterization of PIWI-interacting RNA clusters in a cichlid fish with a B chromosome
Source: BMC Biol. 2022 Sep 21;20:204. doi: 10.1186/s12915-022-01403-2 (PMC9490952; doi:10.1186/s12915-022-01403-2)
Supplement: Supplementary file 1 — Additional file 1. Zipped folder with fasta and interactive html piRNA cluster information for the A. latifasciata genome. The nomenclature is as follows: number-pirna-cluster_sex_B-presence (f, female; m, male; 0b, without B chromosome; 1b, with B chromosome). [file 12915_2022_1403_MOESM1_ESM.zip › 138_m0b.html]

piRNA cluster 138\_m0b 66


Predicted piRNA cluster no. 138\_m0b
  

Show proTRAC run info
Hide proTRAC run info

/\  
                \_\_\_\_\_\_\_\_\_\_\_\_\_\_\_\_\_\_\_\_\_\_\_/\\_\_\_ /  \\_\_\_\_\_\_\_  
               I                      /  \  /    \      I  
               I     pro             /    \/      \     I  
               I        TRAC        /               \   I  
               I   \_\_\_\_\_\_\_\_\_\_\_\_\_\_\_\_/\_\_\_\_\_\_\_\_\_\_\_\_\_\_\_\_\_\\_ I  
               I   \              /                     I  
               I    \            /                      I  
               I     \  /\      /       V.2.4.2         I  
               I      \/  \    /                        I  
               I\_\_\_\_\_\_\_\_\_\_\_\  /\_\_\_\_\_\_\_\_\_\_\_\_\_\_\_\_\_\_\_\_\_\_\_\_\_I  
                            \/  
  
  
================================= proTRAC ====================================  
VERSION: .......... 2.4.2  
LAST MODIFIED: .... 11. May 2018  
  
Please cite:  
Rosenkranz D, Zischler H. proTRAC - a software for probabilistic piRNA cluster  
detection, visualization and analysis. 2012. BMC Bioinformatics 13:5.  
  
  
Contact:  
David Rosenkranz  
Institute of Organismic and Molecular Evolutionary Biology  
Dept. Anthropology, small RNA group  
Johannes Gutenberg University Mainz  
email: rosenkranz@uni-mainz.de  
  
You can find the latest proTRAC version at:  
http://sourceforge.net/projects/protrac/files  
http://www.smallRNAgroup-mainz.de/software  
==============================================================================  
  
PARAMETERS:  
Map file: ...............piwi-machos-0B.fa-collapse.map  
Genome file: ............../../../0B\_ala\_genome.fa  
RepeatMasker annotation: Alatifasciata-all0B-maryan-v2.fa\_corrected.out  
GeneSet:................./guest-storage/Data/annotation/Alatifasciata\_all0B\_maryan-v2\_out2017.gff  
  
Significant (p<=0.01) hit density will be calculated based  
on observed hit distribution.  
  
Sliding window size: ........................................ 5000 bp  
Sliding window increament: .................................. 1000 bp  
Normalize each hit by number of genomic hits: ............... yes  
Normalize each hit by number of sequence reads: ............. yes  
Normalize values (-> per million mapped reads): ............. yes  
Min. fraction of hits with 1T(U) or 10A: .................... 0.75  
Alternatively: Min. fraction of hits with 1T(U) and 10A: .... 0.5  
Min. fraction of hits with typical piRNA length: ............ 0.75  
Typical piRNA length: ....................................... 24-32 nt  
Min. size of a piRNA cluster: ............................... 1000 bp.  
Min. number of hits (absolute): ............................. 0  
Min. number of hits (normalized): ........................... 0  
Min. fraction of hits on the mainstrand: .................... 0.75  
Top fraction of mapped sequences (in terms of read counts): . 1%  
Top fraction accounts for max. n% of sequence reads: ........ 90%  
Min. fraction of hits on each arm of a bidirectional cluster: 0.05  
Output html file for each cluster: .......................... yes  
Output a summary table: ..................................... yes  
Output a FASTA file for each cluster (piRNA sequences): ..... yes  
Output a FASTA file comprising cluster sequences: ........... yes  
Output a GTF file for predicted piRNA clusters: ..............yes  
Search DNA motifs in clusters: .............................. yes  
Output flanking sequences: +/- .............................. 0 bp  
Output ~.pTi file: .......................................... no  
==============================================================================  
  
  
Genome size (without gaps): ............ 758543724 bp  
Gaps (N/X/-): .......................... 417479 bp  
Mapped reads: .......................... 24765598  
Non-identical sequences: ............... 6158275  
Genomic hits: .......................... 53103584  
Significant densitiy of mapped reads: .. 763.098963422187 reads/kb

Show proTRAC cluster info
Hide proTRAC cluster info

|  |  |
| --- | --- |
| Location | NODE\_356908\_length\_2531\_cov\_46.841564 |
| Coordinates | 32-2656 |
| Size [bp] | 2625 |
| Sequence hit loci | 4533 |
| Mapped reads (normalized) | 20681.3 |
| Mapped reads (normalized) per kb | 7878.6 |
| Normalized reads with 1T (1U) | 79.1% |
| Normalized reads with 10A | 35.2% |
| Normalized reads with length 24-32 nt | 98.3% |
| Normalized reads on the main strand(s) | 91.5% |
| Predicted directionality | mono:minus |

100%

0%

1T (1U)  
reads

10A reads

24-32 nt  
reads

reads on mainstrand

**Either the amount of reads with 1T (1U) OR 10A has to exceed 75% (set with option: -1Tor10A)  
Alternatively the amount of reads with 1T (1U) AND 10A has to exceed 50% (set with option: -1Tand10A)  
Minimum amount of reads with preferred size is 75% (set with option: -pisize)  
Minimum amount of reads on the main strand(s) is 75% (set with option: -clstrand)**

Show read coverage
Hide read coverage

WHAT DO I SEE HERE?  
This chart shows the location of mapped sequence reads within a predicted piRNA cluster. The color refers to the number of genomic hits produced by the sequence read in question. A dark red bar indicates that this sequence read produces many other hits elsewhere in the genome. Many adjacent red or yellow bars can indicate the presence of a multi-copy element such as transposons or rRNA genes. A dark green bar indicates that this sequence read maps uniquely to this locus.

1 hit

2-5 hits

6-10 hits

11-20 hits

21-50 hits

51-100 hits

> 100 hits

NODE\_356908\_length\_2531\_cov\_46.841564

32

2656

Gene Set

RepeatMasker

Mapped  
Reads

93.11

plus strand

minus strand

93.11

Region: NODE\_356908\_length\_2531\_cov\_46.841564 12907-34. Max. coverage (+): 0. Max coverage (-): 0.06

Region: NODE\_356908\_length\_2531\_cov\_46.841564 35-39. Max. coverage (+): 0. Max coverage (-): 0.09

Region: NODE\_356908\_length\_2531\_cov\_46.841564 40-45. Max. coverage (+): 0.04. Max coverage (-): 0.81

Region: NODE\_356908\_length\_2531\_cov\_46.841564 46-50. Max. coverage (+): 0.04. Max coverage (-): 1.57

Region: NODE\_356908\_length\_2531\_cov\_46.841564 51-55. Max. coverage (+): 0.08. Max coverage (-): 1.49

Region: NODE\_356908\_length\_2531\_cov\_46.841564 56-60. Max. coverage (+): 0. Max coverage (-): 0.16

Region: NODE\_356908\_length\_2531\_cov\_46.841564 61-66. Max. coverage (+): 0.12. Max coverage (-): 0.83

Region: NODE\_356908\_length\_2531\_cov\_46.841564 67-71. Max. coverage (+): 0.14. Max coverage (-): 0.1

Region: NODE\_356908\_length\_2531\_cov\_46.841564 72-76. Max. coverage (+): 0.04. Max coverage (-): 0.04

Region: NODE\_356908\_length\_2531\_cov\_46.841564 77-81. Max. coverage (+): 0.04. Max coverage (-): 0

Region: NODE\_356908\_length\_2531\_cov\_46.841564 82-87. Max. coverage (+): 0.08. Max coverage (-): 0.4

Region: NODE\_356908\_length\_2531\_cov\_46.841564 88-92. Max. coverage (+): 0.08. Max coverage (-): 0.24

Region: NODE\_356908\_length\_2531\_cov\_46.841564 93-97. Max. coverage (+): 0.04. Max coverage (-): 11.31

Region: NODE\_356908\_length\_2531\_cov\_46.841564 98-102. Max. coverage (+): 0.04. Max coverage (-): 6.78

Region: NODE\_356908\_length\_2531\_cov\_46.841564 103-108. Max. coverage (+): 0. Max coverage (-): 1.21

Region: NODE\_356908\_length\_2531\_cov\_46.841564 109-113. Max. coverage (+): 1.37. Max coverage (-): 0.36

Region: NODE\_356908\_length\_2531\_cov\_46.841564 114-118. Max. coverage (+): 1.53. Max coverage (-): 0.61

Region: NODE\_356908\_length\_2531\_cov\_46.841564 119-123. Max. coverage (+): 0.61. Max coverage (-): 0.12

Region: NODE\_356908\_length\_2531\_cov\_46.841564 124-129. Max. coverage (+): 0.04. Max coverage (-): 0.85

Region: NODE\_356908\_length\_2531\_cov\_46.841564 130-134. Max. coverage (+): 0. Max coverage (-): 69.98

Region: NODE\_356908\_length\_2531\_cov\_46.841564 135-139. Max. coverage (+): 0. Max coverage (-): 93.11

Region: NODE\_356908\_length\_2531\_cov\_46.841564 140-144. Max. coverage (+): 0.16. Max coverage (-): 20.39

Region: NODE\_356908\_length\_2531\_cov\_46.841564 145-150. Max. coverage (+): 0.57. Max coverage (-): 2.91

Region: NODE\_356908\_length\_2531\_cov\_46.841564 151-155. Max. coverage (+): 0.69. Max coverage (-): 0.93

Region: NODE\_356908\_length\_2531\_cov\_46.841564 156-160. Max. coverage (+): 0.12. Max coverage (-): 0.77

Region: NODE\_356908\_length\_2531\_cov\_46.841564 161-165. Max. coverage (+): 0.12. Max coverage (-): 12.52

Region: NODE\_356908\_length\_2531\_cov\_46.841564 166-171. Max. coverage (+): 0. Max coverage (-): 2.14

Region: NODE\_356908\_length\_2531\_cov\_46.841564 172-176. Max. coverage (+): 0. Max coverage (-): 0

Region: NODE\_356908\_length\_2531\_cov\_46.841564 177-181. Max. coverage (+): 0.08. Max coverage (-): 0.04

Region: NODE\_356908\_length\_2531\_cov\_46.841564 182-186. Max. coverage (+): 0.04. Max coverage (-): 1.01

Region: NODE\_356908\_length\_2531\_cov\_46.841564 187-192. Max. coverage (+): 0. Max coverage (-): 0.2

Region: NODE\_356908\_length\_2531\_cov\_46.841564 193-197. Max. coverage (+): 0.08. Max coverage (-): 3.15

Region: NODE\_356908\_length\_2531\_cov\_46.841564 198-202. Max. coverage (+): 0.12. Max coverage (-): 0.81

Region: NODE\_356908\_length\_2531\_cov\_46.841564 203-207. Max. coverage (+): 4.08. Max coverage (-): 0.57

Region: NODE\_356908\_length\_2531\_cov\_46.841564 208-213. Max. coverage (+): 4.16. Max coverage (-): 3.11

Region: NODE\_356908\_length\_2531\_cov\_46.841564 214-218. Max. coverage (+): 0.24. Max coverage (-): 1.09

Region: NODE\_356908\_length\_2531\_cov\_46.841564 219-223. Max. coverage (+): 0.2. Max coverage (-): 1.33

Region: NODE\_356908\_length\_2531\_cov\_46.841564 224-228. Max. coverage (+): 0. Max coverage (-): 5.41

Region: NODE\_356908\_length\_2531\_cov\_46.841564 229-234. Max. coverage (+): 0. Max coverage (-): 4.02

Region: NODE\_356908\_length\_2531\_cov\_46.841564 235-239. Max. coverage (+): 0.02. Max coverage (-): 3.74

Region: NODE\_356908\_length\_2531\_cov\_46.841564 240-244. Max. coverage (+): 2.56. Max coverage (-): 2.75

Region: NODE\_356908\_length\_2531\_cov\_46.841564 245-249. Max. coverage (+): 2.64. Max coverage (-): 2.56

Region: NODE\_356908\_length\_2531\_cov\_46.841564 250-255. Max. coverage (+): 0.2. Max coverage (-): 1.7

Region: NODE\_356908\_length\_2531\_cov\_46.841564 256-260. Max. coverage (+): 0.73. Max coverage (-): 1.05

Region: NODE\_356908\_length\_2531\_cov\_46.841564 261-265. Max. coverage (+): 0. Max coverage (-): 0.73

Region: NODE\_356908\_length\_2531\_cov\_46.841564 266-270. Max. coverage (+): 0.28. Max coverage (-): 1.86

Region: NODE\_356908\_length\_2531\_cov\_46.841564 271-276. Max. coverage (+): 0.12. Max coverage (-): 3.35

Region: NODE\_356908\_length\_2531\_cov\_46.841564 277-281. Max. coverage (+): 0.04. Max coverage (-): 0.2

Region: NODE\_356908\_length\_2531\_cov\_46.841564 282-286. Max. coverage (+): 0.36. Max coverage (-): 1.37

Region: NODE\_356908\_length\_2531\_cov\_46.841564 287-291. Max. coverage (+): 1.33. Max coverage (-): 0.16

Region: NODE\_356908\_length\_2531\_cov\_46.841564 292-297. Max. coverage (+): 0.12. Max coverage (-): 0.12

Region: NODE\_356908\_length\_2531\_cov\_46.841564 298-302. Max. coverage (+): 0.44. Max coverage (-): 0

Region: NODE\_356908\_length\_2531\_cov\_46.841564 303-307. Max. coverage (+): 0. Max coverage (-): 0

Region: NODE\_356908\_length\_2531\_cov\_46.841564 308-312. Max. coverage (+): 0. Max coverage (-): 0.4

Region: NODE\_356908\_length\_2531\_cov\_46.841564 313-318. Max. coverage (+): 0. Max coverage (-): 1.98

Region: NODE\_356908\_length\_2531\_cov\_46.841564 319-323. Max. coverage (+): 0.08. Max coverage (-): 1.86

Region: NODE\_356908\_length\_2531\_cov\_46.841564 324-328. Max. coverage (+): 0. Max coverage (-): 0

Region: NODE\_356908\_length\_2531\_cov\_46.841564 329-333. Max. coverage (+): 0.04. Max coverage (-): 0.24

Region: NODE\_356908\_length\_2531\_cov\_46.841564 334-339. Max. coverage (+): 0.4. Max coverage (-): 0.2

Region: NODE\_356908\_length\_2531\_cov\_46.841564 340-344. Max. coverage (+): 0.16. Max coverage (-): 0.12

Region: NODE\_356908\_length\_2531\_cov\_46.841564 345-349. Max. coverage (+): 0.12. Max coverage (-): 0.04

Region: NODE\_356908\_length\_2531\_cov\_46.841564 350-354. Max. coverage (+): 0.12. Max coverage (-): 1.21

Region: NODE\_356908\_length\_2531\_cov\_46.841564 355-360. Max. coverage (+): 0.04. Max coverage (-): 1.21

Region: NODE\_356908\_length\_2531\_cov\_46.841564 361-365. Max. coverage (+): 0. Max coverage (-): 0.04

Region: NODE\_356908\_length\_2531\_cov\_46.841564 366-370. Max. coverage (+): 0. Max coverage (-): 0

Region: NODE\_356908\_length\_2531\_cov\_46.841564 371-375. Max. coverage (+): 0. Max coverage (-): 0

Region: NODE\_356908\_length\_2531\_cov\_46.841564 376-381. Max. coverage (+): 0.08. Max coverage (-): 0.69

Region: NODE\_356908\_length\_2531\_cov\_46.841564 382-386. Max. coverage (+): 0. Max coverage (-): 2.71

Region: NODE\_356908\_length\_2531\_cov\_46.841564 387-391. Max. coverage (+): 0. Max coverage (-): 2.66

Region: NODE\_356908\_length\_2531\_cov\_46.841564 392-396. Max. coverage (+): 0.04. Max coverage (-): 0.24

Region: NODE\_356908\_length\_2531\_cov\_46.841564 397-402. Max. coverage (+): 0.28. Max coverage (-): 0.04

Region: NODE\_356908\_length\_2531\_cov\_46.841564 403-407. Max. coverage (+): 3.92. Max coverage (-): 1.01

Region: NODE\_356908\_length\_2531\_cov\_46.841564 408-412. Max. coverage (+): 0.24. Max coverage (-): 0.97

Region: NODE\_356908\_length\_2531\_cov\_46.841564 413-417. Max. coverage (+): 0. Max coverage (-): 0.52

Region: NODE\_356908\_length\_2531\_cov\_46.841564 418-423. Max. coverage (+): 0.04. Max coverage (-): 0.52

Region: NODE\_356908\_length\_2531\_cov\_46.841564 424-428. Max. coverage (+): 0.12. Max coverage (-): 0.2

Region: NODE\_356908\_length\_2531\_cov\_46.841564 429-433. Max. coverage (+): 0. Max coverage (-): 0.24

Region: NODE\_356908\_length\_2531\_cov\_46.841564 434-438. Max. coverage (+): 0.16. Max coverage (-): 0.32

Region: NODE\_356908\_length\_2531\_cov\_46.841564 439-444. Max. coverage (+): 0. Max coverage (-): 1.57

Region: NODE\_356908\_length\_2531\_cov\_46.841564 445-449. Max. coverage (+): 0. Max coverage (-): 0.04

Region: NODE\_356908\_length\_2531\_cov\_46.841564 450-454. Max. coverage (+): 0.04. Max coverage (-): 0.36

Region: NODE\_356908\_length\_2531\_cov\_46.841564 455-459. Max. coverage (+): 0.04. Max coverage (-): 1.17

Region: NODE\_356908\_length\_2531\_cov\_46.841564 460-465. Max. coverage (+): 0.04. Max coverage (-): 1.33

Region: NODE\_356908\_length\_2531\_cov\_46.841564 466-470. Max. coverage (+): 0.16. Max coverage (-): 12.72

Region: NODE\_356908\_length\_2531\_cov\_46.841564 471-475. Max. coverage (+): 0. Max coverage (-): 0.12

Region: NODE\_356908\_length\_2531\_cov\_46.841564 476-480. Max. coverage (+): 0. Max coverage (-): 1.21

Region: NODE\_356908\_length\_2531\_cov\_46.841564 481-486. Max. coverage (+): 0. Max coverage (-): 1.29

Region: NODE\_356908\_length\_2531\_cov\_46.841564 487-491. Max. coverage (+): 0. Max coverage (-): 0.48

Region: NODE\_356908\_length\_2531\_cov\_46.841564 492-496. Max. coverage (+): 0. Max coverage (-): 2.1

Region: NODE\_356908\_length\_2531\_cov\_46.841564 497-501. Max. coverage (+): 0.05. Max coverage (-): 2.73

Region: NODE\_356908\_length\_2531\_cov\_46.841564 502-507. Max. coverage (+): 0.01. Max coverage (-): 0.04

Region: NODE\_356908\_length\_2531\_cov\_46.841564 508-512. Max. coverage (+): 0.14. Max coverage (-): 0

Region: NODE\_356908\_length\_2531\_cov\_46.841564 513-517. Max. coverage (+): 0. Max coverage (-): 0.04

Region: NODE\_356908\_length\_2531\_cov\_46.841564 518-522. Max. coverage (+): 0. Max coverage (-): 0.44

Region: NODE\_356908\_length\_2531\_cov\_46.841564 523-528. Max. coverage (+): 0. Max coverage (-): 5.94

Region: NODE\_356908\_length\_2531\_cov\_46.841564 529-533. Max. coverage (+): 0. Max coverage (-): 4.52

Region: NODE\_356908\_length\_2531\_cov\_46.841564 534-538. Max. coverage (+): 0. Max coverage (-): 1.31

Region: NODE\_356908\_length\_2531\_cov\_46.841564 539-543. Max. coverage (+): 0. Max coverage (-): 0

Region: NODE\_356908\_length\_2531\_cov\_46.841564 544-549. Max. coverage (+): 0.2. Max coverage (-): 0.06

Region: NODE\_356908\_length\_2531\_cov\_46.841564 550-554. Max. coverage (+): 0.08. Max coverage (-): 0.08

Region: NODE\_356908\_length\_2531\_cov\_46.841564 555-559. Max. coverage (+): 0. Max coverage (-): 1.33

Region: NODE\_356908\_length\_2531\_cov\_46.841564 560-564. Max. coverage (+): 0. Max coverage (-): 1.13

Region: NODE\_356908\_length\_2531\_cov\_46.841564 565-570. Max. coverage (+): 0. Max coverage (-): 0.65

Region: NODE\_356908\_length\_2531\_cov\_46.841564 571-575. Max. coverage (+): 0. Max coverage (-): 0.08

Region: NODE\_356908\_length\_2531\_cov\_46.841564 576-580. Max. coverage (+): 0. Max coverage (-): 0.46

Region: NODE\_356908\_length\_2531\_cov\_46.841564 581-585. Max. coverage (+): 0. Max coverage (-): 1.21

Region: NODE\_356908\_length\_2531\_cov\_46.841564 586-591. Max. coverage (+): 0. Max coverage (-): 4.72

Region: NODE\_356908\_length\_2531\_cov\_46.841564 592-596. Max. coverage (+): 0. Max coverage (-): 0.76

Region: NODE\_356908\_length\_2531\_cov\_46.841564 597-601. Max. coverage (+): 0.01. Max coverage (-): 1.16

Region: NODE\_356908\_length\_2531\_cov\_46.841564 602-606. Max. coverage (+): 0. Max coverage (-): 1.27

Region: NODE\_356908\_length\_2531\_cov\_46.841564 607-612. Max. coverage (+): 0.04. Max coverage (-): 1.01

Region: NODE\_356908\_length\_2531\_cov\_46.841564 613-617. Max. coverage (+): 0.2. Max coverage (-): 0.16

Region: NODE\_356908\_length\_2531\_cov\_46.841564 618-622. Max. coverage (+): 0.16. Max coverage (-): 2.87

Region: NODE\_356908\_length\_2531\_cov\_46.841564 623-627. Max. coverage (+): 0.08. Max coverage (-): 2.62

Region: NODE\_356908\_length\_2531\_cov\_46.841564 628-633. Max. coverage (+): 0. Max coverage (-): 3.59

Region: NODE\_356908\_length\_2531\_cov\_46.841564 634-638. Max. coverage (+): 0. Max coverage (-): 2.1

Region: NODE\_356908\_length\_2531\_cov\_46.841564 639-643. Max. coverage (+): 0. Max coverage (-): 13.45

Region: NODE\_356908\_length\_2531\_cov\_46.841564 644-648. Max. coverage (+): 0.07. Max coverage (-): 14.25

Region: NODE\_356908\_length\_2531\_cov\_46.841564 649-654. Max. coverage (+): 0. Max coverage (-): 0.98

Region: NODE\_356908\_length\_2531\_cov\_46.841564 655-659. Max. coverage (+): 0.01. Max coverage (-): 1.39

Region: NODE\_356908\_length\_2531\_cov\_46.841564 660-664. Max. coverage (+): 0.01. Max coverage (-): 1.39

Region: NODE\_356908\_length\_2531\_cov\_46.841564 665-669. Max. coverage (+): 0.01. Max coverage (-): 0.07

Region: NODE\_356908\_length\_2531\_cov\_46.841564 670-675. Max. coverage (+): 0.02. Max coverage (-): 0.07

Region: NODE\_356908\_length\_2531\_cov\_46.841564 676-680. Max. coverage (+): 0.06. Max coverage (-): 0.13

Region: NODE\_356908\_length\_2531\_cov\_46.841564 681-685. Max. coverage (+): 0. Max coverage (-): 2.07

Region: NODE\_356908\_length\_2531\_cov\_46.841564 686-690. Max. coverage (+): 0. Max coverage (-): 0.81

Region: NODE\_356908\_length\_2531\_cov\_46.841564 691-696. Max. coverage (+): 0.03. Max coverage (-): 0.38

Region: NODE\_356908\_length\_2531\_cov\_46.841564 697-701. Max. coverage (+): 0. Max coverage (-): 0.03

Region: NODE\_356908\_length\_2531\_cov\_46.841564 702-706. Max. coverage (+): 0. Max coverage (-): 0

Region: NODE\_356908\_length\_2531\_cov\_46.841564 707-711. Max. coverage (+): 0. Max coverage (-): 0

Region: NODE\_356908\_length\_2531\_cov\_46.841564 712-717. Max. coverage (+): 0. Max coverage (-): 0

Region: NODE\_356908\_length\_2531\_cov\_46.841564 718-722. Max. coverage (+): 0. Max coverage (-): 0

Region: NODE\_356908\_length\_2531\_cov\_46.841564 723-727. Max. coverage (+): 0. Max coverage (-): 0

Region: NODE\_356908\_length\_2531\_cov\_46.841564 728-732. Max. coverage (+): 0. Max coverage (-): 0

Region: NODE\_356908\_length\_2531\_cov\_46.841564 733-738. Max. coverage (+): 0. Max coverage (-): 0

Region: NODE\_356908\_length\_2531\_cov\_46.841564 739-743. Max. coverage (+): 0. Max coverage (-): 0

Region: NODE\_356908\_length\_2531\_cov\_46.841564 744-748. Max. coverage (+): 0. Max coverage (-): 0

Region: NODE\_356908\_length\_2531\_cov\_46.841564 749-753. Max. coverage (+): 0. Max coverage (-): 0.08

Region: NODE\_356908\_length\_2531\_cov\_46.841564 754-759. Max. coverage (+): 0. Max coverage (-): 0

Region: NODE\_356908\_length\_2531\_cov\_46.841564 760-764. Max. coverage (+): 0.24. Max coverage (-): 0

Region: NODE\_356908\_length\_2531\_cov\_46.841564 765-769. Max. coverage (+): 0.12. Max coverage (-): 0

Region: NODE\_356908\_length\_2531\_cov\_46.841564 770-774. Max. coverage (+): 0.12. Max coverage (-): 0

Region: NODE\_356908\_length\_2531\_cov\_46.841564 775-780. Max. coverage (+): 0.16. Max coverage (-): 0.12

Region: NODE\_356908\_length\_2531\_cov\_46.841564 781-785. Max. coverage (+): 0. Max coverage (-): 0.06

Region: NODE\_356908\_length\_2531\_cov\_46.841564 786-790. Max. coverage (+): 0. Max coverage (-): 1.27

Region: NODE\_356908\_length\_2531\_cov\_46.841564 791-795. Max. coverage (+): 0. Max coverage (-): 1.29

Region: NODE\_356908\_length\_2531\_cov\_46.841564 796-801. Max. coverage (+): 0. Max coverage (-): 0.52

Region: NODE\_356908\_length\_2531\_cov\_46.841564 802-806. Max. coverage (+): 0. Max coverage (-): 2.73

Region: NODE\_356908\_length\_2531\_cov\_46.841564 807-811. Max. coverage (+): 0.05. Max coverage (-): 1.92

Region: NODE\_356908\_length\_2531\_cov\_46.841564 812-816. Max. coverage (+): 0. Max coverage (-): 0.01

Region: NODE\_356908\_length\_2531\_cov\_46.841564 817-822. Max. coverage (+): 0.14. Max coverage (-): 0.02

Region: NODE\_356908\_length\_2531\_cov\_46.841564 823-827. Max. coverage (+): 0. Max coverage (-): 0.06

Region: NODE\_356908\_length\_2531\_cov\_46.841564 828-832. Max. coverage (+): 0. Max coverage (-): 0.44

Region: NODE\_356908\_length\_2531\_cov\_46.841564 833-837. Max. coverage (+): 0. Max coverage (-): 5.94

Region: NODE\_356908\_length\_2531\_cov\_46.841564 838-843. Max. coverage (+): 0. Max coverage (-): 4.52

Region: NODE\_356908\_length\_2531\_cov\_46.841564 844-848. Max. coverage (+): 0. Max coverage (-): 1.31

Region: NODE\_356908\_length\_2531\_cov\_46.841564 849-853. Max. coverage (+): 0. Max coverage (-): 0

Region: NODE\_356908\_length\_2531\_cov\_46.841564 854-858. Max. coverage (+): 0.34. Max coverage (-): 0.02

Region: NODE\_356908\_length\_2531\_cov\_46.841564 859-864. Max. coverage (+): 0.04. Max coverage (-): 0

Region: NODE\_356908\_length\_2531\_cov\_46.841564 865-869. Max. coverage (+): 0. Max coverage (-): 0

Region: NODE\_356908\_length\_2531\_cov\_46.841564 870-874. Max. coverage (+): 0. Max coverage (-): 0

Region: NODE\_356908\_length\_2531\_cov\_46.841564 875-879. Max. coverage (+): 0. Max coverage (-): 0

Region: NODE\_356908\_length\_2531\_cov\_46.841564 880-885. Max. coverage (+): 0. Max coverage (-): 0

Region: NODE\_356908\_length\_2531\_cov\_46.841564 886-890. Max. coverage (+): 0.04. Max coverage (-): 0

Region: NODE\_356908\_length\_2531\_cov\_46.841564 891-895. Max. coverage (+): 0. Max coverage (-): 0.46

Region: NODE\_356908\_length\_2531\_cov\_46.841564 896-900. Max. coverage (+): 0. Max coverage (-): 1.21

Region: NODE\_356908\_length\_2531\_cov\_46.841564 901-906. Max. coverage (+): 0. Max coverage (-): 4.72

Region: NODE\_356908\_length\_2531\_cov\_46.841564 907-911. Max. coverage (+): 0. Max coverage (-): 0.91

Region: NODE\_356908\_length\_2531\_cov\_46.841564 912-916. Max. coverage (+): 0.01. Max coverage (-): 1.16

Region: NODE\_356908\_length\_2531\_cov\_46.841564 917-921. Max. coverage (+): 0.01. Max coverage (-): 0.82

Region: NODE\_356908\_length\_2531\_cov\_46.841564 922-927. Max. coverage (+): 0.4. Max coverage (-): 0.38

Region: NODE\_356908\_length\_2531\_cov\_46.841564 928-932. Max. coverage (+): 0.12. Max coverage (-): 0

Region: NODE\_356908\_length\_2531\_cov\_46.841564 933-937. Max. coverage (+): 0.24. Max coverage (-): 0

Region: NODE\_356908\_length\_2531\_cov\_46.841564 938-942. Max. coverage (+): 0.08. Max coverage (-): 0

Region: NODE\_356908\_length\_2531\_cov\_46.841564 943-948. Max. coverage (+): 0.04. Max coverage (-): 0

Region: NODE\_356908\_length\_2531\_cov\_46.841564 949-953. Max. coverage (+): 0. Max coverage (-): 0

Region: NODE\_356908\_length\_2531\_cov\_46.841564 954-958. Max. coverage (+): 0. Max coverage (-): 0

Region: NODE\_356908\_length\_2531\_cov\_46.841564 959-963. Max. coverage (+): 0.03. Max coverage (-): 0.85

Region: NODE\_356908\_length\_2531\_cov\_46.841564 964-969. Max. coverage (+): 0.03. Max coverage (-): 0.98

Region: NODE\_356908\_length\_2531\_cov\_46.841564 970-974. Max. coverage (+): 0.01. Max coverage (-): 0.05

Region: NODE\_356908\_length\_2531\_cov\_46.841564 975-979. Max. coverage (+): 0.01. Max coverage (-): 1.39

Region: NODE\_356908\_length\_2531\_cov\_46.841564 980-984. Max. coverage (+): 0.01. Max coverage (-): 0.04

Region: NODE\_356908\_length\_2531\_cov\_46.841564 985-990. Max. coverage (+): 0.02. Max coverage (-): 0.07

Region: NODE\_356908\_length\_2531\_cov\_46.841564 991-995. Max. coverage (+): 0.06. Max coverage (-): 0.04

Region: NODE\_356908\_length\_2531\_cov\_46.841564 996-1000. Max. coverage (+): 0. Max coverage (-): 2.07

Region: NODE\_356908\_length\_2531\_cov\_46.841564 1001-1005. Max. coverage (+): 0. Max coverage (-): 0.82

Region: NODE\_356908\_length\_2531\_cov\_46.841564 1006-1011. Max. coverage (+): 0.03. Max coverage (-): 0.51

Region: NODE\_356908\_length\_2531\_cov\_46.841564 1012-1016. Max. coverage (+): 0.03. Max coverage (-): 0.28

Region: NODE\_356908\_length\_2531\_cov\_46.841564 1017-1021. Max. coverage (+): 0.02. Max coverage (-): 0

Region: NODE\_356908\_length\_2531\_cov\_46.841564 1022-1026. Max. coverage (+): 0.02. Max coverage (-): 0

Region: NODE\_356908\_length\_2531\_cov\_46.841564 1027-1032. Max. coverage (+): 0. Max coverage (-): 0.12

Region: NODE\_356908\_length\_2531\_cov\_46.841564 1033-1037. Max. coverage (+): 0. Max coverage (-): 0.97

Region: NODE\_356908\_length\_2531\_cov\_46.841564 1038-1042. Max. coverage (+): 0.08. Max coverage (-): 0.34

Region: NODE\_356908\_length\_2531\_cov\_46.841564 1043-1047. Max. coverage (+): 0.28. Max coverage (-): 0.1

Region: NODE\_356908\_length\_2531\_cov\_46.841564 1048-1053. Max. coverage (+): 0.3. Max coverage (-): 0.06

Region: NODE\_356908\_length\_2531\_cov\_46.841564 1054-1058. Max. coverage (+): 0.2. Max coverage (-): 0.48

Region: NODE\_356908\_length\_2531\_cov\_46.841564 1059-1063. Max. coverage (+): 0. Max coverage (-): 1.68

Region: NODE\_356908\_length\_2531\_cov\_46.841564 1064-1068. Max. coverage (+): 0. Max coverage (-): 1.64

Region: NODE\_356908\_length\_2531\_cov\_46.841564 1069-1074. Max. coverage (+): 0. Max coverage (-): 0.26

Region: NODE\_356908\_length\_2531\_cov\_46.841564 1075-1079. Max. coverage (+): 0.04. Max coverage (-): 0

Region: NODE\_356908\_length\_2531\_cov\_46.841564 1080-1084. Max. coverage (+): 0.75. Max coverage (-): 0.02

Region: NODE\_356908\_length\_2531\_cov\_46.841564 1085-1089. Max. coverage (+): 0.12. Max coverage (-): 0.02

Region: NODE\_356908\_length\_2531\_cov\_46.841564 1090-1095. Max. coverage (+): 0.04. Max coverage (-): 1.35

Region: NODE\_356908\_length\_2531\_cov\_46.841564 1096-1100. Max. coverage (+): 0. Max coverage (-): 1.35

Region: NODE\_356908\_length\_2531\_cov\_46.841564 1101-1105. Max. coverage (+): 0.04. Max coverage (-): 0.16

Region: NODE\_356908\_length\_2531\_cov\_46.841564 1106-1110. Max. coverage (+): 0.04. Max coverage (-): 0.14

Region: NODE\_356908\_length\_2531\_cov\_46.841564 1111-1116. Max. coverage (+): 0.02. Max coverage (-): 0.04

Region: NODE\_356908\_length\_2531\_cov\_46.841564 1117-1121. Max. coverage (+): 0. Max coverage (-): 0

Region: NODE\_356908\_length\_2531\_cov\_46.841564 1122-1126. Max. coverage (+): 0. Max coverage (-): 0

Region: NODE\_356908\_length\_2531\_cov\_46.841564 1127-1131. Max. coverage (+): 0.02. Max coverage (-): 0.25

Region: NODE\_356908\_length\_2531\_cov\_46.841564 1132-1137. Max. coverage (+): 0.02. Max coverage (-): 0.38

Region: NODE\_356908\_length\_2531\_cov\_46.841564 1138-1142. Max. coverage (+): 0.02. Max coverage (-): 0.44

Region: NODE\_356908\_length\_2531\_cov\_46.841564 1143-1147. Max. coverage (+): 0. Max coverage (-): 1.31

Region: NODE\_356908\_length\_2531\_cov\_46.841564 1148-1152. Max. coverage (+): 0.12. Max coverage (-): 0.16

Region: NODE\_356908\_length\_2531\_cov\_46.841564 1153-1158. Max. coverage (+): 0.16. Max coverage (-): 0.08

Region: NODE\_356908\_length\_2531\_cov\_46.841564 1159-1163. Max. coverage (+): 0.06. Max coverage (-): 0.2

Region: NODE\_356908\_length\_2531\_cov\_46.841564 1164-1168. Max. coverage (+): 0.02. Max coverage (-): 6.9

Region: NODE\_356908\_length\_2531\_cov\_46.841564 1169-1173. Max. coverage (+): 0. Max coverage (-): 22.05

Region: NODE\_356908\_length\_2531\_cov\_46.841564 1174-1179. Max. coverage (+): 0.06. Max coverage (-): 7.91

Region: NODE\_356908\_length\_2531\_cov\_46.841564 1180-1184. Max. coverage (+): 0.93. Max coverage (-): 0.06

Region: NODE\_356908\_length\_2531\_cov\_46.841564 1185-1189. Max. coverage (+): 1.05. Max coverage (-): 0.02

Region: NODE\_356908\_length\_2531\_cov\_46.841564 1190-1194. Max. coverage (+): 0.14. Max coverage (-): 0.2

Region: NODE\_356908\_length\_2531\_cov\_46.841564 1195-1200. Max. coverage (+): 0.06. Max coverage (-): 0.5

Region: NODE\_356908\_length\_2531\_cov\_46.841564 1201-1205. Max. coverage (+): 0. Max coverage (-): 0.1

Region: NODE\_356908\_length\_2531\_cov\_46.841564 1206-1210. Max. coverage (+): 0. Max coverage (-): 1.57

Region: NODE\_356908\_length\_2531\_cov\_46.841564 1211-1215. Max. coverage (+): 0.2. Max coverage (-): 2.25

Region: NODE\_356908\_length\_2531\_cov\_46.841564 1216-1221. Max. coverage (+): 0.03. Max coverage (-): 2.25

Region: NODE\_356908\_length\_2531\_cov\_46.841564 1222-1226. Max. coverage (+): 0.03. Max coverage (-): 2.01

Region: NODE\_356908\_length\_2531\_cov\_46.841564 1227-1231. Max. coverage (+): 0.01. Max coverage (-): 0.87

Region: NODE\_356908\_length\_2531\_cov\_46.841564 1232-1236. Max. coverage (+): 0.02. Max coverage (-): 0.13

Region: NODE\_356908\_length\_2531\_cov\_46.841564 1237-1242. Max. coverage (+): 0.02. Max coverage (-): 1.89

Region: NODE\_356908\_length\_2531\_cov\_46.841564 1243-1247. Max. coverage (+): 0. Max coverage (-): 2.48

Region: NODE\_356908\_length\_2531\_cov\_46.841564 1248-1252. Max. coverage (+): 0. Max coverage (-): 19.68

Region: NODE\_356908\_length\_2531\_cov\_46.841564 1253-1257. Max. coverage (+): 0. Max coverage (-): 18.43

Region: NODE\_356908\_length\_2531\_cov\_46.841564 1258-1263. Max. coverage (+): 0. Max coverage (-): 0.17

Region: NODE\_356908\_length\_2531\_cov\_46.841564 1264-1268. Max. coverage (+): 0.12. Max coverage (-): 0.2

Region: NODE\_356908\_length\_2531\_cov\_46.841564 1269-1273. Max. coverage (+): 0.24. Max coverage (-): 0.36

Region: NODE\_356908\_length\_2531\_cov\_46.841564 1274-1278. Max. coverage (+): 0.28. Max coverage (-): 1.13

Region: NODE\_356908\_length\_2531\_cov\_46.841564 1279-1284. Max. coverage (+): 0.04. Max coverage (-): 2.71

Region: NODE\_356908\_length\_2531\_cov\_46.841564 1285-1289. Max. coverage (+): 0. Max coverage (-): 0.14

Region: NODE\_356908\_length\_2531\_cov\_46.841564 1290-1294. Max. coverage (+): 0. Max coverage (-): 0.3

Region: NODE\_356908\_length\_2531\_cov\_46.841564 1295-1299. Max. coverage (+): 0.02. Max coverage (-): 0.2

Region: NODE\_356908\_length\_2531\_cov\_46.841564 1300-1305. Max. coverage (+): 0.16. Max coverage (-): 0.46

Region: NODE\_356908\_length\_2531\_cov\_46.841564 1306-1310. Max. coverage (+): 0.14. Max coverage (-): 0.48

Region: NODE\_356908\_length\_2531\_cov\_46.841564 1311-1315. Max. coverage (+): 0. Max coverage (-): 5.09

Region: NODE\_356908\_length\_2531\_cov\_46.841564 1316-1320. Max. coverage (+): 0. Max coverage (-): 5.45

Region: NODE\_356908\_length\_2531\_cov\_46.841564 1321-1326. Max. coverage (+): 0. Max coverage (-): 0.36

Region: NODE\_356908\_length\_2531\_cov\_46.841564 1327-1331. Max. coverage (+): 0.4. Max coverage (-): 0.73

Region: NODE\_356908\_length\_2531\_cov\_46.841564 1332-1336. Max. coverage (+): 0.44. Max coverage (-): 0.52

Region: NODE\_356908\_length\_2531\_cov\_46.841564 1337-1341. Max. coverage (+): 0.02. Max coverage (-): 0.04

Region: NODE\_356908\_length\_2531\_cov\_46.841564 1342-1347. Max. coverage (+): 0.19. Max coverage (-): 0.04

Region: NODE\_356908\_length\_2531\_cov\_46.841564 1348-1352. Max. coverage (+): 0.53. Max coverage (-): 0.02

Region: NODE\_356908\_length\_2531\_cov\_46.841564 1353-1357. Max. coverage (+): 0.04. Max coverage (-): 0.14

Region: NODE\_356908\_length\_2531\_cov\_46.841564 1358-1362. Max. coverage (+): 0. Max coverage (-): 10.01

Region: NODE\_356908\_length\_2531\_cov\_46.841564 1363-1368. Max. coverage (+): 0. Max coverage (-): 3.05

Region: NODE\_356908\_length\_2531\_cov\_46.841564 1369-1373. Max. coverage (+): 0. Max coverage (-): 0

Region: NODE\_356908\_length\_2531\_cov\_46.841564 1374-1378. Max. coverage (+): 0. Max coverage (-): 0

Region: NODE\_356908\_length\_2531\_cov\_46.841564 1379-1383. Max. coverage (+): 0. Max coverage (-): 0

Region: NODE\_356908\_length\_2531\_cov\_46.841564 1384-1389. Max. coverage (+): 0. Max coverage (-): 0.09

Region: NODE\_356908\_length\_2531\_cov\_46.841564 1390-1394. Max. coverage (+): 0. Max coverage (-): 1.76

Region: NODE\_356908\_length\_2531\_cov\_46.841564 1395-1399. Max. coverage (+): 0. Max coverage (-): 0.13

Region: NODE\_356908\_length\_2531\_cov\_46.841564 1400-1404. Max. coverage (+): 0.04. Max coverage (-): 0.01

Region: NODE\_356908\_length\_2531\_cov\_46.841564 1405-1410. Max. coverage (+): 0.28. Max coverage (-): 0

Region: NODE\_356908\_length\_2531\_cov\_46.841564 1411-1415. Max. coverage (+): 0.04. Max coverage (-): 0

Region: NODE\_356908\_length\_2531\_cov\_46.841564 1416-1420. Max. coverage (+): 0. Max coverage (-): 0

Region: NODE\_356908\_length\_2531\_cov\_46.841564 1421-1425. Max. coverage (+): 0. Max coverage (-): 0

Region: NODE\_356908\_length\_2531\_cov\_46.841564 1426-1431. Max. coverage (+): 0. Max coverage (-): 0.3

Region: NODE\_356908\_length\_2531\_cov\_46.841564 1432-1436. Max. coverage (+): 0.02. Max coverage (-): 0.2

Region: NODE\_356908\_length\_2531\_cov\_46.841564 1437-1441. Max. coverage (+): 0.16. Max coverage (-): 0.12

Region: NODE\_356908\_length\_2531\_cov\_46.841564 1442-1446. Max. coverage (+): 0.14. Max coverage (-): 0

Region: NODE\_356908\_length\_2531\_cov\_46.841564 1447-1452. Max. coverage (+): 0.24. Max coverage (-): 0.04

Region: NODE\_356908\_length\_2531\_cov\_46.841564 1453-1457. Max. coverage (+): 0.08. Max coverage (-): 0.04

Region: NODE\_356908\_length\_2531\_cov\_46.841564 1458-1462. Max. coverage (+): 0.12. Max coverage (-): 0

Region: NODE\_356908\_length\_2531\_cov\_46.841564 1463-1467. Max. coverage (+): 0.2. Max coverage (-): 0.26

Region: NODE\_356908\_length\_2531\_cov\_46.841564 1468-1473. Max. coverage (+): 0.44. Max coverage (-): 0.52

Region: NODE\_356908\_length\_2531\_cov\_46.841564 1474-1478. Max. coverage (+): 0.02. Max coverage (-): 0.04

Region: NODE\_356908\_length\_2531\_cov\_46.841564 1479-1483. Max. coverage (+): 0.06. Max coverage (-): 0.04

Region: NODE\_356908\_length\_2531\_cov\_46.841564 1484-1488. Max. coverage (+): 0.53. Max coverage (-): 0.02

Region: NODE\_356908\_length\_2531\_cov\_46.841564 1489-1494. Max. coverage (+): 0.36. Max coverage (-): 0.14

Region: NODE\_356908\_length\_2531\_cov\_46.841564 1495-1499. Max. coverage (+): 0. Max coverage (-): 10.01

Region: NODE\_356908\_length\_2531\_cov\_46.841564 1500-1504. Max. coverage (+): 0. Max coverage (-): 3.09

Region: NODE\_356908\_length\_2531\_cov\_46.841564 1505-1509. Max. coverage (+): 0.04. Max coverage (-): 1.09

Region: NODE\_356908\_length\_2531\_cov\_46.841564 1510-1515. Max. coverage (+): 2.16. Max coverage (-): 0.2

Region: NODE\_356908\_length\_2531\_cov\_46.841564 1516-1520. Max. coverage (+): 2.18. Max coverage (-): 0.1

Region: NODE\_356908\_length\_2531\_cov\_46.841564 1521-1525. Max. coverage (+): 0.12. Max coverage (-): 0.12

Region: NODE\_356908\_length\_2531\_cov\_46.841564 1526-1530. Max. coverage (+): 0.16. Max coverage (-): 0.2

Region: NODE\_356908\_length\_2531\_cov\_46.841564 1531-1536. Max. coverage (+): 0. Max coverage (-): 0.81

Region: NODE\_356908\_length\_2531\_cov\_46.841564 1537-1541. Max. coverage (+): 0. Max coverage (-): 0.97

Region: NODE\_356908\_length\_2531\_cov\_46.841564 1542-1546. Max. coverage (+): 0. Max coverage (-): 0.04

Region: NODE\_356908\_length\_2531\_cov\_46.841564 1547-1551. Max. coverage (+): 0. Max coverage (-): 0

Region: NODE\_356908\_length\_2531\_cov\_46.841564 1552-1557. Max. coverage (+): 7.95. Max coverage (-): 0.16

Region: NODE\_356908\_length\_2531\_cov\_46.841564 1558-1562. Max. coverage (+): 0.04. Max coverage (-): 0.24

Region: NODE\_356908\_length\_2531\_cov\_46.841564 1563-1567. Max. coverage (+): 0. Max coverage (-): 0.12

Region: NODE\_356908\_length\_2531\_cov\_46.841564 1568-1572. Max. coverage (+): 0. Max coverage (-): 0.24

Region: NODE\_356908\_length\_2531\_cov\_46.841564 1573-1578. Max. coverage (+): 0. Max coverage (-): 0.2

Region: NODE\_356908\_length\_2531\_cov\_46.841564 1579-1583. Max. coverage (+): 0. Max coverage (-): 0.48

Region: NODE\_356908\_length\_2531\_cov\_46.841564 1584-1588. Max. coverage (+): 0. Max coverage (-): 8.92

Region: NODE\_356908\_length\_2531\_cov\_46.841564 1589-1593. Max. coverage (+): 0.2. Max coverage (-): 14.13

Region: NODE\_356908\_length\_2531\_cov\_46.841564 1594-1599. Max. coverage (+): 0.2. Max coverage (-): 2.06

Region: NODE\_356908\_length\_2531\_cov\_46.841564 1600-1604. Max. coverage (+): 0. Max coverage (-): 0.32

Region: NODE\_356908\_length\_2531\_cov\_46.841564 1605-1609. Max. coverage (+): 0.85. Max coverage (-): 10.14

Region: NODE\_356908\_length\_2531\_cov\_46.841564 1610-1614. Max. coverage (+): 0.77. Max coverage (-): 10.14

Region: NODE\_356908\_length\_2531\_cov\_46.841564 1615-1620. Max. coverage (+): 0. Max coverage (-): 1.86

Region: NODE\_356908\_length\_2531\_cov\_46.841564 1621-1625. Max. coverage (+): 0. Max coverage (-): 2.06

Region: NODE\_356908\_length\_2531\_cov\_46.841564 1626-1630. Max. coverage (+): 0.08. Max coverage (-): 0.08

Region: NODE\_356908\_length\_2531\_cov\_46.841564 1631-1635. Max. coverage (+): 0.08. Max coverage (-): 0

Region: NODE\_356908\_length\_2531\_cov\_46.841564 1636-1641. Max. coverage (+): 0.04. Max coverage (-): 0.08

Region: NODE\_356908\_length\_2531\_cov\_46.841564 1642-1646. Max. coverage (+): 0. Max coverage (-): 0.12

Region: NODE\_356908\_length\_2531\_cov\_46.841564 1647-1651. Max. coverage (+): 0. Max coverage (-): 1.82

Region: NODE\_356908\_length\_2531\_cov\_46.841564 1652-1656. Max. coverage (+): 0.04. Max coverage (-): 1.94

Region: NODE\_356908\_length\_2531\_cov\_46.841564 1657-1662. Max. coverage (+): 0.08. Max coverage (-): 0.52

Region: NODE\_356908\_length\_2531\_cov\_46.841564 1663-1667. Max. coverage (+): 0.4. Max coverage (-): 0.04

Region: NODE\_356908\_length\_2531\_cov\_46.841564 1668-1672. Max. coverage (+): 0.4. Max coverage (-): 0.04

Region: NODE\_356908\_length\_2531\_cov\_46.841564 1673-1677. Max. coverage (+): 0.28. Max coverage (-): 0.28

Region: NODE\_356908\_length\_2531\_cov\_46.841564 1678-1683. Max. coverage (+): 0.04. Max coverage (-): 6.74

Region: NODE\_356908\_length\_2531\_cov\_46.841564 1684-1688. Max. coverage (+): 0.04. Max coverage (-): 0.32

Region: NODE\_356908\_length\_2531\_cov\_46.841564 1689-1693. Max. coverage (+): 0. Max coverage (-): 0.28

Region: NODE\_356908\_length\_2531\_cov\_46.841564 1694-1698. Max. coverage (+): 0.08. Max coverage (-): 4.68

Region: NODE\_356908\_length\_2531\_cov\_46.841564 1699-1704. Max. coverage (+): 0.08. Max coverage (-): 4.89

Region: NODE\_356908\_length\_2531\_cov\_46.841564 1705-1709. Max. coverage (+): 0.04. Max coverage (-): 2.1

Region: NODE\_356908\_length\_2531\_cov\_46.841564 1710-1714. Max. coverage (+): 0.04. Max coverage (-): 0.16

Region: NODE\_356908\_length\_2531\_cov\_46.841564 1715-1719. Max. coverage (+): 0.12. Max coverage (-): 0.24

Region: NODE\_356908\_length\_2531\_cov\_46.841564 1720-1725. Max. coverage (+): 0.16. Max coverage (-): 0.16

Region: NODE\_356908\_length\_2531\_cov\_46.841564 1726-1730. Max. coverage (+): 0.04. Max coverage (-): 1.33

Region: NODE\_356908\_length\_2531\_cov\_46.841564 1731-1735. Max. coverage (+): 0. Max coverage (-): 27.09

Region: NODE\_356908\_length\_2531\_cov\_46.841564 1736-1740. Max. coverage (+): 0. Max coverage (-): 23.18

Region: NODE\_356908\_length\_2531\_cov\_46.841564 1741-1746. Max. coverage (+): 0.81. Max coverage (-): 3.27

Region: NODE\_356908\_length\_2531\_cov\_46.841564 1747-1751. Max. coverage (+): 0.81. Max coverage (-): 3.23

Region: NODE\_356908\_length\_2531\_cov\_46.841564 1752-1756. Max. coverage (+): 0.69. Max coverage (-): 0.77

Region: NODE\_356908\_length\_2531\_cov\_46.841564 1757-1761. Max. coverage (+): 0.04. Max coverage (-): 0.77

Region: NODE\_356908\_length\_2531\_cov\_46.841564 1762-1767. Max. coverage (+): 0.08. Max coverage (-): 0.24

Region: NODE\_356908\_length\_2531\_cov\_46.841564 1768-1772. Max. coverage (+): 0.08. Max coverage (-): 1.05

Region: NODE\_356908\_length\_2531\_cov\_46.841564 1773-1777. Max. coverage (+): 0.08. Max coverage (-): 1.29

Region: NODE\_356908\_length\_2531\_cov\_46.841564 1778-1782. Max. coverage (+): 0. Max coverage (-): 4.48

Region: NODE\_356908\_length\_2531\_cov\_46.841564 1783-1788. Max. coverage (+): 0.12. Max coverage (-): 4.04

Region: NODE\_356908\_length\_2531\_cov\_46.841564 1789-1793. Max. coverage (+): 0.08. Max coverage (-): 1.74

Region: NODE\_356908\_length\_2531\_cov\_46.841564 1794-1798. Max. coverage (+): 0.44. Max coverage (-): 0.12

Region: NODE\_356908\_length\_2531\_cov\_46.841564 1799-1803. Max. coverage (+): 0.48. Max coverage (-): 0.16

Region: NODE\_356908\_length\_2531\_cov\_46.841564 1804-1809. Max. coverage (+): 0.04. Max coverage (-): 3.59

Region: NODE\_356908\_length\_2531\_cov\_46.841564 1810-1814. Max. coverage (+): 0. Max coverage (-): 3.92

Region: NODE\_356908\_length\_2531\_cov\_46.841564 1815-1819. Max. coverage (+): 0. Max coverage (-): 4.85

Region: NODE\_356908\_length\_2531\_cov\_46.841564 1820-1824. Max. coverage (+): 0. Max coverage (-): 0.08

Region: NODE\_356908\_length\_2531\_cov\_46.841564 1825-1830. Max. coverage (+): 0.16. Max coverage (-): 0.04

Region: NODE\_356908\_length\_2531\_cov\_46.841564 1831-1835. Max. coverage (+): 0.2. Max coverage (-): 0.04

Region: NODE\_356908\_length\_2531\_cov\_46.841564 1836-1840. Max. coverage (+): 0.12. Max coverage (-): 0.08

Region: NODE\_356908\_length\_2531\_cov\_46.841564 1841-1845. Max. coverage (+): 0.08. Max coverage (-): 0.16

Region: NODE\_356908\_length\_2531\_cov\_46.841564 1846-1851. Max. coverage (+): 0.16. Max coverage (-): 0.61

Region: NODE\_356908\_length\_2531\_cov\_46.841564 1852-1856. Max. coverage (+): 0.16. Max coverage (-): 0.08

Region: NODE\_356908\_length\_2531\_cov\_46.841564 1857-1861. Max. coverage (+): 0.04. Max coverage (-): 0.2

Region: NODE\_356908\_length\_2531\_cov\_46.841564 1862-1866. Max. coverage (+): 0. Max coverage (-): 9.37

Region: NODE\_356908\_length\_2531\_cov\_46.841564 1867-1872. Max. coverage (+): 0. Max coverage (-): 9.53

Region: NODE\_356908\_length\_2531\_cov\_46.841564 1873-1877. Max. coverage (+): 0. Max coverage (-): 0.04

Region: NODE\_356908\_length\_2531\_cov\_46.841564 1878-1882. Max. coverage (+): 0. Max coverage (-): 0.04

Region: NODE\_356908\_length\_2531\_cov\_46.841564 1883-1887. Max. coverage (+): 0.04. Max coverage (-): 0.04

Region: NODE\_356908\_length\_2531\_cov\_46.841564 1888-1893. Max. coverage (+): 0.04. Max coverage (-): 0.4

Region: NODE\_356908\_length\_2531\_cov\_46.841564 1894-1898. Max. coverage (+): 0. Max coverage (-): 1.05

Region: NODE\_356908\_length\_2531\_cov\_46.841564 1899-1903. Max. coverage (+): 0. Max coverage (-): 0.85

Region: NODE\_356908\_length\_2531\_cov\_46.841564 1904-1908. Max. coverage (+): 0. Max coverage (-): 0.08

Region: NODE\_356908\_length\_2531\_cov\_46.841564 1909-1914. Max. coverage (+): 0.24. Max coverage (-): 0.24

Region: NODE\_356908\_length\_2531\_cov\_46.841564 1915-1919. Max. coverage (+): 0.2. Max coverage (-): 8.52

Region: NODE\_356908\_length\_2531\_cov\_46.841564 1920-1924. Max. coverage (+): 0. Max coverage (-): 17.93

Region: NODE\_356908\_length\_2531\_cov\_46.841564 1925-1929. Max. coverage (+): 1.74. Max coverage (-): 13.69

Region: NODE\_356908\_length\_2531\_cov\_46.841564 1930-1935. Max. coverage (+): 1.78. Max coverage (-): 8.4

Region: NODE\_356908\_length\_2531\_cov\_46.841564 1936-1940. Max. coverage (+): 0. Max coverage (-): 0.48

Region: NODE\_356908\_length\_2531\_cov\_46.841564 1941-1945. Max. coverage (+): 0. Max coverage (-): 0.08

Region: NODE\_356908\_length\_2531\_cov\_46.841564 1946-1950. Max. coverage (+): 0.04. Max coverage (-): 0.2

Region: NODE\_356908\_length\_2531\_cov\_46.841564 1951-1956. Max. coverage (+): 0.08. Max coverage (-): 1.29

Region: NODE\_356908\_length\_2531\_cov\_46.841564 1957-1961. Max. coverage (+): 0. Max coverage (-): 1.53

Region: NODE\_356908\_length\_2531\_cov\_46.841564 1962-1966. Max. coverage (+): 0. Max coverage (-): 0.12

Region: NODE\_356908\_length\_2531\_cov\_46.841564 1967-1971. Max. coverage (+): 0. Max coverage (-): 0.2

Region: NODE\_356908\_length\_2531\_cov\_46.841564 1972-1977. Max. coverage (+): 0.85. Max coverage (-): 1.25

Region: NODE\_356908\_length\_2531\_cov\_46.841564 1978-1982. Max. coverage (+): 0. Max coverage (-): 1.62

Region: NODE\_356908\_length\_2531\_cov\_46.841564 1983-1987. Max. coverage (+): 0. Max coverage (-): 0.52

Region: NODE\_356908\_length\_2531\_cov\_46.841564 1988-1992. Max. coverage (+): 0. Max coverage (-): 0.48

Region: NODE\_356908\_length\_2531\_cov\_46.841564 1993-1998. Max. coverage (+): 0. Max coverage (-): 0.36

Region: NODE\_356908\_length\_2531\_cov\_46.841564 1999-2003. Max. coverage (+): 0. Max coverage (-): 0.77

Region: NODE\_356908\_length\_2531\_cov\_46.841564 2004-2008. Max. coverage (+): 0.2. Max coverage (-): 1.62

Region: NODE\_356908\_length\_2531\_cov\_46.841564 2009-2013. Max. coverage (+): 0.2. Max coverage (-): 0.16

Region: NODE\_356908\_length\_2531\_cov\_46.841564 2014-2019. Max. coverage (+): 0.04. Max coverage (-): 0.48

Region: NODE\_356908\_length\_2531\_cov\_46.841564 2020-2024. Max. coverage (+): 0.04. Max coverage (-): 0.44

Region: NODE\_356908\_length\_2531\_cov\_46.841564 2025-2029. Max. coverage (+): 0.04. Max coverage (-): 1.98

Region: NODE\_356908\_length\_2531\_cov\_46.841564 2030-2034. Max. coverage (+): 0. Max coverage (-): 1.82

Region: NODE\_356908\_length\_2531\_cov\_46.841564 2035-2040. Max. coverage (+): 0.04. Max coverage (-): 0.52

Region: NODE\_356908\_length\_2531\_cov\_46.841564 2041-2045. Max. coverage (+): 0.81. Max coverage (-): 0.57

Region: NODE\_356908\_length\_2531\_cov\_46.841564 2046-2050. Max. coverage (+): 1.01. Max coverage (-): 0.12

Region: NODE\_356908\_length\_2531\_cov\_46.841564 2051-2055. Max. coverage (+): 0.97. Max coverage (-): 0.12

Region: NODE\_356908\_length\_2531\_cov\_46.841564 2056-2061. Max. coverage (+): 0.08. Max coverage (-): 0.16

Region: NODE\_356908\_length\_2531\_cov\_46.841564 2062-2066. Max. coverage (+): 0.04. Max coverage (-): 0.04

Region: NODE\_356908\_length\_2531\_cov\_46.841564 2067-2071. Max. coverage (+): 0.04. Max coverage (-): 0.44

Region: NODE\_356908\_length\_2531\_cov\_46.841564 2072-2076. Max. coverage (+): 0.04. Max coverage (-): 2.83

Region: NODE\_356908\_length\_2531\_cov\_46.841564 2077-2082. Max. coverage (+): 0.04. Max coverage (-): 2.66

Region: NODE\_356908\_length\_2531\_cov\_46.841564 2083-2087. Max. coverage (+): 0. Max coverage (-): 0

Region: NODE\_356908\_length\_2531\_cov\_46.841564 2088-2092. Max. coverage (+): 0. Max coverage (-): 0.08

Region: NODE\_356908\_length\_2531\_cov\_46.841564 2093-2097. Max. coverage (+): 0.85. Max coverage (-): 0.24

Region: NODE\_356908\_length\_2531\_cov\_46.841564 2098-2103. Max. coverage (+): 0.2. Max coverage (-): 0.08

Region: NODE\_356908\_length\_2531\_cov\_46.841564 2104-2108. Max. coverage (+): 0.08. Max coverage (-): 0.12

Region: NODE\_356908\_length\_2531\_cov\_46.841564 2109-2113. Max. coverage (+): 0.08. Max coverage (-): 0.12

Region: NODE\_356908\_length\_2531\_cov\_46.841564 2114-2118. Max. coverage (+): 0. Max coverage (-): 0

Region: NODE\_356908\_length\_2531\_cov\_46.841564 2119-2124. Max. coverage (+): 0. Max coverage (-): 0.04

Region: NODE\_356908\_length\_2531\_cov\_46.841564 2125-2129. Max. coverage (+): 0.2. Max coverage (-): 0

Region: NODE\_356908\_length\_2531\_cov\_46.841564 2130-2134. Max. coverage (+): 0.2. Max coverage (-): 0.57

Region: NODE\_356908\_length\_2531\_cov\_46.841564 2135-2139. Max. coverage (+): 0.04. Max coverage (-): 1.7

Region: NODE\_356908\_length\_2531\_cov\_46.841564 2140-2145. Max. coverage (+): 0.08. Max coverage (-): 2.02

Region: NODE\_356908\_length\_2531\_cov\_46.841564 2146-2150. Max. coverage (+): 0. Max coverage (-): 0.2

Region: NODE\_356908\_length\_2531\_cov\_46.841564 2151-2155. Max. coverage (+): 0.08. Max coverage (-): 0.12

Region: NODE\_356908\_length\_2531\_cov\_46.841564 2156-2160. Max. coverage (+): 0.32. Max coverage (-): 0.16

Region: NODE\_356908\_length\_2531\_cov\_46.841564 2161-2166. Max. coverage (+): 0. Max coverage (-): 0.24

Region: NODE\_356908\_length\_2531\_cov\_46.841564 2167-2171. Max. coverage (+): 0. Max coverage (-): 1.45

Region: NODE\_356908\_length\_2531\_cov\_46.841564 2172-2176. Max. coverage (+): 0. Max coverage (-): 2.14

Region: NODE\_356908\_length\_2531\_cov\_46.841564 2177-2181. Max. coverage (+): 0. Max coverage (-): 2.14

Region: NODE\_356908\_length\_2531\_cov\_46.841564 2182-2187. Max. coverage (+): 2.71. Max coverage (-): 0.12

Region: NODE\_356908\_length\_2531\_cov\_46.841564 2188-2192. Max. coverage (+): 2.71. Max coverage (-): 0.04

Region: NODE\_356908\_length\_2531\_cov\_46.841564 2193-2197. Max. coverage (+): 0.08. Max coverage (-): 0.93

Region: NODE\_356908\_length\_2531\_cov\_46.841564 2198-2202. Max. coverage (+): 0.08. Max coverage (-): 1.66

Region: NODE\_356908\_length\_2531\_cov\_46.841564 2203-2208. Max. coverage (+): 0.04. Max coverage (-): 1.98

Region: NODE\_356908\_length\_2531\_cov\_46.841564 2209-2213. Max. coverage (+): 0. Max coverage (-): 1.74

Region: NODE\_356908\_length\_2531\_cov\_46.841564 2214-2218. Max. coverage (+): 0.65. Max coverage (-): 1.53

Region: NODE\_356908\_length\_2531\_cov\_46.841564 2219-2223. Max. coverage (+): 0.61. Max coverage (-): 2.34

Region: NODE\_356908\_length\_2531\_cov\_46.841564 2224-2229. Max. coverage (+): 0.4. Max coverage (-): 1.41

Region: NODE\_356908\_length\_2531\_cov\_46.841564 2230-2234. Max. coverage (+): 0.24. Max coverage (-): 0.65

Region: NODE\_356908\_length\_2531\_cov\_46.841564 2235-2239. Max. coverage (+): 0.85. Max coverage (-): 3.47

Region: NODE\_356908\_length\_2531\_cov\_46.841564 2240-2244. Max. coverage (+): 0.77. Max coverage (-): 3.11

Region: NODE\_356908\_length\_2531\_cov\_46.841564 2245-2250. Max. coverage (+): 0.2. Max coverage (-): 0.65

Region: NODE\_356908\_length\_2531\_cov\_46.841564 2251-2255. Max. coverage (+): 0.2. Max coverage (-): 0.81

Region: NODE\_356908\_length\_2531\_cov\_46.841564 2256-2260. Max. coverage (+): 0.04. Max coverage (-): 0.12

Region: NODE\_356908\_length\_2531\_cov\_46.841564 2261-2265. Max. coverage (+): 0.08. Max coverage (-): 0.04

Region: NODE\_356908\_length\_2531\_cov\_46.841564 2266-2271. Max. coverage (+): 0.02. Max coverage (-): 6.38

Region: NODE\_356908\_length\_2531\_cov\_46.841564 2272-2276. Max. coverage (+): 0.04. Max coverage (-): 6.22

Region: NODE\_356908\_length\_2531\_cov\_46.841564 2277-2281. Max. coverage (+): 0. Max coverage (-): 0.85

Region: NODE\_356908\_length\_2531\_cov\_46.841564 2282-2286. Max. coverage (+): 0. Max coverage (-): 0

Region: NODE\_356908\_length\_2531\_cov\_46.841564 2287-2292. Max. coverage (+): 0. Max coverage (-): 0.32

Region: NODE\_356908\_length\_2531\_cov\_46.841564 2293-2297. Max. coverage (+): 0.04. Max coverage (-): 0.36

Region: NODE\_356908\_length\_2531\_cov\_46.841564 2298-2302. Max. coverage (+): 0. Max coverage (-): 0.12

Region: NODE\_356908\_length\_2531\_cov\_46.841564 2303-2307. Max. coverage (+): 0.28. Max coverage (-): 0.16

Region: NODE\_356908\_length\_2531\_cov\_46.841564 2308-2313. Max. coverage (+): 0.08. Max coverage (-): 0.24

Region: NODE\_356908\_length\_2531\_cov\_46.841564 2314-2318. Max. coverage (+): 0.12. Max coverage (-): 0.97

Region: NODE\_356908\_length\_2531\_cov\_46.841564 2319-2323. Max. coverage (+): 0.08. Max coverage (-): 0.57

Region: NODE\_356908\_length\_2531\_cov\_46.841564 2324-2328. Max. coverage (+): 0.16. Max coverage (-): 0.32

Region: NODE\_356908\_length\_2531\_cov\_46.841564 2329-2334. Max. coverage (+): 0.04. Max coverage (-): 0.44

Region: NODE\_356908\_length\_2531\_cov\_46.841564 2335-2339. Max. coverage (+): 2.06. Max coverage (-): 2.1

Region: NODE\_356908\_length\_2531\_cov\_46.841564 2340-2344. Max. coverage (+): 2.1. Max coverage (-): 0.4

Region: NODE\_356908\_length\_2531\_cov\_46.841564 2345-2349. Max. coverage (+): 0.36. Max coverage (-): 0.4

Region: NODE\_356908\_length\_2531\_cov\_46.841564 2350-2355. Max. coverage (+): 0.04. Max coverage (-): 8.36

Region: NODE\_356908\_length\_2531\_cov\_46.841564 2356-2360. Max. coverage (+): 0. Max coverage (-): 10.34

Region: NODE\_356908\_length\_2531\_cov\_46.841564 2361-2365. Max. coverage (+): 0.04. Max coverage (-): 0.77

Region: NODE\_356908\_length\_2531\_cov\_46.841564 2366-2370. Max. coverage (+): 0.04. Max coverage (-): 0.65

Region: NODE\_356908\_length\_2531\_cov\_46.841564 2371-2376. Max. coverage (+): 0. Max coverage (-): 0.12

Region: NODE\_356908\_length\_2531\_cov\_46.841564 2377-2381. Max. coverage (+): 0. Max coverage (-): 0.81

Region: NODE\_356908\_length\_2531\_cov\_46.841564 2382-2386. Max. coverage (+): 0. Max coverage (-): 1.62

Region: NODE\_356908\_length\_2531\_cov\_46.841564 2387-2391. Max. coverage (+): 0.04. Max coverage (-): 5.98

Region: NODE\_356908\_length\_2531\_cov\_46.841564 2392-2397. Max. coverage (+): 0.12. Max coverage (-): 2.1

Region: NODE\_356908\_length\_2531\_cov\_46.841564 2398-2402. Max. coverage (+): 0.12. Max coverage (-): 1.82

Region: NODE\_356908\_length\_2531\_cov\_46.841564 2403-2407. Max. coverage (+): 1.29. Max coverage (-): 0.04

Region: NODE\_356908\_length\_2531\_cov\_46.841564 2408-2412. Max. coverage (+): 0.4. Max coverage (-): 0.12

Region: NODE\_356908\_length\_2531\_cov\_46.841564 2413-2418. Max. coverage (+): 0.81. Max coverage (-): 0.12

Region: NODE\_356908\_length\_2531\_cov\_46.841564 2419-2423. Max. coverage (+): 0.57. Max coverage (-): 3.63

Region: NODE\_356908\_length\_2531\_cov\_46.841564 2424-2428. Max. coverage (+): 0.36. Max coverage (-): 14.13

Region: NODE\_356908\_length\_2531\_cov\_46.841564 2429-2433. Max. coverage (+): 0. Max coverage (-): 5.85

Region: NODE\_356908\_length\_2531\_cov\_46.841564 2434-2439. Max. coverage (+): 0. Max coverage (-): 0.81

Region: NODE\_356908\_length\_2531\_cov\_46.841564 2440-2444. Max. coverage (+): 0. Max coverage (-): 0.04

Region: NODE\_356908\_length\_2531\_cov\_46.841564 2445-2449. Max. coverage (+): 0. Max coverage (-): 0.04

Region: NODE\_356908\_length\_2531\_cov\_46.841564 2450-2454. Max. coverage (+): 0.04. Max coverage (-): 0.04

Region: NODE\_356908\_length\_2531\_cov\_46.841564 2455-2460. Max. coverage (+): 0. Max coverage (-): 0.24

Region: NODE\_356908\_length\_2531\_cov\_46.841564 2461-2465. Max. coverage (+): 0. Max coverage (-): 0.44

Region: NODE\_356908\_length\_2531\_cov\_46.841564 2466-2470. Max. coverage (+): 0. Max coverage (-): 1.49

Region: NODE\_356908\_length\_2531\_cov\_46.841564 2471-2475. Max. coverage (+): 0. Max coverage (-): 1.33

Region: NODE\_356908\_length\_2531\_cov\_46.841564 2476-2481. Max. coverage (+): 0.65. Max coverage (-): 0.44

Region: NODE\_356908\_length\_2531\_cov\_46.841564 2482-2486. Max. coverage (+): 0.12. Max coverage (-): 1.74

Region: NODE\_356908\_length\_2531\_cov\_46.841564 2487-2491. Max. coverage (+): 0.12. Max coverage (-): 2.1

Region: NODE\_356908\_length\_2531\_cov\_46.841564 2492-2496. Max. coverage (+): 0.08. Max coverage (-): 1.29

Region: NODE\_356908\_length\_2531\_cov\_46.841564 2497-2502. Max. coverage (+): 0. Max coverage (-): 0.24

Region: NODE\_356908\_length\_2531\_cov\_46.841564 2503-2507. Max. coverage (+): 0. Max coverage (-): 0.16

Region: NODE\_356908\_length\_2531\_cov\_46.841564 2508-2512. Max. coverage (+): 0. Max coverage (-): 0.44

Region: NODE\_356908\_length\_2531\_cov\_46.841564 2513-2517. Max. coverage (+): 0. Max coverage (-): 1.7

Region: NODE\_356908\_length\_2531\_cov\_46.841564 2518-2523. Max. coverage (+): 0. Max coverage (-): 1.78

Region: NODE\_356908\_length\_2531\_cov\_46.841564 2524-2528. Max. coverage (+): 0.08. Max coverage (-): 27.58

Region: NODE\_356908\_length\_2531\_cov\_46.841564 2529-2533. Max. coverage (+): 0.08. Max coverage (-): 1.9

Region: NODE\_356908\_length\_2531\_cov\_46.841564 2534-2538. Max. coverage (+): 0. Max coverage (-): 9.65

Region: NODE\_356908\_length\_2531\_cov\_46.841564 2539-2544. Max. coverage (+): 0.08. Max coverage (-): 0.89

Region: NODE\_356908\_length\_2531\_cov\_46.841564 2545-2549. Max. coverage (+): 0.08. Max coverage (-): 0.2

Region: NODE\_356908\_length\_2531\_cov\_46.841564 2550-2554. Max. coverage (+): 0.08. Max coverage (-): 0.12

Region: NODE\_356908\_length\_2531\_cov\_46.841564 2555-2559. Max. coverage (+): 0. Max coverage (-): 0.2

Region: NODE\_356908\_length\_2531\_cov\_46.841564 2560-2565. Max. coverage (+): 0. Max coverage (-): 0.97

Region: NODE\_356908\_length\_2531\_cov\_46.841564 2566-2570. Max. coverage (+): 0.04. Max coverage (-): 0.04

Region: NODE\_356908\_length\_2531\_cov\_46.841564 2571-2575. Max. coverage (+): 0.04. Max coverage (-): 0.08

Region: NODE\_356908\_length\_2531\_cov\_46.841564 2576-2580. Max. coverage (+): 0.08. Max coverage (-): 0.16

Region: NODE\_356908\_length\_2531\_cov\_46.841564 2581-2586. Max. coverage (+): 0.04. Max coverage (-): 1.05

Region: NODE\_356908\_length\_2531\_cov\_46.841564 2587-2591. Max. coverage (+): 0. Max coverage (-): 4.89

Region: NODE\_356908\_length\_2531\_cov\_46.841564 2592-2596. Max. coverage (+): 0. Max coverage (-): 2.73

Region: NODE\_356908\_length\_2531\_cov\_46.841564 2597-2601. Max. coverage (+): 0. Max coverage (-): 0.01

Region: NODE\_356908\_length\_2531\_cov\_46.841564 2602-2607. Max. coverage (+): 0.03. Max coverage (-): 0.01

Region: NODE\_356908\_length\_2531\_cov\_46.841564 2608-2612. Max. coverage (+): 0.01. Max coverage (-): 0.01

Region: NODE\_356908\_length\_2531\_cov\_46.841564 2613-2617. Max. coverage (+): 0. Max coverage (-): 0.17

Region: NODE\_356908\_length\_2531\_cov\_46.841564 2618-2622. Max. coverage (+): 0. Max coverage (-): 2.66

Region: NODE\_356908\_length\_2531\_cov\_46.841564 2623-2628. Max. coverage (+): 0.04. Max coverage (-): 3.19

Region: NODE\_356908\_length\_2531\_cov\_46.841564 2629-2633. Max. coverage (+): 0.03. Max coverage (-): 0.91

Region: NODE\_356908\_length\_2531\_cov\_46.841564 2634-2638. Max. coverage (+): 0.02. Max coverage (-): 0.15

Region: NODE\_356908\_length\_2531\_cov\_46.841564 2639-2643. Max. coverage (+): 0. Max coverage (-): 0

Region: NODE\_356908\_length\_2531\_cov\_46.841564 2644-2649. Max. coverage (+): 0. Max coverage (-): 0

Region: NODE\_356908\_length\_2531\_cov\_46.841564 2650-2654. Max. coverage (+): 0. Max coverage (-): 0

Region: NODE\_356908\_length\_2531\_cov\_46.841564 2655-. Max. coverage (+): 0. Max coverage (-): 0

RepeatMasker Color Code

**+**

100-98% Identity

<98-95% Identity

<95-90% Identity

<90-85% Identity

<85-80% Identity

<80-75% Identity

<75-70% Identity

<70% Identity

**-**

Gene Set Color Code

**+**

Gene

Pseudogene

Other

**-**

Topology/Coverage Color Code

Coverage Plus Strand

Coverage Minus Strand

Mainstrand: Plus

Mainstrand: Minus

Complementary Strand

Flanking Region  
(if option -flank >0)

Gene Set Annotation  
  
RepeatMasker Annotation  

**1. BEL-9\_GA-I**: 9-512 (+), Divergence to consensus: 33.7%  
**2. BEL-1\_CGi-I**: 893-1380 (+), Divergence to consensus: 39.7%  
**3. BEL36-I\_DR**: 1408-2472 (+), Divergence to consensus: 37.4%

  
Transcription Factor Binding Sites  

**SPZ1** (Sequence: CTCTAACCCT (-): 291)  
**RHOXF1** (Sequence: GGCTTA (-): 353)  
**RHOXF1** (Sequence: GGCTCA (-): 1116)  
**RHOXF1** (Sequence: GGATTA (-): 1272)  
**RHOXF1** (Sequence: AGATTA (-): 1302)  
**RHOXF1** (Sequence: GGATTA (-): 1409)  
**RHOXF1** (Sequence: AGATTA (-): 1439)  
**RHOXF1** (Sequence: TAATCC (+): 1872)  
**RHOXF1** (Sequence: TAATCT (+): 2513)  
**RFX4\_2** (Sequence: GTAACCATG (-): 2258)  
**RFX4\_1** (Sequence: GTTGCCAAG (-): 1181)  
**FOXO3\_hsa** (Sequence: GTAAACAT (+): 2493)  
**SOX9** (Sequence: AACAATGG (-): 266)  
**FOXP1** (Sequence: GTAAACA (+): 2493)  
**FIGLA** (Sequence: TCCAGGTGGT (-): 1165)  
**FOXO3\_mmu** (Sequence: GGTAAACA (+): 2492)  
**Rhox11** (Sequence: TGCTGTTTT (+): 87)  
**Sox5** (Sequence: AACAAT (-): 266)  
**POU5F1** (Sequence: ATGCAAA (+): 2264)
