# Supplementary material for: First characterization of PIWI-interacting RNA clusters in a cichlid fish with a B chromosome
Source: BMC Biol. 2022 Sep 21;20:204. doi: 10.1186/s12915-022-01403-2 (PMC9490952; doi:10.1186/s12915-022-01403-2)
Supplement: Supplementary file 1 — Additional file 1. Zipped folder with fasta and interactive html piRNA cluster information for the A. latifasciata genome. The nomenclature is as follows: number-pirna-cluster_sex_B-presence (f, female; m, male; 0b, without B chromosome; 1b, with B chromosome). [file 12915_2022_1403_MOESM1_ESM.zip › 138_m1b.html]

piRNA cluster 138\_m1b 82


Predicted piRNA cluster no. 138\_m1b
  

Show proTRAC run info
Hide proTRAC run info

/\  
                \_\_\_\_\_\_\_\_\_\_\_\_\_\_\_\_\_\_\_\_\_\_\_/\\_\_\_ /  \\_\_\_\_\_\_\_  
               I                      /  \  /    \      I  
               I     pro             /    \/      \     I  
               I        TRAC        /               \   I  
               I   \_\_\_\_\_\_\_\_\_\_\_\_\_\_\_\_/\_\_\_\_\_\_\_\_\_\_\_\_\_\_\_\_\_\\_ I  
               I   \              /                     I  
               I    \            /                      I  
               I     \  /\      /       V.2.4.2         I  
               I      \/  \    /                        I  
               I\_\_\_\_\_\_\_\_\_\_\_\  /\_\_\_\_\_\_\_\_\_\_\_\_\_\_\_\_\_\_\_\_\_\_\_\_\_I  
                            \/  
  
  
================================= proTRAC ====================================  
VERSION: .......... 2.4.2  
LAST MODIFIED: .... 11. May 2018  
  
Please cite:  
Rosenkranz D, Zischler H. proTRAC - a software for probabilistic piRNA cluster  
detection, visualization and analysis. 2012. BMC Bioinformatics 13:5.  
  
  
Contact:  
David Rosenkranz  
Institute of Organismic and Molecular Evolutionary Biology  
Dept. Anthropology, small RNA group  
Johannes Gutenberg University Mainz  
email: rosenkranz@uni-mainz.de  
  
You can find the latest proTRAC version at:  
http://sourceforge.net/projects/protrac/files  
http://www.smallRNAgroup-mainz.de/software  
==============================================================================  
  
PARAMETERS:  
Map file: ...............piwi-machos-1B.fa-collapse.map  
Genome file: ............../../../0B\_ala\_genome.fa  
RepeatMasker annotation: Alatifasciata-all0B-maryan-v2.fa\_corrected.out  
GeneSet:................./guest-storage/Data/annotation/Alatifasciata\_all0B\_maryan-v2\_out2017.gff  
  
Significant (p<=0.01) hit density will be calculated based  
on observed hit distribution.  
  
Sliding window size: ........................................ 5000 bp  
Sliding window increament: .................................. 1000 bp  
Normalize each hit by number of genomic hits: ............... yes  
Normalize each hit by number of sequence reads: ............. yes  
Normalize values (-> per million mapped reads): ............. yes  
Min. fraction of hits with 1T(U) or 10A: .................... 0.75  
Alternatively: Min. fraction of hits with 1T(U) and 10A: .... 0.5  
Min. fraction of hits with typical piRNA length: ............ 0.75  
Typical piRNA length: ....................................... 24-32 nt  
Min. size of a piRNA cluster: ............................... 1000 bp.  
Min. number of hits (absolute): ............................. 0  
Min. number of hits (normalized): ........................... 0  
Min. fraction of hits on the mainstrand: .................... 0.75  
Top fraction of mapped sequences (in terms of read counts): . 1%  
Top fraction accounts for max. n% of sequence reads: ........ 90%  
Min. fraction of hits on each arm of a bidirectional cluster: 0.05  
Output html file for each cluster: .......................... yes  
Output a summary table: ..................................... yes  
Output a FASTA file for each cluster (piRNA sequences): ..... yes  
Output a FASTA file comprising cluster sequences: ........... yes  
Output a GTF file for predicted piRNA clusters: ..............yes  
Search DNA motifs in clusters: .............................. yes  
Output flanking sequences: +/- .............................. 0 bp  
Output ~.pTi file: .......................................... no  
==============================================================================  
  
  
Genome size (without gaps): ............ 758543724 bp  
Gaps (N/X/-): .......................... 417479 bp  
Mapped reads: .......................... 26973943  
Non-identical sequences: ............... 6209225  
Genomic hits: .......................... 48438990  
Significant densitiy of mapped reads: .. 821.144211136946 reads/kb

Show proTRAC cluster info
Hide proTRAC cluster info

|  |  |
| --- | --- |
| Location | NODE\_356908\_length\_2531\_cov\_46.841564 |
| Coordinates | 4-2656 |
| Size [bp] | 2653 |
| Sequence hit loci | 4350 |
| Mapped reads (normalized) | 19073.2 |
| Mapped reads (normalized) per kb | 7189.3 |
| Normalized reads with 1T (1U) | 79.1% |
| Normalized reads with 10A | 35.1% |
| Normalized reads with length 24-32 nt | 98.2% |
| Normalized reads on the main strand(s) | 90.6% |
| Predicted directionality | mono:minus |

100%

0%

1T (1U)  
reads

10A reads

24-32 nt  
reads

reads on mainstrand

**Either the amount of reads with 1T (1U) OR 10A has to exceed 75% (set with option: -1Tor10A)  
Alternatively the amount of reads with 1T (1U) AND 10A has to exceed 50% (set with option: -1Tand10A)  
Minimum amount of reads with preferred size is 75% (set with option: -pisize)  
Minimum amount of reads on the main strand(s) is 75% (set with option: -clstrand)**

Show read coverage
Hide read coverage

WHAT DO I SEE HERE?  
This chart shows the location of mapped sequence reads within a predicted piRNA cluster. The color refers to the number of genomic hits produced by the sequence read in question. A dark red bar indicates that this sequence read produces many other hits elsewhere in the genome. Many adjacent red or yellow bars can indicate the presence of a multi-copy element such as transposons or rRNA genes. A dark green bar indicates that this sequence read maps uniquely to this locus.

1 hit

2-5 hits

6-10 hits

11-20 hits

21-50 hits

51-100 hits

> 100 hits

NODE\_356908\_length\_2531\_cov\_46.841564

4

2656

Gene Set

RepeatMasker

Mapped  
Reads

82.82

plus strand

minus strand

82.82

Region: NODE\_356908\_length\_2531\_cov\_46.841564 12487-6. Max. coverage (+): 0. Max coverage (-): 0.01

Region: NODE\_356908\_length\_2531\_cov\_46.841564 7-11. Max. coverage (+): 0. Max coverage (-): 0

Region: NODE\_356908\_length\_2531\_cov\_46.841564 12-17. Max. coverage (+): 0. Max coverage (-): 0

Region: NODE\_356908\_length\_2531\_cov\_46.841564 18-22. Max. coverage (+): 0. Max coverage (-): 0

Region: NODE\_356908\_length\_2531\_cov\_46.841564 23-27. Max. coverage (+): 0. Max coverage (-): 0

Region: NODE\_356908\_length\_2531\_cov\_46.841564 28-33. Max. coverage (+): 0. Max coverage (-): 0

Region: NODE\_356908\_length\_2531\_cov\_46.841564 34-38. Max. coverage (+): 0.02. Max coverage (-): 0.12

Region: NODE\_356908\_length\_2531\_cov\_46.841564 39-43. Max. coverage (+): 0. Max coverage (-): 0.96

Region: NODE\_356908\_length\_2531\_cov\_46.841564 44-49. Max. coverage (+): 0. Max coverage (-): 0.89

Region: NODE\_356908\_length\_2531\_cov\_46.841564 50-54. Max. coverage (+): 0.11. Max coverage (-): 0.85

Region: NODE\_356908\_length\_2531\_cov\_46.841564 55-59. Max. coverage (+): 0. Max coverage (-): 0.04

Region: NODE\_356908\_length\_2531\_cov\_46.841564 60-65. Max. coverage (+): 0.09. Max coverage (-): 0.91

Region: NODE\_356908\_length\_2531\_cov\_46.841564 66-70. Max. coverage (+): 0.11. Max coverage (-): 0.15

Region: NODE\_356908\_length\_2531\_cov\_46.841564 71-75. Max. coverage (+): 0.07. Max coverage (-): 0.04

Region: NODE\_356908\_length\_2531\_cov\_46.841564 76-80. Max. coverage (+): 0.11. Max coverage (-): 0.04

Region: NODE\_356908\_length\_2531\_cov\_46.841564 81-86. Max. coverage (+): 0.11. Max coverage (-): 0.3

Region: NODE\_356908\_length\_2531\_cov\_46.841564 87-91. Max. coverage (+): 0.11. Max coverage (-): 0.15

Region: NODE\_356908\_length\_2531\_cov\_46.841564 92-96. Max. coverage (+): 0. Max coverage (-): 2.71

Region: NODE\_356908\_length\_2531\_cov\_46.841564 97-102. Max. coverage (+): 0. Max coverage (-): 7.3

Region: NODE\_356908\_length\_2531\_cov\_46.841564 103-107. Max. coverage (+): 0. Max coverage (-): 1.74

Region: NODE\_356908\_length\_2531\_cov\_46.841564 108-112. Max. coverage (+): 0.96. Max coverage (-): 0.26

Region: NODE\_356908\_length\_2531\_cov\_46.841564 113-118. Max. coverage (+): 1.15. Max coverage (-): 0.85

Region: NODE\_356908\_length\_2531\_cov\_46.841564 119-123. Max. coverage (+): 0.19. Max coverage (-): 0.07

Region: NODE\_356908\_length\_2531\_cov\_46.841564 124-128. Max. coverage (+): 0.07. Max coverage (-): 0.3

Region: NODE\_356908\_length\_2531\_cov\_46.841564 129-133. Max. coverage (+): 0. Max coverage (-): 9.01

Region: NODE\_356908\_length\_2531\_cov\_46.841564 134-139. Max. coverage (+): 0.04. Max coverage (-): 82.82

Region: NODE\_356908\_length\_2531\_cov\_46.841564 140-144. Max. coverage (+): 0.22. Max coverage (-): 17.48

Region: NODE\_356908\_length\_2531\_cov\_46.841564 145-149. Max. coverage (+): 0.44. Max coverage (-): 2.93

Region: NODE\_356908\_length\_2531\_cov\_46.841564 150-155. Max. coverage (+): 0.44. Max coverage (-): 0.74

Region: NODE\_356908\_length\_2531\_cov\_46.841564 156-160. Max. coverage (+): 0.04. Max coverage (-): 0.74

Region: NODE\_356908\_length\_2531\_cov\_46.841564 161-165. Max. coverage (+): 0.07. Max coverage (-): 11.16

Region: NODE\_356908\_length\_2531\_cov\_46.841564 166-171. Max. coverage (+): 0.04. Max coverage (-): 1.41

Region: NODE\_356908\_length\_2531\_cov\_46.841564 172-176. Max. coverage (+): 0. Max coverage (-): 0

Region: NODE\_356908\_length\_2531\_cov\_46.841564 177-181. Max. coverage (+): 0.07. Max coverage (-): 0.04

Region: NODE\_356908\_length\_2531\_cov\_46.841564 182-187. Max. coverage (+): 0.11. Max coverage (-): 0.78

Region: NODE\_356908\_length\_2531\_cov\_46.841564 188-192. Max. coverage (+): 0.04. Max coverage (-): 0.41

Region: NODE\_356908\_length\_2531\_cov\_46.841564 193-197. Max. coverage (+): 0.04. Max coverage (-): 2.52

Region: NODE\_356908\_length\_2531\_cov\_46.841564 198-202. Max. coverage (+): 0.04. Max coverage (-): 1.3

Region: NODE\_356908\_length\_2531\_cov\_46.841564 203-208. Max. coverage (+): 3.45. Max coverage (-): 0.52

Region: NODE\_356908\_length\_2531\_cov\_46.841564 209-213. Max. coverage (+): 1.15. Max coverage (-): 1.56

Region: NODE\_356908\_length\_2531\_cov\_46.841564 214-218. Max. coverage (+): 0.19. Max coverage (-): 1.19

Region: NODE\_356908\_length\_2531\_cov\_46.841564 219-224. Max. coverage (+): 0.11. Max coverage (-): 1.78

Region: NODE\_356908\_length\_2531\_cov\_46.841564 225-229. Max. coverage (+): 0. Max coverage (-): 3.37

Region: NODE\_356908\_length\_2531\_cov\_46.841564 230-234. Max. coverage (+): 0.04. Max coverage (-): 3.04

Region: NODE\_356908\_length\_2531\_cov\_46.841564 235-240. Max. coverage (+): 0.26. Max coverage (-): 2.71

Region: NODE\_356908\_length\_2531\_cov\_46.841564 241-245. Max. coverage (+): 1.74. Max coverage (-): 1.3

Region: NODE\_356908\_length\_2531\_cov\_46.841564 246-250. Max. coverage (+): 1.72. Max coverage (-): 2.06

Region: NODE\_356908\_length\_2531\_cov\_46.841564 251-256. Max. coverage (+): 0.67. Max coverage (-): 0.43

Region: NODE\_356908\_length\_2531\_cov\_46.841564 257-261. Max. coverage (+): 0.82. Max coverage (-): 1.08

Region: NODE\_356908\_length\_2531\_cov\_46.841564 262-266. Max. coverage (+): 0.33. Max coverage (-): 0.56

Region: NODE\_356908\_length\_2531\_cov\_46.841564 267-271. Max. coverage (+): 0.37. Max coverage (-): 1.26

Region: NODE\_356908\_length\_2531\_cov\_46.841564 272-277. Max. coverage (+): 0.04. Max coverage (-): 2.71

Region: NODE\_356908\_length\_2531\_cov\_46.841564 278-282. Max. coverage (+): 0.15. Max coverage (-): 0.59

Region: NODE\_356908\_length\_2531\_cov\_46.841564 283-287. Max. coverage (+): 0.78. Max coverage (-): 1.15

Region: NODE\_356908\_length\_2531\_cov\_46.841564 288-293. Max. coverage (+): 0.74. Max coverage (-): 0.15

Region: NODE\_356908\_length\_2531\_cov\_46.841564 294-298. Max. coverage (+): 0.22. Max coverage (-): 0.04

Region: NODE\_356908\_length\_2531\_cov\_46.841564 299-303. Max. coverage (+): 0.37. Max coverage (-): 0.04

Region: NODE\_356908\_length\_2531\_cov\_46.841564 304-309. Max. coverage (+): 0. Max coverage (-): 0

Region: NODE\_356908\_length\_2531\_cov\_46.841564 310-314. Max. coverage (+): 0. Max coverage (-): 0.48

Region: NODE\_356908\_length\_2531\_cov\_46.841564 315-319. Max. coverage (+): 0. Max coverage (-): 1.48

Region: NODE\_356908\_length\_2531\_cov\_46.841564 320-325. Max. coverage (+): 0. Max coverage (-): 0.37

Region: NODE\_356908\_length\_2531\_cov\_46.841564 326-330. Max. coverage (+): 0. Max coverage (-): 0.04

Region: NODE\_356908\_length\_2531\_cov\_46.841564 331-335. Max. coverage (+): 0. Max coverage (-): 0.3

Region: NODE\_356908\_length\_2531\_cov\_46.841564 336-340. Max. coverage (+): 0.26. Max coverage (-): 0.37

Region: NODE\_356908\_length\_2531\_cov\_46.841564 341-346. Max. coverage (+): 0.07. Max coverage (-): 0.07

Region: NODE\_356908\_length\_2531\_cov\_46.841564 347-351. Max. coverage (+): 0.19. Max coverage (-): 0.11

Region: NODE\_356908\_length\_2531\_cov\_46.841564 352-356. Max. coverage (+): 0. Max coverage (-): 1.04

Region: NODE\_356908\_length\_2531\_cov\_46.841564 357-362. Max. coverage (+): 0. Max coverage (-): 0.19

Region: NODE\_356908\_length\_2531\_cov\_46.841564 363-367. Max. coverage (+): 0. Max coverage (-): 0

Region: NODE\_356908\_length\_2531\_cov\_46.841564 368-372. Max. coverage (+): 0. Max coverage (-): 0

Region: NODE\_356908\_length\_2531\_cov\_46.841564 373-378. Max. coverage (+): 0. Max coverage (-): 0

Region: NODE\_356908\_length\_2531\_cov\_46.841564 379-383. Max. coverage (+): 0. Max coverage (-): 0.33

Region: NODE\_356908\_length\_2531\_cov\_46.841564 384-388. Max. coverage (+): 0. Max coverage (-): 1.63

Region: NODE\_356908\_length\_2531\_cov\_46.841564 389-393. Max. coverage (+): 0. Max coverage (-): 0.3

Region: NODE\_356908\_length\_2531\_cov\_46.841564 394-399. Max. coverage (+): 0.11. Max coverage (-): 0

Region: NODE\_356908\_length\_2531\_cov\_46.841564 400-404. Max. coverage (+): 1.89. Max coverage (-): 0

Region: NODE\_356908\_length\_2531\_cov\_46.841564 405-409. Max. coverage (+): 2. Max coverage (-): 0.63

Region: NODE\_356908\_length\_2531\_cov\_46.841564 410-415. Max. coverage (+): 0. Max coverage (-): 0.63

Region: NODE\_356908\_length\_2531\_cov\_46.841564 416-420. Max. coverage (+): 0.04. Max coverage (-): 0.82

Region: NODE\_356908\_length\_2531\_cov\_46.841564 421-425. Max. coverage (+): 0.52. Max coverage (-): 0.52

Region: NODE\_356908\_length\_2531\_cov\_46.841564 426-431. Max. coverage (+): 0.44. Max coverage (-): 0.11

Region: NODE\_356908\_length\_2531\_cov\_46.841564 432-436. Max. coverage (+): 0.3. Max coverage (-): 0.22

Region: NODE\_356908\_length\_2531\_cov\_46.841564 437-441. Max. coverage (+): 0.3. Max coverage (-): 0.63

Region: NODE\_356908\_length\_2531\_cov\_46.841564 442-447. Max. coverage (+): 0. Max coverage (-): 0.67

Region: NODE\_356908\_length\_2531\_cov\_46.841564 448-452. Max. coverage (+): 0. Max coverage (-): 0.04

Region: NODE\_356908\_length\_2531\_cov\_46.841564 453-457. Max. coverage (+): 0. Max coverage (-): 1.04

Region: NODE\_356908\_length\_2531\_cov\_46.841564 458-462. Max. coverage (+): 0.07. Max coverage (-): 0.63

Region: NODE\_356908\_length\_2531\_cov\_46.841564 463-468. Max. coverage (+): 0.15. Max coverage (-): 12.68

Region: NODE\_356908\_length\_2531\_cov\_46.841564 469-473. Max. coverage (+): 0. Max coverage (-): 0.3

Region: NODE\_356908\_length\_2531\_cov\_46.841564 474-478. Max. coverage (+): 0. Max coverage (-): 0.04

Region: NODE\_356908\_length\_2531\_cov\_46.841564 479-484. Max. coverage (+): 0. Max coverage (-): 1.78

Region: NODE\_356908\_length\_2531\_cov\_46.841564 485-489. Max. coverage (+): 0. Max coverage (-): 0.17

Region: NODE\_356908\_length\_2531\_cov\_46.841564 490-494. Max. coverage (+): 0.02. Max coverage (-): 0.32

Region: NODE\_356908\_length\_2531\_cov\_46.841564 495-500. Max. coverage (+): 0.08. Max coverage (-): 1.59

Region: NODE\_356908\_length\_2531\_cov\_46.841564 501-505. Max. coverage (+): 0.09. Max coverage (-): 0

Region: NODE\_356908\_length\_2531\_cov\_46.841564 506-510. Max. coverage (+): 0.07. Max coverage (-): 0.02

Region: NODE\_356908\_length\_2531\_cov\_46.841564 511-516. Max. coverage (+): 0.06. Max coverage (-): 0.04

Region: NODE\_356908\_length\_2531\_cov\_46.841564 517-521. Max. coverage (+): 0.04. Max coverage (-): 0.37

Region: NODE\_356908\_length\_2531\_cov\_46.841564 522-526. Max. coverage (+): 0.02. Max coverage (-): 3.04

Region: NODE\_356908\_length\_2531\_cov\_46.841564 527-531. Max. coverage (+): 0. Max coverage (-): 3.95

Region: NODE\_356908\_length\_2531\_cov\_46.841564 532-537. Max. coverage (+): 0. Max coverage (-): 1.22

Region: NODE\_356908\_length\_2531\_cov\_46.841564 538-542. Max. coverage (+): 0. Max coverage (-): 0.02

Region: NODE\_356908\_length\_2531\_cov\_46.841564 543-547. Max. coverage (+): 0.22. Max coverage (-): 0.19

Region: NODE\_356908\_length\_2531\_cov\_46.841564 548-553. Max. coverage (+): 0.07. Max coverage (-): 0.09

Region: NODE\_356908\_length\_2531\_cov\_46.841564 554-558. Max. coverage (+): 0. Max coverage (-): 0.82

Region: NODE\_356908\_length\_2531\_cov\_46.841564 559-563. Max. coverage (+): 0. Max coverage (-): 0.41

Region: NODE\_356908\_length\_2531\_cov\_46.841564 564-569. Max. coverage (+): 0. Max coverage (-): 0.52

Region: NODE\_356908\_length\_2531\_cov\_46.841564 570-574. Max. coverage (+): 0. Max coverage (-): 0.11

Region: NODE\_356908\_length\_2531\_cov\_46.841564 575-579. Max. coverage (+): 0.02. Max coverage (-): 0.52

Region: NODE\_356908\_length\_2531\_cov\_46.841564 580-585. Max. coverage (+): 0.02. Max coverage (-): 0.83

Region: NODE\_356908\_length\_2531\_cov\_46.841564 586-590. Max. coverage (+): 0. Max coverage (-): 3.78

Region: NODE\_356908\_length\_2531\_cov\_46.841564 591-595. Max. coverage (+): 0. Max coverage (-): 0.46

Region: NODE\_356908\_length\_2531\_cov\_46.841564 596-600. Max. coverage (+): 0. Max coverage (-): 0.91

Region: NODE\_356908\_length\_2531\_cov\_46.841564 601-606. Max. coverage (+): 0.01. Max coverage (-): 1.22

Region: NODE\_356908\_length\_2531\_cov\_46.841564 607-611. Max. coverage (+): 0.04. Max coverage (-): 0.48

Region: NODE\_356908\_length\_2531\_cov\_46.841564 612-616. Max. coverage (+): 0.04. Max coverage (-): 0.07

Region: NODE\_356908\_length\_2531\_cov\_46.841564 617-622. Max. coverage (+): 0.22. Max coverage (-): 2.19

Region: NODE\_356908\_length\_2531\_cov\_46.841564 623-627. Max. coverage (+): 0. Max coverage (-): 2.11

Region: NODE\_356908\_length\_2531\_cov\_46.841564 628-632. Max. coverage (+): 0.04. Max coverage (-): 1.56

Region: NODE\_356908\_length\_2531\_cov\_46.841564 633-638. Max. coverage (+): 0. Max coverage (-): 1.93

Region: NODE\_356908\_length\_2531\_cov\_46.841564 639-643. Max. coverage (+): 0. Max coverage (-): 17.85

Region: NODE\_356908\_length\_2531\_cov\_46.841564 644-648. Max. coverage (+): 0. Max coverage (-): 18.54

Region: NODE\_356908\_length\_2531\_cov\_46.841564 649-653. Max. coverage (+): 0.01. Max coverage (-): 1.15

Region: NODE\_356908\_length\_2531\_cov\_46.841564 654-659. Max. coverage (+): 0.02. Max coverage (-): 0.83

Region: NODE\_356908\_length\_2531\_cov\_46.841564 660-664. Max. coverage (+): 0. Max coverage (-): 0.83

Region: NODE\_356908\_length\_2531\_cov\_46.841564 665-669. Max. coverage (+): 0. Max coverage (-): 0.04

Region: NODE\_356908\_length\_2531\_cov\_46.841564 670-675. Max. coverage (+): 0.01. Max coverage (-): 0.05

Region: NODE\_356908\_length\_2531\_cov\_46.841564 676-680. Max. coverage (+): 0.06. Max coverage (-): 0.07

Region: NODE\_356908\_length\_2531\_cov\_46.841564 681-685. Max. coverage (+): 0.02. Max coverage (-): 1.88

Region: NODE\_356908\_length\_2531\_cov\_46.841564 686-691. Max. coverage (+): 0.01. Max coverage (-): 0.67

Region: NODE\_356908\_length\_2531\_cov\_46.841564 692-696. Max. coverage (+): 0.02. Max coverage (-): 0.68

Region: NODE\_356908\_length\_2531\_cov\_46.841564 697-701. Max. coverage (+): 0.01. Max coverage (-): 0.01

Region: NODE\_356908\_length\_2531\_cov\_46.841564 702-707. Max. coverage (+): 0. Max coverage (-): 0

Region: NODE\_356908\_length\_2531\_cov\_46.841564 708-712. Max. coverage (+): 0. Max coverage (-): 0

Region: NODE\_356908\_length\_2531\_cov\_46.841564 713-717. Max. coverage (+): 0. Max coverage (-): 0

Region: NODE\_356908\_length\_2531\_cov\_46.841564 718-722. Max. coverage (+): 0. Max coverage (-): 0

Region: NODE\_356908\_length\_2531\_cov\_46.841564 723-728. Max. coverage (+): 0. Max coverage (-): 0

Region: NODE\_356908\_length\_2531\_cov\_46.841564 729-733. Max. coverage (+): 0. Max coverage (-): 0

Region: NODE\_356908\_length\_2531\_cov\_46.841564 734-738. Max. coverage (+): 0. Max coverage (-): 0

Region: NODE\_356908\_length\_2531\_cov\_46.841564 739-744. Max. coverage (+): 0. Max coverage (-): 0

Region: NODE\_356908\_length\_2531\_cov\_46.841564 745-749. Max. coverage (+): 0. Max coverage (-): 0

Region: NODE\_356908\_length\_2531\_cov\_46.841564 750-754. Max. coverage (+): 0. Max coverage (-): 0

Region: NODE\_356908\_length\_2531\_cov\_46.841564 755-760. Max. coverage (+): 0. Max coverage (-): 0

Region: NODE\_356908\_length\_2531\_cov\_46.841564 761-765. Max. coverage (+): 0.11. Max coverage (-): 0

Region: NODE\_356908\_length\_2531\_cov\_46.841564 766-770. Max. coverage (+): 0.19. Max coverage (-): 0

Region: NODE\_356908\_length\_2531\_cov\_46.841564 771-776. Max. coverage (+): 0.3. Max coverage (-): 0.04

Region: NODE\_356908\_length\_2531\_cov\_46.841564 777-781. Max. coverage (+): 0.3. Max coverage (-): 0.09

Region: NODE\_356908\_length\_2531\_cov\_46.841564 782-786. Max. coverage (+): 0. Max coverage (-): 0.04

Region: NODE\_356908\_length\_2531\_cov\_46.841564 787-791. Max. coverage (+): 0. Max coverage (-): 1.78

Region: NODE\_356908\_length\_2531\_cov\_46.841564 792-797. Max. coverage (+): 0. Max coverage (-): 0.48

Region: NODE\_356908\_length\_2531\_cov\_46.841564 798-802. Max. coverage (+): 0.02. Max coverage (-): 0.28

Region: NODE\_356908\_length\_2531\_cov\_46.841564 803-807. Max. coverage (+): 0.02. Max coverage (-): 1.59

Region: NODE\_356908\_length\_2531\_cov\_46.841564 808-813. Max. coverage (+): 0.09. Max coverage (-): 0.57

Region: NODE\_356908\_length\_2531\_cov\_46.841564 814-818. Max. coverage (+): 0.04. Max coverage (-): 0.02

Region: NODE\_356908\_length\_2531\_cov\_46.841564 819-823. Max. coverage (+): 0.07. Max coverage (-): 0.02

Region: NODE\_356908\_length\_2531\_cov\_46.841564 824-829. Max. coverage (+): 0.04. Max coverage (-): 0.09

Region: NODE\_356908\_length\_2531\_cov\_46.841564 830-834. Max. coverage (+): 0.02. Max coverage (-): 2.87

Region: NODE\_356908\_length\_2531\_cov\_46.841564 835-839. Max. coverage (+): 0. Max coverage (-): 3.95

Region: NODE\_356908\_length\_2531\_cov\_46.841564 840-845. Max. coverage (+): 0. Max coverage (-): 1.22

Region: NODE\_356908\_length\_2531\_cov\_46.841564 846-850. Max. coverage (+): 0. Max coverage (-): 0

Region: NODE\_356908\_length\_2531\_cov\_46.841564 851-855. Max. coverage (+): 0.15. Max coverage (-): 0.07

Region: NODE\_356908\_length\_2531\_cov\_46.841564 856-860. Max. coverage (+): 0.41. Max coverage (-): 0.06

Region: NODE\_356908\_length\_2531\_cov\_46.841564 861-866. Max. coverage (+): 0.04. Max coverage (-): 0

Region: NODE\_356908\_length\_2531\_cov\_46.841564 867-871. Max. coverage (+): 0.04. Max coverage (-): 0

Region: NODE\_356908\_length\_2531\_cov\_46.841564 872-876. Max. coverage (+): 0. Max coverage (-): 0

Region: NODE\_356908\_length\_2531\_cov\_46.841564 877-882. Max. coverage (+): 0. Max coverage (-): 0

Region: NODE\_356908\_length\_2531\_cov\_46.841564 883-887. Max. coverage (+): 0. Max coverage (-): 0

Region: NODE\_356908\_length\_2531\_cov\_46.841564 888-892. Max. coverage (+): 0.02. Max coverage (-): 0.15

Region: NODE\_356908\_length\_2531\_cov\_46.841564 893-898. Max. coverage (+): 0.02. Max coverage (-): 0.83

Region: NODE\_356908\_length\_2531\_cov\_46.841564 899-903. Max. coverage (+): 0.02. Max coverage (-): 3.02

Region: NODE\_356908\_length\_2531\_cov\_46.841564 904-908. Max. coverage (+): 0. Max coverage (-): 3.78

Region: NODE\_356908\_length\_2531\_cov\_46.841564 909-913. Max. coverage (+): 0. Max coverage (-): 0.14

Region: NODE\_356908\_length\_2531\_cov\_46.841564 914-919. Max. coverage (+): 0.01. Max coverage (-): 0.91

Region: NODE\_356908\_length\_2531\_cov\_46.841564 920-924. Max. coverage (+): 0.22. Max coverage (-): 0.56

Region: NODE\_356908\_length\_2531\_cov\_46.841564 925-929. Max. coverage (+): 0.22. Max coverage (-): 0

Region: NODE\_356908\_length\_2531\_cov\_46.841564 930-935. Max. coverage (+): 0.15. Max coverage (-): 0

Region: NODE\_356908\_length\_2531\_cov\_46.841564 936-940. Max. coverage (+): 0. Max coverage (-): 0

Region: NODE\_356908\_length\_2531\_cov\_46.841564 941-945. Max. coverage (+): 0. Max coverage (-): 0

Region: NODE\_356908\_length\_2531\_cov\_46.841564 946-951. Max. coverage (+): 0.11. Max coverage (-): 0

Region: NODE\_356908\_length\_2531\_cov\_46.841564 952-956. Max. coverage (+): 0.04. Max coverage (-): 0.04

Region: NODE\_356908\_length\_2531\_cov\_46.841564 957-961. Max. coverage (+): 0.04. Max coverage (-): 0.09

Region: NODE\_356908\_length\_2531\_cov\_46.841564 962-967. Max. coverage (+): 0.04. Max coverage (-): 1.15

Region: NODE\_356908\_length\_2531\_cov\_46.841564 968-972. Max. coverage (+): 0.02. Max coverage (-): 0.1

Region: NODE\_356908\_length\_2531\_cov\_46.841564 973-977. Max. coverage (+): 0.02. Max coverage (-): 0.83

Region: NODE\_356908\_length\_2531\_cov\_46.841564 978-982. Max. coverage (+): 0. Max coverage (-): 0.01

Region: NODE\_356908\_length\_2531\_cov\_46.841564 983-988. Max. coverage (+): 0.01. Max coverage (-): 0.05

Region: NODE\_356908\_length\_2531\_cov\_46.841564 989-993. Max. coverage (+): 0.04. Max coverage (-): 0.03

Region: NODE\_356908\_length\_2531\_cov\_46.841564 994-998. Max. coverage (+): 0.06. Max coverage (-): 1.88

Region: NODE\_356908\_length\_2531\_cov\_46.841564 999-1004. Max. coverage (+): 0.02. Max coverage (-): 1.84

Region: NODE\_356908\_length\_2531\_cov\_46.841564 1005-1009. Max. coverage (+): 0.02. Max coverage (-): 0.74

Region: NODE\_356908\_length\_2531\_cov\_46.841564 1010-1014. Max. coverage (+): 0.02. Max coverage (-): 0.68

Region: NODE\_356908\_length\_2531\_cov\_46.841564 1015-1020. Max. coverage (+): 0.04. Max coverage (-): 0.02

Region: NODE\_356908\_length\_2531\_cov\_46.841564 1021-1025. Max. coverage (+): 0.04. Max coverage (-): 0.02

Region: NODE\_356908\_length\_2531\_cov\_46.841564 1026-1030. Max. coverage (+): 0. Max coverage (-): 0

Region: NODE\_356908\_length\_2531\_cov\_46.841564 1031-1036. Max. coverage (+): 0. Max coverage (-): 0.56

Region: NODE\_356908\_length\_2531\_cov\_46.841564 1037-1041. Max. coverage (+): 0.06. Max coverage (-): 0.48

Region: NODE\_356908\_length\_2531\_cov\_46.841564 1042-1046. Max. coverage (+): 0.26. Max coverage (-): 0.19

Region: NODE\_356908\_length\_2531\_cov\_46.841564 1047-1051. Max. coverage (+): 0.26. Max coverage (-): 0.06

Region: NODE\_356908\_length\_2531\_cov\_46.841564 1052-1057. Max. coverage (+): 0.15. Max coverage (-): 0.3

Region: NODE\_356908\_length\_2531\_cov\_46.841564 1058-1062. Max. coverage (+): 0. Max coverage (-): 0.85

Region: NODE\_356908\_length\_2531\_cov\_46.841564 1063-1067. Max. coverage (+): 0. Max coverage (-): 1.04

Region: NODE\_356908\_length\_2531\_cov\_46.841564 1068-1073. Max. coverage (+): 0.06. Max coverage (-): 0.22

Region: NODE\_356908\_length\_2531\_cov\_46.841564 1074-1078. Max. coverage (+): 0.02. Max coverage (-): 0.02

Region: NODE\_356908\_length\_2531\_cov\_46.841564 1079-1083. Max. coverage (+): 0.89. Max coverage (-): 0.02

Region: NODE\_356908\_length\_2531\_cov\_46.841564 1084-1089. Max. coverage (+): 0.22. Max coverage (-): 0.09

Region: NODE\_356908\_length\_2531\_cov\_46.841564 1090-1094. Max. coverage (+): 0.02. Max coverage (-): 0.83

Region: NODE\_356908\_length\_2531\_cov\_46.841564 1095-1099. Max. coverage (+): 0. Max coverage (-): 0.82

Region: NODE\_356908\_length\_2531\_cov\_46.841564 1100-1104. Max. coverage (+): 0. Max coverage (-): 0.22

Region: NODE\_356908\_length\_2531\_cov\_46.841564 1105-1110. Max. coverage (+): 0.02. Max coverage (-): 0.11

Region: NODE\_356908\_length\_2531\_cov\_46.841564 1111-1115. Max. coverage (+): 0.04. Max coverage (-): 0.02

Region: NODE\_356908\_length\_2531\_cov\_46.841564 1116-1120. Max. coverage (+): 0. Max coverage (-): 0

Region: NODE\_356908\_length\_2531\_cov\_46.841564 1121-1126. Max. coverage (+): 0.07. Max coverage (-): 0

Region: NODE\_356908\_length\_2531\_cov\_46.841564 1127-1131. Max. coverage (+): 0.04. Max coverage (-): 0.37

Region: NODE\_356908\_length\_2531\_cov\_46.841564 1132-1136. Max. coverage (+): 0.02. Max coverage (-): 0.54

Region: NODE\_356908\_length\_2531\_cov\_46.841564 1137-1142. Max. coverage (+): 0.05. Max coverage (-): 0.4

Region: NODE\_356908\_length\_2531\_cov\_46.841564 1143-1147. Max. coverage (+): 0.04. Max coverage (-): 1.45

Region: NODE\_356908\_length\_2531\_cov\_46.841564 1148-1152. Max. coverage (+): 0.26. Max coverage (-): 0.25

Region: NODE\_356908\_length\_2531\_cov\_46.841564 1153-1158. Max. coverage (+): 0.32. Max coverage (-): 0.09

Region: NODE\_356908\_length\_2531\_cov\_46.841564 1159-1163. Max. coverage (+): 0. Max coverage (-): 0.04

Region: NODE\_356908\_length\_2531\_cov\_46.841564 1164-1168. Max. coverage (+): 0. Max coverage (-): 4.12

Region: NODE\_356908\_length\_2531\_cov\_46.841564 1169-1173. Max. coverage (+): 0. Max coverage (-): 20.24

Region: NODE\_356908\_length\_2531\_cov\_46.841564 1174-1179. Max. coverage (+): 0.02. Max coverage (-): 6.75

Region: NODE\_356908\_length\_2531\_cov\_46.841564 1180-1184. Max. coverage (+): 1.09. Max coverage (-): 0.09

Region: NODE\_356908\_length\_2531\_cov\_46.841564 1185-1189. Max. coverage (+): 1.13. Max coverage (-): 0.07

Region: NODE\_356908\_length\_2531\_cov\_46.841564 1190-1195. Max. coverage (+): 0.28. Max coverage (-): 0.43

Region: NODE\_356908\_length\_2531\_cov\_46.841564 1196-1200. Max. coverage (+): 0.06. Max coverage (-): 0.41

Region: NODE\_356908\_length\_2531\_cov\_46.841564 1201-1205. Max. coverage (+): 0.02. Max coverage (-): 0.09

Region: NODE\_356908\_length\_2531\_cov\_46.841564 1206-1211. Max. coverage (+): 0.19. Max coverage (-): 2.73

Region: NODE\_356908\_length\_2531\_cov\_46.841564 1212-1216. Max. coverage (+): 0.2. Max coverage (-): 3.44

Region: NODE\_356908\_length\_2531\_cov\_46.841564 1217-1221. Max. coverage (+): 0.01. Max coverage (-): 1.99

Region: NODE\_356908\_length\_2531\_cov\_46.841564 1222-1227. Max. coverage (+): 0. Max coverage (-): 0.96

Region: NODE\_356908\_length\_2531\_cov\_46.841564 1228-1232. Max. coverage (+): 0. Max coverage (-): 0.43

Region: NODE\_356908\_length\_2531\_cov\_46.841564 1233-1237. Max. coverage (+): 0.01. Max coverage (-): 0.05

Region: NODE\_356908\_length\_2531\_cov\_46.841564 1238-1242. Max. coverage (+): 0.06. Max coverage (-): 2.6

Region: NODE\_356908\_length\_2531\_cov\_46.841564 1243-1248. Max. coverage (+): 0.06. Max coverage (-): 2.8

Region: NODE\_356908\_length\_2531\_cov\_46.841564 1249-1253. Max. coverage (+): 0. Max coverage (-): 23.51

Region: NODE\_356908\_length\_2531\_cov\_46.841564 1254-1258. Max. coverage (+): 0. Max coverage (-): 14.73

Region: NODE\_356908\_length\_2531\_cov\_46.841564 1259-1264. Max. coverage (+): 0. Max coverage (-): 0.12

Region: NODE\_356908\_length\_2531\_cov\_46.841564 1265-1269. Max. coverage (+): 0.26. Max coverage (-): 0.41

Region: NODE\_356908\_length\_2531\_cov\_46.841564 1270-1274. Max. coverage (+): 0.26. Max coverage (-): 0.15

Region: NODE\_356908\_length\_2531\_cov\_46.841564 1275-1280. Max. coverage (+): 0.04. Max coverage (-): 1.15

Region: NODE\_356908\_length\_2531\_cov\_46.841564 1281-1285. Max. coverage (+): 0. Max coverage (-): 1.37

Region: NODE\_356908\_length\_2531\_cov\_46.841564 1286-1290. Max. coverage (+): 0. Max coverage (-): 0.17

Region: NODE\_356908\_length\_2531\_cov\_46.841564 1291-1296. Max. coverage (+): 0. Max coverage (-): 0.22

Region: NODE\_356908\_length\_2531\_cov\_46.841564 1297-1301. Max. coverage (+): 0. Max coverage (-): 0.11

Region: NODE\_356908\_length\_2531\_cov\_46.841564 1302-1306. Max. coverage (+): 0. Max coverage (-): 0.32

Region: NODE\_356908\_length\_2531\_cov\_46.841564 1307-1311. Max. coverage (+): 0.11. Max coverage (-): 0.7

Region: NODE\_356908\_length\_2531\_cov\_46.841564 1312-1317. Max. coverage (+): 0.11. Max coverage (-): 3.23

Region: NODE\_356908\_length\_2531\_cov\_46.841564 1318-1322. Max. coverage (+): 0.04. Max coverage (-): 0.82

Region: NODE\_356908\_length\_2531\_cov\_46.841564 1323-1327. Max. coverage (+): 0. Max coverage (-): 0.11

Region: NODE\_356908\_length\_2531\_cov\_46.841564 1328-1333. Max. coverage (+): 0.35. Max coverage (-): 0.54

Region: NODE\_356908\_length\_2531\_cov\_46.841564 1334-1338. Max. coverage (+): 0.44. Max coverage (-): 0.26

Region: NODE\_356908\_length\_2531\_cov\_46.841564 1339-1343. Max. coverage (+): 0.06. Max coverage (-): 0.06

Region: NODE\_356908\_length\_2531\_cov\_46.841564 1344-1349. Max. coverage (+): 0.75. Max coverage (-): 0

Region: NODE\_356908\_length\_2531\_cov\_46.841564 1350-1354. Max. coverage (+): 0.75. Max coverage (-): 0

Region: NODE\_356908\_length\_2531\_cov\_46.841564 1355-1359. Max. coverage (+): 0. Max coverage (-): 4.3

Region: NODE\_356908\_length\_2531\_cov\_46.841564 1360-1364. Max. coverage (+): 0. Max coverage (-): 5.88

Region: NODE\_356908\_length\_2531\_cov\_46.841564 1365-1370. Max. coverage (+): 0. Max coverage (-): 1.15

Region: NODE\_356908\_length\_2531\_cov\_46.841564 1371-1375. Max. coverage (+): 0. Max coverage (-): 0

Region: NODE\_356908\_length\_2531\_cov\_46.841564 1376-1380. Max. coverage (+): 0. Max coverage (-): 0

Region: NODE\_356908\_length\_2531\_cov\_46.841564 1381-1386. Max. coverage (+): 0. Max coverage (-): 0

Region: NODE\_356908\_length\_2531\_cov\_46.841564 1387-1391. Max. coverage (+): 0. Max coverage (-): 1.2

Region: NODE\_356908\_length\_2531\_cov\_46.841564 1392-1396. Max. coverage (+): 0. Max coverage (-): 1.19

Region: NODE\_356908\_length\_2531\_cov\_46.841564 1397-1402. Max. coverage (+): 0.07. Max coverage (-): 0.02

Region: NODE\_356908\_length\_2531\_cov\_46.841564 1403-1407. Max. coverage (+): 0.3. Max coverage (-): 0

Region: NODE\_356908\_length\_2531\_cov\_46.841564 1408-1412. Max. coverage (+): 0.22. Max coverage (-): 0

Region: NODE\_356908\_length\_2531\_cov\_46.841564 1413-1418. Max. coverage (+): 0.04. Max coverage (-): 0

Region: NODE\_356908\_length\_2531\_cov\_46.841564 1419-1423. Max. coverage (+): 0. Max coverage (-): 0

Region: NODE\_356908\_length\_2531\_cov\_46.841564 1424-1428. Max. coverage (+): 0. Max coverage (-): 0.22

Region: NODE\_356908\_length\_2531\_cov\_46.841564 1429-1433. Max. coverage (+): 0. Max coverage (-): 0.22

Region: NODE\_356908\_length\_2531\_cov\_46.841564 1434-1439. Max. coverage (+): 0. Max coverage (-): 0.11

Region: NODE\_356908\_length\_2531\_cov\_46.841564 1440-1444. Max. coverage (+): 0.11. Max coverage (-): 0.02

Region: NODE\_356908\_length\_2531\_cov\_46.841564 1445-1449. Max. coverage (+): 0.07. Max coverage (-): 0

Region: NODE\_356908\_length\_2531\_cov\_46.841564 1450-1455. Max. coverage (+): 0.11. Max coverage (-): 0.15

Region: NODE\_356908\_length\_2531\_cov\_46.841564 1456-1460. Max. coverage (+): 0.22. Max coverage (-): 0

Region: NODE\_356908\_length\_2531\_cov\_46.841564 1461-1465. Max. coverage (+): 0.19. Max coverage (-): 0

Region: NODE\_356908\_length\_2531\_cov\_46.841564 1466-1471. Max. coverage (+): 0.35. Max coverage (-): 0.54

Region: NODE\_356908\_length\_2531\_cov\_46.841564 1472-1476. Max. coverage (+): 0.44. Max coverage (-): 0.24

Region: NODE\_356908\_length\_2531\_cov\_46.841564 1477-1481. Max. coverage (+): 0.06. Max coverage (-): 0.06

Region: NODE\_356908\_length\_2531\_cov\_46.841564 1482-1487. Max. coverage (+): 0.75. Max coverage (-): 0

Region: NODE\_356908\_length\_2531\_cov\_46.841564 1488-1492. Max. coverage (+): 0.53. Max coverage (-): 0

Region: NODE\_356908\_length\_2531\_cov\_46.841564 1493-1497. Max. coverage (+): 0. Max coverage (-): 4.95

Region: NODE\_356908\_length\_2531\_cov\_46.841564 1498-1502. Max. coverage (+): 0. Max coverage (-): 5.95

Region: NODE\_356908\_length\_2531\_cov\_46.841564 1503-1508. Max. coverage (+): 0.04. Max coverage (-): 1.54

Region: NODE\_356908\_length\_2531\_cov\_46.841564 1509-1513. Max. coverage (+): 1.78. Max coverage (-): 0.41

Region: NODE\_356908\_length\_2531\_cov\_46.841564 1514-1518. Max. coverage (+): 1.89. Max coverage (-): 0.13

Region: NODE\_356908\_length\_2531\_cov\_46.841564 1519-1524. Max. coverage (+): 0.22. Max coverage (-): 0.24

Region: NODE\_356908\_length\_2531\_cov\_46.841564 1525-1529. Max. coverage (+): 0.11. Max coverage (-): 0.06

Region: NODE\_356908\_length\_2531\_cov\_46.841564 1530-1534. Max. coverage (+): 0. Max coverage (-): 0

Region: NODE\_356908\_length\_2531\_cov\_46.841564 1535-1540. Max. coverage (+): 0. Max coverage (-): 0.89

Region: NODE\_356908\_length\_2531\_cov\_46.841564 1541-1545. Max. coverage (+): 0. Max coverage (-): 0.04

Region: NODE\_356908\_length\_2531\_cov\_46.841564 1546-1550. Max. coverage (+): 0.04. Max coverage (-): 0.04

Region: NODE\_356908\_length\_2531\_cov\_46.841564 1551-1556. Max. coverage (+): 7.04. Max coverage (-): 0.04

Region: NODE\_356908\_length\_2531\_cov\_46.841564 1557-1561. Max. coverage (+): 7.04. Max coverage (-): 0.22

Region: NODE\_356908\_length\_2531\_cov\_46.841564 1562-1566. Max. coverage (+): 0. Max coverage (-): 0.04

Region: NODE\_356908\_length\_2531\_cov\_46.841564 1567-1571. Max. coverage (+): 0. Max coverage (-): 0.33

Region: NODE\_356908\_length\_2531\_cov\_46.841564 1572-1577. Max. coverage (+): 0. Max coverage (-): 0.3

Region: NODE\_356908\_length\_2531\_cov\_46.841564 1578-1582. Max. coverage (+): 0.19. Max coverage (-): 0.48

Region: NODE\_356908\_length\_2531\_cov\_46.841564 1583-1587. Max. coverage (+): 0. Max coverage (-): 0.48

Region: NODE\_356908\_length\_2531\_cov\_46.841564 1588-1593. Max. coverage (+): 0.11. Max coverage (-): 16.35

Region: NODE\_356908\_length\_2531\_cov\_46.841564 1594-1598. Max. coverage (+): 0.11. Max coverage (-): 1.37

Region: NODE\_356908\_length\_2531\_cov\_46.841564 1599-1603. Max. coverage (+): 0. Max coverage (-): 0.22

Region: NODE\_356908\_length\_2531\_cov\_46.841564 1604-1609. Max. coverage (+): 0.74. Max coverage (-): 7.82

Region: NODE\_356908\_length\_2531\_cov\_46.841564 1610-1614. Max. coverage (+): 0.74. Max coverage (-): 7.75

Region: NODE\_356908\_length\_2531\_cov\_46.841564 1615-1619. Max. coverage (+): 0.04. Max coverage (-): 1.37

Region: NODE\_356908\_length\_2531\_cov\_46.841564 1620-1624. Max. coverage (+): 0. Max coverage (-): 1.3

Region: NODE\_356908\_length\_2531\_cov\_46.841564 1625-1630. Max. coverage (+): 0.04. Max coverage (-): 0.3

Region: NODE\_356908\_length\_2531\_cov\_46.841564 1631-1635. Max. coverage (+): 0.04. Max coverage (-): 0

Region: NODE\_356908\_length\_2531\_cov\_46.841564 1636-1640. Max. coverage (+): 0. Max coverage (-): 0.07

Region: NODE\_356908\_length\_2531\_cov\_46.841564 1641-1646. Max. coverage (+): 0. Max coverage (-): 0.07

Region: NODE\_356908\_length\_2531\_cov\_46.841564 1647-1651. Max. coverage (+): 0. Max coverage (-): 1.33

Region: NODE\_356908\_length\_2531\_cov\_46.841564 1652-1656. Max. coverage (+): 0. Max coverage (-): 1.15

Region: NODE\_356908\_length\_2531\_cov\_46.841564 1657-1662. Max. coverage (+): 0. Max coverage (-): 0.44

Region: NODE\_356908\_length\_2531\_cov\_46.841564 1663-1667. Max. coverage (+): 0.52. Max coverage (-): 0

Region: NODE\_356908\_length\_2531\_cov\_46.841564 1668-1672. Max. coverage (+): 0.56. Max coverage (-): 0

Region: NODE\_356908\_length\_2531\_cov\_46.841564 1673-1678. Max. coverage (+): 0.11. Max coverage (-): 0.33

Region: NODE\_356908\_length\_2531\_cov\_46.841564 1679-1683. Max. coverage (+): 0. Max coverage (-): 4.97

Region: NODE\_356908\_length\_2531\_cov\_46.841564 1684-1688. Max. coverage (+): 0. Max coverage (-): 0.15

Region: NODE\_356908\_length\_2531\_cov\_46.841564 1689-1693. Max. coverage (+): 0. Max coverage (-): 0.89

Region: NODE\_356908\_length\_2531\_cov\_46.841564 1694-1699. Max. coverage (+): 0.22. Max coverage (-): 3.82

Region: NODE\_356908\_length\_2531\_cov\_46.841564 1700-1704. Max. coverage (+): 0.07. Max coverage (-): 2.67

Region: NODE\_356908\_length\_2531\_cov\_46.841564 1705-1709. Max. coverage (+): 0.07. Max coverage (-): 1.63

Region: NODE\_356908\_length\_2531\_cov\_46.841564 1710-1715. Max. coverage (+): 0.04. Max coverage (-): 0.26

Region: NODE\_356908\_length\_2531\_cov\_46.841564 1716-1720. Max. coverage (+): 0.48. Max coverage (-): 0.11

Region: NODE\_356908\_length\_2531\_cov\_46.841564 1721-1725. Max. coverage (+): 0.22. Max coverage (-): 0.11

Region: NODE\_356908\_length\_2531\_cov\_46.841564 1726-1731. Max. coverage (+): 0.15. Max coverage (-): 1.33

Region: NODE\_356908\_length\_2531\_cov\_46.841564 1732-1736. Max. coverage (+): 0. Max coverage (-): 19.72

Region: NODE\_356908\_length\_2531\_cov\_46.841564 1737-1741. Max. coverage (+): 0.04. Max coverage (-): 13.68

Region: NODE\_356908\_length\_2531\_cov\_46.841564 1742-1747. Max. coverage (+): 0.59. Max coverage (-): 2

Region: NODE\_356908\_length\_2531\_cov\_46.841564 1748-1752. Max. coverage (+): 0.59. Max coverage (-): 1.96

Region: NODE\_356908\_length\_2531\_cov\_46.841564 1753-1757. Max. coverage (+): 0.26. Max coverage (-): 0.41

Region: NODE\_356908\_length\_2531\_cov\_46.841564 1758-1762. Max. coverage (+): 0.07. Max coverage (-): 0.22

Region: NODE\_356908\_length\_2531\_cov\_46.841564 1763-1768. Max. coverage (+): 0.11. Max coverage (-): 0.56

Region: NODE\_356908\_length\_2531\_cov\_46.841564 1769-1773. Max. coverage (+): 0.11. Max coverage (-): 1.78

Region: NODE\_356908\_length\_2531\_cov\_46.841564 1774-1778. Max. coverage (+): 0.04. Max coverage (-): 1.74

Region: NODE\_356908\_length\_2531\_cov\_46.841564 1779-1784. Max. coverage (+): 0.07. Max coverage (-): 4.45

Region: NODE\_356908\_length\_2531\_cov\_46.841564 1785-1789. Max. coverage (+): 0.11. Max coverage (-): 0.78

Region: NODE\_356908\_length\_2531\_cov\_46.841564 1790-1794. Max. coverage (+): 0.07. Max coverage (-): 0.78

Region: NODE\_356908\_length\_2531\_cov\_46.841564 1795-1800. Max. coverage (+): 0.37. Max coverage (-): 0.15

Region: NODE\_356908\_length\_2531\_cov\_46.841564 1801-1805. Max. coverage (+): 0.04. Max coverage (-): 0.41

Region: NODE\_356908\_length\_2531\_cov\_46.841564 1806-1810. Max. coverage (+): 0.04. Max coverage (-): 4.93

Region: NODE\_356908\_length\_2531\_cov\_46.841564 1811-1815. Max. coverage (+): 0.04. Max coverage (-): 4.93

Region: NODE\_356908\_length\_2531\_cov\_46.841564 1816-1821. Max. coverage (+): 0.04. Max coverage (-): 2.15

Region: NODE\_356908\_length\_2531\_cov\_46.841564 1822-1826. Max. coverage (+): 0.04. Max coverage (-): 0.19

Region: NODE\_356908\_length\_2531\_cov\_46.841564 1827-1831. Max. coverage (+): 0.07. Max coverage (-): 0

Region: NODE\_356908\_length\_2531\_cov\_46.841564 1832-1837. Max. coverage (+): 0.11. Max coverage (-): 0.15

Region: NODE\_356908\_length\_2531\_cov\_46.841564 1838-1842. Max. coverage (+): 0.3. Max coverage (-): 0.15

Region: NODE\_356908\_length\_2531\_cov\_46.841564 1843-1847. Max. coverage (+): 0.26. Max coverage (-): 0.74

Region: NODE\_356908\_length\_2531\_cov\_46.841564 1848-1853. Max. coverage (+): 0.11. Max coverage (-): 0.78

Region: NODE\_356908\_length\_2531\_cov\_46.841564 1854-1858. Max. coverage (+): 0.11. Max coverage (-): 0.07

Region: NODE\_356908\_length\_2531\_cov\_46.841564 1859-1863. Max. coverage (+): 0.07. Max coverage (-): 1.26

Region: NODE\_356908\_length\_2531\_cov\_46.841564 1864-1869. Max. coverage (+): 0.04. Max coverage (-): 5.49

Region: NODE\_356908\_length\_2531\_cov\_46.841564 1870-1874. Max. coverage (+): 0. Max coverage (-): 0.74

Region: NODE\_356908\_length\_2531\_cov\_46.841564 1875-1879. Max. coverage (+): 0. Max coverage (-): 0.04

Region: NODE\_356908\_length\_2531\_cov\_46.841564 1880-1884. Max. coverage (+): 0. Max coverage (-): 0.04

Region: NODE\_356908\_length\_2531\_cov\_46.841564 1885-1890. Max. coverage (+): 0.04. Max coverage (-): 0.19

Region: NODE\_356908\_length\_2531\_cov\_46.841564 1891-1895. Max. coverage (+): 0.04. Max coverage (-): 0.74

Region: NODE\_356908\_length\_2531\_cov\_46.841564 1896-1900. Max. coverage (+): 0.04. Max coverage (-): 0.96

Region: NODE\_356908\_length\_2531\_cov\_46.841564 1901-1906. Max. coverage (+): 0. Max coverage (-): 0.15

Region: NODE\_356908\_length\_2531\_cov\_46.841564 1907-1911. Max. coverage (+): 0.22. Max coverage (-): 0.26

Region: NODE\_356908\_length\_2531\_cov\_46.841564 1912-1916. Max. coverage (+): 0.22. Max coverage (-): 0.3

Region: NODE\_356908\_length\_2531\_cov\_46.841564 1917-1922. Max. coverage (+): 0.11. Max coverage (-): 23.54

Region: NODE\_356908\_length\_2531\_cov\_46.841564 1923-1927. Max. coverage (+): 2.04. Max coverage (-): 14.64

Region: NODE\_356908\_length\_2531\_cov\_46.841564 1928-1932. Max. coverage (+): 2.11. Max coverage (-): 10.79

Region: NODE\_356908\_length\_2531\_cov\_46.841564 1933-1938. Max. coverage (+): 0.15. Max coverage (-): 0.96

Region: NODE\_356908\_length\_2531\_cov\_46.841564 1939-1943. Max. coverage (+): 0. Max coverage (-): 0.19

Region: NODE\_356908\_length\_2531\_cov\_46.841564 1944-1948. Max. coverage (+): 0. Max coverage (-): 0.11

Region: NODE\_356908\_length\_2531\_cov\_46.841564 1949-1953. Max. coverage (+): 0.04. Max coverage (-): 0.33

Region: NODE\_356908\_length\_2531\_cov\_46.841564 1954-1959. Max. coverage (+): 0. Max coverage (-): 2.82

Region: NODE\_356908\_length\_2531\_cov\_46.841564 1960-1964. Max. coverage (+): 0. Max coverage (-): 0.89

Region: NODE\_356908\_length\_2531\_cov\_46.841564 1965-1969. Max. coverage (+): 0. Max coverage (-): 0.15

Region: NODE\_356908\_length\_2531\_cov\_46.841564 1970-1975. Max. coverage (+): 1.22. Max coverage (-): 0.93

Region: NODE\_356908\_length\_2531\_cov\_46.841564 1976-1980. Max. coverage (+): 1.22. Max coverage (-): 1.78

Region: NODE\_356908\_length\_2531\_cov\_46.841564 1981-1985. Max. coverage (+): 0. Max coverage (-): 0.59

Region: NODE\_356908\_length\_2531\_cov\_46.841564 1986-1991. Max. coverage (+): 0. Max coverage (-): 0.89

Region: NODE\_356908\_length\_2531\_cov\_46.841564 1992-1996. Max. coverage (+): 0.04. Max coverage (-): 0.22

Region: NODE\_356908\_length\_2531\_cov\_46.841564 1997-2001. Max. coverage (+): 0. Max coverage (-): 0.26

Region: NODE\_356908\_length\_2531\_cov\_46.841564 2002-2007. Max. coverage (+): 0.15. Max coverage (-): 1.04

Region: NODE\_356908\_length\_2531\_cov\_46.841564 2008-2012. Max. coverage (+): 0.15. Max coverage (-): 0.78

Region: NODE\_356908\_length\_2531\_cov\_46.841564 2013-2017. Max. coverage (+): 0.11. Max coverage (-): 0.15

Region: NODE\_356908\_length\_2531\_cov\_46.841564 2018-2022. Max. coverage (+): 0.19. Max coverage (-): 0.19

Region: NODE\_356908\_length\_2531\_cov\_46.841564 2023-2028. Max. coverage (+): 0.04. Max coverage (-): 1.22

Region: NODE\_356908\_length\_2531\_cov\_46.841564 2029-2033. Max. coverage (+): 0. Max coverage (-): 2.71

Region: NODE\_356908\_length\_2531\_cov\_46.841564 2034-2038. Max. coverage (+): 0. Max coverage (-): 0.74

Region: NODE\_356908\_length\_2531\_cov\_46.841564 2039-2044. Max. coverage (+): 0.89. Max coverage (-): 0.67

Region: NODE\_356908\_length\_2531\_cov\_46.841564 2045-2049. Max. coverage (+): 1.04. Max coverage (-): 0.22

Region: NODE\_356908\_length\_2531\_cov\_46.841564 2050-2054. Max. coverage (+): 0.74. Max coverage (-): 0.07

Region: NODE\_356908\_length\_2531\_cov\_46.841564 2055-2060. Max. coverage (+): 0.15. Max coverage (-): 0.07

Region: NODE\_356908\_length\_2531\_cov\_46.841564 2061-2065. Max. coverage (+): 0.19. Max coverage (-): 0.04

Region: NODE\_356908\_length\_2531\_cov\_46.841564 2066-2070. Max. coverage (+): 0. Max coverage (-): 0.11

Region: NODE\_356908\_length\_2531\_cov\_46.841564 2071-2075. Max. coverage (+): 0. Max coverage (-): 1.67

Region: NODE\_356908\_length\_2531\_cov\_46.841564 2076-2081. Max. coverage (+): 0. Max coverage (-): 1.33

Region: NODE\_356908\_length\_2531\_cov\_46.841564 2082-2086. Max. coverage (+): 0.04. Max coverage (-): 0.15

Region: NODE\_356908\_length\_2531\_cov\_46.841564 2087-2091. Max. coverage (+): 0.04. Max coverage (-): 0

Region: NODE\_356908\_length\_2531\_cov\_46.841564 2092-2097. Max. coverage (+): 0.74. Max coverage (-): 0

Region: NODE\_356908\_length\_2531\_cov\_46.841564 2098-2102. Max. coverage (+): 0.15. Max coverage (-): 0

Region: NODE\_356908\_length\_2531\_cov\_46.841564 2103-2107. Max. coverage (+): 0. Max coverage (-): 0.04

Region: NODE\_356908\_length\_2531\_cov\_46.841564 2108-2113. Max. coverage (+): 0.04. Max coverage (-): 0.04

Region: NODE\_356908\_length\_2531\_cov\_46.841564 2114-2118. Max. coverage (+): 0.04. Max coverage (-): 0

Region: NODE\_356908\_length\_2531\_cov\_46.841564 2119-2123. Max. coverage (+): 0. Max coverage (-): 0

Region: NODE\_356908\_length\_2531\_cov\_46.841564 2124-2129. Max. coverage (+): 0.15. Max coverage (-): 0.19

Region: NODE\_356908\_length\_2531\_cov\_46.841564 2130-2134. Max. coverage (+): 0.15. Max coverage (-): 0.67

Region: NODE\_356908\_length\_2531\_cov\_46.841564 2135-2139. Max. coverage (+): 0. Max coverage (-): 1.19

Region: NODE\_356908\_length\_2531\_cov\_46.841564 2140-2144. Max. coverage (+): 0.07. Max coverage (-): 1.11

Region: NODE\_356908\_length\_2531\_cov\_46.841564 2145-2150. Max. coverage (+): 0.04. Max coverage (-): 0.07

Region: NODE\_356908\_length\_2531\_cov\_46.841564 2151-2155. Max. coverage (+): 0.15. Max coverage (-): 0

Region: NODE\_356908\_length\_2531\_cov\_46.841564 2156-2160. Max. coverage (+): 0.37. Max coverage (-): 0.19

Region: NODE\_356908\_length\_2531\_cov\_46.841564 2161-2166. Max. coverage (+): 0. Max coverage (-): 0.44

Region: NODE\_356908\_length\_2531\_cov\_46.841564 2167-2171. Max. coverage (+): 0. Max coverage (-): 0.89

Region: NODE\_356908\_length\_2531\_cov\_46.841564 2172-2176. Max. coverage (+): 0. Max coverage (-): 2

Region: NODE\_356908\_length\_2531\_cov\_46.841564 2177-2182. Max. coverage (+): 0. Max coverage (-): 1.93

Region: NODE\_356908\_length\_2531\_cov\_46.841564 2183-2187. Max. coverage (+): 1.67. Max coverage (-): 0.19

Region: NODE\_356908\_length\_2531\_cov\_46.841564 2188-2192. Max. coverage (+): 1.63. Max coverage (-): 0

Region: NODE\_356908\_length\_2531\_cov\_46.841564 2193-2198. Max. coverage (+): 0.04. Max coverage (-): 1.11

Region: NODE\_356908\_length\_2531\_cov\_46.841564 2199-2203. Max. coverage (+): 0.04. Max coverage (-): 1.56

Region: NODE\_356908\_length\_2531\_cov\_46.841564 2204-2208. Max. coverage (+): 0.04. Max coverage (-): 1.45

Region: NODE\_356908\_length\_2531\_cov\_46.841564 2209-2213. Max. coverage (+): 0. Max coverage (-): 0.93

Region: NODE\_356908\_length\_2531\_cov\_46.841564 2214-2219. Max. coverage (+): 0.41. Max coverage (-): 0.82

Region: NODE\_356908\_length\_2531\_cov\_46.841564 2220-2224. Max. coverage (+): 0.56. Max coverage (-): 1.59

Region: NODE\_356908\_length\_2531\_cov\_46.841564 2225-2229. Max. coverage (+): 0.04. Max coverage (-): 0.11

Region: NODE\_356908\_length\_2531\_cov\_46.841564 2230-2235. Max. coverage (+): 0.11. Max coverage (-): 2.56

Region: NODE\_356908\_length\_2531\_cov\_46.841564 2236-2240. Max. coverage (+): 0.74. Max coverage (-): 3.41

Region: NODE\_356908\_length\_2531\_cov\_46.841564 2241-2245. Max. coverage (+): 0.59. Max coverage (-): 2.15

Region: NODE\_356908\_length\_2531\_cov\_46.841564 2246-2251. Max. coverage (+): 0.15. Max coverage (-): 0.37

Region: NODE\_356908\_length\_2531\_cov\_46.841564 2252-2256. Max. coverage (+): 0.15. Max coverage (-): 0.37

Region: NODE\_356908\_length\_2531\_cov\_46.841564 2257-2261. Max. coverage (+): 0.15. Max coverage (-): 0

Region: NODE\_356908\_length\_2531\_cov\_46.841564 2262-2267. Max. coverage (+): 0. Max coverage (-): 0.04

Region: NODE\_356908\_length\_2531\_cov\_46.841564 2268-2272. Max. coverage (+): 0. Max coverage (-): 4.5

Region: NODE\_356908\_length\_2531\_cov\_46.841564 2273-2277. Max. coverage (+): 0. Max coverage (-): 0.95

Region: NODE\_356908\_length\_2531\_cov\_46.841564 2278-2282. Max. coverage (+): 0. Max coverage (-): 0.74

Region: NODE\_356908\_length\_2531\_cov\_46.841564 2283-2288. Max. coverage (+): 0. Max coverage (-): 0

Region: NODE\_356908\_length\_2531\_cov\_46.841564 2289-2293. Max. coverage (+): 0. Max coverage (-): 0.22

Region: NODE\_356908\_length\_2531\_cov\_46.841564 2294-2298. Max. coverage (+): 0. Max coverage (-): 0.19

Region: NODE\_356908\_length\_2531\_cov\_46.841564 2299-2304. Max. coverage (+): 0.11. Max coverage (-): 0.48

Region: NODE\_356908\_length\_2531\_cov\_46.841564 2305-2309. Max. coverage (+): 0.15. Max coverage (-): 0.48

Region: NODE\_356908\_length\_2531\_cov\_46.841564 2310-2314. Max. coverage (+): 0.11. Max coverage (-): 1.33

Region: NODE\_356908\_length\_2531\_cov\_46.841564 2315-2320. Max. coverage (+): 0.26. Max coverage (-): 1.67

Region: NODE\_356908\_length\_2531\_cov\_46.841564 2321-2325. Max. coverage (+): 0.48. Max coverage (-): 0.44

Region: NODE\_356908\_length\_2531\_cov\_46.841564 2326-2330. Max. coverage (+): 0.48. Max coverage (-): 0.19

Region: NODE\_356908\_length\_2531\_cov\_46.841564 2331-2335. Max. coverage (+): 0.07. Max coverage (-): 1.82

Region: NODE\_356908\_length\_2531\_cov\_46.841564 2336-2341. Max. coverage (+): 2.37. Max coverage (-): 2.19

Region: NODE\_356908\_length\_2531\_cov\_46.841564 2342-2346. Max. coverage (+): 1.67. Max coverage (-): 0.04

Region: NODE\_356908\_length\_2531\_cov\_46.841564 2347-2351. Max. coverage (+): 0.07. Max coverage (-): 0.89

Region: NODE\_356908\_length\_2531\_cov\_46.841564 2352-2357. Max. coverage (+): 0.11. Max coverage (-): 6.75

Region: NODE\_356908\_length\_2531\_cov\_46.841564 2358-2362. Max. coverage (+): 0. Max coverage (-): 1.63

Region: NODE\_356908\_length\_2531\_cov\_46.841564 2363-2367. Max. coverage (+): 0. Max coverage (-): 0.44

Region: NODE\_356908\_length\_2531\_cov\_46.841564 2368-2373. Max. coverage (+): 0.04. Max coverage (-): 0.26

Region: NODE\_356908\_length\_2531\_cov\_46.841564 2374-2378. Max. coverage (+): 0.11. Max coverage (-): 0.41

Region: NODE\_356908\_length\_2531\_cov\_46.841564 2379-2383. Max. coverage (+): 0.15. Max coverage (-): 0.41

Region: NODE\_356908\_length\_2531\_cov\_46.841564 2384-2389. Max. coverage (+): 0.15. Max coverage (-): 2.97

Region: NODE\_356908\_length\_2531\_cov\_46.841564 2390-2394. Max. coverage (+): 0.22. Max coverage (-): 2.34

Region: NODE\_356908\_length\_2531\_cov\_46.841564 2395-2399. Max. coverage (+): 0.19. Max coverage (-): 2.48

Region: NODE\_356908\_length\_2531\_cov\_46.841564 2400-2404. Max. coverage (+): 1.33. Max coverage (-): 0.26

Region: NODE\_356908\_length\_2531\_cov\_46.841564 2405-2410. Max. coverage (+): 1.45. Max coverage (-): 0.22

Region: NODE\_356908\_length\_2531\_cov\_46.841564 2411-2415. Max. coverage (+): 0.67. Max coverage (-): 0.07

Region: NODE\_356908\_length\_2531\_cov\_46.841564 2416-2420. Max. coverage (+): 0.78. Max coverage (-): 1.74

Region: NODE\_356908\_length\_2531\_cov\_46.841564 2421-2426. Max. coverage (+): 1.26. Max coverage (-): 6.34

Region: NODE\_356908\_length\_2531\_cov\_46.841564 2427-2431. Max. coverage (+): 0. Max coverage (-): 5.93

Region: NODE\_356908\_length\_2531\_cov\_46.841564 2432-2436. Max. coverage (+): 0. Max coverage (-): 3.11

Region: NODE\_356908\_length\_2531\_cov\_46.841564 2437-2442. Max. coverage (+): 0. Max coverage (-): 0

Region: NODE\_356908\_length\_2531\_cov\_46.841564 2443-2447. Max. coverage (+): 0.04. Max coverage (-): 0

Region: NODE\_356908\_length\_2531\_cov\_46.841564 2448-2452. Max. coverage (+): 0.04. Max coverage (-): 0

Region: NODE\_356908\_length\_2531\_cov\_46.841564 2453-2458. Max. coverage (+): 0.04. Max coverage (-): 0.11

Region: NODE\_356908\_length\_2531\_cov\_46.841564 2459-2463. Max. coverage (+): 0.04. Max coverage (-): 0.22

Region: NODE\_356908\_length\_2531\_cov\_46.841564 2464-2468. Max. coverage (+): 0.04. Max coverage (-): 0.78

Region: NODE\_356908\_length\_2531\_cov\_46.841564 2469-2473. Max. coverage (+): 0. Max coverage (-): 0.74

Region: NODE\_356908\_length\_2531\_cov\_46.841564 2474-2479. Max. coverage (+): 0.37. Max coverage (-): 0.52

Region: NODE\_356908\_length\_2531\_cov\_46.841564 2480-2484. Max. coverage (+): 0.48. Max coverage (-): 2.26

Region: NODE\_356908\_length\_2531\_cov\_46.841564 2485-2489. Max. coverage (+): 0.11. Max coverage (-): 2.34

Region: NODE\_356908\_length\_2531\_cov\_46.841564 2490-2495. Max. coverage (+): 0.15. Max coverage (-): 1.59

Region: NODE\_356908\_length\_2531\_cov\_46.841564 2496-2500. Max. coverage (+): 0. Max coverage (-): 0.11

Region: NODE\_356908\_length\_2531\_cov\_46.841564 2501-2505. Max. coverage (+): 0. Max coverage (-): 0.11

Region: NODE\_356908\_length\_2531\_cov\_46.841564 2506-2511. Max. coverage (+): 0.04. Max coverage (-): 0.22

Region: NODE\_356908\_length\_2531\_cov\_46.841564 2512-2516. Max. coverage (+): 0. Max coverage (-): 0.96

Region: NODE\_356908\_length\_2531\_cov\_46.841564 2517-2521. Max. coverage (+): 0.04. Max coverage (-): 0.93

Region: NODE\_356908\_length\_2531\_cov\_46.841564 2522-2527. Max. coverage (+): 0. Max coverage (-): 15.83

Region: NODE\_356908\_length\_2531\_cov\_46.841564 2528-2532. Max. coverage (+): 0. Max coverage (-): 15.61

Region: NODE\_356908\_length\_2531\_cov\_46.841564 2533-2537. Max. coverage (+): 0. Max coverage (-): 3.67

Region: NODE\_356908\_length\_2531\_cov\_46.841564 2538-2542. Max. coverage (+): 0. Max coverage (-): 2.37

Region: NODE\_356908\_length\_2531\_cov\_46.841564 2543-2548. Max. coverage (+): 0. Max coverage (-): 0.22

Region: NODE\_356908\_length\_2531\_cov\_46.841564 2549-2553. Max. coverage (+): 0.04. Max coverage (-): 0.04

Region: NODE\_356908\_length\_2531\_cov\_46.841564 2554-2558. Max. coverage (+): 0. Max coverage (-): 0.15

Region: NODE\_356908\_length\_2531\_cov\_46.841564 2559-2564. Max. coverage (+): 0. Max coverage (-): 0.63

Region: NODE\_356908\_length\_2531\_cov\_46.841564 2565-2569. Max. coverage (+): 0. Max coverage (-): 0.07

Region: NODE\_356908\_length\_2531\_cov\_46.841564 2570-2574. Max. coverage (+): 0. Max coverage (-): 0.07

Region: NODE\_356908\_length\_2531\_cov\_46.841564 2575-2580. Max. coverage (+): 0.04. Max coverage (-): 0.11

Region: NODE\_356908\_length\_2531\_cov\_46.841564 2581-2585. Max. coverage (+): 0. Max coverage (-): 0.24

Region: NODE\_356908\_length\_2531\_cov\_46.841564 2586-2590. Max. coverage (+): 0. Max coverage (-): 1.63

Region: NODE\_356908\_length\_2531\_cov\_46.841564 2591-2595. Max. coverage (+): 0. Max coverage (-): 1.82

Region: NODE\_356908\_length\_2531\_cov\_46.841564 2596-2601. Max. coverage (+): 0.01. Max coverage (-): 0.04

Region: NODE\_356908\_length\_2531\_cov\_46.841564 2602-2606. Max. coverage (+): 0.01. Max coverage (-): 0.01

Region: NODE\_356908\_length\_2531\_cov\_46.841564 2607-2611. Max. coverage (+): 0.01. Max coverage (-): 0.01

Region: NODE\_356908\_length\_2531\_cov\_46.841564 2612-2617. Max. coverage (+): 0.01. Max coverage (-): 0.11

Region: NODE\_356908\_length\_2531\_cov\_46.841564 2618-2622. Max. coverage (+): 0.01. Max coverage (-): 1.62

Region: NODE\_356908\_length\_2531\_cov\_46.841564 2623-2627. Max. coverage (+): 0.15. Max coverage (-): 1.78

Region: NODE\_356908\_length\_2531\_cov\_46.841564 2628-2633. Max. coverage (+): 0.14. Max coverage (-): 0.56

Region: NODE\_356908\_length\_2531\_cov\_46.841564 2634-2638. Max. coverage (+): 0.02. Max coverage (-): 0.19

Region: NODE\_356908\_length\_2531\_cov\_46.841564 2639-2643. Max. coverage (+): 0. Max coverage (-): 0

Region: NODE\_356908\_length\_2531\_cov\_46.841564 2644-2649. Max. coverage (+): 0. Max coverage (-): 0

Region: NODE\_356908\_length\_2531\_cov\_46.841564 2650-2654. Max. coverage (+): 0. Max coverage (-): 0

Region: NODE\_356908\_length\_2531\_cov\_46.841564 2655-. Max. coverage (+): 0. Max coverage (-): 0

RepeatMasker Color Code

**+**

100-98% Identity

<98-95% Identity

<95-90% Identity

<90-85% Identity

<85-80% Identity

<80-75% Identity

<75-70% Identity

<70% Identity

**-**

Gene Set Color Code

**+**

Gene

Pseudogene

Other

**-**

Topology/Coverage Color Code

Coverage Plus Strand

Coverage Minus Strand

Mainstrand: Plus

Mainstrand: Minus

Complementary Strand

Flanking Region  
(if option -flank >0)

Gene Set Annotation  
  
RepeatMasker Annotation  

**1. BEL-9\_GA-I**: 9-512 (+), Divergence to consensus: 33.7%  
**2. BEL-1\_CGi-I**: 893-1380 (+), Divergence to consensus: 39.7%  
**3. BEL36-I\_DR**: 1408-2472 (+), Divergence to consensus: 37.4%

  
Transcription Factor Binding Sites  

**SPZ1** (Sequence: CTCTAACCCT (-): 291)  
**RHOXF1** (Sequence: GGCTTA (-): 353)  
**RHOXF1** (Sequence: GGCTCA (-): 1116)  
**RHOXF1** (Sequence: GGATTA (-): 1272)  
**RHOXF1** (Sequence: AGATTA (-): 1302)  
**RHOXF1** (Sequence: GGATTA (-): 1409)  
**RHOXF1** (Sequence: AGATTA (-): 1439)  
**RHOXF1** (Sequence: TAATCC (+): 1872)  
**RHOXF1** (Sequence: TAATCT (+): 2513)  
**RFX4\_2** (Sequence: GTAACCATG (-): 2258)  
**RFX4\_1** (Sequence: GTTGCCAAG (-): 1181)  
**FOXO3\_hsa** (Sequence: GTAAACAT (+): 2493)  
**SOX9** (Sequence: AACAATGG (-): 266)  
**FOXP1** (Sequence: GTAAACA (+): 2493)  
**FIGLA** (Sequence: TCCAGGTGGT (-): 1165)  
**FOXO3\_mmu** (Sequence: GGTAAACA (+): 2492)  
**Rhox11** (Sequence: TGCTGTTTT (+): 87)  
**Sox5** (Sequence: AACAAT (-): 266)  
**POU5F1** (Sequence: ATGCAAA (+): 2264)
