# Supplementary material for: First characterization of PIWI-interacting RNA clusters in a cichlid fish with a B chromosome
Source: BMC Biol. 2022 Sep 21;20:204. doi: 10.1186/s12915-022-01403-2 (PMC9490952; doi:10.1186/s12915-022-01403-2)
Supplement: Supplementary file 1 — Additional file 1. Zipped folder with fasta and interactive html piRNA cluster information for the A. latifasciata genome. The nomenclature is as follows: number-pirna-cluster_sex_B-presence (f, female; m, male; 0b, without B chromosome; 1b, with B chromosome). [file 12915_2022_1403_MOESM1_ESM.zip › 139_f0b.html]

piRNA cluster 139\_f0b 65


Predicted piRNA cluster no. 139\_f0b
  

Show proTRAC run info
Hide proTRAC run info

/\  
                \_\_\_\_\_\_\_\_\_\_\_\_\_\_\_\_\_\_\_\_\_\_\_/\\_\_\_ /  \\_\_\_\_\_\_\_  
               I                      /  \  /    \      I  
               I     pro             /    \/      \     I  
               I        TRAC        /               \   I  
               I   \_\_\_\_\_\_\_\_\_\_\_\_\_\_\_\_/\_\_\_\_\_\_\_\_\_\_\_\_\_\_\_\_\_\\_ I  
               I   \              /                     I  
               I    \            /                      I  
               I     \  /\      /       V.2.4.2         I  
               I      \/  \    /                        I  
               I\_\_\_\_\_\_\_\_\_\_\_\  /\_\_\_\_\_\_\_\_\_\_\_\_\_\_\_\_\_\_\_\_\_\_\_\_\_I  
                            \/  
  
  
================================= proTRAC ====================================  
VERSION: .......... 2.4.2  
LAST MODIFIED: .... 11. May 2018  
  
Please cite:  
Rosenkranz D, Zischler H. proTRAC - a software for probabilistic piRNA cluster  
detection, visualization and analysis. 2012. BMC Bioinformatics 13:5.  
  
  
Contact:  
David Rosenkranz  
Institute of Organismic and Molecular Evolutionary Biology  
Dept. Anthropology, small RNA group  
Johannes Gutenberg University Mainz  
email: rosenkranz@uni-mainz.de  
  
You can find the latest proTRAC version at:  
http://sourceforge.net/projects/protrac/files  
http://www.smallRNAgroup-mainz.de/software  
==============================================================================  
  
PARAMETERS:  
Map file: ...............piwi-femeas-0B.fa-collapse.map  
Genome file: ............../../../0B\_ala\_genome.fa  
RepeatMasker annotation: Alatifasciata-all0B-maryan-v2.fa\_corrected.out  
GeneSet:................./guest-storage/Data/annotation/Alatifasciata\_all0B\_maryan-v2\_out2017.gff  
  
Significant (p<=0.01) hit density will be calculated based  
on observed hit distribution.  
  
Sliding window size: ........................................ 5000 bp  
Sliding window increament: .................................. 1000 bp  
Normalize each hit by number of genomic hits: ............... yes  
Normalize each hit by number of sequence reads: ............. yes  
Normalize values (-> per million mapped reads): ............. yes  
Min. fraction of hits with 1T(U) or 10A: .................... 0.75  
Alternatively: Min. fraction of hits with 1T(U) and 10A: .... 0.5  
Min. fraction of hits with typical piRNA length: ............ 0.75  
Typical piRNA length: ....................................... 24-32 nt  
Min. size of a piRNA cluster: ............................... 1000 bp.  
Min. number of hits (absolute): ............................. 0  
Min. number of hits (normalized): ........................... 0  
Min. fraction of hits on the mainstrand: .................... 0.75  
Top fraction of mapped sequences (in terms of read counts): . 1%  
Top fraction accounts for max. n% of sequence reads: ........ 90%  
Min. fraction of hits on each arm of a bidirectional cluster: 0.05  
Output html file for each cluster: .......................... yes  
Output a summary table: ..................................... yes  
Output a FASTA file for each cluster (piRNA sequences): ..... yes  
Output a FASTA file comprising cluster sequences: ........... yes  
Output a GTF file for predicted piRNA clusters: ..............yes  
Search DNA motifs in clusters: .............................. yes  
Output flanking sequences: +/- .............................. 0 bp  
Output ~.pTi file: .......................................... no  
==============================================================================  
  
  
Genome size (without gaps): ............ 758543724 bp  
Gaps (N/X/-): .......................... 417479 bp  
Mapped reads: .......................... 13052187  
Non-identical sequences: ............... 3338911  
Genomic hits: .......................... 28737726  
Significant densitiy of mapped reads: .. 470.083249848448 reads/kb

Show proTRAC cluster info
Hide proTRAC cluster info

|  |  |
| --- | --- |
| Location | NODE\_360193\_length\_37465\_cov\_31.594688 |
| Coordinates | 31028-36009 |
| Size [bp] | 4982 |
| Sequence hit loci | 895 |
| Mapped reads (normalized) | 2377.6 |
| Mapped reads (normalized) per kb | 477.2 |
| Normalized reads with 1T (1U) | 77.1% |
| Normalized reads with 10A | 44.5% |
| Normalized reads with length 24-32 nt | 98.4% |
| Normalized reads on the main strand(s) | 90.7% |
| Predicted directionality | mono:minus |

100%

0%

1T (1U)  
reads

10A reads

24-32 nt  
reads

reads on mainstrand

**Either the amount of reads with 1T (1U) OR 10A has to exceed 75% (set with option: -1Tor10A)  
Alternatively the amount of reads with 1T (1U) AND 10A has to exceed 50% (set with option: -1Tand10A)  
Minimum amount of reads with preferred size is 75% (set with option: -pisize)  
Minimum amount of reads on the main strand(s) is 75% (set with option: -clstrand)**

Show read coverage
Hide read coverage

WHAT DO I SEE HERE?  
This chart shows the location of mapped sequence reads within a predicted piRNA cluster. The color refers to the number of genomic hits produced by the sequence read in question. A dark red bar indicates that this sequence read produces many other hits elsewhere in the genome. Many adjacent red or yellow bars can indicate the presence of a multi-copy element such as transposons or rRNA genes. A dark green bar indicates that this sequence read maps uniquely to this locus.

1 hit

2-5 hits

6-10 hits

11-20 hits

21-50 hits

51-100 hits

> 100 hits

NODE\_360193\_length\_37465\_cov\_31.594688

31028

36009

Gene Set

RepeatMasker

Mapped  
Reads

39.23

plus strand

minus strand

39.23

Region: NODE\_360193\_length\_37465\_cov\_31.594688 38452-31032. Max. coverage (+): 0. Max coverage (-): 0

Region: NODE\_360193\_length\_37465\_cov\_31.594688 31033-31042. Max. coverage (+): 0.02. Max coverage (-): 0

Region: NODE\_360193\_length\_37465\_cov\_31.594688 31043-31052. Max. coverage (+): 0. Max coverage (-): 0.01

Region: NODE\_360193\_length\_37465\_cov\_31.594688 31053-31062. Max. coverage (+): 0. Max coverage (-): 0.01

Region: NODE\_360193\_length\_37465\_cov\_31.594688 31063-31072. Max. coverage (+): 0.01. Max coverage (-): 0

Region: NODE\_360193\_length\_37465\_cov\_31.594688 31073-31082. Max. coverage (+): 0.01. Max coverage (-): 0

Region: NODE\_360193\_length\_37465\_cov\_31.594688 31083-31092. Max. coverage (+): 0. Max coverage (-): 0

Region: NODE\_360193\_length\_37465\_cov\_31.594688 31093-31102. Max. coverage (+): 0. Max coverage (-): 0

Region: NODE\_360193\_length\_37465\_cov\_31.594688 31103-31112. Max. coverage (+): 0. Max coverage (-): 0

Region: NODE\_360193\_length\_37465\_cov\_31.594688 31113-31122. Max. coverage (+): 0. Max coverage (-): 0

Region: NODE\_360193\_length\_37465\_cov\_31.594688 31123-31132. Max. coverage (+): 0. Max coverage (-): 0

Region: NODE\_360193\_length\_37465\_cov\_31.594688 31133-31142. Max. coverage (+): 0. Max coverage (-): 0

Region: NODE\_360193\_length\_37465\_cov\_31.594688 31143-31152. Max. coverage (+): 0. Max coverage (-): 0

Region: NODE\_360193\_length\_37465\_cov\_31.594688 31153-31162. Max. coverage (+): 0. Max coverage (-): 0

Region: NODE\_360193\_length\_37465\_cov\_31.594688 31163-31172. Max. coverage (+): 0. Max coverage (-): 0.08

Region: NODE\_360193\_length\_37465\_cov\_31.594688 31173-31182. Max. coverage (+): 0. Max coverage (-): 0.08

Region: NODE\_360193\_length\_37465\_cov\_31.594688 31183-31192. Max. coverage (+): 0. Max coverage (-): 0

Region: NODE\_360193\_length\_37465\_cov\_31.594688 31193-31202. Max. coverage (+): 0. Max coverage (-): 0

Region: NODE\_360193\_length\_37465\_cov\_31.594688 31203-31212. Max. coverage (+): 0. Max coverage (-): 0

Region: NODE\_360193\_length\_37465\_cov\_31.594688 31213-31222. Max. coverage (+): 0. Max coverage (-): 0

Region: NODE\_360193\_length\_37465\_cov\_31.594688 31223-31232. Max. coverage (+): 0. Max coverage (-): 0.23

Region: NODE\_360193\_length\_37465\_cov\_31.594688 31233-31242. Max. coverage (+): 0. Max coverage (-): 0.23

Region: NODE\_360193\_length\_37465\_cov\_31.594688 31243-31252. Max. coverage (+): 0.46. Max coverage (-): 0

Region: NODE\_360193\_length\_37465\_cov\_31.594688 31253-31262. Max. coverage (+): 0. Max coverage (-): 0.08

Region: NODE\_360193\_length\_37465\_cov\_31.594688 31263-31272. Max. coverage (+): 0. Max coverage (-): 0

Region: NODE\_360193\_length\_37465\_cov\_31.594688 31273-31282. Max. coverage (+): 0. Max coverage (-): 0.08

Region: NODE\_360193\_length\_37465\_cov\_31.594688 31283-31292. Max. coverage (+): 0. Max coverage (-): 0.15

Region: NODE\_360193\_length\_37465\_cov\_31.594688 31293-31302. Max. coverage (+): 0. Max coverage (-): 0.15

Region: NODE\_360193\_length\_37465\_cov\_31.594688 31303-31311. Max. coverage (+): 0. Max coverage (-): 0

Region: NODE\_360193\_length\_37465\_cov\_31.594688 31312-31321. Max. coverage (+): 0. Max coverage (-): 0.08

Region: NODE\_360193\_length\_37465\_cov\_31.594688 31322-31331. Max. coverage (+): 0. Max coverage (-): 0.08

Region: NODE\_360193\_length\_37465\_cov\_31.594688 31332-31341. Max. coverage (+): 0. Max coverage (-): 0

Region: NODE\_360193\_length\_37465\_cov\_31.594688 31342-31351. Max. coverage (+): 0. Max coverage (-): 0

Region: NODE\_360193\_length\_37465\_cov\_31.594688 31352-31361. Max. coverage (+): 0. Max coverage (-): 0

Region: NODE\_360193\_length\_37465\_cov\_31.594688 31362-31371. Max. coverage (+): 0. Max coverage (-): 0

Region: NODE\_360193\_length\_37465\_cov\_31.594688 31372-31381. Max. coverage (+): 0. Max coverage (-): 0

Region: NODE\_360193\_length\_37465\_cov\_31.594688 31382-31391. Max. coverage (+): 0. Max coverage (-): 0

Region: NODE\_360193\_length\_37465\_cov\_31.594688 31392-31401. Max. coverage (+): 0. Max coverage (-): 0

Region: NODE\_360193\_length\_37465\_cov\_31.594688 31402-31411. Max. coverage (+): 0. Max coverage (-): 0.08

Region: NODE\_360193\_length\_37465\_cov\_31.594688 31412-31421. Max. coverage (+): 0. Max coverage (-): 0.08

Region: NODE\_360193\_length\_37465\_cov\_31.594688 31422-31431. Max. coverage (+): 0. Max coverage (-): 0.08

Region: NODE\_360193\_length\_37465\_cov\_31.594688 31432-31441. Max. coverage (+): 0. Max coverage (-): 0.08

Region: NODE\_360193\_length\_37465\_cov\_31.594688 31442-31451. Max. coverage (+): 0. Max coverage (-): 6.05

Region: NODE\_360193\_length\_37465\_cov\_31.594688 31452-31461. Max. coverage (+): 0.08. Max coverage (-): 0.23

Region: NODE\_360193\_length\_37465\_cov\_31.594688 31462-31471. Max. coverage (+): 0.23. Max coverage (-): 0

Region: NODE\_360193\_length\_37465\_cov\_31.594688 31472-31481. Max. coverage (+): 0.15. Max coverage (-): 0.08

Region: NODE\_360193\_length\_37465\_cov\_31.594688 31482-31491. Max. coverage (+): 0. Max coverage (-): 1.23

Region: NODE\_360193\_length\_37465\_cov\_31.594688 31492-31501. Max. coverage (+): 0. Max coverage (-): 0

Region: NODE\_360193\_length\_37465\_cov\_31.594688 31502-31511. Max. coverage (+): 0. Max coverage (-): 0.08

Region: NODE\_360193\_length\_37465\_cov\_31.594688 31512-31521. Max. coverage (+): 0. Max coverage (-): 1.07

Region: NODE\_360193\_length\_37465\_cov\_31.594688 31522-31531. Max. coverage (+): 0.08. Max coverage (-): 0

Region: NODE\_360193\_length\_37465\_cov\_31.594688 31532-31541. Max. coverage (+): 0.08. Max coverage (-): 0.23

Region: NODE\_360193\_length\_37465\_cov\_31.594688 31542-31551. Max. coverage (+): 0.08. Max coverage (-): 0.23

Region: NODE\_360193\_length\_37465\_cov\_31.594688 31552-31561. Max. coverage (+): 0. Max coverage (-): 0

Region: NODE\_360193\_length\_37465\_cov\_31.594688 31562-31571. Max. coverage (+): 0. Max coverage (-): 0.23

Region: NODE\_360193\_length\_37465\_cov\_31.594688 31572-31581. Max. coverage (+): 0. Max coverage (-): 0.15

Region: NODE\_360193\_length\_37465\_cov\_31.594688 31582-31590. Max. coverage (+): 0.15. Max coverage (-): 0

Region: NODE\_360193\_length\_37465\_cov\_31.594688 31591-31600. Max. coverage (+): 0. Max coverage (-): 0.08

Region: NODE\_360193\_length\_37465\_cov\_31.594688 31601-31610. Max. coverage (+): 0. Max coverage (-): 0.08

Region: NODE\_360193\_length\_37465\_cov\_31.594688 31611-31620. Max. coverage (+): 0. Max coverage (-): 0.08

Region: NODE\_360193\_length\_37465\_cov\_31.594688 31621-31630. Max. coverage (+): 0. Max coverage (-): 0.08

Region: NODE\_360193\_length\_37465\_cov\_31.594688 31631-31640. Max. coverage (+): 0. Max coverage (-): 0.08

Region: NODE\_360193\_length\_37465\_cov\_31.594688 31641-31650. Max. coverage (+): 0.08. Max coverage (-): 0

Region: NODE\_360193\_length\_37465\_cov\_31.594688 31651-31660. Max. coverage (+): 0.08. Max coverage (-): 0.15

Region: NODE\_360193\_length\_37465\_cov\_31.594688 31661-31670. Max. coverage (+): 0. Max coverage (-): 0.23

Region: NODE\_360193\_length\_37465\_cov\_31.594688 31671-31680. Max. coverage (+): 0. Max coverage (-): 0.23

Region: NODE\_360193\_length\_37465\_cov\_31.594688 31681-31690. Max. coverage (+): 0. Max coverage (-): 0

Region: NODE\_360193\_length\_37465\_cov\_31.594688 31691-31700. Max. coverage (+): 0. Max coverage (-): 0

Region: NODE\_360193\_length\_37465\_cov\_31.594688 31701-31710. Max. coverage (+): 0. Max coverage (-): 0.23

Region: NODE\_360193\_length\_37465\_cov\_31.594688 31711-31720. Max. coverage (+): 0.08. Max coverage (-): 0.31

Region: NODE\_360193\_length\_37465\_cov\_31.594688 31721-31730. Max. coverage (+): 0. Max coverage (-): 0.23

Region: NODE\_360193\_length\_37465\_cov\_31.594688 31731-31740. Max. coverage (+): 0. Max coverage (-): 0.54

Region: NODE\_360193\_length\_37465\_cov\_31.594688 31741-31750. Max. coverage (+): 0.08. Max coverage (-): 0.15

Region: NODE\_360193\_length\_37465\_cov\_31.594688 31751-31760. Max. coverage (+): 0.08. Max coverage (-): 0

Region: NODE\_360193\_length\_37465\_cov\_31.594688 31761-31770. Max. coverage (+): 0.08. Max coverage (-): 0.84

Region: NODE\_360193\_length\_37465\_cov\_31.594688 31771-31780. Max. coverage (+): 0.15. Max coverage (-): 0.84

Region: NODE\_360193\_length\_37465\_cov\_31.594688 31781-31790. Max. coverage (+): 0. Max coverage (-): 0.92

Region: NODE\_360193\_length\_37465\_cov\_31.594688 31791-31800. Max. coverage (+): 0. Max coverage (-): 0.92

Region: NODE\_360193\_length\_37465\_cov\_31.594688 31801-31810. Max. coverage (+): 0. Max coverage (-): 0.54

Region: NODE\_360193\_length\_37465\_cov\_31.594688 31811-31820. Max. coverage (+): 0. Max coverage (-): 0.46

Region: NODE\_360193\_length\_37465\_cov\_31.594688 31821-31830. Max. coverage (+): 0. Max coverage (-): 0.54

Region: NODE\_360193\_length\_37465\_cov\_31.594688 31831-31840. Max. coverage (+): 0.08. Max coverage (-): 0.46

Region: NODE\_360193\_length\_37465\_cov\_31.594688 31841-31850. Max. coverage (+): 0.15. Max coverage (-): 0.23

Region: NODE\_360193\_length\_37465\_cov\_31.594688 31851-31859. Max. coverage (+): 0. Max coverage (-): 0

Region: NODE\_360193\_length\_37465\_cov\_31.594688 31860-31869. Max. coverage (+): 0. Max coverage (-): 0

Region: NODE\_360193\_length\_37465\_cov\_31.594688 31870-31879. Max. coverage (+): 0. Max coverage (-): 0

Region: NODE\_360193\_length\_37465\_cov\_31.594688 31880-31889. Max. coverage (+): 0. Max coverage (-): 0

Region: NODE\_360193\_length\_37465\_cov\_31.594688 31890-31899. Max. coverage (+): 0. Max coverage (-): 0

Region: NODE\_360193\_length\_37465\_cov\_31.594688 31900-31909. Max. coverage (+): 0. Max coverage (-): 0

Region: NODE\_360193\_length\_37465\_cov\_31.594688 31910-31919. Max. coverage (+): 0. Max coverage (-): 0

Region: NODE\_360193\_length\_37465\_cov\_31.594688 31920-31929. Max. coverage (+): 0. Max coverage (-): 0

Region: NODE\_360193\_length\_37465\_cov\_31.594688 31930-31939. Max. coverage (+): 0. Max coverage (-): 0

Region: NODE\_360193\_length\_37465\_cov\_31.594688 31940-31949. Max. coverage (+): 0. Max coverage (-): 0

Region: NODE\_360193\_length\_37465\_cov\_31.594688 31950-31959. Max. coverage (+): 0. Max coverage (-): 0

Region: NODE\_360193\_length\_37465\_cov\_31.594688 31960-31969. Max. coverage (+): 0. Max coverage (-): 0

Region: NODE\_360193\_length\_37465\_cov\_31.594688 31970-31979. Max. coverage (+): 0. Max coverage (-): 0

Region: NODE\_360193\_length\_37465\_cov\_31.594688 31980-31989. Max. coverage (+): 0. Max coverage (-): 0

Region: NODE\_360193\_length\_37465\_cov\_31.594688 31990-31999. Max. coverage (+): 0. Max coverage (-): 0

Region: NODE\_360193\_length\_37465\_cov\_31.594688 32000-32009. Max. coverage (+): 0. Max coverage (-): 0

Region: NODE\_360193\_length\_37465\_cov\_31.594688 32010-32019. Max. coverage (+): 0. Max coverage (-): 0

Region: NODE\_360193\_length\_37465\_cov\_31.594688 32020-32029. Max. coverage (+): 0. Max coverage (-): 0

Region: NODE\_360193\_length\_37465\_cov\_31.594688 32030-32039. Max. coverage (+): 0. Max coverage (-): 0

Region: NODE\_360193\_length\_37465\_cov\_31.594688 32040-32049. Max. coverage (+): 0. Max coverage (-): 0

Region: NODE\_360193\_length\_37465\_cov\_31.594688 32050-32059. Max. coverage (+): 0. Max coverage (-): 0

Region: NODE\_360193\_length\_37465\_cov\_31.594688 32060-32069. Max. coverage (+): 0. Max coverage (-): 0

Region: NODE\_360193\_length\_37465\_cov\_31.594688 32070-32079. Max. coverage (+): 0. Max coverage (-): 0

Region: NODE\_360193\_length\_37465\_cov\_31.594688 32080-32089. Max. coverage (+): 0. Max coverage (-): 0

Region: NODE\_360193\_length\_37465\_cov\_31.594688 32090-32099. Max. coverage (+): 0. Max coverage (-): 0

Region: NODE\_360193\_length\_37465\_cov\_31.594688 32100-32109. Max. coverage (+): 0. Max coverage (-): 0

Region: NODE\_360193\_length\_37465\_cov\_31.594688 32110-32119. Max. coverage (+): 0. Max coverage (-): 0

Region: NODE\_360193\_length\_37465\_cov\_31.594688 32120-32129. Max. coverage (+): 0. Max coverage (-): 0.08

Region: NODE\_360193\_length\_37465\_cov\_31.594688 32130-32138. Max. coverage (+): 0. Max coverage (-): 0.15

Region: NODE\_360193\_length\_37465\_cov\_31.594688 32139-32148. Max. coverage (+): 0. Max coverage (-): 0.23

Region: NODE\_360193\_length\_37465\_cov\_31.594688 32149-32158. Max. coverage (+): 0. Max coverage (-): 0.08

Region: NODE\_360193\_length\_37465\_cov\_31.594688 32159-32168. Max. coverage (+): 0.08. Max coverage (-): 0.08

Region: NODE\_360193\_length\_37465\_cov\_31.594688 32169-32178. Max. coverage (+): 0. Max coverage (-): 0.15

Region: NODE\_360193\_length\_37465\_cov\_31.594688 32179-32188. Max. coverage (+): 0.15. Max coverage (-): 0

Region: NODE\_360193\_length\_37465\_cov\_31.594688 32189-32198. Max. coverage (+): 0.54. Max coverage (-): 0

Region: NODE\_360193\_length\_37465\_cov\_31.594688 32199-32208. Max. coverage (+): 0. Max coverage (-): 0.38

Region: NODE\_360193\_length\_37465\_cov\_31.594688 32209-32218. Max. coverage (+): 0. Max coverage (-): 0.08

Region: NODE\_360193\_length\_37465\_cov\_31.594688 32219-32228. Max. coverage (+): 0. Max coverage (-): 0

Region: NODE\_360193\_length\_37465\_cov\_31.594688 32229-32238. Max. coverage (+): 0. Max coverage (-): 0

Region: NODE\_360193\_length\_37465\_cov\_31.594688 32239-32248. Max. coverage (+): 0. Max coverage (-): 0

Region: NODE\_360193\_length\_37465\_cov\_31.594688 32249-32258. Max. coverage (+): 0. Max coverage (-): 0

Region: NODE\_360193\_length\_37465\_cov\_31.594688 32259-32268. Max. coverage (+): 0.15. Max coverage (-): 0.08

Region: NODE\_360193\_length\_37465\_cov\_31.594688 32269-32278. Max. coverage (+): 0. Max coverage (-): 0.08

Region: NODE\_360193\_length\_37465\_cov\_31.594688 32279-32288. Max. coverage (+): 0. Max coverage (-): 0

Region: NODE\_360193\_length\_37465\_cov\_31.594688 32289-32298. Max. coverage (+): 0.08. Max coverage (-): 0.31

Region: NODE\_360193\_length\_37465\_cov\_31.594688 32299-32308. Max. coverage (+): 0.08. Max coverage (-): 0.31

Region: NODE\_360193\_length\_37465\_cov\_31.594688 32309-32318. Max. coverage (+): 0.15. Max coverage (-): 0

Region: NODE\_360193\_length\_37465\_cov\_31.594688 32319-32328. Max. coverage (+): 0.15. Max coverage (-): 0.15

Region: NODE\_360193\_length\_37465\_cov\_31.594688 32329-32338. Max. coverage (+): 0. Max coverage (-): 0.08

Region: NODE\_360193\_length\_37465\_cov\_31.594688 32339-32348. Max. coverage (+): 0. Max coverage (-): 0

Region: NODE\_360193\_length\_37465\_cov\_31.594688 32349-32358. Max. coverage (+): 0. Max coverage (-): 0

Region: NODE\_360193\_length\_37465\_cov\_31.594688 32359-32368. Max. coverage (+): 0. Max coverage (-): 0

Region: NODE\_360193\_length\_37465\_cov\_31.594688 32369-32378. Max. coverage (+): 0. Max coverage (-): 0

Region: NODE\_360193\_length\_37465\_cov\_31.594688 32379-32388. Max. coverage (+): 0. Max coverage (-): 0.15

Region: NODE\_360193\_length\_37465\_cov\_31.594688 32389-32398. Max. coverage (+): 0. Max coverage (-): 1.3

Region: NODE\_360193\_length\_37465\_cov\_31.594688 32399-32408. Max. coverage (+): 0. Max coverage (-): 0

Region: NODE\_360193\_length\_37465\_cov\_31.594688 32409-32417. Max. coverage (+): 0. Max coverage (-): 0

Region: NODE\_360193\_length\_37465\_cov\_31.594688 32418-32427. Max. coverage (+): 0. Max coverage (-): 0

Region: NODE\_360193\_length\_37465\_cov\_31.594688 32428-32437. Max. coverage (+): 0. Max coverage (-): 0.08

Region: NODE\_360193\_length\_37465\_cov\_31.594688 32438-32447. Max. coverage (+): 0. Max coverage (-): 0

Region: NODE\_360193\_length\_37465\_cov\_31.594688 32448-32457. Max. coverage (+): 0. Max coverage (-): 0

Region: NODE\_360193\_length\_37465\_cov\_31.594688 32458-32467. Max. coverage (+): 0. Max coverage (-): 0

Region: NODE\_360193\_length\_37465\_cov\_31.594688 32468-32477. Max. coverage (+): 0. Max coverage (-): 0.15

Region: NODE\_360193\_length\_37465\_cov\_31.594688 32478-32487. Max. coverage (+): 0. Max coverage (-): 1.3

Region: NODE\_360193\_length\_37465\_cov\_31.594688 32488-32497. Max. coverage (+): 0. Max coverage (-): 0.23

Region: NODE\_360193\_length\_37465\_cov\_31.594688 32498-32507. Max. coverage (+): 0.15. Max coverage (-): 0.31

Region: NODE\_360193\_length\_37465\_cov\_31.594688 32508-32517. Max. coverage (+): 0.23. Max coverage (-): 0.08

Region: NODE\_360193\_length\_37465\_cov\_31.594688 32518-32527. Max. coverage (+): 0.08. Max coverage (-): 0.08

Region: NODE\_360193\_length\_37465\_cov\_31.594688 32528-32537. Max. coverage (+): 0. Max coverage (-): 0.08

Region: NODE\_360193\_length\_37465\_cov\_31.594688 32538-32547. Max. coverage (+): 0. Max coverage (-): 0.08

Region: NODE\_360193\_length\_37465\_cov\_31.594688 32548-32557. Max. coverage (+): 0. Max coverage (-): 0.31

Region: NODE\_360193\_length\_37465\_cov\_31.594688 32558-32567. Max. coverage (+): 0.61. Max coverage (-): 0

Region: NODE\_360193\_length\_37465\_cov\_31.594688 32568-32577. Max. coverage (+): 0.69. Max coverage (-): 0

Region: NODE\_360193\_length\_37465\_cov\_31.594688 32578-32587. Max. coverage (+): 0. Max coverage (-): 1.07

Region: NODE\_360193\_length\_37465\_cov\_31.594688 32588-32597. Max. coverage (+): 0. Max coverage (-): 0

Region: NODE\_360193\_length\_37465\_cov\_31.594688 32598-32607. Max. coverage (+): 0. Max coverage (-): 0

Region: NODE\_360193\_length\_37465\_cov\_31.594688 32608-32617. Max. coverage (+): 0. Max coverage (-): 0.61

Region: NODE\_360193\_length\_37465\_cov\_31.594688 32618-32627. Max. coverage (+): 0. Max coverage (-): 0.61

Region: NODE\_360193\_length\_37465\_cov\_31.594688 32628-32637. Max. coverage (+): 0. Max coverage (-): 0

Region: NODE\_360193\_length\_37465\_cov\_31.594688 32638-32647. Max. coverage (+): 0. Max coverage (-): 0.38

Region: NODE\_360193\_length\_37465\_cov\_31.594688 32648-32657. Max. coverage (+): 0. Max coverage (-): 0.38

Region: NODE\_360193\_length\_37465\_cov\_31.594688 32658-32667. Max. coverage (+): 0. Max coverage (-): 0

Region: NODE\_360193\_length\_37465\_cov\_31.594688 32668-32677. Max. coverage (+): 0. Max coverage (-): 0

Region: NODE\_360193\_length\_37465\_cov\_31.594688 32678-32687. Max. coverage (+): 0. Max coverage (-): 0.08

Region: NODE\_360193\_length\_37465\_cov\_31.594688 32688-32696. Max. coverage (+): 0. Max coverage (-): 0.08

Region: NODE\_360193\_length\_37465\_cov\_31.594688 32697-32706. Max. coverage (+): 0. Max coverage (-): 0

Region: NODE\_360193\_length\_37465\_cov\_31.594688 32707-32716. Max. coverage (+): 0. Max coverage (-): 0

Region: NODE\_360193\_length\_37465\_cov\_31.594688 32717-32726. Max. coverage (+): 0. Max coverage (-): 0

Region: NODE\_360193\_length\_37465\_cov\_31.594688 32727-32736. Max. coverage (+): 0. Max coverage (-): 0

Region: NODE\_360193\_length\_37465\_cov\_31.594688 32737-32746. Max. coverage (+): 0.08. Max coverage (-): 0

Region: NODE\_360193\_length\_37465\_cov\_31.594688 32747-32756. Max. coverage (+): 0. Max coverage (-): 0

Region: NODE\_360193\_length\_37465\_cov\_31.594688 32757-32766. Max. coverage (+): 0. Max coverage (-): 0

Region: NODE\_360193\_length\_37465\_cov\_31.594688 32767-32776. Max. coverage (+): 0. Max coverage (-): 0

Region: NODE\_360193\_length\_37465\_cov\_31.594688 32777-32786. Max. coverage (+): 0. Max coverage (-): 0

Region: NODE\_360193\_length\_37465\_cov\_31.594688 32787-32796. Max. coverage (+): 0. Max coverage (-): 0

Region: NODE\_360193\_length\_37465\_cov\_31.594688 32797-32806. Max. coverage (+): 0. Max coverage (-): 0

Region: NODE\_360193\_length\_37465\_cov\_31.594688 32807-32816. Max. coverage (+): 0. Max coverage (-): 0

Region: NODE\_360193\_length\_37465\_cov\_31.594688 32817-32826. Max. coverage (+): 0. Max coverage (-): 0.23

Region: NODE\_360193\_length\_37465\_cov\_31.594688 32827-32836. Max. coverage (+): 0. Max coverage (-): 0.23

Region: NODE\_360193\_length\_37465\_cov\_31.594688 32837-32846. Max. coverage (+): 0. Max coverage (-): 0

Region: NODE\_360193\_length\_37465\_cov\_31.594688 32847-32856. Max. coverage (+): 0. Max coverage (-): 0

Region: NODE\_360193\_length\_37465\_cov\_31.594688 32857-32866. Max. coverage (+): 0. Max coverage (-): 0.31

Region: NODE\_360193\_length\_37465\_cov\_31.594688 32867-32876. Max. coverage (+): 0.08. Max coverage (-): 0

Region: NODE\_360193\_length\_37465\_cov\_31.594688 32877-32886. Max. coverage (+): 0. Max coverage (-): 0.61

Region: NODE\_360193\_length\_37465\_cov\_31.594688 32887-32896. Max. coverage (+): 0.08. Max coverage (-): 0.23

Region: NODE\_360193\_length\_37465\_cov\_31.594688 32897-32906. Max. coverage (+): 0.08. Max coverage (-): 0

Region: NODE\_360193\_length\_37465\_cov\_31.594688 32907-32916. Max. coverage (+): 0. Max coverage (-): 0

Region: NODE\_360193\_length\_37465\_cov\_31.594688 32917-32926. Max. coverage (+): 0. Max coverage (-): 1.15

Region: NODE\_360193\_length\_37465\_cov\_31.594688 32927-32936. Max. coverage (+): 0.23. Max coverage (-): 0.77

Region: NODE\_360193\_length\_37465\_cov\_31.594688 32937-32946. Max. coverage (+): 0.23. Max coverage (-): 0.15

Region: NODE\_360193\_length\_37465\_cov\_31.594688 32947-32956. Max. coverage (+): 0. Max coverage (-): 0.08

Region: NODE\_360193\_length\_37465\_cov\_31.594688 32957-32965. Max. coverage (+): 0. Max coverage (-): 0.15

Region: NODE\_360193\_length\_37465\_cov\_31.594688 32966-32975. Max. coverage (+): 0. Max coverage (-): 0.46

Region: NODE\_360193\_length\_37465\_cov\_31.594688 32976-32985. Max. coverage (+): 0.08. Max coverage (-): 0.46

Region: NODE\_360193\_length\_37465\_cov\_31.594688 32986-32995. Max. coverage (+): 0.08. Max coverage (-): 0.15

Region: NODE\_360193\_length\_37465\_cov\_31.594688 32996-33005. Max. coverage (+): 0.15. Max coverage (-): 0

Region: NODE\_360193\_length\_37465\_cov\_31.594688 33006-33015. Max. coverage (+): 0. Max coverage (-): 0

Region: NODE\_360193\_length\_37465\_cov\_31.594688 33016-33025. Max. coverage (+): 0. Max coverage (-): 0.15

Region: NODE\_360193\_length\_37465\_cov\_31.594688 33026-33035. Max. coverage (+): 0. Max coverage (-): 0.15

Region: NODE\_360193\_length\_37465\_cov\_31.594688 33036-33045. Max. coverage (+): 0. Max coverage (-): 0.23

Region: NODE\_360193\_length\_37465\_cov\_31.594688 33046-33055. Max. coverage (+): 1.46. Max coverage (-): 0.08

Region: NODE\_360193\_length\_37465\_cov\_31.594688 33056-33065. Max. coverage (+): 0. Max coverage (-): 0.08

Region: NODE\_360193\_length\_37465\_cov\_31.594688 33066-33075. Max. coverage (+): 0. Max coverage (-): 0.46

Region: NODE\_360193\_length\_37465\_cov\_31.594688 33076-33085. Max. coverage (+): 0. Max coverage (-): 0.38

Region: NODE\_360193\_length\_37465\_cov\_31.594688 33086-33095. Max. coverage (+): 0.08. Max coverage (-): 0

Region: NODE\_360193\_length\_37465\_cov\_31.594688 33096-33105. Max. coverage (+): 0. Max coverage (-): 0.08

Region: NODE\_360193\_length\_37465\_cov\_31.594688 33106-33115. Max. coverage (+): 0. Max coverage (-): 0.15

Region: NODE\_360193\_length\_37465\_cov\_31.594688 33116-33125. Max. coverage (+): 0. Max coverage (-): 0.08

Region: NODE\_360193\_length\_37465\_cov\_31.594688 33126-33135. Max. coverage (+): 0. Max coverage (-): 0

Region: NODE\_360193\_length\_37465\_cov\_31.594688 33136-33145. Max. coverage (+): 0. Max coverage (-): 0

Region: NODE\_360193\_length\_37465\_cov\_31.594688 33146-33155. Max. coverage (+): 0. Max coverage (-): 0.08

Region: NODE\_360193\_length\_37465\_cov\_31.594688 33156-33165. Max. coverage (+): 0. Max coverage (-): 0.15

Region: NODE\_360193\_length\_37465\_cov\_31.594688 33166-33175. Max. coverage (+): 0. Max coverage (-): 0.08

Region: NODE\_360193\_length\_37465\_cov\_31.594688 33176-33185. Max. coverage (+): 0. Max coverage (-): 0.08

Region: NODE\_360193\_length\_37465\_cov\_31.594688 33186-33195. Max. coverage (+): 0. Max coverage (-): 0

Region: NODE\_360193\_length\_37465\_cov\_31.594688 33196-33205. Max. coverage (+): 0. Max coverage (-): 1

Region: NODE\_360193\_length\_37465\_cov\_31.594688 33206-33215. Max. coverage (+): 0. Max coverage (-): 0.61

Region: NODE\_360193\_length\_37465\_cov\_31.594688 33216-33225. Max. coverage (+): 0.15. Max coverage (-): 0

Region: NODE\_360193\_length\_37465\_cov\_31.594688 33226-33235. Max. coverage (+): 0. Max coverage (-): 0

Region: NODE\_360193\_length\_37465\_cov\_31.594688 33236-33244. Max. coverage (+): 0. Max coverage (-): 0

Region: NODE\_360193\_length\_37465\_cov\_31.594688 33245-33254. Max. coverage (+): 0. Max coverage (-): 0

Region: NODE\_360193\_length\_37465\_cov\_31.594688 33255-33264. Max. coverage (+): 0. Max coverage (-): 0

Region: NODE\_360193\_length\_37465\_cov\_31.594688 33265-33274. Max. coverage (+): 0.08. Max coverage (-): 0.15

Region: NODE\_360193\_length\_37465\_cov\_31.594688 33275-33284. Max. coverage (+): 0.23. Max coverage (-): 0.15

Region: NODE\_360193\_length\_37465\_cov\_31.594688 33285-33294. Max. coverage (+): 0.77. Max coverage (-): 0.08

Region: NODE\_360193\_length\_37465\_cov\_31.594688 33295-33304. Max. coverage (+): 0. Max coverage (-): 0.08

Region: NODE\_360193\_length\_37465\_cov\_31.594688 33305-33314. Max. coverage (+): 0. Max coverage (-): 0.08

Region: NODE\_360193\_length\_37465\_cov\_31.594688 33315-33324. Max. coverage (+): 0. Max coverage (-): 0.08

Region: NODE\_360193\_length\_37465\_cov\_31.594688 33325-33334. Max. coverage (+): 0. Max coverage (-): 0

Region: NODE\_360193\_length\_37465\_cov\_31.594688 33335-33344. Max. coverage (+): 0. Max coverage (-): 0

Region: NODE\_360193\_length\_37465\_cov\_31.594688 33345-33354. Max. coverage (+): 0.31. Max coverage (-): 0.31

Region: NODE\_360193\_length\_37465\_cov\_31.594688 33355-33364. Max. coverage (+): 0.31. Max coverage (-): 13.56

Region: NODE\_360193\_length\_37465\_cov\_31.594688 33365-33374. Max. coverage (+): 0. Max coverage (-): 7.28

Region: NODE\_360193\_length\_37465\_cov\_31.594688 33375-33384. Max. coverage (+): 0. Max coverage (-): 1.46

Region: NODE\_360193\_length\_37465\_cov\_31.594688 33385-33394. Max. coverage (+): 0. Max coverage (-): 0.15

Region: NODE\_360193\_length\_37465\_cov\_31.594688 33395-33404. Max. coverage (+): 0. Max coverage (-): 0

Region: NODE\_360193\_length\_37465\_cov\_31.594688 33405-33414. Max. coverage (+): 0. Max coverage (-): 0.15

Region: NODE\_360193\_length\_37465\_cov\_31.594688 33415-33424. Max. coverage (+): 0.31. Max coverage (-): 0.15

Region: NODE\_360193\_length\_37465\_cov\_31.594688 33425-33434. Max. coverage (+): 0.23. Max coverage (-): 0.08

Region: NODE\_360193\_length\_37465\_cov\_31.594688 33435-33444. Max. coverage (+): 0.23. Max coverage (-): 0.08

Region: NODE\_360193\_length\_37465\_cov\_31.594688 33445-33454. Max. coverage (+): 0. Max coverage (-): 0.23

Region: NODE\_360193\_length\_37465\_cov\_31.594688 33455-33464. Max. coverage (+): 0. Max coverage (-): 0.15

Region: NODE\_360193\_length\_37465\_cov\_31.594688 33465-33474. Max. coverage (+): 0.08. Max coverage (-): 0.23

Region: NODE\_360193\_length\_37465\_cov\_31.594688 33475-33484. Max. coverage (+): 0.08. Max coverage (-): 0.08

Region: NODE\_360193\_length\_37465\_cov\_31.594688 33485-33494. Max. coverage (+): 0.08. Max coverage (-): 0.08

Region: NODE\_360193\_length\_37465\_cov\_31.594688 33495-33504. Max. coverage (+): 0. Max coverage (-): 0.08

Region: NODE\_360193\_length\_37465\_cov\_31.594688 33505-33514. Max. coverage (+): 0. Max coverage (-): 0.38

Region: NODE\_360193\_length\_37465\_cov\_31.594688 33515-33523. Max. coverage (+): 0. Max coverage (-): 1.76

Region: NODE\_360193\_length\_37465\_cov\_31.594688 33524-33533. Max. coverage (+): 0. Max coverage (-): 0.08

Region: NODE\_360193\_length\_37465\_cov\_31.594688 33534-33543. Max. coverage (+): 0. Max coverage (-): 0

Region: NODE\_360193\_length\_37465\_cov\_31.594688 33544-33553. Max. coverage (+): 0. Max coverage (-): 0

Region: NODE\_360193\_length\_37465\_cov\_31.594688 33554-33563. Max. coverage (+): 0. Max coverage (-): 0.15

Region: NODE\_360193\_length\_37465\_cov\_31.594688 33564-33573. Max. coverage (+): 0.15. Max coverage (-): 0.08

Region: NODE\_360193\_length\_37465\_cov\_31.594688 33574-33583. Max. coverage (+): 0.15. Max coverage (-): 0

Region: NODE\_360193\_length\_37465\_cov\_31.594688 33584-33593. Max. coverage (+): 0. Max coverage (-): 0

Region: NODE\_360193\_length\_37465\_cov\_31.594688 33594-33603. Max. coverage (+): 0. Max coverage (-): 0

Region: NODE\_360193\_length\_37465\_cov\_31.594688 33604-33613. Max. coverage (+): 0. Max coverage (-): 0

Region: NODE\_360193\_length\_37465\_cov\_31.594688 33614-33623. Max. coverage (+): 0.08. Max coverage (-): 0

Region: NODE\_360193\_length\_37465\_cov\_31.594688 33624-33633. Max. coverage (+): 0.08. Max coverage (-): 0.84

Region: NODE\_360193\_length\_37465\_cov\_31.594688 33634-33643. Max. coverage (+): 0.08. Max coverage (-): 0.38

Region: NODE\_360193\_length\_37465\_cov\_31.594688 33644-33653. Max. coverage (+): 0. Max coverage (-): 0

Region: NODE\_360193\_length\_37465\_cov\_31.594688 33654-33663. Max. coverage (+): 0. Max coverage (-): 0.08

Region: NODE\_360193\_length\_37465\_cov\_31.594688 33664-33673. Max. coverage (+): 0. Max coverage (-): 0.08

Region: NODE\_360193\_length\_37465\_cov\_31.594688 33674-33683. Max. coverage (+): 0. Max coverage (-): 0

Region: NODE\_360193\_length\_37465\_cov\_31.594688 33684-33693. Max. coverage (+): 0.08. Max coverage (-): 0

Region: NODE\_360193\_length\_37465\_cov\_31.594688 33694-33703. Max. coverage (+): 0. Max coverage (-): 0.46

Region: NODE\_360193\_length\_37465\_cov\_31.594688 33704-33713. Max. coverage (+): 0. Max coverage (-): 0.69

Region: NODE\_360193\_length\_37465\_cov\_31.594688 33714-33723. Max. coverage (+): 0. Max coverage (-): 0.61

Region: NODE\_360193\_length\_37465\_cov\_31.594688 33724-33733. Max. coverage (+): 0. Max coverage (-): 0.61

Region: NODE\_360193\_length\_37465\_cov\_31.594688 33734-33743. Max. coverage (+): 0. Max coverage (-): 0

Region: NODE\_360193\_length\_37465\_cov\_31.594688 33744-33753. Max. coverage (+): 0. Max coverage (-): 0.61

Region: NODE\_360193\_length\_37465\_cov\_31.594688 33754-33763. Max. coverage (+): 0. Max coverage (-): 1

Region: NODE\_360193\_length\_37465\_cov\_31.594688 33764-33773. Max. coverage (+): 0.15. Max coverage (-): 0.15

Region: NODE\_360193\_length\_37465\_cov\_31.594688 33774-33783. Max. coverage (+): 0.15. Max coverage (-): 0

Region: NODE\_360193\_length\_37465\_cov\_31.594688 33784-33793. Max. coverage (+): 0. Max coverage (-): 0

Region: NODE\_360193\_length\_37465\_cov\_31.594688 33794-33802. Max. coverage (+): 0. Max coverage (-): 0

Region: NODE\_360193\_length\_37465\_cov\_31.594688 33803-33812. Max. coverage (+): 0. Max coverage (-): 0

Region: NODE\_360193\_length\_37465\_cov\_31.594688 33813-33822. Max. coverage (+): 0. Max coverage (-): 0

Region: NODE\_360193\_length\_37465\_cov\_31.594688 33823-33832. Max. coverage (+): 0. Max coverage (-): 0

Region: NODE\_360193\_length\_37465\_cov\_31.594688 33833-33842. Max. coverage (+): 0. Max coverage (-): 0

Region: NODE\_360193\_length\_37465\_cov\_31.594688 33843-33852. Max. coverage (+): 0. Max coverage (-): 0

Region: NODE\_360193\_length\_37465\_cov\_31.594688 33853-33862. Max. coverage (+): 0.15. Max coverage (-): 0

Region: NODE\_360193\_length\_37465\_cov\_31.594688 33863-33872. Max. coverage (+): 0. Max coverage (-): 0

Region: NODE\_360193\_length\_37465\_cov\_31.594688 33873-33882. Max. coverage (+): 0. Max coverage (-): 5.98

Region: NODE\_360193\_length\_37465\_cov\_31.594688 33883-33892. Max. coverage (+): 0. Max coverage (-): 5.59

Region: NODE\_360193\_length\_37465\_cov\_31.594688 33893-33902. Max. coverage (+): 0.08. Max coverage (-): 0.46

Region: NODE\_360193\_length\_37465\_cov\_31.594688 33903-33912. Max. coverage (+): 0.08. Max coverage (-): 0.08

Region: NODE\_360193\_length\_37465\_cov\_31.594688 33913-33922. Max. coverage (+): 0. Max coverage (-): 0.08

Region: NODE\_360193\_length\_37465\_cov\_31.594688 33923-33932. Max. coverage (+): 0.15. Max coverage (-): 0

Region: NODE\_360193\_length\_37465\_cov\_31.594688 33933-33942. Max. coverage (+): 0.23. Max coverage (-): 0.08

Region: NODE\_360193\_length\_37465\_cov\_31.594688 33943-33952. Max. coverage (+): 0. Max coverage (-): 0.31

Region: NODE\_360193\_length\_37465\_cov\_31.594688 33953-33962. Max. coverage (+): 0. Max coverage (-): 0.08

Region: NODE\_360193\_length\_37465\_cov\_31.594688 33963-33972. Max. coverage (+): 0. Max coverage (-): 0

Region: NODE\_360193\_length\_37465\_cov\_31.594688 33973-33982. Max. coverage (+): 0. Max coverage (-): 0.08

Region: NODE\_360193\_length\_37465\_cov\_31.594688 33983-33992. Max. coverage (+): 0. Max coverage (-): 0.15

Region: NODE\_360193\_length\_37465\_cov\_31.594688 33993-34002. Max. coverage (+): 0. Max coverage (-): 0.15

Region: NODE\_360193\_length\_37465\_cov\_31.594688 34003-34012. Max. coverage (+): 0.08. Max coverage (-): 0

Region: NODE\_360193\_length\_37465\_cov\_31.594688 34013-34022. Max. coverage (+): 0. Max coverage (-): 0

Region: NODE\_360193\_length\_37465\_cov\_31.594688 34023-34032. Max. coverage (+): 0. Max coverage (-): 0

Region: NODE\_360193\_length\_37465\_cov\_31.594688 34033-34042. Max. coverage (+): 0. Max coverage (-): 0.61

Region: NODE\_360193\_length\_37465\_cov\_31.594688 34043-34052. Max. coverage (+): 0. Max coverage (-): 0.61

Region: NODE\_360193\_length\_37465\_cov\_31.594688 34053-34062. Max. coverage (+): 0.08. Max coverage (-): 0

Region: NODE\_360193\_length\_37465\_cov\_31.594688 34063-34072. Max. coverage (+): 0.08. Max coverage (-): 35.24

Region: NODE\_360193\_length\_37465\_cov\_31.594688 34073-34081. Max. coverage (+): 0. Max coverage (-): 39.23

Region: NODE\_360193\_length\_37465\_cov\_31.594688 34082-34091. Max. coverage (+): 0. Max coverage (-): 0.77

Region: NODE\_360193\_length\_37465\_cov\_31.594688 34092-34101. Max. coverage (+): 0. Max coverage (-): 0.38

Region: NODE\_360193\_length\_37465\_cov\_31.594688 34102-34111. Max. coverage (+): 0. Max coverage (-): 0.46

Region: NODE\_360193\_length\_37465\_cov\_31.594688 34112-34121. Max. coverage (+): 0.08. Max coverage (-): 0.31

Region: NODE\_360193\_length\_37465\_cov\_31.594688 34122-34131. Max. coverage (+): 0.08. Max coverage (-): 0.08

Region: NODE\_360193\_length\_37465\_cov\_31.594688 34132-34141. Max. coverage (+): 0. Max coverage (-): 0.08

Region: NODE\_360193\_length\_37465\_cov\_31.594688 34142-34151. Max. coverage (+): 0.08. Max coverage (-): 0.08

Region: NODE\_360193\_length\_37465\_cov\_31.594688 34152-34161. Max. coverage (+): 0.08. Max coverage (-): 0

Region: NODE\_360193\_length\_37465\_cov\_31.594688 34162-34171. Max. coverage (+): 0. Max coverage (-): 0

Region: NODE\_360193\_length\_37465\_cov\_31.594688 34172-34181. Max. coverage (+): 0. Max coverage (-): 0.54

Region: NODE\_360193\_length\_37465\_cov\_31.594688 34182-34191. Max. coverage (+): 0. Max coverage (-): 2.15

Region: NODE\_360193\_length\_37465\_cov\_31.594688 34192-34201. Max. coverage (+): 0. Max coverage (-): 5.29

Region: NODE\_360193\_length\_37465\_cov\_31.594688 34202-34211. Max. coverage (+): 0. Max coverage (-): 0

Region: NODE\_360193\_length\_37465\_cov\_31.594688 34212-34221. Max. coverage (+): 0. Max coverage (-): 0

Region: NODE\_360193\_length\_37465\_cov\_31.594688 34222-34231. Max. coverage (+): 0. Max coverage (-): 0

Region: NODE\_360193\_length\_37465\_cov\_31.594688 34232-34241. Max. coverage (+): 0.15. Max coverage (-): 0

Region: NODE\_360193\_length\_37465\_cov\_31.594688 34242-34251. Max. coverage (+): 0.08. Max coverage (-): 0.08

Region: NODE\_360193\_length\_37465\_cov\_31.594688 34252-34261. Max. coverage (+): 0. Max coverage (-): 0.38

Region: NODE\_360193\_length\_37465\_cov\_31.594688 34262-34271. Max. coverage (+): 0. Max coverage (-): 0.08

Region: NODE\_360193\_length\_37465\_cov\_31.594688 34272-34281. Max. coverage (+): 0. Max coverage (-): 0.23

Region: NODE\_360193\_length\_37465\_cov\_31.594688 34282-34291. Max. coverage (+): 0. Max coverage (-): 0

Region: NODE\_360193\_length\_37465\_cov\_31.594688 34292-34301. Max. coverage (+): 0. Max coverage (-): 0

Region: NODE\_360193\_length\_37465\_cov\_31.594688 34302-34311. Max. coverage (+): 0.08. Max coverage (-): 0

Region: NODE\_360193\_length\_37465\_cov\_31.594688 34312-34321. Max. coverage (+): 0. Max coverage (-): 0

Region: NODE\_360193\_length\_37465\_cov\_31.594688 34322-34331. Max. coverage (+): 0. Max coverage (-): 0.08

Region: NODE\_360193\_length\_37465\_cov\_31.594688 34332-34341. Max. coverage (+): 0. Max coverage (-): 0

Region: NODE\_360193\_length\_37465\_cov\_31.594688 34342-34350. Max. coverage (+): 0. Max coverage (-): 0

Region: NODE\_360193\_length\_37465\_cov\_31.594688 34351-34360. Max. coverage (+): 0. Max coverage (-): 0

Region: NODE\_360193\_length\_37465\_cov\_31.594688 34361-34370. Max. coverage (+): 0. Max coverage (-): 0.08

Region: NODE\_360193\_length\_37465\_cov\_31.594688 34371-34380. Max. coverage (+): 0. Max coverage (-): 0.08

Region: NODE\_360193\_length\_37465\_cov\_31.594688 34381-34390. Max. coverage (+): 0.15. Max coverage (-): 0

Region: NODE\_360193\_length\_37465\_cov\_31.594688 34391-34400. Max. coverage (+): 0.31. Max coverage (-): 0

Region: NODE\_360193\_length\_37465\_cov\_31.594688 34401-34410. Max. coverage (+): 0. Max coverage (-): 0.31

Region: NODE\_360193\_length\_37465\_cov\_31.594688 34411-34420. Max. coverage (+): 0. Max coverage (-): 0.08

Region: NODE\_360193\_length\_37465\_cov\_31.594688 34421-34430. Max. coverage (+): 0. Max coverage (-): 0

Region: NODE\_360193\_length\_37465\_cov\_31.594688 34431-34440. Max. coverage (+): 0. Max coverage (-): 0

Region: NODE\_360193\_length\_37465\_cov\_31.594688 34441-34450. Max. coverage (+): 0. Max coverage (-): 0.15

Region: NODE\_360193\_length\_37465\_cov\_31.594688 34451-34460. Max. coverage (+): 0. Max coverage (-): 0.23

Region: NODE\_360193\_length\_37465\_cov\_31.594688 34461-34470. Max. coverage (+): 0.15. Max coverage (-): 0.15

Region: NODE\_360193\_length\_37465\_cov\_31.594688 34471-34480. Max. coverage (+): 0.15. Max coverage (-): 0

Region: NODE\_360193\_length\_37465\_cov\_31.594688 34481-34490. Max. coverage (+): 0.08. Max coverage (-): 0.38

Region: NODE\_360193\_length\_37465\_cov\_31.594688 34491-34500. Max. coverage (+): 0. Max coverage (-): 0

Region: NODE\_360193\_length\_37465\_cov\_31.594688 34501-34510. Max. coverage (+): 0. Max coverage (-): 0

Region: NODE\_360193\_length\_37465\_cov\_31.594688 34511-34520. Max. coverage (+): 0. Max coverage (-): 0.15

Region: NODE\_360193\_length\_37465\_cov\_31.594688 34521-34530. Max. coverage (+): 0. Max coverage (-): 0.31

Region: NODE\_360193\_length\_37465\_cov\_31.594688 34531-34540. Max. coverage (+): 0. Max coverage (-): 0.08

Region: NODE\_360193\_length\_37465\_cov\_31.594688 34541-34550. Max. coverage (+): 0.15. Max coverage (-): 0.15

Region: NODE\_360193\_length\_37465\_cov\_31.594688 34551-34560. Max. coverage (+): 0. Max coverage (-): 0.08

Region: NODE\_360193\_length\_37465\_cov\_31.594688 34561-34570. Max. coverage (+): 0. Max coverage (-): 0.08

Region: NODE\_360193\_length\_37465\_cov\_31.594688 34571-34580. Max. coverage (+): 0. Max coverage (-): 0.08

Region: NODE\_360193\_length\_37465\_cov\_31.594688 34581-34590. Max. coverage (+): 0. Max coverage (-): 0

Region: NODE\_360193\_length\_37465\_cov\_31.594688 34591-34600. Max. coverage (+): 0. Max coverage (-): 0.31

Region: NODE\_360193\_length\_37465\_cov\_31.594688 34601-34610. Max. coverage (+): 0. Max coverage (-): 1.15

Region: NODE\_360193\_length\_37465\_cov\_31.594688 34611-34620. Max. coverage (+): 0. Max coverage (-): 1.15

Region: NODE\_360193\_length\_37465\_cov\_31.594688 34621-34629. Max. coverage (+): 0.08. Max coverage (-): 0.08

Region: NODE\_360193\_length\_37465\_cov\_31.594688 34630-34639. Max. coverage (+): 0. Max coverage (-): 0.23

Region: NODE\_360193\_length\_37465\_cov\_31.594688 34640-34649. Max. coverage (+): 0. Max coverage (-): 0.69

Region: NODE\_360193\_length\_37465\_cov\_31.594688 34650-34659. Max. coverage (+): 0.08. Max coverage (-): 2.53

Region: NODE\_360193\_length\_37465\_cov\_31.594688 34660-34669. Max. coverage (+): 0.08. Max coverage (-): 0.61

Region: NODE\_360193\_length\_37465\_cov\_31.594688 34670-34679. Max. coverage (+): 0. Max coverage (-): 0.23

Region: NODE\_360193\_length\_37465\_cov\_31.594688 34680-34689. Max. coverage (+): 0. Max coverage (-): 0.31

Region: NODE\_360193\_length\_37465\_cov\_31.594688 34690-34699. Max. coverage (+): 0. Max coverage (-): 0.23

Region: NODE\_360193\_length\_37465\_cov\_31.594688 34700-34709. Max. coverage (+): 0.08. Max coverage (-): 0.15

Region: NODE\_360193\_length\_37465\_cov\_31.594688 34710-34719. Max. coverage (+): 0.08. Max coverage (-): 0.84

Region: NODE\_360193\_length\_37465\_cov\_31.594688 34720-34729. Max. coverage (+): 0.08. Max coverage (-): 0.08

Region: NODE\_360193\_length\_37465\_cov\_31.594688 34730-34739. Max. coverage (+): 0. Max coverage (-): 0.15

Region: NODE\_360193\_length\_37465\_cov\_31.594688 34740-34749. Max. coverage (+): 0. Max coverage (-): 0.46

Region: NODE\_360193\_length\_37465\_cov\_31.594688 34750-34759. Max. coverage (+): 0. Max coverage (-): 0

Region: NODE\_360193\_length\_37465\_cov\_31.594688 34760-34769. Max. coverage (+): 0. Max coverage (-): 0

Region: NODE\_360193\_length\_37465\_cov\_31.594688 34770-34779. Max. coverage (+): 0. Max coverage (-): 0

Region: NODE\_360193\_length\_37465\_cov\_31.594688 34780-34789. Max. coverage (+): 0. Max coverage (-): 0

Region: NODE\_360193\_length\_37465\_cov\_31.594688 34790-34799. Max. coverage (+): 0. Max coverage (-): 0

Region: NODE\_360193\_length\_37465\_cov\_31.594688 34800-34809. Max. coverage (+): 0. Max coverage (-): 0.15

Region: NODE\_360193\_length\_37465\_cov\_31.594688 34810-34819. Max. coverage (+): 0. Max coverage (-): 0.15

Region: NODE\_360193\_length\_37465\_cov\_31.594688 34820-34829. Max. coverage (+): 0. Max coverage (-): 0.08

Region: NODE\_360193\_length\_37465\_cov\_31.594688 34830-34839. Max. coverage (+): 0. Max coverage (-): 0.08

Region: NODE\_360193\_length\_37465\_cov\_31.594688 34840-34849. Max. coverage (+): 0. Max coverage (-): 0

Region: NODE\_360193\_length\_37465\_cov\_31.594688 34850-34859. Max. coverage (+): 0. Max coverage (-): 1.07

Region: NODE\_360193\_length\_37465\_cov\_31.594688 34860-34869. Max. coverage (+): 0.08. Max coverage (-): 0

Region: NODE\_360193\_length\_37465\_cov\_31.594688 34870-34879. Max. coverage (+): 0.08. Max coverage (-): 0.31

Region: NODE\_360193\_length\_37465\_cov\_31.594688 34880-34889. Max. coverage (+): 0. Max coverage (-): 1.3

Region: NODE\_360193\_length\_37465\_cov\_31.594688 34890-34899. Max. coverage (+): 0. Max coverage (-): 6.13

Region: NODE\_360193\_length\_37465\_cov\_31.594688 34900-34908. Max. coverage (+): 0. Max coverage (-): 0

Region: NODE\_360193\_length\_37465\_cov\_31.594688 34909-34918. Max. coverage (+): 0. Max coverage (-): 0

Region: NODE\_360193\_length\_37465\_cov\_31.594688 34919-34928. Max. coverage (+): 0. Max coverage (-): 0

Region: NODE\_360193\_length\_37465\_cov\_31.594688 34929-34938. Max. coverage (+): 0.15. Max coverage (-): 0.31

Region: NODE\_360193\_length\_37465\_cov\_31.594688 34939-34948. Max. coverage (+): 0.15. Max coverage (-): 0.31

Region: NODE\_360193\_length\_37465\_cov\_31.594688 34949-34958. Max. coverage (+): 0. Max coverage (-): 0.08

Region: NODE\_360193\_length\_37465\_cov\_31.594688 34959-34968. Max. coverage (+): 0. Max coverage (-): 0.08

Region: NODE\_360193\_length\_37465\_cov\_31.594688 34969-34978. Max. coverage (+): 0. Max coverage (-): 0.15

Region: NODE\_360193\_length\_37465\_cov\_31.594688 34979-34988. Max. coverage (+): 0.08. Max coverage (-): 0.61

Region: NODE\_360193\_length\_37465\_cov\_31.594688 34989-34998. Max. coverage (+): 0.08. Max coverage (-): 0.46

Region: NODE\_360193\_length\_37465\_cov\_31.594688 34999-35008. Max. coverage (+): 0. Max coverage (-): 0

Region: NODE\_360193\_length\_37465\_cov\_31.594688 35009-35018. Max. coverage (+): 0. Max coverage (-): 0

Region: NODE\_360193\_length\_37465\_cov\_31.594688 35019-35028. Max. coverage (+): 0. Max coverage (-): 0.08

Region: NODE\_360193\_length\_37465\_cov\_31.594688 35029-35038. Max. coverage (+): 0. Max coverage (-): 0

Region: NODE\_360193\_length\_37465\_cov\_31.594688 35039-35048. Max. coverage (+): 0. Max coverage (-): 0.08

Region: NODE\_360193\_length\_37465\_cov\_31.594688 35049-35058. Max. coverage (+): 0.08. Max coverage (-): 0.08

Region: NODE\_360193\_length\_37465\_cov\_31.594688 35059-35068. Max. coverage (+): 0. Max coverage (-): 0

Region: NODE\_360193\_length\_37465\_cov\_31.594688 35069-35078. Max. coverage (+): 0. Max coverage (-): 0

Region: NODE\_360193\_length\_37465\_cov\_31.594688 35079-35088. Max. coverage (+): 0. Max coverage (-): 0.84

Region: NODE\_360193\_length\_37465\_cov\_31.594688 35089-35098. Max. coverage (+): 0. Max coverage (-): 0.84

Region: NODE\_360193\_length\_37465\_cov\_31.594688 35099-35108. Max. coverage (+): 1.99. Max coverage (-): 0

Region: NODE\_360193\_length\_37465\_cov\_31.594688 35109-35118. Max. coverage (+): 1.38. Max coverage (-): 0

Region: NODE\_360193\_length\_37465\_cov\_31.594688 35119-35128. Max. coverage (+): 0. Max coverage (-): 0

Region: NODE\_360193\_length\_37465\_cov\_31.594688 35129-35138. Max. coverage (+): 0. Max coverage (-): 0.08

Region: NODE\_360193\_length\_37465\_cov\_31.594688 35139-35148. Max. coverage (+): 0.08. Max coverage (-): 0.08

Region: NODE\_360193\_length\_37465\_cov\_31.594688 35149-35158. Max. coverage (+): 0.08. Max coverage (-): 0.08

Region: NODE\_360193\_length\_37465\_cov\_31.594688 35159-35168. Max. coverage (+): 0. Max coverage (-): 0.08

Region: NODE\_360193\_length\_37465\_cov\_31.594688 35169-35178. Max. coverage (+): 0. Max coverage (-): 0

Region: NODE\_360193\_length\_37465\_cov\_31.594688 35179-35187. Max. coverage (+): 0. Max coverage (-): 0.08

Region: NODE\_360193\_length\_37465\_cov\_31.594688 35188-35197. Max. coverage (+): 0. Max coverage (-): 0.15

Region: NODE\_360193\_length\_37465\_cov\_31.594688 35198-35207. Max. coverage (+): 0.08. Max coverage (-): 2.38

Region: NODE\_360193\_length\_37465\_cov\_31.594688 35208-35217. Max. coverage (+): 0.08. Max coverage (-): 1.99

Region: NODE\_360193\_length\_37465\_cov\_31.594688 35218-35227. Max. coverage (+): 0. Max coverage (-): 0.08

Region: NODE\_360193\_length\_37465\_cov\_31.594688 35228-35237. Max. coverage (+): 0. Max coverage (-): 0

Region: NODE\_360193\_length\_37465\_cov\_31.594688 35238-35247. Max. coverage (+): 0.08. Max coverage (-): 0

Region: NODE\_360193\_length\_37465\_cov\_31.594688 35248-35257. Max. coverage (+): 0. Max coverage (-): 0

Region: NODE\_360193\_length\_37465\_cov\_31.594688 35258-35267. Max. coverage (+): 0. Max coverage (-): 0

Region: NODE\_360193\_length\_37465\_cov\_31.594688 35268-35277. Max. coverage (+): 0. Max coverage (-): 0

Region: NODE\_360193\_length\_37465\_cov\_31.594688 35278-35287. Max. coverage (+): 0. Max coverage (-): 0

Region: NODE\_360193\_length\_37465\_cov\_31.594688 35288-35297. Max. coverage (+): 0.08. Max coverage (-): 0

Region: NODE\_360193\_length\_37465\_cov\_31.594688 35298-35307. Max. coverage (+): 0. Max coverage (-): 0

Region: NODE\_360193\_length\_37465\_cov\_31.594688 35308-35317. Max. coverage (+): 0. Max coverage (-): 0

Region: NODE\_360193\_length\_37465\_cov\_31.594688 35318-35327. Max. coverage (+): 0. Max coverage (-): 4.37

Region: NODE\_360193\_length\_37465\_cov\_31.594688 35328-35337. Max. coverage (+): 0.38. Max coverage (-): 6.28

Region: NODE\_360193\_length\_37465\_cov\_31.594688 35338-35347. Max. coverage (+): 0.46. Max coverage (-): 0

Region: NODE\_360193\_length\_37465\_cov\_31.594688 35348-35357. Max. coverage (+): 0.46. Max coverage (-): 0

Region: NODE\_360193\_length\_37465\_cov\_31.594688 35358-35367. Max. coverage (+): 0. Max coverage (-): 0.15

Region: NODE\_360193\_length\_37465\_cov\_31.594688 35368-35377. Max. coverage (+): 0.08. Max coverage (-): 0.23

Region: NODE\_360193\_length\_37465\_cov\_31.594688 35378-35387. Max. coverage (+): 0.08. Max coverage (-): 0

Region: NODE\_360193\_length\_37465\_cov\_31.594688 35388-35397. Max. coverage (+): 0.15. Max coverage (-): 0

Region: NODE\_360193\_length\_37465\_cov\_31.594688 35398-35407. Max. coverage (+): 0. Max coverage (-): 0

Region: NODE\_360193\_length\_37465\_cov\_31.594688 35408-35417. Max. coverage (+): 0. Max coverage (-): 0.23

Region: NODE\_360193\_length\_37465\_cov\_31.594688 35418-35427. Max. coverage (+): 0.15. Max coverage (-): 0

Region: NODE\_360193\_length\_37465\_cov\_31.594688 35428-35437. Max. coverage (+): 0.08. Max coverage (-): 0

Region: NODE\_360193\_length\_37465\_cov\_31.594688 35438-35447. Max. coverage (+): 0. Max coverage (-): 0

Region: NODE\_360193\_length\_37465\_cov\_31.594688 35448-35456. Max. coverage (+): 0. Max coverage (-): 0

Region: NODE\_360193\_length\_37465\_cov\_31.594688 35457-35466. Max. coverage (+): 0. Max coverage (-): 0.08

Region: NODE\_360193\_length\_37465\_cov\_31.594688 35467-35476. Max. coverage (+): 0.08. Max coverage (-): 0.08

Region: NODE\_360193\_length\_37465\_cov\_31.594688 35477-35486. Max. coverage (+): 0. Max coverage (-): 0

Region: NODE\_360193\_length\_37465\_cov\_31.594688 35487-35496. Max. coverage (+): 0. Max coverage (-): 0.15

Region: NODE\_360193\_length\_37465\_cov\_31.594688 35497-35506. Max. coverage (+): 0. Max coverage (-): 0.15

Region: NODE\_360193\_length\_37465\_cov\_31.594688 35507-35516. Max. coverage (+): 0. Max coverage (-): 0

Region: NODE\_360193\_length\_37465\_cov\_31.594688 35517-35526. Max. coverage (+): 0. Max coverage (-): 0.15

Region: NODE\_360193\_length\_37465\_cov\_31.594688 35527-35536. Max. coverage (+): 0. Max coverage (-): 0.08

Region: NODE\_360193\_length\_37465\_cov\_31.594688 35537-35546. Max. coverage (+): 0. Max coverage (-): 0.08

Region: NODE\_360193\_length\_37465\_cov\_31.594688 35547-35556. Max. coverage (+): 0. Max coverage (-): 0

Region: NODE\_360193\_length\_37465\_cov\_31.594688 35557-35566. Max. coverage (+): 0. Max coverage (-): 0

Region: NODE\_360193\_length\_37465\_cov\_31.594688 35567-35576. Max. coverage (+): 0.15. Max coverage (-): 0

Region: NODE\_360193\_length\_37465\_cov\_31.594688 35577-35586. Max. coverage (+): 0. Max coverage (-): 0

Region: NODE\_360193\_length\_37465\_cov\_31.594688 35587-35596. Max. coverage (+): 0. Max coverage (-): 0

Region: NODE\_360193\_length\_37465\_cov\_31.594688 35597-35606. Max. coverage (+): 0. Max coverage (-): 0.15

Region: NODE\_360193\_length\_37465\_cov\_31.594688 35607-35616. Max. coverage (+): 0.31. Max coverage (-): 0.15

Region: NODE\_360193\_length\_37465\_cov\_31.594688 35617-35626. Max. coverage (+): 0.31. Max coverage (-): 0

Region: NODE\_360193\_length\_37465\_cov\_31.594688 35627-35636. Max. coverage (+): 0. Max coverage (-): 0.08

Region: NODE\_360193\_length\_37465\_cov\_31.594688 35637-35646. Max. coverage (+): 0. Max coverage (-): 0.08

Region: NODE\_360193\_length\_37465\_cov\_31.594688 35647-35656. Max. coverage (+): 0. Max coverage (-): 0

Region: NODE\_360193\_length\_37465\_cov\_31.594688 35657-35666. Max. coverage (+): 0. Max coverage (-): 0

Region: NODE\_360193\_length\_37465\_cov\_31.594688 35667-35676. Max. coverage (+): 0. Max coverage (-): 0

Region: NODE\_360193\_length\_37465\_cov\_31.594688 35677-35686. Max. coverage (+): 0. Max coverage (-): 0

Region: NODE\_360193\_length\_37465\_cov\_31.594688 35687-35696. Max. coverage (+): 0. Max coverage (-): 0

Region: NODE\_360193\_length\_37465\_cov\_31.594688 35697-35706. Max. coverage (+): 0.23. Max coverage (-): 0.08

Region: NODE\_360193\_length\_37465\_cov\_31.594688 35707-35716. Max. coverage (+): 0.15. Max coverage (-): 0

Region: NODE\_360193\_length\_37465\_cov\_31.594688 35717-35726. Max. coverage (+): 0. Max coverage (-): 0

Region: NODE\_360193\_length\_37465\_cov\_31.594688 35727-35735. Max. coverage (+): 0. Max coverage (-): 0

Region: NODE\_360193\_length\_37465\_cov\_31.594688 35736-35745. Max. coverage (+): 0. Max coverage (-): 0

Region: NODE\_360193\_length\_37465\_cov\_31.594688 35746-35755. Max. coverage (+): 0. Max coverage (-): 3.6

Region: NODE\_360193\_length\_37465\_cov\_31.594688 35756-35765. Max. coverage (+): 0. Max coverage (-): 2.76

Region: NODE\_360193\_length\_37465\_cov\_31.594688 35766-35775. Max. coverage (+): 0.08. Max coverage (-): 0

Region: NODE\_360193\_length\_37465\_cov\_31.594688 35776-35785. Max. coverage (+): 0.08. Max coverage (-): 0

Region: NODE\_360193\_length\_37465\_cov\_31.594688 35786-35795. Max. coverage (+): 0. Max coverage (-): 0

Region: NODE\_360193\_length\_37465\_cov\_31.594688 35796-35805. Max. coverage (+): 0. Max coverage (-): 0.77

Region: NODE\_360193\_length\_37465\_cov\_31.594688 35806-35815. Max. coverage (+): 0. Max coverage (-): 0.15

Region: NODE\_360193\_length\_37465\_cov\_31.594688 35816-35825. Max. coverage (+): 0. Max coverage (-): 0

Region: NODE\_360193\_length\_37465\_cov\_31.594688 35826-35835. Max. coverage (+): 0. Max coverage (-): 0

Region: NODE\_360193\_length\_37465\_cov\_31.594688 35836-35845. Max. coverage (+): 0. Max coverage (-): 0

Region: NODE\_360193\_length\_37465\_cov\_31.594688 35846-35855. Max. coverage (+): 0. Max coverage (-): 0

Region: NODE\_360193\_length\_37465\_cov\_31.594688 35856-35865. Max. coverage (+): 0. Max coverage (-): 2.6

Region: NODE\_360193\_length\_37465\_cov\_31.594688 35866-35875. Max. coverage (+): 0. Max coverage (-): 6.51

Region: NODE\_360193\_length\_37465\_cov\_31.594688 35876-35885. Max. coverage (+): 0. Max coverage (-): 0

Region: NODE\_360193\_length\_37465\_cov\_31.594688 35886-35895. Max. coverage (+): 0. Max coverage (-): 0.77

Region: NODE\_360193\_length\_37465\_cov\_31.594688 35896-35905. Max. coverage (+): 0. Max coverage (-): 0.38

Region: NODE\_360193\_length\_37465\_cov\_31.594688 35906-35915. Max. coverage (+): 0. Max coverage (-): 0.08

Region: NODE\_360193\_length\_37465\_cov\_31.594688 35916-35925. Max. coverage (+): 0. Max coverage (-): 0.38

Region: NODE\_360193\_length\_37465\_cov\_31.594688 35926-35935. Max. coverage (+): 0. Max coverage (-): 0.31

Region: NODE\_360193\_length\_37465\_cov\_31.594688 35936-35945. Max. coverage (+): 0. Max coverage (-): 0

Region: NODE\_360193\_length\_37465\_cov\_31.594688 35946-35955. Max. coverage (+): 0. Max coverage (-): 0

Region: NODE\_360193\_length\_37465\_cov\_31.594688 35956-35965. Max. coverage (+): 0.23. Max coverage (-): 0

Region: NODE\_360193\_length\_37465\_cov\_31.594688 35966-35975. Max. coverage (+): 0.15. Max coverage (-): 0.23

Region: NODE\_360193\_length\_37465\_cov\_31.594688 35976-35985. Max. coverage (+): 0. Max coverage (-): 0.08

Region: NODE\_360193\_length\_37465\_cov\_31.594688 35986-35995. Max. coverage (+): 0. Max coverage (-): 0

Region: NODE\_360193\_length\_37465\_cov\_31.594688 35996-36005. Max. coverage (+): 0. Max coverage (-): 0

Region: NODE\_360193\_length\_37465\_cov\_31.594688 36006-. Max. coverage (+): 0. Max coverage (-): 0

RepeatMasker Color Code

**+**

100-98% Identity

<98-95% Identity

<95-90% Identity

<90-85% Identity

<85-80% Identity

<80-75% Identity

<75-70% Identity

<70% Identity

**-**

Gene Set Color Code

**+**

Gene

Pseudogene

Other

**-**

Topology/Coverage Color Code

Coverage Plus Strand

Coverage Minus Strand

Mainstrand: Plus

Mainstrand: Minus

Complementary Strand

Flanking Region  
(if option -flank >0)

Gene Set Annotation  
  
RepeatMasker Annotation  

**1. AlRepA-66**: 31025-31090 (+), Divergence to consensus: 3%  
**2. A-rich**: 32096-32125 (+), Divergence to consensus: 15.5%  
**3. (TTA)n**: 32238-32291 (+), Divergence to consensus: 24.4%

  
Transcription Factor Binding Sites  

**RHOXF1** (Sequence: GGATCA (-): 31777)  
**RHOXF1** (Sequence: GGATCA (-): 32967)  
**RHOXF1** (Sequence: AGCTTA (-): 33039)  
**RHOXF1** (Sequence: AGCTCA (-): 33500)  
**RHOXF1** (Sequence: AGCTTA (-): 33517)  
**RHOXF1** (Sequence: AGATTA (-): 34046)  
**RHOXF1** (Sequence: GGATTA (-): 34225)  
**RHOXF1** (Sequence: AGCTTA (-): 34750)  
**RHOXF1** (Sequence: GGCTTA (-): 35810)  
**RHOXF1** (Sequence: TGAGCT (+): 31702)  
**RHOXF1** (Sequence: TAATCC (+): 31752)  
**RHOXF1** (Sequence: TGAGCT (+): 32531)  
**RHOXF1** (Sequence: TGAGCT (+): 33037)  
**RHOXF1** (Sequence: TAAGCT (+): 33515)  
**RHOXF1** (Sequence: TGAGCC (+): 34854)  
**RHOXF1** (Sequence: TGAGCT (+): 34879)  
**Gata4** (Sequence: GTTATCT (+): 32691)  
**POU5F1** (Sequence: TTTGCAT (-): 33851)  
**POU5F1** (Sequence: TTTGCAT (-): 33988)  
**POU5F1** (Sequence: TTTGCAT (-): 34216)  
**POU5F1** (Sequence: TTTGCAT (-): 34772)  
**RFX4\_2** (Sequence: GTAACTAGG (-): 32496)  
**FOXO3\_hsa** (Sequence: GTAAACAA (+): 33925)  
**SOX9** (Sequence: AACAATGG (-): 35654)  
**FOXP1** (Sequence: GTAAACA (+): 33925)  
**FOXO1** (Sequence: CCTGTTTTC (+): 31453)  
**FOXO1** (Sequence: CTTGTTTTT (+): 32270)  
**FOXO1** (Sequence: CTTGTTTAT (+): 34969)  
**FOXO3\_mmu** (Sequence: TGTTTAGC (-): 33495)  
**FOXO3\_mmu** (Sequence: TGTTTTGC (-): 34213)  
**FOXO3\_mmu** (Sequence: TGTTTTCA (-): 34439)  
**FOXO3\_mmu** (Sequence: TGTTTTGC (-): 34576)  
**Sox5** (Sequence: ATTGTT (+): 32255)  
**Sox5** (Sequence: ATTGTT (+): 32258)  
**Sox5** (Sequence: ATTGTT (+): 32261)  
**Sox5** (Sequence: ATTGTT (+): 32264)  
**Sox5** (Sequence: ATTGTT (+): 32325)  
**Sox5** (Sequence: ATTGTT (+): 33173)  
**Sox5** (Sequence: ATTGTT (+): 34387)  
**Sox5** (Sequence: ATTGTT (+): 35131)  
**FIGLA** (Sequence: TACACCTGTT (-): 32985)  
**SOX9** (Sequence: TTATTGTT (+): 32253)  
**SOX9** (Sequence: CTATTGTT (+): 33171)  
**FOXO3\_mmu** (Sequence: TGTAAACA (+): 33924)  
**FOXO3\_mmu** (Sequence: GGAAAACA (+): 35571)  
**Nobox** (Sequence: GGTAATTA (-): 34670)  
**Nobox** (Sequence: GCCAATTA (-): 34958)  
**Nobox** (Sequence: TAATTACC (+): 31403)  
**Nobox** (Sequence: TAATTACT (+): 31673)  
**Rhox11** (Sequence: TGGTGTTTA (+): 32399)  
**Rhox11** (Sequence: TGGTGTTAA (+): 33726)  
**Rhox11** (Sequence: ATAACAGCA (-): 34270)  
**Gata4** (Sequence: AGATAAG (-): 33216)  
**Gata4** (Sequence: AGATAAC (-): 34268)  
**Sox5** (Sequence: AACAAT (-): 34147)  
**Sox5** (Sequence: AACAAT (-): 35489)  
**Sox5** (Sequence: AACAAT (-): 35654)  
**POU5F1** (Sequence: ATGCAAA (+): 33471)
