# Supplementary material for: First characterization of PIWI-interacting RNA clusters in a cichlid fish with a B chromosome
Source: BMC Biol. 2022 Sep 21;20:204. doi: 10.1186/s12915-022-01403-2 (PMC9490952; doi:10.1186/s12915-022-01403-2)
Supplement: Supplementary file 1 — Additional file 1. Zipped folder with fasta and interactive html piRNA cluster information for the A. latifasciata genome. The nomenclature is as follows: number-pirna-cluster_sex_B-presence (f, female; m, male; 0b, without B chromosome; 1b, with B chromosome). [file 12915_2022_1403_MOESM1_ESM.zip › 13_f0b.html]

piRNA cluster 13\_f0b 28


Predicted piRNA cluster no. 13\_f0b
  

Show proTRAC run info
Hide proTRAC run info

/\  
                \_\_\_\_\_\_\_\_\_\_\_\_\_\_\_\_\_\_\_\_\_\_\_/\\_\_\_ /  \\_\_\_\_\_\_\_  
               I                      /  \  /    \      I  
               I     pro             /    \/      \     I  
               I        TRAC        /               \   I  
               I   \_\_\_\_\_\_\_\_\_\_\_\_\_\_\_\_/\_\_\_\_\_\_\_\_\_\_\_\_\_\_\_\_\_\\_ I  
               I   \              /                     I  
               I    \            /                      I  
               I     \  /\      /       V.2.4.2         I  
               I      \/  \    /                        I  
               I\_\_\_\_\_\_\_\_\_\_\_\  /\_\_\_\_\_\_\_\_\_\_\_\_\_\_\_\_\_\_\_\_\_\_\_\_\_I  
                            \/  
  
  
================================= proTRAC ====================================  
VERSION: .......... 2.4.2  
LAST MODIFIED: .... 11. May 2018  
  
Please cite:  
Rosenkranz D, Zischler H. proTRAC - a software for probabilistic piRNA cluster  
detection, visualization and analysis. 2012. BMC Bioinformatics 13:5.  
  
  
Contact:  
David Rosenkranz  
Institute of Organismic and Molecular Evolutionary Biology  
Dept. Anthropology, small RNA group  
Johannes Gutenberg University Mainz  
email: rosenkranz@uni-mainz.de  
  
You can find the latest proTRAC version at:  
http://sourceforge.net/projects/protrac/files  
http://www.smallRNAgroup-mainz.de/software  
==============================================================================  
  
PARAMETERS:  
Map file: ...............piwi-femeas-0B.fa-collapse.map  
Genome file: ............../../../0B\_ala\_genome.fa  
RepeatMasker annotation: Alatifasciata-all0B-maryan-v2.fa\_corrected.out  
GeneSet:................./guest-storage/Data/annotation/Alatifasciata\_all0B\_maryan-v2\_out2017.gff  
  
Significant (p<=0.01) hit density will be calculated based  
on observed hit distribution.  
  
Sliding window size: ........................................ 5000 bp  
Sliding window increament: .................................. 1000 bp  
Normalize each hit by number of genomic hits: ............... yes  
Normalize each hit by number of sequence reads: ............. yes  
Normalize values (-> per million mapped reads): ............. yes  
Min. fraction of hits with 1T(U) or 10A: .................... 0.75  
Alternatively: Min. fraction of hits with 1T(U) and 10A: .... 0.5  
Min. fraction of hits with typical piRNA length: ............ 0.75  
Typical piRNA length: ....................................... 24-32 nt  
Min. size of a piRNA cluster: ............................... 1000 bp.  
Min. number of hits (absolute): ............................. 0  
Min. number of hits (normalized): ........................... 0  
Min. fraction of hits on the mainstrand: .................... 0.75  
Top fraction of mapped sequences (in terms of read counts): . 1%  
Top fraction accounts for max. n% of sequence reads: ........ 90%  
Min. fraction of hits on each arm of a bidirectional cluster: 0.05  
Output html file for each cluster: .......................... yes  
Output a summary table: ..................................... yes  
Output a FASTA file for each cluster (piRNA sequences): ..... yes  
Output a FASTA file comprising cluster sequences: ........... yes  
Output a GTF file for predicted piRNA clusters: ..............yes  
Search DNA motifs in clusters: .............................. yes  
Output flanking sequences: +/- .............................. 0 bp  
Output ~.pTi file: .......................................... no  
==============================================================================  
  
  
Genome size (without gaps): ............ 758543724 bp  
Gaps (N/X/-): .......................... 417479 bp  
Mapped reads: .......................... 13052187  
Non-identical sequences: ............... 3338911  
Genomic hits: .......................... 28737726  
Significant densitiy of mapped reads: .. 470.083249848448 reads/kb

Show proTRAC cluster info
Hide proTRAC cluster info

|  |  |
| --- | --- |
| Location | NODE\_121434\_length\_5572\_cov\_28.541996 |
| Coordinates | 2-5686 |
| Size [bp] | 5685 |
| Sequence hit loci | 2252 |
| Mapped reads (normalized) | 6815.7 |
| Mapped reads (normalized) per kb | 1198.9 |
| Normalized reads with 1T (1U) | 75.2% |
| Normalized reads with 10A | 59.2% |
| Normalized reads with length 24-32 nt | 98.5% |
| Normalized reads on the main strand(s) | 81.4% |
| Predicted directionality | mono:minus |

100%

0%

1T (1U)  
reads

10A reads

24-32 nt  
reads

reads on mainstrand

**Either the amount of reads with 1T (1U) OR 10A has to exceed 75% (set with option: -1Tor10A)  
Alternatively the amount of reads with 1T (1U) AND 10A has to exceed 50% (set with option: -1Tand10A)  
Minimum amount of reads with preferred size is 75% (set with option: -pisize)  
Minimum amount of reads on the main strand(s) is 75% (set with option: -clstrand)**

Show read coverage
Hide read coverage

WHAT DO I SEE HERE?  
This chart shows the location of mapped sequence reads within a predicted piRNA cluster. The color refers to the number of genomic hits produced by the sequence read in question. A dark red bar indicates that this sequence read produces many other hits elsewhere in the genome. Many adjacent red or yellow bars can indicate the presence of a multi-copy element such as transposons or rRNA genes. A dark green bar indicates that this sequence read maps uniquely to this locus.

1 hit

2-5 hits

6-10 hits

11-20 hits

21-50 hits

51-100 hits

> 100 hits

NODE\_121434\_length\_5572\_cov\_28.541996

2

5686

Gene Set

RepeatMasker

Mapped  
Reads

30.88

plus strand

minus strand

30.88

Region: NODE\_121434\_length\_5572\_cov\_28.541996 1655-7. Max. coverage (+): 0.08. Max coverage (-): 0.04

Region: NODE\_121434\_length\_5572\_cov\_28.541996 8-19. Max. coverage (+): 0.04. Max coverage (-): 0.04

Region: NODE\_121434\_length\_5572\_cov\_28.541996 20-30. Max. coverage (+): 0.04. Max coverage (-): 0

Region: NODE\_121434\_length\_5572\_cov\_28.541996 31-41. Max. coverage (+): 0. Max coverage (-): 0.15

Region: NODE\_121434\_length\_5572\_cov\_28.541996 42-53. Max. coverage (+): 0. Max coverage (-): 0

Region: NODE\_121434\_length\_5572\_cov\_28.541996 54-64. Max. coverage (+): 0. Max coverage (-): 0.04

Region: NODE\_121434\_length\_5572\_cov\_28.541996 65-75. Max. coverage (+): 0.34. Max coverage (-): 0.04

Region: NODE\_121434\_length\_5572\_cov\_28.541996 76-87. Max. coverage (+): 0.11. Max coverage (-): 0.08

Region: NODE\_121434\_length\_5572\_cov\_28.541996 88-98. Max. coverage (+): 0. Max coverage (-): 0.11

Region: NODE\_121434\_length\_5572\_cov\_28.541996 99-110. Max. coverage (+): 0. Max coverage (-): 5.02

Region: NODE\_121434\_length\_5572\_cov\_28.541996 111-121. Max. coverage (+): 0. Max coverage (-): 0.19

Region: NODE\_121434\_length\_5572\_cov\_28.541996 122-132. Max. coverage (+): 0. Max coverage (-): 0.11

Region: NODE\_121434\_length\_5572\_cov\_28.541996 133-144. Max. coverage (+): 0. Max coverage (-): 0

Region: NODE\_121434\_length\_5572\_cov\_28.541996 145-155. Max. coverage (+): 0. Max coverage (-): 0

Region: NODE\_121434\_length\_5572\_cov\_28.541996 156-166. Max. coverage (+): 0. Max coverage (-): 0

Region: NODE\_121434\_length\_5572\_cov\_28.541996 167-178. Max. coverage (+): 0. Max coverage (-): 0

Region: NODE\_121434\_length\_5572\_cov\_28.541996 179-189. Max. coverage (+): 0. Max coverage (-): 0

Region: NODE\_121434\_length\_5572\_cov\_28.541996 190-200. Max. coverage (+): 0.46. Max coverage (-): 0

Region: NODE\_121434\_length\_5572\_cov\_28.541996 201-212. Max. coverage (+): 0.38. Max coverage (-): 0.15

Region: NODE\_121434\_length\_5572\_cov\_28.541996 213-223. Max. coverage (+): 0.15. Max coverage (-): 0.38

Region: NODE\_121434\_length\_5572\_cov\_28.541996 224-235. Max. coverage (+): 0.15. Max coverage (-): 0

Region: NODE\_121434\_length\_5572\_cov\_28.541996 236-246. Max. coverage (+): 0. Max coverage (-): 0.69

Region: NODE\_121434\_length\_5572\_cov\_28.541996 247-257. Max. coverage (+): 0. Max coverage (-): 0.38

Region: NODE\_121434\_length\_5572\_cov\_28.541996 258-269. Max. coverage (+): 0. Max coverage (-): 0.08

Region: NODE\_121434\_length\_5572\_cov\_28.541996 270-280. Max. coverage (+): 0.23. Max coverage (-): 0.31

Region: NODE\_121434\_length\_5572\_cov\_28.541996 281-291. Max. coverage (+): 0.23. Max coverage (-): 0.31

Region: NODE\_121434\_length\_5572\_cov\_28.541996 292-303. Max. coverage (+): 0.11. Max coverage (-): 0.04

Region: NODE\_121434\_length\_5572\_cov\_28.541996 304-314. Max. coverage (+): 0.04. Max coverage (-): 0.04

Region: NODE\_121434\_length\_5572\_cov\_28.541996 315-326. Max. coverage (+): 0. Max coverage (-): 0.23

Region: NODE\_121434\_length\_5572\_cov\_28.541996 327-337. Max. coverage (+): 0. Max coverage (-): 0

Region: NODE\_121434\_length\_5572\_cov\_28.541996 338-348. Max. coverage (+): 0. Max coverage (-): 0

Region: NODE\_121434\_length\_5572\_cov\_28.541996 349-360. Max. coverage (+): 0. Max coverage (-): 0

Region: NODE\_121434\_length\_5572\_cov\_28.541996 361-371. Max. coverage (+): 0.08. Max coverage (-): 2.41

Region: NODE\_121434\_length\_5572\_cov\_28.541996 372-382. Max. coverage (+): 0.34. Max coverage (-): 0.46

Region: NODE\_121434\_length\_5572\_cov\_28.541996 383-394. Max. coverage (+): 0.11. Max coverage (-): 0.11

Region: NODE\_121434\_length\_5572\_cov\_28.541996 395-405. Max. coverage (+): 0. Max coverage (-): 0.31

Region: NODE\_121434\_length\_5572\_cov\_28.541996 406-417. Max. coverage (+): 0. Max coverage (-): 5.02

Region: NODE\_121434\_length\_5572\_cov\_28.541996 418-428. Max. coverage (+): 0. Max coverage (-): 0.11

Region: NODE\_121434\_length\_5572\_cov\_28.541996 429-439. Max. coverage (+): 0. Max coverage (-): 0.23

Region: NODE\_121434\_length\_5572\_cov\_28.541996 440-451. Max. coverage (+): 0.23. Max coverage (-): 6.51

Region: NODE\_121434\_length\_5572\_cov\_28.541996 452-462. Max. coverage (+): 0.08. Max coverage (-): 6.59

Region: NODE\_121434\_length\_5572\_cov\_28.541996 463-473. Max. coverage (+): 0.08. Max coverage (-): 0.31

Region: NODE\_121434\_length\_5572\_cov\_28.541996 474-485. Max. coverage (+): 0. Max coverage (-): 0.15

Region: NODE\_121434\_length\_5572\_cov\_28.541996 486-496. Max. coverage (+): 0. Max coverage (-): 0.38

Region: NODE\_121434\_length\_5572\_cov\_28.541996 497-507. Max. coverage (+): 0. Max coverage (-): 0.61

Region: NODE\_121434\_length\_5572\_cov\_28.541996 508-519. Max. coverage (+): 0.15. Max coverage (-): 0.08

Region: NODE\_121434\_length\_5572\_cov\_28.541996 520-530. Max. coverage (+): 0. Max coverage (-): 0

Region: NODE\_121434\_length\_5572\_cov\_28.541996 531-542. Max. coverage (+): 0. Max coverage (-): 0

Region: NODE\_121434\_length\_5572\_cov\_28.541996 543-553. Max. coverage (+): 0. Max coverage (-): 0

Region: NODE\_121434\_length\_5572\_cov\_28.541996 554-564. Max. coverage (+): 0. Max coverage (-): 0.54

Region: NODE\_121434\_length\_5572\_cov\_28.541996 565-576. Max. coverage (+): 0. Max coverage (-): 0.08

Region: NODE\_121434\_length\_5572\_cov\_28.541996 577-587. Max. coverage (+): 0. Max coverage (-): 0.31

Region: NODE\_121434\_length\_5572\_cov\_28.541996 588-598. Max. coverage (+): 0. Max coverage (-): 0.46

Region: NODE\_121434\_length\_5572\_cov\_28.541996 599-610. Max. coverage (+): 0. Max coverage (-): 0.46

Region: NODE\_121434\_length\_5572\_cov\_28.541996 611-621. Max. coverage (+): 1.3. Max coverage (-): 0.15

Region: NODE\_121434\_length\_5572\_cov\_28.541996 622-633. Max. coverage (+): 1.38. Max coverage (-): 12.26

Region: NODE\_121434\_length\_5572\_cov\_28.541996 634-644. Max. coverage (+): 0.08. Max coverage (-): 12.41

Region: NODE\_121434\_length\_5572\_cov\_28.541996 645-655. Max. coverage (+): 0.23. Max coverage (-): 0.31

Region: NODE\_121434\_length\_5572\_cov\_28.541996 656-667. Max. coverage (+): 0.23. Max coverage (-): 1.69

Region: NODE\_121434\_length\_5572\_cov\_28.541996 668-678. Max. coverage (+): 0. Max coverage (-): 0

Region: NODE\_121434\_length\_5572\_cov\_28.541996 679-689. Max. coverage (+): 0.08. Max coverage (-): 0.46

Region: NODE\_121434\_length\_5572\_cov\_28.541996 690-701. Max. coverage (+): 0.08. Max coverage (-): 3.83

Region: NODE\_121434\_length\_5572\_cov\_28.541996 702-712. Max. coverage (+): 0. Max coverage (-): 0

Region: NODE\_121434\_length\_5572\_cov\_28.541996 713-723. Max. coverage (+): 0. Max coverage (-): 0.08

Region: NODE\_121434\_length\_5572\_cov\_28.541996 724-735. Max. coverage (+): 0. Max coverage (-): 0

Region: NODE\_121434\_length\_5572\_cov\_28.541996 736-746. Max. coverage (+): 0. Max coverage (-): 1.38

Region: NODE\_121434\_length\_5572\_cov\_28.541996 747-758. Max. coverage (+): 0.61. Max coverage (-): 0.23

Region: NODE\_121434\_length\_5572\_cov\_28.541996 759-769. Max. coverage (+): 0.61. Max coverage (-): 0.46

Region: NODE\_121434\_length\_5572\_cov\_28.541996 770-780. Max. coverage (+): 0. Max coverage (-): 0.31

Region: NODE\_121434\_length\_5572\_cov\_28.541996 781-792. Max. coverage (+): 0. Max coverage (-): 0.08

Region: NODE\_121434\_length\_5572\_cov\_28.541996 793-803. Max. coverage (+): 0. Max coverage (-): 0

Region: NODE\_121434\_length\_5572\_cov\_28.541996 804-814. Max. coverage (+): 0. Max coverage (-): 0

Region: NODE\_121434\_length\_5572\_cov\_28.541996 815-826. Max. coverage (+): 0. Max coverage (-): 0

Region: NODE\_121434\_length\_5572\_cov\_28.541996 827-837. Max. coverage (+): 0. Max coverage (-): 0.23

Region: NODE\_121434\_length\_5572\_cov\_28.541996 838-849. Max. coverage (+): 1.3. Max coverage (-): 0.15

Region: NODE\_121434\_length\_5572\_cov\_28.541996 850-860. Max. coverage (+): 0.54. Max coverage (-): 0.08

Region: NODE\_121434\_length\_5572\_cov\_28.541996 861-871. Max. coverage (+): 0.15. Max coverage (-): 4.44

Region: NODE\_121434\_length\_5572\_cov\_28.541996 872-883. Max. coverage (+): 0.08. Max coverage (-): 0

Region: NODE\_121434\_length\_5572\_cov\_28.541996 884-894. Max. coverage (+): 0.08. Max coverage (-): 0.08

Region: NODE\_121434\_length\_5572\_cov\_28.541996 895-905. Max. coverage (+): 0. Max coverage (-): 0.31

Region: NODE\_121434\_length\_5572\_cov\_28.541996 906-917. Max. coverage (+): 0. Max coverage (-): 0.23

Region: NODE\_121434\_length\_5572\_cov\_28.541996 918-928. Max. coverage (+): 1.46. Max coverage (-): 0.31

Region: NODE\_121434\_length\_5572\_cov\_28.541996 929-940. Max. coverage (+): 1.38. Max coverage (-): 0.31

Region: NODE\_121434\_length\_5572\_cov\_28.541996 941-951. Max. coverage (+): 0. Max coverage (-): 0

Region: NODE\_121434\_length\_5572\_cov\_28.541996 952-962. Max. coverage (+): 0. Max coverage (-): 1.38

Region: NODE\_121434\_length\_5572\_cov\_28.541996 963-974. Max. coverage (+): 0. Max coverage (-): 0.38

Region: NODE\_121434\_length\_5572\_cov\_28.541996 975-985. Max. coverage (+): 0.08. Max coverage (-): 2.38

Region: NODE\_121434\_length\_5572\_cov\_28.541996 986-996. Max. coverage (+): 0. Max coverage (-): 0.46

Region: NODE\_121434\_length\_5572\_cov\_28.541996 997-1008. Max. coverage (+): 0.92. Max coverage (-): 0.08

Region: NODE\_121434\_length\_5572\_cov\_28.541996 1009-1019. Max. coverage (+): 0.31. Max coverage (-): 0.69

Region: NODE\_121434\_length\_5572\_cov\_28.541996 1020-1030. Max. coverage (+): 0.61. Max coverage (-): 4.44

Region: NODE\_121434\_length\_5572\_cov\_28.541996 1031-1042. Max. coverage (+): 0.69. Max coverage (-): 1.15

Region: NODE\_121434\_length\_5572\_cov\_28.541996 1043-1053. Max. coverage (+): 0. Max coverage (-): 1.53

Region: NODE\_121434\_length\_5572\_cov\_28.541996 1054-1065. Max. coverage (+): 0.23. Max coverage (-): 0.15

Region: NODE\_121434\_length\_5572\_cov\_28.541996 1066-1076. Max. coverage (+): 0.23. Max coverage (-): 0

Region: NODE\_121434\_length\_5572\_cov\_28.541996 1077-1087. Max. coverage (+): 0. Max coverage (-): 2.3

Region: NODE\_121434\_length\_5572\_cov\_28.541996 1088-1099. Max. coverage (+): 0. Max coverage (-): 0.92

Region: NODE\_121434\_length\_5572\_cov\_28.541996 1100-1110. Max. coverage (+): 0.38. Max coverage (-): 0.84

Region: NODE\_121434\_length\_5572\_cov\_28.541996 1111-1121. Max. coverage (+): 0.08. Max coverage (-): 0.23

Region: NODE\_121434\_length\_5572\_cov\_28.541996 1122-1133. Max. coverage (+): 0.08. Max coverage (-): 0.15

Region: NODE\_121434\_length\_5572\_cov\_28.541996 1134-1144. Max. coverage (+): 0.08. Max coverage (-): 0.92

Region: NODE\_121434\_length\_5572\_cov\_28.541996 1145-1156. Max. coverage (+): 0.38. Max coverage (-): 0.08

Region: NODE\_121434\_length\_5572\_cov\_28.541996 1157-1167. Max. coverage (+): 0.08. Max coverage (-): 2.22

Region: NODE\_121434\_length\_5572\_cov\_28.541996 1168-1178. Max. coverage (+): 0. Max coverage (-): 5.98

Region: NODE\_121434\_length\_5572\_cov\_28.541996 1179-1190. Max. coverage (+): 1. Max coverage (-): 0.46

Region: NODE\_121434\_length\_5572\_cov\_28.541996 1191-1201. Max. coverage (+): 0.08. Max coverage (-): 1.07

Region: NODE\_121434\_length\_5572\_cov\_28.541996 1202-1212. Max. coverage (+): 0.08. Max coverage (-): 6.67

Region: NODE\_121434\_length\_5572\_cov\_28.541996 1213-1224. Max. coverage (+): 0. Max coverage (-): 6.59

Region: NODE\_121434\_length\_5572\_cov\_28.541996 1225-1235. Max. coverage (+): 0.15. Max coverage (-): 0.23

Region: NODE\_121434\_length\_5572\_cov\_28.541996 1236-1247. Max. coverage (+): 2.83. Max coverage (-): 0.08

Region: NODE\_121434\_length\_5572\_cov\_28.541996 1248-1258. Max. coverage (+): 0. Max coverage (-): 0.69

Region: NODE\_121434\_length\_5572\_cov\_28.541996 1259-1269. Max. coverage (+): 0. Max coverage (-): 0.08

Region: NODE\_121434\_length\_5572\_cov\_28.541996 1270-1281. Max. coverage (+): 0.23. Max coverage (-): 0.08

Region: NODE\_121434\_length\_5572\_cov\_28.541996 1282-1292. Max. coverage (+): 0. Max coverage (-): 3.37

Region: NODE\_121434\_length\_5572\_cov\_28.541996 1293-1303. Max. coverage (+): 1.46. Max coverage (-): 0

Region: NODE\_121434\_length\_5572\_cov\_28.541996 1304-1315. Max. coverage (+): 1.46. Max coverage (-): 0.08

Region: NODE\_121434\_length\_5572\_cov\_28.541996 1316-1326. Max. coverage (+): 0. Max coverage (-): 2.22

Region: NODE\_121434\_length\_5572\_cov\_28.541996 1327-1337. Max. coverage (+): 0. Max coverage (-): 2.76

Region: NODE\_121434\_length\_5572\_cov\_28.541996 1338-1349. Max. coverage (+): 0.61. Max coverage (-): 0

Region: NODE\_121434\_length\_5572\_cov\_28.541996 1350-1360. Max. coverage (+): 0. Max coverage (-): 2.3

Region: NODE\_121434\_length\_5572\_cov\_28.541996 1361-1372. Max. coverage (+): 0. Max coverage (-): 0.61

Region: NODE\_121434\_length\_5572\_cov\_28.541996 1373-1383. Max. coverage (+): 0.38. Max coverage (-): 0

Region: NODE\_121434\_length\_5572\_cov\_28.541996 1384-1394. Max. coverage (+): 0. Max coverage (-): 24.98

Region: NODE\_121434\_length\_5572\_cov\_28.541996 1395-1406. Max. coverage (+): 0. Max coverage (-): 23.21

Region: NODE\_121434\_length\_5572\_cov\_28.541996 1407-1417. Max. coverage (+): 2.83. Max coverage (-): 0

Region: NODE\_121434\_length\_5572\_cov\_28.541996 1418-1428. Max. coverage (+): 0. Max coverage (-): 0.08

Region: NODE\_121434\_length\_5572\_cov\_28.541996 1429-1440. Max. coverage (+): 0.15. Max coverage (-): 0

Region: NODE\_121434\_length\_5572\_cov\_28.541996 1441-1451. Max. coverage (+): 0.69. Max coverage (-): 0.08

Region: NODE\_121434\_length\_5572\_cov\_28.541996 1452-1463. Max. coverage (+): 0.08. Max coverage (-): 0.38

Region: NODE\_121434\_length\_5572\_cov\_28.541996 1464-1474. Max. coverage (+): 0.92. Max coverage (-): 0.23

Region: NODE\_121434\_length\_5572\_cov\_28.541996 1475-1485. Max. coverage (+): 0.08. Max coverage (-): 1.92

Region: NODE\_121434\_length\_5572\_cov\_28.541996 1486-1497. Max. coverage (+): 0. Max coverage (-): 7.28

Region: NODE\_121434\_length\_5572\_cov\_28.541996 1498-1508. Max. coverage (+): 0.61. Max coverage (-): 0.23

Region: NODE\_121434\_length\_5572\_cov\_28.541996 1509-1519. Max. coverage (+): 0.08. Max coverage (-): 3.75

Region: NODE\_121434\_length\_5572\_cov\_28.541996 1520-1531. Max. coverage (+): 0.46. Max coverage (-): 0.31

Region: NODE\_121434\_length\_5572\_cov\_28.541996 1532-1542. Max. coverage (+): 0.38. Max coverage (-): 0.08

Region: NODE\_121434\_length\_5572\_cov\_28.541996 1543-1554. Max. coverage (+): 0.15. Max coverage (-): 1.61

Region: NODE\_121434\_length\_5572\_cov\_28.541996 1555-1565. Max. coverage (+): 0.23. Max coverage (-): 1.53

Region: NODE\_121434\_length\_5572\_cov\_28.541996 1566-1576. Max. coverage (+): 1. Max coverage (-): 0.15

Region: NODE\_121434\_length\_5572\_cov\_28.541996 1577-1588. Max. coverage (+): 0. Max coverage (-): 0.84

Region: NODE\_121434\_length\_5572\_cov\_28.541996 1589-1599. Max. coverage (+): 0. Max coverage (-): 0.46

Region: NODE\_121434\_length\_5572\_cov\_28.541996 1600-1610. Max. coverage (+): 0. Max coverage (-): 0.08

Region: NODE\_121434\_length\_5572\_cov\_28.541996 1611-1622. Max. coverage (+): 1. Max coverage (-): 0.08

Region: NODE\_121434\_length\_5572\_cov\_28.541996 1623-1633. Max. coverage (+): 0.08. Max coverage (-): 0.23

Region: NODE\_121434\_length\_5572\_cov\_28.541996 1634-1644. Max. coverage (+): 0.08. Max coverage (-): 15.86

Region: NODE\_121434\_length\_5572\_cov\_28.541996 1645-1656. Max. coverage (+): 0.61. Max coverage (-): 0.77

Region: NODE\_121434\_length\_5572\_cov\_28.541996 1657-1667. Max. coverage (+): 0.61. Max coverage (-): 0

Region: NODE\_121434\_length\_5572\_cov\_28.541996 1668-1679. Max. coverage (+): 0. Max coverage (-): 0

Region: NODE\_121434\_length\_5572\_cov\_28.541996 1680-1690. Max. coverage (+): 0. Max coverage (-): 0.15

Region: NODE\_121434\_length\_5572\_cov\_28.541996 1691-1701. Max. coverage (+): 0. Max coverage (-): 6.82

Region: NODE\_121434\_length\_5572\_cov\_28.541996 1702-1713. Max. coverage (+): 0.08. Max coverage (-): 6.74

Region: NODE\_121434\_length\_5572\_cov\_28.541996 1714-1724. Max. coverage (+): 0.23. Max coverage (-): 0.08

Region: NODE\_121434\_length\_5572\_cov\_28.541996 1725-1735. Max. coverage (+): 0. Max coverage (-): 0

Region: NODE\_121434\_length\_5572\_cov\_28.541996 1736-1747. Max. coverage (+): 0. Max coverage (-): 0

Region: NODE\_121434\_length\_5572\_cov\_28.541996 1748-1758. Max. coverage (+): 0. Max coverage (-): 0

Region: NODE\_121434\_length\_5572\_cov\_28.541996 1759-1770. Max. coverage (+): 0. Max coverage (-): 0.08

Region: NODE\_121434\_length\_5572\_cov\_28.541996 1771-1781. Max. coverage (+): 0.15. Max coverage (-): 0.08

Region: NODE\_121434\_length\_5572\_cov\_28.541996 1782-1792. Max. coverage (+): 1.23. Max coverage (-): 0.08

Region: NODE\_121434\_length\_5572\_cov\_28.541996 1793-1804. Max. coverage (+): 0.69. Max coverage (-): 0.31

Region: NODE\_121434\_length\_5572\_cov\_28.541996 1805-1815. Max. coverage (+): 0.08. Max coverage (-): 0.77

Region: NODE\_121434\_length\_5572\_cov\_28.541996 1816-1826. Max. coverage (+): 0.08. Max coverage (-): 0.08

Region: NODE\_121434\_length\_5572\_cov\_28.541996 1827-1838. Max. coverage (+): 0.15. Max coverage (-): 0

Region: NODE\_121434\_length\_5572\_cov\_28.541996 1839-1849. Max. coverage (+): 0. Max coverage (-): 0

Region: NODE\_121434\_length\_5572\_cov\_28.541996 1850-1860. Max. coverage (+): 1.07. Max coverage (-): 0

Region: NODE\_121434\_length\_5572\_cov\_28.541996 1861-1872. Max. coverage (+): 1.07. Max coverage (-): 0

Region: NODE\_121434\_length\_5572\_cov\_28.541996 1873-1883. Max. coverage (+): 0. Max coverage (-): 0.15

Region: NODE\_121434\_length\_5572\_cov\_28.541996 1884-1895. Max. coverage (+): 0. Max coverage (-): 0.54

Region: NODE\_121434\_length\_5572\_cov\_28.541996 1896-1906. Max. coverage (+): 0.15. Max coverage (-): 0

Region: NODE\_121434\_length\_5572\_cov\_28.541996 1907-1917. Max. coverage (+): 0. Max coverage (-): 1.92

Region: NODE\_121434\_length\_5572\_cov\_28.541996 1918-1929. Max. coverage (+): 0. Max coverage (-): 5.21

Region: NODE\_121434\_length\_5572\_cov\_28.541996 1930-1940. Max. coverage (+): 0.23. Max coverage (-): 1

Region: NODE\_121434\_length\_5572\_cov\_28.541996 1941-1951. Max. coverage (+): 1.69. Max coverage (-): 0.23

Region: NODE\_121434\_length\_5572\_cov\_28.541996 1952-1963. Max. coverage (+): 0.69. Max coverage (-): 13.87

Region: NODE\_121434\_length\_5572\_cov\_28.541996 1964-1974. Max. coverage (+): 0. Max coverage (-): 0

Region: NODE\_121434\_length\_5572\_cov\_28.541996 1975-1986. Max. coverage (+): 0.15. Max coverage (-): 1.38

Region: NODE\_121434\_length\_5572\_cov\_28.541996 1987-1997. Max. coverage (+): 0.15. Max coverage (-): 1.38

Region: NODE\_121434\_length\_5572\_cov\_28.541996 1998-2008. Max. coverage (+): 0.31. Max coverage (-): 6.13

Region: NODE\_121434\_length\_5572\_cov\_28.541996 2009-2020. Max. coverage (+): 5.59. Max coverage (-): 0.46

Region: NODE\_121434\_length\_5572\_cov\_28.541996 2021-2031. Max. coverage (+): 5.36. Max coverage (-): 0

Region: NODE\_121434\_length\_5572\_cov\_28.541996 2032-2042. Max. coverage (+): 0.08. Max coverage (-): 0.38

Region: NODE\_121434\_length\_5572\_cov\_28.541996 2043-2054. Max. coverage (+): 0. Max coverage (-): 0.23

Region: NODE\_121434\_length\_5572\_cov\_28.541996 2055-2065. Max. coverage (+): 0.15. Max coverage (-): 0.08

Region: NODE\_121434\_length\_5572\_cov\_28.541996 2066-2077. Max. coverage (+): 0.23. Max coverage (-): 0.31

Region: NODE\_121434\_length\_5572\_cov\_28.541996 2078-2088. Max. coverage (+): 0.84. Max coverage (-): 0.61

Region: NODE\_121434\_length\_5572\_cov\_28.541996 2089-2099. Max. coverage (+): 0.69. Max coverage (-): 0.31

Region: NODE\_121434\_length\_5572\_cov\_28.541996 2100-2111. Max. coverage (+): 0. Max coverage (-): 0.61

Region: NODE\_121434\_length\_5572\_cov\_28.541996 2112-2122. Max. coverage (+): 0. Max coverage (-): 30.88

Region: NODE\_121434\_length\_5572\_cov\_28.541996 2123-2133. Max. coverage (+): 0. Max coverage (-): 1.84

Region: NODE\_121434\_length\_5572\_cov\_28.541996 2134-2145. Max. coverage (+): 1.23. Max coverage (-): 1.61

Region: NODE\_121434\_length\_5572\_cov\_28.541996 2146-2156. Max. coverage (+): 0.08. Max coverage (-): 0.08

Region: NODE\_121434\_length\_5572\_cov\_28.541996 2157-2167. Max. coverage (+): 0. Max coverage (-): 0.38

Region: NODE\_121434\_length\_5572\_cov\_28.541996 2168-2179. Max. coverage (+): 0. Max coverage (-): 0.69

Region: NODE\_121434\_length\_5572\_cov\_28.541996 2180-2190. Max. coverage (+): 2.68. Max coverage (-): 0

Region: NODE\_121434\_length\_5572\_cov\_28.541996 2191-2202. Max. coverage (+): 0.08. Max coverage (-): 0.31

Region: NODE\_121434\_length\_5572\_cov\_28.541996 2203-2213. Max. coverage (+): 0.08. Max coverage (-): 0

Region: NODE\_121434\_length\_5572\_cov\_28.541996 2214-2224. Max. coverage (+): 0. Max coverage (-): 0

Region: NODE\_121434\_length\_5572\_cov\_28.541996 2225-2236. Max. coverage (+): 0.08. Max coverage (-): 0.31

Region: NODE\_121434\_length\_5572\_cov\_28.541996 2237-2247. Max. coverage (+): 0. Max coverage (-): 0.23

Region: NODE\_121434\_length\_5572\_cov\_28.541996 2248-2258. Max. coverage (+): 1.15. Max coverage (-): 0.69

Region: NODE\_121434\_length\_5572\_cov\_28.541996 2259-2270. Max. coverage (+): 0. Max coverage (-): 0.31

Region: NODE\_121434\_length\_5572\_cov\_28.541996 2271-2281. Max. coverage (+): 0. Max coverage (-): 0

Region: NODE\_121434\_length\_5572\_cov\_28.541996 2282-2293. Max. coverage (+): 0.15. Max coverage (-): 3.75

Region: NODE\_121434\_length\_5572\_cov\_28.541996 2294-2304. Max. coverage (+): 0. Max coverage (-): 0

Region: NODE\_121434\_length\_5572\_cov\_28.541996 2305-2315. Max. coverage (+): 0. Max coverage (-): 0.31

Region: NODE\_121434\_length\_5572\_cov\_28.541996 2316-2327. Max. coverage (+): 0. Max coverage (-): 1

Region: NODE\_121434\_length\_5572\_cov\_28.541996 2328-2338. Max. coverage (+): 0.08. Max coverage (-): 4.06

Region: NODE\_121434\_length\_5572\_cov\_28.541996 2339-2349. Max. coverage (+): 0.23. Max coverage (-): 2.45

Region: NODE\_121434\_length\_5572\_cov\_28.541996 2350-2361. Max. coverage (+): 1.3. Max coverage (-): 0.23

Region: NODE\_121434\_length\_5572\_cov\_28.541996 2362-2372. Max. coverage (+): 0.15. Max coverage (-): 4.75

Region: NODE\_121434\_length\_5572\_cov\_28.541996 2373-2384. Max. coverage (+): 0.08. Max coverage (-): 0.23

Region: NODE\_121434\_length\_5572\_cov\_28.541996 2385-2395. Max. coverage (+): 0.38. Max coverage (-): 1.38

Region: NODE\_121434\_length\_5572\_cov\_28.541996 2396-2406. Max. coverage (+): 0. Max coverage (-): 0

Region: NODE\_121434\_length\_5572\_cov\_28.541996 2407-2418. Max. coverage (+): 0.08. Max coverage (-): 1.61

Region: NODE\_121434\_length\_5572\_cov\_28.541996 2419-2429. Max. coverage (+): 0.08. Max coverage (-): 0.08

Region: NODE\_121434\_length\_5572\_cov\_28.541996 2430-2440. Max. coverage (+): 0.08. Max coverage (-): 0.77

Region: NODE\_121434\_length\_5572\_cov\_28.541996 2441-2452. Max. coverage (+): 0.08. Max coverage (-): 0.69

Region: NODE\_121434\_length\_5572\_cov\_28.541996 2453-2463. Max. coverage (+): 0. Max coverage (-): 0.04

Region: NODE\_121434\_length\_5572\_cov\_28.541996 2464-2474. Max. coverage (+): 0.04. Max coverage (-): 0.92

Region: NODE\_121434\_length\_5572\_cov\_28.541996 2475-2486. Max. coverage (+): 0.08. Max coverage (-): 0.84

Region: NODE\_121434\_length\_5572\_cov\_28.541996 2487-2497. Max. coverage (+): 0.34. Max coverage (-): 0.11

Region: NODE\_121434\_length\_5572\_cov\_28.541996 2498-2509. Max. coverage (+): 0.61. Max coverage (-): 9.85

Region: NODE\_121434\_length\_5572\_cov\_28.541996 2510-2520. Max. coverage (+): 0.04. Max coverage (-): 6.21

Region: NODE\_121434\_length\_5572\_cov\_28.541996 2521-2531. Max. coverage (+): 0. Max coverage (-): 0

Region: NODE\_121434\_length\_5572\_cov\_28.541996 2532-2543. Max. coverage (+): 0. Max coverage (-): 0

Region: NODE\_121434\_length\_5572\_cov\_28.541996 2544-2554. Max. coverage (+): 0.08. Max coverage (-): 0.08

Region: NODE\_121434\_length\_5572\_cov\_28.541996 2555-2565. Max. coverage (+): 0. Max coverage (-): 0

Region: NODE\_121434\_length\_5572\_cov\_28.541996 2566-2577. Max. coverage (+): 0. Max coverage (-): 0

Region: NODE\_121434\_length\_5572\_cov\_28.541996 2578-2588. Max. coverage (+): 0. Max coverage (-): 0

Region: NODE\_121434\_length\_5572\_cov\_28.541996 2589-2600. Max. coverage (+): 0. Max coverage (-): 0

Region: NODE\_121434\_length\_5572\_cov\_28.541996 2601-2611. Max. coverage (+): 0. Max coverage (-): 0

Region: NODE\_121434\_length\_5572\_cov\_28.541996 2612-2622. Max. coverage (+): 0. Max coverage (-): 0

Region: NODE\_121434\_length\_5572\_cov\_28.541996 2623-2634. Max. coverage (+): 0. Max coverage (-): 0.04

Region: NODE\_121434\_length\_5572\_cov\_28.541996 2635-2645. Max. coverage (+): 0.08. Max coverage (-): 0.92

Region: NODE\_121434\_length\_5572\_cov\_28.541996 2646-2656. Max. coverage (+): 0. Max coverage (-): 0.23

Region: NODE\_121434\_length\_5572\_cov\_28.541996 2657-2668. Max. coverage (+): 0.61. Max coverage (-): 3.45

Region: NODE\_121434\_length\_5572\_cov\_28.541996 2669-2679. Max. coverage (+): 0.11. Max coverage (-): 9.85

Region: NODE\_121434\_length\_5572\_cov\_28.541996 2680-2691. Max. coverage (+): 0.61. Max coverage (-): 0.57

Region: NODE\_121434\_length\_5572\_cov\_28.541996 2692-2702. Max. coverage (+): 0.38. Max coverage (-): 0

Region: NODE\_121434\_length\_5572\_cov\_28.541996 2703-2713. Max. coverage (+): 0. Max coverage (-): 0.92

Region: NODE\_121434\_length\_5572\_cov\_28.541996 2714-2725. Max. coverage (+): 0.31. Max coverage (-): 0

Region: NODE\_121434\_length\_5572\_cov\_28.541996 2726-2736. Max. coverage (+): 0.77. Max coverage (-): 0.08

Region: NODE\_121434\_length\_5572\_cov\_28.541996 2737-2747. Max. coverage (+): 0. Max coverage (-): 1.07

Region: NODE\_121434\_length\_5572\_cov\_28.541996 2748-2759. Max. coverage (+): 0.84. Max coverage (-): 0.61

Region: NODE\_121434\_length\_5572\_cov\_28.541996 2760-2770. Max. coverage (+): 1.23. Max coverage (-): 0

Region: NODE\_121434\_length\_5572\_cov\_28.541996 2771-2781. Max. coverage (+): 0.08. Max coverage (-): 0

Region: NODE\_121434\_length\_5572\_cov\_28.541996 2782-2793. Max. coverage (+): 0. Max coverage (-): 0.46

Region: NODE\_121434\_length\_5572\_cov\_28.541996 2794-2804. Max. coverage (+): 0.08. Max coverage (-): 0.15

Region: NODE\_121434\_length\_5572\_cov\_28.541996 2805-2816. Max. coverage (+): 2.3. Max coverage (-): 0.46

Region: NODE\_121434\_length\_5572\_cov\_28.541996 2817-2827. Max. coverage (+): 0.08. Max coverage (-): 0.84

Region: NODE\_121434\_length\_5572\_cov\_28.541996 2828-2838. Max. coverage (+): 0.08. Max coverage (-): 0.77

Region: NODE\_121434\_length\_5572\_cov\_28.541996 2839-2850. Max. coverage (+): 0.08. Max coverage (-): 0.15

Region: NODE\_121434\_length\_5572\_cov\_28.541996 2851-2861. Max. coverage (+): 0.46. Max coverage (-): 0.15

Region: NODE\_121434\_length\_5572\_cov\_28.541996 2862-2872. Max. coverage (+): 0.38. Max coverage (-): 1.61

Region: NODE\_121434\_length\_5572\_cov\_28.541996 2873-2884. Max. coverage (+): 2.07. Max coverage (-): 1.53

Region: NODE\_121434\_length\_5572\_cov\_28.541996 2885-2895. Max. coverage (+): 0.38. Max coverage (-): 0.23

Region: NODE\_121434\_length\_5572\_cov\_28.541996 2896-2907. Max. coverage (+): 0.46. Max coverage (-): 0.08

Region: NODE\_121434\_length\_5572\_cov\_28.541996 2908-2918. Max. coverage (+): 0.23. Max coverage (-): 0.08

Region: NODE\_121434\_length\_5572\_cov\_28.541996 2919-2929. Max. coverage (+): 0.15. Max coverage (-): 0.15

Region: NODE\_121434\_length\_5572\_cov\_28.541996 2930-2941. Max. coverage (+): 0.31. Max coverage (-): 0

Region: NODE\_121434\_length\_5572\_cov\_28.541996 2942-2952. Max. coverage (+): 0.08. Max coverage (-): 0

Region: NODE\_121434\_length\_5572\_cov\_28.541996 2953-2963. Max. coverage (+): 0.15. Max coverage (-): 0

Region: NODE\_121434\_length\_5572\_cov\_28.541996 2964-2975. Max. coverage (+): 0.04. Max coverage (-): 0

Region: NODE\_121434\_length\_5572\_cov\_28.541996 2976-2986. Max. coverage (+): 0.04. Max coverage (-): 0

Region: NODE\_121434\_length\_5572\_cov\_28.541996 2987-2997. Max. coverage (+): 0. Max coverage (-): 0.08

Region: NODE\_121434\_length\_5572\_cov\_28.541996 2998-3009. Max. coverage (+): 0. Max coverage (-): 0

Region: NODE\_121434\_length\_5572\_cov\_28.541996 3010-3020. Max. coverage (+): 0. Max coverage (-): 0

Region: NODE\_121434\_length\_5572\_cov\_28.541996 3021-3032. Max. coverage (+): 0. Max coverage (-): 0

Region: NODE\_121434\_length\_5572\_cov\_28.541996 3033-3043. Max. coverage (+): 0.08. Max coverage (-): 0

Region: NODE\_121434\_length\_5572\_cov\_28.541996 3044-3054. Max. coverage (+): 0.08. Max coverage (-): 0.31

Region: NODE\_121434\_length\_5572\_cov\_28.541996 3055-3066. Max. coverage (+): 0.08. Max coverage (-): 0.31

Region: NODE\_121434\_length\_5572\_cov\_28.541996 3067-3077. Max. coverage (+): 0.23. Max coverage (-): 0.31

Region: NODE\_121434\_length\_5572\_cov\_28.541996 3078-3088. Max. coverage (+): 0.46. Max coverage (-): 0.23

Region: NODE\_121434\_length\_5572\_cov\_28.541996 3089-3100. Max. coverage (+): 0.23. Max coverage (-): 0

Region: NODE\_121434\_length\_5572\_cov\_28.541996 3101-3111. Max. coverage (+): 0. Max coverage (-): 0

Region: NODE\_121434\_length\_5572\_cov\_28.541996 3112-3123. Max. coverage (+): 0. Max coverage (-): 0

Region: NODE\_121434\_length\_5572\_cov\_28.541996 3124-3134. Max. coverage (+): 0. Max coverage (-): 0

Region: NODE\_121434\_length\_5572\_cov\_28.541996 3135-3145. Max. coverage (+): 0. Max coverage (-): 0

Region: NODE\_121434\_length\_5572\_cov\_28.541996 3146-3157. Max. coverage (+): 0.61. Max coverage (-): 0.15

Region: NODE\_121434\_length\_5572\_cov\_28.541996 3158-3168. Max. coverage (+): 0.15. Max coverage (-): 0.15

Region: NODE\_121434\_length\_5572\_cov\_28.541996 3169-3179. Max. coverage (+): 0.15. Max coverage (-): 0.08

Region: NODE\_121434\_length\_5572\_cov\_28.541996 3180-3191. Max. coverage (+): 0. Max coverage (-): 0

Region: NODE\_121434\_length\_5572\_cov\_28.541996 3192-3202. Max. coverage (+): 4.9. Max coverage (-): 0.23

Region: NODE\_121434\_length\_5572\_cov\_28.541996 3203-3214. Max. coverage (+): 5.67. Max coverage (-): 0.15

Region: NODE\_121434\_length\_5572\_cov\_28.541996 3215-3225. Max. coverage (+): 0.84. Max coverage (-): 0.61

Region: NODE\_121434\_length\_5572\_cov\_28.541996 3226-3236. Max. coverage (+): 1.99. Max coverage (-): 0.23

Region: NODE\_121434\_length\_5572\_cov\_28.541996 3237-3248. Max. coverage (+): 1.99. Max coverage (-): 0.08

Region: NODE\_121434\_length\_5572\_cov\_28.541996 3249-3259. Max. coverage (+): 0. Max coverage (-): 0.08

Region: NODE\_121434\_length\_5572\_cov\_28.541996 3260-3270. Max. coverage (+): 0.84. Max coverage (-): 0.31

Region: NODE\_121434\_length\_5572\_cov\_28.541996 3271-3282. Max. coverage (+): 0.08. Max coverage (-): 0.08

Region: NODE\_121434\_length\_5572\_cov\_28.541996 3283-3293. Max. coverage (+): 0.08. Max coverage (-): 0.08

Region: NODE\_121434\_length\_5572\_cov\_28.541996 3294-3304. Max. coverage (+): 0.08. Max coverage (-): 0.31

Region: NODE\_121434\_length\_5572\_cov\_28.541996 3305-3316. Max. coverage (+): 0.23. Max coverage (-): 2.38

Region: NODE\_121434\_length\_5572\_cov\_28.541996 3317-3327. Max. coverage (+): 0.15. Max coverage (-): 0.31

Region: NODE\_121434\_length\_5572\_cov\_28.541996 3328-3339. Max. coverage (+): 1.84. Max coverage (-): 0.08

Region: NODE\_121434\_length\_5572\_cov\_28.541996 3340-3350. Max. coverage (+): 0. Max coverage (-): 3.37

Region: NODE\_121434\_length\_5572\_cov\_28.541996 3351-3361. Max. coverage (+): 0. Max coverage (-): 0.69

Region: NODE\_121434\_length\_5572\_cov\_28.541996 3362-3373. Max. coverage (+): 0. Max coverage (-): 0

Region: NODE\_121434\_length\_5572\_cov\_28.541996 3374-3384. Max. coverage (+): 0. Max coverage (-): 2.15

Region: NODE\_121434\_length\_5572\_cov\_28.541996 3385-3395. Max. coverage (+): 0.08. Max coverage (-): 2.15

Region: NODE\_121434\_length\_5572\_cov\_28.541996 3396-3407. Max. coverage (+): 0.46. Max coverage (-): 0

Region: NODE\_121434\_length\_5572\_cov\_28.541996 3408-3418. Max. coverage (+): 0.61. Max coverage (-): 0.15

Region: NODE\_121434\_length\_5572\_cov\_28.541996 3419-3430. Max. coverage (+): 0.15. Max coverage (-): 0.31

Region: NODE\_121434\_length\_5572\_cov\_28.541996 3431-3441. Max. coverage (+): 0.08. Max coverage (-): 0.46

Region: NODE\_121434\_length\_5572\_cov\_28.541996 3442-3452. Max. coverage (+): 0.08. Max coverage (-): 0

Region: NODE\_121434\_length\_5572\_cov\_28.541996 3453-3464. Max. coverage (+): 0. Max coverage (-): 0.15

Region: NODE\_121434\_length\_5572\_cov\_28.541996 3465-3475. Max. coverage (+): 0. Max coverage (-): 0.15

Region: NODE\_121434\_length\_5572\_cov\_28.541996 3476-3486. Max. coverage (+): 0.61. Max coverage (-): 0

Region: NODE\_121434\_length\_5572\_cov\_28.541996 3487-3498. Max. coverage (+): 0. Max coverage (-): 0.08

Region: NODE\_121434\_length\_5572\_cov\_28.541996 3499-3509. Max. coverage (+): 0. Max coverage (-): 0.08

Region: NODE\_121434\_length\_5572\_cov\_28.541996 3510-3521. Max. coverage (+): 0. Max coverage (-): 0.08

Region: NODE\_121434\_length\_5572\_cov\_28.541996 3522-3532. Max. coverage (+): 0. Max coverage (-): 0.31

Region: NODE\_121434\_length\_5572\_cov\_28.541996 3533-3543. Max. coverage (+): 0. Max coverage (-): 0.08

Region: NODE\_121434\_length\_5572\_cov\_28.541996 3544-3555. Max. coverage (+): 0. Max coverage (-): 0

Region: NODE\_121434\_length\_5572\_cov\_28.541996 3556-3566. Max. coverage (+): 0. Max coverage (-): 0.84

Region: NODE\_121434\_length\_5572\_cov\_28.541996 3567-3577. Max. coverage (+): 0. Max coverage (-): 0.31

Region: NODE\_121434\_length\_5572\_cov\_28.541996 3578-3589. Max. coverage (+): 0. Max coverage (-): 0

Region: NODE\_121434\_length\_5572\_cov\_28.541996 3590-3600. Max. coverage (+): 0. Max coverage (-): 0

Region: NODE\_121434\_length\_5572\_cov\_28.541996 3601-3611. Max. coverage (+): 0. Max coverage (-): 0

Region: NODE\_121434\_length\_5572\_cov\_28.541996 3612-3623. Max. coverage (+): 0. Max coverage (-): 0

Region: NODE\_121434\_length\_5572\_cov\_28.541996 3624-3634. Max. coverage (+): 0. Max coverage (-): 0

Region: NODE\_121434\_length\_5572\_cov\_28.541996 3635-3646. Max. coverage (+): 0. Max coverage (-): 0

Region: NODE\_121434\_length\_5572\_cov\_28.541996 3647-3657. Max. coverage (+): 0. Max coverage (-): 0

Region: NODE\_121434\_length\_5572\_cov\_28.541996 3658-3668. Max. coverage (+): 0. Max coverage (-): 0

Region: NODE\_121434\_length\_5572\_cov\_28.541996 3669-3680. Max. coverage (+): 0. Max coverage (-): 0

Region: NODE\_121434\_length\_5572\_cov\_28.541996 3681-3691. Max. coverage (+): 0. Max coverage (-): 0

Region: NODE\_121434\_length\_5572\_cov\_28.541996 3692-3702. Max. coverage (+): 0. Max coverage (-): 0

Region: NODE\_121434\_length\_5572\_cov\_28.541996 3703-3714. Max. coverage (+): 0. Max coverage (-): 0

Region: NODE\_121434\_length\_5572\_cov\_28.541996 3715-3725. Max. coverage (+): 0. Max coverage (-): 0

Region: NODE\_121434\_length\_5572\_cov\_28.541996 3726-3737. Max. coverage (+): 0. Max coverage (-): 0

Region: NODE\_121434\_length\_5572\_cov\_28.541996 3738-3748. Max. coverage (+): 0. Max coverage (-): 0

Region: NODE\_121434\_length\_5572\_cov\_28.541996 3749-3759. Max. coverage (+): 0. Max coverage (-): 0

Region: NODE\_121434\_length\_5572\_cov\_28.541996 3760-3771. Max. coverage (+): 0. Max coverage (-): 0

Region: NODE\_121434\_length\_5572\_cov\_28.541996 3772-3782. Max. coverage (+): 0. Max coverage (-): 0

Region: NODE\_121434\_length\_5572\_cov\_28.541996 3783-3793. Max. coverage (+): 0. Max coverage (-): 0

Region: NODE\_121434\_length\_5572\_cov\_28.541996 3794-3805. Max. coverage (+): 0. Max coverage (-): 0

Region: NODE\_121434\_length\_5572\_cov\_28.541996 3806-3816. Max. coverage (+): 0. Max coverage (-): 0

Region: NODE\_121434\_length\_5572\_cov\_28.541996 3817-3828. Max. coverage (+): 0. Max coverage (-): 0

Region: NODE\_121434\_length\_5572\_cov\_28.541996 3829-3839. Max. coverage (+): 0. Max coverage (-): 0

Region: NODE\_121434\_length\_5572\_cov\_28.541996 3840-3850. Max. coverage (+): 0. Max coverage (-): 0

Region: NODE\_121434\_length\_5572\_cov\_28.541996 3851-3862. Max. coverage (+): 0. Max coverage (-): 0

Region: NODE\_121434\_length\_5572\_cov\_28.541996 3863-3873. Max. coverage (+): 0. Max coverage (-): 0

Region: NODE\_121434\_length\_5572\_cov\_28.541996 3874-3884. Max. coverage (+): 0.08. Max coverage (-): 0

Region: NODE\_121434\_length\_5572\_cov\_28.541996 3885-3896. Max. coverage (+): 0. Max coverage (-): 0

Region: NODE\_121434\_length\_5572\_cov\_28.541996 3897-3907. Max. coverage (+): 0. Max coverage (-): 0

Region: NODE\_121434\_length\_5572\_cov\_28.541996 3908-3918. Max. coverage (+): 0. Max coverage (-): 0

Region: NODE\_121434\_length\_5572\_cov\_28.541996 3919-3930. Max. coverage (+): 0. Max coverage (-): 0.61

Region: NODE\_121434\_length\_5572\_cov\_28.541996 3931-3941. Max. coverage (+): 0. Max coverage (-): 0

Region: NODE\_121434\_length\_5572\_cov\_28.541996 3942-3953. Max. coverage (+): 0. Max coverage (-): 0

Region: NODE\_121434\_length\_5572\_cov\_28.541996 3954-3964. Max. coverage (+): 0. Max coverage (-): 0

Region: NODE\_121434\_length\_5572\_cov\_28.541996 3965-3975. Max. coverage (+): 0. Max coverage (-): 0.08

Region: NODE\_121434\_length\_5572\_cov\_28.541996 3976-3987. Max. coverage (+): 0.08. Max coverage (-): 0

Region: NODE\_121434\_length\_5572\_cov\_28.541996 3988-3998. Max. coverage (+): 0. Max coverage (-): 0

Region: NODE\_121434\_length\_5572\_cov\_28.541996 3999-4009. Max. coverage (+): 0. Max coverage (-): 0

Region: NODE\_121434\_length\_5572\_cov\_28.541996 4010-4021. Max. coverage (+): 0. Max coverage (-): 0

Region: NODE\_121434\_length\_5572\_cov\_28.541996 4022-4032. Max. coverage (+): 0. Max coverage (-): 0

Region: NODE\_121434\_length\_5572\_cov\_28.541996 4033-4044. Max. coverage (+): 0. Max coverage (-): 0

Region: NODE\_121434\_length\_5572\_cov\_28.541996 4045-4055. Max. coverage (+): 0. Max coverage (-): 0.08

Region: NODE\_121434\_length\_5572\_cov\_28.541996 4056-4066. Max. coverage (+): 0. Max coverage (-): 0

Region: NODE\_121434\_length\_5572\_cov\_28.541996 4067-4078. Max. coverage (+): 0.08. Max coverage (-): 0

Region: NODE\_121434\_length\_5572\_cov\_28.541996 4079-4089. Max. coverage (+): 0. Max coverage (-): 0

Region: NODE\_121434\_length\_5572\_cov\_28.541996 4090-4100. Max. coverage (+): 0. Max coverage (-): 0.08

Region: NODE\_121434\_length\_5572\_cov\_28.541996 4101-4112. Max. coverage (+): 0. Max coverage (-): 0.15

Region: NODE\_121434\_length\_5572\_cov\_28.541996 4113-4123. Max. coverage (+): 0. Max coverage (-): 0.15

Region: NODE\_121434\_length\_5572\_cov\_28.541996 4124-4134. Max. coverage (+): 0.15. Max coverage (-): 0

Region: NODE\_121434\_length\_5572\_cov\_28.541996 4135-4146. Max. coverage (+): 0.08. Max coverage (-): 0

Region: NODE\_121434\_length\_5572\_cov\_28.541996 4147-4157. Max. coverage (+): 0.08. Max coverage (-): 0

Region: NODE\_121434\_length\_5572\_cov\_28.541996 4158-4169. Max. coverage (+): 0. Max coverage (-): 0

Region: NODE\_121434\_length\_5572\_cov\_28.541996 4170-4180. Max. coverage (+): 0. Max coverage (-): 0

Region: NODE\_121434\_length\_5572\_cov\_28.541996 4181-4191. Max. coverage (+): 0. Max coverage (-): 0.08

Region: NODE\_121434\_length\_5572\_cov\_28.541996 4192-4203. Max. coverage (+): 0. Max coverage (-): 0

Region: NODE\_121434\_length\_5572\_cov\_28.541996 4204-4214. Max. coverage (+): 0.08. Max coverage (-): 0

Region: NODE\_121434\_length\_5572\_cov\_28.541996 4215-4225. Max. coverage (+): 0. Max coverage (-): 0.77

Region: NODE\_121434\_length\_5572\_cov\_28.541996 4226-4237. Max. coverage (+): 1.15. Max coverage (-): 0.15

Region: NODE\_121434\_length\_5572\_cov\_28.541996 4238-4248. Max. coverage (+): 0.84. Max coverage (-): 1.15

Region: NODE\_121434\_length\_5572\_cov\_28.541996 4249-4260. Max. coverage (+): 0.61. Max coverage (-): 0.84

Region: NODE\_121434\_length\_5572\_cov\_28.541996 4261-4271. Max. coverage (+): 0.77. Max coverage (-): 0

Region: NODE\_121434\_length\_5572\_cov\_28.541996 4272-4282. Max. coverage (+): 0. Max coverage (-): 0

Region: NODE\_121434\_length\_5572\_cov\_28.541996 4283-4294. Max. coverage (+): 0. Max coverage (-): 0

Region: NODE\_121434\_length\_5572\_cov\_28.541996 4295-4305. Max. coverage (+): 0. Max coverage (-): 0

Region: NODE\_121434\_length\_5572\_cov\_28.541996 4306-4316. Max. coverage (+): 0. Max coverage (-): 0.31

Region: NODE\_121434\_length\_5572\_cov\_28.541996 4317-4328. Max. coverage (+): 0.23. Max coverage (-): 0.08

Region: NODE\_121434\_length\_5572\_cov\_28.541996 4329-4339. Max. coverage (+): 0. Max coverage (-): 0

Region: NODE\_121434\_length\_5572\_cov\_28.541996 4340-4351. Max. coverage (+): 0.46. Max coverage (-): 0.08

Region: NODE\_121434\_length\_5572\_cov\_28.541996 4352-4362. Max. coverage (+): 0.38. Max coverage (-): 0.08

Region: NODE\_121434\_length\_5572\_cov\_28.541996 4363-4373. Max. coverage (+): 0. Max coverage (-): 0.46

Region: NODE\_121434\_length\_5572\_cov\_28.541996 4374-4385. Max. coverage (+): 0. Max coverage (-): 0.08

Region: NODE\_121434\_length\_5572\_cov\_28.541996 4386-4396. Max. coverage (+): 0. Max coverage (-): 0

Region: NODE\_121434\_length\_5572\_cov\_28.541996 4397-4407. Max. coverage (+): 0. Max coverage (-): 0

Region: NODE\_121434\_length\_5572\_cov\_28.541996 4408-4419. Max. coverage (+): 0. Max coverage (-): 0

Region: NODE\_121434\_length\_5572\_cov\_28.541996 4420-4430. Max. coverage (+): 0. Max coverage (-): 0

Region: NODE\_121434\_length\_5572\_cov\_28.541996 4431-4441. Max. coverage (+): 0. Max coverage (-): 0

Region: NODE\_121434\_length\_5572\_cov\_28.541996 4442-4453. Max. coverage (+): 0. Max coverage (-): 0.92

Region: NODE\_121434\_length\_5572\_cov\_28.541996 4454-4464. Max. coverage (+): 0. Max coverage (-): 0.84

Region: NODE\_121434\_length\_5572\_cov\_28.541996 4465-4476. Max. coverage (+): 0.61. Max coverage (-): 0.31

Region: NODE\_121434\_length\_5572\_cov\_28.541996 4477-4487. Max. coverage (+): 0. Max coverage (-): 1.15

Region: NODE\_121434\_length\_5572\_cov\_28.541996 4488-4498. Max. coverage (+): 0.08. Max coverage (-): 0.69

Region: NODE\_121434\_length\_5572\_cov\_28.541996 4499-4510. Max. coverage (+): 0.08. Max coverage (-): 0.38

Region: NODE\_121434\_length\_5572\_cov\_28.541996 4511-4521. Max. coverage (+): 0. Max coverage (-): 0.15

Region: NODE\_121434\_length\_5572\_cov\_28.541996 4522-4532. Max. coverage (+): 0. Max coverage (-): 1.84

Region: NODE\_121434\_length\_5572\_cov\_28.541996 4533-4544. Max. coverage (+): 0.38. Max coverage (-): 1.84

Region: NODE\_121434\_length\_5572\_cov\_28.541996 4545-4555. Max. coverage (+): 0. Max coverage (-): 0

Region: NODE\_121434\_length\_5572\_cov\_28.541996 4556-4567. Max. coverage (+): 0. Max coverage (-): 0

Region: NODE\_121434\_length\_5572\_cov\_28.541996 4568-4578. Max. coverage (+): 0. Max coverage (-): 0

Region: NODE\_121434\_length\_5572\_cov\_28.541996 4579-4589. Max. coverage (+): 0. Max coverage (-): 0

Region: NODE\_121434\_length\_5572\_cov\_28.541996 4590-4601. Max. coverage (+): 0. Max coverage (-): 0.08

Region: NODE\_121434\_length\_5572\_cov\_28.541996 4602-4612. Max. coverage (+): 0.08. Max coverage (-): 0.23

Region: NODE\_121434\_length\_5572\_cov\_28.541996 4613-4623. Max. coverage (+): 0. Max coverage (-): 0

Region: NODE\_121434\_length\_5572\_cov\_28.541996 4624-4635. Max. coverage (+): 0. Max coverage (-): 0.38

Region: NODE\_121434\_length\_5572\_cov\_28.541996 4636-4646. Max. coverage (+): 0.08. Max coverage (-): 1.38

Region: NODE\_121434\_length\_5572\_cov\_28.541996 4647-4658. Max. coverage (+): 0.23. Max coverage (-): 0

Region: NODE\_121434\_length\_5572\_cov\_28.541996 4659-4669. Max. coverage (+): 0.15. Max coverage (-): 0.61

Region: NODE\_121434\_length\_5572\_cov\_28.541996 4670-4680. Max. coverage (+): 0.08. Max coverage (-): 1.76

Region: NODE\_121434\_length\_5572\_cov\_28.541996 4681-4692. Max. coverage (+): 0.38. Max coverage (-): 0.38

Region: NODE\_121434\_length\_5572\_cov\_28.541996 4693-4703. Max. coverage (+): 0.15. Max coverage (-): 0

Region: NODE\_121434\_length\_5572\_cov\_28.541996 4704-4714. Max. coverage (+): 0. Max coverage (-): 0

Region: NODE\_121434\_length\_5572\_cov\_28.541996 4715-4726. Max. coverage (+): 0. Max coverage (-): 0

Region: NODE\_121434\_length\_5572\_cov\_28.541996 4727-4737. Max. coverage (+): 0. Max coverage (-): 0

Region: NODE\_121434\_length\_5572\_cov\_28.541996 4738-4748. Max. coverage (+): 0.15. Max coverage (-): 0

Region: NODE\_121434\_length\_5572\_cov\_28.541996 4749-4760. Max. coverage (+): 0. Max coverage (-): 0

Region: NODE\_121434\_length\_5572\_cov\_28.541996 4761-4771. Max. coverage (+): 0. Max coverage (-): 0

Region: NODE\_121434\_length\_5572\_cov\_28.541996 4772-4783. Max. coverage (+): 0. Max coverage (-): 0.61

Region: NODE\_121434\_length\_5572\_cov\_28.541996 4784-4794. Max. coverage (+): 0. Max coverage (-): 0

Region: NODE\_121434\_length\_5572\_cov\_28.541996 4795-4805. Max. coverage (+): 0. Max coverage (-): 0

Region: NODE\_121434\_length\_5572\_cov\_28.541996 4806-4817. Max. coverage (+): 0. Max coverage (-): 0.23

Region: NODE\_121434\_length\_5572\_cov\_28.541996 4818-4828. Max. coverage (+): 0.31. Max coverage (-): 0

Region: NODE\_121434\_length\_5572\_cov\_28.541996 4829-4839. Max. coverage (+): 0. Max coverage (-): 0

Region: NODE\_121434\_length\_5572\_cov\_28.541996 4840-4851. Max. coverage (+): 0. Max coverage (-): 0

Region: NODE\_121434\_length\_5572\_cov\_28.541996 4852-4862. Max. coverage (+): 0.15. Max coverage (-): 0.23

Region: NODE\_121434\_length\_5572\_cov\_28.541996 4863-4874. Max. coverage (+): 0.23. Max coverage (-): 0.23

Region: NODE\_121434\_length\_5572\_cov\_28.541996 4875-4885. Max. coverage (+): 0.23. Max coverage (-): 1.23

Region: NODE\_121434\_length\_5572\_cov\_28.541996 4886-4896. Max. coverage (+): 0.23. Max coverage (-): 0.15

Region: NODE\_121434\_length\_5572\_cov\_28.541996 4897-4908. Max. coverage (+): 0.15. Max coverage (-): 0

Region: NODE\_121434\_length\_5572\_cov\_28.541996 4909-4919. Max. coverage (+): 0. Max coverage (-): 0.08

Region: NODE\_121434\_length\_5572\_cov\_28.541996 4920-4930. Max. coverage (+): 0. Max coverage (-): 0

Region: NODE\_121434\_length\_5572\_cov\_28.541996 4931-4942. Max. coverage (+): 0. Max coverage (-): 0

Region: NODE\_121434\_length\_5572\_cov\_28.541996 4943-4953. Max. coverage (+): 0. Max coverage (-): 0

Region: NODE\_121434\_length\_5572\_cov\_28.541996 4954-4965. Max. coverage (+): 0. Max coverage (-): 0

Region: NODE\_121434\_length\_5572\_cov\_28.541996 4966-4976. Max. coverage (+): 0. Max coverage (-): 0

Region: NODE\_121434\_length\_5572\_cov\_28.541996 4977-4987. Max. coverage (+): 0. Max coverage (-): 0

Region: NODE\_121434\_length\_5572\_cov\_28.541996 4988-4999. Max. coverage (+): 0. Max coverage (-): 0

Region: NODE\_121434\_length\_5572\_cov\_28.541996 5000-5010. Max. coverage (+): 0. Max coverage (-): 0

Region: NODE\_121434\_length\_5572\_cov\_28.541996 5011-5021. Max. coverage (+): 0. Max coverage (-): 0

Region: NODE\_121434\_length\_5572\_cov\_28.541996 5022-5033. Max. coverage (+): 0. Max coverage (-): 0

Region: NODE\_121434\_length\_5572\_cov\_28.541996 5034-5044. Max. coverage (+): 0. Max coverage (-): 0

Region: NODE\_121434\_length\_5572\_cov\_28.541996 5045-5055. Max. coverage (+): 0. Max coverage (-): 0

Region: NODE\_121434\_length\_5572\_cov\_28.541996 5056-5067. Max. coverage (+): 0. Max coverage (-): 0

Region: NODE\_121434\_length\_5572\_cov\_28.541996 5068-5078. Max. coverage (+): 0. Max coverage (-): 0

Region: NODE\_121434\_length\_5572\_cov\_28.541996 5079-5090. Max. coverage (+): 0. Max coverage (-): 0

Region: NODE\_121434\_length\_5572\_cov\_28.541996 5091-5101. Max. coverage (+): 0. Max coverage (-): 0.08

Region: NODE\_121434\_length\_5572\_cov\_28.541996 5102-5112. Max. coverage (+): 0. Max coverage (-): 0

Region: NODE\_121434\_length\_5572\_cov\_28.541996 5113-5124. Max. coverage (+): 0. Max coverage (-): 0.15

Region: NODE\_121434\_length\_5572\_cov\_28.541996 5125-5135. Max. coverage (+): 0. Max coverage (-): 0.15

Region: NODE\_121434\_length\_5572\_cov\_28.541996 5136-5146. Max. coverage (+): 0. Max coverage (-): 0

Region: NODE\_121434\_length\_5572\_cov\_28.541996 5147-5158. Max. coverage (+): 0. Max coverage (-): 0

Region: NODE\_121434\_length\_5572\_cov\_28.541996 5159-5169. Max. coverage (+): 0. Max coverage (-): 0.15

Region: NODE\_121434\_length\_5572\_cov\_28.541996 5170-5181. Max. coverage (+): 0. Max coverage (-): 0

Region: NODE\_121434\_length\_5572\_cov\_28.541996 5182-5192. Max. coverage (+): 0. Max coverage (-): 0.08

Region: NODE\_121434\_length\_5572\_cov\_28.541996 5193-5203. Max. coverage (+): 0. Max coverage (-): 0.08

Region: NODE\_121434\_length\_5572\_cov\_28.541996 5204-5215. Max. coverage (+): 0. Max coverage (-): 0

Region: NODE\_121434\_length\_5572\_cov\_28.541996 5216-5226. Max. coverage (+): 0. Max coverage (-): 0

Region: NODE\_121434\_length\_5572\_cov\_28.541996 5227-5237. Max. coverage (+): 0. Max coverage (-): 0

Region: NODE\_121434\_length\_5572\_cov\_28.541996 5238-5249. Max. coverage (+): 0. Max coverage (-): 0.08

Region: NODE\_121434\_length\_5572\_cov\_28.541996 5250-5260. Max. coverage (+): 0. Max coverage (-): 0.08

Region: NODE\_121434\_length\_5572\_cov\_28.541996 5261-5271. Max. coverage (+): 0. Max coverage (-): 0

Region: NODE\_121434\_length\_5572\_cov\_28.541996 5272-5283. Max. coverage (+): 0. Max coverage (-): 0.08

Region: NODE\_121434\_length\_5572\_cov\_28.541996 5284-5294. Max. coverage (+): 0. Max coverage (-): 0.08

Region: NODE\_121434\_length\_5572\_cov\_28.541996 5295-5306. Max. coverage (+): 0. Max coverage (-): 0

Region: NODE\_121434\_length\_5572\_cov\_28.541996 5307-5317. Max. coverage (+): 0. Max coverage (-): 0.31

Region: NODE\_121434\_length\_5572\_cov\_28.541996 5318-5328. Max. coverage (+): 0. Max coverage (-): 0.92

Region: NODE\_121434\_length\_5572\_cov\_28.541996 5329-5340. Max. coverage (+): 0.08. Max coverage (-): 0

Region: NODE\_121434\_length\_5572\_cov\_28.541996 5341-5351. Max. coverage (+): 0. Max coverage (-): 0

Region: NODE\_121434\_length\_5572\_cov\_28.541996 5352-5362. Max. coverage (+): 0. Max coverage (-): 0

Region: NODE\_121434\_length\_5572\_cov\_28.541996 5363-5374. Max. coverage (+): 0. Max coverage (-): 0

Region: NODE\_121434\_length\_5572\_cov\_28.541996 5375-5385. Max. coverage (+): 0. Max coverage (-): 0

Region: NODE\_121434\_length\_5572\_cov\_28.541996 5386-5397. Max. coverage (+): 0.69. Max coverage (-): 0

Region: NODE\_121434\_length\_5572\_cov\_28.541996 5398-5408. Max. coverage (+): 0. Max coverage (-): 0.54

Region: NODE\_121434\_length\_5572\_cov\_28.541996 5409-5419. Max. coverage (+): 0. Max coverage (-): 0.38

Region: NODE\_121434\_length\_5572\_cov\_28.541996 5420-5431. Max. coverage (+): 1.15. Max coverage (-): 0

Region: NODE\_121434\_length\_5572\_cov\_28.541996 5432-5442. Max. coverage (+): 0.15. Max coverage (-): 1.23

Region: NODE\_121434\_length\_5572\_cov\_28.541996 5443-5453. Max. coverage (+): 0.08. Max coverage (-): 0.61

Region: NODE\_121434\_length\_5572\_cov\_28.541996 5454-5465. Max. coverage (+): 0.31. Max coverage (-): 0

Region: NODE\_121434\_length\_5572\_cov\_28.541996 5466-5476. Max. coverage (+): 0. Max coverage (-): 2.13

Region: NODE\_121434\_length\_5572\_cov\_28.541996 5477-5488. Max. coverage (+): 0.11. Max coverage (-): 0.28

Region: NODE\_121434\_length\_5572\_cov\_28.541996 5489-5499. Max. coverage (+): 0.03. Max coverage (-): 0.03

Region: NODE\_121434\_length\_5572\_cov\_28.541996 5500-5510. Max. coverage (+): 0.01. Max coverage (-): 4.8

Region: NODE\_121434\_length\_5572\_cov\_28.541996 5511-5522. Max. coverage (+): 0. Max coverage (-): 4.54

Region: NODE\_121434\_length\_5572\_cov\_28.541996 5523-5533. Max. coverage (+): 0.14. Max coverage (-): 0

Region: NODE\_121434\_length\_5572\_cov\_28.541996 5534-5544. Max. coverage (+): 0. Max coverage (-): 0

Region: NODE\_121434\_length\_5572\_cov\_28.541996 5545-5556. Max. coverage (+): 0. Max coverage (-): 0.23

Region: NODE\_121434\_length\_5572\_cov\_28.541996 5557-5567. Max. coverage (+): 0. Max coverage (-): 0.05

Region: NODE\_121434\_length\_5572\_cov\_28.541996 5568-5578. Max. coverage (+): 0. Max coverage (-): 0

Region: NODE\_121434\_length\_5572\_cov\_28.541996 5579-5590. Max. coverage (+): 0. Max coverage (-): 0

Region: NODE\_121434\_length\_5572\_cov\_28.541996 5591-5601. Max. coverage (+): 0. Max coverage (-): 0.02

Region: NODE\_121434\_length\_5572\_cov\_28.541996 5602-5613. Max. coverage (+): 0. Max coverage (-): 0.02

Region: NODE\_121434\_length\_5572\_cov\_28.541996 5614-5624. Max. coverage (+): 0. Max coverage (-): 0.02

Region: NODE\_121434\_length\_5572\_cov\_28.541996 5625-5635. Max. coverage (+): 0. Max coverage (-): 0.1

Region: NODE\_121434\_length\_5572\_cov\_28.541996 5636-5647. Max. coverage (+): 0. Max coverage (-): 0.09

Region: NODE\_121434\_length\_5572\_cov\_28.541996 5648-5658. Max. coverage (+): 0. Max coverage (-): 0

Region: NODE\_121434\_length\_5572\_cov\_28.541996 5659-5669. Max. coverage (+): 0. Max coverage (-): 0.02

Region: NODE\_121434\_length\_5572\_cov\_28.541996 5670-5681. Max. coverage (+): 0. Max coverage (-): 0

Region: NODE\_121434\_length\_5572\_cov\_28.541996 5682-. Max. coverage (+): 0. Max coverage (-): 0

RepeatMasker Color Code

**+**

100-98% Identity

<98-95% Identity

<95-90% Identity

<90-85% Identity

<85-80% Identity

<80-75% Identity

<75-70% Identity

<70% Identity

**-**

Gene Set Color Code

**+**

Gene

Pseudogene

Other

**-**

Topology/Coverage Color Code

Coverage Plus Strand

Coverage Minus Strand

Mainstrand: Plus

Mainstrand: Minus

Complementary Strand

Flanking Region  
(if option -flank >0)

Gene Set Annotation  
  
RepeatMasker Annotation  

**1. AlRepD-180**: 1-52 (-), Divergence to consensus: 17.3%  
**2. AlRepC-2154**: 148-195 (+), Divergence to consensus: 4.2%  
**3. AlRepD-180**: 196-376 (-), Divergence to consensus: 37.7%  
**4. AlRepC-905**: 2062-2220 (+), Divergence to consensus: 37.2%  
**5. AlRepC-1227**: 2256-2509 (+), Divergence to consensus: 39.7%  
**6. AlRepC-1227**: 2601-2675 (+), Divergence to consensus: 33.1%  
**7. (AC)n**: 2981-3010 (+), Divergence to consensus: 0%  
**8. AlRepA-24**: 4414-4536 (+), Divergence to consensus: 36.8%  
**9. AlRepA-485**: 4438-4670 (-), Divergence to consensus: 47.3%  
**10. AlRepD-1165**: 4709-4760 (-), Divergence to consensus: 23.1%

  
Transcription Factor Binding Sites  

**RHOXF1** (Sequence: AGATCA (-): 1729)  
**RHOXF1** (Sequence: AGCTTA (-): 1907)  
**RHOXF1** (Sequence: AGATCA (-): 2354)  
**RHOXF1** (Sequence: GGCTTA (-): 2806)  
**RHOXF1** (Sequence: AGATCA (-): 3236)  
**RHOXF1** (Sequence: GGATTA (-): 3610)  
**RHOXF1** (Sequence: AGATTA (-): 3997)  
**RHOXF1** (Sequence: GGCTTA (-): 4002)  
**RHOXF1** (Sequence: AGCTCA (-): 4258)  
**RHOXF1** (Sequence: AGCTCA (-): 5497)  
**RHOXF1** (Sequence: AGCTCA (-): 5512)  
**RHOXF1** (Sequence: GGCTCA (-): 5600)  
**RHOXF1** (Sequence: TAATCT (+): 689)  
**RHOXF1** (Sequence: TAATCC (+): 1122)  
**RHOXF1** (Sequence: TAATCT (+): 1228)  
**RHOXF1** (Sequence: TGAGCT (+): 1958)  
**RHOXF1** (Sequence: TGAGCT (+): 2093)  
**RHOXF1** (Sequence: TGAGCC (+): 2149)  
**RHOXF1** (Sequence: TAAGCT (+): 4153)  
**RHOXF1** (Sequence: TGAGCT (+): 4256)  
**RHOXF1** (Sequence: TAATCC (+): 5477)  
**RHOXF1** (Sequence: TAAGCT (+): 5510)  
**RHOXF1** (Sequence: TGAGCT (+): 5562)  
**Gata4** (Sequence: CTTATCT (+): 2808)  
**Gata4** (Sequence: GTTATCT (+): 5135)  
**Sox5** (Sequence: ATTGTT (+): 925)  
**Sox5** (Sequence: ATTGTT (+): 1871)  
**Sox5** (Sequence: ATTGTT (+): 5334)  
**SOX9** (Sequence: CCATTGTT (+): 923)  
**FOXO3\_mmu** (Sequence: TGAAAACA (+): 751)  
**Nobox** (Sequence: GCCAATTA (-): 1661)  
**FOXO1** (Sequence: AAAAACAAG (-): 909)  
**FOXO1** (Sequence: AAAAACAAC (-): 2180)  
**FOXO1** (Sequence: AAAAACAAC (-): 3340)  
**FOXO1** (Sequence: ATAAACAGC (-): 4161)  
**Nobox** (Sequence: TAATTGCT (+): 1680)  
**POU2F1** (Sequence: ATTTGAATA (-): 5590)  
**Rhox11** (Sequence: TGCTGTAAA (+): 4301)  
**Rhox11** (Sequence: AAAACACCA (-): 351)  
**Rhox11** (Sequence: TAAACAGCA (-): 4162)  
**Gata4** (Sequence: AGATAAC (-): 2260)  
**Sox5** (Sequence: AACAAT (-): 4557)  
**POU2F1** (Sequence: TATGTTAAT (+): 816)  
**POU5F1** (Sequence: ATGCAAA (+): 1776)
