# Supplementary material for: First characterization of PIWI-interacting RNA clusters in a cichlid fish with a B chromosome
Source: BMC Biol. 2022 Sep 21;20:204. doi: 10.1186/s12915-022-01403-2 (PMC9490952; doi:10.1186/s12915-022-01403-2)
Supplement: Supplementary file 1 — Additional file 1. Zipped folder with fasta and interactive html piRNA cluster information for the A. latifasciata genome. The nomenclature is as follows: number-pirna-cluster_sex_B-presence (f, female; m, male; 0b, without B chromosome; 1b, with B chromosome). [file 12915_2022_1403_MOESM1_ESM.zip › 13_f1b.html]

piRNA cluster 13\_f1b 21


Predicted piRNA cluster no. 13\_f1b
  

Show proTRAC run info
Hide proTRAC run info

/\  
                \_\_\_\_\_\_\_\_\_\_\_\_\_\_\_\_\_\_\_\_\_\_\_/\\_\_\_ /  \\_\_\_\_\_\_\_  
               I                      /  \  /    \      I  
               I     pro             /    \/      \     I  
               I        TRAC        /               \   I  
               I   \_\_\_\_\_\_\_\_\_\_\_\_\_\_\_\_/\_\_\_\_\_\_\_\_\_\_\_\_\_\_\_\_\_\\_ I  
               I   \              /                     I  
               I    \            /                      I  
               I     \  /\      /       V.2.4.2         I  
               I      \/  \    /                        I  
               I\_\_\_\_\_\_\_\_\_\_\_\  /\_\_\_\_\_\_\_\_\_\_\_\_\_\_\_\_\_\_\_\_\_\_\_\_\_I  
                            \/  
  
  
================================= proTRAC ====================================  
VERSION: .......... 2.4.2  
LAST MODIFIED: .... 11. May 2018  
  
Please cite:  
Rosenkranz D, Zischler H. proTRAC - a software for probabilistic piRNA cluster  
detection, visualization and analysis. 2012. BMC Bioinformatics 13:5.  
  
  
Contact:  
David Rosenkranz  
Institute of Organismic and Molecular Evolutionary Biology  
Dept. Anthropology, small RNA group  
Johannes Gutenberg University Mainz  
email: rosenkranz@uni-mainz.de  
  
You can find the latest proTRAC version at:  
http://sourceforge.net/projects/protrac/files  
http://www.smallRNAgroup-mainz.de/software  
==============================================================================  
  
PARAMETERS:  
Map file: ...............piwi-femeas-1B.fa-collapse.map  
Genome file: ............../../../0B\_ala\_genome.fa  
RepeatMasker annotation: Alatifasciata-all0B-maryan-v2.fa\_corrected.out  
GeneSet:................./guest-storage/Data/annotation/Alatifasciata\_all0B\_maryan-v2\_out2017.gff  
  
Significant (p<=0.01) hit density will be calculated based  
on observed hit distribution.  
  
Sliding window size: ........................................ 5000 bp  
Sliding window increament: .................................. 1000 bp  
Normalize each hit by number of genomic hits: ............... yes  
Normalize each hit by number of sequence reads: ............. yes  
Normalize values (-> per million mapped reads): ............. yes  
Min. fraction of hits with 1T(U) or 10A: .................... 0.75  
Alternatively: Min. fraction of hits with 1T(U) and 10A: .... 0.5  
Min. fraction of hits with typical piRNA length: ............ 0.75  
Typical piRNA length: ....................................... 24-32 nt  
Min. size of a piRNA cluster: ............................... 1000 bp.  
Min. number of hits (absolute): ............................. 0  
Min. number of hits (normalized): ........................... 0  
Min. fraction of hits on the mainstrand: .................... 0.75  
Top fraction of mapped sequences (in terms of read counts): . 1%  
Top fraction accounts for max. n% of sequence reads: ........ 90%  
Min. fraction of hits on each arm of a bidirectional cluster: 0.05  
Output html file for each cluster: .......................... yes  
Output a summary table: ..................................... yes  
Output a FASTA file for each cluster (piRNA sequences): ..... yes  
Output a FASTA file comprising cluster sequences: ........... yes  
Output a GTF file for predicted piRNA clusters: ..............yes  
Search DNA motifs in clusters: .............................. yes  
Output flanking sequences: +/- .............................. 0 bp  
Output ~.pTi file: .......................................... no  
==============================================================================  
  
  
Genome size (without gaps): ............ 758543724 bp  
Gaps (N/X/-): .......................... 417479 bp  
Mapped reads: .......................... 10641844  
Non-identical sequences: ............... 2832837  
Genomic hits: .......................... 26056853  
Significant densitiy of mapped reads: .. 368.713530323068 reads/kb

Show proTRAC cluster info
Hide proTRAC cluster info

|  |  |
| --- | --- |
| Location | NODE\_121434\_length\_5572\_cov\_28.541996 |
| Coordinates | 2-5660 |
| Size [bp] | 5659 |
| Sequence hit loci | 1919 |
| Mapped reads (normalized) | 4641.7 |
| Mapped reads (normalized) per kb | 820.2 |
| Normalized reads with 1T (1U) | 74.1% |
| Normalized reads with 10A | 57.6% |
| Normalized reads with length 24-32 nt | 99.6% |
| Normalized reads on the main strand(s) | 81.6% |
| Predicted directionality | mono:minus |

100%

0%

1T (1U)  
reads

10A reads

24-32 nt  
reads

reads on mainstrand

**Either the amount of reads with 1T (1U) OR 10A has to exceed 75% (set with option: -1Tor10A)  
Alternatively the amount of reads with 1T (1U) AND 10A has to exceed 50% (set with option: -1Tand10A)  
Minimum amount of reads with preferred size is 75% (set with option: -pisize)  
Minimum amount of reads on the main strand(s) is 75% (set with option: -clstrand)**

Show read coverage
Hide read coverage

WHAT DO I SEE HERE?  
This chart shows the location of mapped sequence reads within a predicted piRNA cluster. The color refers to the number of genomic hits produced by the sequence read in question. A dark red bar indicates that this sequence read produces many other hits elsewhere in the genome. Many adjacent red or yellow bars can indicate the presence of a multi-copy element such as transposons or rRNA genes. A dark green bar indicates that this sequence read maps uniquely to this locus.

1 hit

2-5 hits

6-10 hits

11-20 hits

21-50 hits

51-100 hits

> 100 hits

NODE\_121434\_length\_5572\_cov\_28.541996

2

5660

Gene Set

RepeatMasker

Mapped  
Reads

28.19

plus strand

minus strand

28.19

Region: NODE\_121434\_length\_5572\_cov\_28.541996 15779-7. Max. coverage (+): 0.19. Max coverage (-): 0.09

Region: NODE\_121434\_length\_5572\_cov\_28.541996 8-18. Max. coverage (+): 0.09. Max coverage (-): 0

Region: NODE\_121434\_length\_5572\_cov\_28.541996 19-30. Max. coverage (+): 0. Max coverage (-): 0

Region: NODE\_121434\_length\_5572\_cov\_28.541996 31-41. Max. coverage (+): 0. Max coverage (-): 0.85

Region: NODE\_121434\_length\_5572\_cov\_28.541996 42-52. Max. coverage (+): 0. Max coverage (-): 0

Region: NODE\_121434\_length\_5572\_cov\_28.541996 53-64. Max. coverage (+): 0. Max coverage (-): 0

Region: NODE\_121434\_length\_5572\_cov\_28.541996 65-75. Max. coverage (+): 0.05. Max coverage (-): 0.14

Region: NODE\_121434\_length\_5572\_cov\_28.541996 76-86. Max. coverage (+): 0.05. Max coverage (-): 0.23

Region: NODE\_121434\_length\_5572\_cov\_28.541996 87-98. Max. coverage (+): 0. Max coverage (-): 0.09

Region: NODE\_121434\_length\_5572\_cov\_28.541996 99-109. Max. coverage (+): 0. Max coverage (-): 8.74

Region: NODE\_121434\_length\_5572\_cov\_28.541996 110-120. Max. coverage (+): 0. Max coverage (-): 0.14

Region: NODE\_121434\_length\_5572\_cov\_28.541996 121-132. Max. coverage (+): 0. Max coverage (-): 0.14

Region: NODE\_121434\_length\_5572\_cov\_28.541996 133-143. Max. coverage (+): 0. Max coverage (-): 0

Region: NODE\_121434\_length\_5572\_cov\_28.541996 144-154. Max. coverage (+): 0. Max coverage (-): 0

Region: NODE\_121434\_length\_5572\_cov\_28.541996 155-166. Max. coverage (+): 0. Max coverage (-): 0

Region: NODE\_121434\_length\_5572\_cov\_28.541996 167-177. Max. coverage (+): 0. Max coverage (-): 0

Region: NODE\_121434\_length\_5572\_cov\_28.541996 178-188. Max. coverage (+): 0. Max coverage (-): 0

Region: NODE\_121434\_length\_5572\_cov\_28.541996 189-200. Max. coverage (+): 0.28. Max coverage (-): 0.19

Region: NODE\_121434\_length\_5572\_cov\_28.541996 201-211. Max. coverage (+): 0.19. Max coverage (-): 0.09

Region: NODE\_121434\_length\_5572\_cov\_28.541996 212-222. Max. coverage (+): 0.19. Max coverage (-): 0.19

Region: NODE\_121434\_length\_5572\_cov\_28.541996 223-234. Max. coverage (+): 0. Max coverage (-): 0.19

Region: NODE\_121434\_length\_5572\_cov\_28.541996 235-245. Max. coverage (+): 0. Max coverage (-): 0.19

Region: NODE\_121434\_length\_5572\_cov\_28.541996 246-256. Max. coverage (+): 0. Max coverage (-): 0.09

Region: NODE\_121434\_length\_5572\_cov\_28.541996 257-267. Max. coverage (+): 0. Max coverage (-): 0

Region: NODE\_121434\_length\_5572\_cov\_28.541996 268-279. Max. coverage (+): 0. Max coverage (-): 0.28

Region: NODE\_121434\_length\_5572\_cov\_28.541996 280-290. Max. coverage (+): 0. Max coverage (-): 0.19

Region: NODE\_121434\_length\_5572\_cov\_28.541996 291-301. Max. coverage (+): 0.28. Max coverage (-): 0

Region: NODE\_121434\_length\_5572\_cov\_28.541996 302-313. Max. coverage (+): 0.05. Max coverage (-): 0

Region: NODE\_121434\_length\_5572\_cov\_28.541996 314-324. Max. coverage (+): 0. Max coverage (-): 0.94

Region: NODE\_121434\_length\_5572\_cov\_28.541996 325-335. Max. coverage (+): 0. Max coverage (-): 0.19

Region: NODE\_121434\_length\_5572\_cov\_28.541996 336-347. Max. coverage (+): 0. Max coverage (-): 0

Region: NODE\_121434\_length\_5572\_cov\_28.541996 348-358. Max. coverage (+): 0.19. Max coverage (-): 0

Region: NODE\_121434\_length\_5572\_cov\_28.541996 359-369. Max. coverage (+): 0. Max coverage (-): 2.82

Region: NODE\_121434\_length\_5572\_cov\_28.541996 370-381. Max. coverage (+): 0.09. Max coverage (-): 2.82

Region: NODE\_121434\_length\_5572\_cov\_28.541996 382-392. Max. coverage (+): 0.05. Max coverage (-): 0.23

Region: NODE\_121434\_length\_5572\_cov\_28.541996 393-403. Max. coverage (+): 0. Max coverage (-): 0.14

Region: NODE\_121434\_length\_5572\_cov\_28.541996 404-415. Max. coverage (+): 0. Max coverage (-): 8.74

Region: NODE\_121434\_length\_5572\_cov\_28.541996 416-426. Max. coverage (+): 0. Max coverage (-): 0.14

Region: NODE\_121434\_length\_5572\_cov\_28.541996 427-437. Max. coverage (+): 0. Max coverage (-): 0.14

Region: NODE\_121434\_length\_5572\_cov\_28.541996 438-449. Max. coverage (+): 0.19. Max coverage (-): 2.44

Region: NODE\_121434\_length\_5572\_cov\_28.541996 450-460. Max. coverage (+): 0.19. Max coverage (-): 2.44

Region: NODE\_121434\_length\_5572\_cov\_28.541996 461-471. Max. coverage (+): 0. Max coverage (-): 0.56

Region: NODE\_121434\_length\_5572\_cov\_28.541996 472-483. Max. coverage (+): 0. Max coverage (-): 0.85

Region: NODE\_121434\_length\_5572\_cov\_28.541996 484-494. Max. coverage (+): 0.19. Max coverage (-): 0.66

Region: NODE\_121434\_length\_5572\_cov\_28.541996 495-505. Max. coverage (+): 0.09. Max coverage (-): 0.85

Region: NODE\_121434\_length\_5572\_cov\_28.541996 506-516. Max. coverage (+): 0.28. Max coverage (-): 0.19

Region: NODE\_121434\_length\_5572\_cov\_28.541996 517-528. Max. coverage (+): 0. Max coverage (-): 0

Region: NODE\_121434\_length\_5572\_cov\_28.541996 529-539. Max. coverage (+): 0. Max coverage (-): 0

Region: NODE\_121434\_length\_5572\_cov\_28.541996 540-550. Max. coverage (+): 0. Max coverage (-): 0

Region: NODE\_121434\_length\_5572\_cov\_28.541996 551-562. Max. coverage (+): 0.09. Max coverage (-): 1.69

Region: NODE\_121434\_length\_5572\_cov\_28.541996 563-573. Max. coverage (+): 0.09. Max coverage (-): 0.28

Region: NODE\_121434\_length\_5572\_cov\_28.541996 574-584. Max. coverage (+): 0. Max coverage (-): 0.19

Region: NODE\_121434\_length\_5572\_cov\_28.541996 585-596. Max. coverage (+): 0. Max coverage (-): 0

Region: NODE\_121434\_length\_5572\_cov\_28.541996 597-607. Max. coverage (+): 0. Max coverage (-): 0.85

Region: NODE\_121434\_length\_5572\_cov\_28.541996 608-618. Max. coverage (+): 0.47. Max coverage (-): 0.66

Region: NODE\_121434\_length\_5572\_cov\_28.541996 619-630. Max. coverage (+): 1.22. Max coverage (-): 0.38

Region: NODE\_121434\_length\_5572\_cov\_28.541996 631-641. Max. coverage (+): 0. Max coverage (-): 10.24

Region: NODE\_121434\_length\_5572\_cov\_28.541996 642-652. Max. coverage (+): 0.19. Max coverage (-): 0.09

Region: NODE\_121434\_length\_5572\_cov\_28.541996 653-664. Max. coverage (+): 0.28. Max coverage (-): 1.88

Region: NODE\_121434\_length\_5572\_cov\_28.541996 665-675. Max. coverage (+): 0. Max coverage (-): 0.09

Region: NODE\_121434\_length\_5572\_cov\_28.541996 676-686. Max. coverage (+): 0. Max coverage (-): 0

Region: NODE\_121434\_length\_5572\_cov\_28.541996 687-698. Max. coverage (+): 0.09. Max coverage (-): 1.5

Region: NODE\_121434\_length\_5572\_cov\_28.541996 699-709. Max. coverage (+): 0. Max coverage (-): 0.09

Region: NODE\_121434\_length\_5572\_cov\_28.541996 710-720. Max. coverage (+): 0. Max coverage (-): 0.28

Region: NODE\_121434\_length\_5572\_cov\_28.541996 721-732. Max. coverage (+): 0.19. Max coverage (-): 0.09

Region: NODE\_121434\_length\_5572\_cov\_28.541996 733-743. Max. coverage (+): 0. Max coverage (-): 1.79

Region: NODE\_121434\_length\_5572\_cov\_28.541996 744-754. Max. coverage (+): 0. Max coverage (-): 0.38

Region: NODE\_121434\_length\_5572\_cov\_28.541996 755-765. Max. coverage (+): 1.32. Max coverage (-): 0.19

Region: NODE\_121434\_length\_5572\_cov\_28.541996 766-777. Max. coverage (+): 0.09. Max coverage (-): 0.38

Region: NODE\_121434\_length\_5572\_cov\_28.541996 778-788. Max. coverage (+): 0. Max coverage (-): 0.38

Region: NODE\_121434\_length\_5572\_cov\_28.541996 789-799. Max. coverage (+): 0. Max coverage (-): 0.09

Region: NODE\_121434\_length\_5572\_cov\_28.541996 800-811. Max. coverage (+): 0. Max coverage (-): 0

Region: NODE\_121434\_length\_5572\_cov\_28.541996 812-822. Max. coverage (+): 0. Max coverage (-): 0

Region: NODE\_121434\_length\_5572\_cov\_28.541996 823-833. Max. coverage (+): 0. Max coverage (-): 0

Region: NODE\_121434\_length\_5572\_cov\_28.541996 834-845. Max. coverage (+): 0.66. Max coverage (-): 0.19

Region: NODE\_121434\_length\_5572\_cov\_28.541996 846-856. Max. coverage (+): 0.56. Max coverage (-): 0

Region: NODE\_121434\_length\_5572\_cov\_28.541996 857-867. Max. coverage (+): 0.28. Max coverage (-): 5.54

Region: NODE\_121434\_length\_5572\_cov\_28.541996 868-879. Max. coverage (+): 0. Max coverage (-): 3.01

Region: NODE\_121434\_length\_5572\_cov\_28.541996 880-890. Max. coverage (+): 0.09. Max coverage (-): 0

Region: NODE\_121434\_length\_5572\_cov\_28.541996 891-901. Max. coverage (+): 0. Max coverage (-): 0.56

Region: NODE\_121434\_length\_5572\_cov\_28.541996 902-913. Max. coverage (+): 0. Max coverage (-): 0.47

Region: NODE\_121434\_length\_5572\_cov\_28.541996 914-924. Max. coverage (+): 0. Max coverage (-): 0.19

Region: NODE\_121434\_length\_5572\_cov\_28.541996 925-935. Max. coverage (+): 0.56. Max coverage (-): 0.19

Region: NODE\_121434\_length\_5572\_cov\_28.541996 936-947. Max. coverage (+): 0. Max coverage (-): 0

Region: NODE\_121434\_length\_5572\_cov\_28.541996 948-958. Max. coverage (+): 0.09. Max coverage (-): 1.6

Region: NODE\_121434\_length\_5572\_cov\_28.541996 959-969. Max. coverage (+): 0. Max coverage (-): 1.22

Region: NODE\_121434\_length\_5572\_cov\_28.541996 970-981. Max. coverage (+): 0.47. Max coverage (-): 0.19

Region: NODE\_121434\_length\_5572\_cov\_28.541996 982-992. Max. coverage (+): 0. Max coverage (-): 1.69

Region: NODE\_121434\_length\_5572\_cov\_28.541996 993-1003. Max. coverage (+): 0.38. Max coverage (-): 0.19

Region: NODE\_121434\_length\_5572\_cov\_28.541996 1004-1014. Max. coverage (+): 0.38. Max coverage (-): 0.56

Region: NODE\_121434\_length\_5572\_cov\_28.541996 1015-1026. Max. coverage (+): 0.09. Max coverage (-): 4.23

Region: NODE\_121434\_length\_5572\_cov\_28.541996 1027-1037. Max. coverage (+): 0.75. Max coverage (-): 4.23

Region: NODE\_121434\_length\_5572\_cov\_28.541996 1038-1048. Max. coverage (+): 0.09. Max coverage (-): 2.26

Region: NODE\_121434\_length\_5572\_cov\_28.541996 1049-1060. Max. coverage (+): 0. Max coverage (-): 2.63

Region: NODE\_121434\_length\_5572\_cov\_28.541996 1061-1071. Max. coverage (+): 0.56. Max coverage (-): 0

Region: NODE\_121434\_length\_5572\_cov\_28.541996 1072-1082. Max. coverage (+): 0.09. Max coverage (-): 0.28

Region: NODE\_121434\_length\_5572\_cov\_28.541996 1083-1094. Max. coverage (+): 0. Max coverage (-): 2.35

Region: NODE\_121434\_length\_5572\_cov\_28.541996 1095-1105. Max. coverage (+): 0.56. Max coverage (-): 3.01

Region: NODE\_121434\_length\_5572\_cov\_28.541996 1106-1116. Max. coverage (+): 0.47. Max coverage (-): 2.82

Region: NODE\_121434\_length\_5572\_cov\_28.541996 1117-1128. Max. coverage (+): 0. Max coverage (-): 0.19

Region: NODE\_121434\_length\_5572\_cov\_28.541996 1129-1139. Max. coverage (+): 0.09. Max coverage (-): 0.66

Region: NODE\_121434\_length\_5572\_cov\_28.541996 1140-1150. Max. coverage (+): 0. Max coverage (-): 0.66

Region: NODE\_121434\_length\_5572\_cov\_28.541996 1151-1162. Max. coverage (+): 1.03. Max coverage (-): 0.19

Region: NODE\_121434\_length\_5572\_cov\_28.541996 1163-1173. Max. coverage (+): 0.09. Max coverage (-): 5.07

Region: NODE\_121434\_length\_5572\_cov\_28.541996 1174-1184. Max. coverage (+): 0.19. Max coverage (-): 1.69

Region: NODE\_121434\_length\_5572\_cov\_28.541996 1185-1196. Max. coverage (+): 1.13. Max coverage (-): 0.19

Region: NODE\_121434\_length\_5572\_cov\_28.541996 1197-1207. Max. coverage (+): 0. Max coverage (-): 8.65

Region: NODE\_121434\_length\_5572\_cov\_28.541996 1208-1218. Max. coverage (+): 0. Max coverage (-): 7.99

Region: NODE\_121434\_length\_5572\_cov\_28.541996 1219-1230. Max. coverage (+): 0.09. Max coverage (-): 6.86

Region: NODE\_121434\_length\_5572\_cov\_28.541996 1231-1241. Max. coverage (+): 2.63. Max coverage (-): 0

Region: NODE\_121434\_length\_5572\_cov\_28.541996 1242-1252. Max. coverage (+): 0.09. Max coverage (-): 0.38

Region: NODE\_121434\_length\_5572\_cov\_28.541996 1253-1263. Max. coverage (+): 0.09. Max coverage (-): 0.56

Region: NODE\_121434\_length\_5572\_cov\_28.541996 1264-1275. Max. coverage (+): 0. Max coverage (-): 0.09

Region: NODE\_121434\_length\_5572\_cov\_28.541996 1276-1286. Max. coverage (+): 0. Max coverage (-): 2.82

Region: NODE\_121434\_length\_5572\_cov\_28.541996 1287-1297. Max. coverage (+): 0. Max coverage (-): 3.01

Region: NODE\_121434\_length\_5572\_cov\_28.541996 1298-1309. Max. coverage (+): 1.03. Max coverage (-): 0.09

Region: NODE\_121434\_length\_5572\_cov\_28.541996 1310-1320. Max. coverage (+): 0.09. Max coverage (-): 0.38

Region: NODE\_121434\_length\_5572\_cov\_28.541996 1321-1331. Max. coverage (+): 0.09. Max coverage (-): 1.88

Region: NODE\_121434\_length\_5572\_cov\_28.541996 1332-1343. Max. coverage (+): 1.03. Max coverage (-): 0.85

Region: NODE\_121434\_length\_5572\_cov\_28.541996 1344-1354. Max. coverage (+): 0.47. Max coverage (-): 0

Region: NODE\_121434\_length\_5572\_cov\_28.541996 1355-1365. Max. coverage (+): 0. Max coverage (-): 1.41

Region: NODE\_121434\_length\_5572\_cov\_28.541996 1366-1377. Max. coverage (+): 0.19. Max coverage (-): 0

Region: NODE\_121434\_length\_5572\_cov\_28.541996 1378-1388. Max. coverage (+): 0.09. Max coverage (-): 0.28

Region: NODE\_121434\_length\_5572\_cov\_28.541996 1389-1399. Max. coverage (+): 0. Max coverage (-): 14.28

Region: NODE\_121434\_length\_5572\_cov\_28.541996 1400-1411. Max. coverage (+): 1.6. Max coverage (-): 0

Region: NODE\_121434\_length\_5572\_cov\_28.541996 1412-1422. Max. coverage (+): 1.6. Max coverage (-): 0.09

Region: NODE\_121434\_length\_5572\_cov\_28.541996 1423-1433. Max. coverage (+): 0. Max coverage (-): 0.09

Region: NODE\_121434\_length\_5572\_cov\_28.541996 1434-1445. Max. coverage (+): 0.38. Max coverage (-): 0.09

Region: NODE\_121434\_length\_5572\_cov\_28.541996 1446-1456. Max. coverage (+): 0.09. Max coverage (-): 0.47

Region: NODE\_121434\_length\_5572\_cov\_28.541996 1457-1467. Max. coverage (+): 0. Max coverage (-): 0.38

Region: NODE\_121434\_length\_5572\_cov\_28.541996 1468-1478. Max. coverage (+): 0.75. Max coverage (-): 0.47

Region: NODE\_121434\_length\_5572\_cov\_28.541996 1479-1490. Max. coverage (+): 0.09. Max coverage (-): 5.45

Region: NODE\_121434\_length\_5572\_cov\_28.541996 1491-1501. Max. coverage (+): 0.19. Max coverage (-): 0.19

Region: NODE\_121434\_length\_5572\_cov\_28.541996 1502-1512. Max. coverage (+): 0.19. Max coverage (-): 1.6

Region: NODE\_121434\_length\_5572\_cov\_28.541996 1513-1524. Max. coverage (+): 0. Max coverage (-): 2.73

Region: NODE\_121434\_length\_5572\_cov\_28.541996 1525-1535. Max. coverage (+): 0.19. Max coverage (-): 0.19

Region: NODE\_121434\_length\_5572\_cov\_28.541996 1536-1546. Max. coverage (+): 0. Max coverage (-): 0.09

Region: NODE\_121434\_length\_5572\_cov\_28.541996 1547-1558. Max. coverage (+): 0. Max coverage (-): 2.07

Region: NODE\_121434\_length\_5572\_cov\_28.541996 1559-1569. Max. coverage (+): 0. Max coverage (-): 1.03

Region: NODE\_121434\_length\_5572\_cov\_28.541996 1570-1580. Max. coverage (+): 1.03. Max coverage (-): 0.19

Region: NODE\_121434\_length\_5572\_cov\_28.541996 1581-1592. Max. coverage (+): 0. Max coverage (-): 0.19

Region: NODE\_121434\_length\_5572\_cov\_28.541996 1593-1603. Max. coverage (+): 0.09. Max coverage (-): 0.09

Region: NODE\_121434\_length\_5572\_cov\_28.541996 1604-1614. Max. coverage (+): 0.19. Max coverage (-): 0.09

Region: NODE\_121434\_length\_5572\_cov\_28.541996 1615-1626. Max. coverage (+): 0.19. Max coverage (-): 0.09

Region: NODE\_121434\_length\_5572\_cov\_28.541996 1627-1637. Max. coverage (+): 0.09. Max coverage (-): 4.32

Region: NODE\_121434\_length\_5572\_cov\_28.541996 1638-1648. Max. coverage (+): 0.09. Max coverage (-): 4.51

Region: NODE\_121434\_length\_5572\_cov\_28.541996 1649-1660. Max. coverage (+): 0.56. Max coverage (-): 0

Region: NODE\_121434\_length\_5572\_cov\_28.541996 1661-1671. Max. coverage (+): 0. Max coverage (-): 0

Region: NODE\_121434\_length\_5572\_cov\_28.541996 1672-1682. Max. coverage (+): 0. Max coverage (-): 0

Region: NODE\_121434\_length\_5572\_cov\_28.541996 1683-1694. Max. coverage (+): 0.09. Max coverage (-): 0.28

Region: NODE\_121434\_length\_5572\_cov\_28.541996 1695-1705. Max. coverage (+): 0. Max coverage (-): 7.8

Region: NODE\_121434\_length\_5572\_cov\_28.541996 1706-1716. Max. coverage (+): 0. Max coverage (-): 7.61

Region: NODE\_121434\_length\_5572\_cov\_28.541996 1717-1727. Max. coverage (+): 0.19. Max coverage (-): 0

Region: NODE\_121434\_length\_5572\_cov\_28.541996 1728-1739. Max. coverage (+): 0.19. Max coverage (-): 0

Region: NODE\_121434\_length\_5572\_cov\_28.541996 1740-1750. Max. coverage (+): 0. Max coverage (-): 0

Region: NODE\_121434\_length\_5572\_cov\_28.541996 1751-1761. Max. coverage (+): 0. Max coverage (-): 0

Region: NODE\_121434\_length\_5572\_cov\_28.541996 1762-1773. Max. coverage (+): 0. Max coverage (-): 0

Region: NODE\_121434\_length\_5572\_cov\_28.541996 1774-1784. Max. coverage (+): 0. Max coverage (-): 0

Region: NODE\_121434\_length\_5572\_cov\_28.541996 1785-1795. Max. coverage (+): 0.38. Max coverage (-): 0.09

Region: NODE\_121434\_length\_5572\_cov\_28.541996 1796-1807. Max. coverage (+): 0. Max coverage (-): 0.19

Region: NODE\_121434\_length\_5572\_cov\_28.541996 1808-1818. Max. coverage (+): 0. Max coverage (-): 0.38

Region: NODE\_121434\_length\_5572\_cov\_28.541996 1819-1829. Max. coverage (+): 0.19. Max coverage (-): 0

Region: NODE\_121434\_length\_5572\_cov\_28.541996 1830-1841. Max. coverage (+): 0.09. Max coverage (-): 0

Region: NODE\_121434\_length\_5572\_cov\_28.541996 1842-1852. Max. coverage (+): 0. Max coverage (-): 0

Region: NODE\_121434\_length\_5572\_cov\_28.541996 1853-1863. Max. coverage (+): 1.79. Max coverage (-): 0

Region: NODE\_121434\_length\_5572\_cov\_28.541996 1864-1875. Max. coverage (+): 0.09. Max coverage (-): 0.19

Region: NODE\_121434\_length\_5572\_cov\_28.541996 1876-1886. Max. coverage (+): 0.09. Max coverage (-): 0.66

Region: NODE\_121434\_length\_5572\_cov\_28.541996 1887-1897. Max. coverage (+): 0. Max coverage (-): 0.38

Region: NODE\_121434\_length\_5572\_cov\_28.541996 1898-1909. Max. coverage (+): 0.09. Max coverage (-): 0.09

Region: NODE\_121434\_length\_5572\_cov\_28.541996 1910-1920. Max. coverage (+): 0.09. Max coverage (-): 3.85

Region: NODE\_121434\_length\_5572\_cov\_28.541996 1921-1931. Max. coverage (+): 0. Max coverage (-): 6.39

Region: NODE\_121434\_length\_5572\_cov\_28.541996 1932-1943. Max. coverage (+): 0.66. Max coverage (-): 0.47

Region: NODE\_121434\_length\_5572\_cov\_28.541996 1944-1954. Max. coverage (+): 1.5. Max coverage (-): 0.85

Region: NODE\_121434\_length\_5572\_cov\_28.541996 1955-1965. Max. coverage (+): 0. Max coverage (-): 10.81

Region: NODE\_121434\_length\_5572\_cov\_28.541996 1966-1976. Max. coverage (+): 0.38. Max coverage (-): 0

Region: NODE\_121434\_length\_5572\_cov\_28.541996 1977-1988. Max. coverage (+): 0.38. Max coverage (-): 2.07

Region: NODE\_121434\_length\_5572\_cov\_28.541996 1989-1999. Max. coverage (+): 0.19. Max coverage (-): 0.56

Region: NODE\_121434\_length\_5572\_cov\_28.541996 2000-2010. Max. coverage (+): 0.56. Max coverage (-): 1.5

Region: NODE\_121434\_length\_5572\_cov\_28.541996 2011-2022. Max. coverage (+): 1.03. Max coverage (-): 0.19

Region: NODE\_121434\_length\_5572\_cov\_28.541996 2023-2033. Max. coverage (+): 0. Max coverage (-): 0.28

Region: NODE\_121434\_length\_5572\_cov\_28.541996 2034-2044. Max. coverage (+): 0. Max coverage (-): 1.03

Region: NODE\_121434\_length\_5572\_cov\_28.541996 2045-2056. Max. coverage (+): 0. Max coverage (-): 0

Region: NODE\_121434\_length\_5572\_cov\_28.541996 2057-2067. Max. coverage (+): 0.28. Max coverage (-): 0.28

Region: NODE\_121434\_length\_5572\_cov\_28.541996 2068-2078. Max. coverage (+): 0.09. Max coverage (-): 0.66

Region: NODE\_121434\_length\_5572\_cov\_28.541996 2079-2090. Max. coverage (+): 1.13. Max coverage (-): 0.38

Region: NODE\_121434\_length\_5572\_cov\_28.541996 2091-2101. Max. coverage (+): 0.09. Max coverage (-): 0.09

Region: NODE\_121434\_length\_5572\_cov\_28.541996 2102-2112. Max. coverage (+): 0.09. Max coverage (-): 1.03

Region: NODE\_121434\_length\_5572\_cov\_28.541996 2113-2124. Max. coverage (+): 0.09. Max coverage (-): 28.19

Region: NODE\_121434\_length\_5572\_cov\_28.541996 2125-2135. Max. coverage (+): 0. Max coverage (-): 0.47

Region: NODE\_121434\_length\_5572\_cov\_28.541996 2136-2146. Max. coverage (+): 1.69. Max coverage (-): 1.79

Region: NODE\_121434\_length\_5572\_cov\_28.541996 2147-2158. Max. coverage (+): 0. Max coverage (-): 0.09

Region: NODE\_121434\_length\_5572\_cov\_28.541996 2159-2169. Max. coverage (+): 0. Max coverage (-): 0.94

Region: NODE\_121434\_length\_5572\_cov\_28.541996 2170-2180. Max. coverage (+): 0. Max coverage (-): 0.56

Region: NODE\_121434\_length\_5572\_cov\_28.541996 2181-2192. Max. coverage (+): 1.88. Max coverage (-): 0.38

Region: NODE\_121434\_length\_5572\_cov\_28.541996 2193-2203. Max. coverage (+): 0. Max coverage (-): 0.75

Region: NODE\_121434\_length\_5572\_cov\_28.541996 2204-2214. Max. coverage (+): 0. Max coverage (-): 0

Region: NODE\_121434\_length\_5572\_cov\_28.541996 2215-2225. Max. coverage (+): 0. Max coverage (-): 0

Region: NODE\_121434\_length\_5572\_cov\_28.541996 2226-2237. Max. coverage (+): 0. Max coverage (-): 0.47

Region: NODE\_121434\_length\_5572\_cov\_28.541996 2238-2248. Max. coverage (+): 0. Max coverage (-): 0

Region: NODE\_121434\_length\_5572\_cov\_28.541996 2249-2259. Max. coverage (+): 1.13. Max coverage (-): 1.03

Region: NODE\_121434\_length\_5572\_cov\_28.541996 2260-2271. Max. coverage (+): 0. Max coverage (-): 0.09

Region: NODE\_121434\_length\_5572\_cov\_28.541996 2272-2282. Max. coverage (+): 0.28. Max coverage (-): 0

Region: NODE\_121434\_length\_5572\_cov\_28.541996 2283-2293. Max. coverage (+): 0.66. Max coverage (-): 2.44

Region: NODE\_121434\_length\_5572\_cov\_28.541996 2294-2305. Max. coverage (+): 0.09. Max coverage (-): 0.38

Region: NODE\_121434\_length\_5572\_cov\_28.541996 2306-2316. Max. coverage (+): 0. Max coverage (-): 0.47

Region: NODE\_121434\_length\_5572\_cov\_28.541996 2317-2327. Max. coverage (+): 0. Max coverage (-): 2.07

Region: NODE\_121434\_length\_5572\_cov\_28.541996 2328-2339. Max. coverage (+): 0.28. Max coverage (-): 2.63

Region: NODE\_121434\_length\_5572\_cov\_28.541996 2340-2350. Max. coverage (+): 0.09. Max coverage (-): 2.54

Region: NODE\_121434\_length\_5572\_cov\_28.541996 2351-2361. Max. coverage (+): 0.56. Max coverage (-): 0.09

Region: NODE\_121434\_length\_5572\_cov\_28.541996 2362-2373. Max. coverage (+): 0.09. Max coverage (-): 1.79

Region: NODE\_121434\_length\_5572\_cov\_28.541996 2374-2384. Max. coverage (+): 0. Max coverage (-): 0.28

Region: NODE\_121434\_length\_5572\_cov\_28.541996 2385-2395. Max. coverage (+): 0.09. Max coverage (-): 0.56

Region: NODE\_121434\_length\_5572\_cov\_28.541996 2396-2407. Max. coverage (+): 0. Max coverage (-): 0.09

Region: NODE\_121434\_length\_5572\_cov\_28.541996 2408-2418. Max. coverage (+): 0. Max coverage (-): 0.94

Region: NODE\_121434\_length\_5572\_cov\_28.541996 2419-2429. Max. coverage (+): 0. Max coverage (-): 0.09

Region: NODE\_121434\_length\_5572\_cov\_28.541996 2430-2441. Max. coverage (+): 0.09. Max coverage (-): 0.75

Region: NODE\_121434\_length\_5572\_cov\_28.541996 2442-2452. Max. coverage (+): 0. Max coverage (-): 0.28

Region: NODE\_121434\_length\_5572\_cov\_28.541996 2453-2463. Max. coverage (+): 0.23. Max coverage (-): 0

Region: NODE\_121434\_length\_5572\_cov\_28.541996 2464-2474. Max. coverage (+): 0. Max coverage (-): 0.85

Region: NODE\_121434\_length\_5572\_cov\_28.541996 2475-2486. Max. coverage (+): 0. Max coverage (-): 0.8

Region: NODE\_121434\_length\_5572\_cov\_28.541996 2487-2497. Max. coverage (+): 0.7. Max coverage (-): 0.09

Region: NODE\_121434\_length\_5572\_cov\_28.541996 2498-2508. Max. coverage (+): 0.42. Max coverage (-): 4.42

Region: NODE\_121434\_length\_5572\_cov\_28.541996 2509-2520. Max. coverage (+): 0.05. Max coverage (-): 3.81

Region: NODE\_121434\_length\_5572\_cov\_28.541996 2521-2531. Max. coverage (+): 0. Max coverage (-): 0

Region: NODE\_121434\_length\_5572\_cov\_28.541996 2532-2542. Max. coverage (+): 0. Max coverage (-): 0

Region: NODE\_121434\_length\_5572\_cov\_28.541996 2543-2554. Max. coverage (+): 0. Max coverage (-): 0.14

Region: NODE\_121434\_length\_5572\_cov\_28.541996 2555-2565. Max. coverage (+): 0. Max coverage (-): 0.09

Region: NODE\_121434\_length\_5572\_cov\_28.541996 2566-2576. Max. coverage (+): 0. Max coverage (-): 0

Region: NODE\_121434\_length\_5572\_cov\_28.541996 2577-2588. Max. coverage (+): 0. Max coverage (-): 0

Region: NODE\_121434\_length\_5572\_cov\_28.541996 2589-2599. Max. coverage (+): 0. Max coverage (-): 0

Region: NODE\_121434\_length\_5572\_cov\_28.541996 2600-2610. Max. coverage (+): 0. Max coverage (-): 0.09

Region: NODE\_121434\_length\_5572\_cov\_28.541996 2611-2622. Max. coverage (+): 0. Max coverage (-): 0

Region: NODE\_121434\_length\_5572\_cov\_28.541996 2623-2633. Max. coverage (+): 0.23. Max coverage (-): 0

Region: NODE\_121434\_length\_5572\_cov\_28.541996 2634-2644. Max. coverage (+): 0. Max coverage (-): 0.85

Region: NODE\_121434\_length\_5572\_cov\_28.541996 2645-2656. Max. coverage (+): 0.28. Max coverage (-): 0.14

Region: NODE\_121434\_length\_5572\_cov\_28.541996 2657-2667. Max. coverage (+): 0.7. Max coverage (-): 1.36

Region: NODE\_121434\_length\_5572\_cov\_28.541996 2668-2678. Max. coverage (+): 0.09. Max coverage (-): 4.42

Region: NODE\_121434\_length\_5572\_cov\_28.541996 2679-2690. Max. coverage (+): 0.75. Max coverage (-): 0.7

Region: NODE\_121434\_length\_5572\_cov\_28.541996 2691-2701. Max. coverage (+): 0.47. Max coverage (-): 0.09

Region: NODE\_121434\_length\_5572\_cov\_28.541996 2702-2712. Max. coverage (+): 0. Max coverage (-): 0.66

Region: NODE\_121434\_length\_5572\_cov\_28.541996 2713-2723. Max. coverage (+): 0.09. Max coverage (-): 0.19

Region: NODE\_121434\_length\_5572\_cov\_28.541996 2724-2735. Max. coverage (+): 1.13. Max coverage (-): 0.19

Region: NODE\_121434\_length\_5572\_cov\_28.541996 2736-2746. Max. coverage (+): 0. Max coverage (-): 0.66

Region: NODE\_121434\_length\_5572\_cov\_28.541996 2747-2757. Max. coverage (+): 0.09. Max coverage (-): 0.75

Region: NODE\_121434\_length\_5572\_cov\_28.541996 2758-2769. Max. coverage (+): 0.56. Max coverage (-): 0

Region: NODE\_121434\_length\_5572\_cov\_28.541996 2770-2780. Max. coverage (+): 0.38. Max coverage (-): 0

Region: NODE\_121434\_length\_5572\_cov\_28.541996 2781-2791. Max. coverage (+): 0. Max coverage (-): 0.85

Region: NODE\_121434\_length\_5572\_cov\_28.541996 2792-2803. Max. coverage (+): 0.09. Max coverage (-): 1.13

Region: NODE\_121434\_length\_5572\_cov\_28.541996 2804-2814. Max. coverage (+): 1.97. Max coverage (-): 0.47

Region: NODE\_121434\_length\_5572\_cov\_28.541996 2815-2825. Max. coverage (+): 0.19. Max coverage (-): 1.5

Region: NODE\_121434\_length\_5572\_cov\_28.541996 2826-2837. Max. coverage (+): 0. Max coverage (-): 0.85

Region: NODE\_121434\_length\_5572\_cov\_28.541996 2838-2848. Max. coverage (+): 0. Max coverage (-): 0.28

Region: NODE\_121434\_length\_5572\_cov\_28.541996 2849-2859. Max. coverage (+): 0.75. Max coverage (-): 0.19

Region: NODE\_121434\_length\_5572\_cov\_28.541996 2860-2871. Max. coverage (+): 0.28. Max coverage (-): 0.94

Region: NODE\_121434\_length\_5572\_cov\_28.541996 2872-2882. Max. coverage (+): 1.41. Max coverage (-): 0.94

Region: NODE\_121434\_length\_5572\_cov\_28.541996 2883-2893. Max. coverage (+): 0.56. Max coverage (-): 0.19

Region: NODE\_121434\_length\_5572\_cov\_28.541996 2894-2905. Max. coverage (+): 1.03. Max coverage (-): 0.19

Region: NODE\_121434\_length\_5572\_cov\_28.541996 2906-2916. Max. coverage (+): 0.19. Max coverage (-): 0.28

Region: NODE\_121434\_length\_5572\_cov\_28.541996 2917-2927. Max. coverage (+): 0.19. Max coverage (-): 0.28

Region: NODE\_121434\_length\_5572\_cov\_28.541996 2928-2939. Max. coverage (+): 0.66. Max coverage (-): 0.09

Region: NODE\_121434\_length\_5572\_cov\_28.541996 2940-2950. Max. coverage (+): 1.13. Max coverage (-): 0.19

Region: NODE\_121434\_length\_5572\_cov\_28.541996 2951-2961. Max. coverage (+): 0.19. Max coverage (-): 0.09

Region: NODE\_121434\_length\_5572\_cov\_28.541996 2962-2972. Max. coverage (+): 0.47. Max coverage (-): 0

Region: NODE\_121434\_length\_5572\_cov\_28.541996 2973-2984. Max. coverage (+): 0. Max coverage (-): 0

Region: NODE\_121434\_length\_5572\_cov\_28.541996 2985-2995. Max. coverage (+): 0. Max coverage (-): 0

Region: NODE\_121434\_length\_5572\_cov\_28.541996 2996-3006. Max. coverage (+): 0. Max coverage (-): 0.09

Region: NODE\_121434\_length\_5572\_cov\_28.541996 3007-3018. Max. coverage (+): 0.09. Max coverage (-): 0

Region: NODE\_121434\_length\_5572\_cov\_28.541996 3019-3029. Max. coverage (+): 0. Max coverage (-): 0.09

Region: NODE\_121434\_length\_5572\_cov\_28.541996 3030-3040. Max. coverage (+): 0. Max coverage (-): 0.09

Region: NODE\_121434\_length\_5572\_cov\_28.541996 3041-3052. Max. coverage (+): 0.09. Max coverage (-): 0.09

Region: NODE\_121434\_length\_5572\_cov\_28.541996 3053-3063. Max. coverage (+): 0.09. Max coverage (-): 0.09

Region: NODE\_121434\_length\_5572\_cov\_28.541996 3064-3074. Max. coverage (+): 0.19. Max coverage (-): 0.19

Region: NODE\_121434\_length\_5572\_cov\_28.541996 3075-3086. Max. coverage (+): 0.19. Max coverage (-): 0.47

Region: NODE\_121434\_length\_5572\_cov\_28.541996 3087-3097. Max. coverage (+): 0.19. Max coverage (-): 0

Region: NODE\_121434\_length\_5572\_cov\_28.541996 3098-3108. Max. coverage (+): 0. Max coverage (-): 0

Region: NODE\_121434\_length\_5572\_cov\_28.541996 3109-3120. Max. coverage (+): 0. Max coverage (-): 0

Region: NODE\_121434\_length\_5572\_cov\_28.541996 3121-3131. Max. coverage (+): 0. Max coverage (-): 0

Region: NODE\_121434\_length\_5572\_cov\_28.541996 3132-3142. Max. coverage (+): 0. Max coverage (-): 0

Region: NODE\_121434\_length\_5572\_cov\_28.541996 3143-3154. Max. coverage (+): 0.56. Max coverage (-): 0.19

Region: NODE\_121434\_length\_5572\_cov\_28.541996 3155-3165. Max. coverage (+): 0.38. Max coverage (-): 0.47

Region: NODE\_121434\_length\_5572\_cov\_28.541996 3166-3176. Max. coverage (+): 0.09. Max coverage (-): 0.28

Region: NODE\_121434\_length\_5572\_cov\_28.541996 3177-3188. Max. coverage (+): 0.19. Max coverage (-): 0.09

Region: NODE\_121434\_length\_5572\_cov\_28.541996 3189-3199. Max. coverage (+): 0.09. Max coverage (-): 0.28

Region: NODE\_121434\_length\_5572\_cov\_28.541996 3200-3210. Max. coverage (+): 0.75. Max coverage (-): 0.19

Region: NODE\_121434\_length\_5572\_cov\_28.541996 3211-3221. Max. coverage (+): 1.6. Max coverage (-): 0.66

Region: NODE\_121434\_length\_5572\_cov\_28.541996 3222-3233. Max. coverage (+): 0. Max coverage (-): 0.38

Region: NODE\_121434\_length\_5572\_cov\_28.541996 3234-3244. Max. coverage (+): 0.56. Max coverage (-): 0.28

Region: NODE\_121434\_length\_5572\_cov\_28.541996 3245-3255. Max. coverage (+): 0. Max coverage (-): 0

Region: NODE\_121434\_length\_5572\_cov\_28.541996 3256-3267. Max. coverage (+): 0.47. Max coverage (-): 0.19

Region: NODE\_121434\_length\_5572\_cov\_28.541996 3268-3278. Max. coverage (+): 0.38. Max coverage (-): 0.19

Region: NODE\_121434\_length\_5572\_cov\_28.541996 3279-3289. Max. coverage (+): 0.09. Max coverage (-): 0.28

Region: NODE\_121434\_length\_5572\_cov\_28.541996 3290-3301. Max. coverage (+): 0.28. Max coverage (-): 0.09

Region: NODE\_121434\_length\_5572\_cov\_28.541996 3302-3312. Max. coverage (+): 0.28. Max coverage (-): 2.82

Region: NODE\_121434\_length\_5572\_cov\_28.541996 3313-3323. Max. coverage (+): 0.09. Max coverage (-): 3.66

Region: NODE\_121434\_length\_5572\_cov\_28.541996 3324-3335. Max. coverage (+): 1.22. Max coverage (-): 0

Region: NODE\_121434\_length\_5572\_cov\_28.541996 3336-3346. Max. coverage (+): 0.09. Max coverage (-): 0.28

Region: NODE\_121434\_length\_5572\_cov\_28.541996 3347-3357. Max. coverage (+): 0. Max coverage (-): 3.01

Region: NODE\_121434\_length\_5572\_cov\_28.541996 3358-3369. Max. coverage (+): 0. Max coverage (-): 0.19

Region: NODE\_121434\_length\_5572\_cov\_28.541996 3370-3380. Max. coverage (+): 0.09. Max coverage (-): 0

Region: NODE\_121434\_length\_5572\_cov\_28.541996 3381-3391. Max. coverage (+): 0.09. Max coverage (-): 1.5

Region: NODE\_121434\_length\_5572\_cov\_28.541996 3392-3403. Max. coverage (+): 0.19. Max coverage (-): 0.09

Region: NODE\_121434\_length\_5572\_cov\_28.541996 3404-3414. Max. coverage (+): 0.09. Max coverage (-): 0.09

Region: NODE\_121434\_length\_5572\_cov\_28.541996 3415-3425. Max. coverage (+): 0.09. Max coverage (-): 0.38

Region: NODE\_121434\_length\_5572\_cov\_28.541996 3426-3437. Max. coverage (+): 0. Max coverage (-): 0.09

Region: NODE\_121434\_length\_5572\_cov\_28.541996 3438-3448. Max. coverage (+): 0.09. Max coverage (-): 0.19

Region: NODE\_121434\_length\_5572\_cov\_28.541996 3449-3459. Max. coverage (+): 0.09. Max coverage (-): 0.19

Region: NODE\_121434\_length\_5572\_cov\_28.541996 3460-3470. Max. coverage (+): 0. Max coverage (-): 0.19

Region: NODE\_121434\_length\_5572\_cov\_28.541996 3471-3482. Max. coverage (+): 0.75. Max coverage (-): 0.28

Region: NODE\_121434\_length\_5572\_cov\_28.541996 3483-3493. Max. coverage (+): 0.19. Max coverage (-): 0.09

Region: NODE\_121434\_length\_5572\_cov\_28.541996 3494-3504. Max. coverage (+): 0. Max coverage (-): 0.09

Region: NODE\_121434\_length\_5572\_cov\_28.541996 3505-3516. Max. coverage (+): 0.09. Max coverage (-): 0.09

Region: NODE\_121434\_length\_5572\_cov\_28.541996 3517-3527. Max. coverage (+): 0.09. Max coverage (-): 0

Region: NODE\_121434\_length\_5572\_cov\_28.541996 3528-3538. Max. coverage (+): 0.09. Max coverage (-): 0.09

Region: NODE\_121434\_length\_5572\_cov\_28.541996 3539-3550. Max. coverage (+): 0. Max coverage (-): 0

Region: NODE\_121434\_length\_5572\_cov\_28.541996 3551-3561. Max. coverage (+): 0. Max coverage (-): 0.09

Region: NODE\_121434\_length\_5572\_cov\_28.541996 3562-3572. Max. coverage (+): 0. Max coverage (-): 0.56

Region: NODE\_121434\_length\_5572\_cov\_28.541996 3573-3584. Max. coverage (+): 0.09. Max coverage (-): 0.19

Region: NODE\_121434\_length\_5572\_cov\_28.541996 3585-3595. Max. coverage (+): 0.19. Max coverage (-): 0

Region: NODE\_121434\_length\_5572\_cov\_28.541996 3596-3606. Max. coverage (+): 0. Max coverage (-): 0

Region: NODE\_121434\_length\_5572\_cov\_28.541996 3607-3618. Max. coverage (+): 0. Max coverage (-): 0

Region: NODE\_121434\_length\_5572\_cov\_28.541996 3619-3629. Max. coverage (+): 0. Max coverage (-): 0

Region: NODE\_121434\_length\_5572\_cov\_28.541996 3630-3640. Max. coverage (+): 0. Max coverage (-): 0

Region: NODE\_121434\_length\_5572\_cov\_28.541996 3641-3652. Max. coverage (+): 0. Max coverage (-): 0

Region: NODE\_121434\_length\_5572\_cov\_28.541996 3653-3663. Max. coverage (+): 0. Max coverage (-): 0

Region: NODE\_121434\_length\_5572\_cov\_28.541996 3664-3674. Max. coverage (+): 0. Max coverage (-): 0

Region: NODE\_121434\_length\_5572\_cov\_28.541996 3675-3686. Max. coverage (+): 0. Max coverage (-): 0

Region: NODE\_121434\_length\_5572\_cov\_28.541996 3687-3697. Max. coverage (+): 0. Max coverage (-): 0

Region: NODE\_121434\_length\_5572\_cov\_28.541996 3698-3708. Max. coverage (+): 0. Max coverage (-): 0

Region: NODE\_121434\_length\_5572\_cov\_28.541996 3709-3719. Max. coverage (+): 0. Max coverage (-): 0

Region: NODE\_121434\_length\_5572\_cov\_28.541996 3720-3731. Max. coverage (+): 0. Max coverage (-): 0

Region: NODE\_121434\_length\_5572\_cov\_28.541996 3732-3742. Max. coverage (+): 0. Max coverage (-): 0

Region: NODE\_121434\_length\_5572\_cov\_28.541996 3743-3753. Max. coverage (+): 0. Max coverage (-): 0

Region: NODE\_121434\_length\_5572\_cov\_28.541996 3754-3765. Max. coverage (+): 0. Max coverage (-): 0

Region: NODE\_121434\_length\_5572\_cov\_28.541996 3766-3776. Max. coverage (+): 0. Max coverage (-): 0

Region: NODE\_121434\_length\_5572\_cov\_28.541996 3777-3787. Max. coverage (+): 0. Max coverage (-): 0

Region: NODE\_121434\_length\_5572\_cov\_28.541996 3788-3799. Max. coverage (+): 0. Max coverage (-): 0

Region: NODE\_121434\_length\_5572\_cov\_28.541996 3800-3810. Max. coverage (+): 0. Max coverage (-): 0

Region: NODE\_121434\_length\_5572\_cov\_28.541996 3811-3821. Max. coverage (+): 0. Max coverage (-): 0

Region: NODE\_121434\_length\_5572\_cov\_28.541996 3822-3833. Max. coverage (+): 0. Max coverage (-): 0

Region: NODE\_121434\_length\_5572\_cov\_28.541996 3834-3844. Max. coverage (+): 0. Max coverage (-): 0

Region: NODE\_121434\_length\_5572\_cov\_28.541996 3845-3855. Max. coverage (+): 0. Max coverage (-): 0

Region: NODE\_121434\_length\_5572\_cov\_28.541996 3856-3867. Max. coverage (+): 0. Max coverage (-): 0

Region: NODE\_121434\_length\_5572\_cov\_28.541996 3868-3878. Max. coverage (+): 0.09. Max coverage (-): 0

Region: NODE\_121434\_length\_5572\_cov\_28.541996 3879-3889. Max. coverage (+): 0. Max coverage (-): 0

Region: NODE\_121434\_length\_5572\_cov\_28.541996 3890-3901. Max. coverage (+): 0. Max coverage (-): 0

Region: NODE\_121434\_length\_5572\_cov\_28.541996 3902-3912. Max. coverage (+): 0. Max coverage (-): 0.09

Region: NODE\_121434\_length\_5572\_cov\_28.541996 3913-3923. Max. coverage (+): 0. Max coverage (-): 0

Region: NODE\_121434\_length\_5572\_cov\_28.541996 3924-3935. Max. coverage (+): 0. Max coverage (-): 0.09

Region: NODE\_121434\_length\_5572\_cov\_28.541996 3936-3946. Max. coverage (+): 0. Max coverage (-): 0

Region: NODE\_121434\_length\_5572\_cov\_28.541996 3947-3957. Max. coverage (+): 0. Max coverage (-): 0

Region: NODE\_121434\_length\_5572\_cov\_28.541996 3958-3968. Max. coverage (+): 0. Max coverage (-): 0

Region: NODE\_121434\_length\_5572\_cov\_28.541996 3969-3980. Max. coverage (+): 0. Max coverage (-): 0

Region: NODE\_121434\_length\_5572\_cov\_28.541996 3981-3991. Max. coverage (+): 0.19. Max coverage (-): 0

Region: NODE\_121434\_length\_5572\_cov\_28.541996 3992-4002. Max. coverage (+): 0. Max coverage (-): 0

Region: NODE\_121434\_length\_5572\_cov\_28.541996 4003-4014. Max. coverage (+): 0. Max coverage (-): 0

Region: NODE\_121434\_length\_5572\_cov\_28.541996 4015-4025. Max. coverage (+): 0. Max coverage (-): 0

Region: NODE\_121434\_length\_5572\_cov\_28.541996 4026-4036. Max. coverage (+): 0. Max coverage (-): 0

Region: NODE\_121434\_length\_5572\_cov\_28.541996 4037-4048. Max. coverage (+): 0. Max coverage (-): 0

Region: NODE\_121434\_length\_5572\_cov\_28.541996 4049-4059. Max. coverage (+): 0. Max coverage (-): 0.09

Region: NODE\_121434\_length\_5572\_cov\_28.541996 4060-4070. Max. coverage (+): 0. Max coverage (-): 0

Region: NODE\_121434\_length\_5572\_cov\_28.541996 4071-4082. Max. coverage (+): 0. Max coverage (-): 0

Region: NODE\_121434\_length\_5572\_cov\_28.541996 4083-4093. Max. coverage (+): 0.09. Max coverage (-): 0

Region: NODE\_121434\_length\_5572\_cov\_28.541996 4094-4104. Max. coverage (+): 0.09. Max coverage (-): 0.09

Region: NODE\_121434\_length\_5572\_cov\_28.541996 4105-4116. Max. coverage (+): 0. Max coverage (-): 0.28

Region: NODE\_121434\_length\_5572\_cov\_28.541996 4117-4127. Max. coverage (+): 0.09. Max coverage (-): 0

Region: NODE\_121434\_length\_5572\_cov\_28.541996 4128-4138. Max. coverage (+): 0.09. Max coverage (-): 0

Region: NODE\_121434\_length\_5572\_cov\_28.541996 4139-4150. Max. coverage (+): 0.09. Max coverage (-): 0

Region: NODE\_121434\_length\_5572\_cov\_28.541996 4151-4161. Max. coverage (+): 0. Max coverage (-): 0

Region: NODE\_121434\_length\_5572\_cov\_28.541996 4162-4172. Max. coverage (+): 0. Max coverage (-): 0

Region: NODE\_121434\_length\_5572\_cov\_28.541996 4173-4184. Max. coverage (+): 0. Max coverage (-): 0

Region: NODE\_121434\_length\_5572\_cov\_28.541996 4185-4195. Max. coverage (+): 0. Max coverage (-): 0.09

Region: NODE\_121434\_length\_5572\_cov\_28.541996 4196-4206. Max. coverage (+): 0. Max coverage (-): 0

Region: NODE\_121434\_length\_5572\_cov\_28.541996 4207-4217. Max. coverage (+): 0. Max coverage (-): 0.47

Region: NODE\_121434\_length\_5572\_cov\_28.541996 4218-4229. Max. coverage (+): 0. Max coverage (-): 0.47

Region: NODE\_121434\_length\_5572\_cov\_28.541996 4230-4240. Max. coverage (+): 1.03. Max coverage (-): 0.09

Region: NODE\_121434\_length\_5572\_cov\_28.541996 4241-4251. Max. coverage (+): 0.56. Max coverage (-): 0.56

Region: NODE\_121434\_length\_5572\_cov\_28.541996 4252-4263. Max. coverage (+): 0.66. Max coverage (-): 0

Region: NODE\_121434\_length\_5572\_cov\_28.541996 4264-4274. Max. coverage (+): 0.75. Max coverage (-): 0

Region: NODE\_121434\_length\_5572\_cov\_28.541996 4275-4285. Max. coverage (+): 0. Max coverage (-): 0

Region: NODE\_121434\_length\_5572\_cov\_28.541996 4286-4297. Max. coverage (+): 0. Max coverage (-): 0

Region: NODE\_121434\_length\_5572\_cov\_28.541996 4298-4308. Max. coverage (+): 0. Max coverage (-): 0

Region: NODE\_121434\_length\_5572\_cov\_28.541996 4309-4319. Max. coverage (+): 0.09. Max coverage (-): 0.47

Region: NODE\_121434\_length\_5572\_cov\_28.541996 4320-4331. Max. coverage (+): 0.47. Max coverage (-): 0

Region: NODE\_121434\_length\_5572\_cov\_28.541996 4332-4342. Max. coverage (+): 0. Max coverage (-): 0.09

Region: NODE\_121434\_length\_5572\_cov\_28.541996 4343-4353. Max. coverage (+): 0.19. Max coverage (-): 0.28

Region: NODE\_121434\_length\_5572\_cov\_28.541996 4354-4365. Max. coverage (+): 0.19. Max coverage (-): 0.19

Region: NODE\_121434\_length\_5572\_cov\_28.541996 4366-4376. Max. coverage (+): 0.09. Max coverage (-): 0.09

Region: NODE\_121434\_length\_5572\_cov\_28.541996 4377-4387. Max. coverage (+): 0. Max coverage (-): 0

Region: NODE\_121434\_length\_5572\_cov\_28.541996 4388-4399. Max. coverage (+): 0. Max coverage (-): 0

Region: NODE\_121434\_length\_5572\_cov\_28.541996 4400-4410. Max. coverage (+): 0. Max coverage (-): 0

Region: NODE\_121434\_length\_5572\_cov\_28.541996 4411-4421. Max. coverage (+): 0. Max coverage (-): 0.09

Region: NODE\_121434\_length\_5572\_cov\_28.541996 4422-4432. Max. coverage (+): 0.09. Max coverage (-): 0

Region: NODE\_121434\_length\_5572\_cov\_28.541996 4433-4444. Max. coverage (+): 0.09. Max coverage (-): 0

Region: NODE\_121434\_length\_5572\_cov\_28.541996 4445-4455. Max. coverage (+): 0.09. Max coverage (-): 0.94

Region: NODE\_121434\_length\_5572\_cov\_28.541996 4456-4466. Max. coverage (+): 0. Max coverage (-): 0.38

Region: NODE\_121434\_length\_5572\_cov\_28.541996 4467-4478. Max. coverage (+): 0.28. Max coverage (-): 0.47

Region: NODE\_121434\_length\_5572\_cov\_28.541996 4479-4489. Max. coverage (+): 0. Max coverage (-): 0.66

Region: NODE\_121434\_length\_5572\_cov\_28.541996 4490-4500. Max. coverage (+): 0.09. Max coverage (-): 0.23

Region: NODE\_121434\_length\_5572\_cov\_28.541996 4501-4512. Max. coverage (+): 0. Max coverage (-): 0.09

Region: NODE\_121434\_length\_5572\_cov\_28.541996 4513-4523. Max. coverage (+): 0. Max coverage (-): 0

Region: NODE\_121434\_length\_5572\_cov\_28.541996 4524-4534. Max. coverage (+): 0. Max coverage (-): 1.32

Region: NODE\_121434\_length\_5572\_cov\_28.541996 4535-4546. Max. coverage (+): 0.38. Max coverage (-): 0.19

Region: NODE\_121434\_length\_5572\_cov\_28.541996 4547-4557. Max. coverage (+): 0. Max coverage (-): 0

Region: NODE\_121434\_length\_5572\_cov\_28.541996 4558-4568. Max. coverage (+): 0. Max coverage (-): 0

Region: NODE\_121434\_length\_5572\_cov\_28.541996 4569-4580. Max. coverage (+): 0. Max coverage (-): 0

Region: NODE\_121434\_length\_5572\_cov\_28.541996 4581-4591. Max. coverage (+): 0. Max coverage (-): 0

Region: NODE\_121434\_length\_5572\_cov\_28.541996 4592-4602. Max. coverage (+): 0. Max coverage (-): 0

Region: NODE\_121434\_length\_5572\_cov\_28.541996 4603-4614. Max. coverage (+): 0. Max coverage (-): 0.19

Region: NODE\_121434\_length\_5572\_cov\_28.541996 4615-4625. Max. coverage (+): 0. Max coverage (-): 0

Region: NODE\_121434\_length\_5572\_cov\_28.541996 4626-4636. Max. coverage (+): 0.09. Max coverage (-): 0.47

Region: NODE\_121434\_length\_5572\_cov\_28.541996 4637-4648. Max. coverage (+): 0.19. Max coverage (-): 0.75

Region: NODE\_121434\_length\_5572\_cov\_28.541996 4649-4659. Max. coverage (+): 0. Max coverage (-): 0

Region: NODE\_121434\_length\_5572\_cov\_28.541996 4660-4670. Max. coverage (+): 0. Max coverage (-): 1.13

Region: NODE\_121434\_length\_5572\_cov\_28.541996 4671-4681. Max. coverage (+): 0.09. Max coverage (-): 1.5

Region: NODE\_121434\_length\_5572\_cov\_28.541996 4682-4693. Max. coverage (+): 0. Max coverage (-): 0.56

Region: NODE\_121434\_length\_5572\_cov\_28.541996 4694-4704. Max. coverage (+): 0.09. Max coverage (-): 0

Region: NODE\_121434\_length\_5572\_cov\_28.541996 4705-4715. Max. coverage (+): 0. Max coverage (-): 0

Region: NODE\_121434\_length\_5572\_cov\_28.541996 4716-4727. Max. coverage (+): 0. Max coverage (-): 0

Region: NODE\_121434\_length\_5572\_cov\_28.541996 4728-4738. Max. coverage (+): 0.09. Max coverage (-): 0

Region: NODE\_121434\_length\_5572\_cov\_28.541996 4739-4749. Max. coverage (+): 0.09. Max coverage (-): 0

Region: NODE\_121434\_length\_5572\_cov\_28.541996 4750-4761. Max. coverage (+): 0. Max coverage (-): 0

Region: NODE\_121434\_length\_5572\_cov\_28.541996 4762-4772. Max. coverage (+): 0. Max coverage (-): 0

Region: NODE\_121434\_length\_5572\_cov\_28.541996 4773-4783. Max. coverage (+): 0. Max coverage (-): 0.19

Region: NODE\_121434\_length\_5572\_cov\_28.541996 4784-4795. Max. coverage (+): 0. Max coverage (-): 0

Region: NODE\_121434\_length\_5572\_cov\_28.541996 4796-4806. Max. coverage (+): 0. Max coverage (-): 0.09

Region: NODE\_121434\_length\_5572\_cov\_28.541996 4807-4817. Max. coverage (+): 0. Max coverage (-): 0.28

Region: NODE\_121434\_length\_5572\_cov\_28.541996 4818-4829. Max. coverage (+): 0.38. Max coverage (-): 0

Region: NODE\_121434\_length\_5572\_cov\_28.541996 4830-4840. Max. coverage (+): 0. Max coverage (-): 0

Region: NODE\_121434\_length\_5572\_cov\_28.541996 4841-4851. Max. coverage (+): 0. Max coverage (-): 0

Region: NODE\_121434\_length\_5572\_cov\_28.541996 4852-4863. Max. coverage (+): 0.19. Max coverage (-): 0

Region: NODE\_121434\_length\_5572\_cov\_28.541996 4864-4874. Max. coverage (+): 0.28. Max coverage (-): 0.09

Region: NODE\_121434\_length\_5572\_cov\_28.541996 4875-4885. Max. coverage (+): 0.28. Max coverage (-): 0.94

Region: NODE\_121434\_length\_5572\_cov\_28.541996 4886-4897. Max. coverage (+): 0. Max coverage (-): 0.19

Region: NODE\_121434\_length\_5572\_cov\_28.541996 4898-4908. Max. coverage (+): 0.19. Max coverage (-): 0

Region: NODE\_121434\_length\_5572\_cov\_28.541996 4909-4919. Max. coverage (+): 0.09. Max coverage (-): 0

Region: NODE\_121434\_length\_5572\_cov\_28.541996 4920-4930. Max. coverage (+): 0. Max coverage (-): 0

Region: NODE\_121434\_length\_5572\_cov\_28.541996 4931-4942. Max. coverage (+): 0. Max coverage (-): 0

Region: NODE\_121434\_length\_5572\_cov\_28.541996 4943-4953. Max. coverage (+): 0. Max coverage (-): 0

Region: NODE\_121434\_length\_5572\_cov\_28.541996 4954-4964. Max. coverage (+): 0. Max coverage (-): 0

Region: NODE\_121434\_length\_5572\_cov\_28.541996 4965-4976. Max. coverage (+): 0. Max coverage (-): 0

Region: NODE\_121434\_length\_5572\_cov\_28.541996 4977-4987. Max. coverage (+): 0. Max coverage (-): 0

Region: NODE\_121434\_length\_5572\_cov\_28.541996 4988-4998. Max. coverage (+): 0. Max coverage (-): 0

Region: NODE\_121434\_length\_5572\_cov\_28.541996 4999-5010. Max. coverage (+): 0. Max coverage (-): 0

Region: NODE\_121434\_length\_5572\_cov\_28.541996 5011-5021. Max. coverage (+): 0. Max coverage (-): 0

Region: NODE\_121434\_length\_5572\_cov\_28.541996 5022-5032. Max. coverage (+): 0. Max coverage (-): 0

Region: NODE\_121434\_length\_5572\_cov\_28.541996 5033-5044. Max. coverage (+): 0. Max coverage (-): 0

Region: NODE\_121434\_length\_5572\_cov\_28.541996 5045-5055. Max. coverage (+): 0. Max coverage (-): 0

Region: NODE\_121434\_length\_5572\_cov\_28.541996 5056-5066. Max. coverage (+): 0. Max coverage (-): 0

Region: NODE\_121434\_length\_5572\_cov\_28.541996 5067-5078. Max. coverage (+): 0. Max coverage (-): 0

Region: NODE\_121434\_length\_5572\_cov\_28.541996 5079-5089. Max. coverage (+): 0. Max coverage (-): 0.19

Region: NODE\_121434\_length\_5572\_cov\_28.541996 5090-5100. Max. coverage (+): 0. Max coverage (-): 0.09

Region: NODE\_121434\_length\_5572\_cov\_28.541996 5101-5112. Max. coverage (+): 0. Max coverage (-): 0.09

Region: NODE\_121434\_length\_5572\_cov\_28.541996 5113-5123. Max. coverage (+): 0. Max coverage (-): 0.09

Region: NODE\_121434\_length\_5572\_cov\_28.541996 5124-5134. Max. coverage (+): 0. Max coverage (-): 0

Region: NODE\_121434\_length\_5572\_cov\_28.541996 5135-5146. Max. coverage (+): 0. Max coverage (-): 0.09

Region: NODE\_121434\_length\_5572\_cov\_28.541996 5147-5157. Max. coverage (+): 0. Max coverage (-): 0

Region: NODE\_121434\_length\_5572\_cov\_28.541996 5158-5168. Max. coverage (+): 0. Max coverage (-): 0

Region: NODE\_121434\_length\_5572\_cov\_28.541996 5169-5179. Max. coverage (+): 0. Max coverage (-): 0

Region: NODE\_121434\_length\_5572\_cov\_28.541996 5180-5191. Max. coverage (+): 0. Max coverage (-): 0

Region: NODE\_121434\_length\_5572\_cov\_28.541996 5192-5202. Max. coverage (+): 0. Max coverage (-): 0

Region: NODE\_121434\_length\_5572\_cov\_28.541996 5203-5213. Max. coverage (+): 0. Max coverage (-): 0

Region: NODE\_121434\_length\_5572\_cov\_28.541996 5214-5225. Max. coverage (+): 0. Max coverage (-): 0

Region: NODE\_121434\_length\_5572\_cov\_28.541996 5226-5236. Max. coverage (+): 0. Max coverage (-): 0

Region: NODE\_121434\_length\_5572\_cov\_28.541996 5237-5247. Max. coverage (+): 0. Max coverage (-): 0

Region: NODE\_121434\_length\_5572\_cov\_28.541996 5248-5259. Max. coverage (+): 0. Max coverage (-): 0.09

Region: NODE\_121434\_length\_5572\_cov\_28.541996 5260-5270. Max. coverage (+): 0. Max coverage (-): 0

Region: NODE\_121434\_length\_5572\_cov\_28.541996 5271-5281. Max. coverage (+): 0. Max coverage (-): 0

Region: NODE\_121434\_length\_5572\_cov\_28.541996 5282-5293. Max. coverage (+): 0. Max coverage (-): 0

Region: NODE\_121434\_length\_5572\_cov\_28.541996 5294-5304. Max. coverage (+): 0. Max coverage (-): 0

Region: NODE\_121434\_length\_5572\_cov\_28.541996 5305-5315. Max. coverage (+): 0. Max coverage (-): 0

Region: NODE\_121434\_length\_5572\_cov\_28.541996 5316-5327. Max. coverage (+): 0. Max coverage (-): 0.66

Region: NODE\_121434\_length\_5572\_cov\_28.541996 5328-5338. Max. coverage (+): 0. Max coverage (-): 0

Region: NODE\_121434\_length\_5572\_cov\_28.541996 5339-5349. Max. coverage (+): 0. Max coverage (-): 0

Region: NODE\_121434\_length\_5572\_cov\_28.541996 5350-5361. Max. coverage (+): 0. Max coverage (-): 0

Region: NODE\_121434\_length\_5572\_cov\_28.541996 5362-5372. Max. coverage (+): 0. Max coverage (-): 0.09

Region: NODE\_121434\_length\_5572\_cov\_28.541996 5373-5383. Max. coverage (+): 0. Max coverage (-): 0.09

Region: NODE\_121434\_length\_5572\_cov\_28.541996 5384-5395. Max. coverage (+): 0.38. Max coverage (-): 0

Region: NODE\_121434\_length\_5572\_cov\_28.541996 5396-5406. Max. coverage (+): 0.09. Max coverage (-): 0.09

Region: NODE\_121434\_length\_5572\_cov\_28.541996 5407-5417. Max. coverage (+): 0. Max coverage (-): 0.19

Region: NODE\_121434\_length\_5572\_cov\_28.541996 5418-5428. Max. coverage (+): 0.47. Max coverage (-): 0

Region: NODE\_121434\_length\_5572\_cov\_28.541996 5429-5440. Max. coverage (+): 0.09. Max coverage (-): 1.13

Region: NODE\_121434\_length\_5572\_cov\_28.541996 5441-5451. Max. coverage (+): 0. Max coverage (-): 0.75

Region: NODE\_121434\_length\_5572\_cov\_28.541996 5452-5462. Max. coverage (+): 0.47. Max coverage (-): 0.09

Region: NODE\_121434\_length\_5572\_cov\_28.541996 5463-5474. Max. coverage (+): 0.09. Max coverage (-): 1.22

Region: NODE\_121434\_length\_5572\_cov\_28.541996 5475-5485. Max. coverage (+): 0.02. Max coverage (-): 1.32

Region: NODE\_121434\_length\_5572\_cov\_28.541996 5486-5496. Max. coverage (+): 0.02. Max coverage (-): 0

Region: NODE\_121434\_length\_5572\_cov\_28.541996 5497-5508. Max. coverage (+): 0. Max coverage (-): 3.89

Region: NODE\_121434\_length\_5572\_cov\_28.541996 5509-5519. Max. coverage (+): 0. Max coverage (-): 3.76

Region: NODE\_121434\_length\_5572\_cov\_28.541996 5520-5530. Max. coverage (+): 0.23. Max coverage (-): 0

Region: NODE\_121434\_length\_5572\_cov\_28.541996 5531-5542. Max. coverage (+): 0. Max coverage (-): 0

Region: NODE\_121434\_length\_5572\_cov\_28.541996 5543-5553. Max. coverage (+): 0.01. Max coverage (-): 0.09

Region: NODE\_121434\_length\_5572\_cov\_28.541996 5554-5564. Max. coverage (+): 0.01. Max coverage (-): 0.08

Region: NODE\_121434\_length\_5572\_cov\_28.541996 5565-5576. Max. coverage (+): 0. Max coverage (-): 0.08

Region: NODE\_121434\_length\_5572\_cov\_28.541996 5577-5587. Max. coverage (+): 0. Max coverage (-): 0

Region: NODE\_121434\_length\_5572\_cov\_28.541996 5588-5598. Max. coverage (+): 0. Max coverage (-): 0

Region: NODE\_121434\_length\_5572\_cov\_28.541996 5599-5610. Max. coverage (+): 0. Max coverage (-): 0

Region: NODE\_121434\_length\_5572\_cov\_28.541996 5611-5621. Max. coverage (+): 0.02. Max coverage (-): 0.05

Region: NODE\_121434\_length\_5572\_cov\_28.541996 5622-5632. Max. coverage (+): 0. Max coverage (-): 0.05

Region: NODE\_121434\_length\_5572\_cov\_28.541996 5633-5644. Max. coverage (+): 0. Max coverage (-): 0.17

Region: NODE\_121434\_length\_5572\_cov\_28.541996 5645-5655. Max. coverage (+): 0. Max coverage (-): 0

Region: NODE\_121434\_length\_5572\_cov\_28.541996 5656-. Max. coverage (+): 0. Max coverage (-): 0

RepeatMasker Color Code

**+**

100-98% Identity

<98-95% Identity

<95-90% Identity

<90-85% Identity

<85-80% Identity

<80-75% Identity

<75-70% Identity

<70% Identity

**-**

Gene Set Color Code

**+**

Gene

Pseudogene

Other

**-**

Topology/Coverage Color Code

Coverage Plus Strand

Coverage Minus Strand

Mainstrand: Plus

Mainstrand: Minus

Complementary Strand

Flanking Region  
(if option -flank >0)

Gene Set Annotation  
  
RepeatMasker Annotation  

**1. AlRepD-180**: 1-52 (-), Divergence to consensus: 17.3%  
**2. AlRepC-2154**: 148-195 (+), Divergence to consensus: 4.2%  
**3. AlRepD-180**: 196-376 (-), Divergence to consensus: 37.7%  
**4. AlRepC-905**: 2062-2220 (+), Divergence to consensus: 37.2%  
**5. AlRepC-1227**: 2256-2509 (+), Divergence to consensus: 39.7%  
**6. AlRepC-1227**: 2601-2675 (+), Divergence to consensus: 33.1%  
**7. (AC)n**: 2981-3010 (+), Divergence to consensus: 0%  
**8. AlRepA-24**: 4414-4536 (+), Divergence to consensus: 36.8%  
**9. AlRepA-485**: 4438-4670 (-), Divergence to consensus: 47.3%  
**10. AlRepD-1165**: 4709-4760 (-), Divergence to consensus: 23.1%

  
Transcription Factor Binding Sites  

**RHOXF1** (Sequence: AGATCA (-): 1729)  
**RHOXF1** (Sequence: AGCTTA (-): 1907)  
**RHOXF1** (Sequence: AGATCA (-): 2354)  
**RHOXF1** (Sequence: GGCTTA (-): 2806)  
**RHOXF1** (Sequence: AGATCA (-): 3236)  
**RHOXF1** (Sequence: GGATTA (-): 3610)  
**RHOXF1** (Sequence: AGATTA (-): 3997)  
**RHOXF1** (Sequence: GGCTTA (-): 4002)  
**RHOXF1** (Sequence: AGCTCA (-): 4258)  
**RHOXF1** (Sequence: AGCTCA (-): 5497)  
**RHOXF1** (Sequence: AGCTCA (-): 5512)  
**RHOXF1** (Sequence: GGCTCA (-): 5600)  
**RHOXF1** (Sequence: TAATCT (+): 689)  
**RHOXF1** (Sequence: TAATCC (+): 1122)  
**RHOXF1** (Sequence: TAATCT (+): 1228)  
**RHOXF1** (Sequence: TGAGCT (+): 1958)  
**RHOXF1** (Sequence: TGAGCT (+): 2093)  
**RHOXF1** (Sequence: TGAGCC (+): 2149)  
**RHOXF1** (Sequence: TAAGCT (+): 4153)  
**RHOXF1** (Sequence: TGAGCT (+): 4256)  
**RHOXF1** (Sequence: TAATCC (+): 5477)  
**RHOXF1** (Sequence: TAAGCT (+): 5510)  
**RHOXF1** (Sequence: TGAGCT (+): 5562)  
**Gata4** (Sequence: CTTATCT (+): 2808)  
**Gata4** (Sequence: GTTATCT (+): 5135)  
**Sox5** (Sequence: ATTGTT (+): 925)  
**Sox5** (Sequence: ATTGTT (+): 1871)  
**Sox5** (Sequence: ATTGTT (+): 5334)  
**SOX9** (Sequence: CCATTGTT (+): 923)  
**FOXO3\_mmu** (Sequence: TGAAAACA (+): 751)  
**Nobox** (Sequence: GCCAATTA (-): 1661)  
**FOXO1** (Sequence: AAAAACAAG (-): 909)  
**FOXO1** (Sequence: AAAAACAAC (-): 2180)  
**FOXO1** (Sequence: AAAAACAAC (-): 3340)  
**FOXO1** (Sequence: ATAAACAGC (-): 4161)  
**Nobox** (Sequence: TAATTGCT (+): 1680)  
**POU2F1** (Sequence: ATTTGAATA (-): 5590)  
**Rhox11** (Sequence: TGCTGTAAA (+): 4301)  
**Rhox11** (Sequence: AAAACACCA (-): 351)  
**Rhox11** (Sequence: TAAACAGCA (-): 4162)  
**Gata4** (Sequence: AGATAAC (-): 2260)  
**Sox5** (Sequence: AACAAT (-): 4557)  
**POU2F1** (Sequence: TATGTTAAT (+): 816)  
**POU5F1** (Sequence: ATGCAAA (+): 1776)
