# Supplementary material for: First characterization of PIWI-interacting RNA clusters in a cichlid fish with a B chromosome
Source: BMC Biol. 2022 Sep 21;20:204. doi: 10.1186/s12915-022-01403-2 (PMC9490952; doi:10.1186/s12915-022-01403-2)
Supplement: Supplementary file 1 — Additional file 1. Zipped folder with fasta and interactive html piRNA cluster information for the A. latifasciata genome. The nomenclature is as follows: number-pirna-cluster_sex_B-presence (f, female; m, male; 0b, without B chromosome; 1b, with B chromosome). [file 12915_2022_1403_MOESM1_ESM.zip › 140_f0b.html]

piRNA cluster 140\_f0b 66


Predicted piRNA cluster no. 140\_f0b
  

Show proTRAC run info
Hide proTRAC run info

/\  
                \_\_\_\_\_\_\_\_\_\_\_\_\_\_\_\_\_\_\_\_\_\_\_/\\_\_\_ /  \\_\_\_\_\_\_\_  
               I                      /  \  /    \      I  
               I     pro             /    \/      \     I  
               I        TRAC        /               \   I  
               I   \_\_\_\_\_\_\_\_\_\_\_\_\_\_\_\_/\_\_\_\_\_\_\_\_\_\_\_\_\_\_\_\_\_\\_ I  
               I   \              /                     I  
               I    \            /                      I  
               I     \  /\      /       V.2.4.2         I  
               I      \/  \    /                        I  
               I\_\_\_\_\_\_\_\_\_\_\_\  /\_\_\_\_\_\_\_\_\_\_\_\_\_\_\_\_\_\_\_\_\_\_\_\_\_I  
                            \/  
  
  
================================= proTRAC ====================================  
VERSION: .......... 2.4.2  
LAST MODIFIED: .... 11. May 2018  
  
Please cite:  
Rosenkranz D, Zischler H. proTRAC - a software for probabilistic piRNA cluster  
detection, visualization and analysis. 2012. BMC Bioinformatics 13:5.  
  
  
Contact:  
David Rosenkranz  
Institute of Organismic and Molecular Evolutionary Biology  
Dept. Anthropology, small RNA group  
Johannes Gutenberg University Mainz  
email: rosenkranz@uni-mainz.de  
  
You can find the latest proTRAC version at:  
http://sourceforge.net/projects/protrac/files  
http://www.smallRNAgroup-mainz.de/software  
==============================================================================  
  
PARAMETERS:  
Map file: ...............piwi-femeas-0B.fa-collapse.map  
Genome file: ............../../../0B\_ala\_genome.fa  
RepeatMasker annotation: Alatifasciata-all0B-maryan-v2.fa\_corrected.out  
GeneSet:................./guest-storage/Data/annotation/Alatifasciata\_all0B\_maryan-v2\_out2017.gff  
  
Significant (p<=0.01) hit density will be calculated based  
on observed hit distribution.  
  
Sliding window size: ........................................ 5000 bp  
Sliding window increament: .................................. 1000 bp  
Normalize each hit by number of genomic hits: ............... yes  
Normalize each hit by number of sequence reads: ............. yes  
Normalize values (-> per million mapped reads): ............. yes  
Min. fraction of hits with 1T(U) or 10A: .................... 0.75  
Alternatively: Min. fraction of hits with 1T(U) and 10A: .... 0.5  
Min. fraction of hits with typical piRNA length: ............ 0.75  
Typical piRNA length: ....................................... 24-32 nt  
Min. size of a piRNA cluster: ............................... 1000 bp.  
Min. number of hits (absolute): ............................. 0  
Min. number of hits (normalized): ........................... 0  
Min. fraction of hits on the mainstrand: .................... 0.75  
Top fraction of mapped sequences (in terms of read counts): . 1%  
Top fraction accounts for max. n% of sequence reads: ........ 90%  
Min. fraction of hits on each arm of a bidirectional cluster: 0.05  
Output html file for each cluster: .......................... yes  
Output a summary table: ..................................... yes  
Output a FASTA file for each cluster (piRNA sequences): ..... yes  
Output a FASTA file comprising cluster sequences: ........... yes  
Output a GTF file for predicted piRNA clusters: ..............yes  
Search DNA motifs in clusters: .............................. yes  
Output flanking sequences: +/- .............................. 0 bp  
Output ~.pTi file: .......................................... no  
==============================================================================  
  
  
Genome size (without gaps): ............ 758543724 bp  
Gaps (N/X/-): .......................... 417479 bp  
Mapped reads: .......................... 13052187  
Non-identical sequences: ............... 3338911  
Genomic hits: .......................... 28737726  
Significant densitiy of mapped reads: .. 470.083249848448 reads/kb

Show proTRAC cluster info
Hide proTRAC cluster info

|  |  |
| --- | --- |
| Location | NODE\_360870\_length\_2137\_cov\_16.065512 |
| Coordinates | 10-2197 |
| Size [bp] | 2188 |
| Sequence hit loci | 1056 |
| Mapped reads (normalized) | 10969.1 |
| Mapped reads (normalized) per kb | 5013.3 |
| Normalized reads with 1T (1U) | 85.1% |
| Normalized reads with 10A | 72.5% |
| Normalized reads with length 24-32 nt | 99.4% |
| Normalized reads on the main strand(s) | 90.4% |
| Predicted directionality | bi:plus-minus (split between 1465 and 1470) |

100%

0%

1T (1U)  
reads

10A reads

24-32 nt  
reads

reads on mainstrand

**Either the amount of reads with 1T (1U) OR 10A has to exceed 75% (set with option: -1Tor10A)  
Alternatively the amount of reads with 1T (1U) AND 10A has to exceed 50% (set with option: -1Tand10A)  
Minimum amount of reads with preferred size is 75% (set with option: -pisize)  
Minimum amount of reads on the main strand(s) is 75% (set with option: -clstrand)**

Show read coverage
Hide read coverage

WHAT DO I SEE HERE?  
This chart shows the location of mapped sequence reads within a predicted piRNA cluster. The color refers to the number of genomic hits produced by the sequence read in question. A dark red bar indicates that this sequence read produces many other hits elsewhere in the genome. Many adjacent red or yellow bars can indicate the presence of a multi-copy element such as transposons or rRNA genes. A dark green bar indicates that this sequence read maps uniquely to this locus.

1 hit

2-5 hits

6-10 hits

11-20 hits

21-50 hits

51-100 hits

> 100 hits

NODE\_360870\_length\_2137\_cov\_16.065512

10

2197

Gene Set

RepeatMasker

Mapped  
Reads

626.89

plus strand

minus strand

626.89

Region: NODE\_360870\_length\_2137\_cov\_16.065512 36007-12. Max. coverage (+): 0. Max coverage (-): 0.05

Region: NODE\_360870\_length\_2137\_cov\_16.065512 13-16. Max. coverage (+): 0.03. Max coverage (-): 0

Region: NODE\_360870\_length\_2137\_cov\_16.065512 17-20. Max. coverage (+): 0.03. Max coverage (-): 0

Region: NODE\_360870\_length\_2137\_cov\_16.065512 21-25. Max. coverage (+): 0.03. Max coverage (-): 0

Region: NODE\_360870\_length\_2137\_cov\_16.065512 26-29. Max. coverage (+): 0. Max coverage (-): 0

Region: NODE\_360870\_length\_2137\_cov\_16.065512 30-34. Max. coverage (+): 0. Max coverage (-): 0.03

Region: NODE\_360870\_length\_2137\_cov\_16.065512 35-38. Max. coverage (+): 0. Max coverage (-): 0

Region: NODE\_360870\_length\_2137\_cov\_16.065512 39-42. Max. coverage (+): 0. Max coverage (-): 0

Region: NODE\_360870\_length\_2137\_cov\_16.065512 43-47. Max. coverage (+): 0. Max coverage (-): 0

Region: NODE\_360870\_length\_2137\_cov\_16.065512 48-51. Max. coverage (+): 0.31. Max coverage (-): 0

Region: NODE\_360870\_length\_2137\_cov\_16.065512 52-55. Max. coverage (+): 0. Max coverage (-): 0

Region: NODE\_360870\_length\_2137\_cov\_16.065512 56-60. Max. coverage (+): 0. Max coverage (-): 0

Region: NODE\_360870\_length\_2137\_cov\_16.065512 61-64. Max. coverage (+): 0. Max coverage (-): 0

Region: NODE\_360870\_length\_2137\_cov\_16.065512 65-69. Max. coverage (+): 0. Max coverage (-): 0

Region: NODE\_360870\_length\_2137\_cov\_16.065512 70-73. Max. coverage (+): 0. Max coverage (-): 0

Region: NODE\_360870\_length\_2137\_cov\_16.065512 74-77. Max. coverage (+): 0. Max coverage (-): 0

Region: NODE\_360870\_length\_2137\_cov\_16.065512 78-82. Max. coverage (+): 0. Max coverage (-): 0

Region: NODE\_360870\_length\_2137\_cov\_16.065512 83-86. Max. coverage (+): 0. Max coverage (-): 0

Region: NODE\_360870\_length\_2137\_cov\_16.065512 87-90. Max. coverage (+): 0. Max coverage (-): 0

Region: NODE\_360870\_length\_2137\_cov\_16.065512 91-95. Max. coverage (+): 0. Max coverage (-): 0

Region: NODE\_360870\_length\_2137\_cov\_16.065512 96-99. Max. coverage (+): 0. Max coverage (-): 0

Region: NODE\_360870\_length\_2137\_cov\_16.065512 100-104. Max. coverage (+): 0. Max coverage (-): 0

Region: NODE\_360870\_length\_2137\_cov\_16.065512 105-108. Max. coverage (+): 0. Max coverage (-): 0

Region: NODE\_360870\_length\_2137\_cov\_16.065512 109-112. Max. coverage (+): 0. Max coverage (-): 0

Region: NODE\_360870\_length\_2137\_cov\_16.065512 113-117. Max. coverage (+): 0. Max coverage (-): 0

Region: NODE\_360870\_length\_2137\_cov\_16.065512 118-121. Max. coverage (+): 0. Max coverage (-): 0

Region: NODE\_360870\_length\_2137\_cov\_16.065512 122-125. Max. coverage (+): 0. Max coverage (-): 0

Region: NODE\_360870\_length\_2137\_cov\_16.065512 126-130. Max. coverage (+): 0. Max coverage (-): 0.04

Region: NODE\_360870\_length\_2137\_cov\_16.065512 131-134. Max. coverage (+): 0. Max coverage (-): 0.08

Region: NODE\_360870\_length\_2137\_cov\_16.065512 135-139. Max. coverage (+): 0. Max coverage (-): 0

Region: NODE\_360870\_length\_2137\_cov\_16.065512 140-143. Max. coverage (+): 0. Max coverage (-): 0

Region: NODE\_360870\_length\_2137\_cov\_16.065512 144-147. Max. coverage (+): 0. Max coverage (-): 0

Region: NODE\_360870\_length\_2137\_cov\_16.065512 148-152. Max. coverage (+): 0. Max coverage (-): 0

Region: NODE\_360870\_length\_2137\_cov\_16.065512 153-156. Max. coverage (+): 0. Max coverage (-): 0

Region: NODE\_360870\_length\_2137\_cov\_16.065512 157-160. Max. coverage (+): 0. Max coverage (-): 0.15

Region: NODE\_360870\_length\_2137\_cov\_16.065512 161-165. Max. coverage (+): 0. Max coverage (-): 0.23

Region: NODE\_360870\_length\_2137\_cov\_16.065512 166-169. Max. coverage (+): 0. Max coverage (-): 4.71

Region: NODE\_360870\_length\_2137\_cov\_16.065512 170-174. Max. coverage (+): 0.05. Max coverage (-): 7.47

Region: NODE\_360870\_length\_2137\_cov\_16.065512 175-178. Max. coverage (+): 0.1. Max coverage (-): 0.03

Region: NODE\_360870\_length\_2137\_cov\_16.065512 179-182. Max. coverage (+): 0.59. Max coverage (-): 0

Region: NODE\_360870\_length\_2137\_cov\_16.065512 183-187. Max. coverage (+): 0.08. Max coverage (-): 0

Region: NODE\_360870\_length\_2137\_cov\_16.065512 188-191. Max. coverage (+): 0. Max coverage (-): 0

Region: NODE\_360870\_length\_2137\_cov\_16.065512 192-195. Max. coverage (+): 0.03. Max coverage (-): 0

Region: NODE\_360870\_length\_2137\_cov\_16.065512 196-200. Max. coverage (+): 0. Max coverage (-): 0

Region: NODE\_360870\_length\_2137\_cov\_16.065512 201-204. Max. coverage (+): 0. Max coverage (-): 0

Region: NODE\_360870\_length\_2137\_cov\_16.065512 205-209. Max. coverage (+): 0. Max coverage (-): 0

Region: NODE\_360870\_length\_2137\_cov\_16.065512 210-213. Max. coverage (+): 0. Max coverage (-): 0

Region: NODE\_360870\_length\_2137\_cov\_16.065512 214-217. Max. coverage (+): 0.04. Max coverage (-): 0.04

Region: NODE\_360870\_length\_2137\_cov\_16.065512 218-222. Max. coverage (+): 0.04. Max coverage (-): 0.04

Region: NODE\_360870\_length\_2137\_cov\_16.065512 223-226. Max. coverage (+): 0. Max coverage (-): 0

Region: NODE\_360870\_length\_2137\_cov\_16.065512 227-230. Max. coverage (+): 0. Max coverage (-): 0.04

Region: NODE\_360870\_length\_2137\_cov\_16.065512 231-235. Max. coverage (+): 0. Max coverage (-): 0.04

Region: NODE\_360870\_length\_2137\_cov\_16.065512 236-239. Max. coverage (+): 0. Max coverage (-): 0

Region: NODE\_360870\_length\_2137\_cov\_16.065512 240-244. Max. coverage (+): 0. Max coverage (-): 0

Region: NODE\_360870\_length\_2137\_cov\_16.065512 245-248. Max. coverage (+): 0. Max coverage (-): 0

Region: NODE\_360870\_length\_2137\_cov\_16.065512 249-252. Max. coverage (+): 0. Max coverage (-): 0.08

Region: NODE\_360870\_length\_2137\_cov\_16.065512 253-257. Max. coverage (+): 0. Max coverage (-): 0.11

Region: NODE\_360870\_length\_2137\_cov\_16.065512 258-261. Max. coverage (+): 0. Max coverage (-): 0.15

Region: NODE\_360870\_length\_2137\_cov\_16.065512 262-265. Max. coverage (+): 0. Max coverage (-): 0.15

Region: NODE\_360870\_length\_2137\_cov\_16.065512 266-270. Max. coverage (+): 0. Max coverage (-): 0.08

Region: NODE\_360870\_length\_2137\_cov\_16.065512 271-274. Max. coverage (+): 1.61. Max coverage (-): 0

Region: NODE\_360870\_length\_2137\_cov\_16.065512 275-279. Max. coverage (+): 1.61. Max coverage (-): 0

Region: NODE\_360870\_length\_2137\_cov\_16.065512 280-283. Max. coverage (+): 0. Max coverage (-): 0

Region: NODE\_360870\_length\_2137\_cov\_16.065512 284-287. Max. coverage (+): 0. Max coverage (-): 0

Region: NODE\_360870\_length\_2137\_cov\_16.065512 288-292. Max. coverage (+): 0. Max coverage (-): 0.04

Region: NODE\_360870\_length\_2137\_cov\_16.065512 293-296. Max. coverage (+): 0. Max coverage (-): 0

Region: NODE\_360870\_length\_2137\_cov\_16.065512 297-301. Max. coverage (+): 0. Max coverage (-): 0

Region: NODE\_360870\_length\_2137\_cov\_16.065512 302-305. Max. coverage (+): 0. Max coverage (-): 9.81

Region: NODE\_360870\_length\_2137\_cov\_16.065512 306-309. Max. coverage (+): 0. Max coverage (-): 10.46

Region: NODE\_360870\_length\_2137\_cov\_16.065512 310-314. Max. coverage (+): 0. Max coverage (-): 0.23

Region: NODE\_360870\_length\_2137\_cov\_16.065512 315-318. Max. coverage (+): 0. Max coverage (-): 0

Region: NODE\_360870\_length\_2137\_cov\_16.065512 319-322. Max. coverage (+): 0. Max coverage (-): 0

Region: NODE\_360870\_length\_2137\_cov\_16.065512 323-327. Max. coverage (+): 2.07. Max coverage (-): 0

Region: NODE\_360870\_length\_2137\_cov\_16.065512 328-331. Max. coverage (+): 0. Max coverage (-): 0

Region: NODE\_360870\_length\_2137\_cov\_16.065512 332-336. Max. coverage (+): 0. Max coverage (-): 0.31

Region: NODE\_360870\_length\_2137\_cov\_16.065512 337-340. Max. coverage (+): 0. Max coverage (-): 0.73

Region: NODE\_360870\_length\_2137\_cov\_16.065512 341-344. Max. coverage (+): 0. Max coverage (-): 0.54

Region: NODE\_360870\_length\_2137\_cov\_16.065512 345-349. Max. coverage (+): 0. Max coverage (-): 0

Region: NODE\_360870\_length\_2137\_cov\_16.065512 350-353. Max. coverage (+): 0. Max coverage (-): 0

Region: NODE\_360870\_length\_2137\_cov\_16.065512 354-357. Max. coverage (+): 0. Max coverage (-): 0.03

Region: NODE\_360870\_length\_2137\_cov\_16.065512 358-362. Max. coverage (+): 0. Max coverage (-): 0.08

Region: NODE\_360870\_length\_2137\_cov\_16.065512 363-366. Max. coverage (+): 0.26. Max coverage (-): 0.08

Region: NODE\_360870\_length\_2137\_cov\_16.065512 367-371. Max. coverage (+): 0.28. Max coverage (-): 0

Region: NODE\_360870\_length\_2137\_cov\_16.065512 372-375. Max. coverage (+): 0. Max coverage (-): 0

Region: NODE\_360870\_length\_2137\_cov\_16.065512 376-379. Max. coverage (+): 0.04. Max coverage (-): 0

Region: NODE\_360870\_length\_2137\_cov\_16.065512 380-384. Max. coverage (+): 0. Max coverage (-): 0

Region: NODE\_360870\_length\_2137\_cov\_16.065512 385-388. Max. coverage (+): 0. Max coverage (-): 0

Region: NODE\_360870\_length\_2137\_cov\_16.065512 389-392. Max. coverage (+): 0. Max coverage (-): 0

Region: NODE\_360870\_length\_2137\_cov\_16.065512 393-397. Max. coverage (+): 0. Max coverage (-): 0

Region: NODE\_360870\_length\_2137\_cov\_16.065512 398-401. Max. coverage (+): 0. Max coverage (-): 0

Region: NODE\_360870\_length\_2137\_cov\_16.065512 402-406. Max. coverage (+): 0. Max coverage (-): 0

Region: NODE\_360870\_length\_2137\_cov\_16.065512 407-410. Max. coverage (+): 0.33. Max coverage (-): 0

Region: NODE\_360870\_length\_2137\_cov\_16.065512 411-414. Max. coverage (+): 0.89. Max coverage (-): 0

Region: NODE\_360870\_length\_2137\_cov\_16.065512 415-419. Max. coverage (+): 0.84. Max coverage (-): 0

Region: NODE\_360870\_length\_2137\_cov\_16.065512 420-423. Max. coverage (+): 0. Max coverage (-): 0

Region: NODE\_360870\_length\_2137\_cov\_16.065512 424-427. Max. coverage (+): 0. Max coverage (-): 0

Region: NODE\_360870\_length\_2137\_cov\_16.065512 428-432. Max. coverage (+): 0. Max coverage (-): 0

Region: NODE\_360870\_length\_2137\_cov\_16.065512 433-436. Max. coverage (+): 0. Max coverage (-): 0

Region: NODE\_360870\_length\_2137\_cov\_16.065512 437-441. Max. coverage (+): 0. Max coverage (-): 0

Region: NODE\_360870\_length\_2137\_cov\_16.065512 442-445. Max. coverage (+): 0. Max coverage (-): 0

Region: NODE\_360870\_length\_2137\_cov\_16.065512 446-449. Max. coverage (+): 0. Max coverage (-): 0

Region: NODE\_360870\_length\_2137\_cov\_16.065512 450-454. Max. coverage (+): 0. Max coverage (-): 0

Region: NODE\_360870\_length\_2137\_cov\_16.065512 455-458. Max. coverage (+): 0.08. Max coverage (-): 0

Region: NODE\_360870\_length\_2137\_cov\_16.065512 459-462. Max. coverage (+): 0.08. Max coverage (-): 0

Region: NODE\_360870\_length\_2137\_cov\_16.065512 463-467. Max. coverage (+): 0. Max coverage (-): 0

Region: NODE\_360870\_length\_2137\_cov\_16.065512 468-471. Max. coverage (+): 0. Max coverage (-): 0

Region: NODE\_360870\_length\_2137\_cov\_16.065512 472-476. Max. coverage (+): 0. Max coverage (-): 0

Region: NODE\_360870\_length\_2137\_cov\_16.065512 477-480. Max. coverage (+): 0. Max coverage (-): 0.04

Region: NODE\_360870\_length\_2137\_cov\_16.065512 481-484. Max. coverage (+): 0.04. Max coverage (-): 0.04

Region: NODE\_360870\_length\_2137\_cov\_16.065512 485-489. Max. coverage (+): 0.22. Max coverage (-): 0

Region: NODE\_360870\_length\_2137\_cov\_16.065512 490-493. Max. coverage (+): 0.23. Max coverage (-): 0

Region: NODE\_360870\_length\_2137\_cov\_16.065512 494-497. Max. coverage (+): 0.1. Max coverage (-): 0.05

Region: NODE\_360870\_length\_2137\_cov\_16.065512 498-502. Max. coverage (+): 2.17. Max coverage (-): 0.05

Region: NODE\_360870\_length\_2137\_cov\_16.065512 503-506. Max. coverage (+): 0.23. Max coverage (-): 0

Region: NODE\_360870\_length\_2137\_cov\_16.065512 507-511. Max. coverage (+): 0. Max coverage (-): 0

Region: NODE\_360870\_length\_2137\_cov\_16.065512 512-515. Max. coverage (+): 0. Max coverage (-): 0

Region: NODE\_360870\_length\_2137\_cov\_16.065512 516-519. Max. coverage (+): 0. Max coverage (-): 0

Region: NODE\_360870\_length\_2137\_cov\_16.065512 520-524. Max. coverage (+): 0. Max coverage (-): 0

Region: NODE\_360870\_length\_2137\_cov\_16.065512 525-528. Max. coverage (+): 0. Max coverage (-): 0.08

Region: NODE\_360870\_length\_2137\_cov\_16.065512 529-532. Max. coverage (+): 0. Max coverage (-): 0

Region: NODE\_360870\_length\_2137\_cov\_16.065512 533-537. Max. coverage (+): 0. Max coverage (-): 0

Region: NODE\_360870\_length\_2137\_cov\_16.065512 538-541. Max. coverage (+): 0. Max coverage (-): 0

Region: NODE\_360870\_length\_2137\_cov\_16.065512 542-546. Max. coverage (+): 0. Max coverage (-): 0.34

Region: NODE\_360870\_length\_2137\_cov\_16.065512 547-550. Max. coverage (+): 0. Max coverage (-): 0.94

Region: NODE\_360870\_length\_2137\_cov\_16.065512 551-554. Max. coverage (+): 1.17. Max coverage (-): 0.51

Region: NODE\_360870\_length\_2137\_cov\_16.065512 555-559. Max. coverage (+): 0.03. Max coverage (-): 0

Region: NODE\_360870\_length\_2137\_cov\_16.065512 560-563. Max. coverage (+): 0. Max coverage (-): 0

Region: NODE\_360870\_length\_2137\_cov\_16.065512 564-567. Max. coverage (+): 0.08. Max coverage (-): 0

Region: NODE\_360870\_length\_2137\_cov\_16.065512 568-572. Max. coverage (+): 0.15. Max coverage (-): 0

Region: NODE\_360870\_length\_2137\_cov\_16.065512 573-576. Max. coverage (+): 0.15. Max coverage (-): 0

Region: NODE\_360870\_length\_2137\_cov\_16.065512 577-581. Max. coverage (+): 0. Max coverage (-): 0

Region: NODE\_360870\_length\_2137\_cov\_16.065512 582-585. Max. coverage (+): 0. Max coverage (-): 0

Region: NODE\_360870\_length\_2137\_cov\_16.065512 586-589. Max. coverage (+): 0. Max coverage (-): 0

Region: NODE\_360870\_length\_2137\_cov\_16.065512 590-594. Max. coverage (+): 0. Max coverage (-): 0

Region: NODE\_360870\_length\_2137\_cov\_16.065512 595-598. Max. coverage (+): 0. Max coverage (-): 0

Region: NODE\_360870\_length\_2137\_cov\_16.065512 599-602. Max. coverage (+): 0. Max coverage (-): 0

Region: NODE\_360870\_length\_2137\_cov\_16.065512 603-607. Max. coverage (+): 0. Max coverage (-): 0

Region: NODE\_360870\_length\_2137\_cov\_16.065512 608-611. Max. coverage (+): 0. Max coverage (-): 0

Region: NODE\_360870\_length\_2137\_cov\_16.065512 612-616. Max. coverage (+): 0. Max coverage (-): 0

Region: NODE\_360870\_length\_2137\_cov\_16.065512 617-620. Max. coverage (+): 0.11. Max coverage (-): 0

Region: NODE\_360870\_length\_2137\_cov\_16.065512 621-624. Max. coverage (+): 0. Max coverage (-): 0

Region: NODE\_360870\_length\_2137\_cov\_16.065512 625-629. Max. coverage (+): 0. Max coverage (-): 0

Region: NODE\_360870\_length\_2137\_cov\_16.065512 630-633. Max. coverage (+): 0. Max coverage (-): 0

Region: NODE\_360870\_length\_2137\_cov\_16.065512 634-637. Max. coverage (+): 0. Max coverage (-): 0

Region: NODE\_360870\_length\_2137\_cov\_16.065512 638-642. Max. coverage (+): 0. Max coverage (-): 0

Region: NODE\_360870\_length\_2137\_cov\_16.065512 643-646. Max. coverage (+): 0. Max coverage (-): 0

Region: NODE\_360870\_length\_2137\_cov\_16.065512 647-651. Max. coverage (+): 0. Max coverage (-): 0

Region: NODE\_360870\_length\_2137\_cov\_16.065512 652-655. Max. coverage (+): 0. Max coverage (-): 0

Region: NODE\_360870\_length\_2137\_cov\_16.065512 656-659. Max. coverage (+): 0. Max coverage (-): 0

Region: NODE\_360870\_length\_2137\_cov\_16.065512 660-664. Max. coverage (+): 0. Max coverage (-): 0

Region: NODE\_360870\_length\_2137\_cov\_16.065512 665-668. Max. coverage (+): 0. Max coverage (-): 0

Region: NODE\_360870\_length\_2137\_cov\_16.065512 669-672. Max. coverage (+): 0. Max coverage (-): 0

Region: NODE\_360870\_length\_2137\_cov\_16.065512 673-677. Max. coverage (+): 0. Max coverage (-): 0

Region: NODE\_360870\_length\_2137\_cov\_16.065512 678-681. Max. coverage (+): 0. Max coverage (-): 0

Region: NODE\_360870\_length\_2137\_cov\_16.065512 682-686. Max. coverage (+): 0. Max coverage (-): 0.15

Region: NODE\_360870\_length\_2137\_cov\_16.065512 687-690. Max. coverage (+): 0. Max coverage (-): 0.15

Region: NODE\_360870\_length\_2137\_cov\_16.065512 691-694. Max. coverage (+): 0. Max coverage (-): 0

Region: NODE\_360870\_length\_2137\_cov\_16.065512 695-699. Max. coverage (+): 0. Max coverage (-): 0.04

Region: NODE\_360870\_length\_2137\_cov\_16.065512 700-703. Max. coverage (+): 0. Max coverage (-): 2.57

Region: NODE\_360870\_length\_2137\_cov\_16.065512 704-707. Max. coverage (+): 0. Max coverage (-): 2.87

Region: NODE\_360870\_length\_2137\_cov\_16.065512 708-712. Max. coverage (+): 0. Max coverage (-): 0.27

Region: NODE\_360870\_length\_2137\_cov\_16.065512 713-716. Max. coverage (+): 0.08. Max coverage (-): 0

Region: NODE\_360870\_length\_2137\_cov\_16.065512 717-721. Max. coverage (+): 2.35. Max coverage (-): 0

Region: NODE\_360870\_length\_2137\_cov\_16.065512 722-725. Max. coverage (+): 626.89. Max coverage (-): 0

Region: NODE\_360870\_length\_2137\_cov\_16.065512 726-729. Max. coverage (+): 2.15. Max coverage (-): 0

Region: NODE\_360870\_length\_2137\_cov\_16.065512 730-734. Max. coverage (+): 0.05. Max coverage (-): 0.04

Region: NODE\_360870\_length\_2137\_cov\_16.065512 735-738. Max. coverage (+): 0. Max coverage (-): 0.04

Region: NODE\_360870\_length\_2137\_cov\_16.065512 739-742. Max. coverage (+): 0. Max coverage (-): 0

Region: NODE\_360870\_length\_2137\_cov\_16.065512 743-747. Max. coverage (+): 0.08. Max coverage (-): 0

Region: NODE\_360870\_length\_2137\_cov\_16.065512 748-751. Max. coverage (+): 0.08. Max coverage (-): 0

Region: NODE\_360870\_length\_2137\_cov\_16.065512 752-756. Max. coverage (+): 0. Max coverage (-): 0

Region: NODE\_360870\_length\_2137\_cov\_16.065512 757-760. Max. coverage (+): 0. Max coverage (-): 0

Region: NODE\_360870\_length\_2137\_cov\_16.065512 761-764. Max. coverage (+): 0. Max coverage (-): 0

Region: NODE\_360870\_length\_2137\_cov\_16.065512 765-769. Max. coverage (+): 0. Max coverage (-): 0

Region: NODE\_360870\_length\_2137\_cov\_16.065512 770-773. Max. coverage (+): 0. Max coverage (-): 0

Region: NODE\_360870\_length\_2137\_cov\_16.065512 774-777. Max. coverage (+): 0. Max coverage (-): 0

Region: NODE\_360870\_length\_2137\_cov\_16.065512 778-782. Max. coverage (+): 0. Max coverage (-): 0

Region: NODE\_360870\_length\_2137\_cov\_16.065512 783-786. Max. coverage (+): 0. Max coverage (-): 0

Region: NODE\_360870\_length\_2137\_cov\_16.065512 787-791. Max. coverage (+): 0. Max coverage (-): 0

Region: NODE\_360870\_length\_2137\_cov\_16.065512 792-795. Max. coverage (+): 0. Max coverage (-): 0

Region: NODE\_360870\_length\_2137\_cov\_16.065512 796-799. Max. coverage (+): 0.04. Max coverage (-): 0

Region: NODE\_360870\_length\_2137\_cov\_16.065512 800-804. Max. coverage (+): 13.96. Max coverage (-): 0

Region: NODE\_360870\_length\_2137\_cov\_16.065512 805-808. Max. coverage (+): 14.27. Max coverage (-): 0

Region: NODE\_360870\_length\_2137\_cov\_16.065512 809-812. Max. coverage (+): 0.15. Max coverage (-): 0

Region: NODE\_360870\_length\_2137\_cov\_16.065512 813-817. Max. coverage (+): 0.08. Max coverage (-): 0.08

Region: NODE\_360870\_length\_2137\_cov\_16.065512 818-821. Max. coverage (+): 0. Max coverage (-): 0.03

Region: NODE\_360870\_length\_2137\_cov\_16.065512 822-826. Max. coverage (+): 0. Max coverage (-): 0

Region: NODE\_360870\_length\_2137\_cov\_16.065512 827-830. Max. coverage (+): 0. Max coverage (-): 0

Region: NODE\_360870\_length\_2137\_cov\_16.065512 831-834. Max. coverage (+): 0. Max coverage (-): 0

Region: NODE\_360870\_length\_2137\_cov\_16.065512 835-839. Max. coverage (+): 0. Max coverage (-): 0

Region: NODE\_360870\_length\_2137\_cov\_16.065512 840-843. Max. coverage (+): 0. Max coverage (-): 0

Region: NODE\_360870\_length\_2137\_cov\_16.065512 844-848. Max. coverage (+): 0. Max coverage (-): 0

Region: NODE\_360870\_length\_2137\_cov\_16.065512 849-852. Max. coverage (+): 0. Max coverage (-): 0

Region: NODE\_360870\_length\_2137\_cov\_16.065512 853-856. Max. coverage (+): 0. Max coverage (-): 0.05

Region: NODE\_360870\_length\_2137\_cov\_16.065512 857-861. Max. coverage (+): 0.14. Max coverage (-): 0

Region: NODE\_360870\_length\_2137\_cov\_16.065512 862-865. Max. coverage (+): 0.14. Max coverage (-): 0

Region: NODE\_360870\_length\_2137\_cov\_16.065512 866-869. Max. coverage (+): 0. Max coverage (-): 0

Region: NODE\_360870\_length\_2137\_cov\_16.065512 870-874. Max. coverage (+): 0. Max coverage (-): 1.38

Region: NODE\_360870\_length\_2137\_cov\_16.065512 875-878. Max. coverage (+): 0. Max coverage (-): 1.46

Region: NODE\_360870\_length\_2137\_cov\_16.065512 879-883. Max. coverage (+): 0. Max coverage (-): 0.73

Region: NODE\_360870\_length\_2137\_cov\_16.065512 884-887. Max. coverage (+): 0. Max coverage (-): 0.96

Region: NODE\_360870\_length\_2137\_cov\_16.065512 888-891. Max. coverage (+): 0.18. Max coverage (-): 0.96

Region: NODE\_360870\_length\_2137\_cov\_16.065512 892-896. Max. coverage (+): 0.15. Max coverage (-): 0.08

Region: NODE\_360870\_length\_2137\_cov\_16.065512 897-900. Max. coverage (+): 0. Max coverage (-): 0

Region: NODE\_360870\_length\_2137\_cov\_16.065512 901-904. Max. coverage (+): 0.06. Max coverage (-): 0

Region: NODE\_360870\_length\_2137\_cov\_16.065512 905-909. Max. coverage (+): 0. Max coverage (-): 0

Region: NODE\_360870\_length\_2137\_cov\_16.065512 910-913. Max. coverage (+): 0. Max coverage (-): 0.46

Region: NODE\_360870\_length\_2137\_cov\_16.065512 914-918. Max. coverage (+): 0. Max coverage (-): 0.27

Region: NODE\_360870\_length\_2137\_cov\_16.065512 919-922. Max. coverage (+): 0. Max coverage (-): 0.08

Region: NODE\_360870\_length\_2137\_cov\_16.065512 923-926. Max. coverage (+): 0.03. Max coverage (-): 0.08

Region: NODE\_360870\_length\_2137\_cov\_16.065512 927-931. Max. coverage (+): 0.03. Max coverage (-): 0.04

Region: NODE\_360870\_length\_2137\_cov\_16.065512 932-935. Max. coverage (+): 0. Max coverage (-): 0.04

Region: NODE\_360870\_length\_2137\_cov\_16.065512 936-939. Max. coverage (+): 0. Max coverage (-): 0

Region: NODE\_360870\_length\_2137\_cov\_16.065512 940-944. Max. coverage (+): 0. Max coverage (-): 2.76

Region: NODE\_360870\_length\_2137\_cov\_16.065512 945-948. Max. coverage (+): 0. Max coverage (-): 0.11

Region: NODE\_360870\_length\_2137\_cov\_16.065512 949-953. Max. coverage (+): 0. Max coverage (-): 0.15

Region: NODE\_360870\_length\_2137\_cov\_16.065512 954-957. Max. coverage (+): 0. Max coverage (-): 0.04

Region: NODE\_360870\_length\_2137\_cov\_16.065512 958-961. Max. coverage (+): 0. Max coverage (-): 0.04

Region: NODE\_360870\_length\_2137\_cov\_16.065512 962-966. Max. coverage (+): 0. Max coverage (-): 0.04

Region: NODE\_360870\_length\_2137\_cov\_16.065512 967-970. Max. coverage (+): 0. Max coverage (-): 0.04

Region: NODE\_360870\_length\_2137\_cov\_16.065512 971-974. Max. coverage (+): 0.18. Max coverage (-): 0

Region: NODE\_360870\_length\_2137\_cov\_16.065512 975-979. Max. coverage (+): 0.18. Max coverage (-): 0

Region: NODE\_360870\_length\_2137\_cov\_16.065512 980-983. Max. coverage (+): 0. Max coverage (-): 0

Region: NODE\_360870\_length\_2137\_cov\_16.065512 984-988. Max. coverage (+): 0. Max coverage (-): 0

Region: NODE\_360870\_length\_2137\_cov\_16.065512 989-992. Max. coverage (+): 0.04. Max coverage (-): 0

Region: NODE\_360870\_length\_2137\_cov\_16.065512 993-996. Max. coverage (+): 0.04. Max coverage (-): 0

Region: NODE\_360870\_length\_2137\_cov\_16.065512 997-1001. Max. coverage (+): 0. Max coverage (-): 0.54

Region: NODE\_360870\_length\_2137\_cov\_16.065512 1002-1005. Max. coverage (+): 0. Max coverage (-): 1.61

Region: NODE\_360870\_length\_2137\_cov\_16.065512 1006-1009. Max. coverage (+): 0. Max coverage (-): 1.53

Region: NODE\_360870\_length\_2137\_cov\_16.065512 1010-1014. Max. coverage (+): 0. Max coverage (-): 0

Region: NODE\_360870\_length\_2137\_cov\_16.065512 1015-1018. Max. coverage (+): 0. Max coverage (-): 0

Region: NODE\_360870\_length\_2137\_cov\_16.065512 1019-1023. Max. coverage (+): 0. Max coverage (-): 0

Region: NODE\_360870\_length\_2137\_cov\_16.065512 1024-1027. Max. coverage (+): 0. Max coverage (-): 0

Region: NODE\_360870\_length\_2137\_cov\_16.065512 1028-1031. Max. coverage (+): 0. Max coverage (-): 0

Region: NODE\_360870\_length\_2137\_cov\_16.065512 1032-1036. Max. coverage (+): 0. Max coverage (-): 0.1

Region: NODE\_360870\_length\_2137\_cov\_16.065512 1037-1040. Max. coverage (+): 0.04. Max coverage (-): 0.36

Region: NODE\_360870\_length\_2137\_cov\_16.065512 1041-1044. Max. coverage (+): 0.19. Max coverage (-): 0.28

Region: NODE\_360870\_length\_2137\_cov\_16.065512 1045-1049. Max. coverage (+): 0.08. Max coverage (-): 0

Region: NODE\_360870\_length\_2137\_cov\_16.065512 1050-1053. Max. coverage (+): 0.02. Max coverage (-): 0

Region: NODE\_360870\_length\_2137\_cov\_16.065512 1054-1058. Max. coverage (+): 0. Max coverage (-): 0

Region: NODE\_360870\_length\_2137\_cov\_16.065512 1059-1062. Max. coverage (+): 0. Max coverage (-): 0.09

Region: NODE\_360870\_length\_2137\_cov\_16.065512 1063-1066. Max. coverage (+): 0.04. Max coverage (-): 0.13

Region: NODE\_360870\_length\_2137\_cov\_16.065512 1067-1071. Max. coverage (+): 0.04. Max coverage (-): 0.08

Region: NODE\_360870\_length\_2137\_cov\_16.065512 1072-1075. Max. coverage (+): 0. Max coverage (-): 0

Region: NODE\_360870\_length\_2137\_cov\_16.065512 1076-1079. Max. coverage (+): 0.08. Max coverage (-): 0

Region: NODE\_360870\_length\_2137\_cov\_16.065512 1080-1084. Max. coverage (+): 2.2. Max coverage (-): 0

Region: NODE\_360870\_length\_2137\_cov\_16.065512 1085-1088. Max. coverage (+): 3.57. Max coverage (-): 0

Region: NODE\_360870\_length\_2137\_cov\_16.065512 1089-1093. Max. coverage (+): 2.4. Max coverage (-): 0.08

Region: NODE\_360870\_length\_2137\_cov\_16.065512 1094-1097. Max. coverage (+): 0.15. Max coverage (-): 0.1

Region: NODE\_360870\_length\_2137\_cov\_16.065512 1098-1101. Max. coverage (+): 0.15. Max coverage (-): 0

Region: NODE\_360870\_length\_2137\_cov\_16.065512 1102-1106. Max. coverage (+): 0.19. Max coverage (-): 0

Region: NODE\_360870\_length\_2137\_cov\_16.065512 1107-1110. Max. coverage (+): 0.11. Max coverage (-): 0

Region: NODE\_360870\_length\_2137\_cov\_16.065512 1111-1114. Max. coverage (+): 4.01. Max coverage (-): 0

Region: NODE\_360870\_length\_2137\_cov\_16.065512 1115-1119. Max. coverage (+): 0.08. Max coverage (-): 0

Region: NODE\_360870\_length\_2137\_cov\_16.065512 1120-1123. Max. coverage (+): 0. Max coverage (-): 0.04

Region: NODE\_360870\_length\_2137\_cov\_16.065512 1124-1128. Max. coverage (+): 0. Max coverage (-): 0.23

Region: NODE\_360870\_length\_2137\_cov\_16.065512 1129-1132. Max. coverage (+): 0. Max coverage (-): 14.25

Region: NODE\_360870\_length\_2137\_cov\_16.065512 1133-1136. Max. coverage (+): 0. Max coverage (-): 14.17

Region: NODE\_360870\_length\_2137\_cov\_16.065512 1137-1141. Max. coverage (+): 4.65. Max coverage (-): 0.31

Region: NODE\_360870\_length\_2137\_cov\_16.065512 1142-1145. Max. coverage (+): 21.94. Max coverage (-): 0

Region: NODE\_360870\_length\_2137\_cov\_16.065512 1146-1149. Max. coverage (+): 19.41. Max coverage (-): 0

Region: NODE\_360870\_length\_2137\_cov\_16.065512 1150-1154. Max. coverage (+): 0. Max coverage (-): 0

Region: NODE\_360870\_length\_2137\_cov\_16.065512 1155-1158. Max. coverage (+): 0. Max coverage (-): 0

Region: NODE\_360870\_length\_2137\_cov\_16.065512 1159-1163. Max. coverage (+): 0. Max coverage (-): 0.04

Region: NODE\_360870\_length\_2137\_cov\_16.065512 1164-1167. Max. coverage (+): 0.04. Max coverage (-): 0.04

Region: NODE\_360870\_length\_2137\_cov\_16.065512 1168-1171. Max. coverage (+): 0.04. Max coverage (-): 0.04

Region: NODE\_360870\_length\_2137\_cov\_16.065512 1172-1176. Max. coverage (+): 0. Max coverage (-): 1.07

Region: NODE\_360870\_length\_2137\_cov\_16.065512 1177-1180. Max. coverage (+): 0. Max coverage (-): 19.23

Region: NODE\_360870\_length\_2137\_cov\_16.065512 1181-1184. Max. coverage (+): 0. Max coverage (-): 16.78

Region: NODE\_360870\_length\_2137\_cov\_16.065512 1185-1189. Max. coverage (+): 0. Max coverage (-): 0.11

Region: NODE\_360870\_length\_2137\_cov\_16.065512 1190-1193. Max. coverage (+): 0. Max coverage (-): 0.11

Region: NODE\_360870\_length\_2137\_cov\_16.065512 1194-1198. Max. coverage (+): 0.27. Max coverage (-): 0.31

Region: NODE\_360870\_length\_2137\_cov\_16.065512 1199-1202. Max. coverage (+): 0.27. Max coverage (-): 0.15

Region: NODE\_360870\_length\_2137\_cov\_16.065512 1203-1206. Max. coverage (+): 0.1. Max coverage (-): 0

Region: NODE\_360870\_length\_2137\_cov\_16.065512 1207-1211. Max. coverage (+): 0. Max coverage (-): 0

Region: NODE\_360870\_length\_2137\_cov\_16.065512 1212-1215. Max. coverage (+): 0. Max coverage (-): 0

Region: NODE\_360870\_length\_2137\_cov\_16.065512 1216-1219. Max. coverage (+): 0. Max coverage (-): 0

Region: NODE\_360870\_length\_2137\_cov\_16.065512 1220-1224. Max. coverage (+): 0. Max coverage (-): 0

Region: NODE\_360870\_length\_2137\_cov\_16.065512 1225-1228. Max. coverage (+): 0. Max coverage (-): 0

Region: NODE\_360870\_length\_2137\_cov\_16.065512 1229-1233. Max. coverage (+): 0. Max coverage (-): 0

Region: NODE\_360870\_length\_2137\_cov\_16.065512 1234-1237. Max. coverage (+): 0. Max coverage (-): 0

Region: NODE\_360870\_length\_2137\_cov\_16.065512 1238-1241. Max. coverage (+): 0. Max coverage (-): 0

Region: NODE\_360870\_length\_2137\_cov\_16.065512 1242-1246. Max. coverage (+): 0. Max coverage (-): 0

Region: NODE\_360870\_length\_2137\_cov\_16.065512 1247-1250. Max. coverage (+): 0. Max coverage (-): 0

Region: NODE\_360870\_length\_2137\_cov\_16.065512 1251-1254. Max. coverage (+): 0.08. Max coverage (-): 0.08

Region: NODE\_360870\_length\_2137\_cov\_16.065512 1255-1259. Max. coverage (+): 0.08. Max coverage (-): 0.08

Region: NODE\_360870\_length\_2137\_cov\_16.065512 1260-1263. Max. coverage (+): 0. Max coverage (-): 0.08

Region: NODE\_360870\_length\_2137\_cov\_16.065512 1264-1268. Max. coverage (+): 0. Max coverage (-): 0.08

Region: NODE\_360870\_length\_2137\_cov\_16.065512 1269-1272. Max. coverage (+): 0. Max coverage (-): 0

Region: NODE\_360870\_length\_2137\_cov\_16.065512 1273-1276. Max. coverage (+): 0.04. Max coverage (-): 0

Region: NODE\_360870\_length\_2137\_cov\_16.065512 1277-1281. Max. coverage (+): 0.27. Max coverage (-): 0

Region: NODE\_360870\_length\_2137\_cov\_16.065512 1282-1285. Max. coverage (+): 0. Max coverage (-): 0

Region: NODE\_360870\_length\_2137\_cov\_16.065512 1286-1289. Max. coverage (+): 0. Max coverage (-): 0

Region: NODE\_360870\_length\_2137\_cov\_16.065512 1290-1294. Max. coverage (+): 0. Max coverage (-): 0

Region: NODE\_360870\_length\_2137\_cov\_16.065512 1295-1298. Max. coverage (+): 0. Max coverage (-): 0.1

Region: NODE\_360870\_length\_2137\_cov\_16.065512 1299-1303. Max. coverage (+): 0.69. Max coverage (-): 0.1

Region: NODE\_360870\_length\_2137\_cov\_16.065512 1304-1307. Max. coverage (+): 0.31. Max coverage (-): 0

Region: NODE\_360870\_length\_2137\_cov\_16.065512 1308-1311. Max. coverage (+): 0. Max coverage (-): 0

Region: NODE\_360870\_length\_2137\_cov\_16.065512 1312-1316. Max. coverage (+): 0. Max coverage (-): 0

Region: NODE\_360870\_length\_2137\_cov\_16.065512 1317-1320. Max. coverage (+): 0. Max coverage (-): 0.08

Region: NODE\_360870\_length\_2137\_cov\_16.065512 1321-1324. Max. coverage (+): 0. Max coverage (-): 0.04

Region: NODE\_360870\_length\_2137\_cov\_16.065512 1325-1329. Max. coverage (+): 0.04. Max coverage (-): 0.04

Region: NODE\_360870\_length\_2137\_cov\_16.065512 1330-1333. Max. coverage (+): 0.04. Max coverage (-): 0.23

Region: NODE\_360870\_length\_2137\_cov\_16.065512 1334-1338. Max. coverage (+): 0. Max coverage (-): 0.34

Region: NODE\_360870\_length\_2137\_cov\_16.065512 1339-1342. Max. coverage (+): 0.39. Max coverage (-): 0.26

Region: NODE\_360870\_length\_2137\_cov\_16.065512 1343-1346. Max. coverage (+): 0. Max coverage (-): 0.29

Region: NODE\_360870\_length\_2137\_cov\_16.065512 1347-1351. Max. coverage (+): 0.23. Max coverage (-): 0.02

Region: NODE\_360870\_length\_2137\_cov\_16.065512 1352-1355. Max. coverage (+): 14.02. Max coverage (-): 0.04

Region: NODE\_360870\_length\_2137\_cov\_16.065512 1356-1359. Max. coverage (+): 14.38. Max coverage (-): 0

Region: NODE\_360870\_length\_2137\_cov\_16.065512 1360-1364. Max. coverage (+): 8.93. Max coverage (-): 0

Region: NODE\_360870\_length\_2137\_cov\_16.065512 1365-1368. Max. coverage (+): 0. Max coverage (-): 0

Region: NODE\_360870\_length\_2137\_cov\_16.065512 1369-1373. Max. coverage (+): 0. Max coverage (-): 0

Region: NODE\_360870\_length\_2137\_cov\_16.065512 1374-1377. Max. coverage (+): 0. Max coverage (-): 0

Region: NODE\_360870\_length\_2137\_cov\_16.065512 1378-1381. Max. coverage (+): 0. Max coverage (-): 0

Region: NODE\_360870\_length\_2137\_cov\_16.065512 1382-1386. Max. coverage (+): 0. Max coverage (-): 0.11

Region: NODE\_360870\_length\_2137\_cov\_16.065512 1387-1390. Max. coverage (+): 0. Max coverage (-): 0.08

Region: NODE\_360870\_length\_2137\_cov\_16.065512 1391-1395. Max. coverage (+): 0. Max coverage (-): 0.04

Region: NODE\_360870\_length\_2137\_cov\_16.065512 1396-1399. Max. coverage (+): 0. Max coverage (-): 0

Region: NODE\_360870\_length\_2137\_cov\_16.065512 1400-1403. Max. coverage (+): 0.08. Max coverage (-): 0

Region: NODE\_360870\_length\_2137\_cov\_16.065512 1404-1408. Max. coverage (+): 0.08. Max coverage (-): 0

Region: NODE\_360870\_length\_2137\_cov\_16.065512 1409-1412. Max. coverage (+): 0. Max coverage (-): 0.03

Region: NODE\_360870\_length\_2137\_cov\_16.065512 1413-1416. Max. coverage (+): 0. Max coverage (-): 0.03

Region: NODE\_360870\_length\_2137\_cov\_16.065512 1417-1421. Max. coverage (+): 0. Max coverage (-): 0

Region: NODE\_360870\_length\_2137\_cov\_16.065512 1422-1425. Max. coverage (+): 0. Max coverage (-): 0

Region: NODE\_360870\_length\_2137\_cov\_16.065512 1426-1430. Max. coverage (+): 0. Max coverage (-): 0

Region: NODE\_360870\_length\_2137\_cov\_16.065512 1431-1434. Max. coverage (+): 0.13. Max coverage (-): 0

Region: NODE\_360870\_length\_2137\_cov\_16.065512 1435-1438. Max. coverage (+): 0.13. Max coverage (-): 0

Region: NODE\_360870\_length\_2137\_cov\_16.065512 1439-1443. Max. coverage (+): 0. Max coverage (-): 0

Region: NODE\_360870\_length\_2137\_cov\_16.065512 1444-1447. Max. coverage (+): 0. Max coverage (-): 0

Region: NODE\_360870\_length\_2137\_cov\_16.065512 1448-1451. Max. coverage (+): 0. Max coverage (-): 0

Region: NODE\_360870\_length\_2137\_cov\_16.065512 1452-1456. Max. coverage (+): 0. Max coverage (-): 0

Region: NODE\_360870\_length\_2137\_cov\_16.065512 1457-1460. Max. coverage (+): 0. Max coverage (-): 0

Region: NODE\_360870\_length\_2137\_cov\_16.065512 1461-1465. Max. coverage (+): 0.04. Max coverage (-): 0

Region: NODE\_360870\_length\_2137\_cov\_16.065512 1466-1469. Max. coverage (+): 0.04. Max coverage (-): 0

Region: NODE\_360870\_length\_2137\_cov\_16.065512 1470-1473. Max. coverage (+): 0. Max coverage (-): 0.15

Region: NODE\_360870\_length\_2137\_cov\_16.065512 1474-1478. Max. coverage (+): 0. Max coverage (-): 1.3

Region: NODE\_360870\_length\_2137\_cov\_16.065512 1479-1482. Max. coverage (+): 0. Max coverage (-): 7.66

Region: NODE\_360870\_length\_2137\_cov\_16.065512 1483-1486. Max. coverage (+): 0. Max coverage (-): 3.85

Region: NODE\_360870\_length\_2137\_cov\_16.065512 1487-1491. Max. coverage (+): 0.02. Max coverage (-): 4.33

Region: NODE\_360870\_length\_2137\_cov\_16.065512 1492-1495. Max. coverage (+): 0.1. Max coverage (-): 0.44

Region: NODE\_360870\_length\_2137\_cov\_16.065512 1496-1500. Max. coverage (+): 0.29. Max coverage (-): 0.02

Region: NODE\_360870\_length\_2137\_cov\_16.065512 1501-1504. Max. coverage (+): 0.29. Max coverage (-): 0.02

Region: NODE\_360870\_length\_2137\_cov\_16.065512 1505-1508. Max. coverage (+): 0. Max coverage (-): 0

Region: NODE\_360870\_length\_2137\_cov\_16.065512 1509-1513. Max. coverage (+): 0. Max coverage (-): 0

Region: NODE\_360870\_length\_2137\_cov\_16.065512 1514-1517. Max. coverage (+): 0. Max coverage (-): 0

Region: NODE\_360870\_length\_2137\_cov\_16.065512 1518-1521. Max. coverage (+): 0. Max coverage (-): 0

Region: NODE\_360870\_length\_2137\_cov\_16.065512 1522-1526. Max. coverage (+): 0. Max coverage (-): 0

Region: NODE\_360870\_length\_2137\_cov\_16.065512 1527-1530. Max. coverage (+): 0. Max coverage (-): 0

Region: NODE\_360870\_length\_2137\_cov\_16.065512 1531-1535. Max. coverage (+): 0. Max coverage (-): 0

Region: NODE\_360870\_length\_2137\_cov\_16.065512 1536-1539. Max. coverage (+): 0. Max coverage (-): 0

Region: NODE\_360870\_length\_2137\_cov\_16.065512 1540-1543. Max. coverage (+): 0.08. Max coverage (-): 0

Region: NODE\_360870\_length\_2137\_cov\_16.065512 1544-1548. Max. coverage (+): 0.08. Max coverage (-): 0

Region: NODE\_360870\_length\_2137\_cov\_16.065512 1549-1552. Max. coverage (+): 0.03. Max coverage (-): 0

Region: NODE\_360870\_length\_2137\_cov\_16.065512 1553-1556. Max. coverage (+): 0.03. Max coverage (-): 0

Region: NODE\_360870\_length\_2137\_cov\_16.065512 1557-1561. Max. coverage (+): 0. Max coverage (-): 2.58

Region: NODE\_360870\_length\_2137\_cov\_16.065512 1562-1565. Max. coverage (+): 0. Max coverage (-): 23.43

Region: NODE\_360870\_length\_2137\_cov\_16.065512 1566-1570. Max. coverage (+): 0. Max coverage (-): 23.2

Region: NODE\_360870\_length\_2137\_cov\_16.065512 1571-1574. Max. coverage (+): 0. Max coverage (-): 0.03

Region: NODE\_360870\_length\_2137\_cov\_16.065512 1575-1578. Max. coverage (+): 0. Max coverage (-): 0

Region: NODE\_360870\_length\_2137\_cov\_16.065512 1579-1583. Max. coverage (+): 0.31. Max coverage (-): 0

Region: NODE\_360870\_length\_2137\_cov\_16.065512 1584-1587. Max. coverage (+): 0.38. Max coverage (-): 0

Region: NODE\_360870\_length\_2137\_cov\_16.065512 1588-1591. Max. coverage (+): 0.08. Max coverage (-): 0

Region: NODE\_360870\_length\_2137\_cov\_16.065512 1592-1596. Max. coverage (+): 0. Max coverage (-): 0

Region: NODE\_360870\_length\_2137\_cov\_16.065512 1597-1600. Max. coverage (+): 0. Max coverage (-): 0

Region: NODE\_360870\_length\_2137\_cov\_16.065512 1601-1605. Max. coverage (+): 0. Max coverage (-): 0

Region: NODE\_360870\_length\_2137\_cov\_16.065512 1606-1609. Max. coverage (+): 0.01. Max coverage (-): 0

Region: NODE\_360870\_length\_2137\_cov\_16.065512 1610-1613. Max. coverage (+): 0.05. Max coverage (-): 0

Region: NODE\_360870\_length\_2137\_cov\_16.065512 1614-1618. Max. coverage (+): 0.01. Max coverage (-): 0

Region: NODE\_360870\_length\_2137\_cov\_16.065512 1619-1622. Max. coverage (+): 0. Max coverage (-): 0

Region: NODE\_360870\_length\_2137\_cov\_16.065512 1623-1626. Max. coverage (+): 0. Max coverage (-): 0

Region: NODE\_360870\_length\_2137\_cov\_16.065512 1627-1631. Max. coverage (+): 0. Max coverage (-): 0

Region: NODE\_360870\_length\_2137\_cov\_16.065512 1632-1635. Max. coverage (+): 0. Max coverage (-): 0

Region: NODE\_360870\_length\_2137\_cov\_16.065512 1636-1640. Max. coverage (+): 0. Max coverage (-): 0

Region: NODE\_360870\_length\_2137\_cov\_16.065512 1641-1644. Max. coverage (+): 0. Max coverage (-): 0

Region: NODE\_360870\_length\_2137\_cov\_16.065512 1645-1648. Max. coverage (+): 0.01. Max coverage (-): 0

Region: NODE\_360870\_length\_2137\_cov\_16.065512 1649-1653. Max. coverage (+): 0.01. Max coverage (-): 0

Region: NODE\_360870\_length\_2137\_cov\_16.065512 1654-1657. Max. coverage (+): 0. Max coverage (-): 0

Region: NODE\_360870\_length\_2137\_cov\_16.065512 1658-1661. Max. coverage (+): 0. Max coverage (-): 0

Region: NODE\_360870\_length\_2137\_cov\_16.065512 1662-1666. Max. coverage (+): 0. Max coverage (-): 0

Region: NODE\_360870\_length\_2137\_cov\_16.065512 1667-1670. Max. coverage (+): 0. Max coverage (-): 0

Region: NODE\_360870\_length\_2137\_cov\_16.065512 1671-1675. Max. coverage (+): 0. Max coverage (-): 0.01

Region: NODE\_360870\_length\_2137\_cov\_16.065512 1676-1679. Max. coverage (+): 0. Max coverage (-): 0.01

Region: NODE\_360870\_length\_2137\_cov\_16.065512 1680-1683. Max. coverage (+): 0. Max coverage (-): 0

Region: NODE\_360870\_length\_2137\_cov\_16.065512 1684-1688. Max. coverage (+): 0.01. Max coverage (-): 0.01

Region: NODE\_360870\_length\_2137\_cov\_16.065512 1689-1692. Max. coverage (+): 0.04. Max coverage (-): 0

Region: NODE\_360870\_length\_2137\_cov\_16.065512 1693-1696. Max. coverage (+): 0.04. Max coverage (-): 0

Region: NODE\_360870\_length\_2137\_cov\_16.065512 1697-1701. Max. coverage (+): 0. Max coverage (-): 0.02

Region: NODE\_360870\_length\_2137\_cov\_16.065512 1702-1705. Max. coverage (+): 0. Max coverage (-): 0.02

Region: NODE\_360870\_length\_2137\_cov\_16.065512 1706-1710. Max. coverage (+): 0. Max coverage (-): 0.01

Region: NODE\_360870\_length\_2137\_cov\_16.065512 1711-1714. Max. coverage (+): 0. Max coverage (-): 0.01

Region: NODE\_360870\_length\_2137\_cov\_16.065512 1715-1718. Max. coverage (+): 0. Max coverage (-): 0

Region: NODE\_360870\_length\_2137\_cov\_16.065512 1719-1723. Max. coverage (+): 0.01. Max coverage (-): 0

Region: NODE\_360870\_length\_2137\_cov\_16.065512 1724-1727. Max. coverage (+): 0.01. Max coverage (-): 0

Region: NODE\_360870\_length\_2137\_cov\_16.065512 1728-1731. Max. coverage (+): 0. Max coverage (-): 0

Region: NODE\_360870\_length\_2137\_cov\_16.065512 1732-1736. Max. coverage (+): 0. Max coverage (-): 0.08

Region: NODE\_360870\_length\_2137\_cov\_16.065512 1737-1740. Max. coverage (+): 0. Max coverage (-): 0

Region: NODE\_360870\_length\_2137\_cov\_16.065512 1741-1745. Max. coverage (+): 0. Max coverage (-): 0

Region: NODE\_360870\_length\_2137\_cov\_16.065512 1746-1749. Max. coverage (+): 0. Max coverage (-): 0

Region: NODE\_360870\_length\_2137\_cov\_16.065512 1750-1753. Max. coverage (+): 0. Max coverage (-): 0

Region: NODE\_360870\_length\_2137\_cov\_16.065512 1754-1758. Max. coverage (+): 0. Max coverage (-): 0.02

Region: NODE\_360870\_length\_2137\_cov\_16.065512 1759-1762. Max. coverage (+): 0. Max coverage (-): 0.05

Region: NODE\_360870\_length\_2137\_cov\_16.065512 1763-1766. Max. coverage (+): 0. Max coverage (-): 0.01

Region: NODE\_360870\_length\_2137\_cov\_16.065512 1767-1771. Max. coverage (+): 0. Max coverage (-): 0

Region: NODE\_360870\_length\_2137\_cov\_16.065512 1772-1775. Max. coverage (+): 0. Max coverage (-): 0

Region: NODE\_360870\_length\_2137\_cov\_16.065512 1776-1780. Max. coverage (+): 0. Max coverage (-): 0

Region: NODE\_360870\_length\_2137\_cov\_16.065512 1781-1784. Max. coverage (+): 0. Max coverage (-): 0

Region: NODE\_360870\_length\_2137\_cov\_16.065512 1785-1788. Max. coverage (+): 0. Max coverage (-): 0

Region: NODE\_360870\_length\_2137\_cov\_16.065512 1789-1793. Max. coverage (+): 0. Max coverage (-): 0

Region: NODE\_360870\_length\_2137\_cov\_16.065512 1794-1797. Max. coverage (+): 0. Max coverage (-): 0

Region: NODE\_360870\_length\_2137\_cov\_16.065512 1798-1801. Max. coverage (+): 0.01. Max coverage (-): 0

Region: NODE\_360870\_length\_2137\_cov\_16.065512 1802-1806. Max. coverage (+): 0.02. Max coverage (-): 0

Region: NODE\_360870\_length\_2137\_cov\_16.065512 1807-1810. Max. coverage (+): 0.02. Max coverage (-): 0

Region: NODE\_360870\_length\_2137\_cov\_16.065512 1811-1815. Max. coverage (+): 0. Max coverage (-): 0

Region: NODE\_360870\_length\_2137\_cov\_16.065512 1816-1819. Max. coverage (+): 0.01. Max coverage (-): 0

Region: NODE\_360870\_length\_2137\_cov\_16.065512 1820-1823. Max. coverage (+): 0.01. Max coverage (-): 0

Region: NODE\_360870\_length\_2137\_cov\_16.065512 1824-1828. Max. coverage (+): 0. Max coverage (-): 0

Region: NODE\_360870\_length\_2137\_cov\_16.065512 1829-1832. Max. coverage (+): 0.01. Max coverage (-): 0

Region: NODE\_360870\_length\_2137\_cov\_16.065512 1833-1836. Max. coverage (+): 0. Max coverage (-): 0

Region: NODE\_360870\_length\_2137\_cov\_16.065512 1837-1841. Max. coverage (+): 0. Max coverage (-): 0

Region: NODE\_360870\_length\_2137\_cov\_16.065512 1842-1845. Max. coverage (+): 0. Max coverage (-): 0

Region: NODE\_360870\_length\_2137\_cov\_16.065512 1846-1850. Max. coverage (+): 0. Max coverage (-): 0

Region: NODE\_360870\_length\_2137\_cov\_16.065512 1851-1854. Max. coverage (+): 0. Max coverage (-): 0

Region: NODE\_360870\_length\_2137\_cov\_16.065512 1855-1858. Max. coverage (+): 0. Max coverage (-): 0

Region: NODE\_360870\_length\_2137\_cov\_16.065512 1859-1863. Max. coverage (+): 0.01. Max coverage (-): 0

Region: NODE\_360870\_length\_2137\_cov\_16.065512 1864-1867. Max. coverage (+): 0.12. Max coverage (-): 0

Region: NODE\_360870\_length\_2137\_cov\_16.065512 1868-1871. Max. coverage (+): 0.09. Max coverage (-): 0

Region: NODE\_360870\_length\_2137\_cov\_16.065512 1872-1876. Max. coverage (+): 0.03. Max coverage (-): 0

Region: NODE\_360870\_length\_2137\_cov\_16.065512 1877-1880. Max. coverage (+): 0. Max coverage (-): 0

Region: NODE\_360870\_length\_2137\_cov\_16.065512 1881-1885. Max. coverage (+): 0. Max coverage (-): 0

Region: NODE\_360870\_length\_2137\_cov\_16.065512 1886-1889. Max. coverage (+): 0. Max coverage (-): 0

Region: NODE\_360870\_length\_2137\_cov\_16.065512 1890-1893. Max. coverage (+): 0. Max coverage (-): 0.01

Region: NODE\_360870\_length\_2137\_cov\_16.065512 1894-1898. Max. coverage (+): 0. Max coverage (-): 0

Region: NODE\_360870\_length\_2137\_cov\_16.065512 1899-1902. Max. coverage (+): 0. Max coverage (-): 0

Region: NODE\_360870\_length\_2137\_cov\_16.065512 1903-1906. Max. coverage (+): 0. Max coverage (-): 0

Region: NODE\_360870\_length\_2137\_cov\_16.065512 1907-1911. Max. coverage (+): 0. Max coverage (-): 0

Region: NODE\_360870\_length\_2137\_cov\_16.065512 1912-1915. Max. coverage (+): 0. Max coverage (-): 0

Region: NODE\_360870\_length\_2137\_cov\_16.065512 1916-1920. Max. coverage (+): 0. Max coverage (-): 0

Region: NODE\_360870\_length\_2137\_cov\_16.065512 1921-1924. Max. coverage (+): 0. Max coverage (-): 0

Region: NODE\_360870\_length\_2137\_cov\_16.065512 1925-1928. Max. coverage (+): 0. Max coverage (-): 0

Region: NODE\_360870\_length\_2137\_cov\_16.065512 1929-1933. Max. coverage (+): 0.01. Max coverage (-): 0

Region: NODE\_360870\_length\_2137\_cov\_16.065512 1934-1937. Max. coverage (+): 0.04. Max coverage (-): 0

Region: NODE\_360870\_length\_2137\_cov\_16.065512 1938-1942. Max. coverage (+): 0.04. Max coverage (-): 0

Region: NODE\_360870\_length\_2137\_cov\_16.065512 1943-1946. Max. coverage (+): 0. Max coverage (-): 0

Region: NODE\_360870\_length\_2137\_cov\_16.065512 1947-1950. Max. coverage (+): 0.01. Max coverage (-): 0

Region: NODE\_360870\_length\_2137\_cov\_16.065512 1951-1955. Max. coverage (+): 0. Max coverage (-): 0

Region: NODE\_360870\_length\_2137\_cov\_16.065512 1956-1959. Max. coverage (+): 0. Max coverage (-): 0

Region: NODE\_360870\_length\_2137\_cov\_16.065512 1960-1963. Max. coverage (+): 0. Max coverage (-): 0

Region: NODE\_360870\_length\_2137\_cov\_16.065512 1964-1968. Max. coverage (+): 0. Max coverage (-): 0

Region: NODE\_360870\_length\_2137\_cov\_16.065512 1969-1972. Max. coverage (+): 0. Max coverage (-): 0

Region: NODE\_360870\_length\_2137\_cov\_16.065512 1973-1977. Max. coverage (+): 0. Max coverage (-): 0.04

Region: NODE\_360870\_length\_2137\_cov\_16.065512 1978-1981. Max. coverage (+): 0. Max coverage (-): 0.04

Region: NODE\_360870\_length\_2137\_cov\_16.065512 1982-1985. Max. coverage (+): 0. Max coverage (-): 0.01

Region: NODE\_360870\_length\_2137\_cov\_16.065512 1986-1990. Max. coverage (+): 0. Max coverage (-): 0.01

Region: NODE\_360870\_length\_2137\_cov\_16.065512 1991-1994. Max. coverage (+): 0.01. Max coverage (-): 0

Region: NODE\_360870\_length\_2137\_cov\_16.065512 1995-1998. Max. coverage (+): 0.01. Max coverage (-): 0

Region: NODE\_360870\_length\_2137\_cov\_16.065512 1999-2003. Max. coverage (+): 0. Max coverage (-): 0

Region: NODE\_360870\_length\_2137\_cov\_16.065512 2004-2007. Max. coverage (+): 0. Max coverage (-): 0

Region: NODE\_360870\_length\_2137\_cov\_16.065512 2008-2012. Max. coverage (+): 0. Max coverage (-): 0

Region: NODE\_360870\_length\_2137\_cov\_16.065512 2013-2016. Max. coverage (+): 0. Max coverage (-): 0

Region: NODE\_360870\_length\_2137\_cov\_16.065512 2017-2020. Max. coverage (+): 0. Max coverage (-): 0

Region: NODE\_360870\_length\_2137\_cov\_16.065512 2021-2025. Max. coverage (+): 0. Max coverage (-): 0

Region: NODE\_360870\_length\_2137\_cov\_16.065512 2026-2029. Max. coverage (+): 0. Max coverage (-): 0

Region: NODE\_360870\_length\_2137\_cov\_16.065512 2030-2033. Max. coverage (+): 0. Max coverage (-): 0

Region: NODE\_360870\_length\_2137\_cov\_16.065512 2034-2038. Max. coverage (+): 0. Max coverage (-): 0

Region: NODE\_360870\_length\_2137\_cov\_16.065512 2039-2042. Max. coverage (+): 0. Max coverage (-): 0

Region: NODE\_360870\_length\_2137\_cov\_16.065512 2043-2047. Max. coverage (+): 0. Max coverage (-): 0

Region: NODE\_360870\_length\_2137\_cov\_16.065512 2048-2051. Max. coverage (+): 0. Max coverage (-): 0

Region: NODE\_360870\_length\_2137\_cov\_16.065512 2052-2055. Max. coverage (+): 0. Max coverage (-): 0

Region: NODE\_360870\_length\_2137\_cov\_16.065512 2056-2060. Max. coverage (+): 0.01. Max coverage (-): 0

Region: NODE\_360870\_length\_2137\_cov\_16.065512 2061-2064. Max. coverage (+): 0.01. Max coverage (-): 0

Region: NODE\_360870\_length\_2137\_cov\_16.065512 2065-2068. Max. coverage (+): 0. Max coverage (-): 0

Region: NODE\_360870\_length\_2137\_cov\_16.065512 2069-2073. Max. coverage (+): 0. Max coverage (-): 0

Region: NODE\_360870\_length\_2137\_cov\_16.065512 2074-2077. Max. coverage (+): 0. Max coverage (-): 0

Region: NODE\_360870\_length\_2137\_cov\_16.065512 2078-2082. Max. coverage (+): 0. Max coverage (-): 0

Region: NODE\_360870\_length\_2137\_cov\_16.065512 2083-2086. Max. coverage (+): 0. Max coverage (-): 0

Region: NODE\_360870\_length\_2137\_cov\_16.065512 2087-2090. Max. coverage (+): 0. Max coverage (-): 0

Region: NODE\_360870\_length\_2137\_cov\_16.065512 2091-2095. Max. coverage (+): 0. Max coverage (-): 0

Region: NODE\_360870\_length\_2137\_cov\_16.065512 2096-2099. Max. coverage (+): 0. Max coverage (-): 0

Region: NODE\_360870\_length\_2137\_cov\_16.065512 2100-2103. Max. coverage (+): 0. Max coverage (-): 0

Region: NODE\_360870\_length\_2137\_cov\_16.065512 2104-2108. Max. coverage (+): 0. Max coverage (-): 0

Region: NODE\_360870\_length\_2137\_cov\_16.065512 2109-2112. Max. coverage (+): 0. Max coverage (-): 0

Region: NODE\_360870\_length\_2137\_cov\_16.065512 2113-2117. Max. coverage (+): 0. Max coverage (-): 0

Region: NODE\_360870\_length\_2137\_cov\_16.065512 2118-2121. Max. coverage (+): 0. Max coverage (-): 0

Region: NODE\_360870\_length\_2137\_cov\_16.065512 2122-2125. Max. coverage (+): 0. Max coverage (-): 0

Region: NODE\_360870\_length\_2137\_cov\_16.065512 2126-2130. Max. coverage (+): 0. Max coverage (-): 0

Region: NODE\_360870\_length\_2137\_cov\_16.065512 2131-2134. Max. coverage (+): 0. Max coverage (-): 0

Region: NODE\_360870\_length\_2137\_cov\_16.065512 2135-2138. Max. coverage (+): 0. Max coverage (-): 0

Region: NODE\_360870\_length\_2137\_cov\_16.065512 2139-2143. Max. coverage (+): 1.12. Max coverage (-): 0

Region: NODE\_360870\_length\_2137\_cov\_16.065512 2144-2147. Max. coverage (+): 1.12. Max coverage (-): 0

Region: NODE\_360870\_length\_2137\_cov\_16.065512 2148-2152. Max. coverage (+): 0. Max coverage (-): 0

Region: NODE\_360870\_length\_2137\_cov\_16.065512 2153-2156. Max. coverage (+): 0. Max coverage (-): 0

Region: NODE\_360870\_length\_2137\_cov\_16.065512 2157-2160. Max. coverage (+): 0.08. Max coverage (-): 0

Region: NODE\_360870\_length\_2137\_cov\_16.065512 2161-2165. Max. coverage (+): 0.51. Max coverage (-): 0

Region: NODE\_360870\_length\_2137\_cov\_16.065512 2166-2169. Max. coverage (+): 0.54. Max coverage (-): 0

Region: NODE\_360870\_length\_2137\_cov\_16.065512 2170-2173. Max. coverage (+): 0.13. Max coverage (-): 0

Region: NODE\_360870\_length\_2137\_cov\_16.065512 2174-2178. Max. coverage (+): 0. Max coverage (-): 0

Region: NODE\_360870\_length\_2137\_cov\_16.065512 2179-2182. Max. coverage (+): 0. Max coverage (-): 0

Region: NODE\_360870\_length\_2137\_cov\_16.065512 2183-2187. Max. coverage (+): 0. Max coverage (-): 0

Region: NODE\_360870\_length\_2137\_cov\_16.065512 2188-2191. Max. coverage (+): 0. Max coverage (-): 0

Region: NODE\_360870\_length\_2137\_cov\_16.065512 2192-2195. Max. coverage (+): 0. Max coverage (-): 0

Region: NODE\_360870\_length\_2137\_cov\_16.065512 2196-. Max. coverage (+): 0. Max coverage (-): 0

RepeatMasker Color Code

**+**

100-98% Identity

<98-95% Identity

<95-90% Identity

<90-85% Identity

<85-80% Identity

<80-75% Identity

<75-70% Identity

<70% Identity

**-**

Gene Set Color Code

**+**

Gene

Pseudogene

Other

**-**

Topology/Coverage Color Code

Coverage Plus Strand

Coverage Minus Strand

Mainstrand: Plus

Mainstrand: Minus

Complementary Strand

Flanking Region  
(if option -flank >0)

Gene Set Annotation  
  
RepeatMasker Annotation  

**1. Helitron-2\_DR**: 94-1419 (-), Divergence to consensus: 31.9%  
**2. AlRepB-173**: 1608-2012 (+), Divergence to consensus: 27.7%  
**3. AlRepD-2459**: 2053-2109 (+), Divergence to consensus: 23.1%  
**4. AlRepB-99**: 2064-2111 (-), Divergence to consensus: 16.6%  
**5. Helitron-2\_DR**: 2152-2198 (-), Divergence to consensus: 19.1%

  
Transcription Factor Binding Sites  

**SPZ1** (Sequence: CTCTTACCCT (-): 1779)  
**SPZ1** (Sequence: CTGTAACCCT (-): 2064)  
**RHOXF1** (Sequence: AGCTCA (-): 135)  
**RHOXF1** (Sequence: GGCTCA (-): 1474)  
**RHOXF1** (Sequence: AGCTCA (-): 1893)  
**RHOXF1** (Sequence: GGATCA (-): 2125)  
**RHOXF1** (Sequence: TAATCT (+): 621)  
**FOXO1** (Sequence: GTTGTTTTT (+): 1950)  
**Sox5** (Sequence: ATTGTT (+): 342)  
**FIGLA** (Sequence: TACACGTGGT (-): 516)  
**FIGLA** (Sequence: TCCACCTGTT (-): 770)  
**FIGLA** (Sequence: TCCAGCTGTT (-): 1485)  
**SOX9** (Sequence: TCATTGTT (+): 340)  
**POU2F1** (Sequence: ATTAAAATA (-): 1158)  
**Rhox11** (Sequence: TGGTGTATT (+): 1822)  
**Sox5** (Sequence: AACAAT (-): 698)
