# Supplementary material for: First characterization of PIWI-interacting RNA clusters in a cichlid fish with a B chromosome
Source: BMC Biol. 2022 Sep 21;20:204. doi: 10.1186/s12915-022-01403-2 (PMC9490952; doi:10.1186/s12915-022-01403-2)
Supplement: Supplementary file 1 — Additional file 1. Zipped folder with fasta and interactive html piRNA cluster information for the A. latifasciata genome. The nomenclature is as follows: number-pirna-cluster_sex_B-presence (f, female; m, male; 0b, without B chromosome; 1b, with B chromosome). [file 12915_2022_1403_MOESM1_ESM.zip › 140_f1b.html]

piRNA cluster 140\_f1b 61


Predicted piRNA cluster no. 140\_f1b
  

Show proTRAC run info
Hide proTRAC run info

/\  
                \_\_\_\_\_\_\_\_\_\_\_\_\_\_\_\_\_\_\_\_\_\_\_/\\_\_\_ /  \\_\_\_\_\_\_\_  
               I                      /  \  /    \      I  
               I     pro             /    \/      \     I  
               I        TRAC        /               \   I  
               I   \_\_\_\_\_\_\_\_\_\_\_\_\_\_\_\_/\_\_\_\_\_\_\_\_\_\_\_\_\_\_\_\_\_\\_ I  
               I   \              /                     I  
               I    \            /                      I  
               I     \  /\      /       V.2.4.2         I  
               I      \/  \    /                        I  
               I\_\_\_\_\_\_\_\_\_\_\_\  /\_\_\_\_\_\_\_\_\_\_\_\_\_\_\_\_\_\_\_\_\_\_\_\_\_I  
                            \/  
  
  
================================= proTRAC ====================================  
VERSION: .......... 2.4.2  
LAST MODIFIED: .... 11. May 2018  
  
Please cite:  
Rosenkranz D, Zischler H. proTRAC - a software for probabilistic piRNA cluster  
detection, visualization and analysis. 2012. BMC Bioinformatics 13:5.  
  
  
Contact:  
David Rosenkranz  
Institute of Organismic and Molecular Evolutionary Biology  
Dept. Anthropology, small RNA group  
Johannes Gutenberg University Mainz  
email: rosenkranz@uni-mainz.de  
  
You can find the latest proTRAC version at:  
http://sourceforge.net/projects/protrac/files  
http://www.smallRNAgroup-mainz.de/software  
==============================================================================  
  
PARAMETERS:  
Map file: ...............piwi-femeas-1B.fa-collapse.map  
Genome file: ............../../../0B\_ala\_genome.fa  
RepeatMasker annotation: Alatifasciata-all0B-maryan-v2.fa\_corrected.out  
GeneSet:................./guest-storage/Data/annotation/Alatifasciata\_all0B\_maryan-v2\_out2017.gff  
  
Significant (p<=0.01) hit density will be calculated based  
on observed hit distribution.  
  
Sliding window size: ........................................ 5000 bp  
Sliding window increament: .................................. 1000 bp  
Normalize each hit by number of genomic hits: ............... yes  
Normalize each hit by number of sequence reads: ............. yes  
Normalize values (-> per million mapped reads): ............. yes  
Min. fraction of hits with 1T(U) or 10A: .................... 0.75  
Alternatively: Min. fraction of hits with 1T(U) and 10A: .... 0.5  
Min. fraction of hits with typical piRNA length: ............ 0.75  
Typical piRNA length: ....................................... 24-32 nt  
Min. size of a piRNA cluster: ............................... 1000 bp.  
Min. number of hits (absolute): ............................. 0  
Min. number of hits (normalized): ........................... 0  
Min. fraction of hits on the mainstrand: .................... 0.75  
Top fraction of mapped sequences (in terms of read counts): . 1%  
Top fraction accounts for max. n% of sequence reads: ........ 90%  
Min. fraction of hits on each arm of a bidirectional cluster: 0.05  
Output html file for each cluster: .......................... yes  
Output a summary table: ..................................... yes  
Output a FASTA file for each cluster (piRNA sequences): ..... yes  
Output a FASTA file comprising cluster sequences: ........... yes  
Output a GTF file for predicted piRNA clusters: ..............yes  
Search DNA motifs in clusters: .............................. yes  
Output flanking sequences: +/- .............................. 0 bp  
Output ~.pTi file: .......................................... no  
==============================================================================  
  
  
Genome size (without gaps): ............ 758543724 bp  
Gaps (N/X/-): .......................... 417479 bp  
Mapped reads: .......................... 10641844  
Non-identical sequences: ............... 2832837  
Genomic hits: .......................... 26056853  
Significant densitiy of mapped reads: .. 368.713530323068 reads/kb

Show proTRAC cluster info
Hide proTRAC cluster info

|  |  |
| --- | --- |
| Location | NODE\_360870\_length\_2137\_cov\_16.065512 |
| Coordinates | 14-2195 |
| Size [bp] | 2182 |
| Sequence hit loci | 858 |
| Mapped reads (normalized) | 7032.5 |
| Mapped reads (normalized) per kb | 3223 |
| Normalized reads with 1T (1U) | 87.4% |
| Normalized reads with 10A | 41.2% |
| Normalized reads with length 24-32 nt | 99.6% |
| Normalized reads on the main strand(s) | 93.6% |
| Predicted directionality | bi:plus-minus (split between 1457 and 1466) |

100%

0%

1T (1U)  
reads

10A reads

24-32 nt  
reads

reads on mainstrand

**Either the amount of reads with 1T (1U) OR 10A has to exceed 75% (set with option: -1Tor10A)  
Alternatively the amount of reads with 1T (1U) AND 10A has to exceed 50% (set with option: -1Tand10A)  
Minimum amount of reads with preferred size is 75% (set with option: -pisize)  
Minimum amount of reads on the main strand(s) is 75% (set with option: -clstrand)**

Show read coverage
Hide read coverage

WHAT DO I SEE HERE?  
This chart shows the location of mapped sequence reads within a predicted piRNA cluster. The color refers to the number of genomic hits produced by the sequence read in question. A dark red bar indicates that this sequence read produces many other hits elsewhere in the genome. Many adjacent red or yellow bars can indicate the presence of a multi-copy element such as transposons or rRNA genes. A dark green bar indicates that this sequence read maps uniquely to this locus.

1 hit

2-5 hits

6-10 hits

11-20 hits

21-50 hits

51-100 hits

> 100 hits

NODE\_360870\_length\_2137\_cov\_16.065512

14

2195

Gene Set

RepeatMasker

Mapped  
Reads

515.67

plus strand

minus strand

515.67

Region: NODE\_360870\_length\_2137\_cov\_16.065512 38422-16. Max. coverage (+): 0.02. Max coverage (-): 0

Region: NODE\_360870\_length\_2137\_cov\_16.065512 17-20. Max. coverage (+): 0. Max coverage (-): 0

Region: NODE\_360870\_length\_2137\_cov\_16.065512 21-24. Max. coverage (+): 0. Max coverage (-): 0

Region: NODE\_360870\_length\_2137\_cov\_16.065512 25-29. Max. coverage (+): 0. Max coverage (-): 0

Region: NODE\_360870\_length\_2137\_cov\_16.065512 30-33. Max. coverage (+): 0. Max coverage (-): 0

Region: NODE\_360870\_length\_2137\_cov\_16.065512 34-38. Max. coverage (+): 0. Max coverage (-): 0

Region: NODE\_360870\_length\_2137\_cov\_16.065512 39-42. Max. coverage (+): 0. Max coverage (-): 0

Region: NODE\_360870\_length\_2137\_cov\_16.065512 43-46. Max. coverage (+): 0. Max coverage (-): 0

Region: NODE\_360870\_length\_2137\_cov\_16.065512 47-51. Max. coverage (+): 0.38. Max coverage (-): 0

Region: NODE\_360870\_length\_2137\_cov\_16.065512 52-55. Max. coverage (+): 0. Max coverage (-): 0

Region: NODE\_360870\_length\_2137\_cov\_16.065512 56-59. Max. coverage (+): 0. Max coverage (-): 0

Region: NODE\_360870\_length\_2137\_cov\_16.065512 60-64. Max. coverage (+): 0. Max coverage (-): 0

Region: NODE\_360870\_length\_2137\_cov\_16.065512 65-68. Max. coverage (+): 0. Max coverage (-): 0

Region: NODE\_360870\_length\_2137\_cov\_16.065512 69-72. Max. coverage (+): 0. Max coverage (-): 0

Region: NODE\_360870\_length\_2137\_cov\_16.065512 73-77. Max. coverage (+): 0. Max coverage (-): 0

Region: NODE\_360870\_length\_2137\_cov\_16.065512 78-81. Max. coverage (+): 0.09. Max coverage (-): 0

Region: NODE\_360870\_length\_2137\_cov\_16.065512 82-86. Max. coverage (+): 0. Max coverage (-): 0

Region: NODE\_360870\_length\_2137\_cov\_16.065512 87-90. Max. coverage (+): 0. Max coverage (-): 0

Region: NODE\_360870\_length\_2137\_cov\_16.065512 91-94. Max. coverage (+): 0. Max coverage (-): 0

Region: NODE\_360870\_length\_2137\_cov\_16.065512 95-99. Max. coverage (+): 0. Max coverage (-): 0

Region: NODE\_360870\_length\_2137\_cov\_16.065512 100-103. Max. coverage (+): 0. Max coverage (-): 0

Region: NODE\_360870\_length\_2137\_cov\_16.065512 104-107. Max. coverage (+): 0. Max coverage (-): 0

Region: NODE\_360870\_length\_2137\_cov\_16.065512 108-112. Max. coverage (+): 0. Max coverage (-): 0

Region: NODE\_360870\_length\_2137\_cov\_16.065512 113-116. Max. coverage (+): 0. Max coverage (-): 0

Region: NODE\_360870\_length\_2137\_cov\_16.065512 117-120. Max. coverage (+): 0. Max coverage (-): 0

Region: NODE\_360870\_length\_2137\_cov\_16.065512 121-125. Max. coverage (+): 0. Max coverage (-): 0

Region: NODE\_360870\_length\_2137\_cov\_16.065512 126-129. Max. coverage (+): 0. Max coverage (-): 0

Region: NODE\_360870\_length\_2137\_cov\_16.065512 130-134. Max. coverage (+): 0. Max coverage (-): 0.05

Region: NODE\_360870\_length\_2137\_cov\_16.065512 135-138. Max. coverage (+): 0. Max coverage (-): 0

Region: NODE\_360870\_length\_2137\_cov\_16.065512 139-142. Max. coverage (+): 0. Max coverage (-): 0

Region: NODE\_360870\_length\_2137\_cov\_16.065512 143-147. Max. coverage (+): 0. Max coverage (-): 0

Region: NODE\_360870\_length\_2137\_cov\_16.065512 148-151. Max. coverage (+): 0. Max coverage (-): 0

Region: NODE\_360870\_length\_2137\_cov\_16.065512 152-155. Max. coverage (+): 0. Max coverage (-): 0

Region: NODE\_360870\_length\_2137\_cov\_16.065512 156-160. Max. coverage (+): 0. Max coverage (-): 0.05

Region: NODE\_360870\_length\_2137\_cov\_16.065512 161-164. Max. coverage (+): 0. Max coverage (-): 0.05

Region: NODE\_360870\_length\_2137\_cov\_16.065512 165-168. Max. coverage (+): 0. Max coverage (-): 1.74

Region: NODE\_360870\_length\_2137\_cov\_16.065512 169-173. Max. coverage (+): 0.09. Max coverage (-): 3.05

Region: NODE\_360870\_length\_2137\_cov\_16.065512 174-177. Max. coverage (+): 0.06. Max coverage (-): 0

Region: NODE\_360870\_length\_2137\_cov\_16.065512 178-182. Max. coverage (+): 0.78. Max coverage (-): 0

Region: NODE\_360870\_length\_2137\_cov\_16.065512 183-186. Max. coverage (+): 0.09. Max coverage (-): 0

Region: NODE\_360870\_length\_2137\_cov\_16.065512 187-190. Max. coverage (+): 0. Max coverage (-): 0

Region: NODE\_360870\_length\_2137\_cov\_16.065512 191-195. Max. coverage (+): 0.03. Max coverage (-): 0

Region: NODE\_360870\_length\_2137\_cov\_16.065512 196-199. Max. coverage (+): 0. Max coverage (-): 0

Region: NODE\_360870\_length\_2137\_cov\_16.065512 200-203. Max. coverage (+): 0. Max coverage (-): 0

Region: NODE\_360870\_length\_2137\_cov\_16.065512 204-208. Max. coverage (+): 0. Max coverage (-): 0

Region: NODE\_360870\_length\_2137\_cov\_16.065512 209-212. Max. coverage (+): 0. Max coverage (-): 0

Region: NODE\_360870\_length\_2137\_cov\_16.065512 213-216. Max. coverage (+): 0. Max coverage (-): 0

Region: NODE\_360870\_length\_2137\_cov\_16.065512 217-221. Max. coverage (+): 0. Max coverage (-): 0.05

Region: NODE\_360870\_length\_2137\_cov\_16.065512 222-225. Max. coverage (+): 0. Max coverage (-): 0.05

Region: NODE\_360870\_length\_2137\_cov\_16.065512 226-230. Max. coverage (+): 0. Max coverage (-): 0

Region: NODE\_360870\_length\_2137\_cov\_16.065512 231-234. Max. coverage (+): 0. Max coverage (-): 0

Region: NODE\_360870\_length\_2137\_cov\_16.065512 235-238. Max. coverage (+): 0.05. Max coverage (-): 0

Region: NODE\_360870\_length\_2137\_cov\_16.065512 239-243. Max. coverage (+): 0. Max coverage (-): 0.05

Region: NODE\_360870\_length\_2137\_cov\_16.065512 244-247. Max. coverage (+): 0. Max coverage (-): 0

Region: NODE\_360870\_length\_2137\_cov\_16.065512 248-251. Max. coverage (+): 0. Max coverage (-): 0

Region: NODE\_360870\_length\_2137\_cov\_16.065512 252-256. Max. coverage (+): 0. Max coverage (-): 0.14

Region: NODE\_360870\_length\_2137\_cov\_16.065512 257-260. Max. coverage (+): 0. Max coverage (-): 0.14

Region: NODE\_360870\_length\_2137\_cov\_16.065512 261-264. Max. coverage (+): 0. Max coverage (-): 0

Region: NODE\_360870\_length\_2137\_cov\_16.065512 265-269. Max. coverage (+): 0. Max coverage (-): 0

Region: NODE\_360870\_length\_2137\_cov\_16.065512 270-273. Max. coverage (+): 0.22. Max coverage (-): 0

Region: NODE\_360870\_length\_2137\_cov\_16.065512 274-278. Max. coverage (+): 1.44. Max coverage (-): 0

Region: NODE\_360870\_length\_2137\_cov\_16.065512 279-282. Max. coverage (+): 0. Max coverage (-): 0

Region: NODE\_360870\_length\_2137\_cov\_16.065512 283-286. Max. coverage (+): 0. Max coverage (-): 0

Region: NODE\_360870\_length\_2137\_cov\_16.065512 287-291. Max. coverage (+): 0. Max coverage (-): 0

Region: NODE\_360870\_length\_2137\_cov\_16.065512 292-295. Max. coverage (+): 0. Max coverage (-): 0

Region: NODE\_360870\_length\_2137\_cov\_16.065512 296-299. Max. coverage (+): 0. Max coverage (-): 0

Region: NODE\_360870\_length\_2137\_cov\_16.065512 300-304. Max. coverage (+): 0. Max coverage (-): 0.05

Region: NODE\_360870\_length\_2137\_cov\_16.065512 305-308. Max. coverage (+): 0. Max coverage (-): 5.73

Region: NODE\_360870\_length\_2137\_cov\_16.065512 309-312. Max. coverage (+): 0. Max coverage (-): 0.23

Region: NODE\_360870\_length\_2137\_cov\_16.065512 313-317. Max. coverage (+): 0. Max coverage (-): 0

Region: NODE\_360870\_length\_2137\_cov\_16.065512 318-321. Max. coverage (+): 0. Max coverage (-): 0

Region: NODE\_360870\_length\_2137\_cov\_16.065512 322-326. Max. coverage (+): 1.13. Max coverage (-): 0

Region: NODE\_360870\_length\_2137\_cov\_16.065512 327-330. Max. coverage (+): 0. Max coverage (-): 0

Region: NODE\_360870\_length\_2137\_cov\_16.065512 331-334. Max. coverage (+): 0. Max coverage (-): 0

Region: NODE\_360870\_length\_2137\_cov\_16.065512 335-339. Max. coverage (+): 0. Max coverage (-): 0

Region: NODE\_360870\_length\_2137\_cov\_16.065512 340-343. Max. coverage (+): 0. Max coverage (-): 0

Region: NODE\_360870\_length\_2137\_cov\_16.065512 344-347. Max. coverage (+): 0. Max coverage (-): 0

Region: NODE\_360870\_length\_2137\_cov\_16.065512 348-352. Max. coverage (+): 0. Max coverage (-): 0

Region: NODE\_360870\_length\_2137\_cov\_16.065512 353-356. Max. coverage (+): 0. Max coverage (-): 0

Region: NODE\_360870\_length\_2137\_cov\_16.065512 357-360. Max. coverage (+): 0. Max coverage (-): 0.03

Region: NODE\_360870\_length\_2137\_cov\_16.065512 361-365. Max. coverage (+): 0.5. Max coverage (-): 0.16

Region: NODE\_360870\_length\_2137\_cov\_16.065512 366-369. Max. coverage (+): 0.5. Max coverage (-): 0.13

Region: NODE\_360870\_length\_2137\_cov\_16.065512 370-374. Max. coverage (+): 0. Max coverage (-): 0.03

Region: NODE\_360870\_length\_2137\_cov\_16.065512 375-378. Max. coverage (+): 0.05. Max coverage (-): 0

Region: NODE\_360870\_length\_2137\_cov\_16.065512 379-382. Max. coverage (+): 0.05. Max coverage (-): 0

Region: NODE\_360870\_length\_2137\_cov\_16.065512 383-387. Max. coverage (+): 0. Max coverage (-): 0

Region: NODE\_360870\_length\_2137\_cov\_16.065512 388-391. Max. coverage (+): 0. Max coverage (-): 0

Region: NODE\_360870\_length\_2137\_cov\_16.065512 392-395. Max. coverage (+): 0. Max coverage (-): 0

Region: NODE\_360870\_length\_2137\_cov\_16.065512 396-400. Max. coverage (+): 0. Max coverage (-): 0

Region: NODE\_360870\_length\_2137\_cov\_16.065512 401-404. Max. coverage (+): 0. Max coverage (-): 0

Region: NODE\_360870\_length\_2137\_cov\_16.065512 405-408. Max. coverage (+): 0. Max coverage (-): 0

Region: NODE\_360870\_length\_2137\_cov\_16.065512 409-413. Max. coverage (+): 0.13. Max coverage (-): 0

Region: NODE\_360870\_length\_2137\_cov\_16.065512 414-417. Max. coverage (+): 0.56. Max coverage (-): 0

Region: NODE\_360870\_length\_2137\_cov\_16.065512 418-422. Max. coverage (+): 0. Max coverage (-): 0

Region: NODE\_360870\_length\_2137\_cov\_16.065512 423-426. Max. coverage (+): 0. Max coverage (-): 0

Region: NODE\_360870\_length\_2137\_cov\_16.065512 427-430. Max. coverage (+): 0. Max coverage (-): 0

Region: NODE\_360870\_length\_2137\_cov\_16.065512 431-435. Max. coverage (+): 0. Max coverage (-): 0

Region: NODE\_360870\_length\_2137\_cov\_16.065512 436-439. Max. coverage (+): 0. Max coverage (-): 0

Region: NODE\_360870\_length\_2137\_cov\_16.065512 440-443. Max. coverage (+): 0. Max coverage (-): 0.19

Region: NODE\_360870\_length\_2137\_cov\_16.065512 444-448. Max. coverage (+): 0. Max coverage (-): 0

Region: NODE\_360870\_length\_2137\_cov\_16.065512 449-452. Max. coverage (+): 0. Max coverage (-): 0

Region: NODE\_360870\_length\_2137\_cov\_16.065512 453-456. Max. coverage (+): 0. Max coverage (-): 0

Region: NODE\_360870\_length\_2137\_cov\_16.065512 457-461. Max. coverage (+): 0. Max coverage (-): 0

Region: NODE\_360870\_length\_2137\_cov\_16.065512 462-465. Max. coverage (+): 0. Max coverage (-): 0

Region: NODE\_360870\_length\_2137\_cov\_16.065512 466-470. Max. coverage (+): 0. Max coverage (-): 0

Region: NODE\_360870\_length\_2137\_cov\_16.065512 471-474. Max. coverage (+): 0. Max coverage (-): 0

Region: NODE\_360870\_length\_2137\_cov\_16.065512 475-478. Max. coverage (+): 0. Max coverage (-): 0

Region: NODE\_360870\_length\_2137\_cov\_16.065512 479-483. Max. coverage (+): 0. Max coverage (-): 0

Region: NODE\_360870\_length\_2137\_cov\_16.065512 484-487. Max. coverage (+): 0. Max coverage (-): 0

Region: NODE\_360870\_length\_2137\_cov\_16.065512 488-491. Max. coverage (+): 0.13. Max coverage (-): 0

Region: NODE\_360870\_length\_2137\_cov\_16.065512 492-496. Max. coverage (+): 0.09. Max coverage (-): 0

Region: NODE\_360870\_length\_2137\_cov\_16.065512 497-500. Max. coverage (+): 2.44. Max coverage (-): 0

Region: NODE\_360870\_length\_2137\_cov\_16.065512 501-504. Max. coverage (+): 3.04. Max coverage (-): 0

Region: NODE\_360870\_length\_2137\_cov\_16.065512 505-509. Max. coverage (+): 0.03. Max coverage (-): 0

Region: NODE\_360870\_length\_2137\_cov\_16.065512 510-513. Max. coverage (+): 0. Max coverage (-): 0

Region: NODE\_360870\_length\_2137\_cov\_16.065512 514-518. Max. coverage (+): 0. Max coverage (-): 0

Region: NODE\_360870\_length\_2137\_cov\_16.065512 519-522. Max. coverage (+): 0. Max coverage (-): 0

Region: NODE\_360870\_length\_2137\_cov\_16.065512 523-526. Max. coverage (+): 0. Max coverage (-): 0

Region: NODE\_360870\_length\_2137\_cov\_16.065512 527-531. Max. coverage (+): 0. Max coverage (-): 0

Region: NODE\_360870\_length\_2137\_cov\_16.065512 532-535. Max. coverage (+): 0. Max coverage (-): 0

Region: NODE\_360870\_length\_2137\_cov\_16.065512 536-539. Max. coverage (+): 0. Max coverage (-): 0

Region: NODE\_360870\_length\_2137\_cov\_16.065512 540-544. Max. coverage (+): 0. Max coverage (-): 0

Region: NODE\_360870\_length\_2137\_cov\_16.065512 545-548. Max. coverage (+): 0. Max coverage (-): 0.16

Region: NODE\_360870\_length\_2137\_cov\_16.065512 549-552. Max. coverage (+): 1.5. Max coverage (-): 0.28

Region: NODE\_360870\_length\_2137\_cov\_16.065512 553-557. Max. coverage (+): 1.44. Max coverage (-): 0

Region: NODE\_360870\_length\_2137\_cov\_16.065512 558-561. Max. coverage (+): 0. Max coverage (-): 0

Region: NODE\_360870\_length\_2137\_cov\_16.065512 562-566. Max. coverage (+): 0. Max coverage (-): 0

Region: NODE\_360870\_length\_2137\_cov\_16.065512 567-570. Max. coverage (+): 0. Max coverage (-): 0

Region: NODE\_360870\_length\_2137\_cov\_16.065512 571-574. Max. coverage (+): 0. Max coverage (-): 0

Region: NODE\_360870\_length\_2137\_cov\_16.065512 575-579. Max. coverage (+): 0. Max coverage (-): 0

Region: NODE\_360870\_length\_2137\_cov\_16.065512 580-583. Max. coverage (+): 0. Max coverage (-): 0

Region: NODE\_360870\_length\_2137\_cov\_16.065512 584-587. Max. coverage (+): 0. Max coverage (-): 0

Region: NODE\_360870\_length\_2137\_cov\_16.065512 588-592. Max. coverage (+): 0. Max coverage (-): 0

Region: NODE\_360870\_length\_2137\_cov\_16.065512 593-596. Max. coverage (+): 0. Max coverage (-): 0

Region: NODE\_360870\_length\_2137\_cov\_16.065512 597-600. Max. coverage (+): 0. Max coverage (-): 0

Region: NODE\_360870\_length\_2137\_cov\_16.065512 601-605. Max. coverage (+): 0. Max coverage (-): 0

Region: NODE\_360870\_length\_2137\_cov\_16.065512 606-609. Max. coverage (+): 0. Max coverage (-): 0

Region: NODE\_360870\_length\_2137\_cov\_16.065512 610-614. Max. coverage (+): 0. Max coverage (-): 0

Region: NODE\_360870\_length\_2137\_cov\_16.065512 615-618. Max. coverage (+): 0.09. Max coverage (-): 0

Region: NODE\_360870\_length\_2137\_cov\_16.065512 619-622. Max. coverage (+): 0.09. Max coverage (-): 0

Region: NODE\_360870\_length\_2137\_cov\_16.065512 623-627. Max. coverage (+): 0. Max coverage (-): 0

Region: NODE\_360870\_length\_2137\_cov\_16.065512 628-631. Max. coverage (+): 0. Max coverage (-): 0

Region: NODE\_360870\_length\_2137\_cov\_16.065512 632-635. Max. coverage (+): 0. Max coverage (-): 0

Region: NODE\_360870\_length\_2137\_cov\_16.065512 636-640. Max. coverage (+): 0. Max coverage (-): 0

Region: NODE\_360870\_length\_2137\_cov\_16.065512 641-644. Max. coverage (+): 0. Max coverage (-): 0

Region: NODE\_360870\_length\_2137\_cov\_16.065512 645-648. Max. coverage (+): 0. Max coverage (-): 0

Region: NODE\_360870\_length\_2137\_cov\_16.065512 649-653. Max. coverage (+): 0.09. Max coverage (-): 0

Region: NODE\_360870\_length\_2137\_cov\_16.065512 654-657. Max. coverage (+): 0. Max coverage (-): 0

Region: NODE\_360870\_length\_2137\_cov\_16.065512 658-662. Max. coverage (+): 0. Max coverage (-): 0

Region: NODE\_360870\_length\_2137\_cov\_16.065512 663-666. Max. coverage (+): 0. Max coverage (-): 0

Region: NODE\_360870\_length\_2137\_cov\_16.065512 667-670. Max. coverage (+): 0. Max coverage (-): 0

Region: NODE\_360870\_length\_2137\_cov\_16.065512 671-675. Max. coverage (+): 0. Max coverage (-): 0

Region: NODE\_360870\_length\_2137\_cov\_16.065512 676-679. Max. coverage (+): 0. Max coverage (-): 0

Region: NODE\_360870\_length\_2137\_cov\_16.065512 680-683. Max. coverage (+): 0. Max coverage (-): 0

Region: NODE\_360870\_length\_2137\_cov\_16.065512 684-688. Max. coverage (+): 0. Max coverage (-): 0.14

Region: NODE\_360870\_length\_2137\_cov\_16.065512 689-692. Max. coverage (+): 0. Max coverage (-): 0.05

Region: NODE\_360870\_length\_2137\_cov\_16.065512 693-696. Max. coverage (+): 0. Max coverage (-): 0

Region: NODE\_360870\_length\_2137\_cov\_16.065512 697-701. Max. coverage (+): 0. Max coverage (-): 0.14

Region: NODE\_360870\_length\_2137\_cov\_16.065512 702-705. Max. coverage (+): 0. Max coverage (-): 1.6

Region: NODE\_360870\_length\_2137\_cov\_16.065512 706-710. Max. coverage (+): 0. Max coverage (-): 1.93

Region: NODE\_360870\_length\_2137\_cov\_16.065512 711-714. Max. coverage (+): 0. Max coverage (-): 0

Region: NODE\_360870\_length\_2137\_cov\_16.065512 715-718. Max. coverage (+): 0. Max coverage (-): 0

Region: NODE\_360870\_length\_2137\_cov\_16.065512 719-723. Max. coverage (+): 515.01. Max coverage (-): 0

Region: NODE\_360870\_length\_2137\_cov\_16.065512 724-727. Max. coverage (+): 515.67. Max coverage (-): 0

Region: NODE\_360870\_length\_2137\_cov\_16.065512 728-731. Max. coverage (+): 1.25. Max coverage (-): 0

Region: NODE\_360870\_length\_2137\_cov\_16.065512 732-736. Max. coverage (+): 0. Max coverage (-): 0

Region: NODE\_360870\_length\_2137\_cov\_16.065512 737-740. Max. coverage (+): 0. Max coverage (-): 0

Region: NODE\_360870\_length\_2137\_cov\_16.065512 741-744. Max. coverage (+): 0. Max coverage (-): 0

Region: NODE\_360870\_length\_2137\_cov\_16.065512 745-749. Max. coverage (+): 0. Max coverage (-): 0

Region: NODE\_360870\_length\_2137\_cov\_16.065512 750-753. Max. coverage (+): 0. Max coverage (-): 0

Region: NODE\_360870\_length\_2137\_cov\_16.065512 754-758. Max. coverage (+): 0. Max coverage (-): 0

Region: NODE\_360870\_length\_2137\_cov\_16.065512 759-762. Max. coverage (+): 0. Max coverage (-): 0

Region: NODE\_360870\_length\_2137\_cov\_16.065512 763-766. Max. coverage (+): 0. Max coverage (-): 0

Region: NODE\_360870\_length\_2137\_cov\_16.065512 767-771. Max. coverage (+): 0. Max coverage (-): 0

Region: NODE\_360870\_length\_2137\_cov\_16.065512 772-775. Max. coverage (+): 0. Max coverage (-): 0.05

Region: NODE\_360870\_length\_2137\_cov\_16.065512 776-779. Max. coverage (+): 0. Max coverage (-): 0.05

Region: NODE\_360870\_length\_2137\_cov\_16.065512 780-784. Max. coverage (+): 0. Max coverage (-): 0

Region: NODE\_360870\_length\_2137\_cov\_16.065512 785-788. Max. coverage (+): 0. Max coverage (-): 0

Region: NODE\_360870\_length\_2137\_cov\_16.065512 789-792. Max. coverage (+): 0.28. Max coverage (-): 0

Region: NODE\_360870\_length\_2137\_cov\_16.065512 793-797. Max. coverage (+): 0.33. Max coverage (-): 0

Region: NODE\_360870\_length\_2137\_cov\_16.065512 798-801. Max. coverage (+): 0.14. Max coverage (-): 0

Region: NODE\_360870\_length\_2137\_cov\_16.065512 802-806. Max. coverage (+): 12.97. Max coverage (-): 0.03

Region: NODE\_360870\_length\_2137\_cov\_16.065512 807-810. Max. coverage (+): 0.97. Max coverage (-): 0.03

Region: NODE\_360870\_length\_2137\_cov\_16.065512 811-814. Max. coverage (+): 0.16. Max coverage (-): 0

Region: NODE\_360870\_length\_2137\_cov\_16.065512 815-819. Max. coverage (+): 0. Max coverage (-): 0

Region: NODE\_360870\_length\_2137\_cov\_16.065512 820-823. Max. coverage (+): 0. Max coverage (-): 0

Region: NODE\_360870\_length\_2137\_cov\_16.065512 824-827. Max. coverage (+): 0. Max coverage (-): 0

Region: NODE\_360870\_length\_2137\_cov\_16.065512 828-832. Max. coverage (+): 0. Max coverage (-): 0

Region: NODE\_360870\_length\_2137\_cov\_16.065512 833-836. Max. coverage (+): 0. Max coverage (-): 0

Region: NODE\_360870\_length\_2137\_cov\_16.065512 837-840. Max. coverage (+): 0. Max coverage (-): 0

Region: NODE\_360870\_length\_2137\_cov\_16.065512 841-845. Max. coverage (+): 0. Max coverage (-): 0

Region: NODE\_360870\_length\_2137\_cov\_16.065512 846-849. Max. coverage (+): 0. Max coverage (-): 0

Region: NODE\_360870\_length\_2137\_cov\_16.065512 850-854. Max. coverage (+): 0. Max coverage (-): 0.03

Region: NODE\_360870\_length\_2137\_cov\_16.065512 855-858. Max. coverage (+): 0. Max coverage (-): 0.08

Region: NODE\_360870\_length\_2137\_cov\_16.065512 859-862. Max. coverage (+): 0.03. Max coverage (-): 0.05

Region: NODE\_360870\_length\_2137\_cov\_16.065512 863-867. Max. coverage (+): 0.03. Max coverage (-): 0

Region: NODE\_360870\_length\_2137\_cov\_16.065512 868-871. Max. coverage (+): 0. Max coverage (-): 0

Region: NODE\_360870\_length\_2137\_cov\_16.065512 872-875. Max. coverage (+): 0. Max coverage (-): 0.8

Region: NODE\_360870\_length\_2137\_cov\_16.065512 876-880. Max. coverage (+): 0. Max coverage (-): 0.8

Region: NODE\_360870\_length\_2137\_cov\_16.065512 881-884. Max. coverage (+): 0. Max coverage (-): 0.7

Region: NODE\_360870\_length\_2137\_cov\_16.065512 885-888. Max. coverage (+): 0. Max coverage (-): 1.22

Region: NODE\_360870\_length\_2137\_cov\_16.065512 889-893. Max. coverage (+): 0.06. Max coverage (-): 0.97

Region: NODE\_360870\_length\_2137\_cov\_16.065512 894-897. Max. coverage (+): 0.06. Max coverage (-): 0

Region: NODE\_360870\_length\_2137\_cov\_16.065512 898-902. Max. coverage (+): 0. Max coverage (-): 0

Region: NODE\_360870\_length\_2137\_cov\_16.065512 903-906. Max. coverage (+): 0. Max coverage (-): 0

Region: NODE\_360870\_length\_2137\_cov\_16.065512 907-910. Max. coverage (+): 0. Max coverage (-): 0

Region: NODE\_360870\_length\_2137\_cov\_16.065512 911-915. Max. coverage (+): 0. Max coverage (-): 0.38

Region: NODE\_360870\_length\_2137\_cov\_16.065512 916-919. Max. coverage (+): 0. Max coverage (-): 0

Region: NODE\_360870\_length\_2137\_cov\_16.065512 920-923. Max. coverage (+): 0. Max coverage (-): 0

Region: NODE\_360870\_length\_2137\_cov\_16.065512 924-928. Max. coverage (+): 0. Max coverage (-): 0

Region: NODE\_360870\_length\_2137\_cov\_16.065512 929-932. Max. coverage (+): 0. Max coverage (-): 0

Region: NODE\_360870\_length\_2137\_cov\_16.065512 933-936. Max. coverage (+): 0. Max coverage (-): 0.05

Region: NODE\_360870\_length\_2137\_cov\_16.065512 937-941. Max. coverage (+): 0. Max coverage (-): 1.97

Region: NODE\_360870\_length\_2137\_cov\_16.065512 942-945. Max. coverage (+): 0. Max coverage (-): 1.97

Region: NODE\_360870\_length\_2137\_cov\_16.065512 946-950. Max. coverage (+): 0. Max coverage (-): 0.05

Region: NODE\_360870\_length\_2137\_cov\_16.065512 951-954. Max. coverage (+): 0. Max coverage (-): 0.05

Region: NODE\_360870\_length\_2137\_cov\_16.065512 955-958. Max. coverage (+): 0. Max coverage (-): 0

Region: NODE\_360870\_length\_2137\_cov\_16.065512 959-963. Max. coverage (+): 0.05. Max coverage (-): 0.05

Region: NODE\_360870\_length\_2137\_cov\_16.065512 964-967. Max. coverage (+): 0. Max coverage (-): 0.09

Region: NODE\_360870\_length\_2137\_cov\_16.065512 968-971. Max. coverage (+): 0.03. Max coverage (-): 0.05

Region: NODE\_360870\_length\_2137\_cov\_16.065512 972-976. Max. coverage (+): 0.19. Max coverage (-): 0

Region: NODE\_360870\_length\_2137\_cov\_16.065512 977-980. Max. coverage (+): 0.03. Max coverage (-): 0

Region: NODE\_360870\_length\_2137\_cov\_16.065512 981-984. Max. coverage (+): 0. Max coverage (-): 0

Region: NODE\_360870\_length\_2137\_cov\_16.065512 985-989. Max. coverage (+): 0. Max coverage (-): 0

Region: NODE\_360870\_length\_2137\_cov\_16.065512 990-993. Max. coverage (+): 0.05. Max coverage (-): 0

Region: NODE\_360870\_length\_2137\_cov\_16.065512 994-998. Max. coverage (+): 0. Max coverage (-): 0.05

Region: NODE\_360870\_length\_2137\_cov\_16.065512 999-1002. Max. coverage (+): 0.05. Max coverage (-): 0.61

Region: NODE\_360870\_length\_2137\_cov\_16.065512 1003-1006. Max. coverage (+): 0. Max coverage (-): 0.7

Region: NODE\_360870\_length\_2137\_cov\_16.065512 1007-1011. Max. coverage (+): 0. Max coverage (-): 0.61

Region: NODE\_360870\_length\_2137\_cov\_16.065512 1012-1015. Max. coverage (+): 0. Max coverage (-): 0

Region: NODE\_360870\_length\_2137\_cov\_16.065512 1016-1019. Max. coverage (+): 0. Max coverage (-): 0

Region: NODE\_360870\_length\_2137\_cov\_16.065512 1020-1024. Max. coverage (+): 0. Max coverage (-): 0

Region: NODE\_360870\_length\_2137\_cov\_16.065512 1025-1028. Max. coverage (+): 0. Max coverage (-): 0

Region: NODE\_360870\_length\_2137\_cov\_16.065512 1029-1032. Max. coverage (+): 0. Max coverage (-): 0

Region: NODE\_360870\_length\_2137\_cov\_16.065512 1033-1037. Max. coverage (+): 0. Max coverage (-): 0.06

Region: NODE\_360870\_length\_2137\_cov\_16.065512 1038-1041. Max. coverage (+): 0.16. Max coverage (-): 0.31

Region: NODE\_360870\_length\_2137\_cov\_16.065512 1042-1046. Max. coverage (+): 0.19. Max coverage (-): 0.02

Region: NODE\_360870\_length\_2137\_cov\_16.065512 1047-1050. Max. coverage (+): 0.09. Max coverage (-): 0.02

Region: NODE\_360870\_length\_2137\_cov\_16.065512 1051-1054. Max. coverage (+): 0.05. Max coverage (-): 0

Region: NODE\_360870\_length\_2137\_cov\_16.065512 1055-1059. Max. coverage (+): 0. Max coverage (-): 0.06

Region: NODE\_360870\_length\_2137\_cov\_16.065512 1060-1063. Max. coverage (+): 0. Max coverage (-): 0.23

Region: NODE\_360870\_length\_2137\_cov\_16.065512 1064-1067. Max. coverage (+): 0. Max coverage (-): 0.31

Region: NODE\_360870\_length\_2137\_cov\_16.065512 1068-1072. Max. coverage (+): 0. Max coverage (-): 0

Region: NODE\_360870\_length\_2137\_cov\_16.065512 1073-1076. Max. coverage (+): 0.16. Max coverage (-): 0

Region: NODE\_360870\_length\_2137\_cov\_16.065512 1077-1080. Max. coverage (+): 0.25. Max coverage (-): 0

Region: NODE\_360870\_length\_2137\_cov\_16.065512 1081-1085. Max. coverage (+): 1.13. Max coverage (-): 0

Region: NODE\_360870\_length\_2137\_cov\_16.065512 1086-1089. Max. coverage (+): 2.4. Max coverage (-): 0.07

Region: NODE\_360870\_length\_2137\_cov\_16.065512 1090-1094. Max. coverage (+): 0.6. Max coverage (-): 0.07

Region: NODE\_360870\_length\_2137\_cov\_16.065512 1095-1098. Max. coverage (+): 0.06. Max coverage (-): 0.03

Region: NODE\_360870\_length\_2137\_cov\_16.065512 1099-1102. Max. coverage (+): 0.13. Max coverage (-): 0

Region: NODE\_360870\_length\_2137\_cov\_16.065512 1103-1107. Max. coverage (+): 0. Max coverage (-): 0

Region: NODE\_360870\_length\_2137\_cov\_16.065512 1108-1111. Max. coverage (+): 0.45. Max coverage (-): 0

Region: NODE\_360870\_length\_2137\_cov\_16.065512 1112-1115. Max. coverage (+): 0.45. Max coverage (-): 0

Region: NODE\_360870\_length\_2137\_cov\_16.065512 1116-1120. Max. coverage (+): 0. Max coverage (-): 0

Region: NODE\_360870\_length\_2137\_cov\_16.065512 1121-1124. Max. coverage (+): 0. Max coverage (-): 0.19

Region: NODE\_360870\_length\_2137\_cov\_16.065512 1125-1129. Max. coverage (+): 0. Max coverage (-): 0.09

Region: NODE\_360870\_length\_2137\_cov\_16.065512 1130-1133. Max. coverage (+): 0. Max coverage (-): 9.79

Region: NODE\_360870\_length\_2137\_cov\_16.065512 1134-1137. Max. coverage (+): 0. Max coverage (-): 6.86

Region: NODE\_360870\_length\_2137\_cov\_16.065512 1138-1142. Max. coverage (+): 5.76. Max coverage (-): 0.05

Region: NODE\_360870\_length\_2137\_cov\_16.065512 1143-1146. Max. coverage (+): 16.76. Max coverage (-): 0

Region: NODE\_360870\_length\_2137\_cov\_16.065512 1147-1150. Max. coverage (+): 4.76. Max coverage (-): 0

Region: NODE\_360870\_length\_2137\_cov\_16.065512 1151-1155. Max. coverage (+): 0. Max coverage (-): 0

Region: NODE\_360870\_length\_2137\_cov\_16.065512 1156-1159. Max. coverage (+): 0. Max coverage (-): 0

Region: NODE\_360870\_length\_2137\_cov\_16.065512 1160-1163. Max. coverage (+): 0. Max coverage (-): 0

Region: NODE\_360870\_length\_2137\_cov\_16.065512 1164-1168. Max. coverage (+): 0. Max coverage (-): 0

Region: NODE\_360870\_length\_2137\_cov\_16.065512 1169-1172. Max. coverage (+): 0. Max coverage (-): 0

Region: NODE\_360870\_length\_2137\_cov\_16.065512 1173-1177. Max. coverage (+): 0. Max coverage (-): 0.7

Region: NODE\_360870\_length\_2137\_cov\_16.065512 1178-1181. Max. coverage (+): 0. Max coverage (-): 1.41

Region: NODE\_360870\_length\_2137\_cov\_16.065512 1182-1185. Max. coverage (+): 0. Max coverage (-): 0.8

Region: NODE\_360870\_length\_2137\_cov\_16.065512 1186-1190. Max. coverage (+): 0. Max coverage (-): 0

Region: NODE\_360870\_length\_2137\_cov\_16.065512 1191-1194. Max. coverage (+): 0. Max coverage (-): 0.28

Region: NODE\_360870\_length\_2137\_cov\_16.065512 1195-1198. Max. coverage (+): 0. Max coverage (-): 0.8

Region: NODE\_360870\_length\_2137\_cov\_16.065512 1199-1203. Max. coverage (+): 0. Max coverage (-): 0.85

Region: NODE\_360870\_length\_2137\_cov\_16.065512 1204-1207. Max. coverage (+): 0. Max coverage (-): 0

Region: NODE\_360870\_length\_2137\_cov\_16.065512 1208-1211. Max. coverage (+): 0. Max coverage (-): 0

Region: NODE\_360870\_length\_2137\_cov\_16.065512 1212-1216. Max. coverage (+): 0. Max coverage (-): 0

Region: NODE\_360870\_length\_2137\_cov\_16.065512 1217-1220. Max. coverage (+): 0. Max coverage (-): 0

Region: NODE\_360870\_length\_2137\_cov\_16.065512 1221-1225. Max. coverage (+): 0. Max coverage (-): 0

Region: NODE\_360870\_length\_2137\_cov\_16.065512 1226-1229. Max. coverage (+): 0. Max coverage (-): 0

Region: NODE\_360870\_length\_2137\_cov\_16.065512 1230-1233. Max. coverage (+): 0. Max coverage (-): 0

Region: NODE\_360870\_length\_2137\_cov\_16.065512 1234-1238. Max. coverage (+): 0. Max coverage (-): 0

Region: NODE\_360870\_length\_2137\_cov\_16.065512 1239-1242. Max. coverage (+): 0. Max coverage (-): 0

Region: NODE\_360870\_length\_2137\_cov\_16.065512 1243-1246. Max. coverage (+): 0. Max coverage (-): 0

Region: NODE\_360870\_length\_2137\_cov\_16.065512 1247-1251. Max. coverage (+): 0.05. Max coverage (-): 0

Region: NODE\_360870\_length\_2137\_cov\_16.065512 1252-1255. Max. coverage (+): 0.09. Max coverage (-): 0.05

Region: NODE\_360870\_length\_2137\_cov\_16.065512 1256-1259. Max. coverage (+): 0.09. Max coverage (-): 0

Region: NODE\_360870\_length\_2137\_cov\_16.065512 1260-1264. Max. coverage (+): 0. Max coverage (-): 0.05

Region: NODE\_360870\_length\_2137\_cov\_16.065512 1265-1268. Max. coverage (+): 0. Max coverage (-): 0.08

Region: NODE\_360870\_length\_2137\_cov\_16.065512 1269-1273. Max. coverage (+): 0. Max coverage (-): 0

Region: NODE\_360870\_length\_2137\_cov\_16.065512 1274-1277. Max. coverage (+): 0. Max coverage (-): 0

Region: NODE\_360870\_length\_2137\_cov\_16.065512 1278-1281. Max. coverage (+): 0. Max coverage (-): 0

Region: NODE\_360870\_length\_2137\_cov\_16.065512 1282-1286. Max. coverage (+): 0. Max coverage (-): 0

Region: NODE\_360870\_length\_2137\_cov\_16.065512 1287-1290. Max. coverage (+): 0. Max coverage (-): 0

Region: NODE\_360870\_length\_2137\_cov\_16.065512 1291-1294. Max. coverage (+): 0. Max coverage (-): 0

Region: NODE\_360870\_length\_2137\_cov\_16.065512 1295-1299. Max. coverage (+): 0.25. Max coverage (-): 0.05

Region: NODE\_360870\_length\_2137\_cov\_16.065512 1300-1303. Max. coverage (+): 0.28. Max coverage (-): 0

Region: NODE\_360870\_length\_2137\_cov\_16.065512 1304-1307. Max. coverage (+): 0.06. Max coverage (-): 0

Region: NODE\_360870\_length\_2137\_cov\_16.065512 1308-1312. Max. coverage (+): 0. Max coverage (-): 0

Region: NODE\_360870\_length\_2137\_cov\_16.065512 1313-1316. Max. coverage (+): 0. Max coverage (-): 0.09

Region: NODE\_360870\_length\_2137\_cov\_16.065512 1317-1321. Max. coverage (+): 0.09. Max coverage (-): 0.19

Region: NODE\_360870\_length\_2137\_cov\_16.065512 1322-1325. Max. coverage (+): 0.09. Max coverage (-): 0.05

Region: NODE\_360870\_length\_2137\_cov\_16.065512 1326-1329. Max. coverage (+): 0. Max coverage (-): 0

Region: NODE\_360870\_length\_2137\_cov\_16.065512 1330-1334. Max. coverage (+): 0. Max coverage (-): 0.05

Region: NODE\_360870\_length\_2137\_cov\_16.065512 1335-1338. Max. coverage (+): 0.02. Max coverage (-): 0.14

Region: NODE\_360870\_length\_2137\_cov\_16.065512 1339-1342. Max. coverage (+): 0.16. Max coverage (-): 0.15

Region: NODE\_360870\_length\_2137\_cov\_16.065512 1343-1347. Max. coverage (+): 0. Max coverage (-): 0.16

Region: NODE\_360870\_length\_2137\_cov\_16.065512 1348-1351. Max. coverage (+): 0.05. Max coverage (-): 0.02

Region: NODE\_360870\_length\_2137\_cov\_16.065512 1352-1355. Max. coverage (+): 8.25. Max coverage (-): 0.02

Region: NODE\_360870\_length\_2137\_cov\_16.065512 1356-1360. Max. coverage (+): 8.6. Max coverage (-): 0

Region: NODE\_360870\_length\_2137\_cov\_16.065512 1361-1364. Max. coverage (+): 0.73. Max coverage (-): 0

Region: NODE\_360870\_length\_2137\_cov\_16.065512 1365-1369. Max. coverage (+): 0. Max coverage (-): 0

Region: NODE\_360870\_length\_2137\_cov\_16.065512 1370-1373. Max. coverage (+): 0. Max coverage (-): 0

Region: NODE\_360870\_length\_2137\_cov\_16.065512 1374-1377. Max. coverage (+): 0. Max coverage (-): 0

Region: NODE\_360870\_length\_2137\_cov\_16.065512 1378-1382. Max. coverage (+): 0. Max coverage (-): 0

Region: NODE\_360870\_length\_2137\_cov\_16.065512 1383-1386. Max. coverage (+): 0. Max coverage (-): 0.14

Region: NODE\_360870\_length\_2137\_cov\_16.065512 1387-1390. Max. coverage (+): 0. Max coverage (-): 0.09

Region: NODE\_360870\_length\_2137\_cov\_16.065512 1391-1395. Max. coverage (+): 0. Max coverage (-): 0.09

Region: NODE\_360870\_length\_2137\_cov\_16.065512 1396-1399. Max. coverage (+): 0. Max coverage (-): 0

Region: NODE\_360870\_length\_2137\_cov\_16.065512 1400-1403. Max. coverage (+): 0.09. Max coverage (-): 0

Region: NODE\_360870\_length\_2137\_cov\_16.065512 1404-1408. Max. coverage (+): 0.09. Max coverage (-): 0

Region: NODE\_360870\_length\_2137\_cov\_16.065512 1409-1412. Max. coverage (+): 0. Max coverage (-): 0

Region: NODE\_360870\_length\_2137\_cov\_16.065512 1413-1417. Max. coverage (+): 0.09. Max coverage (-): 0

Region: NODE\_360870\_length\_2137\_cov\_16.065512 1418-1421. Max. coverage (+): 0.09. Max coverage (-): 0

Region: NODE\_360870\_length\_2137\_cov\_16.065512 1422-1425. Max. coverage (+): 0. Max coverage (-): 0

Region: NODE\_360870\_length\_2137\_cov\_16.065512 1426-1430. Max. coverage (+): 0. Max coverage (-): 0

Region: NODE\_360870\_length\_2137\_cov\_16.065512 1431-1434. Max. coverage (+): 0.47. Max coverage (-): 0

Region: NODE\_360870\_length\_2137\_cov\_16.065512 1435-1438. Max. coverage (+): 0.47. Max coverage (-): 0

Region: NODE\_360870\_length\_2137\_cov\_16.065512 1439-1443. Max. coverage (+): 0. Max coverage (-): 0

Region: NODE\_360870\_length\_2137\_cov\_16.065512 1444-1447. Max. coverage (+): 0. Max coverage (-): 0

Region: NODE\_360870\_length\_2137\_cov\_16.065512 1448-1451. Max. coverage (+): 0. Max coverage (-): 0

Region: NODE\_360870\_length\_2137\_cov\_16.065512 1452-1456. Max. coverage (+): 0. Max coverage (-): 0

Region: NODE\_360870\_length\_2137\_cov\_16.065512 1457-1460. Max. coverage (+): 0.09. Max coverage (-): 0

Region: NODE\_360870\_length\_2137\_cov\_16.065512 1461-1465. Max. coverage (+): 0. Max coverage (-): 0

Region: NODE\_360870\_length\_2137\_cov\_16.065512 1466-1469. Max. coverage (+): 0. Max coverage (-): 0.05

Region: NODE\_360870\_length\_2137\_cov\_16.065512 1470-1473. Max. coverage (+): 0. Max coverage (-): 0.05

Region: NODE\_360870\_length\_2137\_cov\_16.065512 1474-1478. Max. coverage (+): 0. Max coverage (-): 0.67

Region: NODE\_360870\_length\_2137\_cov\_16.065512 1479-1482. Max. coverage (+): 0. Max coverage (-): 15.17

Region: NODE\_360870\_length\_2137\_cov\_16.065512 1483-1486. Max. coverage (+): 0. Max coverage (-): 10.83

Region: NODE\_360870\_length\_2137\_cov\_16.065512 1487-1491. Max. coverage (+): 0. Max coverage (-): 2.04

Region: NODE\_360870\_length\_2137\_cov\_16.065512 1492-1495. Max. coverage (+): 0.02. Max coverage (-): 0.09

Region: NODE\_360870\_length\_2137\_cov\_16.065512 1496-1499. Max. coverage (+): 0.94. Max coverage (-): 0.02

Region: NODE\_360870\_length\_2137\_cov\_16.065512 1500-1504. Max. coverage (+): 0.94. Max coverage (-): 0

Region: NODE\_360870\_length\_2137\_cov\_16.065512 1505-1508. Max. coverage (+): 0. Max coverage (-): 0

Region: NODE\_360870\_length\_2137\_cov\_16.065512 1509-1513. Max. coverage (+): 0. Max coverage (-): 0.05

Region: NODE\_360870\_length\_2137\_cov\_16.065512 1514-1517. Max. coverage (+): 0. Max coverage (-): 0

Region: NODE\_360870\_length\_2137\_cov\_16.065512 1518-1521. Max. coverage (+): 0. Max coverage (-): 0

Region: NODE\_360870\_length\_2137\_cov\_16.065512 1522-1526. Max. coverage (+): 0. Max coverage (-): 0

Region: NODE\_360870\_length\_2137\_cov\_16.065512 1527-1530. Max. coverage (+): 0.09. Max coverage (-): 0

Region: NODE\_360870\_length\_2137\_cov\_16.065512 1531-1534. Max. coverage (+): 0.09. Max coverage (-): 0

Region: NODE\_360870\_length\_2137\_cov\_16.065512 1535-1539. Max. coverage (+): 0. Max coverage (-): 0

Region: NODE\_360870\_length\_2137\_cov\_16.065512 1540-1543. Max. coverage (+): 0. Max coverage (-): 0

Region: NODE\_360870\_length\_2137\_cov\_16.065512 1544-1547. Max. coverage (+): 0. Max coverage (-): 0

Region: NODE\_360870\_length\_2137\_cov\_16.065512 1548-1552. Max. coverage (+): 0. Max coverage (-): 0

Region: NODE\_360870\_length\_2137\_cov\_16.065512 1553-1556. Max. coverage (+): 0. Max coverage (-): 0

Region: NODE\_360870\_length\_2137\_cov\_16.065512 1557-1561. Max. coverage (+): 0. Max coverage (-): 0.31

Region: NODE\_360870\_length\_2137\_cov\_16.065512 1562-1565. Max. coverage (+): 0. Max coverage (-): 12.12

Region: NODE\_360870\_length\_2137\_cov\_16.065512 1566-1569. Max. coverage (+): 0. Max coverage (-): 12.37

Region: NODE\_360870\_length\_2137\_cov\_16.065512 1570-1574. Max. coverage (+): 0.02. Max coverage (-): 0.16

Region: NODE\_360870\_length\_2137\_cov\_16.065512 1575-1578. Max. coverage (+): 0. Max coverage (-): 0

Region: NODE\_360870\_length\_2137\_cov\_16.065512 1579-1582. Max. coverage (+): 0.38. Max coverage (-): 0

Region: NODE\_360870\_length\_2137\_cov\_16.065512 1583-1587. Max. coverage (+): 0.38. Max coverage (-): 0

Region: NODE\_360870\_length\_2137\_cov\_16.065512 1588-1591. Max. coverage (+): 0. Max coverage (-): 0

Region: NODE\_360870\_length\_2137\_cov\_16.065512 1592-1595. Max. coverage (+): 0. Max coverage (-): 0

Region: NODE\_360870\_length\_2137\_cov\_16.065512 1596-1600. Max. coverage (+): 0. Max coverage (-): 0

Region: NODE\_360870\_length\_2137\_cov\_16.065512 1601-1604. Max. coverage (+): 0. Max coverage (-): 0

Region: NODE\_360870\_length\_2137\_cov\_16.065512 1605-1609. Max. coverage (+): 0.01. Max coverage (-): 0

Region: NODE\_360870\_length\_2137\_cov\_16.065512 1610-1613. Max. coverage (+): 0.06. Max coverage (-): 0

Region: NODE\_360870\_length\_2137\_cov\_16.065512 1614-1617. Max. coverage (+): 0. Max coverage (-): 0

Region: NODE\_360870\_length\_2137\_cov\_16.065512 1618-1622. Max. coverage (+): 0. Max coverage (-): 0

Region: NODE\_360870\_length\_2137\_cov\_16.065512 1623-1626. Max. coverage (+): 0. Max coverage (-): 0

Region: NODE\_360870\_length\_2137\_cov\_16.065512 1627-1630. Max. coverage (+): 0. Max coverage (-): 0.02

Region: NODE\_360870\_length\_2137\_cov\_16.065512 1631-1635. Max. coverage (+): 0. Max coverage (-): 0.02

Region: NODE\_360870\_length\_2137\_cov\_16.065512 1636-1639. Max. coverage (+): 0. Max coverage (-): 0

Region: NODE\_360870\_length\_2137\_cov\_16.065512 1640-1643. Max. coverage (+): 0. Max coverage (-): 0

Region: NODE\_360870\_length\_2137\_cov\_16.065512 1644-1648. Max. coverage (+): 0.03. Max coverage (-): 0

Region: NODE\_360870\_length\_2137\_cov\_16.065512 1649-1652. Max. coverage (+): 0.03. Max coverage (-): 0

Region: NODE\_360870\_length\_2137\_cov\_16.065512 1653-1657. Max. coverage (+): 0. Max coverage (-): 0

Region: NODE\_360870\_length\_2137\_cov\_16.065512 1658-1661. Max. coverage (+): 0. Max coverage (-): 0

Region: NODE\_360870\_length\_2137\_cov\_16.065512 1662-1665. Max. coverage (+): 0. Max coverage (-): 0

Region: NODE\_360870\_length\_2137\_cov\_16.065512 1666-1670. Max. coverage (+): 0. Max coverage (-): 0

Region: NODE\_360870\_length\_2137\_cov\_16.065512 1671-1674. Max. coverage (+): 0. Max coverage (-): 0.01

Region: NODE\_360870\_length\_2137\_cov\_16.065512 1675-1678. Max. coverage (+): 0. Max coverage (-): 0.02

Region: NODE\_360870\_length\_2137\_cov\_16.065512 1679-1683. Max. coverage (+): 0. Max coverage (-): 0

Region: NODE\_360870\_length\_2137\_cov\_16.065512 1684-1687. Max. coverage (+): 0. Max coverage (-): 0

Region: NODE\_360870\_length\_2137\_cov\_16.065512 1688-1691. Max. coverage (+): 0.02. Max coverage (-): 0

Region: NODE\_360870\_length\_2137\_cov\_16.065512 1692-1696. Max. coverage (+): 0.02. Max coverage (-): 0

Region: NODE\_360870\_length\_2137\_cov\_16.065512 1697-1700. Max. coverage (+): 0. Max coverage (-): 0

Region: NODE\_360870\_length\_2137\_cov\_16.065512 1701-1705. Max. coverage (+): 0. Max coverage (-): 0

Region: NODE\_360870\_length\_2137\_cov\_16.065512 1706-1709. Max. coverage (+): 0.01. Max coverage (-): 0

Region: NODE\_360870\_length\_2137\_cov\_16.065512 1710-1713. Max. coverage (+): 0.01. Max coverage (-): 0.01

Region: NODE\_360870\_length\_2137\_cov\_16.065512 1714-1718. Max. coverage (+): 0. Max coverage (-): 0

Region: NODE\_360870\_length\_2137\_cov\_16.065512 1719-1722. Max. coverage (+): 0.02. Max coverage (-): 0

Region: NODE\_360870\_length\_2137\_cov\_16.065512 1723-1726. Max. coverage (+): 0.03. Max coverage (-): 0

Region: NODE\_360870\_length\_2137\_cov\_16.065512 1727-1731. Max. coverage (+): 0.02. Max coverage (-): 0

Region: NODE\_360870\_length\_2137\_cov\_16.065512 1732-1735. Max. coverage (+): 0. Max coverage (-): 0.06

Region: NODE\_360870\_length\_2137\_cov\_16.065512 1736-1739. Max. coverage (+): 0. Max coverage (-): 0.03

Region: NODE\_360870\_length\_2137\_cov\_16.065512 1740-1744. Max. coverage (+): 0. Max coverage (-): 0

Region: NODE\_360870\_length\_2137\_cov\_16.065512 1745-1748. Max. coverage (+): 0. Max coverage (-): 0

Region: NODE\_360870\_length\_2137\_cov\_16.065512 1749-1753. Max. coverage (+): 0. Max coverage (-): 0

Region: NODE\_360870\_length\_2137\_cov\_16.065512 1754-1757. Max. coverage (+): 0. Max coverage (-): 0.02

Region: NODE\_360870\_length\_2137\_cov\_16.065512 1758-1761. Max. coverage (+): 0. Max coverage (-): 0.07

Region: NODE\_360870\_length\_2137\_cov\_16.065512 1762-1766. Max. coverage (+): 0.02. Max coverage (-): 0.05

Region: NODE\_360870\_length\_2137\_cov\_16.065512 1767-1770. Max. coverage (+): 0. Max coverage (-): 0

Region: NODE\_360870\_length\_2137\_cov\_16.065512 1771-1774. Max. coverage (+): 0.01. Max coverage (-): 0

Region: NODE\_360870\_length\_2137\_cov\_16.065512 1775-1779. Max. coverage (+): 0. Max coverage (-): 0

Region: NODE\_360870\_length\_2137\_cov\_16.065512 1780-1783. Max. coverage (+): 0. Max coverage (-): 0

Region: NODE\_360870\_length\_2137\_cov\_16.065512 1784-1787. Max. coverage (+): 0. Max coverage (-): 0

Region: NODE\_360870\_length\_2137\_cov\_16.065512 1788-1792. Max. coverage (+): 0. Max coverage (-): 0

Region: NODE\_360870\_length\_2137\_cov\_16.065512 1793-1796. Max. coverage (+): 0. Max coverage (-): 0

Region: NODE\_360870\_length\_2137\_cov\_16.065512 1797-1801. Max. coverage (+): 0. Max coverage (-): 0.01

Region: NODE\_360870\_length\_2137\_cov\_16.065512 1802-1805. Max. coverage (+): 0.01. Max coverage (-): 0

Region: NODE\_360870\_length\_2137\_cov\_16.065512 1806-1809. Max. coverage (+): 0.02. Max coverage (-): 0

Region: NODE\_360870\_length\_2137\_cov\_16.065512 1810-1814. Max. coverage (+): 0.01. Max coverage (-): 0

Region: NODE\_360870\_length\_2137\_cov\_16.065512 1815-1818. Max. coverage (+): 0.01. Max coverage (-): 0

Region: NODE\_360870\_length\_2137\_cov\_16.065512 1819-1822. Max. coverage (+): 0.01. Max coverage (-): 0

Region: NODE\_360870\_length\_2137\_cov\_16.065512 1823-1827. Max. coverage (+): 0. Max coverage (-): 0

Region: NODE\_360870\_length\_2137\_cov\_16.065512 1828-1831. Max. coverage (+): 0.01. Max coverage (-): 0

Region: NODE\_360870\_length\_2137\_cov\_16.065512 1832-1835. Max. coverage (+): 0.01. Max coverage (-): 0

Region: NODE\_360870\_length\_2137\_cov\_16.065512 1836-1840. Max. coverage (+): 0. Max coverage (-): 0

Region: NODE\_360870\_length\_2137\_cov\_16.065512 1841-1844. Max. coverage (+): 0. Max coverage (-): 0

Region: NODE\_360870\_length\_2137\_cov\_16.065512 1845-1849. Max. coverage (+): 0. Max coverage (-): 0.01

Region: NODE\_360870\_length\_2137\_cov\_16.065512 1850-1853. Max. coverage (+): 0. Max coverage (-): 0.01

Region: NODE\_360870\_length\_2137\_cov\_16.065512 1854-1857. Max. coverage (+): 0. Max coverage (-): 0

Region: NODE\_360870\_length\_2137\_cov\_16.065512 1858-1862. Max. coverage (+): 0.01. Max coverage (-): 0.01

Region: NODE\_360870\_length\_2137\_cov\_16.065512 1863-1866. Max. coverage (+): 0.05. Max coverage (-): 0

Region: NODE\_360870\_length\_2137\_cov\_16.065512 1867-1870. Max. coverage (+): 0.11. Max coverage (-): 0

Region: NODE\_360870\_length\_2137\_cov\_16.065512 1871-1875. Max. coverage (+): 0.06. Max coverage (-): 0

Region: NODE\_360870\_length\_2137\_cov\_16.065512 1876-1879. Max. coverage (+): 0. Max coverage (-): 0

Region: NODE\_360870\_length\_2137\_cov\_16.065512 1880-1883. Max. coverage (+): 0. Max coverage (-): 0

Region: NODE\_360870\_length\_2137\_cov\_16.065512 1884-1888. Max. coverage (+): 0. Max coverage (-): 0.01

Region: NODE\_360870\_length\_2137\_cov\_16.065512 1889-1892. Max. coverage (+): 0. Max coverage (-): 0.01

Region: NODE\_360870\_length\_2137\_cov\_16.065512 1893-1897. Max. coverage (+): 0. Max coverage (-): 0

Region: NODE\_360870\_length\_2137\_cov\_16.065512 1898-1901. Max. coverage (+): 0. Max coverage (-): 0

Region: NODE\_360870\_length\_2137\_cov\_16.065512 1902-1905. Max. coverage (+): 0. Max coverage (-): 0

Region: NODE\_360870\_length\_2137\_cov\_16.065512 1906-1910. Max. coverage (+): 0. Max coverage (-): 0

Region: NODE\_360870\_length\_2137\_cov\_16.065512 1911-1914. Max. coverage (+): 0. Max coverage (-): 0

Region: NODE\_360870\_length\_2137\_cov\_16.065512 1915-1918. Max. coverage (+): 0. Max coverage (-): 0

Region: NODE\_360870\_length\_2137\_cov\_16.065512 1919-1923. Max. coverage (+): 0. Max coverage (-): 0

Region: NODE\_360870\_length\_2137\_cov\_16.065512 1924-1927. Max. coverage (+): 0. Max coverage (-): 0

Region: NODE\_360870\_length\_2137\_cov\_16.065512 1928-1931. Max. coverage (+): 0.02. Max coverage (-): 0

Region: NODE\_360870\_length\_2137\_cov\_16.065512 1932-1936. Max. coverage (+): 0.09. Max coverage (-): 0

Region: NODE\_360870\_length\_2137\_cov\_16.065512 1937-1940. Max. coverage (+): 0.08. Max coverage (-): 0

Region: NODE\_360870\_length\_2137\_cov\_16.065512 1941-1945. Max. coverage (+): 0.01. Max coverage (-): 0

Region: NODE\_360870\_length\_2137\_cov\_16.065512 1946-1949. Max. coverage (+): 0.01. Max coverage (-): 0

Region: NODE\_360870\_length\_2137\_cov\_16.065512 1950-1953. Max. coverage (+): 0. Max coverage (-): 0

Region: NODE\_360870\_length\_2137\_cov\_16.065512 1954-1958. Max. coverage (+): 0. Max coverage (-): 0

Region: NODE\_360870\_length\_2137\_cov\_16.065512 1959-1962. Max. coverage (+): 0. Max coverage (-): 0

Region: NODE\_360870\_length\_2137\_cov\_16.065512 1963-1966. Max. coverage (+): 0. Max coverage (-): 0

Region: NODE\_360870\_length\_2137\_cov\_16.065512 1967-1971. Max. coverage (+): 0. Max coverage (-): 0

Region: NODE\_360870\_length\_2137\_cov\_16.065512 1972-1975. Max. coverage (+): 0. Max coverage (-): 0

Region: NODE\_360870\_length\_2137\_cov\_16.065512 1976-1979. Max. coverage (+): 0. Max coverage (-): 0.02

Region: NODE\_360870\_length\_2137\_cov\_16.065512 1980-1984. Max. coverage (+): 0. Max coverage (-): 0.01

Region: NODE\_360870\_length\_2137\_cov\_16.065512 1985-1988. Max. coverage (+): 0. Max coverage (-): 0

Region: NODE\_360870\_length\_2137\_cov\_16.065512 1989-1993. Max. coverage (+): 0. Max coverage (-): 0

Region: NODE\_360870\_length\_2137\_cov\_16.065512 1994-1997. Max. coverage (+): 0.01. Max coverage (-): 0

Region: NODE\_360870\_length\_2137\_cov\_16.065512 1998-2001. Max. coverage (+): 0. Max coverage (-): 0

Region: NODE\_360870\_length\_2137\_cov\_16.065512 2002-2006. Max. coverage (+): 0. Max coverage (-): 0

Region: NODE\_360870\_length\_2137\_cov\_16.065512 2007-2010. Max. coverage (+): 0. Max coverage (-): 0

Region: NODE\_360870\_length\_2137\_cov\_16.065512 2011-2014. Max. coverage (+): 0. Max coverage (-): 0

Region: NODE\_360870\_length\_2137\_cov\_16.065512 2015-2019. Max. coverage (+): 0. Max coverage (-): 0

Region: NODE\_360870\_length\_2137\_cov\_16.065512 2020-2023. Max. coverage (+): 0. Max coverage (-): 0

Region: NODE\_360870\_length\_2137\_cov\_16.065512 2024-2027. Max. coverage (+): 0.01. Max coverage (-): 0

Region: NODE\_360870\_length\_2137\_cov\_16.065512 2028-2032. Max. coverage (+): 0. Max coverage (-): 0

Region: NODE\_360870\_length\_2137\_cov\_16.065512 2033-2036. Max. coverage (+): 0. Max coverage (-): 0

Region: NODE\_360870\_length\_2137\_cov\_16.065512 2037-2041. Max. coverage (+): 0. Max coverage (-): 0

Region: NODE\_360870\_length\_2137\_cov\_16.065512 2042-2045. Max. coverage (+): 0. Max coverage (-): 0

Region: NODE\_360870\_length\_2137\_cov\_16.065512 2046-2049. Max. coverage (+): 0. Max coverage (-): 0

Region: NODE\_360870\_length\_2137\_cov\_16.065512 2050-2054. Max. coverage (+): 0. Max coverage (-): 0

Region: NODE\_360870\_length\_2137\_cov\_16.065512 2055-2058. Max. coverage (+): 0. Max coverage (-): 0

Region: NODE\_360870\_length\_2137\_cov\_16.065512 2059-2062. Max. coverage (+): 0. Max coverage (-): 0

Region: NODE\_360870\_length\_2137\_cov\_16.065512 2063-2067. Max. coverage (+): 0. Max coverage (-): 0

Region: NODE\_360870\_length\_2137\_cov\_16.065512 2068-2071. Max. coverage (+): 0. Max coverage (-): 0

Region: NODE\_360870\_length\_2137\_cov\_16.065512 2072-2075. Max. coverage (+): 0. Max coverage (-): 0

Region: NODE\_360870\_length\_2137\_cov\_16.065512 2076-2080. Max. coverage (+): 0. Max coverage (-): 0

Region: NODE\_360870\_length\_2137\_cov\_16.065512 2081-2084. Max. coverage (+): 0. Max coverage (-): 0

Region: NODE\_360870\_length\_2137\_cov\_16.065512 2085-2089. Max. coverage (+): 0. Max coverage (-): 0

Region: NODE\_360870\_length\_2137\_cov\_16.065512 2090-2093. Max. coverage (+): 0. Max coverage (-): 0

Region: NODE\_360870\_length\_2137\_cov\_16.065512 2094-2097. Max. coverage (+): 0. Max coverage (-): 0

Region: NODE\_360870\_length\_2137\_cov\_16.065512 2098-2102. Max. coverage (+): 0. Max coverage (-): 0

Region: NODE\_360870\_length\_2137\_cov\_16.065512 2103-2106. Max. coverage (+): 0. Max coverage (-): 0

Region: NODE\_360870\_length\_2137\_cov\_16.065512 2107-2110. Max. coverage (+): 0. Max coverage (-): 0

Region: NODE\_360870\_length\_2137\_cov\_16.065512 2111-2115. Max. coverage (+): 0. Max coverage (-): 0

Region: NODE\_360870\_length\_2137\_cov\_16.065512 2116-2119. Max. coverage (+): 0. Max coverage (-): 0

Region: NODE\_360870\_length\_2137\_cov\_16.065512 2120-2123. Max. coverage (+): 0. Max coverage (-): 0

Region: NODE\_360870\_length\_2137\_cov\_16.065512 2124-2128. Max. coverage (+): 0. Max coverage (-): 0

Region: NODE\_360870\_length\_2137\_cov\_16.065512 2129-2132. Max. coverage (+): 0. Max coverage (-): 0

Region: NODE\_360870\_length\_2137\_cov\_16.065512 2133-2137. Max. coverage (+): 0. Max coverage (-): 0

Region: NODE\_360870\_length\_2137\_cov\_16.065512 2138-2141. Max. coverage (+): 0.08. Max coverage (-): 0

Region: NODE\_360870\_length\_2137\_cov\_16.065512 2142-2145. Max. coverage (+): 0.38. Max coverage (-): 0

Region: NODE\_360870\_length\_2137\_cov\_16.065512 2146-2150. Max. coverage (+): 0.06. Max coverage (-): 0

Region: NODE\_360870\_length\_2137\_cov\_16.065512 2151-2154. Max. coverage (+): 2.21. Max coverage (-): 0

Region: NODE\_360870\_length\_2137\_cov\_16.065512 2155-2158. Max. coverage (+): 2.21. Max coverage (-): 0

Region: NODE\_360870\_length\_2137\_cov\_16.065512 2159-2163. Max. coverage (+): 0.23. Max coverage (-): 0.05

Region: NODE\_360870\_length\_2137\_cov\_16.065512 2164-2167. Max. coverage (+): 0.47. Max coverage (-): 0

Region: NODE\_360870\_length\_2137\_cov\_16.065512 2168-2171. Max. coverage (+): 0.16. Max coverage (-): 0

Region: NODE\_360870\_length\_2137\_cov\_16.065512 2172-2176. Max. coverage (+): 0. Max coverage (-): 0

Region: NODE\_360870\_length\_2137\_cov\_16.065512 2177-2180. Max. coverage (+): 0. Max coverage (-): 0

Region: NODE\_360870\_length\_2137\_cov\_16.065512 2181-2185. Max. coverage (+): 0. Max coverage (-): 0

Region: NODE\_360870\_length\_2137\_cov\_16.065512 2186-2189. Max. coverage (+): 0. Max coverage (-): 0

Region: NODE\_360870\_length\_2137\_cov\_16.065512 2190-2193. Max. coverage (+): 0. Max coverage (-): 0

Region: NODE\_360870\_length\_2137\_cov\_16.065512 2194-. Max. coverage (+): 0. Max coverage (-): 0

RepeatMasker Color Code

**+**

100-98% Identity

<98-95% Identity

<95-90% Identity

<90-85% Identity

<85-80% Identity

<80-75% Identity

<75-70% Identity

<70% Identity

**-**

Gene Set Color Code

**+**

Gene

Pseudogene

Other

**-**

Topology/Coverage Color Code

Coverage Plus Strand

Coverage Minus Strand

Mainstrand: Plus

Mainstrand: Minus

Complementary Strand

Flanking Region  
(if option -flank >0)

Gene Set Annotation  
  
RepeatMasker Annotation  

**1. Helitron-2\_DR**: 94-1419 (-), Divergence to consensus: 31.9%  
**2. AlRepB-173**: 1608-2012 (+), Divergence to consensus: 27.7%  
**3. AlRepD-2459**: 2053-2109 (+), Divergence to consensus: 23.1%  
**4. AlRepB-99**: 2064-2111 (-), Divergence to consensus: 16.6%  
**5. Helitron-2\_DR**: 2152-2198 (-), Divergence to consensus: 19.1%

  
Transcription Factor Binding Sites  

**SPZ1** (Sequence: CTCTTACCCT (-): 1779)  
**SPZ1** (Sequence: CTGTAACCCT (-): 2064)  
**RHOXF1** (Sequence: AGCTCA (-): 135)  
**RHOXF1** (Sequence: GGCTCA (-): 1474)  
**RHOXF1** (Sequence: AGCTCA (-): 1893)  
**RHOXF1** (Sequence: GGATCA (-): 2125)  
**RHOXF1** (Sequence: TAATCT (+): 621)  
**FOXO1** (Sequence: GTTGTTTTT (+): 1950)  
**Sox5** (Sequence: ATTGTT (+): 342)  
**FIGLA** (Sequence: TACACGTGGT (-): 516)  
**FIGLA** (Sequence: TCCACCTGTT (-): 770)  
**FIGLA** (Sequence: TCCAGCTGTT (-): 1485)  
**SOX9** (Sequence: TCATTGTT (+): 340)  
**POU2F1** (Sequence: ATTAAAATA (-): 1158)  
**Rhox11** (Sequence: TGGTGTATT (+): 1822)  
**Sox5** (Sequence: AACAAT (-): 698)
