# Supplementary material for: First characterization of PIWI-interacting RNA clusters in a cichlid fish with a B chromosome
Source: BMC Biol. 2022 Sep 21;20:204. doi: 10.1186/s12915-022-01403-2 (PMC9490952; doi:10.1186/s12915-022-01403-2)
Supplement: Supplementary file 1 — Additional file 1. Zipped folder with fasta and interactive html piRNA cluster information for the A. latifasciata genome. The nomenclature is as follows: number-pirna-cluster_sex_B-presence (f, female; m, male; 0b, without B chromosome; 1b, with B chromosome). [file 12915_2022_1403_MOESM1_ESM.zip › 141_m0b.html]

piRNA cluster 141\_m0b 67


Predicted piRNA cluster no. 141\_m0b
  

Show proTRAC run info
Hide proTRAC run info

/\  
                \_\_\_\_\_\_\_\_\_\_\_\_\_\_\_\_\_\_\_\_\_\_\_/\\_\_\_ /  \\_\_\_\_\_\_\_  
               I                      /  \  /    \      I  
               I     pro             /    \/      \     I  
               I        TRAC        /               \   I  
               I   \_\_\_\_\_\_\_\_\_\_\_\_\_\_\_\_/\_\_\_\_\_\_\_\_\_\_\_\_\_\_\_\_\_\\_ I  
               I   \              /                     I  
               I    \            /                      I  
               I     \  /\      /       V.2.4.2         I  
               I      \/  \    /                        I  
               I\_\_\_\_\_\_\_\_\_\_\_\  /\_\_\_\_\_\_\_\_\_\_\_\_\_\_\_\_\_\_\_\_\_\_\_\_\_I  
                            \/  
  
  
================================= proTRAC ====================================  
VERSION: .......... 2.4.2  
LAST MODIFIED: .... 11. May 2018  
  
Please cite:  
Rosenkranz D, Zischler H. proTRAC - a software for probabilistic piRNA cluster  
detection, visualization and analysis. 2012. BMC Bioinformatics 13:5.  
  
  
Contact:  
David Rosenkranz  
Institute of Organismic and Molecular Evolutionary Biology  
Dept. Anthropology, small RNA group  
Johannes Gutenberg University Mainz  
email: rosenkranz@uni-mainz.de  
  
You can find the latest proTRAC version at:  
http://sourceforge.net/projects/protrac/files  
http://www.smallRNAgroup-mainz.de/software  
==============================================================================  
  
PARAMETERS:  
Map file: ...............piwi-machos-0B.fa-collapse.map  
Genome file: ............../../../0B\_ala\_genome.fa  
RepeatMasker annotation: Alatifasciata-all0B-maryan-v2.fa\_corrected.out  
GeneSet:................./guest-storage/Data/annotation/Alatifasciata\_all0B\_maryan-v2\_out2017.gff  
  
Significant (p<=0.01) hit density will be calculated based  
on observed hit distribution.  
  
Sliding window size: ........................................ 5000 bp  
Sliding window increament: .................................. 1000 bp  
Normalize each hit by number of genomic hits: ............... yes  
Normalize each hit by number of sequence reads: ............. yes  
Normalize values (-> per million mapped reads): ............. yes  
Min. fraction of hits with 1T(U) or 10A: .................... 0.75  
Alternatively: Min. fraction of hits with 1T(U) and 10A: .... 0.5  
Min. fraction of hits with typical piRNA length: ............ 0.75  
Typical piRNA length: ....................................... 24-32 nt  
Min. size of a piRNA cluster: ............................... 1000 bp.  
Min. number of hits (absolute): ............................. 0  
Min. number of hits (normalized): ........................... 0  
Min. fraction of hits on the mainstrand: .................... 0.75  
Top fraction of mapped sequences (in terms of read counts): . 1%  
Top fraction accounts for max. n% of sequence reads: ........ 90%  
Min. fraction of hits on each arm of a bidirectional cluster: 0.05  
Output html file for each cluster: .......................... yes  
Output a summary table: ..................................... yes  
Output a FASTA file for each cluster (piRNA sequences): ..... yes  
Output a FASTA file comprising cluster sequences: ........... yes  
Output a GTF file for predicted piRNA clusters: ..............yes  
Search DNA motifs in clusters: .............................. yes  
Output flanking sequences: +/- .............................. 0 bp  
Output ~.pTi file: .......................................... no  
==============================================================================  
  
  
Genome size (without gaps): ............ 758543724 bp  
Gaps (N/X/-): .......................... 417479 bp  
Mapped reads: .......................... 24765598  
Non-identical sequences: ............... 6158275  
Genomic hits: .......................... 53103584  
Significant densitiy of mapped reads: .. 763.098963422187 reads/kb

Show proTRAC cluster info
Hide proTRAC cluster info

|  |  |
| --- | --- |
| Location | NODE\_360916\_length\_1940\_cov\_14.107732 |
| Coordinates | 1-1930 |
| Size [bp] | 1930 |
| Sequence hit loci | 2362 |
| Mapped reads (normalized) | 4332.8 |
| Mapped reads (normalized) per kb | 2245 |
| Normalized reads with 1T (1U) | 75.7% |
| Normalized reads with 10A | 52.8% |
| Normalized reads with length 24-32 nt | 99.2% |
| Normalized reads on the main strand(s) | 80.8% |
| Predicted directionality | mono:plus |

100%

0%

1T (1U)  
reads

10A reads

24-32 nt  
reads

reads on mainstrand

**Either the amount of reads with 1T (1U) OR 10A has to exceed 75% (set with option: -1Tor10A)  
Alternatively the amount of reads with 1T (1U) AND 10A has to exceed 50% (set with option: -1Tand10A)  
Minimum amount of reads with preferred size is 75% (set with option: -pisize)  
Minimum amount of reads on the main strand(s) is 75% (set with option: -clstrand)**

Show read coverage
Hide read coverage

WHAT DO I SEE HERE?  
This chart shows the location of mapped sequence reads within a predicted piRNA cluster. The color refers to the number of genomic hits produced by the sequence read in question. A dark red bar indicates that this sequence read produces many other hits elsewhere in the genome. Many adjacent red or yellow bars can indicate the presence of a multi-copy element such as transposons or rRNA genes. A dark green bar indicates that this sequence read maps uniquely to this locus.

1 hit

2-5 hits

6-10 hits

11-20 hits

21-50 hits

51-100 hits

> 100 hits

NODE\_360916\_length\_1940\_cov\_14.107732

1

1930

Gene Set

RepeatMasker

Mapped  
Reads

32.3

plus strand

minus strand

32.3

Region: NODE\_360916\_length\_1940\_cov\_14.107732 2656-2. Max. coverage (+): 0.01. Max coverage (-): 0.01

Region: NODE\_360916\_length\_1940\_cov\_14.107732 3-6. Max. coverage (+): 0.01. Max coverage (-): 0

Region: NODE\_360916\_length\_1940\_cov\_14.107732 7-10. Max. coverage (+): 0.01. Max coverage (-): 0

Region: NODE\_360916\_length\_1940\_cov\_14.107732 11-14. Max. coverage (+): 0. Max coverage (-): 0.01

Region: NODE\_360916\_length\_1940\_cov\_14.107732 15-18. Max. coverage (+): 0. Max coverage (-): 0.01

Region: NODE\_360916\_length\_1940\_cov\_14.107732 19-22. Max. coverage (+): 0. Max coverage (-): 0.02

Region: NODE\_360916\_length\_1940\_cov\_14.107732 23-26. Max. coverage (+): 0. Max coverage (-): 0.02

Region: NODE\_360916\_length\_1940\_cov\_14.107732 27-29. Max. coverage (+): 0.04. Max coverage (-): 0

Region: NODE\_360916\_length\_1940\_cov\_14.107732 30-33. Max. coverage (+): 0.13. Max coverage (-): 0

Region: NODE\_360916\_length\_1940\_cov\_14.107732 34-37. Max. coverage (+): 0.01. Max coverage (-): 0

Region: NODE\_360916\_length\_1940\_cov\_14.107732 38-41. Max. coverage (+): 0. Max coverage (-): 0

Region: NODE\_360916\_length\_1940\_cov\_14.107732 42-45. Max. coverage (+): 0. Max coverage (-): 0

Region: NODE\_360916\_length\_1940\_cov\_14.107732 46-49. Max. coverage (+): 0. Max coverage (-): 0

Region: NODE\_360916\_length\_1940\_cov\_14.107732 50-53. Max. coverage (+): 0. Max coverage (-): 0

Region: NODE\_360916\_length\_1940\_cov\_14.107732 54-56. Max. coverage (+): 0. Max coverage (-): 0

Region: NODE\_360916\_length\_1940\_cov\_14.107732 57-60. Max. coverage (+): 0. Max coverage (-): 0

Region: NODE\_360916\_length\_1940\_cov\_14.107732 61-64. Max. coverage (+): 0. Max coverage (-): 0

Region: NODE\_360916\_length\_1940\_cov\_14.107732 65-68. Max. coverage (+): 0. Max coverage (-): 0

Region: NODE\_360916\_length\_1940\_cov\_14.107732 69-72. Max. coverage (+): 0. Max coverage (-): 0

Region: NODE\_360916\_length\_1940\_cov\_14.107732 73-76. Max. coverage (+): 0. Max coverage (-): 0.04

Region: NODE\_360916\_length\_1940\_cov\_14.107732 77-80. Max. coverage (+): 0. Max coverage (-): 0.04

Region: NODE\_360916\_length\_1940\_cov\_14.107732 81-83. Max. coverage (+): 0. Max coverage (-): 0

Region: NODE\_360916\_length\_1940\_cov\_14.107732 84-87. Max. coverage (+): 0. Max coverage (-): 0

Region: NODE\_360916\_length\_1940\_cov\_14.107732 88-91. Max. coverage (+): 0. Max coverage (-): 0

Region: NODE\_360916\_length\_1940\_cov\_14.107732 92-95. Max. coverage (+): 0. Max coverage (-): 0.3

Region: NODE\_360916\_length\_1940\_cov\_14.107732 96-99. Max. coverage (+): 0. Max coverage (-): 0.06

Region: NODE\_360916\_length\_1940\_cov\_14.107732 100-103. Max. coverage (+): 0.02. Max coverage (-): 0.1

Region: NODE\_360916\_length\_1940\_cov\_14.107732 104-107. Max. coverage (+): 0.02. Max coverage (-): 0.1

Region: NODE\_360916\_length\_1940\_cov\_14.107732 108-111. Max. coverage (+): 0. Max coverage (-): 0.06

Region: NODE\_360916\_length\_1940\_cov\_14.107732 112-114. Max. coverage (+): 0. Max coverage (-): 0.02

Region: NODE\_360916\_length\_1940\_cov\_14.107732 115-118. Max. coverage (+): 0. Max coverage (-): 1.07

Region: NODE\_360916\_length\_1940\_cov\_14.107732 119-122. Max. coverage (+): 0. Max coverage (-): 0.26

Region: NODE\_360916\_length\_1940\_cov\_14.107732 123-126. Max. coverage (+): 0. Max coverage (-): 0.04

Region: NODE\_360916\_length\_1940\_cov\_14.107732 127-130. Max. coverage (+): 0. Max coverage (-): 0.02

Region: NODE\_360916\_length\_1940\_cov\_14.107732 131-134. Max. coverage (+): 0.49. Max coverage (-): 0

Region: NODE\_360916\_length\_1940\_cov\_14.107732 135-138. Max. coverage (+): 0.46. Max coverage (-): 0.05

Region: NODE\_360916\_length\_1940\_cov\_14.107732 139-141. Max. coverage (+): 0.2. Max coverage (-): 0.04

Region: NODE\_360916\_length\_1940\_cov\_14.107732 142-145. Max. coverage (+): 1.79. Max coverage (-): 0.02

Region: NODE\_360916\_length\_1940\_cov\_14.107732 146-149. Max. coverage (+): 1.61. Max coverage (-): 0.01

Region: NODE\_360916\_length\_1940\_cov\_14.107732 150-153. Max. coverage (+): 0.07. Max coverage (-): 0.01

Region: NODE\_360916\_length\_1940\_cov\_14.107732 154-157. Max. coverage (+): 0.01. Max coverage (-): 0.01

Region: NODE\_360916\_length\_1940\_cov\_14.107732 158-161. Max. coverage (+): 0. Max coverage (-): 0

Region: NODE\_360916\_length\_1940\_cov\_14.107732 162-165. Max. coverage (+): 0. Max coverage (-): 0

Region: NODE\_360916\_length\_1940\_cov\_14.107732 166-168. Max. coverage (+): 0.07. Max coverage (-): 0

Region: NODE\_360916\_length\_1940\_cov\_14.107732 169-172. Max. coverage (+): 0.16. Max coverage (-): 0

Region: NODE\_360916\_length\_1940\_cov\_14.107732 173-176. Max. coverage (+): 0.02. Max coverage (-): 0

Region: NODE\_360916\_length\_1940\_cov\_14.107732 177-180. Max. coverage (+): 0.02. Max coverage (-): 0.12

Region: NODE\_360916\_length\_1940\_cov\_14.107732 181-184. Max. coverage (+): 0.01. Max coverage (-): 0.57

Region: NODE\_360916\_length\_1940\_cov\_14.107732 185-188. Max. coverage (+): 0.02. Max coverage (-): 0.07

Region: NODE\_360916\_length\_1940\_cov\_14.107732 189-192. Max. coverage (+): 0.01. Max coverage (-): 0.02

Region: NODE\_360916\_length\_1940\_cov\_14.107732 193-195. Max. coverage (+): 0. Max coverage (-): 0

Region: NODE\_360916\_length\_1940\_cov\_14.107732 196-199. Max. coverage (+): 0.02. Max coverage (-): 0

Region: NODE\_360916\_length\_1940\_cov\_14.107732 200-203. Max. coverage (+): 0.09. Max coverage (-): 0.02

Region: NODE\_360916\_length\_1940\_cov\_14.107732 204-207. Max. coverage (+): 0. Max coverage (-): 0.02

Region: NODE\_360916\_length\_1940\_cov\_14.107732 208-211. Max. coverage (+): 0. Max coverage (-): 0.01

Region: NODE\_360916\_length\_1940\_cov\_14.107732 212-215. Max. coverage (+): 0.04. Max coverage (-): 0

Region: NODE\_360916\_length\_1940\_cov\_14.107732 216-219. Max. coverage (+): 0.08. Max coverage (-): 0

Region: NODE\_360916\_length\_1940\_cov\_14.107732 220-222. Max. coverage (+): 0.08. Max coverage (-): 0

Region: NODE\_360916\_length\_1940\_cov\_14.107732 223-226. Max. coverage (+): 0.08. Max coverage (-): 0

Region: NODE\_360916\_length\_1940\_cov\_14.107732 227-230. Max. coverage (+): 0.2. Max coverage (-): 0.01

Region: NODE\_360916\_length\_1940\_cov\_14.107732 231-234. Max. coverage (+): 0.73. Max coverage (-): 0.01

Region: NODE\_360916\_length\_1940\_cov\_14.107732 235-238. Max. coverage (+): 0.34. Max coverage (-): 0.03

Region: NODE\_360916\_length\_1940\_cov\_14.107732 239-242. Max. coverage (+): 0.05. Max coverage (-): 0.02

Region: NODE\_360916\_length\_1940\_cov\_14.107732 243-246. Max. coverage (+): 0.01. Max coverage (-): 0

Region: NODE\_360916\_length\_1940\_cov\_14.107732 247-249. Max. coverage (+): 0. Max coverage (-): 0.01

Region: NODE\_360916\_length\_1940\_cov\_14.107732 250-253. Max. coverage (+): 0. Max coverage (-): 0.01

Region: NODE\_360916\_length\_1940\_cov\_14.107732 254-257. Max. coverage (+): 0.01. Max coverage (-): 0.02

Region: NODE\_360916\_length\_1940\_cov\_14.107732 258-261. Max. coverage (+): 0.03. Max coverage (-): 0.02

Region: NODE\_360916\_length\_1940\_cov\_14.107732 262-265. Max. coverage (+): 0.02. Max coverage (-): 0

Region: NODE\_360916\_length\_1940\_cov\_14.107732 266-269. Max. coverage (+): 0.01. Max coverage (-): 0.13

Region: NODE\_360916\_length\_1940\_cov\_14.107732 270-273. Max. coverage (+): 0.01. Max coverage (-): 0.81

Region: NODE\_360916\_length\_1940\_cov\_14.107732 274-276. Max. coverage (+): 0. Max coverage (-): 0.77

Region: NODE\_360916\_length\_1940\_cov\_14.107732 277-280. Max. coverage (+): 0. Max coverage (-): 0

Region: NODE\_360916\_length\_1940\_cov\_14.107732 281-284. Max. coverage (+): 0.01. Max coverage (-): 0

Region: NODE\_360916\_length\_1940\_cov\_14.107732 285-288. Max. coverage (+): 1.68. Max coverage (-): 0

Region: NODE\_360916\_length\_1940\_cov\_14.107732 289-292. Max. coverage (+): 1.7. Max coverage (-): 0

Region: NODE\_360916\_length\_1940\_cov\_14.107732 293-296. Max. coverage (+): 0.04. Max coverage (-): 0.06

Region: NODE\_360916\_length\_1940\_cov\_14.107732 297-300. Max. coverage (+): 0.02. Max coverage (-): 0.06

Region: NODE\_360916\_length\_1940\_cov\_14.107732 301-304. Max. coverage (+): 0. Max coverage (-): 0

Region: NODE\_360916\_length\_1940\_cov\_14.107732 305-307. Max. coverage (+): 0.02. Max coverage (-): 0

Region: NODE\_360916\_length\_1940\_cov\_14.107732 308-311. Max. coverage (+): 0. Max coverage (-): 0

Region: NODE\_360916\_length\_1940\_cov\_14.107732 312-315. Max. coverage (+): 0. Max coverage (-): 0.01

Region: NODE\_360916\_length\_1940\_cov\_14.107732 316-319. Max. coverage (+): 0.08. Max coverage (-): 0.03

Region: NODE\_360916\_length\_1940\_cov\_14.107732 320-323. Max. coverage (+): 0.16. Max coverage (-): 0.03

Region: NODE\_360916\_length\_1940\_cov\_14.107732 324-327. Max. coverage (+): 0.04. Max coverage (-): 0

Region: NODE\_360916\_length\_1940\_cov\_14.107732 328-331. Max. coverage (+): 0.1. Max coverage (-): 0.04

Region: NODE\_360916\_length\_1940\_cov\_14.107732 332-334. Max. coverage (+): 0.42. Max coverage (-): 0.1

Region: NODE\_360916\_length\_1940\_cov\_14.107732 335-338. Max. coverage (+): 0.24. Max coverage (-): 0

Region: NODE\_360916\_length\_1940\_cov\_14.107732 339-342. Max. coverage (+): 0.24. Max coverage (-): 0

Region: NODE\_360916\_length\_1940\_cov\_14.107732 343-346. Max. coverage (+): 0.01. Max coverage (-): 0.02

Region: NODE\_360916\_length\_1940\_cov\_14.107732 347-350. Max. coverage (+): 0.25. Max coverage (-): 0.02

Region: NODE\_360916\_length\_1940\_cov\_14.107732 351-354. Max. coverage (+): 0.25. Max coverage (-): 0

Region: NODE\_360916\_length\_1940\_cov\_14.107732 355-358. Max. coverage (+): 0.11. Max coverage (-): 0

Region: NODE\_360916\_length\_1940\_cov\_14.107732 359-361. Max. coverage (+): 0.02. Max coverage (-): 0

Region: NODE\_360916\_length\_1940\_cov\_14.107732 362-365. Max. coverage (+): 0.01. Max coverage (-): 0

Region: NODE\_360916\_length\_1940\_cov\_14.107732 366-369. Max. coverage (+): 0.01. Max coverage (-): 0

Region: NODE\_360916\_length\_1940\_cov\_14.107732 370-373. Max. coverage (+): 0.03. Max coverage (-): 0

Region: NODE\_360916\_length\_1940\_cov\_14.107732 374-377. Max. coverage (+): 0.01. Max coverage (-): 0

Region: NODE\_360916\_length\_1940\_cov\_14.107732 378-381. Max. coverage (+): 0.01. Max coverage (-): 0.04

Region: NODE\_360916\_length\_1940\_cov\_14.107732 382-385. Max. coverage (+): 0.02. Max coverage (-): 0.08

Region: NODE\_360916\_length\_1940\_cov\_14.107732 386-388. Max. coverage (+): 0. Max coverage (-): 0

Region: NODE\_360916\_length\_1940\_cov\_14.107732 389-392. Max. coverage (+): 0. Max coverage (-): 0.79

Region: NODE\_360916\_length\_1940\_cov\_14.107732 393-396. Max. coverage (+): 0. Max coverage (-): 1.45

Region: NODE\_360916\_length\_1940\_cov\_14.107732 397-400. Max. coverage (+): 0. Max coverage (-): 0.85

Region: NODE\_360916\_length\_1940\_cov\_14.107732 401-404. Max. coverage (+): 0.11. Max coverage (-): 0.85

Region: NODE\_360916\_length\_1940\_cov\_14.107732 405-408. Max. coverage (+): 0.13. Max coverage (-): 0.12

Region: NODE\_360916\_length\_1940\_cov\_14.107732 409-412. Max. coverage (+): 0.08. Max coverage (-): 0.1

Region: NODE\_360916\_length\_1940\_cov\_14.107732 413-415. Max. coverage (+): 0.18. Max coverage (-): 0.08

Region: NODE\_360916\_length\_1940\_cov\_14.107732 416-419. Max. coverage (+): 0.22. Max coverage (-): 0

Region: NODE\_360916\_length\_1940\_cov\_14.107732 420-423. Max. coverage (+): 0.03. Max coverage (-): 0.15

Region: NODE\_360916\_length\_1940\_cov\_14.107732 424-427. Max. coverage (+): 0.04. Max coverage (-): 0.09

Region: NODE\_360916\_length\_1940\_cov\_14.107732 428-431. Max. coverage (+): 0.01. Max coverage (-): 0.09

Region: NODE\_360916\_length\_1940\_cov\_14.107732 432-435. Max. coverage (+): 0.01. Max coverage (-): 0.21

Region: NODE\_360916\_length\_1940\_cov\_14.107732 436-439. Max. coverage (+): 0. Max coverage (-): 0.11

Region: NODE\_360916\_length\_1940\_cov\_14.107732 440-442. Max. coverage (+): 0. Max coverage (-): 0.02

Region: NODE\_360916\_length\_1940\_cov\_14.107732 443-446. Max. coverage (+): 0. Max coverage (-): 0

Region: NODE\_360916\_length\_1940\_cov\_14.107732 447-450. Max. coverage (+): 0.01. Max coverage (-): 0

Region: NODE\_360916\_length\_1940\_cov\_14.107732 451-454. Max. coverage (+): 0.04. Max coverage (-): 0.02

Region: NODE\_360916\_length\_1940\_cov\_14.107732 455-458. Max. coverage (+): 0. Max coverage (-): 0.31

Region: NODE\_360916\_length\_1940\_cov\_14.107732 459-462. Max. coverage (+): 0. Max coverage (-): 0.57

Region: NODE\_360916\_length\_1940\_cov\_14.107732 463-466. Max. coverage (+): 0.01. Max coverage (-): 0.59

Region: NODE\_360916\_length\_1940\_cov\_14.107732 467-469. Max. coverage (+): 0.01. Max coverage (-): 0.01

Region: NODE\_360916\_length\_1940\_cov\_14.107732 470-473. Max. coverage (+): 0.06. Max coverage (-): 0.02

Region: NODE\_360916\_length\_1940\_cov\_14.107732 474-477. Max. coverage (+): 0.05. Max coverage (-): 0

Region: NODE\_360916\_length\_1940\_cov\_14.107732 478-481. Max. coverage (+): 0.07. Max coverage (-): 0

Region: NODE\_360916\_length\_1940\_cov\_14.107732 482-485. Max. coverage (+): 0.07. Max coverage (-): 0

Region: NODE\_360916\_length\_1940\_cov\_14.107732 486-489. Max. coverage (+): 0.08. Max coverage (-): 0.04

Region: NODE\_360916\_length\_1940\_cov\_14.107732 490-493. Max. coverage (+): 0.07. Max coverage (-): 0.08

Region: NODE\_360916\_length\_1940\_cov\_14.107732 494-497. Max. coverage (+): 0.04. Max coverage (-): 0.1

Region: NODE\_360916\_length\_1940\_cov\_14.107732 498-500. Max. coverage (+): 0.04. Max coverage (-): 0.11

Region: NODE\_360916\_length\_1940\_cov\_14.107732 501-504. Max. coverage (+): 0.11. Max coverage (-): 0.11

Region: NODE\_360916\_length\_1940\_cov\_14.107732 505-508. Max. coverage (+): 0.1. Max coverage (-): 0.11

Region: NODE\_360916\_length\_1940\_cov\_14.107732 509-512. Max. coverage (+): 0.55. Max coverage (-): 0.07

Region: NODE\_360916\_length\_1940\_cov\_14.107732 513-516. Max. coverage (+): 0.97. Max coverage (-): 0

Region: NODE\_360916\_length\_1940\_cov\_14.107732 517-520. Max. coverage (+): 0.44. Max coverage (-): 0.01

Region: NODE\_360916\_length\_1940\_cov\_14.107732 521-524. Max. coverage (+): 0.52. Max coverage (-): 0

Region: NODE\_360916\_length\_1940\_cov\_14.107732 525-527. Max. coverage (+): 0.18. Max coverage (-): 0

Region: NODE\_360916\_length\_1940\_cov\_14.107732 528-531. Max. coverage (+): 0.04. Max coverage (-): 0

Region: NODE\_360916\_length\_1940\_cov\_14.107732 532-535. Max. coverage (+): 0.05. Max coverage (-): 0

Region: NODE\_360916\_length\_1940\_cov\_14.107732 536-539. Max. coverage (+): 0.01. Max coverage (-): 0.01

Region: NODE\_360916\_length\_1940\_cov\_14.107732 540-543. Max. coverage (+): 0.03. Max coverage (-): 0.01

Region: NODE\_360916\_length\_1940\_cov\_14.107732 544-547. Max. coverage (+): 0.32. Max coverage (-): 0.03

Region: NODE\_360916\_length\_1940\_cov\_14.107732 548-551. Max. coverage (+): 0.18. Max coverage (-): 0.07

Region: NODE\_360916\_length\_1940\_cov\_14.107732 552-554. Max. coverage (+): 0.05. Max coverage (-): 0.08

Region: NODE\_360916\_length\_1940\_cov\_14.107732 555-558. Max. coverage (+): 0.06. Max coverage (-): 0.02

Region: NODE\_360916\_length\_1940\_cov\_14.107732 559-562. Max. coverage (+): 0.04. Max coverage (-): 0.01

Region: NODE\_360916\_length\_1940\_cov\_14.107732 563-566. Max. coverage (+): 0.08. Max coverage (-): 0.06

Region: NODE\_360916\_length\_1940\_cov\_14.107732 567-570. Max. coverage (+): 0.09. Max coverage (-): 0.04

Region: NODE\_360916\_length\_1940\_cov\_14.107732 571-574. Max. coverage (+): 0.08. Max coverage (-): 0.04

Region: NODE\_360916\_length\_1940\_cov\_14.107732 575-578. Max. coverage (+): 0.08. Max coverage (-): 0.04

Region: NODE\_360916\_length\_1940\_cov\_14.107732 579-581. Max. coverage (+): 0.08. Max coverage (-): 0.04

Region: NODE\_360916\_length\_1940\_cov\_14.107732 582-585. Max. coverage (+): 0.12. Max coverage (-): 0.08

Region: NODE\_360916\_length\_1940\_cov\_14.107732 586-589. Max. coverage (+): 0.16. Max coverage (-): 0.08

Region: NODE\_360916\_length\_1940\_cov\_14.107732 590-593. Max. coverage (+): 0.36. Max coverage (-): 0.77

Region: NODE\_360916\_length\_1940\_cov\_14.107732 594-597. Max. coverage (+): 0.23. Max coverage (-): 0.13

Region: NODE\_360916\_length\_1940\_cov\_14.107732 598-601. Max. coverage (+): 0.01. Max coverage (-): 0

Region: NODE\_360916\_length\_1940\_cov\_14.107732 602-605. Max. coverage (+): 0.08. Max coverage (-): 0.24

Region: NODE\_360916\_length\_1940\_cov\_14.107732 606-608. Max. coverage (+): 10.54. Max coverage (-): 0.65

Region: NODE\_360916\_length\_1940\_cov\_14.107732 609-612. Max. coverage (+): 32.3. Max coverage (-): 0.2

Region: NODE\_360916\_length\_1940\_cov\_14.107732 613-616. Max. coverage (+): 3.51. Max coverage (-): 0

Region: NODE\_360916\_length\_1940\_cov\_14.107732 617-620. Max. coverage (+): 0.36. Max coverage (-): 2.5

Region: NODE\_360916\_length\_1940\_cov\_14.107732 621-624. Max. coverage (+): 0.32. Max coverage (-): 1.82

Region: NODE\_360916\_length\_1940\_cov\_14.107732 625-628. Max. coverage (+): 0.08. Max coverage (-): 0.04

Region: NODE\_360916\_length\_1940\_cov\_14.107732 629-632. Max. coverage (+): 0.08. Max coverage (-): 0.21

Region: NODE\_360916\_length\_1940\_cov\_14.107732 633-635. Max. coverage (+): 0.09. Max coverage (-): 0.03

Region: NODE\_360916\_length\_1940\_cov\_14.107732 636-639. Max. coverage (+): 0.57. Max coverage (-): 0.01

Region: NODE\_360916\_length\_1940\_cov\_14.107732 640-643. Max. coverage (+): 0.15. Max coverage (-): 0.01

Region: NODE\_360916\_length\_1940\_cov\_14.107732 644-647. Max. coverage (+): 0.48. Max coverage (-): 0

Region: NODE\_360916\_length\_1940\_cov\_14.107732 648-651. Max. coverage (+): 0.17. Max coverage (-): 0

Region: NODE\_360916\_length\_1940\_cov\_14.107732 652-655. Max. coverage (+): 0. Max coverage (-): 0.02

Region: NODE\_360916\_length\_1940\_cov\_14.107732 656-659. Max. coverage (+): 0. Max coverage (-): 0.03

Region: NODE\_360916\_length\_1940\_cov\_14.107732 660-662. Max. coverage (+): 0.01. Max coverage (-): 0.12

Region: NODE\_360916\_length\_1940\_cov\_14.107732 663-666. Max. coverage (+): 0.01. Max coverage (-): 0.09

Region: NODE\_360916\_length\_1940\_cov\_14.107732 667-670. Max. coverage (+): 0.32. Max coverage (-): 0.01

Region: NODE\_360916\_length\_1940\_cov\_14.107732 671-674. Max. coverage (+): 0.31. Max coverage (-): 0.02

Region: NODE\_360916\_length\_1940\_cov\_14.107732 675-678. Max. coverage (+): 0.19. Max coverage (-): 0.03

Region: NODE\_360916\_length\_1940\_cov\_14.107732 679-682. Max. coverage (+): 0.01. Max coverage (-): 0.1

Region: NODE\_360916\_length\_1940\_cov\_14.107732 683-686. Max. coverage (+): 0.63. Max coverage (-): 0.15

Region: NODE\_360916\_length\_1940\_cov\_14.107732 687-690. Max. coverage (+): 0.74. Max coverage (-): 0.08

Region: NODE\_360916\_length\_1940\_cov\_14.107732 691-693. Max. coverage (+): 0.3. Max coverage (-): 0.01

Region: NODE\_360916\_length\_1940\_cov\_14.107732 694-697. Max. coverage (+): 0.3. Max coverage (-): 0

Region: NODE\_360916\_length\_1940\_cov\_14.107732 698-701. Max. coverage (+): 1.42. Max coverage (-): 0

Region: NODE\_360916\_length\_1940\_cov\_14.107732 702-705. Max. coverage (+): 1.03. Max coverage (-): 0.02

Region: NODE\_360916\_length\_1940\_cov\_14.107732 706-709. Max. coverage (+): 0.59. Max coverage (-): 0.02

Region: NODE\_360916\_length\_1940\_cov\_14.107732 710-713. Max. coverage (+): 0.03. Max coverage (-): 0.02

Region: NODE\_360916\_length\_1940\_cov\_14.107732 714-717. Max. coverage (+): 0.01. Max coverage (-): 0.02

Region: NODE\_360916\_length\_1940\_cov\_14.107732 718-720. Max. coverage (+): 0.01. Max coverage (-): 0

Region: NODE\_360916\_length\_1940\_cov\_14.107732 721-724. Max. coverage (+): 0.01. Max coverage (-): 0.02

Region: NODE\_360916\_length\_1940\_cov\_14.107732 725-728. Max. coverage (+): 0. Max coverage (-): 0.01

Region: NODE\_360916\_length\_1940\_cov\_14.107732 729-732. Max. coverage (+): 0.02. Max coverage (-): 0.09

Region: NODE\_360916\_length\_1940\_cov\_14.107732 733-736. Max. coverage (+): 0.01. Max coverage (-): 0.02

Region: NODE\_360916\_length\_1940\_cov\_14.107732 737-740. Max. coverage (+): 0.03. Max coverage (-): 0

Region: NODE\_360916\_length\_1940\_cov\_14.107732 741-744. Max. coverage (+): 0.02. Max coverage (-): 0

Region: NODE\_360916\_length\_1940\_cov\_14.107732 745-747. Max. coverage (+): 0.05. Max coverage (-): 0.02

Region: NODE\_360916\_length\_1940\_cov\_14.107732 748-751. Max. coverage (+): 0.04. Max coverage (-): 0.01

Region: NODE\_360916\_length\_1940\_cov\_14.107732 752-755. Max. coverage (+): 0.03. Max coverage (-): 0.03

Region: NODE\_360916\_length\_1940\_cov\_14.107732 756-759. Max. coverage (+): 0.07. Max coverage (-): 0.03

Region: NODE\_360916\_length\_1940\_cov\_14.107732 760-763. Max. coverage (+): 19.42. Max coverage (-): 0.02

Region: NODE\_360916\_length\_1940\_cov\_14.107732 764-767. Max. coverage (+): 18.62. Max coverage (-): 0.11

Region: NODE\_360916\_length\_1940\_cov\_14.107732 768-771. Max. coverage (+): 0.33. Max coverage (-): 0.01

Region: NODE\_360916\_length\_1940\_cov\_14.107732 772-774. Max. coverage (+): 0.24. Max coverage (-): 0

Region: NODE\_360916\_length\_1940\_cov\_14.107732 775-778. Max. coverage (+): 0.06. Max coverage (-): 0.03

Region: NODE\_360916\_length\_1940\_cov\_14.107732 779-782. Max. coverage (+): 0. Max coverage (-): 0.04

Region: NODE\_360916\_length\_1940\_cov\_14.107732 783-786. Max. coverage (+): 0.05. Max coverage (-): 0.05

Region: NODE\_360916\_length\_1940\_cov\_14.107732 787-790. Max. coverage (+): 0.12. Max coverage (-): 0

Region: NODE\_360916\_length\_1940\_cov\_14.107732 791-794. Max. coverage (+): 0.31. Max coverage (-): 0

Region: NODE\_360916\_length\_1940\_cov\_14.107732 795-798. Max. coverage (+): 0.5. Max coverage (-): 0.08

Region: NODE\_360916\_length\_1940\_cov\_14.107732 799-801. Max. coverage (+): 0.07. Max coverage (-): 0.09

Region: NODE\_360916\_length\_1940\_cov\_14.107732 802-805. Max. coverage (+): 0.02. Max coverage (-): 0.01

Region: NODE\_360916\_length\_1940\_cov\_14.107732 806-809. Max. coverage (+): 0.05. Max coverage (-): 0.02

Region: NODE\_360916\_length\_1940\_cov\_14.107732 810-813. Max. coverage (+): 0.13. Max coverage (-): 0.01

Region: NODE\_360916\_length\_1940\_cov\_14.107732 814-817. Max. coverage (+): 0.93. Max coverage (-): 0.03

Region: NODE\_360916\_length\_1940\_cov\_14.107732 818-821. Max. coverage (+): 0.15. Max coverage (-): 0

Region: NODE\_360916\_length\_1940\_cov\_14.107732 822-825. Max. coverage (+): 0.15. Max coverage (-): 0.15

Region: NODE\_360916\_length\_1940\_cov\_14.107732 826-828. Max. coverage (+): 0.06. Max coverage (-): 0.22

Region: NODE\_360916\_length\_1940\_cov\_14.107732 829-832. Max. coverage (+): 0.12. Max coverage (-): 0.2

Region: NODE\_360916\_length\_1940\_cov\_14.107732 833-836. Max. coverage (+): 0.1. Max coverage (-): 0

Region: NODE\_360916\_length\_1940\_cov\_14.107732 837-840. Max. coverage (+): 0.89. Max coverage (-): 0.1

Region: NODE\_360916\_length\_1940\_cov\_14.107732 841-844. Max. coverage (+): 0.94. Max coverage (-): 0.04

Region: NODE\_360916\_length\_1940\_cov\_14.107732 845-848. Max. coverage (+): 0.39. Max coverage (-): 0.04

Region: NODE\_360916\_length\_1940\_cov\_14.107732 849-852. Max. coverage (+): 0.01. Max coverage (-): 0.07

Region: NODE\_360916\_length\_1940\_cov\_14.107732 853-855. Max. coverage (+): 0.11. Max coverage (-): 0.04

Region: NODE\_360916\_length\_1940\_cov\_14.107732 856-859. Max. coverage (+): 0.12. Max coverage (-): 0.51

Region: NODE\_360916\_length\_1940\_cov\_14.107732 860-863. Max. coverage (+): 0.22. Max coverage (-): 0.43

Region: NODE\_360916\_length\_1940\_cov\_14.107732 864-867. Max. coverage (+): 0.11. Max coverage (-): 0

Region: NODE\_360916\_length\_1940\_cov\_14.107732 868-871. Max. coverage (+): 0.08. Max coverage (-): 0.02

Region: NODE\_360916\_length\_1940\_cov\_14.107732 872-875. Max. coverage (+): 0.05. Max coverage (-): 0.51

Region: NODE\_360916\_length\_1940\_cov\_14.107732 876-879. Max. coverage (+): 0.32. Max coverage (-): 1.21

Region: NODE\_360916\_length\_1940\_cov\_14.107732 880-883. Max. coverage (+): 0.57. Max coverage (-): 1.68

Region: NODE\_360916\_length\_1940\_cov\_14.107732 884-886. Max. coverage (+): 0.34. Max coverage (-): 0.26

Region: NODE\_360916\_length\_1940\_cov\_14.107732 887-890. Max. coverage (+): 0.09. Max coverage (-): 0.01

Region: NODE\_360916\_length\_1940\_cov\_14.107732 891-894. Max. coverage (+): 0.44. Max coverage (-): 0.08

Region: NODE\_360916\_length\_1940\_cov\_14.107732 895-898. Max. coverage (+): 1.87. Max coverage (-): 0.07

Region: NODE\_360916\_length\_1940\_cov\_14.107732 899-902. Max. coverage (+): 0.42. Max coverage (-): 0.07

Region: NODE\_360916\_length\_1940\_cov\_14.107732 903-906. Max. coverage (+): 0.01. Max coverage (-): 0

Region: NODE\_360916\_length\_1940\_cov\_14.107732 907-910. Max. coverage (+): 0.02. Max coverage (-): 0

Region: NODE\_360916\_length\_1940\_cov\_14.107732 911-913. Max. coverage (+): 0.38. Max coverage (-): 0

Region: NODE\_360916\_length\_1940\_cov\_14.107732 914-917. Max. coverage (+): 0.12. Max coverage (-): 0.04

Region: NODE\_360916\_length\_1940\_cov\_14.107732 918-921. Max. coverage (+): 0. Max coverage (-): 0.04

Region: NODE\_360916\_length\_1940\_cov\_14.107732 922-925. Max. coverage (+): 0.44. Max coverage (-): 0

Region: NODE\_360916\_length\_1940\_cov\_14.107732 926-929. Max. coverage (+): 1.37. Max coverage (-): 0

Region: NODE\_360916\_length\_1940\_cov\_14.107732 930-933. Max. coverage (+): 0.69. Max coverage (-): 0

Region: NODE\_360916\_length\_1940\_cov\_14.107732 934-937. Max. coverage (+): 0.32. Max coverage (-): 0.08

Region: NODE\_360916\_length\_1940\_cov\_14.107732 938-940. Max. coverage (+): 0.08. Max coverage (-): 1.25

Region: NODE\_360916\_length\_1940\_cov\_14.107732 941-944. Max. coverage (+): 1.62. Max coverage (-): 5.42

Region: NODE\_360916\_length\_1940\_cov\_14.107732 945-948. Max. coverage (+): 2.66. Max coverage (-): 2.99

Region: NODE\_360916\_length\_1940\_cov\_14.107732 949-952. Max. coverage (+): 0.08. Max coverage (-): 0.04

Region: NODE\_360916\_length\_1940\_cov\_14.107732 953-956. Max. coverage (+): 1.08. Max coverage (-): 0

Region: NODE\_360916\_length\_1940\_cov\_14.107732 957-960. Max. coverage (+): 1.2. Max coverage (-): 0

Region: NODE\_360916\_length\_1940\_cov\_14.107732 961-964. Max. coverage (+): 1.21. Max coverage (-): 0

Region: NODE\_360916\_length\_1940\_cov\_14.107732 965-967. Max. coverage (+): 0.1. Max coverage (-): 0.01

Region: NODE\_360916\_length\_1940\_cov\_14.107732 968-971. Max. coverage (+): 0.06. Max coverage (-): 0.02

Region: NODE\_360916\_length\_1940\_cov\_14.107732 972-975. Max. coverage (+): 0.03. Max coverage (-): 0.02

Region: NODE\_360916\_length\_1940\_cov\_14.107732 976-979. Max. coverage (+): 0.01. Max coverage (-): 0.01

Region: NODE\_360916\_length\_1940\_cov\_14.107732 980-983. Max. coverage (+): 0.01. Max coverage (-): 0.01

Region: NODE\_360916\_length\_1940\_cov\_14.107732 984-987. Max. coverage (+): 0.27. Max coverage (-): 0.02

Region: NODE\_360916\_length\_1940\_cov\_14.107732 988-991. Max. coverage (+): 1.16. Max coverage (-): 0.02

Region: NODE\_360916\_length\_1940\_cov\_14.107732 992-994. Max. coverage (+): 0.36. Max coverage (-): 0

Region: NODE\_360916\_length\_1940\_cov\_14.107732 995-998. Max. coverage (+): 0.02. Max coverage (-): 0.01

Region: NODE\_360916\_length\_1940\_cov\_14.107732 999-1002. Max. coverage (+): 0. Max coverage (-): 0.01

Region: NODE\_360916\_length\_1940\_cov\_14.107732 1003-1006. Max. coverage (+): 0. Max coverage (-): 0

Region: NODE\_360916\_length\_1940\_cov\_14.107732 1007-1010. Max. coverage (+): 0. Max coverage (-): 0

Region: NODE\_360916\_length\_1940\_cov\_14.107732 1011-1014. Max. coverage (+): 0. Max coverage (-): 0.01

Region: NODE\_360916\_length\_1940\_cov\_14.107732 1015-1018. Max. coverage (+): 0. Max coverage (-): 0.01

Region: NODE\_360916\_length\_1940\_cov\_14.107732 1019-1021. Max. coverage (+): 0.21. Max coverage (-): 0

Region: NODE\_360916\_length\_1940\_cov\_14.107732 1022-1025. Max. coverage (+): 0.21. Max coverage (-): 0

Region: NODE\_360916\_length\_1940\_cov\_14.107732 1026-1029. Max. coverage (+): 0.04. Max coverage (-): 0

Region: NODE\_360916\_length\_1940\_cov\_14.107732 1030-1033. Max. coverage (+): 0.04. Max coverage (-): 0.08

Region: NODE\_360916\_length\_1940\_cov\_14.107732 1034-1037. Max. coverage (+): 0. Max coverage (-): 0.06

Region: NODE\_360916\_length\_1940\_cov\_14.107732 1038-1041. Max. coverage (+): 0. Max coverage (-): 0.02

Region: NODE\_360916\_length\_1940\_cov\_14.107732 1042-1045. Max. coverage (+): 0.02. Max coverage (-): 0

Region: NODE\_360916\_length\_1940\_cov\_14.107732 1046-1048. Max. coverage (+): 0.15. Max coverage (-): 0

Region: NODE\_360916\_length\_1940\_cov\_14.107732 1049-1052. Max. coverage (+): 0.15. Max coverage (-): 0

Region: NODE\_360916\_length\_1940\_cov\_14.107732 1053-1056. Max. coverage (+): 0. Max coverage (-): 0

Region: NODE\_360916\_length\_1940\_cov\_14.107732 1057-1060. Max. coverage (+): 0. Max coverage (-): 0.04

Region: NODE\_360916\_length\_1940\_cov\_14.107732 1061-1064. Max. coverage (+): 0. Max coverage (-): 0.2

Region: NODE\_360916\_length\_1940\_cov\_14.107732 1065-1068. Max. coverage (+): 0.03. Max coverage (-): 0.26

Region: NODE\_360916\_length\_1940\_cov\_14.107732 1069-1072. Max. coverage (+): 0.05. Max coverage (-): 0.03

Region: NODE\_360916\_length\_1940\_cov\_14.107732 1073-1076. Max. coverage (+): 0.04. Max coverage (-): 0

Region: NODE\_360916\_length\_1940\_cov\_14.107732 1077-1079. Max. coverage (+): 0.04. Max coverage (-): 0

Region: NODE\_360916\_length\_1940\_cov\_14.107732 1080-1083. Max. coverage (+): 27.5. Max coverage (-): 0

Region: NODE\_360916\_length\_1940\_cov\_14.107732 1084-1087. Max. coverage (+): 27.58. Max coverage (-): 0

Region: NODE\_360916\_length\_1940\_cov\_14.107732 1088-1091. Max. coverage (+): 0.2. Max coverage (-): 0

Region: NODE\_360916\_length\_1940\_cov\_14.107732 1092-1095. Max. coverage (+): 0.04. Max coverage (-): 0

Region: NODE\_360916\_length\_1940\_cov\_14.107732 1096-1099. Max. coverage (+): 0. Max coverage (-): 0

Region: NODE\_360916\_length\_1940\_cov\_14.107732 1100-1103. Max. coverage (+): 0.03. Max coverage (-): 0

Region: NODE\_360916\_length\_1940\_cov\_14.107732 1104-1106. Max. coverage (+): 0.03. Max coverage (-): 0

Region: NODE\_360916\_length\_1940\_cov\_14.107732 1107-1110. Max. coverage (+): 0. Max coverage (-): 0

Region: NODE\_360916\_length\_1940\_cov\_14.107732 1111-1114. Max. coverage (+): 0.2. Max coverage (-): 0.04

Region: NODE\_360916\_length\_1940\_cov\_14.107732 1115-1118. Max. coverage (+): 0.24. Max coverage (-): 0.04

Region: NODE\_360916\_length\_1940\_cov\_14.107732 1119-1122. Max. coverage (+): 0.24. Max coverage (-): 0

Region: NODE\_360916\_length\_1940\_cov\_14.107732 1123-1126. Max. coverage (+): 0.57. Max coverage (-): 0.04

Region: NODE\_360916\_length\_1940\_cov\_14.107732 1127-1130. Max. coverage (+): 0.4. Max coverage (-): 0.08

Region: NODE\_360916\_length\_1940\_cov\_14.107732 1131-1133. Max. coverage (+): 0.04. Max coverage (-): 0.12

Region: NODE\_360916\_length\_1940\_cov\_14.107732 1134-1137. Max. coverage (+): 0.12. Max coverage (-): 0.12

Region: NODE\_360916\_length\_1940\_cov\_14.107732 1138-1141. Max. coverage (+): 0.24. Max coverage (-): 0.12

Region: NODE\_360916\_length\_1940\_cov\_14.107732 1142-1145. Max. coverage (+): 6.42. Max coverage (-): 0.04

Region: NODE\_360916\_length\_1940\_cov\_14.107732 1146-1149. Max. coverage (+): 6.42. Max coverage (-): 0.28

Region: NODE\_360916\_length\_1940\_cov\_14.107732 1150-1153. Max. coverage (+): 0.08. Max coverage (-): 0.28

Region: NODE\_360916\_length\_1940\_cov\_14.107732 1154-1157. Max. coverage (+): 0.16. Max coverage (-): 0.97

Region: NODE\_360916\_length\_1940\_cov\_14.107732 1158-1160. Max. coverage (+): 0.05. Max coverage (-): 1.45

Region: NODE\_360916\_length\_1940\_cov\_14.107732 1161-1164. Max. coverage (+): 0.05. Max coverage (-): 0.86

Region: NODE\_360916\_length\_1940\_cov\_14.107732 1165-1168. Max. coverage (+): 0.01. Max coverage (-): 0.03

Region: NODE\_360916\_length\_1940\_cov\_14.107732 1169-1172. Max. coverage (+): 0.06. Max coverage (-): 0.52

Region: NODE\_360916\_length\_1940\_cov\_14.107732 1173-1176. Max. coverage (+): 0.3. Max coverage (-): 0.78

Region: NODE\_360916\_length\_1940\_cov\_14.107732 1177-1180. Max. coverage (+): 0.09. Max coverage (-): 0.06

Region: NODE\_360916\_length\_1940\_cov\_14.107732 1181-1184. Max. coverage (+): 0. Max coverage (-): 0.01

Region: NODE\_360916\_length\_1940\_cov\_14.107732 1185-1187. Max. coverage (+): 0. Max coverage (-): 0.01

Region: NODE\_360916\_length\_1940\_cov\_14.107732 1188-1191. Max. coverage (+): 0. Max coverage (-): 0

Region: NODE\_360916\_length\_1940\_cov\_14.107732 1192-1195. Max. coverage (+): 0.12. Max coverage (-): 0

Region: NODE\_360916\_length\_1940\_cov\_14.107732 1196-1199. Max. coverage (+): 0.16. Max coverage (-): 0.04

Region: NODE\_360916\_length\_1940\_cov\_14.107732 1200-1203. Max. coverage (+): 3.63. Max coverage (-): 0.04

Region: NODE\_360916\_length\_1940\_cov\_14.107732 1204-1207. Max. coverage (+): 3.19. Max coverage (-): 0

Region: NODE\_360916\_length\_1940\_cov\_14.107732 1208-1211. Max. coverage (+): 0.61. Max coverage (-): 0.04

Region: NODE\_360916\_length\_1940\_cov\_14.107732 1212-1214. Max. coverage (+): 0.28. Max coverage (-): 0

Region: NODE\_360916\_length\_1940\_cov\_14.107732 1215-1218. Max. coverage (+): 0.24. Max coverage (-): 0

Region: NODE\_360916\_length\_1940\_cov\_14.107732 1219-1222. Max. coverage (+): 0. Max coverage (-): 0

Region: NODE\_360916\_length\_1940\_cov\_14.107732 1223-1226. Max. coverage (+): 0. Max coverage (-): 0

Region: NODE\_360916\_length\_1940\_cov\_14.107732 1227-1230. Max. coverage (+): 0.01. Max coverage (-): 0

Region: NODE\_360916\_length\_1940\_cov\_14.107732 1231-1234. Max. coverage (+): 0.08. Max coverage (-): 0

Region: NODE\_360916\_length\_1940\_cov\_14.107732 1235-1238. Max. coverage (+): 0.09. Max coverage (-): 0

Region: NODE\_360916\_length\_1940\_cov\_14.107732 1239-1241. Max. coverage (+): 0.13. Max coverage (-): 0.02

Region: NODE\_360916\_length\_1940\_cov\_14.107732 1242-1245. Max. coverage (+): 0.13. Max coverage (-): 0.03

Region: NODE\_360916\_length\_1940\_cov\_14.107732 1246-1249. Max. coverage (+): 0.02. Max coverage (-): 0.01

Region: NODE\_360916\_length\_1940\_cov\_14.107732 1250-1253. Max. coverage (+): 0.03. Max coverage (-): 0.01

Region: NODE\_360916\_length\_1940\_cov\_14.107732 1254-1257. Max. coverage (+): 0.01. Max coverage (-): 0

Region: NODE\_360916\_length\_1940\_cov\_14.107732 1258-1261. Max. coverage (+): 0.08. Max coverage (-): 0

Region: NODE\_360916\_length\_1940\_cov\_14.107732 1262-1265. Max. coverage (+): 0.08. Max coverage (-): 0

Region: NODE\_360916\_length\_1940\_cov\_14.107732 1266-1269. Max. coverage (+): 0.04. Max coverage (-): 0.04

Region: NODE\_360916\_length\_1940\_cov\_14.107732 1270-1272. Max. coverage (+): 0.04. Max coverage (-): 0

Region: NODE\_360916\_length\_1940\_cov\_14.107732 1273-1276. Max. coverage (+): 0. Max coverage (-): 0

Region: NODE\_360916\_length\_1940\_cov\_14.107732 1277-1280. Max. coverage (+): 0.08. Max coverage (-): 0

Region: NODE\_360916\_length\_1940\_cov\_14.107732 1281-1284. Max. coverage (+): 0.08. Max coverage (-): 0

Region: NODE\_360916\_length\_1940\_cov\_14.107732 1285-1288. Max. coverage (+): 0.02. Max coverage (-): 0.03

Region: NODE\_360916\_length\_1940\_cov\_14.107732 1289-1292. Max. coverage (+): 0.06. Max coverage (-): 0.09

Region: NODE\_360916\_length\_1940\_cov\_14.107732 1293-1296. Max. coverage (+): 0.01. Max coverage (-): 0.03

Region: NODE\_360916\_length\_1940\_cov\_14.107732 1297-1299. Max. coverage (+): 0.02. Max coverage (-): 0.02

Region: NODE\_360916\_length\_1940\_cov\_14.107732 1300-1303. Max. coverage (+): 0.01. Max coverage (-): 0.03

Region: NODE\_360916\_length\_1940\_cov\_14.107732 1304-1307. Max. coverage (+): 0.07. Max coverage (-): 0

Region: NODE\_360916\_length\_1940\_cov\_14.107732 1308-1311. Max. coverage (+): 0.11. Max coverage (-): 0

Region: NODE\_360916\_length\_1940\_cov\_14.107732 1312-1315. Max. coverage (+): 0.24. Max coverage (-): 0

Region: NODE\_360916\_length\_1940\_cov\_14.107732 1316-1319. Max. coverage (+): 0.23. Max coverage (-): 0

Region: NODE\_360916\_length\_1940\_cov\_14.107732 1320-1323. Max. coverage (+): 0.09. Max coverage (-): 0

Region: NODE\_360916\_length\_1940\_cov\_14.107732 1324-1326. Max. coverage (+): 0.04. Max coverage (-): 0

Region: NODE\_360916\_length\_1940\_cov\_14.107732 1327-1330. Max. coverage (+): 0.04. Max coverage (-): 0

Region: NODE\_360916\_length\_1940\_cov\_14.107732 1331-1334. Max. coverage (+): 0.04. Max coverage (-): 0

Region: NODE\_360916\_length\_1940\_cov\_14.107732 1335-1338. Max. coverage (+): 0.04. Max coverage (-): 0

Region: NODE\_360916\_length\_1940\_cov\_14.107732 1339-1342. Max. coverage (+): 0. Max coverage (-): 0

Region: NODE\_360916\_length\_1940\_cov\_14.107732 1343-1346. Max. coverage (+): 0. Max coverage (-): 0

Region: NODE\_360916\_length\_1940\_cov\_14.107732 1347-1350. Max. coverage (+): 0. Max coverage (-): 0

Region: NODE\_360916\_length\_1940\_cov\_14.107732 1351-1353. Max. coverage (+): 0. Max coverage (-): 0

Region: NODE\_360916\_length\_1940\_cov\_14.107732 1354-1357. Max. coverage (+): 0. Max coverage (-): 0

Region: NODE\_360916\_length\_1940\_cov\_14.107732 1358-1361. Max. coverage (+): 0. Max coverage (-): 0

Region: NODE\_360916\_length\_1940\_cov\_14.107732 1362-1365. Max. coverage (+): 0. Max coverage (-): 0

Region: NODE\_360916\_length\_1940\_cov\_14.107732 1366-1369. Max. coverage (+): 0. Max coverage (-): 0

Region: NODE\_360916\_length\_1940\_cov\_14.107732 1370-1373. Max. coverage (+): 0.04. Max coverage (-): 0.12

Region: NODE\_360916\_length\_1940\_cov\_14.107732 1374-1377. Max. coverage (+): 0.08. Max coverage (-): 0.12

Region: NODE\_360916\_length\_1940\_cov\_14.107732 1378-1380. Max. coverage (+): 0.04. Max coverage (-): 0

Region: NODE\_360916\_length\_1940\_cov\_14.107732 1381-1384. Max. coverage (+): 0.04. Max coverage (-): 0.01

Region: NODE\_360916\_length\_1940\_cov\_14.107732 1385-1388. Max. coverage (+): 0. Max coverage (-): 0.01

Region: NODE\_360916\_length\_1940\_cov\_14.107732 1389-1392. Max. coverage (+): 0. Max coverage (-): 0

Region: NODE\_360916\_length\_1940\_cov\_14.107732 1393-1396. Max. coverage (+): 0. Max coverage (-): 0

Region: NODE\_360916\_length\_1940\_cov\_14.107732 1397-1400. Max. coverage (+): 0. Max coverage (-): 0

Region: NODE\_360916\_length\_1940\_cov\_14.107732 1401-1404. Max. coverage (+): 0. Max coverage (-): 0

Region: NODE\_360916\_length\_1940\_cov\_14.107732 1405-1407. Max. coverage (+): 0. Max coverage (-): 0

Region: NODE\_360916\_length\_1940\_cov\_14.107732 1408-1411. Max. coverage (+): 0. Max coverage (-): 0

Region: NODE\_360916\_length\_1940\_cov\_14.107732 1412-1415. Max. coverage (+): 0. Max coverage (-): 0

Region: NODE\_360916\_length\_1940\_cov\_14.107732 1416-1419. Max. coverage (+): 0. Max coverage (-): 0

Region: NODE\_360916\_length\_1940\_cov\_14.107732 1420-1423. Max. coverage (+): 0. Max coverage (-): 0

Region: NODE\_360916\_length\_1940\_cov\_14.107732 1424-1427. Max. coverage (+): 0. Max coverage (-): 0

Region: NODE\_360916\_length\_1940\_cov\_14.107732 1428-1431. Max. coverage (+): 0. Max coverage (-): 0

Region: NODE\_360916\_length\_1940\_cov\_14.107732 1432-1434. Max. coverage (+): 0. Max coverage (-): 0

Region: NODE\_360916\_length\_1940\_cov\_14.107732 1435-1438. Max. coverage (+): 0. Max coverage (-): 0

Region: NODE\_360916\_length\_1940\_cov\_14.107732 1439-1442. Max. coverage (+): 0. Max coverage (-): 0

Region: NODE\_360916\_length\_1940\_cov\_14.107732 1443-1446. Max. coverage (+): 0. Max coverage (-): 0

Region: NODE\_360916\_length\_1940\_cov\_14.107732 1447-1450. Max. coverage (+): 0.01. Max coverage (-): 0

Region: NODE\_360916\_length\_1940\_cov\_14.107732 1451-1454. Max. coverage (+): 0. Max coverage (-): 0

Region: NODE\_360916\_length\_1940\_cov\_14.107732 1455-1458. Max. coverage (+): 0. Max coverage (-): 0

Region: NODE\_360916\_length\_1940\_cov\_14.107732 1459-1462. Max. coverage (+): 0.01. Max coverage (-): 0

Region: NODE\_360916\_length\_1940\_cov\_14.107732 1463-1465. Max. coverage (+): 0.01. Max coverage (-): 0

Region: NODE\_360916\_length\_1940\_cov\_14.107732 1466-1469. Max. coverage (+): 0. Max coverage (-): 0

Region: NODE\_360916\_length\_1940\_cov\_14.107732 1470-1473. Max. coverage (+): 0. Max coverage (-): 0

Region: NODE\_360916\_length\_1940\_cov\_14.107732 1474-1477. Max. coverage (+): 0. Max coverage (-): 0

Region: NODE\_360916\_length\_1940\_cov\_14.107732 1478-1481. Max. coverage (+): 0. Max coverage (-): 0

Region: NODE\_360916\_length\_1940\_cov\_14.107732 1482-1485. Max. coverage (+): 0. Max coverage (-): 0

Region: NODE\_360916\_length\_1940\_cov\_14.107732 1486-1489. Max. coverage (+): 0. Max coverage (-): 0

Region: NODE\_360916\_length\_1940\_cov\_14.107732 1490-1492. Max. coverage (+): 0. Max coverage (-): 0

Region: NODE\_360916\_length\_1940\_cov\_14.107732 1493-1496. Max. coverage (+): 0.09. Max coverage (-): 0

Region: NODE\_360916\_length\_1940\_cov\_14.107732 1497-1500. Max. coverage (+): 0.09. Max coverage (-): 0

Region: NODE\_360916\_length\_1940\_cov\_14.107732 1501-1504. Max. coverage (+): 0.02. Max coverage (-): 0

Region: NODE\_360916\_length\_1940\_cov\_14.107732 1505-1508. Max. coverage (+): 0.01. Max coverage (-): 0

Region: NODE\_360916\_length\_1940\_cov\_14.107732 1509-1512. Max. coverage (+): 0.01. Max coverage (-): 0

Region: NODE\_360916\_length\_1940\_cov\_14.107732 1513-1516. Max. coverage (+): 0. Max coverage (-): 0

Region: NODE\_360916\_length\_1940\_cov\_14.107732 1517-1519. Max. coverage (+): 0.01. Max coverage (-): 0

Region: NODE\_360916\_length\_1940\_cov\_14.107732 1520-1523. Max. coverage (+): 0.01. Max coverage (-): 0

Region: NODE\_360916\_length\_1940\_cov\_14.107732 1524-1527. Max. coverage (+): 0.08. Max coverage (-): 0

Region: NODE\_360916\_length\_1940\_cov\_14.107732 1528-1531. Max. coverage (+): 0.09. Max coverage (-): 0

Region: NODE\_360916\_length\_1940\_cov\_14.107732 1532-1535. Max. coverage (+): 0.13. Max coverage (-): 0.02

Region: NODE\_360916\_length\_1940\_cov\_14.107732 1536-1539. Max. coverage (+): 0.13. Max coverage (-): 0.03

Region: NODE\_360916\_length\_1940\_cov\_14.107732 1540-1543. Max. coverage (+): 0.02. Max coverage (-): 0.01

Region: NODE\_360916\_length\_1940\_cov\_14.107732 1544-1546. Max. coverage (+): 0.03. Max coverage (-): 0.01

Region: NODE\_360916\_length\_1940\_cov\_14.107732 1547-1550. Max. coverage (+): 0.01. Max coverage (-): 0

Region: NODE\_360916\_length\_1940\_cov\_14.107732 1551-1554. Max. coverage (+): 0. Max coverage (-): 0

Region: NODE\_360916\_length\_1940\_cov\_14.107732 1555-1558. Max. coverage (+): 0.04. Max coverage (-): 0

Region: NODE\_360916\_length\_1940\_cov\_14.107732 1559-1562. Max. coverage (+): 0.04. Max coverage (-): 0

Region: NODE\_360916\_length\_1940\_cov\_14.107732 1563-1566. Max. coverage (+): 0.08. Max coverage (-): 0

Region: NODE\_360916\_length\_1940\_cov\_14.107732 1567-1570. Max. coverage (+): 0.08. Max coverage (-): 0.04

Region: NODE\_360916\_length\_1940\_cov\_14.107732 1571-1573. Max. coverage (+): 0.2. Max coverage (-): 0.04

Region: NODE\_360916\_length\_1940\_cov\_14.107732 1574-1577. Max. coverage (+): 0.12. Max coverage (-): 0.04

Region: NODE\_360916\_length\_1940\_cov\_14.107732 1578-1581. Max. coverage (+): 0.12. Max coverage (-): 0.02

Region: NODE\_360916\_length\_1940\_cov\_14.107732 1582-1585. Max. coverage (+): 0.1. Max coverage (-): 0.09

Region: NODE\_360916\_length\_1940\_cov\_14.107732 1586-1589. Max. coverage (+): 0.05. Max coverage (-): 0.06

Region: NODE\_360916\_length\_1940\_cov\_14.107732 1590-1593. Max. coverage (+): 0.02. Max coverage (-): 0.02

Region: NODE\_360916\_length\_1940\_cov\_14.107732 1594-1597. Max. coverage (+): 0.01. Max coverage (-): 0.03

Region: NODE\_360916\_length\_1940\_cov\_14.107732 1598-1600. Max. coverage (+): 0.02. Max coverage (-): 0

Region: NODE\_360916\_length\_1940\_cov\_14.107732 1601-1604. Max. coverage (+): 0.11. Max coverage (-): 0

Region: NODE\_360916\_length\_1940\_cov\_14.107732 1605-1608. Max. coverage (+): 0.24. Max coverage (-): 0

Region: NODE\_360916\_length\_1940\_cov\_14.107732 1609-1612. Max. coverage (+): 0.23. Max coverage (-): 0

Region: NODE\_360916\_length\_1940\_cov\_14.107732 1613-1616. Max. coverage (+): 0.11. Max coverage (-): 0

Region: NODE\_360916\_length\_1940\_cov\_14.107732 1617-1620. Max. coverage (+): 0. Max coverage (-): 0

Region: NODE\_360916\_length\_1940\_cov\_14.107732 1621-1624. Max. coverage (+): 0. Max coverage (-): 0

Region: NODE\_360916\_length\_1940\_cov\_14.107732 1625-1627. Max. coverage (+): 0. Max coverage (-): 0

Region: NODE\_360916\_length\_1940\_cov\_14.107732 1628-1631. Max. coverage (+): 0. Max coverage (-): 0

Region: NODE\_360916\_length\_1940\_cov\_14.107732 1632-1635. Max. coverage (+): 0. Max coverage (-): 0

Region: NODE\_360916\_length\_1940\_cov\_14.107732 1636-1639. Max. coverage (+): 0. Max coverage (-): 0

Region: NODE\_360916\_length\_1940\_cov\_14.107732 1640-1643. Max. coverage (+): 0. Max coverage (-): 0

Region: NODE\_360916\_length\_1940\_cov\_14.107732 1644-1647. Max. coverage (+): 0. Max coverage (-): 0

Region: NODE\_360916\_length\_1940\_cov\_14.107732 1648-1651. Max. coverage (+): 0. Max coverage (-): 0

Region: NODE\_360916\_length\_1940\_cov\_14.107732 1652-1655. Max. coverage (+): 0. Max coverage (-): 0

Region: NODE\_360916\_length\_1940\_cov\_14.107732 1656-1658. Max. coverage (+): 0. Max coverage (-): 0

Region: NODE\_360916\_length\_1940\_cov\_14.107732 1659-1662. Max. coverage (+): 0.04. Max coverage (-): 0

Region: NODE\_360916\_length\_1940\_cov\_14.107732 1663-1666. Max. coverage (+): 0.04. Max coverage (-): 0.01

Region: NODE\_360916\_length\_1940\_cov\_14.107732 1667-1670. Max. coverage (+): 0. Max coverage (-): 0.02

Region: NODE\_360916\_length\_1940\_cov\_14.107732 1671-1674. Max. coverage (+): 0. Max coverage (-): 0

Region: NODE\_360916\_length\_1940\_cov\_14.107732 1675-1678. Max. coverage (+): 0. Max coverage (-): 0

Region: NODE\_360916\_length\_1940\_cov\_14.107732 1679-1682. Max. coverage (+): 0.09. Max coverage (-): 0

Region: NODE\_360916\_length\_1940\_cov\_14.107732 1683-1685. Max. coverage (+): 0.1. Max coverage (-): 0

Region: NODE\_360916\_length\_1940\_cov\_14.107732 1686-1689. Max. coverage (+): 0.03. Max coverage (-): 0

Region: NODE\_360916\_length\_1940\_cov\_14.107732 1690-1693. Max. coverage (+): 0. Max coverage (-): 0

Region: NODE\_360916\_length\_1940\_cov\_14.107732 1694-1697. Max. coverage (+): 0. Max coverage (-): 0

Region: NODE\_360916\_length\_1940\_cov\_14.107732 1698-1701. Max. coverage (+): 0. Max coverage (-): 0.02

Region: NODE\_360916\_length\_1940\_cov\_14.107732 1702-1705. Max. coverage (+): 0. Max coverage (-): 0.02

Region: NODE\_360916\_length\_1940\_cov\_14.107732 1706-1709. Max. coverage (+): 0. Max coverage (-): 0

Region: NODE\_360916\_length\_1940\_cov\_14.107732 1710-1712. Max. coverage (+): 0. Max coverage (-): 0

Region: NODE\_360916\_length\_1940\_cov\_14.107732 1713-1716. Max. coverage (+): 0.16. Max coverage (-): 0

Region: NODE\_360916\_length\_1940\_cov\_14.107732 1717-1720. Max. coverage (+): 0. Max coverage (-): 0

Region: NODE\_360916\_length\_1940\_cov\_14.107732 1721-1724. Max. coverage (+): 0. Max coverage (-): 0

Region: NODE\_360916\_length\_1940\_cov\_14.107732 1725-1728. Max. coverage (+): 0. Max coverage (-): 0.03

Region: NODE\_360916\_length\_1940\_cov\_14.107732 1729-1732. Max. coverage (+): 0. Max coverage (-): 0.03

Region: NODE\_360916\_length\_1940\_cov\_14.107732 1733-1736. Max. coverage (+): 0. Max coverage (-): 0.02

Region: NODE\_360916\_length\_1940\_cov\_14.107732 1737-1739. Max. coverage (+): 0. Max coverage (-): 0

Region: NODE\_360916\_length\_1940\_cov\_14.107732 1740-1743. Max. coverage (+): 0.04. Max coverage (-): 0

Region: NODE\_360916\_length\_1940\_cov\_14.107732 1744-1747. Max. coverage (+): 0. Max coverage (-): 0

Region: NODE\_360916\_length\_1940\_cov\_14.107732 1748-1751. Max. coverage (+): 0. Max coverage (-): 0.01

Region: NODE\_360916\_length\_1940\_cov\_14.107732 1752-1755. Max. coverage (+): 0. Max coverage (-): 0.02

Region: NODE\_360916\_length\_1940\_cov\_14.107732 1756-1759. Max. coverage (+): 0. Max coverage (-): 0.08

Region: NODE\_360916\_length\_1940\_cov\_14.107732 1760-1763. Max. coverage (+): 0. Max coverage (-): 0.06

Region: NODE\_360916\_length\_1940\_cov\_14.107732 1764-1766. Max. coverage (+): 0. Max coverage (-): 0

Region: NODE\_360916\_length\_1940\_cov\_14.107732 1767-1770. Max. coverage (+): 0. Max coverage (-): 0

Region: NODE\_360916\_length\_1940\_cov\_14.107732 1771-1774. Max. coverage (+): 0. Max coverage (-): 0

Region: NODE\_360916\_length\_1940\_cov\_14.107732 1775-1778. Max. coverage (+): 0. Max coverage (-): 0

Region: NODE\_360916\_length\_1940\_cov\_14.107732 1779-1782. Max. coverage (+): 0. Max coverage (-): 0

Region: NODE\_360916\_length\_1940\_cov\_14.107732 1783-1786. Max. coverage (+): 0. Max coverage (-): 0

Region: NODE\_360916\_length\_1940\_cov\_14.107732 1787-1790. Max. coverage (+): 0. Max coverage (-): 0

Region: NODE\_360916\_length\_1940\_cov\_14.107732 1791-1793. Max. coverage (+): 0. Max coverage (-): 0

Region: NODE\_360916\_length\_1940\_cov\_14.107732 1794-1797. Max. coverage (+): 0. Max coverage (-): 0

Region: NODE\_360916\_length\_1940\_cov\_14.107732 1798-1801. Max. coverage (+): 0. Max coverage (-): 0

Region: NODE\_360916\_length\_1940\_cov\_14.107732 1802-1805. Max. coverage (+): 0. Max coverage (-): 0

Region: NODE\_360916\_length\_1940\_cov\_14.107732 1806-1809. Max. coverage (+): 0. Max coverage (-): 0

Region: NODE\_360916\_length\_1940\_cov\_14.107732 1810-1813. Max. coverage (+): 0. Max coverage (-): 0

Region: NODE\_360916\_length\_1940\_cov\_14.107732 1814-1817. Max. coverage (+): 0. Max coverage (-): 0

Region: NODE\_360916\_length\_1940\_cov\_14.107732 1818-1820. Max. coverage (+): 0. Max coverage (-): 0

Region: NODE\_360916\_length\_1940\_cov\_14.107732 1821-1824. Max. coverage (+): 0. Max coverage (-): 0

Region: NODE\_360916\_length\_1940\_cov\_14.107732 1825-1828. Max. coverage (+): 0. Max coverage (-): 0

Region: NODE\_360916\_length\_1940\_cov\_14.107732 1829-1832. Max. coverage (+): 0. Max coverage (-): 0

Region: NODE\_360916\_length\_1940\_cov\_14.107732 1833-1836. Max. coverage (+): 0. Max coverage (-): 0

Region: NODE\_360916\_length\_1940\_cov\_14.107732 1837-1840. Max. coverage (+): 0. Max coverage (-): 0

Region: NODE\_360916\_length\_1940\_cov\_14.107732 1841-1844. Max. coverage (+): 0. Max coverage (-): 0

Region: NODE\_360916\_length\_1940\_cov\_14.107732 1845-1848. Max. coverage (+): 0. Max coverage (-): 0

Region: NODE\_360916\_length\_1940\_cov\_14.107732 1849-1851. Max. coverage (+): 0. Max coverage (-): 0

Region: NODE\_360916\_length\_1940\_cov\_14.107732 1852-1855. Max. coverage (+): 0. Max coverage (-): 0

Region: NODE\_360916\_length\_1940\_cov\_14.107732 1856-1859. Max. coverage (+): 0. Max coverage (-): 0

Region: NODE\_360916\_length\_1940\_cov\_14.107732 1860-1863. Max. coverage (+): 0. Max coverage (-): 0

Region: NODE\_360916\_length\_1940\_cov\_14.107732 1864-1867. Max. coverage (+): 0. Max coverage (-): 0.04

Region: NODE\_360916\_length\_1940\_cov\_14.107732 1868-1871. Max. coverage (+): 0. Max coverage (-): 0.03

Region: NODE\_360916\_length\_1940\_cov\_14.107732 1872-1875. Max. coverage (+): 0. Max coverage (-): 0

Region: NODE\_360916\_length\_1940\_cov\_14.107732 1876-1878. Max. coverage (+): 0. Max coverage (-): 0

Region: NODE\_360916\_length\_1940\_cov\_14.107732 1879-1882. Max. coverage (+): 0. Max coverage (-): 0

Region: NODE\_360916\_length\_1940\_cov\_14.107732 1883-1886. Max. coverage (+): 0. Max coverage (-): 0

Region: NODE\_360916\_length\_1940\_cov\_14.107732 1887-1890. Max. coverage (+): 0. Max coverage (-): 0

Region: NODE\_360916\_length\_1940\_cov\_14.107732 1891-1894. Max. coverage (+): 0. Max coverage (-): 0

Region: NODE\_360916\_length\_1940\_cov\_14.107732 1895-1898. Max. coverage (+): 0. Max coverage (-): 0

Region: NODE\_360916\_length\_1940\_cov\_14.107732 1899-1902. Max. coverage (+): 0. Max coverage (-): 0

Region: NODE\_360916\_length\_1940\_cov\_14.107732 1903-1905. Max. coverage (+): 0. Max coverage (-): 0

Region: NODE\_360916\_length\_1940\_cov\_14.107732 1906-1909. Max. coverage (+): 0. Max coverage (-): 0

Region: NODE\_360916\_length\_1940\_cov\_14.107732 1910-1913. Max. coverage (+): 0. Max coverage (-): 0

Region: NODE\_360916\_length\_1940\_cov\_14.107732 1914-1917. Max. coverage (+): 0. Max coverage (-): 0

Region: NODE\_360916\_length\_1940\_cov\_14.107732 1918-1921. Max. coverage (+): 0. Max coverage (-): 0

Region: NODE\_360916\_length\_1940\_cov\_14.107732 1922-1925. Max. coverage (+): 0. Max coverage (-): 0

Region: NODE\_360916\_length\_1940\_cov\_14.107732 1926-1929. Max. coverage (+): 0. Max coverage (-): 0

Region: NODE\_360916\_length\_1940\_cov\_14.107732 1930-. Max. coverage (+): 0. Max coverage (-): 0

RepeatMasker Color Code

**+**

100-98% Identity

<98-95% Identity

<95-90% Identity

<90-85% Identity

<85-80% Identity

<80-75% Identity

<75-70% Identity

<70% Identity

**-**

Gene Set Color Code

**+**

Gene

Pseudogene

Other

**-**

Topology/Coverage Color Code

Coverage Plus Strand

Coverage Minus Strand

Mainstrand: Plus

Mainstrand: Minus

Complementary Strand

Flanking Region  
(if option -flank >0)

Gene Set Annotation  
  
RepeatMasker Annotation  

**1. A-rich**: 59-94 (+), Divergence to consensus: 22.5%  
**2. (AACA)n**: 209-237 (+), Divergence to consensus: 14.6%  
**3. Tx1-4\_DR**: 312-1079 (-), Divergence to consensus: 40.1%  
**4. AlRepB-727**: 1380-1486 (+), Divergence to consensus: 3.7%  
**5. AlRepB-727**: 1655-1784 (+), Divergence to consensus: 11%  
**6. AlRepB-727**: 1845-1930 (+), Divergence to consensus: 9.6%

  
Transcription Factor Binding Sites  

**RHOXF1** (Sequence: AGCTTA (-): 14)  
**RHOXF1** (Sequence: AGCTTA (-): 94)  
**RHOXF1** (Sequence: GGATCA (-): 294)  
**RHOXF1** (Sequence: AGCTTA (-): 1024)  
**RHOXF1** (Sequence: AGCTCA (-): 1304)  
**RHOXF1** (Sequence: AGCTCA (-): 1598)  
**RHOXF1** (Sequence: AGCTCA (-): 1706)  
**RHOXF1** (Sequence: TAATCT (+): 255)  
**RHOXF1** (Sequence: TGATCT (+): 632)  
**SOX9** (Sequence: AACAATGG (-): 1718)  
**FOXO1** (Sequence: CTTGTTTTC (+): 365)  
**Nobox** (Sequence: ACTAATTA (-): 48)  
**POU2F1** (Sequence: ATTTAAATA (-): 39)  
**Sox5** (Sequence: AACAAT (-): 1718)
