# Supplementary material for: First characterization of PIWI-interacting RNA clusters in a cichlid fish with a B chromosome
Source: BMC Biol. 2022 Sep 21;20:204. doi: 10.1186/s12915-022-01403-2 (PMC9490952; doi:10.1186/s12915-022-01403-2)
Supplement: Supplementary file 1 — Additional file 1. Zipped folder with fasta and interactive html piRNA cluster information for the A. latifasciata genome. The nomenclature is as follows: number-pirna-cluster_sex_B-presence (f, female; m, male; 0b, without B chromosome; 1b, with B chromosome). [file 12915_2022_1403_MOESM1_ESM.zip › 142_m0b.html]

piRNA cluster 142\_m0b 68


Predicted piRNA cluster no. 142\_m0b
  

Show proTRAC run info
Hide proTRAC run info

/\  
                \_\_\_\_\_\_\_\_\_\_\_\_\_\_\_\_\_\_\_\_\_\_\_/\\_\_\_ /  \\_\_\_\_\_\_\_  
               I                      /  \  /    \      I  
               I     pro             /    \/      \     I  
               I        TRAC        /               \   I  
               I   \_\_\_\_\_\_\_\_\_\_\_\_\_\_\_\_/\_\_\_\_\_\_\_\_\_\_\_\_\_\_\_\_\_\\_ I  
               I   \              /                     I  
               I    \            /                      I  
               I     \  /\      /       V.2.4.2         I  
               I      \/  \    /                        I  
               I\_\_\_\_\_\_\_\_\_\_\_\  /\_\_\_\_\_\_\_\_\_\_\_\_\_\_\_\_\_\_\_\_\_\_\_\_\_I  
                            \/  
  
  
================================= proTRAC ====================================  
VERSION: .......... 2.4.2  
LAST MODIFIED: .... 11. May 2018  
  
Please cite:  
Rosenkranz D, Zischler H. proTRAC - a software for probabilistic piRNA cluster  
detection, visualization and analysis. 2012. BMC Bioinformatics 13:5.  
  
  
Contact:  
David Rosenkranz  
Institute of Organismic and Molecular Evolutionary Biology  
Dept. Anthropology, small RNA group  
Johannes Gutenberg University Mainz  
email: rosenkranz@uni-mainz.de  
  
You can find the latest proTRAC version at:  
http://sourceforge.net/projects/protrac/files  
http://www.smallRNAgroup-mainz.de/software  
==============================================================================  
  
PARAMETERS:  
Map file: ...............piwi-machos-0B.fa-collapse.map  
Genome file: ............../../../0B\_ala\_genome.fa  
RepeatMasker annotation: Alatifasciata-all0B-maryan-v2.fa\_corrected.out  
GeneSet:................./guest-storage/Data/annotation/Alatifasciata\_all0B\_maryan-v2\_out2017.gff  
  
Significant (p<=0.01) hit density will be calculated based  
on observed hit distribution.  
  
Sliding window size: ........................................ 5000 bp  
Sliding window increament: .................................. 1000 bp  
Normalize each hit by number of genomic hits: ............... yes  
Normalize each hit by number of sequence reads: ............. yes  
Normalize values (-> per million mapped reads): ............. yes  
Min. fraction of hits with 1T(U) or 10A: .................... 0.75  
Alternatively: Min. fraction of hits with 1T(U) and 10A: .... 0.5  
Min. fraction of hits with typical piRNA length: ............ 0.75  
Typical piRNA length: ....................................... 24-32 nt  
Min. size of a piRNA cluster: ............................... 1000 bp.  
Min. number of hits (absolute): ............................. 0  
Min. number of hits (normalized): ........................... 0  
Min. fraction of hits on the mainstrand: .................... 0.75  
Top fraction of mapped sequences (in terms of read counts): . 1%  
Top fraction accounts for max. n% of sequence reads: ........ 90%  
Min. fraction of hits on each arm of a bidirectional cluster: 0.05  
Output html file for each cluster: .......................... yes  
Output a summary table: ..................................... yes  
Output a FASTA file for each cluster (piRNA sequences): ..... yes  
Output a FASTA file comprising cluster sequences: ........... yes  
Output a GTF file for predicted piRNA clusters: ..............yes  
Search DNA motifs in clusters: .............................. yes  
Output flanking sequences: +/- .............................. 0 bp  
Output ~.pTi file: .......................................... no  
==============================================================================  
  
  
Genome size (without gaps): ............ 758543724 bp  
Gaps (N/X/-): .......................... 417479 bp  
Mapped reads: .......................... 24765598  
Non-identical sequences: ............... 6158275  
Genomic hits: .......................... 53103584  
Significant densitiy of mapped reads: .. 763.098963422187 reads/kb

Show proTRAC cluster info
Hide proTRAC cluster info

|  |  |
| --- | --- |
| Location | NODE\_369549\_length\_1565\_cov\_30.364218 |
| Coordinates | 2-1626 |
| Size [bp] | 1625 |
| Sequence hit loci | 3819 |
| Mapped reads (normalized) | 15091.7 |
| Mapped reads (normalized) per kb | 9287.2 |
| Normalized reads with 1T (1U) | 66.2% |
| Normalized reads with 10A | 53.5% |
| Normalized reads with length 24-32 nt | 98.1% |
| Normalized reads on the main strand(s) | 80.5% |
| Predicted directionality | mono:plus |

100%

0%

1T (1U)  
reads

10A reads

24-32 nt  
reads

reads on mainstrand

**Either the amount of reads with 1T (1U) OR 10A has to exceed 75% (set with option: -1Tor10A)  
Alternatively the amount of reads with 1T (1U) AND 10A has to exceed 50% (set with option: -1Tand10A)  
Minimum amount of reads with preferred size is 75% (set with option: -pisize)  
Minimum amount of reads on the main strand(s) is 75% (set with option: -clstrand)**

Show read coverage
Hide read coverage

WHAT DO I SEE HERE?  
This chart shows the location of mapped sequence reads within a predicted piRNA cluster. The color refers to the number of genomic hits produced by the sequence read in question. A dark red bar indicates that this sequence read produces many other hits elsewhere in the genome. Many adjacent red or yellow bars can indicate the presence of a multi-copy element such as transposons or rRNA genes. A dark green bar indicates that this sequence read maps uniquely to this locus.

1 hit

2-5 hits

6-10 hits

11-20 hits

21-50 hits

51-100 hits

> 100 hits

NODE\_369549\_length\_1565\_cov\_30.364218

2

1626

Gene Set

RepeatMasker

Mapped  
Reads

34.97

plus strand

minus strand

34.97

Region: NODE\_369549\_length\_1565\_cov\_30.364218 1931-3. Max. coverage (+): 0.03. Max coverage (-): 0

Region: NODE\_369549\_length\_1565\_cov\_30.364218 4-6. Max. coverage (+): 0.09. Max coverage (-): 0.01

Region: NODE\_369549\_length\_1565\_cov\_30.364218 7-10. Max. coverage (+): 0.74. Max coverage (-): 0.11

Region: NODE\_369549\_length\_1565\_cov\_30.364218 11-13. Max. coverage (+): 0.79. Max coverage (-): 0.23

Region: NODE\_369549\_length\_1565\_cov\_30.364218 14-16. Max. coverage (+): 0.04. Max coverage (-): 0.14

Region: NODE\_369549\_length\_1565\_cov\_30.364218 17-19. Max. coverage (+): 0.79. Max coverage (-): 0.02

Region: NODE\_369549\_length\_1565\_cov\_30.364218 20-23. Max. coverage (+): 0.83. Max coverage (-): 0.08

Region: NODE\_369549\_length\_1565\_cov\_30.364218 24-26. Max. coverage (+): 0.11. Max coverage (-): 0.01

Region: NODE\_369549\_length\_1565\_cov\_30.364218 27-29. Max. coverage (+): 0.03. Max coverage (-): 0.01

Region: NODE\_369549\_length\_1565\_cov\_30.364218 30-32. Max. coverage (+): 0.29. Max coverage (-): 0

Region: NODE\_369549\_length\_1565\_cov\_30.364218 33-36. Max. coverage (+): 0.17. Max coverage (-): 0.05

Region: NODE\_369549\_length\_1565\_cov\_30.364218 37-39. Max. coverage (+): 0.17. Max coverage (-): 0.15

Region: NODE\_369549\_length\_1565\_cov\_30.364218 40-42. Max. coverage (+): 0.9. Max coverage (-): 0.04

Region: NODE\_369549\_length\_1565\_cov\_30.364218 43-45. Max. coverage (+): 0.08. Max coverage (-): 0.08

Region: NODE\_369549\_length\_1565\_cov\_30.364218 46-49. Max. coverage (+): 0.04. Max coverage (-): 0.08

Region: NODE\_369549\_length\_1565\_cov\_30.364218 50-52. Max. coverage (+): 0.4. Max coverage (-): 1.13

Region: NODE\_369549\_length\_1565\_cov\_30.364218 53-55. Max. coverage (+): 0.04. Max coverage (-): 0.85

Region: NODE\_369549\_length\_1565\_cov\_30.364218 56-58. Max. coverage (+): 0. Max coverage (-): 0.04

Region: NODE\_369549\_length\_1565\_cov\_30.364218 59-62. Max. coverage (+): 0.2. Max coverage (-): 0.61

Region: NODE\_369549\_length\_1565\_cov\_30.364218 63-65. Max. coverage (+): 0.2. Max coverage (-): 0.12

Region: NODE\_369549\_length\_1565\_cov\_30.364218 66-68. Max. coverage (+): 0.2. Max coverage (-): 0.1

Region: NODE\_369549\_length\_1565\_cov\_30.364218 69-71. Max. coverage (+): 0.08. Max coverage (-): 0.35

Region: NODE\_369549\_length\_1565\_cov\_30.364218 72-75. Max. coverage (+): 0.13. Max coverage (-): 0.09

Region: NODE\_369549\_length\_1565\_cov\_30.364218 76-78. Max. coverage (+): 0.15. Max coverage (-): 0.03

Region: NODE\_369549\_length\_1565\_cov\_30.364218 79-81. Max. coverage (+): 0.04. Max coverage (-): 0.01

Region: NODE\_369549\_length\_1565\_cov\_30.364218 82-84. Max. coverage (+): 0.16. Max coverage (-): 0

Region: NODE\_369549\_length\_1565\_cov\_30.364218 85-88. Max. coverage (+): 0.93. Max coverage (-): 0.01

Region: NODE\_369549\_length\_1565\_cov\_30.364218 89-91. Max. coverage (+): 0.36. Max coverage (-): 0

Region: NODE\_369549\_length\_1565\_cov\_30.364218 92-94. Max. coverage (+): 0.08. Max coverage (-): 0

Region: NODE\_369549\_length\_1565\_cov\_30.364218 95-97. Max. coverage (+): 0.04. Max coverage (-): 0.04

Region: NODE\_369549\_length\_1565\_cov\_30.364218 98-101. Max. coverage (+): 0. Max coverage (-): 0.04

Region: NODE\_369549\_length\_1565\_cov\_30.364218 102-104. Max. coverage (+): 0. Max coverage (-): 0.08

Region: NODE\_369549\_length\_1565\_cov\_30.364218 105-107. Max. coverage (+): 0. Max coverage (-): 0.24

Region: NODE\_369549\_length\_1565\_cov\_30.364218 108-110. Max. coverage (+): 0.04. Max coverage (-): 0.65

Region: NODE\_369549\_length\_1565\_cov\_30.364218 111-114. Max. coverage (+): 0.28. Max coverage (-): 1.37

Region: NODE\_369549\_length\_1565\_cov\_30.364218 115-117. Max. coverage (+): 0.36. Max coverage (-): 0.93

Region: NODE\_369549\_length\_1565\_cov\_30.364218 118-120. Max. coverage (+): 0.28. Max coverage (-): 0

Region: NODE\_369549\_length\_1565\_cov\_30.364218 121-123. Max. coverage (+): 0.08. Max coverage (-): 0

Region: NODE\_369549\_length\_1565\_cov\_30.364218 124-127. Max. coverage (+): 0.04. Max coverage (-): 0

Region: NODE\_369549\_length\_1565\_cov\_30.364218 128-130. Max. coverage (+): 6.06. Max coverage (-): 0

Region: NODE\_369549\_length\_1565\_cov\_30.364218 131-133. Max. coverage (+): 0.28. Max coverage (-): 0.08

Region: NODE\_369549\_length\_1565\_cov\_30.364218 134-136. Max. coverage (+): 0.04. Max coverage (-): 0.04

Region: NODE\_369549\_length\_1565\_cov\_30.364218 137-140. Max. coverage (+): 0.04. Max coverage (-): 0

Region: NODE\_369549\_length\_1565\_cov\_30.364218 141-143. Max. coverage (+): 0. Max coverage (-): 0.03

Region: NODE\_369549\_length\_1565\_cov\_30.364218 144-146. Max. coverage (+): 0. Max coverage (-): 0.12

Region: NODE\_369549\_length\_1565\_cov\_30.364218 147-149. Max. coverage (+): 0. Max coverage (-): 0.46

Region: NODE\_369549\_length\_1565\_cov\_30.364218 150-153. Max. coverage (+): 0.05. Max coverage (-): 0.36

Region: NODE\_369549\_length\_1565\_cov\_30.364218 154-156. Max. coverage (+): 0.05. Max coverage (-): 0.05

Region: NODE\_369549\_length\_1565\_cov\_30.364218 157-159. Max. coverage (+): 0.04. Max coverage (-): 0.05

Region: NODE\_369549\_length\_1565\_cov\_30.364218 160-162. Max. coverage (+): 0.03. Max coverage (-): 0.09

Region: NODE\_369549\_length\_1565\_cov\_30.364218 163-166. Max. coverage (+): 0.08. Max coverage (-): 0.09

Region: NODE\_369549\_length\_1565\_cov\_30.364218 167-169. Max. coverage (+): 0.12. Max coverage (-): 0.08

Region: NODE\_369549\_length\_1565\_cov\_30.364218 170-172. Max. coverage (+): 0.04. Max coverage (-): 0.12

Region: NODE\_369549\_length\_1565\_cov\_30.364218 173-175. Max. coverage (+): 0.36. Max coverage (-): 0.16

Region: NODE\_369549\_length\_1565\_cov\_30.364218 176-179. Max. coverage (+): 0.2. Max coverage (-): 0.2

Region: NODE\_369549\_length\_1565\_cov\_30.364218 180-182. Max. coverage (+): 0.24. Max coverage (-): 0.24

Region: NODE\_369549\_length\_1565\_cov\_30.364218 183-185. Max. coverage (+): 0.36. Max coverage (-): 0.16

Region: NODE\_369549\_length\_1565\_cov\_30.364218 186-188. Max. coverage (+): 0.36. Max coverage (-): 0

Region: NODE\_369549\_length\_1565\_cov\_30.364218 189-192. Max. coverage (+): 2.42. Max coverage (-): 0

Region: NODE\_369549\_length\_1565\_cov\_30.364218 193-195. Max. coverage (+): 0.16. Max coverage (-): 0.04

Region: NODE\_369549\_length\_1565\_cov\_30.364218 196-198. Max. coverage (+): 0.04. Max coverage (-): 0.04

Region: NODE\_369549\_length\_1565\_cov\_30.364218 199-201. Max. coverage (+): 0.04. Max coverage (-): 0.04

Region: NODE\_369549\_length\_1565\_cov\_30.364218 202-205. Max. coverage (+): 0.04. Max coverage (-): 0

Region: NODE\_369549\_length\_1565\_cov\_30.364218 206-208. Max. coverage (+): 0.12. Max coverage (-): 0.04

Region: NODE\_369549\_length\_1565\_cov\_30.364218 209-211. Max. coverage (+): 0.03. Max coverage (-): 0

Region: NODE\_369549\_length\_1565\_cov\_30.364218 212-214. Max. coverage (+): 0.01. Max coverage (-): 0

Region: NODE\_369549\_length\_1565\_cov\_30.364218 215-218. Max. coverage (+): 0. Max coverage (-): 0

Region: NODE\_369549\_length\_1565\_cov\_30.364218 219-221. Max. coverage (+): 0.18. Max coverage (-): 0.01

Region: NODE\_369549\_length\_1565\_cov\_30.364218 222-224. Max. coverage (+): 0.29. Max coverage (-): 0.19

Region: NODE\_369549\_length\_1565\_cov\_30.364218 225-227. Max. coverage (+): 0.25. Max coverage (-): 0.18

Region: NODE\_369549\_length\_1565\_cov\_30.364218 228-231. Max. coverage (+): 0.07. Max coverage (-): 4.2

Region: NODE\_369549\_length\_1565\_cov\_30.364218 232-234. Max. coverage (+): 0.2. Max coverage (-): 4.44

Region: NODE\_369549\_length\_1565\_cov\_30.364218 235-237. Max. coverage (+): 0.04. Max coverage (-): 3.03

Region: NODE\_369549\_length\_1565\_cov\_30.364218 238-240. Max. coverage (+): 0. Max coverage (-): 0.69

Region: NODE\_369549\_length\_1565\_cov\_30.364218 241-244. Max. coverage (+): 0.65. Max coverage (-): 0.12

Region: NODE\_369549\_length\_1565\_cov\_30.364218 245-247. Max. coverage (+): 0.97. Max coverage (-): 0

Region: NODE\_369549\_length\_1565\_cov\_30.364218 248-250. Max. coverage (+): 0.81. Max coverage (-): 0

Region: NODE\_369549\_length\_1565\_cov\_30.364218 251-253. Max. coverage (+): 5.13. Max coverage (-): 0

Region: NODE\_369549\_length\_1565\_cov\_30.364218 254-257. Max. coverage (+): 3.55. Max coverage (-): 0.2

Region: NODE\_369549\_length\_1565\_cov\_30.364218 258-260. Max. coverage (+): 0.16. Max coverage (-): 0.2

Region: NODE\_369549\_length\_1565\_cov\_30.364218 261-263. Max. coverage (+): 0.07. Max coverage (-): 1.71

Region: NODE\_369549\_length\_1565\_cov\_30.364218 264-266. Max. coverage (+): 0.03. Max coverage (-): 1.51

Region: NODE\_369549\_length\_1565\_cov\_30.364218 267-270. Max. coverage (+): 0.04. Max coverage (-): 0.77

Region: NODE\_369549\_length\_1565\_cov\_30.364218 271-273. Max. coverage (+): 0.04. Max coverage (-): 0.97

Region: NODE\_369549\_length\_1565\_cov\_30.364218 274-276. Max. coverage (+): 0.04. Max coverage (-): 1.05

Region: NODE\_369549\_length\_1565\_cov\_30.364218 277-279. Max. coverage (+): 0. Max coverage (-): 0.08

Region: NODE\_369549\_length\_1565\_cov\_30.364218 280-283. Max. coverage (+): 0.4. Max coverage (-): 0.08

Region: NODE\_369549\_length\_1565\_cov\_30.364218 284-286. Max. coverage (+): 0.44. Max coverage (-): 0.04

Region: NODE\_369549\_length\_1565\_cov\_30.364218 287-289. Max. coverage (+): 0.16. Max coverage (-): 0.32

Region: NODE\_369549\_length\_1565\_cov\_30.364218 290-292. Max. coverage (+): 0.08. Max coverage (-): 1.09

Region: NODE\_369549\_length\_1565\_cov\_30.364218 293-296. Max. coverage (+): 1.66. Max coverage (-): 1.41

Region: NODE\_369549\_length\_1565\_cov\_30.364218 297-299. Max. coverage (+): 1.01. Max coverage (-): 0.97

Region: NODE\_369549\_length\_1565\_cov\_30.364218 300-302. Max. coverage (+): 0.97. Max coverage (-): 0.85

Region: NODE\_369549\_length\_1565\_cov\_30.364218 303-305. Max. coverage (+): 0.08. Max coverage (-): 0.47

Region: NODE\_369549\_length\_1565\_cov\_30.364218 306-309. Max. coverage (+): 0.44. Max coverage (-): 0.11

Region: NODE\_369549\_length\_1565\_cov\_30.364218 310-312. Max. coverage (+): 0.87. Max coverage (-): 0.01

Region: NODE\_369549\_length\_1565\_cov\_30.364218 313-315. Max. coverage (+): 0.67. Max coverage (-): 0.01

Region: NODE\_369549\_length\_1565\_cov\_30.364218 316-318. Max. coverage (+): 0.65. Max coverage (-): 0

Region: NODE\_369549\_length\_1565\_cov\_30.364218 319-322. Max. coverage (+): 9.85. Max coverage (-): 0.04

Region: NODE\_369549\_length\_1565\_cov\_30.364218 323-325. Max. coverage (+): 3.31. Max coverage (-): 0.04

Region: NODE\_369549\_length\_1565\_cov\_30.364218 326-328. Max. coverage (+): 1.13. Max coverage (-): 0

Region: NODE\_369549\_length\_1565\_cov\_30.364218 329-331. Max. coverage (+): 0.28. Max coverage (-): 0

Region: NODE\_369549\_length\_1565\_cov\_30.364218 332-335. Max. coverage (+): 5.33. Max coverage (-): 0.12

Region: NODE\_369549\_length\_1565\_cov\_30.364218 336-338. Max. coverage (+): 7.19. Max coverage (-): 0.04

Region: NODE\_369549\_length\_1565\_cov\_30.364218 339-341. Max. coverage (+): 0.48. Max coverage (-): 0

Region: NODE\_369549\_length\_1565\_cov\_30.364218 342-344. Max. coverage (+): 0.97. Max coverage (-): 0

Region: NODE\_369549\_length\_1565\_cov\_30.364218 345-348. Max. coverage (+): 0.77. Max coverage (-): 1.66

Region: NODE\_369549\_length\_1565\_cov\_30.364218 349-351. Max. coverage (+): 2.14. Max coverage (-): 1.66

Region: NODE\_369549\_length\_1565\_cov\_30.364218 352-354. Max. coverage (+): 1.01. Max coverage (-): 0.36

Region: NODE\_369549\_length\_1565\_cov\_30.364218 355-357. Max. coverage (+): 0.04. Max coverage (-): 3.63

Region: NODE\_369549\_length\_1565\_cov\_30.364218 358-361. Max. coverage (+): 0.2. Max coverage (-): 4.04

Region: NODE\_369549\_length\_1565\_cov\_30.364218 362-364. Max. coverage (+): 2.1. Max coverage (-): 0.16

Region: NODE\_369549\_length\_1565\_cov\_30.364218 365-367. Max. coverage (+): 2.18. Max coverage (-): 0

Region: NODE\_369549\_length\_1565\_cov\_30.364218 368-370. Max. coverage (+): 0.05. Max coverage (-): 0

Region: NODE\_369549\_length\_1565\_cov\_30.364218 371-374. Max. coverage (+): 0.34. Max coverage (-): 0.02

Region: NODE\_369549\_length\_1565\_cov\_30.364218 375-377. Max. coverage (+): 0.44. Max coverage (-): 0.02

Region: NODE\_369549\_length\_1565\_cov\_30.364218 378-380. Max. coverage (+): 0.02. Max coverage (-): 0.02

Region: NODE\_369549\_length\_1565\_cov\_30.364218 381-383. Max. coverage (+): 0.05. Max coverage (-): 0.01

Region: NODE\_369549\_length\_1565\_cov\_30.364218 384-387. Max. coverage (+): 0.03. Max coverage (-): 0.18

Region: NODE\_369549\_length\_1565\_cov\_30.364218 388-390. Max. coverage (+): 0.02. Max coverage (-): 0.52

Region: NODE\_369549\_length\_1565\_cov\_30.364218 391-393. Max. coverage (+): 0.04. Max coverage (-): 0.46

Region: NODE\_369549\_length\_1565\_cov\_30.364218 394-396. Max. coverage (+): 0.32. Max coverage (-): 0.89

Region: NODE\_369549\_length\_1565\_cov\_30.364218 397-400. Max. coverage (+): 6.1. Max coverage (-): 0

Region: NODE\_369549\_length\_1565\_cov\_30.364218 401-403. Max. coverage (+): 6.7. Max coverage (-): 0

Region: NODE\_369549\_length\_1565\_cov\_30.364218 404-406. Max. coverage (+): 2.1. Max coverage (-): 0.12

Region: NODE\_369549\_length\_1565\_cov\_30.364218 407-409. Max. coverage (+): 3.11. Max coverage (-): 0.16

Region: NODE\_369549\_length\_1565\_cov\_30.364218 410-413. Max. coverage (+): 3.47. Max coverage (-): 0.65

Region: NODE\_369549\_length\_1565\_cov\_30.364218 414-416. Max. coverage (+): 0.65. Max coverage (-): 1.01

Region: NODE\_369549\_length\_1565\_cov\_30.364218 417-419. Max. coverage (+): 0.4. Max coverage (-): 1.49

Region: NODE\_369549\_length\_1565\_cov\_30.364218 420-422. Max. coverage (+): 0.12. Max coverage (-): 3.03

Region: NODE\_369549\_length\_1565\_cov\_30.364218 423-426. Max. coverage (+): 0.08. Max coverage (-): 0.04

Region: NODE\_369549\_length\_1565\_cov\_30.364218 427-429. Max. coverage (+): 0.16. Max coverage (-): 0.08

Region: NODE\_369549\_length\_1565\_cov\_30.364218 430-432. Max. coverage (+): 0.52. Max coverage (-): 0.69

Region: NODE\_369549\_length\_1565\_cov\_30.364218 433-435. Max. coverage (+): 0.69. Max coverage (-): 0.61

Region: NODE\_369549\_length\_1565\_cov\_30.364218 436-439. Max. coverage (+): 0.24. Max coverage (-): 0.36

Region: NODE\_369549\_length\_1565\_cov\_30.364218 440-442. Max. coverage (+): 1.21. Max coverage (-): 0.32

Region: NODE\_369549\_length\_1565\_cov\_30.364218 443-445. Max. coverage (+): 1.05. Max coverage (-): 0.32

Region: NODE\_369549\_length\_1565\_cov\_30.364218 446-448. Max. coverage (+): 0.06. Max coverage (-): 0.38

Region: NODE\_369549\_length\_1565\_cov\_30.364218 449-452. Max. coverage (+): 0.12. Max coverage (-): 0.84

Region: NODE\_369549\_length\_1565\_cov\_30.364218 453-455. Max. coverage (+): 0.2. Max coverage (-): 0.73

Region: NODE\_369549\_length\_1565\_cov\_30.364218 456-458. Max. coverage (+): 0.19. Max coverage (-): 0.16

Region: NODE\_369549\_length\_1565\_cov\_30.364218 459-461. Max. coverage (+): 0.19. Max coverage (-): 0.08

Region: NODE\_369549\_length\_1565\_cov\_30.364218 462-465. Max. coverage (+): 2.89. Max coverage (-): 0

Region: NODE\_369549\_length\_1565\_cov\_30.364218 466-468. Max. coverage (+): 0.24. Max coverage (-): 0.04

Region: NODE\_369549\_length\_1565\_cov\_30.364218 469-471. Max. coverage (+): 0.12. Max coverage (-): 0.04

Region: NODE\_369549\_length\_1565\_cov\_30.364218 472-474. Max. coverage (+): 0.08. Max coverage (-): 0.12

Region: NODE\_369549\_length\_1565\_cov\_30.364218 475-478. Max. coverage (+): 0.16. Max coverage (-): 0.97

Region: NODE\_369549\_length\_1565\_cov\_30.364218 479-481. Max. coverage (+): 0.04. Max coverage (-): 0.28

Region: NODE\_369549\_length\_1565\_cov\_30.364218 482-484. Max. coverage (+): 0.08. Max coverage (-): 0.85

Region: NODE\_369549\_length\_1565\_cov\_30.364218 485-487. Max. coverage (+): 1.09. Max coverage (-): 0.89

Region: NODE\_369549\_length\_1565\_cov\_30.364218 488-491. Max. coverage (+): 3.88. Max coverage (-): 0.57

Region: NODE\_369549\_length\_1565\_cov\_30.364218 492-494. Max. coverage (+): 3.63. Max coverage (-): 0.08

Region: NODE\_369549\_length\_1565\_cov\_30.364218 495-497. Max. coverage (+): 0.08. Max coverage (-): 0.04

Region: NODE\_369549\_length\_1565\_cov\_30.364218 498-500. Max. coverage (+): 0.02. Max coverage (-): 0.08

Region: NODE\_369549\_length\_1565\_cov\_30.364218 501-504. Max. coverage (+): 0.67. Max coverage (-): 0.1

Region: NODE\_369549\_length\_1565\_cov\_30.364218 505-507. Max. coverage (+): 0.42. Max coverage (-): 0.52

Region: NODE\_369549\_length\_1565\_cov\_30.364218 508-510. Max. coverage (+): 0.81. Max coverage (-): 0.59

Region: NODE\_369549\_length\_1565\_cov\_30.364218 511-513. Max. coverage (+): 2.2. Max coverage (-): 0.02

Region: NODE\_369549\_length\_1565\_cov\_30.364218 514-517. Max. coverage (+): 1.88. Max coverage (-): 0

Region: NODE\_369549\_length\_1565\_cov\_30.364218 518-520. Max. coverage (+): 0.04. Max coverage (-): 0

Region: NODE\_369549\_length\_1565\_cov\_30.364218 521-523. Max. coverage (+): 0.08. Max coverage (-): 0.04

Region: NODE\_369549\_length\_1565\_cov\_30.364218 524-526. Max. coverage (+): 0.12. Max coverage (-): 0.04

Region: NODE\_369549\_length\_1565\_cov\_30.364218 527-530. Max. coverage (+): 0.69. Max coverage (-): 0.04

Region: NODE\_369549\_length\_1565\_cov\_30.364218 531-533. Max. coverage (+): 0.12. Max coverage (-): 0.04

Region: NODE\_369549\_length\_1565\_cov\_30.364218 534-536. Max. coverage (+): 0. Max coverage (-): 0.36

Region: NODE\_369549\_length\_1565\_cov\_30.364218 537-539. Max. coverage (+): 0. Max coverage (-): 0.16

Region: NODE\_369549\_length\_1565\_cov\_30.364218 540-543. Max. coverage (+): 0. Max coverage (-): 0.32

Region: NODE\_369549\_length\_1565\_cov\_30.364218 544-546. Max. coverage (+): 0. Max coverage (-): 0.16

Region: NODE\_369549\_length\_1565\_cov\_30.364218 547-549. Max. coverage (+): 0.04. Max coverage (-): 0.16

Region: NODE\_369549\_length\_1565\_cov\_30.364218 550-552. Max. coverage (+): 0.12. Max coverage (-): 0.08

Region: NODE\_369549\_length\_1565\_cov\_30.364218 553-556. Max. coverage (+): 1.43. Max coverage (-): 0.04

Region: NODE\_369549\_length\_1565\_cov\_30.364218 557-559. Max. coverage (+): 0.65. Max coverage (-): 0.04

Region: NODE\_369549\_length\_1565\_cov\_30.364218 560-562. Max. coverage (+): 0.32. Max coverage (-): 0

Region: NODE\_369549\_length\_1565\_cov\_30.364218 563-565. Max. coverage (+): 0.71. Max coverage (-): 0.08

Region: NODE\_369549\_length\_1565\_cov\_30.364218 566-569. Max. coverage (+): 2.62. Max coverage (-): 0.52

Region: NODE\_369549\_length\_1565\_cov\_30.364218 570-572. Max. coverage (+): 0.14. Max coverage (-): 0.48

Region: NODE\_369549\_length\_1565\_cov\_30.364218 573-575. Max. coverage (+): 0.3. Max coverage (-): 0.04

Region: NODE\_369549\_length\_1565\_cov\_30.364218 576-578. Max. coverage (+): 0.28. Max coverage (-): 0

Region: NODE\_369549\_length\_1565\_cov\_30.364218 579-582. Max. coverage (+): 0.22. Max coverage (-): 0

Region: NODE\_369549\_length\_1565\_cov\_30.364218 583-585. Max. coverage (+): 4.42. Max coverage (-): 0

Region: NODE\_369549\_length\_1565\_cov\_30.364218 586-588. Max. coverage (+): 4.52. Max coverage (-): 0

Region: NODE\_369549\_length\_1565\_cov\_30.364218 589-591. Max. coverage (+): 0.28. Max coverage (-): 0

Region: NODE\_369549\_length\_1565\_cov\_30.364218 592-595. Max. coverage (+): 0.57. Max coverage (-): 0.16

Region: NODE\_369549\_length\_1565\_cov\_30.364218 596-598. Max. coverage (+): 0.57. Max coverage (-): 0.16

Region: NODE\_369549\_length\_1565\_cov\_30.364218 599-601. Max. coverage (+): 0.12. Max coverage (-): 0.2

Region: NODE\_369549\_length\_1565\_cov\_30.364218 602-604. Max. coverage (+): 0.04. Max coverage (-): 0.2

Region: NODE\_369549\_length\_1565\_cov\_30.364218 605-608. Max. coverage (+): 0. Max coverage (-): 0.08

Region: NODE\_369549\_length\_1565\_cov\_30.364218 609-611. Max. coverage (+): 0.52. Max coverage (-): 0.04

Region: NODE\_369549\_length\_1565\_cov\_30.364218 612-614. Max. coverage (+): 28.63. Max coverage (-): 0.04

Region: NODE\_369549\_length\_1565\_cov\_30.364218 615-617. Max. coverage (+): 30.69. Max coverage (-): 0.12

Region: NODE\_369549\_length\_1565\_cov\_30.364218 618-621. Max. coverage (+): 0.08. Max coverage (-): 0.16

Region: NODE\_369549\_length\_1565\_cov\_30.364218 622-624. Max. coverage (+): 0. Max coverage (-): 0

Region: NODE\_369549\_length\_1565\_cov\_30.364218 625-627. Max. coverage (+): 0.02. Max coverage (-): 0.02

Region: NODE\_369549\_length\_1565\_cov\_30.364218 628-630. Max. coverage (+): 0. Max coverage (-): 0.1

Region: NODE\_369549\_length\_1565\_cov\_30.364218 631-634. Max. coverage (+): 0.56. Max coverage (-): 0.11

Region: NODE\_369549\_length\_1565\_cov\_30.364218 635-637. Max. coverage (+): 0.62. Max coverage (-): 0.03

Region: NODE\_369549\_length\_1565\_cov\_30.364218 638-640. Max. coverage (+): 0.02. Max coverage (-): 0

Region: NODE\_369549\_length\_1565\_cov\_30.364218 641-643. Max. coverage (+): 0.02. Max coverage (-): 0

Region: NODE\_369549\_length\_1565\_cov\_30.364218 644-647. Max. coverage (+): 0. Max coverage (-): 0

Region: NODE\_369549\_length\_1565\_cov\_30.364218 648-650. Max. coverage (+): 0. Max coverage (-): 0

Region: NODE\_369549\_length\_1565\_cov\_30.364218 651-653. Max. coverage (+): 0. Max coverage (-): 0.12

Region: NODE\_369549\_length\_1565\_cov\_30.364218 654-656. Max. coverage (+): 0. Max coverage (-): 0.08

Region: NODE\_369549\_length\_1565\_cov\_30.364218 657-660. Max. coverage (+): 0.04. Max coverage (-): 0.08

Region: NODE\_369549\_length\_1565\_cov\_30.364218 661-663. Max. coverage (+): 0.08. Max coverage (-): 0.08

Region: NODE\_369549\_length\_1565\_cov\_30.364218 664-666. Max. coverage (+): 0. Max coverage (-): 0

Region: NODE\_369549\_length\_1565\_cov\_30.364218 667-669. Max. coverage (+): 0.04. Max coverage (-): 0.08

Region: NODE\_369549\_length\_1565\_cov\_30.364218 670-673. Max. coverage (+): 0.08. Max coverage (-): 0.12

Region: NODE\_369549\_length\_1565\_cov\_30.364218 674-676. Max. coverage (+): 0.28. Max coverage (-): 0

Region: NODE\_369549\_length\_1565\_cov\_30.364218 677-679. Max. coverage (+): 0.16. Max coverage (-): 0.57

Region: NODE\_369549\_length\_1565\_cov\_30.364218 680-682. Max. coverage (+): 0. Max coverage (-): 0.57

Region: NODE\_369549\_length\_1565\_cov\_30.364218 683-686. Max. coverage (+): 0. Max coverage (-): 0.28

Region: NODE\_369549\_length\_1565\_cov\_30.364218 687-689. Max. coverage (+): 0. Max coverage (-): 0.16

Region: NODE\_369549\_length\_1565\_cov\_30.364218 690-692. Max. coverage (+): 0. Max coverage (-): 0.28

Region: NODE\_369549\_length\_1565\_cov\_30.364218 693-695. Max. coverage (+): 0. Max coverage (-): 0.2

Region: NODE\_369549\_length\_1565\_cov\_30.364218 696-699. Max. coverage (+): 1.9. Max coverage (-): 0.12

Region: NODE\_369549\_length\_1565\_cov\_30.364218 700-702. Max. coverage (+): 2.06. Max coverage (-): 0.44

Region: NODE\_369549\_length\_1565\_cov\_30.364218 703-705. Max. coverage (+): 0.45. Max coverage (-): 0.44

Region: NODE\_369549\_length\_1565\_cov\_30.364218 706-708. Max. coverage (+): 0.15. Max coverage (-): 0.04

Region: NODE\_369549\_length\_1565\_cov\_30.364218 709-712. Max. coverage (+): 0.35. Max coverage (-): 0.02

Region: NODE\_369549\_length\_1565\_cov\_30.364218 713-715. Max. coverage (+): 0.06. Max coverage (-): 0.05

Region: NODE\_369549\_length\_1565\_cov\_30.364218 716-718. Max. coverage (+): 0.16. Max coverage (-): 0.05

Region: NODE\_369549\_length\_1565\_cov\_30.364218 719-721. Max. coverage (+): 0.19. Max coverage (-): 0

Region: NODE\_369549\_length\_1565\_cov\_30.364218 722-725. Max. coverage (+): 0.22. Max coverage (-): 0.05

Region: NODE\_369549\_length\_1565\_cov\_30.364218 726-728. Max. coverage (+): 0.1. Max coverage (-): 0.19

Region: NODE\_369549\_length\_1565\_cov\_30.364218 729-731. Max. coverage (+): 0.05. Max coverage (-): 0.04

Region: NODE\_369549\_length\_1565\_cov\_30.364218 732-734. Max. coverage (+): 0.02. Max coverage (-): 0.04

Region: NODE\_369549\_length\_1565\_cov\_30.364218 735-738. Max. coverage (+): 0.09. Max coverage (-): 0.02

Region: NODE\_369549\_length\_1565\_cov\_30.364218 739-741. Max. coverage (+): 0.42. Max coverage (-): 0.04

Region: NODE\_369549\_length\_1565\_cov\_30.364218 742-744. Max. coverage (+): 0.32. Max coverage (-): 0.04

Region: NODE\_369549\_length\_1565\_cov\_30.364218 745-747. Max. coverage (+): 0. Max coverage (-): 0.12

Region: NODE\_369549\_length\_1565\_cov\_30.364218 748-751. Max. coverage (+): 0.08. Max coverage (-): 0.08

Region: NODE\_369549\_length\_1565\_cov\_30.364218 752-754. Max. coverage (+): 4. Max coverage (-): 0.04

Region: NODE\_369549\_length\_1565\_cov\_30.364218 755-757. Max. coverage (+): 2.02. Max coverage (-): 0.12

Region: NODE\_369549\_length\_1565\_cov\_30.364218 758-760. Max. coverage (+): 0.32. Max coverage (-): 0.36

Region: NODE\_369549\_length\_1565\_cov\_30.364218 761-764. Max. coverage (+): 2.26. Max coverage (-): 0.2

Region: NODE\_369549\_length\_1565\_cov\_30.364218 765-767. Max. coverage (+): 0.17. Max coverage (-): 0.07

Region: NODE\_369549\_length\_1565\_cov\_30.364218 768-770. Max. coverage (+): 0.5. Max coverage (-): 0.07

Region: NODE\_369549\_length\_1565\_cov\_30.364218 771-773. Max. coverage (+): 4.4. Max coverage (-): 0.05

Region: NODE\_369549\_length\_1565\_cov\_30.364218 774-777. Max. coverage (+): 2.73. Max coverage (-): 0.02

Region: NODE\_369549\_length\_1565\_cov\_30.364218 778-780. Max. coverage (+): 4.07. Max coverage (-): 0.01

Region: NODE\_369549\_length\_1565\_cov\_30.364218 781-783. Max. coverage (+): 3.83. Max coverage (-): 0.04

Region: NODE\_369549\_length\_1565\_cov\_30.364218 784-786. Max. coverage (+): 1.49. Max coverage (-): 0.04

Region: NODE\_369549\_length\_1565\_cov\_30.364218 787-790. Max. coverage (+): 0.44. Max coverage (-): 0.08

Region: NODE\_369549\_length\_1565\_cov\_30.364218 791-793. Max. coverage (+): 0.12. Max coverage (-): 0.12

Region: NODE\_369549\_length\_1565\_cov\_30.364218 794-796. Max. coverage (+): 0.16. Max coverage (-): 0.04

Region: NODE\_369549\_length\_1565\_cov\_30.364218 797-799. Max. coverage (+): 1.37. Max coverage (-): 0.2

Region: NODE\_369549\_length\_1565\_cov\_30.364218 800-803. Max. coverage (+): 2.18. Max coverage (-): 0.65

Region: NODE\_369549\_length\_1565\_cov\_30.364218 804-806. Max. coverage (+): 1.01. Max coverage (-): 0.52

Region: NODE\_369549\_length\_1565\_cov\_30.364218 807-809. Max. coverage (+): 0.89. Max coverage (-): 0

Region: NODE\_369549\_length\_1565\_cov\_30.364218 810-812. Max. coverage (+): 0. Max coverage (-): 0

Region: NODE\_369549\_length\_1565\_cov\_30.364218 813-816. Max. coverage (+): 0.35. Max coverage (-): 0.08

Region: NODE\_369549\_length\_1565\_cov\_30.364218 817-819. Max. coverage (+): 24.25. Max coverage (-): 0.22

Region: NODE\_369549\_length\_1565\_cov\_30.364218 820-822. Max. coverage (+): 2.57. Max coverage (-): 0.04

Region: NODE\_369549\_length\_1565\_cov\_30.364218 823-825. Max. coverage (+): 2.48. Max coverage (-): 0

Region: NODE\_369549\_length\_1565\_cov\_30.364218 826-829. Max. coverage (+): 0.03. Max coverage (-): 0.03

Region: NODE\_369549\_length\_1565\_cov\_30.364218 830-832. Max. coverage (+): 0.01. Max coverage (-): 0.36

Region: NODE\_369549\_length\_1565\_cov\_30.364218 833-835. Max. coverage (+): 0. Max coverage (-): 0.34

Region: NODE\_369549\_length\_1565\_cov\_30.364218 836-838. Max. coverage (+): 0. Max coverage (-): 0.02

Region: NODE\_369549\_length\_1565\_cov\_30.364218 839-842. Max. coverage (+): 0. Max coverage (-): 0.22

Region: NODE\_369549\_length\_1565\_cov\_30.364218 843-845. Max. coverage (+): 0. Max coverage (-): 0.38

Region: NODE\_369549\_length\_1565\_cov\_30.364218 846-848. Max. coverage (+): 0. Max coverage (-): 0.44

Region: NODE\_369549\_length\_1565\_cov\_30.364218 849-851. Max. coverage (+): 0.24. Max coverage (-): 0

Region: NODE\_369549\_length\_1565\_cov\_30.364218 852-855. Max. coverage (+): 5.81. Max coverage (-): 0

Region: NODE\_369549\_length\_1565\_cov\_30.364218 856-858. Max. coverage (+): 6.32. Max coverage (-): 0

Region: NODE\_369549\_length\_1565\_cov\_30.364218 859-861. Max. coverage (+): 17.54. Max coverage (-): 0.06

Region: NODE\_369549\_length\_1565\_cov\_30.364218 862-864. Max. coverage (+): 12.44. Max coverage (-): 0.06

Region: NODE\_369549\_length\_1565\_cov\_30.364218 865-868. Max. coverage (+): 0.31. Max coverage (-): 0

Region: NODE\_369549\_length\_1565\_cov\_30.364218 869-871. Max. coverage (+): 0. Max coverage (-): 0

Region: NODE\_369549\_length\_1565\_cov\_30.364218 872-874. Max. coverage (+): 0.02. Max coverage (-): 0.18

Region: NODE\_369549\_length\_1565\_cov\_30.364218 875-877. Max. coverage (+): 0.02. Max coverage (-): 0.33

Region: NODE\_369549\_length\_1565\_cov\_30.364218 878-881. Max. coverage (+): 0.02. Max coverage (-): 0.69

Region: NODE\_369549\_length\_1565\_cov\_30.364218 882-884. Max. coverage (+): 0.85. Max coverage (-): 1.05

Region: NODE\_369549\_length\_1565\_cov\_30.364218 885-887. Max. coverage (+): 0.93. Max coverage (-): 0.08

Region: NODE\_369549\_length\_1565\_cov\_30.364218 888-890. Max. coverage (+): 2.22. Max coverage (-): 0.08

Region: NODE\_369549\_length\_1565\_cov\_30.364218 891-894. Max. coverage (+): 1.13. Max coverage (-): 0

Region: NODE\_369549\_length\_1565\_cov\_30.364218 895-897. Max. coverage (+): 0.89. Max coverage (-): 0

Region: NODE\_369549\_length\_1565\_cov\_30.364218 898-900. Max. coverage (+): 0.85. Max coverage (-): 0.04

Region: NODE\_369549\_length\_1565\_cov\_30.364218 901-903. Max. coverage (+): 1.9. Max coverage (-): 0.04

Region: NODE\_369549\_length\_1565\_cov\_30.364218 904-907. Max. coverage (+): 0.32. Max coverage (-): 0.07

Region: NODE\_369549\_length\_1565\_cov\_30.364218 908-910. Max. coverage (+): 0.33. Max coverage (-): 0.17

Region: NODE\_369549\_length\_1565\_cov\_30.364218 911-913. Max. coverage (+): 0.33. Max coverage (-): 0.04

Region: NODE\_369549\_length\_1565\_cov\_30.364218 914-916. Max. coverage (+): 0.4. Max coverage (-): 0.04

Region: NODE\_369549\_length\_1565\_cov\_30.364218 917-920. Max. coverage (+): 0.28. Max coverage (-): 0.12

Region: NODE\_369549\_length\_1565\_cov\_30.364218 921-923. Max. coverage (+): 6.3. Max coverage (-): 0.08

Region: NODE\_369549\_length\_1565\_cov\_30.364218 924-926. Max. coverage (+): 6.5. Max coverage (-): 0

Region: NODE\_369549\_length\_1565\_cov\_30.364218 927-929. Max. coverage (+): 0.24. Max coverage (-): 0

Region: NODE\_369549\_length\_1565\_cov\_30.364218 930-933. Max. coverage (+): 0.52. Max coverage (-): 0.28

Region: NODE\_369549\_length\_1565\_cov\_30.364218 934-936. Max. coverage (+): 0.12. Max coverage (-): 0.16

Region: NODE\_369549\_length\_1565\_cov\_30.364218 937-939. Max. coverage (+): 0.07. Max coverage (-): 0.17

Region: NODE\_369549\_length\_1565\_cov\_30.364218 940-942. Max. coverage (+): 0.08. Max coverage (-): 0.32

Region: NODE\_369549\_length\_1565\_cov\_30.364218 943-946. Max. coverage (+): 0.19. Max coverage (-): 0.01

Region: NODE\_369549\_length\_1565\_cov\_30.364218 947-949. Max. coverage (+): 0.06. Max coverage (-): 0.04

Region: NODE\_369549\_length\_1565\_cov\_30.364218 950-952. Max. coverage (+): 0.41. Max coverage (-): 0.12

Region: NODE\_369549\_length\_1565\_cov\_30.364218 953-955. Max. coverage (+): 0.76. Max coverage (-): 0.01

Region: NODE\_369549\_length\_1565\_cov\_30.364218 956-959. Max. coverage (+): 11.27. Max coverage (-): 0.04

Region: NODE\_369549\_length\_1565\_cov\_30.364218 960-962. Max. coverage (+): 2.38. Max coverage (-): 0.08

Region: NODE\_369549\_length\_1565\_cov\_30.364218 963-965. Max. coverage (+): 0.28. Max coverage (-): 0

Region: NODE\_369549\_length\_1565\_cov\_30.364218 966-968. Max. coverage (+): 0.12. Max coverage (-): 0

Region: NODE\_369549\_length\_1565\_cov\_30.364218 969-972. Max. coverage (+): 0. Max coverage (-): 0

Region: NODE\_369549\_length\_1565\_cov\_30.364218 973-975. Max. coverage (+): 1.33. Max coverage (-): 0

Region: NODE\_369549\_length\_1565\_cov\_30.364218 976-978. Max. coverage (+): 3.23. Max coverage (-): 0.08

Region: NODE\_369549\_length\_1565\_cov\_30.364218 979-981. Max. coverage (+): 2.14. Max coverage (-): 0.97

Region: NODE\_369549\_length\_1565\_cov\_30.364218 982-985. Max. coverage (+): 0.08. Max coverage (-): 0.97

Region: NODE\_369549\_length\_1565\_cov\_30.364218 986-988. Max. coverage (+): 0.16. Max coverage (-): 0.36

Region: NODE\_369549\_length\_1565\_cov\_30.364218 989-991. Max. coverage (+): 0.16. Max coverage (-): 0.24

Region: NODE\_369549\_length\_1565\_cov\_30.364218 992-994. Max. coverage (+): 0.05. Max coverage (-): 0.07

Region: NODE\_369549\_length\_1565\_cov\_30.364218 995-998. Max. coverage (+): 0.55. Max coverage (-): 0.01

Region: NODE\_369549\_length\_1565\_cov\_30.364218 999-1001. Max. coverage (+): 0.54. Max coverage (-): 0.03

Region: NODE\_369549\_length\_1565\_cov\_30.364218 1002-1004. Max. coverage (+): 0.07. Max coverage (-): 0.04

Region: NODE\_369549\_length\_1565\_cov\_30.364218 1005-1007. Max. coverage (+): 0.95. Max coverage (-): 0.03

Region: NODE\_369549\_length\_1565\_cov\_30.364218 1008-1011. Max. coverage (+): 3.26. Max coverage (-): 0.04

Region: NODE\_369549\_length\_1565\_cov\_30.364218 1012-1014. Max. coverage (+): 3.31. Max coverage (-): 0.05

Region: NODE\_369549\_length\_1565\_cov\_30.364218 1015-1017. Max. coverage (+): 0.24. Max coverage (-): 0.08

Region: NODE\_369549\_length\_1565\_cov\_30.364218 1018-1020. Max. coverage (+): 0.77. Max coverage (-): 0.08

Region: NODE\_369549\_length\_1565\_cov\_30.364218 1021-1024. Max. coverage (+): 0.77. Max coverage (-): 29.23

Region: NODE\_369549\_length\_1565\_cov\_30.364218 1025-1027. Max. coverage (+): 0.04. Max coverage (-): 30.08

Region: NODE\_369549\_length\_1565\_cov\_30.364218 1028-1030. Max. coverage (+): 0.69. Max coverage (-): 2.08

Region: NODE\_369549\_length\_1565\_cov\_30.364218 1031-1033. Max. coverage (+): 4.68. Max coverage (-): 1.21

Region: NODE\_369549\_length\_1565\_cov\_30.364218 1034-1037. Max. coverage (+): 10.78. Max coverage (-): 0.4

Region: NODE\_369549\_length\_1565\_cov\_30.364218 1038-1040. Max. coverage (+): 6.95. Max coverage (-): 0.08

Region: NODE\_369549\_length\_1565\_cov\_30.364218 1041-1043. Max. coverage (+): 34. Max coverage (-): 0.08

Region: NODE\_369549\_length\_1565\_cov\_30.364218 1044-1046. Max. coverage (+): 34.97. Max coverage (-): 0.08

Region: NODE\_369549\_length\_1565\_cov\_30.364218 1047-1050. Max. coverage (+): 29.23. Max coverage (-): 0.04

Region: NODE\_369549\_length\_1565\_cov\_30.364218 1051-1053. Max. coverage (+): 4.36. Max coverage (-): 0.12

Region: NODE\_369549\_length\_1565\_cov\_30.364218 1054-1056. Max. coverage (+): 0.61. Max coverage (-): 0.28

Region: NODE\_369549\_length\_1565\_cov\_30.364218 1057-1059. Max. coverage (+): 0.57. Max coverage (-): 0.57

Region: NODE\_369549\_length\_1565\_cov\_30.364218 1060-1063. Max. coverage (+): 0.28. Max coverage (-): 2.22

Region: NODE\_369549\_length\_1565\_cov\_30.364218 1064-1066. Max. coverage (+): 0.28. Max coverage (-): 0.71

Region: NODE\_369549\_length\_1565\_cov\_30.364218 1067-1069. Max. coverage (+): 0. Max coverage (-): 0.69

Region: NODE\_369549\_length\_1565\_cov\_30.364218 1070-1072. Max. coverage (+): 0.4. Max coverage (-): 0.89

Region: NODE\_369549\_length\_1565\_cov\_30.364218 1073-1076. Max. coverage (+): 1.21. Max coverage (-): 0.85

Region: NODE\_369549\_length\_1565\_cov\_30.364218 1077-1079. Max. coverage (+): 1.17. Max coverage (-): 0

Region: NODE\_369549\_length\_1565\_cov\_30.364218 1080-1082. Max. coverage (+): 1.21. Max coverage (-): 0

Region: NODE\_369549\_length\_1565\_cov\_30.364218 1083-1085. Max. coverage (+): 0.93. Max coverage (-): 0

Region: NODE\_369549\_length\_1565\_cov\_30.364218 1086-1089. Max. coverage (+): 2.95. Max coverage (-): 0

Region: NODE\_369549\_length\_1565\_cov\_30.364218 1090-1092. Max. coverage (+): 0.36. Max coverage (-): 0

Region: NODE\_369549\_length\_1565\_cov\_30.364218 1093-1095. Max. coverage (+): 0.04. Max coverage (-): 0

Region: NODE\_369549\_length\_1565\_cov\_30.364218 1096-1098. Max. coverage (+): 0. Max coverage (-): 0.04

Region: NODE\_369549\_length\_1565\_cov\_30.364218 1099-1102. Max. coverage (+): 0. Max coverage (-): 1.01

Region: NODE\_369549\_length\_1565\_cov\_30.364218 1103-1105. Max. coverage (+): 0.2. Max coverage (-): 1.21

Region: NODE\_369549\_length\_1565\_cov\_30.364218 1106-1108. Max. coverage (+): 0.48. Max coverage (-): 0.85

Region: NODE\_369549\_length\_1565\_cov\_30.364218 1109-1111. Max. coverage (+): 0.44. Max coverage (-): 0.85

Region: NODE\_369549\_length\_1565\_cov\_30.364218 1112-1115. Max. coverage (+): 0.44. Max coverage (-): 0.57

Region: NODE\_369549\_length\_1565\_cov\_30.364218 1116-1118. Max. coverage (+): 1.13. Max coverage (-): 0.36

Region: NODE\_369549\_length\_1565\_cov\_30.364218 1119-1121. Max. coverage (+): 4.12. Max coverage (-): 0.04

Region: NODE\_369549\_length\_1565\_cov\_30.364218 1122-1124. Max. coverage (+): 7.79. Max coverage (-): 0.04

Region: NODE\_369549\_length\_1565\_cov\_30.364218 1125-1128. Max. coverage (+): 4.89. Max coverage (-): 0.04

Region: NODE\_369549\_length\_1565\_cov\_30.364218 1129-1131. Max. coverage (+): 2.87. Max coverage (-): 0.12

Region: NODE\_369549\_length\_1565\_cov\_30.364218 1132-1134. Max. coverage (+): 1.94. Max coverage (-): 0.32

Region: NODE\_369549\_length\_1565\_cov\_30.364218 1135-1137. Max. coverage (+): 0.36. Max coverage (-): 0.28

Region: NODE\_369549\_length\_1565\_cov\_30.364218 1138-1141. Max. coverage (+): 0.2. Max coverage (-): 0.16

Region: NODE\_369549\_length\_1565\_cov\_30.364218 1142-1144. Max. coverage (+): 0.16. Max coverage (-): 0

Region: NODE\_369549\_length\_1565\_cov\_30.364218 1145-1147. Max. coverage (+): 0.85. Max coverage (-): 0

Region: NODE\_369549\_length\_1565\_cov\_30.364218 1148-1150. Max. coverage (+): 0.89. Max coverage (-): 0.04

Region: NODE\_369549\_length\_1565\_cov\_30.364218 1151-1154. Max. coverage (+): 0.48. Max coverage (-): 0.04

Region: NODE\_369549\_length\_1565\_cov\_30.364218 1155-1157. Max. coverage (+): 0.28. Max coverage (-): 0.04

Region: NODE\_369549\_length\_1565\_cov\_30.364218 1158-1160. Max. coverage (+): 0.2. Max coverage (-): 0

Region: NODE\_369549\_length\_1565\_cov\_30.364218 1161-1163. Max. coverage (+): 0. Max coverage (-): 0

Region: NODE\_369549\_length\_1565\_cov\_30.364218 1164-1167. Max. coverage (+): 0. Max coverage (-): 0

Region: NODE\_369549\_length\_1565\_cov\_30.364218 1168-1170. Max. coverage (+): 0. Max coverage (-): 0.08

Region: NODE\_369549\_length\_1565\_cov\_30.364218 1171-1173. Max. coverage (+): 0.12. Max coverage (-): 0.08

Region: NODE\_369549\_length\_1565\_cov\_30.364218 1174-1176. Max. coverage (+): 0.13. Max coverage (-): 0.05

Region: NODE\_369549\_length\_1565\_cov\_30.364218 1177-1180. Max. coverage (+): 0.04. Max coverage (-): 0.04

Region: NODE\_369549\_length\_1565\_cov\_30.364218 1181-1183. Max. coverage (+): 4. Max coverage (-): 0

Region: NODE\_369549\_length\_1565\_cov\_30.364218 1184-1186. Max. coverage (+): 4.06. Max coverage (-): 0

Region: NODE\_369549\_length\_1565\_cov\_30.364218 1187-1189. Max. coverage (+): 0.2. Max coverage (-): 0

Region: NODE\_369549\_length\_1565\_cov\_30.364218 1190-1193. Max. coverage (+): 0.24. Max coverage (-): 0

Region: NODE\_369549\_length\_1565\_cov\_30.364218 1194-1196. Max. coverage (+): 0.1. Max coverage (-): 0

Region: NODE\_369549\_length\_1565\_cov\_30.364218 1197-1199. Max. coverage (+): 0.12. Max coverage (-): 0

Region: NODE\_369549\_length\_1565\_cov\_30.364218 1200-1202. Max. coverage (+): 0.04. Max coverage (-): 0

Region: NODE\_369549\_length\_1565\_cov\_30.364218 1203-1206. Max. coverage (+): 0. Max coverage (-): 0

Region: NODE\_369549\_length\_1565\_cov\_30.364218 1207-1209. Max. coverage (+): 0. Max coverage (-): 0

Region: NODE\_369549\_length\_1565\_cov\_30.364218 1210-1212. Max. coverage (+): 0.08. Max coverage (-): 0

Region: NODE\_369549\_length\_1565\_cov\_30.364218 1213-1215. Max. coverage (+): 0.36. Max coverage (-): 0

Region: NODE\_369549\_length\_1565\_cov\_30.364218 1216-1219. Max. coverage (+): 3.23. Max coverage (-): 0.04

Region: NODE\_369549\_length\_1565\_cov\_30.364218 1220-1222. Max. coverage (+): 10.98. Max coverage (-): 0.04

Region: NODE\_369549\_length\_1565\_cov\_30.364218 1223-1225. Max. coverage (+): 9.09. Max coverage (-): 0.04

Region: NODE\_369549\_length\_1565\_cov\_30.364218 1226-1228. Max. coverage (+): 0.4. Max coverage (-): 0.04

Region: NODE\_369549\_length\_1565\_cov\_30.364218 1229-1232. Max. coverage (+): 7.07. Max coverage (-): 0

Region: NODE\_369549\_length\_1565\_cov\_30.364218 1233-1235. Max. coverage (+): 10.74. Max coverage (-): 0.04

Region: NODE\_369549\_length\_1565\_cov\_30.364218 1236-1238. Max. coverage (+): 28.67. Max coverage (-): 0.04

Region: NODE\_369549\_length\_1565\_cov\_30.364218 1239-1241. Max. coverage (+): 24.75. Max coverage (-): 0.04

Region: NODE\_369549\_length\_1565\_cov\_30.364218 1242-1245. Max. coverage (+): 0.2. Max coverage (-): 0.04

Region: NODE\_369549\_length\_1565\_cov\_30.364218 1246-1248. Max. coverage (+): 0. Max coverage (-): 0.08

Region: NODE\_369549\_length\_1565\_cov\_30.364218 1249-1251. Max. coverage (+): 0. Max coverage (-): 0.28

Region: NODE\_369549\_length\_1565\_cov\_30.364218 1252-1254. Max. coverage (+): 0.08. Max coverage (-): 0.2

Region: NODE\_369549\_length\_1565\_cov\_30.364218 1255-1258. Max. coverage (+): 0.24. Max coverage (-): 0

Region: NODE\_369549\_length\_1565\_cov\_30.364218 1259-1261. Max. coverage (+): 0.55. Max coverage (-): 0

Region: NODE\_369549\_length\_1565\_cov\_30.364218 1262-1264. Max. coverage (+): 1.55. Max coverage (-): 0

Region: NODE\_369549\_length\_1565\_cov\_30.364218 1265-1267. Max. coverage (+): 1.39. Max coverage (-): 0

Region: NODE\_369549\_length\_1565\_cov\_30.364218 1268-1271. Max. coverage (+): 0.52. Max coverage (-): 0

Region: NODE\_369549\_length\_1565\_cov\_30.364218 1272-1274. Max. coverage (+): 0.34. Max coverage (-): 0.06

Region: NODE\_369549\_length\_1565\_cov\_30.364218 1275-1277. Max. coverage (+): 0.69. Max coverage (-): 0.12

Region: NODE\_369549\_length\_1565\_cov\_30.364218 1278-1280. Max. coverage (+): 1.01. Max coverage (-): 0.28

Region: NODE\_369549\_length\_1565\_cov\_30.364218 1281-1284. Max. coverage (+): 0.4. Max coverage (-): 0.97

Region: NODE\_369549\_length\_1565\_cov\_30.364218 1285-1287. Max. coverage (+): 0. Max coverage (-): 0.93

Region: NODE\_369549\_length\_1565\_cov\_30.364218 1288-1290. Max. coverage (+): 0. Max coverage (-): 0.61

Region: NODE\_369549\_length\_1565\_cov\_30.364218 1291-1293. Max. coverage (+): 0. Max coverage (-): 0.52

Region: NODE\_369549\_length\_1565\_cov\_30.364218 1294-1297. Max. coverage (+): 1.25. Max coverage (-): 0.04

Region: NODE\_369549\_length\_1565\_cov\_30.364218 1298-1300. Max. coverage (+): 6.02. Max coverage (-): 0

Region: NODE\_369549\_length\_1565\_cov\_30.364218 1301-1303. Max. coverage (+): 5.13. Max coverage (-): 0

Region: NODE\_369549\_length\_1565\_cov\_30.364218 1304-1306. Max. coverage (+): 3.67. Max coverage (-): 0

Region: NODE\_369549\_length\_1565\_cov\_30.364218 1307-1310. Max. coverage (+): 1.82. Max coverage (-): 0.12

Region: NODE\_369549\_length\_1565\_cov\_30.364218 1311-1313. Max. coverage (+): 0.24. Max coverage (-): 0.14

Region: NODE\_369549\_length\_1565\_cov\_30.364218 1314-1316. Max. coverage (+): 0.74. Max coverage (-): 0.03

Region: NODE\_369549\_length\_1565\_cov\_30.364218 1317-1319. Max. coverage (+): 1.48. Max coverage (-): 0.01

Region: NODE\_369549\_length\_1565\_cov\_30.364218 1320-1323. Max. coverage (+): 1.27. Max coverage (-): 0.16

Region: NODE\_369549\_length\_1565\_cov\_30.364218 1324-1326. Max. coverage (+): 0.97. Max coverage (-): 0.97

Region: NODE\_369549\_length\_1565\_cov\_30.364218 1327-1329. Max. coverage (+): 1.05. Max coverage (-): 1.05

Region: NODE\_369549\_length\_1565\_cov\_30.364218 1330-1332. Max. coverage (+): 1.09. Max coverage (-): 0.28

Region: NODE\_369549\_length\_1565\_cov\_30.364218 1333-1336. Max. coverage (+): 0.24. Max coverage (-): 0.2

Region: NODE\_369549\_length\_1565\_cov\_30.364218 1337-1339. Max. coverage (+): 0.2. Max coverage (-): 0.12

Region: NODE\_369549\_length\_1565\_cov\_30.364218 1340-1342. Max. coverage (+): 0.12. Max coverage (-): 0

Region: NODE\_369549\_length\_1565\_cov\_30.364218 1343-1345. Max. coverage (+): 0.12. Max coverage (-): 0

Region: NODE\_369549\_length\_1565\_cov\_30.364218 1346-1349. Max. coverage (+): 0.24. Max coverage (-): 0

Region: NODE\_369549\_length\_1565\_cov\_30.364218 1350-1352. Max. coverage (+): 0.08. Max coverage (-): 0

Region: NODE\_369549\_length\_1565\_cov\_30.364218 1353-1355. Max. coverage (+): 0.04. Max coverage (-): 0

Region: NODE\_369549\_length\_1565\_cov\_30.364218 1356-1358. Max. coverage (+): 0.04. Max coverage (-): 0

Region: NODE\_369549\_length\_1565\_cov\_30.364218 1359-1362. Max. coverage (+): 0. Max coverage (-): 0

Region: NODE\_369549\_length\_1565\_cov\_30.364218 1363-1365. Max. coverage (+): 0. Max coverage (-): 0

Region: NODE\_369549\_length\_1565\_cov\_30.364218 1366-1368. Max. coverage (+): 0. Max coverage (-): 0

Region: NODE\_369549\_length\_1565\_cov\_30.364218 1369-1371. Max. coverage (+): 0. Max coverage (-): 0

Region: NODE\_369549\_length\_1565\_cov\_30.364218 1372-1375. Max. coverage (+): 0.04. Max coverage (-): 0

Region: NODE\_369549\_length\_1565\_cov\_30.364218 1376-1378. Max. coverage (+): 0.04. Max coverage (-): 0

Region: NODE\_369549\_length\_1565\_cov\_30.364218 1379-1381. Max. coverage (+): 0. Max coverage (-): 0

Region: NODE\_369549\_length\_1565\_cov\_30.364218 1382-1384. Max. coverage (+): 0.12. Max coverage (-): 0

Region: NODE\_369549\_length\_1565\_cov\_30.364218 1385-1388. Max. coverage (+): 0.12. Max coverage (-): 0.04

Region: NODE\_369549\_length\_1565\_cov\_30.364218 1389-1391. Max. coverage (+): 0.04. Max coverage (-): 0.04

Region: NODE\_369549\_length\_1565\_cov\_30.364218 1392-1394. Max. coverage (+): 0. Max coverage (-): 0

Region: NODE\_369549\_length\_1565\_cov\_30.364218 1395-1397. Max. coverage (+): 0. Max coverage (-): 0

Region: NODE\_369549\_length\_1565\_cov\_30.364218 1398-1401. Max. coverage (+): 0.08. Max coverage (-): 0

Region: NODE\_369549\_length\_1565\_cov\_30.364218 1402-1404. Max. coverage (+): 1.17. Max coverage (-): 0

Region: NODE\_369549\_length\_1565\_cov\_30.364218 1405-1407. Max. coverage (+): 8.72. Max coverage (-): 0

Region: NODE\_369549\_length\_1565\_cov\_30.364218 1408-1410. Max. coverage (+): 7.79. Max coverage (-): 0

Region: NODE\_369549\_length\_1565\_cov\_30.364218 1411-1414. Max. coverage (+): 0.12. Max coverage (-): 0

Region: NODE\_369549\_length\_1565\_cov\_30.364218 1415-1417. Max. coverage (+): 0.16. Max coverage (-): 0

Region: NODE\_369549\_length\_1565\_cov\_30.364218 1418-1420. Max. coverage (+): 0.12. Max coverage (-): 0.04

Region: NODE\_369549\_length\_1565\_cov\_30.364218 1421-1423. Max. coverage (+): 0.16. Max coverage (-): 0.61

Region: NODE\_369549\_length\_1565\_cov\_30.364218 1424-1427. Max. coverage (+): 0.04. Max coverage (-): 5.57

Region: NODE\_369549\_length\_1565\_cov\_30.364218 1428-1430. Max. coverage (+): 0.04. Max coverage (-): 4.97

Region: NODE\_369549\_length\_1565\_cov\_30.364218 1431-1433. Max. coverage (+): 0.08. Max coverage (-): 1.33

Region: NODE\_369549\_length\_1565\_cov\_30.364218 1434-1436. Max. coverage (+): 0.04. Max coverage (-): 0.89

Region: NODE\_369549\_length\_1565\_cov\_30.364218 1437-1440. Max. coverage (+): 0. Max coverage (-): 0.65

Region: NODE\_369549\_length\_1565\_cov\_30.364218 1441-1443. Max. coverage (+): 0.44. Max coverage (-): 0

Region: NODE\_369549\_length\_1565\_cov\_30.364218 1444-1446. Max. coverage (+): 1.49. Max coverage (-): 0

Region: NODE\_369549\_length\_1565\_cov\_30.364218 1447-1449. Max. coverage (+): 7.67. Max coverage (-): 0

Region: NODE\_369549\_length\_1565\_cov\_30.364218 1450-1453. Max. coverage (+): 8.92. Max coverage (-): 0

Region: NODE\_369549\_length\_1565\_cov\_30.364218 1454-1456. Max. coverage (+): 5.81. Max coverage (-): 0

Region: NODE\_369549\_length\_1565\_cov\_30.364218 1457-1459. Max. coverage (+): 0. Max coverage (-): 0

Region: NODE\_369549\_length\_1565\_cov\_30.364218 1460-1462. Max. coverage (+): 0. Max coverage (-): 0

Region: NODE\_369549\_length\_1565\_cov\_30.364218 1463-1466. Max. coverage (+): 0. Max coverage (-): 0.08

Region: NODE\_369549\_length\_1565\_cov\_30.364218 1467-1469. Max. coverage (+): 0.04. Max coverage (-): 0.08

Region: NODE\_369549\_length\_1565\_cov\_30.364218 1470-1472. Max. coverage (+): 0.12. Max coverage (-): 0

Region: NODE\_369549\_length\_1565\_cov\_30.364218 1473-1475. Max. coverage (+): 0.12. Max coverage (-): 0.04

Region: NODE\_369549\_length\_1565\_cov\_30.364218 1476-1479. Max. coverage (+): 0.52. Max coverage (-): 0.08

Region: NODE\_369549\_length\_1565\_cov\_30.364218 1480-1482. Max. coverage (+): 0.85. Max coverage (-): 0.2

Region: NODE\_369549\_length\_1565\_cov\_30.364218 1483-1485. Max. coverage (+): 0.57. Max coverage (-): 0.16

Region: NODE\_369549\_length\_1565\_cov\_30.364218 1486-1488. Max. coverage (+): 0.08. Max coverage (-): 0.04

Region: NODE\_369549\_length\_1565\_cov\_30.364218 1489-1492. Max. coverage (+): 0. Max coverage (-): 0.04

Region: NODE\_369549\_length\_1565\_cov\_30.364218 1493-1495. Max. coverage (+): 0.08. Max coverage (-): 0.04

Region: NODE\_369549\_length\_1565\_cov\_30.364218 1496-1498. Max. coverage (+): 0.48. Max coverage (-): 0.77

Region: NODE\_369549\_length\_1565\_cov\_30.364218 1499-1501. Max. coverage (+): 1.17. Max coverage (-): 1.17

Region: NODE\_369549\_length\_1565\_cov\_30.364218 1502-1505. Max. coverage (+): 0.85. Max coverage (-): 0.4

Region: NODE\_369549\_length\_1565\_cov\_30.364218 1506-1508. Max. coverage (+): 14.17. Max coverage (-): 0.04

Region: NODE\_369549\_length\_1565\_cov\_30.364218 1509-1511. Max. coverage (+): 14.25. Max coverage (-): 0.04

Region: NODE\_369549\_length\_1565\_cov\_30.364218 1512-1514. Max. coverage (+): 3.15. Max coverage (-): 0.08

Region: NODE\_369549\_length\_1565\_cov\_30.364218 1515-1518. Max. coverage (+): 3.31. Max coverage (-): 0.12

Region: NODE\_369549\_length\_1565\_cov\_30.364218 1519-1521. Max. coverage (+): 0.16. Max coverage (-): 0.04

Region: NODE\_369549\_length\_1565\_cov\_30.364218 1522-1524. Max. coverage (+): 0. Max coverage (-): 0.04

Region: NODE\_369549\_length\_1565\_cov\_30.364218 1525-1527. Max. coverage (+): 0.2. Max coverage (-): 0.16

Region: NODE\_369549\_length\_1565\_cov\_30.364218 1528-1531. Max. coverage (+): 0.28. Max coverage (-): 0.2

Region: NODE\_369549\_length\_1565\_cov\_30.364218 1532-1534. Max. coverage (+): 0.38. Max coverage (-): 0.08

Region: NODE\_369549\_length\_1565\_cov\_30.364218 1535-1537. Max. coverage (+): 6.56. Max coverage (-): 0.12

Region: NODE\_369549\_length\_1565\_cov\_30.364218 1538-1540. Max. coverage (+): 9.17. Max coverage (-): 0.1

Region: NODE\_369549\_length\_1565\_cov\_30.364218 1541-1544. Max. coverage (+): 3.47. Max coverage (-): 0.16

Region: NODE\_369549\_length\_1565\_cov\_30.364218 1545-1547. Max. coverage (+): 1.25. Max coverage (-): 0.16

Region: NODE\_369549\_length\_1565\_cov\_30.364218 1548-1550. Max. coverage (+): 0.69. Max coverage (-): 0.12

Region: NODE\_369549\_length\_1565\_cov\_30.364218 1551-1553. Max. coverage (+): 0.32. Max coverage (-): 0.16

Region: NODE\_369549\_length\_1565\_cov\_30.364218 1554-1557. Max. coverage (+): 0. Max coverage (-): 0.24

Region: NODE\_369549\_length\_1565\_cov\_30.364218 1558-1560. Max. coverage (+): 0.01. Max coverage (-): 0.14

Region: NODE\_369549\_length\_1565\_cov\_30.364218 1561-1563. Max. coverage (+): 0.01. Max coverage (-): 0.14

Region: NODE\_369549\_length\_1565\_cov\_30.364218 1564-1566. Max. coverage (+): 0. Max coverage (-): 0.14

Region: NODE\_369549\_length\_1565\_cov\_30.364218 1567-1570. Max. coverage (+): 0.01. Max coverage (-): 0.04

Region: NODE\_369549\_length\_1565\_cov\_30.364218 1571-1573. Max. coverage (+): 0.08. Max coverage (-): 0

Region: NODE\_369549\_length\_1565\_cov\_30.364218 1574-1576. Max. coverage (+): 0.08. Max coverage (-): 0

Region: NODE\_369549\_length\_1565\_cov\_30.364218 1577-1579. Max. coverage (+): 0.01. Max coverage (-): 0

Region: NODE\_369549\_length\_1565\_cov\_30.364218 1580-1583. Max. coverage (+): 0.01. Max coverage (-): 0

Region: NODE\_369549\_length\_1565\_cov\_30.364218 1584-1586. Max. coverage (+): 0.01. Max coverage (-): 0.01

Region: NODE\_369549\_length\_1565\_cov\_30.364218 1587-1589. Max. coverage (+): 0. Max coverage (-): 0.01

Region: NODE\_369549\_length\_1565\_cov\_30.364218 1590-1592. Max. coverage (+): 0. Max coverage (-): 0.19

Region: NODE\_369549\_length\_1565\_cov\_30.364218 1593-1596. Max. coverage (+): 0. Max coverage (-): 1.12

Region: NODE\_369549\_length\_1565\_cov\_30.364218 1597-1599. Max. coverage (+): 0. Max coverage (-): 1.11

Region: NODE\_369549\_length\_1565\_cov\_30.364218 1600-1602. Max. coverage (+): 0. Max coverage (-): 0.03

Region: NODE\_369549\_length\_1565\_cov\_30.364218 1603-1605. Max. coverage (+): 0. Max coverage (-): 0.01

Region: NODE\_369549\_length\_1565\_cov\_30.364218 1606-1609. Max. coverage (+): 0. Max coverage (-): 0

Region: NODE\_369549\_length\_1565\_cov\_30.364218 1610-1612. Max. coverage (+): 0. Max coverage (-): 0

Region: NODE\_369549\_length\_1565\_cov\_30.364218 1613-1615. Max. coverage (+): 0. Max coverage (-): 0

Region: NODE\_369549\_length\_1565\_cov\_30.364218 1616-1618. Max. coverage (+): 0. Max coverage (-): 0

Region: NODE\_369549\_length\_1565\_cov\_30.364218 1619-1622. Max. coverage (+): 0. Max coverage (-): 0

Region: NODE\_369549\_length\_1565\_cov\_30.364218 1623-1625. Max. coverage (+): 0. Max coverage (-): 0

Region: NODE\_369549\_length\_1565\_cov\_30.364218 1626-. Max. coverage (+): 0. Max coverage (-): 0

RepeatMasker Color Code

**+**

100-98% Identity

<98-95% Identity

<95-90% Identity

<90-85% Identity

<85-80% Identity

<80-75% Identity

<75-70% Identity

<70% Identity

**-**

Gene Set Color Code

**+**

Gene

Pseudogene

Other

**-**

Topology/Coverage Color Code

Coverage Plus Strand

Coverage Minus Strand

Mainstrand: Plus

Mainstrand: Minus

Complementary Strand

Flanking Region  
(if option -flank >0)

Gene Set Annotation  
  
RepeatMasker Annotation  

**1. L1-16\_DR**: 163-1053 (-), Divergence to consensus: 38.1%  
**2. (A)n**: 1364-1388 (+), Divergence to consensus: 8.7%  
**3. AlRepD-382**: 1566-1629 (-), Divergence to consensus: 3.1%

  
Transcription Factor Binding Sites  

**RHOXF1** (Sequence: GGATCA (-): 637)  
**RHOXF1** (Sequence: AGATCA (-): 700)  
**RHOXF1** (Sequence: GGATCA (-): 814)  
**RHOXF1** (Sequence: AGATCA (-): 1510)  
**RHOXF1** (Sequence: TAATCT (+): 155)  
**RHOXF1** (Sequence: TGATCT (+): 190)  
**RHOXF1** (Sequence: TAATCT (+): 465)  
**RHOXF1** (Sequence: TAAGCC (+): 1033)  
**SOX9** (Sequence: AACAATGA (-): 1494)  
**SOX9** (Sequence: AACAATAG (-): 1497)  
**Sox5** (Sequence: ATTGTT (+): 573)  
**Sox5** (Sequence: ATTGTT (+): 1090)  
**Nobox** (Sequence: ACCAATTA (-): 663)  
**Sox5** (Sequence: AACAAT (-): 1494)
