# Supplementary material for: First characterization of PIWI-interacting RNA clusters in a cichlid fish with a B chromosome
Source: BMC Biol. 2022 Sep 21;20:204. doi: 10.1186/s12915-022-01403-2 (PMC9490952; doi:10.1186/s12915-022-01403-2)
Supplement: Supplementary file 1 — Additional file 1. Zipped folder with fasta and interactive html piRNA cluster information for the A. latifasciata genome. The nomenclature is as follows: number-pirna-cluster_sex_B-presence (f, female; m, male; 0b, without B chromosome; 1b, with B chromosome). [file 12915_2022_1403_MOESM1_ESM.zip › 142_m1b.html]

piRNA cluster 142\_m1b 83


Predicted piRNA cluster no. 142\_m1b
  

Show proTRAC run info
Hide proTRAC run info

/\  
                \_\_\_\_\_\_\_\_\_\_\_\_\_\_\_\_\_\_\_\_\_\_\_/\\_\_\_ /  \\_\_\_\_\_\_\_  
               I                      /  \  /    \      I  
               I     pro             /    \/      \     I  
               I        TRAC        /               \   I  
               I   \_\_\_\_\_\_\_\_\_\_\_\_\_\_\_\_/\_\_\_\_\_\_\_\_\_\_\_\_\_\_\_\_\_\\_ I  
               I   \              /                     I  
               I    \            /                      I  
               I     \  /\      /       V.2.4.2         I  
               I      \/  \    /                        I  
               I\_\_\_\_\_\_\_\_\_\_\_\  /\_\_\_\_\_\_\_\_\_\_\_\_\_\_\_\_\_\_\_\_\_\_\_\_\_I  
                            \/  
  
  
================================= proTRAC ====================================  
VERSION: .......... 2.4.2  
LAST MODIFIED: .... 11. May 2018  
  
Please cite:  
Rosenkranz D, Zischler H. proTRAC - a software for probabilistic piRNA cluster  
detection, visualization and analysis. 2012. BMC Bioinformatics 13:5.  
  
  
Contact:  
David Rosenkranz  
Institute of Organismic and Molecular Evolutionary Biology  
Dept. Anthropology, small RNA group  
Johannes Gutenberg University Mainz  
email: rosenkranz@uni-mainz.de  
  
You can find the latest proTRAC version at:  
http://sourceforge.net/projects/protrac/files  
http://www.smallRNAgroup-mainz.de/software  
==============================================================================  
  
PARAMETERS:  
Map file: ...............piwi-machos-1B.fa-collapse.map  
Genome file: ............../../../0B\_ala\_genome.fa  
RepeatMasker annotation: Alatifasciata-all0B-maryan-v2.fa\_corrected.out  
GeneSet:................./guest-storage/Data/annotation/Alatifasciata\_all0B\_maryan-v2\_out2017.gff  
  
Significant (p<=0.01) hit density will be calculated based  
on observed hit distribution.  
  
Sliding window size: ........................................ 5000 bp  
Sliding window increament: .................................. 1000 bp  
Normalize each hit by number of genomic hits: ............... yes  
Normalize each hit by number of sequence reads: ............. yes  
Normalize values (-> per million mapped reads): ............. yes  
Min. fraction of hits with 1T(U) or 10A: .................... 0.75  
Alternatively: Min. fraction of hits with 1T(U) and 10A: .... 0.5  
Min. fraction of hits with typical piRNA length: ............ 0.75  
Typical piRNA length: ....................................... 24-32 nt  
Min. size of a piRNA cluster: ............................... 1000 bp.  
Min. number of hits (absolute): ............................. 0  
Min. number of hits (normalized): ........................... 0  
Min. fraction of hits on the mainstrand: .................... 0.75  
Top fraction of mapped sequences (in terms of read counts): . 1%  
Top fraction accounts for max. n% of sequence reads: ........ 90%  
Min. fraction of hits on each arm of a bidirectional cluster: 0.05  
Output html file for each cluster: .......................... yes  
Output a summary table: ..................................... yes  
Output a FASTA file for each cluster (piRNA sequences): ..... yes  
Output a FASTA file comprising cluster sequences: ........... yes  
Output a GTF file for predicted piRNA clusters: ..............yes  
Search DNA motifs in clusters: .............................. yes  
Output flanking sequences: +/- .............................. 0 bp  
Output ~.pTi file: .......................................... no  
==============================================================================  
  
  
Genome size (without gaps): ............ 758543724 bp  
Gaps (N/X/-): .......................... 417479 bp  
Mapped reads: .......................... 26973943  
Non-identical sequences: ............... 6209225  
Genomic hits: .......................... 48438990  
Significant densitiy of mapped reads: .. 821.144211136946 reads/kb

Show proTRAC cluster info
Hide proTRAC cluster info

|  |  |
| --- | --- |
| Location | NODE\_369549\_length\_1565\_cov\_30.364218 |
| Coordinates | 1-1629 |
| Size [bp] | 1629 |
| Sequence hit loci | 3676 |
| Mapped reads (normalized) | 14337.2 |
| Mapped reads (normalized) per kb | 8801.2 |
| Normalized reads with 1T (1U) | 69.4% |
| Normalized reads with 10A | 50.7% |
| Normalized reads with length 24-32 nt | 98.4% |
| Normalized reads on the main strand(s) | 85.3% |
| Predicted directionality | mono:plus |

100%

0%

1T (1U)  
reads

10A reads

24-32 nt  
reads

reads on mainstrand

**Either the amount of reads with 1T (1U) OR 10A has to exceed 75% (set with option: -1Tor10A)  
Alternatively the amount of reads with 1T (1U) AND 10A has to exceed 50% (set with option: -1Tand10A)  
Minimum amount of reads with preferred size is 75% (set with option: -pisize)  
Minimum amount of reads on the main strand(s) is 75% (set with option: -clstrand)**

Show read coverage
Hide read coverage

WHAT DO I SEE HERE?  
This chart shows the location of mapped sequence reads within a predicted piRNA cluster. The color refers to the number of genomic hits produced by the sequence read in question. A dark red bar indicates that this sequence read produces many other hits elsewhere in the genome. Many adjacent red or yellow bars can indicate the presence of a multi-copy element such as transposons or rRNA genes. A dark green bar indicates that this sequence read maps uniquely to this locus.

1 hit

2-5 hits

6-10 hits

11-20 hits

21-50 hits

51-100 hits

> 100 hits

NODE\_369549\_length\_1565\_cov\_30.364218

1

1629

Gene Set

RepeatMasker

Mapped  
Reads

35.63

plus strand

minus strand

35.63

Region: NODE\_369549\_length\_1565\_cov\_30.364218 2656-2. Max. coverage (+): 0.01. Max coverage (-): 0

Region: NODE\_369549\_length\_1565\_cov\_30.364218 3-5. Max. coverage (+): 0.06. Max coverage (-): 0.01

Region: NODE\_369549\_length\_1565\_cov\_30.364218 6-9. Max. coverage (+): 0.05. Max coverage (-): 0.05

Region: NODE\_369549\_length\_1565\_cov\_30.364218 10-12. Max. coverage (+): 0.41. Max coverage (-): 0.11

Region: NODE\_369549\_length\_1565\_cov\_30.364218 13-15. Max. coverage (+): 0.06. Max coverage (-): 0.29

Region: NODE\_369549\_length\_1565\_cov\_30.364218 16-18. Max. coverage (+): 0.07. Max coverage (-): 0

Region: NODE\_369549\_length\_1565\_cov\_30.364218 19-22. Max. coverage (+): 0.56. Max coverage (-): 0.06

Region: NODE\_369549\_length\_1565\_cov\_30.364218 23-25. Max. coverage (+): 0.19. Max coverage (-): 0.04

Region: NODE\_369549\_length\_1565\_cov\_30.364218 26-28. Max. coverage (+): 0.04. Max coverage (-): 0

Region: NODE\_369549\_length\_1565\_cov\_30.364218 29-31. Max. coverage (+): 0.18. Max coverage (-): 0

Region: NODE\_369549\_length\_1565\_cov\_30.364218 32-35. Max. coverage (+): 0.39. Max coverage (-): 0.01

Region: NODE\_369549\_length\_1565\_cov\_30.364218 36-38. Max. coverage (+): 0.04. Max coverage (-): 0.37

Region: NODE\_369549\_length\_1565\_cov\_30.364218 39-41. Max. coverage (+): 0.27. Max coverage (-): 0.19

Region: NODE\_369549\_length\_1565\_cov\_30.364218 42-44. Max. coverage (+): 0.15. Max coverage (-): 0

Region: NODE\_369549\_length\_1565\_cov\_30.364218 45-48. Max. coverage (+): 0. Max coverage (-): 0

Region: NODE\_369549\_length\_1565\_cov\_30.364218 49-51. Max. coverage (+): 0.3. Max coverage (-): 0.22

Region: NODE\_369549\_length\_1565\_cov\_30.364218 52-54. Max. coverage (+): 0.04. Max coverage (-): 0.37

Region: NODE\_369549\_length\_1565\_cov\_30.364218 55-58. Max. coverage (+): 0.15. Max coverage (-): 0.3

Region: NODE\_369549\_length\_1565\_cov\_30.364218 59-61. Max. coverage (+): 0.3. Max coverage (-): 0.37

Region: NODE\_369549\_length\_1565\_cov\_30.364218 62-64. Max. coverage (+): 0.11. Max coverage (-): 0.07

Region: NODE\_369549\_length\_1565\_cov\_30.364218 65-67. Max. coverage (+): 0.07. Max coverage (-): 0.06

Region: NODE\_369549\_length\_1565\_cov\_30.364218 68-71. Max. coverage (+): 0.07. Max coverage (-): 0.15

Region: NODE\_369549\_length\_1565\_cov\_30.364218 72-74. Max. coverage (+): 0.01. Max coverage (-): 0.23

Region: NODE\_369549\_length\_1565\_cov\_30.364218 75-77. Max. coverage (+): 0.09. Max coverage (-): 0.21

Region: NODE\_369549\_length\_1565\_cov\_30.364218 78-80. Max. coverage (+): 0.01. Max coverage (-): 0.02

Region: NODE\_369549\_length\_1565\_cov\_30.364218 81-84. Max. coverage (+): 0.19. Max coverage (-): 0.01

Region: NODE\_369549\_length\_1565\_cov\_30.364218 85-87. Max. coverage (+): 0.73. Max coverage (-): 0.01

Region: NODE\_369549\_length\_1565\_cov\_30.364218 88-90. Max. coverage (+): 0.85. Max coverage (-): 0.02

Region: NODE\_369549\_length\_1565\_cov\_30.364218 91-93. Max. coverage (+): 0.06. Max coverage (-): 0

Region: NODE\_369549\_length\_1565\_cov\_30.364218 94-97. Max. coverage (+): 0.01. Max coverage (-): 0

Region: NODE\_369549\_length\_1565\_cov\_30.364218 98-100. Max. coverage (+): 0.04. Max coverage (-): 0.04

Region: NODE\_369549\_length\_1565\_cov\_30.364218 101-103. Max. coverage (+): 0. Max coverage (-): 0.04

Region: NODE\_369549\_length\_1565\_cov\_30.364218 104-106. Max. coverage (+): 0. Max coverage (-): 0.07

Region: NODE\_369549\_length\_1565\_cov\_30.364218 107-110. Max. coverage (+): 0.04. Max coverage (-): 0.48

Region: NODE\_369549\_length\_1565\_cov\_30.364218 111-113. Max. coverage (+): 0.33. Max coverage (-): 0.78

Region: NODE\_369549\_length\_1565\_cov\_30.364218 114-116. Max. coverage (+): 0.19. Max coverage (-): 0.48

Region: NODE\_369549\_length\_1565\_cov\_30.364218 117-119. Max. coverage (+): 0.56. Max coverage (-): 0.07

Region: NODE\_369549\_length\_1565\_cov\_30.364218 120-123. Max. coverage (+): 0.52. Max coverage (-): 0.04

Region: NODE\_369549\_length\_1565\_cov\_30.364218 124-126. Max. coverage (+): 0.07. Max coverage (-): 0

Region: NODE\_369549\_length\_1565\_cov\_30.364218 127-129. Max. coverage (+): 5.23. Max coverage (-): 0.07

Region: NODE\_369549\_length\_1565\_cov\_30.364218 130-132. Max. coverage (+): 5.08. Max coverage (-): 0.07

Region: NODE\_369549\_length\_1565\_cov\_30.364218 133-136. Max. coverage (+): 0.15. Max coverage (-): 0.04

Region: NODE\_369549\_length\_1565\_cov\_30.364218 137-139. Max. coverage (+): 0.04. Max coverage (-): 0.04

Region: NODE\_369549\_length\_1565\_cov\_30.364218 140-142. Max. coverage (+): 0.04. Max coverage (-): 0

Region: NODE\_369549\_length\_1565\_cov\_30.364218 143-145. Max. coverage (+): 0. Max coverage (-): 0.06

Region: NODE\_369549\_length\_1565\_cov\_30.364218 146-149. Max. coverage (+): 0. Max coverage (-): 0.31

Region: NODE\_369549\_length\_1565\_cov\_30.364218 150-152. Max. coverage (+): 0. Max coverage (-): 0.26

Region: NODE\_369549\_length\_1565\_cov\_30.364218 153-155. Max. coverage (+): 0. Max coverage (-): 0.05

Region: NODE\_369549\_length\_1565\_cov\_30.364218 156-159. Max. coverage (+): 0. Max coverage (-): 0

Region: NODE\_369549\_length\_1565\_cov\_30.364218 160-162. Max. coverage (+): 0.01. Max coverage (-): 0.28

Region: NODE\_369549\_length\_1565\_cov\_30.364218 163-165. Max. coverage (+): 0. Max coverage (-): 0.28

Region: NODE\_369549\_length\_1565\_cov\_30.364218 166-168. Max. coverage (+): 0.3. Max coverage (-): 0.04

Region: NODE\_369549\_length\_1565\_cov\_30.364218 169-172. Max. coverage (+): 0.26. Max coverage (-): 0

Region: NODE\_369549\_length\_1565\_cov\_30.364218 173-175. Max. coverage (+): 0.48. Max coverage (-): 0.04

Region: NODE\_369549\_length\_1565\_cov\_30.364218 176-178. Max. coverage (+): 0.15. Max coverage (-): 0.11

Region: NODE\_369549\_length\_1565\_cov\_30.364218 179-181. Max. coverage (+): 0.22. Max coverage (-): 0.11

Region: NODE\_369549\_length\_1565\_cov\_30.364218 182-185. Max. coverage (+): 0.63. Max coverage (-): 0.04

Region: NODE\_369549\_length\_1565\_cov\_30.364218 186-188. Max. coverage (+): 0.67. Max coverage (-): 0

Region: NODE\_369549\_length\_1565\_cov\_30.364218 189-191. Max. coverage (+): 1.74. Max coverage (-): 0

Region: NODE\_369549\_length\_1565\_cov\_30.364218 192-194. Max. coverage (+): 1.59. Max coverage (-): 0

Region: NODE\_369549\_length\_1565\_cov\_30.364218 195-198. Max. coverage (+): 0.15. Max coverage (-): 0

Region: NODE\_369549\_length\_1565\_cov\_30.364218 199-201. Max. coverage (+): 0. Max coverage (-): 0.04

Region: NODE\_369549\_length\_1565\_cov\_30.364218 202-204. Max. coverage (+): 0. Max coverage (-): 0

Region: NODE\_369549\_length\_1565\_cov\_30.364218 205-207. Max. coverage (+): 0.07. Max coverage (-): 0.04

Region: NODE\_369549\_length\_1565\_cov\_30.364218 208-211. Max. coverage (+): 0.07. Max coverage (-): 0.04

Region: NODE\_369549\_length\_1565\_cov\_30.364218 212-214. Max. coverage (+): 0. Max coverage (-): 0

Region: NODE\_369549\_length\_1565\_cov\_30.364218 215-217. Max. coverage (+): 0. Max coverage (-): 0

Region: NODE\_369549\_length\_1565\_cov\_30.364218 218-220. Max. coverage (+): 0. Max coverage (-): 0.02

Region: NODE\_369549\_length\_1565\_cov\_30.364218 221-224. Max. coverage (+): 0.34. Max coverage (-): 0.16

Region: NODE\_369549\_length\_1565\_cov\_30.364218 225-227. Max. coverage (+): 0.17. Max coverage (-): 0.16

Region: NODE\_369549\_length\_1565\_cov\_30.364218 228-230. Max. coverage (+): 0.19. Max coverage (-): 1.82

Region: NODE\_369549\_length\_1565\_cov\_30.364218 231-233. Max. coverage (+): 0.38. Max coverage (-): 2.04

Region: NODE\_369549\_length\_1565\_cov\_30.364218 234-237. Max. coverage (+): 0.34. Max coverage (-): 1.96

Region: NODE\_369549\_length\_1565\_cov\_30.364218 238-240. Max. coverage (+): 0.07. Max coverage (-): 0.52

Region: NODE\_369549\_length\_1565\_cov\_30.364218 241-243. Max. coverage (+): 0.37. Max coverage (-): 0.04

Region: NODE\_369549\_length\_1565\_cov\_30.364218 244-246. Max. coverage (+): 0.7. Max coverage (-): 0.07

Region: NODE\_369549\_length\_1565\_cov\_30.364218 247-250. Max. coverage (+): 0.96. Max coverage (-): 0.04

Region: NODE\_369549\_length\_1565\_cov\_30.364218 251-253. Max. coverage (+): 4.63. Max coverage (-): 0

Region: NODE\_369549\_length\_1565\_cov\_30.364218 254-256. Max. coverage (+): 3.74. Max coverage (-): 0.07

Region: NODE\_369549\_length\_1565\_cov\_30.364218 257-260. Max. coverage (+): 0.11. Max coverage (-): 0.04

Region: NODE\_369549\_length\_1565\_cov\_30.364218 261-263. Max. coverage (+): 0.15. Max coverage (-): 1.41

Region: NODE\_369549\_length\_1565\_cov\_30.364218 264-266. Max. coverage (+): 0.04. Max coverage (-): 1.33

Region: NODE\_369549\_length\_1565\_cov\_30.364218 267-269. Max. coverage (+): 0.02. Max coverage (-): 0.57

Region: NODE\_369549\_length\_1565\_cov\_30.364218 270-273. Max. coverage (+): 0.02. Max coverage (-): 0.39

Region: NODE\_369549\_length\_1565\_cov\_30.364218 274-276. Max. coverage (+): 0.06. Max coverage (-): 0.39

Region: NODE\_369549\_length\_1565\_cov\_30.364218 277-279. Max. coverage (+): 0.04. Max coverage (-): 0.07

Region: NODE\_369549\_length\_1565\_cov\_30.364218 280-282. Max. coverage (+): 0.15. Max coverage (-): 0.15

Region: NODE\_369549\_length\_1565\_cov\_30.364218 283-286. Max. coverage (+): 0.15. Max coverage (-): 0.11

Region: NODE\_369549\_length\_1565\_cov\_30.364218 287-289. Max. coverage (+): 0.04. Max coverage (-): 0.15

Region: NODE\_369549\_length\_1565\_cov\_30.364218 290-292. Max. coverage (+): 0.15. Max coverage (-): 0.44

Region: NODE\_369549\_length\_1565\_cov\_30.364218 293-295. Max. coverage (+): 1.65. Max coverage (-): 0.63

Region: NODE\_369549\_length\_1565\_cov\_30.364218 296-299. Max. coverage (+): 1.76. Max coverage (-): 0.67

Region: NODE\_369549\_length\_1565\_cov\_30.364218 300-302. Max. coverage (+): 0.82. Max coverage (-): 0.33

Region: NODE\_369549\_length\_1565\_cov\_30.364218 303-305. Max. coverage (+): 0.19. Max coverage (-): 0.22

Region: NODE\_369549\_length\_1565\_cov\_30.364218 306-308. Max. coverage (+): 0.23. Max coverage (-): 0.05

Region: NODE\_369549\_length\_1565\_cov\_30.364218 309-312. Max. coverage (+): 1.06. Max coverage (-): 0.02

Region: NODE\_369549\_length\_1565\_cov\_30.364218 313-315. Max. coverage (+): 0.68. Max coverage (-): 0.02

Region: NODE\_369549\_length\_1565\_cov\_30.364218 316-318. Max. coverage (+): 0.7. Max coverage (-): 0

Region: NODE\_369549\_length\_1565\_cov\_30.364218 319-321. Max. coverage (+): 4.91. Max coverage (-): 0

Region: NODE\_369549\_length\_1565\_cov\_30.364218 322-325. Max. coverage (+): 5.1. Max coverage (-): 0

Region: NODE\_369549\_length\_1565\_cov\_30.364218 326-328. Max. coverage (+): 0.63. Max coverage (-): 0

Region: NODE\_369549\_length\_1565\_cov\_30.364218 329-331. Max. coverage (+): 0.26. Max coverage (-): 0

Region: NODE\_369549\_length\_1565\_cov\_30.364218 332-334. Max. coverage (+): 0.52. Max coverage (-): 0.07

Region: NODE\_369549\_length\_1565\_cov\_30.364218 335-338. Max. coverage (+): 5.86. Max coverage (-): 0.07

Region: NODE\_369549\_length\_1565\_cov\_30.364218 339-341. Max. coverage (+): 0.56. Max coverage (-): 0.04

Region: NODE\_369549\_length\_1565\_cov\_30.364218 342-344. Max. coverage (+): 0.78. Max coverage (-): 0.04

Region: NODE\_369549\_length\_1565\_cov\_30.364218 345-347. Max. coverage (+): 0.52. Max coverage (-): 0.22

Region: NODE\_369549\_length\_1565\_cov\_30.364218 348-351. Max. coverage (+): 1.96. Max coverage (-): 1.33

Region: NODE\_369549\_length\_1565\_cov\_30.364218 352-354. Max. coverage (+): 0.85. Max coverage (-): 0.52

Region: NODE\_369549\_length\_1565\_cov\_30.364218 355-357. Max. coverage (+): 0.07. Max coverage (-): 2.89

Region: NODE\_369549\_length\_1565\_cov\_30.364218 358-361. Max. coverage (+): 0.11. Max coverage (-): 3.15

Region: NODE\_369549\_length\_1565\_cov\_30.364218 362-364. Max. coverage (+): 1.85. Max coverage (-): 0.04

Region: NODE\_369549\_length\_1565\_cov\_30.364218 365-367. Max. coverage (+): 1.85. Max coverage (-): 0

Region: NODE\_369549\_length\_1565\_cov\_30.364218 368-370. Max. coverage (+): 0.03. Max coverage (-): 0

Region: NODE\_369549\_length\_1565\_cov\_30.364218 371-374. Max. coverage (+): 0.19. Max coverage (-): 0

Region: NODE\_369549\_length\_1565\_cov\_30.364218 375-377. Max. coverage (+): 0.39. Max coverage (-): 0

Region: NODE\_369549\_length\_1565\_cov\_30.364218 378-380. Max. coverage (+): 0.07. Max coverage (-): 0.01

Region: NODE\_369549\_length\_1565\_cov\_30.364218 381-383. Max. coverage (+): 0.01. Max coverage (-): 0.01

Region: NODE\_369549\_length\_1565\_cov\_30.364218 384-387. Max. coverage (+): 0.01. Max coverage (-): 0.06

Region: NODE\_369549\_length\_1565\_cov\_30.364218 388-390. Max. coverage (+): 0.01. Max coverage (-): 0.59

Region: NODE\_369549\_length\_1565\_cov\_30.364218 391-393. Max. coverage (+): 0.04. Max coverage (-): 0.33

Region: NODE\_369549\_length\_1565\_cov\_30.364218 394-396. Max. coverage (+): 0.15. Max coverage (-): 0.49

Region: NODE\_369549\_length\_1565\_cov\_30.364218 397-400. Max. coverage (+): 3.63. Max coverage (-): 0

Region: NODE\_369549\_length\_1565\_cov\_30.364218 401-403. Max. coverage (+): 4.08. Max coverage (-): 0

Region: NODE\_369549\_length\_1565\_cov\_30.364218 404-406. Max. coverage (+): 2. Max coverage (-): 0.04

Region: NODE\_369549\_length\_1565\_cov\_30.364218 407-409. Max. coverage (+): 3.52. Max coverage (-): 0.07

Region: NODE\_369549\_length\_1565\_cov\_30.364218 410-413. Max. coverage (+): 3.52. Max coverage (-): 0.26

Region: NODE\_369549\_length\_1565\_cov\_30.364218 414-416. Max. coverage (+): 0.41. Max coverage (-): 0.37

Region: NODE\_369549\_length\_1565\_cov\_30.364218 417-419. Max. coverage (+): 0.59. Max coverage (-): 0.67

Region: NODE\_369549\_length\_1565\_cov\_30.364218 420-422. Max. coverage (+): 0.26. Max coverage (-): 1.37

Region: NODE\_369549\_length\_1565\_cov\_30.364218 423-426. Max. coverage (+): 0.07. Max coverage (-): 0.04

Region: NODE\_369549\_length\_1565\_cov\_30.364218 427-429. Max. coverage (+): 0.19. Max coverage (-): 0.11

Region: NODE\_369549\_length\_1565\_cov\_30.364218 430-432. Max. coverage (+): 0.19. Max coverage (-): 0.85

Region: NODE\_369549\_length\_1565\_cov\_30.364218 433-435. Max. coverage (+): 0.33. Max coverage (-): 0.67

Region: NODE\_369549\_length\_1565\_cov\_30.364218 436-439. Max. coverage (+): 0.33. Max coverage (-): 0.33

Region: NODE\_369549\_length\_1565\_cov\_30.364218 440-442. Max. coverage (+): 1.45. Max coverage (-): 0.26

Region: NODE\_369549\_length\_1565\_cov\_30.364218 443-445. Max. coverage (+): 1.02. Max coverage (-): 0.17

Region: NODE\_369549\_length\_1565\_cov\_30.364218 446-448. Max. coverage (+): 0.17. Max coverage (-): 0.15

Region: NODE\_369549\_length\_1565\_cov\_30.364218 449-452. Max. coverage (+): 0.07. Max coverage (-): 0.61

Region: NODE\_369549\_length\_1565\_cov\_30.364218 453-455. Max. coverage (+): 0.11. Max coverage (-): 0.56

Region: NODE\_369549\_length\_1565\_cov\_30.364218 456-458. Max. coverage (+): 0.12. Max coverage (-): 0.2

Region: NODE\_369549\_length\_1565\_cov\_30.364218 459-462. Max. coverage (+): 2.78. Max coverage (-): 0.19

Region: NODE\_369549\_length\_1565\_cov\_30.364218 463-465. Max. coverage (+): 2.74. Max coverage (-): 0

Region: NODE\_369549\_length\_1565\_cov\_30.364218 466-468. Max. coverage (+): 0.11. Max coverage (-): 0

Region: NODE\_369549\_length\_1565\_cov\_30.364218 469-471. Max. coverage (+): 0.22. Max coverage (-): 0

Region: NODE\_369549\_length\_1565\_cov\_30.364218 472-475. Max. coverage (+): 0.15. Max coverage (-): 0.26

Region: NODE\_369549\_length\_1565\_cov\_30.364218 476-478. Max. coverage (+): 0.07. Max coverage (-): 0.7

Region: NODE\_369549\_length\_1565\_cov\_30.364218 479-481. Max. coverage (+): 0. Max coverage (-): 0.22

Region: NODE\_369549\_length\_1565\_cov\_30.364218 482-484. Max. coverage (+): 0.07. Max coverage (-): 1

Region: NODE\_369549\_length\_1565\_cov\_30.364218 485-488. Max. coverage (+): 0.74. Max coverage (-): 1.08

Region: NODE\_369549\_length\_1565\_cov\_30.364218 489-491. Max. coverage (+): 2.93. Max coverage (-): 0.44

Region: NODE\_369549\_length\_1565\_cov\_30.364218 492-494. Max. coverage (+): 2.74. Max coverage (-): 0.04

Region: NODE\_369549\_length\_1565\_cov\_30.364218 495-497. Max. coverage (+): 0.07. Max coverage (-): 0.04

Region: NODE\_369549\_length\_1565\_cov\_30.364218 498-501. Max. coverage (+): 0.02. Max coverage (-): 0.11

Region: NODE\_369549\_length\_1565\_cov\_30.364218 502-504. Max. coverage (+): 1.11. Max coverage (-): 0.19

Region: NODE\_369549\_length\_1565\_cov\_30.364218 505-507. Max. coverage (+): 0.95. Max coverage (-): 0.26

Region: NODE\_369549\_length\_1565\_cov\_30.364218 508-510. Max. coverage (+): 0.59. Max coverage (-): 0.33

Region: NODE\_369549\_length\_1565\_cov\_30.364218 511-514. Max. coverage (+): 2.06. Max coverage (-): 0

Region: NODE\_369549\_length\_1565\_cov\_30.364218 515-517. Max. coverage (+): 0.33. Max coverage (-): 0

Region: NODE\_369549\_length\_1565\_cov\_30.364218 518-520. Max. coverage (+): 0. Max coverage (-): 0

Region: NODE\_369549\_length\_1565\_cov\_30.364218 521-523. Max. coverage (+): 0.01. Max coverage (-): 0

Region: NODE\_369549\_length\_1565\_cov\_30.364218 524-527. Max. coverage (+): 0.16. Max coverage (-): 0

Region: NODE\_369549\_length\_1565\_cov\_30.364218 528-530. Max. coverage (+): 0.56. Max coverage (-): 0.07

Region: NODE\_369549\_length\_1565\_cov\_30.364218 531-533. Max. coverage (+): 0.26. Max coverage (-): 0.04

Region: NODE\_369549\_length\_1565\_cov\_30.364218 534-536. Max. coverage (+): 0. Max coverage (-): 0.19

Region: NODE\_369549\_length\_1565\_cov\_30.364218 537-540. Max. coverage (+): 0. Max coverage (-): 0.26

Region: NODE\_369549\_length\_1565\_cov\_30.364218 541-543. Max. coverage (+): 0. Max coverage (-): 0.26

Region: NODE\_369549\_length\_1565\_cov\_30.364218 544-546. Max. coverage (+): 0.07. Max coverage (-): 0.07

Region: NODE\_369549\_length\_1565\_cov\_30.364218 547-549. Max. coverage (+): 0.11. Max coverage (-): 0.07

Region: NODE\_369549\_length\_1565\_cov\_30.364218 550-553. Max. coverage (+): 0.15. Max coverage (-): 0.04

Region: NODE\_369549\_length\_1565\_cov\_30.364218 554-556. Max. coverage (+): 0.96. Max coverage (-): 0

Region: NODE\_369549\_length\_1565\_cov\_30.364218 557-559. Max. coverage (+): 0.82. Max coverage (-): 0

Region: NODE\_369549\_length\_1565\_cov\_30.364218 560-563. Max. coverage (+): 0.33. Max coverage (-): 0.04

Region: NODE\_369549\_length\_1565\_cov\_30.364218 564-566. Max. coverage (+): 0.85. Max coverage (-): 0.06

Region: NODE\_369549\_length\_1565\_cov\_30.364218 567-569. Max. coverage (+): 2.85. Max coverage (-): 0.35

Region: NODE\_369549\_length\_1565\_cov\_30.364218 570-572. Max. coverage (+): 0.15. Max coverage (-): 0.2

Region: NODE\_369549\_length\_1565\_cov\_30.364218 573-576. Max. coverage (+): 0.33. Max coverage (-): 0.04

Region: NODE\_369549\_length\_1565\_cov\_30.364218 577-579. Max. coverage (+): 0.09. Max coverage (-): 0.02

Region: NODE\_369549\_length\_1565\_cov\_30.364218 580-582. Max. coverage (+): 0.33. Max coverage (-): 0.02

Region: NODE\_369549\_length\_1565\_cov\_30.364218 583-585. Max. coverage (+): 3.48. Max coverage (-): 0

Region: NODE\_369549\_length\_1565\_cov\_30.364218 586-589. Max. coverage (+): 3.39. Max coverage (-): 0

Region: NODE\_369549\_length\_1565\_cov\_30.364218 590-592. Max. coverage (+): 0.15. Max coverage (-): 0

Region: NODE\_369549\_length\_1565\_cov\_30.364218 593-595. Max. coverage (+): 0.15. Max coverage (-): 0.02

Region: NODE\_369549\_length\_1565\_cov\_30.364218 596-598. Max. coverage (+): 0.13. Max coverage (-): 0.04

Region: NODE\_369549\_length\_1565\_cov\_30.364218 599-602. Max. coverage (+): 0.11. Max coverage (-): 0.07

Region: NODE\_369549\_length\_1565\_cov\_30.364218 603-605. Max. coverage (+): 0.04. Max coverage (-): 0.07

Region: NODE\_369549\_length\_1565\_cov\_30.364218 606-608. Max. coverage (+): 0.04. Max coverage (-): 0.19

Region: NODE\_369549\_length\_1565\_cov\_30.364218 609-611. Max. coverage (+): 0.26. Max coverage (-): 0

Region: NODE\_369549\_length\_1565\_cov\_30.364218 612-615. Max. coverage (+): 24.65. Max coverage (-): 0.04

Region: NODE\_369549\_length\_1565\_cov\_30.364218 616-618. Max. coverage (+): 20.72. Max coverage (-): 0.11

Region: NODE\_369549\_length\_1565\_cov\_30.364218 619-621. Max. coverage (+): 0.04. Max coverage (-): 0.11

Region: NODE\_369549\_length\_1565\_cov\_30.364218 622-624. Max. coverage (+): 0.04. Max coverage (-): 0

Region: NODE\_369549\_length\_1565\_cov\_30.364218 625-628. Max. coverage (+): 0.04. Max coverage (-): 0.04

Region: NODE\_369549\_length\_1565\_cov\_30.364218 629-631. Max. coverage (+): 0. Max coverage (-): 0.04

Region: NODE\_369549\_length\_1565\_cov\_30.364218 632-634. Max. coverage (+): 0.28. Max coverage (-): 0.06

Region: NODE\_369549\_length\_1565\_cov\_30.364218 635-637. Max. coverage (+): 0.3. Max coverage (-): 0

Region: NODE\_369549\_length\_1565\_cov\_30.364218 638-641. Max. coverage (+): 0. Max coverage (-): 0

Region: NODE\_369549\_length\_1565\_cov\_30.364218 642-644. Max. coverage (+): 0. Max coverage (-): 0

Region: NODE\_369549\_length\_1565\_cov\_30.364218 645-647. Max. coverage (+): 0. Max coverage (-): 0

Region: NODE\_369549\_length\_1565\_cov\_30.364218 648-650. Max. coverage (+): 0. Max coverage (-): 0

Region: NODE\_369549\_length\_1565\_cov\_30.364218 651-654. Max. coverage (+): 0. Max coverage (-): 0.15

Region: NODE\_369549\_length\_1565\_cov\_30.364218 655-657. Max. coverage (+): 0. Max coverage (-): 0.11

Region: NODE\_369549\_length\_1565\_cov\_30.364218 658-660. Max. coverage (+): 0.07. Max coverage (-): 0.07

Region: NODE\_369549\_length\_1565\_cov\_30.364218 661-664. Max. coverage (+): 0.15. Max coverage (-): 0.11

Region: NODE\_369549\_length\_1565\_cov\_30.364218 665-667. Max. coverage (+): 0. Max coverage (-): 0

Region: NODE\_369549\_length\_1565\_cov\_30.364218 668-670. Max. coverage (+): 0.15. Max coverage (-): 0

Region: NODE\_369549\_length\_1565\_cov\_30.364218 671-673. Max. coverage (+): 0.15. Max coverage (-): 0

Region: NODE\_369549\_length\_1565\_cov\_30.364218 674-677. Max. coverage (+): 0.33. Max coverage (-): 0.04

Region: NODE\_369549\_length\_1565\_cov\_30.364218 678-680. Max. coverage (+): 0.04. Max coverage (-): 0.26

Region: NODE\_369549\_length\_1565\_cov\_30.364218 681-683. Max. coverage (+): 0. Max coverage (-): 0.3

Region: NODE\_369549\_length\_1565\_cov\_30.364218 684-686. Max. coverage (+): 0. Max coverage (-): 0.15

Region: NODE\_369549\_length\_1565\_cov\_30.364218 687-690. Max. coverage (+): 0.04. Max coverage (-): 0.19

Region: NODE\_369549\_length\_1565\_cov\_30.364218 691-693. Max. coverage (+): 0. Max coverage (-): 0.15

Region: NODE\_369549\_length\_1565\_cov\_30.364218 694-696. Max. coverage (+): 0.04. Max coverage (-): 0.15

Region: NODE\_369549\_length\_1565\_cov\_30.364218 697-699. Max. coverage (+): 1.3. Max coverage (-): 0.07

Region: NODE\_369549\_length\_1565\_cov\_30.364218 700-703. Max. coverage (+): 1.37. Max coverage (-): 0.22

Region: NODE\_369549\_length\_1565\_cov\_30.364218 704-706. Max. coverage (+): 0.15. Max coverage (-): 0.01

Region: NODE\_369549\_length\_1565\_cov\_30.364218 707-709. Max. coverage (+): 0.2. Max coverage (-): 0.02

Region: NODE\_369549\_length\_1565\_cov\_30.364218 710-712. Max. coverage (+): 0.51. Max coverage (-): 0.02

Region: NODE\_369549\_length\_1565\_cov\_30.364218 713-716. Max. coverage (+): 0.11. Max coverage (-): 0.01

Region: NODE\_369549\_length\_1565\_cov\_30.364218 717-719. Max. coverage (+): 0.21. Max coverage (-): 0.01

Region: NODE\_369549\_length\_1565\_cov\_30.364218 720-722. Max. coverage (+): 0.15. Max coverage (-): 0.07

Region: NODE\_369549\_length\_1565\_cov\_30.364218 723-725. Max. coverage (+): 0.06. Max coverage (-): 0.07

Region: NODE\_369549\_length\_1565\_cov\_30.364218 726-729. Max. coverage (+): 0.04. Max coverage (-): 0.09

Region: NODE\_369549\_length\_1565\_cov\_30.364218 730-732. Max. coverage (+): 0.03. Max coverage (-): 0.01

Region: NODE\_369549\_length\_1565\_cov\_30.364218 733-735. Max. coverage (+): 0.01. Max coverage (-): 0.02

Region: NODE\_369549\_length\_1565\_cov\_30.364218 736-738. Max. coverage (+): 0.01. Max coverage (-): 0.02

Region: NODE\_369549\_length\_1565\_cov\_30.364218 739-742. Max. coverage (+): 0.28. Max coverage (-): 0

Region: NODE\_369549\_length\_1565\_cov\_30.364218 743-745. Max. coverage (+): 0.11. Max coverage (-): 0.04

Region: NODE\_369549\_length\_1565\_cov\_30.364218 746-748. Max. coverage (+): 0.04. Max coverage (-): 0.15

Region: NODE\_369549\_length\_1565\_cov\_30.364218 749-751. Max. coverage (+): 0.07. Max coverage (-): 0.04

Region: NODE\_369549\_length\_1565\_cov\_30.364218 752-755. Max. coverage (+): 3.71. Max coverage (-): 0

Region: NODE\_369549\_length\_1565\_cov\_30.364218 756-758. Max. coverage (+): 1.82. Max coverage (-): 0.3

Region: NODE\_369549\_length\_1565\_cov\_30.364218 759-761. Max. coverage (+): 0.56. Max coverage (-): 0.3

Region: NODE\_369549\_length\_1565\_cov\_30.364218 762-765. Max. coverage (+): 1.93. Max coverage (-): 0.04

Region: NODE\_369549\_length\_1565\_cov\_30.364218 766-768. Max. coverage (+): 0.18. Max coverage (-): 0.06

Region: NODE\_369549\_length\_1565\_cov\_30.364218 769-771. Max. coverage (+): 1.75. Max coverage (-): 0

Region: NODE\_369549\_length\_1565\_cov\_30.364218 772-774. Max. coverage (+): 2.98. Max coverage (-): 0.01

Region: NODE\_369549\_length\_1565\_cov\_30.364218 775-778. Max. coverage (+): 2.06. Max coverage (-): 0.01

Region: NODE\_369549\_length\_1565\_cov\_30.364218 779-781. Max. coverage (+): 4.06. Max coverage (-): 0.02

Region: NODE\_369549\_length\_1565\_cov\_30.364218 782-784. Max. coverage (+): 2.36. Max coverage (-): 0.02

Region: NODE\_369549\_length\_1565\_cov\_30.364218 785-787. Max. coverage (+): 0.3. Max coverage (-): 0.01

Region: NODE\_369549\_length\_1565\_cov\_30.364218 788-791. Max. coverage (+): 0.48. Max coverage (-): 0

Region: NODE\_369549\_length\_1565\_cov\_30.364218 792-794. Max. coverage (+): 0.11. Max coverage (-): 0

Region: NODE\_369549\_length\_1565\_cov\_30.364218 795-797. Max. coverage (+): 0.15. Max coverage (-): 0.04

Region: NODE\_369549\_length\_1565\_cov\_30.364218 798-800. Max. coverage (+): 2.41. Max coverage (-): 0.22

Region: NODE\_369549\_length\_1565\_cov\_30.364218 801-804. Max. coverage (+): 2.74. Max coverage (-): 0.41

Region: NODE\_369549\_length\_1565\_cov\_30.364218 805-807. Max. coverage (+): 0.44. Max coverage (-): 0.11

Region: NODE\_369549\_length\_1565\_cov\_30.364218 808-810. Max. coverage (+): 0.28. Max coverage (-): 0.07

Region: NODE\_369549\_length\_1565\_cov\_30.364218 811-813. Max. coverage (+): 0.02. Max coverage (-): 0

Region: NODE\_369549\_length\_1565\_cov\_30.364218 814-817. Max. coverage (+): 19.35. Max coverage (-): 0.11

Region: NODE\_369549\_length\_1565\_cov\_30.364218 818-820. Max. coverage (+): 19.88. Max coverage (-): 0.09

Region: NODE\_369549\_length\_1565\_cov\_30.364218 821-823. Max. coverage (+): 1.98. Max coverage (-): 0.01

Region: NODE\_369549\_length\_1565\_cov\_30.364218 824-826. Max. coverage (+): 0.1. Max coverage (-): 0.02

Region: NODE\_369549\_length\_1565\_cov\_30.364218 827-830. Max. coverage (+): 0.04. Max coverage (-): 0.08

Region: NODE\_369549\_length\_1565\_cov\_30.364218 831-833. Max. coverage (+): 0. Max coverage (-): 0.35

Region: NODE\_369549\_length\_1565\_cov\_30.364218 834-836. Max. coverage (+): 0. Max coverage (-): 0.12

Region: NODE\_369549\_length\_1565\_cov\_30.364218 837-839. Max. coverage (+): 0. Max coverage (-): 0.06

Region: NODE\_369549\_length\_1565\_cov\_30.364218 840-843. Max. coverage (+): 0. Max coverage (-): 0.24

Region: NODE\_369549\_length\_1565\_cov\_30.364218 844-846. Max. coverage (+): 0. Max coverage (-): 0.35

Region: NODE\_369549\_length\_1565\_cov\_30.364218 847-849. Max. coverage (+): 0. Max coverage (-): 0.22

Region: NODE\_369549\_length\_1565\_cov\_30.364218 850-852. Max. coverage (+): 2.22. Max coverage (-): 0.02

Region: NODE\_369549\_length\_1565\_cov\_30.364218 853-856. Max. coverage (+): 5.65. Max coverage (-): 0

Region: NODE\_369549\_length\_1565\_cov\_30.364218 857-859. Max. coverage (+): 9.77. Max coverage (-): 0.02

Region: NODE\_369549\_length\_1565\_cov\_30.364218 860-862. Max. coverage (+): 23.41. Max coverage (-): 0.03

Region: NODE\_369549\_length\_1565\_cov\_30.364218 863-865. Max. coverage (+): 0.32. Max coverage (-): 0.03

Region: NODE\_369549\_length\_1565\_cov\_30.364218 866-869. Max. coverage (+): 0.11. Max coverage (-): 0

Region: NODE\_369549\_length\_1565\_cov\_30.364218 870-872. Max. coverage (+): 0. Max coverage (-): 0

Region: NODE\_369549\_length\_1565\_cov\_30.364218 873-875. Max. coverage (+): 0. Max coverage (-): 0.12

Region: NODE\_369549\_length\_1565\_cov\_30.364218 876-879. Max. coverage (+): 0.07. Max coverage (-): 0.12

Region: NODE\_369549\_length\_1565\_cov\_30.364218 880-882. Max. coverage (+): 0.15. Max coverage (-): 0.67

Region: NODE\_369549\_length\_1565\_cov\_30.364218 883-885. Max. coverage (+): 0.63. Max coverage (-): 0.3

Region: NODE\_369549\_length\_1565\_cov\_30.364218 886-888. Max. coverage (+): 2.56. Max coverage (-): 0.04

Region: NODE\_369549\_length\_1565\_cov\_30.364218 889-892. Max. coverage (+): 3.19. Max coverage (-): 0

Region: NODE\_369549\_length\_1565\_cov\_30.364218 893-895. Max. coverage (+): 0.19. Max coverage (-): 0

Region: NODE\_369549\_length\_1565\_cov\_30.364218 896-898. Max. coverage (+): 0.85. Max coverage (-): 0

Region: NODE\_369549\_length\_1565\_cov\_30.364218 899-901. Max. coverage (+): 1.78. Max coverage (-): 0

Region: NODE\_369549\_length\_1565\_cov\_30.364218 902-905. Max. coverage (+): 1.74. Max coverage (-): 0.01

Region: NODE\_369549\_length\_1565\_cov\_30.364218 906-908. Max. coverage (+): 0.21. Max coverage (-): 0.05

Region: NODE\_369549\_length\_1565\_cov\_30.364218 909-911. Max. coverage (+): 0.16. Max coverage (-): 0.04

Region: NODE\_369549\_length\_1565\_cov\_30.364218 912-914. Max. coverage (+): 0.26. Max coverage (-): 0.04

Region: NODE\_369549\_length\_1565\_cov\_30.364218 915-918. Max. coverage (+): 0.7. Max coverage (-): 0.15

Region: NODE\_369549\_length\_1565\_cov\_30.364218 919-921. Max. coverage (+): 0.22. Max coverage (-): 0.04

Region: NODE\_369549\_length\_1565\_cov\_30.364218 922-924. Max. coverage (+): 4.93. Max coverage (-): 0

Region: NODE\_369549\_length\_1565\_cov\_30.364218 925-927. Max. coverage (+): 0.37. Max coverage (-): 0

Region: NODE\_369549\_length\_1565\_cov\_30.364218 928-931. Max. coverage (+): 0.3. Max coverage (-): 0.19

Region: NODE\_369549\_length\_1565\_cov\_30.364218 932-934. Max. coverage (+): 0.22. Max coverage (-): 0.11

Region: NODE\_369549\_length\_1565\_cov\_30.364218 935-937. Max. coverage (+): 0. Max coverage (-): 0

Region: NODE\_369549\_length\_1565\_cov\_30.364218 938-940. Max. coverage (+): 0.06. Max coverage (-): 0.14

Region: NODE\_369549\_length\_1565\_cov\_30.364218 941-944. Max. coverage (+): 0.36. Max coverage (-): 0.06

Region: NODE\_369549\_length\_1565\_cov\_30.364218 945-947. Max. coverage (+): 0.36. Max coverage (-): 0

Region: NODE\_369549\_length\_1565\_cov\_30.364218 948-950. Max. coverage (+): 0.12. Max coverage (-): 0.01

Region: NODE\_369549\_length\_1565\_cov\_30.364218 951-953. Max. coverage (+): 0.26. Max coverage (-): 0.01

Region: NODE\_369549\_length\_1565\_cov\_30.364218 954-957. Max. coverage (+): 8.34. Max coverage (-): 0

Region: NODE\_369549\_length\_1565\_cov\_30.364218 958-960. Max. coverage (+): 9.79. Max coverage (-): 0.04

Region: NODE\_369549\_length\_1565\_cov\_30.364218 961-963. Max. coverage (+): 0.26. Max coverage (-): 0.04

Region: NODE\_369549\_length\_1565\_cov\_30.364218 964-966. Max. coverage (+): 0.41. Max coverage (-): 0

Region: NODE\_369549\_length\_1565\_cov\_30.364218 967-970. Max. coverage (+): 0.04. Max coverage (-): 0

Region: NODE\_369549\_length\_1565\_cov\_30.364218 971-973. Max. coverage (+): 0. Max coverage (-): 0

Region: NODE\_369549\_length\_1565\_cov\_30.364218 974-976. Max. coverage (+): 0.3. Max coverage (-): 0

Region: NODE\_369549\_length\_1565\_cov\_30.364218 977-980. Max. coverage (+): 2.3. Max coverage (-): 0.3

Region: NODE\_369549\_length\_1565\_cov\_30.364218 981-983. Max. coverage (+): 0.15. Max coverage (-): 0.3

Region: NODE\_369549\_length\_1565\_cov\_30.364218 984-986. Max. coverage (+): 0. Max coverage (-): 0.11

Region: NODE\_369549\_length\_1565\_cov\_30.364218 987-989. Max. coverage (+): 0.21. Max coverage (-): 0.12

Region: NODE\_369549\_length\_1565\_cov\_30.364218 990-993. Max. coverage (+): 0.11. Max coverage (-): 0.1

Region: NODE\_369549\_length\_1565\_cov\_30.364218 994-996. Max. coverage (+): 0.11. Max coverage (-): 0.01

Region: NODE\_369549\_length\_1565\_cov\_30.364218 997-999. Max. coverage (+): 0.36. Max coverage (-): 0.01

Region: NODE\_369549\_length\_1565\_cov\_30.364218 1000-1002. Max. coverage (+): 0.52. Max coverage (-): 0.01

Region: NODE\_369549\_length\_1565\_cov\_30.364218 1003-1006. Max. coverage (+): 0.69. Max coverage (-): 0.06

Region: NODE\_369549\_length\_1565\_cov\_30.364218 1007-1009. Max. coverage (+): 0.74. Max coverage (-): 0.07

Region: NODE\_369549\_length\_1565\_cov\_30.364218 1010-1012. Max. coverage (+): 3.03. Max coverage (-): 0.05

Region: NODE\_369549\_length\_1565\_cov\_30.364218 1013-1015. Max. coverage (+): 3.07. Max coverage (-): 0.04

Region: NODE\_369549\_length\_1565\_cov\_30.364218 1016-1019. Max. coverage (+): 0.88. Max coverage (-): 0.01

Region: NODE\_369549\_length\_1565\_cov\_30.364218 1020-1022. Max. coverage (+): 1.08. Max coverage (-): 4.12

Region: NODE\_369549\_length\_1565\_cov\_30.364218 1023-1025. Max. coverage (+): 0.3. Max coverage (-): 19.13

Region: NODE\_369549\_length\_1565\_cov\_30.364218 1026-1028. Max. coverage (+): 0.04. Max coverage (-): 15.42

Region: NODE\_369549\_length\_1565\_cov\_30.364218 1029-1032. Max. coverage (+): 0.59. Max coverage (-): 1.08

Region: NODE\_369549\_length\_1565\_cov\_30.364218 1033-1035. Max. coverage (+): 6.08. Max coverage (-): 0.19

Region: NODE\_369549\_length\_1565\_cov\_30.364218 1036-1038. Max. coverage (+): 9.05. Max coverage (-): 0.22

Region: NODE\_369549\_length\_1565\_cov\_30.364218 1039-1041. Max. coverage (+): 34.77. Max coverage (-): 0.07

Region: NODE\_369549\_length\_1565\_cov\_30.364218 1042-1045. Max. coverage (+): 34.18. Max coverage (-): 0

Region: NODE\_369549\_length\_1565\_cov\_30.364218 1046-1048. Max. coverage (+): 35.63. Max coverage (-): 0

Region: NODE\_369549\_length\_1565\_cov\_30.364218 1049-1051. Max. coverage (+): 34.7. Max coverage (-): 0.07

Region: NODE\_369549\_length\_1565\_cov\_30.364218 1052-1054. Max. coverage (+): 1.08. Max coverage (-): 0.19

Region: NODE\_369549\_length\_1565\_cov\_30.364218 1055-1058. Max. coverage (+): 0.56. Max coverage (-): 0.41

Region: NODE\_369549\_length\_1565\_cov\_30.364218 1059-1061. Max. coverage (+): 0.15. Max coverage (-): 2.24

Region: NODE\_369549\_length\_1565\_cov\_30.364218 1062-1064. Max. coverage (+): 0.56. Max coverage (-): 1.98

Region: NODE\_369549\_length\_1565\_cov\_30.364218 1065-1067. Max. coverage (+): 0.56. Max coverage (-): 0.56

Region: NODE\_369549\_length\_1565\_cov\_30.364218 1068-1071. Max. coverage (+): 0.04. Max coverage (-): 0.67

Region: NODE\_369549\_length\_1565\_cov\_30.364218 1072-1074. Max. coverage (+): 0.41. Max coverage (-): 0.82

Region: NODE\_369549\_length\_1565\_cov\_30.364218 1075-1077. Max. coverage (+): 1.82. Max coverage (-): 0.19

Region: NODE\_369549\_length\_1565\_cov\_30.364218 1078-1081. Max. coverage (+): 2.26. Max coverage (-): 0.04

Region: NODE\_369549\_length\_1565\_cov\_30.364218 1082-1084. Max. coverage (+): 0.85. Max coverage (-): 0.04

Region: NODE\_369549\_length\_1565\_cov\_30.364218 1085-1087. Max. coverage (+): 4.41. Max coverage (-): 0

Region: NODE\_369549\_length\_1565\_cov\_30.364218 1088-1090. Max. coverage (+): 4.37. Max coverage (-): 0

Region: NODE\_369549\_length\_1565\_cov\_30.364218 1091-1094. Max. coverage (+): 0.26. Max coverage (-): 0

Region: NODE\_369549\_length\_1565\_cov\_30.364218 1095-1097. Max. coverage (+): 0. Max coverage (-): 0.11

Region: NODE\_369549\_length\_1565\_cov\_30.364218 1098-1100. Max. coverage (+): 0. Max coverage (-): 0.11

Region: NODE\_369549\_length\_1565\_cov\_30.364218 1101-1103. Max. coverage (+): 0. Max coverage (-): 0.3

Region: NODE\_369549\_length\_1565\_cov\_30.364218 1104-1107. Max. coverage (+): 0.56. Max coverage (-): 0.59

Region: NODE\_369549\_length\_1565\_cov\_30.364218 1108-1110. Max. coverage (+): 0.44. Max coverage (-): 0.89

Region: NODE\_369549\_length\_1565\_cov\_30.364218 1111-1113. Max. coverage (+): 0.33. Max coverage (-): 0.41

Region: NODE\_369549\_length\_1565\_cov\_30.364218 1114-1116. Max. coverage (+): 0.26. Max coverage (-): 0.48

Region: NODE\_369549\_length\_1565\_cov\_30.364218 1117-1120. Max. coverage (+): 2.93. Max coverage (-): 0.07

Region: NODE\_369549\_length\_1565\_cov\_30.364218 1121-1123. Max. coverage (+): 7.12. Max coverage (-): 0

Region: NODE\_369549\_length\_1565\_cov\_30.364218 1124-1126. Max. coverage (+): 4.82. Max coverage (-): 0

Region: NODE\_369549\_length\_1565\_cov\_30.364218 1127-1129. Max. coverage (+): 6.52. Max coverage (-): 0

Region: NODE\_369549\_length\_1565\_cov\_30.364218 1130-1133. Max. coverage (+): 6.52. Max coverage (-): 0.22

Region: NODE\_369549\_length\_1565\_cov\_30.364218 1134-1136. Max. coverage (+): 0.19. Max coverage (-): 0.15

Region: NODE\_369549\_length\_1565\_cov\_30.364218 1137-1139. Max. coverage (+): 0.22. Max coverage (-): 0.15

Region: NODE\_369549\_length\_1565\_cov\_30.364218 1140-1142. Max. coverage (+): 0.04. Max coverage (-): 0.04

Region: NODE\_369549\_length\_1565\_cov\_30.364218 1143-1146. Max. coverage (+): 0.3. Max coverage (-): 0

Region: NODE\_369549\_length\_1565\_cov\_30.364218 1147-1149. Max. coverage (+): 0.59. Max coverage (-): 0

Region: NODE\_369549\_length\_1565\_cov\_30.364218 1150-1152. Max. coverage (+): 0.59. Max coverage (-): 0.04

Region: NODE\_369549\_length\_1565\_cov\_30.364218 1153-1155. Max. coverage (+): 0.3. Max coverage (-): 0.04

Region: NODE\_369549\_length\_1565\_cov\_30.364218 1156-1159. Max. coverage (+): 0.19. Max coverage (-): 0

Region: NODE\_369549\_length\_1565\_cov\_30.364218 1160-1162. Max. coverage (+): 0.11. Max coverage (-): 0

Region: NODE\_369549\_length\_1565\_cov\_30.364218 1163-1165. Max. coverage (+): 0. Max coverage (-): 0.04

Region: NODE\_369549\_length\_1565\_cov\_30.364218 1166-1168. Max. coverage (+): 0. Max coverage (-): 0.04

Region: NODE\_369549\_length\_1565\_cov\_30.364218 1169-1172. Max. coverage (+): 0.08. Max coverage (-): 0.07

Region: NODE\_369549\_length\_1565\_cov\_30.364218 1173-1175. Max. coverage (+): 0.08. Max coverage (-): 0.02

Region: NODE\_369549\_length\_1565\_cov\_30.364218 1176-1178. Max. coverage (+): 0.05. Max coverage (-): 0.02

Region: NODE\_369549\_length\_1565\_cov\_30.364218 1179-1182. Max. coverage (+): 0.45. Max coverage (-): 0

Region: NODE\_369549\_length\_1565\_cov\_30.364218 1183-1185. Max. coverage (+): 4.47. Max coverage (-): 0

Region: NODE\_369549\_length\_1565\_cov\_30.364218 1186-1188. Max. coverage (+): 4.14. Max coverage (-): 0

Region: NODE\_369549\_length\_1565\_cov\_30.364218 1189-1191. Max. coverage (+): 0.19. Max coverage (-): 0

Region: NODE\_369549\_length\_1565\_cov\_30.364218 1192-1195. Max. coverage (+): 0.09. Max coverage (-): 0

Region: NODE\_369549\_length\_1565\_cov\_30.364218 1196-1198. Max. coverage (+): 0.09. Max coverage (-): 0

Region: NODE\_369549\_length\_1565\_cov\_30.364218 1199-1201. Max. coverage (+): 0.06. Max coverage (-): 0

Region: NODE\_369549\_length\_1565\_cov\_30.364218 1202-1204. Max. coverage (+): 0. Max coverage (-): 0

Region: NODE\_369549\_length\_1565\_cov\_30.364218 1205-1208. Max. coverage (+): 0. Max coverage (-): 0

Region: NODE\_369549\_length\_1565\_cov\_30.364218 1209-1211. Max. coverage (+): 0.07. Max coverage (-): 0

Region: NODE\_369549\_length\_1565\_cov\_30.364218 1212-1214. Max. coverage (+): 0.26. Max coverage (-): 0

Region: NODE\_369549\_length\_1565\_cov\_30.364218 1215-1217. Max. coverage (+): 0.41. Max coverage (-): 0

Region: NODE\_369549\_length\_1565\_cov\_30.364218 1218-1221. Max. coverage (+): 8.49. Max coverage (-): 0

Region: NODE\_369549\_length\_1565\_cov\_30.364218 1222-1224. Max. coverage (+): 8.68. Max coverage (-): 0

Region: NODE\_369549\_length\_1565\_cov\_30.364218 1225-1227. Max. coverage (+): 1.22. Max coverage (-): 0.04

Region: NODE\_369549\_length\_1565\_cov\_30.364218 1228-1230. Max. coverage (+): 0.96. Max coverage (-): 0.04

Region: NODE\_369549\_length\_1565\_cov\_30.364218 1231-1234. Max. coverage (+): 8.71. Max coverage (-): 0.04

Region: NODE\_369549\_length\_1565\_cov\_30.364218 1235-1237. Max. coverage (+): 19.83. Max coverage (-): 0.04

Region: NODE\_369549\_length\_1565\_cov\_30.364218 1238-1240. Max. coverage (+): 17.65. Max coverage (-): 0

Region: NODE\_369549\_length\_1565\_cov\_30.364218 1241-1243. Max. coverage (+): 0.63. Max coverage (-): 0

Region: NODE\_369549\_length\_1565\_cov\_30.364218 1244-1247. Max. coverage (+): 0.04. Max coverage (-): 0.04

Region: NODE\_369549\_length\_1565\_cov\_30.364218 1248-1250. Max. coverage (+): 0. Max coverage (-): 0.19

Region: NODE\_369549\_length\_1565\_cov\_30.364218 1251-1253. Max. coverage (+): 0.04. Max coverage (-): 0.15

Region: NODE\_369549\_length\_1565\_cov\_30.364218 1254-1256. Max. coverage (+): 0.19. Max coverage (-): 0

Region: NODE\_369549\_length\_1565\_cov\_30.364218 1257-1260. Max. coverage (+): 0.54. Max coverage (-): 0

Region: NODE\_369549\_length\_1565\_cov\_30.364218 1261-1263. Max. coverage (+): 0.76. Max coverage (-): 0.02

Region: NODE\_369549\_length\_1565\_cov\_30.364218 1264-1266. Max. coverage (+): 1.59. Max coverage (-): 0.02

Region: NODE\_369549\_length\_1565\_cov\_30.364218 1267-1269. Max. coverage (+): 1.32. Max coverage (-): 0

Region: NODE\_369549\_length\_1565\_cov\_30.364218 1270-1273. Max. coverage (+): 0.26. Max coverage (-): 0

Region: NODE\_369549\_length\_1565\_cov\_30.364218 1274-1276. Max. coverage (+): 0.39. Max coverage (-): 0.17

Region: NODE\_369549\_length\_1565\_cov\_30.364218 1277-1279. Max. coverage (+): 0.54. Max coverage (-): 0.3

Region: NODE\_369549\_length\_1565\_cov\_30.364218 1280-1283. Max. coverage (+): 0.22. Max coverage (-): 1

Region: NODE\_369549\_length\_1565\_cov\_30.364218 1284-1286. Max. coverage (+): 0. Max coverage (-): 0.96

Region: NODE\_369549\_length\_1565\_cov\_30.364218 1287-1289. Max. coverage (+): 0. Max coverage (-): 0.19

Region: NODE\_369549\_length\_1565\_cov\_30.364218 1290-1292. Max. coverage (+): 0.04. Max coverage (-): 0.19

Region: NODE\_369549\_length\_1565\_cov\_30.364218 1293-1296. Max. coverage (+): 0.89. Max coverage (-): 0.07

Region: NODE\_369549\_length\_1565\_cov\_30.364218 1297-1299. Max. coverage (+): 5.6. Max coverage (-): 0

Region: NODE\_369549\_length\_1565\_cov\_30.364218 1300-1302. Max. coverage (+): 4.89. Max coverage (-): 0.04

Region: NODE\_369549\_length\_1565\_cov\_30.364218 1303-1305. Max. coverage (+): 2.19. Max coverage (-): 0.04

Region: NODE\_369549\_length\_1565\_cov\_30.364218 1306-1309. Max. coverage (+): 3.67. Max coverage (-): 0

Region: NODE\_369549\_length\_1565\_cov\_30.364218 1310-1312. Max. coverage (+): 0.44. Max coverage (-): 0.03

Region: NODE\_369549\_length\_1565\_cov\_30.364218 1313-1315. Max. coverage (+): 0.61. Max coverage (-): 0.07

Region: NODE\_369549\_length\_1565\_cov\_30.364218 1316-1318. Max. coverage (+): 1.95. Max coverage (-): 0.06

Region: NODE\_369549\_length\_1565\_cov\_30.364218 1319-1322. Max. coverage (+): 2.21. Max coverage (-): 0

Region: NODE\_369549\_length\_1565\_cov\_30.364218 1323-1325. Max. coverage (+): 1.57. Max coverage (-): 0.44

Region: NODE\_369549\_length\_1565\_cov\_30.364218 1326-1328. Max. coverage (+): 0.33. Max coverage (-): 0.59

Region: NODE\_369549\_length\_1565\_cov\_30.364218 1329-1331. Max. coverage (+): 1.56. Max coverage (-): 0.26

Region: NODE\_369549\_length\_1565\_cov\_30.364218 1332-1335. Max. coverage (+): 1.52. Max coverage (-): 0.04

Region: NODE\_369549\_length\_1565\_cov\_30.364218 1336-1338. Max. coverage (+): 0.22. Max coverage (-): 0.07

Region: NODE\_369549\_length\_1565\_cov\_30.364218 1339-1341. Max. coverage (+): 0.11. Max coverage (-): 0.07

Region: NODE\_369549\_length\_1565\_cov\_30.364218 1342-1344. Max. coverage (+): 0.19. Max coverage (-): 0

Region: NODE\_369549\_length\_1565\_cov\_30.364218 1345-1348. Max. coverage (+): 0.19. Max coverage (-): 0

Region: NODE\_369549\_length\_1565\_cov\_30.364218 1349-1351. Max. coverage (+): 0.15. Max coverage (-): 0

Region: NODE\_369549\_length\_1565\_cov\_30.364218 1352-1354. Max. coverage (+): 0.15. Max coverage (-): 0

Region: NODE\_369549\_length\_1565\_cov\_30.364218 1355-1357. Max. coverage (+): 0.04. Max coverage (-): 0

Region: NODE\_369549\_length\_1565\_cov\_30.364218 1358-1361. Max. coverage (+): 0. Max coverage (-): 0

Region: NODE\_369549\_length\_1565\_cov\_30.364218 1362-1364. Max. coverage (+): 0. Max coverage (-): 0

Region: NODE\_369549\_length\_1565\_cov\_30.364218 1365-1367. Max. coverage (+): 0. Max coverage (-): 0

Region: NODE\_369549\_length\_1565\_cov\_30.364218 1368-1370. Max. coverage (+): 0. Max coverage (-): 0

Region: NODE\_369549\_length\_1565\_cov\_30.364218 1371-1374. Max. coverage (+): 0.07. Max coverage (-): 0

Region: NODE\_369549\_length\_1565\_cov\_30.364218 1375-1377. Max. coverage (+): 0.15. Max coverage (-): 0

Region: NODE\_369549\_length\_1565\_cov\_30.364218 1378-1380. Max. coverage (+): 0.15. Max coverage (-): 0

Region: NODE\_369549\_length\_1565\_cov\_30.364218 1381-1384. Max. coverage (+): 0.07. Max coverage (-): 0

Region: NODE\_369549\_length\_1565\_cov\_30.364218 1385-1387. Max. coverage (+): 0.15. Max coverage (-): 0.04

Region: NODE\_369549\_length\_1565\_cov\_30.364218 1388-1390. Max. coverage (+): 0.07. Max coverage (-): 0.07

Region: NODE\_369549\_length\_1565\_cov\_30.364218 1391-1393. Max. coverage (+): 0. Max coverage (-): 0.04

Region: NODE\_369549\_length\_1565\_cov\_30.364218 1394-1397. Max. coverage (+): 0. Max coverage (-): 0.07

Region: NODE\_369549\_length\_1565\_cov\_30.364218 1398-1400. Max. coverage (+): 0.11. Max coverage (-): 0.07

Region: NODE\_369549\_length\_1565\_cov\_30.364218 1401-1403. Max. coverage (+): 0.89. Max coverage (-): 0

Region: NODE\_369549\_length\_1565\_cov\_30.364218 1404-1406. Max. coverage (+): 9.56. Max coverage (-): 0

Region: NODE\_369549\_length\_1565\_cov\_30.364218 1407-1410. Max. coverage (+): 8.97. Max coverage (-): 0

Region: NODE\_369549\_length\_1565\_cov\_30.364218 1411-1413. Max. coverage (+): 0.44. Max coverage (-): 0

Region: NODE\_369549\_length\_1565\_cov\_30.364218 1414-1416. Max. coverage (+): 0.56. Max coverage (-): 0

Region: NODE\_369549\_length\_1565\_cov\_30.364218 1417-1419. Max. coverage (+): 0.11. Max coverage (-): 0.04

Region: NODE\_369549\_length\_1565\_cov\_30.364218 1420-1423. Max. coverage (+): 0.07. Max coverage (-): 0.11

Region: NODE\_369549\_length\_1565\_cov\_30.364218 1424-1426. Max. coverage (+): 0.07. Max coverage (-): 2.63

Region: NODE\_369549\_length\_1565\_cov\_30.364218 1427-1429. Max. coverage (+): 0.04. Max coverage (-): 4.71

Region: NODE\_369549\_length\_1565\_cov\_30.364218 1430-1432. Max. coverage (+): 0.07. Max coverage (-): 3.04

Region: NODE\_369549\_length\_1565\_cov\_30.364218 1433-1436. Max. coverage (+): 0.04. Max coverage (-): 0.78

Region: NODE\_369549\_length\_1565\_cov\_30.364218 1437-1439. Max. coverage (+): 0. Max coverage (-): 0.63

Region: NODE\_369549\_length\_1565\_cov\_30.364218 1440-1442. Max. coverage (+): 0. Max coverage (-): 0.04

Region: NODE\_369549\_length\_1565\_cov\_30.364218 1443-1445. Max. coverage (+): 1.63. Max coverage (-): 0

Region: NODE\_369549\_length\_1565\_cov\_30.364218 1446-1449. Max. coverage (+): 6.23. Max coverage (-): 0

Region: NODE\_369549\_length\_1565\_cov\_30.364218 1450-1452. Max. coverage (+): 8.01. Max coverage (-): 0

Region: NODE\_369549\_length\_1565\_cov\_30.364218 1453-1455. Max. coverage (+): 6.75. Max coverage (-): 0.04

Region: NODE\_369549\_length\_1565\_cov\_30.364218 1456-1458. Max. coverage (+): 0. Max coverage (-): 0.07

Region: NODE\_369549\_length\_1565\_cov\_30.364218 1459-1462. Max. coverage (+): 0. Max coverage (-): 0.04

Region: NODE\_369549\_length\_1565\_cov\_30.364218 1463-1465. Max. coverage (+): 0. Max coverage (-): 0.04

Region: NODE\_369549\_length\_1565\_cov\_30.364218 1466-1468. Max. coverage (+): 0.04. Max coverage (-): 0.04

Region: NODE\_369549\_length\_1565\_cov\_30.364218 1469-1471. Max. coverage (+): 0.04. Max coverage (-): 0

Region: NODE\_369549\_length\_1565\_cov\_30.364218 1472-1475. Max. coverage (+): 0.07. Max coverage (-): 0.04

Region: NODE\_369549\_length\_1565\_cov\_30.364218 1476-1478. Max. coverage (+): 0.48. Max coverage (-): 0.04

Region: NODE\_369549\_length\_1565\_cov\_30.364218 1479-1481. Max. coverage (+): 0.82. Max coverage (-): 0.04

Region: NODE\_369549\_length\_1565\_cov\_30.364218 1482-1485. Max. coverage (+): 0.37. Max coverage (-): 0.04

Region: NODE\_369549\_length\_1565\_cov\_30.364218 1486-1488. Max. coverage (+): 0.11. Max coverage (-): 0.04

Region: NODE\_369549\_length\_1565\_cov\_30.364218 1489-1491. Max. coverage (+): 0.04. Max coverage (-): 0

Region: NODE\_369549\_length\_1565\_cov\_30.364218 1492-1494. Max. coverage (+): 0.07. Max coverage (-): 0

Region: NODE\_369549\_length\_1565\_cov\_30.364218 1495-1498. Max. coverage (+): 1.45. Max coverage (-): 0.41

Region: NODE\_369549\_length\_1565\_cov\_30.364218 1499-1501. Max. coverage (+): 1.82. Max coverage (-): 0.63

Region: NODE\_369549\_length\_1565\_cov\_30.364218 1502-1504. Max. coverage (+): 0.48. Max coverage (-): 0.22

Region: NODE\_369549\_length\_1565\_cov\_30.364218 1505-1507. Max. coverage (+): 1.45. Max coverage (-): 0

Region: NODE\_369549\_length\_1565\_cov\_30.364218 1508-1511. Max. coverage (+): 10.2. Max coverage (-): 0

Region: NODE\_369549\_length\_1565\_cov\_30.364218 1512-1514. Max. coverage (+): 2.3. Max coverage (-): 0

Region: NODE\_369549\_length\_1565\_cov\_30.364218 1515-1517. Max. coverage (+): 2.41. Max coverage (-): 0.11

Region: NODE\_369549\_length\_1565\_cov\_30.364218 1518-1520. Max. coverage (+): 0.22. Max coverage (-): 0.11

Region: NODE\_369549\_length\_1565\_cov\_30.364218 1521-1524. Max. coverage (+): 0. Max coverage (-): 0

Region: NODE\_369549\_length\_1565\_cov\_30.364218 1525-1527. Max. coverage (+): 0.07. Max coverage (-): 0.06

Region: NODE\_369549\_length\_1565\_cov\_30.364218 1528-1530. Max. coverage (+): 0.17. Max coverage (-): 0.06

Region: NODE\_369549\_length\_1565\_cov\_30.364218 1531-1533. Max. coverage (+): 0.39. Max coverage (-): 0.02

Region: NODE\_369549\_length\_1565\_cov\_30.364218 1534-1537. Max. coverage (+): 6.1. Max coverage (-): 0.09

Region: NODE\_369549\_length\_1565\_cov\_30.364218 1538-1540. Max. coverage (+): 8.99. Max coverage (-): 0.09

Region: NODE\_369549\_length\_1565\_cov\_30.364218 1541-1543. Max. coverage (+): 4.06. Max coverage (-): 0.19

Region: NODE\_369549\_length\_1565\_cov\_30.364218 1544-1546. Max. coverage (+): 0.82. Max coverage (-): 0.22

Region: NODE\_369549\_length\_1565\_cov\_30.364218 1547-1550. Max. coverage (+): 0.44. Max coverage (-): 0.07

Region: NODE\_369549\_length\_1565\_cov\_30.364218 1551-1553. Max. coverage (+): 0.11. Max coverage (-): 0.11

Region: NODE\_369549\_length\_1565\_cov\_30.364218 1554-1556. Max. coverage (+): 0.11. Max coverage (-): 0.19

Region: NODE\_369549\_length\_1565\_cov\_30.364218 1557-1559. Max. coverage (+): 0.11. Max coverage (-): 0.15

Region: NODE\_369549\_length\_1565\_cov\_30.364218 1560-1563. Max. coverage (+): 0.01. Max coverage (-): 0.08

Region: NODE\_369549\_length\_1565\_cov\_30.364218 1564-1566. Max. coverage (+): 0. Max coverage (-): 0.09

Region: NODE\_369549\_length\_1565\_cov\_30.364218 1567-1569. Max. coverage (+): 0. Max coverage (-): 0.02

Region: NODE\_369549\_length\_1565\_cov\_30.364218 1570-1572. Max. coverage (+): 0.06. Max coverage (-): 0

Region: NODE\_369549\_length\_1565\_cov\_30.364218 1573-1576. Max. coverage (+): 0.06. Max coverage (-): 0

Region: NODE\_369549\_length\_1565\_cov\_30.364218 1577-1579. Max. coverage (+): 0.01. Max coverage (-): 0

Region: NODE\_369549\_length\_1565\_cov\_30.364218 1580-1582. Max. coverage (+): 0.01. Max coverage (-): 0

Region: NODE\_369549\_length\_1565\_cov\_30.364218 1583-1586. Max. coverage (+): 0.01. Max coverage (-): 0

Region: NODE\_369549\_length\_1565\_cov\_30.364218 1587-1589. Max. coverage (+): 0. Max coverage (-): 0.01

Region: NODE\_369549\_length\_1565\_cov\_30.364218 1590-1592. Max. coverage (+): 0. Max coverage (-): 0.13

Region: NODE\_369549\_length\_1565\_cov\_30.364218 1593-1595. Max. coverage (+): 0. Max coverage (-): 0.77

Region: NODE\_369549\_length\_1565\_cov\_30.364218 1596-1599. Max. coverage (+): 0. Max coverage (-): 0.78

Region: NODE\_369549\_length\_1565\_cov\_30.364218 1600-1602. Max. coverage (+): 0. Max coverage (-): 0.01

Region: NODE\_369549\_length\_1565\_cov\_30.364218 1603-1605. Max. coverage (+): 0. Max coverage (-): 0

Region: NODE\_369549\_length\_1565\_cov\_30.364218 1606-1608. Max. coverage (+): 0. Max coverage (-): 0

Region: NODE\_369549\_length\_1565\_cov\_30.364218 1609-1612. Max. coverage (+): 0. Max coverage (-): 0

Region: NODE\_369549\_length\_1565\_cov\_30.364218 1613-1615. Max. coverage (+): 0. Max coverage (-): 0

Region: NODE\_369549\_length\_1565\_cov\_30.364218 1616-1618. Max. coverage (+): 0. Max coverage (-): 0

Region: NODE\_369549\_length\_1565\_cov\_30.364218 1619-1621. Max. coverage (+): 0. Max coverage (-): 0

Region: NODE\_369549\_length\_1565\_cov\_30.364218 1622-1625. Max. coverage (+): 0. Max coverage (-): 0

Region: NODE\_369549\_length\_1565\_cov\_30.364218 1626-1628. Max. coverage (+): 0. Max coverage (-): 0

Region: NODE\_369549\_length\_1565\_cov\_30.364218 1629-. Max. coverage (+): 0. Max coverage (-): 0

RepeatMasker Color Code

**+**

100-98% Identity

<98-95% Identity

<95-90% Identity

<90-85% Identity

<85-80% Identity

<80-75% Identity

<75-70% Identity

<70% Identity

**-**

Gene Set Color Code

**+**

Gene

Pseudogene

Other

**-**

Topology/Coverage Color Code

Coverage Plus Strand

Coverage Minus Strand

Mainstrand: Plus

Mainstrand: Minus

Complementary Strand

Flanking Region  
(if option -flank >0)

Gene Set Annotation  
  
RepeatMasker Annotation  

**1. L1-16\_DR**: 163-1053 (-), Divergence to consensus: 38.1%  
**2. (A)n**: 1364-1388 (+), Divergence to consensus: 8.7%  
**3. AlRepD-382**: 1566-1629 (-), Divergence to consensus: 3.1%

  
Transcription Factor Binding Sites  

**RHOXF1** (Sequence: GGATCA (-): 637)  
**RHOXF1** (Sequence: AGATCA (-): 700)  
**RHOXF1** (Sequence: GGATCA (-): 814)  
**RHOXF1** (Sequence: AGATCA (-): 1510)  
**RHOXF1** (Sequence: TAATCT (+): 155)  
**RHOXF1** (Sequence: TGATCT (+): 190)  
**RHOXF1** (Sequence: TAATCT (+): 465)  
**RHOXF1** (Sequence: TAAGCC (+): 1033)  
**SOX9** (Sequence: AACAATGA (-): 1494)  
**SOX9** (Sequence: AACAATAG (-): 1497)  
**Sox5** (Sequence: ATTGTT (+): 573)  
**Sox5** (Sequence: ATTGTT (+): 1090)  
**Nobox** (Sequence: ACCAATTA (-): 663)  
**Sox5** (Sequence: AACAAT (-): 1494)
