# Supplementary material for: First characterization of PIWI-interacting RNA clusters in a cichlid fish with a B chromosome
Source: BMC Biol. 2022 Sep 21;20:204. doi: 10.1186/s12915-022-01403-2 (PMC9490952; doi:10.1186/s12915-022-01403-2)
Supplement: Supplementary file 1 — Additional file 1. Zipped folder with fasta and interactive html piRNA cluster information for the A. latifasciata genome. The nomenclature is as follows: number-pirna-cluster_sex_B-presence (f, female; m, male; 0b, without B chromosome; 1b, with B chromosome). [file 12915_2022_1403_MOESM1_ESM.zip › 143_m0b.html]

piRNA cluster 143\_m0b 69


Predicted piRNA cluster no. 143\_m0b
  

Show proTRAC run info
Hide proTRAC run info

/\  
                \_\_\_\_\_\_\_\_\_\_\_\_\_\_\_\_\_\_\_\_\_\_\_/\\_\_\_ /  \\_\_\_\_\_\_\_  
               I                      /  \  /    \      I  
               I     pro             /    \/      \     I  
               I        TRAC        /               \   I  
               I   \_\_\_\_\_\_\_\_\_\_\_\_\_\_\_\_/\_\_\_\_\_\_\_\_\_\_\_\_\_\_\_\_\_\\_ I  
               I   \              /                     I  
               I    \            /                      I  
               I     \  /\      /       V.2.4.2         I  
               I      \/  \    /                        I  
               I\_\_\_\_\_\_\_\_\_\_\_\  /\_\_\_\_\_\_\_\_\_\_\_\_\_\_\_\_\_\_\_\_\_\_\_\_\_I  
                            \/  
  
  
================================= proTRAC ====================================  
VERSION: .......... 2.4.2  
LAST MODIFIED: .... 11. May 2018  
  
Please cite:  
Rosenkranz D, Zischler H. proTRAC - a software for probabilistic piRNA cluster  
detection, visualization and analysis. 2012. BMC Bioinformatics 13:5.  
  
  
Contact:  
David Rosenkranz  
Institute of Organismic and Molecular Evolutionary Biology  
Dept. Anthropology, small RNA group  
Johannes Gutenberg University Mainz  
email: rosenkranz@uni-mainz.de  
  
You can find the latest proTRAC version at:  
http://sourceforge.net/projects/protrac/files  
http://www.smallRNAgroup-mainz.de/software  
==============================================================================  
  
PARAMETERS:  
Map file: ...............piwi-machos-0B.fa-collapse.map  
Genome file: ............../../../0B\_ala\_genome.fa  
RepeatMasker annotation: Alatifasciata-all0B-maryan-v2.fa\_corrected.out  
GeneSet:................./guest-storage/Data/annotation/Alatifasciata\_all0B\_maryan-v2\_out2017.gff  
  
Significant (p<=0.01) hit density will be calculated based  
on observed hit distribution.  
  
Sliding window size: ........................................ 5000 bp  
Sliding window increament: .................................. 1000 bp  
Normalize each hit by number of genomic hits: ............... yes  
Normalize each hit by number of sequence reads: ............. yes  
Normalize values (-> per million mapped reads): ............. yes  
Min. fraction of hits with 1T(U) or 10A: .................... 0.75  
Alternatively: Min. fraction of hits with 1T(U) and 10A: .... 0.5  
Min. fraction of hits with typical piRNA length: ............ 0.75  
Typical piRNA length: ....................................... 24-32 nt  
Min. size of a piRNA cluster: ............................... 1000 bp.  
Min. number of hits (absolute): ............................. 0  
Min. number of hits (normalized): ........................... 0  
Min. fraction of hits on the mainstrand: .................... 0.75  
Top fraction of mapped sequences (in terms of read counts): . 1%  
Top fraction accounts for max. n% of sequence reads: ........ 90%  
Min. fraction of hits on each arm of a bidirectional cluster: 0.05  
Output html file for each cluster: .......................... yes  
Output a summary table: ..................................... yes  
Output a FASTA file for each cluster (piRNA sequences): ..... yes  
Output a FASTA file comprising cluster sequences: ........... yes  
Output a GTF file for predicted piRNA clusters: ..............yes  
Search DNA motifs in clusters: .............................. yes  
Output flanking sequences: +/- .............................. 0 bp  
Output ~.pTi file: .......................................... no  
==============================================================================  
  
  
Genome size (without gaps): ............ 758543724 bp  
Gaps (N/X/-): .......................... 417479 bp  
Mapped reads: .......................... 24765598  
Non-identical sequences: ............... 6158275  
Genomic hits: .......................... 53103584  
Significant densitiy of mapped reads: .. 763.098963422187 reads/kb

Show proTRAC cluster info
Hide proTRAC cluster info

|  |  |
| --- | --- |
| Location | NODE\_371225\_length\_8483\_cov\_20.365791 |
| Coordinates | 7-8804 |
| Size [bp] | 8798 |
| Sequence hit loci | 3563 |
| Mapped reads (normalized) | 9478.4 |
| Mapped reads (normalized) per kb | 1077.3 |
| Normalized reads with 1T (1U) | 74.1% |
| Normalized reads with 10A | 51.6% |
| Normalized reads with length 24-32 nt | 99% |
| Normalized reads on the main strand(s) | 78.3% |
| Predicted directionality | mono:minus |

100%

0%

1T (1U)  
reads

10A reads

24-32 nt  
reads

reads on mainstrand

**Either the amount of reads with 1T (1U) OR 10A has to exceed 75% (set with option: -1Tor10A)  
Alternatively the amount of reads with 1T (1U) AND 10A has to exceed 50% (set with option: -1Tand10A)  
Minimum amount of reads with preferred size is 75% (set with option: -pisize)  
Minimum amount of reads on the main strand(s) is 75% (set with option: -clstrand)**

Show read coverage
Hide read coverage

WHAT DO I SEE HERE?  
This chart shows the location of mapped sequence reads within a predicted piRNA cluster. The color refers to the number of genomic hits produced by the sequence read in question. A dark red bar indicates that this sequence read produces many other hits elsewhere in the genome. Many adjacent red or yellow bars can indicate the presence of a multi-copy element such as transposons or rRNA genes. A dark green bar indicates that this sequence read maps uniquely to this locus.

1 hit

2-5 hits

6-10 hits

11-20 hits

21-50 hits

51-100 hits

> 100 hits

NODE\_371225\_length\_8483\_cov\_20.365791

7

8804

Gene Set

RepeatMasker

Mapped  
Reads

83.07

plus strand

minus strand

83.07

Region: NODE\_371225\_length\_8483\_cov\_20.365791 1627-15. Max. coverage (+): 0. Max coverage (-): 0.04

Region: NODE\_371225\_length\_8483\_cov\_20.365791 16-33. Max. coverage (+): 1.7. Max coverage (-): 0

Region: NODE\_371225\_length\_8483\_cov\_20.365791 34-50. Max. coverage (+): 0.12. Max coverage (-): 0.06

Region: NODE\_371225\_length\_8483\_cov\_20.365791 51-68. Max. coverage (+): 0.04. Max coverage (-): 0.04

Region: NODE\_371225\_length\_8483\_cov\_20.365791 69-86. Max. coverage (+): 0.08. Max coverage (-): 0

Region: NODE\_371225\_length\_8483\_cov\_20.365791 87-103. Max. coverage (+): 0.04. Max coverage (-): 0.04

Region: NODE\_371225\_length\_8483\_cov\_20.365791 104-121. Max. coverage (+): 0. Max coverage (-): 0.04

Region: NODE\_371225\_length\_8483\_cov\_20.365791 122-138. Max. coverage (+): 0.2. Max coverage (-): 0.04

Region: NODE\_371225\_length\_8483\_cov\_20.365791 139-156. Max. coverage (+): 0. Max coverage (-): 0

Region: NODE\_371225\_length\_8483\_cov\_20.365791 157-174. Max. coverage (+): 1.53. Max coverage (-): 0.04

Region: NODE\_371225\_length\_8483\_cov\_20.365791 175-191. Max. coverage (+): 0. Max coverage (-): 0.24

Region: NODE\_371225\_length\_8483\_cov\_20.365791 192-209. Max. coverage (+): 0.32. Max coverage (-): 0.08

Region: NODE\_371225\_length\_8483\_cov\_20.365791 210-226. Max. coverage (+): 0.04. Max coverage (-): 0

Region: NODE\_371225\_length\_8483\_cov\_20.365791 227-244. Max. coverage (+): 0. Max coverage (-): 0

Region: NODE\_371225\_length\_8483\_cov\_20.365791 245-262. Max. coverage (+): 0. Max coverage (-): 0

Region: NODE\_371225\_length\_8483\_cov\_20.365791 263-279. Max. coverage (+): 0.02. Max coverage (-): 0

Region: NODE\_371225\_length\_8483\_cov\_20.365791 280-297. Max. coverage (+): 0.04. Max coverage (-): 0.26

Region: NODE\_371225\_length\_8483\_cov\_20.365791 298-314. Max. coverage (+): 0. Max coverage (-): 0.06

Region: NODE\_371225\_length\_8483\_cov\_20.365791 315-332. Max. coverage (+): 0. Max coverage (-): 0.26

Region: NODE\_371225\_length\_8483\_cov\_20.365791 333-350. Max. coverage (+): 0.91. Max coverage (-): 0.16

Region: NODE\_371225\_length\_8483\_cov\_20.365791 351-367. Max. coverage (+): 0.06. Max coverage (-): 0

Region: NODE\_371225\_length\_8483\_cov\_20.365791 368-385. Max. coverage (+): 0. Max coverage (-): 0

Region: NODE\_371225\_length\_8483\_cov\_20.365791 386-402. Max. coverage (+): 0. Max coverage (-): 0

Region: NODE\_371225\_length\_8483\_cov\_20.365791 403-420. Max. coverage (+): 0. Max coverage (-): 0

Region: NODE\_371225\_length\_8483\_cov\_20.365791 421-438. Max. coverage (+): 0.13. Max coverage (-): 0.02

Region: NODE\_371225\_length\_8483\_cov\_20.365791 439-455. Max. coverage (+): 0.02. Max coverage (-): 0.02

Region: NODE\_371225\_length\_8483\_cov\_20.365791 456-473. Max. coverage (+): 0. Max coverage (-): 0

Region: NODE\_371225\_length\_8483\_cov\_20.365791 474-490. Max. coverage (+): 0. Max coverage (-): 0.04

Region: NODE\_371225\_length\_8483\_cov\_20.365791 491-508. Max. coverage (+): 0. Max coverage (-): 0

Region: NODE\_371225\_length\_8483\_cov\_20.365791 509-526. Max. coverage (+): 0. Max coverage (-): 0.16

Region: NODE\_371225\_length\_8483\_cov\_20.365791 527-543. Max. coverage (+): 0.04. Max coverage (-): 0.02

Region: NODE\_371225\_length\_8483\_cov\_20.365791 544-561. Max. coverage (+): 0.04. Max coverage (-): 0.04

Region: NODE\_371225\_length\_8483\_cov\_20.365791 562-578. Max. coverage (+): 0. Max coverage (-): 0

Region: NODE\_371225\_length\_8483\_cov\_20.365791 579-596. Max. coverage (+): 0.02. Max coverage (-): 0

Region: NODE\_371225\_length\_8483\_cov\_20.365791 597-614. Max. coverage (+): 0. Max coverage (-): 0

Region: NODE\_371225\_length\_8483\_cov\_20.365791 615-631. Max. coverage (+): 0. Max coverage (-): 0.04

Region: NODE\_371225\_length\_8483\_cov\_20.365791 632-649. Max. coverage (+): 0.04. Max coverage (-): 0.04

Region: NODE\_371225\_length\_8483\_cov\_20.365791 650-666. Max. coverage (+): 0. Max coverage (-): 0.04

Region: NODE\_371225\_length\_8483\_cov\_20.365791 667-684. Max. coverage (+): 0. Max coverage (-): 0

Region: NODE\_371225\_length\_8483\_cov\_20.365791 685-702. Max. coverage (+): 0.04. Max coverage (-): 0.06

Region: NODE\_371225\_length\_8483\_cov\_20.365791 703-719. Max. coverage (+): 0.12. Max coverage (-): 0.1

Region: NODE\_371225\_length\_8483\_cov\_20.365791 720-737. Max. coverage (+): 0.04. Max coverage (-): 0

Region: NODE\_371225\_length\_8483\_cov\_20.365791 738-754. Max. coverage (+): 0. Max coverage (-): 0.04

Region: NODE\_371225\_length\_8483\_cov\_20.365791 755-772. Max. coverage (+): 0. Max coverage (-): 0.02

Region: NODE\_371225\_length\_8483\_cov\_20.365791 773-790. Max. coverage (+): 0.04. Max coverage (-): 0.04

Region: NODE\_371225\_length\_8483\_cov\_20.365791 791-807. Max. coverage (+): 0.04. Max coverage (-): 0

Region: NODE\_371225\_length\_8483\_cov\_20.365791 808-825. Max. coverage (+): 0.16. Max coverage (-): 0.02

Region: NODE\_371225\_length\_8483\_cov\_20.365791 826-842. Max. coverage (+): 0.12. Max coverage (-): 0.04

Region: NODE\_371225\_length\_8483\_cov\_20.365791 843-860. Max. coverage (+): 0.02. Max coverage (-): 0.1

Region: NODE\_371225\_length\_8483\_cov\_20.365791 861-878. Max. coverage (+): 0.2. Max coverage (-): 0

Region: NODE\_371225\_length\_8483\_cov\_20.365791 879-895. Max. coverage (+): 0.02. Max coverage (-): 0

Region: NODE\_371225\_length\_8483\_cov\_20.365791 896-913. Max. coverage (+): 0. Max coverage (-): 0

Region: NODE\_371225\_length\_8483\_cov\_20.365791 914-930. Max. coverage (+): 0.04. Max coverage (-): 0.02

Region: NODE\_371225\_length\_8483\_cov\_20.365791 931-948. Max. coverage (+): 0.02. Max coverage (-): 0.02

Region: NODE\_371225\_length\_8483\_cov\_20.365791 949-965. Max. coverage (+): 0. Max coverage (-): 0.28

Region: NODE\_371225\_length\_8483\_cov\_20.365791 966-983. Max. coverage (+): 0. Max coverage (-): 0.16

Region: NODE\_371225\_length\_8483\_cov\_20.365791 984-1001. Max. coverage (+): 0. Max coverage (-): 0.06

Region: NODE\_371225\_length\_8483\_cov\_20.365791 1002-1018. Max. coverage (+): 0.42. Max coverage (-): 0.02

Region: NODE\_371225\_length\_8483\_cov\_20.365791 1019-1036. Max. coverage (+): 0.04. Max coverage (-): 0

Region: NODE\_371225\_length\_8483\_cov\_20.365791 1037-1053. Max. coverage (+): 0.02. Max coverage (-): 0.08

Region: NODE\_371225\_length\_8483\_cov\_20.365791 1054-1071. Max. coverage (+): 0.04. Max coverage (-): 0.06

Region: NODE\_371225\_length\_8483\_cov\_20.365791 1072-1089. Max. coverage (+): 0.04. Max coverage (-): 0.06

Region: NODE\_371225\_length\_8483\_cov\_20.365791 1090-1106. Max. coverage (+): 0. Max coverage (-): 0.02

Region: NODE\_371225\_length\_8483\_cov\_20.365791 1107-1124. Max. coverage (+): 0. Max coverage (-): 0

Region: NODE\_371225\_length\_8483\_cov\_20.365791 1125-1141. Max. coverage (+): 0.01. Max coverage (-): 0

Region: NODE\_371225\_length\_8483\_cov\_20.365791 1142-1159. Max. coverage (+): 0.02. Max coverage (-): 0.05

Region: NODE\_371225\_length\_8483\_cov\_20.365791 1160-1177. Max. coverage (+): 0.06. Max coverage (-): 0

Region: NODE\_371225\_length\_8483\_cov\_20.365791 1178-1194. Max. coverage (+): 0.18. Max coverage (-): 0.02

Region: NODE\_371225\_length\_8483\_cov\_20.365791 1195-1212. Max. coverage (+): 0. Max coverage (-): 0

Region: NODE\_371225\_length\_8483\_cov\_20.365791 1213-1229. Max. coverage (+): 0.04. Max coverage (-): 0

Region: NODE\_371225\_length\_8483\_cov\_20.365791 1230-1247. Max. coverage (+): 0. Max coverage (-): 0

Region: NODE\_371225\_length\_8483\_cov\_20.365791 1248-1265. Max. coverage (+): 0. Max coverage (-): 0

Region: NODE\_371225\_length\_8483\_cov\_20.365791 1266-1282. Max. coverage (+): 0.02. Max coverage (-): 0.02

Region: NODE\_371225\_length\_8483\_cov\_20.365791 1283-1300. Max. coverage (+): 0.02. Max coverage (-): 0.04

Region: NODE\_371225\_length\_8483\_cov\_20.365791 1301-1317. Max. coverage (+): 0.02. Max coverage (-): 0.04

Region: NODE\_371225\_length\_8483\_cov\_20.365791 1318-1335. Max. coverage (+): 0.02. Max coverage (-): 0.04

Region: NODE\_371225\_length\_8483\_cov\_20.365791 1336-1353. Max. coverage (+): 0.04. Max coverage (-): 0.04

Region: NODE\_371225\_length\_8483\_cov\_20.365791 1354-1370. Max. coverage (+): 0. Max coverage (-): 0

Region: NODE\_371225\_length\_8483\_cov\_20.365791 1371-1388. Max. coverage (+): 0.12. Max coverage (-): 0

Region: NODE\_371225\_length\_8483\_cov\_20.365791 1389-1405. Max. coverage (+): 0.12. Max coverage (-): 0.14

Region: NODE\_371225\_length\_8483\_cov\_20.365791 1406-1423. Max. coverage (+): 0.02. Max coverage (-): 0.14

Region: NODE\_371225\_length\_8483\_cov\_20.365791 1424-1441. Max. coverage (+): 0. Max coverage (-): 0

Region: NODE\_371225\_length\_8483\_cov\_20.365791 1442-1458. Max. coverage (+): 0. Max coverage (-): 0

Region: NODE\_371225\_length\_8483\_cov\_20.365791 1459-1476. Max. coverage (+): 0.06. Max coverage (-): 0.04

Region: NODE\_371225\_length\_8483\_cov\_20.365791 1477-1493. Max. coverage (+): 0.02. Max coverage (-): 0

Region: NODE\_371225\_length\_8483\_cov\_20.365791 1494-1511. Max. coverage (+): 0.06. Max coverage (-): 0.02

Region: NODE\_371225\_length\_8483\_cov\_20.365791 1512-1529. Max. coverage (+): 0.06. Max coverage (-): 0.04

Region: NODE\_371225\_length\_8483\_cov\_20.365791 1530-1546. Max. coverage (+): 0. Max coverage (-): 0

Region: NODE\_371225\_length\_8483\_cov\_20.365791 1547-1564. Max. coverage (+): 0.08. Max coverage (-): 0.04

Region: NODE\_371225\_length\_8483\_cov\_20.365791 1565-1581. Max. coverage (+): 0.06. Max coverage (-): 0.04

Region: NODE\_371225\_length\_8483\_cov\_20.365791 1582-1599. Max. coverage (+): 0.38. Max coverage (-): 0

Region: NODE\_371225\_length\_8483\_cov\_20.365791 1600-1617. Max. coverage (+): 0.02. Max coverage (-): 0.1

Region: NODE\_371225\_length\_8483\_cov\_20.365791 1618-1634. Max. coverage (+): 0.08. Max coverage (-): 0.04

Region: NODE\_371225\_length\_8483\_cov\_20.365791 1635-1652. Max. coverage (+): 0.04. Max coverage (-): 0

Region: NODE\_371225\_length\_8483\_cov\_20.365791 1653-1669. Max. coverage (+): 0.04. Max coverage (-): 0

Region: NODE\_371225\_length\_8483\_cov\_20.365791 1670-1687. Max. coverage (+): 0. Max coverage (-): 0.04

Region: NODE\_371225\_length\_8483\_cov\_20.365791 1688-1705. Max. coverage (+): 0. Max coverage (-): 0

Region: NODE\_371225\_length\_8483\_cov\_20.365791 1706-1722. Max. coverage (+): 0.04. Max coverage (-): 0

Region: NODE\_371225\_length\_8483\_cov\_20.365791 1723-1740. Max. coverage (+): 0.02. Max coverage (-): 0

Region: NODE\_371225\_length\_8483\_cov\_20.365791 1741-1757. Max. coverage (+): 0. Max coverage (-): 0

Region: NODE\_371225\_length\_8483\_cov\_20.365791 1758-1775. Max. coverage (+): 0.02. Max coverage (-): 0.04

Region: NODE\_371225\_length\_8483\_cov\_20.365791 1776-1792. Max. coverage (+): 0. Max coverage (-): 0.2

Region: NODE\_371225\_length\_8483\_cov\_20.365791 1793-1810. Max. coverage (+): 0. Max coverage (-): 0

Region: NODE\_371225\_length\_8483\_cov\_20.365791 1811-1828. Max. coverage (+): 0.04. Max coverage (-): 0.04

Region: NODE\_371225\_length\_8483\_cov\_20.365791 1829-1845. Max. coverage (+): 0.04. Max coverage (-): 0.1

Region: NODE\_371225\_length\_8483\_cov\_20.365791 1846-1863. Max. coverage (+): 0.12. Max coverage (-): 0.69

Region: NODE\_371225\_length\_8483\_cov\_20.365791 1864-1880. Max. coverage (+): 0.13. Max coverage (-): 0.04

Region: NODE\_371225\_length\_8483\_cov\_20.365791 1881-1898. Max. coverage (+): 0.01. Max coverage (-): 0.2

Region: NODE\_371225\_length\_8483\_cov\_20.365791 1899-1916. Max. coverage (+): 0.72. Max coverage (-): 0.04

Region: NODE\_371225\_length\_8483\_cov\_20.365791 1917-1933. Max. coverage (+): 0. Max coverage (-): 0.59

Region: NODE\_371225\_length\_8483\_cov\_20.365791 1934-1951. Max. coverage (+): 0.08. Max coverage (-): 0.16

Region: NODE\_371225\_length\_8483\_cov\_20.365791 1952-1968. Max. coverage (+): 0. Max coverage (-): 2.91

Region: NODE\_371225\_length\_8483\_cov\_20.365791 1969-1986. Max. coverage (+): 0. Max coverage (-): 6.95

Region: NODE\_371225\_length\_8483\_cov\_20.365791 1987-2004. Max. coverage (+): 0.08. Max coverage (-): 0.08

Region: NODE\_371225\_length\_8483\_cov\_20.365791 2005-2021. Max. coverage (+): 0. Max coverage (-): 2.48

Region: NODE\_371225\_length\_8483\_cov\_20.365791 2022-2039. Max. coverage (+): 0.13. Max coverage (-): 0.12

Region: NODE\_371225\_length\_8483\_cov\_20.365791 2040-2056. Max. coverage (+): 0.01. Max coverage (-): 0.01

Region: NODE\_371225\_length\_8483\_cov\_20.365791 2057-2074. Max. coverage (+): 0. Max coverage (-): 0.22

Region: NODE\_371225\_length\_8483\_cov\_20.365791 2075-2092. Max. coverage (+): 2.78. Max coverage (-): 0.09

Region: NODE\_371225\_length\_8483\_cov\_20.365791 2093-2109. Max. coverage (+): 0. Max coverage (-): 0.22

Region: NODE\_371225\_length\_8483\_cov\_20.365791 2110-2127. Max. coverage (+): 0. Max coverage (-): 0.08

Region: NODE\_371225\_length\_8483\_cov\_20.365791 2128-2144. Max. coverage (+): 0.04. Max coverage (-): 0.61

Region: NODE\_371225\_length\_8483\_cov\_20.365791 2145-2162. Max. coverage (+): 1.13. Max coverage (-): 0.69

Region: NODE\_371225\_length\_8483\_cov\_20.365791 2163-2180. Max. coverage (+): 0.26. Max coverage (-): 0.26

Region: NODE\_371225\_length\_8483\_cov\_20.365791 2181-2197. Max. coverage (+): 0.09. Max coverage (-): 0.59

Region: NODE\_371225\_length\_8483\_cov\_20.365791 2198-2215. Max. coverage (+): 0.06. Max coverage (-): 0.99

Region: NODE\_371225\_length\_8483\_cov\_20.365791 2216-2232. Max. coverage (+): 0.03. Max coverage (-): 0.7

Region: NODE\_371225\_length\_8483\_cov\_20.365791 2233-2250. Max. coverage (+): 0.01. Max coverage (-): 0.03

Region: NODE\_371225\_length\_8483\_cov\_20.365791 2251-2268. Max. coverage (+): 0. Max coverage (-): 0

Region: NODE\_371225\_length\_8483\_cov\_20.365791 2269-2285. Max. coverage (+): 0. Max coverage (-): 0

Region: NODE\_371225\_length\_8483\_cov\_20.365791 2286-2303. Max. coverage (+): 0. Max coverage (-): 0

Region: NODE\_371225\_length\_8483\_cov\_20.365791 2304-2320. Max. coverage (+): 0. Max coverage (-): 0.02

Region: NODE\_371225\_length\_8483\_cov\_20.365791 2321-2338. Max. coverage (+): 0. Max coverage (-): 0

Region: NODE\_371225\_length\_8483\_cov\_20.365791 2339-2356. Max. coverage (+): 0.02. Max coverage (-): 0.14

Region: NODE\_371225\_length\_8483\_cov\_20.365791 2357-2373. Max. coverage (+): 0.02. Max coverage (-): 0

Region: NODE\_371225\_length\_8483\_cov\_20.365791 2374-2391. Max. coverage (+): 0.03. Max coverage (-): 0

Region: NODE\_371225\_length\_8483\_cov\_20.365791 2392-2408. Max. coverage (+): 0.01. Max coverage (-): 0

Region: NODE\_371225\_length\_8483\_cov\_20.365791 2409-2426. Max. coverage (+): 0.06. Max coverage (-): 0

Region: NODE\_371225\_length\_8483\_cov\_20.365791 2427-2444. Max. coverage (+): 0.02. Max coverage (-): 0

Region: NODE\_371225\_length\_8483\_cov\_20.365791 2445-2461. Max. coverage (+): 0. Max coverage (-): 0

Region: NODE\_371225\_length\_8483\_cov\_20.365791 2462-2479. Max. coverage (+): 0. Max coverage (-): 0

Region: NODE\_371225\_length\_8483\_cov\_20.365791 2480-2496. Max. coverage (+): 0. Max coverage (-): 0.08

Region: NODE\_371225\_length\_8483\_cov\_20.365791 2497-2514. Max. coverage (+): 0. Max coverage (-): 0

Region: NODE\_371225\_length\_8483\_cov\_20.365791 2515-2532. Max. coverage (+): 0. Max coverage (-): 0.04

Region: NODE\_371225\_length\_8483\_cov\_20.365791 2533-2549. Max. coverage (+): 0.04. Max coverage (-): 0.06

Region: NODE\_371225\_length\_8483\_cov\_20.365791 2550-2567. Max. coverage (+): 0. Max coverage (-): 0

Region: NODE\_371225\_length\_8483\_cov\_20.365791 2568-2584. Max. coverage (+): 0.06. Max coverage (-): 0.02

Region: NODE\_371225\_length\_8483\_cov\_20.365791 2585-2602. Max. coverage (+): 0. Max coverage (-): 0

Region: NODE\_371225\_length\_8483\_cov\_20.365791 2603-2620. Max. coverage (+): 0. Max coverage (-): 0

Region: NODE\_371225\_length\_8483\_cov\_20.365791 2621-2637. Max. coverage (+): 0. Max coverage (-): 0

Region: NODE\_371225\_length\_8483\_cov\_20.365791 2638-2655. Max. coverage (+): 0. Max coverage (-): 0

Region: NODE\_371225\_length\_8483\_cov\_20.365791 2656-2672. Max. coverage (+): 0.08. Max coverage (-): 0.02

Region: NODE\_371225\_length\_8483\_cov\_20.365791 2673-2690. Max. coverage (+): 0. Max coverage (-): 0

Region: NODE\_371225\_length\_8483\_cov\_20.365791 2691-2707. Max. coverage (+): 0. Max coverage (-): 0.04

Region: NODE\_371225\_length\_8483\_cov\_20.365791 2708-2725. Max. coverage (+): 0. Max coverage (-): 0

Region: NODE\_371225\_length\_8483\_cov\_20.365791 2726-2743. Max. coverage (+): 0. Max coverage (-): 0

Region: NODE\_371225\_length\_8483\_cov\_20.365791 2744-2760. Max. coverage (+): 0. Max coverage (-): 0

Region: NODE\_371225\_length\_8483\_cov\_20.365791 2761-2778. Max. coverage (+): 0. Max coverage (-): 0.04

Region: NODE\_371225\_length\_8483\_cov\_20.365791 2779-2795. Max. coverage (+): 0. Max coverage (-): 0.04

Region: NODE\_371225\_length\_8483\_cov\_20.365791 2796-2813. Max. coverage (+): 0.04. Max coverage (-): 0

Region: NODE\_371225\_length\_8483\_cov\_20.365791 2814-2831. Max. coverage (+): 0.04. Max coverage (-): 0.02

Region: NODE\_371225\_length\_8483\_cov\_20.365791 2832-2848. Max. coverage (+): 0.02. Max coverage (-): 0.02

Region: NODE\_371225\_length\_8483\_cov\_20.365791 2849-2866. Max. coverage (+): 0.01. Max coverage (-): 0.06

Region: NODE\_371225\_length\_8483\_cov\_20.365791 2867-2883. Max. coverage (+): 0.06. Max coverage (-): 0.1

Region: NODE\_371225\_length\_8483\_cov\_20.365791 2884-2901. Max. coverage (+): 0.04. Max coverage (-): 0.18

Region: NODE\_371225\_length\_8483\_cov\_20.365791 2902-2919. Max. coverage (+): 0.06. Max coverage (-): 0

Region: NODE\_371225\_length\_8483\_cov\_20.365791 2920-2936. Max. coverage (+): 0.04. Max coverage (-): 0.01

Region: NODE\_371225\_length\_8483\_cov\_20.365791 2937-2954. Max. coverage (+): 0. Max coverage (-): 0.01

Region: NODE\_371225\_length\_8483\_cov\_20.365791 2955-2971. Max. coverage (+): 0.16. Max coverage (-): 0.04

Region: NODE\_371225\_length\_8483\_cov\_20.365791 2972-2989. Max. coverage (+): 0.04. Max coverage (-): 0.02

Region: NODE\_371225\_length\_8483\_cov\_20.365791 2990-3007. Max. coverage (+): 0. Max coverage (-): 0

Region: NODE\_371225\_length\_8483\_cov\_20.365791 3008-3024. Max. coverage (+): 0. Max coverage (-): 0.02

Region: NODE\_371225\_length\_8483\_cov\_20.365791 3025-3042. Max. coverage (+): 0.08. Max coverage (-): 0

Region: NODE\_371225\_length\_8483\_cov\_20.365791 3043-3059. Max. coverage (+): 0.02. Max coverage (-): 0.04

Region: NODE\_371225\_length\_8483\_cov\_20.365791 3060-3077. Max. coverage (+): 0. Max coverage (-): 0.02

Region: NODE\_371225\_length\_8483\_cov\_20.365791 3078-3095. Max. coverage (+): 0. Max coverage (-): 0

Region: NODE\_371225\_length\_8483\_cov\_20.365791 3096-3112. Max. coverage (+): 0. Max coverage (-): 0

Region: NODE\_371225\_length\_8483\_cov\_20.365791 3113-3130. Max. coverage (+): 0.02. Max coverage (-): 0

Region: NODE\_371225\_length\_8483\_cov\_20.365791 3131-3147. Max. coverage (+): 0.02. Max coverage (-): 0

Region: NODE\_371225\_length\_8483\_cov\_20.365791 3148-3165. Max. coverage (+): 0. Max coverage (-): 0

Region: NODE\_371225\_length\_8483\_cov\_20.365791 3166-3183. Max. coverage (+): 0. Max coverage (-): 0.08

Region: NODE\_371225\_length\_8483\_cov\_20.365791 3184-3200. Max. coverage (+): 0.02. Max coverage (-): 0.02

Region: NODE\_371225\_length\_8483\_cov\_20.365791 3201-3218. Max. coverage (+): 0.02. Max coverage (-): 0.02

Region: NODE\_371225\_length\_8483\_cov\_20.365791 3219-3235. Max. coverage (+): 0. Max coverage (-): 0

Region: NODE\_371225\_length\_8483\_cov\_20.365791 3236-3253. Max. coverage (+): 0. Max coverage (-): 0

Region: NODE\_371225\_length\_8483\_cov\_20.365791 3254-3271. Max. coverage (+): 0. Max coverage (-): 1.21

Region: NODE\_371225\_length\_8483\_cov\_20.365791 3272-3288. Max. coverage (+): 0.89. Max coverage (-): 3.76

Region: NODE\_371225\_length\_8483\_cov\_20.365791 3289-3306. Max. coverage (+): 0.16. Max coverage (-): 0.2

Region: NODE\_371225\_length\_8483\_cov\_20.365791 3307-3323. Max. coverage (+): 0.22. Max coverage (-): 1.13

Region: NODE\_371225\_length\_8483\_cov\_20.365791 3324-3341. Max. coverage (+): 1. Max coverage (-): 0.94

Region: NODE\_371225\_length\_8483\_cov\_20.365791 3342-3359. Max. coverage (+): 0.98. Max coverage (-): 0

Region: NODE\_371225\_length\_8483\_cov\_20.365791 3360-3376. Max. coverage (+): 0.15. Max coverage (-): 0.93

Region: NODE\_371225\_length\_8483\_cov\_20.365791 3377-3394. Max. coverage (+): 0.47. Max coverage (-): 0.03

Region: NODE\_371225\_length\_8483\_cov\_20.365791 3395-3411. Max. coverage (+): 0.01. Max coverage (-): 0.03

Region: NODE\_371225\_length\_8483\_cov\_20.365791 3412-3429. Max. coverage (+): 0.04. Max coverage (-): 0.2

Region: NODE\_371225\_length\_8483\_cov\_20.365791 3430-3447. Max. coverage (+): 6.92. Max coverage (-): 0.22

Region: NODE\_371225\_length\_8483\_cov\_20.365791 3448-3464. Max. coverage (+): 0.08. Max coverage (-): 3.19

Region: NODE\_371225\_length\_8483\_cov\_20.365791 3465-3482. Max. coverage (+): 0.01. Max coverage (-): 0.89

Region: NODE\_371225\_length\_8483\_cov\_20.365791 3483-3499. Max. coverage (+): 0. Max coverage (-): 9.13

Region: NODE\_371225\_length\_8483\_cov\_20.365791 3500-3517. Max. coverage (+): 0. Max coverage (-): 0.69

Region: NODE\_371225\_length\_8483\_cov\_20.365791 3518-3534. Max. coverage (+): 0. Max coverage (-): 0.65

Region: NODE\_371225\_length\_8483\_cov\_20.365791 3535-3552. Max. coverage (+): 0.04. Max coverage (-): 0.07

Region: NODE\_371225\_length\_8483\_cov\_20.365791 3553-3570. Max. coverage (+): 0.63. Max coverage (-): 1.71

Region: NODE\_371225\_length\_8483\_cov\_20.365791 3571-3587. Max. coverage (+): 1.13. Max coverage (-): 0.59

Region: NODE\_371225\_length\_8483\_cov\_20.365791 3588-3605. Max. coverage (+): 0. Max coverage (-): 0.71

Region: NODE\_371225\_length\_8483\_cov\_20.365791 3606-3622. Max. coverage (+): 0.08. Max coverage (-): 1.76

Region: NODE\_371225\_length\_8483\_cov\_20.365791 3623-3640. Max. coverage (+): 2.95. Max coverage (-): 0.12

Region: NODE\_371225\_length\_8483\_cov\_20.365791 3641-3658. Max. coverage (+): 0. Max coverage (-): 0.73

Region: NODE\_371225\_length\_8483\_cov\_20.365791 3659-3675. Max. coverage (+): 0.2. Max coverage (-): 0

Region: NODE\_371225\_length\_8483\_cov\_20.365791 3676-3693. Max. coverage (+): 0. Max coverage (-): 0.28

Region: NODE\_371225\_length\_8483\_cov\_20.365791 3694-3710. Max. coverage (+): 0.04. Max coverage (-): 5.09

Region: NODE\_371225\_length\_8483\_cov\_20.365791 3711-3728. Max. coverage (+): 0.12. Max coverage (-): 5.45

Region: NODE\_371225\_length\_8483\_cov\_20.365791 3729-3746. Max. coverage (+): 0.12. Max coverage (-): 2.34

Region: NODE\_371225\_length\_8483\_cov\_20.365791 3747-3763. Max. coverage (+): 0. Max coverage (-): 0.04

Region: NODE\_371225\_length\_8483\_cov\_20.365791 3764-3781. Max. coverage (+): 0.01. Max coverage (-): 4.02

Region: NODE\_371225\_length\_8483\_cov\_20.365791 3782-3798. Max. coverage (+): 0. Max coverage (-): 0.95

Region: NODE\_371225\_length\_8483\_cov\_20.365791 3799-3816. Max. coverage (+): 0. Max coverage (-): 1.01

Region: NODE\_371225\_length\_8483\_cov\_20.365791 3817-3834. Max. coverage (+): 0.32. Max coverage (-): 1.29

Region: NODE\_371225\_length\_8483\_cov\_20.365791 3835-3851. Max. coverage (+): 0. Max coverage (-): 0

Region: NODE\_371225\_length\_8483\_cov\_20.365791 3852-3869. Max. coverage (+): 0. Max coverage (-): 0.61

Region: NODE\_371225\_length\_8483\_cov\_20.365791 3870-3886. Max. coverage (+): 0.36. Max coverage (-): 0.61

Region: NODE\_371225\_length\_8483\_cov\_20.365791 3887-3904. Max. coverage (+): 0. Max coverage (-): 2.02

Region: NODE\_371225\_length\_8483\_cov\_20.365791 3905-3922. Max. coverage (+): 0. Max coverage (-): 1.37

Region: NODE\_371225\_length\_8483\_cov\_20.365791 3923-3939. Max. coverage (+): 1.31. Max coverage (-): 1.09

Region: NODE\_371225\_length\_8483\_cov\_20.365791 3940-3957. Max. coverage (+): 1.49. Max coverage (-): 0.26

Region: NODE\_371225\_length\_8483\_cov\_20.365791 3958-3974. Max. coverage (+): 0. Max coverage (-): 0.08

Region: NODE\_371225\_length\_8483\_cov\_20.365791 3975-3992. Max. coverage (+): 0.59. Max coverage (-): 2.58

Region: NODE\_371225\_length\_8483\_cov\_20.365791 3993-4010. Max. coverage (+): 0.61. Max coverage (-): 0.04

Region: NODE\_371225\_length\_8483\_cov\_20.365791 4011-4027. Max. coverage (+): 0.12. Max coverage (-): 0.71

Region: NODE\_371225\_length\_8483\_cov\_20.365791 4028-4045. Max. coverage (+): 0.04. Max coverage (-): 5.57

Region: NODE\_371225\_length\_8483\_cov\_20.365791 4046-4062. Max. coverage (+): 0.04. Max coverage (-): 2.42

Region: NODE\_371225\_length\_8483\_cov\_20.365791 4063-4080. Max. coverage (+): 0.28. Max coverage (-): 0.71

Region: NODE\_371225\_length\_8483\_cov\_20.365791 4081-4098. Max. coverage (+): 0.04. Max coverage (-): 0.4

Region: NODE\_371225\_length\_8483\_cov\_20.365791 4099-4115. Max. coverage (+): 0.04. Max coverage (-): 1.13

Region: NODE\_371225\_length\_8483\_cov\_20.365791 4116-4133. Max. coverage (+): 0. Max coverage (-): 0.04

Region: NODE\_371225\_length\_8483\_cov\_20.365791 4134-4150. Max. coverage (+): 0.14. Max coverage (-): 0.65

Region: NODE\_371225\_length\_8483\_cov\_20.365791 4151-4168. Max. coverage (+): 0.04. Max coverage (-): 0.16

Region: NODE\_371225\_length\_8483\_cov\_20.365791 4169-4186. Max. coverage (+): 0.12. Max coverage (-): 0.12

Region: NODE\_371225\_length\_8483\_cov\_20.365791 4187-4203. Max. coverage (+): 0.4. Max coverage (-): 1.05

Region: NODE\_371225\_length\_8483\_cov\_20.365791 4204-4221. Max. coverage (+): 1.68. Max coverage (-): 0.18

Region: NODE\_371225\_length\_8483\_cov\_20.365791 4222-4238. Max. coverage (+): 0.06. Max coverage (-): 0.02

Region: NODE\_371225\_length\_8483\_cov\_20.365791 4239-4256. Max. coverage (+): 0. Max coverage (-): 3.39

Region: NODE\_371225\_length\_8483\_cov\_20.365791 4257-4274. Max. coverage (+): 0.02. Max coverage (-): 0.3

Region: NODE\_371225\_length\_8483\_cov\_20.365791 4275-4291. Max. coverage (+): 0.02. Max coverage (-): 0.52

Region: NODE\_371225\_length\_8483\_cov\_20.365791 4292-4309. Max. coverage (+): 1.01. Max coverage (-): 0.55

Region: NODE\_371225\_length\_8483\_cov\_20.365791 4310-4326. Max. coverage (+): 0.18. Max coverage (-): 0.38

Region: NODE\_371225\_length\_8483\_cov\_20.365791 4327-4344. Max. coverage (+): 0. Max coverage (-): 0.75

Region: NODE\_371225\_length\_8483\_cov\_20.365791 4345-4362. Max. coverage (+): 0. Max coverage (-): 0

Region: NODE\_371225\_length\_8483\_cov\_20.365791 4363-4379. Max. coverage (+): 0.12. Max coverage (-): 1.05

Region: NODE\_371225\_length\_8483\_cov\_20.365791 4380-4397. Max. coverage (+): 0.12. Max coverage (-): 0

Region: NODE\_371225\_length\_8483\_cov\_20.365791 4398-4414. Max. coverage (+): 0. Max coverage (-): 0.4

Region: NODE\_371225\_length\_8483\_cov\_20.365791 4415-4432. Max. coverage (+): 0.08. Max coverage (-): 0.08

Region: NODE\_371225\_length\_8483\_cov\_20.365791 4433-4449. Max. coverage (+): 0. Max coverage (-): 0.16

Region: NODE\_371225\_length\_8483\_cov\_20.365791 4450-4467. Max. coverage (+): 0. Max coverage (-): 0.04

Region: NODE\_371225\_length\_8483\_cov\_20.365791 4468-4485. Max. coverage (+): 0. Max coverage (-): 2.02

Region: NODE\_371225\_length\_8483\_cov\_20.365791 4486-4502. Max. coverage (+): 0. Max coverage (-): 0.24

Region: NODE\_371225\_length\_8483\_cov\_20.365791 4503-4520. Max. coverage (+): 0. Max coverage (-): 0.24

Region: NODE\_371225\_length\_8483\_cov\_20.365791 4521-4537. Max. coverage (+): 0.08. Max coverage (-): 1.94

Region: NODE\_371225\_length\_8483\_cov\_20.365791 4538-4555. Max. coverage (+): 0.12. Max coverage (-): 0.89

Region: NODE\_371225\_length\_8483\_cov\_20.365791 4556-4573. Max. coverage (+): 0. Max coverage (-): 0.95

Region: NODE\_371225\_length\_8483\_cov\_20.365791 4574-4590. Max. coverage (+): 0.85. Max coverage (-): 0.89

Region: NODE\_371225\_length\_8483\_cov\_20.365791 4591-4608. Max. coverage (+): 0.75. Max coverage (-): 0.44

Region: NODE\_371225\_length\_8483\_cov\_20.365791 4609-4625. Max. coverage (+): 0. Max coverage (-): 4.64

Region: NODE\_371225\_length\_8483\_cov\_20.365791 4626-4643. Max. coverage (+): 6.7. Max coverage (-): 0.04

Region: NODE\_371225\_length\_8483\_cov\_20.365791 4644-4661. Max. coverage (+): 0. Max coverage (-): 0.52

Region: NODE\_371225\_length\_8483\_cov\_20.365791 4662-4678. Max. coverage (+): 0.04. Max coverage (-): 0.48

Region: NODE\_371225\_length\_8483\_cov\_20.365791 4679-4696. Max. coverage (+): 0.28. Max coverage (-): 0.22

Region: NODE\_371225\_length\_8483\_cov\_20.365791 4697-4713. Max. coverage (+): 0. Max coverage (-): 0

Region: NODE\_371225\_length\_8483\_cov\_20.365791 4714-4731. Max. coverage (+): 0.1. Max coverage (-): 0.44

Region: NODE\_371225\_length\_8483\_cov\_20.365791 4732-4749. Max. coverage (+): 0.08. Max coverage (-): 0.05

Region: NODE\_371225\_length\_8483\_cov\_20.365791 4750-4766. Max. coverage (+): 0.16. Max coverage (-): 0.22

Region: NODE\_371225\_length\_8483\_cov\_20.365791 4767-4784. Max. coverage (+): 1.09. Max coverage (-): 0.12

Region: NODE\_371225\_length\_8483\_cov\_20.365791 4785-4801. Max. coverage (+): 0.08. Max coverage (-): 4.75

Region: NODE\_371225\_length\_8483\_cov\_20.365791 4802-4819. Max. coverage (+): 10.36. Max coverage (-): 0.61

Region: NODE\_371225\_length\_8483\_cov\_20.365791 4820-4837. Max. coverage (+): 10.04. Max coverage (-): 0.06

Region: NODE\_371225\_length\_8483\_cov\_20.365791 4838-4854. Max. coverage (+): 0.01. Max coverage (-): 0.75

Region: NODE\_371225\_length\_8483\_cov\_20.365791 4855-4872. Max. coverage (+): 0.28. Max coverage (-): 0.08

Region: NODE\_371225\_length\_8483\_cov\_20.365791 4873-4889. Max. coverage (+): 0.05. Max coverage (-): 82.38

Region: NODE\_371225\_length\_8483\_cov\_20.365791 4890-4907. Max. coverage (+): 0.16. Max coverage (-): 83.07

Region: NODE\_371225\_length\_8483\_cov\_20.365791 4908-4925. Max. coverage (+): 0.02. Max coverage (-): 0.57

Region: NODE\_371225\_length\_8483\_cov\_20.365791 4926-4942. Max. coverage (+): 0.04. Max coverage (-): 1.78

Region: NODE\_371225\_length\_8483\_cov\_20.365791 4943-4960. Max. coverage (+): 0. Max coverage (-): 9.41

Region: NODE\_371225\_length\_8483\_cov\_20.365791 4961-4977. Max. coverage (+): 0. Max coverage (-): 2.42

Region: NODE\_371225\_length\_8483\_cov\_20.365791 4978-4995. Max. coverage (+): 1.01. Max coverage (-): 1.94

Region: NODE\_371225\_length\_8483\_cov\_20.365791 4996-5013. Max. coverage (+): 0.02. Max coverage (-): 0.4

Region: NODE\_371225\_length\_8483\_cov\_20.365791 5014-5030. Max. coverage (+): 0. Max coverage (-): 0.32

Region: NODE\_371225\_length\_8483\_cov\_20.365791 5031-5048. Max. coverage (+): 0. Max coverage (-): 1.57

Region: NODE\_371225\_length\_8483\_cov\_20.365791 5049-5065. Max. coverage (+): 0.06. Max coverage (-): 0.44

Region: NODE\_371225\_length\_8483\_cov\_20.365791 5066-5083. Max. coverage (+): 0. Max coverage (-): 1.05

Region: NODE\_371225\_length\_8483\_cov\_20.365791 5084-5101. Max. coverage (+): 0. Max coverage (-): 0.5

Region: NODE\_371225\_length\_8483\_cov\_20.365791 5102-5118. Max. coverage (+): 0.26. Max coverage (-): 0.71

Region: NODE\_371225\_length\_8483\_cov\_20.365791 5119-5136. Max. coverage (+): 0.47. Max coverage (-): 0.16

Region: NODE\_371225\_length\_8483\_cov\_20.365791 5137-5153. Max. coverage (+): 0. Max coverage (-): 0.77

Region: NODE\_371225\_length\_8483\_cov\_20.365791 5154-5171. Max. coverage (+): 0. Max coverage (-): 4.64

Region: NODE\_371225\_length\_8483\_cov\_20.365791 5172-5189. Max. coverage (+): 0.02. Max coverage (-): 0.04

Region: NODE\_371225\_length\_8483\_cov\_20.365791 5190-5206. Max. coverage (+): 0. Max coverage (-): 0.2

Region: NODE\_371225\_length\_8483\_cov\_20.365791 5207-5224. Max. coverage (+): 1.17. Max coverage (-): 3.39

Region: NODE\_371225\_length\_8483\_cov\_20.365791 5225-5241. Max. coverage (+): 1.7. Max coverage (-): 0.48

Region: NODE\_371225\_length\_8483\_cov\_20.365791 5242-5259. Max. coverage (+): 0. Max coverage (-): 0.73

Region: NODE\_371225\_length\_8483\_cov\_20.365791 5260-5277. Max. coverage (+): 0.93. Max coverage (-): 0.28

Region: NODE\_371225\_length\_8483\_cov\_20.365791 5278-5294. Max. coverage (+): 1.01. Max coverage (-): 0.73

Region: NODE\_371225\_length\_8483\_cov\_20.365791 5295-5312. Max. coverage (+): 0.3. Max coverage (-): 1.74

Region: NODE\_371225\_length\_8483\_cov\_20.365791 5313-5329. Max. coverage (+): 0.3. Max coverage (-): 0.2

Region: NODE\_371225\_length\_8483\_cov\_20.365791 5330-5347. Max. coverage (+): 0.08. Max coverage (-): 0.12

Region: NODE\_371225\_length\_8483\_cov\_20.365791 5348-5364. Max. coverage (+): 0.16. Max coverage (-): 4.72

Region: NODE\_371225\_length\_8483\_cov\_20.365791 5365-5382. Max. coverage (+): 0.04. Max coverage (-): 4.81

Region: NODE\_371225\_length\_8483\_cov\_20.365791 5383-5400. Max. coverage (+): 0.08. Max coverage (-): 3.96

Region: NODE\_371225\_length\_8483\_cov\_20.365791 5401-5417. Max. coverage (+): 0.08. Max coverage (-): 0.2

Region: NODE\_371225\_length\_8483\_cov\_20.365791 5418-5435. Max. coverage (+): 0.04. Max coverage (-): 0.2

Region: NODE\_371225\_length\_8483\_cov\_20.365791 5436-5452. Max. coverage (+): 0.04. Max coverage (-): 1.01

Region: NODE\_371225\_length\_8483\_cov\_20.365791 5453-5470. Max. coverage (+): 0.04. Max coverage (-): 1.39

Region: NODE\_371225\_length\_8483\_cov\_20.365791 5471-5488. Max. coverage (+): 3.45. Max coverage (-): 1.41

Region: NODE\_371225\_length\_8483\_cov\_20.365791 5489-5505. Max. coverage (+): 1.62. Max coverage (-): 0.04

Region: NODE\_371225\_length\_8483\_cov\_20.365791 5506-5523. Max. coverage (+): 0.14. Max coverage (-): 0.04

Region: NODE\_371225\_length\_8483\_cov\_20.365791 5524-5540. Max. coverage (+): 0. Max coverage (-): 0.32

Region: NODE\_371225\_length\_8483\_cov\_20.365791 5541-5558. Max. coverage (+): 0.04. Max coverage (-): 2.06

Region: NODE\_371225\_length\_8483\_cov\_20.365791 5559-5576. Max. coverage (+): 0.04. Max coverage (-): 0.44

Region: NODE\_371225\_length\_8483\_cov\_20.365791 5577-5593. Max. coverage (+): 0.08. Max coverage (-): 2.34

Region: NODE\_371225\_length\_8483\_cov\_20.365791 5594-5611. Max. coverage (+): 0.04. Max coverage (-): 0.01

Region: NODE\_371225\_length\_8483\_cov\_20.365791 5612-5628. Max. coverage (+): 0.02. Max coverage (-): 0.36

Region: NODE\_371225\_length\_8483\_cov\_20.365791 5629-5646. Max. coverage (+): 0.05. Max coverage (-): 0.2

Region: NODE\_371225\_length\_8483\_cov\_20.365791 5647-5664. Max. coverage (+): 0.08. Max coverage (-): 0.65

Region: NODE\_371225\_length\_8483\_cov\_20.365791 5665-5681. Max. coverage (+): 0.08. Max coverage (-): 0.04

Region: NODE\_371225\_length\_8483\_cov\_20.365791 5682-5699. Max. coverage (+): 0. Max coverage (-): 0.04

Region: NODE\_371225\_length\_8483\_cov\_20.365791 5700-5716. Max. coverage (+): 0. Max coverage (-): 1.33

Region: NODE\_371225\_length\_8483\_cov\_20.365791 5717-5734. Max. coverage (+): 0.02. Max coverage (-): 0.24

Region: NODE\_371225\_length\_8483\_cov\_20.365791 5735-5752. Max. coverage (+): 0.02. Max coverage (-): 0.44

Region: NODE\_371225\_length\_8483\_cov\_20.365791 5753-5769. Max. coverage (+): 0.04. Max coverage (-): 0

Region: NODE\_371225\_length\_8483\_cov\_20.365791 5770-5787. Max. coverage (+): 0. Max coverage (-): 0.04

Region: NODE\_371225\_length\_8483\_cov\_20.365791 5788-5804. Max. coverage (+): 0.01. Max coverage (-): 0

Region: NODE\_371225\_length\_8483\_cov\_20.365791 5805-5822. Max. coverage (+): 0. Max coverage (-): 0.04

Region: NODE\_371225\_length\_8483\_cov\_20.365791 5823-5840. Max. coverage (+): 0. Max coverage (-): 0.46

Region: NODE\_371225\_length\_8483\_cov\_20.365791 5841-5857. Max. coverage (+): 0. Max coverage (-): 0.42

Region: NODE\_371225\_length\_8483\_cov\_20.365791 5858-5875. Max. coverage (+): 0.01. Max coverage (-): 0

Region: NODE\_371225\_length\_8483\_cov\_20.365791 5876-5892. Max. coverage (+): 0.01. Max coverage (-): 0.03

Region: NODE\_371225\_length\_8483\_cov\_20.365791 5893-5910. Max. coverage (+): 0.02. Max coverage (-): 0.02

Region: NODE\_371225\_length\_8483\_cov\_20.365791 5911-5928. Max. coverage (+): 0.01. Max coverage (-): 0

Region: NODE\_371225\_length\_8483\_cov\_20.365791 5929-5945. Max. coverage (+): 0. Max coverage (-): 0.02

Region: NODE\_371225\_length\_8483\_cov\_20.365791 5946-5963. Max. coverage (+): 0. Max coverage (-): 0

Region: NODE\_371225\_length\_8483\_cov\_20.365791 5964-5980. Max. coverage (+): 0. Max coverage (-): 0.02

Region: NODE\_371225\_length\_8483\_cov\_20.365791 5981-5998. Max. coverage (+): 0.02. Max coverage (-): 0

Region: NODE\_371225\_length\_8483\_cov\_20.365791 5999-6016. Max. coverage (+): 0.01. Max coverage (-): 0

Region: NODE\_371225\_length\_8483\_cov\_20.365791 6017-6033. Max. coverage (+): 0. Max coverage (-): 0

Region: NODE\_371225\_length\_8483\_cov\_20.365791 6034-6051. Max. coverage (+): 0. Max coverage (-): 0

Region: NODE\_371225\_length\_8483\_cov\_20.365791 6052-6068. Max. coverage (+): 0. Max coverage (-): 0.02

Region: NODE\_371225\_length\_8483\_cov\_20.365791 6069-6086. Max. coverage (+): 0. Max coverage (-): 0.04

Region: NODE\_371225\_length\_8483\_cov\_20.365791 6087-6104. Max. coverage (+): 0. Max coverage (-): 0.12

Region: NODE\_371225\_length\_8483\_cov\_20.365791 6105-6121. Max. coverage (+): 0. Max coverage (-): 0.12

Region: NODE\_371225\_length\_8483\_cov\_20.365791 6122-6139. Max. coverage (+): 0. Max coverage (-): 0.48

Region: NODE\_371225\_length\_8483\_cov\_20.365791 6140-6156. Max. coverage (+): 0. Max coverage (-): 0

Region: NODE\_371225\_length\_8483\_cov\_20.365791 6157-6174. Max. coverage (+): 0. Max coverage (-): 0

Region: NODE\_371225\_length\_8483\_cov\_20.365791 6175-6191. Max. coverage (+): 0. Max coverage (-): 0

Region: NODE\_371225\_length\_8483\_cov\_20.365791 6192-6209. Max. coverage (+): 0. Max coverage (-): 0

Region: NODE\_371225\_length\_8483\_cov\_20.365791 6210-6227. Max. coverage (+): 0. Max coverage (-): 0

Region: NODE\_371225\_length\_8483\_cov\_20.365791 6228-6244. Max. coverage (+): 0. Max coverage (-): 0.1

Region: NODE\_371225\_length\_8483\_cov\_20.365791 6245-6262. Max. coverage (+): 0.04. Max coverage (-): 0.04

Region: NODE\_371225\_length\_8483\_cov\_20.365791 6263-6279. Max. coverage (+): 0. Max coverage (-): 1.17

Region: NODE\_371225\_length\_8483\_cov\_20.365791 6280-6297. Max. coverage (+): 0. Max coverage (-): 1.17

Region: NODE\_371225\_length\_8483\_cov\_20.365791 6298-6315. Max. coverage (+): 0. Max coverage (-): 0

Region: NODE\_371225\_length\_8483\_cov\_20.365791 6316-6332. Max. coverage (+): 0. Max coverage (-): 0.08

Region: NODE\_371225\_length\_8483\_cov\_20.365791 6333-6350. Max. coverage (+): 0.02. Max coverage (-): 0.12

Region: NODE\_371225\_length\_8483\_cov\_20.365791 6351-6367. Max. coverage (+): 0.08. Max coverage (-): 0.01

Region: NODE\_371225\_length\_8483\_cov\_20.365791 6368-6385. Max. coverage (+): 0. Max coverage (-): 0.03

Region: NODE\_371225\_length\_8483\_cov\_20.365791 6386-6403. Max. coverage (+): 0.02. Max coverage (-): 0

Region: NODE\_371225\_length\_8483\_cov\_20.365791 6404-6420. Max. coverage (+): 0. Max coverage (-): 0.04

Region: NODE\_371225\_length\_8483\_cov\_20.365791 6421-6438. Max. coverage (+): 0.08. Max coverage (-): 0

Region: NODE\_371225\_length\_8483\_cov\_20.365791 6439-6455. Max. coverage (+): 0. Max coverage (-): 0.02

Region: NODE\_371225\_length\_8483\_cov\_20.365791 6456-6473. Max. coverage (+): 0.03. Max coverage (-): 0

Region: NODE\_371225\_length\_8483\_cov\_20.365791 6474-6491. Max. coverage (+): 0. Max coverage (-): 0

Region: NODE\_371225\_length\_8483\_cov\_20.365791 6492-6508. Max. coverage (+): 0. Max coverage (-): 0

Region: NODE\_371225\_length\_8483\_cov\_20.365791 6509-6526. Max. coverage (+): 0. Max coverage (-): 0.55

Region: NODE\_371225\_length\_8483\_cov\_20.365791 6527-6543. Max. coverage (+): 0.02. Max coverage (-): 0

Region: NODE\_371225\_length\_8483\_cov\_20.365791 6544-6561. Max. coverage (+): 0. Max coverage (-): 0

Region: NODE\_371225\_length\_8483\_cov\_20.365791 6562-6579. Max. coverage (+): 0. Max coverage (-): 0.04

Region: NODE\_371225\_length\_8483\_cov\_20.365791 6580-6596. Max. coverage (+): 0. Max coverage (-): 0.14

Region: NODE\_371225\_length\_8483\_cov\_20.365791 6597-6614. Max. coverage (+): 0.01. Max coverage (-): 0.01

Region: NODE\_371225\_length\_8483\_cov\_20.365791 6615-6631. Max. coverage (+): 0.1. Max coverage (-): 0.02

Region: NODE\_371225\_length\_8483\_cov\_20.365791 6632-6649. Max. coverage (+): 0.11. Max coverage (-): 0

Region: NODE\_371225\_length\_8483\_cov\_20.365791 6650-6667. Max. coverage (+): 0. Max coverage (-): 0

Region: NODE\_371225\_length\_8483\_cov\_20.365791 6668-6684. Max. coverage (+): 0.11. Max coverage (-): 0.01

Region: NODE\_371225\_length\_8483\_cov\_20.365791 6685-6702. Max. coverage (+): 0.01. Max coverage (-): 0

Region: NODE\_371225\_length\_8483\_cov\_20.365791 6703-6719. Max. coverage (+): 0. Max coverage (-): 0

Region: NODE\_371225\_length\_8483\_cov\_20.365791 6720-6737. Max. coverage (+): 0. Max coverage (-): 0

Region: NODE\_371225\_length\_8483\_cov\_20.365791 6738-6755. Max. coverage (+): 0. Max coverage (-): 0.04

Region: NODE\_371225\_length\_8483\_cov\_20.365791 6756-6772. Max. coverage (+): 0. Max coverage (-): 0

Region: NODE\_371225\_length\_8483\_cov\_20.365791 6773-6790. Max. coverage (+): 0. Max coverage (-): 0

Region: NODE\_371225\_length\_8483\_cov\_20.365791 6791-6807. Max. coverage (+): 0. Max coverage (-): 0.01

Region: NODE\_371225\_length\_8483\_cov\_20.365791 6808-6825. Max. coverage (+): 0. Max coverage (-): 0

Region: NODE\_371225\_length\_8483\_cov\_20.365791 6826-6843. Max. coverage (+): 0.01. Max coverage (-): 0

Region: NODE\_371225\_length\_8483\_cov\_20.365791 6844-6860. Max. coverage (+): 0.01. Max coverage (-): 0

Region: NODE\_371225\_length\_8483\_cov\_20.365791 6861-6878. Max. coverage (+): 0. Max coverage (-): 0.02

Region: NODE\_371225\_length\_8483\_cov\_20.365791 6879-6895. Max. coverage (+): 0. Max coverage (-): 0

Region: NODE\_371225\_length\_8483\_cov\_20.365791 6896-6913. Max. coverage (+): 0. Max coverage (-): 0

Region: NODE\_371225\_length\_8483\_cov\_20.365791 6914-6931. Max. coverage (+): 0. Max coverage (-): 0

Region: NODE\_371225\_length\_8483\_cov\_20.365791 6932-6948. Max. coverage (+): 0. Max coverage (-): 0

Region: NODE\_371225\_length\_8483\_cov\_20.365791 6949-6966. Max. coverage (+): 0. Max coverage (-): 0

Region: NODE\_371225\_length\_8483\_cov\_20.365791 6967-6983. Max. coverage (+): 0. Max coverage (-): 0

Region: NODE\_371225\_length\_8483\_cov\_20.365791 6984-7001. Max. coverage (+): 0.07. Max coverage (-): 0

Region: NODE\_371225\_length\_8483\_cov\_20.365791 7002-7019. Max. coverage (+): 0.01. Max coverage (-): 0

Region: NODE\_371225\_length\_8483\_cov\_20.365791 7020-7036. Max. coverage (+): 0.01. Max coverage (-): 0

Region: NODE\_371225\_length\_8483\_cov\_20.365791 7037-7054. Max. coverage (+): 0. Max coverage (-): 0

Region: NODE\_371225\_length\_8483\_cov\_20.365791 7055-7071. Max. coverage (+): 0.01. Max coverage (-): 0.01

Region: NODE\_371225\_length\_8483\_cov\_20.365791 7072-7089. Max. coverage (+): 0. Max coverage (-): 0

Region: NODE\_371225\_length\_8483\_cov\_20.365791 7090-7106. Max. coverage (+): 0. Max coverage (-): 0

Region: NODE\_371225\_length\_8483\_cov\_20.365791 7107-7124. Max. coverage (+): 0. Max coverage (-): 0.02

Region: NODE\_371225\_length\_8483\_cov\_20.365791 7125-7142. Max. coverage (+): 0. Max coverage (-): 0.02

Region: NODE\_371225\_length\_8483\_cov\_20.365791 7143-7159. Max. coverage (+): 0. Max coverage (-): 0

Region: NODE\_371225\_length\_8483\_cov\_20.365791 7160-7177. Max. coverage (+): 0. Max coverage (-): 0

Region: NODE\_371225\_length\_8483\_cov\_20.365791 7178-7194. Max. coverage (+): 0. Max coverage (-): 0

Region: NODE\_371225\_length\_8483\_cov\_20.365791 7195-7212. Max. coverage (+): 0. Max coverage (-): 0

Region: NODE\_371225\_length\_8483\_cov\_20.365791 7213-7230. Max. coverage (+): 0. Max coverage (-): 0

Region: NODE\_371225\_length\_8483\_cov\_20.365791 7231-7247. Max. coverage (+): 0.01. Max coverage (-): 0

Region: NODE\_371225\_length\_8483\_cov\_20.365791 7248-7265. Max. coverage (+): 0. Max coverage (-): 0

Region: NODE\_371225\_length\_8483\_cov\_20.365791 7266-7282. Max. coverage (+): 0. Max coverage (-): 0

Region: NODE\_371225\_length\_8483\_cov\_20.365791 7283-7300. Max. coverage (+): 0. Max coverage (-): 0

Region: NODE\_371225\_length\_8483\_cov\_20.365791 7301-7318. Max. coverage (+): 0. Max coverage (-): 0

Region: NODE\_371225\_length\_8483\_cov\_20.365791 7319-7335. Max. coverage (+): 0. Max coverage (-): 0

Region: NODE\_371225\_length\_8483\_cov\_20.365791 7336-7353. Max. coverage (+): 0. Max coverage (-): 0

Region: NODE\_371225\_length\_8483\_cov\_20.365791 7354-7370. Max. coverage (+): 0.08. Max coverage (-): 0

Region: NODE\_371225\_length\_8483\_cov\_20.365791 7371-7388. Max. coverage (+): 0.01. Max coverage (-): 0

Region: NODE\_371225\_length\_8483\_cov\_20.365791 7389-7406. Max. coverage (+): 0.01. Max coverage (-): 0.01

Region: NODE\_371225\_length\_8483\_cov\_20.365791 7407-7423. Max. coverage (+): 0. Max coverage (-): 0.17

Region: NODE\_371225\_length\_8483\_cov\_20.365791 7424-7441. Max. coverage (+): 0. Max coverage (-): 0.05

Region: NODE\_371225\_length\_8483\_cov\_20.365791 7442-7458. Max. coverage (+): 0.11. Max coverage (-): 0.02

Region: NODE\_371225\_length\_8483\_cov\_20.365791 7459-7476. Max. coverage (+): 0.08. Max coverage (-): 0.01

Region: NODE\_371225\_length\_8483\_cov\_20.365791 7477-7494. Max. coverage (+): 0.11. Max coverage (-): 0

Region: NODE\_371225\_length\_8483\_cov\_20.365791 7495-7511. Max. coverage (+): 0.01. Max coverage (-): 0.02

Region: NODE\_371225\_length\_8483\_cov\_20.365791 7512-7529. Max. coverage (+): 0.02. Max coverage (-): 0.04

Region: NODE\_371225\_length\_8483\_cov\_20.365791 7530-7546. Max. coverage (+): 0.01. Max coverage (-): 0.01

Region: NODE\_371225\_length\_8483\_cov\_20.365791 7547-7564. Max. coverage (+): 0. Max coverage (-): 0

Region: NODE\_371225\_length\_8483\_cov\_20.365791 7565-7582. Max. coverage (+): 0. Max coverage (-): 0

Region: NODE\_371225\_length\_8483\_cov\_20.365791 7583-7599. Max. coverage (+): 0. Max coverage (-): 0.02

Region: NODE\_371225\_length\_8483\_cov\_20.365791 7600-7617. Max. coverage (+): 0.01. Max coverage (-): 0

Region: NODE\_371225\_length\_8483\_cov\_20.365791 7618-7634. Max. coverage (+): 0. Max coverage (-): 0

Region: NODE\_371225\_length\_8483\_cov\_20.365791 7635-7652. Max. coverage (+): 0. Max coverage (-): 0

Region: NODE\_371225\_length\_8483\_cov\_20.365791 7653-7670. Max. coverage (+): 0. Max coverage (-): 0

Region: NODE\_371225\_length\_8483\_cov\_20.365791 7671-7687. Max. coverage (+): 0. Max coverage (-): 0

Region: NODE\_371225\_length\_8483\_cov\_20.365791 7688-7705. Max. coverage (+): 0. Max coverage (-): 0

Region: NODE\_371225\_length\_8483\_cov\_20.365791 7706-7722. Max. coverage (+): 0. Max coverage (-): 0

Region: NODE\_371225\_length\_8483\_cov\_20.365791 7723-7740. Max. coverage (+): 0. Max coverage (-): 0

Region: NODE\_371225\_length\_8483\_cov\_20.365791 7741-7758. Max. coverage (+): 0. Max coverage (-): 0

Region: NODE\_371225\_length\_8483\_cov\_20.365791 7759-7775. Max. coverage (+): 0. Max coverage (-): 0

Region: NODE\_371225\_length\_8483\_cov\_20.365791 7776-7793. Max. coverage (+): 0. Max coverage (-): 0

Region: NODE\_371225\_length\_8483\_cov\_20.365791 7794-7810. Max. coverage (+): 0. Max coverage (-): 0

Region: NODE\_371225\_length\_8483\_cov\_20.365791 7811-7828. Max. coverage (+): 0. Max coverage (-): 0

Region: NODE\_371225\_length\_8483\_cov\_20.365791 7829-7846. Max. coverage (+): 0. Max coverage (-): 0

Region: NODE\_371225\_length\_8483\_cov\_20.365791 7847-7863. Max. coverage (+): 0. Max coverage (-): 0

Region: NODE\_371225\_length\_8483\_cov\_20.365791 7864-7881. Max. coverage (+): 0. Max coverage (-): 0

Region: NODE\_371225\_length\_8483\_cov\_20.365791 7882-7898. Max. coverage (+): 0. Max coverage (-): 0

Region: NODE\_371225\_length\_8483\_cov\_20.365791 7899-7916. Max. coverage (+): 0. Max coverage (-): 0

Region: NODE\_371225\_length\_8483\_cov\_20.365791 7917-7933. Max. coverage (+): 0. Max coverage (-): 0

Region: NODE\_371225\_length\_8483\_cov\_20.365791 7934-7951. Max. coverage (+): 0. Max coverage (-): 0

Region: NODE\_371225\_length\_8483\_cov\_20.365791 7952-7969. Max. coverage (+): 0. Max coverage (-): 0

Region: NODE\_371225\_length\_8483\_cov\_20.365791 7970-7986. Max. coverage (+): 0. Max coverage (-): 0

Region: NODE\_371225\_length\_8483\_cov\_20.365791 7987-8004. Max. coverage (+): 0. Max coverage (-): 0

Region: NODE\_371225\_length\_8483\_cov\_20.365791 8005-8021. Max. coverage (+): 0. Max coverage (-): 0

Region: NODE\_371225\_length\_8483\_cov\_20.365791 8022-8039. Max. coverage (+): 0. Max coverage (-): 0

Region: NODE\_371225\_length\_8483\_cov\_20.365791 8040-8057. Max. coverage (+): 0. Max coverage (-): 0

Region: NODE\_371225\_length\_8483\_cov\_20.365791 8058-8074. Max. coverage (+): 0. Max coverage (-): 0

Region: NODE\_371225\_length\_8483\_cov\_20.365791 8075-8092. Max. coverage (+): 0. Max coverage (-): 0

Region: NODE\_371225\_length\_8483\_cov\_20.365791 8093-8109. Max. coverage (+): 0. Max coverage (-): 0

Region: NODE\_371225\_length\_8483\_cov\_20.365791 8110-8127. Max. coverage (+): 0. Max coverage (-): 0.04

Region: NODE\_371225\_length\_8483\_cov\_20.365791 8128-8145. Max. coverage (+): 0.08. Max coverage (-): 0

Region: NODE\_371225\_length\_8483\_cov\_20.365791 8146-8162. Max. coverage (+): 0.65. Max coverage (-): 0.04

Region: NODE\_371225\_length\_8483\_cov\_20.365791 8163-8180. Max. coverage (+): 0. Max coverage (-): 0

Region: NODE\_371225\_length\_8483\_cov\_20.365791 8181-8197. Max. coverage (+): 0. Max coverage (-): 0.01

Region: NODE\_371225\_length\_8483\_cov\_20.365791 8198-8215. Max. coverage (+): 0. Max coverage (-): 0.17

Region: NODE\_371225\_length\_8483\_cov\_20.365791 8216-8233. Max. coverage (+): 0. Max coverage (-): 0.02

Region: NODE\_371225\_length\_8483\_cov\_20.365791 8234-8250. Max. coverage (+): 0.11. Max coverage (-): 0.01

Region: NODE\_371225\_length\_8483\_cov\_20.365791 8251-8268. Max. coverage (+): 0.11. Max coverage (-): 0

Region: NODE\_371225\_length\_8483\_cov\_20.365791 8269-8285. Max. coverage (+): 0.07. Max coverage (-): 0.01

Region: NODE\_371225\_length\_8483\_cov\_20.365791 8286-8303. Max. coverage (+): 0.01. Max coverage (-): 0.02

Region: NODE\_371225\_length\_8483\_cov\_20.365791 8304-8321. Max. coverage (+): 0.02. Max coverage (-): 0.04

Region: NODE\_371225\_length\_8483\_cov\_20.365791 8322-8338. Max. coverage (+): 0.01. Max coverage (-): 0.1

Region: NODE\_371225\_length\_8483\_cov\_20.365791 8339-8356. Max. coverage (+): 0.02. Max coverage (-): 0.1

Region: NODE\_371225\_length\_8483\_cov\_20.365791 8357-8373. Max. coverage (+): 0.02. Max coverage (-): 0.01

Region: NODE\_371225\_length\_8483\_cov\_20.365791 8374-8391. Max. coverage (+): 0. Max coverage (-): 0.02

Region: NODE\_371225\_length\_8483\_cov\_20.365791 8392-8409. Max. coverage (+): 0.01. Max coverage (-): 0

Region: NODE\_371225\_length\_8483\_cov\_20.365791 8410-8426. Max. coverage (+): 0. Max coverage (-): 0

Region: NODE\_371225\_length\_8483\_cov\_20.365791 8427-8444. Max. coverage (+): 0. Max coverage (-): 0

Region: NODE\_371225\_length\_8483\_cov\_20.365791 8445-8461. Max. coverage (+): 0. Max coverage (-): 0

Region: NODE\_371225\_length\_8483\_cov\_20.365791 8462-8479. Max. coverage (+): 0. Max coverage (-): 0.04

Region: NODE\_371225\_length\_8483\_cov\_20.365791 8480-8497. Max. coverage (+): 0. Max coverage (-): 0

Region: NODE\_371225\_length\_8483\_cov\_20.365791 8498-8514. Max. coverage (+): 0.01. Max coverage (-): 0

Region: NODE\_371225\_length\_8483\_cov\_20.365791 8515-8532. Max. coverage (+): 0. Max coverage (-): 0

Region: NODE\_371225\_length\_8483\_cov\_20.365791 8533-8549. Max. coverage (+): 0. Max coverage (-): 0

Region: NODE\_371225\_length\_8483\_cov\_20.365791 8550-8567. Max. coverage (+): 0.03. Max coverage (-): 0.07

Region: NODE\_371225\_length\_8483\_cov\_20.365791 8568-8585. Max. coverage (+): 0.16. Max coverage (-): 0.09

Region: NODE\_371225\_length\_8483\_cov\_20.365791 8586-8602. Max. coverage (+): 0. Max coverage (-): 0.04

Region: NODE\_371225\_length\_8483\_cov\_20.365791 8603-8620. Max. coverage (+): 0. Max coverage (-): 0.04

Region: NODE\_371225\_length\_8483\_cov\_20.365791 8621-8637. Max. coverage (+): 0.04. Max coverage (-): 0

Region: NODE\_371225\_length\_8483\_cov\_20.365791 8638-8655. Max. coverage (+): 0. Max coverage (-): 0

Region: NODE\_371225\_length\_8483\_cov\_20.365791 8656-8673. Max. coverage (+): 0. Max coverage (-): 0

Region: NODE\_371225\_length\_8483\_cov\_20.365791 8674-8690. Max. coverage (+): 0. Max coverage (-): 0

Region: NODE\_371225\_length\_8483\_cov\_20.365791 8691-8708. Max. coverage (+): 0. Max coverage (-): 0

Region: NODE\_371225\_length\_8483\_cov\_20.365791 8709-8725. Max. coverage (+): 0. Max coverage (-): 0

Region: NODE\_371225\_length\_8483\_cov\_20.365791 8726-8743. Max. coverage (+): 0. Max coverage (-): 0

Region: NODE\_371225\_length\_8483\_cov\_20.365791 8744-8761. Max. coverage (+): 0. Max coverage (-): 0.03

Region: NODE\_371225\_length\_8483\_cov\_20.365791 8762-8778. Max. coverage (+): 0.08. Max coverage (-): 0

Region: NODE\_371225\_length\_8483\_cov\_20.365791 8779-8796. Max. coverage (+): 0.04. Max coverage (-): 0

Region: NODE\_371225\_length\_8483\_cov\_20.365791 8797-. Max. coverage (+): 0. Max coverage (-): 0

RepeatMasker Color Code

**+**

100-98% Identity

<98-95% Identity

<95-90% Identity

<90-85% Identity

<85-80% Identity

<80-75% Identity

<75-70% Identity

<70% Identity

**-**

Gene Set Color Code

**+**

Gene

Pseudogene

Other

**-**

Topology/Coverage Color Code

Coverage Plus Strand

Coverage Minus Strand

Mainstrand: Plus

Mainstrand: Minus

Complementary Strand

Flanking Region  
(if option -flank >0)

Gene Set Annotation  

**1. unknown (unknownunknown) Tr:unknown**: 4662-5381 (+)  
**2. unknown (unknownunknown) Tr:unknown**: 5466-6131 (+)  
**3. unknown (unknownunknown) Tr:unknown**: 3814-4524 (+)

  
RepeatMasker Annotation  

**1. Gypsy4-I\_DR**: 228-396 (-), Divergence to consensus: 40.1%  
**2. Gypsy4-I\_DR**: 491-556 (-), Divergence to consensus: 19.7%  
**3. Gypsy4-I\_DR**: 579-1901 (-), Divergence to consensus: 31.4%  
**4. Gypsy4-I\_DR**: 2441-3061 (-), Divergence to consensus: 36.5%  
**5. Gypsy4-I\_DR**: 6253-6593 (-), Divergence to consensus: 36.8%  
**6. Gypsy4-LTR\_DR**: 6594-6803 (-), Divergence to consensus: 34.5%  
**7. Gypsy4-LTR\_DR**: 7404-7494 (-), Divergence to consensus: 28.9%  
**8. Mariner-16\_DF**: 7705-7761 (+), Divergence to consensus: 29.7%  
**9. Ginger1-5\_HM**: 7724-7765 (-), Divergence to consensus: 21.4%  
**10. AlRepE-1229**: 7758-8067 (+), Divergence to consensus: 42.7%  
**11. Gypsy4-LTR\_DR**: 8191-8492 (-), Divergence to consensus: 43.4%  
**12. Gypsy4-I\_DR**: 8493-8808 (-), Divergence to consensus: 44.7%

  
Transcription Factor Binding Sites  

**RHOXF1** (Sequence: GGCTCA (-): 262)  
**RHOXF1** (Sequence: AGATCA (-): 682)  
**RHOXF1** (Sequence: AGCTTA (-): 1282)  
**RHOXF1** (Sequence: AGCTTA (-): 1654)  
**RHOXF1** (Sequence: AGATCA (-): 1911)  
**RHOXF1** (Sequence: AGATCA (-): 2079)  
**RHOXF1** (Sequence: GGCTTA (-): 3273)  
**RHOXF1** (Sequence: AGCTTA (-): 3779)  
**RHOXF1** (Sequence: AGCTCA (-): 4515)  
**RHOXF1** (Sequence: AGATCA (-): 4681)  
**RHOXF1** (Sequence: AGATCA (-): 4915)  
**RHOXF1** (Sequence: GGATCA (-): 5599)  
**RHOXF1** (Sequence: AGCTTA (-): 5713)  
**RHOXF1** (Sequence: AGATCA (-): 6722)  
**RHOXF1** (Sequence: AGCTTA (-): 6954)  
**RHOXF1** (Sequence: AGATTA (-): 6994)  
**RHOXF1** (Sequence: AGCTTA (-): 7061)  
**RHOXF1** (Sequence: TGATCC (+): 661)  
**RHOXF1** (Sequence: TGATCT (+): 926)  
**RHOXF1** (Sequence: TGAGCC (+): 1494)  
**RHOXF1** (Sequence: TGAGCT (+): 1685)  
**RHOXF1** (Sequence: TAATCT (+): 2514)  
**RHOXF1** (Sequence: TAATCT (+): 2958)  
**RHOXF1** (Sequence: TAATCT (+): 3084)  
**RHOXF1** (Sequence: TAAGCT (+): 3404)  
**RHOXF1** (Sequence: TGAGCT (+): 3524)  
**RHOXF1** (Sequence: TGAGCT (+): 3945)  
**RHOXF1** (Sequence: TAAGCT (+): 4014)  
**RHOXF1** (Sequence: TGAGCT (+): 4131)  
**RHOXF1** (Sequence: TAATCT (+): 4266)  
**RHOXF1** (Sequence: TGATCT (+): 4301)  
**RHOXF1** (Sequence: TGATCC (+): 4431)  
**RHOXF1** (Sequence: TGATCC (+): 5102)  
**RHOXF1** (Sequence: TAATCC (+): 5192)  
**RHOXF1** (Sequence: TGAGCT (+): 5711)  
**RHOXF1** (Sequence: TGAGCC (+): 5936)  
**RHOXF1** (Sequence: TGAGCT (+): 6233)  
**RHOXF1** (Sequence: TGAGCC (+): 8793)  
**Lhx8** (Sequence: TTAATTAG (-): 2333)  
**Gata4** (Sequence: CTTATCT (+): 3781)  
**POU5F1** (Sequence: TTTGCAT (-): 3230)  
**POU5F1** (Sequence: TTTGCAT (-): 7930)  
**RFX4\_2** (Sequence: GTATCTATG (-): 6815)  
**FOXP1** (Sequence: GTAAACA (+): 8145)  
**FOXO3\_mmu** (Sequence: TGTTTTCA (-): 6749)  
**Sox5** (Sequence: ATTGTT (+): 2390)  
**Sox5** (Sequence: ATTGTT (+): 2403)  
**Sox5** (Sequence: ATTGTT (+): 2554)  
**Sox5** (Sequence: ATTGTT (+): 7182)  
**Sox5** (Sequence: ATTGTT (+): 7588)  
**Sox5** (Sequence: ATTGTT (+): 8375)  
**FIGLA** (Sequence: TACAGCTGTT (-): 1577)  
**FOXO3\_mmu** (Sequence: TGAAAACA (+): 6303)  
**FOXO1** (Sequence: GAAAACAAG (-): 6304)  
**FOXP1** (Sequence: TGTTTAC (-): 5033)  
**Nobox** (Sequence: TAATTGCT (+): 7632)  
**Nobox** (Sequence: TAATTGCT (+): 8419)  
**Rhox11** (Sequence: TGGTGTTAA (+): 1587)  
**Rhox11** (Sequence: TAAACACCG (-): 8146)  
**Sox5** (Sequence: AACAAT (-): 2917)  
**Sox5** (Sequence: AACAAT (-): 5065)
