# Supplementary material for: First characterization of PIWI-interacting RNA clusters in a cichlid fish with a B chromosome
Source: BMC Biol. 2022 Sep 21;20:204. doi: 10.1186/s12915-022-01403-2 (PMC9490952; doi:10.1186/s12915-022-01403-2)
Supplement: Supplementary file 1 — Additional file 1. Zipped folder with fasta and interactive html piRNA cluster information for the A. latifasciata genome. The nomenclature is as follows: number-pirna-cluster_sex_B-presence (f, female; m, male; 0b, without B chromosome; 1b, with B chromosome). [file 12915_2022_1403_MOESM1_ESM.zip › 143_m1b.html]

piRNA cluster 143\_m1b 84


Predicted piRNA cluster no. 143\_m1b
  

Show proTRAC run info
Hide proTRAC run info

/\  
                \_\_\_\_\_\_\_\_\_\_\_\_\_\_\_\_\_\_\_\_\_\_\_/\\_\_\_ /  \\_\_\_\_\_\_\_  
               I                      /  \  /    \      I  
               I     pro             /    \/      \     I  
               I        TRAC        /               \   I  
               I   \_\_\_\_\_\_\_\_\_\_\_\_\_\_\_\_/\_\_\_\_\_\_\_\_\_\_\_\_\_\_\_\_\_\\_ I  
               I   \              /                     I  
               I    \            /                      I  
               I     \  /\      /       V.2.4.2         I  
               I      \/  \    /                        I  
               I\_\_\_\_\_\_\_\_\_\_\_\  /\_\_\_\_\_\_\_\_\_\_\_\_\_\_\_\_\_\_\_\_\_\_\_\_\_I  
                            \/  
  
  
================================= proTRAC ====================================  
VERSION: .......... 2.4.2  
LAST MODIFIED: .... 11. May 2018  
  
Please cite:  
Rosenkranz D, Zischler H. proTRAC - a software for probabilistic piRNA cluster  
detection, visualization and analysis. 2012. BMC Bioinformatics 13:5.  
  
  
Contact:  
David Rosenkranz  
Institute of Organismic and Molecular Evolutionary Biology  
Dept. Anthropology, small RNA group  
Johannes Gutenberg University Mainz  
email: rosenkranz@uni-mainz.de  
  
You can find the latest proTRAC version at:  
http://sourceforge.net/projects/protrac/files  
http://www.smallRNAgroup-mainz.de/software  
==============================================================================  
  
PARAMETERS:  
Map file: ...............piwi-machos-1B.fa-collapse.map  
Genome file: ............../../../0B\_ala\_genome.fa  
RepeatMasker annotation: Alatifasciata-all0B-maryan-v2.fa\_corrected.out  
GeneSet:................./guest-storage/Data/annotation/Alatifasciata\_all0B\_maryan-v2\_out2017.gff  
  
Significant (p<=0.01) hit density will be calculated based  
on observed hit distribution.  
  
Sliding window size: ........................................ 5000 bp  
Sliding window increament: .................................. 1000 bp  
Normalize each hit by number of genomic hits: ............... yes  
Normalize each hit by number of sequence reads: ............. yes  
Normalize values (-> per million mapped reads): ............. yes  
Min. fraction of hits with 1T(U) or 10A: .................... 0.75  
Alternatively: Min. fraction of hits with 1T(U) and 10A: .... 0.5  
Min. fraction of hits with typical piRNA length: ............ 0.75  
Typical piRNA length: ....................................... 24-32 nt  
Min. size of a piRNA cluster: ............................... 1000 bp.  
Min. number of hits (absolute): ............................. 0  
Min. number of hits (normalized): ........................... 0  
Min. fraction of hits on the mainstrand: .................... 0.75  
Top fraction of mapped sequences (in terms of read counts): . 1%  
Top fraction accounts for max. n% of sequence reads: ........ 90%  
Min. fraction of hits on each arm of a bidirectional cluster: 0.05  
Output html file for each cluster: .......................... yes  
Output a summary table: ..................................... yes  
Output a FASTA file for each cluster (piRNA sequences): ..... yes  
Output a FASTA file comprising cluster sequences: ........... yes  
Output a GTF file for predicted piRNA clusters: ..............yes  
Search DNA motifs in clusters: .............................. yes  
Output flanking sequences: +/- .............................. 0 bp  
Output ~.pTi file: .......................................... no  
==============================================================================  
  
  
Genome size (without gaps): ............ 758543724 bp  
Gaps (N/X/-): .......................... 417479 bp  
Mapped reads: .......................... 26973943  
Non-identical sequences: ............... 6209225  
Genomic hits: .......................... 48438990  
Significant densitiy of mapped reads: .. 821.144211136946 reads/kb

Show proTRAC cluster info
Hide proTRAC cluster info

|  |  |
| --- | --- |
| Location | NODE\_371225\_length\_8483\_cov\_20.365791 |
| Coordinates | 21-8805 |
| Size [bp] | 8785 |
| Sequence hit loci | 3316 |
| Mapped reads (normalized) | 8967.5 |
| Mapped reads (normalized) per kb | 1020.8 |
| Normalized reads with 1T (1U) | 77.2% |
| Normalized reads with 10A | 54.9% |
| Normalized reads with length 24-32 nt | 99% |
| Normalized reads on the main strand(s) | 81.6% |
| Predicted directionality | mono:minus |

100%

0%

1T (1U)  
reads

10A reads

24-32 nt  
reads

reads on mainstrand

**Either the amount of reads with 1T (1U) OR 10A has to exceed 75% (set with option: -1Tor10A)  
Alternatively the amount of reads with 1T (1U) AND 10A has to exceed 50% (set with option: -1Tand10A)  
Minimum amount of reads with preferred size is 75% (set with option: -pisize)  
Minimum amount of reads on the main strand(s) is 75% (set with option: -clstrand)**

Show read coverage
Hide read coverage

WHAT DO I SEE HERE?  
This chart shows the location of mapped sequence reads within a predicted piRNA cluster. The color refers to the number of genomic hits produced by the sequence read in question. A dark red bar indicates that this sequence read produces many other hits elsewhere in the genome. Many adjacent red or yellow bars can indicate the presence of a multi-copy element such as transposons or rRNA genes. A dark green bar indicates that this sequence read maps uniquely to this locus.

1 hit

2-5 hits

6-10 hits

11-20 hits

21-50 hits

51-100 hits

> 100 hits

NODE\_371225\_length\_8483\_cov\_20.365791

21

8805

Gene Set

RepeatMasker

Mapped  
Reads

88.08

plus strand

minus strand

88.08

Region: NODE\_371225\_length\_8483\_cov\_20.365791 1630-29. Max. coverage (+): 0.87. Max coverage (-): 0

Region: NODE\_371225\_length\_8483\_cov\_20.365791 30-47. Max. coverage (+): 0.59. Max coverage (-): 0.06

Region: NODE\_371225\_length\_8483\_cov\_20.365791 48-64. Max. coverage (+): 0. Max coverage (-): 0.04

Region: NODE\_371225\_length\_8483\_cov\_20.365791 65-82. Max. coverage (+): 0.11. Max coverage (-): 0.04

Region: NODE\_371225\_length\_8483\_cov\_20.365791 83-100. Max. coverage (+): 0.04. Max coverage (-): 0

Region: NODE\_371225\_length\_8483\_cov\_20.365791 101-117. Max. coverage (+): 0. Max coverage (-): 0

Region: NODE\_371225\_length\_8483\_cov\_20.365791 118-135. Max. coverage (+): 0.37. Max coverage (-): 0

Region: NODE\_371225\_length\_8483\_cov\_20.365791 136-152. Max. coverage (+): 0. Max coverage (-): 0

Region: NODE\_371225\_length\_8483\_cov\_20.365791 153-170. Max. coverage (+): 0.37. Max coverage (-): 0

Region: NODE\_371225\_length\_8483\_cov\_20.365791 171-187. Max. coverage (+): 0.04. Max coverage (-): 0.11

Region: NODE\_371225\_length\_8483\_cov\_20.365791 188-205. Max. coverage (+): 0.07. Max coverage (-): 0.04

Region: NODE\_371225\_length\_8483\_cov\_20.365791 206-223. Max. coverage (+): 0.26. Max coverage (-): 0

Region: NODE\_371225\_length\_8483\_cov\_20.365791 224-240. Max. coverage (+): 0. Max coverage (-): 0

Region: NODE\_371225\_length\_8483\_cov\_20.365791 241-258. Max. coverage (+): 0. Max coverage (-): 0

Region: NODE\_371225\_length\_8483\_cov\_20.365791 259-275. Max. coverage (+): 0.04. Max coverage (-): 0.04

Region: NODE\_371225\_length\_8483\_cov\_20.365791 276-293. Max. coverage (+): 0.02. Max coverage (-): 0.07

Region: NODE\_371225\_length\_8483\_cov\_20.365791 294-310. Max. coverage (+): 0.02. Max coverage (-): 0.04

Region: NODE\_371225\_length\_8483\_cov\_20.365791 311-328. Max. coverage (+): 0.02. Max coverage (-): 0.19

Region: NODE\_371225\_length\_8483\_cov\_20.365791 329-346. Max. coverage (+): 1.13. Max coverage (-): 0.02

Region: NODE\_371225\_length\_8483\_cov\_20.365791 347-363. Max. coverage (+): 0.09. Max coverage (-): 0.02

Region: NODE\_371225\_length\_8483\_cov\_20.365791 364-381. Max. coverage (+): 0. Max coverage (-): 0

Region: NODE\_371225\_length\_8483\_cov\_20.365791 382-398. Max. coverage (+): 0. Max coverage (-): 0

Region: NODE\_371225\_length\_8483\_cov\_20.365791 399-416. Max. coverage (+): 0.04. Max coverage (-): 0.04

Region: NODE\_371225\_length\_8483\_cov\_20.365791 417-433. Max. coverage (+): 0.03. Max coverage (-): 0

Region: NODE\_371225\_length\_8483\_cov\_20.365791 434-451. Max. coverage (+): 0. Max coverage (-): 0.02

Region: NODE\_371225\_length\_8483\_cov\_20.365791 452-469. Max. coverage (+): 0. Max coverage (-): 0

Region: NODE\_371225\_length\_8483\_cov\_20.365791 470-486. Max. coverage (+): 0. Max coverage (-): 0

Region: NODE\_371225\_length\_8483\_cov\_20.365791 487-504. Max. coverage (+): 0.04. Max coverage (-): 0

Region: NODE\_371225\_length\_8483\_cov\_20.365791 505-521. Max. coverage (+): 0. Max coverage (-): 0.06

Region: NODE\_371225\_length\_8483\_cov\_20.365791 522-539. Max. coverage (+): 0.06. Max coverage (-): 0.04

Region: NODE\_371225\_length\_8483\_cov\_20.365791 540-556. Max. coverage (+): 0.07. Max coverage (-): 0

Region: NODE\_371225\_length\_8483\_cov\_20.365791 557-574. Max. coverage (+): 0. Max coverage (-): 0

Region: NODE\_371225\_length\_8483\_cov\_20.365791 575-592. Max. coverage (+): 0.02. Max coverage (-): 0

Region: NODE\_371225\_length\_8483\_cov\_20.365791 593-609. Max. coverage (+): 0. Max coverage (-): 0

Region: NODE\_371225\_length\_8483\_cov\_20.365791 610-627. Max. coverage (+): 0. Max coverage (-): 0

Region: NODE\_371225\_length\_8483\_cov\_20.365791 628-644. Max. coverage (+): 0. Max coverage (-): 0

Region: NODE\_371225\_length\_8483\_cov\_20.365791 645-662. Max. coverage (+): 0. Max coverage (-): 0.04

Region: NODE\_371225\_length\_8483\_cov\_20.365791 663-679. Max. coverage (+): 0.04. Max coverage (-): 0

Region: NODE\_371225\_length\_8483\_cov\_20.365791 680-697. Max. coverage (+): 0.07. Max coverage (-): 0.04

Region: NODE\_371225\_length\_8483\_cov\_20.365791 698-715. Max. coverage (+): 0.07. Max coverage (-): 0.04

Region: NODE\_371225\_length\_8483\_cov\_20.365791 716-732. Max. coverage (+): 0.07. Max coverage (-): 0

Region: NODE\_371225\_length\_8483\_cov\_20.365791 733-750. Max. coverage (+): 0. Max coverage (-): 0

Region: NODE\_371225\_length\_8483\_cov\_20.365791 751-767. Max. coverage (+): 0. Max coverage (-): 0

Region: NODE\_371225\_length\_8483\_cov\_20.365791 768-785. Max. coverage (+): 0.02. Max coverage (-): 0.04

Region: NODE\_371225\_length\_8483\_cov\_20.365791 786-802. Max. coverage (+): 0.02. Max coverage (-): 0

Region: NODE\_371225\_length\_8483\_cov\_20.365791 803-820. Max. coverage (+): 0.15. Max coverage (-): 0.02

Region: NODE\_371225\_length\_8483\_cov\_20.365791 821-838. Max. coverage (+): 0.17. Max coverage (-): 0.04

Region: NODE\_371225\_length\_8483\_cov\_20.365791 839-855. Max. coverage (+): 0.07. Max coverage (-): 0.04

Region: NODE\_371225\_length\_8483\_cov\_20.365791 856-873. Max. coverage (+): 0.19. Max coverage (-): 0

Region: NODE\_371225\_length\_8483\_cov\_20.365791 874-890. Max. coverage (+): 0.02. Max coverage (-): 0

Region: NODE\_371225\_length\_8483\_cov\_20.365791 891-908. Max. coverage (+): 0. Max coverage (-): 0.04

Region: NODE\_371225\_length\_8483\_cov\_20.365791 909-925. Max. coverage (+): 0.04. Max coverage (-): 0.04

Region: NODE\_371225\_length\_8483\_cov\_20.365791 926-943. Max. coverage (+): 0. Max coverage (-): 0.02

Region: NODE\_371225\_length\_8483\_cov\_20.365791 944-960. Max. coverage (+): 0. Max coverage (-): 0.11

Region: NODE\_371225\_length\_8483\_cov\_20.365791 961-978. Max. coverage (+): 0. Max coverage (-): 0.22

Region: NODE\_371225\_length\_8483\_cov\_20.365791 979-996. Max. coverage (+): 0. Max coverage (-): 0.07

Region: NODE\_371225\_length\_8483\_cov\_20.365791 997-1013. Max. coverage (+): 0.28. Max coverage (-): 0.06

Region: NODE\_371225\_length\_8483\_cov\_20.365791 1014-1031. Max. coverage (+): 0.04. Max coverage (-): 0

Region: NODE\_371225\_length\_8483\_cov\_20.365791 1032-1048. Max. coverage (+): 0.04. Max coverage (-): 0.04

Region: NODE\_371225\_length\_8483\_cov\_20.365791 1049-1066. Max. coverage (+): 0.06. Max coverage (-): 0.09

Region: NODE\_371225\_length\_8483\_cov\_20.365791 1067-1083. Max. coverage (+): 0.04. Max coverage (-): 0.02

Region: NODE\_371225\_length\_8483\_cov\_20.365791 1084-1101. Max. coverage (+): 0.02. Max coverage (-): 0.02

Region: NODE\_371225\_length\_8483\_cov\_20.365791 1102-1119. Max. coverage (+): 0. Max coverage (-): 0

Region: NODE\_371225\_length\_8483\_cov\_20.365791 1120-1136. Max. coverage (+): 0.01. Max coverage (-): 0.01

Region: NODE\_371225\_length\_8483\_cov\_20.365791 1137-1154. Max. coverage (+): 0.02. Max coverage (-): 0.05

Region: NODE\_371225\_length\_8483\_cov\_20.365791 1155-1171. Max. coverage (+): 0.06. Max coverage (-): 0.02

Region: NODE\_371225\_length\_8483\_cov\_20.365791 1172-1189. Max. coverage (+): 0.09. Max coverage (-): 0

Region: NODE\_371225\_length\_8483\_cov\_20.365791 1190-1206. Max. coverage (+): 0. Max coverage (-): 0

Region: NODE\_371225\_length\_8483\_cov\_20.365791 1207-1224. Max. coverage (+): 0. Max coverage (-): 0.04

Region: NODE\_371225\_length\_8483\_cov\_20.365791 1225-1242. Max. coverage (+): 0.04. Max coverage (-): 0

Region: NODE\_371225\_length\_8483\_cov\_20.365791 1243-1259. Max. coverage (+): 0. Max coverage (-): 0

Region: NODE\_371225\_length\_8483\_cov\_20.365791 1260-1277. Max. coverage (+): 0. Max coverage (-): 0

Region: NODE\_371225\_length\_8483\_cov\_20.365791 1278-1294. Max. coverage (+): 0.04. Max coverage (-): 0.02

Region: NODE\_371225\_length\_8483\_cov\_20.365791 1295-1312. Max. coverage (+): 0.02. Max coverage (-): 0.04

Region: NODE\_371225\_length\_8483\_cov\_20.365791 1313-1329. Max. coverage (+): 0.02. Max coverage (-): 0.02

Region: NODE\_371225\_length\_8483\_cov\_20.365791 1330-1347. Max. coverage (+): 0.04. Max coverage (-): 0.04

Region: NODE\_371225\_length\_8483\_cov\_20.365791 1348-1365. Max. coverage (+): 0.04. Max coverage (-): 0

Region: NODE\_371225\_length\_8483\_cov\_20.365791 1366-1382. Max. coverage (+): 0.04. Max coverage (-): 0

Region: NODE\_371225\_length\_8483\_cov\_20.365791 1383-1400. Max. coverage (+): 0.04. Max coverage (-): 0.02

Region: NODE\_371225\_length\_8483\_cov\_20.365791 1401-1417. Max. coverage (+): 0.02. Max coverage (-): 0.07

Region: NODE\_371225\_length\_8483\_cov\_20.365791 1418-1435. Max. coverage (+): 0.02. Max coverage (-): 0.04

Region: NODE\_371225\_length\_8483\_cov\_20.365791 1436-1452. Max. coverage (+): 0. Max coverage (-): 0

Region: NODE\_371225\_length\_8483\_cov\_20.365791 1453-1470. Max. coverage (+): 0.02. Max coverage (-): 0.02

Region: NODE\_371225\_length\_8483\_cov\_20.365791 1471-1488. Max. coverage (+): 0. Max coverage (-): 0

Region: NODE\_371225\_length\_8483\_cov\_20.365791 1489-1505. Max. coverage (+): 0. Max coverage (-): 0.04

Region: NODE\_371225\_length\_8483\_cov\_20.365791 1506-1523. Max. coverage (+): 0.06. Max coverage (-): 0.04

Region: NODE\_371225\_length\_8483\_cov\_20.365791 1524-1540. Max. coverage (+): 0. Max coverage (-): 0

Region: NODE\_371225\_length\_8483\_cov\_20.365791 1541-1558. Max. coverage (+): 0.04. Max coverage (-): 0

Region: NODE\_371225\_length\_8483\_cov\_20.365791 1559-1575. Max. coverage (+): 0.04. Max coverage (-): 0.06

Region: NODE\_371225\_length\_8483\_cov\_20.365791 1576-1593. Max. coverage (+): 0.32. Max coverage (-): 0.02

Region: NODE\_371225\_length\_8483\_cov\_20.365791 1594-1611. Max. coverage (+): 0.02. Max coverage (-): 0.04

Region: NODE\_371225\_length\_8483\_cov\_20.365791 1612-1628. Max. coverage (+): 0.07. Max coverage (-): 0.02

Region: NODE\_371225\_length\_8483\_cov\_20.365791 1629-1646. Max. coverage (+): 0. Max coverage (-): 0

Region: NODE\_371225\_length\_8483\_cov\_20.365791 1647-1663. Max. coverage (+): 0.11. Max coverage (-): 0

Region: NODE\_371225\_length\_8483\_cov\_20.365791 1664-1681. Max. coverage (+): 0. Max coverage (-): 0

Region: NODE\_371225\_length\_8483\_cov\_20.365791 1682-1698. Max. coverage (+): 0. Max coverage (-): 0

Region: NODE\_371225\_length\_8483\_cov\_20.365791 1699-1716. Max. coverage (+): 0.02. Max coverage (-): 0

Region: NODE\_371225\_length\_8483\_cov\_20.365791 1717-1734. Max. coverage (+): 0. Max coverage (-): 0

Region: NODE\_371225\_length\_8483\_cov\_20.365791 1735-1751. Max. coverage (+): 0. Max coverage (-): 0

Region: NODE\_371225\_length\_8483\_cov\_20.365791 1752-1769. Max. coverage (+): 0. Max coverage (-): 0.04

Region: NODE\_371225\_length\_8483\_cov\_20.365791 1770-1786. Max. coverage (+): 0. Max coverage (-): 0.07

Region: NODE\_371225\_length\_8483\_cov\_20.365791 1787-1804. Max. coverage (+): 0. Max coverage (-): 0.07

Region: NODE\_371225\_length\_8483\_cov\_20.365791 1805-1821. Max. coverage (+): 0.04. Max coverage (-): 0.02

Region: NODE\_371225\_length\_8483\_cov\_20.365791 1822-1839. Max. coverage (+): 0.04. Max coverage (-): 0

Region: NODE\_371225\_length\_8483\_cov\_20.365791 1840-1857. Max. coverage (+): 0. Max coverage (-): 0.41

Region: NODE\_371225\_length\_8483\_cov\_20.365791 1858-1874. Max. coverage (+): 0.31. Max coverage (-): 0.04

Region: NODE\_371225\_length\_8483\_cov\_20.365791 1875-1892. Max. coverage (+): 0.02. Max coverage (-): 0.15

Region: NODE\_371225\_length\_8483\_cov\_20.365791 1893-1909. Max. coverage (+): 0.29. Max coverage (-): 0.15

Region: NODE\_371225\_length\_8483\_cov\_20.365791 1910-1927. Max. coverage (+): 0.29. Max coverage (-): 0.11

Region: NODE\_371225\_length\_8483\_cov\_20.365791 1928-1944. Max. coverage (+): 0. Max coverage (-): 0.78

Region: NODE\_371225\_length\_8483\_cov\_20.365791 1945-1962. Max. coverage (+): 0.04. Max coverage (-): 3

Region: NODE\_371225\_length\_8483\_cov\_20.365791 1963-1980. Max. coverage (+): 0. Max coverage (-): 5.15

Region: NODE\_371225\_length\_8483\_cov\_20.365791 1981-1997. Max. coverage (+): 0. Max coverage (-): 1.85

Region: NODE\_371225\_length\_8483\_cov\_20.365791 1998-2015. Max. coverage (+): 0.04. Max coverage (-): 2.11

Region: NODE\_371225\_length\_8483\_cov\_20.365791 2016-2032. Max. coverage (+): 0.1. Max coverage (-): 1.69

Region: NODE\_371225\_length\_8483\_cov\_20.365791 2033-2050. Max. coverage (+): 0.12. Max coverage (-): 0.01

Region: NODE\_371225\_length\_8483\_cov\_20.365791 2051-2067. Max. coverage (+): 0. Max coverage (-): 0.22

Region: NODE\_371225\_length\_8483\_cov\_20.365791 2068-2085. Max. coverage (+): 1.45. Max coverage (-): 0.03

Region: NODE\_371225\_length\_8483\_cov\_20.365791 2086-2103. Max. coverage (+): 0. Max coverage (-): 0.14

Region: NODE\_371225\_length\_8483\_cov\_20.365791 2104-2120. Max. coverage (+): 0. Max coverage (-): 0

Region: NODE\_371225\_length\_8483\_cov\_20.365791 2121-2138. Max. coverage (+): 0. Max coverage (-): 0.89

Region: NODE\_371225\_length\_8483\_cov\_20.365791 2139-2155. Max. coverage (+): 1.11. Max coverage (-): 0.15

Region: NODE\_371225\_length\_8483\_cov\_20.365791 2156-2173. Max. coverage (+): 0.13. Max coverage (-): 0.19

Region: NODE\_371225\_length\_8483\_cov\_20.365791 2174-2190. Max. coverage (+): 0.15. Max coverage (-): 0.45

Region: NODE\_371225\_length\_8483\_cov\_20.365791 2191-2208. Max. coverage (+): 0.22. Max coverage (-): 0.06

Region: NODE\_371225\_length\_8483\_cov\_20.365791 2209-2226. Max. coverage (+): 0. Max coverage (-): 0.46

Region: NODE\_371225\_length\_8483\_cov\_20.365791 2227-2243. Max. coverage (+): 0.01. Max coverage (-): 0.04

Region: NODE\_371225\_length\_8483\_cov\_20.365791 2244-2261. Max. coverage (+): 0. Max coverage (-): 0

Region: NODE\_371225\_length\_8483\_cov\_20.365791 2262-2278. Max. coverage (+): 0. Max coverage (-): 0

Region: NODE\_371225\_length\_8483\_cov\_20.365791 2279-2296. Max. coverage (+): 0. Max coverage (-): 0

Region: NODE\_371225\_length\_8483\_cov\_20.365791 2297-2313. Max. coverage (+): 0.01. Max coverage (-): 0.01

Region: NODE\_371225\_length\_8483\_cov\_20.365791 2314-2331. Max. coverage (+): 0. Max coverage (-): 0

Region: NODE\_371225\_length\_8483\_cov\_20.365791 2332-2349. Max. coverage (+): 0. Max coverage (-): 0.11

Region: NODE\_371225\_length\_8483\_cov\_20.365791 2350-2366. Max. coverage (+): 0.04. Max coverage (-): 0.11

Region: NODE\_371225\_length\_8483\_cov\_20.365791 2367-2384. Max. coverage (+): 0.02. Max coverage (-): 0

Region: NODE\_371225\_length\_8483\_cov\_20.365791 2385-2401. Max. coverage (+): 0.1. Max coverage (-): 0.01

Region: NODE\_371225\_length\_8483\_cov\_20.365791 2402-2419. Max. coverage (+): 0. Max coverage (-): 0

Region: NODE\_371225\_length\_8483\_cov\_20.365791 2420-2436. Max. coverage (+): 0.04. Max coverage (-): 0

Region: NODE\_371225\_length\_8483\_cov\_20.365791 2437-2454. Max. coverage (+): 0.02. Max coverage (-): 0

Region: NODE\_371225\_length\_8483\_cov\_20.365791 2455-2472. Max. coverage (+): 0. Max coverage (-): 0

Region: NODE\_371225\_length\_8483\_cov\_20.365791 2473-2489. Max. coverage (+): 0. Max coverage (-): 0.04

Region: NODE\_371225\_length\_8483\_cov\_20.365791 2490-2507. Max. coverage (+): 0.09. Max coverage (-): 0.04

Region: NODE\_371225\_length\_8483\_cov\_20.365791 2508-2524. Max. coverage (+): 0.02. Max coverage (-): 0

Region: NODE\_371225\_length\_8483\_cov\_20.365791 2525-2542. Max. coverage (+): 0.04. Max coverage (-): 0.09

Region: NODE\_371225\_length\_8483\_cov\_20.365791 2543-2559. Max. coverage (+): 0.04. Max coverage (-): 0

Region: NODE\_371225\_length\_8483\_cov\_20.365791 2560-2577. Max. coverage (+): 0.02. Max coverage (-): 0

Region: NODE\_371225\_length\_8483\_cov\_20.365791 2578-2595. Max. coverage (+): 0.02. Max coverage (-): 0

Region: NODE\_371225\_length\_8483\_cov\_20.365791 2596-2612. Max. coverage (+): 0. Max coverage (-): 0

Region: NODE\_371225\_length\_8483\_cov\_20.365791 2613-2630. Max. coverage (+): 0. Max coverage (-): 0

Region: NODE\_371225\_length\_8483\_cov\_20.365791 2631-2647. Max. coverage (+): 0. Max coverage (-): 0

Region: NODE\_371225\_length\_8483\_cov\_20.365791 2648-2665. Max. coverage (+): 0.07. Max coverage (-): 0

Region: NODE\_371225\_length\_8483\_cov\_20.365791 2666-2682. Max. coverage (+): 0.06. Max coverage (-): 0

Region: NODE\_371225\_length\_8483\_cov\_20.365791 2683-2700. Max. coverage (+): 0. Max coverage (-): 0

Region: NODE\_371225\_length\_8483\_cov\_20.365791 2701-2717. Max. coverage (+): 0.02. Max coverage (-): 0

Region: NODE\_371225\_length\_8483\_cov\_20.365791 2718-2735. Max. coverage (+): 0. Max coverage (-): 0

Region: NODE\_371225\_length\_8483\_cov\_20.365791 2736-2753. Max. coverage (+): 0. Max coverage (-): 0

Region: NODE\_371225\_length\_8483\_cov\_20.365791 2754-2770. Max. coverage (+): 0. Max coverage (-): 0

Region: NODE\_371225\_length\_8483\_cov\_20.365791 2771-2788. Max. coverage (+): 0. Max coverage (-): 0.07

Region: NODE\_371225\_length\_8483\_cov\_20.365791 2789-2805. Max. coverage (+): 0. Max coverage (-): 0

Region: NODE\_371225\_length\_8483\_cov\_20.365791 2806-2823. Max. coverage (+): 0.02. Max coverage (-): 0

Region: NODE\_371225\_length\_8483\_cov\_20.365791 2824-2840. Max. coverage (+): 0.04. Max coverage (-): 0.01

Region: NODE\_371225\_length\_8483\_cov\_20.365791 2841-2858. Max. coverage (+): 0.06. Max coverage (-): 0.05

Region: NODE\_371225\_length\_8483\_cov\_20.365791 2859-2876. Max. coverage (+): 0.07. Max coverage (-): 0.06

Region: NODE\_371225\_length\_8483\_cov\_20.365791 2877-2893. Max. coverage (+): 0.02. Max coverage (-): 0.24

Region: NODE\_371225\_length\_8483\_cov\_20.365791 2894-2911. Max. coverage (+): 0.06. Max coverage (-): 0.02

Region: NODE\_371225\_length\_8483\_cov\_20.365791 2912-2928. Max. coverage (+): 0. Max coverage (-): 0

Region: NODE\_371225\_length\_8483\_cov\_20.365791 2929-2946. Max. coverage (+): 0.01. Max coverage (-): 0

Region: NODE\_371225\_length\_8483\_cov\_20.365791 2947-2963. Max. coverage (+): 0.07. Max coverage (-): 0

Region: NODE\_371225\_length\_8483\_cov\_20.365791 2964-2981. Max. coverage (+): 0.06. Max coverage (-): 0.04

Region: NODE\_371225\_length\_8483\_cov\_20.365791 2982-2999. Max. coverage (+): 0.04. Max coverage (-): 0

Region: NODE\_371225\_length\_8483\_cov\_20.365791 3000-3016. Max. coverage (+): 0. Max coverage (-): 0

Region: NODE\_371225\_length\_8483\_cov\_20.365791 3017-3034. Max. coverage (+): 0.02. Max coverage (-): 0.02

Region: NODE\_371225\_length\_8483\_cov\_20.365791 3035-3051. Max. coverage (+): 0. Max coverage (-): 0

Region: NODE\_371225\_length\_8483\_cov\_20.365791 3052-3069. Max. coverage (+): 0.09. Max coverage (-): 0.02

Region: NODE\_371225\_length\_8483\_cov\_20.365791 3070-3086. Max. coverage (+): 0.02. Max coverage (-): 0.02

Region: NODE\_371225\_length\_8483\_cov\_20.365791 3087-3104. Max. coverage (+): 0. Max coverage (-): 0

Region: NODE\_371225\_length\_8483\_cov\_20.365791 3105-3122. Max. coverage (+): 0. Max coverage (-): 0

Region: NODE\_371225\_length\_8483\_cov\_20.365791 3123-3139. Max. coverage (+): 0.02. Max coverage (-): 0.02

Region: NODE\_371225\_length\_8483\_cov\_20.365791 3140-3157. Max. coverage (+): 0. Max coverage (-): 0

Region: NODE\_371225\_length\_8483\_cov\_20.365791 3158-3174. Max. coverage (+): 0. Max coverage (-): 0.07

Region: NODE\_371225\_length\_8483\_cov\_20.365791 3175-3192. Max. coverage (+): 0.02. Max coverage (-): 0.07

Region: NODE\_371225\_length\_8483\_cov\_20.365791 3193-3209. Max. coverage (+): 0.02. Max coverage (-): 0.02

Region: NODE\_371225\_length\_8483\_cov\_20.365791 3210-3227. Max. coverage (+): 0.02. Max coverage (-): 0

Region: NODE\_371225\_length\_8483\_cov\_20.365791 3228-3245. Max. coverage (+): 0.04. Max coverage (-): 0

Region: NODE\_371225\_length\_8483\_cov\_20.365791 3246-3262. Max. coverage (+): 0. Max coverage (-): 0

Region: NODE\_371225\_length\_8483\_cov\_20.365791 3263-3280. Max. coverage (+): 0.15. Max coverage (-): 4.8

Region: NODE\_371225\_length\_8483\_cov\_20.365791 3281-3297. Max. coverage (+): 0.22. Max coverage (-): 0.26

Region: NODE\_371225\_length\_8483\_cov\_20.365791 3298-3315. Max. coverage (+): 0.04. Max coverage (-): 0.05

Region: NODE\_371225\_length\_8483\_cov\_20.365791 3316-3332. Max. coverage (+): 0.09. Max coverage (-): 0.62

Region: NODE\_371225\_length\_8483\_cov\_20.365791 3333-3350. Max. coverage (+): 0.8. Max coverage (-): 0.02

Region: NODE\_371225\_length\_8483\_cov\_20.365791 3351-3368. Max. coverage (+): 0.07. Max coverage (-): 0.7

Region: NODE\_371225\_length\_8483\_cov\_20.365791 3369-3385. Max. coverage (+): 0.26. Max coverage (-): 0.76

Region: NODE\_371225\_length\_8483\_cov\_20.365791 3386-3403. Max. coverage (+): 0.26. Max coverage (-): 0.06

Region: NODE\_371225\_length\_8483\_cov\_20.365791 3404-3420. Max. coverage (+): 0.02. Max coverage (-): 0.11

Region: NODE\_371225\_length\_8483\_cov\_20.365791 3421-3438. Max. coverage (+): 4.73. Max coverage (-): 0.06

Region: NODE\_371225\_length\_8483\_cov\_20.365791 3439-3455. Max. coverage (+): 0.37. Max coverage (-): 0.22

Region: NODE\_371225\_length\_8483\_cov\_20.365791 3456-3473. Max. coverage (+): 0. Max coverage (-): 3.97

Region: NODE\_371225\_length\_8483\_cov\_20.365791 3474-3491. Max. coverage (+): 0. Max coverage (-): 4.93

Region: NODE\_371225\_length\_8483\_cov\_20.365791 3492-3508. Max. coverage (+): 0. Max coverage (-): 0.15

Region: NODE\_371225\_length\_8483\_cov\_20.365791 3509-3526. Max. coverage (+): 0. Max coverage (-): 0.19

Region: NODE\_371225\_length\_8483\_cov\_20.365791 3527-3543. Max. coverage (+): 0. Max coverage (-): 0.07

Region: NODE\_371225\_length\_8483\_cov\_20.365791 3544-3561. Max. coverage (+): 0.18. Max coverage (-): 0.76

Region: NODE\_371225\_length\_8483\_cov\_20.365791 3562-3578. Max. coverage (+): 0.3. Max coverage (-): 1.07

Region: NODE\_371225\_length\_8483\_cov\_20.365791 3579-3596. Max. coverage (+): 0.06. Max coverage (-): 0.15

Region: NODE\_371225\_length\_8483\_cov\_20.365791 3597-3614. Max. coverage (+): 0. Max coverage (-): 1

Region: NODE\_371225\_length\_8483\_cov\_20.365791 3615-3631. Max. coverage (+): 0.15. Max coverage (-): 1.13

Region: NODE\_371225\_length\_8483\_cov\_20.365791 3632-3649. Max. coverage (+): 2.15. Max coverage (-): 0.19

Region: NODE\_371225\_length\_8483\_cov\_20.365791 3650-3666. Max. coverage (+): 0. Max coverage (-): 0.67

Region: NODE\_371225\_length\_8483\_cov\_20.365791 3667-3684. Max. coverage (+): 0.15. Max coverage (-): 0.26

Region: NODE\_371225\_length\_8483\_cov\_20.365791 3685-3701. Max. coverage (+): 0.07. Max coverage (-): 0.04

Region: NODE\_371225\_length\_8483\_cov\_20.365791 3702-3719. Max. coverage (+): 0.01. Max coverage (-): 4.34

Region: NODE\_371225\_length\_8483\_cov\_20.365791 3720-3737. Max. coverage (+): 0.06. Max coverage (-): 0.04

Region: NODE\_371225\_length\_8483\_cov\_20.365791 3738-3754. Max. coverage (+): 0. Max coverage (-): 1.45

Region: NODE\_371225\_length\_8483\_cov\_20.365791 3755-3772. Max. coverage (+): 0.01. Max coverage (-): 3.36

Region: NODE\_371225\_length\_8483\_cov\_20.365791 3773-3789. Max. coverage (+): 0. Max coverage (-): 1.28

Region: NODE\_371225\_length\_8483\_cov\_20.365791 3790-3807. Max. coverage (+): 0. Max coverage (-): 0.26

Region: NODE\_371225\_length\_8483\_cov\_20.365791 3808-3824. Max. coverage (+): 0. Max coverage (-): 1.04

Region: NODE\_371225\_length\_8483\_cov\_20.365791 3825-3842. Max. coverage (+): 0.32. Max coverage (-): 0.04

Region: NODE\_371225\_length\_8483\_cov\_20.365791 3843-3860. Max. coverage (+): 0. Max coverage (-): 0.74

Region: NODE\_371225\_length\_8483\_cov\_20.365791 3861-3877. Max. coverage (+): 0.3. Max coverage (-): 0.11

Region: NODE\_371225\_length\_8483\_cov\_20.365791 3878-3895. Max. coverage (+): 0. Max coverage (-): 1.48

Region: NODE\_371225\_length\_8483\_cov\_20.365791 3896-3912. Max. coverage (+): 0. Max coverage (-): 1.48

Region: NODE\_371225\_length\_8483\_cov\_20.365791 3913-3930. Max. coverage (+): 0.02. Max coverage (-): 2.41

Region: NODE\_371225\_length\_8483\_cov\_20.365791 3931-3947. Max. coverage (+): 1.32. Max coverage (-): 0.02

Region: NODE\_371225\_length\_8483\_cov\_20.365791 3948-3965. Max. coverage (+): 0.04. Max coverage (-): 0.2

Region: NODE\_371225\_length\_8483\_cov\_20.365791 3966-3983. Max. coverage (+): 0. Max coverage (-): 1.78

Region: NODE\_371225\_length\_8483\_cov\_20.365791 3984-4000. Max. coverage (+): 0.54. Max coverage (-): 1.08

Region: NODE\_371225\_length\_8483\_cov\_20.365791 4001-4018. Max. coverage (+): 0.22. Max coverage (-): 0.11

Region: NODE\_371225\_length\_8483\_cov\_20.365791 4019-4035. Max. coverage (+): 0.02. Max coverage (-): 0.76

Region: NODE\_371225\_length\_8483\_cov\_20.365791 4036-4053. Max. coverage (+): 0. Max coverage (-): 3.82

Region: NODE\_371225\_length\_8483\_cov\_20.365791 4054-4070. Max. coverage (+): 0.17. Max coverage (-): 0.56

Region: NODE\_371225\_length\_8483\_cov\_20.365791 4071-4088. Max. coverage (+): 0.07. Max coverage (-): 0.32

Region: NODE\_371225\_length\_8483\_cov\_20.365791 4089-4106. Max. coverage (+): 0. Max coverage (-): 0.3

Region: NODE\_371225\_length\_8483\_cov\_20.365791 4107-4123. Max. coverage (+): 0. Max coverage (-): 0.44

Region: NODE\_371225\_length\_8483\_cov\_20.365791 4124-4141. Max. coverage (+): 0.02. Max coverage (-): 0.52

Region: NODE\_371225\_length\_8483\_cov\_20.365791 4142-4158. Max. coverage (+): 0.13. Max coverage (-): 0.24

Region: NODE\_371225\_length\_8483\_cov\_20.365791 4159-4176. Max. coverage (+): 0.04. Max coverage (-): 0.06

Region: NODE\_371225\_length\_8483\_cov\_20.365791 4177-4193. Max. coverage (+): 0.07. Max coverage (-): 0.57

Region: NODE\_371225\_length\_8483\_cov\_20.365791 4194-4211. Max. coverage (+): 1.69. Max coverage (-): 0.39

Region: NODE\_371225\_length\_8483\_cov\_20.365791 4212-4229. Max. coverage (+): 0.06. Max coverage (-): 0.06

Region: NODE\_371225\_length\_8483\_cov\_20.365791 4230-4246. Max. coverage (+): 0. Max coverage (-): 0.04

Region: NODE\_371225\_length\_8483\_cov\_20.365791 4247-4264. Max. coverage (+): 0. Max coverage (-): 8.42

Region: NODE\_371225\_length\_8483\_cov\_20.365791 4265-4281. Max. coverage (+): 0. Max coverage (-): 0.19

Region: NODE\_371225\_length\_8483\_cov\_20.365791 4282-4299. Max. coverage (+): 0.04. Max coverage (-): 0.56

Region: NODE\_371225\_length\_8483\_cov\_20.365791 4300-4316. Max. coverage (+): 0.67. Max coverage (-): 0.11

Region: NODE\_371225\_length\_8483\_cov\_20.365791 4317-4334. Max. coverage (+): 0.09. Max coverage (-): 0.54

Region: NODE\_371225\_length\_8483\_cov\_20.365791 4335-4352. Max. coverage (+): 0. Max coverage (-): 0.74

Region: NODE\_371225\_length\_8483\_cov\_20.365791 4353-4369. Max. coverage (+): 0. Max coverage (-): 0.11

Region: NODE\_371225\_length\_8483\_cov\_20.365791 4370-4387. Max. coverage (+): 0.07. Max coverage (-): 0.63

Region: NODE\_371225\_length\_8483\_cov\_20.365791 4388-4404. Max. coverage (+): 0. Max coverage (-): 0.07

Region: NODE\_371225\_length\_8483\_cov\_20.365791 4405-4422. Max. coverage (+): 0.02. Max coverage (-): 0.46

Region: NODE\_371225\_length\_8483\_cov\_20.365791 4423-4439. Max. coverage (+): 0. Max coverage (-): 0.07

Region: NODE\_371225\_length\_8483\_cov\_20.365791 4440-4457. Max. coverage (+): 0. Max coverage (-): 0.19

Region: NODE\_371225\_length\_8483\_cov\_20.365791 4458-4474. Max. coverage (+): 0. Max coverage (-): 1.3

Region: NODE\_371225\_length\_8483\_cov\_20.365791 4475-4492. Max. coverage (+): 0. Max coverage (-): 2.11

Region: NODE\_371225\_length\_8483\_cov\_20.365791 4493-4510. Max. coverage (+): 0.04. Max coverage (-): 0.11

Region: NODE\_371225\_length\_8483\_cov\_20.365791 4511-4527. Max. coverage (+): 0. Max coverage (-): 3.04

Region: NODE\_371225\_length\_8483\_cov\_20.365791 4528-4545. Max. coverage (+): 0.07. Max coverage (-): 1.56

Region: NODE\_371225\_length\_8483\_cov\_20.365791 4546-4562. Max. coverage (+): 0.07. Max coverage (-): 0.37

Region: NODE\_371225\_length\_8483\_cov\_20.365791 4563-4580. Max. coverage (+): 0.06. Max coverage (-): 0.44

Region: NODE\_371225\_length\_8483\_cov\_20.365791 4581-4597. Max. coverage (+): 0.59. Max coverage (-): 0.54

Region: NODE\_371225\_length\_8483\_cov\_20.365791 4598-4615. Max. coverage (+): 0.31. Max coverage (-): 3.74

Region: NODE\_371225\_length\_8483\_cov\_20.365791 4616-4633. Max. coverage (+): 7.1. Max coverage (-): 0.67

Region: NODE\_371225\_length\_8483\_cov\_20.365791 4634-4650. Max. coverage (+): 0.04. Max coverage (-): 0

Region: NODE\_371225\_length\_8483\_cov\_20.365791 4651-4668. Max. coverage (+): 0. Max coverage (-): 0.85

Region: NODE\_371225\_length\_8483\_cov\_20.365791 4669-4685. Max. coverage (+): 0.28. Max coverage (-): 0.31

Region: NODE\_371225\_length\_8483\_cov\_20.365791 4686-4703. Max. coverage (+): 0.07. Max coverage (-): 0.11

Region: NODE\_371225\_length\_8483\_cov\_20.365791 4704-4720. Max. coverage (+): 0. Max coverage (-): 0.67

Region: NODE\_371225\_length\_8483\_cov\_20.365791 4721-4738. Max. coverage (+): 0.11. Max coverage (-): 0.05

Region: NODE\_371225\_length\_8483\_cov\_20.365791 4739-4756. Max. coverage (+): 0. Max coverage (-): 0.27

Region: NODE\_371225\_length\_8483\_cov\_20.365791 4757-4773. Max. coverage (+): 1.41. Max coverage (-): 0.26

Region: NODE\_371225\_length\_8483\_cov\_20.365791 4774-4791. Max. coverage (+): 0.12. Max coverage (-): 0.02

Region: NODE\_371225\_length\_8483\_cov\_20.365791 4792-4808. Max. coverage (+): 0.08. Max coverage (-): 1.9

Region: NODE\_371225\_length\_8483\_cov\_20.365791 4809-4826. Max. coverage (+): 6.59. Max coverage (-): 0.03

Region: NODE\_371225\_length\_8483\_cov\_20.365791 4827-4843. Max. coverage (+): 0.02. Max coverage (-): 0.09

Region: NODE\_371225\_length\_8483\_cov\_20.365791 4844-4861. Max. coverage (+): 0.02. Max coverage (-): 0.99

Region: NODE\_371225\_length\_8483\_cov\_20.365791 4862-4879. Max. coverage (+): 0.19. Max coverage (-): 0.64

Region: NODE\_371225\_length\_8483\_cov\_20.365791 4880-4896. Max. coverage (+): 0.04. Max coverage (-): 88.08

Region: NODE\_371225\_length\_8483\_cov\_20.365791 4897-4914. Max. coverage (+): 0.07. Max coverage (-): 0.11

Region: NODE\_371225\_length\_8483\_cov\_20.365791 4915-4931. Max. coverage (+): 0. Max coverage (-): 0.22

Region: NODE\_371225\_length\_8483\_cov\_20.365791 4932-4949. Max. coverage (+): 0.04. Max coverage (-): 10.57

Region: NODE\_371225\_length\_8483\_cov\_20.365791 4950-4966. Max. coverage (+): 0. Max coverage (-): 10.38

Region: NODE\_371225\_length\_8483\_cov\_20.365791 4967-4984. Max. coverage (+): 0.02. Max coverage (-): 1.33

Region: NODE\_371225\_length\_8483\_cov\_20.365791 4985-5002. Max. coverage (+): 0.8. Max coverage (-): 0.3

Region: NODE\_371225\_length\_8483\_cov\_20.365791 5003-5019. Max. coverage (+): 0.04. Max coverage (-): 0.3

Region: NODE\_371225\_length\_8483\_cov\_20.365791 5020-5037. Max. coverage (+): 0. Max coverage (-): 1.11

Region: NODE\_371225\_length\_8483\_cov\_20.365791 5038-5054. Max. coverage (+): 0.09. Max coverage (-): 0.07

Region: NODE\_371225\_length\_8483\_cov\_20.365791 5055-5072. Max. coverage (+): 0.02. Max coverage (-): 0.7

Region: NODE\_371225\_length\_8483\_cov\_20.365791 5073-5089. Max. coverage (+): 0. Max coverage (-): 0.37

Region: NODE\_371225\_length\_8483\_cov\_20.365791 5090-5107. Max. coverage (+): 0.02. Max coverage (-): 0.76

Region: NODE\_371225\_length\_8483\_cov\_20.365791 5108-5125. Max. coverage (+): 0.44. Max coverage (-): 0

Region: NODE\_371225\_length\_8483\_cov\_20.365791 5126-5142. Max. coverage (+): 0.01. Max coverage (-): 0.22

Region: NODE\_371225\_length\_8483\_cov\_20.365791 5143-5160. Max. coverage (+): 0. Max coverage (-): 4

Region: NODE\_371225\_length\_8483\_cov\_20.365791 5161-5177. Max. coverage (+): 0. Max coverage (-): 0.11

Region: NODE\_371225\_length\_8483\_cov\_20.365791 5178-5195. Max. coverage (+): 0. Max coverage (-): 0.78

Region: NODE\_371225\_length\_8483\_cov\_20.365791 5196-5212. Max. coverage (+): 0. Max coverage (-): 1.2

Region: NODE\_371225\_length\_8483\_cov\_20.365791 5213-5230. Max. coverage (+): 1.59. Max coverage (-): 3.04

Region: NODE\_371225\_length\_8483\_cov\_20.365791 5231-5248. Max. coverage (+): 1.46. Max coverage (-): 0.15

Region: NODE\_371225\_length\_8483\_cov\_20.365791 5249-5265. Max. coverage (+): 0. Max coverage (-): 0.22

Region: NODE\_371225\_length\_8483\_cov\_20.365791 5266-5283. Max. coverage (+): 1.08. Max coverage (-): 0.15

Region: NODE\_371225\_length\_8483\_cov\_20.365791 5284-5300. Max. coverage (+): 0.2. Max coverage (-): 0.7

Region: NODE\_371225\_length\_8483\_cov\_20.365791 5301-5318. Max. coverage (+): 0.14. Max coverage (-): 0.04

Region: NODE\_371225\_length\_8483\_cov\_20.365791 5319-5335. Max. coverage (+): 0. Max coverage (-): 0.22

Region: NODE\_371225\_length\_8483\_cov\_20.365791 5336-5353. Max. coverage (+): 0.13. Max coverage (-): 0.07

Region: NODE\_371225\_length\_8483\_cov\_20.365791 5354-5371. Max. coverage (+): 0. Max coverage (-): 1.67

Region: NODE\_371225\_length\_8483\_cov\_20.365791 5372-5388. Max. coverage (+): 0.07. Max coverage (-): 3.04

Region: NODE\_371225\_length\_8483\_cov\_20.365791 5389-5406. Max. coverage (+): 0.11. Max coverage (-): 2.93

Region: NODE\_371225\_length\_8483\_cov\_20.365791 5407-5423. Max. coverage (+): 0.04. Max coverage (-): 0.15

Region: NODE\_371225\_length\_8483\_cov\_20.365791 5424-5441. Max. coverage (+): 0.04. Max coverage (-): 0.13

Region: NODE\_371225\_length\_8483\_cov\_20.365791 5442-5458. Max. coverage (+): 0. Max coverage (-): 0.52

Region: NODE\_371225\_length\_8483\_cov\_20.365791 5459-5476. Max. coverage (+): 0.04. Max coverage (-): 0.76

Region: NODE\_371225\_length\_8483\_cov\_20.365791 5477-5494. Max. coverage (+): 1.5. Max coverage (-): 0.09

Region: NODE\_371225\_length\_8483\_cov\_20.365791 5495-5511. Max. coverage (+): 0.13. Max coverage (-): 0.02

Region: NODE\_371225\_length\_8483\_cov\_20.365791 5512-5529. Max. coverage (+): 0.02. Max coverage (-): 0.37

Region: NODE\_371225\_length\_8483\_cov\_20.365791 5530-5546. Max. coverage (+): 0.04. Max coverage (-): 0.11

Region: NODE\_371225\_length\_8483\_cov\_20.365791 5547-5564. Max. coverage (+): 0.02. Max coverage (-): 1.38

Region: NODE\_371225\_length\_8483\_cov\_20.365791 5565-5581. Max. coverage (+): 0. Max coverage (-): 1.15

Region: NODE\_371225\_length\_8483\_cov\_20.365791 5582-5599. Max. coverage (+): 0.01. Max coverage (-): 0.59

Region: NODE\_371225\_length\_8483\_cov\_20.365791 5600-5617. Max. coverage (+): 0.02. Max coverage (-): 0.04

Region: NODE\_371225\_length\_8483\_cov\_20.365791 5618-5634. Max. coverage (+): 0. Max coverage (-): 0.2

Region: NODE\_371225\_length\_8483\_cov\_20.365791 5635-5652. Max. coverage (+): 0. Max coverage (-): 0.15

Region: NODE\_371225\_length\_8483\_cov\_20.365791 5653-5669. Max. coverage (+): 0.07. Max coverage (-): 0.37

Region: NODE\_371225\_length\_8483\_cov\_20.365791 5670-5687. Max. coverage (+): 0. Max coverage (-): 0

Region: NODE\_371225\_length\_8483\_cov\_20.365791 5688-5704. Max. coverage (+): 0. Max coverage (-): 0.74

Region: NODE\_371225\_length\_8483\_cov\_20.365791 5705-5722. Max. coverage (+): 0. Max coverage (-): 0.7

Region: NODE\_371225\_length\_8483\_cov\_20.365791 5723-5740. Max. coverage (+): 0. Max coverage (-): 0.26

Region: NODE\_371225\_length\_8483\_cov\_20.365791 5741-5757. Max. coverage (+): 0. Max coverage (-): 0.02

Region: NODE\_371225\_length\_8483\_cov\_20.365791 5758-5775. Max. coverage (+): 0. Max coverage (-): 0.03

Region: NODE\_371225\_length\_8483\_cov\_20.365791 5776-5792. Max. coverage (+): 0. Max coverage (-): 0

Region: NODE\_371225\_length\_8483\_cov\_20.365791 5793-5810. Max. coverage (+): 0. Max coverage (-): 0

Region: NODE\_371225\_length\_8483\_cov\_20.365791 5811-5827. Max. coverage (+): 0. Max coverage (-): 0.05

Region: NODE\_371225\_length\_8483\_cov\_20.365791 5828-5845. Max. coverage (+): 0. Max coverage (-): 0.31

Region: NODE\_371225\_length\_8483\_cov\_20.365791 5846-5863. Max. coverage (+): 0.01. Max coverage (-): 0.01

Region: NODE\_371225\_length\_8483\_cov\_20.365791 5864-5880. Max. coverage (+): 0. Max coverage (-): 0.01

Region: NODE\_371225\_length\_8483\_cov\_20.365791 5881-5898. Max. coverage (+): 0.02. Max coverage (-): 0.02

Region: NODE\_371225\_length\_8483\_cov\_20.365791 5899-5915. Max. coverage (+): 0.04. Max coverage (-): 0

Region: NODE\_371225\_length\_8483\_cov\_20.365791 5916-5933. Max. coverage (+): 0. Max coverage (-): 0.02

Region: NODE\_371225\_length\_8483\_cov\_20.365791 5934-5950. Max. coverage (+): 0. Max coverage (-): 0.04

Region: NODE\_371225\_length\_8483\_cov\_20.365791 5951-5968. Max. coverage (+): 0. Max coverage (-): 0.01

Region: NODE\_371225\_length\_8483\_cov\_20.365791 5969-5986. Max. coverage (+): 0.01. Max coverage (-): 0.01

Region: NODE\_371225\_length\_8483\_cov\_20.365791 5987-6003. Max. coverage (+): 0. Max coverage (-): 0

Region: NODE\_371225\_length\_8483\_cov\_20.365791 6004-6021. Max. coverage (+): 0. Max coverage (-): 0

Region: NODE\_371225\_length\_8483\_cov\_20.365791 6022-6038. Max. coverage (+): 0. Max coverage (-): 0

Region: NODE\_371225\_length\_8483\_cov\_20.365791 6039-6056. Max. coverage (+): 0. Max coverage (-): 0

Region: NODE\_371225\_length\_8483\_cov\_20.365791 6057-6073. Max. coverage (+): 0. Max coverage (-): 0

Region: NODE\_371225\_length\_8483\_cov\_20.365791 6074-6091. Max. coverage (+): 0. Max coverage (-): 0

Region: NODE\_371225\_length\_8483\_cov\_20.365791 6092-6109. Max. coverage (+): 0. Max coverage (-): 0.04

Region: NODE\_371225\_length\_8483\_cov\_20.365791 6110-6126. Max. coverage (+): 0. Max coverage (-): 0.22

Region: NODE\_371225\_length\_8483\_cov\_20.365791 6127-6144. Max. coverage (+): 0. Max coverage (-): 0.3

Region: NODE\_371225\_length\_8483\_cov\_20.365791 6145-6161. Max. coverage (+): 0. Max coverage (-): 0

Region: NODE\_371225\_length\_8483\_cov\_20.365791 6162-6179. Max. coverage (+): 0. Max coverage (-): 0

Region: NODE\_371225\_length\_8483\_cov\_20.365791 6180-6196. Max. coverage (+): 0. Max coverage (-): 0

Region: NODE\_371225\_length\_8483\_cov\_20.365791 6197-6214. Max. coverage (+): 0. Max coverage (-): 0

Region: NODE\_371225\_length\_8483\_cov\_20.365791 6215-6231. Max. coverage (+): 0. Max coverage (-): 0

Region: NODE\_371225\_length\_8483\_cov\_20.365791 6232-6249. Max. coverage (+): 0. Max coverage (-): 0

Region: NODE\_371225\_length\_8483\_cov\_20.365791 6250-6267. Max. coverage (+): 0.15. Max coverage (-): 0

Region: NODE\_371225\_length\_8483\_cov\_20.365791 6268-6284. Max. coverage (+): 0. Max coverage (-): 1.74

Region: NODE\_371225\_length\_8483\_cov\_20.365791 6285-6302. Max. coverage (+): 0. Max coverage (-): 0.01

Region: NODE\_371225\_length\_8483\_cov\_20.365791 6303-6319. Max. coverage (+): 0. Max coverage (-): 0

Region: NODE\_371225\_length\_8483\_cov\_20.365791 6320-6337. Max. coverage (+): 0.04. Max coverage (-): 0.04

Region: NODE\_371225\_length\_8483\_cov\_20.365791 6338-6354. Max. coverage (+): 0.02. Max coverage (-): 0.41

Region: NODE\_371225\_length\_8483\_cov\_20.365791 6355-6372. Max. coverage (+): 0.06. Max coverage (-): 0.02

Region: NODE\_371225\_length\_8483\_cov\_20.365791 6373-6390. Max. coverage (+): 0.02. Max coverage (-): 0.02

Region: NODE\_371225\_length\_8483\_cov\_20.365791 6391-6407. Max. coverage (+): 0. Max coverage (-): 0

Region: NODE\_371225\_length\_8483\_cov\_20.365791 6408-6425. Max. coverage (+): 0.02. Max coverage (-): 0

Region: NODE\_371225\_length\_8483\_cov\_20.365791 6426-6442. Max. coverage (+): 0.09. Max coverage (-): 0

Region: NODE\_371225\_length\_8483\_cov\_20.365791 6443-6460. Max. coverage (+): 0. Max coverage (-): 0

Region: NODE\_371225\_length\_8483\_cov\_20.365791 6461-6477. Max. coverage (+): 0.01. Max coverage (-): 0

Region: NODE\_371225\_length\_8483\_cov\_20.365791 6478-6495. Max. coverage (+): 0. Max coverage (-): 0

Region: NODE\_371225\_length\_8483\_cov\_20.365791 6496-6513. Max. coverage (+): 0. Max coverage (-): 0.48

Region: NODE\_371225\_length\_8483\_cov\_20.365791 6514-6530. Max. coverage (+): 0.28. Max coverage (-): 0.8

Region: NODE\_371225\_length\_8483\_cov\_20.365791 6531-6548. Max. coverage (+): 0.26. Max coverage (-): 0

Region: NODE\_371225\_length\_8483\_cov\_20.365791 6549-6565. Max. coverage (+): 0. Max coverage (-): 0

Region: NODE\_371225\_length\_8483\_cov\_20.365791 6566-6583. Max. coverage (+): 0. Max coverage (-): 0

Region: NODE\_371225\_length\_8483\_cov\_20.365791 6584-6600. Max. coverage (+): 0. Max coverage (-): 0.19

Region: NODE\_371225\_length\_8483\_cov\_20.365791 6601-6618. Max. coverage (+): 0.01. Max coverage (-): 0.02

Region: NODE\_371225\_length\_8483\_cov\_20.365791 6619-6636. Max. coverage (+): 0.1. Max coverage (-): 0

Region: NODE\_371225\_length\_8483\_cov\_20.365791 6637-6653. Max. coverage (+): 0.01. Max coverage (-): 0

Region: NODE\_371225\_length\_8483\_cov\_20.365791 6654-6671. Max. coverage (+): 0.1. Max coverage (-): 0

Region: NODE\_371225\_length\_8483\_cov\_20.365791 6672-6688. Max. coverage (+): 0.11. Max coverage (-): 0

Region: NODE\_371225\_length\_8483\_cov\_20.365791 6689-6706. Max. coverage (+): 0.01. Max coverage (-): 0

Region: NODE\_371225\_length\_8483\_cov\_20.365791 6707-6723. Max. coverage (+): 0. Max coverage (-): 0

Region: NODE\_371225\_length\_8483\_cov\_20.365791 6724-6741. Max. coverage (+): 0. Max coverage (-): 0

Region: NODE\_371225\_length\_8483\_cov\_20.365791 6742-6759. Max. coverage (+): 0.01. Max coverage (-): 0.01

Region: NODE\_371225\_length\_8483\_cov\_20.365791 6760-6776. Max. coverage (+): 0. Max coverage (-): 0

Region: NODE\_371225\_length\_8483\_cov\_20.365791 6777-6794. Max. coverage (+): 0.01. Max coverage (-): 0

Region: NODE\_371225\_length\_8483\_cov\_20.365791 6795-6811. Max. coverage (+): 0.01. Max coverage (-): 0

Region: NODE\_371225\_length\_8483\_cov\_20.365791 6812-6829. Max. coverage (+): 0. Max coverage (-): 0

Region: NODE\_371225\_length\_8483\_cov\_20.365791 6830-6846. Max. coverage (+): 0. Max coverage (-): 0

Region: NODE\_371225\_length\_8483\_cov\_20.365791 6847-6864. Max. coverage (+): 0. Max coverage (-): 0

Region: NODE\_371225\_length\_8483\_cov\_20.365791 6865-6882. Max. coverage (+): 0. Max coverage (-): 0

Region: NODE\_371225\_length\_8483\_cov\_20.365791 6883-6899. Max. coverage (+): 0. Max coverage (-): 0

Region: NODE\_371225\_length\_8483\_cov\_20.365791 6900-6917. Max. coverage (+): 0. Max coverage (-): 0

Region: NODE\_371225\_length\_8483\_cov\_20.365791 6918-6934. Max. coverage (+): 0. Max coverage (-): 0

Region: NODE\_371225\_length\_8483\_cov\_20.365791 6935-6952. Max. coverage (+): 0. Max coverage (-): 0.01

Region: NODE\_371225\_length\_8483\_cov\_20.365791 6953-6969. Max. coverage (+): 0.01. Max coverage (-): 0

Region: NODE\_371225\_length\_8483\_cov\_20.365791 6970-6987. Max. coverage (+): 0.01. Max coverage (-): 0.01

Region: NODE\_371225\_length\_8483\_cov\_20.365791 6988-7005. Max. coverage (+): 0.04. Max coverage (-): 0

Region: NODE\_371225\_length\_8483\_cov\_20.365791 7006-7022. Max. coverage (+): 0.05. Max coverage (-): 0

Region: NODE\_371225\_length\_8483\_cov\_20.365791 7023-7040. Max. coverage (+): 0. Max coverage (-): 0

Region: NODE\_371225\_length\_8483\_cov\_20.365791 7041-7057. Max. coverage (+): 0.01. Max coverage (-): 0

Region: NODE\_371225\_length\_8483\_cov\_20.365791 7058-7075. Max. coverage (+): 0.01. Max coverage (-): 0.01

Region: NODE\_371225\_length\_8483\_cov\_20.365791 7076-7092. Max. coverage (+): 0.01. Max coverage (-): 0.01

Region: NODE\_371225\_length\_8483\_cov\_20.365791 7093-7110. Max. coverage (+): 0. Max coverage (-): 0

Region: NODE\_371225\_length\_8483\_cov\_20.365791 7111-7128. Max. coverage (+): 0. Max coverage (-): 0

Region: NODE\_371225\_length\_8483\_cov\_20.365791 7129-7145. Max. coverage (+): 0. Max coverage (-): 0.01

Region: NODE\_371225\_length\_8483\_cov\_20.365791 7146-7163. Max. coverage (+): 0. Max coverage (-): 0

Region: NODE\_371225\_length\_8483\_cov\_20.365791 7164-7180. Max. coverage (+): 0. Max coverage (-): 0

Region: NODE\_371225\_length\_8483\_cov\_20.365791 7181-7198. Max. coverage (+): 0. Max coverage (-): 0

Region: NODE\_371225\_length\_8483\_cov\_20.365791 7199-7215. Max. coverage (+): 0. Max coverage (-): 0

Region: NODE\_371225\_length\_8483\_cov\_20.365791 7216-7233. Max. coverage (+): 0. Max coverage (-): 0

Region: NODE\_371225\_length\_8483\_cov\_20.365791 7234-7251. Max. coverage (+): 0. Max coverage (-): 0

Region: NODE\_371225\_length\_8483\_cov\_20.365791 7252-7268. Max. coverage (+): 0. Max coverage (-): 0

Region: NODE\_371225\_length\_8483\_cov\_20.365791 7269-7286. Max. coverage (+): 0. Max coverage (-): 0

Region: NODE\_371225\_length\_8483\_cov\_20.365791 7287-7303. Max. coverage (+): 0. Max coverage (-): 0

Region: NODE\_371225\_length\_8483\_cov\_20.365791 7304-7321. Max. coverage (+): 0. Max coverage (-): 0

Region: NODE\_371225\_length\_8483\_cov\_20.365791 7322-7338. Max. coverage (+): 0. Max coverage (-): 0.01

Region: NODE\_371225\_length\_8483\_cov\_20.365791 7339-7356. Max. coverage (+): 0.04. Max coverage (-): 0.01

Region: NODE\_371225\_length\_8483\_cov\_20.365791 7357-7374. Max. coverage (+): 0.15. Max coverage (-): 0

Region: NODE\_371225\_length\_8483\_cov\_20.365791 7375-7391. Max. coverage (+): 0.01. Max coverage (-): 0.02

Region: NODE\_371225\_length\_8483\_cov\_20.365791 7392-7409. Max. coverage (+): 0.01. Max coverage (-): 0.01

Region: NODE\_371225\_length\_8483\_cov\_20.365791 7410-7426. Max. coverage (+): 0. Max coverage (-): 0.1

Region: NODE\_371225\_length\_8483\_cov\_20.365791 7427-7444. Max. coverage (+): 0.01. Max coverage (-): 0.02

Region: NODE\_371225\_length\_8483\_cov\_20.365791 7445-7461. Max. coverage (+): 0.11. Max coverage (-): 0.01

Region: NODE\_371225\_length\_8483\_cov\_20.365791 7462-7479. Max. coverage (+): 0.11. Max coverage (-): 0

Region: NODE\_371225\_length\_8483\_cov\_20.365791 7480-7497. Max. coverage (+): 0.05. Max coverage (-): 0

Region: NODE\_371225\_length\_8483\_cov\_20.365791 7498-7514. Max. coverage (+): 0.01. Max coverage (-): 0.02

Region: NODE\_371225\_length\_8483\_cov\_20.365791 7515-7532. Max. coverage (+): 0.02. Max coverage (-): 0.02

Region: NODE\_371225\_length\_8483\_cov\_20.365791 7533-7549. Max. coverage (+): 0. Max coverage (-): 0

Region: NODE\_371225\_length\_8483\_cov\_20.365791 7550-7567. Max. coverage (+): 0. Max coverage (-): 0

Region: NODE\_371225\_length\_8483\_cov\_20.365791 7568-7584. Max. coverage (+): 0. Max coverage (-): 0

Region: NODE\_371225\_length\_8483\_cov\_20.365791 7585-7602. Max. coverage (+): 0. Max coverage (-): 0.01

Region: NODE\_371225\_length\_8483\_cov\_20.365791 7603-7620. Max. coverage (+): 0.01. Max coverage (-): 0

Region: NODE\_371225\_length\_8483\_cov\_20.365791 7621-7637. Max. coverage (+): 0. Max coverage (-): 0

Region: NODE\_371225\_length\_8483\_cov\_20.365791 7638-7655. Max. coverage (+): 0. Max coverage (-): 0

Region: NODE\_371225\_length\_8483\_cov\_20.365791 7656-7672. Max. coverage (+): 0.02. Max coverage (-): 0

Region: NODE\_371225\_length\_8483\_cov\_20.365791 7673-7690. Max. coverage (+): 0. Max coverage (-): 0

Region: NODE\_371225\_length\_8483\_cov\_20.365791 7691-7707. Max. coverage (+): 0. Max coverage (-): 0

Region: NODE\_371225\_length\_8483\_cov\_20.365791 7708-7725. Max. coverage (+): 0. Max coverage (-): 0

Region: NODE\_371225\_length\_8483\_cov\_20.365791 7726-7743. Max. coverage (+): 0. Max coverage (-): 0

Region: NODE\_371225\_length\_8483\_cov\_20.365791 7744-7760. Max. coverage (+): 0. Max coverage (-): 0

Region: NODE\_371225\_length\_8483\_cov\_20.365791 7761-7778. Max. coverage (+): 0. Max coverage (-): 0

Region: NODE\_371225\_length\_8483\_cov\_20.365791 7779-7795. Max. coverage (+): 0. Max coverage (-): 0

Region: NODE\_371225\_length\_8483\_cov\_20.365791 7796-7813. Max. coverage (+): 0. Max coverage (-): 0

Region: NODE\_371225\_length\_8483\_cov\_20.365791 7814-7830. Max. coverage (+): 0. Max coverage (-): 0

Region: NODE\_371225\_length\_8483\_cov\_20.365791 7831-7848. Max. coverage (+): 0. Max coverage (-): 0

Region: NODE\_371225\_length\_8483\_cov\_20.365791 7849-7866. Max. coverage (+): 0. Max coverage (-): 0

Region: NODE\_371225\_length\_8483\_cov\_20.365791 7867-7883. Max. coverage (+): 0. Max coverage (-): 0

Region: NODE\_371225\_length\_8483\_cov\_20.365791 7884-7901. Max. coverage (+): 0. Max coverage (-): 0

Region: NODE\_371225\_length\_8483\_cov\_20.365791 7902-7918. Max. coverage (+): 0. Max coverage (-): 0

Region: NODE\_371225\_length\_8483\_cov\_20.365791 7919-7936. Max. coverage (+): 0. Max coverage (-): 0

Region: NODE\_371225\_length\_8483\_cov\_20.365791 7937-7953. Max. coverage (+): 0. Max coverage (-): 0

Region: NODE\_371225\_length\_8483\_cov\_20.365791 7954-7971. Max. coverage (+): 0. Max coverage (-): 0

Region: NODE\_371225\_length\_8483\_cov\_20.365791 7972-7988. Max. coverage (+): 0. Max coverage (-): 0

Region: NODE\_371225\_length\_8483\_cov\_20.365791 7989-8006. Max. coverage (+): 0. Max coverage (-): 0

Region: NODE\_371225\_length\_8483\_cov\_20.365791 8007-8024. Max. coverage (+): 0. Max coverage (-): 0

Region: NODE\_371225\_length\_8483\_cov\_20.365791 8025-8041. Max. coverage (+): 0. Max coverage (-): 0

Region: NODE\_371225\_length\_8483\_cov\_20.365791 8042-8059. Max. coverage (+): 0. Max coverage (-): 0

Region: NODE\_371225\_length\_8483\_cov\_20.365791 8060-8076. Max. coverage (+): 0. Max coverage (-): 0

Region: NODE\_371225\_length\_8483\_cov\_20.365791 8077-8094. Max. coverage (+): 0. Max coverage (-): 0

Region: NODE\_371225\_length\_8483\_cov\_20.365791 8095-8111. Max. coverage (+): 0. Max coverage (-): 0

Region: NODE\_371225\_length\_8483\_cov\_20.365791 8112-8129. Max. coverage (+): 0. Max coverage (-): 0.01

Region: NODE\_371225\_length\_8483\_cov\_20.365791 8130-8147. Max. coverage (+): 0.09. Max coverage (-): 0

Region: NODE\_371225\_length\_8483\_cov\_20.365791 8148-8164. Max. coverage (+): 0.48. Max coverage (-): 0

Region: NODE\_371225\_length\_8483\_cov\_20.365791 8165-8182. Max. coverage (+): 0. Max coverage (-): 0

Region: NODE\_371225\_length\_8483\_cov\_20.365791 8183-8199. Max. coverage (+): 0.01. Max coverage (-): 0.01

Region: NODE\_371225\_length\_8483\_cov\_20.365791 8200-8217. Max. coverage (+): 0. Max coverage (-): 0.1

Region: NODE\_371225\_length\_8483\_cov\_20.365791 8218-8234. Max. coverage (+): 0.01. Max coverage (-): 0.02

Region: NODE\_371225\_length\_8483\_cov\_20.365791 8235-8252. Max. coverage (+): 0.11. Max coverage (-): 0.01

Region: NODE\_371225\_length\_8483\_cov\_20.365791 8253-8270. Max. coverage (+): 0.11. Max coverage (-): 0

Region: NODE\_371225\_length\_8483\_cov\_20.365791 8271-8287. Max. coverage (+): 0.05. Max coverage (-): 0.02

Region: NODE\_371225\_length\_8483\_cov\_20.365791 8288-8305. Max. coverage (+): 0.02. Max coverage (-): 0.02

Region: NODE\_371225\_length\_8483\_cov\_20.365791 8306-8322. Max. coverage (+): 0.02. Max coverage (-): 0.02

Region: NODE\_371225\_length\_8483\_cov\_20.365791 8323-8340. Max. coverage (+): 0.01. Max coverage (-): 0.05

Region: NODE\_371225\_length\_8483\_cov\_20.365791 8341-8357. Max. coverage (+): 0.01. Max coverage (-): 0.01

Region: NODE\_371225\_length\_8483\_cov\_20.365791 8358-8375. Max. coverage (+): 0.02. Max coverage (-): 0.03

Region: NODE\_371225\_length\_8483\_cov\_20.365791 8376-8393. Max. coverage (+): 0.01. Max coverage (-): 0.01

Region: NODE\_371225\_length\_8483\_cov\_20.365791 8394-8410. Max. coverage (+): 0.01. Max coverage (-): 0

Region: NODE\_371225\_length\_8483\_cov\_20.365791 8411-8428. Max. coverage (+): 0. Max coverage (-): 0

Region: NODE\_371225\_length\_8483\_cov\_20.365791 8429-8445. Max. coverage (+): 0. Max coverage (-): 0

Region: NODE\_371225\_length\_8483\_cov\_20.365791 8446-8463. Max. coverage (+): 0. Max coverage (-): 0

Region: NODE\_371225\_length\_8483\_cov\_20.365791 8464-8480. Max. coverage (+): 0. Max coverage (-): 0

Region: NODE\_371225\_length\_8483\_cov\_20.365791 8481-8498. Max. coverage (+): 0. Max coverage (-): 0

Region: NODE\_371225\_length\_8483\_cov\_20.365791 8499-8516. Max. coverage (+): 0.01. Max coverage (-): 0

Region: NODE\_371225\_length\_8483\_cov\_20.365791 8517-8533. Max. coverage (+): 0. Max coverage (-): 0

Region: NODE\_371225\_length\_8483\_cov\_20.365791 8534-8551. Max. coverage (+): 0. Max coverage (-): 0

Region: NODE\_371225\_length\_8483\_cov\_20.365791 8552-8568. Max. coverage (+): 0.01. Max coverage (-): 0.01

Region: NODE\_371225\_length\_8483\_cov\_20.365791 8569-8586. Max. coverage (+): 0.04. Max coverage (-): 0.01

Region: NODE\_371225\_length\_8483\_cov\_20.365791 8587-8603. Max. coverage (+): 0. Max coverage (-): 0

Region: NODE\_371225\_length\_8483\_cov\_20.365791 8604-8621. Max. coverage (+): 0. Max coverage (-): 0

Region: NODE\_371225\_length\_8483\_cov\_20.365791 8622-8639. Max. coverage (+): 0.07. Max coverage (-): 0

Region: NODE\_371225\_length\_8483\_cov\_20.365791 8640-8656. Max. coverage (+): 0. Max coverage (-): 0

Region: NODE\_371225\_length\_8483\_cov\_20.365791 8657-8674. Max. coverage (+): 0. Max coverage (-): 0

Region: NODE\_371225\_length\_8483\_cov\_20.365791 8675-8691. Max. coverage (+): 0. Max coverage (-): 0

Region: NODE\_371225\_length\_8483\_cov\_20.365791 8692-8709. Max. coverage (+): 0. Max coverage (-): 0

Region: NODE\_371225\_length\_8483\_cov\_20.365791 8710-8726. Max. coverage (+): 0. Max coverage (-): 0

Region: NODE\_371225\_length\_8483\_cov\_20.365791 8727-8744. Max. coverage (+): 0. Max coverage (-): 0

Region: NODE\_371225\_length\_8483\_cov\_20.365791 8745-8762. Max. coverage (+): 0. Max coverage (-): 0.01

Region: NODE\_371225\_length\_8483\_cov\_20.365791 8763-8779. Max. coverage (+): 0.05. Max coverage (-): 0

Region: NODE\_371225\_length\_8483\_cov\_20.365791 8780-8797. Max. coverage (+): 0.04. Max coverage (-): 0

Region: NODE\_371225\_length\_8483\_cov\_20.365791 8798-. Max. coverage (+): 0. Max coverage (-): 0

RepeatMasker Color Code

**+**

100-98% Identity

<98-95% Identity

<95-90% Identity

<90-85% Identity

<85-80% Identity

<80-75% Identity

<75-70% Identity

<70% Identity

**-**

Gene Set Color Code

**+**

Gene

Pseudogene

Other

**-**

Topology/Coverage Color Code

Coverage Plus Strand

Coverage Minus Strand

Mainstrand: Plus

Mainstrand: Minus

Complementary Strand

Flanking Region  
(if option -flank >0)

Gene Set Annotation  

**1. unknown (unknownunknown) Tr:unknown**: 4662-5381 (+)  
**2. unknown (unknownunknown) Tr:unknown**: 5466-6131 (+)  
**3. unknown (unknownunknown) Tr:unknown**: 3814-4524 (+)

  
RepeatMasker Annotation  

**1. Gypsy4-I\_DR**: 228-396 (-), Divergence to consensus: 40.1%  
**2. Gypsy4-I\_DR**: 491-556 (-), Divergence to consensus: 19.7%  
**3. Gypsy4-I\_DR**: 579-1901 (-), Divergence to consensus: 31.4%  
**4. Gypsy4-I\_DR**: 2441-3061 (-), Divergence to consensus: 36.5%  
**5. Gypsy4-I\_DR**: 6253-6593 (-), Divergence to consensus: 36.8%  
**6. Gypsy4-LTR\_DR**: 6594-6803 (-), Divergence to consensus: 34.5%  
**7. Gypsy4-LTR\_DR**: 7404-7494 (-), Divergence to consensus: 28.9%  
**8. Mariner-16\_DF**: 7705-7761 (+), Divergence to consensus: 29.7%  
**9. Ginger1-5\_HM**: 7724-7765 (-), Divergence to consensus: 21.4%  
**10. AlRepE-1229**: 7758-8067 (+), Divergence to consensus: 42.7%  
**11. Gypsy4-LTR\_DR**: 8191-8492 (-), Divergence to consensus: 43.4%  
**12. Gypsy4-I\_DR**: 8493-8808 (-), Divergence to consensus: 44.7%

  
Transcription Factor Binding Sites  

**RHOXF1** (Sequence: GGCTCA (-): 262)  
**RHOXF1** (Sequence: AGATCA (-): 682)  
**RHOXF1** (Sequence: AGCTTA (-): 1282)  
**RHOXF1** (Sequence: AGCTTA (-): 1654)  
**RHOXF1** (Sequence: AGATCA (-): 1911)  
**RHOXF1** (Sequence: AGATCA (-): 2079)  
**RHOXF1** (Sequence: GGCTTA (-): 3273)  
**RHOXF1** (Sequence: AGCTTA (-): 3779)  
**RHOXF1** (Sequence: AGCTCA (-): 4515)  
**RHOXF1** (Sequence: AGATCA (-): 4681)  
**RHOXF1** (Sequence: AGATCA (-): 4915)  
**RHOXF1** (Sequence: GGATCA (-): 5599)  
**RHOXF1** (Sequence: AGCTTA (-): 5713)  
**RHOXF1** (Sequence: AGATCA (-): 6722)  
**RHOXF1** (Sequence: AGCTTA (-): 6954)  
**RHOXF1** (Sequence: AGATTA (-): 6994)  
**RHOXF1** (Sequence: AGCTTA (-): 7061)  
**RHOXF1** (Sequence: TGATCC (+): 661)  
**RHOXF1** (Sequence: TGATCT (+): 926)  
**RHOXF1** (Sequence: TGAGCC (+): 1494)  
**RHOXF1** (Sequence: TGAGCT (+): 1685)  
**RHOXF1** (Sequence: TAATCT (+): 2514)  
**RHOXF1** (Sequence: TAATCT (+): 2958)  
**RHOXF1** (Sequence: TAATCT (+): 3084)  
**RHOXF1** (Sequence: TAAGCT (+): 3404)  
**RHOXF1** (Sequence: TGAGCT (+): 3524)  
**RHOXF1** (Sequence: TGAGCT (+): 3945)  
**RHOXF1** (Sequence: TAAGCT (+): 4014)  
**RHOXF1** (Sequence: TGAGCT (+): 4131)  
**RHOXF1** (Sequence: TAATCT (+): 4266)  
**RHOXF1** (Sequence: TGATCT (+): 4301)  
**RHOXF1** (Sequence: TGATCC (+): 4431)  
**RHOXF1** (Sequence: TGATCC (+): 5102)  
**RHOXF1** (Sequence: TAATCC (+): 5192)  
**RHOXF1** (Sequence: TGAGCT (+): 5711)  
**RHOXF1** (Sequence: TGAGCC (+): 5936)  
**RHOXF1** (Sequence: TGAGCT (+): 6233)  
**RHOXF1** (Sequence: TGAGCC (+): 8793)  
**Lhx8** (Sequence: TTAATTAG (-): 2333)  
**Gata4** (Sequence: CTTATCT (+): 3781)  
**POU5F1** (Sequence: TTTGCAT (-): 3230)  
**POU5F1** (Sequence: TTTGCAT (-): 7930)  
**RFX4\_2** (Sequence: GTATCTATG (-): 6815)  
**FOXP1** (Sequence: GTAAACA (+): 8145)  
**FOXO3\_mmu** (Sequence: TGTTTTCA (-): 6749)  
**Sox5** (Sequence: ATTGTT (+): 2390)  
**Sox5** (Sequence: ATTGTT (+): 2403)  
**Sox5** (Sequence: ATTGTT (+): 2554)  
**Sox5** (Sequence: ATTGTT (+): 7182)  
**Sox5** (Sequence: ATTGTT (+): 7588)  
**Sox5** (Sequence: ATTGTT (+): 8375)  
**FIGLA** (Sequence: TACAGCTGTT (-): 1577)  
**FOXO3\_mmu** (Sequence: TGAAAACA (+): 6303)  
**FOXO1** (Sequence: GAAAACAAG (-): 6304)  
**FOXP1** (Sequence: TGTTTAC (-): 5033)  
**Nobox** (Sequence: TAATTGCT (+): 7632)  
**Nobox** (Sequence: TAATTGCT (+): 8419)  
**Rhox11** (Sequence: TGGTGTTAA (+): 1587)  
**Rhox11** (Sequence: TAAACACCG (-): 8146)  
**Sox5** (Sequence: AACAAT (-): 2917)  
**Sox5** (Sequence: AACAAT (-): 5065)
