# Supplementary material for: First characterization of PIWI-interacting RNA clusters in a cichlid fish with a B chromosome
Source: BMC Biol. 2022 Sep 21;20:204. doi: 10.1186/s12915-022-01403-2 (PMC9490952; doi:10.1186/s12915-022-01403-2)
Supplement: Supplementary file 1 — Additional file 1. Zipped folder with fasta and interactive html piRNA cluster information for the A. latifasciata genome. The nomenclature is as follows: number-pirna-cluster_sex_B-presence (f, female; m, male; 0b, without B chromosome; 1b, with B chromosome). [file 12915_2022_1403_MOESM1_ESM.zip › 144_m0b.html]

piRNA cluster 144\_m0b 70


Predicted piRNA cluster no. 144\_m0b
  

Show proTRAC run info
Hide proTRAC run info

/\  
                \_\_\_\_\_\_\_\_\_\_\_\_\_\_\_\_\_\_\_\_\_\_\_/\\_\_\_ /  \\_\_\_\_\_\_\_  
               I                      /  \  /    \      I  
               I     pro             /    \/      \     I  
               I        TRAC        /               \   I  
               I   \_\_\_\_\_\_\_\_\_\_\_\_\_\_\_\_/\_\_\_\_\_\_\_\_\_\_\_\_\_\_\_\_\_\\_ I  
               I   \              /                     I  
               I    \            /                      I  
               I     \  /\      /       V.2.4.2         I  
               I      \/  \    /                        I  
               I\_\_\_\_\_\_\_\_\_\_\_\  /\_\_\_\_\_\_\_\_\_\_\_\_\_\_\_\_\_\_\_\_\_\_\_\_\_I  
                            \/  
  
  
================================= proTRAC ====================================  
VERSION: .......... 2.4.2  
LAST MODIFIED: .... 11. May 2018  
  
Please cite:  
Rosenkranz D, Zischler H. proTRAC - a software for probabilistic piRNA cluster  
detection, visualization and analysis. 2012. BMC Bioinformatics 13:5.  
  
  
Contact:  
David Rosenkranz  
Institute of Organismic and Molecular Evolutionary Biology  
Dept. Anthropology, small RNA group  
Johannes Gutenberg University Mainz  
email: rosenkranz@uni-mainz.de  
  
You can find the latest proTRAC version at:  
http://sourceforge.net/projects/protrac/files  
http://www.smallRNAgroup-mainz.de/software  
==============================================================================  
  
PARAMETERS:  
Map file: ...............piwi-machos-0B.fa-collapse.map  
Genome file: ............../../../0B\_ala\_genome.fa  
RepeatMasker annotation: Alatifasciata-all0B-maryan-v2.fa\_corrected.out  
GeneSet:................./guest-storage/Data/annotation/Alatifasciata\_all0B\_maryan-v2\_out2017.gff  
  
Significant (p<=0.01) hit density will be calculated based  
on observed hit distribution.  
  
Sliding window size: ........................................ 5000 bp  
Sliding window increament: .................................. 1000 bp  
Normalize each hit by number of genomic hits: ............... yes  
Normalize each hit by number of sequence reads: ............. yes  
Normalize values (-> per million mapped reads): ............. yes  
Min. fraction of hits with 1T(U) or 10A: .................... 0.75  
Alternatively: Min. fraction of hits with 1T(U) and 10A: .... 0.5  
Min. fraction of hits with typical piRNA length: ............ 0.75  
Typical piRNA length: ....................................... 24-32 nt  
Min. size of a piRNA cluster: ............................... 1000 bp.  
Min. number of hits (absolute): ............................. 0  
Min. number of hits (normalized): ........................... 0  
Min. fraction of hits on the mainstrand: .................... 0.75  
Top fraction of mapped sequences (in terms of read counts): . 1%  
Top fraction accounts for max. n% of sequence reads: ........ 90%  
Min. fraction of hits on each arm of a bidirectional cluster: 0.05  
Output html file for each cluster: .......................... yes  
Output a summary table: ..................................... yes  
Output a FASTA file for each cluster (piRNA sequences): ..... yes  
Output a FASTA file comprising cluster sequences: ........... yes  
Output a GTF file for predicted piRNA clusters: ..............yes  
Search DNA motifs in clusters: .............................. yes  
Output flanking sequences: +/- .............................. 0 bp  
Output ~.pTi file: .......................................... no  
==============================================================================  
  
  
Genome size (without gaps): ............ 758543724 bp  
Gaps (N/X/-): .......................... 417479 bp  
Mapped reads: .......................... 24765598  
Non-identical sequences: ............... 6158275  
Genomic hits: .......................... 53103584  
Significant densitiy of mapped reads: .. 763.098963422187 reads/kb

Show proTRAC cluster info
Hide proTRAC cluster info

|  |  |
| --- | --- |
| Location | NODE\_372714\_length\_2623\_cov\_15.020205 |
| Coordinates | 3-2675 |
| Size [bp] | 2673 |
| Sequence hit loci | 2492 |
| Mapped reads (normalized) | 4582.8 |
| Mapped reads (normalized) per kb | 1714.5 |
| Normalized reads with 1T (1U) | 77.8% |
| Normalized reads with 10A | 48.7% |
| Normalized reads with length 24-32 nt | 98.8% |
| Normalized reads on the main strand(s) | 82.4% |
| Predicted directionality | bi:minus-plus (split between 506 and 510) |

100%

0%

1T (1U)  
reads

10A reads

24-32 nt  
reads

reads on mainstrand

**Either the amount of reads with 1T (1U) OR 10A has to exceed 75% (set with option: -1Tor10A)  
Alternatively the amount of reads with 1T (1U) AND 10A has to exceed 50% (set with option: -1Tand10A)  
Minimum amount of reads with preferred size is 75% (set with option: -pisize)  
Minimum amount of reads on the main strand(s) is 75% (set with option: -clstrand)**

Show read coverage
Hide read coverage

WHAT DO I SEE HERE?  
This chart shows the location of mapped sequence reads within a predicted piRNA cluster. The color refers to the number of genomic hits produced by the sequence read in question. A dark red bar indicates that this sequence read produces many other hits elsewhere in the genome. Many adjacent red or yellow bars can indicate the presence of a multi-copy element such as transposons or rRNA genes. A dark green bar indicates that this sequence read maps uniquely to this locus.

1 hit

2-5 hits

6-10 hits

11-20 hits

21-50 hits

51-100 hits

> 100 hits

NODE\_372714\_length\_2623\_cov\_15.020205

3

2675

Gene Set

RepeatMasker

Mapped  
Reads

42.4

plus strand

minus strand

42.4

Region: NODE\_372714\_length\_2623\_cov\_15.020205 8798-5. Max. coverage (+): 0. Max coverage (-): 0.04

Region: NODE\_372714\_length\_2623\_cov\_15.020205 6-11. Max. coverage (+): 0.04. Max coverage (-): 0.04

Region: NODE\_372714\_length\_2623\_cov\_15.020205 12-16. Max. coverage (+): 0. Max coverage (-): 0.04

Region: NODE\_372714\_length\_2623\_cov\_15.020205 17-21. Max. coverage (+): 0. Max coverage (-): 0.04

Region: NODE\_372714\_length\_2623\_cov\_15.020205 22-27. Max. coverage (+): 0. Max coverage (-): 0

Region: NODE\_372714\_length\_2623\_cov\_15.020205 28-32. Max. coverage (+): 0. Max coverage (-): 0.04

Region: NODE\_372714\_length\_2623\_cov\_15.020205 33-37. Max. coverage (+): 0.04. Max coverage (-): 0

Region: NODE\_372714\_length\_2623\_cov\_15.020205 38-43. Max. coverage (+): 0. Max coverage (-): 0.08

Region: NODE\_372714\_length\_2623\_cov\_15.020205 44-48. Max. coverage (+): 0.12. Max coverage (-): 0

Region: NODE\_372714\_length\_2623\_cov\_15.020205 49-53. Max. coverage (+): 0. Max coverage (-): 0.04

Region: NODE\_372714\_length\_2623\_cov\_15.020205 54-59. Max. coverage (+): 0.04. Max coverage (-): 0.12

Region: NODE\_372714\_length\_2623\_cov\_15.020205 60-64. Max. coverage (+): 0.16. Max coverage (-): 0.04

Region: NODE\_372714\_length\_2623\_cov\_15.020205 65-69. Max. coverage (+): 0. Max coverage (-): 0

Region: NODE\_372714\_length\_2623\_cov\_15.020205 70-75. Max. coverage (+): 0.02. Max coverage (-): 0.02

Region: NODE\_372714\_length\_2623\_cov\_15.020205 76-80. Max. coverage (+): 0. Max coverage (-): 0.2

Region: NODE\_372714\_length\_2623\_cov\_15.020205 81-85. Max. coverage (+): 0. Max coverage (-): 0.04

Region: NODE\_372714\_length\_2623\_cov\_15.020205 86-91. Max. coverage (+): 0. Max coverage (-): 0

Region: NODE\_372714\_length\_2623\_cov\_15.020205 92-96. Max. coverage (+): 0.57. Max coverage (-): 0

Region: NODE\_372714\_length\_2623\_cov\_15.020205 97-101. Max. coverage (+): 0.57. Max coverage (-): 0

Region: NODE\_372714\_length\_2623\_cov\_15.020205 102-107. Max. coverage (+): 0.02. Max coverage (-): 0.06

Region: NODE\_372714\_length\_2623\_cov\_15.020205 108-112. Max. coverage (+): 0. Max coverage (-): 0.52

Region: NODE\_372714\_length\_2623\_cov\_15.020205 113-117. Max. coverage (+): 0. Max coverage (-): 0.18

Region: NODE\_372714\_length\_2623\_cov\_15.020205 118-123. Max. coverage (+): 0.04. Max coverage (-): 0.14

Region: NODE\_372714\_length\_2623\_cov\_15.020205 124-128. Max. coverage (+): 0.04. Max coverage (-): 0.32

Region: NODE\_372714\_length\_2623\_cov\_15.020205 129-133. Max. coverage (+): 0.08. Max coverage (-): 0.32

Region: NODE\_372714\_length\_2623\_cov\_15.020205 134-139. Max. coverage (+): 0.89. Max coverage (-): 1.05

Region: NODE\_372714\_length\_2623\_cov\_15.020205 140-144. Max. coverage (+): 0.73. Max coverage (-): 0.2

Region: NODE\_372714\_length\_2623\_cov\_15.020205 145-150. Max. coverage (+): 0.65. Max coverage (-): 1.33

Region: NODE\_372714\_length\_2623\_cov\_15.020205 151-155. Max. coverage (+): 0.01. Max coverage (-): 0.09

Region: NODE\_372714\_length\_2623\_cov\_15.020205 156-160. Max. coverage (+): 0.01. Max coverage (-): 0.03

Region: NODE\_372714\_length\_2623\_cov\_15.020205 161-166. Max. coverage (+): 0.01. Max coverage (-): 0

Region: NODE\_372714\_length\_2623\_cov\_15.020205 167-171. Max. coverage (+): 0. Max coverage (-): 0.12

Region: NODE\_372714\_length\_2623\_cov\_15.020205 172-176. Max. coverage (+): 0. Max coverage (-): 0.27

Region: NODE\_372714\_length\_2623\_cov\_15.020205 177-182. Max. coverage (+): 0. Max coverage (-): 0.16

Region: NODE\_372714\_length\_2623\_cov\_15.020205 183-187. Max. coverage (+): 0. Max coverage (-): 0

Region: NODE\_372714\_length\_2623\_cov\_15.020205 188-192. Max. coverage (+): 0. Max coverage (-): 0.04

Region: NODE\_372714\_length\_2623\_cov\_15.020205 193-198. Max. coverage (+): 0.03. Max coverage (-): 0

Region: NODE\_372714\_length\_2623\_cov\_15.020205 199-203. Max. coverage (+): 0.01. Max coverage (-): 0

Region: NODE\_372714\_length\_2623\_cov\_15.020205 204-208. Max. coverage (+): 0. Max coverage (-): 0

Region: NODE\_372714\_length\_2623\_cov\_15.020205 209-214. Max. coverage (+): 0. Max coverage (-): 0

Region: NODE\_372714\_length\_2623\_cov\_15.020205 215-219. Max. coverage (+): 0. Max coverage (-): 0

Region: NODE\_372714\_length\_2623\_cov\_15.020205 220-224. Max. coverage (+): 0. Max coverage (-): 0

Region: NODE\_372714\_length\_2623\_cov\_15.020205 225-230. Max. coverage (+): 0. Max coverage (-): 0.03

Region: NODE\_372714\_length\_2623\_cov\_15.020205 231-235. Max. coverage (+): 0.01. Max coverage (-): 0.04

Region: NODE\_372714\_length\_2623\_cov\_15.020205 236-240. Max. coverage (+): 0. Max coverage (-): 0.08

Region: NODE\_372714\_length\_2623\_cov\_15.020205 241-246. Max. coverage (+): 0.01. Max coverage (-): 0.04

Region: NODE\_372714\_length\_2623\_cov\_15.020205 247-251. Max. coverage (+): 0.01. Max coverage (-): 0.01

Region: NODE\_372714\_length\_2623\_cov\_15.020205 252-256. Max. coverage (+): 0.04. Max coverage (-): 0.07

Region: NODE\_372714\_length\_2623\_cov\_15.020205 257-262. Max. coverage (+): 0.01. Max coverage (-): 0.01

Region: NODE\_372714\_length\_2623\_cov\_15.020205 263-267. Max. coverage (+): 0. Max coverage (-): 0.03

Region: NODE\_372714\_length\_2623\_cov\_15.020205 268-272. Max. coverage (+): 0.01. Max coverage (-): 0.01

Region: NODE\_372714\_length\_2623\_cov\_15.020205 273-278. Max. coverage (+): 0. Max coverage (-): 0

Region: NODE\_372714\_length\_2623\_cov\_15.020205 279-283. Max. coverage (+): 0.08. Max coverage (-): 0.08

Region: NODE\_372714\_length\_2623\_cov\_15.020205 284-289. Max. coverage (+): 0. Max coverage (-): 0.08

Region: NODE\_372714\_length\_2623\_cov\_15.020205 290-294. Max. coverage (+): 0. Max coverage (-): 0

Region: NODE\_372714\_length\_2623\_cov\_15.020205 295-299. Max. coverage (+): 0. Max coverage (-): 0.08

Region: NODE\_372714\_length\_2623\_cov\_15.020205 300-305. Max. coverage (+): 0. Max coverage (-): 0.16

Region: NODE\_372714\_length\_2623\_cov\_15.020205 306-310. Max. coverage (+): 0.02. Max coverage (-): 0.02

Region: NODE\_372714\_length\_2623\_cov\_15.020205 311-315. Max. coverage (+): 0.02. Max coverage (-): 0.24

Region: NODE\_372714\_length\_2623\_cov\_15.020205 316-321. Max. coverage (+): 0.34. Max coverage (-): 0.38

Region: NODE\_372714\_length\_2623\_cov\_15.020205 322-326. Max. coverage (+): 0.32. Max coverage (-): 0.44

Region: NODE\_372714\_length\_2623\_cov\_15.020205 327-331. Max. coverage (+): 0.04. Max coverage (-): 0

Region: NODE\_372714\_length\_2623\_cov\_15.020205 332-337. Max. coverage (+): 0.48. Max coverage (-): 0.32

Region: NODE\_372714\_length\_2623\_cov\_15.020205 338-342. Max. coverage (+): 2.42. Max coverage (-): 0.32

Region: NODE\_372714\_length\_2623\_cov\_15.020205 343-347. Max. coverage (+): 0.28. Max coverage (-): 0.24

Region: NODE\_372714\_length\_2623\_cov\_15.020205 348-353. Max. coverage (+): 0. Max coverage (-): 0.24

Region: NODE\_372714\_length\_2623\_cov\_15.020205 354-358. Max. coverage (+): 0.02. Max coverage (-): 0.18

Region: NODE\_372714\_length\_2623\_cov\_15.020205 359-363. Max. coverage (+): 0.02. Max coverage (-): 0

Region: NODE\_372714\_length\_2623\_cov\_15.020205 364-369. Max. coverage (+): 0.06. Max coverage (-): 0.02

Region: NODE\_372714\_length\_2623\_cov\_15.020205 370-374. Max. coverage (+): 0.06. Max coverage (-): 0

Region: NODE\_372714\_length\_2623\_cov\_15.020205 375-379. Max. coverage (+): 0.14. Max coverage (-): 0.02

Region: NODE\_372714\_length\_2623\_cov\_15.020205 380-385. Max. coverage (+): 0. Max coverage (-): 2.85

Region: NODE\_372714\_length\_2623\_cov\_15.020205 386-390. Max. coverage (+): 0. Max coverage (-): 9.89

Region: NODE\_372714\_length\_2623\_cov\_15.020205 391-395. Max. coverage (+): 0. Max coverage (-): 0.26

Region: NODE\_372714\_length\_2623\_cov\_15.020205 396-401. Max. coverage (+): 0.04. Max coverage (-): 0.06

Region: NODE\_372714\_length\_2623\_cov\_15.020205 402-406. Max. coverage (+): 0.18. Max coverage (-): 0.08

Region: NODE\_372714\_length\_2623\_cov\_15.020205 407-411. Max. coverage (+): 0.06. Max coverage (-): 0.06

Region: NODE\_372714\_length\_2623\_cov\_15.020205 412-417. Max. coverage (+): 0. Max coverage (-): 0.02

Region: NODE\_372714\_length\_2623\_cov\_15.020205 418-422. Max. coverage (+): 0.02. Max coverage (-): 0.04

Region: NODE\_372714\_length\_2623\_cov\_15.020205 423-428. Max. coverage (+): 0.04. Max coverage (-): 0

Region: NODE\_372714\_length\_2623\_cov\_15.020205 429-433. Max. coverage (+): 0.02. Max coverage (-): 0

Region: NODE\_372714\_length\_2623\_cov\_15.020205 434-438. Max. coverage (+): 0. Max coverage (-): 0.06

Region: NODE\_372714\_length\_2623\_cov\_15.020205 439-444. Max. coverage (+): 0.04. Max coverage (-): 0

Region: NODE\_372714\_length\_2623\_cov\_15.020205 445-449. Max. coverage (+): 0. Max coverage (-): 0.06

Region: NODE\_372714\_length\_2623\_cov\_15.020205 450-454. Max. coverage (+): 0. Max coverage (-): 0.08

Region: NODE\_372714\_length\_2623\_cov\_15.020205 455-460. Max. coverage (+): 0.02. Max coverage (-): 0.02

Region: NODE\_372714\_length\_2623\_cov\_15.020205 461-465. Max. coverage (+): 0.22. Max coverage (-): 0.06

Region: NODE\_372714\_length\_2623\_cov\_15.020205 466-470. Max. coverage (+): 0.24. Max coverage (-): 0.08

Region: NODE\_372714\_length\_2623\_cov\_15.020205 471-476. Max. coverage (+): 0. Max coverage (-): 0

Region: NODE\_372714\_length\_2623\_cov\_15.020205 477-481. Max. coverage (+): 0.06. Max coverage (-): 0.12

Region: NODE\_372714\_length\_2623\_cov\_15.020205 482-486. Max. coverage (+): 0.02. Max coverage (-): 0.08

Region: NODE\_372714\_length\_2623\_cov\_15.020205 487-492. Max. coverage (+): 0.02. Max coverage (-): 0.3

Region: NODE\_372714\_length\_2623\_cov\_15.020205 493-497. Max. coverage (+): 0. Max coverage (-): 0.81

Region: NODE\_372714\_length\_2623\_cov\_15.020205 498-502. Max. coverage (+): 0. Max coverage (-): 0.22

Region: NODE\_372714\_length\_2623\_cov\_15.020205 503-508. Max. coverage (+): 0. Max coverage (-): 0.22

Region: NODE\_372714\_length\_2623\_cov\_15.020205 509-513. Max. coverage (+): 0.34. Max coverage (-): 0

Region: NODE\_372714\_length\_2623\_cov\_15.020205 514-518. Max. coverage (+): 0.61. Max coverage (-): 0.02

Region: NODE\_372714\_length\_2623\_cov\_15.020205 519-524. Max. coverage (+): 0.02. Max coverage (-): 0.02

Region: NODE\_372714\_length\_2623\_cov\_15.020205 525-529. Max. coverage (+): 0. Max coverage (-): 0

Region: NODE\_372714\_length\_2623\_cov\_15.020205 530-534. Max. coverage (+): 0. Max coverage (-): 0

Region: NODE\_372714\_length\_2623\_cov\_15.020205 535-540. Max. coverage (+): 0. Max coverage (-): 0.04

Region: NODE\_372714\_length\_2623\_cov\_15.020205 541-545. Max. coverage (+): 0.08. Max coverage (-): 0.22

Region: NODE\_372714\_length\_2623\_cov\_15.020205 546-550. Max. coverage (+): 0.2. Max coverage (-): 0

Region: NODE\_372714\_length\_2623\_cov\_15.020205 551-556. Max. coverage (+): 0.24. Max coverage (-): 0.06

Region: NODE\_372714\_length\_2623\_cov\_15.020205 557-561. Max. coverage (+): 0.02. Max coverage (-): 0.06

Region: NODE\_372714\_length\_2623\_cov\_15.020205 562-567. Max. coverage (+): 0.04. Max coverage (-): 0.06

Region: NODE\_372714\_length\_2623\_cov\_15.020205 568-572. Max. coverage (+): 0. Max coverage (-): 0.16

Region: NODE\_372714\_length\_2623\_cov\_15.020205 573-577. Max. coverage (+): 0. Max coverage (-): 0.52

Region: NODE\_372714\_length\_2623\_cov\_15.020205 578-583. Max. coverage (+): 0.12. Max coverage (-): 0.36

Region: NODE\_372714\_length\_2623\_cov\_15.020205 584-588. Max. coverage (+): 0. Max coverage (-): 0.32

Region: NODE\_372714\_length\_2623\_cov\_15.020205 589-593. Max. coverage (+): 2.91. Max coverage (-): 0.2

Region: NODE\_372714\_length\_2623\_cov\_15.020205 594-599. Max. coverage (+): 2.95. Max coverage (-): 0.08

Region: NODE\_372714\_length\_2623\_cov\_15.020205 600-604. Max. coverage (+): 0. Max coverage (-): 0.12

Region: NODE\_372714\_length\_2623\_cov\_15.020205 605-609. Max. coverage (+): 0. Max coverage (-): 0.04

Region: NODE\_372714\_length\_2623\_cov\_15.020205 610-615. Max. coverage (+): 0. Max coverage (-): 0

Region: NODE\_372714\_length\_2623\_cov\_15.020205 616-620. Max. coverage (+): 0. Max coverage (-): 0

Region: NODE\_372714\_length\_2623\_cov\_15.020205 621-625. Max. coverage (+): 0.04. Max coverage (-): 0

Region: NODE\_372714\_length\_2623\_cov\_15.020205 626-631. Max. coverage (+): 0.04. Max coverage (-): 0.14

Region: NODE\_372714\_length\_2623\_cov\_15.020205 632-636. Max. coverage (+): 0.02. Max coverage (-): 0.14

Region: NODE\_372714\_length\_2623\_cov\_15.020205 637-641. Max. coverage (+): 0. Max coverage (-): 0.06

Region: NODE\_372714\_length\_2623\_cov\_15.020205 642-647. Max. coverage (+): 0.2. Max coverage (-): 0

Region: NODE\_372714\_length\_2623\_cov\_15.020205 648-652. Max. coverage (+): 1.66. Max coverage (-): 0.04

Region: NODE\_372714\_length\_2623\_cov\_15.020205 653-657. Max. coverage (+): 0.04. Max coverage (-): 0

Region: NODE\_372714\_length\_2623\_cov\_15.020205 658-663. Max. coverage (+): 0. Max coverage (-): 0

Region: NODE\_372714\_length\_2623\_cov\_15.020205 664-668. Max. coverage (+): 0.04. Max coverage (-): 0

Region: NODE\_372714\_length\_2623\_cov\_15.020205 669-673. Max. coverage (+): 0.04. Max coverage (-): 0.13

Region: NODE\_372714\_length\_2623\_cov\_15.020205 674-679. Max. coverage (+): 0.17. Max coverage (-): 0.01

Region: NODE\_372714\_length\_2623\_cov\_15.020205 680-684. Max. coverage (+): 0.24. Max coverage (-): 0

Region: NODE\_372714\_length\_2623\_cov\_15.020205 685-689. Max. coverage (+): 0.2. Max coverage (-): 0

Region: NODE\_372714\_length\_2623\_cov\_15.020205 690-695. Max. coverage (+): 0.16. Max coverage (-): 0

Region: NODE\_372714\_length\_2623\_cov\_15.020205 696-700. Max. coverage (+): 0. Max coverage (-): 0.04

Region: NODE\_372714\_length\_2623\_cov\_15.020205 701-705. Max. coverage (+): 0. Max coverage (-): 0.2

Region: NODE\_372714\_length\_2623\_cov\_15.020205 706-711. Max. coverage (+): 0.03. Max coverage (-): 0.13

Region: NODE\_372714\_length\_2623\_cov\_15.020205 712-716. Max. coverage (+): 0. Max coverage (-): 0

Region: NODE\_372714\_length\_2623\_cov\_15.020205 717-722. Max. coverage (+): 0.05. Max coverage (-): 0.01

Region: NODE\_372714\_length\_2623\_cov\_15.020205 723-727. Max. coverage (+): 0.13. Max coverage (-): 0.01

Region: NODE\_372714\_length\_2623\_cov\_15.020205 728-732. Max. coverage (+): 0.55. Max coverage (-): 0.01

Region: NODE\_372714\_length\_2623\_cov\_15.020205 733-738. Max. coverage (+): 0.11. Max coverage (-): 0

Region: NODE\_372714\_length\_2623\_cov\_15.020205 739-743. Max. coverage (+): 0. Max coverage (-): 0

Region: NODE\_372714\_length\_2623\_cov\_15.020205 744-748. Max. coverage (+): 0. Max coverage (-): 0.02

Region: NODE\_372714\_length\_2623\_cov\_15.020205 749-754. Max. coverage (+): 0.01. Max coverage (-): 0.1

Region: NODE\_372714\_length\_2623\_cov\_15.020205 755-759. Max. coverage (+): 0. Max coverage (-): 0.13

Region: NODE\_372714\_length\_2623\_cov\_15.020205 760-764. Max. coverage (+): 0.01. Max coverage (-): 0.01

Region: NODE\_372714\_length\_2623\_cov\_15.020205 765-770. Max. coverage (+): 0.02. Max coverage (-): 0

Region: NODE\_372714\_length\_2623\_cov\_15.020205 771-775. Max. coverage (+): 0.04. Max coverage (-): 0.01

Region: NODE\_372714\_length\_2623\_cov\_15.020205 776-780. Max. coverage (+): 0. Max coverage (-): 0

Region: NODE\_372714\_length\_2623\_cov\_15.020205 781-786. Max. coverage (+): 0. Max coverage (-): 0

Region: NODE\_372714\_length\_2623\_cov\_15.020205 787-791. Max. coverage (+): 0. Max coverage (-): 0

Region: NODE\_372714\_length\_2623\_cov\_15.020205 792-796. Max. coverage (+): 0. Max coverage (-): 0

Region: NODE\_372714\_length\_2623\_cov\_15.020205 797-802. Max. coverage (+): 0. Max coverage (-): 0

Region: NODE\_372714\_length\_2623\_cov\_15.020205 803-807. Max. coverage (+): 0. Max coverage (-): 0

Region: NODE\_372714\_length\_2623\_cov\_15.020205 808-812. Max. coverage (+): 0. Max coverage (-): 0.03

Region: NODE\_372714\_length\_2623\_cov\_15.020205 813-818. Max. coverage (+): 0.22. Max coverage (-): 0

Region: NODE\_372714\_length\_2623\_cov\_15.020205 819-823. Max. coverage (+): 0.12. Max coverage (-): 0.04

Region: NODE\_372714\_length\_2623\_cov\_15.020205 824-828. Max. coverage (+): 0.04. Max coverage (-): 0

Region: NODE\_372714\_length\_2623\_cov\_15.020205 829-834. Max. coverage (+): 0.04. Max coverage (-): 0

Region: NODE\_372714\_length\_2623\_cov\_15.020205 835-839. Max. coverage (+): 0. Max coverage (-): 0

Region: NODE\_372714\_length\_2623\_cov\_15.020205 840-844. Max. coverage (+): 0. Max coverage (-): 0.16

Region: NODE\_372714\_length\_2623\_cov\_15.020205 845-850. Max. coverage (+): 0.03. Max coverage (-): 0.02

Region: NODE\_372714\_length\_2623\_cov\_15.020205 851-855. Max. coverage (+): 0. Max coverage (-): 0

Region: NODE\_372714\_length\_2623\_cov\_15.020205 856-861. Max. coverage (+): 0.05. Max coverage (-): 0.01

Region: NODE\_372714\_length\_2623\_cov\_15.020205 862-866. Max. coverage (+): 0.55. Max coverage (-): 0.01

Region: NODE\_372714\_length\_2623\_cov\_15.020205 867-871. Max. coverage (+): 0.51. Max coverage (-): 0.01

Region: NODE\_372714\_length\_2623\_cov\_15.020205 872-877. Max. coverage (+): 0.04. Max coverage (-): 0

Region: NODE\_372714\_length\_2623\_cov\_15.020205 878-882. Max. coverage (+): 0. Max coverage (-): 0.01

Region: NODE\_372714\_length\_2623\_cov\_15.020205 883-887. Max. coverage (+): 0. Max coverage (-): 0.02

Region: NODE\_372714\_length\_2623\_cov\_15.020205 888-893. Max. coverage (+): 0.01. Max coverage (-): 0.11

Region: NODE\_372714\_length\_2623\_cov\_15.020205 894-898. Max. coverage (+): 0.01. Max coverage (-): 0.13

Region: NODE\_372714\_length\_2623\_cov\_15.020205 899-903. Max. coverage (+): 0.01. Max coverage (-): 0.01

Region: NODE\_372714\_length\_2623\_cov\_15.020205 904-909. Max. coverage (+): 0.02. Max coverage (-): 0.01

Region: NODE\_372714\_length\_2623\_cov\_15.020205 910-914. Max. coverage (+): 0.04. Max coverage (-): 0.01

Region: NODE\_372714\_length\_2623\_cov\_15.020205 915-919. Max. coverage (+): 0. Max coverage (-): 0

Region: NODE\_372714\_length\_2623\_cov\_15.020205 920-925. Max. coverage (+): 0. Max coverage (-): 0.34

Region: NODE\_372714\_length\_2623\_cov\_15.020205 926-930. Max. coverage (+): 0.09. Max coverage (-): 0.35

Region: NODE\_372714\_length\_2623\_cov\_15.020205 931-935. Max. coverage (+): 0.35. Max coverage (-): 0

Region: NODE\_372714\_length\_2623\_cov\_15.020205 936-941. Max. coverage (+): 0.05. Max coverage (-): 0

Region: NODE\_372714\_length\_2623\_cov\_15.020205 942-946. Max. coverage (+): 0.13. Max coverage (-): 0

Region: NODE\_372714\_length\_2623\_cov\_15.020205 947-951. Max. coverage (+): 0.04. Max coverage (-): 0

Region: NODE\_372714\_length\_2623\_cov\_15.020205 952-957. Max. coverage (+): 0.03. Max coverage (-): 0.01

Region: NODE\_372714\_length\_2623\_cov\_15.020205 958-962. Max. coverage (+): 0.05. Max coverage (-): 0.01

Region: NODE\_372714\_length\_2623\_cov\_15.020205 963-967. Max. coverage (+): 0.08. Max coverage (-): 0.03

Region: NODE\_372714\_length\_2623\_cov\_15.020205 968-973. Max. coverage (+): 0.22. Max coverage (-): 0.03

Region: NODE\_372714\_length\_2623\_cov\_15.020205 974-978. Max. coverage (+): 0.17. Max coverage (-): 0

Region: NODE\_372714\_length\_2623\_cov\_15.020205 979-983. Max. coverage (+): 0.04. Max coverage (-): 0

Region: NODE\_372714\_length\_2623\_cov\_15.020205 984-989. Max. coverage (+): 0.12. Max coverage (-): 0

Region: NODE\_372714\_length\_2623\_cov\_15.020205 990-994. Max. coverage (+): 0.12. Max coverage (-): 0.04

Region: NODE\_372714\_length\_2623\_cov\_15.020205 995-1000. Max. coverage (+): 0.16. Max coverage (-): 0.61

Region: NODE\_372714\_length\_2623\_cov\_15.020205 1001-1005. Max. coverage (+): 0.04. Max coverage (-): 0.04

Region: NODE\_372714\_length\_2623\_cov\_15.020205 1006-1010. Max. coverage (+): 0. Max coverage (-): 0

Region: NODE\_372714\_length\_2623\_cov\_15.020205 1011-1016. Max. coverage (+): 0. Max coverage (-): 0.08

Region: NODE\_372714\_length\_2623\_cov\_15.020205 1017-1021. Max. coverage (+): 0. Max coverage (-): 0.04

Region: NODE\_372714\_length\_2623\_cov\_15.020205 1022-1026. Max. coverage (+): 0. Max coverage (-): 0

Region: NODE\_372714\_length\_2623\_cov\_15.020205 1027-1032. Max. coverage (+): 0. Max coverage (-): 0

Region: NODE\_372714\_length\_2623\_cov\_15.020205 1033-1037. Max. coverage (+): 0. Max coverage (-): 0

Region: NODE\_372714\_length\_2623\_cov\_15.020205 1038-1042. Max. coverage (+): 0. Max coverage (-): 0

Region: NODE\_372714\_length\_2623\_cov\_15.020205 1043-1048. Max. coverage (+): 0. Max coverage (-): 0

Region: NODE\_372714\_length\_2623\_cov\_15.020205 1049-1053. Max. coverage (+): 0. Max coverage (-): 0.07

Region: NODE\_372714\_length\_2623\_cov\_15.020205 1054-1058. Max. coverage (+): 0. Max coverage (-): 0.09

Region: NODE\_372714\_length\_2623\_cov\_15.020205 1059-1064. Max. coverage (+): 0. Max coverage (-): 0.01

Region: NODE\_372714\_length\_2623\_cov\_15.020205 1065-1069. Max. coverage (+): 0. Max coverage (-): 0

Region: NODE\_372714\_length\_2623\_cov\_15.020205 1070-1074. Max. coverage (+): 0. Max coverage (-): 0

Region: NODE\_372714\_length\_2623\_cov\_15.020205 1075-1080. Max. coverage (+): 0. Max coverage (-): 0

Region: NODE\_372714\_length\_2623\_cov\_15.020205 1081-1085. Max. coverage (+): 0. Max coverage (-): 0

Region: NODE\_372714\_length\_2623\_cov\_15.020205 1086-1090. Max. coverage (+): 0.01. Max coverage (-): 0.01

Region: NODE\_372714\_length\_2623\_cov\_15.020205 1091-1096. Max. coverage (+): 0.01. Max coverage (-): 0.15

Region: NODE\_372714\_length\_2623\_cov\_15.020205 1097-1101. Max. coverage (+): 0.01. Max coverage (-): 0.27

Region: NODE\_372714\_length\_2623\_cov\_15.020205 1102-1106. Max. coverage (+): 0.01. Max coverage (-): 0.22

Region: NODE\_372714\_length\_2623\_cov\_15.020205 1107-1112. Max. coverage (+): 0. Max coverage (-): 0.01

Region: NODE\_372714\_length\_2623\_cov\_15.020205 1113-1117. Max. coverage (+): 0.01. Max coverage (-): 0

Region: NODE\_372714\_length\_2623\_cov\_15.020205 1118-1122. Max. coverage (+): 0.01. Max coverage (-): 0

Region: NODE\_372714\_length\_2623\_cov\_15.020205 1123-1128. Max. coverage (+): 0. Max coverage (-): 0.01

Region: NODE\_372714\_length\_2623\_cov\_15.020205 1129-1133. Max. coverage (+): 0. Max coverage (-): 0.04

Region: NODE\_372714\_length\_2623\_cov\_15.020205 1134-1139. Max. coverage (+): 0.06. Max coverage (-): 1.03

Region: NODE\_372714\_length\_2623\_cov\_15.020205 1140-1144. Max. coverage (+): 0.12. Max coverage (-): 0.97

Region: NODE\_372714\_length\_2623\_cov\_15.020205 1145-1149. Max. coverage (+): 0.02. Max coverage (-): 0

Region: NODE\_372714\_length\_2623\_cov\_15.020205 1150-1155. Max. coverage (+): 0.37. Max coverage (-): 0.01

Region: NODE\_372714\_length\_2623\_cov\_15.020205 1156-1160. Max. coverage (+): 0.07. Max coverage (-): 0.05

Region: NODE\_372714\_length\_2623\_cov\_15.020205 1161-1165. Max. coverage (+): 0.04. Max coverage (-): 0.12

Region: NODE\_372714\_length\_2623\_cov\_15.020205 1166-1171. Max. coverage (+): 0.02. Max coverage (-): 0.12

Region: NODE\_372714\_length\_2623\_cov\_15.020205 1172-1176. Max. coverage (+): 0. Max coverage (-): 0.12

Region: NODE\_372714\_length\_2623\_cov\_15.020205 1177-1181. Max. coverage (+): 0. Max coverage (-): 0.04

Region: NODE\_372714\_length\_2623\_cov\_15.020205 1182-1187. Max. coverage (+): 0. Max coverage (-): 0.04

Region: NODE\_372714\_length\_2623\_cov\_15.020205 1188-1192. Max. coverage (+): 0.02. Max coverage (-): 0

Region: NODE\_372714\_length\_2623\_cov\_15.020205 1193-1197. Max. coverage (+): 0.16. Max coverage (-): 0

Region: NODE\_372714\_length\_2623\_cov\_15.020205 1198-1203. Max. coverage (+): 0. Max coverage (-): 0

Region: NODE\_372714\_length\_2623\_cov\_15.020205 1204-1208. Max. coverage (+): 0. Max coverage (-): 0

Region: NODE\_372714\_length\_2623\_cov\_15.020205 1209-1213. Max. coverage (+): 0.03. Max coverage (-): 0

Region: NODE\_372714\_length\_2623\_cov\_15.020205 1214-1219. Max. coverage (+): 5.13. Max coverage (-): 0

Region: NODE\_372714\_length\_2623\_cov\_15.020205 1220-1224. Max. coverage (+): 0.46. Max coverage (-): 0.03

Region: NODE\_372714\_length\_2623\_cov\_15.020205 1225-1229. Max. coverage (+): 0.12. Max coverage (-): 0.04

Region: NODE\_372714\_length\_2623\_cov\_15.020205 1230-1235. Max. coverage (+): 0.08. Max coverage (-): 0.67

Region: NODE\_372714\_length\_2623\_cov\_15.020205 1236-1240. Max. coverage (+): 0.08. Max coverage (-): 0.65

Region: NODE\_372714\_length\_2623\_cov\_15.020205 1241-1245. Max. coverage (+): 0.07. Max coverage (-): 0

Region: NODE\_372714\_length\_2623\_cov\_15.020205 1246-1251. Max. coverage (+): 0.69. Max coverage (-): 0

Region: NODE\_372714\_length\_2623\_cov\_15.020205 1252-1256. Max. coverage (+): 0.66. Max coverage (-): 0

Region: NODE\_372714\_length\_2623\_cov\_15.020205 1257-1261. Max. coverage (+): 0. Max coverage (-): 0.04

Region: NODE\_372714\_length\_2623\_cov\_15.020205 1262-1267. Max. coverage (+): 0. Max coverage (-): 0.22

Region: NODE\_372714\_length\_2623\_cov\_15.020205 1268-1272. Max. coverage (+): 0.01. Max coverage (-): 0.31

Region: NODE\_372714\_length\_2623\_cov\_15.020205 1273-1278. Max. coverage (+): 0.08. Max coverage (-): 0.04

Region: NODE\_372714\_length\_2623\_cov\_15.020205 1279-1283. Max. coverage (+): 0.09. Max coverage (-): 0.01

Region: NODE\_372714\_length\_2623\_cov\_15.020205 1284-1288. Max. coverage (+): 0.17. Max coverage (-): 0.15

Region: NODE\_372714\_length\_2623\_cov\_15.020205 1289-1294. Max. coverage (+): 0.83. Max coverage (-): 0.03

Region: NODE\_372714\_length\_2623\_cov\_15.020205 1295-1299. Max. coverage (+): 0.19. Max coverage (-): 0.03

Region: NODE\_372714\_length\_2623\_cov\_15.020205 1300-1304. Max. coverage (+): 0.22. Max coverage (-): 0.07

Region: NODE\_372714\_length\_2623\_cov\_15.020205 1305-1310. Max. coverage (+): 0.15. Max coverage (-): 0.04

Region: NODE\_372714\_length\_2623\_cov\_15.020205 1311-1315. Max. coverage (+): 0.08. Max coverage (-): 0

Region: NODE\_372714\_length\_2623\_cov\_15.020205 1316-1320. Max. coverage (+): 0.4. Max coverage (-): 0.01

Region: NODE\_372714\_length\_2623\_cov\_15.020205 1321-1326. Max. coverage (+): 0.51. Max coverage (-): 0.01

Region: NODE\_372714\_length\_2623\_cov\_15.020205 1327-1331. Max. coverage (+): 0.23. Max coverage (-): 0.09

Region: NODE\_372714\_length\_2623\_cov\_15.020205 1332-1336. Max. coverage (+): 0.05. Max coverage (-): 0.22

Region: NODE\_372714\_length\_2623\_cov\_15.020205 1337-1342. Max. coverage (+): 0. Max coverage (-): 0.01

Region: NODE\_372714\_length\_2623\_cov\_15.020205 1343-1347. Max. coverage (+): 0.17. Max coverage (-): 0.04

Region: NODE\_372714\_length\_2623\_cov\_15.020205 1348-1352. Max. coverage (+): 0.24. Max coverage (-): 0.08

Region: NODE\_372714\_length\_2623\_cov\_15.020205 1353-1358. Max. coverage (+): 0.16. Max coverage (-): 0.08

Region: NODE\_372714\_length\_2623\_cov\_15.020205 1359-1363. Max. coverage (+): 0.08. Max coverage (-): 0.32

Region: NODE\_372714\_length\_2623\_cov\_15.020205 1364-1368. Max. coverage (+): 0.28. Max coverage (-): 0.44

Region: NODE\_372714\_length\_2623\_cov\_15.020205 1369-1374. Max. coverage (+): 0.28. Max coverage (-): 0.2

Region: NODE\_372714\_length\_2623\_cov\_15.020205 1375-1379. Max. coverage (+): 0.07. Max coverage (-): 0.19

Region: NODE\_372714\_length\_2623\_cov\_15.020205 1380-1384. Max. coverage (+): 0.34. Max coverage (-): 0.01

Region: NODE\_372714\_length\_2623\_cov\_15.020205 1385-1390. Max. coverage (+): 0.82. Max coverage (-): 0.05

Region: NODE\_372714\_length\_2623\_cov\_15.020205 1391-1395. Max. coverage (+): 0.13. Max coverage (-): 0

Region: NODE\_372714\_length\_2623\_cov\_15.020205 1396-1400. Max. coverage (+): 0.01. Max coverage (-): 0

Region: NODE\_372714\_length\_2623\_cov\_15.020205 1401-1406. Max. coverage (+): 0. Max coverage (-): 0.01

Region: NODE\_372714\_length\_2623\_cov\_15.020205 1407-1411. Max. coverage (+): 0. Max coverage (-): 0

Region: NODE\_372714\_length\_2623\_cov\_15.020205 1412-1417. Max. coverage (+): 0.65. Max coverage (-): 0.03

Region: NODE\_372714\_length\_2623\_cov\_15.020205 1418-1422. Max. coverage (+): 1.75. Max coverage (-): 0.03

Region: NODE\_372714\_length\_2623\_cov\_15.020205 1423-1427. Max. coverage (+): 0.26. Max coverage (-): 0

Region: NODE\_372714\_length\_2623\_cov\_15.020205 1428-1433. Max. coverage (+): 0.17. Max coverage (-): 0.01

Region: NODE\_372714\_length\_2623\_cov\_15.020205 1434-1438. Max. coverage (+): 0. Max coverage (-): 0.36

Region: NODE\_372714\_length\_2623\_cov\_15.020205 1439-1443. Max. coverage (+): 0. Max coverage (-): 0.11

Region: NODE\_372714\_length\_2623\_cov\_15.020205 1444-1449. Max. coverage (+): 0. Max coverage (-): 0.01

Region: NODE\_372714\_length\_2623\_cov\_15.020205 1450-1454. Max. coverage (+): 0.34. Max coverage (-): 0.01

Region: NODE\_372714\_length\_2623\_cov\_15.020205 1455-1459. Max. coverage (+): 0.44. Max coverage (-): 0.01

Region: NODE\_372714\_length\_2623\_cov\_15.020205 1460-1465. Max. coverage (+): 0.39. Max coverage (-): 0.03

Region: NODE\_372714\_length\_2623\_cov\_15.020205 1466-1470. Max. coverage (+): 0. Max coverage (-): 0.27

Region: NODE\_372714\_length\_2623\_cov\_15.020205 1471-1475. Max. coverage (+): 0. Max coverage (-): 0.57

Region: NODE\_372714\_length\_2623\_cov\_15.020205 1476-1481. Max. coverage (+): 0.04. Max coverage (-): 0.08

Region: NODE\_372714\_length\_2623\_cov\_15.020205 1482-1486. Max. coverage (+): 0.03. Max coverage (-): 0

Region: NODE\_372714\_length\_2623\_cov\_15.020205 1487-1491. Max. coverage (+): 0.09. Max coverage (-): 0

Region: NODE\_372714\_length\_2623\_cov\_15.020205 1492-1497. Max. coverage (+): 0.03. Max coverage (-): 0

Region: NODE\_372714\_length\_2623\_cov\_15.020205 1498-1502. Max. coverage (+): 0. Max coverage (-): 0

Region: NODE\_372714\_length\_2623\_cov\_15.020205 1503-1507. Max. coverage (+): 0. Max coverage (-): 0

Region: NODE\_372714\_length\_2623\_cov\_15.020205 1508-1513. Max. coverage (+): 0. Max coverage (-): 0.05

Region: NODE\_372714\_length\_2623\_cov\_15.020205 1514-1518. Max. coverage (+): 0.01. Max coverage (-): 0.05

Region: NODE\_372714\_length\_2623\_cov\_15.020205 1519-1523. Max. coverage (+): 0. Max coverage (-): 0.03

Region: NODE\_372714\_length\_2623\_cov\_15.020205 1524-1529. Max. coverage (+): 0.01. Max coverage (-): 0.05

Region: NODE\_372714\_length\_2623\_cov\_15.020205 1530-1534. Max. coverage (+): 0.01. Max coverage (-): 0

Region: NODE\_372714\_length\_2623\_cov\_15.020205 1535-1539. Max. coverage (+): 0.11. Max coverage (-): 0

Region: NODE\_372714\_length\_2623\_cov\_15.020205 1540-1545. Max. coverage (+): 0.08. Max coverage (-): 0

Region: NODE\_372714\_length\_2623\_cov\_15.020205 1546-1550. Max. coverage (+): 0.01. Max coverage (-): 0

Region: NODE\_372714\_length\_2623\_cov\_15.020205 1551-1556. Max. coverage (+): 0.01. Max coverage (-): 0.03

Region: NODE\_372714\_length\_2623\_cov\_15.020205 1557-1561. Max. coverage (+): 0. Max coverage (-): 0.03

Region: NODE\_372714\_length\_2623\_cov\_15.020205 1562-1566. Max. coverage (+): 0. Max coverage (-): 0

Region: NODE\_372714\_length\_2623\_cov\_15.020205 1567-1572. Max. coverage (+): 0. Max coverage (-): 0

Region: NODE\_372714\_length\_2623\_cov\_15.020205 1573-1577. Max. coverage (+): 0. Max coverage (-): 0

Region: NODE\_372714\_length\_2623\_cov\_15.020205 1578-1582. Max. coverage (+): 0. Max coverage (-): 0

Region: NODE\_372714\_length\_2623\_cov\_15.020205 1583-1588. Max. coverage (+): 0. Max coverage (-): 0.01

Region: NODE\_372714\_length\_2623\_cov\_15.020205 1589-1593. Max. coverage (+): 0. Max coverage (-): 0.04

Region: NODE\_372714\_length\_2623\_cov\_15.020205 1594-1598. Max. coverage (+): 0.36. Max coverage (-): 0

Region: NODE\_372714\_length\_2623\_cov\_15.020205 1599-1604. Max. coverage (+): 0.28. Max coverage (-): 0.04

Region: NODE\_372714\_length\_2623\_cov\_15.020205 1605-1609. Max. coverage (+): 0.04. Max coverage (-): 0.04

Region: NODE\_372714\_length\_2623\_cov\_15.020205 1610-1614. Max. coverage (+): 0.04. Max coverage (-): 0.04

Region: NODE\_372714\_length\_2623\_cov\_15.020205 1615-1620. Max. coverage (+): 0.04. Max coverage (-): 0.04

Region: NODE\_372714\_length\_2623\_cov\_15.020205 1621-1625. Max. coverage (+): 0.04. Max coverage (-): 0.16

Region: NODE\_372714\_length\_2623\_cov\_15.020205 1626-1630. Max. coverage (+): 0.16. Max coverage (-): 0

Region: NODE\_372714\_length\_2623\_cov\_15.020205 1631-1636. Max. coverage (+): 0.02. Max coverage (-): 0

Region: NODE\_372714\_length\_2623\_cov\_15.020205 1637-1641. Max. coverage (+): 2.02. Max coverage (-): 0

Region: NODE\_372714\_length\_2623\_cov\_15.020205 1642-1646. Max. coverage (+): 2.5. Max coverage (-): 0

Region: NODE\_372714\_length\_2623\_cov\_15.020205 1647-1652. Max. coverage (+): 0.28. Max coverage (-): 0

Region: NODE\_372714\_length\_2623\_cov\_15.020205 1653-1657. Max. coverage (+): 1.29. Max coverage (-): 0

Region: NODE\_372714\_length\_2623\_cov\_15.020205 1658-1662. Max. coverage (+): 0.83. Max coverage (-): 0

Region: NODE\_372714\_length\_2623\_cov\_15.020205 1663-1668. Max. coverage (+): 0.08. Max coverage (-): 0

Region: NODE\_372714\_length\_2623\_cov\_15.020205 1669-1673. Max. coverage (+): 0. Max coverage (-): 0

Region: NODE\_372714\_length\_2623\_cov\_15.020205 1674-1678. Max. coverage (+): 0.04. Max coverage (-): 0

Region: NODE\_372714\_length\_2623\_cov\_15.020205 1679-1684. Max. coverage (+): 0.48. Max coverage (-): 0

Region: NODE\_372714\_length\_2623\_cov\_15.020205 1685-1689. Max. coverage (+): 0.48. Max coverage (-): 0.02

Region: NODE\_372714\_length\_2623\_cov\_15.020205 1690-1695. Max. coverage (+): 0.18. Max coverage (-): 0.04

Region: NODE\_372714\_length\_2623\_cov\_15.020205 1696-1700. Max. coverage (+): 0.12. Max coverage (-): 0.06

Region: NODE\_372714\_length\_2623\_cov\_15.020205 1701-1705. Max. coverage (+): 0.08. Max coverage (-): 0.12

Region: NODE\_372714\_length\_2623\_cov\_15.020205 1706-1711. Max. coverage (+): 0.18. Max coverage (-): 0.06

Region: NODE\_372714\_length\_2623\_cov\_15.020205 1712-1716. Max. coverage (+): 0.32. Max coverage (-): 0

Region: NODE\_372714\_length\_2623\_cov\_15.020205 1717-1721. Max. coverage (+): 0.2. Max coverage (-): 0.65

Region: NODE\_372714\_length\_2623\_cov\_15.020205 1722-1727. Max. coverage (+): 1.33. Max coverage (-): 0.65

Region: NODE\_372714\_length\_2623\_cov\_15.020205 1728-1732. Max. coverage (+): 0.32. Max coverage (-): 0

Region: NODE\_372714\_length\_2623\_cov\_15.020205 1733-1737. Max. coverage (+): 0.16. Max coverage (-): 0.2

Region: NODE\_372714\_length\_2623\_cov\_15.020205 1738-1743. Max. coverage (+): 0.57. Max coverage (-): 0.2

Region: NODE\_372714\_length\_2623\_cov\_15.020205 1744-1748. Max. coverage (+): 0.42. Max coverage (-): 0.06

Region: NODE\_372714\_length\_2623\_cov\_15.020205 1749-1753. Max. coverage (+): 0.16. Max coverage (-): 0.02

Region: NODE\_372714\_length\_2623\_cov\_15.020205 1754-1759. Max. coverage (+): 0.24. Max coverage (-): 0.02

Region: NODE\_372714\_length\_2623\_cov\_15.020205 1760-1764. Max. coverage (+): 0.08. Max coverage (-): 0.02

Region: NODE\_372714\_length\_2623\_cov\_15.020205 1765-1769. Max. coverage (+): 0.08. Max coverage (-): 0.02

Region: NODE\_372714\_length\_2623\_cov\_15.020205 1770-1775. Max. coverage (+): 0.04. Max coverage (-): 0

Region: NODE\_372714\_length\_2623\_cov\_15.020205 1776-1780. Max. coverage (+): 0.16. Max coverage (-): 0

Region: NODE\_372714\_length\_2623\_cov\_15.020205 1781-1785. Max. coverage (+): 0.12. Max coverage (-): 0

Region: NODE\_372714\_length\_2623\_cov\_15.020205 1786-1791. Max. coverage (+): 0.12. Max coverage (-): 0

Region: NODE\_372714\_length\_2623\_cov\_15.020205 1792-1796. Max. coverage (+): 0. Max coverage (-): 0

Region: NODE\_372714\_length\_2623\_cov\_15.020205 1797-1801. Max. coverage (+): 0.36. Max coverage (-): 0

Region: NODE\_372714\_length\_2623\_cov\_15.020205 1802-1807. Max. coverage (+): 0.81. Max coverage (-): 0

Region: NODE\_372714\_length\_2623\_cov\_15.020205 1808-1812. Max. coverage (+): 2.14. Max coverage (-): 0

Region: NODE\_372714\_length\_2623\_cov\_15.020205 1813-1817. Max. coverage (+): 0.24. Max coverage (-): 0

Region: NODE\_372714\_length\_2623\_cov\_15.020205 1818-1823. Max. coverage (+): 0.12. Max coverage (-): 0.08

Region: NODE\_372714\_length\_2623\_cov\_15.020205 1824-1828. Max. coverage (+): 0. Max coverage (-): 0.16

Region: NODE\_372714\_length\_2623\_cov\_15.020205 1829-1834. Max. coverage (+): 0.04. Max coverage (-): 0.04

Region: NODE\_372714\_length\_2623\_cov\_15.020205 1835-1839. Max. coverage (+): 0.08. Max coverage (-): 0.04

Region: NODE\_372714\_length\_2623\_cov\_15.020205 1840-1844. Max. coverage (+): 0. Max coverage (-): 0.04

Region: NODE\_372714\_length\_2623\_cov\_15.020205 1845-1850. Max. coverage (+): 0. Max coverage (-): 0

Region: NODE\_372714\_length\_2623\_cov\_15.020205 1851-1855. Max. coverage (+): 0. Max coverage (-): 0

Region: NODE\_372714\_length\_2623\_cov\_15.020205 1856-1860. Max. coverage (+): 0.01. Max coverage (-): 0

Region: NODE\_372714\_length\_2623\_cov\_15.020205 1861-1866. Max. coverage (+): 0.06. Max coverage (-): 0

Region: NODE\_372714\_length\_2623\_cov\_15.020205 1867-1871. Max. coverage (+): 0.06. Max coverage (-): 0.04

Region: NODE\_372714\_length\_2623\_cov\_15.020205 1872-1876. Max. coverage (+): 0.28. Max coverage (-): 0.1

Region: NODE\_372714\_length\_2623\_cov\_15.020205 1877-1882. Max. coverage (+): 0.65. Max coverage (-): 0.08

Region: NODE\_372714\_length\_2623\_cov\_15.020205 1883-1887. Max. coverage (+): 0.24. Max coverage (-): 0.12

Region: NODE\_372714\_length\_2623\_cov\_15.020205 1888-1892. Max. coverage (+): 0.4. Max coverage (-): 0

Region: NODE\_372714\_length\_2623\_cov\_15.020205 1893-1898. Max. coverage (+): 6.3. Max coverage (-): 0

Region: NODE\_372714\_length\_2623\_cov\_15.020205 1899-1903. Max. coverage (+): 0.48. Max coverage (-): 0.12

Region: NODE\_372714\_length\_2623\_cov\_15.020205 1904-1908. Max. coverage (+): 0.61. Max coverage (-): 0.08

Region: NODE\_372714\_length\_2623\_cov\_15.020205 1909-1914. Max. coverage (+): 0.04. Max coverage (-): 0

Region: NODE\_372714\_length\_2623\_cov\_15.020205 1915-1919. Max. coverage (+): 0.2. Max coverage (-): 0.02

Region: NODE\_372714\_length\_2623\_cov\_15.020205 1920-1924. Max. coverage (+): 0.18. Max coverage (-): 0

Region: NODE\_372714\_length\_2623\_cov\_15.020205 1925-1930. Max. coverage (+): 0.02. Max coverage (-): 0

Region: NODE\_372714\_length\_2623\_cov\_15.020205 1931-1935. Max. coverage (+): 0.12. Max coverage (-): 0

Region: NODE\_372714\_length\_2623\_cov\_15.020205 1936-1940. Max. coverage (+): 0.28. Max coverage (-): 0.32

Region: NODE\_372714\_length\_2623\_cov\_15.020205 1941-1946. Max. coverage (+): 0.16. Max coverage (-): 0.32

Region: NODE\_372714\_length\_2623\_cov\_15.020205 1947-1951. Max. coverage (+): 0.69. Max coverage (-): 0.77

Region: NODE\_372714\_length\_2623\_cov\_15.020205 1952-1956. Max. coverage (+): 2.06. Max coverage (-): 0.16

Region: NODE\_372714\_length\_2623\_cov\_15.020205 1957-1962. Max. coverage (+): 4.81. Max coverage (-): 0.36

Region: NODE\_372714\_length\_2623\_cov\_15.020205 1963-1967. Max. coverage (+): 0.42. Max coverage (-): 0.28

Region: NODE\_372714\_length\_2623\_cov\_15.020205 1968-1973. Max. coverage (+): 0.4. Max coverage (-): 0.16

Region: NODE\_372714\_length\_2623\_cov\_15.020205 1974-1978. Max. coverage (+): 42.4. Max coverage (-): 0.04

Region: NODE\_372714\_length\_2623\_cov\_15.020205 1979-1983. Max. coverage (+): 10.78. Max coverage (-): 0

Region: NODE\_372714\_length\_2623\_cov\_15.020205 1984-1989. Max. coverage (+): 0.04. Max coverage (-): 0

Region: NODE\_372714\_length\_2623\_cov\_15.020205 1990-1994. Max. coverage (+): 0.08. Max coverage (-): 0

Region: NODE\_372714\_length\_2623\_cov\_15.020205 1995-1999. Max. coverage (+): 0.97. Max coverage (-): 0

Region: NODE\_372714\_length\_2623\_cov\_15.020205 2000-2005. Max. coverage (+): 0.93. Max coverage (-): 0.04

Region: NODE\_372714\_length\_2623\_cov\_15.020205 2006-2010. Max. coverage (+): 0. Max coverage (-): 0.04

Region: NODE\_372714\_length\_2623\_cov\_15.020205 2011-2015. Max. coverage (+): 0.1. Max coverage (-): 0.24

Region: NODE\_372714\_length\_2623\_cov\_15.020205 2016-2021. Max. coverage (+): 0.12. Max coverage (-): 1.84

Region: NODE\_372714\_length\_2623\_cov\_15.020205 2022-2026. Max. coverage (+): 0.22. Max coverage (-): 0.16

Region: NODE\_372714\_length\_2623\_cov\_15.020205 2027-2031. Max. coverage (+): 0.87. Max coverage (-): 0.04

Region: NODE\_372714\_length\_2623\_cov\_15.020205 2032-2037. Max. coverage (+): 2.56. Max coverage (-): 0

Region: NODE\_372714\_length\_2623\_cov\_15.020205 2038-2042. Max. coverage (+): 0.81. Max coverage (-): 0.02

Region: NODE\_372714\_length\_2623\_cov\_15.020205 2043-2047. Max. coverage (+): 0.06. Max coverage (-): 0.16

Region: NODE\_372714\_length\_2623\_cov\_15.020205 2048-2053. Max. coverage (+): 0.12. Max coverage (-): 0.2

Region: NODE\_372714\_length\_2623\_cov\_15.020205 2054-2058. Max. coverage (+): 0.81. Max coverage (-): 0.12

Region: NODE\_372714\_length\_2623\_cov\_15.020205 2059-2063. Max. coverage (+): 0.52. Max coverage (-): 0

Region: NODE\_372714\_length\_2623\_cov\_15.020205 2064-2069. Max. coverage (+): 0.12. Max coverage (-): 0

Region: NODE\_372714\_length\_2623\_cov\_15.020205 2070-2074. Max. coverage (+): 0.3. Max coverage (-): 0

Region: NODE\_372714\_length\_2623\_cov\_15.020205 2075-2079. Max. coverage (+): 0.85. Max coverage (-): 0

Region: NODE\_372714\_length\_2623\_cov\_15.020205 2080-2085. Max. coverage (+): 0.03. Max coverage (-): 0

Region: NODE\_372714\_length\_2623\_cov\_15.020205 2086-2090. Max. coverage (+): 0. Max coverage (-): 0.01

Region: NODE\_372714\_length\_2623\_cov\_15.020205 2091-2095. Max. coverage (+): 0.01. Max coverage (-): 0.01

Region: NODE\_372714\_length\_2623\_cov\_15.020205 2096-2101. Max. coverage (+): 0.08. Max coverage (-): 0

Region: NODE\_372714\_length\_2623\_cov\_15.020205 2102-2106. Max. coverage (+): 0.11. Max coverage (-): 0.07

Region: NODE\_372714\_length\_2623\_cov\_15.020205 2107-2111. Max. coverage (+): 0.01. Max coverage (-): 0.07

Region: NODE\_372714\_length\_2623\_cov\_15.020205 2112-2117. Max. coverage (+): 0.31. Max coverage (-): 0.11

Region: NODE\_372714\_length\_2623\_cov\_15.020205 2118-2122. Max. coverage (+): 0.63. Max coverage (-): 0.01

Region: NODE\_372714\_length\_2623\_cov\_15.020205 2123-2128. Max. coverage (+): 0.63. Max coverage (-): 0.01

Region: NODE\_372714\_length\_2623\_cov\_15.020205 2129-2133. Max. coverage (+): 0.11. Max coverage (-): 0

Region: NODE\_372714\_length\_2623\_cov\_15.020205 2134-2138. Max. coverage (+): 0.04. Max coverage (-): 0

Region: NODE\_372714\_length\_2623\_cov\_15.020205 2139-2144. Max. coverage (+): 0. Max coverage (-): 0.01

Region: NODE\_372714\_length\_2623\_cov\_15.020205 2145-2149. Max. coverage (+): 0.01. Max coverage (-): 0

Region: NODE\_372714\_length\_2623\_cov\_15.020205 2150-2154. Max. coverage (+): 0.04. Max coverage (-): 0

Region: NODE\_372714\_length\_2623\_cov\_15.020205 2155-2160. Max. coverage (+): 0.01. Max coverage (-): 0

Region: NODE\_372714\_length\_2623\_cov\_15.020205 2161-2165. Max. coverage (+): 0. Max coverage (-): 0

Region: NODE\_372714\_length\_2623\_cov\_15.020205 2166-2170. Max. coverage (+): 0.01. Max coverage (-): 0

Region: NODE\_372714\_length\_2623\_cov\_15.020205 2171-2176. Max. coverage (+): 0.08. Max coverage (-): 0.01

Region: NODE\_372714\_length\_2623\_cov\_15.020205 2177-2181. Max. coverage (+): 0.42. Max coverage (-): 0

Region: NODE\_372714\_length\_2623\_cov\_15.020205 2182-2186. Max. coverage (+): 0.11. Max coverage (-): 0

Region: NODE\_372714\_length\_2623\_cov\_15.020205 2187-2192. Max. coverage (+): 0.15. Max coverage (-): 0

Region: NODE\_372714\_length\_2623\_cov\_15.020205 2193-2197. Max. coverage (+): 0. Max coverage (-): 0.03

Region: NODE\_372714\_length\_2623\_cov\_15.020205 2198-2202. Max. coverage (+): 0.03. Max coverage (-): 0.04

Region: NODE\_372714\_length\_2623\_cov\_15.020205 2203-2208. Max. coverage (+): 0.04. Max coverage (-): 0.24

Region: NODE\_372714\_length\_2623\_cov\_15.020205 2209-2213. Max. coverage (+): 0.3. Max coverage (-): 0.09

Region: NODE\_372714\_length\_2623\_cov\_15.020205 2214-2218. Max. coverage (+): 0.32. Max coverage (-): 0.03

Region: NODE\_372714\_length\_2623\_cov\_15.020205 2219-2224. Max. coverage (+): 0.26. Max coverage (-): 0.04

Region: NODE\_372714\_length\_2623\_cov\_15.020205 2225-2229. Max. coverage (+): 1.01. Max coverage (-): 0

Region: NODE\_372714\_length\_2623\_cov\_15.020205 2230-2234. Max. coverage (+): 0.87. Max coverage (-): 0

Region: NODE\_372714\_length\_2623\_cov\_15.020205 2235-2240. Max. coverage (+): 0.51. Max coverage (-): 0.03

Region: NODE\_372714\_length\_2623\_cov\_15.020205 2241-2245. Max. coverage (+): 0.08. Max coverage (-): 0.03

Region: NODE\_372714\_length\_2623\_cov\_15.020205 2246-2250. Max. coverage (+): 0.08. Max coverage (-): 0

Region: NODE\_372714\_length\_2623\_cov\_15.020205 2251-2256. Max. coverage (+): 0.05. Max coverage (-): 0

Region: NODE\_372714\_length\_2623\_cov\_15.020205 2257-2261. Max. coverage (+): 0.01. Max coverage (-): 0.05

Region: NODE\_372714\_length\_2623\_cov\_15.020205 2262-2267. Max. coverage (+): 0.31. Max coverage (-): 0.27

Region: NODE\_372714\_length\_2623\_cov\_15.020205 2268-2272. Max. coverage (+): 0.27. Max coverage (-): 0.05

Region: NODE\_372714\_length\_2623\_cov\_15.020205 2273-2277. Max. coverage (+): 0.23. Max coverage (-): 0.09

Region: NODE\_372714\_length\_2623\_cov\_15.020205 2278-2283. Max. coverage (+): 0.05. Max coverage (-): 0.09

Region: NODE\_372714\_length\_2623\_cov\_15.020205 2284-2288. Max. coverage (+): 0.01. Max coverage (-): 0.01

Region: NODE\_372714\_length\_2623\_cov\_15.020205 2289-2293. Max. coverage (+): 0.03. Max coverage (-): 0.01

Region: NODE\_372714\_length\_2623\_cov\_15.020205 2294-2299. Max. coverage (+): 0.01. Max coverage (-): 0

Region: NODE\_372714\_length\_2623\_cov\_15.020205 2300-2304. Max. coverage (+): 0. Max coverage (-): 0

Region: NODE\_372714\_length\_2623\_cov\_15.020205 2305-2309. Max. coverage (+): 0. Max coverage (-): 0

Region: NODE\_372714\_length\_2623\_cov\_15.020205 2310-2315. Max. coverage (+): 0. Max coverage (-): 0

Region: NODE\_372714\_length\_2623\_cov\_15.020205 2316-2320. Max. coverage (+): 0. Max coverage (-): 0

Region: NODE\_372714\_length\_2623\_cov\_15.020205 2321-2325. Max. coverage (+): 0.04. Max coverage (-): 0.16

Region: NODE\_372714\_length\_2623\_cov\_15.020205 2326-2331. Max. coverage (+): 0.04. Max coverage (-): 0.12

Region: NODE\_372714\_length\_2623\_cov\_15.020205 2332-2336. Max. coverage (+): 0.77. Max coverage (-): 0

Region: NODE\_372714\_length\_2623\_cov\_15.020205 2337-2341. Max. coverage (+): 0.31. Max coverage (-): 0

Region: NODE\_372714\_length\_2623\_cov\_15.020205 2342-2347. Max. coverage (+): 0.13. Max coverage (-): 0

Region: NODE\_372714\_length\_2623\_cov\_15.020205 2348-2352. Max. coverage (+): 0. Max coverage (-): 0.01

Region: NODE\_372714\_length\_2623\_cov\_15.020205 2353-2357. Max. coverage (+): 0. Max coverage (-): 0.07

Region: NODE\_372714\_length\_2623\_cov\_15.020205 2358-2363. Max. coverage (+): 0. Max coverage (-): 0.05

Region: NODE\_372714\_length\_2623\_cov\_15.020205 2364-2368. Max. coverage (+): 0.03. Max coverage (-): 0

Region: NODE\_372714\_length\_2623\_cov\_15.020205 2369-2373. Max. coverage (+): 0.31. Max coverage (-): 0.01

Region: NODE\_372714\_length\_2623\_cov\_15.020205 2374-2379. Max. coverage (+): 0.34. Max coverage (-): 0.03

Region: NODE\_372714\_length\_2623\_cov\_15.020205 2380-2384. Max. coverage (+): 0.4. Max coverage (-): 0

Region: NODE\_372714\_length\_2623\_cov\_15.020205 2385-2389. Max. coverage (+): 0.04. Max coverage (-): 0.08

Region: NODE\_372714\_length\_2623\_cov\_15.020205 2390-2395. Max. coverage (+): 0.38. Max coverage (-): 0.36

Region: NODE\_372714\_length\_2623\_cov\_15.020205 2396-2400. Max. coverage (+): 0.36. Max coverage (-): 0.44

Region: NODE\_372714\_length\_2623\_cov\_15.020205 2401-2406. Max. coverage (+): 0.46. Max coverage (-): 0.03

Region: NODE\_372714\_length\_2623\_cov\_15.020205 2407-2411. Max. coverage (+): 0.03. Max coverage (-): 0

Region: NODE\_372714\_length\_2623\_cov\_15.020205 2412-2416. Max. coverage (+): 0. Max coverage (-): 0

Region: NODE\_372714\_length\_2623\_cov\_15.020205 2417-2422. Max. coverage (+): 0. Max coverage (-): 0

Region: NODE\_372714\_length\_2623\_cov\_15.020205 2423-2427. Max. coverage (+): 0. Max coverage (-): 0

Region: NODE\_372714\_length\_2623\_cov\_15.020205 2428-2432. Max. coverage (+): 0.01. Max coverage (-): 0

Region: NODE\_372714\_length\_2623\_cov\_15.020205 2433-2438. Max. coverage (+): 0.08. Max coverage (-): 0.03

Region: NODE\_372714\_length\_2623\_cov\_15.020205 2439-2443. Max. coverage (+): 0.03. Max coverage (-): 0.03

Region: NODE\_372714\_length\_2623\_cov\_15.020205 2444-2448. Max. coverage (+): 0.92. Max coverage (-): 0.07

Region: NODE\_372714\_length\_2623\_cov\_15.020205 2449-2454. Max. coverage (+): 0.83. Max coverage (-): 0.12

Region: NODE\_372714\_length\_2623\_cov\_15.020205 2455-2459. Max. coverage (+): 1.02. Max coverage (-): 0.08

Region: NODE\_372714\_length\_2623\_cov\_15.020205 2460-2464. Max. coverage (+): 0.16. Max coverage (-): 0.04

Region: NODE\_372714\_length\_2623\_cov\_15.020205 2465-2470. Max. coverage (+): 0.17. Max coverage (-): 0

Region: NODE\_372714\_length\_2623\_cov\_15.020205 2471-2475. Max. coverage (+): 0.13. Max coverage (-): 0.01

Region: NODE\_372714\_length\_2623\_cov\_15.020205 2476-2480. Max. coverage (+): 0.36. Max coverage (-): 0

Region: NODE\_372714\_length\_2623\_cov\_15.020205 2481-2486. Max. coverage (+): 0.09. Max coverage (-): 0

Region: NODE\_372714\_length\_2623\_cov\_15.020205 2487-2491. Max. coverage (+): 0.07. Max coverage (-): 0

Region: NODE\_372714\_length\_2623\_cov\_15.020205 2492-2496. Max. coverage (+): 0.09. Max coverage (-): 0.01

Region: NODE\_372714\_length\_2623\_cov\_15.020205 2497-2502. Max. coverage (+): 0.23. Max coverage (-): 0.01

Region: NODE\_372714\_length\_2623\_cov\_15.020205 2503-2507. Max. coverage (+): 0.42. Max coverage (-): 0.05

Region: NODE\_372714\_length\_2623\_cov\_15.020205 2508-2512. Max. coverage (+): 0.15. Max coverage (-): 0.17

Region: NODE\_372714\_length\_2623\_cov\_15.020205 2513-2518. Max. coverage (+): 0.04. Max coverage (-): 0.04

Region: NODE\_372714\_length\_2623\_cov\_15.020205 2519-2523. Max. coverage (+): 0.92. Max coverage (-): 0.03

Region: NODE\_372714\_length\_2623\_cov\_15.020205 2524-2528. Max. coverage (+): 0.8. Max coverage (-): 0.01

Region: NODE\_372714\_length\_2623\_cov\_15.020205 2529-2534. Max. coverage (+): 0.09. Max coverage (-): 0

Region: NODE\_372714\_length\_2623\_cov\_15.020205 2535-2539. Max. coverage (+): 0. Max coverage (-): 0.04

Region: NODE\_372714\_length\_2623\_cov\_15.020205 2540-2545. Max. coverage (+): 0. Max coverage (-): 0.12

Region: NODE\_372714\_length\_2623\_cov\_15.020205 2546-2550. Max. coverage (+): 0. Max coverage (-): 0.57

Region: NODE\_372714\_length\_2623\_cov\_15.020205 2551-2555. Max. coverage (+): 0. Max coverage (-): 0.81

Region: NODE\_372714\_length\_2623\_cov\_15.020205 2556-2561. Max. coverage (+): 0.97. Max coverage (-): 0

Region: NODE\_372714\_length\_2623\_cov\_15.020205 2562-2566. Max. coverage (+): 2.14. Max coverage (-): 0

Region: NODE\_372714\_length\_2623\_cov\_15.020205 2567-2571. Max. coverage (+): 2.16. Max coverage (-): 0.02

Region: NODE\_372714\_length\_2623\_cov\_15.020205 2572-2577. Max. coverage (+): 0.38. Max coverage (-): 0.02

Region: NODE\_372714\_length\_2623\_cov\_15.020205 2578-2582. Max. coverage (+): 0. Max coverage (-): 0.06

Region: NODE\_372714\_length\_2623\_cov\_15.020205 2583-2587. Max. coverage (+): 0. Max coverage (-): 0.02

Region: NODE\_372714\_length\_2623\_cov\_15.020205 2588-2593. Max. coverage (+): 0.73. Max coverage (-): 0

Region: NODE\_372714\_length\_2623\_cov\_15.020205 2594-2598. Max. coverage (+): 0.81. Max coverage (-): 0

Region: NODE\_372714\_length\_2623\_cov\_15.020205 2599-2603. Max. coverage (+): 0.12. Max coverage (-): 0.04

Region: NODE\_372714\_length\_2623\_cov\_15.020205 2604-2609. Max. coverage (+): 0.04. Max coverage (-): 0.08

Region: NODE\_372714\_length\_2623\_cov\_15.020205 2610-2614. Max. coverage (+): 0. Max coverage (-): 0.08

Region: NODE\_372714\_length\_2623\_cov\_15.020205 2615-2619. Max. coverage (+): 0. Max coverage (-): 1.43

Region: NODE\_372714\_length\_2623\_cov\_15.020205 2620-2625. Max. coverage (+): 0.06. Max coverage (-): 0.87

Region: NODE\_372714\_length\_2623\_cov\_15.020205 2626-2630. Max. coverage (+): 0.63. Max coverage (-): 0.34

Region: NODE\_372714\_length\_2623\_cov\_15.020205 2631-2635. Max. coverage (+): 2.77. Max coverage (-): 0.02

Region: NODE\_372714\_length\_2623\_cov\_15.020205 2636-2641. Max. coverage (+): 3.53. Max coverage (-): 0.02

Region: NODE\_372714\_length\_2623\_cov\_15.020205 2642-2646. Max. coverage (+): 0.08. Max coverage (-): 0.02

Region: NODE\_372714\_length\_2623\_cov\_15.020205 2647-2651. Max. coverage (+): 0.02. Max coverage (-): 0.06

Region: NODE\_372714\_length\_2623\_cov\_15.020205 2652-2657. Max. coverage (+): 0. Max coverage (-): 0.06

Region: NODE\_372714\_length\_2623\_cov\_15.020205 2658-2662. Max. coverage (+): 0. Max coverage (-): 0

Region: NODE\_372714\_length\_2623\_cov\_15.020205 2663-2667. Max. coverage (+): 0. Max coverage (-): 0

Region: NODE\_372714\_length\_2623\_cov\_15.020205 2668-2673. Max. coverage (+): 0. Max coverage (-): 0

Region: NODE\_372714\_length\_2623\_cov\_15.020205 2674-. Max. coverage (+): 0. Max coverage (-): 0

RepeatMasker Color Code

**+**

100-98% Identity

<98-95% Identity

<95-90% Identity

<90-85% Identity

<85-80% Identity

<80-75% Identity

<75-70% Identity

<70% Identity

**-**

Gene Set Color Code

**+**

Gene

Pseudogene

Other

**-**

Topology/Coverage Color Code

Coverage Plus Strand

Coverage Minus Strand

Mainstrand: Plus

Mainstrand: Minus

Complementary Strand

Flanking Region  
(if option -flank >0)

Gene Set Annotation  
  
RepeatMasker Annotation  

**1. AlRepD-2080**: 250-668 (+), Divergence to consensus: 37.3%  
**2. AlRepD-2080**: 678-758 (+), Divergence to consensus: 32.1%  
**3. AlRepD-2080**: 808-1207 (+), Divergence to consensus: 37.7%  
**4. (TGTT)n**: 1846-1895 (+), Divergence to consensus: 14.2%  
**5. AlRepC-312**: 2120-2173 (+), Divergence to consensus: 20.4%  
**6. AlRepC-312**: 2169-2347 (-), Divergence to consensus: 48%  
**7. AlRepE-294**: 2497-2659 (+), Divergence to consensus: 24.8%

  
Transcription Factor Binding Sites  

**RHOXF1** (Sequence: GGATCA (-): 362)  
**RHOXF1** (Sequence: GGCTTA (-): 1228)  
**RHOXF1** (Sequence: GGCTCA (-): 1270)  
**RHOXF1** (Sequence: GGCTCA (-): 1372)  
**RHOXF1** (Sequence: AGATTA (-): 1725)  
**RHOXF1** (Sequence: AGCTCA (-): 2087)  
**RHOXF1** (Sequence: TGATCT (+): 299)  
**RHOXF1** (Sequence: TAAGCT (+): 1211)  
**RHOXF1** (Sequence: TAATCC (+): 1755)  
**Gata4** (Sequence: GTTATCT (+): 1320)  
**RFX4\_1** (Sequence: GTTGCTATG (-): 2182)  
**SOX9** (Sequence: AACAATGA (-): 729)  
**SOX9** (Sequence: AACAATGA (-): 867)  
**FOXO1** (Sequence: CCTGTTTTC (+): 2662)  
**FOXO3\_mmu** (Sequence: TGTTTTGA (-): 1891)  
**FIGLA** (Sequence: TCCAGCTGTT (-): 2217)  
**FOXO3\_mmu** (Sequence: TCAAAACA (+): 2377)  
**FOXO1** (Sequence: ATAAACAGG (-): 1221)  
**Sox5** (Sequence: AACAAT (-): 729)  
**Sox5** (Sequence: AACAAT (-): 867)  
**Sox5** (Sequence: AACAAT (-): 1128)  
**Sox5** (Sequence: AACAAT (-): 2160)  
**POU5F1** (Sequence: ATGCAAA (+): 2203)
