# Supplementary material for: First characterization of PIWI-interacting RNA clusters in a cichlid fish with a B chromosome
Source: BMC Biol. 2022 Sep 21;20:204. doi: 10.1186/s12915-022-01403-2 (PMC9490952; doi:10.1186/s12915-022-01403-2)
Supplement: Supplementary file 1 — Additional file 1. Zipped folder with fasta and interactive html piRNA cluster information for the A. latifasciata genome. The nomenclature is as follows: number-pirna-cluster_sex_B-presence (f, female; m, male; 0b, without B chromosome; 1b, with B chromosome). [file 12915_2022_1403_MOESM1_ESM.zip › 144_m1b.html]

piRNA cluster 144\_m1b 85


Predicted piRNA cluster no. 144\_m1b
  

Show proTRAC run info
Hide proTRAC run info

/\  
                \_\_\_\_\_\_\_\_\_\_\_\_\_\_\_\_\_\_\_\_\_\_\_/\\_\_\_ /  \\_\_\_\_\_\_\_  
               I                      /  \  /    \      I  
               I     pro             /    \/      \     I  
               I        TRAC        /               \   I  
               I   \_\_\_\_\_\_\_\_\_\_\_\_\_\_\_\_/\_\_\_\_\_\_\_\_\_\_\_\_\_\_\_\_\_\\_ I  
               I   \              /                     I  
               I    \            /                      I  
               I     \  /\      /       V.2.4.2         I  
               I      \/  \    /                        I  
               I\_\_\_\_\_\_\_\_\_\_\_\  /\_\_\_\_\_\_\_\_\_\_\_\_\_\_\_\_\_\_\_\_\_\_\_\_\_I  
                            \/  
  
  
================================= proTRAC ====================================  
VERSION: .......... 2.4.2  
LAST MODIFIED: .... 11. May 2018  
  
Please cite:  
Rosenkranz D, Zischler H. proTRAC - a software for probabilistic piRNA cluster  
detection, visualization and analysis. 2012. BMC Bioinformatics 13:5.  
  
  
Contact:  
David Rosenkranz  
Institute of Organismic and Molecular Evolutionary Biology  
Dept. Anthropology, small RNA group  
Johannes Gutenberg University Mainz  
email: rosenkranz@uni-mainz.de  
  
You can find the latest proTRAC version at:  
http://sourceforge.net/projects/protrac/files  
http://www.smallRNAgroup-mainz.de/software  
==============================================================================  
  
PARAMETERS:  
Map file: ...............piwi-machos-1B.fa-collapse.map  
Genome file: ............../../../0B\_ala\_genome.fa  
RepeatMasker annotation: Alatifasciata-all0B-maryan-v2.fa\_corrected.out  
GeneSet:................./guest-storage/Data/annotation/Alatifasciata\_all0B\_maryan-v2\_out2017.gff  
  
Significant (p<=0.01) hit density will be calculated based  
on observed hit distribution.  
  
Sliding window size: ........................................ 5000 bp  
Sliding window increament: .................................. 1000 bp  
Normalize each hit by number of genomic hits: ............... yes  
Normalize each hit by number of sequence reads: ............. yes  
Normalize values (-> per million mapped reads): ............. yes  
Min. fraction of hits with 1T(U) or 10A: .................... 0.75  
Alternatively: Min. fraction of hits with 1T(U) and 10A: .... 0.5  
Min. fraction of hits with typical piRNA length: ............ 0.75  
Typical piRNA length: ....................................... 24-32 nt  
Min. size of a piRNA cluster: ............................... 1000 bp.  
Min. number of hits (absolute): ............................. 0  
Min. number of hits (normalized): ........................... 0  
Min. fraction of hits on the mainstrand: .................... 0.75  
Top fraction of mapped sequences (in terms of read counts): . 1%  
Top fraction accounts for max. n% of sequence reads: ........ 90%  
Min. fraction of hits on each arm of a bidirectional cluster: 0.05  
Output html file for each cluster: .......................... yes  
Output a summary table: ..................................... yes  
Output a FASTA file for each cluster (piRNA sequences): ..... yes  
Output a FASTA file comprising cluster sequences: ........... yes  
Output a GTF file for predicted piRNA clusters: ..............yes  
Search DNA motifs in clusters: .............................. yes  
Output flanking sequences: +/- .............................. 0 bp  
Output ~.pTi file: .......................................... no  
==============================================================================  
  
  
Genome size (without gaps): ............ 758543724 bp  
Gaps (N/X/-): .......................... 417479 bp  
Mapped reads: .......................... 26973943  
Non-identical sequences: ............... 6209225  
Genomic hits: .......................... 48438990  
Significant densitiy of mapped reads: .. 821.144211136946 reads/kb

Show proTRAC cluster info
Hide proTRAC cluster info

|  |  |
| --- | --- |
| Location | NODE\_372714\_length\_2623\_cov\_15.020205 |
| Coordinates | 2-2675 |
| Size [bp] | 2674 |
| Sequence hit loci | 2943 |
| Mapped reads (normalized) | 8087.3 |
| Mapped reads (normalized) per kb | 3024.4 |
| Normalized reads with 1T (1U) | 85.5% |
| Normalized reads with 10A | 59.6% |
| Normalized reads with length 24-32 nt | 99.1% |
| Normalized reads on the main strand(s) | 88.6% |
| Predicted directionality | bi:minus-plus (split between 588 and 589) |

100%

0%

1T (1U)  
reads

10A reads

24-32 nt  
reads

reads on mainstrand

**Either the amount of reads with 1T (1U) OR 10A has to exceed 75% (set with option: -1Tor10A)  
Alternatively the amount of reads with 1T (1U) AND 10A has to exceed 50% (set with option: -1Tand10A)  
Minimum amount of reads with preferred size is 75% (set with option: -pisize)  
Minimum amount of reads on the main strand(s) is 75% (set with option: -clstrand)**

Show read coverage
Hide read coverage

WHAT DO I SEE HERE?  
This chart shows the location of mapped sequence reads within a predicted piRNA cluster. The color refers to the number of genomic hits produced by the sequence read in question. A dark red bar indicates that this sequence read produces many other hits elsewhere in the genome. Many adjacent red or yellow bars can indicate the presence of a multi-copy element such as transposons or rRNA genes. A dark green bar indicates that this sequence read maps uniquely to this locus.

1 hit

2-5 hits

6-10 hits

11-20 hits

21-50 hits

51-100 hits

> 100 hits

NODE\_372714\_length\_2623\_cov\_15.020205

2

2675

Gene Set

RepeatMasker

Mapped  
Reads

123.6

plus strand

minus strand

123.6

Region: NODE\_372714\_length\_2623\_cov\_15.020205 8799-4. Max. coverage (+): 0. Max coverage (-): 0.04

Region: NODE\_372714\_length\_2623\_cov\_15.020205 5-10. Max. coverage (+): 0. Max coverage (-): 0.04

Region: NODE\_372714\_length\_2623\_cov\_15.020205 11-15. Max. coverage (+): 0.04. Max coverage (-): 0

Region: NODE\_372714\_length\_2623\_cov\_15.020205 16-20. Max. coverage (+): 0. Max coverage (-): 0.07

Region: NODE\_372714\_length\_2623\_cov\_15.020205 21-26. Max. coverage (+): 0. Max coverage (-): 0.07

Region: NODE\_372714\_length\_2623\_cov\_15.020205 27-31. Max. coverage (+): 0. Max coverage (-): 0

Region: NODE\_372714\_length\_2623\_cov\_15.020205 32-36. Max. coverage (+): 0.15. Max coverage (-): 0

Region: NODE\_372714\_length\_2623\_cov\_15.020205 37-42. Max. coverage (+): 0.15. Max coverage (-): 0.19

Region: NODE\_372714\_length\_2623\_cov\_15.020205 43-47. Max. coverage (+): 0.15. Max coverage (-): 0

Region: NODE\_372714\_length\_2623\_cov\_15.020205 48-52. Max. coverage (+): 0. Max coverage (-): 0

Region: NODE\_372714\_length\_2623\_cov\_15.020205 53-58. Max. coverage (+): 0. Max coverage (-): 0.07

Region: NODE\_372714\_length\_2623\_cov\_15.020205 59-63. Max. coverage (+): 0. Max coverage (-): 0.07

Region: NODE\_372714\_length\_2623\_cov\_15.020205 64-68. Max. coverage (+): 0. Max coverage (-): 0.07

Region: NODE\_372714\_length\_2623\_cov\_15.020205 69-74. Max. coverage (+): 0. Max coverage (-): 0

Region: NODE\_372714\_length\_2623\_cov\_15.020205 75-79. Max. coverage (+): 0. Max coverage (-): 0.19

Region: NODE\_372714\_length\_2623\_cov\_15.020205 80-84. Max. coverage (+): 0. Max coverage (-): 0.19

Region: NODE\_372714\_length\_2623\_cov\_15.020205 85-90. Max. coverage (+): 0. Max coverage (-): 0.07

Region: NODE\_372714\_length\_2623\_cov\_15.020205 91-95. Max. coverage (+): 0.07. Max coverage (-): 0

Region: NODE\_372714\_length\_2623\_cov\_15.020205 96-100. Max. coverage (+): 0.37. Max coverage (-): 0

Region: NODE\_372714\_length\_2623\_cov\_15.020205 101-106. Max. coverage (+): 0. Max coverage (-): 0.04

Region: NODE\_372714\_length\_2623\_cov\_15.020205 107-111. Max. coverage (+): 0. Max coverage (-): 0.83

Region: NODE\_372714\_length\_2623\_cov\_15.020205 112-116. Max. coverage (+): 0. Max coverage (-): 0.22

Region: NODE\_372714\_length\_2623\_cov\_15.020205 117-122. Max. coverage (+): 0.04. Max coverage (-): 0.13

Region: NODE\_372714\_length\_2623\_cov\_15.020205 123-127. Max. coverage (+): 0.04. Max coverage (-): 0

Region: NODE\_372714\_length\_2623\_cov\_15.020205 128-133. Max. coverage (+): 0.11. Max coverage (-): 0.48

Region: NODE\_372714\_length\_2623\_cov\_15.020205 134-138. Max. coverage (+): 0.74. Max coverage (-): 0.93

Region: NODE\_372714\_length\_2623\_cov\_15.020205 139-143. Max. coverage (+): 1.22. Max coverage (-): 0.26

Region: NODE\_372714\_length\_2623\_cov\_15.020205 144-149. Max. coverage (+): 0.48. Max coverage (-): 1.15

Region: NODE\_372714\_length\_2623\_cov\_15.020205 150-154. Max. coverage (+): 0.04. Max coverage (-): 1.04

Region: NODE\_372714\_length\_2623\_cov\_15.020205 155-159. Max. coverage (+): 0. Max coverage (-): 0.02

Region: NODE\_372714\_length\_2623\_cov\_15.020205 160-165. Max. coverage (+): 0.01. Max coverage (-): 0.01

Region: NODE\_372714\_length\_2623\_cov\_15.020205 166-170. Max. coverage (+): 0. Max coverage (-): 0.2

Region: NODE\_372714\_length\_2623\_cov\_15.020205 171-175. Max. coverage (+): 0. Max coverage (-): 0.53

Region: NODE\_372714\_length\_2623\_cov\_15.020205 176-181. Max. coverage (+): 0.01. Max coverage (-): 0.46

Region: NODE\_372714\_length\_2623\_cov\_15.020205 182-186. Max. coverage (+): 0. Max coverage (-): 0.04

Region: NODE\_372714\_length\_2623\_cov\_15.020205 187-191. Max. coverage (+): 0. Max coverage (-): 0.01

Region: NODE\_372714\_length\_2623\_cov\_15.020205 192-197. Max. coverage (+): 0.01. Max coverage (-): 0.01

Region: NODE\_372714\_length\_2623\_cov\_15.020205 198-202. Max. coverage (+): 0. Max coverage (-): 0.01

Region: NODE\_372714\_length\_2623\_cov\_15.020205 203-207. Max. coverage (+): 0. Max coverage (-): 0.01

Region: NODE\_372714\_length\_2623\_cov\_15.020205 208-213. Max. coverage (+): 0. Max coverage (-): 0

Region: NODE\_372714\_length\_2623\_cov\_15.020205 214-218. Max. coverage (+): 0. Max coverage (-): 0

Region: NODE\_372714\_length\_2623\_cov\_15.020205 219-223. Max. coverage (+): 0. Max coverage (-): 0.01

Region: NODE\_372714\_length\_2623\_cov\_15.020205 224-229. Max. coverage (+): 0. Max coverage (-): 0.1

Region: NODE\_372714\_length\_2623\_cov\_15.020205 230-234. Max. coverage (+): 0.01. Max coverage (-): 0.05

Region: NODE\_372714\_length\_2623\_cov\_15.020205 235-239. Max. coverage (+): 0.01. Max coverage (-): 0.07

Region: NODE\_372714\_length\_2623\_cov\_15.020205 240-245. Max. coverage (+): 0.05. Max coverage (-): 0.06

Region: NODE\_372714\_length\_2623\_cov\_15.020205 246-250. Max. coverage (+): 0. Max coverage (-): 0.04

Region: NODE\_372714\_length\_2623\_cov\_15.020205 251-256. Max. coverage (+): 0. Max coverage (-): 0.05

Region: NODE\_372714\_length\_2623\_cov\_15.020205 257-261. Max. coverage (+): 0. Max coverage (-): 0

Region: NODE\_372714\_length\_2623\_cov\_15.020205 262-266. Max. coverage (+): 0.01. Max coverage (-): 0.01

Region: NODE\_372714\_length\_2623\_cov\_15.020205 267-272. Max. coverage (+): 0.01. Max coverage (-): 0.11

Region: NODE\_372714\_length\_2623\_cov\_15.020205 273-277. Max. coverage (+): 0. Max coverage (-): 0.04

Region: NODE\_372714\_length\_2623\_cov\_15.020205 278-282. Max. coverage (+): 0. Max coverage (-): 0.07

Region: NODE\_372714\_length\_2623\_cov\_15.020205 283-288. Max. coverage (+): 0.04. Max coverage (-): 0.11

Region: NODE\_372714\_length\_2623\_cov\_15.020205 289-293. Max. coverage (+): 0.04. Max coverage (-): 0

Region: NODE\_372714\_length\_2623\_cov\_15.020205 294-298. Max. coverage (+): 0. Max coverage (-): 0

Region: NODE\_372714\_length\_2623\_cov\_15.020205 299-304. Max. coverage (+): 0. Max coverage (-): 0.17

Region: NODE\_372714\_length\_2623\_cov\_15.020205 305-309. Max. coverage (+): 0. Max coverage (-): 0.02

Region: NODE\_372714\_length\_2623\_cov\_15.020205 310-314. Max. coverage (+): 0. Max coverage (-): 0.28

Region: NODE\_372714\_length\_2623\_cov\_15.020205 315-320. Max. coverage (+): 0.13. Max coverage (-): 0.41

Region: NODE\_372714\_length\_2623\_cov\_15.020205 321-325. Max. coverage (+): 0.19. Max coverage (-): 0.8

Region: NODE\_372714\_length\_2623\_cov\_15.020205 326-330. Max. coverage (+): 0.04. Max coverage (-): 0.04

Region: NODE\_372714\_length\_2623\_cov\_15.020205 331-336. Max. coverage (+): 0.04. Max coverage (-): 0

Region: NODE\_372714\_length\_2623\_cov\_15.020205 337-341. Max. coverage (+): 1.52. Max coverage (-): 0.56

Region: NODE\_372714\_length\_2623\_cov\_15.020205 342-346. Max. coverage (+): 0.15. Max coverage (-): 0.41

Region: NODE\_372714\_length\_2623\_cov\_15.020205 347-352. Max. coverage (+): 0.02. Max coverage (-): 0.44

Region: NODE\_372714\_length\_2623\_cov\_15.020205 353-357. Max. coverage (+): 0.02. Max coverage (-): 0.28

Region: NODE\_372714\_length\_2623\_cov\_15.020205 358-362. Max. coverage (+): 0.04. Max coverage (-): 0.04

Region: NODE\_372714\_length\_2623\_cov\_15.020205 363-368. Max. coverage (+): 0.04. Max coverage (-): 0.02

Region: NODE\_372714\_length\_2623\_cov\_15.020205 369-373. Max. coverage (+): 0.11. Max coverage (-): 0.04

Region: NODE\_372714\_length\_2623\_cov\_15.020205 374-379. Max. coverage (+): 0.11. Max coverage (-): 0.04

Region: NODE\_372714\_length\_2623\_cov\_15.020205 380-384. Max. coverage (+): 0. Max coverage (-): 2.35

Region: NODE\_372714\_length\_2623\_cov\_15.020205 385-389. Max. coverage (+): 0. Max coverage (-): 13.23

Region: NODE\_372714\_length\_2623\_cov\_15.020205 390-395. Max. coverage (+): 0. Max coverage (-): 12.2

Region: NODE\_372714\_length\_2623\_cov\_15.020205 396-400. Max. coverage (+): 0.07. Max coverage (-): 0.04

Region: NODE\_372714\_length\_2623\_cov\_15.020205 401-405. Max. coverage (+): 0.19. Max coverage (-): 0.2

Region: NODE\_372714\_length\_2623\_cov\_15.020205 406-411. Max. coverage (+): 0.19. Max coverage (-): 0.19

Region: NODE\_372714\_length\_2623\_cov\_15.020205 412-416. Max. coverage (+): 0. Max coverage (-): 0.04

Region: NODE\_372714\_length\_2623\_cov\_15.020205 417-421. Max. coverage (+): 0. Max coverage (-): 0.09

Region: NODE\_372714\_length\_2623\_cov\_15.020205 422-427. Max. coverage (+): 0. Max coverage (-): 0

Region: NODE\_372714\_length\_2623\_cov\_15.020205 428-432. Max. coverage (+): 0.02. Max coverage (-): 0

Region: NODE\_372714\_length\_2623\_cov\_15.020205 433-437. Max. coverage (+): 0.02. Max coverage (-): 0.04

Region: NODE\_372714\_length\_2623\_cov\_15.020205 438-443. Max. coverage (+): 0.02. Max coverage (-): 0.04

Region: NODE\_372714\_length\_2623\_cov\_15.020205 444-448. Max. coverage (+): 0. Max coverage (-): 0.02

Region: NODE\_372714\_length\_2623\_cov\_15.020205 449-453. Max. coverage (+): 0. Max coverage (-): 0.17

Region: NODE\_372714\_length\_2623\_cov\_15.020205 454-459. Max. coverage (+): 0.02. Max coverage (-): 0.09

Region: NODE\_372714\_length\_2623\_cov\_15.020205 460-464. Max. coverage (+): 0.07. Max coverage (-): 0.09

Region: NODE\_372714\_length\_2623\_cov\_15.020205 465-469. Max. coverage (+): 0.06. Max coverage (-): 0.02

Region: NODE\_372714\_length\_2623\_cov\_15.020205 470-475. Max. coverage (+): 0.02. Max coverage (-): 0.02

Region: NODE\_372714\_length\_2623\_cov\_15.020205 476-480. Max. coverage (+): 0. Max coverage (-): 0.13

Region: NODE\_372714\_length\_2623\_cov\_15.020205 481-485. Max. coverage (+): 0.02. Max coverage (-): 0.04

Region: NODE\_372714\_length\_2623\_cov\_15.020205 486-491. Max. coverage (+): 0.04. Max coverage (-): 0.46

Region: NODE\_372714\_length\_2623\_cov\_15.020205 492-496. Max. coverage (+): 0. Max coverage (-): 1.71

Region: NODE\_372714\_length\_2623\_cov\_15.020205 497-502. Max. coverage (+): 0. Max coverage (-): 1.71

Region: NODE\_372714\_length\_2623\_cov\_15.020205 503-507. Max. coverage (+): 0. Max coverage (-): 0.17

Region: NODE\_372714\_length\_2623\_cov\_15.020205 508-512. Max. coverage (+): 0.09. Max coverage (-): 0.07

Region: NODE\_372714\_length\_2623\_cov\_15.020205 513-518. Max. coverage (+): 0.39. Max coverage (-): 0.07

Region: NODE\_372714\_length\_2623\_cov\_15.020205 519-523. Max. coverage (+): 0. Max coverage (-): 0.02

Region: NODE\_372714\_length\_2623\_cov\_15.020205 524-528. Max. coverage (+): 0. Max coverage (-): 0

Region: NODE\_372714\_length\_2623\_cov\_15.020205 529-534. Max. coverage (+): 0. Max coverage (-): 0.02

Region: NODE\_372714\_length\_2623\_cov\_15.020205 535-539. Max. coverage (+): 0.02. Max coverage (-): 0.02

Region: NODE\_372714\_length\_2623\_cov\_15.020205 540-544. Max. coverage (+): 0.04. Max coverage (-): 0.15

Region: NODE\_372714\_length\_2623\_cov\_15.020205 545-550. Max. coverage (+): 0.2. Max coverage (-): 0.11

Region: NODE\_372714\_length\_2623\_cov\_15.020205 551-555. Max. coverage (+): 0.15. Max coverage (-): 0

Region: NODE\_372714\_length\_2623\_cov\_15.020205 556-560. Max. coverage (+): 0.06. Max coverage (-): 0.06

Region: NODE\_372714\_length\_2623\_cov\_15.020205 561-566. Max. coverage (+): 0.02. Max coverage (-): 0.04

Region: NODE\_372714\_length\_2623\_cov\_15.020205 567-571. Max. coverage (+): 0. Max coverage (-): 0.06

Region: NODE\_372714\_length\_2623\_cov\_15.020205 572-576. Max. coverage (+): 0. Max coverage (-): 0.7

Region: NODE\_372714\_length\_2623\_cov\_15.020205 577-582. Max. coverage (+): 0.15. Max coverage (-): 0.69

Region: NODE\_372714\_length\_2623\_cov\_15.020205 583-587. Max. coverage (+): 0.15. Max coverage (-): 0.37

Region: NODE\_372714\_length\_2623\_cov\_15.020205 588-592. Max. coverage (+): 0.48. Max coverage (-): 0.33

Region: NODE\_372714\_length\_2623\_cov\_15.020205 593-598. Max. coverage (+): 3.08. Max coverage (-): 0.22

Region: NODE\_372714\_length\_2623\_cov\_15.020205 599-603. Max. coverage (+): 0. Max coverage (-): 0.19

Region: NODE\_372714\_length\_2623\_cov\_15.020205 604-608. Max. coverage (+): 0. Max coverage (-): 0.11

Region: NODE\_372714\_length\_2623\_cov\_15.020205 609-614. Max. coverage (+): 0. Max coverage (-): 0

Region: NODE\_372714\_length\_2623\_cov\_15.020205 615-619. Max. coverage (+): 0. Max coverage (-): 0.02

Region: NODE\_372714\_length\_2623\_cov\_15.020205 620-625. Max. coverage (+): 0.02. Max coverage (-): 0

Region: NODE\_372714\_length\_2623\_cov\_15.020205 626-630. Max. coverage (+): 0.02. Max coverage (-): 0.07

Region: NODE\_372714\_length\_2623\_cov\_15.020205 631-635. Max. coverage (+): 0.02. Max coverage (-): 0.15

Region: NODE\_372714\_length\_2623\_cov\_15.020205 636-641. Max. coverage (+): 0.02. Max coverage (-): 0.15

Region: NODE\_372714\_length\_2623\_cov\_15.020205 642-646. Max. coverage (+): 0.04. Max coverage (-): 0

Region: NODE\_372714\_length\_2623\_cov\_15.020205 647-651. Max. coverage (+): 1.74. Max coverage (-): 0.04

Region: NODE\_372714\_length\_2623\_cov\_15.020205 652-657. Max. coverage (+): 0. Max coverage (-): 0

Region: NODE\_372714\_length\_2623\_cov\_15.020205 658-662. Max. coverage (+): 0.07. Max coverage (-): 0

Region: NODE\_372714\_length\_2623\_cov\_15.020205 663-667. Max. coverage (+): 0.04. Max coverage (-): 0

Region: NODE\_372714\_length\_2623\_cov\_15.020205 668-673. Max. coverage (+): 0.01. Max coverage (-): 0.19

Region: NODE\_372714\_length\_2623\_cov\_15.020205 674-678. Max. coverage (+): 0.44. Max coverage (-): 0.04

Region: NODE\_372714\_length\_2623\_cov\_15.020205 679-683. Max. coverage (+): 0.5. Max coverage (-): 0.01

Region: NODE\_372714\_length\_2623\_cov\_15.020205 684-689. Max. coverage (+): 0.11. Max coverage (-): 0

Region: NODE\_372714\_length\_2623\_cov\_15.020205 690-694. Max. coverage (+): 0.07. Max coverage (-): 0

Region: NODE\_372714\_length\_2623\_cov\_15.020205 695-699. Max. coverage (+): 0. Max coverage (-): 0.04

Region: NODE\_372714\_length\_2623\_cov\_15.020205 700-705. Max. coverage (+): 0. Max coverage (-): 0.11

Region: NODE\_372714\_length\_2623\_cov\_15.020205 706-710. Max. coverage (+): 0.01. Max coverage (-): 0.03

Region: NODE\_372714\_length\_2623\_cov\_15.020205 711-715. Max. coverage (+): 0. Max coverage (-): 0.02

Region: NODE\_372714\_length\_2623\_cov\_15.020205 716-721. Max. coverage (+): 0.04. Max coverage (-): 0.03

Region: NODE\_372714\_length\_2623\_cov\_15.020205 722-726. Max. coverage (+): 0.06. Max coverage (-): 0.01

Region: NODE\_372714\_length\_2623\_cov\_15.020205 727-732. Max. coverage (+): 0.29. Max coverage (-): 0.01

Region: NODE\_372714\_length\_2623\_cov\_15.020205 733-737. Max. coverage (+): 0.04. Max coverage (-): 0

Region: NODE\_372714\_length\_2623\_cov\_15.020205 738-742. Max. coverage (+): 0. Max coverage (-): 0.01

Region: NODE\_372714\_length\_2623\_cov\_15.020205 743-748. Max. coverage (+): 0. Max coverage (-): 0.01

Region: NODE\_372714\_length\_2623\_cov\_15.020205 749-753. Max. coverage (+): 0.01. Max coverage (-): 0.03

Region: NODE\_372714\_length\_2623\_cov\_15.020205 754-758. Max. coverage (+): 0.01. Max coverage (-): 0.17

Region: NODE\_372714\_length\_2623\_cov\_15.020205 759-764. Max. coverage (+): 0. Max coverage (-): 0.04

Region: NODE\_372714\_length\_2623\_cov\_15.020205 765-769. Max. coverage (+): 0. Max coverage (-): 0

Region: NODE\_372714\_length\_2623\_cov\_15.020205 770-774. Max. coverage (+): 0.02. Max coverage (-): 0

Region: NODE\_372714\_length\_2623\_cov\_15.020205 775-780. Max. coverage (+): 0. Max coverage (-): 0

Region: NODE\_372714\_length\_2623\_cov\_15.020205 781-785. Max. coverage (+): 0. Max coverage (-): 0

Region: NODE\_372714\_length\_2623\_cov\_15.020205 786-790. Max. coverage (+): 0. Max coverage (-): 0

Region: NODE\_372714\_length\_2623\_cov\_15.020205 791-796. Max. coverage (+): 0. Max coverage (-): 0

Region: NODE\_372714\_length\_2623\_cov\_15.020205 797-801. Max. coverage (+): 0. Max coverage (-): 0

Region: NODE\_372714\_length\_2623\_cov\_15.020205 802-806. Max. coverage (+): 0. Max coverage (-): 0

Region: NODE\_372714\_length\_2623\_cov\_15.020205 807-812. Max. coverage (+): 0.03. Max coverage (-): 0.04

Region: NODE\_372714\_length\_2623\_cov\_15.020205 813-817. Max. coverage (+): 0.11. Max coverage (-): 0.01

Region: NODE\_372714\_length\_2623\_cov\_15.020205 818-822. Max. coverage (+): 0.14. Max coverage (-): 0.11

Region: NODE\_372714\_length\_2623\_cov\_15.020205 823-828. Max. coverage (+): 0.04. Max coverage (-): 0

Region: NODE\_372714\_length\_2623\_cov\_15.020205 829-833. Max. coverage (+): 0. Max coverage (-): 0

Region: NODE\_372714\_length\_2623\_cov\_15.020205 834-838. Max. coverage (+): 0. Max coverage (-): 0

Region: NODE\_372714\_length\_2623\_cov\_15.020205 839-844. Max. coverage (+): 0. Max coverage (-): 0.17

Region: NODE\_372714\_length\_2623\_cov\_15.020205 845-849. Max. coverage (+): 0.01. Max coverage (-): 0.03

Region: NODE\_372714\_length\_2623\_cov\_15.020205 850-855. Max. coverage (+): 0. Max coverage (-): 0.02

Region: NODE\_372714\_length\_2623\_cov\_15.020205 856-860. Max. coverage (+): 0.04. Max coverage (-): 0.03

Region: NODE\_372714\_length\_2623\_cov\_15.020205 861-865. Max. coverage (+): 0.06. Max coverage (-): 0.01

Region: NODE\_372714\_length\_2623\_cov\_15.020205 866-871. Max. coverage (+): 0.29. Max coverage (-): 0

Region: NODE\_372714\_length\_2623\_cov\_15.020205 872-876. Max. coverage (+): 0.03. Max coverage (-): 0

Region: NODE\_372714\_length\_2623\_cov\_15.020205 877-881. Max. coverage (+): 0. Max coverage (-): 0.01

Region: NODE\_372714\_length\_2623\_cov\_15.020205 882-887. Max. coverage (+): 0. Max coverage (-): 0.01

Region: NODE\_372714\_length\_2623\_cov\_15.020205 888-892. Max. coverage (+): 0.01. Max coverage (-): 0.13

Region: NODE\_372714\_length\_2623\_cov\_15.020205 893-897. Max. coverage (+): 0.01. Max coverage (-): 0.17

Region: NODE\_372714\_length\_2623\_cov\_15.020205 898-903. Max. coverage (+): 0. Max coverage (-): 0.03

Region: NODE\_372714\_length\_2623\_cov\_15.020205 904-908. Max. coverage (+): 0. Max coverage (-): 0

Region: NODE\_372714\_length\_2623\_cov\_15.020205 909-913. Max. coverage (+): 0.02. Max coverage (-): 0

Region: NODE\_372714\_length\_2623\_cov\_15.020205 914-919. Max. coverage (+): 0. Max coverage (-): 0

Region: NODE\_372714\_length\_2623\_cov\_15.020205 920-924. Max. coverage (+): 0. Max coverage (-): 0.26

Region: NODE\_372714\_length\_2623\_cov\_15.020205 925-929. Max. coverage (+): 0.01. Max coverage (-): 0.26

Region: NODE\_372714\_length\_2623\_cov\_15.020205 930-935. Max. coverage (+): 0.2. Max coverage (-): 0

Region: NODE\_372714\_length\_2623\_cov\_15.020205 936-940. Max. coverage (+): 0.01. Max coverage (-): 0

Region: NODE\_372714\_length\_2623\_cov\_15.020205 941-945. Max. coverage (+): 0.2. Max coverage (-): 0.01

Region: NODE\_372714\_length\_2623\_cov\_15.020205 946-951. Max. coverage (+): 0.15. Max coverage (-): 0.01

Region: NODE\_372714\_length\_2623\_cov\_15.020205 952-956. Max. coverage (+): 0.01. Max coverage (-): 0

Region: NODE\_372714\_length\_2623\_cov\_15.020205 957-961. Max. coverage (+): 0.04. Max coverage (-): 0.02

Region: NODE\_372714\_length\_2623\_cov\_15.020205 962-967. Max. coverage (+): 0.05. Max coverage (-): 0.06

Region: NODE\_372714\_length\_2623\_cov\_15.020205 968-972. Max. coverage (+): 0.04. Max coverage (-): 0.05

Region: NODE\_372714\_length\_2623\_cov\_15.020205 973-978. Max. coverage (+): 0.07. Max coverage (-): 0.01

Region: NODE\_372714\_length\_2623\_cov\_15.020205 979-983. Max. coverage (+): 0.07. Max coverage (-): 0

Region: NODE\_372714\_length\_2623\_cov\_15.020205 984-988. Max. coverage (+): 0.01. Max coverage (-): 0

Region: NODE\_372714\_length\_2623\_cov\_15.020205 989-994. Max. coverage (+): 0.04. Max coverage (-): 0.04

Region: NODE\_372714\_length\_2623\_cov\_15.020205 995-999. Max. coverage (+): 0.04. Max coverage (-): 0.7

Region: NODE\_372714\_length\_2623\_cov\_15.020205 1000-1004. Max. coverage (+): 0.04. Max coverage (-): 0.48

Region: NODE\_372714\_length\_2623\_cov\_15.020205 1005-1010. Max. coverage (+): 0.04. Max coverage (-): 0.04

Region: NODE\_372714\_length\_2623\_cov\_15.020205 1011-1015. Max. coverage (+): 0. Max coverage (-): 0

Region: NODE\_372714\_length\_2623\_cov\_15.020205 1016-1020. Max. coverage (+): 0. Max coverage (-): 0.07

Region: NODE\_372714\_length\_2623\_cov\_15.020205 1021-1026. Max. coverage (+): 0. Max coverage (-): 0.11

Region: NODE\_372714\_length\_2623\_cov\_15.020205 1027-1031. Max. coverage (+): 0. Max coverage (-): 0

Region: NODE\_372714\_length\_2623\_cov\_15.020205 1032-1036. Max. coverage (+): 0. Max coverage (-): 0

Region: NODE\_372714\_length\_2623\_cov\_15.020205 1037-1042. Max. coverage (+): 0. Max coverage (-): 0

Region: NODE\_372714\_length\_2623\_cov\_15.020205 1043-1047. Max. coverage (+): 0. Max coverage (-): 0

Region: NODE\_372714\_length\_2623\_cov\_15.020205 1048-1052. Max. coverage (+): 0.01. Max coverage (-): 0

Region: NODE\_372714\_length\_2623\_cov\_15.020205 1053-1058. Max. coverage (+): 0.01. Max coverage (-): 0.06

Region: NODE\_372714\_length\_2623\_cov\_15.020205 1059-1063. Max. coverage (+): 0.01. Max coverage (-): 0

Region: NODE\_372714\_length\_2623\_cov\_15.020205 1064-1068. Max. coverage (+): 0.01. Max coverage (-): 0

Region: NODE\_372714\_length\_2623\_cov\_15.020205 1069-1074. Max. coverage (+): 0.01. Max coverage (-): 0

Region: NODE\_372714\_length\_2623\_cov\_15.020205 1075-1079. Max. coverage (+): 0. Max coverage (-): 0

Region: NODE\_372714\_length\_2623\_cov\_15.020205 1080-1084. Max. coverage (+): 0. Max coverage (-): 0

Region: NODE\_372714\_length\_2623\_cov\_15.020205 1085-1090. Max. coverage (+): 0.02. Max coverage (-): 0.02

Region: NODE\_372714\_length\_2623\_cov\_15.020205 1091-1095. Max. coverage (+): 0.02. Max coverage (-): 0.15

Region: NODE\_372714\_length\_2623\_cov\_15.020205 1096-1101. Max. coverage (+): 0.01. Max coverage (-): 0.4

Region: NODE\_372714\_length\_2623\_cov\_15.020205 1102-1106. Max. coverage (+): 0.05. Max coverage (-): 0.4

Region: NODE\_372714\_length\_2623\_cov\_15.020205 1107-1111. Max. coverage (+): 0.06. Max coverage (-): 0

Region: NODE\_372714\_length\_2623\_cov\_15.020205 1112-1117. Max. coverage (+): 0. Max coverage (-): 0

Region: NODE\_372714\_length\_2623\_cov\_15.020205 1118-1122. Max. coverage (+): 0. Max coverage (-): 0.01

Region: NODE\_372714\_length\_2623\_cov\_15.020205 1123-1127. Max. coverage (+): 0. Max coverage (-): 0.05

Region: NODE\_372714\_length\_2623\_cov\_15.020205 1128-1133. Max. coverage (+): 0.02. Max coverage (-): 0.3

Region: NODE\_372714\_length\_2623\_cov\_15.020205 1134-1138. Max. coverage (+): 0.02. Max coverage (-): 1.32

Region: NODE\_372714\_length\_2623\_cov\_15.020205 1139-1143. Max. coverage (+): 0. Max coverage (-): 1.24

Region: NODE\_372714\_length\_2623\_cov\_15.020205 1144-1149. Max. coverage (+): 0. Max coverage (-): 0

Region: NODE\_372714\_length\_2623\_cov\_15.020205 1150-1154. Max. coverage (+): 0.31. Max coverage (-): 0.01

Region: NODE\_372714\_length\_2623\_cov\_15.020205 1155-1159. Max. coverage (+): 0.01. Max coverage (-): 0.05

Region: NODE\_372714\_length\_2623\_cov\_15.020205 1160-1165. Max. coverage (+): 0.02. Max coverage (-): 0.11

Region: NODE\_372714\_length\_2623\_cov\_15.020205 1166-1170. Max. coverage (+): 0.04. Max coverage (-): 0.07

Region: NODE\_372714\_length\_2623\_cov\_15.020205 1171-1175. Max. coverage (+): 0.07. Max coverage (-): 0

Region: NODE\_372714\_length\_2623\_cov\_15.020205 1176-1181. Max. coverage (+): 0. Max coverage (-): 0

Region: NODE\_372714\_length\_2623\_cov\_15.020205 1182-1186. Max. coverage (+): 0. Max coverage (-): 0

Region: NODE\_372714\_length\_2623\_cov\_15.020205 1187-1191. Max. coverage (+): 0.04. Max coverage (-): 0

Region: NODE\_372714\_length\_2623\_cov\_15.020205 1192-1197. Max. coverage (+): 0.04. Max coverage (-): 0

Region: NODE\_372714\_length\_2623\_cov\_15.020205 1198-1202. Max. coverage (+): 0.02. Max coverage (-): 0

Region: NODE\_372714\_length\_2623\_cov\_15.020205 1203-1207. Max. coverage (+): 0. Max coverage (-): 0

Region: NODE\_372714\_length\_2623\_cov\_15.020205 1208-1213. Max. coverage (+): 0.01. Max coverage (-): 0

Region: NODE\_372714\_length\_2623\_cov\_15.020205 1214-1218. Max. coverage (+): 4.5. Max coverage (-): 0

Region: NODE\_372714\_length\_2623\_cov\_15.020205 1219-1224. Max. coverage (+): 3.08. Max coverage (-): 0

Region: NODE\_372714\_length\_2623\_cov\_15.020205 1225-1229. Max. coverage (+): 0.2. Max coverage (-): 0.1

Region: NODE\_372714\_length\_2623\_cov\_15.020205 1230-1234. Max. coverage (+): 0.05. Max coverage (-): 0.83

Region: NODE\_372714\_length\_2623\_cov\_15.020205 1235-1240. Max. coverage (+): 0.06. Max coverage (-): 0.83

Region: NODE\_372714\_length\_2623\_cov\_15.020205 1241-1245. Max. coverage (+): 0.05. Max coverage (-): 0

Region: NODE\_372714\_length\_2623\_cov\_15.020205 1246-1250. Max. coverage (+): 0.82. Max coverage (-): 0.01

Region: NODE\_372714\_length\_2623\_cov\_15.020205 1251-1256. Max. coverage (+): 0.84. Max coverage (-): 0.01

Region: NODE\_372714\_length\_2623\_cov\_15.020205 1257-1261. Max. coverage (+): 0. Max coverage (-): 0.01

Region: NODE\_372714\_length\_2623\_cov\_15.020205 1262-1266. Max. coverage (+): 0. Max coverage (-): 0.1

Region: NODE\_372714\_length\_2623\_cov\_15.020205 1267-1272. Max. coverage (+): 0.01. Max coverage (-): 0.4

Region: NODE\_372714\_length\_2623\_cov\_15.020205 1273-1277. Max. coverage (+): 0.01. Max coverage (-): 0.01

Region: NODE\_372714\_length\_2623\_cov\_15.020205 1278-1282. Max. coverage (+): 0.02. Max coverage (-): 0

Region: NODE\_372714\_length\_2623\_cov\_15.020205 1283-1288. Max. coverage (+): 0.05. Max coverage (-): 0.12

Region: NODE\_372714\_length\_2623\_cov\_15.020205 1289-1293. Max. coverage (+): 0.38. Max coverage (-): 0.01

Region: NODE\_372714\_length\_2623\_cov\_15.020205 1294-1298. Max. coverage (+): 0.02. Max coverage (-): 0.01

Region: NODE\_372714\_length\_2623\_cov\_15.020205 1299-1304. Max. coverage (+): 0.31. Max coverage (-): 0.09

Region: NODE\_372714\_length\_2623\_cov\_15.020205 1305-1309. Max. coverage (+): 0.07. Max coverage (-): 0.07

Region: NODE\_372714\_length\_2623\_cov\_15.020205 1310-1314. Max. coverage (+): 0.09. Max coverage (-): 0.01

Region: NODE\_372714\_length\_2623\_cov\_15.020205 1315-1320. Max. coverage (+): 0.65. Max coverage (-): 0.09

Region: NODE\_372714\_length\_2623\_cov\_15.020205 1321-1325. Max. coverage (+): 0.46. Max coverage (-): 0.07

Region: NODE\_372714\_length\_2623\_cov\_15.020205 1326-1330. Max. coverage (+): 0.33. Max coverage (-): 0.17

Region: NODE\_372714\_length\_2623\_cov\_15.020205 1331-1336. Max. coverage (+): 0.16. Max coverage (-): 0.22

Region: NODE\_372714\_length\_2623\_cov\_15.020205 1337-1341. Max. coverage (+): 0.06. Max coverage (-): 0.01

Region: NODE\_372714\_length\_2623\_cov\_15.020205 1342-1347. Max. coverage (+): 0.23. Max coverage (-): 0.05

Region: NODE\_372714\_length\_2623\_cov\_15.020205 1348-1352. Max. coverage (+): 0.23. Max coverage (-): 0.04

Region: NODE\_372714\_length\_2623\_cov\_15.020205 1353-1357. Max. coverage (+): 0.07. Max coverage (-): 0.04

Region: NODE\_372714\_length\_2623\_cov\_15.020205 1358-1363. Max. coverage (+): 0.11. Max coverage (-): 0.33

Region: NODE\_372714\_length\_2623\_cov\_15.020205 1364-1368. Max. coverage (+): 0.11. Max coverage (-): 0.33

Region: NODE\_372714\_length\_2623\_cov\_15.020205 1369-1373. Max. coverage (+): 0.07. Max coverage (-): 0.49

Region: NODE\_372714\_length\_2623\_cov\_15.020205 1374-1379. Max. coverage (+): 0.09. Max coverage (-): 0.47

Region: NODE\_372714\_length\_2623\_cov\_15.020205 1380-1384. Max. coverage (+): 0.22. Max coverage (-): 0.01

Region: NODE\_372714\_length\_2623\_cov\_15.020205 1385-1389. Max. coverage (+): 0.59. Max coverage (-): 0.04

Region: NODE\_372714\_length\_2623\_cov\_15.020205 1390-1395. Max. coverage (+): 0.22. Max coverage (-): 0

Region: NODE\_372714\_length\_2623\_cov\_15.020205 1396-1400. Max. coverage (+): 0. Max coverage (-): 0

Region: NODE\_372714\_length\_2623\_cov\_15.020205 1401-1405. Max. coverage (+): 0. Max coverage (-): 0

Region: NODE\_372714\_length\_2623\_cov\_15.020205 1406-1411. Max. coverage (+): 0.05. Max coverage (-): 0.04

Region: NODE\_372714\_length\_2623\_cov\_15.020205 1412-1416. Max. coverage (+): 0.05. Max coverage (-): 0.15

Region: NODE\_372714\_length\_2623\_cov\_15.020205 1417-1421. Max. coverage (+): 1.66. Max coverage (-): 0.05

Region: NODE\_372714\_length\_2623\_cov\_15.020205 1422-1427. Max. coverage (+): 1.16. Max coverage (-): 0.04

Region: NODE\_372714\_length\_2623\_cov\_15.020205 1428-1432. Max. coverage (+): 0.19. Max coverage (-): 0.04

Region: NODE\_372714\_length\_2623\_cov\_15.020205 1433-1437. Max. coverage (+): 0.04. Max coverage (-): 0.41

Region: NODE\_372714\_length\_2623\_cov\_15.020205 1438-1443. Max. coverage (+): 0.01. Max coverage (-): 0.22

Region: NODE\_372714\_length\_2623\_cov\_15.020205 1444-1448. Max. coverage (+): 0. Max coverage (-): 0.09

Region: NODE\_372714\_length\_2623\_cov\_15.020205 1449-1453. Max. coverage (+): 0.25. Max coverage (-): 0

Region: NODE\_372714\_length\_2623\_cov\_15.020205 1454-1459. Max. coverage (+): 0.46. Max coverage (-): 0.01

Region: NODE\_372714\_length\_2623\_cov\_15.020205 1460-1464. Max. coverage (+): 0.42. Max coverage (-): 0.1

Region: NODE\_372714\_length\_2623\_cov\_15.020205 1465-1470. Max. coverage (+): 0. Max coverage (-): 0.33

Region: NODE\_372714\_length\_2623\_cov\_15.020205 1471-1475. Max. coverage (+): 0. Max coverage (-): 0.7

Region: NODE\_372714\_length\_2623\_cov\_15.020205 1476-1480. Max. coverage (+): 0.06. Max coverage (-): 0.14

Region: NODE\_372714\_length\_2623\_cov\_15.020205 1481-1486. Max. coverage (+): 0.07. Max coverage (-): 0.04

Region: NODE\_372714\_length\_2623\_cov\_15.020205 1487-1491. Max. coverage (+): 0.11. Max coverage (-): 0

Region: NODE\_372714\_length\_2623\_cov\_15.020205 1492-1496. Max. coverage (+): 0.04. Max coverage (-): 0

Region: NODE\_372714\_length\_2623\_cov\_15.020205 1497-1502. Max. coverage (+): 0. Max coverage (-): 0

Region: NODE\_372714\_length\_2623\_cov\_15.020205 1503-1507. Max. coverage (+): 0. Max coverage (-): 0

Region: NODE\_372714\_length\_2623\_cov\_15.020205 1508-1512. Max. coverage (+): 0. Max coverage (-): 0.02

Region: NODE\_372714\_length\_2623\_cov\_15.020205 1513-1518. Max. coverage (+): 0.01. Max coverage (-): 0.09

Region: NODE\_372714\_length\_2623\_cov\_15.020205 1519-1523. Max. coverage (+): 0.01. Max coverage (-): 0

Region: NODE\_372714\_length\_2623\_cov\_15.020205 1524-1528. Max. coverage (+): 0. Max coverage (-): 0.02

Region: NODE\_372714\_length\_2623\_cov\_15.020205 1529-1534. Max. coverage (+): 0.02. Max coverage (-): 0.02

Region: NODE\_372714\_length\_2623\_cov\_15.020205 1535-1539. Max. coverage (+): 0.07. Max coverage (-): 0

Region: NODE\_372714\_length\_2623\_cov\_15.020205 1540-1544. Max. coverage (+): 0.06. Max coverage (-): 0

Region: NODE\_372714\_length\_2623\_cov\_15.020205 1545-1550. Max. coverage (+): 0.01. Max coverage (-): 0

Region: NODE\_372714\_length\_2623\_cov\_15.020205 1551-1555. Max. coverage (+): 0.01. Max coverage (-): 0

Region: NODE\_372714\_length\_2623\_cov\_15.020205 1556-1560. Max. coverage (+): 0. Max coverage (-): 0

Region: NODE\_372714\_length\_2623\_cov\_15.020205 1561-1566. Max. coverage (+): 0. Max coverage (-): 0

Region: NODE\_372714\_length\_2623\_cov\_15.020205 1567-1571. Max. coverage (+): 0.01. Max coverage (-): 0

Region: NODE\_372714\_length\_2623\_cov\_15.020205 1572-1576. Max. coverage (+): 0. Max coverage (-): 0

Region: NODE\_372714\_length\_2623\_cov\_15.020205 1577-1582. Max. coverage (+): 0. Max coverage (-): 0

Region: NODE\_372714\_length\_2623\_cov\_15.020205 1583-1587. Max. coverage (+): 0. Max coverage (-): 0.01

Region: NODE\_372714\_length\_2623\_cov\_15.020205 1588-1593. Max. coverage (+): 0. Max coverage (-): 0.01

Region: NODE\_372714\_length\_2623\_cov\_15.020205 1594-1598. Max. coverage (+): 0.16. Max coverage (-): 0

Region: NODE\_372714\_length\_2623\_cov\_15.020205 1599-1603. Max. coverage (+): 0.14. Max coverage (-): 0.04

Region: NODE\_372714\_length\_2623\_cov\_15.020205 1604-1609. Max. coverage (+): 0.11. Max coverage (-): 0.19

Region: NODE\_372714\_length\_2623\_cov\_15.020205 1610-1614. Max. coverage (+): 0.11. Max coverage (-): 0

Region: NODE\_372714\_length\_2623\_cov\_15.020205 1615-1619. Max. coverage (+): 0.07. Max coverage (-): 0.04

Region: NODE\_372714\_length\_2623\_cov\_15.020205 1620-1625. Max. coverage (+): 0.11. Max coverage (-): 0.22

Region: NODE\_372714\_length\_2623\_cov\_15.020205 1626-1630. Max. coverage (+): 0.11. Max coverage (-): 0

Region: NODE\_372714\_length\_2623\_cov\_15.020205 1631-1635. Max. coverage (+): 0.04. Max coverage (-): 0

Region: NODE\_372714\_length\_2623\_cov\_15.020205 1636-1641. Max. coverage (+): 2.34. Max coverage (-): 0

Region: NODE\_372714\_length\_2623\_cov\_15.020205 1642-1646. Max. coverage (+): 3.04. Max coverage (-): 0.02

Region: NODE\_372714\_length\_2623\_cov\_15.020205 1647-1651. Max. coverage (+): 0.35. Max coverage (-): 0

Region: NODE\_372714\_length\_2623\_cov\_15.020205 1652-1657. Max. coverage (+): 1.33. Max coverage (-): 0

Region: NODE\_372714\_length\_2623\_cov\_15.020205 1658-1662. Max. coverage (+): 0.89. Max coverage (-): 0

Region: NODE\_372714\_length\_2623\_cov\_15.020205 1663-1667. Max. coverage (+): 0.41. Max coverage (-): 0

Region: NODE\_372714\_length\_2623\_cov\_15.020205 1668-1673. Max. coverage (+): 0.04. Max coverage (-): 0

Region: NODE\_372714\_length\_2623\_cov\_15.020205 1674-1678. Max. coverage (+): 0.07. Max coverage (-): 0

Region: NODE\_372714\_length\_2623\_cov\_15.020205 1679-1683. Max. coverage (+): 0.33. Max coverage (-): 0

Region: NODE\_372714\_length\_2623\_cov\_15.020205 1684-1689. Max. coverage (+): 0.33. Max coverage (-): 0.06

Region: NODE\_372714\_length\_2623\_cov\_15.020205 1690-1694. Max. coverage (+): 0.11. Max coverage (-): 0.07

Region: NODE\_372714\_length\_2623\_cov\_15.020205 1695-1699. Max. coverage (+): 0.06. Max coverage (-): 0.07

Region: NODE\_372714\_length\_2623\_cov\_15.020205 1700-1705. Max. coverage (+): 0.06. Max coverage (-): 0.28

Region: NODE\_372714\_length\_2623\_cov\_15.020205 1706-1710. Max. coverage (+): 0.07. Max coverage (-): 0.11

Region: NODE\_372714\_length\_2623\_cov\_15.020205 1711-1716. Max. coverage (+): 0.2. Max coverage (-): 0

Region: NODE\_372714\_length\_2623\_cov\_15.020205 1717-1721. Max. coverage (+): 0.22. Max coverage (-): 1.61

Region: NODE\_372714\_length\_2623\_cov\_15.020205 1722-1726. Max. coverage (+): 2.37. Max coverage (-): 1.61

Region: NODE\_372714\_length\_2623\_cov\_15.020205 1727-1732. Max. coverage (+): 0.26. Max coverage (-): 0

Region: NODE\_372714\_length\_2623\_cov\_15.020205 1733-1737. Max. coverage (+): 0.19. Max coverage (-): 0.26

Region: NODE\_372714\_length\_2623\_cov\_15.020205 1738-1742. Max. coverage (+): 0.7. Max coverage (-): 0.22

Region: NODE\_372714\_length\_2623\_cov\_15.020205 1743-1748. Max. coverage (+): 0.43. Max coverage (-): 0.02

Region: NODE\_372714\_length\_2623\_cov\_15.020205 1749-1753. Max. coverage (+): 0.13. Max coverage (-): 0.02

Region: NODE\_372714\_length\_2623\_cov\_15.020205 1754-1758. Max. coverage (+): 0.22. Max coverage (-): 0.02

Region: NODE\_372714\_length\_2623\_cov\_15.020205 1759-1764. Max. coverage (+): 0.02. Max coverage (-): 0.04

Region: NODE\_372714\_length\_2623\_cov\_15.020205 1765-1769. Max. coverage (+): 0.09. Max coverage (-): 0

Region: NODE\_372714\_length\_2623\_cov\_15.020205 1770-1774. Max. coverage (+): 0.02. Max coverage (-): 0

Region: NODE\_372714\_length\_2623\_cov\_15.020205 1775-1780. Max. coverage (+): 0.11. Max coverage (-): 0.04

Region: NODE\_372714\_length\_2623\_cov\_15.020205 1781-1785. Max. coverage (+): 0.07. Max coverage (-): 0.07

Region: NODE\_372714\_length\_2623\_cov\_15.020205 1786-1790. Max. coverage (+): 0.19. Max coverage (-): 0.04

Region: NODE\_372714\_length\_2623\_cov\_15.020205 1791-1796. Max. coverage (+): 0.19. Max coverage (-): 0.04

Region: NODE\_372714\_length\_2623\_cov\_15.020205 1797-1801. Max. coverage (+): 1.26. Max coverage (-): 0

Region: NODE\_372714\_length\_2623\_cov\_15.020205 1802-1806. Max. coverage (+): 1.45. Max coverage (-): 0

Region: NODE\_372714\_length\_2623\_cov\_15.020205 1807-1812. Max. coverage (+): 3.11. Max coverage (-): 0

Region: NODE\_372714\_length\_2623\_cov\_15.020205 1813-1817. Max. coverage (+): 0.19. Max coverage (-): 0

Region: NODE\_372714\_length\_2623\_cov\_15.020205 1818-1822. Max. coverage (+): 0.11. Max coverage (-): 0.15

Region: NODE\_372714\_length\_2623\_cov\_15.020205 1823-1828. Max. coverage (+): 0. Max coverage (-): 0.3

Region: NODE\_372714\_length\_2623\_cov\_15.020205 1829-1833. Max. coverage (+): 0. Max coverage (-): 0.15

Region: NODE\_372714\_length\_2623\_cov\_15.020205 1834-1839. Max. coverage (+): 0. Max coverage (-): 0

Region: NODE\_372714\_length\_2623\_cov\_15.020205 1840-1844. Max. coverage (+): 0. Max coverage (-): 0

Region: NODE\_372714\_length\_2623\_cov\_15.020205 1845-1849. Max. coverage (+): 0. Max coverage (-): 0

Region: NODE\_372714\_length\_2623\_cov\_15.020205 1850-1855. Max. coverage (+): 0. Max coverage (-): 0

Region: NODE\_372714\_length\_2623\_cov\_15.020205 1856-1860. Max. coverage (+): 0.04. Max coverage (-): 0.04

Region: NODE\_372714\_length\_2623\_cov\_15.020205 1861-1865. Max. coverage (+): 0.07. Max coverage (-): 0.11

Region: NODE\_372714\_length\_2623\_cov\_15.020205 1866-1871. Max. coverage (+): 0.09. Max coverage (-): 0.13

Region: NODE\_372714\_length\_2623\_cov\_15.020205 1872-1876. Max. coverage (+): 0.15. Max coverage (-): 0.11

Region: NODE\_372714\_length\_2623\_cov\_15.020205 1877-1881. Max. coverage (+): 2. Max coverage (-): 0.15

Region: NODE\_372714\_length\_2623\_cov\_15.020205 1882-1887. Max. coverage (+): 1.08. Max coverage (-): 0.15

Region: NODE\_372714\_length\_2623\_cov\_15.020205 1888-1892. Max. coverage (+): 0.52. Max coverage (-): 0.07

Region: NODE\_372714\_length\_2623\_cov\_15.020205 1893-1897. Max. coverage (+): 6.82. Max coverage (-): 0

Region: NODE\_372714\_length\_2623\_cov\_15.020205 1898-1903. Max. coverage (+): 2.85. Max coverage (-): 0.09

Region: NODE\_372714\_length\_2623\_cov\_15.020205 1904-1908. Max. coverage (+): 0.98. Max coverage (-): 0.04

Region: NODE\_372714\_length\_2623\_cov\_15.020205 1909-1913. Max. coverage (+): 0.09. Max coverage (-): 0

Region: NODE\_372714\_length\_2623\_cov\_15.020205 1914-1919. Max. coverage (+): 0.67. Max coverage (-): 0.06

Region: NODE\_372714\_length\_2623\_cov\_15.020205 1920-1924. Max. coverage (+): 0.65. Max coverage (-): 0.06

Region: NODE\_372714\_length\_2623\_cov\_15.020205 1925-1929. Max. coverage (+): 0.04. Max coverage (-): 0.02

Region: NODE\_372714\_length\_2623\_cov\_15.020205 1930-1935. Max. coverage (+): 0.19. Max coverage (-): 0.04

Region: NODE\_372714\_length\_2623\_cov\_15.020205 1936-1940. Max. coverage (+): 0.44. Max coverage (-): 0.3

Region: NODE\_372714\_length\_2623\_cov\_15.020205 1941-1945. Max. coverage (+): 0.15. Max coverage (-): 0.3

Region: NODE\_372714\_length\_2623\_cov\_15.020205 1946-1951. Max. coverage (+): 0.44. Max coverage (-): 0.56

Region: NODE\_372714\_length\_2623\_cov\_15.020205 1952-1956. Max. coverage (+): 3. Max coverage (-): 0.22

Region: NODE\_372714\_length\_2623\_cov\_15.020205 1957-1962. Max. coverage (+): 7.27. Max coverage (-): 0.5

Region: NODE\_372714\_length\_2623\_cov\_15.020205 1963-1967. Max. coverage (+): 0.32. Max coverage (-): 0.24

Region: NODE\_372714\_length\_2623\_cov\_15.020205 1968-1972. Max. coverage (+): 0.26. Max coverage (-): 0.11

Region: NODE\_372714\_length\_2623\_cov\_15.020205 1973-1978. Max. coverage (+): 123.6. Max coverage (-): 0.04

Region: NODE\_372714\_length\_2623\_cov\_15.020205 1979-1983. Max. coverage (+): 14.2. Max coverage (-): 0

Region: NODE\_372714\_length\_2623\_cov\_15.020205 1984-1988. Max. coverage (+): 0.11. Max coverage (-): 0

Region: NODE\_372714\_length\_2623\_cov\_15.020205 1989-1994. Max. coverage (+): 0.04. Max coverage (-): 0.04

Region: NODE\_372714\_length\_2623\_cov\_15.020205 1995-1999. Max. coverage (+): 0.74. Max coverage (-): 0.07

Region: NODE\_372714\_length\_2623\_cov\_15.020205 2000-2004. Max. coverage (+): 0.67. Max coverage (-): 0.37

Region: NODE\_372714\_length\_2623\_cov\_15.020205 2005-2010. Max. coverage (+): 0. Max coverage (-): 0.41

Region: NODE\_372714\_length\_2623\_cov\_15.020205 2011-2015. Max. coverage (+): 0. Max coverage (-): 0.24

Region: NODE\_372714\_length\_2623\_cov\_15.020205 2016-2020. Max. coverage (+): 0.11. Max coverage (-): 1.72

Region: NODE\_372714\_length\_2623\_cov\_15.020205 2021-2026. Max. coverage (+): 0.69. Max coverage (-): 0.78

Region: NODE\_372714\_length\_2623\_cov\_15.020205 2027-2031. Max. coverage (+): 1.04. Max coverage (-): 0.02

Region: NODE\_372714\_length\_2623\_cov\_15.020205 2032-2036. Max. coverage (+): 4.45. Max coverage (-): 0.02

Region: NODE\_372714\_length\_2623\_cov\_15.020205 2037-2042. Max. coverage (+): 2.17. Max coverage (-): 0.02

Region: NODE\_372714\_length\_2623\_cov\_15.020205 2043-2047. Max. coverage (+): 0. Max coverage (-): 0.04

Region: NODE\_372714\_length\_2623\_cov\_15.020205 2048-2052. Max. coverage (+): 0.04. Max coverage (-): 0.04

Region: NODE\_372714\_length\_2623\_cov\_15.020205 2053-2058. Max. coverage (+): 1.45. Max coverage (-): 0

Region: NODE\_372714\_length\_2623\_cov\_15.020205 2059-2063. Max. coverage (+): 0.82. Max coverage (-): 0.04

Region: NODE\_372714\_length\_2623\_cov\_15.020205 2064-2069. Max. coverage (+): 0.07. Max coverage (-): 0

Region: NODE\_372714\_length\_2623\_cov\_15.020205 2070-2074. Max. coverage (+): 0.46. Max coverage (-): 0

Region: NODE\_372714\_length\_2623\_cov\_15.020205 2075-2079. Max. coverage (+): 2.16. Max coverage (-): 0.01

Region: NODE\_372714\_length\_2623\_cov\_15.020205 2080-2085. Max. coverage (+): 0.06. Max coverage (-): 0.04

Region: NODE\_372714\_length\_2623\_cov\_15.020205 2086-2090. Max. coverage (+): 0. Max coverage (-): 0

Region: NODE\_372714\_length\_2623\_cov\_15.020205 2091-2095. Max. coverage (+): 0.01. Max coverage (-): 0

Region: NODE\_372714\_length\_2623\_cov\_15.020205 2096-2101. Max. coverage (+): 0.1. Max coverage (-): 0.01

Region: NODE\_372714\_length\_2623\_cov\_15.020205 2102-2106. Max. coverage (+): 0.15. Max coverage (-): 0.14

Region: NODE\_372714\_length\_2623\_cov\_15.020205 2107-2111. Max. coverage (+): 0.09. Max coverage (-): 0.14

Region: NODE\_372714\_length\_2623\_cov\_15.020205 2112-2117. Max. coverage (+): 0.63. Max coverage (-): 0.12

Region: NODE\_372714\_length\_2623\_cov\_15.020205 2118-2122. Max. coverage (+): 1.06. Max coverage (-): 0.07

Region: NODE\_372714\_length\_2623\_cov\_15.020205 2123-2127. Max. coverage (+): 1.09. Max coverage (-): 0

Region: NODE\_372714\_length\_2623\_cov\_15.020205 2128-2133. Max. coverage (+): 0.16. Max coverage (-): 0

Region: NODE\_372714\_length\_2623\_cov\_15.020205 2134-2138. Max. coverage (+): 0.01. Max coverage (-): 0

Region: NODE\_372714\_length\_2623\_cov\_15.020205 2139-2143. Max. coverage (+): 0. Max coverage (-): 0.02

Region: NODE\_372714\_length\_2623\_cov\_15.020205 2144-2149. Max. coverage (+): 0.04. Max coverage (-): 0.02

Region: NODE\_372714\_length\_2623\_cov\_15.020205 2150-2154. Max. coverage (+): 0.02. Max coverage (-): 0.01

Region: NODE\_372714\_length\_2623\_cov\_15.020205 2155-2159. Max. coverage (+): 0.01. Max coverage (-): 0.01

Region: NODE\_372714\_length\_2623\_cov\_15.020205 2160-2165. Max. coverage (+): 0.01. Max coverage (-): 0

Region: NODE\_372714\_length\_2623\_cov\_15.020205 2166-2170. Max. coverage (+): 0. Max coverage (-): 0

Region: NODE\_372714\_length\_2623\_cov\_15.020205 2171-2175. Max. coverage (+): 0. Max coverage (-): 0.02

Region: NODE\_372714\_length\_2623\_cov\_15.020205 2176-2181. Max. coverage (+): 0.54. Max coverage (-): 0.05

Region: NODE\_372714\_length\_2623\_cov\_15.020205 2182-2186. Max. coverage (+): 0.22. Max coverage (-): 0.01

Region: NODE\_372714\_length\_2623\_cov\_15.020205 2187-2192. Max. coverage (+): 0.42. Max coverage (-): 0

Region: NODE\_372714\_length\_2623\_cov\_15.020205 2193-2197. Max. coverage (+): 0.05. Max coverage (-): 0.01

Region: NODE\_372714\_length\_2623\_cov\_15.020205 2198-2202. Max. coverage (+): 0.07. Max coverage (-): 0.04

Region: NODE\_372714\_length\_2623\_cov\_15.020205 2203-2208. Max. coverage (+): 0.01. Max coverage (-): 0.35

Region: NODE\_372714\_length\_2623\_cov\_15.020205 2209-2213. Max. coverage (+): 0.52. Max coverage (-): 0.09

Region: NODE\_372714\_length\_2623\_cov\_15.020205 2214-2218. Max. coverage (+): 0.59. Max coverage (-): 0.1

Region: NODE\_372714\_length\_2623\_cov\_15.020205 2219-2224. Max. coverage (+): 0.54. Max coverage (-): 0.05

Region: NODE\_372714\_length\_2623\_cov\_15.020205 2225-2229. Max. coverage (+): 1.3. Max coverage (-): 0

Region: NODE\_372714\_length\_2623\_cov\_15.020205 2230-2234. Max. coverage (+): 1.15. Max coverage (-): 0.01

Region: NODE\_372714\_length\_2623\_cov\_15.020205 2235-2240. Max. coverage (+): 0.88. Max coverage (-): 0.05

Region: NODE\_372714\_length\_2623\_cov\_15.020205 2241-2245. Max. coverage (+): 0.07. Max coverage (-): 0.06

Region: NODE\_372714\_length\_2623\_cov\_15.020205 2246-2250. Max. coverage (+): 0.06. Max coverage (-): 0.01

Region: NODE\_372714\_length\_2623\_cov\_15.020205 2251-2256. Max. coverage (+): 0.02. Max coverage (-): 0.02

Region: NODE\_372714\_length\_2623\_cov\_15.020205 2257-2261. Max. coverage (+): 0.04. Max coverage (-): 0.1

Region: NODE\_372714\_length\_2623\_cov\_15.020205 2262-2266. Max. coverage (+): 0.47. Max coverage (-): 0.31

Region: NODE\_372714\_length\_2623\_cov\_15.020205 2267-2272. Max. coverage (+): 0.35. Max coverage (-): 0.1

Region: NODE\_372714\_length\_2623\_cov\_15.020205 2273-2277. Max. coverage (+): 0.25. Max coverage (-): 0.07

Region: NODE\_372714\_length\_2623\_cov\_15.020205 2278-2282. Max. coverage (+): 0.09. Max coverage (-): 0.06

Region: NODE\_372714\_length\_2623\_cov\_15.020205 2283-2288. Max. coverage (+): 0.07. Max coverage (-): 0.04

Region: NODE\_372714\_length\_2623\_cov\_15.020205 2289-2293. Max. coverage (+): 0.05. Max coverage (-): 0

Region: NODE\_372714\_length\_2623\_cov\_15.020205 2294-2298. Max. coverage (+): 0.04. Max coverage (-): 0

Region: NODE\_372714\_length\_2623\_cov\_15.020205 2299-2304. Max. coverage (+): 0. Max coverage (-): 0

Region: NODE\_372714\_length\_2623\_cov\_15.020205 2305-2309. Max. coverage (+): 0. Max coverage (-): 0

Region: NODE\_372714\_length\_2623\_cov\_15.020205 2310-2315. Max. coverage (+): 0.01. Max coverage (-): 0

Region: NODE\_372714\_length\_2623\_cov\_15.020205 2316-2320. Max. coverage (+): 0. Max coverage (-): 0

Region: NODE\_372714\_length\_2623\_cov\_15.020205 2321-2325. Max. coverage (+): 0.02. Max coverage (-): 0.3

Region: NODE\_372714\_length\_2623\_cov\_15.020205 2326-2331. Max. coverage (+): 0.04. Max coverage (-): 0.3

Region: NODE\_372714\_length\_2623\_cov\_15.020205 2332-2336. Max. coverage (+): 1.87. Max coverage (-): 0

Region: NODE\_372714\_length\_2623\_cov\_15.020205 2337-2341. Max. coverage (+): 1.1. Max coverage (-): 0

Region: NODE\_372714\_length\_2623\_cov\_15.020205 2342-2347. Max. coverage (+): 0.23. Max coverage (-): 0.02

Region: NODE\_372714\_length\_2623\_cov\_15.020205 2348-2352. Max. coverage (+): 0. Max coverage (-): 0.02

Region: NODE\_372714\_length\_2623\_cov\_15.020205 2353-2357. Max. coverage (+): 0. Max coverage (-): 0.09

Region: NODE\_372714\_length\_2623\_cov\_15.020205 2358-2363. Max. coverage (+): 0.05. Max coverage (-): 0.05

Region: NODE\_372714\_length\_2623\_cov\_15.020205 2364-2368. Max. coverage (+): 0.06. Max coverage (-): 0.01

Region: NODE\_372714\_length\_2623\_cov\_15.020205 2369-2373. Max. coverage (+): 0.33. Max coverage (-): 0

Region: NODE\_372714\_length\_2623\_cov\_15.020205 2374-2379. Max. coverage (+): 0.85. Max coverage (-): 0.02

Region: NODE\_372714\_length\_2623\_cov\_15.020205 2380-2384. Max. coverage (+): 0.95. Max coverage (-): 0.01

Region: NODE\_372714\_length\_2623\_cov\_15.020205 2385-2389. Max. coverage (+): 0.05. Max coverage (-): 0.05

Region: NODE\_372714\_length\_2623\_cov\_15.020205 2390-2395. Max. coverage (+): 0.53. Max coverage (-): 0.15

Region: NODE\_372714\_length\_2623\_cov\_15.020205 2396-2400. Max. coverage (+): 0.56. Max coverage (-): 0.17

Region: NODE\_372714\_length\_2623\_cov\_15.020205 2401-2405. Max. coverage (+): 0.65. Max coverage (-): 0.01

Region: NODE\_372714\_length\_2623\_cov\_15.020205 2406-2411. Max. coverage (+): 0.04. Max coverage (-): 0

Region: NODE\_372714\_length\_2623\_cov\_15.020205 2412-2416. Max. coverage (+): 0. Max coverage (-): 0

Region: NODE\_372714\_length\_2623\_cov\_15.020205 2417-2421. Max. coverage (+): 0. Max coverage (-): 0

Region: NODE\_372714\_length\_2623\_cov\_15.020205 2422-2427. Max. coverage (+): 0. Max coverage (-): 0

Region: NODE\_372714\_length\_2623\_cov\_15.020205 2428-2432. Max. coverage (+): 0.11. Max coverage (-): 0

Region: NODE\_372714\_length\_2623\_cov\_15.020205 2433-2438. Max. coverage (+): 0.02. Max coverage (-): 0

Region: NODE\_372714\_length\_2623\_cov\_15.020205 2439-2443. Max. coverage (+): 0.02. Max coverage (-): 0

Region: NODE\_372714\_length\_2623\_cov\_15.020205 2444-2448. Max. coverage (+): 1.32. Max coverage (-): 0.23

Region: NODE\_372714\_length\_2623\_cov\_15.020205 2449-2454. Max. coverage (+): 1.53. Max coverage (-): 0.25

Region: NODE\_372714\_length\_2623\_cov\_15.020205 2455-2459. Max. coverage (+): 1.8. Max coverage (-): 0.05

Region: NODE\_372714\_length\_2623\_cov\_15.020205 2460-2464. Max. coverage (+): 0.23. Max coverage (-): 0.04

Region: NODE\_372714\_length\_2623\_cov\_15.020205 2465-2470. Max. coverage (+): 0.26. Max coverage (-): 0.02

Region: NODE\_372714\_length\_2623\_cov\_15.020205 2471-2475. Max. coverage (+): 0.17. Max coverage (-): 0.01

Region: NODE\_372714\_length\_2623\_cov\_15.020205 2476-2480. Max. coverage (+): 0.41. Max coverage (-): 0

Region: NODE\_372714\_length\_2623\_cov\_15.020205 2481-2486. Max. coverage (+): 0.17. Max coverage (-): 0

Region: NODE\_372714\_length\_2623\_cov\_15.020205 2487-2491. Max. coverage (+): 0.12. Max coverage (-): 0.01

Region: NODE\_372714\_length\_2623\_cov\_15.020205 2492-2496. Max. coverage (+): 0.19. Max coverage (-): 0

Region: NODE\_372714\_length\_2623\_cov\_15.020205 2497-2502. Max. coverage (+): 0.38. Max coverage (-): 0.02

Region: NODE\_372714\_length\_2623\_cov\_15.020205 2503-2507. Max. coverage (+): 0.64. Max coverage (-): 0.1

Region: NODE\_372714\_length\_2623\_cov\_15.020205 2508-2512. Max. coverage (+): 0.2. Max coverage (-): 0.2

Region: NODE\_372714\_length\_2623\_cov\_15.020205 2513-2518. Max. coverage (+): 0.05. Max coverage (-): 0.01

Region: NODE\_372714\_length\_2623\_cov\_15.020205 2519-2523. Max. coverage (+): 0.65. Max coverage (-): 0

Region: NODE\_372714\_length\_2623\_cov\_15.020205 2524-2528. Max. coverage (+): 0.61. Max coverage (-): 0

Region: NODE\_372714\_length\_2623\_cov\_15.020205 2529-2534. Max. coverage (+): 0.19. Max coverage (-): 0

Region: NODE\_372714\_length\_2623\_cov\_15.020205 2535-2539. Max. coverage (+): 0.04. Max coverage (-): 0.04

Region: NODE\_372714\_length\_2623\_cov\_15.020205 2540-2544. Max. coverage (+): 0.04. Max coverage (-): 0.11

Region: NODE\_372714\_length\_2623\_cov\_15.020205 2545-2550. Max. coverage (+): 0.15. Max coverage (-): 0.33

Region: NODE\_372714\_length\_2623\_cov\_15.020205 2551-2555. Max. coverage (+): 0.07. Max coverage (-): 0.56

Region: NODE\_372714\_length\_2623\_cov\_15.020205 2556-2561. Max. coverage (+): 0.72. Max coverage (-): 0.09

Region: NODE\_372714\_length\_2623\_cov\_15.020205 2562-2566. Max. coverage (+): 2.93. Max coverage (-): 0

Region: NODE\_372714\_length\_2623\_cov\_15.020205 2567-2571. Max. coverage (+): 3.15. Max coverage (-): 0

Region: NODE\_372714\_length\_2623\_cov\_15.020205 2572-2577. Max. coverage (+): 0.39. Max coverage (-): 0.02

Region: NODE\_372714\_length\_2623\_cov\_15.020205 2578-2582. Max. coverage (+): 0. Max coverage (-): 0.07

Region: NODE\_372714\_length\_2623\_cov\_15.020205 2583-2587. Max. coverage (+): 0.02. Max coverage (-): 0.04

Region: NODE\_372714\_length\_2623\_cov\_15.020205 2588-2593. Max. coverage (+): 0.59. Max coverage (-): 0.02

Region: NODE\_372714\_length\_2623\_cov\_15.020205 2594-2598. Max. coverage (+): 0.63. Max coverage (-): 0

Region: NODE\_372714\_length\_2623\_cov\_15.020205 2599-2603. Max. coverage (+): 0.26. Max coverage (-): 0

Region: NODE\_372714\_length\_2623\_cov\_15.020205 2604-2609. Max. coverage (+): 0.07. Max coverage (-): 0

Region: NODE\_372714\_length\_2623\_cov\_15.020205 2610-2614. Max. coverage (+): 0. Max coverage (-): 0.04

Region: NODE\_372714\_length\_2623\_cov\_15.020205 2615-2619. Max. coverage (+): 0.02. Max coverage (-): 1.15

Region: NODE\_372714\_length\_2623\_cov\_15.020205 2620-2625. Max. coverage (+): 0.04. Max coverage (-): 0.8

Region: NODE\_372714\_length\_2623\_cov\_15.020205 2626-2630. Max. coverage (+): 0.96. Max coverage (-): 0.22

Region: NODE\_372714\_length\_2623\_cov\_15.020205 2631-2635. Max. coverage (+): 2.97. Max coverage (-): 0

Region: NODE\_372714\_length\_2623\_cov\_15.020205 2636-2641. Max. coverage (+): 4.21. Max coverage (-): 0

Region: NODE\_372714\_length\_2623\_cov\_15.020205 2642-2646. Max. coverage (+): 0.02. Max coverage (-): 0

Region: NODE\_372714\_length\_2623\_cov\_15.020205 2647-2651. Max. coverage (+): 0. Max coverage (-): 0.04

Region: NODE\_372714\_length\_2623\_cov\_15.020205 2652-2657. Max. coverage (+): 0. Max coverage (-): 0.04

Region: NODE\_372714\_length\_2623\_cov\_15.020205 2658-2662. Max. coverage (+): 0. Max coverage (-): 0

Region: NODE\_372714\_length\_2623\_cov\_15.020205 2663-2667. Max. coverage (+): 0. Max coverage (-): 0

Region: NODE\_372714\_length\_2623\_cov\_15.020205 2668-2673. Max. coverage (+): 0. Max coverage (-): 0

Region: NODE\_372714\_length\_2623\_cov\_15.020205 2674-. Max. coverage (+): 0. Max coverage (-): 0

RepeatMasker Color Code

**+**

100-98% Identity

<98-95% Identity

<95-90% Identity

<90-85% Identity

<85-80% Identity

<80-75% Identity

<75-70% Identity

<70% Identity

**-**

Gene Set Color Code

**+**

Gene

Pseudogene

Other

**-**

Topology/Coverage Color Code

Coverage Plus Strand

Coverage Minus Strand

Mainstrand: Plus

Mainstrand: Minus

Complementary Strand

Flanking Region  
(if option -flank >0)

Gene Set Annotation  
  
RepeatMasker Annotation  

**1. AlRepD-2080**: 250-668 (+), Divergence to consensus: 37.3%  
**2. AlRepD-2080**: 678-758 (+), Divergence to consensus: 32.1%  
**3. AlRepD-2080**: 808-1207 (+), Divergence to consensus: 37.7%  
**4. (TGTT)n**: 1846-1895 (+), Divergence to consensus: 14.2%  
**5. AlRepC-312**: 2120-2173 (+), Divergence to consensus: 20.4%  
**6. AlRepC-312**: 2169-2347 (-), Divergence to consensus: 48%  
**7. AlRepE-294**: 2497-2659 (+), Divergence to consensus: 24.8%

  
Transcription Factor Binding Sites  

**RHOXF1** (Sequence: GGATCA (-): 362)  
**RHOXF1** (Sequence: GGCTTA (-): 1228)  
**RHOXF1** (Sequence: GGCTCA (-): 1270)  
**RHOXF1** (Sequence: GGCTCA (-): 1372)  
**RHOXF1** (Sequence: AGATTA (-): 1725)  
**RHOXF1** (Sequence: AGCTCA (-): 2087)  
**RHOXF1** (Sequence: TGATCT (+): 299)  
**RHOXF1** (Sequence: TAAGCT (+): 1211)  
**RHOXF1** (Sequence: TAATCC (+): 1755)  
**Gata4** (Sequence: GTTATCT (+): 1320)  
**RFX4\_1** (Sequence: GTTGCTATG (-): 2182)  
**SOX9** (Sequence: AACAATGA (-): 729)  
**SOX9** (Sequence: AACAATGA (-): 867)  
**FOXO1** (Sequence: CCTGTTTTC (+): 2662)  
**FOXO3\_mmu** (Sequence: TGTTTTGA (-): 1891)  
**FIGLA** (Sequence: TCCAGCTGTT (-): 2217)  
**FOXO3\_mmu** (Sequence: TCAAAACA (+): 2377)  
**FOXO1** (Sequence: ATAAACAGG (-): 1221)  
**Sox5** (Sequence: AACAAT (-): 729)  
**Sox5** (Sequence: AACAAT (-): 867)  
**Sox5** (Sequence: AACAAT (-): 1128)  
**Sox5** (Sequence: AACAAT (-): 2160)  
**POU5F1** (Sequence: ATGCAAA (+): 2203)
