# Supplementary material for: First characterization of PIWI-interacting RNA clusters in a cichlid fish with a B chromosome
Source: BMC Biol. 2022 Sep 21;20:204. doi: 10.1186/s12915-022-01403-2 (PMC9490952; doi:10.1186/s12915-022-01403-2)
Supplement: Supplementary file 1 — Additional file 1. Zipped folder with fasta and interactive html piRNA cluster information for the A. latifasciata genome. The nomenclature is as follows: number-pirna-cluster_sex_B-presence (f, female; m, male; 0b, without B chromosome; 1b, with B chromosome). [file 12915_2022_1403_MOESM1_ESM.zip › 145_f0b.html]

piRNA cluster 145\_f0b 67


Predicted piRNA cluster no. 145\_f0b
  

Show proTRAC run info
Hide proTRAC run info

/\  
                \_\_\_\_\_\_\_\_\_\_\_\_\_\_\_\_\_\_\_\_\_\_\_/\\_\_\_ /  \\_\_\_\_\_\_\_  
               I                      /  \  /    \      I  
               I     pro             /    \/      \     I  
               I        TRAC        /               \   I  
               I   \_\_\_\_\_\_\_\_\_\_\_\_\_\_\_\_/\_\_\_\_\_\_\_\_\_\_\_\_\_\_\_\_\_\\_ I  
               I   \              /                     I  
               I    \            /                      I  
               I     \  /\      /       V.2.4.2         I  
               I      \/  \    /                        I  
               I\_\_\_\_\_\_\_\_\_\_\_\  /\_\_\_\_\_\_\_\_\_\_\_\_\_\_\_\_\_\_\_\_\_\_\_\_\_I  
                            \/  
  
  
================================= proTRAC ====================================  
VERSION: .......... 2.4.2  
LAST MODIFIED: .... 11. May 2018  
  
Please cite:  
Rosenkranz D, Zischler H. proTRAC - a software for probabilistic piRNA cluster  
detection, visualization and analysis. 2012. BMC Bioinformatics 13:5.  
  
  
Contact:  
David Rosenkranz  
Institute of Organismic and Molecular Evolutionary Biology  
Dept. Anthropology, small RNA group  
Johannes Gutenberg University Mainz  
email: rosenkranz@uni-mainz.de  
  
You can find the latest proTRAC version at:  
http://sourceforge.net/projects/protrac/files  
http://www.smallRNAgroup-mainz.de/software  
==============================================================================  
  
PARAMETERS:  
Map file: ...............piwi-femeas-0B.fa-collapse.map  
Genome file: ............../../../0B\_ala\_genome.fa  
RepeatMasker annotation: Alatifasciata-all0B-maryan-v2.fa\_corrected.out  
GeneSet:................./guest-storage/Data/annotation/Alatifasciata\_all0B\_maryan-v2\_out2017.gff  
  
Significant (p<=0.01) hit density will be calculated based  
on observed hit distribution.  
  
Sliding window size: ........................................ 5000 bp  
Sliding window increament: .................................. 1000 bp  
Normalize each hit by number of genomic hits: ............... yes  
Normalize each hit by number of sequence reads: ............. yes  
Normalize values (-> per million mapped reads): ............. yes  
Min. fraction of hits with 1T(U) or 10A: .................... 0.75  
Alternatively: Min. fraction of hits with 1T(U) and 10A: .... 0.5  
Min. fraction of hits with typical piRNA length: ............ 0.75  
Typical piRNA length: ....................................... 24-32 nt  
Min. size of a piRNA cluster: ............................... 1000 bp.  
Min. number of hits (absolute): ............................. 0  
Min. number of hits (normalized): ........................... 0  
Min. fraction of hits on the mainstrand: .................... 0.75  
Top fraction of mapped sequences (in terms of read counts): . 1%  
Top fraction accounts for max. n% of sequence reads: ........ 90%  
Min. fraction of hits on each arm of a bidirectional cluster: 0.05  
Output html file for each cluster: .......................... yes  
Output a summary table: ..................................... yes  
Output a FASTA file for each cluster (piRNA sequences): ..... yes  
Output a FASTA file comprising cluster sequences: ........... yes  
Output a GTF file for predicted piRNA clusters: ..............yes  
Search DNA motifs in clusters: .............................. yes  
Output flanking sequences: +/- .............................. 0 bp  
Output ~.pTi file: .......................................... no  
==============================================================================  
  
  
Genome size (without gaps): ............ 758543724 bp  
Gaps (N/X/-): .......................... 417479 bp  
Mapped reads: .......................... 13052187  
Non-identical sequences: ............... 3338911  
Genomic hits: .......................... 28737726  
Significant densitiy of mapped reads: .. 470.083249848448 reads/kb

Show proTRAC cluster info
Hide proTRAC cluster info

|  |  |
| --- | --- |
| Location | NODE\_372995\_length\_1546\_cov\_22.922380 |
| Coordinates | 12-1609 |
| Size [bp] | 1598 |
| Sequence hit loci | 858 |
| Mapped reads (normalized) | 3469.5 |
| Mapped reads (normalized) per kb | 2171.2 |
| Normalized reads with 1T (1U) | 85.1% |
| Normalized reads with 10A | 19% |
| Normalized reads with length 24-32 nt | 98.8% |
| Normalized reads on the main strand(s) | 95.1% |
| Predicted directionality | mono:plus |

100%

0%

1T (1U)  
reads

10A reads

24-32 nt  
reads

reads on mainstrand

**Either the amount of reads with 1T (1U) OR 10A has to exceed 75% (set with option: -1Tor10A)  
Alternatively the amount of reads with 1T (1U) AND 10A has to exceed 50% (set with option: -1Tand10A)  
Minimum amount of reads with preferred size is 75% (set with option: -pisize)  
Minimum amount of reads on the main strand(s) is 75% (set with option: -clstrand)**

Show read coverage
Hide read coverage

WHAT DO I SEE HERE?  
This chart shows the location of mapped sequence reads within a predicted piRNA cluster. The color refers to the number of genomic hits produced by the sequence read in question. A dark red bar indicates that this sequence read produces many other hits elsewhere in the genome. Many adjacent red or yellow bars can indicate the presence of a multi-copy element such as transposons or rRNA genes. A dark green bar indicates that this sequence read maps uniquely to this locus.

1 hit

2-5 hits

6-10 hits

11-20 hits

21-50 hits

51-100 hits

> 100 hits

NODE\_372995\_length\_1546\_cov\_22.922380

12

1609

Gene Set

RepeatMasker

Mapped  
Reads

101.36

plus strand

minus strand

101.36

Region: NODE\_372995\_length\_1546\_cov\_22.922380 2197-13. Max. coverage (+): 0.31. Max coverage (-): 0

Region: NODE\_372995\_length\_1546\_cov\_22.922380 14-16. Max. coverage (+): 0.15. Max coverage (-): 0

Region: NODE\_372995\_length\_1546\_cov\_22.922380 17-19. Max. coverage (+): 0.15. Max coverage (-): 0

Region: NODE\_372995\_length\_1546\_cov\_22.922380 20-23. Max. coverage (+): 0. Max coverage (-): 0

Region: NODE\_372995\_length\_1546\_cov\_22.922380 24-26. Max. coverage (+): 0. Max coverage (-): 0

Region: NODE\_372995\_length\_1546\_cov\_22.922380 27-29. Max. coverage (+): 0. Max coverage (-): 0

Region: NODE\_372995\_length\_1546\_cov\_22.922380 30-32. Max. coverage (+): 0. Max coverage (-): 0

Region: NODE\_372995\_length\_1546\_cov\_22.922380 33-35. Max. coverage (+): 0. Max coverage (-): 0

Region: NODE\_372995\_length\_1546\_cov\_22.922380 36-39. Max. coverage (+): 0. Max coverage (-): 0

Region: NODE\_372995\_length\_1546\_cov\_22.922380 40-42. Max. coverage (+): 0. Max coverage (-): 0

Region: NODE\_372995\_length\_1546\_cov\_22.922380 43-45. Max. coverage (+): 0. Max coverage (-): 0.08

Region: NODE\_372995\_length\_1546\_cov\_22.922380 46-48. Max. coverage (+): 1.3. Max coverage (-): 0.08

Region: NODE\_372995\_length\_1546\_cov\_22.922380 49-51. Max. coverage (+): 0.69. Max coverage (-): 0.08

Region: NODE\_372995\_length\_1546\_cov\_22.922380 52-55. Max. coverage (+): 0.46. Max coverage (-): 0.31

Region: NODE\_372995\_length\_1546\_cov\_22.922380 56-58. Max. coverage (+): 0. Max coverage (-): 0

Region: NODE\_372995\_length\_1546\_cov\_22.922380 59-61. Max. coverage (+): 0. Max coverage (-): 0

Region: NODE\_372995\_length\_1546\_cov\_22.922380 62-64. Max. coverage (+): 0. Max coverage (-): 0

Region: NODE\_372995\_length\_1546\_cov\_22.922380 65-67. Max. coverage (+): 0.02. Max coverage (-): 0

Region: NODE\_372995\_length\_1546\_cov\_22.922380 68-71. Max. coverage (+): 0.02. Max coverage (-): 0

Region: NODE\_372995\_length\_1546\_cov\_22.922380 72-74. Max. coverage (+): 0.26. Max coverage (-): 0

Region: NODE\_372995\_length\_1546\_cov\_22.922380 75-77. Max. coverage (+): 0.02. Max coverage (-): 0

Region: NODE\_372995\_length\_1546\_cov\_22.922380 78-80. Max. coverage (+): 0. Max coverage (-): 0

Region: NODE\_372995\_length\_1546\_cov\_22.922380 81-83. Max. coverage (+): 0. Max coverage (-): 0

Region: NODE\_372995\_length\_1546\_cov\_22.922380 84-87. Max. coverage (+): 0. Max coverage (-): 0

Region: NODE\_372995\_length\_1546\_cov\_22.922380 88-90. Max. coverage (+): 0.01. Max coverage (-): 0

Region: NODE\_372995\_length\_1546\_cov\_22.922380 91-93. Max. coverage (+): 0. Max coverage (-): 0

Region: NODE\_372995\_length\_1546\_cov\_22.922380 94-96. Max. coverage (+): 0. Max coverage (-): 0

Region: NODE\_372995\_length\_1546\_cov\_22.922380 97-99. Max. coverage (+): 0. Max coverage (-): 0

Region: NODE\_372995\_length\_1546\_cov\_22.922380 100-103. Max. coverage (+): 0.01. Max coverage (-): 0.03

Region: NODE\_372995\_length\_1546\_cov\_22.922380 104-106. Max. coverage (+): 0.03. Max coverage (-): 0.02

Region: NODE\_372995\_length\_1546\_cov\_22.922380 107-109. Max. coverage (+): 0.03. Max coverage (-): 0

Region: NODE\_372995\_length\_1546\_cov\_22.922380 110-112. Max. coverage (+): 0.02. Max coverage (-): 0

Region: NODE\_372995\_length\_1546\_cov\_22.922380 113-115. Max. coverage (+): 0. Max coverage (-): 0

Region: NODE\_372995\_length\_1546\_cov\_22.922380 116-119. Max. coverage (+): 0. Max coverage (-): 0

Region: NODE\_372995\_length\_1546\_cov\_22.922380 120-122. Max. coverage (+): 0. Max coverage (-): 0

Region: NODE\_372995\_length\_1546\_cov\_22.922380 123-125. Max. coverage (+): 0. Max coverage (-): 0

Region: NODE\_372995\_length\_1546\_cov\_22.922380 126-128. Max. coverage (+): 0. Max coverage (-): 0

Region: NODE\_372995\_length\_1546\_cov\_22.922380 129-131. Max. coverage (+): 0. Max coverage (-): 0.01

Region: NODE\_372995\_length\_1546\_cov\_22.922380 132-135. Max. coverage (+): 0. Max coverage (-): 0.01

Region: NODE\_372995\_length\_1546\_cov\_22.922380 136-138. Max. coverage (+): 0. Max coverage (-): 0

Region: NODE\_372995\_length\_1546\_cov\_22.922380 139-141. Max. coverage (+): 0. Max coverage (-): 0

Region: NODE\_372995\_length\_1546\_cov\_22.922380 142-144. Max. coverage (+): 0. Max coverage (-): 0

Region: NODE\_372995\_length\_1546\_cov\_22.922380 145-147. Max. coverage (+): 0. Max coverage (-): 0

Region: NODE\_372995\_length\_1546\_cov\_22.922380 148-151. Max. coverage (+): 0. Max coverage (-): 0

Region: NODE\_372995\_length\_1546\_cov\_22.922380 152-154. Max. coverage (+): 0.08. Max coverage (-): 0

Region: NODE\_372995\_length\_1546\_cov\_22.922380 155-157. Max. coverage (+): 0.08. Max coverage (-): 0.69

Region: NODE\_372995\_length\_1546\_cov\_22.922380 158-160. Max. coverage (+): 0. Max coverage (-): 0.69

Region: NODE\_372995\_length\_1546\_cov\_22.922380 161-163. Max. coverage (+): 0. Max coverage (-): 0

Region: NODE\_372995\_length\_1546\_cov\_22.922380 164-167. Max. coverage (+): 0.01. Max coverage (-): 0

Region: NODE\_372995\_length\_1546\_cov\_22.922380 168-170. Max. coverage (+): 0.01. Max coverage (-): 0

Region: NODE\_372995\_length\_1546\_cov\_22.922380 171-173. Max. coverage (+): 0.42. Max coverage (-): 0

Region: NODE\_372995\_length\_1546\_cov\_22.922380 174-176. Max. coverage (+): 0.43. Max coverage (-): 0

Region: NODE\_372995\_length\_1546\_cov\_22.922380 177-179. Max. coverage (+): 0. Max coverage (-): 0

Region: NODE\_372995\_length\_1546\_cov\_22.922380 180-182. Max. coverage (+): 0. Max coverage (-): 0

Region: NODE\_372995\_length\_1546\_cov\_22.922380 183-186. Max. coverage (+): 0. Max coverage (-): 0

Region: NODE\_372995\_length\_1546\_cov\_22.922380 187-189. Max. coverage (+): 0. Max coverage (-): 0

Region: NODE\_372995\_length\_1546\_cov\_22.922380 190-192. Max. coverage (+): 0. Max coverage (-): 0.04

Region: NODE\_372995\_length\_1546\_cov\_22.922380 193-195. Max. coverage (+): 0. Max coverage (-): 0.05

Region: NODE\_372995\_length\_1546\_cov\_22.922380 196-198. Max. coverage (+): 0. Max coverage (-): 0

Region: NODE\_372995\_length\_1546\_cov\_22.922380 199-202. Max. coverage (+): 0. Max coverage (-): 0

Region: NODE\_372995\_length\_1546\_cov\_22.922380 203-205. Max. coverage (+): 0. Max coverage (-): 0

Region: NODE\_372995\_length\_1546\_cov\_22.922380 206-208. Max. coverage (+): 0. Max coverage (-): 0

Region: NODE\_372995\_length\_1546\_cov\_22.922380 209-211. Max. coverage (+): 0. Max coverage (-): 0

Region: NODE\_372995\_length\_1546\_cov\_22.922380 212-214. Max. coverage (+): 0. Max coverage (-): 0

Region: NODE\_372995\_length\_1546\_cov\_22.922380 215-218. Max. coverage (+): 0. Max coverage (-): 0

Region: NODE\_372995\_length\_1546\_cov\_22.922380 219-221. Max. coverage (+): 0. Max coverage (-): 0

Region: NODE\_372995\_length\_1546\_cov\_22.922380 222-224. Max. coverage (+): 0. Max coverage (-): 0

Region: NODE\_372995\_length\_1546\_cov\_22.922380 225-227. Max. coverage (+): 0. Max coverage (-): 0

Region: NODE\_372995\_length\_1546\_cov\_22.922380 228-230. Max. coverage (+): 0. Max coverage (-): 0

Region: NODE\_372995\_length\_1546\_cov\_22.922380 231-234. Max. coverage (+): 0. Max coverage (-): 0

Region: NODE\_372995\_length\_1546\_cov\_22.922380 235-237. Max. coverage (+): 0. Max coverage (-): 0

Region: NODE\_372995\_length\_1546\_cov\_22.922380 238-240. Max. coverage (+): 0. Max coverage (-): 0

Region: NODE\_372995\_length\_1546\_cov\_22.922380 241-243. Max. coverage (+): 0. Max coverage (-): 0

Region: NODE\_372995\_length\_1546\_cov\_22.922380 244-246. Max. coverage (+): 0. Max coverage (-): 0

Region: NODE\_372995\_length\_1546\_cov\_22.922380 247-250. Max. coverage (+): 0. Max coverage (-): 0

Region: NODE\_372995\_length\_1546\_cov\_22.922380 251-253. Max. coverage (+): 0. Max coverage (-): 0

Region: NODE\_372995\_length\_1546\_cov\_22.922380 254-256. Max. coverage (+): 0. Max coverage (-): 0

Region: NODE\_372995\_length\_1546\_cov\_22.922380 257-259. Max. coverage (+): 0. Max coverage (-): 0

Region: NODE\_372995\_length\_1546\_cov\_22.922380 260-262. Max. coverage (+): 0. Max coverage (-): 0

Region: NODE\_372995\_length\_1546\_cov\_22.922380 263-266. Max. coverage (+): 0. Max coverage (-): 0

Region: NODE\_372995\_length\_1546\_cov\_22.922380 267-269. Max. coverage (+): 0. Max coverage (-): 0

Region: NODE\_372995\_length\_1546\_cov\_22.922380 270-272. Max. coverage (+): 0. Max coverage (-): 0

Region: NODE\_372995\_length\_1546\_cov\_22.922380 273-275. Max. coverage (+): 0. Max coverage (-): 0

Region: NODE\_372995\_length\_1546\_cov\_22.922380 276-278. Max. coverage (+): 0. Max coverage (-): 0

Region: NODE\_372995\_length\_1546\_cov\_22.922380 279-282. Max. coverage (+): 0. Max coverage (-): 0

Region: NODE\_372995\_length\_1546\_cov\_22.922380 283-285. Max. coverage (+): 0. Max coverage (-): 0

Region: NODE\_372995\_length\_1546\_cov\_22.922380 286-288. Max. coverage (+): 0. Max coverage (-): 0

Region: NODE\_372995\_length\_1546\_cov\_22.922380 289-291. Max. coverage (+): 0. Max coverage (-): 0

Region: NODE\_372995\_length\_1546\_cov\_22.922380 292-294. Max. coverage (+): 0. Max coverage (-): 0

Region: NODE\_372995\_length\_1546\_cov\_22.922380 295-298. Max. coverage (+): 0. Max coverage (-): 0

Region: NODE\_372995\_length\_1546\_cov\_22.922380 299-301. Max. coverage (+): 0. Max coverage (-): 0

Region: NODE\_372995\_length\_1546\_cov\_22.922380 302-304. Max. coverage (+): 0. Max coverage (-): 0

Region: NODE\_372995\_length\_1546\_cov\_22.922380 305-307. Max. coverage (+): 0. Max coverage (-): 0

Region: NODE\_372995\_length\_1546\_cov\_22.922380 308-310. Max. coverage (+): 0. Max coverage (-): 0

Region: NODE\_372995\_length\_1546\_cov\_22.922380 311-314. Max. coverage (+): 0. Max coverage (-): 0

Region: NODE\_372995\_length\_1546\_cov\_22.922380 315-317. Max. coverage (+): 0.04. Max coverage (-): 0

Region: NODE\_372995\_length\_1546\_cov\_22.922380 318-320. Max. coverage (+): 0.02. Max coverage (-): 0

Region: NODE\_372995\_length\_1546\_cov\_22.922380 321-323. Max. coverage (+): 0. Max coverage (-): 0

Region: NODE\_372995\_length\_1546\_cov\_22.922380 324-326. Max. coverage (+): 0. Max coverage (-): 0

Region: NODE\_372995\_length\_1546\_cov\_22.922380 327-330. Max. coverage (+): 0.08. Max coverage (-): 0.15

Region: NODE\_372995\_length\_1546\_cov\_22.922380 331-333. Max. coverage (+): 0. Max coverage (-): 0.15

Region: NODE\_372995\_length\_1546\_cov\_22.922380 334-336. Max. coverage (+): 0. Max coverage (-): 0

Region: NODE\_372995\_length\_1546\_cov\_22.922380 337-339. Max. coverage (+): 0. Max coverage (-): 0

Region: NODE\_372995\_length\_1546\_cov\_22.922380 340-342. Max. coverage (+): 0. Max coverage (-): 0

Region: NODE\_372995\_length\_1546\_cov\_22.922380 343-345. Max. coverage (+): 0. Max coverage (-): 0

Region: NODE\_372995\_length\_1546\_cov\_22.922380 346-349. Max. coverage (+): 0.54. Max coverage (-): 0

Region: NODE\_372995\_length\_1546\_cov\_22.922380 350-352. Max. coverage (+): 0.46. Max coverage (-): 0

Region: NODE\_372995\_length\_1546\_cov\_22.922380 353-355. Max. coverage (+): 0. Max coverage (-): 0

Region: NODE\_372995\_length\_1546\_cov\_22.922380 356-358. Max. coverage (+): 0. Max coverage (-): 0

Region: NODE\_372995\_length\_1546\_cov\_22.922380 359-361. Max. coverage (+): 0. Max coverage (-): 0

Region: NODE\_372995\_length\_1546\_cov\_22.922380 362-365. Max. coverage (+): 0. Max coverage (-): 0

Region: NODE\_372995\_length\_1546\_cov\_22.922380 366-368. Max. coverage (+): 0. Max coverage (-): 0

Region: NODE\_372995\_length\_1546\_cov\_22.922380 369-371. Max. coverage (+): 0.08. Max coverage (-): 0.09

Region: NODE\_372995\_length\_1546\_cov\_22.922380 372-374. Max. coverage (+): 0.08. Max coverage (-): 0

Region: NODE\_372995\_length\_1546\_cov\_22.922380 375-377. Max. coverage (+): 0. Max coverage (-): 0

Region: NODE\_372995\_length\_1546\_cov\_22.922380 378-381. Max. coverage (+): 0.01. Max coverage (-): 0.01

Region: NODE\_372995\_length\_1546\_cov\_22.922380 382-384. Max. coverage (+): 0. Max coverage (-): 0

Region: NODE\_372995\_length\_1546\_cov\_22.922380 385-387. Max. coverage (+): 0. Max coverage (-): 0

Region: NODE\_372995\_length\_1546\_cov\_22.922380 388-390. Max. coverage (+): 0.06. Max coverage (-): 0

Region: NODE\_372995\_length\_1546\_cov\_22.922380 391-393. Max. coverage (+): 0.02. Max coverage (-): 0

Region: NODE\_372995\_length\_1546\_cov\_22.922380 394-397. Max. coverage (+): 0.02. Max coverage (-): 0

Region: NODE\_372995\_length\_1546\_cov\_22.922380 398-400. Max. coverage (+): 0.03. Max coverage (-): 0

Region: NODE\_372995\_length\_1546\_cov\_22.922380 401-403. Max. coverage (+): 0.03. Max coverage (-): 0

Region: NODE\_372995\_length\_1546\_cov\_22.922380 404-406. Max. coverage (+): 0. Max coverage (-): 0.04

Region: NODE\_372995\_length\_1546\_cov\_22.922380 407-409. Max. coverage (+): 0. Max coverage (-): 0.03

Region: NODE\_372995\_length\_1546\_cov\_22.922380 410-413. Max. coverage (+): 0. Max coverage (-): 0.07

Region: NODE\_372995\_length\_1546\_cov\_22.922380 414-416. Max. coverage (+): 0. Max coverage (-): 0

Region: NODE\_372995\_length\_1546\_cov\_22.922380 417-419. Max. coverage (+): 0. Max coverage (-): 0

Region: NODE\_372995\_length\_1546\_cov\_22.922380 420-422. Max. coverage (+): 0. Max coverage (-): 0

Region: NODE\_372995\_length\_1546\_cov\_22.922380 423-425. Max. coverage (+): 0.01. Max coverage (-): 0

Region: NODE\_372995\_length\_1546\_cov\_22.922380 426-429. Max. coverage (+): 0.11. Max coverage (-): 0

Region: NODE\_372995\_length\_1546\_cov\_22.922380 430-432. Max. coverage (+): 0. Max coverage (-): 0

Region: NODE\_372995\_length\_1546\_cov\_22.922380 433-435. Max. coverage (+): 0. Max coverage (-): 0

Region: NODE\_372995\_length\_1546\_cov\_22.922380 436-438. Max. coverage (+): 0. Max coverage (-): 0

Region: NODE\_372995\_length\_1546\_cov\_22.922380 439-441. Max. coverage (+): 0. Max coverage (-): 0

Region: NODE\_372995\_length\_1546\_cov\_22.922380 442-445. Max. coverage (+): 0. Max coverage (-): 0

Region: NODE\_372995\_length\_1546\_cov\_22.922380 446-448. Max. coverage (+): 0. Max coverage (-): 0

Region: NODE\_372995\_length\_1546\_cov\_22.922380 449-451. Max. coverage (+): 0.02. Max coverage (-): 0

Region: NODE\_372995\_length\_1546\_cov\_22.922380 452-454. Max. coverage (+): 0.01. Max coverage (-): 0

Region: NODE\_372995\_length\_1546\_cov\_22.922380 455-457. Max. coverage (+): 0.01. Max coverage (-): 0

Region: NODE\_372995\_length\_1546\_cov\_22.922380 458-461. Max. coverage (+): 0.12. Max coverage (-): 0

Region: NODE\_372995\_length\_1546\_cov\_22.922380 462-464. Max. coverage (+): 0.46. Max coverage (-): 0

Region: NODE\_372995\_length\_1546\_cov\_22.922380 465-467. Max. coverage (+): 0.38. Max coverage (-): 0

Region: NODE\_372995\_length\_1546\_cov\_22.922380 468-470. Max. coverage (+): 0. Max coverage (-): 0

Region: NODE\_372995\_length\_1546\_cov\_22.922380 471-473. Max. coverage (+): 0.23. Max coverage (-): 0.08

Region: NODE\_372995\_length\_1546\_cov\_22.922380 474-477. Max. coverage (+): 0.46. Max coverage (-): 0.08

Region: NODE\_372995\_length\_1546\_cov\_22.922380 478-480. Max. coverage (+): 0.38. Max coverage (-): 0.15

Region: NODE\_372995\_length\_1546\_cov\_22.922380 481-483. Max. coverage (+): 0. Max coverage (-): 0.08

Region: NODE\_372995\_length\_1546\_cov\_22.922380 484-486. Max. coverage (+): 0. Max coverage (-): 0

Region: NODE\_372995\_length\_1546\_cov\_22.922380 487-489. Max. coverage (+): 0. Max coverage (-): 0

Region: NODE\_372995\_length\_1546\_cov\_22.922380 490-492. Max. coverage (+): 0.02. Max coverage (-): 0.02

Region: NODE\_372995\_length\_1546\_cov\_22.922380 493-496. Max. coverage (+): 0.05. Max coverage (-): 0.03

Region: NODE\_372995\_length\_1546\_cov\_22.922380 497-499. Max. coverage (+): 0.08. Max coverage (-): 0

Region: NODE\_372995\_length\_1546\_cov\_22.922380 500-502. Max. coverage (+): 0.55. Max coverage (-): 0.04

Region: NODE\_372995\_length\_1546\_cov\_22.922380 503-505. Max. coverage (+): 1.3. Max coverage (-): 0.08

Region: NODE\_372995\_length\_1546\_cov\_22.922380 506-508. Max. coverage (+): 0.61. Max coverage (-): 0.08

Region: NODE\_372995\_length\_1546\_cov\_22.922380 509-512. Max. coverage (+): 1.38. Max coverage (-): 0

Region: NODE\_372995\_length\_1546\_cov\_22.922380 513-515. Max. coverage (+): 0. Max coverage (-): 0

Region: NODE\_372995\_length\_1546\_cov\_22.922380 516-518. Max. coverage (+): 0. Max coverage (-): 0

Region: NODE\_372995\_length\_1546\_cov\_22.922380 519-521. Max. coverage (+): 0. Max coverage (-): 0

Region: NODE\_372995\_length\_1546\_cov\_22.922380 522-524. Max. coverage (+): 0. Max coverage (-): 0

Region: NODE\_372995\_length\_1546\_cov\_22.922380 525-528. Max. coverage (+): 0. Max coverage (-): 0

Region: NODE\_372995\_length\_1546\_cov\_22.922380 529-531. Max. coverage (+): 0. Max coverage (-): 0

Region: NODE\_372995\_length\_1546\_cov\_22.922380 532-534. Max. coverage (+): 0. Max coverage (-): 0

Region: NODE\_372995\_length\_1546\_cov\_22.922380 535-537. Max. coverage (+): 0. Max coverage (-): 0

Region: NODE\_372995\_length\_1546\_cov\_22.922380 538-540. Max. coverage (+): 0. Max coverage (-): 0

Region: NODE\_372995\_length\_1546\_cov\_22.922380 541-544. Max. coverage (+): 0. Max coverage (-): 0

Region: NODE\_372995\_length\_1546\_cov\_22.922380 545-547. Max. coverage (+): 0. Max coverage (-): 0

Region: NODE\_372995\_length\_1546\_cov\_22.922380 548-550. Max. coverage (+): 0. Max coverage (-): 0

Region: NODE\_372995\_length\_1546\_cov\_22.922380 551-553. Max. coverage (+): 0. Max coverage (-): 0

Region: NODE\_372995\_length\_1546\_cov\_22.922380 554-556. Max. coverage (+): 0. Max coverage (-): 0

Region: NODE\_372995\_length\_1546\_cov\_22.922380 557-560. Max. coverage (+): 0. Max coverage (-): 0

Region: NODE\_372995\_length\_1546\_cov\_22.922380 561-563. Max. coverage (+): 0. Max coverage (-): 0

Region: NODE\_372995\_length\_1546\_cov\_22.922380 564-566. Max. coverage (+): 0.04. Max coverage (-): 0

Region: NODE\_372995\_length\_1546\_cov\_22.922380 567-569. Max. coverage (+): 0.04. Max coverage (-): 0

Region: NODE\_372995\_length\_1546\_cov\_22.922380 570-572. Max. coverage (+): 0. Max coverage (-): 0

Region: NODE\_372995\_length\_1546\_cov\_22.922380 573-576. Max. coverage (+): 0. Max coverage (-): 0.05

Region: NODE\_372995\_length\_1546\_cov\_22.922380 577-579. Max. coverage (+): 0. Max coverage (-): 0.05

Region: NODE\_372995\_length\_1546\_cov\_22.922380 580-582. Max. coverage (+): 0. Max coverage (-): 0

Region: NODE\_372995\_length\_1546\_cov\_22.922380 583-585. Max. coverage (+): 0. Max coverage (-): 0

Region: NODE\_372995\_length\_1546\_cov\_22.922380 586-588. Max. coverage (+): 0. Max coverage (-): 0.01

Region: NODE\_372995\_length\_1546\_cov\_22.922380 589-592. Max. coverage (+): 0. Max coverage (-): 0.01

Region: NODE\_372995\_length\_1546\_cov\_22.922380 593-595. Max. coverage (+): 0. Max coverage (-): 0

Region: NODE\_372995\_length\_1546\_cov\_22.922380 596-598. Max. coverage (+): 0. Max coverage (-): 0.01

Region: NODE\_372995\_length\_1546\_cov\_22.922380 599-601. Max. coverage (+): 0. Max coverage (-): 0.02

Region: NODE\_372995\_length\_1546\_cov\_22.922380 602-604. Max. coverage (+): 0. Max coverage (-): 0.02

Region: NODE\_372995\_length\_1546\_cov\_22.922380 605-608. Max. coverage (+): 0.08. Max coverage (-): 0

Region: NODE\_372995\_length\_1546\_cov\_22.922380 609-611. Max. coverage (+): 0.23. Max coverage (-): 0

Region: NODE\_372995\_length\_1546\_cov\_22.922380 612-614. Max. coverage (+): 0.23. Max coverage (-): 0.08

Region: NODE\_372995\_length\_1546\_cov\_22.922380 615-617. Max. coverage (+): 0.61. Max coverage (-): 0

Region: NODE\_372995\_length\_1546\_cov\_22.922380 618-620. Max. coverage (+): 0.61. Max coverage (-): 0

Region: NODE\_372995\_length\_1546\_cov\_22.922380 621-624. Max. coverage (+): 0.08. Max coverage (-): 0

Region: NODE\_372995\_length\_1546\_cov\_22.922380 625-627. Max. coverage (+): 0. Max coverage (-): 0

Region: NODE\_372995\_length\_1546\_cov\_22.922380 628-630. Max. coverage (+): 0. Max coverage (-): 0.08

Region: NODE\_372995\_length\_1546\_cov\_22.922380 631-633. Max. coverage (+): 0.08. Max coverage (-): 0.23

Region: NODE\_372995\_length\_1546\_cov\_22.922380 634-636. Max. coverage (+): 0. Max coverage (-): 0.31

Region: NODE\_372995\_length\_1546\_cov\_22.922380 637-640. Max. coverage (+): 0. Max coverage (-): 0.46

Region: NODE\_372995\_length\_1546\_cov\_22.922380 641-643. Max. coverage (+): 0. Max coverage (-): 0.23

Region: NODE\_372995\_length\_1546\_cov\_22.922380 644-646. Max. coverage (+): 0.23. Max coverage (-): 0

Region: NODE\_372995\_length\_1546\_cov\_22.922380 647-649. Max. coverage (+): 0.31. Max coverage (-): 0

Region: NODE\_372995\_length\_1546\_cov\_22.922380 650-652. Max. coverage (+): 3.37. Max coverage (-): 0

Region: NODE\_372995\_length\_1546\_cov\_22.922380 653-655. Max. coverage (+): 3.37. Max coverage (-): 0

Region: NODE\_372995\_length\_1546\_cov\_22.922380 656-659. Max. coverage (+): 3.22. Max coverage (-): 0

Region: NODE\_372995\_length\_1546\_cov\_22.922380 660-662. Max. coverage (+): 0. Max coverage (-): 0

Region: NODE\_372995\_length\_1546\_cov\_22.922380 663-665. Max. coverage (+): 0. Max coverage (-): 0

Region: NODE\_372995\_length\_1546\_cov\_22.922380 666-668. Max. coverage (+): 0.08. Max coverage (-): 0

Region: NODE\_372995\_length\_1546\_cov\_22.922380 669-671. Max. coverage (+): 0. Max coverage (-): 0

Region: NODE\_372995\_length\_1546\_cov\_22.922380 672-675. Max. coverage (+): 0. Max coverage (-): 0

Region: NODE\_372995\_length\_1546\_cov\_22.922380 676-678. Max. coverage (+): 0. Max coverage (-): 0

Region: NODE\_372995\_length\_1546\_cov\_22.922380 679-681. Max. coverage (+): 0. Max coverage (-): 0

Region: NODE\_372995\_length\_1546\_cov\_22.922380 682-684. Max. coverage (+): 0. Max coverage (-): 0

Region: NODE\_372995\_length\_1546\_cov\_22.922380 685-687. Max. coverage (+): 0. Max coverage (-): 0.08

Region: NODE\_372995\_length\_1546\_cov\_22.922380 688-691. Max. coverage (+): 0.08. Max coverage (-): 0.08

Region: NODE\_372995\_length\_1546\_cov\_22.922380 692-694. Max. coverage (+): 0.08. Max coverage (-): 0

Region: NODE\_372995\_length\_1546\_cov\_22.922380 695-697. Max. coverage (+): 0. Max coverage (-): 0

Region: NODE\_372995\_length\_1546\_cov\_22.922380 698-700. Max. coverage (+): 0. Max coverage (-): 0

Region: NODE\_372995\_length\_1546\_cov\_22.922380 701-703. Max. coverage (+): 0. Max coverage (-): 0

Region: NODE\_372995\_length\_1546\_cov\_22.922380 704-707. Max. coverage (+): 0. Max coverage (-): 0

Region: NODE\_372995\_length\_1546\_cov\_22.922380 708-710. Max. coverage (+): 0. Max coverage (-): 0

Region: NODE\_372995\_length\_1546\_cov\_22.922380 711-713. Max. coverage (+): 0. Max coverage (-): 0

Region: NODE\_372995\_length\_1546\_cov\_22.922380 714-716. Max. coverage (+): 3.06. Max coverage (-): 0

Region: NODE\_372995\_length\_1546\_cov\_22.922380 717-719. Max. coverage (+): 3.06. Max coverage (-): 0

Region: NODE\_372995\_length\_1546\_cov\_22.922380 720-723. Max. coverage (+): 0.08. Max coverage (-): 0

Region: NODE\_372995\_length\_1546\_cov\_22.922380 724-726. Max. coverage (+): 0. Max coverage (-): 0

Region: NODE\_372995\_length\_1546\_cov\_22.922380 727-729. Max. coverage (+): 0. Max coverage (-): 0

Region: NODE\_372995\_length\_1546\_cov\_22.922380 730-732. Max. coverage (+): 0. Max coverage (-): 0

Region: NODE\_372995\_length\_1546\_cov\_22.922380 733-735. Max. coverage (+): 0.08. Max coverage (-): 0

Region: NODE\_372995\_length\_1546\_cov\_22.922380 736-739. Max. coverage (+): 0.15. Max coverage (-): 0.08

Region: NODE\_372995\_length\_1546\_cov\_22.922380 740-742. Max. coverage (+): 0.15. Max coverage (-): 0.08

Region: NODE\_372995\_length\_1546\_cov\_22.922380 743-745. Max. coverage (+): 0.08. Max coverage (-): 0

Region: NODE\_372995\_length\_1546\_cov\_22.922380 746-748. Max. coverage (+): 0.08. Max coverage (-): 0

Region: NODE\_372995\_length\_1546\_cov\_22.922380 749-751. Max. coverage (+): 0.08. Max coverage (-): 0

Region: NODE\_372995\_length\_1546\_cov\_22.922380 752-755. Max. coverage (+): 0.61. Max coverage (-): 0

Region: NODE\_372995\_length\_1546\_cov\_22.922380 756-758. Max. coverage (+): 0.61. Max coverage (-): 0

Region: NODE\_372995\_length\_1546\_cov\_22.922380 759-761. Max. coverage (+): 0. Max coverage (-): 0

Region: NODE\_372995\_length\_1546\_cov\_22.922380 762-764. Max. coverage (+): 0. Max coverage (-): 0

Region: NODE\_372995\_length\_1546\_cov\_22.922380 765-767. Max. coverage (+): 0. Max coverage (-): 0

Region: NODE\_372995\_length\_1546\_cov\_22.922380 768-771. Max. coverage (+): 0. Max coverage (-): 0

Region: NODE\_372995\_length\_1546\_cov\_22.922380 772-774. Max. coverage (+): 0. Max coverage (-): 0

Region: NODE\_372995\_length\_1546\_cov\_22.922380 775-777. Max. coverage (+): 0. Max coverage (-): 0

Region: NODE\_372995\_length\_1546\_cov\_22.922380 778-780. Max. coverage (+): 0. Max coverage (-): 0.69

Region: NODE\_372995\_length\_1546\_cov\_22.922380 781-783. Max. coverage (+): 0. Max coverage (-): 0.77

Region: NODE\_372995\_length\_1546\_cov\_22.922380 784-787. Max. coverage (+): 0. Max coverage (-): 0

Region: NODE\_372995\_length\_1546\_cov\_22.922380 788-790. Max. coverage (+): 0. Max coverage (-): 0

Region: NODE\_372995\_length\_1546\_cov\_22.922380 791-793. Max. coverage (+): 0.15. Max coverage (-): 0

Region: NODE\_372995\_length\_1546\_cov\_22.922380 794-796. Max. coverage (+): 0.31. Max coverage (-): 0

Region: NODE\_372995\_length\_1546\_cov\_22.922380 797-799. Max. coverage (+): 0.38. Max coverage (-): 0

Region: NODE\_372995\_length\_1546\_cov\_22.922380 800-803. Max. coverage (+): 101.36. Max coverage (-): 0

Region: NODE\_372995\_length\_1546\_cov\_22.922380 804-806. Max. coverage (+): 2.15. Max coverage (-): 0

Region: NODE\_372995\_length\_1546\_cov\_22.922380 807-809. Max. coverage (+): 0.84. Max coverage (-): 0

Region: NODE\_372995\_length\_1546\_cov\_22.922380 810-812. Max. coverage (+): 0. Max coverage (-): 0

Region: NODE\_372995\_length\_1546\_cov\_22.922380 813-815. Max. coverage (+): 0. Max coverage (-): 0

Region: NODE\_372995\_length\_1546\_cov\_22.922380 816-818. Max. coverage (+): 0. Max coverage (-): 0

Region: NODE\_372995\_length\_1546\_cov\_22.922380 819-822. Max. coverage (+): 0. Max coverage (-): 0

Region: NODE\_372995\_length\_1546\_cov\_22.922380 823-825. Max. coverage (+): 0. Max coverage (-): 0

Region: NODE\_372995\_length\_1546\_cov\_22.922380 826-828. Max. coverage (+): 0. Max coverage (-): 0

Region: NODE\_372995\_length\_1546\_cov\_22.922380 829-831. Max. coverage (+): 0. Max coverage (-): 0

Region: NODE\_372995\_length\_1546\_cov\_22.922380 832-834. Max. coverage (+): 0. Max coverage (-): 0

Region: NODE\_372995\_length\_1546\_cov\_22.922380 835-838. Max. coverage (+): 0.31. Max coverage (-): 0

Region: NODE\_372995\_length\_1546\_cov\_22.922380 839-841. Max. coverage (+): 0.84. Max coverage (-): 0

Region: NODE\_372995\_length\_1546\_cov\_22.922380 842-844. Max. coverage (+): 0.46. Max coverage (-): 0

Region: NODE\_372995\_length\_1546\_cov\_22.922380 845-847. Max. coverage (+): 1.15. Max coverage (-): 0

Region: NODE\_372995\_length\_1546\_cov\_22.922380 848-850. Max. coverage (+): 0.92. Max coverage (-): 0

Region: NODE\_372995\_length\_1546\_cov\_22.922380 851-854. Max. coverage (+): 0.31. Max coverage (-): 0

Region: NODE\_372995\_length\_1546\_cov\_22.922380 855-857. Max. coverage (+): 0. Max coverage (-): 0

Region: NODE\_372995\_length\_1546\_cov\_22.922380 858-860. Max. coverage (+): 0.69. Max coverage (-): 0

Region: NODE\_372995\_length\_1546\_cov\_22.922380 861-863. Max. coverage (+): 0.69. Max coverage (-): 0.23

Region: NODE\_372995\_length\_1546\_cov\_22.922380 864-866. Max. coverage (+): 0. Max coverage (-): 0.15

Region: NODE\_372995\_length\_1546\_cov\_22.922380 867-870. Max. coverage (+): 0. Max coverage (-): 0.08

Region: NODE\_372995\_length\_1546\_cov\_22.922380 871-873. Max. coverage (+): 0. Max coverage (-): 0

Region: NODE\_372995\_length\_1546\_cov\_22.922380 874-876. Max. coverage (+): 0.08. Max coverage (-): 0

Region: NODE\_372995\_length\_1546\_cov\_22.922380 877-879. Max. coverage (+): 0.31. Max coverage (-): 0

Region: NODE\_372995\_length\_1546\_cov\_22.922380 880-882. Max. coverage (+): 0.31. Max coverage (-): 0.15

Region: NODE\_372995\_length\_1546\_cov\_22.922380 883-886. Max. coverage (+): 1.3. Max coverage (-): 0.15

Region: NODE\_372995\_length\_1546\_cov\_22.922380 887-889. Max. coverage (+): 0. Max coverage (-): 0

Region: NODE\_372995\_length\_1546\_cov\_22.922380 890-892. Max. coverage (+): 0.08. Max coverage (-): 0

Region: NODE\_372995\_length\_1546\_cov\_22.922380 893-895. Max. coverage (+): 0.08. Max coverage (-): 0

Region: NODE\_372995\_length\_1546\_cov\_22.922380 896-898. Max. coverage (+): 0.15. Max coverage (-): 0

Region: NODE\_372995\_length\_1546\_cov\_22.922380 899-902. Max. coverage (+): 0.23. Max coverage (-): 0

Region: NODE\_372995\_length\_1546\_cov\_22.922380 903-905. Max. coverage (+): 0. Max coverage (-): 0

Region: NODE\_372995\_length\_1546\_cov\_22.922380 906-908. Max. coverage (+): 0. Max coverage (-): 0

Region: NODE\_372995\_length\_1546\_cov\_22.922380 909-911. Max. coverage (+): 0. Max coverage (-): 0

Region: NODE\_372995\_length\_1546\_cov\_22.922380 912-914. Max. coverage (+): 0.08. Max coverage (-): 0

Region: NODE\_372995\_length\_1546\_cov\_22.922380 915-918. Max. coverage (+): 0.08. Max coverage (-): 0.38

Region: NODE\_372995\_length\_1546\_cov\_22.922380 919-921. Max. coverage (+): 0.08. Max coverage (-): 0.08

Region: NODE\_372995\_length\_1546\_cov\_22.922380 922-924. Max. coverage (+): 0.84. Max coverage (-): 0

Region: NODE\_372995\_length\_1546\_cov\_22.922380 925-927. Max. coverage (+): 1.92. Max coverage (-): 0

Region: NODE\_372995\_length\_1546\_cov\_22.922380 928-930. Max. coverage (+): 0.92. Max coverage (-): 0

Region: NODE\_372995\_length\_1546\_cov\_22.922380 931-934. Max. coverage (+): 0.92. Max coverage (-): 0

Region: NODE\_372995\_length\_1546\_cov\_22.922380 935-937. Max. coverage (+): 0.92. Max coverage (-): 0

Region: NODE\_372995\_length\_1546\_cov\_22.922380 938-940. Max. coverage (+): 0.08. Max coverage (-): 0

Region: NODE\_372995\_length\_1546\_cov\_22.922380 941-943. Max. coverage (+): 0.08. Max coverage (-): 0

Region: NODE\_372995\_length\_1546\_cov\_22.922380 944-946. Max. coverage (+): 0. Max coverage (-): 0.08

Region: NODE\_372995\_length\_1546\_cov\_22.922380 947-950. Max. coverage (+): 0.08. Max coverage (-): 0.08

Region: NODE\_372995\_length\_1546\_cov\_22.922380 951-953. Max. coverage (+): 0.08. Max coverage (-): 0

Region: NODE\_372995\_length\_1546\_cov\_22.922380 954-956. Max. coverage (+): 0. Max coverage (-): 0

Region: NODE\_372995\_length\_1546\_cov\_22.922380 957-959. Max. coverage (+): 0.77. Max coverage (-): 0.08

Region: NODE\_372995\_length\_1546\_cov\_22.922380 960-962. Max. coverage (+): 5.75. Max coverage (-): 0.08

Region: NODE\_372995\_length\_1546\_cov\_22.922380 963-966. Max. coverage (+): 11.11. Max coverage (-): 0

Region: NODE\_372995\_length\_1546\_cov\_22.922380 967-969. Max. coverage (+): 1.15. Max coverage (-): 0.08

Region: NODE\_372995\_length\_1546\_cov\_22.922380 970-972. Max. coverage (+): 0. Max coverage (-): 0.08

Region: NODE\_372995\_length\_1546\_cov\_22.922380 973-975. Max. coverage (+): 0. Max coverage (-): 0.15

Region: NODE\_372995\_length\_1546\_cov\_22.922380 976-978. Max. coverage (+): 0.15. Max coverage (-): 0.08

Region: NODE\_372995\_length\_1546\_cov\_22.922380 979-981. Max. coverage (+): 0.15. Max coverage (-): 0.31

Region: NODE\_372995\_length\_1546\_cov\_22.922380 982-985. Max. coverage (+): 0. Max coverage (-): 3.37

Region: NODE\_372995\_length\_1546\_cov\_22.922380 986-988. Max. coverage (+): 0.08. Max coverage (-): 3.14

Region: NODE\_372995\_length\_1546\_cov\_22.922380 989-991. Max. coverage (+): 0.69. Max coverage (-): 0

Region: NODE\_372995\_length\_1546\_cov\_22.922380 992-994. Max. coverage (+): 0.54. Max coverage (-): 0

Region: NODE\_372995\_length\_1546\_cov\_22.922380 995-997. Max. coverage (+): 0.54. Max coverage (-): 0

Region: NODE\_372995\_length\_1546\_cov\_22.922380 998-1001. Max. coverage (+): 0.08. Max coverage (-): 0.08

Region: NODE\_372995\_length\_1546\_cov\_22.922380 1002-1004. Max. coverage (+): 2.3. Max coverage (-): 0

Region: NODE\_372995\_length\_1546\_cov\_22.922380 1005-1007. Max. coverage (+): 3.37. Max coverage (-): 0

Region: NODE\_372995\_length\_1546\_cov\_22.922380 1008-1010. Max. coverage (+): 1.38. Max coverage (-): 0.08

Region: NODE\_372995\_length\_1546\_cov\_22.922380 1011-1013. Max. coverage (+): 0.23. Max coverage (-): 0.08

Region: NODE\_372995\_length\_1546\_cov\_22.922380 1014-1017. Max. coverage (+): 4.37. Max coverage (-): 0

Region: NODE\_372995\_length\_1546\_cov\_22.922380 1018-1020. Max. coverage (+): 0.84. Max coverage (-): 0

Region: NODE\_372995\_length\_1546\_cov\_22.922380 1021-1023. Max. coverage (+): 0.31. Max coverage (-): 0

Region: NODE\_372995\_length\_1546\_cov\_22.922380 1024-1026. Max. coverage (+): 0.31. Max coverage (-): 0

Region: NODE\_372995\_length\_1546\_cov\_22.922380 1027-1029. Max. coverage (+): 3.22. Max coverage (-): 0

Region: NODE\_372995\_length\_1546\_cov\_22.922380 1030-1033. Max. coverage (+): 4.37. Max coverage (-): 0.92

Region: NODE\_372995\_length\_1546\_cov\_22.922380 1034-1036. Max. coverage (+): 2.38. Max coverage (-): 0.92

Region: NODE\_372995\_length\_1546\_cov\_22.922380 1037-1039. Max. coverage (+): 2.38. Max coverage (-): 0

Region: NODE\_372995\_length\_1546\_cov\_22.922380 1040-1042. Max. coverage (+): 0.08. Max coverage (-): 0

Region: NODE\_372995\_length\_1546\_cov\_22.922380 1043-1045. Max. coverage (+): 0.08. Max coverage (-): 0.15

Region: NODE\_372995\_length\_1546\_cov\_22.922380 1046-1049. Max. coverage (+): 0.31. Max coverage (-): 0.15

Region: NODE\_372995\_length\_1546\_cov\_22.922380 1050-1052. Max. coverage (+): 1.46. Max coverage (-): 0

Region: NODE\_372995\_length\_1546\_cov\_22.922380 1053-1055. Max. coverage (+): 1.38. Max coverage (-): 0

Region: NODE\_372995\_length\_1546\_cov\_22.922380 1056-1058. Max. coverage (+): 2.22. Max coverage (-): 0

Region: NODE\_372995\_length\_1546\_cov\_22.922380 1059-1061. Max. coverage (+): 8.27. Max coverage (-): 0.31

Region: NODE\_372995\_length\_1546\_cov\_22.922380 1062-1065. Max. coverage (+): 11.95. Max coverage (-): 0.38

Region: NODE\_372995\_length\_1546\_cov\_22.922380 1066-1068. Max. coverage (+): 4.83. Max coverage (-): 0.46

Region: NODE\_372995\_length\_1546\_cov\_22.922380 1069-1071. Max. coverage (+): 0.15. Max coverage (-): 0.46

Region: NODE\_372995\_length\_1546\_cov\_22.922380 1072-1074. Max. coverage (+): 0.15. Max coverage (-): 0.23

Region: NODE\_372995\_length\_1546\_cov\_22.922380 1075-1077. Max. coverage (+): 0. Max coverage (-): 0

Region: NODE\_372995\_length\_1546\_cov\_22.922380 1078-1081. Max. coverage (+): 0.84. Max coverage (-): 0

Region: NODE\_372995\_length\_1546\_cov\_22.922380 1082-1084. Max. coverage (+): 0. Max coverage (-): 0.08

Region: NODE\_372995\_length\_1546\_cov\_22.922380 1085-1087. Max. coverage (+): 0.08. Max coverage (-): 0.08

Region: NODE\_372995\_length\_1546\_cov\_22.922380 1088-1090. Max. coverage (+): 4.83. Max coverage (-): 0

Region: NODE\_372995\_length\_1546\_cov\_22.922380 1091-1093. Max. coverage (+): 29.34. Max coverage (-): 0.08

Region: NODE\_372995\_length\_1546\_cov\_22.922380 1094-1097. Max. coverage (+): 24.59. Max coverage (-): 0.15

Region: NODE\_372995\_length\_1546\_cov\_22.922380 1098-1100. Max. coverage (+): 1.84. Max coverage (-): 0

Region: NODE\_372995\_length\_1546\_cov\_22.922380 1101-1103. Max. coverage (+): 2.15. Max coverage (-): 0.15

Region: NODE\_372995\_length\_1546\_cov\_22.922380 1104-1106. Max. coverage (+): 0.31. Max coverage (-): 0.23

Region: NODE\_372995\_length\_1546\_cov\_22.922380 1107-1109. Max. coverage (+): 7.66. Max coverage (-): 0.15

Region: NODE\_372995\_length\_1546\_cov\_22.922380 1110-1113. Max. coverage (+): 8.81. Max coverage (-): 0.15

Region: NODE\_372995\_length\_1546\_cov\_22.922380 1114-1116. Max. coverage (+): 3.52. Max coverage (-): 0.23

Region: NODE\_372995\_length\_1546\_cov\_22.922380 1117-1119. Max. coverage (+): 1. Max coverage (-): 0.31

Region: NODE\_372995\_length\_1546\_cov\_22.922380 1120-1122. Max. coverage (+): 0.31. Max coverage (-): 0.31

Region: NODE\_372995\_length\_1546\_cov\_22.922380 1123-1125. Max. coverage (+): 0.23. Max coverage (-): 0

Region: NODE\_372995\_length\_1546\_cov\_22.922380 1126-1129. Max. coverage (+): 0.38. Max coverage (-): 0

Region: NODE\_372995\_length\_1546\_cov\_22.922380 1130-1132. Max. coverage (+): 2.07. Max coverage (-): 0

Region: NODE\_372995\_length\_1546\_cov\_22.922380 1133-1135. Max. coverage (+): 2.07. Max coverage (-): 0

Region: NODE\_372995\_length\_1546\_cov\_22.922380 1136-1138. Max. coverage (+): 0.31. Max coverage (-): 0

Region: NODE\_372995\_length\_1546\_cov\_22.922380 1139-1141. Max. coverage (+): 0.38. Max coverage (-): 0

Region: NODE\_372995\_length\_1546\_cov\_22.922380 1142-1144. Max. coverage (+): 0.15. Max coverage (-): 0

Region: NODE\_372995\_length\_1546\_cov\_22.922380 1145-1148. Max. coverage (+): 0. Max coverage (-): 0

Region: NODE\_372995\_length\_1546\_cov\_22.922380 1149-1151. Max. coverage (+): 0. Max coverage (-): 0

Region: NODE\_372995\_length\_1546\_cov\_22.922380 1152-1154. Max. coverage (+): 0.08. Max coverage (-): 0

Region: NODE\_372995\_length\_1546\_cov\_22.922380 1155-1157. Max. coverage (+): 0.31. Max coverage (-): 0

Region: NODE\_372995\_length\_1546\_cov\_22.922380 1158-1160. Max. coverage (+): 5.06. Max coverage (-): 0

Region: NODE\_372995\_length\_1546\_cov\_22.922380 1161-1164. Max. coverage (+): 7.58. Max coverage (-): 0

Region: NODE\_372995\_length\_1546\_cov\_22.922380 1165-1167. Max. coverage (+): 0.46. Max coverage (-): 0

Region: NODE\_372995\_length\_1546\_cov\_22.922380 1168-1170. Max. coverage (+): 0.08. Max coverage (-): 0.08

Region: NODE\_372995\_length\_1546\_cov\_22.922380 1171-1173. Max. coverage (+): 0.38. Max coverage (-): 0.08

Region: NODE\_372995\_length\_1546\_cov\_22.922380 1174-1176. Max. coverage (+): 0.31. Max coverage (-): 0

Region: NODE\_372995\_length\_1546\_cov\_22.922380 1177-1180. Max. coverage (+): 0. Max coverage (-): 0

Region: NODE\_372995\_length\_1546\_cov\_22.922380 1181-1183. Max. coverage (+): 1.53. Max coverage (-): 0

Region: NODE\_372995\_length\_1546\_cov\_22.922380 1184-1186. Max. coverage (+): 2.15. Max coverage (-): 0

Region: NODE\_372995\_length\_1546\_cov\_22.922380 1187-1189. Max. coverage (+): 0.77. Max coverage (-): 0

Region: NODE\_372995\_length\_1546\_cov\_22.922380 1190-1192. Max. coverage (+): 0.69. Max coverage (-): 0

Region: NODE\_372995\_length\_1546\_cov\_22.922380 1193-1196. Max. coverage (+): 0.31. Max coverage (-): 0

Region: NODE\_372995\_length\_1546\_cov\_22.922380 1197-1199. Max. coverage (+): 0. Max coverage (-): 0

Region: NODE\_372995\_length\_1546\_cov\_22.922380 1200-1202. Max. coverage (+): 0. Max coverage (-): 0

Region: NODE\_372995\_length\_1546\_cov\_22.922380 1203-1205. Max. coverage (+): 0. Max coverage (-): 0

Region: NODE\_372995\_length\_1546\_cov\_22.922380 1206-1208. Max. coverage (+): 0.08. Max coverage (-): 0

Region: NODE\_372995\_length\_1546\_cov\_22.922380 1209-1212. Max. coverage (+): 0.15. Max coverage (-): 0

Region: NODE\_372995\_length\_1546\_cov\_22.922380 1213-1215. Max. coverage (+): 2.15. Max coverage (-): 0

Region: NODE\_372995\_length\_1546\_cov\_22.922380 1216-1218. Max. coverage (+): 8.35. Max coverage (-): 0

Region: NODE\_372995\_length\_1546\_cov\_22.922380 1219-1221. Max. coverage (+): 8.43. Max coverage (-): 0

Region: NODE\_372995\_length\_1546\_cov\_22.922380 1222-1224. Max. coverage (+): 0.46. Max coverage (-): 0

Region: NODE\_372995\_length\_1546\_cov\_22.922380 1225-1228. Max. coverage (+): 0.46. Max coverage (-): 0

Region: NODE\_372995\_length\_1546\_cov\_22.922380 1229-1231. Max. coverage (+): 0. Max coverage (-): 0

Region: NODE\_372995\_length\_1546\_cov\_22.922380 1232-1234. Max. coverage (+): 0. Max coverage (-): 0

Region: NODE\_372995\_length\_1546\_cov\_22.922380 1235-1237. Max. coverage (+): 0. Max coverage (-): 0

Region: NODE\_372995\_length\_1546\_cov\_22.922380 1238-1240. Max. coverage (+): 0. Max coverage (-): 0

Region: NODE\_372995\_length\_1546\_cov\_22.922380 1241-1244. Max. coverage (+): 0. Max coverage (-): 0

Region: NODE\_372995\_length\_1546\_cov\_22.922380 1245-1247. Max. coverage (+): 0. Max coverage (-): 0

Region: NODE\_372995\_length\_1546\_cov\_22.922380 1248-1250. Max. coverage (+): 0. Max coverage (-): 0

Region: NODE\_372995\_length\_1546\_cov\_22.922380 1251-1253. Max. coverage (+): 0. Max coverage (-): 0

Region: NODE\_372995\_length\_1546\_cov\_22.922380 1254-1256. Max. coverage (+): 0. Max coverage (-): 0

Region: NODE\_372995\_length\_1546\_cov\_22.922380 1257-1260. Max. coverage (+): 0. Max coverage (-): 0

Region: NODE\_372995\_length\_1546\_cov\_22.922380 1261-1263. Max. coverage (+): 0. Max coverage (-): 0

Region: NODE\_372995\_length\_1546\_cov\_22.922380 1264-1266. Max. coverage (+): 0. Max coverage (-): 0

Region: NODE\_372995\_length\_1546\_cov\_22.922380 1267-1269. Max. coverage (+): 0. Max coverage (-): 0.15

Region: NODE\_372995\_length\_1546\_cov\_22.922380 1270-1272. Max. coverage (+): 0.08. Max coverage (-): 0.15

Region: NODE\_372995\_length\_1546\_cov\_22.922380 1273-1276. Max. coverage (+): 1.23. Max coverage (-): 0

Region: NODE\_372995\_length\_1546\_cov\_22.922380 1277-1279. Max. coverage (+): 1.23. Max coverage (-): 0

Region: NODE\_372995\_length\_1546\_cov\_22.922380 1280-1282. Max. coverage (+): 0.15. Max coverage (-): 0

Region: NODE\_372995\_length\_1546\_cov\_22.922380 1283-1285. Max. coverage (+): 0.15. Max coverage (-): 0

Region: NODE\_372995\_length\_1546\_cov\_22.922380 1286-1288. Max. coverage (+): 0. Max coverage (-): 0

Region: NODE\_372995\_length\_1546\_cov\_22.922380 1289-1291. Max. coverage (+): 0. Max coverage (-): 0

Region: NODE\_372995\_length\_1546\_cov\_22.922380 1292-1295. Max. coverage (+): 0. Max coverage (-): 0

Region: NODE\_372995\_length\_1546\_cov\_22.922380 1296-1298. Max. coverage (+): 0. Max coverage (-): 0

Region: NODE\_372995\_length\_1546\_cov\_22.922380 1299-1301. Max. coverage (+): 0. Max coverage (-): 0

Region: NODE\_372995\_length\_1546\_cov\_22.922380 1302-1304. Max. coverage (+): 0. Max coverage (-): 0

Region: NODE\_372995\_length\_1546\_cov\_22.922380 1305-1307. Max. coverage (+): 0. Max coverage (-): 0

Region: NODE\_372995\_length\_1546\_cov\_22.922380 1308-1311. Max. coverage (+): 0. Max coverage (-): 0

Region: NODE\_372995\_length\_1546\_cov\_22.922380 1312-1314. Max. coverage (+): 0. Max coverage (-): 0

Region: NODE\_372995\_length\_1546\_cov\_22.922380 1315-1317. Max. coverage (+): 0. Max coverage (-): 0

Region: NODE\_372995\_length\_1546\_cov\_22.922380 1318-1320. Max. coverage (+): 0. Max coverage (-): 0

Region: NODE\_372995\_length\_1546\_cov\_22.922380 1321-1323. Max. coverage (+): 0. Max coverage (-): 0

Region: NODE\_372995\_length\_1546\_cov\_22.922380 1324-1327. Max. coverage (+): 0. Max coverage (-): 0

Region: NODE\_372995\_length\_1546\_cov\_22.922380 1328-1330. Max. coverage (+): 0. Max coverage (-): 0

Region: NODE\_372995\_length\_1546\_cov\_22.922380 1331-1333. Max. coverage (+): 0. Max coverage (-): 0

Region: NODE\_372995\_length\_1546\_cov\_22.922380 1334-1336. Max. coverage (+): 0. Max coverage (-): 0

Region: NODE\_372995\_length\_1546\_cov\_22.922380 1337-1339. Max. coverage (+): 0. Max coverage (-): 0

Region: NODE\_372995\_length\_1546\_cov\_22.922380 1340-1343. Max. coverage (+): 0. Max coverage (-): 0.01

Region: NODE\_372995\_length\_1546\_cov\_22.922380 1344-1346. Max. coverage (+): 0. Max coverage (-): 0.01

Region: NODE\_372995\_length\_1546\_cov\_22.922380 1347-1349. Max. coverage (+): 0. Max coverage (-): 0

Region: NODE\_372995\_length\_1546\_cov\_22.922380 1350-1352. Max. coverage (+): 0. Max coverage (-): 0

Region: NODE\_372995\_length\_1546\_cov\_22.922380 1353-1355. Max. coverage (+): 0. Max coverage (-): 0

Region: NODE\_372995\_length\_1546\_cov\_22.922380 1356-1359. Max. coverage (+): 0.03. Max coverage (-): 0

Region: NODE\_372995\_length\_1546\_cov\_22.922380 1360-1362. Max. coverage (+): 0.03. Max coverage (-): 0

Region: NODE\_372995\_length\_1546\_cov\_22.922380 1363-1365. Max. coverage (+): 0. Max coverage (-): 0

Region: NODE\_372995\_length\_1546\_cov\_22.922380 1366-1368. Max. coverage (+): 0. Max coverage (-): 0

Region: NODE\_372995\_length\_1546\_cov\_22.922380 1369-1371. Max. coverage (+): 0. Max coverage (-): 0

Region: NODE\_372995\_length\_1546\_cov\_22.922380 1372-1375. Max. coverage (+): 0. Max coverage (-): 0

Region: NODE\_372995\_length\_1546\_cov\_22.922380 1376-1378. Max. coverage (+): 0. Max coverage (-): 0

Region: NODE\_372995\_length\_1546\_cov\_22.922380 1379-1381. Max. coverage (+): 0. Max coverage (-): 0

Region: NODE\_372995\_length\_1546\_cov\_22.922380 1382-1384. Max. coverage (+): 0. Max coverage (-): 0

Region: NODE\_372995\_length\_1546\_cov\_22.922380 1385-1387. Max. coverage (+): 0. Max coverage (-): 0

Region: NODE\_372995\_length\_1546\_cov\_22.922380 1388-1391. Max. coverage (+): 0. Max coverage (-): 0

Region: NODE\_372995\_length\_1546\_cov\_22.922380 1392-1394. Max. coverage (+): 0. Max coverage (-): 0

Region: NODE\_372995\_length\_1546\_cov\_22.922380 1395-1397. Max. coverage (+): 0. Max coverage (-): 0

Region: NODE\_372995\_length\_1546\_cov\_22.922380 1398-1400. Max. coverage (+): 0. Max coverage (-): 0

Region: NODE\_372995\_length\_1546\_cov\_22.922380 1401-1403. Max. coverage (+): 0. Max coverage (-): 0

Region: NODE\_372995\_length\_1546\_cov\_22.922380 1404-1407. Max. coverage (+): 0. Max coverage (-): 0

Region: NODE\_372995\_length\_1546\_cov\_22.922380 1408-1410. Max. coverage (+): 0. Max coverage (-): 0

Region: NODE\_372995\_length\_1546\_cov\_22.922380 1411-1413. Max. coverage (+): 0. Max coverage (-): 0

Region: NODE\_372995\_length\_1546\_cov\_22.922380 1414-1416. Max. coverage (+): 0. Max coverage (-): 0

Region: NODE\_372995\_length\_1546\_cov\_22.922380 1417-1419. Max. coverage (+): 0. Max coverage (-): 0

Region: NODE\_372995\_length\_1546\_cov\_22.922380 1420-1423. Max. coverage (+): 0. Max coverage (-): 0

Region: NODE\_372995\_length\_1546\_cov\_22.922380 1424-1426. Max. coverage (+): 0. Max coverage (-): 0

Region: NODE\_372995\_length\_1546\_cov\_22.922380 1427-1429. Max. coverage (+): 0. Max coverage (-): 0

Region: NODE\_372995\_length\_1546\_cov\_22.922380 1430-1432. Max. coverage (+): 0. Max coverage (-): 0

Region: NODE\_372995\_length\_1546\_cov\_22.922380 1433-1435. Max. coverage (+): 0. Max coverage (-): 0

Region: NODE\_372995\_length\_1546\_cov\_22.922380 1436-1439. Max. coverage (+): 0. Max coverage (-): 0

Region: NODE\_372995\_length\_1546\_cov\_22.922380 1440-1442. Max. coverage (+): 0. Max coverage (-): 0

Region: NODE\_372995\_length\_1546\_cov\_22.922380 1443-1445. Max. coverage (+): 0. Max coverage (-): 0

Region: NODE\_372995\_length\_1546\_cov\_22.922380 1446-1448. Max. coverage (+): 0. Max coverage (-): 0

Region: NODE\_372995\_length\_1546\_cov\_22.922380 1449-1451. Max. coverage (+): 0. Max coverage (-): 0

Region: NODE\_372995\_length\_1546\_cov\_22.922380 1452-1454. Max. coverage (+): 0. Max coverage (-): 0

Region: NODE\_372995\_length\_1546\_cov\_22.922380 1455-1458. Max. coverage (+): 0. Max coverage (-): 0

Region: NODE\_372995\_length\_1546\_cov\_22.922380 1459-1461. Max. coverage (+): 0. Max coverage (-): 0

Region: NODE\_372995\_length\_1546\_cov\_22.922380 1462-1464. Max. coverage (+): 0. Max coverage (-): 0

Region: NODE\_372995\_length\_1546\_cov\_22.922380 1465-1467. Max. coverage (+): 0. Max coverage (-): 0

Region: NODE\_372995\_length\_1546\_cov\_22.922380 1468-1470. Max. coverage (+): 0. Max coverage (-): 0

Region: NODE\_372995\_length\_1546\_cov\_22.922380 1471-1474. Max. coverage (+): 0. Max coverage (-): 0

Region: NODE\_372995\_length\_1546\_cov\_22.922380 1475-1477. Max. coverage (+): 0. Max coverage (-): 0

Region: NODE\_372995\_length\_1546\_cov\_22.922380 1478-1480. Max. coverage (+): 0. Max coverage (-): 0

Region: NODE\_372995\_length\_1546\_cov\_22.922380 1481-1483. Max. coverage (+): 0. Max coverage (-): 0

Region: NODE\_372995\_length\_1546\_cov\_22.922380 1484-1486. Max. coverage (+): 0. Max coverage (-): 0

Region: NODE\_372995\_length\_1546\_cov\_22.922380 1487-1490. Max. coverage (+): 0. Max coverage (-): 0

Region: NODE\_372995\_length\_1546\_cov\_22.922380 1491-1493. Max. coverage (+): 0. Max coverage (-): 0

Region: NODE\_372995\_length\_1546\_cov\_22.922380 1494-1496. Max. coverage (+): 0. Max coverage (-): 0

Region: NODE\_372995\_length\_1546\_cov\_22.922380 1497-1499. Max. coverage (+): 0. Max coverage (-): 0

Region: NODE\_372995\_length\_1546\_cov\_22.922380 1500-1502. Max. coverage (+): 0. Max coverage (-): 0

Region: NODE\_372995\_length\_1546\_cov\_22.922380 1503-1506. Max. coverage (+): 0. Max coverage (-): 0

Region: NODE\_372995\_length\_1546\_cov\_22.922380 1507-1509. Max. coverage (+): 0. Max coverage (-): 0

Region: NODE\_372995\_length\_1546\_cov\_22.922380 1510-1512. Max. coverage (+): 0. Max coverage (-): 0

Region: NODE\_372995\_length\_1546\_cov\_22.922380 1513-1515. Max. coverage (+): 0. Max coverage (-): 0

Region: NODE\_372995\_length\_1546\_cov\_22.922380 1516-1518. Max. coverage (+): 0. Max coverage (-): 0.02

Region: NODE\_372995\_length\_1546\_cov\_22.922380 1519-1522. Max. coverage (+): 0. Max coverage (-): 0.02

Region: NODE\_372995\_length\_1546\_cov\_22.922380 1523-1525. Max. coverage (+): 0. Max coverage (-): 0

Region: NODE\_372995\_length\_1546\_cov\_22.922380 1526-1528. Max. coverage (+): 0. Max coverage (-): 0

Region: NODE\_372995\_length\_1546\_cov\_22.922380 1529-1531. Max. coverage (+): 0. Max coverage (-): 0

Region: NODE\_372995\_length\_1546\_cov\_22.922380 1532-1534. Max. coverage (+): 0. Max coverage (-): 0

Region: NODE\_372995\_length\_1546\_cov\_22.922380 1535-1538. Max. coverage (+): 0. Max coverage (-): 0

Region: NODE\_372995\_length\_1546\_cov\_22.922380 1539-1541. Max. coverage (+): 0.15. Max coverage (-): 0

Region: NODE\_372995\_length\_1546\_cov\_22.922380 1542-1544. Max. coverage (+): 0.15. Max coverage (-): 0

Region: NODE\_372995\_length\_1546\_cov\_22.922380 1545-1547. Max. coverage (+): 0. Max coverage (-): 0

Region: NODE\_372995\_length\_1546\_cov\_22.922380 1548-1550. Max. coverage (+): 0. Max coverage (-): 0

Region: NODE\_372995\_length\_1546\_cov\_22.922380 1551-1554. Max. coverage (+): 0. Max coverage (-): 0

Region: NODE\_372995\_length\_1546\_cov\_22.922380 1555-1557. Max. coverage (+): 0. Max coverage (-): 0

Region: NODE\_372995\_length\_1546\_cov\_22.922380 1558-1560. Max. coverage (+): 0. Max coverage (-): 0

Region: NODE\_372995\_length\_1546\_cov\_22.922380 1561-1563. Max. coverage (+): 0. Max coverage (-): 0

Region: NODE\_372995\_length\_1546\_cov\_22.922380 1564-1566. Max. coverage (+): 0.01. Max coverage (-): 0

Region: NODE\_372995\_length\_1546\_cov\_22.922380 1567-1570. Max. coverage (+): 0. Max coverage (-): 0

Region: NODE\_372995\_length\_1546\_cov\_22.922380 1571-1573. Max. coverage (+): 0. Max coverage (-): 0

Region: NODE\_372995\_length\_1546\_cov\_22.922380 1574-1576. Max. coverage (+): 0. Max coverage (-): 0

Region: NODE\_372995\_length\_1546\_cov\_22.922380 1577-1579. Max. coverage (+): 0. Max coverage (-): 0

Region: NODE\_372995\_length\_1546\_cov\_22.922380 1580-1582. Max. coverage (+): 0. Max coverage (-): 0

Region: NODE\_372995\_length\_1546\_cov\_22.922380 1583-1586. Max. coverage (+): 0. Max coverage (-): 0.02

Region: NODE\_372995\_length\_1546\_cov\_22.922380 1587-1589. Max. coverage (+): 0. Max coverage (-): 0.01

Region: NODE\_372995\_length\_1546\_cov\_22.922380 1590-1592. Max. coverage (+): 0. Max coverage (-): 0

Region: NODE\_372995\_length\_1546\_cov\_22.922380 1593-1595. Max. coverage (+): 0. Max coverage (-): 0

Region: NODE\_372995\_length\_1546\_cov\_22.922380 1596-1598. Max. coverage (+): 0. Max coverage (-): 0

Region: NODE\_372995\_length\_1546\_cov\_22.922380 1599-1602. Max. coverage (+): 0. Max coverage (-): 0

Region: NODE\_372995\_length\_1546\_cov\_22.922380 1603-1605. Max. coverage (+): 0. Max coverage (-): 0

Region: NODE\_372995\_length\_1546\_cov\_22.922380 1606-1608. Max. coverage (+): 0. Max coverage (-): 0

Region: NODE\_372995\_length\_1546\_cov\_22.922380 1609-. Max. coverage (+): 0. Max coverage (-): 0

RepeatMasker Color Code

**+**

100-98% Identity

<98-95% Identity

<95-90% Identity

<90-85% Identity

<85-80% Identity

<80-75% Identity

<75-70% Identity

<70% Identity

**-**

Gene Set Color Code

**+**

Gene

Pseudogene

Other

**-**

Topology/Coverage Color Code

Coverage Plus Strand

Coverage Minus Strand

Mainstrand: Plus

Mainstrand: Minus

Complementary Strand

Flanking Region  
(if option -flank >0)

Gene Set Annotation  
  
RepeatMasker Annotation  

**1. AlRepB-356**: 487-567 (+), Divergence to consensus: 40.7%  
**2. AlRepB-923**: 572-682 (+), Divergence to consensus: 13.6%  
**3. Tc1-2\_FR**: 1300-1400 (-), Divergence to consensus: 9.9%  
**4. Tc1-2\_FR**: 1508-1603 (-), Divergence to consensus: 2.2%

  
Transcription Factor Binding Sites  

**RHOXF1** (Sequence: TGAGCT (+): 558)  
**FOXO1** (Sequence: CCTGTTTTC (+): 1234)  
**FOXO3\_mmu** (Sequence: TGTTTTCA (-): 1236)  
**Nobox** (Sequence: ACCAATTA (-): 1153)  
**FOXO1** (Sequence: AAAAACAAC (-): 890)  
**FOXO1** (Sequence: AAAAACAAC (-): 1247)  
**Sox5** (Sequence: AACAAT (-): 871)
