# Supplementary material for: First characterization of PIWI-interacting RNA clusters in a cichlid fish with a B chromosome
Source: BMC Biol. 2022 Sep 21;20:204. doi: 10.1186/s12915-022-01403-2 (PMC9490952; doi:10.1186/s12915-022-01403-2)
Supplement: Supplementary file 1 — Additional file 1. Zipped folder with fasta and interactive html piRNA cluster information for the A. latifasciata genome. The nomenclature is as follows: number-pirna-cluster_sex_B-presence (f, female; m, male; 0b, without B chromosome; 1b, with B chromosome). [file 12915_2022_1403_MOESM1_ESM.zip › 146_f0b.html]

piRNA cluster 146\_f0b 68


Predicted piRNA cluster no. 146\_f0b
  

Show proTRAC run info
Hide proTRAC run info

/\  
                \_\_\_\_\_\_\_\_\_\_\_\_\_\_\_\_\_\_\_\_\_\_\_/\\_\_\_ /  \\_\_\_\_\_\_\_  
               I                      /  \  /    \      I  
               I     pro             /    \/      \     I  
               I        TRAC        /               \   I  
               I   \_\_\_\_\_\_\_\_\_\_\_\_\_\_\_\_/\_\_\_\_\_\_\_\_\_\_\_\_\_\_\_\_\_\\_ I  
               I   \              /                     I  
               I    \            /                      I  
               I     \  /\      /       V.2.4.2         I  
               I      \/  \    /                        I  
               I\_\_\_\_\_\_\_\_\_\_\_\  /\_\_\_\_\_\_\_\_\_\_\_\_\_\_\_\_\_\_\_\_\_\_\_\_\_I  
                            \/  
  
  
================================= proTRAC ====================================  
VERSION: .......... 2.4.2  
LAST MODIFIED: .... 11. May 2018  
  
Please cite:  
Rosenkranz D, Zischler H. proTRAC - a software for probabilistic piRNA cluster  
detection, visualization and analysis. 2012. BMC Bioinformatics 13:5.  
  
  
Contact:  
David Rosenkranz  
Institute of Organismic and Molecular Evolutionary Biology  
Dept. Anthropology, small RNA group  
Johannes Gutenberg University Mainz  
email: rosenkranz@uni-mainz.de  
  
You can find the latest proTRAC version at:  
http://sourceforge.net/projects/protrac/files  
http://www.smallRNAgroup-mainz.de/software  
==============================================================================  
  
PARAMETERS:  
Map file: ...............piwi-femeas-0B.fa-collapse.map  
Genome file: ............../../../0B\_ala\_genome.fa  
RepeatMasker annotation: Alatifasciata-all0B-maryan-v2.fa\_corrected.out  
GeneSet:................./guest-storage/Data/annotation/Alatifasciata\_all0B\_maryan-v2\_out2017.gff  
  
Significant (p<=0.01) hit density will be calculated based  
on observed hit distribution.  
  
Sliding window size: ........................................ 5000 bp  
Sliding window increament: .................................. 1000 bp  
Normalize each hit by number of genomic hits: ............... yes  
Normalize each hit by number of sequence reads: ............. yes  
Normalize values (-> per million mapped reads): ............. yes  
Min. fraction of hits with 1T(U) or 10A: .................... 0.75  
Alternatively: Min. fraction of hits with 1T(U) and 10A: .... 0.5  
Min. fraction of hits with typical piRNA length: ............ 0.75  
Typical piRNA length: ....................................... 24-32 nt  
Min. size of a piRNA cluster: ............................... 1000 bp.  
Min. number of hits (absolute): ............................. 0  
Min. number of hits (normalized): ........................... 0  
Min. fraction of hits on the mainstrand: .................... 0.75  
Top fraction of mapped sequences (in terms of read counts): . 1%  
Top fraction accounts for max. n% of sequence reads: ........ 90%  
Min. fraction of hits on each arm of a bidirectional cluster: 0.05  
Output html file for each cluster: .......................... yes  
Output a summary table: ..................................... yes  
Output a FASTA file for each cluster (piRNA sequences): ..... yes  
Output a FASTA file comprising cluster sequences: ........... yes  
Output a GTF file for predicted piRNA clusters: ..............yes  
Search DNA motifs in clusters: .............................. yes  
Output flanking sequences: +/- .............................. 0 bp  
Output ~.pTi file: .......................................... no  
==============================================================================  
  
  
Genome size (without gaps): ............ 758543724 bp  
Gaps (N/X/-): .......................... 417479 bp  
Mapped reads: .......................... 13052187  
Non-identical sequences: ............... 3338911  
Genomic hits: .......................... 28737726  
Significant densitiy of mapped reads: .. 470.083249848448 reads/kb

Show proTRAC cluster info
Hide proTRAC cluster info

|  |  |
| --- | --- |
| Location | NODE\_374673\_length\_37756\_cov\_28.992161 |
| Coordinates | 35407-37031 |
| Size [bp] | 1625 |
| Sequence hit loci | 122 |
| Mapped reads (normalized) | 48315 |
| Mapped reads (normalized) per kb | 29732.3 |
| Normalized reads with 1T (1U) | 99.9% |
| Normalized reads with 10A | 100% |
| Normalized reads with length 24-32 nt | 100% |
| Normalized reads on the main strand(s) | 100% |
| Predicted directionality | mono:plus |

100%

0%

1T (1U)  
reads

10A reads

24-32 nt  
reads

reads on mainstrand

**Either the amount of reads with 1T (1U) OR 10A has to exceed 75% (set with option: -1Tor10A)  
Alternatively the amount of reads with 1T (1U) AND 10A has to exceed 50% (set with option: -1Tand10A)  
Minimum amount of reads with preferred size is 75% (set with option: -pisize)  
Minimum amount of reads on the main strand(s) is 75% (set with option: -clstrand)**

Show read coverage
Hide read coverage

WHAT DO I SEE HERE?  
This chart shows the location of mapped sequence reads within a predicted piRNA cluster. The color refers to the number of genomic hits produced by the sequence read in question. A dark red bar indicates that this sequence read produces many other hits elsewhere in the genome. Many adjacent red or yellow bars can indicate the presence of a multi-copy element such as transposons or rRNA genes. A dark green bar indicates that this sequence read maps uniquely to this locus.

1 hit

2-5 hits

6-10 hits

11-20 hits

21-50 hits

51-100 hits

> 100 hits

NODE\_374673\_length\_37756\_cov\_28.992161

35407

37031

Gene Set

RepeatMasker

Mapped  
Reads

3699.99

plus strand

minus strand

3699.99

Region: NODE\_374673\_length\_37756\_cov\_28.992161 1610-35408. Max. coverage (+): 0.08. Max coverage (-): 0

Region: NODE\_374673\_length\_37756\_cov\_28.992161 35409-35411. Max. coverage (+): 0.08. Max coverage (-): 0

Region: NODE\_374673\_length\_37756\_cov\_28.992161 35412-35415. Max. coverage (+): 0. Max coverage (-): 0

Region: NODE\_374673\_length\_37756\_cov\_28.992161 35416-35418. Max. coverage (+): 0. Max coverage (-): 0

Region: NODE\_374673\_length\_37756\_cov\_28.992161 35419-35421. Max. coverage (+): 0. Max coverage (-): 0

Region: NODE\_374673\_length\_37756\_cov\_28.992161 35422-35424. Max. coverage (+): 0. Max coverage (-): 0

Region: NODE\_374673\_length\_37756\_cov\_28.992161 35425-35428. Max. coverage (+): 0. Max coverage (-): 0

Region: NODE\_374673\_length\_37756\_cov\_28.992161 35429-35431. Max. coverage (+): 0. Max coverage (-): 0

Region: NODE\_374673\_length\_37756\_cov\_28.992161 35432-35434. Max. coverage (+): 0. Max coverage (-): 0

Region: NODE\_374673\_length\_37756\_cov\_28.992161 35435-35437. Max. coverage (+): 0. Max coverage (-): 0

Region: NODE\_374673\_length\_37756\_cov\_28.992161 35438-35441. Max. coverage (+): 0. Max coverage (-): 0

Region: NODE\_374673\_length\_37756\_cov\_28.992161 35442-35444. Max. coverage (+): 0. Max coverage (-): 0

Region: NODE\_374673\_length\_37756\_cov\_28.992161 35445-35447. Max. coverage (+): 0. Max coverage (-): 0

Region: NODE\_374673\_length\_37756\_cov\_28.992161 35448-35450. Max. coverage (+): 0. Max coverage (-): 0

Region: NODE\_374673\_length\_37756\_cov\_28.992161 35451-35454. Max. coverage (+): 0. Max coverage (-): 0

Region: NODE\_374673\_length\_37756\_cov\_28.992161 35455-35457. Max. coverage (+): 0. Max coverage (-): 0

Region: NODE\_374673\_length\_37756\_cov\_28.992161 35458-35460. Max. coverage (+): 0. Max coverage (-): 0

Region: NODE\_374673\_length\_37756\_cov\_28.992161 35461-35463. Max. coverage (+): 0. Max coverage (-): 0

Region: NODE\_374673\_length\_37756\_cov\_28.992161 35464-35467. Max. coverage (+): 0. Max coverage (-): 0

Region: NODE\_374673\_length\_37756\_cov\_28.992161 35468-35470. Max. coverage (+): 0. Max coverage (-): 0

Region: NODE\_374673\_length\_37756\_cov\_28.992161 35471-35473. Max. coverage (+): 0. Max coverage (-): 0

Region: NODE\_374673\_length\_37756\_cov\_28.992161 35474-35476. Max. coverage (+): 0. Max coverage (-): 0

Region: NODE\_374673\_length\_37756\_cov\_28.992161 35477-35480. Max. coverage (+): 0. Max coverage (-): 0

Region: NODE\_374673\_length\_37756\_cov\_28.992161 35481-35483. Max. coverage (+): 0. Max coverage (-): 0

Region: NODE\_374673\_length\_37756\_cov\_28.992161 35484-35486. Max. coverage (+): 0. Max coverage (-): 0

Region: NODE\_374673\_length\_37756\_cov\_28.992161 35487-35489. Max. coverage (+): 0. Max coverage (-): 0

Region: NODE\_374673\_length\_37756\_cov\_28.992161 35490-35493. Max. coverage (+): 0. Max coverage (-): 0

Region: NODE\_374673\_length\_37756\_cov\_28.992161 35494-35496. Max. coverage (+): 0. Max coverage (-): 0

Region: NODE\_374673\_length\_37756\_cov\_28.992161 35497-35499. Max. coverage (+): 0. Max coverage (-): 0

Region: NODE\_374673\_length\_37756\_cov\_28.992161 35500-35502. Max. coverage (+): 0. Max coverage (-): 0

Region: NODE\_374673\_length\_37756\_cov\_28.992161 35503-35506. Max. coverage (+): 0. Max coverage (-): 0

Region: NODE\_374673\_length\_37756\_cov\_28.992161 35507-35509. Max. coverage (+): 0. Max coverage (-): 0

Region: NODE\_374673\_length\_37756\_cov\_28.992161 35510-35512. Max. coverage (+): 0. Max coverage (-): 0

Region: NODE\_374673\_length\_37756\_cov\_28.992161 35513-35515. Max. coverage (+): 0. Max coverage (-): 0

Region: NODE\_374673\_length\_37756\_cov\_28.992161 35516-35519. Max. coverage (+): 0. Max coverage (-): 0

Region: NODE\_374673\_length\_37756\_cov\_28.992161 35520-35522. Max. coverage (+): 0. Max coverage (-): 0

Region: NODE\_374673\_length\_37756\_cov\_28.992161 35523-35525. Max. coverage (+): 0. Max coverage (-): 0

Region: NODE\_374673\_length\_37756\_cov\_28.992161 35526-35528. Max. coverage (+): 0. Max coverage (-): 0

Region: NODE\_374673\_length\_37756\_cov\_28.992161 35529-35532. Max. coverage (+): 0. Max coverage (-): 0

Region: NODE\_374673\_length\_37756\_cov\_28.992161 35533-35535. Max. coverage (+): 0. Max coverage (-): 0

Region: NODE\_374673\_length\_37756\_cov\_28.992161 35536-35538. Max. coverage (+): 0. Max coverage (-): 0

Region: NODE\_374673\_length\_37756\_cov\_28.992161 35539-35541. Max. coverage (+): 0. Max coverage (-): 0

Region: NODE\_374673\_length\_37756\_cov\_28.992161 35542-35545. Max. coverage (+): 0. Max coverage (-): 0

Region: NODE\_374673\_length\_37756\_cov\_28.992161 35546-35548. Max. coverage (+): 0. Max coverage (-): 0

Region: NODE\_374673\_length\_37756\_cov\_28.992161 35549-35551. Max. coverage (+): 0. Max coverage (-): 0

Region: NODE\_374673\_length\_37756\_cov\_28.992161 35552-35554. Max. coverage (+): 0. Max coverage (-): 0

Region: NODE\_374673\_length\_37756\_cov\_28.992161 35555-35558. Max. coverage (+): 0. Max coverage (-): 0

Region: NODE\_374673\_length\_37756\_cov\_28.992161 35559-35561. Max. coverage (+): 0. Max coverage (-): 0

Region: NODE\_374673\_length\_37756\_cov\_28.992161 35562-35564. Max. coverage (+): 0. Max coverage (-): 0

Region: NODE\_374673\_length\_37756\_cov\_28.992161 35565-35567. Max. coverage (+): 0. Max coverage (-): 0

Region: NODE\_374673\_length\_37756\_cov\_28.992161 35568-35571. Max. coverage (+): 0. Max coverage (-): 0

Region: NODE\_374673\_length\_37756\_cov\_28.992161 35572-35574. Max. coverage (+): 0. Max coverage (-): 0

Region: NODE\_374673\_length\_37756\_cov\_28.992161 35575-35577. Max. coverage (+): 0. Max coverage (-): 0

Region: NODE\_374673\_length\_37756\_cov\_28.992161 35578-35580. Max. coverage (+): 0. Max coverage (-): 0

Region: NODE\_374673\_length\_37756\_cov\_28.992161 35581-35584. Max. coverage (+): 0. Max coverage (-): 0

Region: NODE\_374673\_length\_37756\_cov\_28.992161 35585-35587. Max. coverage (+): 0. Max coverage (-): 0

Region: NODE\_374673\_length\_37756\_cov\_28.992161 35588-35590. Max. coverage (+): 0. Max coverage (-): 0

Region: NODE\_374673\_length\_37756\_cov\_28.992161 35591-35593. Max. coverage (+): 0. Max coverage (-): 0

Region: NODE\_374673\_length\_37756\_cov\_28.992161 35594-35597. Max. coverage (+): 0. Max coverage (-): 0

Region: NODE\_374673\_length\_37756\_cov\_28.992161 35598-35600. Max. coverage (+): 0. Max coverage (-): 0

Region: NODE\_374673\_length\_37756\_cov\_28.992161 35601-35603. Max. coverage (+): 0. Max coverage (-): 0

Region: NODE\_374673\_length\_37756\_cov\_28.992161 35604-35606. Max. coverage (+): 0. Max coverage (-): 0

Region: NODE\_374673\_length\_37756\_cov\_28.992161 35607-35610. Max. coverage (+): 0. Max coverage (-): 0

Region: NODE\_374673\_length\_37756\_cov\_28.992161 35611-35613. Max. coverage (+): 0. Max coverage (-): 0

Region: NODE\_374673\_length\_37756\_cov\_28.992161 35614-35616. Max. coverage (+): 0. Max coverage (-): 0

Region: NODE\_374673\_length\_37756\_cov\_28.992161 35617-35619. Max. coverage (+): 0. Max coverage (-): 0

Region: NODE\_374673\_length\_37756\_cov\_28.992161 35620-35623. Max. coverage (+): 0. Max coverage (-): 0

Region: NODE\_374673\_length\_37756\_cov\_28.992161 35624-35626. Max. coverage (+): 0. Max coverage (-): 0

Region: NODE\_374673\_length\_37756\_cov\_28.992161 35627-35629. Max. coverage (+): 0. Max coverage (-): 0

Region: NODE\_374673\_length\_37756\_cov\_28.992161 35630-35632. Max. coverage (+): 0. Max coverage (-): 0

Region: NODE\_374673\_length\_37756\_cov\_28.992161 35633-35636. Max. coverage (+): 0. Max coverage (-): 0

Region: NODE\_374673\_length\_37756\_cov\_28.992161 35637-35639. Max. coverage (+): 0. Max coverage (-): 0

Region: NODE\_374673\_length\_37756\_cov\_28.992161 35640-35642. Max. coverage (+): 0. Max coverage (-): 0

Region: NODE\_374673\_length\_37756\_cov\_28.992161 35643-35645. Max. coverage (+): 0. Max coverage (-): 0

Region: NODE\_374673\_length\_37756\_cov\_28.992161 35646-35649. Max. coverage (+): 0. Max coverage (-): 0

Region: NODE\_374673\_length\_37756\_cov\_28.992161 35650-35652. Max. coverage (+): 0. Max coverage (-): 0

Region: NODE\_374673\_length\_37756\_cov\_28.992161 35653-35655. Max. coverage (+): 0. Max coverage (-): 0

Region: NODE\_374673\_length\_37756\_cov\_28.992161 35656-35658. Max. coverage (+): 0. Max coverage (-): 0

Region: NODE\_374673\_length\_37756\_cov\_28.992161 35659-35662. Max. coverage (+): 0. Max coverage (-): 0

Region: NODE\_374673\_length\_37756\_cov\_28.992161 35663-35665. Max. coverage (+): 0. Max coverage (-): 0

Region: NODE\_374673\_length\_37756\_cov\_28.992161 35666-35668. Max. coverage (+): 0. Max coverage (-): 0

Region: NODE\_374673\_length\_37756\_cov\_28.992161 35669-35671. Max. coverage (+): 0. Max coverage (-): 0

Region: NODE\_374673\_length\_37756\_cov\_28.992161 35672-35675. Max. coverage (+): 0. Max coverage (-): 0

Region: NODE\_374673\_length\_37756\_cov\_28.992161 35676-35678. Max. coverage (+): 0. Max coverage (-): 0

Region: NODE\_374673\_length\_37756\_cov\_28.992161 35679-35681. Max. coverage (+): 0. Max coverage (-): 0

Region: NODE\_374673\_length\_37756\_cov\_28.992161 35682-35684. Max. coverage (+): 0. Max coverage (-): 0

Region: NODE\_374673\_length\_37756\_cov\_28.992161 35685-35688. Max. coverage (+): 0. Max coverage (-): 0

Region: NODE\_374673\_length\_37756\_cov\_28.992161 35689-35691. Max. coverage (+): 0. Max coverage (-): 0

Region: NODE\_374673\_length\_37756\_cov\_28.992161 35692-35694. Max. coverage (+): 0. Max coverage (-): 0

Region: NODE\_374673\_length\_37756\_cov\_28.992161 35695-35697. Max. coverage (+): 0. Max coverage (-): 0

Region: NODE\_374673\_length\_37756\_cov\_28.992161 35698-35701. Max. coverage (+): 0. Max coverage (-): 0

Region: NODE\_374673\_length\_37756\_cov\_28.992161 35702-35704. Max. coverage (+): 0. Max coverage (-): 0

Region: NODE\_374673\_length\_37756\_cov\_28.992161 35705-35707. Max. coverage (+): 0. Max coverage (-): 0

Region: NODE\_374673\_length\_37756\_cov\_28.992161 35708-35710. Max. coverage (+): 0. Max coverage (-): 0

Region: NODE\_374673\_length\_37756\_cov\_28.992161 35711-35714. Max. coverage (+): 0. Max coverage (-): 0

Region: NODE\_374673\_length\_37756\_cov\_28.992161 35715-35717. Max. coverage (+): 0. Max coverage (-): 0

Region: NODE\_374673\_length\_37756\_cov\_28.992161 35718-35720. Max. coverage (+): 0. Max coverage (-): 0

Region: NODE\_374673\_length\_37756\_cov\_28.992161 35721-35723. Max. coverage (+): 0. Max coverage (-): 0

Region: NODE\_374673\_length\_37756\_cov\_28.992161 35724-35727. Max. coverage (+): 0. Max coverage (-): 0

Region: NODE\_374673\_length\_37756\_cov\_28.992161 35728-35730. Max. coverage (+): 0. Max coverage (-): 0

Region: NODE\_374673\_length\_37756\_cov\_28.992161 35731-35733. Max. coverage (+): 0. Max coverage (-): 0

Region: NODE\_374673\_length\_37756\_cov\_28.992161 35734-35736. Max. coverage (+): 0. Max coverage (-): 0

Region: NODE\_374673\_length\_37756\_cov\_28.992161 35737-35740. Max. coverage (+): 0. Max coverage (-): 0

Region: NODE\_374673\_length\_37756\_cov\_28.992161 35741-35743. Max. coverage (+): 0. Max coverage (-): 0

Region: NODE\_374673\_length\_37756\_cov\_28.992161 35744-35746. Max. coverage (+): 0. Max coverage (-): 0

Region: NODE\_374673\_length\_37756\_cov\_28.992161 35747-35749. Max. coverage (+): 0. Max coverage (-): 0

Region: NODE\_374673\_length\_37756\_cov\_28.992161 35750-35753. Max. coverage (+): 0. Max coverage (-): 0

Region: NODE\_374673\_length\_37756\_cov\_28.992161 35754-35756. Max. coverage (+): 0. Max coverage (-): 0

Region: NODE\_374673\_length\_37756\_cov\_28.992161 35757-35759. Max. coverage (+): 0. Max coverage (-): 0

Region: NODE\_374673\_length\_37756\_cov\_28.992161 35760-35762. Max. coverage (+): 0. Max coverage (-): 0

Region: NODE\_374673\_length\_37756\_cov\_28.992161 35763-35766. Max. coverage (+): 0. Max coverage (-): 0

Region: NODE\_374673\_length\_37756\_cov\_28.992161 35767-35769. Max. coverage (+): 0. Max coverage (-): 0

Region: NODE\_374673\_length\_37756\_cov\_28.992161 35770-35772. Max. coverage (+): 0. Max coverage (-): 0

Region: NODE\_374673\_length\_37756\_cov\_28.992161 35773-35775. Max. coverage (+): 0. Max coverage (-): 0

Region: NODE\_374673\_length\_37756\_cov\_28.992161 35776-35779. Max. coverage (+): 0. Max coverage (-): 0

Region: NODE\_374673\_length\_37756\_cov\_28.992161 35780-35782. Max. coverage (+): 0. Max coverage (-): 0

Region: NODE\_374673\_length\_37756\_cov\_28.992161 35783-35785. Max. coverage (+): 0. Max coverage (-): 0

Region: NODE\_374673\_length\_37756\_cov\_28.992161 35786-35788. Max. coverage (+): 0. Max coverage (-): 0

Region: NODE\_374673\_length\_37756\_cov\_28.992161 35789-35792. Max. coverage (+): 0. Max coverage (-): 0

Region: NODE\_374673\_length\_37756\_cov\_28.992161 35793-35795. Max. coverage (+): 0. Max coverage (-): 0

Region: NODE\_374673\_length\_37756\_cov\_28.992161 35796-35798. Max. coverage (+): 0. Max coverage (-): 0

Region: NODE\_374673\_length\_37756\_cov\_28.992161 35799-35801. Max. coverage (+): 0. Max coverage (-): 0

Region: NODE\_374673\_length\_37756\_cov\_28.992161 35802-35805. Max. coverage (+): 0. Max coverage (-): 0

Region: NODE\_374673\_length\_37756\_cov\_28.992161 35806-35808. Max. coverage (+): 0. Max coverage (-): 0

Region: NODE\_374673\_length\_37756\_cov\_28.992161 35809-35811. Max. coverage (+): 0. Max coverage (-): 0

Region: NODE\_374673\_length\_37756\_cov\_28.992161 35812-35814. Max. coverage (+): 0. Max coverage (-): 0

Region: NODE\_374673\_length\_37756\_cov\_28.992161 35815-35818. Max. coverage (+): 0. Max coverage (-): 0

Region: NODE\_374673\_length\_37756\_cov\_28.992161 35819-35821. Max. coverage (+): 0. Max coverage (-): 0

Region: NODE\_374673\_length\_37756\_cov\_28.992161 35822-35824. Max. coverage (+): 0. Max coverage (-): 0

Region: NODE\_374673\_length\_37756\_cov\_28.992161 35825-35827. Max. coverage (+): 0. Max coverage (-): 0

Region: NODE\_374673\_length\_37756\_cov\_28.992161 35828-35831. Max. coverage (+): 0. Max coverage (-): 0

Region: NODE\_374673\_length\_37756\_cov\_28.992161 35832-35834. Max. coverage (+): 0. Max coverage (-): 0

Region: NODE\_374673\_length\_37756\_cov\_28.992161 35835-35837. Max. coverage (+): 0. Max coverage (-): 0

Region: NODE\_374673\_length\_37756\_cov\_28.992161 35838-35840. Max. coverage (+): 0. Max coverage (-): 0

Region: NODE\_374673\_length\_37756\_cov\_28.992161 35841-35844. Max. coverage (+): 0. Max coverage (-): 0

Region: NODE\_374673\_length\_37756\_cov\_28.992161 35845-35847. Max. coverage (+): 0. Max coverage (-): 0

Region: NODE\_374673\_length\_37756\_cov\_28.992161 35848-35850. Max. coverage (+): 0. Max coverage (-): 0

Region: NODE\_374673\_length\_37756\_cov\_28.992161 35851-35853. Max. coverage (+): 0. Max coverage (-): 0

Region: NODE\_374673\_length\_37756\_cov\_28.992161 35854-35857. Max. coverage (+): 0. Max coverage (-): 0

Region: NODE\_374673\_length\_37756\_cov\_28.992161 35858-35860. Max. coverage (+): 0. Max coverage (-): 0

Region: NODE\_374673\_length\_37756\_cov\_28.992161 35861-35863. Max. coverage (+): 0. Max coverage (-): 0

Region: NODE\_374673\_length\_37756\_cov\_28.992161 35864-35866. Max. coverage (+): 0. Max coverage (-): 0

Region: NODE\_374673\_length\_37756\_cov\_28.992161 35867-35870. Max. coverage (+): 0. Max coverage (-): 0

Region: NODE\_374673\_length\_37756\_cov\_28.992161 35871-35873. Max. coverage (+): 0. Max coverage (-): 0

Region: NODE\_374673\_length\_37756\_cov\_28.992161 35874-35876. Max. coverage (+): 0. Max coverage (-): 0

Region: NODE\_374673\_length\_37756\_cov\_28.992161 35877-35879. Max. coverage (+): 0. Max coverage (-): 0

Region: NODE\_374673\_length\_37756\_cov\_28.992161 35880-35883. Max. coverage (+): 0. Max coverage (-): 0

Region: NODE\_374673\_length\_37756\_cov\_28.992161 35884-35886. Max. coverage (+): 0. Max coverage (-): 0

Region: NODE\_374673\_length\_37756\_cov\_28.992161 35887-35889. Max. coverage (+): 0. Max coverage (-): 0

Region: NODE\_374673\_length\_37756\_cov\_28.992161 35890-35892. Max. coverage (+): 0. Max coverage (-): 0

Region: NODE\_374673\_length\_37756\_cov\_28.992161 35893-35896. Max. coverage (+): 0. Max coverage (-): 0

Region: NODE\_374673\_length\_37756\_cov\_28.992161 35897-35899. Max. coverage (+): 0. Max coverage (-): 0

Region: NODE\_374673\_length\_37756\_cov\_28.992161 35900-35902. Max. coverage (+): 0. Max coverage (-): 0

Region: NODE\_374673\_length\_37756\_cov\_28.992161 35903-35905. Max. coverage (+): 0. Max coverage (-): 0

Region: NODE\_374673\_length\_37756\_cov\_28.992161 35906-35909. Max. coverage (+): 0. Max coverage (-): 0

Region: NODE\_374673\_length\_37756\_cov\_28.992161 35910-35912. Max. coverage (+): 0. Max coverage (-): 0

Region: NODE\_374673\_length\_37756\_cov\_28.992161 35913-35915. Max. coverage (+): 0. Max coverage (-): 0

Region: NODE\_374673\_length\_37756\_cov\_28.992161 35916-35918. Max. coverage (+): 0. Max coverage (-): 0

Region: NODE\_374673\_length\_37756\_cov\_28.992161 35919-35922. Max. coverage (+): 0. Max coverage (-): 0

Region: NODE\_374673\_length\_37756\_cov\_28.992161 35923-35925. Max. coverage (+): 0. Max coverage (-): 0

Region: NODE\_374673\_length\_37756\_cov\_28.992161 35926-35928. Max. coverage (+): 0. Max coverage (-): 0

Region: NODE\_374673\_length\_37756\_cov\_28.992161 35929-35931. Max. coverage (+): 0. Max coverage (-): 0

Region: NODE\_374673\_length\_37756\_cov\_28.992161 35932-35935. Max. coverage (+): 0. Max coverage (-): 0

Region: NODE\_374673\_length\_37756\_cov\_28.992161 35936-35938. Max. coverage (+): 0. Max coverage (-): 0

Region: NODE\_374673\_length\_37756\_cov\_28.992161 35939-35941. Max. coverage (+): 0.08. Max coverage (-): 0

Region: NODE\_374673\_length\_37756\_cov\_28.992161 35942-35944. Max. coverage (+): 0.08. Max coverage (-): 0

Region: NODE\_374673\_length\_37756\_cov\_28.992161 35945-35948. Max. coverage (+): 0. Max coverage (-): 0

Region: NODE\_374673\_length\_37756\_cov\_28.992161 35949-35951. Max. coverage (+): 0. Max coverage (-): 0

Region: NODE\_374673\_length\_37756\_cov\_28.992161 35952-35954. Max. coverage (+): 0. Max coverage (-): 0

Region: NODE\_374673\_length\_37756\_cov\_28.992161 35955-35957. Max. coverage (+): 0. Max coverage (-): 0

Region: NODE\_374673\_length\_37756\_cov\_28.992161 35958-35961. Max. coverage (+): 0. Max coverage (-): 0

Region: NODE\_374673\_length\_37756\_cov\_28.992161 35962-35964. Max. coverage (+): 0. Max coverage (-): 0

Region: NODE\_374673\_length\_37756\_cov\_28.992161 35965-35967. Max. coverage (+): 0. Max coverage (-): 0

Region: NODE\_374673\_length\_37756\_cov\_28.992161 35968-35970. Max. coverage (+): 0. Max coverage (-): 0

Region: NODE\_374673\_length\_37756\_cov\_28.992161 35971-35974. Max. coverage (+): 0. Max coverage (-): 0

Region: NODE\_374673\_length\_37756\_cov\_28.992161 35975-35977. Max. coverage (+): 0. Max coverage (-): 0

Region: NODE\_374673\_length\_37756\_cov\_28.992161 35978-35980. Max. coverage (+): 0. Max coverage (-): 0

Region: NODE\_374673\_length\_37756\_cov\_28.992161 35981-35983. Max. coverage (+): 0. Max coverage (-): 0

Region: NODE\_374673\_length\_37756\_cov\_28.992161 35984-35987. Max. coverage (+): 0. Max coverage (-): 0

Region: NODE\_374673\_length\_37756\_cov\_28.992161 35988-35990. Max. coverage (+): 0. Max coverage (-): 0

Region: NODE\_374673\_length\_37756\_cov\_28.992161 35991-35993. Max. coverage (+): 0. Max coverage (-): 0

Region: NODE\_374673\_length\_37756\_cov\_28.992161 35994-35996. Max. coverage (+): 0. Max coverage (-): 0

Region: NODE\_374673\_length\_37756\_cov\_28.992161 35997-36000. Max. coverage (+): 0. Max coverage (-): 0

Region: NODE\_374673\_length\_37756\_cov\_28.992161 36001-36003. Max. coverage (+): 0. Max coverage (-): 0

Region: NODE\_374673\_length\_37756\_cov\_28.992161 36004-36006. Max. coverage (+): 0. Max coverage (-): 0

Region: NODE\_374673\_length\_37756\_cov\_28.992161 36007-36009. Max. coverage (+): 0. Max coverage (-): 0

Region: NODE\_374673\_length\_37756\_cov\_28.992161 36010-36013. Max. coverage (+): 0. Max coverage (-): 0

Region: NODE\_374673\_length\_37756\_cov\_28.992161 36014-36016. Max. coverage (+): 0. Max coverage (-): 0

Region: NODE\_374673\_length\_37756\_cov\_28.992161 36017-36019. Max. coverage (+): 0. Max coverage (-): 0

Region: NODE\_374673\_length\_37756\_cov\_28.992161 36020-36022. Max. coverage (+): 0. Max coverage (-): 0

Region: NODE\_374673\_length\_37756\_cov\_28.992161 36023-36026. Max. coverage (+): 0. Max coverage (-): 0

Region: NODE\_374673\_length\_37756\_cov\_28.992161 36027-36029. Max. coverage (+): 0. Max coverage (-): 0

Region: NODE\_374673\_length\_37756\_cov\_28.992161 36030-36032. Max. coverage (+): 0. Max coverage (-): 0

Region: NODE\_374673\_length\_37756\_cov\_28.992161 36033-36035. Max. coverage (+): 0. Max coverage (-): 0

Region: NODE\_374673\_length\_37756\_cov\_28.992161 36036-36039. Max. coverage (+): 0.08. Max coverage (-): 0

Region: NODE\_374673\_length\_37756\_cov\_28.992161 36040-36042. Max. coverage (+): 0.08. Max coverage (-): 0

Region: NODE\_374673\_length\_37756\_cov\_28.992161 36043-36045. Max. coverage (+): 0. Max coverage (-): 0

Region: NODE\_374673\_length\_37756\_cov\_28.992161 36046-36048. Max. coverage (+): 0. Max coverage (-): 0

Region: NODE\_374673\_length\_37756\_cov\_28.992161 36049-36052. Max. coverage (+): 0. Max coverage (-): 0

Region: NODE\_374673\_length\_37756\_cov\_28.992161 36053-36055. Max. coverage (+): 0. Max coverage (-): 0

Region: NODE\_374673\_length\_37756\_cov\_28.992161 36056-36058. Max. coverage (+): 0. Max coverage (-): 0

Region: NODE\_374673\_length\_37756\_cov\_28.992161 36059-36061. Max. coverage (+): 0. Max coverage (-): 0

Region: NODE\_374673\_length\_37756\_cov\_28.992161 36062-36065. Max. coverage (+): 0. Max coverage (-): 0

Region: NODE\_374673\_length\_37756\_cov\_28.992161 36066-36068. Max. coverage (+): 0. Max coverage (-): 0

Region: NODE\_374673\_length\_37756\_cov\_28.992161 36069-36071. Max. coverage (+): 0. Max coverage (-): 0

Region: NODE\_374673\_length\_37756\_cov\_28.992161 36072-36074. Max. coverage (+): 0. Max coverage (-): 0

Region: NODE\_374673\_length\_37756\_cov\_28.992161 36075-36078. Max. coverage (+): 0. Max coverage (-): 0

Region: NODE\_374673\_length\_37756\_cov\_28.992161 36079-36081. Max. coverage (+): 0. Max coverage (-): 0

Region: NODE\_374673\_length\_37756\_cov\_28.992161 36082-36084. Max. coverage (+): 0. Max coverage (-): 0

Region: NODE\_374673\_length\_37756\_cov\_28.992161 36085-36087. Max. coverage (+): 0. Max coverage (-): 0

Region: NODE\_374673\_length\_37756\_cov\_28.992161 36088-36091. Max. coverage (+): 0. Max coverage (-): 0

Region: NODE\_374673\_length\_37756\_cov\_28.992161 36092-36094. Max. coverage (+): 0. Max coverage (-): 0

Region: NODE\_374673\_length\_37756\_cov\_28.992161 36095-36097. Max. coverage (+): 0. Max coverage (-): 0

Region: NODE\_374673\_length\_37756\_cov\_28.992161 36098-36100. Max. coverage (+): 0. Max coverage (-): 0

Region: NODE\_374673\_length\_37756\_cov\_28.992161 36101-36104. Max. coverage (+): 0. Max coverage (-): 0

Region: NODE\_374673\_length\_37756\_cov\_28.992161 36105-36107. Max. coverage (+): 0. Max coverage (-): 0

Region: NODE\_374673\_length\_37756\_cov\_28.992161 36108-36110. Max. coverage (+): 0.08. Max coverage (-): 0

Region: NODE\_374673\_length\_37756\_cov\_28.992161 36111-36113. Max. coverage (+): 0.08. Max coverage (-): 0

Region: NODE\_374673\_length\_37756\_cov\_28.992161 36114-36117. Max. coverage (+): 0.08. Max coverage (-): 0

Region: NODE\_374673\_length\_37756\_cov\_28.992161 36118-36120. Max. coverage (+): 0. Max coverage (-): 0

Region: NODE\_374673\_length\_37756\_cov\_28.992161 36121-36123. Max. coverage (+): 0. Max coverage (-): 0

Region: NODE\_374673\_length\_37756\_cov\_28.992161 36124-36126. Max. coverage (+): 0. Max coverage (-): 0

Region: NODE\_374673\_length\_37756\_cov\_28.992161 36127-36130. Max. coverage (+): 0. Max coverage (-): 0

Region: NODE\_374673\_length\_37756\_cov\_28.992161 36131-36133. Max. coverage (+): 0. Max coverage (-): 0

Region: NODE\_374673\_length\_37756\_cov\_28.992161 36134-36136. Max. coverage (+): 0. Max coverage (-): 0

Region: NODE\_374673\_length\_37756\_cov\_28.992161 36137-36139. Max. coverage (+): 0. Max coverage (-): 0

Region: NODE\_374673\_length\_37756\_cov\_28.992161 36140-36143. Max. coverage (+): 0. Max coverage (-): 0

Region: NODE\_374673\_length\_37756\_cov\_28.992161 36144-36146. Max. coverage (+): 0. Max coverage (-): 0

Region: NODE\_374673\_length\_37756\_cov\_28.992161 36147-36149. Max. coverage (+): 0. Max coverage (-): 0

Region: NODE\_374673\_length\_37756\_cov\_28.992161 36150-36152. Max. coverage (+): 0. Max coverage (-): 0

Region: NODE\_374673\_length\_37756\_cov\_28.992161 36153-36156. Max. coverage (+): 0. Max coverage (-): 0

Region: NODE\_374673\_length\_37756\_cov\_28.992161 36157-36159. Max. coverage (+): 0. Max coverage (-): 0

Region: NODE\_374673\_length\_37756\_cov\_28.992161 36160-36162. Max. coverage (+): 0. Max coverage (-): 0

Region: NODE\_374673\_length\_37756\_cov\_28.992161 36163-36165. Max. coverage (+): 0. Max coverage (-): 0

Region: NODE\_374673\_length\_37756\_cov\_28.992161 36166-36169. Max. coverage (+): 0. Max coverage (-): 0

Region: NODE\_374673\_length\_37756\_cov\_28.992161 36170-36172. Max. coverage (+): 0. Max coverage (-): 0

Region: NODE\_374673\_length\_37756\_cov\_28.992161 36173-36175. Max. coverage (+): 0. Max coverage (-): 0

Region: NODE\_374673\_length\_37756\_cov\_28.992161 36176-36178. Max. coverage (+): 0. Max coverage (-): 0

Region: NODE\_374673\_length\_37756\_cov\_28.992161 36179-36182. Max. coverage (+): 0. Max coverage (-): 0

Region: NODE\_374673\_length\_37756\_cov\_28.992161 36183-36185. Max. coverage (+): 0. Max coverage (-): 0

Region: NODE\_374673\_length\_37756\_cov\_28.992161 36186-36188. Max. coverage (+): 0. Max coverage (-): 0

Region: NODE\_374673\_length\_37756\_cov\_28.992161 36189-36191. Max. coverage (+): 0. Max coverage (-): 0

Region: NODE\_374673\_length\_37756\_cov\_28.992161 36192-36195. Max. coverage (+): 0. Max coverage (-): 0

Region: NODE\_374673\_length\_37756\_cov\_28.992161 36196-36198. Max. coverage (+): 0. Max coverage (-): 0

Region: NODE\_374673\_length\_37756\_cov\_28.992161 36199-36201. Max. coverage (+): 0. Max coverage (-): 0

Region: NODE\_374673\_length\_37756\_cov\_28.992161 36202-36204. Max. coverage (+): 0. Max coverage (-): 0

Region: NODE\_374673\_length\_37756\_cov\_28.992161 36205-36208. Max. coverage (+): 0. Max coverage (-): 0

Region: NODE\_374673\_length\_37756\_cov\_28.992161 36209-36211. Max. coverage (+): 0. Max coverage (-): 0

Region: NODE\_374673\_length\_37756\_cov\_28.992161 36212-36214. Max. coverage (+): 0. Max coverage (-): 0

Region: NODE\_374673\_length\_37756\_cov\_28.992161 36215-36217. Max. coverage (+): 0. Max coverage (-): 0

Region: NODE\_374673\_length\_37756\_cov\_28.992161 36218-36221. Max. coverage (+): 0. Max coverage (-): 0

Region: NODE\_374673\_length\_37756\_cov\_28.992161 36222-36224. Max. coverage (+): 0. Max coverage (-): 0

Region: NODE\_374673\_length\_37756\_cov\_28.992161 36225-36227. Max. coverage (+): 0. Max coverage (-): 0

Region: NODE\_374673\_length\_37756\_cov\_28.992161 36228-36230. Max. coverage (+): 0. Max coverage (-): 0

Region: NODE\_374673\_length\_37756\_cov\_28.992161 36231-36234. Max. coverage (+): 0. Max coverage (-): 0

Region: NODE\_374673\_length\_37756\_cov\_28.992161 36235-36237. Max. coverage (+): 0. Max coverage (-): 0

Region: NODE\_374673\_length\_37756\_cov\_28.992161 36238-36240. Max. coverage (+): 0. Max coverage (-): 0

Region: NODE\_374673\_length\_37756\_cov\_28.992161 36241-36243. Max. coverage (+): 0. Max coverage (-): 0

Region: NODE\_374673\_length\_37756\_cov\_28.992161 36244-36247. Max. coverage (+): 0. Max coverage (-): 0

Region: NODE\_374673\_length\_37756\_cov\_28.992161 36248-36250. Max. coverage (+): 0. Max coverage (-): 0

Region: NODE\_374673\_length\_37756\_cov\_28.992161 36251-36253. Max. coverage (+): 0. Max coverage (-): 0

Region: NODE\_374673\_length\_37756\_cov\_28.992161 36254-36256. Max. coverage (+): 0. Max coverage (-): 0

Region: NODE\_374673\_length\_37756\_cov\_28.992161 36257-36260. Max. coverage (+): 0. Max coverage (-): 0

Region: NODE\_374673\_length\_37756\_cov\_28.992161 36261-36263. Max. coverage (+): 0. Max coverage (-): 0

Region: NODE\_374673\_length\_37756\_cov\_28.992161 36264-36266. Max. coverage (+): 0. Max coverage (-): 0

Region: NODE\_374673\_length\_37756\_cov\_28.992161 36267-36269. Max. coverage (+): 0. Max coverage (-): 0

Region: NODE\_374673\_length\_37756\_cov\_28.992161 36270-36273. Max. coverage (+): 0. Max coverage (-): 0

Region: NODE\_374673\_length\_37756\_cov\_28.992161 36274-36276. Max. coverage (+): 0. Max coverage (-): 0

Region: NODE\_374673\_length\_37756\_cov\_28.992161 36277-36279. Max. coverage (+): 0. Max coverage (-): 0

Region: NODE\_374673\_length\_37756\_cov\_28.992161 36280-36282. Max. coverage (+): 0. Max coverage (-): 0

Region: NODE\_374673\_length\_37756\_cov\_28.992161 36283-36286. Max. coverage (+): 0. Max coverage (-): 0

Region: NODE\_374673\_length\_37756\_cov\_28.992161 36287-36289. Max. coverage (+): 0. Max coverage (-): 0

Region: NODE\_374673\_length\_37756\_cov\_28.992161 36290-36292. Max. coverage (+): 0. Max coverage (-): 0

Region: NODE\_374673\_length\_37756\_cov\_28.992161 36293-36295. Max. coverage (+): 0. Max coverage (-): 0

Region: NODE\_374673\_length\_37756\_cov\_28.992161 36296-36299. Max. coverage (+): 0. Max coverage (-): 0

Region: NODE\_374673\_length\_37756\_cov\_28.992161 36300-36302. Max. coverage (+): 0. Max coverage (-): 0

Region: NODE\_374673\_length\_37756\_cov\_28.992161 36303-36305. Max. coverage (+): 0. Max coverage (-): 0

Region: NODE\_374673\_length\_37756\_cov\_28.992161 36306-36308. Max. coverage (+): 0. Max coverage (-): 0

Region: NODE\_374673\_length\_37756\_cov\_28.992161 36309-36312. Max. coverage (+): 0. Max coverage (-): 0

Region: NODE\_374673\_length\_37756\_cov\_28.992161 36313-36315. Max. coverage (+): 0. Max coverage (-): 0

Region: NODE\_374673\_length\_37756\_cov\_28.992161 36316-36318. Max. coverage (+): 0. Max coverage (-): 0

Region: NODE\_374673\_length\_37756\_cov\_28.992161 36319-36321. Max. coverage (+): 0. Max coverage (-): 0

Region: NODE\_374673\_length\_37756\_cov\_28.992161 36322-36325. Max. coverage (+): 0. Max coverage (-): 0

Region: NODE\_374673\_length\_37756\_cov\_28.992161 36326-36328. Max. coverage (+): 0. Max coverage (-): 0

Region: NODE\_374673\_length\_37756\_cov\_28.992161 36329-36331. Max. coverage (+): 0. Max coverage (-): 0

Region: NODE\_374673\_length\_37756\_cov\_28.992161 36332-36334. Max. coverage (+): 0. Max coverage (-): 0

Region: NODE\_374673\_length\_37756\_cov\_28.992161 36335-36338. Max. coverage (+): 0. Max coverage (-): 0

Region: NODE\_374673\_length\_37756\_cov\_28.992161 36339-36341. Max. coverage (+): 0. Max coverage (-): 0

Region: NODE\_374673\_length\_37756\_cov\_28.992161 36342-36344. Max. coverage (+): 0. Max coverage (-): 0

Region: NODE\_374673\_length\_37756\_cov\_28.992161 36345-36347. Max. coverage (+): 0. Max coverage (-): 0

Region: NODE\_374673\_length\_37756\_cov\_28.992161 36348-36351. Max. coverage (+): 0. Max coverage (-): 0

Region: NODE\_374673\_length\_37756\_cov\_28.992161 36352-36354. Max. coverage (+): 0. Max coverage (-): 0

Region: NODE\_374673\_length\_37756\_cov\_28.992161 36355-36357. Max. coverage (+): 0. Max coverage (-): 0

Region: NODE\_374673\_length\_37756\_cov\_28.992161 36358-36360. Max. coverage (+): 0. Max coverage (-): 0

Region: NODE\_374673\_length\_37756\_cov\_28.992161 36361-36364. Max. coverage (+): 0. Max coverage (-): 0

Region: NODE\_374673\_length\_37756\_cov\_28.992161 36365-36367. Max. coverage (+): 0. Max coverage (-): 0

Region: NODE\_374673\_length\_37756\_cov\_28.992161 36368-36370. Max. coverage (+): 0. Max coverage (-): 0

Region: NODE\_374673\_length\_37756\_cov\_28.992161 36371-36373. Max. coverage (+): 0. Max coverage (-): 0

Region: NODE\_374673\_length\_37756\_cov\_28.992161 36374-36377. Max. coverage (+): 0. Max coverage (-): 0

Region: NODE\_374673\_length\_37756\_cov\_28.992161 36378-36380. Max. coverage (+): 0. Max coverage (-): 0

Region: NODE\_374673\_length\_37756\_cov\_28.992161 36381-36383. Max. coverage (+): 0. Max coverage (-): 0

Region: NODE\_374673\_length\_37756\_cov\_28.992161 36384-36386. Max. coverage (+): 0. Max coverage (-): 0

Region: NODE\_374673\_length\_37756\_cov\_28.992161 36387-36390. Max. coverage (+): 0. Max coverage (-): 0

Region: NODE\_374673\_length\_37756\_cov\_28.992161 36391-36393. Max. coverage (+): 0. Max coverage (-): 0

Region: NODE\_374673\_length\_37756\_cov\_28.992161 36394-36396. Max. coverage (+): 0. Max coverage (-): 0

Region: NODE\_374673\_length\_37756\_cov\_28.992161 36397-36399. Max. coverage (+): 0. Max coverage (-): 0

Region: NODE\_374673\_length\_37756\_cov\_28.992161 36400-36403. Max. coverage (+): 0. Max coverage (-): 0

Region: NODE\_374673\_length\_37756\_cov\_28.992161 36404-36406. Max. coverage (+): 0. Max coverage (-): 0

Region: NODE\_374673\_length\_37756\_cov\_28.992161 36407-36409. Max. coverage (+): 0. Max coverage (-): 0

Region: NODE\_374673\_length\_37756\_cov\_28.992161 36410-36412. Max. coverage (+): 0. Max coverage (-): 0

Region: NODE\_374673\_length\_37756\_cov\_28.992161 36413-36416. Max. coverage (+): 0. Max coverage (-): 0

Region: NODE\_374673\_length\_37756\_cov\_28.992161 36417-36419. Max. coverage (+): 0. Max coverage (-): 0

Region: NODE\_374673\_length\_37756\_cov\_28.992161 36420-36422. Max. coverage (+): 0. Max coverage (-): 0

Region: NODE\_374673\_length\_37756\_cov\_28.992161 36423-36425. Max. coverage (+): 0. Max coverage (-): 0

Region: NODE\_374673\_length\_37756\_cov\_28.992161 36426-36429. Max. coverage (+): 0. Max coverage (-): 0

Region: NODE\_374673\_length\_37756\_cov\_28.992161 36430-36432. Max. coverage (+): 0. Max coverage (-): 0

Region: NODE\_374673\_length\_37756\_cov\_28.992161 36433-36435. Max. coverage (+): 0. Max coverage (-): 0

Region: NODE\_374673\_length\_37756\_cov\_28.992161 36436-36438. Max. coverage (+): 0. Max coverage (-): 0

Region: NODE\_374673\_length\_37756\_cov\_28.992161 36439-36442. Max. coverage (+): 0. Max coverage (-): 0

Region: NODE\_374673\_length\_37756\_cov\_28.992161 36443-36445. Max. coverage (+): 0. Max coverage (-): 0

Region: NODE\_374673\_length\_37756\_cov\_28.992161 36446-36448. Max. coverage (+): 0. Max coverage (-): 0

Region: NODE\_374673\_length\_37756\_cov\_28.992161 36449-36451. Max. coverage (+): 0. Max coverage (-): 0

Region: NODE\_374673\_length\_37756\_cov\_28.992161 36452-36455. Max. coverage (+): 0. Max coverage (-): 0

Region: NODE\_374673\_length\_37756\_cov\_28.992161 36456-36458. Max. coverage (+): 0. Max coverage (-): 0

Region: NODE\_374673\_length\_37756\_cov\_28.992161 36459-36461. Max. coverage (+): 0. Max coverage (-): 0

Region: NODE\_374673\_length\_37756\_cov\_28.992161 36462-36464. Max. coverage (+): 0. Max coverage (-): 0

Region: NODE\_374673\_length\_37756\_cov\_28.992161 36465-36468. Max. coverage (+): 0. Max coverage (-): 0

Region: NODE\_374673\_length\_37756\_cov\_28.992161 36469-36471. Max. coverage (+): 0. Max coverage (-): 0

Region: NODE\_374673\_length\_37756\_cov\_28.992161 36472-36474. Max. coverage (+): 0. Max coverage (-): 0

Region: NODE\_374673\_length\_37756\_cov\_28.992161 36475-36477. Max. coverage (+): 0. Max coverage (-): 0

Region: NODE\_374673\_length\_37756\_cov\_28.992161 36478-36481. Max. coverage (+): 3699.07. Max coverage (-): 0

Region: NODE\_374673\_length\_37756\_cov\_28.992161 36482-36484. Max. coverage (+): 3699.99. Max coverage (-): 0

Region: NODE\_374673\_length\_37756\_cov\_28.992161 36485-36487. Max. coverage (+): 3699.84. Max coverage (-): 0

Region: NODE\_374673\_length\_37756\_cov\_28.992161 36488-36490. Max. coverage (+): 0. Max coverage (-): 0

Region: NODE\_374673\_length\_37756\_cov\_28.992161 36491-36494. Max. coverage (+): 0. Max coverage (-): 0

Region: NODE\_374673\_length\_37756\_cov\_28.992161 36495-36497. Max. coverage (+): 0. Max coverage (-): 0

Region: NODE\_374673\_length\_37756\_cov\_28.992161 36498-36500. Max. coverage (+): 0. Max coverage (-): 0

Region: NODE\_374673\_length\_37756\_cov\_28.992161 36501-36503. Max. coverage (+): 0. Max coverage (-): 0

Region: NODE\_374673\_length\_37756\_cov\_28.992161 36504-36507. Max. coverage (+): 0.08. Max coverage (-): 0

Region: NODE\_374673\_length\_37756\_cov\_28.992161 36508-36510. Max. coverage (+): 0.08. Max coverage (-): 0

Region: NODE\_374673\_length\_37756\_cov\_28.992161 36511-36513. Max. coverage (+): 0.08. Max coverage (-): 0

Region: NODE\_374673\_length\_37756\_cov\_28.992161 36514-36516. Max. coverage (+): 0. Max coverage (-): 0

Region: NODE\_374673\_length\_37756\_cov\_28.992161 36517-36520. Max. coverage (+): 0.69. Max coverage (-): 0

Region: NODE\_374673\_length\_37756\_cov\_28.992161 36521-36523. Max. coverage (+): 1.3. Max coverage (-): 0

Region: NODE\_374673\_length\_37756\_cov\_28.992161 36524-36526. Max. coverage (+): 1.3. Max coverage (-): 0

Region: NODE\_374673\_length\_37756\_cov\_28.992161 36527-36529. Max. coverage (+): 0. Max coverage (-): 0

Region: NODE\_374673\_length\_37756\_cov\_28.992161 36530-36533. Max. coverage (+): 0. Max coverage (-): 0

Region: NODE\_374673\_length\_37756\_cov\_28.992161 36534-36536. Max. coverage (+): 0. Max coverage (-): 0

Region: NODE\_374673\_length\_37756\_cov\_28.992161 36537-36539. Max. coverage (+): 0. Max coverage (-): 0

Region: NODE\_374673\_length\_37756\_cov\_28.992161 36540-36542. Max. coverage (+): 0. Max coverage (-): 0

Region: NODE\_374673\_length\_37756\_cov\_28.992161 36543-36546. Max. coverage (+): 0. Max coverage (-): 0

Region: NODE\_374673\_length\_37756\_cov\_28.992161 36547-36549. Max. coverage (+): 0. Max coverage (-): 0

Region: NODE\_374673\_length\_37756\_cov\_28.992161 36550-36552. Max. coverage (+): 0. Max coverage (-): 0

Region: NODE\_374673\_length\_37756\_cov\_28.992161 36553-36555. Max. coverage (+): 0. Max coverage (-): 0

Region: NODE\_374673\_length\_37756\_cov\_28.992161 36556-36559. Max. coverage (+): 0. Max coverage (-): 0

Region: NODE\_374673\_length\_37756\_cov\_28.992161 36560-36562. Max. coverage (+): 0. Max coverage (-): 0

Region: NODE\_374673\_length\_37756\_cov\_28.992161 36563-36565. Max. coverage (+): 0. Max coverage (-): 0

Region: NODE\_374673\_length\_37756\_cov\_28.992161 36566-36568. Max. coverage (+): 0. Max coverage (-): 0

Region: NODE\_374673\_length\_37756\_cov\_28.992161 36569-36572. Max. coverage (+): 0. Max coverage (-): 0

Region: NODE\_374673\_length\_37756\_cov\_28.992161 36573-36575. Max. coverage (+): 0. Max coverage (-): 0

Region: NODE\_374673\_length\_37756\_cov\_28.992161 36576-36578. Max. coverage (+): 0. Max coverage (-): 0

Region: NODE\_374673\_length\_37756\_cov\_28.992161 36579-36581. Max. coverage (+): 0. Max coverage (-): 0

Region: NODE\_374673\_length\_37756\_cov\_28.992161 36582-36585. Max. coverage (+): 0. Max coverage (-): 0

Region: NODE\_374673\_length\_37756\_cov\_28.992161 36586-36588. Max. coverage (+): 0. Max coverage (-): 0

Region: NODE\_374673\_length\_37756\_cov\_28.992161 36589-36591. Max. coverage (+): 0. Max coverage (-): 0

Region: NODE\_374673\_length\_37756\_cov\_28.992161 36592-36594. Max. coverage (+): 0. Max coverage (-): 0

Region: NODE\_374673\_length\_37756\_cov\_28.992161 36595-36598. Max. coverage (+): 0. Max coverage (-): 0

Region: NODE\_374673\_length\_37756\_cov\_28.992161 36599-36601. Max. coverage (+): 0. Max coverage (-): 0

Region: NODE\_374673\_length\_37756\_cov\_28.992161 36602-36604. Max. coverage (+): 0. Max coverage (-): 0

Region: NODE\_374673\_length\_37756\_cov\_28.992161 36605-36607. Max. coverage (+): 0. Max coverage (-): 0

Region: NODE\_374673\_length\_37756\_cov\_28.992161 36608-36611. Max. coverage (+): 0. Max coverage (-): 0

Region: NODE\_374673\_length\_37756\_cov\_28.992161 36612-36614. Max. coverage (+): 0. Max coverage (-): 0

Region: NODE\_374673\_length\_37756\_cov\_28.992161 36615-36617. Max. coverage (+): 0. Max coverage (-): 0

Region: NODE\_374673\_length\_37756\_cov\_28.992161 36618-36620. Max. coverage (+): 0. Max coverage (-): 0

Region: NODE\_374673\_length\_37756\_cov\_28.992161 36621-36624. Max. coverage (+): 0. Max coverage (-): 0

Region: NODE\_374673\_length\_37756\_cov\_28.992161 36625-36627. Max. coverage (+): 0. Max coverage (-): 0

Region: NODE\_374673\_length\_37756\_cov\_28.992161 36628-36630. Max. coverage (+): 0. Max coverage (-): 0

Region: NODE\_374673\_length\_37756\_cov\_28.992161 36631-36633. Max. coverage (+): 0. Max coverage (-): 0

Region: NODE\_374673\_length\_37756\_cov\_28.992161 36634-36637. Max. coverage (+): 0. Max coverage (-): 0

Region: NODE\_374673\_length\_37756\_cov\_28.992161 36638-36640. Max. coverage (+): 0. Max coverage (-): 0

Region: NODE\_374673\_length\_37756\_cov\_28.992161 36641-36643. Max. coverage (+): 0. Max coverage (-): 0

Region: NODE\_374673\_length\_37756\_cov\_28.992161 36644-36646. Max. coverage (+): 0. Max coverage (-): 0

Region: NODE\_374673\_length\_37756\_cov\_28.992161 36647-36650. Max. coverage (+): 0. Max coverage (-): 0

Region: NODE\_374673\_length\_37756\_cov\_28.992161 36651-36653. Max. coverage (+): 0. Max coverage (-): 0

Region: NODE\_374673\_length\_37756\_cov\_28.992161 36654-36656. Max. coverage (+): 0. Max coverage (-): 0

Region: NODE\_374673\_length\_37756\_cov\_28.992161 36657-36659. Max. coverage (+): 0. Max coverage (-): 0

Region: NODE\_374673\_length\_37756\_cov\_28.992161 36660-36663. Max. coverage (+): 0. Max coverage (-): 0

Region: NODE\_374673\_length\_37756\_cov\_28.992161 36664-36666. Max. coverage (+): 0. Max coverage (-): 0

Region: NODE\_374673\_length\_37756\_cov\_28.992161 36667-36669. Max. coverage (+): 0. Max coverage (-): 0

Region: NODE\_374673\_length\_37756\_cov\_28.992161 36670-36672. Max. coverage (+): 0. Max coverage (-): 0

Region: NODE\_374673\_length\_37756\_cov\_28.992161 36673-36676. Max. coverage (+): 0. Max coverage (-): 0

Region: NODE\_374673\_length\_37756\_cov\_28.992161 36677-36679. Max. coverage (+): 0. Max coverage (-): 0

Region: NODE\_374673\_length\_37756\_cov\_28.992161 36680-36682. Max. coverage (+): 0. Max coverage (-): 0

Region: NODE\_374673\_length\_37756\_cov\_28.992161 36683-36685. Max. coverage (+): 0. Max coverage (-): 0

Region: NODE\_374673\_length\_37756\_cov\_28.992161 36686-36689. Max. coverage (+): 0. Max coverage (-): 0

Region: NODE\_374673\_length\_37756\_cov\_28.992161 36690-36692. Max. coverage (+): 0. Max coverage (-): 0

Region: NODE\_374673\_length\_37756\_cov\_28.992161 36693-36695. Max. coverage (+): 0. Max coverage (-): 0

Region: NODE\_374673\_length\_37756\_cov\_28.992161 36696-36698. Max. coverage (+): 0. Max coverage (-): 0

Region: NODE\_374673\_length\_37756\_cov\_28.992161 36699-36702. Max. coverage (+): 0. Max coverage (-): 0

Region: NODE\_374673\_length\_37756\_cov\_28.992161 36703-36705. Max. coverage (+): 0. Max coverage (-): 0

Region: NODE\_374673\_length\_37756\_cov\_28.992161 36706-36708. Max. coverage (+): 0. Max coverage (-): 0

Region: NODE\_374673\_length\_37756\_cov\_28.992161 36709-36711. Max. coverage (+): 0. Max coverage (-): 0

Region: NODE\_374673\_length\_37756\_cov\_28.992161 36712-36715. Max. coverage (+): 0. Max coverage (-): 0

Region: NODE\_374673\_length\_37756\_cov\_28.992161 36716-36718. Max. coverage (+): 0. Max coverage (-): 0

Region: NODE\_374673\_length\_37756\_cov\_28.992161 36719-36721. Max. coverage (+): 0. Max coverage (-): 0

Region: NODE\_374673\_length\_37756\_cov\_28.992161 36722-36724. Max. coverage (+): 0. Max coverage (-): 0

Region: NODE\_374673\_length\_37756\_cov\_28.992161 36725-36728. Max. coverage (+): 0. Max coverage (-): 0

Region: NODE\_374673\_length\_37756\_cov\_28.992161 36729-36731. Max. coverage (+): 0. Max coverage (-): 0

Region: NODE\_374673\_length\_37756\_cov\_28.992161 36732-36734. Max. coverage (+): 0. Max coverage (-): 0

Region: NODE\_374673\_length\_37756\_cov\_28.992161 36735-36737. Max. coverage (+): 0. Max coverage (-): 0

Region: NODE\_374673\_length\_37756\_cov\_28.992161 36738-36741. Max. coverage (+): 0. Max coverage (-): 0

Region: NODE\_374673\_length\_37756\_cov\_28.992161 36742-36744. Max. coverage (+): 0. Max coverage (-): 0

Region: NODE\_374673\_length\_37756\_cov\_28.992161 36745-36747. Max. coverage (+): 0. Max coverage (-): 0

Region: NODE\_374673\_length\_37756\_cov\_28.992161 36748-36750. Max. coverage (+): 0. Max coverage (-): 0

Region: NODE\_374673\_length\_37756\_cov\_28.992161 36751-36754. Max. coverage (+): 0. Max coverage (-): 0

Region: NODE\_374673\_length\_37756\_cov\_28.992161 36755-36757. Max. coverage (+): 0. Max coverage (-): 0

Region: NODE\_374673\_length\_37756\_cov\_28.992161 36758-36760. Max. coverage (+): 0. Max coverage (-): 0

Region: NODE\_374673\_length\_37756\_cov\_28.992161 36761-36763. Max. coverage (+): 0. Max coverage (-): 0

Region: NODE\_374673\_length\_37756\_cov\_28.992161 36764-36767. Max. coverage (+): 0. Max coverage (-): 0

Region: NODE\_374673\_length\_37756\_cov\_28.992161 36768-36770. Max. coverage (+): 0. Max coverage (-): 0

Region: NODE\_374673\_length\_37756\_cov\_28.992161 36771-36773. Max. coverage (+): 0. Max coverage (-): 0

Region: NODE\_374673\_length\_37756\_cov\_28.992161 36774-36776. Max. coverage (+): 0. Max coverage (-): 0

Region: NODE\_374673\_length\_37756\_cov\_28.992161 36777-36780. Max. coverage (+): 0. Max coverage (-): 0

Region: NODE\_374673\_length\_37756\_cov\_28.992161 36781-36783. Max. coverage (+): 0. Max coverage (-): 0

Region: NODE\_374673\_length\_37756\_cov\_28.992161 36784-36786. Max. coverage (+): 0. Max coverage (-): 0

Region: NODE\_374673\_length\_37756\_cov\_28.992161 36787-36789. Max. coverage (+): 0. Max coverage (-): 0

Region: NODE\_374673\_length\_37756\_cov\_28.992161 36790-36793. Max. coverage (+): 0. Max coverage (-): 0

Region: NODE\_374673\_length\_37756\_cov\_28.992161 36794-36796. Max. coverage (+): 0. Max coverage (-): 0

Region: NODE\_374673\_length\_37756\_cov\_28.992161 36797-36799. Max. coverage (+): 0. Max coverage (-): 0

Region: NODE\_374673\_length\_37756\_cov\_28.992161 36800-36802. Max. coverage (+): 0. Max coverage (-): 0

Region: NODE\_374673\_length\_37756\_cov\_28.992161 36803-36806. Max. coverage (+): 0. Max coverage (-): 0

Region: NODE\_374673\_length\_37756\_cov\_28.992161 36807-36809. Max. coverage (+): 0. Max coverage (-): 0

Region: NODE\_374673\_length\_37756\_cov\_28.992161 36810-36812. Max. coverage (+): 0. Max coverage (-): 0

Region: NODE\_374673\_length\_37756\_cov\_28.992161 36813-36815. Max. coverage (+): 0. Max coverage (-): 0

Region: NODE\_374673\_length\_37756\_cov\_28.992161 36816-36819. Max. coverage (+): 0. Max coverage (-): 0

Region: NODE\_374673\_length\_37756\_cov\_28.992161 36820-36822. Max. coverage (+): 0. Max coverage (-): 0

Region: NODE\_374673\_length\_37756\_cov\_28.992161 36823-36825. Max. coverage (+): 0. Max coverage (-): 0

Region: NODE\_374673\_length\_37756\_cov\_28.992161 36826-36828. Max. coverage (+): 0. Max coverage (-): 0

Region: NODE\_374673\_length\_37756\_cov\_28.992161 36829-36832. Max. coverage (+): 0. Max coverage (-): 0

Region: NODE\_374673\_length\_37756\_cov\_28.992161 36833-36835. Max. coverage (+): 0. Max coverage (-): 0

Region: NODE\_374673\_length\_37756\_cov\_28.992161 36836-36838. Max. coverage (+): 0. Max coverage (-): 0

Region: NODE\_374673\_length\_37756\_cov\_28.992161 36839-36841. Max. coverage (+): 0. Max coverage (-): 0

Region: NODE\_374673\_length\_37756\_cov\_28.992161 36842-36845. Max. coverage (+): 0. Max coverage (-): 0

Region: NODE\_374673\_length\_37756\_cov\_28.992161 36846-36848. Max. coverage (+): 0. Max coverage (-): 0

Region: NODE\_374673\_length\_37756\_cov\_28.992161 36849-36851. Max. coverage (+): 0. Max coverage (-): 0

Region: NODE\_374673\_length\_37756\_cov\_28.992161 36852-36854. Max. coverage (+): 0. Max coverage (-): 0

Region: NODE\_374673\_length\_37756\_cov\_28.992161 36855-36858. Max. coverage (+): 0. Max coverage (-): 0

Region: NODE\_374673\_length\_37756\_cov\_28.992161 36859-36861. Max. coverage (+): 0. Max coverage (-): 0

Region: NODE\_374673\_length\_37756\_cov\_28.992161 36862-36864. Max. coverage (+): 0. Max coverage (-): 0

Region: NODE\_374673\_length\_37756\_cov\_28.992161 36865-36867. Max. coverage (+): 0. Max coverage (-): 0

Region: NODE\_374673\_length\_37756\_cov\_28.992161 36868-36871. Max. coverage (+): 0. Max coverage (-): 0

Region: NODE\_374673\_length\_37756\_cov\_28.992161 36872-36874. Max. coverage (+): 0. Max coverage (-): 0

Region: NODE\_374673\_length\_37756\_cov\_28.992161 36875-36877. Max. coverage (+): 0. Max coverage (-): 0

Region: NODE\_374673\_length\_37756\_cov\_28.992161 36878-36880. Max. coverage (+): 0. Max coverage (-): 0

Region: NODE\_374673\_length\_37756\_cov\_28.992161 36881-36884. Max. coverage (+): 0. Max coverage (-): 0

Region: NODE\_374673\_length\_37756\_cov\_28.992161 36885-36887. Max. coverage (+): 0. Max coverage (-): 0

Region: NODE\_374673\_length\_37756\_cov\_28.992161 36888-36890. Max. coverage (+): 0. Max coverage (-): 0

Region: NODE\_374673\_length\_37756\_cov\_28.992161 36891-36893. Max. coverage (+): 0. Max coverage (-): 0

Region: NODE\_374673\_length\_37756\_cov\_28.992161 36894-36897. Max. coverage (+): 0. Max coverage (-): 0

Region: NODE\_374673\_length\_37756\_cov\_28.992161 36898-36900. Max. coverage (+): 0. Max coverage (-): 0

Region: NODE\_374673\_length\_37756\_cov\_28.992161 36901-36903. Max. coverage (+): 0. Max coverage (-): 0

Region: NODE\_374673\_length\_37756\_cov\_28.992161 36904-36906. Max. coverage (+): 0. Max coverage (-): 0

Region: NODE\_374673\_length\_37756\_cov\_28.992161 36907-36910. Max. coverage (+): 0. Max coverage (-): 0

Region: NODE\_374673\_length\_37756\_cov\_28.992161 36911-36913. Max. coverage (+): 0. Max coverage (-): 0

Region: NODE\_374673\_length\_37756\_cov\_28.992161 36914-36916. Max. coverage (+): 0. Max coverage (-): 0

Region: NODE\_374673\_length\_37756\_cov\_28.992161 36917-36919. Max. coverage (+): 0. Max coverage (-): 0

Region: NODE\_374673\_length\_37756\_cov\_28.992161 36920-36923. Max. coverage (+): 0. Max coverage (-): 0

Region: NODE\_374673\_length\_37756\_cov\_28.992161 36924-36926. Max. coverage (+): 0. Max coverage (-): 0

Region: NODE\_374673\_length\_37756\_cov\_28.992161 36927-36929. Max. coverage (+): 0. Max coverage (-): 0

Region: NODE\_374673\_length\_37756\_cov\_28.992161 36930-36932. Max. coverage (+): 0. Max coverage (-): 0

Region: NODE\_374673\_length\_37756\_cov\_28.992161 36933-36936. Max. coverage (+): 0. Max coverage (-): 0

Region: NODE\_374673\_length\_37756\_cov\_28.992161 36937-36939. Max. coverage (+): 0. Max coverage (-): 0

Region: NODE\_374673\_length\_37756\_cov\_28.992161 36940-36942. Max. coverage (+): 0. Max coverage (-): 0

Region: NODE\_374673\_length\_37756\_cov\_28.992161 36943-36945. Max. coverage (+): 0. Max coverage (-): 0

Region: NODE\_374673\_length\_37756\_cov\_28.992161 36946-36949. Max. coverage (+): 0. Max coverage (-): 0

Region: NODE\_374673\_length\_37756\_cov\_28.992161 36950-36952. Max. coverage (+): 0. Max coverage (-): 0

Region: NODE\_374673\_length\_37756\_cov\_28.992161 36953-36955. Max. coverage (+): 0. Max coverage (-): 0

Region: NODE\_374673\_length\_37756\_cov\_28.992161 36956-36958. Max. coverage (+): 0. Max coverage (-): 0

Region: NODE\_374673\_length\_37756\_cov\_28.992161 36959-36962. Max. coverage (+): 0. Max coverage (-): 0

Region: NODE\_374673\_length\_37756\_cov\_28.992161 36963-36965. Max. coverage (+): 0. Max coverage (-): 0

Region: NODE\_374673\_length\_37756\_cov\_28.992161 36966-36968. Max. coverage (+): 0. Max coverage (-): 0

Region: NODE\_374673\_length\_37756\_cov\_28.992161 36969-36971. Max. coverage (+): 0. Max coverage (-): 0

Region: NODE\_374673\_length\_37756\_cov\_28.992161 36972-36975. Max. coverage (+): 0. Max coverage (-): 0

Region: NODE\_374673\_length\_37756\_cov\_28.992161 36976-36978. Max. coverage (+): 0. Max coverage (-): 0

Region: NODE\_374673\_length\_37756\_cov\_28.992161 36979-36981. Max. coverage (+): 0. Max coverage (-): 0

Region: NODE\_374673\_length\_37756\_cov\_28.992161 36982-36984. Max. coverage (+): 0. Max coverage (-): 0

Region: NODE\_374673\_length\_37756\_cov\_28.992161 36985-36988. Max. coverage (+): 0. Max coverage (-): 0

Region: NODE\_374673\_length\_37756\_cov\_28.992161 36989-36991. Max. coverage (+): 0. Max coverage (-): 0

Region: NODE\_374673\_length\_37756\_cov\_28.992161 36992-36994. Max. coverage (+): 0. Max coverage (-): 0

Region: NODE\_374673\_length\_37756\_cov\_28.992161 36995-36997. Max. coverage (+): 0. Max coverage (-): 0

Region: NODE\_374673\_length\_37756\_cov\_28.992161 36998-37001. Max. coverage (+): 0. Max coverage (-): 0

Region: NODE\_374673\_length\_37756\_cov\_28.992161 37002-37004. Max. coverage (+): 0. Max coverage (-): 0

Region: NODE\_374673\_length\_37756\_cov\_28.992161 37005-37007. Max. coverage (+): 0. Max coverage (-): 0

Region: NODE\_374673\_length\_37756\_cov\_28.992161 37008-37010. Max. coverage (+): 0. Max coverage (-): 0

Region: NODE\_374673\_length\_37756\_cov\_28.992161 37011-37014. Max. coverage (+): 0. Max coverage (-): 0

Region: NODE\_374673\_length\_37756\_cov\_28.992161 37015-37017. Max. coverage (+): 0. Max coverage (-): 0

Region: NODE\_374673\_length\_37756\_cov\_28.992161 37018-37020. Max. coverage (+): 0. Max coverage (-): 0

Region: NODE\_374673\_length\_37756\_cov\_28.992161 37021-37023. Max. coverage (+): 0. Max coverage (-): 0

Region: NODE\_374673\_length\_37756\_cov\_28.992161 37024-37027. Max. coverage (+): 0. Max coverage (-): 0

Region: NODE\_374673\_length\_37756\_cov\_28.992161 37028-37030. Max. coverage (+): 0. Max coverage (-): 0

Region: NODE\_374673\_length\_37756\_cov\_28.992161 37031-. Max. coverage (+): 0. Max coverage (-): 0

RepeatMasker Color Code

**+**

100-98% Identity

<98-95% Identity

<95-90% Identity

<90-85% Identity

<85-80% Identity

<80-75% Identity

<75-70% Identity

<70% Identity

**-**

Gene Set Color Code

**+**

Gene

Pseudogene

Other

**-**

Topology/Coverage Color Code

Coverage Plus Strand

Coverage Minus Strand

Mainstrand: Plus

Mainstrand: Minus

Complementary Strand

Flanking Region  
(if option -flank >0)

Gene Set Annotation  
  
RepeatMasker Annotation  

**1. (AC)n**: 36967-36989 (+), Divergence to consensus: 4.5%  
**2. (TG)n**: 36992-37033 (+), Divergence to consensus: 2.4%

  
Transcription Factor Binding Sites  

**RHOXF1** (Sequence: GGCTCA (-): 36528)  
**RHOXF1** (Sequence: TGATCC (+): 35957)  
**RHOXF1** (Sequence: TAATCC (+): 36138)  
**RHOXF1** (Sequence: TGAGCT (+): 36405)  
**RHOXF1** (Sequence: TAAGCT (+): 36933)  
**Lhx8** (Sequence: TTAATTAG (-): 36371)  
**Lhx8** (Sequence: TTAATTAA (-): 36806)  
**Sox5** (Sequence: ATTGTT (+): 36758)  
**Rhox11** (Sequence: TGGTGTTAA (+): 36624)  
**Rhox11** (Sequence: TTAACAGCA (-): 36075)  
**Gata4** (Sequence: AGATAAC (-): 35569)
