# Supplementary material for: First characterization of PIWI-interacting RNA clusters in a cichlid fish with a B chromosome
Source: BMC Biol. 2022 Sep 21;20:204. doi: 10.1186/s12915-022-01403-2 (PMC9490952; doi:10.1186/s12915-022-01403-2)
Supplement: Supplementary file 1 — Additional file 1. Zipped folder with fasta and interactive html piRNA cluster information for the A. latifasciata genome. The nomenclature is as follows: number-pirna-cluster_sex_B-presence (f, female; m, male; 0b, without B chromosome; 1b, with B chromosome). [file 12915_2022_1403_MOESM1_ESM.zip › 146_f1b.html]

piRNA cluster 146\_f1b 63


Predicted piRNA cluster no. 146\_f1b
  

Show proTRAC run info
Hide proTRAC run info

/\  
                \_\_\_\_\_\_\_\_\_\_\_\_\_\_\_\_\_\_\_\_\_\_\_/\\_\_\_ /  \\_\_\_\_\_\_\_  
               I                      /  \  /    \      I  
               I     pro             /    \/      \     I  
               I        TRAC        /               \   I  
               I   \_\_\_\_\_\_\_\_\_\_\_\_\_\_\_\_/\_\_\_\_\_\_\_\_\_\_\_\_\_\_\_\_\_\\_ I  
               I   \              /                     I  
               I    \            /                      I  
               I     \  /\      /       V.2.4.2         I  
               I      \/  \    /                        I  
               I\_\_\_\_\_\_\_\_\_\_\_\  /\_\_\_\_\_\_\_\_\_\_\_\_\_\_\_\_\_\_\_\_\_\_\_\_\_I  
                            \/  
  
  
================================= proTRAC ====================================  
VERSION: .......... 2.4.2  
LAST MODIFIED: .... 11. May 2018  
  
Please cite:  
Rosenkranz D, Zischler H. proTRAC - a software for probabilistic piRNA cluster  
detection, visualization and analysis. 2012. BMC Bioinformatics 13:5.  
  
  
Contact:  
David Rosenkranz  
Institute of Organismic and Molecular Evolutionary Biology  
Dept. Anthropology, small RNA group  
Johannes Gutenberg University Mainz  
email: rosenkranz@uni-mainz.de  
  
You can find the latest proTRAC version at:  
http://sourceforge.net/projects/protrac/files  
http://www.smallRNAgroup-mainz.de/software  
==============================================================================  
  
PARAMETERS:  
Map file: ...............piwi-femeas-1B.fa-collapse.map  
Genome file: ............../../../0B\_ala\_genome.fa  
RepeatMasker annotation: Alatifasciata-all0B-maryan-v2.fa\_corrected.out  
GeneSet:................./guest-storage/Data/annotation/Alatifasciata\_all0B\_maryan-v2\_out2017.gff  
  
Significant (p<=0.01) hit density will be calculated based  
on observed hit distribution.  
  
Sliding window size: ........................................ 5000 bp  
Sliding window increament: .................................. 1000 bp  
Normalize each hit by number of genomic hits: ............... yes  
Normalize each hit by number of sequence reads: ............. yes  
Normalize values (-> per million mapped reads): ............. yes  
Min. fraction of hits with 1T(U) or 10A: .................... 0.75  
Alternatively: Min. fraction of hits with 1T(U) and 10A: .... 0.5  
Min. fraction of hits with typical piRNA length: ............ 0.75  
Typical piRNA length: ....................................... 24-32 nt  
Min. size of a piRNA cluster: ............................... 1000 bp.  
Min. number of hits (absolute): ............................. 0  
Min. number of hits (normalized): ........................... 0  
Min. fraction of hits on the mainstrand: .................... 0.75  
Top fraction of mapped sequences (in terms of read counts): . 1%  
Top fraction accounts for max. n% of sequence reads: ........ 90%  
Min. fraction of hits on each arm of a bidirectional cluster: 0.05  
Output html file for each cluster: .......................... yes  
Output a summary table: ..................................... yes  
Output a FASTA file for each cluster (piRNA sequences): ..... yes  
Output a FASTA file comprising cluster sequences: ........... yes  
Output a GTF file for predicted piRNA clusters: ..............yes  
Search DNA motifs in clusters: .............................. yes  
Output flanking sequences: +/- .............................. 0 bp  
Output ~.pTi file: .......................................... no  
==============================================================================  
  
  
Genome size (without gaps): ............ 758543724 bp  
Gaps (N/X/-): .......................... 417479 bp  
Mapped reads: .......................... 10641844  
Non-identical sequences: ............... 2832837  
Genomic hits: .......................... 26056853  
Significant densitiy of mapped reads: .. 368.713530323068 reads/kb

Show proTRAC cluster info
Hide proTRAC cluster info

|  |  |
| --- | --- |
| Location | NODE\_374673\_length\_37756\_cov\_28.992161 |
| Coordinates | 35743-37015 |
| Size [bp] | 1273 |
| Sequence hit loci | 135 |
| Mapped reads (normalized) | 56323 |
| Mapped reads (normalized) per kb | 44244.3 |
| Normalized reads with 1T (1U) | 99.9% |
| Normalized reads with 10A | 99.9% |
| Normalized reads with length 24-32 nt | 99.9% |
| Normalized reads on the main strand(s) | 100% |
| Predicted directionality | mono:plus |

100%

0%

1T (1U)  
reads

10A reads

24-32 nt  
reads

reads on mainstrand

**Either the amount of reads with 1T (1U) OR 10A has to exceed 75% (set with option: -1Tor10A)  
Alternatively the amount of reads with 1T (1U) AND 10A has to exceed 50% (set with option: -1Tand10A)  
Minimum amount of reads with preferred size is 75% (set with option: -pisize)  
Minimum amount of reads on the main strand(s) is 75% (set with option: -clstrand)**

Show read coverage
Hide read coverage

WHAT DO I SEE HERE?  
This chart shows the location of mapped sequence reads within a predicted piRNA cluster. The color refers to the number of genomic hits produced by the sequence read in question. A dark red bar indicates that this sequence read produces many other hits elsewhere in the genome. Many adjacent red or yellow bars can indicate the presence of a multi-copy element such as transposons or rRNA genes. A dark green bar indicates that this sequence read maps uniquely to this locus.

1 hit

2-5 hits

6-10 hits

11-20 hits

21-50 hits

51-100 hits

> 100 hits

NODE\_374673\_length\_37756\_cov\_28.992161

35743

37015

Gene Set

RepeatMasker

Mapped  
Reads

5286.49

plus strand

minus strand

5286.49

Region: NODE\_374673\_length\_37756\_cov\_28.992161 1610-35744. Max. coverage (+): 0.09. Max coverage (-): 0

Region: NODE\_374673\_length\_37756\_cov\_28.992161 35745-35746. Max. coverage (+): 0.09. Max coverage (-): 0

Region: NODE\_374673\_length\_37756\_cov\_28.992161 35747-35749. Max. coverage (+): 0.09. Max coverage (-): 0

Region: NODE\_374673\_length\_37756\_cov\_28.992161 35750-35751. Max. coverage (+): 0. Max coverage (-): 0

Region: NODE\_374673\_length\_37756\_cov\_28.992161 35752-35754. Max. coverage (+): 0. Max coverage (-): 0

Region: NODE\_374673\_length\_37756\_cov\_28.992161 35755-35757. Max. coverage (+): 0. Max coverage (-): 0

Region: NODE\_374673\_length\_37756\_cov\_28.992161 35758-35759. Max. coverage (+): 0. Max coverage (-): 0

Region: NODE\_374673\_length\_37756\_cov\_28.992161 35760-35762. Max. coverage (+): 0. Max coverage (-): 0

Region: NODE\_374673\_length\_37756\_cov\_28.992161 35763-35764. Max. coverage (+): 0. Max coverage (-): 0

Region: NODE\_374673\_length\_37756\_cov\_28.992161 35765-35767. Max. coverage (+): 0. Max coverage (-): 0

Region: NODE\_374673\_length\_37756\_cov\_28.992161 35768-35769. Max. coverage (+): 0. Max coverage (-): 0

Region: NODE\_374673\_length\_37756\_cov\_28.992161 35770-35772. Max. coverage (+): 0. Max coverage (-): 0

Region: NODE\_374673\_length\_37756\_cov\_28.992161 35773-35774. Max. coverage (+): 0. Max coverage (-): 0

Region: NODE\_374673\_length\_37756\_cov\_28.992161 35775-35777. Max. coverage (+): 0. Max coverage (-): 0

Region: NODE\_374673\_length\_37756\_cov\_28.992161 35778-35779. Max. coverage (+): 0. Max coverage (-): 0

Region: NODE\_374673\_length\_37756\_cov\_28.992161 35780-35782. Max. coverage (+): 0. Max coverage (-): 0

Region: NODE\_374673\_length\_37756\_cov\_28.992161 35783-35785. Max. coverage (+): 0. Max coverage (-): 0

Region: NODE\_374673\_length\_37756\_cov\_28.992161 35786-35787. Max. coverage (+): 0. Max coverage (-): 0

Region: NODE\_374673\_length\_37756\_cov\_28.992161 35788-35790. Max. coverage (+): 0. Max coverage (-): 0

Region: NODE\_374673\_length\_37756\_cov\_28.992161 35791-35792. Max. coverage (+): 0. Max coverage (-): 0

Region: NODE\_374673\_length\_37756\_cov\_28.992161 35793-35795. Max. coverage (+): 0. Max coverage (-): 0

Region: NODE\_374673\_length\_37756\_cov\_28.992161 35796-35797. Max. coverage (+): 0. Max coverage (-): 0

Region: NODE\_374673\_length\_37756\_cov\_28.992161 35798-35800. Max. coverage (+): 0. Max coverage (-): 0

Region: NODE\_374673\_length\_37756\_cov\_28.992161 35801-35802. Max. coverage (+): 0. Max coverage (-): 0

Region: NODE\_374673\_length\_37756\_cov\_28.992161 35803-35805. Max. coverage (+): 0. Max coverage (-): 0

Region: NODE\_374673\_length\_37756\_cov\_28.992161 35806-35807. Max. coverage (+): 0. Max coverage (-): 0

Region: NODE\_374673\_length\_37756\_cov\_28.992161 35808-35810. Max. coverage (+): 0. Max coverage (-): 0

Region: NODE\_374673\_length\_37756\_cov\_28.992161 35811-35813. Max. coverage (+): 0. Max coverage (-): 0

Region: NODE\_374673\_length\_37756\_cov\_28.992161 35814-35815. Max. coverage (+): 0. Max coverage (-): 0

Region: NODE\_374673\_length\_37756\_cov\_28.992161 35816-35818. Max. coverage (+): 0. Max coverage (-): 0

Region: NODE\_374673\_length\_37756\_cov\_28.992161 35819-35820. Max. coverage (+): 0. Max coverage (-): 0

Region: NODE\_374673\_length\_37756\_cov\_28.992161 35821-35823. Max. coverage (+): 0. Max coverage (-): 0

Region: NODE\_374673\_length\_37756\_cov\_28.992161 35824-35825. Max. coverage (+): 0. Max coverage (-): 0

Region: NODE\_374673\_length\_37756\_cov\_28.992161 35826-35828. Max. coverage (+): 0. Max coverage (-): 0

Region: NODE\_374673\_length\_37756\_cov\_28.992161 35829-35830. Max. coverage (+): 0. Max coverage (-): 0

Region: NODE\_374673\_length\_37756\_cov\_28.992161 35831-35833. Max. coverage (+): 0. Max coverage (-): 0

Region: NODE\_374673\_length\_37756\_cov\_28.992161 35834-35835. Max. coverage (+): 0. Max coverage (-): 0

Region: NODE\_374673\_length\_37756\_cov\_28.992161 35836-35838. Max. coverage (+): 0. Max coverage (-): 0

Region: NODE\_374673\_length\_37756\_cov\_28.992161 35839-35841. Max. coverage (+): 0. Max coverage (-): 0

Region: NODE\_374673\_length\_37756\_cov\_28.992161 35842-35843. Max. coverage (+): 0. Max coverage (-): 0

Region: NODE\_374673\_length\_37756\_cov\_28.992161 35844-35846. Max. coverage (+): 0. Max coverage (-): 0

Region: NODE\_374673\_length\_37756\_cov\_28.992161 35847-35848. Max. coverage (+): 0. Max coverage (-): 0

Region: NODE\_374673\_length\_37756\_cov\_28.992161 35849-35851. Max. coverage (+): 0. Max coverage (-): 0

Region: NODE\_374673\_length\_37756\_cov\_28.992161 35852-35853. Max. coverage (+): 0. Max coverage (-): 0

Region: NODE\_374673\_length\_37756\_cov\_28.992161 35854-35856. Max. coverage (+): 0. Max coverage (-): 0

Region: NODE\_374673\_length\_37756\_cov\_28.992161 35857-35858. Max. coverage (+): 0. Max coverage (-): 0

Region: NODE\_374673\_length\_37756\_cov\_28.992161 35859-35861. Max. coverage (+): 0. Max coverage (-): 0

Region: NODE\_374673\_length\_37756\_cov\_28.992161 35862-35863. Max. coverage (+): 0. Max coverage (-): 0

Region: NODE\_374673\_length\_37756\_cov\_28.992161 35864-35866. Max. coverage (+): 0. Max coverage (-): 0

Region: NODE\_374673\_length\_37756\_cov\_28.992161 35867-35869. Max. coverage (+): 0. Max coverage (-): 0

Region: NODE\_374673\_length\_37756\_cov\_28.992161 35870-35871. Max. coverage (+): 0. Max coverage (-): 0

Region: NODE\_374673\_length\_37756\_cov\_28.992161 35872-35874. Max. coverage (+): 0. Max coverage (-): 0

Region: NODE\_374673\_length\_37756\_cov\_28.992161 35875-35876. Max. coverage (+): 0. Max coverage (-): 0

Region: NODE\_374673\_length\_37756\_cov\_28.992161 35877-35879. Max. coverage (+): 0. Max coverage (-): 0

Region: NODE\_374673\_length\_37756\_cov\_28.992161 35880-35881. Max. coverage (+): 0. Max coverage (-): 0

Region: NODE\_374673\_length\_37756\_cov\_28.992161 35882-35884. Max. coverage (+): 0. Max coverage (-): 0

Region: NODE\_374673\_length\_37756\_cov\_28.992161 35885-35886. Max. coverage (+): 0. Max coverage (-): 0

Region: NODE\_374673\_length\_37756\_cov\_28.992161 35887-35889. Max. coverage (+): 0. Max coverage (-): 0

Region: NODE\_374673\_length\_37756\_cov\_28.992161 35890-35891. Max. coverage (+): 0. Max coverage (-): 0

Region: NODE\_374673\_length\_37756\_cov\_28.992161 35892-35894. Max. coverage (+): 0. Max coverage (-): 0

Region: NODE\_374673\_length\_37756\_cov\_28.992161 35895-35897. Max. coverage (+): 0. Max coverage (-): 0

Region: NODE\_374673\_length\_37756\_cov\_28.992161 35898-35899. Max. coverage (+): 0. Max coverage (-): 0

Region: NODE\_374673\_length\_37756\_cov\_28.992161 35900-35902. Max. coverage (+): 0. Max coverage (-): 0

Region: NODE\_374673\_length\_37756\_cov\_28.992161 35903-35904. Max. coverage (+): 0. Max coverage (-): 0

Region: NODE\_374673\_length\_37756\_cov\_28.992161 35905-35907. Max. coverage (+): 0. Max coverage (-): 0

Region: NODE\_374673\_length\_37756\_cov\_28.992161 35908-35909. Max. coverage (+): 0. Max coverage (-): 0

Region: NODE\_374673\_length\_37756\_cov\_28.992161 35910-35912. Max. coverage (+): 0. Max coverage (-): 0

Region: NODE\_374673\_length\_37756\_cov\_28.992161 35913-35914. Max. coverage (+): 0. Max coverage (-): 0

Region: NODE\_374673\_length\_37756\_cov\_28.992161 35915-35917. Max. coverage (+): 0. Max coverage (-): 0

Region: NODE\_374673\_length\_37756\_cov\_28.992161 35918-35919. Max. coverage (+): 0. Max coverage (-): 0

Region: NODE\_374673\_length\_37756\_cov\_28.992161 35920-35922. Max. coverage (+): 0. Max coverage (-): 0

Region: NODE\_374673\_length\_37756\_cov\_28.992161 35923-35925. Max. coverage (+): 0. Max coverage (-): 0

Region: NODE\_374673\_length\_37756\_cov\_28.992161 35926-35927. Max. coverage (+): 0. Max coverage (-): 0

Region: NODE\_374673\_length\_37756\_cov\_28.992161 35928-35930. Max. coverage (+): 0. Max coverage (-): 0

Region: NODE\_374673\_length\_37756\_cov\_28.992161 35931-35932. Max. coverage (+): 0. Max coverage (-): 0

Region: NODE\_374673\_length\_37756\_cov\_28.992161 35933-35935. Max. coverage (+): 0. Max coverage (-): 0

Region: NODE\_374673\_length\_37756\_cov\_28.992161 35936-35937. Max. coverage (+): 0. Max coverage (-): 0

Region: NODE\_374673\_length\_37756\_cov\_28.992161 35938-35940. Max. coverage (+): 0. Max coverage (-): 0

Region: NODE\_374673\_length\_37756\_cov\_28.992161 35941-35942. Max. coverage (+): 0. Max coverage (-): 0

Region: NODE\_374673\_length\_37756\_cov\_28.992161 35943-35945. Max. coverage (+): 0. Max coverage (-): 0

Region: NODE\_374673\_length\_37756\_cov\_28.992161 35946-35947. Max. coverage (+): 0. Max coverage (-): 0

Region: NODE\_374673\_length\_37756\_cov\_28.992161 35948-35950. Max. coverage (+): 0. Max coverage (-): 0

Region: NODE\_374673\_length\_37756\_cov\_28.992161 35951-35953. Max. coverage (+): 0. Max coverage (-): 0

Region: NODE\_374673\_length\_37756\_cov\_28.992161 35954-35955. Max. coverage (+): 0. Max coverage (-): 0

Region: NODE\_374673\_length\_37756\_cov\_28.992161 35956-35958. Max. coverage (+): 0. Max coverage (-): 0

Region: NODE\_374673\_length\_37756\_cov\_28.992161 35959-35960. Max. coverage (+): 0. Max coverage (-): 0

Region: NODE\_374673\_length\_37756\_cov\_28.992161 35961-35963. Max. coverage (+): 0. Max coverage (-): 0

Region: NODE\_374673\_length\_37756\_cov\_28.992161 35964-35965. Max. coverage (+): 0. Max coverage (-): 0

Region: NODE\_374673\_length\_37756\_cov\_28.992161 35966-35968. Max. coverage (+): 0. Max coverage (-): 0

Region: NODE\_374673\_length\_37756\_cov\_28.992161 35969-35970. Max. coverage (+): 0. Max coverage (-): 0

Region: NODE\_374673\_length\_37756\_cov\_28.992161 35971-35973. Max. coverage (+): 0. Max coverage (-): 0

Region: NODE\_374673\_length\_37756\_cov\_28.992161 35974-35975. Max. coverage (+): 0. Max coverage (-): 0

Region: NODE\_374673\_length\_37756\_cov\_28.992161 35976-35978. Max. coverage (+): 0. Max coverage (-): 0

Region: NODE\_374673\_length\_37756\_cov\_28.992161 35979-35981. Max. coverage (+): 0. Max coverage (-): 0

Region: NODE\_374673\_length\_37756\_cov\_28.992161 35982-35983. Max. coverage (+): 0. Max coverage (-): 0

Region: NODE\_374673\_length\_37756\_cov\_28.992161 35984-35986. Max. coverage (+): 0. Max coverage (-): 0

Region: NODE\_374673\_length\_37756\_cov\_28.992161 35987-35988. Max. coverage (+): 0. Max coverage (-): 0

Region: NODE\_374673\_length\_37756\_cov\_28.992161 35989-35991. Max. coverage (+): 0. Max coverage (-): 0

Region: NODE\_374673\_length\_37756\_cov\_28.992161 35992-35993. Max. coverage (+): 0. Max coverage (-): 0

Region: NODE\_374673\_length\_37756\_cov\_28.992161 35994-35996. Max. coverage (+): 0. Max coverage (-): 0

Region: NODE\_374673\_length\_37756\_cov\_28.992161 35997-35998. Max. coverage (+): 0. Max coverage (-): 0

Region: NODE\_374673\_length\_37756\_cov\_28.992161 35999-36001. Max. coverage (+): 0. Max coverage (-): 0

Region: NODE\_374673\_length\_37756\_cov\_28.992161 36002-36003. Max. coverage (+): 0. Max coverage (-): 0

Region: NODE\_374673\_length\_37756\_cov\_28.992161 36004-36006. Max. coverage (+): 0. Max coverage (-): 0

Region: NODE\_374673\_length\_37756\_cov\_28.992161 36007-36009. Max. coverage (+): 0. Max coverage (-): 0

Region: NODE\_374673\_length\_37756\_cov\_28.992161 36010-36011. Max. coverage (+): 0. Max coverage (-): 0

Region: NODE\_374673\_length\_37756\_cov\_28.992161 36012-36014. Max. coverage (+): 0. Max coverage (-): 0

Region: NODE\_374673\_length\_37756\_cov\_28.992161 36015-36016. Max. coverage (+): 0. Max coverage (-): 0

Region: NODE\_374673\_length\_37756\_cov\_28.992161 36017-36019. Max. coverage (+): 0. Max coverage (-): 0

Region: NODE\_374673\_length\_37756\_cov\_28.992161 36020-36021. Max. coverage (+): 0. Max coverage (-): 0

Region: NODE\_374673\_length\_37756\_cov\_28.992161 36022-36024. Max. coverage (+): 0. Max coverage (-): 0

Region: NODE\_374673\_length\_37756\_cov\_28.992161 36025-36026. Max. coverage (+): 0. Max coverage (-): 0

Region: NODE\_374673\_length\_37756\_cov\_28.992161 36027-36029. Max. coverage (+): 0. Max coverage (-): 0

Region: NODE\_374673\_length\_37756\_cov\_28.992161 36030-36031. Max. coverage (+): 0. Max coverage (-): 0

Region: NODE\_374673\_length\_37756\_cov\_28.992161 36032-36034. Max. coverage (+): 0. Max coverage (-): 0

Region: NODE\_374673\_length\_37756\_cov\_28.992161 36035-36037. Max. coverage (+): 0. Max coverage (-): 0

Region: NODE\_374673\_length\_37756\_cov\_28.992161 36038-36039. Max. coverage (+): 0. Max coverage (-): 0

Region: NODE\_374673\_length\_37756\_cov\_28.992161 36040-36042. Max. coverage (+): 0. Max coverage (-): 0

Region: NODE\_374673\_length\_37756\_cov\_28.992161 36043-36044. Max. coverage (+): 0. Max coverage (-): 0

Region: NODE\_374673\_length\_37756\_cov\_28.992161 36045-36047. Max. coverage (+): 0. Max coverage (-): 0

Region: NODE\_374673\_length\_37756\_cov\_28.992161 36048-36049. Max. coverage (+): 0. Max coverage (-): 0

Region: NODE\_374673\_length\_37756\_cov\_28.992161 36050-36052. Max. coverage (+): 0. Max coverage (-): 0

Region: NODE\_374673\_length\_37756\_cov\_28.992161 36053-36054. Max. coverage (+): 0. Max coverage (-): 0

Region: NODE\_374673\_length\_37756\_cov\_28.992161 36055-36057. Max. coverage (+): 0. Max coverage (-): 0

Region: NODE\_374673\_length\_37756\_cov\_28.992161 36058-36059. Max. coverage (+): 0. Max coverage (-): 0

Region: NODE\_374673\_length\_37756\_cov\_28.992161 36060-36062. Max. coverage (+): 0. Max coverage (-): 0

Region: NODE\_374673\_length\_37756\_cov\_28.992161 36063-36065. Max. coverage (+): 0. Max coverage (-): 0

Region: NODE\_374673\_length\_37756\_cov\_28.992161 36066-36067. Max. coverage (+): 0. Max coverage (-): 0

Region: NODE\_374673\_length\_37756\_cov\_28.992161 36068-36070. Max. coverage (+): 0. Max coverage (-): 0

Region: NODE\_374673\_length\_37756\_cov\_28.992161 36071-36072. Max. coverage (+): 0. Max coverage (-): 0

Region: NODE\_374673\_length\_37756\_cov\_28.992161 36073-36075. Max. coverage (+): 0. Max coverage (-): 0

Region: NODE\_374673\_length\_37756\_cov\_28.992161 36076-36077. Max. coverage (+): 0. Max coverage (-): 0

Region: NODE\_374673\_length\_37756\_cov\_28.992161 36078-36080. Max. coverage (+): 0. Max coverage (-): 0

Region: NODE\_374673\_length\_37756\_cov\_28.992161 36081-36082. Max. coverage (+): 0. Max coverage (-): 0

Region: NODE\_374673\_length\_37756\_cov\_28.992161 36083-36085. Max. coverage (+): 0. Max coverage (-): 0

Region: NODE\_374673\_length\_37756\_cov\_28.992161 36086-36087. Max. coverage (+): 0. Max coverage (-): 0

Region: NODE\_374673\_length\_37756\_cov\_28.992161 36088-36090. Max. coverage (+): 0. Max coverage (-): 0

Region: NODE\_374673\_length\_37756\_cov\_28.992161 36091-36093. Max. coverage (+): 0. Max coverage (-): 0

Region: NODE\_374673\_length\_37756\_cov\_28.992161 36094-36095. Max. coverage (+): 0. Max coverage (-): 0

Region: NODE\_374673\_length\_37756\_cov\_28.992161 36096-36098. Max. coverage (+): 0. Max coverage (-): 0

Region: NODE\_374673\_length\_37756\_cov\_28.992161 36099-36100. Max. coverage (+): 0. Max coverage (-): 0

Region: NODE\_374673\_length\_37756\_cov\_28.992161 36101-36103. Max. coverage (+): 0. Max coverage (-): 0

Region: NODE\_374673\_length\_37756\_cov\_28.992161 36104-36105. Max. coverage (+): 0. Max coverage (-): 0

Region: NODE\_374673\_length\_37756\_cov\_28.992161 36106-36108. Max. coverage (+): 0. Max coverage (-): 0

Region: NODE\_374673\_length\_37756\_cov\_28.992161 36109-36110. Max. coverage (+): 0. Max coverage (-): 0

Region: NODE\_374673\_length\_37756\_cov\_28.992161 36111-36113. Max. coverage (+): 0. Max coverage (-): 0

Region: NODE\_374673\_length\_37756\_cov\_28.992161 36114-36115. Max. coverage (+): 0. Max coverage (-): 0

Region: NODE\_374673\_length\_37756\_cov\_28.992161 36116-36118. Max. coverage (+): 0. Max coverage (-): 0

Region: NODE\_374673\_length\_37756\_cov\_28.992161 36119-36121. Max. coverage (+): 0. Max coverage (-): 0

Region: NODE\_374673\_length\_37756\_cov\_28.992161 36122-36123. Max. coverage (+): 0. Max coverage (-): 0

Region: NODE\_374673\_length\_37756\_cov\_28.992161 36124-36126. Max. coverage (+): 0. Max coverage (-): 0

Region: NODE\_374673\_length\_37756\_cov\_28.992161 36127-36128. Max. coverage (+): 0. Max coverage (-): 0

Region: NODE\_374673\_length\_37756\_cov\_28.992161 36129-36131. Max. coverage (+): 0. Max coverage (-): 0

Region: NODE\_374673\_length\_37756\_cov\_28.992161 36132-36133. Max. coverage (+): 0. Max coverage (-): 0

Region: NODE\_374673\_length\_37756\_cov\_28.992161 36134-36136. Max. coverage (+): 0. Max coverage (-): 0

Region: NODE\_374673\_length\_37756\_cov\_28.992161 36137-36138. Max. coverage (+): 0. Max coverage (-): 0

Region: NODE\_374673\_length\_37756\_cov\_28.992161 36139-36141. Max. coverage (+): 0. Max coverage (-): 0

Region: NODE\_374673\_length\_37756\_cov\_28.992161 36142-36143. Max. coverage (+): 0. Max coverage (-): 0

Region: NODE\_374673\_length\_37756\_cov\_28.992161 36144-36146. Max. coverage (+): 0. Max coverage (-): 0

Region: NODE\_374673\_length\_37756\_cov\_28.992161 36147-36149. Max. coverage (+): 0. Max coverage (-): 0

Region: NODE\_374673\_length\_37756\_cov\_28.992161 36150-36151. Max. coverage (+): 0. Max coverage (-): 0

Region: NODE\_374673\_length\_37756\_cov\_28.992161 36152-36154. Max. coverage (+): 0. Max coverage (-): 0

Region: NODE\_374673\_length\_37756\_cov\_28.992161 36155-36156. Max. coverage (+): 0. Max coverage (-): 0

Region: NODE\_374673\_length\_37756\_cov\_28.992161 36157-36159. Max. coverage (+): 0. Max coverage (-): 0

Region: NODE\_374673\_length\_37756\_cov\_28.992161 36160-36161. Max. coverage (+): 0. Max coverage (-): 0

Region: NODE\_374673\_length\_37756\_cov\_28.992161 36162-36164. Max. coverage (+): 0. Max coverage (-): 0

Region: NODE\_374673\_length\_37756\_cov\_28.992161 36165-36166. Max. coverage (+): 0. Max coverage (-): 0

Region: NODE\_374673\_length\_37756\_cov\_28.992161 36167-36169. Max. coverage (+): 0. Max coverage (-): 0

Region: NODE\_374673\_length\_37756\_cov\_28.992161 36170-36172. Max. coverage (+): 0. Max coverage (-): 0

Region: NODE\_374673\_length\_37756\_cov\_28.992161 36173-36174. Max. coverage (+): 0. Max coverage (-): 0

Region: NODE\_374673\_length\_37756\_cov\_28.992161 36175-36177. Max. coverage (+): 0. Max coverage (-): 0

Region: NODE\_374673\_length\_37756\_cov\_28.992161 36178-36179. Max. coverage (+): 0. Max coverage (-): 0

Region: NODE\_374673\_length\_37756\_cov\_28.992161 36180-36182. Max. coverage (+): 0. Max coverage (-): 0

Region: NODE\_374673\_length\_37756\_cov\_28.992161 36183-36184. Max. coverage (+): 0. Max coverage (-): 0

Region: NODE\_374673\_length\_37756\_cov\_28.992161 36185-36187. Max. coverage (+): 0. Max coverage (-): 0

Region: NODE\_374673\_length\_37756\_cov\_28.992161 36188-36189. Max. coverage (+): 0. Max coverage (-): 0

Region: NODE\_374673\_length\_37756\_cov\_28.992161 36190-36192. Max. coverage (+): 0. Max coverage (-): 0

Region: NODE\_374673\_length\_37756\_cov\_28.992161 36193-36194. Max. coverage (+): 0. Max coverage (-): 0

Region: NODE\_374673\_length\_37756\_cov\_28.992161 36195-36197. Max. coverage (+): 0. Max coverage (-): 0

Region: NODE\_374673\_length\_37756\_cov\_28.992161 36198-36200. Max. coverage (+): 0. Max coverage (-): 0

Region: NODE\_374673\_length\_37756\_cov\_28.992161 36201-36202. Max. coverage (+): 0. Max coverage (-): 0

Region: NODE\_374673\_length\_37756\_cov\_28.992161 36203-36205. Max. coverage (+): 0. Max coverage (-): 0

Region: NODE\_374673\_length\_37756\_cov\_28.992161 36206-36207. Max. coverage (+): 0. Max coverage (-): 0

Region: NODE\_374673\_length\_37756\_cov\_28.992161 36208-36210. Max. coverage (+): 0. Max coverage (-): 0

Region: NODE\_374673\_length\_37756\_cov\_28.992161 36211-36212. Max. coverage (+): 0. Max coverage (-): 0

Region: NODE\_374673\_length\_37756\_cov\_28.992161 36213-36215. Max. coverage (+): 0. Max coverage (-): 0

Region: NODE\_374673\_length\_37756\_cov\_28.992161 36216-36217. Max. coverage (+): 0. Max coverage (-): 0

Region: NODE\_374673\_length\_37756\_cov\_28.992161 36218-36220. Max. coverage (+): 0. Max coverage (-): 0

Region: NODE\_374673\_length\_37756\_cov\_28.992161 36221-36222. Max. coverage (+): 0. Max coverage (-): 0

Region: NODE\_374673\_length\_37756\_cov\_28.992161 36223-36225. Max. coverage (+): 0. Max coverage (-): 0

Region: NODE\_374673\_length\_37756\_cov\_28.992161 36226-36228. Max. coverage (+): 0. Max coverage (-): 0

Region: NODE\_374673\_length\_37756\_cov\_28.992161 36229-36230. Max. coverage (+): 0. Max coverage (-): 0

Region: NODE\_374673\_length\_37756\_cov\_28.992161 36231-36233. Max. coverage (+): 0. Max coverage (-): 0

Region: NODE\_374673\_length\_37756\_cov\_28.992161 36234-36235. Max. coverage (+): 0. Max coverage (-): 0

Region: NODE\_374673\_length\_37756\_cov\_28.992161 36236-36238. Max. coverage (+): 0. Max coverage (-): 0

Region: NODE\_374673\_length\_37756\_cov\_28.992161 36239-36240. Max. coverage (+): 0. Max coverage (-): 0

Region: NODE\_374673\_length\_37756\_cov\_28.992161 36241-36243. Max. coverage (+): 0. Max coverage (-): 0

Region: NODE\_374673\_length\_37756\_cov\_28.992161 36244-36245. Max. coverage (+): 0. Max coverage (-): 0

Region: NODE\_374673\_length\_37756\_cov\_28.992161 36246-36248. Max. coverage (+): 0. Max coverage (-): 0

Region: NODE\_374673\_length\_37756\_cov\_28.992161 36249-36250. Max. coverage (+): 0. Max coverage (-): 0

Region: NODE\_374673\_length\_37756\_cov\_28.992161 36251-36253. Max. coverage (+): 0. Max coverage (-): 0

Region: NODE\_374673\_length\_37756\_cov\_28.992161 36254-36256. Max. coverage (+): 0. Max coverage (-): 0

Region: NODE\_374673\_length\_37756\_cov\_28.992161 36257-36258. Max. coverage (+): 0. Max coverage (-): 0

Region: NODE\_374673\_length\_37756\_cov\_28.992161 36259-36261. Max. coverage (+): 0. Max coverage (-): 0

Region: NODE\_374673\_length\_37756\_cov\_28.992161 36262-36263. Max. coverage (+): 0. Max coverage (-): 0

Region: NODE\_374673\_length\_37756\_cov\_28.992161 36264-36266. Max. coverage (+): 0. Max coverage (-): 0

Region: NODE\_374673\_length\_37756\_cov\_28.992161 36267-36268. Max. coverage (+): 0. Max coverage (-): 0

Region: NODE\_374673\_length\_37756\_cov\_28.992161 36269-36271. Max. coverage (+): 0. Max coverage (-): 0

Region: NODE\_374673\_length\_37756\_cov\_28.992161 36272-36273. Max. coverage (+): 0. Max coverage (-): 0

Region: NODE\_374673\_length\_37756\_cov\_28.992161 36274-36276. Max. coverage (+): 0.09. Max coverage (-): 0

Region: NODE\_374673\_length\_37756\_cov\_28.992161 36277-36278. Max. coverage (+): 0.09. Max coverage (-): 0

Region: NODE\_374673\_length\_37756\_cov\_28.992161 36279-36281. Max. coverage (+): 0.09. Max coverage (-): 0

Region: NODE\_374673\_length\_37756\_cov\_28.992161 36282-36284. Max. coverage (+): 0. Max coverage (-): 0

Region: NODE\_374673\_length\_37756\_cov\_28.992161 36285-36286. Max. coverage (+): 0. Max coverage (-): 0

Region: NODE\_374673\_length\_37756\_cov\_28.992161 36287-36289. Max. coverage (+): 0. Max coverage (-): 0

Region: NODE\_374673\_length\_37756\_cov\_28.992161 36290-36291. Max. coverage (+): 0. Max coverage (-): 0

Region: NODE\_374673\_length\_37756\_cov\_28.992161 36292-36294. Max. coverage (+): 0. Max coverage (-): 0

Region: NODE\_374673\_length\_37756\_cov\_28.992161 36295-36296. Max. coverage (+): 0. Max coverage (-): 0

Region: NODE\_374673\_length\_37756\_cov\_28.992161 36297-36299. Max. coverage (+): 0. Max coverage (-): 0

Region: NODE\_374673\_length\_37756\_cov\_28.992161 36300-36301. Max. coverage (+): 0. Max coverage (-): 0

Region: NODE\_374673\_length\_37756\_cov\_28.992161 36302-36304. Max. coverage (+): 0. Max coverage (-): 0

Region: NODE\_374673\_length\_37756\_cov\_28.992161 36305-36306. Max. coverage (+): 0. Max coverage (-): 0

Region: NODE\_374673\_length\_37756\_cov\_28.992161 36307-36309. Max. coverage (+): 0. Max coverage (-): 0

Region: NODE\_374673\_length\_37756\_cov\_28.992161 36310-36312. Max. coverage (+): 0. Max coverage (-): 0

Region: NODE\_374673\_length\_37756\_cov\_28.992161 36313-36314. Max. coverage (+): 0. Max coverage (-): 0

Region: NODE\_374673\_length\_37756\_cov\_28.992161 36315-36317. Max. coverage (+): 0. Max coverage (-): 0

Region: NODE\_374673\_length\_37756\_cov\_28.992161 36318-36319. Max. coverage (+): 0. Max coverage (-): 0

Region: NODE\_374673\_length\_37756\_cov\_28.992161 36320-36322. Max. coverage (+): 0. Max coverage (-): 0

Region: NODE\_374673\_length\_37756\_cov\_28.992161 36323-36324. Max. coverage (+): 0. Max coverage (-): 0

Region: NODE\_374673\_length\_37756\_cov\_28.992161 36325-36327. Max. coverage (+): 0. Max coverage (-): 0

Region: NODE\_374673\_length\_37756\_cov\_28.992161 36328-36329. Max. coverage (+): 0. Max coverage (-): 0

Region: NODE\_374673\_length\_37756\_cov\_28.992161 36330-36332. Max. coverage (+): 0. Max coverage (-): 0

Region: NODE\_374673\_length\_37756\_cov\_28.992161 36333-36334. Max. coverage (+): 0. Max coverage (-): 0

Region: NODE\_374673\_length\_37756\_cov\_28.992161 36335-36337. Max. coverage (+): 0. Max coverage (-): 0

Region: NODE\_374673\_length\_37756\_cov\_28.992161 36338-36340. Max. coverage (+): 0. Max coverage (-): 0

Region: NODE\_374673\_length\_37756\_cov\_28.992161 36341-36342. Max. coverage (+): 0. Max coverage (-): 0

Region: NODE\_374673\_length\_37756\_cov\_28.992161 36343-36345. Max. coverage (+): 0. Max coverage (-): 0

Region: NODE\_374673\_length\_37756\_cov\_28.992161 36346-36347. Max. coverage (+): 0. Max coverage (-): 0

Region: NODE\_374673\_length\_37756\_cov\_28.992161 36348-36350. Max. coverage (+): 0. Max coverage (-): 0

Region: NODE\_374673\_length\_37756\_cov\_28.992161 36351-36352. Max. coverage (+): 0. Max coverage (-): 0

Region: NODE\_374673\_length\_37756\_cov\_28.992161 36353-36355. Max. coverage (+): 0. Max coverage (-): 0

Region: NODE\_374673\_length\_37756\_cov\_28.992161 36356-36357. Max. coverage (+): 0. Max coverage (-): 0

Region: NODE\_374673\_length\_37756\_cov\_28.992161 36358-36360. Max. coverage (+): 0. Max coverage (-): 0

Region: NODE\_374673\_length\_37756\_cov\_28.992161 36361-36362. Max. coverage (+): 0. Max coverage (-): 0

Region: NODE\_374673\_length\_37756\_cov\_28.992161 36363-36365. Max. coverage (+): 0. Max coverage (-): 0

Region: NODE\_374673\_length\_37756\_cov\_28.992161 36366-36368. Max. coverage (+): 0. Max coverage (-): 0

Region: NODE\_374673\_length\_37756\_cov\_28.992161 36369-36370. Max. coverage (+): 0. Max coverage (-): 0

Region: NODE\_374673\_length\_37756\_cov\_28.992161 36371-36373. Max. coverage (+): 0. Max coverage (-): 0

Region: NODE\_374673\_length\_37756\_cov\_28.992161 36374-36375. Max. coverage (+): 0. Max coverage (-): 0

Region: NODE\_374673\_length\_37756\_cov\_28.992161 36376-36378. Max. coverage (+): 0. Max coverage (-): 0

Region: NODE\_374673\_length\_37756\_cov\_28.992161 36379-36380. Max. coverage (+): 0. Max coverage (-): 0

Region: NODE\_374673\_length\_37756\_cov\_28.992161 36381-36383. Max. coverage (+): 0. Max coverage (-): 0

Region: NODE\_374673\_length\_37756\_cov\_28.992161 36384-36385. Max. coverage (+): 0. Max coverage (-): 0

Region: NODE\_374673\_length\_37756\_cov\_28.992161 36386-36388. Max. coverage (+): 0. Max coverage (-): 0

Region: NODE\_374673\_length\_37756\_cov\_28.992161 36389-36390. Max. coverage (+): 0. Max coverage (-): 0

Region: NODE\_374673\_length\_37756\_cov\_28.992161 36391-36393. Max. coverage (+): 0. Max coverage (-): 0

Region: NODE\_374673\_length\_37756\_cov\_28.992161 36394-36396. Max. coverage (+): 0. Max coverage (-): 0

Region: NODE\_374673\_length\_37756\_cov\_28.992161 36397-36398. Max. coverage (+): 0. Max coverage (-): 0

Region: NODE\_374673\_length\_37756\_cov\_28.992161 36399-36401. Max. coverage (+): 0. Max coverage (-): 0

Region: NODE\_374673\_length\_37756\_cov\_28.992161 36402-36403. Max. coverage (+): 0. Max coverage (-): 0

Region: NODE\_374673\_length\_37756\_cov\_28.992161 36404-36406. Max. coverage (+): 0. Max coverage (-): 0

Region: NODE\_374673\_length\_37756\_cov\_28.992161 36407-36408. Max. coverage (+): 0. Max coverage (-): 0

Region: NODE\_374673\_length\_37756\_cov\_28.992161 36409-36411. Max. coverage (+): 0. Max coverage (-): 0

Region: NODE\_374673\_length\_37756\_cov\_28.992161 36412-36413. Max. coverage (+): 0. Max coverage (-): 0

Region: NODE\_374673\_length\_37756\_cov\_28.992161 36414-36416. Max. coverage (+): 0. Max coverage (-): 0

Region: NODE\_374673\_length\_37756\_cov\_28.992161 36417-36418. Max. coverage (+): 0. Max coverage (-): 0

Region: NODE\_374673\_length\_37756\_cov\_28.992161 36419-36421. Max. coverage (+): 0. Max coverage (-): 0

Region: NODE\_374673\_length\_37756\_cov\_28.992161 36422-36424. Max. coverage (+): 0. Max coverage (-): 0

Region: NODE\_374673\_length\_37756\_cov\_28.992161 36425-36426. Max. coverage (+): 0. Max coverage (-): 0

Region: NODE\_374673\_length\_37756\_cov\_28.992161 36427-36429. Max. coverage (+): 0. Max coverage (-): 0

Region: NODE\_374673\_length\_37756\_cov\_28.992161 36430-36431. Max. coverage (+): 0. Max coverage (-): 0

Region: NODE\_374673\_length\_37756\_cov\_28.992161 36432-36434. Max. coverage (+): 0. Max coverage (-): 0

Region: NODE\_374673\_length\_37756\_cov\_28.992161 36435-36436. Max. coverage (+): 0. Max coverage (-): 0

Region: NODE\_374673\_length\_37756\_cov\_28.992161 36437-36439. Max. coverage (+): 0. Max coverage (-): 0

Region: NODE\_374673\_length\_37756\_cov\_28.992161 36440-36441. Max. coverage (+): 0. Max coverage (-): 0

Region: NODE\_374673\_length\_37756\_cov\_28.992161 36442-36444. Max. coverage (+): 0. Max coverage (-): 0

Region: NODE\_374673\_length\_37756\_cov\_28.992161 36445-36446. Max. coverage (+): 0. Max coverage (-): 0

Region: NODE\_374673\_length\_37756\_cov\_28.992161 36447-36449. Max. coverage (+): 0. Max coverage (-): 0

Region: NODE\_374673\_length\_37756\_cov\_28.992161 36450-36452. Max. coverage (+): 0. Max coverage (-): 0

Region: NODE\_374673\_length\_37756\_cov\_28.992161 36453-36454. Max. coverage (+): 0. Max coverage (-): 0

Region: NODE\_374673\_length\_37756\_cov\_28.992161 36455-36457. Max. coverage (+): 0. Max coverage (-): 0

Region: NODE\_374673\_length\_37756\_cov\_28.992161 36458-36459. Max. coverage (+): 0. Max coverage (-): 0

Region: NODE\_374673\_length\_37756\_cov\_28.992161 36460-36462. Max. coverage (+): 0. Max coverage (-): 0

Region: NODE\_374673\_length\_37756\_cov\_28.992161 36463-36464. Max. coverage (+): 0. Max coverage (-): 0

Region: NODE\_374673\_length\_37756\_cov\_28.992161 36465-36467. Max. coverage (+): 0. Max coverage (-): 0

Region: NODE\_374673\_length\_37756\_cov\_28.992161 36468-36469. Max. coverage (+): 0. Max coverage (-): 0

Region: NODE\_374673\_length\_37756\_cov\_28.992161 36470-36472. Max. coverage (+): 0. Max coverage (-): 0

Region: NODE\_374673\_length\_37756\_cov\_28.992161 36473-36474. Max. coverage (+): 0. Max coverage (-): 0

Region: NODE\_374673\_length\_37756\_cov\_28.992161 36475-36477. Max. coverage (+): 0. Max coverage (-): 0

Region: NODE\_374673\_length\_37756\_cov\_28.992161 36478-36480. Max. coverage (+): 0.38. Max coverage (-): 0

Region: NODE\_374673\_length\_37756\_cov\_28.992161 36481-36482. Max. coverage (+): 5286.3. Max coverage (-): 0

Region: NODE\_374673\_length\_37756\_cov\_28.992161 36483-36485. Max. coverage (+): 5286.49. Max coverage (-): 0

Region: NODE\_374673\_length\_37756\_cov\_28.992161 36486-36487. Max. coverage (+): 1.32. Max coverage (-): 0

Region: NODE\_374673\_length\_37756\_cov\_28.992161 36488-36490. Max. coverage (+): 0. Max coverage (-): 0

Region: NODE\_374673\_length\_37756\_cov\_28.992161 36491-36492. Max. coverage (+): 0. Max coverage (-): 0

Region: NODE\_374673\_length\_37756\_cov\_28.992161 36493-36495. Max. coverage (+): 0. Max coverage (-): 0

Region: NODE\_374673\_length\_37756\_cov\_28.992161 36496-36497. Max. coverage (+): 0. Max coverage (-): 0

Region: NODE\_374673\_length\_37756\_cov\_28.992161 36498-36500. Max. coverage (+): 0. Max coverage (-): 0

Region: NODE\_374673\_length\_37756\_cov\_28.992161 36501-36502. Max. coverage (+): 0. Max coverage (-): 0

Region: NODE\_374673\_length\_37756\_cov\_28.992161 36503-36505. Max. coverage (+): 0. Max coverage (-): 0

Region: NODE\_374673\_length\_37756\_cov\_28.992161 36506-36508. Max. coverage (+): 0. Max coverage (-): 0

Region: NODE\_374673\_length\_37756\_cov\_28.992161 36509-36510. Max. coverage (+): 0.09. Max coverage (-): 0

Region: NODE\_374673\_length\_37756\_cov\_28.992161 36511-36513. Max. coverage (+): 0.09. Max coverage (-): 0

Region: NODE\_374673\_length\_37756\_cov\_28.992161 36514-36515. Max. coverage (+): 0. Max coverage (-): 0

Region: NODE\_374673\_length\_37756\_cov\_28.992161 36516-36518. Max. coverage (+): 0. Max coverage (-): 0

Region: NODE\_374673\_length\_37756\_cov\_28.992161 36519-36520. Max. coverage (+): 4.23. Max coverage (-): 0

Region: NODE\_374673\_length\_37756\_cov\_28.992161 36521-36523. Max. coverage (+): 5.73. Max coverage (-): 0

Region: NODE\_374673\_length\_37756\_cov\_28.992161 36524-36525. Max. coverage (+): 5.54. Max coverage (-): 0

Region: NODE\_374673\_length\_37756\_cov\_28.992161 36526-36528. Max. coverage (+): 0. Max coverage (-): 0

Region: NODE\_374673\_length\_37756\_cov\_28.992161 36529-36530. Max. coverage (+): 0. Max coverage (-): 0

Region: NODE\_374673\_length\_37756\_cov\_28.992161 36531-36533. Max. coverage (+): 0. Max coverage (-): 0

Region: NODE\_374673\_length\_37756\_cov\_28.992161 36534-36536. Max. coverage (+): 0. Max coverage (-): 0

Region: NODE\_374673\_length\_37756\_cov\_28.992161 36537-36538. Max. coverage (+): 0. Max coverage (-): 0

Region: NODE\_374673\_length\_37756\_cov\_28.992161 36539-36541. Max. coverage (+): 0. Max coverage (-): 0

Region: NODE\_374673\_length\_37756\_cov\_28.992161 36542-36543. Max. coverage (+): 0. Max coverage (-): 0

Region: NODE\_374673\_length\_37756\_cov\_28.992161 36544-36546. Max. coverage (+): 0. Max coverage (-): 0

Region: NODE\_374673\_length\_37756\_cov\_28.992161 36547-36548. Max. coverage (+): 0. Max coverage (-): 0

Region: NODE\_374673\_length\_37756\_cov\_28.992161 36549-36551. Max. coverage (+): 0. Max coverage (-): 0

Region: NODE\_374673\_length\_37756\_cov\_28.992161 36552-36553. Max. coverage (+): 0. Max coverage (-): 0

Region: NODE\_374673\_length\_37756\_cov\_28.992161 36554-36556. Max. coverage (+): 0. Max coverage (-): 0

Region: NODE\_374673\_length\_37756\_cov\_28.992161 36557-36558. Max. coverage (+): 0. Max coverage (-): 0

Region: NODE\_374673\_length\_37756\_cov\_28.992161 36559-36561. Max. coverage (+): 0. Max coverage (-): 0

Region: NODE\_374673\_length\_37756\_cov\_28.992161 36562-36564. Max. coverage (+): 0. Max coverage (-): 0

Region: NODE\_374673\_length\_37756\_cov\_28.992161 36565-36566. Max. coverage (+): 0. Max coverage (-): 0

Region: NODE\_374673\_length\_37756\_cov\_28.992161 36567-36569. Max. coverage (+): 0. Max coverage (-): 0

Region: NODE\_374673\_length\_37756\_cov\_28.992161 36570-36571. Max. coverage (+): 0. Max coverage (-): 0

Region: NODE\_374673\_length\_37756\_cov\_28.992161 36572-36574. Max. coverage (+): 0. Max coverage (-): 0

Region: NODE\_374673\_length\_37756\_cov\_28.992161 36575-36576. Max. coverage (+): 0. Max coverage (-): 0

Region: NODE\_374673\_length\_37756\_cov\_28.992161 36577-36579. Max. coverage (+): 0. Max coverage (-): 0

Region: NODE\_374673\_length\_37756\_cov\_28.992161 36580-36581. Max. coverage (+): 0. Max coverage (-): 0

Region: NODE\_374673\_length\_37756\_cov\_28.992161 36582-36584. Max. coverage (+): 0. Max coverage (-): 0

Region: NODE\_374673\_length\_37756\_cov\_28.992161 36585-36586. Max. coverage (+): 0. Max coverage (-): 0

Region: NODE\_374673\_length\_37756\_cov\_28.992161 36587-36589. Max. coverage (+): 0. Max coverage (-): 0

Region: NODE\_374673\_length\_37756\_cov\_28.992161 36590-36592. Max. coverage (+): 0. Max coverage (-): 0

Region: NODE\_374673\_length\_37756\_cov\_28.992161 36593-36594. Max. coverage (+): 0. Max coverage (-): 0

Region: NODE\_374673\_length\_37756\_cov\_28.992161 36595-36597. Max. coverage (+): 0. Max coverage (-): 0

Region: NODE\_374673\_length\_37756\_cov\_28.992161 36598-36599. Max. coverage (+): 0. Max coverage (-): 0

Region: NODE\_374673\_length\_37756\_cov\_28.992161 36600-36602. Max. coverage (+): 0. Max coverage (-): 0

Region: NODE\_374673\_length\_37756\_cov\_28.992161 36603-36604. Max. coverage (+): 0. Max coverage (-): 0

Region: NODE\_374673\_length\_37756\_cov\_28.992161 36605-36607. Max. coverage (+): 0. Max coverage (-): 0

Region: NODE\_374673\_length\_37756\_cov\_28.992161 36608-36609. Max. coverage (+): 0. Max coverage (-): 0

Region: NODE\_374673\_length\_37756\_cov\_28.992161 36610-36612. Max. coverage (+): 0. Max coverage (-): 0

Region: NODE\_374673\_length\_37756\_cov\_28.992161 36613-36615. Max. coverage (+): 0. Max coverage (-): 0

Region: NODE\_374673\_length\_37756\_cov\_28.992161 36616-36617. Max. coverage (+): 0. Max coverage (-): 0

Region: NODE\_374673\_length\_37756\_cov\_28.992161 36618-36620. Max. coverage (+): 0. Max coverage (-): 0

Region: NODE\_374673\_length\_37756\_cov\_28.992161 36621-36622. Max. coverage (+): 0. Max coverage (-): 0

Region: NODE\_374673\_length\_37756\_cov\_28.992161 36623-36625. Max. coverage (+): 0. Max coverage (-): 0

Region: NODE\_374673\_length\_37756\_cov\_28.992161 36626-36627. Max. coverage (+): 0. Max coverage (-): 0

Region: NODE\_374673\_length\_37756\_cov\_28.992161 36628-36630. Max. coverage (+): 0. Max coverage (-): 0

Region: NODE\_374673\_length\_37756\_cov\_28.992161 36631-36632. Max. coverage (+): 0. Max coverage (-): 0

Region: NODE\_374673\_length\_37756\_cov\_28.992161 36633-36635. Max. coverage (+): 0. Max coverage (-): 0

Region: NODE\_374673\_length\_37756\_cov\_28.992161 36636-36637. Max. coverage (+): 0. Max coverage (-): 0

Region: NODE\_374673\_length\_37756\_cov\_28.992161 36638-36640. Max. coverage (+): 0.09. Max coverage (-): 0

Region: NODE\_374673\_length\_37756\_cov\_28.992161 36641-36643. Max. coverage (+): 0.09. Max coverage (-): 0

Region: NODE\_374673\_length\_37756\_cov\_28.992161 36644-36645. Max. coverage (+): 0. Max coverage (-): 0

Region: NODE\_374673\_length\_37756\_cov\_28.992161 36646-36648. Max. coverage (+): 0. Max coverage (-): 0

Region: NODE\_374673\_length\_37756\_cov\_28.992161 36649-36650. Max. coverage (+): 0. Max coverage (-): 0

Region: NODE\_374673\_length\_37756\_cov\_28.992161 36651-36653. Max. coverage (+): 0. Max coverage (-): 0

Region: NODE\_374673\_length\_37756\_cov\_28.992161 36654-36655. Max. coverage (+): 0. Max coverage (-): 0

Region: NODE\_374673\_length\_37756\_cov\_28.992161 36656-36658. Max. coverage (+): 0. Max coverage (-): 0

Region: NODE\_374673\_length\_37756\_cov\_28.992161 36659-36660. Max. coverage (+): 0. Max coverage (-): 0

Region: NODE\_374673\_length\_37756\_cov\_28.992161 36661-36663. Max. coverage (+): 0. Max coverage (-): 0

Region: NODE\_374673\_length\_37756\_cov\_28.992161 36664-36665. Max. coverage (+): 0. Max coverage (-): 0

Region: NODE\_374673\_length\_37756\_cov\_28.992161 36666-36668. Max. coverage (+): 0. Max coverage (-): 0

Region: NODE\_374673\_length\_37756\_cov\_28.992161 36669-36671. Max. coverage (+): 0. Max coverage (-): 0

Region: NODE\_374673\_length\_37756\_cov\_28.992161 36672-36673. Max. coverage (+): 0. Max coverage (-): 0

Region: NODE\_374673\_length\_37756\_cov\_28.992161 36674-36676. Max. coverage (+): 0. Max coverage (-): 0

Region: NODE\_374673\_length\_37756\_cov\_28.992161 36677-36678. Max. coverage (+): 0. Max coverage (-): 0

Region: NODE\_374673\_length\_37756\_cov\_28.992161 36679-36681. Max. coverage (+): 0. Max coverage (-): 0

Region: NODE\_374673\_length\_37756\_cov\_28.992161 36682-36683. Max. coverage (+): 0. Max coverage (-): 0

Region: NODE\_374673\_length\_37756\_cov\_28.992161 36684-36686. Max. coverage (+): 0. Max coverage (-): 0

Region: NODE\_374673\_length\_37756\_cov\_28.992161 36687-36688. Max. coverage (+): 0. Max coverage (-): 0

Region: NODE\_374673\_length\_37756\_cov\_28.992161 36689-36691. Max. coverage (+): 0. Max coverage (-): 0

Region: NODE\_374673\_length\_37756\_cov\_28.992161 36692-36693. Max. coverage (+): 0. Max coverage (-): 0

Region: NODE\_374673\_length\_37756\_cov\_28.992161 36694-36696. Max. coverage (+): 0. Max coverage (-): 0

Region: NODE\_374673\_length\_37756\_cov\_28.992161 36697-36699. Max. coverage (+): 0. Max coverage (-): 0

Region: NODE\_374673\_length\_37756\_cov\_28.992161 36700-36701. Max. coverage (+): 0. Max coverage (-): 0

Region: NODE\_374673\_length\_37756\_cov\_28.992161 36702-36704. Max. coverage (+): 0. Max coverage (-): 0

Region: NODE\_374673\_length\_37756\_cov\_28.992161 36705-36706. Max. coverage (+): 0. Max coverage (-): 0

Region: NODE\_374673\_length\_37756\_cov\_28.992161 36707-36709. Max. coverage (+): 0. Max coverage (-): 0

Region: NODE\_374673\_length\_37756\_cov\_28.992161 36710-36711. Max. coverage (+): 0. Max coverage (-): 0

Region: NODE\_374673\_length\_37756\_cov\_28.992161 36712-36714. Max. coverage (+): 0. Max coverage (-): 0

Region: NODE\_374673\_length\_37756\_cov\_28.992161 36715-36716. Max. coverage (+): 0. Max coverage (-): 0

Region: NODE\_374673\_length\_37756\_cov\_28.992161 36717-36719. Max. coverage (+): 0. Max coverage (-): 0

Region: NODE\_374673\_length\_37756\_cov\_28.992161 36720-36721. Max. coverage (+): 0. Max coverage (-): 0

Region: NODE\_374673\_length\_37756\_cov\_28.992161 36722-36724. Max. coverage (+): 0. Max coverage (-): 0

Region: NODE\_374673\_length\_37756\_cov\_28.992161 36725-36727. Max. coverage (+): 0. Max coverage (-): 0

Region: NODE\_374673\_length\_37756\_cov\_28.992161 36728-36729. Max. coverage (+): 0. Max coverage (-): 0

Region: NODE\_374673\_length\_37756\_cov\_28.992161 36730-36732. Max. coverage (+): 0. Max coverage (-): 0

Region: NODE\_374673\_length\_37756\_cov\_28.992161 36733-36734. Max. coverage (+): 0. Max coverage (-): 0

Region: NODE\_374673\_length\_37756\_cov\_28.992161 36735-36737. Max. coverage (+): 0. Max coverage (-): 0

Region: NODE\_374673\_length\_37756\_cov\_28.992161 36738-36739. Max. coverage (+): 0. Max coverage (-): 0

Region: NODE\_374673\_length\_37756\_cov\_28.992161 36740-36742. Max. coverage (+): 0. Max coverage (-): 0

Region: NODE\_374673\_length\_37756\_cov\_28.992161 36743-36744. Max. coverage (+): 0. Max coverage (-): 0

Region: NODE\_374673\_length\_37756\_cov\_28.992161 36745-36747. Max. coverage (+): 0. Max coverage (-): 0

Region: NODE\_374673\_length\_37756\_cov\_28.992161 36748-36749. Max. coverage (+): 0. Max coverage (-): 0

Region: NODE\_374673\_length\_37756\_cov\_28.992161 36750-36752. Max. coverage (+): 0. Max coverage (-): 0

Region: NODE\_374673\_length\_37756\_cov\_28.992161 36753-36755. Max. coverage (+): 0. Max coverage (-): 0

Region: NODE\_374673\_length\_37756\_cov\_28.992161 36756-36757. Max. coverage (+): 0. Max coverage (-): 0

Region: NODE\_374673\_length\_37756\_cov\_28.992161 36758-36760. Max. coverage (+): 0. Max coverage (-): 0

Region: NODE\_374673\_length\_37756\_cov\_28.992161 36761-36762. Max. coverage (+): 0. Max coverage (-): 0

Region: NODE\_374673\_length\_37756\_cov\_28.992161 36763-36765. Max. coverage (+): 0. Max coverage (-): 0

Region: NODE\_374673\_length\_37756\_cov\_28.992161 36766-36767. Max. coverage (+): 0. Max coverage (-): 0

Region: NODE\_374673\_length\_37756\_cov\_28.992161 36768-36770. Max. coverage (+): 0. Max coverage (-): 0

Region: NODE\_374673\_length\_37756\_cov\_28.992161 36771-36772. Max. coverage (+): 0. Max coverage (-): 0

Region: NODE\_374673\_length\_37756\_cov\_28.992161 36773-36775. Max. coverage (+): 0. Max coverage (-): 0

Region: NODE\_374673\_length\_37756\_cov\_28.992161 36776-36777. Max. coverage (+): 0. Max coverage (-): 0

Region: NODE\_374673\_length\_37756\_cov\_28.992161 36778-36780. Max. coverage (+): 0. Max coverage (-): 0

Region: NODE\_374673\_length\_37756\_cov\_28.992161 36781-36783. Max. coverage (+): 0. Max coverage (-): 0

Region: NODE\_374673\_length\_37756\_cov\_28.992161 36784-36785. Max. coverage (+): 0. Max coverage (-): 0

Region: NODE\_374673\_length\_37756\_cov\_28.992161 36786-36788. Max. coverage (+): 0. Max coverage (-): 0

Region: NODE\_374673\_length\_37756\_cov\_28.992161 36789-36790. Max. coverage (+): 0. Max coverage (-): 0

Region: NODE\_374673\_length\_37756\_cov\_28.992161 36791-36793. Max. coverage (+): 0. Max coverage (-): 0

Region: NODE\_374673\_length\_37756\_cov\_28.992161 36794-36795. Max. coverage (+): 0. Max coverage (-): 0

Region: NODE\_374673\_length\_37756\_cov\_28.992161 36796-36798. Max. coverage (+): 0. Max coverage (-): 0

Region: NODE\_374673\_length\_37756\_cov\_28.992161 36799-36800. Max. coverage (+): 0. Max coverage (-): 0

Region: NODE\_374673\_length\_37756\_cov\_28.992161 36801-36803. Max. coverage (+): 0. Max coverage (-): 0

Region: NODE\_374673\_length\_37756\_cov\_28.992161 36804-36805. Max. coverage (+): 0. Max coverage (-): 0

Region: NODE\_374673\_length\_37756\_cov\_28.992161 36806-36808. Max. coverage (+): 0. Max coverage (-): 0

Region: NODE\_374673\_length\_37756\_cov\_28.992161 36809-36811. Max. coverage (+): 0. Max coverage (-): 0

Region: NODE\_374673\_length\_37756\_cov\_28.992161 36812-36813. Max. coverage (+): 0. Max coverage (-): 0

Region: NODE\_374673\_length\_37756\_cov\_28.992161 36814-36816. Max. coverage (+): 0. Max coverage (-): 0

Region: NODE\_374673\_length\_37756\_cov\_28.992161 36817-36818. Max. coverage (+): 0. Max coverage (-): 0

Region: NODE\_374673\_length\_37756\_cov\_28.992161 36819-36821. Max. coverage (+): 0. Max coverage (-): 0

Region: NODE\_374673\_length\_37756\_cov\_28.992161 36822-36823. Max. coverage (+): 0. Max coverage (-): 0

Region: NODE\_374673\_length\_37756\_cov\_28.992161 36824-36826. Max. coverage (+): 0. Max coverage (-): 0

Region: NODE\_374673\_length\_37756\_cov\_28.992161 36827-36828. Max. coverage (+): 0. Max coverage (-): 0

Region: NODE\_374673\_length\_37756\_cov\_28.992161 36829-36831. Max. coverage (+): 0. Max coverage (-): 0

Region: NODE\_374673\_length\_37756\_cov\_28.992161 36832-36833. Max. coverage (+): 0. Max coverage (-): 0

Region: NODE\_374673\_length\_37756\_cov\_28.992161 36834-36836. Max. coverage (+): 0. Max coverage (-): 0

Region: NODE\_374673\_length\_37756\_cov\_28.992161 36837-36839. Max. coverage (+): 0. Max coverage (-): 0

Region: NODE\_374673\_length\_37756\_cov\_28.992161 36840-36841. Max. coverage (+): 0. Max coverage (-): 0

Region: NODE\_374673\_length\_37756\_cov\_28.992161 36842-36844. Max. coverage (+): 0. Max coverage (-): 0

Region: NODE\_374673\_length\_37756\_cov\_28.992161 36845-36846. Max. coverage (+): 0. Max coverage (-): 0

Region: NODE\_374673\_length\_37756\_cov\_28.992161 36847-36849. Max. coverage (+): 0. Max coverage (-): 0

Region: NODE\_374673\_length\_37756\_cov\_28.992161 36850-36851. Max. coverage (+): 0. Max coverage (-): 0

Region: NODE\_374673\_length\_37756\_cov\_28.992161 36852-36854. Max. coverage (+): 0. Max coverage (-): 0

Region: NODE\_374673\_length\_37756\_cov\_28.992161 36855-36856. Max. coverage (+): 0. Max coverage (-): 0

Region: NODE\_374673\_length\_37756\_cov\_28.992161 36857-36859. Max. coverage (+): 0. Max coverage (-): 0

Region: NODE\_374673\_length\_37756\_cov\_28.992161 36860-36861. Max. coverage (+): 0. Max coverage (-): 0

Region: NODE\_374673\_length\_37756\_cov\_28.992161 36862-36864. Max. coverage (+): 0. Max coverage (-): 0

Region: NODE\_374673\_length\_37756\_cov\_28.992161 36865-36867. Max. coverage (+): 0. Max coverage (-): 0

Region: NODE\_374673\_length\_37756\_cov\_28.992161 36868-36869. Max. coverage (+): 0. Max coverage (-): 0

Region: NODE\_374673\_length\_37756\_cov\_28.992161 36870-36872. Max. coverage (+): 0. Max coverage (-): 0

Region: NODE\_374673\_length\_37756\_cov\_28.992161 36873-36874. Max. coverage (+): 0. Max coverage (-): 0

Region: NODE\_374673\_length\_37756\_cov\_28.992161 36875-36877. Max. coverage (+): 0. Max coverage (-): 0

Region: NODE\_374673\_length\_37756\_cov\_28.992161 36878-36879. Max. coverage (+): 0. Max coverage (-): 0

Region: NODE\_374673\_length\_37756\_cov\_28.992161 36880-36882. Max. coverage (+): 0. Max coverage (-): 0

Region: NODE\_374673\_length\_37756\_cov\_28.992161 36883-36884. Max. coverage (+): 0. Max coverage (-): 0

Region: NODE\_374673\_length\_37756\_cov\_28.992161 36885-36887. Max. coverage (+): 0. Max coverage (-): 0

Region: NODE\_374673\_length\_37756\_cov\_28.992161 36888-36889. Max. coverage (+): 0. Max coverage (-): 0

Region: NODE\_374673\_length\_37756\_cov\_28.992161 36890-36892. Max. coverage (+): 0. Max coverage (-): 0

Region: NODE\_374673\_length\_37756\_cov\_28.992161 36893-36895. Max. coverage (+): 0. Max coverage (-): 0

Region: NODE\_374673\_length\_37756\_cov\_28.992161 36896-36897. Max. coverage (+): 0. Max coverage (-): 0

Region: NODE\_374673\_length\_37756\_cov\_28.992161 36898-36900. Max. coverage (+): 0. Max coverage (-): 0

Region: NODE\_374673\_length\_37756\_cov\_28.992161 36901-36902. Max. coverage (+): 0. Max coverage (-): 0

Region: NODE\_374673\_length\_37756\_cov\_28.992161 36903-36905. Max. coverage (+): 0. Max coverage (-): 0

Region: NODE\_374673\_length\_37756\_cov\_28.992161 36906-36907. Max. coverage (+): 0. Max coverage (-): 0

Region: NODE\_374673\_length\_37756\_cov\_28.992161 36908-36910. Max. coverage (+): 0. Max coverage (-): 0

Region: NODE\_374673\_length\_37756\_cov\_28.992161 36911-36912. Max. coverage (+): 0. Max coverage (-): 0

Region: NODE\_374673\_length\_37756\_cov\_28.992161 36913-36915. Max. coverage (+): 0. Max coverage (-): 0

Region: NODE\_374673\_length\_37756\_cov\_28.992161 36916-36917. Max. coverage (+): 0. Max coverage (-): 0

Region: NODE\_374673\_length\_37756\_cov\_28.992161 36918-36920. Max. coverage (+): 0. Max coverage (-): 0

Region: NODE\_374673\_length\_37756\_cov\_28.992161 36921-36923. Max. coverage (+): 0. Max coverage (-): 0

Region: NODE\_374673\_length\_37756\_cov\_28.992161 36924-36925. Max. coverage (+): 0. Max coverage (-): 0

Region: NODE\_374673\_length\_37756\_cov\_28.992161 36926-36928. Max. coverage (+): 0. Max coverage (-): 0

Region: NODE\_374673\_length\_37756\_cov\_28.992161 36929-36930. Max. coverage (+): 0. Max coverage (-): 0

Region: NODE\_374673\_length\_37756\_cov\_28.992161 36931-36933. Max. coverage (+): 0. Max coverage (-): 0

Region: NODE\_374673\_length\_37756\_cov\_28.992161 36934-36935. Max. coverage (+): 0. Max coverage (-): 0

Region: NODE\_374673\_length\_37756\_cov\_28.992161 36936-36938. Max. coverage (+): 0. Max coverage (-): 0

Region: NODE\_374673\_length\_37756\_cov\_28.992161 36939-36940. Max. coverage (+): 0. Max coverage (-): 0

Region: NODE\_374673\_length\_37756\_cov\_28.992161 36941-36943. Max. coverage (+): 0. Max coverage (-): 0

Region: NODE\_374673\_length\_37756\_cov\_28.992161 36944-36945. Max. coverage (+): 0. Max coverage (-): 0

Region: NODE\_374673\_length\_37756\_cov\_28.992161 36946-36948. Max. coverage (+): 0. Max coverage (-): 0

Region: NODE\_374673\_length\_37756\_cov\_28.992161 36949-36951. Max. coverage (+): 0. Max coverage (-): 0

Region: NODE\_374673\_length\_37756\_cov\_28.992161 36952-36953. Max. coverage (+): 0. Max coverage (-): 0

Region: NODE\_374673\_length\_37756\_cov\_28.992161 36954-36956. Max. coverage (+): 0. Max coverage (-): 0

Region: NODE\_374673\_length\_37756\_cov\_28.992161 36957-36958. Max. coverage (+): 0. Max coverage (-): 0

Region: NODE\_374673\_length\_37756\_cov\_28.992161 36959-36961. Max. coverage (+): 0. Max coverage (-): 0

Region: NODE\_374673\_length\_37756\_cov\_28.992161 36962-36963. Max. coverage (+): 0. Max coverage (-): 0

Region: NODE\_374673\_length\_37756\_cov\_28.992161 36964-36966. Max. coverage (+): 0. Max coverage (-): 0

Region: NODE\_374673\_length\_37756\_cov\_28.992161 36967-36968. Max. coverage (+): 0. Max coverage (-): 0

Region: NODE\_374673\_length\_37756\_cov\_28.992161 36969-36971. Max. coverage (+): 0. Max coverage (-): 0

Region: NODE\_374673\_length\_37756\_cov\_28.992161 36972-36973. Max. coverage (+): 0. Max coverage (-): 0

Region: NODE\_374673\_length\_37756\_cov\_28.992161 36974-36976. Max. coverage (+): 0. Max coverage (-): 0

Region: NODE\_374673\_length\_37756\_cov\_28.992161 36977-36979. Max. coverage (+): 0. Max coverage (-): 0

Region: NODE\_374673\_length\_37756\_cov\_28.992161 36980-36981. Max. coverage (+): 0. Max coverage (-): 0

Region: NODE\_374673\_length\_37756\_cov\_28.992161 36982-36984. Max. coverage (+): 0. Max coverage (-): 0

Region: NODE\_374673\_length\_37756\_cov\_28.992161 36985-36986. Max. coverage (+): 0. Max coverage (-): 0

Region: NODE\_374673\_length\_37756\_cov\_28.992161 36987-36989. Max. coverage (+): 0. Max coverage (-): 0

Region: NODE\_374673\_length\_37756\_cov\_28.992161 36990-36991. Max. coverage (+): 0. Max coverage (-): 0

Region: NODE\_374673\_length\_37756\_cov\_28.992161 36992-36994. Max. coverage (+): 0. Max coverage (-): 0

Region: NODE\_374673\_length\_37756\_cov\_28.992161 36995-36996. Max. coverage (+): 0. Max coverage (-): 0

Region: NODE\_374673\_length\_37756\_cov\_28.992161 36997-36999. Max. coverage (+): 0. Max coverage (-): 0

Region: NODE\_374673\_length\_37756\_cov\_28.992161 37000-37001. Max. coverage (+): 0. Max coverage (-): 0

Region: NODE\_374673\_length\_37756\_cov\_28.992161 37002-37004. Max. coverage (+): 0. Max coverage (-): 0

Region: NODE\_374673\_length\_37756\_cov\_28.992161 37005-37007. Max. coverage (+): 0. Max coverage (-): 0

Region: NODE\_374673\_length\_37756\_cov\_28.992161 37008-37009. Max. coverage (+): 0. Max coverage (-): 0

Region: NODE\_374673\_length\_37756\_cov\_28.992161 37010-37012. Max. coverage (+): 0. Max coverage (-): 0

Region: NODE\_374673\_length\_37756\_cov\_28.992161 37013-37014. Max. coverage (+): 0. Max coverage (-): 0

Region: NODE\_374673\_length\_37756\_cov\_28.992161 37015-. Max. coverage (+): 0. Max coverage (-): 0

RepeatMasker Color Code

**+**

100-98% Identity

<98-95% Identity

<95-90% Identity

<90-85% Identity

<85-80% Identity

<80-75% Identity

<75-70% Identity

<70% Identity

**-**

Gene Set Color Code

**+**

Gene

Pseudogene

Other

**-**

Topology/Coverage Color Code

Coverage Plus Strand

Coverage Minus Strand

Mainstrand: Plus

Mainstrand: Minus

Complementary Strand

Flanking Region  
(if option -flank >0)

Gene Set Annotation  
  
RepeatMasker Annotation  

**1. (AC)n**: 36967-36989 (+), Divergence to consensus: 4.5%  
**2. (TG)n**: 36992-37033 (+), Divergence to consensus: 2.4%

  
Transcription Factor Binding Sites  

**RHOXF1** (Sequence: GGCTCA (-): 36528)  
**RHOXF1** (Sequence: TGATCC (+): 35957)  
**RHOXF1** (Sequence: TAATCC (+): 36138)  
**RHOXF1** (Sequence: TGAGCT (+): 36405)  
**RHOXF1** (Sequence: TAAGCT (+): 36933)  
**Lhx8** (Sequence: TTAATTAG (-): 36371)  
**Lhx8** (Sequence: TTAATTAA (-): 36806)  
**Sox5** (Sequence: ATTGTT (+): 36758)  
**Rhox11** (Sequence: TGGTGTTAA (+): 36624)  
**Rhox11** (Sequence: TTAACAGCA (-): 36075)
