# Supplementary material for: First characterization of PIWI-interacting RNA clusters in a cichlid fish with a B chromosome
Source: BMC Biol. 2022 Sep 21;20:204. doi: 10.1186/s12915-022-01403-2 (PMC9490952; doi:10.1186/s12915-022-01403-2)
Supplement: Supplementary file 1 — Additional file 1. Zipped folder with fasta and interactive html piRNA cluster information for the A. latifasciata genome. The nomenclature is as follows: number-pirna-cluster_sex_B-presence (f, female; m, male; 0b, without B chromosome; 1b, with B chromosome). [file 12915_2022_1403_MOESM1_ESM.zip › 146_m0b.html]

piRNA cluster 146\_m0b 71


Predicted piRNA cluster no. 146\_m0b
  

Show proTRAC run info
Hide proTRAC run info

/\  
                \_\_\_\_\_\_\_\_\_\_\_\_\_\_\_\_\_\_\_\_\_\_\_/\\_\_\_ /  \\_\_\_\_\_\_\_  
               I                      /  \  /    \      I  
               I     pro             /    \/      \     I  
               I        TRAC        /               \   I  
               I   \_\_\_\_\_\_\_\_\_\_\_\_\_\_\_\_/\_\_\_\_\_\_\_\_\_\_\_\_\_\_\_\_\_\\_ I  
               I   \              /                     I  
               I    \            /                      I  
               I     \  /\      /       V.2.4.2         I  
               I      \/  \    /                        I  
               I\_\_\_\_\_\_\_\_\_\_\_\  /\_\_\_\_\_\_\_\_\_\_\_\_\_\_\_\_\_\_\_\_\_\_\_\_\_I  
                            \/  
  
  
================================= proTRAC ====================================  
VERSION: .......... 2.4.2  
LAST MODIFIED: .... 11. May 2018  
  
Please cite:  
Rosenkranz D, Zischler H. proTRAC - a software for probabilistic piRNA cluster  
detection, visualization and analysis. 2012. BMC Bioinformatics 13:5.  
  
  
Contact:  
David Rosenkranz  
Institute of Organismic and Molecular Evolutionary Biology  
Dept. Anthropology, small RNA group  
Johannes Gutenberg University Mainz  
email: rosenkranz@uni-mainz.de  
  
You can find the latest proTRAC version at:  
http://sourceforge.net/projects/protrac/files  
http://www.smallRNAgroup-mainz.de/software  
==============================================================================  
  
PARAMETERS:  
Map file: ...............piwi-machos-0B.fa-collapse.map  
Genome file: ............../../../0B\_ala\_genome.fa  
RepeatMasker annotation: Alatifasciata-all0B-maryan-v2.fa\_corrected.out  
GeneSet:................./guest-storage/Data/annotation/Alatifasciata\_all0B\_maryan-v2\_out2017.gff  
  
Significant (p<=0.01) hit density will be calculated based  
on observed hit distribution.  
  
Sliding window size: ........................................ 5000 bp  
Sliding window increament: .................................. 1000 bp  
Normalize each hit by number of genomic hits: ............... yes  
Normalize each hit by number of sequence reads: ............. yes  
Normalize values (-> per million mapped reads): ............. yes  
Min. fraction of hits with 1T(U) or 10A: .................... 0.75  
Alternatively: Min. fraction of hits with 1T(U) and 10A: .... 0.5  
Min. fraction of hits with typical piRNA length: ............ 0.75  
Typical piRNA length: ....................................... 24-32 nt  
Min. size of a piRNA cluster: ............................... 1000 bp.  
Min. number of hits (absolute): ............................. 0  
Min. number of hits (normalized): ........................... 0  
Min. fraction of hits on the mainstrand: .................... 0.75  
Top fraction of mapped sequences (in terms of read counts): . 1%  
Top fraction accounts for max. n% of sequence reads: ........ 90%  
Min. fraction of hits on each arm of a bidirectional cluster: 0.05  
Output html file for each cluster: .......................... yes  
Output a summary table: ..................................... yes  
Output a FASTA file for each cluster (piRNA sequences): ..... yes  
Output a FASTA file comprising cluster sequences: ........... yes  
Output a GTF file for predicted piRNA clusters: ..............yes  
Search DNA motifs in clusters: .............................. yes  
Output flanking sequences: +/- .............................. 0 bp  
Output ~.pTi file: .......................................... no  
==============================================================================  
  
  
Genome size (without gaps): ............ 758543724 bp  
Gaps (N/X/-): .......................... 417479 bp  
Mapped reads: .......................... 24765598  
Non-identical sequences: ............... 6158275  
Genomic hits: .......................... 53103584  
Significant densitiy of mapped reads: .. 763.098963422187 reads/kb

Show proTRAC cluster info
Hide proTRAC cluster info

|  |  |
| --- | --- |
| Location | NODE\_374673\_length\_37756\_cov\_28.992161 |
| Coordinates | 35825-37015 |
| Size [bp] | 1191 |
| Sequence hit loci | 71 |
| Mapped reads (normalized) | 6125 |
| Mapped reads (normalized) per kb | 5142.7 |
| Normalized reads with 1T (1U) | 99.8% |
| Normalized reads with 10A | 99.9% |
| Normalized reads with length 24-32 nt | 99.8% |
| Normalized reads on the main strand(s) | 100% |
| Predicted directionality | mono:plus |

100%

0%

1T (1U)  
reads

10A reads

24-32 nt  
reads

reads on mainstrand

**Either the amount of reads with 1T (1U) OR 10A has to exceed 75% (set with option: -1Tor10A)  
Alternatively the amount of reads with 1T (1U) AND 10A has to exceed 50% (set with option: -1Tand10A)  
Minimum amount of reads with preferred size is 75% (set with option: -pisize)  
Minimum amount of reads on the main strand(s) is 75% (set with option: -clstrand)**

Show read coverage
Hide read coverage

WHAT DO I SEE HERE?  
This chart shows the location of mapped sequence reads within a predicted piRNA cluster. The color refers to the number of genomic hits produced by the sequence read in question. A dark red bar indicates that this sequence read produces many other hits elsewhere in the genome. Many adjacent red or yellow bars can indicate the presence of a multi-copy element such as transposons or rRNA genes. A dark green bar indicates that this sequence read maps uniquely to this locus.

1 hit

2-5 hits

6-10 hits

11-20 hits

21-50 hits

51-100 hits

> 100 hits

NODE\_374673\_length\_37756\_cov\_28.992161

35825

37015

Gene Set

RepeatMasker

Mapped  
Reads

246.96

plus strand

minus strand

246.96

Region: NODE\_374673\_length\_37756\_cov\_28.992161 2675-35826. Max. coverage (+): 0.04. Max coverage (-): 0

Region: NODE\_374673\_length\_37756\_cov\_28.992161 35827-35828. Max. coverage (+): 0.04. Max coverage (-): 0

Region: NODE\_374673\_length\_37756\_cov\_28.992161 35829-35830. Max. coverage (+): 0.04. Max coverage (-): 0

Region: NODE\_374673\_length\_37756\_cov\_28.992161 35831-35833. Max. coverage (+): 0. Max coverage (-): 0

Region: NODE\_374673\_length\_37756\_cov\_28.992161 35834-35835. Max. coverage (+): 0. Max coverage (-): 0

Region: NODE\_374673\_length\_37756\_cov\_28.992161 35836-35838. Max. coverage (+): 0. Max coverage (-): 0

Region: NODE\_374673\_length\_37756\_cov\_28.992161 35839-35840. Max. coverage (+): 0. Max coverage (-): 0

Region: NODE\_374673\_length\_37756\_cov\_28.992161 35841-35842. Max. coverage (+): 0. Max coverage (-): 0

Region: NODE\_374673\_length\_37756\_cov\_28.992161 35843-35845. Max. coverage (+): 0. Max coverage (-): 0

Region: NODE\_374673\_length\_37756\_cov\_28.992161 35846-35847. Max. coverage (+): 0. Max coverage (-): 0

Region: NODE\_374673\_length\_37756\_cov\_28.992161 35848-35850. Max. coverage (+): 0. Max coverage (-): 0

Region: NODE\_374673\_length\_37756\_cov\_28.992161 35851-35852. Max. coverage (+): 0. Max coverage (-): 0

Region: NODE\_374673\_length\_37756\_cov\_28.992161 35853-35854. Max. coverage (+): 0. Max coverage (-): 0

Region: NODE\_374673\_length\_37756\_cov\_28.992161 35855-35857. Max. coverage (+): 0. Max coverage (-): 0

Region: NODE\_374673\_length\_37756\_cov\_28.992161 35858-35859. Max. coverage (+): 0. Max coverage (-): 0

Region: NODE\_374673\_length\_37756\_cov\_28.992161 35860-35861. Max. coverage (+): 0. Max coverage (-): 0

Region: NODE\_374673\_length\_37756\_cov\_28.992161 35862-35864. Max. coverage (+): 0. Max coverage (-): 0

Region: NODE\_374673\_length\_37756\_cov\_28.992161 35865-35866. Max. coverage (+): 0. Max coverage (-): 0

Region: NODE\_374673\_length\_37756\_cov\_28.992161 35867-35869. Max. coverage (+): 0. Max coverage (-): 0

Region: NODE\_374673\_length\_37756\_cov\_28.992161 35870-35871. Max. coverage (+): 0. Max coverage (-): 0

Region: NODE\_374673\_length\_37756\_cov\_28.992161 35872-35873. Max. coverage (+): 0. Max coverage (-): 0

Region: NODE\_374673\_length\_37756\_cov\_28.992161 35874-35876. Max. coverage (+): 0. Max coverage (-): 0

Region: NODE\_374673\_length\_37756\_cov\_28.992161 35877-35878. Max. coverage (+): 0. Max coverage (-): 0

Region: NODE\_374673\_length\_37756\_cov\_28.992161 35879-35880. Max. coverage (+): 0. Max coverage (-): 0

Region: NODE\_374673\_length\_37756\_cov\_28.992161 35881-35883. Max. coverage (+): 0. Max coverage (-): 0

Region: NODE\_374673\_length\_37756\_cov\_28.992161 35884-35885. Max. coverage (+): 0. Max coverage (-): 0

Region: NODE\_374673\_length\_37756\_cov\_28.992161 35886-35888. Max. coverage (+): 0. Max coverage (-): 0

Region: NODE\_374673\_length\_37756\_cov\_28.992161 35889-35890. Max. coverage (+): 0. Max coverage (-): 0

Region: NODE\_374673\_length\_37756\_cov\_28.992161 35891-35892. Max. coverage (+): 0. Max coverage (-): 0

Region: NODE\_374673\_length\_37756\_cov\_28.992161 35893-35895. Max. coverage (+): 0. Max coverage (-): 0

Region: NODE\_374673\_length\_37756\_cov\_28.992161 35896-35897. Max. coverage (+): 0. Max coverage (-): 0

Region: NODE\_374673\_length\_37756\_cov\_28.992161 35898-35900. Max. coverage (+): 0. Max coverage (-): 0

Region: NODE\_374673\_length\_37756\_cov\_28.992161 35901-35902. Max. coverage (+): 0. Max coverage (-): 0

Region: NODE\_374673\_length\_37756\_cov\_28.992161 35903-35904. Max. coverage (+): 0. Max coverage (-): 0

Region: NODE\_374673\_length\_37756\_cov\_28.992161 35905-35907. Max. coverage (+): 0. Max coverage (-): 0

Region: NODE\_374673\_length\_37756\_cov\_28.992161 35908-35909. Max. coverage (+): 0. Max coverage (-): 0

Region: NODE\_374673\_length\_37756\_cov\_28.992161 35910-35911. Max. coverage (+): 0. Max coverage (-): 0

Region: NODE\_374673\_length\_37756\_cov\_28.992161 35912-35914. Max. coverage (+): 0. Max coverage (-): 0

Region: NODE\_374673\_length\_37756\_cov\_28.992161 35915-35916. Max. coverage (+): 0. Max coverage (-): 0

Region: NODE\_374673\_length\_37756\_cov\_28.992161 35917-35919. Max. coverage (+): 0. Max coverage (-): 0

Region: NODE\_374673\_length\_37756\_cov\_28.992161 35920-35921. Max. coverage (+): 0. Max coverage (-): 0

Region: NODE\_374673\_length\_37756\_cov\_28.992161 35922-35923. Max. coverage (+): 0. Max coverage (-): 0

Region: NODE\_374673\_length\_37756\_cov\_28.992161 35924-35926. Max. coverage (+): 0. Max coverage (-): 0

Region: NODE\_374673\_length\_37756\_cov\_28.992161 35927-35928. Max. coverage (+): 0. Max coverage (-): 0

Region: NODE\_374673\_length\_37756\_cov\_28.992161 35929-35930. Max. coverage (+): 0. Max coverage (-): 0

Region: NODE\_374673\_length\_37756\_cov\_28.992161 35931-35933. Max. coverage (+): 0. Max coverage (-): 0

Region: NODE\_374673\_length\_37756\_cov\_28.992161 35934-35935. Max. coverage (+): 0. Max coverage (-): 0

Region: NODE\_374673\_length\_37756\_cov\_28.992161 35936-35938. Max. coverage (+): 0. Max coverage (-): 0

Region: NODE\_374673\_length\_37756\_cov\_28.992161 35939-35940. Max. coverage (+): 0. Max coverage (-): 0

Region: NODE\_374673\_length\_37756\_cov\_28.992161 35941-35942. Max. coverage (+): 0. Max coverage (-): 0

Region: NODE\_374673\_length\_37756\_cov\_28.992161 35943-35945. Max. coverage (+): 0. Max coverage (-): 0

Region: NODE\_374673\_length\_37756\_cov\_28.992161 35946-35947. Max. coverage (+): 0. Max coverage (-): 0

Region: NODE\_374673\_length\_37756\_cov\_28.992161 35948-35950. Max. coverage (+): 0. Max coverage (-): 0

Region: NODE\_374673\_length\_37756\_cov\_28.992161 35951-35952. Max. coverage (+): 0. Max coverage (-): 0

Region: NODE\_374673\_length\_37756\_cov\_28.992161 35953-35954. Max. coverage (+): 0. Max coverage (-): 0

Region: NODE\_374673\_length\_37756\_cov\_28.992161 35955-35957. Max. coverage (+): 0. Max coverage (-): 0

Region: NODE\_374673\_length\_37756\_cov\_28.992161 35958-35959. Max. coverage (+): 0. Max coverage (-): 0

Region: NODE\_374673\_length\_37756\_cov\_28.992161 35960-35961. Max. coverage (+): 0. Max coverage (-): 0

Region: NODE\_374673\_length\_37756\_cov\_28.992161 35962-35964. Max. coverage (+): 0. Max coverage (-): 0

Region: NODE\_374673\_length\_37756\_cov\_28.992161 35965-35966. Max. coverage (+): 0. Max coverage (-): 0

Region: NODE\_374673\_length\_37756\_cov\_28.992161 35967-35969. Max. coverage (+): 0. Max coverage (-): 0

Region: NODE\_374673\_length\_37756\_cov\_28.992161 35970-35971. Max. coverage (+): 0. Max coverage (-): 0

Region: NODE\_374673\_length\_37756\_cov\_28.992161 35972-35973. Max. coverage (+): 0. Max coverage (-): 0

Region: NODE\_374673\_length\_37756\_cov\_28.992161 35974-35976. Max. coverage (+): 0. Max coverage (-): 0

Region: NODE\_374673\_length\_37756\_cov\_28.992161 35977-35978. Max. coverage (+): 0. Max coverage (-): 0

Region: NODE\_374673\_length\_37756\_cov\_28.992161 35979-35981. Max. coverage (+): 0. Max coverage (-): 0

Region: NODE\_374673\_length\_37756\_cov\_28.992161 35982-35983. Max. coverage (+): 0. Max coverage (-): 0

Region: NODE\_374673\_length\_37756\_cov\_28.992161 35984-35985. Max. coverage (+): 0. Max coverage (-): 0

Region: NODE\_374673\_length\_37756\_cov\_28.992161 35986-35988. Max. coverage (+): 0. Max coverage (-): 0

Region: NODE\_374673\_length\_37756\_cov\_28.992161 35989-35990. Max. coverage (+): 0. Max coverage (-): 0

Region: NODE\_374673\_length\_37756\_cov\_28.992161 35991-35992. Max. coverage (+): 0. Max coverage (-): 0

Region: NODE\_374673\_length\_37756\_cov\_28.992161 35993-35995. Max. coverage (+): 0. Max coverage (-): 0

Region: NODE\_374673\_length\_37756\_cov\_28.992161 35996-35997. Max. coverage (+): 0. Max coverage (-): 0

Region: NODE\_374673\_length\_37756\_cov\_28.992161 35998-36000. Max. coverage (+): 0. Max coverage (-): 0

Region: NODE\_374673\_length\_37756\_cov\_28.992161 36001-36002. Max. coverage (+): 0. Max coverage (-): 0

Region: NODE\_374673\_length\_37756\_cov\_28.992161 36003-36004. Max. coverage (+): 0. Max coverage (-): 0

Region: NODE\_374673\_length\_37756\_cov\_28.992161 36005-36007. Max. coverage (+): 0. Max coverage (-): 0

Region: NODE\_374673\_length\_37756\_cov\_28.992161 36008-36009. Max. coverage (+): 0. Max coverage (-): 0

Region: NODE\_374673\_length\_37756\_cov\_28.992161 36010-36011. Max. coverage (+): 0. Max coverage (-): 0

Region: NODE\_374673\_length\_37756\_cov\_28.992161 36012-36014. Max. coverage (+): 0. Max coverage (-): 0

Region: NODE\_374673\_length\_37756\_cov\_28.992161 36015-36016. Max. coverage (+): 0. Max coverage (-): 0

Region: NODE\_374673\_length\_37756\_cov\_28.992161 36017-36019. Max. coverage (+): 0. Max coverage (-): 0

Region: NODE\_374673\_length\_37756\_cov\_28.992161 36020-36021. Max. coverage (+): 0. Max coverage (-): 0

Region: NODE\_374673\_length\_37756\_cov\_28.992161 36022-36023. Max. coverage (+): 0. Max coverage (-): 0

Region: NODE\_374673\_length\_37756\_cov\_28.992161 36024-36026. Max. coverage (+): 0. Max coverage (-): 0

Region: NODE\_374673\_length\_37756\_cov\_28.992161 36027-36028. Max. coverage (+): 0. Max coverage (-): 0

Region: NODE\_374673\_length\_37756\_cov\_28.992161 36029-36031. Max. coverage (+): 0. Max coverage (-): 0

Region: NODE\_374673\_length\_37756\_cov\_28.992161 36032-36033. Max. coverage (+): 0. Max coverage (-): 0

Region: NODE\_374673\_length\_37756\_cov\_28.992161 36034-36035. Max. coverage (+): 0. Max coverage (-): 0

Region: NODE\_374673\_length\_37756\_cov\_28.992161 36036-36038. Max. coverage (+): 0. Max coverage (-): 0

Region: NODE\_374673\_length\_37756\_cov\_28.992161 36039-36040. Max. coverage (+): 0. Max coverage (-): 0

Region: NODE\_374673\_length\_37756\_cov\_28.992161 36041-36042. Max. coverage (+): 0. Max coverage (-): 0

Region: NODE\_374673\_length\_37756\_cov\_28.992161 36043-36045. Max. coverage (+): 0. Max coverage (-): 0

Region: NODE\_374673\_length\_37756\_cov\_28.992161 36046-36047. Max. coverage (+): 0. Max coverage (-): 0

Region: NODE\_374673\_length\_37756\_cov\_28.992161 36048-36050. Max. coverage (+): 0. Max coverage (-): 0

Region: NODE\_374673\_length\_37756\_cov\_28.992161 36051-36052. Max. coverage (+): 0. Max coverage (-): 0

Region: NODE\_374673\_length\_37756\_cov\_28.992161 36053-36054. Max. coverage (+): 0. Max coverage (-): 0

Region: NODE\_374673\_length\_37756\_cov\_28.992161 36055-36057. Max. coverage (+): 0. Max coverage (-): 0

Region: NODE\_374673\_length\_37756\_cov\_28.992161 36058-36059. Max. coverage (+): 0. Max coverage (-): 0

Region: NODE\_374673\_length\_37756\_cov\_28.992161 36060-36062. Max. coverage (+): 0. Max coverage (-): 0

Region: NODE\_374673\_length\_37756\_cov\_28.992161 36063-36064. Max. coverage (+): 0. Max coverage (-): 0

Region: NODE\_374673\_length\_37756\_cov\_28.992161 36065-36066. Max. coverage (+): 0. Max coverage (-): 0

Region: NODE\_374673\_length\_37756\_cov\_28.992161 36067-36069. Max. coverage (+): 0. Max coverage (-): 0

Region: NODE\_374673\_length\_37756\_cov\_28.992161 36070-36071. Max. coverage (+): 0. Max coverage (-): 0

Region: NODE\_374673\_length\_37756\_cov\_28.992161 36072-36073. Max. coverage (+): 0. Max coverage (-): 0

Region: NODE\_374673\_length\_37756\_cov\_28.992161 36074-36076. Max. coverage (+): 0. Max coverage (-): 0

Region: NODE\_374673\_length\_37756\_cov\_28.992161 36077-36078. Max. coverage (+): 0. Max coverage (-): 0

Region: NODE\_374673\_length\_37756\_cov\_28.992161 36079-36081. Max. coverage (+): 0. Max coverage (-): 0

Region: NODE\_374673\_length\_37756\_cov\_28.992161 36082-36083. Max. coverage (+): 0. Max coverage (-): 0

Region: NODE\_374673\_length\_37756\_cov\_28.992161 36084-36085. Max. coverage (+): 0. Max coverage (-): 0

Region: NODE\_374673\_length\_37756\_cov\_28.992161 36086-36088. Max. coverage (+): 0. Max coverage (-): 0

Region: NODE\_374673\_length\_37756\_cov\_28.992161 36089-36090. Max. coverage (+): 0. Max coverage (-): 0

Region: NODE\_374673\_length\_37756\_cov\_28.992161 36091-36092. Max. coverage (+): 0. Max coverage (-): 0

Region: NODE\_374673\_length\_37756\_cov\_28.992161 36093-36095. Max. coverage (+): 0. Max coverage (-): 0

Region: NODE\_374673\_length\_37756\_cov\_28.992161 36096-36097. Max. coverage (+): 0. Max coverage (-): 0

Region: NODE\_374673\_length\_37756\_cov\_28.992161 36098-36100. Max. coverage (+): 0. Max coverage (-): 0

Region: NODE\_374673\_length\_37756\_cov\_28.992161 36101-36102. Max. coverage (+): 0. Max coverage (-): 0

Region: NODE\_374673\_length\_37756\_cov\_28.992161 36103-36104. Max. coverage (+): 0. Max coverage (-): 0

Region: NODE\_374673\_length\_37756\_cov\_28.992161 36105-36107. Max. coverage (+): 0. Max coverage (-): 0

Region: NODE\_374673\_length\_37756\_cov\_28.992161 36108-36109. Max. coverage (+): 0. Max coverage (-): 0

Region: NODE\_374673\_length\_37756\_cov\_28.992161 36110-36112. Max. coverage (+): 0. Max coverage (-): 0

Region: NODE\_374673\_length\_37756\_cov\_28.992161 36113-36114. Max. coverage (+): 0. Max coverage (-): 0

Region: NODE\_374673\_length\_37756\_cov\_28.992161 36115-36116. Max. coverage (+): 0. Max coverage (-): 0

Region: NODE\_374673\_length\_37756\_cov\_28.992161 36117-36119. Max. coverage (+): 0. Max coverage (-): 0

Region: NODE\_374673\_length\_37756\_cov\_28.992161 36120-36121. Max. coverage (+): 0. Max coverage (-): 0

Region: NODE\_374673\_length\_37756\_cov\_28.992161 36122-36123. Max. coverage (+): 0. Max coverage (-): 0

Region: NODE\_374673\_length\_37756\_cov\_28.992161 36124-36126. Max. coverage (+): 0. Max coverage (-): 0

Region: NODE\_374673\_length\_37756\_cov\_28.992161 36127-36128. Max. coverage (+): 0. Max coverage (-): 0

Region: NODE\_374673\_length\_37756\_cov\_28.992161 36129-36131. Max. coverage (+): 0. Max coverage (-): 0

Region: NODE\_374673\_length\_37756\_cov\_28.992161 36132-36133. Max. coverage (+): 0. Max coverage (-): 0

Region: NODE\_374673\_length\_37756\_cov\_28.992161 36134-36135. Max. coverage (+): 0. Max coverage (-): 0

Region: NODE\_374673\_length\_37756\_cov\_28.992161 36136-36138. Max. coverage (+): 0. Max coverage (-): 0

Region: NODE\_374673\_length\_37756\_cov\_28.992161 36139-36140. Max. coverage (+): 0. Max coverage (-): 0

Region: NODE\_374673\_length\_37756\_cov\_28.992161 36141-36142. Max. coverage (+): 0. Max coverage (-): 0

Region: NODE\_374673\_length\_37756\_cov\_28.992161 36143-36145. Max. coverage (+): 0. Max coverage (-): 0

Region: NODE\_374673\_length\_37756\_cov\_28.992161 36146-36147. Max. coverage (+): 0. Max coverage (-): 0

Region: NODE\_374673\_length\_37756\_cov\_28.992161 36148-36150. Max. coverage (+): 0. Max coverage (-): 0

Region: NODE\_374673\_length\_37756\_cov\_28.992161 36151-36152. Max. coverage (+): 0. Max coverage (-): 0

Region: NODE\_374673\_length\_37756\_cov\_28.992161 36153-36154. Max. coverage (+): 0. Max coverage (-): 0

Region: NODE\_374673\_length\_37756\_cov\_28.992161 36155-36157. Max. coverage (+): 0. Max coverage (-): 0

Region: NODE\_374673\_length\_37756\_cov\_28.992161 36158-36159. Max. coverage (+): 0. Max coverage (-): 0

Region: NODE\_374673\_length\_37756\_cov\_28.992161 36160-36162. Max. coverage (+): 0. Max coverage (-): 0

Region: NODE\_374673\_length\_37756\_cov\_28.992161 36163-36164. Max. coverage (+): 0. Max coverage (-): 0

Region: NODE\_374673\_length\_37756\_cov\_28.992161 36165-36166. Max. coverage (+): 0. Max coverage (-): 0

Region: NODE\_374673\_length\_37756\_cov\_28.992161 36167-36169. Max. coverage (+): 0. Max coverage (-): 0

Region: NODE\_374673\_length\_37756\_cov\_28.992161 36170-36171. Max. coverage (+): 0. Max coverage (-): 0

Region: NODE\_374673\_length\_37756\_cov\_28.992161 36172-36173. Max. coverage (+): 0. Max coverage (-): 0

Region: NODE\_374673\_length\_37756\_cov\_28.992161 36174-36176. Max. coverage (+): 0. Max coverage (-): 0

Region: NODE\_374673\_length\_37756\_cov\_28.992161 36177-36178. Max. coverage (+): 0. Max coverage (-): 0

Region: NODE\_374673\_length\_37756\_cov\_28.992161 36179-36181. Max. coverage (+): 0. Max coverage (-): 0

Region: NODE\_374673\_length\_37756\_cov\_28.992161 36182-36183. Max. coverage (+): 0. Max coverage (-): 0

Region: NODE\_374673\_length\_37756\_cov\_28.992161 36184-36185. Max. coverage (+): 0. Max coverage (-): 0

Region: NODE\_374673\_length\_37756\_cov\_28.992161 36186-36188. Max. coverage (+): 0. Max coverage (-): 0

Region: NODE\_374673\_length\_37756\_cov\_28.992161 36189-36190. Max. coverage (+): 0. Max coverage (-): 0

Region: NODE\_374673\_length\_37756\_cov\_28.992161 36191-36193. Max. coverage (+): 0. Max coverage (-): 0

Region: NODE\_374673\_length\_37756\_cov\_28.992161 36194-36195. Max. coverage (+): 0. Max coverage (-): 0

Region: NODE\_374673\_length\_37756\_cov\_28.992161 36196-36197. Max. coverage (+): 0. Max coverage (-): 0

Region: NODE\_374673\_length\_37756\_cov\_28.992161 36198-36200. Max. coverage (+): 0. Max coverage (-): 0

Region: NODE\_374673\_length\_37756\_cov\_28.992161 36201-36202. Max. coverage (+): 0. Max coverage (-): 0

Region: NODE\_374673\_length\_37756\_cov\_28.992161 36203-36204. Max. coverage (+): 0. Max coverage (-): 0

Region: NODE\_374673\_length\_37756\_cov\_28.992161 36205-36207. Max. coverage (+): 0. Max coverage (-): 0

Region: NODE\_374673\_length\_37756\_cov\_28.992161 36208-36209. Max. coverage (+): 0. Max coverage (-): 0

Region: NODE\_374673\_length\_37756\_cov\_28.992161 36210-36212. Max. coverage (+): 0. Max coverage (-): 0

Region: NODE\_374673\_length\_37756\_cov\_28.992161 36213-36214. Max. coverage (+): 0. Max coverage (-): 0

Region: NODE\_374673\_length\_37756\_cov\_28.992161 36215-36216. Max. coverage (+): 0. Max coverage (-): 0

Region: NODE\_374673\_length\_37756\_cov\_28.992161 36217-36219. Max. coverage (+): 0. Max coverage (-): 0

Region: NODE\_374673\_length\_37756\_cov\_28.992161 36220-36221. Max. coverage (+): 0. Max coverage (-): 0

Region: NODE\_374673\_length\_37756\_cov\_28.992161 36222-36223. Max. coverage (+): 0. Max coverage (-): 0

Region: NODE\_374673\_length\_37756\_cov\_28.992161 36224-36226. Max. coverage (+): 0. Max coverage (-): 0

Region: NODE\_374673\_length\_37756\_cov\_28.992161 36227-36228. Max. coverage (+): 0. Max coverage (-): 0

Region: NODE\_374673\_length\_37756\_cov\_28.992161 36229-36231. Max. coverage (+): 0. Max coverage (-): 0

Region: NODE\_374673\_length\_37756\_cov\_28.992161 36232-36233. Max. coverage (+): 0. Max coverage (-): 0

Region: NODE\_374673\_length\_37756\_cov\_28.992161 36234-36235. Max. coverage (+): 0. Max coverage (-): 0

Region: NODE\_374673\_length\_37756\_cov\_28.992161 36236-36238. Max. coverage (+): 0. Max coverage (-): 0

Region: NODE\_374673\_length\_37756\_cov\_28.992161 36239-36240. Max. coverage (+): 0. Max coverage (-): 0

Region: NODE\_374673\_length\_37756\_cov\_28.992161 36241-36243. Max. coverage (+): 0. Max coverage (-): 0

Region: NODE\_374673\_length\_37756\_cov\_28.992161 36244-36245. Max. coverage (+): 0. Max coverage (-): 0

Region: NODE\_374673\_length\_37756\_cov\_28.992161 36246-36247. Max. coverage (+): 0. Max coverage (-): 0

Region: NODE\_374673\_length\_37756\_cov\_28.992161 36248-36250. Max. coverage (+): 0. Max coverage (-): 0

Region: NODE\_374673\_length\_37756\_cov\_28.992161 36251-36252. Max. coverage (+): 0. Max coverage (-): 0

Region: NODE\_374673\_length\_37756\_cov\_28.992161 36253-36254. Max. coverage (+): 0. Max coverage (-): 0

Region: NODE\_374673\_length\_37756\_cov\_28.992161 36255-36257. Max. coverage (+): 0. Max coverage (-): 0

Region: NODE\_374673\_length\_37756\_cov\_28.992161 36258-36259. Max. coverage (+): 0. Max coverage (-): 0

Region: NODE\_374673\_length\_37756\_cov\_28.992161 36260-36262. Max. coverage (+): 0. Max coverage (-): 0

Region: NODE\_374673\_length\_37756\_cov\_28.992161 36263-36264. Max. coverage (+): 0. Max coverage (-): 0

Region: NODE\_374673\_length\_37756\_cov\_28.992161 36265-36266. Max. coverage (+): 0. Max coverage (-): 0

Region: NODE\_374673\_length\_37756\_cov\_28.992161 36267-36269. Max. coverage (+): 0. Max coverage (-): 0

Region: NODE\_374673\_length\_37756\_cov\_28.992161 36270-36271. Max. coverage (+): 0. Max coverage (-): 0

Region: NODE\_374673\_length\_37756\_cov\_28.992161 36272-36274. Max. coverage (+): 0. Max coverage (-): 0

Region: NODE\_374673\_length\_37756\_cov\_28.992161 36275-36276. Max. coverage (+): 0. Max coverage (-): 0

Region: NODE\_374673\_length\_37756\_cov\_28.992161 36277-36278. Max. coverage (+): 0. Max coverage (-): 0

Region: NODE\_374673\_length\_37756\_cov\_28.992161 36279-36281. Max. coverage (+): 0. Max coverage (-): 0

Region: NODE\_374673\_length\_37756\_cov\_28.992161 36282-36283. Max. coverage (+): 0. Max coverage (-): 0

Region: NODE\_374673\_length\_37756\_cov\_28.992161 36284-36285. Max. coverage (+): 0. Max coverage (-): 0

Region: NODE\_374673\_length\_37756\_cov\_28.992161 36286-36288. Max. coverage (+): 0. Max coverage (-): 0

Region: NODE\_374673\_length\_37756\_cov\_28.992161 36289-36290. Max. coverage (+): 0. Max coverage (-): 0

Region: NODE\_374673\_length\_37756\_cov\_28.992161 36291-36293. Max. coverage (+): 0. Max coverage (-): 0

Region: NODE\_374673\_length\_37756\_cov\_28.992161 36294-36295. Max. coverage (+): 0. Max coverage (-): 0

Region: NODE\_374673\_length\_37756\_cov\_28.992161 36296-36297. Max. coverage (+): 0. Max coverage (-): 0

Region: NODE\_374673\_length\_37756\_cov\_28.992161 36298-36300. Max. coverage (+): 0. Max coverage (-): 0

Region: NODE\_374673\_length\_37756\_cov\_28.992161 36301-36302. Max. coverage (+): 0. Max coverage (-): 0

Region: NODE\_374673\_length\_37756\_cov\_28.992161 36303-36304. Max. coverage (+): 0. Max coverage (-): 0

Region: NODE\_374673\_length\_37756\_cov\_28.992161 36305-36307. Max. coverage (+): 0. Max coverage (-): 0

Region: NODE\_374673\_length\_37756\_cov\_28.992161 36308-36309. Max. coverage (+): 0. Max coverage (-): 0

Region: NODE\_374673\_length\_37756\_cov\_28.992161 36310-36312. Max. coverage (+): 0. Max coverage (-): 0

Region: NODE\_374673\_length\_37756\_cov\_28.992161 36313-36314. Max. coverage (+): 0. Max coverage (-): 0

Region: NODE\_374673\_length\_37756\_cov\_28.992161 36315-36316. Max. coverage (+): 0. Max coverage (-): 0

Region: NODE\_374673\_length\_37756\_cov\_28.992161 36317-36319. Max. coverage (+): 0. Max coverage (-): 0

Region: NODE\_374673\_length\_37756\_cov\_28.992161 36320-36321. Max. coverage (+): 0. Max coverage (-): 0

Region: NODE\_374673\_length\_37756\_cov\_28.992161 36322-36324. Max. coverage (+): 0. Max coverage (-): 0

Region: NODE\_374673\_length\_37756\_cov\_28.992161 36325-36326. Max. coverage (+): 0. Max coverage (-): 0

Region: NODE\_374673\_length\_37756\_cov\_28.992161 36327-36328. Max. coverage (+): 0. Max coverage (-): 0

Region: NODE\_374673\_length\_37756\_cov\_28.992161 36329-36331. Max. coverage (+): 0. Max coverage (-): 0

Region: NODE\_374673\_length\_37756\_cov\_28.992161 36332-36333. Max. coverage (+): 0. Max coverage (-): 0

Region: NODE\_374673\_length\_37756\_cov\_28.992161 36334-36335. Max. coverage (+): 0. Max coverage (-): 0

Region: NODE\_374673\_length\_37756\_cov\_28.992161 36336-36338. Max. coverage (+): 0. Max coverage (-): 0

Region: NODE\_374673\_length\_37756\_cov\_28.992161 36339-36340. Max. coverage (+): 0. Max coverage (-): 0

Region: NODE\_374673\_length\_37756\_cov\_28.992161 36341-36343. Max. coverage (+): 0. Max coverage (-): 0

Region: NODE\_374673\_length\_37756\_cov\_28.992161 36344-36345. Max. coverage (+): 0. Max coverage (-): 0

Region: NODE\_374673\_length\_37756\_cov\_28.992161 36346-36347. Max. coverage (+): 0. Max coverage (-): 0

Region: NODE\_374673\_length\_37756\_cov\_28.992161 36348-36350. Max. coverage (+): 0. Max coverage (-): 0

Region: NODE\_374673\_length\_37756\_cov\_28.992161 36351-36352. Max. coverage (+): 0. Max coverage (-): 0

Region: NODE\_374673\_length\_37756\_cov\_28.992161 36353-36354. Max. coverage (+): 0. Max coverage (-): 0

Region: NODE\_374673\_length\_37756\_cov\_28.992161 36355-36357. Max. coverage (+): 0. Max coverage (-): 0

Region: NODE\_374673\_length\_37756\_cov\_28.992161 36358-36359. Max. coverage (+): 0. Max coverage (-): 0

Region: NODE\_374673\_length\_37756\_cov\_28.992161 36360-36362. Max. coverage (+): 0. Max coverage (-): 0

Region: NODE\_374673\_length\_37756\_cov\_28.992161 36363-36364. Max. coverage (+): 0. Max coverage (-): 0

Region: NODE\_374673\_length\_37756\_cov\_28.992161 36365-36366. Max. coverage (+): 0. Max coverage (-): 0

Region: NODE\_374673\_length\_37756\_cov\_28.992161 36367-36369. Max. coverage (+): 0. Max coverage (-): 0

Region: NODE\_374673\_length\_37756\_cov\_28.992161 36370-36371. Max. coverage (+): 0. Max coverage (-): 0

Region: NODE\_374673\_length\_37756\_cov\_28.992161 36372-36374. Max. coverage (+): 0. Max coverage (-): 0

Region: NODE\_374673\_length\_37756\_cov\_28.992161 36375-36376. Max. coverage (+): 0. Max coverage (-): 0

Region: NODE\_374673\_length\_37756\_cov\_28.992161 36377-36378. Max. coverage (+): 0. Max coverage (-): 0

Region: NODE\_374673\_length\_37756\_cov\_28.992161 36379-36381. Max. coverage (+): 0. Max coverage (-): 0

Region: NODE\_374673\_length\_37756\_cov\_28.992161 36382-36383. Max. coverage (+): 0. Max coverage (-): 0

Region: NODE\_374673\_length\_37756\_cov\_28.992161 36384-36385. Max. coverage (+): 0. Max coverage (-): 0

Region: NODE\_374673\_length\_37756\_cov\_28.992161 36386-36388. Max. coverage (+): 0. Max coverage (-): 0

Region: NODE\_374673\_length\_37756\_cov\_28.992161 36389-36390. Max. coverage (+): 0. Max coverage (-): 0

Region: NODE\_374673\_length\_37756\_cov\_28.992161 36391-36393. Max. coverage (+): 0. Max coverage (-): 0

Region: NODE\_374673\_length\_37756\_cov\_28.992161 36394-36395. Max. coverage (+): 0. Max coverage (-): 0

Region: NODE\_374673\_length\_37756\_cov\_28.992161 36396-36397. Max. coverage (+): 0. Max coverage (-): 0

Region: NODE\_374673\_length\_37756\_cov\_28.992161 36398-36400. Max. coverage (+): 0. Max coverage (-): 0

Region: NODE\_374673\_length\_37756\_cov\_28.992161 36401-36402. Max. coverage (+): 0. Max coverage (-): 0

Region: NODE\_374673\_length\_37756\_cov\_28.992161 36403-36405. Max. coverage (+): 0. Max coverage (-): 0

Region: NODE\_374673\_length\_37756\_cov\_28.992161 36406-36407. Max. coverage (+): 0. Max coverage (-): 0

Region: NODE\_374673\_length\_37756\_cov\_28.992161 36408-36409. Max. coverage (+): 0. Max coverage (-): 0

Region: NODE\_374673\_length\_37756\_cov\_28.992161 36410-36412. Max. coverage (+): 0. Max coverage (-): 0

Region: NODE\_374673\_length\_37756\_cov\_28.992161 36413-36414. Max. coverage (+): 0. Max coverage (-): 0

Region: NODE\_374673\_length\_37756\_cov\_28.992161 36415-36416. Max. coverage (+): 0. Max coverage (-): 0

Region: NODE\_374673\_length\_37756\_cov\_28.992161 36417-36419. Max. coverage (+): 0. Max coverage (-): 0

Region: NODE\_374673\_length\_37756\_cov\_28.992161 36420-36421. Max. coverage (+): 0. Max coverage (-): 0

Region: NODE\_374673\_length\_37756\_cov\_28.992161 36422-36424. Max. coverage (+): 0. Max coverage (-): 0

Region: NODE\_374673\_length\_37756\_cov\_28.992161 36425-36426. Max. coverage (+): 0. Max coverage (-): 0

Region: NODE\_374673\_length\_37756\_cov\_28.992161 36427-36428. Max. coverage (+): 0. Max coverage (-): 0

Region: NODE\_374673\_length\_37756\_cov\_28.992161 36429-36431. Max. coverage (+): 0. Max coverage (-): 0

Region: NODE\_374673\_length\_37756\_cov\_28.992161 36432-36433. Max. coverage (+): 0. Max coverage (-): 0

Region: NODE\_374673\_length\_37756\_cov\_28.992161 36434-36435. Max. coverage (+): 0. Max coverage (-): 0

Region: NODE\_374673\_length\_37756\_cov\_28.992161 36436-36438. Max. coverage (+): 0. Max coverage (-): 0

Region: NODE\_374673\_length\_37756\_cov\_28.992161 36439-36440. Max. coverage (+): 0. Max coverage (-): 0

Region: NODE\_374673\_length\_37756\_cov\_28.992161 36441-36443. Max. coverage (+): 0. Max coverage (-): 0

Region: NODE\_374673\_length\_37756\_cov\_28.992161 36444-36445. Max. coverage (+): 0. Max coverage (-): 0

Region: NODE\_374673\_length\_37756\_cov\_28.992161 36446-36447. Max. coverage (+): 0. Max coverage (-): 0

Region: NODE\_374673\_length\_37756\_cov\_28.992161 36448-36450. Max. coverage (+): 0. Max coverage (-): 0

Region: NODE\_374673\_length\_37756\_cov\_28.992161 36451-36452. Max. coverage (+): 0. Max coverage (-): 0

Region: NODE\_374673\_length\_37756\_cov\_28.992161 36453-36455. Max. coverage (+): 0. Max coverage (-): 0

Region: NODE\_374673\_length\_37756\_cov\_28.992161 36456-36457. Max. coverage (+): 0. Max coverage (-): 0

Region: NODE\_374673\_length\_37756\_cov\_28.992161 36458-36459. Max. coverage (+): 0. Max coverage (-): 0

Region: NODE\_374673\_length\_37756\_cov\_28.992161 36460-36462. Max. coverage (+): 0. Max coverage (-): 0

Region: NODE\_374673\_length\_37756\_cov\_28.992161 36463-36464. Max. coverage (+): 0. Max coverage (-): 0

Region: NODE\_374673\_length\_37756\_cov\_28.992161 36465-36466. Max. coverage (+): 0. Max coverage (-): 0

Region: NODE\_374673\_length\_37756\_cov\_28.992161 36467-36469. Max. coverage (+): 0. Max coverage (-): 0

Region: NODE\_374673\_length\_37756\_cov\_28.992161 36470-36471. Max. coverage (+): 0. Max coverage (-): 0

Region: NODE\_374673\_length\_37756\_cov\_28.992161 36472-36474. Max. coverage (+): 0. Max coverage (-): 0

Region: NODE\_374673\_length\_37756\_cov\_28.992161 36475-36476. Max. coverage (+): 0. Max coverage (-): 0

Region: NODE\_374673\_length\_37756\_cov\_28.992161 36477-36478. Max. coverage (+): 0. Max coverage (-): 0

Region: NODE\_374673\_length\_37756\_cov\_28.992161 36479-36481. Max. coverage (+): 246.67. Max coverage (-): 0

Region: NODE\_374673\_length\_37756\_cov\_28.992161 36482-36483. Max. coverage (+): 246.96. Max coverage (-): 0

Region: NODE\_374673\_length\_37756\_cov\_28.992161 36484-36486. Max. coverage (+): 246.96. Max coverage (-): 0

Region: NODE\_374673\_length\_37756\_cov\_28.992161 36487-36488. Max. coverage (+): 0. Max coverage (-): 0

Region: NODE\_374673\_length\_37756\_cov\_28.992161 36489-36490. Max. coverage (+): 0. Max coverage (-): 0

Region: NODE\_374673\_length\_37756\_cov\_28.992161 36491-36493. Max. coverage (+): 0. Max coverage (-): 0

Region: NODE\_374673\_length\_37756\_cov\_28.992161 36494-36495. Max. coverage (+): 0. Max coverage (-): 0

Region: NODE\_374673\_length\_37756\_cov\_28.992161 36496-36497. Max. coverage (+): 0. Max coverage (-): 0

Region: NODE\_374673\_length\_37756\_cov\_28.992161 36498-36500. Max. coverage (+): 0. Max coverage (-): 0

Region: NODE\_374673\_length\_37756\_cov\_28.992161 36501-36502. Max. coverage (+): 0. Max coverage (-): 0

Region: NODE\_374673\_length\_37756\_cov\_28.992161 36503-36505. Max. coverage (+): 0. Max coverage (-): 0

Region: NODE\_374673\_length\_37756\_cov\_28.992161 36506-36507. Max. coverage (+): 0. Max coverage (-): 0

Region: NODE\_374673\_length\_37756\_cov\_28.992161 36508-36509. Max. coverage (+): 0. Max coverage (-): 0

Region: NODE\_374673\_length\_37756\_cov\_28.992161 36510-36512. Max. coverage (+): 0. Max coverage (-): 0

Region: NODE\_374673\_length\_37756\_cov\_28.992161 36513-36514. Max. coverage (+): 0. Max coverage (-): 0

Region: NODE\_374673\_length\_37756\_cov\_28.992161 36515-36516. Max. coverage (+): 0. Max coverage (-): 0

Region: NODE\_374673\_length\_37756\_cov\_28.992161 36517-36519. Max. coverage (+): 0.04. Max coverage (-): 0

Region: NODE\_374673\_length\_37756\_cov\_28.992161 36520-36521. Max. coverage (+): 0.28. Max coverage (-): 0

Region: NODE\_374673\_length\_37756\_cov\_28.992161 36522-36524. Max. coverage (+): 0.32. Max coverage (-): 0

Region: NODE\_374673\_length\_37756\_cov\_28.992161 36525-36526. Max. coverage (+): 0.12. Max coverage (-): 0

Region: NODE\_374673\_length\_37756\_cov\_28.992161 36527-36528. Max. coverage (+): 0. Max coverage (-): 0

Region: NODE\_374673\_length\_37756\_cov\_28.992161 36529-36531. Max. coverage (+): 0. Max coverage (-): 0

Region: NODE\_374673\_length\_37756\_cov\_28.992161 36532-36533. Max. coverage (+): 0. Max coverage (-): 0

Region: NODE\_374673\_length\_37756\_cov\_28.992161 36534-36536. Max. coverage (+): 0. Max coverage (-): 0

Region: NODE\_374673\_length\_37756\_cov\_28.992161 36537-36538. Max. coverage (+): 0. Max coverage (-): 0

Region: NODE\_374673\_length\_37756\_cov\_28.992161 36539-36540. Max. coverage (+): 0. Max coverage (-): 0

Region: NODE\_374673\_length\_37756\_cov\_28.992161 36541-36543. Max. coverage (+): 0. Max coverage (-): 0

Region: NODE\_374673\_length\_37756\_cov\_28.992161 36544-36545. Max. coverage (+): 0. Max coverage (-): 0

Region: NODE\_374673\_length\_37756\_cov\_28.992161 36546-36547. Max. coverage (+): 0. Max coverage (-): 0

Region: NODE\_374673\_length\_37756\_cov\_28.992161 36548-36550. Max. coverage (+): 0. Max coverage (-): 0

Region: NODE\_374673\_length\_37756\_cov\_28.992161 36551-36552. Max. coverage (+): 0. Max coverage (-): 0

Region: NODE\_374673\_length\_37756\_cov\_28.992161 36553-36555. Max. coverage (+): 0. Max coverage (-): 0

Region: NODE\_374673\_length\_37756\_cov\_28.992161 36556-36557. Max. coverage (+): 0. Max coverage (-): 0

Region: NODE\_374673\_length\_37756\_cov\_28.992161 36558-36559. Max. coverage (+): 0. Max coverage (-): 0

Region: NODE\_374673\_length\_37756\_cov\_28.992161 36560-36562. Max. coverage (+): 0. Max coverage (-): 0

Region: NODE\_374673\_length\_37756\_cov\_28.992161 36563-36564. Max. coverage (+): 0. Max coverage (-): 0

Region: NODE\_374673\_length\_37756\_cov\_28.992161 36565-36566. Max. coverage (+): 0. Max coverage (-): 0

Region: NODE\_374673\_length\_37756\_cov\_28.992161 36567-36569. Max. coverage (+): 0. Max coverage (-): 0

Region: NODE\_374673\_length\_37756\_cov\_28.992161 36570-36571. Max. coverage (+): 0. Max coverage (-): 0

Region: NODE\_374673\_length\_37756\_cov\_28.992161 36572-36574. Max. coverage (+): 0. Max coverage (-): 0

Region: NODE\_374673\_length\_37756\_cov\_28.992161 36575-36576. Max. coverage (+): 0. Max coverage (-): 0

Region: NODE\_374673\_length\_37756\_cov\_28.992161 36577-36578. Max. coverage (+): 0. Max coverage (-): 0

Region: NODE\_374673\_length\_37756\_cov\_28.992161 36579-36581. Max. coverage (+): 0. Max coverage (-): 0

Region: NODE\_374673\_length\_37756\_cov\_28.992161 36582-36583. Max. coverage (+): 0. Max coverage (-): 0

Region: NODE\_374673\_length\_37756\_cov\_28.992161 36584-36586. Max. coverage (+): 0. Max coverage (-): 0

Region: NODE\_374673\_length\_37756\_cov\_28.992161 36587-36588. Max. coverage (+): 0. Max coverage (-): 0

Region: NODE\_374673\_length\_37756\_cov\_28.992161 36589-36590. Max. coverage (+): 0. Max coverage (-): 0

Region: NODE\_374673\_length\_37756\_cov\_28.992161 36591-36593. Max. coverage (+): 0. Max coverage (-): 0

Region: NODE\_374673\_length\_37756\_cov\_28.992161 36594-36595. Max. coverage (+): 0. Max coverage (-): 0

Region: NODE\_374673\_length\_37756\_cov\_28.992161 36596-36597. Max. coverage (+): 0. Max coverage (-): 0

Region: NODE\_374673\_length\_37756\_cov\_28.992161 36598-36600. Max. coverage (+): 0. Max coverage (-): 0

Region: NODE\_374673\_length\_37756\_cov\_28.992161 36601-36602. Max. coverage (+): 0. Max coverage (-): 0

Region: NODE\_374673\_length\_37756\_cov\_28.992161 36603-36605. Max. coverage (+): 0. Max coverage (-): 0

Region: NODE\_374673\_length\_37756\_cov\_28.992161 36606-36607. Max. coverage (+): 0. Max coverage (-): 0

Region: NODE\_374673\_length\_37756\_cov\_28.992161 36608-36609. Max. coverage (+): 0. Max coverage (-): 0

Region: NODE\_374673\_length\_37756\_cov\_28.992161 36610-36612. Max. coverage (+): 0. Max coverage (-): 0

Region: NODE\_374673\_length\_37756\_cov\_28.992161 36613-36614. Max. coverage (+): 0. Max coverage (-): 0

Region: NODE\_374673\_length\_37756\_cov\_28.992161 36615-36617. Max. coverage (+): 0. Max coverage (-): 0

Region: NODE\_374673\_length\_37756\_cov\_28.992161 36618-36619. Max. coverage (+): 0. Max coverage (-): 0

Region: NODE\_374673\_length\_37756\_cov\_28.992161 36620-36621. Max. coverage (+): 0. Max coverage (-): 0

Region: NODE\_374673\_length\_37756\_cov\_28.992161 36622-36624. Max. coverage (+): 0. Max coverage (-): 0

Region: NODE\_374673\_length\_37756\_cov\_28.992161 36625-36626. Max. coverage (+): 0. Max coverage (-): 0

Region: NODE\_374673\_length\_37756\_cov\_28.992161 36627-36628. Max. coverage (+): 0. Max coverage (-): 0

Region: NODE\_374673\_length\_37756\_cov\_28.992161 36629-36631. Max. coverage (+): 0. Max coverage (-): 0

Region: NODE\_374673\_length\_37756\_cov\_28.992161 36632-36633. Max. coverage (+): 0. Max coverage (-): 0

Region: NODE\_374673\_length\_37756\_cov\_28.992161 36634-36636. Max. coverage (+): 0. Max coverage (-): 0

Region: NODE\_374673\_length\_37756\_cov\_28.992161 36637-36638. Max. coverage (+): 0. Max coverage (-): 0

Region: NODE\_374673\_length\_37756\_cov\_28.992161 36639-36640. Max. coverage (+): 0. Max coverage (-): 0

Region: NODE\_374673\_length\_37756\_cov\_28.992161 36641-36643. Max. coverage (+): 0. Max coverage (-): 0

Region: NODE\_374673\_length\_37756\_cov\_28.992161 36644-36645. Max. coverage (+): 0. Max coverage (-): 0

Region: NODE\_374673\_length\_37756\_cov\_28.992161 36646-36647. Max. coverage (+): 0. Max coverage (-): 0

Region: NODE\_374673\_length\_37756\_cov\_28.992161 36648-36650. Max. coverage (+): 0. Max coverage (-): 0

Region: NODE\_374673\_length\_37756\_cov\_28.992161 36651-36652. Max. coverage (+): 0. Max coverage (-): 0

Region: NODE\_374673\_length\_37756\_cov\_28.992161 36653-36655. Max. coverage (+): 0. Max coverage (-): 0

Region: NODE\_374673\_length\_37756\_cov\_28.992161 36656-36657. Max. coverage (+): 0. Max coverage (-): 0

Region: NODE\_374673\_length\_37756\_cov\_28.992161 36658-36659. Max. coverage (+): 0. Max coverage (-): 0

Region: NODE\_374673\_length\_37756\_cov\_28.992161 36660-36662. Max. coverage (+): 0. Max coverage (-): 0

Region: NODE\_374673\_length\_37756\_cov\_28.992161 36663-36664. Max. coverage (+): 0. Max coverage (-): 0

Region: NODE\_374673\_length\_37756\_cov\_28.992161 36665-36667. Max. coverage (+): 0. Max coverage (-): 0

Region: NODE\_374673\_length\_37756\_cov\_28.992161 36668-36669. Max. coverage (+): 0. Max coverage (-): 0

Region: NODE\_374673\_length\_37756\_cov\_28.992161 36670-36671. Max. coverage (+): 0. Max coverage (-): 0

Region: NODE\_374673\_length\_37756\_cov\_28.992161 36672-36674. Max. coverage (+): 0. Max coverage (-): 0

Region: NODE\_374673\_length\_37756\_cov\_28.992161 36675-36676. Max. coverage (+): 0. Max coverage (-): 0

Region: NODE\_374673\_length\_37756\_cov\_28.992161 36677-36678. Max. coverage (+): 0. Max coverage (-): 0

Region: NODE\_374673\_length\_37756\_cov\_28.992161 36679-36681. Max. coverage (+): 0. Max coverage (-): 0

Region: NODE\_374673\_length\_37756\_cov\_28.992161 36682-36683. Max. coverage (+): 0. Max coverage (-): 0

Region: NODE\_374673\_length\_37756\_cov\_28.992161 36684-36686. Max. coverage (+): 0. Max coverage (-): 0

Region: NODE\_374673\_length\_37756\_cov\_28.992161 36687-36688. Max. coverage (+): 0. Max coverage (-): 0

Region: NODE\_374673\_length\_37756\_cov\_28.992161 36689-36690. Max. coverage (+): 0. Max coverage (-): 0

Region: NODE\_374673\_length\_37756\_cov\_28.992161 36691-36693. Max. coverage (+): 0. Max coverage (-): 0

Region: NODE\_374673\_length\_37756\_cov\_28.992161 36694-36695. Max. coverage (+): 0. Max coverage (-): 0

Region: NODE\_374673\_length\_37756\_cov\_28.992161 36696-36698. Max. coverage (+): 0. Max coverage (-): 0

Region: NODE\_374673\_length\_37756\_cov\_28.992161 36699-36700. Max. coverage (+): 0. Max coverage (-): 0

Region: NODE\_374673\_length\_37756\_cov\_28.992161 36701-36702. Max. coverage (+): 0. Max coverage (-): 0

Region: NODE\_374673\_length\_37756\_cov\_28.992161 36703-36705. Max. coverage (+): 0. Max coverage (-): 0

Region: NODE\_374673\_length\_37756\_cov\_28.992161 36706-36707. Max. coverage (+): 0. Max coverage (-): 0

Region: NODE\_374673\_length\_37756\_cov\_28.992161 36708-36709. Max. coverage (+): 0. Max coverage (-): 0

Region: NODE\_374673\_length\_37756\_cov\_28.992161 36710-36712. Max. coverage (+): 0. Max coverage (-): 0

Region: NODE\_374673\_length\_37756\_cov\_28.992161 36713-36714. Max. coverage (+): 0. Max coverage (-): 0

Region: NODE\_374673\_length\_37756\_cov\_28.992161 36715-36717. Max. coverage (+): 0. Max coverage (-): 0

Region: NODE\_374673\_length\_37756\_cov\_28.992161 36718-36719. Max. coverage (+): 0. Max coverage (-): 0

Region: NODE\_374673\_length\_37756\_cov\_28.992161 36720-36721. Max. coverage (+): 0. Max coverage (-): 0

Region: NODE\_374673\_length\_37756\_cov\_28.992161 36722-36724. Max. coverage (+): 0. Max coverage (-): 0

Region: NODE\_374673\_length\_37756\_cov\_28.992161 36725-36726. Max. coverage (+): 0. Max coverage (-): 0

Region: NODE\_374673\_length\_37756\_cov\_28.992161 36727-36728. Max. coverage (+): 0. Max coverage (-): 0

Region: NODE\_374673\_length\_37756\_cov\_28.992161 36729-36731. Max. coverage (+): 0. Max coverage (-): 0

Region: NODE\_374673\_length\_37756\_cov\_28.992161 36732-36733. Max. coverage (+): 0. Max coverage (-): 0

Region: NODE\_374673\_length\_37756\_cov\_28.992161 36734-36736. Max. coverage (+): 0. Max coverage (-): 0

Region: NODE\_374673\_length\_37756\_cov\_28.992161 36737-36738. Max. coverage (+): 0. Max coverage (-): 0

Region: NODE\_374673\_length\_37756\_cov\_28.992161 36739-36740. Max. coverage (+): 0. Max coverage (-): 0

Region: NODE\_374673\_length\_37756\_cov\_28.992161 36741-36743. Max. coverage (+): 0. Max coverage (-): 0

Region: NODE\_374673\_length\_37756\_cov\_28.992161 36744-36745. Max. coverage (+): 0. Max coverage (-): 0

Region: NODE\_374673\_length\_37756\_cov\_28.992161 36746-36748. Max. coverage (+): 0. Max coverage (-): 0

Region: NODE\_374673\_length\_37756\_cov\_28.992161 36749-36750. Max. coverage (+): 0. Max coverage (-): 0

Region: NODE\_374673\_length\_37756\_cov\_28.992161 36751-36752. Max. coverage (+): 0. Max coverage (-): 0

Region: NODE\_374673\_length\_37756\_cov\_28.992161 36753-36755. Max. coverage (+): 0. Max coverage (-): 0

Region: NODE\_374673\_length\_37756\_cov\_28.992161 36756-36757. Max. coverage (+): 0. Max coverage (-): 0

Region: NODE\_374673\_length\_37756\_cov\_28.992161 36758-36759. Max. coverage (+): 0. Max coverage (-): 0

Region: NODE\_374673\_length\_37756\_cov\_28.992161 36760-36762. Max. coverage (+): 0. Max coverage (-): 0

Region: NODE\_374673\_length\_37756\_cov\_28.992161 36763-36764. Max. coverage (+): 0. Max coverage (-): 0

Region: NODE\_374673\_length\_37756\_cov\_28.992161 36765-36767. Max. coverage (+): 0. Max coverage (-): 0

Region: NODE\_374673\_length\_37756\_cov\_28.992161 36768-36769. Max. coverage (+): 0. Max coverage (-): 0

Region: NODE\_374673\_length\_37756\_cov\_28.992161 36770-36771. Max. coverage (+): 0. Max coverage (-): 0

Region: NODE\_374673\_length\_37756\_cov\_28.992161 36772-36774. Max. coverage (+): 0. Max coverage (-): 0

Region: NODE\_374673\_length\_37756\_cov\_28.992161 36775-36776. Max. coverage (+): 0. Max coverage (-): 0

Region: NODE\_374673\_length\_37756\_cov\_28.992161 36777-36778. Max. coverage (+): 0. Max coverage (-): 0

Region: NODE\_374673\_length\_37756\_cov\_28.992161 36779-36781. Max. coverage (+): 0. Max coverage (-): 0

Region: NODE\_374673\_length\_37756\_cov\_28.992161 36782-36783. Max. coverage (+): 0. Max coverage (-): 0

Region: NODE\_374673\_length\_37756\_cov\_28.992161 36784-36786. Max. coverage (+): 0. Max coverage (-): 0

Region: NODE\_374673\_length\_37756\_cov\_28.992161 36787-36788. Max. coverage (+): 0. Max coverage (-): 0

Region: NODE\_374673\_length\_37756\_cov\_28.992161 36789-36790. Max. coverage (+): 0. Max coverage (-): 0

Region: NODE\_374673\_length\_37756\_cov\_28.992161 36791-36793. Max. coverage (+): 0. Max coverage (-): 0

Region: NODE\_374673\_length\_37756\_cov\_28.992161 36794-36795. Max. coverage (+): 0. Max coverage (-): 0

Region: NODE\_374673\_length\_37756\_cov\_28.992161 36796-36798. Max. coverage (+): 0. Max coverage (-): 0

Region: NODE\_374673\_length\_37756\_cov\_28.992161 36799-36800. Max. coverage (+): 0. Max coverage (-): 0

Region: NODE\_374673\_length\_37756\_cov\_28.992161 36801-36802. Max. coverage (+): 0. Max coverage (-): 0

Region: NODE\_374673\_length\_37756\_cov\_28.992161 36803-36805. Max. coverage (+): 0. Max coverage (-): 0

Region: NODE\_374673\_length\_37756\_cov\_28.992161 36806-36807. Max. coverage (+): 0. Max coverage (-): 0

Region: NODE\_374673\_length\_37756\_cov\_28.992161 36808-36809. Max. coverage (+): 0. Max coverage (-): 0

Region: NODE\_374673\_length\_37756\_cov\_28.992161 36810-36812. Max. coverage (+): 0. Max coverage (-): 0

Region: NODE\_374673\_length\_37756\_cov\_28.992161 36813-36814. Max. coverage (+): 0. Max coverage (-): 0

Region: NODE\_374673\_length\_37756\_cov\_28.992161 36815-36817. Max. coverage (+): 0. Max coverage (-): 0

Region: NODE\_374673\_length\_37756\_cov\_28.992161 36818-36819. Max. coverage (+): 0. Max coverage (-): 0

Region: NODE\_374673\_length\_37756\_cov\_28.992161 36820-36821. Max. coverage (+): 0. Max coverage (-): 0

Region: NODE\_374673\_length\_37756\_cov\_28.992161 36822-36824. Max. coverage (+): 0. Max coverage (-): 0

Region: NODE\_374673\_length\_37756\_cov\_28.992161 36825-36826. Max. coverage (+): 0. Max coverage (-): 0

Region: NODE\_374673\_length\_37756\_cov\_28.992161 36827-36829. Max. coverage (+): 0. Max coverage (-): 0

Region: NODE\_374673\_length\_37756\_cov\_28.992161 36830-36831. Max. coverage (+): 0. Max coverage (-): 0

Region: NODE\_374673\_length\_37756\_cov\_28.992161 36832-36833. Max. coverage (+): 0. Max coverage (-): 0

Region: NODE\_374673\_length\_37756\_cov\_28.992161 36834-36836. Max. coverage (+): 0. Max coverage (-): 0

Region: NODE\_374673\_length\_37756\_cov\_28.992161 36837-36838. Max. coverage (+): 0. Max coverage (-): 0

Region: NODE\_374673\_length\_37756\_cov\_28.992161 36839-36840. Max. coverage (+): 0. Max coverage (-): 0

Region: NODE\_374673\_length\_37756\_cov\_28.992161 36841-36843. Max. coverage (+): 0. Max coverage (-): 0

Region: NODE\_374673\_length\_37756\_cov\_28.992161 36844-36845. Max. coverage (+): 0. Max coverage (-): 0

Region: NODE\_374673\_length\_37756\_cov\_28.992161 36846-36848. Max. coverage (+): 0. Max coverage (-): 0

Region: NODE\_374673\_length\_37756\_cov\_28.992161 36849-36850. Max. coverage (+): 0. Max coverage (-): 0

Region: NODE\_374673\_length\_37756\_cov\_28.992161 36851-36852. Max. coverage (+): 0. Max coverage (-): 0

Region: NODE\_374673\_length\_37756\_cov\_28.992161 36853-36855. Max. coverage (+): 0. Max coverage (-): 0

Region: NODE\_374673\_length\_37756\_cov\_28.992161 36856-36857. Max. coverage (+): 0. Max coverage (-): 0

Region: NODE\_374673\_length\_37756\_cov\_28.992161 36858-36859. Max. coverage (+): 0. Max coverage (-): 0

Region: NODE\_374673\_length\_37756\_cov\_28.992161 36860-36862. Max. coverage (+): 0. Max coverage (-): 0

Region: NODE\_374673\_length\_37756\_cov\_28.992161 36863-36864. Max. coverage (+): 0. Max coverage (-): 0

Region: NODE\_374673\_length\_37756\_cov\_28.992161 36865-36867. Max. coverage (+): 0. Max coverage (-): 0

Region: NODE\_374673\_length\_37756\_cov\_28.992161 36868-36869. Max. coverage (+): 0. Max coverage (-): 0

Region: NODE\_374673\_length\_37756\_cov\_28.992161 36870-36871. Max. coverage (+): 0. Max coverage (-): 0

Region: NODE\_374673\_length\_37756\_cov\_28.992161 36872-36874. Max. coverage (+): 0. Max coverage (-): 0

Region: NODE\_374673\_length\_37756\_cov\_28.992161 36875-36876. Max. coverage (+): 0. Max coverage (-): 0

Region: NODE\_374673\_length\_37756\_cov\_28.992161 36877-36879. Max. coverage (+): 0. Max coverage (-): 0

Region: NODE\_374673\_length\_37756\_cov\_28.992161 36880-36881. Max. coverage (+): 0. Max coverage (-): 0

Region: NODE\_374673\_length\_37756\_cov\_28.992161 36882-36883. Max. coverage (+): 0. Max coverage (-): 0

Region: NODE\_374673\_length\_37756\_cov\_28.992161 36884-36886. Max. coverage (+): 0. Max coverage (-): 0

Region: NODE\_374673\_length\_37756\_cov\_28.992161 36887-36888. Max. coverage (+): 0. Max coverage (-): 0

Region: NODE\_374673\_length\_37756\_cov\_28.992161 36889-36890. Max. coverage (+): 0. Max coverage (-): 0

Region: NODE\_374673\_length\_37756\_cov\_28.992161 36891-36893. Max. coverage (+): 0. Max coverage (-): 0

Region: NODE\_374673\_length\_37756\_cov\_28.992161 36894-36895. Max. coverage (+): 0. Max coverage (-): 0

Region: NODE\_374673\_length\_37756\_cov\_28.992161 36896-36898. Max. coverage (+): 0. Max coverage (-): 0

Region: NODE\_374673\_length\_37756\_cov\_28.992161 36899-36900. Max. coverage (+): 0. Max coverage (-): 0

Region: NODE\_374673\_length\_37756\_cov\_28.992161 36901-36902. Max. coverage (+): 0. Max coverage (-): 0

Region: NODE\_374673\_length\_37756\_cov\_28.992161 36903-36905. Max. coverage (+): 0. Max coverage (-): 0

Region: NODE\_374673\_length\_37756\_cov\_28.992161 36906-36907. Max. coverage (+): 0. Max coverage (-): 0

Region: NODE\_374673\_length\_37756\_cov\_28.992161 36908-36910. Max. coverage (+): 0. Max coverage (-): 0

Region: NODE\_374673\_length\_37756\_cov\_28.992161 36911-36912. Max. coverage (+): 0. Max coverage (-): 0

Region: NODE\_374673\_length\_37756\_cov\_28.992161 36913-36914. Max. coverage (+): 0. Max coverage (-): 0

Region: NODE\_374673\_length\_37756\_cov\_28.992161 36915-36917. Max. coverage (+): 0. Max coverage (-): 0

Region: NODE\_374673\_length\_37756\_cov\_28.992161 36918-36919. Max. coverage (+): 0. Max coverage (-): 0

Region: NODE\_374673\_length\_37756\_cov\_28.992161 36920-36921. Max. coverage (+): 0. Max coverage (-): 0

Region: NODE\_374673\_length\_37756\_cov\_28.992161 36922-36924. Max. coverage (+): 0. Max coverage (-): 0

Region: NODE\_374673\_length\_37756\_cov\_28.992161 36925-36926. Max. coverage (+): 0. Max coverage (-): 0

Region: NODE\_374673\_length\_37756\_cov\_28.992161 36927-36929. Max. coverage (+): 0. Max coverage (-): 0

Region: NODE\_374673\_length\_37756\_cov\_28.992161 36930-36931. Max. coverage (+): 0. Max coverage (-): 0

Region: NODE\_374673\_length\_37756\_cov\_28.992161 36932-36933. Max. coverage (+): 0. Max coverage (-): 0

Region: NODE\_374673\_length\_37756\_cov\_28.992161 36934-36936. Max. coverage (+): 0. Max coverage (-): 0

Region: NODE\_374673\_length\_37756\_cov\_28.992161 36937-36938. Max. coverage (+): 0. Max coverage (-): 0

Region: NODE\_374673\_length\_37756\_cov\_28.992161 36939-36940. Max. coverage (+): 0. Max coverage (-): 0

Region: NODE\_374673\_length\_37756\_cov\_28.992161 36941-36943. Max. coverage (+): 0. Max coverage (-): 0

Region: NODE\_374673\_length\_37756\_cov\_28.992161 36944-36945. Max. coverage (+): 0. Max coverage (-): 0

Region: NODE\_374673\_length\_37756\_cov\_28.992161 36946-36948. Max. coverage (+): 0. Max coverage (-): 0

Region: NODE\_374673\_length\_37756\_cov\_28.992161 36949-36950. Max. coverage (+): 0. Max coverage (-): 0

Region: NODE\_374673\_length\_37756\_cov\_28.992161 36951-36952. Max. coverage (+): 0. Max coverage (-): 0

Region: NODE\_374673\_length\_37756\_cov\_28.992161 36953-36955. Max. coverage (+): 0. Max coverage (-): 0

Region: NODE\_374673\_length\_37756\_cov\_28.992161 36956-36957. Max. coverage (+): 0. Max coverage (-): 0

Region: NODE\_374673\_length\_37756\_cov\_28.992161 36958-36960. Max. coverage (+): 0. Max coverage (-): 0

Region: NODE\_374673\_length\_37756\_cov\_28.992161 36961-36962. Max. coverage (+): 0. Max coverage (-): 0

Region: NODE\_374673\_length\_37756\_cov\_28.992161 36963-36964. Max. coverage (+): 0. Max coverage (-): 0

Region: NODE\_374673\_length\_37756\_cov\_28.992161 36965-36967. Max. coverage (+): 0. Max coverage (-): 0

Region: NODE\_374673\_length\_37756\_cov\_28.992161 36968-36969. Max. coverage (+): 0. Max coverage (-): 0

Region: NODE\_374673\_length\_37756\_cov\_28.992161 36970-36971. Max. coverage (+): 0. Max coverage (-): 0

Region: NODE\_374673\_length\_37756\_cov\_28.992161 36972-36974. Max. coverage (+): 0. Max coverage (-): 0

Region: NODE\_374673\_length\_37756\_cov\_28.992161 36975-36976. Max. coverage (+): 0. Max coverage (-): 0

Region: NODE\_374673\_length\_37756\_cov\_28.992161 36977-36979. Max. coverage (+): 0. Max coverage (-): 0

Region: NODE\_374673\_length\_37756\_cov\_28.992161 36980-36981. Max. coverage (+): 0. Max coverage (-): 0

Region: NODE\_374673\_length\_37756\_cov\_28.992161 36982-36983. Max. coverage (+): 0. Max coverage (-): 0

Region: NODE\_374673\_length\_37756\_cov\_28.992161 36984-36986. Max. coverage (+): 0. Max coverage (-): 0

Region: NODE\_374673\_length\_37756\_cov\_28.992161 36987-36988. Max. coverage (+): 0. Max coverage (-): 0

Region: NODE\_374673\_length\_37756\_cov\_28.992161 36989-36990. Max. coverage (+): 0. Max coverage (-): 0

Region: NODE\_374673\_length\_37756\_cov\_28.992161 36991-36993. Max. coverage (+): 0. Max coverage (-): 0

Region: NODE\_374673\_length\_37756\_cov\_28.992161 36994-36995. Max. coverage (+): 0. Max coverage (-): 0

Region: NODE\_374673\_length\_37756\_cov\_28.992161 36996-36998. Max. coverage (+): 0. Max coverage (-): 0

Region: NODE\_374673\_length\_37756\_cov\_28.992161 36999-37000. Max. coverage (+): 0. Max coverage (-): 0

Region: NODE\_374673\_length\_37756\_cov\_28.992161 37001-37002. Max. coverage (+): 0. Max coverage (-): 0

Region: NODE\_374673\_length\_37756\_cov\_28.992161 37003-37005. Max. coverage (+): 0. Max coverage (-): 0

Region: NODE\_374673\_length\_37756\_cov\_28.992161 37006-37007. Max. coverage (+): 0. Max coverage (-): 0

Region: NODE\_374673\_length\_37756\_cov\_28.992161 37008-37010. Max. coverage (+): 0. Max coverage (-): 0

Region: NODE\_374673\_length\_37756\_cov\_28.992161 37011-37012. Max. coverage (+): 0. Max coverage (-): 0

Region: NODE\_374673\_length\_37756\_cov\_28.992161 37013-37014. Max. coverage (+): 0. Max coverage (-): 0

Region: NODE\_374673\_length\_37756\_cov\_28.992161 37015-. Max. coverage (+): 0. Max coverage (-): 0

RepeatMasker Color Code

**+**

100-98% Identity

<98-95% Identity

<95-90% Identity

<90-85% Identity

<85-80% Identity

<80-75% Identity

<75-70% Identity

<70% Identity

**-**

Gene Set Color Code

**+**

Gene

Pseudogene

Other

**-**

Topology/Coverage Color Code

Coverage Plus Strand

Coverage Minus Strand

Mainstrand: Plus

Mainstrand: Minus

Complementary Strand

Flanking Region  
(if option -flank >0)

Gene Set Annotation  
  
RepeatMasker Annotation  

**1. (AC)n**: 36967-36989 (+), Divergence to consensus: 4.5%  
**2. (TG)n**: 36992-37033 (+), Divergence to consensus: 2.4%

  
Transcription Factor Binding Sites  

**RHOXF1** (Sequence: GGCTCA (-): 36528)  
**RHOXF1** (Sequence: TGATCC (+): 35957)  
**RHOXF1** (Sequence: TAATCC (+): 36138)  
**RHOXF1** (Sequence: TGAGCT (+): 36405)  
**RHOXF1** (Sequence: TAAGCT (+): 36933)  
**Lhx8** (Sequence: TTAATTAG (-): 36371)  
**Lhx8** (Sequence: TTAATTAA (-): 36806)  
**Sox5** (Sequence: ATTGTT (+): 36758)  
**Rhox11** (Sequence: TGGTGTTAA (+): 36624)  
**Rhox11** (Sequence: TTAACAGCA (-): 36075)
