# Supplementary material for: First characterization of PIWI-interacting RNA clusters in a cichlid fish with a B chromosome
Source: BMC Biol. 2022 Sep 21;20:204. doi: 10.1186/s12915-022-01403-2 (PMC9490952; doi:10.1186/s12915-022-01403-2)
Supplement: Supplementary file 1 — Additional file 1. Zipped folder with fasta and interactive html piRNA cluster information for the A. latifasciata genome. The nomenclature is as follows: number-pirna-cluster_sex_B-presence (f, female; m, male; 0b, without B chromosome; 1b, with B chromosome). [file 12915_2022_1403_MOESM1_ESM.zip › 147_m0b.html]

piRNA cluster 147\_m0b 72


Predicted piRNA cluster no. 147\_m0b
  

Show proTRAC run info
Hide proTRAC run info

/\  
                \_\_\_\_\_\_\_\_\_\_\_\_\_\_\_\_\_\_\_\_\_\_\_/\\_\_\_ /  \\_\_\_\_\_\_\_  
               I                      /  \  /    \      I  
               I     pro             /    \/      \     I  
               I        TRAC        /               \   I  
               I   \_\_\_\_\_\_\_\_\_\_\_\_\_\_\_\_/\_\_\_\_\_\_\_\_\_\_\_\_\_\_\_\_\_\\_ I  
               I   \              /                     I  
               I    \            /                      I  
               I     \  /\      /       V.2.4.2         I  
               I      \/  \    /                        I  
               I\_\_\_\_\_\_\_\_\_\_\_\  /\_\_\_\_\_\_\_\_\_\_\_\_\_\_\_\_\_\_\_\_\_\_\_\_\_I  
                            \/  
  
  
================================= proTRAC ====================================  
VERSION: .......... 2.4.2  
LAST MODIFIED: .... 11. May 2018  
  
Please cite:  
Rosenkranz D, Zischler H. proTRAC - a software for probabilistic piRNA cluster  
detection, visualization and analysis. 2012. BMC Bioinformatics 13:5.  
  
  
Contact:  
David Rosenkranz  
Institute of Organismic and Molecular Evolutionary Biology  
Dept. Anthropology, small RNA group  
Johannes Gutenberg University Mainz  
email: rosenkranz@uni-mainz.de  
  
You can find the latest proTRAC version at:  
http://sourceforge.net/projects/protrac/files  
http://www.smallRNAgroup-mainz.de/software  
==============================================================================  
  
PARAMETERS:  
Map file: ...............piwi-machos-0B.fa-collapse.map  
Genome file: ............../../../0B\_ala\_genome.fa  
RepeatMasker annotation: Alatifasciata-all0B-maryan-v2.fa\_corrected.out  
GeneSet:................./guest-storage/Data/annotation/Alatifasciata\_all0B\_maryan-v2\_out2017.gff  
  
Significant (p<=0.01) hit density will be calculated based  
on observed hit distribution.  
  
Sliding window size: ........................................ 5000 bp  
Sliding window increament: .................................. 1000 bp  
Normalize each hit by number of genomic hits: ............... yes  
Normalize each hit by number of sequence reads: ............. yes  
Normalize values (-> per million mapped reads): ............. yes  
Min. fraction of hits with 1T(U) or 10A: .................... 0.75  
Alternatively: Min. fraction of hits with 1T(U) and 10A: .... 0.5  
Min. fraction of hits with typical piRNA length: ............ 0.75  
Typical piRNA length: ....................................... 24-32 nt  
Min. size of a piRNA cluster: ............................... 1000 bp.  
Min. number of hits (absolute): ............................. 0  
Min. number of hits (normalized): ........................... 0  
Min. fraction of hits on the mainstrand: .................... 0.75  
Top fraction of mapped sequences (in terms of read counts): . 1%  
Top fraction accounts for max. n% of sequence reads: ........ 90%  
Min. fraction of hits on each arm of a bidirectional cluster: 0.05  
Output html file for each cluster: .......................... yes  
Output a summary table: ..................................... yes  
Output a FASTA file for each cluster (piRNA sequences): ..... yes  
Output a FASTA file comprising cluster sequences: ........... yes  
Output a GTF file for predicted piRNA clusters: ..............yes  
Search DNA motifs in clusters: .............................. yes  
Output flanking sequences: +/- .............................. 0 bp  
Output ~.pTi file: .......................................... no  
==============================================================================  
  
  
Genome size (without gaps): ............ 758543724 bp  
Gaps (N/X/-): .......................... 417479 bp  
Mapped reads: .......................... 24765598  
Non-identical sequences: ............... 6158275  
Genomic hits: .......................... 53103584  
Significant densitiy of mapped reads: .. 763.098963422187 reads/kb

Show proTRAC cluster info
Hide proTRAC cluster info

|  |  |
| --- | --- |
| Location | NODE\_382453\_length\_1102\_cov\_120.509071 |
| Coordinates | 10-1164 |
| Size [bp] | 1155 |
| Sequence hit loci | 3182 |
| Mapped reads (normalized) | 25125.7 |
| Mapped reads (normalized) per kb | 21753.9 |
| Normalized reads with 1T (1U) | 76.9% |
| Normalized reads with 10A | 46.3% |
| Normalized reads with length 24-32 nt | 98.7% |
| Normalized reads on the main strand(s) | 91.4% |
| Predicted directionality | mono:plus |

100%

0%

1T (1U)  
reads

10A reads

24-32 nt  
reads

reads on mainstrand

**Either the amount of reads with 1T (1U) OR 10A has to exceed 75% (set with option: -1Tor10A)  
Alternatively the amount of reads with 1T (1U) AND 10A has to exceed 50% (set with option: -1Tand10A)  
Minimum amount of reads with preferred size is 75% (set with option: -pisize)  
Minimum amount of reads on the main strand(s) is 75% (set with option: -clstrand)**

Show read coverage
Hide read coverage

WHAT DO I SEE HERE?  
This chart shows the location of mapped sequence reads within a predicted piRNA cluster. The color refers to the number of genomic hits produced by the sequence read in question. A dark red bar indicates that this sequence read produces many other hits elsewhere in the genome. Many adjacent red or yellow bars can indicate the presence of a multi-copy element such as transposons or rRNA genes. A dark green bar indicates that this sequence read maps uniquely to this locus.

1 hit

2-5 hits

6-10 hits

11-20 hits

21-50 hits

51-100 hits

> 100 hits

NODE\_382453\_length\_1102\_cov\_120.509071

10

1164

Gene Set

RepeatMasker

Mapped  
Reads

165.43

plus strand

minus strand

165.43

Region: NODE\_382453\_length\_1102\_cov\_120.509071 37016-11. Max. coverage (+): 0.01. Max coverage (-): 0

Region: NODE\_382453\_length\_1102\_cov\_120.509071 12-13. Max. coverage (+): 0.13. Max coverage (-): 0

Region: NODE\_382453\_length\_1102\_cov\_120.509071 14-15. Max. coverage (+): 0.13. Max coverage (-): 0

Region: NODE\_382453\_length\_1102\_cov\_120.509071 16-18. Max. coverage (+): 0.03. Max coverage (-): 0.04

Region: NODE\_382453\_length\_1102\_cov\_120.509071 19-20. Max. coverage (+): 0. Max coverage (-): 0.05

Region: NODE\_382453\_length\_1102\_cov\_120.509071 21-22. Max. coverage (+): 0.08. Max coverage (-): 0.07

Region: NODE\_382453\_length\_1102\_cov\_120.509071 23-25. Max. coverage (+): 0.11. Max coverage (-): 0.16

Region: NODE\_382453\_length\_1102\_cov\_120.509071 26-27. Max. coverage (+): 0.01. Max coverage (-): 0.22

Region: NODE\_382453\_length\_1102\_cov\_120.509071 28-29. Max. coverage (+): 0.01. Max coverage (-): 0.05

Region: NODE\_382453\_length\_1102\_cov\_120.509071 30-31. Max. coverage (+): 0.01. Max coverage (-): 0.04

Region: NODE\_382453\_length\_1102\_cov\_120.509071 32-34. Max. coverage (+): 0.09. Max coverage (-): 0.19

Region: NODE\_382453\_length\_1102\_cov\_120.509071 35-36. Max. coverage (+): 0.09. Max coverage (-): 0.13

Region: NODE\_382453\_length\_1102\_cov\_120.509071 37-38. Max. coverage (+): 0.81. Max coverage (-): 0.98

Region: NODE\_382453\_length\_1102\_cov\_120.509071 39-41. Max. coverage (+): 1.9. Max coverage (-): 0.86

Region: NODE\_382453\_length\_1102\_cov\_120.509071 42-43. Max. coverage (+): 0.24. Max coverage (-): 0.24

Region: NODE\_382453\_length\_1102\_cov\_120.509071 44-45. Max. coverage (+): 0.65. Max coverage (-): 0.32

Region: NODE\_382453\_length\_1102\_cov\_120.509071 46-48. Max. coverage (+): 1.21. Max coverage (-): 0.08

Region: NODE\_382453\_length\_1102\_cov\_120.509071 49-50. Max. coverage (+): 1.25. Max coverage (-): 0.04

Region: NODE\_382453\_length\_1102\_cov\_120.509071 51-52. Max. coverage (+): 0.24. Max coverage (-): 0.04

Region: NODE\_382453\_length\_1102\_cov\_120.509071 53-55. Max. coverage (+): 0.24. Max coverage (-): 0.04

Region: NODE\_382453\_length\_1102\_cov\_120.509071 56-57. Max. coverage (+): 0.2. Max coverage (-): 0.12

Region: NODE\_382453\_length\_1102\_cov\_120.509071 58-59. Max. coverage (+): 0.04. Max coverage (-): 0.08

Region: NODE\_382453\_length\_1102\_cov\_120.509071 60-61. Max. coverage (+): 0.16. Max coverage (-): 0.12

Region: NODE\_382453\_length\_1102\_cov\_120.509071 62-64. Max. coverage (+): 0.48. Max coverage (-): 0.08

Region: NODE\_382453\_length\_1102\_cov\_120.509071 65-66. Max. coverage (+): 0.04. Max coverage (-): 0.16

Region: NODE\_382453\_length\_1102\_cov\_120.509071 67-68. Max. coverage (+): 0. Max coverage (-): 0.12

Region: NODE\_382453\_length\_1102\_cov\_120.509071 69-71. Max. coverage (+): 0. Max coverage (-): 1.29

Region: NODE\_382453\_length\_1102\_cov\_120.509071 72-73. Max. coverage (+): 0. Max coverage (-): 1.94

Region: NODE\_382453\_length\_1102\_cov\_120.509071 74-75. Max. coverage (+): 0.08. Max coverage (-): 2.42

Region: NODE\_382453\_length\_1102\_cov\_120.509071 76-78. Max. coverage (+): 1.86. Max coverage (-): 0.52

Region: NODE\_382453\_length\_1102\_cov\_120.509071 79-80. Max. coverage (+): 1.9. Max coverage (-): 0.08

Region: NODE\_382453\_length\_1102\_cov\_120.509071 81-82. Max. coverage (+): 1.86. Max coverage (-): 0.12

Region: NODE\_382453\_length\_1102\_cov\_120.509071 83-85. Max. coverage (+): 0.04. Max coverage (-): 0.97

Region: NODE\_382453\_length\_1102\_cov\_120.509071 86-87. Max. coverage (+): 1.09. Max coverage (-): 0.12

Region: NODE\_382453\_length\_1102\_cov\_120.509071 88-89. Max. coverage (+): 4.28. Max coverage (-): 0.08

Region: NODE\_382453\_length\_1102\_cov\_120.509071 90-92. Max. coverage (+): 2.99. Max coverage (-): 0.2

Region: NODE\_382453\_length\_1102\_cov\_120.509071 93-94. Max. coverage (+): 0.52. Max coverage (-): 0.16

Region: NODE\_382453\_length\_1102\_cov\_120.509071 95-96. Max. coverage (+): 0.08. Max coverage (-): 0.4

Region: NODE\_382453\_length\_1102\_cov\_120.509071 97-98. Max. coverage (+): 0.77. Max coverage (-): 0.12

Region: NODE\_382453\_length\_1102\_cov\_120.509071 99-101. Max. coverage (+): 0.85. Max coverage (-): 1.17

Region: NODE\_382453\_length\_1102\_cov\_120.509071 102-103. Max. coverage (+): 0.08. Max coverage (-): 1.21

Region: NODE\_382453\_length\_1102\_cov\_120.509071 104-105. Max. coverage (+): 0.08. Max coverage (-): 0.08

Region: NODE\_382453\_length\_1102\_cov\_120.509071 106-108. Max. coverage (+): 1.9. Max coverage (-): 0.16

Region: NODE\_382453\_length\_1102\_cov\_120.509071 109-110. Max. coverage (+): 3.59. Max coverage (-): 0.2

Region: NODE\_382453\_length\_1102\_cov\_120.509071 111-112. Max. coverage (+): 2.83. Max coverage (-): 0.12

Region: NODE\_382453\_length\_1102\_cov\_120.509071 113-115. Max. coverage (+): 1.37. Max coverage (-): 0.08

Region: NODE\_382453\_length\_1102\_cov\_120.509071 116-117. Max. coverage (+): 0.93. Max coverage (-): 0

Region: NODE\_382453\_length\_1102\_cov\_120.509071 118-119. Max. coverage (+): 0.57. Max coverage (-): 0.04

Region: NODE\_382453\_length\_1102\_cov\_120.509071 120-122. Max. coverage (+): 1.09. Max coverage (-): 0.04

Region: NODE\_382453\_length\_1102\_cov\_120.509071 123-124. Max. coverage (+): 0.85. Max coverage (-): 0.2

Region: NODE\_382453\_length\_1102\_cov\_120.509071 125-126. Max. coverage (+): 0.24. Max coverage (-): 0.44

Region: NODE\_382453\_length\_1102\_cov\_120.509071 127-128. Max. coverage (+): 0.12. Max coverage (-): 0.52

Region: NODE\_382453\_length\_1102\_cov\_120.509071 129-131. Max. coverage (+): 0.08. Max coverage (-): 0.4

Region: NODE\_382453\_length\_1102\_cov\_120.509071 132-133. Max. coverage (+): 0.32. Max coverage (-): 0.08

Region: NODE\_382453\_length\_1102\_cov\_120.509071 134-135. Max. coverage (+): 0.36. Max coverage (-): 0.08

Region: NODE\_382453\_length\_1102\_cov\_120.509071 136-138. Max. coverage (+): 0.44. Max coverage (-): 0.85

Region: NODE\_382453\_length\_1102\_cov\_120.509071 139-140. Max. coverage (+): 0.36. Max coverage (-): 0.89

Region: NODE\_382453\_length\_1102\_cov\_120.509071 141-142. Max. coverage (+): 0.44. Max coverage (-): 0.04

Region: NODE\_382453\_length\_1102\_cov\_120.509071 143-145. Max. coverage (+): 7.27. Max coverage (-): 0

Region: NODE\_382453\_length\_1102\_cov\_120.509071 146-147. Max. coverage (+): 7.31. Max coverage (-): 0.08

Region: NODE\_382453\_length\_1102\_cov\_120.509071 148-149. Max. coverage (+): 5.37. Max coverage (-): 0.08

Region: NODE\_382453\_length\_1102\_cov\_120.509071 150-152. Max. coverage (+): 2.18. Max coverage (-): 0

Region: NODE\_382453\_length\_1102\_cov\_120.509071 153-154. Max. coverage (+): 3.96. Max coverage (-): 0

Region: NODE\_382453\_length\_1102\_cov\_120.509071 155-156. Max. coverage (+): 1.94. Max coverage (-): 0.2

Region: NODE\_382453\_length\_1102\_cov\_120.509071 157-158. Max. coverage (+): 0.04. Max coverage (-): 0.24

Region: NODE\_382453\_length\_1102\_cov\_120.509071 159-161. Max. coverage (+): 0.04. Max coverage (-): 0.08

Region: NODE\_382453\_length\_1102\_cov\_120.509071 162-163. Max. coverage (+): 0.04. Max coverage (-): 0.24

Region: NODE\_382453\_length\_1102\_cov\_120.509071 164-165. Max. coverage (+): 0.04. Max coverage (-): 0.28

Region: NODE\_382453\_length\_1102\_cov\_120.509071 166-168. Max. coverage (+): 0.08. Max coverage (-): 0.12

Region: NODE\_382453\_length\_1102\_cov\_120.509071 169-170. Max. coverage (+): 0.2. Max coverage (-): 0.08

Region: NODE\_382453\_length\_1102\_cov\_120.509071 171-172. Max. coverage (+): 0.36. Max coverage (-): 0.08

Region: NODE\_382453\_length\_1102\_cov\_120.509071 173-175. Max. coverage (+): 1.13. Max coverage (-): 0.24

Region: NODE\_382453\_length\_1102\_cov\_120.509071 176-177. Max. coverage (+): 0.61. Max coverage (-): 0.24

Region: NODE\_382453\_length\_1102\_cov\_120.509071 178-179. Max. coverage (+): 0.61. Max coverage (-): 0.16

Region: NODE\_382453\_length\_1102\_cov\_120.509071 180-182. Max. coverage (+): 0.28. Max coverage (-): 0.04

Region: NODE\_382453\_length\_1102\_cov\_120.509071 183-184. Max. coverage (+): 0.24. Max coverage (-): 0.08

Region: NODE\_382453\_length\_1102\_cov\_120.509071 185-186. Max. coverage (+): 0.04. Max coverage (-): 0.04

Region: NODE\_382453\_length\_1102\_cov\_120.509071 187-189. Max. coverage (+): 0. Max coverage (-): 0.2

Region: NODE\_382453\_length\_1102\_cov\_120.509071 190-191. Max. coverage (+): 0. Max coverage (-): 0

Region: NODE\_382453\_length\_1102\_cov\_120.509071 192-193. Max. coverage (+): 0.04. Max coverage (-): 0.24

Region: NODE\_382453\_length\_1102\_cov\_120.509071 194-195. Max. coverage (+): 0.2. Max coverage (-): 0.24

Region: NODE\_382453\_length\_1102\_cov\_120.509071 196-198. Max. coverage (+): 0.32. Max coverage (-): 0.08

Region: NODE\_382453\_length\_1102\_cov\_120.509071 199-200. Max. coverage (+): 0. Max coverage (-): 0.16

Region: NODE\_382453\_length\_1102\_cov\_120.509071 201-202. Max. coverage (+): 0.65. Max coverage (-): 0.16

Region: NODE\_382453\_length\_1102\_cov\_120.509071 203-205. Max. coverage (+): 0.65. Max coverage (-): 1.9

Region: NODE\_382453\_length\_1102\_cov\_120.509071 206-207. Max. coverage (+): 0.04. Max coverage (-): 0.08

Region: NODE\_382453\_length\_1102\_cov\_120.509071 208-209. Max. coverage (+): 0. Max coverage (-): 0

Region: NODE\_382453\_length\_1102\_cov\_120.509071 210-212. Max. coverage (+): 0. Max coverage (-): 0.12

Region: NODE\_382453\_length\_1102\_cov\_120.509071 213-214. Max. coverage (+): 0.04. Max coverage (-): 0.12

Region: NODE\_382453\_length\_1102\_cov\_120.509071 215-216. Max. coverage (+): 0.2. Max coverage (-): 0

Region: NODE\_382453\_length\_1102\_cov\_120.509071 217-219. Max. coverage (+): 0.36. Max coverage (-): 0

Region: NODE\_382453\_length\_1102\_cov\_120.509071 220-221. Max. coverage (+): 0.61. Max coverage (-): 0

Region: NODE\_382453\_length\_1102\_cov\_120.509071 222-223. Max. coverage (+): 0.73. Max coverage (-): 0

Region: NODE\_382453\_length\_1102\_cov\_120.509071 224-225. Max. coverage (+): 0.24. Max coverage (-): 0

Region: NODE\_382453\_length\_1102\_cov\_120.509071 226-228. Max. coverage (+): 0.24. Max coverage (-): 0

Region: NODE\_382453\_length\_1102\_cov\_120.509071 229-230. Max. coverage (+): 0.04. Max coverage (-): 0.04

Region: NODE\_382453\_length\_1102\_cov\_120.509071 231-232. Max. coverage (+): 0.08. Max coverage (-): 0.04

Region: NODE\_382453\_length\_1102\_cov\_120.509071 233-235. Max. coverage (+): 0.08. Max coverage (-): 0

Region: NODE\_382453\_length\_1102\_cov\_120.509071 236-237. Max. coverage (+): 0.04. Max coverage (-): 0.12

Region: NODE\_382453\_length\_1102\_cov\_120.509071 238-239. Max. coverage (+): 0. Max coverage (-): 0.2

Region: NODE\_382453\_length\_1102\_cov\_120.509071 240-242. Max. coverage (+): 0. Max coverage (-): 0.2

Region: NODE\_382453\_length\_1102\_cov\_120.509071 243-244. Max. coverage (+): 0. Max coverage (-): 0.04

Region: NODE\_382453\_length\_1102\_cov\_120.509071 245-246. Max. coverage (+): 0. Max coverage (-): 0

Region: NODE\_382453\_length\_1102\_cov\_120.509071 247-249. Max. coverage (+): 0. Max coverage (-): 0

Region: NODE\_382453\_length\_1102\_cov\_120.509071 250-251. Max. coverage (+): 0. Max coverage (-): 0

Region: NODE\_382453\_length\_1102\_cov\_120.509071 252-253. Max. coverage (+): 0.44. Max coverage (-): 0.08

Region: NODE\_382453\_length\_1102\_cov\_120.509071 254-256. Max. coverage (+): 2.54. Max coverage (-): 0.08

Region: NODE\_382453\_length\_1102\_cov\_120.509071 257-258. Max. coverage (+): 2.62. Max coverage (-): 0.44

Region: NODE\_382453\_length\_1102\_cov\_120.509071 259-260. Max. coverage (+): 0.52. Max coverage (-): 0.48

Region: NODE\_382453\_length\_1102\_cov\_120.509071 261-262. Max. coverage (+): 0.28. Max coverage (-): 0.16

Region: NODE\_382453\_length\_1102\_cov\_120.509071 263-265. Max. coverage (+): 0.08. Max coverage (-): 0.2

Region: NODE\_382453\_length\_1102\_cov\_120.509071 266-267. Max. coverage (+): 0.08. Max coverage (-): 0.32

Region: NODE\_382453\_length\_1102\_cov\_120.509071 268-269. Max. coverage (+): 0.4. Max coverage (-): 0.73

Region: NODE\_382453\_length\_1102\_cov\_120.509071 270-272. Max. coverage (+): 0.36. Max coverage (-): 0.81

Region: NODE\_382453\_length\_1102\_cov\_120.509071 273-274. Max. coverage (+): 0.16. Max coverage (-): 0.61

Region: NODE\_382453\_length\_1102\_cov\_120.509071 275-276. Max. coverage (+): 9.21. Max coverage (-): 0.16

Region: NODE\_382453\_length\_1102\_cov\_120.509071 277-279. Max. coverage (+): 29.92. Max coverage (-): 2.62

Region: NODE\_382453\_length\_1102\_cov\_120.509071 280-281. Max. coverage (+): 33.23. Max coverage (-): 2.26

Region: NODE\_382453\_length\_1102\_cov\_120.509071 282-283. Max. coverage (+): 34.64. Max coverage (-): 1.01

Region: NODE\_382453\_length\_1102\_cov\_120.509071 284-286. Max. coverage (+): 137. Max coverage (-): 0.57

Region: NODE\_382453\_length\_1102\_cov\_120.509071 287-288. Max. coverage (+): 146.78. Max coverage (-): 0.04

Region: NODE\_382453\_length\_1102\_cov\_120.509071 289-290. Max. coverage (+): 15.14. Max coverage (-): 0.04

Region: NODE\_382453\_length\_1102\_cov\_120.509071 291-292. Max. coverage (+): 2.22. Max coverage (-): 0.04

Region: NODE\_382453\_length\_1102\_cov\_120.509071 293-295. Max. coverage (+): 2.87. Max coverage (-): 0

Region: NODE\_382453\_length\_1102\_cov\_120.509071 296-297. Max. coverage (+): 2.26. Max coverage (-): 0

Region: NODE\_382453\_length\_1102\_cov\_120.509071 298-299. Max. coverage (+): 2.95. Max coverage (-): 0

Region: NODE\_382453\_length\_1102\_cov\_120.509071 300-302. Max. coverage (+): 1.05. Max coverage (-): 0

Region: NODE\_382453\_length\_1102\_cov\_120.509071 303-304. Max. coverage (+): 1.17. Max coverage (-): 0

Region: NODE\_382453\_length\_1102\_cov\_120.509071 305-306. Max. coverage (+): 2.14. Max coverage (-): 0.04

Region: NODE\_382453\_length\_1102\_cov\_120.509071 307-309. Max. coverage (+): 1.37. Max coverage (-): 0.04

Region: NODE\_382453\_length\_1102\_cov\_120.509071 310-311. Max. coverage (+): 0.65. Max coverage (-): 0

Region: NODE\_382453\_length\_1102\_cov\_120.509071 312-313. Max. coverage (+): 0.48. Max coverage (-): 0

Region: NODE\_382453\_length\_1102\_cov\_120.509071 314-316. Max. coverage (+): 3.19. Max coverage (-): 0

Region: NODE\_382453\_length\_1102\_cov\_120.509071 317-318. Max. coverage (+): 0.28. Max coverage (-): 0.04

Region: NODE\_382453\_length\_1102\_cov\_120.509071 319-320. Max. coverage (+): 1.01. Max coverage (-): 0.04

Region: NODE\_382453\_length\_1102\_cov\_120.509071 321-323. Max. coverage (+): 6.78. Max coverage (-): 0.08

Region: NODE\_382453\_length\_1102\_cov\_120.509071 324-325. Max. coverage (+): 9.17. Max coverage (-): 0.12

Region: NODE\_382453\_length\_1102\_cov\_120.509071 326-327. Max. coverage (+): 5.25. Max coverage (-): 0.16

Region: NODE\_382453\_length\_1102\_cov\_120.509071 328-329. Max. coverage (+): 5.37. Max coverage (-): 0.57

Region: NODE\_382453\_length\_1102\_cov\_120.509071 330-332. Max. coverage (+): 5.53. Max coverage (-): 0.44

Region: NODE\_382453\_length\_1102\_cov\_120.509071 333-334. Max. coverage (+): 2.3. Max coverage (-): 0.44

Region: NODE\_382453\_length\_1102\_cov\_120.509071 335-336. Max. coverage (+): 2.5. Max coverage (-): 0.08

Region: NODE\_382453\_length\_1102\_cov\_120.509071 337-339. Max. coverage (+): 1.13. Max coverage (-): 0.16

Region: NODE\_382453\_length\_1102\_cov\_120.509071 340-341. Max. coverage (+): 2.22. Max coverage (-): 0.16

Region: NODE\_382453\_length\_1102\_cov\_120.509071 342-343. Max. coverage (+): 1.17. Max coverage (-): 0

Region: NODE\_382453\_length\_1102\_cov\_120.509071 344-346. Max. coverage (+): 2.54. Max coverage (-): 0

Region: NODE\_382453\_length\_1102\_cov\_120.509071 347-348. Max. coverage (+): 30.12. Max coverage (-): 0

Region: NODE\_382453\_length\_1102\_cov\_120.509071 349-350. Max. coverage (+): 28.39. Max coverage (-): 0.04

Region: NODE\_382453\_length\_1102\_cov\_120.509071 351-353. Max. coverage (+): 0.85. Max coverage (-): 0.04

Region: NODE\_382453\_length\_1102\_cov\_120.509071 354-355. Max. coverage (+): 0.16. Max coverage (-): 0

Region: NODE\_382453\_length\_1102\_cov\_120.509071 356-357. Max. coverage (+): 0.04. Max coverage (-): 0

Region: NODE\_382453\_length\_1102\_cov\_120.509071 358-359. Max. coverage (+): 0. Max coverage (-): 0

Region: NODE\_382453\_length\_1102\_cov\_120.509071 360-362. Max. coverage (+): 0.04. Max coverage (-): 0

Region: NODE\_382453\_length\_1102\_cov\_120.509071 363-364. Max. coverage (+): 0.04. Max coverage (-): 0

Region: NODE\_382453\_length\_1102\_cov\_120.509071 365-366. Max. coverage (+): 0.04. Max coverage (-): 0.08

Region: NODE\_382453\_length\_1102\_cov\_120.509071 367-369. Max. coverage (+): 0.04. Max coverage (-): 0.12

Region: NODE\_382453\_length\_1102\_cov\_120.509071 370-371. Max. coverage (+): 0.12. Max coverage (-): 0.08

Region: NODE\_382453\_length\_1102\_cov\_120.509071 372-373. Max. coverage (+): 0.08. Max coverage (-): 0.12

Region: NODE\_382453\_length\_1102\_cov\_120.509071 374-376. Max. coverage (+): 0.04. Max coverage (-): 0.08

Region: NODE\_382453\_length\_1102\_cov\_120.509071 377-378. Max. coverage (+): 0.12. Max coverage (-): 0.04

Region: NODE\_382453\_length\_1102\_cov\_120.509071 379-380. Max. coverage (+): 0.24. Max coverage (-): 0.04

Region: NODE\_382453\_length\_1102\_cov\_120.509071 381-383. Max. coverage (+): 2.71. Max coverage (-): 0.08

Region: NODE\_382453\_length\_1102\_cov\_120.509071 384-385. Max. coverage (+): 5.45. Max coverage (-): 0.08

Region: NODE\_382453\_length\_1102\_cov\_120.509071 386-387. Max. coverage (+): 18.33. Max coverage (-): 0

Region: NODE\_382453\_length\_1102\_cov\_120.509071 388-389. Max. coverage (+): 19.14. Max coverage (-): 0

Region: NODE\_382453\_length\_1102\_cov\_120.509071 390-392. Max. coverage (+): 7.07. Max coverage (-): 0

Region: NODE\_382453\_length\_1102\_cov\_120.509071 393-394. Max. coverage (+): 0.32. Max coverage (-): 0.24

Region: NODE\_382453\_length\_1102\_cov\_120.509071 395-396. Max. coverage (+): 0.08. Max coverage (-): 0.28

Region: NODE\_382453\_length\_1102\_cov\_120.509071 397-399. Max. coverage (+): 0.57. Max coverage (-): 0.08

Region: NODE\_382453\_length\_1102\_cov\_120.509071 400-401. Max. coverage (+): 0.65. Max coverage (-): 0.04

Region: NODE\_382453\_length\_1102\_cov\_120.509071 402-403. Max. coverage (+): 0.08. Max coverage (-): 0

Region: NODE\_382453\_length\_1102\_cov\_120.509071 404-406. Max. coverage (+): 0.04. Max coverage (-): 0.16

Region: NODE\_382453\_length\_1102\_cov\_120.509071 407-408. Max. coverage (+): 0.04. Max coverage (-): 0.08

Region: NODE\_382453\_length\_1102\_cov\_120.509071 409-410. Max. coverage (+): 0.04. Max coverage (-): 0

Region: NODE\_382453\_length\_1102\_cov\_120.509071 411-413. Max. coverage (+): 0. Max coverage (-): 0

Region: NODE\_382453\_length\_1102\_cov\_120.509071 414-415. Max. coverage (+): 0.08. Max coverage (-): 0

Region: NODE\_382453\_length\_1102\_cov\_120.509071 416-417. Max. coverage (+): 0.16. Max coverage (-): 0

Region: NODE\_382453\_length\_1102\_cov\_120.509071 418-420. Max. coverage (+): 0.97. Max coverage (-): 0.04

Region: NODE\_382453\_length\_1102\_cov\_120.509071 421-422. Max. coverage (+): 2.02. Max coverage (-): 0.08

Region: NODE\_382453\_length\_1102\_cov\_120.509071 423-424. Max. coverage (+): 3.39. Max coverage (-): 0.04

Region: NODE\_382453\_length\_1102\_cov\_120.509071 425-426. Max. coverage (+): 2.54. Max coverage (-): 0.12

Region: NODE\_382453\_length\_1102\_cov\_120.509071 427-429. Max. coverage (+): 0.32. Max coverage (-): 0.16

Region: NODE\_382453\_length\_1102\_cov\_120.509071 430-431. Max. coverage (+): 0.04. Max coverage (-): 0.16

Region: NODE\_382453\_length\_1102\_cov\_120.509071 432-433. Max. coverage (+): 0.77. Max coverage (-): 0.16

Region: NODE\_382453\_length\_1102\_cov\_120.509071 434-436. Max. coverage (+): 0.77. Max coverage (-): 0.12

Region: NODE\_382453\_length\_1102\_cov\_120.509071 437-438. Max. coverage (+): 0. Max coverage (-): 0.81

Region: NODE\_382453\_length\_1102\_cov\_120.509071 439-440. Max. coverage (+): 0. Max coverage (-): 0.93

Region: NODE\_382453\_length\_1102\_cov\_120.509071 441-443. Max. coverage (+): 0.08. Max coverage (-): 0.44

Region: NODE\_382453\_length\_1102\_cov\_120.509071 444-445. Max. coverage (+): 0.12. Max coverage (-): 0.57

Region: NODE\_382453\_length\_1102\_cov\_120.509071 446-447. Max. coverage (+): 0.24. Max coverage (-): 0.4

Region: NODE\_382453\_length\_1102\_cov\_120.509071 448-450. Max. coverage (+): 0.24. Max coverage (-): 0.4

Region: NODE\_382453\_length\_1102\_cov\_120.509071 451-452. Max. coverage (+): 0.32. Max coverage (-): 0.04

Region: NODE\_382453\_length\_1102\_cov\_120.509071 453-454. Max. coverage (+): 0.48. Max coverage (-): 0

Region: NODE\_382453\_length\_1102\_cov\_120.509071 455-456. Max. coverage (+): 0.2. Max coverage (-): 0

Region: NODE\_382453\_length\_1102\_cov\_120.509071 457-459. Max. coverage (+): 0.04. Max coverage (-): 0

Region: NODE\_382453\_length\_1102\_cov\_120.509071 460-461. Max. coverage (+): 0. Max coverage (-): 0

Region: NODE\_382453\_length\_1102\_cov\_120.509071 462-463. Max. coverage (+): 0. Max coverage (-): 0

Region: NODE\_382453\_length\_1102\_cov\_120.509071 464-466. Max. coverage (+): 0. Max coverage (-): 0

Region: NODE\_382453\_length\_1102\_cov\_120.509071 467-468. Max. coverage (+): 0. Max coverage (-): 0

Region: NODE\_382453\_length\_1102\_cov\_120.509071 469-470. Max. coverage (+): 0. Max coverage (-): 0

Region: NODE\_382453\_length\_1102\_cov\_120.509071 471-473. Max. coverage (+): 0. Max coverage (-): 0

Region: NODE\_382453\_length\_1102\_cov\_120.509071 474-475. Max. coverage (+): 0. Max coverage (-): 0

Region: NODE\_382453\_length\_1102\_cov\_120.509071 476-477. Max. coverage (+): 0. Max coverage (-): 0

Region: NODE\_382453\_length\_1102\_cov\_120.509071 478-480. Max. coverage (+): 0. Max coverage (-): 0

Region: NODE\_382453\_length\_1102\_cov\_120.509071 481-482. Max. coverage (+): 0. Max coverage (-): 0

Region: NODE\_382453\_length\_1102\_cov\_120.509071 483-484. Max. coverage (+): 0. Max coverage (-): 0

Region: NODE\_382453\_length\_1102\_cov\_120.509071 485-487. Max. coverage (+): 0. Max coverage (-): 0

Region: NODE\_382453\_length\_1102\_cov\_120.509071 488-489. Max. coverage (+): 0. Max coverage (-): 0

Region: NODE\_382453\_length\_1102\_cov\_120.509071 490-491. Max. coverage (+): 0. Max coverage (-): 0

Region: NODE\_382453\_length\_1102\_cov\_120.509071 492-493. Max. coverage (+): 0. Max coverage (-): 0

Region: NODE\_382453\_length\_1102\_cov\_120.509071 494-496. Max. coverage (+): 0. Max coverage (-): 0

Region: NODE\_382453\_length\_1102\_cov\_120.509071 497-498. Max. coverage (+): 0. Max coverage (-): 0

Region: NODE\_382453\_length\_1102\_cov\_120.509071 499-500. Max. coverage (+): 0. Max coverage (-): 0

Region: NODE\_382453\_length\_1102\_cov\_120.509071 501-503. Max. coverage (+): 0.08. Max coverage (-): 0

Region: NODE\_382453\_length\_1102\_cov\_120.509071 504-505. Max. coverage (+): 0.28. Max coverage (-): 0

Region: NODE\_382453\_length\_1102\_cov\_120.509071 506-507. Max. coverage (+): 0.36. Max coverage (-): 0.04

Region: NODE\_382453\_length\_1102\_cov\_120.509071 508-510. Max. coverage (+): 0.08. Max coverage (-): 0.04

Region: NODE\_382453\_length\_1102\_cov\_120.509071 511-512. Max. coverage (+): 0. Max coverage (-): 0.52

Region: NODE\_382453\_length\_1102\_cov\_120.509071 513-514. Max. coverage (+): 0. Max coverage (-): 0.61

Region: NODE\_382453\_length\_1102\_cov\_120.509071 515-517. Max. coverage (+): 0.32. Max coverage (-): 0.65

Region: NODE\_382453\_length\_1102\_cov\_120.509071 518-519. Max. coverage (+): 0.32. Max coverage (-): 0.61

Region: NODE\_382453\_length\_1102\_cov\_120.509071 520-521. Max. coverage (+): 0.16. Max coverage (-): 0.28

Region: NODE\_382453\_length\_1102\_cov\_120.509071 522-523. Max. coverage (+): 0.12. Max coverage (-): 0.97

Region: NODE\_382453\_length\_1102\_cov\_120.509071 524-526. Max. coverage (+): 0.4. Max coverage (-): 0.73

Region: NODE\_382453\_length\_1102\_cov\_120.509071 527-528. Max. coverage (+): 0.04. Max coverage (-): 1.29

Region: NODE\_382453\_length\_1102\_cov\_120.509071 529-530. Max. coverage (+): 0.2. Max coverage (-): 1.09

Region: NODE\_382453\_length\_1102\_cov\_120.509071 531-533. Max. coverage (+): 0.44. Max coverage (-): 0.28

Region: NODE\_382453\_length\_1102\_cov\_120.509071 534-535. Max. coverage (+): 1.29. Max coverage (-): 0

Region: NODE\_382453\_length\_1102\_cov\_120.509071 536-537. Max. coverage (+): 6.02. Max coverage (-): 0.04

Region: NODE\_382453\_length\_1102\_cov\_120.509071 538-540. Max. coverage (+): 5.13. Max coverage (-): 0.04

Region: NODE\_382453\_length\_1102\_cov\_120.509071 541-542. Max. coverage (+): 1.09. Max coverage (-): 0

Region: NODE\_382453\_length\_1102\_cov\_120.509071 543-544. Max. coverage (+): 0.61. Max coverage (-): 0

Region: NODE\_382453\_length\_1102\_cov\_120.509071 545-547. Max. coverage (+): 0.93. Max coverage (-): 0

Region: NODE\_382453\_length\_1102\_cov\_120.509071 548-549. Max. coverage (+): 1.33. Max coverage (-): 0

Region: NODE\_382453\_length\_1102\_cov\_120.509071 550-551. Max. coverage (+): 0.52. Max coverage (-): 0

Region: NODE\_382453\_length\_1102\_cov\_120.509071 552-554. Max. coverage (+): 0. Max coverage (-): 0

Region: NODE\_382453\_length\_1102\_cov\_120.509071 555-556. Max. coverage (+): 0. Max coverage (-): 0

Region: NODE\_382453\_length\_1102\_cov\_120.509071 557-558. Max. coverage (+): 0. Max coverage (-): 0

Region: NODE\_382453\_length\_1102\_cov\_120.509071 559-560. Max. coverage (+): 0. Max coverage (-): 0.04

Region: NODE\_382453\_length\_1102\_cov\_120.509071 561-563. Max. coverage (+): 0. Max coverage (-): 0.32

Region: NODE\_382453\_length\_1102\_cov\_120.509071 564-565. Max. coverage (+): 0. Max coverage (-): 1.25

Region: NODE\_382453\_length\_1102\_cov\_120.509071 566-567. Max. coverage (+): 0.04. Max coverage (-): 1.53

Region: NODE\_382453\_length\_1102\_cov\_120.509071 568-570. Max. coverage (+): 0.08. Max coverage (-): 0.44

Region: NODE\_382453\_length\_1102\_cov\_120.509071 571-572. Max. coverage (+): 0. Max coverage (-): 0.04

Region: NODE\_382453\_length\_1102\_cov\_120.509071 573-574. Max. coverage (+): 0.12. Max coverage (-): 0

Region: NODE\_382453\_length\_1102\_cov\_120.509071 575-577. Max. coverage (+): 26.53. Max coverage (-): 0.04

Region: NODE\_382453\_length\_1102\_cov\_120.509071 578-579. Max. coverage (+): 45.35. Max coverage (-): 0.04

Region: NODE\_382453\_length\_1102\_cov\_120.509071 580-581. Max. coverage (+): 19.14. Max coverage (-): 0

Region: NODE\_382453\_length\_1102\_cov\_120.509071 582-584. Max. coverage (+): 13.49. Max coverage (-): 0.04

Region: NODE\_382453\_length\_1102\_cov\_120.509071 585-586. Max. coverage (+): 0.97. Max coverage (-): 0.04

Region: NODE\_382453\_length\_1102\_cov\_120.509071 587-588. Max. coverage (+): 0.52. Max coverage (-): 0

Region: NODE\_382453\_length\_1102\_cov\_120.509071 589-590. Max. coverage (+): 0.08. Max coverage (-): 0

Region: NODE\_382453\_length\_1102\_cov\_120.509071 591-593. Max. coverage (+): 0.04. Max coverage (-): 0.24

Region: NODE\_382453\_length\_1102\_cov\_120.509071 594-595. Max. coverage (+): 0. Max coverage (-): 0.12

Region: NODE\_382453\_length\_1102\_cov\_120.509071 596-597. Max. coverage (+): 0. Max coverage (-): 0.16

Region: NODE\_382453\_length\_1102\_cov\_120.509071 598-600. Max. coverage (+): 0. Max coverage (-): 0.16

Region: NODE\_382453\_length\_1102\_cov\_120.509071 601-602. Max. coverage (+): 0. Max coverage (-): 0.16

Region: NODE\_382453\_length\_1102\_cov\_120.509071 603-604. Max. coverage (+): 0. Max coverage (-): 0.12

Region: NODE\_382453\_length\_1102\_cov\_120.509071 605-607. Max. coverage (+): 0. Max coverage (-): 0.04

Region: NODE\_382453\_length\_1102\_cov\_120.509071 608-609. Max. coverage (+): 0.12. Max coverage (-): 0

Region: NODE\_382453\_length\_1102\_cov\_120.509071 610-611. Max. coverage (+): 0.12. Max coverage (-): 0

Region: NODE\_382453\_length\_1102\_cov\_120.509071 612-614. Max. coverage (+): 0.08. Max coverage (-): 0.06

Region: NODE\_382453\_length\_1102\_cov\_120.509071 615-616. Max. coverage (+): 0.08. Max coverage (-): 0.06

Region: NODE\_382453\_length\_1102\_cov\_120.509071 617-618. Max. coverage (+): 0.61. Max coverage (-): 0

Region: NODE\_382453\_length\_1102\_cov\_120.509071 619-620. Max. coverage (+): 0.74. Max coverage (-): 0

Region: NODE\_382453\_length\_1102\_cov\_120.509071 621-623. Max. coverage (+): 0.21. Max coverage (-): 0

Region: NODE\_382453\_length\_1102\_cov\_120.509071 624-625. Max. coverage (+): 0.12. Max coverage (-): 0

Region: NODE\_382453\_length\_1102\_cov\_120.509071 626-627. Max. coverage (+): 0.24. Max coverage (-): 0

Region: NODE\_382453\_length\_1102\_cov\_120.509071 628-630. Max. coverage (+): 0.48. Max coverage (-): 0.2

Region: NODE\_382453\_length\_1102\_cov\_120.509071 631-632. Max. coverage (+): 0.3. Max coverage (-): 0.08

Region: NODE\_382453\_length\_1102\_cov\_120.509071 633-634. Max. coverage (+): 0.17. Max coverage (-): 0

Region: NODE\_382453\_length\_1102\_cov\_120.509071 635-637. Max. coverage (+): 0.03. Max coverage (-): 0

Region: NODE\_382453\_length\_1102\_cov\_120.509071 638-639. Max. coverage (+): 0. Max coverage (-): 0

Region: NODE\_382453\_length\_1102\_cov\_120.509071 640-641. Max. coverage (+): 0. Max coverage (-): 0.04

Region: NODE\_382453\_length\_1102\_cov\_120.509071 642-644. Max. coverage (+): 0. Max coverage (-): 0.2

Region: NODE\_382453\_length\_1102\_cov\_120.509071 645-646. Max. coverage (+): 0.04. Max coverage (-): 0.08

Region: NODE\_382453\_length\_1102\_cov\_120.509071 647-648. Max. coverage (+): 0.04. Max coverage (-): 0

Region: NODE\_382453\_length\_1102\_cov\_120.509071 649-651. Max. coverage (+): 1.62. Max coverage (-): 0.04

Region: NODE\_382453\_length\_1102\_cov\_120.509071 652-653. Max. coverage (+): 2.18. Max coverage (-): 0.04

Region: NODE\_382453\_length\_1102\_cov\_120.509071 654-655. Max. coverage (+): 0.81. Max coverage (-): 0

Region: NODE\_382453\_length\_1102\_cov\_120.509071 656-657. Max. coverage (+): 0.04. Max coverage (-): 0.32

Region: NODE\_382453\_length\_1102\_cov\_120.509071 658-660. Max. coverage (+): 0. Max coverage (-): 0.36

Region: NODE\_382453\_length\_1102\_cov\_120.509071 661-662. Max. coverage (+): 0. Max coverage (-): 0.87

Region: NODE\_382453\_length\_1102\_cov\_120.509071 663-664. Max. coverage (+): 0.87. Max coverage (-): 2.5

Region: NODE\_382453\_length\_1102\_cov\_120.509071 665-667. Max. coverage (+): 0.96. Max coverage (-): 2.33

Region: NODE\_382453\_length\_1102\_cov\_120.509071 668-669. Max. coverage (+): 0.04. Max coverage (-): 1.66

Region: NODE\_382453\_length\_1102\_cov\_120.509071 670-671. Max. coverage (+): 0.16. Max coverage (-): 0.16

Region: NODE\_382453\_length\_1102\_cov\_120.509071 672-674. Max. coverage (+): 0.2. Max coverage (-): 0.04

Region: NODE\_382453\_length\_1102\_cov\_120.509071 675-676. Max. coverage (+): 164.99. Max coverage (-): 0.04

Region: NODE\_382453\_length\_1102\_cov\_120.509071 677-678. Max. coverage (+): 165.43. Max coverage (-): 0

Region: NODE\_382453\_length\_1102\_cov\_120.509071 679-681. Max. coverage (+): 7.79. Max coverage (-): 0.04

Region: NODE\_382453\_length\_1102\_cov\_120.509071 682-683. Max. coverage (+): 1.33. Max coverage (-): 0

Region: NODE\_382453\_length\_1102\_cov\_120.509071 684-685. Max. coverage (+): 0.08. Max coverage (-): 0.12

Region: NODE\_382453\_length\_1102\_cov\_120.509071 686-687. Max. coverage (+): 0.24. Max coverage (-): 0.24

Region: NODE\_382453\_length\_1102\_cov\_120.509071 688-690. Max. coverage (+): 0.36. Max coverage (-): 0.89

Region: NODE\_382453\_length\_1102\_cov\_120.509071 691-692. Max. coverage (+): 0.08. Max coverage (-): 0.48

Region: NODE\_382453\_length\_1102\_cov\_120.509071 693-694. Max. coverage (+): 0. Max coverage (-): 0.08

Region: NODE\_382453\_length\_1102\_cov\_120.509071 695-697. Max. coverage (+): 0. Max coverage (-): 0

Region: NODE\_382453\_length\_1102\_cov\_120.509071 698-699. Max. coverage (+): 0. Max coverage (-): 0

Region: NODE\_382453\_length\_1102\_cov\_120.509071 700-701. Max. coverage (+): 0. Max coverage (-): 0

Region: NODE\_382453\_length\_1102\_cov\_120.509071 702-704. Max. coverage (+): 0.2. Max coverage (-): 0

Region: NODE\_382453\_length\_1102\_cov\_120.509071 705-706. Max. coverage (+): 0.24. Max coverage (-): 0

Region: NODE\_382453\_length\_1102\_cov\_120.509071 707-708. Max. coverage (+): 0.52. Max coverage (-): 0.04

Region: NODE\_382453\_length\_1102\_cov\_120.509071 709-711. Max. coverage (+): 1.09. Max coverage (-): 0.04

Region: NODE\_382453\_length\_1102\_cov\_120.509071 712-713. Max. coverage (+): 0.44. Max coverage (-): 0.04

Region: NODE\_382453\_length\_1102\_cov\_120.509071 714-715. Max. coverage (+): 0.04. Max coverage (-): 0.04

Region: NODE\_382453\_length\_1102\_cov\_120.509071 716-718. Max. coverage (+): 0. Max coverage (-): 0

Region: NODE\_382453\_length\_1102\_cov\_120.509071 719-720. Max. coverage (+): 0. Max coverage (-): 0.04

Region: NODE\_382453\_length\_1102\_cov\_120.509071 721-722. Max. coverage (+): 0. Max coverage (-): 0.36

Region: NODE\_382453\_length\_1102\_cov\_120.509071 723-724. Max. coverage (+): 0. Max coverage (-): 0.48

Region: NODE\_382453\_length\_1102\_cov\_120.509071 725-727. Max. coverage (+): 0. Max coverage (-): 0.73

Region: NODE\_382453\_length\_1102\_cov\_120.509071 728-729. Max. coverage (+): 0. Max coverage (-): 0.93

Region: NODE\_382453\_length\_1102\_cov\_120.509071 730-731. Max. coverage (+): 0. Max coverage (-): 0.77

Region: NODE\_382453\_length\_1102\_cov\_120.509071 732-734. Max. coverage (+): 1.57. Max coverage (-): 0.48

Region: NODE\_382453\_length\_1102\_cov\_120.509071 735-736. Max. coverage (+): 1.9. Max coverage (-): 0.12

Region: NODE\_382453\_length\_1102\_cov\_120.509071 737-738. Max. coverage (+): 9.37. Max coverage (-): 0.16

Region: NODE\_382453\_length\_1102\_cov\_120.509071 739-741. Max. coverage (+): 12.52. Max coverage (-): 0.08

Region: NODE\_382453\_length\_1102\_cov\_120.509071 742-743. Max. coverage (+): 0.55. Max coverage (-): 0.04

Region: NODE\_382453\_length\_1102\_cov\_120.509071 744-745. Max. coverage (+): 3.41. Max coverage (-): 0.02

Region: NODE\_382453\_length\_1102\_cov\_120.509071 746-748. Max. coverage (+): 3.25. Max coverage (-): 0.18

Region: NODE\_382453\_length\_1102\_cov\_120.509071 749-750. Max. coverage (+): 0. Max coverage (-): 1.49

Region: NODE\_382453\_length\_1102\_cov\_120.509071 751-752. Max. coverage (+): 0. Max coverage (-): 1.41

Region: NODE\_382453\_length\_1102\_cov\_120.509071 753-754. Max. coverage (+): 0. Max coverage (-): 0

Region: NODE\_382453\_length\_1102\_cov\_120.509071 755-757. Max. coverage (+): 0. Max coverage (-): 0.04

Region: NODE\_382453\_length\_1102\_cov\_120.509071 758-759. Max. coverage (+): 0. Max coverage (-): 0

Region: NODE\_382453\_length\_1102\_cov\_120.509071 760-761. Max. coverage (+): 0.08. Max coverage (-): 0

Region: NODE\_382453\_length\_1102\_cov\_120.509071 762-764. Max. coverage (+): 0.32. Max coverage (-): 0

Region: NODE\_382453\_length\_1102\_cov\_120.509071 765-766. Max. coverage (+): 0.2. Max coverage (-): 0

Region: NODE\_382453\_length\_1102\_cov\_120.509071 767-768. Max. coverage (+): 0.08. Max coverage (-): 0

Region: NODE\_382453\_length\_1102\_cov\_120.509071 769-771. Max. coverage (+): 1.13. Max coverage (-): 0.04

Region: NODE\_382453\_length\_1102\_cov\_120.509071 772-773. Max. coverage (+): 0.32. Max coverage (-): 0

Region: NODE\_382453\_length\_1102\_cov\_120.509071 774-775. Max. coverage (+): 0.4. Max coverage (-): 0

Region: NODE\_382453\_length\_1102\_cov\_120.509071 776-778. Max. coverage (+): 0.24. Max coverage (-): 0.08

Region: NODE\_382453\_length\_1102\_cov\_120.509071 779-780. Max. coverage (+): 0.08. Max coverage (-): 0.24

Region: NODE\_382453\_length\_1102\_cov\_120.509071 781-782. Max. coverage (+): 0. Max coverage (-): 0.2

Region: NODE\_382453\_length\_1102\_cov\_120.509071 783-785. Max. coverage (+): 0. Max coverage (-): 0.48

Region: NODE\_382453\_length\_1102\_cov\_120.509071 786-787. Max. coverage (+): 0.08. Max coverage (-): 0.2

Region: NODE\_382453\_length\_1102\_cov\_120.509071 788-789. Max. coverage (+): 0.08. Max coverage (-): 0.16

Region: NODE\_382453\_length\_1102\_cov\_120.509071 790-791. Max. coverage (+): 0. Max coverage (-): 0.2

Region: NODE\_382453\_length\_1102\_cov\_120.509071 792-794. Max. coverage (+): 0. Max coverage (-): 0.12

Region: NODE\_382453\_length\_1102\_cov\_120.509071 795-796. Max. coverage (+): 0. Max coverage (-): 0

Region: NODE\_382453\_length\_1102\_cov\_120.509071 797-798. Max. coverage (+): 0. Max coverage (-): 0

Region: NODE\_382453\_length\_1102\_cov\_120.509071 799-801. Max. coverage (+): 0. Max coverage (-): 0

Region: NODE\_382453\_length\_1102\_cov\_120.509071 802-803. Max. coverage (+): 0. Max coverage (-): 0

Region: NODE\_382453\_length\_1102\_cov\_120.509071 804-805. Max. coverage (+): 0. Max coverage (-): 0

Region: NODE\_382453\_length\_1102\_cov\_120.509071 806-808. Max. coverage (+): 0. Max coverage (-): 0

Region: NODE\_382453\_length\_1102\_cov\_120.509071 809-810. Max. coverage (+): 0. Max coverage (-): 0

Region: NODE\_382453\_length\_1102\_cov\_120.509071 811-812. Max. coverage (+): 0. Max coverage (-): 0

Region: NODE\_382453\_length\_1102\_cov\_120.509071 813-815. Max. coverage (+): 0. Max coverage (-): 0

Region: NODE\_382453\_length\_1102\_cov\_120.509071 816-817. Max. coverage (+): 0. Max coverage (-): 0

Region: NODE\_382453\_length\_1102\_cov\_120.509071 818-819. Max. coverage (+): 0. Max coverage (-): 0

Region: NODE\_382453\_length\_1102\_cov\_120.509071 820-821. Max. coverage (+): 0. Max coverage (-): 0

Region: NODE\_382453\_length\_1102\_cov\_120.509071 822-824. Max. coverage (+): 0. Max coverage (-): 0.04

Region: NODE\_382453\_length\_1102\_cov\_120.509071 825-826. Max. coverage (+): 0. Max coverage (-): 0.08

Region: NODE\_382453\_length\_1102\_cov\_120.509071 827-828. Max. coverage (+): 0. Max coverage (-): 0.2

Region: NODE\_382453\_length\_1102\_cov\_120.509071 829-831. Max. coverage (+): 0. Max coverage (-): 0.12

Region: NODE\_382453\_length\_1102\_cov\_120.509071 832-833. Max. coverage (+): 0.04. Max coverage (-): 0.12

Region: NODE\_382453\_length\_1102\_cov\_120.509071 834-835. Max. coverage (+): 0.48. Max coverage (-): 0.08

Region: NODE\_382453\_length\_1102\_cov\_120.509071 836-838. Max. coverage (+): 1.01. Max coverage (-): 0.04

Region: NODE\_382453\_length\_1102\_cov\_120.509071 839-840. Max. coverage (+): 0.48. Max coverage (-): 0.04

Region: NODE\_382453\_length\_1102\_cov\_120.509071 841-842. Max. coverage (+): 0. Max coverage (-): 0

Region: NODE\_382453\_length\_1102\_cov\_120.509071 843-845. Max. coverage (+): 0.04. Max coverage (-): 0

Region: NODE\_382453\_length\_1102\_cov\_120.509071 846-847. Max. coverage (+): 0.04. Max coverage (-): 0.04

Region: NODE\_382453\_length\_1102\_cov\_120.509071 848-849. Max. coverage (+): 0. Max coverage (-): 0.08

Region: NODE\_382453\_length\_1102\_cov\_120.509071 850-851. Max. coverage (+): 0. Max coverage (-): 0.04

Region: NODE\_382453\_length\_1102\_cov\_120.509071 852-854. Max. coverage (+): 0. Max coverage (-): 0

Region: NODE\_382453\_length\_1102\_cov\_120.509071 855-856. Max. coverage (+): 0.01. Max coverage (-): 0

Region: NODE\_382453\_length\_1102\_cov\_120.509071 857-858. Max. coverage (+): 0.1. Max coverage (-): 0

Region: NODE\_382453\_length\_1102\_cov\_120.509071 859-861. Max. coverage (+): 0.24. Max coverage (-): 0.24

Region: NODE\_382453\_length\_1102\_cov\_120.509071 862-863. Max. coverage (+): 0.94. Max coverage (-): 0.12

Region: NODE\_382453\_length\_1102\_cov\_120.509071 864-865. Max. coverage (+): 3.46. Max coverage (-): 0.32

Region: NODE\_382453\_length\_1102\_cov\_120.509071 866-868. Max. coverage (+): 2.87. Max coverage (-): 0.44

Region: NODE\_382453\_length\_1102\_cov\_120.509071 869-870. Max. coverage (+): 1.01. Max coverage (-): 0

Region: NODE\_382453\_length\_1102\_cov\_120.509071 871-872. Max. coverage (+): 1.06. Max coverage (-): 0.04

Region: NODE\_382453\_length\_1102\_cov\_120.509071 873-875. Max. coverage (+): 0.42. Max coverage (-): 0.04

Region: NODE\_382453\_length\_1102\_cov\_120.509071 876-877. Max. coverage (+): 0.9. Max coverage (-): 0.04

Region: NODE\_382453\_length\_1102\_cov\_120.509071 878-879. Max. coverage (+): 12.28. Max coverage (-): 0.04

Region: NODE\_382453\_length\_1102\_cov\_120.509071 880-882. Max. coverage (+): 15.38. Max coverage (-): 0.04

Region: NODE\_382453\_length\_1102\_cov\_120.509071 883-884. Max. coverage (+): 0.65. Max coverage (-): 0.04

Region: NODE\_382453\_length\_1102\_cov\_120.509071 885-886. Max. coverage (+): 0.28. Max coverage (-): 0.08

Region: NODE\_382453\_length\_1102\_cov\_120.509071 887-888. Max. coverage (+): 0.2. Max coverage (-): 0.08

Region: NODE\_382453\_length\_1102\_cov\_120.509071 889-891. Max. coverage (+): 3.23. Max coverage (-): 0.08

Region: NODE\_382453\_length\_1102\_cov\_120.509071 892-893. Max. coverage (+): 4.56. Max coverage (-): 0.04

Region: NODE\_382453\_length\_1102\_cov\_120.509071 894-895. Max. coverage (+): 2.34. Max coverage (-): 0.48

Region: NODE\_382453\_length\_1102\_cov\_120.509071 896-898. Max. coverage (+): 0.77. Max coverage (-): 0.48

Region: NODE\_382453\_length\_1102\_cov\_120.509071 899-900. Max. coverage (+): 0.93. Max coverage (-): 0.36

Region: NODE\_382453\_length\_1102\_cov\_120.509071 901-902. Max. coverage (+): 1.17. Max coverage (-): 0.32

Region: NODE\_382453\_length\_1102\_cov\_120.509071 903-905. Max. coverage (+): 1.41. Max coverage (-): 0.2

Region: NODE\_382453\_length\_1102\_cov\_120.509071 906-907. Max. coverage (+): 0.89. Max coverage (-): 0.61

Region: NODE\_382453\_length\_1102\_cov\_120.509071 908-909. Max. coverage (+): 2.18. Max coverage (-): 4.48

Region: NODE\_382453\_length\_1102\_cov\_120.509071 910-912. Max. coverage (+): 24.19. Max coverage (-): 4.6

Region: NODE\_382453\_length\_1102\_cov\_120.509071 913-914. Max. coverage (+): 22.73. Max coverage (-): 0.69

Region: NODE\_382453\_length\_1102\_cov\_120.509071 915-916. Max. coverage (+): 2.54. Max coverage (-): 0.61

Region: NODE\_382453\_length\_1102\_cov\_120.509071 917-918. Max. coverage (+): 1.13. Max coverage (-): 0.61

Region: NODE\_382453\_length\_1102\_cov\_120.509071 919-921. Max. coverage (+): 0.81. Max coverage (-): 0.28

Region: NODE\_382453\_length\_1102\_cov\_120.509071 922-923. Max. coverage (+): 0.2. Max coverage (-): 0

Region: NODE\_382453\_length\_1102\_cov\_120.509071 924-925. Max. coverage (+): 0.65. Max coverage (-): 0

Region: NODE\_382453\_length\_1102\_cov\_120.509071 926-928. Max. coverage (+): 0.65. Max coverage (-): 0

Region: NODE\_382453\_length\_1102\_cov\_120.509071 929-930. Max. coverage (+): 0. Max coverage (-): 0

Region: NODE\_382453\_length\_1102\_cov\_120.509071 931-932. Max. coverage (+): 0. Max coverage (-): 0.08

Region: NODE\_382453\_length\_1102\_cov\_120.509071 933-935. Max. coverage (+): 0. Max coverage (-): 0.24

Region: NODE\_382453\_length\_1102\_cov\_120.509071 936-937. Max. coverage (+): 0. Max coverage (-): 0.32

Region: NODE\_382453\_length\_1102\_cov\_120.509071 938-939. Max. coverage (+): 0.04. Max coverage (-): 0.2

Region: NODE\_382453\_length\_1102\_cov\_120.509071 940-942. Max. coverage (+): 0.04. Max coverage (-): 0.04

Region: NODE\_382453\_length\_1102\_cov\_120.509071 943-944. Max. coverage (+): 0. Max coverage (-): 0.04

Region: NODE\_382453\_length\_1102\_cov\_120.509071 945-946. Max. coverage (+): 0. Max coverage (-): 0

Region: NODE\_382453\_length\_1102\_cov\_120.509071 947-949. Max. coverage (+): 0. Max coverage (-): 0.04

Region: NODE\_382453\_length\_1102\_cov\_120.509071 950-951. Max. coverage (+): 0. Max coverage (-): 0.04

Region: NODE\_382453\_length\_1102\_cov\_120.509071 952-953. Max. coverage (+): 0. Max coverage (-): 0.04

Region: NODE\_382453\_length\_1102\_cov\_120.509071 954-955. Max. coverage (+): 0.04. Max coverage (-): 0.04

Region: NODE\_382453\_length\_1102\_cov\_120.509071 956-958. Max. coverage (+): 0.61. Max coverage (-): 0.04

Region: NODE\_382453\_length\_1102\_cov\_120.509071 959-960. Max. coverage (+): 0.48. Max coverage (-): 0.08

Region: NODE\_382453\_length\_1102\_cov\_120.509071 961-962. Max. coverage (+): 1.05. Max coverage (-): 0.04

Region: NODE\_382453\_length\_1102\_cov\_120.509071 963-965. Max. coverage (+): 1.05. Max coverage (-): 0

Region: NODE\_382453\_length\_1102\_cov\_120.509071 966-967. Max. coverage (+): 1.09. Max coverage (-): 0.24

Region: NODE\_382453\_length\_1102\_cov\_120.509071 968-969. Max. coverage (+): 0.4. Max coverage (-): 0.24

Region: NODE\_382453\_length\_1102\_cov\_120.509071 970-972. Max. coverage (+): 0.12. Max coverage (-): 0

Region: NODE\_382453\_length\_1102\_cov\_120.509071 973-974. Max. coverage (+): 0. Max coverage (-): 0.04

Region: NODE\_382453\_length\_1102\_cov\_120.509071 975-976. Max. coverage (+): 0. Max coverage (-): 0.04

Region: NODE\_382453\_length\_1102\_cov\_120.509071 977-979. Max. coverage (+): 1.37. Max coverage (-): 0.08

Region: NODE\_382453\_length\_1102\_cov\_120.509071 980-981. Max. coverage (+): 27.5. Max coverage (-): 0.08

Region: NODE\_382453\_length\_1102\_cov\_120.509071 982-983. Max. coverage (+): 26.53. Max coverage (-): 0.04

Region: NODE\_382453\_length\_1102\_cov\_120.509071 984-985. Max. coverage (+): 0.4. Max coverage (-): 0.04

Region: NODE\_382453\_length\_1102\_cov\_120.509071 986-988. Max. coverage (+): 0.04. Max coverage (-): 0.04

Region: NODE\_382453\_length\_1102\_cov\_120.509071 989-990. Max. coverage (+): 0. Max coverage (-): 0.57

Region: NODE\_382453\_length\_1102\_cov\_120.509071 991-992. Max. coverage (+): 0. Max coverage (-): 3.92

Region: NODE\_382453\_length\_1102\_cov\_120.509071 993-995. Max. coverage (+): 0.04. Max coverage (-): 3.43

Region: NODE\_382453\_length\_1102\_cov\_120.509071 996-997. Max. coverage (+): 0. Max coverage (-): 1.09

Region: NODE\_382453\_length\_1102\_cov\_120.509071 998-999. Max. coverage (+): 0. Max coverage (-): 0.61

Region: NODE\_382453\_length\_1102\_cov\_120.509071 1000-1002. Max. coverage (+): 0. Max coverage (-): 0.04

Region: NODE\_382453\_length\_1102\_cov\_120.509071 1003-1004. Max. coverage (+): 0. Max coverage (-): 0.2

Region: NODE\_382453\_length\_1102\_cov\_120.509071 1005-1006. Max. coverage (+): 0.12. Max coverage (-): 0.2

Region: NODE\_382453\_length\_1102\_cov\_120.509071 1007-1009. Max. coverage (+): 0.61. Max coverage (-): 0.2

Region: NODE\_382453\_length\_1102\_cov\_120.509071 1010-1011. Max. coverage (+): 3.8. Max coverage (-): 0.04

Region: NODE\_382453\_length\_1102\_cov\_120.509071 1012-1013. Max. coverage (+): 76.8. Max coverage (-): 0

Region: NODE\_382453\_length\_1102\_cov\_120.509071 1014-1016. Max. coverage (+): 81.81. Max coverage (-): 0.04

Region: NODE\_382453\_length\_1102\_cov\_120.509071 1017-1018. Max. coverage (+): 10.86. Max coverage (-): 0.04

Region: NODE\_382453\_length\_1102\_cov\_120.509071 1019-1020. Max. coverage (+): 5.25. Max coverage (-): 0.04

Region: NODE\_382453\_length\_1102\_cov\_120.509071 1021-1022. Max. coverage (+): 0.69. Max coverage (-): 0.08

Region: NODE\_382453\_length\_1102\_cov\_120.509071 1023-1025. Max. coverage (+): 0.57. Max coverage (-): 4.68

Region: NODE\_382453\_length\_1102\_cov\_120.509071 1026-1027. Max. coverage (+): 0. Max coverage (-): 4.72

Region: NODE\_382453\_length\_1102\_cov\_120.509071 1028-1029. Max. coverage (+): 0.04. Max coverage (-): 0.44

Region: NODE\_382453\_length\_1102\_cov\_120.509071 1030-1032. Max. coverage (+): 0.12. Max coverage (-): 0.85

Region: NODE\_382453\_length\_1102\_cov\_120.509071 1033-1034. Max. coverage (+): 0.08. Max coverage (-): 0.48

Region: NODE\_382453\_length\_1102\_cov\_120.509071 1035-1036. Max. coverage (+): 0.08. Max coverage (-): 0

Region: NODE\_382453\_length\_1102\_cov\_120.509071 1037-1039. Max. coverage (+): 0.08. Max coverage (-): 0

Region: NODE\_382453\_length\_1102\_cov\_120.509071 1040-1041. Max. coverage (+): 0. Max coverage (-): 0

Region: NODE\_382453\_length\_1102\_cov\_120.509071 1042-1043. Max. coverage (+): 2.79. Max coverage (-): 0

Region: NODE\_382453\_length\_1102\_cov\_120.509071 1044-1046. Max. coverage (+): 8.48. Max coverage (-): 0.16

Region: NODE\_382453\_length\_1102\_cov\_120.509071 1047-1048. Max. coverage (+): 9.97. Max coverage (-): 0.16

Region: NODE\_382453\_length\_1102\_cov\_120.509071 1049-1050. Max. coverage (+): 13.37. Max coverage (-): 0.08

Region: NODE\_382453\_length\_1102\_cov\_120.509071 1051-1052. Max. coverage (+): 6.1. Max coverage (-): 0.48

Region: NODE\_382453\_length\_1102\_cov\_120.509071 1053-1055. Max. coverage (+): 1.41. Max coverage (-): 0.52

Region: NODE\_382453\_length\_1102\_cov\_120.509071 1056-1057. Max. coverage (+): 3.23. Max coverage (-): 3.31

Region: NODE\_382453\_length\_1102\_cov\_120.509071 1058-1059. Max. coverage (+): 4.48. Max coverage (-): 4.48

Region: NODE\_382453\_length\_1102\_cov\_120.509071 1060-1062. Max. coverage (+): 2.58. Max coverage (-): 1.78

Region: NODE\_382453\_length\_1102\_cov\_120.509071 1063-1064. Max. coverage (+): 0.12. Max coverage (-): 0.81

Region: NODE\_382453\_length\_1102\_cov\_120.509071 1065-1066. Max. coverage (+): 0.32. Max coverage (-): 0.61

Region: NODE\_382453\_length\_1102\_cov\_120.509071 1067-1069. Max. coverage (+): 0.4. Max coverage (-): 0.94

Region: NODE\_382453\_length\_1102\_cov\_120.509071 1070-1071. Max. coverage (+): 0.24. Max coverage (-): 0.92

Region: NODE\_382453\_length\_1102\_cov\_120.509071 1072-1073. Max. coverage (+): 0.15. Max coverage (-): 0.5

Region: NODE\_382453\_length\_1102\_cov\_120.509071 1074-1076. Max. coverage (+): 0.84. Max coverage (-): 0.44

Region: NODE\_382453\_length\_1102\_cov\_120.509071 1077-1078. Max. coverage (+): 2.35. Max coverage (-): 0.23

Region: NODE\_382453\_length\_1102\_cov\_120.509071 1079-1080. Max. coverage (+): 3.44. Max coverage (-): 0.36

Region: NODE\_382453\_length\_1102\_cov\_120.509071 1081-1082. Max. coverage (+): 6.88. Max coverage (-): 0.36

Region: NODE\_382453\_length\_1102\_cov\_120.509071 1083-1085. Max. coverage (+): 18.61. Max coverage (-): 1.01

Region: NODE\_382453\_length\_1102\_cov\_120.509071 1086-1087. Max. coverage (+): 14.62. Max coverage (-): 4.08

Region: NODE\_382453\_length\_1102\_cov\_120.509071 1088-1089. Max. coverage (+): 14.5. Max coverage (-): 4.12

Region: NODE\_382453\_length\_1102\_cov\_120.509071 1090-1092. Max. coverage (+): 10.01. Max coverage (-): 4

Region: NODE\_382453\_length\_1102\_cov\_120.509071 1093-1094. Max. coverage (+): 6.38. Max coverage (-): 2.02

Region: NODE\_382453\_length\_1102\_cov\_120.509071 1095-1096. Max. coverage (+): 0.24. Max coverage (-): 1.86

Region: NODE\_382453\_length\_1102\_cov\_120.509071 1097-1099. Max. coverage (+): 0. Max coverage (-): 0.12

Region: NODE\_382453\_length\_1102\_cov\_120.509071 1100-1101. Max. coverage (+): 0. Max coverage (-): 0.48

Region: NODE\_382453\_length\_1102\_cov\_120.509071 1102-1103. Max. coverage (+): 0. Max coverage (-): 0.52

Region: NODE\_382453\_length\_1102\_cov\_120.509071 1104-1106. Max. coverage (+): 0.43. Max coverage (-): 0.42

Region: NODE\_382453\_length\_1102\_cov\_120.509071 1107-1108. Max. coverage (+): 0.82. Max coverage (-): 0.46

Region: NODE\_382453\_length\_1102\_cov\_120.509071 1109-1110. Max. coverage (+): 1.25. Max coverage (-): 0.22

Region: NODE\_382453\_length\_1102\_cov\_120.509071 1111-1113. Max. coverage (+): 1.43. Max coverage (-): 0.13

Region: NODE\_382453\_length\_1102\_cov\_120.509071 1114-1115. Max. coverage (+): 1.05. Max coverage (-): 0.11

Region: NODE\_382453\_length\_1102\_cov\_120.509071 1116-1117. Max. coverage (+): 0.57. Max coverage (-): 0.03

Region: NODE\_382453\_length\_1102\_cov\_120.509071 1118-1119. Max. coverage (+): 1.32. Max coverage (-): 0.03

Region: NODE\_382453\_length\_1102\_cov\_120.509071 1120-1122. Max. coverage (+): 8.09. Max coverage (-): 0.04

Region: NODE\_382453\_length\_1102\_cov\_120.509071 1123-1124. Max. coverage (+): 6.95. Max coverage (-): 0.19

Region: NODE\_382453\_length\_1102\_cov\_120.509071 1125-1126. Max. coverage (+): 0.05. Max coverage (-): 0.17

Region: NODE\_382453\_length\_1102\_cov\_120.509071 1127-1129. Max. coverage (+): 2.68. Max coverage (-): 0.15

Region: NODE\_382453\_length\_1102\_cov\_120.509071 1130-1131. Max. coverage (+): 2.68. Max coverage (-): 0.01

Region: NODE\_382453\_length\_1102\_cov\_120.509071 1132-1133. Max. coverage (+): 5.26. Max coverage (-): 0.01

Region: NODE\_382453\_length\_1102\_cov\_120.509071 1134-1136. Max. coverage (+): 5.57. Max coverage (-): 0.01

Region: NODE\_382453\_length\_1102\_cov\_120.509071 1137-1138. Max. coverage (+): 1.01. Max coverage (-): 0

Region: NODE\_382453\_length\_1102\_cov\_120.509071 1139-1140. Max. coverage (+): 0.44. Max coverage (-): 0

Region: NODE\_382453\_length\_1102\_cov\_120.509071 1141-1143. Max. coverage (+): 0.16. Max coverage (-): 0

Region: NODE\_382453\_length\_1102\_cov\_120.509071 1144-1145. Max. coverage (+): 0. Max coverage (-): 0

Region: NODE\_382453\_length\_1102\_cov\_120.509071 1146-1147. Max. coverage (+): 0. Max coverage (-): 0

Region: NODE\_382453\_length\_1102\_cov\_120.509071 1148-1149. Max. coverage (+): 0. Max coverage (-): 0

Region: NODE\_382453\_length\_1102\_cov\_120.509071 1150-1152. Max. coverage (+): 0. Max coverage (-): 0

Region: NODE\_382453\_length\_1102\_cov\_120.509071 1153-1154. Max. coverage (+): 0. Max coverage (-): 0

Region: NODE\_382453\_length\_1102\_cov\_120.509071 1155-1156. Max. coverage (+): 0. Max coverage (-): 0

Region: NODE\_382453\_length\_1102\_cov\_120.509071 1157-1159. Max. coverage (+): 0. Max coverage (-): 0

Region: NODE\_382453\_length\_1102\_cov\_120.509071 1160-1161. Max. coverage (+): 0. Max coverage (-): 0

Region: NODE\_382453\_length\_1102\_cov\_120.509071 1162-1163. Max. coverage (+): 0. Max coverage (-): 0

Region: NODE\_382453\_length\_1102\_cov\_120.509071 1164-. Max. coverage (+): 0. Max coverage (-): 0

RepeatMasker Color Code

**+**

100-98% Identity

<98-95% Identity

<95-90% Identity

<90-85% Identity

<85-80% Identity

<80-75% Identity

<75-70% Identity

<70% Identity

**-**

Gene Set Color Code

**+**

Gene

Pseudogene

Other

**-**

Topology/Coverage Color Code

Coverage Plus Strand

Coverage Minus Strand

Mainstrand: Plus

Mainstrand: Minus

Complementary Strand

Flanking Region  
(if option -flank >0)

Gene Set Annotation  
  
RepeatMasker Annotation  

**1. AlRepB-14**: 1-34 (+), Divergence to consensus: 5.9%  
**2. (AT)n**: 487-517 (+), Divergence to consensus: 17.1%  
**3. AlRepD-1165**: 523-737 (-), Divergence to consensus: 23.8%

  
Transcription Factor Binding Sites  

**RHOXF1** (Sequence: AGATCA (-): 198)  
**RHOXF1** (Sequence: AGCTCA (-): 283)  
**RHOXF1** (Sequence: GGCTCA (-): 689)  
**RHOXF1** (Sequence: TGATCC (+): 608)  
**Sox5** (Sequence: ATTGTT (+): 474)  
**SOX9** (Sequence: TTATTGTT (+): 472)  
**FOXO1** (Sequence: AAAAACAAG (-): 435)  
**Sox5** (Sequence: AACAAT (-): 572)  
**Sox5** (Sequence: AACAAT (-): 598)
