# Supplementary material for: First characterization of PIWI-interacting RNA clusters in a cichlid fish with a B chromosome
Source: BMC Biol. 2022 Sep 21;20:204. doi: 10.1186/s12915-022-01403-2 (PMC9490952; doi:10.1186/s12915-022-01403-2)
Supplement: Supplementary file 1 — Additional file 1. Zipped folder with fasta and interactive html piRNA cluster information for the A. latifasciata genome. The nomenclature is as follows: number-pirna-cluster_sex_B-presence (f, female; m, male; 0b, without B chromosome; 1b, with B chromosome). [file 12915_2022_1403_MOESM1_ESM.zip › 147_m1b.html]

piRNA cluster 147\_m1b 86


Predicted piRNA cluster no. 147\_m1b
  

Show proTRAC run info
Hide proTRAC run info

/\  
                \_\_\_\_\_\_\_\_\_\_\_\_\_\_\_\_\_\_\_\_\_\_\_/\\_\_\_ /  \\_\_\_\_\_\_\_  
               I                      /  \  /    \      I  
               I     pro             /    \/      \     I  
               I        TRAC        /               \   I  
               I   \_\_\_\_\_\_\_\_\_\_\_\_\_\_\_\_/\_\_\_\_\_\_\_\_\_\_\_\_\_\_\_\_\_\\_ I  
               I   \              /                     I  
               I    \            /                      I  
               I     \  /\      /       V.2.4.2         I  
               I      \/  \    /                        I  
               I\_\_\_\_\_\_\_\_\_\_\_\  /\_\_\_\_\_\_\_\_\_\_\_\_\_\_\_\_\_\_\_\_\_\_\_\_\_I  
                            \/  
  
  
================================= proTRAC ====================================  
VERSION: .......... 2.4.2  
LAST MODIFIED: .... 11. May 2018  
  
Please cite:  
Rosenkranz D, Zischler H. proTRAC - a software for probabilistic piRNA cluster  
detection, visualization and analysis. 2012. BMC Bioinformatics 13:5.  
  
  
Contact:  
David Rosenkranz  
Institute of Organismic and Molecular Evolutionary Biology  
Dept. Anthropology, small RNA group  
Johannes Gutenberg University Mainz  
email: rosenkranz@uni-mainz.de  
  
You can find the latest proTRAC version at:  
http://sourceforge.net/projects/protrac/files  
http://www.smallRNAgroup-mainz.de/software  
==============================================================================  
  
PARAMETERS:  
Map file: ...............piwi-machos-1B.fa-collapse.map  
Genome file: ............../../../0B\_ala\_genome.fa  
RepeatMasker annotation: Alatifasciata-all0B-maryan-v2.fa\_corrected.out  
GeneSet:................./guest-storage/Data/annotation/Alatifasciata\_all0B\_maryan-v2\_out2017.gff  
  
Significant (p<=0.01) hit density will be calculated based  
on observed hit distribution.  
  
Sliding window size: ........................................ 5000 bp  
Sliding window increament: .................................. 1000 bp  
Normalize each hit by number of genomic hits: ............... yes  
Normalize each hit by number of sequence reads: ............. yes  
Normalize values (-> per million mapped reads): ............. yes  
Min. fraction of hits with 1T(U) or 10A: .................... 0.75  
Alternatively: Min. fraction of hits with 1T(U) and 10A: .... 0.5  
Min. fraction of hits with typical piRNA length: ............ 0.75  
Typical piRNA length: ....................................... 24-32 nt  
Min. size of a piRNA cluster: ............................... 1000 bp.  
Min. number of hits (absolute): ............................. 0  
Min. number of hits (normalized): ........................... 0  
Min. fraction of hits on the mainstrand: .................... 0.75  
Top fraction of mapped sequences (in terms of read counts): . 1%  
Top fraction accounts for max. n% of sequence reads: ........ 90%  
Min. fraction of hits on each arm of a bidirectional cluster: 0.05  
Output html file for each cluster: .......................... yes  
Output a summary table: ..................................... yes  
Output a FASTA file for each cluster (piRNA sequences): ..... yes  
Output a FASTA file comprising cluster sequences: ........... yes  
Output a GTF file for predicted piRNA clusters: ..............yes  
Search DNA motifs in clusters: .............................. yes  
Output flanking sequences: +/- .............................. 0 bp  
Output ~.pTi file: .......................................... no  
==============================================================================  
  
  
Genome size (without gaps): ............ 758543724 bp  
Gaps (N/X/-): .......................... 417479 bp  
Mapped reads: .......................... 26973943  
Non-identical sequences: ............... 6209225  
Genomic hits: .......................... 48438990  
Significant densitiy of mapped reads: .. 821.144211136946 reads/kb

Show proTRAC cluster info
Hide proTRAC cluster info

|  |  |
| --- | --- |
| Location | NODE\_382453\_length\_1102\_cov\_120.509071 |
| Coordinates | 10-1166 |
| Size [bp] | 1157 |
| Sequence hit loci | 3084 |
| Mapped reads (normalized) | 22107.2 |
| Mapped reads (normalized) per kb | 19107.3 |
| Normalized reads with 1T (1U) | 78.8% |
| Normalized reads with 10A | 47.2% |
| Normalized reads with length 24-32 nt | 98.7% |
| Normalized reads on the main strand(s) | 92.1% |
| Predicted directionality | mono:plus |

100%

0%

1T (1U)  
reads

10A reads

24-32 nt  
reads

reads on mainstrand

**Either the amount of reads with 1T (1U) OR 10A has to exceed 75% (set with option: -1Tor10A)  
Alternatively the amount of reads with 1T (1U) AND 10A has to exceed 50% (set with option: -1Tand10A)  
Minimum amount of reads with preferred size is 75% (set with option: -pisize)  
Minimum amount of reads on the main strand(s) is 75% (set with option: -clstrand)**

Show read coverage
Hide read coverage

WHAT DO I SEE HERE?  
This chart shows the location of mapped sequence reads within a predicted piRNA cluster. The color refers to the number of genomic hits produced by the sequence read in question. A dark red bar indicates that this sequence read produces many other hits elsewhere in the genome. Many adjacent red or yellow bars can indicate the presence of a multi-copy element such as transposons or rRNA genes. A dark green bar indicates that this sequence read maps uniquely to this locus.

1 hit

2-5 hits

6-10 hits

11-20 hits

21-50 hits

51-100 hits

> 100 hits

NODE\_382453\_length\_1102\_cov\_120.509071

10

1166

Gene Set

RepeatMasker

Mapped  
Reads

133.76

plus strand

minus strand

133.76

Region: NODE\_382453\_length\_1102\_cov\_120.509071 2675-11. Max. coverage (+): 0.02. Max coverage (-): 0.01

Region: NODE\_382453\_length\_1102\_cov\_120.509071 12-13. Max. coverage (+): 0.19. Max coverage (-): 0

Region: NODE\_382453\_length\_1102\_cov\_120.509071 14-15. Max. coverage (+): 0.2. Max coverage (-): 0

Region: NODE\_382453\_length\_1102\_cov\_120.509071 16-18. Max. coverage (+): 0.01. Max coverage (-): 0.07

Region: NODE\_382453\_length\_1102\_cov\_120.509071 19-20. Max. coverage (+): 0. Max coverage (-): 0.05

Region: NODE\_382453\_length\_1102\_cov\_120.509071 21-22. Max. coverage (+): 0.01. Max coverage (-): 0.02

Region: NODE\_382453\_length\_1102\_cov\_120.509071 23-25. Max. coverage (+): 0.04. Max coverage (-): 0.05

Region: NODE\_382453\_length\_1102\_cov\_120.509071 26-27. Max. coverage (+): 0.01. Max coverage (-): 0.1

Region: NODE\_382453\_length\_1102\_cov\_120.509071 28-29. Max. coverage (+): 0. Max coverage (-): 0.05

Region: NODE\_382453\_length\_1102\_cov\_120.509071 30-31. Max. coverage (+): 0. Max coverage (-): 0.07

Region: NODE\_382453\_length\_1102\_cov\_120.509071 32-34. Max. coverage (+): 0.17. Max coverage (-): 0.11

Region: NODE\_382453\_length\_1102\_cov\_120.509071 35-36. Max. coverage (+): 0.17. Max coverage (-): 0.07

Region: NODE\_382453\_length\_1102\_cov\_120.509071 37-38. Max. coverage (+): 0.48. Max coverage (-): 0.48

Region: NODE\_382453\_length\_1102\_cov\_120.509071 39-41. Max. coverage (+): 1.32. Max coverage (-): 0.44

Region: NODE\_382453\_length\_1102\_cov\_120.509071 42-43. Max. coverage (+): 0. Max coverage (-): 0.22

Region: NODE\_382453\_length\_1102\_cov\_120.509071 44-45. Max. coverage (+): 0.48. Max coverage (-): 0.33

Region: NODE\_382453\_length\_1102\_cov\_120.509071 46-48. Max. coverage (+): 0.41. Max coverage (-): 0.04

Region: NODE\_382453\_length\_1102\_cov\_120.509071 49-50. Max. coverage (+): 0.48. Max coverage (-): 0.07

Region: NODE\_382453\_length\_1102\_cov\_120.509071 51-52. Max. coverage (+): 0.26. Max coverage (-): 0.07

Region: NODE\_382453\_length\_1102\_cov\_120.509071 53-55. Max. coverage (+): 0.26. Max coverage (-): 0.07

Region: NODE\_382453\_length\_1102\_cov\_120.509071 56-57. Max. coverage (+): 0.22. Max coverage (-): 0.07

Region: NODE\_382453\_length\_1102\_cov\_120.509071 58-59. Max. coverage (+): 0.04. Max coverage (-): 0.07

Region: NODE\_382453\_length\_1102\_cov\_120.509071 60-62. Max. coverage (+): 0.26. Max coverage (-): 0.11

Region: NODE\_382453\_length\_1102\_cov\_120.509071 63-64. Max. coverage (+): 0.3. Max coverage (-): 0.04

Region: NODE\_382453\_length\_1102\_cov\_120.509071 65-66. Max. coverage (+): 0.19. Max coverage (-): 0

Region: NODE\_382453\_length\_1102\_cov\_120.509071 67-69. Max. coverage (+): 0. Max coverage (-): 0.82

Region: NODE\_382453\_length\_1102\_cov\_120.509071 70-71. Max. coverage (+): 0. Max coverage (-): 0.82

Region: NODE\_382453\_length\_1102\_cov\_120.509071 72-73. Max. coverage (+): 0. Max coverage (-): 1.33

Region: NODE\_382453\_length\_1102\_cov\_120.509071 74-75. Max. coverage (+): 0.04. Max coverage (-): 1.71

Region: NODE\_382453\_length\_1102\_cov\_120.509071 76-78. Max. coverage (+): 1.26. Max coverage (-): 0.3

Region: NODE\_382453\_length\_1102\_cov\_120.509071 79-80. Max. coverage (+): 1.3. Max coverage (-): 0.11

Region: NODE\_382453\_length\_1102\_cov\_120.509071 81-82. Max. coverage (+): 1.45. Max coverage (-): 0.11

Region: NODE\_382453\_length\_1102\_cov\_120.509071 83-85. Max. coverage (+): 0.11. Max coverage (-): 0.93

Region: NODE\_382453\_length\_1102\_cov\_120.509071 86-87. Max. coverage (+): 0.63. Max coverage (-): 0.07

Region: NODE\_382453\_length\_1102\_cov\_120.509071 88-89. Max. coverage (+): 3.3. Max coverage (-): 0.04

Region: NODE\_382453\_length\_1102\_cov\_120.509071 90-92. Max. coverage (+): 2.22. Max coverage (-): 0.11

Region: NODE\_382453\_length\_1102\_cov\_120.509071 93-94. Max. coverage (+): 0.56. Max coverage (-): 0.11

Region: NODE\_382453\_length\_1102\_cov\_120.509071 95-96. Max. coverage (+): 0.04. Max coverage (-): 0.19

Region: NODE\_382453\_length\_1102\_cov\_120.509071 97-99. Max. coverage (+): 1.33. Max coverage (-): 0.07

Region: NODE\_382453\_length\_1102\_cov\_120.509071 100-101. Max. coverage (+): 0.26. Max coverage (-): 0.85

Region: NODE\_382453\_length\_1102\_cov\_120.509071 102-103. Max. coverage (+): 0. Max coverage (-): 0.85

Region: NODE\_382453\_length\_1102\_cov\_120.509071 104-106. Max. coverage (+): 0.04. Max coverage (-): 0

Region: NODE\_382453\_length\_1102\_cov\_120.509071 107-108. Max. coverage (+): 0.78. Max coverage (-): 0.07

Region: NODE\_382453\_length\_1102\_cov\_120.509071 109-110. Max. coverage (+): 1.19. Max coverage (-): 0.07

Region: NODE\_382453\_length\_1102\_cov\_120.509071 111-112. Max. coverage (+): 1.26. Max coverage (-): 0

Region: NODE\_382453\_length\_1102\_cov\_120.509071 113-115. Max. coverage (+): 0.78. Max coverage (-): 0

Region: NODE\_382453\_length\_1102\_cov\_120.509071 116-117. Max. coverage (+): 0.59. Max coverage (-): 0

Region: NODE\_382453\_length\_1102\_cov\_120.509071 118-119. Max. coverage (+): 0.37. Max coverage (-): 0

Region: NODE\_382453\_length\_1102\_cov\_120.509071 120-122. Max. coverage (+): 0.74. Max coverage (-): 0.07

Region: NODE\_382453\_length\_1102\_cov\_120.509071 123-124. Max. coverage (+): 0.48. Max coverage (-): 0.11

Region: NODE\_382453\_length\_1102\_cov\_120.509071 125-126. Max. coverage (+): 0.04. Max coverage (-): 0.15

Region: NODE\_382453\_length\_1102\_cov\_120.509071 127-129. Max. coverage (+): 0. Max coverage (-): 0.19

Region: NODE\_382453\_length\_1102\_cov\_120.509071 130-131. Max. coverage (+): 0. Max coverage (-): 0.11

Region: NODE\_382453\_length\_1102\_cov\_120.509071 132-133. Max. coverage (+): 0.19. Max coverage (-): 0

Region: NODE\_382453\_length\_1102\_cov\_120.509071 134-136. Max. coverage (+): 0.33. Max coverage (-): 0

Region: NODE\_382453\_length\_1102\_cov\_120.509071 137-138. Max. coverage (+): 0.56. Max coverage (-): 0.52

Region: NODE\_382453\_length\_1102\_cov\_120.509071 139-140. Max. coverage (+): 0.22. Max coverage (-): 0.52

Region: NODE\_382453\_length\_1102\_cov\_120.509071 141-143. Max. coverage (+): 4. Max coverage (-): 0

Region: NODE\_382453\_length\_1102\_cov\_120.509071 144-145. Max. coverage (+): 5.64. Max coverage (-): 0

Region: NODE\_382453\_length\_1102\_cov\_120.509071 146-147. Max. coverage (+): 6.52. Max coverage (-): 0.11

Region: NODE\_382453\_length\_1102\_cov\_120.509071 148-149. Max. coverage (+): 5.04. Max coverage (-): 0.11

Region: NODE\_382453\_length\_1102\_cov\_120.509071 150-152. Max. coverage (+): 1.33. Max coverage (-): 0

Region: NODE\_382453\_length\_1102\_cov\_120.509071 153-154. Max. coverage (+): 2.48. Max coverage (-): 0.07

Region: NODE\_382453\_length\_1102\_cov\_120.509071 155-156. Max. coverage (+): 1.26. Max coverage (-): 0.19

Region: NODE\_382453\_length\_1102\_cov\_120.509071 157-159. Max. coverage (+): 0.07. Max coverage (-): 0.19

Region: NODE\_382453\_length\_1102\_cov\_120.509071 160-161. Max. coverage (+): 0.07. Max coverage (-): 0.19

Region: NODE\_382453\_length\_1102\_cov\_120.509071 162-163. Max. coverage (+): 0.04. Max coverage (-): 0.15

Region: NODE\_382453\_length\_1102\_cov\_120.509071 164-166. Max. coverage (+): 0.11. Max coverage (-): 0.11

Region: NODE\_382453\_length\_1102\_cov\_120.509071 167-168. Max. coverage (+): 0.11. Max coverage (-): 0.04

Region: NODE\_382453\_length\_1102\_cov\_120.509071 169-170. Max. coverage (+): 0.19. Max coverage (-): 0.04

Region: NODE\_382453\_length\_1102\_cov\_120.509071 171-173. Max. coverage (+): 1.22. Max coverage (-): 0.04

Region: NODE\_382453\_length\_1102\_cov\_120.509071 174-175. Max. coverage (+): 1.37. Max coverage (-): 0.04

Region: NODE\_382453\_length\_1102\_cov\_120.509071 176-177. Max. coverage (+): 0.74. Max coverage (-): 0.04

Region: NODE\_382453\_length\_1102\_cov\_120.509071 178-180. Max. coverage (+): 0.52. Max coverage (-): 0.11

Region: NODE\_382453\_length\_1102\_cov\_120.509071 181-182. Max. coverage (+): 0.11. Max coverage (-): 0.11

Region: NODE\_382453\_length\_1102\_cov\_120.509071 183-184. Max. coverage (+): 0.07. Max coverage (-): 0.15

Region: NODE\_382453\_length\_1102\_cov\_120.509071 185-187. Max. coverage (+): 0. Max coverage (-): 0.04

Region: NODE\_382453\_length\_1102\_cov\_120.509071 188-189. Max. coverage (+): 0.07. Max coverage (-): 0.07

Region: NODE\_382453\_length\_1102\_cov\_120.509071 190-191. Max. coverage (+): 0.07. Max coverage (-): 0.07

Region: NODE\_382453\_length\_1102\_cov\_120.509071 192-193. Max. coverage (+): 0. Max coverage (-): 0.04

Region: NODE\_382453\_length\_1102\_cov\_120.509071 194-196. Max. coverage (+): 0.33. Max coverage (-): 0.11

Region: NODE\_382453\_length\_1102\_cov\_120.509071 197-198. Max. coverage (+): 0.26. Max coverage (-): 0.07

Region: NODE\_382453\_length\_1102\_cov\_120.509071 199-200. Max. coverage (+): 0. Max coverage (-): 0.04

Region: NODE\_382453\_length\_1102\_cov\_120.509071 201-203. Max. coverage (+): 0.3. Max coverage (-): 1.3

Region: NODE\_382453\_length\_1102\_cov\_120.509071 204-205. Max. coverage (+): 0.04. Max coverage (-): 1.41

Region: NODE\_382453\_length\_1102\_cov\_120.509071 206-207. Max. coverage (+): 0.04. Max coverage (-): 0.15

Region: NODE\_382453\_length\_1102\_cov\_120.509071 208-210. Max. coverage (+): 0.04. Max coverage (-): 0.11

Region: NODE\_382453\_length\_1102\_cov\_120.509071 211-212. Max. coverage (+): 0. Max coverage (-): 0.04

Region: NODE\_382453\_length\_1102\_cov\_120.509071 213-214. Max. coverage (+): 0.07. Max coverage (-): 0.04

Region: NODE\_382453\_length\_1102\_cov\_120.509071 215-217. Max. coverage (+): 0.26. Max coverage (-): 0.04

Region: NODE\_382453\_length\_1102\_cov\_120.509071 218-219. Max. coverage (+): 0.22. Max coverage (-): 0

Region: NODE\_382453\_length\_1102\_cov\_120.509071 220-221. Max. coverage (+): 0.3. Max coverage (-): 0

Region: NODE\_382453\_length\_1102\_cov\_120.509071 222-224. Max. coverage (+): 0.33. Max coverage (-): 0

Region: NODE\_382453\_length\_1102\_cov\_120.509071 225-226. Max. coverage (+): 0.33. Max coverage (-): 0

Region: NODE\_382453\_length\_1102\_cov\_120.509071 227-228. Max. coverage (+): 0.33. Max coverage (-): 0

Region: NODE\_382453\_length\_1102\_cov\_120.509071 229-230. Max. coverage (+): 0.11. Max coverage (-): 0

Region: NODE\_382453\_length\_1102\_cov\_120.509071 231-233. Max. coverage (+): 0. Max coverage (-): 0

Region: NODE\_382453\_length\_1102\_cov\_120.509071 234-235. Max. coverage (+): 0. Max coverage (-): 0.04

Region: NODE\_382453\_length\_1102\_cov\_120.509071 236-237. Max. coverage (+): 0. Max coverage (-): 0.07

Region: NODE\_382453\_length\_1102\_cov\_120.509071 238-240. Max. coverage (+): 0. Max coverage (-): 0.11

Region: NODE\_382453\_length\_1102\_cov\_120.509071 241-242. Max. coverage (+): 0. Max coverage (-): 0.04

Region: NODE\_382453\_length\_1102\_cov\_120.509071 243-244. Max. coverage (+): 0. Max coverage (-): 0

Region: NODE\_382453\_length\_1102\_cov\_120.509071 245-247. Max. coverage (+): 0. Max coverage (-): 0

Region: NODE\_382453\_length\_1102\_cov\_120.509071 248-249. Max. coverage (+): 0. Max coverage (-): 0

Region: NODE\_382453\_length\_1102\_cov\_120.509071 250-251. Max. coverage (+): 0. Max coverage (-): 0

Region: NODE\_382453\_length\_1102\_cov\_120.509071 252-254. Max. coverage (+): 0.48. Max coverage (-): 0

Region: NODE\_382453\_length\_1102\_cov\_120.509071 255-256. Max. coverage (+): 1.71. Max coverage (-): 0

Region: NODE\_382453\_length\_1102\_cov\_120.509071 257-258. Max. coverage (+): 1.67. Max coverage (-): 0.07

Region: NODE\_382453\_length\_1102\_cov\_120.509071 259-261. Max. coverage (+): 0.19. Max coverage (-): 0.11

Region: NODE\_382453\_length\_1102\_cov\_120.509071 262-263. Max. coverage (+): 0.11. Max coverage (-): 0.04

Region: NODE\_382453\_length\_1102\_cov\_120.509071 264-265. Max. coverage (+): 0.04. Max coverage (-): 0.04

Region: NODE\_382453\_length\_1102\_cov\_120.509071 266-268. Max. coverage (+): 0.33. Max coverage (-): 0.07

Region: NODE\_382453\_length\_1102\_cov\_120.509071 269-270. Max. coverage (+): 0.44. Max coverage (-): 0.41

Region: NODE\_382453\_length\_1102\_cov\_120.509071 271-272. Max. coverage (+): 0.26. Max coverage (-): 0.44

Region: NODE\_382453\_length\_1102\_cov\_120.509071 273-274. Max. coverage (+): 0.11. Max coverage (-): 0.33

Region: NODE\_382453\_length\_1102\_cov\_120.509071 275-277. Max. coverage (+): 11.23. Max coverage (-): 0.37

Region: NODE\_382453\_length\_1102\_cov\_120.509071 278-279. Max. coverage (+): 27.4. Max coverage (-): 1.48

Region: NODE\_382453\_length\_1102\_cov\_120.509071 280-281. Max. coverage (+): 29.25. Max coverage (-): 1.45

Region: NODE\_382453\_length\_1102\_cov\_120.509071 282-284. Max. coverage (+): 24.32. Max coverage (-): 0.33

Region: NODE\_382453\_length\_1102\_cov\_120.509071 285-286. Max. coverage (+): 101.99. Max coverage (-): 0.37

Region: NODE\_382453\_length\_1102\_cov\_120.509071 287-288. Max. coverage (+): 108.55. Max coverage (-): 0.07

Region: NODE\_382453\_length\_1102\_cov\_120.509071 289-291. Max. coverage (+): 10.68. Max coverage (-): 0.04

Region: NODE\_382453\_length\_1102\_cov\_120.509071 292-293. Max. coverage (+): 2.74. Max coverage (-): 0.04

Region: NODE\_382453\_length\_1102\_cov\_120.509071 294-295. Max. coverage (+): 3.11. Max coverage (-): 0

Region: NODE\_382453\_length\_1102\_cov\_120.509071 296-298. Max. coverage (+): 2.41. Max coverage (-): 0

Region: NODE\_382453\_length\_1102\_cov\_120.509071 299-300. Max. coverage (+): 1.67. Max coverage (-): 0

Region: NODE\_382453\_length\_1102\_cov\_120.509071 301-302. Max. coverage (+): 0.48. Max coverage (-): 0

Region: NODE\_382453\_length\_1102\_cov\_120.509071 303-305. Max. coverage (+): 0.85. Max coverage (-): 0

Region: NODE\_382453\_length\_1102\_cov\_120.509071 306-307. Max. coverage (+): 1.26. Max coverage (-): 0

Region: NODE\_382453\_length\_1102\_cov\_120.509071 308-309. Max. coverage (+): 0.96. Max coverage (-): 0

Region: NODE\_382453\_length\_1102\_cov\_120.509071 310-311. Max. coverage (+): 0.89. Max coverage (-): 0

Region: NODE\_382453\_length\_1102\_cov\_120.509071 312-314. Max. coverage (+): 2.71. Max coverage (-): 0

Region: NODE\_382453\_length\_1102\_cov\_120.509071 315-316. Max. coverage (+): 2.74. Max coverage (-): 0

Region: NODE\_382453\_length\_1102\_cov\_120.509071 317-318. Max. coverage (+): 0.48. Max coverage (-): 0.07

Region: NODE\_382453\_length\_1102\_cov\_120.509071 319-321. Max. coverage (+): 1.08. Max coverage (-): 0.15

Region: NODE\_382453\_length\_1102\_cov\_120.509071 322-323. Max. coverage (+): 5.6. Max coverage (-): 0.19

Region: NODE\_382453\_length\_1102\_cov\_120.509071 324-325. Max. coverage (+): 6.97. Max coverage (-): 0.07

Region: NODE\_382453\_length\_1102\_cov\_120.509071 326-328. Max. coverage (+): 3.08. Max coverage (-): 0.04

Region: NODE\_382453\_length\_1102\_cov\_120.509071 329-330. Max. coverage (+): 3.56. Max coverage (-): 0.04

Region: NODE\_382453\_length\_1102\_cov\_120.509071 331-332. Max. coverage (+): 2.93. Max coverage (-): 0.19

Region: NODE\_382453\_length\_1102\_cov\_120.509071 333-335. Max. coverage (+): 0.93. Max coverage (-): 0.33

Region: NODE\_382453\_length\_1102\_cov\_120.509071 336-337. Max. coverage (+): 1.04. Max coverage (-): 0.04

Region: NODE\_382453\_length\_1102\_cov\_120.509071 338-339. Max. coverage (+): 0.41. Max coverage (-): 0.11

Region: NODE\_382453\_length\_1102\_cov\_120.509071 340-342. Max. coverage (+): 0.85. Max coverage (-): 0.07

Region: NODE\_382453\_length\_1102\_cov\_120.509071 343-344. Max. coverage (+): 0.04. Max coverage (-): 0

Region: NODE\_382453\_length\_1102\_cov\_120.509071 345-346. Max. coverage (+): 2.48. Max coverage (-): 0

Region: NODE\_382453\_length\_1102\_cov\_120.509071 347-349. Max. coverage (+): 32.77. Max coverage (-): 0

Region: NODE\_382453\_length\_1102\_cov\_120.509071 350-351. Max. coverage (+): 3.19. Max coverage (-): 0.04

Region: NODE\_382453\_length\_1102\_cov\_120.509071 352-353. Max. coverage (+): 0.19. Max coverage (-): 0.04

Region: NODE\_382453\_length\_1102\_cov\_120.509071 354-355. Max. coverage (+): 0.04. Max coverage (-): 0

Region: NODE\_382453\_length\_1102\_cov\_120.509071 356-358. Max. coverage (+): 0. Max coverage (-): 0

Region: NODE\_382453\_length\_1102\_cov\_120.509071 359-360. Max. coverage (+): 0. Max coverage (-): 0

Region: NODE\_382453\_length\_1102\_cov\_120.509071 361-362. Max. coverage (+): 0. Max coverage (-): 0

Region: NODE\_382453\_length\_1102\_cov\_120.509071 363-365. Max. coverage (+): 0. Max coverage (-): 0.04

Region: NODE\_382453\_length\_1102\_cov\_120.509071 366-367. Max. coverage (+): 0. Max coverage (-): 0.04

Region: NODE\_382453\_length\_1102\_cov\_120.509071 368-369. Max. coverage (+): 0.04. Max coverage (-): 0

Region: NODE\_382453\_length\_1102\_cov\_120.509071 370-372. Max. coverage (+): 0.07. Max coverage (-): 0.07

Region: NODE\_382453\_length\_1102\_cov\_120.509071 373-374. Max. coverage (+): 0.07. Max coverage (-): 0.07

Region: NODE\_382453\_length\_1102\_cov\_120.509071 375-376. Max. coverage (+): 0.07. Max coverage (-): 0.07

Region: NODE\_382453\_length\_1102\_cov\_120.509071 377-379. Max. coverage (+): 0.11. Max coverage (-): 0.04

Region: NODE\_382453\_length\_1102\_cov\_120.509071 380-381. Max. coverage (+): 0.26. Max coverage (-): 0

Region: NODE\_382453\_length\_1102\_cov\_120.509071 382-383. Max. coverage (+): 2.04. Max coverage (-): 0

Region: NODE\_382453\_length\_1102\_cov\_120.509071 384-386. Max. coverage (+): 20.06. Max coverage (-): 0.07

Region: NODE\_382453\_length\_1102\_cov\_120.509071 387-388. Max. coverage (+): 21.02. Max coverage (-): 0.07

Region: NODE\_382453\_length\_1102\_cov\_120.509071 389-390. Max. coverage (+): 6.56. Max coverage (-): 0.07

Region: NODE\_382453\_length\_1102\_cov\_120.509071 391-392. Max. coverage (+): 3.48. Max coverage (-): 0.04

Region: NODE\_382453\_length\_1102\_cov\_120.509071 393-395. Max. coverage (+): 0.44. Max coverage (-): 0.3

Region: NODE\_382453\_length\_1102\_cov\_120.509071 396-397. Max. coverage (+): 0. Max coverage (-): 0.37

Region: NODE\_382453\_length\_1102\_cov\_120.509071 398-399. Max. coverage (+): 0. Max coverage (-): 0.07

Region: NODE\_382453\_length\_1102\_cov\_120.509071 400-402. Max. coverage (+): 0. Max coverage (-): 0.07

Region: NODE\_382453\_length\_1102\_cov\_120.509071 403-404. Max. coverage (+): 0.04. Max coverage (-): 0.07

Region: NODE\_382453\_length\_1102\_cov\_120.509071 405-406. Max. coverage (+): 0.04. Max coverage (-): 0.19

Region: NODE\_382453\_length\_1102\_cov\_120.509071 407-409. Max. coverage (+): 0. Max coverage (-): 0.11

Region: NODE\_382453\_length\_1102\_cov\_120.509071 410-411. Max. coverage (+): 0. Max coverage (-): 0

Region: NODE\_382453\_length\_1102\_cov\_120.509071 412-413. Max. coverage (+): 0. Max coverage (-): 0.04

Region: NODE\_382453\_length\_1102\_cov\_120.509071 414-416. Max. coverage (+): 0.15. Max coverage (-): 0.04

Region: NODE\_382453\_length\_1102\_cov\_120.509071 417-418. Max. coverage (+): 0.19. Max coverage (-): 0

Region: NODE\_382453\_length\_1102\_cov\_120.509071 419-420. Max. coverage (+): 1. Max coverage (-): 0

Region: NODE\_382453\_length\_1102\_cov\_120.509071 421-423. Max. coverage (+): 1.67. Max coverage (-): 0.04

Region: NODE\_382453\_length\_1102\_cov\_120.509071 424-425. Max. coverage (+): 3. Max coverage (-): 0.04

Region: NODE\_382453\_length\_1102\_cov\_120.509071 426-427. Max. coverage (+): 2.37. Max coverage (-): 0.07

Region: NODE\_382453\_length\_1102\_cov\_120.509071 428-429. Max. coverage (+): 0.26. Max coverage (-): 0.07

Region: NODE\_382453\_length\_1102\_cov\_120.509071 430-432. Max. coverage (+): 0.37. Max coverage (-): 0.22

Region: NODE\_382453\_length\_1102\_cov\_120.509071 433-434. Max. coverage (+): 0.33. Max coverage (-): 0

Region: NODE\_382453\_length\_1102\_cov\_120.509071 435-436. Max. coverage (+): 0. Max coverage (-): 0

Region: NODE\_382453\_length\_1102\_cov\_120.509071 437-439. Max. coverage (+): 0. Max coverage (-): 0.82

Region: NODE\_382453\_length\_1102\_cov\_120.509071 440-441. Max. coverage (+): 0. Max coverage (-): 0.82

Region: NODE\_382453\_length\_1102\_cov\_120.509071 442-443. Max. coverage (+): 0. Max coverage (-): 0.15

Region: NODE\_382453\_length\_1102\_cov\_120.509071 444-446. Max. coverage (+): 0.26. Max coverage (-): 0.15

Region: NODE\_382453\_length\_1102\_cov\_120.509071 447-448. Max. coverage (+): 0.33. Max coverage (-): 0.63

Region: NODE\_382453\_length\_1102\_cov\_120.509071 449-450. Max. coverage (+): 0.07. Max coverage (-): 0.74

Region: NODE\_382453\_length\_1102\_cov\_120.509071 451-453. Max. coverage (+): 0.26. Max coverage (-): 0.19

Region: NODE\_382453\_length\_1102\_cov\_120.509071 454-455. Max. coverage (+): 0.04. Max coverage (-): 0

Region: NODE\_382453\_length\_1102\_cov\_120.509071 456-457. Max. coverage (+): 0.07. Max coverage (-): 0

Region: NODE\_382453\_length\_1102\_cov\_120.509071 458-460. Max. coverage (+): 0.07. Max coverage (-): 0

Region: NODE\_382453\_length\_1102\_cov\_120.509071 461-462. Max. coverage (+): 0. Max coverage (-): 0

Region: NODE\_382453\_length\_1102\_cov\_120.509071 463-464. Max. coverage (+): 0. Max coverage (-): 0

Region: NODE\_382453\_length\_1102\_cov\_120.509071 465-467. Max. coverage (+): 0. Max coverage (-): 0

Region: NODE\_382453\_length\_1102\_cov\_120.509071 468-469. Max. coverage (+): 0. Max coverage (-): 0

Region: NODE\_382453\_length\_1102\_cov\_120.509071 470-471. Max. coverage (+): 0. Max coverage (-): 0

Region: NODE\_382453\_length\_1102\_cov\_120.509071 472-473. Max. coverage (+): 0. Max coverage (-): 0

Region: NODE\_382453\_length\_1102\_cov\_120.509071 474-476. Max. coverage (+): 0. Max coverage (-): 0

Region: NODE\_382453\_length\_1102\_cov\_120.509071 477-478. Max. coverage (+): 0. Max coverage (-): 0

Region: NODE\_382453\_length\_1102\_cov\_120.509071 479-480. Max. coverage (+): 0. Max coverage (-): 0

Region: NODE\_382453\_length\_1102\_cov\_120.509071 481-483. Max. coverage (+): 0. Max coverage (-): 0

Region: NODE\_382453\_length\_1102\_cov\_120.509071 484-485. Max. coverage (+): 0. Max coverage (-): 0

Region: NODE\_382453\_length\_1102\_cov\_120.509071 486-487. Max. coverage (+): 0. Max coverage (-): 0

Region: NODE\_382453\_length\_1102\_cov\_120.509071 488-490. Max. coverage (+): 0. Max coverage (-): 0

Region: NODE\_382453\_length\_1102\_cov\_120.509071 491-492. Max. coverage (+): 0. Max coverage (-): 0

Region: NODE\_382453\_length\_1102\_cov\_120.509071 493-494. Max. coverage (+): 0. Max coverage (-): 0

Region: NODE\_382453\_length\_1102\_cov\_120.509071 495-497. Max. coverage (+): 0. Max coverage (-): 0

Region: NODE\_382453\_length\_1102\_cov\_120.509071 498-499. Max. coverage (+): 0. Max coverage (-): 0

Region: NODE\_382453\_length\_1102\_cov\_120.509071 500-501. Max. coverage (+): 0. Max coverage (-): 0

Region: NODE\_382453\_length\_1102\_cov\_120.509071 502-504. Max. coverage (+): 0.04. Max coverage (-): 0.04

Region: NODE\_382453\_length\_1102\_cov\_120.509071 505-506. Max. coverage (+): 0.33. Max coverage (-): 0.07

Region: NODE\_382453\_length\_1102\_cov\_120.509071 507-508. Max. coverage (+): 0.3. Max coverage (-): 0.04

Region: NODE\_382453\_length\_1102\_cov\_120.509071 509-510. Max. coverage (+): 0. Max coverage (-): 0.07

Region: NODE\_382453\_length\_1102\_cov\_120.509071 511-513. Max. coverage (+): 0.04. Max coverage (-): 0.26

Region: NODE\_382453\_length\_1102\_cov\_120.509071 514-515. Max. coverage (+): 0.07. Max coverage (-): 0.11

Region: NODE\_382453\_length\_1102\_cov\_120.509071 516-517. Max. coverage (+): 0.37. Max coverage (-): 0.37

Region: NODE\_382453\_length\_1102\_cov\_120.509071 518-520. Max. coverage (+): 0.33. Max coverage (-): 0.56

Region: NODE\_382453\_length\_1102\_cov\_120.509071 521-522. Max. coverage (+): 0.07. Max coverage (-): 0.7

Region: NODE\_382453\_length\_1102\_cov\_120.509071 523-524. Max. coverage (+): 0.37. Max coverage (-): 0.7

Region: NODE\_382453\_length\_1102\_cov\_120.509071 525-527. Max. coverage (+): 0.44. Max coverage (-): 0.67

Region: NODE\_382453\_length\_1102\_cov\_120.509071 528-529. Max. coverage (+): 0.19. Max coverage (-): 0.78

Region: NODE\_382453\_length\_1102\_cov\_120.509071 530-531. Max. coverage (+): 0.15. Max coverage (-): 0.33

Region: NODE\_382453\_length\_1102\_cov\_120.509071 532-534. Max. coverage (+): 0.63. Max coverage (-): 0.11

Region: NODE\_382453\_length\_1102\_cov\_120.509071 535-536. Max. coverage (+): 3.74. Max coverage (-): 0

Region: NODE\_382453\_length\_1102\_cov\_120.509071 537-538. Max. coverage (+): 3.41. Max coverage (-): 0.04

Region: NODE\_382453\_length\_1102\_cov\_120.509071 539-541. Max. coverage (+): 0.44. Max coverage (-): 0.04

Region: NODE\_382453\_length\_1102\_cov\_120.509071 542-543. Max. coverage (+): 0.41. Max coverage (-): 0

Region: NODE\_382453\_length\_1102\_cov\_120.509071 544-545. Max. coverage (+): 0.56. Max coverage (-): 0

Region: NODE\_382453\_length\_1102\_cov\_120.509071 546-548. Max. coverage (+): 0.89. Max coverage (-): 0

Region: NODE\_382453\_length\_1102\_cov\_120.509071 549-550. Max. coverage (+): 0.78. Max coverage (-): 0

Region: NODE\_382453\_length\_1102\_cov\_120.509071 551-552. Max. coverage (+): 0. Max coverage (-): 0

Region: NODE\_382453\_length\_1102\_cov\_120.509071 553-554. Max. coverage (+): 0. Max coverage (-): 0

Region: NODE\_382453\_length\_1102\_cov\_120.509071 555-557. Max. coverage (+): 0.04. Max coverage (-): 0

Region: NODE\_382453\_length\_1102\_cov\_120.509071 558-559. Max. coverage (+): 0. Max coverage (-): 0.07

Region: NODE\_382453\_length\_1102\_cov\_120.509071 560-561. Max. coverage (+): 0. Max coverage (-): 0.07

Region: NODE\_382453\_length\_1102\_cov\_120.509071 562-564. Max. coverage (+): 0.11. Max coverage (-): 0.48

Region: NODE\_382453\_length\_1102\_cov\_120.509071 565-566. Max. coverage (+): 0.15. Max coverage (-): 1.19

Region: NODE\_382453\_length\_1102\_cov\_120.509071 567-568. Max. coverage (+): 0.15. Max coverage (-): 1.04

Region: NODE\_382453\_length\_1102\_cov\_120.509071 569-571. Max. coverage (+): 0.15. Max coverage (-): 0.11

Region: NODE\_382453\_length\_1102\_cov\_120.509071 572-573. Max. coverage (+): 0.04. Max coverage (-): 0.11

Region: NODE\_382453\_length\_1102\_cov\_120.509071 574-575. Max. coverage (+): 0.19. Max coverage (-): 0.04

Region: NODE\_382453\_length\_1102\_cov\_120.509071 576-578. Max. coverage (+): 26.69. Max coverage (-): 0

Region: NODE\_382453\_length\_1102\_cov\_120.509071 579-580. Max. coverage (+): 35.55. Max coverage (-): 0

Region: NODE\_382453\_length\_1102\_cov\_120.509071 581-582. Max. coverage (+): 12.83. Max coverage (-): 0

Region: NODE\_382453\_length\_1102\_cov\_120.509071 583-585. Max. coverage (+): 12.9. Max coverage (-): 0

Region: NODE\_382453\_length\_1102\_cov\_120.509071 586-587. Max. coverage (+): 0.63. Max coverage (-): 0

Region: NODE\_382453\_length\_1102\_cov\_120.509071 588-589. Max. coverage (+): 0.07. Max coverage (-): 0.04

Region: NODE\_382453\_length\_1102\_cov\_120.509071 590-591. Max. coverage (+): 0. Max coverage (-): 0.07

Region: NODE\_382453\_length\_1102\_cov\_120.509071 592-594. Max. coverage (+): 0.04. Max coverage (-): 0.3

Region: NODE\_382453\_length\_1102\_cov\_120.509071 595-596. Max. coverage (+): 0.04. Max coverage (-): 0.19

Region: NODE\_382453\_length\_1102\_cov\_120.509071 597-598. Max. coverage (+): 0.04. Max coverage (-): 0.04

Region: NODE\_382453\_length\_1102\_cov\_120.509071 599-601. Max. coverage (+): 0. Max coverage (-): 0.15

Region: NODE\_382453\_length\_1102\_cov\_120.509071 602-603. Max. coverage (+): 0. Max coverage (-): 0.22

Region: NODE\_382453\_length\_1102\_cov\_120.509071 604-605. Max. coverage (+): 0. Max coverage (-): 0.07

Region: NODE\_382453\_length\_1102\_cov\_120.509071 606-608. Max. coverage (+): 0.11. Max coverage (-): 0.07

Region: NODE\_382453\_length\_1102\_cov\_120.509071 609-610. Max. coverage (+): 0.11. Max coverage (-): 0.04

Region: NODE\_382453\_length\_1102\_cov\_120.509071 611-612. Max. coverage (+): 0.11. Max coverage (-): 0

Region: NODE\_382453\_length\_1102\_cov\_120.509071 613-615. Max. coverage (+): 0.07. Max coverage (-): 0

Region: NODE\_382453\_length\_1102\_cov\_120.509071 616-617. Max. coverage (+): 0.37. Max coverage (-): 0.22

Region: NODE\_382453\_length\_1102\_cov\_120.509071 618-619. Max. coverage (+): 0.7. Max coverage (-): 0.26

Region: NODE\_382453\_length\_1102\_cov\_120.509071 620-622. Max. coverage (+): 0.33. Max coverage (-): 0.09

Region: NODE\_382453\_length\_1102\_cov\_120.509071 623-624. Max. coverage (+): 0.07. Max coverage (-): 0.06

Region: NODE\_382453\_length\_1102\_cov\_120.509071 625-626. Max. coverage (+): 0.07. Max coverage (-): 0

Region: NODE\_382453\_length\_1102\_cov\_120.509071 627-628. Max. coverage (+): 0.44. Max coverage (-): 0.07

Region: NODE\_382453\_length\_1102\_cov\_120.509071 629-631. Max. coverage (+): 0.67. Max coverage (-): 0.07

Region: NODE\_382453\_length\_1102\_cov\_120.509071 632-633. Max. coverage (+): 0.44. Max coverage (-): 0

Region: NODE\_382453\_length\_1102\_cov\_120.509071 634-635. Max. coverage (+): 0.35. Max coverage (-): 0

Region: NODE\_382453\_length\_1102\_cov\_120.509071 636-638. Max. coverage (+): 0.13. Max coverage (-): 0

Region: NODE\_382453\_length\_1102\_cov\_120.509071 639-640. Max. coverage (+): 0.04. Max coverage (-): 0.04

Region: NODE\_382453\_length\_1102\_cov\_120.509071 641-642. Max. coverage (+): 0.04. Max coverage (-): 0.04

Region: NODE\_382453\_length\_1102\_cov\_120.509071 643-645. Max. coverage (+): 0.07. Max coverage (-): 0.11

Region: NODE\_382453\_length\_1102\_cov\_120.509071 646-647. Max. coverage (+): 0.07. Max coverage (-): 0

Region: NODE\_382453\_length\_1102\_cov\_120.509071 648-649. Max. coverage (+): 0.07. Max coverage (-): 0

Region: NODE\_382453\_length\_1102\_cov\_120.509071 650-652. Max. coverage (+): 1.33. Max coverage (-): 0

Region: NODE\_382453\_length\_1102\_cov\_120.509071 653-654. Max. coverage (+): 1. Max coverage (-): 0

Region: NODE\_382453\_length\_1102\_cov\_120.509071 655-656. Max. coverage (+): 0.11. Max coverage (-): 0

Region: NODE\_382453\_length\_1102\_cov\_120.509071 657-659. Max. coverage (+): 0.04. Max coverage (-): 0.19

Region: NODE\_382453\_length\_1102\_cov\_120.509071 660-661. Max. coverage (+): 0.07. Max coverage (-): 0.44

Region: NODE\_382453\_length\_1102\_cov\_120.509071 662-663. Max. coverage (+): 0.21. Max coverage (-): 1.57

Region: NODE\_382453\_length\_1102\_cov\_120.509071 664-666. Max. coverage (+): 0.53. Max coverage (-): 1.89

Region: NODE\_382453\_length\_1102\_cov\_120.509071 667-668. Max. coverage (+): 0.04. Max coverage (-): 1.11

Region: NODE\_382453\_length\_1102\_cov\_120.509071 669-670. Max. coverage (+): 0.04. Max coverage (-): 1.08

Region: NODE\_382453\_length\_1102\_cov\_120.509071 671-672. Max. coverage (+): 0.15. Max coverage (-): 0.07

Region: NODE\_382453\_length\_1102\_cov\_120.509071 673-675. Max. coverage (+): 54.9. Max coverage (-): 0.15

Region: NODE\_382453\_length\_1102\_cov\_120.509071 676-677. Max. coverage (+): 133.76. Max coverage (-): 0.11

Region: NODE\_382453\_length\_1102\_cov\_120.509071 678-679. Max. coverage (+): 81.37. Max coverage (-): 0

Region: NODE\_382453\_length\_1102\_cov\_120.509071 680-682. Max. coverage (+): 6.67. Max coverage (-): 0.04

Region: NODE\_382453\_length\_1102\_cov\_120.509071 683-684. Max. coverage (+): 0.43. Max coverage (-): 0.07

Region: NODE\_382453\_length\_1102\_cov\_120.509071 685-686. Max. coverage (+): 0.17. Max coverage (-): 0.32

Region: NODE\_382453\_length\_1102\_cov\_120.509071 687-689. Max. coverage (+): 0.19. Max coverage (-): 0.56

Region: NODE\_382453\_length\_1102\_cov\_120.509071 690-691. Max. coverage (+): 0.04. Max coverage (-): 0.56

Region: NODE\_382453\_length\_1102\_cov\_120.509071 692-693. Max. coverage (+): 0. Max coverage (-): 0.04

Region: NODE\_382453\_length\_1102\_cov\_120.509071 694-696. Max. coverage (+): 0. Max coverage (-): 0

Region: NODE\_382453\_length\_1102\_cov\_120.509071 697-698. Max. coverage (+): 0. Max coverage (-): 0

Region: NODE\_382453\_length\_1102\_cov\_120.509071 699-700. Max. coverage (+): 0. Max coverage (-): 0.04

Region: NODE\_382453\_length\_1102\_cov\_120.509071 701-703. Max. coverage (+): 0. Max coverage (-): 0.11

Region: NODE\_382453\_length\_1102\_cov\_120.509071 704-705. Max. coverage (+): 0.15. Max coverage (-): 0

Region: NODE\_382453\_length\_1102\_cov\_120.509071 706-707. Max. coverage (+): 0.37. Max coverage (-): 0

Region: NODE\_382453\_length\_1102\_cov\_120.509071 708-709. Max. coverage (+): 1.52. Max coverage (-): 0.04

Region: NODE\_382453\_length\_1102\_cov\_120.509071 710-712. Max. coverage (+): 1.41. Max coverage (-): 0.04

Region: NODE\_382453\_length\_1102\_cov\_120.509071 713-714. Max. coverage (+): 0.22. Max coverage (-): 0

Region: NODE\_382453\_length\_1102\_cov\_120.509071 715-716. Max. coverage (+): 0. Max coverage (-): 0

Region: NODE\_382453\_length\_1102\_cov\_120.509071 717-719. Max. coverage (+): 0. Max coverage (-): 0.07

Region: NODE\_382453\_length\_1102\_cov\_120.509071 720-721. Max. coverage (+): 0. Max coverage (-): 0.22

Region: NODE\_382453\_length\_1102\_cov\_120.509071 722-723. Max. coverage (+): 0. Max coverage (-): 0.33

Region: NODE\_382453\_length\_1102\_cov\_120.509071 724-726. Max. coverage (+): 0. Max coverage (-): 0.52

Region: NODE\_382453\_length\_1102\_cov\_120.509071 727-728. Max. coverage (+): 0. Max coverage (-): 0.85

Region: NODE\_382453\_length\_1102\_cov\_120.509071 729-730. Max. coverage (+): 0. Max coverage (-): 0.89

Region: NODE\_382453\_length\_1102\_cov\_120.509071 731-733. Max. coverage (+): 0.56. Max coverage (-): 0.41

Region: NODE\_382453\_length\_1102\_cov\_120.509071 734-735. Max. coverage (+): 0.7. Max coverage (-): 0.04

Region: NODE\_382453\_length\_1102\_cov\_120.509071 736-737. Max. coverage (+): 3.93. Max coverage (-): 0.15

Region: NODE\_382453\_length\_1102\_cov\_120.509071 738-740. Max. coverage (+): 9.64. Max coverage (-): 0.11

Region: NODE\_382453\_length\_1102\_cov\_120.509071 741-742. Max. coverage (+): 3.82. Max coverage (-): 0.02

Region: NODE\_382453\_length\_1102\_cov\_120.509071 743-744. Max. coverage (+): 1.87. Max coverage (-): 0.04

Region: NODE\_382453\_length\_1102\_cov\_120.509071 745-747. Max. coverage (+): 2.35. Max coverage (-): 0.2

Region: NODE\_382453\_length\_1102\_cov\_120.509071 748-749. Max. coverage (+): 0. Max coverage (-): 1.08

Region: NODE\_382453\_length\_1102\_cov\_120.509071 750-751. Max. coverage (+): 0. Max coverage (-): 1.08

Region: NODE\_382453\_length\_1102\_cov\_120.509071 752-753. Max. coverage (+): 0. Max coverage (-): 0

Region: NODE\_382453\_length\_1102\_cov\_120.509071 754-756. Max. coverage (+): 0. Max coverage (-): 0.04

Region: NODE\_382453\_length\_1102\_cov\_120.509071 757-758. Max. coverage (+): 0. Max coverage (-): 0

Region: NODE\_382453\_length\_1102\_cov\_120.509071 759-760. Max. coverage (+): 0. Max coverage (-): 0

Region: NODE\_382453\_length\_1102\_cov\_120.509071 761-763. Max. coverage (+): 0.15. Max coverage (-): 0.04

Region: NODE\_382453\_length\_1102\_cov\_120.509071 764-765. Max. coverage (+): 0.26. Max coverage (-): 0.04

Region: NODE\_382453\_length\_1102\_cov\_120.509071 766-767. Max. coverage (+): 0.11. Max coverage (-): 0

Region: NODE\_382453\_length\_1102\_cov\_120.509071 768-770. Max. coverage (+): 0.96. Max coverage (-): 0

Region: NODE\_382453\_length\_1102\_cov\_120.509071 771-772. Max. coverage (+): 0.89. Max coverage (-): 0

Region: NODE\_382453\_length\_1102\_cov\_120.509071 773-774. Max. coverage (+): 0.33. Max coverage (-): 0.04

Region: NODE\_382453\_length\_1102\_cov\_120.509071 775-777. Max. coverage (+): 0.15. Max coverage (-): 0.07

Region: NODE\_382453\_length\_1102\_cov\_120.509071 778-779. Max. coverage (+): 0.07. Max coverage (-): 0.07

Region: NODE\_382453\_length\_1102\_cov\_120.509071 780-781. Max. coverage (+): 0. Max coverage (-): 0.11

Region: NODE\_382453\_length\_1102\_cov\_120.509071 782-784. Max. coverage (+): 0. Max coverage (-): 0.37

Region: NODE\_382453\_length\_1102\_cov\_120.509071 785-786. Max. coverage (+): 0.04. Max coverage (-): 0.3

Region: NODE\_382453\_length\_1102\_cov\_120.509071 787-788. Max. coverage (+): 0.04. Max coverage (-): 0.07

Region: NODE\_382453\_length\_1102\_cov\_120.509071 789-790. Max. coverage (+): 0. Max coverage (-): 0.07

Region: NODE\_382453\_length\_1102\_cov\_120.509071 791-793. Max. coverage (+): 0. Max coverage (-): 0

Region: NODE\_382453\_length\_1102\_cov\_120.509071 794-795. Max. coverage (+): 0. Max coverage (-): 0

Region: NODE\_382453\_length\_1102\_cov\_120.509071 796-797. Max. coverage (+): 0. Max coverage (-): 0.04

Region: NODE\_382453\_length\_1102\_cov\_120.509071 798-800. Max. coverage (+): 0. Max coverage (-): 0.04

Region: NODE\_382453\_length\_1102\_cov\_120.509071 801-802. Max. coverage (+): 0. Max coverage (-): 0

Region: NODE\_382453\_length\_1102\_cov\_120.509071 803-804. Max. coverage (+): 0. Max coverage (-): 0.04

Region: NODE\_382453\_length\_1102\_cov\_120.509071 805-807. Max. coverage (+): 0.04. Max coverage (-): 0.07

Region: NODE\_382453\_length\_1102\_cov\_120.509071 808-809. Max. coverage (+): 0.04. Max coverage (-): 0

Region: NODE\_382453\_length\_1102\_cov\_120.509071 810-811. Max. coverage (+): 0. Max coverage (-): 0

Region: NODE\_382453\_length\_1102\_cov\_120.509071 812-814. Max. coverage (+): 0. Max coverage (-): 0

Region: NODE\_382453\_length\_1102\_cov\_120.509071 815-816. Max. coverage (+): 0. Max coverage (-): 0

Region: NODE\_382453\_length\_1102\_cov\_120.509071 817-818. Max. coverage (+): 0. Max coverage (-): 0.04

Region: NODE\_382453\_length\_1102\_cov\_120.509071 819-821. Max. coverage (+): 0. Max coverage (-): 0.11

Region: NODE\_382453\_length\_1102\_cov\_120.509071 822-823. Max. coverage (+): 0. Max coverage (-): 0.11

Region: NODE\_382453\_length\_1102\_cov\_120.509071 824-825. Max. coverage (+): 0. Max coverage (-): 0

Region: NODE\_382453\_length\_1102\_cov\_120.509071 826-827. Max. coverage (+): 0. Max coverage (-): 0

Region: NODE\_382453\_length\_1102\_cov\_120.509071 828-830. Max. coverage (+): 0. Max coverage (-): 0

Region: NODE\_382453\_length\_1102\_cov\_120.509071 831-832. Max. coverage (+): 0.04. Max coverage (-): 0.07

Region: NODE\_382453\_length\_1102\_cov\_120.509071 833-834. Max. coverage (+): 0.26. Max coverage (-): 0.11

Region: NODE\_382453\_length\_1102\_cov\_120.509071 835-837. Max. coverage (+): 0.74. Max coverage (-): 0.04

Region: NODE\_382453\_length\_1102\_cov\_120.509071 838-839. Max. coverage (+): 0.63. Max coverage (-): 0

Region: NODE\_382453\_length\_1102\_cov\_120.509071 840-841. Max. coverage (+): 0. Max coverage (-): 0

Region: NODE\_382453\_length\_1102\_cov\_120.509071 842-844. Max. coverage (+): 0.11. Max coverage (-): 0

Region: NODE\_382453\_length\_1102\_cov\_120.509071 845-846. Max. coverage (+): 0.04. Max coverage (-): 0

Region: NODE\_382453\_length\_1102\_cov\_120.509071 847-848. Max. coverage (+): 0.04. Max coverage (-): 0.07

Region: NODE\_382453\_length\_1102\_cov\_120.509071 849-851. Max. coverage (+): 0. Max coverage (-): 0.07

Region: NODE\_382453\_length\_1102\_cov\_120.509071 852-853. Max. coverage (+): 0. Max coverage (-): 0

Region: NODE\_382453\_length\_1102\_cov\_120.509071 854-855. Max. coverage (+): 0. Max coverage (-): 0

Region: NODE\_382453\_length\_1102\_cov\_120.509071 856-858. Max. coverage (+): 0.15. Max coverage (-): 0

Region: NODE\_382453\_length\_1102\_cov\_120.509071 859-860. Max. coverage (+): 0.12. Max coverage (-): 0.11

Region: NODE\_382453\_length\_1102\_cov\_120.509071 861-862. Max. coverage (+): 0.16. Max coverage (-): 0.11

Region: NODE\_382453\_length\_1102\_cov\_120.509071 863-865. Max. coverage (+): 1.73. Max coverage (-): 0.57

Region: NODE\_382453\_length\_1102\_cov\_120.509071 866-867. Max. coverage (+): 1.63. Max coverage (-): 0.59

Region: NODE\_382453\_length\_1102\_cov\_120.509071 868-869. Max. coverage (+): 0.33. Max coverage (-): 0.04

Region: NODE\_382453\_length\_1102\_cov\_120.509071 870-871. Max. coverage (+): 0.82. Max coverage (-): 0

Region: NODE\_382453\_length\_1102\_cov\_120.509071 872-874. Max. coverage (+): 0.68. Max coverage (-): 0

Region: NODE\_382453\_length\_1102\_cov\_120.509071 875-876. Max. coverage (+): 0.45. Max coverage (-): 0

Region: NODE\_382453\_length\_1102\_cov\_120.509071 877-878. Max. coverage (+): 2.45. Max coverage (-): 0

Region: NODE\_382453\_length\_1102\_cov\_120.509071 879-881. Max. coverage (+): 14.2. Max coverage (-): 0.11

Region: NODE\_382453\_length\_1102\_cov\_120.509071 882-883. Max. coverage (+): 4.08. Max coverage (-): 0.11

Region: NODE\_382453\_length\_1102\_cov\_120.509071 884-885. Max. coverage (+): 0.33. Max coverage (-): 0

Region: NODE\_382453\_length\_1102\_cov\_120.509071 886-888. Max. coverage (+): 0.41. Max coverage (-): 0.04

Region: NODE\_382453\_length\_1102\_cov\_120.509071 889-890. Max. coverage (+): 1.52. Max coverage (-): 0.11

Region: NODE\_382453\_length\_1102\_cov\_120.509071 891-892. Max. coverage (+): 2.78. Max coverage (-): 0.11

Region: NODE\_382453\_length\_1102\_cov\_120.509071 893-895. Max. coverage (+): 2.85. Max coverage (-): 0.22

Region: NODE\_382453\_length\_1102\_cov\_120.509071 896-897. Max. coverage (+): 0.82. Max coverage (-): 0.22

Region: NODE\_382453\_length\_1102\_cov\_120.509071 898-899. Max. coverage (+): 0.89. Max coverage (-): 0.22

Region: NODE\_382453\_length\_1102\_cov\_120.509071 900-902. Max. coverage (+): 1.41. Max coverage (-): 0.3

Region: NODE\_382453\_length\_1102\_cov\_120.509071 903-904. Max. coverage (+): 1.45. Max coverage (-): 0.19

Region: NODE\_382453\_length\_1102\_cov\_120.509071 905-906. Max. coverage (+): 1.04. Max coverage (-): 0.44

Region: NODE\_382453\_length\_1102\_cov\_120.509071 907-908. Max. coverage (+): 2.08. Max coverage (-): 2.78

Region: NODE\_382453\_length\_1102\_cov\_120.509071 909-911. Max. coverage (+): 17.5. Max coverage (-): 3.3

Region: NODE\_382453\_length\_1102\_cov\_120.509071 912-913. Max. coverage (+): 19.09. Max coverage (-): 0.56

Region: NODE\_382453\_length\_1102\_cov\_120.509071 914-915. Max. coverage (+): 4.04. Max coverage (-): 0.63

Region: NODE\_382453\_length\_1102\_cov\_120.509071 916-918. Max. coverage (+): 1.19. Max coverage (-): 0.56

Region: NODE\_382453\_length\_1102\_cov\_120.509071 919-920. Max. coverage (+): 0.85. Max coverage (-): 0.19

Region: NODE\_382453\_length\_1102\_cov\_120.509071 921-922. Max. coverage (+): 0.33. Max coverage (-): 0

Region: NODE\_382453\_length\_1102\_cov\_120.509071 923-925. Max. coverage (+): 1.22. Max coverage (-): 0

Region: NODE\_382453\_length\_1102\_cov\_120.509071 926-927. Max. coverage (+): 1.37. Max coverage (-): 0

Region: NODE\_382453\_length\_1102\_cov\_120.509071 928-929. Max. coverage (+): 0.15. Max coverage (-): 0

Region: NODE\_382453\_length\_1102\_cov\_120.509071 930-932. Max. coverage (+): 0. Max coverage (-): 0.04

Region: NODE\_382453\_length\_1102\_cov\_120.509071 933-934. Max. coverage (+): 0. Max coverage (-): 0.04

Region: NODE\_382453\_length\_1102\_cov\_120.509071 935-936. Max. coverage (+): 0. Max coverage (-): 0.04

Region: NODE\_382453\_length\_1102\_cov\_120.509071 937-939. Max. coverage (+): 0. Max coverage (-): 0.19

Region: NODE\_382453\_length\_1102\_cov\_120.509071 940-941. Max. coverage (+): 0.04. Max coverage (-): 0.19

Region: NODE\_382453\_length\_1102\_cov\_120.509071 942-943. Max. coverage (+): 0.04. Max coverage (-): 0

Region: NODE\_382453\_length\_1102\_cov\_120.509071 944-946. Max. coverage (+): 0. Max coverage (-): 0

Region: NODE\_382453\_length\_1102\_cov\_120.509071 947-948. Max. coverage (+): 0. Max coverage (-): 0

Region: NODE\_382453\_length\_1102\_cov\_120.509071 949-950. Max. coverage (+): 0. Max coverage (-): 0

Region: NODE\_382453\_length\_1102\_cov\_120.509071 951-952. Max. coverage (+): 0.04. Max coverage (-): 0

Region: NODE\_382453\_length\_1102\_cov\_120.509071 953-955. Max. coverage (+): 0.07. Max coverage (-): 0

Region: NODE\_382453\_length\_1102\_cov\_120.509071 956-957. Max. coverage (+): 0.56. Max coverage (-): 0

Region: NODE\_382453\_length\_1102\_cov\_120.509071 958-959. Max. coverage (+): 0.56. Max coverage (-): 0

Region: NODE\_382453\_length\_1102\_cov\_120.509071 960-962. Max. coverage (+): 0.59. Max coverage (-): 0.04

Region: NODE\_382453\_length\_1102\_cov\_120.509071 963-964. Max. coverage (+): 0.63. Max coverage (-): 0.04

Region: NODE\_382453\_length\_1102\_cov\_120.509071 965-966. Max. coverage (+): 1.37. Max coverage (-): 0.04

Region: NODE\_382453\_length\_1102\_cov\_120.509071 967-969. Max. coverage (+): 1. Max coverage (-): 0.3

Region: NODE\_382453\_length\_1102\_cov\_120.509071 970-971. Max. coverage (+): 0.11. Max coverage (-): 0

Region: NODE\_382453\_length\_1102\_cov\_120.509071 972-973. Max. coverage (+): 0.11. Max coverage (-): 0

Region: NODE\_382453\_length\_1102\_cov\_120.509071 974-976. Max. coverage (+): 0.22. Max coverage (-): 0

Region: NODE\_382453\_length\_1102\_cov\_120.509071 977-978. Max. coverage (+): 0.04. Max coverage (-): 0.15

Region: NODE\_382453\_length\_1102\_cov\_120.509071 979-980. Max. coverage (+): 6.19. Max coverage (-): 0.15

Region: NODE\_382453\_length\_1102\_cov\_120.509071 981-983. Max. coverage (+): 24.88. Max coverage (-): 0

Region: NODE\_382453\_length\_1102\_cov\_120.509071 984-985. Max. coverage (+): 0.37. Max coverage (-): 0

Region: NODE\_382453\_length\_1102\_cov\_120.509071 986-987. Max. coverage (+): 0.04. Max coverage (-): 0

Region: NODE\_382453\_length\_1102\_cov\_120.509071 988-989. Max. coverage (+): 0. Max coverage (-): 0.04

Region: NODE\_382453\_length\_1102\_cov\_120.509071 990-992. Max. coverage (+): 0. Max coverage (-): 2.22

Region: NODE\_382453\_length\_1102\_cov\_120.509071 993-994. Max. coverage (+): 0.04. Max coverage (-): 1.82

Region: NODE\_382453\_length\_1102\_cov\_120.509071 995-996. Max. coverage (+): 0.04. Max coverage (-): 1

Region: NODE\_382453\_length\_1102\_cov\_120.509071 997-999. Max. coverage (+): 0. Max coverage (-): 0.89

Region: NODE\_382453\_length\_1102\_cov\_120.509071 1000-1001. Max. coverage (+): 0. Max coverage (-): 0

Region: NODE\_382453\_length\_1102\_cov\_120.509071 1002-1003. Max. coverage (+): 0. Max coverage (-): 0.15

Region: NODE\_382453\_length\_1102\_cov\_120.509071 1004-1006. Max. coverage (+): 0.15. Max coverage (-): 0.22

Region: NODE\_382453\_length\_1102\_cov\_120.509071 1007-1008. Max. coverage (+): 0.15. Max coverage (-): 0.15

Region: NODE\_382453\_length\_1102\_cov\_120.509071 1009-1010. Max. coverage (+): 0.67. Max coverage (-): 0.07

Region: NODE\_382453\_length\_1102\_cov\_120.509071 1011-1013. Max. coverage (+): 52.05. Max coverage (-): 0

Region: NODE\_382453\_length\_1102\_cov\_120.509071 1014-1015. Max. coverage (+): 54.79. Max coverage (-): 0.04

Region: NODE\_382453\_length\_1102\_cov\_120.509071 1016-1017. Max. coverage (+): 55.76. Max coverage (-): 0.04

Region: NODE\_382453\_length\_1102\_cov\_120.509071 1018-1020. Max. coverage (+): 3.97. Max coverage (-): 0.15

Region: NODE\_382453\_length\_1102\_cov\_120.509071 1021-1022. Max. coverage (+): 0.7. Max coverage (-): 0.19

Region: NODE\_382453\_length\_1102\_cov\_120.509071 1023-1024. Max. coverage (+): 0.63. Max coverage (-): 4.34

Region: NODE\_382453\_length\_1102\_cov\_120.509071 1025-1027. Max. coverage (+): 0.11. Max coverage (-): 4.41

Region: NODE\_382453\_length\_1102\_cov\_120.509071 1028-1029. Max. coverage (+): 0.22. Max coverage (-): 0.41

Region: NODE\_382453\_length\_1102\_cov\_120.509071 1030-1031. Max. coverage (+): 0.19. Max coverage (-): 0.59

Region: NODE\_382453\_length\_1102\_cov\_120.509071 1032-1033. Max. coverage (+): 0.19. Max coverage (-): 0.37

Region: NODE\_382453\_length\_1102\_cov\_120.509071 1034-1036. Max. coverage (+): 0.07. Max coverage (-): 0.04

Region: NODE\_382453\_length\_1102\_cov\_120.509071 1037-1038. Max. coverage (+): 0.07. Max coverage (-): 0.04

Region: NODE\_382453\_length\_1102\_cov\_120.509071 1039-1040. Max. coverage (+): 0. Max coverage (-): 0.04

Region: NODE\_382453\_length\_1102\_cov\_120.509071 1041-1043. Max. coverage (+): 2.56. Max coverage (-): 0.11

Region: NODE\_382453\_length\_1102\_cov\_120.509071 1044-1045. Max. coverage (+): 3.11. Max coverage (-): 0.37

Region: NODE\_382453\_length\_1102\_cov\_120.509071 1046-1047. Max. coverage (+): 5.08. Max coverage (-): 0.41

Region: NODE\_382453\_length\_1102\_cov\_120.509071 1048-1050. Max. coverage (+): 9.9. Max coverage (-): 0.15

Region: NODE\_382453\_length\_1102\_cov\_120.509071 1051-1052. Max. coverage (+): 5.64. Max coverage (-): 0.15

Region: NODE\_382453\_length\_1102\_cov\_120.509071 1053-1054. Max. coverage (+): 1.3. Max coverage (-): 0.22

Region: NODE\_382453\_length\_1102\_cov\_120.509071 1055-1057. Max. coverage (+): 3.6. Max coverage (-): 2.85

Region: NODE\_382453\_length\_1102\_cov\_120.509071 1058-1059. Max. coverage (+): 5.19. Max coverage (-): 3.78

Region: NODE\_382453\_length\_1102\_cov\_120.509071 1060-1061. Max. coverage (+): 2.74. Max coverage (-): 1.74

Region: NODE\_382453\_length\_1102\_cov\_120.509071 1062-1064. Max. coverage (+): 0.33. Max coverage (-): 0.89

Region: NODE\_382453\_length\_1102\_cov\_120.509071 1065-1066. Max. coverage (+): 0.41. Max coverage (-): 0.44

Region: NODE\_382453\_length\_1102\_cov\_120.509071 1067-1068. Max. coverage (+): 0.58. Max coverage (-): 1.08

Region: NODE\_382453\_length\_1102\_cov\_120.509071 1069-1070. Max. coverage (+): 0.49. Max coverage (-): 1.14

Region: NODE\_382453\_length\_1102\_cov\_120.509071 1071-1073. Max. coverage (+): 0.27. Max coverage (-): 0.89

Region: NODE\_382453\_length\_1102\_cov\_120.509071 1074-1075. Max. coverage (+): 0.95. Max coverage (-): 0.53

Region: NODE\_382453\_length\_1102\_cov\_120.509071 1076-1077. Max. coverage (+): 2.43. Max coverage (-): 0.36

Region: NODE\_382453\_length\_1102\_cov\_120.509071 1078-1080. Max. coverage (+): 2.58. Max coverage (-): 0.43

Region: NODE\_382453\_length\_1102\_cov\_120.509071 1081-1082. Max. coverage (+): 6.04. Max coverage (-): 0.43

Region: NODE\_382453\_length\_1102\_cov\_120.509071 1083-1084. Max. coverage (+): 5.52. Max coverage (-): 0.82

Region: NODE\_382453\_length\_1102\_cov\_120.509071 1085-1087. Max. coverage (+): 20.17. Max coverage (-): 2.78

Region: NODE\_382453\_length\_1102\_cov\_120.509071 1088-1089. Max. coverage (+): 16.16. Max coverage (-): 2.85

Region: NODE\_382453\_length\_1102\_cov\_120.509071 1090-1091. Max. coverage (+): 7.49. Max coverage (-): 2.6

Region: NODE\_382453\_length\_1102\_cov\_120.509071 1092-1094. Max. coverage (+): 7.34. Max coverage (-): 1.56

Region: NODE\_382453\_length\_1102\_cov\_120.509071 1095-1096. Max. coverage (+): 0.26. Max coverage (-): 1.48

Region: NODE\_382453\_length\_1102\_cov\_120.509071 1097-1098. Max. coverage (+): 0.04. Max coverage (-): 0.26

Region: NODE\_382453\_length\_1102\_cov\_120.509071 1099-1101. Max. coverage (+): 0.04. Max coverage (-): 0.41

Region: NODE\_382453\_length\_1102\_cov\_120.509071 1102-1103. Max. coverage (+): 0. Max coverage (-): 0.42

Region: NODE\_382453\_length\_1102\_cov\_120.509071 1104-1105. Max. coverage (+): 0.37. Max coverage (-): 0.16

Region: NODE\_382453\_length\_1102\_cov\_120.509071 1106-1107. Max. coverage (+): 0.94. Max coverage (-): 0.38

Region: NODE\_382453\_length\_1102\_cov\_120.509071 1108-1110. Max. coverage (+): 1.01. Max coverage (-): 0.41

Region: NODE\_382453\_length\_1102\_cov\_120.509071 1111-1112. Max. coverage (+): 0.85. Max coverage (-): 0.11

Region: NODE\_382453\_length\_1102\_cov\_120.509071 1113-1114. Max. coverage (+): 0.88. Max coverage (-): 0.09

Region: NODE\_382453\_length\_1102\_cov\_120.509071 1115-1117. Max. coverage (+): 0.78. Max coverage (-): 0.02

Region: NODE\_382453\_length\_1102\_cov\_120.509071 1118-1119. Max. coverage (+): 1.33. Max coverage (-): 0.02

Region: NODE\_382453\_length\_1102\_cov\_120.509071 1120-1121. Max. coverage (+): 7.12. Max coverage (-): 0

Region: NODE\_382453\_length\_1102\_cov\_120.509071 1122-1124. Max. coverage (+): 6.18. Max coverage (-): 0.12

Region: NODE\_382453\_length\_1102\_cov\_120.509071 1125-1126. Max. coverage (+): 0.05. Max coverage (-): 0.12

Region: NODE\_382453\_length\_1102\_cov\_120.509071 1127-1128. Max. coverage (+): 2.56. Max coverage (-): 0.04

Region: NODE\_382453\_length\_1102\_cov\_120.509071 1129-1131. Max. coverage (+): 2.94. Max coverage (-): 0.01

Region: NODE\_382453\_length\_1102\_cov\_120.509071 1132-1133. Max. coverage (+): 8.19. Max coverage (-): 0.01

Region: NODE\_382453\_length\_1102\_cov\_120.509071 1134-1135. Max. coverage (+): 8.49. Max coverage (-): 0.01

Region: NODE\_382453\_length\_1102\_cov\_120.509071 1136-1138. Max. coverage (+): 7.33. Max coverage (-): 0

Region: NODE\_382453\_length\_1102\_cov\_120.509071 1139-1140. Max. coverage (+): 0.35. Max coverage (-): 0

Region: NODE\_382453\_length\_1102\_cov\_120.509071 1141-1142. Max. coverage (+): 0.04. Max coverage (-): 0

Region: NODE\_382453\_length\_1102\_cov\_120.509071 1143-1145. Max. coverage (+): 0. Max coverage (-): 0.01

Region: NODE\_382453\_length\_1102\_cov\_120.509071 1146-1147. Max. coverage (+): 0. Max coverage (-): 0.01

Region: NODE\_382453\_length\_1102\_cov\_120.509071 1148-1149. Max. coverage (+): 0. Max coverage (-): 0

Region: NODE\_382453\_length\_1102\_cov\_120.509071 1150-1151. Max. coverage (+): 0. Max coverage (-): 0

Region: NODE\_382453\_length\_1102\_cov\_120.509071 1152-1154. Max. coverage (+): 0. Max coverage (-): 0

Region: NODE\_382453\_length\_1102\_cov\_120.509071 1155-1156. Max. coverage (+): 0. Max coverage (-): 0

Region: NODE\_382453\_length\_1102\_cov\_120.509071 1157-1158. Max. coverage (+): 0. Max coverage (-): 0

Region: NODE\_382453\_length\_1102\_cov\_120.509071 1159-1161. Max. coverage (+): 0. Max coverage (-): 0

Region: NODE\_382453\_length\_1102\_cov\_120.509071 1162-1163. Max. coverage (+): 0. Max coverage (-): 0

Region: NODE\_382453\_length\_1102\_cov\_120.509071 1164-1165. Max. coverage (+): 0. Max coverage (-): 0

Region: NODE\_382453\_length\_1102\_cov\_120.509071 1166-. Max. coverage (+): 0. Max coverage (-): 0

RepeatMasker Color Code

**+**

100-98% Identity

<98-95% Identity

<95-90% Identity

<90-85% Identity

<85-80% Identity

<80-75% Identity

<75-70% Identity

<70% Identity

**-**

Gene Set Color Code

**+**

Gene

Pseudogene

Other

**-**

Topology/Coverage Color Code

Coverage Plus Strand

Coverage Minus Strand

Mainstrand: Plus

Mainstrand: Minus

Complementary Strand

Flanking Region  
(if option -flank >0)

Gene Set Annotation  
  
RepeatMasker Annotation  

**1. AlRepB-14**: 1-34 (+), Divergence to consensus: 5.9%  
**2. (AT)n**: 487-517 (+), Divergence to consensus: 17.1%  
**3. AlRepD-1165**: 523-737 (-), Divergence to consensus: 23.8%

  
Transcription Factor Binding Sites  

**RHOXF1** (Sequence: AGATCA (-): 198)  
**RHOXF1** (Sequence: AGCTCA (-): 283)  
**RHOXF1** (Sequence: GGCTCA (-): 689)  
**RHOXF1** (Sequence: TGATCC (+): 608)  
**Sox5** (Sequence: ATTGTT (+): 474)  
**SOX9** (Sequence: TTATTGTT (+): 472)  
**FOXO1** (Sequence: AAAAACAAG (-): 435)  
**Sox5** (Sequence: AACAAT (-): 572)  
**Sox5** (Sequence: AACAAT (-): 598)
