# Supplementary material for: First characterization of PIWI-interacting RNA clusters in a cichlid fish with a B chromosome
Source: BMC Biol. 2022 Sep 21;20:204. doi: 10.1186/s12915-022-01403-2 (PMC9490952; doi:10.1186/s12915-022-01403-2)
Supplement: Supplementary file 1 — Additional file 1. Zipped folder with fasta and interactive html piRNA cluster information for the A. latifasciata genome. The nomenclature is as follows: number-pirna-cluster_sex_B-presence (f, female; m, male; 0b, without B chromosome; 1b, with B chromosome). [file 12915_2022_1403_MOESM1_ESM.zip › 148_m0b.html]

piRNA cluster 148\_m0b 73


Predicted piRNA cluster no. 148\_m0b
  

Show proTRAC run info
Hide proTRAC run info

/\  
                \_\_\_\_\_\_\_\_\_\_\_\_\_\_\_\_\_\_\_\_\_\_\_/\\_\_\_ /  \\_\_\_\_\_\_\_  
               I                      /  \  /    \      I  
               I     pro             /    \/      \     I  
               I        TRAC        /               \   I  
               I   \_\_\_\_\_\_\_\_\_\_\_\_\_\_\_\_/\_\_\_\_\_\_\_\_\_\_\_\_\_\_\_\_\_\\_ I  
               I   \              /                     I  
               I    \            /                      I  
               I     \  /\      /       V.2.4.2         I  
               I      \/  \    /                        I  
               I\_\_\_\_\_\_\_\_\_\_\_\  /\_\_\_\_\_\_\_\_\_\_\_\_\_\_\_\_\_\_\_\_\_\_\_\_\_I  
                            \/  
  
  
================================= proTRAC ====================================  
VERSION: .......... 2.4.2  
LAST MODIFIED: .... 11. May 2018  
  
Please cite:  
Rosenkranz D, Zischler H. proTRAC - a software for probabilistic piRNA cluster  
detection, visualization and analysis. 2012. BMC Bioinformatics 13:5.  
  
  
Contact:  
David Rosenkranz  
Institute of Organismic and Molecular Evolutionary Biology  
Dept. Anthropology, small RNA group  
Johannes Gutenberg University Mainz  
email: rosenkranz@uni-mainz.de  
  
You can find the latest proTRAC version at:  
http://sourceforge.net/projects/protrac/files  
http://www.smallRNAgroup-mainz.de/software  
==============================================================================  
  
PARAMETERS:  
Map file: ...............piwi-machos-0B.fa-collapse.map  
Genome file: ............../../../0B\_ala\_genome.fa  
RepeatMasker annotation: Alatifasciata-all0B-maryan-v2.fa\_corrected.out  
GeneSet:................./guest-storage/Data/annotation/Alatifasciata\_all0B\_maryan-v2\_out2017.gff  
  
Significant (p<=0.01) hit density will be calculated based  
on observed hit distribution.  
  
Sliding window size: ........................................ 5000 bp  
Sliding window increament: .................................. 1000 bp  
Normalize each hit by number of genomic hits: ............... yes  
Normalize each hit by number of sequence reads: ............. yes  
Normalize values (-> per million mapped reads): ............. yes  
Min. fraction of hits with 1T(U) or 10A: .................... 0.75  
Alternatively: Min. fraction of hits with 1T(U) and 10A: .... 0.5  
Min. fraction of hits with typical piRNA length: ............ 0.75  
Typical piRNA length: ....................................... 24-32 nt  
Min. size of a piRNA cluster: ............................... 1000 bp.  
Min. number of hits (absolute): ............................. 0  
Min. number of hits (normalized): ........................... 0  
Min. fraction of hits on the mainstrand: .................... 0.75  
Top fraction of mapped sequences (in terms of read counts): . 1%  
Top fraction accounts for max. n% of sequence reads: ........ 90%  
Min. fraction of hits on each arm of a bidirectional cluster: 0.05  
Output html file for each cluster: .......................... yes  
Output a summary table: ..................................... yes  
Output a FASTA file for each cluster (piRNA sequences): ..... yes  
Output a FASTA file comprising cluster sequences: ........... yes  
Output a GTF file for predicted piRNA clusters: ..............yes  
Search DNA motifs in clusters: .............................. yes  
Output flanking sequences: +/- .............................. 0 bp  
Output ~.pTi file: .......................................... no  
==============================================================================  
  
  
Genome size (without gaps): ............ 758543724 bp  
Gaps (N/X/-): .......................... 417479 bp  
Mapped reads: .......................... 24765598  
Non-identical sequences: ............... 6158275  
Genomic hits: .......................... 53103584  
Significant densitiy of mapped reads: .. 763.098963422187 reads/kb

Show proTRAC cluster info
Hide proTRAC cluster info

|  |  |
| --- | --- |
| Location | NODE\_382493\_length\_8613\_cov\_31.659817 |
| Coordinates | 3-8740 |
| Size [bp] | 8738 |
| Sequence hit loci | 17704 |
| Mapped reads (normalized) | 131902.5 |
| Mapped reads (normalized) per kb | 15095.3 |
| Normalized reads with 1T (1U) | 78.4% |
| Normalized reads with 10A | 43.6% |
| Normalized reads with length 24-32 nt | 99% |
| Normalized reads on the main strand(s) | 93.1% |
| Predicted directionality | mono:plus |

100%

0%

1T (1U)  
reads

10A reads

24-32 nt  
reads

reads on mainstrand

**Either the amount of reads with 1T (1U) OR 10A has to exceed 75% (set with option: -1Tor10A)  
Alternatively the amount of reads with 1T (1U) AND 10A has to exceed 50% (set with option: -1Tand10A)  
Minimum amount of reads with preferred size is 75% (set with option: -pisize)  
Minimum amount of reads on the main strand(s) is 75% (set with option: -clstrand)**

Show read coverage
Hide read coverage

WHAT DO I SEE HERE?  
This chart shows the location of mapped sequence reads within a predicted piRNA cluster. The color refers to the number of genomic hits produced by the sequence read in question. A dark red bar indicates that this sequence read produces many other hits elsewhere in the genome. Many adjacent red or yellow bars can indicate the presence of a multi-copy element such as transposons or rRNA genes. A dark green bar indicates that this sequence read maps uniquely to this locus.

1 hit

2-5 hits

6-10 hits

11-20 hits

21-50 hits

51-100 hits

> 100 hits

NODE\_382493\_length\_8613\_cov\_31.659817

3

8740

Gene Set

RepeatMasker

Mapped  
Reads

369.5

plus strand

minus strand

369.5

Region: NODE\_382493\_length\_8613\_cov\_31.659817 1165-11. Max. coverage (+): 0. Max coverage (-): 0

Region: NODE\_382493\_length\_8613\_cov\_31.659817 12-29. Max. coverage (+): 0.08. Max coverage (-): 0.2

Region: NODE\_382493\_length\_8613\_cov\_31.659817 30-46. Max. coverage (+): 0.09. Max coverage (-): 0.08

Region: NODE\_382493\_length\_8613\_cov\_31.659817 47-64. Max. coverage (+): 0.04. Max coverage (-): 0.28

Region: NODE\_382493\_length\_8613\_cov\_31.659817 65-81. Max. coverage (+): 0.27. Max coverage (-): 0.04

Region: NODE\_382493\_length\_8613\_cov\_31.659817 82-99. Max. coverage (+): 0.24. Max coverage (-): 0.02

Region: NODE\_382493\_length\_8613\_cov\_31.659817 100-116. Max. coverage (+): 0.08. Max coverage (-): 0.01

Region: NODE\_382493\_length\_8613\_cov\_31.659817 117-134. Max. coverage (+): 0.12. Max coverage (-): 0

Region: NODE\_382493\_length\_8613\_cov\_31.659817 135-151. Max. coverage (+): 0.03. Max coverage (-): 0.03

Region: NODE\_382493\_length\_8613\_cov\_31.659817 152-169. Max. coverage (+): 0.15. Max coverage (-): 0.32

Region: NODE\_382493\_length\_8613\_cov\_31.659817 170-186. Max. coverage (+): 0.04. Max coverage (-): 0.4

Region: NODE\_382493\_length\_8613\_cov\_31.659817 187-203. Max. coverage (+): 0.02. Max coverage (-): 0

Region: NODE\_382493\_length\_8613\_cov\_31.659817 204-221. Max. coverage (+): 0.08. Max coverage (-): 0

Region: NODE\_382493\_length\_8613\_cov\_31.659817 222-238. Max. coverage (+): 0.08. Max coverage (-): 0.08

Region: NODE\_382493\_length\_8613\_cov\_31.659817 239-256. Max. coverage (+): 0.01. Max coverage (-): 0

Region: NODE\_382493\_length\_8613\_cov\_31.659817 257-273. Max. coverage (+): 0. Max coverage (-): 0.07

Region: NODE\_382493\_length\_8613\_cov\_31.659817 274-291. Max. coverage (+): 0.1. Max coverage (-): 0.01

Region: NODE\_382493\_length\_8613\_cov\_31.659817 292-308. Max. coverage (+): 0. Max coverage (-): 0.04

Region: NODE\_382493\_length\_8613\_cov\_31.659817 309-326. Max. coverage (+): 0.18. Max coverage (-): 0

Region: NODE\_382493\_length\_8613\_cov\_31.659817 327-343. Max. coverage (+): 0.06. Max coverage (-): 0.01

Region: NODE\_382493\_length\_8613\_cov\_31.659817 344-361. Max. coverage (+): 0.03. Max coverage (-): 0.01

Region: NODE\_382493\_length\_8613\_cov\_31.659817 362-378. Max. coverage (+): 0. Max coverage (-): 0.01

Region: NODE\_382493\_length\_8613\_cov\_31.659817 379-396. Max. coverage (+): 0.18. Max coverage (-): 0.01

Region: NODE\_382493\_length\_8613\_cov\_31.659817 397-413. Max. coverage (+): 0.08. Max coverage (-): 0.08

Region: NODE\_382493\_length\_8613\_cov\_31.659817 414-431. Max. coverage (+): 0.02. Max coverage (-): 0.16

Region: NODE\_382493\_length\_8613\_cov\_31.659817 432-448. Max. coverage (+): 0.12. Max coverage (-): 0

Region: NODE\_382493\_length\_8613\_cov\_31.659817 449-466. Max. coverage (+): 0. Max coverage (-): 0.16

Region: NODE\_382493\_length\_8613\_cov\_31.659817 467-483. Max. coverage (+): 0.01. Max coverage (-): 0.07

Region: NODE\_382493\_length\_8613\_cov\_31.659817 484-501. Max. coverage (+): 0. Max coverage (-): 0.01

Region: NODE\_382493\_length\_8613\_cov\_31.659817 502-518. Max. coverage (+): 0.01. Max coverage (-): 0

Region: NODE\_382493\_length\_8613\_cov\_31.659817 519-536. Max. coverage (+): 0.01. Max coverage (-): 0.04

Region: NODE\_382493\_length\_8613\_cov\_31.659817 537-553. Max. coverage (+): 0.2. Max coverage (-): 0.07

Region: NODE\_382493\_length\_8613\_cov\_31.659817 554-570. Max. coverage (+): 0.12. Max coverage (-): 0

Region: NODE\_382493\_length\_8613\_cov\_31.659817 571-588. Max. coverage (+): 0.89. Max coverage (-): 1.45

Region: NODE\_382493\_length\_8613\_cov\_31.659817 589-605. Max. coverage (+): 0.36. Max coverage (-): 0.32

Region: NODE\_382493\_length\_8613\_cov\_31.659817 606-623. Max. coverage (+): 0.05. Max coverage (-): 0.03

Region: NODE\_382493\_length\_8613\_cov\_31.659817 624-640. Max. coverage (+): 0.01. Max coverage (-): 0.01

Region: NODE\_382493\_length\_8613\_cov\_31.659817 641-658. Max. coverage (+): 0.01. Max coverage (-): 0.03

Region: NODE\_382493\_length\_8613\_cov\_31.659817 659-675. Max. coverage (+): 0.02. Max coverage (-): 0

Region: NODE\_382493\_length\_8613\_cov\_31.659817 676-693. Max. coverage (+): 0. Max coverage (-): 0.04

Region: NODE\_382493\_length\_8613\_cov\_31.659817 694-710. Max. coverage (+): 0.06. Max coverage (-): 0

Region: NODE\_382493\_length\_8613\_cov\_31.659817 711-728. Max. coverage (+): 0. Max coverage (-): 0.02

Region: NODE\_382493\_length\_8613\_cov\_31.659817 729-745. Max. coverage (+): 0. Max coverage (-): 0

Region: NODE\_382493\_length\_8613\_cov\_31.659817 746-763. Max. coverage (+): 0. Max coverage (-): 0

Region: NODE\_382493\_length\_8613\_cov\_31.659817 764-780. Max. coverage (+): 0.01. Max coverage (-): 0.02

Region: NODE\_382493\_length\_8613\_cov\_31.659817 781-798. Max. coverage (+): 0.04. Max coverage (-): 0.02

Region: NODE\_382493\_length\_8613\_cov\_31.659817 799-815. Max. coverage (+): 0.2. Max coverage (-): 0.63

Region: NODE\_382493\_length\_8613\_cov\_31.659817 816-833. Max. coverage (+): 0.32. Max coverage (-): 0.28

Region: NODE\_382493\_length\_8613\_cov\_31.659817 834-850. Max. coverage (+): 0.08. Max coverage (-): 0.2

Region: NODE\_382493\_length\_8613\_cov\_31.659817 851-868. Max. coverage (+): 0.09. Max coverage (-): 0.04

Region: NODE\_382493\_length\_8613\_cov\_31.659817 869-885. Max. coverage (+): 0.02. Max coverage (-): 0.04

Region: NODE\_382493\_length\_8613\_cov\_31.659817 886-903. Max. coverage (+): 0. Max coverage (-): 0

Region: NODE\_382493\_length\_8613\_cov\_31.659817 904-920. Max. coverage (+): 0. Max coverage (-): 0.01

Region: NODE\_382493\_length\_8613\_cov\_31.659817 921-937. Max. coverage (+): 0.01. Max coverage (-): 0.01

Region: NODE\_382493\_length\_8613\_cov\_31.659817 938-955. Max. coverage (+): 0. Max coverage (-): 0.12

Region: NODE\_382493\_length\_8613\_cov\_31.659817 956-972. Max. coverage (+): 0.16. Max coverage (-): 0.14

Region: NODE\_382493\_length\_8613\_cov\_31.659817 973-990. Max. coverage (+): 0.02. Max coverage (-): 0.67

Region: NODE\_382493\_length\_8613\_cov\_31.659817 991-1007. Max. coverage (+): 0.06. Max coverage (-): 0.04

Region: NODE\_382493\_length\_8613\_cov\_31.659817 1008-1025. Max. coverage (+): 0.2. Max coverage (-): 0

Region: NODE\_382493\_length\_8613\_cov\_31.659817 1026-1042. Max. coverage (+): 0.04. Max coverage (-): 0.48

Region: NODE\_382493\_length\_8613\_cov\_31.659817 1043-1060. Max. coverage (+): 0.69. Max coverage (-): 0.44

Region: NODE\_382493\_length\_8613\_cov\_31.659817 1061-1077. Max. coverage (+): 1.57. Max coverage (-): 0.24

Region: NODE\_382493\_length\_8613\_cov\_31.659817 1078-1095. Max. coverage (+): 0.04. Max coverage (-): 0

Region: NODE\_382493\_length\_8613\_cov\_31.659817 1096-1112. Max. coverage (+): 0. Max coverage (-): 0

Region: NODE\_382493\_length\_8613\_cov\_31.659817 1113-1130. Max. coverage (+): 0. Max coverage (-): 0

Region: NODE\_382493\_length\_8613\_cov\_31.659817 1131-1147. Max. coverage (+): 0.12. Max coverage (-): 0.04

Region: NODE\_382493\_length\_8613\_cov\_31.659817 1148-1165. Max. coverage (+): 0.24. Max coverage (-): 0

Region: NODE\_382493\_length\_8613\_cov\_31.659817 1166-1182. Max. coverage (+): 0.02. Max coverage (-): 0.65

Region: NODE\_382493\_length\_8613\_cov\_31.659817 1183-1200. Max. coverage (+): 0.04. Max coverage (-): 0.65

Region: NODE\_382493\_length\_8613\_cov\_31.659817 1201-1217. Max. coverage (+): 0.08. Max coverage (-): 0.32

Region: NODE\_382493\_length\_8613\_cov\_31.659817 1218-1235. Max. coverage (+): 0.22. Max coverage (-): 0.04

Region: NODE\_382493\_length\_8613\_cov\_31.659817 1236-1252. Max. coverage (+): 0.24. Max coverage (-): 0.12

Region: NODE\_382493\_length\_8613\_cov\_31.659817 1253-1270. Max. coverage (+): 0.03. Max coverage (-): 0.03

Region: NODE\_382493\_length\_8613\_cov\_31.659817 1271-1287. Max. coverage (+): 0.02. Max coverage (-): 0.13

Region: NODE\_382493\_length\_8613\_cov\_31.659817 1288-1304. Max. coverage (+): 3.23. Max coverage (-): 0.45

Region: NODE\_382493\_length\_8613\_cov\_31.659817 1305-1322. Max. coverage (+): 8.56. Max coverage (-): 0.06

Region: NODE\_382493\_length\_8613\_cov\_31.659817 1323-1339. Max. coverage (+): 0.12. Max coverage (-): 0.2

Region: NODE\_382493\_length\_8613\_cov\_31.659817 1340-1357. Max. coverage (+): 0.15. Max coverage (-): 0.1

Region: NODE\_382493\_length\_8613\_cov\_31.659817 1358-1374. Max. coverage (+): 0.24. Max coverage (-): 0.04

Region: NODE\_382493\_length\_8613\_cov\_31.659817 1375-1392. Max. coverage (+): 0.08. Max coverage (-): 0.08

Region: NODE\_382493\_length\_8613\_cov\_31.659817 1393-1409. Max. coverage (+): 0. Max coverage (-): 0.08

Region: NODE\_382493\_length\_8613\_cov\_31.659817 1410-1427. Max. coverage (+): 0.22. Max coverage (-): 0.23

Region: NODE\_382493\_length\_8613\_cov\_31.659817 1428-1444. Max. coverage (+): 0.17. Max coverage (-): 0.48

Region: NODE\_382493\_length\_8613\_cov\_31.659817 1445-1462. Max. coverage (+): 0.69. Max coverage (-): 0.16

Region: NODE\_382493\_length\_8613\_cov\_31.659817 1463-1479. Max. coverage (+): 0.12. Max coverage (-): 0.04

Region: NODE\_382493\_length\_8613\_cov\_31.659817 1480-1497. Max. coverage (+): 0.3. Max coverage (-): 0.15

Region: NODE\_382493\_length\_8613\_cov\_31.659817 1498-1514. Max. coverage (+): 1.03. Max coverage (-): 0.07

Region: NODE\_382493\_length\_8613\_cov\_31.659817 1515-1532. Max. coverage (+): 0.45. Max coverage (-): 0.44

Region: NODE\_382493\_length\_8613\_cov\_31.659817 1533-1549. Max. coverage (+): 1.47. Max coverage (-): 1.82

Region: NODE\_382493\_length\_8613\_cov\_31.659817 1550-1567. Max. coverage (+): 1.45. Max coverage (-): 0.55

Region: NODE\_382493\_length\_8613\_cov\_31.659817 1568-1584. Max. coverage (+): 0.52. Max coverage (-): 0.67

Region: NODE\_382493\_length\_8613\_cov\_31.659817 1585-1602. Max. coverage (+): 0.97. Max coverage (-): 0.89

Region: NODE\_382493\_length\_8613\_cov\_31.659817 1603-1619. Max. coverage (+): 0.04. Max coverage (-): 0.02

Region: NODE\_382493\_length\_8613\_cov\_31.659817 1620-1637. Max. coverage (+): 0. Max coverage (-): 0

Region: NODE\_382493\_length\_8613\_cov\_31.659817 1638-1654. Max. coverage (+): 0.16. Max coverage (-): 0

Region: NODE\_382493\_length\_8613\_cov\_31.659817 1655-1671. Max. coverage (+): 0.12. Max coverage (-): 0

Region: NODE\_382493\_length\_8613\_cov\_31.659817 1672-1689. Max. coverage (+): 0.12. Max coverage (-): 0.2

Region: NODE\_382493\_length\_8613\_cov\_31.659817 1690-1706. Max. coverage (+): 0.31. Max coverage (-): 0.22

Region: NODE\_382493\_length\_8613\_cov\_31.659817 1707-1724. Max. coverage (+): 0.04. Max coverage (-): 0.36

Region: NODE\_382493\_length\_8613\_cov\_31.659817 1725-1741. Max. coverage (+): 0.69. Max coverage (-): 1.94

Region: NODE\_382493\_length\_8613\_cov\_31.659817 1742-1759. Max. coverage (+): 0.65. Max coverage (-): 0.85

Region: NODE\_382493\_length\_8613\_cov\_31.659817 1760-1776. Max. coverage (+): 6.14. Max coverage (-): 0.89

Region: NODE\_382493\_length\_8613\_cov\_31.659817 1777-1794. Max. coverage (+): 7.23. Max coverage (-): 0.89

Region: NODE\_382493\_length\_8613\_cov\_31.659817 1795-1811. Max. coverage (+): 0.28. Max coverage (-): 0.17

Region: NODE\_382493\_length\_8613\_cov\_31.659817 1812-1829. Max. coverage (+): 0.12. Max coverage (-): 0.03

Region: NODE\_382493\_length\_8613\_cov\_31.659817 1830-1846. Max. coverage (+): 1.75. Max coverage (-): 1.81

Region: NODE\_382493\_length\_8613\_cov\_31.659817 1847-1864. Max. coverage (+): 1.7. Max coverage (-): 0.1

Region: NODE\_382493\_length\_8613\_cov\_31.659817 1865-1881. Max. coverage (+): 5.49. Max coverage (-): 0.32

Region: NODE\_382493\_length\_8613\_cov\_31.659817 1882-1899. Max. coverage (+): 19.95. Max coverage (-): 0.24

Region: NODE\_382493\_length\_8613\_cov\_31.659817 1900-1916. Max. coverage (+): 17.06. Max coverage (-): 0.2

Region: NODE\_382493\_length\_8613\_cov\_31.659817 1917-1934. Max. coverage (+): 0.5. Max coverage (-): 0.02

Region: NODE\_382493\_length\_8613\_cov\_31.659817 1935-1951. Max. coverage (+): 1.47. Max coverage (-): 0.02

Region: NODE\_382493\_length\_8613\_cov\_31.659817 1952-1969. Max. coverage (+): 0.24. Max coverage (-): 0.65

Region: NODE\_382493\_length\_8613\_cov\_31.659817 1970-1986. Max. coverage (+): 1.41. Max coverage (-): 0.44

Region: NODE\_382493\_length\_8613\_cov\_31.659817 1987-2004. Max. coverage (+): 0.97. Max coverage (-): 0.52

Region: NODE\_382493\_length\_8613\_cov\_31.659817 2005-2021. Max. coverage (+): 0.57. Max coverage (-): 0.02

Region: NODE\_382493\_length\_8613\_cov\_31.659817 2022-2038. Max. coverage (+): 1.07. Max coverage (-): 0.1

Region: NODE\_382493\_length\_8613\_cov\_31.659817 2039-2056. Max. coverage (+): 3.11. Max coverage (-): 6.3

Region: NODE\_382493\_length\_8613\_cov\_31.659817 2057-2073. Max. coverage (+): 7.51. Max coverage (-): 0.2

Region: NODE\_382493\_length\_8613\_cov\_31.659817 2074-2091. Max. coverage (+): 1.53. Max coverage (-): 0.81

Region: NODE\_382493\_length\_8613\_cov\_31.659817 2092-2108. Max. coverage (+): 42.4. Max coverage (-): 0.12

Region: NODE\_382493\_length\_8613\_cov\_31.659817 2109-2126. Max. coverage (+): 4.32. Max coverage (-): 1.9

Region: NODE\_382493\_length\_8613\_cov\_31.659817 2127-2143. Max. coverage (+): 0.73. Max coverage (-): 1.25

Region: NODE\_382493\_length\_8613\_cov\_31.659817 2144-2161. Max. coverage (+): 0.73. Max coverage (-): 0.08

Region: NODE\_382493\_length\_8613\_cov\_31.659817 2162-2178. Max. coverage (+): 6.34. Max coverage (-): 0

Region: NODE\_382493\_length\_8613\_cov\_31.659817 2179-2196. Max. coverage (+): 7.11. Max coverage (-): 0.4

Region: NODE\_382493\_length\_8613\_cov\_31.659817 2197-2213. Max. coverage (+): 9. Max coverage (-): 1.01

Region: NODE\_382493\_length\_8613\_cov\_31.659817 2214-2231. Max. coverage (+): 8.88. Max coverage (-): 2.18

Region: NODE\_382493\_length\_8613\_cov\_31.659817 2232-2248. Max. coverage (+): 1.9. Max coverage (-): 0.08

Region: NODE\_382493\_length\_8613\_cov\_31.659817 2249-2266. Max. coverage (+): 0.44. Max coverage (-): 0.32

Region: NODE\_382493\_length\_8613\_cov\_31.659817 2267-2283. Max. coverage (+): 3.11. Max coverage (-): 0.12

Region: NODE\_382493\_length\_8613\_cov\_31.659817 2284-2301. Max. coverage (+): 2.22. Max coverage (-): 0.81

Region: NODE\_382493\_length\_8613\_cov\_31.659817 2302-2318. Max. coverage (+): 54.35. Max coverage (-): 0.2

Region: NODE\_382493\_length\_8613\_cov\_31.659817 2319-2336. Max. coverage (+): 17.4. Max coverage (-): 0.4

Region: NODE\_382493\_length\_8613\_cov\_31.659817 2337-2353. Max. coverage (+): 4.44. Max coverage (-): 1.86

Region: NODE\_382493\_length\_8613\_cov\_31.659817 2354-2370. Max. coverage (+): 6.74. Max coverage (-): 1.66

Region: NODE\_382493\_length\_8613\_cov\_31.659817 2371-2388. Max. coverage (+): 143.26. Max coverage (-): 1.13

Region: NODE\_382493\_length\_8613\_cov\_31.659817 2389-2405. Max. coverage (+): 4.56. Max coverage (-): 0.52

Region: NODE\_382493\_length\_8613\_cov\_31.659817 2406-2423. Max. coverage (+): 22.41. Max coverage (-): 0.57

Region: NODE\_382493\_length\_8613\_cov\_31.659817 2424-2440. Max. coverage (+): 19.34. Max coverage (-): 1.7

Region: NODE\_382493\_length\_8613\_cov\_31.659817 2441-2458. Max. coverage (+): 7.07. Max coverage (-): 0.48

Region: NODE\_382493\_length\_8613\_cov\_31.659817 2459-2475. Max. coverage (+): 0.36. Max coverage (-): 0.04

Region: NODE\_382493\_length\_8613\_cov\_31.659817 2476-2493. Max. coverage (+): 13.32. Max coverage (-): 0.04

Region: NODE\_382493\_length\_8613\_cov\_31.659817 2494-2510. Max. coverage (+): 2.91. Max coverage (-): 0.08

Region: NODE\_382493\_length\_8613\_cov\_31.659817 2511-2528. Max. coverage (+): 13.08. Max coverage (-): 0.08

Region: NODE\_382493\_length\_8613\_cov\_31.659817 2529-2545. Max. coverage (+): 5.69. Max coverage (-): 0.08

Region: NODE\_382493\_length\_8613\_cov\_31.659817 2546-2563. Max. coverage (+): 0.2. Max coverage (-): 0.04

Region: NODE\_382493\_length\_8613\_cov\_31.659817 2564-2580. Max. coverage (+): 0.4. Max coverage (-): 0.32

Region: NODE\_382493\_length\_8613\_cov\_31.659817 2581-2598. Max. coverage (+): 22.57. Max coverage (-): 0.65

Region: NODE\_382493\_length\_8613\_cov\_31.659817 2599-2615. Max. coverage (+): 4.24. Max coverage (-): 0.28

Region: NODE\_382493\_length\_8613\_cov\_31.659817 2616-2633. Max. coverage (+): 1.25. Max coverage (-): 2.26

Region: NODE\_382493\_length\_8613\_cov\_31.659817 2634-2650. Max. coverage (+): 2.06. Max coverage (-): 3.88

Region: NODE\_382493\_length\_8613\_cov\_31.659817 2651-2668. Max. coverage (+): 4.6. Max coverage (-): 1.01

Region: NODE\_382493\_length\_8613\_cov\_31.659817 2669-2685. Max. coverage (+): 3.39. Max coverage (-): 0.2

Region: NODE\_382493\_length\_8613\_cov\_31.659817 2686-2703. Max. coverage (+): 4.06. Max coverage (-): 0.36

Region: NODE\_382493\_length\_8613\_cov\_31.659817 2704-2720. Max. coverage (+): 52.43. Max coverage (-): 0.24

Region: NODE\_382493\_length\_8613\_cov\_31.659817 2721-2737. Max. coverage (+): 2.62. Max coverage (-): 0.36

Region: NODE\_382493\_length\_8613\_cov\_31.659817 2738-2755. Max. coverage (+): 4.76. Max coverage (-): 1.98

Region: NODE\_382493\_length\_8613\_cov\_31.659817 2756-2772. Max. coverage (+): 11.14. Max coverage (-): 0.28

Region: NODE\_382493\_length\_8613\_cov\_31.659817 2773-2790. Max. coverage (+): 30.84. Max coverage (-): 0.4

Region: NODE\_382493\_length\_8613\_cov\_31.659817 2791-2807. Max. coverage (+): 30.74. Max coverage (-): 1.62

Region: NODE\_382493\_length\_8613\_cov\_31.659817 2808-2825. Max. coverage (+): 3.43. Max coverage (-): 0.16

Region: NODE\_382493\_length\_8613\_cov\_31.659817 2826-2842. Max. coverage (+): 1.37. Max coverage (-): 1.09

Region: NODE\_382493\_length\_8613\_cov\_31.659817 2843-2860. Max. coverage (+): 5.77. Max coverage (-): 0.2

Region: NODE\_382493\_length\_8613\_cov\_31.659817 2861-2877. Max. coverage (+): 0.59. Max coverage (-): 0.04

Region: NODE\_382493\_length\_8613\_cov\_31.659817 2878-2895. Max. coverage (+): 2.23. Max coverage (-): 0.05

Region: NODE\_382493\_length\_8613\_cov\_31.659817 2896-2912. Max. coverage (+): 3.41. Max coverage (-): 0.09

Region: NODE\_382493\_length\_8613\_cov\_31.659817 2913-2930. Max. coverage (+): 1.7. Max coverage (-): 0.57

Region: NODE\_382493\_length\_8613\_cov\_31.659817 2931-2947. Max. coverage (+): 16.58. Max coverage (-): 0.5

Region: NODE\_382493\_length\_8613\_cov\_31.659817 2948-2965. Max. coverage (+): 281.5. Max coverage (-): 0.4

Region: NODE\_382493\_length\_8613\_cov\_31.659817 2966-2982. Max. coverage (+): 4.12. Max coverage (-): 1.53

Region: NODE\_382493\_length\_8613\_cov\_31.659817 2983-3000. Max. coverage (+): 2.02. Max coverage (-): 1.29

Region: NODE\_382493\_length\_8613\_cov\_31.659817 3001-3017. Max. coverage (+): 3.19. Max coverage (-): 0.77

Region: NODE\_382493\_length\_8613\_cov\_31.659817 3018-3035. Max. coverage (+): 4.72. Max coverage (-): 0.16

Region: NODE\_382493\_length\_8613\_cov\_31.659817 3036-3052. Max. coverage (+): 0.28. Max coverage (-): 0.44

Region: NODE\_382493\_length\_8613\_cov\_31.659817 3053-3070. Max. coverage (+): 3.92. Max coverage (-): 0.4

Region: NODE\_382493\_length\_8613\_cov\_31.659817 3071-3087. Max. coverage (+): 4.55. Max coverage (-): 0.08

Region: NODE\_382493\_length\_8613\_cov\_31.659817 3088-3104. Max. coverage (+): 2.27. Max coverage (-): 0.04

Region: NODE\_382493\_length\_8613\_cov\_31.659817 3105-3122. Max. coverage (+): 16.96. Max coverage (-): 0.12

Region: NODE\_382493\_length\_8613\_cov\_31.659817 3123-3139. Max. coverage (+): 22.41. Max coverage (-): 0.28

Region: NODE\_382493\_length\_8613\_cov\_31.659817 3140-3157. Max. coverage (+): 5.49. Max coverage (-): 0.85

Region: NODE\_382493\_length\_8613\_cov\_31.659817 3158-3174. Max. coverage (+): 369.5. Max coverage (-): 0.28

Region: NODE\_382493\_length\_8613\_cov\_31.659817 3175-3192. Max. coverage (+): 16.92. Max coverage (-): 0.52

Region: NODE\_382493\_length\_8613\_cov\_31.659817 3193-3209. Max. coverage (+): 8.44. Max coverage (-): 0.65

Region: NODE\_382493\_length\_8613\_cov\_31.659817 3210-3227. Max. coverage (+): 9.85. Max coverage (-): 4.4

Region: NODE\_382493\_length\_8613\_cov\_31.659817 3228-3244. Max. coverage (+): 9.17. Max coverage (-): 5.77

Region: NODE\_382493\_length\_8613\_cov\_31.659817 3245-3262. Max. coverage (+): 56.97. Max coverage (-): 4.81

Region: NODE\_382493\_length\_8613\_cov\_31.659817 3263-3279. Max. coverage (+): 7.39. Max coverage (-): 0.89

Region: NODE\_382493\_length\_8613\_cov\_31.659817 3280-3297. Max. coverage (+): 8.96. Max coverage (-): 0.81

Region: NODE\_382493\_length\_8613\_cov\_31.659817 3298-3314. Max. coverage (+): 0.77. Max coverage (-): 0.2

Region: NODE\_382493\_length\_8613\_cov\_31.659817 3315-3332. Max. coverage (+): 0.04. Max coverage (-): 0.12

Region: NODE\_382493\_length\_8613\_cov\_31.659817 3333-3349. Max. coverage (+): 11.59. Max coverage (-): 0.08

Region: NODE\_382493\_length\_8613\_cov\_31.659817 3350-3367. Max. coverage (+): 7.23. Max coverage (-): 0.38

Region: NODE\_382493\_length\_8613\_cov\_31.659817 3368-3384. Max. coverage (+): 62.63. Max coverage (-): 0.4

Region: NODE\_382493\_length\_8613\_cov\_31.659817 3385-3402. Max. coverage (+): 3.88. Max coverage (-): 0.12

Region: NODE\_382493\_length\_8613\_cov\_31.659817 3403-3419. Max. coverage (+): 208.35. Max coverage (-): 5.57

Region: NODE\_382493\_length\_8613\_cov\_31.659817 3420-3437. Max. coverage (+): 70.1. Max coverage (-): 0.65

Region: NODE\_382493\_length\_8613\_cov\_31.659817 3438-3454. Max. coverage (+): 5.81. Max coverage (-): 0.61

Region: NODE\_382493\_length\_8613\_cov\_31.659817 3455-3471. Max. coverage (+): 0.97. Max coverage (-): 0.52

Region: NODE\_382493\_length\_8613\_cov\_31.659817 3472-3489. Max. coverage (+): 45.14. Max coverage (-): 0.48

Region: NODE\_382493\_length\_8613\_cov\_31.659817 3490-3506. Max. coverage (+): 2.18. Max coverage (-): 0.4

Region: NODE\_382493\_length\_8613\_cov\_31.659817 3507-3524. Max. coverage (+): 3.65. Max coverage (-): 1.17

Region: NODE\_382493\_length\_8613\_cov\_31.659817 3525-3541. Max. coverage (+): 0.4. Max coverage (-): 0.12

Region: NODE\_382493\_length\_8613\_cov\_31.659817 3542-3559. Max. coverage (+): 0.48. Max coverage (-): 0

Region: NODE\_382493\_length\_8613\_cov\_31.659817 3560-3576. Max. coverage (+): 0.73. Max coverage (-): 0

Region: NODE\_382493\_length\_8613\_cov\_31.659817 3577-3594. Max. coverage (+): 0.08. Max coverage (-): 0

Region: NODE\_382493\_length\_8613\_cov\_31.659817 3595-3611. Max. coverage (+): 2.62. Max coverage (-): 0

Region: NODE\_382493\_length\_8613\_cov\_31.659817 3612-3629. Max. coverage (+): 2.34. Max coverage (-): 2.1

Region: NODE\_382493\_length\_8613\_cov\_31.659817 3630-3646. Max. coverage (+): 60.24. Max coverage (-): 0.08

Region: NODE\_382493\_length\_8613\_cov\_31.659817 3647-3664. Max. coverage (+): 22.73. Max coverage (-): 0

Region: NODE\_382493\_length\_8613\_cov\_31.659817 3665-3681. Max. coverage (+): 12.72. Max coverage (-): 0.12

Region: NODE\_382493\_length\_8613\_cov\_31.659817 3682-3699. Max. coverage (+): 0.97. Max coverage (-): 0.69

Region: NODE\_382493\_length\_8613\_cov\_31.659817 3700-3716. Max. coverage (+): 25.84. Max coverage (-): 0.24

Region: NODE\_382493\_length\_8613\_cov\_31.659817 3717-3734. Max. coverage (+): 16.96. Max coverage (-): 0.69

Region: NODE\_382493\_length\_8613\_cov\_31.659817 3735-3751. Max. coverage (+): 8.24. Max coverage (-): 0.28

Region: NODE\_382493\_length\_8613\_cov\_31.659817 3752-3769. Max. coverage (+): 8.28. Max coverage (-): 0.08

Region: NODE\_382493\_length\_8613\_cov\_31.659817 3770-3786. Max. coverage (+): 9.29. Max coverage (-): 0

Region: NODE\_382493\_length\_8613\_cov\_31.659817 3787-3804. Max. coverage (+): 1.05. Max coverage (-): 2.3

Region: NODE\_382493\_length\_8613\_cov\_31.659817 3805-3821. Max. coverage (+): 41.79. Max coverage (-): 0.16

Region: NODE\_382493\_length\_8613\_cov\_31.659817 3822-3838. Max. coverage (+): 0.48. Max coverage (-): 3.15

Region: NODE\_382493\_length\_8613\_cov\_31.659817 3839-3856. Max. coverage (+): 10.14. Max coverage (-): 0.16

Region: NODE\_382493\_length\_8613\_cov\_31.659817 3857-3873. Max. coverage (+): 5.09. Max coverage (-): 0.12

Region: NODE\_382493\_length\_8613\_cov\_31.659817 3874-3891. Max. coverage (+): 1.62. Max coverage (-): 0.04

Region: NODE\_382493\_length\_8613\_cov\_31.659817 3892-3908. Max. coverage (+): 17.04. Max coverage (-): 0.2

Region: NODE\_382493\_length\_8613\_cov\_31.659817 3909-3926. Max. coverage (+): 32.34. Max coverage (-): 1.94

Region: NODE\_382493\_length\_8613\_cov\_31.659817 3927-3943. Max. coverage (+): 4.08. Max coverage (-): 2.06

Region: NODE\_382493\_length\_8613\_cov\_31.659817 3944-3961. Max. coverage (+): 10.3. Max coverage (-): 0.24

Region: NODE\_382493\_length\_8613\_cov\_31.659817 3962-3978. Max. coverage (+): 9.81. Max coverage (-): 11.47

Region: NODE\_382493\_length\_8613\_cov\_31.659817 3979-3996. Max. coverage (+): 13.73. Max coverage (-): 0.57

Region: NODE\_382493\_length\_8613\_cov\_31.659817 3997-4013. Max. coverage (+): 15.34. Max coverage (-): 0.04

Region: NODE\_382493\_length\_8613\_cov\_31.659817 4014-4031. Max. coverage (+): 6.3. Max coverage (-): 0.77

Region: NODE\_382493\_length\_8613\_cov\_31.659817 4032-4048. Max. coverage (+): 8.4. Max coverage (-): 0.52

Region: NODE\_382493\_length\_8613\_cov\_31.659817 4049-4066. Max. coverage (+): 1.13. Max coverage (-): 14.37

Region: NODE\_382493\_length\_8613\_cov\_31.659817 4067-4083. Max. coverage (+): 1.01. Max coverage (-): 0.4

Region: NODE\_382493\_length\_8613\_cov\_31.659817 4084-4101. Max. coverage (+): 0.36. Max coverage (-): 12.07

Region: NODE\_382493\_length\_8613\_cov\_31.659817 4102-4118. Max. coverage (+): 8.92. Max coverage (-): 0

Region: NODE\_382493\_length\_8613\_cov\_31.659817 4119-4136. Max. coverage (+): 0.44. Max coverage (-): 0.48

Region: NODE\_382493\_length\_8613\_cov\_31.659817 4137-4153. Max. coverage (+): 44.19. Max coverage (-): 0.04

Region: NODE\_382493\_length\_8613\_cov\_31.659817 4154-4171. Max. coverage (+): 4.52. Max coverage (-): 0.2

Region: NODE\_382493\_length\_8613\_cov\_31.659817 4172-4188. Max. coverage (+): 8.88. Max coverage (-): 0.44

Region: NODE\_382493\_length\_8613\_cov\_31.659817 4189-4205. Max. coverage (+): 2.75. Max coverage (-): 0.28

Region: NODE\_382493\_length\_8613\_cov\_31.659817 4206-4223. Max. coverage (+): 3.23. Max coverage (-): 0.12

Region: NODE\_382493\_length\_8613\_cov\_31.659817 4224-4240. Max. coverage (+): 1.9. Max coverage (-): 0.32

Region: NODE\_382493\_length\_8613\_cov\_31.659817 4241-4258. Max. coverage (+): 11.59. Max coverage (-): 0.65

Region: NODE\_382493\_length\_8613\_cov\_31.659817 4259-4275. Max. coverage (+): 63.31. Max coverage (-): 0.77

Region: NODE\_382493\_length\_8613\_cov\_31.659817 4276-4293. Max. coverage (+): 22.41. Max coverage (-): 0.77

Region: NODE\_382493\_length\_8613\_cov\_31.659817 4294-4310. Max. coverage (+): 13.97. Max coverage (-): 3.76

Region: NODE\_382493\_length\_8613\_cov\_31.659817 4311-4328. Max. coverage (+): 16.37. Max coverage (-): 0.24

Region: NODE\_382493\_length\_8613\_cov\_31.659817 4329-4345. Max. coverage (+): 9.49. Max coverage (-): 0.17

Region: NODE\_382493\_length\_8613\_cov\_31.659817 4346-4363. Max. coverage (+): 3.39. Max coverage (-): 3.15

Region: NODE\_382493\_length\_8613\_cov\_31.659817 4364-4380. Max. coverage (+): 2.75. Max coverage (-): 0.65

Region: NODE\_382493\_length\_8613\_cov\_31.659817 4381-4398. Max. coverage (+): 1.57. Max coverage (-): 6.3

Region: NODE\_382493\_length\_8613\_cov\_31.659817 4399-4415. Max. coverage (+): 32.59. Max coverage (-): 4.85

Region: NODE\_382493\_length\_8613\_cov\_31.659817 4416-4433. Max. coverage (+): 1.92. Max coverage (-): 1.8

Region: NODE\_382493\_length\_8613\_cov\_31.659817 4434-4450. Max. coverage (+): 3.8. Max coverage (-): 0.1

Region: NODE\_382493\_length\_8613\_cov\_31.659817 4451-4468. Max. coverage (+): 7.31. Max coverage (-): 1.17

Region: NODE\_382493\_length\_8613\_cov\_31.659817 4469-4485. Max. coverage (+): 11.47. Max coverage (-): 0.44

Region: NODE\_382493\_length\_8613\_cov\_31.659817 4486-4503. Max. coverage (+): 2.87. Max coverage (-): 0.14

Region: NODE\_382493\_length\_8613\_cov\_31.659817 4504-4520. Max. coverage (+): 4.28. Max coverage (-): 0.12

Region: NODE\_382493\_length\_8613\_cov\_31.659817 4521-4538. Max. coverage (+): 7.59. Max coverage (-): 0

Region: NODE\_382493\_length\_8613\_cov\_31.659817 4539-4555. Max. coverage (+): 6.3. Max coverage (-): 0.04

Region: NODE\_382493\_length\_8613\_cov\_31.659817 4556-4572. Max. coverage (+): 6.58. Max coverage (-): 0.61

Region: NODE\_382493\_length\_8613\_cov\_31.659817 4573-4590. Max. coverage (+): 65.49. Max coverage (-): 0.08

Region: NODE\_382493\_length\_8613\_cov\_31.659817 4591-4607. Max. coverage (+): 2.38. Max coverage (-): 0.2

Region: NODE\_382493\_length\_8613\_cov\_31.659817 4608-4625. Max. coverage (+): 5.02. Max coverage (-): 0.02

Region: NODE\_382493\_length\_8613\_cov\_31.659817 4626-4642. Max. coverage (+): 3.88. Max coverage (-): 0.48

Region: NODE\_382493\_length\_8613\_cov\_31.659817 4643-4660. Max. coverage (+): 5.13. Max coverage (-): 0.12

Region: NODE\_382493\_length\_8613\_cov\_31.659817 4661-4677. Max. coverage (+): 6.86. Max coverage (-): 0.16

Region: NODE\_382493\_length\_8613\_cov\_31.659817 4678-4695. Max. coverage (+): 1.29. Max coverage (-): 1.37

Region: NODE\_382493\_length\_8613\_cov\_31.659817 4696-4712. Max. coverage (+): 1.13. Max coverage (-): 1.98

Region: NODE\_382493\_length\_8613\_cov\_31.659817 4713-4730. Max. coverage (+): 0.81. Max coverage (-): 0.57

Region: NODE\_382493\_length\_8613\_cov\_31.659817 4731-4747. Max. coverage (+): 0.97. Max coverage (-): 0.52

Region: NODE\_382493\_length\_8613\_cov\_31.659817 4748-4765. Max. coverage (+): 79.02. Max coverage (-): 3.11

Region: NODE\_382493\_length\_8613\_cov\_31.659817 4766-4782. Max. coverage (+): 37.75. Max coverage (-): 1.53

Region: NODE\_382493\_length\_8613\_cov\_31.659817 4783-4800. Max. coverage (+): 1.05. Max coverage (-): 0

Region: NODE\_382493\_length\_8613\_cov\_31.659817 4801-4817. Max. coverage (+): 0.57. Max coverage (-): 0.08

Region: NODE\_382493\_length\_8613\_cov\_31.659817 4818-4835. Max. coverage (+): 0.57. Max coverage (-): 0.16

Region: NODE\_382493\_length\_8613\_cov\_31.659817 4836-4852. Max. coverage (+): 0.24. Max coverage (-): 0.04

Region: NODE\_382493\_length\_8613\_cov\_31.659817 4853-4870. Max. coverage (+): 5.25. Max coverage (-): 0.04

Region: NODE\_382493\_length\_8613\_cov\_31.659817 4871-4887. Max. coverage (+): 0.85. Max coverage (-): 0.32

Region: NODE\_382493\_length\_8613\_cov\_31.659817 4888-4905. Max. coverage (+): 1.86. Max coverage (-): 0.16

Region: NODE\_382493\_length\_8613\_cov\_31.659817 4906-4922. Max. coverage (+): 7.23. Max coverage (-): 0.16

Region: NODE\_382493\_length\_8613\_cov\_31.659817 4923-4939. Max. coverage (+): 0.04. Max coverage (-): 0.12

Region: NODE\_382493\_length\_8613\_cov\_31.659817 4940-4957. Max. coverage (+): 5.13. Max coverage (-): 0.44

Region: NODE\_382493\_length\_8613\_cov\_31.659817 4958-4974. Max. coverage (+): 3.15. Max coverage (-): 0.04

Region: NODE\_382493\_length\_8613\_cov\_31.659817 4975-4992. Max. coverage (+): 1.82. Max coverage (-): 0.89

Region: NODE\_382493\_length\_8613\_cov\_31.659817 4993-5009. Max. coverage (+): 62.83. Max coverage (-): 0.52

Region: NODE\_382493\_length\_8613\_cov\_31.659817 5010-5027. Max. coverage (+): 18.01. Max coverage (-): 0.08

Region: NODE\_382493\_length\_8613\_cov\_31.659817 5028-5044. Max. coverage (+): 15.67. Max coverage (-): 3.96

Region: NODE\_382493\_length\_8613\_cov\_31.659817 5045-5062. Max. coverage (+): 1.41. Max coverage (-): 2.79

Region: NODE\_382493\_length\_8613\_cov\_31.659817 5063-5079. Max. coverage (+): 1.17. Max coverage (-): 0.16

Region: NODE\_382493\_length\_8613\_cov\_31.659817 5080-5097. Max. coverage (+): 0.48. Max coverage (-): 1.01

Region: NODE\_382493\_length\_8613\_cov\_31.659817 5098-5114. Max. coverage (+): 2.99. Max coverage (-): 0.04

Region: NODE\_382493\_length\_8613\_cov\_31.659817 5115-5132. Max. coverage (+): 0.85. Max coverage (-): 0.24

Region: NODE\_382493\_length\_8613\_cov\_31.659817 5133-5149. Max. coverage (+): 7.27. Max coverage (-): 0.24

Region: NODE\_382493\_length\_8613\_cov\_31.659817 5150-5167. Max. coverage (+): 6.02. Max coverage (-): 0.61

Region: NODE\_382493\_length\_8613\_cov\_31.659817 5168-5184. Max. coverage (+): 3.11. Max coverage (-): 1.01

Region: NODE\_382493\_length\_8613\_cov\_31.659817 5185-5202. Max. coverage (+): 1.82. Max coverage (-): 0

Region: NODE\_382493\_length\_8613\_cov\_31.659817 5203-5219. Max. coverage (+): 0.73. Max coverage (-): 2.06

Region: NODE\_382493\_length\_8613\_cov\_31.659817 5220-5237. Max. coverage (+): 10.3. Max coverage (-): 0.4

Region: NODE\_382493\_length\_8613\_cov\_31.659817 5238-5254. Max. coverage (+): 0.28. Max coverage (-): 0.77

Region: NODE\_382493\_length\_8613\_cov\_31.659817 5255-5272. Max. coverage (+): 0.28. Max coverage (-): 0.24

Region: NODE\_382493\_length\_8613\_cov\_31.659817 5273-5289. Max. coverage (+): 0.12. Max coverage (-): 3.23

Region: NODE\_382493\_length\_8613\_cov\_31.659817 5290-5306. Max. coverage (+): 0.61. Max coverage (-): 0.12

Region: NODE\_382493\_length\_8613\_cov\_31.659817 5307-5324. Max. coverage (+): 0.52. Max coverage (-): 0.48

Region: NODE\_382493\_length\_8613\_cov\_31.659817 5325-5341. Max. coverage (+): 1.62. Max coverage (-): 0.16

Region: NODE\_382493\_length\_8613\_cov\_31.659817 5342-5359. Max. coverage (+): 14.05. Max coverage (-): 1.57

Region: NODE\_382493\_length\_8613\_cov\_31.659817 5360-5376. Max. coverage (+): 21.89. Max coverage (-): 0.2

Region: NODE\_382493\_length\_8613\_cov\_31.659817 5377-5394. Max. coverage (+): 1.37. Max coverage (-): 0.2

Region: NODE\_382493\_length\_8613\_cov\_31.659817 5395-5411. Max. coverage (+): 0.4. Max coverage (-): 0.12

Region: NODE\_382493\_length\_8613\_cov\_31.659817 5412-5429. Max. coverage (+): 227.09. Max coverage (-): 0.16

Region: NODE\_382493\_length\_8613\_cov\_31.659817 5430-5446. Max. coverage (+): 2.22. Max coverage (-): 6.95

Region: NODE\_382493\_length\_8613\_cov\_31.659817 5447-5464. Max. coverage (+): 0.61. Max coverage (-): 0.16

Region: NODE\_382493\_length\_8613\_cov\_31.659817 5465-5481. Max. coverage (+): 0.44. Max coverage (-): 0.65

Region: NODE\_382493\_length\_8613\_cov\_31.659817 5482-5499. Max. coverage (+): 0.2. Max coverage (-): 0.04

Region: NODE\_382493\_length\_8613\_cov\_31.659817 5500-5516. Max. coverage (+): 0.32. Max coverage (-): 4

Region: NODE\_382493\_length\_8613\_cov\_31.659817 5517-5534. Max. coverage (+): 1.05. Max coverage (-): 0.16

Region: NODE\_382493\_length\_8613\_cov\_31.659817 5535-5551. Max. coverage (+): 1.7. Max coverage (-): 0.32

Region: NODE\_382493\_length\_8613\_cov\_31.659817 5552-5569. Max. coverage (+): 36.06. Max coverage (-): 0.12

Region: NODE\_382493\_length\_8613\_cov\_31.659817 5570-5586. Max. coverage (+): 7.95. Max coverage (-): 0.4

Region: NODE\_382493\_length\_8613\_cov\_31.659817 5587-5604. Max. coverage (+): 2.54. Max coverage (-): 0.16

Region: NODE\_382493\_length\_8613\_cov\_31.659817 5605-5621. Max. coverage (+): 0.28. Max coverage (-): 0.08

Region: NODE\_382493\_length\_8613\_cov\_31.659817 5622-5639. Max. coverage (+): 1.33. Max coverage (-): 0.08

Region: NODE\_382493\_length\_8613\_cov\_31.659817 5640-5656. Max. coverage (+): 1.17. Max coverage (-): 0.12

Region: NODE\_382493\_length\_8613\_cov\_31.659817 5657-5673. Max. coverage (+): 3.63. Max coverage (-): 0.12

Region: NODE\_382493\_length\_8613\_cov\_31.659817 5674-5691. Max. coverage (+): 13. Max coverage (-): 1.57

Region: NODE\_382493\_length\_8613\_cov\_31.659817 5692-5708. Max. coverage (+): 24.31. Max coverage (-): 0.24

Region: NODE\_382493\_length\_8613\_cov\_31.659817 5709-5726. Max. coverage (+): 20.15. Max coverage (-): 0.28

Region: NODE\_382493\_length\_8613\_cov\_31.659817 5727-5743. Max. coverage (+): 0.57. Max coverage (-): 0.2

Region: NODE\_382493\_length\_8613\_cov\_31.659817 5744-5761. Max. coverage (+): 0.57. Max coverage (-): 1.86

Region: NODE\_382493\_length\_8613\_cov\_31.659817 5762-5778. Max. coverage (+): 3.27. Max coverage (-): 0.44

Region: NODE\_382493\_length\_8613\_cov\_31.659817 5779-5796. Max. coverage (+): 1.01. Max coverage (-): 0.2

Region: NODE\_382493\_length\_8613\_cov\_31.659817 5797-5813. Max. coverage (+): 1.29. Max coverage (-): 0.48

Region: NODE\_382493\_length\_8613\_cov\_31.659817 5814-5831. Max. coverage (+): 2.71. Max coverage (-): 0.77

Region: NODE\_382493\_length\_8613\_cov\_31.659817 5832-5848. Max. coverage (+): 3.96. Max coverage (-): 0.08

Region: NODE\_382493\_length\_8613\_cov\_31.659817 5849-5866. Max. coverage (+): 4.97. Max coverage (-): 0.28

Region: NODE\_382493\_length\_8613\_cov\_31.659817 5867-5883. Max. coverage (+): 41.55. Max coverage (-): 0.04

Region: NODE\_382493\_length\_8613\_cov\_31.659817 5884-5901. Max. coverage (+): 0.4. Max coverage (-): 0.36

Region: NODE\_382493\_length\_8613\_cov\_31.659817 5902-5918. Max. coverage (+): 8.76. Max coverage (-): 0.32

Region: NODE\_382493\_length\_8613\_cov\_31.659817 5919-5936. Max. coverage (+): 3.23. Max coverage (-): 1.7

Region: NODE\_382493\_length\_8613\_cov\_31.659817 5937-5953. Max. coverage (+): 5.61. Max coverage (-): 0.77

Region: NODE\_382493\_length\_8613\_cov\_31.659817 5954-5971. Max. coverage (+): 0.97. Max coverage (-): 0.69

Region: NODE\_382493\_length\_8613\_cov\_31.659817 5972-5988. Max. coverage (+): 0.93. Max coverage (-): 0.4

Region: NODE\_382493\_length\_8613\_cov\_31.659817 5989-6006. Max. coverage (+): 1.86. Max coverage (-): 0.16

Region: NODE\_382493\_length\_8613\_cov\_31.659817 6007-6023. Max. coverage (+): 3.43. Max coverage (-): 0.24

Region: NODE\_382493\_length\_8613\_cov\_31.659817 6024-6040. Max. coverage (+): 1.68. Max coverage (-): 0.12

Region: NODE\_382493\_length\_8613\_cov\_31.659817 6041-6058. Max. coverage (+): 2.62. Max coverage (-): 0.2

Region: NODE\_382493\_length\_8613\_cov\_31.659817 6059-6075. Max. coverage (+): 0.89. Max coverage (-): 0.12

Region: NODE\_382493\_length\_8613\_cov\_31.659817 6076-6093. Max. coverage (+): 0.44. Max coverage (-): 0.77

Region: NODE\_382493\_length\_8613\_cov\_31.659817 6094-6110. Max. coverage (+): 12.07. Max coverage (-): 0.73

Region: NODE\_382493\_length\_8613\_cov\_31.659817 6111-6128. Max. coverage (+): 11.47. Max coverage (-): 0.04

Region: NODE\_382493\_length\_8613\_cov\_31.659817 6129-6145. Max. coverage (+): 0.61. Max coverage (-): 0.04

Region: NODE\_382493\_length\_8613\_cov\_31.659817 6146-6163. Max. coverage (+): 0.04. Max coverage (-): 0

Region: NODE\_382493\_length\_8613\_cov\_31.659817 6164-6180. Max. coverage (+): 0.12. Max coverage (-): 0

Region: NODE\_382493\_length\_8613\_cov\_31.659817 6181-6198. Max. coverage (+): 0.08. Max coverage (-): 0

Region: NODE\_382493\_length\_8613\_cov\_31.659817 6199-6215. Max. coverage (+): 0.16. Max coverage (-): 0.2

Region: NODE\_382493\_length\_8613\_cov\_31.659817 6216-6233. Max. coverage (+): 0.65. Max coverage (-): 0.2

Region: NODE\_382493\_length\_8613\_cov\_31.659817 6234-6250. Max. coverage (+): 0.08. Max coverage (-): 0.04

Region: NODE\_382493\_length\_8613\_cov\_31.659817 6251-6268. Max. coverage (+): 0.28. Max coverage (-): 0.04

Region: NODE\_382493\_length\_8613\_cov\_31.659817 6269-6285. Max. coverage (+): 0.65. Max coverage (-): 0.36

Region: NODE\_382493\_length\_8613\_cov\_31.659817 6286-6303. Max. coverage (+): 1.82. Max coverage (-): 0.12

Region: NODE\_382493\_length\_8613\_cov\_31.659817 6304-6320. Max. coverage (+): 2.18. Max coverage (-): 0.12

Region: NODE\_382493\_length\_8613\_cov\_31.659817 6321-6338. Max. coverage (+): 2.38. Max coverage (-): 2.46

Region: NODE\_382493\_length\_8613\_cov\_31.659817 6339-6355. Max. coverage (+): 42.56. Max coverage (-): 1.25

Region: NODE\_382493\_length\_8613\_cov\_31.659817 6356-6373. Max. coverage (+): 0.08. Max coverage (-): 0.69

Region: NODE\_382493\_length\_8613\_cov\_31.659817 6374-6390. Max. coverage (+): 0.12. Max coverage (-): 0.04

Region: NODE\_382493\_length\_8613\_cov\_31.659817 6391-6407. Max. coverage (+): 0. Max coverage (-): 0.04

Region: NODE\_382493\_length\_8613\_cov\_31.659817 6408-6425. Max. coverage (+): 3.55. Max coverage (-): 0.28

Region: NODE\_382493\_length\_8613\_cov\_31.659817 6426-6442. Max. coverage (+): 3.47. Max coverage (-): 0

Region: NODE\_382493\_length\_8613\_cov\_31.659817 6443-6460. Max. coverage (+): 26.45. Max coverage (-): 0.08

Region: NODE\_382493\_length\_8613\_cov\_31.659817 6461-6477. Max. coverage (+): 37.11. Max coverage (-): 0.08

Region: NODE\_382493\_length\_8613\_cov\_31.659817 6478-6495. Max. coverage (+): 0.2. Max coverage (-): 0

Region: NODE\_382493\_length\_8613\_cov\_31.659817 6496-6512. Max. coverage (+): 0.12. Max coverage (-): 0.2

Region: NODE\_382493\_length\_8613\_cov\_31.659817 6513-6530. Max. coverage (+): 3.55. Max coverage (-): 0.04

Region: NODE\_382493\_length\_8613\_cov\_31.659817 6531-6547. Max. coverage (+): 0.57. Max coverage (-): 0.12

Region: NODE\_382493\_length\_8613\_cov\_31.659817 6548-6565. Max. coverage (+): 0.85. Max coverage (-): 0.16

Region: NODE\_382493\_length\_8613\_cov\_31.659817 6566-6582. Max. coverage (+): 1.17. Max coverage (-): 0.08

Region: NODE\_382493\_length\_8613\_cov\_31.659817 6583-6600. Max. coverage (+): 13.73. Max coverage (-): 0.04

Region: NODE\_382493\_length\_8613\_cov\_31.659817 6601-6617. Max. coverage (+): 14.62. Max coverage (-): 0.04

Region: NODE\_382493\_length\_8613\_cov\_31.659817 6618-6635. Max. coverage (+): 0.69. Max coverage (-): 0

Region: NODE\_382493\_length\_8613\_cov\_31.659817 6636-6652. Max. coverage (+): 4.56. Max coverage (-): 0

Region: NODE\_382493\_length\_8613\_cov\_31.659817 6653-6670. Max. coverage (+): 0.16. Max coverage (-): 0.44

Region: NODE\_382493\_length\_8613\_cov\_31.659817 6671-6687. Max. coverage (+): 0.16. Max coverage (-): 0.08

Region: NODE\_382493\_length\_8613\_cov\_31.659817 6688-6705. Max. coverage (+): 0.83. Max coverage (-): 0

Region: NODE\_382493\_length\_8613\_cov\_31.659817 6706-6722. Max. coverage (+): 0.61. Max coverage (-): 0

Region: NODE\_382493\_length\_8613\_cov\_31.659817 6723-6739. Max. coverage (+): 0.32. Max coverage (-): 0.12

Region: NODE\_382493\_length\_8613\_cov\_31.659817 6740-6757. Max. coverage (+): 0.28. Max coverage (-): 0.08

Region: NODE\_382493\_length\_8613\_cov\_31.659817 6758-6774. Max. coverage (+): 1.86. Max coverage (-): 0.36

Region: NODE\_382493\_length\_8613\_cov\_31.659817 6775-6792. Max. coverage (+): 0.4. Max coverage (-): 0.28

Region: NODE\_382493\_length\_8613\_cov\_31.659817 6793-6809. Max. coverage (+): 6.99. Max coverage (-): 0.61

Region: NODE\_382493\_length\_8613\_cov\_31.659817 6810-6827. Max. coverage (+): 4.2. Max coverage (-): 0.57

Region: NODE\_382493\_length\_8613\_cov\_31.659817 6828-6844. Max. coverage (+): 1.37. Max coverage (-): 0.04

Region: NODE\_382493\_length\_8613\_cov\_31.659817 6845-6862. Max. coverage (+): 0.32. Max coverage (-): 0.28

Region: NODE\_382493\_length\_8613\_cov\_31.659817 6863-6879. Max. coverage (+): 3.39. Max coverage (-): 0.2

Region: NODE\_382493\_length\_8613\_cov\_31.659817 6880-6897. Max. coverage (+): 0.4. Max coverage (-): 0.08

Region: NODE\_382493\_length\_8613\_cov\_31.659817 6898-6914. Max. coverage (+): 0.08. Max coverage (-): 0.28

Region: NODE\_382493\_length\_8613\_cov\_31.659817 6915-6932. Max. coverage (+): 0.08. Max coverage (-): 0.08

Region: NODE\_382493\_length\_8613\_cov\_31.659817 6933-6949. Max. coverage (+): 2.1. Max coverage (-): 0.06

Region: NODE\_382493\_length\_8613\_cov\_31.659817 6950-6967. Max. coverage (+): 16.88. Max coverage (-): 0.1

Region: NODE\_382493\_length\_8613\_cov\_31.659817 6968-6984. Max. coverage (+): 0.2. Max coverage (-): 0

Region: NODE\_382493\_length\_8613\_cov\_31.659817 6985-7002. Max. coverage (+): 1.47. Max coverage (-): 0.02

Region: NODE\_382493\_length\_8613\_cov\_31.659817 7003-7019. Max. coverage (+): 0.69. Max coverage (-): 0.2

Region: NODE\_382493\_length\_8613\_cov\_31.659817 7020-7037. Max. coverage (+): 1.39. Max coverage (-): 0.2

Region: NODE\_382493\_length\_8613\_cov\_31.659817 7038-7054. Max. coverage (+): 0.97. Max coverage (-): 0.52

Region: NODE\_382493\_length\_8613\_cov\_31.659817 7055-7072. Max. coverage (+): 0.57. Max coverage (-): 0.06

Region: NODE\_382493\_length\_8613\_cov\_31.659817 7073-7089. Max. coverage (+): 1.07. Max coverage (-): 0.02

Region: NODE\_382493\_length\_8613\_cov\_31.659817 7090-7106. Max. coverage (+): 2.75. Max coverage (-): 0.2

Region: NODE\_382493\_length\_8613\_cov\_31.659817 7107-7124. Max. coverage (+): 0.36. Max coverage (-): 0.2

Region: NODE\_382493\_length\_8613\_cov\_31.659817 7125-7141. Max. coverage (+): 0.52. Max coverage (-): 0.4

Region: NODE\_382493\_length\_8613\_cov\_31.659817 7142-7159. Max. coverage (+): 0.87. Max coverage (-): 0.16

Region: NODE\_382493\_length\_8613\_cov\_31.659817 7160-7176. Max. coverage (+): 4.89. Max coverage (-): 0.04

Region: NODE\_382493\_length\_8613\_cov\_31.659817 7177-7194. Max. coverage (+): 0.89. Max coverage (-): 0.16

Region: NODE\_382493\_length\_8613\_cov\_31.659817 7195-7211. Max. coverage (+): 0.89. Max coverage (-): 0

Region: NODE\_382493\_length\_8613\_cov\_31.659817 7212-7229. Max. coverage (+): 0.08. Max coverage (-): 0.12

Region: NODE\_382493\_length\_8613\_cov\_31.659817 7230-7246. Max. coverage (+): 2.1. Max coverage (-): 0.04

Region: NODE\_382493\_length\_8613\_cov\_31.659817 7247-7264. Max. coverage (+): 0.16. Max coverage (-): 0.16

Region: NODE\_382493\_length\_8613\_cov\_31.659817 7265-7281. Max. coverage (+): 0.61. Max coverage (-): 0

Region: NODE\_382493\_length\_8613\_cov\_31.659817 7282-7299. Max. coverage (+): 1.62. Max coverage (-): 1.7

Region: NODE\_382493\_length\_8613\_cov\_31.659817 7300-7316. Max. coverage (+): 3.07. Max coverage (-): 1.66

Region: NODE\_382493\_length\_8613\_cov\_31.659817 7317-7334. Max. coverage (+): 0.16. Max coverage (-): 0.73

Region: NODE\_382493\_length\_8613\_cov\_31.659817 7335-7351. Max. coverage (+): 0.16. Max coverage (-): 0.16

Region: NODE\_382493\_length\_8613\_cov\_31.659817 7352-7369. Max. coverage (+): 0.52. Max coverage (-): 0.08

Region: NODE\_382493\_length\_8613\_cov\_31.659817 7370-7386. Max. coverage (+): 1.21. Max coverage (-): 0

Region: NODE\_382493\_length\_8613\_cov\_31.659817 7387-7404. Max. coverage (+): 0.44. Max coverage (-): 5.65

Region: NODE\_382493\_length\_8613\_cov\_31.659817 7405-7421. Max. coverage (+): 0.73. Max coverage (-): 0.16

Region: NODE\_382493\_length\_8613\_cov\_31.659817 7422-7439. Max. coverage (+): 2.34. Max coverage (-): 0.2

Region: NODE\_382493\_length\_8613\_cov\_31.659817 7440-7456. Max. coverage (+): 2.26. Max coverage (-): 0.2

Region: NODE\_382493\_length\_8613\_cov\_31.659817 7457-7473. Max. coverage (+): 0.2. Max coverage (-): 0.93

Region: NODE\_382493\_length\_8613\_cov\_31.659817 7474-7491. Max. coverage (+): 0.4. Max coverage (-): 0.61

Region: NODE\_382493\_length\_8613\_cov\_31.659817 7492-7508. Max. coverage (+): 9.37. Max coverage (-): 0.12

Region: NODE\_382493\_length\_8613\_cov\_31.659817 7509-7526. Max. coverage (+): 3.15. Max coverage (-): 0

Region: NODE\_382493\_length\_8613\_cov\_31.659817 7527-7543. Max. coverage (+): 3.71. Max coverage (-): 0.08

Region: NODE\_382493\_length\_8613\_cov\_31.659817 7544-7561. Max. coverage (+): 13.61. Max coverage (-): 0.24

Region: NODE\_382493\_length\_8613\_cov\_31.659817 7562-7578. Max. coverage (+): 0.4. Max coverage (-): 0.12

Region: NODE\_382493\_length\_8613\_cov\_31.659817 7579-7596. Max. coverage (+): 4.6. Max coverage (-): 1.53

Region: NODE\_382493\_length\_8613\_cov\_31.659817 7597-7613. Max. coverage (+): 9.65. Max coverage (-): 0.36

Region: NODE\_382493\_length\_8613\_cov\_31.659817 7614-7631. Max. coverage (+): 16.23. Max coverage (-): 0.36

Region: NODE\_382493\_length\_8613\_cov\_31.659817 7632-7648. Max. coverage (+): 16.8. Max coverage (-): 0.2

Region: NODE\_382493\_length\_8613\_cov\_31.659817 7649-7666. Max. coverage (+): 25.84. Max coverage (-): 0.2

Region: NODE\_382493\_length\_8613\_cov\_31.659817 7667-7683. Max. coverage (+): 5.77. Max coverage (-): 0.48

Region: NODE\_382493\_length\_8613\_cov\_31.659817 7684-7701. Max. coverage (+): 24.39. Max coverage (-): 0.12

Region: NODE\_382493\_length\_8613\_cov\_31.659817 7702-7718. Max. coverage (+): 2.14. Max coverage (-): 0.32

Region: NODE\_382493\_length\_8613\_cov\_31.659817 7719-7736. Max. coverage (+): 3.59. Max coverage (-): 1.01

Region: NODE\_382493\_length\_8613\_cov\_31.659817 7737-7753. Max. coverage (+): 1.78. Max coverage (-): 0.57

Region: NODE\_382493\_length\_8613\_cov\_31.659817 7754-7771. Max. coverage (+): 3.51. Max coverage (-): 0.65

Region: NODE\_382493\_length\_8613\_cov\_31.659817 7772-7788. Max. coverage (+): 1.37. Max coverage (-): 0.08

Region: NODE\_382493\_length\_8613\_cov\_31.659817 7789-7806. Max. coverage (+): 0.81. Max coverage (-): 0

Region: NODE\_382493\_length\_8613\_cov\_31.659817 7807-7823. Max. coverage (+): 0.04. Max coverage (-): 0.04

Region: NODE\_382493\_length\_8613\_cov\_31.659817 7824-7840. Max. coverage (+): 0.08. Max coverage (-): 0.04

Region: NODE\_382493\_length\_8613\_cov\_31.659817 7841-7858. Max. coverage (+): 0.69. Max coverage (-): 0.04

Region: NODE\_382493\_length\_8613\_cov\_31.659817 7859-7875. Max. coverage (+): 10.05. Max coverage (-): 0.04

Region: NODE\_382493\_length\_8613\_cov\_31.659817 7876-7893. Max. coverage (+): 7.91. Max coverage (-): 0.12

Region: NODE\_382493\_length\_8613\_cov\_31.659817 7894-7910. Max. coverage (+): 0.12. Max coverage (-): 0.44

Region: NODE\_382493\_length\_8613\_cov\_31.659817 7911-7928. Max. coverage (+): 4.32. Max coverage (-): 0.28

Region: NODE\_382493\_length\_8613\_cov\_31.659817 7929-7945. Max. coverage (+): 7.19. Max coverage (-): 0.65

Region: NODE\_382493\_length\_8613\_cov\_31.659817 7946-7963. Max. coverage (+): 0.61. Max coverage (-): 0.77

Region: NODE\_382493\_length\_8613\_cov\_31.659817 7964-7980. Max. coverage (+): 0.77. Max coverage (-): 0.04

Region: NODE\_382493\_length\_8613\_cov\_31.659817 7981-7998. Max. coverage (+): 1.33. Max coverage (-): 0.16

Region: NODE\_382493\_length\_8613\_cov\_31.659817 7999-8015. Max. coverage (+): 8.88. Max coverage (-): 0.12

Region: NODE\_382493\_length\_8613\_cov\_31.659817 8016-8033. Max. coverage (+): 1.9. Max coverage (-): 0

Region: NODE\_382493\_length\_8613\_cov\_31.659817 8034-8050. Max. coverage (+): 0.36. Max coverage (-): 0.16

Region: NODE\_382493\_length\_8613\_cov\_31.659817 8051-8068. Max. coverage (+): 23.98. Max coverage (-): 0.12

Region: NODE\_382493\_length\_8613\_cov\_31.659817 8069-8085. Max. coverage (+): 0.32. Max coverage (-): 0.61

Region: NODE\_382493\_length\_8613\_cov\_31.659817 8086-8103. Max. coverage (+): 31.29. Max coverage (-): 0.04

Region: NODE\_382493\_length\_8613\_cov\_31.659817 8104-8120. Max. coverage (+): 13.77. Max coverage (-): 0.24

Region: NODE\_382493\_length\_8613\_cov\_31.659817 8121-8138. Max. coverage (+): 0.16. Max coverage (-): 0.2

Region: NODE\_382493\_length\_8613\_cov\_31.659817 8139-8155. Max. coverage (+): 1.33. Max coverage (-): 0.81

Region: NODE\_382493\_length\_8613\_cov\_31.659817 8156-8173. Max. coverage (+): 7.47. Max coverage (-): 0.4

Region: NODE\_382493\_length\_8613\_cov\_31.659817 8174-8190. Max. coverage (+): 1.45. Max coverage (-): 0.2

Region: NODE\_382493\_length\_8613\_cov\_31.659817 8191-8207. Max. coverage (+): 1.7. Max coverage (-): 0.16

Region: NODE\_382493\_length\_8613\_cov\_31.659817 8208-8225. Max. coverage (+): 0.81. Max coverage (-): 0.08

Region: NODE\_382493\_length\_8613\_cov\_31.659817 8226-8242. Max. coverage (+): 0.57. Max coverage (-): 0.04

Region: NODE\_382493\_length\_8613\_cov\_31.659817 8243-8260. Max. coverage (+): 0.2. Max coverage (-): 1.09

Region: NODE\_382493\_length\_8613\_cov\_31.659817 8261-8277. Max. coverage (+): 13.41. Max coverage (-): 0.08

Region: NODE\_382493\_length\_8613\_cov\_31.659817 8278-8295. Max. coverage (+): 0.36. Max coverage (-): 1.53

Region: NODE\_382493\_length\_8613\_cov\_31.659817 8296-8312. Max. coverage (+): 4.24. Max coverage (-): 0.12

Region: NODE\_382493\_length\_8613\_cov\_31.659817 8313-8330. Max. coverage (+): 143.95. Max coverage (-): 0.48

Region: NODE\_382493\_length\_8613\_cov\_31.659817 8331-8347. Max. coverage (+): 151.38. Max coverage (-): 0

Region: NODE\_382493\_length\_8613\_cov\_31.659817 8348-8365. Max. coverage (+): 0.08. Max coverage (-): 1.45

Region: NODE\_382493\_length\_8613\_cov\_31.659817 8366-8382. Max. coverage (+): 32.46. Max coverage (-): 0.04

Region: NODE\_382493\_length\_8613\_cov\_31.659817 8383-8400. Max. coverage (+): 0.08. Max coverage (-): 0.28

Region: NODE\_382493\_length\_8613\_cov\_31.659817 8401-8417. Max. coverage (+): 3.15. Max coverage (-): 0

Region: NODE\_382493\_length\_8613\_cov\_31.659817 8418-8435. Max. coverage (+): 0.69. Max coverage (-): 0.89

Region: NODE\_382493\_length\_8613\_cov\_31.659817 8436-8452. Max. coverage (+): 47.44. Max coverage (-): 0.85

Region: NODE\_382493\_length\_8613\_cov\_31.659817 8453-8470. Max. coverage (+): 30.81. Max coverage (-): 1.37

Region: NODE\_382493\_length\_8613\_cov\_31.659817 8471-8487. Max. coverage (+): 11.47. Max coverage (-): 0.12

Region: NODE\_382493\_length\_8613\_cov\_31.659817 8488-8505. Max. coverage (+): 0.69. Max coverage (-): 0.16

Region: NODE\_382493\_length\_8613\_cov\_31.659817 8506-8522. Max. coverage (+): 1.45. Max coverage (-): 0.04

Region: NODE\_382493\_length\_8613\_cov\_31.659817 8523-8540. Max. coverage (+): 5.65. Max coverage (-): 1.78

Region: NODE\_382493\_length\_8613\_cov\_31.659817 8541-8557. Max. coverage (+): 1.25. Max coverage (-): 0.12

Region: NODE\_382493\_length\_8613\_cov\_31.659817 8558-8574. Max. coverage (+): 0.32. Max coverage (-): 0.52

Region: NODE\_382493\_length\_8613\_cov\_31.659817 8575-8592. Max. coverage (+): 28.71. Max coverage (-): 1.29

Region: NODE\_382493\_length\_8613\_cov\_31.659817 8593-8609. Max. coverage (+): 2.99. Max coverage (-): 3.31

Region: NODE\_382493\_length\_8613\_cov\_31.659817 8610-8627. Max. coverage (+): 2.38. Max coverage (-): 0.12

Region: NODE\_382493\_length\_8613\_cov\_31.659817 8628-8644. Max. coverage (+): 1.05. Max coverage (-): 0.04

Region: NODE\_382493\_length\_8613\_cov\_31.659817 8645-8662. Max. coverage (+): 0.61. Max coverage (-): 0

Region: NODE\_382493\_length\_8613\_cov\_31.659817 8663-8679. Max. coverage (+): 0.44. Max coverage (-): 0.04

Region: NODE\_382493\_length\_8613\_cov\_31.659817 8680-8697. Max. coverage (+): 0.16. Max coverage (-): 0.02

Region: NODE\_382493\_length\_8613\_cov\_31.659817 8698-8714. Max. coverage (+): 0.01. Max coverage (-): 0

Region: NODE\_382493\_length\_8613\_cov\_31.659817 8715-8732. Max. coverage (+): 0. Max coverage (-): 0

Region: NODE\_382493\_length\_8613\_cov\_31.659817 8733-. Max. coverage (+): 0. Max coverage (-): 0

RepeatMasker Color Code

**+**

100-98% Identity

<98-95% Identity

<95-90% Identity

<90-85% Identity

<85-80% Identity

<80-75% Identity

<75-70% Identity

<70% Identity

**-**

Gene Set Color Code

**+**

Gene

Pseudogene

Other

**-**

Topology/Coverage Color Code

Coverage Plus Strand

Coverage Minus Strand

Mainstrand: Plus

Mainstrand: Minus

Complementary Strand

Flanking Region  
(if option -flank >0)

Gene Set Annotation  
  
RepeatMasker Annotation  

**1. AlRepC-2145**: 1-169 (+), Divergence to consensus: 16.8%  
**2. AlRepA-66**: 170-261 (-), Divergence to consensus: 27.4%  
**3. AlRepB-731**: 270-521 (+), Divergence to consensus: 16.5%  
**4. AlRepC-625**: 522-900 (+), Divergence to consensus: 7.8%  
**5. hAT-N74\_DR**: 901-963 (+), Divergence to consensus: 11.9%  
**6. AlRepB-193**: 1286-1334 (+), Divergence to consensus: 8.2%  
**7. Polinton-2\_CI**: 1741-1793 (+), Divergence to consensus: 26.7%  
**8. AlRepB-569**: 1909-1947 (+), Divergence to consensus: 12.9%  
**9. AlRepD-1254**: 2481-2615 (+), Divergence to consensus: 41.5%  
**10. AlRepD-1254**: 2617-2661 (+), Divergence to consensus: 20%  
**11. AlRepC-847**: 2675-3124 (-), Divergence to consensus: 39.2%  
**12. AlRepD-1254**: 3366-3501 (+), Divergence to consensus: 25.2%  
**13. hAT-24N1\_DR**: 3476-3642 (+), Divergence to consensus: 34.9%  
**14. AlRepC-743**: 5011-5236 (-), Divergence to consensus: 35.8%  
**15. AlRepA-93**: 5541-5649 (-), Divergence to consensus: 19.2%  
**16. AlRepB-269**: 5680-5823 (-), Divergence to consensus: 37.8%  
**17. AlRepB-269**: 5978-6059 (-), Divergence to consensus: 17.1%  
**18. (AC)n**: 6146-6165 (+), Divergence to consensus: 0%  
**19. AlRepC-670**: 6529-6593 (-), Divergence to consensus: 16.9%  
**20. AlRepB-738**: 6596-6749 (+), Divergence to consensus: 16.2%  
**21. AlRepB-103**: 6761-6987 (+), Divergence to consensus: 29.6%  
**22. AlRepB-569**: 6965-7003 (+), Divergence to consensus: 12.9%  
**23. AlRepC-905**: 7619-7701 (+), Divergence to consensus: 35%  
**24. AlRepC-373**: 7639-7748 (-), Divergence to consensus: 33.6%  
**25. L1-1\_AFC**: 8652-8680 (+), Divergence to consensus: 3.5%  
**26. L1-1\_AFC**: 8681-8743 (+), Divergence to consensus: 0%

  
Transcription Factor Binding Sites  

**RHOXF1** (Sequence: AGCTCA (-): 1982)  
**RHOXF1** (Sequence: AGCTCA (-): 2402)  
**RHOXF1** (Sequence: GGCTCA (-): 2838)  
**RHOXF1** (Sequence: AGCTTA (-): 4852)  
**RHOXF1** (Sequence: AGCTCA (-): 5423)  
**RHOXF1** (Sequence: GGATTA (-): 5994)  
**RHOXF1** (Sequence: AGATCA (-): 6087)  
**RHOXF1** (Sequence: GGATTA (-): 6715)  
**RHOXF1** (Sequence: AGCTCA (-): 6909)  
**RHOXF1** (Sequence: AGCTCA (-): 7038)  
**RHOXF1** (Sequence: TAAGCT (+): 410)  
**RHOXF1** (Sequence: TGAGCT (+): 683)  
**RHOXF1** (Sequence: TGATCT (+): 1566)  
**RHOXF1** (Sequence: TAAGCT (+): 1943)  
**RHOXF1** (Sequence: TGATCC (+): 2627)  
**RHOXF1** (Sequence: TAATCT (+): 2642)  
**RHOXF1** (Sequence: TAATCT (+): 3518)  
**RHOXF1** (Sequence: TGAGCC (+): 4431)  
**RHOXF1** (Sequence: TAAGCT (+): 4540)  
**RHOXF1** (Sequence: TAATCT (+): 4992)  
**RHOXF1** (Sequence: TGAGCT (+): 5096)  
**RHOXF1** (Sequence: TAATCT (+): 5154)  
**RHOXF1** (Sequence: TAATCT (+): 5299)  
**RHOXF1** (Sequence: TAAGCT (+): 6186)  
**RHOXF1** (Sequence: TAAGCT (+): 6999)  
**RHOXF1** (Sequence: TAAGCC (+): 7948)  
**RHOXF1** (Sequence: TGATCT (+): 8305)  
**Gata4** (Sequence: CTTATCT (+): 5939)  
**Gata4** (Sequence: CTTATCT (+): 7828)  
**POU5F1** (Sequence: TTTGCAT (-): 3391)  
**RFX4\_2** (Sequence: GTAACTAGG (-): 8175)  
**SOX9** (Sequence: AACAATAA (-): 2120)  
**SOX9** (Sequence: AACAATGA (-): 3771)  
**SOX9** (Sequence: AACAATAA (-): 8563)  
**FOXP1** (Sequence: GTAAACA (+): 4310)  
**FOXO3\_mmu** (Sequence: TGTTTTCA (-): 4384)  
**FOXO3\_mmu** (Sequence: TGTTTTCC (-): 6522)  
**Sox5** (Sequence: ATTGTT (+): 1651)  
**Sox5** (Sequence: ATTGTT (+): 4707)  
**Sox5** (Sequence: ATTGTT (+): 6428)  
**Sox5** (Sequence: ATTGTT (+): 7928)  
**FOXO3\_mmu** (Sequence: TCAAAACA (+): 4094)  
**FOXO3\_mmu** (Sequence: TGTAAACA (+): 4309)  
**FOXO3\_mmu** (Sequence: TCTAAACA (+): 6265)  
**Nobox** (Sequence: GGTAATTA (-): 5282)  
**Nobox** (Sequence: AGCAATTA (-): 7294)  
**POU2F1** (Sequence: ATTTACATA (-): 6443)  
**POU2F1** (Sequence: ATTTACATA (-): 7489)  
**POU2F1** (Sequence: ATTAAAATA (-): 8640)  
**Rhox11** (Sequence: ATTACAGCG (-): 2281)  
**Rhox11** (Sequence: TAAACACCA (-): 4311)  
**Gata4** (Sequence: AGATAAC (-): 1696)  
**Gata4** (Sequence: AGATAAG (-): 2254)  
**Sox5** (Sequence: AACAAT (-): 1118)  
**Sox5** (Sequence: AACAAT (-): 2120)  
**Sox5** (Sequence: AACAAT (-): 2127)  
**Sox5** (Sequence: AACAAT (-): 2297)  
**Sox5** (Sequence: AACAAT (-): 3771)  
**Sox5** (Sequence: AACAAT (-): 7396)  
**Sox5** (Sequence: AACAAT (-): 7576)  
**Sox5** (Sequence: AACAAT (-): 8563)  
**POU2F1** (Sequence: TATGTTAAT (+): 799)  
**POU2F1** (Sequence: TATGTAAAT (+): 6368)  
**POU2F1** (Sequence: TATTTAAAT (+): 6627)  
**POU2F1** (Sequence: TATGCAAAT (+): 7921)  
**POU2F1** (Sequence: TATTCAAAT (+): 8608)  
**POU5F1** (Sequence: ATGCAAA (+): 2703)  
**POU5F1** (Sequence: ATGCAAA (+): 4753)  
**POU5F1** (Sequence: ATGCAAA (+): 6634)  
**POU5F1** (Sequence: ATGCAAA (+): 7922)
