# Supplementary material for: First characterization of PIWI-interacting RNA clusters in a cichlid fish with a B chromosome
Source: BMC Biol. 2022 Sep 21;20:204. doi: 10.1186/s12915-022-01403-2 (PMC9490952; doi:10.1186/s12915-022-01403-2)
Supplement: Supplementary file 1 — Additional file 1. Zipped folder with fasta and interactive html piRNA cluster information for the A. latifasciata genome. The nomenclature is as follows: number-pirna-cluster_sex_B-presence (f, female; m, male; 0b, without B chromosome; 1b, with B chromosome). [file 12915_2022_1403_MOESM1_ESM.zip › 148_m1b.html]

piRNA cluster 148\_m1b 87


Predicted piRNA cluster no. 148\_m1b
  

Show proTRAC run info
Hide proTRAC run info

/\  
                \_\_\_\_\_\_\_\_\_\_\_\_\_\_\_\_\_\_\_\_\_\_\_/\\_\_\_ /  \\_\_\_\_\_\_\_  
               I                      /  \  /    \      I  
               I     pro             /    \/      \     I  
               I        TRAC        /               \   I  
               I   \_\_\_\_\_\_\_\_\_\_\_\_\_\_\_\_/\_\_\_\_\_\_\_\_\_\_\_\_\_\_\_\_\_\\_ I  
               I   \              /                     I  
               I    \            /                      I  
               I     \  /\      /       V.2.4.2         I  
               I      \/  \    /                        I  
               I\_\_\_\_\_\_\_\_\_\_\_\  /\_\_\_\_\_\_\_\_\_\_\_\_\_\_\_\_\_\_\_\_\_\_\_\_\_I  
                            \/  
  
  
================================= proTRAC ====================================  
VERSION: .......... 2.4.2  
LAST MODIFIED: .... 11. May 2018  
  
Please cite:  
Rosenkranz D, Zischler H. proTRAC - a software for probabilistic piRNA cluster  
detection, visualization and analysis. 2012. BMC Bioinformatics 13:5.  
  
  
Contact:  
David Rosenkranz  
Institute of Organismic and Molecular Evolutionary Biology  
Dept. Anthropology, small RNA group  
Johannes Gutenberg University Mainz  
email: rosenkranz@uni-mainz.de  
  
You can find the latest proTRAC version at:  
http://sourceforge.net/projects/protrac/files  
http://www.smallRNAgroup-mainz.de/software  
==============================================================================  
  
PARAMETERS:  
Map file: ...............piwi-machos-1B.fa-collapse.map  
Genome file: ............../../../0B\_ala\_genome.fa  
RepeatMasker annotation: Alatifasciata-all0B-maryan-v2.fa\_corrected.out  
GeneSet:................./guest-storage/Data/annotation/Alatifasciata\_all0B\_maryan-v2\_out2017.gff  
  
Significant (p<=0.01) hit density will be calculated based  
on observed hit distribution.  
  
Sliding window size: ........................................ 5000 bp  
Sliding window increament: .................................. 1000 bp  
Normalize each hit by number of genomic hits: ............... yes  
Normalize each hit by number of sequence reads: ............. yes  
Normalize values (-> per million mapped reads): ............. yes  
Min. fraction of hits with 1T(U) or 10A: .................... 0.75  
Alternatively: Min. fraction of hits with 1T(U) and 10A: .... 0.5  
Min. fraction of hits with typical piRNA length: ............ 0.75  
Typical piRNA length: ....................................... 24-32 nt  
Min. size of a piRNA cluster: ............................... 1000 bp.  
Min. number of hits (absolute): ............................. 0  
Min. number of hits (normalized): ........................... 0  
Min. fraction of hits on the mainstrand: .................... 0.75  
Top fraction of mapped sequences (in terms of read counts): . 1%  
Top fraction accounts for max. n% of sequence reads: ........ 90%  
Min. fraction of hits on each arm of a bidirectional cluster: 0.05  
Output html file for each cluster: .......................... yes  
Output a summary table: ..................................... yes  
Output a FASTA file for each cluster (piRNA sequences): ..... yes  
Output a FASTA file comprising cluster sequences: ........... yes  
Output a GTF file for predicted piRNA clusters: ..............yes  
Search DNA motifs in clusters: .............................. yes  
Output flanking sequences: +/- .............................. 0 bp  
Output ~.pTi file: .......................................... no  
==============================================================================  
  
  
Genome size (without gaps): ............ 758543724 bp  
Gaps (N/X/-): .......................... 417479 bp  
Mapped reads: .......................... 26973943  
Non-identical sequences: ............... 6209225  
Genomic hits: .......................... 48438990  
Significant densitiy of mapped reads: .. 821.144211136946 reads/kb

Show proTRAC cluster info
Hide proTRAC cluster info

|  |  |
| --- | --- |
| Location | NODE\_382493\_length\_8613\_cov\_31.659817 |
| Coordinates | 4-8741 |
| Size [bp] | 8738 |
| Sequence hit loci | 20754 |
| Mapped reads (normalized) | 174221.5 |
| Mapped reads (normalized) per kb | 19938.4 |
| Normalized reads with 1T (1U) | 78.7% |
| Normalized reads with 10A | 42.8% |
| Normalized reads with length 24-32 nt | 98.9% |
| Normalized reads on the main strand(s) | 93.5% |
| Predicted directionality | mono:plus |

100%

0%

1T (1U)  
reads

10A reads

24-32 nt  
reads

reads on mainstrand

**Either the amount of reads with 1T (1U) OR 10A has to exceed 75% (set with option: -1Tor10A)  
Alternatively the amount of reads with 1T (1U) AND 10A has to exceed 50% (set with option: -1Tand10A)  
Minimum amount of reads with preferred size is 75% (set with option: -pisize)  
Minimum amount of reads on the main strand(s) is 75% (set with option: -clstrand)**

Show read coverage
Hide read coverage

WHAT DO I SEE HERE?  
This chart shows the location of mapped sequence reads within a predicted piRNA cluster. The color refers to the number of genomic hits produced by the sequence read in question. A dark red bar indicates that this sequence read produces many other hits elsewhere in the genome. Many adjacent red or yellow bars can indicate the presence of a multi-copy element such as transposons or rRNA genes. A dark green bar indicates that this sequence read maps uniquely to this locus.

1 hit

2-5 hits

6-10 hits

11-20 hits

21-50 hits

51-100 hits

> 100 hits

NODE\_382493\_length\_8613\_cov\_31.659817

4

8741

Gene Set

RepeatMasker

Mapped  
Reads

371.51

plus strand

minus strand

371.51

Region: NODE\_382493\_length\_8613\_cov\_31.659817 1167-12. Max. coverage (+): 0. Max coverage (-): 0

Region: NODE\_382493\_length\_8613\_cov\_31.659817 13-30. Max. coverage (+): 0.02. Max coverage (-): 0.15

Region: NODE\_382493\_length\_8613\_cov\_31.659817 31-47. Max. coverage (+): 0.12. Max coverage (-): 0.06

Region: NODE\_382493\_length\_8613\_cov\_31.659817 48-65. Max. coverage (+): 0.04. Max coverage (-): 0.24

Region: NODE\_382493\_length\_8613\_cov\_31.659817 66-82. Max. coverage (+): 0.28. Max coverage (-): 0.04

Region: NODE\_382493\_length\_8613\_cov\_31.659817 83-100. Max. coverage (+): 0.07. Max coverage (-): 0.07

Region: NODE\_382493\_length\_8613\_cov\_31.659817 101-117. Max. coverage (+): 0.05. Max coverage (-): 0.02

Region: NODE\_382493\_length\_8613\_cov\_31.659817 118-135. Max. coverage (+): 0.04. Max coverage (-): 0

Region: NODE\_382493\_length\_8613\_cov\_31.659817 136-152. Max. coverage (+): 0.08. Max coverage (-): 0.02

Region: NODE\_382493\_length\_8613\_cov\_31.659817 153-170. Max. coverage (+): 0.14. Max coverage (-): 0.22

Region: NODE\_382493\_length\_8613\_cov\_31.659817 171-187. Max. coverage (+): 0.04. Max coverage (-): 0.24

Region: NODE\_382493\_length\_8613\_cov\_31.659817 188-204. Max. coverage (+): 0.01. Max coverage (-): 0

Region: NODE\_382493\_length\_8613\_cov\_31.659817 205-222. Max. coverage (+): 0.07. Max coverage (-): 0.07

Region: NODE\_382493\_length\_8613\_cov\_31.659817 223-239. Max. coverage (+): 0. Max coverage (-): 0.04

Region: NODE\_382493\_length\_8613\_cov\_31.659817 240-257. Max. coverage (+): 0.02. Max coverage (-): 0

Region: NODE\_382493\_length\_8613\_cov\_31.659817 258-274. Max. coverage (+): 0.01. Max coverage (-): 0.04

Region: NODE\_382493\_length\_8613\_cov\_31.659817 275-292. Max. coverage (+): 0.08. Max coverage (-): 0.01

Region: NODE\_382493\_length\_8613\_cov\_31.659817 293-309. Max. coverage (+): 0. Max coverage (-): 0.02

Region: NODE\_382493\_length\_8613\_cov\_31.659817 310-327. Max. coverage (+): 0.17. Max coverage (-): 0

Region: NODE\_382493\_length\_8613\_cov\_31.659817 328-344. Max. coverage (+): 0.1. Max coverage (-): 0.01

Region: NODE\_382493\_length\_8613\_cov\_31.659817 345-362. Max. coverage (+): 0.02. Max coverage (-): 0.01

Region: NODE\_382493\_length\_8613\_cov\_31.659817 363-379. Max. coverage (+): 0.01. Max coverage (-): 0.01

Region: NODE\_382493\_length\_8613\_cov\_31.659817 380-397. Max. coverage (+): 0.22. Max coverage (-): 0.01

Region: NODE\_382493\_length\_8613\_cov\_31.659817 398-414. Max. coverage (+): 0.22. Max coverage (-): 0.15

Region: NODE\_382493\_length\_8613\_cov\_31.659817 415-432. Max. coverage (+): 0.04. Max coverage (-): 0.07

Region: NODE\_382493\_length\_8613\_cov\_31.659817 433-449. Max. coverage (+): 0.07. Max coverage (-): 0

Region: NODE\_382493\_length\_8613\_cov\_31.659817 450-467. Max. coverage (+): 0.04. Max coverage (-): 0.22

Region: NODE\_382493\_length\_8613\_cov\_31.659817 468-484. Max. coverage (+): 0.01. Max coverage (-): 0.01

Region: NODE\_382493\_length\_8613\_cov\_31.659817 485-502. Max. coverage (+): 0. Max coverage (-): 0.01

Region: NODE\_382493\_length\_8613\_cov\_31.659817 503-519. Max. coverage (+): 0.01. Max coverage (-): 0

Region: NODE\_382493\_length\_8613\_cov\_31.659817 520-537. Max. coverage (+): 0.02. Max coverage (-): 0.03

Region: NODE\_382493\_length\_8613\_cov\_31.659817 538-554. Max. coverage (+): 0.13. Max coverage (-): 0.06

Region: NODE\_382493\_length\_8613\_cov\_31.659817 555-571. Max. coverage (+): 0.05. Max coverage (-): 0.02

Region: NODE\_382493\_length\_8613\_cov\_31.659817 572-589. Max. coverage (+): 0.39. Max coverage (-): 2.31

Region: NODE\_382493\_length\_8613\_cov\_31.659817 590-606. Max. coverage (+): 0.22. Max coverage (-): 0.04

Region: NODE\_382493\_length\_8613\_cov\_31.659817 607-624. Max. coverage (+): 0.01. Max coverage (-): 0.02

Region: NODE\_382493\_length\_8613\_cov\_31.659817 625-641. Max. coverage (+): 0. Max coverage (-): 0.01

Region: NODE\_382493\_length\_8613\_cov\_31.659817 642-659. Max. coverage (+): 0.01. Max coverage (-): 0.01

Region: NODE\_382493\_length\_8613\_cov\_31.659817 660-676. Max. coverage (+): 0.04. Max coverage (-): 0

Region: NODE\_382493\_length\_8613\_cov\_31.659817 677-694. Max. coverage (+): 0. Max coverage (-): 0.02

Region: NODE\_382493\_length\_8613\_cov\_31.659817 695-711. Max. coverage (+): 0.06. Max coverage (-): 0.04

Region: NODE\_382493\_length\_8613\_cov\_31.659817 712-729. Max. coverage (+): 0. Max coverage (-): 0.04

Region: NODE\_382493\_length\_8613\_cov\_31.659817 730-746. Max. coverage (+): 0. Max coverage (-): 0

Region: NODE\_382493\_length\_8613\_cov\_31.659817 747-764. Max. coverage (+): 0. Max coverage (-): 0

Region: NODE\_382493\_length\_8613\_cov\_31.659817 765-781. Max. coverage (+): 0.01. Max coverage (-): 0.06

Region: NODE\_382493\_length\_8613\_cov\_31.659817 782-799. Max. coverage (+): 0.2. Max coverage (-): 0.35

Region: NODE\_382493\_length\_8613\_cov\_31.659817 800-816. Max. coverage (+): 0.2. Max coverage (-): 0.52

Region: NODE\_382493\_length\_8613\_cov\_31.659817 817-834. Max. coverage (+): 0.26. Max coverage (-): 0.16

Region: NODE\_382493\_length\_8613\_cov\_31.659817 835-851. Max. coverage (+): 0.08. Max coverage (-): 0.17

Region: NODE\_382493\_length\_8613\_cov\_31.659817 852-869. Max. coverage (+): 0.08. Max coverage (-): 0.07

Region: NODE\_382493\_length\_8613\_cov\_31.659817 870-886. Max. coverage (+): 0.05. Max coverage (-): 0.04

Region: NODE\_382493\_length\_8613\_cov\_31.659817 887-904. Max. coverage (+): 0. Max coverage (-): 0

Region: NODE\_382493\_length\_8613\_cov\_31.659817 905-921. Max. coverage (+): 0.01. Max coverage (-): 0

Region: NODE\_382493\_length\_8613\_cov\_31.659817 922-938. Max. coverage (+): 0.01. Max coverage (-): 0.02

Region: NODE\_382493\_length\_8613\_cov\_31.659817 939-956. Max. coverage (+): 0. Max coverage (-): 0.04

Region: NODE\_382493\_length\_8613\_cov\_31.659817 957-973. Max. coverage (+): 0.2. Max coverage (-): 0.13

Region: NODE\_382493\_length\_8613\_cov\_31.659817 974-991. Max. coverage (+): 0.02. Max coverage (-): 0.7

Region: NODE\_382493\_length\_8613\_cov\_31.659817 992-1008. Max. coverage (+): 0.04. Max coverage (-): 0.09

Region: NODE\_382493\_length\_8613\_cov\_31.659817 1009-1026. Max. coverage (+): 0.24. Max coverage (-): 0.02

Region: NODE\_382493\_length\_8613\_cov\_31.659817 1027-1043. Max. coverage (+): 0.04. Max coverage (-): 0.44

Region: NODE\_382493\_length\_8613\_cov\_31.659817 1044-1061. Max. coverage (+): 0.48. Max coverage (-): 0.3

Region: NODE\_382493\_length\_8613\_cov\_31.659817 1062-1078. Max. coverage (+): 0.85. Max coverage (-): 0.09

Region: NODE\_382493\_length\_8613\_cov\_31.659817 1079-1096. Max. coverage (+): 0. Max coverage (-): 0

Region: NODE\_382493\_length\_8613\_cov\_31.659817 1097-1113. Max. coverage (+): 0. Max coverage (-): 0

Region: NODE\_382493\_length\_8613\_cov\_31.659817 1114-1131. Max. coverage (+): 0. Max coverage (-): 0

Region: NODE\_382493\_length\_8613\_cov\_31.659817 1132-1148. Max. coverage (+): 0.37. Max coverage (-): 0.04

Region: NODE\_382493\_length\_8613\_cov\_31.659817 1149-1166. Max. coverage (+): 0.39. Max coverage (-): 0

Region: NODE\_382493\_length\_8613\_cov\_31.659817 1167-1183. Max. coverage (+): 0. Max coverage (-): 0.95

Region: NODE\_382493\_length\_8613\_cov\_31.659817 1184-1201. Max. coverage (+): 0.15. Max coverage (-): 0.26

Region: NODE\_382493\_length\_8613\_cov\_31.659817 1202-1218. Max. coverage (+): 0.06. Max coverage (-): 0.48

Region: NODE\_382493\_length\_8613\_cov\_31.659817 1219-1236. Max. coverage (+): 0.37. Max coverage (-): 0.04

Region: NODE\_382493\_length\_8613\_cov\_31.659817 1237-1253. Max. coverage (+): 0.11. Max coverage (-): 0.04

Region: NODE\_382493\_length\_8613\_cov\_31.659817 1254-1271. Max. coverage (+): 0.09. Max coverage (-): 0.01

Region: NODE\_382493\_length\_8613\_cov\_31.659817 1272-1288. Max. coverage (+): 0.06. Max coverage (-): 0.11

Region: NODE\_382493\_length\_8613\_cov\_31.659817 1289-1305. Max. coverage (+): 6.88. Max coverage (-): 0.33

Region: NODE\_382493\_length\_8613\_cov\_31.659817 1306-1323. Max. coverage (+): 6.77. Max coverage (-): 0.07

Region: NODE\_382493\_length\_8613\_cov\_31.659817 1324-1340. Max. coverage (+): 0.22. Max coverage (-): 0.22

Region: NODE\_382493\_length\_8613\_cov\_31.659817 1341-1358. Max. coverage (+): 0.09. Max coverage (-): 0.04

Region: NODE\_382493\_length\_8613\_cov\_31.659817 1359-1375. Max. coverage (+): 0.11. Max coverage (-): 0.04

Region: NODE\_382493\_length\_8613\_cov\_31.659817 1376-1393. Max. coverage (+): 0.04. Max coverage (-): 0.07

Region: NODE\_382493\_length\_8613\_cov\_31.659817 1394-1410. Max. coverage (+): 0. Max coverage (-): 0.04

Region: NODE\_382493\_length\_8613\_cov\_31.659817 1411-1428. Max. coverage (+): 0.11. Max coverage (-): 0.15

Region: NODE\_382493\_length\_8613\_cov\_31.659817 1429-1445. Max. coverage (+): 0.11. Max coverage (-): 0.3

Region: NODE\_382493\_length\_8613\_cov\_31.659817 1446-1463. Max. coverage (+): 0.3. Max coverage (-): 0.11

Region: NODE\_382493\_length\_8613\_cov\_31.659817 1464-1480. Max. coverage (+): 0.15. Max coverage (-): 0.04

Region: NODE\_382493\_length\_8613\_cov\_31.659817 1481-1498. Max. coverage (+): 0.43. Max coverage (-): 0.15

Region: NODE\_382493\_length\_8613\_cov\_31.659817 1499-1515. Max. coverage (+): 0.46. Max coverage (-): 0.03

Region: NODE\_382493\_length\_8613\_cov\_31.659817 1516-1533. Max. coverage (+): 0.65. Max coverage (-): 0.15

Region: NODE\_382493\_length\_8613\_cov\_31.659817 1534-1550. Max. coverage (+): 0.68. Max coverage (-): 0.3

Region: NODE\_382493\_length\_8613\_cov\_31.659817 1551-1568. Max. coverage (+): 0.24. Max coverage (-): 0.33

Region: NODE\_382493\_length\_8613\_cov\_31.659817 1569-1585. Max. coverage (+): 0.37. Max coverage (-): 0.35

Region: NODE\_382493\_length\_8613\_cov\_31.659817 1586-1603. Max. coverage (+): 0.56. Max coverage (-): 0.36

Region: NODE\_382493\_length\_8613\_cov\_31.659817 1604-1620. Max. coverage (+): 0.02. Max coverage (-): 0.06

Region: NODE\_382493\_length\_8613\_cov\_31.659817 1621-1638. Max. coverage (+): 0.06. Max coverage (-): 0.02

Region: NODE\_382493\_length\_8613\_cov\_31.659817 1639-1655. Max. coverage (+): 0.07. Max coverage (-): 0

Region: NODE\_382493\_length\_8613\_cov\_31.659817 1656-1672. Max. coverage (+): 0.15. Max coverage (-): 0

Region: NODE\_382493\_length\_8613\_cov\_31.659817 1673-1690. Max. coverage (+): 0.19. Max coverage (-): 0.33

Region: NODE\_382493\_length\_8613\_cov\_31.659817 1691-1707. Max. coverage (+): 0.26. Max coverage (-): 0.12

Region: NODE\_382493\_length\_8613\_cov\_31.659817 1708-1725. Max. coverage (+): 0.11. Max coverage (-): 0.07

Region: NODE\_382493\_length\_8613\_cov\_31.659817 1726-1742. Max. coverage (+): 0.56. Max coverage (-): 0.67

Region: NODE\_382493\_length\_8613\_cov\_31.659817 1743-1760. Max. coverage (+): 0.26. Max coverage (-): 0.19

Region: NODE\_382493\_length\_8613\_cov\_31.659817 1761-1777. Max. coverage (+): 3.97. Max coverage (-): 0.33

Region: NODE\_382493\_length\_8613\_cov\_31.659817 1778-1795. Max. coverage (+): 3.86. Max coverage (-): 0.19

Region: NODE\_382493\_length\_8613\_cov\_31.659817 1796-1812. Max. coverage (+): 0.29. Max coverage (-): 0.15

Region: NODE\_382493\_length\_8613\_cov\_31.659817 1813-1830. Max. coverage (+): 0. Max coverage (-): 0.04

Region: NODE\_382493\_length\_8613\_cov\_31.659817 1831-1847. Max. coverage (+): 0.97. Max coverage (-): 1.14

Region: NODE\_382493\_length\_8613\_cov\_31.659817 1848-1865. Max. coverage (+): 1.24. Max coverage (-): 0.19

Region: NODE\_382493\_length\_8613\_cov\_31.659817 1866-1882. Max. coverage (+): 4.56. Max coverage (-): 0.19

Region: NODE\_382493\_length\_8613\_cov\_31.659817 1883-1900. Max. coverage (+): 18.11. Max coverage (-): 0.26

Region: NODE\_382493\_length\_8613\_cov\_31.659817 1901-1917. Max. coverage (+): 14.64. Max coverage (-): 0.11

Region: NODE\_382493\_length\_8613\_cov\_31.659817 1918-1935. Max. coverage (+): 0.37. Max coverage (-): 0.04

Region: NODE\_382493\_length\_8613\_cov\_31.659817 1936-1952. Max. coverage (+): 1.56. Max coverage (-): 0.04

Region: NODE\_382493\_length\_8613\_cov\_31.659817 1953-1970. Max. coverage (+): 0.3. Max coverage (-): 0.96

Region: NODE\_382493\_length\_8613\_cov\_31.659817 1971-1987. Max. coverage (+): 1.22. Max coverage (-): 0.22

Region: NODE\_382493\_length\_8613\_cov\_31.659817 1988-2005. Max. coverage (+): 1.06. Max coverage (-): 0.35

Region: NODE\_382493\_length\_8613\_cov\_31.659817 2006-2022. Max. coverage (+): 0.67. Max coverage (-): 0.04

Region: NODE\_382493\_length\_8613\_cov\_31.659817 2023-2039. Max. coverage (+): 1.46. Max coverage (-): 0.04

Region: NODE\_382493\_length\_8613\_cov\_31.659817 2040-2057. Max. coverage (+): 2.17. Max coverage (-): 4.56

Region: NODE\_382493\_length\_8613\_cov\_31.659817 2058-2074. Max. coverage (+): 5.41. Max coverage (-): 0.41

Region: NODE\_382493\_length\_8613\_cov\_31.659817 2075-2092. Max. coverage (+): 1.89. Max coverage (-): 0.19

Region: NODE\_382493\_length\_8613\_cov\_31.659817 2093-2109. Max. coverage (+): 53.46. Max coverage (-): 0.09

Region: NODE\_382493\_length\_8613\_cov\_31.659817 2110-2127. Max. coverage (+): 2.11. Max coverage (-): 1.71

Region: NODE\_382493\_length\_8613\_cov\_31.659817 2128-2144. Max. coverage (+): 0.82. Max coverage (-): 0.78

Region: NODE\_382493\_length\_8613\_cov\_31.659817 2145-2162. Max. coverage (+): 0.37. Max coverage (-): 0.11

Region: NODE\_382493\_length\_8613\_cov\_31.659817 2163-2179. Max. coverage (+): 6.56. Max coverage (-): 0.04

Region: NODE\_382493\_length\_8613\_cov\_31.659817 2180-2197. Max. coverage (+): 11.53. Max coverage (-): 1.37

Region: NODE\_382493\_length\_8613\_cov\_31.659817 2198-2214. Max. coverage (+): 6.93. Max coverage (-): 1.37

Region: NODE\_382493\_length\_8613\_cov\_31.659817 2215-2232. Max. coverage (+): 2.22. Max coverage (-): 2.63

Region: NODE\_382493\_length\_8613\_cov\_31.659817 2233-2249. Max. coverage (+): 1.85. Max coverage (-): 0.07

Region: NODE\_382493\_length\_8613\_cov\_31.659817 2250-2267. Max. coverage (+): 0.59. Max coverage (-): 0.33

Region: NODE\_382493\_length\_8613\_cov\_31.659817 2268-2284. Max. coverage (+): 4.15. Max coverage (-): 0.15

Region: NODE\_382493\_length\_8613\_cov\_31.659817 2285-2302. Max. coverage (+): 2.93. Max coverage (-): 1

Region: NODE\_382493\_length\_8613\_cov\_31.659817 2303-2319. Max. coverage (+): 57.17. Max coverage (-): 0.3

Region: NODE\_382493\_length\_8613\_cov\_31.659817 2320-2337. Max. coverage (+): 19.02. Max coverage (-): 0.48

Region: NODE\_382493\_length\_8613\_cov\_31.659817 2338-2354. Max. coverage (+): 4.26. Max coverage (-): 2.15

Region: NODE\_382493\_length\_8613\_cov\_31.659817 2355-2371. Max. coverage (+): 128.75. Max coverage (-): 2.08

Region: NODE\_382493\_length\_8613\_cov\_31.659817 2372-2389. Max. coverage (+): 118.89. Max coverage (-): 1.26

Region: NODE\_382493\_length\_8613\_cov\_31.659817 2390-2406. Max. coverage (+): 3.93. Max coverage (-): 1.08

Region: NODE\_382493\_length\_8613\_cov\_31.659817 2407-2424. Max. coverage (+): 36.48. Max coverage (-): 1

Region: NODE\_382493\_length\_8613\_cov\_31.659817 2425-2441. Max. coverage (+): 23.76. Max coverage (-): 1.56

Region: NODE\_382493\_length\_8613\_cov\_31.659817 2442-2459. Max. coverage (+): 7.9. Max coverage (-): 0.44

Region: NODE\_382493\_length\_8613\_cov\_31.659817 2460-2476. Max. coverage (+): 0.56. Max coverage (-): 0.07

Region: NODE\_382493\_length\_8613\_cov\_31.659817 2477-2494. Max. coverage (+): 18.05. Max coverage (-): 0.07

Region: NODE\_382493\_length\_8613\_cov\_31.659817 2495-2511. Max. coverage (+): 10.2. Max coverage (-): 0.07

Region: NODE\_382493\_length\_8613\_cov\_31.659817 2512-2529. Max. coverage (+): 13.12. Max coverage (-): 0.04

Region: NODE\_382493\_length\_8613\_cov\_31.659817 2530-2546. Max. coverage (+): 8.27. Max coverage (-): 0.07

Region: NODE\_382493\_length\_8613\_cov\_31.659817 2547-2564. Max. coverage (+): 0.33. Max coverage (-): 0.07

Region: NODE\_382493\_length\_8613\_cov\_31.659817 2565-2581. Max. coverage (+): 0.85. Max coverage (-): 0.22

Region: NODE\_382493\_length\_8613\_cov\_31.659817 2582-2599. Max. coverage (+): 30.25. Max coverage (-): 0.78

Region: NODE\_382493\_length\_8613\_cov\_31.659817 2600-2616. Max. coverage (+): 4.86. Max coverage (-): 0.67

Region: NODE\_382493\_length\_8613\_cov\_31.659817 2617-2634. Max. coverage (+): 2.45. Max coverage (-): 2.57

Region: NODE\_382493\_length\_8613\_cov\_31.659817 2635-2651. Max. coverage (+): 1.11. Max coverage (-): 4.93

Region: NODE\_382493\_length\_8613\_cov\_31.659817 2652-2669. Max. coverage (+): 4.97. Max coverage (-): 1.67

Region: NODE\_382493\_length\_8613\_cov\_31.659817 2670-2686. Max. coverage (+): 3.6. Max coverage (-): 0.4

Region: NODE\_382493\_length\_8613\_cov\_31.659817 2687-2704. Max. coverage (+): 48.64. Max coverage (-): 0.57

Region: NODE\_382493\_length\_8613\_cov\_31.659817 2705-2721. Max. coverage (+): 48.86. Max coverage (-): 0.22

Region: NODE\_382493\_length\_8613\_cov\_31.659817 2722-2738. Max. coverage (+): 1.59. Max coverage (-): 0.44

Region: NODE\_382493\_length\_8613\_cov\_31.659817 2739-2756. Max. coverage (+): 8.3. Max coverage (-): 1.85

Region: NODE\_382493\_length\_8613\_cov\_31.659817 2757-2773. Max. coverage (+): 11.79. Max coverage (-): 0.26

Region: NODE\_382493\_length\_8613\_cov\_31.659817 2774-2791. Max. coverage (+): 28.78. Max coverage (-): 0.35

Region: NODE\_382493\_length\_8613\_cov\_31.659817 2792-2808. Max. coverage (+): 28.73. Max coverage (-): 1.56

Region: NODE\_382493\_length\_8613\_cov\_31.659817 2809-2826. Max. coverage (+): 2.6. Max coverage (-): 0.15

Region: NODE\_382493\_length\_8613\_cov\_31.659817 2827-2843. Max. coverage (+): 0.67. Max coverage (-): 0.85

Region: NODE\_382493\_length\_8613\_cov\_31.659817 2844-2861. Max. coverage (+): 2.34. Max coverage (-): 0.22

Region: NODE\_382493\_length\_8613\_cov\_31.659817 2862-2878. Max. coverage (+): 0.44. Max coverage (-): 0.04

Region: NODE\_382493\_length\_8613\_cov\_31.659817 2879-2896. Max. coverage (+): 0.56. Max coverage (-): 0.03

Region: NODE\_382493\_length\_8613\_cov\_31.659817 2897-2913. Max. coverage (+): 2.78. Max coverage (-): 0.05

Region: NODE\_382493\_length\_8613\_cov\_31.659817 2914-2931. Max. coverage (+): 0.63. Max coverage (-): 0.34

Region: NODE\_382493\_length\_8613\_cov\_31.659817 2932-2948. Max. coverage (+): 20.97. Max coverage (-): 0.27

Region: NODE\_382493\_length\_8613\_cov\_31.659817 2949-2966. Max. coverage (+): 121.73. Max coverage (-): 0.44

Region: NODE\_382493\_length\_8613\_cov\_31.659817 2967-2983. Max. coverage (+): 4.52. Max coverage (-): 0.37

Region: NODE\_382493\_length\_8613\_cov\_31.659817 2984-3001. Max. coverage (+): 1. Max coverage (-): 2.04

Region: NODE\_382493\_length\_8613\_cov\_31.659817 3002-3018. Max. coverage (+): 4.49. Max coverage (-): 0.19

Region: NODE\_382493\_length\_8613\_cov\_31.659817 3019-3036. Max. coverage (+): 0.33. Max coverage (-): 0.04

Region: NODE\_382493\_length\_8613\_cov\_31.659817 3037-3053. Max. coverage (+): 1.19. Max coverage (-): 0.19

Region: NODE\_382493\_length\_8613\_cov\_31.659817 3054-3071. Max. coverage (+): 2.48. Max coverage (-): 0.37

Region: NODE\_382493\_length\_8613\_cov\_31.659817 3072-3088. Max. coverage (+): 1.43. Max coverage (-): 0.06

Region: NODE\_382493\_length\_8613\_cov\_31.659817 3089-3105. Max. coverage (+): 3.31. Max coverage (-): 0

Region: NODE\_382493\_length\_8613\_cov\_31.659817 3106-3123. Max. coverage (+): 14.94. Max coverage (-): 0.19

Region: NODE\_382493\_length\_8613\_cov\_31.659817 3124-3140. Max. coverage (+): 13.2. Max coverage (-): 0.52

Region: NODE\_382493\_length\_8613\_cov\_31.659817 3141-3158. Max. coverage (+): 5.93. Max coverage (-): 0.85

Region: NODE\_382493\_length\_8613\_cov\_31.659817 3159-3175. Max. coverage (+): 371.51. Max coverage (-): 0.37

Region: NODE\_382493\_length\_8613\_cov\_31.659817 3176-3193. Max. coverage (+): 22.39. Max coverage (-): 0.52

Region: NODE\_382493\_length\_8613\_cov\_31.659817 3194-3210. Max. coverage (+): 15.31. Max coverage (-): 0.48

Region: NODE\_382493\_length\_8613\_cov\_31.659817 3211-3228. Max. coverage (+): 9.86. Max coverage (-): 10.94

Region: NODE\_382493\_length\_8613\_cov\_31.659817 3229-3245. Max. coverage (+): 7.34. Max coverage (-): 7.71

Region: NODE\_382493\_length\_8613\_cov\_31.659817 3246-3263. Max. coverage (+): 65.8. Max coverage (-): 4.04

Region: NODE\_382493\_length\_8613\_cov\_31.659817 3264-3280. Max. coverage (+): 9.38. Max coverage (-): 0.85

Region: NODE\_382493\_length\_8613\_cov\_31.659817 3281-3298. Max. coverage (+): 8.04. Max coverage (-): 0.3

Region: NODE\_382493\_length\_8613\_cov\_31.659817 3299-3315. Max. coverage (+): 0.89. Max coverage (-): 0.3

Region: NODE\_382493\_length\_8613\_cov\_31.659817 3316-3333. Max. coverage (+): 0.33. Max coverage (-): 0.15

Region: NODE\_382493\_length\_8613\_cov\_31.659817 3334-3350. Max. coverage (+): 24.28. Max coverage (-): 0.04

Region: NODE\_382493\_length\_8613\_cov\_31.659817 3351-3368. Max. coverage (+): 12.38. Max coverage (-): 0.59

Region: NODE\_382493\_length\_8613\_cov\_31.659817 3369-3385. Max. coverage (+): 92.76. Max coverage (-): 0.22

Region: NODE\_382493\_length\_8613\_cov\_31.659817 3386-3403. Max. coverage (+): 4.52. Max coverage (-): 0.19

Region: NODE\_382493\_length\_8613\_cov\_31.659817 3404-3420. Max. coverage (+): 292.32. Max coverage (-): 6.82

Region: NODE\_382493\_length\_8613\_cov\_31.659817 3421-3438. Max. coverage (+): 92.5. Max coverage (-): 0.74

Region: NODE\_382493\_length\_8613\_cov\_31.659817 3439-3455. Max. coverage (+): 9.16. Max coverage (-): 0.67

Region: NODE\_382493\_length\_8613\_cov\_31.659817 3456-3472. Max. coverage (+): 1.56. Max coverage (-): 0.52

Region: NODE\_382493\_length\_8613\_cov\_31.659817 3473-3490. Max. coverage (+): 75.41. Max coverage (-): 0.41

Region: NODE\_382493\_length\_8613\_cov\_31.659817 3491-3507. Max. coverage (+): 3.11. Max coverage (-): 0.3

Region: NODE\_382493\_length\_8613\_cov\_31.659817 3508-3525. Max. coverage (+): 5.84. Max coverage (-): 1.41

Region: NODE\_382493\_length\_8613\_cov\_31.659817 3526-3542. Max. coverage (+): 1.11. Max coverage (-): 0.11

Region: NODE\_382493\_length\_8613\_cov\_31.659817 3543-3560. Max. coverage (+): 0.48. Max coverage (-): 0

Region: NODE\_382493\_length\_8613\_cov\_31.659817 3561-3577. Max. coverage (+): 1.56. Max coverage (-): 0

Region: NODE\_382493\_length\_8613\_cov\_31.659817 3578-3595. Max. coverage (+): 0.07. Max coverage (-): 0

Region: NODE\_382493\_length\_8613\_cov\_31.659817 3596-3612. Max. coverage (+): 3.37. Max coverage (-): 0

Region: NODE\_382493\_length\_8613\_cov\_31.659817 3613-3630. Max. coverage (+): 2.08. Max coverage (-): 2.37

Region: NODE\_382493\_length\_8613\_cov\_31.659817 3631-3647. Max. coverage (+): 76.7. Max coverage (-): 0.19

Region: NODE\_382493\_length\_8613\_cov\_31.659817 3648-3665. Max. coverage (+): 13.72. Max coverage (-): 0.07

Region: NODE\_382493\_length\_8613\_cov\_31.659817 3666-3682. Max. coverage (+): 30.88. Max coverage (-): 0.11

Region: NODE\_382493\_length\_8613\_cov\_31.659817 3683-3700. Max. coverage (+): 1.3. Max coverage (-): 0.56

Region: NODE\_382493\_length\_8613\_cov\_31.659817 3701-3717. Max. coverage (+): 14.16. Max coverage (-): 0.07

Region: NODE\_382493\_length\_8613\_cov\_31.659817 3718-3735. Max. coverage (+): 24.62. Max coverage (-): 0.74

Region: NODE\_382493\_length\_8613\_cov\_31.659817 3736-3752. Max. coverage (+): 11.94. Max coverage (-): 0.59

Region: NODE\_382493\_length\_8613\_cov\_31.659817 3753-3770. Max. coverage (+): 7.49. Max coverage (-): 0.11

Region: NODE\_382493\_length\_8613\_cov\_31.659817 3771-3787. Max. coverage (+): 13.83. Max coverage (-): 0

Region: NODE\_382493\_length\_8613\_cov\_31.659817 3788-3805. Max. coverage (+): 2.63. Max coverage (-): 2.63

Region: NODE\_382493\_length\_8613\_cov\_31.659817 3806-3822. Max. coverage (+): 50.38. Max coverage (-): 0.07

Region: NODE\_382493\_length\_8613\_cov\_31.659817 3823-3839. Max. coverage (+): 0.67. Max coverage (-): 2.78

Region: NODE\_382493\_length\_8613\_cov\_31.659817 3840-3857. Max. coverage (+): 12.68. Max coverage (-): 0.22

Region: NODE\_382493\_length\_8613\_cov\_31.659817 3858-3874. Max. coverage (+): 2.56. Max coverage (-): 0.11

Region: NODE\_382493\_length\_8613\_cov\_31.659817 3875-3892. Max. coverage (+): 1.82. Max coverage (-): 0.15

Region: NODE\_382493\_length\_8613\_cov\_31.659817 3893-3909. Max. coverage (+): 46.19. Max coverage (-): 0.3

Region: NODE\_382493\_length\_8613\_cov\_31.659817 3910-3927. Max. coverage (+): 46.53. Max coverage (-): 1.67

Region: NODE\_382493\_length\_8613\_cov\_31.659817 3928-3944. Max. coverage (+): 5.64. Max coverage (-): 0.63

Region: NODE\_382493\_length\_8613\_cov\_31.659817 3945-3962. Max. coverage (+): 15.09. Max coverage (-): 0.15

Region: NODE\_382493\_length\_8613\_cov\_31.659817 3963-3979. Max. coverage (+): 12.79. Max coverage (-): 12.53

Region: NODE\_382493\_length\_8613\_cov\_31.659817 3980-3997. Max. coverage (+): 21.39. Max coverage (-): 0.63

Region: NODE\_382493\_length\_8613\_cov\_31.659817 3998-4014. Max. coverage (+): 18.76. Max coverage (-): 0.11

Region: NODE\_382493\_length\_8613\_cov\_31.659817 4015-4032. Max. coverage (+): 7.38. Max coverage (-): 1.04

Region: NODE\_382493\_length\_8613\_cov\_31.659817 4033-4049. Max. coverage (+): 11.94. Max coverage (-): 0.74

Region: NODE\_382493\_length\_8613\_cov\_31.659817 4050-4067. Max. coverage (+): 2.04. Max coverage (-): 18.83

Region: NODE\_382493\_length\_8613\_cov\_31.659817 4068-4084. Max. coverage (+): 0.74. Max coverage (-): 0.15

Region: NODE\_382493\_length\_8613\_cov\_31.659817 4085-4102. Max. coverage (+): 0.44. Max coverage (-): 14.5

Region: NODE\_382493\_length\_8613\_cov\_31.659817 4103-4119. Max. coverage (+): 11.34. Max coverage (-): 0.04

Region: NODE\_382493\_length\_8613\_cov\_31.659817 4120-4137. Max. coverage (+): 0.41. Max coverage (-): 0.41

Region: NODE\_382493\_length\_8613\_cov\_31.659817 4138-4154. Max. coverage (+): 62.23. Max coverage (-): 0.04

Region: NODE\_382493\_length\_8613\_cov\_31.659817 4155-4172. Max. coverage (+): 4.93. Max coverage (-): 0.33

Region: NODE\_382493\_length\_8613\_cov\_31.659817 4173-4189. Max. coverage (+): 9.97. Max coverage (-): 0.48

Region: NODE\_382493\_length\_8613\_cov\_31.659817 4190-4206. Max. coverage (+): 4.52. Max coverage (-): 0.22

Region: NODE\_382493\_length\_8613\_cov\_31.659817 4207-4224. Max. coverage (+): 4.93. Max coverage (-): 0.04

Region: NODE\_382493\_length\_8613\_cov\_31.659817 4225-4241. Max. coverage (+): 3.15. Max coverage (-): 0.33

Region: NODE\_382493\_length\_8613\_cov\_31.659817 4242-4259. Max. coverage (+): 22.1. Max coverage (-): 1

Region: NODE\_382493\_length\_8613\_cov\_31.659817 4260-4276. Max. coverage (+): 93.76. Max coverage (-): 1.22

Region: NODE\_382493\_length\_8613\_cov\_31.659817 4277-4294. Max. coverage (+): 25.36. Max coverage (-): 0.48

Region: NODE\_382493\_length\_8613\_cov\_31.659817 4295-4311. Max. coverage (+): 20.06. Max coverage (-): 5.62

Region: NODE\_382493\_length\_8613\_cov\_31.659817 4312-4329. Max. coverage (+): 19.57. Max coverage (-): 0.34

Region: NODE\_382493\_length\_8613\_cov\_31.659817 4330-4346. Max. coverage (+): 11.73. Max coverage (-): 0.32

Region: NODE\_382493\_length\_8613\_cov\_31.659817 4347-4364. Max. coverage (+): 2.93. Max coverage (-): 4

Region: NODE\_382493\_length\_8613\_cov\_31.659817 4365-4381. Max. coverage (+): 2.85. Max coverage (-): 0.44

Region: NODE\_382493\_length\_8613\_cov\_31.659817 4382-4399. Max. coverage (+): 1.59. Max coverage (-): 10.6

Region: NODE\_382493\_length\_8613\_cov\_31.659817 4400-4416. Max. coverage (+): 52.57. Max coverage (-): 3

Region: NODE\_382493\_length\_8613\_cov\_31.659817 4417-4434. Max. coverage (+): 2. Max coverage (-): 1.76

Region: NODE\_382493\_length\_8613\_cov\_31.659817 4435-4451. Max. coverage (+): 5.21. Max coverage (-): 0.07

Region: NODE\_382493\_length\_8613\_cov\_31.659817 4452-4469. Max. coverage (+): 7.67. Max coverage (-): 1.41

Region: NODE\_382493\_length\_8613\_cov\_31.659817 4470-4486. Max. coverage (+): 10.07. Max coverage (-): 0.33

Region: NODE\_382493\_length\_8613\_cov\_31.659817 4487-4504. Max. coverage (+): 4.08. Max coverage (-): 0.22

Region: NODE\_382493\_length\_8613\_cov\_31.659817 4505-4521. Max. coverage (+): 4.71. Max coverage (-): 0.07

Region: NODE\_382493\_length\_8613\_cov\_31.659817 4522-4539. Max. coverage (+): 9.05. Max coverage (-): 0

Region: NODE\_382493\_length\_8613\_cov\_31.659817 4540-4556. Max. coverage (+): 5.56. Max coverage (-): 0.15

Region: NODE\_382493\_length\_8613\_cov\_31.659817 4557-4573. Max. coverage (+): 7.12. Max coverage (-): 0.56

Region: NODE\_382493\_length\_8613\_cov\_31.659817 4574-4591. Max. coverage (+): 65.92. Max coverage (-): 0.04

Region: NODE\_382493\_length\_8613\_cov\_31.659817 4592-4608. Max. coverage (+): 3.89. Max coverage (-): 0.19

Region: NODE\_382493\_length\_8613\_cov\_31.659817 4609-4626. Max. coverage (+): 6.75. Max coverage (-): 0.04

Region: NODE\_382493\_length\_8613\_cov\_31.659817 4627-4643. Max. coverage (+): 5.08. Max coverage (-): 0.37

Region: NODE\_382493\_length\_8613\_cov\_31.659817 4644-4661. Max. coverage (+): 6.19. Max coverage (-): 0.22

Region: NODE\_382493\_length\_8613\_cov\_31.659817 4662-4678. Max. coverage (+): 10.27. Max coverage (-): 0.15

Region: NODE\_382493\_length\_8613\_cov\_31.659817 4679-4696. Max. coverage (+): 0.82. Max coverage (-): 1.59

Region: NODE\_382493\_length\_8613\_cov\_31.659817 4697-4713. Max. coverage (+): 1.82. Max coverage (-): 2

Region: NODE\_382493\_length\_8613\_cov\_31.659817 4714-4731. Max. coverage (+): 1.41. Max coverage (-): 0.56

Region: NODE\_382493\_length\_8613\_cov\_31.659817 4732-4748. Max. coverage (+): 0.78. Max coverage (-): 1.11

Region: NODE\_382493\_length\_8613\_cov\_31.659817 4749-4766. Max. coverage (+): 75.37. Max coverage (-): 2.93

Region: NODE\_382493\_length\_8613\_cov\_31.659817 4767-4783. Max. coverage (+): 7.93. Max coverage (-): 1.19

Region: NODE\_382493\_length\_8613\_cov\_31.659817 4784-4801. Max. coverage (+): 1.74. Max coverage (-): 0

Region: NODE\_382493\_length\_8613\_cov\_31.659817 4802-4818. Max. coverage (+): 0.56. Max coverage (-): 0.04

Region: NODE\_382493\_length\_8613\_cov\_31.659817 4819-4836. Max. coverage (+): 0.63. Max coverage (-): 0.07

Region: NODE\_382493\_length\_8613\_cov\_31.659817 4837-4853. Max. coverage (+): 0.44. Max coverage (-): 0.04

Region: NODE\_382493\_length\_8613\_cov\_31.659817 4854-4871. Max. coverage (+): 6.41. Max coverage (-): 0.07

Region: NODE\_382493\_length\_8613\_cov\_31.659817 4872-4888. Max. coverage (+): 1.56. Max coverage (-): 0.74

Region: NODE\_382493\_length\_8613\_cov\_31.659817 4889-4906. Max. coverage (+): 8.01. Max coverage (-): 0.11

Region: NODE\_382493\_length\_8613\_cov\_31.659817 4907-4923. Max. coverage (+): 8.16. Max coverage (-): 0.19

Region: NODE\_382493\_length\_8613\_cov\_31.659817 4924-4940. Max. coverage (+): 0.15. Max coverage (-): 0.3

Region: NODE\_382493\_length\_8613\_cov\_31.659817 4941-4958. Max. coverage (+): 7.71. Max coverage (-): 0.59

Region: NODE\_382493\_length\_8613\_cov\_31.659817 4959-4975. Max. coverage (+): 2.67. Max coverage (-): 0.04

Region: NODE\_382493\_length\_8613\_cov\_31.659817 4976-4993. Max. coverage (+): 2.11. Max coverage (-): 0.82

Region: NODE\_382493\_length\_8613\_cov\_31.659817 4994-5010. Max. coverage (+): 74.85. Max coverage (-): 0.41

Region: NODE\_382493\_length\_8613\_cov\_31.659817 5011-5028. Max. coverage (+): 18.87. Max coverage (-): 0.07

Region: NODE\_382493\_length\_8613\_cov\_31.659817 5029-5045. Max. coverage (+): 22.84. Max coverage (-): 5.34

Region: NODE\_382493\_length\_8613\_cov\_31.659817 5046-5063. Max. coverage (+): 3.15. Max coverage (-): 1.82

Region: NODE\_382493\_length\_8613\_cov\_31.659817 5064-5080. Max. coverage (+): 1.93. Max coverage (-): 0.19

Region: NODE\_382493\_length\_8613\_cov\_31.659817 5081-5098. Max. coverage (+): 0.59. Max coverage (-): 1.33

Region: NODE\_382493\_length\_8613\_cov\_31.659817 5099-5115. Max. coverage (+): 4.23. Max coverage (-): 0.07

Region: NODE\_382493\_length\_8613\_cov\_31.659817 5116-5133. Max. coverage (+): 1.04. Max coverage (-): 0.44

Region: NODE\_382493\_length\_8613\_cov\_31.659817 5134-5150. Max. coverage (+): 11.16. Max coverage (-): 0.15

Region: NODE\_382493\_length\_8613\_cov\_31.659817 5151-5168. Max. coverage (+): 9.27. Max coverage (-): 1.37

Region: NODE\_382493\_length\_8613\_cov\_31.659817 5169-5185. Max. coverage (+): 5.86. Max coverage (-): 1.04

Region: NODE\_382493\_length\_8613\_cov\_31.659817 5186-5203. Max. coverage (+): 2.48. Max coverage (-): 0

Region: NODE\_382493\_length\_8613\_cov\_31.659817 5204-5220. Max. coverage (+): 11.27. Max coverage (-): 2.04

Region: NODE\_382493\_length\_8613\_cov\_31.659817 5221-5238. Max. coverage (+): 15.46. Max coverage (-): 0.44

Region: NODE\_382493\_length\_8613\_cov\_31.659817 5239-5255. Max. coverage (+): 0.11. Max coverage (-): 1.04

Region: NODE\_382493\_length\_8613\_cov\_31.659817 5256-5273. Max. coverage (+): 0.15. Max coverage (-): 0.26

Region: NODE\_382493\_length\_8613\_cov\_31.659817 5274-5290. Max. coverage (+): 0.22. Max coverage (-): 2.48

Region: NODE\_382493\_length\_8613\_cov\_31.659817 5291-5307. Max. coverage (+): 1.04. Max coverage (-): 0.59

Region: NODE\_382493\_length\_8613\_cov\_31.659817 5308-5325. Max. coverage (+): 0.89. Max coverage (-): 0.59

Region: NODE\_382493\_length\_8613\_cov\_31.659817 5326-5342. Max. coverage (+): 1.82. Max coverage (-): 0.52

Region: NODE\_382493\_length\_8613\_cov\_31.659817 5343-5360. Max. coverage (+): 19.98. Max coverage (-): 2.11

Region: NODE\_382493\_length\_8613\_cov\_31.659817 5361-5377. Max. coverage (+): 29.25. Max coverage (-): 0.15

Region: NODE\_382493\_length\_8613\_cov\_31.659817 5378-5395. Max. coverage (+): 2.26. Max coverage (-): 0.19

Region: NODE\_382493\_length\_8613\_cov\_31.659817 5396-5412. Max. coverage (+): 0.56. Max coverage (-): 0.19

Region: NODE\_382493\_length\_8613\_cov\_31.659817 5413-5430. Max. coverage (+): 316.08. Max coverage (-): 0.22

Region: NODE\_382493\_length\_8613\_cov\_31.659817 5431-5447. Max. coverage (+): 1.45. Max coverage (-): 8.86

Region: NODE\_382493\_length\_8613\_cov\_31.659817 5448-5465. Max. coverage (+): 0.89. Max coverage (-): 0.15

Region: NODE\_382493\_length\_8613\_cov\_31.659817 5466-5482. Max. coverage (+): 0.74. Max coverage (-): 0.67

Region: NODE\_382493\_length\_8613\_cov\_31.659817 5483-5500. Max. coverage (+): 0.33. Max coverage (-): 0.07

Region: NODE\_382493\_length\_8613\_cov\_31.659817 5501-5517. Max. coverage (+): 0.59. Max coverage (-): 5.41

Region: NODE\_382493\_length\_8613\_cov\_31.659817 5518-5535. Max. coverage (+): 1.08. Max coverage (-): 0.3

Region: NODE\_382493\_length\_8613\_cov\_31.659817 5536-5552. Max. coverage (+): 2.63. Max coverage (-): 0.22

Region: NODE\_382493\_length\_8613\_cov\_31.659817 5553-5570. Max. coverage (+): 38.11. Max coverage (-): 0.11

Region: NODE\_382493\_length\_8613\_cov\_31.659817 5571-5587. Max. coverage (+): 9.23. Max coverage (-): 0.48

Region: NODE\_382493\_length\_8613\_cov\_31.659817 5588-5605. Max. coverage (+): 3.08. Max coverage (-): 0.44

Region: NODE\_382493\_length\_8613\_cov\_31.659817 5606-5622. Max. coverage (+): 0.2. Max coverage (-): 0.07

Region: NODE\_382493\_length\_8613\_cov\_31.659817 5623-5640. Max. coverage (+): 1.74. Max coverage (-): 0.07

Region: NODE\_382493\_length\_8613\_cov\_31.659817 5641-5657. Max. coverage (+): 1.41. Max coverage (-): 0.11

Region: NODE\_382493\_length\_8613\_cov\_31.659817 5658-5674. Max. coverage (+): 6.12. Max coverage (-): 0.52

Region: NODE\_382493\_length\_8613\_cov\_31.659817 5675-5692. Max. coverage (+): 20.69. Max coverage (-): 2

Region: NODE\_382493\_length\_8613\_cov\_31.659817 5693-5709. Max. coverage (+): 31.44. Max coverage (-): 0.37

Region: NODE\_382493\_length\_8613\_cov\_31.659817 5710-5727. Max. coverage (+): 31.25. Max coverage (-): 0.26

Region: NODE\_382493\_length\_8613\_cov\_31.659817 5728-5744. Max. coverage (+): 0.56. Max coverage (-): 0.11

Region: NODE\_382493\_length\_8613\_cov\_31.659817 5745-5762. Max. coverage (+): 0.7. Max coverage (-): 1.59

Region: NODE\_382493\_length\_8613\_cov\_31.659817 5763-5779. Max. coverage (+): 2.63. Max coverage (-): 0.96

Region: NODE\_382493\_length\_8613\_cov\_31.659817 5780-5797. Max. coverage (+): 2.6. Max coverage (-): 0.26

Region: NODE\_382493\_length\_8613\_cov\_31.659817 5798-5814. Max. coverage (+): 4.52. Max coverage (-): 0.52

Region: NODE\_382493\_length\_8613\_cov\_31.659817 5815-5832. Max. coverage (+): 7.56. Max coverage (-): 0.63

Region: NODE\_382493\_length\_8613\_cov\_31.659817 5833-5849. Max. coverage (+): 6.78. Max coverage (-): 0.11

Region: NODE\_382493\_length\_8613\_cov\_31.659817 5850-5867. Max. coverage (+): 8.71. Max coverage (-): 0.44

Region: NODE\_382493\_length\_8613\_cov\_31.659817 5868-5884. Max. coverage (+): 47.82. Max coverage (-): 0.04

Region: NODE\_382493\_length\_8613\_cov\_31.659817 5885-5902. Max. coverage (+): 0.33. Max coverage (-): 0.3

Region: NODE\_382493\_length\_8613\_cov\_31.659817 5903-5919. Max. coverage (+): 11.31. Max coverage (-): 0.19

Region: NODE\_382493\_length\_8613\_cov\_31.659817 5920-5937. Max. coverage (+): 8.45. Max coverage (-): 1.19

Region: NODE\_382493\_length\_8613\_cov\_31.659817 5938-5954. Max. coverage (+): 4.71. Max coverage (-): 0.33

Region: NODE\_382493\_length\_8613\_cov\_31.659817 5955-5972. Max. coverage (+): 1.3. Max coverage (-): 0.48

Region: NODE\_382493\_length\_8613\_cov\_31.659817 5973-5989. Max. coverage (+): 2.08. Max coverage (-): 0.3

Region: NODE\_382493\_length\_8613\_cov\_31.659817 5990-6007. Max. coverage (+): 3.04. Max coverage (-): 0.11

Region: NODE\_382493\_length\_8613\_cov\_31.659817 6008-6024. Max. coverage (+): 5.19. Max coverage (-): 0.26

Region: NODE\_382493\_length\_8613\_cov\_31.659817 6025-6041. Max. coverage (+): 2.22. Max coverage (-): 0.33

Region: NODE\_382493\_length\_8613\_cov\_31.659817 6042-6059. Max. coverage (+): 3.26. Max coverage (-): 0.52

Region: NODE\_382493\_length\_8613\_cov\_31.659817 6060-6076. Max. coverage (+): 0.59. Max coverage (-): 0.37

Region: NODE\_382493\_length\_8613\_cov\_31.659817 6077-6094. Max. coverage (+): 0.74. Max coverage (-): 0.67

Region: NODE\_382493\_length\_8613\_cov\_31.659817 6095-6111. Max. coverage (+): 22.28. Max coverage (-): 0.7

Region: NODE\_382493\_length\_8613\_cov\_31.659817 6112-6129. Max. coverage (+): 13.42. Max coverage (-): 0.19

Region: NODE\_382493\_length\_8613\_cov\_31.659817 6130-6146. Max. coverage (+): 0.37. Max coverage (-): 0

Region: NODE\_382493\_length\_8613\_cov\_31.659817 6147-6164. Max. coverage (+): 0.11. Max coverage (-): 0

Region: NODE\_382493\_length\_8613\_cov\_31.659817 6165-6181. Max. coverage (+): 0.33. Max coverage (-): 0

Region: NODE\_382493\_length\_8613\_cov\_31.659817 6182-6199. Max. coverage (+): 0.07. Max coverage (-): 0.04

Region: NODE\_382493\_length\_8613\_cov\_31.659817 6200-6216. Max. coverage (+): 0.19. Max coverage (-): 0.19

Region: NODE\_382493\_length\_8613\_cov\_31.659817 6217-6234. Max. coverage (+): 1.15. Max coverage (-): 0.07

Region: NODE\_382493\_length\_8613\_cov\_31.659817 6235-6251. Max. coverage (+): 0.22. Max coverage (-): 0

Region: NODE\_382493\_length\_8613\_cov\_31.659817 6252-6269. Max. coverage (+): 0.56. Max coverage (-): 0.22

Region: NODE\_382493\_length\_8613\_cov\_31.659817 6270-6286. Max. coverage (+): 1.37. Max coverage (-): 0.48

Region: NODE\_382493\_length\_8613\_cov\_31.659817 6287-6304. Max. coverage (+): 2.74. Max coverage (-): 0.04

Region: NODE\_382493\_length\_8613\_cov\_31.659817 6305-6321. Max. coverage (+): 3.11. Max coverage (-): 0.15

Region: NODE\_382493\_length\_8613\_cov\_31.659817 6322-6339. Max. coverage (+): 1.04. Max coverage (-): 2

Region: NODE\_382493\_length\_8613\_cov\_31.659817 6340-6356. Max. coverage (+): 53.53. Max coverage (-): 0.33

Region: NODE\_382493\_length\_8613\_cov\_31.659817 6357-6374. Max. coverage (+): 0.33. Max coverage (-): 0.82

Region: NODE\_382493\_length\_8613\_cov\_31.659817 6375-6391. Max. coverage (+): 0.3. Max coverage (-): 0

Region: NODE\_382493\_length\_8613\_cov\_31.659817 6392-6408. Max. coverage (+): 0.15. Max coverage (-): 0.04

Region: NODE\_382493\_length\_8613\_cov\_31.659817 6409-6426. Max. coverage (+): 4.67. Max coverage (-): 0.15

Region: NODE\_382493\_length\_8613\_cov\_31.659817 6427-6443. Max. coverage (+): 0.78. Max coverage (-): 0.07

Region: NODE\_382493\_length\_8613\_cov\_31.659817 6444-6461. Max. coverage (+): 42.56. Max coverage (-): 0.07

Region: NODE\_382493\_length\_8613\_cov\_31.659817 6462-6478. Max. coverage (+): 58.06. Max coverage (-): 0.07

Region: NODE\_382493\_length\_8613\_cov\_31.659817 6479-6496. Max. coverage (+): 0.48. Max coverage (-): 0.04

Region: NODE\_382493\_length\_8613\_cov\_31.659817 6497-6513. Max. coverage (+): 0.22. Max coverage (-): 0.37

Region: NODE\_382493\_length\_8613\_cov\_31.659817 6514-6531. Max. coverage (+): 5.52. Max coverage (-): 0.11

Region: NODE\_382493\_length\_8613\_cov\_31.659817 6532-6548. Max. coverage (+): 0.33. Max coverage (-): 0.22

Region: NODE\_382493\_length\_8613\_cov\_31.659817 6549-6566. Max. coverage (+): 1.26. Max coverage (-): 0.19

Region: NODE\_382493\_length\_8613\_cov\_31.659817 6567-6583. Max. coverage (+): 3.48. Max coverage (-): 0.22

Region: NODE\_382493\_length\_8613\_cov\_31.659817 6584-6601. Max. coverage (+): 23.47. Max coverage (-): 0.26

Region: NODE\_382493\_length\_8613\_cov\_31.659817 6602-6618. Max. coverage (+): 22.32. Max coverage (-): 0.07

Region: NODE\_382493\_length\_8613\_cov\_31.659817 6619-6636. Max. coverage (+): 1.63. Max coverage (-): 0.04

Region: NODE\_382493\_length\_8613\_cov\_31.659817 6637-6653. Max. coverage (+): 9.16. Max coverage (-): 0.07

Region: NODE\_382493\_length\_8613\_cov\_31.659817 6654-6671. Max. coverage (+): 0.19. Max coverage (-): 0.56

Region: NODE\_382493\_length\_8613\_cov\_31.659817 6672-6688. Max. coverage (+): 0.15. Max coverage (-): 0.04

Region: NODE\_382493\_length\_8613\_cov\_31.659817 6689-6706. Max. coverage (+): 0.75. Max coverage (-): 0

Region: NODE\_382493\_length\_8613\_cov\_31.659817 6707-6723. Max. coverage (+): 0.59. Max coverage (-): 0.11

Region: NODE\_382493\_length\_8613\_cov\_31.659817 6724-6740. Max. coverage (+): 0.33. Max coverage (-): 0.07

Region: NODE\_382493\_length\_8613\_cov\_31.659817 6741-6758. Max. coverage (+): 0.37. Max coverage (-): 0.04

Region: NODE\_382493\_length\_8613\_cov\_31.659817 6759-6775. Max. coverage (+): 1.78. Max coverage (-): 0.41

Region: NODE\_382493\_length\_8613\_cov\_31.659817 6776-6793. Max. coverage (+): 0.19. Max coverage (-): 0.33

Region: NODE\_382493\_length\_8613\_cov\_31.659817 6794-6810. Max. coverage (+): 13.01. Max coverage (-): 0.48

Region: NODE\_382493\_length\_8613\_cov\_31.659817 6811-6828. Max. coverage (+): 4.37. Max coverage (-): 0.85

Region: NODE\_382493\_length\_8613\_cov\_31.659817 6829-6845. Max. coverage (+): 1.3. Max coverage (-): 0.11

Region: NODE\_382493\_length\_8613\_cov\_31.659817 6846-6863. Max. coverage (+): 2.6. Max coverage (-): 0.19

Region: NODE\_382493\_length\_8613\_cov\_31.659817 6864-6880. Max. coverage (+): 3.52. Max coverage (-): 0.11

Region: NODE\_382493\_length\_8613\_cov\_31.659817 6881-6898. Max. coverage (+): 0.59. Max coverage (-): 0

Region: NODE\_382493\_length\_8613\_cov\_31.659817 6899-6915. Max. coverage (+): 0.22. Max coverage (-): 0.44

Region: NODE\_382493\_length\_8613\_cov\_31.659817 6916-6933. Max. coverage (+): 0.22. Max coverage (-): 0.11

Region: NODE\_382493\_length\_8613\_cov\_31.659817 6934-6950. Max. coverage (+): 2.47. Max coverage (-): 0.06

Region: NODE\_382493\_length\_8613\_cov\_31.659817 6951-6968. Max. coverage (+): 14.85. Max coverage (-): 0.13

Region: NODE\_382493\_length\_8613\_cov\_31.659817 6969-6985. Max. coverage (+): 0.24. Max coverage (-): 0.04

Region: NODE\_382493\_length\_8613\_cov\_31.659817 6986-7003. Max. coverage (+): 1.56. Max coverage (-): 0.04

Region: NODE\_382493\_length\_8613\_cov\_31.659817 7004-7020. Max. coverage (+): 1.19. Max coverage (-): 0.33

Region: NODE\_382493\_length\_8613\_cov\_31.659817 7021-7038. Max. coverage (+): 1.09. Max coverage (-): 0.3

Region: NODE\_382493\_length\_8613\_cov\_31.659817 7039-7055. Max. coverage (+): 1.06. Max coverage (-): 0.35

Region: NODE\_382493\_length\_8613\_cov\_31.659817 7056-7073. Max. coverage (+): 0.67. Max coverage (-): 0.07

Region: NODE\_382493\_length\_8613\_cov\_31.659817 7074-7090. Max. coverage (+): 1.46. Max coverage (-): 0.04

Region: NODE\_382493\_length\_8613\_cov\_31.659817 7091-7107. Max. coverage (+): 2.72. Max coverage (-): 0.19

Region: NODE\_382493\_length\_8613\_cov\_31.659817 7108-7125. Max. coverage (+): 0.59. Max coverage (-): 0.26

Region: NODE\_382493\_length\_8613\_cov\_31.659817 7126-7142. Max. coverage (+): 0.59. Max coverage (-): 0.48

Region: NODE\_382493\_length\_8613\_cov\_31.659817 7143-7160. Max. coverage (+): 1.71. Max coverage (-): 0.33

Region: NODE\_382493\_length\_8613\_cov\_31.659817 7161-7177. Max. coverage (+): 6.56. Max coverage (-): 0.11

Region: NODE\_382493\_length\_8613\_cov\_31.659817 7178-7195. Max. coverage (+): 2.19. Max coverage (-): 0.22

Region: NODE\_382493\_length\_8613\_cov\_31.659817 7196-7212. Max. coverage (+): 0.63. Max coverage (-): 0

Region: NODE\_382493\_length\_8613\_cov\_31.659817 7213-7230. Max. coverage (+): 0.26. Max coverage (-): 0.22

Region: NODE\_382493\_length\_8613\_cov\_31.659817 7231-7247. Max. coverage (+): 2.19. Max coverage (-): 0

Region: NODE\_382493\_length\_8613\_cov\_31.659817 7248-7265. Max. coverage (+): 0.44. Max coverage (-): 0.3

Region: NODE\_382493\_length\_8613\_cov\_31.659817 7266-7282. Max. coverage (+): 1.04. Max coverage (-): 0.04

Region: NODE\_382493\_length\_8613\_cov\_31.659817 7283-7300. Max. coverage (+): 3.48. Max coverage (-): 2.78

Region: NODE\_382493\_length\_8613\_cov\_31.659817 7301-7317. Max. coverage (+): 3.26. Max coverage (-): 0.78

Region: NODE\_382493\_length\_8613\_cov\_31.659817 7318-7335. Max. coverage (+): 0.22. Max coverage (-): 0.59

Region: NODE\_382493\_length\_8613\_cov\_31.659817 7336-7352. Max. coverage (+): 0.85. Max coverage (-): 0.22

Region: NODE\_382493\_length\_8613\_cov\_31.659817 7353-7370. Max. coverage (+): 1. Max coverage (-): 0.22

Region: NODE\_382493\_length\_8613\_cov\_31.659817 7371-7387. Max. coverage (+): 0.96. Max coverage (-): 0.19

Region: NODE\_382493\_length\_8613\_cov\_31.659817 7388-7405. Max. coverage (+): 0.74. Max coverage (-): 7.3

Region: NODE\_382493\_length\_8613\_cov\_31.659817 7406-7422. Max. coverage (+): 1.33. Max coverage (-): 0.11

Region: NODE\_382493\_length\_8613\_cov\_31.659817 7423-7440. Max. coverage (+): 2.52. Max coverage (-): 0.22

Region: NODE\_382493\_length\_8613\_cov\_31.659817 7441-7457. Max. coverage (+): 1.26. Max coverage (-): 0.59

Region: NODE\_382493\_length\_8613\_cov\_31.659817 7458-7474. Max. coverage (+): 0.3. Max coverage (-): 0.63

Region: NODE\_382493\_length\_8613\_cov\_31.659817 7475-7492. Max. coverage (+): 0.78. Max coverage (-): 0.63

Region: NODE\_382493\_length\_8613\_cov\_31.659817 7493-7509. Max. coverage (+): 22.87. Max coverage (-): 0.04

Region: NODE\_382493\_length\_8613\_cov\_31.659817 7510-7527. Max. coverage (+): 3.67. Max coverage (-): 0.04

Region: NODE\_382493\_length\_8613\_cov\_31.659817 7528-7544. Max. coverage (+): 7.93. Max coverage (-): 0.15

Region: NODE\_382493\_length\_8613\_cov\_31.659817 7545-7562. Max. coverage (+): 36.26. Max coverage (-): 0.26

Region: NODE\_382493\_length\_8613\_cov\_31.659817 7563-7579. Max. coverage (+): 0.59. Max coverage (-): 0.11

Region: NODE\_382493\_length\_8613\_cov\_31.659817 7580-7597. Max. coverage (+): 6.23. Max coverage (-): 1.96

Region: NODE\_382493\_length\_8613\_cov\_31.659817 7598-7614. Max. coverage (+): 20.58. Max coverage (-): 0.48

Region: NODE\_382493\_length\_8613\_cov\_31.659817 7615-7632. Max. coverage (+): 23.58. Max coverage (-): 0.11

Region: NODE\_382493\_length\_8613\_cov\_31.659817 7633-7649. Max. coverage (+): 23.58. Max coverage (-): 0.59

Region: NODE\_382493\_length\_8613\_cov\_31.659817 7650-7667. Max. coverage (+): 33.18. Max coverage (-): 0.26

Region: NODE\_382493\_length\_8613\_cov\_31.659817 7668-7684. Max. coverage (+): 10.31. Max coverage (-): 0.78

Region: NODE\_382493\_length\_8613\_cov\_31.659817 7685-7702. Max. coverage (+): 22.21. Max coverage (-): 0.22

Region: NODE\_382493\_length\_8613\_cov\_31.659817 7703-7719. Max. coverage (+): 3.78. Max coverage (-): 0.48

Region: NODE\_382493\_length\_8613\_cov\_31.659817 7720-7737. Max. coverage (+): 6.97. Max coverage (-): 1.37

Region: NODE\_382493\_length\_8613\_cov\_31.659817 7738-7754. Max. coverage (+): 2.67. Max coverage (-): 0.56

Region: NODE\_382493\_length\_8613\_cov\_31.659817 7755-7772. Max. coverage (+): 3.93. Max coverage (-): 0.78

Region: NODE\_382493\_length\_8613\_cov\_31.659817 7773-7789. Max. coverage (+): 1.96. Max coverage (-): 0.11

Region: NODE\_382493\_length\_8613\_cov\_31.659817 7790-7807. Max. coverage (+): 1.19. Max coverage (-): 0

Region: NODE\_382493\_length\_8613\_cov\_31.659817 7808-7824. Max. coverage (+): 0.04. Max coverage (-): 0.04

Region: NODE\_382493\_length\_8613\_cov\_31.659817 7825-7841. Max. coverage (+): 0.07. Max coverage (-): 0.04

Region: NODE\_382493\_length\_8613\_cov\_31.659817 7842-7859. Max. coverage (+): 0.74. Max coverage (-): 0.07

Region: NODE\_382493\_length\_8613\_cov\_31.659817 7860-7876. Max. coverage (+): 19.76. Max coverage (-): 0.07

Region: NODE\_382493\_length\_8613\_cov\_31.659817 7877-7894. Max. coverage (+): 11.23. Max coverage (-): 0.19

Region: NODE\_382493\_length\_8613\_cov\_31.659817 7895-7911. Max. coverage (+): 0.11. Max coverage (-): 1.22

Region: NODE\_382493\_length\_8613\_cov\_31.659817 7912-7929. Max. coverage (+): 8.86. Max coverage (-): 0.33

Region: NODE\_382493\_length\_8613\_cov\_31.659817 7930-7946. Max. coverage (+): 8.93. Max coverage (-): 1.26

Region: NODE\_382493\_length\_8613\_cov\_31.659817 7947-7964. Max. coverage (+): 1.63. Max coverage (-): 0.11

Region: NODE\_382493\_length\_8613\_cov\_31.659817 7965-7981. Max. coverage (+): 1.37. Max coverage (-): 0.04

Region: NODE\_382493\_length\_8613\_cov\_31.659817 7982-7999. Max. coverage (+): 4.3. Max coverage (-): 0.11

Region: NODE\_382493\_length\_8613\_cov\_31.659817 8000-8016. Max. coverage (+): 18.09. Max coverage (-): 0.04

Region: NODE\_382493\_length\_8613\_cov\_31.659817 8017-8034. Max. coverage (+): 5.12. Max coverage (-): 0.04

Region: NODE\_382493\_length\_8613\_cov\_31.659817 8035-8051. Max. coverage (+): 0.33. Max coverage (-): 0.26

Region: NODE\_382493\_length\_8613\_cov\_31.659817 8052-8069. Max. coverage (+): 36.03. Max coverage (-): 0.33

Region: NODE\_382493\_length\_8613\_cov\_31.659817 8070-8086. Max. coverage (+): 0.19. Max coverage (-): 0.89

Region: NODE\_382493\_length\_8613\_cov\_31.659817 8087-8104. Max. coverage (+): 61.87. Max coverage (-): 0.11

Region: NODE\_382493\_length\_8613\_cov\_31.659817 8105-8121. Max. coverage (+): 36.26. Max coverage (-): 0.19

Region: NODE\_382493\_length\_8613\_cov\_31.659817 8122-8139. Max. coverage (+): 0.52. Max coverage (-): 0.44

Region: NODE\_382493\_length\_8613\_cov\_31.659817 8140-8156. Max. coverage (+): 3.41. Max coverage (-): 0.78

Region: NODE\_382493\_length\_8613\_cov\_31.659817 8157-8174. Max. coverage (+): 10.05. Max coverage (-): 0.89

Region: NODE\_382493\_length\_8613\_cov\_31.659817 8175-8191. Max. coverage (+): 1.96. Max coverage (-): 0.33

Region: NODE\_382493\_length\_8613\_cov\_31.659817 8192-8208. Max. coverage (+): 2.67. Max coverage (-): 0.37

Region: NODE\_382493\_length\_8613\_cov\_31.659817 8209-8226. Max. coverage (+): 2.22. Max coverage (-): 0

Region: NODE\_382493\_length\_8613\_cov\_31.659817 8227-8243. Max. coverage (+): 0.67. Max coverage (-): 0.04

Region: NODE\_382493\_length\_8613\_cov\_31.659817 8244-8261. Max. coverage (+): 0.59. Max coverage (-): 2.19

Region: NODE\_382493\_length\_8613\_cov\_31.659817 8262-8278. Max. coverage (+): 20.54. Max coverage (-): 0.07

Region: NODE\_382493\_length\_8613\_cov\_31.659817 8279-8296. Max. coverage (+): 0.3. Max coverage (-): 1.78

Region: NODE\_382493\_length\_8613\_cov\_31.659817 8297-8313. Max. coverage (+): 7.01. Max coverage (-): 0.19

Region: NODE\_382493\_length\_8613\_cov\_31.659817 8314-8331. Max. coverage (+): 163.53. Max coverage (-): 0.44

Region: NODE\_382493\_length\_8613\_cov\_31.659817 8332-8348. Max. coverage (+): 170.39. Max coverage (-): 0.22

Region: NODE\_382493\_length\_8613\_cov\_31.659817 8349-8366. Max. coverage (+): 0.15. Max coverage (-): 1.63

Region: NODE\_382493\_length\_8613\_cov\_31.659817 8367-8383. Max. coverage (+): 52.12. Max coverage (-): 0

Region: NODE\_382493\_length\_8613\_cov\_31.659817 8384-8401. Max. coverage (+): 0.11. Max coverage (-): 0.07

Region: NODE\_382493\_length\_8613\_cov\_31.659817 8402-8418. Max. coverage (+): 4.37. Max coverage (-): 0.07

Region: NODE\_382493\_length\_8613\_cov\_31.659817 8419-8436. Max. coverage (+): 1.45. Max coverage (-): 0.67

Region: NODE\_382493\_length\_8613\_cov\_31.659817 8437-8453. Max. coverage (+): 60.28. Max coverage (-): 0.93

Region: NODE\_382493\_length\_8613\_cov\_31.659817 8454-8471. Max. coverage (+): 7.6. Max coverage (-): 1.37

Region: NODE\_382493\_length\_8613\_cov\_31.659817 8472-8488. Max. coverage (+): 20.06. Max coverage (-): 0.07

Region: NODE\_382493\_length\_8613\_cov\_31.659817 8489-8506. Max. coverage (+): 3. Max coverage (-): 0.19

Region: NODE\_382493\_length\_8613\_cov\_31.659817 8507-8523. Max. coverage (+): 3. Max coverage (-): 0.11

Region: NODE\_382493\_length\_8613\_cov\_31.659817 8524-8541. Max. coverage (+): 9.53. Max coverage (-): 2.22

Region: NODE\_382493\_length\_8613\_cov\_31.659817 8542-8558. Max. coverage (+): 1.26. Max coverage (-): 0.15

Region: NODE\_382493\_length\_8613\_cov\_31.659817 8559-8575. Max. coverage (+): 0.48. Max coverage (-): 0.41

Region: NODE\_382493\_length\_8613\_cov\_31.659817 8576-8593. Max. coverage (+): 42.78. Max coverage (-): 2.74

Region: NODE\_382493\_length\_8613\_cov\_31.659817 8594-8610. Max. coverage (+): 8.45. Max coverage (-): 2.78

Region: NODE\_382493\_length\_8613\_cov\_31.659817 8611-8628. Max. coverage (+): 2.71. Max coverage (-): 0.11

Region: NODE\_382493\_length\_8613\_cov\_31.659817 8629-8645. Max. coverage (+): 3.63. Max coverage (-): 0.11

Region: NODE\_382493\_length\_8613\_cov\_31.659817 8646-8663. Max. coverage (+): 0.89. Max coverage (-): 0

Region: NODE\_382493\_length\_8613\_cov\_31.659817 8664-8680. Max. coverage (+): 0.82. Max coverage (-): 0.04

Region: NODE\_382493\_length\_8613\_cov\_31.659817 8681-8698. Max. coverage (+): 0.37. Max coverage (-): 0.04

Region: NODE\_382493\_length\_8613\_cov\_31.659817 8699-8715. Max. coverage (+): 0.02. Max coverage (-): 0

Region: NODE\_382493\_length\_8613\_cov\_31.659817 8716-8733. Max. coverage (+): 0. Max coverage (-): 0

Region: NODE\_382493\_length\_8613\_cov\_31.659817 8734-. Max. coverage (+): 0. Max coverage (-): 0

RepeatMasker Color Code

**+**

100-98% Identity

<98-95% Identity

<95-90% Identity

<90-85% Identity

<85-80% Identity

<80-75% Identity

<75-70% Identity

<70% Identity

**-**

Gene Set Color Code

**+**

Gene

Pseudogene

Other

**-**

Topology/Coverage Color Code

Coverage Plus Strand

Coverage Minus Strand

Mainstrand: Plus

Mainstrand: Minus

Complementary Strand

Flanking Region  
(if option -flank >0)

Gene Set Annotation  
  
RepeatMasker Annotation  

**1. AlRepC-2145**: 1-169 (+), Divergence to consensus: 16.8%  
**2. AlRepA-66**: 170-261 (-), Divergence to consensus: 27.4%  
**3. AlRepB-731**: 270-521 (+), Divergence to consensus: 16.5%  
**4. AlRepC-625**: 522-900 (+), Divergence to consensus: 7.8%  
**5. hAT-N74\_DR**: 901-963 (+), Divergence to consensus: 11.9%  
**6. AlRepB-193**: 1286-1334 (+), Divergence to consensus: 8.2%  
**7. Polinton-2\_CI**: 1741-1793 (+), Divergence to consensus: 26.7%  
**8. AlRepB-569**: 1909-1947 (+), Divergence to consensus: 12.9%  
**9. AlRepD-1254**: 2481-2615 (+), Divergence to consensus: 41.5%  
**10. AlRepD-1254**: 2617-2661 (+), Divergence to consensus: 20%  
**11. AlRepC-847**: 2675-3124 (-), Divergence to consensus: 39.2%  
**12. AlRepD-1254**: 3366-3501 (+), Divergence to consensus: 25.2%  
**13. hAT-24N1\_DR**: 3476-3642 (+), Divergence to consensus: 34.9%  
**14. AlRepC-743**: 5011-5236 (-), Divergence to consensus: 35.8%  
**15. AlRepA-93**: 5541-5649 (-), Divergence to consensus: 19.2%  
**16. AlRepB-269**: 5680-5823 (-), Divergence to consensus: 37.8%  
**17. AlRepB-269**: 5978-6059 (-), Divergence to consensus: 17.1%  
**18. (AC)n**: 6146-6165 (+), Divergence to consensus: 0%  
**19. AlRepC-670**: 6529-6593 (-), Divergence to consensus: 16.9%  
**20. AlRepB-738**: 6596-6749 (+), Divergence to consensus: 16.2%  
**21. AlRepB-103**: 6761-6987 (+), Divergence to consensus: 29.6%  
**22. AlRepB-569**: 6965-7003 (+), Divergence to consensus: 12.9%  
**23. AlRepC-905**: 7619-7701 (+), Divergence to consensus: 35%  
**24. AlRepC-373**: 7639-7748 (-), Divergence to consensus: 33.6%  
**25. L1-1\_AFC**: 8652-8680 (+), Divergence to consensus: 3.5%  
**26. L1-1\_AFC**: 8681-8743 (+), Divergence to consensus: 0%

  
Transcription Factor Binding Sites  

**RHOXF1** (Sequence: AGCTCA (-): 1982)  
**RHOXF1** (Sequence: AGCTCA (-): 2402)  
**RHOXF1** (Sequence: GGCTCA (-): 2838)  
**RHOXF1** (Sequence: AGCTTA (-): 4852)  
**RHOXF1** (Sequence: AGCTCA (-): 5423)  
**RHOXF1** (Sequence: GGATTA (-): 5994)  
**RHOXF1** (Sequence: AGATCA (-): 6087)  
**RHOXF1** (Sequence: GGATTA (-): 6715)  
**RHOXF1** (Sequence: AGCTCA (-): 6909)  
**RHOXF1** (Sequence: AGCTCA (-): 7038)  
**RHOXF1** (Sequence: TAAGCT (+): 410)  
**RHOXF1** (Sequence: TGAGCT (+): 683)  
**RHOXF1** (Sequence: TGATCT (+): 1566)  
**RHOXF1** (Sequence: TAAGCT (+): 1943)  
**RHOXF1** (Sequence: TGATCC (+): 2627)  
**RHOXF1** (Sequence: TAATCT (+): 2642)  
**RHOXF1** (Sequence: TAATCT (+): 3518)  
**RHOXF1** (Sequence: TGAGCC (+): 4431)  
**RHOXF1** (Sequence: TAAGCT (+): 4540)  
**RHOXF1** (Sequence: TAATCT (+): 4992)  
**RHOXF1** (Sequence: TGAGCT (+): 5096)  
**RHOXF1** (Sequence: TAATCT (+): 5154)  
**RHOXF1** (Sequence: TAATCT (+): 5299)  
**RHOXF1** (Sequence: TAAGCT (+): 6186)  
**RHOXF1** (Sequence: TAAGCT (+): 6999)  
**RHOXF1** (Sequence: TAAGCC (+): 7948)  
**RHOXF1** (Sequence: TGATCT (+): 8305)  
**Gata4** (Sequence: CTTATCT (+): 5939)  
**Gata4** (Sequence: CTTATCT (+): 7828)  
**POU5F1** (Sequence: TTTGCAT (-): 3391)  
**RFX4\_2** (Sequence: GTAACTAGG (-): 8175)  
**SOX9** (Sequence: AACAATAA (-): 2120)  
**SOX9** (Sequence: AACAATGA (-): 3771)  
**SOX9** (Sequence: AACAATAA (-): 8563)  
**FOXP1** (Sequence: GTAAACA (+): 4310)  
**FOXO3\_mmu** (Sequence: TGTTTTCA (-): 4384)  
**FOXO3\_mmu** (Sequence: TGTTTTCC (-): 6522)  
**Sox5** (Sequence: ATTGTT (+): 1651)  
**Sox5** (Sequence: ATTGTT (+): 4707)  
**Sox5** (Sequence: ATTGTT (+): 6428)  
**Sox5** (Sequence: ATTGTT (+): 7928)  
**FOXO3\_mmu** (Sequence: TCAAAACA (+): 4094)  
**FOXO3\_mmu** (Sequence: TGTAAACA (+): 4309)  
**FOXO3\_mmu** (Sequence: TCTAAACA (+): 6265)  
**Nobox** (Sequence: GGTAATTA (-): 5282)  
**Nobox** (Sequence: AGCAATTA (-): 7294)  
**POU2F1** (Sequence: ATTTACATA (-): 6443)  
**POU2F1** (Sequence: ATTTACATA (-): 7489)  
**POU2F1** (Sequence: ATTAAAATA (-): 8640)  
**Rhox11** (Sequence: ATTACAGCG (-): 2281)  
**Rhox11** (Sequence: TAAACACCA (-): 4311)  
**Gata4** (Sequence: AGATAAC (-): 1696)  
**Gata4** (Sequence: AGATAAG (-): 2254)  
**Sox5** (Sequence: AACAAT (-): 1118)  
**Sox5** (Sequence: AACAAT (-): 2120)  
**Sox5** (Sequence: AACAAT (-): 2127)  
**Sox5** (Sequence: AACAAT (-): 2297)  
**Sox5** (Sequence: AACAAT (-): 3771)  
**Sox5** (Sequence: AACAAT (-): 7396)  
**Sox5** (Sequence: AACAAT (-): 7576)  
**Sox5** (Sequence: AACAAT (-): 8563)  
**POU2F1** (Sequence: TATGTTAAT (+): 799)  
**POU2F1** (Sequence: TATGTAAAT (+): 6368)  
**POU2F1** (Sequence: TATTTAAAT (+): 6627)  
**POU2F1** (Sequence: TATGCAAAT (+): 7921)  
**POU2F1** (Sequence: TATTCAAAT (+): 8608)  
**POU5F1** (Sequence: ATGCAAA (+): 2703)  
**POU5F1** (Sequence: ATGCAAA (+): 4753)  
**POU5F1** (Sequence: ATGCAAA (+): 6634)  
**POU5F1** (Sequence: ATGCAAA (+): 7922)
