# Supplementary material for: First characterization of PIWI-interacting RNA clusters in a cichlid fish with a B chromosome
Source: BMC Biol. 2022 Sep 21;20:204. doi: 10.1186/s12915-022-01403-2 (PMC9490952; doi:10.1186/s12915-022-01403-2)
Supplement: Supplementary file 1 — Additional file 1. Zipped folder with fasta and interactive html piRNA cluster information for the A. latifasciata genome. The nomenclature is as follows: number-pirna-cluster_sex_B-presence (f, female; m, male; 0b, without B chromosome; 1b, with B chromosome). [file 12915_2022_1403_MOESM1_ESM.zip › 149_m1b.html]

piRNA cluster 149\_m1b 88


Predicted piRNA cluster no. 149\_m1b
  

Show proTRAC run info
Hide proTRAC run info

/\  
                \_\_\_\_\_\_\_\_\_\_\_\_\_\_\_\_\_\_\_\_\_\_\_/\\_\_\_ /  \\_\_\_\_\_\_\_  
               I                      /  \  /    \      I  
               I     pro             /    \/      \     I  
               I        TRAC        /               \   I  
               I   \_\_\_\_\_\_\_\_\_\_\_\_\_\_\_\_/\_\_\_\_\_\_\_\_\_\_\_\_\_\_\_\_\_\\_ I  
               I   \              /                     I  
               I    \            /                      I  
               I     \  /\      /       V.2.4.2         I  
               I      \/  \    /                        I  
               I\_\_\_\_\_\_\_\_\_\_\_\  /\_\_\_\_\_\_\_\_\_\_\_\_\_\_\_\_\_\_\_\_\_\_\_\_\_I  
                            \/  
  
  
================================= proTRAC ====================================  
VERSION: .......... 2.4.2  
LAST MODIFIED: .... 11. May 2018  
  
Please cite:  
Rosenkranz D, Zischler H. proTRAC - a software for probabilistic piRNA cluster  
detection, visualization and analysis. 2012. BMC Bioinformatics 13:5.  
  
  
Contact:  
David Rosenkranz  
Institute of Organismic and Molecular Evolutionary Biology  
Dept. Anthropology, small RNA group  
Johannes Gutenberg University Mainz  
email: rosenkranz@uni-mainz.de  
  
You can find the latest proTRAC version at:  
http://sourceforge.net/projects/protrac/files  
http://www.smallRNAgroup-mainz.de/software  
==============================================================================  
  
PARAMETERS:  
Map file: ...............piwi-machos-1B.fa-collapse.map  
Genome file: ............../../../0B\_ala\_genome.fa  
RepeatMasker annotation: Alatifasciata-all0B-maryan-v2.fa\_corrected.out  
GeneSet:................./guest-storage/Data/annotation/Alatifasciata\_all0B\_maryan-v2\_out2017.gff  
  
Significant (p<=0.01) hit density will be calculated based  
on observed hit distribution.  
  
Sliding window size: ........................................ 5000 bp  
Sliding window increament: .................................. 1000 bp  
Normalize each hit by number of genomic hits: ............... yes  
Normalize each hit by number of sequence reads: ............. yes  
Normalize values (-> per million mapped reads): ............. yes  
Min. fraction of hits with 1T(U) or 10A: .................... 0.75  
Alternatively: Min. fraction of hits with 1T(U) and 10A: .... 0.5  
Min. fraction of hits with typical piRNA length: ............ 0.75  
Typical piRNA length: ....................................... 24-32 nt  
Min. size of a piRNA cluster: ............................... 1000 bp.  
Min. number of hits (absolute): ............................. 0  
Min. number of hits (normalized): ........................... 0  
Min. fraction of hits on the mainstrand: .................... 0.75  
Top fraction of mapped sequences (in terms of read counts): . 1%  
Top fraction accounts for max. n% of sequence reads: ........ 90%  
Min. fraction of hits on each arm of a bidirectional cluster: 0.05  
Output html file for each cluster: .......................... yes  
Output a summary table: ..................................... yes  
Output a FASTA file for each cluster (piRNA sequences): ..... yes  
Output a FASTA file comprising cluster sequences: ........... yes  
Output a GTF file for predicted piRNA clusters: ..............yes  
Search DNA motifs in clusters: .............................. yes  
Output flanking sequences: +/- .............................. 0 bp  
Output ~.pTi file: .......................................... no  
==============================================================================  
  
  
Genome size (without gaps): ............ 758543724 bp  
Gaps (N/X/-): .......................... 417479 bp  
Mapped reads: .......................... 26973943  
Non-identical sequences: ............... 6209225  
Genomic hits: .......................... 48438990  
Significant densitiy of mapped reads: .. 821.144211136946 reads/kb

Show proTRAC cluster info
Hide proTRAC cluster info

|  |  |
| --- | --- |
| Location | NODE\_385231\_length\_5676\_cov\_22.431114 |
| Coordinates | 1-5799 |
| Size [bp] | 5799 |
| Sequence hit loci | 5923 |
| Mapped reads (normalized) | 20481 |
| Mapped reads (normalized) per kb | 3531.8 |
| Normalized reads with 1T (1U) | 81.1% |
| Normalized reads with 10A | 43.6% |
| Normalized reads with length 24-32 nt | 98.3% |
| Normalized reads on the main strand(s) | 96.1% |
| Predicted directionality | mono:plus |

100%

0%

1T (1U)  
reads

10A reads

24-32 nt  
reads

reads on mainstrand

**Either the amount of reads with 1T (1U) OR 10A has to exceed 75% (set with option: -1Tor10A)  
Alternatively the amount of reads with 1T (1U) AND 10A has to exceed 50% (set with option: -1Tand10A)  
Minimum amount of reads with preferred size is 75% (set with option: -pisize)  
Minimum amount of reads on the main strand(s) is 75% (set with option: -clstrand)**

Show read coverage
Hide read coverage

WHAT DO I SEE HERE?  
This chart shows the location of mapped sequence reads within a predicted piRNA cluster. The color refers to the number of genomic hits produced by the sequence read in question. A dark red bar indicates that this sequence read produces many other hits elsewhere in the genome. Many adjacent red or yellow bars can indicate the presence of a multi-copy element such as transposons or rRNA genes. A dark green bar indicates that this sequence read maps uniquely to this locus.

1 hit

2-5 hits

6-10 hits

11-20 hits

21-50 hits

51-100 hits

> 100 hits

NODE\_385231\_length\_5676\_cov\_22.431114

1

5799

Gene Set

RepeatMasker

Mapped  
Reads

112.55

plus strand

minus strand

112.55

Region: NODE\_385231\_length\_5676\_cov\_22.431114 8735-6. Max. coverage (+): 0.2. Max coverage (-): 0.01

Region: NODE\_385231\_length\_5676\_cov\_22.431114 7-18. Max. coverage (+): 0. Max coverage (-): 0

Region: NODE\_385231\_length\_5676\_cov\_22.431114 19-29. Max. coverage (+): 0. Max coverage (-): 0

Region: NODE\_385231\_length\_5676\_cov\_22.431114 30-41. Max. coverage (+): 0.01. Max coverage (-): 0

Region: NODE\_385231\_length\_5676\_cov\_22.431114 42-53. Max. coverage (+): 0.04. Max coverage (-): 0

Region: NODE\_385231\_length\_5676\_cov\_22.431114 54-64. Max. coverage (+): 0. Max coverage (-): 0

Region: NODE\_385231\_length\_5676\_cov\_22.431114 65-76. Max. coverage (+): 0. Max coverage (-): 0

Region: NODE\_385231\_length\_5676\_cov\_22.431114 77-87. Max. coverage (+): 0.01. Max coverage (-): 0.02

Region: NODE\_385231\_length\_5676\_cov\_22.431114 88-99. Max. coverage (+): 0.02. Max coverage (-): 0

Region: NODE\_385231\_length\_5676\_cov\_22.431114 100-111. Max. coverage (+): 0.02. Max coverage (-): 0.02

Region: NODE\_385231\_length\_5676\_cov\_22.431114 112-122. Max. coverage (+): 0.05. Max coverage (-): 0

Region: NODE\_385231\_length\_5676\_cov\_22.431114 123-134. Max. coverage (+): 0.04. Max coverage (-): 0.01

Region: NODE\_385231\_length\_5676\_cov\_22.431114 135-145. Max. coverage (+): 0. Max coverage (-): 0

Region: NODE\_385231\_length\_5676\_cov\_22.431114 146-157. Max. coverage (+): 0. Max coverage (-): 0.04

Region: NODE\_385231\_length\_5676\_cov\_22.431114 158-169. Max. coverage (+): 0.12. Max coverage (-): 0

Region: NODE\_385231\_length\_5676\_cov\_22.431114 170-180. Max. coverage (+): 0.02. Max coverage (-): 0

Region: NODE\_385231\_length\_5676\_cov\_22.431114 181-192. Max. coverage (+): 0.04. Max coverage (-): 0

Region: NODE\_385231\_length\_5676\_cov\_22.431114 193-203. Max. coverage (+): 0.04. Max coverage (-): 0.01

Region: NODE\_385231\_length\_5676\_cov\_22.431114 204-215. Max. coverage (+): 0.02. Max coverage (-): 0.02

Region: NODE\_385231\_length\_5676\_cov\_22.431114 216-227. Max. coverage (+): 0.05. Max coverage (-): 0.01

Region: NODE\_385231\_length\_5676\_cov\_22.431114 228-238. Max. coverage (+): 0. Max coverage (-): 0

Region: NODE\_385231\_length\_5676\_cov\_22.431114 239-250. Max. coverage (+): 0.04. Max coverage (-): 0

Region: NODE\_385231\_length\_5676\_cov\_22.431114 251-261. Max. coverage (+): 0.07. Max coverage (-): 0

Region: NODE\_385231\_length\_5676\_cov\_22.431114 262-273. Max. coverage (+): 0.06. Max coverage (-): 0

Region: NODE\_385231\_length\_5676\_cov\_22.431114 274-285. Max. coverage (+): 0.04. Max coverage (-): 0

Region: NODE\_385231\_length\_5676\_cov\_22.431114 286-296. Max. coverage (+): 0.17. Max coverage (-): 0

Region: NODE\_385231\_length\_5676\_cov\_22.431114 297-308. Max. coverage (+): 0.3. Max coverage (-): 0

Region: NODE\_385231\_length\_5676\_cov\_22.431114 309-319. Max. coverage (+): 0.22. Max coverage (-): 0

Region: NODE\_385231\_length\_5676\_cov\_22.431114 320-331. Max. coverage (+): 0.26. Max coverage (-): 0.04

Region: NODE\_385231\_length\_5676\_cov\_22.431114 332-343. Max. coverage (+): 0.11. Max coverage (-): 0.07

Region: NODE\_385231\_length\_5676\_cov\_22.431114 344-354. Max. coverage (+): 0.41. Max coverage (-): 0.07

Region: NODE\_385231\_length\_5676\_cov\_22.431114 355-366. Max. coverage (+): 0.04. Max coverage (-): 0

Region: NODE\_385231\_length\_5676\_cov\_22.431114 367-377. Max. coverage (+): 1.89. Max coverage (-): 0

Region: NODE\_385231\_length\_5676\_cov\_22.431114 378-389. Max. coverage (+): 2.6. Max coverage (-): 0.07

Region: NODE\_385231\_length\_5676\_cov\_22.431114 390-401. Max. coverage (+): 0.19. Max coverage (-): 0.11

Region: NODE\_385231\_length\_5676\_cov\_22.431114 402-412. Max. coverage (+): 0.44. Max coverage (-): 0.07

Region: NODE\_385231\_length\_5676\_cov\_22.431114 413-424. Max. coverage (+): 0.07. Max coverage (-): 0

Region: NODE\_385231\_length\_5676\_cov\_22.431114 425-435. Max. coverage (+): 0.09. Max coverage (-): 0.04

Region: NODE\_385231\_length\_5676\_cov\_22.431114 436-447. Max. coverage (+): 0.11. Max coverage (-): 0.04

Region: NODE\_385231\_length\_5676\_cov\_22.431114 448-459. Max. coverage (+): 0.19. Max coverage (-): 0.02

Region: NODE\_385231\_length\_5676\_cov\_22.431114 460-470. Max. coverage (+): 0. Max coverage (-): 0

Region: NODE\_385231\_length\_5676\_cov\_22.431114 471-482. Max. coverage (+): 0.3. Max coverage (-): 0

Region: NODE\_385231\_length\_5676\_cov\_22.431114 483-493. Max. coverage (+): 0.04. Max coverage (-): 0.01

Region: NODE\_385231\_length\_5676\_cov\_22.431114 494-505. Max. coverage (+): 4.91. Max coverage (-): 0.09

Region: NODE\_385231\_length\_5676\_cov\_22.431114 506-517. Max. coverage (+): 0.74. Max coverage (-): 0.04

Region: NODE\_385231\_length\_5676\_cov\_22.431114 518-528. Max. coverage (+): 0.19. Max coverage (-): 0

Region: NODE\_385231\_length\_5676\_cov\_22.431114 529-540. Max. coverage (+): 0.2. Max coverage (-): 0

Region: NODE\_385231\_length\_5676\_cov\_22.431114 541-551. Max. coverage (+): 0.09. Max coverage (-): 0.01

Region: NODE\_385231\_length\_5676\_cov\_22.431114 552-563. Max. coverage (+): 0.37. Max coverage (-): 0.05

Region: NODE\_385231\_length\_5676\_cov\_22.431114 564-575. Max. coverage (+): 0.41. Max coverage (-): 0

Region: NODE\_385231\_length\_5676\_cov\_22.431114 576-586. Max. coverage (+): 0.07. Max coverage (-): 0

Region: NODE\_385231\_length\_5676\_cov\_22.431114 587-598. Max. coverage (+): 0.06. Max coverage (-): 0.01

Region: NODE\_385231\_length\_5676\_cov\_22.431114 599-609. Max. coverage (+): 0.02. Max coverage (-): 0.04

Region: NODE\_385231\_length\_5676\_cov\_22.431114 610-621. Max. coverage (+): 0.67. Max coverage (-): 0.01

Region: NODE\_385231\_length\_5676\_cov\_22.431114 622-633. Max. coverage (+): 0.36. Max coverage (-): 0

Region: NODE\_385231\_length\_5676\_cov\_22.431114 634-644. Max. coverage (+): 0.04. Max coverage (-): 0.09

Region: NODE\_385231\_length\_5676\_cov\_22.431114 645-656. Max. coverage (+): 0.33. Max coverage (-): 0.02

Region: NODE\_385231\_length\_5676\_cov\_22.431114 657-667. Max. coverage (+): 0. Max coverage (-): 0

Region: NODE\_385231\_length\_5676\_cov\_22.431114 668-679. Max. coverage (+): 0.06. Max coverage (-): 0.03

Region: NODE\_385231\_length\_5676\_cov\_22.431114 680-691. Max. coverage (+): 0.33. Max coverage (-): 0.15

Region: NODE\_385231\_length\_5676\_cov\_22.431114 692-702. Max. coverage (+): 0.3. Max coverage (-): 0.07

Region: NODE\_385231\_length\_5676\_cov\_22.431114 703-714. Max. coverage (+): 3.6. Max coverage (-): 0.07

Region: NODE\_385231\_length\_5676\_cov\_22.431114 715-725. Max. coverage (+): 0.07. Max coverage (-): 0.01

Region: NODE\_385231\_length\_5676\_cov\_22.431114 726-737. Max. coverage (+): 0.11. Max coverage (-): 0.02

Region: NODE\_385231\_length\_5676\_cov\_22.431114 738-749. Max. coverage (+): 2.39. Max coverage (-): 0.06

Region: NODE\_385231\_length\_5676\_cov\_22.431114 750-760. Max. coverage (+): 2.28. Max coverage (-): 0

Region: NODE\_385231\_length\_5676\_cov\_22.431114 761-772. Max. coverage (+): 0.41. Max coverage (-): 0.11

Region: NODE\_385231\_length\_5676\_cov\_22.431114 773-783. Max. coverage (+): 6.3. Max coverage (-): 0

Region: NODE\_385231\_length\_5676\_cov\_22.431114 784-795. Max. coverage (+): 3.45. Max coverage (-): 0.07

Region: NODE\_385231\_length\_5676\_cov\_22.431114 796-807. Max. coverage (+): 2.84. Max coverage (-): 0.19

Region: NODE\_385231\_length\_5676\_cov\_22.431114 808-818. Max. coverage (+): 0.94. Max coverage (-): 0

Region: NODE\_385231\_length\_5676\_cov\_22.431114 819-830. Max. coverage (+): 0.08. Max coverage (-): 0.12

Region: NODE\_385231\_length\_5676\_cov\_22.431114 831-841. Max. coverage (+): 0.12. Max coverage (-): 0.11

Region: NODE\_385231\_length\_5676\_cov\_22.431114 842-853. Max. coverage (+): 1.2. Max coverage (-): 0

Region: NODE\_385231\_length\_5676\_cov\_22.431114 854-865. Max. coverage (+): 0. Max coverage (-): 0.06

Region: NODE\_385231\_length\_5676\_cov\_22.431114 866-876. Max. coverage (+): 0.03. Max coverage (-): 0.04

Region: NODE\_385231\_length\_5676\_cov\_22.431114 877-888. Max. coverage (+): 0. Max coverage (-): 0

Region: NODE\_385231\_length\_5676\_cov\_22.431114 889-899. Max. coverage (+): 0. Max coverage (-): 0.1

Region: NODE\_385231\_length\_5676\_cov\_22.431114 900-911. Max. coverage (+): 0.1. Max coverage (-): 0.01

Region: NODE\_385231\_length\_5676\_cov\_22.431114 912-923. Max. coverage (+): 0.29. Max coverage (-): 0.41

Region: NODE\_385231\_length\_5676\_cov\_22.431114 924-934. Max. coverage (+): 1.26. Max coverage (-): 0.01

Region: NODE\_385231\_length\_5676\_cov\_22.431114 935-946. Max. coverage (+): 0.43. Max coverage (-): 0.04

Region: NODE\_385231\_length\_5676\_cov\_22.431114 947-957. Max. coverage (+): 0.15. Max coverage (-): 0.04

Region: NODE\_385231\_length\_5676\_cov\_22.431114 958-969. Max. coverage (+): 1.26. Max coverage (-): 0.56

Region: NODE\_385231\_length\_5676\_cov\_22.431114 970-981. Max. coverage (+): 4.09. Max coverage (-): 0

Region: NODE\_385231\_length\_5676\_cov\_22.431114 982-992. Max. coverage (+): 2.34. Max coverage (-): 0.07

Region: NODE\_385231\_length\_5676\_cov\_22.431114 993-1004. Max. coverage (+): 0.15. Max coverage (-): 0

Region: NODE\_385231\_length\_5676\_cov\_22.431114 1005-1015. Max. coverage (+): 4.96. Max coverage (-): 0.07

Region: NODE\_385231\_length\_5676\_cov\_22.431114 1016-1027. Max. coverage (+): 13.98. Max coverage (-): 0.07

Region: NODE\_385231\_length\_5676\_cov\_22.431114 1028-1039. Max. coverage (+): 3.48. Max coverage (-): 0

Region: NODE\_385231\_length\_5676\_cov\_22.431114 1040-1050. Max. coverage (+): 1.78. Max coverage (-): 0

Region: NODE\_385231\_length\_5676\_cov\_22.431114 1051-1062. Max. coverage (+): 3.48. Max coverage (-): 0.48

Region: NODE\_385231\_length\_5676\_cov\_22.431114 1063-1073. Max. coverage (+): 2.71. Max coverage (-): 0.89

Region: NODE\_385231\_length\_5676\_cov\_22.431114 1074-1085. Max. coverage (+): 0.35. Max coverage (-): 0.11

Region: NODE\_385231\_length\_5676\_cov\_22.431114 1086-1097. Max. coverage (+): 0.37. Max coverage (-): 0.04

Region: NODE\_385231\_length\_5676\_cov\_22.431114 1098-1108. Max. coverage (+): 0.01. Max coverage (-): 0.04

Region: NODE\_385231\_length\_5676\_cov\_22.431114 1109-1120. Max. coverage (+): 0.15. Max coverage (-): 0.03

Region: NODE\_385231\_length\_5676\_cov\_22.431114 1121-1131. Max. coverage (+): 1.08. Max coverage (-): 0.19

Region: NODE\_385231\_length\_5676\_cov\_22.431114 1132-1143. Max. coverage (+): 3.11. Max coverage (-): 0.85

Region: NODE\_385231\_length\_5676\_cov\_22.431114 1144-1155. Max. coverage (+): 0.06. Max coverage (-): 0

Region: NODE\_385231\_length\_5676\_cov\_22.431114 1156-1166. Max. coverage (+): 0.15. Max coverage (-): 0

Region: NODE\_385231\_length\_5676\_cov\_22.431114 1167-1178. Max. coverage (+): 0.7. Max coverage (-): 0.07

Region: NODE\_385231\_length\_5676\_cov\_22.431114 1179-1189. Max. coverage (+): 0.44. Max coverage (-): 0.07

Region: NODE\_385231\_length\_5676\_cov\_22.431114 1190-1201. Max. coverage (+): 0.15. Max coverage (-): 0.04

Region: NODE\_385231\_length\_5676\_cov\_22.431114 1202-1212. Max. coverage (+): 0.13. Max coverage (-): 0.03

Region: NODE\_385231\_length\_5676\_cov\_22.431114 1213-1224. Max. coverage (+): 0.01. Max coverage (-): 0.13

Region: NODE\_385231\_length\_5676\_cov\_22.431114 1225-1236. Max. coverage (+): 0.02. Max coverage (-): 0.1

Region: NODE\_385231\_length\_5676\_cov\_22.431114 1237-1247. Max. coverage (+): 0.64. Max coverage (-): 0.02

Region: NODE\_385231\_length\_5676\_cov\_22.431114 1248-1259. Max. coverage (+): 0.74. Max coverage (-): 0.11

Region: NODE\_385231\_length\_5676\_cov\_22.431114 1260-1270. Max. coverage (+): 0.26. Max coverage (-): 0

Region: NODE\_385231\_length\_5676\_cov\_22.431114 1271-1282. Max. coverage (+): 0.19. Max coverage (-): 0

Region: NODE\_385231\_length\_5676\_cov\_22.431114 1283-1294. Max. coverage (+): 0.78. Max coverage (-): 0

Region: NODE\_385231\_length\_5676\_cov\_22.431114 1295-1305. Max. coverage (+): 1.26. Max coverage (-): 0.07

Region: NODE\_385231\_length\_5676\_cov\_22.431114 1306-1317. Max. coverage (+): 0.48. Max coverage (-): 0.07

Region: NODE\_385231\_length\_5676\_cov\_22.431114 1318-1328. Max. coverage (+): 4.63. Max coverage (-): 0.04

Region: NODE\_385231\_length\_5676\_cov\_22.431114 1329-1340. Max. coverage (+): 3.84. Max coverage (-): 0.04

Region: NODE\_385231\_length\_5676\_cov\_22.431114 1341-1352. Max. coverage (+): 0.26. Max coverage (-): 0.07

Region: NODE\_385231\_length\_5676\_cov\_22.431114 1353-1363. Max. coverage (+): 0. Max coverage (-): 0.04

Region: NODE\_385231\_length\_5676\_cov\_22.431114 1364-1375. Max. coverage (+): 0.17. Max coverage (-): 0

Region: NODE\_385231\_length\_5676\_cov\_22.431114 1376-1386. Max. coverage (+): 0.01. Max coverage (-): 0

Region: NODE\_385231\_length\_5676\_cov\_22.431114 1387-1398. Max. coverage (+): 0.07. Max coverage (-): 0.06

Region: NODE\_385231\_length\_5676\_cov\_22.431114 1399-1410. Max. coverage (+): 0.67. Max coverage (-): 0.06

Region: NODE\_385231\_length\_5676\_cov\_22.431114 1411-1421. Max. coverage (+): 0.41. Max coverage (-): 0.15

Region: NODE\_385231\_length\_5676\_cov\_22.431114 1422-1433. Max. coverage (+): 0. Max coverage (-): 1.56

Region: NODE\_385231\_length\_5676\_cov\_22.431114 1434-1444. Max. coverage (+): 0.52. Max coverage (-): 0.82

Region: NODE\_385231\_length\_5676\_cov\_22.431114 1445-1456. Max. coverage (+): 1.96. Max coverage (-): 0.07

Region: NODE\_385231\_length\_5676\_cov\_22.431114 1457-1468. Max. coverage (+): 0.26. Max coverage (-): 0.19

Region: NODE\_385231\_length\_5676\_cov\_22.431114 1469-1479. Max. coverage (+): 0.52. Max coverage (-): 0.26

Region: NODE\_385231\_length\_5676\_cov\_22.431114 1480-1491. Max. coverage (+): 2.04. Max coverage (-): 0.07

Region: NODE\_385231\_length\_5676\_cov\_22.431114 1492-1502. Max. coverage (+): 0.96. Max coverage (-): 0

Region: NODE\_385231\_length\_5676\_cov\_22.431114 1503-1514. Max. coverage (+): 1.52. Max coverage (-): 0

Region: NODE\_385231\_length\_5676\_cov\_22.431114 1515-1526. Max. coverage (+): 1.63. Max coverage (-): 0

Region: NODE\_385231\_length\_5676\_cov\_22.431114 1527-1537. Max. coverage (+): 0.41. Max coverage (-): 0

Region: NODE\_385231\_length\_5676\_cov\_22.431114 1538-1549. Max. coverage (+): 0.41. Max coverage (-): 0.07

Region: NODE\_385231\_length\_5676\_cov\_22.431114 1550-1560. Max. coverage (+): 0.56. Max coverage (-): 0.82

Region: NODE\_385231\_length\_5676\_cov\_22.431114 1561-1572. Max. coverage (+): 0.11. Max coverage (-): 0.3

Region: NODE\_385231\_length\_5676\_cov\_22.431114 1573-1584. Max. coverage (+): 0.48. Max coverage (-): 0

Region: NODE\_385231\_length\_5676\_cov\_22.431114 1585-1595. Max. coverage (+): 0.33. Max coverage (-): 0

Region: NODE\_385231\_length\_5676\_cov\_22.431114 1596-1607. Max. coverage (+): 0.22. Max coverage (-): 0

Region: NODE\_385231\_length\_5676\_cov\_22.431114 1608-1618. Max. coverage (+): 2.6. Max coverage (-): 0.04

Region: NODE\_385231\_length\_5676\_cov\_22.431114 1619-1630. Max. coverage (+): 1.52. Max coverage (-): 0.04

Region: NODE\_385231\_length\_5676\_cov\_22.431114 1631-1642. Max. coverage (+): 1.59. Max coverage (-): 0

Region: NODE\_385231\_length\_5676\_cov\_22.431114 1643-1653. Max. coverage (+): 0.05. Max coverage (-): 0

Region: NODE\_385231\_length\_5676\_cov\_22.431114 1654-1665. Max. coverage (+): 0.02. Max coverage (-): 0

Region: NODE\_385231\_length\_5676\_cov\_22.431114 1666-1676. Max. coverage (+): 0. Max coverage (-): 0

Region: NODE\_385231\_length\_5676\_cov\_22.431114 1677-1688. Max. coverage (+): 0. Max coverage (-): 0

Region: NODE\_385231\_length\_5676\_cov\_22.431114 1689-1700. Max. coverage (+): 0. Max coverage (-): 0

Region: NODE\_385231\_length\_5676\_cov\_22.431114 1701-1711. Max. coverage (+): 0.1. Max coverage (-): 0.06

Region: NODE\_385231\_length\_5676\_cov\_22.431114 1712-1723. Max. coverage (+): 0.4. Max coverage (-): 0.02

Region: NODE\_385231\_length\_5676\_cov\_22.431114 1724-1734. Max. coverage (+): 0.61. Max coverage (-): 0.01

Region: NODE\_385231\_length\_5676\_cov\_22.431114 1735-1746. Max. coverage (+): 0.21. Max coverage (-): 0.01

Region: NODE\_385231\_length\_5676\_cov\_22.431114 1747-1758. Max. coverage (+): 0.77. Max coverage (-): 0.02

Region: NODE\_385231\_length\_5676\_cov\_22.431114 1759-1769. Max. coverage (+): 0.74. Max coverage (-): 0.05

Region: NODE\_385231\_length\_5676\_cov\_22.431114 1770-1781. Max. coverage (+): 0.35. Max coverage (-): 0.04

Region: NODE\_385231\_length\_5676\_cov\_22.431114 1782-1792. Max. coverage (+): 0.3. Max coverage (-): 0.03

Region: NODE\_385231\_length\_5676\_cov\_22.431114 1793-1804. Max. coverage (+): 0.06. Max coverage (-): 0.11

Region: NODE\_385231\_length\_5676\_cov\_22.431114 1805-1816. Max. coverage (+): 1.19. Max coverage (-): 0.04

Region: NODE\_385231\_length\_5676\_cov\_22.431114 1817-1827. Max. coverage (+): 0. Max coverage (-): 0.04

Region: NODE\_385231\_length\_5676\_cov\_22.431114 1828-1839. Max. coverage (+): 0.48. Max coverage (-): 0.19

Region: NODE\_385231\_length\_5676\_cov\_22.431114 1840-1850. Max. coverage (+): 0.15. Max coverage (-): 0.28

Region: NODE\_385231\_length\_5676\_cov\_22.431114 1851-1862. Max. coverage (+): 14.71. Max coverage (-): 0.02

Region: NODE\_385231\_length\_5676\_cov\_22.431114 1863-1874. Max. coverage (+): 15.05. Max coverage (-): 0.04

Region: NODE\_385231\_length\_5676\_cov\_22.431114 1875-1885. Max. coverage (+): 0.19. Max coverage (-): 0.04

Region: NODE\_385231\_length\_5676\_cov\_22.431114 1886-1897. Max. coverage (+): 1.52. Max coverage (-): 0

Region: NODE\_385231\_length\_5676\_cov\_22.431114 1898-1908. Max. coverage (+): 0.15. Max coverage (-): 0.04

Region: NODE\_385231\_length\_5676\_cov\_22.431114 1909-1920. Max. coverage (+): 0.59. Max coverage (-): 0

Region: NODE\_385231\_length\_5676\_cov\_22.431114 1921-1932. Max. coverage (+): 0.3. Max coverage (-): 0.07

Region: NODE\_385231\_length\_5676\_cov\_22.431114 1933-1943. Max. coverage (+): 37.6. Max coverage (-): 0.06

Region: NODE\_385231\_length\_5676\_cov\_22.431114 1944-1955. Max. coverage (+): 0.4. Max coverage (-): 0.11

Region: NODE\_385231\_length\_5676\_cov\_22.431114 1956-1966. Max. coverage (+): 0.3. Max coverage (-): 0.48

Region: NODE\_385231\_length\_5676\_cov\_22.431114 1967-1978. Max. coverage (+): 11.79. Max coverage (-): 0.41

Region: NODE\_385231\_length\_5676\_cov\_22.431114 1979-1990. Max. coverage (+): 2.08. Max coverage (-): 0

Region: NODE\_385231\_length\_5676\_cov\_22.431114 1991-2001. Max. coverage (+): 0.06. Max coverage (-): 0.32

Region: NODE\_385231\_length\_5676\_cov\_22.431114 2002-2013. Max. coverage (+): 1.8. Max coverage (-): 0.19

Region: NODE\_385231\_length\_5676\_cov\_22.431114 2014-2024. Max. coverage (+): 1.8. Max coverage (-): 0.04

Region: NODE\_385231\_length\_5676\_cov\_22.431114 2025-2036. Max. coverage (+): 1.65. Max coverage (-): 0.11

Region: NODE\_385231\_length\_5676\_cov\_22.431114 2037-2048. Max. coverage (+): 0.15. Max coverage (-): 0.11

Region: NODE\_385231\_length\_5676\_cov\_22.431114 2049-2059. Max. coverage (+): 6.67. Max coverage (-): 0

Region: NODE\_385231\_length\_5676\_cov\_22.431114 2060-2071. Max. coverage (+): 0.37. Max coverage (-): 0.11

Region: NODE\_385231\_length\_5676\_cov\_22.431114 2072-2082. Max. coverage (+): 0.26. Max coverage (-): 0.06

Region: NODE\_385231\_length\_5676\_cov\_22.431114 2083-2094. Max. coverage (+): 0.22. Max coverage (-): 0

Region: NODE\_385231\_length\_5676\_cov\_22.431114 2095-2106. Max. coverage (+): 0.41. Max coverage (-): 0.15

Region: NODE\_385231\_length\_5676\_cov\_22.431114 2107-2117. Max. coverage (+): 1.87. Max coverage (-): 0.07

Region: NODE\_385231\_length\_5676\_cov\_22.431114 2118-2129. Max. coverage (+): 3.04. Max coverage (-): 0

Region: NODE\_385231\_length\_5676\_cov\_22.431114 2130-2140. Max. coverage (+): 2.93. Max coverage (-): 0

Region: NODE\_385231\_length\_5676\_cov\_22.431114 2141-2152. Max. coverage (+): 0.43. Max coverage (-): 0.07

Region: NODE\_385231\_length\_5676\_cov\_22.431114 2153-2164. Max. coverage (+): 1.89. Max coverage (-): 0.13

Region: NODE\_385231\_length\_5676\_cov\_22.431114 2165-2175. Max. coverage (+): 0.63. Max coverage (-): 0

Region: NODE\_385231\_length\_5676\_cov\_22.431114 2176-2187. Max. coverage (+): 0.22. Max coverage (-): 0.07

Region: NODE\_385231\_length\_5676\_cov\_22.431114 2188-2198. Max. coverage (+): 0.06. Max coverage (-): 0.07

Region: NODE\_385231\_length\_5676\_cov\_22.431114 2199-2210. Max. coverage (+): 0.52. Max coverage (-): 0

Region: NODE\_385231\_length\_5676\_cov\_22.431114 2211-2222. Max. coverage (+): 0.07. Max coverage (-): 0.07

Region: NODE\_385231\_length\_5676\_cov\_22.431114 2223-2233. Max. coverage (+): 0.07. Max coverage (-): 0.04

Region: NODE\_385231\_length\_5676\_cov\_22.431114 2234-2245. Max. coverage (+): 0.11. Max coverage (-): 0.07

Region: NODE\_385231\_length\_5676\_cov\_22.431114 2246-2256. Max. coverage (+): 0.3. Max coverage (-): 0.07

Region: NODE\_385231\_length\_5676\_cov\_22.431114 2257-2268. Max. coverage (+): 0.22. Max coverage (-): 0.15

Region: NODE\_385231\_length\_5676\_cov\_22.431114 2269-2280. Max. coverage (+): 0.22. Max coverage (-): 0.11

Region: NODE\_385231\_length\_5676\_cov\_22.431114 2281-2291. Max. coverage (+): 19.56. Max coverage (-): 0

Region: NODE\_385231\_length\_5676\_cov\_22.431114 2292-2303. Max. coverage (+): 0.09. Max coverage (-): 0.08

Region: NODE\_385231\_length\_5676\_cov\_22.431114 2304-2314. Max. coverage (+): 0.02. Max coverage (-): 0.12

Region: NODE\_385231\_length\_5676\_cov\_22.431114 2315-2326. Max. coverage (+): 19.71. Max coverage (-): 0

Region: NODE\_385231\_length\_5676\_cov\_22.431114 2327-2337. Max. coverage (+): 0.05. Max coverage (-): 0

Region: NODE\_385231\_length\_5676\_cov\_22.431114 2338-2349. Max. coverage (+): 0. Max coverage (-): 0

Region: NODE\_385231\_length\_5676\_cov\_22.431114 2350-2361. Max. coverage (+): 0.02. Max coverage (-): 0.01

Region: NODE\_385231\_length\_5676\_cov\_22.431114 2362-2372. Max. coverage (+): 0.02. Max coverage (-): 0.05

Region: NODE\_385231\_length\_5676\_cov\_22.431114 2373-2384. Max. coverage (+): 0.04. Max coverage (-): 0.06

Region: NODE\_385231\_length\_5676\_cov\_22.431114 2385-2395. Max. coverage (+): 0.09. Max coverage (-): 0.04

Region: NODE\_385231\_length\_5676\_cov\_22.431114 2396-2407. Max. coverage (+): 0.09. Max coverage (-): 0

Region: NODE\_385231\_length\_5676\_cov\_22.431114 2408-2419. Max. coverage (+): 0.07. Max coverage (-): 0

Region: NODE\_385231\_length\_5676\_cov\_22.431114 2420-2430. Max. coverage (+): 0.11. Max coverage (-): 0

Region: NODE\_385231\_length\_5676\_cov\_22.431114 2431-2442. Max. coverage (+): 0. Max coverage (-): 0

Region: NODE\_385231\_length\_5676\_cov\_22.431114 2443-2453. Max. coverage (+): 1. Max coverage (-): 0

Region: NODE\_385231\_length\_5676\_cov\_22.431114 2454-2465. Max. coverage (+): 0.24. Max coverage (-): 0.04

Region: NODE\_385231\_length\_5676\_cov\_22.431114 2466-2477. Max. coverage (+): 5.08. Max coverage (-): 0.06

Region: NODE\_385231\_length\_5676\_cov\_22.431114 2478-2488. Max. coverage (+): 0.02. Max coverage (-): 0.03

Region: NODE\_385231\_length\_5676\_cov\_22.431114 2489-2500. Max. coverage (+): 0.23. Max coverage (-): 0.05

Region: NODE\_385231\_length\_5676\_cov\_22.431114 2501-2511. Max. coverage (+): 0.23. Max coverage (-): 0.01

Region: NODE\_385231\_length\_5676\_cov\_22.431114 2512-2523. Max. coverage (+): 0.01. Max coverage (-): 0

Region: NODE\_385231\_length\_5676\_cov\_22.431114 2524-2535. Max. coverage (+): 0.01. Max coverage (-): 0

Region: NODE\_385231\_length\_5676\_cov\_22.431114 2536-2546. Max. coverage (+): 0. Max coverage (-): 0

Region: NODE\_385231\_length\_5676\_cov\_22.431114 2547-2558. Max. coverage (+): 0.06. Max coverage (-): 0

Region: NODE\_385231\_length\_5676\_cov\_22.431114 2559-2569. Max. coverage (+): 0.06. Max coverage (-): 0.02

Region: NODE\_385231\_length\_5676\_cov\_22.431114 2570-2581. Max. coverage (+): 0.67. Max coverage (-): 0

Region: NODE\_385231\_length\_5676\_cov\_22.431114 2582-2593. Max. coverage (+): 0.74. Max coverage (-): 0.06

Region: NODE\_385231\_length\_5676\_cov\_22.431114 2594-2604. Max. coverage (+): 0.05. Max coverage (-): 0.03

Region: NODE\_385231\_length\_5676\_cov\_22.431114 2605-2616. Max. coverage (+): 0.15. Max coverage (-): 0.04

Region: NODE\_385231\_length\_5676\_cov\_22.431114 2617-2627. Max. coverage (+): 0.22. Max coverage (-): 0

Region: NODE\_385231\_length\_5676\_cov\_22.431114 2628-2639. Max. coverage (+): 0.02. Max coverage (-): 0.06

Region: NODE\_385231\_length\_5676\_cov\_22.431114 2640-2651. Max. coverage (+): 0.02. Max coverage (-): 0.59

Region: NODE\_385231\_length\_5676\_cov\_22.431114 2652-2662. Max. coverage (+): 1.91. Max coverage (-): 0

Region: NODE\_385231\_length\_5676\_cov\_22.431114 2663-2674. Max. coverage (+): 1.91. Max coverage (-): 0.09

Region: NODE\_385231\_length\_5676\_cov\_22.431114 2675-2685. Max. coverage (+): 0.37. Max coverage (-): 0

Region: NODE\_385231\_length\_5676\_cov\_22.431114 2686-2697. Max. coverage (+): 0.11. Max coverage (-): 0

Region: NODE\_385231\_length\_5676\_cov\_22.431114 2698-2709. Max. coverage (+): 0.04. Max coverage (-): 0.11

Region: NODE\_385231\_length\_5676\_cov\_22.431114 2710-2720. Max. coverage (+): 0. Max coverage (-): 0.06

Region: NODE\_385231\_length\_5676\_cov\_22.431114 2721-2732. Max. coverage (+): 3.97. Max coverage (-): 0.02

Region: NODE\_385231\_length\_5676\_cov\_22.431114 2733-2743. Max. coverage (+): 0.22. Max coverage (-): 0

Region: NODE\_385231\_length\_5676\_cov\_22.431114 2744-2755. Max. coverage (+): 1.52. Max coverage (-): 0

Region: NODE\_385231\_length\_5676\_cov\_22.431114 2756-2767. Max. coverage (+): 1.11. Max coverage (-): 0.04

Region: NODE\_385231\_length\_5676\_cov\_22.431114 2768-2778. Max. coverage (+): 0.22. Max coverage (-): 0.22

Region: NODE\_385231\_length\_5676\_cov\_22.431114 2779-2790. Max. coverage (+): 9.99. Max coverage (-): 0

Region: NODE\_385231\_length\_5676\_cov\_22.431114 2791-2801. Max. coverage (+): 1. Max coverage (-): 0.02

Region: NODE\_385231\_length\_5676\_cov\_22.431114 2802-2813. Max. coverage (+): 0.2. Max coverage (-): 0.01

Region: NODE\_385231\_length\_5676\_cov\_22.431114 2814-2825. Max. coverage (+): 2.03. Max coverage (-): 0

Region: NODE\_385231\_length\_5676\_cov\_22.431114 2826-2836. Max. coverage (+): 0.07. Max coverage (-): 0.07

Region: NODE\_385231\_length\_5676\_cov\_22.431114 2837-2848. Max. coverage (+): 0.1. Max coverage (-): 0.02

Region: NODE\_385231\_length\_5676\_cov\_22.431114 2849-2859. Max. coverage (+): 0.16. Max coverage (-): 0

Region: NODE\_385231\_length\_5676\_cov\_22.431114 2860-2871. Max. coverage (+): 0.26. Max coverage (-): 0

Region: NODE\_385231\_length\_5676\_cov\_22.431114 2872-2883. Max. coverage (+): 0.3. Max coverage (-): 0

Region: NODE\_385231\_length\_5676\_cov\_22.431114 2884-2894. Max. coverage (+): 0.56. Max coverage (-): 0.11

Region: NODE\_385231\_length\_5676\_cov\_22.431114 2895-2906. Max. coverage (+): 0.24. Max coverage (-): 0.07

Region: NODE\_385231\_length\_5676\_cov\_22.431114 2907-2917. Max. coverage (+): 2.78. Max coverage (-): 0

Region: NODE\_385231\_length\_5676\_cov\_22.431114 2918-2929. Max. coverage (+): 0.07. Max coverage (-): 0

Region: NODE\_385231\_length\_5676\_cov\_22.431114 2930-2941. Max. coverage (+): 0.15. Max coverage (-): 0.07

Region: NODE\_385231\_length\_5676\_cov\_22.431114 2942-2952. Max. coverage (+): 0.37. Max coverage (-): 0.07

Region: NODE\_385231\_length\_5676\_cov\_22.431114 2953-2964. Max. coverage (+): 2.78. Max coverage (-): 0.02

Region: NODE\_385231\_length\_5676\_cov\_22.431114 2965-2975. Max. coverage (+): 0.02. Max coverage (-): 0

Region: NODE\_385231\_length\_5676\_cov\_22.431114 2976-2987. Max. coverage (+): 0. Max coverage (-): 0

Region: NODE\_385231\_length\_5676\_cov\_22.431114 2988-2999. Max. coverage (+): 0.63. Max coverage (-): 0.04

Region: NODE\_385231\_length\_5676\_cov\_22.431114 3000-3010. Max. coverage (+): 0.54. Max coverage (-): 0

Region: NODE\_385231\_length\_5676\_cov\_22.431114 3011-3022. Max. coverage (+): 0.54. Max coverage (-): 0

Region: NODE\_385231\_length\_5676\_cov\_22.431114 3023-3033. Max. coverage (+): 0.06. Max coverage (-): 0.01

Region: NODE\_385231\_length\_5676\_cov\_22.431114 3034-3045. Max. coverage (+): 0.06. Max coverage (-): 0.24

Region: NODE\_385231\_length\_5676\_cov\_22.431114 3046-3057. Max. coverage (+): 0.15. Max coverage (-): 0.17

Region: NODE\_385231\_length\_5676\_cov\_22.431114 3058-3068. Max. coverage (+): 0.07. Max coverage (-): 0

Region: NODE\_385231\_length\_5676\_cov\_22.431114 3069-3080. Max. coverage (+): 0.15. Max coverage (-): 0

Region: NODE\_385231\_length\_5676\_cov\_22.431114 3081-3091. Max. coverage (+): 0.19. Max coverage (-): 0

Region: NODE\_385231\_length\_5676\_cov\_22.431114 3092-3103. Max. coverage (+): 0.26. Max coverage (-): 0.04

Region: NODE\_385231\_length\_5676\_cov\_22.431114 3104-3115. Max. coverage (+): 0.07. Max coverage (-): 0.15

Region: NODE\_385231\_length\_5676\_cov\_22.431114 3116-3126. Max. coverage (+): 1.48. Max coverage (-): 0.04

Region: NODE\_385231\_length\_5676\_cov\_22.431114 3127-3138. Max. coverage (+): 1.41. Max coverage (-): 0

Region: NODE\_385231\_length\_5676\_cov\_22.431114 3139-3149. Max. coverage (+): 0.04. Max coverage (-): 0.06

Region: NODE\_385231\_length\_5676\_cov\_22.431114 3150-3161. Max. coverage (+): 0.37. Max coverage (-): 0.04

Region: NODE\_385231\_length\_5676\_cov\_22.431114 3162-3173. Max. coverage (+): 0.15. Max coverage (-): 0.07

Region: NODE\_385231\_length\_5676\_cov\_22.431114 3174-3184. Max. coverage (+): 0.54. Max coverage (-): 0.11

Region: NODE\_385231\_length\_5676\_cov\_22.431114 3185-3196. Max. coverage (+): 0.24. Max coverage (-): 0.06

Region: NODE\_385231\_length\_5676\_cov\_22.431114 3197-3207. Max. coverage (+): 0.02. Max coverage (-): 0

Region: NODE\_385231\_length\_5676\_cov\_22.431114 3208-3219. Max. coverage (+): 0.59. Max coverage (-): 0.04

Region: NODE\_385231\_length\_5676\_cov\_22.431114 3220-3231. Max. coverage (+): 0.61. Max coverage (-): 0

Region: NODE\_385231\_length\_5676\_cov\_22.431114 3232-3242. Max. coverage (+): 0.43. Max coverage (-): 0

Region: NODE\_385231\_length\_5676\_cov\_22.431114 3243-3254. Max. coverage (+): 0. Max coverage (-): 0

Region: NODE\_385231\_length\_5676\_cov\_22.431114 3255-3265. Max. coverage (+): 0. Max coverage (-): 0.24

Region: NODE\_385231\_length\_5676\_cov\_22.431114 3266-3277. Max. coverage (+): 0.93. Max coverage (-): 0.3

Region: NODE\_385231\_length\_5676\_cov\_22.431114 3278-3289. Max. coverage (+): 5.3. Max coverage (-): 0.02

Region: NODE\_385231\_length\_5676\_cov\_22.431114 3290-3300. Max. coverage (+): 2.87. Max coverage (-): 0

Region: NODE\_385231\_length\_5676\_cov\_22.431114 3301-3312. Max. coverage (+): 0.11. Max coverage (-): 0.02

Region: NODE\_385231\_length\_5676\_cov\_22.431114 3313-3323. Max. coverage (+): 3.6. Max coverage (-): 0.02

Region: NODE\_385231\_length\_5676\_cov\_22.431114 3324-3335. Max. coverage (+): 10.34. Max coverage (-): 0

Region: NODE\_385231\_length\_5676\_cov\_22.431114 3336-3347. Max. coverage (+): 0.82. Max coverage (-): 0

Region: NODE\_385231\_length\_5676\_cov\_22.431114 3348-3358. Max. coverage (+): 0. Max coverage (-): 0

Region: NODE\_385231\_length\_5676\_cov\_22.431114 3359-3370. Max. coverage (+): 4.62. Max coverage (-): 0

Region: NODE\_385231\_length\_5676\_cov\_22.431114 3371-3381. Max. coverage (+): 4.28. Max coverage (-): 0.02

Region: NODE\_385231\_length\_5676\_cov\_22.431114 3382-3393. Max. coverage (+): 0.02. Max coverage (-): 0.02

Region: NODE\_385231\_length\_5676\_cov\_22.431114 3394-3405. Max. coverage (+): 0.17. Max coverage (-): 0.02

Region: NODE\_385231\_length\_5676\_cov\_22.431114 3406-3416. Max. coverage (+): 0.07. Max coverage (-): 0

Region: NODE\_385231\_length\_5676\_cov\_22.431114 3417-3428. Max. coverage (+): 1.89. Max coverage (-): 0

Region: NODE\_385231\_length\_5676\_cov\_22.431114 3429-3439. Max. coverage (+): 0.04. Max coverage (-): 0.04

Region: NODE\_385231\_length\_5676\_cov\_22.431114 3440-3451. Max. coverage (+): 0.04. Max coverage (-): 0.15

Region: NODE\_385231\_length\_5676\_cov\_22.431114 3452-3463. Max. coverage (+): 0.06. Max coverage (-): 0

Region: NODE\_385231\_length\_5676\_cov\_22.431114 3464-3474. Max. coverage (+): 0.2. Max coverage (-): 0

Region: NODE\_385231\_length\_5676\_cov\_22.431114 3475-3486. Max. coverage (+): 0.22. Max coverage (-): 0

Region: NODE\_385231\_length\_5676\_cov\_22.431114 3487-3497. Max. coverage (+): 0.33. Max coverage (-): 0.07

Region: NODE\_385231\_length\_5676\_cov\_22.431114 3498-3509. Max. coverage (+): 0.37. Max coverage (-): 0.07

Region: NODE\_385231\_length\_5676\_cov\_22.431114 3510-3520. Max. coverage (+): 8.04. Max coverage (-): 0.04

Region: NODE\_385231\_length\_5676\_cov\_22.431114 3521-3532. Max. coverage (+): 4. Max coverage (-): 0

Region: NODE\_385231\_length\_5676\_cov\_22.431114 3533-3544. Max. coverage (+): 0.13. Max coverage (-): 0

Region: NODE\_385231\_length\_5676\_cov\_22.431114 3545-3555. Max. coverage (+): 0.85. Max coverage (-): 0.43

Region: NODE\_385231\_length\_5676\_cov\_22.431114 3556-3567. Max. coverage (+): 3.48. Max coverage (-): 0.04

Region: NODE\_385231\_length\_5676\_cov\_22.431114 3568-3578. Max. coverage (+): 6.97. Max coverage (-): 0.04

Region: NODE\_385231\_length\_5676\_cov\_22.431114 3579-3590. Max. coverage (+): 0.11. Max coverage (-): 0.2

Region: NODE\_385231\_length\_5676\_cov\_22.431114 3591-3602. Max. coverage (+): 0.35. Max coverage (-): 0.06

Region: NODE\_385231\_length\_5676\_cov\_22.431114 3603-3613. Max. coverage (+): 2.52. Max coverage (-): 0

Region: NODE\_385231\_length\_5676\_cov\_22.431114 3614-3625. Max. coverage (+): 1.04. Max coverage (-): 0.02

Region: NODE\_385231\_length\_5676\_cov\_22.431114 3626-3636. Max. coverage (+): 0.04. Max coverage (-): 0.04

Region: NODE\_385231\_length\_5676\_cov\_22.431114 3637-3648. Max. coverage (+): 1.5. Max coverage (-): 0

Region: NODE\_385231\_length\_5676\_cov\_22.431114 3649-3660. Max. coverage (+): 0.37. Max coverage (-): 0.09

Region: NODE\_385231\_length\_5676\_cov\_22.431114 3661-3671. Max. coverage (+): 0.09. Max coverage (-): 0.06

Region: NODE\_385231\_length\_5676\_cov\_22.431114 3672-3683. Max. coverage (+): 0.63. Max coverage (-): 0

Region: NODE\_385231\_length\_5676\_cov\_22.431114 3684-3694. Max. coverage (+): 0.91. Max coverage (-): 0

Region: NODE\_385231\_length\_5676\_cov\_22.431114 3695-3706. Max. coverage (+): 1.24. Max coverage (-): 0.04

Region: NODE\_385231\_length\_5676\_cov\_22.431114 3707-3718. Max. coverage (+): 0.02. Max coverage (-): 0

Region: NODE\_385231\_length\_5676\_cov\_22.431114 3719-3729. Max. coverage (+): 0.15. Max coverage (-): 0

Region: NODE\_385231\_length\_5676\_cov\_22.431114 3730-3741. Max. coverage (+): 0.02. Max coverage (-): 0

Region: NODE\_385231\_length\_5676\_cov\_22.431114 3742-3752. Max. coverage (+): 0. Max coverage (-): 0.02

Region: NODE\_385231\_length\_5676\_cov\_22.431114 3753-3764. Max. coverage (+): 0.22. Max coverage (-): 0.06

Region: NODE\_385231\_length\_5676\_cov\_22.431114 3765-3776. Max. coverage (+): 0.15. Max coverage (-): 0.04

Region: NODE\_385231\_length\_5676\_cov\_22.431114 3777-3787. Max. coverage (+): 1.65. Max coverage (-): 0

Region: NODE\_385231\_length\_5676\_cov\_22.431114 3788-3799. Max. coverage (+): 0.09. Max coverage (-): 0.06

Region: NODE\_385231\_length\_5676\_cov\_22.431114 3800-3810. Max. coverage (+): 0.04. Max coverage (-): 0.19

Region: NODE\_385231\_length\_5676\_cov\_22.431114 3811-3822. Max. coverage (+): 0.13. Max coverage (-): 0.11

Region: NODE\_385231\_length\_5676\_cov\_22.431114 3823-3834. Max. coverage (+): 0.11. Max coverage (-): 0

Region: NODE\_385231\_length\_5676\_cov\_22.431114 3835-3845. Max. coverage (+): 0.02. Max coverage (-): 0.07

Region: NODE\_385231\_length\_5676\_cov\_22.431114 3846-3857. Max. coverage (+): 0.52. Max coverage (-): 0.02

Region: NODE\_385231\_length\_5676\_cov\_22.431114 3858-3868. Max. coverage (+): 0.39. Max coverage (-): 0

Region: NODE\_385231\_length\_5676\_cov\_22.431114 3869-3880. Max. coverage (+): 0.3. Max coverage (-): 0.04

Region: NODE\_385231\_length\_5676\_cov\_22.431114 3881-3892. Max. coverage (+): 1.19. Max coverage (-): 0.04

Region: NODE\_385231\_length\_5676\_cov\_22.431114 3893-3903. Max. coverage (+): 0.07. Max coverage (-): 0.04

Region: NODE\_385231\_length\_5676\_cov\_22.431114 3904-3915. Max. coverage (+): 0.19. Max coverage (-): 0.07

Region: NODE\_385231\_length\_5676\_cov\_22.431114 3916-3926. Max. coverage (+): 2.74. Max coverage (-): 0.04

Region: NODE\_385231\_length\_5676\_cov\_22.431114 3927-3938. Max. coverage (+): 2. Max coverage (-): 0

Region: NODE\_385231\_length\_5676\_cov\_22.431114 3939-3950. Max. coverage (+): 0.11. Max coverage (-): 0

Region: NODE\_385231\_length\_5676\_cov\_22.431114 3951-3961. Max. coverage (+): 0.06. Max coverage (-): 0

Region: NODE\_385231\_length\_5676\_cov\_22.431114 3962-3973. Max. coverage (+): 0.91. Max coverage (-): 0

Region: NODE\_385231\_length\_5676\_cov\_22.431114 3974-3984. Max. coverage (+): 0.44. Max coverage (-): 0

Region: NODE\_385231\_length\_5676\_cov\_22.431114 3985-3996. Max. coverage (+): 9.49. Max coverage (-): 0

Region: NODE\_385231\_length\_5676\_cov\_22.431114 3997-4008. Max. coverage (+): 5.12. Max coverage (-): 0.15

Region: NODE\_385231\_length\_5676\_cov\_22.431114 4009-4019. Max. coverage (+): 0.51. Max coverage (-): 0

Region: NODE\_385231\_length\_5676\_cov\_22.431114 4020-4031. Max. coverage (+): 0.07. Max coverage (-): 0.05

Region: NODE\_385231\_length\_5676\_cov\_22.431114 4032-4042. Max. coverage (+): 5.04. Max coverage (-): 0.05

Region: NODE\_385231\_length\_5676\_cov\_22.431114 4043-4054. Max. coverage (+): 5.2. Max coverage (-): 0

Region: NODE\_385231\_length\_5676\_cov\_22.431114 4055-4066. Max. coverage (+): 0.09. Max coverage (-): 0.11

Region: NODE\_385231\_length\_5676\_cov\_22.431114 4067-4077. Max. coverage (+): 0.04. Max coverage (-): 0.15

Region: NODE\_385231\_length\_5676\_cov\_22.431114 4078-4089. Max. coverage (+): 0.07. Max coverage (-): 0.07

Region: NODE\_385231\_length\_5676\_cov\_22.431114 4090-4100. Max. coverage (+): 0.22. Max coverage (-): 0.07

Region: NODE\_385231\_length\_5676\_cov\_22.431114 4101-4112. Max. coverage (+): 0.56. Max coverage (-): 0.04

Region: NODE\_385231\_length\_5676\_cov\_22.431114 4113-4124. Max. coverage (+): 1.41. Max coverage (-): 0.26

Region: NODE\_385231\_length\_5676\_cov\_22.431114 4125-4135. Max. coverage (+): 0.74. Max coverage (-): 0.22

Region: NODE\_385231\_length\_5676\_cov\_22.431114 4136-4147. Max. coverage (+): 0.33. Max coverage (-): 0.04

Region: NODE\_385231\_length\_5676\_cov\_22.431114 4148-4158. Max. coverage (+): 1.67. Max coverage (-): 0.04

Region: NODE\_385231\_length\_5676\_cov\_22.431114 4159-4170. Max. coverage (+): 0.04. Max coverage (-): 0

Region: NODE\_385231\_length\_5676\_cov\_22.431114 4171-4182. Max. coverage (+): 0. Max coverage (-): 0.04

Region: NODE\_385231\_length\_5676\_cov\_22.431114 4183-4193. Max. coverage (+): 0.22. Max coverage (-): 0.15

Region: NODE\_385231\_length\_5676\_cov\_22.431114 4194-4205. Max. coverage (+): 0.39. Max coverage (-): 0.02

Region: NODE\_385231\_length\_5676\_cov\_22.431114 4206-4216. Max. coverage (+): 0.04. Max coverage (-): 0.02

Region: NODE\_385231\_length\_5676\_cov\_22.431114 4217-4228. Max. coverage (+): 0.15. Max coverage (-): 0

Region: NODE\_385231\_length\_5676\_cov\_22.431114 4229-4240. Max. coverage (+): 0.04. Max coverage (-): 0

Region: NODE\_385231\_length\_5676\_cov\_22.431114 4241-4251. Max. coverage (+): 0. Max coverage (-): 0

Region: NODE\_385231\_length\_5676\_cov\_22.431114 4252-4263. Max. coverage (+): 0.15. Max coverage (-): 0.02

Region: NODE\_385231\_length\_5676\_cov\_22.431114 4264-4274. Max. coverage (+): 0.11. Max coverage (-): 0.03

Region: NODE\_385231\_length\_5676\_cov\_22.431114 4275-4286. Max. coverage (+): 5.67. Max coverage (-): 0

Region: NODE\_385231\_length\_5676\_cov\_22.431114 4287-4298. Max. coverage (+): 0.52. Max coverage (-): 0.02

Region: NODE\_385231\_length\_5676\_cov\_22.431114 4299-4309. Max. coverage (+): 0.03. Max coverage (-): 0.04

Region: NODE\_385231\_length\_5676\_cov\_22.431114 4310-4321. Max. coverage (+): 0.7. Max coverage (-): 0

Region: NODE\_385231\_length\_5676\_cov\_22.431114 4322-4332. Max. coverage (+): 0.37. Max coverage (-): 0

Region: NODE\_385231\_length\_5676\_cov\_22.431114 4333-4344. Max. coverage (+): 0.11. Max coverage (-): 0

Region: NODE\_385231\_length\_5676\_cov\_22.431114 4345-4356. Max. coverage (+): 0.11. Max coverage (-): 0.04

Region: NODE\_385231\_length\_5676\_cov\_22.431114 4357-4367. Max. coverage (+): 0.04. Max coverage (-): 0.04

Region: NODE\_385231\_length\_5676\_cov\_22.431114 4368-4379. Max. coverage (+): 4.75. Max coverage (-): 0.04

Region: NODE\_385231\_length\_5676\_cov\_22.431114 4380-4390. Max. coverage (+): 0.04. Max coverage (-): 0

Region: NODE\_385231\_length\_5676\_cov\_22.431114 4391-4402. Max. coverage (+): 0.19. Max coverage (-): 0.04

Region: NODE\_385231\_length\_5676\_cov\_22.431114 4403-4414. Max. coverage (+): 1.85. Max coverage (-): 0

Region: NODE\_385231\_length\_5676\_cov\_22.431114 4415-4425. Max. coverage (+): 0.26. Max coverage (-): 0

Region: NODE\_385231\_length\_5676\_cov\_22.431114 4426-4437. Max. coverage (+): 0.15. Max coverage (-): 0.07

Region: NODE\_385231\_length\_5676\_cov\_22.431114 4438-4448. Max. coverage (+): 3.86. Max coverage (-): 0

Region: NODE\_385231\_length\_5676\_cov\_22.431114 4449-4460. Max. coverage (+): 3.67. Max coverage (-): 0.07

Region: NODE\_385231\_length\_5676\_cov\_22.431114 4461-4472. Max. coverage (+): 0.57. Max coverage (-): 0.02

Region: NODE\_385231\_length\_5676\_cov\_22.431114 4473-4483. Max. coverage (+): 0.59. Max coverage (-): 0.13

Region: NODE\_385231\_length\_5676\_cov\_22.431114 4484-4495. Max. coverage (+): 2.89. Max coverage (-): 0.07

Region: NODE\_385231\_length\_5676\_cov\_22.431114 4496-4506. Max. coverage (+): 0.89. Max coverage (-): 0

Region: NODE\_385231\_length\_5676\_cov\_22.431114 4507-4518. Max. coverage (+): 0.74. Max coverage (-): 0.07

Region: NODE\_385231\_length\_5676\_cov\_22.431114 4519-4530. Max. coverage (+): 1.19. Max coverage (-): 0.04

Region: NODE\_385231\_length\_5676\_cov\_22.431114 4531-4541. Max. coverage (+): 2.97. Max coverage (-): 0.07

Region: NODE\_385231\_length\_5676\_cov\_22.431114 4542-4553. Max. coverage (+): 3.37. Max coverage (-): 0.06

Region: NODE\_385231\_length\_5676\_cov\_22.431114 4554-4564. Max. coverage (+): 0.74. Max coverage (-): 0

Region: NODE\_385231\_length\_5676\_cov\_22.431114 4565-4576. Max. coverage (+): 0.56. Max coverage (-): 0

Region: NODE\_385231\_length\_5676\_cov\_22.431114 4577-4588. Max. coverage (+): 2.41. Max coverage (-): 0

Region: NODE\_385231\_length\_5676\_cov\_22.431114 4589-4599. Max. coverage (+): 1. Max coverage (-): 0

Region: NODE\_385231\_length\_5676\_cov\_22.431114 4600-4611. Max. coverage (+): 0.7. Max coverage (-): 0

Region: NODE\_385231\_length\_5676\_cov\_22.431114 4612-4622. Max. coverage (+): 0.37. Max coverage (-): 0

Region: NODE\_385231\_length\_5676\_cov\_22.431114 4623-4634. Max. coverage (+): 0.15. Max coverage (-): 0

Region: NODE\_385231\_length\_5676\_cov\_22.431114 4635-4645. Max. coverage (+): 0.7. Max coverage (-): 0.11

Region: NODE\_385231\_length\_5676\_cov\_22.431114 4646-4657. Max. coverage (+): 1.67. Max coverage (-): 0.04

Region: NODE\_385231\_length\_5676\_cov\_22.431114 4658-4669. Max. coverage (+): 2.3. Max coverage (-): 0.04

Region: NODE\_385231\_length\_5676\_cov\_22.431114 4670-4680. Max. coverage (+): 0.15. Max coverage (-): 0

Region: NODE\_385231\_length\_5676\_cov\_22.431114 4681-4692. Max. coverage (+): 0.28. Max coverage (-): 0.02

Region: NODE\_385231\_length\_5676\_cov\_22.431114 4693-4703. Max. coverage (+): 0.43. Max coverage (-): 0

Region: NODE\_385231\_length\_5676\_cov\_22.431114 4704-4715. Max. coverage (+): 0.19. Max coverage (-): 0

Region: NODE\_385231\_length\_5676\_cov\_22.431114 4716-4727. Max. coverage (+): 0.56. Max coverage (-): 0

Region: NODE\_385231\_length\_5676\_cov\_22.431114 4728-4738. Max. coverage (+): 0.78. Max coverage (-): 0

Region: NODE\_385231\_length\_5676\_cov\_22.431114 4739-4750. Max. coverage (+): 0.56. Max coverage (-): 0

Region: NODE\_385231\_length\_5676\_cov\_22.431114 4751-4761. Max. coverage (+): 0.35. Max coverage (-): 0.01

Region: NODE\_385231\_length\_5676\_cov\_22.431114 4762-4773. Max. coverage (+): 0.35. Max coverage (-): 0.07

Region: NODE\_385231\_length\_5676\_cov\_22.431114 4774-4785. Max. coverage (+): 0.44. Max coverage (-): 0.01

Region: NODE\_385231\_length\_5676\_cov\_22.431114 4786-4796. Max. coverage (+): 0.52. Max coverage (-): 0

Region: NODE\_385231\_length\_5676\_cov\_22.431114 4797-4808. Max. coverage (+): 0.37. Max coverage (-): 0

Region: NODE\_385231\_length\_5676\_cov\_22.431114 4809-4819. Max. coverage (+): 0.09. Max coverage (-): 0.01

Region: NODE\_385231\_length\_5676\_cov\_22.431114 4820-4831. Max. coverage (+): 0.01. Max coverage (-): 0.15

Region: NODE\_385231\_length\_5676\_cov\_22.431114 4832-4843. Max. coverage (+): 7.71. Max coverage (-): 0

Region: NODE\_385231\_length\_5676\_cov\_22.431114 4844-4854. Max. coverage (+): 7.19. Max coverage (-): 0

Region: NODE\_385231\_length\_5676\_cov\_22.431114 4855-4866. Max. coverage (+): 0.04. Max coverage (-): 0.04

Region: NODE\_385231\_length\_5676\_cov\_22.431114 4867-4877. Max. coverage (+): 0.7. Max coverage (-): 0.02

Region: NODE\_385231\_length\_5676\_cov\_22.431114 4878-4889. Max. coverage (+): 0.15. Max coverage (-): 0.04

Region: NODE\_385231\_length\_5676\_cov\_22.431114 4890-4901. Max. coverage (+): 12.2. Max coverage (-): 0.04

Region: NODE\_385231\_length\_5676\_cov\_22.431114 4902-4912. Max. coverage (+): 0.19. Max coverage (-): 0.46

Region: NODE\_385231\_length\_5676\_cov\_22.431114 4913-4924. Max. coverage (+): 1.14. Max coverage (-): 0.04

Region: NODE\_385231\_length\_5676\_cov\_22.431114 4925-4935. Max. coverage (+): 2.45. Max coverage (-): 0

Region: NODE\_385231\_length\_5676\_cov\_22.431114 4936-4947. Max. coverage (+): 2.37. Max coverage (-): 0.06

Region: NODE\_385231\_length\_5676\_cov\_22.431114 4948-4959. Max. coverage (+): 0.7. Max coverage (-): 0

Region: NODE\_385231\_length\_5676\_cov\_22.431114 4960-4970. Max. coverage (+): 0.33. Max coverage (-): 0.07

Region: NODE\_385231\_length\_5676\_cov\_22.431114 4971-4982. Max. coverage (+): 0.52. Max coverage (-): 0

Region: NODE\_385231\_length\_5676\_cov\_22.431114 4983-4993. Max. coverage (+): 1.11. Max coverage (-): 0.17

Region: NODE\_385231\_length\_5676\_cov\_22.431114 4994-5005. Max. coverage (+): 0.05. Max coverage (-): 0.12

Region: NODE\_385231\_length\_5676\_cov\_22.431114 5006-5017. Max. coverage (+): 0.96. Max coverage (-): 0.07

Region: NODE\_385231\_length\_5676\_cov\_22.431114 5018-5028. Max. coverage (+): 0.96. Max coverage (-): 0.15

Region: NODE\_385231\_length\_5676\_cov\_22.431114 5029-5040. Max. coverage (+): 0.26. Max coverage (-): 0.15

Region: NODE\_385231\_length\_5676\_cov\_22.431114 5041-5051. Max. coverage (+): 2.15. Max coverage (-): 0.1

Region: NODE\_385231\_length\_5676\_cov\_22.431114 5052-5063. Max. coverage (+): 0.22. Max coverage (-): 0

Region: NODE\_385231\_length\_5676\_cov\_22.431114 5064-5075. Max. coverage (+): 0.04. Max coverage (-): 0.11

Region: NODE\_385231\_length\_5676\_cov\_22.431114 5076-5086. Max. coverage (+): 0.06. Max coverage (-): 0.12

Region: NODE\_385231\_length\_5676\_cov\_22.431114 5087-5098. Max. coverage (+): 0.65. Max coverage (-): 0.02

Region: NODE\_385231\_length\_5676\_cov\_22.431114 5099-5109. Max. coverage (+): 0.07. Max coverage (-): 0.2

Region: NODE\_385231\_length\_5676\_cov\_22.431114 5110-5121. Max. coverage (+): 0.61. Max coverage (-): 0.01

Region: NODE\_385231\_length\_5676\_cov\_22.431114 5122-5133. Max. coverage (+): 0.5. Max coverage (-): 0.01

Region: NODE\_385231\_length\_5676\_cov\_22.431114 5134-5144. Max. coverage (+): 0.41. Max coverage (-): 0.04

Region: NODE\_385231\_length\_5676\_cov\_22.431114 5145-5156. Max. coverage (+): 3.11. Max coverage (-): 0.04

Region: NODE\_385231\_length\_5676\_cov\_22.431114 5157-5167. Max. coverage (+): 0.07. Max coverage (-): 0

Region: NODE\_385231\_length\_5676\_cov\_22.431114 5168-5179. Max. coverage (+): 0. Max coverage (-): 0

Region: NODE\_385231\_length\_5676\_cov\_22.431114 5180-5191. Max. coverage (+): 0. Max coverage (-): 0

Region: NODE\_385231\_length\_5676\_cov\_22.431114 5192-5202. Max. coverage (+): 0.05. Max coverage (-): 0

Region: NODE\_385231\_length\_5676\_cov\_22.431114 5203-5214. Max. coverage (+): 0.06. Max coverage (-): 0.01

Region: NODE\_385231\_length\_5676\_cov\_22.431114 5215-5225. Max. coverage (+): 4.62. Max coverage (-): 0

Region: NODE\_385231\_length\_5676\_cov\_22.431114 5226-5237. Max. coverage (+): 8.26. Max coverage (-): 0

Region: NODE\_385231\_length\_5676\_cov\_22.431114 5238-5249. Max. coverage (+): 0.85. Max coverage (-): 0

Region: NODE\_385231\_length\_5676\_cov\_22.431114 5250-5260. Max. coverage (+): 0.19. Max coverage (-): 0

Region: NODE\_385231\_length\_5676\_cov\_22.431114 5261-5272. Max. coverage (+): 0.11. Max coverage (-): 0

Region: NODE\_385231\_length\_5676\_cov\_22.431114 5273-5283. Max. coverage (+): 0.07. Max coverage (-): 0

Region: NODE\_385231\_length\_5676\_cov\_22.431114 5284-5295. Max. coverage (+): 1.87. Max coverage (-): 0

Region: NODE\_385231\_length\_5676\_cov\_22.431114 5296-5307. Max. coverage (+): 0.07. Max coverage (-): 0

Region: NODE\_385231\_length\_5676\_cov\_22.431114 5308-5318. Max. coverage (+): 0.04. Max coverage (-): 0.15

Region: NODE\_385231\_length\_5676\_cov\_22.431114 5319-5330. Max. coverage (+): 7.97. Max coverage (-): 0.07

Region: NODE\_385231\_length\_5676\_cov\_22.431114 5331-5341. Max. coverage (+): 112.55. Max coverage (-): 0

Region: NODE\_385231\_length\_5676\_cov\_22.431114 5342-5353. Max. coverage (+): 0.15. Max coverage (-): 0

Region: NODE\_385231\_length\_5676\_cov\_22.431114 5354-5365. Max. coverage (+): 0.15. Max coverage (-): 0.04

Region: NODE\_385231\_length\_5676\_cov\_22.431114 5366-5376. Max. coverage (+): 0.04. Max coverage (-): 0.22

Region: NODE\_385231\_length\_5676\_cov\_22.431114 5377-5388. Max. coverage (+): 0.32. Max coverage (-): 0.11

Region: NODE\_385231\_length\_5676\_cov\_22.431114 5389-5399. Max. coverage (+): 4.65. Max coverage (-): 0.01

Region: NODE\_385231\_length\_5676\_cov\_22.431114 5400-5411. Max. coverage (+): 0.01. Max coverage (-): 0.01

Region: NODE\_385231\_length\_5676\_cov\_22.431114 5412-5423. Max. coverage (+): 0.13. Max coverage (-): 0.01

Region: NODE\_385231\_length\_5676\_cov\_22.431114 5424-5434. Max. coverage (+): 0.12. Max coverage (-): 0.08

Region: NODE\_385231\_length\_5676\_cov\_22.431114 5435-5446. Max. coverage (+): 0. Max coverage (-): 0.11

Region: NODE\_385231\_length\_5676\_cov\_22.431114 5447-5457. Max. coverage (+): 15.35. Max coverage (-): 0

Region: NODE\_385231\_length\_5676\_cov\_22.431114 5458-5469. Max. coverage (+): 0.02. Max coverage (-): 0.03

Region: NODE\_385231\_length\_5676\_cov\_22.431114 5470-5481. Max. coverage (+): 1.31. Max coverage (-): 0.04

Region: NODE\_385231\_length\_5676\_cov\_22.431114 5482-5492. Max. coverage (+): 5.59. Max coverage (-): 0

Region: NODE\_385231\_length\_5676\_cov\_22.431114 5493-5504. Max. coverage (+): 0.9. Max coverage (-): 0

Region: NODE\_385231\_length\_5676\_cov\_22.431114 5505-5515. Max. coverage (+): 0. Max coverage (-): 0.13

Region: NODE\_385231\_length\_5676\_cov\_22.431114 5516-5527. Max. coverage (+): 0. Max coverage (-): 0.2

Region: NODE\_385231\_length\_5676\_cov\_22.431114 5528-5539. Max. coverage (+): 0.17. Max coverage (-): 0.01

Region: NODE\_385231\_length\_5676\_cov\_22.431114 5540-5550. Max. coverage (+): 0.08. Max coverage (-): 0.01

Region: NODE\_385231\_length\_5676\_cov\_22.431114 5551-5562. Max. coverage (+): 0.03. Max coverage (-): 0.01

Region: NODE\_385231\_length\_5676\_cov\_22.431114 5563-5573. Max. coverage (+): 0.06. Max coverage (-): 0

Region: NODE\_385231\_length\_5676\_cov\_22.431114 5574-5585. Max. coverage (+): 0.23. Max coverage (-): 0.01

Region: NODE\_385231\_length\_5676\_cov\_22.431114 5586-5597. Max. coverage (+): 0.2. Max coverage (-): 0.02

Region: NODE\_385231\_length\_5676\_cov\_22.431114 5598-5608. Max. coverage (+): 0.05. Max coverage (-): 0.02

Region: NODE\_385231\_length\_5676\_cov\_22.431114 5609-5620. Max. coverage (+): 1. Max coverage (-): 0

Region: NODE\_385231\_length\_5676\_cov\_22.431114 5621-5631. Max. coverage (+): 0.22. Max coverage (-): 0

Region: NODE\_385231\_length\_5676\_cov\_22.431114 5632-5643. Max. coverage (+): 0.31. Max coverage (-): 0.06

Region: NODE\_385231\_length\_5676\_cov\_22.431114 5644-5655. Max. coverage (+): 1.84. Max coverage (-): 0.01

Region: NODE\_385231\_length\_5676\_cov\_22.431114 5656-5666. Max. coverage (+): 1.84. Max coverage (-): 0.01

Region: NODE\_385231\_length\_5676\_cov\_22.431114 5667-5678. Max. coverage (+): 0.11. Max coverage (-): 0.01

Region: NODE\_385231\_length\_5676\_cov\_22.431114 5679-5689. Max. coverage (+): 0.02. Max coverage (-): 0.19

Region: NODE\_385231\_length\_5676\_cov\_22.431114 5690-5701. Max. coverage (+): 0.07. Max coverage (-): 0.01

Region: NODE\_385231\_length\_5676\_cov\_22.431114 5702-5713. Max. coverage (+): 0.07. Max coverage (-): 0

Region: NODE\_385231\_length\_5676\_cov\_22.431114 5714-5724. Max. coverage (+): 0.04. Max coverage (-): 0

Region: NODE\_385231\_length\_5676\_cov\_22.431114 5725-5736. Max. coverage (+): 0.37. Max coverage (-): 0

Region: NODE\_385231\_length\_5676\_cov\_22.431114 5737-5747. Max. coverage (+): 0.07. Max coverage (-): 0

Region: NODE\_385231\_length\_5676\_cov\_22.431114 5748-5759. Max. coverage (+): 0.06. Max coverage (-): 0.01

Region: NODE\_385231\_length\_5676\_cov\_22.431114 5760-5771. Max. coverage (+): 0.01. Max coverage (-): 0

Region: NODE\_385231\_length\_5676\_cov\_22.431114 5772-5782. Max. coverage (+): 0.01. Max coverage (-): 0

Region: NODE\_385231\_length\_5676\_cov\_22.431114 5783-5794. Max. coverage (+): 0. Max coverage (-): 0

Region: NODE\_385231\_length\_5676\_cov\_22.431114 5795-. Max. coverage (+): 0. Max coverage (-): 0

RepeatMasker Color Code

**+**

100-98% Identity

<98-95% Identity

<95-90% Identity

<90-85% Identity

<85-80% Identity

<80-75% Identity

<75-70% Identity

<70% Identity

**-**

Gene Set Color Code

**+**

Gene

Pseudogene

Other

**-**

Topology/Coverage Color Code

Coverage Plus Strand

Coverage Minus Strand

Mainstrand: Plus

Mainstrand: Minus

Complementary Strand

Flanking Region  
(if option -flank >0)

Gene Set Annotation  

**1. unknown (unknownunknown) Tr:unknown**: 560-1074 (+)  
**2. unknown (unknownunknown) Tr:unknown**: 1483-2097 (+)  
**3. unknown (unknownunknown) Tr:unknown**: 2732-2881 (+)  
**4. unknown (unknownunknown) Tr:unknown**: 4269-4713 (+)  
**5. unknown (unknownunknown) Tr:unknown UTR**: 560-561 (+)  
**6. unknown (unknownunknown) Tr:unknown UTR**: 4632-4713 (+)

  
RepeatMasker Annotation  

**1. AlRepC-696**: 61-133 (+), Divergence to consensus: 16.9%  
**2. AlRepD-2553**: 158-205 (-), Divergence to consensus: 14.9%  
**3. AlRepD-2553**: 209-259 (-), Divergence to consensus: 7.9%  
**4. AlRepB-221**: 287-375 (+), Divergence to consensus: 25.6%  
**5. AlRepA-102**: 386-467 (+), Divergence to consensus: 20.1%  
**6. AlRepC-693**: 2113-2233 (+), Divergence to consensus: 22.4%  
**7. AlRepB-26**: 2244-2359 (-), Divergence to consensus: 33.4%  
**8. AlRepE-277**: 3266-3314 (+), Divergence to consensus: 18.4%  
**9. AlRepC-328**: 3858-3907 (+), Divergence to consensus: 16%  
**10. AlRepC-687**: 4008-4174 (-), Divergence to consensus: 38.2%  
**11. AlRepE-1436**: 4980-5039 (-), Divergence to consensus: 18.3%

  
Transcription Factor Binding Sites  

**RHOXF1** (Sequence: AGCTTA (-): 58)  
**RHOXF1** (Sequence: AGATTA (-): 208)  
**RHOXF1** (Sequence: GGCTCA (-): 718)  
**RHOXF1** (Sequence: GGATTA (-): 1019)  
**RHOXF1** (Sequence: GGATCA (-): 2866)  
**RHOXF1** (Sequence: GGCTTA (-): 2953)  
**RHOXF1** (Sequence: GGCTTA (-): 3567)  
**RHOXF1** (Sequence: AGATCA (-): 4062)  
**RHOXF1** (Sequence: GGCTTA (-): 4310)  
**RHOXF1** (Sequence: GGATTA (-): 4599)  
**RHOXF1** (Sequence: AGATCA (-): 4897)  
**RHOXF1** (Sequence: AGCTTA (-): 5111)  
**RHOXF1** (Sequence: TGATCC (+): 1143)  
**RHOXF1** (Sequence: TGAGCT (+): 1536)  
**RHOXF1** (Sequence: TGAGCC (+): 1701)  
**RHOXF1** (Sequence: TAAGCC (+): 3281)  
**RHOXF1** (Sequence: TAATCT (+): 3601)  
**RHOXF1** (Sequence: TGATCT (+): 3881)  
**RHOXF1** (Sequence: TGAGCC (+): 4247)  
**RHOXF1** (Sequence: TGAGCT (+): 4516)  
**RHOXF1** (Sequence: TAAGCC (+): 4906)  
**POU5F1** (Sequence: TTTGCAT (-): 2492)  
**RFX4\_2** (Sequence: GTAACCAAG (-): 5591)  
**RFX4\_1** (Sequence: GTTGCTAGG (-): 2187)  
**FOXO3\_hsa** (Sequence: GTAAACAA (+): 4076)  
**SOX9** (Sequence: AACAATAG (-): 177)  
**SOX9** (Sequence: AACAATAA (-): 4812)  
**FOXP1** (Sequence: GTAAACA (+): 4076)  
**FOXP1** (Sequence: GTAAACA (+): 4940)  
**FOXO3\_mmu** (Sequence: TGTTTAGA (-): 403)  
**FOXO3\_mmu** (Sequence: TGTTTTCA (-): 2400)  
**FOXO3\_mmu** (Sequence: TGTTTTCA (-): 2723)  
**FOXO3\_mmu** (Sequence: TGTTTTGC (-): 3373)  
**Sox5** (Sequence: ATTGTT (+): 2671)  
**FIGLA** (Sequence: AACACGTGGA (-): 4329)  
**FOXO3\_mmu** (Sequence: GGAAAACA (+): 173)  
**FOXO3\_mmu** (Sequence: TGAAAACA (+): 3682)  
**FOXO3\_mmu** (Sequence: GGTAAACA (+): 4075)  
**FOXO3\_mmu** (Sequence: GGTAAACA (+): 4939)  
**Rhox11** (Sequence: TGCTGTATA (+): 191)  
**Rhox11** (Sequence: ATAACAGCA (-): 3981)  
**Rhox11** (Sequence: TAAACACCA (-): 4941)  
**Sox5** (Sequence: AACAAT (-): 177)  
**Sox5** (Sequence: AACAAT (-): 517)  
**Sox5** (Sequence: AACAAT (-): 4079)  
**Sox5** (Sequence: AACAAT (-): 4812)  
**POU5F1** (Sequence: ATGCAAA (+): 2299)  
**POU5F1** (Sequence: ATGCAAA (+): 3923)
