# Supplementary material for: First characterization of PIWI-interacting RNA clusters in a cichlid fish with a B chromosome
Source: BMC Biol. 2022 Sep 21;20:204. doi: 10.1186/s12915-022-01403-2 (PMC9490952; doi:10.1186/s12915-022-01403-2)
Supplement: Supplementary file 1 — Additional file 1. Zipped folder with fasta and interactive html piRNA cluster information for the A. latifasciata genome. The nomenclature is as follows: number-pirna-cluster_sex_B-presence (f, female; m, male; 0b, without B chromosome; 1b, with B chromosome). [file 12915_2022_1403_MOESM1_ESM.zip › 14_m1b.html]

piRNA cluster 14\_m1b 27


Predicted piRNA cluster no. 14\_m1b
  

Show proTRAC run info
Hide proTRAC run info

/\  
                \_\_\_\_\_\_\_\_\_\_\_\_\_\_\_\_\_\_\_\_\_\_\_/\\_\_\_ /  \\_\_\_\_\_\_\_  
               I                      /  \  /    \      I  
               I     pro             /    \/      \     I  
               I        TRAC        /               \   I  
               I   \_\_\_\_\_\_\_\_\_\_\_\_\_\_\_\_/\_\_\_\_\_\_\_\_\_\_\_\_\_\_\_\_\_\\_ I  
               I   \              /                     I  
               I    \            /                      I  
               I     \  /\      /       V.2.4.2         I  
               I      \/  \    /                        I  
               I\_\_\_\_\_\_\_\_\_\_\_\  /\_\_\_\_\_\_\_\_\_\_\_\_\_\_\_\_\_\_\_\_\_\_\_\_\_I  
                            \/  
  
  
================================= proTRAC ====================================  
VERSION: .......... 2.4.2  
LAST MODIFIED: .... 11. May 2018  
  
Please cite:  
Rosenkranz D, Zischler H. proTRAC - a software for probabilistic piRNA cluster  
detection, visualization and analysis. 2012. BMC Bioinformatics 13:5.  
  
  
Contact:  
David Rosenkranz  
Institute of Organismic and Molecular Evolutionary Biology  
Dept. Anthropology, small RNA group  
Johannes Gutenberg University Mainz  
email: rosenkranz@uni-mainz.de  
  
You can find the latest proTRAC version at:  
http://sourceforge.net/projects/protrac/files  
http://www.smallRNAgroup-mainz.de/software  
==============================================================================  
  
PARAMETERS:  
Map file: ...............piwi-machos-1B.fa-collapse.map  
Genome file: ............../../../0B\_ala\_genome.fa  
RepeatMasker annotation: Alatifasciata-all0B-maryan-v2.fa\_corrected.out  
GeneSet:................./guest-storage/Data/annotation/Alatifasciata\_all0B\_maryan-v2\_out2017.gff  
  
Significant (p<=0.01) hit density will be calculated based  
on observed hit distribution.  
  
Sliding window size: ........................................ 5000 bp  
Sliding window increament: .................................. 1000 bp  
Normalize each hit by number of genomic hits: ............... yes  
Normalize each hit by number of sequence reads: ............. yes  
Normalize values (-> per million mapped reads): ............. yes  
Min. fraction of hits with 1T(U) or 10A: .................... 0.75  
Alternatively: Min. fraction of hits with 1T(U) and 10A: .... 0.5  
Min. fraction of hits with typical piRNA length: ............ 0.75  
Typical piRNA length: ....................................... 24-32 nt  
Min. size of a piRNA cluster: ............................... 1000 bp.  
Min. number of hits (absolute): ............................. 0  
Min. number of hits (normalized): ........................... 0  
Min. fraction of hits on the mainstrand: .................... 0.75  
Top fraction of mapped sequences (in terms of read counts): . 1%  
Top fraction accounts for max. n% of sequence reads: ........ 90%  
Min. fraction of hits on each arm of a bidirectional cluster: 0.05  
Output html file for each cluster: .......................... yes  
Output a summary table: ..................................... yes  
Output a FASTA file for each cluster (piRNA sequences): ..... yes  
Output a FASTA file comprising cluster sequences: ........... yes  
Output a GTF file for predicted piRNA clusters: ..............yes  
Search DNA motifs in clusters: .............................. yes  
Output flanking sequences: +/- .............................. 0 bp  
Output ~.pTi file: .......................................... no  
==============================================================================  
  
  
Genome size (without gaps): ............ 758543724 bp  
Gaps (N/X/-): .......................... 417479 bp  
Mapped reads: .......................... 26973943  
Non-identical sequences: ............... 6209225  
Genomic hits: .......................... 48438990  
Significant densitiy of mapped reads: .. 821.144211136946 reads/kb

Show proTRAC cluster info
Hide proTRAC cluster info

|  |  |
| --- | --- |
| Location | NODE\_122013\_length\_9657\_cov\_34.346691 |
| Coordinates | 4022-9718 |
| Size [bp] | 5697 |
| Sequence hit loci | 3339 |
| Mapped reads (normalized) | 9148.4 |
| Mapped reads (normalized) per kb | 1605.8 |
| Normalized reads with 1T (1U) | 76% |
| Normalized reads with 10A | 33.2% |
| Normalized reads with length 24-32 nt | 99.4% |
| Normalized reads on the main strand(s) | 94.2% |
| Predicted directionality | mono:minus |

100%

0%

1T (1U)  
reads

10A reads

24-32 nt  
reads

reads on mainstrand

**Either the amount of reads with 1T (1U) OR 10A has to exceed 75% (set with option: -1Tor10A)  
Alternatively the amount of reads with 1T (1U) AND 10A has to exceed 50% (set with option: -1Tand10A)  
Minimum amount of reads with preferred size is 75% (set with option: -pisize)  
Minimum amount of reads on the main strand(s) is 75% (set with option: -clstrand)**

Show read coverage
Hide read coverage

WHAT DO I SEE HERE?  
This chart shows the location of mapped sequence reads within a predicted piRNA cluster. The color refers to the number of genomic hits produced by the sequence read in question. A dark red bar indicates that this sequence read produces many other hits elsewhere in the genome. Many adjacent red or yellow bars can indicate the presence of a multi-copy element such as transposons or rRNA genes. A dark green bar indicates that this sequence read maps uniquely to this locus.

1 hit

2-5 hits

6-10 hits

11-20 hits

21-50 hits

51-100 hits

> 100 hits

NODE\_122013\_length\_9657\_cov\_34.346691

4022

9718

Gene Set

RepeatMasker

Mapped  
Reads

51.01

plus strand

minus strand

51.01

Region: NODE\_122013\_length\_9657\_cov\_34.346691 1021-4027. Max. coverage (+): 0.04. Max coverage (-): 0.04

Region: NODE\_122013\_length\_9657\_cov\_34.346691 4028-4039. Max. coverage (+): 0. Max coverage (-): 0.07

Region: NODE\_122013\_length\_9657\_cov\_34.346691 4040-4050. Max. coverage (+): 0. Max coverage (-): 0

Region: NODE\_122013\_length\_9657\_cov\_34.346691 4051-4061. Max. coverage (+): 0. Max coverage (-): 0

Region: NODE\_122013\_length\_9657\_cov\_34.346691 4062-4073. Max. coverage (+): 0. Max coverage (-): 0

Region: NODE\_122013\_length\_9657\_cov\_34.346691 4074-4084. Max. coverage (+): 0. Max coverage (-): 0.04

Region: NODE\_122013\_length\_9657\_cov\_34.346691 4085-4096. Max. coverage (+): 0. Max coverage (-): 0.07

Region: NODE\_122013\_length\_9657\_cov\_34.346691 4097-4107. Max. coverage (+): 0. Max coverage (-): 0

Region: NODE\_122013\_length\_9657\_cov\_34.346691 4108-4118. Max. coverage (+): 0. Max coverage (-): 0

Region: NODE\_122013\_length\_9657\_cov\_34.346691 4119-4130. Max. coverage (+): 0. Max coverage (-): 0

Region: NODE\_122013\_length\_9657\_cov\_34.346691 4131-4141. Max. coverage (+): 0. Max coverage (-): 0

Region: NODE\_122013\_length\_9657\_cov\_34.346691 4142-4153. Max. coverage (+): 0. Max coverage (-): 0

Region: NODE\_122013\_length\_9657\_cov\_34.346691 4154-4164. Max. coverage (+): 0. Max coverage (-): 0

Region: NODE\_122013\_length\_9657\_cov\_34.346691 4165-4175. Max. coverage (+): 0. Max coverage (-): 0

Region: NODE\_122013\_length\_9657\_cov\_34.346691 4176-4187. Max. coverage (+): 0. Max coverage (-): 0

Region: NODE\_122013\_length\_9657\_cov\_34.346691 4188-4198. Max. coverage (+): 0. Max coverage (-): 0

Region: NODE\_122013\_length\_9657\_cov\_34.346691 4199-4210. Max. coverage (+): 0. Max coverage (-): 0

Region: NODE\_122013\_length\_9657\_cov\_34.346691 4211-4221. Max. coverage (+): 0. Max coverage (-): 0

Region: NODE\_122013\_length\_9657\_cov\_34.346691 4222-4232. Max. coverage (+): 0. Max coverage (-): 0

Region: NODE\_122013\_length\_9657\_cov\_34.346691 4233-4244. Max. coverage (+): 0. Max coverage (-): 0

Region: NODE\_122013\_length\_9657\_cov\_34.346691 4245-4255. Max. coverage (+): 0. Max coverage (-): 0

Region: NODE\_122013\_length\_9657\_cov\_34.346691 4256-4266. Max. coverage (+): 0.04. Max coverage (-): 0

Region: NODE\_122013\_length\_9657\_cov\_34.346691 4267-4278. Max. coverage (+): 0. Max coverage (-): 0

Region: NODE\_122013\_length\_9657\_cov\_34.346691 4279-4289. Max. coverage (+): 0. Max coverage (-): 0

Region: NODE\_122013\_length\_9657\_cov\_34.346691 4290-4301. Max. coverage (+): 0. Max coverage (-): 0

Region: NODE\_122013\_length\_9657\_cov\_34.346691 4302-4312. Max. coverage (+): 0. Max coverage (-): 0

Region: NODE\_122013\_length\_9657\_cov\_34.346691 4313-4323. Max. coverage (+): 0. Max coverage (-): 0.11

Region: NODE\_122013\_length\_9657\_cov\_34.346691 4324-4335. Max. coverage (+): 0. Max coverage (-): 0.04

Region: NODE\_122013\_length\_9657\_cov\_34.346691 4336-4346. Max. coverage (+): 0. Max coverage (-): 0.04

Region: NODE\_122013\_length\_9657\_cov\_34.346691 4347-4358. Max. coverage (+): 0. Max coverage (-): 0

Region: NODE\_122013\_length\_9657\_cov\_34.346691 4359-4369. Max. coverage (+): 0. Max coverage (-): 0.04

Region: NODE\_122013\_length\_9657\_cov\_34.346691 4370-4380. Max. coverage (+): 0. Max coverage (-): 0

Region: NODE\_122013\_length\_9657\_cov\_34.346691 4381-4392. Max. coverage (+): 0. Max coverage (-): 0

Region: NODE\_122013\_length\_9657\_cov\_34.346691 4393-4403. Max. coverage (+): 0. Max coverage (-): 0.04

Region: NODE\_122013\_length\_9657\_cov\_34.346691 4404-4415. Max. coverage (+): 0. Max coverage (-): 0

Region: NODE\_122013\_length\_9657\_cov\_34.346691 4416-4426. Max. coverage (+): 0. Max coverage (-): 0

Region: NODE\_122013\_length\_9657\_cov\_34.346691 4427-4437. Max. coverage (+): 0. Max coverage (-): 0

Region: NODE\_122013\_length\_9657\_cov\_34.346691 4438-4449. Max. coverage (+): 0. Max coverage (-): 0

Region: NODE\_122013\_length\_9657\_cov\_34.346691 4450-4460. Max. coverage (+): 0. Max coverage (-): 0

Region: NODE\_122013\_length\_9657\_cov\_34.346691 4461-4472. Max. coverage (+): 0. Max coverage (-): 0

Region: NODE\_122013\_length\_9657\_cov\_34.346691 4473-4483. Max. coverage (+): 0. Max coverage (-): 0

Region: NODE\_122013\_length\_9657\_cov\_34.346691 4484-4494. Max. coverage (+): 0. Max coverage (-): 0

Region: NODE\_122013\_length\_9657\_cov\_34.346691 4495-4506. Max. coverage (+): 0. Max coverage (-): 0

Region: NODE\_122013\_length\_9657\_cov\_34.346691 4507-4517. Max. coverage (+): 0. Max coverage (-): 0

Region: NODE\_122013\_length\_9657\_cov\_34.346691 4518-4529. Max. coverage (+): 0. Max coverage (-): 0

Region: NODE\_122013\_length\_9657\_cov\_34.346691 4530-4540. Max. coverage (+): 0. Max coverage (-): 0

Region: NODE\_122013\_length\_9657\_cov\_34.346691 4541-4551. Max. coverage (+): 0. Max coverage (-): 0

Region: NODE\_122013\_length\_9657\_cov\_34.346691 4552-4563. Max. coverage (+): 0. Max coverage (-): 0

Region: NODE\_122013\_length\_9657\_cov\_34.346691 4564-4574. Max. coverage (+): 0. Max coverage (-): 0

Region: NODE\_122013\_length\_9657\_cov\_34.346691 4575-4586. Max. coverage (+): 0. Max coverage (-): 0

Region: NODE\_122013\_length\_9657\_cov\_34.346691 4587-4597. Max. coverage (+): 0. Max coverage (-): 0

Region: NODE\_122013\_length\_9657\_cov\_34.346691 4598-4608. Max. coverage (+): 0. Max coverage (-): 0

Region: NODE\_122013\_length\_9657\_cov\_34.346691 4609-4620. Max. coverage (+): 0. Max coverage (-): 0

Region: NODE\_122013\_length\_9657\_cov\_34.346691 4621-4631. Max. coverage (+): 0. Max coverage (-): 0

Region: NODE\_122013\_length\_9657\_cov\_34.346691 4632-4642. Max. coverage (+): 0. Max coverage (-): 0

Region: NODE\_122013\_length\_9657\_cov\_34.346691 4643-4654. Max. coverage (+): 0. Max coverage (-): 0

Region: NODE\_122013\_length\_9657\_cov\_34.346691 4655-4665. Max. coverage (+): 0. Max coverage (-): 0

Region: NODE\_122013\_length\_9657\_cov\_34.346691 4666-4677. Max. coverage (+): 0. Max coverage (-): 0.04

Region: NODE\_122013\_length\_9657\_cov\_34.346691 4678-4688. Max. coverage (+): 0. Max coverage (-): 0

Region: NODE\_122013\_length\_9657\_cov\_34.346691 4689-4699. Max. coverage (+): 0. Max coverage (-): 0

Region: NODE\_122013\_length\_9657\_cov\_34.346691 4700-4711. Max. coverage (+): 0. Max coverage (-): 0

Region: NODE\_122013\_length\_9657\_cov\_34.346691 4712-4722. Max. coverage (+): 0. Max coverage (-): 0

Region: NODE\_122013\_length\_9657\_cov\_34.346691 4723-4734. Max. coverage (+): 0. Max coverage (-): 0.07

Region: NODE\_122013\_length\_9657\_cov\_34.346691 4735-4745. Max. coverage (+): 0. Max coverage (-): 0

Region: NODE\_122013\_length\_9657\_cov\_34.346691 4746-4756. Max. coverage (+): 0. Max coverage (-): 0

Region: NODE\_122013\_length\_9657\_cov\_34.346691 4757-4768. Max. coverage (+): 0. Max coverage (-): 0

Region: NODE\_122013\_length\_9657\_cov\_34.346691 4769-4779. Max. coverage (+): 0. Max coverage (-): 0

Region: NODE\_122013\_length\_9657\_cov\_34.346691 4780-4791. Max. coverage (+): 0. Max coverage (-): 0

Region: NODE\_122013\_length\_9657\_cov\_34.346691 4792-4802. Max. coverage (+): 0. Max coverage (-): 0

Region: NODE\_122013\_length\_9657\_cov\_34.346691 4803-4813. Max. coverage (+): 0. Max coverage (-): 0

Region: NODE\_122013\_length\_9657\_cov\_34.346691 4814-4825. Max. coverage (+): 0.04. Max coverage (-): 0

Region: NODE\_122013\_length\_9657\_cov\_34.346691 4826-4836. Max. coverage (+): 0.04. Max coverage (-): 0

Region: NODE\_122013\_length\_9657\_cov\_34.346691 4837-4848. Max. coverage (+): 0. Max coverage (-): 0.11

Region: NODE\_122013\_length\_9657\_cov\_34.346691 4849-4859. Max. coverage (+): 0. Max coverage (-): 0

Region: NODE\_122013\_length\_9657\_cov\_34.346691 4860-4870. Max. coverage (+): 0. Max coverage (-): 0.04

Region: NODE\_122013\_length\_9657\_cov\_34.346691 4871-4882. Max. coverage (+): 0. Max coverage (-): 0

Region: NODE\_122013\_length\_9657\_cov\_34.346691 4883-4893. Max. coverage (+): 0. Max coverage (-): 0

Region: NODE\_122013\_length\_9657\_cov\_34.346691 4894-4905. Max. coverage (+): 0. Max coverage (-): 0

Region: NODE\_122013\_length\_9657\_cov\_34.346691 4906-4916. Max. coverage (+): 0. Max coverage (-): 0

Region: NODE\_122013\_length\_9657\_cov\_34.346691 4917-4927. Max. coverage (+): 0. Max coverage (-): 0

Region: NODE\_122013\_length\_9657\_cov\_34.346691 4928-4939. Max. coverage (+): 0. Max coverage (-): 0

Region: NODE\_122013\_length\_9657\_cov\_34.346691 4940-4950. Max. coverage (+): 0. Max coverage (-): 0

Region: NODE\_122013\_length\_9657\_cov\_34.346691 4951-4962. Max. coverage (+): 0. Max coverage (-): 0

Region: NODE\_122013\_length\_9657\_cov\_34.346691 4963-4973. Max. coverage (+): 0. Max coverage (-): 0

Region: NODE\_122013\_length\_9657\_cov\_34.346691 4974-4984. Max. coverage (+): 0. Max coverage (-): 0

Region: NODE\_122013\_length\_9657\_cov\_34.346691 4985-4996. Max. coverage (+): 0. Max coverage (-): 0

Region: NODE\_122013\_length\_9657\_cov\_34.346691 4997-5007. Max. coverage (+): 0. Max coverage (-): 0

Region: NODE\_122013\_length\_9657\_cov\_34.346691 5008-5018. Max. coverage (+): 0. Max coverage (-): 0

Region: NODE\_122013\_length\_9657\_cov\_34.346691 5019-5030. Max. coverage (+): 0. Max coverage (-): 0

Region: NODE\_122013\_length\_9657\_cov\_34.346691 5031-5041. Max. coverage (+): 0. Max coverage (-): 0

Region: NODE\_122013\_length\_9657\_cov\_34.346691 5042-5053. Max. coverage (+): 0. Max coverage (-): 0

Region: NODE\_122013\_length\_9657\_cov\_34.346691 5054-5064. Max. coverage (+): 0. Max coverage (-): 0

Region: NODE\_122013\_length\_9657\_cov\_34.346691 5065-5075. Max. coverage (+): 0. Max coverage (-): 0

Region: NODE\_122013\_length\_9657\_cov\_34.346691 5076-5087. Max. coverage (+): 0. Max coverage (-): 0

Region: NODE\_122013\_length\_9657\_cov\_34.346691 5088-5098. Max. coverage (+): 0. Max coverage (-): 0

Region: NODE\_122013\_length\_9657\_cov\_34.346691 5099-5110. Max. coverage (+): 0. Max coverage (-): 0

Region: NODE\_122013\_length\_9657\_cov\_34.346691 5111-5121. Max. coverage (+): 0. Max coverage (-): 0

Region: NODE\_122013\_length\_9657\_cov\_34.346691 5122-5132. Max. coverage (+): 0. Max coverage (-): 0

Region: NODE\_122013\_length\_9657\_cov\_34.346691 5133-5144. Max. coverage (+): 0. Max coverage (-): 0

Region: NODE\_122013\_length\_9657\_cov\_34.346691 5145-5155. Max. coverage (+): 0.04. Max coverage (-): 0

Region: NODE\_122013\_length\_9657\_cov\_34.346691 5156-5167. Max. coverage (+): 0.04. Max coverage (-): 0

Region: NODE\_122013\_length\_9657\_cov\_34.346691 5168-5178. Max. coverage (+): 0. Max coverage (-): 0.04

Region: NODE\_122013\_length\_9657\_cov\_34.346691 5179-5189. Max. coverage (+): 0. Max coverage (-): 0

Region: NODE\_122013\_length\_9657\_cov\_34.346691 5190-5201. Max. coverage (+): 0. Max coverage (-): 0

Region: NODE\_122013\_length\_9657\_cov\_34.346691 5202-5212. Max. coverage (+): 0. Max coverage (-): 0

Region: NODE\_122013\_length\_9657\_cov\_34.346691 5213-5224. Max. coverage (+): 0. Max coverage (-): 0

Region: NODE\_122013\_length\_9657\_cov\_34.346691 5225-5235. Max. coverage (+): 0. Max coverage (-): 0

Region: NODE\_122013\_length\_9657\_cov\_34.346691 5236-5246. Max. coverage (+): 0. Max coverage (-): 0

Region: NODE\_122013\_length\_9657\_cov\_34.346691 5247-5258. Max. coverage (+): 0. Max coverage (-): 0

Region: NODE\_122013\_length\_9657\_cov\_34.346691 5259-5269. Max. coverage (+): 0. Max coverage (-): 0

Region: NODE\_122013\_length\_9657\_cov\_34.346691 5270-5281. Max. coverage (+): 0. Max coverage (-): 0

Region: NODE\_122013\_length\_9657\_cov\_34.346691 5282-5292. Max. coverage (+): 0. Max coverage (-): 0

Region: NODE\_122013\_length\_9657\_cov\_34.346691 5293-5303. Max. coverage (+): 0. Max coverage (-): 0

Region: NODE\_122013\_length\_9657\_cov\_34.346691 5304-5315. Max. coverage (+): 0. Max coverage (-): 0

Region: NODE\_122013\_length\_9657\_cov\_34.346691 5316-5326. Max. coverage (+): 0. Max coverage (-): 0.19

Region: NODE\_122013\_length\_9657\_cov\_34.346691 5327-5338. Max. coverage (+): 0. Max coverage (-): 0

Region: NODE\_122013\_length\_9657\_cov\_34.346691 5339-5349. Max. coverage (+): 0. Max coverage (-): 0

Region: NODE\_122013\_length\_9657\_cov\_34.346691 5350-5360. Max. coverage (+): 0. Max coverage (-): 0

Region: NODE\_122013\_length\_9657\_cov\_34.346691 5361-5372. Max. coverage (+): 0. Max coverage (-): 0

Region: NODE\_122013\_length\_9657\_cov\_34.346691 5373-5383. Max. coverage (+): 0. Max coverage (-): 0

Region: NODE\_122013\_length\_9657\_cov\_34.346691 5384-5394. Max. coverage (+): 0. Max coverage (-): 0.04

Region: NODE\_122013\_length\_9657\_cov\_34.346691 5395-5406. Max. coverage (+): 0. Max coverage (-): 0.04

Region: NODE\_122013\_length\_9657\_cov\_34.346691 5407-5417. Max. coverage (+): 0.04. Max coverage (-): 0

Region: NODE\_122013\_length\_9657\_cov\_34.346691 5418-5429. Max. coverage (+): 0.07. Max coverage (-): 0

Region: NODE\_122013\_length\_9657\_cov\_34.346691 5430-5440. Max. coverage (+): 0. Max coverage (-): 0

Region: NODE\_122013\_length\_9657\_cov\_34.346691 5441-5451. Max. coverage (+): 0. Max coverage (-): 0

Region: NODE\_122013\_length\_9657\_cov\_34.346691 5452-5463. Max. coverage (+): 0. Max coverage (-): 0

Region: NODE\_122013\_length\_9657\_cov\_34.346691 5464-5474. Max. coverage (+): 0. Max coverage (-): 0

Region: NODE\_122013\_length\_9657\_cov\_34.346691 5475-5486. Max. coverage (+): 0. Max coverage (-): 0

Region: NODE\_122013\_length\_9657\_cov\_34.346691 5487-5497. Max. coverage (+): 0.02. Max coverage (-): 0

Region: NODE\_122013\_length\_9657\_cov\_34.346691 5498-5508. Max. coverage (+): 0.16. Max coverage (-): 0

Region: NODE\_122013\_length\_9657\_cov\_34.346691 5509-5520. Max. coverage (+): 0. Max coverage (-): 0.04

Region: NODE\_122013\_length\_9657\_cov\_34.346691 5521-5531. Max. coverage (+): 0. Max coverage (-): 0.1

Region: NODE\_122013\_length\_9657\_cov\_34.346691 5532-5543. Max. coverage (+): 0. Max coverage (-): 0.1

Region: NODE\_122013\_length\_9657\_cov\_34.346691 5544-5554. Max. coverage (+): 0. Max coverage (-): 0

Region: NODE\_122013\_length\_9657\_cov\_34.346691 5555-5565. Max. coverage (+): 0. Max coverage (-): 0

Region: NODE\_122013\_length\_9657\_cov\_34.346691 5566-5577. Max. coverage (+): 0.01. Max coverage (-): 0

Region: NODE\_122013\_length\_9657\_cov\_34.346691 5578-5588. Max. coverage (+): 0. Max coverage (-): 0.11

Region: NODE\_122013\_length\_9657\_cov\_34.346691 5589-5600. Max. coverage (+): 0. Max coverage (-): 0.15

Region: NODE\_122013\_length\_9657\_cov\_34.346691 5601-5611. Max. coverage (+): 0.04. Max coverage (-): 0

Region: NODE\_122013\_length\_9657\_cov\_34.346691 5612-5622. Max. coverage (+): 0. Max coverage (-): 0.02

Region: NODE\_122013\_length\_9657\_cov\_34.346691 5623-5634. Max. coverage (+): 0. Max coverage (-): 0

Region: NODE\_122013\_length\_9657\_cov\_34.346691 5635-5645. Max. coverage (+): 0. Max coverage (-): 0

Region: NODE\_122013\_length\_9657\_cov\_34.346691 5646-5657. Max. coverage (+): 0. Max coverage (-): 0

Region: NODE\_122013\_length\_9657\_cov\_34.346691 5658-5668. Max. coverage (+): 0. Max coverage (-): 0

Region: NODE\_122013\_length\_9657\_cov\_34.346691 5669-5679. Max. coverage (+): 0. Max coverage (-): 0

Region: NODE\_122013\_length\_9657\_cov\_34.346691 5680-5691. Max. coverage (+): 0. Max coverage (-): 0

Region: NODE\_122013\_length\_9657\_cov\_34.346691 5692-5702. Max. coverage (+): 0. Max coverage (-): 0

Region: NODE\_122013\_length\_9657\_cov\_34.346691 5703-5714. Max. coverage (+): 0. Max coverage (-): 0

Region: NODE\_122013\_length\_9657\_cov\_34.346691 5715-5725. Max. coverage (+): 0. Max coverage (-): 0

Region: NODE\_122013\_length\_9657\_cov\_34.346691 5726-5736. Max. coverage (+): 0. Max coverage (-): 0

Region: NODE\_122013\_length\_9657\_cov\_34.346691 5737-5748. Max. coverage (+): 0. Max coverage (-): 0

Region: NODE\_122013\_length\_9657\_cov\_34.346691 5749-5759. Max. coverage (+): 0. Max coverage (-): 0.02

Region: NODE\_122013\_length\_9657\_cov\_34.346691 5760-5770. Max. coverage (+): 0. Max coverage (-): 0

Region: NODE\_122013\_length\_9657\_cov\_34.346691 5771-5782. Max. coverage (+): 0. Max coverage (-): 0

Region: NODE\_122013\_length\_9657\_cov\_34.346691 5783-5793. Max. coverage (+): 0. Max coverage (-): 0

Region: NODE\_122013\_length\_9657\_cov\_34.346691 5794-5805. Max. coverage (+): 0. Max coverage (-): 0

Region: NODE\_122013\_length\_9657\_cov\_34.346691 5806-5816. Max. coverage (+): 0. Max coverage (-): 0.02

Region: NODE\_122013\_length\_9657\_cov\_34.346691 5817-5827. Max. coverage (+): 0. Max coverage (-): 0

Region: NODE\_122013\_length\_9657\_cov\_34.346691 5828-5839. Max. coverage (+): 0. Max coverage (-): 0

Region: NODE\_122013\_length\_9657\_cov\_34.346691 5840-5850. Max. coverage (+): 0. Max coverage (-): 0

Region: NODE\_122013\_length\_9657\_cov\_34.346691 5851-5862. Max. coverage (+): 0. Max coverage (-): 0

Region: NODE\_122013\_length\_9657\_cov\_34.346691 5863-5873. Max. coverage (+): 0. Max coverage (-): 0

Region: NODE\_122013\_length\_9657\_cov\_34.346691 5874-5884. Max. coverage (+): 0.01. Max coverage (-): 0

Region: NODE\_122013\_length\_9657\_cov\_34.346691 5885-5896. Max. coverage (+): 0.01. Max coverage (-): 0

Region: NODE\_122013\_length\_9657\_cov\_34.346691 5897-5907. Max. coverage (+): 0. Max coverage (-): 0

Region: NODE\_122013\_length\_9657\_cov\_34.346691 5908-5919. Max. coverage (+): 0. Max coverage (-): 0

Region: NODE\_122013\_length\_9657\_cov\_34.346691 5920-5930. Max. coverage (+): 0. Max coverage (-): 0.74

Region: NODE\_122013\_length\_9657\_cov\_34.346691 5931-5941. Max. coverage (+): 0.04. Max coverage (-): 0.48

Region: NODE\_122013\_length\_9657\_cov\_34.346691 5942-5953. Max. coverage (+): 0. Max coverage (-): 0

Region: NODE\_122013\_length\_9657\_cov\_34.346691 5954-5964. Max. coverage (+): 0. Max coverage (-): 0

Region: NODE\_122013\_length\_9657\_cov\_34.346691 5965-5976. Max. coverage (+): 0. Max coverage (-): 0

Region: NODE\_122013\_length\_9657\_cov\_34.346691 5977-5987. Max. coverage (+): 0. Max coverage (-): 0.05

Region: NODE\_122013\_length\_9657\_cov\_34.346691 5988-5998. Max. coverage (+): 0. Max coverage (-): 0

Region: NODE\_122013\_length\_9657\_cov\_34.346691 5999-6010. Max. coverage (+): 0. Max coverage (-): 0.01

Region: NODE\_122013\_length\_9657\_cov\_34.346691 6011-6021. Max. coverage (+): 0. Max coverage (-): 0

Region: NODE\_122013\_length\_9657\_cov\_34.346691 6022-6033. Max. coverage (+): 0.02. Max coverage (-): 0

Region: NODE\_122013\_length\_9657\_cov\_34.346691 6034-6044. Max. coverage (+): 0. Max coverage (-): 0

Region: NODE\_122013\_length\_9657\_cov\_34.346691 6045-6055. Max. coverage (+): 0. Max coverage (-): 0

Region: NODE\_122013\_length\_9657\_cov\_34.346691 6056-6067. Max. coverage (+): 0. Max coverage (-): 0

Region: NODE\_122013\_length\_9657\_cov\_34.346691 6068-6078. Max. coverage (+): 0. Max coverage (-): 0

Region: NODE\_122013\_length\_9657\_cov\_34.346691 6079-6090. Max. coverage (+): 0. Max coverage (-): 0

Region: NODE\_122013\_length\_9657\_cov\_34.346691 6091-6101. Max. coverage (+): 0. Max coverage (-): 0

Region: NODE\_122013\_length\_9657\_cov\_34.346691 6102-6112. Max. coverage (+): 0. Max coverage (-): 0

Region: NODE\_122013\_length\_9657\_cov\_34.346691 6113-6124. Max. coverage (+): 0. Max coverage (-): 0

Region: NODE\_122013\_length\_9657\_cov\_34.346691 6125-6135. Max. coverage (+): 0. Max coverage (-): 0

Region: NODE\_122013\_length\_9657\_cov\_34.346691 6136-6146. Max. coverage (+): 0. Max coverage (-): 0

Region: NODE\_122013\_length\_9657\_cov\_34.346691 6147-6158. Max. coverage (+): 0. Max coverage (-): 0

Region: NODE\_122013\_length\_9657\_cov\_34.346691 6159-6169. Max. coverage (+): 0. Max coverage (-): 0

Region: NODE\_122013\_length\_9657\_cov\_34.346691 6170-6181. Max. coverage (+): 0. Max coverage (-): 0

Region: NODE\_122013\_length\_9657\_cov\_34.346691 6182-6192. Max. coverage (+): 0. Max coverage (-): 0

Region: NODE\_122013\_length\_9657\_cov\_34.346691 6193-6203. Max. coverage (+): 0. Max coverage (-): 0

Region: NODE\_122013\_length\_9657\_cov\_34.346691 6204-6215. Max. coverage (+): 0. Max coverage (-): 0

Region: NODE\_122013\_length\_9657\_cov\_34.346691 6216-6226. Max. coverage (+): 0. Max coverage (-): 0

Region: NODE\_122013\_length\_9657\_cov\_34.346691 6227-6238. Max. coverage (+): 0. Max coverage (-): 0

Region: NODE\_122013\_length\_9657\_cov\_34.346691 6239-6249. Max. coverage (+): 0. Max coverage (-): 0

Region: NODE\_122013\_length\_9657\_cov\_34.346691 6250-6260. Max. coverage (+): 0. Max coverage (-): 0

Region: NODE\_122013\_length\_9657\_cov\_34.346691 6261-6272. Max. coverage (+): 0. Max coverage (-): 0.06

Region: NODE\_122013\_length\_9657\_cov\_34.346691 6273-6283. Max. coverage (+): 0. Max coverage (-): 0

Region: NODE\_122013\_length\_9657\_cov\_34.346691 6284-6295. Max. coverage (+): 0. Max coverage (-): 0

Region: NODE\_122013\_length\_9657\_cov\_34.346691 6296-6306. Max. coverage (+): 0. Max coverage (-): 0

Region: NODE\_122013\_length\_9657\_cov\_34.346691 6307-6317. Max. coverage (+): 0.04. Max coverage (-): 0.11

Region: NODE\_122013\_length\_9657\_cov\_34.346691 6318-6329. Max. coverage (+): 0.04. Max coverage (-): 0.11

Region: NODE\_122013\_length\_9657\_cov\_34.346691 6330-6340. Max. coverage (+): 0. Max coverage (-): 0.01

Region: NODE\_122013\_length\_9657\_cov\_34.346691 6341-6352. Max. coverage (+): 0. Max coverage (-): 0.03

Region: NODE\_122013\_length\_9657\_cov\_34.346691 6353-6363. Max. coverage (+): 0. Max coverage (-): 0

Region: NODE\_122013\_length\_9657\_cov\_34.346691 6364-6374. Max. coverage (+): 0. Max coverage (-): 0

Region: NODE\_122013\_length\_9657\_cov\_34.346691 6375-6386. Max. coverage (+): 0. Max coverage (-): 0

Region: NODE\_122013\_length\_9657\_cov\_34.346691 6387-6397. Max. coverage (+): 0. Max coverage (-): 0

Region: NODE\_122013\_length\_9657\_cov\_34.346691 6398-6409. Max. coverage (+): 0. Max coverage (-): 0.07

Region: NODE\_122013\_length\_9657\_cov\_34.346691 6410-6420. Max. coverage (+): 0. Max coverage (-): 0.07

Region: NODE\_122013\_length\_9657\_cov\_34.346691 6421-6431. Max. coverage (+): 0. Max coverage (-): 0

Region: NODE\_122013\_length\_9657\_cov\_34.346691 6432-6443. Max. coverage (+): 0. Max coverage (-): 0.04

Region: NODE\_122013\_length\_9657\_cov\_34.346691 6444-6454. Max. coverage (+): 0. Max coverage (-): 0

Region: NODE\_122013\_length\_9657\_cov\_34.346691 6455-6466. Max. coverage (+): 0.02. Max coverage (-): 0

Region: NODE\_122013\_length\_9657\_cov\_34.346691 6467-6477. Max. coverage (+): 0.02. Max coverage (-): 0

Region: NODE\_122013\_length\_9657\_cov\_34.346691 6478-6488. Max. coverage (+): 0.01. Max coverage (-): 0

Region: NODE\_122013\_length\_9657\_cov\_34.346691 6489-6500. Max. coverage (+): 0. Max coverage (-): 0.04

Region: NODE\_122013\_length\_9657\_cov\_34.346691 6501-6511. Max. coverage (+): 0. Max coverage (-): 0.02

Region: NODE\_122013\_length\_9657\_cov\_34.346691 6512-6522. Max. coverage (+): 0. Max coverage (-): 0.02

Region: NODE\_122013\_length\_9657\_cov\_34.346691 6523-6534. Max. coverage (+): 0. Max coverage (-): 0

Region: NODE\_122013\_length\_9657\_cov\_34.346691 6535-6545. Max. coverage (+): 0. Max coverage (-): 0

Region: NODE\_122013\_length\_9657\_cov\_34.346691 6546-6557. Max. coverage (+): 0. Max coverage (-): 0

Region: NODE\_122013\_length\_9657\_cov\_34.346691 6558-6568. Max. coverage (+): 0. Max coverage (-): 0

Region: NODE\_122013\_length\_9657\_cov\_34.346691 6569-6579. Max. coverage (+): 0. Max coverage (-): 0

Region: NODE\_122013\_length\_9657\_cov\_34.346691 6580-6591. Max. coverage (+): 0. Max coverage (-): 0.15

Region: NODE\_122013\_length\_9657\_cov\_34.346691 6592-6602. Max. coverage (+): 0.07. Max coverage (-): 0.04

Region: NODE\_122013\_length\_9657\_cov\_34.346691 6603-6614. Max. coverage (+): 0. Max coverage (-): 0.04

Region: NODE\_122013\_length\_9657\_cov\_34.346691 6615-6625. Max. coverage (+): 0. Max coverage (-): 0.04

Region: NODE\_122013\_length\_9657\_cov\_34.346691 6626-6636. Max. coverage (+): 0. Max coverage (-): 0

Region: NODE\_122013\_length\_9657\_cov\_34.346691 6637-6648. Max. coverage (+): 0. Max coverage (-): 0

Region: NODE\_122013\_length\_9657\_cov\_34.346691 6649-6659. Max. coverage (+): 0.04. Max coverage (-): 0.06

Region: NODE\_122013\_length\_9657\_cov\_34.346691 6660-6671. Max. coverage (+): 0.02. Max coverage (-): 0.05

Region: NODE\_122013\_length\_9657\_cov\_34.346691 6672-6682. Max. coverage (+): 0.02. Max coverage (-): 0.04

Region: NODE\_122013\_length\_9657\_cov\_34.346691 6683-6693. Max. coverage (+): 0. Max coverage (-): 0.02

Region: NODE\_122013\_length\_9657\_cov\_34.346691 6694-6705. Max. coverage (+): 0. Max coverage (-): 0.04

Region: NODE\_122013\_length\_9657\_cov\_34.346691 6706-6716. Max. coverage (+): 0. Max coverage (-): 0

Region: NODE\_122013\_length\_9657\_cov\_34.346691 6717-6728. Max. coverage (+): 0.07. Max coverage (-): 0.44

Region: NODE\_122013\_length\_9657\_cov\_34.346691 6729-6739. Max. coverage (+): 0.04. Max coverage (-): 0.7

Region: NODE\_122013\_length\_9657\_cov\_34.346691 6740-6750. Max. coverage (+): 0.02. Max coverage (-): 0.44

Region: NODE\_122013\_length\_9657\_cov\_34.346691 6751-6762. Max. coverage (+): 0. Max coverage (-): 0

Region: NODE\_122013\_length\_9657\_cov\_34.346691 6763-6773. Max. coverage (+): 0. Max coverage (-): 0

Region: NODE\_122013\_length\_9657\_cov\_34.346691 6774-6785. Max. coverage (+): 0. Max coverage (-): 0.04

Region: NODE\_122013\_length\_9657\_cov\_34.346691 6786-6796. Max. coverage (+): 0.07. Max coverage (-): 0.04

Region: NODE\_122013\_length\_9657\_cov\_34.346691 6797-6807. Max. coverage (+): 0.04. Max coverage (-): 0.04

Region: NODE\_122013\_length\_9657\_cov\_34.346691 6808-6819. Max. coverage (+): 0. Max coverage (-): 0.33

Region: NODE\_122013\_length\_9657\_cov\_34.346691 6820-6830. Max. coverage (+): 0. Max coverage (-): 0.37

Region: NODE\_122013\_length\_9657\_cov\_34.346691 6831-6842. Max. coverage (+): 0.02. Max coverage (-): 0

Region: NODE\_122013\_length\_9657\_cov\_34.346691 6843-6853. Max. coverage (+): 0.02. Max coverage (-): 0.06

Region: NODE\_122013\_length\_9657\_cov\_34.346691 6854-6864. Max. coverage (+): 0. Max coverage (-): 0.15

Region: NODE\_122013\_length\_9657\_cov\_34.346691 6865-6876. Max. coverage (+): 0. Max coverage (-): 0.22

Region: NODE\_122013\_length\_9657\_cov\_34.346691 6877-6887. Max. coverage (+): 0. Max coverage (-): 0.04

Region: NODE\_122013\_length\_9657\_cov\_34.346691 6888-6898. Max. coverage (+): 0. Max coverage (-): 0

Region: NODE\_122013\_length\_9657\_cov\_34.346691 6899-6910. Max. coverage (+): 0. Max coverage (-): 0

Region: NODE\_122013\_length\_9657\_cov\_34.346691 6911-6921. Max. coverage (+): 0. Max coverage (-): 0

Region: NODE\_122013\_length\_9657\_cov\_34.346691 6922-6933. Max. coverage (+): 0. Max coverage (-): 0

Region: NODE\_122013\_length\_9657\_cov\_34.346691 6934-6944. Max. coverage (+): 0.04. Max coverage (-): 0.22

Region: NODE\_122013\_length\_9657\_cov\_34.346691 6945-6955. Max. coverage (+): 0. Max coverage (-): 0

Region: NODE\_122013\_length\_9657\_cov\_34.346691 6956-6967. Max. coverage (+): 0.04. Max coverage (-): 0.3

Region: NODE\_122013\_length\_9657\_cov\_34.346691 6968-6978. Max. coverage (+): 0. Max coverage (-): 1.52

Region: NODE\_122013\_length\_9657\_cov\_34.346691 6979-6990. Max. coverage (+): 0. Max coverage (-): 0.19

Region: NODE\_122013\_length\_9657\_cov\_34.346691 6991-7001. Max. coverage (+): 0. Max coverage (-): 0.3

Region: NODE\_122013\_length\_9657\_cov\_34.346691 7002-7012. Max. coverage (+): 0. Max coverage (-): 0.26

Region: NODE\_122013\_length\_9657\_cov\_34.346691 7013-7024. Max. coverage (+): 0.04. Max coverage (-): 1.11

Region: NODE\_122013\_length\_9657\_cov\_34.346691 7025-7035. Max. coverage (+): 0. Max coverage (-): 0.15

Region: NODE\_122013\_length\_9657\_cov\_34.346691 7036-7047. Max. coverage (+): 0.04. Max coverage (-): 0.7

Region: NODE\_122013\_length\_9657\_cov\_34.346691 7048-7058. Max. coverage (+): 0. Max coverage (-): 0.19

Region: NODE\_122013\_length\_9657\_cov\_34.346691 7059-7069. Max. coverage (+): 0. Max coverage (-): 0.22

Region: NODE\_122013\_length\_9657\_cov\_34.346691 7070-7081. Max. coverage (+): 0.04. Max coverage (-): 0.33

Region: NODE\_122013\_length\_9657\_cov\_34.346691 7082-7092. Max. coverage (+): 0.04. Max coverage (-): 0.56

Region: NODE\_122013\_length\_9657\_cov\_34.346691 7093-7104. Max. coverage (+): 0. Max coverage (-): 0

Region: NODE\_122013\_length\_9657\_cov\_34.346691 7105-7115. Max. coverage (+): 0.04. Max coverage (-): 2.22

Region: NODE\_122013\_length\_9657\_cov\_34.346691 7116-7126. Max. coverage (+): 0.07. Max coverage (-): 3

Region: NODE\_122013\_length\_9657\_cov\_34.346691 7127-7138. Max. coverage (+): 0.04. Max coverage (-): 0.3

Region: NODE\_122013\_length\_9657\_cov\_34.346691 7139-7149. Max. coverage (+): 0. Max coverage (-): 0.37

Region: NODE\_122013\_length\_9657\_cov\_34.346691 7150-7161. Max. coverage (+): 0.07. Max coverage (-): 0.04

Region: NODE\_122013\_length\_9657\_cov\_34.346691 7162-7172. Max. coverage (+): 0. Max coverage (-): 0.41

Region: NODE\_122013\_length\_9657\_cov\_34.346691 7173-7183. Max. coverage (+): 0.04. Max coverage (-): 0.11

Region: NODE\_122013\_length\_9657\_cov\_34.346691 7184-7195. Max. coverage (+): 0. Max coverage (-): 0.93

Region: NODE\_122013\_length\_9657\_cov\_34.346691 7196-7206. Max. coverage (+): 0. Max coverage (-): 1

Region: NODE\_122013\_length\_9657\_cov\_34.346691 7207-7218. Max. coverage (+): 0. Max coverage (-): 0.11

Region: NODE\_122013\_length\_9657\_cov\_34.346691 7219-7229. Max. coverage (+): 0.04. Max coverage (-): 0.7

Region: NODE\_122013\_length\_9657\_cov\_34.346691 7230-7240. Max. coverage (+): 0. Max coverage (-): 0.78

Region: NODE\_122013\_length\_9657\_cov\_34.346691 7241-7252. Max. coverage (+): 0. Max coverage (-): 1.37

Region: NODE\_122013\_length\_9657\_cov\_34.346691 7253-7263. Max. coverage (+): 0. Max coverage (-): 0.3

Region: NODE\_122013\_length\_9657\_cov\_34.346691 7264-7274. Max. coverage (+): 0.04. Max coverage (-): 0.41

Region: NODE\_122013\_length\_9657\_cov\_34.346691 7275-7286. Max. coverage (+): 0.37. Max coverage (-): 1.67

Region: NODE\_122013\_length\_9657\_cov\_34.346691 7287-7297. Max. coverage (+): 0. Max coverage (-): 10.42

Region: NODE\_122013\_length\_9657\_cov\_34.346691 7298-7309. Max. coverage (+): 0.04. Max coverage (-): 0.48

Region: NODE\_122013\_length\_9657\_cov\_34.346691 7310-7320. Max. coverage (+): 0.04. Max coverage (-): 0.11

Region: NODE\_122013\_length\_9657\_cov\_34.346691 7321-7331. Max. coverage (+): 0.07. Max coverage (-): 0.37

Region: NODE\_122013\_length\_9657\_cov\_34.346691 7332-7343. Max. coverage (+): 0. Max coverage (-): 0.11

Region: NODE\_122013\_length\_9657\_cov\_34.346691 7344-7354. Max. coverage (+): 0. Max coverage (-): 0.04

Region: NODE\_122013\_length\_9657\_cov\_34.346691 7355-7366. Max. coverage (+): 0. Max coverage (-): 0.44

Region: NODE\_122013\_length\_9657\_cov\_34.346691 7367-7377. Max. coverage (+): 0.11. Max coverage (-): 0.82

Region: NODE\_122013\_length\_9657\_cov\_34.346691 7378-7388. Max. coverage (+): 0.07. Max coverage (-): 0.07

Region: NODE\_122013\_length\_9657\_cov\_34.346691 7389-7400. Max. coverage (+): 0.04. Max coverage (-): 0.11

Region: NODE\_122013\_length\_9657\_cov\_34.346691 7401-7411. Max. coverage (+): 0. Max coverage (-): 7.38

Region: NODE\_122013\_length\_9657\_cov\_34.346691 7412-7423. Max. coverage (+): 0. Max coverage (-): 0.22

Region: NODE\_122013\_length\_9657\_cov\_34.346691 7424-7434. Max. coverage (+): 0. Max coverage (-): 0.44

Region: NODE\_122013\_length\_9657\_cov\_34.346691 7435-7445. Max. coverage (+): 0.04. Max coverage (-): 0.33

Region: NODE\_122013\_length\_9657\_cov\_34.346691 7446-7457. Max. coverage (+): 1.26. Max coverage (-): 0.15

Region: NODE\_122013\_length\_9657\_cov\_34.346691 7458-7468. Max. coverage (+): 0. Max coverage (-): 0.15

Region: NODE\_122013\_length\_9657\_cov\_34.346691 7469-7480. Max. coverage (+): 0.04. Max coverage (-): 1.22

Region: NODE\_122013\_length\_9657\_cov\_34.346691 7481-7491. Max. coverage (+): 0.04. Max coverage (-): 0.15

Region: NODE\_122013\_length\_9657\_cov\_34.346691 7492-7502. Max. coverage (+): 0.26. Max coverage (-): 0.19

Region: NODE\_122013\_length\_9657\_cov\_34.346691 7503-7514. Max. coverage (+): 0. Max coverage (-): 11.9

Region: NODE\_122013\_length\_9657\_cov\_34.346691 7515-7525. Max. coverage (+): 0. Max coverage (-): 2.19

Region: NODE\_122013\_length\_9657\_cov\_34.346691 7526-7537. Max. coverage (+): 0.07. Max coverage (-): 0.04

Region: NODE\_122013\_length\_9657\_cov\_34.346691 7538-7548. Max. coverage (+): 0. Max coverage (-): 0.41

Region: NODE\_122013\_length\_9657\_cov\_34.346691 7549-7559. Max. coverage (+): 0. Max coverage (-): 2.34

Region: NODE\_122013\_length\_9657\_cov\_34.346691 7560-7571. Max. coverage (+): 0. Max coverage (-): 0.52

Region: NODE\_122013\_length\_9657\_cov\_34.346691 7572-7582. Max. coverage (+): 0.11. Max coverage (-): 0.7

Region: NODE\_122013\_length\_9657\_cov\_34.346691 7583-7594. Max. coverage (+): 0. Max coverage (-): 2.82

Region: NODE\_122013\_length\_9657\_cov\_34.346691 7595-7605. Max. coverage (+): 0.07. Max coverage (-): 0.11

Region: NODE\_122013\_length\_9657\_cov\_34.346691 7606-7616. Max. coverage (+): 0. Max coverage (-): 0.85

Region: NODE\_122013\_length\_9657\_cov\_34.346691 7617-7628. Max. coverage (+): 0.04. Max coverage (-): 0.93

Region: NODE\_122013\_length\_9657\_cov\_34.346691 7629-7639. Max. coverage (+): 0. Max coverage (-): 0.3

Region: NODE\_122013\_length\_9657\_cov\_34.346691 7640-7650. Max. coverage (+): 0. Max coverage (-): 0.3

Region: NODE\_122013\_length\_9657\_cov\_34.346691 7651-7662. Max. coverage (+): 0.04. Max coverage (-): 0.26

Region: NODE\_122013\_length\_9657\_cov\_34.346691 7663-7673. Max. coverage (+): 0.26. Max coverage (-): 0.11

Region: NODE\_122013\_length\_9657\_cov\_34.346691 7674-7685. Max. coverage (+): 0. Max coverage (-): 0.3

Region: NODE\_122013\_length\_9657\_cov\_34.346691 7686-7696. Max. coverage (+): 0.07. Max coverage (-): 0.15

Region: NODE\_122013\_length\_9657\_cov\_34.346691 7697-7707. Max. coverage (+): 0. Max coverage (-): 0.04

Region: NODE\_122013\_length\_9657\_cov\_34.346691 7708-7719. Max. coverage (+): 0. Max coverage (-): 0.04

Region: NODE\_122013\_length\_9657\_cov\_34.346691 7720-7730. Max. coverage (+): 0. Max coverage (-): 1.26

Region: NODE\_122013\_length\_9657\_cov\_34.346691 7731-7742. Max. coverage (+): 0. Max coverage (-): 0.93

Region: NODE\_122013\_length\_9657\_cov\_34.346691 7743-7753. Max. coverage (+): 0. Max coverage (-): 0.67

Region: NODE\_122013\_length\_9657\_cov\_34.346691 7754-7764. Max. coverage (+): 0.04. Max coverage (-): 0.04

Region: NODE\_122013\_length\_9657\_cov\_34.346691 7765-7776. Max. coverage (+): 0. Max coverage (-): 0.07

Region: NODE\_122013\_length\_9657\_cov\_34.346691 7777-7787. Max. coverage (+): 0. Max coverage (-): 0.19

Region: NODE\_122013\_length\_9657\_cov\_34.346691 7788-7799. Max. coverage (+): 0. Max coverage (-): 9.53

Region: NODE\_122013\_length\_9657\_cov\_34.346691 7800-7810. Max. coverage (+): 0. Max coverage (-): 10.42

Region: NODE\_122013\_length\_9657\_cov\_34.346691 7811-7821. Max. coverage (+): 0. Max coverage (-): 0.22

Region: NODE\_122013\_length\_9657\_cov\_34.346691 7822-7833. Max. coverage (+): 0. Max coverage (-): 0.41

Region: NODE\_122013\_length\_9657\_cov\_34.346691 7834-7844. Max. coverage (+): 0.15. Max coverage (-): 0.33

Region: NODE\_122013\_length\_9657\_cov\_34.346691 7845-7856. Max. coverage (+): 0.04. Max coverage (-): 0.89

Region: NODE\_122013\_length\_9657\_cov\_34.346691 7857-7867. Max. coverage (+): 0.07. Max coverage (-): 0.67

Region: NODE\_122013\_length\_9657\_cov\_34.346691 7868-7878. Max. coverage (+): 0. Max coverage (-): 0.11

Region: NODE\_122013\_length\_9657\_cov\_34.346691 7879-7890. Max. coverage (+): 0. Max coverage (-): 0.96

Region: NODE\_122013\_length\_9657\_cov\_34.346691 7891-7901. Max. coverage (+): 0. Max coverage (-): 0.74

Region: NODE\_122013\_length\_9657\_cov\_34.346691 7902-7913. Max. coverage (+): 0.07. Max coverage (-): 0.11

Region: NODE\_122013\_length\_9657\_cov\_34.346691 7914-7924. Max. coverage (+): 0.04. Max coverage (-): 0.22

Region: NODE\_122013\_length\_9657\_cov\_34.346691 7925-7935. Max. coverage (+): 0.15. Max coverage (-): 0.15

Region: NODE\_122013\_length\_9657\_cov\_34.346691 7936-7947. Max. coverage (+): 0.07. Max coverage (-): 0.11

Region: NODE\_122013\_length\_9657\_cov\_34.346691 7948-7958. Max. coverage (+): 0.07. Max coverage (-): 0.78

Region: NODE\_122013\_length\_9657\_cov\_34.346691 7959-7970. Max. coverage (+): 0. Max coverage (-): 0.74

Region: NODE\_122013\_length\_9657\_cov\_34.346691 7971-7981. Max. coverage (+): 0. Max coverage (-): 0.3

Region: NODE\_122013\_length\_9657\_cov\_34.346691 7982-7992. Max. coverage (+): 0.04. Max coverage (-): 0.56

Region: NODE\_122013\_length\_9657\_cov\_34.346691 7993-8004. Max. coverage (+): 0.04. Max coverage (-): 0.82

Region: NODE\_122013\_length\_9657\_cov\_34.346691 8005-8015. Max. coverage (+): 0.11. Max coverage (-): 0.04

Region: NODE\_122013\_length\_9657\_cov\_34.346691 8016-8026. Max. coverage (+): 0.04. Max coverage (-): 0.15

Region: NODE\_122013\_length\_9657\_cov\_34.346691 8027-8038. Max. coverage (+): 0.04. Max coverage (-): 0.19

Region: NODE\_122013\_length\_9657\_cov\_34.346691 8039-8049. Max. coverage (+): 0. Max coverage (-): 0.15

Region: NODE\_122013\_length\_9657\_cov\_34.346691 8050-8061. Max. coverage (+): 0.07. Max coverage (-): 0.85

Region: NODE\_122013\_length\_9657\_cov\_34.346691 8062-8072. Max. coverage (+): 0.07. Max coverage (-): 1

Region: NODE\_122013\_length\_9657\_cov\_34.346691 8073-8083. Max. coverage (+): 0.04. Max coverage (-): 1.52

Region: NODE\_122013\_length\_9657\_cov\_34.346691 8084-8095. Max. coverage (+): 0.19. Max coverage (-): 1.41

Region: NODE\_122013\_length\_9657\_cov\_34.346691 8096-8106. Max. coverage (+): 0. Max coverage (-): 0.3

Region: NODE\_122013\_length\_9657\_cov\_34.346691 8107-8118. Max. coverage (+): 0.04. Max coverage (-): 0

Region: NODE\_122013\_length\_9657\_cov\_34.346691 8119-8129. Max. coverage (+): 0.04. Max coverage (-): 0

Region: NODE\_122013\_length\_9657\_cov\_34.346691 8130-8140. Max. coverage (+): 0.04. Max coverage (-): 0.04

Region: NODE\_122013\_length\_9657\_cov\_34.346691 8141-8152. Max. coverage (+): 0.07. Max coverage (-): 0.48

Region: NODE\_122013\_length\_9657\_cov\_34.346691 8153-8163. Max. coverage (+): 0.04. Max coverage (-): 1.82

Region: NODE\_122013\_length\_9657\_cov\_34.346691 8164-8175. Max. coverage (+): 0. Max coverage (-): 1.71

Region: NODE\_122013\_length\_9657\_cov\_34.346691 8176-8186. Max. coverage (+): 0. Max coverage (-): 22.69

Region: NODE\_122013\_length\_9657\_cov\_34.346691 8187-8197. Max. coverage (+): 0.04. Max coverage (-): 0.52

Region: NODE\_122013\_length\_9657\_cov\_34.346691 8198-8209. Max. coverage (+): 0.11. Max coverage (-): 1.08

Region: NODE\_122013\_length\_9657\_cov\_34.346691 8210-8220. Max. coverage (+): 0.33. Max coverage (-): 0.33

Region: NODE\_122013\_length\_9657\_cov\_34.346691 8221-8232. Max. coverage (+): 0.37. Max coverage (-): 0.19

Region: NODE\_122013\_length\_9657\_cov\_34.346691 8233-8243. Max. coverage (+): 0.04. Max coverage (-): 1.08

Region: NODE\_122013\_length\_9657\_cov\_34.346691 8244-8254. Max. coverage (+): 0.19. Max coverage (-): 2.08

Region: NODE\_122013\_length\_9657\_cov\_34.346691 8255-8266. Max. coverage (+): 1.15. Max coverage (-): 0.22

Region: NODE\_122013\_length\_9657\_cov\_34.346691 8267-8277. Max. coverage (+): 0.19. Max coverage (-): 0.37

Region: NODE\_122013\_length\_9657\_cov\_34.346691 8278-8289. Max. coverage (+): 0.07. Max coverage (-): 0.56

Region: NODE\_122013\_length\_9657\_cov\_34.346691 8290-8300. Max. coverage (+): 0.44. Max coverage (-): 0.67

Region: NODE\_122013\_length\_9657\_cov\_34.346691 8301-8311. Max. coverage (+): 0.04. Max coverage (-): 0.89

Region: NODE\_122013\_length\_9657\_cov\_34.346691 8312-8323. Max. coverage (+): 0. Max coverage (-): 0.89

Region: NODE\_122013\_length\_9657\_cov\_34.346691 8324-8334. Max. coverage (+): 0. Max coverage (-): 0.07

Region: NODE\_122013\_length\_9657\_cov\_34.346691 8335-8346. Max. coverage (+): 0.07. Max coverage (-): 0.04

Region: NODE\_122013\_length\_9657\_cov\_34.346691 8347-8357. Max. coverage (+): 0. Max coverage (-): 0.15

Region: NODE\_122013\_length\_9657\_cov\_34.346691 8358-8368. Max. coverage (+): 0. Max coverage (-): 1.08

Region: NODE\_122013\_length\_9657\_cov\_34.346691 8369-8380. Max. coverage (+): 0.04. Max coverage (-): 0.63

Region: NODE\_122013\_length\_9657\_cov\_34.346691 8381-8391. Max. coverage (+): 0.19. Max coverage (-): 1.19

Region: NODE\_122013\_length\_9657\_cov\_34.346691 8392-8402. Max. coverage (+): 0.19. Max coverage (-): 0.07

Region: NODE\_122013\_length\_9657\_cov\_34.346691 8403-8414. Max. coverage (+): 0.04. Max coverage (-): 0

Region: NODE\_122013\_length\_9657\_cov\_34.346691 8415-8425. Max. coverage (+): 0. Max coverage (-): 0.22

Region: NODE\_122013\_length\_9657\_cov\_34.346691 8426-8437. Max. coverage (+): 0. Max coverage (-): 3.04

Region: NODE\_122013\_length\_9657\_cov\_34.346691 8438-8448. Max. coverage (+): 0. Max coverage (-): 0.52

Region: NODE\_122013\_length\_9657\_cov\_34.346691 8449-8459. Max. coverage (+): 0. Max coverage (-): 0.52

Region: NODE\_122013\_length\_9657\_cov\_34.346691 8460-8471. Max. coverage (+): 0.04. Max coverage (-): 0.07

Region: NODE\_122013\_length\_9657\_cov\_34.346691 8472-8482. Max. coverage (+): 0.04. Max coverage (-): 4.52

Region: NODE\_122013\_length\_9657\_cov\_34.346691 8483-8494. Max. coverage (+): 0. Max coverage (-): 5

Region: NODE\_122013\_length\_9657\_cov\_34.346691 8495-8505. Max. coverage (+): 0. Max coverage (-): 0.04

Region: NODE\_122013\_length\_9657\_cov\_34.346691 8506-8516. Max. coverage (+): 0.19. Max coverage (-): 0.09

Region: NODE\_122013\_length\_9657\_cov\_34.346691 8517-8528. Max. coverage (+): 0.01. Max coverage (-): 0.34

Region: NODE\_122013\_length\_9657\_cov\_34.346691 8529-8539. Max. coverage (+): 0.01. Max coverage (-): 0

Region: NODE\_122013\_length\_9657\_cov\_34.346691 8540-8551. Max. coverage (+): 0. Max coverage (-): 0.19

Region: NODE\_122013\_length\_9657\_cov\_34.346691 8552-8562. Max. coverage (+): 0. Max coverage (-): 0.41

Region: NODE\_122013\_length\_9657\_cov\_34.346691 8563-8573. Max. coverage (+): 0.07. Max coverage (-): 0.3

Region: NODE\_122013\_length\_9657\_cov\_34.346691 8574-8585. Max. coverage (+): 0.15. Max coverage (-): 0.3

Region: NODE\_122013\_length\_9657\_cov\_34.346691 8586-8596. Max. coverage (+): 0. Max coverage (-): 0.48

Region: NODE\_122013\_length\_9657\_cov\_34.346691 8597-8608. Max. coverage (+): 0.19. Max coverage (-): 0.15

Region: NODE\_122013\_length\_9657\_cov\_34.346691 8609-8619. Max. coverage (+): 0.07. Max coverage (-): 0.48

Region: NODE\_122013\_length\_9657\_cov\_34.346691 8620-8630. Max. coverage (+): 0. Max coverage (-): 2.34

Region: NODE\_122013\_length\_9657\_cov\_34.346691 8631-8642. Max. coverage (+): 0.11. Max coverage (-): 5.26

Region: NODE\_122013\_length\_9657\_cov\_34.346691 8643-8653. Max. coverage (+): 0. Max coverage (-): 1.37

Region: NODE\_122013\_length\_9657\_cov\_34.346691 8654-8665. Max. coverage (+): 0. Max coverage (-): 0.07

Region: NODE\_122013\_length\_9657\_cov\_34.346691 8666-8676. Max. coverage (+): 0. Max coverage (-): 1.74

Region: NODE\_122013\_length\_9657\_cov\_34.346691 8677-8687. Max. coverage (+): 0.04. Max coverage (-): 0.11

Region: NODE\_122013\_length\_9657\_cov\_34.346691 8688-8699. Max. coverage (+): 0.04. Max coverage (-): 0.22

Region: NODE\_122013\_length\_9657\_cov\_34.346691 8700-8710. Max. coverage (+): 0.07. Max coverage (-): 1.08

Region: NODE\_122013\_length\_9657\_cov\_34.346691 8711-8722. Max. coverage (+): 0.04. Max coverage (-): 1.26

Region: NODE\_122013\_length\_9657\_cov\_34.346691 8723-8733. Max. coverage (+): 0.44. Max coverage (-): 0.19

Region: NODE\_122013\_length\_9657\_cov\_34.346691 8734-8744. Max. coverage (+): 0.07. Max coverage (-): 0.11

Region: NODE\_122013\_length\_9657\_cov\_34.346691 8745-8756. Max. coverage (+): 0.04. Max coverage (-): 0.78

Region: NODE\_122013\_length\_9657\_cov\_34.346691 8757-8767. Max. coverage (+): 0. Max coverage (-): 0.89

Region: NODE\_122013\_length\_9657\_cov\_34.346691 8768-8778. Max. coverage (+): 0.07. Max coverage (-): 0.04

Region: NODE\_122013\_length\_9657\_cov\_34.346691 8779-8790. Max. coverage (+): 0. Max coverage (-): 1.22

Region: NODE\_122013\_length\_9657\_cov\_34.346691 8791-8801. Max. coverage (+): 0.04. Max coverage (-): 0.04

Region: NODE\_122013\_length\_9657\_cov\_34.346691 8802-8813. Max. coverage (+): 0.07. Max coverage (-): 0.11

Region: NODE\_122013\_length\_9657\_cov\_34.346691 8814-8824. Max. coverage (+): 0. Max coverage (-): 0.22

Region: NODE\_122013\_length\_9657\_cov\_34.346691 8825-8835. Max. coverage (+): 0.04. Max coverage (-): 1.96

Region: NODE\_122013\_length\_9657\_cov\_34.346691 8836-8847. Max. coverage (+): 0.22. Max coverage (-): 0.82

Region: NODE\_122013\_length\_9657\_cov\_34.346691 8848-8858. Max. coverage (+): 0.19. Max coverage (-): 0.26

Region: NODE\_122013\_length\_9657\_cov\_34.346691 8859-8870. Max. coverage (+): 0.67. Max coverage (-): 0.89

Region: NODE\_122013\_length\_9657\_cov\_34.346691 8871-8881. Max. coverage (+): 0.07. Max coverage (-): 2.37

Region: NODE\_122013\_length\_9657\_cov\_34.346691 8882-8892. Max. coverage (+): 0.07. Max coverage (-): 2.08

Region: NODE\_122013\_length\_9657\_cov\_34.346691 8893-8904. Max. coverage (+): 0. Max coverage (-): 5.41

Region: NODE\_122013\_length\_9657\_cov\_34.346691 8905-8915. Max. coverage (+): 0.04. Max coverage (-): 0.41

Region: NODE\_122013\_length\_9657\_cov\_34.346691 8916-8927. Max. coverage (+): 0.04. Max coverage (-): 0

Region: NODE\_122013\_length\_9657\_cov\_34.346691 8928-8938. Max. coverage (+): 0. Max coverage (-): 0.56

Region: NODE\_122013\_length\_9657\_cov\_34.346691 8939-8949. Max. coverage (+): 0.04. Max coverage (-): 0.11

Region: NODE\_122013\_length\_9657\_cov\_34.346691 8950-8961. Max. coverage (+): 0. Max coverage (-): 3.93

Region: NODE\_122013\_length\_9657\_cov\_34.346691 8962-8972. Max. coverage (+): 0.04. Max coverage (-): 51.01

Region: NODE\_122013\_length\_9657\_cov\_34.346691 8973-8984. Max. coverage (+): 0.04. Max coverage (-): 0

Region: NODE\_122013\_length\_9657\_cov\_34.346691 8985-8995. Max. coverage (+): 0.07. Max coverage (-): 7.56

Region: NODE\_122013\_length\_9657\_cov\_34.346691 8996-9006. Max. coverage (+): 0.04. Max coverage (-): 6.04

Region: NODE\_122013\_length\_9657\_cov\_34.346691 9007-9018. Max. coverage (+): 0.07. Max coverage (-): 2.34

Region: NODE\_122013\_length\_9657\_cov\_34.346691 9019-9029. Max. coverage (+): 0. Max coverage (-): 0.07

Region: NODE\_122013\_length\_9657\_cov\_34.346691 9030-9041. Max. coverage (+): 0.19. Max coverage (-): 0.11

Region: NODE\_122013\_length\_9657\_cov\_34.346691 9042-9052. Max. coverage (+): 0.33. Max coverage (-): 0.15

Region: NODE\_122013\_length\_9657\_cov\_34.346691 9053-9063. Max. coverage (+): 0.11. Max coverage (-): 0.52

Region: NODE\_122013\_length\_9657\_cov\_34.346691 9064-9075. Max. coverage (+): 0.11. Max coverage (-): 0.11

Region: NODE\_122013\_length\_9657\_cov\_34.346691 9076-9086. Max. coverage (+): 0.11. Max coverage (-): 0.19

Region: NODE\_122013\_length\_9657\_cov\_34.346691 9087-9098. Max. coverage (+): 0. Max coverage (-): 0.19

Region: NODE\_122013\_length\_9657\_cov\_34.346691 9099-9109. Max. coverage (+): 0. Max coverage (-): 1.04

Region: NODE\_122013\_length\_9657\_cov\_34.346691 9110-9120. Max. coverage (+): 0.04. Max coverage (-): 1.33

Region: NODE\_122013\_length\_9657\_cov\_34.346691 9121-9132. Max. coverage (+): 0.19. Max coverage (-): 3.74

Region: NODE\_122013\_length\_9657\_cov\_34.346691 9133-9143. Max. coverage (+): 0.19. Max coverage (-): 0.59

Region: NODE\_122013\_length\_9657\_cov\_34.346691 9144-9154. Max. coverage (+): 0.11. Max coverage (-): 3.67

Region: NODE\_122013\_length\_9657\_cov\_34.346691 9155-9166. Max. coverage (+): 0.7. Max coverage (-): 0.82

Region: NODE\_122013\_length\_9657\_cov\_34.346691 9167-9177. Max. coverage (+): 0.33. Max coverage (-): 1.33

Region: NODE\_122013\_length\_9657\_cov\_34.346691 9178-9189. Max. coverage (+): 0.15. Max coverage (-): 1.33

Region: NODE\_122013\_length\_9657\_cov\_34.346691 9190-9200. Max. coverage (+): 0.07. Max coverage (-): 0.07

Region: NODE\_122013\_length\_9657\_cov\_34.346691 9201-9211. Max. coverage (+): 0.11. Max coverage (-): 0.33

Region: NODE\_122013\_length\_9657\_cov\_34.346691 9212-9223. Max. coverage (+): 0.11. Max coverage (-): 0.11

Region: NODE\_122013\_length\_9657\_cov\_34.346691 9224-9234. Max. coverage (+): 0. Max coverage (-): 0.04

Region: NODE\_122013\_length\_9657\_cov\_34.346691 9235-9246. Max. coverage (+): 0.11. Max coverage (-): 5.49

Region: NODE\_122013\_length\_9657\_cov\_34.346691 9247-9257. Max. coverage (+): 0.15. Max coverage (-): 0.82

Region: NODE\_122013\_length\_9657\_cov\_34.346691 9258-9268. Max. coverage (+): 0.04. Max coverage (-): 0.89

Region: NODE\_122013\_length\_9657\_cov\_34.346691 9269-9280. Max. coverage (+): 0. Max coverage (-): 0.04

Region: NODE\_122013\_length\_9657\_cov\_34.346691 9281-9291. Max. coverage (+): 0.04. Max coverage (-): 0.56

Region: NODE\_122013\_length\_9657\_cov\_34.346691 9292-9303. Max. coverage (+): 0.04. Max coverage (-): 0.59

Region: NODE\_122013\_length\_9657\_cov\_34.346691 9304-9314. Max. coverage (+): 0.15. Max coverage (-): 0.44

Region: NODE\_122013\_length\_9657\_cov\_34.346691 9315-9325. Max. coverage (+): 0.04. Max coverage (-): 0.63

Region: NODE\_122013\_length\_9657\_cov\_34.346691 9326-9337. Max. coverage (+): 0.04. Max coverage (-): 0.19

Region: NODE\_122013\_length\_9657\_cov\_34.346691 9338-9348. Max. coverage (+): 0. Max coverage (-): 0.48

Region: NODE\_122013\_length\_9657\_cov\_34.346691 9349-9360. Max. coverage (+): 0.19. Max coverage (-): 0.11

Region: NODE\_122013\_length\_9657\_cov\_34.346691 9361-9371. Max. coverage (+): 0.15. Max coverage (-): 0

Region: NODE\_122013\_length\_9657\_cov\_34.346691 9372-9382. Max. coverage (+): 0. Max coverage (-): 0.07

Region: NODE\_122013\_length\_9657\_cov\_34.346691 9383-9394. Max. coverage (+): 0.04. Max coverage (-): 0.3

Region: NODE\_122013\_length\_9657\_cov\_34.346691 9395-9405. Max. coverage (+): 0.11. Max coverage (-): 0.15

Region: NODE\_122013\_length\_9657\_cov\_34.346691 9406-9417. Max. coverage (+): 0.11. Max coverage (-): 0

Region: NODE\_122013\_length\_9657\_cov\_34.346691 9418-9428. Max. coverage (+): 0. Max coverage (-): 0

Region: NODE\_122013\_length\_9657\_cov\_34.346691 9429-9439. Max. coverage (+): 0.04. Max coverage (-): 0.15

Region: NODE\_122013\_length\_9657\_cov\_34.346691 9440-9451. Max. coverage (+): 0.15. Max coverage (-): 0.19

Region: NODE\_122013\_length\_9657\_cov\_34.346691 9452-9462. Max. coverage (+): 0.07. Max coverage (-): 0.22

Region: NODE\_122013\_length\_9657\_cov\_34.346691 9463-9474. Max. coverage (+): 0.15. Max coverage (-): 7.16

Region: NODE\_122013\_length\_9657\_cov\_34.346691 9475-9485. Max. coverage (+): 0.04. Max coverage (-): 0.63

Region: NODE\_122013\_length\_9657\_cov\_34.346691 9486-9496. Max. coverage (+): 0. Max coverage (-): 1.08

Region: NODE\_122013\_length\_9657\_cov\_34.346691 9497-9508. Max. coverage (+): 0. Max coverage (-): 1.48

Region: NODE\_122013\_length\_9657\_cov\_34.346691 9509-9519. Max. coverage (+): 0.04. Max coverage (-): 0

Region: NODE\_122013\_length\_9657\_cov\_34.346691 9520-9530. Max. coverage (+): 0.19. Max coverage (-): 0.37

Region: NODE\_122013\_length\_9657\_cov\_34.346691 9531-9542. Max. coverage (+): 0.07. Max coverage (-): 0.04

Region: NODE\_122013\_length\_9657\_cov\_34.346691 9543-9553. Max. coverage (+): 0. Max coverage (-): 0.07

Region: NODE\_122013\_length\_9657\_cov\_34.346691 9554-9565. Max. coverage (+): 0. Max coverage (-): 0.07

Region: NODE\_122013\_length\_9657\_cov\_34.346691 9566-9576. Max. coverage (+): 0. Max coverage (-): 0.07

Region: NODE\_122013\_length\_9657\_cov\_34.346691 9577-9587. Max. coverage (+): 0. Max coverage (-): 0

Region: NODE\_122013\_length\_9657\_cov\_34.346691 9588-9599. Max. coverage (+): 0.04. Max coverage (-): 0.26

Region: NODE\_122013\_length\_9657\_cov\_34.346691 9600-9610. Max. coverage (+): 0.11. Max coverage (-): 0.33

Region: NODE\_122013\_length\_9657\_cov\_34.346691 9611-9622. Max. coverage (+): 0.04. Max coverage (-): 0.22

Region: NODE\_122013\_length\_9657\_cov\_34.346691 9623-9633. Max. coverage (+): 0.04. Max coverage (-): 0

Region: NODE\_122013\_length\_9657\_cov\_34.346691 9634-9644. Max. coverage (+): 0. Max coverage (-): 0

Region: NODE\_122013\_length\_9657\_cov\_34.346691 9645-9656. Max. coverage (+): 0.04. Max coverage (-): 0.07

Region: NODE\_122013\_length\_9657\_cov\_34.346691 9657-9667. Max. coverage (+): 0.02. Max coverage (-): 0.07

Region: NODE\_122013\_length\_9657\_cov\_34.346691 9668-9679. Max. coverage (+): 0.26. Max coverage (-): 0.62

Region: NODE\_122013\_length\_9657\_cov\_34.346691 9680-9690. Max. coverage (+): 0.63. Max coverage (-): 0.55

Region: NODE\_122013\_length\_9657\_cov\_34.346691 9691-9701. Max. coverage (+): 0.07. Max coverage (-): 0

Region: NODE\_122013\_length\_9657\_cov\_34.346691 9702-9713. Max. coverage (+): 0. Max coverage (-): 0

Region: NODE\_122013\_length\_9657\_cov\_34.346691 9714-. Max. coverage (+): 0. Max coverage (-): 0

RepeatMasker Color Code

**+**

100-98% Identity

<98-95% Identity

<95-90% Identity

<90-85% Identity

<85-80% Identity

<80-75% Identity

<75-70% Identity

<70% Identity

**-**

Gene Set Color Code

**+**

Gene

Pseudogene

Other

**-**

Topology/Coverage Color Code

Coverage Plus Strand

Coverage Minus Strand

Mainstrand: Plus

Mainstrand: Minus

Complementary Strand

Flanking Region  
(if option -flank >0)

Gene Set Annotation  
  
RepeatMasker Annotation  

**1. (TATC)n**: 4485-4612 (+), Divergence to consensus: 1.6%  
**2. (TG)n**: 4638-4657 (+), Divergence to consensus: 0%  
**3. AlRepB-59**: 5438-5581 (-), Divergence to consensus: 14.6%  
**4. AlRepB-59**: 5557-5635 (-), Divergence to consensus: 14%  
**5. AlRepB-60**: 5636-5789 (-), Divergence to consensus: 8.2%  
**6. AlRepB-60**: 5784-6096 (-), Divergence to consensus: 18.9%  
**7. AlRepB-60**: 6181-6617 (-), Divergence to consensus: 15.8%  
**8. AlRepC-300**: 6593-6690 (-), Divergence to consensus: 15.6%  
**9. AlRepE-4385**: 6666-6772 (-), Divergence to consensus: 15.9%  
**10. AlRepB-60**: 6692-6964 (-), Divergence to consensus: 22.1%  
**11. AlRepD-756**: 7673-7749 (-), Divergence to consensus: 26%  
**12. L2-14\_DRe**: 9658-9721 (+), Divergence to consensus: 7.8%

  
Transcription Factor Binding Sites  

**RHOXF1** (Sequence: GGATTA (-): 4123)  
**RHOXF1** (Sequence: AGATCA (-): 4811)  
**RHOXF1** (Sequence: AGCTTA (-): 6237)  
**RHOXF1** (Sequence: AGCTCA (-): 7810)  
**RHOXF1** (Sequence: AGATCA (-): 8157)  
**RHOXF1** (Sequence: AGCTCA (-): 8183)  
**RHOXF1** (Sequence: AGCTCA (-): 8272)  
**RHOXF1** (Sequence: GGATTA (-): 8588)  
**RHOXF1** (Sequence: AGATTA (-): 9074)  
**RHOXF1** (Sequence: GGATTA (-): 9266)  
**RHOXF1** (Sequence: TGATCT (+): 4818)  
**RHOXF1** (Sequence: TAATCT (+): 4967)  
**RHOXF1** (Sequence: TAATCC (+): 5687)  
**RHOXF1** (Sequence: TGAGCT (+): 6840)  
**RHOXF1** (Sequence: TAATCT (+): 8131)  
**RHOXF1** (Sequence: TAAGCT (+): 8181)  
**RHOXF1** (Sequence: TAATCC (+): 8682)  
**RHOXF1** (Sequence: TAAGCT (+): 9430)  
**FOXO3\_hsa** (Sequence: GTAAACAA (+): 7164)  
**FOXP1** (Sequence: GTAAACA (+): 7164)  
**FOXP1** (Sequence: GTAAACA (+): 9298)  
**FOXO1** (Sequence: CCTGTTTAT (+): 4132)  
**FOXO3\_mmu** (Sequence: TGTTTTGC (-): 5126)  
**FOXO3\_mmu** (Sequence: TGTTTAGC (-): 6256)  
**FOXO3\_mmu** (Sequence: TGTTTTCC (-): 6275)  
**FOXO3\_mmu** (Sequence: TGTTTTCA (-): 7619)  
**FOXO3\_mmu** (Sequence: TGTTTTCA (-): 9572)  
**Sox5** (Sequence: ATTGTT (+): 4916)  
**Sox5** (Sequence: ATTGTT (+): 6889)  
**Sox5** (Sequence: ATTGTT (+): 8882)  
**SOX9** (Sequence: TCATTGTT (+): 6887)  
**SOX9** (Sequence: TCATTGTT (+): 8880)  
**FOXO3\_mmu** (Sequence: TCAAAACA (+): 4344)  
**FOXO3\_mmu** (Sequence: TGAAAACA (+): 5506)  
**FOXO3\_mmu** (Sequence: TGAAAACA (+): 5510)  
**FOXO3\_mmu** (Sequence: TGTAAACA (+): 7163)  
**FOXO3\_mmu** (Sequence: TCAAAACA (+): 8406)  
**FOXO3\_mmu** (Sequence: GGTAAACA (+): 9297)  
**FOXO1** (Sequence: ATAAACAAG (-): 6305)  
**Nobox** (Sequence: TAATTGCT (+): 4023)  
**Nobox** (Sequence: TAATTGGC (+): 7143)  
**Rhox11** (Sequence: TGGTGTTAT (+): 5773)  
**Rhox11** (Sequence: TGGTGTTAT (+): 5828)  
**Rhox11** (Sequence: TGGTGTAAA (+): 8468)  
**Rhox11** (Sequence: TTAACAGCA (-): 4989)  
**Rhox11** (Sequence: TTTACACCA (-): 5573)  
**Rhox11** (Sequence: TATACAGCG (-): 8244)  
**Sox5** (Sequence: AACAAT (-): 5261)  
**Sox5** (Sequence: AACAAT (-): 7167)  
**POU2F1** (Sequence: TATGCAAAT (+): 8366)  
**POU5F1** (Sequence: ATGCAAA (+): 7442)  
**POU5F1** (Sequence: ATGCAAA (+): 8367)
