# Supplementary material for: First characterization of PIWI-interacting RNA clusters in a cichlid fish with a B chromosome
Source: BMC Biol. 2022 Sep 21;20:204. doi: 10.1186/s12915-022-01403-2 (PMC9490952; doi:10.1186/s12915-022-01403-2)
Supplement: Supplementary file 1 — Additional file 1. Zipped folder with fasta and interactive html piRNA cluster information for the A. latifasciata genome. The nomenclature is as follows: number-pirna-cluster_sex_B-presence (f, female; m, male; 0b, without B chromosome; 1b, with B chromosome). [file 12915_2022_1403_MOESM1_ESM.zip › 150_f0b.html]

piRNA cluster 150\_f0b 69


Predicted piRNA cluster no. 150\_f0b
  

Show proTRAC run info
Hide proTRAC run info

/\  
                \_\_\_\_\_\_\_\_\_\_\_\_\_\_\_\_\_\_\_\_\_\_\_/\\_\_\_ /  \\_\_\_\_\_\_\_  
               I                      /  \  /    \      I  
               I     pro             /    \/      \     I  
               I        TRAC        /               \   I  
               I   \_\_\_\_\_\_\_\_\_\_\_\_\_\_\_\_/\_\_\_\_\_\_\_\_\_\_\_\_\_\_\_\_\_\\_ I  
               I   \              /                     I  
               I    \            /                      I  
               I     \  /\      /       V.2.4.2         I  
               I      \/  \    /                        I  
               I\_\_\_\_\_\_\_\_\_\_\_\  /\_\_\_\_\_\_\_\_\_\_\_\_\_\_\_\_\_\_\_\_\_\_\_\_\_I  
                            \/  
  
  
================================= proTRAC ====================================  
VERSION: .......... 2.4.2  
LAST MODIFIED: .... 11. May 2018  
  
Please cite:  
Rosenkranz D, Zischler H. proTRAC - a software for probabilistic piRNA cluster  
detection, visualization and analysis. 2012. BMC Bioinformatics 13:5.  
  
  
Contact:  
David Rosenkranz  
Institute of Organismic and Molecular Evolutionary Biology  
Dept. Anthropology, small RNA group  
Johannes Gutenberg University Mainz  
email: rosenkranz@uni-mainz.de  
  
You can find the latest proTRAC version at:  
http://sourceforge.net/projects/protrac/files  
http://www.smallRNAgroup-mainz.de/software  
==============================================================================  
  
PARAMETERS:  
Map file: ...............piwi-femeas-0B.fa-collapse.map  
Genome file: ............../../../0B\_ala\_genome.fa  
RepeatMasker annotation: Alatifasciata-all0B-maryan-v2.fa\_corrected.out  
GeneSet:................./guest-storage/Data/annotation/Alatifasciata\_all0B\_maryan-v2\_out2017.gff  
  
Significant (p<=0.01) hit density will be calculated based  
on observed hit distribution.  
  
Sliding window size: ........................................ 5000 bp  
Sliding window increament: .................................. 1000 bp  
Normalize each hit by number of genomic hits: ............... yes  
Normalize each hit by number of sequence reads: ............. yes  
Normalize values (-> per million mapped reads): ............. yes  
Min. fraction of hits with 1T(U) or 10A: .................... 0.75  
Alternatively: Min. fraction of hits with 1T(U) and 10A: .... 0.5  
Min. fraction of hits with typical piRNA length: ............ 0.75  
Typical piRNA length: ....................................... 24-32 nt  
Min. size of a piRNA cluster: ............................... 1000 bp.  
Min. number of hits (absolute): ............................. 0  
Min. number of hits (normalized): ........................... 0  
Min. fraction of hits on the mainstrand: .................... 0.75  
Top fraction of mapped sequences (in terms of read counts): . 1%  
Top fraction accounts for max. n% of sequence reads: ........ 90%  
Min. fraction of hits on each arm of a bidirectional cluster: 0.05  
Output html file for each cluster: .......................... yes  
Output a summary table: ..................................... yes  
Output a FASTA file for each cluster (piRNA sequences): ..... yes  
Output a FASTA file comprising cluster sequences: ........... yes  
Output a GTF file for predicted piRNA clusters: ..............yes  
Search DNA motifs in clusters: .............................. yes  
Output flanking sequences: +/- .............................. 0 bp  
Output ~.pTi file: .......................................... no  
==============================================================================  
  
  
Genome size (without gaps): ............ 758543724 bp  
Gaps (N/X/-): .......................... 417479 bp  
Mapped reads: .......................... 13052187  
Non-identical sequences: ............... 3338911  
Genomic hits: .......................... 28737726  
Significant densitiy of mapped reads: .. 470.083249848448 reads/kb

Show proTRAC cluster info
Hide proTRAC cluster info

|  |  |
| --- | --- |
| Location | NODE\_387203\_length\_2970\_cov\_21.957239 |
| Coordinates | 4-3070 |
| Size [bp] | 3067 |
| Sequence hit loci | 1695 |
| Mapped reads (normalized) | 5056.3 |
| Mapped reads (normalized) per kb | 1648.6 |
| Normalized reads with 1T (1U) | 85% |
| Normalized reads with 10A | 34.9% |
| Normalized reads with length 24-32 nt | 99.2% |
| Normalized reads on the main strand(s) | 92.1% |
| Predicted directionality | mono:plus |

100%

0%

1T (1U)  
reads

10A reads

24-32 nt  
reads

reads on mainstrand

**Either the amount of reads with 1T (1U) OR 10A has to exceed 75% (set with option: -1Tor10A)  
Alternatively the amount of reads with 1T (1U) AND 10A has to exceed 50% (set with option: -1Tand10A)  
Minimum amount of reads with preferred size is 75% (set with option: -pisize)  
Minimum amount of reads on the main strand(s) is 75% (set with option: -clstrand)**

Show read coverage
Hide read coverage

WHAT DO I SEE HERE?  
This chart shows the location of mapped sequence reads within a predicted piRNA cluster. The color refers to the number of genomic hits produced by the sequence read in question. A dark red bar indicates that this sequence read produces many other hits elsewhere in the genome. Many adjacent red or yellow bars can indicate the presence of a multi-copy element such as transposons or rRNA genes. A dark green bar indicates that this sequence read maps uniquely to this locus.

1 hit

2-5 hits

6-10 hits

11-20 hits

21-50 hits

51-100 hits

> 100 hits

NODE\_387203\_length\_2970\_cov\_21.957239

4

3070

Gene Set

RepeatMasker

Mapped  
Reads

68.11

plus strand

minus strand

68.11

Region: NODE\_387203\_length\_2970\_cov\_21.957239 37032-7. Max. coverage (+): 0.03. Max coverage (-): 0.02

Region: NODE\_387203\_length\_2970\_cov\_21.957239 8-13. Max. coverage (+): 0.08. Max coverage (-): 0

Region: NODE\_387203\_length\_2970\_cov\_21.957239 14-19. Max. coverage (+): 0. Max coverage (-): 0.36

Region: NODE\_387203\_length\_2970\_cov\_21.957239 20-25. Max. coverage (+): 0. Max coverage (-): 0.41

Region: NODE\_387203\_length\_2970\_cov\_21.957239 26-31. Max. coverage (+): 0. Max coverage (-): 0

Region: NODE\_387203\_length\_2970\_cov\_21.957239 32-37. Max. coverage (+): 3.45. Max coverage (-): 0

Region: NODE\_387203\_length\_2970\_cov\_21.957239 38-43. Max. coverage (+): 0. Max coverage (-): 0

Region: NODE\_387203\_length\_2970\_cov\_21.957239 44-50. Max. coverage (+): 0. Max coverage (-): 0

Region: NODE\_387203\_length\_2970\_cov\_21.957239 51-56. Max. coverage (+): 0. Max coverage (-): 0

Region: NODE\_387203\_length\_2970\_cov\_21.957239 57-62. Max. coverage (+): 0. Max coverage (-): 0

Region: NODE\_387203\_length\_2970\_cov\_21.957239 63-68. Max. coverage (+): 0. Max coverage (-): 0

Region: NODE\_387203\_length\_2970\_cov\_21.957239 69-74. Max. coverage (+): 0. Max coverage (-): 0

Region: NODE\_387203\_length\_2970\_cov\_21.957239 75-80. Max. coverage (+): 0. Max coverage (-): 0

Region: NODE\_387203\_length\_2970\_cov\_21.957239 81-86. Max. coverage (+): 0. Max coverage (-): 0

Region: NODE\_387203\_length\_2970\_cov\_21.957239 87-92. Max. coverage (+): 0. Max coverage (-): 0

Region: NODE\_387203\_length\_2970\_cov\_21.957239 93-99. Max. coverage (+): 0. Max coverage (-): 0

Region: NODE\_387203\_length\_2970\_cov\_21.957239 100-105. Max. coverage (+): 0. Max coverage (-): 0

Region: NODE\_387203\_length\_2970\_cov\_21.957239 106-111. Max. coverage (+): 0. Max coverage (-): 0

Region: NODE\_387203\_length\_2970\_cov\_21.957239 112-117. Max. coverage (+): 0. Max coverage (-): 0

Region: NODE\_387203\_length\_2970\_cov\_21.957239 118-123. Max. coverage (+): 0. Max coverage (-): 0

Region: NODE\_387203\_length\_2970\_cov\_21.957239 124-129. Max. coverage (+): 0. Max coverage (-): 0

Region: NODE\_387203\_length\_2970\_cov\_21.957239 130-135. Max. coverage (+): 0.04. Max coverage (-): 0

Region: NODE\_387203\_length\_2970\_cov\_21.957239 136-142. Max. coverage (+): 0.08. Max coverage (-): 0

Region: NODE\_387203\_length\_2970\_cov\_21.957239 143-148. Max. coverage (+): 0.08. Max coverage (-): 0.04

Region: NODE\_387203\_length\_2970\_cov\_21.957239 149-154. Max. coverage (+): 0.08. Max coverage (-): 0

Region: NODE\_387203\_length\_2970\_cov\_21.957239 155-160. Max. coverage (+): 0. Max coverage (-): 0

Region: NODE\_387203\_length\_2970\_cov\_21.957239 161-166. Max. coverage (+): 0.31. Max coverage (-): 0

Region: NODE\_387203\_length\_2970\_cov\_21.957239 167-172. Max. coverage (+): 0.08. Max coverage (-): 0

Region: NODE\_387203\_length\_2970\_cov\_21.957239 173-178. Max. coverage (+): 0. Max coverage (-): 0

Region: NODE\_387203\_length\_2970\_cov\_21.957239 179-184. Max. coverage (+): 0. Max coverage (-): 0.28

Region: NODE\_387203\_length\_2970\_cov\_21.957239 185-191. Max. coverage (+): 0. Max coverage (-): 0

Region: NODE\_387203\_length\_2970\_cov\_21.957239 192-197. Max. coverage (+): 0. Max coverage (-): 0

Region: NODE\_387203\_length\_2970\_cov\_21.957239 198-203. Max. coverage (+): 0. Max coverage (-): 0

Region: NODE\_387203\_length\_2970\_cov\_21.957239 204-209. Max. coverage (+): 0. Max coverage (-): 0

Region: NODE\_387203\_length\_2970\_cov\_21.957239 210-215. Max. coverage (+): 0. Max coverage (-): 0

Region: NODE\_387203\_length\_2970\_cov\_21.957239 216-221. Max. coverage (+): 0. Max coverage (-): 0

Region: NODE\_387203\_length\_2970\_cov\_21.957239 222-227. Max. coverage (+): 0. Max coverage (-): 0

Region: NODE\_387203\_length\_2970\_cov\_21.957239 228-234. Max. coverage (+): 0. Max coverage (-): 0

Region: NODE\_387203\_length\_2970\_cov\_21.957239 235-240. Max. coverage (+): 0. Max coverage (-): 0

Region: NODE\_387203\_length\_2970\_cov\_21.957239 241-246. Max. coverage (+): 0. Max coverage (-): 0

Region: NODE\_387203\_length\_2970\_cov\_21.957239 247-252. Max. coverage (+): 0. Max coverage (-): 0

Region: NODE\_387203\_length\_2970\_cov\_21.957239 253-258. Max. coverage (+): 0. Max coverage (-): 0

Region: NODE\_387203\_length\_2970\_cov\_21.957239 259-264. Max. coverage (+): 0. Max coverage (-): 0

Region: NODE\_387203\_length\_2970\_cov\_21.957239 265-270. Max. coverage (+): 0. Max coverage (-): 0

Region: NODE\_387203\_length\_2970\_cov\_21.957239 271-276. Max. coverage (+): 0. Max coverage (-): 0

Region: NODE\_387203\_length\_2970\_cov\_21.957239 277-283. Max. coverage (+): 0.04. Max coverage (-): 0

Region: NODE\_387203\_length\_2970\_cov\_21.957239 284-289. Max. coverage (+): 0.23. Max coverage (-): 0

Region: NODE\_387203\_length\_2970\_cov\_21.957239 290-295. Max. coverage (+): 0.23. Max coverage (-): 0

Region: NODE\_387203\_length\_2970\_cov\_21.957239 296-301. Max. coverage (+): 0.08. Max coverage (-): 0

Region: NODE\_387203\_length\_2970\_cov\_21.957239 302-307. Max. coverage (+): 0.04. Max coverage (-): 0.08

Region: NODE\_387203\_length\_2970\_cov\_21.957239 308-313. Max. coverage (+): 0. Max coverage (-): 0

Region: NODE\_387203\_length\_2970\_cov\_21.957239 314-319. Max. coverage (+): 0.04. Max coverage (-): 0.04

Region: NODE\_387203\_length\_2970\_cov\_21.957239 320-326. Max. coverage (+): 0.15. Max coverage (-): 0

Region: NODE\_387203\_length\_2970\_cov\_21.957239 327-332. Max. coverage (+): 0. Max coverage (-): 0

Region: NODE\_387203\_length\_2970\_cov\_21.957239 333-338. Max. coverage (+): 0. Max coverage (-): 0

Region: NODE\_387203\_length\_2970\_cov\_21.957239 339-344. Max. coverage (+): 0.04. Max coverage (-): 0

Region: NODE\_387203\_length\_2970\_cov\_21.957239 345-350. Max. coverage (+): 0.11. Max coverage (-): 0

Region: NODE\_387203\_length\_2970\_cov\_21.957239 351-356. Max. coverage (+): 0.08. Max coverage (-): 0

Region: NODE\_387203\_length\_2970\_cov\_21.957239 357-362. Max. coverage (+): 0. Max coverage (-): 0

Region: NODE\_387203\_length\_2970\_cov\_21.957239 363-368. Max. coverage (+): 0. Max coverage (-): 0

Region: NODE\_387203\_length\_2970\_cov\_21.957239 369-375. Max. coverage (+): 0. Max coverage (-): 0

Region: NODE\_387203\_length\_2970\_cov\_21.957239 376-381. Max. coverage (+): 0. Max coverage (-): 0

Region: NODE\_387203\_length\_2970\_cov\_21.957239 382-387. Max. coverage (+): 3.95. Max coverage (-): 0

Region: NODE\_387203\_length\_2970\_cov\_21.957239 388-393. Max. coverage (+): 0.15. Max coverage (-): 0

Region: NODE\_387203\_length\_2970\_cov\_21.957239 394-399. Max. coverage (+): 0. Max coverage (-): 0

Region: NODE\_387203\_length\_2970\_cov\_21.957239 400-405. Max. coverage (+): 0. Max coverage (-): 0

Region: NODE\_387203\_length\_2970\_cov\_21.957239 406-411. Max. coverage (+): 0. Max coverage (-): 0

Region: NODE\_387203\_length\_2970\_cov\_21.957239 412-418. Max. coverage (+): 0.04. Max coverage (-): 0

Region: NODE\_387203\_length\_2970\_cov\_21.957239 419-424. Max. coverage (+): 0.04. Max coverage (-): 0

Region: NODE\_387203\_length\_2970\_cov\_21.957239 425-430. Max. coverage (+): 0.19. Max coverage (-): 0.04

Region: NODE\_387203\_length\_2970\_cov\_21.957239 431-436. Max. coverage (+): 0.04. Max coverage (-): 0

Region: NODE\_387203\_length\_2970\_cov\_21.957239 437-442. Max. coverage (+): 0.11. Max coverage (-): 0.04

Region: NODE\_387203\_length\_2970\_cov\_21.957239 443-448. Max. coverage (+): 0.92. Max coverage (-): 0.04

Region: NODE\_387203\_length\_2970\_cov\_21.957239 449-454. Max. coverage (+): 0.96. Max coverage (-): 0.04

Region: NODE\_387203\_length\_2970\_cov\_21.957239 455-460. Max. coverage (+): 0.04. Max coverage (-): 0

Region: NODE\_387203\_length\_2970\_cov\_21.957239 461-467. Max. coverage (+): 0.08. Max coverage (-): 0

Region: NODE\_387203\_length\_2970\_cov\_21.957239 468-473. Max. coverage (+): 0. Max coverage (-): 0

Region: NODE\_387203\_length\_2970\_cov\_21.957239 474-479. Max. coverage (+): 0. Max coverage (-): 0

Region: NODE\_387203\_length\_2970\_cov\_21.957239 480-485. Max. coverage (+): 0. Max coverage (-): 0

Region: NODE\_387203\_length\_2970\_cov\_21.957239 486-491. Max. coverage (+): 0. Max coverage (-): 0.04

Region: NODE\_387203\_length\_2970\_cov\_21.957239 492-497. Max. coverage (+): 0.11. Max coverage (-): 0.04

Region: NODE\_387203\_length\_2970\_cov\_21.957239 498-503. Max. coverage (+): 1.23. Max coverage (-): 0

Region: NODE\_387203\_length\_2970\_cov\_21.957239 504-510. Max. coverage (+): 1.49. Max coverage (-): 0

Region: NODE\_387203\_length\_2970\_cov\_21.957239 511-516. Max. coverage (+): 0.15. Max coverage (-): 0

Region: NODE\_387203\_length\_2970\_cov\_21.957239 517-522. Max. coverage (+): 0. Max coverage (-): 0

Region: NODE\_387203\_length\_2970\_cov\_21.957239 523-528. Max. coverage (+): 0. Max coverage (-): 0

Region: NODE\_387203\_length\_2970\_cov\_21.957239 529-534. Max. coverage (+): 0. Max coverage (-): 0

Region: NODE\_387203\_length\_2970\_cov\_21.957239 535-540. Max. coverage (+): 0. Max coverage (-): 0

Region: NODE\_387203\_length\_2970\_cov\_21.957239 541-546. Max. coverage (+): 0. Max coverage (-): 0

Region: NODE\_387203\_length\_2970\_cov\_21.957239 547-552. Max. coverage (+): 0. Max coverage (-): 0

Region: NODE\_387203\_length\_2970\_cov\_21.957239 553-559. Max. coverage (+): 0. Max coverage (-): 0

Region: NODE\_387203\_length\_2970\_cov\_21.957239 560-565. Max. coverage (+): 0. Max coverage (-): 0

Region: NODE\_387203\_length\_2970\_cov\_21.957239 566-571. Max. coverage (+): 0. Max coverage (-): 0

Region: NODE\_387203\_length\_2970\_cov\_21.957239 572-577. Max. coverage (+): 0. Max coverage (-): 0

Region: NODE\_387203\_length\_2970\_cov\_21.957239 578-583. Max. coverage (+): 0. Max coverage (-): 0

Region: NODE\_387203\_length\_2970\_cov\_21.957239 584-589. Max. coverage (+): 0.23. Max coverage (-): 0

Region: NODE\_387203\_length\_2970\_cov\_21.957239 590-595. Max. coverage (+): 1.34. Max coverage (-): 0

Region: NODE\_387203\_length\_2970\_cov\_21.957239 596-602. Max. coverage (+): 0.08. Max coverage (-): 0

Region: NODE\_387203\_length\_2970\_cov\_21.957239 603-608. Max. coverage (+): 0. Max coverage (-): 0

Region: NODE\_387203\_length\_2970\_cov\_21.957239 609-614. Max. coverage (+): 0. Max coverage (-): 0

Region: NODE\_387203\_length\_2970\_cov\_21.957239 615-620. Max. coverage (+): 0. Max coverage (-): 0

Region: NODE\_387203\_length\_2970\_cov\_21.957239 621-626. Max. coverage (+): 0. Max coverage (-): 0

Region: NODE\_387203\_length\_2970\_cov\_21.957239 627-632. Max. coverage (+): 18.2. Max coverage (-): 0

Region: NODE\_387203\_length\_2970\_cov\_21.957239 633-638. Max. coverage (+): 13.02. Max coverage (-): 0

Region: NODE\_387203\_length\_2970\_cov\_21.957239 639-645. Max. coverage (+): 0. Max coverage (-): 0

Region: NODE\_387203\_length\_2970\_cov\_21.957239 646-651. Max. coverage (+): 0. Max coverage (-): 0

Region: NODE\_387203\_length\_2970\_cov\_21.957239 652-657. Max. coverage (+): 0. Max coverage (-): 0

Region: NODE\_387203\_length\_2970\_cov\_21.957239 658-663. Max. coverage (+): 0. Max coverage (-): 0

Region: NODE\_387203\_length\_2970\_cov\_21.957239 664-669. Max. coverage (+): 0. Max coverage (-): 0

Region: NODE\_387203\_length\_2970\_cov\_21.957239 670-675. Max. coverage (+): 0. Max coverage (-): 0

Region: NODE\_387203\_length\_2970\_cov\_21.957239 676-681. Max. coverage (+): 9.19. Max coverage (-): 0

Region: NODE\_387203\_length\_2970\_cov\_21.957239 682-687. Max. coverage (+): 0.34. Max coverage (-): 0.73

Region: NODE\_387203\_length\_2970\_cov\_21.957239 688-694. Max. coverage (+): 0.11. Max coverage (-): 0.57

Region: NODE\_387203\_length\_2970\_cov\_21.957239 695-700. Max. coverage (+): 0.04. Max coverage (-): 0.04

Region: NODE\_387203\_length\_2970\_cov\_21.957239 701-706. Max. coverage (+): 0. Max coverage (-): 0

Region: NODE\_387203\_length\_2970\_cov\_21.957239 707-712. Max. coverage (+): 0. Max coverage (-): 0

Region: NODE\_387203\_length\_2970\_cov\_21.957239 713-718. Max. coverage (+): 0. Max coverage (-): 0

Region: NODE\_387203\_length\_2970\_cov\_21.957239 719-724. Max. coverage (+): 0. Max coverage (-): 0

Region: NODE\_387203\_length\_2970\_cov\_21.957239 725-730. Max. coverage (+): 0. Max coverage (-): 0

Region: NODE\_387203\_length\_2970\_cov\_21.957239 731-737. Max. coverage (+): 0. Max coverage (-): 0

Region: NODE\_387203\_length\_2970\_cov\_21.957239 738-743. Max. coverage (+): 0. Max coverage (-): 0

Region: NODE\_387203\_length\_2970\_cov\_21.957239 744-749. Max. coverage (+): 0. Max coverage (-): 0

Region: NODE\_387203\_length\_2970\_cov\_21.957239 750-755. Max. coverage (+): 31.03. Max coverage (-): 0

Region: NODE\_387203\_length\_2970\_cov\_21.957239 756-761. Max. coverage (+): 0.08. Max coverage (-): 0

Region: NODE\_387203\_length\_2970\_cov\_21.957239 762-767. Max. coverage (+): 0.08. Max coverage (-): 0.08

Region: NODE\_387203\_length\_2970\_cov\_21.957239 768-773. Max. coverage (+): 0. Max coverage (-): 0

Region: NODE\_387203\_length\_2970\_cov\_21.957239 774-779. Max. coverage (+): 0. Max coverage (-): 0

Region: NODE\_387203\_length\_2970\_cov\_21.957239 780-786. Max. coverage (+): 0.08. Max coverage (-): 0

Region: NODE\_387203\_length\_2970\_cov\_21.957239 787-792. Max. coverage (+): 0. Max coverage (-): 0

Region: NODE\_387203\_length\_2970\_cov\_21.957239 793-798. Max. coverage (+): 0. Max coverage (-): 0

Region: NODE\_387203\_length\_2970\_cov\_21.957239 799-804. Max. coverage (+): 0. Max coverage (-): 0

Region: NODE\_387203\_length\_2970\_cov\_21.957239 805-810. Max. coverage (+): 0.04. Max coverage (-): 0

Region: NODE\_387203\_length\_2970\_cov\_21.957239 811-816. Max. coverage (+): 0.57. Max coverage (-): 0

Region: NODE\_387203\_length\_2970\_cov\_21.957239 817-822. Max. coverage (+): 0.77. Max coverage (-): 0.04

Region: NODE\_387203\_length\_2970\_cov\_21.957239 823-829. Max. coverage (+): 0.77. Max coverage (-): 0.04

Region: NODE\_387203\_length\_2970\_cov\_21.957239 830-835. Max. coverage (+): 0.34. Max coverage (-): 0.04

Region: NODE\_387203\_length\_2970\_cov\_21.957239 836-841. Max. coverage (+): 2.8. Max coverage (-): 0.04

Region: NODE\_387203\_length\_2970\_cov\_21.957239 842-847. Max. coverage (+): 2.03. Max coverage (-): 0

Region: NODE\_387203\_length\_2970\_cov\_21.957239 848-853. Max. coverage (+): 0.46. Max coverage (-): 0.08

Region: NODE\_387203\_length\_2970\_cov\_21.957239 854-859. Max. coverage (+): 1.11. Max coverage (-): 0.04

Region: NODE\_387203\_length\_2970\_cov\_21.957239 860-865. Max. coverage (+): 0. Max coverage (-): 0

Region: NODE\_387203\_length\_2970\_cov\_21.957239 866-871. Max. coverage (+): 1.72. Max coverage (-): 0.04

Region: NODE\_387203\_length\_2970\_cov\_21.957239 872-878. Max. coverage (+): 2.07. Max coverage (-): 0.04

Region: NODE\_387203\_length\_2970\_cov\_21.957239 879-884. Max. coverage (+): 0.73. Max coverage (-): 0

Region: NODE\_387203\_length\_2970\_cov\_21.957239 885-890. Max. coverage (+): 0.69. Max coverage (-): 0.11

Region: NODE\_387203\_length\_2970\_cov\_21.957239 891-896. Max. coverage (+): 0.15. Max coverage (-): 0.19

Region: NODE\_387203\_length\_2970\_cov\_21.957239 897-902. Max. coverage (+): 0.19. Max coverage (-): 0.08

Region: NODE\_387203\_length\_2970\_cov\_21.957239 903-908. Max. coverage (+): 11.8. Max coverage (-): 0

Region: NODE\_387203\_length\_2970\_cov\_21.957239 909-914. Max. coverage (+): 0.69. Max coverage (-): 0

Region: NODE\_387203\_length\_2970\_cov\_21.957239 915-921. Max. coverage (+): 0. Max coverage (-): 0

Region: NODE\_387203\_length\_2970\_cov\_21.957239 922-927. Max. coverage (+): 0.8. Max coverage (-): 0.61

Region: NODE\_387203\_length\_2970\_cov\_21.957239 928-933. Max. coverage (+): 0.19. Max coverage (-): 0

Region: NODE\_387203\_length\_2970\_cov\_21.957239 934-939. Max. coverage (+): 1.76. Max coverage (-): 0

Region: NODE\_387203\_length\_2970\_cov\_21.957239 940-945. Max. coverage (+): 8.01. Max coverage (-): 0.08

Region: NODE\_387203\_length\_2970\_cov\_21.957239 946-951. Max. coverage (+): 0.15. Max coverage (-): 0.08

Region: NODE\_387203\_length\_2970\_cov\_21.957239 952-957. Max. coverage (+): 5.17. Max coverage (-): 0.04

Region: NODE\_387203\_length\_2970\_cov\_21.957239 958-963. Max. coverage (+): 5.17. Max coverage (-): 0.04

Region: NODE\_387203\_length\_2970\_cov\_21.957239 964-970. Max. coverage (+): 0.04. Max coverage (-): 0.27

Region: NODE\_387203\_length\_2970\_cov\_21.957239 971-976. Max. coverage (+): 0. Max coverage (-): 0.27

Region: NODE\_387203\_length\_2970\_cov\_21.957239 977-982. Max. coverage (+): 0. Max coverage (-): 0

Region: NODE\_387203\_length\_2970\_cov\_21.957239 983-988. Max. coverage (+): 0.31. Max coverage (-): 0

Region: NODE\_387203\_length\_2970\_cov\_21.957239 989-994. Max. coverage (+): 0.34. Max coverage (-): 0

Region: NODE\_387203\_length\_2970\_cov\_21.957239 995-1000. Max. coverage (+): 0. Max coverage (-): 0

Region: NODE\_387203\_length\_2970\_cov\_21.957239 1001-1006. Max. coverage (+): 0. Max coverage (-): 0

Region: NODE\_387203\_length\_2970\_cov\_21.957239 1007-1013. Max. coverage (+): 0. Max coverage (-): 0

Region: NODE\_387203\_length\_2970\_cov\_21.957239 1014-1019. Max. coverage (+): 0. Max coverage (-): 0

Region: NODE\_387203\_length\_2970\_cov\_21.957239 1020-1025. Max. coverage (+): 0. Max coverage (-): 0

Region: NODE\_387203\_length\_2970\_cov\_21.957239 1026-1031. Max. coverage (+): 0.19. Max coverage (-): 0

Region: NODE\_387203\_length\_2970\_cov\_21.957239 1032-1037. Max. coverage (+): 0.04. Max coverage (-): 0

Region: NODE\_387203\_length\_2970\_cov\_21.957239 1038-1043. Max. coverage (+): 0.04. Max coverage (-): 0

Region: NODE\_387203\_length\_2970\_cov\_21.957239 1044-1049. Max. coverage (+): 0.04. Max coverage (-): 0

Region: NODE\_387203\_length\_2970\_cov\_21.957239 1050-1055. Max. coverage (+): 0. Max coverage (-): 0

Region: NODE\_387203\_length\_2970\_cov\_21.957239 1056-1062. Max. coverage (+): 0. Max coverage (-): 0

Region: NODE\_387203\_length\_2970\_cov\_21.957239 1063-1068. Max. coverage (+): 0. Max coverage (-): 0

Region: NODE\_387203\_length\_2970\_cov\_21.957239 1069-1074. Max. coverage (+): 0. Max coverage (-): 0

Region: NODE\_387203\_length\_2970\_cov\_21.957239 1075-1080. Max. coverage (+): 0. Max coverage (-): 0

Region: NODE\_387203\_length\_2970\_cov\_21.957239 1081-1086. Max. coverage (+): 0. Max coverage (-): 0

Region: NODE\_387203\_length\_2970\_cov\_21.957239 1087-1092. Max. coverage (+): 0. Max coverage (-): 0

Region: NODE\_387203\_length\_2970\_cov\_21.957239 1093-1098. Max. coverage (+): 0.23. Max coverage (-): 0

Region: NODE\_387203\_length\_2970\_cov\_21.957239 1099-1105. Max. coverage (+): 0.15. Max coverage (-): 0

Region: NODE\_387203\_length\_2970\_cov\_21.957239 1106-1111. Max. coverage (+): 0.27. Max coverage (-): 0

Region: NODE\_387203\_length\_2970\_cov\_21.957239 1112-1117. Max. coverage (+): 0.31. Max coverage (-): 0

Region: NODE\_387203\_length\_2970\_cov\_21.957239 1118-1123. Max. coverage (+): 0. Max coverage (-): 0

Region: NODE\_387203\_length\_2970\_cov\_21.957239 1124-1129. Max. coverage (+): 0. Max coverage (-): 0

Region: NODE\_387203\_length\_2970\_cov\_21.957239 1130-1135. Max. coverage (+): 0. Max coverage (-): 0

Region: NODE\_387203\_length\_2970\_cov\_21.957239 1136-1141. Max. coverage (+): 0. Max coverage (-): 0

Region: NODE\_387203\_length\_2970\_cov\_21.957239 1142-1147. Max. coverage (+): 0.08. Max coverage (-): 0

Region: NODE\_387203\_length\_2970\_cov\_21.957239 1148-1154. Max. coverage (+): 5.63. Max coverage (-): 0.84

Region: NODE\_387203\_length\_2970\_cov\_21.957239 1155-1160. Max. coverage (+): 0.42. Max coverage (-): 3.64

Region: NODE\_387203\_length\_2970\_cov\_21.957239 1161-1166. Max. coverage (+): 0.04. Max coverage (-): 3.49

Region: NODE\_387203\_length\_2970\_cov\_21.957239 1167-1172. Max. coverage (+): 0.11. Max coverage (-): 0

Region: NODE\_387203\_length\_2970\_cov\_21.957239 1173-1178. Max. coverage (+): 1.92. Max coverage (-): 0

Region: NODE\_387203\_length\_2970\_cov\_21.957239 1179-1184. Max. coverage (+): 0.54. Max coverage (-): 0

Region: NODE\_387203\_length\_2970\_cov\_21.957239 1185-1190. Max. coverage (+): 1.69. Max coverage (-): 0

Region: NODE\_387203\_length\_2970\_cov\_21.957239 1191-1197. Max. coverage (+): 0.08. Max coverage (-): 0

Region: NODE\_387203\_length\_2970\_cov\_21.957239 1198-1203. Max. coverage (+): 0.31. Max coverage (-): 0

Region: NODE\_387203\_length\_2970\_cov\_21.957239 1204-1209. Max. coverage (+): 0.34. Max coverage (-): 0

Region: NODE\_387203\_length\_2970\_cov\_21.957239 1210-1215. Max. coverage (+): 0. Max coverage (-): 0

Region: NODE\_387203\_length\_2970\_cov\_21.957239 1216-1221. Max. coverage (+): 0. Max coverage (-): 0

Region: NODE\_387203\_length\_2970\_cov\_21.957239 1222-1227. Max. coverage (+): 0. Max coverage (-): 0

Region: NODE\_387203\_length\_2970\_cov\_21.957239 1228-1233. Max. coverage (+): 0. Max coverage (-): 0

Region: NODE\_387203\_length\_2970\_cov\_21.957239 1234-1240. Max. coverage (+): 0. Max coverage (-): 0

Region: NODE\_387203\_length\_2970\_cov\_21.957239 1241-1246. Max. coverage (+): 0. Max coverage (-): 0

Region: NODE\_387203\_length\_2970\_cov\_21.957239 1247-1252. Max. coverage (+): 0. Max coverage (-): 0

Region: NODE\_387203\_length\_2970\_cov\_21.957239 1253-1258. Max. coverage (+): 0. Max coverage (-): 0

Region: NODE\_387203\_length\_2970\_cov\_21.957239 1259-1264. Max. coverage (+): 0. Max coverage (-): 0

Region: NODE\_387203\_length\_2970\_cov\_21.957239 1265-1270. Max. coverage (+): 0. Max coverage (-): 0

Region: NODE\_387203\_length\_2970\_cov\_21.957239 1271-1276. Max. coverage (+): 0. Max coverage (-): 0

Region: NODE\_387203\_length\_2970\_cov\_21.957239 1277-1282. Max. coverage (+): 0. Max coverage (-): 0

Region: NODE\_387203\_length\_2970\_cov\_21.957239 1283-1289. Max. coverage (+): 0. Max coverage (-): 0

Region: NODE\_387203\_length\_2970\_cov\_21.957239 1290-1295. Max. coverage (+): 0. Max coverage (-): 0

Region: NODE\_387203\_length\_2970\_cov\_21.957239 1296-1301. Max. coverage (+): 0. Max coverage (-): 0

Region: NODE\_387203\_length\_2970\_cov\_21.957239 1302-1307. Max. coverage (+): 0. Max coverage (-): 0

Region: NODE\_387203\_length\_2970\_cov\_21.957239 1308-1313. Max. coverage (+): 0. Max coverage (-): 0

Region: NODE\_387203\_length\_2970\_cov\_21.957239 1314-1319. Max. coverage (+): 0. Max coverage (-): 0

Region: NODE\_387203\_length\_2970\_cov\_21.957239 1320-1325. Max. coverage (+): 0. Max coverage (-): 0

Region: NODE\_387203\_length\_2970\_cov\_21.957239 1326-1332. Max. coverage (+): 0.08. Max coverage (-): 0

Region: NODE\_387203\_length\_2970\_cov\_21.957239 1333-1338. Max. coverage (+): 0.08. Max coverage (-): 0

Region: NODE\_387203\_length\_2970\_cov\_21.957239 1339-1344. Max. coverage (+): 0.04. Max coverage (-): 0

Region: NODE\_387203\_length\_2970\_cov\_21.957239 1345-1350. Max. coverage (+): 0. Max coverage (-): 0

Region: NODE\_387203\_length\_2970\_cov\_21.957239 1351-1356. Max. coverage (+): 0. Max coverage (-): 0

Region: NODE\_387203\_length\_2970\_cov\_21.957239 1357-1362. Max. coverage (+): 0. Max coverage (-): 0

Region: NODE\_387203\_length\_2970\_cov\_21.957239 1363-1368. Max. coverage (+): 1.95. Max coverage (-): 0

Region: NODE\_387203\_length\_2970\_cov\_21.957239 1369-1374. Max. coverage (+): 2.34. Max coverage (-): 0.04

Region: NODE\_387203\_length\_2970\_cov\_21.957239 1375-1381. Max. coverage (+): 0.04. Max coverage (-): 0.04

Region: NODE\_387203\_length\_2970\_cov\_21.957239 1382-1387. Max. coverage (+): 0. Max coverage (-): 3.68

Region: NODE\_387203\_length\_2970\_cov\_21.957239 1388-1393. Max. coverage (+): 0.04. Max coverage (-): 1.26

Region: NODE\_387203\_length\_2970\_cov\_21.957239 1394-1399. Max. coverage (+): 7.36. Max coverage (-): 0

Region: NODE\_387203\_length\_2970\_cov\_21.957239 1400-1405. Max. coverage (+): 8.12. Max coverage (-): 0.08

Region: NODE\_387203\_length\_2970\_cov\_21.957239 1406-1411. Max. coverage (+): 1.76. Max coverage (-): 0.08

Region: NODE\_387203\_length\_2970\_cov\_21.957239 1412-1417. Max. coverage (+): 0.27. Max coverage (-): 0.15

Region: NODE\_387203\_length\_2970\_cov\_21.957239 1418-1424. Max. coverage (+): 0. Max coverage (-): 0.27

Region: NODE\_387203\_length\_2970\_cov\_21.957239 1425-1430. Max. coverage (+): 0. Max coverage (-): 0

Region: NODE\_387203\_length\_2970\_cov\_21.957239 1431-1436. Max. coverage (+): 0. Max coverage (-): 0

Region: NODE\_387203\_length\_2970\_cov\_21.957239 1437-1442. Max. coverage (+): 0. Max coverage (-): 0

Region: NODE\_387203\_length\_2970\_cov\_21.957239 1443-1448. Max. coverage (+): 0. Max coverage (-): 0

Region: NODE\_387203\_length\_2970\_cov\_21.957239 1449-1454. Max. coverage (+): 0. Max coverage (-): 0

Region: NODE\_387203\_length\_2970\_cov\_21.957239 1455-1460. Max. coverage (+): 0.11. Max coverage (-): 0

Region: NODE\_387203\_length\_2970\_cov\_21.957239 1461-1466. Max. coverage (+): 0.11. Max coverage (-): 0

Region: NODE\_387203\_length\_2970\_cov\_21.957239 1467-1473. Max. coverage (+): 0. Max coverage (-): 0

Region: NODE\_387203\_length\_2970\_cov\_21.957239 1474-1479. Max. coverage (+): 0. Max coverage (-): 0

Region: NODE\_387203\_length\_2970\_cov\_21.957239 1480-1485. Max. coverage (+): 0. Max coverage (-): 0

Region: NODE\_387203\_length\_2970\_cov\_21.957239 1486-1491. Max. coverage (+): 0. Max coverage (-): 0

Region: NODE\_387203\_length\_2970\_cov\_21.957239 1492-1497. Max. coverage (+): 0. Max coverage (-): 0

Region: NODE\_387203\_length\_2970\_cov\_21.957239 1498-1503. Max. coverage (+): 0.04. Max coverage (-): 0

Region: NODE\_387203\_length\_2970\_cov\_21.957239 1504-1509. Max. coverage (+): 0.08. Max coverage (-): 0

Region: NODE\_387203\_length\_2970\_cov\_21.957239 1510-1516. Max. coverage (+): 0. Max coverage (-): 0

Region: NODE\_387203\_length\_2970\_cov\_21.957239 1517-1522. Max. coverage (+): 0. Max coverage (-): 0

Region: NODE\_387203\_length\_2970\_cov\_21.957239 1523-1528. Max. coverage (+): 0. Max coverage (-): 0

Region: NODE\_387203\_length\_2970\_cov\_21.957239 1529-1534. Max. coverage (+): 0. Max coverage (-): 0

Region: NODE\_387203\_length\_2970\_cov\_21.957239 1535-1540. Max. coverage (+): 0. Max coverage (-): 0

Region: NODE\_387203\_length\_2970\_cov\_21.957239 1541-1546. Max. coverage (+): 0. Max coverage (-): 0

Region: NODE\_387203\_length\_2970\_cov\_21.957239 1547-1552. Max. coverage (+): 0. Max coverage (-): 0

Region: NODE\_387203\_length\_2970\_cov\_21.957239 1553-1558. Max. coverage (+): 0. Max coverage (-): 0

Region: NODE\_387203\_length\_2970\_cov\_21.957239 1559-1565. Max. coverage (+): 0. Max coverage (-): 0

Region: NODE\_387203\_length\_2970\_cov\_21.957239 1566-1571. Max. coverage (+): 0. Max coverage (-): 0

Region: NODE\_387203\_length\_2970\_cov\_21.957239 1572-1577. Max. coverage (+): 0. Max coverage (-): 0

Region: NODE\_387203\_length\_2970\_cov\_21.957239 1578-1583. Max. coverage (+): 0. Max coverage (-): 0

Region: NODE\_387203\_length\_2970\_cov\_21.957239 1584-1589. Max. coverage (+): 0. Max coverage (-): 0

Region: NODE\_387203\_length\_2970\_cov\_21.957239 1590-1595. Max. coverage (+): 0. Max coverage (-): 0

Region: NODE\_387203\_length\_2970\_cov\_21.957239 1596-1601. Max. coverage (+): 0. Max coverage (-): 0

Region: NODE\_387203\_length\_2970\_cov\_21.957239 1602-1608. Max. coverage (+): 0. Max coverage (-): 0

Region: NODE\_387203\_length\_2970\_cov\_21.957239 1609-1614. Max. coverage (+): 0.42. Max coverage (-): 0

Region: NODE\_387203\_length\_2970\_cov\_21.957239 1615-1620. Max. coverage (+): 0. Max coverage (-): 0

Region: NODE\_387203\_length\_2970\_cov\_21.957239 1621-1626. Max. coverage (+): 0.04. Max coverage (-): 0

Region: NODE\_387203\_length\_2970\_cov\_21.957239 1627-1632. Max. coverage (+): 0. Max coverage (-): 0

Region: NODE\_387203\_length\_2970\_cov\_21.957239 1633-1638. Max. coverage (+): 0. Max coverage (-): 0

Region: NODE\_387203\_length\_2970\_cov\_21.957239 1639-1644. Max. coverage (+): 0. Max coverage (-): 0

Region: NODE\_387203\_length\_2970\_cov\_21.957239 1645-1650. Max. coverage (+): 3.26. Max coverage (-): 0.08

Region: NODE\_387203\_length\_2970\_cov\_21.957239 1651-1657. Max. coverage (+): 1.72. Max coverage (-): 0

Region: NODE\_387203\_length\_2970\_cov\_21.957239 1658-1663. Max. coverage (+): 0.42. Max coverage (-): 0

Region: NODE\_387203\_length\_2970\_cov\_21.957239 1664-1669. Max. coverage (+): 0.38. Max coverage (-): 0

Region: NODE\_387203\_length\_2970\_cov\_21.957239 1670-1675. Max. coverage (+): 0.04. Max coverage (-): 0

Region: NODE\_387203\_length\_2970\_cov\_21.957239 1676-1681. Max. coverage (+): 0. Max coverage (-): 0

Region: NODE\_387203\_length\_2970\_cov\_21.957239 1682-1687. Max. coverage (+): 0. Max coverage (-): 0

Region: NODE\_387203\_length\_2970\_cov\_21.957239 1688-1693. Max. coverage (+): 0.15. Max coverage (-): 0

Region: NODE\_387203\_length\_2970\_cov\_21.957239 1694-1700. Max. coverage (+): 0.15. Max coverage (-): 0

Region: NODE\_387203\_length\_2970\_cov\_21.957239 1701-1706. Max. coverage (+): 0. Max coverage (-): 0

Region: NODE\_387203\_length\_2970\_cov\_21.957239 1707-1712. Max. coverage (+): 0. Max coverage (-): 0

Region: NODE\_387203\_length\_2970\_cov\_21.957239 1713-1718. Max. coverage (+): 0. Max coverage (-): 0

Region: NODE\_387203\_length\_2970\_cov\_21.957239 1719-1724. Max. coverage (+): 0. Max coverage (-): 0

Region: NODE\_387203\_length\_2970\_cov\_21.957239 1725-1730. Max. coverage (+): 0. Max coverage (-): 0

Region: NODE\_387203\_length\_2970\_cov\_21.957239 1731-1736. Max. coverage (+): 0. Max coverage (-): 0

Region: NODE\_387203\_length\_2970\_cov\_21.957239 1737-1742. Max. coverage (+): 0.04. Max coverage (-): 0

Region: NODE\_387203\_length\_2970\_cov\_21.957239 1743-1749. Max. coverage (+): 0.31. Max coverage (-): 0.04

Region: NODE\_387203\_length\_2970\_cov\_21.957239 1750-1755. Max. coverage (+): 2.18. Max coverage (-): 0

Region: NODE\_387203\_length\_2970\_cov\_21.957239 1756-1761. Max. coverage (+): 0.11. Max coverage (-): 0.08

Region: NODE\_387203\_length\_2970\_cov\_21.957239 1762-1767. Max. coverage (+): 0.54. Max coverage (-): 0.11

Region: NODE\_387203\_length\_2970\_cov\_21.957239 1768-1773. Max. coverage (+): 0.08. Max coverage (-): 0.08

Region: NODE\_387203\_length\_2970\_cov\_21.957239 1774-1779. Max. coverage (+): 0.04. Max coverage (-): 0.08

Region: NODE\_387203\_length\_2970\_cov\_21.957239 1780-1785. Max. coverage (+): 1.38. Max coverage (-): 0

Region: NODE\_387203\_length\_2970\_cov\_21.957239 1786-1792. Max. coverage (+): 2.68. Max coverage (-): 0

Region: NODE\_387203\_length\_2970\_cov\_21.957239 1793-1798. Max. coverage (+): 1.42. Max coverage (-): 0.11

Region: NODE\_387203\_length\_2970\_cov\_21.957239 1799-1804. Max. coverage (+): 0.31. Max coverage (-): 0.11

Region: NODE\_387203\_length\_2970\_cov\_21.957239 1805-1810. Max. coverage (+): 1.34. Max coverage (-): 0.15

Region: NODE\_387203\_length\_2970\_cov\_21.957239 1811-1816. Max. coverage (+): 1.34. Max coverage (-): 0.15

Region: NODE\_387203\_length\_2970\_cov\_21.957239 1817-1822. Max. coverage (+): 0.84. Max coverage (-): 0

Region: NODE\_387203\_length\_2970\_cov\_21.957239 1823-1828. Max. coverage (+): 0. Max coverage (-): 0

Region: NODE\_387203\_length\_2970\_cov\_21.957239 1829-1834. Max. coverage (+): 0. Max coverage (-): 0

Region: NODE\_387203\_length\_2970\_cov\_21.957239 1835-1841. Max. coverage (+): 0. Max coverage (-): 0

Region: NODE\_387203\_length\_2970\_cov\_21.957239 1842-1847. Max. coverage (+): 0. Max coverage (-): 0

Region: NODE\_387203\_length\_2970\_cov\_21.957239 1848-1853. Max. coverage (+): 0. Max coverage (-): 0.04

Region: NODE\_387203\_length\_2970\_cov\_21.957239 1854-1859. Max. coverage (+): 0.04. Max coverage (-): 0.04

Region: NODE\_387203\_length\_2970\_cov\_21.957239 1860-1865. Max. coverage (+): 0.11. Max coverage (-): 0

Region: NODE\_387203\_length\_2970\_cov\_21.957239 1866-1871. Max. coverage (+): 3.75. Max coverage (-): 0.04

Region: NODE\_387203\_length\_2970\_cov\_21.957239 1872-1877. Max. coverage (+): 3.26. Max coverage (-): 0

Region: NODE\_387203\_length\_2970\_cov\_21.957239 1878-1884. Max. coverage (+): 1.95. Max coverage (-): 0

Region: NODE\_387203\_length\_2970\_cov\_21.957239 1885-1890. Max. coverage (+): 0.69. Max coverage (-): 0.04

Region: NODE\_387203\_length\_2970\_cov\_21.957239 1891-1896. Max. coverage (+): 0.61. Max coverage (-): 0.08

Region: NODE\_387203\_length\_2970\_cov\_21.957239 1897-1902. Max. coverage (+): 0.57. Max coverage (-): 0.11

Region: NODE\_387203\_length\_2970\_cov\_21.957239 1903-1908. Max. coverage (+): 0.65. Max coverage (-): 0.04

Region: NODE\_387203\_length\_2970\_cov\_21.957239 1909-1914. Max. coverage (+): 0.42. Max coverage (-): 0

Region: NODE\_387203\_length\_2970\_cov\_21.957239 1915-1920. Max. coverage (+): 4.21. Max coverage (-): 0.08

Region: NODE\_387203\_length\_2970\_cov\_21.957239 1921-1927. Max. coverage (+): 0.08. Max coverage (-): 0.08

Region: NODE\_387203\_length\_2970\_cov\_21.957239 1928-1933. Max. coverage (+): 0. Max coverage (-): 2.15

Region: NODE\_387203\_length\_2970\_cov\_21.957239 1934-1939. Max. coverage (+): 0.04. Max coverage (-): 6.05

Region: NODE\_387203\_length\_2970\_cov\_21.957239 1940-1945. Max. coverage (+): 1.15. Max coverage (-): 0.46

Region: NODE\_387203\_length\_2970\_cov\_21.957239 1946-1951. Max. coverage (+): 2.68. Max coverage (-): 0.19

Region: NODE\_387203\_length\_2970\_cov\_21.957239 1952-1957. Max. coverage (+): 8.27. Max coverage (-): 0.04

Region: NODE\_387203\_length\_2970\_cov\_21.957239 1958-1963. Max. coverage (+): 2.3. Max coverage (-): 0.04

Region: NODE\_387203\_length\_2970\_cov\_21.957239 1964-1969. Max. coverage (+): 0.46. Max coverage (-): 0.04

Region: NODE\_387203\_length\_2970\_cov\_21.957239 1970-1976. Max. coverage (+): 0.31. Max coverage (-): 0

Region: NODE\_387203\_length\_2970\_cov\_21.957239 1977-1982. Max. coverage (+): 3.37. Max coverage (-): 0

Region: NODE\_387203\_length\_2970\_cov\_21.957239 1983-1988. Max. coverage (+): 4.87. Max coverage (-): 0

Region: NODE\_387203\_length\_2970\_cov\_21.957239 1989-1994. Max. coverage (+): 2.15. Max coverage (-): 0

Region: NODE\_387203\_length\_2970\_cov\_21.957239 1995-2000. Max. coverage (+): 0.04. Max coverage (-): 0

Region: NODE\_387203\_length\_2970\_cov\_21.957239 2001-2006. Max. coverage (+): 0.04. Max coverage (-): 0.34

Region: NODE\_387203\_length\_2970\_cov\_21.957239 2007-2012. Max. coverage (+): 0.11. Max coverage (-): 0.04

Region: NODE\_387203\_length\_2970\_cov\_21.957239 2013-2019. Max. coverage (+): 12.99. Max coverage (-): 0

Region: NODE\_387203\_length\_2970\_cov\_21.957239 2020-2025. Max. coverage (+): 13.64. Max coverage (-): 0

Region: NODE\_387203\_length\_2970\_cov\_21.957239 2026-2031. Max. coverage (+): 2.64. Max coverage (-): 0

Region: NODE\_387203\_length\_2970\_cov\_21.957239 2032-2037. Max. coverage (+): 0.96. Max coverage (-): 0.04

Region: NODE\_387203\_length\_2970\_cov\_21.957239 2038-2043. Max. coverage (+): 0.27. Max coverage (-): 0.04

Region: NODE\_387203\_length\_2970\_cov\_21.957239 2044-2049. Max. coverage (+): 0.08. Max coverage (-): 0.27

Region: NODE\_387203\_length\_2970\_cov\_21.957239 2050-2055. Max. coverage (+): 0.08. Max coverage (-): 0

Region: NODE\_387203\_length\_2970\_cov\_21.957239 2056-2061. Max. coverage (+): 0.34. Max coverage (-): 0

Region: NODE\_387203\_length\_2970\_cov\_21.957239 2062-2068. Max. coverage (+): 1.34. Max coverage (-): 0

Region: NODE\_387203\_length\_2970\_cov\_21.957239 2069-2074. Max. coverage (+): 0.04. Max coverage (-): 0.08

Region: NODE\_387203\_length\_2970\_cov\_21.957239 2075-2080. Max. coverage (+): 0. Max coverage (-): 0.19

Region: NODE\_387203\_length\_2970\_cov\_21.957239 2081-2086. Max. coverage (+): 0.11. Max coverage (-): 0.08

Region: NODE\_387203\_length\_2970\_cov\_21.957239 2087-2092. Max. coverage (+): 1.3. Max coverage (-): 0

Region: NODE\_387203\_length\_2970\_cov\_21.957239 2093-2098. Max. coverage (+): 1.61. Max coverage (-): 0

Region: NODE\_387203\_length\_2970\_cov\_21.957239 2099-2104. Max. coverage (+): 0.23. Max coverage (-): 0

Region: NODE\_387203\_length\_2970\_cov\_21.957239 2105-2111. Max. coverage (+): 0.23. Max coverage (-): 0.04

Region: NODE\_387203\_length\_2970\_cov\_21.957239 2112-2117. Max. coverage (+): 0.19. Max coverage (-): 0.04

Region: NODE\_387203\_length\_2970\_cov\_21.957239 2118-2123. Max. coverage (+): 4.44. Max coverage (-): 0.04

Region: NODE\_387203\_length\_2970\_cov\_21.957239 2124-2129. Max. coverage (+): 68.11. Max coverage (-): 0

Region: NODE\_387203\_length\_2970\_cov\_21.957239 2130-2135. Max. coverage (+): 66.58. Max coverage (-): 0

Region: NODE\_387203\_length\_2970\_cov\_21.957239 2136-2141. Max. coverage (+): 1.19. Max coverage (-): 0.27

Region: NODE\_387203\_length\_2970\_cov\_21.957239 2142-2147. Max. coverage (+): 0. Max coverage (-): 0.23

Region: NODE\_387203\_length\_2970\_cov\_21.957239 2148-2153. Max. coverage (+): 0.11. Max coverage (-): 0.23

Region: NODE\_387203\_length\_2970\_cov\_21.957239 2154-2160. Max. coverage (+): 0.11. Max coverage (-): 0.11

Region: NODE\_387203\_length\_2970\_cov\_21.957239 2161-2166. Max. coverage (+): 2.3. Max coverage (-): 0.69

Region: NODE\_387203\_length\_2970\_cov\_21.957239 2167-2172. Max. coverage (+): 2.22. Max coverage (-): 0.11

Region: NODE\_387203\_length\_2970\_cov\_21.957239 2173-2178. Max. coverage (+): 0.77. Max coverage (-): 0

Region: NODE\_387203\_length\_2970\_cov\_21.957239 2179-2184. Max. coverage (+): 1.95. Max coverage (-): 0

Region: NODE\_387203\_length\_2970\_cov\_21.957239 2185-2190. Max. coverage (+): 0.08. Max coverage (-): 0

Region: NODE\_387203\_length\_2970\_cov\_21.957239 2191-2196. Max. coverage (+): 0.15. Max coverage (-): 0

Region: NODE\_387203\_length\_2970\_cov\_21.957239 2197-2203. Max. coverage (+): 0.54. Max coverage (-): 0

Region: NODE\_387203\_length\_2970\_cov\_21.957239 2204-2209. Max. coverage (+): 0. Max coverage (-): 0

Region: NODE\_387203\_length\_2970\_cov\_21.957239 2210-2215. Max. coverage (+): 0. Max coverage (-): 0

Region: NODE\_387203\_length\_2970\_cov\_21.957239 2216-2221. Max. coverage (+): 0. Max coverage (-): 0

Region: NODE\_387203\_length\_2970\_cov\_21.957239 2222-2227. Max. coverage (+): 0. Max coverage (-): 0

Region: NODE\_387203\_length\_2970\_cov\_21.957239 2228-2233. Max. coverage (+): 5.94. Max coverage (-): 0

Region: NODE\_387203\_length\_2970\_cov\_21.957239 2234-2239. Max. coverage (+): 5.63. Max coverage (-): 0

Region: NODE\_387203\_length\_2970\_cov\_21.957239 2240-2245. Max. coverage (+): 0.96. Max coverage (-): 0.08

Region: NODE\_387203\_length\_2970\_cov\_21.957239 2246-2252. Max. coverage (+): 1.23. Max coverage (-): 0.08

Region: NODE\_387203\_length\_2970\_cov\_21.957239 2253-2258. Max. coverage (+): 0.04. Max coverage (-): 0

Region: NODE\_387203\_length\_2970\_cov\_21.957239 2259-2264. Max. coverage (+): 0. Max coverage (-): 0

Region: NODE\_387203\_length\_2970\_cov\_21.957239 2265-2270. Max. coverage (+): 0. Max coverage (-): 0

Region: NODE\_387203\_length\_2970\_cov\_21.957239 2271-2276. Max. coverage (+): 0. Max coverage (-): 0

Region: NODE\_387203\_length\_2970\_cov\_21.957239 2277-2282. Max. coverage (+): 0.08. Max coverage (-): 0

Region: NODE\_387203\_length\_2970\_cov\_21.957239 2283-2288. Max. coverage (+): 0.04. Max coverage (-): 0.04

Region: NODE\_387203\_length\_2970\_cov\_21.957239 2289-2295. Max. coverage (+): 0.08. Max coverage (-): 0.19

Region: NODE\_387203\_length\_2970\_cov\_21.957239 2296-2301. Max. coverage (+): 0.04. Max coverage (-): 0.15

Region: NODE\_387203\_length\_2970\_cov\_21.957239 2302-2307. Max. coverage (+): 3.87. Max coverage (-): 0

Region: NODE\_387203\_length\_2970\_cov\_21.957239 2308-2313. Max. coverage (+): 3.03. Max coverage (-): 0

Region: NODE\_387203\_length\_2970\_cov\_21.957239 2314-2319. Max. coverage (+): 1.38. Max coverage (-): 0

Region: NODE\_387203\_length\_2970\_cov\_21.957239 2320-2325. Max. coverage (+): 0.04. Max coverage (-): 0

Region: NODE\_387203\_length\_2970\_cov\_21.957239 2326-2331. Max. coverage (+): 0. Max coverage (-): 0

Region: NODE\_387203\_length\_2970\_cov\_21.957239 2332-2337. Max. coverage (+): 0. Max coverage (-): 0

Region: NODE\_387203\_length\_2970\_cov\_21.957239 2338-2344. Max. coverage (+): 0. Max coverage (-): 0.15

Region: NODE\_387203\_length\_2970\_cov\_21.957239 2345-2350. Max. coverage (+): 0.11. Max coverage (-): 0.23

Region: NODE\_387203\_length\_2970\_cov\_21.957239 2351-2356. Max. coverage (+): 0.27. Max coverage (-): 0

Region: NODE\_387203\_length\_2970\_cov\_21.957239 2357-2362. Max. coverage (+): 9.04. Max coverage (-): 0

Region: NODE\_387203\_length\_2970\_cov\_21.957239 2363-2368. Max. coverage (+): 0.15. Max coverage (-): 0

Region: NODE\_387203\_length\_2970\_cov\_21.957239 2369-2374. Max. coverage (+): 0.36. Max coverage (-): 0

Region: NODE\_387203\_length\_2970\_cov\_21.957239 2375-2380. Max. coverage (+): 0.74. Max coverage (-): 0

Region: NODE\_387203\_length\_2970\_cov\_21.957239 2381-2387. Max. coverage (+): 1.58. Max coverage (-): 0

Region: NODE\_387203\_length\_2970\_cov\_21.957239 2388-2393. Max. coverage (+): 7.27. Max coverage (-): 0

Region: NODE\_387203\_length\_2970\_cov\_21.957239 2394-2399. Max. coverage (+): 0.15. Max coverage (-): 0

Region: NODE\_387203\_length\_2970\_cov\_21.957239 2400-2405. Max. coverage (+): 0.05. Max coverage (-): 0

Region: NODE\_387203\_length\_2970\_cov\_21.957239 2406-2411. Max. coverage (+): 0. Max coverage (-): 0

Region: NODE\_387203\_length\_2970\_cov\_21.957239 2412-2417. Max. coverage (+): 0. Max coverage (-): 0

Region: NODE\_387203\_length\_2970\_cov\_21.957239 2418-2423. Max. coverage (+): 0.14. Max coverage (-): 0

Region: NODE\_387203\_length\_2970\_cov\_21.957239 2424-2429. Max. coverage (+): 1.09. Max coverage (-): 0

Region: NODE\_387203\_length\_2970\_cov\_21.957239 2430-2436. Max. coverage (+): 0.43. Max coverage (-): 0

Region: NODE\_387203\_length\_2970\_cov\_21.957239 2437-2442. Max. coverage (+): 0.02. Max coverage (-): 0

Region: NODE\_387203\_length\_2970\_cov\_21.957239 2443-2448. Max. coverage (+): 0. Max coverage (-): 0

Region: NODE\_387203\_length\_2970\_cov\_21.957239 2449-2454. Max. coverage (+): 0. Max coverage (-): 0

Region: NODE\_387203\_length\_2970\_cov\_21.957239 2455-2460. Max. coverage (+): 0. Max coverage (-): 0

Region: NODE\_387203\_length\_2970\_cov\_21.957239 2461-2466. Max. coverage (+): 0. Max coverage (-): 0

Region: NODE\_387203\_length\_2970\_cov\_21.957239 2467-2472. Max. coverage (+): 0. Max coverage (-): 0

Region: NODE\_387203\_length\_2970\_cov\_21.957239 2473-2479. Max. coverage (+): 0. Max coverage (-): 0

Region: NODE\_387203\_length\_2970\_cov\_21.957239 2480-2485. Max. coverage (+): 0. Max coverage (-): 0

Region: NODE\_387203\_length\_2970\_cov\_21.957239 2486-2491. Max. coverage (+): 0.01. Max coverage (-): 0

Region: NODE\_387203\_length\_2970\_cov\_21.957239 2492-2497. Max. coverage (+): 0.04. Max coverage (-): 0

Region: NODE\_387203\_length\_2970\_cov\_21.957239 2498-2503. Max. coverage (+): 0.03. Max coverage (-): 0

Region: NODE\_387203\_length\_2970\_cov\_21.957239 2504-2509. Max. coverage (+): 0. Max coverage (-): 0

Region: NODE\_387203\_length\_2970\_cov\_21.957239 2510-2515. Max. coverage (+): 0. Max coverage (-): 0

Region: NODE\_387203\_length\_2970\_cov\_21.957239 2516-2522. Max. coverage (+): 0. Max coverage (-): 0

Region: NODE\_387203\_length\_2970\_cov\_21.957239 2523-2528. Max. coverage (+): 0.08. Max coverage (-): 0

Region: NODE\_387203\_length\_2970\_cov\_21.957239 2529-2534. Max. coverage (+): 0.08. Max coverage (-): 0

Region: NODE\_387203\_length\_2970\_cov\_21.957239 2535-2540. Max. coverage (+): 0.04. Max coverage (-): 0

Region: NODE\_387203\_length\_2970\_cov\_21.957239 2541-2546. Max. coverage (+): 0.04. Max coverage (-): 0

Region: NODE\_387203\_length\_2970\_cov\_21.957239 2547-2552. Max. coverage (+): 0.04. Max coverage (-): 0

Region: NODE\_387203\_length\_2970\_cov\_21.957239 2553-2558. Max. coverage (+): 0. Max coverage (-): 0

Region: NODE\_387203\_length\_2970\_cov\_21.957239 2559-2564. Max. coverage (+): 0.54. Max coverage (-): 0

Region: NODE\_387203\_length\_2970\_cov\_21.957239 2565-2571. Max. coverage (+): 0.69. Max coverage (-): 0.04

Region: NODE\_387203\_length\_2970\_cov\_21.957239 2572-2577. Max. coverage (+): 0.11. Max coverage (-): 0.04

Region: NODE\_387203\_length\_2970\_cov\_21.957239 2578-2583. Max. coverage (+): 0.19. Max coverage (-): 0.04

Region: NODE\_387203\_length\_2970\_cov\_21.957239 2584-2589. Max. coverage (+): 0.15. Max coverage (-): 0

Region: NODE\_387203\_length\_2970\_cov\_21.957239 2590-2595. Max. coverage (+): 0.08. Max coverage (-): 0

Region: NODE\_387203\_length\_2970\_cov\_21.957239 2596-2601. Max. coverage (+): 0. Max coverage (-): 0

Region: NODE\_387203\_length\_2970\_cov\_21.957239 2602-2607. Max. coverage (+): 0. Max coverage (-): 1.57

Region: NODE\_387203\_length\_2970\_cov\_21.957239 2608-2614. Max. coverage (+): 0.42. Max coverage (-): 1.57

Region: NODE\_387203\_length\_2970\_cov\_21.957239 2615-2620. Max. coverage (+): 0.1. Max coverage (-): 0.03

Region: NODE\_387203\_length\_2970\_cov\_21.957239 2621-2626. Max. coverage (+): 6.08. Max coverage (-): 0.03

Region: NODE\_387203\_length\_2970\_cov\_21.957239 2627-2632. Max. coverage (+): 6.05. Max coverage (-): 0.05

Region: NODE\_387203\_length\_2970\_cov\_21.957239 2633-2638. Max. coverage (+): 0.05. Max coverage (-): 0.49

Region: NODE\_387203\_length\_2970\_cov\_21.957239 2639-2644. Max. coverage (+): 0. Max coverage (-): 0.46

Region: NODE\_387203\_length\_2970\_cov\_21.957239 2645-2650. Max. coverage (+): 0. Max coverage (-): 0

Region: NODE\_387203\_length\_2970\_cov\_21.957239 2651-2656. Max. coverage (+): 0. Max coverage (-): 0

Region: NODE\_387203\_length\_2970\_cov\_21.957239 2657-2663. Max. coverage (+): 0. Max coverage (-): 0

Region: NODE\_387203\_length\_2970\_cov\_21.957239 2664-2669. Max. coverage (+): 0. Max coverage (-): 0.08

Region: NODE\_387203\_length\_2970\_cov\_21.957239 2670-2675. Max. coverage (+): 0.05. Max coverage (-): 0.73

Region: NODE\_387203\_length\_2970\_cov\_21.957239 2676-2681. Max. coverage (+): 0.03. Max coverage (-): 0.46

Region: NODE\_387203\_length\_2970\_cov\_21.957239 2682-2687. Max. coverage (+): 0. Max coverage (-): 0

Region: NODE\_387203\_length\_2970\_cov\_21.957239 2688-2693. Max. coverage (+): 3.01. Max coverage (-): 0

Region: NODE\_387203\_length\_2970\_cov\_21.957239 2694-2699. Max. coverage (+): 2.76. Max coverage (-): 0

Region: NODE\_387203\_length\_2970\_cov\_21.957239 2700-2706. Max. coverage (+): 0.04. Max coverage (-): 0

Region: NODE\_387203\_length\_2970\_cov\_21.957239 2707-2712. Max. coverage (+): 0. Max coverage (-): 0

Region: NODE\_387203\_length\_2970\_cov\_21.957239 2713-2718. Max. coverage (+): 0. Max coverage (-): 0

Region: NODE\_387203\_length\_2970\_cov\_21.957239 2719-2724. Max. coverage (+): 0.73. Max coverage (-): 0

Region: NODE\_387203\_length\_2970\_cov\_21.957239 2725-2730. Max. coverage (+): 1.34. Max coverage (-): 0

Region: NODE\_387203\_length\_2970\_cov\_21.957239 2731-2736. Max. coverage (+): 0.27. Max coverage (-): 0.04

Region: NODE\_387203\_length\_2970\_cov\_21.957239 2737-2742. Max. coverage (+): 0.04. Max coverage (-): 0

Region: NODE\_387203\_length\_2970\_cov\_21.957239 2743-2748. Max. coverage (+): 0.08. Max coverage (-): 0

Region: NODE\_387203\_length\_2970\_cov\_21.957239 2749-2755. Max. coverage (+): 0.08. Max coverage (-): 0

Region: NODE\_387203\_length\_2970\_cov\_21.957239 2756-2761. Max. coverage (+): 0.04. Max coverage (-): 0

Region: NODE\_387203\_length\_2970\_cov\_21.957239 2762-2767. Max. coverage (+): 0.11. Max coverage (-): 0

Region: NODE\_387203\_length\_2970\_cov\_21.957239 2768-2773. Max. coverage (+): 0.23. Max coverage (-): 0

Region: NODE\_387203\_length\_2970\_cov\_21.957239 2774-2779. Max. coverage (+): 0. Max coverage (-): 0

Region: NODE\_387203\_length\_2970\_cov\_21.957239 2780-2785. Max. coverage (+): 0. Max coverage (-): 0

Region: NODE\_387203\_length\_2970\_cov\_21.957239 2786-2791. Max. coverage (+): 0. Max coverage (-): 0

Region: NODE\_387203\_length\_2970\_cov\_21.957239 2792-2798. Max. coverage (+): 0. Max coverage (-): 0.11

Region: NODE\_387203\_length\_2970\_cov\_21.957239 2799-2804. Max. coverage (+): 0.08. Max coverage (-): 0

Region: NODE\_387203\_length\_2970\_cov\_21.957239 2805-2810. Max. coverage (+): 0.05. Max coverage (-): 0

Region: NODE\_387203\_length\_2970\_cov\_21.957239 2811-2816. Max. coverage (+): 0. Max coverage (-): 0.74

Region: NODE\_387203\_length\_2970\_cov\_21.957239 2817-2822. Max. coverage (+): 0. Max coverage (-): 1.23

Region: NODE\_387203\_length\_2970\_cov\_21.957239 2823-2828. Max. coverage (+): 0. Max coverage (-): 0.05

Region: NODE\_387203\_length\_2970\_cov\_21.957239 2829-2834. Max. coverage (+): 0.05. Max coverage (-): 0.05

Region: NODE\_387203\_length\_2970\_cov\_21.957239 2835-2840. Max. coverage (+): 0.13. Max coverage (-): 0.03

Region: NODE\_387203\_length\_2970\_cov\_21.957239 2841-2847. Max. coverage (+): 0.15. Max coverage (-): 0.08

Region: NODE\_387203\_length\_2970\_cov\_21.957239 2848-2853. Max. coverage (+): 0.08. Max coverage (-): 0

Region: NODE\_387203\_length\_2970\_cov\_21.957239 2854-2859. Max. coverage (+): 0.08. Max coverage (-): 0

Region: NODE\_387203\_length\_2970\_cov\_21.957239 2860-2865. Max. coverage (+): 0.08. Max coverage (-): 0.04

Region: NODE\_387203\_length\_2970\_cov\_21.957239 2866-2871. Max. coverage (+): 0.08. Max coverage (-): 0.04

Region: NODE\_387203\_length\_2970\_cov\_21.957239 2872-2877. Max. coverage (+): 0.08. Max coverage (-): 0

Region: NODE\_387203\_length\_2970\_cov\_21.957239 2878-2883. Max. coverage (+): 0. Max coverage (-): 0

Region: NODE\_387203\_length\_2970\_cov\_21.957239 2884-2890. Max. coverage (+): 0. Max coverage (-): 0

Region: NODE\_387203\_length\_2970\_cov\_21.957239 2891-2896. Max. coverage (+): 0.05. Max coverage (-): 0

Region: NODE\_387203\_length\_2970\_cov\_21.957239 2897-2902. Max. coverage (+): 0.03. Max coverage (-): 0

Region: NODE\_387203\_length\_2970\_cov\_21.957239 2903-2908. Max. coverage (+): 0.05. Max coverage (-): 0

Region: NODE\_387203\_length\_2970\_cov\_21.957239 2909-2914. Max. coverage (+): 0.33. Max coverage (-): 0

Region: NODE\_387203\_length\_2970\_cov\_21.957239 2915-2920. Max. coverage (+): 0.23. Max coverage (-): 0

Region: NODE\_387203\_length\_2970\_cov\_21.957239 2921-2926. Max. coverage (+): 0. Max coverage (-): 0

Region: NODE\_387203\_length\_2970\_cov\_21.957239 2927-2932. Max. coverage (+): 0. Max coverage (-): 0

Region: NODE\_387203\_length\_2970\_cov\_21.957239 2933-2939. Max. coverage (+): 0. Max coverage (-): 0

Region: NODE\_387203\_length\_2970\_cov\_21.957239 2940-2945. Max. coverage (+): 0. Max coverage (-): 0

Region: NODE\_387203\_length\_2970\_cov\_21.957239 2946-2951. Max. coverage (+): 0. Max coverage (-): 0

Region: NODE\_387203\_length\_2970\_cov\_21.957239 2952-2957. Max. coverage (+): 0. Max coverage (-): 0

Region: NODE\_387203\_length\_2970\_cov\_21.957239 2958-2963. Max. coverage (+): 0. Max coverage (-): 0

Region: NODE\_387203\_length\_2970\_cov\_21.957239 2964-2969. Max. coverage (+): 0. Max coverage (-): 0

Region: NODE\_387203\_length\_2970\_cov\_21.957239 2970-2975. Max. coverage (+): 0. Max coverage (-): 0

Region: NODE\_387203\_length\_2970\_cov\_21.957239 2976-2982. Max. coverage (+): 0. Max coverage (-): 0

Region: NODE\_387203\_length\_2970\_cov\_21.957239 2983-2988. Max. coverage (+): 0. Max coverage (-): 0

Region: NODE\_387203\_length\_2970\_cov\_21.957239 2989-2994. Max. coverage (+): 0. Max coverage (-): 0

Region: NODE\_387203\_length\_2970\_cov\_21.957239 2995-3000. Max. coverage (+): 0. Max coverage (-): 0

Region: NODE\_387203\_length\_2970\_cov\_21.957239 3001-3006. Max. coverage (+): 0. Max coverage (-): 0

Region: NODE\_387203\_length\_2970\_cov\_21.957239 3007-3012. Max. coverage (+): 0. Max coverage (-): 0

Region: NODE\_387203\_length\_2970\_cov\_21.957239 3013-3018. Max. coverage (+): 0. Max coverage (-): 0

Region: NODE\_387203\_length\_2970\_cov\_21.957239 3019-3024. Max. coverage (+): 0. Max coverage (-): 0

Region: NODE\_387203\_length\_2970\_cov\_21.957239 3025-3031. Max. coverage (+): 0. Max coverage (-): 0

Region: NODE\_387203\_length\_2970\_cov\_21.957239 3032-3037. Max. coverage (+): 0. Max coverage (-): 0

Region: NODE\_387203\_length\_2970\_cov\_21.957239 3038-3043. Max. coverage (+): 0.03. Max coverage (-): 0

Region: NODE\_387203\_length\_2970\_cov\_21.957239 3044-3049. Max. coverage (+): 0.03. Max coverage (-): 0

Region: NODE\_387203\_length\_2970\_cov\_21.957239 3050-3055. Max. coverage (+): 0. Max coverage (-): 0

Region: NODE\_387203\_length\_2970\_cov\_21.957239 3056-3061. Max. coverage (+): 0. Max coverage (-): 0

Region: NODE\_387203\_length\_2970\_cov\_21.957239 3062-3067. Max. coverage (+): 0. Max coverage (-): 0

Region: NODE\_387203\_length\_2970\_cov\_21.957239 3068-. Max. coverage (+): 0. Max coverage (-): 0

RepeatMasker Color Code

**+**

100-98% Identity

<98-95% Identity

<95-90% Identity

<90-85% Identity

<85-80% Identity

<80-75% Identity

<75-70% Identity

<70% Identity

**-**

Gene Set Color Code

**+**

Gene

Pseudogene

Other

**-**

Topology/Coverage Color Code

Coverage Plus Strand

Coverage Minus Strand

Mainstrand: Plus

Mainstrand: Minus

Complementary Strand

Flanking Region  
(if option -flank >0)

Gene Set Annotation  
  
RepeatMasker Annotation  

**1. EnSpm-15\_DR**: 913-1142 (-), Divergence to consensus: 31.6%  
**2. EnSpm-15\_DR**: 1350-2349 (-), Divergence to consensus: 27.8%  
**3. EnSpm-15\_DR**: 2508-2710 (-), Divergence to consensus: 23.3%  
**4. EnSpm-15\_DR**: 2857-3084 (-), Divergence to consensus: 23.7%

  
Transcription Factor Binding Sites  

**RHOXF1** (Sequence: AGCTTA (-): 1309)  
**RHOXF1** (Sequence: GGATCA (-): 2196)  
**RHOXF1** (Sequence: GGATCA (-): 2250)  
**RHOXF1** (Sequence: AGATTA (-): 2427)  
**RHOXF1** (Sequence: AGATCA (-): 2579)  
**RHOXF1** (Sequence: GGATTA (-): 2658)  
**RHOXF1** (Sequence: TAAGCC (+): 215)  
**RHOXF1** (Sequence: TAAGCT (+): 413)  
**RHOXF1** (Sequence: TAAGCT (+): 488)  
**RHOXF1** (Sequence: TAAGCT (+): 873)  
**RHOXF1** (Sequence: TAATCC (+): 1053)  
**RHOXF1** (Sequence: TGATCC (+): 1966)  
**RHOXF1** (Sequence: TGATCT (+): 2797)  
**Lhx8** (Sequence: CTAATTAG (-): 626)  
**Gata4** (Sequence: GTTATCT (+): 2290)  
**POU5F1** (Sequence: TTTGCAT (-): 1728)  
**SOX9** (Sequence: AACAATGA (-): 1363)  
**SOX9** (Sequence: AACAATAG (-): 2421)  
**FOXO1** (Sequence: GCTGTTTAT (+): 1744)  
**Sox5** (Sequence: ATTGTT (+): 736)  
**FOXO3\_mmu** (Sequence: GCAAAACA (+): 354)  
**FOXO3\_mmu** (Sequence: GGAAAACA (+): 462)  
**FOXO3\_mmu** (Sequence: TGAAAACA (+): 831)  
**Nobox** (Sequence: GCTAATTA (-): 625)  
**FOXO1** (Sequence: ATAAACAAC (-): 838)  
**FOXP1** (Sequence: TGTTTAC (-): 159)  
**Rhox11** (Sequence: TGCTGTTTA (+): 575)  
**Rhox11** (Sequence: CGCTGTTTA (+): 1743)  
**Rhox11** (Sequence: TGCTGTAAA (+): 2401)  
**Rhox11** (Sequence: TTTACACCA (-): 2758)  
**Sox5** (Sequence: AACAAT (-): 716)  
**Sox5** (Sequence: AACAAT (-): 1363)  
**Sox5** (Sequence: AACAAT (-): 2409)  
**Sox5** (Sequence: AACAAT (-): 2421)  
**POU2F1** (Sequence: TATTCAAAT (+): 1248)  
**POU5F1** (Sequence: ATGCAAA (+): 1180)
