# Supplementary material for: First characterization of PIWI-interacting RNA clusters in a cichlid fish with a B chromosome
Source: BMC Biol. 2022 Sep 21;20:204. doi: 10.1186/s12915-022-01403-2 (PMC9490952; doi:10.1186/s12915-022-01403-2)
Supplement: Supplementary file 1 — Additional file 1. Zipped folder with fasta and interactive html piRNA cluster information for the A. latifasciata genome. The nomenclature is as follows: number-pirna-cluster_sex_B-presence (f, female; m, male; 0b, without B chromosome; 1b, with B chromosome). [file 12915_2022_1403_MOESM1_ESM.zip › 150_f1b.html]

piRNA cluster 150\_f1b 64


Predicted piRNA cluster no. 150\_f1b
  

Show proTRAC run info
Hide proTRAC run info

/\  
                \_\_\_\_\_\_\_\_\_\_\_\_\_\_\_\_\_\_\_\_\_\_\_/\\_\_\_ /  \\_\_\_\_\_\_\_  
               I                      /  \  /    \      I  
               I     pro             /    \/      \     I  
               I        TRAC        /               \   I  
               I   \_\_\_\_\_\_\_\_\_\_\_\_\_\_\_\_/\_\_\_\_\_\_\_\_\_\_\_\_\_\_\_\_\_\\_ I  
               I   \              /                     I  
               I    \            /                      I  
               I     \  /\      /       V.2.4.2         I  
               I      \/  \    /                        I  
               I\_\_\_\_\_\_\_\_\_\_\_\  /\_\_\_\_\_\_\_\_\_\_\_\_\_\_\_\_\_\_\_\_\_\_\_\_\_I  
                            \/  
  
  
================================= proTRAC ====================================  
VERSION: .......... 2.4.2  
LAST MODIFIED: .... 11. May 2018  
  
Please cite:  
Rosenkranz D, Zischler H. proTRAC - a software for probabilistic piRNA cluster  
detection, visualization and analysis. 2012. BMC Bioinformatics 13:5.  
  
  
Contact:  
David Rosenkranz  
Institute of Organismic and Molecular Evolutionary Biology  
Dept. Anthropology, small RNA group  
Johannes Gutenberg University Mainz  
email: rosenkranz@uni-mainz.de  
  
You can find the latest proTRAC version at:  
http://sourceforge.net/projects/protrac/files  
http://www.smallRNAgroup-mainz.de/software  
==============================================================================  
  
PARAMETERS:  
Map file: ...............piwi-femeas-1B.fa-collapse.map  
Genome file: ............../../../0B\_ala\_genome.fa  
RepeatMasker annotation: Alatifasciata-all0B-maryan-v2.fa\_corrected.out  
GeneSet:................./guest-storage/Data/annotation/Alatifasciata\_all0B\_maryan-v2\_out2017.gff  
  
Significant (p<=0.01) hit density will be calculated based  
on observed hit distribution.  
  
Sliding window size: ........................................ 5000 bp  
Sliding window increament: .................................. 1000 bp  
Normalize each hit by number of genomic hits: ............... yes  
Normalize each hit by number of sequence reads: ............. yes  
Normalize values (-> per million mapped reads): ............. yes  
Min. fraction of hits with 1T(U) or 10A: .................... 0.75  
Alternatively: Min. fraction of hits with 1T(U) and 10A: .... 0.5  
Min. fraction of hits with typical piRNA length: ............ 0.75  
Typical piRNA length: ....................................... 24-32 nt  
Min. size of a piRNA cluster: ............................... 1000 bp.  
Min. number of hits (absolute): ............................. 0  
Min. number of hits (normalized): ........................... 0  
Min. fraction of hits on the mainstrand: .................... 0.75  
Top fraction of mapped sequences (in terms of read counts): . 1%  
Top fraction accounts for max. n% of sequence reads: ........ 90%  
Min. fraction of hits on each arm of a bidirectional cluster: 0.05  
Output html file for each cluster: .......................... yes  
Output a summary table: ..................................... yes  
Output a FASTA file for each cluster (piRNA sequences): ..... yes  
Output a FASTA file comprising cluster sequences: ........... yes  
Output a GTF file for predicted piRNA clusters: ..............yes  
Search DNA motifs in clusters: .............................. yes  
Output flanking sequences: +/- .............................. 0 bp  
Output ~.pTi file: .......................................... no  
==============================================================================  
  
  
Genome size (without gaps): ............ 758543724 bp  
Gaps (N/X/-): .......................... 417479 bp  
Mapped reads: .......................... 10641844  
Non-identical sequences: ............... 2832837  
Genomic hits: .......................... 26056853  
Significant densitiy of mapped reads: .. 368.713530323068 reads/kb

Show proTRAC cluster info
Hide proTRAC cluster info

|  |  |
| --- | --- |
| Location | NODE\_387203\_length\_2970\_cov\_21.957239 |
| Coordinates | 7-3072 |
| Size [bp] | 3066 |
| Sequence hit loci | 1445 |
| Mapped reads (normalized) | 3051.9 |
| Mapped reads (normalized) per kb | 995.4 |
| Normalized reads with 1T (1U) | 81.2% |
| Normalized reads with 10A | 34.8% |
| Normalized reads with length 24-32 nt | 99.4% |
| Normalized reads on the main strand(s) | 91.1% |
| Predicted directionality | mono:plus |

100%

0%

1T (1U)  
reads

10A reads

24-32 nt  
reads

reads on mainstrand

**Either the amount of reads with 1T (1U) OR 10A has to exceed 75% (set with option: -1Tor10A)  
Alternatively the amount of reads with 1T (1U) AND 10A has to exceed 50% (set with option: -1Tand10A)  
Minimum amount of reads with preferred size is 75% (set with option: -pisize)  
Minimum amount of reads on the main strand(s) is 75% (set with option: -clstrand)**

Show read coverage
Hide read coverage

WHAT DO I SEE HERE?  
This chart shows the location of mapped sequence reads within a predicted piRNA cluster. The color refers to the number of genomic hits produced by the sequence read in question. A dark red bar indicates that this sequence read produces many other hits elsewhere in the genome. Many adjacent red or yellow bars can indicate the presence of a multi-copy element such as transposons or rRNA genes. A dark green bar indicates that this sequence read maps uniquely to this locus.

1 hit

2-5 hits

6-10 hits

11-20 hits

21-50 hits

51-100 hits

> 100 hits

NODE\_387203\_length\_2970\_cov\_21.957239

7

3072

Gene Set

RepeatMasker

Mapped  
Reads

38.1

plus strand

minus strand

38.1

Region: NODE\_387203\_length\_2970\_cov\_21.957239 37016-10. Max. coverage (+): 0.06. Max coverage (-): 0.03

Region: NODE\_387203\_length\_2970\_cov\_21.957239 11-16. Max. coverage (+): 0.06. Max coverage (-): 0.06

Region: NODE\_387203\_length\_2970\_cov\_21.957239 17-22. Max. coverage (+): 0. Max coverage (-): 0.25

Region: NODE\_387203\_length\_2970\_cov\_21.957239 23-28. Max. coverage (+): 0. Max coverage (-): 0

Region: NODE\_387203\_length\_2970\_cov\_21.957239 29-34. Max. coverage (+): 3.01. Max coverage (-): 0

Region: NODE\_387203\_length\_2970\_cov\_21.957239 35-40. Max. coverage (+): 3.01. Max coverage (-): 0

Region: NODE\_387203\_length\_2970\_cov\_21.957239 41-46. Max. coverage (+): 0. Max coverage (-): 0

Region: NODE\_387203\_length\_2970\_cov\_21.957239 47-52. Max. coverage (+): 0. Max coverage (-): 0

Region: NODE\_387203\_length\_2970\_cov\_21.957239 53-59. Max. coverage (+): 0. Max coverage (-): 0

Region: NODE\_387203\_length\_2970\_cov\_21.957239 60-65. Max. coverage (+): 0. Max coverage (-): 0

Region: NODE\_387203\_length\_2970\_cov\_21.957239 66-71. Max. coverage (+): 0. Max coverage (-): 0

Region: NODE\_387203\_length\_2970\_cov\_21.957239 72-77. Max. coverage (+): 0. Max coverage (-): 0

Region: NODE\_387203\_length\_2970\_cov\_21.957239 78-83. Max. coverage (+): 0. Max coverage (-): 0

Region: NODE\_387203\_length\_2970\_cov\_21.957239 84-89. Max. coverage (+): 0. Max coverage (-): 0

Region: NODE\_387203\_length\_2970\_cov\_21.957239 90-95. Max. coverage (+): 0. Max coverage (-): 0

Region: NODE\_387203\_length\_2970\_cov\_21.957239 96-102. Max. coverage (+): 0. Max coverage (-): 0

Region: NODE\_387203\_length\_2970\_cov\_21.957239 103-108. Max. coverage (+): 0. Max coverage (-): 0

Region: NODE\_387203\_length\_2970\_cov\_21.957239 109-114. Max. coverage (+): 0. Max coverage (-): 0

Region: NODE\_387203\_length\_2970\_cov\_21.957239 115-120. Max. coverage (+): 0. Max coverage (-): 0

Region: NODE\_387203\_length\_2970\_cov\_21.957239 121-126. Max. coverage (+): 0. Max coverage (-): 0

Region: NODE\_387203\_length\_2970\_cov\_21.957239 127-132. Max. coverage (+): 0. Max coverage (-): 0

Region: NODE\_387203\_length\_2970\_cov\_21.957239 133-138. Max. coverage (+): 0.09. Max coverage (-): 0

Region: NODE\_387203\_length\_2970\_cov\_21.957239 139-144. Max. coverage (+): 0.05. Max coverage (-): 0

Region: NODE\_387203\_length\_2970\_cov\_21.957239 145-151. Max. coverage (+): 0.14. Max coverage (-): 0

Region: NODE\_387203\_length\_2970\_cov\_21.957239 152-157. Max. coverage (+): 0.05. Max coverage (-): 0

Region: NODE\_387203\_length\_2970\_cov\_21.957239 158-163. Max. coverage (+): 0.38. Max coverage (-): 0

Region: NODE\_387203\_length\_2970\_cov\_21.957239 164-169. Max. coverage (+): 0.42. Max coverage (-): 0

Region: NODE\_387203\_length\_2970\_cov\_21.957239 170-175. Max. coverage (+): 0.16. Max coverage (-): 0

Region: NODE\_387203\_length\_2970\_cov\_21.957239 176-181. Max. coverage (+): 0. Max coverage (-): 0.28

Region: NODE\_387203\_length\_2970\_cov\_21.957239 182-187. Max. coverage (+): 0. Max coverage (-): 0.06

Region: NODE\_387203\_length\_2970\_cov\_21.957239 188-194. Max. coverage (+): 0. Max coverage (-): 0

Region: NODE\_387203\_length\_2970\_cov\_21.957239 195-200. Max. coverage (+): 0. Max coverage (-): 0

Region: NODE\_387203\_length\_2970\_cov\_21.957239 201-206. Max. coverage (+): 0. Max coverage (-): 0

Region: NODE\_387203\_length\_2970\_cov\_21.957239 207-212. Max. coverage (+): 0. Max coverage (-): 0

Region: NODE\_387203\_length\_2970\_cov\_21.957239 213-218. Max. coverage (+): 0. Max coverage (-): 0

Region: NODE\_387203\_length\_2970\_cov\_21.957239 219-224. Max. coverage (+): 0. Max coverage (-): 0

Region: NODE\_387203\_length\_2970\_cov\_21.957239 225-230. Max. coverage (+): 0. Max coverage (-): 0

Region: NODE\_387203\_length\_2970\_cov\_21.957239 231-236. Max. coverage (+): 0. Max coverage (-): 0

Region: NODE\_387203\_length\_2970\_cov\_21.957239 237-243. Max. coverage (+): 0. Max coverage (-): 0

Region: NODE\_387203\_length\_2970\_cov\_21.957239 244-249. Max. coverage (+): 0. Max coverage (-): 0

Region: NODE\_387203\_length\_2970\_cov\_21.957239 250-255. Max. coverage (+): 0. Max coverage (-): 0

Region: NODE\_387203\_length\_2970\_cov\_21.957239 256-261. Max. coverage (+): 0. Max coverage (-): 0

Region: NODE\_387203\_length\_2970\_cov\_21.957239 262-267. Max. coverage (+): 0. Max coverage (-): 0

Region: NODE\_387203\_length\_2970\_cov\_21.957239 268-273. Max. coverage (+): 0. Max coverage (-): 0

Region: NODE\_387203\_length\_2970\_cov\_21.957239 274-279. Max. coverage (+): 0. Max coverage (-): 0

Region: NODE\_387203\_length\_2970\_cov\_21.957239 280-286. Max. coverage (+): 0.05. Max coverage (-): 0

Region: NODE\_387203\_length\_2970\_cov\_21.957239 287-292. Max. coverage (+): 0.05. Max coverage (-): 0.05

Region: NODE\_387203\_length\_2970\_cov\_21.957239 293-298. Max. coverage (+): 0. Max coverage (-): 0

Region: NODE\_387203\_length\_2970\_cov\_21.957239 299-304. Max. coverage (+): 0.05. Max coverage (-): 0.05

Region: NODE\_387203\_length\_2970\_cov\_21.957239 305-310. Max. coverage (+): 0. Max coverage (-): 0.05

Region: NODE\_387203\_length\_2970\_cov\_21.957239 311-316. Max. coverage (+): 0. Max coverage (-): 0

Region: NODE\_387203\_length\_2970\_cov\_21.957239 317-322. Max. coverage (+): 0.05. Max coverage (-): 0

Region: NODE\_387203\_length\_2970\_cov\_21.957239 323-328. Max. coverage (+): 0. Max coverage (-): 0

Region: NODE\_387203\_length\_2970\_cov\_21.957239 329-335. Max. coverage (+): 0. Max coverage (-): 0

Region: NODE\_387203\_length\_2970\_cov\_21.957239 336-341. Max. coverage (+): 0. Max coverage (-): 0

Region: NODE\_387203\_length\_2970\_cov\_21.957239 342-347. Max. coverage (+): 0. Max coverage (-): 0

Region: NODE\_387203\_length\_2970\_cov\_21.957239 348-353. Max. coverage (+): 0.05. Max coverage (-): 0

Region: NODE\_387203\_length\_2970\_cov\_21.957239 354-359. Max. coverage (+): 0. Max coverage (-): 0

Region: NODE\_387203\_length\_2970\_cov\_21.957239 360-365. Max. coverage (+): 0. Max coverage (-): 0

Region: NODE\_387203\_length\_2970\_cov\_21.957239 366-371. Max. coverage (+): 0. Max coverage (-): 0

Region: NODE\_387203\_length\_2970\_cov\_21.957239 372-377. Max. coverage (+): 0.05. Max coverage (-): 0

Region: NODE\_387203\_length\_2970\_cov\_21.957239 378-384. Max. coverage (+): 0.14. Max coverage (-): 0

Region: NODE\_387203\_length\_2970\_cov\_21.957239 385-390. Max. coverage (+): 0.8. Max coverage (-): 0.05

Region: NODE\_387203\_length\_2970\_cov\_21.957239 391-396. Max. coverage (+): 0. Max coverage (-): 0

Region: NODE\_387203\_length\_2970\_cov\_21.957239 397-402. Max. coverage (+): 0. Max coverage (-): 0

Region: NODE\_387203\_length\_2970\_cov\_21.957239 403-408. Max. coverage (+): 0. Max coverage (-): 0

Region: NODE\_387203\_length\_2970\_cov\_21.957239 409-414. Max. coverage (+): 0. Max coverage (-): 0

Region: NODE\_387203\_length\_2970\_cov\_21.957239 415-420. Max. coverage (+): 0. Max coverage (-): 0

Region: NODE\_387203\_length\_2970\_cov\_21.957239 421-427. Max. coverage (+): 0.05. Max coverage (-): 0

Region: NODE\_387203\_length\_2970\_cov\_21.957239 428-433. Max. coverage (+): 0.05. Max coverage (-): 0

Region: NODE\_387203\_length\_2970\_cov\_21.957239 434-439. Max. coverage (+): 0. Max coverage (-): 0

Region: NODE\_387203\_length\_2970\_cov\_21.957239 440-445. Max. coverage (+): 0. Max coverage (-): 0.05

Region: NODE\_387203\_length\_2970\_cov\_21.957239 446-451. Max. coverage (+): 1.88. Max coverage (-): 0

Region: NODE\_387203\_length\_2970\_cov\_21.957239 452-457. Max. coverage (+): 0.42. Max coverage (-): 0

Region: NODE\_387203\_length\_2970\_cov\_21.957239 458-463. Max. coverage (+): 0. Max coverage (-): 0

Region: NODE\_387203\_length\_2970\_cov\_21.957239 464-469. Max. coverage (+): 0. Max coverage (-): 0

Region: NODE\_387203\_length\_2970\_cov\_21.957239 470-476. Max. coverage (+): 0. Max coverage (-): 0

Region: NODE\_387203\_length\_2970\_cov\_21.957239 477-482. Max. coverage (+): 0. Max coverage (-): 0

Region: NODE\_387203\_length\_2970\_cov\_21.957239 483-488. Max. coverage (+): 0. Max coverage (-): 0

Region: NODE\_387203\_length\_2970\_cov\_21.957239 489-494. Max. coverage (+): 0. Max coverage (-): 0

Region: NODE\_387203\_length\_2970\_cov\_21.957239 495-500. Max. coverage (+): 0. Max coverage (-): 0.05

Region: NODE\_387203\_length\_2970\_cov\_21.957239 501-506. Max. coverage (+): 0.52. Max coverage (-): 0.05

Region: NODE\_387203\_length\_2970\_cov\_21.957239 507-512. Max. coverage (+): 0.56. Max coverage (-): 0

Region: NODE\_387203\_length\_2970\_cov\_21.957239 513-519. Max. coverage (+): 0. Max coverage (-): 0

Region: NODE\_387203\_length\_2970\_cov\_21.957239 520-525. Max. coverage (+): 0. Max coverage (-): 0

Region: NODE\_387203\_length\_2970\_cov\_21.957239 526-531. Max. coverage (+): 0. Max coverage (-): 0

Region: NODE\_387203\_length\_2970\_cov\_21.957239 532-537. Max. coverage (+): 0. Max coverage (-): 0

Region: NODE\_387203\_length\_2970\_cov\_21.957239 538-543. Max. coverage (+): 0. Max coverage (-): 0

Region: NODE\_387203\_length\_2970\_cov\_21.957239 544-549. Max. coverage (+): 0. Max coverage (-): 0

Region: NODE\_387203\_length\_2970\_cov\_21.957239 550-555. Max. coverage (+): 0. Max coverage (-): 0

Region: NODE\_387203\_length\_2970\_cov\_21.957239 556-561. Max. coverage (+): 0. Max coverage (-): 0

Region: NODE\_387203\_length\_2970\_cov\_21.957239 562-568. Max. coverage (+): 0. Max coverage (-): 0

Region: NODE\_387203\_length\_2970\_cov\_21.957239 569-574. Max. coverage (+): 0. Max coverage (-): 0

Region: NODE\_387203\_length\_2970\_cov\_21.957239 575-580. Max. coverage (+): 0. Max coverage (-): 0

Region: NODE\_387203\_length\_2970\_cov\_21.957239 581-586. Max. coverage (+): 0.14. Max coverage (-): 0

Region: NODE\_387203\_length\_2970\_cov\_21.957239 587-592. Max. coverage (+): 0.38. Max coverage (-): 0

Region: NODE\_387203\_length\_2970\_cov\_21.957239 593-598. Max. coverage (+): 0.42. Max coverage (-): 0

Region: NODE\_387203\_length\_2970\_cov\_21.957239 599-604. Max. coverage (+): 0.05. Max coverage (-): 0.09

Region: NODE\_387203\_length\_2970\_cov\_21.957239 605-611. Max. coverage (+): 0. Max coverage (-): 0

Region: NODE\_387203\_length\_2970\_cov\_21.957239 612-617. Max. coverage (+): 0. Max coverage (-): 0

Region: NODE\_387203\_length\_2970\_cov\_21.957239 618-623. Max. coverage (+): 0. Max coverage (-): 0

Region: NODE\_387203\_length\_2970\_cov\_21.957239 624-629. Max. coverage (+): 1.27. Max coverage (-): 0

Region: NODE\_387203\_length\_2970\_cov\_21.957239 630-635. Max. coverage (+): 5.83. Max coverage (-): 0

Region: NODE\_387203\_length\_2970\_cov\_21.957239 636-641. Max. coverage (+): 0. Max coverage (-): 0

Region: NODE\_387203\_length\_2970\_cov\_21.957239 642-647. Max. coverage (+): 0. Max coverage (-): 0

Region: NODE\_387203\_length\_2970\_cov\_21.957239 648-653. Max. coverage (+): 0. Max coverage (-): 0

Region: NODE\_387203\_length\_2970\_cov\_21.957239 654-660. Max. coverage (+): 0. Max coverage (-): 0

Region: NODE\_387203\_length\_2970\_cov\_21.957239 661-666. Max. coverage (+): 0. Max coverage (-): 0

Region: NODE\_387203\_length\_2970\_cov\_21.957239 667-672. Max. coverage (+): 0. Max coverage (-): 0

Region: NODE\_387203\_length\_2970\_cov\_21.957239 673-678. Max. coverage (+): 2.77. Max coverage (-): 0

Region: NODE\_387203\_length\_2970\_cov\_21.957239 679-684. Max. coverage (+): 0.8. Max coverage (-): 0

Region: NODE\_387203\_length\_2970\_cov\_21.957239 685-690. Max. coverage (+): 0.56. Max coverage (-): 0.09

Region: NODE\_387203\_length\_2970\_cov\_21.957239 691-696. Max. coverage (+): 0.19. Max coverage (-): 0

Region: NODE\_387203\_length\_2970\_cov\_21.957239 697-702. Max. coverage (+): 0.05. Max coverage (-): 0

Region: NODE\_387203\_length\_2970\_cov\_21.957239 703-709. Max. coverage (+): 0. Max coverage (-): 0

Region: NODE\_387203\_length\_2970\_cov\_21.957239 710-715. Max. coverage (+): 0. Max coverage (-): 0

Region: NODE\_387203\_length\_2970\_cov\_21.957239 716-721. Max. coverage (+): 0. Max coverage (-): 0

Region: NODE\_387203\_length\_2970\_cov\_21.957239 722-727. Max. coverage (+): 0. Max coverage (-): 0

Region: NODE\_387203\_length\_2970\_cov\_21.957239 728-733. Max. coverage (+): 0. Max coverage (-): 0

Region: NODE\_387203\_length\_2970\_cov\_21.957239 734-739. Max. coverage (+): 0. Max coverage (-): 0

Region: NODE\_387203\_length\_2970\_cov\_21.957239 740-745. Max. coverage (+): 0. Max coverage (-): 0

Region: NODE\_387203\_length\_2970\_cov\_21.957239 746-752. Max. coverage (+): 11.98. Max coverage (-): 0

Region: NODE\_387203\_length\_2970\_cov\_21.957239 753-758. Max. coverage (+): 0.66. Max coverage (-): 0

Region: NODE\_387203\_length\_2970\_cov\_21.957239 759-764. Max. coverage (+): 0.61. Max coverage (-): 0

Region: NODE\_387203\_length\_2970\_cov\_21.957239 765-770. Max. coverage (+): 0.14. Max coverage (-): 0

Region: NODE\_387203\_length\_2970\_cov\_21.957239 771-776. Max. coverage (+): 0. Max coverage (-): 0

Region: NODE\_387203\_length\_2970\_cov\_21.957239 777-782. Max. coverage (+): 0. Max coverage (-): 0

Region: NODE\_387203\_length\_2970\_cov\_21.957239 783-788. Max. coverage (+): 0. Max coverage (-): 0

Region: NODE\_387203\_length\_2970\_cov\_21.957239 789-794. Max. coverage (+): 0. Max coverage (-): 0

Region: NODE\_387203\_length\_2970\_cov\_21.957239 795-801. Max. coverage (+): 0. Max coverage (-): 0

Region: NODE\_387203\_length\_2970\_cov\_21.957239 802-807. Max. coverage (+): 0. Max coverage (-): 0

Region: NODE\_387203\_length\_2970\_cov\_21.957239 808-813. Max. coverage (+): 0. Max coverage (-): 0

Region: NODE\_387203\_length\_2970\_cov\_21.957239 814-819. Max. coverage (+): 0.61. Max coverage (-): 0

Region: NODE\_387203\_length\_2970\_cov\_21.957239 820-825. Max. coverage (+): 0.23. Max coverage (-): 0.09

Region: NODE\_387203\_length\_2970\_cov\_21.957239 826-831. Max. coverage (+): 0.23. Max coverage (-): 0

Region: NODE\_387203\_length\_2970\_cov\_21.957239 832-837. Max. coverage (+): 0.14. Max coverage (-): 0

Region: NODE\_387203\_length\_2970\_cov\_21.957239 838-844. Max. coverage (+): 1.17. Max coverage (-): 0.05

Region: NODE\_387203\_length\_2970\_cov\_21.957239 845-850. Max. coverage (+): 1.13. Max coverage (-): 0

Region: NODE\_387203\_length\_2970\_cov\_21.957239 851-856. Max. coverage (+): 0.89. Max coverage (-): 0

Region: NODE\_387203\_length\_2970\_cov\_21.957239 857-862. Max. coverage (+): 0.75. Max coverage (-): 0

Region: NODE\_387203\_length\_2970\_cov\_21.957239 863-868. Max. coverage (+): 1.08. Max coverage (-): 0

Region: NODE\_387203\_length\_2970\_cov\_21.957239 869-874. Max. coverage (+): 1.46. Max coverage (-): 0

Region: NODE\_387203\_length\_2970\_cov\_21.957239 875-880. Max. coverage (+): 0.56. Max coverage (-): 0

Region: NODE\_387203\_length\_2970\_cov\_21.957239 881-886. Max. coverage (+): 1.36. Max coverage (-): 0.05

Region: NODE\_387203\_length\_2970\_cov\_21.957239 887-893. Max. coverage (+): 0.14. Max coverage (-): 0.14

Region: NODE\_387203\_length\_2970\_cov\_21.957239 894-899. Max. coverage (+): 0. Max coverage (-): 0.09

Region: NODE\_387203\_length\_2970\_cov\_21.957239 900-905. Max. coverage (+): 0.23. Max coverage (-): 0

Region: NODE\_387203\_length\_2970\_cov\_21.957239 906-911. Max. coverage (+): 6.95. Max coverage (-): 0

Region: NODE\_387203\_length\_2970\_cov\_21.957239 912-917. Max. coverage (+): 0. Max coverage (-): 0

Region: NODE\_387203\_length\_2970\_cov\_21.957239 918-923. Max. coverage (+): 0. Max coverage (-): 0.61

Region: NODE\_387203\_length\_2970\_cov\_21.957239 924-929. Max. coverage (+): 0.56. Max coverage (-): 0.66

Region: NODE\_387203\_length\_2970\_cov\_21.957239 930-935. Max. coverage (+): 0.23. Max coverage (-): 0.05

Region: NODE\_387203\_length\_2970\_cov\_21.957239 936-942. Max. coverage (+): 6.2. Max coverage (-): 0

Region: NODE\_387203\_length\_2970\_cov\_21.957239 943-948. Max. coverage (+): 0.14. Max coverage (-): 0

Region: NODE\_387203\_length\_2970\_cov\_21.957239 949-954. Max. coverage (+): 0.14. Max coverage (-): 0.14

Region: NODE\_387203\_length\_2970\_cov\_21.957239 955-960. Max. coverage (+): 2.11. Max coverage (-): 0.14

Region: NODE\_387203\_length\_2970\_cov\_21.957239 961-966. Max. coverage (+): 0.28. Max coverage (-): 0.14

Region: NODE\_387203\_length\_2970\_cov\_21.957239 967-972. Max. coverage (+): 0.19. Max coverage (-): 0

Region: NODE\_387203\_length\_2970\_cov\_21.957239 973-978. Max. coverage (+): 0.28. Max coverage (-): 0

Region: NODE\_387203\_length\_2970\_cov\_21.957239 979-985. Max. coverage (+): 0.14. Max coverage (-): 0

Region: NODE\_387203\_length\_2970\_cov\_21.957239 986-991. Max. coverage (+): 0.05. Max coverage (-): 0

Region: NODE\_387203\_length\_2970\_cov\_21.957239 992-997. Max. coverage (+): 0. Max coverage (-): 0

Region: NODE\_387203\_length\_2970\_cov\_21.957239 998-1003. Max. coverage (+): 0. Max coverage (-): 0

Region: NODE\_387203\_length\_2970\_cov\_21.957239 1004-1009. Max. coverage (+): 0. Max coverage (-): 0

Region: NODE\_387203\_length\_2970\_cov\_21.957239 1010-1015. Max. coverage (+): 0. Max coverage (-): 0

Region: NODE\_387203\_length\_2970\_cov\_21.957239 1016-1021. Max. coverage (+): 0. Max coverage (-): 0

Region: NODE\_387203\_length\_2970\_cov\_21.957239 1022-1027. Max. coverage (+): 0. Max coverage (-): 0

Region: NODE\_387203\_length\_2970\_cov\_21.957239 1028-1034. Max. coverage (+): 0.05. Max coverage (-): 0

Region: NODE\_387203\_length\_2970\_cov\_21.957239 1035-1040. Max. coverage (+): 0.05. Max coverage (-): 0

Region: NODE\_387203\_length\_2970\_cov\_21.957239 1041-1046. Max. coverage (+): 0. Max coverage (-): 0

Region: NODE\_387203\_length\_2970\_cov\_21.957239 1047-1052. Max. coverage (+): 0. Max coverage (-): 0

Region: NODE\_387203\_length\_2970\_cov\_21.957239 1053-1058. Max. coverage (+): 0. Max coverage (-): 0

Region: NODE\_387203\_length\_2970\_cov\_21.957239 1059-1064. Max. coverage (+): 0. Max coverage (-): 0

Region: NODE\_387203\_length\_2970\_cov\_21.957239 1065-1070. Max. coverage (+): 0. Max coverage (-): 0

Region: NODE\_387203\_length\_2970\_cov\_21.957239 1071-1077. Max. coverage (+): 0. Max coverage (-): 0

Region: NODE\_387203\_length\_2970\_cov\_21.957239 1078-1083. Max. coverage (+): 0. Max coverage (-): 0

Region: NODE\_387203\_length\_2970\_cov\_21.957239 1084-1089. Max. coverage (+): 0. Max coverage (-): 0

Region: NODE\_387203\_length\_2970\_cov\_21.957239 1090-1095. Max. coverage (+): 0. Max coverage (-): 0

Region: NODE\_387203\_length\_2970\_cov\_21.957239 1096-1101. Max. coverage (+): 0. Max coverage (-): 0

Region: NODE\_387203\_length\_2970\_cov\_21.957239 1102-1107. Max. coverage (+): 0. Max coverage (-): 0

Region: NODE\_387203\_length\_2970\_cov\_21.957239 1108-1113. Max. coverage (+): 0.38. Max coverage (-): 0

Region: NODE\_387203\_length\_2970\_cov\_21.957239 1114-1119. Max. coverage (+): 0.23. Max coverage (-): 0

Region: NODE\_387203\_length\_2970\_cov\_21.957239 1120-1126. Max. coverage (+): 0. Max coverage (-): 0

Region: NODE\_387203\_length\_2970\_cov\_21.957239 1127-1132. Max. coverage (+): 0. Max coverage (-): 0

Region: NODE\_387203\_length\_2970\_cov\_21.957239 1133-1138. Max. coverage (+): 0. Max coverage (-): 0

Region: NODE\_387203\_length\_2970\_cov\_21.957239 1139-1144. Max. coverage (+): 0.05. Max coverage (-): 0

Region: NODE\_387203\_length\_2970\_cov\_21.957239 1145-1150. Max. coverage (+): 1.97. Max coverage (-): 0

Region: NODE\_387203\_length\_2970\_cov\_21.957239 1151-1156. Max. coverage (+): 2.26. Max coverage (-): 2.73

Region: NODE\_387203\_length\_2970\_cov\_21.957239 1157-1162. Max. coverage (+): 0.05. Max coverage (-): 1.55

Region: NODE\_387203\_length\_2970\_cov\_21.957239 1163-1169. Max. coverage (+): 0.19. Max coverage (-): 0.05

Region: NODE\_387203\_length\_2970\_cov\_21.957239 1170-1175. Max. coverage (+): 1.27. Max coverage (-): 0

Region: NODE\_387203\_length\_2970\_cov\_21.957239 1176-1181. Max. coverage (+): 1.36. Max coverage (-): 0

Region: NODE\_387203\_length\_2970\_cov\_21.957239 1182-1187. Max. coverage (+): 1.27. Max coverage (-): 0

Region: NODE\_387203\_length\_2970\_cov\_21.957239 1188-1193. Max. coverage (+): 1.13. Max coverage (-): 0

Region: NODE\_387203\_length\_2970\_cov\_21.957239 1194-1199. Max. coverage (+): 0.23. Max coverage (-): 0

Region: NODE\_387203\_length\_2970\_cov\_21.957239 1200-1205. Max. coverage (+): 0.42. Max coverage (-): 0

Region: NODE\_387203\_length\_2970\_cov\_21.957239 1206-1211. Max. coverage (+): 0.09. Max coverage (-): 0

Region: NODE\_387203\_length\_2970\_cov\_21.957239 1212-1218. Max. coverage (+): 0. Max coverage (-): 0

Region: NODE\_387203\_length\_2970\_cov\_21.957239 1219-1224. Max. coverage (+): 0. Max coverage (-): 0

Region: NODE\_387203\_length\_2970\_cov\_21.957239 1225-1230. Max. coverage (+): 0. Max coverage (-): 0

Region: NODE\_387203\_length\_2970\_cov\_21.957239 1231-1236. Max. coverage (+): 0. Max coverage (-): 0

Region: NODE\_387203\_length\_2970\_cov\_21.957239 1237-1242. Max. coverage (+): 0. Max coverage (-): 0

Region: NODE\_387203\_length\_2970\_cov\_21.957239 1243-1248. Max. coverage (+): 0. Max coverage (-): 0

Region: NODE\_387203\_length\_2970\_cov\_21.957239 1249-1254. Max. coverage (+): 0. Max coverage (-): 0

Region: NODE\_387203\_length\_2970\_cov\_21.957239 1255-1260. Max. coverage (+): 0. Max coverage (-): 0

Region: NODE\_387203\_length\_2970\_cov\_21.957239 1261-1267. Max. coverage (+): 0. Max coverage (-): 0

Region: NODE\_387203\_length\_2970\_cov\_21.957239 1268-1273. Max. coverage (+): 0. Max coverage (-): 0

Region: NODE\_387203\_length\_2970\_cov\_21.957239 1274-1279. Max. coverage (+): 0. Max coverage (-): 0

Region: NODE\_387203\_length\_2970\_cov\_21.957239 1280-1285. Max. coverage (+): 0. Max coverage (-): 0

Region: NODE\_387203\_length\_2970\_cov\_21.957239 1286-1291. Max. coverage (+): 0. Max coverage (-): 0

Region: NODE\_387203\_length\_2970\_cov\_21.957239 1292-1297. Max. coverage (+): 0. Max coverage (-): 0

Region: NODE\_387203\_length\_2970\_cov\_21.957239 1298-1303. Max. coverage (+): 0. Max coverage (-): 0

Region: NODE\_387203\_length\_2970\_cov\_21.957239 1304-1310. Max. coverage (+): 0. Max coverage (-): 0

Region: NODE\_387203\_length\_2970\_cov\_21.957239 1311-1316. Max. coverage (+): 0. Max coverage (-): 0

Region: NODE\_387203\_length\_2970\_cov\_21.957239 1317-1322. Max. coverage (+): 0. Max coverage (-): 0

Region: NODE\_387203\_length\_2970\_cov\_21.957239 1323-1328. Max. coverage (+): 0. Max coverage (-): 0

Region: NODE\_387203\_length\_2970\_cov\_21.957239 1329-1334. Max. coverage (+): 0.05. Max coverage (-): 0

Region: NODE\_387203\_length\_2970\_cov\_21.957239 1335-1340. Max. coverage (+): 0.05. Max coverage (-): 0

Region: NODE\_387203\_length\_2970\_cov\_21.957239 1341-1346. Max. coverage (+): 0. Max coverage (-): 0

Region: NODE\_387203\_length\_2970\_cov\_21.957239 1347-1352. Max. coverage (+): 0. Max coverage (-): 0

Region: NODE\_387203\_length\_2970\_cov\_21.957239 1353-1359. Max. coverage (+): 0. Max coverage (-): 0

Region: NODE\_387203\_length\_2970\_cov\_21.957239 1360-1365. Max. coverage (+): 0. Max coverage (-): 0

Region: NODE\_387203\_length\_2970\_cov\_21.957239 1366-1371. Max. coverage (+): 1.55. Max coverage (-): 0

Region: NODE\_387203\_length\_2970\_cov\_21.957239 1372-1377. Max. coverage (+): 0.94. Max coverage (-): 0

Region: NODE\_387203\_length\_2970\_cov\_21.957239 1378-1383. Max. coverage (+): 0.05. Max coverage (-): 0.7

Region: NODE\_387203\_length\_2970\_cov\_21.957239 1384-1389. Max. coverage (+): 0.09. Max coverage (-): 2.16

Region: NODE\_387203\_length\_2970\_cov\_21.957239 1390-1395. Max. coverage (+): 0.14. Max coverage (-): 0.09

Region: NODE\_387203\_length\_2970\_cov\_21.957239 1396-1402. Max. coverage (+): 6.25. Max coverage (-): 0

Region: NODE\_387203\_length\_2970\_cov\_21.957239 1403-1408. Max. coverage (+): 1.22. Max coverage (-): 0.23

Region: NODE\_387203\_length\_2970\_cov\_21.957239 1409-1414. Max. coverage (+): 1.41. Max coverage (-): 0

Region: NODE\_387203\_length\_2970\_cov\_21.957239 1415-1420. Max. coverage (+): 0. Max coverage (-): 0.33

Region: NODE\_387203\_length\_2970\_cov\_21.957239 1421-1426. Max. coverage (+): 0. Max coverage (-): 0.19

Region: NODE\_387203\_length\_2970\_cov\_21.957239 1427-1432. Max. coverage (+): 0. Max coverage (-): 0

Region: NODE\_387203\_length\_2970\_cov\_21.957239 1433-1438. Max. coverage (+): 0. Max coverage (-): 0

Region: NODE\_387203\_length\_2970\_cov\_21.957239 1439-1444. Max. coverage (+): 0. Max coverage (-): 0

Region: NODE\_387203\_length\_2970\_cov\_21.957239 1445-1451. Max. coverage (+): 0. Max coverage (-): 0

Region: NODE\_387203\_length\_2970\_cov\_21.957239 1452-1457. Max. coverage (+): 0. Max coverage (-): 0

Region: NODE\_387203\_length\_2970\_cov\_21.957239 1458-1463. Max. coverage (+): 0. Max coverage (-): 0

Region: NODE\_387203\_length\_2970\_cov\_21.957239 1464-1469. Max. coverage (+): 0. Max coverage (-): 0

Region: NODE\_387203\_length\_2970\_cov\_21.957239 1470-1475. Max. coverage (+): 0. Max coverage (-): 0

Region: NODE\_387203\_length\_2970\_cov\_21.957239 1476-1481. Max. coverage (+): 0. Max coverage (-): 0

Region: NODE\_387203\_length\_2970\_cov\_21.957239 1482-1487. Max. coverage (+): 0. Max coverage (-): 0

Region: NODE\_387203\_length\_2970\_cov\_21.957239 1488-1494. Max. coverage (+): 0. Max coverage (-): 0

Region: NODE\_387203\_length\_2970\_cov\_21.957239 1495-1500. Max. coverage (+): 0.05. Max coverage (-): 0

Region: NODE\_387203\_length\_2970\_cov\_21.957239 1501-1506. Max. coverage (+): 0.05. Max coverage (-): 0

Region: NODE\_387203\_length\_2970\_cov\_21.957239 1507-1512. Max. coverage (+): 0.05. Max coverage (-): 0

Region: NODE\_387203\_length\_2970\_cov\_21.957239 1513-1518. Max. coverage (+): 0. Max coverage (-): 0

Region: NODE\_387203\_length\_2970\_cov\_21.957239 1519-1524. Max. coverage (+): 0. Max coverage (-): 0

Region: NODE\_387203\_length\_2970\_cov\_21.957239 1525-1530. Max. coverage (+): 0. Max coverage (-): 0

Region: NODE\_387203\_length\_2970\_cov\_21.957239 1531-1536. Max. coverage (+): 0. Max coverage (-): 0

Region: NODE\_387203\_length\_2970\_cov\_21.957239 1537-1543. Max. coverage (+): 0. Max coverage (-): 0

Region: NODE\_387203\_length\_2970\_cov\_21.957239 1544-1549. Max. coverage (+): 0. Max coverage (-): 0

Region: NODE\_387203\_length\_2970\_cov\_21.957239 1550-1555. Max. coverage (+): 0. Max coverage (-): 0

Region: NODE\_387203\_length\_2970\_cov\_21.957239 1556-1561. Max. coverage (+): 0. Max coverage (-): 0

Region: NODE\_387203\_length\_2970\_cov\_21.957239 1562-1567. Max. coverage (+): 0. Max coverage (-): 0

Region: NODE\_387203\_length\_2970\_cov\_21.957239 1568-1573. Max. coverage (+): 0. Max coverage (-): 0

Region: NODE\_387203\_length\_2970\_cov\_21.957239 1574-1579. Max. coverage (+): 0. Max coverage (-): 0

Region: NODE\_387203\_length\_2970\_cov\_21.957239 1580-1585. Max. coverage (+): 0. Max coverage (-): 0

Region: NODE\_387203\_length\_2970\_cov\_21.957239 1586-1592. Max. coverage (+): 0. Max coverage (-): 0

Region: NODE\_387203\_length\_2970\_cov\_21.957239 1593-1598. Max. coverage (+): 0. Max coverage (-): 0

Region: NODE\_387203\_length\_2970\_cov\_21.957239 1599-1604. Max. coverage (+): 0. Max coverage (-): 0

Region: NODE\_387203\_length\_2970\_cov\_21.957239 1605-1610. Max. coverage (+): 0.23. Max coverage (-): 0

Region: NODE\_387203\_length\_2970\_cov\_21.957239 1611-1616. Max. coverage (+): 0.33. Max coverage (-): 0

Region: NODE\_387203\_length\_2970\_cov\_21.957239 1617-1622. Max. coverage (+): 0. Max coverage (-): 0

Region: NODE\_387203\_length\_2970\_cov\_21.957239 1623-1628. Max. coverage (+): 0. Max coverage (-): 0

Region: NODE\_387203\_length\_2970\_cov\_21.957239 1629-1635. Max. coverage (+): 0. Max coverage (-): 0

Region: NODE\_387203\_length\_2970\_cov\_21.957239 1636-1641. Max. coverage (+): 0. Max coverage (-): 0

Region: NODE\_387203\_length\_2970\_cov\_21.957239 1642-1647. Max. coverage (+): 4.32. Max coverage (-): 0.05

Region: NODE\_387203\_length\_2970\_cov\_21.957239 1648-1653. Max. coverage (+): 4.37. Max coverage (-): 0.09

Region: NODE\_387203\_length\_2970\_cov\_21.957239 1654-1659. Max. coverage (+): 2.96. Max coverage (-): 0

Region: NODE\_387203\_length\_2970\_cov\_21.957239 1660-1665. Max. coverage (+): 0.38. Max coverage (-): 0

Region: NODE\_387203\_length\_2970\_cov\_21.957239 1666-1671. Max. coverage (+): 0.19. Max coverage (-): 0

Region: NODE\_387203\_length\_2970\_cov\_21.957239 1672-1677. Max. coverage (+): 0. Max coverage (-): 0

Region: NODE\_387203\_length\_2970\_cov\_21.957239 1678-1684. Max. coverage (+): 0. Max coverage (-): 0

Region: NODE\_387203\_length\_2970\_cov\_21.957239 1685-1690. Max. coverage (+): 0. Max coverage (-): 0

Region: NODE\_387203\_length\_2970\_cov\_21.957239 1691-1696. Max. coverage (+): 0. Max coverage (-): 0

Region: NODE\_387203\_length\_2970\_cov\_21.957239 1697-1702. Max. coverage (+): 0. Max coverage (-): 0

Region: NODE\_387203\_length\_2970\_cov\_21.957239 1703-1708. Max. coverage (+): 0. Max coverage (-): 0

Region: NODE\_387203\_length\_2970\_cov\_21.957239 1709-1714. Max. coverage (+): 0. Max coverage (-): 0

Region: NODE\_387203\_length\_2970\_cov\_21.957239 1715-1720. Max. coverage (+): 0. Max coverage (-): 0

Region: NODE\_387203\_length\_2970\_cov\_21.957239 1721-1727. Max. coverage (+): 0. Max coverage (-): 0

Region: NODE\_387203\_length\_2970\_cov\_21.957239 1728-1733. Max. coverage (+): 0. Max coverage (-): 0

Region: NODE\_387203\_length\_2970\_cov\_21.957239 1734-1739. Max. coverage (+): 0. Max coverage (-): 0

Region: NODE\_387203\_length\_2970\_cov\_21.957239 1740-1745. Max. coverage (+): 0.19. Max coverage (-): 0

Region: NODE\_387203\_length\_2970\_cov\_21.957239 1746-1751. Max. coverage (+): 1.69. Max coverage (-): 0.05

Region: NODE\_387203\_length\_2970\_cov\_21.957239 1752-1757. Max. coverage (+): 1.79. Max coverage (-): 0.05

Region: NODE\_387203\_length\_2970\_cov\_21.957239 1758-1763. Max. coverage (+): 0.8. Max coverage (-): 0.28

Region: NODE\_387203\_length\_2970\_cov\_21.957239 1764-1769. Max. coverage (+): 0.89. Max coverage (-): 0.28

Region: NODE\_387203\_length\_2970\_cov\_21.957239 1770-1776. Max. coverage (+): 0.05. Max coverage (-): 0.09

Region: NODE\_387203\_length\_2970\_cov\_21.957239 1777-1782. Max. coverage (+): 0.09. Max coverage (-): 0.09

Region: NODE\_387203\_length\_2970\_cov\_21.957239 1783-1788. Max. coverage (+): 2.73. Max coverage (-): 0

Region: NODE\_387203\_length\_2970\_cov\_21.957239 1789-1794. Max. coverage (+): 1.64. Max coverage (-): 0

Region: NODE\_387203\_length\_2970\_cov\_21.957239 1795-1800. Max. coverage (+): 1.32. Max coverage (-): 0.19

Region: NODE\_387203\_length\_2970\_cov\_21.957239 1801-1806. Max. coverage (+): 1.97. Max coverage (-): 0.14

Region: NODE\_387203\_length\_2970\_cov\_21.957239 1807-1812. Max. coverage (+): 1.97. Max coverage (-): 0.19

Region: NODE\_387203\_length\_2970\_cov\_21.957239 1813-1819. Max. coverage (+): 0.8. Max coverage (-): 0.19

Region: NODE\_387203\_length\_2970\_cov\_21.957239 1820-1825. Max. coverage (+): 0. Max coverage (-): 0

Region: NODE\_387203\_length\_2970\_cov\_21.957239 1826-1831. Max. coverage (+): 0. Max coverage (-): 0

Region: NODE\_387203\_length\_2970\_cov\_21.957239 1832-1837. Max. coverage (+): 0. Max coverage (-): 0

Region: NODE\_387203\_length\_2970\_cov\_21.957239 1838-1843. Max. coverage (+): 0. Max coverage (-): 0

Region: NODE\_387203\_length\_2970\_cov\_21.957239 1844-1849. Max. coverage (+): 0. Max coverage (-): 0

Region: NODE\_387203\_length\_2970\_cov\_21.957239 1850-1855. Max. coverage (+): 0. Max coverage (-): 0

Region: NODE\_387203\_length\_2970\_cov\_21.957239 1856-1861. Max. coverage (+): 0.14. Max coverage (-): 0

Region: NODE\_387203\_length\_2970\_cov\_21.957239 1862-1868. Max. coverage (+): 0.66. Max coverage (-): 0.05

Region: NODE\_387203\_length\_2970\_cov\_21.957239 1869-1874. Max. coverage (+): 2.44. Max coverage (-): 0.09

Region: NODE\_387203\_length\_2970\_cov\_21.957239 1875-1880. Max. coverage (+): 1.97. Max coverage (-): 0

Region: NODE\_387203\_length\_2970\_cov\_21.957239 1881-1886. Max. coverage (+): 0.33. Max coverage (-): 0

Region: NODE\_387203\_length\_2970\_cov\_21.957239 1887-1892. Max. coverage (+): 0.47. Max coverage (-): 0.09

Region: NODE\_387203\_length\_2970\_cov\_21.957239 1893-1898. Max. coverage (+): 0.14. Max coverage (-): 0.09

Region: NODE\_387203\_length\_2970\_cov\_21.957239 1899-1904. Max. coverage (+): 0.38. Max coverage (-): 0.09

Region: NODE\_387203\_length\_2970\_cov\_21.957239 1905-1910. Max. coverage (+): 0.09. Max coverage (-): 0

Region: NODE\_387203\_length\_2970\_cov\_21.957239 1911-1917. Max. coverage (+): 3.85. Max coverage (-): 0

Region: NODE\_387203\_length\_2970\_cov\_21.957239 1918-1923. Max. coverage (+): 2.91. Max coverage (-): 0

Region: NODE\_387203\_length\_2970\_cov\_21.957239 1924-1929. Max. coverage (+): 0. Max coverage (-): 0.09

Region: NODE\_387203\_length\_2970\_cov\_21.957239 1930-1935. Max. coverage (+): 0.05. Max coverage (-): 3.57

Region: NODE\_387203\_length\_2970\_cov\_21.957239 1936-1941. Max. coverage (+): 0. Max coverage (-): 3.62

Region: NODE\_387203\_length\_2970\_cov\_21.957239 1942-1947. Max. coverage (+): 2.73. Max coverage (-): 0.42

Region: NODE\_387203\_length\_2970\_cov\_21.957239 1948-1953. Max. coverage (+): 4.89. Max coverage (-): 0.38

Region: NODE\_387203\_length\_2970\_cov\_21.957239 1954-1960. Max. coverage (+): 5.54. Max coverage (-): 0

Region: NODE\_387203\_length\_2970\_cov\_21.957239 1961-1966. Max. coverage (+): 0.19. Max coverage (-): 0

Region: NODE\_387203\_length\_2970\_cov\_21.957239 1967-1972. Max. coverage (+): 0.23. Max coverage (-): 0

Region: NODE\_387203\_length\_2970\_cov\_21.957239 1973-1978. Max. coverage (+): 1.83. Max coverage (-): 0.05

Region: NODE\_387203\_length\_2970\_cov\_21.957239 1979-1984. Max. coverage (+): 3.48. Max coverage (-): 0

Region: NODE\_387203\_length\_2970\_cov\_21.957239 1985-1990. Max. coverage (+): 3.05. Max coverage (-): 0

Region: NODE\_387203\_length\_2970\_cov\_21.957239 1991-1996. Max. coverage (+): 1.36. Max coverage (-): 0

Region: NODE\_387203\_length\_2970\_cov\_21.957239 1997-2002. Max. coverage (+): 0.05. Max coverage (-): 0.85

Region: NODE\_387203\_length\_2970\_cov\_21.957239 2003-2009. Max. coverage (+): 0.09. Max coverage (-): 0.89

Region: NODE\_387203\_length\_2970\_cov\_21.957239 2010-2015. Max. coverage (+): 0. Max coverage (-): 0

Region: NODE\_387203\_length\_2970\_cov\_21.957239 2016-2021. Max. coverage (+): 16.96. Max coverage (-): 0

Region: NODE\_387203\_length\_2970\_cov\_21.957239 2022-2027. Max. coverage (+): 16.96. Max coverage (-): 0

Region: NODE\_387203\_length\_2970\_cov\_21.957239 2028-2033. Max. coverage (+): 2.96. Max coverage (-): 0

Region: NODE\_387203\_length\_2970\_cov\_21.957239 2034-2039. Max. coverage (+): 0.33. Max coverage (-): 0

Region: NODE\_387203\_length\_2970\_cov\_21.957239 2040-2045. Max. coverage (+): 0.33. Max coverage (-): 0.19

Region: NODE\_387203\_length\_2970\_cov\_21.957239 2046-2052. Max. coverage (+): 0. Max coverage (-): 0.28

Region: NODE\_387203\_length\_2970\_cov\_21.957239 2053-2058. Max. coverage (+): 0.28. Max coverage (-): 0

Region: NODE\_387203\_length\_2970\_cov\_21.957239 2059-2064. Max. coverage (+): 0.56. Max coverage (-): 0

Region: NODE\_387203\_length\_2970\_cov\_21.957239 2065-2070. Max. coverage (+): 0.8. Max coverage (-): 0

Region: NODE\_387203\_length\_2970\_cov\_21.957239 2071-2076. Max. coverage (+): 0.09. Max coverage (-): 0.05

Region: NODE\_387203\_length\_2970\_cov\_21.957239 2077-2082. Max. coverage (+): 0.05. Max coverage (-): 0.05

Region: NODE\_387203\_length\_2970\_cov\_21.957239 2083-2088. Max. coverage (+): 0.19. Max coverage (-): 0

Region: NODE\_387203\_length\_2970\_cov\_21.957239 2089-2094. Max. coverage (+): 1.69. Max coverage (-): 0

Region: NODE\_387203\_length\_2970\_cov\_21.957239 2095-2101. Max. coverage (+): 1.22. Max coverage (-): 0

Region: NODE\_387203\_length\_2970\_cov\_21.957239 2102-2107. Max. coverage (+): 0.33. Max coverage (-): 0

Region: NODE\_387203\_length\_2970\_cov\_21.957239 2108-2113. Max. coverage (+): 0.09. Max coverage (-): 0.05

Region: NODE\_387203\_length\_2970\_cov\_21.957239 2114-2119. Max. coverage (+): 0.42. Max coverage (-): 0.09

Region: NODE\_387203\_length\_2970\_cov\_21.957239 2120-2125. Max. coverage (+): 12.73. Max coverage (-): 0.09

Region: NODE\_387203\_length\_2970\_cov\_21.957239 2126-2131. Max. coverage (+): 38.1. Max coverage (-): 0.05

Region: NODE\_387203\_length\_2970\_cov\_21.957239 2132-2137. Max. coverage (+): 3.85. Max coverage (-): 0.23

Region: NODE\_387203\_length\_2970\_cov\_21.957239 2138-2144. Max. coverage (+): 0.52. Max coverage (-): 0.23

Region: NODE\_387203\_length\_2970\_cov\_21.957239 2145-2150. Max. coverage (+): 0.05. Max coverage (-): 0.23

Region: NODE\_387203\_length\_2970\_cov\_21.957239 2151-2156. Max. coverage (+): 0.52. Max coverage (-): 0.14

Region: NODE\_387203\_length\_2970\_cov\_21.957239 2157-2162. Max. coverage (+): 0.05. Max coverage (-): 1.32

Region: NODE\_387203\_length\_2970\_cov\_21.957239 2163-2168. Max. coverage (+): 1.32. Max coverage (-): 1.08

Region: NODE\_387203\_length\_2970\_cov\_21.957239 2169-2174. Max. coverage (+): 0.14. Max coverage (-): 0.05

Region: NODE\_387203\_length\_2970\_cov\_21.957239 2175-2180. Max. coverage (+): 1.55. Max coverage (-): 0.05

Region: NODE\_387203\_length\_2970\_cov\_21.957239 2181-2186. Max. coverage (+): 1.46. Max coverage (-): 0.05

Region: NODE\_387203\_length\_2970\_cov\_21.957239 2187-2193. Max. coverage (+): 0.19. Max coverage (-): 0

Region: NODE\_387203\_length\_2970\_cov\_21.957239 2194-2199. Max. coverage (+): 0.28. Max coverage (-): 0.05

Region: NODE\_387203\_length\_2970\_cov\_21.957239 2200-2205. Max. coverage (+): 0.23. Max coverage (-): 0.05

Region: NODE\_387203\_length\_2970\_cov\_21.957239 2206-2211. Max. coverage (+): 0. Max coverage (-): 0

Region: NODE\_387203\_length\_2970\_cov\_21.957239 2212-2217. Max. coverage (+): 0. Max coverage (-): 0

Region: NODE\_387203\_length\_2970\_cov\_21.957239 2218-2223. Max. coverage (+): 0. Max coverage (-): 0

Region: NODE\_387203\_length\_2970\_cov\_21.957239 2224-2229. Max. coverage (+): 2.49. Max coverage (-): 0

Region: NODE\_387203\_length\_2970\_cov\_21.957239 2230-2235. Max. coverage (+): 8.6. Max coverage (-): 0

Region: NODE\_387203\_length\_2970\_cov\_21.957239 2236-2242. Max. coverage (+): 2.77. Max coverage (-): 0

Region: NODE\_387203\_length\_2970\_cov\_21.957239 2243-2248. Max. coverage (+): 0.7. Max coverage (-): 0.09

Region: NODE\_387203\_length\_2970\_cov\_21.957239 2249-2254. Max. coverage (+): 0.33. Max coverage (-): 0.09

Region: NODE\_387203\_length\_2970\_cov\_21.957239 2255-2260. Max. coverage (+): 0. Max coverage (-): 0

Region: NODE\_387203\_length\_2970\_cov\_21.957239 2261-2266. Max. coverage (+): 0. Max coverage (-): 0

Region: NODE\_387203\_length\_2970\_cov\_21.957239 2267-2272. Max. coverage (+): 0. Max coverage (-): 0

Region: NODE\_387203\_length\_2970\_cov\_21.957239 2273-2278. Max. coverage (+): 0.05. Max coverage (-): 0

Region: NODE\_387203\_length\_2970\_cov\_21.957239 2279-2285. Max. coverage (+): 0.09. Max coverage (-): 0

Region: NODE\_387203\_length\_2970\_cov\_21.957239 2286-2291. Max. coverage (+): 0.05. Max coverage (-): 0.09

Region: NODE\_387203\_length\_2970\_cov\_21.957239 2292-2297. Max. coverage (+): 0.19. Max coverage (-): 0.19

Region: NODE\_387203\_length\_2970\_cov\_21.957239 2298-2303. Max. coverage (+): 0.61. Max coverage (-): 0.14

Region: NODE\_387203\_length\_2970\_cov\_21.957239 2304-2309. Max. coverage (+): 2.87. Max coverage (-): 0.14

Region: NODE\_387203\_length\_2970\_cov\_21.957239 2310-2315. Max. coverage (+): 1.27. Max coverage (-): 0

Region: NODE\_387203\_length\_2970\_cov\_21.957239 2316-2321. Max. coverage (+): 0.14. Max coverage (-): 0

Region: NODE\_387203\_length\_2970\_cov\_21.957239 2322-2327. Max. coverage (+): 0. Max coverage (-): 0

Region: NODE\_387203\_length\_2970\_cov\_21.957239 2328-2334. Max. coverage (+): 0. Max coverage (-): 0

Region: NODE\_387203\_length\_2970\_cov\_21.957239 2335-2340. Max. coverage (+): 0.05. Max coverage (-): 0

Region: NODE\_387203\_length\_2970\_cov\_21.957239 2341-2346. Max. coverage (+): 0.09. Max coverage (-): 0.05

Region: NODE\_387203\_length\_2970\_cov\_21.957239 2347-2352. Max. coverage (+): 0.33. Max coverage (-): 0

Region: NODE\_387203\_length\_2970\_cov\_21.957239 2353-2358. Max. coverage (+): 1.1. Max coverage (-): 0

Region: NODE\_387203\_length\_2970\_cov\_21.957239 2359-2364. Max. coverage (+): 6.08. Max coverage (-): 0.03

Region: NODE\_387203\_length\_2970\_cov\_21.957239 2365-2370. Max. coverage (+): 1.06. Max coverage (-): 0.03

Region: NODE\_387203\_length\_2970\_cov\_21.957239 2371-2377. Max. coverage (+): 1.28. Max coverage (-): 0

Region: NODE\_387203\_length\_2970\_cov\_21.957239 2378-2383. Max. coverage (+): 0.5. Max coverage (-): 0

Region: NODE\_387203\_length\_2970\_cov\_21.957239 2384-2389. Max. coverage (+): 5.95. Max coverage (-): 0

Region: NODE\_387203\_length\_2970\_cov\_21.957239 2390-2395. Max. coverage (+): 6.01. Max coverage (-): 0

Region: NODE\_387203\_length\_2970\_cov\_21.957239 2396-2401. Max. coverage (+): 0.17. Max coverage (-): 0

Region: NODE\_387203\_length\_2970\_cov\_21.957239 2402-2407. Max. coverage (+): 0. Max coverage (-): 0

Region: NODE\_387203\_length\_2970\_cov\_21.957239 2408-2413. Max. coverage (+): 0. Max coverage (-): 0

Region: NODE\_387203\_length\_2970\_cov\_21.957239 2414-2419. Max. coverage (+): 0. Max coverage (-): 0

Region: NODE\_387203\_length\_2970\_cov\_21.957239 2420-2426. Max. coverage (+): 0.34. Max coverage (-): 0

Region: NODE\_387203\_length\_2970\_cov\_21.957239 2427-2432. Max. coverage (+): 0.53. Max coverage (-): 0

Region: NODE\_387203\_length\_2970\_cov\_21.957239 2433-2438. Max. coverage (+): 0.04. Max coverage (-): 0

Region: NODE\_387203\_length\_2970\_cov\_21.957239 2439-2444. Max. coverage (+): 0. Max coverage (-): 0

Region: NODE\_387203\_length\_2970\_cov\_21.957239 2445-2450. Max. coverage (+): 0. Max coverage (-): 0

Region: NODE\_387203\_length\_2970\_cov\_21.957239 2451-2456. Max. coverage (+): 0. Max coverage (-): 0

Region: NODE\_387203\_length\_2970\_cov\_21.957239 2457-2462. Max. coverage (+): 0. Max coverage (-): 0

Region: NODE\_387203\_length\_2970\_cov\_21.957239 2463-2468. Max. coverage (+): 0. Max coverage (-): 0

Region: NODE\_387203\_length\_2970\_cov\_21.957239 2469-2475. Max. coverage (+): 0. Max coverage (-): 0

Region: NODE\_387203\_length\_2970\_cov\_21.957239 2476-2481. Max. coverage (+): 0. Max coverage (-): 0

Region: NODE\_387203\_length\_2970\_cov\_21.957239 2482-2487. Max. coverage (+): 0. Max coverage (-): 0

Region: NODE\_387203\_length\_2970\_cov\_21.957239 2488-2493. Max. coverage (+): 0. Max coverage (-): 0

Region: NODE\_387203\_length\_2970\_cov\_21.957239 2494-2499. Max. coverage (+): 0.05. Max coverage (-): 0

Region: NODE\_387203\_length\_2970\_cov\_21.957239 2500-2505. Max. coverage (+): 0.05. Max coverage (-): 0

Region: NODE\_387203\_length\_2970\_cov\_21.957239 2506-2511. Max. coverage (+): 0. Max coverage (-): 0

Region: NODE\_387203\_length\_2970\_cov\_21.957239 2512-2518. Max. coverage (+): 0. Max coverage (-): 0

Region: NODE\_387203\_length\_2970\_cov\_21.957239 2519-2524. Max. coverage (+): 0. Max coverage (-): 0

Region: NODE\_387203\_length\_2970\_cov\_21.957239 2525-2530. Max. coverage (+): 0.05. Max coverage (-): 0

Region: NODE\_387203\_length\_2970\_cov\_21.957239 2531-2536. Max. coverage (+): 0. Max coverage (-): 0

Region: NODE\_387203\_length\_2970\_cov\_21.957239 2537-2542. Max. coverage (+): 0. Max coverage (-): 0.09

Region: NODE\_387203\_length\_2970\_cov\_21.957239 2543-2548. Max. coverage (+): 0. Max coverage (-): 0.14

Region: NODE\_387203\_length\_2970\_cov\_21.957239 2549-2554. Max. coverage (+): 0. Max coverage (-): 0

Region: NODE\_387203\_length\_2970\_cov\_21.957239 2555-2560. Max. coverage (+): 0.33. Max coverage (-): 0

Region: NODE\_387203\_length\_2970\_cov\_21.957239 2561-2567. Max. coverage (+): 0.52. Max coverage (-): 0

Region: NODE\_387203\_length\_2970\_cov\_21.957239 2568-2573. Max. coverage (+): 0.61. Max coverage (-): 0.09

Region: NODE\_387203\_length\_2970\_cov\_21.957239 2574-2579. Max. coverage (+): 0. Max coverage (-): 0

Region: NODE\_387203\_length\_2970\_cov\_21.957239 2580-2585. Max. coverage (+): 0.19. Max coverage (-): 0

Region: NODE\_387203\_length\_2970\_cov\_21.957239 2586-2591. Max. coverage (+): 0.05. Max coverage (-): 0

Region: NODE\_387203\_length\_2970\_cov\_21.957239 2592-2597. Max. coverage (+): 0. Max coverage (-): 0

Region: NODE\_387203\_length\_2970\_cov\_21.957239 2598-2603. Max. coverage (+): 0. Max coverage (-): 0

Region: NODE\_387203\_length\_2970\_cov\_21.957239 2604-2610. Max. coverage (+): 0.05. Max coverage (-): 0.66

Region: NODE\_387203\_length\_2970\_cov\_21.957239 2611-2616. Max. coverage (+): 0.09. Max coverage (-): 0.05

Region: NODE\_387203\_length\_2970\_cov\_21.957239 2617-2622. Max. coverage (+): 0.09. Max coverage (-): 0.03

Region: NODE\_387203\_length\_2970\_cov\_21.957239 2623-2628. Max. coverage (+): 6.08. Max coverage (-): 0.03

Region: NODE\_387203\_length\_2970\_cov\_21.957239 2629-2634. Max. coverage (+): 6.01. Max coverage (-): 0.05

Region: NODE\_387203\_length\_2970\_cov\_21.957239 2635-2640. Max. coverage (+): 0.16. Max coverage (-): 0.25

Region: NODE\_387203\_length\_2970\_cov\_21.957239 2641-2646. Max. coverage (+): 0. Max coverage (-): 0

Region: NODE\_387203\_length\_2970\_cov\_21.957239 2647-2652. Max. coverage (+): 0. Max coverage (-): 0

Region: NODE\_387203\_length\_2970\_cov\_21.957239 2653-2659. Max. coverage (+): 0. Max coverage (-): 0

Region: NODE\_387203\_length\_2970\_cov\_21.957239 2660-2665. Max. coverage (+): 0. Max coverage (-): 0

Region: NODE\_387203\_length\_2970\_cov\_21.957239 2666-2671. Max. coverage (+): 0. Max coverage (-): 0.16

Region: NODE\_387203\_length\_2970\_cov\_21.957239 2672-2677. Max. coverage (+): 0. Max coverage (-): 0.88

Region: NODE\_387203\_length\_2970\_cov\_21.957239 2678-2683. Max. coverage (+): 0.06. Max coverage (-): 0.06

Region: NODE\_387203\_length\_2970\_cov\_21.957239 2684-2689. Max. coverage (+): 0.09. Max coverage (-): 0

Region: NODE\_387203\_length\_2970\_cov\_21.957239 2690-2695. Max. coverage (+): 2.76. Max coverage (-): 0

Region: NODE\_387203\_length\_2970\_cov\_21.957239 2696-2702. Max. coverage (+): 0. Max coverage (-): 0

Region: NODE\_387203\_length\_2970\_cov\_21.957239 2703-2708. Max. coverage (+): 0. Max coverage (-): 0.09

Region: NODE\_387203\_length\_2970\_cov\_21.957239 2709-2714. Max. coverage (+): 0. Max coverage (-): 0.09

Region: NODE\_387203\_length\_2970\_cov\_21.957239 2715-2720. Max. coverage (+): 0. Max coverage (-): 0

Region: NODE\_387203\_length\_2970\_cov\_21.957239 2721-2726. Max. coverage (+): 0.7. Max coverage (-): 0

Region: NODE\_387203\_length\_2970\_cov\_21.957239 2727-2732. Max. coverage (+): 0.8. Max coverage (-): 0

Region: NODE\_387203\_length\_2970\_cov\_21.957239 2733-2738. Max. coverage (+): 0.05. Max coverage (-): 0

Region: NODE\_387203\_length\_2970\_cov\_21.957239 2739-2744. Max. coverage (+): 0.05. Max coverage (-): 0

Region: NODE\_387203\_length\_2970\_cov\_21.957239 2745-2751. Max. coverage (+): 0.05. Max coverage (-): 0

Region: NODE\_387203\_length\_2970\_cov\_21.957239 2752-2757. Max. coverage (+): 0. Max coverage (-): 0

Region: NODE\_387203\_length\_2970\_cov\_21.957239 2758-2763. Max. coverage (+): 0. Max coverage (-): 0

Region: NODE\_387203\_length\_2970\_cov\_21.957239 2764-2769. Max. coverage (+): 0.19. Max coverage (-): 0

Region: NODE\_387203\_length\_2970\_cov\_21.957239 2770-2775. Max. coverage (+): 0.19. Max coverage (-): 0.05

Region: NODE\_387203\_length\_2970\_cov\_21.957239 2776-2781. Max. coverage (+): 0.09. Max coverage (-): 0

Region: NODE\_387203\_length\_2970\_cov\_21.957239 2782-2787. Max. coverage (+): 0.05. Max coverage (-): 0

Region: NODE\_387203\_length\_2970\_cov\_21.957239 2788-2793. Max. coverage (+): 0. Max coverage (-): 0

Region: NODE\_387203\_length\_2970\_cov\_21.957239 2794-2800. Max. coverage (+): 0. Max coverage (-): 0.05

Region: NODE\_387203\_length\_2970\_cov\_21.957239 2801-2806. Max. coverage (+): 0. Max coverage (-): 0

Region: NODE\_387203\_length\_2970\_cov\_21.957239 2807-2812. Max. coverage (+): 0. Max coverage (-): 0

Region: NODE\_387203\_length\_2970\_cov\_21.957239 2813-2818. Max. coverage (+): 0. Max coverage (-): 0.6

Region: NODE\_387203\_length\_2970\_cov\_21.957239 2819-2824. Max. coverage (+): 0. Max coverage (-): 0.34

Region: NODE\_387203\_length\_2970\_cov\_21.957239 2825-2830. Max. coverage (+): 0.03. Max coverage (-): 0.03

Region: NODE\_387203\_length\_2970\_cov\_21.957239 2831-2836. Max. coverage (+): 0.16. Max coverage (-): 0

Region: NODE\_387203\_length\_2970\_cov\_21.957239 2837-2843. Max. coverage (+): 0.16. Max coverage (-): 0.06

Region: NODE\_387203\_length\_2970\_cov\_21.957239 2844-2849. Max. coverage (+): 0.09. Max coverage (-): 0.06

Region: NODE\_387203\_length\_2970\_cov\_21.957239 2850-2855. Max. coverage (+): 0.09. Max coverage (-): 0.09

Region: NODE\_387203\_length\_2970\_cov\_21.957239 2856-2861. Max. coverage (+): 0.05. Max coverage (-): 0.05

Region: NODE\_387203\_length\_2970\_cov\_21.957239 2862-2867. Max. coverage (+): 0.19. Max coverage (-): 0

Region: NODE\_387203\_length\_2970\_cov\_21.957239 2868-2873. Max. coverage (+): 0.19. Max coverage (-): 0

Region: NODE\_387203\_length\_2970\_cov\_21.957239 2874-2879. Max. coverage (+): 0.09. Max coverage (-): 0

Region: NODE\_387203\_length\_2970\_cov\_21.957239 2880-2885. Max. coverage (+): 0. Max coverage (-): 0

Region: NODE\_387203\_length\_2970\_cov\_21.957239 2886-2892. Max. coverage (+): 0. Max coverage (-): 0

Region: NODE\_387203\_length\_2970\_cov\_21.957239 2893-2898. Max. coverage (+): 0.06. Max coverage (-): 0

Region: NODE\_387203\_length\_2970\_cov\_21.957239 2899-2904. Max. coverage (+): 0.03. Max coverage (-): 0.03

Region: NODE\_387203\_length\_2970\_cov\_21.957239 2905-2910. Max. coverage (+): 0.19. Max coverage (-): 0.03

Region: NODE\_387203\_length\_2970\_cov\_21.957239 2911-2916. Max. coverage (+): 0.38. Max coverage (-): 0

Region: NODE\_387203\_length\_2970\_cov\_21.957239 2917-2922. Max. coverage (+): 0. Max coverage (-): 0

Region: NODE\_387203\_length\_2970\_cov\_21.957239 2923-2928. Max. coverage (+): 0. Max coverage (-): 0

Region: NODE\_387203\_length\_2970\_cov\_21.957239 2929-2935. Max. coverage (+): 0. Max coverage (-): 0

Region: NODE\_387203\_length\_2970\_cov\_21.957239 2936-2941. Max. coverage (+): 0. Max coverage (-): 0

Region: NODE\_387203\_length\_2970\_cov\_21.957239 2942-2947. Max. coverage (+): 0. Max coverage (-): 0

Region: NODE\_387203\_length\_2970\_cov\_21.957239 2948-2953. Max. coverage (+): 0. Max coverage (-): 0

Region: NODE\_387203\_length\_2970\_cov\_21.957239 2954-2959. Max. coverage (+): 0. Max coverage (-): 0

Region: NODE\_387203\_length\_2970\_cov\_21.957239 2960-2965. Max. coverage (+): 0. Max coverage (-): 0

Region: NODE\_387203\_length\_2970\_cov\_21.957239 2966-2971. Max. coverage (+): 0. Max coverage (-): 0

Region: NODE\_387203\_length\_2970\_cov\_21.957239 2972-2977. Max. coverage (+): 0. Max coverage (-): 0

Region: NODE\_387203\_length\_2970\_cov\_21.957239 2978-2984. Max. coverage (+): 0. Max coverage (-): 0

Region: NODE\_387203\_length\_2970\_cov\_21.957239 2985-2990. Max. coverage (+): 0. Max coverage (-): 0

Region: NODE\_387203\_length\_2970\_cov\_21.957239 2991-2996. Max. coverage (+): 0. Max coverage (-): 0

Region: NODE\_387203\_length\_2970\_cov\_21.957239 2997-3002. Max. coverage (+): 0. Max coverage (-): 0

Region: NODE\_387203\_length\_2970\_cov\_21.957239 3003-3008. Max. coverage (+): 0. Max coverage (-): 0

Region: NODE\_387203\_length\_2970\_cov\_21.957239 3009-3014. Max. coverage (+): 0. Max coverage (-): 0

Region: NODE\_387203\_length\_2970\_cov\_21.957239 3015-3020. Max. coverage (+): 0. Max coverage (-): 0

Region: NODE\_387203\_length\_2970\_cov\_21.957239 3021-3027. Max. coverage (+): 0. Max coverage (-): 0

Region: NODE\_387203\_length\_2970\_cov\_21.957239 3028-3033. Max. coverage (+): 0. Max coverage (-): 0

Region: NODE\_387203\_length\_2970\_cov\_21.957239 3034-3039. Max. coverage (+): 0. Max coverage (-): 0

Region: NODE\_387203\_length\_2970\_cov\_21.957239 3040-3045. Max. coverage (+): 0.06. Max coverage (-): 0

Region: NODE\_387203\_length\_2970\_cov\_21.957239 3046-3051. Max. coverage (+): 0.03. Max coverage (-): 0

Region: NODE\_387203\_length\_2970\_cov\_21.957239 3052-3057. Max. coverage (+): 0. Max coverage (-): 0

Region: NODE\_387203\_length\_2970\_cov\_21.957239 3058-3063. Max. coverage (+): 0. Max coverage (-): 0

Region: NODE\_387203\_length\_2970\_cov\_21.957239 3064-3069. Max. coverage (+): 0. Max coverage (-): 0

Region: NODE\_387203\_length\_2970\_cov\_21.957239 3070-. Max. coverage (+): 0. Max coverage (-): 0

RepeatMasker Color Code

**+**

100-98% Identity

<98-95% Identity

<95-90% Identity

<90-85% Identity

<85-80% Identity

<80-75% Identity

<75-70% Identity

<70% Identity

**-**

Gene Set Color Code

**+**

Gene

Pseudogene

Other

**-**

Topology/Coverage Color Code

Coverage Plus Strand

Coverage Minus Strand

Mainstrand: Plus

Mainstrand: Minus

Complementary Strand

Flanking Region  
(if option -flank >0)

Gene Set Annotation  
  
RepeatMasker Annotation  

**1. EnSpm-15\_DR**: 913-1142 (-), Divergence to consensus: 31.6%  
**2. EnSpm-15\_DR**: 1350-2349 (-), Divergence to consensus: 27.8%  
**3. EnSpm-15\_DR**: 2508-2710 (-), Divergence to consensus: 23.3%  
**4. EnSpm-15\_DR**: 2857-3084 (-), Divergence to consensus: 23.7%

  
Transcription Factor Binding Sites  

**RHOXF1** (Sequence: AGCTTA (-): 1309)  
**RHOXF1** (Sequence: GGATCA (-): 2196)  
**RHOXF1** (Sequence: GGATCA (-): 2250)  
**RHOXF1** (Sequence: AGATTA (-): 2427)  
**RHOXF1** (Sequence: AGATCA (-): 2579)  
**RHOXF1** (Sequence: GGATTA (-): 2658)  
**RHOXF1** (Sequence: TAAGCC (+): 215)  
**RHOXF1** (Sequence: TAAGCT (+): 413)  
**RHOXF1** (Sequence: TAAGCT (+): 488)  
**RHOXF1** (Sequence: TAAGCT (+): 873)  
**RHOXF1** (Sequence: TAATCC (+): 1053)  
**RHOXF1** (Sequence: TGATCC (+): 1966)  
**RHOXF1** (Sequence: TGATCT (+): 2797)  
**Lhx8** (Sequence: CTAATTAG (-): 626)  
**Gata4** (Sequence: GTTATCT (+): 2290)  
**POU5F1** (Sequence: TTTGCAT (-): 1728)  
**SOX9** (Sequence: AACAATGA (-): 1363)  
**SOX9** (Sequence: AACAATAG (-): 2421)  
**FOXO1** (Sequence: GCTGTTTAT (+): 1744)  
**Sox5** (Sequence: ATTGTT (+): 736)  
**FOXO3\_mmu** (Sequence: GCAAAACA (+): 354)  
**FOXO3\_mmu** (Sequence: GGAAAACA (+): 462)  
**FOXO3\_mmu** (Sequence: TGAAAACA (+): 831)  
**Nobox** (Sequence: GCTAATTA (-): 625)  
**FOXO1** (Sequence: ATAAACAAC (-): 838)  
**FOXP1** (Sequence: TGTTTAC (-): 159)  
**Rhox11** (Sequence: TGCTGTTTA (+): 575)  
**Rhox11** (Sequence: CGCTGTTTA (+): 1743)  
**Rhox11** (Sequence: TGCTGTAAA (+): 2401)  
**Rhox11** (Sequence: TTTACACCA (-): 2758)  
**Sox5** (Sequence: AACAAT (-): 716)  
**Sox5** (Sequence: AACAAT (-): 1363)  
**Sox5** (Sequence: AACAAT (-): 2409)  
**Sox5** (Sequence: AACAAT (-): 2421)  
**POU2F1** (Sequence: TATTCAAAT (+): 1248)  
**POU5F1** (Sequence: ATGCAAA (+): 1180)
