# Supplementary material for: First characterization of PIWI-interacting RNA clusters in a cichlid fish with a B chromosome
Source: BMC Biol. 2022 Sep 21;20:204. doi: 10.1186/s12915-022-01403-2 (PMC9490952; doi:10.1186/s12915-022-01403-2)
Supplement: Supplementary file 1 — Additional file 1. Zipped folder with fasta and interactive html piRNA cluster information for the A. latifasciata genome. The nomenclature is as follows: number-pirna-cluster_sex_B-presence (f, female; m, male; 0b, without B chromosome; 1b, with B chromosome). [file 12915_2022_1403_MOESM1_ESM.zip › 151_m1b.html]

piRNA cluster 151\_m1b 89


Predicted piRNA cluster no. 151\_m1b
  

Show proTRAC run info
Hide proTRAC run info

/\  
                \_\_\_\_\_\_\_\_\_\_\_\_\_\_\_\_\_\_\_\_\_\_\_/\\_\_\_ /  \\_\_\_\_\_\_\_  
               I                      /  \  /    \      I  
               I     pro             /    \/      \     I  
               I        TRAC        /               \   I  
               I   \_\_\_\_\_\_\_\_\_\_\_\_\_\_\_\_/\_\_\_\_\_\_\_\_\_\_\_\_\_\_\_\_\_\\_ I  
               I   \              /                     I  
               I    \            /                      I  
               I     \  /\      /       V.2.4.2         I  
               I      \/  \    /                        I  
               I\_\_\_\_\_\_\_\_\_\_\_\  /\_\_\_\_\_\_\_\_\_\_\_\_\_\_\_\_\_\_\_\_\_\_\_\_\_I  
                            \/  
  
  
================================= proTRAC ====================================  
VERSION: .......... 2.4.2  
LAST MODIFIED: .... 11. May 2018  
  
Please cite:  
Rosenkranz D, Zischler H. proTRAC - a software for probabilistic piRNA cluster  
detection, visualization and analysis. 2012. BMC Bioinformatics 13:5.  
  
  
Contact:  
David Rosenkranz  
Institute of Organismic and Molecular Evolutionary Biology  
Dept. Anthropology, small RNA group  
Johannes Gutenberg University Mainz  
email: rosenkranz@uni-mainz.de  
  
You can find the latest proTRAC version at:  
http://sourceforge.net/projects/protrac/files  
http://www.smallRNAgroup-mainz.de/software  
==============================================================================  
  
PARAMETERS:  
Map file: ...............piwi-machos-1B.fa-collapse.map  
Genome file: ............../../../0B\_ala\_genome.fa  
RepeatMasker annotation: Alatifasciata-all0B-maryan-v2.fa\_corrected.out  
GeneSet:................./guest-storage/Data/annotation/Alatifasciata\_all0B\_maryan-v2\_out2017.gff  
  
Significant (p<=0.01) hit density will be calculated based  
on observed hit distribution.  
  
Sliding window size: ........................................ 5000 bp  
Sliding window increament: .................................. 1000 bp  
Normalize each hit by number of genomic hits: ............... yes  
Normalize each hit by number of sequence reads: ............. yes  
Normalize values (-> per million mapped reads): ............. yes  
Min. fraction of hits with 1T(U) or 10A: .................... 0.75  
Alternatively: Min. fraction of hits with 1T(U) and 10A: .... 0.5  
Min. fraction of hits with typical piRNA length: ............ 0.75  
Typical piRNA length: ....................................... 24-32 nt  
Min. size of a piRNA cluster: ............................... 1000 bp.  
Min. number of hits (absolute): ............................. 0  
Min. number of hits (normalized): ........................... 0  
Min. fraction of hits on the mainstrand: .................... 0.75  
Top fraction of mapped sequences (in terms of read counts): . 1%  
Top fraction accounts for max. n% of sequence reads: ........ 90%  
Min. fraction of hits on each arm of a bidirectional cluster: 0.05  
Output html file for each cluster: .......................... yes  
Output a summary table: ..................................... yes  
Output a FASTA file for each cluster (piRNA sequences): ..... yes  
Output a FASTA file comprising cluster sequences: ........... yes  
Output a GTF file for predicted piRNA clusters: ..............yes  
Search DNA motifs in clusters: .............................. yes  
Output flanking sequences: +/- .............................. 0 bp  
Output ~.pTi file: .......................................... no  
==============================================================================  
  
  
Genome size (without gaps): ............ 758543724 bp  
Gaps (N/X/-): .......................... 417479 bp  
Mapped reads: .......................... 26973943  
Non-identical sequences: ............... 6209225  
Genomic hits: .......................... 48438990  
Significant densitiy of mapped reads: .. 821.144211136946 reads/kb

Show proTRAC cluster info
Hide proTRAC cluster info

|  |  |
| --- | --- |
| Location | NODE\_388156\_length\_1812\_cov\_24.704746 |
| Coordinates | 1-1860 |
| Size [bp] | 1860 |
| Sequence hit loci | 2621 |
| Mapped reads (normalized) | 4348.6 |
| Mapped reads (normalized) per kb | 2338 |
| Normalized reads with 1T (1U) | 67.1% |
| Normalized reads with 10A | 58.2% |
| Normalized reads with length 24-32 nt | 99.1% |
| Normalized reads on the main strand(s) | 80.2% |
| Predicted directionality | bi:minus-plus (split between 730 and 731) |

100%

0%

1T (1U)  
reads

10A reads

24-32 nt  
reads

reads on mainstrand

**Either the amount of reads with 1T (1U) OR 10A has to exceed 75% (set with option: -1Tor10A)  
Alternatively the amount of reads with 1T (1U) AND 10A has to exceed 50% (set with option: -1Tand10A)  
Minimum amount of reads with preferred size is 75% (set with option: -pisize)  
Minimum amount of reads on the main strand(s) is 75% (set with option: -clstrand)**

Show read coverage
Hide read coverage

WHAT DO I SEE HERE?  
This chart shows the location of mapped sequence reads within a predicted piRNA cluster. The color refers to the number of genomic hits produced by the sequence read in question. A dark red bar indicates that this sequence read produces many other hits elsewhere in the genome. Many adjacent red or yellow bars can indicate the presence of a multi-copy element such as transposons or rRNA genes. A dark green bar indicates that this sequence read maps uniquely to this locus.

1 hit

2-5 hits

6-10 hits

11-20 hits

21-50 hits

51-100 hits

> 100 hits

NODE\_388156\_length\_1812\_cov\_24.704746

1

1860

Gene Set

RepeatMasker

Mapped  
Reads

24.73

plus strand

minus strand

24.73

Region: NODE\_388156\_length\_1812\_cov\_24.704746 5796-2. Max. coverage (+): 0.04. Max coverage (-): 0.04

Region: NODE\_388156\_length\_1812\_cov\_24.704746 3-6. Max. coverage (+): 0. Max coverage (-): 0.04

Region: NODE\_388156\_length\_1812\_cov\_24.704746 7-10. Max. coverage (+): 0. Max coverage (-): 0.11

Region: NODE\_388156\_length\_1812\_cov\_24.704746 11-14. Max. coverage (+): 0.04. Max coverage (-): 0.74

Region: NODE\_388156\_length\_1812\_cov\_24.704746 15-17. Max. coverage (+): 0.07. Max coverage (-): 0.26

Region: NODE\_388156\_length\_1812\_cov\_24.704746 18-21. Max. coverage (+): 0. Max coverage (-): 0.63

Region: NODE\_388156\_length\_1812\_cov\_24.704746 22-25. Max. coverage (+): 0. Max coverage (-): 0.97

Region: NODE\_388156\_length\_1812\_cov\_24.704746 26-28. Max. coverage (+): 0.01. Max coverage (-): 0.21

Region: NODE\_388156\_length\_1812\_cov\_24.704746 29-32. Max. coverage (+): 0.01. Max coverage (-): 0.07

Region: NODE\_388156\_length\_1812\_cov\_24.704746 33-36. Max. coverage (+): 0.04. Max coverage (-): 0.07

Region: NODE\_388156\_length\_1812\_cov\_24.704746 37-40. Max. coverage (+): 0.04. Max coverage (-): 0.3

Region: NODE\_388156\_length\_1812\_cov\_24.704746 41-43. Max. coverage (+): 0.07. Max coverage (-): 0.33

Region: NODE\_388156\_length\_1812\_cov\_24.704746 44-47. Max. coverage (+): 0.04. Max coverage (-): 2.3

Region: NODE\_388156\_length\_1812\_cov\_24.704746 48-51. Max. coverage (+): 0.15. Max coverage (-): 4.37

Region: NODE\_388156\_length\_1812\_cov\_24.704746 52-54. Max. coverage (+): 0.15. Max coverage (-): 0.44

Region: NODE\_388156\_length\_1812\_cov\_24.704746 55-58. Max. coverage (+): 0.04. Max coverage (-): 0.33

Region: NODE\_388156\_length\_1812\_cov\_24.704746 59-62. Max. coverage (+): 0.04. Max coverage (-): 0.07

Region: NODE\_388156\_length\_1812\_cov\_24.704746 63-66. Max. coverage (+): 0.78. Max coverage (-): 0.33

Region: NODE\_388156\_length\_1812\_cov\_24.704746 67-69. Max. coverage (+): 0.17. Max coverage (-): 0.12

Region: NODE\_388156\_length\_1812\_cov\_24.704746 70-73. Max. coverage (+): 0.07. Max coverage (-): 0.12

Region: NODE\_388156\_length\_1812\_cov\_24.704746 74-77. Max. coverage (+): 0.01. Max coverage (-): 0.14

Region: NODE\_388156\_length\_1812\_cov\_24.704746 78-80. Max. coverage (+): 0. Max coverage (-): 0

Region: NODE\_388156\_length\_1812\_cov\_24.704746 81-84. Max. coverage (+): 0. Max coverage (-): 0.3

Region: NODE\_388156\_length\_1812\_cov\_24.704746 85-88. Max. coverage (+): 0.07. Max coverage (-): 0.32

Region: NODE\_388156\_length\_1812\_cov\_24.704746 89-92. Max. coverage (+): 0.15. Max coverage (-): 0.07

Region: NODE\_388156\_length\_1812\_cov\_24.704746 93-95. Max. coverage (+): 0.06. Max coverage (-): 0.32

Region: NODE\_388156\_length\_1812\_cov\_24.704746 96-99. Max. coverage (+): 0. Max coverage (-): 2.17

Region: NODE\_388156\_length\_1812\_cov\_24.704746 100-103. Max. coverage (+): 0.04. Max coverage (-): 1.85

Region: NODE\_388156\_length\_1812\_cov\_24.704746 104-107. Max. coverage (+): 0. Max coverage (-): 0.09

Region: NODE\_388156\_length\_1812\_cov\_24.704746 108-110. Max. coverage (+): 0. Max coverage (-): 0.13

Region: NODE\_388156\_length\_1812\_cov\_24.704746 111-114. Max. coverage (+): 0. Max coverage (-): 0.13

Region: NODE\_388156\_length\_1812\_cov\_24.704746 115-118. Max. coverage (+): 0.03. Max coverage (-): 0.07

Region: NODE\_388156\_length\_1812\_cov\_24.704746 119-121. Max. coverage (+): 0. Max coverage (-): 0.02

Region: NODE\_388156\_length\_1812\_cov\_24.704746 122-125. Max. coverage (+): 0. Max coverage (-): 0

Region: NODE\_388156\_length\_1812\_cov\_24.704746 126-129. Max. coverage (+): 0. Max coverage (-): 0.01

Region: NODE\_388156\_length\_1812\_cov\_24.704746 130-133. Max. coverage (+): 0.11. Max coverage (-): 0.01

Region: NODE\_388156\_length\_1812\_cov\_24.704746 134-136. Max. coverage (+): 0.1. Max coverage (-): 0

Region: NODE\_388156\_length\_1812\_cov\_24.704746 137-140. Max. coverage (+): 0. Max coverage (-): 0

Region: NODE\_388156\_length\_1812\_cov\_24.704746 141-144. Max. coverage (+): 0. Max coverage (-): 0.01

Region: NODE\_388156\_length\_1812\_cov\_24.704746 145-147. Max. coverage (+): 0.01. Max coverage (-): 0.01

Region: NODE\_388156\_length\_1812\_cov\_24.704746 148-151. Max. coverage (+): 0.01. Max coverage (-): 0.01

Region: NODE\_388156\_length\_1812\_cov\_24.704746 152-155. Max. coverage (+): 0. Max coverage (-): 0.01

Region: NODE\_388156\_length\_1812\_cov\_24.704746 156-159. Max. coverage (+): 0.01. Max coverage (-): 0.02

Region: NODE\_388156\_length\_1812\_cov\_24.704746 160-162. Max. coverage (+): 0.01. Max coverage (-): 0.02

Region: NODE\_388156\_length\_1812\_cov\_24.704746 163-166. Max. coverage (+): 0.01. Max coverage (-): 0.02

Region: NODE\_388156\_length\_1812\_cov\_24.704746 167-170. Max. coverage (+): 0.01. Max coverage (-): 0.27

Region: NODE\_388156\_length\_1812\_cov\_24.704746 171-173. Max. coverage (+): 0.01. Max coverage (-): 0.15

Region: NODE\_388156\_length\_1812\_cov\_24.704746 174-177. Max. coverage (+): 0.01. Max coverage (-): 0.03

Region: NODE\_388156\_length\_1812\_cov\_24.704746 178-181. Max. coverage (+): 0. Max coverage (-): 0

Region: NODE\_388156\_length\_1812\_cov\_24.704746 182-185. Max. coverage (+): 0. Max coverage (-): 0.02

Region: NODE\_388156\_length\_1812\_cov\_24.704746 186-188. Max. coverage (+): 0.04. Max coverage (-): 0

Region: NODE\_388156\_length\_1812\_cov\_24.704746 189-192. Max. coverage (+): 0.02. Max coverage (-): 0

Region: NODE\_388156\_length\_1812\_cov\_24.704746 193-196. Max. coverage (+): 0.06. Max coverage (-): 0

Region: NODE\_388156\_length\_1812\_cov\_24.704746 197-200. Max. coverage (+): 0.02. Max coverage (-): 0.04

Region: NODE\_388156\_length\_1812\_cov\_24.704746 201-203. Max. coverage (+): 0.02. Max coverage (-): 0

Region: NODE\_388156\_length\_1812\_cov\_24.704746 204-207. Max. coverage (+): 0. Max coverage (-): 0.44

Region: NODE\_388156\_length\_1812\_cov\_24.704746 208-211. Max. coverage (+): 0. Max coverage (-): 0.2

Region: NODE\_388156\_length\_1812\_cov\_24.704746 212-214. Max. coverage (+): 0.04. Max coverage (-): 0.01

Region: NODE\_388156\_length\_1812\_cov\_24.704746 215-218. Max. coverage (+): 1.2. Max coverage (-): 0.04

Region: NODE\_388156\_length\_1812\_cov\_24.704746 219-222. Max. coverage (+): 1.34. Max coverage (-): 0.06

Region: NODE\_388156\_length\_1812\_cov\_24.704746 223-226. Max. coverage (+): 0.01. Max coverage (-): 0.02

Region: NODE\_388156\_length\_1812\_cov\_24.704746 227-229. Max. coverage (+): 0. Max coverage (-): 0.19

Region: NODE\_388156\_length\_1812\_cov\_24.704746 230-233. Max. coverage (+): 0. Max coverage (-): 0.19

Region: NODE\_388156\_length\_1812\_cov\_24.704746 234-237. Max. coverage (+): 0. Max coverage (-): 0

Region: NODE\_388156\_length\_1812\_cov\_24.704746 238-240. Max. coverage (+): 0. Max coverage (-): 0.04

Region: NODE\_388156\_length\_1812\_cov\_24.704746 241-244. Max. coverage (+): 0.06. Max coverage (-): 0.65

Region: NODE\_388156\_length\_1812\_cov\_24.704746 245-248. Max. coverage (+): 0.06. Max coverage (-): 0.43

Region: NODE\_388156\_length\_1812\_cov\_24.704746 249-252. Max. coverage (+): 0.02. Max coverage (-): 0.26

Region: NODE\_388156\_length\_1812\_cov\_24.704746 253-255. Max. coverage (+): 0.02. Max coverage (-): 0.74

Region: NODE\_388156\_length\_1812\_cov\_24.704746 256-259. Max. coverage (+): 0.02. Max coverage (-): 1.86

Region: NODE\_388156\_length\_1812\_cov\_24.704746 260-263. Max. coverage (+): 0.07. Max coverage (-): 1.12

Region: NODE\_388156\_length\_1812\_cov\_24.704746 264-266. Max. coverage (+): 0.06. Max coverage (-): 0.05

Region: NODE\_388156\_length\_1812\_cov\_24.704746 267-270. Max. coverage (+): 0.07. Max coverage (-): 0.01

Region: NODE\_388156\_length\_1812\_cov\_24.704746 271-274. Max. coverage (+): 0.1. Max coverage (-): 0.12

Region: NODE\_388156\_length\_1812\_cov\_24.704746 275-278. Max. coverage (+): 0.02. Max coverage (-): 0.11

Region: NODE\_388156\_length\_1812\_cov\_24.704746 279-281. Max. coverage (+): 0.01. Max coverage (-): 0.02

Region: NODE\_388156\_length\_1812\_cov\_24.704746 282-285. Max. coverage (+): 0. Max coverage (-): 0.25

Region: NODE\_388156\_length\_1812\_cov\_24.704746 286-289. Max. coverage (+): 0. Max coverage (-): 0.48

Region: NODE\_388156\_length\_1812\_cov\_24.704746 290-293. Max. coverage (+): 0. Max coverage (-): 1.24

Region: NODE\_388156\_length\_1812\_cov\_24.704746 294-296. Max. coverage (+): 0.03. Max coverage (-): 1.02

Region: NODE\_388156\_length\_1812\_cov\_24.704746 297-300. Max. coverage (+): 0. Max coverage (-): 0.86

Region: NODE\_388156\_length\_1812\_cov\_24.704746 301-304. Max. coverage (+): 0.06. Max coverage (-): 0.14

Region: NODE\_388156\_length\_1812\_cov\_24.704746 305-307. Max. coverage (+): 0.54. Max coverage (-): 0.01

Region: NODE\_388156\_length\_1812\_cov\_24.704746 308-311. Max. coverage (+): 0.58. Max coverage (-): 0.01

Region: NODE\_388156\_length\_1812\_cov\_24.704746 312-315. Max. coverage (+): 0.05. Max coverage (-): 0.47

Region: NODE\_388156\_length\_1812\_cov\_24.704746 316-319. Max. coverage (+): 0. Max coverage (-): 0.89

Region: NODE\_388156\_length\_1812\_cov\_24.704746 320-322. Max. coverage (+): 0.15. Max coverage (-): 0.75

Region: NODE\_388156\_length\_1812\_cov\_24.704746 323-326. Max. coverage (+): 0.24. Max coverage (-): 0.09

Region: NODE\_388156\_length\_1812\_cov\_24.704746 327-330. Max. coverage (+): 0.26. Max coverage (-): 0.09

Region: NODE\_388156\_length\_1812\_cov\_24.704746 331-333. Max. coverage (+): 0.28. Max coverage (-): 0.02

Region: NODE\_388156\_length\_1812\_cov\_24.704746 334-337. Max. coverage (+): 0.63. Max coverage (-): 0.26

Region: NODE\_388156\_length\_1812\_cov\_24.704746 338-341. Max. coverage (+): 0.61. Max coverage (-): 2.04

Region: NODE\_388156\_length\_1812\_cov\_24.704746 342-345. Max. coverage (+): 0.06. Max coverage (-): 1.26

Region: NODE\_388156\_length\_1812\_cov\_24.704746 346-348. Max. coverage (+): 0.01. Max coverage (-): 0.13

Region: NODE\_388156\_length\_1812\_cov\_24.704746 349-352. Max. coverage (+): 0.01. Max coverage (-): 0.75

Region: NODE\_388156\_length\_1812\_cov\_24.704746 353-356. Max. coverage (+): 0.02. Max coverage (-): 0.75

Region: NODE\_388156\_length\_1812\_cov\_24.704746 357-359. Max. coverage (+): 0.35. Max coverage (-): 0.21

Region: NODE\_388156\_length\_1812\_cov\_24.704746 360-363. Max. coverage (+): 0.3. Max coverage (-): 0.16

Region: NODE\_388156\_length\_1812\_cov\_24.704746 364-367. Max. coverage (+): 0.08. Max coverage (-): 0.16

Region: NODE\_388156\_length\_1812\_cov\_24.704746 368-371. Max. coverage (+): 1.32. Max coverage (-): 0.18

Region: NODE\_388156\_length\_1812\_cov\_24.704746 372-374. Max. coverage (+): 0.61. Max coverage (-): 0.15

Region: NODE\_388156\_length\_1812\_cov\_24.704746 375-378. Max. coverage (+): 0.52. Max coverage (-): 0.06

Region: NODE\_388156\_length\_1812\_cov\_24.704746 379-382. Max. coverage (+): 0. Max coverage (-): 0.02

Region: NODE\_388156\_length\_1812\_cov\_24.704746 383-386. Max. coverage (+): 0. Max coverage (-): 0

Region: NODE\_388156\_length\_1812\_cov\_24.704746 387-389. Max. coverage (+): 0. Max coverage (-): 0

Region: NODE\_388156\_length\_1812\_cov\_24.704746 390-393. Max. coverage (+): 0.04. Max coverage (-): 0

Region: NODE\_388156\_length\_1812\_cov\_24.704746 394-397. Max. coverage (+): 0.07. Max coverage (-): 0

Region: NODE\_388156\_length\_1812\_cov\_24.704746 398-400. Max. coverage (+): 0. Max coverage (-): 0.04

Region: NODE\_388156\_length\_1812\_cov\_24.704746 401-404. Max. coverage (+): 0. Max coverage (-): 0.3

Region: NODE\_388156\_length\_1812\_cov\_24.704746 405-408. Max. coverage (+): 0.11. Max coverage (-): 0.3

Region: NODE\_388156\_length\_1812\_cov\_24.704746 409-412. Max. coverage (+): 0.74. Max coverage (-): 0.07

Region: NODE\_388156\_length\_1812\_cov\_24.704746 413-415. Max. coverage (+): 0.67. Max coverage (-): 0.15

Region: NODE\_388156\_length\_1812\_cov\_24.704746 416-419. Max. coverage (+): 0.22. Max coverage (-): 0.7

Region: NODE\_388156\_length\_1812\_cov\_24.704746 420-423. Max. coverage (+): 0.3. Max coverage (-): 0.56

Region: NODE\_388156\_length\_1812\_cov\_24.704746 424-426. Max. coverage (+): 0.04. Max coverage (-): 0

Region: NODE\_388156\_length\_1812\_cov\_24.704746 427-430. Max. coverage (+): 0.01. Max coverage (-): 0.01

Region: NODE\_388156\_length\_1812\_cov\_24.704746 431-434. Max. coverage (+): 0.01. Max coverage (-): 0.17

Region: NODE\_388156\_length\_1812\_cov\_24.704746 435-438. Max. coverage (+): 0.01. Max coverage (-): 0.09

Region: NODE\_388156\_length\_1812\_cov\_24.704746 439-441. Max. coverage (+): 0.01. Max coverage (-): 0.03

Region: NODE\_388156\_length\_1812\_cov\_24.704746 442-445. Max. coverage (+): 0.72. Max coverage (-): 0.03

Region: NODE\_388156\_length\_1812\_cov\_24.704746 446-449. Max. coverage (+): 0.66. Max coverage (-): 0.02

Region: NODE\_388156\_length\_1812\_cov\_24.704746 450-452. Max. coverage (+): 0.02. Max coverage (-): 0

Region: NODE\_388156\_length\_1812\_cov\_24.704746 453-456. Max. coverage (+): 0.01. Max coverage (-): 0.03

Region: NODE\_388156\_length\_1812\_cov\_24.704746 457-460. Max. coverage (+): 0.01. Max coverage (-): 0.07

Region: NODE\_388156\_length\_1812\_cov\_24.704746 461-464. Max. coverage (+): 0.01. Max coverage (-): 0.11

Region: NODE\_388156\_length\_1812\_cov\_24.704746 465-467. Max. coverage (+): 0. Max coverage (-): 0.09

Region: NODE\_388156\_length\_1812\_cov\_24.704746 468-471. Max. coverage (+): 0.04. Max coverage (-): 0.26

Region: NODE\_388156\_length\_1812\_cov\_24.704746 472-475. Max. coverage (+): 0. Max coverage (-): 0.7

Region: NODE\_388156\_length\_1812\_cov\_24.704746 476-479. Max. coverage (+): 0. Max coverage (-): 6.45

Region: NODE\_388156\_length\_1812\_cov\_24.704746 480-482. Max. coverage (+): 0.11. Max coverage (-): 3.08

Region: NODE\_388156\_length\_1812\_cov\_24.704746 483-486. Max. coverage (+): 0.19. Max coverage (-): 0.3

Region: NODE\_388156\_length\_1812\_cov\_24.704746 487-490. Max. coverage (+): 0.59. Max coverage (-): 0.15

Region: NODE\_388156\_length\_1812\_cov\_24.704746 491-493. Max. coverage (+): 0.15. Max coverage (-): 0.15

Region: NODE\_388156\_length\_1812\_cov\_24.704746 494-497. Max. coverage (+): 0.07. Max coverage (-): 0.22

Region: NODE\_388156\_length\_1812\_cov\_24.704746 498-501. Max. coverage (+): 0.04. Max coverage (-): 0.48

Region: NODE\_388156\_length\_1812\_cov\_24.704746 502-505. Max. coverage (+): 0.11. Max coverage (-): 0

Region: NODE\_388156\_length\_1812\_cov\_24.704746 506-508. Max. coverage (+): 0.19. Max coverage (-): 0.11

Region: NODE\_388156\_length\_1812\_cov\_24.704746 509-512. Max. coverage (+): 0.19. Max coverage (-): 0.07

Region: NODE\_388156\_length\_1812\_cov\_24.704746 513-516. Max. coverage (+): 0. Max coverage (-): 0.63

Region: NODE\_388156\_length\_1812\_cov\_24.704746 517-519. Max. coverage (+): 0. Max coverage (-): 0.07

Region: NODE\_388156\_length\_1812\_cov\_24.704746 520-523. Max. coverage (+): 0.04. Max coverage (-): 0.04

Region: NODE\_388156\_length\_1812\_cov\_24.704746 524-527. Max. coverage (+): 0.04. Max coverage (-): 0.15

Region: NODE\_388156\_length\_1812\_cov\_24.704746 528-531. Max. coverage (+): 0.15. Max coverage (-): 0.22

Region: NODE\_388156\_length\_1812\_cov\_24.704746 532-534. Max. coverage (+): 0.16. Max coverage (-): 0.07

Region: NODE\_388156\_length\_1812\_cov\_24.704746 535-538. Max. coverage (+): 0. Max coverage (-): 0.07

Region: NODE\_388156\_length\_1812\_cov\_24.704746 539-542. Max. coverage (+): 0. Max coverage (-): 0.04

Region: NODE\_388156\_length\_1812\_cov\_24.704746 543-545. Max. coverage (+): 0. Max coverage (-): 0

Region: NODE\_388156\_length\_1812\_cov\_24.704746 546-549. Max. coverage (+): 0. Max coverage (-): 0.01

Region: NODE\_388156\_length\_1812\_cov\_24.704746 550-553. Max. coverage (+): 0. Max coverage (-): 0

Region: NODE\_388156\_length\_1812\_cov\_24.704746 554-557. Max. coverage (+): 0. Max coverage (-): 0

Region: NODE\_388156\_length\_1812\_cov\_24.704746 558-560. Max. coverage (+): 0. Max coverage (-): 0

Region: NODE\_388156\_length\_1812\_cov\_24.704746 561-564. Max. coverage (+): 0. Max coverage (-): 0.02

Region: NODE\_388156\_length\_1812\_cov\_24.704746 565-568. Max. coverage (+): 0. Max coverage (-): 0.01

Region: NODE\_388156\_length\_1812\_cov\_24.704746 569-572. Max. coverage (+): 0. Max coverage (-): 0

Region: NODE\_388156\_length\_1812\_cov\_24.704746 573-575. Max. coverage (+): 0.04. Max coverage (-): 0

Region: NODE\_388156\_length\_1812\_cov\_24.704746 576-579. Max. coverage (+): 0. Max coverage (-): 0.11

Region: NODE\_388156\_length\_1812\_cov\_24.704746 580-583. Max. coverage (+): 0. Max coverage (-): 0.26

Region: NODE\_388156\_length\_1812\_cov\_24.704746 584-586. Max. coverage (+): 0. Max coverage (-): 0.07

Region: NODE\_388156\_length\_1812\_cov\_24.704746 587-590. Max. coverage (+): 0.11. Max coverage (-): 0.04

Region: NODE\_388156\_length\_1812\_cov\_24.704746 591-594. Max. coverage (+): 0.07. Max coverage (-): 0.07

Region: NODE\_388156\_length\_1812\_cov\_24.704746 595-598. Max. coverage (+): 0.01. Max coverage (-): 0.07

Region: NODE\_388156\_length\_1812\_cov\_24.704746 599-601. Max. coverage (+): 0.01. Max coverage (-): 0

Region: NODE\_388156\_length\_1812\_cov\_24.704746 602-605. Max. coverage (+): 0.05. Max coverage (-): 0.03

Region: NODE\_388156\_length\_1812\_cov\_24.704746 606-609. Max. coverage (+): 0.04. Max coverage (-): 0.04

Region: NODE\_388156\_length\_1812\_cov\_24.704746 610-612. Max. coverage (+): 0.11. Max coverage (-): 0.07

Region: NODE\_388156\_length\_1812\_cov\_24.704746 613-616. Max. coverage (+): 0.59. Max coverage (-): 0.19

Region: NODE\_388156\_length\_1812\_cov\_24.704746 617-620. Max. coverage (+): 1.33. Max coverage (-): 0.04

Region: NODE\_388156\_length\_1812\_cov\_24.704746 621-624. Max. coverage (+): 0.7. Max coverage (-): 0.11

Region: NODE\_388156\_length\_1812\_cov\_24.704746 625-627. Max. coverage (+): 0. Max coverage (-): 0.19

Region: NODE\_388156\_length\_1812\_cov\_24.704746 628-631. Max. coverage (+): 0. Max coverage (-): 24.69

Region: NODE\_388156\_length\_1812\_cov\_24.704746 632-635. Max. coverage (+): 0. Max coverage (-): 24.73

Region: NODE\_388156\_length\_1812\_cov\_24.704746 636-638. Max. coverage (+): 0.05. Max coverage (-): 2.91

Region: NODE\_388156\_length\_1812\_cov\_24.704746 639-642. Max. coverage (+): 0.28. Max coverage (-): 0.28

Region: NODE\_388156\_length\_1812\_cov\_24.704746 643-646. Max. coverage (+): 0.1. Max coverage (-): 0.01

Region: NODE\_388156\_length\_1812\_cov\_24.704746 647-650. Max. coverage (+): 0.01. Max coverage (-): 0.03

Region: NODE\_388156\_length\_1812\_cov\_24.704746 651-653. Max. coverage (+): 0.01. Max coverage (-): 0

Region: NODE\_388156\_length\_1812\_cov\_24.704746 654-657. Max. coverage (+): 0.02. Max coverage (-): 0

Region: NODE\_388156\_length\_1812\_cov\_24.704746 658-661. Max. coverage (+): 0.01. Max coverage (-): 0.01

Region: NODE\_388156\_length\_1812\_cov\_24.704746 662-665. Max. coverage (+): 0. Max coverage (-): 0.01

Region: NODE\_388156\_length\_1812\_cov\_24.704746 666-668. Max. coverage (+): 0. Max coverage (-): 0.12

Region: NODE\_388156\_length\_1812\_cov\_24.704746 669-672. Max. coverage (+): 0. Max coverage (-): 0.14

Region: NODE\_388156\_length\_1812\_cov\_24.704746 673-676. Max. coverage (+): 0. Max coverage (-): 0.08

Region: NODE\_388156\_length\_1812\_cov\_24.704746 677-679. Max. coverage (+): 0.04. Max coverage (-): 0

Region: NODE\_388156\_length\_1812\_cov\_24.704746 680-683. Max. coverage (+): 0.04. Max coverage (-): 0.04

Region: NODE\_388156\_length\_1812\_cov\_24.704746 684-687. Max. coverage (+): 0. Max coverage (-): 0.04

Region: NODE\_388156\_length\_1812\_cov\_24.704746 688-691. Max. coverage (+): 0. Max coverage (-): 0.11

Region: NODE\_388156\_length\_1812\_cov\_24.704746 692-694. Max. coverage (+): 0.06. Max coverage (-): 0.02

Region: NODE\_388156\_length\_1812\_cov\_24.704746 695-698. Max. coverage (+): 0.07. Max coverage (-): 0.2

Region: NODE\_388156\_length\_1812\_cov\_24.704746 699-702. Max. coverage (+): 0.04. Max coverage (-): 0.26

Region: NODE\_388156\_length\_1812\_cov\_24.704746 703-705. Max. coverage (+): 0.02. Max coverage (-): 0.24

Region: NODE\_388156\_length\_1812\_cov\_24.704746 706-709. Max. coverage (+): 0.02. Max coverage (-): 0

Region: NODE\_388156\_length\_1812\_cov\_24.704746 710-713. Max. coverage (+): 0.01. Max coverage (-): 0.07

Region: NODE\_388156\_length\_1812\_cov\_24.704746 714-717. Max. coverage (+): 0.06. Max coverage (-): 0.05

Region: NODE\_388156\_length\_1812\_cov\_24.704746 718-720. Max. coverage (+): 0.11. Max coverage (-): 0.06

Region: NODE\_388156\_length\_1812\_cov\_24.704746 721-724. Max. coverage (+): 0.07. Max coverage (-): 0.43

Region: NODE\_388156\_length\_1812\_cov\_24.704746 725-728. Max. coverage (+): 0.04. Max coverage (-): 0.05

Region: NODE\_388156\_length\_1812\_cov\_24.704746 729-731. Max. coverage (+): 0.03. Max coverage (-): 0.04

Region: NODE\_388156\_length\_1812\_cov\_24.704746 732-735. Max. coverage (+): 0.06. Max coverage (-): 0.02

Region: NODE\_388156\_length\_1812\_cov\_24.704746 736-739. Max. coverage (+): 0.08. Max coverage (-): 0.02

Region: NODE\_388156\_length\_1812\_cov\_24.704746 740-743. Max. coverage (+): 0.32. Max coverage (-): 0

Region: NODE\_388156\_length\_1812\_cov\_24.704746 744-746. Max. coverage (+): 0.27. Max coverage (-): 0.01

Region: NODE\_388156\_length\_1812\_cov\_24.704746 747-750. Max. coverage (+): 0.17. Max coverage (-): 0

Region: NODE\_388156\_length\_1812\_cov\_24.704746 751-754. Max. coverage (+): 0. Max coverage (-): 0

Region: NODE\_388156\_length\_1812\_cov\_24.704746 755-758. Max. coverage (+): 0. Max coverage (-): 0.02

Region: NODE\_388156\_length\_1812\_cov\_24.704746 759-761. Max. coverage (+): 0. Max coverage (-): 0

Region: NODE\_388156\_length\_1812\_cov\_24.704746 762-765. Max. coverage (+): 0. Max coverage (-): 0

Region: NODE\_388156\_length\_1812\_cov\_24.704746 766-769. Max. coverage (+): 0. Max coverage (-): 0

Region: NODE\_388156\_length\_1812\_cov\_24.704746 770-772. Max. coverage (+): 0.07. Max coverage (-): 0.16

Region: NODE\_388156\_length\_1812\_cov\_24.704746 773-776. Max. coverage (+): 0.78. Max coverage (-): 0.16

Region: NODE\_388156\_length\_1812\_cov\_24.704746 777-780. Max. coverage (+): 4.45. Max coverage (-): 0.33

Region: NODE\_388156\_length\_1812\_cov\_24.704746 781-784. Max. coverage (+): 0.26. Max coverage (-): 0.33

Region: NODE\_388156\_length\_1812\_cov\_24.704746 785-787. Max. coverage (+): 0.11. Max coverage (-): 0.22

Region: NODE\_388156\_length\_1812\_cov\_24.704746 788-791. Max. coverage (+): 0.07. Max coverage (-): 0.3

Region: NODE\_388156\_length\_1812\_cov\_24.704746 792-795. Max. coverage (+): 0.04. Max coverage (-): 0.04

Region: NODE\_388156\_length\_1812\_cov\_24.704746 796-798. Max. coverage (+): 0. Max coverage (-): 0

Region: NODE\_388156\_length\_1812\_cov\_24.704746 799-802. Max. coverage (+): 0.01. Max coverage (-): 0

Region: NODE\_388156\_length\_1812\_cov\_24.704746 803-806. Max. coverage (+): 0. Max coverage (-): 0

Region: NODE\_388156\_length\_1812\_cov\_24.704746 807-810. Max. coverage (+): 0. Max coverage (-): 0

Region: NODE\_388156\_length\_1812\_cov\_24.704746 811-813. Max. coverage (+): 0. Max coverage (-): 0

Region: NODE\_388156\_length\_1812\_cov\_24.704746 814-817. Max. coverage (+): 0. Max coverage (-): 0

Region: NODE\_388156\_length\_1812\_cov\_24.704746 818-821. Max. coverage (+): 0. Max coverage (-): 0

Region: NODE\_388156\_length\_1812\_cov\_24.704746 822-824. Max. coverage (+): 0. Max coverage (-): 0

Region: NODE\_388156\_length\_1812\_cov\_24.704746 825-828. Max. coverage (+): 0.04. Max coverage (-): 0

Region: NODE\_388156\_length\_1812\_cov\_24.704746 829-832. Max. coverage (+): 0.07. Max coverage (-): 0.15

Region: NODE\_388156\_length\_1812\_cov\_24.704746 833-836. Max. coverage (+): 0.11. Max coverage (-): 0.19

Region: NODE\_388156\_length\_1812\_cov\_24.704746 837-839. Max. coverage (+): 0.26. Max coverage (-): 0

Region: NODE\_388156\_length\_1812\_cov\_24.704746 840-843. Max. coverage (+): 0.07. Max coverage (-): 0

Region: NODE\_388156\_length\_1812\_cov\_24.704746 844-847. Max. coverage (+): 0.07. Max coverage (-): 0

Region: NODE\_388156\_length\_1812\_cov\_24.704746 848-851. Max. coverage (+): 0.22. Max coverage (-): 0

Region: NODE\_388156\_length\_1812\_cov\_24.704746 852-854. Max. coverage (+): 0.15. Max coverage (-): 0

Region: NODE\_388156\_length\_1812\_cov\_24.704746 855-858. Max. coverage (+): 0.15. Max coverage (-): 0.04

Region: NODE\_388156\_length\_1812\_cov\_24.704746 859-862. Max. coverage (+): 0.04. Max coverage (-): 0.04

Region: NODE\_388156\_length\_1812\_cov\_24.704746 863-865. Max. coverage (+): 0. Max coverage (-): 0.11

Region: NODE\_388156\_length\_1812\_cov\_24.704746 866-869. Max. coverage (+): 0.04. Max coverage (-): 0.07

Region: NODE\_388156\_length\_1812\_cov\_24.704746 870-873. Max. coverage (+): 0.04. Max coverage (-): 0

Region: NODE\_388156\_length\_1812\_cov\_24.704746 874-877. Max. coverage (+): 0.07. Max coverage (-): 0

Region: NODE\_388156\_length\_1812\_cov\_24.704746 878-880. Max. coverage (+): 0.19. Max coverage (-): 0

Region: NODE\_388156\_length\_1812\_cov\_24.704746 881-884. Max. coverage (+): 0.11. Max coverage (-): 0

Region: NODE\_388156\_length\_1812\_cov\_24.704746 885-888. Max. coverage (+): 0. Max coverage (-): 0.04

Region: NODE\_388156\_length\_1812\_cov\_24.704746 889-891. Max. coverage (+): 0. Max coverage (-): 0.04

Region: NODE\_388156\_length\_1812\_cov\_24.704746 892-895. Max. coverage (+): 0.04. Max coverage (-): 0.04

Region: NODE\_388156\_length\_1812\_cov\_24.704746 896-899. Max. coverage (+): 0.04. Max coverage (-): 0

Region: NODE\_388156\_length\_1812\_cov\_24.704746 900-903. Max. coverage (+): 0.15. Max coverage (-): 0.19

Region: NODE\_388156\_length\_1812\_cov\_24.704746 904-906. Max. coverage (+): 0.15. Max coverage (-): 0.26

Region: NODE\_388156\_length\_1812\_cov\_24.704746 907-910. Max. coverage (+): 0.11. Max coverage (-): 0.19

Region: NODE\_388156\_length\_1812\_cov\_24.704746 911-914. Max. coverage (+): 0.59. Max coverage (-): 0.19

Region: NODE\_388156\_length\_1812\_cov\_24.704746 915-917. Max. coverage (+): 0.56. Max coverage (-): 0

Region: NODE\_388156\_length\_1812\_cov\_24.704746 918-921. Max. coverage (+): 0.41. Max coverage (-): 0.04

Region: NODE\_388156\_length\_1812\_cov\_24.704746 922-925. Max. coverage (+): 0.26. Max coverage (-): 0.07

Region: NODE\_388156\_length\_1812\_cov\_24.704746 926-929. Max. coverage (+): 0.22. Max coverage (-): 0

Region: NODE\_388156\_length\_1812\_cov\_24.704746 930-932. Max. coverage (+): 0.3. Max coverage (-): 0.07

Region: NODE\_388156\_length\_1812\_cov\_24.704746 933-936. Max. coverage (+): 0.04. Max coverage (-): 0.11

Region: NODE\_388156\_length\_1812\_cov\_24.704746 937-940. Max. coverage (+): 0.19. Max coverage (-): 0.11

Region: NODE\_388156\_length\_1812\_cov\_24.704746 941-944. Max. coverage (+): 0.41. Max coverage (-): 0.04

Region: NODE\_388156\_length\_1812\_cov\_24.704746 945-947. Max. coverage (+): 0.26. Max coverage (-): 0.3

Region: NODE\_388156\_length\_1812\_cov\_24.704746 948-951. Max. coverage (+): 0.07. Max coverage (-): 0.02

Region: NODE\_388156\_length\_1812\_cov\_24.704746 952-955. Max. coverage (+): 0.06. Max coverage (-): 0

Region: NODE\_388156\_length\_1812\_cov\_24.704746 956-958. Max. coverage (+): 0.13. Max coverage (-): 0.02

Region: NODE\_388156\_length\_1812\_cov\_24.704746 959-962. Max. coverage (+): 0.09. Max coverage (-): 0.04

Region: NODE\_388156\_length\_1812\_cov\_24.704746 963-966. Max. coverage (+): 0.09. Max coverage (-): 0.06

Region: NODE\_388156\_length\_1812\_cov\_24.704746 967-970. Max. coverage (+): 0.04. Max coverage (-): 0.41

Region: NODE\_388156\_length\_1812\_cov\_24.704746 971-973. Max. coverage (+): 0. Max coverage (-): 0.04

Region: NODE\_388156\_length\_1812\_cov\_24.704746 974-977. Max. coverage (+): 0.07. Max coverage (-): 0.11

Region: NODE\_388156\_length\_1812\_cov\_24.704746 978-981. Max. coverage (+): 0.22. Max coverage (-): 0.26

Region: NODE\_388156\_length\_1812\_cov\_24.704746 982-984. Max. coverage (+): 0.04. Max coverage (-): 0.12

Region: NODE\_388156\_length\_1812\_cov\_24.704746 985-988. Max. coverage (+): 0.06. Max coverage (-): 0.01

Region: NODE\_388156\_length\_1812\_cov\_24.704746 989-992. Max. coverage (+): 0.02. Max coverage (-): 0

Region: NODE\_388156\_length\_1812\_cov\_24.704746 993-996. Max. coverage (+): 0.01. Max coverage (-): 0.03

Region: NODE\_388156\_length\_1812\_cov\_24.704746 997-999. Max. coverage (+): 0. Max coverage (-): 0.04

Region: NODE\_388156\_length\_1812\_cov\_24.704746 1000-1003. Max. coverage (+): 0.04. Max coverage (-): 0.04

Region: NODE\_388156\_length\_1812\_cov\_24.704746 1004-1007. Max. coverage (+): 0.07. Max coverage (-): 0

Region: NODE\_388156\_length\_1812\_cov\_24.704746 1008-1010. Max. coverage (+): 0. Max coverage (-): 0

Region: NODE\_388156\_length\_1812\_cov\_24.704746 1011-1014. Max. coverage (+): 0.07. Max coverage (-): 0.15

Region: NODE\_388156\_length\_1812\_cov\_24.704746 1015-1018. Max. coverage (+): 0.11. Max coverage (-): 0.85

Region: NODE\_388156\_length\_1812\_cov\_24.704746 1019-1022. Max. coverage (+): 0.22. Max coverage (-): 0.96

Region: NODE\_388156\_length\_1812\_cov\_24.704746 1023-1025. Max. coverage (+): 0.15. Max coverage (-): 0.78

Region: NODE\_388156\_length\_1812\_cov\_24.704746 1026-1029. Max. coverage (+): 0.04. Max coverage (-): 0.04

Region: NODE\_388156\_length\_1812\_cov\_24.704746 1030-1033. Max. coverage (+): 0.11. Max coverage (-): 0

Region: NODE\_388156\_length\_1812\_cov\_24.704746 1034-1037. Max. coverage (+): 0.67. Max coverage (-): 0

Region: NODE\_388156\_length\_1812\_cov\_24.704746 1038-1040. Max. coverage (+): 0.7. Max coverage (-): 0.11

Region: NODE\_388156\_length\_1812\_cov\_24.704746 1041-1044. Max. coverage (+): 0.04. Max coverage (-): 0.11

Region: NODE\_388156\_length\_1812\_cov\_24.704746 1045-1048. Max. coverage (+): 0.04. Max coverage (-): 0.11

Region: NODE\_388156\_length\_1812\_cov\_24.704746 1049-1051. Max. coverage (+): 0. Max coverage (-): 0.15

Region: NODE\_388156\_length\_1812\_cov\_24.704746 1052-1055. Max. coverage (+): 0. Max coverage (-): 0.04

Region: NODE\_388156\_length\_1812\_cov\_24.704746 1056-1059. Max. coverage (+): 0.07. Max coverage (-): 0.04

Region: NODE\_388156\_length\_1812\_cov\_24.704746 1060-1063. Max. coverage (+): 0. Max coverage (-): 2.22

Region: NODE\_388156\_length\_1812\_cov\_24.704746 1064-1066. Max. coverage (+): 0.22. Max coverage (-): 1

Region: NODE\_388156\_length\_1812\_cov\_24.704746 1067-1070. Max. coverage (+): 0.26. Max coverage (-): 0.22

Region: NODE\_388156\_length\_1812\_cov\_24.704746 1071-1074. Max. coverage (+): 0.07. Max coverage (-): 0.15

Region: NODE\_388156\_length\_1812\_cov\_24.704746 1075-1077. Max. coverage (+): 0.11. Max coverage (-): 0

Region: NODE\_388156\_length\_1812\_cov\_24.704746 1078-1081. Max. coverage (+): 0.11. Max coverage (-): 0

Region: NODE\_388156\_length\_1812\_cov\_24.704746 1082-1085. Max. coverage (+): 1.08. Max coverage (-): 0.33

Region: NODE\_388156\_length\_1812\_cov\_24.704746 1086-1089. Max. coverage (+): 0.85. Max coverage (-): 0.3

Region: NODE\_388156\_length\_1812\_cov\_24.704746 1090-1092. Max. coverage (+): 0.37. Max coverage (-): 0

Region: NODE\_388156\_length\_1812\_cov\_24.704746 1093-1096. Max. coverage (+): 0.04. Max coverage (-): 0

Region: NODE\_388156\_length\_1812\_cov\_24.704746 1097-1100. Max. coverage (+): 0.04. Max coverage (-): 0.04

Region: NODE\_388156\_length\_1812\_cov\_24.704746 1101-1103. Max. coverage (+): 0. Max coverage (-): 0.06

Region: NODE\_388156\_length\_1812\_cov\_24.704746 1104-1107. Max. coverage (+): 0. Max coverage (-): 0.02

Region: NODE\_388156\_length\_1812\_cov\_24.704746 1108-1111. Max. coverage (+): 0. Max coverage (-): 0

Region: NODE\_388156\_length\_1812\_cov\_24.704746 1112-1115. Max. coverage (+): 0.07. Max coverage (-): 0

Region: NODE\_388156\_length\_1812\_cov\_24.704746 1116-1118. Max. coverage (+): 1.41. Max coverage (-): 0.04

Region: NODE\_388156\_length\_1812\_cov\_24.704746 1119-1122. Max. coverage (+): 1.85. Max coverage (-): 0.04

Region: NODE\_388156\_length\_1812\_cov\_24.704746 1123-1126. Max. coverage (+): 0.07. Max coverage (-): 0

Region: NODE\_388156\_length\_1812\_cov\_24.704746 1127-1130. Max. coverage (+): 0.19. Max coverage (-): 0.06

Region: NODE\_388156\_length\_1812\_cov\_24.704746 1131-1133. Max. coverage (+): 0.19. Max coverage (-): 0.07

Region: NODE\_388156\_length\_1812\_cov\_24.704746 1134-1137. Max. coverage (+): 0.04. Max coverage (-): 0.17

Region: NODE\_388156\_length\_1812\_cov\_24.704746 1138-1141. Max. coverage (+): 0.01. Max coverage (-): 0.06

Region: NODE\_388156\_length\_1812\_cov\_24.704746 1142-1144. Max. coverage (+): 0. Max coverage (-): 0

Region: NODE\_388156\_length\_1812\_cov\_24.704746 1145-1148. Max. coverage (+): 0.09. Max coverage (-): 0.02

Region: NODE\_388156\_length\_1812\_cov\_24.704746 1149-1152. Max. coverage (+): 0.61. Max coverage (-): 0.02

Region: NODE\_388156\_length\_1812\_cov\_24.704746 1153-1156. Max. coverage (+): 0.52. Max coverage (-): 0.01

Region: NODE\_388156\_length\_1812\_cov\_24.704746 1157-1159. Max. coverage (+): 0.05. Max coverage (-): 0

Region: NODE\_388156\_length\_1812\_cov\_24.704746 1160-1163. Max. coverage (+): 0.06. Max coverage (-): 0

Region: NODE\_388156\_length\_1812\_cov\_24.704746 1164-1167. Max. coverage (+): 0.06. Max coverage (-): 0

Region: NODE\_388156\_length\_1812\_cov\_24.704746 1168-1170. Max. coverage (+): 0.08. Max coverage (-): 0

Region: NODE\_388156\_length\_1812\_cov\_24.704746 1171-1174. Max. coverage (+): 0.09. Max coverage (-): 0.02

Region: NODE\_388156\_length\_1812\_cov\_24.704746 1175-1178. Max. coverage (+): 0.37. Max coverage (-): 0.06

Region: NODE\_388156\_length\_1812\_cov\_24.704746 1179-1182. Max. coverage (+): 0.65. Max coverage (-): 0.07

Region: NODE\_388156\_length\_1812\_cov\_24.704746 1183-1185. Max. coverage (+): 0.46. Max coverage (-): 0.02

Region: NODE\_388156\_length\_1812\_cov\_24.704746 1186-1189. Max. coverage (+): 0.07. Max coverage (-): 0

Region: NODE\_388156\_length\_1812\_cov\_24.704746 1190-1193. Max. coverage (+): 0.07. Max coverage (-): 0.04

Region: NODE\_388156\_length\_1812\_cov\_24.704746 1194-1196. Max. coverage (+): 0.07. Max coverage (-): 0.15

Region: NODE\_388156\_length\_1812\_cov\_24.704746 1197-1200. Max. coverage (+): 0.09. Max coverage (-): 0.15

Region: NODE\_388156\_length\_1812\_cov\_24.704746 1201-1204. Max. coverage (+): 0.02. Max coverage (-): 0.03

Region: NODE\_388156\_length\_1812\_cov\_24.704746 1205-1208. Max. coverage (+): 0.17. Max coverage (-): 0.03

Region: NODE\_388156\_length\_1812\_cov\_24.704746 1209-1211. Max. coverage (+): 1.69. Max coverage (-): 0

Region: NODE\_388156\_length\_1812\_cov\_24.704746 1212-1215. Max. coverage (+): 1.56. Max coverage (-): 0.04

Region: NODE\_388156\_length\_1812\_cov\_24.704746 1216-1219. Max. coverage (+): 0.3. Max coverage (-): 0.07

Region: NODE\_388156\_length\_1812\_cov\_24.704746 1220-1223. Max. coverage (+): 0.07. Max coverage (-): 0.07

Region: NODE\_388156\_length\_1812\_cov\_24.704746 1224-1226. Max. coverage (+): 0.04. Max coverage (-): 0.48

Region: NODE\_388156\_length\_1812\_cov\_24.704746 1227-1230. Max. coverage (+): 0. Max coverage (-): 0.44

Region: NODE\_388156\_length\_1812\_cov\_24.704746 1231-1234. Max. coverage (+): 0. Max coverage (-): 0.19

Region: NODE\_388156\_length\_1812\_cov\_24.704746 1235-1237. Max. coverage (+): 0.01. Max coverage (-): 0.07

Region: NODE\_388156\_length\_1812\_cov\_24.704746 1238-1241. Max. coverage (+): 0.08. Max coverage (-): 0.1

Region: NODE\_388156\_length\_1812\_cov\_24.704746 1242-1245. Max. coverage (+): 0.07. Max coverage (-): 0.22

Region: NODE\_388156\_length\_1812\_cov\_24.704746 1246-1249. Max. coverage (+): 0.09. Max coverage (-): 0.21

Region: NODE\_388156\_length\_1812\_cov\_24.704746 1250-1252. Max. coverage (+): 0.09. Max coverage (-): 0.09

Region: NODE\_388156\_length\_1812\_cov\_24.704746 1253-1256. Max. coverage (+): 0.1. Max coverage (-): 0.04

Region: NODE\_388156\_length\_1812\_cov\_24.704746 1257-1260. Max. coverage (+): 0.33. Max coverage (-): 0.07

Region: NODE\_388156\_length\_1812\_cov\_24.704746 1261-1263. Max. coverage (+): 0.22. Max coverage (-): 0

Region: NODE\_388156\_length\_1812\_cov\_24.704746 1264-1267. Max. coverage (+): 0.22. Max coverage (-): 0.07

Region: NODE\_388156\_length\_1812\_cov\_24.704746 1268-1271. Max. coverage (+): 0.37. Max coverage (-): 0.07

Region: NODE\_388156\_length\_1812\_cov\_24.704746 1272-1275. Max. coverage (+): 1.22. Max coverage (-): 0.15

Region: NODE\_388156\_length\_1812\_cov\_24.704746 1276-1278. Max. coverage (+): 1.15. Max coverage (-): 0.04

Region: NODE\_388156\_length\_1812\_cov\_24.704746 1279-1282. Max. coverage (+): 0.15. Max coverage (-): 0

Region: NODE\_388156\_length\_1812\_cov\_24.704746 1283-1286. Max. coverage (+): 0.01. Max coverage (-): 0

Region: NODE\_388156\_length\_1812\_cov\_24.704746 1287-1289. Max. coverage (+): 2.09. Max coverage (-): 0

Region: NODE\_388156\_length\_1812\_cov\_24.704746 1290-1293. Max. coverage (+): 8.8. Max coverage (-): 0

Region: NODE\_388156\_length\_1812\_cov\_24.704746 1294-1297. Max. coverage (+): 2.26. Max coverage (-): 0

Region: NODE\_388156\_length\_1812\_cov\_24.704746 1298-1301. Max. coverage (+): 0.63. Max coverage (-): 0

Region: NODE\_388156\_length\_1812\_cov\_24.704746 1302-1304. Max. coverage (+): 0.63. Max coverage (-): 0

Region: NODE\_388156\_length\_1812\_cov\_24.704746 1305-1308. Max. coverage (+): 0.41. Max coverage (-): 0

Region: NODE\_388156\_length\_1812\_cov\_24.704746 1309-1312. Max. coverage (+): 0. Max coverage (-): 0

Region: NODE\_388156\_length\_1812\_cov\_24.704746 1313-1316. Max. coverage (+): 0.07. Max coverage (-): 0.04

Region: NODE\_388156\_length\_1812\_cov\_24.704746 1317-1319. Max. coverage (+): 0.07. Max coverage (-): 0

Region: NODE\_388156\_length\_1812\_cov\_24.704746 1320-1323. Max. coverage (+): 0. Max coverage (-): 0

Region: NODE\_388156\_length\_1812\_cov\_24.704746 1324-1327. Max. coverage (+): 0.02. Max coverage (-): 0

Region: NODE\_388156\_length\_1812\_cov\_24.704746 1328-1330. Max. coverage (+): 0.01. Max coverage (-): 0.02

Region: NODE\_388156\_length\_1812\_cov\_24.704746 1331-1334. Max. coverage (+): 0.02. Max coverage (-): 0.01

Region: NODE\_388156\_length\_1812\_cov\_24.704746 1335-1338. Max. coverage (+): 0.02. Max coverage (-): 0

Region: NODE\_388156\_length\_1812\_cov\_24.704746 1339-1342. Max. coverage (+): 0.01. Max coverage (-): 0

Region: NODE\_388156\_length\_1812\_cov\_24.704746 1343-1345. Max. coverage (+): 0.08. Max coverage (-): 0

Region: NODE\_388156\_length\_1812\_cov\_24.704746 1346-1349. Max. coverage (+): 0.14. Max coverage (-): 0.01

Region: NODE\_388156\_length\_1812\_cov\_24.704746 1350-1353. Max. coverage (+): 0.14. Max coverage (-): 0.03

Region: NODE\_388156\_length\_1812\_cov\_24.704746 1354-1356. Max. coverage (+): 0.06. Max coverage (-): 0.06

Region: NODE\_388156\_length\_1812\_cov\_24.704746 1357-1360. Max. coverage (+): 0.06. Max coverage (-): 0.06

Region: NODE\_388156\_length\_1812\_cov\_24.704746 1361-1364. Max. coverage (+): 0. Max coverage (-): 0

Region: NODE\_388156\_length\_1812\_cov\_24.704746 1365-1368. Max. coverage (+): 0.02. Max coverage (-): 0

Region: NODE\_388156\_length\_1812\_cov\_24.704746 1369-1371. Max. coverage (+): 0.02. Max coverage (-): 0

Region: NODE\_388156\_length\_1812\_cov\_24.704746 1372-1375. Max. coverage (+): 0.01. Max coverage (-): 0.01

Region: NODE\_388156\_length\_1812\_cov\_24.704746 1376-1379. Max. coverage (+): 0.04. Max coverage (-): 0.01

Region: NODE\_388156\_length\_1812\_cov\_24.704746 1380-1382. Max. coverage (+): 0.03. Max coverage (-): 0.04

Region: NODE\_388156\_length\_1812\_cov\_24.704746 1383-1386. Max. coverage (+): 0.02. Max coverage (-): 0.11

Region: NODE\_388156\_length\_1812\_cov\_24.704746 1387-1390. Max. coverage (+): 0.11. Max coverage (-): 0.01

Region: NODE\_388156\_length\_1812\_cov\_24.704746 1391-1394. Max. coverage (+): 0.15. Max coverage (-): 0.02

Region: NODE\_388156\_length\_1812\_cov\_24.704746 1395-1397. Max. coverage (+): 1.06. Max coverage (-): 0.01

Region: NODE\_388156\_length\_1812\_cov\_24.704746 1398-1401. Max. coverage (+): 1. Max coverage (-): 0.02

Region: NODE\_388156\_length\_1812\_cov\_24.704746 1402-1405. Max. coverage (+): 0.02. Max coverage (-): 0.01

Region: NODE\_388156\_length\_1812\_cov\_24.704746 1406-1409. Max. coverage (+): 0.05. Max coverage (-): 0.09

Region: NODE\_388156\_length\_1812\_cov\_24.704746 1410-1412. Max. coverage (+): 0. Max coverage (-): 0.06

Region: NODE\_388156\_length\_1812\_cov\_24.704746 1413-1416. Max. coverage (+): 0.04. Max coverage (-): 0

Region: NODE\_388156\_length\_1812\_cov\_24.704746 1417-1420. Max. coverage (+): 0.04. Max coverage (-): 0

Region: NODE\_388156\_length\_1812\_cov\_24.704746 1421-1423. Max. coverage (+): 0.04. Max coverage (-): 0

Region: NODE\_388156\_length\_1812\_cov\_24.704746 1424-1427. Max. coverage (+): 0.19. Max coverage (-): 0

Region: NODE\_388156\_length\_1812\_cov\_24.704746 1428-1431. Max. coverage (+): 0.67. Max coverage (-): 0

Region: NODE\_388156\_length\_1812\_cov\_24.704746 1432-1435. Max. coverage (+): 0.78. Max coverage (-): 0

Region: NODE\_388156\_length\_1812\_cov\_24.704746 1436-1438. Max. coverage (+): 0.22. Max coverage (-): 0.04

Region: NODE\_388156\_length\_1812\_cov\_24.704746 1439-1442. Max. coverage (+): 0.07. Max coverage (-): 0.56

Region: NODE\_388156\_length\_1812\_cov\_24.704746 1443-1446. Max. coverage (+): 0.07. Max coverage (-): 0.63

Region: NODE\_388156\_length\_1812\_cov\_24.704746 1447-1449. Max. coverage (+): 0.04. Max coverage (-): 0.82

Region: NODE\_388156\_length\_1812\_cov\_24.704746 1450-1453. Max. coverage (+): 0.17. Max coverage (-): 0.74

Region: NODE\_388156\_length\_1812\_cov\_24.704746 1454-1457. Max. coverage (+): 0.22. Max coverage (-): 0.02

Region: NODE\_388156\_length\_1812\_cov\_24.704746 1458-1461. Max. coverage (+): 2.04. Max coverage (-): 0.04

Region: NODE\_388156\_length\_1812\_cov\_24.704746 1462-1464. Max. coverage (+): 1.95. Max coverage (-): 0

Region: NODE\_388156\_length\_1812\_cov\_24.704746 1465-1468. Max. coverage (+): 1.52. Max coverage (-): 0.06

Region: NODE\_388156\_length\_1812\_cov\_24.704746 1469-1472. Max. coverage (+): 0.04. Max coverage (-): 0.11

Region: NODE\_388156\_length\_1812\_cov\_24.704746 1473-1475. Max. coverage (+): 0.04. Max coverage (-): 0.19

Region: NODE\_388156\_length\_1812\_cov\_24.704746 1476-1479. Max. coverage (+): 0.04. Max coverage (-): 0.07

Region: NODE\_388156\_length\_1812\_cov\_24.704746 1480-1483. Max. coverage (+): 0. Max coverage (-): 0.04

Region: NODE\_388156\_length\_1812\_cov\_24.704746 1484-1487. Max. coverage (+): 0. Max coverage (-): 0.07

Region: NODE\_388156\_length\_1812\_cov\_24.704746 1488-1490. Max. coverage (+): 0.04. Max coverage (-): 0.04

Region: NODE\_388156\_length\_1812\_cov\_24.704746 1491-1494. Max. coverage (+): 0.04. Max coverage (-): 0

Region: NODE\_388156\_length\_1812\_cov\_24.704746 1495-1498. Max. coverage (+): 0. Max coverage (-): 0

Region: NODE\_388156\_length\_1812\_cov\_24.704746 1499-1502. Max. coverage (+): 0.22. Max coverage (-): 0

Region: NODE\_388156\_length\_1812\_cov\_24.704746 1503-1505. Max. coverage (+): 3. Max coverage (-): 0

Region: NODE\_388156\_length\_1812\_cov\_24.704746 1506-1509. Max. coverage (+): 5.08. Max coverage (-): 0

Region: NODE\_388156\_length\_1812\_cov\_24.704746 1510-1513. Max. coverage (+): 3.37. Max coverage (-): 0

Region: NODE\_388156\_length\_1812\_cov\_24.704746 1514-1516. Max. coverage (+): 0.26. Max coverage (-): 0.07

Region: NODE\_388156\_length\_1812\_cov\_24.704746 1517-1520. Max. coverage (+): 0.33. Max coverage (-): 0.11

Region: NODE\_388156\_length\_1812\_cov\_24.704746 1521-1524. Max. coverage (+): 1.37. Max coverage (-): 0.11

Region: NODE\_388156\_length\_1812\_cov\_24.704746 1525-1528. Max. coverage (+): 1. Max coverage (-): 0.01

Region: NODE\_388156\_length\_1812\_cov\_24.704746 1529-1531. Max. coverage (+): 0. Max coverage (-): 0.05

Region: NODE\_388156\_length\_1812\_cov\_24.704746 1532-1535. Max. coverage (+): 0.04. Max coverage (-): 0.04

Region: NODE\_388156\_length\_1812\_cov\_24.704746 1536-1539. Max. coverage (+): 0.01. Max coverage (-): 0.08

Region: NODE\_388156\_length\_1812\_cov\_24.704746 1540-1542. Max. coverage (+): 0.01. Max coverage (-): 0.06

Region: NODE\_388156\_length\_1812\_cov\_24.704746 1543-1546. Max. coverage (+): 0.01. Max coverage (-): 0.01

Region: NODE\_388156\_length\_1812\_cov\_24.704746 1547-1550. Max. coverage (+): 0.53. Max coverage (-): 0.01

Region: NODE\_388156\_length\_1812\_cov\_24.704746 1551-1554. Max. coverage (+): 0.68. Max coverage (-): 0.01

Region: NODE\_388156\_length\_1812\_cov\_24.704746 1555-1557. Max. coverage (+): 0.32. Max coverage (-): 0.07

Region: NODE\_388156\_length\_1812\_cov\_24.704746 1558-1561. Max. coverage (+): 0.19. Max coverage (-): 0.07

Region: NODE\_388156\_length\_1812\_cov\_24.704746 1562-1565. Max. coverage (+): 0.19. Max coverage (-): 0.07

Region: NODE\_388156\_length\_1812\_cov\_24.704746 1566-1568. Max. coverage (+): 0.07. Max coverage (-): 0

Region: NODE\_388156\_length\_1812\_cov\_24.704746 1569-1572. Max. coverage (+): 0.04. Max coverage (-): 0.15

Region: NODE\_388156\_length\_1812\_cov\_24.704746 1573-1576. Max. coverage (+): 0.07. Max coverage (-): 0

Region: NODE\_388156\_length\_1812\_cov\_24.704746 1577-1580. Max. coverage (+): 0.15. Max coverage (-): 0

Region: NODE\_388156\_length\_1812\_cov\_24.704746 1581-1583. Max. coverage (+): 0.19. Max coverage (-): 0

Region: NODE\_388156\_length\_1812\_cov\_24.704746 1584-1587. Max. coverage (+): 0.07. Max coverage (-): 0

Region: NODE\_388156\_length\_1812\_cov\_24.704746 1588-1591. Max. coverage (+): 0.04. Max coverage (-): 0

Region: NODE\_388156\_length\_1812\_cov\_24.704746 1592-1595. Max. coverage (+): 0. Max coverage (-): 0

Region: NODE\_388156\_length\_1812\_cov\_24.704746 1596-1598. Max. coverage (+): 0. Max coverage (-): 0

Region: NODE\_388156\_length\_1812\_cov\_24.704746 1599-1602. Max. coverage (+): 0. Max coverage (-): 0

Region: NODE\_388156\_length\_1812\_cov\_24.704746 1603-1606. Max. coverage (+): 0. Max coverage (-): 0

Region: NODE\_388156\_length\_1812\_cov\_24.704746 1607-1609. Max. coverage (+): 0. Max coverage (-): 0

Region: NODE\_388156\_length\_1812\_cov\_24.704746 1610-1613. Max. coverage (+): 0. Max coverage (-): 0

Region: NODE\_388156\_length\_1812\_cov\_24.704746 1614-1617. Max. coverage (+): 0. Max coverage (-): 0

Region: NODE\_388156\_length\_1812\_cov\_24.704746 1618-1621. Max. coverage (+): 0. Max coverage (-): 0

Region: NODE\_388156\_length\_1812\_cov\_24.704746 1622-1624. Max. coverage (+): 0. Max coverage (-): 0

Region: NODE\_388156\_length\_1812\_cov\_24.704746 1625-1628. Max. coverage (+): 0. Max coverage (-): 0

Region: NODE\_388156\_length\_1812\_cov\_24.704746 1629-1632. Max. coverage (+): 0. Max coverage (-): 0

Region: NODE\_388156\_length\_1812\_cov\_24.704746 1633-1635. Max. coverage (+): 0. Max coverage (-): 0

Region: NODE\_388156\_length\_1812\_cov\_24.704746 1636-1639. Max. coverage (+): 0. Max coverage (-): 0

Region: NODE\_388156\_length\_1812\_cov\_24.704746 1640-1643. Max. coverage (+): 0.01. Max coverage (-): 0

Region: NODE\_388156\_length\_1812\_cov\_24.704746 1644-1647. Max. coverage (+): 0.01. Max coverage (-): 0

Region: NODE\_388156\_length\_1812\_cov\_24.704746 1648-1650. Max. coverage (+): 0. Max coverage (-): 0

Region: NODE\_388156\_length\_1812\_cov\_24.704746 1651-1654. Max. coverage (+): 0. Max coverage (-): 0

Region: NODE\_388156\_length\_1812\_cov\_24.704746 1655-1658. Max. coverage (+): 0. Max coverage (-): 0

Region: NODE\_388156\_length\_1812\_cov\_24.704746 1659-1661. Max. coverage (+): 0. Max coverage (-): 0.04

Region: NODE\_388156\_length\_1812\_cov\_24.704746 1662-1665. Max. coverage (+): 0. Max coverage (-): 0.04

Region: NODE\_388156\_length\_1812\_cov\_24.704746 1666-1669. Max. coverage (+): 0. Max coverage (-): 0

Region: NODE\_388156\_length\_1812\_cov\_24.704746 1670-1673. Max. coverage (+): 0. Max coverage (-): 0

Region: NODE\_388156\_length\_1812\_cov\_24.704746 1674-1676. Max. coverage (+): 0. Max coverage (-): 0

Region: NODE\_388156\_length\_1812\_cov\_24.704746 1677-1680. Max. coverage (+): 0. Max coverage (-): 0

Region: NODE\_388156\_length\_1812\_cov\_24.704746 1681-1684. Max. coverage (+): 0.35. Max coverage (-): 0

Region: NODE\_388156\_length\_1812\_cov\_24.704746 1685-1688. Max. coverage (+): 0.35. Max coverage (-): 0.01

Region: NODE\_388156\_length\_1812\_cov\_24.704746 1689-1691. Max. coverage (+): 0. Max coverage (-): 0

Region: NODE\_388156\_length\_1812\_cov\_24.704746 1692-1695. Max. coverage (+): 0. Max coverage (-): 0.01

Region: NODE\_388156\_length\_1812\_cov\_24.704746 1696-1699. Max. coverage (+): 0. Max coverage (-): 0.06

Region: NODE\_388156\_length\_1812\_cov\_24.704746 1700-1702. Max. coverage (+): 0. Max coverage (-): 0.04

Region: NODE\_388156\_length\_1812\_cov\_24.704746 1703-1706. Max. coverage (+): 0.01. Max coverage (-): 0

Region: NODE\_388156\_length\_1812\_cov\_24.704746 1707-1710. Max. coverage (+): 0.01. Max coverage (-): 0

Region: NODE\_388156\_length\_1812\_cov\_24.704746 1711-1714. Max. coverage (+): 0.04. Max coverage (-): 0

Region: NODE\_388156\_length\_1812\_cov\_24.704746 1715-1717. Max. coverage (+): 0.02. Max coverage (-): 0

Region: NODE\_388156\_length\_1812\_cov\_24.704746 1718-1721. Max. coverage (+): 0.01. Max coverage (-): 0

Region: NODE\_388156\_length\_1812\_cov\_24.704746 1722-1725. Max. coverage (+): 0.01. Max coverage (-): 0

Region: NODE\_388156\_length\_1812\_cov\_24.704746 1726-1728. Max. coverage (+): 0.01. Max coverage (-): 0.01

Region: NODE\_388156\_length\_1812\_cov\_24.704746 1729-1732. Max. coverage (+): 0.03. Max coverage (-): 0.01

Region: NODE\_388156\_length\_1812\_cov\_24.704746 1733-1736. Max. coverage (+): 0.05. Max coverage (-): 0

Region: NODE\_388156\_length\_1812\_cov\_24.704746 1737-1740. Max. coverage (+): 0.17. Max coverage (-): 0

Region: NODE\_388156\_length\_1812\_cov\_24.704746 1741-1743. Max. coverage (+): 0.03. Max coverage (-): 0

Region: NODE\_388156\_length\_1812\_cov\_24.704746 1744-1747. Max. coverage (+): 0. Max coverage (-): 0

Region: NODE\_388156\_length\_1812\_cov\_24.704746 1748-1751. Max. coverage (+): 0. Max coverage (-): 0.03

Region: NODE\_388156\_length\_1812\_cov\_24.704746 1752-1754. Max. coverage (+): 0.02. Max coverage (-): 0.04

Region: NODE\_388156\_length\_1812\_cov\_24.704746 1755-1758. Max. coverage (+): 0.04. Max coverage (-): 0.01

Region: NODE\_388156\_length\_1812\_cov\_24.704746 1759-1762. Max. coverage (+): 0.03. Max coverage (-): 0.01

Region: NODE\_388156\_length\_1812\_cov\_24.704746 1763-1766. Max. coverage (+): 0.02. Max coverage (-): 0.01

Region: NODE\_388156\_length\_1812\_cov\_24.704746 1767-1769. Max. coverage (+): 0.02. Max coverage (-): 0

Region: NODE\_388156\_length\_1812\_cov\_24.704746 1770-1773. Max. coverage (+): 0.04. Max coverage (-): 0

Region: NODE\_388156\_length\_1812\_cov\_24.704746 1774-1777. Max. coverage (+): 0.04. Max coverage (-): 0

Region: NODE\_388156\_length\_1812\_cov\_24.704746 1778-1781. Max. coverage (+): 0.01. Max coverage (-): 0

Region: NODE\_388156\_length\_1812\_cov\_24.704746 1782-1784. Max. coverage (+): 0.01. Max coverage (-): 0

Region: NODE\_388156\_length\_1812\_cov\_24.704746 1785-1788. Max. coverage (+): 0.02. Max coverage (-): 0

Region: NODE\_388156\_length\_1812\_cov\_24.704746 1789-1792. Max. coverage (+): 0.01. Max coverage (-): 0

Region: NODE\_388156\_length\_1812\_cov\_24.704746 1793-1795. Max. coverage (+): 0.01. Max coverage (-): 0

Region: NODE\_388156\_length\_1812\_cov\_24.704746 1796-1799. Max. coverage (+): 0. Max coverage (-): 0.01

Region: NODE\_388156\_length\_1812\_cov\_24.704746 1800-1803. Max. coverage (+): 0. Max coverage (-): 0.01

Region: NODE\_388156\_length\_1812\_cov\_24.704746 1804-1807. Max. coverage (+): 0. Max coverage (-): 0

Region: NODE\_388156\_length\_1812\_cov\_24.704746 1808-1810. Max. coverage (+): 0. Max coverage (-): 0

Region: NODE\_388156\_length\_1812\_cov\_24.704746 1811-1814. Max. coverage (+): 0. Max coverage (-): 0

Region: NODE\_388156\_length\_1812\_cov\_24.704746 1815-1818. Max. coverage (+): 0. Max coverage (-): 0.01

Region: NODE\_388156\_length\_1812\_cov\_24.704746 1819-1821. Max. coverage (+): 0. Max coverage (-): 0.01

Region: NODE\_388156\_length\_1812\_cov\_24.704746 1822-1825. Max. coverage (+): 0. Max coverage (-): 0

Region: NODE\_388156\_length\_1812\_cov\_24.704746 1826-1829. Max. coverage (+): 0. Max coverage (-): 0

Region: NODE\_388156\_length\_1812\_cov\_24.704746 1830-1833. Max. coverage (+): 0.01. Max coverage (-): 0

Region: NODE\_388156\_length\_1812\_cov\_24.704746 1834-1836. Max. coverage (+): 0.01. Max coverage (-): 0

Region: NODE\_388156\_length\_1812\_cov\_24.704746 1837-1840. Max. coverage (+): 0. Max coverage (-): 0

Region: NODE\_388156\_length\_1812\_cov\_24.704746 1841-1844. Max. coverage (+): 0. Max coverage (-): 0

Region: NODE\_388156\_length\_1812\_cov\_24.704746 1845-1847. Max. coverage (+): 0. Max coverage (-): 0

Region: NODE\_388156\_length\_1812\_cov\_24.704746 1848-1851. Max. coverage (+): 0. Max coverage (-): 0

Region: NODE\_388156\_length\_1812\_cov\_24.704746 1852-1855. Max. coverage (+): 0. Max coverage (-): 0

Region: NODE\_388156\_length\_1812\_cov\_24.704746 1856-1859. Max. coverage (+): 0. Max coverage (-): 0

Region: NODE\_388156\_length\_1812\_cov\_24.704746 1860-. Max. coverage (+): 0. Max coverage (-): 0

RepeatMasker Color Code

**+**

100-98% Identity

<98-95% Identity

<95-90% Identity

<90-85% Identity

<85-80% Identity

<80-75% Identity

<75-70% Identity

<70% Identity

**-**

Gene Set Color Code

**+**

Gene

Pseudogene

Other

**-**

Topology/Coverage Color Code

Coverage Plus Strand

Coverage Minus Strand

Mainstrand: Plus

Mainstrand: Minus

Complementary Strand

Flanking Region  
(if option -flank >0)

Gene Set Annotation  
  
RepeatMasker Annotation  

**1. Gypsy-42\_GA-I**: 66-415 (+), Divergence to consensus: 36.7%  
**2. AlRepE-1201**: 425-527 (-), Divergence to consensus: 34.1%  
**3. AlRepE-1331**: 1076-1415 (-), Divergence to consensus: 32.5%  
**4. AlRepD-1055**: 1452-1573 (-), Divergence to consensus: 18.9%  
**5. AlRepB-356**: 1585-1740 (+), Divergence to consensus: 10.3%  
**6. AlRepB-356**: 1755-1826 (+), Divergence to consensus: 8.2%

  
Transcription Factor Binding Sites  

**RHOXF1** (Sequence: AGATCA (-): 57)  
**RHOXF1** (Sequence: GGATCA (-): 994)  
**RHOXF1** (Sequence: AGATTA (-): 1582)  
**RHOXF1** (Sequence: TGATCC (+): 236)  
**RHOXF1** (Sequence: TAATCT (+): 1237)  
**RHOXF1** (Sequence: TAATCC (+): 1451)  
**RHOXF1** (Sequence: TGATCC (+): 1574)  
**RFX4\_2** (Sequence: GTAACCATG (-): 1180)  
**FOXP1** (Sequence: GTAAACA (+): 796)  
**FOXO1** (Sequence: GTTGTTTAT (+): 870)  
**FOXO3\_mmu** (Sequence: TGTAAACA (+): 795)  
**FOXO3\_mmu** (Sequence: TGAAAACA (+): 1103)  
**Nobox** (Sequence: AGCAATTA (-): 755)  
**POU2F1** (Sequence: ATTAAAATA (-): 759)  
**Sox5** (Sequence: AACAAT (-): 1320)
