# Supplementary material for: First characterization of PIWI-interacting RNA clusters in a cichlid fish with a B chromosome
Source: BMC Biol. 2022 Sep 21;20:204. doi: 10.1186/s12915-022-01403-2 (PMC9490952; doi:10.1186/s12915-022-01403-2)
Supplement: Supplementary file 1 — Additional file 1. Zipped folder with fasta and interactive html piRNA cluster information for the A. latifasciata genome. The nomenclature is as follows: number-pirna-cluster_sex_B-presence (f, female; m, male; 0b, without B chromosome; 1b, with B chromosome). [file 12915_2022_1403_MOESM1_ESM.zip › 152_m0b.html]

piRNA cluster 152\_m0b 74


Predicted piRNA cluster no. 152\_m0b
  

Show proTRAC run info
Hide proTRAC run info

/\  
                \_\_\_\_\_\_\_\_\_\_\_\_\_\_\_\_\_\_\_\_\_\_\_/\\_\_\_ /  \\_\_\_\_\_\_\_  
               I                      /  \  /    \      I  
               I     pro             /    \/      \     I  
               I        TRAC        /               \   I  
               I   \_\_\_\_\_\_\_\_\_\_\_\_\_\_\_\_/\_\_\_\_\_\_\_\_\_\_\_\_\_\_\_\_\_\\_ I  
               I   \              /                     I  
               I    \            /                      I  
               I     \  /\      /       V.2.4.2         I  
               I      \/  \    /                        I  
               I\_\_\_\_\_\_\_\_\_\_\_\  /\_\_\_\_\_\_\_\_\_\_\_\_\_\_\_\_\_\_\_\_\_\_\_\_\_I  
                            \/  
  
  
================================= proTRAC ====================================  
VERSION: .......... 2.4.2  
LAST MODIFIED: .... 11. May 2018  
  
Please cite:  
Rosenkranz D, Zischler H. proTRAC - a software for probabilistic piRNA cluster  
detection, visualization and analysis. 2012. BMC Bioinformatics 13:5.  
  
  
Contact:  
David Rosenkranz  
Institute of Organismic and Molecular Evolutionary Biology  
Dept. Anthropology, small RNA group  
Johannes Gutenberg University Mainz  
email: rosenkranz@uni-mainz.de  
  
You can find the latest proTRAC version at:  
http://sourceforge.net/projects/protrac/files  
http://www.smallRNAgroup-mainz.de/software  
==============================================================================  
  
PARAMETERS:  
Map file: ...............piwi-machos-0B.fa-collapse.map  
Genome file: ............../../../0B\_ala\_genome.fa  
RepeatMasker annotation: Alatifasciata-all0B-maryan-v2.fa\_corrected.out  
GeneSet:................./guest-storage/Data/annotation/Alatifasciata\_all0B\_maryan-v2\_out2017.gff  
  
Significant (p<=0.01) hit density will be calculated based  
on observed hit distribution.  
  
Sliding window size: ........................................ 5000 bp  
Sliding window increament: .................................. 1000 bp  
Normalize each hit by number of genomic hits: ............... yes  
Normalize each hit by number of sequence reads: ............. yes  
Normalize values (-> per million mapped reads): ............. yes  
Min. fraction of hits with 1T(U) or 10A: .................... 0.75  
Alternatively: Min. fraction of hits with 1T(U) and 10A: .... 0.5  
Min. fraction of hits with typical piRNA length: ............ 0.75  
Typical piRNA length: ....................................... 24-32 nt  
Min. size of a piRNA cluster: ............................... 1000 bp.  
Min. number of hits (absolute): ............................. 0  
Min. number of hits (normalized): ........................... 0  
Min. fraction of hits on the mainstrand: .................... 0.75  
Top fraction of mapped sequences (in terms of read counts): . 1%  
Top fraction accounts for max. n% of sequence reads: ........ 90%  
Min. fraction of hits on each arm of a bidirectional cluster: 0.05  
Output html file for each cluster: .......................... yes  
Output a summary table: ..................................... yes  
Output a FASTA file for each cluster (piRNA sequences): ..... yes  
Output a FASTA file comprising cluster sequences: ........... yes  
Output a GTF file for predicted piRNA clusters: ..............yes  
Search DNA motifs in clusters: .............................. yes  
Output flanking sequences: +/- .............................. 0 bp  
Output ~.pTi file: .......................................... no  
==============================================================================  
  
  
Genome size (without gaps): ............ 758543724 bp  
Gaps (N/X/-): .......................... 417479 bp  
Mapped reads: .......................... 24765598  
Non-identical sequences: ............... 6158275  
Genomic hits: .......................... 53103584  
Significant densitiy of mapped reads: .. 763.098963422187 reads/kb

Show proTRAC cluster info
Hide proTRAC cluster info

|  |  |
| --- | --- |
| Location | NODE\_389151\_length\_73825\_cov\_29.945791 |
| Coordinates | 19195-23544 |
| Size [bp] | 4350 |
| Sequence hit loci | 2039 |
| Mapped reads (normalized) | 7368.7 |
| Mapped reads (normalized) per kb | 1693.9 |
| Normalized reads with 1T (1U) | 80.9% |
| Normalized reads with 10A | 38.8% |
| Normalized reads with length 24-32 nt | 99.2% |
| Normalized reads on the main strand(s) | 90.6% |
| Predicted directionality | mono:minus |

100%

0%

1T (1U)  
reads

10A reads

24-32 nt  
reads

reads on mainstrand

**Either the amount of reads with 1T (1U) OR 10A has to exceed 75% (set with option: -1Tor10A)  
Alternatively the amount of reads with 1T (1U) AND 10A has to exceed 50% (set with option: -1Tand10A)  
Minimum amount of reads with preferred size is 75% (set with option: -pisize)  
Minimum amount of reads on the main strand(s) is 75% (set with option: -clstrand)**

Show read coverage
Hide read coverage

WHAT DO I SEE HERE?  
This chart shows the location of mapped sequence reads within a predicted piRNA cluster. The color refers to the number of genomic hits produced by the sequence read in question. A dark red bar indicates that this sequence read produces many other hits elsewhere in the genome. Many adjacent red or yellow bars can indicate the presence of a multi-copy element such as transposons or rRNA genes. A dark green bar indicates that this sequence read maps uniquely to this locus.

1 hit

2-5 hits

6-10 hits

11-20 hits

21-50 hits

51-100 hits

> 100 hits

NODE\_389151\_length\_73825\_cov\_29.945791

19195

23544

Gene Set

RepeatMasker

Mapped  
Reads

26.47

plus strand

minus strand

26.47

Region: NODE\_389151\_length\_73825\_cov\_29.945791 8734-19199. Max. coverage (+): 0.04. Max coverage (-): 0

Region: NODE\_389151\_length\_73825\_cov\_29.945791 19200-19208. Max. coverage (+): 0. Max coverage (-): 0

Region: NODE\_389151\_length\_73825\_cov\_29.945791 19209-19216. Max. coverage (+): 0. Max coverage (-): 0

Region: NODE\_389151\_length\_73825\_cov\_29.945791 19217-19225. Max. coverage (+): 0. Max coverage (-): 0

Region: NODE\_389151\_length\_73825\_cov\_29.945791 19226-19234. Max. coverage (+): 0. Max coverage (-): 0

Region: NODE\_389151\_length\_73825\_cov\_29.945791 19235-19242. Max. coverage (+): 0. Max coverage (-): 0

Region: NODE\_389151\_length\_73825\_cov\_29.945791 19243-19251. Max. coverage (+): 0. Max coverage (-): 0

Region: NODE\_389151\_length\_73825\_cov\_29.945791 19252-19260. Max. coverage (+): 0. Max coverage (-): 0

Region: NODE\_389151\_length\_73825\_cov\_29.945791 19261-19268. Max. coverage (+): 0. Max coverage (-): 0

Region: NODE\_389151\_length\_73825\_cov\_29.945791 19269-19277. Max. coverage (+): 0. Max coverage (-): 0

Region: NODE\_389151\_length\_73825\_cov\_29.945791 19278-19286. Max. coverage (+): 0. Max coverage (-): 0

Region: NODE\_389151\_length\_73825\_cov\_29.945791 19287-19295. Max. coverage (+): 0. Max coverage (-): 0

Region: NODE\_389151\_length\_73825\_cov\_29.945791 19296-19303. Max. coverage (+): 0. Max coverage (-): 0

Region: NODE\_389151\_length\_73825\_cov\_29.945791 19304-19312. Max. coverage (+): 0. Max coverage (-): 0

Region: NODE\_389151\_length\_73825\_cov\_29.945791 19313-19321. Max. coverage (+): 0. Max coverage (-): 0

Region: NODE\_389151\_length\_73825\_cov\_29.945791 19322-19329. Max. coverage (+): 0. Max coverage (-): 0

Region: NODE\_389151\_length\_73825\_cov\_29.945791 19330-19338. Max. coverage (+): 0. Max coverage (-): 0

Region: NODE\_389151\_length\_73825\_cov\_29.945791 19339-19347. Max. coverage (+): 0. Max coverage (-): 0

Region: NODE\_389151\_length\_73825\_cov\_29.945791 19348-19355. Max. coverage (+): 0. Max coverage (-): 0

Region: NODE\_389151\_length\_73825\_cov\_29.945791 19356-19364. Max. coverage (+): 0. Max coverage (-): 0

Region: NODE\_389151\_length\_73825\_cov\_29.945791 19365-19373. Max. coverage (+): 0. Max coverage (-): 0

Region: NODE\_389151\_length\_73825\_cov\_29.945791 19374-19382. Max. coverage (+): 0. Max coverage (-): 0

Region: NODE\_389151\_length\_73825\_cov\_29.945791 19383-19390. Max. coverage (+): 0. Max coverage (-): 0

Region: NODE\_389151\_length\_73825\_cov\_29.945791 19391-19399. Max. coverage (+): 0. Max coverage (-): 0

Region: NODE\_389151\_length\_73825\_cov\_29.945791 19400-19408. Max. coverage (+): 0. Max coverage (-): 0

Region: NODE\_389151\_length\_73825\_cov\_29.945791 19409-19416. Max. coverage (+): 0. Max coverage (-): 0

Region: NODE\_389151\_length\_73825\_cov\_29.945791 19417-19425. Max. coverage (+): 0. Max coverage (-): 0.04

Region: NODE\_389151\_length\_73825\_cov\_29.945791 19426-19434. Max. coverage (+): 0. Max coverage (-): 0.08

Region: NODE\_389151\_length\_73825\_cov\_29.945791 19435-19442. Max. coverage (+): 0. Max coverage (-): 0

Region: NODE\_389151\_length\_73825\_cov\_29.945791 19443-19451. Max. coverage (+): 0. Max coverage (-): 0

Region: NODE\_389151\_length\_73825\_cov\_29.945791 19452-19460. Max. coverage (+): 0. Max coverage (-): 0

Region: NODE\_389151\_length\_73825\_cov\_29.945791 19461-19469. Max. coverage (+): 0. Max coverage (-): 0

Region: NODE\_389151\_length\_73825\_cov\_29.945791 19470-19477. Max. coverage (+): 0. Max coverage (-): 0

Region: NODE\_389151\_length\_73825\_cov\_29.945791 19478-19486. Max. coverage (+): 0. Max coverage (-): 0

Region: NODE\_389151\_length\_73825\_cov\_29.945791 19487-19495. Max. coverage (+): 0. Max coverage (-): 0

Region: NODE\_389151\_length\_73825\_cov\_29.945791 19496-19503. Max. coverage (+): 0. Max coverage (-): 0

Region: NODE\_389151\_length\_73825\_cov\_29.945791 19504-19512. Max. coverage (+): 0. Max coverage (-): 0

Region: NODE\_389151\_length\_73825\_cov\_29.945791 19513-19521. Max. coverage (+): 0. Max coverage (-): 0

Region: NODE\_389151\_length\_73825\_cov\_29.945791 19522-19529. Max. coverage (+): 0. Max coverage (-): 0

Region: NODE\_389151\_length\_73825\_cov\_29.945791 19530-19538. Max. coverage (+): 0. Max coverage (-): 0

Region: NODE\_389151\_length\_73825\_cov\_29.945791 19539-19547. Max. coverage (+): 0. Max coverage (-): 0

Region: NODE\_389151\_length\_73825\_cov\_29.945791 19548-19556. Max. coverage (+): 0. Max coverage (-): 0

Region: NODE\_389151\_length\_73825\_cov\_29.945791 19557-19564. Max. coverage (+): 0. Max coverage (-): 0

Region: NODE\_389151\_length\_73825\_cov\_29.945791 19565-19573. Max. coverage (+): 0. Max coverage (-): 0

Region: NODE\_389151\_length\_73825\_cov\_29.945791 19574-19582. Max. coverage (+): 0. Max coverage (-): 0

Region: NODE\_389151\_length\_73825\_cov\_29.945791 19583-19590. Max. coverage (+): 0. Max coverage (-): 0

Region: NODE\_389151\_length\_73825\_cov\_29.945791 19591-19599. Max. coverage (+): 0. Max coverage (-): 0

Region: NODE\_389151\_length\_73825\_cov\_29.945791 19600-19608. Max. coverage (+): 0. Max coverage (-): 0

Region: NODE\_389151\_length\_73825\_cov\_29.945791 19609-19616. Max. coverage (+): 0. Max coverage (-): 0

Region: NODE\_389151\_length\_73825\_cov\_29.945791 19617-19625. Max. coverage (+): 0. Max coverage (-): 0

Region: NODE\_389151\_length\_73825\_cov\_29.945791 19626-19634. Max. coverage (+): 0. Max coverage (-): 0.02

Region: NODE\_389151\_length\_73825\_cov\_29.945791 19635-19643. Max. coverage (+): 0. Max coverage (-): 0.1

Region: NODE\_389151\_length\_73825\_cov\_29.945791 19644-19651. Max. coverage (+): 0. Max coverage (-): 0.2

Region: NODE\_389151\_length\_73825\_cov\_29.945791 19652-19660. Max. coverage (+): 0.02. Max coverage (-): 13.55

Region: NODE\_389151\_length\_73825\_cov\_29.945791 19661-19669. Max. coverage (+): 0.02. Max coverage (-): 3.39

Region: NODE\_389151\_length\_73825\_cov\_29.945791 19670-19677. Max. coverage (+): 0.1. Max coverage (-): 1.01

Region: NODE\_389151\_length\_73825\_cov\_29.945791 19678-19686. Max. coverage (+): 0. Max coverage (-): 1.33

Region: NODE\_389151\_length\_73825\_cov\_29.945791 19687-19695. Max. coverage (+): 0.02. Max coverage (-): 1.19

Region: NODE\_389151\_length\_73825\_cov\_29.945791 19696-19703. Max. coverage (+): 0.16. Max coverage (-): 0.04

Region: NODE\_389151\_length\_73825\_cov\_29.945791 19704-19712. Max. coverage (+): 0.14. Max coverage (-): 0.04

Region: NODE\_389151\_length\_73825\_cov\_29.945791 19713-19721. Max. coverage (+): 0. Max coverage (-): 0

Region: NODE\_389151\_length\_73825\_cov\_29.945791 19722-19730. Max. coverage (+): 0.02. Max coverage (-): 0

Region: NODE\_389151\_length\_73825\_cov\_29.945791 19731-19738. Max. coverage (+): 0.04. Max coverage (-): 0.03

Region: NODE\_389151\_length\_73825\_cov\_29.945791 19739-19747. Max. coverage (+): 0.08. Max coverage (-): 0.04

Region: NODE\_389151\_length\_73825\_cov\_29.945791 19748-19756. Max. coverage (+): 0.77. Max coverage (-): 0.08

Region: NODE\_389151\_length\_73825\_cov\_29.945791 19757-19764. Max. coverage (+): 0.4. Max coverage (-): 0.08

Region: NODE\_389151\_length\_73825\_cov\_29.945791 19765-19773. Max. coverage (+): 0. Max coverage (-): 0.04

Region: NODE\_389151\_length\_73825\_cov\_29.945791 19774-19782. Max. coverage (+): 0. Max coverage (-): 0.04

Region: NODE\_389151\_length\_73825\_cov\_29.945791 19783-19790. Max. coverage (+): 0. Max coverage (-): 0.04

Region: NODE\_389151\_length\_73825\_cov\_29.945791 19791-19799. Max. coverage (+): 0.08. Max coverage (-): 3.43

Region: NODE\_389151\_length\_73825\_cov\_29.945791 19800-19808. Max. coverage (+): 0.12. Max coverage (-): 2.06

Region: NODE\_389151\_length\_73825\_cov\_29.945791 19809-19817. Max. coverage (+): 0.08. Max coverage (-): 0.52

Region: NODE\_389151\_length\_73825\_cov\_29.945791 19818-19825. Max. coverage (+): 0.12. Max coverage (-): 0

Region: NODE\_389151\_length\_73825\_cov\_29.945791 19826-19834. Max. coverage (+): 0.04. Max coverage (-): 11.06

Region: NODE\_389151\_length\_73825\_cov\_29.945791 19835-19843. Max. coverage (+): 0.04. Max coverage (-): 10.34

Region: NODE\_389151\_length\_73825\_cov\_29.945791 19844-19851. Max. coverage (+): 0.12. Max coverage (-): 1.13

Region: NODE\_389151\_length\_73825\_cov\_29.945791 19852-19860. Max. coverage (+): 0.16. Max coverage (-): 11.63

Region: NODE\_389151\_length\_73825\_cov\_29.945791 19861-19869. Max. coverage (+): 0. Max coverage (-): 0.4

Region: NODE\_389151\_length\_73825\_cov\_29.945791 19870-19877. Max. coverage (+): 0.08. Max coverage (-): 0.52

Region: NODE\_389151\_length\_73825\_cov\_29.945791 19878-19886. Max. coverage (+): 0.08. Max coverage (-): 0.08

Region: NODE\_389151\_length\_73825\_cov\_29.945791 19887-19895. Max. coverage (+): 0.16. Max coverage (-): 0.2

Region: NODE\_389151\_length\_73825\_cov\_29.945791 19896-19904. Max. coverage (+): 0.12. Max coverage (-): 0.24

Region: NODE\_389151\_length\_73825\_cov\_29.945791 19905-19912. Max. coverage (+): 0.08. Max coverage (-): 1.57

Region: NODE\_389151\_length\_73825\_cov\_29.945791 19913-19921. Max. coverage (+): 0.12. Max coverage (-): 2.46

Region: NODE\_389151\_length\_73825\_cov\_29.945791 19922-19930. Max. coverage (+): 0.08. Max coverage (-): 2.5

Region: NODE\_389151\_length\_73825\_cov\_29.945791 19931-19938. Max. coverage (+): 0.04. Max coverage (-): 1.7

Region: NODE\_389151\_length\_73825\_cov\_29.945791 19939-19947. Max. coverage (+): 0.12. Max coverage (-): 0.52

Region: NODE\_389151\_length\_73825\_cov\_29.945791 19948-19956. Max. coverage (+): 0.08. Max coverage (-): 0.08

Region: NODE\_389151\_length\_73825\_cov\_29.945791 19957-19964. Max. coverage (+): 0.04. Max coverage (-): 0.08

Region: NODE\_389151\_length\_73825\_cov\_29.945791 19965-19973. Max. coverage (+): 0. Max coverage (-): 1.74

Region: NODE\_389151\_length\_73825\_cov\_29.945791 19974-19982. Max. coverage (+): 0. Max coverage (-): 1.74

Region: NODE\_389151\_length\_73825\_cov\_29.945791 19983-19991. Max. coverage (+): 0. Max coverage (-): 0.04

Region: NODE\_389151\_length\_73825\_cov\_29.945791 19992-19999. Max. coverage (+): 0.16. Max coverage (-): 8.48

Region: NODE\_389151\_length\_73825\_cov\_29.945791 20000-20008. Max. coverage (+): 0.32. Max coverage (-): 8.24

Region: NODE\_389151\_length\_73825\_cov\_29.945791 20009-20017. Max. coverage (+): 0.12. Max coverage (-): 0.08

Region: NODE\_389151\_length\_73825\_cov\_29.945791 20018-20025. Max. coverage (+): 0.08. Max coverage (-): 0.04

Region: NODE\_389151\_length\_73825\_cov\_29.945791 20026-20034. Max. coverage (+): 0. Max coverage (-): 0.08

Region: NODE\_389151\_length\_73825\_cov\_29.945791 20035-20043. Max. coverage (+): 0. Max coverage (-): 0

Region: NODE\_389151\_length\_73825\_cov\_29.945791 20044-20051. Max. coverage (+): 0. Max coverage (-): 0

Region: NODE\_389151\_length\_73825\_cov\_29.945791 20052-20060. Max. coverage (+): 0. Max coverage (-): 0

Region: NODE\_389151\_length\_73825\_cov\_29.945791 20061-20069. Max. coverage (+): 0. Max coverage (-): 0.04

Region: NODE\_389151\_length\_73825\_cov\_29.945791 20070-20078. Max. coverage (+): 0. Max coverage (-): 0

Region: NODE\_389151\_length\_73825\_cov\_29.945791 20079-20086. Max. coverage (+): 0. Max coverage (-): 0

Region: NODE\_389151\_length\_73825\_cov\_29.945791 20087-20095. Max. coverage (+): 0. Max coverage (-): 0.36

Region: NODE\_389151\_length\_73825\_cov\_29.945791 20096-20104. Max. coverage (+): 0. Max coverage (-): 1.01

Region: NODE\_389151\_length\_73825\_cov\_29.945791 20105-20112. Max. coverage (+): 0.12. Max coverage (-): 0.89

Region: NODE\_389151\_length\_73825\_cov\_29.945791 20113-20121. Max. coverage (+): 0.12. Max coverage (-): 0.16

Region: NODE\_389151\_length\_73825\_cov\_29.945791 20122-20130. Max. coverage (+): 0. Max coverage (-): 1.7

Region: NODE\_389151\_length\_73825\_cov\_29.945791 20131-20138. Max. coverage (+): 0.04. Max coverage (-): 1.82

Region: NODE\_389151\_length\_73825\_cov\_29.945791 20139-20147. Max. coverage (+): 0.69. Max coverage (-): 0.24

Region: NODE\_389151\_length\_73825\_cov\_29.945791 20148-20156. Max. coverage (+): 0.24. Max coverage (-): 0.2

Region: NODE\_389151\_length\_73825\_cov\_29.945791 20157-20165. Max. coverage (+): 0.44. Max coverage (-): 0.12

Region: NODE\_389151\_length\_73825\_cov\_29.945791 20166-20173. Max. coverage (+): 0.08. Max coverage (-): 0.57

Region: NODE\_389151\_length\_73825\_cov\_29.945791 20174-20182. Max. coverage (+): 0.08. Max coverage (-): 0.65

Region: NODE\_389151\_length\_73825\_cov\_29.945791 20183-20191. Max. coverage (+): 0.28. Max coverage (-): 1.13

Region: NODE\_389151\_length\_73825\_cov\_29.945791 20192-20199. Max. coverage (+): 0.36. Max coverage (-): 1.21

Region: NODE\_389151\_length\_73825\_cov\_29.945791 20200-20208. Max. coverage (+): 0.04. Max coverage (-): 0.52

Region: NODE\_389151\_length\_73825\_cov\_29.945791 20209-20217. Max. coverage (+): 0. Max coverage (-): 0.73

Region: NODE\_389151\_length\_73825\_cov\_29.945791 20218-20225. Max. coverage (+): 0.08. Max coverage (-): 0

Region: NODE\_389151\_length\_73825\_cov\_29.945791 20226-20234. Max. coverage (+): 0. Max coverage (-): 0

Region: NODE\_389151\_length\_73825\_cov\_29.945791 20235-20243. Max. coverage (+): 0. Max coverage (-): 0.04

Region: NODE\_389151\_length\_73825\_cov\_29.945791 20244-20252. Max. coverage (+): 0. Max coverage (-): 0

Region: NODE\_389151\_length\_73825\_cov\_29.945791 20253-20260. Max. coverage (+): 0. Max coverage (-): 0

Region: NODE\_389151\_length\_73825\_cov\_29.945791 20261-20269. Max. coverage (+): 0. Max coverage (-): 1.13

Region: NODE\_389151\_length\_73825\_cov\_29.945791 20270-20278. Max. coverage (+): 0. Max coverage (-): 1.53

Region: NODE\_389151\_length\_73825\_cov\_29.945791 20279-20286. Max. coverage (+): 0. Max coverage (-): 0.16

Region: NODE\_389151\_length\_73825\_cov\_29.945791 20287-20295. Max. coverage (+): 0. Max coverage (-): 0.16

Region: NODE\_389151\_length\_73825\_cov\_29.945791 20296-20304. Max. coverage (+): 0. Max coverage (-): 0.12

Region: NODE\_389151\_length\_73825\_cov\_29.945791 20305-20312. Max. coverage (+): 0.04. Max coverage (-): 0.4

Region: NODE\_389151\_length\_73825\_cov\_29.945791 20313-20321. Max. coverage (+): 0.08. Max coverage (-): 0.24

Region: NODE\_389151\_length\_73825\_cov\_29.945791 20322-20330. Max. coverage (+): 0.04. Max coverage (-): 0.24

Region: NODE\_389151\_length\_73825\_cov\_29.945791 20331-20339. Max. coverage (+): 0. Max coverage (-): 0.61

Region: NODE\_389151\_length\_73825\_cov\_29.945791 20340-20347. Max. coverage (+): 0. Max coverage (-): 0.24

Region: NODE\_389151\_length\_73825\_cov\_29.945791 20348-20356. Max. coverage (+): 0.04. Max coverage (-): 0.04

Region: NODE\_389151\_length\_73825\_cov\_29.945791 20357-20365. Max. coverage (+): 0. Max coverage (-): 0.2

Region: NODE\_389151\_length\_73825\_cov\_29.945791 20366-20373. Max. coverage (+): 0.08. Max coverage (-): 5.09

Region: NODE\_389151\_length\_73825\_cov\_29.945791 20374-20382. Max. coverage (+): 0.48. Max coverage (-): 4.36

Region: NODE\_389151\_length\_73825\_cov\_29.945791 20383-20391. Max. coverage (+): 0.36. Max coverage (-): 0.69

Region: NODE\_389151\_length\_73825\_cov\_29.945791 20392-20399. Max. coverage (+): 0.24. Max coverage (-): 1.74

Region: NODE\_389151\_length\_73825\_cov\_29.945791 20400-20408. Max. coverage (+): 0.08. Max coverage (-): 5.21

Region: NODE\_389151\_length\_73825\_cov\_29.945791 20409-20417. Max. coverage (+): 0.16. Max coverage (-): 0.32

Region: NODE\_389151\_length\_73825\_cov\_29.945791 20418-20426. Max. coverage (+): 0.24. Max coverage (-): 0.61

Region: NODE\_389151\_length\_73825\_cov\_29.945791 20427-20434. Max. coverage (+): 0. Max coverage (-): 0.08

Region: NODE\_389151\_length\_73825\_cov\_29.945791 20435-20443. Max. coverage (+): 0. Max coverage (-): 0.32

Region: NODE\_389151\_length\_73825\_cov\_29.945791 20444-20452. Max. coverage (+): 0.08. Max coverage (-): 3.35

Region: NODE\_389151\_length\_73825\_cov\_29.945791 20453-20460. Max. coverage (+): 0.12. Max coverage (-): 2.14

Region: NODE\_389151\_length\_73825\_cov\_29.945791 20461-20469. Max. coverage (+): 0. Max coverage (-): 1.86

Region: NODE\_389151\_length\_73825\_cov\_29.945791 20470-20478. Max. coverage (+): 0.12. Max coverage (-): 2.99

Region: NODE\_389151\_length\_73825\_cov\_29.945791 20479-20486. Max. coverage (+): 0.16. Max coverage (-): 1.09

Region: NODE\_389151\_length\_73825\_cov\_29.945791 20487-20495. Max. coverage (+): 0.08. Max coverage (-): 0.12

Region: NODE\_389151\_length\_73825\_cov\_29.945791 20496-20504. Max. coverage (+): 0.04. Max coverage (-): 3.62

Region: NODE\_389151\_length\_73825\_cov\_29.945791 20505-20513. Max. coverage (+): 0.04. Max coverage (-): 4.94

Region: NODE\_389151\_length\_73825\_cov\_29.945791 20514-20521. Max. coverage (+): 0.24. Max coverage (-): 0.65

Region: NODE\_389151\_length\_73825\_cov\_29.945791 20522-20530. Max. coverage (+): 0.08. Max coverage (-): 0.4

Region: NODE\_389151\_length\_73825\_cov\_29.945791 20531-20539. Max. coverage (+): 0.04. Max coverage (-): 0.32

Region: NODE\_389151\_length\_73825\_cov\_29.945791 20540-20547. Max. coverage (+): 0.04. Max coverage (-): 0.52

Region: NODE\_389151\_length\_73825\_cov\_29.945791 20548-20556. Max. coverage (+): 0.04. Max coverage (-): 0.12

Region: NODE\_389151\_length\_73825\_cov\_29.945791 20557-20565. Max. coverage (+): 0. Max coverage (-): 0.08

Region: NODE\_389151\_length\_73825\_cov\_29.945791 20566-20573. Max. coverage (+): 0. Max coverage (-): 0.04

Region: NODE\_389151\_length\_73825\_cov\_29.945791 20574-20582. Max. coverage (+): 0. Max coverage (-): 8.96

Region: NODE\_389151\_length\_73825\_cov\_29.945791 20583-20591. Max. coverage (+): 0. Max coverage (-): 5.57

Region: NODE\_389151\_length\_73825\_cov\_29.945791 20592-20600. Max. coverage (+): 0. Max coverage (-): 0.28

Region: NODE\_389151\_length\_73825\_cov\_29.945791 20601-20608. Max. coverage (+): 0. Max coverage (-): 0.12

Region: NODE\_389151\_length\_73825\_cov\_29.945791 20609-20617. Max. coverage (+): 0.04. Max coverage (-): 0.81

Region: NODE\_389151\_length\_73825\_cov\_29.945791 20618-20626. Max. coverage (+): 0.12. Max coverage (-): 0.16

Region: NODE\_389151\_length\_73825\_cov\_29.945791 20627-20634. Max. coverage (+): 0.18. Max coverage (-): 11.89

Region: NODE\_389151\_length\_73825\_cov\_29.945791 20635-20643. Max. coverage (+): 0. Max coverage (-): 1.8

Region: NODE\_389151\_length\_73825\_cov\_29.945791 20644-20652. Max. coverage (+): 0.08. Max coverage (-): 3.71

Region: NODE\_389151\_length\_73825\_cov\_29.945791 20653-20660. Max. coverage (+): 0. Max coverage (-): 6.66

Region: NODE\_389151\_length\_73825\_cov\_29.945791 20661-20669. Max. coverage (+): 0. Max coverage (-): 0.93

Region: NODE\_389151\_length\_73825\_cov\_29.945791 20670-20678. Max. coverage (+): 0.1. Max coverage (-): 3.82

Region: NODE\_389151\_length\_73825\_cov\_29.945791 20679-20687. Max. coverage (+): 0.12. Max coverage (-): 1.19

Region: NODE\_389151\_length\_73825\_cov\_29.945791 20688-20695. Max. coverage (+): 2.12. Max coverage (-): 1.84

Region: NODE\_389151\_length\_73825\_cov\_29.945791 20696-20704. Max. coverage (+): 0.04. Max coverage (-): 8.72

Region: NODE\_389151\_length\_73825\_cov\_29.945791 20705-20713. Max. coverage (+): 2.42. Max coverage (-): 8.76

Region: NODE\_389151\_length\_73825\_cov\_29.945791 20714-20721. Max. coverage (+): 2.99. Max coverage (-): 0.85

Region: NODE\_389151\_length\_73825\_cov\_29.945791 20722-20730. Max. coverage (+): 2.87. Max coverage (-): 0.24

Region: NODE\_389151\_length\_73825\_cov\_29.945791 20731-20739. Max. coverage (+): 0.32. Max coverage (-): 4.04

Region: NODE\_389151\_length\_73825\_cov\_29.945791 20740-20747. Max. coverage (+): 0.12. Max coverage (-): 4.12

Region: NODE\_389151\_length\_73825\_cov\_29.945791 20748-20756. Max. coverage (+): 0.12. Max coverage (-): 4.2

Region: NODE\_389151\_length\_73825\_cov\_29.945791 20757-20765. Max. coverage (+): 0.4. Max coverage (-): 0.69

Region: NODE\_389151\_length\_73825\_cov\_29.945791 20766-20774. Max. coverage (+): 0.36. Max coverage (-): 0.44

Region: NODE\_389151\_length\_73825\_cov\_29.945791 20775-20782. Max. coverage (+): 0.2. Max coverage (-): 0.65

Region: NODE\_389151\_length\_73825\_cov\_29.945791 20783-20791. Max. coverage (+): 0.26. Max coverage (-): 0.86

Region: NODE\_389151\_length\_73825\_cov\_29.945791 20792-20800. Max. coverage (+): 0.32. Max coverage (-): 2.13

Region: NODE\_389151\_length\_73825\_cov\_29.945791 20801-20808. Max. coverage (+): 0.18. Max coverage (-): 4.07

Region: NODE\_389151\_length\_73825\_cov\_29.945791 20809-20817. Max. coverage (+): 0.16. Max coverage (-): 0.36

Region: NODE\_389151\_length\_73825\_cov\_29.945791 20818-20826. Max. coverage (+): 0.04. Max coverage (-): 0.08

Region: NODE\_389151\_length\_73825\_cov\_29.945791 20827-20834. Max. coverage (+): 0.04. Max coverage (-): 0

Region: NODE\_389151\_length\_73825\_cov\_29.945791 20835-20843. Max. coverage (+): 0.12. Max coverage (-): 1.53

Region: NODE\_389151\_length\_73825\_cov\_29.945791 20844-20852. Max. coverage (+): 0.97. Max coverage (-): 1.29

Region: NODE\_389151\_length\_73825\_cov\_29.945791 20853-20861. Max. coverage (+): 0.77. Max coverage (-): 6.62

Region: NODE\_389151\_length\_73825\_cov\_29.945791 20862-20869. Max. coverage (+): 0.24. Max coverage (-): 5.17

Region: NODE\_389151\_length\_73825\_cov\_29.945791 20870-20878. Max. coverage (+): 0.44. Max coverage (-): 0

Region: NODE\_389151\_length\_73825\_cov\_29.945791 20879-20887. Max. coverage (+): 0.48. Max coverage (-): 0.04

Region: NODE\_389151\_length\_73825\_cov\_29.945791 20888-20895. Max. coverage (+): 0.04. Max coverage (-): 0.08

Region: NODE\_389151\_length\_73825\_cov\_29.945791 20896-20904. Max. coverage (+): 0.04. Max coverage (-): 0.24

Region: NODE\_389151\_length\_73825\_cov\_29.945791 20905-20913. Max. coverage (+): 0.04. Max coverage (-): 0.04

Region: NODE\_389151\_length\_73825\_cov\_29.945791 20914-20921. Max. coverage (+): 0.04. Max coverage (-): 0

Region: NODE\_389151\_length\_73825\_cov\_29.945791 20922-20930. Max. coverage (+): 0.04. Max coverage (-): 1.49

Region: NODE\_389151\_length\_73825\_cov\_29.945791 20931-20939. Max. coverage (+): 0. Max coverage (-): 5.13

Region: NODE\_389151\_length\_73825\_cov\_29.945791 20940-20948. Max. coverage (+): 0.04. Max coverage (-): 0.24

Region: NODE\_389151\_length\_73825\_cov\_29.945791 20949-20956. Max. coverage (+): 0.36. Max coverage (-): 0.12

Region: NODE\_389151\_length\_73825\_cov\_29.945791 20957-20965. Max. coverage (+): 0. Max coverage (-): 0.52

Region: NODE\_389151\_length\_73825\_cov\_29.945791 20966-20974. Max. coverage (+): 0. Max coverage (-): 26.47

Region: NODE\_389151\_length\_73825\_cov\_29.945791 20975-20982. Max. coverage (+): 0. Max coverage (-): 0.02

Region: NODE\_389151\_length\_73825\_cov\_29.945791 20983-20991. Max. coverage (+): 0.04. Max coverage (-): 0.1

Region: NODE\_389151\_length\_73825\_cov\_29.945791 20992-21000. Max. coverage (+): 0. Max coverage (-): 0.12

Region: NODE\_389151\_length\_73825\_cov\_29.945791 21001-21008. Max. coverage (+): 0. Max coverage (-): 0.12

Region: NODE\_389151\_length\_73825\_cov\_29.945791 21009-21017. Max. coverage (+): 0.08. Max coverage (-): 0.12

Region: NODE\_389151\_length\_73825\_cov\_29.945791 21018-21026. Max. coverage (+): 0.52. Max coverage (-): 0.04

Region: NODE\_389151\_length\_73825\_cov\_29.945791 21027-21035. Max. coverage (+): 0.08. Max coverage (-): 0.04

Region: NODE\_389151\_length\_73825\_cov\_29.945791 21036-21043. Max. coverage (+): 0. Max coverage (-): 0.04

Region: NODE\_389151\_length\_73825\_cov\_29.945791 21044-21052. Max. coverage (+): 0. Max coverage (-): 0.04

Region: NODE\_389151\_length\_73825\_cov\_29.945791 21053-21061. Max. coverage (+): 0. Max coverage (-): 0

Region: NODE\_389151\_length\_73825\_cov\_29.945791 21062-21069. Max. coverage (+): 0. Max coverage (-): 0.12

Region: NODE\_389151\_length\_73825\_cov\_29.945791 21070-21078. Max. coverage (+): 0. Max coverage (-): 0.65

Region: NODE\_389151\_length\_73825\_cov\_29.945791 21079-21087. Max. coverage (+): 0.04. Max coverage (-): 0.77

Region: NODE\_389151\_length\_73825\_cov\_29.945791 21088-21095. Max. coverage (+): 0.12. Max coverage (-): 0.85

Region: NODE\_389151\_length\_73825\_cov\_29.945791 21096-21104. Max. coverage (+): 0. Max coverage (-): 0.04

Region: NODE\_389151\_length\_73825\_cov\_29.945791 21105-21113. Max. coverage (+): 0. Max coverage (-): 0.02

Region: NODE\_389151\_length\_73825\_cov\_29.945791 21114-21122. Max. coverage (+): 0. Max coverage (-): 0.1

Region: NODE\_389151\_length\_73825\_cov\_29.945791 21123-21130. Max. coverage (+): 0. Max coverage (-): 0.34

Region: NODE\_389151\_length\_73825\_cov\_29.945791 21131-21139. Max. coverage (+): 0.02. Max coverage (-): 13.55

Region: NODE\_389151\_length\_73825\_cov\_29.945791 21140-21148. Max. coverage (+): 0.02. Max coverage (-): 1.53

Region: NODE\_389151\_length\_73825\_cov\_29.945791 21149-21156. Max. coverage (+): 0.1. Max coverage (-): 1.01

Region: NODE\_389151\_length\_73825\_cov\_29.945791 21157-21165. Max. coverage (+): 0. Max coverage (-): 1.33

Region: NODE\_389151\_length\_73825\_cov\_29.945791 21166-21174. Max. coverage (+): 0.02. Max coverage (-): 1.15

Region: NODE\_389151\_length\_73825\_cov\_29.945791 21175-21182. Max. coverage (+): 0.22. Max coverage (-): 0.04

Region: NODE\_389151\_length\_73825\_cov\_29.945791 21183-21191. Max. coverage (+): 0.36. Max coverage (-): 0.04

Region: NODE\_389151\_length\_73825\_cov\_29.945791 21192-21200. Max. coverage (+): 0. Max coverage (-): 0.32

Region: NODE\_389151\_length\_73825\_cov\_29.945791 21201-21209. Max. coverage (+): 0. Max coverage (-): 0.4

Region: NODE\_389151\_length\_73825\_cov\_29.945791 21210-21217. Max. coverage (+): 0. Max coverage (-): 0.16

Region: NODE\_389151\_length\_73825\_cov\_29.945791 21218-21226. Max. coverage (+): 0.04. Max coverage (-): 0.16

Region: NODE\_389151\_length\_73825\_cov\_29.945791 21227-21235. Max. coverage (+): 0.12. Max coverage (-): 0.73

Region: NODE\_389151\_length\_73825\_cov\_29.945791 21236-21243. Max. coverage (+): 0. Max coverage (-): 0.77

Region: NODE\_389151\_length\_73825\_cov\_29.945791 21244-21252. Max. coverage (+): 0.04. Max coverage (-): 0.16

Region: NODE\_389151\_length\_73825\_cov\_29.945791 21253-21261. Max. coverage (+): 0.12. Max coverage (-): 0.97

Region: NODE\_389151\_length\_73825\_cov\_29.945791 21262-21269. Max. coverage (+): 0. Max coverage (-): 0.93

Region: NODE\_389151\_length\_73825\_cov\_29.945791 21270-21278. Max. coverage (+): 0. Max coverage (-): 0

Region: NODE\_389151\_length\_73825\_cov\_29.945791 21279-21287. Max. coverage (+): 0. Max coverage (-): 0

Region: NODE\_389151\_length\_73825\_cov\_29.945791 21288-21296. Max. coverage (+): 0. Max coverage (-): 0.16

Region: NODE\_389151\_length\_73825\_cov\_29.945791 21297-21304. Max. coverage (+): 0. Max coverage (-): 0.12

Region: NODE\_389151\_length\_73825\_cov\_29.945791 21305-21313. Max. coverage (+): 0. Max coverage (-): 0

Region: NODE\_389151\_length\_73825\_cov\_29.945791 21314-21322. Max. coverage (+): 0.08. Max coverage (-): 0.93

Region: NODE\_389151\_length\_73825\_cov\_29.945791 21323-21330. Max. coverage (+): 0. Max coverage (-): 6.46

Region: NODE\_389151\_length\_73825\_cov\_29.945791 21331-21339. Max. coverage (+): 0. Max coverage (-): 6.34

Region: NODE\_389151\_length\_73825\_cov\_29.945791 21340-21348. Max. coverage (+): 0. Max coverage (-): 0

Region: NODE\_389151\_length\_73825\_cov\_29.945791 21349-21356. Max. coverage (+): 0. Max coverage (-): 0

Region: NODE\_389151\_length\_73825\_cov\_29.945791 21357-21365. Max. coverage (+): 0. Max coverage (-): 0

Region: NODE\_389151\_length\_73825\_cov\_29.945791 21366-21374. Max. coverage (+): 0. Max coverage (-): 0

Region: NODE\_389151\_length\_73825\_cov\_29.945791 21375-21383. Max. coverage (+): 0. Max coverage (-): 0

Region: NODE\_389151\_length\_73825\_cov\_29.945791 21384-21391. Max. coverage (+): 0. Max coverage (-): 0

Region: NODE\_389151\_length\_73825\_cov\_29.945791 21392-21400. Max. coverage (+): 0. Max coverage (-): 0

Region: NODE\_389151\_length\_73825\_cov\_29.945791 21401-21409. Max. coverage (+): 0. Max coverage (-): 0

Region: NODE\_389151\_length\_73825\_cov\_29.945791 21410-21417. Max. coverage (+): 0. Max coverage (-): 0.04

Region: NODE\_389151\_length\_73825\_cov\_29.945791 21418-21426. Max. coverage (+): 0. Max coverage (-): 0.04

Region: NODE\_389151\_length\_73825\_cov\_29.945791 21427-21435. Max. coverage (+): 0.02. Max coverage (-): 0

Region: NODE\_389151\_length\_73825\_cov\_29.945791 21436-21443. Max. coverage (+): 0. Max coverage (-): 0

Region: NODE\_389151\_length\_73825\_cov\_29.945791 21444-21452. Max. coverage (+): 0. Max coverage (-): 0

Region: NODE\_389151\_length\_73825\_cov\_29.945791 21453-21461. Max. coverage (+): 0. Max coverage (-): 0

Region: NODE\_389151\_length\_73825\_cov\_29.945791 21462-21470. Max. coverage (+): 0. Max coverage (-): 0

Region: NODE\_389151\_length\_73825\_cov\_29.945791 21471-21478. Max. coverage (+): 0. Max coverage (-): 0

Region: NODE\_389151\_length\_73825\_cov\_29.945791 21479-21487. Max. coverage (+): 0. Max coverage (-): 0

Region: NODE\_389151\_length\_73825\_cov\_29.945791 21488-21496. Max. coverage (+): 0. Max coverage (-): 0

Region: NODE\_389151\_length\_73825\_cov\_29.945791 21497-21504. Max. coverage (+): 0. Max coverage (-): 0

Region: NODE\_389151\_length\_73825\_cov\_29.945791 21505-21513. Max. coverage (+): 0. Max coverage (-): 0

Region: NODE\_389151\_length\_73825\_cov\_29.945791 21514-21522. Max. coverage (+): 0. Max coverage (-): 0

Region: NODE\_389151\_length\_73825\_cov\_29.945791 21523-21530. Max. coverage (+): 0. Max coverage (-): 0

Region: NODE\_389151\_length\_73825\_cov\_29.945791 21531-21539. Max. coverage (+): 0. Max coverage (-): 0

Region: NODE\_389151\_length\_73825\_cov\_29.945791 21540-21548. Max. coverage (+): 0. Max coverage (-): 0

Region: NODE\_389151\_length\_73825\_cov\_29.945791 21549-21557. Max. coverage (+): 0. Max coverage (-): 0

Region: NODE\_389151\_length\_73825\_cov\_29.945791 21558-21565. Max. coverage (+): 0. Max coverage (-): 0

Region: NODE\_389151\_length\_73825\_cov\_29.945791 21566-21574. Max. coverage (+): 0. Max coverage (-): 0

Region: NODE\_389151\_length\_73825\_cov\_29.945791 21575-21583. Max. coverage (+): 0. Max coverage (-): 0

Region: NODE\_389151\_length\_73825\_cov\_29.945791 21584-21591. Max. coverage (+): 0. Max coverage (-): 0

Region: NODE\_389151\_length\_73825\_cov\_29.945791 21592-21600. Max. coverage (+): 0. Max coverage (-): 0.04

Region: NODE\_389151\_length\_73825\_cov\_29.945791 21601-21609. Max. coverage (+): 0. Max coverage (-): 0.04

Region: NODE\_389151\_length\_73825\_cov\_29.945791 21610-21617. Max. coverage (+): 0. Max coverage (-): 0

Region: NODE\_389151\_length\_73825\_cov\_29.945791 21618-21626. Max. coverage (+): 0. Max coverage (-): 0

Region: NODE\_389151\_length\_73825\_cov\_29.945791 21627-21635. Max. coverage (+): 0. Max coverage (-): 0

Region: NODE\_389151\_length\_73825\_cov\_29.945791 21636-21644. Max. coverage (+): 0. Max coverage (-): 0

Region: NODE\_389151\_length\_73825\_cov\_29.945791 21645-21652. Max. coverage (+): 0. Max coverage (-): 0

Region: NODE\_389151\_length\_73825\_cov\_29.945791 21653-21661. Max. coverage (+): 0. Max coverage (-): 0

Region: NODE\_389151\_length\_73825\_cov\_29.945791 21662-21670. Max. coverage (+): 0. Max coverage (-): 0

Region: NODE\_389151\_length\_73825\_cov\_29.945791 21671-21678. Max. coverage (+): 0.01. Max coverage (-): 0

Region: NODE\_389151\_length\_73825\_cov\_29.945791 21679-21687. Max. coverage (+): 0. Max coverage (-): 0

Region: NODE\_389151\_length\_73825\_cov\_29.945791 21688-21696. Max. coverage (+): 0. Max coverage (-): 0

Region: NODE\_389151\_length\_73825\_cov\_29.945791 21697-21704. Max. coverage (+): 0. Max coverage (-): 0

Region: NODE\_389151\_length\_73825\_cov\_29.945791 21705-21713. Max. coverage (+): 0. Max coverage (-): 0

Region: NODE\_389151\_length\_73825\_cov\_29.945791 21714-21722. Max. coverage (+): 0. Max coverage (-): 0

Region: NODE\_389151\_length\_73825\_cov\_29.945791 21723-21731. Max. coverage (+): 0. Max coverage (-): 0

Region: NODE\_389151\_length\_73825\_cov\_29.945791 21732-21739. Max. coverage (+): 0. Max coverage (-): 0

Region: NODE\_389151\_length\_73825\_cov\_29.945791 21740-21748. Max. coverage (+): 0. Max coverage (-): 0

Region: NODE\_389151\_length\_73825\_cov\_29.945791 21749-21757. Max. coverage (+): 0. Max coverage (-): 0

Region: NODE\_389151\_length\_73825\_cov\_29.945791 21758-21765. Max. coverage (+): 0. Max coverage (-): 0

Region: NODE\_389151\_length\_73825\_cov\_29.945791 21766-21774. Max. coverage (+): 0. Max coverage (-): 0

Region: NODE\_389151\_length\_73825\_cov\_29.945791 21775-21783. Max. coverage (+): 0. Max coverage (-): 0

Region: NODE\_389151\_length\_73825\_cov\_29.945791 21784-21791. Max. coverage (+): 0. Max coverage (-): 0

Region: NODE\_389151\_length\_73825\_cov\_29.945791 21792-21800. Max. coverage (+): 0. Max coverage (-): 0

Region: NODE\_389151\_length\_73825\_cov\_29.945791 21801-21809. Max. coverage (+): 0. Max coverage (-): 0

Region: NODE\_389151\_length\_73825\_cov\_29.945791 21810-21818. Max. coverage (+): 0. Max coverage (-): 0

Region: NODE\_389151\_length\_73825\_cov\_29.945791 21819-21826. Max. coverage (+): 0. Max coverage (-): 0

Region: NODE\_389151\_length\_73825\_cov\_29.945791 21827-21835. Max. coverage (+): 0. Max coverage (-): 0

Region: NODE\_389151\_length\_73825\_cov\_29.945791 21836-21844. Max. coverage (+): 0. Max coverage (-): 0

Region: NODE\_389151\_length\_73825\_cov\_29.945791 21845-21852. Max. coverage (+): 0. Max coverage (-): 0

Region: NODE\_389151\_length\_73825\_cov\_29.945791 21853-21861. Max. coverage (+): 0. Max coverage (-): 0.04

Region: NODE\_389151\_length\_73825\_cov\_29.945791 21862-21870. Max. coverage (+): 0. Max coverage (-): 0

Region: NODE\_389151\_length\_73825\_cov\_29.945791 21871-21878. Max. coverage (+): 0. Max coverage (-): 0

Region: NODE\_389151\_length\_73825\_cov\_29.945791 21879-21887. Max. coverage (+): 0. Max coverage (-): 0

Region: NODE\_389151\_length\_73825\_cov\_29.945791 21888-21896. Max. coverage (+): 0. Max coverage (-): 0

Region: NODE\_389151\_length\_73825\_cov\_29.945791 21897-21905. Max. coverage (+): 0. Max coverage (-): 0

Region: NODE\_389151\_length\_73825\_cov\_29.945791 21906-21913. Max. coverage (+): 0. Max coverage (-): 0

Region: NODE\_389151\_length\_73825\_cov\_29.945791 21914-21922. Max. coverage (+): 0. Max coverage (-): 0

Region: NODE\_389151\_length\_73825\_cov\_29.945791 21923-21931. Max. coverage (+): 0. Max coverage (-): 0

Region: NODE\_389151\_length\_73825\_cov\_29.945791 21932-21939. Max. coverage (+): 2.89. Max coverage (-): 0.01

Region: NODE\_389151\_length\_73825\_cov\_29.945791 21940-21948. Max. coverage (+): 0.01. Max coverage (-): 0

Region: NODE\_389151\_length\_73825\_cov\_29.945791 21949-21957. Max. coverage (+): 0. Max coverage (-): 0

Region: NODE\_389151\_length\_73825\_cov\_29.945791 21958-21965. Max. coverage (+): 0. Max coverage (-): 0

Region: NODE\_389151\_length\_73825\_cov\_29.945791 21966-21974. Max. coverage (+): 0. Max coverage (-): 0

Region: NODE\_389151\_length\_73825\_cov\_29.945791 21975-21983. Max. coverage (+): 0. Max coverage (-): 0

Region: NODE\_389151\_length\_73825\_cov\_29.945791 21984-21992. Max. coverage (+): 0. Max coverage (-): 0

Region: NODE\_389151\_length\_73825\_cov\_29.945791 21993-22000. Max. coverage (+): 0. Max coverage (-): 0.01

Region: NODE\_389151\_length\_73825\_cov\_29.945791 22001-22009. Max. coverage (+): 0. Max coverage (-): 0

Region: NODE\_389151\_length\_73825\_cov\_29.945791 22010-22018. Max. coverage (+): 0. Max coverage (-): 0

Region: NODE\_389151\_length\_73825\_cov\_29.945791 22019-22026. Max. coverage (+): 0. Max coverage (-): 0

Region: NODE\_389151\_length\_73825\_cov\_29.945791 22027-22035. Max. coverage (+): 0. Max coverage (-): 0

Region: NODE\_389151\_length\_73825\_cov\_29.945791 22036-22044. Max. coverage (+): 0. Max coverage (-): 0

Region: NODE\_389151\_length\_73825\_cov\_29.945791 22045-22052. Max. coverage (+): 0. Max coverage (-): 0

Region: NODE\_389151\_length\_73825\_cov\_29.945791 22053-22061. Max. coverage (+): 0. Max coverage (-): 0

Region: NODE\_389151\_length\_73825\_cov\_29.945791 22062-22070. Max. coverage (+): 0. Max coverage (-): 0

Region: NODE\_389151\_length\_73825\_cov\_29.945791 22071-22079. Max. coverage (+): 0. Max coverage (-): 0

Region: NODE\_389151\_length\_73825\_cov\_29.945791 22080-22087. Max. coverage (+): 0. Max coverage (-): 0

Region: NODE\_389151\_length\_73825\_cov\_29.945791 22088-22096. Max. coverage (+): 0. Max coverage (-): 0

Region: NODE\_389151\_length\_73825\_cov\_29.945791 22097-22105. Max. coverage (+): 0. Max coverage (-): 0

Region: NODE\_389151\_length\_73825\_cov\_29.945791 22106-22113. Max. coverage (+): 0. Max coverage (-): 0

Region: NODE\_389151\_length\_73825\_cov\_29.945791 22114-22122. Max. coverage (+): 0. Max coverage (-): 0

Region: NODE\_389151\_length\_73825\_cov\_29.945791 22123-22131. Max. coverage (+): 0. Max coverage (-): 0

Region: NODE\_389151\_length\_73825\_cov\_29.945791 22132-22139. Max. coverage (+): 0. Max coverage (-): 0

Region: NODE\_389151\_length\_73825\_cov\_29.945791 22140-22148. Max. coverage (+): 0. Max coverage (-): 0

Region: NODE\_389151\_length\_73825\_cov\_29.945791 22149-22157. Max. coverage (+): 0. Max coverage (-): 0

Region: NODE\_389151\_length\_73825\_cov\_29.945791 22158-22166. Max. coverage (+): 0. Max coverage (-): 0

Region: NODE\_389151\_length\_73825\_cov\_29.945791 22167-22174. Max. coverage (+): 0.04. Max coverage (-): 0

Region: NODE\_389151\_length\_73825\_cov\_29.945791 22175-22183. Max. coverage (+): 0. Max coverage (-): 0

Region: NODE\_389151\_length\_73825\_cov\_29.945791 22184-22192. Max. coverage (+): 0. Max coverage (-): 0

Region: NODE\_389151\_length\_73825\_cov\_29.945791 22193-22200. Max. coverage (+): 0.01. Max coverage (-): 0

Region: NODE\_389151\_length\_73825\_cov\_29.945791 22201-22209. Max. coverage (+): 0.01. Max coverage (-): 0

Region: NODE\_389151\_length\_73825\_cov\_29.945791 22210-22218. Max. coverage (+): 0.01. Max coverage (-): 0

Region: NODE\_389151\_length\_73825\_cov\_29.945791 22219-22226. Max. coverage (+): 0. Max coverage (-): 0

Region: NODE\_389151\_length\_73825\_cov\_29.945791 22227-22235. Max. coverage (+): 0. Max coverage (-): 0

Region: NODE\_389151\_length\_73825\_cov\_29.945791 22236-22244. Max. coverage (+): 0. Max coverage (-): 0

Region: NODE\_389151\_length\_73825\_cov\_29.945791 22245-22253. Max. coverage (+): 0. Max coverage (-): 0

Region: NODE\_389151\_length\_73825\_cov\_29.945791 22254-22261. Max. coverage (+): 0. Max coverage (-): 0

Region: NODE\_389151\_length\_73825\_cov\_29.945791 22262-22270. Max. coverage (+): 0. Max coverage (-): 0.01

Region: NODE\_389151\_length\_73825\_cov\_29.945791 22271-22279. Max. coverage (+): 0. Max coverage (-): 0.03

Region: NODE\_389151\_length\_73825\_cov\_29.945791 22280-22287. Max. coverage (+): 0.02. Max coverage (-): 0.01

Region: NODE\_389151\_length\_73825\_cov\_29.945791 22288-22296. Max. coverage (+): 0.02. Max coverage (-): 0

Region: NODE\_389151\_length\_73825\_cov\_29.945791 22297-22305. Max. coverage (+): 0.01. Max coverage (-): 0

Region: NODE\_389151\_length\_73825\_cov\_29.945791 22306-22313. Max. coverage (+): 0. Max coverage (-): 0

Region: NODE\_389151\_length\_73825\_cov\_29.945791 22314-22322. Max. coverage (+): 0. Max coverage (-): 0

Region: NODE\_389151\_length\_73825\_cov\_29.945791 22323-22331. Max. coverage (+): 0.04. Max coverage (-): 0

Region: NODE\_389151\_length\_73825\_cov\_29.945791 22332-22340. Max. coverage (+): 0. Max coverage (-): 0

Region: NODE\_389151\_length\_73825\_cov\_29.945791 22341-22348. Max. coverage (+): 0. Max coverage (-): 0

Region: NODE\_389151\_length\_73825\_cov\_29.945791 22349-22357. Max. coverage (+): 0. Max coverage (-): 0

Region: NODE\_389151\_length\_73825\_cov\_29.945791 22358-22366. Max. coverage (+): 0. Max coverage (-): 0

Region: NODE\_389151\_length\_73825\_cov\_29.945791 22367-22374. Max. coverage (+): 0. Max coverage (-): 0

Region: NODE\_389151\_length\_73825\_cov\_29.945791 22375-22383. Max. coverage (+): 0.01. Max coverage (-): 0

Region: NODE\_389151\_length\_73825\_cov\_29.945791 22384-22392. Max. coverage (+): 0. Max coverage (-): 0.04

Region: NODE\_389151\_length\_73825\_cov\_29.945791 22393-22400. Max. coverage (+): 0. Max coverage (-): 0.12

Region: NODE\_389151\_length\_73825\_cov\_29.945791 22401-22409. Max. coverage (+): 0. Max coverage (-): 0.04

Region: NODE\_389151\_length\_73825\_cov\_29.945791 22410-22418. Max. coverage (+): 0. Max coverage (-): 0

Region: NODE\_389151\_length\_73825\_cov\_29.945791 22419-22427. Max. coverage (+): 0. Max coverage (-): 0

Region: NODE\_389151\_length\_73825\_cov\_29.945791 22428-22435. Max. coverage (+): 0. Max coverage (-): 0

Region: NODE\_389151\_length\_73825\_cov\_29.945791 22436-22444. Max. coverage (+): 0. Max coverage (-): 0

Region: NODE\_389151\_length\_73825\_cov\_29.945791 22445-22453. Max. coverage (+): 0. Max coverage (-): 0

Region: NODE\_389151\_length\_73825\_cov\_29.945791 22454-22461. Max. coverage (+): 0. Max coverage (-): 0

Region: NODE\_389151\_length\_73825\_cov\_29.945791 22462-22470. Max. coverage (+): 0. Max coverage (-): 0.01

Region: NODE\_389151\_length\_73825\_cov\_29.945791 22471-22479. Max. coverage (+): 0. Max coverage (-): 0

Region: NODE\_389151\_length\_73825\_cov\_29.945791 22480-22487. Max. coverage (+): 0. Max coverage (-): 0

Region: NODE\_389151\_length\_73825\_cov\_29.945791 22488-22496. Max. coverage (+): 0. Max coverage (-): 0

Region: NODE\_389151\_length\_73825\_cov\_29.945791 22497-22505. Max. coverage (+): 0. Max coverage (-): 0

Region: NODE\_389151\_length\_73825\_cov\_29.945791 22506-22514. Max. coverage (+): 0. Max coverage (-): 0

Region: NODE\_389151\_length\_73825\_cov\_29.945791 22515-22522. Max. coverage (+): 0. Max coverage (-): 0

Region: NODE\_389151\_length\_73825\_cov\_29.945791 22523-22531. Max. coverage (+): 0. Max coverage (-): 0

Region: NODE\_389151\_length\_73825\_cov\_29.945791 22532-22540. Max. coverage (+): 0. Max coverage (-): 0

Region: NODE\_389151\_length\_73825\_cov\_29.945791 22541-22548. Max. coverage (+): 0. Max coverage (-): 0

Region: NODE\_389151\_length\_73825\_cov\_29.945791 22549-22557. Max. coverage (+): 0. Max coverage (-): 0

Region: NODE\_389151\_length\_73825\_cov\_29.945791 22558-22566. Max. coverage (+): 0. Max coverage (-): 0

Region: NODE\_389151\_length\_73825\_cov\_29.945791 22567-22574. Max. coverage (+): 0. Max coverage (-): 0

Region: NODE\_389151\_length\_73825\_cov\_29.945791 22575-22583. Max. coverage (+): 0. Max coverage (-): 0

Region: NODE\_389151\_length\_73825\_cov\_29.945791 22584-22592. Max. coverage (+): 0. Max coverage (-): 0

Region: NODE\_389151\_length\_73825\_cov\_29.945791 22593-22601. Max. coverage (+): 0.01. Max coverage (-): 0

Region: NODE\_389151\_length\_73825\_cov\_29.945791 22602-22609. Max. coverage (+): 0. Max coverage (-): 0.02

Region: NODE\_389151\_length\_73825\_cov\_29.945791 22610-22618. Max. coverage (+): 0. Max coverage (-): 0.02

Region: NODE\_389151\_length\_73825\_cov\_29.945791 22619-22627. Max. coverage (+): 0. Max coverage (-): 0

Region: NODE\_389151\_length\_73825\_cov\_29.945791 22628-22635. Max. coverage (+): 0. Max coverage (-): 0

Region: NODE\_389151\_length\_73825\_cov\_29.945791 22636-22644. Max. coverage (+): 0. Max coverage (-): 0

Region: NODE\_389151\_length\_73825\_cov\_29.945791 22645-22653. Max. coverage (+): 0. Max coverage (-): 0

Region: NODE\_389151\_length\_73825\_cov\_29.945791 22654-22661. Max. coverage (+): 0. Max coverage (-): 0

Region: NODE\_389151\_length\_73825\_cov\_29.945791 22662-22670. Max. coverage (+): 0. Max coverage (-): 0

Region: NODE\_389151\_length\_73825\_cov\_29.945791 22671-22679. Max. coverage (+): 0. Max coverage (-): 0

Region: NODE\_389151\_length\_73825\_cov\_29.945791 22680-22688. Max. coverage (+): 0. Max coverage (-): 0

Region: NODE\_389151\_length\_73825\_cov\_29.945791 22689-22696. Max. coverage (+): 0. Max coverage (-): 0

Region: NODE\_389151\_length\_73825\_cov\_29.945791 22697-22705. Max. coverage (+): 0. Max coverage (-): 0

Region: NODE\_389151\_length\_73825\_cov\_29.945791 22706-22714. Max. coverage (+): 0. Max coverage (-): 0

Region: NODE\_389151\_length\_73825\_cov\_29.945791 22715-22722. Max. coverage (+): 0. Max coverage (-): 0

Region: NODE\_389151\_length\_73825\_cov\_29.945791 22723-22731. Max. coverage (+): 0. Max coverage (-): 0

Region: NODE\_389151\_length\_73825\_cov\_29.945791 22732-22740. Max. coverage (+): 0. Max coverage (-): 0

Region: NODE\_389151\_length\_73825\_cov\_29.945791 22741-22748. Max. coverage (+): 0. Max coverage (-): 0

Region: NODE\_389151\_length\_73825\_cov\_29.945791 22749-22757. Max. coverage (+): 0. Max coverage (-): 0

Region: NODE\_389151\_length\_73825\_cov\_29.945791 22758-22766. Max. coverage (+): 0. Max coverage (-): 0

Region: NODE\_389151\_length\_73825\_cov\_29.945791 22767-22775. Max. coverage (+): 0. Max coverage (-): 0

Region: NODE\_389151\_length\_73825\_cov\_29.945791 22776-22783. Max. coverage (+): 0. Max coverage (-): 0

Region: NODE\_389151\_length\_73825\_cov\_29.945791 22784-22792. Max. coverage (+): 0. Max coverage (-): 0

Region: NODE\_389151\_length\_73825\_cov\_29.945791 22793-22801. Max. coverage (+): 0. Max coverage (-): 0

Region: NODE\_389151\_length\_73825\_cov\_29.945791 22802-22809. Max. coverage (+): 0. Max coverage (-): 0

Region: NODE\_389151\_length\_73825\_cov\_29.945791 22810-22818. Max. coverage (+): 0. Max coverage (-): 0

Region: NODE\_389151\_length\_73825\_cov\_29.945791 22819-22827. Max. coverage (+): 0. Max coverage (-): 0

Region: NODE\_389151\_length\_73825\_cov\_29.945791 22828-22835. Max. coverage (+): 0. Max coverage (-): 0

Region: NODE\_389151\_length\_73825\_cov\_29.945791 22836-22844. Max. coverage (+): 0. Max coverage (-): 0

Region: NODE\_389151\_length\_73825\_cov\_29.945791 22845-22853. Max. coverage (+): 0. Max coverage (-): 0

Region: NODE\_389151\_length\_73825\_cov\_29.945791 22854-22862. Max. coverage (+): 0. Max coverage (-): 0

Region: NODE\_389151\_length\_73825\_cov\_29.945791 22863-22870. Max. coverage (+): 0. Max coverage (-): 0

Region: NODE\_389151\_length\_73825\_cov\_29.945791 22871-22879. Max. coverage (+): 0. Max coverage (-): 0

Region: NODE\_389151\_length\_73825\_cov\_29.945791 22880-22888. Max. coverage (+): 0. Max coverage (-): 0

Region: NODE\_389151\_length\_73825\_cov\_29.945791 22889-22896. Max. coverage (+): 0. Max coverage (-): 0

Region: NODE\_389151\_length\_73825\_cov\_29.945791 22897-22905. Max. coverage (+): 0.04. Max coverage (-): 0

Region: NODE\_389151\_length\_73825\_cov\_29.945791 22906-22914. Max. coverage (+): 0. Max coverage (-): 0

Region: NODE\_389151\_length\_73825\_cov\_29.945791 22915-22922. Max. coverage (+): 0. Max coverage (-): 0

Region: NODE\_389151\_length\_73825\_cov\_29.945791 22923-22931. Max. coverage (+): 0. Max coverage (-): 0

Region: NODE\_389151\_length\_73825\_cov\_29.945791 22932-22940. Max. coverage (+): 0. Max coverage (-): 0

Region: NODE\_389151\_length\_73825\_cov\_29.945791 22941-22949. Max. coverage (+): 0. Max coverage (-): 0

Region: NODE\_389151\_length\_73825\_cov\_29.945791 22950-22957. Max. coverage (+): 0.04. Max coverage (-): 0

Region: NODE\_389151\_length\_73825\_cov\_29.945791 22958-22966. Max. coverage (+): 0.04. Max coverage (-): 0

Region: NODE\_389151\_length\_73825\_cov\_29.945791 22967-22975. Max. coverage (+): 0. Max coverage (-): 0

Region: NODE\_389151\_length\_73825\_cov\_29.945791 22976-22983. Max. coverage (+): 0. Max coverage (-): 0

Region: NODE\_389151\_length\_73825\_cov\_29.945791 22984-22992. Max. coverage (+): 0. Max coverage (-): 0

Region: NODE\_389151\_length\_73825\_cov\_29.945791 22993-23001. Max. coverage (+): 0. Max coverage (-): 0

Region: NODE\_389151\_length\_73825\_cov\_29.945791 23002-23009. Max. coverage (+): 0. Max coverage (-): 0

Region: NODE\_389151\_length\_73825\_cov\_29.945791 23010-23018. Max. coverage (+): 0. Max coverage (-): 0

Region: NODE\_389151\_length\_73825\_cov\_29.945791 23019-23027. Max. coverage (+): 0. Max coverage (-): 0

Region: NODE\_389151\_length\_73825\_cov\_29.945791 23028-23036. Max. coverage (+): 0. Max coverage (-): 0

Region: NODE\_389151\_length\_73825\_cov\_29.945791 23037-23044. Max. coverage (+): 0. Max coverage (-): 0.04

Region: NODE\_389151\_length\_73825\_cov\_29.945791 23045-23053. Max. coverage (+): 0. Max coverage (-): 0

Region: NODE\_389151\_length\_73825\_cov\_29.945791 23054-23062. Max. coverage (+): 0.01. Max coverage (-): 0

Region: NODE\_389151\_length\_73825\_cov\_29.945791 23063-23070. Max. coverage (+): 0.04. Max coverage (-): 0

Region: NODE\_389151\_length\_73825\_cov\_29.945791 23071-23079. Max. coverage (+): 0.04. Max coverage (-): 0

Region: NODE\_389151\_length\_73825\_cov\_29.945791 23080-23088. Max. coverage (+): 0. Max coverage (-): 0

Region: NODE\_389151\_length\_73825\_cov\_29.945791 23089-23096. Max. coverage (+): 0. Max coverage (-): 0.01

Region: NODE\_389151\_length\_73825\_cov\_29.945791 23097-23105. Max. coverage (+): 0. Max coverage (-): 0.01

Region: NODE\_389151\_length\_73825\_cov\_29.945791 23106-23114. Max. coverage (+): 0. Max coverage (-): 0.01

Region: NODE\_389151\_length\_73825\_cov\_29.945791 23115-23123. Max. coverage (+): 0. Max coverage (-): 0

Region: NODE\_389151\_length\_73825\_cov\_29.945791 23124-23131. Max. coverage (+): 0. Max coverage (-): 0

Region: NODE\_389151\_length\_73825\_cov\_29.945791 23132-23140. Max. coverage (+): 0. Max coverage (-): 0

Region: NODE\_389151\_length\_73825\_cov\_29.945791 23141-23149. Max. coverage (+): 0. Max coverage (-): 0

Region: NODE\_389151\_length\_73825\_cov\_29.945791 23150-23157. Max. coverage (+): 0. Max coverage (-): 0

Region: NODE\_389151\_length\_73825\_cov\_29.945791 23158-23166. Max. coverage (+): 0. Max coverage (-): 0

Region: NODE\_389151\_length\_73825\_cov\_29.945791 23167-23175. Max. coverage (+): 0. Max coverage (-): 0

Region: NODE\_389151\_length\_73825\_cov\_29.945791 23176-23183. Max. coverage (+): 0. Max coverage (-): 0

Region: NODE\_389151\_length\_73825\_cov\_29.945791 23184-23192. Max. coverage (+): 0. Max coverage (-): 0

Region: NODE\_389151\_length\_73825\_cov\_29.945791 23193-23201. Max. coverage (+): 0. Max coverage (-): 0

Region: NODE\_389151\_length\_73825\_cov\_29.945791 23202-23210. Max. coverage (+): 0. Max coverage (-): 0

Region: NODE\_389151\_length\_73825\_cov\_29.945791 23211-23218. Max. coverage (+): 0. Max coverage (-): 0

Region: NODE\_389151\_length\_73825\_cov\_29.945791 23219-23227. Max. coverage (+): 0. Max coverage (-): 0

Region: NODE\_389151\_length\_73825\_cov\_29.945791 23228-23236. Max. coverage (+): 0. Max coverage (-): 0

Region: NODE\_389151\_length\_73825\_cov\_29.945791 23237-23244. Max. coverage (+): 0. Max coverage (-): 0

Region: NODE\_389151\_length\_73825\_cov\_29.945791 23245-23253. Max. coverage (+): 0. Max coverage (-): 0

Region: NODE\_389151\_length\_73825\_cov\_29.945791 23254-23262. Max. coverage (+): 0. Max coverage (-): 0

Region: NODE\_389151\_length\_73825\_cov\_29.945791 23263-23270. Max. coverage (+): 0. Max coverage (-): 0

Region: NODE\_389151\_length\_73825\_cov\_29.945791 23271-23279. Max. coverage (+): 0.04. Max coverage (-): 0

Region: NODE\_389151\_length\_73825\_cov\_29.945791 23280-23288. Max. coverage (+): 0.04. Max coverage (-): 0

Region: NODE\_389151\_length\_73825\_cov\_29.945791 23289-23297. Max. coverage (+): 0. Max coverage (-): 0

Region: NODE\_389151\_length\_73825\_cov\_29.945791 23298-23305. Max. coverage (+): 0. Max coverage (-): 0

Region: NODE\_389151\_length\_73825\_cov\_29.945791 23306-23314. Max. coverage (+): 0. Max coverage (-): 0

Region: NODE\_389151\_length\_73825\_cov\_29.945791 23315-23323. Max. coverage (+): 0. Max coverage (-): 0

Region: NODE\_389151\_length\_73825\_cov\_29.945791 23324-23331. Max. coverage (+): 0. Max coverage (-): 0

Region: NODE\_389151\_length\_73825\_cov\_29.945791 23332-23340. Max. coverage (+): 0. Max coverage (-): 0

Region: NODE\_389151\_length\_73825\_cov\_29.945791 23341-23349. Max. coverage (+): 0. Max coverage (-): 0

Region: NODE\_389151\_length\_73825\_cov\_29.945791 23350-23357. Max. coverage (+): 0. Max coverage (-): 0

Region: NODE\_389151\_length\_73825\_cov\_29.945791 23358-23366. Max. coverage (+): 0. Max coverage (-): 0

Region: NODE\_389151\_length\_73825\_cov\_29.945791 23367-23375. Max. coverage (+): 0. Max coverage (-): 0

Region: NODE\_389151\_length\_73825\_cov\_29.945791 23376-23384. Max. coverage (+): 0. Max coverage (-): 0

Region: NODE\_389151\_length\_73825\_cov\_29.945791 23385-23392. Max. coverage (+): 0. Max coverage (-): 0

Region: NODE\_389151\_length\_73825\_cov\_29.945791 23393-23401. Max. coverage (+): 0. Max coverage (-): 0

Region: NODE\_389151\_length\_73825\_cov\_29.945791 23402-23410. Max. coverage (+): 0. Max coverage (-): 0

Region: NODE\_389151\_length\_73825\_cov\_29.945791 23411-23418. Max. coverage (+): 0. Max coverage (-): 0

Region: NODE\_389151\_length\_73825\_cov\_29.945791 23419-23427. Max. coverage (+): 0. Max coverage (-): 0

Region: NODE\_389151\_length\_73825\_cov\_29.945791 23428-23436. Max. coverage (+): 0. Max coverage (-): 0

Region: NODE\_389151\_length\_73825\_cov\_29.945791 23437-23444. Max. coverage (+): 0. Max coverage (-): 0

Region: NODE\_389151\_length\_73825\_cov\_29.945791 23445-23453. Max. coverage (+): 0. Max coverage (-): 0

Region: NODE\_389151\_length\_73825\_cov\_29.945791 23454-23462. Max. coverage (+): 0. Max coverage (-): 0

Region: NODE\_389151\_length\_73825\_cov\_29.945791 23463-23471. Max. coverage (+): 0. Max coverage (-): 0

Region: NODE\_389151\_length\_73825\_cov\_29.945791 23472-23479. Max. coverage (+): 0. Max coverage (-): 0

Region: NODE\_389151\_length\_73825\_cov\_29.945791 23480-23488. Max. coverage (+): 0. Max coverage (-): 0

Region: NODE\_389151\_length\_73825\_cov\_29.945791 23489-23497. Max. coverage (+): 0. Max coverage (-): 0

Region: NODE\_389151\_length\_73825\_cov\_29.945791 23498-23505. Max. coverage (+): 0. Max coverage (-): 0

Region: NODE\_389151\_length\_73825\_cov\_29.945791 23506-23514. Max. coverage (+): 0. Max coverage (-): 0

Region: NODE\_389151\_length\_73825\_cov\_29.945791 23515-23523. Max. coverage (+): 0.04. Max coverage (-): 0

Region: NODE\_389151\_length\_73825\_cov\_29.945791 23524-23531. Max. coverage (+): 0. Max coverage (-): 0

Region: NODE\_389151\_length\_73825\_cov\_29.945791 23532-23540. Max. coverage (+): 0. Max coverage (-): 0

Region: NODE\_389151\_length\_73825\_cov\_29.945791 23541-. Max. coverage (+): 0. Max coverage (-): 0

RepeatMasker Color Code

**+**

100-98% Identity

<98-95% Identity

<95-90% Identity

<90-85% Identity

<85-80% Identity

<80-75% Identity

<75-70% Identity

<70% Identity

**-**

Gene Set Color Code

**+**

Gene

Pseudogene

Other

**-**

Topology/Coverage Color Code

Coverage Plus Strand

Coverage Minus Strand

Mainstrand: Plus

Mainstrand: Minus

Complementary Strand

Flanking Region  
(if option -flank >0)

Gene Set Annotation  
  
RepeatMasker Annotation  

**1. (TTATT)n**: 21030-21085 (+), Divergence to consensus: 29%  
**2. AlRepC-98**: 21417-21727 (-), Divergence to consensus: 12.9%  
**3. AlRepC-911**: 21795-21869 (-), Divergence to consensus: 22.9%  
**4. AlRepC-239**: 21973-22688 (+), Divergence to consensus: 36.6%  
**5. AlRepC-911**: 22689-23211 (-), Divergence to consensus: 26.6%  
**6. TC1DR3**: 23220-23590 (-), Divergence to consensus: 24.1%

  
Transcription Factor Binding Sites  

**SPZ1** (Sequence: CTCTAACCCC (-): 19774)  
**RHOXF1** (Sequence: AGATTA (-): 19639)  
**RHOXF1** (Sequence: AGCTCA (-): 20266)  
**RHOXF1** (Sequence: GGCTCA (-): 20702)  
**RHOXF1** (Sequence: AGATCA (-): 20857)  
**RHOXF1** (Sequence: AGATTA (-): 21117)  
**RHOXF1** (Sequence: AGCTCA (-): 21643)  
**RHOXF1** (Sequence: AGATTA (-): 21786)  
**RHOXF1** (Sequence: AGATCA (-): 22152)  
**RHOXF1** (Sequence: GGATTA (-): 22373)  
**RHOXF1** (Sequence: AGCTTA (-): 22466)  
**RHOXF1** (Sequence: TGATCC (+): 19286)  
**RHOXF1** (Sequence: TGAGCT (+): 19424)  
**RHOXF1** (Sequence: TGAGCC (+): 20160)  
**RHOXF1** (Sequence: TGATCT (+): 21375)  
**RHOXF1** (Sequence: TAATCC (+): 21943)  
**RHOXF1** (Sequence: TGATCT (+): 22259)  
**SOX9** (Sequence: AACAATGA (-): 22022)  
**FOXO1** (Sequence: CTTGTTTAC (+): 21520)  
**FOXO1** (Sequence: GTTGTTTAT (+): 22991)  
**FOXO3\_mmu** (Sequence: TGTTTACA (-): 21522)  
**Sox5** (Sequence: ATTGTT (+): 20959)  
**Sox5** (Sequence: ATTGTT (+): 21029)  
**FOXO1** (Sequence: AAAAACAGC (-): 20584)  
**FOXO1** (Sequence: ATAAACAGG (-): 22740)  
**FOXO3\_hsa** (Sequence: TTGTTTAC (-): 21521)  
**FOXP1** (Sequence: TGTTTAC (-): 20822)  
**FOXP1** (Sequence: TGTTTAC (-): 21522)  
**Rhox11** (Sequence: TGGTGTTTA (+): 20819)  
**Rhox11** (Sequence: TGCTGTTAA (+): 21685)  
**Rhox11** (Sequence: AATACAGCA (-): 21949)  
**Sox5** (Sequence: AACAAT (-): 19343)  
**Sox5** (Sequence: AACAAT (-): 20955)  
**Sox5** (Sequence: AACAAT (-): 22022)  
**POU2F1** (Sequence: TATTTTAAT (+): 21046)
