# Supplementary material for: First characterization of PIWI-interacting RNA clusters in a cichlid fish with a B chromosome
Source: BMC Biol. 2022 Sep 21;20:204. doi: 10.1186/s12915-022-01403-2 (PMC9490952; doi:10.1186/s12915-022-01403-2)
Supplement: Supplementary file 1 — Additional file 1. Zipped folder with fasta and interactive html piRNA cluster information for the A. latifasciata genome. The nomenclature is as follows: number-pirna-cluster_sex_B-presence (f, female; m, male; 0b, without B chromosome; 1b, with B chromosome). [file 12915_2022_1403_MOESM1_ESM.zip › 152_m1b.html]

piRNA cluster 152\_m1b 90


Predicted piRNA cluster no. 152\_m1b
  

Show proTRAC run info
Hide proTRAC run info

/\  
                \_\_\_\_\_\_\_\_\_\_\_\_\_\_\_\_\_\_\_\_\_\_\_/\\_\_\_ /  \\_\_\_\_\_\_\_  
               I                      /  \  /    \      I  
               I     pro             /    \/      \     I  
               I        TRAC        /               \   I  
               I   \_\_\_\_\_\_\_\_\_\_\_\_\_\_\_\_/\_\_\_\_\_\_\_\_\_\_\_\_\_\_\_\_\_\\_ I  
               I   \              /                     I  
               I    \            /                      I  
               I     \  /\      /       V.2.4.2         I  
               I      \/  \    /                        I  
               I\_\_\_\_\_\_\_\_\_\_\_\  /\_\_\_\_\_\_\_\_\_\_\_\_\_\_\_\_\_\_\_\_\_\_\_\_\_I  
                            \/  
  
  
================================= proTRAC ====================================  
VERSION: .......... 2.4.2  
LAST MODIFIED: .... 11. May 2018  
  
Please cite:  
Rosenkranz D, Zischler H. proTRAC - a software for probabilistic piRNA cluster  
detection, visualization and analysis. 2012. BMC Bioinformatics 13:5.  
  
  
Contact:  
David Rosenkranz  
Institute of Organismic and Molecular Evolutionary Biology  
Dept. Anthropology, small RNA group  
Johannes Gutenberg University Mainz  
email: rosenkranz@uni-mainz.de  
  
You can find the latest proTRAC version at:  
http://sourceforge.net/projects/protrac/files  
http://www.smallRNAgroup-mainz.de/software  
==============================================================================  
  
PARAMETERS:  
Map file: ...............piwi-machos-1B.fa-collapse.map  
Genome file: ............../../../0B\_ala\_genome.fa  
RepeatMasker annotation: Alatifasciata-all0B-maryan-v2.fa\_corrected.out  
GeneSet:................./guest-storage/Data/annotation/Alatifasciata\_all0B\_maryan-v2\_out2017.gff  
  
Significant (p<=0.01) hit density will be calculated based  
on observed hit distribution.  
  
Sliding window size: ........................................ 5000 bp  
Sliding window increament: .................................. 1000 bp  
Normalize each hit by number of genomic hits: ............... yes  
Normalize each hit by number of sequence reads: ............. yes  
Normalize values (-> per million mapped reads): ............. yes  
Min. fraction of hits with 1T(U) or 10A: .................... 0.75  
Alternatively: Min. fraction of hits with 1T(U) and 10A: .... 0.5  
Min. fraction of hits with typical piRNA length: ............ 0.75  
Typical piRNA length: ....................................... 24-32 nt  
Min. size of a piRNA cluster: ............................... 1000 bp.  
Min. number of hits (absolute): ............................. 0  
Min. number of hits (normalized): ........................... 0  
Min. fraction of hits on the mainstrand: .................... 0.75  
Top fraction of mapped sequences (in terms of read counts): . 1%  
Top fraction accounts for max. n% of sequence reads: ........ 90%  
Min. fraction of hits on each arm of a bidirectional cluster: 0.05  
Output html file for each cluster: .......................... yes  
Output a summary table: ..................................... yes  
Output a FASTA file for each cluster (piRNA sequences): ..... yes  
Output a FASTA file comprising cluster sequences: ........... yes  
Output a GTF file for predicted piRNA clusters: ..............yes  
Search DNA motifs in clusters: .............................. yes  
Output flanking sequences: +/- .............................. 0 bp  
Output ~.pTi file: .......................................... no  
==============================================================================  
  
  
Genome size (without gaps): ............ 758543724 bp  
Gaps (N/X/-): .......................... 417479 bp  
Mapped reads: .......................... 26973943  
Non-identical sequences: ............... 6209225  
Genomic hits: .......................... 48438990  
Significant densitiy of mapped reads: .. 821.144211136946 reads/kb

Show proTRAC cluster info
Hide proTRAC cluster info

|  |  |
| --- | --- |
| Location | NODE\_389151\_length\_73825\_cov\_29.945791 |
| Coordinates | 18511-23757 |
| Size [bp] | 5247 |
| Sequence hit loci | 2583 |
| Mapped reads (normalized) | 11618.7 |
| Mapped reads (normalized) per kb | 2214.4 |
| Normalized reads with 1T (1U) | 82.3% |
| Normalized reads with 10A | 34.3% |
| Normalized reads with length 24-32 nt | 99.2% |
| Normalized reads on the main strand(s) | 93% |
| Predicted directionality | mono:minus |

100%

0%

1T (1U)  
reads

10A reads

24-32 nt  
reads

reads on mainstrand

**Either the amount of reads with 1T (1U) OR 10A has to exceed 75% (set with option: -1Tor10A)  
Alternatively the amount of reads with 1T (1U) AND 10A has to exceed 50% (set with option: -1Tand10A)  
Minimum amount of reads with preferred size is 75% (set with option: -pisize)  
Minimum amount of reads on the main strand(s) is 75% (set with option: -clstrand)**

Show read coverage
Hide read coverage

WHAT DO I SEE HERE?  
This chart shows the location of mapped sequence reads within a predicted piRNA cluster. The color refers to the number of genomic hits produced by the sequence read in question. A dark red bar indicates that this sequence read produces many other hits elsewhere in the genome. Many adjacent red or yellow bars can indicate the presence of a multi-copy element such as transposons or rRNA genes. A dark green bar indicates that this sequence read maps uniquely to this locus.

1 hit

2-5 hits

6-10 hits

11-20 hits

21-50 hits

51-100 hits

> 100 hits

NODE\_389151\_length\_73825\_cov\_29.945791

18511

23757

Gene Set

RepeatMasker

Mapped  
Reads

25.78

plus strand

minus strand

25.78

Region: NODE\_389151\_length\_73825\_cov\_29.945791 1861-18516. Max. coverage (+): 0.04. Max coverage (-): 0

Region: NODE\_389151\_length\_73825\_cov\_29.945791 18517-18526. Max. coverage (+): 0. Max coverage (-): 0

Region: NODE\_389151\_length\_73825\_cov\_29.945791 18527-18537. Max. coverage (+): 0. Max coverage (-): 0

Region: NODE\_389151\_length\_73825\_cov\_29.945791 18538-18547. Max. coverage (+): 0. Max coverage (-): 0

Region: NODE\_389151\_length\_73825\_cov\_29.945791 18548-18558. Max. coverage (+): 0. Max coverage (-): 0

Region: NODE\_389151\_length\_73825\_cov\_29.945791 18559-18568. Max. coverage (+): 0. Max coverage (-): 0

Region: NODE\_389151\_length\_73825\_cov\_29.945791 18569-18579. Max. coverage (+): 0. Max coverage (-): 0

Region: NODE\_389151\_length\_73825\_cov\_29.945791 18580-18589. Max. coverage (+): 0. Max coverage (-): 0

Region: NODE\_389151\_length\_73825\_cov\_29.945791 18590-18600. Max. coverage (+): 0. Max coverage (-): 0

Region: NODE\_389151\_length\_73825\_cov\_29.945791 18601-18610. Max. coverage (+): 0. Max coverage (-): 0

Region: NODE\_389151\_length\_73825\_cov\_29.945791 18611-18621. Max. coverage (+): 0. Max coverage (-): 0

Region: NODE\_389151\_length\_73825\_cov\_29.945791 18622-18631. Max. coverage (+): 0. Max coverage (-): 0

Region: NODE\_389151\_length\_73825\_cov\_29.945791 18632-18642. Max. coverage (+): 0. Max coverage (-): 0

Region: NODE\_389151\_length\_73825\_cov\_29.945791 18643-18652. Max. coverage (+): 0. Max coverage (-): 0

Region: NODE\_389151\_length\_73825\_cov\_29.945791 18653-18663. Max. coverage (+): 0. Max coverage (-): 0

Region: NODE\_389151\_length\_73825\_cov\_29.945791 18664-18673. Max. coverage (+): 0. Max coverage (-): 0

Region: NODE\_389151\_length\_73825\_cov\_29.945791 18674-18684. Max. coverage (+): 0. Max coverage (-): 0

Region: NODE\_389151\_length\_73825\_cov\_29.945791 18685-18694. Max. coverage (+): 0. Max coverage (-): 0

Region: NODE\_389151\_length\_73825\_cov\_29.945791 18695-18705. Max. coverage (+): 0. Max coverage (-): 0

Region: NODE\_389151\_length\_73825\_cov\_29.945791 18706-18715. Max. coverage (+): 0. Max coverage (-): 0

Region: NODE\_389151\_length\_73825\_cov\_29.945791 18716-18726. Max. coverage (+): 0. Max coverage (-): 0

Region: NODE\_389151\_length\_73825\_cov\_29.945791 18727-18736. Max. coverage (+): 0. Max coverage (-): 0

Region: NODE\_389151\_length\_73825\_cov\_29.945791 18737-18747. Max. coverage (+): 0. Max coverage (-): 0

Region: NODE\_389151\_length\_73825\_cov\_29.945791 18748-18757. Max. coverage (+): 0. Max coverage (-): 0

Region: NODE\_389151\_length\_73825\_cov\_29.945791 18758-18768. Max. coverage (+): 0. Max coverage (-): 0

Region: NODE\_389151\_length\_73825\_cov\_29.945791 18769-18778. Max. coverage (+): 0. Max coverage (-): 0

Region: NODE\_389151\_length\_73825\_cov\_29.945791 18779-18789. Max. coverage (+): 0. Max coverage (-): 0

Region: NODE\_389151\_length\_73825\_cov\_29.945791 18790-18799. Max. coverage (+): 0. Max coverage (-): 0

Region: NODE\_389151\_length\_73825\_cov\_29.945791 18800-18810. Max. coverage (+): 0. Max coverage (-): 0

Region: NODE\_389151\_length\_73825\_cov\_29.945791 18811-18820. Max. coverage (+): 0. Max coverage (-): 0

Region: NODE\_389151\_length\_73825\_cov\_29.945791 18821-18831. Max. coverage (+): 0. Max coverage (-): 0

Region: NODE\_389151\_length\_73825\_cov\_29.945791 18832-18841. Max. coverage (+): 0. Max coverage (-): 0

Region: NODE\_389151\_length\_73825\_cov\_29.945791 18842-18852. Max. coverage (+): 0. Max coverage (-): 0

Region: NODE\_389151\_length\_73825\_cov\_29.945791 18853-18862. Max. coverage (+): 0. Max coverage (-): 0

Region: NODE\_389151\_length\_73825\_cov\_29.945791 18863-18873. Max. coverage (+): 0. Max coverage (-): 0

Region: NODE\_389151\_length\_73825\_cov\_29.945791 18874-18883. Max. coverage (+): 0. Max coverage (-): 0

Region: NODE\_389151\_length\_73825\_cov\_29.945791 18884-18894. Max. coverage (+): 0. Max coverage (-): 0

Region: NODE\_389151\_length\_73825\_cov\_29.945791 18895-18904. Max. coverage (+): 0. Max coverage (-): 0

Region: NODE\_389151\_length\_73825\_cov\_29.945791 18905-18915. Max. coverage (+): 0. Max coverage (-): 0

Region: NODE\_389151\_length\_73825\_cov\_29.945791 18916-18925. Max. coverage (+): 0. Max coverage (-): 0

Region: NODE\_389151\_length\_73825\_cov\_29.945791 18926-18936. Max. coverage (+): 0. Max coverage (-): 0

Region: NODE\_389151\_length\_73825\_cov\_29.945791 18937-18946. Max. coverage (+): 0. Max coverage (-): 0

Region: NODE\_389151\_length\_73825\_cov\_29.945791 18947-18956. Max. coverage (+): 0. Max coverage (-): 0

Region: NODE\_389151\_length\_73825\_cov\_29.945791 18957-18967. Max. coverage (+): 0. Max coverage (-): 0

Region: NODE\_389151\_length\_73825\_cov\_29.945791 18968-18977. Max. coverage (+): 0. Max coverage (-): 0

Region: NODE\_389151\_length\_73825\_cov\_29.945791 18978-18988. Max. coverage (+): 0. Max coverage (-): 0

Region: NODE\_389151\_length\_73825\_cov\_29.945791 18989-18998. Max. coverage (+): 0. Max coverage (-): 0

Region: NODE\_389151\_length\_73825\_cov\_29.945791 18999-19009. Max. coverage (+): 0. Max coverage (-): 0

Region: NODE\_389151\_length\_73825\_cov\_29.945791 19010-19019. Max. coverage (+): 0. Max coverage (-): 0

Region: NODE\_389151\_length\_73825\_cov\_29.945791 19020-19030. Max. coverage (+): 0. Max coverage (-): 0

Region: NODE\_389151\_length\_73825\_cov\_29.945791 19031-19040. Max. coverage (+): 0. Max coverage (-): 0

Region: NODE\_389151\_length\_73825\_cov\_29.945791 19041-19051. Max. coverage (+): 0. Max coverage (-): 0

Region: NODE\_389151\_length\_73825\_cov\_29.945791 19052-19061. Max. coverage (+): 0. Max coverage (-): 0

Region: NODE\_389151\_length\_73825\_cov\_29.945791 19062-19072. Max. coverage (+): 0. Max coverage (-): 0

Region: NODE\_389151\_length\_73825\_cov\_29.945791 19073-19082. Max. coverage (+): 0. Max coverage (-): 0

Region: NODE\_389151\_length\_73825\_cov\_29.945791 19083-19093. Max. coverage (+): 0. Max coverage (-): 0

Region: NODE\_389151\_length\_73825\_cov\_29.945791 19094-19103. Max. coverage (+): 0. Max coverage (-): 0

Region: NODE\_389151\_length\_73825\_cov\_29.945791 19104-19114. Max. coverage (+): 0. Max coverage (-): 0

Region: NODE\_389151\_length\_73825\_cov\_29.945791 19115-19124. Max. coverage (+): 0. Max coverage (-): 0

Region: NODE\_389151\_length\_73825\_cov\_29.945791 19125-19135. Max. coverage (+): 0. Max coverage (-): 0

Region: NODE\_389151\_length\_73825\_cov\_29.945791 19136-19145. Max. coverage (+): 0. Max coverage (-): 0

Region: NODE\_389151\_length\_73825\_cov\_29.945791 19146-19156. Max. coverage (+): 0. Max coverage (-): 0

Region: NODE\_389151\_length\_73825\_cov\_29.945791 19157-19166. Max. coverage (+): 0. Max coverage (-): 0

Region: NODE\_389151\_length\_73825\_cov\_29.945791 19167-19177. Max. coverage (+): 0. Max coverage (-): 0

Region: NODE\_389151\_length\_73825\_cov\_29.945791 19178-19187. Max. coverage (+): 0. Max coverage (-): 0

Region: NODE\_389151\_length\_73825\_cov\_29.945791 19188-19198. Max. coverage (+): 0. Max coverage (-): 0

Region: NODE\_389151\_length\_73825\_cov\_29.945791 19199-19208. Max. coverage (+): 0. Max coverage (-): 0

Region: NODE\_389151\_length\_73825\_cov\_29.945791 19209-19219. Max. coverage (+): 0. Max coverage (-): 0

Region: NODE\_389151\_length\_73825\_cov\_29.945791 19220-19229. Max. coverage (+): 0. Max coverage (-): 0

Region: NODE\_389151\_length\_73825\_cov\_29.945791 19230-19240. Max. coverage (+): 0. Max coverage (-): 0

Region: NODE\_389151\_length\_73825\_cov\_29.945791 19241-19250. Max. coverage (+): 0. Max coverage (-): 0

Region: NODE\_389151\_length\_73825\_cov\_29.945791 19251-19261. Max. coverage (+): 0. Max coverage (-): 0

Region: NODE\_389151\_length\_73825\_cov\_29.945791 19262-19271. Max. coverage (+): 0. Max coverage (-): 0

Region: NODE\_389151\_length\_73825\_cov\_29.945791 19272-19282. Max. coverage (+): 0. Max coverage (-): 0

Region: NODE\_389151\_length\_73825\_cov\_29.945791 19283-19292. Max. coverage (+): 0. Max coverage (-): 0

Region: NODE\_389151\_length\_73825\_cov\_29.945791 19293-19303. Max. coverage (+): 0. Max coverage (-): 0

Region: NODE\_389151\_length\_73825\_cov\_29.945791 19304-19313. Max. coverage (+): 0. Max coverage (-): 0

Region: NODE\_389151\_length\_73825\_cov\_29.945791 19314-19324. Max. coverage (+): 0. Max coverage (-): 0

Region: NODE\_389151\_length\_73825\_cov\_29.945791 19325-19334. Max. coverage (+): 0. Max coverage (-): 0

Region: NODE\_389151\_length\_73825\_cov\_29.945791 19335-19345. Max. coverage (+): 0. Max coverage (-): 0

Region: NODE\_389151\_length\_73825\_cov\_29.945791 19346-19355. Max. coverage (+): 0. Max coverage (-): 0

Region: NODE\_389151\_length\_73825\_cov\_29.945791 19356-19366. Max. coverage (+): 0. Max coverage (-): 0

Region: NODE\_389151\_length\_73825\_cov\_29.945791 19367-19376. Max. coverage (+): 0. Max coverage (-): 0

Region: NODE\_389151\_length\_73825\_cov\_29.945791 19377-19387. Max. coverage (+): 0. Max coverage (-): 0

Region: NODE\_389151\_length\_73825\_cov\_29.945791 19388-19397. Max. coverage (+): 0. Max coverage (-): 0

Region: NODE\_389151\_length\_73825\_cov\_29.945791 19398-19408. Max. coverage (+): 0. Max coverage (-): 0

Region: NODE\_389151\_length\_73825\_cov\_29.945791 19409-19418. Max. coverage (+): 0. Max coverage (-): 0

Region: NODE\_389151\_length\_73825\_cov\_29.945791 19419-19429. Max. coverage (+): 0. Max coverage (-): 0

Region: NODE\_389151\_length\_73825\_cov\_29.945791 19430-19439. Max. coverage (+): 0. Max coverage (-): 0

Region: NODE\_389151\_length\_73825\_cov\_29.945791 19440-19450. Max. coverage (+): 0. Max coverage (-): 0

Region: NODE\_389151\_length\_73825\_cov\_29.945791 19451-19460. Max. coverage (+): 0. Max coverage (-): 0

Region: NODE\_389151\_length\_73825\_cov\_29.945791 19461-19471. Max. coverage (+): 0. Max coverage (-): 0

Region: NODE\_389151\_length\_73825\_cov\_29.945791 19472-19481. Max. coverage (+): 0. Max coverage (-): 0

Region: NODE\_389151\_length\_73825\_cov\_29.945791 19482-19492. Max. coverage (+): 0. Max coverage (-): 0

Region: NODE\_389151\_length\_73825\_cov\_29.945791 19493-19502. Max. coverage (+): 0. Max coverage (-): 0

Region: NODE\_389151\_length\_73825\_cov\_29.945791 19503-19513. Max. coverage (+): 0. Max coverage (-): 0

Region: NODE\_389151\_length\_73825\_cov\_29.945791 19514-19523. Max. coverage (+): 0. Max coverage (-): 0

Region: NODE\_389151\_length\_73825\_cov\_29.945791 19524-19534. Max. coverage (+): 0. Max coverage (-): 0

Region: NODE\_389151\_length\_73825\_cov\_29.945791 19535-19544. Max. coverage (+): 0. Max coverage (-): 0

Region: NODE\_389151\_length\_73825\_cov\_29.945791 19545-19555. Max. coverage (+): 0. Max coverage (-): 0

Region: NODE\_389151\_length\_73825\_cov\_29.945791 19556-19565. Max. coverage (+): 0. Max coverage (-): 0

Region: NODE\_389151\_length\_73825\_cov\_29.945791 19566-19576. Max. coverage (+): 0. Max coverage (-): 0

Region: NODE\_389151\_length\_73825\_cov\_29.945791 19577-19586. Max. coverage (+): 0. Max coverage (-): 0

Region: NODE\_389151\_length\_73825\_cov\_29.945791 19587-19597. Max. coverage (+): 0. Max coverage (-): 0

Region: NODE\_389151\_length\_73825\_cov\_29.945791 19598-19607. Max. coverage (+): 0. Max coverage (-): 0

Region: NODE\_389151\_length\_73825\_cov\_29.945791 19608-19618. Max. coverage (+): 0. Max coverage (-): 0

Region: NODE\_389151\_length\_73825\_cov\_29.945791 19619-19628. Max. coverage (+): 0. Max coverage (-): 0

Region: NODE\_389151\_length\_73825\_cov\_29.945791 19629-19639. Max. coverage (+): 0. Max coverage (-): 0.09

Region: NODE\_389151\_length\_73825\_cov\_29.945791 19640-19649. Max. coverage (+): 0. Max coverage (-): 0.13

Region: NODE\_389151\_length\_73825\_cov\_29.945791 19650-19660. Max. coverage (+): 0. Max coverage (-): 24.19

Region: NODE\_389151\_length\_73825\_cov\_29.945791 19661-19670. Max. coverage (+): 0. Max coverage (-): 7.1

Region: NODE\_389151\_length\_73825\_cov\_29.945791 19671-19681. Max. coverage (+): 0.32. Max coverage (-): 0.5

Region: NODE\_389151\_length\_73825\_cov\_29.945791 19682-19691. Max. coverage (+): 0.04. Max coverage (-): 1.67

Region: NODE\_389151\_length\_73825\_cov\_29.945791 19692-19702. Max. coverage (+): 0.15. Max coverage (-): 0.17

Region: NODE\_389151\_length\_73825\_cov\_29.945791 19703-19712. Max. coverage (+): 0.11. Max coverage (-): 0.17

Region: NODE\_389151\_length\_73825\_cov\_29.945791 19713-19723. Max. coverage (+): 0. Max coverage (-): 0

Region: NODE\_389151\_length\_73825\_cov\_29.945791 19724-19733. Max. coverage (+): 0.02. Max coverage (-): 0.03

Region: NODE\_389151\_length\_73825\_cov\_29.945791 19734-19744. Max. coverage (+): 0.02. Max coverage (-): 0.11

Region: NODE\_389151\_length\_73825\_cov\_29.945791 19745-19754. Max. coverage (+): 0.59. Max coverage (-): 0.15

Region: NODE\_389151\_length\_73825\_cov\_29.945791 19755-19765. Max. coverage (+): 0.59. Max coverage (-): 0

Region: NODE\_389151\_length\_73825\_cov\_29.945791 19766-19775. Max. coverage (+): 0. Max coverage (-): 0.07

Region: NODE\_389151\_length\_73825\_cov\_29.945791 19776-19786. Max. coverage (+): 0.04. Max coverage (-): 0.11

Region: NODE\_389151\_length\_73825\_cov\_29.945791 19787-19796. Max. coverage (+): 0. Max coverage (-): 4.04

Region: NODE\_389151\_length\_73825\_cov\_29.945791 19797-19807. Max. coverage (+): 0.04. Max coverage (-): 4.08

Region: NODE\_389151\_length\_73825\_cov\_29.945791 19808-19817. Max. coverage (+): 0. Max coverage (-): 1.85

Region: NODE\_389151\_length\_73825\_cov\_29.945791 19818-19827. Max. coverage (+): 0.04. Max coverage (-): 0.04

Region: NODE\_389151\_length\_73825\_cov\_29.945791 19828-19838. Max. coverage (+): 0.07. Max coverage (-): 16.42

Region: NODE\_389151\_length\_73825\_cov\_29.945791 19839-19848. Max. coverage (+): 0.04. Max coverage (-): 3.63

Region: NODE\_389151\_length\_73825\_cov\_29.945791 19849-19859. Max. coverage (+): 0.11. Max coverage (-): 16.91

Region: NODE\_389151\_length\_73825\_cov\_29.945791 19860-19869. Max. coverage (+): 0.04. Max coverage (-): 1

Region: NODE\_389151\_length\_73825\_cov\_29.945791 19870-19880. Max. coverage (+): 0.15. Max coverage (-): 1.37

Region: NODE\_389151\_length\_73825\_cov\_29.945791 19881-19890. Max. coverage (+): 0.19. Max coverage (-): 0.26

Region: NODE\_389151\_length\_73825\_cov\_29.945791 19891-19901. Max. coverage (+): 0.11. Max coverage (-): 0.22

Region: NODE\_389151\_length\_73825\_cov\_29.945791 19902-19911. Max. coverage (+): 0.04. Max coverage (-): 2.3

Region: NODE\_389151\_length\_73825\_cov\_29.945791 19912-19922. Max. coverage (+): 0.15. Max coverage (-): 4.23

Region: NODE\_389151\_length\_73825\_cov\_29.945791 19923-19932. Max. coverage (+): 0.04. Max coverage (-): 3.56

Region: NODE\_389151\_length\_73825\_cov\_29.945791 19933-19943. Max. coverage (+): 0.04. Max coverage (-): 2.22

Region: NODE\_389151\_length\_73825\_cov\_29.945791 19944-19953. Max. coverage (+): 0.3. Max coverage (-): 0.15

Region: NODE\_389151\_length\_73825\_cov\_29.945791 19954-19964. Max. coverage (+): 0.3. Max coverage (-): 0.11

Region: NODE\_389151\_length\_73825\_cov\_29.945791 19965-19974. Max. coverage (+): 0.15. Max coverage (-): 3.23

Region: NODE\_389151\_length\_73825\_cov\_29.945791 19975-19985. Max. coverage (+): 0. Max coverage (-): 2.78

Region: NODE\_389151\_length\_73825\_cov\_29.945791 19986-19995. Max. coverage (+): 0.11. Max coverage (-): 1.04

Region: NODE\_389151\_length\_73825\_cov\_29.945791 19996-20006. Max. coverage (+): 0.22. Max coverage (-): 11.08

Region: NODE\_389151\_length\_73825\_cov\_29.945791 20007-20016. Max. coverage (+): 0.22. Max coverage (-): 0.15

Region: NODE\_389151\_length\_73825\_cov\_29.945791 20017-20027. Max. coverage (+): 0. Max coverage (-): 0.19

Region: NODE\_389151\_length\_73825\_cov\_29.945791 20028-20037. Max. coverage (+): 0. Max coverage (-): 0.22

Region: NODE\_389151\_length\_73825\_cov\_29.945791 20038-20048. Max. coverage (+): 0.07. Max coverage (-): 0

Region: NODE\_389151\_length\_73825\_cov\_29.945791 20049-20058. Max. coverage (+): 0.07. Max coverage (-): 0.04

Region: NODE\_389151\_length\_73825\_cov\_29.945791 20059-20069. Max. coverage (+): 0.04. Max coverage (-): 0.04

Region: NODE\_389151\_length\_73825\_cov\_29.945791 20070-20079. Max. coverage (+): 0. Max coverage (-): 0

Region: NODE\_389151\_length\_73825\_cov\_29.945791 20080-20090. Max. coverage (+): 0.04. Max coverage (-): 0.07

Region: NODE\_389151\_length\_73825\_cov\_29.945791 20091-20100. Max. coverage (+): 0. Max coverage (-): 0.74

Region: NODE\_389151\_length\_73825\_cov\_29.945791 20101-20111. Max. coverage (+): 0.07. Max coverage (-): 2.45

Region: NODE\_389151\_length\_73825\_cov\_29.945791 20112-20121. Max. coverage (+): 0.11. Max coverage (-): 0.41

Region: NODE\_389151\_length\_73825\_cov\_29.945791 20122-20132. Max. coverage (+): 0.07. Max coverage (-): 4.49

Region: NODE\_389151\_length\_73825\_cov\_29.945791 20133-20142. Max. coverage (+): 2.19. Max coverage (-): 4.78

Region: NODE\_389151\_length\_73825\_cov\_29.945791 20143-20153. Max. coverage (+): 2.19. Max coverage (-): 0.74

Region: NODE\_389151\_length\_73825\_cov\_29.945791 20154-20163. Max. coverage (+): 0.41. Max coverage (-): 0.7

Region: NODE\_389151\_length\_73825\_cov\_29.945791 20164-20174. Max. coverage (+): 0. Max coverage (-): 1.56

Region: NODE\_389151\_length\_73825\_cov\_29.945791 20175-20184. Max. coverage (+): 0.04. Max coverage (-): 2.89

Region: NODE\_389151\_length\_73825\_cov\_29.945791 20185-20195. Max. coverage (+): 0.33. Max coverage (-): 2.85

Region: NODE\_389151\_length\_73825\_cov\_29.945791 20196-20205. Max. coverage (+): 0.04. Max coverage (-): 0.3

Region: NODE\_389151\_length\_73825\_cov\_29.945791 20206-20216. Max. coverage (+): 0. Max coverage (-): 1

Region: NODE\_389151\_length\_73825\_cov\_29.945791 20217-20226. Max. coverage (+): 0.11. Max coverage (-): 0.15

Region: NODE\_389151\_length\_73825\_cov\_29.945791 20227-20237. Max. coverage (+): 0. Max coverage (-): 0

Region: NODE\_389151\_length\_73825\_cov\_29.945791 20238-20247. Max. coverage (+): 0. Max coverage (-): 0

Region: NODE\_389151\_length\_73825\_cov\_29.945791 20248-20258. Max. coverage (+): 0. Max coverage (-): 0

Region: NODE\_389151\_length\_73825\_cov\_29.945791 20259-20268. Max. coverage (+): 0. Max coverage (-): 2.37

Region: NODE\_389151\_length\_73825\_cov\_29.945791 20269-20279. Max. coverage (+): 0. Max coverage (-): 2.78

Region: NODE\_389151\_length\_73825\_cov\_29.945791 20280-20289. Max. coverage (+): 0.07. Max coverage (-): 0.56

Region: NODE\_389151\_length\_73825\_cov\_29.945791 20290-20300. Max. coverage (+): 0.04. Max coverage (-): 0.22

Region: NODE\_389151\_length\_73825\_cov\_29.945791 20301-20310. Max. coverage (+): 0. Max coverage (-): 0.63

Region: NODE\_389151\_length\_73825\_cov\_29.945791 20311-20321. Max. coverage (+): 0. Max coverage (-): 0.63

Region: NODE\_389151\_length\_73825\_cov\_29.945791 20322-20331. Max. coverage (+): 0. Max coverage (-): 0.74

Region: NODE\_389151\_length\_73825\_cov\_29.945791 20332-20342. Max. coverage (+): 0. Max coverage (-): 1.11

Region: NODE\_389151\_length\_73825\_cov\_29.945791 20343-20352. Max. coverage (+): 0. Max coverage (-): 0.11

Region: NODE\_389151\_length\_73825\_cov\_29.945791 20353-20363. Max. coverage (+): 0. Max coverage (-): 0.19

Region: NODE\_389151\_length\_73825\_cov\_29.945791 20364-20373. Max. coverage (+): 0. Max coverage (-): 8.16

Region: NODE\_389151\_length\_73825\_cov\_29.945791 20374-20384. Max. coverage (+): 0.37. Max coverage (-): 6.78

Region: NODE\_389151\_length\_73825\_cov\_29.945791 20385-20394. Max. coverage (+): 0.37. Max coverage (-): 1.48

Region: NODE\_389151\_length\_73825\_cov\_29.945791 20395-20405. Max. coverage (+): 0.33. Max coverage (-): 8.34

Region: NODE\_389151\_length\_73825\_cov\_29.945791 20406-20415. Max. coverage (+): 0.22. Max coverage (-): 1.11

Region: NODE\_389151\_length\_73825\_cov\_29.945791 20416-20426. Max. coverage (+): 0.22. Max coverage (-): 0.89

Region: NODE\_389151\_length\_73825\_cov\_29.945791 20427-20436. Max. coverage (+): 0. Max coverage (-): 0.07

Region: NODE\_389151\_length\_73825\_cov\_29.945791 20437-20447. Max. coverage (+): 0. Max coverage (-): 4

Region: NODE\_389151\_length\_73825\_cov\_29.945791 20448-20457. Max. coverage (+): 0.07. Max coverage (-): 6.15

Region: NODE\_389151\_length\_73825\_cov\_29.945791 20458-20468. Max. coverage (+): 0.04. Max coverage (-): 3.19

Region: NODE\_389151\_length\_73825\_cov\_29.945791 20469-20478. Max. coverage (+): 0.04. Max coverage (-): 3.97

Region: NODE\_389151\_length\_73825\_cov\_29.945791 20479-20489. Max. coverage (+): 0.3. Max coverage (-): 2.15

Region: NODE\_389151\_length\_73825\_cov\_29.945791 20490-20499. Max. coverage (+): 0.15. Max coverage (-): 0.7

Region: NODE\_389151\_length\_73825\_cov\_29.945791 20500-20510. Max. coverage (+): 0.02. Max coverage (-): 6.9

Region: NODE\_389151\_length\_73825\_cov\_29.945791 20511-20520. Max. coverage (+): 0.19. Max coverage (-): 1.37

Region: NODE\_389151\_length\_73825\_cov\_29.945791 20521-20531. Max. coverage (+): 0. Max coverage (-): 0.52

Region: NODE\_389151\_length\_73825\_cov\_29.945791 20532-20541. Max. coverage (+): 0.04. Max coverage (-): 1.11

Region: NODE\_389151\_length\_73825\_cov\_29.945791 20542-20552. Max. coverage (+): 0. Max coverage (-): 0.37

Region: NODE\_389151\_length\_73825\_cov\_29.945791 20553-20562. Max. coverage (+): 0. Max coverage (-): 0.3

Region: NODE\_389151\_length\_73825\_cov\_29.945791 20563-20573. Max. coverage (+): 0. Max coverage (-): 0.07

Region: NODE\_389151\_length\_73825\_cov\_29.945791 20574-20583. Max. coverage (+): 0.04. Max coverage (-): 12.98

Region: NODE\_389151\_length\_73825\_cov\_29.945791 20584-20594. Max. coverage (+): 0. Max coverage (-): 4.37

Region: NODE\_389151\_length\_73825\_cov\_29.945791 20595-20604. Max. coverage (+): 0. Max coverage (-): 0.22

Region: NODE\_389151\_length\_73825\_cov\_29.945791 20605-20615. Max. coverage (+): 0. Max coverage (-): 1.59

Region: NODE\_389151\_length\_73825\_cov\_29.945791 20616-20625. Max. coverage (+): 0.13. Max coverage (-): 1.45

Region: NODE\_389151\_length\_73825\_cov\_29.945791 20626-20636. Max. coverage (+): 0.35. Max coverage (-): 10.42

Region: NODE\_389151\_length\_73825\_cov\_29.945791 20637-20646. Max. coverage (+): 0. Max coverage (-): 1.67

Region: NODE\_389151\_length\_73825\_cov\_29.945791 20647-20657. Max. coverage (+): 0.07. Max coverage (-): 8.45

Region: NODE\_389151\_length\_73825\_cov\_29.945791 20658-20667. Max. coverage (+): 0.07. Max coverage (-): 5.86

Region: NODE\_389151\_length\_73825\_cov\_29.945791 20668-20678. Max. coverage (+): 0.04. Max coverage (-): 5.25

Region: NODE\_389151\_length\_73825\_cov\_29.945791 20679-20688. Max. coverage (+): 0.15. Max coverage (-): 1.72

Region: NODE\_389151\_length\_73825\_cov\_29.945791 20689-20698. Max. coverage (+): 2.21. Max coverage (-): 1.93

Region: NODE\_389151\_length\_73825\_cov\_29.945791 20699-20709. Max. coverage (+): 0.3. Max coverage (-): 16.05

Region: NODE\_389151\_length\_73825\_cov\_29.945791 20710-20719. Max. coverage (+): 4.08. Max coverage (-): 0.93

Region: NODE\_389151\_length\_73825\_cov\_29.945791 20720-20730. Max. coverage (+): 4. Max coverage (-): 0.41

Region: NODE\_389151\_length\_73825\_cov\_29.945791 20731-20740. Max. coverage (+): 0.33. Max coverage (-): 6.6

Region: NODE\_389151\_length\_73825\_cov\_29.945791 20741-20751. Max. coverage (+): 0. Max coverage (-): 6.38

Region: NODE\_389151\_length\_73825\_cov\_29.945791 20752-20761. Max. coverage (+): 0.56. Max coverage (-): 7.04

Region: NODE\_389151\_length\_73825\_cov\_29.945791 20762-20772. Max. coverage (+): 0.48. Max coverage (-): 0.74

Region: NODE\_389151\_length\_73825\_cov\_29.945791 20773-20782. Max. coverage (+): 0.37. Max coverage (-): 0.74

Region: NODE\_389151\_length\_73825\_cov\_29.945791 20783-20793. Max. coverage (+): 0.2. Max coverage (-): 1.38

Region: NODE\_389151\_length\_73825\_cov\_29.945791 20794-20803. Max. coverage (+): 0.25. Max coverage (-): 6.97

Region: NODE\_389151\_length\_73825\_cov\_29.945791 20804-20814. Max. coverage (+): 0.25. Max coverage (-): 6.36

Region: NODE\_389151\_length\_73825\_cov\_29.945791 20815-20824. Max. coverage (+): 0.15. Max coverage (-): 0.07

Region: NODE\_389151\_length\_73825\_cov\_29.945791 20825-20835. Max. coverage (+): 0.04. Max coverage (-): 0.11

Region: NODE\_389151\_length\_73825\_cov\_29.945791 20836-20845. Max. coverage (+): 0.3. Max coverage (-): 2.78

Region: NODE\_389151\_length\_73825\_cov\_29.945791 20846-20856. Max. coverage (+): 0.78. Max coverage (-): 2.19

Region: NODE\_389151\_length\_73825\_cov\_29.945791 20857-20866. Max. coverage (+): 0.37. Max coverage (-): 7.9

Region: NODE\_389151\_length\_73825\_cov\_29.945791 20867-20877. Max. coverage (+): 0.26. Max coverage (-): 0.37

Region: NODE\_389151\_length\_73825\_cov\_29.945791 20878-20887. Max. coverage (+): 0.3. Max coverage (-): 0.07

Region: NODE\_389151\_length\_73825\_cov\_29.945791 20888-20898. Max. coverage (+): 0. Max coverage (-): 0.19

Region: NODE\_389151\_length\_73825\_cov\_29.945791 20899-20908. Max. coverage (+): 0.04. Max coverage (-): 0.19

Region: NODE\_389151\_length\_73825\_cov\_29.945791 20909-20919. Max. coverage (+): 0. Max coverage (-): 0.07

Region: NODE\_389151\_length\_73825\_cov\_29.945791 20920-20929. Max. coverage (+): 0. Max coverage (-): 2.41

Region: NODE\_389151\_length\_73825\_cov\_29.945791 20930-20940. Max. coverage (+): 0. Max coverage (-): 8.38

Region: NODE\_389151\_length\_73825\_cov\_29.945791 20941-20950. Max. coverage (+): 0.93. Max coverage (-): 0.22

Region: NODE\_389151\_length\_73825\_cov\_29.945791 20951-20961. Max. coverage (+): 0.89. Max coverage (-): 0.93

Region: NODE\_389151\_length\_73825\_cov\_29.945791 20962-20971. Max. coverage (+): 0. Max coverage (-): 25.78

Region: NODE\_389151\_length\_73825\_cov\_29.945791 20972-20982. Max. coverage (+): 0. Max coverage (-): 0.52

Region: NODE\_389151\_length\_73825\_cov\_29.945791 20983-20992. Max. coverage (+): 0.19. Max coverage (-): 0.09

Region: NODE\_389151\_length\_73825\_cov\_29.945791 20993-21003. Max. coverage (+): 0. Max coverage (-): 0.04

Region: NODE\_389151\_length\_73825\_cov\_29.945791 21004-21013. Max. coverage (+): 0. Max coverage (-): 0.04

Region: NODE\_389151\_length\_73825\_cov\_29.945791 21014-21024. Max. coverage (+): 0.59. Max coverage (-): 0.04

Region: NODE\_389151\_length\_73825\_cov\_29.945791 21025-21034. Max. coverage (+): 0.04. Max coverage (-): 0

Region: NODE\_389151\_length\_73825\_cov\_29.945791 21035-21045. Max. coverage (+): 0. Max coverage (-): 0.15

Region: NODE\_389151\_length\_73825\_cov\_29.945791 21046-21055. Max. coverage (+): 0. Max coverage (-): 0.15

Region: NODE\_389151\_length\_73825\_cov\_29.945791 21056-21066. Max. coverage (+): 0. Max coverage (-): 0.04

Region: NODE\_389151\_length\_73825\_cov\_29.945791 21067-21076. Max. coverage (+): 0. Max coverage (-): 2.11

Region: NODE\_389151\_length\_73825\_cov\_29.945791 21077-21087. Max. coverage (+): 0. Max coverage (-): 1.3

Region: NODE\_389151\_length\_73825\_cov\_29.945791 21088-21097. Max. coverage (+): 0.11. Max coverage (-): 1.15

Region: NODE\_389151\_length\_73825\_cov\_29.945791 21098-21108. Max. coverage (+): 0. Max coverage (-): 0.04

Region: NODE\_389151\_length\_73825\_cov\_29.945791 21109-21118. Max. coverage (+): 0. Max coverage (-): 0.13

Region: NODE\_389151\_length\_73825\_cov\_29.945791 21119-21129. Max. coverage (+): 0. Max coverage (-): 0.26

Region: NODE\_389151\_length\_73825\_cov\_29.945791 21130-21139. Max. coverage (+): 0. Max coverage (-): 24.19

Region: NODE\_389151\_length\_73825\_cov\_29.945791 21140-21150. Max. coverage (+): 0. Max coverage (-): 3.71

Region: NODE\_389151\_length\_73825\_cov\_29.945791 21151-21160. Max. coverage (+): 0.32. Max coverage (-): 0.5

Region: NODE\_389151\_length\_73825\_cov\_29.945791 21161-21171. Max. coverage (+): 0.04. Max coverage (-): 1.67

Region: NODE\_389151\_length\_73825\_cov\_29.945791 21172-21181. Max. coverage (+): 0.19. Max coverage (-): 0.17

Region: NODE\_389151\_length\_73825\_cov\_29.945791 21182-21192. Max. coverage (+): 0.33. Max coverage (-): 0.11

Region: NODE\_389151\_length\_73825\_cov\_29.945791 21193-21202. Max. coverage (+): 0. Max coverage (-): 0.33

Region: NODE\_389151\_length\_73825\_cov\_29.945791 21203-21213. Max. coverage (+): 0.07. Max coverage (-): 1

Region: NODE\_389151\_length\_73825\_cov\_29.945791 21214-21223. Max. coverage (+): 0.15. Max coverage (-): 0.56

Region: NODE\_389151\_length\_73825\_cov\_29.945791 21224-21234. Max. coverage (+): 0.22. Max coverage (-): 1.41

Region: NODE\_389151\_length\_73825\_cov\_29.945791 21235-21244. Max. coverage (+): 0.04. Max coverage (-): 1.22

Region: NODE\_389151\_length\_73825\_cov\_29.945791 21245-21255. Max. coverage (+): 0.33. Max coverage (-): 0.3

Region: NODE\_389151\_length\_73825\_cov\_29.945791 21256-21265. Max. coverage (+): 0.11. Max coverage (-): 1.3

Region: NODE\_389151\_length\_73825\_cov\_29.945791 21266-21276. Max. coverage (+): 0. Max coverage (-): 0.26

Region: NODE\_389151\_length\_73825\_cov\_29.945791 21277-21286. Max. coverage (+): 0. Max coverage (-): 0

Region: NODE\_389151\_length\_73825\_cov\_29.945791 21287-21297. Max. coverage (+): 0. Max coverage (-): 0.19

Region: NODE\_389151\_length\_73825\_cov\_29.945791 21298-21307. Max. coverage (+): 0.07. Max coverage (-): 0.19

Region: NODE\_389151\_length\_73825\_cov\_29.945791 21308-21318. Max. coverage (+): 0.07. Max coverage (-): 0.59

Region: NODE\_389151\_length\_73825\_cov\_29.945791 21319-21328. Max. coverage (+): 0. Max coverage (-): 8.9

Region: NODE\_389151\_length\_73825\_cov\_29.945791 21329-21339. Max. coverage (+): 0. Max coverage (-): 8.9

Region: NODE\_389151\_length\_73825\_cov\_29.945791 21340-21349. Max. coverage (+): 0.04. Max coverage (-): 0

Region: NODE\_389151\_length\_73825\_cov\_29.945791 21350-21360. Max. coverage (+): 0. Max coverage (-): 0

Region: NODE\_389151\_length\_73825\_cov\_29.945791 21361-21370. Max. coverage (+): 0. Max coverage (-): 0

Region: NODE\_389151\_length\_73825\_cov\_29.945791 21371-21381. Max. coverage (+): 0. Max coverage (-): 0

Region: NODE\_389151\_length\_73825\_cov\_29.945791 21382-21391. Max. coverage (+): 0. Max coverage (-): 0

Region: NODE\_389151\_length\_73825\_cov\_29.945791 21392-21402. Max. coverage (+): 0. Max coverage (-): 0

Region: NODE\_389151\_length\_73825\_cov\_29.945791 21403-21412. Max. coverage (+): 0. Max coverage (-): 0

Region: NODE\_389151\_length\_73825\_cov\_29.945791 21413-21423. Max. coverage (+): 0. Max coverage (-): 0

Region: NODE\_389151\_length\_73825\_cov\_29.945791 21424-21433. Max. coverage (+): 0. Max coverage (-): 0

Region: NODE\_389151\_length\_73825\_cov\_29.945791 21434-21444. Max. coverage (+): 0. Max coverage (-): 0

Region: NODE\_389151\_length\_73825\_cov\_29.945791 21445-21454. Max. coverage (+): 0. Max coverage (-): 0

Region: NODE\_389151\_length\_73825\_cov\_29.945791 21455-21465. Max. coverage (+): 0. Max coverage (-): 0

Region: NODE\_389151\_length\_73825\_cov\_29.945791 21466-21475. Max. coverage (+): 0. Max coverage (-): 0

Region: NODE\_389151\_length\_73825\_cov\_29.945791 21476-21486. Max. coverage (+): 0. Max coverage (-): 0.04

Region: NODE\_389151\_length\_73825\_cov\_29.945791 21487-21496. Max. coverage (+): 0. Max coverage (-): 0.04

Region: NODE\_389151\_length\_73825\_cov\_29.945791 21497-21507. Max. coverage (+): 0. Max coverage (-): 0

Region: NODE\_389151\_length\_73825\_cov\_29.945791 21508-21517. Max. coverage (+): 0. Max coverage (-): 0

Region: NODE\_389151\_length\_73825\_cov\_29.945791 21518-21528. Max. coverage (+): 0. Max coverage (-): 0

Region: NODE\_389151\_length\_73825\_cov\_29.945791 21529-21538. Max. coverage (+): 0. Max coverage (-): 0

Region: NODE\_389151\_length\_73825\_cov\_29.945791 21539-21549. Max. coverage (+): 0. Max coverage (-): 0

Region: NODE\_389151\_length\_73825\_cov\_29.945791 21550-21559. Max. coverage (+): 0. Max coverage (-): 0

Region: NODE\_389151\_length\_73825\_cov\_29.945791 21560-21570. Max. coverage (+): 0. Max coverage (-): 0

Region: NODE\_389151\_length\_73825\_cov\_29.945791 21571-21580. Max. coverage (+): 0. Max coverage (-): 0

Region: NODE\_389151\_length\_73825\_cov\_29.945791 21581-21590. Max. coverage (+): 0. Max coverage (-): 0

Region: NODE\_389151\_length\_73825\_cov\_29.945791 21591-21601. Max. coverage (+): 0. Max coverage (-): 0

Region: NODE\_389151\_length\_73825\_cov\_29.945791 21602-21611. Max. coverage (+): 0. Max coverage (-): 0

Region: NODE\_389151\_length\_73825\_cov\_29.945791 21612-21622. Max. coverage (+): 0. Max coverage (-): 0

Region: NODE\_389151\_length\_73825\_cov\_29.945791 21623-21632. Max. coverage (+): 0. Max coverage (-): 0

Region: NODE\_389151\_length\_73825\_cov\_29.945791 21633-21643. Max. coverage (+): 0. Max coverage (-): 0

Region: NODE\_389151\_length\_73825\_cov\_29.945791 21644-21653. Max. coverage (+): 0. Max coverage (-): 0

Region: NODE\_389151\_length\_73825\_cov\_29.945791 21654-21664. Max. coverage (+): 0. Max coverage (-): 0

Region: NODE\_389151\_length\_73825\_cov\_29.945791 21665-21674. Max. coverage (+): 0. Max coverage (-): 0

Region: NODE\_389151\_length\_73825\_cov\_29.945791 21675-21685. Max. coverage (+): 0. Max coverage (-): 0

Region: NODE\_389151\_length\_73825\_cov\_29.945791 21686-21695. Max. coverage (+): 0. Max coverage (-): 0

Region: NODE\_389151\_length\_73825\_cov\_29.945791 21696-21706. Max. coverage (+): 0. Max coverage (-): 0

Region: NODE\_389151\_length\_73825\_cov\_29.945791 21707-21716. Max. coverage (+): 0. Max coverage (-): 0

Region: NODE\_389151\_length\_73825\_cov\_29.945791 21717-21727. Max. coverage (+): 0. Max coverage (-): 0

Region: NODE\_389151\_length\_73825\_cov\_29.945791 21728-21737. Max. coverage (+): 0. Max coverage (-): 0

Region: NODE\_389151\_length\_73825\_cov\_29.945791 21738-21748. Max. coverage (+): 0. Max coverage (-): 0

Region: NODE\_389151\_length\_73825\_cov\_29.945791 21749-21758. Max. coverage (+): 0. Max coverage (-): 0

Region: NODE\_389151\_length\_73825\_cov\_29.945791 21759-21769. Max. coverage (+): 0. Max coverage (-): 0

Region: NODE\_389151\_length\_73825\_cov\_29.945791 21770-21779. Max. coverage (+): 0. Max coverage (-): 0

Region: NODE\_389151\_length\_73825\_cov\_29.945791 21780-21790. Max. coverage (+): 0. Max coverage (-): 0

Region: NODE\_389151\_length\_73825\_cov\_29.945791 21791-21800. Max. coverage (+): 0. Max coverage (-): 0

Region: NODE\_389151\_length\_73825\_cov\_29.945791 21801-21811. Max. coverage (+): 0. Max coverage (-): 0.04

Region: NODE\_389151\_length\_73825\_cov\_29.945791 21812-21821. Max. coverage (+): 0. Max coverage (-): 0

Region: NODE\_389151\_length\_73825\_cov\_29.945791 21822-21832. Max. coverage (+): 0. Max coverage (-): 0

Region: NODE\_389151\_length\_73825\_cov\_29.945791 21833-21842. Max. coverage (+): 0. Max coverage (-): 0

Region: NODE\_389151\_length\_73825\_cov\_29.945791 21843-21853. Max. coverage (+): 0. Max coverage (-): 0

Region: NODE\_389151\_length\_73825\_cov\_29.945791 21854-21863. Max. coverage (+): 0. Max coverage (-): 0

Region: NODE\_389151\_length\_73825\_cov\_29.945791 21864-21874. Max. coverage (+): 0. Max coverage (-): 0

Region: NODE\_389151\_length\_73825\_cov\_29.945791 21875-21884. Max. coverage (+): 0. Max coverage (-): 0

Region: NODE\_389151\_length\_73825\_cov\_29.945791 21885-21895. Max. coverage (+): 0. Max coverage (-): 0

Region: NODE\_389151\_length\_73825\_cov\_29.945791 21896-21905. Max. coverage (+): 0. Max coverage (-): 0

Region: NODE\_389151\_length\_73825\_cov\_29.945791 21906-21916. Max. coverage (+): 0. Max coverage (-): 0

Region: NODE\_389151\_length\_73825\_cov\_29.945791 21917-21926. Max. coverage (+): 0. Max coverage (-): 0

Region: NODE\_389151\_length\_73825\_cov\_29.945791 21927-21937. Max. coverage (+): 1.95. Max coverage (-): 0

Region: NODE\_389151\_length\_73825\_cov\_29.945791 21938-21947. Max. coverage (+): 1.2. Max coverage (-): 0.01

Region: NODE\_389151\_length\_73825\_cov\_29.945791 21948-21958. Max. coverage (+): 0. Max coverage (-): 0

Region: NODE\_389151\_length\_73825\_cov\_29.945791 21959-21968. Max. coverage (+): 0. Max coverage (-): 0

Region: NODE\_389151\_length\_73825\_cov\_29.945791 21969-21979. Max. coverage (+): 0. Max coverage (-): 0

Region: NODE\_389151\_length\_73825\_cov\_29.945791 21980-21989. Max. coverage (+): 0. Max coverage (-): 0

Region: NODE\_389151\_length\_73825\_cov\_29.945791 21990-22000. Max. coverage (+): 0. Max coverage (-): 0

Region: NODE\_389151\_length\_73825\_cov\_29.945791 22001-22010. Max. coverage (+): 0. Max coverage (-): 0

Region: NODE\_389151\_length\_73825\_cov\_29.945791 22011-22021. Max. coverage (+): 0. Max coverage (-): 0

Region: NODE\_389151\_length\_73825\_cov\_29.945791 22022-22031. Max. coverage (+): 0. Max coverage (-): 0

Region: NODE\_389151\_length\_73825\_cov\_29.945791 22032-22042. Max. coverage (+): 0. Max coverage (-): 0

Region: NODE\_389151\_length\_73825\_cov\_29.945791 22043-22052. Max. coverage (+): 0. Max coverage (-): 0

Region: NODE\_389151\_length\_73825\_cov\_29.945791 22053-22063. Max. coverage (+): 0.04. Max coverage (-): 0

Region: NODE\_389151\_length\_73825\_cov\_29.945791 22064-22073. Max. coverage (+): 0.04. Max coverage (-): 0

Region: NODE\_389151\_length\_73825\_cov\_29.945791 22074-22084. Max. coverage (+): 0. Max coverage (-): 0

Region: NODE\_389151\_length\_73825\_cov\_29.945791 22085-22094. Max. coverage (+): 0. Max coverage (-): 0

Region: NODE\_389151\_length\_73825\_cov\_29.945791 22095-22105. Max. coverage (+): 0. Max coverage (-): 0

Region: NODE\_389151\_length\_73825\_cov\_29.945791 22106-22115. Max. coverage (+): 0. Max coverage (-): 0

Region: NODE\_389151\_length\_73825\_cov\_29.945791 22116-22126. Max. coverage (+): 0. Max coverage (-): 0

Region: NODE\_389151\_length\_73825\_cov\_29.945791 22127-22136. Max. coverage (+): 0. Max coverage (-): 0

Region: NODE\_389151\_length\_73825\_cov\_29.945791 22137-22147. Max. coverage (+): 0. Max coverage (-): 0

Region: NODE\_389151\_length\_73825\_cov\_29.945791 22148-22157. Max. coverage (+): 0. Max coverage (-): 0

Region: NODE\_389151\_length\_73825\_cov\_29.945791 22158-22168. Max. coverage (+): 0. Max coverage (-): 0

Region: NODE\_389151\_length\_73825\_cov\_29.945791 22169-22178. Max. coverage (+): 0. Max coverage (-): 0

Region: NODE\_389151\_length\_73825\_cov\_29.945791 22179-22189. Max. coverage (+): 0. Max coverage (-): 0

Region: NODE\_389151\_length\_73825\_cov\_29.945791 22190-22199. Max. coverage (+): 0. Max coverage (-): 0

Region: NODE\_389151\_length\_73825\_cov\_29.945791 22200-22210. Max. coverage (+): 0. Max coverage (-): 0

Region: NODE\_389151\_length\_73825\_cov\_29.945791 22211-22220. Max. coverage (+): 0. Max coverage (-): 0

Region: NODE\_389151\_length\_73825\_cov\_29.945791 22221-22231. Max. coverage (+): 0. Max coverage (-): 0.04

Region: NODE\_389151\_length\_73825\_cov\_29.945791 22232-22241. Max. coverage (+): 0. Max coverage (-): 0.04

Region: NODE\_389151\_length\_73825\_cov\_29.945791 22242-22252. Max. coverage (+): 0. Max coverage (-): 0.02

Region: NODE\_389151\_length\_73825\_cov\_29.945791 22253-22262. Max. coverage (+): 0. Max coverage (-): 0.02

Region: NODE\_389151\_length\_73825\_cov\_29.945791 22263-22273. Max. coverage (+): 0. Max coverage (-): 0

Region: NODE\_389151\_length\_73825\_cov\_29.945791 22274-22283. Max. coverage (+): 0. Max coverage (-): 0.01

Region: NODE\_389151\_length\_73825\_cov\_29.945791 22284-22294. Max. coverage (+): 0. Max coverage (-): 0

Region: NODE\_389151\_length\_73825\_cov\_29.945791 22295-22304. Max. coverage (+): 0. Max coverage (-): 0

Region: NODE\_389151\_length\_73825\_cov\_29.945791 22305-22315. Max. coverage (+): 0. Max coverage (-): 0

Region: NODE\_389151\_length\_73825\_cov\_29.945791 22316-22325. Max. coverage (+): 0.02. Max coverage (-): 0

Region: NODE\_389151\_length\_73825\_cov\_29.945791 22326-22336. Max. coverage (+): 0.02. Max coverage (-): 0.01

Region: NODE\_389151\_length\_73825\_cov\_29.945791 22337-22346. Max. coverage (+): 0.01. Max coverage (-): 0

Region: NODE\_389151\_length\_73825\_cov\_29.945791 22347-22357. Max. coverage (+): 0. Max coverage (-): 0

Region: NODE\_389151\_length\_73825\_cov\_29.945791 22358-22367. Max. coverage (+): 0. Max coverage (-): 0.01

Region: NODE\_389151\_length\_73825\_cov\_29.945791 22368-22378. Max. coverage (+): 0. Max coverage (-): 0

Region: NODE\_389151\_length\_73825\_cov\_29.945791 22379-22388. Max. coverage (+): 0. Max coverage (-): 0

Region: NODE\_389151\_length\_73825\_cov\_29.945791 22389-22399. Max. coverage (+): 0. Max coverage (-): 0.07

Region: NODE\_389151\_length\_73825\_cov\_29.945791 22400-22409. Max. coverage (+): 0. Max coverage (-): 0.04

Region: NODE\_389151\_length\_73825\_cov\_29.945791 22410-22420. Max. coverage (+): 0. Max coverage (-): 0

Region: NODE\_389151\_length\_73825\_cov\_29.945791 22421-22430. Max. coverage (+): 0. Max coverage (-): 0

Region: NODE\_389151\_length\_73825\_cov\_29.945791 22431-22441. Max. coverage (+): 0. Max coverage (-): 0

Region: NODE\_389151\_length\_73825\_cov\_29.945791 22442-22451. Max. coverage (+): 0. Max coverage (-): 0

Region: NODE\_389151\_length\_73825\_cov\_29.945791 22452-22461. Max. coverage (+): 0. Max coverage (-): 0

Region: NODE\_389151\_length\_73825\_cov\_29.945791 22462-22472. Max. coverage (+): 0. Max coverage (-): 0

Region: NODE\_389151\_length\_73825\_cov\_29.945791 22473-22482. Max. coverage (+): 0. Max coverage (-): 0

Region: NODE\_389151\_length\_73825\_cov\_29.945791 22483-22493. Max. coverage (+): 0. Max coverage (-): 0

Region: NODE\_389151\_length\_73825\_cov\_29.945791 22494-22503. Max. coverage (+): 0. Max coverage (-): 0

Region: NODE\_389151\_length\_73825\_cov\_29.945791 22504-22514. Max. coverage (+): 0. Max coverage (-): 0

Region: NODE\_389151\_length\_73825\_cov\_29.945791 22515-22524. Max. coverage (+): 0. Max coverage (-): 0

Region: NODE\_389151\_length\_73825\_cov\_29.945791 22525-22535. Max. coverage (+): 0. Max coverage (-): 0.02

Region: NODE\_389151\_length\_73825\_cov\_29.945791 22536-22545. Max. coverage (+): 0. Max coverage (-): 0

Region: NODE\_389151\_length\_73825\_cov\_29.945791 22546-22556. Max. coverage (+): 0. Max coverage (-): 0

Region: NODE\_389151\_length\_73825\_cov\_29.945791 22557-22566. Max. coverage (+): 0. Max coverage (-): 0

Region: NODE\_389151\_length\_73825\_cov\_29.945791 22567-22577. Max. coverage (+): 0. Max coverage (-): 0

Region: NODE\_389151\_length\_73825\_cov\_29.945791 22578-22587. Max. coverage (+): 0. Max coverage (-): 0

Region: NODE\_389151\_length\_73825\_cov\_29.945791 22588-22598. Max. coverage (+): 0. Max coverage (-): 0.01

Region: NODE\_389151\_length\_73825\_cov\_29.945791 22599-22608. Max. coverage (+): 0. Max coverage (-): 0

Region: NODE\_389151\_length\_73825\_cov\_29.945791 22609-22619. Max. coverage (+): 0. Max coverage (-): 0

Region: NODE\_389151\_length\_73825\_cov\_29.945791 22620-22629. Max. coverage (+): 0. Max coverage (-): 0

Region: NODE\_389151\_length\_73825\_cov\_29.945791 22630-22640. Max. coverage (+): 0. Max coverage (-): 0

Region: NODE\_389151\_length\_73825\_cov\_29.945791 22641-22650. Max. coverage (+): 0. Max coverage (-): 0

Region: NODE\_389151\_length\_73825\_cov\_29.945791 22651-22661. Max. coverage (+): 0. Max coverage (-): 0

Region: NODE\_389151\_length\_73825\_cov\_29.945791 22662-22671. Max. coverage (+): 0. Max coverage (-): 0

Region: NODE\_389151\_length\_73825\_cov\_29.945791 22672-22682. Max. coverage (+): 0. Max coverage (-): 0

Region: NODE\_389151\_length\_73825\_cov\_29.945791 22683-22692. Max. coverage (+): 0. Max coverage (-): 0

Region: NODE\_389151\_length\_73825\_cov\_29.945791 22693-22703. Max. coverage (+): 0. Max coverage (-): 0

Region: NODE\_389151\_length\_73825\_cov\_29.945791 22704-22713. Max. coverage (+): 0. Max coverage (-): 0

Region: NODE\_389151\_length\_73825\_cov\_29.945791 22714-22724. Max. coverage (+): 0. Max coverage (-): 0

Region: NODE\_389151\_length\_73825\_cov\_29.945791 22725-22734. Max. coverage (+): 0. Max coverage (-): 0

Region: NODE\_389151\_length\_73825\_cov\_29.945791 22735-22745. Max. coverage (+): 0. Max coverage (-): 0

Region: NODE\_389151\_length\_73825\_cov\_29.945791 22746-22755. Max. coverage (+): 0. Max coverage (-): 0

Region: NODE\_389151\_length\_73825\_cov\_29.945791 22756-22766. Max. coverage (+): 0. Max coverage (-): 0

Region: NODE\_389151\_length\_73825\_cov\_29.945791 22767-22776. Max. coverage (+): 0. Max coverage (-): 0

Region: NODE\_389151\_length\_73825\_cov\_29.945791 22777-22787. Max. coverage (+): 0. Max coverage (-): 0

Region: NODE\_389151\_length\_73825\_cov\_29.945791 22788-22797. Max. coverage (+): 0. Max coverage (-): 0

Region: NODE\_389151\_length\_73825\_cov\_29.945791 22798-22808. Max. coverage (+): 0. Max coverage (-): 0

Region: NODE\_389151\_length\_73825\_cov\_29.945791 22809-22818. Max. coverage (+): 0. Max coverage (-): 0

Region: NODE\_389151\_length\_73825\_cov\_29.945791 22819-22829. Max. coverage (+): 0. Max coverage (-): 0

Region: NODE\_389151\_length\_73825\_cov\_29.945791 22830-22839. Max. coverage (+): 0. Max coverage (-): 0

Region: NODE\_389151\_length\_73825\_cov\_29.945791 22840-22850. Max. coverage (+): 0. Max coverage (-): 0

Region: NODE\_389151\_length\_73825\_cov\_29.945791 22851-22860. Max. coverage (+): 0. Max coverage (-): 0

Region: NODE\_389151\_length\_73825\_cov\_29.945791 22861-22871. Max. coverage (+): 0. Max coverage (-): 0

Region: NODE\_389151\_length\_73825\_cov\_29.945791 22872-22881. Max. coverage (+): 0. Max coverage (-): 0

Region: NODE\_389151\_length\_73825\_cov\_29.945791 22882-22892. Max. coverage (+): 0. Max coverage (-): 0

Region: NODE\_389151\_length\_73825\_cov\_29.945791 22893-22902. Max. coverage (+): 0. Max coverage (-): 0

Region: NODE\_389151\_length\_73825\_cov\_29.945791 22903-22913. Max. coverage (+): 0. Max coverage (-): 0

Region: NODE\_389151\_length\_73825\_cov\_29.945791 22914-22923. Max. coverage (+): 0. Max coverage (-): 0

Region: NODE\_389151\_length\_73825\_cov\_29.945791 22924-22934. Max. coverage (+): 0. Max coverage (-): 0

Region: NODE\_389151\_length\_73825\_cov\_29.945791 22935-22944. Max. coverage (+): 0. Max coverage (-): 0

Region: NODE\_389151\_length\_73825\_cov\_29.945791 22945-22955. Max. coverage (+): 0.04. Max coverage (-): 0

Region: NODE\_389151\_length\_73825\_cov\_29.945791 22956-22965. Max. coverage (+): 0.04. Max coverage (-): 0

Region: NODE\_389151\_length\_73825\_cov\_29.945791 22966-22976. Max. coverage (+): 0. Max coverage (-): 0

Region: NODE\_389151\_length\_73825\_cov\_29.945791 22977-22986. Max. coverage (+): 0. Max coverage (-): 0

Region: NODE\_389151\_length\_73825\_cov\_29.945791 22987-22997. Max. coverage (+): 0. Max coverage (-): 0

Region: NODE\_389151\_length\_73825\_cov\_29.945791 22998-23007. Max. coverage (+): 0. Max coverage (-): 0

Region: NODE\_389151\_length\_73825\_cov\_29.945791 23008-23018. Max. coverage (+): 0. Max coverage (-): 0

Region: NODE\_389151\_length\_73825\_cov\_29.945791 23019-23028. Max. coverage (+): 0. Max coverage (-): 0

Region: NODE\_389151\_length\_73825\_cov\_29.945791 23029-23039. Max. coverage (+): 0. Max coverage (-): 0

Region: NODE\_389151\_length\_73825\_cov\_29.945791 23040-23049. Max. coverage (+): 0. Max coverage (-): 0

Region: NODE\_389151\_length\_73825\_cov\_29.945791 23050-23060. Max. coverage (+): 0. Max coverage (-): 0

Region: NODE\_389151\_length\_73825\_cov\_29.945791 23061-23070. Max. coverage (+): 0. Max coverage (-): 0

Region: NODE\_389151\_length\_73825\_cov\_29.945791 23071-23081. Max. coverage (+): 0. Max coverage (-): 0

Region: NODE\_389151\_length\_73825\_cov\_29.945791 23082-23091. Max. coverage (+): 0.01. Max coverage (-): 0

Region: NODE\_389151\_length\_73825\_cov\_29.945791 23092-23102. Max. coverage (+): 0. Max coverage (-): 0

Region: NODE\_389151\_length\_73825\_cov\_29.945791 23103-23112. Max. coverage (+): 0.02. Max coverage (-): 0

Region: NODE\_389151\_length\_73825\_cov\_29.945791 23113-23123. Max. coverage (+): 0. Max coverage (-): 0

Region: NODE\_389151\_length\_73825\_cov\_29.945791 23124-23133. Max. coverage (+): 0. Max coverage (-): 0

Region: NODE\_389151\_length\_73825\_cov\_29.945791 23134-23144. Max. coverage (+): 0. Max coverage (-): 0

Region: NODE\_389151\_length\_73825\_cov\_29.945791 23145-23154. Max. coverage (+): 0. Max coverage (-): 0

Region: NODE\_389151\_length\_73825\_cov\_29.945791 23155-23165. Max. coverage (+): 0. Max coverage (-): 0

Region: NODE\_389151\_length\_73825\_cov\_29.945791 23166-23175. Max. coverage (+): 0. Max coverage (-): 0

Region: NODE\_389151\_length\_73825\_cov\_29.945791 23176-23186. Max. coverage (+): 0. Max coverage (-): 0

Region: NODE\_389151\_length\_73825\_cov\_29.945791 23187-23196. Max. coverage (+): 0. Max coverage (-): 0

Region: NODE\_389151\_length\_73825\_cov\_29.945791 23197-23207. Max. coverage (+): 0. Max coverage (-): 0

Region: NODE\_389151\_length\_73825\_cov\_29.945791 23208-23217. Max. coverage (+): 0. Max coverage (-): 0

Region: NODE\_389151\_length\_73825\_cov\_29.945791 23218-23228. Max. coverage (+): 0. Max coverage (-): 0

Region: NODE\_389151\_length\_73825\_cov\_29.945791 23229-23238. Max. coverage (+): 0.07. Max coverage (-): 0

Region: NODE\_389151\_length\_73825\_cov\_29.945791 23239-23249. Max. coverage (+): 0.04. Max coverage (-): 0

Region: NODE\_389151\_length\_73825\_cov\_29.945791 23250-23259. Max. coverage (+): 0. Max coverage (-): 0

Region: NODE\_389151\_length\_73825\_cov\_29.945791 23260-23270. Max. coverage (+): 0. Max coverage (-): 0

Region: NODE\_389151\_length\_73825\_cov\_29.945791 23271-23280. Max. coverage (+): 0. Max coverage (-): 0

Region: NODE\_389151\_length\_73825\_cov\_29.945791 23281-23291. Max. coverage (+): 0. Max coverage (-): 0

Region: NODE\_389151\_length\_73825\_cov\_29.945791 23292-23301. Max. coverage (+): 0. Max coverage (-): 0

Region: NODE\_389151\_length\_73825\_cov\_29.945791 23302-23312. Max. coverage (+): 0.04. Max coverage (-): 0

Region: NODE\_389151\_length\_73825\_cov\_29.945791 23313-23322. Max. coverage (+): 0.04. Max coverage (-): 0

Region: NODE\_389151\_length\_73825\_cov\_29.945791 23323-23332. Max. coverage (+): 0. Max coverage (-): 0

Region: NODE\_389151\_length\_73825\_cov\_29.945791 23333-23343. Max. coverage (+): 0. Max coverage (-): 0

Region: NODE\_389151\_length\_73825\_cov\_29.945791 23344-23353. Max. coverage (+): 0. Max coverage (-): 0

Region: NODE\_389151\_length\_73825\_cov\_29.945791 23354-23364. Max. coverage (+): 0. Max coverage (-): 0

Region: NODE\_389151\_length\_73825\_cov\_29.945791 23365-23374. Max. coverage (+): 0. Max coverage (-): 0

Region: NODE\_389151\_length\_73825\_cov\_29.945791 23375-23385. Max. coverage (+): 0. Max coverage (-): 0

Region: NODE\_389151\_length\_73825\_cov\_29.945791 23386-23395. Max. coverage (+): 0. Max coverage (-): 0

Region: NODE\_389151\_length\_73825\_cov\_29.945791 23396-23406. Max. coverage (+): 0. Max coverage (-): 0

Region: NODE\_389151\_length\_73825\_cov\_29.945791 23407-23416. Max. coverage (+): 0. Max coverage (-): 0

Region: NODE\_389151\_length\_73825\_cov\_29.945791 23417-23427. Max. coverage (+): 0. Max coverage (-): 0

Region: NODE\_389151\_length\_73825\_cov\_29.945791 23428-23437. Max. coverage (+): 0.01. Max coverage (-): 0

Region: NODE\_389151\_length\_73825\_cov\_29.945791 23438-23448. Max. coverage (+): 0.01. Max coverage (-): 0

Region: NODE\_389151\_length\_73825\_cov\_29.945791 23449-23458. Max. coverage (+): 0. Max coverage (-): 0

Region: NODE\_389151\_length\_73825\_cov\_29.945791 23459-23469. Max. coverage (+): 0. Max coverage (-): 0

Region: NODE\_389151\_length\_73825\_cov\_29.945791 23470-23479. Max. coverage (+): 0. Max coverage (-): 0

Region: NODE\_389151\_length\_73825\_cov\_29.945791 23480-23490. Max. coverage (+): 0. Max coverage (-): 0

Region: NODE\_389151\_length\_73825\_cov\_29.945791 23491-23500. Max. coverage (+): 0. Max coverage (-): 0

Region: NODE\_389151\_length\_73825\_cov\_29.945791 23501-23511. Max. coverage (+): 0. Max coverage (-): 0

Region: NODE\_389151\_length\_73825\_cov\_29.945791 23512-23521. Max. coverage (+): 0. Max coverage (-): 0

Region: NODE\_389151\_length\_73825\_cov\_29.945791 23522-23532. Max. coverage (+): 0. Max coverage (-): 0

Region: NODE\_389151\_length\_73825\_cov\_29.945791 23533-23542. Max. coverage (+): 0. Max coverage (-): 0

Region: NODE\_389151\_length\_73825\_cov\_29.945791 23543-23553. Max. coverage (+): 0. Max coverage (-): 0

Region: NODE\_389151\_length\_73825\_cov\_29.945791 23554-23563. Max. coverage (+): 0. Max coverage (-): 0

Region: NODE\_389151\_length\_73825\_cov\_29.945791 23564-23574. Max. coverage (+): 0. Max coverage (-): 0

Region: NODE\_389151\_length\_73825\_cov\_29.945791 23575-23584. Max. coverage (+): 0. Max coverage (-): 0

Region: NODE\_389151\_length\_73825\_cov\_29.945791 23585-23595. Max. coverage (+): 0. Max coverage (-): 0

Region: NODE\_389151\_length\_73825\_cov\_29.945791 23596-23605. Max. coverage (+): 0. Max coverage (-): 0

Region: NODE\_389151\_length\_73825\_cov\_29.945791 23606-23616. Max. coverage (+): 0. Max coverage (-): 0

Region: NODE\_389151\_length\_73825\_cov\_29.945791 23617-23626. Max. coverage (+): 0. Max coverage (-): 0

Region: NODE\_389151\_length\_73825\_cov\_29.945791 23627-23637. Max. coverage (+): 0. Max coverage (-): 0

Region: NODE\_389151\_length\_73825\_cov\_29.945791 23638-23647. Max. coverage (+): 0. Max coverage (-): 0

Region: NODE\_389151\_length\_73825\_cov\_29.945791 23648-23658. Max. coverage (+): 0. Max coverage (-): 0

Region: NODE\_389151\_length\_73825\_cov\_29.945791 23659-23668. Max. coverage (+): 0. Max coverage (-): 0

Region: NODE\_389151\_length\_73825\_cov\_29.945791 23669-23679. Max. coverage (+): 0. Max coverage (-): 0

Region: NODE\_389151\_length\_73825\_cov\_29.945791 23680-23689. Max. coverage (+): 0. Max coverage (-): 0

Region: NODE\_389151\_length\_73825\_cov\_29.945791 23690-23700. Max. coverage (+): 0. Max coverage (-): 0

Region: NODE\_389151\_length\_73825\_cov\_29.945791 23701-23710. Max. coverage (+): 0. Max coverage (-): 0

Region: NODE\_389151\_length\_73825\_cov\_29.945791 23711-23721. Max. coverage (+): 0. Max coverage (-): 0

Region: NODE\_389151\_length\_73825\_cov\_29.945791 23722-23731. Max. coverage (+): 0. Max coverage (-): 0

Region: NODE\_389151\_length\_73825\_cov\_29.945791 23732-23742. Max. coverage (+): 0. Max coverage (-): 0.02

Region: NODE\_389151\_length\_73825\_cov\_29.945791 23743-23752. Max. coverage (+): 0. Max coverage (-): 0

Region: NODE\_389151\_length\_73825\_cov\_29.945791 23753-. Max. coverage (+): 0. Max coverage (-): 0

RepeatMasker Color Code

**+**

100-98% Identity

<98-95% Identity

<95-90% Identity

<90-85% Identity

<85-80% Identity

<80-75% Identity

<75-70% Identity

<70% Identity

**-**

Gene Set Color Code

**+**

Gene

Pseudogene

Other

**-**

Topology/Coverage Color Code

Coverage Plus Strand

Coverage Minus Strand

Mainstrand: Plus

Mainstrand: Minus

Complementary Strand

Flanking Region  
(if option -flank >0)

Gene Set Annotation  
  
RepeatMasker Annotation  

**1. (TTATT)n**: 21030-21085 (+), Divergence to consensus: 29%  
**2. AlRepC-98**: 21417-21727 (-), Divergence to consensus: 12.9%  
**3. AlRepC-911**: 21795-21869 (-), Divergence to consensus: 22.9%  
**4. AlRepC-239**: 21973-22688 (+), Divergence to consensus: 36.6%  
**5. AlRepC-911**: 22689-23211 (-), Divergence to consensus: 26.6%  
**6. TC1DR3**: 23220-23590 (-), Divergence to consensus: 24.1%  
**7. TC1DR3**: 23608-23779 (-), Divergence to consensus: 29.8%

  
Transcription Factor Binding Sites  

**SPZ1** (Sequence: CTCTAACCCC (-): 19774)  
**RHOXF1** (Sequence: AGCTCA (-): 18931)  
**RHOXF1** (Sequence: AGATTA (-): 19639)  
**RHOXF1** (Sequence: AGCTCA (-): 20266)  
**RHOXF1** (Sequence: GGCTCA (-): 20702)  
**RHOXF1** (Sequence: AGATCA (-): 20857)  
**RHOXF1** (Sequence: AGATTA (-): 21117)  
**RHOXF1** (Sequence: AGCTCA (-): 21643)  
**RHOXF1** (Sequence: AGATTA (-): 21786)  
**RHOXF1** (Sequence: AGATCA (-): 22152)  
**RHOXF1** (Sequence: GGATTA (-): 22373)  
**RHOXF1** (Sequence: AGCTTA (-): 22466)  
**RHOXF1** (Sequence: TGAGCT (+): 19085)  
**RHOXF1** (Sequence: TGATCC (+): 19286)  
**RHOXF1** (Sequence: TGAGCT (+): 19424)  
**RHOXF1** (Sequence: TGAGCC (+): 20160)  
**RHOXF1** (Sequence: TGATCT (+): 21375)  
**RHOXF1** (Sequence: TAATCC (+): 21943)  
**RHOXF1** (Sequence: TGATCT (+): 22259)  
**RHOXF1** (Sequence: TGAGCT (+): 23697)  
**SOX9** (Sequence: AACAATGA (-): 22022)  
**FOXO1** (Sequence: CTTGTTTAC (+): 21520)  
**FOXO1** (Sequence: GTTGTTTAT (+): 22991)  
**FOXO3\_mmu** (Sequence: TGTTTACA (-): 21522)  
**Sox5** (Sequence: ATTGTT (+): 20959)  
**Sox5** (Sequence: ATTGTT (+): 21029)  
**FOXO1** (Sequence: AAAAACAGC (-): 20584)  
**FOXO1** (Sequence: ATAAACAGG (-): 22740)  
**FOXO3\_hsa** (Sequence: TTGTTTAC (-): 21521)  
**FOXP1** (Sequence: TGTTTAC (-): 20822)  
**FOXP1** (Sequence: TGTTTAC (-): 21522)  
**Rhox11** (Sequence: TGGTGTTTA (+): 20819)  
**Rhox11** (Sequence: TGCTGTTAA (+): 21685)  
**Rhox11** (Sequence: TTTACAGCA (-): 18784)  
**Rhox11** (Sequence: AATACAGCA (-): 21949)  
**Gata4** (Sequence: AGATAAG (-): 18734)  
**Sox5** (Sequence: AACAAT (-): 19343)  
**Sox5** (Sequence: AACAAT (-): 20955)  
**Sox5** (Sequence: AACAAT (-): 22022)  
**Sox5** (Sequence: AACAAT (-): 23581)  
**POU2F1** (Sequence: TATGTAAAT (+): 19147)  
**POU2F1** (Sequence: TATTTTAAT (+): 21046)
