# Supplementary material for: First characterization of PIWI-interacting RNA clusters in a cichlid fish with a B chromosome
Source: BMC Biol. 2022 Sep 21;20:204. doi: 10.1186/s12915-022-01403-2 (PMC9490952; doi:10.1186/s12915-022-01403-2)
Supplement: Supplementary file 1 — Additional file 1. Zipped folder with fasta and interactive html piRNA cluster information for the A. latifasciata genome. The nomenclature is as follows: number-pirna-cluster_sex_B-presence (f, female; m, male; 0b, without B chromosome; 1b, with B chromosome). [file 12915_2022_1403_MOESM1_ESM.zip › 153_m1b.html]

piRNA cluster 153\_m1b 91


Predicted piRNA cluster no. 153\_m1b
  

Show proTRAC run info
Hide proTRAC run info

/\  
                \_\_\_\_\_\_\_\_\_\_\_\_\_\_\_\_\_\_\_\_\_\_\_/\\_\_\_ /  \\_\_\_\_\_\_\_  
               I                      /  \  /    \      I  
               I     pro             /    \/      \     I  
               I        TRAC        /               \   I  
               I   \_\_\_\_\_\_\_\_\_\_\_\_\_\_\_\_/\_\_\_\_\_\_\_\_\_\_\_\_\_\_\_\_\_\\_ I  
               I   \              /                     I  
               I    \            /                      I  
               I     \  /\      /       V.2.4.2         I  
               I      \/  \    /                        I  
               I\_\_\_\_\_\_\_\_\_\_\_\  /\_\_\_\_\_\_\_\_\_\_\_\_\_\_\_\_\_\_\_\_\_\_\_\_\_I  
                            \/  
  
  
================================= proTRAC ====================================  
VERSION: .......... 2.4.2  
LAST MODIFIED: .... 11. May 2018  
  
Please cite:  
Rosenkranz D, Zischler H. proTRAC - a software for probabilistic piRNA cluster  
detection, visualization and analysis. 2012. BMC Bioinformatics 13:5.  
  
  
Contact:  
David Rosenkranz  
Institute of Organismic and Molecular Evolutionary Biology  
Dept. Anthropology, small RNA group  
Johannes Gutenberg University Mainz  
email: rosenkranz@uni-mainz.de  
  
You can find the latest proTRAC version at:  
http://sourceforge.net/projects/protrac/files  
http://www.smallRNAgroup-mainz.de/software  
==============================================================================  
  
PARAMETERS:  
Map file: ...............piwi-machos-1B.fa-collapse.map  
Genome file: ............../../../0B\_ala\_genome.fa  
RepeatMasker annotation: Alatifasciata-all0B-maryan-v2.fa\_corrected.out  
GeneSet:................./guest-storage/Data/annotation/Alatifasciata\_all0B\_maryan-v2\_out2017.gff  
  
Significant (p<=0.01) hit density will be calculated based  
on observed hit distribution.  
  
Sliding window size: ........................................ 5000 bp  
Sliding window increament: .................................. 1000 bp  
Normalize each hit by number of genomic hits: ............... yes  
Normalize each hit by number of sequence reads: ............. yes  
Normalize values (-> per million mapped reads): ............. yes  
Min. fraction of hits with 1T(U) or 10A: .................... 0.75  
Alternatively: Min. fraction of hits with 1T(U) and 10A: .... 0.5  
Min. fraction of hits with typical piRNA length: ............ 0.75  
Typical piRNA length: ....................................... 24-32 nt  
Min. size of a piRNA cluster: ............................... 1000 bp.  
Min. number of hits (absolute): ............................. 0  
Min. number of hits (normalized): ........................... 0  
Min. fraction of hits on the mainstrand: .................... 0.75  
Top fraction of mapped sequences (in terms of read counts): . 1%  
Top fraction accounts for max. n% of sequence reads: ........ 90%  
Min. fraction of hits on each arm of a bidirectional cluster: 0.05  
Output html file for each cluster: .......................... yes  
Output a summary table: ..................................... yes  
Output a FASTA file for each cluster (piRNA sequences): ..... yes  
Output a FASTA file comprising cluster sequences: ........... yes  
Output a GTF file for predicted piRNA clusters: ..............yes  
Search DNA motifs in clusters: .............................. yes  
Output flanking sequences: +/- .............................. 0 bp  
Output ~.pTi file: .......................................... no  
==============================================================================  
  
  
Genome size (without gaps): ............ 758543724 bp  
Gaps (N/X/-): .......................... 417479 bp  
Mapped reads: .......................... 26973943  
Non-identical sequences: ............... 6209225  
Genomic hits: .......................... 48438990  
Significant densitiy of mapped reads: .. 821.144211136946 reads/kb

Show proTRAC cluster info
Hide proTRAC cluster info

|  |  |
| --- | --- |
| Location | NODE\_400925\_length\_4705\_cov\_23.806589 |
| Coordinates | 1-4769 |
| Size [bp] | 4769 |
| Sequence hit loci | 2593 |
| Mapped reads (normalized) | 11355.8 |
| Mapped reads (normalized) per kb | 2381.2 |
| Normalized reads with 1T (1U) | 76.3% |
| Normalized reads with 10A | 38.7% |
| Normalized reads with length 24-32 nt | 99.2% |
| Normalized reads on the main strand(s) | 97.6% |
| Predicted directionality | mono:minus |

100%

0%

1T (1U)  
reads

10A reads

24-32 nt  
reads

reads on mainstrand

**Either the amount of reads with 1T (1U) OR 10A has to exceed 75% (set with option: -1Tor10A)  
Alternatively the amount of reads with 1T (1U) AND 10A has to exceed 50% (set with option: -1Tand10A)  
Minimum amount of reads with preferred size is 75% (set with option: -pisize)  
Minimum amount of reads on the main strand(s) is 75% (set with option: -clstrand)**

Show read coverage
Hide read coverage

WHAT DO I SEE HERE?  
This chart shows the location of mapped sequence reads within a predicted piRNA cluster. The color refers to the number of genomic hits produced by the sequence read in question. A dark red bar indicates that this sequence read produces many other hits elsewhere in the genome. Many adjacent red or yellow bars can indicate the presence of a multi-copy element such as transposons or rRNA genes. A dark green bar indicates that this sequence read maps uniquely to this locus.

1 hit

2-5 hits

6-10 hits

11-20 hits

21-50 hits

51-100 hits

> 100 hits

NODE\_400925\_length\_4705\_cov\_23.806589

1

4769

Gene Set

RepeatMasker

Mapped  
Reads

59.32

plus strand

minus strand

59.32

Region: NODE\_400925\_length\_4705\_cov\_23.806589 23754-5. Max. coverage (+): 0. Max coverage (-): 0

Region: NODE\_400925\_length\_4705\_cov\_23.806589 6-15. Max. coverage (+): 0. Max coverage (-): 0

Region: NODE\_400925\_length\_4705\_cov\_23.806589 16-24. Max. coverage (+): 0. Max coverage (-): 0.01

Region: NODE\_400925\_length\_4705\_cov\_23.806589 25-34. Max. coverage (+): 0.01. Max coverage (-): 0

Region: NODE\_400925\_length\_4705\_cov\_23.806589 35-43. Max. coverage (+): 0.01. Max coverage (-): 0.06

Region: NODE\_400925\_length\_4705\_cov\_23.806589 44-53. Max. coverage (+): 0. Max coverage (-): 0

Region: NODE\_400925\_length\_4705\_cov\_23.806589 54-62. Max. coverage (+): 0.02. Max coverage (-): 0.01

Region: NODE\_400925\_length\_4705\_cov\_23.806589 63-72. Max. coverage (+): 0.02. Max coverage (-): 0

Region: NODE\_400925\_length\_4705\_cov\_23.806589 73-82. Max. coverage (+): 0. Max coverage (-): 0

Region: NODE\_400925\_length\_4705\_cov\_23.806589 83-91. Max. coverage (+): 0. Max coverage (-): 0

Region: NODE\_400925\_length\_4705\_cov\_23.806589 92-101. Max. coverage (+): 0. Max coverage (-): 0

Region: NODE\_400925\_length\_4705\_cov\_23.806589 102-110. Max. coverage (+): 0. Max coverage (-): 0

Region: NODE\_400925\_length\_4705\_cov\_23.806589 111-120. Max. coverage (+): 0. Max coverage (-): 0

Region: NODE\_400925\_length\_4705\_cov\_23.806589 121-129. Max. coverage (+): 0. Max coverage (-): 0

Region: NODE\_400925\_length\_4705\_cov\_23.806589 130-139. Max. coverage (+): 0. Max coverage (-): 0

Region: NODE\_400925\_length\_4705\_cov\_23.806589 140-148. Max. coverage (+): 0. Max coverage (-): 0

Region: NODE\_400925\_length\_4705\_cov\_23.806589 149-158. Max. coverage (+): 0. Max coverage (-): 0

Region: NODE\_400925\_length\_4705\_cov\_23.806589 159-167. Max. coverage (+): 0. Max coverage (-): 0

Region: NODE\_400925\_length\_4705\_cov\_23.806589 168-177. Max. coverage (+): 0. Max coverage (-): 0

Region: NODE\_400925\_length\_4705\_cov\_23.806589 178-186. Max. coverage (+): 0. Max coverage (-): 0

Region: NODE\_400925\_length\_4705\_cov\_23.806589 187-196. Max. coverage (+): 0. Max coverage (-): 0

Region: NODE\_400925\_length\_4705\_cov\_23.806589 197-206. Max. coverage (+): 0. Max coverage (-): 0

Region: NODE\_400925\_length\_4705\_cov\_23.806589 207-215. Max. coverage (+): 0. Max coverage (-): 0

Region: NODE\_400925\_length\_4705\_cov\_23.806589 216-225. Max. coverage (+): 0. Max coverage (-): 0

Region: NODE\_400925\_length\_4705\_cov\_23.806589 226-234. Max. coverage (+): 0. Max coverage (-): 0

Region: NODE\_400925\_length\_4705\_cov\_23.806589 235-244. Max. coverage (+): 0. Max coverage (-): 0

Region: NODE\_400925\_length\_4705\_cov\_23.806589 245-253. Max. coverage (+): 0. Max coverage (-): 0

Region: NODE\_400925\_length\_4705\_cov\_23.806589 254-263. Max. coverage (+): 0. Max coverage (-): 0

Region: NODE\_400925\_length\_4705\_cov\_23.806589 264-272. Max. coverage (+): 0. Max coverage (-): 0

Region: NODE\_400925\_length\_4705\_cov\_23.806589 273-282. Max. coverage (+): 0. Max coverage (-): 0

Region: NODE\_400925\_length\_4705\_cov\_23.806589 283-291. Max. coverage (+): 0. Max coverage (-): 0

Region: NODE\_400925\_length\_4705\_cov\_23.806589 292-301. Max. coverage (+): 0. Max coverage (-): 0

Region: NODE\_400925\_length\_4705\_cov\_23.806589 302-310. Max. coverage (+): 0. Max coverage (-): 0

Region: NODE\_400925\_length\_4705\_cov\_23.806589 311-320. Max. coverage (+): 0. Max coverage (-): 0

Region: NODE\_400925\_length\_4705\_cov\_23.806589 321-330. Max. coverage (+): 0. Max coverage (-): 0

Region: NODE\_400925\_length\_4705\_cov\_23.806589 331-339. Max. coverage (+): 0. Max coverage (-): 0

Region: NODE\_400925\_length\_4705\_cov\_23.806589 340-349. Max. coverage (+): 0. Max coverage (-): 0

Region: NODE\_400925\_length\_4705\_cov\_23.806589 350-358. Max. coverage (+): 0. Max coverage (-): 0

Region: NODE\_400925\_length\_4705\_cov\_23.806589 359-368. Max. coverage (+): 0. Max coverage (-): 0

Region: NODE\_400925\_length\_4705\_cov\_23.806589 369-377. Max. coverage (+): 0. Max coverage (-): 0

Region: NODE\_400925\_length\_4705\_cov\_23.806589 378-387. Max. coverage (+): 0. Max coverage (-): 0

Region: NODE\_400925\_length\_4705\_cov\_23.806589 388-396. Max. coverage (+): 0. Max coverage (-): 0

Region: NODE\_400925\_length\_4705\_cov\_23.806589 397-406. Max. coverage (+): 0. Max coverage (-): 0

Region: NODE\_400925\_length\_4705\_cov\_23.806589 407-415. Max. coverage (+): 0. Max coverage (-): 0

Region: NODE\_400925\_length\_4705\_cov\_23.806589 416-425. Max. coverage (+): 0. Max coverage (-): 0

Region: NODE\_400925\_length\_4705\_cov\_23.806589 426-434. Max. coverage (+): 0. Max coverage (-): 0

Region: NODE\_400925\_length\_4705\_cov\_23.806589 435-444. Max. coverage (+): 0. Max coverage (-): 0

Region: NODE\_400925\_length\_4705\_cov\_23.806589 445-454. Max. coverage (+): 0. Max coverage (-): 0

Region: NODE\_400925\_length\_4705\_cov\_23.806589 455-463. Max. coverage (+): 0. Max coverage (-): 0

Region: NODE\_400925\_length\_4705\_cov\_23.806589 464-473. Max. coverage (+): 0. Max coverage (-): 0

Region: NODE\_400925\_length\_4705\_cov\_23.806589 474-482. Max. coverage (+): 0. Max coverage (-): 0

Region: NODE\_400925\_length\_4705\_cov\_23.806589 483-492. Max. coverage (+): 0. Max coverage (-): 0

Region: NODE\_400925\_length\_4705\_cov\_23.806589 493-501. Max. coverage (+): 0. Max coverage (-): 0

Region: NODE\_400925\_length\_4705\_cov\_23.806589 502-511. Max. coverage (+): 0. Max coverage (-): 0.07

Region: NODE\_400925\_length\_4705\_cov\_23.806589 512-520. Max. coverage (+): 0.04. Max coverage (-): 0

Region: NODE\_400925\_length\_4705\_cov\_23.806589 521-530. Max. coverage (+): 0. Max coverage (-): 0

Region: NODE\_400925\_length\_4705\_cov\_23.806589 531-539. Max. coverage (+): 0. Max coverage (-): 0.11

Region: NODE\_400925\_length\_4705\_cov\_23.806589 540-549. Max. coverage (+): 0. Max coverage (-): 0

Region: NODE\_400925\_length\_4705\_cov\_23.806589 550-558. Max. coverage (+): 0. Max coverage (-): 0

Region: NODE\_400925\_length\_4705\_cov\_23.806589 559-568. Max. coverage (+): 0. Max coverage (-): 0

Region: NODE\_400925\_length\_4705\_cov\_23.806589 569-578. Max. coverage (+): 0. Max coverage (-): 0

Region: NODE\_400925\_length\_4705\_cov\_23.806589 579-587. Max. coverage (+): 0. Max coverage (-): 0

Region: NODE\_400925\_length\_4705\_cov\_23.806589 588-597. Max. coverage (+): 0. Max coverage (-): 0.02

Region: NODE\_400925\_length\_4705\_cov\_23.806589 598-606. Max. coverage (+): 0. Max coverage (-): 0

Region: NODE\_400925\_length\_4705\_cov\_23.806589 607-616. Max. coverage (+): 0. Max coverage (-): 0

Region: NODE\_400925\_length\_4705\_cov\_23.806589 617-625. Max. coverage (+): 0. Max coverage (-): 0

Region: NODE\_400925\_length\_4705\_cov\_23.806589 626-635. Max. coverage (+): 0. Max coverage (-): 0

Region: NODE\_400925\_length\_4705\_cov\_23.806589 636-644. Max. coverage (+): 0. Max coverage (-): 0

Region: NODE\_400925\_length\_4705\_cov\_23.806589 645-654. Max. coverage (+): 0. Max coverage (-): 0

Region: NODE\_400925\_length\_4705\_cov\_23.806589 655-663. Max. coverage (+): 0. Max coverage (-): 0

Region: NODE\_400925\_length\_4705\_cov\_23.806589 664-673. Max. coverage (+): 0. Max coverage (-): 0

Region: NODE\_400925\_length\_4705\_cov\_23.806589 674-682. Max. coverage (+): 0. Max coverage (-): 0

Region: NODE\_400925\_length\_4705\_cov\_23.806589 683-692. Max. coverage (+): 0. Max coverage (-): 0

Region: NODE\_400925\_length\_4705\_cov\_23.806589 693-702. Max. coverage (+): 0. Max coverage (-): 0

Region: NODE\_400925\_length\_4705\_cov\_23.806589 703-711. Max. coverage (+): 0. Max coverage (-): 0

Region: NODE\_400925\_length\_4705\_cov\_23.806589 712-721. Max. coverage (+): 0. Max coverage (-): 0

Region: NODE\_400925\_length\_4705\_cov\_23.806589 722-730. Max. coverage (+): 0. Max coverage (-): 0

Region: NODE\_400925\_length\_4705\_cov\_23.806589 731-740. Max. coverage (+): 0. Max coverage (-): 0

Region: NODE\_400925\_length\_4705\_cov\_23.806589 741-749. Max. coverage (+): 0. Max coverage (-): 0

Region: NODE\_400925\_length\_4705\_cov\_23.806589 750-759. Max. coverage (+): 0. Max coverage (-): 0

Region: NODE\_400925\_length\_4705\_cov\_23.806589 760-768. Max. coverage (+): 0. Max coverage (-): 0.01

Region: NODE\_400925\_length\_4705\_cov\_23.806589 769-778. Max. coverage (+): 0. Max coverage (-): 0.06

Region: NODE\_400925\_length\_4705\_cov\_23.806589 779-787. Max. coverage (+): 0. Max coverage (-): 0.07

Region: NODE\_400925\_length\_4705\_cov\_23.806589 788-797. Max. coverage (+): 0. Max coverage (-): 0.02

Region: NODE\_400925\_length\_4705\_cov\_23.806589 798-806. Max. coverage (+): 0. Max coverage (-): 0

Region: NODE\_400925\_length\_4705\_cov\_23.806589 807-816. Max. coverage (+): 0. Max coverage (-): 0

Region: NODE\_400925\_length\_4705\_cov\_23.806589 817-826. Max. coverage (+): 0. Max coverage (-): 0.04

Region: NODE\_400925\_length\_4705\_cov\_23.806589 827-835. Max. coverage (+): 0. Max coverage (-): 0.04

Region: NODE\_400925\_length\_4705\_cov\_23.806589 836-845. Max. coverage (+): 0. Max coverage (-): 0

Region: NODE\_400925\_length\_4705\_cov\_23.806589 846-854. Max. coverage (+): 0. Max coverage (-): 0

Region: NODE\_400925\_length\_4705\_cov\_23.806589 855-864. Max. coverage (+): 0. Max coverage (-): 0.07

Region: NODE\_400925\_length\_4705\_cov\_23.806589 865-873. Max. coverage (+): 0. Max coverage (-): 0

Region: NODE\_400925\_length\_4705\_cov\_23.806589 874-883. Max. coverage (+): 0. Max coverage (-): 0

Region: NODE\_400925\_length\_4705\_cov\_23.806589 884-892. Max. coverage (+): 0. Max coverage (-): 0.07

Region: NODE\_400925\_length\_4705\_cov\_23.806589 893-902. Max. coverage (+): 0. Max coverage (-): 0.18

Region: NODE\_400925\_length\_4705\_cov\_23.806589 903-911. Max. coverage (+): 0. Max coverage (-): 0.03

Region: NODE\_400925\_length\_4705\_cov\_23.806589 912-921. Max. coverage (+): 0.09. Max coverage (-): 0.01

Region: NODE\_400925\_length\_4705\_cov\_23.806589 922-930. Max. coverage (+): 0.01. Max coverage (-): 0.07

Region: NODE\_400925\_length\_4705\_cov\_23.806589 931-940. Max. coverage (+): 0. Max coverage (-): 0.19

Region: NODE\_400925\_length\_4705\_cov\_23.806589 941-950. Max. coverage (+): 0. Max coverage (-): 0.2

Region: NODE\_400925\_length\_4705\_cov\_23.806589 951-959. Max. coverage (+): 0.04. Max coverage (-): 0

Region: NODE\_400925\_length\_4705\_cov\_23.806589 960-969. Max. coverage (+): 0. Max coverage (-): 0.11

Region: NODE\_400925\_length\_4705\_cov\_23.806589 970-978. Max. coverage (+): 0. Max coverage (-): 0.07

Region: NODE\_400925\_length\_4705\_cov\_23.806589 979-988. Max. coverage (+): 0. Max coverage (-): 0

Region: NODE\_400925\_length\_4705\_cov\_23.806589 989-997. Max. coverage (+): 0. Max coverage (-): 0

Region: NODE\_400925\_length\_4705\_cov\_23.806589 998-1007. Max. coverage (+): 0. Max coverage (-): 0

Region: NODE\_400925\_length\_4705\_cov\_23.806589 1008-1016. Max. coverage (+): 0. Max coverage (-): 0

Region: NODE\_400925\_length\_4705\_cov\_23.806589 1017-1026. Max. coverage (+): 0. Max coverage (-): 0

Region: NODE\_400925\_length\_4705\_cov\_23.806589 1027-1035. Max. coverage (+): 0. Max coverage (-): 0.01

Region: NODE\_400925\_length\_4705\_cov\_23.806589 1036-1045. Max. coverage (+): 0. Max coverage (-): 0

Region: NODE\_400925\_length\_4705\_cov\_23.806589 1046-1054. Max. coverage (+): 0. Max coverage (-): 0

Region: NODE\_400925\_length\_4705\_cov\_23.806589 1055-1064. Max. coverage (+): 0. Max coverage (-): 0

Region: NODE\_400925\_length\_4705\_cov\_23.806589 1065-1074. Max. coverage (+): 0. Max coverage (-): 0

Region: NODE\_400925\_length\_4705\_cov\_23.806589 1075-1083. Max. coverage (+): 0. Max coverage (-): 0.11

Region: NODE\_400925\_length\_4705\_cov\_23.806589 1084-1093. Max. coverage (+): 0.11. Max coverage (-): 0.67

Region: NODE\_400925\_length\_4705\_cov\_23.806589 1094-1102. Max. coverage (+): 0. Max coverage (-): 0.33

Region: NODE\_400925\_length\_4705\_cov\_23.806589 1103-1112. Max. coverage (+): 0.02. Max coverage (-): 0.07

Region: NODE\_400925\_length\_4705\_cov\_23.806589 1113-1121. Max. coverage (+): 0.04. Max coverage (-): 0

Region: NODE\_400925\_length\_4705\_cov\_23.806589 1122-1131. Max. coverage (+): 0. Max coverage (-): 0.07

Region: NODE\_400925\_length\_4705\_cov\_23.806589 1132-1140. Max. coverage (+): 0. Max coverage (-): 0.16

Region: NODE\_400925\_length\_4705\_cov\_23.806589 1141-1150. Max. coverage (+): 0. Max coverage (-): 0

Region: NODE\_400925\_length\_4705\_cov\_23.806589 1151-1159. Max. coverage (+): 0. Max coverage (-): 0

Region: NODE\_400925\_length\_4705\_cov\_23.806589 1160-1169. Max. coverage (+): 0.04. Max coverage (-): 0.07

Region: NODE\_400925\_length\_4705\_cov\_23.806589 1170-1178. Max. coverage (+): 0.04. Max coverage (-): 0.04

Region: NODE\_400925\_length\_4705\_cov\_23.806589 1179-1188. Max. coverage (+): 0.02. Max coverage (-): 0.04

Region: NODE\_400925\_length\_4705\_cov\_23.806589 1189-1198. Max. coverage (+): 0.02. Max coverage (-): 0.03

Region: NODE\_400925\_length\_4705\_cov\_23.806589 1199-1207. Max. coverage (+): 0. Max coverage (-): 0.05

Region: NODE\_400925\_length\_4705\_cov\_23.806589 1208-1217. Max. coverage (+): 0.02. Max coverage (-): 0.02

Region: NODE\_400925\_length\_4705\_cov\_23.806589 1218-1226. Max. coverage (+): 0.02. Max coverage (-): 0.02

Region: NODE\_400925\_length\_4705\_cov\_23.806589 1227-1236. Max. coverage (+): 0. Max coverage (-): 0.07

Region: NODE\_400925\_length\_4705\_cov\_23.806589 1237-1245. Max. coverage (+): 0. Max coverage (-): 0.15

Region: NODE\_400925\_length\_4705\_cov\_23.806589 1246-1255. Max. coverage (+): 0. Max coverage (-): 0.11

Region: NODE\_400925\_length\_4705\_cov\_23.806589 1256-1264. Max. coverage (+): 0. Max coverage (-): 0

Region: NODE\_400925\_length\_4705\_cov\_23.806589 1265-1274. Max. coverage (+): 0. Max coverage (-): 0

Region: NODE\_400925\_length\_4705\_cov\_23.806589 1275-1283. Max. coverage (+): 0. Max coverage (-): 0

Region: NODE\_400925\_length\_4705\_cov\_23.806589 1284-1293. Max. coverage (+): 0. Max coverage (-): 0

Region: NODE\_400925\_length\_4705\_cov\_23.806589 1294-1302. Max. coverage (+): 0. Max coverage (-): 0

Region: NODE\_400925\_length\_4705\_cov\_23.806589 1303-1312. Max. coverage (+): 0.01. Max coverage (-): 0

Region: NODE\_400925\_length\_4705\_cov\_23.806589 1313-1322. Max. coverage (+): 0. Max coverage (-): 0

Region: NODE\_400925\_length\_4705\_cov\_23.806589 1323-1331. Max. coverage (+): 0. Max coverage (-): 0

Region: NODE\_400925\_length\_4705\_cov\_23.806589 1332-1341. Max. coverage (+): 0. Max coverage (-): 0

Region: NODE\_400925\_length\_4705\_cov\_23.806589 1342-1350. Max. coverage (+): 0. Max coverage (-): 0

Region: NODE\_400925\_length\_4705\_cov\_23.806589 1351-1360. Max. coverage (+): 0. Max coverage (-): 0

Region: NODE\_400925\_length\_4705\_cov\_23.806589 1361-1369. Max. coverage (+): 0.04. Max coverage (-): 0.11

Region: NODE\_400925\_length\_4705\_cov\_23.806589 1370-1379. Max. coverage (+): 0. Max coverage (-): 0.37

Region: NODE\_400925\_length\_4705\_cov\_23.806589 1380-1388. Max. coverage (+): 0. Max coverage (-): 0.26

Region: NODE\_400925\_length\_4705\_cov\_23.806589 1389-1398. Max. coverage (+): 0. Max coverage (-): 0.07

Region: NODE\_400925\_length\_4705\_cov\_23.806589 1399-1407. Max. coverage (+): 0. Max coverage (-): 0.11

Region: NODE\_400925\_length\_4705\_cov\_23.806589 1408-1417. Max. coverage (+): 0. Max coverage (-): 0.07

Region: NODE\_400925\_length\_4705\_cov\_23.806589 1418-1426. Max. coverage (+): 0. Max coverage (-): 0.19

Region: NODE\_400925\_length\_4705\_cov\_23.806589 1427-1436. Max. coverage (+): 0. Max coverage (-): 0.07

Region: NODE\_400925\_length\_4705\_cov\_23.806589 1437-1446. Max. coverage (+): 0. Max coverage (-): 0.11

Region: NODE\_400925\_length\_4705\_cov\_23.806589 1447-1455. Max. coverage (+): 0. Max coverage (-): 0.04

Region: NODE\_400925\_length\_4705\_cov\_23.806589 1456-1465. Max. coverage (+): 0. Max coverage (-): 0.07

Region: NODE\_400925\_length\_4705\_cov\_23.806589 1466-1474. Max. coverage (+): 0.04. Max coverage (-): 0.33

Region: NODE\_400925\_length\_4705\_cov\_23.806589 1475-1484. Max. coverage (+): 0. Max coverage (-): 0.33

Region: NODE\_400925\_length\_4705\_cov\_23.806589 1485-1493. Max. coverage (+): 0. Max coverage (-): 0.04

Region: NODE\_400925\_length\_4705\_cov\_23.806589 1494-1503. Max. coverage (+): 0. Max coverage (-): 0.11

Region: NODE\_400925\_length\_4705\_cov\_23.806589 1504-1512. Max. coverage (+): 0.04. Max coverage (-): 0.19

Region: NODE\_400925\_length\_4705\_cov\_23.806589 1513-1522. Max. coverage (+): 0. Max coverage (-): 0.33

Region: NODE\_400925\_length\_4705\_cov\_23.806589 1523-1531. Max. coverage (+): 0. Max coverage (-): 0.41

Region: NODE\_400925\_length\_4705\_cov\_23.806589 1532-1541. Max. coverage (+): 0. Max coverage (-): 0.07

Region: NODE\_400925\_length\_4705\_cov\_23.806589 1542-1550. Max. coverage (+): 0. Max coverage (-): 0.11

Region: NODE\_400925\_length\_4705\_cov\_23.806589 1551-1560. Max. coverage (+): 0. Max coverage (-): 0.82

Region: NODE\_400925\_length\_4705\_cov\_23.806589 1561-1570. Max. coverage (+): 0. Max coverage (-): 0.26

Region: NODE\_400925\_length\_4705\_cov\_23.806589 1571-1579. Max. coverage (+): 0. Max coverage (-): 0.07

Region: NODE\_400925\_length\_4705\_cov\_23.806589 1580-1589. Max. coverage (+): 0.22. Max coverage (-): 0.41

Region: NODE\_400925\_length\_4705\_cov\_23.806589 1590-1598. Max. coverage (+): 0.07. Max coverage (-): 3.67

Region: NODE\_400925\_length\_4705\_cov\_23.806589 1599-1608. Max. coverage (+): 0. Max coverage (-): 5.78

Region: NODE\_400925\_length\_4705\_cov\_23.806589 1609-1617. Max. coverage (+): 0. Max coverage (-): 9.38

Region: NODE\_400925\_length\_4705\_cov\_23.806589 1618-1627. Max. coverage (+): 0.11. Max coverage (-): 0.07

Region: NODE\_400925\_length\_4705\_cov\_23.806589 1628-1636. Max. coverage (+): 0. Max coverage (-): 0.04

Region: NODE\_400925\_length\_4705\_cov\_23.806589 1637-1646. Max. coverage (+): 0. Max coverage (-): 0

Region: NODE\_400925\_length\_4705\_cov\_23.806589 1647-1655. Max. coverage (+): 0. Max coverage (-): 0.07

Region: NODE\_400925\_length\_4705\_cov\_23.806589 1656-1665. Max. coverage (+): 0. Max coverage (-): 0.07

Region: NODE\_400925\_length\_4705\_cov\_23.806589 1666-1674. Max. coverage (+): 0. Max coverage (-): 0.07

Region: NODE\_400925\_length\_4705\_cov\_23.806589 1675-1684. Max. coverage (+): 0.07. Max coverage (-): 0.48

Region: NODE\_400925\_length\_4705\_cov\_23.806589 1685-1693. Max. coverage (+): 0.07. Max coverage (-): 0.48

Region: NODE\_400925\_length\_4705\_cov\_23.806589 1694-1703. Max. coverage (+): 0.04. Max coverage (-): 0.3

Region: NODE\_400925\_length\_4705\_cov\_23.806589 1704-1713. Max. coverage (+): 0.04. Max coverage (-): 0.11

Region: NODE\_400925\_length\_4705\_cov\_23.806589 1714-1722. Max. coverage (+): 0. Max coverage (-): 0.19

Region: NODE\_400925\_length\_4705\_cov\_23.806589 1723-1732. Max. coverage (+): 0. Max coverage (-): 0.44

Region: NODE\_400925\_length\_4705\_cov\_23.806589 1733-1741. Max. coverage (+): 0. Max coverage (-): 0.7

Region: NODE\_400925\_length\_4705\_cov\_23.806589 1742-1751. Max. coverage (+): 0.04. Max coverage (-): 0.33

Region: NODE\_400925\_length\_4705\_cov\_23.806589 1752-1760. Max. coverage (+): 0.04. Max coverage (-): 0.04

Region: NODE\_400925\_length\_4705\_cov\_23.806589 1761-1770. Max. coverage (+): 0.04. Max coverage (-): 0.15

Region: NODE\_400925\_length\_4705\_cov\_23.806589 1771-1779. Max. coverage (+): 0. Max coverage (-): 0.11

Region: NODE\_400925\_length\_4705\_cov\_23.806589 1780-1789. Max. coverage (+): 0.04. Max coverage (-): 0.26

Region: NODE\_400925\_length\_4705\_cov\_23.806589 1790-1798. Max. coverage (+): 0.04. Max coverage (-): 1.08

Region: NODE\_400925\_length\_4705\_cov\_23.806589 1799-1808. Max. coverage (+): 0. Max coverage (-): 1.15

Region: NODE\_400925\_length\_4705\_cov\_23.806589 1809-1817. Max. coverage (+): 0. Max coverage (-): 2.15

Region: NODE\_400925\_length\_4705\_cov\_23.806589 1818-1827. Max. coverage (+): 0.07. Max coverage (-): 0.22

Region: NODE\_400925\_length\_4705\_cov\_23.806589 1828-1837. Max. coverage (+): 0.07. Max coverage (-): 0.15

Region: NODE\_400925\_length\_4705\_cov\_23.806589 1838-1846. Max. coverage (+): 0.07. Max coverage (-): 0.04

Region: NODE\_400925\_length\_4705\_cov\_23.806589 1847-1856. Max. coverage (+): 0. Max coverage (-): 0.85

Region: NODE\_400925\_length\_4705\_cov\_23.806589 1857-1865. Max. coverage (+): 0. Max coverage (-): 59.32

Region: NODE\_400925\_length\_4705\_cov\_23.806589 1866-1875. Max. coverage (+): 0. Max coverage (-): 22.61

Region: NODE\_400925\_length\_4705\_cov\_23.806589 1876-1884. Max. coverage (+): 0.11. Max coverage (-): 0.7

Region: NODE\_400925\_length\_4705\_cov\_23.806589 1885-1894. Max. coverage (+): 0. Max coverage (-): 0

Region: NODE\_400925\_length\_4705\_cov\_23.806589 1895-1903. Max. coverage (+): 0.04. Max coverage (-): 0

Region: NODE\_400925\_length\_4705\_cov\_23.806589 1904-1913. Max. coverage (+): 0.04. Max coverage (-): 0.89

Region: NODE\_400925\_length\_4705\_cov\_23.806589 1914-1922. Max. coverage (+): 0. Max coverage (-): 0.78

Region: NODE\_400925\_length\_4705\_cov\_23.806589 1923-1932. Max. coverage (+): 0.04. Max coverage (-): 5.78

Region: NODE\_400925\_length\_4705\_cov\_23.806589 1933-1941. Max. coverage (+): 0. Max coverage (-): 0.22

Region: NODE\_400925\_length\_4705\_cov\_23.806589 1942-1951. Max. coverage (+): 0. Max coverage (-): 4.23

Region: NODE\_400925\_length\_4705\_cov\_23.806589 1952-1961. Max. coverage (+): 0.04. Max coverage (-): 1.37

Region: NODE\_400925\_length\_4705\_cov\_23.806589 1962-1970. Max. coverage (+): 0. Max coverage (-): 0.41

Region: NODE\_400925\_length\_4705\_cov\_23.806589 1971-1980. Max. coverage (+): 0.07. Max coverage (-): 0.44

Region: NODE\_400925\_length\_4705\_cov\_23.806589 1981-1989. Max. coverage (+): 0.19. Max coverage (-): 0.3

Region: NODE\_400925\_length\_4705\_cov\_23.806589 1990-1999. Max. coverage (+): 0.19. Max coverage (-): 0.37

Region: NODE\_400925\_length\_4705\_cov\_23.806589 2000-2008. Max. coverage (+): 0. Max coverage (-): 0.33

Region: NODE\_400925\_length\_4705\_cov\_23.806589 2009-2018. Max. coverage (+): 0.04. Max coverage (-): 0.93

Region: NODE\_400925\_length\_4705\_cov\_23.806589 2019-2027. Max. coverage (+): 0.15. Max coverage (-): 0.59

Region: NODE\_400925\_length\_4705\_cov\_23.806589 2028-2037. Max. coverage (+): 0.04. Max coverage (-): 0

Region: NODE\_400925\_length\_4705\_cov\_23.806589 2038-2046. Max. coverage (+): 0. Max coverage (-): 0.04

Region: NODE\_400925\_length\_4705\_cov\_23.806589 2047-2056. Max. coverage (+): 0.19. Max coverage (-): 2

Region: NODE\_400925\_length\_4705\_cov\_23.806589 2057-2065. Max. coverage (+): 0.19. Max coverage (-): 0.15

Region: NODE\_400925\_length\_4705\_cov\_23.806589 2066-2075. Max. coverage (+): 0. Max coverage (-): 0.07

Region: NODE\_400925\_length\_4705\_cov\_23.806589 2076-2085. Max. coverage (+): 0.07. Max coverage (-): 0.07

Region: NODE\_400925\_length\_4705\_cov\_23.806589 2086-2094. Max. coverage (+): 0. Max coverage (-): 1.71

Region: NODE\_400925\_length\_4705\_cov\_23.806589 2095-2104. Max. coverage (+): 0. Max coverage (-): 1.78

Region: NODE\_400925\_length\_4705\_cov\_23.806589 2105-2113. Max. coverage (+): 0.11. Max coverage (-): 5.26

Region: NODE\_400925\_length\_4705\_cov\_23.806589 2114-2123. Max. coverage (+): 0.04. Max coverage (-): 0.44

Region: NODE\_400925\_length\_4705\_cov\_23.806589 2124-2132. Max. coverage (+): 0. Max coverage (-): 6.08

Region: NODE\_400925\_length\_4705\_cov\_23.806589 2133-2142. Max. coverage (+): 0. Max coverage (-): 0.26

Region: NODE\_400925\_length\_4705\_cov\_23.806589 2143-2151. Max. coverage (+): 0.15. Max coverage (-): 0.15

Region: NODE\_400925\_length\_4705\_cov\_23.806589 2152-2161. Max. coverage (+): 0.04. Max coverage (-): 0.04

Region: NODE\_400925\_length\_4705\_cov\_23.806589 2162-2170. Max. coverage (+): 0. Max coverage (-): 0.07

Region: NODE\_400925\_length\_4705\_cov\_23.806589 2171-2180. Max. coverage (+): 0. Max coverage (-): 18.87

Region: NODE\_400925\_length\_4705\_cov\_23.806589 2181-2189. Max. coverage (+): 0. Max coverage (-): 18.91

Region: NODE\_400925\_length\_4705\_cov\_23.806589 2190-2199. Max. coverage (+): 0.04. Max coverage (-): 0.41

Region: NODE\_400925\_length\_4705\_cov\_23.806589 2200-2209. Max. coverage (+): 0.04. Max coverage (-): 47.75

Region: NODE\_400925\_length\_4705\_cov\_23.806589 2210-2218. Max. coverage (+): 0. Max coverage (-): 0.48

Region: NODE\_400925\_length\_4705\_cov\_23.806589 2219-2228. Max. coverage (+): 0.19. Max coverage (-): 0.33

Region: NODE\_400925\_length\_4705\_cov\_23.806589 2229-2237. Max. coverage (+): 0.04. Max coverage (-): 0.3

Region: NODE\_400925\_length\_4705\_cov\_23.806589 2238-2247. Max. coverage (+): 0. Max coverage (-): 5.41

Region: NODE\_400925\_length\_4705\_cov\_23.806589 2248-2256. Max. coverage (+): 0.19. Max coverage (-): 6.23

Region: NODE\_400925\_length\_4705\_cov\_23.806589 2257-2266. Max. coverage (+): 0.15. Max coverage (-): 0.11

Region: NODE\_400925\_length\_4705\_cov\_23.806589 2267-2275. Max. coverage (+): 0. Max coverage (-): 0

Region: NODE\_400925\_length\_4705\_cov\_23.806589 2276-2285. Max. coverage (+): 0. Max coverage (-): 0.15

Region: NODE\_400925\_length\_4705\_cov\_23.806589 2286-2294. Max. coverage (+): 0. Max coverage (-): 0.11

Region: NODE\_400925\_length\_4705\_cov\_23.806589 2295-2304. Max. coverage (+): 0. Max coverage (-): 0.04

Region: NODE\_400925\_length\_4705\_cov\_23.806589 2305-2313. Max. coverage (+): 0. Max coverage (-): 0.02

Region: NODE\_400925\_length\_4705\_cov\_23.806589 2314-2323. Max. coverage (+): 0. Max coverage (-): 0.07

Region: NODE\_400925\_length\_4705\_cov\_23.806589 2324-2333. Max. coverage (+): 0.04. Max coverage (-): 0.48

Region: NODE\_400925\_length\_4705\_cov\_23.806589 2334-2342. Max. coverage (+): 0. Max coverage (-): 0.41

Region: NODE\_400925\_length\_4705\_cov\_23.806589 2343-2352. Max. coverage (+): 0. Max coverage (-): 4.19

Region: NODE\_400925\_length\_4705\_cov\_23.806589 2353-2361. Max. coverage (+): 0.37. Max coverage (-): 0.48

Region: NODE\_400925\_length\_4705\_cov\_23.806589 2362-2371. Max. coverage (+): 0.37. Max coverage (-): 0.44

Region: NODE\_400925\_length\_4705\_cov\_23.806589 2372-2380. Max. coverage (+): 0.07. Max coverage (-): 0.37

Region: NODE\_400925\_length\_4705\_cov\_23.806589 2381-2390. Max. coverage (+): 0. Max coverage (-): 1.22

Region: NODE\_400925\_length\_4705\_cov\_23.806589 2391-2399. Max. coverage (+): 0. Max coverage (-): 0.96

Region: NODE\_400925\_length\_4705\_cov\_23.806589 2400-2409. Max. coverage (+): 0.19. Max coverage (-): 0.3

Region: NODE\_400925\_length\_4705\_cov\_23.806589 2410-2418. Max. coverage (+): 0.15. Max coverage (-): 0.3

Region: NODE\_400925\_length\_4705\_cov\_23.806589 2419-2428. Max. coverage (+): 0. Max coverage (-): 0.26

Region: NODE\_400925\_length\_4705\_cov\_23.806589 2429-2437. Max. coverage (+): 0. Max coverage (-): 0

Region: NODE\_400925\_length\_4705\_cov\_23.806589 2438-2447. Max. coverage (+): 0. Max coverage (-): 0.15

Region: NODE\_400925\_length\_4705\_cov\_23.806589 2448-2457. Max. coverage (+): 0.04. Max coverage (-): 8.16

Region: NODE\_400925\_length\_4705\_cov\_23.806589 2458-2466. Max. coverage (+): 0. Max coverage (-): 1.04

Region: NODE\_400925\_length\_4705\_cov\_23.806589 2467-2476. Max. coverage (+): 0. Max coverage (-): 1.56

Region: NODE\_400925\_length\_4705\_cov\_23.806589 2477-2485. Max. coverage (+): 0. Max coverage (-): 0.11

Region: NODE\_400925\_length\_4705\_cov\_23.806589 2486-2495. Max. coverage (+): 0.04. Max coverage (-): 0.07

Region: NODE\_400925\_length\_4705\_cov\_23.806589 2496-2504. Max. coverage (+): 0. Max coverage (-): 0.07

Region: NODE\_400925\_length\_4705\_cov\_23.806589 2505-2514. Max. coverage (+): 0. Max coverage (-): 0.19

Region: NODE\_400925\_length\_4705\_cov\_23.806589 2515-2523. Max. coverage (+): 0. Max coverage (-): 0.07

Region: NODE\_400925\_length\_4705\_cov\_23.806589 2524-2533. Max. coverage (+): 0. Max coverage (-): 7.3

Region: NODE\_400925\_length\_4705\_cov\_23.806589 2534-2542. Max. coverage (+): 0. Max coverage (-): 7.82

Region: NODE\_400925\_length\_4705\_cov\_23.806589 2543-2552. Max. coverage (+): 0.04. Max coverage (-): 0.11

Region: NODE\_400925\_length\_4705\_cov\_23.806589 2553-2561. Max. coverage (+): 0.04. Max coverage (-): 0.11

Region: NODE\_400925\_length\_4705\_cov\_23.806589 2562-2571. Max. coverage (+): 0.04. Max coverage (-): 2.89

Region: NODE\_400925\_length\_4705\_cov\_23.806589 2572-2581. Max. coverage (+): 0. Max coverage (-): 0.96

Region: NODE\_400925\_length\_4705\_cov\_23.806589 2582-2590. Max. coverage (+): 0.04. Max coverage (-): 0.07

Region: NODE\_400925\_length\_4705\_cov\_23.806589 2591-2600. Max. coverage (+): 0. Max coverage (-): 0

Region: NODE\_400925\_length\_4705\_cov\_23.806589 2601-2609. Max. coverage (+): 0. Max coverage (-): 0.04

Region: NODE\_400925\_length\_4705\_cov\_23.806589 2610-2619. Max. coverage (+): 0. Max coverage (-): 0.3

Region: NODE\_400925\_length\_4705\_cov\_23.806589 2620-2628. Max. coverage (+): 0. Max coverage (-): 0.41

Region: NODE\_400925\_length\_4705\_cov\_23.806589 2629-2638. Max. coverage (+): 0. Max coverage (-): 0.04

Region: NODE\_400925\_length\_4705\_cov\_23.806589 2639-2647. Max. coverage (+): 0.04. Max coverage (-): 0.04

Region: NODE\_400925\_length\_4705\_cov\_23.806589 2648-2657. Max. coverage (+): 0.04. Max coverage (-): 0.07

Region: NODE\_400925\_length\_4705\_cov\_23.806589 2658-2666. Max. coverage (+): 0. Max coverage (-): 0.07

Region: NODE\_400925\_length\_4705\_cov\_23.806589 2667-2676. Max. coverage (+): 0. Max coverage (-): 1.78

Region: NODE\_400925\_length\_4705\_cov\_23.806589 2677-2685. Max. coverage (+): 0. Max coverage (-): 0.56

Region: NODE\_400925\_length\_4705\_cov\_23.806589 2686-2695. Max. coverage (+): 0. Max coverage (-): 0.3

Region: NODE\_400925\_length\_4705\_cov\_23.806589 2696-2705. Max. coverage (+): 0. Max coverage (-): 2

Region: NODE\_400925\_length\_4705\_cov\_23.806589 2706-2714. Max. coverage (+): 0.04. Max coverage (-): 0.33

Region: NODE\_400925\_length\_4705\_cov\_23.806589 2715-2724. Max. coverage (+): 0. Max coverage (-): 0.74

Region: NODE\_400925\_length\_4705\_cov\_23.806589 2725-2733. Max. coverage (+): 0. Max coverage (-): 0.37

Region: NODE\_400925\_length\_4705\_cov\_23.806589 2734-2743. Max. coverage (+): 0. Max coverage (-): 0.63

Region: NODE\_400925\_length\_4705\_cov\_23.806589 2744-2752. Max. coverage (+): 0.04. Max coverage (-): 0.11

Region: NODE\_400925\_length\_4705\_cov\_23.806589 2753-2762. Max. coverage (+): 0.11. Max coverage (-): 0.26

Region: NODE\_400925\_length\_4705\_cov\_23.806589 2763-2771. Max. coverage (+): 0.11. Max coverage (-): 0.82

Region: NODE\_400925\_length\_4705\_cov\_23.806589 2772-2781. Max. coverage (+): 0.04. Max coverage (-): 0.48

Region: NODE\_400925\_length\_4705\_cov\_23.806589 2782-2790. Max. coverage (+): 1.19. Max coverage (-): 0.78

Region: NODE\_400925\_length\_4705\_cov\_23.806589 2791-2800. Max. coverage (+): 0.04. Max coverage (-): 0.15

Region: NODE\_400925\_length\_4705\_cov\_23.806589 2801-2809. Max. coverage (+): 0. Max coverage (-): 0

Region: NODE\_400925\_length\_4705\_cov\_23.806589 2810-2819. Max. coverage (+): 0. Max coverage (-): 0.04

Region: NODE\_400925\_length\_4705\_cov\_23.806589 2820-2829. Max. coverage (+): 0. Max coverage (-): 0.11

Region: NODE\_400925\_length\_4705\_cov\_23.806589 2830-2838. Max. coverage (+): 0. Max coverage (-): 0.67

Region: NODE\_400925\_length\_4705\_cov\_23.806589 2839-2848. Max. coverage (+): 0.15. Max coverage (-): 0.33

Region: NODE\_400925\_length\_4705\_cov\_23.806589 2849-2857. Max. coverage (+): 0. Max coverage (-): 0.19

Region: NODE\_400925\_length\_4705\_cov\_23.806589 2858-2867. Max. coverage (+): 0.11. Max coverage (-): 0.19

Region: NODE\_400925\_length\_4705\_cov\_23.806589 2868-2876. Max. coverage (+): 0. Max coverage (-): 0.15

Region: NODE\_400925\_length\_4705\_cov\_23.806589 2877-2886. Max. coverage (+): 0. Max coverage (-): 0.96

Region: NODE\_400925\_length\_4705\_cov\_23.806589 2887-2895. Max. coverage (+): 0. Max coverage (-): 0.56

Region: NODE\_400925\_length\_4705\_cov\_23.806589 2896-2905. Max. coverage (+): 0.15. Max coverage (-): 0.59

Region: NODE\_400925\_length\_4705\_cov\_23.806589 2906-2914. Max. coverage (+): 0.04. Max coverage (-): 0

Region: NODE\_400925\_length\_4705\_cov\_23.806589 2915-2924. Max. coverage (+): 0.04. Max coverage (-): 6.08

Region: NODE\_400925\_length\_4705\_cov\_23.806589 2925-2933. Max. coverage (+): 0. Max coverage (-): 5.82

Region: NODE\_400925\_length\_4705\_cov\_23.806589 2934-2943. Max. coverage (+): 0. Max coverage (-): 0.19

Region: NODE\_400925\_length\_4705\_cov\_23.806589 2944-2953. Max. coverage (+): 0.04. Max coverage (-): 0.19

Region: NODE\_400925\_length\_4705\_cov\_23.806589 2954-2962. Max. coverage (+): 0. Max coverage (-): 0.19

Region: NODE\_400925\_length\_4705\_cov\_23.806589 2963-2972. Max. coverage (+): 0. Max coverage (-): 0.15

Region: NODE\_400925\_length\_4705\_cov\_23.806589 2973-2981. Max. coverage (+): 0.04. Max coverage (-): 0.11

Region: NODE\_400925\_length\_4705\_cov\_23.806589 2982-2991. Max. coverage (+): 0.04. Max coverage (-): 1.78

Region: NODE\_400925\_length\_4705\_cov\_23.806589 2992-3000. Max. coverage (+): 0.04. Max coverage (-): 0.89

Region: NODE\_400925\_length\_4705\_cov\_23.806589 3001-3010. Max. coverage (+): 0.48. Max coverage (-): 0.63

Region: NODE\_400925\_length\_4705\_cov\_23.806589 3011-3019. Max. coverage (+): 0.07. Max coverage (-): 0.15

Region: NODE\_400925\_length\_4705\_cov\_23.806589 3020-3029. Max. coverage (+): 0. Max coverage (-): 0.07

Region: NODE\_400925\_length\_4705\_cov\_23.806589 3030-3038. Max. coverage (+): 0. Max coverage (-): 0.11

Region: NODE\_400925\_length\_4705\_cov\_23.806589 3039-3048. Max. coverage (+): 0. Max coverage (-): 0.74

Region: NODE\_400925\_length\_4705\_cov\_23.806589 3049-3057. Max. coverage (+): 0. Max coverage (-): 0.96

Region: NODE\_400925\_length\_4705\_cov\_23.806589 3058-3067. Max. coverage (+): 0.04. Max coverage (-): 0.11

Region: NODE\_400925\_length\_4705\_cov\_23.806589 3068-3077. Max. coverage (+): 0.07. Max coverage (-): 0.3

Region: NODE\_400925\_length\_4705\_cov\_23.806589 3078-3086. Max. coverage (+): 0.04. Max coverage (-): 0.37

Region: NODE\_400925\_length\_4705\_cov\_23.806589 3087-3096. Max. coverage (+): 0.15. Max coverage (-): 0.15

Region: NODE\_400925\_length\_4705\_cov\_23.806589 3097-3105. Max. coverage (+): 0.15. Max coverage (-): 0

Region: NODE\_400925\_length\_4705\_cov\_23.806589 3106-3115. Max. coverage (+): 0.04. Max coverage (-): 0.07

Region: NODE\_400925\_length\_4705\_cov\_23.806589 3116-3124. Max. coverage (+): 0. Max coverage (-): 13.64

Region: NODE\_400925\_length\_4705\_cov\_23.806589 3125-3134. Max. coverage (+): 0. Max coverage (-): 13.68

Region: NODE\_400925\_length\_4705\_cov\_23.806589 3135-3143. Max. coverage (+): 0. Max coverage (-): 0.85

Region: NODE\_400925\_length\_4705\_cov\_23.806589 3144-3153. Max. coverage (+): 0. Max coverage (-): 5.56

Region: NODE\_400925\_length\_4705\_cov\_23.806589 3154-3162. Max. coverage (+): 0. Max coverage (-): 20.54

Region: NODE\_400925\_length\_4705\_cov\_23.806589 3163-3172. Max. coverage (+): 0.04. Max coverage (-): 1.15

Region: NODE\_400925\_length\_4705\_cov\_23.806589 3173-3181. Max. coverage (+): 0.3. Max coverage (-): 0.56

Region: NODE\_400925\_length\_4705\_cov\_23.806589 3182-3191. Max. coverage (+): 0.11. Max coverage (-): 2.78

Region: NODE\_400925\_length\_4705\_cov\_23.806589 3192-3200. Max. coverage (+): 0.04. Max coverage (-): 1.74

Region: NODE\_400925\_length\_4705\_cov\_23.806589 3201-3210. Max. coverage (+): 0.04. Max coverage (-): 0.56

Region: NODE\_400925\_length\_4705\_cov\_23.806589 3211-3220. Max. coverage (+): 0. Max coverage (-): 2.04

Region: NODE\_400925\_length\_4705\_cov\_23.806589 3221-3229. Max. coverage (+): 0. Max coverage (-): 1.04

Region: NODE\_400925\_length\_4705\_cov\_23.806589 3230-3239. Max. coverage (+): 0. Max coverage (-): 1.67

Region: NODE\_400925\_length\_4705\_cov\_23.806589 3240-3248. Max. coverage (+): 0.04. Max coverage (-): 0.15

Region: NODE\_400925\_length\_4705\_cov\_23.806589 3249-3258. Max. coverage (+): 0. Max coverage (-): 14.72

Region: NODE\_400925\_length\_4705\_cov\_23.806589 3259-3267. Max. coverage (+): 0. Max coverage (-): 0.44

Region: NODE\_400925\_length\_4705\_cov\_23.806589 3268-3277. Max. coverage (+): 0. Max coverage (-): 0.04

Region: NODE\_400925\_length\_4705\_cov\_23.806589 3278-3286. Max. coverage (+): 0. Max coverage (-): 0.04

Region: NODE\_400925\_length\_4705\_cov\_23.806589 3287-3296. Max. coverage (+): 0. Max coverage (-): 0.37

Region: NODE\_400925\_length\_4705\_cov\_23.806589 3297-3305. Max. coverage (+): 0. Max coverage (-): 0

Region: NODE\_400925\_length\_4705\_cov\_23.806589 3306-3315. Max. coverage (+): 0.04. Max coverage (-): 2.04

Region: NODE\_400925\_length\_4705\_cov\_23.806589 3316-3324. Max. coverage (+): 0.04. Max coverage (-): 2.37

Region: NODE\_400925\_length\_4705\_cov\_23.806589 3325-3334. Max. coverage (+): 0.07. Max coverage (-): 0.78

Region: NODE\_400925\_length\_4705\_cov\_23.806589 3335-3344. Max. coverage (+): 0.17. Max coverage (-): 0.11

Region: NODE\_400925\_length\_4705\_cov\_23.806589 3345-3353. Max. coverage (+): 0.16. Max coverage (-): 0.63

Region: NODE\_400925\_length\_4705\_cov\_23.806589 3354-3363. Max. coverage (+): 0. Max coverage (-): 0.74

Region: NODE\_400925\_length\_4705\_cov\_23.806589 3364-3372. Max. coverage (+): 0. Max coverage (-): 0

Region: NODE\_400925\_length\_4705\_cov\_23.806589 3373-3382. Max. coverage (+): 0. Max coverage (-): 0.07

Region: NODE\_400925\_length\_4705\_cov\_23.806589 3383-3391. Max. coverage (+): 0.04. Max coverage (-): 0.15

Region: NODE\_400925\_length\_4705\_cov\_23.806589 3392-3401. Max. coverage (+): 0.04. Max coverage (-): 0.22

Region: NODE\_400925\_length\_4705\_cov\_23.806589 3402-3410. Max. coverage (+): 0. Max coverage (-): 0

Region: NODE\_400925\_length\_4705\_cov\_23.806589 3411-3420. Max. coverage (+): 0.07. Max coverage (-): 0

Region: NODE\_400925\_length\_4705\_cov\_23.806589 3421-3429. Max. coverage (+): 0. Max coverage (-): 0.15

Region: NODE\_400925\_length\_4705\_cov\_23.806589 3430-3439. Max. coverage (+): 0. Max coverage (-): 0.15

Region: NODE\_400925\_length\_4705\_cov\_23.806589 3440-3448. Max. coverage (+): 0. Max coverage (-): 0.06

Region: NODE\_400925\_length\_4705\_cov\_23.806589 3449-3458. Max. coverage (+): 0.04. Max coverage (-): 0.11

Region: NODE\_400925\_length\_4705\_cov\_23.806589 3459-3468. Max. coverage (+): 0.07. Max coverage (-): 0.26

Region: NODE\_400925\_length\_4705\_cov\_23.806589 3469-3477. Max. coverage (+): 0.07. Max coverage (-): 0.26

Region: NODE\_400925\_length\_4705\_cov\_23.806589 3478-3487. Max. coverage (+): 0. Max coverage (-): 0.04

Region: NODE\_400925\_length\_4705\_cov\_23.806589 3488-3496. Max. coverage (+): 0. Max coverage (-): 0.11

Region: NODE\_400925\_length\_4705\_cov\_23.806589 3497-3506. Max. coverage (+): 0. Max coverage (-): 0.41

Region: NODE\_400925\_length\_4705\_cov\_23.806589 3507-3515. Max. coverage (+): 0. Max coverage (-): 0.04

Region: NODE\_400925\_length\_4705\_cov\_23.806589 3516-3525. Max. coverage (+): 0. Max coverage (-): 0.04

Region: NODE\_400925\_length\_4705\_cov\_23.806589 3526-3534. Max. coverage (+): 0. Max coverage (-): 0.15

Region: NODE\_400925\_length\_4705\_cov\_23.806589 3535-3544. Max. coverage (+): 0. Max coverage (-): 0.19

Region: NODE\_400925\_length\_4705\_cov\_23.806589 3545-3553. Max. coverage (+): 0.04. Max coverage (-): 0.04

Region: NODE\_400925\_length\_4705\_cov\_23.806589 3554-3563. Max. coverage (+): 0.04. Max coverage (-): 0.07

Region: NODE\_400925\_length\_4705\_cov\_23.806589 3564-3572. Max. coverage (+): 0. Max coverage (-): 0.04

Region: NODE\_400925\_length\_4705\_cov\_23.806589 3573-3582. Max. coverage (+): 0. Max coverage (-): 0

Region: NODE\_400925\_length\_4705\_cov\_23.806589 3583-3592. Max. coverage (+): 0. Max coverage (-): 0

Region: NODE\_400925\_length\_4705\_cov\_23.806589 3593-3601. Max. coverage (+): 0. Max coverage (-): 0

Region: NODE\_400925\_length\_4705\_cov\_23.806589 3602-3611. Max. coverage (+): 0. Max coverage (-): 0

Region: NODE\_400925\_length\_4705\_cov\_23.806589 3612-3620. Max. coverage (+): 0. Max coverage (-): 0

Region: NODE\_400925\_length\_4705\_cov\_23.806589 3621-3630. Max. coverage (+): 0.07. Max coverage (-): 0.04

Region: NODE\_400925\_length\_4705\_cov\_23.806589 3631-3639. Max. coverage (+): 0.07. Max coverage (-): 0.11

Region: NODE\_400925\_length\_4705\_cov\_23.806589 3640-3649. Max. coverage (+): 0.07. Max coverage (-): 1

Region: NODE\_400925\_length\_4705\_cov\_23.806589 3650-3658. Max. coverage (+): 0.04. Max coverage (-): 0

Region: NODE\_400925\_length\_4705\_cov\_23.806589 3659-3668. Max. coverage (+): 0. Max coverage (-): 0.04

Region: NODE\_400925\_length\_4705\_cov\_23.806589 3669-3677. Max. coverage (+): 0.02. Max coverage (-): 0.06

Region: NODE\_400925\_length\_4705\_cov\_23.806589 3678-3687. Max. coverage (+): 0.15. Max coverage (-): 0.04

Region: NODE\_400925\_length\_4705\_cov\_23.806589 3688-3696. Max. coverage (+): 0.15. Max coverage (-): 0.04

Region: NODE\_400925\_length\_4705\_cov\_23.806589 3697-3706. Max. coverage (+): 0. Max coverage (-): 0

Region: NODE\_400925\_length\_4705\_cov\_23.806589 3707-3716. Max. coverage (+): 0. Max coverage (-): 0

Region: NODE\_400925\_length\_4705\_cov\_23.806589 3717-3725. Max. coverage (+): 0. Max coverage (-): 0.04

Region: NODE\_400925\_length\_4705\_cov\_23.806589 3726-3735. Max. coverage (+): 0. Max coverage (-): 0.04

Region: NODE\_400925\_length\_4705\_cov\_23.806589 3736-3744. Max. coverage (+): 0. Max coverage (-): 0.07

Region: NODE\_400925\_length\_4705\_cov\_23.806589 3745-3754. Max. coverage (+): 0. Max coverage (-): 0.04

Region: NODE\_400925\_length\_4705\_cov\_23.806589 3755-3763. Max. coverage (+): 0. Max coverage (-): 0.04

Region: NODE\_400925\_length\_4705\_cov\_23.806589 3764-3773. Max. coverage (+): 0. Max coverage (-): 5.52

Region: NODE\_400925\_length\_4705\_cov\_23.806589 3774-3782. Max. coverage (+): 0.04. Max coverage (-): 4.56

Region: NODE\_400925\_length\_4705\_cov\_23.806589 3783-3792. Max. coverage (+): 0.32. Max coverage (-): 0.04

Region: NODE\_400925\_length\_4705\_cov\_23.806589 3793-3801. Max. coverage (+): 0.07. Max coverage (-): 0

Region: NODE\_400925\_length\_4705\_cov\_23.806589 3802-3811. Max. coverage (+): 0. Max coverage (-): 0.63

Region: NODE\_400925\_length\_4705\_cov\_23.806589 3812-3820. Max. coverage (+): 0. Max coverage (-): 0.11

Region: NODE\_400925\_length\_4705\_cov\_23.806589 3821-3830. Max. coverage (+): 0.11. Max coverage (-): 0

Region: NODE\_400925\_length\_4705\_cov\_23.806589 3831-3840. Max. coverage (+): 0. Max coverage (-): 0

Region: NODE\_400925\_length\_4705\_cov\_23.806589 3841-3849. Max. coverage (+): 0. Max coverage (-): 0.67

Region: NODE\_400925\_length\_4705\_cov\_23.806589 3850-3859. Max. coverage (+): 0. Max coverage (-): 0.67

Region: NODE\_400925\_length\_4705\_cov\_23.806589 3860-3868. Max. coverage (+): 0.04. Max coverage (-): 0

Region: NODE\_400925\_length\_4705\_cov\_23.806589 3869-3878. Max. coverage (+): 0.04. Max coverage (-): 0.41

Region: NODE\_400925\_length\_4705\_cov\_23.806589 3879-3887. Max. coverage (+): 0. Max coverage (-): 1.67

Region: NODE\_400925\_length\_4705\_cov\_23.806589 3888-3897. Max. coverage (+): 0. Max coverage (-): 0.59

Region: NODE\_400925\_length\_4705\_cov\_23.806589 3898-3906. Max. coverage (+): 0. Max coverage (-): 0.07

Region: NODE\_400925\_length\_4705\_cov\_23.806589 3907-3916. Max. coverage (+): 0.02. Max coverage (-): 0.02

Region: NODE\_400925\_length\_4705\_cov\_23.806589 3917-3925. Max. coverage (+): 0. Max coverage (-): 0.04

Region: NODE\_400925\_length\_4705\_cov\_23.806589 3926-3935. Max. coverage (+): 0. Max coverage (-): 0.02

Region: NODE\_400925\_length\_4705\_cov\_23.806589 3936-3944. Max. coverage (+): 0.07. Max coverage (-): 0

Region: NODE\_400925\_length\_4705\_cov\_23.806589 3945-3954. Max. coverage (+): 0.07. Max coverage (-): 0

Region: NODE\_400925\_length\_4705\_cov\_23.806589 3955-3964. Max. coverage (+): 0. Max coverage (-): 0

Region: NODE\_400925\_length\_4705\_cov\_23.806589 3965-3973. Max. coverage (+): 0. Max coverage (-): 0

Region: NODE\_400925\_length\_4705\_cov\_23.806589 3974-3983. Max. coverage (+): 0. Max coverage (-): 0

Region: NODE\_400925\_length\_4705\_cov\_23.806589 3984-3992. Max. coverage (+): 0. Max coverage (-): 0.07

Region: NODE\_400925\_length\_4705\_cov\_23.806589 3993-4002. Max. coverage (+): 0. Max coverage (-): 0.07

Region: NODE\_400925\_length\_4705\_cov\_23.806589 4003-4011. Max. coverage (+): 0. Max coverage (-): 0

Region: NODE\_400925\_length\_4705\_cov\_23.806589 4012-4021. Max. coverage (+): 0. Max coverage (-): 0

Region: NODE\_400925\_length\_4705\_cov\_23.806589 4022-4030. Max. coverage (+): 0. Max coverage (-): 0

Region: NODE\_400925\_length\_4705\_cov\_23.806589 4031-4040. Max. coverage (+): 0. Max coverage (-): 0.04

Region: NODE\_400925\_length\_4705\_cov\_23.806589 4041-4049. Max. coverage (+): 0. Max coverage (-): 0.04

Region: NODE\_400925\_length\_4705\_cov\_23.806589 4050-4059. Max. coverage (+): 0. Max coverage (-): 0

Region: NODE\_400925\_length\_4705\_cov\_23.806589 4060-4068. Max. coverage (+): 0. Max coverage (-): 0.04

Region: NODE\_400925\_length\_4705\_cov\_23.806589 4069-4078. Max. coverage (+): 0. Max coverage (-): 0

Region: NODE\_400925\_length\_4705\_cov\_23.806589 4079-4088. Max. coverage (+): 0. Max coverage (-): 0

Region: NODE\_400925\_length\_4705\_cov\_23.806589 4089-4097. Max. coverage (+): 0. Max coverage (-): 0

Region: NODE\_400925\_length\_4705\_cov\_23.806589 4098-4107. Max. coverage (+): 0. Max coverage (-): 0

Region: NODE\_400925\_length\_4705\_cov\_23.806589 4108-4116. Max. coverage (+): 0. Max coverage (-): 0

Region: NODE\_400925\_length\_4705\_cov\_23.806589 4117-4126. Max. coverage (+): 0. Max coverage (-): 0

Region: NODE\_400925\_length\_4705\_cov\_23.806589 4127-4135. Max. coverage (+): 0. Max coverage (-): 0

Region: NODE\_400925\_length\_4705\_cov\_23.806589 4136-4145. Max. coverage (+): 0. Max coverage (-): 0

Region: NODE\_400925\_length\_4705\_cov\_23.806589 4146-4154. Max. coverage (+): 0. Max coverage (-): 0

Region: NODE\_400925\_length\_4705\_cov\_23.806589 4155-4164. Max. coverage (+): 0. Max coverage (-): 0.15

Region: NODE\_400925\_length\_4705\_cov\_23.806589 4165-4173. Max. coverage (+): 0. Max coverage (-): 0.04

Region: NODE\_400925\_length\_4705\_cov\_23.806589 4174-4183. Max. coverage (+): 0.04. Max coverage (-): 0.22

Region: NODE\_400925\_length\_4705\_cov\_23.806589 4184-4192. Max. coverage (+): 0. Max coverage (-): 0.04

Region: NODE\_400925\_length\_4705\_cov\_23.806589 4193-4202. Max. coverage (+): 0. Max coverage (-): 0.07

Region: NODE\_400925\_length\_4705\_cov\_23.806589 4203-4212. Max. coverage (+): 0. Max coverage (-): 0.04

Region: NODE\_400925\_length\_4705\_cov\_23.806589 4213-4221. Max. coverage (+): 0. Max coverage (-): 0

Region: NODE\_400925\_length\_4705\_cov\_23.806589 4222-4231. Max. coverage (+): 0. Max coverage (-): 0.52

Region: NODE\_400925\_length\_4705\_cov\_23.806589 4232-4240. Max. coverage (+): 0. Max coverage (-): 0.82

Region: NODE\_400925\_length\_4705\_cov\_23.806589 4241-4250. Max. coverage (+): 0.04. Max coverage (-): 0.04

Region: NODE\_400925\_length\_4705\_cov\_23.806589 4251-4259. Max. coverage (+): 0.07. Max coverage (-): 0

Region: NODE\_400925\_length\_4705\_cov\_23.806589 4260-4269. Max. coverage (+): 0. Max coverage (-): 0

Region: NODE\_400925\_length\_4705\_cov\_23.806589 4270-4278. Max. coverage (+): 0. Max coverage (-): 0

Region: NODE\_400925\_length\_4705\_cov\_23.806589 4279-4288. Max. coverage (+): 0. Max coverage (-): 0

Region: NODE\_400925\_length\_4705\_cov\_23.806589 4289-4297. Max. coverage (+): 0. Max coverage (-): 0

Region: NODE\_400925\_length\_4705\_cov\_23.806589 4298-4307. Max. coverage (+): 0. Max coverage (-): 0

Region: NODE\_400925\_length\_4705\_cov\_23.806589 4308-4316. Max. coverage (+): 0. Max coverage (-): 0

Region: NODE\_400925\_length\_4705\_cov\_23.806589 4317-4326. Max. coverage (+): 0. Max coverage (-): 0.07

Region: NODE\_400925\_length\_4705\_cov\_23.806589 4327-4336. Max. coverage (+): 0. Max coverage (-): 0.04

Region: NODE\_400925\_length\_4705\_cov\_23.806589 4337-4345. Max. coverage (+): 0.04. Max coverage (-): 0.04

Region: NODE\_400925\_length\_4705\_cov\_23.806589 4346-4355. Max. coverage (+): 0.04. Max coverage (-): 0.04

Region: NODE\_400925\_length\_4705\_cov\_23.806589 4356-4364. Max. coverage (+): 0. Max coverage (-): 0

Region: NODE\_400925\_length\_4705\_cov\_23.806589 4365-4374. Max. coverage (+): 0. Max coverage (-): 0

Region: NODE\_400925\_length\_4705\_cov\_23.806589 4375-4383. Max. coverage (+): 0. Max coverage (-): 0

Region: NODE\_400925\_length\_4705\_cov\_23.806589 4384-4393. Max. coverage (+): 0. Max coverage (-): 0

Region: NODE\_400925\_length\_4705\_cov\_23.806589 4394-4402. Max. coverage (+): 0. Max coverage (-): 0.04

Region: NODE\_400925\_length\_4705\_cov\_23.806589 4403-4412. Max. coverage (+): 0. Max coverage (-): 0.04

Region: NODE\_400925\_length\_4705\_cov\_23.806589 4413-4421. Max. coverage (+): 0. Max coverage (-): 0.07

Region: NODE\_400925\_length\_4705\_cov\_23.806589 4422-4431. Max. coverage (+): 0. Max coverage (-): 0

Region: NODE\_400925\_length\_4705\_cov\_23.806589 4432-4440. Max. coverage (+): 0. Max coverage (-): 0

Region: NODE\_400925\_length\_4705\_cov\_23.806589 4441-4450. Max. coverage (+): 0. Max coverage (-): 0

Region: NODE\_400925\_length\_4705\_cov\_23.806589 4451-4460. Max. coverage (+): 0. Max coverage (-): 0

Region: NODE\_400925\_length\_4705\_cov\_23.806589 4461-4469. Max. coverage (+): 0. Max coverage (-): 0

Region: NODE\_400925\_length\_4705\_cov\_23.806589 4470-4479. Max. coverage (+): 0.04. Max coverage (-): 0

Region: NODE\_400925\_length\_4705\_cov\_23.806589 4480-4488. Max. coverage (+): 0. Max coverage (-): 0

Region: NODE\_400925\_length\_4705\_cov\_23.806589 4489-4498. Max. coverage (+): 0. Max coverage (-): 0

Region: NODE\_400925\_length\_4705\_cov\_23.806589 4499-4507. Max. coverage (+): 0. Max coverage (-): 0.26

Region: NODE\_400925\_length\_4705\_cov\_23.806589 4508-4517. Max. coverage (+): 0. Max coverage (-): 0.04

Region: NODE\_400925\_length\_4705\_cov\_23.806589 4518-4526. Max. coverage (+): 0. Max coverage (-): 0

Region: NODE\_400925\_length\_4705\_cov\_23.806589 4527-4536. Max. coverage (+): 0. Max coverage (-): 0

Region: NODE\_400925\_length\_4705\_cov\_23.806589 4537-4545. Max. coverage (+): 0. Max coverage (-): 0.04

Region: NODE\_400925\_length\_4705\_cov\_23.806589 4546-4555. Max. coverage (+): 0.04. Max coverage (-): 0

Region: NODE\_400925\_length\_4705\_cov\_23.806589 4556-4564. Max. coverage (+): 0.04. Max coverage (-): 0.07

Region: NODE\_400925\_length\_4705\_cov\_23.806589 4565-4574. Max. coverage (+): 0. Max coverage (-): 0.33

Region: NODE\_400925\_length\_4705\_cov\_23.806589 4575-4584. Max. coverage (+): 0. Max coverage (-): 0.09

Region: NODE\_400925\_length\_4705\_cov\_23.806589 4585-4593. Max. coverage (+): 0. Max coverage (-): 0

Region: NODE\_400925\_length\_4705\_cov\_23.806589 4594-4603. Max. coverage (+): 0. Max coverage (-): 0

Region: NODE\_400925\_length\_4705\_cov\_23.806589 4604-4612. Max. coverage (+): 0. Max coverage (-): 0.01

Region: NODE\_400925\_length\_4705\_cov\_23.806589 4613-4622. Max. coverage (+): 0. Max coverage (-): 0.01

Region: NODE\_400925\_length\_4705\_cov\_23.806589 4623-4631. Max. coverage (+): 0. Max coverage (-): 0

Region: NODE\_400925\_length\_4705\_cov\_23.806589 4632-4641. Max. coverage (+): 0. Max coverage (-): 0

Region: NODE\_400925\_length\_4705\_cov\_23.806589 4642-4650. Max. coverage (+): 0. Max coverage (-): 0

Region: NODE\_400925\_length\_4705\_cov\_23.806589 4651-4660. Max. coverage (+): 0. Max coverage (-): 0

Region: NODE\_400925\_length\_4705\_cov\_23.806589 4661-4669. Max. coverage (+): 0. Max coverage (-): 0

Region: NODE\_400925\_length\_4705\_cov\_23.806589 4670-4679. Max. coverage (+): 0. Max coverage (-): 0

Region: NODE\_400925\_length\_4705\_cov\_23.806589 4680-4688. Max. coverage (+): 0. Max coverage (-): 0

Region: NODE\_400925\_length\_4705\_cov\_23.806589 4689-4698. Max. coverage (+): 0. Max coverage (-): 0

Region: NODE\_400925\_length\_4705\_cov\_23.806589 4699-4708. Max. coverage (+): 0. Max coverage (-): 0.04

Region: NODE\_400925\_length\_4705\_cov\_23.806589 4709-4717. Max. coverage (+): 0. Max coverage (-): 0

Region: NODE\_400925\_length\_4705\_cov\_23.806589 4718-4727. Max. coverage (+): 0. Max coverage (-): 0

Region: NODE\_400925\_length\_4705\_cov\_23.806589 4728-4736. Max. coverage (+): 0. Max coverage (-): 0

Region: NODE\_400925\_length\_4705\_cov\_23.806589 4737-4746. Max. coverage (+): 0. Max coverage (-): 0

Region: NODE\_400925\_length\_4705\_cov\_23.806589 4747-4755. Max. coverage (+): 0. Max coverage (-): 0

Region: NODE\_400925\_length\_4705\_cov\_23.806589 4756-4765. Max. coverage (+): 0. Max coverage (-): 0

Region: NODE\_400925\_length\_4705\_cov\_23.806589 4766-. Max. coverage (+): 0. Max coverage (-): 0

RepeatMasker Color Code

**+**

100-98% Identity

<98-95% Identity

<95-90% Identity

<90-85% Identity

<85-80% Identity

<80-75% Identity

<75-70% Identity

<70% Identity

**-**

Gene Set Color Code

**+**

Gene

Pseudogene

Other

**-**

Topology/Coverage Color Code

Coverage Plus Strand

Coverage Minus Strand

Mainstrand: Plus

Mainstrand: Minus

Complementary Strand

Flanking Region  
(if option -flank >0)

Gene Set Annotation  
  
RepeatMasker Annotation  

**1. AlRepB-358**: 1-129 (-), Divergence to consensus: 2.4%  
**2. AlRepB-358**: 509-661 (-), Divergence to consensus: 4%  
**3. AlRepB-358**: 738-967 (-), Divergence to consensus: 12.3%  
**4. AlRepC-235**: 986-1367 (+), Divergence to consensus: 6.6%  
**5. AlRepD-2195**: 1896-1982 (-), Divergence to consensus: 32.6%  
**6. (CCT)n**: 2307-2335 (+), Divergence to consensus: 7.3%  
**7. (GGA)n**: 2817-2837 (+), Divergence to consensus: 4.8%  
**8. TC1\_FR3**: 3209-3759 (-), Divergence to consensus: 31.5%  
**9. TC1\_FR3**: 3783-3835 (-), Divergence to consensus: 28.3%  
**10. AlRepD-1165**: 3828-4044 (+), Divergence to consensus: 18.4%  
**11. AlRepB-44**: 4341-4414 (-), Divergence to consensus: 24%  
**12. Mariner-N1\_AAe**: 4371-4426 (+), Divergence to consensus: 25%  
**13. Expander**: 4490-4561 (-), Divergence to consensus: 23.9%  
**14. REX1-3\_AFC**: 4576-4769 (-), Divergence to consensus: 11.9%

  
Transcription Factor Binding Sites  

**RFX4\_1** (Sequence: CTTGGCAAC (+): 3662)  
**RHOXF1** (Sequence: AGCTTA (-): 564)  
**RHOXF1** (Sequence: AGATCA (-): 1013)  
**RHOXF1** (Sequence: AGCTTA (-): 1212)  
**RHOXF1** (Sequence: AGCTCA (-): 1471)  
**RHOXF1** (Sequence: GGCTCA (-): 1495)  
**RHOXF1** (Sequence: GGATTA (-): 2364)  
**RHOXF1** (Sequence: AGCTCA (-): 3190)  
**RHOXF1** (Sequence: GGCTCA (-): 3405)  
**RHOXF1** (Sequence: GGATTA (-): 4226)  
**RHOXF1** (Sequence: AGCTCA (-): 4525)  
**RHOXF1** (Sequence: AGATCA (-): 4564)  
**RHOXF1** (Sequence: TAATCT (+): 652)  
**RHOXF1** (Sequence: TAATCT (+): 758)  
**RHOXF1** (Sequence: TGATCT (+): 981)  
**RHOXF1** (Sequence: TGATCC (+): 1304)  
**RHOXF1** (Sequence: TGATCT (+): 1330)  
**RHOXF1** (Sequence: TGAGCC (+): 3570)  
**RHOXF1** (Sequence: TAATCC (+): 3603)  
**RHOXF1** (Sequence: TAATCC (+): 4234)  
**POU5F1** (Sequence: TTTGCAT (-): 594)  
**POU5F1** (Sequence: TTTGCAT (-): 1519)  
**POU5F1** (Sequence: TTTGCAT (-): 1662)  
**RFX4\_1** (Sequence: GTTGCCATG (-): 1970)  
**Sox5** (Sequence: ATTGTT (+): 1065)  
**Sox5** (Sequence: ATTGTT (+): 3932)  
**Sox5** (Sequence: ATTGTT (+): 3955)  
**SOX9** (Sequence: TCATTGTT (+): 3953)  
**Nobox** (Sequence: AGCAATTA (-): 2883)  
**Rhox11** (Sequence: TGGTGTTAT (+): 4012)  
**Sox5** (Sequence: AACAAT (-): 4355)  
**Sox5** (Sequence: AACAAT (-): 4421)
