# Supplementary material for: First characterization of PIWI-interacting RNA clusters in a cichlid fish with a B chromosome
Source: BMC Biol. 2022 Sep 21;20:204. doi: 10.1186/s12915-022-01403-2 (PMC9490952; doi:10.1186/s12915-022-01403-2)
Supplement: Supplementary file 2 — Additional file 2. PDF file containing reverse transcriptase and transposase alignment information. [file 12915_2022_1403_MOESM2_ESM.pdf]

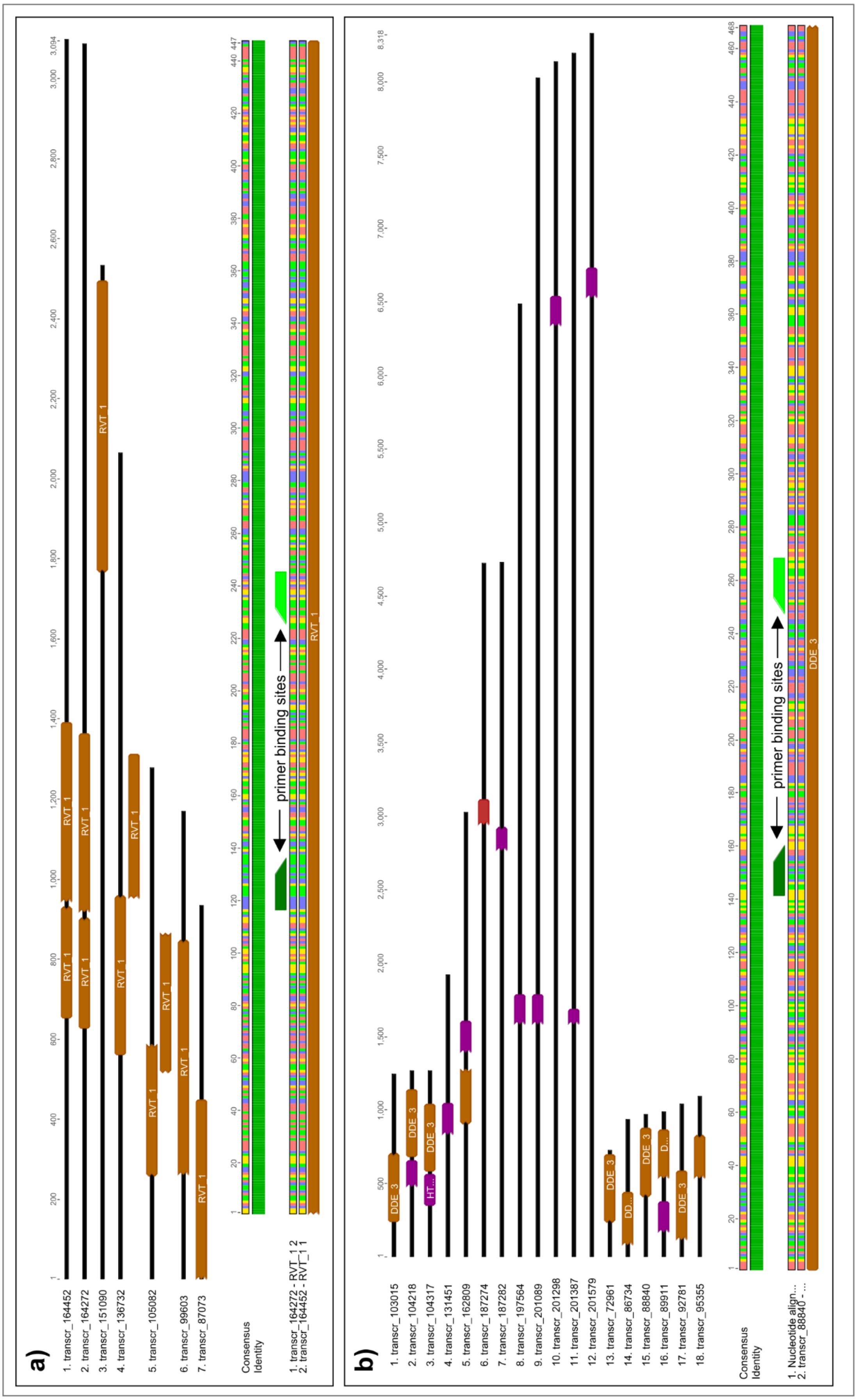

Additional File 2 – *Reverse transcriptase* and *transposase* transcripts alignments. a) reverse transcriptase transcripts: the RVT domain from Pfam is highlighted by the orange boxes. Below the transcript annotation, there are consensus sequences used to primer binding, highlighted by the arrows. b) transposase transcripts: the domains DDE (orange boxes) and HTH (purple and red boxes) are highlighted in the sequences. The consensus sequences below the annotation shows the RT-PCR primer binding sites.
