# Supplementary material for: First characterization of PIWI-interacting RNA clusters in a cichlid fish with a B chromosome
Source: BMC Biol. 2022 Sep 21;20:204. doi: 10.1186/s12915-022-01403-2 (PMC9490952; doi:10.1186/s12915-022-01403-2)
Supplement: Supplementary file 3 — Additional file 3. PDF file containing the pld6 B-mutation alignment. [file 12915_2022_1403_MOESM3_ESM.pdf]

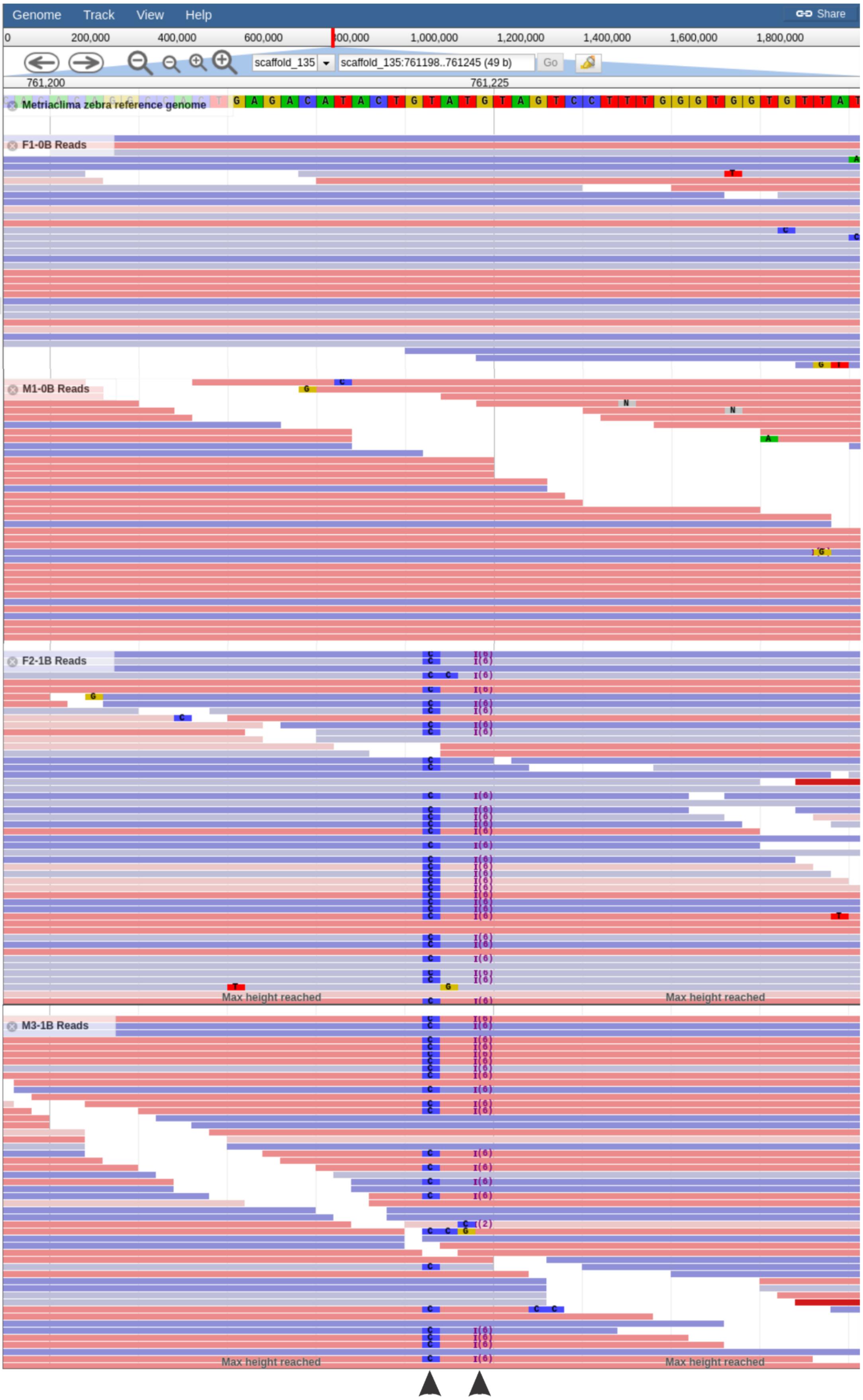


**Additional File 3 –** *Pld6* B specific mutation. Genomic sequencing reads of female B- (F1-0B Reads), male B- (M1-0B Reads), female B+ (F2-1B Reads), male B+ (M3-1B Reads) samples aligned against *M. zebra* scaffold. The arrows bellow to alignments highlighted the B-specific mutations used to B-pld6 primer described in Figure 5c. The B specific mutations at this point are: a transition of thymine to cytosine and a insertion of 6 bases (CCGGTA).
