# Supplementary material for: First characterization of PIWI-interacting RNA clusters in a cichlid fish with a B chromosome
Source: BMC Biol. 2022 Sep 21;20:204. doi: 10.1186/s12915-022-01403-2 (PMC9490952; doi:10.1186/s12915-022-01403-2)
Supplement: Supplementary file 5 — Additional file 5. PDF file containing Table S01 Small RNA sequencing Table S02 Primer sequences. [file 12915_2022_1403_MOESM5_ESM.pdf]

Additional File 5 – Table S01 small RNA sequencing

Library	Sample	Raw reads	After filtering (by length and quality)
Female B-	1	11075285	4886497
	2	11342803	3758188
	3	15910422	6262122
Female B+	1	16160535	2977051
	2	19471118	6689944
	3	11240729	3819732
Male B-	1	12462763	8512392
	2	15851784	11510138
	3	12930943	8940430
Male B+	1	12914266	9235521
	2	13043032	9329908
	3	19914188	12903238

Additional File 5 – Table S02 Primer sequence list

Primer name	Primer sequence
biwi1-RT-FW	GGGGACTTGATGCATTGAGAGA
biwi1-RT-RV	CAGCAAGCAATCCAAGATAAACG
biwi2-RT-FW	ACACATGAAACAGTCCCTGACC
biwi2-RT-RV	ATGAGCTGGGATGGATTTCTGA
biwi3-RT-FW	GTGTGAAAAGTGGTCAATAAAGAGA
biwi3-RT-RV	CGAGACTTTCGACCCCAGAT
pld6-RT-FW	CCTCAGTAAGCCATGTCTGCTC
pld6-RT-RV	CGAGTAGACCTTGTCAGTGAGGA
pld6-gDNA-AeB-FW	CTCATTGCCTCATGGTGTAGA
pld6-gDNA-A-RV	CAATAACACCACCCAAAGGACTAC
pld6-gDNA-Bmut-RV	CACCACCCAAAGGACTACCG
RVT-RT-fw	CCCCTTTTGCCTCTTCTTTT
RVT-RT-rv	ATGTCGTCCGCAAATAATCC
DDE_3-RT-fw	GGTTGCTTCTCATCCAAGG
DDE_3-RT-rv	CACCAAACTGTTGTTGCATTG
UBCE_Fw	GTCCGTTTCAATCCCAACTT
UBCE_Rv	GTTCTCCGTCATCAGAGACT
HPRT-p2F	GACATCATGGATGACATGGGGG
HPRT-p1R	CTTTCAGCACACACAGAGCC
Genotipagem 1	GGAGTGAATTGTGATGGT
Genotipagem 2	AGAATGGTCCAAGGAAGG
Genotipagem 3	CCATCAGAACCAGCATTA
Piwi1_Ala_F	CGTCAGCGTTCCTCTGTATATTAG
Piwi1_Ala_R	CCTAACAGGCTTGACTGATAAGATG
Piwi2_Ala_F	ACTCCGTTCCCGTCAGAGAG
Piwi2_Ala_R	CAGAGGCAGCAGTTTCCTTG
