# Supplementary material for: First characterization of PIWI-interacting RNA clusters in a cichlid fish with a B chromosome
Source: BMC Biol. 2022 Sep 21;20:204. doi: 10.1186/s12915-022-01403-2 (PMC9490952; doi:10.1186/s12915-022-01403-2)
Supplement: Supplementary file 6 — Additional file 6. PDF file containing Table S03Pld6 NCBI ID accession numbers. [file 12915_2022_1403_MOESM6_ESM.pdf]

Additional File 6

Table S03 - *Pld6* NCBI ID access

Species	NCBI access	group
Amblyraja radiata	XM_033040771.1	Chondrichthyes
Callorhinchus milii	XM_007893529.1	Chondrichthyes
Carcharodon carcharias	XM_041206335.1	Chondrichthyes
Scyliorhinus canicula	XM_038821098.1	Chondrichthyes
Drosophila melanogaster	NM_135686.3	fly
Canis lupus familiaris	XM_038665822.1	mammal
Equus caballus	XM_023653687.1	mammal
Homo sapiens	NM_178836.3	mammal
Mus musculus	NM_001290283.1	mammal
Ornithorhynchus anatinus	XM_029058309.2	mammal
Pan troglodytes	NM_001246461.1	mammal
Pteropus giganteus	XM_039847592.1	mammal
Acanthochromis polyacanthus	XM_022193845.1	teleost
Anabas testudineus	XM_026360256.1	teleost
Anarrhichthys ocellatus	XM_031872056.1	teleost
Astatotilapia calliptera	XM_026170637.1	teleost
Astyanax mexicanus	XM_022675028.1	teleost
Boleophthalmus pectinirostris	XM_020938480.1	teleost
Chanos chanos	XM_030780291.1	teleost
Cottoperca gobio	XM_029433102.1	teleost
Cynoglossus semilaevis	XM_008317564.3	teleost
Danio rerio	NM_001089414.1	teleost
Gadus morhua	XM_030351978.1	teleost
Gouania willdenowi	XM_028458385.1	teleost
Haplochromis burtoni	XM_005936420.1	teleost
Hippocampus comes	XM_019862566.1	teleost
Metriaclima zebra	XM_004567832.2	teleost
Monopterus albus	XM_020597006.1	teleost
Nothobranchius furzeri	XM_015954278.1	teleost
Notothenia coriiceps	XM_010777047.1	teleost
Oreochromis niloticus	XM_003447935.4	teleost
Oryzias latipes	XM_004065686.4	teleost
Paralichthys olivaceus	XM_020084343.1	teleost
Paramormyrops kingsleyae	XM_023791931.1	teleost
Poecilia formosa	XM_007551629.2	teleost
Salarias fasciatus	XM_030092971.1	teleost
Scleropages formosus	XM_018734810.2	teleost
Takifugu rubripes	XM_029851242.1	teleost
Xiphophorus maculatus	XM_023334031.1	teleost
